# Supplementary material for: A novel Drosophila injury model reveals severed axons are cleared through a Draper/MMP-1 signaling cascade
Source: eLife. 2017 Aug 21;6:e23611. doi: 10.7554/eLife.23611 (PMC5565368; doi:10.7554/eLife.23611)
Supplement: Table 1—source code 3. — DOI: http://dx.doi.org/10.7554/eLife.23611.015 [file elife-23611-table1-code3.zip › Table 1 - Source Data 9 - human_annokey_report.html]

 
 
 
 
 Annokey Search Report 
 
 
 
 
 Annokey Search Report 
 
 
  annokey version:  1.0.2 
  annokey homepage:   http://bjpop.github.io/annokey/  
  hostname:  barcoo 
  directory:  /vlsci/VLSCI/bjpop/scratch/annokey_sean_speese/human 
  command:  annokey --email bjpope@unimelb.edu.au --terms ../search_terms.csv --genes human_genes_unique.csv --log log --pubmed 
  date:  29 Apr 2017 
 
  
 MTHFD2 
 
  NCBI Gene  
  GeneRIF  
  Pubmed  
 
 
  Search Term  Rank  Fields  
  [Mm]igration  6  PubMed(1)  
  [Ee]xtracellular [Mm]atrix  8  PubMed(1)  
  [Ii]nvasive  10  PubMed(2)  
  [Mm]etastasis  12  PubMed(1)  
  [Cc]ell [Ii]nvasion  13  PubMed(1)  
 
 
 hide/show details for MTHFD2 
  [Mm]igration  PubMed  
 High-throughput RNAi screening for novel modulators of vimentin expression identifies MTHFD2 as a regulator of breast cancer cell   migration   and invasion. Vimentin is an intermediate filament protein, with a key role in the epithelial to mesenchymal transition as well as cell invasion, and it is often upregulated during cancer progression. However, relatively little is known about its regulation in cancer cells. Here, we performed an RNA interference screen followed by protein lysate microarray analysis in bone metastatic MDA-MB-231(SA) breast cancer cells to identify novel regulators of vimentin expression. Out of the 596 genes investigated, three novel vimentin regulators EPHB4, WIPF2 and MTHFD2 were identified. The reduced vimentin expression in response to EPHB4, WIPF2 and MTHFD2 silencing was observed at mRNA and protein levels. Bioinformatic analysis of gene expression data across cancers indicated overexpression of EPHB4 and MTHFD2 in breast cancer and high expression associated with poor clinical characteristics. Analysis of 96 cDNA samples derived from both normal and malignant human tissues suggested putative association with metastatic disease. MTHFD2 knockdown resulted in impaired cell   migration   and invasion into extracellular matrix as well as decreased the fraction of cells with a high CD44 expression, a marker of cancer stem cells. Furthermore, MTHFD2 expression was induced in response to TGF-β stimulation in breast cancer cells. Our results show that MTHFD2 is overexpressed in breast cancer, associates with poor clinical characteristics and promotes cellular features connected with metastatic disease, thus implicating MTHFD2 as a potential drug target to block breast cancer cell   migratio  n and invasion. 
  [Ee]xtracellular [Mm]atrix  PubMed  
 High-throughput RNAi screening for novel modulators of vimentin expression identifies MTHFD2 as a regulator of breast cancer cell migration and invasion. Vimentin is an intermediate filament protein, with a key role in the epithelial to mesenchymal transition as well as cell invasion, and it is often upregulated during cancer progression. However, relatively little is known about its regulation in cancer cells. Here, we performed an RNA interference screen followed by protein lysate microarray analysis in bone metastatic MDA-MB-231(SA) breast cancer cells to identify novel regulators of vimentin expression. Out of the 596 genes investigated, three novel vimentin regulators EPHB4, WIPF2 and MTHFD2 were identified. The reduced vimentin expression in response to EPHB4, WIPF2 and MTHFD2 silencing was observed at mRNA and protein levels. Bioinformatic analysis of gene expression data across cancers indicated overexpression of EPHB4 and MTHFD2 in breast cancer and high expression associated with poor clinical characteristics. Analysis of 96 cDNA samples derived from both normal and malignant human tissues suggested putative association with metastatic disease. MTHFD2 knockdown resulted in impaired cell migration and invasion into   extracellular matrix   as well as decreased the fraction of cells with a high CD44 expression, a marker of cancer stem cells. Furthermore, MTHFD2 expression was induced in response to TGF-β stimulation in breast cancer cells. Our results show that MTHFD2 is overexpressed in breast cancer, associates with poor clinical characteristics and promotes cellular features connected with metastatic disease, thus implicating MTHFD2 as a potential drug target to block breast cancer cell migration and invasion. 
  [Ii]nvasive  PubMed  
 Comparison of tear protein levels in breast cancer patients and healthy controls using a de novo proteomic approach. Non  invasive   biomarkers are urgently needed for early detection of breast cancer since the risk of recurrence, morbidity and mortality are closely related to disease stage at the time of primary surgery. In the past decade, many proteomics-based approaches were developed that utilize the protein profiling of human body fluids or identification of putative biomarkers to obtain more knowledge on the effects of cancer emergence and progression. Herein, we report on an analysis of proteins in the tear fluid from breast carcinoma patients and healthy women using a de novo proteomic approach and 25 mixed samples from each group. This study included 25 patients with primary   invasiv  e breast carcinoma and 25 age-matched healthy controls. We performed a MALDI-TOF-TOF-driven semi-quantitative comparison of tear protein levels in cancer (CA) and control (CTRL) using a de novo approach in pooled samples. Over 150 proteins in the tear fluid of CTRL and CA were identified. Using an in-house-developed algorithm we found more than 20 proteins distinctly upregulated or downregulated in the CTRL and CA groups. We identified several proteins that had modified expression in breast cancer patients. These proteins are involved in host immune system pathways (e.g., C1Q1 or S100A8) and different metabolic cascades (ALDH3A or TPI). Further validation of the results in an independent population combined with individual protein profiling of participants is needed to confirm the specificity of our findings and may lead to a better understanding of the pathological mechanism of breast cancer. 
 Genetic susceptibility to distinct bladder cancer subphenotypes. Clinical, pathologic, and molecular evidence indicate that bladder cancer is heterogeneous with pathologic/molecular features that define distinct subphenotypes with different prognoses. It is conceivable that specific patterns of genetic susceptibility are associated with particular subphenotypes.To examine evidence for the contribution of germline genetic variation to bladder cancer heterogeneity.The Spanish Bladder Cancer/EPICURO Study is a case-control study based in 18 hospitals located in five areas in Spain. Cases were patients with a newly diagnosed, histologically confirmed, urothelial cell carcinoma of the bladder from 1998 to 2001. Case diagnoses were reviewed and uniformly classified by pathologists following the World Health Organisation/International Society of Urological Pathology 1999 criteria. Controls were hospital-matched patients (n=1149).A total of 1526 candidate variants in 423 candidate genes were analysed. Three distinct subphenotypes were defined according to stage and grade: low-grade nonmuscle   invasive   (n=586), high-grade nonmuscle   invasive   (n=219), and muscle   invasive   (n=246). The association between each variant and subphenotype was assessed by polytomous risk models adjusting for potential confounders. Heterogeneity in genetic susceptibility among subphenotypes was also tested.Two established bladder cancer susceptibility genotypes, NAT2 slow-acetylation and GSTM1-null, exhibited similar associations among the subphenotypes, as did VEGF-rs25648, which was previously identified in our study. Other variants conferred risks for specific tumour subphenotypes such as PMS2-rs6463524 and CD4-rs3213427 (respective heterogeneity p values of 0.006 and 0.004), which were associated with muscle-  invasive   tumours (per-allele odds ratios [95% confidence interval] of 0.56 [0.41-0.77] and 0.71 [0.57-0.88], respectively) but not with non-muscle-  invasive   tumours. Heterogeneity p values were not robust in multiple testing according to their false-discovery rate.These exploratory analyses suggest that genetic susceptibility loci might be related to the molecular/pathologic diversity of bladder cancer. Validation through large-scale replication studies and the study of additional genes and single nucleotide polymorphisms are required. 
  [Mm]etastasis  PubMed  
 Increased MTHFD2 expression is associated with poor prognosis in breast cancer. The aim of this study was to investigate the expression levels of methylenetetrahydrofolate dehydrogenase (NADP + -dependent) 2 (MTHFD2) and the associated clinical implications in breast cancer. MTHFD2 expression was measured by Western blot and immunohistochemistry in 698 tissue sections taken from breast cancer patients. The relationship between MTHFD2 expression, clinicopathological parameters, and the prognosis of breast cancer was subsequently determined. In comparison with para-carcinoma tissue specimens, an enhanced expression of MTHFD2 was observed in breast cancer tissue specimens (P < 0.05). In total, 41.12 % (287/698) of breast cancer tissue specimens had high levels of MTHFD2. After universal and Spearman regression correlation analyses, MTHFD2 expression was found to correlate with tumor size, histological grade, lym  ph node me  tastasis, and distant metastases (P = 0.001, 0.002, 0.001, and 0.001, respectively). Furthermore, patients with MTHFD2-expressing tumors had a significantly poorer prognosis than those with no or low MTHFD2 expression. (P = 0.002). Using the Cox regression test, MTHFD2 was identified as an independent prognostic factor (P = 0.001). MTHFD2 was differentially expressed in breast cancer tissue. Therefore, this protein may be an independent prognostic factor and a potential therapeutic target for future breast cancer treatments. 
  [Cc]ell [Ii]nvasion  PubMed  
 High-throughput RNAi screening for novel modulators of vimentin expression identifies MTHFD2 as a regulator of breast cancer cell migration and invasion. Vimentin is an intermediate filament protein, with a key role in the epithelial to mesenchymal transition as well as   cell invasion  , and it is often upregulated during cancer progression. However, relatively little is known about its regulation in cancer cells. Here, we performed an RNA interference screen followed by protein lysate microarray analysis in bone metastatic MDA-MB-231(SA) breast cancer cells to identify novel regulators of vimentin expression. Out of the 596 genes investigated, three novel vimentin regulators EPHB4, WIPF2 and MTHFD2 were identified. The reduced vimentin expression in response to EPHB4, WIPF2 and MTHFD2 silencing was observed at mRNA and protein levels. Bioinformatic analysis of gene expression data across cancers indicated overexpression of EPHB4 and MTHFD2 in breast cancer and high expression associated with poor clinical characteristics. Analysis of 96 cDNA samples derived from both normal and malignant human tissues suggested putative association with metastatic disease. MTHFD2 knockdown resulted in impaired cell migration and invasion into extracellular matrix as well as decreased the fraction of cells with a high CD44 expression, a marker of cancer stem cells. Furthermore, MTHFD2 expression was induced in response to TGF-β stimulation in breast cancer cells. Our results show that MTHFD2 is overexpressed in breast cancer, associates with poor clinical characteristics and promotes cellular features connected with metastatic disease, thus implicating MTHFD2 as a potential drug target to block breast cancer cell migration and invasion. 
   
  
 TINAGL1 
 
  NCBI Gene  
  GeneRIF  
  Pubmed  
 
 
  Search Term  Rank  Fields  
  ECM  7  PubMed(2)  
  [Ee]xtracellular [Mm]atrix  8  Function(1); PubMed(5); Component(2)  
  [Mm]etastasis  12  PubMed(1)  
 
 
 hide/show details for TINAGL1 
  ECM  PubMed  
 Proteomics analysis of cardiac extracellular matrix remodeling in a porcine model of ischemia/reperfusion injury. After myocardial ischemia, extracellular matrix (  ECM  ) deposition occurs at the site of the focal injury and at the border region.We have applied a novel proteomic method for the analysis of   ECM   in cardiovascular tissues to a porcine model of ischemia/reperfusion injury.   ECM   proteins were sequentially extracted and identified by liquid chromatography tandem mass spectrometry. For the first time,   ECM   proteins such as cartilage intermediate layer protein 1, matrilin-4, extracellular adipocyte enhancer binding protein 1, collagen α-1(XIV), and several members of the small leucine-rich proteoglycan family, including asporin and prolargin, were shown to contribute to cardiac remodeling. A comparison in 2 distinct cardiac regions (the focal injury in the left ventricle and the border region close to the occluded coronary artery) revealed a discordant regulation of protein and mRNA levels; although gene expression for selected   EC  M proteins was similar in both regions, the corresponding protein levels were much higher in the focal lesion. Further analysis based on >100   EC  M proteins delineated a signature of early- and late-stage cardiac remodeling with transforming growth factor-β1 signaling at the center of the interaction network. Finally, novel cardia  c E  CM proteins identified by proteomics were validated in human left ventricular tissue acquired from ischemic cardiomyopathy patients at cardiac transplantation.Our findings reveal a biosignature of early- and late-stag  e E  CM remodeling after myocardial ischemia/reperfusion injury, which may have clinical utility as a prognostic marker and modifiable target for drug discovery. 
 Proteomics characterization of extracellular space components in the human aorta. The vascular extracellular matrix (  ECM  ) is essential for the structural integrity of the vessel wall and also serves as a substrate for the binding and retention of secreted products of vascular cells as well as molecules coming from the circulation. Although proteomics has been previously applied to vascular tissues, few studies have specifically targeted the vascular   ECM   and its associated proteins. Thus, its detailed composition remains to be characterized. In this study, we describe a methodology for the extraction of extracellular proteins from human aortas and their identification by proteomics. The approach is based on (a) effective decellularization to enrich for scarce extracellular proteins, (b) successful solubilization and deglycosylation of   ECM   proteins, and (c) relative estimation of protein abundance using spectral counting. Our three-step extraction approach resulted in the identification of 103 extracellular proteins of which one-third have never been reported in the proteomics literature of vascular tissues. In particular, three glycoproteins (podocan, sclerostin, and agrin) were identified for the first time in human aortas at the protein level. We also identified extracellular adipocyte enhancer-binding protein 1, the cartilage glycoprotein asporin, and a previously hypothetical protein, retinal pigment epithelium (RPE) spondin. Moreover, our methodology allowed us to screen for proteolysis in the aortic samples based on the identification of proteolytic enzymes and their corresponding degradation products. For instance, we were able to detect matrix metalloproteinase-9 by mass spectrometry and relate its presence to degradation of fibronectin in a clinical specimen. We expect this proteomics methodology to further our understanding of the composition of the vascular extracellular environment, shed light on   ECM   remodeling and degradation, and provide insights into important pathological processes, such as plaque rupture, aneurysm formation, and restenosis. 
  [Ee]xtracellular [Mm]atrix  Function  
   extracellular matrix   structural constituent 
  PubMed  
 SILAC-based proteomics of human primary endothelial cell morphogenesis unveils tumor angiogenic markers. Proteomics has been successfully used for cell culture on dishes, but more complex cellular systems have proven to be challenging and so far poorly approached with proteomics. Because of the complexity of the angiogenic program, we still do not have a complete understanding of the molecular mechanisms involved in this process, and there have been no in depth quantitative proteomic studies. Plating endothelial cells on matrigel recapitulates aspects of vessel growth, and here we investigate this mechanism by using a spike-in SILAC quantitative proteomic approach. By comparing proteomic changes in primary human endothelial cells morphogenesis on matrigel to general adhesion mechanisms in cells spreading on culture dish, we pinpoint pathways and proteins modulated by endothelial cells. The cell-  extracellular matrix   adhesion proteome depends on the adhesion substrate, and a detailed proteomic profile of the   extracellular matrix   secreted by endothelial cells identified CLEC14A as a matrix component, which binds to MMRN2. We verify deregulated levels of these proteins during tumor angiogenesis in models of multistage carcinogenesis. This is the most in depth quantitative proteomic study of endothelial cell morphogenesis, which shows the potential of applying high accuracy quantitative proteomics to in vitro models of vessel growth to shed new light on mechanisms that accompany pathological angiogenesis. The mass spectrometry proteomics data have been deposited to the ProteomeXchange Consortium with the data set identifier PXD000359. 
 Proteomics analysis of cardiac   extracellular matrix   remodeling in a porcine model of ischemia/reperfusion injury. After myocardial ischemia,   extracellular matrix   (ECM) deposition occurs at the site of the focal injury and at the border region.We have applied a novel proteomic method for the analysis of ECM in cardiovascular tissues to a porcine model of ischemia/reperfusion injury. ECM proteins were sequentially extracted and identified by liquid chromatography tandem mass spectrometry. For the first time, ECM proteins such as cartilage intermediate layer protein 1, matrilin-4, extracellular adipocyte enhancer binding protein 1, collagen α-1(XIV), and several members of the small leucine-rich proteoglycan family, including asporin and prolargin, were shown to contribute to cardiac remodeling. A comparison in 2 distinct cardiac regions (the focal injury in the left ventricle and the border region close to the occluded coronary artery) revealed a discordant regulation of protein and mRNA levels; although gene expression for selected ECM proteins was similar in both regions, the corresponding protein levels were much higher in the focal lesion. Further analysis based on >100 ECM proteins delineated a signature of early- and late-stage cardiac remodeling with transforming growth factor-β1 signaling at the center of the interaction network. Finally, novel cardiac ECM proteins identified by proteomics were validated in human left ventricular tissue acquired from ischemic cardiomyopathy patients at cardiac transplantation.Our findings reveal a biosignature of early- and late-stage ECM remodeling after myocardial ischemia/reperfusion injury, which may have clinical utility as a prognostic marker and modifiable target for drug discovery. 
 Cloning, characterization, and expression of the human TIN-ag-RP gene encoding a novel putative   extracellular matrix   protein. The human gene encoding a novel tubulointerstitial nephritis antigen (TIN-ag)-related protein (TIN-ag-RP) was isolated, and its genomic organization was determined. BLAST searches revealed the highest degree of homology to several mammalian TIN-ag orthologues, and a weak homology to cathepsin B-like proteases. The 12 kb gene was mapped by fluorescence in situ hybridization to chromosome 1p34.2-3, a locus neither related to that of the human TIN-ag (6p11.2-12) nor to that of cathepsin B (8p22-23.1). The TIN-ag-RP is encoded in ten exons with introns ranging from 83 bp to 4 kb. In addition, the gene contained one exon in the 5'UTR, but none in the 3'UTR. Five of the 10 splice sites of the TIN-ag-RP gene were fully conserved when compared to a related gene of C. elegans, whereas only one splice site was identical to those found in cathepsin B genes. Furthermore, human TIN-ag-RP tagged with the T7-epitope, was expressed in HeLa cells, and was found to be localized in vesicular compartments as well as secreted into the medium suggesting the involvement of the endosomal trafficking pathway. Based on the high degree of homology of the amino acid sequences and genomic organization between TIN-ag-RP and TIN-ag, we suggest that both molecules may form a distinct group or family of TIN-ag-like proteins. 
 Proteomics characterization of extracellular space components in the human aorta. The vascular   extracellular matrix   (ECM) is essential for the structural integrity of the vessel wall and also serves as a substrate for the binding and retention of secreted products of vascular cells as well as molecules coming from the circulation. Although proteomics has been previously applied to vascular tissues, few studies have specifically targeted the vascular ECM and its associated proteins. Thus, its detailed composition remains to be characterized. In this study, we describe a methodology for the extraction of extracellular proteins from human aortas and their identification by proteomics. The approach is based on (a) effective decellularization to enrich for scarce extracellular proteins, (b) successful solubilization and deglycosylation of ECM proteins, and (c) relative estimation of protein abundance using spectral counting. Our three-step extraction approach resulted in the identification of 103 extracellular proteins of which one-third have never been reported in the proteomics literature of vascular tissues. In particular, three glycoproteins (podocan, sclerostin, and agrin) were identified for the first time in human aortas at the protein level. We also identified extracellular adipocyte enhancer-binding protein 1, the cartilage glycoprotein asporin, and a previously hypothetical protein, retinal pigment epithelium (RPE) spondin. Moreover, our methodology allowed us to screen for proteolysis in the aortic samples based on the identification of proteolytic enzymes and their corresponding degradation products. For instance, we were able to detect matrix metalloproteinase-9 by mass spectrometry and relate its presence to degradation of fibronectin in a clinical specimen. We expect this proteomics methodology to further our understanding of the composition of the vascular extracellular environment, shed light on ECM remodeling and degradation, and provide insights into important pathological processes, such as plaque rupture, aneurysm formation, and restenosis. 
 Proteomic analysis of human parotid gland exosomes by multidimensional protein identification technology (MudPIT). Human ductal saliva contributes over a thousand unique proteins to whole oral fluids. The mechanism by which most of these proteins are secreted by salivary glands remains to be determined. The present study used a mass spectrometry-based, shotgun proteomics approach to explore the possibility that a subset of the proteins found in saliva are derived from exosomes, membrane-bound vesicles of endosomal origin within multivesicular endosomes. Using MudPIT (multidimensional protein identification technology) mass spectrometry, we catalogued 491 proteins in the exosome fraction of human parotid saliva. Many of these proteins were previously observed in ductal saliva from parotid glands (265 proteins). Furthermore, 72 of the proteins in parotid exosomes overlap with those previously identified as urinary exosome proteins, proteins which are also frequently associated with exosomes from other tissues and cell types. Gene Ontology (GO) and KEGG pathway analyses found that cytosolic proteins comprise the largest category of proteins in parotid exosomes (43%), involved in such processes as phosphatidylinositol signaling system, calcium signaling pathway, inositol metabolism, protein export, and signal transduction, among others; whereas the integral plasma membrane proteins and associated/peripheral plasma membrane proteins (26%) were associated with   extracellular matrix  -receptor interaction, epithelial cell signaling, T-cell and B-cell receptor signaling, cytokine receptor interaction, and antigen processing and presentation, among other biological functions. In addition, these putative saliva exosomal proteins were linked to specific diseases (e.g., neurodegenerative disorders, prion disease, cancers, type I and II diabetes). Consequently, parotid glands secrete exosomes that reflect the metabolic and functional status of the gland and may also carry informative protein markers useful in the diagnosis and treatment of systemic diseases. 
  Component  
   extracellular matrix   
   extracellular matrix   
  [Mm]etastasis  PubMed  
 TINAGL1 and B3GALNT1 are potential therapy target genes to suppress   metastasis   in non-small cell lung cancer. Non-small cell lung cancer (NSCLC) remains lethal despite the development of numerous drug therapy technologies. About 85% to 90% of lung cancers are NSCLC and the 5-year survival rate is at best still below 50%. Thus, it is important to find drugable target genes for NSCLC to develop an effective therapy for NSCLC.Integrated analysis of publically available gene expression and promoter methylation patterns of two highly aggressive NSCLC cell lines generated by in vivo selection was performed. We selected eleven critical genes that may mediate   metastasis   using recently proposed principal component analysis based unsupervised feature extraction. The eleven selected genes were significantly related to cancer diagnosis. The tertiary protein structure of the selected genes was inferred by Full Automatic Modeling System, a profile-based protein structure inference software, to determine protein functions and to specify genes that could be potential drug targets.We identified eleven potentially critical genes that may mediate NSCLC   metastasis   using bioinformatic analysis of publically available data sets. These genes are potential target genes for the therapy of NSCLC. Among the eleven genes, TINAGL1 and B3GALNT1 are possible candidates for drug compounds that inhibit their gene expression. 
   
  
 MYO1D 
 
  NCBI Gene  
  GeneRIF  
  Pubmed  
 
 
  Search Term  Rank  Fields  
  [Mm]igration  6  PubMed(1); GeneRIFs(1)  
  [Ee]xtracellular [Mm]atrix  8  PubMed(2)  
  [Ii]nvasive  10  PubMed(1)  
 
 
 hide/show details for MYO1D 
  [Mm]igration  PubMed  
 Genome-wide association identifies regulatory Loci associated with distinct local histogram emphysema patterns. Emphysema is a heritable trait that occurs in smokers with and without chronic obstructive pulmonary disease. Emphysema occurs in distinct pathologic patterns, but the genetic determinants of these patterns are unknown.To identify genetic loci associated with distinct patterns of emphysema in smokers and investigate the regulatory function of these loci.Quantitative measures of distinct emphysema patterns were generated from computed tomography scans from smokers in the COPDGene Study using the local histogram emphysema quantification method. Genome-wide association studies (GWAS) were performed in 9,614 subjects for five emphysema patterns, and the results were referenced against enhancer and DNase I hypersensitive regions from ENCODE and Roadmap Epigenomics cell lines.Genome-wide significant associations were identified for seven loci. Two are novel associations (top single-nucleotide polymorphism rs379123 in MYO1D and rs9590614 in VMA8) located within genes that function in cell-cell signaling and cell   migration  , and five are in loci previously associated with chronic obstructive pulmonary disease susceptibility (HHIP, IREB2/CHRNA3, CYP2A6/ADCK, TGFB2, and MMP12). Five of these seven loci lay within enhancer or DNase I hypersensitivity regions in lung fibroblasts or small airway epithelial cells, respectively. Enhancer enrichment analysis for top GWAS associations (single-nucleotide polymorphisms associated at P < 5 × 10(-6)) identified multiple cell lines with significant enhancer enrichment among top GWAS loci, including lung fibroblasts.This study demonstrates for the first time genetic associations with distinct patterns of pulmonary emphysema quantified by computed tomography scan. Enhancer regions are significantly enriched among these GWAS results, with pulmonary fibroblasts among the cell types showing the strongest enrichment. 
  GeneRIFs  
 Top single-nucleotide polymorphism rs379123 in MYO1D is located within genes that function in cell-cell signaling and cell   migration  . 
  [Ee]xtracellular [Mm]atrix  PubMed  
 Proteomic analysis of human parotid gland exosomes by multidimensional protein identification technology (MudPIT). Human ductal saliva contributes over a thousand unique proteins to whole oral fluids. The mechanism by which most of these proteins are secreted by salivary glands remains to be determined. The present study used a mass spectrometry-based, shotgun proteomics approach to explore the possibility that a subset of the proteins found in saliva are derived from exosomes, membrane-bound vesicles of endosomal origin within multivesicular endosomes. Using MudPIT (multidimensional protein identification technology) mass spectrometry, we catalogued 491 proteins in the exosome fraction of human parotid saliva. Many of these proteins were previously observed in ductal saliva from parotid glands (265 proteins). Furthermore, 72 of the proteins in parotid exosomes overlap with those previously identified as urinary exosome proteins, proteins which are also frequently associated with exosomes from other tissues and cell types. Gene Ontology (GO) and KEGG pathway analyses found that cytosolic proteins comprise the largest category of proteins in parotid exosomes (43%), involved in such processes as phosphatidylinositol signaling system, calcium signaling pathway, inositol metabolism, protein export, and signal transduction, among others; whereas the integral plasma membrane proteins and associated/peripheral plasma membrane proteins (26%) were associated with   extracellular matrix  -receptor interaction, epithelial cell signaling, T-cell and B-cell receptor signaling, cytokine receptor interaction, and antigen processing and presentation, among other biological functions. In addition, these putative saliva exosomal proteins were linked to specific diseases (e.g., neurodegenerative disorders, prion disease, cancers, type I and II diabetes). Consequently, parotid glands secrete exosomes that reflect the metabolic and functional status of the gland and may also carry informative protein markers useful in the diagnosis and treatment of systemic diseases. 
 Genome-wide meta-analyses of multiancestry cohorts identify multiple new susceptibility loci for refractive error and myopia. Refractive error is the most common eye disorder worldwide and is a prominent cause of blindness. Myopia affects over 30% of Western populations and up to 80% of Asians. The CREAM consortium conducted genome-wide meta-analyses, including 37,382 individuals from 27 studies of European ancestry and 8,376 from 5 Asian cohorts. We identified 16 new loci for refractive error in individuals of European ancestry, of which 8 were shared with Asians. Combined analysis identified 8 additional associated loci. The new loci include candidate genes with functions in neurotransmission (GRIA4), ion transport (KCNQ5), retinoic acid metabolism (RDH5),   extracellular matrix   remodeling (LAMA2 and BMP2) and eye development (SIX6 and PRSS56). We also confirmed previously reported associations with GJD2 and RASGRF1. Risk score analysis using associated SNPs showed a tenfold increased risk of myopia for individuals carrying the highest genetic load. Our results, based on a large meta-analysis across independent multiancestry studies, considerably advance understanding of the mechanisms involved in refractive error and myopia. 
  [Ii]nvasive  PubMed  
 Genome-wide association study of pancreatic cancer in Japanese population. Pancreatic cancer shows very poor prognosis and is the fifth leading cause of cancer death in Japan. Previous studies indicated some genetic factors contributing to the development and progression of pancreatic cancer; however, there are limited reports for common genetic variants to be associated with this disease, especially in the Asian population. We have conducted a genome-wide association study (GWAS) using 991   invasive   pancreatic ductal adenocarcinoma cases and 5,209 controls, and identified three loci showing significant association (P-value<5x10(-7)) with susceptibility to pancreatic cancer. The SNPs that showed significant association carried estimated odds ratios of 1.29, 1.32, and 3.73 with 95% confidence intervals of 1.17-1.43, 1.19-1.47, and 2.24-6.21; P-value of 3.30x10(-7), 3.30x10(-7), and 4.41x10(-7); located on chromosomes 6p25.3, 12p11.21 and 7q36.2, respectively. These associated SNPs are located within linkage disequilibrium blocks containing genes that have been implicated some roles in the oncogenesis of pancreatic cancer. 
   
  
 EFHD2 
 
  NCBI Gene  
  GeneRIF  
  Pubmed  
 
 
  Search Term  Rank  Fields  
  [Mm]igration  6  PubMed(1)  
 
 
 hide/show details for EFHD2 
  [Mm]igration  PubMed  
 Swiprosin-1 is a novel actin bundling protein that regulates cell spreading and   migration  . Protein functions are often revealed by their localization to specialized cellular sites. Recent reports demonstrated that swiprosin-1 is found together with actin and actin-binding proteins in the cytoskeleton fraction of human mast cells and NK-like cells. However, direct evidence of whether swiprosin-1 regulates actin dynamics is currently lacking. We found that swiprosin-1 localizes to microvilli-like membrane protrusions and lamellipodia and exhibits actin-binding activity. Overexpression of swiprosin-1 enhanced lamellipodia formation and cell spreading. In contrast, swiprosin-1 knockdown showed reduced cell spreading and   migration  . Swiprosin-1 induced actin bundling in the presence of Ca(2+), and deletion of the EF-hand motifs partially reduced bundling activity. Swiprosin-1 dimerized in the presence of Ca(2+) via its coiled-coil domain, and a lysine (Lys)-rich region in the coiled-coil domain was essential for regulation of actin bundling. Consistent with these observations, mutations of the EF-hand motifs and coiled-coil region significantly reduced cell spreading and lamellipodia formation. We provide new evidence of how swiprosin-1 influences cytoskeleton reorganization and cell spreading. 
   
  
 DARS 
 
  NCBI Gene  
  GeneRIF  
  Pubmed  
 
 
  Search Term  Rank  Fields  
  [Mm]etastasis  12  PubMed(1)  
 
 
 hide/show details for DARS 
  [Mm]etastasis  PubMed  
 Proteomics analysis of the interactome of N-myc downstream regulated gene 1 and its interactions with the androgen response program in prostate cancer cells. NDRG1 is known to play important roles in both androgen-induced cell differentiation and inhibition of prostate cancer   metastasis  . However, the proteins associated with NDRG1 function are not fully enumerated. Using coimmunoprecipitation and mass spectrometry analysis, we identified 58 proteins that interact with NDRG1 in prostate cancer cells. These proteins include nuclear proteins, adhesion molecules, endoplasmic reticulum (ER) chaperons, proteasome subunits, and signaling proteins. Integration of our data with protein-protein interaction data from the Human Proteome Reference Database allowed us to build a comprehensive interactome map of NDRG1. This interactome map consists of several modules such as a nuclear module and a cell membrane module; these modules explain the reported versatile functions of NDRG1. We also determined that serine 330 and threonine 366 of NDRG1 were phosphorylated and demonstrated that the phosphorylation of NDRG1 was prominently mediated by protein kinase A (PKA). Further, we showed that NDRG1 directly binds to beta-catenin and E-cadherin. However, the phosphorylation of NDRG1 did not interrupt the binding of NDRG1 to E-cadherin and beta-catenin. Finally, we showed that the inhibition of NDRG1 expression by RNA interference decreased the ER inducible chaperon GRP94 expression, directly proving that NDRG1 is involved in the ER stress response. Intriguingly, we observed that many members of the NDRG1 interactome are androgen-regulated and that the NDRG1 interactome links to the androgen response network through common interactions with beta-catenin and heat shock protein 90. Therefore we overlaid the transcriptomic expression changes in the NDRG1 interactome in response to androgen treatment and built a dual dynamic picture of the NDRG1 interactome in response to androgen. This interactome map provides the first road map for understanding the functions of NDRG1 in cells and its roles in human diseases, such as prostate cancer, which can progress from androgen-dependent curable stages to androgen-independent incurable stages. 
   
  
 GUSB 
 
  NCBI Gene  
  GeneRIF  
  Pubmed  
 
 
  Search Term  Rank  Fields  
  [Mm]etastasis  12  PubMed(1); GeneRIFs(1)  
 
 
 hide/show details for GUSB 
  [Mm]etastasis  PubMed  
 Clinical significance of TGF- beta1 and beta-glucuronidase synchronous detection in human pancreatic cancer. To investigate the relation of transfer growth factor (TGF-beta1) and beta-glucuronidase (beta-GCD) on the occurrence and progress of pancreatic cancer.The expression of TGF-beta1 and beta-GCD in the pancreatic cancer tissue and normal pancreatic tissue was determined synchronously using ABC method of immunohistochemistry.The percentage of TGF-beta1 positive cells was significantly higher in pancreatic cancer tissue (43.8%+/-5.2%) than in adjacent pancreatic tissue (28.7%+/-3.6%, P<0.01). The worse the cancer cells differentiated and lymph nodes   metastasis  , the more over-expression of TGF-beta1. The percentage of beta-GCD positive cells was also significantly higher in the pancreatic cancer tissue (62.5%+/-4.1%) than in the adjacent pancreatic tissue (33.5%+/-2.8%, P<0.01). The degree of over-expression of beta-GCD was related to the degree of cancer cells differentiation, but not to the lymph nodes   metastasis  . The expression of TGF-beta1 was significantly correlated with the expression of beta-GCD in pancreatic cancer tissue.The genesis of pancreatic cancer results from multi-factor, multi-step and multi-gene variation. The synchronous detection of TGF-beta1 and beta-GCD helps to determine the malignant degree of tumors and the prognosis of patients with such disease. 
  GeneRIFs  
 Over-expression of beta-glucuronidase was related to the degree of cancer differentiation, but not to lymph node   metastasis  . 
   
  
 CORO1C 
 
  NCBI Gene  
  GeneRIF  
  Pubmed  
 
 
  Search Term  Rank  Fields  
  [Mm]igration  6  Process(4); PubMed(7); GeneRIFs(3)  
  [Ee]xtracellular [Mm]atrix  8  PubMed(1)  
  [Ii]nvasive  10  PubMed(1)  
  [Mm]etastasis  12  PubMed(3); GeneRIFs(1)  
 
 
 hide/show details for CORO1C 
  [Mm]igration  Process  
 negative regulation of epithelial cell   migration   
 neural crest cell   migration   
 regulation of epithelial cell   migration   
 regulation of fibroblast   migration   
  PubMed  
 Knockdown of Coronin-1C disrupts Rac1 activation and impairs tumorigenic potential in hepatocellular carcinoma cells. Coronin-1C is an important F-actin binding protein which is critical for cell motility. Furthermore, the expression of this protein was found to be increased in diffuse tumors and was correlated with the degree of tumor malignancy. However, the mechanism(s) through which this protein enhances malignancy in hepatocellular carcinoma (HCC) is poorly understood. In this study, we found that Coronin-1C was overexpressed in human HCC tissues compared with the adjacent non-tumor tissues. Overexpression of Coronin-1C enhanced the cell   migration   in the human HCC cell line BEL-7402, whereas suppressed cell   migration   and proliferation were observed in Coronin-1C-knockdown BEL-7402 cells together with impaired cell polarity, disrupted cytoskeleton and decreased Rac-1 activation. Moreover, the Coronin-1C knockdown cells displayed a lower degree of malignancy by inducing smaller tumors in nude mice. Thus, we demonstrated a relationship between Coronin-1C overexpression and human HCC growth through enhancement of tumor cell proliferation and   migration  , which are correlated with Rac-1 activation. 
 Downregulation of the microRNA-1/133a cluster enhances cancer cell   migration   and invasion in lung-squamous cell carcinoma via regulation of Coronin1C. Lung cancer is clearly the primary cause of cancer-related deaths worldwide. Recent molecular-targeted strategy has contributed to improvement of the curative effect of adenocarcinoma of the lung. However, such current treatment has not been developed for squamous cell carcinoma (SCC) of the disease. The new genome-wide RNA analysis of lung-SCC may provide new avenues for research and the development of the disease. Our recent microRNA (miRNA) expression signatures of lung-SCC revealed that clustered miRNAs miR-1/133a were significantly reduced in cancer tissues. Here, we found that restoration of both mature miR-1 and miR-133a significantly inhibited cancer cell proliferation,   migration   and invasion. Coronin-1C (CORO1C) was a common target gene of the miR-1/133a cluster, as shown by the genome-wide gene expression analysis and the luciferase reporter assay. Silencing of CORO1C gene expression inhibited cancer cell proliferation,   migration   and invasion. Furthermore, CORO1C-regulated molecular pathways were categorized by using si-CORO1C transfectants. Further analysis of novel cancer signaling pathways modulated by the tumor-suppressive cluster miR-1/133a will provide insights into the molecular mechanisms of lung-SCC oncogenesis and metastasis. 
 Coronin 1C negatively regulates cell-matrix adhesion and motility of intestinal epithelial cells. Coronins, WD-repeat actin-binding proteins, are known to regulate cell motility by coordinating actin filament turnover in lamellipodia of migrating cell. Here we report a novel mechanism of Coronin 1C-mediated cell motility that involves regulation of cell-matrix adhesion. RNAi silencing of Coronin 1C in intestinal epithelial cells enhanced cell   migration   and modulated lamellipodia dynamics by increasing the persistence of lamellipodial protrusion. Coronin 1C-depleted cells showed increased cell-matrix adhesions and enhanced cell spreading compared to control cells, while over-expression of Coronin 1C antagonized cell adhesion and spreading. Enhanced cell-matrix adhesion of coronin-deficient cells correlated with hyperphosphorylation of focal adhesion kinase (FAK) and paxillin, and an increase in number of focal adhesions and their redistribution at the cell periphery. siRNA depletion of FAK in coronin-deficient cells rescued the effects of Coronin 1C depletion on motility, cell-matrix adhesion, and spreading. Thus, our findings provide the first evidence that Coronin 1C negatively regulates epithelial cell   migration   via FAK-mediated inhibition of cell-matrix adhesion. 
 Analysis of the myosin-II-responsive focal adhesion proteome reveals a role for β-Pix in negative regulation of focal adhesion maturation. Focal adhesions undergo myosin-II-mediated maturation wherein they grow and change composition to modulate integrin signalling for cell   migratio  n, growth and differentiation. To determine how focal adhesion composition is affected by myosin II activity, we performed proteomic analysis of isolated focal adhesions and compared protein abundance in focal adhesions from cells with and without myosin II inhibition. We identified 905 focal adhesion proteins, 459 of which changed in abundance with myosin II inhibition, defining the myosin-II-responsive focal adhesion proteome. The abundance of 73% of the proteins in the myosin-II-responsive focal adhesion proteome was enhanced by contractility, including proteins involved in Rho-mediated focal adhesion maturation and endocytosis- and calpain-dependent focal adhesion disassembly. During myosin II inhibition, 27% of proteins in the myosin-II-responsive focal adhesion proteome, including proteins involved in Rac-mediated lamellipodial protrusion, were enriched in focal adhesions, establishing that focal adhesion protein recruitment is also negatively regulated by contractility. We focused on the Rac guanine nucleotide exchange factor β-Pix, documenting its role in the negative regulation of focal adhesion maturation and the promotion of lamellipodial protrusion and focal adhesion turnover to drive cel  l migrati  on. 
 Coronin-1C and RCC2 guide mesenchymal   migration   by trafficking Rac1 and controlling GEF exposure. Sustained forward   migration   through a fibrillar extracellular matrix requires localization of protrusive signals. Contact with fibronectin at the tip of a cell protrusion activates Rac1, and for linear   migration   it is necessary to dampen Rac1 activity in off-axial positions and redistribute Rac1 from non-protrusive membrane to the leading edge. Here, we identify interactions between coronin-1C (Coro1C), RCC2 and Rac1 that focus active Rac1 to a single protrusion. Coro1C mediates release of inactive Rac1 from non-protrusive membrane and is necessary for Rac1 redistribution to a protrusive tip and fibronectin-dependent Rac1 activation. The second component, RCC2, attenuates Rac1 activation outside the protrusive tip by binding to the Rac1 switch regions and competitively inhibiting GEF action, thus preventing off-axial protrusion. Depletion of Coro1C or RCC2 by RNA interference causes loss of cell polarity that results in shunting   migration   in 1D or 3D culture systems. Furthermore, morpholinos against Coro1C or RCC2, or mutation of any of the binding sites in the Rac1-RCC2-Coro1C complex delays the arrival of neural crest derivatives at the correct location in developing zebrafish, demonstrating the crucial role in   migration   guidance in vivo. 
 Coronin 3 promotes gastric cancer metastasis via the up-regulation of MMP-9 and cathepsin K. Coronins are a family of highly evolutionary conserved proteins reportedly involved in the regulation of actin cytoskeletal dynamics, although only coronin 3 has been shown to be related to cancer cell   migration  . In glioblastoma cells, the knockdown of coronin 3 inhibits cell proliferation and invasion. Coronin 3 is also associated with the aggression and metastasis of hepatocellular carcinoma. In this paper, we analyze the   migration  , invasion and metastasis abilities of gastric cancer cells after up- or down-regulation of coronin 3, and explore the mechanism of coronin 3 in the process of gastric cancer metastasis.The expression of coronin 3 was higher in the highly metastatic sub-cell line MKN28-M, which we established in our laboratory. We also demonstrated that the expression of coronin 3 was remarkably higher in lymph lode metastases than in primary gastric cancer tissues, and over-expression of coronin 3 was correlated with the increased clinical stage and lymph lode metastasis. Recombinant lentiviral vectors encoding shRNAs were designed to down-regulate coronin 3 expression in gastric cancer cell lines. Stable knockdown of coronin 3 by this lentiviral vector could efficiently inhibit the   migration   and invasion of MKN45 gastric cancer cells. In contrast, up-regulation of coronin 3 significantly enhanced   migration   and invasion of MKN28-NM cells. In addition, knockdown of coronin 3 significantly reduced liver metastasis in mice after tail vein injection of gastric cancer cells. The Human Tumor Metastasis PCR Array was used to screen the metastasis-associated genes identified by the down-regulation of coronin 3, and the results suggested that, following the knockdown of coronin 3, the tumor cell   migration   and invasion were inhibited by the reduced expression of MMP-9 and cathepsin K.Coronin 3 is highly expressed in gastric cancer metastases and can promote the metastatic behaviors of gastric cancer cells, including their   migration   and invasion. 
 Primary effusion lymphoma: genomic profiling revealed amplification of SELPLG and CORO1C encoding for proteins important for cell   migration  . Primary effusion lymphoma (PEL) is associated with Kaposi sarcoma herpesvirus (KSHV) but its pathogenesis is poorly understood. Many KSHV-associated products can deregulate cellular pathways commonly targeted in cancer. However, KSHV infection alone is insufficient for malignant transformation. PEL also lacks the chromosomal translocations seen in other lymphoma subtypes. We investigated 28 PELs and ten PEL cell lines by 1 Mb resolution array comparative genomic hybridization (CGH) and found frequent gains of 1q21-41 (47%), 4q28.3-35 (29%), 7q (58%), 8q (63%), 11 (32%), 12 (61%), 17q (29%), 19p (34%), and 20q (34%), and losses of 4q (32%), 11q25 (29%), and 14q32 (63%). Recurrent focal amplification was seen at several regions on chromosomes 7, 8, and 12. High-resolution chromosome-specific tile-path array CGH confirmed these findings, and identified selectin-P ligand (SELPLG) and coronin-1C (CORO1C) as the targets of a cryptic amplification at 12q24.11. Interphase FISH and quantitative PCR showed SELPLG/CORO1C amplification (>4 extra copies) and low levels of copy number gain (1-4 extra copies) in 23% of PELs, respectively. Immunohistochemistry revealed strong expression of both SELPLG and coronin-1C in the majority of PELs, irrespective of their gene dosage. SELPLG is critical for cell   migration   and chemotaxis, while CORO1C regulates actin-dependent processes, thus important for cell motility. Their overexpression in PEL is expected to play an important role in its pathogenesis. 
  GeneRIFs  
 Coronin-1C overexpression is associated withand hepatocellular carcinoma growth through enhancement of tumor cell proliferation and   migration  , which are correlated with Rac-1 activation. 
 There is a strong expression of both SELPLG and coronin-1C in the majority of primary effusion lymphomas, irrespective of their gene dosage. SELPLG is critical for cell   migration   and chemotaxis, while CORO1C regulates actin-dependent processes. 
 Coronin 1C negatively regulates epithelial cell   migration   via FAK-mediated inhibition of cell-matrix adhesion. 
  [Ee]xtracellular [Mm]atrix  PubMed  
 Coronin-1C and RCC2 guide mesenchymal migration by trafficking Rac1 and controlling GEF exposure. Sustained forward migration through a fibrillar   extracellular matrix   requires localization of protrusive signals. Contact with fibronectin at the tip of a cell protrusion activates Rac1, and for linear migration it is necessary to dampen Rac1 activity in off-axial positions and redistribute Rac1 from non-protrusive membrane to the leading edge. Here, we identify interactions between coronin-1C (Coro1C), RCC2 and Rac1 that focus active Rac1 to a single protrusion. Coro1C mediates release of inactive Rac1 from non-protrusive membrane and is necessary for Rac1 redistribution to a protrusive tip and fibronectin-dependent Rac1 activation. The second component, RCC2, attenuates Rac1 activation outside the protrusive tip by binding to the Rac1 switch regions and competitively inhibiting GEF action, thus preventing off-axial protrusion. Depletion of Coro1C or RCC2 by RNA interference causes loss of cell polarity that results in shunting migration in 1D or 3D culture systems. Furthermore, morpholinos against Coro1C or RCC2, or mutation of any of the binding sites in the Rac1-RCC2-Coro1C complex delays the arrival of neural crest derivatives at the correct location in developing zebrafish, demonstrating the crucial role in migration guidance in vivo. 
  [Ii]nvasive  PubMed  
 [Increased coronin-1C expression is related to hepatocellular carcinoma invasion and metastasis]. To search for hepatocellular carcinoma (HCC) invasion related biomarkers using the cell membrane proteomics approaches, and to validate the markers using experimental and clinical specimens.The HCCLM9 and MHCC97L cells with a similar genetic background and remarkably different metastasis behaviors were used for comparative membrane proteome profiling using sodium dodecyl sulfate polyacrylamide gel electrophoresis and electrospray ionization mass spectrometry technologies. Candidate protein makers were further validated by western blot on cells, immunohistochemistry (IHC) on animal tumor tissues, and tissue micro-array on clinical specimens.The membrane proteins of MHCC97L and HCCLM9 cells were compared by sodium dodecyl sulfate polyacrylamide gel electrophoresis analyses. 14 proteins were identified by ESI-MS/MS among the differential bands. Coronin-1C was overexpressed in HCCLM9 (7.31+/-0.73) versus MHCC97L (2.84+/-0.99) validated by western blot. Elevated coronin-1C expression was observed in liver cancer tissues of HCCLM9 nude mice. IHC study in 115 human HCC specimens demonstrated that patients with higher coronin-1C expression had more advanced stage.The study suggests that coronin-1C could be a potential molecule to predict HCC   invasive   behavior. 
  [Mm]etastasis  PubMed  
 Downregulation of the microRNA-1/133a cluster enhances cancer cell migration and invasion in lung-squamous cell carcinoma via regulation of Coronin1C. Lung cancer is clearly the primary cause of cancer-related deaths worldwide. Recent molecular-targeted strategy has contributed to improvement of the curative effect of adenocarcinoma of the lung. However, such current treatment has not been developed for squamous cell carcinoma (SCC) of the disease. The new genome-wide RNA analysis of lung-SCC may provide new avenues for research and the development of the disease. Our recent microRNA (miRNA) expression signatures of lung-SCC revealed that clustered miRNAs miR-1/133a were significantly reduced in cancer tissues. Here, we found that restoration of both mature miR-1 and miR-133a significantly inhibited cancer cell proliferation, migration and invasion. Coronin-1C (CORO1C) was a common target gene of the miR-1/133a cluster, as shown by the genome-wide gene expression analysis and the luciferase reporter assay. Silencing of CORO1C gene expression inhibited cancer cell proliferation, migration and invasion. Furthermore, CORO1C-regulated molecular pathways were categorized by using si-CORO1C transfectants. Further analysis of novel cancer signaling pathways modulated by the tumor-suppressive cluster miR-1/133a will provide insights into the molecular mechanisms of lung-SCC oncogenesis and   metastasis  . 
 Coronin 3 promotes gastric cancer   metastasis   via the up-regulation of MMP-9 and cathepsin K. Coronins are a family of highly evolutionary conserved proteins reportedly involved in the regulation of actin cytoskeletal dynamics, although only coronin 3 has been shown to be related to cancer cell migration. In glioblastoma cells, the knockdown of coronin 3 inhibits cell proliferation and invasion. Coronin 3 is also associated with the aggression and   metastasis   of hepatocellular carcinoma. In this paper, we analyze the migration, invasion and   metastasis   abilities of gastric cancer cells after up- or down-regulation of coronin 3, and explore the mechanism of coronin 3 in the process of gastric cancer   metastasis  .The expression of coronin 3 was higher in the highly metastatic sub-cell line MKN28-M, which we established in our laboratory. We also demonstrated that the expression of coronin 3 was remarkably higher in lymph lode metastases than in primary gastric cancer tissues, and over-expression of coronin 3 was correlated with the increased clinical stage and lymph lode   metastasis  . Recombinant lentiviral vectors encoding shRNAs were designed to down-regulate coronin 3 expression in gastric cancer cell lines. Stable knockdown of coronin 3 by this lentiviral vector could efficiently inhibit the migration and invasion of MKN45 gastric cancer cells. In contrast, up-regulation of coronin 3 significantly enhanced migration and invasion of MKN28-NM cells. In addition, knockdown of coronin 3 significantly reduced liver   metastasis   in mice after tail vein injection of gastric cancer cells. The Human Tumor   Metastasis   PCR Array was used to screen the   metastasis  -associated genes identified by the down-regulation of coronin 3, and the results suggested that, following the knockdown of coronin 3, the tumor cell migration and invasion were inhibited by the reduced expression of MMP-9 and cathepsin K.Coronin 3 is highly expressed in gastric cancer metastases and can promote the metastatic behaviors of gastric cancer cells, including their migration and invasion. 
 [Increased coronin-1C expression is related to hepatocellular carcinoma invasion and   metastasis  ]. To search for hepatocellular carcinoma (HCC) invasion related biomarkers using the cell membrane proteomics approaches, and to validate the markers using experimental and clinical specimens.The HCCLM9 and MHCC97L cells with a similar genetic background and remarkably different   metastasis   behaviors were used for comparative membrane proteome profiling using sodium dodecyl sulfate polyacrylamide gel electrophoresis and electrospray ionization mass spectrometry technologies. Candidate protein makers were further validated by western blot on cells, immunohistochemistry (IHC) on animal tumor tissues, and tissue micro-array on clinical specimens.The membrane proteins of MHCC97L and HCCLM9 cells were compared by sodium dodecyl sulfate polyacrylamide gel electrophoresis analyses. 14 proteins were identified by ESI-MS/MS among the differential bands. Coronin-1C was overexpressed in HCCLM9 (7.31+/-0.73) versus MHCC97L (2.84+/-0.99) validated by western blot. Elevated coronin-1C expression was observed in liver cancer tissues of HCCLM9 nude mice. IHC study in 115 human HCC specimens demonstrated that patients with higher coronin-1C expression had more advanced stage.The study suggests that coronin-1C could be a potential molecule to predict HCC invasive behavior. 
  GeneRIFs  
 Coronin 3 promotes gastric cancer   metastasis   via the up-regulation of MMP-9 and cathepsin K. 
   
  
 LDHA 
 
  NCBI Gene  
  GeneRIF  
  Pubmed  
 
 
  Search Term  Rank  Fields  
  [Mm]igration  6  PubMed(10); GeneRIFs(1)  
  [Ee]xtracellular [Mm]atrix  8  PubMed(2)  
  [Ii]nvasive  10  PubMed(3); GeneRIFs(1)  
  [Mm]etastasize  11  PubMed(2)  
  [Mm]etastasis  12  PubMed(13); GeneRIFs(4)  
 
 
 hide/show details for LDHA 
  [Mm]igration  PubMed  
 LDH-A promotes malignant progression via activation of epithelial-to-mesenchymal transition and conferring stemness in muscle-invasive bladder cancer. Lactate dehydrogenase-A(LDH-A) is an important rate-limiting enzyme in the Warburg effect. Survival analysis indicated poor clinical outcomes in MIBC with high LDH-A expression. The results of in vitro experiment indicated that LDH-A promotes MIBC cells proliferation, invasion and   migration  . The positive relationship between LDH-A expression and CSC/EMT markers was confirmed both in invasive bladder cell line and in 136 MIBC specimens. Thus, we conclude that LDH-A may be a promising target for MIBC. 
 Lactate dehydrogenase inhibitors can reverse inflammation induced changes in colon cancer cells. The inflammatory microenvironment is an essential component of neoplastic lesions and can significantly impact on tumor progression. Besides facilitating invasive growth, inflammatory cytokines were also found to reprogram cancer cell metabolism and to induce aerobic glycolysis. Previous studies did not consider the possible contribution played in these changes by lactate dehydrogenase (LDH). The A isoform of LDH (LDH-A) is the master regulator of aerobic glycolysis; it actively reduces pyruvate and causes enhanced lactate levels in tumor tissues. In cancer cells, lactate was recently found to directly increase   migration   ability; moreover, when released in the microenvironment, it can facilitate matrix remodeling. In this paper, we illustrate that treatment of human colon adenocarcinoma cells with TNF-α and IL-17, two pro-inflammatory cytokines, modifies LDH activity, causing a shift toward the A isoform which results in increased lactate production. At the same time, the two cytokines appeared to induce features of epithelial-mesenchymal transition in the treated cells, such as reduction of E-cadherin levels and increased secretion of metalloproteinases. Noteworthy, oxamate and galloflavin, two inhibitors of LDH activity which reduce lactate production in cells, were found to relieve the inflammation-induced effects. These results suggest LDH-A and/or lactate as common elements at the cross-road between cancer cell metabolism, tumor progression and inflammation. At present, LDH inhibitors suitable for clinical use are actively searched as possible anti-proliferative agents; our data lead to hypothesize for these compounds a wider potential in anticancer treatment. 
 Lysine-5 acetylation negatively regulates lactate dehydrogenase A and is decreased in pancreatic cancer. Tumor cells commonly have increased glucose uptake and lactate accumulation. Lactate is produced from pyruvate by lactate dehydrogenase A (LDH-A), which is frequently overexpressed in tumor cells and is important for cell growth. Elevated transcription by c-Myc or HIF1α may contribute to increased LDH-A in some cancer types. Here, we show that LDH-A is acetylated at lysine 5 (K5) and that this acetylation inhibits LDH-A activity. Furthermore, the K5-acetylated LDH-A is recognized by the HSC70 chaperone and delivered to lysosomes for degradation. Replacement of endogenous LDH-A with an acetylation mimetic mutant decreases cell proliferation and   migratio  n. Importantly, K5 acetylation of LDH-A is reduced in human pancreatic cancers. Our study reveals a mechanism of LDH-A upregulation in pancreatic cancers. 
 LDHA is necessary for the tumorigenicity of esophageal squamous cell carcinoma. Esophageal squamous cell carcinoma (ESCC) is one of the most common lethal tumors in the world, and the development of new therapeutic targets is needed. Recent studies have shown that aerobic glycolysis, also known as the Warburg effect, mediated the anti-apoptotic effects in cancer cells. Lactate dehydrogenase A (LDHA) which executed the final step of aerobic lactate production has been reported to be involved in the tumor progression. However, the function of LDHA in ESCC has not been investigated. In this study, it was found that LDHA was up-regulated in ESCC clinical samples. Knockdown of the expression of LDHA inhibited cell growth and cell   migration   in vitro as well as tumorigenesis in vivo. With regard to the molecular mechanism, silencing the expression of LDHA was related to decreased AKT activation and cyclin D1 expression and increased cleavage of PARP and caspase 8. Taken together, our findings suggest that LDHA plays an important role in the progression of ESCC by modulating cell growth, and LDHA might be a potential therapeutic target in ESCC. 
 Effects of the suppression of lactate dehydrogenase A on the growth and invasion of human gastric cancer cells. Lactate dehydrogenase A (LDH-A), which regulates glycolytic flux by catalyzing pyruvate to lactate in the cytoplasm, is believed to be one of the highly attractive therapeutic targets for cancers. Firstly, we detected the expression of LDH-A in gastric cancer (GC) cells. LDH-A inhibitor oxamate was then used to suppress the LDH-A activity in GC cells. Cell proliferation, lactic acid production, Transwell   migration   assay and apoptosis were assessed, respectively. The results showed that inhibition of LDH-A by oxamate decreased the lactate production. In the presence of glucose, oxamate inhibited cell proliferation in a dose-dependent manner. Flow cytometry assay further confirmed a pro-apoptotic effect of oxamate, and this was likely through increased expression of Bax, activated caspase-3, and decreased expression of Bcl-2. Therefore, we believe that oxamate inhibits cell growth, suppresses tumor invasion, and induces apoptosis in GC cells. LDH-A may be a potential therapeutic target for GC. 
 Analysis of genetic mutations in human lactate dehydrogenase-A(M) deficiency using DNA conformation polymorphism in combination with polyacrylamide gradient gel and silver staining. Human lactate dehydrogenase (LDH)-A mutant gene was analyzed by polymerase chain reaction - DNA conformation polymorphism (DCP). We used polyacrylamide gradient gel and silver staining procedures for DCP analysis and observed abnormal   migration   patterns in individuals heterozygous for LDH-A deficiency. Further sequence determination of the mutant alleles consistently resulted in detection of base substitutions, a G to T transversion at codon 328 (GAG----TAG), and synonymous substitutions at codon 115, 160 and 172. Such mutations were easily detectable using the DCP technique. The DCP technique using the polyacrylamide gradient gel and silver staining method seems likely to be useful for the rapid screening of mutations and for further genotype detection. 
 FOXM1-LDHA signaling promoted gastric cancer glycolytic phenotype and progression. The oncogenic transcription factor forkhead box protein M1 (FOXM1) plays critical roles in gastric cancer (GC) development and progression. However, the underlying mechanisms has not fully demonstrated. Lactate dehydrogenase A (LDHA) is widely overexpressed in a series of cancers and is one of the two subunits of Lactate dehydrogenase (LDH), which is the key glycolytic enzyme and catalyzes the interconversion of pyruvate and lactate. In this study, we characterized the regulation of aerobic glycolysis by FOXM1 via transactivation of LDHA in GC. We found that LDHA was overexpressed GC cells, and the expression of LDHA was transcriptionally regulated by FOXM1. Furthermore, FOXM1 regulated GC cells glycolytic phenotype, proliferation,   migration   and invasion via LDHA. Thus, FOXM1-LDHA signaling functioned as a stimulator of glycolysis and promoted GC progression. 
 Fast-type electrophoretic variant of lactate dehydrogenase M(A) and comparison with other missense mutations in lactate dehydrogenase M(A) and H(B) genes. An electrophoretic variant of lactate dehydrogenase (LD) M(A) subunit was discovered in a female patient with chest pain. Her LD activity in serum was within the normal reference interval, and analysis of her LD isoenzyme pattern showed an abnormal   migration   indicating a fast-type LD-M(A) subunit variant. DNA analysis of the mutant LD-M gene detected a single base substitution, an A to G transition at codon 220 (AAA-->GAA). This mutation resulted in the replacement of a lysine by a glutamic acid (mutation K220E) and produced a subunit variant (electrophoretic fast type). This missense mutation was also observed in the patient's son, and genotypes of mother and son were consistent with their biochemical phenotypes, as evaluated by LD isoenzyme analysis. 
 Inhibition of LDHA suppresses tumor progression in prostate cancer. A key hallmark of cancer cells is their altered metabolism, known as Warburg effect. Lactate dehydrogenase A (LDHA) executes the final step of aerobic glycolysis and has been reported to be involved in the tumor progression. However, the function of LDHA in prostate cancer has not been studied. In current study, we observed overexpression of LDHA in the clinical prostate cancer samples compared with benign prostate hyperplasia tissues as demonstrated by immunohistochemistry and real-time qPCR. Attenuated expression of LDHA by siRNA or inhibition of LDHA activities by FX11 inhibited cell proliferation,   migration  , invasion, and promoted cell apoptosis of PC-3 and DU145 cells. Mechanistically, decreased Warburg effect as demonstrated by reduced glucose consumption and lactate secretion and reduced expression of MMP-9, PLAU, and cathepsin B were found after LDHA knockdown or FX11 treatment in PC-3 and DU145 cells. Taken together, our study revealed the oncogenic role of LDHA in prostate cancer and suggested that LDHA might be a potential therapeutic target. 
 Attenuation of LDHA expression in cancer cells leads to redox-dependent alterations in cytoskeletal structure and cell   migration  . Aerobic glycolysis, the preferential use of glycolysis even in the presence of oxygen to meet cellular metabolic demands, is a near universal feature of cancer. This unique type of metabolism is thought to protect cancer cells from damaging reactive oxygen species (ROS) produced in the mitochondria. Using the cancer cell line MDA-MB-435 it is shown that shRNA mediated knockdown of lactate dehydrogenase A (LDHA), a key mediator of aerobic glycolysis, results in elevated mitochondrial ROS production and a concomitant decrease in cell proliferation and motility. Redox-sensitive proteins affected by oxidative stress associated with LDHA knockdown were identified by Redox 2D-PAGE and mass spectrometry. In particular, tropomyosin (Tm) isoforms Tm4, Tm5NM1 and Tm5NM5, proteins involved in cell   migration   and cytoskeletal dynamics, exhibited changes in disulfide bonding and co-localized with peri-nuclear actin aggregates in LDHA knockdown cells. In contrast, treatment with the thiol-based antioxidant N-acetylcysteine promoted the relocalization of Tms to cortical actin microfilaments and partially rescued the   migration   defects associated with attenuated LDHA expression. These results suggest that aerobic glycolysis and reduced mitochondrial ROS production create an environment conducive to cytoskeletal remodeling; key events linked to the high cell motility associated with cancer. 
  GeneRIFs  
 LDHA expression is elevated in gastric cancer cells and antagonism with oxamic acid inhibited cell proliferation and   migration  . 
  [Ee]xtracellular [Mm]atrix  PubMed  
 Ionizing radiation induces myofibroblast differentiation via lactate dehydrogenase. Pulmonary fibrosis is a common and dose-limiting side-effect of ionizing radiation used to treat cancers of the thoracic region. Few effective therapies are available for this disease. Pulmonary fibrosis is characterized by an accumulation of myofibroblasts and excess deposition of   extracellular matrix   proteins. Although prior studies have reported that ionizing radiation induces fibroblast to myofibroblast differentiation and collagen production, the mechanism remains unclear. Transforming growth factor-β (TGF-β) is a key profibrotic cytokine that drives myofibroblast differentiation an  d extracellular matr  ix production. However, its activation and precise role in radiation-induced fibrosis are poorly understood. Recently, we reported that lactate activates latent TGF-β through a pH-dependent mechanism. Here, we wanted to test the hypothesis that ionizing radiation leads to excessive lactate production via expression of the enzyme lactate dehydrogenase-A (LDHA) to promote myofibroblast differentiation. We found that LDHA expression is increased in human and animal lung tissue exposed to ionizing radiation. We demonstrate that ionizing radiation induces LDHA, lactate production, and extracellular acidification in primary human lung fibroblasts in a dose-dependent manner. We also demonstrate that genetic and pharmacologic inhibition of LDHA protects against radiation-induced myofibroblast differentiation. Furthermore, LDHA inhibition protects from radiation-induced activation of TGF-β. We propose a profibrotic feed forward loop, in which radiation induces LDHA expression and lactate production, which can lead to further activation of TGF-β to drive the fibrotic process. These studies support the concept of LDHA as an important therapeutic target in radiation-induced pulmonary fibrosis. 
 Identification of breast cancer-restricted antigens by antibody screening of SKBR3 cDNA library using a preselected patient's serum. Screening of a breast cancer cDNA library from SKBR3 human breast cancer cells by SEREX (serological analysis of cDNA expression library) using a preselected serum from a breast cancer patient revealed 13 genes, two of which, INT-MI-1 and INT-MI-2, encode novel gene products, while the remaining 11 genes and their products are identical with or highly homologous to known GenBank entries. Immunoscreening of the 13 clones using 20 allogeneic sera from breast cancer patients and 20 samples from age- and gender-matched healthy donors showed that lactate dehydrogenase-A (LDH-A), lactate dehydrogenase-B (LDH-B), fibulin-1, and thyroid hormone-binding protein (THBP) were recognized principally by the breast cancer patient sera, indicating the immunogenicity of these molecules in vivo. The other antigens were similarly recognized by normal and patients sera, and thus not tumor-restricted immunologically. RT-PCR analysis revealed strong expression of fibulin-1 in tumor cell lines and surgical specimen whereas in the same experimental conditions, normal tissues scored negative. Also THBP expression was found in various tumors whereas in normal tissues, its expression is restricted to the testis and, at lower levels, in ovary, liver, and spleen. In contrast, LDH-A and LDH-B were ubiquitously expressed in normal and tumor tissues, with LDH-B levels considerably lower and heterogeneous in normal samples compared to those expressed in tumor cell lines. The differential expression of fibulin-1 between the normal tissues and breast carcinoma cell lines (5/6) and surgical specimens (5/6) suggests the possible involvement of the overexpression of this   extracellular matrix  -associated glycoprotein in the pathogenesis of this neoplasm. 
  [Ii]nvasive  PubMed  
 LDH-A promotes malignant progression via activation of epithelial-to-mesenchymal transition and conferring stemness in muscle-  invasive   bladder cancer. Lactate dehydrogenase-A(LDH-A) is an important rate-limiting enzyme in the Warburg effect. Survival analysis indicated poor clinical outcomes in MIBC with high LDH-A expression. The results of in vitro experiment indicated that LDH-A promotes MIBC cells proliferation, invasion and migration. The positive relationship between LDH-A expression and CSC/EMT markers was confirmed both in   invasive   bladder cell line and in 136 MIBC specimens. Thus, we conclude that LDH-A may be a promising target for MIBC. 
 Lactate dehydrogenase inhibitors can reverse inflammation induced changes in colon cancer cells. The inflammatory microenvironment is an essential component of neoplastic lesions and can significantly impact on tumor progression. Besides facilitating   invasive   growth, inflammatory cytokines were also found to reprogram cancer cell metabolism and to induce aerobic glycolysis. Previous studies did not consider the possible contribution played in these changes by lactate dehydrogenase (LDH). The A isoform of LDH (LDH-A) is the master regulator of aerobic glycolysis; it actively reduces pyruvate and causes enhanced lactate levels in tumor tissues. In cancer cells, lactate was recently found to directly increase migration ability; moreover, when released in the microenvironment, it can facilitate matrix remodeling. In this paper, we illustrate that treatment of human colon adenocarcinoma cells with TNF-α and IL-17, two pro-inflammatory cytokines, modifies LDH activity, causing a shift toward the A isoform which results in increased lactate production. At the same time, the two cytokines appeared to induce features of epithelial-mesenchymal transition in the treated cells, such as reduction of E-cadherin levels and increased secretion of metalloproteinases. Noteworthy, oxamate and galloflavin, two inhibitors of LDH activity which reduce lactate production in cells, were found to relieve the inflammation-induced effects. These results suggest LDH-A and/or lactate as common elements at the cross-road between cancer cell metabolism, tumor progression and inflammation. At present, LDH inhibitors suitable for clinical use are actively searched as possible anti-proliferative agents; our data lead to hypothesize for these compounds a wider potential in anticancer treatment. 
 Suppression of lactate dehydrogenase A compromises tumor progression by downregulation of the Warburg effect in glioblastoma. Reprogrammed glucose metabolism is an emerging hallmark of cancer cells, which show a unique metabolic phenotype known as the Warburg effect. Lactate dehydrogenase A (LDHA), a key enzyme in the glycolytic process, executes the final step by conversion of lactate into pyruvate. However, little is known about the roles of LDHA in human glioblastoma (GBM). In this study, we aimed to determine the effects of LDHA and elucidate related underlying mechanisms. Data derived from Oncomine database showed that LDHA is commonly upregulated in GBM tissues in comparison with corresponding normal controls. Silencing of LDHA expression resulted in reduced glycolysis, decreased cell growth, increased cell apoptosis, and attenuated   invasive   ability. In the presence of 2-deoxyglucose, a glycolysis inhibitor, the oncogenic activities of LDHA were completely blocked. These findings provide evidence of the cellular functions of LDHA in the progression of GBM and suggest that LDHA might act as a potential therapeutic target for GBM treatment. 
  GeneRIFs  
 positive relationship between LDH-A expression and CSC/EMT markers was confirmed both in   invasive   bladder cell line and in 136 MIBC specimens. 
  [Mm]etastasize  PubMed  
 Increased glyceraldehyde-3-phosphate dehydrogenase expression in renal cell carcinoma identified by RNA-based, arbitrarily primed polymerase chain reaction. Renal cell carcinoma (RCC) comprises 85% of renal tumors and displays a great capacity to   metastasize  . The lack of diagnostic and prognostic markers complicates its early detection and in the majority of cases metastases are present at the time of diagnosis.The current study reports on the identification of differentially expressed genes in RCC using random arbitrarily primed polymerase chain reaction (RAP-PCR).Four genes were identified, including glyceraldehyde-3-phosphate dehydrogenase (GAPDH), lactate dehydrogenase A (LDH A), human leukocyte antigen A (HLA A), and ferritin. GAPDH and HLA A were found to be overexpressed in 100% of the tumors and LDH A was increased in > 85% of the tumors analyzed compared with normal kidney counterparts. For GAPDH and LDH A higher protein levels in the tumors also were determined by Western blot analysis. Differential expression did not appear to correlate with gene amplification events as demonstrated by Southern blot analysis, indicating that regulatory mechanisms controlling the expression of these genes were altered. Finally, ferritin was judged to have a variable expression because it was decreased in approximately 50% of the tumors and augmented in 20%. The implications in proliferation and differentiation of all these genes were analyzed in RCC cell lines grown at different stages of confluency and additional information was obtained regarding expression of the GAPDH gene in proliferating primary cultures of normal and tumor cells derived from the same kidney samples.The authors conclude that RAP-PCR is a useful technique with which to identify rapidly differentially expressed genes in a given system. In addition, they also conclude that GAPDH is a potent marker of cell proliferation in kidney tumor cells whose overexpression appears to be a late event in the development of RCC. 
 Increased LDH5 expression is associated with lymph node metastasis and outcome in oral squamous cell carcinoma. Oral squamous cell carcinoma (OSCC) account for more than 90 % of all oral malignant lesions. Lactate dehydrogenase 5 (LDH5) has the highest efficiency among all other isoenzymes to catalyse pyruvate transformation to lactate and is significantly overexpressed in several different tumour entities. LDH5 overexpression confers an advantage on malignant cells, allows them to grow faster, and to   metastasiz  e. No data regarding LDH5 expression and OSCC outcome are available. Expression of LDH5 was analysed in OSCC specimen (n = 191) and cancer cell lines (BICR3, BICR56) by immunohistochemistry, real-time quantitative reverse transcription-PCR (RT-PCR) analysis, and western blotting. Scanned images were digitally analysed using ImageJ and the immunomembrane plug-in. LDH5 expression on protein level was correlated with clinicopathological characteristics and impact on survival. LDH5 was co-labelled with glucose transporter-1 (GLUT-1), Ki-67, and hypoxia inducible factor 1 (HIF-1α) in immunohistochemical double staining experiments. Expression subgroups were identified by receiver operating characteristics analysis. LDH5 expression was significantly associated with tumour progression, and recurrence of the tumour. Multivariate analysis demonstrated LDH5 expression as an independent prognostic factor (p < 0.0001). Immunohistochemical double staining experiments revealed LDH5 expression by cancer cells in association with glucose uptake (GLUT-1), proliferation (Ki-67), and hypoxia (HIF-1α). LDH5 specificity was confirmed by western blot and RT-PCR analysis. For the first time, this study provides evidence that LDH5 expression in OSCC might be associated with tumour formation and metastasis in a large patient cohort. Therefore, adjuvant therapies targeting glucose metabolism might be promising for therapy of OSCC. 
  [Mm]etastasis  PubMed  
 Proteomics analysis of the interactome of N-myc downstream regulated gene 1 and its interactions with the androgen response program in prostate cancer cells. NDRG1 is known to play important roles in both androgen-induced cell differentiation and inhibition of prostate cancer   metastasis  . However, the proteins associated with NDRG1 function are not fully enumerated. Using coimmunoprecipitation and mass spectrometry analysis, we identified 58 proteins that interact with NDRG1 in prostate cancer cells. These proteins include nuclear proteins, adhesion molecules, endoplasmic reticulum (ER) chaperons, proteasome subunits, and signaling proteins. Integration of our data with protein-protein interaction data from the Human Proteome Reference Database allowed us to build a comprehensive interactome map of NDRG1. This interactome map consists of several modules such as a nuclear module and a cell membrane module; these modules explain the reported versatile functions of NDRG1. We also determined that serine 330 and threonine 366 of NDRG1 were phosphorylated and demonstrated that the phosphorylation of NDRG1 was prominently mediated by protein kinase A (PKA). Further, we showed that NDRG1 directly binds to beta-catenin and E-cadherin. However, the phosphorylation of NDRG1 did not interrupt the binding of NDRG1 to E-cadherin and beta-catenin. Finally, we showed that the inhibition of NDRG1 expression by RNA interference decreased the ER inducible chaperon GRP94 expression, directly proving that NDRG1 is involved in the ER stress response. Intriguingly, we observed that many members of the NDRG1 interactome are androgen-regulated and that the NDRG1 interactome links to the androgen response network through common interactions with beta-catenin and heat shock protein 90. Therefore we overlaid the transcriptomic expression changes in the NDRG1 interactome in response to androgen treatment and built a dual dynamic picture of the NDRG1 interactome in response to androgen. This interactome map provides the first road map for understanding the functions of NDRG1 in cells and its roles in human diseases, such as prostate cancer, which can progress from androgen-dependent curable stages to androgen-independent incurable stages. 
 Lactate dehydrogenase A is a potential prognostic marker in clear cell renal cell carcinoma. Over 90% of cancer-related deaths in clear cell renal cell carcinoma (RCC) are caused by tumor relapse and   metastasis  . Thus, there is an urgent need for new molecular markers that can potentiate the efficacy of the current clinical-based models of prognosis assessment. The objective of this study is to evaluate the potential significance of lactate dehydrogenase A (LDHA), assessed by immunohistochemical staining, as a prognostic marker in clear cell renal cell carcinoma in relation to clinicopathological features and clinical outcome.We assessed the expression of LDHA at the protein level, by immunohistochemistry, and correlated its expression with multiple clinicopathological features including tumor size, clinical stage, histological grade, disease-free and overall survival in 385 patients with primary clear cell renal cell carcinoma. We also correlated the LDHA expression with overall survival, at mRNA level, in an independent data set of 170 clear cell renal cell carcinoma cases from The Cancer Genome Atlas databases. Cox proportional hazards models adjusted for the potential clinicopathological factors were used to test for associations between the LDHA expression and both disease-free survival and overall survival.There is statistically significant positive correlation between LDHA level of expression and tumor size, clinical stage and histological grade. Moreover, LDHA expression shows significantly inverse correlation with both disease-free survival and overall survival in patients with clear cell renal cell carcinoma. Our results are validated by examining LDHA expression, at the mRNA level, in the independent data set of clear cell renal cell carcinoma cases from The Cancer Genome Atlas databases which also shows that higher lactate dehydrogenase A expression is associated with significantly shorter overall survival.Our results indicate that LDHA up-regulation can be a predictor of poor prognosis in clear cell renal cell carcinoma. Thus, it represents a potential prognostic biomarker that can boost the accuracy of other prognostic models in patients with clear cell renal cell carcinoma. 
 Knockdown of lactate dehydrogenase A suppresses tumor growth and   metastasis   of human hepatocellular carcinoma. In previous studies, lactate dehydrogenase A (LDHA) was identified as one of the leading genes that promote the proliferative and tumorigenic potential of malignancies. However, less definitive evidence was reported in hepatocellular carcinoma (HCC) cells. Furthermore, the role of LDHA in promoting   metastasis   of HCC, and its possible mechanism, is not clear. In this study, RNA interference (RNAi) mediated by lentiviral vectors (which induce strong down-regulation of gene expression) was used to analyze the role of LDHA in tumor growth and   metastasis   in HCC. We performed transient and stable RNAi knockdowns of LDHA in HCCLM3 cells, a line that over-expresses LDHA and has a high metastatic potential. Our studies reveal that previously unidentified effects of LHDA may mediate tumor growth and metastasic effects in HCC. First, HCC cell lines over-express LDHA. Second, LDHA inhibition results in increased apoptosis via production of reactive oxygen species in HCCLM3 cells. Thus, LDHA knockdown resulted in significant reduction in metastatic potential in a xenograft mouse model. Furthermore, we found that FAK, MMP-2, VEGF and E-cadherin proteins contribute to inhibitory effects on   metastasis   in HCC cells. These studies have important implications for understanding the mechanisms by which LDHA promotes tumor growth and   metastasis  . 
 Melanoma brain   metastasis   is independent of lactate dehydrogenase A expression. The key metabolic enzyme lactate dehydrogenase A (LDHA) is overexpressed in many cancers, and several preclinical studies have shown encouraging results of targeted inhibition. However, the mechanistic importance of LDHA in melanoma is largely unknown and hitherto unexplored in brain   metastasis  .We investigated the spatial, temporal, and functional features of LDHA expression in melanoma brain   metastasis   across multiple in vitro assays, in a robust and predictive animal model employing MRI and PET imaging, and in a unique cohort of 80 operated patients. We further assessed the genomic and proteomic landscapes of LDHA in different cancers, particularly melanomas.LDHA expression was especially strong in early and small brain metastases in vivo and related to intratumoral hypoxia in late and large brain metastases in vivo and in patients. However, LDHA expression in human brain metastases was not associated with the number of tumors, BRAF(V600E) status, or survival. Moreover, LDHA depletion by small hairpin RNA interference did not affect cell proliferation or 3D tumorsphere growth in vitro or brain   metastasis   formation or survival in vivo. Integrated analyses of the genomic and proteomic landscapes of LDHA indicated that LDHA is present but not imperative for tumor progression within the CNS, or predictive of survival in melanoma patients.In a large patient cohort and in a robust animal model, we show that although LDHA expression varies biphasically during melanoma brain   metastasis   formation, tumor progression and survival seem to be functionally independent of LDHA. 
 The miR-34a-LDHA axis regulates glucose metabolism and tumor growth in breast cancer. Lactate dehydrogenase A (LDHA) is involved in a variety of cancers. The purpose of this study was to investigate the expression, prognostic roles and function of LDHA in breast cancer. We found that LDHA was upregulated in both breast cancer cell lines and clinical specimens using quantitative real-time PCR (qRT-PCR). Immunohistochemistry (IHC) analysis of tissue microarrays (TMAs) showed that high LDHA expression was associated with cell proliferation,   metastasis   and poor patient overall survival (OS) and disease free survival (DFS). Furthermore, we found that LDHA promoted glycolysis and cell proliferation in vitro and in vivo. We also performed luciferase reporter assays and found that LDHA was a direct target of miR-34a. Repression of LDHA by miR-34a suppressed glycolysis and cell proliferation in breast cancer cells in vitro. Our findings provide clues regarding the role of miR-34a as a tumor suppressor in breast cancer through the inhibition of LDHA both in vitro and in vivo. Targeting LDHA through miR-34a could be a potential therapeutic strategy in breast cancer. 
 Prognostic implications of dynamic serum lactate dehydrogenase assessments in nasopharyngeal carcinoma patients treated with intensity-modulated radiotherapy. The prognostic value of dynamic serum lactate dehydrogenase (LDH) levels in patients with nasopharyngeal carcinoma (NPC) treated with intensity-modulated radiotherapy (IMRT) hasn't been explored. We retrospectively analyzed 1,428 cases of NPC treated with IMRT with or without chemotherapy. Elevated pre- and/or post-treatment LDH levels were found to be associated with unfavorable overall survival (OS), disease-free survival (DFS) and distant   metastasis  -free survival (DMFS), but not with local relapse-free survival (LRFS). The dynamic variations in LDH levels were prognostic factors for OS, DFS and DMFS, but not for LRFS. Multivariate analysis revealed that the N category, T category, post-treatment serum LDH level and age were independent prognostic factors for OS. Our results demonstrated that dynamic variations in LDH levels were associated with risk of distant failure and death, which may shed light on the dynamics of the disease and the response to therapy. We consider that LDH measurements will be of great clinical importance in the management of NPC, especially, when considering "decision points" in treatment algorithms. Therefore, we strongly recommend that LDH levels should be determined before and after treatment in NPC patients and the results integrated into decisions regarding treatment strategies. 
 FOXM1 promotes the warburg effect and pancreatic cancer progression via transactivation of LDHA expression. The transcription factor Forkhead box protein M1 (FOXM1) plays critical roles in cancer development and progression. However, the regulatory role and underlying mechanisms of FOXM1 in cancer metabolism are unknown. In this study, we characterized the regulation of aerobic glycolysis by FOXM1 and its impact on pancreatic cancer metabolism.The effect of altered expression of FOXM1 on expression of glycolytic enzymes and tumor development and progression was examined using animal models of pancreatic cancer. Also, the underlying mechanisms of altered pancreatic cancer glycolysis were analyzed using in vitro molecular biology. The clinical relevance of aberrant metabolism caused by dysregulated FOXM1 signaling was determined using pancreatic tumor and normal pancreatic tissue specimens.We found that FOXM1 did not markedly change the expression of most glycolytic enzymes except for phosphoglycerate kinase 1 (PGK-1) and lactate dehydrogenase A (LDHA). FOXM1 and LDHA were overexpressed concomitantly in pancreatic tumors and cancer cell lines. Increased expression of FOXM1 upregulated the expression of LDHA at both the mRNA and protein level and elevated LDH activity, lactate production, and glucose utilization, whereas reduced expression of FOXM1 did the opposite. Further studies demonstrated that FOXM1 bound directly to the LDHA promoter region and regulated the expression of the LDHA gene at the transcriptional level. Also, elevated FOXM1-LDHA signaling increased the pancreatic cancer cell growth and   metastasis  .Dysregulated expression and activation of FOXM1 play important roles in aerobic glycolysis and tumorigenesis in patients with pancreatic cancer via transcriptional regulation of LDHA expression. 
 Lactate dehydrogenase 5 (LDH5) relates to up-regulated hypoxia inducible factor pathway and   metastasis   in colorectal cancer. Lactate dehydrogenase 5 (LDH5) is one of the five LDH isoenzymes and, apparently, the most important for promoting anaerobic glycolysis. LDH5 is transcriptionally regulated by the hypoxia inducible factors (HIF) 1alpha and 2alpha. In this study, the possible aggressive advantages that colorectal tumours may gain from a high LDH5 content was investigated. To this end, 75 colorectal adenocarcinomas were studied immunohistochemically for the expression of LDH5, and the results were related to tumor differentiation, lymph node and distant metastases, the expression of HIF1alpha and HIF2alpha, vascular density (VD) and vascular endothelial growth factor (VEGF). A high LDH5 content was noted in 51 of 75 (68%) colorectal adenocarcinomas. The reactivity was nuclear and/or cytoplasmic. Nuclear LDH5 reactivity was correlated with lymph node involvement and distant metastases. There was a direct association between LDH5 up-regulation and HIF1alpha and HIF2alpha accumulation. HIF1alpha was linked with VEGF, VD and also with extramural invasion, nodal and distant metastases. It is concluded that a high LDH5 content in tumor cells is directly related to an up-regulated HIF pathway and is linked with an aggressive phenotype in colorectal adenocarcinomas. 
 Lactate dehydrogenase 5 expression in squamous cell head and neck cancer relates to prognosis following radical or postoperative radiotherapy. We assessed the expression and the prognostic role of lactate dehydrogenase 5 (LDH5, the major LDH isoenzyme involved in anaerobic glycolysis) in patients with squamous cell head and neck cancer (SCHNC).LDH5 was assessed immunohistochemically in whole tissue sections from 141 patients with SCHNC. Of these, 102 were subjected to surgery with (90 patients) or without (12 patients) postoperative radiotherapy (group A), while 39 patients were treated with radical radiotherapy (group B).Mixed nuclear/cytoplasmic LDH5 expression was detected in 72.5% of group A and 61.5% of group B patients. This was significantly related to T4-stage (p = 0.04) and hypoxia-inducible factor-1alpha (HIF-1alpha) expression (p = 0.002). In group A, high LDH5 was linked with poorer distant   metastasis  -free survival (p = 0.01) and disease-specific overall survival (OS; p = 0.009). In multivariate analysis, LDH5 (p = 0.002) and HIF-1alpha (p = 0.01) were independently linked with distant   metastasis  . LDH5 was also linked with death events (p = 0.005). In group B, high LDH5 expression was significantly associated with poorer local relapse-free survival (p = 0.009) and OS (p = 0.01). In multivariate analysis, only T stage was a significant predictor of death events (p = 0.04).LDH5 is highly expressed in SCHNC and is linked with local relapse, survival and distant   metastasis  , suggesting that LDH5 is a marker of radioresistance and a target for therapeutic interventions. 
 Overexpression of metabolic markers PKM2 and LDH5 correlates with aggressive clinicopathological features and adverse patient prognosis in tongue cancer. Pyruvate kinase M2 (PKM2) and lactate dehydrogenase 5 (LDH5) are two metabolic and oncogenic markers of cancer. In this study, we sought to investigate their expression patterns and prognostic value in tongue squamous cell carcinoma (TSCC).The expression and subcellular localization of PKM2 and LDH5 in TSCC cell lines were determined by Western blot and immunofluorescence. PKM2 and LDH5 abundance was examined by immunohistochemistry in 63 TSCC tumour specimens; their association with multiple clinicopathological parameters and overall patient survival was assessed. The protein levels of PKM2 and LDH5 were both significantly higher in TSCC cells than in an immortalized oral epithelial cell line. Overexpression of PKM2 associated significantly with cervical node   metastasis   (P = 0.0373), while elevated LDH5 levels correlated significantly with tumour size (P = 0.0094), pathological grade (P = 0.0052), cervical   node meta  stasis (P = 0.0023) and clinical stage (P = 0.0024). Patients with tumours showing an increase in either PKM2 or LDH5 expression displayed significantly reduced overall survival, while patients with tumours overexpressing both proteins showed the worst prognosis with lowest overall survival. Furthermore, PKM2 and LDH5 were identified as independent prognostic predictors for overall patient survival in TSCC.Our data indicate that overexpression of PKM2 and LDH5 associates with key clinicopathological features and unfavourable prognosis in TSCC. 
 Increased LDH5 expression is associated with lymph node   metastasis   and outcome in oral squamous cell carcinoma. Oral squamous cell carcinoma (OSCC) account for more than 90 % of all oral malignant lesions. Lactate dehydrogenase 5 (LDH5) has the highest efficiency among all other isoenzymes to catalyse pyruvate transformation to lactate and is significantly overexpressed in several different tumour entities. LDH5 overexpression confers an advantage on malignant cells, allows them to grow faster, and to metastasize. No data regarding LDH5 expression and OSCC outcome are available. Expression of LDH5 was analysed in OSCC specimen (n = 191) and cancer cell lines (BICR3, BICR56) by immunohistochemistry, real-time quantitative reverse transcription-PCR (RT-PCR) analysis, and western blotting. Scanned images were digitally analysed using ImageJ and the immunomembrane plug-in. LDH5 expression on protein level was correlated with clinicopathological characteristics and impact on survival. LDH5 was co-labelled with glucose transporter-1 (GLUT-1), Ki-67, and hypoxia inducible factor 1 (HIF-1α) in immunohistochemical double staining experiments. Expression subgroups were identified by receiver operating characteristics analysis. LDH5 expression was significantly associated with tumour progression, and recurrence of the tumour. Multivariate analysis demonstrated LDH5 expression as an independent prognostic factor (p < 0.0001). Immunohistochemical double staining experiments revealed LDH5 expression by cancer cells in association with glucose uptake (GLUT-1), proliferation (Ki-67), and hypoxia (HIF-1α). LDH5 specificity was confirmed by western blot and RT-PCR analysis. For the first time, this study provides evidence that LDH5 expression in OSCC might be associated with tumour formati  on and met  astasis in a large patient cohort. Therefore, adjuvant therapies targeting glucose metabolism might be promising for therapy of OSCC. 
 Relationship between 18F-FDG accumulation and lactate dehydrogenase A expression in lung adenocarcinomas. (18)F-FDG PET has been widely used in the management of malignant tumors. Lactate dehydrogenase A (LDHA) plays an important role in the development, invasion, and   metastasis   of malignancies. However, the relationship between (18)F-FDG accumulation and LDHA expression has not been investigated.Retrospective analysis was conducted for 51 patients with lung adenocarcinomas who underwent (18)F-FDG PET. The relationship between maximum standardized uptake value and the expression of LDHA, glucose transporter 1 (GLUT1), and hexokinase 2 (HK2) were examined. RNA interference was used to analyze the role of LDHA in tumor metabolism and growth in A549 cells. The AKT, also known as protein kinase B, pathway was also investigated to evaluate the molecular mechanisms of the relationship between LDHA expression and (18)F-FDG uptake.Maximum standardized uptake value was significantly higher in the LDHA high-expression group than the LDHA low-expression group (P = 0.018). GLUT1 expression in lung adenocarcinomas was positively correlated with (18)F-FDG accumulation and LDHA expression whereas HK2 expression was not. Knockdown of LDHA led to a significant decrease in GLUT1 expression, (18)F-FDG uptake, and cell proliferation. The activated form of AKT was also decreased after LDHA knockdown.LDHA increases (18)F-FDG accumulation into non-small cell lung cancer, possibly by upregulation of GLUT1 expression but not HK2 expression. LDHA may modulate (18)F-FDG uptake in lung adenocarcinomas via the AKT-GLUT1 pathway. These results indicate that (18)F-FDG PET/CT may predict LDHA expression levels and response to anti-LDHA therapy in lung adenocarcinomas. 
 High lactate dehydrogenase 5 expression correlates with high tumoral and stromal vascular endothelial growth factor expression in gastric cancer. Lactate dehydrogenase 5 (LDH5) is a major lactate dehydrogenase isoenzyme catalyzing the transformation of pyruvate to lactate to provide anaerobic energy. Vascular endothelial growth factor (VEGF) is expressed in both tumor and stromal cells in gastric cancer. Our aim was to study the prognostic effect of LDH5, and tumoral and stromal expression of the angiogenic factor VEGF in gastric cancer, and the intercorrelation of tissue expression of both factors.Tissue microarray analysis of 382 consecutive gastric cancer resection specimens was used for immunohistochemistry of LDH5 and VEGF, and expression of LDH5, tumoral VEGF, and stromal VEGF was categorized into low and high groups.High expression was observed for LDH5 in 57.9% (219/378), tumoral VEGF in 35.7% (136/381), and stromal VEGF in 58.5% (223/381) of the specimens. Regarding high expression of LDH5 and VEGF, significant associations with intestinal type, advanced gastric cancer, lymph node   metastasis  , higher TNM stage, and upper-third location were noted. Positive intercorrelations occurred among the expression of LDH5 and VEGF. Results of survival analyses revealed a significant association of high expression of LDH5 and VEGF with lower survival (overall and disease-free survival). Five-year survival rates were significantly lower in tumors with high LDH5 and tumoral VEGF expression in diffuse- or mixed-type cancers and high expression of stromal VEGF in intestinal-type cancer.The results of our study showed that high LDH5 and VEGF expression in both tumor and stroma was a prognostic factor for patients with gastric cancers, especially diffuse- or mixed-type cancers. Therefore, LDH5 expression may play a role in the regulation of tumoral and stromal VEGF expression in gastric cancer. Our results suggest the potential use of LDH5 expression as a biomarker for response to VEGF-targeted therapy. 
  GeneRIFs  
 Although lactate dehydrogenase A (LDHA) expression varies biphasically during melanoma brain   metastasis   formation, tumor progression and survival seem to be functionally independent of LDHA. 
 Increased LDH5 expression is associated with lymph node   metastasis   in oral squamous cell carcinoma. 
 Studies indicate the mechanisms by which lactate dehydrogenase A (LDHA) promotes tumor growth and   metastasis  . 
 LDH5 is highly expressed in squamous cell head and neck cancer and is linked with local relapse, survival and distant   metastasis  . 
   
  
 LDHB 
 
  NCBI Gene  
  GeneRIF  
  Pubmed  
 
 
  Search Term  Rank  Fields  
  [Mm]igration  6  PubMed(2)  
  [Ee]xtracellular [Mm]atrix  8  PubMed(1)  
  [Ii]nvasive  10  PubMed(1); GeneRIFs(1)  
  [Mm]etastasis  12  PubMed(4)  
 
 
 hide/show details for LDHB 
  [Mm]igration  PubMed  
 Analysis of a genetic mutation in an electrophoretic variant of the human lactate dehydrogenase-B(H) subunit. An electrophoretic variant of the lactate dehydrogenase (LDH)-B(H) subunit was discovered in a patient with diabetes mellitus. His LDH activity in serum was slightly lower than normal and the LDH isozyme pattern showed an abnormal   migration   indicating an LDH-B subunit variant of the fast type. The LDH containing the variant subunit revealed a decreased heat stability. DNA analysis of the variant allele detected a base substitution, an A to G transition, at codon 6 (AAA-->GAA). The mutation resulted in the replacement of a lysine by a glutamic acid (K6E). The change may cause the heat instability and affect the net charge of the variant subunit, resulting in an electrophoretic LDH-B subunit variant of the fast type. 
 Detection and characterization of new genetic mutations in individuals heterozygous for lactate dehydrogenase-B(H) deficiency using DNA conformation polymorphism analysis and silver staining. Human lactate dehydrogenase (LDH)--B(H) mutant genes were analyzed by polymerase chain reaction (PCR) and DNA conformation polymorphism. We used polyacrylamide gradient gel and silver staining procedures for DCP analysis, and observed abnormal   migration   patterns in individuals heterozygous for the LDH-B deficiency. Subsequent sequence determination of the mutant alleles consistently resulted in detection of three single base substitutions (transversions), viz., a C to A at residue "35" (GCG, Ala-->GAG, Glu), a T to G at residue "172" (TTT, Phe-->GTT, Val), and an A to T at residue "176" (ATG, Met-->TTG, Leu). Furthermore, mismatched PCR or amplification refractory mutation system was developed for the rapid screening and confirmation of these mutations. These amino acid replacements may cause conformational changes in neighboring residues; this probably affects the active site arrangement and results in the loss of enzyme activity. 
  [Ee]xtracellular [Mm]atrix  PubMed  
 Identification of breast cancer-restricted antigens by antibody screening of SKBR3 cDNA library using a preselected patient's serum. Screening of a breast cancer cDNA library from SKBR3 human breast cancer cells by SEREX (serological analysis of cDNA expression library) using a preselected serum from a breast cancer patient revealed 13 genes, two of which, INT-MI-1 and INT-MI-2, encode novel gene products, while the remaining 11 genes and their products are identical with or highly homologous to known GenBank entries. Immunoscreening of the 13 clones using 20 allogeneic sera from breast cancer patients and 20 samples from age- and gender-matched healthy donors showed that lactate dehydrogenase-A (LDH-A), lactate dehydrogenase-B (LDH-B), fibulin-1, and thyroid hormone-binding protein (THBP) were recognized principally by the breast cancer patient sera, indicating the immunogenicity of these molecules in vivo. The other antigens were similarly recognized by normal and patients sera, and thus not tumor-restricted immunologically. RT-PCR analysis revealed strong expression of fibulin-1 in tumor cell lines and surgical specimen whereas in the same experimental conditions, normal tissues scored negative. Also THBP expression was found in various tumors whereas in normal tissues, its expression is restricted to the testis and, at lower levels, in ovary, liver, and spleen. In contrast, LDH-A and LDH-B were ubiquitously expressed in normal and tumor tissues, with LDH-B levels considerably lower and heterogeneous in normal samples compared to those expressed in tumor cell lines. The differential expression of fibulin-1 between the normal tissues and breast carcinoma cell lines (5/6) and surgical specimens (5/6) suggests the possible involvement of the overexpression of this   extracellular matrix  -associated glycoprotein in the pathogenesis of this neoplasm. 
  [Ii]nvasive  PubMed  
 Decreased lactate dehydrogenase B expression enhances claudin 1-mediated hepatoma cell   invasive  ness via mitochondrial defects. Aerobic lactate production of which the final step is executed by lactate dehydrogenase (LDH) is one of the typical phenotypes in   invasive   tumor development. However, detailed mechanism of how LDH links to cancer cell   invasive  ness remains unclear. This study shows that suppressed LDHB expression plays a critical role in hepatoma cell   invasive  ness by inducing claudin-1 (Cln-1), a tight junction protein, via mitochondrial respiratory defects. First, we found that all the SNU human hepatoma cells with increased glycolytic lactate production have the defective mitochondrial respiratory activity and the Cln-1-mediated high   invasive   activity. Similar results were also obtained with human hepatocellular carcinoma tissues. Unexpectedly, the increased lactate production was due to LDH isozyme shifts to LDH5 by LDHB down-expression rather than LDHA induction, implying the importance of LDHB modulation. Second, LDHB knockdown did not only trigger Cln-1 induction at the transcriptional level, but also induced respiratory impairment. Interestingly, most respiratory inhibitors except KCN induced Cln-1 expression although complex I inhibition by rotenone was most effective on Cln-1 induction. Respiratory defect-mediated Cln-1 induction was further confirmed by knockdown of NDUFA9, one of complex I subunits. Finally, ectopic expression of LDHB attenuated the   invasive  ness of both SNU 354 and 449 cells whereas LDHB knockdown significantly augmented the   invasive  ness of Chang cells with Cln-1induction. The increased   invasive   activity by LDHB modulation was clearly reversed by knocking-down Cln-1. Taken together, our results suggest that LDHB suppression plays an important role in triggering or maintaining the mitochondrial defects and then contributes to cancer cell   invasive  ness by inducing Cln-1 protein. 
  GeneRIFs  
 Results suggest that lactate dehydrogenase B suppression plays an important role in triggering or maintaining the mitochondrial defects and then contributes to cancer cell   invasive  ness by inducing claudin-1 protein. 
  [Mm]etastasis  PubMed  
 Molecular risk stratification of medulloblastoma patients based on immunohistochemical analysis of MYC, LDHB, and CCNB1 expression. Medulloblastoma is the most common malignant embryonal brain tumor in children. The current clinical risk stratification to select treatment modalities is not optimal because it does not identify the standard-risk patients with resistant disease or the unknown number of high-risk patients who might be overtreated with current protocols. The aim of this study is to improve the risk stratification of medulloblastoma patients by using the expression of multiple prognostic markers in combination with current clinical parameters.Candidate prognostic markers were selected from literature or from medulloblastoma expression data. Selected genes were immunohistochemically analyzed for their prognostic value using medulloblastoma tissue arrays containing 124 well-characterized patient samples.Protein expression analyses showed that the combined expression of three genes was able to predict survival in medulloblastoma patients. Low MYC expression identified medulloblastoma patients with a very good outcome. In contrast, concomitant expression of LDHB and CCNB1 characterized patients with a very poor outcome. Multivariate analyses showed that both expression of MYC and the LDHB/CCNB1 gene signature were strong prognostic markers independent of the clinical parameters   metastasis   and residual disease. Combined analysis of clinical and molecular markers enabled greater resolution of disease risk than clinical factors alone.A molecular risk stratification model for medulloblastoma patients is proposed based on the signature of MYC, LDHB, and CCNB1 expression. Combined with clinical variables, the model may provide a more accurate basis for targeting therapy in children with this disease. 
 Serum S100B and LDH are not useful in predicting the sentinel node status in melanoma patients. Serum S100B and LDH, as well as the status of the sentinel node, have been reported as prognostic markers in melanoma patients. The purpose of this study was to determine the value of serum S-100B and LDH in melanoma patients prior to sentinel lymph node dissection (SLND) with respect to the clinical outcome.Serum S100B and LDH were measured prior to SLND in 259 melanoma patients between 2000 and 2006. Upper institutional limits were 0.12 microg/l for S100B and 240U/l for LDH.The median follow-up time was 27.1 months. The median S-100B value for SN-negative and SN-positive patients was 0.06 microg/l and 0.05 microg/l, respectively (p=0.291). Similarly for LDH, the values were 171.5 U/l and 166.5 U/l, respectively (p=0.763). Neither of the proposed markers were a statistically significant prognostic parameter for disease-free survival (DFS), distant   metastasis  -free survival (DMFS) and overall survival (OS).In the present study neither serum S100B nor LDH prior to SLND were useful in predicting the histopathological status of the sentinel node. None of them correlated with DFS, DMFS or OS. 
 Identification of novel molecular targets regulated by tumor suppressive miR-375 induced by histone acetylation in esophageal squamous cell carcinoma. The aim of this study was to determine whether histone acetylation regulates tumor suppressive microRNAs (miRNAs) in esophageal squamous cell carcinoma (ESCC) and to identify genes which are regulated by these miRNAs. We identified a miRNA that was highly upregulated in an ESCC cell line by cyclic hydroxamic acid-containing peptide 31 (CHAP31), one of the histone deacetylase inhibitors (HDACIs), using a miRNA array analysis. miR-375 was strongly upregulated by CHAP31 treatment in an ESCC cell line. The expression levels of the most upregulated miRNA, miR-375 were analyzed by quantitative real-time PCR in human ESCC specimens. The tumor suppressive function of miR-375 was revealed by restoration of miR-375 in ESCC cell lines. We performed a microarray analysis to identify target genes of miR-375. The mRNA and protein expression levels of these genes were verified in ESCC clinical specimens. LDHB and AEG-1/MTDH were detected as miR‑375-targeted genes. The restoration of miR-375 suppressed the expression of LDHB and AEG-1/MTDH. The ESCC clinical specimens exhibited a high level of LDHB expression at both the mRNA and protein levels. A loss-of-function assay using a siRNA analysis was performed to examine the oncogenic function of the gene. Knockdown of LDHB by RNAi showed a tumor suppressive function in the ESCC cells. The correlation between gene expression and clinicopathological features was investigated by immunohistochemistry for 94 cases of ESCC. The positive staining of LDHB correlated significantly with lymph nod  e metastas  is and tumor stage. It also had a tendency to be associated with a poor prognosis. Our results indicate that HDACIs upregulate miRNAs, at least some of which act as tumor suppressors. LDHB, which is regulated by the tumor suppressive miR-375, may therefore act as an oncogene in ESCC. 
 Loss of lactate dehydrogenase B subunit expression is correlated with tumour progression and independently predicts inferior disease-specific survival in urinary bladder urothelial carcinoma. In our previous studies, comparative proteomics demonstrated that lactate dehydrogenase B subunit (LDH-B) is down-regulated in high grade compared to non-high grade urinary bladder urothelial carcinoma (UBUC). However, this finding has not been validated by clinical cohort investigation. Therefore, in the present study, 269 primary localised UBUC specimens were examined for LDH-B expression to clarify the relevance of LDH-B expression level to UBUC progression.Immunohistochemistry (IHC) was implemented to investigate LDH-B protein expression in 269 primary localised UBUC specimens and to evaluate the association with tumour progression and prognosis.Our data demonstrated that dwindled LDH-B expression level was strongly associated with increment of primary tumour status (p < 0.0001), higher histological grade (p = 0.0024), and the presence of vascular (p = 0.0118) as well as perineurial (p = 0.0094) invasion, suggesting that LDH-B might be related to tumour progression. At the univariate level, low LDH-B expression is one of many parameters which significantly predicted both disease-specific survival (DSS) (  p = 0.  0001) and metastasis-free survival (MeFS) (p = 0.0024). In Cox multivariate regression model, higher pT status was the strongest independent prognosticator for both DSS (p = 0.0006) and MeFS (p = 0.0067) while low LDH-B expression remained prognostically significant for DSS (p = 0.0401).The above results confirmed the prognostic roles of LDH-B in UBUC. 
   
  
 FLNA 
 
  NCBI Gene  
  GeneRIF  
  Pubmed  
 
 
  Search Term  Rank  Fields  
  [Ff]ilopodia  1  PubMed(2)  
  [Pp]odosome(s)?  5  PubMed(1); GeneRIFs(1)  
  [Mm]igration  6  Process(2); PubMed(55); GeneRIFs(8); Summary(1)  
  ECM  7  PubMed(1)  
  [Ee]xtracellular [Mm]atrix  8  PubMed(14); Component(1); GeneRIFs(1)  
  [Ii]nvasive  10  PubMed(7)  
  [Mm]etastasis  12  PubMed(15); GeneRIFs(2)  
  [Cc]ell [Ii]nvasion  13  PubMed(1)  
 
 
 hide/show details for FLNA 
  [Ff]ilopodia  PubMed  
 Localization and enhanced current density of the Kv4.2 potassium channel by interaction with the actin-binding protein filamin. Kv4.2 potassium channels play a critical role in postsynaptic excitability. Immunocytochemical studies reveal a somatodendritic Kv4.2 expression pattern, with the channels concentrated mainly at dendritic spines. The molecular mechanism that underlies the localization of Kv4.2 to this subcellular region is unknown. We used the yeast two-hybrid system to identify the Kv4.2-associated proteins that are involved in channel localization. Here we demonstrate a direct interaction between Kv4.2 and the actin-binding protein, filamin. We show that Kv4.2 and filamin can be coimmunoprecipitated both in vitro and in brain and that Kv4.2 and filamin share an overlapping expression pattern in the cerebellum and cultured hippocampal neurons. To examine the functional consequences of this interaction, we expressed Kv4.2 in filamin(+) and filamin(-) cells and performed immunocytochemical and electrophysiological analyses. Our results indicate that Kv4.2 colocalizes with filamin at   filopodia  l roots in filamin(+) cells but shows a nonspecific expression pattern in filamin(-) cells, with no localization to   filopodia  l roots. Furthermore, the magnitude of whole-cell Kv4.2 current density is approximately 2.7-fold larger in filamin(+) cells as compared with these currents in filamin(-) cells. We propose that filamin may function as a scaffold protein in the postsynaptic density, mediating a direct link between Kv4.2 and the actin cytoskeleton, and that this interaction is essential for the generation of appropriate Kv4.2 current densities. 
 The small GTPase RalA targets filamin to induce   filopodia  . The Ras-related small GTPases Rac, Rho, Cdc42, and RalA bind filamin, an actin filament-crosslinking protein that also links membrane and other intracellular proteins to actin. Of these GTPases only RalA binds filamin in a GTP-specific manner, and GTP-RalA elicits actin-rich filopods on surfaces of Swiss 3T3 cells and recruits filamin into the   filopodia  l cytoskeleton. Either a dominant negative RalA construct or the RalA-binding domain of filamin 1 specifically block Cdc42-induced filopod formation, but a Cdc42 inhibitor does not impair RalA's effects, which, unlike Cdc42, are Rac independent. RalA does not generate   filopodia   in filamin-deficient human melanoma cells, whereas transfection of filamin 1 restores the functional response. RalA therefore is a downstream intermediate in Cdc42-mediated filopod production and uses filamin in this pathway. 
  [Pp]odosome(s)?  PubMed  
 Macrophage mesenchymal migration requires   podosome   stabilization by filamin A. Filamin A (FLNa) is a cross-linker of actin filaments and serves as a scaffold protein mostly involved in the regulation of actin polymerization. It is distributed ubiquitously, and null mutations have strong consequences on embryonic development in humans, with organ defects which suggest deficiencies in cell migration. We have reported previously that macrophages, the archetypal migratory cells, use the protease- and   podosome  -dependent mesenchymal migration mode in dense three-dimensional environments, whereas they use the protease- and   podosome  -independent amoeboid mode in more porous matrices. Because FLNa has been shown to localize to   podosomes  , we hypothesized that the defects seen in patients carrying FLNa mutations could be related to the capacity of certain cell types to form   podosomes  . Using strategies based on FLNa knock-out, knockdown, and rescue, we show that FLNa (i) is involved in   podosome   stability and their organization as rosettes and three-dimensional   podosomes  , (ii) regulates the proteolysis of the matrix mediated by   podosomes   in macrophages, (iii) is required for   podosome   rosette formation triggered by Hck, and (iv) is necessary for mesenchymal migration but dispensable for amoeboid migration. These new functions assigned to FLNa, particularly its role in mesenchymal migration, could be directly related to the defects in cell migration described during the embryonic development in FLNa-defective patients. 
  GeneRIFs  
 FLNa is involved in   podosome   stability and their organization as rosettes and three-dimensional   podosomes   in macrophages. 
  [Mm]igration  Process  
 positive regulation of neuron   migration   
 regulation of cell   migration   
  PubMed  
 Analysis of the myosin-II-responsive focal adhesion proteome reveals a role for β-Pix in negative regulation of focal adhesion maturation. Focal adhesions undergo myosin-II-mediated maturation wherein they grow and change composition to modulate integrin signalling for cell   migratio  n, growth and differentiation. To determine how focal adhesion composition is affected by myosin II activity, we performed proteomic analysis of isolated focal adhesions and compared protein abundance in focal adhesions from cells with and without myosin II inhibition. We identified 905 focal adhesion proteins, 459 of which changed in abundance with myosin II inhibition, defining the myosin-II-responsive focal adhesion proteome. The abundance of 73% of the proteins in the myosin-II-responsive focal adhesion proteome was enhanced by contractility, including proteins involved in Rho-mediated focal adhesion maturation and endocytosis- and calpain-dependent focal adhesion disassembly. During myosin II inhibition, 27% of proteins in the myosin-II-responsive focal adhesion proteome, including proteins involved in Rac-mediated lamellipodial protrusion, were enriched in focal adhesions, establishing that focal adhesion protein recruitment is also negatively regulated by contractility. We focused on the Rac guanine nucleotide exchange factor β-Pix, documenting its role in the negative regulation of focal adhesion maturation and the promotion of lamellipodial protrusion and focal adhesion turnover to drive cel  l migrati  on. 
 Grb7 and Filamin-a associate and are colocalized to cell membrane ruffles upon EGF stimulation. Grb7 is an adaptor molecule mediating signal transduction from multiple cell surface receptors to diverse downstream pathways. Grb7, along with Grb10 and Grb14, make up the Grb7 protein family. This protein family has been shown to be overexpressed in certain cancers and cancer cell lines. Grb7 and a receptor tyrosine kinase, ErbB2, are overexpressed in 20-30% of breast cancers. Grb7 overexpression has been linked to enhanced cell   migration   and metastasis, although the participants in these pathways have not been fully determined. In this study, we report the Grb7 protein interacts with Filamin-a, an actin-crosslinking component of the cell cytoskeleton. Additionally, we have demonstrated the interaction between Grb7 and Flna is specific to the RA-PH domains of Grb7, and the immunoglobulin-like repeat 16-19 domains of Flna. We demonstrate that full-length Grb7 and Flna interact in the mammalian cellular environment, as well as in vitro. Immunofluorescent microscopy shows potential co-localization of Grb7 and Flna in membrane ruffles upon epidermal growth factor stimulation. These studies are amongst the first to establish a clear connection between Grb7 signaling and cytoskeletal remodeling. 
 Filamin A and Filamin B are co-expressed within neurons during periods of neuronal   migration   and can physically interact. Mutations in the X-linked gene Filamin A (FLNA) lead to the human neurological disorder, periventricular heterotopia (PH). Although PH is characterized by a failure in neuronal   migration   into the cerebral cortex with consequent formation of nodules in the ventricular and subventricular zones, many neurons appear to migrate normally, even in males, suggesting compensatory mechanisms. Here we characterize expression patterns for FlnA and a highly homologous protein Filamin B (FlnB) within the nervous system, in order to better understand their potential roles in cortical development. FlnA mRNA was widely expressed in all cortical layers while FlnB mRNA was most highly expressed in the ventricular and subventricular zones during development. In adulthood, widespread but reduced expression of FlnA and FlnB persisted throughout the cerebral cortex. FlnA and FlnB proteins were highly expressed in both the leading processes and somata of migratory neurons during corticogenesis. Postnatally, FlnA immunoreactivity was largely localized to the cell body with FlnB in the soma and neuropil during neuronal differentiation. In adulthood, diminished expression of both proteins localized to the cell soma and nucleus. Moreover, the putative FLNB homodimerization domain strongly interacted with itself or the corresponding homologous region of FLNA by yeast two-hybrid interaction, the two proteins co-localized within neuronal precursors by immunocytochemistry and the existence of FLNA-FLNB heterodimers could be detected by co-immunoprecipitation. These results suggest that FLNA and FLNB may form both homodimers and heterodimers and that their interaction could potentially compensate for the loss of FLNA function during cortical development within PH individuals. 
 Ribosomal S6 kinase (RSK) regulates phosphorylation of filamin A on an important regulatory site. The Ras-mitogen-activated protein (Ras-MAP) kinase pathway regulates various cellular processes, including gene expression, cell proliferation, and survival. Ribosomal S6 kinase (RSK), a key player in this pathway, modulates the activities of several cytoplasmic and nuclear proteins via phosphorylation. Here we report the characterization of the cytoskeletal protein filamin A (FLNa) as a membrane-associated RSK target. We show that the N-terminal kinase domain of RSK phosphorylates FLNa on Ser(2152) in response to mitogens. Inhibition of MAP kinase signaling with UO126 or mutation of Ser(2152) to Ala on FLNa prevents epidermal growth factor (EGF)-stimulated phosphorylation of FLNa in vivo. Furthermore, phosphorylation of FLNa on Ser(2152) is significantly enhanced by the expression of wild-type RSK and antagonized by kinase-inactive RSK or specific reduction of endogenous RSK. Strikingly, EGF-induced, FLNa-dependent   migration   of human melanoma cells is significantly reduced by UO126 treatment. Together, these data provide substantial evidence that RSK phosphorylates FLNa on Ser(2152) in vivo. Given that phosphorylation of FLNa on Ser(2152) is required for Pak1-mediated membrane ruffling, our results suggest a novel role for RSK in the regulation of the actin cytoskeleton. 
 Filamin A-interacting protein (FILIP) regulates cortical cell   migration   out of the ventricular zone. Precisely regulated radial   migration   out of the ventricular zone is essential for corticogenesis. Here, we identify a mechanism that can tether ventricular zone cells in situ. FILIP interacts with Filamin A, an indispensable actin-binding protein that is required for cell motility, and induces its degradation in COS-7 cells. Degradation of Filamin A is identified in the cortical ventricular zone, where filip mRNA is localized. Furthermore, most ventricular zone cells that overexpress FILIP fail to migrate in explants. These results demonstrate that FILIP functions through a Filamin A F-actin axis to control the start of neocortical cell   migration   from the ventricular zone. 
 Filamin a mediates HGF/c-MET signaling in tumor cell   migration  . Deregulated hepatocyte growth factor (HGF)/c-MET axis has been correlated with poor clinical outcome and drug resistance in many human cancers. Identification of novel regulatory mechanisms influencing HGF/c-MET signaling may therefore be necessary to develop more effective cancer therapies. In our study, we show that multiple human cancer tissues and cells express filamin A (FLNA), a large cytoskeletal actin-binding protein, and expression of c-MET is significantly reduced in human tumor cells deficient for FLNA. The FLNA-deficient tumor cells exhibited poor migrative and invasive ability in response to HGF. On the other hand, the anchorage-dependent and independent tumor cell proliferation was not altered by HGF. The FLNA-deficiency specifically attenuated the activation of the c-MET downstream signaling molecule AKT in response to HGF stimulation. Furthermore, FLNA enhanced c-MET promoter activity by its binding to SMAD2. The impact of FLNA deficiency on c-MET expression and HGF-mediated cell   migration   in human tumor cells was confirmed in primary mouse embryonic fibroblasts deficient for Flna. These data suggest that FLNA is one of the important regulators of c-MET signaling and HGF-induced tumor cell   migration  . 
 FLNA p.V528M substitution is neither associated with bilateral periventricular nodular heterotopia nor with macrothrombocytopenia. Filamin A is encoded by the FLNA gene on chromosome Xq28 and functions in cross-linking actin filaments into orthogonal networks in the cortical cytoplasm. FLNA p.V528M was initially detected in a female autopsy case of X-linked bilateral periventricular nodular heterotopia (BPNH), a neuronal   migration   disorder characterized by subependymal nodules of gray matter. During our mutation analysis of FLNA in a boy with apparent X-linked thrombocytopenia, we detected the p.V528M variant. The patient, mother and sister, who were heterozygous for the substitution, did not have BPNH. We observed an allele frequency of 4.8% in healthy control Japanese, but did not observe the variant in Caucasian subjects. Hemizygous controls had a normal platelet count and size. We suggest that p.V528M is neither associated with BPNH nor with thrombocytopenia and giant platelets, and represents a functional polymorphism. 
 Macrophage mesenchymal   migration   requires podosome stabilization by filamin A. Filamin A (FLNa) is a cross-linker of actin filaments and serves as a scaffold protein mostly involved in the regulation of actin polymerization. It is distributed ubiquitously, and null mutations have strong consequences on embryonic development in humans, with organ defects which suggest deficiencies in cell   migration  . We have reported previously that macrophages, the archetypal migratory cells, use the protease- and podosome-dependent mesenchymal   migration   mode in dense three-dimensional environments, whereas they use the protease- and podosome-independent amoeboid mode in more porous matrices. Because FLNa has been shown to localize to podosomes, we hypothesized that the defects seen in patients carrying FLNa mutations could be related to the capacity of certain cell types to form podosomes. Using strategies based on FLNa knock-out, knockdown, and rescue, we show that FLNa (i) is involved in podosome stability and their organization as rosettes and three-dimensional podosomes, (ii) regulates the proteolysis of the matrix mediated by podosomes in macrophages, (iii) is required for podosome rosette formation triggered by Hck, and (iv) is necessary for mesenchymal   migration   but dispensable for amoeboid   migration  . These new functions assigned to FLNa, particularly its role in mesenchymal   migration  , could be directly related to the defects in cell   migration   described during the embryonic development in FLNa-defective patients. 
 Nephrin regulates lamellipodia formation by assembling a protein complex that includes Ship2, filamin and lamellipodin. Actin dynamics has emerged at the forefront of podocyte biology. Slit diaphragm junctional adhesion protein Nephrin is necessary for development of the podocyte morphology and transduces phosphorylation-dependent signals that regulate cytoskeletal dynamics. The present study extends our understanding of Nephrin function by showing in cultured podocytes that Nephrin activation induced actin dynamics is necessary for lamellipodia formation. Upon activation Nephrin recruits and regulates a protein complex that includes Ship2 (SH2 domain containing 5' inositol phosphatase), Filamin and Lamellipodin, proteins important in regulation of actin and focal adhesion dynamics, as well as lamellipodia formation. Using the previously described CD16-Nephrin clustering system, Nephrin ligation or activation resulted in phosphorylation of the actin crosslinking protein Filamin in a p21 activated kinase dependent manner. Nephrin activation in cell culture results in formation of lamellipodia, a process that requires specialized actin dynamics at the leading edge of the cell along with focal adhesion turnover. In the CD16-Nephrin clustering model, Nephrin ligation resulted in abnormal morphology of actin tails in human podocytes when Ship2, Filamin or Lamellipodin were individually knocked down. We also observed decreased lamellipodia formation and cell   migration   in these knock down cells. These data provide evidence that Nephrin not only initiates actin polymerization but also assembles a protein complex that is necessary to regulate the architecture of the generated actin filament network and focal adhesion dynamics. 
 Mutations in the X-linked filamin 1 gene cause periventricular nodular heterotopia in males as well as in females. Periventricular heterotopia (PH) is a human neuronal   migration   disorder in which many neurons destined for the cerebral cortex fail to migrate. Previous analysis showed heterozygous mutations in the X-linked gene filamin 1 (FLN1), but examined only the first six (of 48) coding exons of the gene and hence did not assess the incidence and functional consequences of FLN1 mutations. Here we perform single-strand conformation polymorphism (SSCP) analysis of FLN1 throughout its entire coding region in six PH pedigrees, 31 sporadic female PH patients and 24 sporadic male PH patients. We detected FLN1 mutations by SSCP in 83% of PH pedigrees and 19% of sporadic females with PH. Moreover, no PH females (0/7 tested) with atypical radiographic features showed FLN1 mutations, suggesting that other genes may cause atypical PH. Surprisingly, 2/24 males analyzed with PH (9%) also carried FLN1 mutations. Whereas FLN1 mutations in PH pedigrees caused severe predicted loss of FLN1 protein function, both male FLN1 mutations were consistent with partial loss of function of the protein. Moreover, sporadic female FLN1 mutations associated with PH appear to cause either severe or partial loss of function. Neither male could be shown to be mosaic for the FLN1 mutation in peripheral blood lymphocytes, suggesting that some neurons in the intact cortex of PH males may be mutant for FLN1 but migrate adequately. These results demonstrate the sensitivity and specificity of DNA testing for FLN1 mutations and have important functional implications for models of FLN1 protein function in neuronal   migration  . 
 Filamin A mediates interactions between cytoskeletal proteins that control cell adhesion. Cell adhesion, spreading and   migration   on extracellular matrices are regulated by complex processes that involve the cytoskeleton and a large array of adhesion receptors, including the β1 integrin. Filamin A is a large, multi-domain, homodimeric actin binding protein that contributes to the mechanical stability of cells and interacts with several proteins that regulate cell adhesion including β1 integrin and several protein kinases. Here we review current data on the structure, mechanical properties and intracellular signaling functions of filamin that regulate cell adhesion. We also consider new data showing that interactions of filamin A with intermediate filaments and protein kinase C enable tight regulation of β1 integrin function and consequently early events in cell adhesion a  nd migrat  ion on extracellular matrix proteins. 
 Filamin A mutation associated with normal reading skills and dyslexia in a family with periventricular heterotopia. Periventricular heterotopia (PH) is a disorder of neuronal   migration   during fetal development that is characterized by morphologically normal neurons being located in an anatomically abnormal position in the mature brain. PH is usually diagnosed in patients presenting with a seizure disorder, when neuroimaging demonstrates the ectopically placed nodules of neurons. PH is a genetically and phenotypically heterogeneous disorder. The most commonly identified genetic cause is the X-linked dominant inheritance of mutations in the Filamin A (FLNA) gene. Multiple lines of evidence support the contribution of genetic factors in dyslexia. As dyslexia does not show a single-gene pattern of inheritance, it is classified as a complex genetic disorder. We have recently identified a specific reading fluency deficit in a variable group of patients with PH, in the context of normal intelligence. Here, we present a study of a mother-daughter pair who share bilateral widespread gray matter heterotopia caused by a novel mutation in FLNA and the same pattern of X-chromosome inactivation but who exhibit divergent reading and cognitive profiles. This novel observation highlights the uncertainty of using heterotopia anatomy in clinical practice to predict behavioral outcome. 
 Filamin-A is required to mediate SST2 effects in pancreatic neuroendocrine tumours. Somatostatin receptor type 2 (SST2) is the main pharmacological target of somatostatin (SS) analogues widely used in patients with pancreatic neuroendocrine tumours (P-NETs), this treatment being ineffective in a subset of patients. Since it has been demonstrated that Filamin A (FLNA) is involved in mediating GPCR expression, membrane anchoring and signalling, we investigated the role of this cytoskeleton protein in SST2 expression and signalling, angiogenesis, cell adhesion and cell   migration   in human P-NETs and in QGP1 cell line. We demonstrated that FLNA silencing was not able to affect SST2 expression in P-NET cells in basal conditions. Conversely, a significant reduction in SST2 expression (-43 ± 21%, P < 0.05 vs untreated cells) was observed in FLNA silenced QGP1 cells after long term SST2 activation with BIM23120. Moreover, the inhibitory effect of BIM23120 on cyclin D1 expression (-46 ± 18%, P < 0.05 vs untreated cells), P-ERK1/2 levels (-42 ± 14%; P < 0.05 vs untreated cells), cAMP accumulation (-24 ± 3%, P < 0.05 vs untreated cells), VEGF expression (-31 ± 5%, P < 0.01 vs untreated cells) and in vitro release (-40 ± 24%, P < 0.05 vs untreated cells) was completely lost after FLNA silencing. Interestingly, BIM23120 promoted cell adhesion (+86 ± 45%, P < 0.05 vs untreated cells) and inhibite  d cell mi  gration (-24 ± 2%, P < 0.00001 vs untreated cells) in P-NETs cells and these effects were abolished in FLNA silenced cells. In conclusion, we demonstrated that FLNA plays a crucial role in SST2 expression and signalling, angiogenesis, cell adhesion a  nd cell m  igration in P-NETs and in QGP1 cell line, suggesting a possible role of FLNA in determining the different responsiveness to SS analogues observed in P-NET patients. 
 Pro-prion binds filamin A, facilitating its interaction with integrin beta1, and contributes to melanomagenesis. Filamin A (FLNA) is an integrator of cell mechanics and signaling. The spreading and   migration   observed in FLNA sufficient A7 melanoma cells but not in the parental FLNA deficient M2 cells have been attributed to FLNA. In A7 and M2 cells, the normal prion (PrP) exists as pro-PrP, retaining its glycosylphosphatidyl-inositol (GPI) anchor peptide signal sequence (GPI-PSS). The GPI-PSS of PrP has a FLNA binding motif and binds FLNA. Reducing PrP expression in A7 cells alters the spatial distribution of FLNA and organization of actin and diminishes cell spreading and   migration  . Integrin β1 also binds FLNA. In A7 cells, FLNA, PrP, and integrin β1 exist as two independent, yet functionally linked, complexes; they are FLNA with PrP or FLNA with integrin β1. Reducing PrP expression in A7 cells decreases the amount of integrin β1 bound to FLNA. A PrP GPI-PSS synthetic peptide that crosses the cell membrane inhibits A7 cell spreading   and migra  tion. Thus, in A7 cells FLNA does not act alone; the binding of pro-PrP enhances association between FLNA and integrin β1, which then promotes cell spreading   and migr  ation. Pro-PrP is detected in melanoma in situ but not in melanocyte. Invasive melanoma has more pro-PrP. The binding of pro-PrP to FLNA, therefore, contributes to melanomagenesis. 
 Filamin A interacts with the coactivator MKL1 to promote the activity of the transcription factor SRF and cell   migration  . Megakaryoblastic leukemia 1 (MKL1) is a coactivator of serum response factor (SRF) that promotes the expression of genes associated with cell proliferation, motility, adhesion, and differentiation-processes that also involve dynamic cytoskeletal changes in the cell. MKL1 is inactive when bound to monomeric globular actin (G-actin), but signals that activate the small guanosine triphosphatase RhoA cause actin polymerization and MKL1 dissociation from G-actin. We found a new mechanism of MKL1 activation that is mediated through its binding to filamin A (FLNA), a protein that binds filamentous actin (F-actin). The interaction of FLNA and MKL1 was required for the expression of MKL1 target genes in primary fibroblasts, melanoma, mammary and hepatocellular carcinoma cells. We identified the regions of interaction between MKL1 and FLNA, and cells expressing an MKL1 mutant that was unable to bind FLNA exhibited impaired cell   migration   and reduced expression of MKL1-SRF target genes. Induction and repression of MKL1-SRF target genes correlated with increased or decreased MKL1-FLNA interaction, respectively. Lysophosphatidic acid-induced RhoA activation in primary human fibroblasts promoted the association of endogenous MKL1 with FLNA, whereas exposure to an actin polymerization inhibitor dissociated MKL1 from FLNA and decreased MKL1-SRF target gene expression in melanoma cells. Thus, FLNA functions as a positive cellular transducer linking actin polymerization to MKL1-SRF activity, counteracting the known repressive complex of MKL1 and monomeric G-actin. 
 Androgen-induced cell   migration  : role of androgen receptor/filamin A association. Androgen receptor (AR) controls male morphogenesis, gametogenesis and prostate growth as well as development of prostate cancer. These findings support a role for AR in cell   migration   and invasiveness. However, the molecular mechanism involved in AR-mediated cell   migration   still remains elusive.Mouse embryo NIH3T3 fibroblasts and highly metastatic human fibrosarcoma HT1080 cells harbor low levels of transcriptionally incompetent AR. We now report that, through extra nuclear action, AR triggers   migration   of both cell types upon stimulation with physiological concentrations of the androgen R1881. We analyzed the initial events leading to androgen-induced cell   migration   and observed that challenging NIH3T3 cells with 10 nM R1881 rapidly induces interaction of AR with filamin A (FlnA) at cytoskeleton. AR/FlnA complex recruits integrin beta 1, thus activating its dependent cascade. Silencing of AR, FlnA and integrin beta 1 shows that this ternary complex controls focal adhesion kinase (FAK), paxillin and Rac, thereby driving cell   migration  . FAK-null fibroblasts migrate poorly and Rac inhibition by EHT impairs motility of androgen-treated NIH3T3 cells. Interestingly, FAK and Rac activation by androgens are independent of each other. Findings in human fibrosarcoma HT1080 cells strengthen the role of Rac in androgen signaling. The Rac inhibitor significantly impairs androgen-induced   migration   in these cells. A mutant AR, deleted of the sequence interacting with FlnA, fails to mediate FAK activation and paxillin tyrosine phosphorylation in androgen-stimulated cells, further reinforcing the role of AR/FlnA interaction in androgen-mediated motility.The present report, for the first time, indicates that the extra nuclear AR/FlnA/integrin beta 1 complex is the key by which androgen activates signaling leading to cell   migration  . Assembly of this ternary complex may control organ development and prostate cancer metastasis. 
 Novel X-linked syndrome of cardiac valvulopathy, keloid scarring, and reduced joint mobility due to filamin A substitution G1576R. Filamin A (FLNA) is known to be involved in intracellular actin binding, cell   migration  , scaffolding, and signaling. We report a novel X-linked syndrome characterized by cardiac valvular disease, keloid scarring and reduced joint mobility in male second cousins due to a previously unreported mutation in FLNA. Whole exome sequencing was performed using standard methods and segregation analysis was performed in affected and non-affected family members. A novel hemizygous c.4726G>A (p.G1576R) mutation in FLNA was detected. Segregation analysis performed on multiple maternal family members showed c.4726G>A (p.G1576R) segregated with disease in an X-linked inheritance pattern. The findings in these cases are distinct from previously described FLNA related disorders by virtue of decreased joint mobility and spontaneous keloid scarring. They occur in association with a novel mutation and represent a novel genetic syndrome. 
 Filamin A is mutated in X-linked chronic idiopathic intestinal pseudo-obstruction with central nervous system involvement. We have previously reported that an X-linked recessive form of chronic idiopathic intestinal pseudo-obstruction (CIIPX) maps to Xq28. To select candidate genes for the disease, we analyzed the expression in murine fetal brain and intestine of 56 genes from the critical region. We selected and sequenced seven genes and found that one affected male from a large CIIPX-affected kindred bears a 2-bp deletion in exon 2 of the FLNA gene that is present at the heterozygous state in the carrier females of the family. The frameshift mutation is located between two close methionines at the filamin N terminus and is predicted to produce a protein truncated shortly after the first predicted methionine. Loss-of-function FLNA mutations have been associated with X-linked dominant nodular ventricular heterotopia (PVNH), a central nervous system (CNS)   migration   defect that presents with seizures in females and lethality in males. Notably, the affected male bearing the FLNA deletion had signs of CNS involvement and potentially has PVNH. To understand how the severe frameshift mutation we found can explain the CIIPX phenotype and its X-linked recessive inheritance, we transiently expressed both the wild- type and mutant filamin in cell culture and found that filamin translation can start from either of the two initial methionines in these conditions. Therefore, translation of a normal shorter filamin can occur in vitro from the second methionine downstream of the 2-bp insertion we found. We confirmed this, demonstrating that the filamin protein is present in the patient's lymphoblastoid cell line that shows abnormal cytoskeletal actin organization compared with normal lymphoblasts. We conclude that the filamin N terminal region between the initial two methionines is crucial for proper enteric neuron development. 
 Filamin A promotes dynamin-dependent internalization of hyperpolarization-activated cyclic nucleotide-gated type 1 (HCN1) channels and restricts Ih in hippocampal neurons. The actin-binding protein filamin A (FLNa) regulates neuronal   migration   during development, yet its roles in the mature brain remain largely obscure. Here, we probed the effects of FLNa on the regulation of ion channels that influence neuronal properties. We focused on the HCN1 channels that conduct Ih, a hyperpolarization-activated current crucial for shaping intrinsic neuronal properties. Whereas regulation of HCN1 channels by FLNa has been observed in melanoma cell lines, its physiological relevance to neuronal function and the underlying cellular pathways that govern this regulation remain unknown. Using a combination of mutational, pharmacological, and imaging approaches, we find here that FLNa facilitates a selective and reversible dynamin-dependent internalization of HCN1 channels in HEK293 cells. This internalization is accompanied by a redistribution of HCN1 channels on the cell surface, by accumulation of the channels in endosomal compartments, and by reduced Ih density. In hippocampal neurons, expression of a truncated dominant-negative FLNa enhances the expression of native HCN1. Furthermore, acute abrogation of HCN1-FLNa interaction in neurons, with the use of decoy peptides that mimic the FLNa-binding domain of HCN1, abolishes the punctate distribution of HCN1 channels in neuronal cell bodies, augments endogenous Ih, and enhances the rebound-response ("voltage-sag") of the neuronal membrane to transient hyperpolarizing events. Together, these results support a major function of FLNa in modulating ion channel abundance and membrane trafficking in neurons, thereby shaping their biophysical properties and function. 
 The cytoplasmic domain of neuropilin-1 regulates focal adhesion turnover. Though the vascular endothelial growth factor coreceptor neuropilin-1 (Nrp1) plays a critical role in vascular development, its precise function is not fully understood. We identified a group of novel binding partners of the cytoplasmic domain of Nrp1 that includes the focal adhesion regulator, Filamin A (FlnA). Endothelial cells (ECs) expressing a Nrp1 mutant devoid of the cytoplasmic domain (nrp1(cyto)(Δ/Δ)) migrated significantly slower in response to VEGF relative to the cells expressing wild-type Nrp1 (nrp1(+/+) cells). The rate of FA turnover in VEGF-treated nrp1(cyto)(Δ/Δ) ECs was an order of magnitude lower in comparison to nrp1(+/+) ECs, thus accounting for the slo  wer migra  tion rate of the nrp1(cyto)(Δ/Δ) ECs. 
 Significance of filamin A in mTORC2 function in glioblastoma. Glioblastoma multiforme (GBM) is one of the most highly metastatic cancers. GBM has been associated with a high level of the mechanistic target of rapamycin complex 2 (mTORC2) activity. We aimed to observe roles of mTORC2 in GBM cells especially on actin cytoskeleton reorganization, cell   migration   and invasion, and further determine new important players involved in the regulation of these cellular processes.To further investigate the significance of mTORC2 in GBM, we treated GBM cells with PP242, an ATP-competitive inhibitor of mTOR, and used RICTOR siRNA to knock down mTORC2 activity. Effects on actin cytoskeleton, focal adhesion,   migration  , and invasion of GBM cells were examined. To gain insight into molecular basis of the mTORC2 effects on cellular cytoskeletal arrangement and motility/invasion, we affinity purified mTORC2 from GBM cells and identified proteins of interest by mass spectrometry. Characterization of the protein of interest was performed.In addition to the inhibition of mTORC2 activity, we demonstrated significant alteration of actin distribution as revealed by the use of phalloidin staining. Furthermore, vinculin staining was altered which suggests changes in focal adhesion. Inhibition of cell   migration   and invasion was observed with PP242. Two major proteins that are associated with this mTORC2 multiprotein complex were found. Mass spectrometry identified one of them as Filamin A (FLNA). Association of FLNA with RICTOR but not mTOR was demonstrated. Moreover, in vitro, purified mTORC2 can phosphorylate FLNA likewise its known substrate, AKT. In GBM cells, colocalization of FLNA with RICTOR was observed, and the overall amounts of FLNA protein as well as phosphorylated FLNA are high. Upon treatments of RICTOR siRNA or PP242, phosphorylated FLNA levels at the regulatory residue (Ser2152) decreased. This treatment also disrupted colocalization of Actin filaments and FLNA.Our results support FLNA as a new downstream effector of mTORC2 controlling GBM cell motility. This new mTORC2-FLNA signaling pathway plays important roles in motility and invasion of glioblastoma cells. 
 The filamins: organizers of cell structure and function. Filamin A (FLNa), the first non-muscle actin filament cross-linking protein, was identified in 1975. Thirty five years of FLNa research has revealed its structure in great detail, discovered its isoforms (FLNb and c), and identified over 90 binding partners including channels, receptors, intracellular signaling molecules, and even transcription factors. Due to this diversity, mutations in human FLN genes result in a wide range of anomalies with moderate to lethal consequences. This review focuses on the structure and functions of FLNa in cell   migration   and adhesion. 
 Filamin A expression correlates with proliferation and invasive properties of human metastatic melanoma tumors: implications for survival in patients. Filamin A (FLNa) cross-links actin filaments into dynamic orthogonal networks and interacts with binding proteins of diverse cellular functions that are implicated in cell growth and motility regulation. Here, we tested the hypothesis that FLNa plays a role in cancer proliferation and metastasis via the regulation of epidermal growth factor receptor (EGFR) function.Ectopic expression and knockdown of FLNa in human melanoma cell lines was performed to investigate changes in cellular proliferation,   migration   and invasion in vitro and tumor growth in a xenograft model in the mouse. The role of FLNa in EGFR expression and signaling was evaluated by Western blot. Immunohistochemistry was performed on histological sections of human melanoma tumors to determine whether an association existed between FLNa and overall survival.The depletion of FLNa significantly reduced the proliferation,   migration   and invasion of two melanoma cell lines in vitro and was associated with smaller tumors in a xenograft model in vivo. EGF-induced phosphorylation of EGFR and activation of the Raf-MEK-ERK cascade was negatively affected by the silencing of FLNa both in vitro and in vivo. Cancer patients with low melanoma tumor FLNa expression have improved survival benefit.These data indicate that enhanced tumorigenesis occurs through increase in EGF-induced EGFR activation in FLNa-expressing melanoma cells and that high FLNa levels are predictors of negative outcome for patients with melanoma tumors. 
 Familial periventricular nodular heterotopia, epilepsy and Melnick-Needles Syndrome caused by a single FLNA mutation with combined gain-of-function and loss-of-function effects. Loss-of-function mutations of the FLNA gene cause a neuronal   migration   disorder defined as X-linked periventricular nodular heterotopia (PNH); gain-of-function mutations are associated with a group of X-linked skeletal dysplasias designed as otopalatodigital (OPD) spectrum. We describe a family in which a woman and her three daughters exhibited a complex phenotype combining PNH, epilepsy and Melnick-Needles syndrome (MNS), a skeletal disorder assigned to the OPD spectrum. All four individuals harboured a novel non-conservative missense mutation in FLNA exon 3.In all affected family members, we performed mutation analysis of the FLNA gene, RT-PCR, ultradeep sequencing analysis in FLNA cDNAs and western blot in lymphocyte cells to further characterise the mutation. We also assessed the effects on RT-PCR products of treatment of patients' lymphocytes with cycloheximide, a nonsense mediated mRNA decay (NMD) inhibitor.We identified a novel c.622G>C change in FLNA exon 3, leading to the substitution of a highly conserved aminoacid (p.Gly208Arg). Gel electrophoresis and ultradeep sequencing revealed the missense mutation as well as retention of intron 3. Cycloheximide treatment demonstrated that the aberrant mRNA transcript-retaining intron 3 is subjected to NMD. Western blot analysis confirmed reduced FLNA levels in lymphocyte cells.The novel c.622G>C substitution leads to two aberrant FLNA transcripts, one of which carries the missense mutation, plus a longer transcript resulting from intron 3 retention. We propose that the exceptional co-occurrence of PNH and MNS, two otherwise mutually exclusive allelic phenotypes, is the consequence of a single mutational event resulting in co-occurring gain-of-function and loss-of-function effects. 
 Interactions between filamin A and MMP-9 regulate proliferation and invasion in renal cell carcinoma. This study aimed to analyze the expression, clinical significance of filamin A (FLNA) in renal cell carcinoma (RCC) and biological effects in a cell line by regulating FLNA expression. Immunohistochemistry and Western blotting were used to analyze FLNA protein expression in 70 cases of RCC and normal tissues to study the relationship with clinical factors. FLNA lentiviral and empty vectors were transfected into RCC to study the influence of up-regulated expression of FLNA. FLNA siRNA was transiently transfected into ACHN kidney carcinoma cells by a liposome-mediated method and protein was detected by Western blotting. The level of expression was found to be significantly lower in RCC than normal tissues (p<0.05). No correlation was noted with gender, age, tumor size or pathological types (p>0.05), but links with lymph node metastasis, clinic stage and histological grade were noted (p<0.05). Loss of FLNA expression correlated significantly with poor overall survival time by Kaplan-Meier analysis (p<0.05). Results for biological function showed that ACHN cells transfected with FLNA had a lower survival fraction, significant decrease in   migration   and invasion, higher cell apoptosis, higher percentage of the G0/G1 phases, and lower MMP-9 protein expression compared with ACHN cells untransfected with FLNA (p<0.05). However, renal 786-0 cells transfected with FLNA siRNA had a higher survival fraction, significant increase in   migration   and invasion, and higher MMP-9 protein expression compared (p<0.05). In conclusion, FLNA expression was decreased in RCC and correlated significantly with lymph node metastasis, clinic stage, histological grade and poor overall survival, suggesting that FLNA may play important roles as a a tumor suppressor in RCC by promoting degradation of MMP-9. 
 Trouble making the first move: interpreting arrested neuronal   migration   in the cerebral cortex. Postmitotic cortical neurons that fail to initiate   migration   can remain near their site of origin and form persistent periventricular nodular heterotopia (PH). In human telencephalon, this malformation is most commonly associated with Filamin-A (FLNa) mutations. The lack of genetic animal models that reliably produce PH has delayed our understanding of the underlying molecular mechanisms. This review examines PH pathogenesis using a new mouse model. Although PH have not been observed in Flna-deficient mice generated thus far, the loss of MEKK4, a regulator of Flna, produces striking PH in mice and offers insight into the mechanisms involved in neuronal   migration   initiation. Elucidating the basic functions of FLNa and associated molecules is crucial for understanding the causes of PH and for developing prevention for at-risk patients. 
 Diverse phenotypic consequences of mutations affecting the C-terminus of FLNA. Filamin A, the filamentous protein encoded by the X-linked gene FLNA, cross-links cytoskeletal actin into three-dimensional networks, facilitating its role as a signalling scaffold and a mechanosensor of extrinsic shear forces. Central to these functions is the ability of FLNA to form V-shaped homodimers through its C-terminal located filamin repeat 24. Additionally, many proteins that interact with FLNA have a binding site that includes the C-terminus of the protein. Here, a cohort of patients with mutations affecting this region of the protein is studied, with particular emphasis on the phenotype of male hemizygotes. Seven unrelated families are reported, with five exhibiting a typical female presentation of periventricular heterotopia (PH), a neuronal   migration   disorder typically caused by loss-of-function mutations in FLNA. One male presents with widespread PH consistent with previous male phenotypes attributable to hypomorphic mutations in FLNA. In stark contrast, two brothers are described with a mild PH presentation, due to a missense mutation (p.Gly2593Glu) inserting a large negatively charged amino acid into the hydrophobic dimerisation interface of FLNA. Co-immunoprecipitation, in vitro cross-linking studies and gel filtration chromatography all demonstrated that homodimerisation of isolated FLNA repeat 24 is abolished by this p.Gly2593Glu substitution but that extended FLNA(Gly2593Glu) repeat 16-24 constructs exhibit dimerisation. These observations imply that other interactions apart from those mediated by the canonical repeat 24 dimerisation interface contribute to FLNA homodimerisation and that mutations affecting this region of the protein can have broad phenotypic effects.• Mutations in the X-linked gene FLNA cause a spectrum of syndromes. • Genotype-phenotype correlations are emerging but still remain unclear. • C-term mutations can confer male lethality, survival or connective tissue defects. • Mutations leading to the latter affect filamin dimerisation. • This deficit is compensated for by remotely acting domains elsewhere in FLNA. 
 Absent expression of FLNA is correlated with poor prognosis of nasopharyngeal cancer. This study aimed to analyze the expression, clinical significance of filamin A (FLNA) in nasopharyngeal carcinoma, and the biological effect in its cell line by FLNA overexpression. Immunohistochemistry and western blot were used to analyze FLNA protein expression in 63 cases of nasopharyngeal cancer and 21 cases of normal tissues to study the relationship between FLNA expression and clinical factors. FLNA lentiviral vector and empty vector were respectively transfected into nasopharyngeal cancer CNE2 cell line. Reverse transcription-polymerase chain reaction (RT-PCR) and western blot were used to detect the mRNA level and protein of FLNA. 3-[4,5-Dimethylthiazol-2-yl]-2,5-diphenyltetrazolium bromide,   migration  , and invasion assays were also conducted as to the influence of the upregulated expression of FLNA that might be found on CNE2 cell biological effect. Immunohistochemistry: the level of FLNA protein expression was found to be significantly lower in nasopharyngeal cancer tissue than normal tissues (P < 0.05). Western blot: the relative amount of FLNA protein in nasopharyngeal cancer tissue was found to be significantly lower than in normal tissues (P < 0.05). The level of FLNA protein expression was correlated with T stages, lymph node metastasis, clinic stage, and histological grade (P < 0.05). Loss of FLNA expression correlated significantly with poor overall survival time by Kaplan-Meier analysis (P < 0.05). The result of biological function has shown that CNE2 cell-transfected FLNA had a lower survival fraction, significant decrease in   migration   and invasion, and lower matrix metallopeptidase 9 (MMP-9) protein expression compared with CNE2 cell-untransfected FLNA (P < 0.05). FLNA expression decreased in nasopharyngeal cancer and correlated significantly lymph node metastasis, clinic stage, histological grade, and poor overall survival, suggesting that FLNA may play important roles as a negative regulator to nasopharyngeal cancer CNE2 cell by promoting degradation of MMP-9. 
 Filamin a binds to CCR2B and regulates its internalization. The chemokine (C-C motif) receptor 2B (CCR2B) is one of the two isoforms of the receptor for monocyte chemoattractant protein-1 (CCL2), the major chemoattractant for monocytes, involved in an array of chronic inflammatory diseases. Employing the yeast two-hybrid system, we identified the actin-binding protein filamin A (FLNa) as a protein that associates with the carboxyl-terminal tail of CCR2B. Co-immunoprecipitation experiments and in vitro pull down assays demonstrated that FLNa binds constitutively to CCR2B. The colocalization of endogenous CCR2B and filamin A was detected at the surface and in internalized vesicles of THP-1 cells. In addition, CCR2B and FLNa were colocalized in lamellipodia structures of CCR2B-expressing A7 cells. Expression of the receptor in filamin-deficient M2 cells together with siRNA experiments knocking down FLNa in HEK293 cells, demonstrated that lack of FLNa delays the internalization of the receptor. Furthermore, depletion of FLNa in THP-1 monocytes by RNA interference reduced the   migration   of cells in response to MCP-1. Therefore, FLNa emerges as an important protein for controlling the internalization and spatial localization of the CCR2B receptor in different dynamic membrane structures. 
 Ehlers-Danlos syndrome and periventricular nodular heterotopia in a Spanish family with a single FLNA mutation. The Ehlers-Danlos syndrome (EDS) comprises a group of hereditary connective tissue disorders. Periventricular nodular heterotopia (PNH) is a human neuronal   migration   disorder characterised by seizures and conglomerates of neural cells around the lateral ventricles of the brain, caused by FLNA mutations. FLNA encodes filamin A, an actin binding protein involved in cytoskeletal organisation. The amino-terminal actin binding domain (ABD) of filamins contains two tandem calponin homology domains, CHD1 and CHD2.To report clinical and genetic analyses in a Spanish family affected by a connective tissue disorder suggestive of EDS type III and PNH.A clinical and molecular study was undertaken in the three affected women. Clinical histories, physical and neurological examinations, brain magnetic resonance imaging studies, and skin biopsies were done. Genetic analysis of the FLNA gene was undertaken by direct sequencing and restriction fragment length polymorphism analysis.Mutation analysis of the FLNA gene resulted in the identification of a novel mutation in exon 3 (c.383C-->T) segregating with the combination of both syndromes. This mutation results in a substitution of an alanine residue (A128V) in CHD1.The findings suggest that the Ala128Val mutation causes the dual EDS-PNH phenotype. This association constitutes a new variant within the EDS spectrum. This is the first description of a familial EDS-PNH association with a mutation in FLNA. 
 Filamin A is a novel caveolin-1-dependent target in IGF-I-stimulated cancer cell   migration  . Caveolin-1 is an essential structural constituent of caveolae which is involved in regulation of mitogenic signaling and oncogenesis. Caveolin-1 has been implicated in cell   migration   but its exact role and mechanism of action in this process remained obscure. We have previously reported that expression of caveolin-1 in stably transfected MCF-7 human breast cancer (MCF-7/Cav1) cells up-regulates phosphorylation of a putative Akt substrate protein, designated pp340 [D. Ravid, S. Maor, H. Werner, M. Liscovitch, Caveolin-1 inhibits cell detachment-induced p53 activation and anoikis by upregulation of insulin-like growth factor-I receptors and signaling, Oncogene 24 (2005) 1338-1347.]. We now show, using differential detergent extraction, SDS-PAGE and mass spectrometry, that the major protein in the pp340 band is the actin filament cross-linking protein filamin A. The identity of pp340 as filamin A was confirmed by immunoprecipitation of pp340 with specific filamin A antibodies. RT-PCR, flow cytometry and Western blot analyses show that filamin A mRNA and protein levels are respectively 3.5- and 2.5-fold higher in MCF-7/Cav1 cells than in MCF-7 cells. Basal filamin A phosphorylation on Ser-2152, normalized to total filamin A levels, is 7.8-fold higher in MCF-7/Cav1 than in MCF-7 cells. Insulin-like growth factor-I (IGF-I) stimulates phosphorylation of filamin A on Ser-2152 in MCF-7 cells and further enhances Ser-2152 phosphorylation over its already high basal level in MCF-7/Cav1 cells. The effect of IGF-I is inhibited by the PI3K inhibitor wortmannin, indicating that IGF-I-stimulated phosphorylation of filamin A occurs via the PI3K/Akt pathway. Co-immunoprecipitation experiments have confirmed a previous report showing that filamin A and caveolin-1 co-exist in a complex and have revealed the presence of active phospho-Akt in this complex. Ser-2152 phosphorylation of filamin A has been implicated in cancer cell   migration  . Accordingly, caveolin-1 expression dramatically enhances IGF-I-dependent MCF-7 cell   migration  . These data indicate that caveolin-1 specifies filamin A as a novel target for Akt-mediated filamin A Ser-2152 phosphorylation thus mediating the effects of caveolin-1 on IGF-I-induced cancer cell   migration  . 
 Mammalian target of rapamycin (mTOR) complex 2 regulates filamin A-dependent focal adhesion dynamics and cell   migration  . The serine/threonine kinase mTOR forms two distinct complexes, mTORC1 and mTORC2, and controls a number of biological processes, including proliferation, survival and autophagy. Although the function of mTORC1 has been extensively studied, the mTORC2 signaling pathway largely remains to be elucidated. Here, we have shown that mTORC2 phosphorylates filamin A, an actin cross-linking protein, at serine 2152 (S2152) both in vivo and in living cells. Treatment of HeLa cells with Torin1 (an mTORC1/mTORC2 inhibitor), but not rapamycin (an mTORC1 inhibitor), suppressed the phosphorylation of filamin A, which decreased the binding of filamin A with β7-integrin cytoplasmic tail. Torin1 also inhibited focal adhesion formation and cel  l migrati  on in A7 filamin A-replete melanoma cells but not in M2 filamin A-deficient cells, suggesting a pivotal role for mTORC2 in filamin A function. Finally, reduced focal adhesion formation in M2 cells was significantly rescued by expressing wild type but not S2152A nonphosphorylatable mutant of filamin A. Taken together, our results indicate that mTORC2 regulates filamin A-dependent focal adhesions and cel  l migrati  on. 
 Tyrosyl phosphorylated PAK1 regulates breast cancer cell motility in response to prolactin through filamin A. The p21-activated serine-threonine kinase (PAK1) is activated by small GTPase-dependent and -independent mechanisms and regulates cell motility. Both PAK1 and the hormone prolactin (PRL) have been implicated in breast cancer by numerous studies. We have previously shown that the PRL-activated tyrosine kinase JAK2 (Janus tyrosine kinase 2) phosphorylates PAK1 in vivo and identified tyrosines (Tyr) 153, 201, and 285 in the PAK1 molecule as sites of JAK2 tyrosyl phosphorylation. Here, we have used human breast cancer T47D cells stably overexpressing PAK1 wild type or PAK1 Y3F mutant in which Tyr(s) 153, 201, and 285 were mutated to phenylalanines to demonstrate that phosphorylation of these three tyrosines are required for maximal PRL-dependent ruffling. In addition, phosphorylation of these three tyrosines is required for increased   migration   of T47D cells in response to PRL as assessed by two independent motility assays. Finally, we show that PAK1 phosphorylates serine (Ser) 2152 of the actin-binding protein filamin A to a greater extent when PAK1 is tyrosyl phosphorylated by JAK2. Down-regulation of PAK1 or filamin A abolishes the effect of PRL on cell   migration  . Thus, our data presented here bring some insight into the mechanism of PRL-stimulated motility of breast cancer cells. 
 Bilateral periventricular nodular heterotopia due to filamin 1 gene mutation: widespread glomeruloid microvascular anomaly and dysplastic cytoarchitecture in the cerebral cortex. Bilateral periventricular nodular heterotopia (BPNH) is a neuronal   migration   disorder that is characterized by subependymal nodules of gray matter. Recently, a causative gene for BPNH, filamin 1, has been identified, and possible roles of the translated protein in cell   migration   and blood vessel development have been proposed. We report here the histopathological features of an autopsy case of BPNH with widespread glomeruloid microvascular anomaly and dysplastic cytoarchitecture in the cerebral cortex, in whom we found a novel exon 11 (Val528Met) filamin 1 mutation. Within the periventricular nodules, well-differentiated pyramidal neurons were randomly oriented. A small proportion of neurons were immunolabeled with antibodies raised against calbindin D-28k, parvalbumin, or calretinin. We used a carbocyanine dye (DiI) tracing technique to investigate the extent of fiber projections within and outside the nodules. The labeled fibers formed bundles that extended into the surrounding white matter. Connections between adjacent nodules were evident. Connections between the nodules and the cerebral cortex were also seen, with a small number of labeled fibers reaching the cortex. In the cerebral cortex, small closely packed vessels ran in a parallel fashion throughout all of the layers. Immunohistochemically, the inner rim of individual vessel lumina was labeled by an antibody against factor VIII, and the vessel walls were labeled by antibodies against actin and laminin. Astrocyte processes, labeled with an antibody to glial fibrillary acidic protein, invaded these vascular channels. Ultrastructurally, a network of basal lamina-like materials lined with endothelial cells was evident. The cytoarchitecture of the cerebral cortex was disturbed, in that the columnar neuronal arrangement was distorted around the malformed vessels. This case appears to represent an example of BPNH manifesting widespread developmental anomalies within the blood vessels and the cortical cytoarchitecture in the cerebrum. 
 Filamin A regulates MMP-9 expression and suppresses prostate cancer cell   migration   and invasion. This study aims to analyze the expression and clinical significance of Filamin A (FLNA) in prostate carcinoma and the biological effect in its cell line by FLNA overexpression. Immunohistochemistry and Western blot were used to analyze FLNA protein expression in 68 cases of prostate cancer and 37 cases of normal tissues to study the influence of the upregulated expression of FLNA that might be found on PC-3 cell biological effect. In the immunohistochemical analysis, the level of FLNA protein expression was found to be significantly lower in prostate cancer tissue than in normal tissues (P < 0.05). In the Western blot analysis, the relative amount of FLNA protein in prostate cancer tissue was found to be significantly lower than in normal tissues (P < 0.05). The level of FLNA protein expression was not correlated with age and PSA concentration (P > 0.05), but it was correlated with T stages, lymph node metastasis, clinic stage, and Gleason score (P < 0.05). The result of biological function showed that PC-3 cell transfected FLNA had a lower survival fraction, a significant decrease in   migration   and invasion, and a lower matrix metallopeptidase 9 (MMP-9) protein expression compared with PC-3 cell untransfected FLNA (P < 0.05). FLNA expression decreased in prostate cancer and correlated significantly with T stages, lymph node metastasis, clinic stage, and Gleason score, suggesting that FLNA may play important roles as a negative regulator to prostate cancer PC-3 cell by promoting the degradation of MMP-9. 
 Valvular dystrophy associated filamin A mutations reveal a new role of its first repeats in small-GTPase regulation. Filamin A (FlnA) is a ubiquitous actin binding protein which anchors various transmembrane proteins to the cell cytoskeleton and provides a scaffold to many cytoplasmic signaling proteins involved in actin cytoskeleton remodeling in response to mechanical stress and cytokines stimulation. Although the vast majority of FlnA binding partners interact with the carboxy-terminal immunoglobulin like (Igl) repeats of FlnA, little is known on the role of the amino-N-terminal repeats. Here, using cardiac mitral valvular dystrophy associated FlnA-G288R and P637Q mutations located in the N-terminal Igl repeat 1 and 4 respectively as a model, we identified a new role of FlnA N-terminal repeats in small Rho-GTPases regulation. Using FlnA-deficient melanoma and HT1080 cell lines as expression systems we showed that FlnA mutations reduce cell spreading and   migration   capacities. Furthermore, we defined a signaling network in which FlnA mutations alter the balance between RhoA and Rac1 GTPases activities in favor of RhoA and provided evidences for a role of the Rac1 specific GTPase activating protein FilGAP in this process. Together our work ascribed a new role to the N-terminal repeats of FlnA in Small GTPases regulation and supports a conceptual framework for the role of FlnA mutations in cardiac valve diseases centered around signaling molecules regulating cellular actin cytoskeleton in response to mechanical stress. 
 A role for tissue factor in cell adhesion and   migration   mediated by interaction with actin-binding protein 280. Tissue factor (TF), the protease receptor initiating the coagulation system, functions in vascular development, angiogenesis, and tumor cell metastasis by poorly defined molecular mechanisms. We demonstrate that immobilized ligands for TF specifically support cell adhesion,   migration  , spreading, and intracellular signaling, which are not inhibited by RGD peptides. Two-hybrid screening identified actin-binding protein 280 (ABP-280) as ligand for the TF cytoplasmic domain. Extracellular ligation of TF is necessary for ABP-280 binding. ABP-280 recruitment to TF adhesion contacts is associated with reorganization of actin filaments, but cytoskeletal adaptor molecules typically found in integrin-mediated focal contacts are not associated with TF. Chimeric molecules of the TF cytoplasmic domain and an unrelated extracellular domain support cell spreading and   migration  , demonstrating that the extracellular domain of TF is not involved in the recruitment of accessory molecules that influence adhesive functions. Replacement of TF's cytoplasmic Ser residues with Asp to mimic phosphorylation enhances the interaction with ABP-280, whereas Ala mutations abolish coprecipitation of ABP-280 with immobilized TF cytoplasmic domain, and severely reduce cell spreading. The specific interaction of the TF cytoplasmic domain with ABP-280 provides a molecular pathway by which TF supports tumor cell metastasis and vascular remodeling. 
 A glial origin for periventricular nodular heterotopia caused by impaired expression of Filamin-A. Periventricular nodular heterotopia (PH) is a human brain malformation caused by defective neuronal   migration   that results in ectopic neuronal nodules lining the lateral ventricles beneath a normal appearing cortex. Most affected patients have seizures and their cognitive level varies from normal to severely impaired. Mutations in the Filamin-A (or FLNA) gene are the main cause of PH, but the underlying pathological mechanism remains unknown. Although two FlnA knockout mouse strains have been generated, none of them showed the presence of ectopic nodules. To recapitulate the loss of FlnA function in the developing rat brain, we used an in utero RNA interference-mediated knockdown approach and successfully reproduced a PH phenotype in rats comparable with that observed in human patients. In FlnA-knockdown rats, we report that PH results from a disruption of the polarized radial glial scaffold in the ventricular zone altering progression of neural progenitors through the cell cycle and impairing   migration   of neurons into the cortical plate. Similar alterations of radial glia are observed in human PH brains of a 35-week fetus and a 3-month-old child, harboring distinct FLNA mutations not previously reported. Finally, juvenile FlnA-knockdown rats are highly susceptible to seizures, confirming the reliability of this novel animal model of PH. Our findings suggest that the disorganization of radial glia is the leading cause of PH pathogenesis associated with FLNA mutations. Rattus norvegicus FlnA mRNA (GenBank accession number FJ416060). 
 An antiproliferative gene FLNA regulates   migration   and invasion of gastric carcinoma cell in vitro and its clinical significance. This study aimed to analyze the expression and clinical significance of filamin A (FLNA) in gastric carcinoma and the biological effect in its cell line by FLNA overexpression. Immunohistochemistry and western blot were used to analyze FLNA protein expression in 47 cases of gastric cancer and 47 cases of normal tissues to study the relationship between FLNA expression and clinical factors. FLNA lentiviral vector and empty vector were respectively transfected into gastric cancer SGC-7901 cell line. Reverse transcription-polymerase chain reaction (RT-PCR) and western blot were used to detect the mRNA level and protein of FLNA. 3-[4,5-Dimethylthiazol-2-yl]-2,5-diphenyltetrazolium bromide (MTT) assay and   migration   and invasion assays were also conducted to determine the influence of the upregulated expression of FLNA that might be found on SGC-7901 cell biological effect. Immunohistochemistry: The level of FLNA protein expression was found to be significantly lower in gastric cancer tissue than normal tissues (P < 0.05). Western blot: The relative amount of FLNA protein in gastric cancer tissue was found to be significantly lower than in normal tissues (P < 0.05). The level of FLNA protein expression was not correlated with gender, age, and tumor invasion (P > 0.05), but it was correlated with lymph node metastasis, clinic stage, and histological grade (P < 0.05). Loss of FLNA expression correlated significantly with poor overall survival time by Kaplan-Meier analysis (P < 0.05). The result of biological function showed that SGC-7901 cell transfected FLNA had a lower survival fraction, sign  ificant d  ecrease in migration and invasion, and lower matrix metallopeptidase 9 (MMP-9) protein expression compared with SGC-7901 cell untransfected FLNA (P < 0.05). FLNA expression decreased in gastric cancer and correlated significantly with lymph node metastasis, clinic stage, histological grade, and poor overall survival, suggesting that FLNA may play important roles as a negative regulator to gastric cancer SGC-7901 cell by promoting degradation of MMP-9. 
 CEACAM1 functionally interacts with filamin A and exerts a dual role in the regulation of cell   migration  . The carcinoembryonic antigen-related cell adhesion molecule CEACAM1 (CD66a) and the scaffolding protein filamin A have both been implicated in tumor cell   migration  . In the present study we identified filamin A as a novel binding partner for the CEACAM1-L cytoplasmic domain in a yeast two-hybrid screen. Direct binding was shown by surface plasmon resonance analysis and by affinity precipitation assays. The association was shown for human and rodent CEACAM1-L in endogenous CEACAM1-L expressing cells. To address functional aspects of the interaction, we used a well-established melanoma cell system. We found in different   migration   studies that the interaction of CEACAM1-L and filamin A drastically reduced   migration   and cell scattering, whereas each of these proteins when expressed alone, acted promigratory. CEACAM1-L binding to filamin A reduced the interaction of the latter with RalA, a member of the Ras-family of GTPases. Furthermore, co-expression of CEACAM1-L and filamin A led to a reduced focal adhesion turnover. Independent of the presence of filamin A, the expression of CEACAM1-L led to an increased phosphorylation of focal adhesions and to altered cytoskeletal rearrangements during monolayer wound healing assays. Together, our data demonstrate a novel mechanism for how CEACAM1-L regulates cell   migration   via its interaction with filamin A. 
 Interaction with BRCA2 suggests a role for filamin-1 (hsFLNa) in DNA damage response. The BRCA2 tumor suppressor plays significant roles in DNA damage response. The human actin binding protein filamin-1 (hsFLNa, also known as ABP-280) participates in orthogonal actin network, cellular stress responses, signal transduction, and cell   migration  . Through a yeast two-hybrid system, an in vitro binding assay, and in vivo co-immunoprecipitations, we identified an interaction between BRCA2 and hsFLNa. The hsFLNa binding domain of BRCA2 was mapped to an internal conserved region, and the BRCA2-interacting domain of hsFLNa was mapped to its C terminus. Although hsFLNa is known for its cytoplasmic functions in cell   migration   and signal transduction, some hsFLNa resides in the nucleus, raising the possibility that it participates in DNA damage response through a nuclear interaction with BRCA2. Lack of hsFLNa renders a human melanoma cell line (M2) more sensitive to several genotoxic agents including gamma irradiation, bleomycin, and ultraviolet-c light. These results suggest that BRCA2/hsFLNa interaction may serve to connect cytoskeletal signal transduction to DNA damage response pathways. 
 Role of non-genomic androgen signalling in suppressing proliferation of fibroblasts and fibrosarcoma cells. The functions of androgen receptor (AR) in stromal cells are still debated in spite of the demonstrated importance of these cells in organ development and diseases. Here, we show that physiological androgen concentration (10 nM R1881 or DHT) fails to induce DNA synthesis, while it consistently stimulates cel  l migrati  on in mesenchymal and transformed mesenchymal cells. Ten nanomolar R1881 triggers p27 Ser10 phosphorylation and its stabilization in NIH3T3 fibroblasts. Activation of Rac and its downstream effector DYRK 1B is responsible for p27 Ser10 phosphorylation and cell quiescence. Ten nanomolar androgen also inhibits transformation induced by oncogenic Ras in NIH3T3 fibroblasts. Overexpression of an AR mutant unable to interact with filamin A, use of a small peptide displacing AR/filamin A interaction, and filamin A knockdown indicate that the androgen-triggered AR/filamin A complex regulates the pathway leading to p27 Ser10 phosphorylation and cell cycle arrest. As the AR/filamin A complex is also responsible fo  r migrati  on stimulated by 10 nM androgen, our report shows that the androgen-triggered AR/filamin A complex controls, through Rac 1, the decision of cells to halt cell cycle   and migra  tion. This study reveals a new and unexpected role of androgen/AR signalling in coordinating stromal cell functions. 
 Adapter protein SH2B1beta binds filamin A to regulate prolactin-dependent cytoskeletal reorganization and cell motility. Prolactin (PRL) regulates cytoskeletal rearrangement and cell motility. PRL-activated Janus tyrosine kinase 2 (JAK2) phosphorylates the p21-activated serine-threonine kinase (PAK)1 and the Src homology 2 (SH2) domain-containing adapter protein SH2B1β. SH2B1β is an actin-binding protein that cross-links actin filaments, whereas PAK1 regulates the actin cytoskeleton by different mechanisms, including direct phosphorylation of the actin-binding protein filamin A (FLNa). Here, we have used a FLNa-deficient human melanoma cell line (M2) and its derivative line (A7) that stably expresses FLNa to demonstrate that SH2B1β and FLNa are required for maximal PRL-dependent cell ruffling. We have found that in addition to two actin-binding domains, SH2B1β has a FLNa-binding domain (amino acids 200-260) that binds directly to repeats 17-23 of FLNa. The SH2B1β-FLNa interaction participates in PRL-dependent actin rearrangement. We also show that phosphorylation of the three tyrosines of PAK1 by JAK2, as well as the presence of FLNa, play a role in PRL-dependent cell ruffling. Finally, we show that the actin- and FLNa-binding-deficient mutant of SH2B1β (SH2B1β 3Δ) abolished PRL-dependent ruffling and PRL-depende  nt cell m  igration when expressed along with PAK1 Y3F (JAK2 tyrosyl-phosphorylation-deficient mutant). Together, these data provide insight into a novel mechanism of PRL-stimulated regulation of the actin cytoskeleton and cell motility via JAK2 signaling through FLNa, PAK1, and SH2B1β. We propose a model for PRL-dependent regulation of the actin cytoskeleton that integrates our findings with previous studies. 
 Increased filamin binding to beta-integrin cytoplasmic domains inhibits cell   migration  . Multicellular animal development depends on integrins. These adhesion receptors link to the actin cytoskeleton, transmitting biochemical signals and force during cell   migration   and interactions with the extracellular matrix. Many integrin-cytoskeleton connections are formed by filamins and talin. The beta7 integrin tail binds strongly to filamin and supports less   migration  , fibronectin matrix assembly and focal adhesion formation than either the beta1D tail, which binds strongly to talin, or the beta1A tail, which binds modestly to both filamin and talin. To probe the role of filamin binding, we mapped the filamin-binding site of integrin tails and identified amino acid substitutions that led to selective loss of filamin binding to the beta7 tail and gain of filamin binding to the beta1A tail. These changes affected cell   migration   and membrane protrusions but not fibronectin matrix assembly or focal adhesion formation. Thus, tight filamin binding restricts integrin-dependent cell   migration   by inhibiting transient membrane protrusion and cell polarization. 
 Mosaic mutations of the FLN1 gene cause a mild phenotype in patients with periventricular heterotopia. X-linked periventricular nodular heterotopia (PNH) (OMIM 300049) is a neuronal   migration   disorder, associated with mutations of the FLN1 gene (Xq28), accompanied by severe epilepsy and normal to mildly impaired cognitive function in affected women. The recurrence risk has been estimated to be 50% in daughters of affected women, with early post-natal lethality in boys. Mutation analysis [denaturing high-performance liquid chromatography (DHPLC) and sequencing], performed in a woman and a man with PNH, was suggestive of somatic mosaicism. Both patients were investigated using single nucleotide primer extension (SNuPE) and DHPLC. To better characterize mosaicism in the affected man, SNuPE-DHPLC analysis was also performed on a pool of hair roots and single hair roots. The affected woman had features of PNH on magnetic resonance imaging. She had well-controlled epilepsy and normal cognitive function. She was mosaic for a nucleotide insertion (c.568_569ingG). SNuPE-DHPLC findings showed 17% of mutant allele. The affected man had classical PNH and was mosaic for an A>G substitution (intron 11 acceptor splice site). SNuPE-DHPLC on both leukocyte and hair root DNA revealed 42% and 69% of mutant allele. Single hair root analysis confirmed that this patient did not harbor the mutation in all ectodermal derivative cells. His daughter had not inherited the mutation. Phenotypic heterogeneity associated with X-linked PNH may depend on the type of mutation, its location on the protein, as well as on somatic mosaicism. Mosaicism can influence the recurrence risk rates in affected women. Mosaic mutations in men may not be transmitted to their daughters, masking the X-linked nature of the disorder. 
 Mutations in filamin 1 prevent   migration   of cerebral cortical neurons in human periventricular heterotopia. Long-range, directed   migration   is particularly dramatic in the cerebral cortex, where postmitotic neurons generated deep in the brain migrate to form layers with distinct form and function. In the X-linked dominant human disorder periventricular heterotopia (PH), many neurons fail to migrate and persist as nodules lining the ventricular surface. Females with PH present with epilepsy and other signs, including patent ductus arteriosus and coagulopathy, while hemizygous males die embryonically. We have identified the PH gene as filamin 1 (FLN1), which encodes an actin-cross-linking phosphoprotein that transduces ligand-receptor binding into actin reorganization, and which is required for locomotion of many cell types. FLN1 shows previously unrecognized, high-level expression in the developing cortex, is required for neuronal   migration   to the cortex, and is essential for embryogenesis. 
 R-Ras regulates   migration   through an interaction with filamin A in melanoma cells. Changes in cell adhesion and   migration   in the tumor microenvironment are key in the initiation and progression of metastasis. R-Ras is one of several small GTPases that regulate cell adhesion and   migration   on the extracellular matrix, however the mechanism has not been completely elucidated. Using a yeast two-hybrid approach we sought to identify novel R-Ras binding proteins that might mediate its effects on integrins.We identified Filamin A (FLNa) as a candidate interacting protein. FLNa is an actin-binding scaffold protein that also binds to integrin beta1, beta2 and beta7 tails and is associated with diverse cell processes including cell   migration  . Indeed, M2 melanoma cells require FLNa for motility. We further show that R-Ras and FLNa interact in co-immunoprecipitations and pull-down assays. Deletion of FLNa repeat 3 (FLNaDelta3) abrogated this interaction. In M2 melanoma cells active R-Ras co-localized with FLNa but did not co-localize with FLNa lacking repeat 3. Thus, activated R-Ras binds repeat 3 of FLNa. The functional consequence of this interaction was that active R-Ras and FLNa coordinately increased cell   migration  . In contrast, co-expression of R-Ras and FLNaDelta3 had a significantly reduced effect on   migration  . While there was enhancement of integrin activation and fibronectin matrix assembly, cell adhesion was not altered. Finally, siRNA knockdown of endogenous R-Ras impaired FLNa-dependent fibronectin matrix assembly.These data support a model in which R-Ras functionally associates with FLNa and thereby regulates integrin-dependent   migration  . Thus in melanoma cells R-Ras and FLNa may cooperatively promote metastasis by enhancing cell   migration  . 
 Expression of FLNa in human melanoma cells regulates the function of integrin α1β1 and phosphorylation and localisation of PKB/AKT/ERK1/2 kinases. FLNa is a ubiquitous cytoskeletal protein that links transmembrane receptors, including integrins, to F-actin and functions as a signalling intermediate. We investigated FLNa's role in the function of integrin-type collagen receptors, EGF-EGFR signalling and regulation of PKB/Akt and ERK1/2. Using FLNa-deficient M2 human melanoma cells, and same cells expressing EGFP-FLNa (M2F) or its Ig-like repeats 1-8+24, 8-15+24 and 16-24, we found that in M2F and M2 8-15+24 cells, EGF induced the increased phosphorylation of PKB/Akt and ERK1/2. In M2F cells EGF induced the localisation of these kinases to cell nucleus and lamellipodia, respectively, and the ERK1/2 phosphorylation-dependent co-immunoprecipitation of FLNa with ERK1/2. Only M2F and M2 8-15+24 cells adhered to and spread on type I collagen whereas on fibronectin all cells behaved similarly. α1β1 and α2β1 were the integrin-type collagen receptors expressed on these cells with primarily α1β1 localising to focal contacts and affecting cell adhes  ion and m  igration in a manner dependent on FLNa or its Ig-like repeats 8-15. Our results suggest a role for FLNa repeats 8-15 in the α1-subunit-dependent regulation of integrin α1β1 function, EGF-EGFR signalling to PKB/Akt and ERK1/2, identify ERK1/2 in EGF-induced FLNa-associated protein complexes, and show that the function of different integrins is subjected to differential regulation by FLNa. 
 The regulation mechanism for the auto-inhibition of binding of human filamin A to integrin. The ability of adhesion receptors to transmit biochemical signals and mechanical force across cell membranes depends on interactions with the actin cytoskeleton. Human filamins are large actin cross-linking proteins that connect integrins to the cytoskeleton. Filamin binding to the cytoplasmic tail of beta integrins has been shown to prevent integrin activation in cells, which is important for controlling cell adhesion and   migration  . The molecular-level mechanism for filamin binding to integrin has been unclear, however, as it was recently demonstrated that filamin undergoes intramolecular auto-inhibition of integrin binding. In this study, using steered molecular dynamics simulations, we found that mechanical force applied to filamin can expose cryptic integrin binding sites. The forces required for this are considerably lower than those for filamin immunoglobulin domain unfolding. The mechanical-force-induced unfolding of filamin and exposure of integrin binding sites occur through stable intermediates where integrin binding is possible. Accordingly, our results support filamin's role as a mechanotransducer, since force-induced conformational changes allow binding of integrin and other transmembrane and intracellular proteins. This observed force-induced conformational change can also be one of possible mechanisms involved in the regulation of integrin activation. 
 Phosphorylation of filamin A by Cdk1 regulates filamin A localization and daughter cell separation. In cell culture, many adherent mammalian cells undergo substantial actin cytoskeleton rearrangement prior to mitosis as they detach from the extracellular matrix and become spherical. At the end of mitosis, the actin cytoskeleton is required for cytokinesis and the reassembly of interphase structures as cells spread and reattach to substrate. To understand the processes regulating mitotic cytoskeletal remodeling, we studied how mitotic phosphorylation regulates filamin A (FLNa). FLNa is an actin-crosslinking protein that was previously identified as a cyclin-dependent kinase 1 (Cdk1) binding partner and substrate in vitro. Using quantitative label-based mass spectrometry, we find that FLNa serines 1084, 1459 and 1533 are phosphorylated in mitotic HeLa cells and all three sites match the phosphorylation consensus sequence of Cdk1. To investigate the functional role of mitotic FLNa phosphorylation, we mutated serines 1084, 1459 and 1533 to nonphosphorylatable alanine residues and expressed GFP-tagged FLNa(S1084A,S1459A,S1533A) (FLNa-AAA GFP) in a FLNa-deficient human melanoma cell line called M2. M2 cells expressing FLNa-AAA GFP have enhanced FLNa-AAA GFP and actin localization at sites of contact between daughter cells, impaired post-mitotic daughter cell separation and defects in cell   migration  . Therefore, mitotic phosphorylation of FLNa is important for successful cell division and interphase cell behavior. 
 Localized mutations in the gene encoding the cytoskeletal protein filamin A cause diverse malformations in humans. Remodeling of the cytoskeleton is central to the modulation of cell shape and   migration  . Filamin A, encoded by the gene FLNA, is a widely expressed protein that regulates re-organization of the actin cytoskeleton by interacting with integrins, transmembrane receptor complexes and second messengers. We identified localized mutations in FLNA that conserve the reading frame and lead to a broad range of congenital malformations, affecting craniofacial structures, skeleton, brain, viscera and urogenital tract, in four X-linked human disorders: otopalatodigital syndrome types 1 (OPD1; OMIM 311300) and 2 (OPD2; OMIM 304120), frontometaphyseal dysplasia (FMD; OMIM 305620) and Melnick-Needles syndrome (MNS; OMIM 309350). Several mutations are recurrent, and all are clustered into four regions of the gene: the actin-binding domain and rod domain repeats 3, 10 and 14/15. Our findings contrast with previous observations that loss of function of FLNA is embryonic lethal in males but manifests in females as a localized neuronal   migration   disorder, called periventricular nodular heterotopia (PVNH; refs. 3-6). The patterns of mutation, X-chromosome inactivation and phenotypic manifestations in the newly described mutations indicate that they have gain-of-function effects, implicating filamin A in signaling pathways that mediate organogenesis in multiple systems during embryonic development. 
 Skeletal dysplasias due to filamin A mutations result from a gain-of-function mechanism distinct from allelic neurological disorders. Filamin A (FLNA) crosslinks F-actin and binds proteins consistent with roles integrating cell signalling and the cytoskeleton. FLNA missense mutations are associated with the otopalatodigital syndrome (OPD) spectrum of skeletal disorders, clustering in discrete domains. One cluster is found in the second calponin homology domain of the FLNA actin-binding domain (ABD), implicating this region as essential for mediating correct function. Here we show that OPD (FLNA E254K) fibroblast lysates have equivalent concentrations of FLNA compared with controls and that recombinant FLNA E254K ABD has increased in vitro F-actin binding (K(d) 13 microm) compared with wild type (WT; K(d) 48 microm). These observations are consistent with a gain-of-function mechanism for OPD. We have determined the crystal structures of the WT and E254K FLNA ABDs at 2.3 A resolution, revealing that they adopt similar closed conformations. The E254K mutation removes a conserved salt bridge but does not disrupt the ABD structure. The solution structures are also equivalent as determined by circular dichroism spectroscopy, but differential scanning fluorimetry denaturation showed reduced stability (decreased T(m) of 5.6 degrees C) for E254K relative to WT. Ex vivo characterization of E254K OPD patient fibroblasts revealed they have similar motility and adhesion as control cells, implying that many core functions mediated by FLNA are unaffected, consistent with OPD only affecting specific tissues despite FLNA being widely expressed. These data provide the first biochemical evidence for a gain-of-function mechanism for the OPD disorders, and mechanistically distinguishes them from the loss-of-function phenotypes that manifest as disorders of neuronal   migration  . 
 Filamin A links sphingosine kinase 1 and sphingosine-1-phosphate receptor 1 at lamellipodia to orchestrate cell   migration  . Sphingosine kinase 1 (SphK1) catalyzes the phosphorylation of sphingosine to produce the potent lipid mediator sphingosine-1-phosphate (S1P), which plays a critical role in cell motility via its cell surface receptors. Here, we have identified filamin A (FLNa), an actin-cross-linking protein involved in cell movement, as a bona fide SphK1-interacting protein. Heregulin stimulated SphK1 activity only in FLNa-expressing A7 melanoma cells but not in FLNa-deficient cells and induced its translocation and colocalization with FLNa at lamellipodia. SphK1 was required for heregulin-induced   migration  , lamellipodia formation, activation of PAK1, and subsequent FLNa phosphorylation. S1P directly stimulated PAK1 kinase, suggesting that it may be a target of intracellularly generated S1P. Heregulin also induced colocalization of S1P(1) (promotility S1P receptor) but not S1P(2), with SphK1 and FLNa at membrane ruffles. Moreover, an S1P(1) antagonist inhibited the lamellipodia formation induced by heregulin. Hence, FLNa links SphK1 and S1P(1) to locally influence the dynamics of actin cytoskeletal structures by orchestrating the concerted actions of the triumvirate of SphK1, FLNa, and PAK1, each of which requires and/or regulates the actions of the others, at lamellipodia to promote cell movement. 
 A meckelin-filamin A interaction mediates ciliogenesis. MKS3, encoding the transmembrane receptor meckelin, is mutated in Meckel-Gruber syndrome (MKS), an autosomal-recessive ciliopathy. Meckelin localizes to the primary cilium, basal body and elsewhere within the cell. Here, we found that the cytoplasmic domain of meckelin directly interacts with the actin-binding protein filamin A, potentially at the apical cell surface associated with the basal body. Mutations in FLNA, the gene for filamin A, cause periventricular heterotopias. We identified a single consanguineous patient with an MKS-like ciliopathy that presented with both MKS and cerebellar heterotopia, caused by an unusual in-frame deletion mutation in the meckelin C-terminus at the region of interaction with filamin A. We modelled this mutation and found it to abrogate the meckelin-filamin A interaction. Furthermore, we found that loss of filamin A by siRNA knockdown, in patient cells, and in tissues from Flna(Dilp2) null mouse embryos results in cellular phenotypes identical to those caused by meckelin loss, namely basal body positioning and ciliogenesis defects. In addition, morpholino knockdown of flna in zebrafish embryos significantly increases the frequency of dysmorphology and severity of ciliopathy developmental defects caused by mks3 knockdown. Our results suggest that meckelin forms a functional complex with filamin A that is disrupted in MKS and causes defects in neuronal   migration   and Wnt signalling. Furthermore, filamin A has a crucial role in the normal processes of ciliogenesis and basal body positioning. Concurrent with these processes, the meckelin-filamin A signalling axis may be a key regulator in maintaining correct, normal levels of Wnt signalling. 
 Filamin A regulates focal adhesion disassembly and suppresses breast cancer cell   migration   and invasion. The actin cross-linking protein filamin A (FLNa) functions as a scaffolding protein and couples cell cytoskeleton to extracellular matrix and integrin receptor signaling. In this study, we report that FLNa suppresses invasion of breast cancer cells and regulates focal adhesion (FA) turnover. Two large progression tissue microarrays from breast cancer patients revealed a significant decrease of FLNa levels in tissues from invasive breast cancer compared with benign disease and in lymph node-positive compared with lymph node-negative breast cancer. In breast cancer cells and orthotopic mouse breast cancer models, down-regulation of FLNa stimulated cancer cell   migration  , invasion, and metastasis formation. Time-lapse microscopy and biochemical assays after FLNa silencing and rescue with wild-type or mutant protein resistant to calpain cleavage revealed that FLNa regulates FA disassembly at the leading edge of motile cells. Moreover, FLNa down-regulation enhanced calpain activity through the mitogen-activated protein kinase-extracellular signal-regulated kinase cascade and stimulated the cleavage of FA proteins. These results document a regulation of FA dynamics by FLNa in breast cancer cells. 
  GeneRIFs  
 Results indicate that mTORC2 regulates filamin A-dependent focal adhesions and cell   migration  . 
 The androgen-triggered AR/filamin A complex controls, through Rac 1, the decision of cells to halt cell cycle and   migration  . 
 data highlight the critical role of FLNA in radial glia organization and function for neurogenesis and   migration   and also provide insight into the molecular pathogenesis of human periventricular nodular heterotopia 
 The extra nuclear AR/FlnA/integrin beta 1 complex is the key by which androgen activates signaling leading to cell   migration  . Assembly of this ternary complex may control organ development and prostate cancer metastasis. 
 Studies indicate that interactions of filamin A with intermediate filaments and protein kinase C enable tight regulation of beta1 integrin function and consequently early events in cell adhesion and   migration   on extracellular matrix proteins. 
 High FLNA is associated with tumor cell   migration  . 
 data support a model in which R-Ras functionally associates with FLNa and thereby regulates integrin-dependent   migration   
 These data indicate that caveolin-1 specifies filamin A as a novel target for Akt-mediated filamin A Ser-2152 phosphorylation thus mediating the effects of caveolin-1 on IGF-I-induced cancer cell   migration  . 
  Summary  
 The protein encoded by this gene is an actin-binding protein that crosslinks actin filaments and links actin filaments to membrane glycoproteins. The encoded protein is involved in remodeling the cytoskeleton to effect changes in cell shape and   migration  . This protein interacts with integrins, transmembrane receptor complexes, and second messengers. Defects in this gene are a cause of several syndromes, including periventricular nodular heterotopias (PVNH1, PVNH4), otopalatodigital syndromes (OPD1, OPD2), frontometaphyseal dysplasia (FMD), Melnick-Needles syndrome (MNS), and X-linked congenital idiopathic intestinal pseudoobstruction (CIIPX). Two transcript variants encoding different isoforms have been found for this gene.[provided by RefSeq, Mar 2009] 
  ECM  PubMed  
 Filamin A-beta1 integrin complex tunes epithelial cell response to matrix tension. The physical properties of the extracellular matrix (  ECM  ) regulate the behavior of several cell types; yet, mechanisms by which cells recognize and respond to changes in these properties are not clear. For example, breast epithelial cells undergo ductal morphogenesis only when cultured in a compliant collagen matrix, but not when the tension of the matrix is increased by loading collagen gels or by increasing collagen density. We report that the actin-binding protein filamin A (FLNa) is necessary for cells to contract collagen gels, and pull on collagen fibrils, which leads to collagen remodeling and morphogenesis in compliant, low-density gels. In stiffer, high-density gels, cells are not able to contract and remodel the matrix, and morphogenesis does not occur. However, increased FLNa-beta1 integrin interactions rescue gel contraction and remodeling in high-density gels, resulting in branching morphogenesis. These results suggest morphogenesis can be "tuned" by the balance between cell-generated contractility and opposing matrix stiffness. Our findings support a role for FLNa-beta1 integrin as a mechanosensitive complex that bidirectionally senses the tension of the matrix and, in turn, regulates cellular contractility and response to this matrix tension. 
  [Ee]xtracellular [Mm]atrix  PubMed  
 SILAC-based proteomics of human primary endothelial cell morphogenesis unveils tumor angiogenic markers. Proteomics has been successfully used for cell culture on dishes, but more complex cellular systems have proven to be challenging and so far poorly approached with proteomics. Because of the complexity of the angiogenic program, we still do not have a complete understanding of the molecular mechanisms involved in this process, and there have been no in depth quantitative proteomic studies. Plating endothelial cells on matrigel recapitulates aspects of vessel growth, and here we investigate this mechanism by using a spike-in SILAC quantitative proteomic approach. By comparing proteomic changes in primary human endothelial cells morphogenesis on matrigel to general adhesion mechanisms in cells spreading on culture dish, we pinpoint pathways and proteins modulated by endothelial cells. The cell-  extracellular matrix   adhesion proteome depends on the adhesion substrate, and a detailed proteomic profile of the   extracellular matrix   secreted by endothelial cells identified CLEC14A as a matrix component, which binds to MMRN2. We verify deregulated levels of these proteins during tumor angiogenesis in models of multistage carcinogenesis. This is the most in depth quantitative proteomic study of endothelial cell morphogenesis, which shows the potential of applying high accuracy quantitative proteomics to in vitro models of vessel growth to shed new light on mechanisms that accompany pathological angiogenesis. The mass spectrometry proteomics data have been deposited to the ProteomeXchange Consortium with the data set identifier PXD000359. 
 Filamin A mediates interactions between cytoskeletal proteins that control cell adhesion. Cell adhesion, spreading and migration on extracellular matrices are regulated by complex processes that involve the cytoskeleton and a large array of adhesion receptors, including the β1 integrin. Filamin A is a large, multi-domain, homodimeric actin binding protein that contributes to the mechanical stability of cells and interacts with several proteins that regulate cell adhesion including β1 integrin and several protein kinases. Here we review current data on the structure, mechanical properties and intracellular signaling functions of filamin that regulate cell adhesion. We also consider new data showing that interactions of filamin A with intermediate filaments and protein kinase C enable tight regulation of β1 integrin function and consequently early events in cell adhesion and migration   on extracellular mat  rix proteins. 
 Different splice variants of filamin-B affect myogenesis, subcellular distribution, and determine binding to integrin [beta] subunits. Integrins connect the   extracellular matrix   with the cell interior, and transduce signals through interactions of their cytoplasmic tails with cytoskeletal and signaling proteins. Using the yeast two-hybrid system, we isolated a novel splice variant (filamin-Bvar-1) of the filamentous actin cross-linking protein, filamin-B, that interacts with the cytoplasmic domain of the integrin beta1A and beta1D subunits. RT-PCR analysis showed weak, but wide, expression of filamin-Bvar-1 and a similar splice variant of filamin-A (filamin-Avar-1) in human tissues. Furthermore, alternative splice variants of filamin-B and filamin-C, from which the flexible hinge-1 region is deleted (DeltaH1), were induced during in vitro differentiation of C2C12 mouse myoblasts. We show that both filamin-Avar-1 and filamin-Bvar-1 bind more strongly than their wild-type isoforms to different integrin beta subunits. The mere presence of the high-affinity binding site for beta1A is not sufficient for targeting the filamin-Bvar-1 construct to focal contacts. Interestingly, the simultaneous deletion of the H1 region is required for the localization of filamin-B at the tips of actin stress fibers. When expressed in C2C12 cells, filamin-Bvar-1(DeltaH1) accelerates their differentiation into myotubes. Furthermore, filamin-B variants lacking the H1 region induce the formation of thinner myotubes than those in cells containing variants with this region. These findings suggest that specific combinations of filamin mRNA splicing events modulate the organization of the actin cytoskeleton and the binding affinity for integrins. 
 Filamin A regulates cell spreading and survival via beta1 integrins. Cell spreading and exploration of topographically complex substrates require tightly-regulated interactions between   extracellular matrix   receptors and the cytoskeleton, but the molecular determinants of these interactions are not defined. We examined whether the actin-binding proteins cortactin, vinculin and filamin A are involved in the formation of the earliest extensions of cells spreading over collagen or poly-L-lysine-coated smooth and beaded substrates. Spreading of human gingival fibroblasts was substantially reduced on beaded or poly-L-lysine-coated substrates. Filamin A, vinculin and cortactin were found in cell extensions on smooth collagen. HEK-293 cells also spread rapidly on smooth collagen and formed numerous cell extensions enriched with filamin A. Knockdown of filamin A in HEK-293 cells by short hairpin RNA reduced spreading and the number of cell extensions. Blocking beta1 integrin function significantly reduced cell spreading and localization of filamin A to cell extensions. Conversely, filamin A-knockdown reduced beta1 integrin-collagen binding as measured by 12G10 antibody, suggesting co-dependence between filamin A and beta1 integrin functions. TUNEL staining showed higher percentages of apoptosis after filamin A-knockdown in spreading cells. Chelation of [Ca2+]i with BAPTA/AM reduced spreading of wild-type and filamin A-knockdown cells, however wild-type cells showed recruitment of filamin A to the subcortex, indicating independent roles of filamin A and [Ca2+]i in cell spreading. We conclude that filamin A integrates with beta1 integrins to mediate cell spreading and prevent apoptosis. 
 Structural basis of the migfilin-filamin interaction and competition with integrin beta tails. A link between sites of cell adhesion and the cytoskeleton is essential for regulation of cell shape, motility, and signaling. Migfilin is a recently identified adaptor protein that localizes at cell-cell and cell-  extracellular matrix   adhesion sites, where it is thought to provide a link to the cytoskeleton by interacting with the actin cross-linking protein filamin. Here we have used x-ray crystallography, NMR spectroscopy, and protein-protein interaction studies to investigate the molecular basis of migfilin binding to filamin. We report that the N-terminal portion of migfilin can bind all three human filamins (FLNa, -b, or -c) and that there are multiple migfilin-binding sites in FLNa. Human filamins are composed of an N-terminal actin-binding domain followed by 24 immunoglobulin-like (IgFLN) domains and we find that migfilin binds preferentially to IgFLNa21 and more weakly to IgFLNa19 and -22. The filamin-binding site in migfilin is localized between Pro(5) and Pro(19) and binds to the CD face of the IgFLNa21 beta-sandwich. This interaction is similar to the previously characterized beta 7 integrin-IgFLNa21 interaction and migfilin and integrin beta tails can compete with one another for binding to IgFLNa21. This suggests that competition between filamin ligands for common binding sites on IgFLN domains may provide a general means of modulating filamin interactions and signaling. In this specific case, displacement of integrin tails from filamin by migfilin may provide a mechanism for switching between different integrin-cytoskeleton linkages. 
 Filamin A-beta1 integrin complex tunes epithelial cell response to matrix tension. The physical properties of the   extracellular matrix   (ECM) regulate the behavior of several cell types; yet, mechanisms by which cells recognize and respond to changes in these properties are not clear. For example, breast epithelial cells undergo ductal morphogenesis only when cultured in a compliant collagen matrix, but not when the tension of the matrix is increased by loading collagen gels or by increasing collagen density. We report that the actin-binding protein filamin A (FLNa) is necessary for cells to contract collagen gels, and pull on collagen fibrils, which leads to collagen remodeling and morphogenesis in compliant, low-density gels. In stiffer, high-density gels, cells are not able to contract and remodel the matrix, and morphogenesis does not occur. However, increased FLNa-beta1 integrin interactions rescue gel contraction and remodeling in high-density gels, resulting in branching morphogenesis. These results suggest morphogenesis can be "tuned" by the balance between cell-generated contractility and opposing matrix stiffness. Our findings support a role for FLNa-beta1 integrin as a mechanosensitive complex that bidirectionally senses the tension of the matrix and, in turn, regulates cellular contractility and response to this matrix tension. 
 Leucine-rich repeat region of decorin binds to filamin-A. Decorin is a member of the family of small leucine-rich proteoglycans found in the   extracellular matrix   and has an important role in promoting fiber formation and in controlling cell proliferation. Here, we have investigated whether the leucine-rich repeat (LRR) region of decorin interacts with proteins from human lung fibroblasts by using a yeast two-hybrid assay. We report that the LRR region of decorin interacts with the cytoskeletal protein, filamin-A (ABP-280), a peripheral cytoplasmic protein. This interaction is dependent on the 288 carboxyl-terminal amino acids of filamin-A, which correspond to repeats 22-24 of its conserved beta-sheet structure. We also show that the recombinant LRR region of decorin binds to filamin-A in vitro, and that the deglycosylated core protein of decorin coprecipitates with filamin-A, whereas intact decorin does not. Together, these results suggest that proteins containing the LRR motif that interact with filamin-A may be present in the cytoplasm or at the plasma membrane. 
 Migfilin and Mig-2 link focal adhesions to filamin and the actin cytoskeleton and function in cell shape modulation. Cell-  extracellular matrix   adhesion is an important determinant of cell morphology. We show here that migfilin, a LIM-containing protein, localizes to cell-matrix adhesions, associates with actin filaments, and is essential for cell shape modulation. Migfilin interacts with the cell-matrix adhesion protein Mig-2 (mitogen inducible gene-2), a mammalian homolog of UNC-112, and the actin binding protein filamin through its C- and N-terminal domains, respectively. Loss of Mig-2 or migfilin impairs cell shape modulation. Mig-2 recruits migfilin to cell-matrix adhesions, while the interaction with filamin mediates the association of migfilin with actin filaments. Migfilin therefore functions as an important scaffold at cell-matrix adhesions. Together, Mig-2, migfilin and filamin define a connection between cell matrix adhesions and the actin cytoskeleton and participate in the orchestration of actin assembly and cell shape modulation. 
 Epidermal growth factor (EGF) regulates α5β1 integrin activation state in human cancer cell lines through the p90RSK-dependent phosphorylation of filamin A. Regulation of integrin activation has important implications for tumor cell invasion and metastasis.EGF activates ERK/p90RSK and Rho/Rho kinase signaling in A431 and DiFi colon cancer cells, leading to phosphorylation of filamin A (FLNa) and inactivation of the α5β1 integrin receptor.EGF promotes α5β1 inactivation through the p90RSK-dependent phosphorylation of FLNa.We have identified a novel EGF-dependent mechanism controlling the α5β1 integrin activation state. Cell adhesion, motility, and invasion are regulated by the ligand-binding activity of integrin receptors, transmembrane proteins that bind   to the extracellula  r matrix. Integrins whose conformation allows for ligand binding and appropriate functional activity are said to be in an active state. Integrin activation and subsequent ligand binding are dynamically regulated by the association of cytoplasmic proteins with integrin intracellular domains. In this study, we evaluated the role of EGF in the regulation of the activation state of the α5β1 integrin receptor for fibronectin. The addition of EGF to either A431 squamous carcinoma cells or DiFi colon cancer cells resulted in loss of α5β1-dependent adhesion to fibronectin but no loss of integrin from the cell surface. EGF activated the EGF receptor/ERK/p90RSK and Rho/Rho kinase signaling pathways. Blocking either pathway inhibited EGF-mediated loss of adhesion, suggesting that they work in parallel to regulate integrin function. EGF treatment also resulted in phosphorylation of filamin A (FLNa), which binds and inactivates β1 integrins. EGF-mediated FLNa phosphorylation was completely blocked by an inhibitor of p90RSK and partially attenuated by an inhibitor of Rho kinase, suggesting that both pathways converge on FLNa to regulate integrin function. A431 clonal cell lines expressing non-phosphorylated dominant-negative FLNa were resistant to the inhibitory effects of EGF on integrin function, whereas clonal cell lines overexpressing wild-type FLNa were more sensitive to the inhibitory effect of EGF. These data suggest that EGF-dependent inactivation of α5β1 integrin is regulated through FLNa phosphorylation and cellular contractility. 
 Increased filamin binding to beta-integrin cytoplasmic domains inhibits cell migration. Multicellular animal development depends on integrins. These adhesion receptors link to the actin cytoskeleton, transmitting biochemical signals and force during cell migration and interactions with the   extracellular matrix  . Many integrin-cytoskeleton connections are formed by filamins and talin. The beta7 integrin tail binds strongly to filamin and supports less migration, fibronectin matrix assembly and focal adhesion formation than either the beta1D tail, which binds strongly to talin, or the beta1A tail, which binds modestly to both filamin and talin. To probe the role of filamin binding, we mapped the filamin-binding site of integrin tails and identified amino acid substitutions that led to selective loss of filamin binding to the beta7 tail and gain of filamin binding to the beta1A tail. These changes affected cell migration and membrane protrusions but not fibronectin matrix assembly or focal adhesion formation. Thus, tight filamin binding restricts integrin-dependent cell migration by inhibiting transient membrane protrusion and cell polarization. 
 R-Ras regulates migration through an interaction with filamin A in melanoma cells. Changes in cell adhesion and migration in the tumor microenvironment are key in the initiation and progression of metastasis. R-Ras is one of several small GTPases that regulate cell adhesion and migration on the   extracellular matrix  , however the mechanism has not been completely elucidated. Using a yeast two-hybrid approach we sought to identify novel R-Ras binding proteins that might mediate its effects on integrins.We identified Filamin A (FLNa) as a candidate interacting protein. FLNa is an actin-binding scaffold protein that also binds to integrin beta1, beta2 and beta7 tails and is associated with diverse cell processes including cell migration. Indeed, M2 melanoma cells require FLNa for motility. We further show that R-Ras and FLNa interact in co-immunoprecipitations and pull-down assays. Deletion of FLNa repeat 3 (FLNaDelta3) abrogated this interaction. In M2 melanoma cells active R-Ras co-localized with FLNa but did not co-localize with FLNa lacking repeat 3. Thus, activated R-Ras binds repeat 3 of FLNa. The functional consequence of this interaction was that active R-Ras and FLNa coordinately increased cell migration. In contrast, co-expression of R-Ras and FLNaDelta3 had a significantly reduced effect on migration. While there was enhancement of integrin activation and fibronectin matrix assembly, cell adhesion was not altered. Finally, siRNA knockdown of endogenous R-Ras impaired FLNa-dependent fibronectin matrix assembly.These data support a model in which R-Ras functionally associates with FLNa and thereby regulates integrin-dependent migration. Thus in melanoma cells R-Ras and FLNa may cooperatively promote metastasis by enhancing cell migration. 
 Filamin A is required for vimentin-mediated cell adhesion and spreading. Cell adhesion and spreading are regulated by complex interactions involving the cytoskeleton and   extracellular matrix   proteins. We examined the interaction of the intermediate filament protein vimentin with the actin cross-linking protein filamin A in regulation of spreading in HEK-293 and 3T3 cells. Filamin A and vimentin-expressing cells were well spread on collagen and exhibited numerous cell extensions enriched with filamin A and vimentin. By contrast, cells treated with small interfering RNA (siRNA) to knock down filamin A or vimentin were poorly spread; both of these cell populations exhibited >50% reductions of cell adhesion, cell surface beta1 integrin expression, and beta1 integrin activation. Knockdown of filamin A reduced vimentin phosphorylation and blocked recruitment of vimentin to cell extensions, whereas knockdown of filamin and/or vimentin inhibited the formation of cell extensions. Reduced vimentin phosphorylation, cell spreading, and beta1 integrin surface expression, and activation were phenocopied in cells treated with the protein kinase C inhibitor bisindolylmaleimide; cell spreading was also reduced by siRNA knockdown of protein kinase C-epsilon. By immunoprecipitation of cell lysates and by pull-down assays using purified proteins, we found an association between filamin A and vimentin. Filamin A also associated with protein kinase C-epsilon, which was enriched in cell extensions. These data indicate that filamin A associates with vimentin and to protein kinase C-epsilon, thereby enabling vimentin phosphorylation, which is important for beta1 integrin activation and cell spreading on collagen. 
 Phosphorylation of filamin A by Cdk1 regulates filamin A localization and daughter cell separation. In cell culture, many adherent mammalian cells undergo substantial actin cytoskeleton rearrangement prior to mitosis as they detach from the   extracellular matrix   and become spherical. At the end of mitosis, the actin cytoskeleton is required for cytokinesis and the reassembly of interphase structures as cells spread and reattach to substrate. To understand the processes regulating mitotic cytoskeletal remodeling, we studied how mitotic phosphorylation regulates filamin A (FLNa). FLNa is an actin-crosslinking protein that was previously identified as a cyclin-dependent kinase 1 (Cdk1) binding partner and substrate in vitro. Using quantitative label-based mass spectrometry, we find that FLNa serines 1084, 1459 and 1533 are phosphorylated in mitotic HeLa cells and all three sites match the phosphorylation consensus sequence of Cdk1. To investigate the functional role of mitotic FLNa phosphorylation, we mutated serines 1084, 1459 and 1533 to nonphosphorylatable alanine residues and expressed GFP-tagged FLNa(S1084A,S1459A,S1533A) (FLNa-AAA GFP) in a FLNa-deficient human melanoma cell line called M2. M2 cells expressing FLNa-AAA GFP have enhanced FLNa-AAA GFP and actin localization at sites of contact between daughter cells, impaired post-mitotic daughter cell separation and defects in cell migration. Therefore, mitotic phosphorylation of FLNa is important for successful cell division and interphase cell behavior. 
 Filamin A regulates focal adhesion disassembly and suppresses breast cancer cell migration and invasion. The actin cross-linking protein filamin A (FLNa) functions as a scaffolding protein and couples cell cytoskeleton to   extracellular matrix   and integrin receptor signaling. In this study, we report that FLNa suppresses invasion of breast cancer cells and regulates focal adhesion (FA) turnover. Two large progression tissue microarrays from breast cancer patients revealed a significant decrease of FLNa levels in tissues from invasive breast cancer compared with benign disease and in lymph node-positive compared with lymph node-negative breast cancer. In breast cancer cells and orthotopic mouse breast cancer models, down-regulation of FLNa stimulated cancer cell migration, invasion, and metastasis formation. Time-lapse microscopy and biochemical assays after FLNa silencing and rescue with wild-type or mutant protein resistant to calpain cleavage revealed that FLNa regulates FA disassembly at the leading edge of motile cells. Moreover, FLNa down-regulation enhanced calpain activity through the mitogen-activated protein kinase-extracellular signal-regulated kinase cascade and stimulated the cleavage of FA proteins. These results document a regulation of FA dynamics by FLNa in breast cancer cells. 
  Component  
   extracellular matrix   
  GeneRIFs  
 Studies indicate that interactions of filamin A with intermediate filaments and protein kinase C enable tight regulation of beta1 integrin function and consequently early events in cell adhesion and migration on   extracellular matrix   proteins. 
  [Ii]nvasive  PubMed  
 Filamin a mediates HGF/c-MET signaling in tumor cell migration. Deregulated hepatocyte growth factor (HGF)/c-MET axis has been correlated with poor clinical outcome and drug resistance in many human cancers. Identification of novel regulatory mechanisms influencing HGF/c-MET signaling may therefore be necessary to develop more effective cancer therapies. In our study, we show that multiple human cancer tissues and cells express filamin A (FLNA), a large cytoskeletal actin-binding protein, and expression of c-MET is significantly reduced in human tumor cells deficient for FLNA. The FLNA-deficient tumor cells exhibited poor migrative and   invasive   ability in response to HGF. On the other hand, the anchorage-dependent and independent tumor cell proliferation was not altered by HGF. The FLNA-deficiency specifically attenuated the activation of the c-MET downstream signaling molecule AKT in response to HGF stimulation. Furthermore, FLNA enhanced c-MET promoter activity by its binding to SMAD2. The impact of FLNA deficiency on c-MET expression and HGF-mediated cell migration in human tumor cells was confirmed in primary mouse embryonic fibroblasts deficient for Flna. These data suggest that FLNA is one of the important regulators of c-MET signaling and HGF-induced tumor cell migration. 
 Pro-prion binds filamin A, facilitating its interaction with integrin beta1, and contributes to melanomagenesis. Filamin A (FLNA) is an integrator of cell mechanics and signaling. The spreading and migration observed in FLNA sufficient A7 melanoma cells but not in the parental FLNA deficient M2 cells have been attributed to FLNA. In A7 and M2 cells, the normal prion (PrP) exists as pro-PrP, retaining its glycosylphosphatidyl-inositol (GPI) anchor peptide signal sequence (GPI-PSS). The GPI-PSS of PrP has a FLNA binding motif and binds FLNA. Reducing PrP expression in A7 cells alters the spatial distribution of FLNA and organization of actin and diminishes cell spreading and migration. Integrin β1 also binds FLNA. In A7 cells, FLNA, PrP, and integrin β1 exist as two independent, yet functionally linked, complexes; they are FLNA with PrP or FLNA with integrin β1. Reducing PrP expression in A7 cells decreases the amount of integrin β1 bound to FLNA. A PrP GPI-PSS synthetic peptide that crosses the cell membrane inhibits A7 cell spreading and migration. Thus, in A7 cells FLNA does not act alone; the binding of pro-PrP enhances association between FLNA and integrin β1, which then promotes cell spreading and migration. Pro-PrP is detected in melanoma in situ but not in melanoc  yte. Inv  asive melanoma has more pro-PrP. The binding of pro-PrP to FLNA, therefore, contributes to melanomagenesis. 
 Androgen-induced cell migration: role of androgen receptor/filamin A association. Androgen receptor (AR) controls male morphogenesis, gametogenesis and prostate growth as well as development of prostate cancer. These findings support a role for AR in cell migration and   invasive  ness. However, the molecular mechanism involved in AR-mediated cell migration still remains elusive.Mouse embryo NIH3T3 fibroblasts and highly metastatic human fibrosarcoma HT1080 cells harbor low levels of transcriptionally incompetent AR. We now report that, through extra nuclear action, AR triggers migration of both cell types upon stimulation with physiological concentrations of the androgen R1881. We analyzed the initial events leading to androgen-induced cell migration and observed that challenging NIH3T3 cells with 10 nM R1881 rapidly induces interaction of AR with filamin A (FlnA) at cytoskeleton. AR/FlnA complex recruits integrin beta 1, thus activating its dependent cascade. Silencing of AR, FlnA and integrin beta 1 shows that this ternary complex controls focal adhesion kinase (FAK), paxillin and Rac, thereby driving cell migration. FAK-null fibroblasts migrate poorly and Rac inhibition by EHT impairs motility of androgen-treated NIH3T3 cells. Interestingly, FAK and Rac activation by androgens are independent of each other. Findings in human fibrosarcoma HT1080 cells strengthen the role of Rac in androgen signaling. The Rac inhibitor significantly impairs androgen-induced migration in these cells. A mutant AR, deleted of the sequence interacting with FlnA, fails to mediate FAK activation and paxillin tyrosine phosphorylation in androgen-stimulated cells, further reinforcing the role of AR/FlnA interaction in androgen-mediated motility.The present report, for the first time, indicates that the extra nuclear AR/FlnA/integrin beta 1 complex is the key by which androgen activates signaling leading to cell migration. Assembly of this ternary complex may control organ development and prostate cancer metastasis. 
 Filamin A expression correlates with proliferation and   invasive   properties of human metastatic melanoma tumors: implications for survival in patients. Filamin A (FLNa) cross-links actin filaments into dynamic orthogonal networks and interacts with binding proteins of diverse cellular functions that are implicated in cell growth and motility regulation. Here, we tested the hypothesis that FLNa plays a role in cancer proliferation and metastasis via the regulation of epidermal growth factor receptor (EGFR) function.Ectopic expression and knockdown of FLNa in human melanoma cell lines was performed to investigate changes in cellular proliferation, migration and invasion in vitro and tumor growth in a xenograft model in the mouse. The role of FLNa in EGFR expression and signaling was evaluated by Western blot. Immunohistochemistry was performed on histological sections of human melanoma tumors to determine whether an association existed between FLNa and overall survival.The depletion of FLNa significantly reduced the proliferation, migration and invasion of two melanoma cell lines in vitro and was associated with smaller tumors in a xenograft model in vivo. EGF-induced phosphorylation of EGFR and activation of the Raf-MEK-ERK cascade was negatively affected by the silencing of FLNa both in vitro and in vivo. Cancer patients with low melanoma tumor FLNa expression have improved survival benefit.These data indicate that enhanced tumorigenesis occurs through increase in EGF-induced EGFR activation in FLNa-expressing melanoma cells and that high FLNa levels are predictors of negative outcome for patients with melanoma tumors. 
 Binding of pro-prion to filamin A disrupts cytoskeleton and correlates with poor prognosis in pancreatic cancer. The cellular prion protein (PrP) is a highly conserved, widely expressed, glycosylphosphatidylinositol-anchored (GPI-anchored) cell surface glycoprotein. Since its discovery, most studies on PrP have focused on its role in neurodegenerative prion diseases, whereas its function outside the nervous system remains unclear. Here, we report that human pancreatic ductal adenocarcinoma (PDAC) cell lines expressed PrP. However, the PrP was neither glycosylated nor GPI-anchored, existing as pro-PrP and retaining its GPI anchor peptide signal sequence (GPI-PSS). We also showed that the PrP GPI-PSS has a filamin A-binding (FLNa-binding) motif and interacted with FLNa, an actin-associated protein that integrates cell mechanics and signaling. Binding of pro-PrP to FLNa disrupted cytoskeletal organization. Inhibition of PrP expression by shRNA in the PDAC cell lines altered the cytoskeleton and expression of multiple signaling proteins; it also reduced cellular proliferation and   invasive  ness in vitro as well as tumor growth in vivo. A subgroup of human patients with pancreatic cancer was found to have tumors that expressed pro-PrP. Most importantly, PrP expression in tumors correlated with a marked decrease in patient survival. We propose that binding of pro-PrP to FLNa perturbs FLNa function, thus contributing to the aggressiveness of PDAC. Prevention of this interaction could provide an attractive target for therapeutic intervention in human PDAC. 
 Systematic mapping and functional analysis of a family of human epididymal secretory sperm-located proteins. The mammalian spermatozoon has many cellular compartments, such as head and tail, permitting it to interact with the female reproductive tract and fertilize the egg. It acquires this fertilizing potential during transit through the epididymis, which secretes proteins that coat different sperm domains. Optimal levels of these proteins provide the spermatozoon with its ability to move to, bind to, fuse with, and penetrate the egg; otherwise male infertility results. As few human epididymal proteins have been characterized, this work was performed to generate a database of human epididymal sperm-located proteins involved in maturation. Two-dimensional gel electrophoresis of epididymal tissue and luminal fluid proteins, followed by identification using MALDI-TOF/MS or MALDI-TOF/TOF, revealed over a thousand spots in gels comprising 745 abundant nonstructural proteins, 408 in luminal fluids, of which 207 were present on spermatozoa. Antibodies raised to 619 recombinant or synthetic peptides, used in Western blots, histological sections, and washed sperm preparations to confirm antibody quality and protein expression, indicated their regional location in the epididymal epithelium and highly specific locations on washed functional spermatozoa. Sperm function tests suggested the role of some proteins in motility and protection against oxidative attack. A large database of these proteins, characterized by size, pI, chromosomal location, and function, was given a unified terminology reflecting their sperm domain location. These novel, secreted human epididymal proteins are potential targets for a posttesticular contraceptive acting to provide rapid, reversible, functional sterility in men and they are also biomarkers that could be used in non  invasive   assessments of male fertility. 
 Filamin A regulates focal adhesion disassembly and suppresses breast cancer cell migration and invasion. The actin cross-linking protein filamin A (FLNa) functions as a scaffolding protein and couples cell cytoskeleton to extracellular matrix and integrin receptor signaling. In this study, we report that FLNa suppresses invasion of breast cancer cells and regulates focal adhesion (FA) turnover. Two large progression tissue microarrays from breast cancer patients revealed a significant decrease of FLNa levels in tissues from   invasive   breast cancer compared with benign disease and in lymph node-positive compared with lymph node-negative breast cancer. In breast cancer cells and orthotopic mouse breast cancer models, down-regulation of FLNa stimulated cancer cell migration, invasion, and metastasis formation. Time-lapse microscopy and biochemical assays after FLNa silencing and rescue with wild-type or mutant protein resistant to calpain cleavage revealed that FLNa regulates FA disassembly at the leading edge of motile cells. Moreover, FLNa down-regulation enhanced calpain activity through the mitogen-activated protein kinase-extracellular signal-regulated kinase cascade and stimulated the cleavage of FA proteins. These results document a regulation of FA dynamics by FLNa in breast cancer cells. 
  [Mm]etastasis  PubMed  
 Grb7 and Filamin-a associate and are colocalized to cell membrane ruffles upon EGF stimulation. Grb7 is an adaptor molecule mediating signal transduction from multiple cell surface receptors to diverse downstream pathways. Grb7, along with Grb10 and Grb14, make up the Grb7 protein family. This protein family has been shown to be overexpressed in certain cancers and cancer cell lines. Grb7 and a receptor tyrosine kinase, ErbB2, are overexpressed in 20-30% of breast cancers. Grb7 overexpression has been linked to enhanced cell migration and   metastasis  , although the participants in these pathways have not been fully determined. In this study, we report the Grb7 protein interacts with Filamin-a, an actin-crosslinking component of the cell cytoskeleton. Additionally, we have demonstrated the interaction between Grb7 and Flna is specific to the RA-PH domains of Grb7, and the immunoglobulin-like repeat 16-19 domains of Flna. We demonstrate that full-length Grb7 and Flna interact in the mammalian cellular environment, as well as in vitro. Immunofluorescent microscopy shows potential co-localization of Grb7 and Flna in membrane ruffles upon epidermal growth factor stimulation. These studies are amongst the first to establish a clear connection between Grb7 signaling and cytoskeletal remodeling. 
 [Filamin A as a mediator of alterations in cancer cells]. Filamin A (FLNA, filamin-1) is a homodimeric protein, commonly expressed in animal organisms. Its basic function in the cell is actin crosslinking and forming 3D cytoskeleton structure. Filamin-1 interacts with more than 60 different proteins with various functions such as: cell membrane and cytoskeleton formation, maintaining cell shape, intracellular signaling, nuclear functions or GTP-binding proteins regulation. FLNA interactions with oncogenesis- and   metastasis  -related proteins, such as K-RAS, TRAF2 or NIK indicate its crucial role in cancer progression. Filamin-1 undergoes proteolytic fragmentation producing products, translocation of which to the nucleus may be related to alterations in the cell metastatic ability. It was also demonstrated that FLNA dysfunctions can lead to sensitization of cells to ionizing irradiation or common chemotherapeutics: bleomycin and cisplatin. These findings indicate that FLNA can be considered as a novel target in anti-cancer therapy. 
 Androgen-induced cell migration: role of androgen receptor/filamin A association. Androgen receptor (AR) controls male morphogenesis, gametogenesis and prostate growth as well as development of prostate cancer. These findings support a role for AR in cell migration and invasiveness. However, the molecular mechanism involved in AR-mediated cell migration still remains elusive.Mouse embryo NIH3T3 fibroblasts and highly metastatic human fibrosarcoma HT1080 cells harbor low levels of transcriptionally incompetent AR. We now report that, through extra nuclear action, AR triggers migration of both cell types upon stimulation with physiological concentrations of the androgen R1881. We analyzed the initial events leading to androgen-induced cell migration and observed that challenging NIH3T3 cells with 10 nM R1881 rapidly induces interaction of AR with filamin A (FlnA) at cytoskeleton. AR/FlnA complex recruits integrin beta 1, thus activating its dependent cascade. Silencing of AR, FlnA and integrin beta 1 shows that this ternary complex controls focal adhesion kinase (FAK), paxillin and Rac, thereby driving cell migration. FAK-null fibroblasts migrate poorly and Rac inhibition by EHT impairs motility of androgen-treated NIH3T3 cells. Interestingly, FAK and Rac activation by androgens are independent of each other. Findings in human fibrosarcoma HT1080 cells strengthen the role of Rac in androgen signaling. The Rac inhibitor significantly impairs androgen-induced migration in these cells. A mutant AR, deleted of the sequence interacting with FlnA, fails to mediate FAK activation and paxillin tyrosine phosphorylation in androgen-stimulated cells, further reinforcing the role of AR/FlnA interaction in androgen-mediated motility.The present report, for the first time, indicates that the extra nuclear AR/FlnA/integrin beta 1 complex is the key by which androgen activates signaling leading to cell migration. Assembly of this ternary complex may control organ development and prostate cancer   metastasis  . 
 An adventitious interaction of filamin A with RhoGDI2(Tyr153Glu). Filamin A (FLNA) is an actin filament crosslinking protein with multiple intracellular binding partners. Mechanical force exposes cryptic FLNA binding sites for some of these ligands. To identify new force-dependent binding interactions, we used a fusion construct composed of two FLNA domains, one of which was previously identified as containing a force-dependent binding site as a bait in a yeast two-hybrid system and identified the Rho dissociation inhibitor 2 (RhoGDI2) as a potential interacting partner. A RhoGDI2 truncate with 81 N-terminal amino acid residues and a phosphomimetic mutant, RhoGDI(Tyr153Glu) interacted with the FLNA construct. However, neither wild-type or full-length RhoGDI2 phosphorylated at Y153 interacted with FLNA. Our interpretation of these contradictions is that truncation and/or mutation of RhoGDI2 perturbs its conformation to expose a site that adventitiously binds FLNA and is not a bona-fide interaction. Therefore, previous studies reporting that a RhoGDI(Y153E) mutant suppresses the   metastasi  s of human bladder cancer cells must be reinvestigated in light of artificial interaction of this point mutant with FLNA. 
 Filamin A expression correlates with proliferation and invasive properties of human metastatic melanoma tumors: implications for survival in patients. Filamin A (FLNa) cross-links actin filaments into dynamic orthogonal networks and interacts with binding proteins of diverse cellular functions that are implicated in cell growth and motility regulation. Here, we tested the hypothesis that FLNa plays a role in cancer proliferation and   metastasis   via the regulation of epidermal growth factor receptor (EGFR) function.Ectopic expression and knockdown of FLNa in human melanoma cell lines was performed to investigate changes in cellular proliferation, migration and invasion in vitro and tumor growth in a xenograft model in the mouse. The role of FLNa in EGFR expression and signaling was evaluated by Western blot. Immunohistochemistry was performed on histological sections of human melanoma tumors to determine whether an association existed between FLNa and overall survival.The depletion of FLNa significantly reduced the proliferation, migration and invasion of two melanoma cell lines in vitro and was associated with smaller tumors in a xenograft model in vivo. EGF-induced phosphorylation of EGFR and activation of the Raf-MEK-ERK cascade was negatively affected by the silencing of FLNa both in vitro and in vivo. Cancer patients with low melanoma tumor FLNa expression have improved survival benefit.These data indicate that enhanced tumorigenesis occurs through increase in EGF-induced EGFR activation in FLNa-expressing melanoma cells and that high FLNa levels are predictors of negative outcome for patients with melanoma tumors. 
 Interactions between filamin A and MMP-9 regulate proliferation and invasion in renal cell carcinoma. This study aimed to analyze the expression, clinical significance of filamin A (FLNA) in renal cell carcinoma (RCC) and biological effects in a cell line by regulating FLNA expression. Immunohistochemistry and Western blotting were used to analyze FLNA protein expression in 70 cases of RCC and normal tissues to study the relationship with clinical factors. FLNA lentiviral and empty vectors were transfected into RCC to study the influence of up-regulated expression of FLNA. FLNA siRNA was transiently transfected into ACHN kidney carcinoma cells by a liposome-mediated method and protein was detected by Western blotting. The level of expression was found to be significantly lower in RCC than normal tissues (p<0.05). No correlation was noted with gender, age, tumor size or pathological types (p>0.05), but links with lymph node   metastasis  , clinic stage and histological grade were noted (p<0.05). Loss of FLNA expression correlated significantly with poor overall survival time by Kaplan-Meier analysis (p<0.05). Results for biological function showed that ACHN cells transfected with FLNA had a lower survival fraction, significant decrease in migration and invasion, higher cell apoptosis, higher percentage of the G0/G1 phases, and lower MMP-9 protein expression compared with ACHN cells untransfected with FLNA (p<0.05). However, renal 786-0 cells transfected with FLNA siRNA had a higher survival fraction, significant increase in migration and invasion, and higher MMP-9 protein expression compared (p<0.05). In conclusion, FLNA expression was decreased in RCC and correlated significantly with lymph node   metastasis  , clinic stage, histological grade and poor overall survival, suggesting that FLNA may play important roles as a a tumor suppressor in RCC by promoting degradation of MMP-9. 
 Absent expression of FLNA is correlated with poor prognosis of nasopharyngeal cancer. This study aimed to analyze the expression, clinical significance of filamin A (FLNA) in nasopharyngeal carcinoma, and the biological effect in its cell line by FLNA overexpression. Immunohistochemistry and western blot were used to analyze FLNA protein expression in 63 cases of nasopharyngeal cancer and 21 cases of normal tissues to study the relationship between FLNA expression and clinical factors. FLNA lentiviral vector and empty vector were respectively transfected into nasopharyngeal cancer CNE2 cell line. Reverse transcription-polymerase chain reaction (RT-PCR) and western blot were used to detect the mRNA level and protein of FLNA. 3-[4,5-Dimethylthiazol-2-yl]-2,5-diphenyltetrazolium bromide, migration, and invasion assays were also conducted as to the influence of the upregulated expression of FLNA that might be found on CNE2 cell biological effect. Immunohistochemistry: the level of FLNA protein expression was found to be significantly lower in nasopharyngeal cancer tissue than normal tissues (P < 0.05). Western blot: the relative amount of FLNA protein in nasopharyngeal cancer tissue was found to be significantly lower than in normal tissues (P < 0.05). The level of FLNA protein expression was correlated with T stages, lymph node   metastasis  , clinic stage, and histological grade (P < 0.05). Loss of FLNA expression correlated significantly with poor overall survival time by Kaplan-Meier analysis (P < 0.05). The result of biological function has shown that CNE2 cell-transfected FLNA had a lower survival fraction, significant decrease in migration and invasion, and lower matrix metallopeptidase 9 (MMP-9) protein expression compared with CNE2 cell-untransfected FLNA (P < 0.05). FLNA expression decreased in nasopharyngeal cancer and correlated significantly lymph node   metastasis  , clinic stage, histological grade, and poor overall survival, suggesting that FLNA may play important roles as a negative regulator to nasopharyngeal cancer CNE2 cell by promoting degradation of MMP-9. 
 Filamin A regulates MMP-9 expression and suppresses prostate cancer cell migration and invasion. This study aims to analyze the expression and clinical significance of Filamin A (FLNA) in prostate carcinoma and the biological effect in its cell line by FLNA overexpression. Immunohistochemistry and Western blot were used to analyze FLNA protein expression in 68 cases of prostate cancer and 37 cases of normal tissues to study the influence of the upregulated expression of FLNA that might be found on PC-3 cell biological effect. In the immunohistochemical analysis, the level of FLNA protein expression was found to be significantly lower in prostate cancer tissue than in normal tissues (P < 0.05). In the Western blot analysis, the relative amount of FLNA protein in prostate cancer tissue was found to be significantly lower than in normal tissues (P < 0.05). The level of FLNA protein expression was not correlated with age and PSA concentration (P > 0.05), but it was correlated with T stages, lymph node   metastasis  , clinic stage, and Gleason score (P < 0.05). The result of biological function showed that PC-3 cell transfected FLNA had a lower survival fraction, a significant decrease in migration and invasion, and a lower matrix metallopeptidase 9 (MMP-9) protein expression compared with PC-3 cell untransfected FLNA (P < 0.05). FLNA expression decreased in prostate cancer and correlated significantly with T stages, lymph node   metastasis  , clinic stage, and Gleason score, suggesting that FLNA may play important roles as a negative regulator to prostate cancer PC-3 cell by promoting the degradation of MMP-9. 
 A role for tissue factor in cell adhesion and migration mediated by interaction with actin-binding protein 280. Tissue factor (TF), the protease receptor initiating the coagulation system, functions in vascular development, angiogenesis, and tumor cell   metastasis   by poorly defined molecular mechanisms. We demonstrate that immobilized ligands for TF specifically support cell adhesion, migration, spreading, and intracellular signaling, which are not inhibited by RGD peptides. Two-hybrid screening identified actin-binding protein 280 (ABP-280) as ligand for the TF cytoplasmic domain. Extracellular ligation of TF is necessary for ABP-280 binding. ABP-280 recruitment to TF adhesion contacts is associated with reorganization of actin filaments, but cytoskeletal adaptor molecules typically found in integrin-mediated focal contacts are not associated with TF. Chimeric molecules of the TF cytoplasmic domain and an unrelated extracellular domain support cell spreading and migration, demonstrating that the extracellular domain of TF is not involved in the recruitment of accessory molecules that influence adhesive functions. Replacement of TF's cytoplasmic Ser residues with Asp to mimic phosphorylation enhances the interaction with ABP-280, whereas Ala mutations abolish coprecipitation of ABP-280 with immobilized TF cytoplasmic domain, and severely reduce cell spreading. The specific interaction of the TF cytoplasmic domain with ABP-280 provides a molecular pathway by which TF supports tumor cell   metastasis   and vascular remodeling. 
 An antiproliferative gene FLNA regulates migration and invasion of gastric carcinoma cell in vitro and its clinical significance. This study aimed to analyze the expression and clinical significance of filamin A (FLNA) in gastric carcinoma and the biological effect in its cell line by FLNA overexpression. Immunohistochemistry and western blot were used to analyze FLNA protein expression in 47 cases of gastric cancer and 47 cases of normal tissues to study the relationship between FLNA expression and clinical factors. FLNA lentiviral vector and empty vector were respectively transfected into gastric cancer SGC-7901 cell line. Reverse transcription-polymerase chain reaction (RT-PCR) and western blot were used to detect the mRNA level and protein of FLNA. 3-[4,5-Dimethylthiazol-2-yl]-2,5-diphenyltetrazolium bromide (MTT) assay and migration and invasion assays were also conducted to determine the influence of the upregulated expression of FLNA that might be found on SGC-7901 cell biological effect. Immunohistochemistry: The level of FLNA protein expression was found to be significantly lower in gastric cancer tissue than normal tissues (P < 0.05). Western blot: The relative amount of FLNA protein in gastric cancer tissue was found to be significantly lower than in normal tissues (P < 0.05). The level of FLNA protein expression was not correlated with gender, age, and tumor invasion (P > 0.05), but it was correlated with   lymph nod  e metastasis, clinic stage, and histological grade (P < 0.05). Loss of FLNA expression correlated significantly with poor overall survival time by Kaplan-Meier analysis (P < 0.05). The result of biological function showed that SGC-7901 cell transfected FLNA had a lower survival fraction, significant decrease in migration and invasion, and lower matrix metallopeptidase 9 (MMP-9) protein expression compared with SGC-7901 cell untransfected FLNA (P < 0.05). FLNA expression decreased in gastric cancer and correlated signif  icantly wi  th lymph node metastasis, clinic stage, histological grade, and poor overall survival, suggesting that FLNA may play important roles as a negative regulator to gastric cancer SGC-7901 cell by promoting degradation of MMP-9. 
 Epidermal growth factor (EGF) regulates α5β1 integrin activation state in human cancer cell lines through the p90RSK-dependent phosphorylation of filamin A. Regulation of integrin activation has important implications for tumor cell invasion an  d metastas  is.EGF activates ERK/p90RSK and Rho/Rho kinase signaling in A431 and DiFi colon cancer cells, leading to phosphorylation of filamin A (FLNa) and inactivation of the α5β1 integrin receptor.EGF promotes α5β1 inactivation through the p90RSK-dependent phosphorylation of FLNa.We have identified a novel EGF-dependent mechanism controlling the α5β1 integrin activation state. Cell adhesion, motility, and invasion are regulated by the ligand-binding activity of integrin receptors, transmembrane proteins that bind to the extracellular matrix. Integrins whose conformation allows for ligand binding and appropriate functional activity are said to be in an active state. Integrin activation and subsequent ligand binding are dynamically regulated by the association of cytoplasmic proteins with integrin intracellular domains. In this study, we evaluated the role of EGF in the regulation of the activation state of the α5β1 integrin receptor for fibronectin. The addition of EGF to either A431 squamous carcinoma cells or DiFi colon cancer cells resulted in loss of α5β1-dependent adhesion to fibronectin but no loss of integrin from the cell surface. EGF activated the EGF receptor/ERK/p90RSK and Rho/Rho kinase signaling pathways. Blocking either pathway inhibited EGF-mediated loss of adhesion, suggesting that they work in parallel to regulate integrin function. EGF treatment also resulted in phosphorylation of filamin A (FLNa), which binds and inactivates β1 integrins. EGF-mediated FLNa phosphorylation was completely blocked by an inhibitor of p90RSK and partially attenuated by an inhibitor of Rho kinase, suggesting that both pathways converge on FLNa to regulate integrin function. A431 clonal cell lines expressing non-phosphorylated dominant-negative FLNa were resistant to the inhibitory effects of EGF on integrin function, whereas clonal cell lines overexpressing wild-type FLNa were more sensitive to the inhibitory effect of EGF. These data suggest that EGF-dependent inactivation of α5β1 integrin is regulated through FLNa phosphorylation and cellular contractility. 
 A positive relationship between filamin and VEGF in patients with lung cancer. Induction of angiogenic responses by multiple factors, a crucial step in tumor growth and   metastasis  , is not completely understood. Recently, involvement of the cytoskeletal actin-binding proteins in angiogenesis has been suggested as a target for anti-neovascular cancer therapy in vitro. In this study, the expression of filamin A (FLNA) and vascular endothelial growth factor (VEGF) in paraffin-embedded tumor samples from patients with well-characterized lung tumors was immunohistochemically analyzed and compared with clinical variables and survival outcome. A positive expression of FLNA and VEGF was detected in the cytoplasm of tumor cells in 66 (48.2%) and 69 (50.4%) of the 137 patients with lung cancer, respectively (p<0.0001). A significant difference was observed between FLNA expression and VEGF expression. Although our findings do not suggest that the expression of FLNA alone plays an independent prognostic role, the angiogenesis pathway mediated by FLNA appears to be responsible for controlling the growth of lung tumors. 
 R-Ras regulates migration through an interaction with filamin A in melanoma cells. Changes in cell adhesion and migration in the tumor microenvironment are key in the initiation and progression of   metastasis  . R-Ras is one of several small GTPases that regulate cell adhesion and migration on the extracellular matrix, however the mechanism has not been completely elucidated. Using a yeast two-hybrid approach we sought to identify novel R-Ras binding proteins that might mediate its effects on integrins.We identified Filamin A (FLNa) as a candidate interacting protein. FLNa is an actin-binding scaffold protein that also binds to integrin beta1, beta2 and beta7 tails and is associated with diverse cell processes including cell migration. Indeed, M2 melanoma cells require FLNa for motility. We further show that R-Ras and FLNa interact in co-immunoprecipitations and pull-down assays. Deletion of FLNa repeat 3 (FLNaDelta3) abrogated this interaction. In M2 melanoma cells active R-Ras co-localized with FLNa but did not co-localize with FLNa lacking repeat 3. Thus, activated R-Ras binds repeat 3 of FLNa. The functional consequence of this interaction was that active R-Ras and FLNa coordinately increased cell migration. In contrast, co-expression of R-Ras and FLNaDelta3 had a significantly reduced effect on migration. While there was enhancement of integrin activation and fibronectin matrix assembly, cell adhesion was not altered. Finally, siRNA knockdown of endogenous R-Ras impaired FLNa-dependent fibronectin matrix assembly.These data support a model in which R-Ras functionally associates with FLNa and thereby regulates integrin-dependent migration. Thus in melanoma cells R-Ras and FLNa may cooperatively promote   metastasis   by enhancing cell migration. 
 New cancer suppressor gene for colorectal adenocarcinoma: filamin A. To determine the expression and significance of filamin A (FLNa) in colorectal adenocarcinoma tissue.The expression of FLNa in 46 colorectal cancer tissues and normal tissues was detected by immunohistochemistry, reverse transcription polymerase chain reaction (RT-PCR) and Western blotting, and its relationship with clinical parameters and prognosis was analyzed.The positive expression of FLNa in cancer tissues was lower than that in normal mucosa, and the difference was statistically significant. The expression of FLNa correlated with liver   metastasis  , lymph node   metastasis   and rectal invasion depth, regardless of sex, age, tumor location, tumor size, gross shape and histological type of colorectal carcinoma. Multivariate analysis showed that FLNa was an independent risk factor for postoperative survival of patients with colorectal adenocarcinoma. Moreover, survival analysis showed that the expression level of FLNa was closely related with survival of patients with colorectal adenocarcinoma. The results of RT-PCR and Western blotting were consistent with those of immunohistochemistry.FLNa showed low expression in colorectal adenocarcinoma, high correlation with the incidence and development of colorectal cancer, and was considered an indicator of prognosis. 
 Filamin A regulates focal adhesion disassembly and suppresses breast cancer cell migration and invasion. The actin cross-linking protein filamin A (FLNa) functions as a scaffolding protein and couples cell cytoskeleton to extracellular matrix and integrin receptor signaling. In this study, we report that FLNa suppresses invasion of breast cancer cells and regulates focal adhesion (FA) turnover. Two large progression tissue microarrays from breast cancer patients revealed a significant decrease of FLNa levels in tissues from invasive breast cancer compared with benign disease and in lymph node-positive compared with lymph node-negative breast cancer. In breast cancer cells and orthotopic mouse breast cancer models, down-regulation of FLNa stimulated cancer cell migration, invasion, and   metastasis   formation. Time-lapse microscopy and biochemical assays after FLNa silencing and rescue with wild-type or mutant protein resistant to calpain cleavage revealed that FLNa regulates FA disassembly at the leading edge of motile cells. Moreover, FLNa down-regulation enhanced calpain activity through the mitogen-activated protein kinase-extracellular signal-regulated kinase cascade and stimulated the cleavage of FA proteins. These results document a regulation of FA dynamics by FLNa in breast cancer cells. 
  GeneRIFs  
 FLNA expression decreased in gastric cancer and correlated significantly with lymph node   metastasis  , clinic stage, histological grade, and poor overall survival 
 The extra nuclear AR/FlnA/integrin beta 1 complex is the key by which androgen activates signaling leading to cell migration. Assembly of this ternary complex may control organ development and prostate cancer   metastasis  . 
  [Cc]ell [Ii]nvasion  PubMed  
 Epidermal growth factor (EGF) regulates α5β1 integrin activation state in human cancer cell lines through the p90RSK-dependent phosphorylation of filamin A. Regulation of integrin activation has important implications for tumo  r cell invasi  on and metastasis.EGF activates ERK/p90RSK and Rho/Rho kinase signaling in A431 and DiFi colon cancer cells, leading to phosphorylation of filamin A (FLNa) and inactivation of the α5β1 integrin receptor.EGF promotes α5β1 inactivation through the p90RSK-dependent phosphorylation of FLNa.We have identified a novel EGF-dependent mechanism controlling the α5β1 integrin activation state. Cell adhesion, motility, and invasion are regulated by the ligand-binding activity of integrin receptors, transmembrane proteins that bind to the extracellular matrix. Integrins whose conformation allows for ligand binding and appropriate functional activity are said to be in an active state. Integrin activation and subsequent ligand binding are dynamically regulated by the association of cytoplasmic proteins with integrin intracellular domains. In this study, we evaluated the role of EGF in the regulation of the activation state of the α5β1 integrin receptor for fibronectin. The addition of EGF to either A431 squamous carcinoma cells or DiFi colon cancer cells resulted in loss of α5β1-dependent adhesion to fibronectin but no loss of integrin from the cell surface. EGF activated the EGF receptor/ERK/p90RSK and Rho/Rho kinase signaling pathways. Blocking either pathway inhibited EGF-mediated loss of adhesion, suggesting that they work in parallel to regulate integrin function. EGF treatment also resulted in phosphorylation of filamin A (FLNa), which binds and inactivates β1 integrins. EGF-mediated FLNa phosphorylation was completely blocked by an inhibitor of p90RSK and partially attenuated by an inhibitor of Rho kinase, suggesting that both pathways converge on FLNa to regulate integrin function. A431 clonal cell lines expressing non-phosphorylated dominant-negative FLNa were resistant to the inhibitory effects of EGF on integrin function, whereas clonal cell lines overexpressing wild-type FLNa were more sensitive to the inhibitory effect of EGF. These data suggest that EGF-dependent inactivation of α5β1 integrin is regulated through FLNa phosphorylation and cellular contractility. 
   
  
 SMPD1 
 
  NCBI Gene  
  GeneRIF  
  Pubmed  
 
 
  Search Term  Rank  Fields  
  [Ff]ilopodia  1  PubMed(1)  
  ECM  7  PubMed(1)  
  [Ee]xtracellular [Mm]atrix  8  PubMed(1)  
  [Ii]nvasive  10  PubMed(2); GeneRIFs(1)  
 
 
 hide/show details for SMPD1 
  [Ff]ilopodia  PubMed  
 Remodeling of cellular cytoskeleton by the acid sphingomyelinase/ceramide pathway. The chemotherapeutic agent cisplatin is widely used in treatment of solid tumors. In breast cancer cells, cisplatin produces early and marked changes in cell morphology and the actin cytoskeleton. These changes manifest as loss of lamellipodia/  filopodia   and appearance of membrane ruffles. Furthermore, cisplatin induces dephosphorylation of the actin-binding protein ezrin, and its relocation from membrane protrusions to the cytosol. Because cisplatin activates acid sphingomyelinase (ASMase), we investigate here the role of the ASMase/ceramide (Cer) pathway in mediating these morphological changes. We find that cisplatin induces a transient elevation in ASMase activity and its redistribution to the plasma membrane. This translocation is blocked upon overexpression of a dominant-negative (DN) ASMase(S508A) mutant and by a DN PKCdelta. Importantly; knockdown of ASMase protects MCF-7 cells from cisplatin-induced cytoskeletal changes including ezrin dephosphorylation. Reciprocally, exogenous delivery of D-e-C16-Cer, but not dihydro-C16-Cer, recapitulates the morphotropic effects of cisplatin. Collectively, these results highlight a novel tumor suppressor property for Cer and a function for ASMase in cisplatin-induced cytoskeletal remodeling. 
  ECM  PubMed  
 Acid sphingomyelinase deficiency contributes to resistance of scleroderma fibroblasts to Fas-mediated apoptosis. Scleroderma (SSc) is characterized by excess production and deposition of extracellular matrix (  ECM  ) proteins. Activated fibroblasts play a key role in fibrosis in SSc and are resistant to Fas-mediated apoptosis. Acid sphingomyelinase (ASMase), a major sphingolipid enzyme, plays an important role in the Fas-mediated apoptosis.We investigated whether dysregulation of ASMase contributes to Fas-mediated apoptosis resistance in SSc fibroblasts.Fibroblasts were isolated from SSc patients and healthy controls. Western blot was performed to analyze protein levels and quantitative real time RT-PCR was used to determine mRNA expression. Cells were transiently transfected with siRNA oligos against ASMase or transduced with adenoviruses overexpressing ASMase. Apoptosis was induced using anti-Fas antibody (1 μg/mL) and analyzed using caspase-3 antibody or Cell Death Detection ELISA.SSc fibroblasts showed increased resistance to Fas-mediated apoptosis. ASMase expression was decreased in SSc fibroblasts and Transforming Growth Factor beta (TGFβ), the major fibrogenic cytokine involved in the pathogenesis of SSc, downregulated ASMase in normal fibroblasts. Forced expression of ASMase in SSc fibroblasts restored sensitivity of these cells to Fas-mediated apoptosis while blockade of ASMase was sufficient to induce partial resistance to Fas-induced apoptosis in normal fibroblasts. In addition, ASMase blockade decreased activity of protein phosphatase 2A (PP2A) through phosphorylation on Tyr(307) and resulted in activation of extracellular regulated kinase 1/2 (Erk1/2) and protein kinase B (Akt/PKB).In conclusion, this study suggests that ASMase deficiency promotes apoptosis resistance and contributes to activation of profibrotic signaling in SSc fibroblasts. 
  [Ee]xtracellular [Mm]atrix  PubMed  
 Acid sphingomyelinase deficiency contributes to resistance of scleroderma fibroblasts to Fas-mediated apoptosis. Scleroderma (SSc) is characterized by excess production and deposition of   extracellular matrix   (ECM) proteins. Activated fibroblasts play a key role in fibrosis in SSc and are resistant to Fas-mediated apoptosis. Acid sphingomyelinase (ASMase), a major sphingolipid enzyme, plays an important role in the Fas-mediated apoptosis.We investigated whether dysregulation of ASMase contributes to Fas-mediated apoptosis resistance in SSc fibroblasts.Fibroblasts were isolated from SSc patients and healthy controls. Western blot was performed to analyze protein levels and quantitative real time RT-PCR was used to determine mRNA expression. Cells were transiently transfected with siRNA oligos against ASMase or transduced with adenoviruses overexpressing ASMase. Apoptosis was induced using anti-Fas antibody (1 μg/mL) and analyzed using caspase-3 antibody or Cell Death Detection ELISA.SSc fibroblasts showed increased resistance to Fas-mediated apoptosis. ASMase expression was decreased in SSc fibroblasts and Transforming Growth Factor beta (TGFβ), the major fibrogenic cytokine involved in the pathogenesis of SSc, downregulated ASMase in normal fibroblasts. Forced expression of ASMase in SSc fibroblasts restored sensitivity of these cells to Fas-mediated apoptosis while blockade of ASMase was sufficient to induce partial resistance to Fas-induced apoptosis in normal fibroblasts. In addition, ASMase blockade decreased activity of protein phosphatase 2A (PP2A) through phosphorylation on Tyr(307) and resulted in activation of extracellular regulated kinase 1/2 (Erk1/2) and protein kinase B (Akt/PKB).In conclusion, this study suggests that ASMase deficiency promotes apoptosis resistance and contributes to activation of profibrotic signaling in SSc fibroblasts. 
  [Ii]nvasive  PubMed  
 Differential activation of acid sphingomyelinase and ceramide release determines   invasive  ness of Neisseria meningitidis into brain endothelial cells. The interaction with brain endothelial cells is central to the pathogenicity of Neisseria meningitidis infections. Here, we show that N. meningitidis causes transient activation of acid sphingomyelinase (ASM) followed by ceramide release in brain endothelial cells. In response to N. meningitidis infection, ASM and ceramide are displayed at the outer leaflet of the cell membrane and condense into large membrane platforms which also concentrate the ErbB2 receptor. The outer membrane protein Opc and phosphatidylcholine-specific phospholipase C that is activated upon binding of the pathogen to heparan sulfate proteoglycans, are required for N. meningitidis-mediated ASM activation. Pharmacologic or genetic ablation of ASM abrogated meningococcal internalization without affecting bacterial adherence. In accordance, the restricted   invasive  ness of a defined set of pathogenic isolates of the ST-11/ST-8 clonal complex into brain endothelial cells directly correlated with their restricted ability to induce ASM and ceramide release. In conclusion, ASM activation and ceramide release are essential for internalization of Opc-expressing meningococci into brain endothelial cells, and this segregates with   invasive  ness of N. meningitidis strains. 
 Comprehensive Evaluation of Plasma 7-Ketocholesterol and Cholestan-3β,5α,6β-Triol in an Italian Cohort of Patients Affected by Niemann-Pick Disease due to NPC1 and SMPD1 Mutations. Niemann-Pick C disease (NPCD) is a rare autosomal recessive neurovisceral disorder with a heterogeneous clinical presentation. Cholestan-3β,5α,6β-triol and 7-ketocholesterol have been proposed as biomarkers for the screening of NPCD. In this work, we assessed oxysterols levels in a cohort of Italian patients affected by NPCD and analyzed the obtained results in the context of the clinical, biochemical and molecular data. In addition, a group of patients affected by Niemann-Pick B disease (NPBD) were also analyzed. NPC patients presented levels of both oxysterols way above the cut off value, except for 5 siblings presenting the variant biochemical phenotype who displayed levels of 3β,5α,6β-triol below or just above the cut-off value; 2 of them presented also normal levels of 7-KC. Both oxysterols were extremely high in a patient presenting the neonatal systemic lethal phenotype. All NPB patients showed increased oxysterols levels. In conclusion, the reported LC-MS/MS assay provides a ro  bust non  -invasive screening tool for NPCD. However, false negative results can be obtained in patients expressing the variant biochemical phenotype. These data strengthen the concept that the results should always be interpreted in the context of the patients' clinical picture and filipin staining and/or genetic studies might still be undertaken in patients with normal levels of oxysterols if symptoms are highly suggestive of NPCD. Both oxysterols are significantly elevated in NPB patients; thus a differential diagnosis should always be performed in patients presenting isolated hepatosplenomegaly, a common clinical sign of both NPCD and NPBD. 
  GeneRIFs  
 Data suggest   invasive  ness of Neisseria meningitidis depends on activation of SMPD1 and up-regulation of ceramide release to form ceramide-enriched platforms in cell membrane of endothelium of brain microvessels upon attachment of N. meningitidis. 
   
  
 PTPRB 
 
  NCBI Gene  
  GeneRIF  
  Pubmed  
 
 
  Search Term  Rank  Fields  
  [Ff]ilopodia  1  PubMed(1)  
  [Mm]igration  6  PubMed(1); GeneRIFs(1)  
  ECM  7  PubMed(1)  
  [Ee]xtracellular [Mm]atrix  8  PubMed(4)  
 
 
 hide/show details for PTPRB 
  [Ff]ilopodia  PubMed  
 Promotion of cell spreading and migration by vascular endothelial-protein tyrosine phosphatase (VE-PTP) in cooperation with integrins. Vascular endothelial-protein tyrosine phosphatase (VE-PTP) is a receptor-type protein tyrosine phosphatase with a single catalytic domain in its cytoplasmic region and multiple fibronectin type III-like domains in its extracellular region. VE-PTP is expressed specifically in endothelial cells and is implicated in regulation of angiogenesis. The molecular basis for such regulation by VE-PTP has remained largely unknown, however. We now show that forced expression of VE-PTP promoted cell spreading as well as formation of lamellipodia and   filopodia   in cultured fibroblasts plated on fibronectin. These effects of VE-PTP on cell morphology required its catalytic activity as well as activation of integrins and Ras. In addition, VE-PTP-induced cell spreading and lamellipodium formation were prevented by inhibition of Src family kinases or of Rac or Cdc42. Indeed, forced expression of VE-PTP increased the level of c-Src phosphorylation at tyrosine-416. Moreover, the VE-PTP-induced changes in cell morphology were suppressed by expression of dominant negative forms of FRG or Vav2, both of which are guanine nucleotide exchange factors for Rho family proteins and are activated by tyrosine phosphorylation. Forced expression of VE-PTP also enhanced fibronectin-dependent migration of cultured fibroblasts. Conversely, depletion of VE-PTP by RNA interference in human umbilical vein endothelial cells or mouse endothelioma cells inhibited cell spreading on fibronectin. These results suggest that VE-PTP, in cooperation with integrins, regulates the spreading and migration of endothelial cells during angiogenesis. 
  [Mm]igration  PubMed  
 Promotion of cell spreading and   migration   by vascular endothelial-protein tyrosine phosphatase (VE-PTP) in cooperation with integrins. Vascular endothelial-protein tyrosine phosphatase (VE-PTP) is a receptor-type protein tyrosine phosphatase with a single catalytic domain in its cytoplasmic region and multiple fibronectin type III-like domains in its extracellular region. VE-PTP is expressed specifically in endothelial cells and is implicated in regulation of angiogenesis. The molecular basis for such regulation by VE-PTP has remained largely unknown, however. We now show that forced expression of VE-PTP promoted cell spreading as well as formation of lamellipodia and filopodia in cultured fibroblasts plated on fibronectin. These effects of VE-PTP on cell morphology required its catalytic activity as well as activation of integrins and Ras. In addition, VE-PTP-induced cell spreading and lamellipodium formation were prevented by inhibition of Src family kinases or of Rac or Cdc42. Indeed, forced expression of VE-PTP increased the level of c-Src phosphorylation at tyrosine-416. Moreover, the VE-PTP-induced changes in cell morphology were suppressed by expression of dominant negative forms of FRG or Vav2, both of which are guanine nucleotide exchange factors for Rho family proteins and are activated by tyrosine phosphorylation. Forced expression of VE-PTP also enhanced fibronectin-dependent   migration   of cultured fibroblasts. Conversely, depletion of VE-PTP by RNA interference in human umbilical vein endothelial cells or mouse endothelioma cells inhibited cell spreading on fibronectin. These results suggest that VE-PTP, in cooperation with integrins, regulates the spreading and   migration   of endothelial cells during angiogenesis. 
  GeneRIFs  
 Suggest that VE-PTP, in cooperation with integrins, regulates the spreading and   migration   of endothelial cells during angiogenesis. 
  ECM  PubMed  
 Multi-ligand interactions with receptor-like protein tyrosine phosphatase beta: implications for intercellular signaling. Receptor-like protein tyrosine phosphatase beta (RPTP beta) shows structural and functional similarity to cell adhesion molecules (CAMs). It binds to several neuronal CAMs and extracellular matrix (  ECM  ) proteins that combine to form cell-recognition complexes. Here, the authors discuss the implications of such complexes for intercellular signaling, and the regulation of RPTP activity by cell-cell and cell-  ECM   contact. 
  [Ee]xtracellular [Mm]atrix  PubMed  
 Shear stress-induced redistribution of vascular endothelial-protein-tyrosine phosphatase (VE-PTP) in endothelial cells and its role in cell elongation. Vascular endothelial cells (ECs) are continuously exposed to shear stress (SS) generated by blood flow. Such stress plays a key role in regulation of various aspects of EC function including cell proliferation and motility as well as changes in cell morphology. Vascular endothelial-protein-tyrosine phosphatase (VE-PTP) is an R3-subtype PTP that possesses multiple fibronectin type III-like domains in its extracellular region and is expressed specifically in ECs. The role of VE-PTP in EC responses to SS has remained unknown, however. Here we show that VE-PTP is diffusely localized in ECs maintained under static culture conditions, whereas it undergoes rapid accumulation at the downstream edge of the cells relative to the direction of flow in response to SS. This redistribution of VE-PTP triggered by SS was found to require its extracellular and transmembrane regions and was promoted by integrin engagement of   extracellular matrix   ligands. Inhibition of actin polymerization or of Cdc42, Rab5, or Arf6 activities attenuated the SS-induced redistribution of VE-PTP. VE-PTP also underwent endocytosis in the static and SS conditions. SS induced the polarized distribution of internalized VE-PTP. Such an effect was promoted by integrin engagement of fibronectin but prevented by inhibition of Cdc42 activity or of actin polymerization. In addition, depletion of VE-PTP by RNA interference in human umbilical vein ECs blocked cell elongation in the direction of flow induced by SS. Our results suggest that the polarized redistribution of VE-PTP in response to SS plays an important role in the regulation of EC function by blood flow. 
 Phosphacan short isoform, a novel non-proteoglycan variant of phosphacan/receptor protein tyrosine phosphatase-beta, interacts with neuronal receptors and promotes neurite outgrowth. Phosphacan, one of the principal proteoglycans in the   extracellular matrix   of the central nervous system, is implicated in neuron-glia interactions associated with neuronal differentiation and myelination. We report here the identification of a novel truncated form of phosphacan, phosphacan short isoform (PSI), that corresponds to the N-terminal carbonic anhydrase- and fibronectin type III-like domains and half of the spacer region. The novel cDNA transcript was isolated by screening of a neonatal brain cDNA expression library using a polyclonal antibody raised against phosphacan. Expression of this transcript in vivo was confirmed by Northern blot hybridization. Analysis of brain protein extracts reveals the presence of a 90-kDa glycosylated protein in the phosphate-buffered saline-insoluble 100000 x g fraction that reacts with antisera against both phosphacan and a recombinant PSI protein and that has the predicted N-terminal sequence. This protein is post-translationally modified with oligosaccharides, including the HNK-1 epitope, but, unlike phosphacan, it is not a proteoglycan. The expression of the PSI protein varies during central nervous system development in a fashion similar to that observed for phosphacan, being first detected around embryonic day 16 and then showing a dramatic increase in expression to plateau around the second week post-natal. Both the native and recombinant PSI protein can interact with the Ig cell adhesion molecules, F3/contactin and L1, and in neurite outgrowth assays, the PSI protein can promote outgrowth of cortical neurons when used as a coated substrate. Hence, the identification of this novel isoform of phosphacan/receptor protein tyrosine phosphatase-beta provides a new component in cell-cell and cell-  extracellular matrix   signaling events in which these proteins have been implicated. 
 Multi-ligand interactions with receptor-like protein tyrosine phosphatase beta: implications for intercellular signaling. Receptor-like protein tyrosine phosphatase beta (RPTP beta) shows structural and functional similarity to cell adhesion molecules (CAMs). It binds to several neuronal CAMs and   extracellular matrix   (ECM) proteins that combine to form cell-recognition complexes. Here, the authors discuss the implications of such complexes for intercellular signaling, and the regulation of RPTP activity by cell-cell and cell-ECM contact. 
 Glial tumor cell adhesion is mediated by binding of the FNIII domain of receptor protein tyrosine phosphatase beta (RPTPbeta) to tenascin C. The extracellular domain of receptor protein tyrosine phosphatase beta (RPTPbeta) is composed of several domains which mediate its interactions with distinct ligands present on the surface of either neurons or glial cells. Here, we demonstrate that the fibronectin type III domain (FNIII) of RPTPbeta binds to glial tumor-derived cell lines and primary astrocytes. We used affinity purification to isolate several proteins that specifically bind to the FNIII domain of RPTPbeta. One of these, a 240 kDa protein that was purified from U118MG glioblastoma cell, was identified as tenascin C based on the amino acid sequence of several tryptic peptides. The interaction of RPTPbeta with tenascin C was found to mediate cell adhesion. Adhesion and spreading of SF763T astrocytoma cells expressing RPTPbeta on tenascin C was specifically abolished by the addition of a soluble fragment containing the FNIII domain of the receptor. RPTPbeta-dependent cell adhesion was mediated by binding to the alternatively spliced FNIII repeats A1,2,4 (TnfnA1,2,4) of tenascin C. Furthermore, COS cells expressing RPTPbeta adhere to TnfnA1,2,4, while the parental cells did not. These results demonstrate that the FNIII domain of RPTPbeta binds to tenascin C and suggest that RPTPbeta present on glial tumor cells is a primary adhesion receptor system to the   extracellular matrix  . 
   
  
 ITGB1 
 
  NCBI Gene  
  GeneRIF  
  Pubmed  
 
 
  Search Term  Rank  Fields  
  [Ff]ilopodia  1  PubMed(5); GeneRIFs(1)  
  [Ff]ilopodium  2  PubMed(1); Component(1)  
  [Ii]nvadopodia  3  PubMed(4); GeneRIFs(2)  
  [Ii]nvadopodium  4  PubMed(1); Component(1)  
  [Mm]igration  6  Process(4); PubMed(302); GeneRIFs(89)  
  ECM  7  PubMed(80); GeneRIFs(6)  
  [Ee]xtracellular [Mm]atrix  8  Process(1); PubMed(215); GeneRIFs(16)  
  [Ii]nvasive  10  PubMed(87); GeneRIFs(22)  
  [Mm]etastasize  11  PubMed(3)  
  [Mm]etastasis  12  PubMed(134); GeneRIFs(30)  
  [Cc]ell [Ii]nvasion  13  PubMed(55); GeneRIFs(5)  
 
 
 hide/show details for ITGB1 
  [Ff]ilopodia  PubMed  
 Functional relevance of urinary-type plasminogen activator receptor-alpha3beta1 integrin association in proteinase regulatory pathways. Squamous cell carcinoma of the oral cavity is characterized by persistent, disorganized expression of integrin alpha3beta1 and enhanced production of urinary-type plasminogen activator (uPA) and its receptor (uPAR) relative to normal oral mucosa. Because multivalent aggregation of alpha3beta1 integrin up-regulates uPA and induces a dramatic co-clustering of uPAR, we explored the hypothesis that lateral ligation of alpha3beta1 integrin by uPAR contributes to uPA regulation in oral mucosal cells. To investigate mechanisms by which uPAR/alpha3beta1 binding enhances uPA expression, integrin-dependent signal activation was assessed. Both Src and ERK1/2 were phosphorylated in response to integrin aggregation, and blocking Src kinase activity completely abrogated ERK1/2 activation and uPA induction, whereas inhibition of epidermal growth factor receptor tyrosine kinase activity did not alter uPA expression. Proteinase up-regulation occurred at the transcriptional level and mutation of the AP1 (-1967) site in the uPA promoter blocked the uPAR/integrin-mediated transcriptional activation. Because uPAR is redistributed to clustered alpha3beta1 integrins, the requirement for uPAR/alpha3beta1 interaction in uPA regulation was assessed. Clustering of alpha3beta1 in the presence of a peptide (alpha325) that disrupts uPAR/alpha3beta1 integrin binding prevented uPA induction. Depletion of cell surface uPAR using small interfering RNA also blocked uPA induction following integrin alpha3beta1 clustering. These results were confirmed using a genetic strategy in which alpha3 null epithelial cells reconstituted with wild type alpha3 integrin, but not a mutant alpha3 unable to bind uPAR, induced uPA expression upon integrin clustering, confirming the critical role of uPAR in integrin-regulated proteinase expression. Disruption of uPAR/alpha3beta1 binding using peptide alpha325 or small interfering RNA blocked   filopodia   formation and matrix invasion, indicating that this interaction stimulates invasive behavior. Together these data support a model wherein matrix-induced clustering ofalpha3beta1 integrin promotes uPAR/alpha3beta1 interaction, thereby potentiating cellular signal transduction pathways culminating in activation of uPA expression and enhanced uPA-dependent invasive behavior. 
 α5β1 integrin recycling promotes Arp2/3-independent cancer cell invasion via the formin FHOD3. Invasive migration in 3D extracellular matrix (ECM) is crucial to cancer metastasis, yet little is known of the molecular mechanisms that drive reorganization of the cytoskeleton as cancer cells disseminate in vivo. 2D Rac-driven lamellipodial migration is well understood, but how these features apply to 3D migration is not clear. We find that lamellipodia-like protrusions and retrograde actin flow are indeed observed in cells moving in 3D ECM. However, Rab-coupling protein (RCP)-driven endocytic recycling of α5β1 integrin enhances invasive migration of cancer cells into fibronectin-rich 3D ECM, driven by RhoA   and filop  odial spike-based protrusions, not lamellipodia. Furthermore, we show that actin spike protrusions are Arp2/3-independent. Dynamic actin spike assembly in cells invading in vitro and in vivo is regulated by Formin homology-2 domain containing 3 (FHOD3), which is activated by RhoA/ROCK, establishing a novel mechanism through which the RCP-α5β1 pathway reprograms the actin cytoskeleton to promote invasive migration and local invasion in vivo. 
 EWI-2 regulates alpha3beta1 integrin-dependent cell functions on laminin-5. EWI-2, a cell surface immunoglobulin SF protein of unknown function, associates with tetraspanins CD9 and CD81 with high stoichiometry. Overexpression of EWI-2 in A431 epidermoid carcinoma cells did not alter cell adhesion or spreading on laminin-5, and had no effect on reaggregation of cells plated on collagen I (alpha2beta1 integrin ligand). However, on laminin-5 (alpha3beta1 integrin ligand), A431 cell reaggregation and motility functions were markedly impaired. Immunodepletion and reexpression experiments revealed that tetraspanins CD9 and CD81 physically link EWI-2 to alpha3beta1 integrin, but not to other integrins. CD81 also controlled EWI-2 maturation and cell surface localization. EWI-2 overexpression not only suppressed cell migration, but also redirected CD81 to cell   filopodia   and enhanced alpha3beta1-CD81 complex formation. In contrast, an EWI-2 chimeric mutant failed to suppress cell migration, redirect CD81 to   filopodia  , or enhance alpha3beta1-CD81 complex formation. These results show how laterally associated EWI-2 might regulate alpha3beta1 function in disease and development, and demonstrate how tetraspanin proteins can assemble multiple nontetraspanin proteins into functional complexes. 
 Structural basis of cargo recognition by the myosin-X MyTH4-FERM domain. Myosin-X is an important unconventional myosin that is critical for cargo transportation to   filopodia   tips and is also utilized in spindle assembly by interacting with microtubules. We present a series of structural and biochemical studies of the myosin-X tail domain cassette, consisting of myosin tail homology 4 (MyTH4) and FERM domains in complex with its specific cargo, a netrin receptor DCC (deleted in colorectal cancer). The MyTH4 domain is folded into a helical VHS-like structure and is associated with the FERM domain. We found an unexpected binding mode of the DCC peptide to the subdomain C groove of the FERM domain, which is distinct from previously reported β-β associations found in radixin-adhesion molecule complexes. We also revealed direct interactions between the MyTH4-FERM cassette and tubulin C-terminal acidic tails, and identified a positively charged patch of the MyTH4 domain, which is involved in tubulin binding. We demonstrated that both DCC and integrin bindings interfere with microtubule binding and that DCC binding interferes with integrin binding. Our results provide the molecular basis by which myosin-X facilitates alternative dual binding to cargos and microtubules. 
 The integrin alpha6beta1 modulation of PI3K and Cdc42 activities induces dynamic filopodium formation in human platelets. Platelets are an ideal model for studying a rapid morphological change in response to various signal transduction systems. Morphological changes via the activation of integrin alphaIIbbeta3 in platelets have been investigated intensively. In contrast, activation via integrin alpha6beta1 is less well studied. Here, we provide the first biochemical evidence that integrins alpha6beta1 and alphaIIbbeta3 of platelets are associated with different membrane proteins. We also demonstrate that platelets activated by integrin alpha6beta1 show dynamic change by actively forming   filopodia   and never fully spreading over a period of more than an hour. In addition, platelets activated by integrin alpha6beta1 are different from those activated by integrin alphaIIbbeta3 in terms of cell-substrate contact and in their distribution pattern of actin, Arp2/3 and various phosphotyrosine proteins. The morphological appearance of platelets produced through integrin alpha6beta1 activation is highly dependent on PI3 kinase (PI3K) but less dependent on Src kinase. Suppression of PI3K activity in integrin alpha6beta1 activated platelets induces an increase in Cdc42 activity and more filopodium formation. However, both Cdc42 and PI3K activity are higher in platelets activated by integrin alpha6beta1 than in those activated by integrin alphaIIbbeta3. Taken together, this study demonstrates that the signals induced by integrin alpha6beta1 modulate at the level of PI3K and Cdc42 activity to allow platelets to actively form   filopodia  . 
  GeneRIFs  
 the signals induced by integrin alpha6beta1 modulate at the level of PI3K and Cdc42 activity to allow platelets to actively form   filopodia   
  [Ff]ilopodium  PubMed  
 The integrin alpha6beta1 modulation of PI3K and Cdc42 activities induces dynamic   filopodium   formation in human platelets. Platelets are an ideal model for studying a rapid morphological change in response to various signal transduction systems. Morphological changes via the activation of integrin alphaIIbbeta3 in platelets have been investigated intensively. In contrast, activation via integrin alpha6beta1 is less well studied. Here, we provide the first biochemical evidence that integrins alpha6beta1 and alphaIIbbeta3 of platelets are associated with different membrane proteins. We also demonstrate that platelets activated by integrin alpha6beta1 show dynamic change by actively forming filopodia and never fully spreading over a period of more than an hour. In addition, platelets activated by integrin alpha6beta1 are different from those activated by integrin alphaIIbbeta3 in terms of cell-substrate contact and in their distribution pattern of actin, Arp2/3 and various phosphotyrosine proteins. The morphological appearance of platelets produced through integrin alpha6beta1 activation is highly dependent on PI3 kinase (PI3K) but less dependent on Src kinase. Suppression of PI3K activity in integrin alpha6beta1 activated platelets induces an increase in Cdc42 activity and more   filopodium   formation. However, both Cdc42 and PI3K activity are higher in platelets activated by integrin alpha6beta1 than in those activated by integrin alphaIIbbeta3. Taken together, this study demonstrates that the signals induced by integrin alpha6beta1 modulate at the level of PI3K and Cdc42 activity to allow platelets to actively form filopodia. 
  Component  
   filopodium   
  [Ii]nvadopodia  PubMed  
 A novel protease-docking function of integrin at   invadopodia  .   Invadopodia   are membrane extensions of aggressive tumor cells that function in the activation of membrane-bound proteases occurring during tumor cell invasion. We explore a novel and provocative activity of integrins in docking proteases to sites of invasion, termed   invadopodia  . In the absence of collagen, alpha(3)beta(1) integrin and the gelatinolytic enzyme, seprase, exist as nonassociating membrane proteins. Type I collagen substratum induces the association of alpha(3)beta(1) integrin with seprase as a complex on   invadopodia  . The results show that alpha(3)beta(1) integrin is a docking protein for seprase to form functional   invadopodia  . In addition, alpha(5)beta(1) integrin may participate in the adhesion process necessary for   invadopodia  l formation. Thus, alpha(3)beta(1) and alpha(5)beta(1) integrins play major organizational roles in the adhesion and formation of   invadopodia  , promoting invasive cell behavior. 
 β1 integrin regulates Arg to promote   invadopodi  al maturation and matrix degradation. β1 integrin has been shown to promote metastasis in a number of tumor models, including breast, ovarian, pancreatic, and skin cancer; however, the mechanism by which it does so is poorly understood. Invasive membrane protrusions calle  d invadopod  ia are believed to facilitate extracellular matrix degradation and intravasation during metastasis. Previous work showed that β1 integrin localizes   to invadopo  dia, but its role in regulati  ng invadopo  dial function has not been well characterized. We find that β1 integrin is required for the formation of mature, degradation-compet  ent invadop  odia in both two- and three-dimensional matrices but is dispensable for invadopodium precursor formation in metastatic human breast cancer cells. β1 integrin is activated during invadopodium precursor maturation, and forced β1 integrin activation enhances the ra  te of invad  opodial matrix proteolysis. Furthermore, β1 integrin interacts with the tyrosine kinase Arg and stimulates Arg-dependent phosphorylation of cortactin on tyrosine 421. Silencing β1 integrin with small interfering RNA completely abrogates Arg-dependent cortactin phosphorylation and cofilin-dependent barbed-end forma  tion at inv  adopodia, leading to a significant decrease in the number and stability of   mature inv  adopodia. These results describe a fundamental role for β1 integrin in controlling actin polymerization-d  ependent in  vadopodial maturation and matrix degradation in metastatic tumor cells. 
 Molecular proximity of seprase and the urokinase-type plasminogen activator receptor on malignant melanoma cell membranes: dependence on beta1 integrins and the cytoskeleton. Previous studies have shown that several proteolytic enzymes are associated with membrane protrusions at the leading edge of migrating tumor cells. In this study we demonstrate that seprase and the urokinase plasminogen activator receptor (uPAR), co-localize in the plasma membrane of LOX malignant melanoma cells. Cells were labeled with fluorochrome-conjugated monoclonal antibodies (mAb) directed against seprase and uPAR. Proximity between these two molecules was detected with resonance energy transfer (RET) imaging, single-cell emission spectrophotometry, and single-cell excitation spectrophotometry. Significant RET signals were detected on LOX cells when adherent to uncoated and extracellular matrix (ECM)-coated surfaces. This indicates that seprase and uPAR are within approximately 7 nm in the plasma membrane of LOX cells. When LOX cells adhered to a 3D extracellular-like matrix, seprase-uPAR complexes were found to be associated with   invadopodia  . Further microscopy experiments demonstrated gelatinolytic activity, a functional attribute of seprase, in association with seprase-uPAR membrane domains. Formation of seprase-uPAR membrane complexes is dependent upon both the cytoskeleton and integrins. Specifically, the involvement of beta(1)-integrins was demonstrated by the inhibition of RET by an inhibitory anti-beta(1)-integrin mAb. Based on these findings, we speculate that formation of heterogeneous lytic domains in the invading membranes of LOX cells increases the efficiency of directed pericellular proteolysis. 
 ß1 integrin binding phosphorylates ezrin at T567 to activate a lipid raft signalsome driving   invadopodi  a activity and invasion. Extracellular matrix (ECM) degradation is a critical process in tumor cell invasion and requires matrix degrading protrusions called   invadopodi  a. The Na(+)/H(+) exchanger (NHE1) has recently been shown to be fundamental in the regulation of   invadopodi  a actin cytoskeleton dynamics and activity. However, the structural link between the   invadopodi  a cytoskeleton and NHE1 is still unknown. A candidate could be ezrin, a linker between the NHE1 and the actin cytoskeleton known to play a pivotal role in invasion and metastasis. However, the mechanistic basis for its role remains unknown. Here, we demonstrate that ezrin phosphorylated at T567 is highly overexpressed in the membrane of human breast tumors and positively associated with invasive growth and HER2 overexpression. Further, in the metastatic cell line, MDA-MB-231, p-ezrin was almost exclusively expressed in   invadopodi  a lipid rafts where it co-localized in a functional complex with NHE1, EGFR, ß1-integrin and phosphorylated-NHERF1. Manipulation by mutation of ezrins T567 phosphorylation state and/or PIP2 binding capacity or of NHE1s binding to ezrin or PIP2 demonstrated that p-ezrin expression and binding to PIP2 are required fo  r invadopod  ia-mediated ECM degradation and invasion and identified NHE1 as the membrane protein that p-ezrin regulates to induc  e invadopod  ia formation and activity. 
  GeneRIFs  
 ss1 integrin binding phosphorylates ezrin at T567 to activate a lipid raft signalsome driving   invadopodia   activity and invasion. 
 Beta1 integrin regulates Arg to promote   invadopodia  l maturation and matrix degradation. 
  [Ii]nvadopodium  PubMed  
 β1 integrin regulates Arg to promote invadopodial maturation and matrix degradation. β1 integrin has been shown to promote metastasis in a number of tumor models, including breast, ovarian, pancreatic, and skin cancer; however, the mechanism by which it does so is poorly understood. Invasive membrane protrusions called invadopodia are believed to facilitate extracellular matrix degradation and intravasation during metastasis. Previous work showed that β1 integrin localizes to invadopodia, but its role in regulating invadopodial function has not been well characterized. We find that β1 integrin is required for the formation of mature, degradation-competent invadopodia in both two- and three-dimensional matrices but is dispensable   for invadopo  dium precursor formation in metastatic human breast cancer cells. β1 integrin is activated du  ring invadop  odium precursor maturation, and forced β1 integrin activation enhances the rate of invadopodial matrix proteolysis. Furthermore, β1 integrin interacts with the tyrosine kinase Arg and stimulates Arg-dependent phosphorylation of cortactin on tyrosine 421. Silencing β1 integrin with small interfering RNA completely abrogates Arg-dependent cortactin phosphorylation and cofilin-dependent barbed-end formation at invadopodia, leading to a significant decrease in the number and stability of mature invadopodia. These results describe a fundamental role for β1 integrin in controlling actin polymerization-dependent invadopodial maturation and matrix degradation in metastatic tumor cells. 
  Component  
   invadopodium   membrane 
  [Mm]igration  Process  
 cell   migration   
 cell   migration   involved in sprouting angiogenesis 
 germ cell   migration   
 leukocyte   migration   
  PubMed  
 Pro-prion binds filamin A, facilitating its interaction with integrin beta1, and contributes to melanomagenesis. Filamin A (FLNA) is an integrator of cell mechanics and signaling. The spreading and   migration   observed in FLNA sufficient A7 melanoma cells but not in the parental FLNA deficient M2 cells have been attributed to FLNA. In A7 and M2 cells, the normal prion (PrP) exists as pro-PrP, retaining its glycosylphosphatidyl-inositol (GPI) anchor peptide signal sequence (GPI-PSS). The GPI-PSS of PrP has a FLNA binding motif and binds FLNA. Reducing PrP expression in A7 cells alters the spatial distribution of FLNA and organization of actin and diminishes cell spreading and   migration  . Integrin β1 also binds FLNA. In A7 cells, FLNA, PrP, and integrin β1 exist as two independent, yet functionally linked, complexes; they are FLNA with PrP or FLNA with integrin β1. Reducing PrP expression in A7 cells decreases the amount of integrin β1 bound to FLNA. A PrP GPI-PSS synthetic peptide that crosses the cell membrane inhibits A7 cell spreading   and migra  tion. Thus, in A7 cells FLNA does not act alone; the binding of pro-PrP enhances association between FLNA and integrin β1, which then promotes cell spreading   and migr  ation. Pro-PrP is detected in melanoma in situ but not in melanocyte. Invasive melanoma has more pro-PrP. The binding of pro-PrP to FLNA, therefore, contributes to melanomagenesis. 
 Androgen-induced cell   migration  : role of androgen receptor/filamin A association. Androgen receptor (AR) controls male morphogenesis, gametogenesis and prostate growth as well as development of prostate cancer. These findings support a role for AR in cell   migration   and invasiveness. However, the molecular mechanism involved in AR-mediated cell   migration   still remains elusive.Mouse embryo NIH3T3 fibroblasts and highly metastatic human fibrosarcoma HT1080 cells harbor low levels of transcriptionally incompetent AR. We now report that, through extra nuclear action, AR triggers   migration   of both cell types upon stimulation with physiological concentrations of the androgen R1881. We analyzed the initial events leading to androgen-induced cell   migration   and observed that challenging NIH3T3 cells with 10 nM R1881 rapidly induces interaction of AR with filamin A (FlnA) at cytoskeleton. AR/FlnA complex recruits integrin beta 1, thus activating its dependent cascade. Silencing of AR, FlnA and integrin beta 1 shows that this ternary complex controls focal adhesion kinase (FAK), paxillin and Rac, thereby driving cell   migration  . FAK-null fibroblasts migrate poorly and Rac inhibition by EHT impairs motility of androgen-treated NIH3T3 cells. Interestingly, FAK and Rac activation by androgens are independent of each other. Findings in human fibrosarcoma HT1080 cells strengthen the role of Rac in androgen signaling. The Rac inhibitor significantly impairs androgen-induced   migration   in these cells. A mutant AR, deleted of the sequence interacting with FlnA, fails to mediate FAK activation and paxillin tyrosine phosphorylation in androgen-stimulated cells, further reinforcing the role of AR/FlnA interaction in androgen-mediated motility.The present report, for the first time, indicates that the extra nuclear AR/FlnA/integrin beta 1 complex is the key by which androgen activates signaling leading to cell   migration  . Assembly of this ternary complex may control organ development and prostate cancer metastasis. 
 Analysis of the myosin-II-responsive focal adhesion proteome reveals a role for β-Pix in negative regulation of focal adhesion maturation. Focal adhesions undergo myosin-II-mediated maturation wherein they grow and change composition to modulate integrin signalling for cell   migratio  n, growth and differentiation. To determine how focal adhesion composition is affected by myosin II activity, we performed proteomic analysis of isolated focal adhesions and compared protein abundance in focal adhesions from cells with and without myosin II inhibition. We identified 905 focal adhesion proteins, 459 of which changed in abundance with myosin II inhibition, defining the myosin-II-responsive focal adhesion proteome. The abundance of 73% of the proteins in the myosin-II-responsive focal adhesion proteome was enhanced by contractility, including proteins involved in Rho-mediated focal adhesion maturation and endocytosis- and calpain-dependent focal adhesion disassembly. During myosin II inhibition, 27% of proteins in the myosin-II-responsive focal adhesion proteome, including proteins involved in Rac-mediated lamellipodial protrusion, were enriched in focal adhesions, establishing that focal adhesion protein recruitment is also negatively regulated by contractility. We focused on the Rac guanine nucleotide exchange factor β-Pix, documenting its role in the negative regulation of focal adhesion maturation and the promotion of lamellipodial protrusion and focal adhesion turnover to drive cel  l migrati  on. 
 Androgen-induced cell   migration  : role of androgen receptor/filamin A association. Androgen receptor (AR) controls male morphogenesis, gametogenesis and prostate growth as well as development of prostate cancer. These findings support a role for AR in cell   migration   and invasiveness. However, the molecular mechanism involved in AR-mediated cell   migration   still remains elusive.Mouse embryo NIH3T3 fibroblasts and highly metastatic human fibrosarcoma HT1080 cells harbor low levels of transcriptionally incompetent AR. We now report that, through extra nuclear action, AR triggers   migration   of both cell types upon stimulation with physiological concentrations of the androgen R1881. We analyzed the initial events leading to androgen-induced cell   migration   and observed that challenging NIH3T3 cells with 10 nM R1881 rapidly induces interaction of AR with filamin A (FlnA) at cytoskeleton. AR/FlnA complex recruits integrin beta 1, thus activating its dependent cascade. Silencing of AR, FlnA and integrin beta 1 shows that this ternary complex controls focal adhesion kinase (FAK), paxillin and Rac, thereby driving cell   migration  . FAK-null fibroblasts migrate poorly and Rac inhibition by EHT impairs motility of androgen-treated NIH3T3 cells. Interestingly, FAK and Rac activation by androgens are independent of each other. Findings in human fibrosarcoma HT1080 cells strengthen the role of Rac in androgen signaling. The Rac inhibitor significantly impairs androgen-induced   migration   in these cells. A mutant AR, deleted of the sequence interacting with FlnA, fails to mediate FAK activation and paxillin tyrosine phosphorylation in androgen-stimulated cells, further reinforcing the role of AR/FlnA interaction in androgen-mediated motility.The present report, for the first time, indicates that the extra nuclear AR/FlnA/integrin beta 1 complex is the key by which androgen activates signaling leading to cell   migration  . Assembly of this ternary complex may control organ development and prostate cancer metastasis. 
 Pro-prion binds filamin A, facilitating its interaction with integrin beta1, and contributes to melanomagenesis. Filamin A (FLNA) is an integrator of cell mechanics and signaling. The spreading and   migration   observed in FLNA sufficient A7 melanoma cells but not in the parental FLNA deficient M2 cells have been attributed to FLNA. In A7 and M2 cells, the normal prion (PrP) exists as pro-PrP, retaining its glycosylphosphatidyl-inositol (GPI) anchor peptide signal sequence (GPI-PSS). The GPI-PSS of PrP has a FLNA binding motif and binds FLNA. Reducing PrP expression in A7 cells alters the spatial distribution of FLNA and organization of actin and diminishes cell spreading and   migration  . Integrin β1 also binds FLNA. In A7 cells, FLNA, PrP, and integrin β1 exist as two independent, yet functionally linked, complexes; they are FLNA with PrP or FLNA with integrin β1. Reducing PrP expression in A7 cells decreases the amount of integrin β1 bound to FLNA. A PrP GPI-PSS synthetic peptide that crosses the cell membrane inhibits A7 cell spreading   and migra  tion. Thus, in A7 cells FLNA does not act alone; the binding of pro-PrP enhances association between FLNA and integrin β1, which then promotes cell spreading   and migr  ation. Pro-PrP is detected in melanoma in situ but not in melanocyte. Invasive melanoma has more pro-PrP. The binding of pro-PrP to FLNA, therefore, contributes to melanomagenesis. 
 Caveolin-1-dependent beta1 integrin endocytosis is a critical regulator of fibronectin turnover. beta1 integrins are major cell surface receptors for fibronectin. Some integrins, including beta1 integrins, are known to undergo constitutive endocytosis and recycling. Integrin endocytosis/recycling has been implicated in the regulation of cell   migration  . However, the mechanisms by which integrin endocytosis/recycling regulates cell   migration  , and other biological consequences of integrin trafficking are not completely understood. We previously showed that turnover of extracellular matrix (ECM) fibronectin occurs via receptor-mediated endocytosis. Here, we investigate the biological relevance of beta1 integrin endocytosis to fibronectin matrix turnover. First, we demonstrate that beta1 integrins, including alpha5beta1 play an important role in endocytosis and turnover of matrix fibronectin. Second, we show that caveolin-1 constitutively regulates endocytosis of alpha5beta1 integrins, and that alpha5beta1 integrin endocytosis can occur in the absence of fibronectin and fibronectin matrix. We also show that downregulation of caveolin-1 expression by siRNA results in marked reduction of beta1 integrin and fibronectin endocytosis. Hence, caveolin-1-dependent beta1 integrin and fibronectin endocytosis plays a critical role in fibronectin matrix turnover, and may contribute to abnormal ECM remodeling that occurs in fibrotic disorders. 
 Focal adhesion kinase modulates cell adhesion strengthening via integrin activation. Focal adhesion kinase (FAK) is an essential nonreceptor tyrosine kinase regulating cell   migration  , adhesive signaling, and mechanosensing. Using FAK-null cells expressing FAK under an inducible promoter, we demonstrate that FAK regulates the time-dependent generation of adhesive forces. During the early stages of adhesion, FAK expression in FAK-null cells enhances integrin activation to promote integrin binding and, hence, the adhesion strengthening rate. Importantly, FAK expression regulated integrin activation, and talin was required for the FAK-dependent effects. A role for FAK in integrin activation was confirmed in human fibroblasts with knocked-down FAK expression. The FAK autophosphorylation Y397 site was required for the enhancements in adhesion strengthening and integrin-binding responses. This work demonstrates a novel role for FAK in integrin activation and the time-dependent generation of cell-ECM forces. 
 Urokinase-type plasminogen activator receptor (uPAR) ligation induces a raft-localized integrin signaling switch that mediates the hypermotile phenotype of fibrotic fibroblasts. The urokinase-type plasminogen activator receptor (uPAR) is a glycosylphosphatidylinositol-linked membrane protein with no cytosolic domain that localizes to lipid raft microdomains. Our laboratory and others have documented that lung fibroblasts from patients with idiopathic pulmonary fibrosis (IPF) exhibit a hypermotile phenotype. This study was undertaken to elucidate the molecular mechanism whereby uPAR ligation with its cognate ligand, urokinase, induces a motile phenotype in human lung fibroblasts. We found that uPAR ligation with the urokinase receptor binding domain (amino-terminal fragment) leads to enhanced   migration   of fibroblasts on fibronectin in a protease-independent, lipid raft-dependent manner. Ligation of uPAR with the amino-terminal fragment recruited α5β1 integrin and the acylated form of the Src family kinase, Fyn, to lipid rafts. The biological consequences of this translocation were an increase in fibroblast motility and a switch of the integrin-initiated signal pathway fo  r migrati  on away from the lipid raft-independent focal adhesion kinase pathway and toward a lipid raft-dependent caveolin-Fyn-Shc pathway. Furthermore, an integrin homologous peptide as well as an antibody that competes with β1 for uPAR binding have the ability to block this effect. In addition, its relative insensitivity to cholesterol depletion suggests that the interactions of α5β1 integrin and uPAR drive the translocation of α5β1 integrin-acylated Fyn signaling complexes into lipid rafts upon uPAR ligation through protein-protein interactions. This signal switch is a novel pathway leading to the hypermotile phenotype of IPF patient-derived fibroblasts, seen with uPAR ligation. This uPAR dependent, fibrotic matrix-selective, and profibrotic fibroblast phenotype may be amenable to targeted therapeutics designed to ameliorate IPF. 
 The diacylglycerol kinase α/atypical PKC/β1 integrin pathway in SDF-1α mammary carcinoma invasiveness. Diacylglycerol kinase α (DGKα), by phosphorylating diacylglycerol into phosphatidic acid, provides a key signal driving   cell migr  ation and matrix invasion. We previously demonstrated that in epithelial cells activation of DGKα activity promotes cytoskeletal remodeling and matrix invasion by recruiting atypical PKC at ruffling sites and by promoting RCP-mediated recycling of α5β1 integrin to the tip of pseudopods. In here we investigate the signaling pathway by which DGKα mediates SDF-1α-induced matrix invasion of MDA-MB-231 invasive breast carcinoma cells. Indeed we showed that, following SDF-1α stimulation, DGKα is activated and localized at cell protrusion, thus promoting their elongation and mediating SDF-1α induced MMP-9 metalloproteinase secretion and matrix invasion. Phosphatidic acid generated by DGKα promotes localization at cell protrusions of atypical PKCs which play an essential role downstream of DGKα by promoting Rac-mediated protrusion elongation and localized recruitment of β1 integrin and MMP-9. We finally demonstrate that activation of DGKα, atypical PKCs signaling and β1 integrin are all essential for MDA-MB-231 invasiveness. These data indicates the existence of a SDF-1α induced DGKα - atypical PKC - β1 integrin signaling pathway, which is essential for matrix invasion of carcinoma cells. 
 Mammary-derived growth inhibitor (MDGI) interacts with integrin α-subunits and suppresses integrin activity and invasion. The majority of mortality associated with cancer is due to formation of metastases from the primary tumor. Adhesion mediated by different integrin heterodimers has an important role during cell   migratio  n and invasion. Protein interactions with the β1-integrin cytoplasmic tail are known to influence integrin affinity for extracellular ligands, but regulating binding partners for the α-subunit cytoplasmic tails have remained elusive. In this study, we show that mammary-derived growth inhibitor (MDGI) (also known as FABP-3 or H-FABP) binds directly to the cytoplasmic tail of integrin α-subunits and its expression inhibits integrin activity. In breast cancer cell lines, MDGI expression correlates with suppression of the active conformation of integrins. This results in reduced integrin adhesion to type I collagen and fibronectin and inhibition of c  ell migra  tion and invasion. In tissue microarray of 1331 breast cancer patients, patients with MDGI-positive tumors had more favorable 10-year distant disease-free survival compared with patients with MDGI-negative tumors. Our data indicate that MDGI is a novel interacting partner for integrin α-subunits, and its expression modulates integrin activity and suppresses cell invasion in breast cancer patients. Retained MDGI expression is associated with favorable prognosis. 
 Junctional adhesion molecule A interacts with Afadin and PDZ-GEF2 to activate Rap1A, regulate beta1 integrin levels, and enhance cell   migration  . Junctional adhesion molecule-A (JAM-A) is a transmembrane tight junction protein that has been shown to regulate barrier function and cell   migration   through incompletely understood mechanisms. We have previously demonstrated that JAM-A regulates cell   migration   by dimerization of the membrane-distal immunoglobulin-like loop and a C-terminal postsynaptic density 95/disc-large/zona occludens (PDZ) binding motif. Disruption of dimerization resulted in decreased epithelial cell   migration   secondary to diminished levels of beta1 integrin and active Rap1. Here, we report that JAM-A is physically and functionally associated with the PDZ domain-containing molecules Afadin and PDZ-guanine nucleotide exchange factor (GEF) 2, but not zonula occludens (ZO)-1, in epithelial cells, and these interactions mediate outside-in signaling events. Both Afadin and PDZ-GEF2 colocalized and coimmunoprecipitated with JAM-A. Furthermore, association of PDZ-GEF2 with Afadin was dependent on the expression of JAM-A. Loss of JAM-A, Afadin, or PDZ-GEF2, but not ZO-1 or PDZ-GEF1, similarly decreased cellular levels of activated Rap1, beta1 integrin protein, and epithelial cell   migration  . The functional effects observed were secondary to decreased levels of Rap1A because knockdown of Rap1A, but not Rap1B, resulted in decreased beta1 integrin levels and reduced cell   migration  . These findings suggest that JAM-A dimerization facilitates formation of a complex with Afadin and PDZ-GEF2 that activates Rap1A, which regulates beta1 integrin levels and cell   migration  . 
 Gln-362 of angiopoietin-2 mediates   migration   of tumor and endothelial cells through association with α5β1 integrin. Angiopoietin-2 (Ang-2) not only regulates angiogenesis by binding to its well known receptor Tie2 on endothelial cells but also controls sprouting of Tie2-negative angiogenic endothelial cells and invasion of Tie2-negative non-endothelial cells by binding to integrins. However, the molecular mechanism of the Ang-2/integrin association has been unclear. In this study, we found that the Gln-362 residue of Ang-2 was essential for binding to α5β1 integrin. A Q362E Ang-2 mutant, which still bound to Tie2, failed to associate with α5β1 integrin and was unable to activate the integrin downstream signaling of focal adhesion kinase. In addition, unlike wild-type Ang-2, the Q362E Ang-2 mutant was defective in mediating invasion of Tie2-negative glioma or Tie2-positive endothelial cells. Furthermore, the tailpiece domain of the α5 subunit in α5β1 integrin was critical for binding to Ang-2. Taken together, these results provide a novel insight into the mechanism of integrin regulation by Ang-2, which contributes to tumor invasion and endothel  ial cell   migration in a Tie2-independent manner. 
 Mesenchymal stem cell   migration   is regulated by fibronectin through α5β1-integrin-mediated activation of PDGFR-β and potentiation of growth factor signals. Ce  ll migrat  ion during vascular remodelling is regulated by crosstalk between growth factor receptors and integrin receptors, which together coordinate cytoskeletal and motogenic changes. Here, we report extracellular matrix (ECM)-directed crosstalk between platelet-derived growth factor receptor (PDGFR)-β and α5β1-integrin, which control  s the mig  ration of mesenchymal stem (stromal) cells (MSCs). Cell adhesion to fibronectin induced α5β1-integrin-dependent phosphorylation of PDGFR-β in the absence of growth factor stimulation. Phosphorylated PDGFR-β co-immunoprecipitated with α5-integrin and colocalised with α5β1-integrin in the transient tidemarks of focal adhesions. Adhesion to fibronectin also strongly potentiated PDGF-BB-induced PDGFR-β phosphorylation and focal adhesion kinase (FAK) activity, in an α5β1-integrin-dependent manner. PDGFR-β-induced phosphoinositide 3-kinase (PI3K) and Akt activity, actin reorgan  isation a  nd cell migration were all regulated by fibronectin and α5β1-integrin. This synergistic relationship between α5β1-integrin and PDGFR-β is a fundamenta  l determi  nant of cell migration. Thus, fibronectin-rich matrices can prime PDGFR-β to recruit mesenchymal cells at sites of vascular remodelling. 
 Trisomy 12 chronic lymphocytic leukemia cells exhibit upregulation of integrin signaling that is modulated by NOTCH1 mutations. The leukocyte adhesion cascade is important in chronic lymphocytic leukemia (CLL), as it controls   migration   of malignant cells into the pro-survival lymph node microenvironment. Circulating trisomy 12 CLL cells have increased expression of the integrins CD11a and CD49d, as well as CD38, but the tissue expression of these and other molecules, and the functional and clinical sequelae of these changes have not been described. Here, we demonstrate that circulating trisomy 12 CLL cells also have increased expression of the integrins CD11b, CD18, CD29, and ITGB7, and the adhesion molecule CD323. Notably, there was reduced expression of CD11a, CD11b, and CD18 in trisomy 12 cases with NOTCH1 mutations compared with wild type. Trisomy 12 cells also exhibit upregulation of intracellular integrin signaling molecules CALDAG-GEFI, RAP1B, and Ras-related protein ligand, resulting in enhanced very late antigen-4 [VLA-4] directed adhesion and motility. CD38 expression in CLL has prognostic significance, but the increased CD38 expression in trisomy 12 CLL cells must be taken into account in this subgroup, and the threshold of CD38 positivity should be raised to 40% for this marker to retain its prognostic value. In conclusion, trisomy 12 CLL cells exhibit functional upregulation of integrin signaling, with β2-integrin expression being modulated by NOTCH1 mutation status. 
 Changes in adhesive and migratory characteristics of hepatocellular carcinoma (HCC) cells induced by expression of alpha3beta1 integrin. The invasive and metastatic potentials of hepatocellular carcinoma are positively correlated with the expression level of alpha3beta1 integrin, a high-affinity adhesion receptor for laminin isoforms including laminin-5. In this study, we investigated changes in the adhesive and invasive behaviors of human HCC HepG2 cells after transfection with cDNA for alpha3 integrin in order to elucidate the direct involvement of this integrin in these cellular processes. We introduced cDNA for splice variants of alpha3 integrin (alpha3A and alpha3B) into the cells, and selected two transfectant clones (HepG2-3A and HepG2-3B), which express the alpha3A and alpha3B integrins, respectively. Both transfectant cells adhered almost equally to laminin-5-coated plates in an alpha3 integrin-dependent manner, indicating that transfected alpha3Abeta1 and alpha3Bbeta1 integrins were functionally active in these cells. The migratory and invasive potentials of the transfectant cells were assessed by scratch wound assay and in vitro chemoinvasion assay. The results demonstrated that the   migration   of HepG2-3A and HepG2-3B cells but not of mock transfectant (HepG2-M) cells was stimulated on the plates coated with laminin-5. Furthermore, HepG2-3A and HepG2-3B cells were found to be more invasive into laminin-5-containing matrices than were HepG2-M cells. These results strongly suggest that enhanced expression of alpha3beta1 integrin on HCC cells is directly involved in their malignant phenotypes such as invasion and metastasis. 
 The integrin receptor alpha 8 beta 1 mediates interactions of embryonic chick motor and sensory neurons with tenascin-C. This paper identifies a neuronal receptor for tenascin-C (tenascin/cytotactin), an extracellular matrix protein that has previously been detected in developing sensory and motor neuron pathways and has been shown to regulate cell   migration   in the developing CNS. Antibodies specific for each subunit of the integrin alpha 8 beta 1 are used to demonstrate that alpha 8 beta 1 mediates neurite outgrowth of embryonic sensory and motor neurons on this extracellular matrix protein. In addition, expression of alpha 8 in K562 cells results in surface expression of alpha 8 beta 1 heterodimers that are shown to promote attachment of this cell line to tenascin. The major domain in tenascin that mediates neurite outgrowth is shown to be localized to fibronectin type III repeats 6-8. 
 Polymeric osteopontin employs integrin alpha9beta1 as a receptor and attracts neutrophils by presenting a de novo binding site. Osteopontin (OPN) is a cytokine and ligand for multiple members of the integrin family. OPN undergoes the in vivo polymerization catalyzed by cross-linking enzyme transglutaminase 2, which consequently increases the bioactivity through enhanced interaction with integrins. The integrin alpha9beta1, highly expressed on neutrophils, binds to the sequence SVVYGLR only after intact OPN is cleaved by thrombin. The SVVYGLR sequence appears to be cryptic in intact OPN because alpha9beta1 does not recognize intact OPN. Because transglutaminase 2-catalyzed polymers change their physical and chemical properties, we hypothesized that the SVVYGLR site might also be exposed on polymeric OPN. As expected, alpha9beta1 turned into a receptor for polymeric OPN, a result obtained by cell adhesion and   migration   assays with alpha9-transfected cells and by detection of direct binding of recombinant soluble alpha9beta1 with colorimetry and surface plasmon resonance analysis. Because the N-terminal fragment of thrombin-cleaved OPN, a ligand for alpha9beta1, has been reported to attract neutrophils, we next examined   migration   of neutrophils to polymeric OPN using time-lapse microscopy. Polymeric OPN showed potent neutrophil chemotactic activity, which was clearly inhibited by anti-alpha9beta1 antibody. Unexpectedly, mutagenesis studies showed that alpha9beta1 bound to polymeric OPN independently of the SVVYGLR sequence, and further, SVVYGLR sequence of polymeric OPN was cryptic because SVVYGLR-specific antibody did not recognize polymeric OPN. These results demonstrate that polymerization of OPN generates a novel alpha9beta1-binding site and that the interaction of this site with the alpha9beta1 integrin is critical to the neutrophil chemotaxis induced by polymeric OPN. 
 CBAP functions as a novel component in chemokine-induced ZAP70-mediated T-cell adhesion and   migration  . Activated chemokine receptor initiates inside-out signaling to transiently trigger activation of integrins, a process involving multiple components that have not been fully characterized. Here we report that GM-CSF/IL-3/IL-5 receptor common beta-chain-associated protein (CBAP) is required to optimize this inside-out signaling and activation of integrins. First, knockdown of CBAP expression in human Jurkat T cells caused attenuated CXC chemokine ligand-12 (CXCL12)-induced cell   migration   and integrin α4β1- and αLβ2-mediated cell adhesion in vitro, which could be rescued sufficiently upon expression of murine CBAP proteins. Freshly isolated CBAP-deficient primary T cells also exhibited diminution of chemotaxis toward CC chemokine ligand-21 (CCL21) and CXCL12, and these chemokines-induced T-cell adhesions in vitro. Adoptive transfer of isolated naive T cells demonstrated that CBAP deficiency significantly reduced lymph node homing ability in vivo. Final  ly, migra  tion of T cell-receptor-activated T cells induced by inflammatory chemokines was also attenuated in CBAP-deficient cells. Further analyses revealed that CBAP constitutively associated with both integrin β1 and ZAP70 and that CBAP is required for chemokine-induced initial binding of the talin-Vav1 complex to integrin β1 and to facilitate subsequent ZAP70-mediated dissociation of the talin-Vav1 complex and Vav1 phosphorylation. Within such an integrin signaling complex, CBAP likely functions as an adaptor and ultimately leads to activation of both integrin α4β1 and Rac1. Taken together, our data suggest that CBAP indeed can function as a novel signaling component within the ZAP70/Vav1/talin complex and plays an important role in regulating chemokine-promoted T-cell trafficking. 
 beta1 and beta2 integrins activate different signalling pathways in monocytes. Integrin-mediated signals play an important but poorly understood role in regulating many leucocyte functions. In monocytes and macrophages, integrins of the beta2 subfamily are involved in cell-cell interactions that are important for   migration   of the cells through the endothelium and also for phagocytosis. On the other hand, in the same cells, beta1 integrin-mediated adhesion to extracellular matrix proteins results in a strong induction of immediate early genes that are important in inflammation. To investigate the signalling pathways from these two types of integrin in monocytic cells, THP-1 cells were selectively stimulated via beta1 or beta2 integrins by cross-linking each type of receptor with specific monoclonal antibodies or their natural ligands. The involvement of extracellular signal-regulated kinase (ERK), Syk and phosphoinositide 3-kinase (PI-3K) was then analysed. Nuclear factor kappaB (NF-kappaB) activation was also detected in THP-1 cells transiently transfected with an NF-kappaB-driven luciferase reporter gene. We found that binding of both types of integrin to their natural ligands activated ERK in a Syk- and PI-3K-dependent manner. Yet, cross-linking of integrins by anti-beta1 antibodies caused activation of ERK while that by anti-beta2 antibodies did not. Also both types of integrin activated NF-kappaB. However, PI-3K was required for beta1 integrin-, but not beta2 integrin-, mediated NF-kappaB activation. In addition, inhibition of PI-3K with wortmannin and LY294002 blocked beta1 integrin-mediated NF-kappaB activation, but did not affect that mediated by beta2 integrin. These data suggest that distinct integrins activate different signalling pathways in monocytic cells. 
 Dependence of fibroblast infiltration in tumor stroma on type IV collagen-initiated integrin signal through induction of platelet-derived growth factor. Cancer-associated fibroblasts play a crucial role in accelerating tumor progression, but there is a knowledge gap regarding the chemotactic signal activated in a tumor microenvironment. In this study, the expression of type IV collagen was knocked down using a lentiviral-mediated short hairpin RNA strategy. Although there was no obvious effect on cell growth in vitro, silencing the Col4-α1 gene decreased the tumorigenicity of B16F10 in C57BL/6 mice, which was accompanied by a reduction in the infiltration of alpha-smooth muscle actin-positive (α-SMA+) fibroblasts. Silencing the Col4-α1 gene or disrupting integrin engagement by blocking the antibody reduced the expression of platelet-derived growth factor A (PDGF-A), a potent chemotactic factor for fibroblasts. Furthermore, ectopic expression of the autoclustering integrin mutant significantly stimulated PDGF-A expression in murine B16F10 and human U118MG and Huh7 cells. PDGF-A-specific sh-RNA and neutralizing anti-PDGF-A antibody effectively inhibited the transwe  ll migrat  ion of fibroblasts. Adding recombinant PDGF-A back to shCol cell-conditioned media restored the fibroblast-attraction ability indicating that PDGF-A is a major chemotactic factor for fibroblasts in the current study model. The integrin-associated PDGF-A production correlated with the activation of Src and ERK. High type IV collagen staining intensity colocalized with elevated PDGF-A expression was observed in tumor tissues obtained from hepatoma and glioma patients. The integrin signal pathway was activated by collagen engagement through Src and ERK, leading to enhanced PDGF-A production, which serves as a key regulator of fibroblast recruitment. 
 Real-time analysis of very late antigen-4 affinity modulation by shear. Shear promotes endothelial recruitment of leukocytes, cell activation, and trans  migration  . Mechanical stress on cells caused by shear can induce a rapid integrin conformational change and activation, followed by an increase in binding to the extracellular matrix. The molecular mechanism of increased avidity is unknown. We have shown previously that the affinity of the alpha(4)beta(1) integrin, very late antigen-4 (VLA-4), measured with an LDV-containing small molecule, varies with cellular avidity, measured from cell disaggregation rates. In this study, we measured in real time affinity changes of VLA-4 in response to shear. The resulting affinity was comparable with the state mediated by receptor signaling and corresponded in time with intracellular Ca(2+) responses. Ca(2+) ionophores and N,N'-[1,2-ethanediyl-bis(oxy-2,1-phenylene)]bis[N-[2-[(acetyloxy)methoxy]-2-oxoethyl]]-, bis[(acetyloxy)methyl]ester demonstrate that the affinity regulation of VLA-4 in the presence of shear was related to Ca(2+) signaling. Pertussis toxin treatment implicates G(i) in an unknown pathway that connects shear, Ca(2+) elevation, VLA-4 affinity, and cell avidity. 
 Integrin beta cytoplasmic domain interactions with phosphotyrosine-binding domains: a structural prototype for diversity in integrin signaling. The cytoplasmic domains (tails) of heterodimeric integrin adhesion receptors mediate integrins' biological functions by binding to cytoplasmic proteins. Most integrin beta tails contain one or two NPXYF motifs that can form beta turns. These motifs are part of a canonical recognition sequence for phosphotyrosine-binding (PTB) domains, protein modules that are present in a wide variety of signaling and cytoskeletal proteins. Indeed, talin and ICAP1-alpha bind to integrin beta tails by means of a PTB domain-NPXY ligand interaction. To assess the generality of this interaction we examined the binding of a series of recombinant PTB domains to a panel of short integrin beta tails. In addition to the known integrin-binding proteins, we found that Numb (a negative regulator of Notch signaling) and Dok-1 (a signaling adaptor involved in cell   migration  ) and their isolated PTB domain bound to integrin tails. Furthermore, Dok-1 physically associated with integrin alpha IIb beta 3. Mutations of the integrin beta tails confirmed that these interactions are canonical PTB domain-ligand interactions. First, the interactions were blocked by mutation of an NPXY motif in the integrin tail. Second, integrin class-specific interactions were observed with the PTB domains of Dab, EPS8, and tensin. We used this specificity, and a molecular model of an integrin beta tail-PTB domain interaction to predict critical interacting residues. The importance of these residues was confirmed by generation of gain- and loss-of-function mutations in beta 7 and beta 3 tails. These data establish that short integrin beta tails interact with a large number of PTB domain-containing proteins through a structurally conserved mechanism. 
 Adhesion molecule mechanisms mediating monocyte   migration   through synovial fibroblast and endothelium barriers: role for CD11/CD18, very late antigen-4 (CD49d/CD29), very late antigen-5 (CD49e/CD29), and vascular cell adhesion molecule-1 (CD106). Monocytes migrate through vascular endothelium, and then in connective tissue. As a model of this process, we investigated adhesion molecules involved in monocyte   migration   through HUVEC and a barrier of human synovial fibroblasts (HSF). Minimal spontaneous monocyte   migration   (6-7%) occurred through either cell barrier, but this increased markedly (27-35% of added monocytes) when a C5a chemotactic gradient was present.   Migration   across unstimulated HUVEC was partially inhibited (40%) by mAb to CD18 (beta2 integrin) and completely blocked by anti-CD18 plus anti-alpha4 (CD49d; very late Ag-4 (VLA-4)) mAbs. In contrast,   migration   across HSF induced by C5a or monocyte chemoattractant protein-1 was not inhibited by mAb to CD18 and was only partially inhibited (33%) in combination with anti-alpha4 mAb. The CD18- and VLA-4-independent   migration   across HSF was completely inhibited by mAb to alpha5 of VLA-5. The inhibitory effect of mAbs to VLA-4 and VLA-5 was on the monocyte and required blockade of CD11/CD18 to be observed. In contrast to HSF, no role for VLA-5 in monocyte transendothelial   migration   was detected. Both HSF and IL-1-stimulated HUVEC expressed vascular cell adhesion molecule-1 (VCAM-1). However, VLA-4-mediated monocyte   migration   across HSF was only partially dependent on VCAM-1, in contrast to transendothelial   migration  , which was completely blocked by anti-VCAM-1 mAbs. In conclusion, unlike transendothelial   migration  , for which VLA-4 is the alternative mechanism to CD11/CD18 on monocytes, both VLA-4 and VLA-5 can mediate monocyte   migration   through fibroblast barriers. In addition to VCAM-1, other ligand(s) on HSF are also involved in the VLA-4-mediated   migration  . 
 β-1,4-Galactosyltransferase III enhances invasive phenotypes via β1-integrin and predicts poor prognosis in neuroblastoma. Neuroblastoma (NB) is a neural crest-derived tumor that commonly occurs in childhood. β-1,4-Galactosyltransferase III (B4GALT3) is highly expressed in human fetal brain and is responsible for the generation of poly-N-acetyllactosamine, which plays a critical role in tumor progression. We therefore investigated the expression and role of B4GALT3 in NB.We examined B4GALT3 expression in tumor specimens from 101 NB patients by immunohistochemistry and analyzed the correlation between B4GALT3 expression and clinicopathologic factors or survival. The functional role of B4GALT3 expression was investigated by overexpression or knockdown of B4GALT3 in NB cells for in vitro and in vivo studies.We found that B4GALT3 expression correlated with advanced clinical stages (P = 0.040), unfavorable Shimada histology (P < 0.001), and lower survival rate (P < 0.001). Multivariate analysis showed that B4GALT3 expression is an independent prognostic factor for poor survival of NB patients. B4GALT3 overexpression increas  ed migrat  ion, invasion, and tumor growth of NB cells, whereas B4GALT3 knockdown suppressed the malignant phenotypes of NB cells. Mechanistic investigation showed that B4GALT3-enhanc  ed migrat  ion and invasion were significantly suppressed by β1-integrin blocking antibody. Furthermore, B4GALT3 overexpression increased lactosamine glycans on β1-integrin, increased expression of mature β1-integrin via delayed degradation, and enhanced phosphorylation of focal adhesion kinase. Conversely, these properties were decreased by knockdown of B4GALT3 in NB cells.Our findings suggest that B4GALT3 predicts an unfavorable prognosis for NB and may regulate invasive phenotypes through modulating glycosylation, degradation, and signaling of β1-integrin in NB cells. 
 Integrins as receptors for laminins. Laminins are a family of trimeric glycoproteins present in the extracellular matrix and the major constituents of basement membranes. Integrins are alpha beta transmembrane receptors that play critical roles in both cell-matrix and cell-cell adhesion. Several members of the integrin family, including alpha 1 beta 1, alpha 2 beta 1, alpha 3 beta 1, alpha 6 beta 1, alpha 7 beta 1 and alpha 6 beta 4 heterodimers serve as laminin receptors on a variety of cell types. This review summarizes recent advances in understanding the involvement of individual integrins in cell interactions with laminins and the roles of laminin-binding integrins in adhesion-mediated events in vertebrates, including embryonic development, cell   migration   and tumor cell invasiveness, cell proliferation and differentiation, as well as basement membrane assembly. We discuss the regulation of integrin function via alternative splicing of cytoplasmic domains of alpha and beta subunits of the integrin receptors for laminins and present examples of functional collaboration between laminin-binding integrins and non-integrin laminin receptors. Advances in our understanding of the laminin-binding integrins continue to demonstrate the essential roles these receptors play in maintaining cell polarity and tissue architecture. 
 Interleukin-1alpha enhances the aggressive behavior of pancreatic cancer cells by regulating the alpha6beta1-integrin and urokinase plasminogen activator receptor expression. In human pancreatic cancer progression, the alpha6beta1-integrin is expressed on cancer cell surface during invasion and metastasis formation. In this study, we investigated whether interleukin (IL)-1alpha induces the alterations of integrin subunits and urokinase plasminogen activator/urokinase plasminogen activator receptor (uPA/uPAR) expression in pancreatic cancer cells. We hypothesize that the alterations of integrin subunits and uPA/uPAR expression make an important role in signaling pathways responsible for biological behavior of pancreatic cancer cells.IL-1alpha upregulated the expression of alpha6 and beta1 integrins without any alterations of alpha5 and alphav integrins expression. IL-1alpha also induced enhancement in the expression of uPA/uPAR in pancreatic cancer cells. IL-1alpha enhanced the proliferation, adhesion, and   migration   in pancreatic cancer cells, and IL-1alpha-induced alterations of uPA/uPAR expression correlated with the increased the   migration   of pancreatic cancer cells. Upregulation of alpha6 integrin subunit and uPA/uPAR correlated with the activation of Ras and downstream extracellular signal-regulated kinase (ERK) pathways. IL-1alpha-induced activation of Ras and downstream ERK can be inhibited by using inhibitory antibodies against alpha6 and beta1 integrin and uPAR, consistent with the inhibition of proliferation, adhesion and   migration   of pancreatic cancer cells. Immunohistochemical analysis demonstrated a significant association between strong expressions of alpha6 integrin with uPAR in pancreatic cancer specimens. Furthermore, the strong expression of alpha6 integrin and uPAR was found to be independent prognosticator in pancreatic cancer patients.Based on these findings, we conclude that IL-1alpha can induce selective upregulation of alpha6beta1-integrin and uPA/uPAR in pancreatic cancer cells and these changes may modulate the aggressive functions of pancreatic cancer. 
 Extracellular membrane-proximal domain of HAb18G/CD147 binds to metal ion-dependent adhesion site (MIDAS) motif of integrin β1 to modulate malignant properties of hepatoma cells. Several lines of evidence suggest that HAb18G/CD147 interacts with the integrin variants α3β1 and α6β1. However, the mechanism of the interaction remains largely unknown. In this study, mammalian protein-protein interaction trap (MAPPIT), a mammalian two-hybrid method, was used to study the CD147-integrin β1 subunit interaction. CD147 in human hepatocellular carcinoma (HCC) cells was interfered with by small hairpin RNA. Nude mouse xenograft model and metastatic model of HCC were used to detect the role of CD147 in carcinogenesis and metastasis. We found that the extracellular membrane-proximal domain of HAb18G/CD147 (I-type domain) binds at the metal ion-dependent adhesion site in the βA domain of the integrin β1 subunit, and Asp(179) in the I-type domain of HAb18G/CD147 plays an important role in the interaction. The levels of the proteins that act downstream of integrin, including focal adhesion kinase (FAK) and phospho-FAK, were decreased, and the cytoskeletal structures of HCC cells were rearranged bearing the HAb18G/CD147 deletion. Simultaneous  ly, the m  igration and invasion capacities, secretion of matrix metalloproteinases, colony formation rate in vitro, and tumor growth and metastatic potential in vivo were decreased. These results indicate that the interaction of HAb18G/CD147 extracellular I-type domain with the integrin β1 metal ion-dependent adhesion site motif activates the downstream FAK signaling pathway, subsequently enhancing the malignant properties of HCC cells. 
 Intensified antineoplastic effect by combining an HDAC-inhibitor, an mTOR-inhibitor and low dosed interferon alpha in prostate cancer cells. A significant proportion of men diagnosed with prostate cancer (PCa) eventually develop metastatic disease, which progresses to castration resistance, despite initial response to androgen deprivation. As anticancer therapy has become increasingly effective, acquired drug resistance has emerged, limiting efficacy. Combination treatment, utilizing different drug classes, exemplifies a possible strategy to foil resistance development. The effects of the triple application of the histone deacetylase (HDAC) inhibitor valproic acid (VPA), the mammalian target of rapamycin inhibitor everolimus and low dosed interferon alpha (IFNα) on PCa cell growth and dissemination capacity were investigated. For that purpose, the human PCa cell lines, PC-3, DU-145 and LNCaP were treated with the combined regimen or separate single agents. Cell growth was investigated by the MTT dye reduction assay. Flow cytometry served to analyse cell cycle progression. Adhesion to vascular endothelium or immobilized collagen, fibronectin and laminin was quantified.   Migratio  n and invasion characteristics were determined by the modified Boyden chamber assay. Integrin α and β subtypes were investigated by flow cytometry, western blotting and RT-PCR. Integrin related signalling, Epidermal Growth Factor Receptor (EGFr), Akt, p70S6kinase and extracellular signal-regulated kinases (ERK)1/2 activation were also assessed. The triple application of VPA, everolimus and low dosed IFNα blocked tumour cell growth and dissemination significantly better than any agent alone. Antitumour effects were associated with pronounced alteration in the cell cycle machinery, intracellular signalling and integrin expression profile. Combining VPA, everolimus and low dosed IFNα might be a promising option to counteract resistance development and improve outcome in PCa patients. 
 Overexpression of malignancy-associated laminins and laminin receptors by angiotropic human melanoma cells in a chick chorioallantoic membrane model. As distinct from intravascular/lymphatic dissemination, extravascular migratory metastasis (EVMM) has been described as a potential additional mechanism of melanoma spread in which tumor cells migrate along the external surfaces of vessels. Angiotropic melanoma cells are linked to the endothelium by a matrix containing laminin. In addition, it has been shown that C16 laminin-derived peptide increases extravascular   migration   of human green fluorescent protein (GFP) melanoma cells along vessels in a chicken chorioallantoic membrane model (CAM). In this study, we have tested the hypothesis that expression levels of some genes related to lamimin and metastasis are differentially expressed in vascularized angiotropic melanoma areas vs. avascular melanoma areas from the same tumor.C8161 human melanoma cells in a shell-less chick CAM assay were used to study EVMM associated with the presence of vascularized angiotropic melanoma areas. For both high-quality histomorphology and RNA preservation in paraffin-embedded tissue, we used a methanol-based fixative coupled with microwave-assisted rapid tissue processing as previously described. Using laser capture microdissection, angiotropic melanoma areas as well as avascular areas were microdissected. Using quantitative real time polymerase chain reaction (QRT-PCR), six genes have been studied: LAMC2 (laminin gamma2 chain), LAMA4 (laminin alpha4 chain), ITGB1 (integrin beta1), ITGB3 (integrin beta3), RSPA (ribosomal protein), and MMP2 (matrix metallopeptidase 2). QRT-PCR data were normalized to human GAPDH housekeeping gene and values were compared against Human Total RNA. Final results were expressed as percentage of expression.All tumors demonstrated a similar pattern, i.e. EVMM of angiotropic melanoma cells. The microdissected histopathological sections presented both angiotropic areas and avascular areas. All genes were overexpressed in angiotropic melanoma areas vs. avascular melanoma areas, especially LAMC2, LAMA4 and ITGB3 (respectively, 165.18, 208.86, and 483.69%).This study shows that several genes related to laminin are overexpressed in angiotropic melanoma areas vs. avascular melanoma areas. Since extravascular   migration   of melanoma cells along vessels has been demonstrated in the CAM model, taken together these results suggests that some laminins and laminin receptors may play a role in extravascular migratory metastasis. This model may represent a promising strategy to analyze differential gene expression in EVMM. 
 Upregulation of Eps8 in oral squamous cell carcinoma promotes cell   migration   and invasion through integrin-dependent Rac1 activation. Oral squamous cell carcinoma (OSCC) is a lethal disease and early death usually occurs as a result of local invasion and regional lymph node metastases. Current treatment regimens are, to a certain degree, inadequate, with a 5-year mortality rate of around 50% and novel therapeutic targets are urgently required. Using expression microarrays, we identified the eps8 gene as being overexpressed in OSCC cell lines relative to normal oral keratinocytes, and confirmed these findings using RT-PCR and western blotting. In human tissues, we found that Eps8 was upregulated in OSCC (32% of primary tumors) compared with normal oral mucosa, and that expression correlated significantly with lymph node metastasis (P=0.032), suggesting a disease-promoting effect. Using OSCC cell lines, we assessed the functional role of Eps8 in tumor cells. Although suppression of Eps8 produced no effect on cell proliferation, both cell spreading and   migration   were markedly inhibited. The latter cell functions may be modulated through the small GTP-ase, Rac1 and we used pull-down assays to investigate the role of Eps8 in Rac1 signaling. We found that alphavbeta6- and alpha5beta1-integrin-dependent activation of Rac1 was mediated through Eps8. Knockdown of either Eps8 or Rac1, inhibited integrin-dependent cell   migration   similarly and transient expression of constitutively active Rac1 restored   migration   of cells in which Eps8 expression had been suppressed. We also showed that knockdown of Eps8 inhibited tumor cell invasion in an organotypic model of OSCC. These data suggest that Eps8 and Rac1 are part of an integrated signaling pathway modulating integrin-dependent tumour cell motility and identify Eps8 as a possible therapeutic target. 
 alpha4beta1 integrin regulates lamellipodia protrusion via a focal complex/focal adhesion-independent mechanism. alpha4beta1 integrin plays an important role in cell   migration  . We show that when ectopically expressed in Chinese hamster ovary cells, alpha4beta1 is sufficient and required for promoting protrusion of broad lamellipodia in response to scratch-wounding, whereas alpha5beta1 does not have this effect. By time-lapse microscopy of cells expressing an alpha4/green fluorescent protein fusion protein, we show that alpha4beta1 forms transient puncta at the leading edge of cells that begin to protrude lamellipodia in response to scratch-wounding. The cells expressing a mutant alpha4/green fluorescent protein that binds paxillin at a reduced level had a faster response to scratch-wounding, forming alpha4-positive puncta and protruding lamellipodia much earlier. While enhancing lamellipodia protrusion, this mutation reduces random motility of the cells in Transwell assays, indicating that lamellipodia protrusion and random motility are distinct types of motile activities that are differentially regulated by interactions between alpha4beta1 and paxillin. Finally, we show that, at the leading edge, alpha4-positive puncta and paxillin-positive focal complexes/adhesions do not colocalize, but alpha4beta1 and paxillin colocalize partially in ruffles. These findings provide evidence for a specific role of alpha4beta1 in lamellipodia protrusion that is distinct from the motility-promoting functions of alpha5beta1 and other integrins that mediate cell adhesion and signaling events through focal complexes and focal adhesions. 
 The LIM-only proteins FHL2 and FHL3 interact with alpha- and beta-subunits of the muscle alpha7beta1 integrin receptor. FHL1, FHL2, and FHL3 are members of the four and one-half LIM domain protein subclass that are expressed in striated muscles. Here we show that FHL2 and FHL3 are novel alpha(7)beta(1) integrin-interacting proteins. They bind both the alpha- and the beta-subunit as well as different splice isoforms. The minimal binding sites for FHL2 and FHL3 on beta(1A)-chain overlap, whereas on alpha(7A) and alpha(7B) subunits they are situated adjacent. Determining the binding sites for integrins on FHL2 or FHL3 revealed that the suprastructure of the whole molecule is important for these associations, rather than any single LIM domain. Immunofluorescence studies with cells expressing full-length FHL proteins or their deletion mutants showed that FHL2 and FHL3 but not FHL1 colocalize with integrins at cell adhesion sites. Further, their recruitment to the membrane results from binding to either the alpha- or the beta-chain of the integrin receptor. The association of FHL2 or FHL3 with integrin receptors neither influences attachment of cells to different substrates nor changes their   migration   capacity. However, in cardiac and skeletal muscles, FHL2 and FHL3, respectively, are colocalized with alpha(7)beta(1) integrin receptor at the periphery of Z-discs, suggesting a role in mechanical stabilization of muscle cells. 
 Effects of integrin α6β1 o  n migrati  on of hepatocellular carcinoma cells. In this study, we applied specific blocking antibodies for integrin α6 or β1 subunit, and evaluated the in vitro effects of integrins α6β1 on the adhesion, chemotaxi  s and mig  ration of hepatocellular carcinoma (HCC) cell line SMMC-7721 to type IV collagen. The adhesion force and   cell mig  ration, as measured by a micropipette aspiration system and Boyden chamber assay respectively, was dramatically reduced when either integrin subunits was blocked. The chemotaxis, as determined using a dual-micropipette system, was only affected by the antibody against β1 subunit. This study suggests that integrin α6β1 is an important cell surface receptor that mediates the adhesion of SMMC-7721 to type IV collagen. But the α6 subunit has minimal effect on pseudopod formation in response to type IV collagen. Therefore, the integrin α6β1-me  diated ce  ll migration is, at least in part, through the regulation on the cell adhesion step. 
 Human neutrophil integrin alpha9beta1: up-regulation by cell activation and synergy with beta2 integrins during adhesion to endothelium under flow. Neutrophil beta1 integrin expression and contribution to cell adhesion were revisited in this study. alpha9beta1 and alpha5beta1 appeared here as the main beta1 integrins expressed on the membrane of resting platelet-depleted neutrophils-alpha6beta1 representing <15% and alpha2beta1 undetectable. Neutrophil activation slightly enhanced alpha5 expression, did not change alpha6, but resulted in a two- to threefold increase of alpha9beta1, which then became the major beta1 integrin of the neutrophil membrane. alpha9beta1 was the only beta1 integrin to be up-regulated after transendothelial   migration   across TNF-alpha-activated HUVECs. As alpha9beta1 binds VCAM-1, we analyzed its participation to neutrophil adhesion to TNF-alpha-activated endothelial cells. Blocking anti-alpha9 mAb had little effect on neutrophil static adhesion, contrasting with the strong inhibition by anti-beta2 mAb. Under flow conditions, the anti-alpha9 mAb had no effect by itself on neutrophil adhesion to activated HUVECs but enhanced the blocking effect of anti-beta2 antibodies significantly and further enhanced the velocity of beta2-blocked rolling neutrophils. In conclusion, we describe here for the first time a nearly exclusive up-regulation of alpha9beta1 expression among all beta1 integrins during neutrophil activation and transendothelial   migration   and a possibly important synergy between alpha9beta1 and beta2 integrins in stabilizing neutrophil adhesion to endothelium under flow conditions. 
 Insulin-Like Growth Factor Binding Protein-2 Promotes Adhesion of Endothelial Progenitor Cells to Endothelial Cells via Integrin α5β1. The contribution of endothelial progenitor cells (EPCs) to new vessel formation has been studied in different physiological and pathological conditions for decades. As previously suggested, insulin-like growth factor binding protein-2 (IGFBP-2) may interact with integrins and promote cel  l migrati  on. However, the role of IGFBP-2 in regulation of EPC functions remains largely unknown. In this present study, we found that overexpression of IGFBP-2 in human umbilical vein endothelial cells (HUVECs) promoted EPC-endothelial adhesion. Conversely, siRNA-mediated depletion of IGFBP-2 inhibited oxygen-glucose deprivation (OGD)-induced EPC-endothelial adhesion. Further, we demonstrated that the arginine-glycine-aspartic acid (RGD) motif in its C-domain is required for interaction with integrin α5β1. In addition, treatment with IGFBP-2 significantly enhanced incorporation of EPCs into tubule networks formed by HUVECs. Thus, our findings suggest that exogenous administration of IGFBP-2 may facilitate neovascularization and improve treatment of ischemic conditions. 
 [Effect of integrin alpha2beta1 on invasion and   migration   of neuroblastoma cells]. To study the effect of integrin alpha2beta1 on invasion and   migration   of SK-N-SH neuroblastoma cells.Neuroblastoma SK-N-SH cell line was cultured in the modified eagle's medium. The effects of monoclonal antibodies to integrin alpha2 and integrin beta1 on   migration   and invasion were measured by inclined test and polycarbonate filters incorporated in modified Transwell chambers respectively. The   migration   and invasion cells were stained with Gimsa staining and counted under a 200 multiplied microscope. The blocking rate of   migration   and invasion of cells was calculated.The number of migrated SK-N-SH cells in the anti-alpha2 and anti-beta1 treatment groups (50.9+/-10.5 and 54.3+/-9.0 respectively) was significantly less than that in the control group without monoclonal antibody treatment (98.1+/-7.4) (P<0.01), with a blocking rate of cell   migration   of 48.1% and 44.5% respectively. The invasion to matrigel of SK-N-SH cells exposed monoclonal antibodies to integrin alpha2 and integrin beta1 was significantly blocked compared with the control SK-N-SH cells, with the number of invasion cells in the anti-alpha2 and anti-beta1 treatment groups of 25.3 +/- 4.4 and 18.8 +/- 3.9 respectively vs 41.5 +/- 4.8 in the control group (P<0.01). The blocking rate of cell invasion in the anti-alpha2 and anti-beta1 treatment groups was 39.0% and 54.7% respectively.Integrin alpha2beta1 may promote   migration   and invasion of neuroblastoma cells. 
 α3β1 integrins regulate CD151 complex assembly and membrane dynamics in carcinoma cells within 3D environments. Integrins are extracellular matrix (ECM) receptors that are key players in the regulation of tumour cell invasion. The laminin-binding integrin α3β1 has previously been shown to regulate adhesion   and migra  tion of carcinoma cells in part through co-operative signalling with the tetraspanin family of transmembrane proteins. However, the spatial and temporal regulation of crosstalk between these families of transmembrane proteins in intact cells remains poorly understood. Here we have used fluorescence resonance energy transfer (FRET) to demonstrate for the first time that α3β1 and the tetraspanin CD151 directly associate at the front and retracting rear of polarised migrating breast carcinoma cells in both two-dimentional (2D) and three-dimentional (3D)matrices. Furthermore, localised α3β1-CD151 binding correlates with lower CD151 homodimerisation in cells migrating on laminin or within matrigel. Loss of α3β1 integrin leads to increased CD151 homodimer formation, increased activation of Rho GTPase, loss of cell polarity and decreased invasion in 3D ECM. As a result, α3-silenced cells show decreased actin-based membrane protrusion and retraction in both 2D and 3D environments. These data demonstrate that associations between α3β1 and CD151 occur dynamically within discrete subcellular compartments and act to establish local GTPase signalling to promote tumour cell invasion. These novel findings shed light on the complex crosstalk and switching between receptor complexes in response to different extracellular cues during cell invasion in 3D environments. 
 Coculture with endothelial cells enhances vascular smooth muscle cell adhesion and spreading via activation of beta1-integrin and phosphatidylinositol 3-kinase/Akt. The interactions between endothelial cells (ECs) and vascular smooth muscle cells (VSMCs) play significant roles in the homeostasis of the blood vessel during vascular remodeling. Cell adhesion and spreading are an essential process for VSMC   migration  , survival and proliferation in the events of vascular physiology and pathophysiology. However, effects of ECs on adhesion and spreading of VSMCs have not been characterized yet. Here, the interaction of ECs and VSMCs on adhesion and spreading of VSMCs were investigated by using a coculture system. The results showed that VSMCs cocultured with ECs exhibited a significant increase in the number of adherent and spreading cells, and much more mRNA (twofold, P<0.01) and protein (threefold, P<0.05) expression of beta(1)-integrin comparing to the control, i.e., VSMCs cultured alone. Furthermore, the enhanced functional activity of beta(1)-integrin expression was confirmed by FACS. A beta(1)-integrin blocking antibody (P5D2) could inhibit the EC-induced VSMC adhesion and spreading. It was demonstrated that in correspondence with enhanced cell adhesion, ECs also prompted focal adhesion complex assembly and stress fiber formation of VSMCs. The phosphatidylinositol 3-kinase (PI3K)/Akt pathway was more pronouncedly activated in response to VSMC attachment. Our results for the first time show that coculture with ECs enhances VSMC adhesion and spreading by up-regulating beta(1)-integrin expression and activating the PI3K/Akt pathway, suggesting that the interaction between ECs and VSMCs serves an important role in vascular homeostasis and remodeling. 
 Interaction of monocarboxylate transporter 4 with beta1-integrin and its role in cell   migration  . Monocarboxylate transporter (MCT) 4 is a heteromeric proton-coupled lactate transporter that is noncovalently linked to the extracellular matrix metalloproteinase inducer CD147 and is typically expressed in glycolytic tissues. There is increasing evidence to suggest that ion transporters are part of macromolecular complexes involved in regulating beta(1)-integrin adhesion and cell movement. In the present study we examined whether MCTs play a role in cell   migration   through their interaction with beta(1)-integrin. Using reciprocal coimmunoprecipitation assays, we found that beta(1)-integrin selectively associated with MCT4 in ARPE-19 and MDCK cells, two epithelial cell lines that express both MCT1 and MCT4. In polarized monolayers of ARPE-19 cells, MCT4 and beta(1)-integrin colocalized to the basolateral membrane, while both proteins were found in the leading edge lamellapodia of migrating cells. In scratch-wound assays, MCT4 knockdown slowed   migration   and increased focal adhesion size. In contrast, silencing MCT1 did not alter the rate of cell   migration   or focal adhesion size. Taken together, our findings suggest that the specific interaction of MCT4 with beta(1)-integrin may regulate cell   migration   through modulation of focal adhesions. 
 Fibulin-1 suppression of fibronectin-regulated cell adhesion and motility. Fibulin-1 is an extracellular matrix protein often associated with fibronectin (FN) in vivo. In this study, the ability of fibulin-1 to modulate adhesion, spreading and motility-promoting activities of FN was investigated. Fibulin-1 was found to have pronounced inhibitory effects on the cell attachment and spreading promoted by FN. Fibulin-1 was also found to inhibit the motility of a variety of cell types on FN substrata. For example, the FN-dependent haptotactic motility of breast carcinoma (MDA MB231) cells, epidermal carcinoma (A431), melanoma (A375 SM), rat pulmonary aortic smooth muscle cells (PAC1) and Chinese hamster ovary (CHO) cells was inhibited by the presence of fibulin-1 bound to FN-coated Boyden chamber membranes. Cells transfected to overproduce fibulin-1 displayed reduced velocity, distance of movement and persistence time on FN substrata. Similarly, the incorporation of fibulin-1 into FN-containing type I collagen gels inhibited the invasion of endocardial cushion mesenchymal cells migrating from cultured embryonic heart explants. By contrast, incorporation of fibulin-1 into collagen gels lacking FN had no effect on the   migration   of endocardial cushion cells. These results suggest that the motility-suppressive effects of fibulin-1 might be FN specific. Furthermore, such effects are cell-type specific, in that the   migration   of gingival fibroblasts and endothelial cells on FN substrata is not responsive to fibulin-1. Additional studies found that the mechanism for the motility-suppressive effects of fibulin-1 does not involve perturbations of interactions between alpha5beta1 or alpha4 integrins, or heparan sulfate proteoglycans with FN. However, fibulin-1 was found to inhibit extracellular signal regulated kinase (ERK) activation and to suppress phosphorylation of myosin heavy chain. This ability to influence signal transduction cascades that modulate the actin-myosin motor complex might be the basis for the effects of fibulin-1 on adhesion and motility. 
 IGF-I enhances α5β1 integrin expression and cell motility in human chondrosarcoma cells. Chondrosarcoma is a type of highly malignant tumor with a potent capacity to invade locally and cause distant metastasis. Chondrosarcoma shows a predilection for metastasis to the lungs. Integrins are the major adhesive molecules in mammalian cells and have been associated with metastasis of cancer cells. Insulin-like growth factor-I (IGF)-I plays an important role in regulating cell growth, proliferation, survival, and metabolism. However, the effects of IGF-I i  n migrati  on and integrin expression in chondrosarcoma cells are largely unknown. In this study, we found that IGF-I increased th  e migrati  on and the expression of α5β1 integrin in human chondrosarcoma cells. Pretreatment of cells with IGF-I receptor antibody reduced IGF-I-induced c  ell migra  tion and integrin expression. Activations of phosphatidylinositol 3-kinase (PI3K), Akt, and nuclear factor-κB (NF-κB) pathways after IGF-I treatment were demonstrated, and IGF-I-induced expression of integri  n and mig  ration activity was inhibited by the specific inhibitor and mutant of PI3K, Akt, and NF-κB cascades. Taken together, our results indicated that IGF-I enhanc  es the mi  gration of chondrosarcoma cells by increasing α5β1 integrin expression through the IGF-I receptor/PI3K/Akt/NF-κB signal transduction pathway. 
 Platelet binding to monocytes increases the adhesive properties of monocytes by up-regulating the expression and functionality of beta1 and beta2 integrins. Human monocytes adhere to activated platelets, resulting in the formation of platelet-monocyte complexes (PMC). Complex formation depends on the interaction between platelet-displayed P-selectin and the specific ligand for P-selectin on leukocytes, P-selectin glycoprotein ligand-1 (PSGL-1). We have recently shown that monocytes within PMC have increased adhesive capacity to the activated endothelium. To better understand the effect of platelet binding on the capacity of monocytes to adhere to activated endothelium, the P-selectin-PSGL-1 interaction-induced changes in integrin functionality were studied. The binding of platelets to monocytes via P-selectin-PSGL-1 interactions was shown to increase expression and activity of alpha4beta1 and alphaMbeta2integrin, with a concomitant decrease in L-selectin expression. Furthermore, the binding of platelets to monocytes resulted in increased monocyte adhesion to intercellular adhesion molecule-1, vascular cell adhesion molecule-1, and fibronectin. Platelet binding was also responsible for an increase in monocyte transendothelial   migration  . Similar effects were observed after engagement of PSGL-1 with specific antibodies or with P-selectin immunoglobulin protein. Our data suggest that platelets, by binding via P-selectin to PSGL-1 on monocytes, induce up-regulation and activation of beta1 and beta2integrins and increased adhesion of monocytes to activated endothelium. Hence, monocytes within PMC are in a higher state of activation and may have, therefore, an increased atherogenic capacity. 
 AMPA receptors promote perivascular glioma invasion via beta1 integrin-dependent adhesion to the extracellular matrix. High-grade gliomas release excitotoxic concentrations of glutamate, which has been shown to enhance tumor proliferation and   migration  . alpha-Amino-3-hydroxy-5-methylisoxazole-4-propionic acid (AMPA) glutamate receptors are abundantly expressed at the invading edge of glioblastoma specimens, suggesting they may play an important biologic role in tumor invasion. In this study, we examined potential mechanisms by which AMPA receptor (AMPAR) expression and stimulation promote glioma cell   migration   and invasion. Overexpression of GluR1, the most abundant AMPAR subunit in gliomas, positively correlated with glioma cell adhesion to type I and type IV collagen, which was decreased in cells with knockdown of GluR1 and with blocking antibodies to beta1 integrin. Furthermore, stimulation of the AMPAR led to detachment of cells from the extracellular matrix (ECM). Immunoprecipitation studies showed that GluR1 associated with the actin cytoskeleton-linked protein band 4.1B (brain type), which may serve as a link between GluR1 and integrins. Overexpression of GluR1 correlated with increased cell-surface expression of beta1 integrin, increased phosphorylation of focal adhesion kinase (FAK-Y397), and enhanced numbers of focal adhesion (FA) complexes. Cells overexpressing GluR1 had increased colocalization of actin and paxillin at FAs and, in several glioma cell lines, significantly increased invasion in an in vitro Matrigel transwell assay. Likewise, in an intracranial xenograft model, overexpression of GluR1 led to perivascular and subependymal glioma cell invasion similar to patterns of tumor dissemination described in human glioblastoma. Together, these results suggest that AMPARs may link signals from the ECM to sites of FA, where signal integration promotes tumor invasion. 
 Decreased expression of alpha3 and beta1 integrin subunits is responsible for differentiation-associated changes in cells behavior in terminally differentiated human oral keratinocytes. Primary normal human oral keratinocytes (NHOKs) terminally differentiate in serial subculture. To investigate whether this subculture-induced differentiation of NHOKs affects integrin expression and cell-matrix interaction, we studied the expression levels of integrin subunits and cellular response to the extracellular matrix (ECM) proteins in NHOKs at different population doublings. The phosphorylation statuses of focal adhesion kinase (FAK), extracellular signal regulated kinase (ERK), p38, and c-Jun amino-terminal kinase (JNK) were also determined in NHOK cells cultured on ECM proteins, to evaluate the functions of integrins with respect to cellular responses to ECM proteins. The expression levels of alpha3 and beta1 integrin subunits progressively decreased in NHOKs undergoing terminal differentiation. The ability of NHOKs to spread upon laminin and type I collagen significantly decreased in terminally differentiated oral keratinocytes. Keratinocyte   migration   was significantly increased on type I collagen for terminally differentiated NHOKs. Similar results were seen following preincubation of rapidly proliferating NHOKs with function-blocking antibodies to alpha3 or beta1 integrin subunit. In contrast, fibronectin had no effect on cellular responses in NHOKs, which were almost negligible in the expression levels of alpha5 integrin subunits. The extent of FAK phosphorylation in terminally differentiated NHOKs was notably lower than that of rapidly proliferating cells, but was enhanced in terminally differentiated cells that were cultured on type I collagen. Our results indicate that decreased expression of alpha3 and beta1 integrin subunits is responsible for differentiation-associated changes in cells behavior in terminally differentiated oral keratinocytes. Our data also show that the abrogation of the alpha5beta1 integrin function caused by omitting alpha5 subunit is linked to the loss of a cell-fibronectin interaction in human oral keratinocytes. 
 Trop-2 promotes prostate cancer metastasis by modulating β(1) integrin functions. The molecular mechanisms underlying metastatic dissemination are still not completely understood. We have recently shown that β(1) integrin-dependent cell adhesion to fibronectin and signaling is affected by a transmembrane molecule, Trop-2, which is frequently upregulated in human carcinomas. Here, we report that Trop-2 promotes metastatic dissemination of prostate cancer cells in vivo and is abundantly expressed in metastasis from human prostate cancer. We also show here that Trop-2 promotes prostate cancer cel  l migrati  on on fibronectin, a phenomenon dependent on β(1) integrins. Mechanistically, we demonstrate that Trop-2 and the α(5)β(1) integrin associate through their extracellular domains, causing relocalization of α(5)β(1) and the β(1)-associated molecule talin from focal adhesions to the leading edges. Trop-2 effect is specific as this molecule does not m  odulate m  igration on vitronectin, does not associate with the major vitronectin receptor, α(v)β(3) integrin, and does not affect localization of α(v)β(3) integrin as well as vinculin in focal adhesions. We show that Trop-2 enhances directional prostate   cancer ce  ll migration, through modulation of Rac1 GTPase activity. Finally, we show that Trop-2 induces activation of PAK4, a kinase that has been reported to mediate   cancer ce  ll migration. In conclusion, we provide the first evidence that β(1) integrin-dependent migratory and metastatic competence of prostate cancer cells is enhanced by Trop-2. 
 Prostaglandin E2 stimulates β1-integrin expression in hepatocellular carcinoma through the EP1 receptor/PKC/NF-κB pathway. Prostaglandin E2 (PGE2) has been implicated in cell invasion in hepatocellular carcinoma (HCC), via increased β1-integrin expression and ce  ll migrat  ion; however, the mechanism remains unclear. PGE2 exerts its effects via four subtypes of the E prostanoid receptor (EP receptor 1-4). The present study investigated the effect of EP1 receptor activation on β1-integrin expression and c  ell migra  tion in HCC. C  ell migra  tion increased by 60% in cells treated with 17-PT-PGE2 (EP1 agonist), which was suppressed by pretreatment with a β1-integrin polyclonal antibody. PGE2 increased β1-integrin expression by approximately 2-fold. EP1 receptor transfection or treatment with 17-PT-PGE2 mimicked the effect of PGE2 treatment. EP1 siRNA blocked PGE2-mediated β1-integrin expression. 17-PT-PGE2 treatment induced PKC and NF-κB activation; PKC and NF-κB inhibitors suppressed 17-PT-PGE2-mediated β1-integrin expression. FoxC2, a β1-integrin transcription factor, was also upregulated by 17-PT-PGE2. NF-κB inhibitor suppressed 17-PT-PGE2-mediated FoxC2 upregulation. Immunohistochemistry showed p65, FoxC2, EP1 receptor and β1-integrin were all highly expressed in the HCC cases. This study suggested that PGE2 upregulates β1-integrin expres  sion and   cell migration in HCC cells by activating the PKC/NF-κB signaling pathway. Targeting PGE2/EP1/PKC/NF-κB/FoxC2/β1-integrin pathway may represent a new therapeutic strategy for the prevention and treatment of this cancer. 
 Targeting of integrin beta1 and kinesin 2alpha by microRNA 183. MicroRNA 183 (miR-183) has been reported to inhibit tumor invasiveness and is believed to be involved in the development and function of ciliated neurosensory organs. We have recently found that expression of miR-183 increased after the induction of cellular senescence by exposure to H(2)O(2). To gain insight into the biological roles of miR-183 we investigated two potential novel targets: integrin beta1 (ITGB1) and kinesin 2alpha (KIF2A). miR-183 significantly decreased the expression of ITGB1 and KIF2A measured by Western blot. Targeting of the 3'-untranslated region (3'-UTR) of ITGB1 and KIF2A by miR-183 was confirmed by luciferase assay. Transfection with miR-183 led to a significant decrease in cell invasion and   migration   capacities of HeLa cells that could be rescued by expression of ITGB1 lacking the 3'-UTR. Although miR-183 had no effects on cell adhesion in HeLa cells, it significantly decreased adhesion to laminin, gelatin, and collagen type I in normal human diploid fibroblasts and human trabecular meshwork cells. These effects were also rescued by expression of ITGB1 lacking the 3'-UTR. Targeting of KIF2A by miR-183 resulted in some increase in the formation of cells with monopolar spindles in HeLa cells but not in human diploid fibroblast or human trabecular meshwork cells. The regulation of ITGB1 expression by miR-183 provides a new mechanism for the anti-metastatic role of miR-183 and suggests that this miRNA could influence the development and function in neurosensory organs, and contribute to functional alterations associated with cellular senescence in human diploid fibroblasts and human trabecular meshwork cells. 
 β-1,4-Galactosyltransferase III suppresses extravillous trophoblast invasion through modifying β1-integrin glycosylation. Glycosylation controls diverse protein functions and regulates various cellular phenotypes. Trophoblast invasion is essential for normal placental development. However, the role of glycosylation in human placenta throughout pregnancy is still unclear. The β-1,4-galactosyltransferase III (B4GALT3) has been found to regulate cancer cell invasion. We therefore investigated the expression of B4GALT3 in placenta and its roles in trophoblast.B4GALT3 protein expression was examined by quantitative Western blotting analysis in human placentas. For identification of B4GALT3-positive cells in normal human placenta, immunohistochemistry and immunofluorescence methods were used. To investigate effects of B4GALT3 on extravillous trophoblast (EVT)-like cell and primary EVT cells, we analyzed cell growth, adhesio  n, migrat  ion, and invasion in mock and B4GALT3-transfected cell.B4GALT3 expression significantly increased in third trimester human placenta. Immunostaining revealed that B4GALT3 expressed in placental villous cytotrophoblast, syncytiotrophoblast, and a subpopulation of EVT cells throughout pregnancy. Interestingly, we found increases in the expression level and percentage of B4GALT3-positive cells in third trimester EVT, but not in syncytiotrophoblasts and cytotrophoblasts of placental villi. Overexpression of B4GALT3 in HTR8/SVneo cells and primary trophoblast cells significantly suppressed ce  ll migrat  ion. In addition, B4GALT3 suppressed cell invasion, and enhanced cell adhesion to laminin in HTR8/SVneo cells. Notably, we found that B4GALT3 modified glycans on β1-integrin, suppressed focal adhesion kinase (FAK) signaling, and enhanced β1-integrin degradation.We propose that B4GALT3-mediated glycosylation change not only enhances β1-integrin binding to laminin, but also attenuates β1-integrin stability. Our findings suggest that B4GALT3 is a critical regulator for suppressing EVT invasion in the late stages of pregnancy. 
 Analysis of conserved residues in the betapat-3 cytoplasmic tail reveals important functions of integrin in multiple tissues. Integrin cytoplasmic tails contain motifs that link extracellular information to cell behavior such as cell   migration   and contraction. To investigate the cell functions mediated by the conserved motifs, we created mutations in the Caenorhabditis elegans betapat-3 cytoplasmic tail. The beta1D (799FK800), NPXY, tryptophan (784W), and threonine (797TT798) motifs were disrupted to identify their functions in vivo. Animals expressing integrins with disrupted NPXY motifs were viable, but displayed distal tip cell   migration   and ovulation defects. The conserved threonines were required for gonad   migration   and contraction as well as tail morphogenesis, whereas disruption of the beta1D and tryptophan motifs produced only mild defects. To abolish multiple conserved motifs, a beta1C-like variant, which results in a frameshift, was constructed. The betapat-3(beta1C) transgenic animals showed cold-sensitive larval arrests and defective muscle structure and gonad   migration   and contraction. Our study suggests that the conserved NPXY and TT motifs play important roles in the tissue-specific function of integrin. 
 Activated Notch4 inhibits angiogenesis: role of beta 1-integrin activation. Notch4 is a member of the Notch family of transmembrane receptors that is expressed primarily on endothelial cells. Activation of Notch in various cell systems has been shown to regulate cell fate decisions. The sprouting of endothelial cells from microvessels, or angiogenesis, involves the modulation of the endothelial cell phenotype. Based on the function of other Notch family members and the expression pattern of Notch4, we postulated that Notch4 activation would modulate angiogenesis. Using an in vitro endothelial-sprouting assay, we show that expression of constitutively active Notch4 in human dermal microvascular endothelial cells (HMEC-1) inhibits endothelial sprouting. We also show that activated Notch4 inhibits vascular endothelial growth factor (VEGF)-induced angiogenesis in the chick chorioallantoic membrane in vivo. Activated Notch4 does not inhibit HMEC-1 proliferation or   migration   through fibrinogen. However,   migration   through collagen is inhibited. Our data show that Notch4 cells exhibit increased beta1-integrin-mediated adhesion to collagen. HMEC-1 expressing activated Notch4 do not have increased surface expression of beta 1-integrins. Rather, we demonstrate that Notch4-expressing cells display beta1-integrin in an active, high-affinity conformation. Furthermore, using function-activating beta 1-integrin antibodies, we demonstrate that activation of beta1-integrins is sufficient to inhibit VEGF-induced endothelial sprouting in vitro and angiogenesis in vivo. Our findings suggest that constitutive Notch4 activation in endothelial cells inhibits angiogenesis in part by promoting beta 1-integrin-mediated adhesion to the underlying matrix. 
 Association of CD98, integrin β1, integrin β3 and Fak with the progression and liver metastases of colorectal cancer. CD98-mediated β1 and β3 integrins activation can induce Fak phosphorylation which eventually promotes cell survival, proliferation,   and migra  tion. We evaluated the expression of CD98, integrin β1, integrin β3 and Fak in 45 cases of matched colorectal cancer (CRC) and liver metastases as well as 35 cases of CRC without liver metastases. There was a gradual increase of the expression of CD98, integrin β1, integrin β3 and Fak as tumor progressed from normal colon to carcinoma to budding tumor cells at the invasive front and to liver metastases. The expression of CD98 and integrin β1 in CRC with liver metastases was significantly higher than that in CRC without liver metastases. Furthermore, for those liver metastases with desmoplastic growth pattern, expression of CD98, integrin β1, integrin β3 and Fak at the metastases center was as strong as that at the metastases periphery. For those liver metastases with pushing or replacement growth patterns, more intense expression of these markers was found at the metastases center than the periphery. Overexpression of CD98, integrin β1, integrin β3 and Fak is associated with the progression and liver metastases of CRC. Overexpression of these markers in liver metastases requires direct contact between tumor cells and the stroma. 
 Expression and correlation of Lewis y antigen and integrins α5 and β1 in ovarian serous and mucinous carcinoma. This study investigates the expression and the clinical significance of Lewis y and integrins α5 and β1 in serous and mucinous ovarian tumors and then evaluates the association between them.Lewis y and integrin α5 and β1 expression are detected on tissues from malignant, borderline, and benign ovarian serous and mucinous tumors and normal tissues. Their expression and relationship are assessed in paraffin sections using immunohistochemistry and double-labeling immunofluorescence method.Lewis y was mainly expressed in ovarian serous and mucinous cancers (88.33%); its positive rate was obviously higher than rates in the borderline (60.00%, P < 0.05) and benign ovarian tumors (35.00%, P < 0.01) and normal ovarian tissues (0, P < 0.01) and was not associated with clinicopathological characteristics. Integrins α5 (85.00%) and β1 (81.67%) were also mainly expressed in ovarian serous and mucinous cancers; their positive rates were all obviously higher than those in benign ovarian tumors (60.00% and 55.00%, respectively; all P < 0.05) and normal tissues (40.00% and 30.00%, respectively; all P < 0.01). Increased expression of integrins α5 and β1 correlated with higher clinical stage (P < 0.05) but were not associated with histological types, differentiation degree, and lymphatic metastasis (P > 0.05). The expression intensity of Lewis y and integrins α5 and β1 was significant with clinical stage and differentiation degree (all P < 0.05) in ovarian cancer; positive significant correlation between Lewis y antigen and integrins α5 and β1 was observed in serous and mucinous ovarian cancer tissues.A close correlation between Lewis y, integrins α5 and β1, and ovarian cancer was observed. Lewis y can influence the biological behavior of a tumor cell as an important composition of integrins α5 and β1 by some signal pathway, such as promoting   cell adhe  sion and migration, and this study provides theoretical evidence of ovarian cancer biological treatment. 
 ECM regulates MT1-MMP localization with beta1 or alphavbeta3 integrins at distinct cell compartments modulating its internalization and activity on human endothelial cells. Regulation of membrane-type 1 matrix metalloproteinase (MT1-MMP) by different extracellular matrices (ECMs) on human endothelial cells (ECs) has been investigated. First, MT1-MMP is found at the intercellular contacts of confluent ECs grown on beta1 integrin-dependent matrix such as type 1 collagen (COL I), fibronectin (FN), or fibrinogen (FG), but not on gelatin (GEL) or vitronectin (VN). The novel localization of MT1-MMP at cell-cell contacts is assessed by confocal videomicroscopy of MT1-MMP-GFP-transfected ECs. Moreover, MT1-MMP colocalizes with beta1 integrins at the intercellular contacts, whereas it is preferentially found with alphavbeta3 integrin at motility-associated structures on migrating ECs. In addition, clustered integrins recruit MT1-MMP and neutralizing anti-beta1 or anti-alphav integrin mAb displace MT1-MMP from its specific sites, pointing to a biochemical association that is finally demonstrated by coimmunoprecipitation assays. On the other hand, COL I, FN, or FG up-regulate cell surface MT1-MMP on confluent ECs by an impairment of its internalization, whereas expression and internalization are not modified on GEL or VN. In addition, MT1-MMP activity is diminished in confluent ECs on COL I, FN, or FG. Finally, MT1-MMP participates and cooperates with beta1 and alphavbeta3 integrins in the   migration   of ECs on different ECM. These data show a novel mechanism by which ECM regulates MT1-MMP association with beta1 or alphavbeta3 integrins at distinct cellular compartments, thus modulating its internalization, activity, and function on human ECs. 
 Rab25 associates with alpha5beta1 integrin to promote invasive   migration   in 3D microenvironments. Here, we report a direct interaction between the beta1 integrin cytoplasmic tail and Rab25, a GTPase that has been linked to tumor aggressiveness and metastasis. Rab25 promotes a mode of   migration   on 3D matrices that is characterized by the extension of long pseudopodia, and the association of the GTPase with alpha5beta1 promotes localization of vesicles that deliver integrin to the plasma membrane at pseudopodial tips as well as the retention of a pool of cycling alpha5beta1 at the cell front. Furthermore, Rab25-driven tumor-cell invasion into a 3D extracellular matrix environment is strongly dependent on ligation of fibronectin by alpha5beta1 integrin and the capacity of Rab25 to interact with beta1 integrin. These data indicate that Rab25 contributes to tumor progression by directing the localization of integrin-recycling vesicles and thereby enhancing the ability of tumor cells to invade the extracellular matrix. 
 Alpha9beta1 integrin in melanoma cells can signal different adhesion states for   migration   and anchorage. Cell surface integrins are the primary receptors for cell   migration   on extracellular matrix, and exist in several activation states regulated in part by ectodomain conformation. The alpha9 integrin subunit, which pairs only with beta1, has specific roles in the immune system and may regulate cell   migration  . Melanoma cells express abundant alpha9beta1 integrin, and its role in cell   migration   was assessed. Ligands derived from Tenascin-C and ADAM12 supported alpha9beta1 integrin-mediated cell attachment and GTP-Rac dependent   migration  , but not focal adhesion formation. Manganese ions induced alpha9beta1 integrin- and Rho kinase-dependent focal adhesion and stress fibre formation, suggesting that the activation status of alpha9beta1 integrin was altered. The effect of manganese ions in promoting focal adhesion formation was reproduced by beta1 integrin activating antibody. The alpha9beta1 integrin translocated to focal adhesions, where active beta1 integrin was also detected by conformation-specific antibodies. Focal adhesion assembly was commensurate with reduced cell   migration  . Endogenous alpha9beta1 integrin-mediated adhesion was sensitive to the PP1 chemical inhibitor and an inhibitor of endosomal vesicle recycling, but not inhibitors of protein kinase C or the small GTPase Rho. Our results demonstrated that although alpha9beta1 integrin can induce and localise to focal adhesions in a high activation state, its intermediate activity state normally supports cell adhesion consistent with   migration  . 
 Bombesin-dependent pro-MMP-9 activation in prostatic cancer cells requires beta1 integrin engagement. Bombesin-like peptides, including the mammalian homologue gastrin-releasing peptide, are highly expressed and secreted by neuroendocrine cells in prostate carcinoma tissues and are likely to be related to the progression of this neoplastic disease. Previously, we demonstrated that bombesin increased   migration   and protease expression in androgen-independent cells. In this work we show that bombesin is able to activate pro-MMP-9 through a mechanism involving the beta1 integrin subunit. In fact, MMP-9 processing was evident only when beta1 integrin was engaged with specific adhesive substrates, such as type I collagen, or when cells were seeded on dishes coated with antibodies against beta1 integrin, resulting in activation of the surface ligand. When exogenous pro-MMP-9 was added to PC3 cells, MMP-9 active forms were produced within 30 min by bombesin-treated cultures while control cultures expressed activated forms only after a longer time and at lower levels. MMP-9 activation required cytoskeleton integrity since this effect was abolished by cytochalasin D. Engagement of beta1 integrin caused an increased membrane-linked uPA activity which was required for MMP-9 activation. The cross talk between bombesin- and beta1-integrin-engaged signals seems to be crucial for the modulation of both membrane-linked uPA activity and MMP-9 activation and triggers complex intracellular signaling pathways requiring activation of tyrosine kinase activity, including that of src and PI3K. The beta1 integrin may be considered an important mechanism by which bombesin induces MMP-9 activation. This finding supports the idea that cellular responses to growth factors may be driven by cell-matrix interactions and stresses the role of neuroendocrine factors in prostate carcinoma progression. 
 Core3 O-glycan synthase suppresses tumor formation and metastasis of prostate carcinoma PC3 and LNCaP cells through down-regulation of alpha2beta1 integrin complex. Although there are numerous reports of carbohydrates enriched in cancer cells, very few studies have addressed the functions of carbohydrates present in normal cells that decrease in cancer cells. It has been reported that core3 O-glycans are synthesized in normal gastrointestinal cells but are down-regulated in cancer cells. To determine the roles of core3 O-glycans, we transfected PC3 and LNCaP prostate cancer cells with beta3-N-acetylglucosaminyltransferase-6 (core3 synthase) required to synthesize core3 O-glycans. Both engineered cell lines exhibited reduced   migration   and invasion through extracellular matrix components compared with mock-transfected cells. Moreover we found that alpha2beta1 integrin acquired core3 O-glycans in cells expressing core3 synthase with decreased maturation of beta1 integrin, leading to decreased levels of the alpha2beta1 integrin complex, decreased activation of focal adhesion kinase, and reduced lamellipodia formation. Upon inoculation into the prostate of nude mice, PC3 cells expressing core3 O-glycans produced much smaller tumors without metastasis to the surrounding lymph nodes in contrast to robust tumor formation and metastasis seen in mock-transfected PC3 cells. Similarly LNCaP cells expressing core3 O-glycans barely produced subcutaneous tumors in contrast to robust tumor formation by mock-transfected LNCaP cells. These findings indicate that addition of core3 O-glycans to beta1 and alpha2 integrin subunits in prostate cancer cells suppresses tumor formation and tumor metastasis. 
 Citrullination of collagen II affects integrin-mediated cell adhesion in a receptor-specific manner. Citrullinated collagen II (CII) is a well-known autoantigen in rheumatoid arthritis (RA). However, the direct effects of CII citrullination on cell behavior have not been described. To study whether citrullination of CII could affect cellular functions, we measured the adhesion of 3 different cell types (human Saos2 osteosarcoma cells, human synovial fibroblasts, and rat mesenchymal stem cells) with impedance-based technology. The binding of different collagen receptor integrins to citrullinated collagen was studied by CHO cell lines, each overexpressing 1 of the 4 human collagen receptors on the cell surface, and with solid-phase binding assays, using the recombinant human integrin α1I, α2I, α10I, and α11I domains. Collagen citrullination decreased the adhesion of synovial fibroblasts ∼50% (P<0.05) and mesenchymal stem cells ∼40% (P<0.05) by specifically decreasing the binding of integrins α10β1 and α11β1 to arginine-containing motifs, such as GFOGER. In contrast, citrullination had only a minor effect on the function of α1β1 and α2β1 integrins, which have been reported to play a critical role in regulating leukocyte function. Molecular modeling was used to explain the detected functional differences at the structural level. Given that the integrins regulate cell metabolism, pro  liferatio  n, and migration, we suggest that collagen citrullination modifies the pathogenesis of RA. Here, CII citrullination was shown to decrease the survival of mesenchymal stem cells. 
 β1 integrin controls EGFR signaling and tumorigenic properties of lung cancer cells. Lung cancer is the leading cause of cancer death worldwide. The epidermal growth factor receptor (EGFR) represents the main target for non-small cell lung cancer (NSCLC) therapy, as its overexpression or constitutive activation contributes to malignancy and correlates with poor prognosis. Our previous work demonstrated that in epithelial cells β1 integrin is required for propagating EGFR signaling from the plasma membrane to the nucleus. In this study, we silenced β1 integrin in human NSCLC A549 cells. The β1 integrin-silenced cells show a defective activation of the EGFR signaling cascade, leading to decreased in vitro proliferation, enhanced sensitivity to cisplatin and Gefitinib, impai  red migra  tion and invasive behavior. Inhibitory effects on tumor growth and on the EGFR pathway were also observed in in vivo experiments. Moreover, β1 integrin silencing increases the amount of EGFR on the cell surface, suggesting that β1 integrin is required for efficient constitutive EGFR turnover at the cell membrane. Although the rate of EGF internalization and recycling is not affected in silenced cells, EGFR signaling is recovered only by expression of the Rab-coupling protein RCP, indicating that β1 integrin sustains the endocytic machinery required for EGFR signaling. Overall, these results show that β1 integrin is an essential regulator of EGFR signaling and tumorigenic properties of lung cancer cells, and that its silencing might represent an adjuvant approach to anti-EGFR therapy. 
 Cleavage of ST6Gal I by radiation-induced BACE1 inhibits golgi-anchored ST6Gal I-mediated sialylation of integrin β1 and   migratio  n in colon cancer cells. Previously, we found that β-galactoside α2,6-sialyltransferase (ST6Gal I), an enzyme that adds sialic acids to N-linked oligosaccharides of glycoproteins and is frequently overexpressed in cancer cells, is up-regulated by ionizing radiation (IR) and cleaved to a form possessing catalytic activity comparable to that of the Golgi-localized enzyme. Moreover, this soluble form is secreted into the culture media. Induction of ST6Gal I significantly increased t  he migrat  ion of colon cancer cells via sialylation of integrin β1. Here, we further investigated the mechanisms underlying ST6Gal I cleavage, solubilization and release from cells, and addressed its functions, focusing primarily on cancer c  ell migra  tion.We performed immunoblotting and lectin affinity assay to analyze the expression of ST6 Gal I and level of sialylated integrin β1. After ionizing radiat  ion, migr  ation of cells was measured by in v  itro migr  ation assay. α2, 6 sialylation level of cell surface was analyzed by flow cytometry. Cell culture media were concentrated and then analyzed for soluble ST6Gal I levels using an α2, 6 sialyltransferase sandwich ELISA.We found that ST6Gal I was cleaved by BACE1 (β-site amyloid precursor protein-cleaving enzyme), which was specifically overexpressed in response to IR. The soluble form of ST6Gal I, which also has sialyltransferase enzymatic activity, was cleaved from the Golgi membrane and then released into the culture media. Both non-cleaved and cleaved forms of ST6Gal I significantly increased colon canc  er cell m  igration in a sialylation-dependent manner. The pro-migratory effect of the non-cleaved form of ST6Gal I was dependent on integrin β1 sialylation, whereas that of the cleaved form of ST6Gal I was not, suggesting that other intracellular sialylated molecules apart from cell surface molecules such as integrin β1 might be involved in mediating the pro-migratory effects of the soluble form of ST6Gal I. Moreover, production of soluble form ST6Gal I by BACE 1 inhibited integrin β1 sialy  lation an  d migration by Golgi-anchored form of ST6Gal I.Our results suggest that soluble ST6Gal I, possibly in cooperation with the Golgi-bound form, may participate in cancer progression and metastasis prior to being secreted from cancer cells. 
 Differential expression of beta 1c integrin messenger ribonucleic acid and protein levels in human endometrium and decidua during the menstrual cycle and pregnancy. beta(1C) and beta(1A) integrins are alternatively spliced variants of the human beta(1)-subunit; the former has been shown to inhibit cell proliferation, and the latter to promote it. Although some components of the beta(1) integrin subfamily are expressed in human endometrial and decidual cells during the menstrual cycle and early pregnancy, to date no information is available about the expression of beta(1C) integrin in endometrial and decidual tissues and its possible roles during implantation and pregnancy. To gain further insight on this subject, we have explored beta(1C) integrin expression in endometrial (proliferative, secretory, and atrophic) and decidual (from the first and third trimesters of pregnancy) tissue samples at both gene and protein levels by Northern and Western blotting analyses and by immunohistochemistry. beta(1A) protein levels were also measured in the same tissues as a control. The results of this study demonstrate that both beta(1C)- and beta(1A)-subunits are expressed in the endometrium and decidua. In the former, maximal beta(1C) expression was detected in atrophic endometria, whereas beta(1A) expression levels were increased in secretive and decreased in atrophic endometrial tissues compared with proliferative endometria. In addition, whereas beta(1A) levels were significantly increased in decidual tissues, compared with proliferative endometria, beta(1C) expression was dramatically reduced in the same tissues, thus pointing to selective down-regulation of beta(1C) expression in the decidua. These data suggest that the expression of beta(1C) integrin, a very efficient inhibitor of cell proliferation, may be modulated by the maternal microenvironment and may play a fundamental role in mediating trophoblast outgrowth and   migration   during pregnancy. 
 Galectin-1 interacts with beta-1 subunit of integrin. Galectin-1, a beta-galactoside-binding dimeric lectin, is involved in adhesion,   migration  , and proliferation of vascular smooth muscle cells (SMC), the key steps in the development of atherosclerosis and restenosis. Here we investigated the molecular basis of the interactions between galectin-1 and SMCs. Galectin-1 modulated SMC attachment in a dose- and beta-galactoside-dependent manner. Direct binding of galectin-1 to beta1 integrin was detected by the immune precipitation of beta1 integrin after chemical cross-linking of 125I-labelled galectin-1 to the cell surface proteins. Galectin-1 transiently increased availability of beta1 integrins on the cell surface to antibodies against beta1 integrin. Incubation of SMCs with galectin-1 transiently increased the amount of the active form of beta1 integrin and tyrosine phosphorylation of two cytoskeleton-associated proteins; one of them coincided with focal adhesion kinase (FAK). Galectin-1 is likely to affect SMC adhesion by interacting with beta1 integrin on the cell surface of SMCs and inducing outside-in signalling. 
 Silencing profilin-1 inhibits gastric cancer progression via integrin β1/focal adhesion kinase pathway modulation. To investigate the role of profilin-1 (PFN1) in gastric cancer and the underlying mechanisms.Immunohistochemical analysis, quantitative real-time polymerase chain reaction (qRT-PCR) and Western blot were performed to detect PFN1 expression in clinical gastric carcinoma and adjacent tissues, and the association of PFN1 expression with patient clinicopathological characteristics was analyzed. PFN1 was knocked down to investigate the role of this protein in cell proliferation and metastasis in the SGC-7901 cell line. To explore the underlying mechanisms, the expression of integrin β1 and the activity of focal adhesion kinase (FAK) and the downstream proteins extracellular-regulated kinase (ERK)1/2, P38 mitogen-activated protein kinase (MAPK), phosphatidylinositol 3-kinase (PI3K), AKT and mammalian target of rapamycin (mTOR) were measured through Western blot or qRT-PCR analysis. Fibronectin (FN), a ligand of integrin β1, was used to verify the correlation between alterations in the integrin β1/FAK pathway and changes in tumor cell aggressiveness upon PFN1 perturbation.Immunohistochemical, Western blot and qRT-PCR analyses revealed that PFN1 expression was higher at both the protein and mRNA levels in gastric carcinoma tissues compared with the adjacent tissues. In addition, high PFN1 expression (53/75, 70.4%) was correlated with tumor infiltration, lymph node metastasis and TNM stage in gastric cancer, but not with gender, age, location, tumor size, or histological differentiation. In vitro experiments showed that PFN1 knockdown inhibited the proliferation of SGC-7901 cells through the induction G0/G1 arrest. Silencing PFN1 inhibited c  ell migra  tion and invasion and down-regulated the expression of matrix metalloproteinase (MMP)-2 and MMP9. Moreover, silencing PFN1 reduced the expression of integrin β1 at the protein level and inhibited the activity of FAK, and the downstream effectors ERK1/2, P38MAPK, PI3K, AKT and mTOR. FN-promoted cell proliferation and metastasis via the integrin β1/FAK pathway was ameliorated by PFN1 silencing.These findings suggest that PFN1 plays a critical role in gastric carcinoma progression, and these effects are likely mediated through the integrin β1/FAK pathway. 
 CD98 modulates integrin beta1 function in polarized epithelial cells. The type II transmembrane protein CD98, best known as the heavy chain of the heterodimeric amino acid transporters (HAT), is required for the surface expression and basolateral localization of this transporter complex in polarized epithelial cells. CD98 also interacts with beta1 integrins resulting in an increase in their affinity for ligand. In this study we explored the role of the transmembrane and cytoplasmic domains of CD98 on integrin-dependent cell adhesion and   migration   in polarized renal epithelial cells. We demonstrate that the transmembrane domain of CD98 was sufficient, whereas the five N-terminal amino acids of this domain were required for CD98 interactions with beta1 integrins. Overexpression of either full-length CD98 or CD98 lacking its cytoplasmic tail increased cell adhesion and   migration  , whereas deletion of the five N-terminal amino acids of the transmembrane domain of CD98 abrogated this effect. CD98 and mutants that interacted with beta1 integrins increased both focal adhesion formation and FAK and AKT phosphorylation. CD98-induced cell adhesion and   migration   was inhibited by addition of phosphoinositol 3-OH kinase (PI3-K) inhibitors suggesting these cell functions are PI3-K-dependent. Finally, CD98 and mutants that interacted with beta1, induced marked changes in polarized renal epithelial cell branching morphogenesis in collagen gels. Thus, in polarized renal epithelial cells, CD98 might be viewed as a scaffolding protein that interacts with basolaterally expressed amino acid transporters and beta1 integrins and can alter diverse cellular functions such as amino acid transport as well as cell adhesion,   migration   and branching morphogenesis. 
 Mycobacterium tuberculosis infection of human dendritic cells decreases integrin expression, adhesion and   migration   to chemokines. Tuberculosis (TB) remains a major global health problem accounting for millions of deaths annually. Approximately one-third of the world's population is infected with the causative agent Mycobacterium tuberculosis. The onset of an adaptive immune response to M. tuberculosis is delayed compared with other microbial infections. This delay permits bacterial growth and dissemination. The precise mechanism(s) responsible for this delay have remained obscure. T-cell activation is preceded by dendritic cell (DC)   migration   from infected lungs to local lymph nodes and synapsis with T cells. We hypothesized that M. tuberculosis may impede the ability of DCs to reach lymph nodes and initiate an adaptive immune response. We used primary human DCs to determine the effect of M. tuberculosis on expression of heterodimeric integrins involved in cellular adhesion and   migration  . We also evaluated the ability of infected DCs to adhere to and migrate through lung endothelial cells, which is necessary to reach lymph nodes. We show by flow cytometry and confocal microscopy that M. tuberculosis-infected DCs exhibit a significant reduction in surface expression of the β(2) (CD18) integrin. Distribution of integrin β(2) is also markedly altered in M. tuberculosis-infected DCs. A corresponding reduction in the αL (CD11a) and αM (CD11b) subunits that associate with integrin β(2) was also observed. Consistent with reduced integrin surface expression, we show a significant reduction in adherence to lung endothelial cell monolayers   and migr  ation towards lymphatic chemokines when DCs are infected with M. tuberculosis. These findings suggest that M. tuberculosis modulates DC adhesion   and migr  ation to increase the time required to initiate an adaptive immune response. 
 JAML, a novel protein with characteristics of a junctional adhesion molecule, is induced during differentiation of myeloid leukemia cells. Retinoic acid induces clinical remission in acute promyelocytic leukemia (APL) by triggering differentiation of leukemia promyelocytes. Here, we have characterized a gene encoding a member of the immunoglobulin superfamily, among novel retinoic acid-induced genes identified in APL cells. This protein, which was named JAML (junctional adhesion molecule-like), contains 2 extracellular immunoglobulin-like domains, a transmembrane segment, and a cytoplasmic tail. JAML mRNA is expressed in hematopoietic tissues and is prominently expressed in granulocytes. The fact that JAML protein is localized at the cell plasma membrane in the areas of cell-cell contacts, whereas it is not detected at free cell borders, suggests that JAML is engaged in homophilic interactions. Furthermore, a conserved dimerization motif among JAM members was shown to be important for JAML localization at the cell membrane. Finally, exogenous expression of JAML in myeloid leukemia cells resulted in enhanced cell adhesion to endothelial cells. Altogether, our results point to JAML as a novel member of the JAM family expressed on leukocytes with a possible role in leukocyte trans  migration  . 
 Glia Maturation Factor-γ Regulates Monocyte   Migratio  n through Modulation of β1-Integrin. Monocyt  e migrati  on requires the dynamic redistribution of integrins through a regulated endo-exocytosis cycle, but the complex molecular mechanisms underlying this process have not been fully elucidated. Glia maturation factor-γ (GMFG), a novel regulator of the Arp2/3 complex, has been shown to regulate direction  al migrat  ion of neutrophils and T-lymphocytes. In this study, we explored the important role of GMFG in monocyte chemotaxis, adhesion, and β1-integrin turnover. We found that knockdown of GMFG in monocytes resulted in impaired chemotac  tic migra  tion toward formyl-Met-Leu-Phe (fMLP) and stromal cell-derived factor 1α (SDF-1α) as well as decreased α5β1-integrin-mediated chemoattractant-stimulated adhesion. These GMFG knockdown impaired effects could be reversed by cotransfection of GFP-tagged full-length GMFG. GMFG knockdown cells reduced the cell surface and total protein levels of α5β1-integrin and increased its degradation. Importantly, we demonstrate that GMFG mediates the ubiquitination of β1-integrin through knockdown or overexpression of GMFG. Moreover, GMFG knockdown retarded the efficient recycling of β1-integrin back to the plasma membrane following normal endocytosis of α5β1-integrin, suggesting that the involvement of GMFG in maintaining α5β1-integrin stability may occur in part by preventing ubiquitin-mediated degradation and promoting β1-integrin recycling. Furthermore, we observed that GMFG interacted with syntaxin 4 (STX4) and syntaxin-binding protein 4 (STXBP4); however, only knockdown of STXBP4, but not STX4,   reduced m  onocyte migration and decreased β1-integrin cell surface expression. Knockdown of STXBP4 also substantially inhibited β1-integrin recycling in human monocytes. These results indicate that the effects o  f GMFG on   monocyte migration and adhesion probably occur through preventing ubiquitin-mediated proteasome degradation of α5β1-integrin and facilitating effective β1-integrin recycling back to the plasma membrane. 
 Human tumstatin and human endostatin exhibit distinct antiangiogenic activities mediated by alpha v beta 3 and alpha 5 beta 1 integrins. Tumstatin and endostatin are two inhibitors of angiogenesis derived from precursor human collagen molecules known as alpha 3 chain of type IV collagen and alpha1 chain of type XVIII collagen, respectively. Although both these inhibitors are noncollagenous (NC1) domain fragments of collagens, they only share a 14% amino acid homology. In the present study we evaluated the functional receptors, mechanism of action, and intracellular signaling induced by these two collagen-derived inhibitors. Human tumstatin prevents angiogenesis via inhibition of endothelial cell proliferation and promotion of apoptosis with no effect on   migration  , whereas human endostatin prevents endothelial cell   migration   with no effect on proliferation. We demonstrate that human tumstatin binds to alpha v beta 3 integrin in a vitronectin/fibronectin/RGD cyclic peptide independent manner, whereas human endostatin competes with fibronectin/RGD cyclic peptide to bind alpha 5 beta 1 integrin. The activity of human tumstatin is mediated by alpha v beta 3 integrin, whereas the activity of human endostatin is mediated by alpha 5 beta 1 integrin. Additionally, although human tumstatin binding to alpha v beta 3 integrin leads to the inhibition of Cap-dependent translation (protein synthesis) mediated by focal adhesion kinase/phosphatidylinositol 3-kinase/Akt/mTOR/4E-BP1 pathway, human endostatin binding to alpha 5 beta 1 integrin leads to the inhibition of focal adhesion kinase/c-Raf/MEK1/2/p38/ERK1 mitogen-activated protein kinase pathway, with no effect on phosphatidylinositol 3-kinase/Akt/mTOR/4E-BP1 and Cap-dependent translation. Collectively, such distinct properties of human tumstatin and human endostatin provide the first insight into their diverse antiangiogenic actions and argue for combining them for targeting tumor angiogenesis. 
 Hyperhomocysteinemia suppresses bone marrow CD34+/VEGF receptor 2+ cells and inhibits progenitor cell mobilization and homing to injured vasculature-a role of β1-integrin in progenitor cell   migratio  n and adhesion. Hyperhomocysteinemia (HHcy) impairs re-endothelialization and accelerates vascular remodeling. The role of CD34(+)/VEGF receptor (VEGFR) 2(+) progenitor cells (PCs) in vascular repair in HHcy is unknown. We studied the effect of HHcy on PCs and its role in vascular repair in severe HHcy (∼150 μM), which was induced in cystathionine-β synthase heterozygous mice fed a high-methionine diet for 8 weeks. Vascular injury was introduced by carotid air-dry endothelium denudation. CD34(+)/VEGFR2(+) cells were examined by flow cytometry. HHcy reduced bone marrow (BM) CD34(+)/VEGFR2(+) cells and suppressed replenishment of postinjury CD34(+)/VEGFR2(+) cells in peripheral blood (PB). Donor green fluorescent protein-positive PC homing to the injured vessel was reduced in HHcy after CD34(+) PCs from enhanced green fluorescent protein mice were adoptively transferred following carotid injury. CD34(+) PC transfusion partially reversed HHcy-suppressed re-endothelialization and HHcy-induced neointimal formation. Furthermore, homocysteine (Hcy) inhibited proliferation, adhesion,   and migr  ation and suppressed β1-integrin expression and activity in human CD34(+) endothelial colony-forming cells (ECFCs) isolated from PBs in a dose-dependent manner. A functional-activating β1-integrin antibody rescued Hcy-suppressed adhesi  on and mi  gration in CD34(+) ECFCs. In conclusion, HHcy reduces BM CD34(+)/VEGFR2(+) generation and suppresses CD34(+)/VEGFR2(+) cell mobilization and homing to the injured vessel via β1-integrin inhibition, which partially contributes to impaired re-endothelialization and vascular remodeling. 
 Galectin-3 promotes lamellipodia formation in epithelial cells by interacting with complex N-glycans on alpha3beta1 integrin. Recent studies have shown that galectin-3 (Gal-3; also known as LGALS3), a beta-galactoside-binding lectin, promotes cell   migration   during re-epithelialization of corneal wounds. The goal of this study was to characterize the molecular mechanism by which Gal-3 stimulates cell   migration  . We demonstrate here that exogenous Gal-3, but not Gal-1 or Gal-8, promotes cell scattering and formation of lamellipodia in human corneal epithelial cells in a beta-lactose-inhibitable manner. alpha3beta1 integrin was identified as the major Gal-3-binding protein in corneal epithelial cells by affinity chromatography of cell lysates on a Gal-3-Sepharose column. Preincubation of cells with anti-alpha3 integrin function-blocking antibody significantly inhibited the induction of lamellipodia by Gal-3. Furthermore, exogenous Gal-3 activated both focal adhesion kinase, a key regulator of integrin-dependent intracellular signaling, and Rac1 GTPase, a member of the family of Rho GTPases, well known for its role in the reorganization of the actin cytoskeleton and formation of lamellipodial extensions. Experiments involving knockdown of beta-1,6-N-acetylglucosaminytransferase V, an enzyme that synthesizes high-affinity glycan ligands for Gal-3, revealed that carbohydrate-mediated interaction between Gal-3 and complex N-glycans on alpha3beta1 integrin plays a key role in Gal-3-induced lamellipodia formation. We propose that Gal-3 promotes epithelial cell   migration   by cross-linking MGAT5-modified complex N-glycans on alpha3beta1 integrin and subsequently activating alpha3beta1-integrin-Rac1 signaling to promote lamellipodia formation. 
 The metastasis-promoting phosphatase PRL-3 shows activity toward phosphoinositides. Phosphatase of regenerating liver 3 (PRL-3) is suggested as a biomarker and therapeutic target in several cancers. It has a well-established causative role in cancer metastasis. However, little is known about its natural substrates, pathways, and biological functions, and only a few protein substrates have been suggested so far. To improve our understanding of the substrate specificity and molecular determinants of PRL-3 activity, the wild-type (WT) protein, two supposedly catalytically inactive mutants D72A and C104S, and the reported hyperactive mutant A111S were tested in vitro for substrate specificity and activity toward phosphopeptides and phosphoinositides (PIPs), their structural stability, and their ability to promote cell   migration   using stable HEK293 cell lines. We discovered that WT PRL-3 does not dephosphorylate the tested phosphopeptides in vitro. However, as shown by two complementary biochemical assays, PRL-3 is active toward the phosphoinositide PI(4,5)P(2). Our experimental results substantiated by molecular docking studies suggest that PRL-3 is a phosphatidylinositol 5-phosphatase. The C104S variant was shown to be not only catalytically inactive but also structurally destabilized and unable to promote cell   migration  , whereas WT PRL-3 promotes cell   migration  . The D72A mutant is structurally stable and does not dephosphorylate the unnatural substrate 3-O-methylfluorescein phosphate (OMFP). However, we observed residual in vitro activity of D72A against PI(4,5)P(2), and in accordance with this, it exhibits the same cellular phenotype as WT PRL-3. Our analysis of the A111S variant shows that the hyperactivity toward the unnatural OMFP substrate is not apparent in dephosphorylation assays with phosphoinositides: the mutant is completely inactive against PIPs. We observed significant structural destabilization of this variant. The cellular phenotype of this mutant equals that of the catalytically inactive C104S mutant. These results provide a possible explanation for the absence of the conserved Ser of the PTP catalytic motif in the PRL family. The correlation of the phosphatase activity toward PI(4,5)P(2) with the observed phenotypes for WT PRL-3 and the mutants suggests a link between the PI(4,5)P(2) dephosphorylation by PRL-3 and its role in cell   migration  . 
 Maspin, the molecular bridge between the plasminogen activator system and beta1 integrin that facilitates cell adhesion. Maspin is a non-inhibitory serine protease inhibitor (serpin) that influences many cellular functions including adhesion,   migration  , and invasion. The underlying molecular mechanisms that facilitate these actions are still being elucidated. In this study we determined the mechanism by which maspin mediates increased MCF10A cell adhesion. Utilizing competition peptides and mutation analyses, we discovered two unique regions (amino acid residues 190-202 and 260-275) involved in facilitating the increased adhesion function of maspin. In addition, we demonstrate that the urokinase-type plasminogen activator (uPA)/uPA receptor (uPAR) complex is required for the localization and adhesion function of maspin. Finally, we showed that maspin, uPAR, and β1 integrin co-immunoprecipitate, suggesting a novel maspin-uPA-uPAR-β1 integrin mega-complex that regulates mammary epithelial cell adhesion. 
 Expression of laminin-5 and integrins in actinic cheilitis and superficially invasive squamous cell carcinomas of the lip. The progression of carcinogenesis entails the detachment of cells, invasion and   migration   of neoplastic cells. Alterations in epithelial adhesion and basement membrane proteins might mediate the early stages of carcinogenesis. This study investigated the expression of adhesion molecules and the basement membrane protein laminin-5 in actinic cheilitis (AC) and incipient squamous cell carcinoma of the lower lip to understand early photocarcinogenesis. Ln-5γ2 chain as well as α3, β1 subunits of α3β1 heterodimer and β4 subunit of integrin α6β4 were evaluated by immunohistochemistry in 16 cases of AC and 16 cases of superficially invasive squamous cell carcinoma (SISCC). Most AC cases showed reduced expression of β1, β4 and α3 integrins, and SISCCs lacked β1, β4 and α3 integrins in the invasive front. AC cases were negative for the Ln-5γ2 chain. Five cases of SISCC (31%) showed heterogeneous Ln-5γ2 chain expression in the invasive front of the tumor. Integrin β1, β4 and α3 expression is lost during the early stages of lip carcinogenesis. Expression of Ln-5γ2 in the invasive front in cases and its correlation with tumor progression suggest that it mediates the acquisition of the migrating and invading epithelial cell phenotype. 
 Reduced podocyte expression of alpha3beta1 integrins and podocyte depletion in patients with primary focal segmental glomerulosclerosis and chronic PAN-treated rats. Integrins attach cells to extracellular matrix (ECM) and mediate signals from ECM to cells or from cells to ECM. They regulate cell functions, including adhesion,   migration  , cell cycle regulation, and differentiation. Podocytes may detach from the glomerular basement membrane (GBM) and be excreted in the urine, and proteinuria is found in patients with primary focal segmental glomerulosclerosis (FSGS); both may be associated with loss of alpha3beta1integrins. In this study, we have examined the podocyte number in patients with primary FSGS and normal controls, and the alpha3- and beta1-integrin subunits expression of podocytes in patients with primary FSGS and chronic puromycin aminonucleoside (PAN)-treated rats by the morphometric, immunoperoxidase histochemical, and immunoelectron microscopic examination. We also measured their expression serially in rats that received repeated PAN injection. The results showed that the podocyte number was significantly decreased in patients with primary FSGS than in normal control (P < 0.05). The immunostaining score showed that both alpha3- and beta1-integrin subunits on podocytes in patients with primary FSGS were significantly lower than in normal controls (both P < 0.01). The number of immuno-gold particles of alpha3- and beta1-integrins at the effaced foot process area of patients with primary FSGS were also significantly decreased than that of normal controls (both P < 0.05). The immunostaining score of both alpha3- and beta1-integrin subunits was negatively correlated with the degree of glomerular sclerosing score and the amount of daily protein loss, and they were positively correlated with the number of podocytes. Chronic 12-week PAN-treated rats showed similar findings with decreased immunostaining expression and immuno-gold particles of alpha3-integrin on podocytes than in normal control (both P < 0.05). The chronic PAN-treated rats also showed a trend toward gradually decreased immunostaining expression of alpha3-integrin subunit on podocyte during the progress from normal to FSGS state. These studies indicate that podocyte expression of alpha3- and beta1-integrin subunits is significantly reduced in humans with primary FSGS and chronic PAN-treated rats, before the morphological changes of FSGS are observed. The decreased podocyte expression of alpha3beta1 integrins is closely related with podocyte depletion, glomerular sclerosis, and daily protein loss in patients with primary FSGS. 
 CCN3 (NOV) is a novel angiogenic regulator of the CCN protein family. CCN3 (NOV) is a matricellular protein of the CCN family, which also includes CCN1 (CYR61), CCN2 (CTGF), CCN4 (WISP-1), CCN5 (WISP-2), and CCN6 (WISP-3). During development, CCN3 is expressed widely in derivatives of all three germ layers, and high levels of expression are observed in smooth muscle cells of the arterial vessel wall. Altered expression of CCN3 has been observed in a variety of tumors, including hepatocellular carcinomas, Wilm's tumors, Ewing's sarcomas, gliomas, rhabdomyosarcomas, and adrenocortical carcinomas. To understand its biological functions, we have investigated the activities of purified recombinant CCN3. We show that in endothelial cells, CCN3 supports cell adhesion, induces directed cell   migration   (chemotaxis), and promotes cell survival. Mechanistically, CCN3 supports human umbilical vein endothelial cell adhesion through multiple cell surface receptors, including integrins alphavbeta3, alpha5beta1, alpha6beta1, and heparan sulfate proteoglycans. In contrast, CCN3-induced cell   migration   is dependent on integrins alphavbeta3 and alpha5beta1, whereas alpha6beta1 does not play a role in this process. Although CCN3 does not contain a RGD sequence, it binds directly to immobilized integrins alphavbeta3 and alpha5beta1, with half-maximal binding occurring at 10 nm and 50 nm CCN3, respectively. Furthermore, CCN3 induces neovascularization when implanted in rat cornea, demonstrating that it is a novel angiogenic inducer. Together, these findings show that CCN3 is a ligand of integrins alphavbeta3 and alpha5beta1, acts directly upon endothelial cells to stimulate pro-angiogenic activities, and induces angiogenesis in vivo. 
 β1 integrin signaling in asymmetric   migratio  n of keratinocytes under mechanical stretch in a co-cultured wound repair model. Keratinocyte (KC)   migratio  n in re-epithelization is crucial in repairing injured skin. But the mechanisms of how mechanical stimuli regulate the   migratio  n of keratinocytes have been poorly understood.Human immortalized keratinocyte HaCaT cells were co-cultured with skin fibroblasts on PDMS membranes and transferred to the static stretch device developed in-house for additional 6 day culture under mechanical stretch to mimic surface tension in skin. To detect the expression of proteins on different position at different time points and the effect of β1 integrin mechanotransduction on HaC  aT migrat  ion, Immunofluorescence, Reverse transcription-polymerase chain reaction, Flow cytometry, Western blotting assays were applied.Mechanical receptor of β1 integrin that recognizes its ligand of collagen I was found to be strongly associated w  ith migra  tion of HaCaT cells since the knockdown of β1 integrin via RNA silence eliminated the key protein expression dynamically. Here the expression of vinculin was lower but that of Cdc42 was higher for the cells at outward edge than those at inward edge, respectively, supporting that   the migr  ation capability of keratinocytes is inversely correlated with the formation of focal adhesion complexes but positively related to the lamellipodia formation. This asymmetric expression feature was further confirmed by high or low expression of PI3K for outward- or inward-migrating cells. And ERK1/2 phosphorylation was up-regulated by mechanical stretch.We reported here, a novel mechanotransduction signaling pathways were β1 integrin-dependent pattern of keratino  cytes mig  ration under static stretch in an in vitro co-culture model. These results provided an insight into underlying molecular mechanisms of keratin  ocyte mig  ration under mechanical stimuli. 
 Immediate early response protein 2 regulates hepatocellular carcinoma cell adhesion and motility via integrin β1-mediated signaling pathway. Human immediate early response 2 (IER2) has been reported to function as a potential transcriptional factor or transcriptional co‑activator and seems to play a pivotal role in tumor cell motility and metastasis, however, its role and underlying mechanisms in hepatocellular carcinoma (HCC) remain unknown. Herein, we demonstrated that overexpression of IER2 in HCC cells increased cell adhesion to fibron  ectin, mi  gration and invasion, whereas knockdown of IER2 displayed the opposite effects. In agreement with this phenotype, IER2 expression was positively correlated with the metastatic potential and integrin β1 (ITGB1) expression in HCC cell lines. Moreover, we demonstrated a critical role for IER2 in regulation of HCC cell‑extracellular matrix (ECM) adhesion and motility by the transcriptionally promoted ITGB1. Furthermore, we showed that ITGB1‑focal adhesion kinase (FAK)‑Src‑paxillin signal pathway activated by IER2 may contribute to the HCC cell‑ECM adhesion and motility. These results demonstrated that IER2 promoted HCC cell adhesion and motility probably by directly increasing ITGB1 expression and subsequently activating the ITGB1‑FAK‑Src‑paxillin signal pathway. 
 α5β1 integrin recycling promotes Arp2/3-independent cancer cell invasion via the formin FHOD3. Invasiv  e migrati  on in 3D extracellular matrix (ECM) is crucial to cancer metastasis, yet little is known of the molecular mechanisms that drive reorganization of the cytoskeleton as cancer cells disseminate in vivo. 2D Rac-driven lamellipodia  l migrati  on is well understood, but how these features apply to 3  D migrati  on is not clear. We find that lamellipodia-like protrusions and retrograde actin flow are indeed observed in cells moving in 3D ECM. However, Rab-coupling protein (RCP)-driven endocytic recycling of α5β1 integrin enhances invas  ive migra  tion of cancer cells into fibronectin-rich 3D ECM, driven by RhoA and filopodial spike-based protrusions, not lamellipodia. Furthermore, we show that actin spike protrusions are Arp2/3-independent. Dynamic actin spike assembly in cells invading in vitro and in vivo is regulated by Formin homology-2 domain containing 3 (FHOD3), which is activated by RhoA/ROCK, establishing a novel mechanism through which the RCP-α5β1 pathway reprograms the actin cytoskeleton to promote inv  asive mig  ration and local invasion in vivo. 
 CCR7/CCL21   migration   on fibronectin is mediated by phospholipase Cgamma1 and ERK1/2 in primary T lymphocytes. CCR7 binds to its cognate ligand, CCL21, to mediate the   migration   of circulating naive T lymphocytes to the lymph nodes. T lymphocytes can bind to fibronectin, a constituent of lymph nodes, via their β1 integrins, which is a primary mechanism of T lymphocyte   migratio  n; however, the signaling pathways involved are unclear. We report that rapid (within 2 min) and transient phosphorylation of ERK1/2 is required for T cell   migratio  n on fibronectin in response to CCL21. Conversely, prevention of ERK1/2 phosphorylation by inhibition of its kinase, MAPK/MEK, prevented T lymphocyte   migratio  n. Previous studies have suggested that phospholipase Cγ1 (PLCγ1) can mediate phosphorylation of ERK1/2, which is required for β1 integrin activation. Paradoxically, we found that inhibition of PLCγ1 phosphorylation by the general PLC inhibitor U73122 was associated with a delayed and reduced phosphorylation of ERK1/2 and red  uced migr  ation of T lymphocytes on fibronectin. To further characterize the relationship between ERK1/2 and PLCγ1, we reduced PLCγ1 levels by 85% using shRNA and observed a reduced phosphorylation of ERK1/2 and a significant loss of CCR7-me  diated mi  gration of T lymphocytes on fibronectin. In addition, we found that inhibition of ERK1/2 phosphorylation by U0126 resulted in a decreased phosphorylation of PLCγ1, suggesting a feedback loop between ERK1/2 and PLCγ1. Overall, these results suggest that the CCR7 signaling pathway leading to T ly  mphocyte   migration on fibronectin is a β1 integrin-dependent pathway involving transient ERK1/2 phosphorylation, which is modulated by PLCγ1. 
 Angiogenesis in differentiated placental multipotent mesenchymal stromal cells is dependent on integrin alpha5beta1. Human placental multipotent mesenchymal stromal cells (hPMSCs) can be isolated from term placenta, but their angiogenic ability and the regulatory pathways involved are not known. hPMSCs were shown to express integrins alpha(v), alpha(4), alpha(5), beta(1), beta(3), and beta(5) and could be induced to differentiate into cells expressing endothelial markers. Increases in cell surface integrins alpha(5) and beta(1), but not alpha(4), alpha(v)beta(3), or alpha(v)beta(5), accompanied endothelial differentiation. Vascular endothelial growth factor-A augmented the effect of fibronectin in enhancing adhesion and   migration   of differentiated hPMSC through integrin alpha(5)beta(1), but not alpha(v)beta(3) or alpha(v)beta(5). Formation of capillary-like structures in vitro from differentiated cells was inhibited by pre-treatment with function-blocking antibodies to integrins alpha(5) and beta(1). When hPMSCs were seeded onto chick chorioallantoic membranes (CAM), human von Willebrand factor-positive cells were observed to engraft in the chick endothelium. CAMs transplanted with differentiated hPMSCs had a greater number of vessels containing human cells and more incorporated cells per vessel compared to CAMs transplanted with undifferentiated hPMSCs, and overall angiogenesis was enhanced more by the differentiated cells. Function-blocking antibodies to integrins alpha(5) and beta(1) inhibited angiogenesis in the CAM assay. These results suggest that differentiated hPMSCs may contribute to blood vessel formation, and this activity depends on integrin alpha(5)beta(1). 
 Integrin beta1 over-expression associates with resistance to tyrosine kinase inhibitor gefitinib in non-small cell lung cancer. The epidermal growth factor receptor tyrosine kinase inhibitors (EGFR TKIs) such as gefitinib and erlotinib have been widely used in treating patients with advanced non-small cell lung cancer (NSCLC). However, acquired resistance to EGFR TKI almost occurs in every patient eventually. To identify its potential mechanism, we established a human NSCLC cell line PC9/AB2 which was 576-fold decrease in gefitinib sensitivity compared with its parental PC9 cell lines. No EGFR-T790M mutation or abnormal expression of c-Met protein was found in PC9/AB2 cells. Over-expression of integrin β1 was found, accompanied with increase of the cells' adhesion and   migratio  n. To further confirm the role of integrin β1 in gefitinib acquired resistance, we transferred its siRNA-expressing plasmid and its whole cDNA expressing plasmid into PC9/AB2 and into PC9 cells, respectively. The sensitivity of NSCLC cells to gefitinib was negatively correlated with integrin β1 expression levels. All these data suggest that up-regulation of integrin β1 might be an important factor for gefitinib resistance in NSCLC cell line PC9/AB2. 
 Expression profiles of adhesion molecules on naïve T cells in bone marrow grafts of healthy donors treated with granulocyte colony-stimulating factor. Adhesion molecules on T cells were implicated in the process of leukocyte   migratio  n and GVHD. The aim of this study was to investigate the profiles of adhesion molecule expression on naïve T cells in bone marrow grafts of healthy donors treated with G-CSF.The expression of four adhesion molecules, including VLA-4, ICAM-1, L-selectin, and LFA-1, on naïve T cells in G-CSF-primed bone marrow grafts (G-BM) from 35 healthy donors was analyzed using flow cytometry.G-CSF administration significantly decreased the expression of VLA-4, ICAM-1, L-selectin, and LFA-1 on naïve CD4+ and CD8+ T cells in bone marrow grafts. Treating healthy donors with G-CSF significantly increased the percentages of interleukin-4 (IL-4) positive cells in naïve CD4+ and CD8+ T cell subsets, leading to polarization of bone marrow naïve CD4+ and CD8+ T cells from Th1 to Th2 phenotype.Our results suggest that CD4+ and CD8+ naïve T cells in G-BM have lower intensities of expression of several adhesion molecules. The clinical significance of these alteration warrants further study. 
 EWI-2 regulates alpha3beta1 integrin-dependent cell functions on laminin-5. EWI-2, a cell surface immunoglobulin SF protein of unknown function, associates with tetraspanins CD9 and CD81 with high stoichiometry. Overexpression of EWI-2 in A431 epidermoid carcinoma cells did not alter cell adhesion or spreading on laminin-5, and had no effect on reaggregation of cells plated on collagen I (alpha2beta1 integrin ligand). However, on laminin-5 (alpha3beta1 integrin ligand), A431 cell reaggregation and motility functions were markedly impaired. Immunodepletion and reexpression experiments revealed that tetraspanins CD9 and CD81 physically link EWI-2 to alpha3beta1 integrin, but not to other integrins. CD81 also controlled EWI-2 maturation and cell surface localization. EWI-2 overexpression not only suppressed cell   migration  , but also redirected CD81 to cell filopodia and enhanced alpha3beta1-CD81 complex formation. In contrast, an EWI-2 chimeric mutant failed to suppress cell   migration  , redirect CD81 to filopodia, or enhance alpha3beta1-CD81 complex formation. These results show how laterally associated EWI-2 might regulate alpha3beta1 function in disease and development, and demonstrate how tetraspanin proteins can assemble multiple nontetraspanin proteins into functional complexes. 
 High affinity interaction of integrin alpha4beta1 (VLA-4) and vascular cell adhesion molecule 1 (VCAM-1) enhances   migration   of human melanoma cells across activated endothelial cell layers. The capacity of tumor cells to form metastatic foci correlates with their ability to interact with and migrate through endothelial cell layers. This process involves multiple adhesive interactions between tumor cells and the endothelium. Only little is known about the molecular nature of these interactions during extravasation of tumor cells. In human melanoma cells, the integrin alphavbeta3 is involved in transendothelial   migration   and its expression correlates with metastasis. However, many human melanoma cells do not express beta3 integrins. Therefore, it remained unclear how these cells undergo transendothelial   migration  . In this study we show that human melanoma cells with different metastatic potency, which do not express beta2 or beta3 integrins, express the VCAM-1 receptor alpha4beta1. VCAM-1 is up-regulated on activated endothelial cells and is known to promote transendothelial   migration   of leukocytes. Interestingly, despite comparable cell surface levels of alpha4beta1, only the highly metastatic melanoma cell lines MV3 and BLM, but not the low metastatic cell lines IF6 and 530, bind VCAM-1 with high affinity without further stimulation, and are therefore able to adhere to and migrate on isolated VCAM-1. Moreover, we demonstrate that function-blocking antibodies against the integrin alpha4beta1, as well as siRNA-mediated knock-down of the alpha4 subunit in these highly metastatic human melanoma cells reduce their transendothelial   migration  . These data imply that only high affinity interactions between the integrin alpha4beta1 on melanoma cells and VCAM-1 on activated endothelial cells may enhance the metastatic capacity of human beta2/beta3-negative melanoma cells. 
 Insulin-like growth factor-independent insulin-like growth factor binding protein 3 promotes cell   migration   and lymph node metastasis of oral squamous cell carcinoma cells by requirement of integrin β1. Frequent metastasis to the cervical lymph nodes leads to poor survival of patients with oral squamous cell carcinoma (OSCC). To understand the underlying mechanisms of lymph node metastasis, two sublines were successfully isolated from cervical lymph nodes of nude mice through in vivo selection, and identified as originating from poorly metastatic parental cells. These two sublines specifically metastasized to cervical lymph nodes in 83% of mice, whereas OEC-M1 cells did not metastasize after injection into the oral cavity. After gene expression analysis, we identified insulin-like growth factor binding protein 3 (IGFBP3) as one of the significantly up-regulated genes in the sublines in comparison with their parental cells. Consistently, meta-analysis of the public microarray datasets and IGFBP3 immunohistochemical analysis revealed increased both levels of IGFBP3 mRNA and protein in human OSCC tissues when compared to normal oral or adjacent nontumorous tissues. Interestingly, the up-regulated IGFBP3 mRNA expression was significantly associated with OSCC patients with lymph node metastasis. IGFBP3 knockdown in the sublines impaired and ectopic IGFBP3 expression in the parental cells promoted   migratio  n, transendothelial   migratio  n and lymph node metastasis of orthotopic transplantation. Additionally, ectopic expression of IGFBP3 with an IGF-binding defect sustained the IGFBP3-enhanced biological functions. Results indicated that IGFBP3 regulates metastasis-related functions of OSCC cells through an IGF-independent mechanism. Furthermore, exogenous IGFBP3 was sufficient to induce cell motility and extracellular signal-regulated kinase (ERK) activation. The silencing of integrin β1 was able to impair exogenous IGFBP3-mediate  d migrati  on and ERK phosphorylation, suggesting a critical role of integrin β1 in IGFBP3-enchanced functions. 
 Vascular endothelial growth factor A (VEGF-A) induces endothelial and cancer cell   migration   through direct binding to integrin {alpha}9{beta}1: identification of a specific {alpha}9{beta}1 binding site. Integrin α9β1 mediates accelerated cell adhesion an  d migrati  on through interactions with a number of diverse extracellular ligands. We have shown previously that it directly binds the vascular endothelial growth factors (VEGF) A, C, and D and contributes to VEGF-induced angiogenesis and lymphangiogenesis. Until now, the α9β1 binding site in VEGF has not been identified. Here, we report that the three-amino acid sequence, EYP, encoded by exon 3 of VEGF-A is essential for binding of VEGF to integrin α9β1 and induces adhesio  n and mig  ration of endothelial and cancer cells. EYP is specific for α9β1 binding and neither requires nor activates VEGFR-2, the cognate receptor for VEGF-A. Following binding to EYP, integrin α9β1 transd  uces cell   migration through direct activation of the integrin signaling intermediates Src and focal adhesion kinase. This interaction is biologically important because it mediates in vitro endothelial cell tube formation, wound healing, and cancer cell invasion. These novel findings identify EYP as a potential site for directed pharmacotherapy. 
 Fibronectin induces endothelial cell   migration   through β1 integrin and Src-dependent phosphorylation of fibroblast growth factor receptor-1 at tyrosines 653/654 and 766. The extracellular matrix microenvironment regulates cell phenotype and function. One mechanism by which this is achieved is the transactivation of receptor tyrosine kinases by specific matrix molecules. Here, we demonstrate that the provisional matrix protein, fibronectin (FN), activates fibroblast growth factor (FGF) receptor-1 (FGFR1) independent of FGF ligand in liver endothelial cells. FN activation of FGFR1 requires β1 integrin, as evidenced by neutralizing antibody and siRNA-based studies. Complementary genetic and pharmacologic approaches identify that the non-receptor tyrosine kinase Src is required for FN transactivation of FGFR1. Whereas FGF ligand-induced phosphorylation of FGFR1 preferentially activates ERK, FN-induced phosphorylation of FGFR1 preferentially activates AKT, indicating differential downstream signaling of FGFR1 in response to alternate stimuli. Mutation analysis of known tyrosine residues of FGFR1 reveals that tyrosine 653/654 and 766 residues are required for FN-FGFR1 activation of AKT and chemotaxis. Thus, our study mechanistically dissects a new signaling pathway by which FN achieves endothelial cell chemotaxis, demonstrates how differential phosphorylation profiles of FGFR1 can achieve alternate downstream signals, and, more broadly, highlights the diversity of mechanisms by which the extracellular matrix microenvironment regulates cell behavior through transactivation of receptor tyrosine kinases. 
 CCL25 and CCL28 promote alpha4 beta7-integrin-dependent adhesion of lymphocytes to MAdCAM-1 under shear flow. Inflammatory bowel disease is characterized by the recruitment of lymphocytes to the gut via mucosal vessels. Chemokines are believed to trigger alpha(4)beta(1)- and alpha(4)beta(7)-integrin-mediated adhesion to vascular cell adhesion molecule-1 (VCAM-1) and mucosal addressin cell adhesion molecule-1 (MAdCAM-1) on mucosal vessels, although the contribution of each pathway and the chemokines involved are not well characterized. These interactions occur under conditions of hemodynamic shear, which is critical in determining how lymphocytes integrate chemokine signals to promote trans  migration  . To define the role of specific chemokines in mediating lymphocyte adhesion to VCAM-1 and MAdCAM-1, we studied the ability of immobilized chemokines to activate adhesion of human lymphocytes in a flow-based adhesion assay. Adhesion to immobilized MAdCAM-1 was alpha(4)beta(7) dependent, with no contribution from alpha(4)beta(1), whereas alpha(4)beta(1) mediated rolling and static adhesion on VCAM-1. Immobilized CC-chemokine ligand (CCL) 25 and CCL28 were both able to trigger alpha(4)beta(7)-dependent lymphocyte arrest on MAdCAM-1 under shear, highlighting a potential role for these chemokines in the arrest of lymphocytes on postcapillary venules in the gut. Neither had any effect on adhesion to VCAM-1, suggesting that they selectively trigger alpha(4)beta(7)-mediated adhesion. Immobilized CCL21, CCL25, CCL28, and CXC-chemokine ligand (CXCL) 12 all converted rolling adhesion to static arrest on MAdCAM-1 by activating lymphocyte integrins, but only CCL21 and CXCL12 also triggered a motile phenotype characterized by lamelipodia and uropod formation. Thus alpha(4)beta(1)/VCAM-1 and alpha(4)beta(7)/MAdCAM-1 operate independently to support lymphocyte adhesion from flow, and chemokines may act in concert with one chemokine triggering integrin-mediated arrest and a second chemokine promoting motility and transendothelial   migration  . 
 Netrin-4 activates endothelial integrin {alpha}6{beta}1. Netrin-4 regulates vascular development. Identity of netrin-4 endothelial receptor and its subsequent cell functions is controversial. We previously demonstrated that the inhibition of netrin-1 canonical receptors, Unc5B and neogenin, expressed by lymphatic endothelial cells, do not suppress netrin-4-induced cell signaling and functions. Netrin family members were shown to signal through a range of receptors, including integrins (such as α3β1, α6β1, and α6β4) in nonendothelial cells.We tested whether integrins are netrin-4 receptors in the endothelium.The α6β1 integrin is expressed by endothelial cells, and binds netrin-4 in a dose-dependent manner. Inhibition of α6 or β1 integrin subunits suppresses netrin-4-induced endothe  lial cell   migration, adhesion, and focal adhesion contact. Netrin-4-stimulated phosphorylation of Src kinase family, effectors of endothe  lial cell   migration, is also abolished by α6 or β1 inhibition. Finally, netrin-4 and α6β1 integrin expression colocalize in mouse embryonic, intestine, and tumor vasculature.The α6β1 integrin is a netrin-4 receptor in lymphatic endothelium and consequently represents a potential target to inhibit netrin-4-induced metastatic dissemination. 
 Autocrine semaphorin3A stimulates alpha2 beta1 integrin expression/function in breast tumor cells. The axon repulsion factor semaphorin3A (SEMA3A) and its receptor neuropilin-1 (NP-1) are expressed in breast tumor cells, and function as suppressors of tumor cell   migration  . Based on the knowledge that both SEMA3A and the alpha2beta1 integrin suppress breast tumor cell   migration  , we studied the impact of SEMA3A signaling on alpha2beta1 integrin expression/function. The incubation of breast tumor cells with SEMA3A increased alpha2 and beta1 integrin levels, and stimulated tumor cell adhesion to the alpha2beta1-binding matrix protein collagen I. Conversely, reducing SEMA3A expression in breast tumor cells decreased alpha2beta1 levels and collagen adhesion. The ability of SEMA3A to increase tumor cell adhesion to collagen was dependent on both the SEMA3A receptor NP-1 and the glycogen synthase kinase-3. The incubation of breast tumor cells with SEMA3A disrupted the actin cytoskeleton, and reduced both tumor cell migratory and invasive behavior. Importantly, using an alpha2beta1-neutralizing antibody, we demonstrated that SEMA3A suppression of tumor cell   migration   is dependent on alpha2beta1. Our studies indicate that expression of the alpha2beta1 integrin, a suppressor of metastatic breast tumor growth, is stimulated in breast tumor cells by an autocrine SEMA3A pathway. 
 17-β-Estradiol counteracts the effects of high frequency electromagnetic fields on trophoblastic connexins and integrins. We investigated the effect of high-frequency electromagnetic fields (HF-EMFs) and 17-β-estradiol on connexins (Cxs), integrins (Ints), and estrogen receptor (ER) expression, as well as on ultrastructure of trophoblast-derived HTR-8/SVneo cells. HF-EMF, 17-β-estradiol, and their combination induced an increase of Cx40 and Cx43 mRNA expression. HF-EMF decreased Int alpha1 and β 1 mRNA levels but enhanced Int alpha5 mRNA expression. All the Ints mRNA expressions were increased by 17-β-estradiol and exposure to both stimuli. ER-β mRNA was reduced by HF-EMF but augmented by 17-β-estradiol alone or with HF-EMF. ER-β immunofluorescence showed a cytoplasmic localization in sham and HF-EMF exposed cells which became nuclear after treatment with hormone or both stimuli. Electron microscopy evidenced a loss of cellular contact in exposed cells which appeared counteracted by 17-β-estradiol. We demonstrate that 17-β-estradiol modulates Cxs and Ints as well as ER-β expression induced by HF-EMF, suggesting an influence of both stimuli on trophoblast different  iation an  d migration. 
 Genome-wide screening identified that miR-134 acts as a metastasis suppressor by targeting integrin β1 in hepatocellular carcinoma. MicroRNAs (miRNAs) are small, single-stranded, non-coding RNAs that play pivotal roles in human cancer development and progression, such as tumor metastasis. Here, we identified the miRNAs that regulate hepatocellular carcinoma (HCC) cell   migratio  n by a high-throughput screening method using the classical wound-healing assay with time-lapse video microscopy and validation with a transwell   migratio  n assay. Eleven miRNAs (miR-134, -146b-3p, -188-3p, -525-3p, -661, -767-5p, -891a, -891b, -1244, -1247 and miR-1471) were found to promote or inhibit HCC cell   migratio  n. Further investigation revealed that miR-134 suppressed the invasion and metastasis of HCC cells in vitro and in vivo, and integrin beta 1 (ITGB1) was a direct and functional target gene of miR-134. Moreover, miR-134 inhibited the phosphorylation of focal adhesion kinase (FAK) and the activation of RhoA downstream of the ITGB1 pathway, thereby decreasing stress fiber formation and cell adhesion in HCC cells. In conclusion, we demonstrated that miR-134 is a novel metastasis suppressor in HCC and could be a potential therapeutic target for the treatment of HCC. 
 Quantitative proteomics identifies a Dab2/integrin module regulating cell   migration  . Clathrin-associated endocytic adapters recruit cargoes to coated pits as a first step in endocytosis. We developed an unbiased quantitative proteomics approach to identify and quantify glycoprotein cargoes for an endocytic adapter, Dab2. Surface levels of integrins beta1, alpha1, alpha2, and alpha3 but not alpha5 or alphav chains were specifically increased on Dab2-deficient HeLa cells. Dab2 colocalizes with integrin beta1 in coated pits that are dispersed over the cell surface, suggesting that it regulates bulk endocytosis of inactive integrins. Depletion of Dab2 inhibits cell   migration   and polarized movement of integrin beta1 and vinculin to the leading edge. By manipulating intracellular and surface integrin beta1 levels, we show that   migration   speed correlates with the intracellular integrin pool but not the surface level. Together, these results suggest that Dab2 internalizes integrins freely diffusing on the cell surface and that Dab2 regulates   migration  , perhaps by maintaining an internal pool of integrins that can be recycled to create new adhesions at the leading edge. 
 High-level β1-integrin expression in a subpopulation of highly tumorigenic oral cancer cells. The β1 integrin (CD29) is a putative marker for cancerous epithelial stem cells. Cancer stem cells are essential to drive tumor growth, recurrence, and metastasis. We investigated the role of β1-integrin expression in the development of malignant phenotypes of oral squamous cell carcinoma (OSCC).Immunostaining was used to analyze the expression levels of β1 integrins in different types of cell colonies and tumor spheres. The results of cell viability   and migra  tion assays with and without siRNA knockdown of β1-integrin expression were compared. Cells expressing β1 integrins were evaluated for their tumorigenicity in mice. The expression of β1 integrins in human specimens of oral cancers at different clinical stages was semiquantified based on immunohistochemical staining of the β1-integrin protein.The expression level of β1 integrins in Meng-1 oral epidermoid carcinoma cells (OECM-1) cells was significantly higher in holoclonal colonies and tumor spheres compared to control cells. The knockdown of β1-integrin expression in OECM-1 cells reduced cell proli  feration,   migration, and tumor sphere formation. Beta-1 integrin (+) cells were more tumorigenic in the mouse xenograft model than β1 integrin (-) cells. In the human specimens, the expression level of the β1-integrin protein positively correlated with the clinical stage.The expression of β1 integrin in OECM-1 cells is involved in the development of malignant phenotypes of OSCC.Inhibitors for β1-integrin signaling may be suitable to become target-specific therapies for OSCC. 
 Spatiotemporally-regulated interaction between β1 integrin and ErbB4 that is involved in fibronectin-dependent cell   migratio  n. Integrins are widely expressed cell surface molecules that mediate cell attachment to extracellular matrix (ECM) proteins. They also interact with molecules on their own membranes, and these cis-interactions play a crucial role in integrin-dependent cellular responses. We herein analysed what molecules interact with β1 integrin during biological events induced by cell attachment to different ECM proteins, using a recently established reaction, the enzyme-mediated activation of radical sources (EMARS). The interactions between β1 integrin and receptor tyrosine kinases including EGFR and ErbB4 reached a peak at 2 h after seeding HeLa S3 cells onto the ECM proteins. The peak of phosphorylation of ErbB4 (at 2 h after seeding the cells onto fibronectin) coincided with the peak of the interaction with β1 integrin, while that of EGFR (at 1 day) did not. Accompanying with these findings, suppression of c  ell migra  tion by a pharmacological inhibitor of the ErbB family receptors, PD168393 and an anti-ErbB4 neutralizing antibody, 12D8 was observed at 2 h after seeding. Taken together, it is deduced that interactions between β1 integrin and ErbB4 occur in a spatiotemporally-regulated manner, and such interaction contributes to the integrin-dependent   cell migr  ation. 
 Homeobox D1 regulates angiogenic functions of endothelial cells via integrin β1 expression. Homeobox (HOX) family genes, major transcription factors for embryonic development, have been also implicated in vascular development and angiogenesis, particularly with regulation of genes involved in cell-cell or cell-extracellular matrix (ECM) interactions. However, the cellular and molecular functions of HOXD1 in endothelial cells (ECs) are yet to be explored. We here report that HOXD1 is prominently expressed in human ECs and regulates angiogenic activities. Knockdown of HOXD1 in ECs resulted in significant inhibition of   migratio  n and adhesion as well as tube like structure formation. These effects were correlated with the reduced expression of integrin β1 (ITGB1), an important signaling component of angiogenesis. Consistently, ITGB1 promoter activity was decreased by HOXD1 knockdown in ECs. Furthermore, we identified the putative HOXD1-binding sites in the promoter region of ITGB1. Together, these findings suggest that HOXD1 plays a significant role in EC functions by regulating the expression of ITGB1. 
 Integrin beta1-mediated signaling is involved in transforming growth factor-beta2-promoted   migration   in human lens epithelial cells. Transforming growth factor-beta 2 (TGF-beta2) is a potent growth inhibitor and apoptosis inducer. However, typically TGF-beta2 loses its growth-inhibitory and apoptosis-inducing effects but stimulates the migratory capacity of epithelial cells. In this study, we investigate the possible involvement of integrin and integrin-mediated signaling in TGF-beta2-promoted   migration   and adhesion in human lens epithelial cells.A human lens epithelial cell line (HLE B-3) was treated with 100 pg/ml TGF-beta2 for 6 h, 12 h, and 24 h. In vitro wound healing assay and cell adhesion assay were performed to detect the effect of TGF-beta2 on HLEC adhesion and   migration  . Integrin beta1 expression changes in HLECs during the treatment of TGF-beta2 were detected in protein levels and mRNA levels using confocal microscopy, flow cytometric analysis, and real time quantitative reverse transcription polymerase chain reaction (RT-PCR). Focal adhesion kinase (FAK) activity was examined by FAK phosphorylation and total tyrosine phosphorylation during treatment with TGF-beta2 in HLEC. Production of endogenous TGF-beta2 was measured by ELISA assay.In this study, we found that TGF-beta2 significantly stimulated cell adhesion and   migration   in HLECs. By immunofluorescence staining and western blotting, we observed that TGF-beta2 markedly enhanced the expression of integrin beta1 and the Tyr-phosphorylation of focal adhesion kinase (FAK). Real time quantitative RT-PCR also showed the mRNA level of integrin beta1 was upregulated. Neutralizing anti-integrin beta1 monoclonal antibody significantly (p<0.05) inhibited TGF-beta2-promoted HLEC adhesion and   migration  .TGF-beta2 promoted HLEC adhesion and   migration   in vitro. Integrin beta1 and integrin-mediated signaling are necessary for TGF-beta2-promoted adhesion and   migration   in human lens epithelial cells. 
 Formin-like 2 Promotes β1-Integrin Trafficking and Invasive Motility Downstream of PKCα. Regulated turnover of integrin receptors is essential for cell adhesion an  d migrati  on. Pathways selectively regulating β1-integrin recycling are implicated in cancer invasion and metastasis, yet proteins required for the internalization of this pro-invasive integrin remain to be identified. Here, we uncover formin-like 2 (FMNL2) as a critical regulator of β1-integrin internalization downstream of protein kinase C (PKC). PKCα associates with and phosphorylates FMNL2 at S1072 within its Diaphanous autoregulatory region, leading to the release of formin autoinhibition. Phosphorylation of FMNL2 triggers its rapid relocation and promotes its interaction with the cytoplasmic tails of the α-integrin subunits for β1-integrin endocytosis. FMNL2 drives β1-integrin internalization and invasive motility in a phosphorylation-dependent manner, while a FMNL2 mutant defective in actin assembly interferes with β1-integrin endocytosis and cancer cell invasion. Our data establish a role for FMNL2 in the regulation of β1-integrin and provide a mechanistic understanding of the function of FMNL2 in cancer invasiveness. 
 Under-expression of α8 integrin aggravates experimental atherosclerosis. Integrins play an important role in vascular biology. The α8 integrin chain attenuates smooth muscle cel  l migrati  on but its functional role in the development of atherosclerosis is unclear. Therefore, we studied the contribution of α8 integrin to atherosclerosis and vascular remodelling. We hypothesized that α8 integrin expression is reduced in atherosclerotic lesions, and that its under-expression leads to a more severe course of atherosclerosis. α8 Integrin was detected by immunohistochemistry and qPCR and α8 integrin-deficient mice were used to induce two models of atherosclerotic lesions. First, ligation of the carotid artery led to medial thickening and neointima formation, which was quantified in carotid cross-sections. Second, after crossing into ApoE-deficient mice, the formation of advanced vascular lesions with atherosclerotic plaques was quantified in aortic en face preparations stained with Sudan IV. Parameters of renal physiology and histopathology were assessed: α8 integrin was detected in the media of human and murine vascular tissue and was down-regulated in arteries with advanced atherosclerotic lesions. In α8 integrin-deficient mice (α8(-/-) ) as well as α8(+/-) and α8(+/+) littermates, carotid artery ligation increased media:lumen ratios in all genotypes, with higher values in ligated α8(-/-) and α8(+/-) compared to ligated α8(+/+) animals. Carotid artery ligation increased smooth muscle cell number in the media of α8(+/+) mice and, more prominently, of α8(-/-) or α8(+/-) mice. On an ApoE(-/-) background, α8(+/-) and α8(-/-) mice developed more atherosclerotic plaques than α8(+/+) mice. α8 Integrin expression was reduced in α8(+/-) animals. Renal damage with increased serum creatinine and glomerulosclerosis was detected in α8(-/-) mice only. Thus, under-expression of α8 integrin aggravates vascular lesions, while a complete loss of α8 integrin results in reduced renal mass and additional renal disease in the presence of generalized atherosclerosis. Our data support the hypothesis that integrin α8β1 has a protective role in arterial remodelling and atherosclerosis. 
 Slug regulates integrin expression and cell proliferation in human epidermal keratinocytes. The human epidermis is a self-renewing epithelial tissue composed of several layers of keratinocytes. Within the epidermis there exists a complex array of cell adhesion structures, and many of the cellular events within the epidermis (differentiation, proliferation, and   migration  ) require that these adhesion structures be remodeled. The link between cell adhesion, proliferation, and differentiation within the epidermis is well established, and in particular, there is strong evidence to link the process of terminal differentiation to integrin adhesion molecule expression and function. In this paper, we have analyzed the role of a transcriptional repressor called Slug in the regulation of adhesion molecule expression and function in epidermal keratinocytes. We report that activation of Slug, which is expressed predominantly in the basal layer of the epidermis, results in down-regulation of a number of cell adhesion molecules, including E-cadherin, and several integrins, including alpha3, beta1, and beta4. We demonstrate that Slug binds to the alpha3 promoter and that repression of alpha3 transcription by Slug is dependent on an E-box sequence within the promoter. This reduction in integrin expression is reflected in decreased cell adhesion to fibronectin and laminin-5. Despite the reduction in integrin expression and function, we do not observe any increase in differentiation. We do, however, find that activation of Slug results in a significant reduction in keratinocyte proliferation. 
 Plasmin-induced   migration   requires signaling through protease-activated receptor 1 and integrin alpha(9)beta(1). Plasmin is a major extracellular protease that elicits intracellular signals to mediate platelet aggregation, chemotaxis of peripheral blood monocytes, and release of arachidonate and leukotriene from several cell types in a G protein-dependent manner. Angiostatin, a fragment of plasmin(ogen), is a ligand and an antagonist for integrin alpha(9)beta(1). Here we report that plasmin specifically interacts with alpha(9)beta(1) and that plasmin induces of cells expressing   migration   recombinant alpha(9)beta(1) (alpha(9)-Chinese hamster ovary (CHO) cells).   Migration   was dependent on an interaction of the kringle domains of plasmin with alpha(9)beta(1) as well as the catalytic activity of plasmin. Angiostatin, representing the kringle domains of plasmin, alone did not induce the   migration   of alpha(9)-CHO cells, but simultaneous activation of the G protein-coupled protease-activated receptor (PAR)-1 with an agonist peptide induced the   migration   on angiostatin, whereas PAR-2 or PAR-4 agonist peptides were without effect. Furthermore, a small chemical inhibitor of PAR-1 (RWJ 58259) and a palmitoylated PAR-1-blocking peptide inhibited plasmin-induced   migration   of alpha(9)-CHO cells. These results suggest that plasmin induces   migration   by kringle-mediated binding to alpha(9)beta(1) and simultaneous proteolytic activation of PAR-1. 
 [Effect of integrin beta1 on adhesion and   migration   of human trophoblast cells]. Although the mechanism by which migratory trophoblasts reach the spiral arteries is currently obscure, yet the process has been noted to involve the attachment, adhesion and   migration   of trophoblasts on the blood vessel walls. To test this, micropipette and flow chamber were used to measure quantitatively the adhesion forces and   migration   of early gestation human trophoblast cells (TCs) cultured on the glass slides coated with type I rat collagen or cultured with human umbilical vein endothelial cells (HUVECs). The results showed that the interdiction of integrin beta1 interaction remarkably reduced the adhesion forces of TCs to type I rat collagen or endothelial cells, and remarkably resisted the displacement of TCs induced by shear stress. By contact between TCs and endothelial cells, the TCs' adhesion force and TCs' resistance to shear stress were significantly enhanced. The results indicated that the contacts of TCs with endothelial cells enhanced the adhesion forces of human TCs, and regulated the   migration   of human TCs by shear stress. 
 New mechanistic insights of integrin β1 in breast cancer bone colonization. Bone metastasis is a frequent and life-threatening complication of breast cancer. The molecular mechanisms supporting the establishment of breast cancer cells in the skeleton are still not fully understood, which may be attributed to the lack of suitable models that interrogate interactions between human breast cancer cells and the bone microenvironment. Although it is well-known that integrins mediate adhesion of malignant cells to bone extracellular matrix, their role during bone colonization remains unclear. Here, the role of β1 integrins in bone colonization was investigated using tissue-engineered humanized in vitro and in vivo bone models. In vitro, bone-metastatic breast cancer cells with suppressed integrin β1 expression showed reduced attachment, spreading, a  nd migrat  ion within human bone matrix compared to control cells. Cell proliferation in vitro was not affected by β1 integrin knockdown, yet tumor growth in vivo within humanized bone microenvironments was significantly inhibited upon β1 integrin suppression, as revealed by quantitative in/ex vivo fluorescence imaging and histological analysis. Tumor cells invaded bone marrow spaces in the humanized bone and formed osteolytic lesions; osteoclastic bone resorption was, however, not reduced by β1 integrin knockdown. Taken together, we demonstrate that β1 integrins have a pivotal role in bone colonization using unique tissue-engineered humanized bone models. 
 Computational analysis of mRNA expression profiles identifies the ITG family and PIK3R3 as crucial genes for regulating triple negative breast cancer cell   migration  . Triple-negative breast cancer (TNBC) is an aggressive type of breast cancer that does not express estrogen receptor (ER), progesterone receptor (PR), and human epidermal growth factor receptor (Her2/neu). TNBC has worse clinical outcomes than other breast cancer subtypes. However, the key molecules and mechanisms of TNBC   migration   remain unclear. In this study, we compared two normalized microarray datasets from GEO database between Asian (GSE33926) and non-Asian populations (GSE46581) to determine the molecules and common pathways in TNBC   migration  . We demonstrated that 16 genes in non-Asian samples and 9 genes in Asian samples are related to TNBC   migration  . In addition, our analytic results showed that 4 genes, PIK3R3, ITGB1, ITGAL, and ITGA6, were involved in the regulation of actin cytoskeleton. Our results indicated potential genes that link to TNBC   migration  . This study may help identify novel therapeutic targets for drug development in cancer therapy. 
 Pro-angiogenic activities of CYR61 (CCN1) mediated through integrins alphavbeta3 and alpha6beta1 in human umbilical vein endothelial cells. CYR61 (CCN1) is an extracellular matrix-associated protein of the CCN family, which also includes CTGF (CCN2), NOV (CCN3), WISP-1 (CCN4), WISP-2 (CCN5), and WISP-3 (CCN6). Purified CYR61 induces neovascularization in corneal implants, and Cyr61-null mice suffer embryonic death due to vascular defects, thus establishing that CYR61 is an important regulator of angiogenesis. Aberrant expression of Cyr61 is associated with breast cancer, wound healing, and vascular diseases such as atherosclerosis and restenosis. In culture, CYR61 functions through integrin-mediated pathways to promote cell adhesion,   migration  , and proliferation. Here we show that CYR61 can also promote cell survival and tubule formation in human umbilical vein endothelial cells. Furthermore, we have dissected the integrin receptor requirements of CYR61 with respect to its pro-angiogenic activities. Thus, CYR61-induced cell adhesion and tubule formation occur through interaction with integrin alpha(6)beta(1) in early passage endothelial cells in which integrins have not been activated. By contrast, in endothelial cells in which integrins are activated by phorbol ester or vascular endothelial growth factor, CYR61-promoted cell adhesion,   migration  , survival, growth factor-induced mitogenesis, and endothelial tubule formation are all mediated through integrin alpha(v)beta(3). These findings indicate that CYR61 is an activation-dependent ligand of integrin alpha(v)beta(3) and an activation-independent ligand of integrin alpha(6)beta(1) and that these integrins differentially mediate the pro-angiogenic activities of CYR61. These findings help to define the mechanisms by which CYR61 acts as an angiogenic regulator, provide a molecular interpretation for the loss of vascular integrity and increased apoptosis of vascular cells in Cyr61-null mice, and underscore the importance of CYR61 in the development and homeostasis of the vascular system. 
 Binding of paxillin to alpha4 integrins modifies integrin-dependent biological responses. The alpha4 integrins are indispensable for embryogenesis, haematopoiesis and immune responses, possibly because alpha4 regulates cellular functions differently from other integrins through its cytoplasmic tail. We used novel mimics of the alpha4 tail to identify molecules that could account for alpha4-specific signalling. Here we report that the alpha4 tail, but not several other alpha-subunit tails, binds tightly to the signalling adaptor paxillin. Paxillin physically associated with alpha4 integrins in Jurkat T cells at high stoichiometry, and joining the alpha4 tail to alphaIIb resulted in a complex of integrin alphaIIbbeta3 with paxillin. This association markedly enhanced the rates of alphaIIbbeta3-dependent phosphorylation of focal adhesion kinase and cell   migration  . It also reduced cell spreading, focal adhesion and stress fibre formation. A point mutation within the alpha4 tail that disrupts paxillin binding reversed all of these effects. Furthermore, alpha4beta1-dependent adhesion to VCAM-1 led to spreading of mouse embryonic fibroblasts derived from paxillin-null but not from wild-type mice. Thus, the tight association of paxillin with the alpha4 tail leads to distinct biochemical and biological responses to integrin-mediated cell adhesion. 
 Cdc42 promotes transendothelial   migration   of cancer cells through β1 integrin. Cancer cells interact with endothelial cells during the process of metastatic spreading. Here, we use a small interfering RNA screen targeting Rho GTPases in cancer cells to identify Cdc42 as a critical regulator of cancer cell-endothelial cell interactions and transendothelial   migratio  n. We find that Cdc42 regulates β1 integrin expression at the transcriptional level via the transcription factor serum response factor (SRF). β1 integrin is the main target for Cdc42-mediating interaction of cancer cells with endothelial cells and the underlying extracellular matrix, as exogenous β1 integrin expression was sufficient to rescue the Cdc42-silencing phenotype. We show that Cdc42 was required in vivo for cancer cell spreading and protrusion extension along blood vessels and retention in the lungs. Interestingly, transient Cdc42 depletion was sufficient to decrease experimental lung metastases, which suggests that its role in endothelial attachment is important for metastasis. By identifying β1 integrin as a transcriptional target of Cdc42, our results provide new insight into Cdc42 function. 
 HAb18G/CD147 promotes radioresistance in hepatocellular carcinoma cells: a potential role for integrin β1 signaling. Radiotherapy has played a limited role in the treatment of hepatocellular carcinoma (HCC) due to the risk of tumor radioresistance. A previous study in our laboratory confirmed that CD147 interacts with integrin β1 and plays an important role in modulating the malignant properties of HCC cells. In this study, we further evaluated the role of CD147 in the radioresistance of HCC and as a potential target for improving radiosensitivity. Upon irradiation, the colony formation, apoptosis, cell-cycle distribution  , migrati  on, and invasion of SMMC-7721, CD147-knockout SMMC-7721, HepG2, and CD147-knockdown HepG2 cells were determined. A nude mouse xenograft model and a metastatic model of HCC were used to detect the role of CD147 in radioresistance in vivo. Deletion of HAb18G/CD147 significantly enhanced the radiosensitivity of SMMC-7721 and HepG2 cells, and knocking out HAb18G/CD147 in SMMC-7721 cells attenuated irradiation-enhance  d migrati  on and invasion. The knockout and antibody blockade of CD147 decreased the tumor growth and metastatic potentials of HCC cells under irradiation. CD147-deleted SMMC-7721 cells showed diminished levels of calpain, cleaved talin, active integrin β1, and decreased p-FAK (Tyr397) and p-Akt (Ser473) levels. FAK and PI3K inhibitors, as well as integrin β1 antibodies, increased the radiation-induced apoptosis of SMMC-7721 cells. Our data provide evidence for CD147 as an important determinant of radioresistance via the regulation of integrin β1 signaling. Inhibition of the HAb18G/CD147 integrin interaction may improve the efficiency of radiosensitivity and provide a potential new approach for HCC therapy. 
 CD73 and CD29 concurrently mediate the mechanically induced decrease of migratory capacity of mesenchymal stromal cells. e assumption that mesenchymal stromal cell (MSC)-based-therapies are capable of augmenting physiological regeneration processes has fostered intensive basic and clinical research activities. However, to achieve sustained therapeutic success in vivo, not only the biological, but also the mechanical microenvironment of MSCs during these regeneration processes needs to be taken into account. This is especially important for e.g., bone fracture repair, since MSCs present at the fracture site undergo significant biomechanical stimulation. This study has therefore investigated cellular characteristics and the functional behaviour of MSCs in response to mechanical loading. Our results demonstrated a reduced expression of MSC surface markers CD73 (ecto-5'-nucleotidase) and CD29 (integrin β1) after loading. On the functional level, loading led to a reduced   migratio  n of MSCs. Both effects persisted for a week after the removal of the loading stimulus. Specific inhibition of CD73/CD29 demonstrated their substrate dependent involvement in MSC   migratio  n after loading. These results were supported by scanning electron microscopy images and phalloidin staining of actin filaments displaying less cell spreading, lamellipodia formation and actin accumulations. Moreover, focal adhesion kinase and Src-family kinases were identified as candidate downstream targets of CD73/CD29 that might contribute to the mechanically induced decrease in MSC   migratio  n. These results suggest that MSC   migratio  n is controlled by CD73/CD29, which in turn are regulated by mechanical stimulation of cells. We therefore speculate that MSCs migrate into the fracture site, become mechanically entrapped, and thereby accumulate to fulfil their regenerative functions. 
 Regulation of melanoma cell   migration   and invasion by laminin-5 and alpha3beta1 integrin (VLA-3). We evaluated the role of soluble factors produced from epidermal cells in melanoma cell motility by using the Boyden chamber chemoinvasion system. The   migration   of two melanoma cell lines, A375 and Mewo, was potentiated by conditioned media of A431 epidermoid cells in a concentration-dependent manner. The enhancement of A375 melanoma cell motility induced by the conditioned medium was blocked by antibodies against either alpha3 or beta1 integrin subunit. The motility-stimulating activity was recovered in the same fraction as the alpha3 integrin-dependent adhesion-promoting activity in a high-molecular-weight (>200 kDa) fraction on Superose 12 gel chromatography, and adsorbed with an anti-laminin-5 antibody. Purified laminin-5 was capable of potentiating melanoma cell   migration   as measured in either the chemotaxis assay with a soluble form of laminin-5 or the haptotaxis assay with membranes coated with a mixture of laminin-5 and Matrigel. Furthermore, immobilized laminin-5 induced A375 melanoma cells to secrete matrix metalloproteinase-9 (type IV collagenase) into the culture medium. These results strongly suggest that the interaction of laminin-5 produced in the epidermis with alpha3beta1 integrin on melanoma cells is involved in cell   migration  , invasion, and degradation of extracellular matrix proteins. 
 ACAP4 protein cooperates with Grb2 protein to orchestrate epidermal growth factor-stimulated integrin β1 recycling in cell   migratio  n. ARF6 GTPase is an important regulator of membrane trafficking and actin-based cytoskeleton dynamics active at the leading edge of migrating cells. The integrin family heterodimeric transmembrane proteins serve as major receptors for extracellular matrix proteins, which play essential roles in cell adhesion and   migratio  n. Our recent proteomic analyses of ARF6 effectors have identified a novel ARF6 GTPase-activating protein, ACAP4, essential for EGF-induced cell   migratio  n. However, molecular mechanisms underlying ACAP4-mediated cell   migratio  n have remained elusive. Here, we show that ACAP4 regulates integrin β1 dynamics during EGF-stimulated cel  l migrati  on by interaction with Grb2. Our biochemical study shows that EGF stimulation induces phosphorylation of tyrosine 733, which enables ACAP4 to bind Grb2. This interaction of ACAP4 with Grb2 regulates integrin β1 recycling to the plasma membrane. Importantly, knockdown of ACAP4 by siRNA or overexpression of ACAP4 decreased recycling of integrin β1 to the plasma membrane and reduced integrin-mediated c  ell migra  tion. Taken together, these results suggest a novel function for ACAP4 in the regulation of c  ell migra  tion through controlling integrin β1 dynamics. 
 Laminin isoforms and their integrin receptors in glioma cell   migration   and invasiveness: Evidence for a role of alpha5-laminin(s) and alpha3beta1 integrin. Glioma cell infiltration of brain tissue often occurs along the basement membrane (BM) of blood vessels. In the present study we have investigated the role of laminins, major structural components of BMs and strong promoters of cell   migration  . Immunohistochemical studies of glioma tumor tissue demonstrated expression of alpha2-, alpha3-, alpha4- and alpha5-, but not alpha1-, laminins by the tumor vasculature. In functional assays, alpha3 (Lm-332/laminin-5)- and alpha5 (Lm-511/laminin-10)-laminins strongly promoted   migration   of all glioma cell lines tested. alpha1-Laminin (Lm-111/laminin-1) displayed lower activity, whereas alpha2 (Lm-211/laminin-2)- and alpha4 (Lm-411/laminin-8)-laminins were practically inactive. Global integrin phenotyping identified alpha3beta1 as the most abundant integrin in all the glioma cell lines, and this laminin-binding integrin exclusively or largely mediate the cell   migration  . Moreover, pretreatment of U251 glioma cells with blocking antibodies to alpha3beta1 integrin followed by intracerebral injection into nude mice inhibited invasion of the tumor cells into the brain tissue. The cell lines secreted Lm-211, Lm-411 and Lm-511, at different ratios. The results indicate that glioma cells secrete alpha2-, alpha4- and alpha5-laminins and that alpha3- and alpha5-laminins, found in brain vasculature, selectively promote glioma cell   migration  . They identify alpha3beta1 as the predominant integrin and laminin receptor in glioma cells, and as a brain invasion-mediating integrin. 
 Cell contact-dependent activation of alpha3beta1 integrin modulates endothelial cell responses to thrombospondin-1. Thrombospondin-1 (TSP1) can inhibit angiogenesis by interacting with endothelial cell CD36 or proteoglycan receptors. We have now identified alpha3beta1 integrin as an additional receptor for TSP1 that modulates angiogenesis and the in vitro behavior of endothelial cells. Recognition of TSP1 and an alpha3beta1 integrin-binding peptide from TSP1 by normal endothelial cells is induced after loss of cell-cell contact or ligation of CD98. Although confluent endothelial cells do not spread on a TSP1 substrate, alpha3beta1 integrin mediates efficient spreading on TSP1 substrates of endothelial cells deprived of cell-cell contact or vascular endothelial cadherin signaling. Activation of this integrin is independent of proliferation, but ligation of the alpha3beta1 integrin modulates endothelial cell proliferation. In solution, both intact TSP1 and the alpha3beta1 integrin-binding peptide from TSP1 inhibit proliferation of sparse endothelial cell cultures independent of their CD36 expression. However, TSP1 or the same peptide immobilized on the substratum promotes their proliferation. The TSP1 peptide, when added in solution, specifically inhibits endothelial cell   migration   and inhibits angiogenesis in the chick chorioallantoic membrane, whereas a fragment of TSP1 containing this sequence stimulates angiogenesis. Therefore, recognition of immobilized TSP1 by alpha3beta1 integrin may stimulate endothelial cell proliferation and angiogenesis. Peptides that inhibit this interaction are a novel class of angiogenesis inhibitors. 
 The MSP receptor regulates alpha6beta4 and alpha3beta1 integrins via 14-3-3 proteins in keratinocyte   migration  . Growth factors, integrins, and the extracellular matrix (ECM) are known to play key roles in epidermal wound healing, although the interplay between these proteins is not fully understood. We show that growth factor macrophage stimulating protein (MSP)- and its receptor Ron-mediated PI3K activation in keratinocytes induces phosphorylation of both Ron and alpha6beta4 integrin at specific 14-3-3 binding sites. Consequently, a Ron/alpha6beta4 complex formed via 14-3-3 binding displaces alpha6beta4 from its location at hemidesmosomes (structures supporting cell adhesion) and relocalizes it to lamellipodia. Concomitant activation of alpha3beta1 and keratinocyte spreading/  migration   on laminin-5 occurs. Further, MSP-dependent beta4 tyrosine phosphorylation evokes p38 and NF-kappaB signaling required for keratinocyte wound closure. Based on these results, we propose a mechanism based on MSP-Ron-dependent phosphorylation and 14-3-3 association, whereby the function of alpha6beta4 switches from a mechanical adhesive device into a signaling component, and might be critically involved in human epidermal wound healing. 
 VLA-4 blockade promotes differential routes into human CNS involving PSGL-1 rolling of T cells and MCAM-adhesion of TH17 cells. The focus of this study is the characterization of human T cell blood-brain barrier   migration   and corresponding molecular trafficking signatures. We examined peripheral blood and cerebrospinal fluid immune cells from patients under long-term anti-very late antigen-4 (VLA-4)/natalizumab therapy (LTNT) and from CNS specimens. LTNT patients' cerebrospinal fluid T cells exhibited healthy central-/effector-memory ratios, but lacked CD49d and showed enhanced myeloma cell adhesion molecule (MCAM) expression. LTNT led to an increase of PSGL-1 expression on peripheral T cells. Although vascular cell adhesion molecule-1 (VLA-4 receptor) was expressed at all CNS barriers, P-selectin (PSGL-1-receptor) was mainly detected at the choroid plexus. Accordingly, in vitro experiments under physiological flow conditions using primary human endothelial cells and LTNT patients' T cells showed increased PSGL-1-mediated rolling and residual adhesion, even under VLA-4 blockade. Adhesion of MCAM(+)/TH17 cells was not affected by VLA-4 blocking alone, but was abrogated when both VLA-4 and MCAM were inhibited. Consistent with these data, MCAM(+) cells were detected in white matter lesions, and in gray matter of multiple sclerosis patients. Our data indicate that lymphocyte trafficking into the CNS under VLA-4 blockade can occur by using the alternative adhesion molecules, PSGL-1 and MCAM, the latter representing an exclusive pathway for TH17 cells to migrate over the blood-brain barrier. 
 The involvement of integrin β1 signaling in the   migratio  n and myofibroblastic differentiation of skin fibroblasts on anisotropic collagen-containing nanofibers. Utilization of nanofibrous matrices for skin wound repair holds great promise due to their morphological and dimensional similarity to native extracellular matrix (ECM). It becomes highly desired to understand how various nanofibrous matrices regulate skin cell behaviors and intracellular signaling pathways, important to tuning the functionality of tissue-engineered skin grafts and affecting the wound healing process. In this study, the phenotypic expressions of normal human dermal fibroblasts (NHDFs) on collagen-containing nanofibrous matrices with either isotropic (i.e., fibers collected randomly with no alignment) or anisotropic (i.e., fibers collected with alignment) fiber organizations were studied by immunostaining,   migratio  n assay and molecular analyses. Results showed that both nanofibrous matrices supported the attachment and growth of NHDFs similarly, while showing different cell morphology with distinct variation in focal adhesion formation and distribution. Anisotropic nanofibers significantly triggered the integrin β1 signaling pathway in NHDFs as evidenced by an increase of active integrin β1 (130 kD mature form) and phosphorylation of focal adhesion kinase (FAK) at Tyr-397. Anisotropic matrices also promoted t  he migrat  ion of NHDFs along the fibers, while neutralization of the integrin β1 activity abolished this promotion. Moreover, the fibroblast-to-myofibroblast differentiation was greatly enhanced for the NHDFs cultured on anisotropic nanofibrous matrices over a period of 48 h. Inhibition of cellular integrin β1 activity by neutralizing antibody eliminated this enhancement. These findings suggest the important role of integrin β1 signaling pathway in regulating the nanofiber-induced fibroblast phenotypic alteration and providing insightful understanding of the possible application of collagen-containing nanofibrous matrices for skin regeneration. 
 Integrin alpha8beta1 regulates adhesion,   migration   and proliferation of human intestinal crypt cells via a predominant RhoA/ROCK-dependent mechanism. Integrins are transmembrane alphabeta heterodimer receptors that function as structural and functional bridges between the cytoskeleton and ECM (extracellular matrix) molecules. The RGD (arginine-glycine-aspartate tripeptide motif)-dependent integrin alpha8beta1 has been shown to be involved in various cell functions in neuronal and mesenchymal-derived cell types. Its role in epithelial cells remains unknown.Integrin alpha8beta1 was found to be expressed in the crypt cell population of the human intestine but was absent from differentiating and mature epithelial cells of the villus. The function of alpha8beta1 in epithelial crypt cells was investigated at the cellular level using normal HIECs (human intestinal epithelial cells). Specific knockdown of alpha8 subunit expression using an shRNA (small-hairpin RNA) approach showed that alpha8beta1 plays important roles in RGD-dependent cell adhesion,   migration   and proliferation via a RhoA/ROCK (Rho-associated kinase)-dependent mechanism as demonstrated by active RhoA quantification and pharmacological inhibition of ROCK. Moreover, loss of alpha8beta1, through RhoA/ROCK, impairs FA (focal adhesion) complex integrity as demonstrated by faulty vinculin recruitment.Integrin alpha8beta1 is expressed in epithelial cells. In intestinal crypt cells, alpha8beta1 is closely involved in the regulation of adhesion,   migration   and cell proliferation via a predominant RhoA/ROCK-dependent mechanism. These results suggest an important role for this integrin in intestinal crypt cell homoeostasis. 
 Kaposi's sarcoma-associated herpesvirus activation of vascular endothelial growth factor receptor 3 alters endothelial function and enhances infection. Kaposi's sarcoma-associated herpesvirus (KSHV; also known as human herpesvirus 8) is the etiologic agent of Kaposi's sarcoma, an endothelial neoplasm. This gamma-herpesvirus encodes for several unique proteins that alter target cell function, including the virion envelope-associated glycoprotein B (gB). Glycoprotein B has an RGD (Arg-Gly-Asp) motif at the extracellular amino terminus region and binds to the alpha3beta1 surface integrin, which enhances virus entry. We now report that gB can activate the vascular endothelial growth factor receptor 3 (VEGFR-3) on the surface of microvascular endothelial cells and trigger receptor signaling, which can modulate endothelial   migration   and proliferation. Furthermore, we observed that VEGFR-3 expression and activation enhance KSHV infection and participate in KSHV-mediated transformation. These functional changes in the endothelium may contribute to the pathogenesis of Kaposi's sarcoma and suggest that interventions that inhibit gB activation of VEGFR-3 could be useful in the treatment of this neoplasm. 
 LOXL2 status correlates with tumor stage and regulates integrin levels to promote tumor progression in ccRCC. Clear cell renal cell carcinoma (ccRCC) is the most common histologically defined subtype of renal cell carcinoma (RCC). To define the molecular mechanism in the progression of ccRCC, we focused on LOX-like protein 2 (LOXL2), which is critical for the first step in collagen and elastin cross-linking. Using exon array analysis and quantitative validation, LOXL2 was shown to be significantly upregulated in clinical specimens of human ccRCC tumor tissues, compared with adjacent noncancerous renal tissues, and this elevated expression correlated with the pathologic stages of ccRCC. RNAi-mediated knockdown of LOXL2 resulted in marked suppression of stress-fiber and focal adhesion formation in ccRCC cells. Moreover, LOXL2 siRNA knockdown significantly inhibited cell growth,   migration  , and invasion. Mechanistically, LOXL2 regulated the degradation of both integrins α5 (ITGAV5) and β1 (ITGB1) via protease- and proteasome-dependent systems. In clinical ccRCC specimens, the expression levels of LOXL2 and integrin α5 correlated with the pathologic tumor grades. In conclusion, LOXL2 is a potent regulator of integrin α5 and integrin β1 protein levels and functions in a tumor-promoting capacity in ccRCC.This is the first report demonstrating that LOXL2 is highly expressed and involved in ccRCC progression by regulating the levels of integrins α5 and β1. 
 Autophagy modulates cell   migration   and β1 integrin membrane recycling. Cell   migratio  n is dependent on a series of integrated cellular events including the membrane recycling of the extracellular matrix receptor integrins. In this paper, we investigate the role of autophagy in regulating cell   migratio  n. In a wound-healing assay, we observed that autophagy was reduced in cells at the leading edge than in cells located rearward. These differences in autophagy were correlated with the robustness of MTOR activity. The spatial difference in the accumulation of autophagic structures was not detected in rapamycin-treated cells, which had less   migratio  n capacity than untreated cells. In contrast, the knockdown of the autophagic protein ATG7 stimulated cell   migratio  n of HeLa cells. Accordingly, atg3(-/-) and atg5(-/-) MEFs have greater cell   migratio  n properties than their wild-type counterparts. Stimulation of autophagy increased the co-localization of β1 integrin-containing vesicles with LC3-stained autophagic vacuoles. Moreover, inhibition of autophagy slowed down the lysosomal degradation of internalized β1 integrins and promoted its membrane recycling. From these findings, we conclude that autophagy regulates ce  ll migrat  ion, a central mechanism in cell development, angiogenesis, and tumor progression, by mitigating the cell surface expression of β1 integrins. 
 Integrative analyses identify osteopontin, LAMB3 and ITGB1 as critical pro-metastatic genes for lung cancer. To explore the key regulatory genes associated with lung cancer in order to reduce its occurrence and progress through silencing these key genes.To identify the key regulatory genes involved in lung cancer, we performed a combination of gene array and bioinformatics analyses to compare gene transcription profiles in 3 monoclonal cell strains with high, medium or low metastatic abilities, which were separated from the SPC-A-1sci and SPC-A-1 cell lines by limiting dilution monoclone assay. We then analyzed those genes' biological activities by knocking down their expression in SPC-A-1sci cells using siRNA and lenti-viral shRNA vectors, followed by determinations of the invasion and   migration   capabilities of the resulting cell lines in vitro as well as their potential for inducing occurrence and metastasis of lung cancer in vivo. To examine the clinical relevance of these findings, we analyzed the expression levels of the identified genes in human lung cancer tissues (n = 135) and matched adjacent normal tissues by immunohistochemical (IHC) staining.Three monoclonal cell strains characterized with high, medium or low metastatic abilities were successfully selected. Gene array and bioinformatics analyses implied that osteopontin, LAMB3 and ITGB1 were key genes involved in lung cancer. Knockdown of these genes suppressed human lung cancer cell invasion and metastasis in vitro and in vivo. Clinical sample analyses indicated that osteopontin, LAMB3 and ITGB1 protein expression levels were higher in lung cancer patients, compared to non-cancerous adjacent tissues, and correlated with lymphatic metastasis.We confirmed that osteopontin, LAMB3 and ITGB1 played important roles in the occurrence and metastasis of lung cancer, thus provided important clues to understanding the molecular mechanism of metastasis and contributing to the therapeutic treatment of lung cancer. 
 Metastatic properties of prostate cancer cells are controlled by VEGF. Mechanisms of metastasis, the major complication of prostate cancer, are poorly understood. In this study, we define molecular mechanisms that may contribute to the highly invasive potential of prostate cancer cells. Vascular endothelial growth factor (VEGF), its receptors (VEGFRs), and alpha5beta1 integrin were expressed by prostate cancer cells in vitro and by prostate tumors in vivo, and their expression was elevated at sites of bone metastasis compared to original prostate tumor. VEGF, through interaction with its receptors, regulated adhesive and migratory properties of the cancer cells. Specifically, the highly metastatic prostate cancer cell subline LNCaP-C4-2 showed a decreased adhesive but an enhanced migratory response to fibronectin, a ligand for alpha5beta1 integrin, compared to its nonmetastatic counterpart. A similar pattern was also observed when bone sialoprotein was used as a ligand in   migration   assays. Increased   migration   of metastatic prostate cancer cells to fibronectin and bone sialoprotein was regulated by VEGF via VEGFR-2. Tumor suppressor PTEN was involved in control of VEGF/VEGFR-2 stimulated prostate cancer cell adhesion as well as proliferation. 
 Integrin alpha4beta1 signaling is required for lymphangiogenesis and tumor metastasis. Recent studies have shown that lymphangiogenesis or the growth of lymphatic vessels at the periphery of tumors promotes tumor metastasis to lymph nodes. We show here that the fibronectin-binding integrin alpha4beta1 and its ligand fibronectin are novel functional markers of proliferative lymphatic endothelium. Tumors and lymphangiogenic growth factors, such as vascular endothelial growth factor-C (VEGF-C) and VEGF-A, induce lymphatic vessel expression of integrin alpha4beta1. Integrin alpha4beta1 then promotes growth factor and tumor-induced lymphangiogenesis, as genetic loss of integrin alpha4beta1 expression in Tie2Cre+ alpha4(loxp/loxp) mice or genetic loss of alpha4 signaling in alpha4Y991A knock-in mice blocks growth factor and tumor-induced lymphangiogenesis, as well as tumor metastasis to lymph nodes. In addition, antagonists of integrin alpha4beta1 suppress lymphangiogenesis and tumor metastasis. Our studies show that integrin alpha4beta1 and the signals it transduces regulate the adhesion,   migration  , invasion, and survival of proliferating lymphatic endothelial cells. As suppression of alpha4beta1 expression, signal transduction, or function in tumor lymphatic endothelium not only inhibits tumor lymphangiogenesis but also prevents metastatic disease, these results show that integrin alpha4beta1-mediated tumor lymphangiogenesis promotes metastasis and is a useful target for the suppression of metastatic disease. 
 N-glycosylation of the I-like domain of beta1 integrin is essential for beta1 integrin expression and biological function: identification of the minimal N-glycosylation requirement for alpha5beta1. N-Glycosylation of integrin alpha5beta1 plays a crucial role in cell spreading, cell   migration  , ligand binding, and dimer formation, but the detailed mechanisms by which N-glycosylation mediates these functions remain unclear. In a previous study, we showed that three potential N-glycosylation sites (alpha5S3-5) on the beta-propeller of the alpha5 subunit are essential to the functional expression of the subunit. In particular, site 5 (alpha5S5) is the most important for its expression on the cell surface. In this study, the function of the N-glycans on the integrin beta1 subunit was investigated using sequential site-directed mutagenesis to remove the combined putative N-glycosylation sites. Removal of the N-glycosylation sites on the I-like domain of the beta1 subunit (i.e. the Delta4-6 mutant) decreased both the level of expression and heterodimeric formation, resulting in inhibition of cell spreading. Interestingly, cell spreading was observed only when the beta1 subunit possessed these three N-glycosylation sites (i.e. the S4-6 mutant). Furthermore, the S4-6 mutant could form heterodimers with either alpha5S3-5 or alpha5S5 mutant of the alpha5 subunit. Taken together, the results of the present study reveal for the first time that N-glycosylation of the I-like domain of the beta1 subunit is essential to both the heterodimer formation and biological function of the subunit. Moreover, because the alpha5S3-5/beta1S4-6 mutant represents the minimal N-glycosylation required for functional expression of the beta1 subunit, it might also be useful for the study of molecular structures. 
 N-acetylglucosaminyltransferase V regulates extravillous trophoblast invasion through glycosylation of alpha5beta1 integrin. For successful human placentation, invasion of trophoblast cells into the uterus and its associated vasculature is essential, and the regulation of this process is controlled by many factors at the fetal-maternal interface. N-acetylglucosaminyltransferase V (GnT-V) is a key enzyme that catalyzes beta1, 6-N-acetylglucosamine (beta1-6GlcNAc) branching on asparagine-linked oligosaccharides of cell proteins. GnT-V and its product, beta1-6GlcNAc, are known to regulate cellular transformation and correlate with the metastatic potential of various cancer cells. The aim of the present study was to determine whether extravillous trophoblast (EVT) expressed this molecule and examine the role of GnT-V in the regulation of human trophoblast invasion. Immunohistochemistry showed that GnT-V was strongly expressed within the cytoplasm of EVT in the anchoring villi; this expression was down-regulated in EVTs invading the decidua. Suppression of beta1-6GlcNAc glycosylation by swainsonine enhanced the migratory potential and invasive capability of both primary EVTs and the EVT cell line, HTR-8/SVneo. Down-regulation of GnT-V expression by small interfering RNA in the choriocarcinoma cell line Jar consistently enhanced the   migration   and invasive capacity of these cells and elevated cellular adhesion to extracellular matrix proteins, such as fibronectin and collagen type I/IV. The extent of beta1-6 branching of alpha5beta1 integrin was significantly reduced in small interfering GnT-V-transfected Jar cells compared with mock transfectants, although the expression of alpha1, alpha5, alpha6, and beta1 integrin on the cell surface was not changed. These results suggest that GnT-V is expressed in human EVT and is involved in regulating trophoblast invasion through modifications of the oligosaccharide chains of alpha5beta1 integrin. 
 Regulation of urokinase receptor function and pericellular proteolysis by the integrin alpha(5)beta(1). Interactions between the uPA receptor (uPAR) and various integrins, including alpha(5)beta(1), are known to modulate integrin-dependent cell adhesion, and we have shown that the integrin-associated tetraspanin protein CD82 down-regulates uPAR-dependent plasminogen activation by affecting alpha(5)beta(1) cellular localisation. Here we have investigated whether overexpression of alpha(5)beta(1) directly affects uPAR-dependent pericellular proteolysis. CHO cells overexpressing alpha(5)beta(1) were found to activate plasminogen at a rate up to 18-fold faster than B2CHO cells which are alpha(5)-deficient. This effect was dependent on the activation state of alpha(5)beta(1), as it was maximal in the presence of Mn(2+). To determine the role of uPAR-alpha(5)beta(1) interactions in this effect, we determined the adhesion of these cells to immobilised soluble uPAR (suPAR). Neither cell-type was found to adhere to suPAR, but both cell types were found to adhere to an anti-uPAR monoclonal antibody in a uPAR- and integrin-dependent manner. This adhesion was 10-fold greater in the absence of alpha(5)beta(1), possibly implicating the involvement of non-alpha(5)-integrins. Soluble forms of the various components were used to investigate the molecular basis of these effects, but no direct interactions could be demonstrated between alpha(5)beta(1) and either uPAR, uPA or uPA-uPAR complex. This suggests that assembly of these components on the plasma membrane is required to influence uPAR function, increasing uPAR-dependent pericellular proteolysis and decreasing uPAR-dependent cell adhesion. These interactions may be modified by other integrins, suggesting a complex interplay between uPAR and integrins on the cell surface with the potential to regulate invasive cell   migration  . 
 CD9 of mouse brain is implicated in neurite outgrowth and cell   migration   in vitro and is associated with the alpha 6/beta 1 integrin and the neural adhesion molecule L1. We describe here a novel monoclonal antibody (mab H6) which recognizes CD9, an integral cell surface constituent previously described in cells of the hematopoietic lineage and involved in the aggregation of platelets. Mab H6 was raised against membranes of immature mouse astrocytes and reacted with a protein of 25-27 kD in detergent extracts of adult mouse brain membranes. Sequence analysis of the N-terminal amino acids revealed an identity of 96% with CD9 from mouse kidney. CD9 was localized in the central and peripheral mouse nervous systems: in the spinal cord of 11-day-old mouse embryos, CD9 was strongly expressed in the floor and roof plates. In the adult mouse sciatic nerve, myelin sheaths were highly CD9-immunoreactive. Mab H6 reacted with the cell surfaces of both glial cells and neurons in culture and inhibited   migration   of neuronal cell bodies, neurite fasciculation and outgrowth of astrocytic processes from cerebellar microexplants. Neurite outgrowth from isolated small cerebellar neurons was increased in the presence of mab H6 on substrate-coated laminin, but not on substrate-coated poly-L-lysine. Addition of mab H6 elicited an increase in intracellular Ca2+ concentration in these cells on substrate-coated laminin. Immunoprecipitates of CD9 from cultured mouse neuroblastoma N2A cells contained the alpha 6/beta 1 integrin. Moreover, preparations of CD9 immunoaffinity-purified from adult mouse brain using a mab H6 column contained the neural adhesion molecule L1, but not other neural adhesion molecules. CD9 bound to L1, but not to NCAM or MAG. Both the alpha 6/beta 1 integrin and L1 could be induced to coredistribute with CD9 on the surface of cultured neuroblastoma N2A cells. The combined observations suggest that CD9 can associate with L1 and alpha 6/beta 1 integrin to influence neural cell interactions in vitro. 
 Stimulation of beta 1 integrin induces tyrosine phosphorylation of vascular endothelial growth factor receptor-3 and modulates cell   migration  . Interactions between integrins and tyrosine kinase receptors can modulate a variety of cell functions. We observed a cooperative interaction between the beta(1) integrin and vascular endothelial growth factor receptor-3 (VEGFR-3 or Flt4) that appeared to be required for cell   migration  . By using VEGFR-3-transfected 293 cells (293/VEGFR-3) or primary dermal microvascular endothelial cells (DMEC), we found that stimulation with either soluble or immobilized extracellular matrix (ECM) proteins, collagen or fibronectin (FN), resulted in the increased tyrosine phosphorylation of VEGFR-3 in the absence of a cognate ligand. This increased tyrosine phosphorylation of VEGFR-3 was diminished by pretreatment with a blocking antibody against the beta(1) integrin. Cross-linking with anti-beta(1) integrin antibody induced a similar degree of tyrosine phosphorylation of VEGFR-3. Stimulation with collagen or FN induced an association between beta(1) integrin and VEGFR-3 in both 293/VEGFR-3 and primary DMEC cells. Collagen or FN-induced tyrosine phosphorylation of VEGFR-3 was inhibited by treatment with cytochalasin D, an inhibitor of actin polymerization. Collagen or FN was able to induce the   migration   of 293/VEGFR-3 or DMEC cells to a limited extent. However,   migration   was dramatically enhanced when a gradient of the cognate ligand, VEGF-D, was added. VEGF-D failed to induce cell   migration   in the absence of ECM proteins. Introducing a mutation at the kinase domain of VEGFR-3 or treatment with blocking antibody against either VEGFR-3 or beta(1) integrin inhibited cell   migration   induced by ECM and VEGF-D, indicating that signals from both beta(1) integrin and VEGFR-3 are required for this cell function. 
 Human CMV infection of endothelial cells induces an angiogenic response through viral binding to EGF receptor and beta1 and beta3 integrins. Human cytomegalovirus (HCMV) infection is associated with atherosclerosis, transplant vascular sclerosis, and coronary restenosis. A common theme in these vascular diseases is an increased rate of angiogenesis. Angiogenesis is a complex biological process mediated by endothelial cell (EC) proliferation,   migration  , and morphogenesis. Although angiogenesis is a normal process in the host, its dysregulation, after viral infection or injury to the vessel wall, is associated with plaque development in atherosclerotic patients. We now document that HCMV infection results in increased EC proliferation, motility, and capillary tube formation. The observed HCMV-induced angiogenic response depended on viral binding to and signaling through the beta(1) and beta(3) integrins and the epidermal growth factor receptor, via their ability to activate the phosphatidylinositol 3-kinase and the mitogen-activated protein kinase signaling pathways. Because a proangiogenic response drives the neovascularization observed in atherosclerotic disease, our findings identify a possible mechanism for how HCMV infection contributes to vascular disease. 
 Ionizing radiation induces up-regulation of functional beta1-integrin in human lung tumour cell lines in vitro. Cell-matrix interactions are in part mediated through the beta1-integrin pathway regulating cell survival, proliferation, adhesion and   migration  . This study was performed to elucidate alterations of expression of the beta1-integrin and its co-localized protein kinase, integrin-linked kinase (ILK), after exposure to ionizing radiation in two lung carcinoma cell lines in the presence or absence of different beta1-integrin-dependent matrix proteins.Exponentially growing A549 and SKMES1 cells grown on fibronectin, laminin, BSA or plastic were exposed to 2 Gy or 6 Gy. Besides colony formation assays (0.5-8 Gy) and immediate plating experiments, flow cytometry (for beta1-integrin) and immunoblotting (for beta1-integrin and ILK) were carried out to analyze the protein expression. The localization of both proteins plus filamentous (f-) actin was further examined by immunofluorescence staining and laser confocal scanning microscopy. Functionality of the beta1 receptor subunit after irradiation was investigated in adhesion assays.A549 and SKMES1 cells grown on fibronectin or laminin demonstrated a significant increase in cell survival after irradiation compared to cells grown on BSA or plastic. Immediate plating of cells after irradiation on fibronectin did not show an improved survival. Flow cytometric and Western blot data showed a dose- and matrix-dependent induction of beta1-integrin and ILK expression after irradiation within 48 h. Adhesion to fibronectin or laminin compared to BSA or plastic was increased by 10-fold after irradiation, demonstrating these specific cell surface receptors to be functional. The staining of beta1-integrin and ILK in A549 cells confirmed the radiation-induced up-regulation of both proteins. Additionally, beta1-integrin and ILK co-localized with accumulated actin fibers at the cytoplasmic face of the cell membrane at confined areas.Ionizing radiation strongly induced the expression of functional beta1-integrin and ILK in the two lung cancer cell lines, A549 and SKMES1, dependent on different matrices used. Additionally, the subcellular localization of both proteins was altered by irradiation, and the individual cellular radiosensitivity was reduced in the presence of an extracellular matrix. On the one hand, this may result in aggravated therapeutic approaches and on the other hand, cells could adhere more strongly in their environment by the increase in functional surface receptor density preventing metastasis. Concerning intravascular located tumour cells, beta1-integrin up-regulation might enable these cells to adhere to the endothelium, which represents a prerequisite for metastatic disease. Identification of such mechanisms will provide considerable insights into the understanding of tumorigenicity and metastatic phenotypes, possibly leading to new, optimized radiochemotherapeutic regimens. 
 The tetraspanin CD151 regulates cell morphology and intracellular signaling on laminin-511. The tetraspanin CD151 forms a stable complex with integrin alpha3beta1, a widely expressed laminin receptor, and is implicated in the regulation of integrin alpha3beta1-mediated cellular responses, including cell attachment, spreading and   migration  . However, the molecular mechanism by which CD151 regulates integrin alpha3beta1 functions remains unclear. To address this issue, we knocked down CD151 expression in A549 human lung adenocarcinoma cells by RNA interference. When plated on laminin-511 (laminin-10), the CD151-knocked-down cells showed aberrant membrane protrusions and exhibited reductions in the tyrosine phosphorylation of focal adhesion kinase, Src, p130Cas and paxillin. The formation of membrane protrusions was attenuated when the cells were either plated on surfaces coated with higher concentrations of laminin-511 or treated with the integrin beta1-activating mAb TS2/16; however, neither treatment could rescue the reduced tyrosine phosphorylation. These results indicate that CD151 knockdown weakens the integrin alpha3beta1-mediated adhesion to laminin-511 and thereby provokes an aberrant morphology, but this reduced adhesive activity is not involved in the decline of signaling events in CD151-knocked-down cells. Thus, our results suggest that CD151 regulates integrin alpha3beta1 functions in two independent aspects: potentiation of integrin alpha3beta1-mediated cell adhesion and promotion of integrin alpha3beta1-stimulated signaling events involving tyrosine phosphorylation. 
 Functional live-cell imaging demonstrates that beta1-integrin promotes type IV collagen degradation by breast and prostate cancer cells. The ability of tumor cells to adhere to, migrate on, and remodel extracellular matrices is mediated by cell surface receptors such as beta1-integrins. Here we conducted functional live-cell imaging in real time to investigate the effects of modulating beta1-integrin expression and function on proteolytic remodeling of the extracellular matrix. Human breast and prostate cancer cells were grown on reconstituted basement membrane containing a quenched fluorescent form of collagen IV. Generation of cleavage products and the resulting increases in fluorescence were imaged and quantified. Decreases in the expression and activity of beta1-integrin reduced digestion of quenched fluorescent-collagen IV by the breast and prostate cancer cells and correspondingly their invasion through and   migration   on reconstituted basement membrane. Decreased extracellular matrix degradation also was associated with changes in the constituents of proteolytic pathways: decreases in secretion of the cysteine protease cathepsin B, the matrix metalloproteinase (MMP)-13, and tissue inhibitors of metalloproteinases (TIMP)-1 and 2; a decrease in expression of MMP-14 or membrane type 1 MMP; and an increase in secretion of TIMP-3. This is the first study to demonstrate through functional live-cell imaging that downregulation of beta1-integrin expression and function reduces proteolysis of collagen IV by breast and prostate cancer cells. 
 Extracellular matrix regulates endothelial functions through interaction of VEGFR-3 and integrin alpha5beta1. Endothelium extracellular matrix (ECM) interactions can provide distinct spatial and molecular signals which control cellular proliferation,   migration  , and differentiation. Here, we investigated the role of fibronectin (FN), a major ECM protein, on the functions of lymphatic endothelial cells (LEC). We observed that FN, the ligand for integrin alpha5beta1, selectively promoted the growth of LEC as compared with vitronectin (VN) in the presence of the ligand for vascular endothelial growth factor receptor 3 [VEGFR-3 (VEGF-C156S)]. Upon investigating the mechanisms whereby ECM components regulate VEGFR-3 signaling, we found that FN transactivated VEGFR-3 and significantly enhanced the phosphorylation of VEGFR-3 induced by VEGF-C156S as compared to VN. An enhanced association of the integrin subunit alpha5 or beta1 with VEGFR-3, after stimulation with VEGF-C156S, was observed by co-immunoprecipitation. While blockade of integrin alpha5beta1 inhibited the VEGF-C156S-induced phosphorylation of VEGFR-3, no similar effect was obtained by blocking integrin alphavbeta3. FN also protected the endothelial cells from serum deprivation-induced apoptosis. Moreover, while the specific PI3 kinase inhibitor, LY294002, abolished this FN-mediated cell survival, the MAPK kinase inhibitor, PD98059, had no significant effect. Furthermore, a dominant-negative mutant of VEGFR-3 (G857R) reduced VEGF-C156S or FN-mediated cell survival, as well as the activities of PI3 kinase/Akt. Our results indicate that integrin alpha5beta1 participates in the activation of both VEGFR-3 and its downstream PI3 kinase/Akt signaling pathway, which is essential for FN-mediated lymphatic endothelial cell survival and proliferation. 
 Brag2 differentially regulates β1- and β3-integrin-dependent adhesion in endothelial cells and is involved in developmental and pathological angiogenesis. β1-Integrins are essential for angiogenesis. The mechanisms regulating integrin function in endothelial cells (EC) and their contribution to angiogenesis remain elusive. Brag2 is a guanine nucleotide exchange factor for the small Arf-GTPases Arf5 and Arf6. The role of Brag2 in EC and angiogenesis and the underlying molecular mechanisms remain unclear. siRNA-mediated Brag2-silencing reduced EC angiogenic sprouting a  nd migrat  ion. Brag2-siRNA transfection differentially affected α5β1- and αVβ3-integrin function: specifically, Brag2-silencing increased focal/fibrillar adhesions and adhesion on β1-integrin ligands (fibronectin and collagen), while reducing the adhesion on the αVβ3-integrin ligand, vitronectin. Consistent with these results, Brag2-silencing enhanced surface expression of α5β1-integrin, while reducing surface expression of αVβ3-integrin. Mechanistically, Brag2-mediated αVβ3-integrin-recycling and β1-integrin endocytosis and specifically of the active/matrix-bound α5β1-integrin present in fibrillar/focal adhesions (FA), suggesting that Brag2 contributes to the disassembly of FA via β1-integrin endocytosis. Arf5 and Arf6 are promoting downstream of Brag2 angiogenic sprouting, β1-integrin endocytosis and the regulation of FA. In vivo silencing of the Brag2-orthologues in zebrafish embryos using morpholinos perturbed vascular development. Furthermore, in vivo intravitreal injection of plasmids containing Brag2-shRNA reduced pathological ischemia-induced retinal and choroidal neovascularization. These data reveal that Brag2 is essential for developmental and pathological angiogenesis by promoting EC sprouting through regulation of adhesion by mediating β1-integrin internalization and link for the first time the process of β1-integrin endocytosis with angiogenesis. 
 CD47 plays a critical role in T-cell recruitment by regulation of LFA-1 and VLA-4 integrin adhesive functions. CD47 plays an important but incompletely understood role in the innate and adaptive immune responses. CD47, also called integrin-associated protein, has been demonstrated to associate in cis with β1 and β3 integrins. Here we test the hypothesis that CD47 regulates adhesive functions of T-cell α4β1 (VLA-4) and αLβ2 (LFA-1) in in vivo and in vitro models of inflammation. Intravital microscopy studies reveal that CD47(-/-) Th1 cells exhibit reduced interactions with wild-type (WT) inflamed cremaster muscle microvessels. Similarly, murine CD47(-/-) Th1 cells, as compared with WT, showed defects in adhesion and   transmig  ration across tumor necrosis factor-α (TNF-α)-activated murine endothelium and in adhesion to immobilized intercellular adhesion molecule 1 (ICAM-1) and vascular cell adhesion protein 1 (VCAM-1) under flow conditions. Human Jurkat T-cells lacking CD47 also showed reduced adhesion to TNF-α-activated endothelium and ICAM-1 and VCAM-1. In cis interactions between Jurkat T-cell β2 integrins and CD47 were detected by fluorescence lifetime imaging microscopy. Unexpectedly, Jurkat CD47 null cells exhibited a striking defect in β1 and β2 integrin activation in response to Mn(2+) or Mg(2+)/ethylene glycol tetraacetic acid treatment. Our results demonstrate that CD47 associates with β2 integrins and is necessary to induce high-affinity conformations of LFA-1 and VLA-4 that recognize their endothelial cell ligands and support leukocyte adhesion and tran  sendothel  ial migration. 
 C-terminal COOH of integrin β1 is necessary for β1 association with the kindlin-2 adapter protein. Protein-protein interactions are driving forces in cellular processes. As a prime example, transmembrane integrins link extracellular matrix and intracellular proteins, resulting in bidirectional signaling that regulates cel  l migrati  on, proliferation, differentiation, and survival. Here we provide the first evidence that interaction between the integrin β1 cytoplasmic tail and kindlin-2, a member of a family of adapters implicated in human disease pathogenesis, is mainly governed by the β1 C-terminal carboxylate moiety and is required for laterality organ development in zebrafish. Affinity measurements indicate that this unusual protein-protein interaction mode is coordinated by a putative carboxylate-binding motif in the kindlin-2 FERM subdomain F3. Contrary to the C terminus of proteins that engage PDZ domains, the C-terminal three residues of β1, per se, do not contribute to kindlin-2 binding or to laterality organ development. Thus, by employing zebrafish as an in situ physiological tool to correlate protein structure and function, we have discovered an unexpected association chemistry between an integrin and a key adapter involved in integrin signaling. 
 [Effect of Integrin α5β1-mediated ERK signal pathway on proliferation an  d migrati  on of A549 cells]. Recent studies have shown that integrin α5β1 as a core in the integrin family plays an important role in metastasis, invasion and poor difference of non-small cell lung cancer. In this study, A549 cells were cultured and treated with integrin α5β1 small interfering RNA (siRNA) and extracelluar signal-regulated protein kinase (ERK) inhibitor PD98059 to investigate the effect of integrin α5β1 on proliferat  ion and m  igration of A549 cells and explore its signal transduction mechanism.A549 cells were divided into four groups: Untransfection, Lipofectamine, Integrin α5β1 siRNA-transfected group and PD98059 group. The protein expression levels of integrin α5β1 were detected by Western blot analysis and the expression levels of integrin α5β1 mRNA was measured by reverse transcription-polymerase chain reaction (RT-PCR). The protein expression level of ERK1/2, MMP-9 and caspase-3 were measured by Western blot analysis. The proliferation and apoptosis of A549 cells were measured by MTT assay and Annexin-V FITC PI double staining.Integrin α5β1 siRNA could inhibit the phosphorylated ratio of ERK by down-regulate the expression of ERK 1/2 proteins. In addition, integrin α5β1 siRNA or PD98059 could inhibit the proliferation of A549 cells, induce the apoptosis of A549 cells, up-regulate the expression of caspase-3 and down-regulate the expression of MMP-9.Integrin α5β1 might involves the abnorma  l prolife  ration and migration of A549 cells through mediating ERK signal transduction pathway. 
 Amphiregulin enhances alpha6beta1 integrin expression and cell motility in human chondrosarcoma cells through Ras/Raf/MEK/ERK/AP-1 pathway. Chondrosarcoma is a malignant tumor that produces cartilage matrix. The most lethal aspect is its metastatic property. We demonstrated that amphiregulin (AR) is significantly upregulated in highly aggressive cells. AR silencing markedly suppressed cell   migration  . Exogenous AR markedly increased cell   migration   by transactivation of α6β1 integrin expression. A neutralizing α6β1 integrin antibody can abolish AR-induced cell motility. Knockdown of AR inhibits metastasis of cells to the lung in vivo. Furthermore, elevated AR expression is positively correlated with α6β1 integrin levels and higher grades in patients. These findings can potentially serve as biomarker and therapeutic approach for controlling chondrosarcoma metastasis. 
 [Expression of E-cadherin and beta1-integrin mRNA in endometrial cancer]. The metastatic ability of tumors is characteristic for malignant neoplasms and constitutes the main cause of therapeutics failures. Metastasis formation involves the sequence of processes such as proteolytic degradation of the basement membrane,   migration  , intravasation, extravasation, proliferation and angiogenesis. Cadherins and integrins are groups of proteins directly involved in these processes. In the present study we analyzed the mRNA expression of CDH1 and ITGB1 genes by real-time polymerase chain reaction (RT-PCR). The study included 106 endometrial carcinomas. CDH1 and ITGB1 mRNA expression was found in all of the studied samples. Generally the CDH1 and ITGB1 mRNA expression was significantly higher in well-differentiated rather than poorly differentiated tumors.The mRNA expression levels of CDH1 and ITGB1 in series of 83 samples of endometrial carcinoma were studied by real time RT-PCR method. Statistical analysis of the obtained results was performed.CDH1 and ITGB1 gene expression was observed in all examined tissues and was correlated with cancer malignancy (G). In high grade malignant tumors (G1), CDH1 and ITGB1 gene expression was the highest, in G2 and G3 tumors the expression of both genes was gradually lowering. Moreover the statistically significant correlation between CDH1 and ITGB1 gene expression was observed. (Spearman test, r=0.29, p<O.01).CDH1 and ITGB1 mRNA expression seems to be an important marker of cancer progression and metastases in endometrial malignant tumors. 
 Role of ADAM-9 disintegrin-cysteine-rich domains in human keratinocyte   migration  . ADAM-9 belongs to a family of transmembrane, disintegrin-containing metalloproteinases involved in protein ectodomain shedding and cell-cell and cell-matrix interactions. The aim of this study was to analyze the expression of ADAM-9 in skin and to assess the role of this proteolytic/adhesive protein in skin physiology. In normal skin, ADAM-9 expression was detected in both the epidermis and dermis and in vitro in keratinocytes and fibroblasts. Here we report that ADAM-9 functions as a cell adhesion molecule via its disintegrin-cysteine-rich domain. Using solid phase binding assays and antibody inhibition experiments, we demonstrated that the recombinant disintegrin-cysteine-rich domain of ADAM-9 specifically interacts with the beta1 integrin subunit on keratinocytes. This was corroborated by co-immunoprecipitation. In addition, engagement of integrin receptors by the disintegrin-cysteine-rich domain resulted in ERK phosphorylation and increased MMP-9 synthesis. Treatment with the ERK inhibitor PD98059 inhibited MMP-9 induction. Furthermore, the presence of the soluble disintegrin-cysteine-rich domain did not interfere with cell   migration   on different substrates. However, keratinocytes adhering to the immobilized disintegrin-cysteine-rich domain showed increased motility, which was partially due to the induction of MMP-9 secretion. In summary, our results indicate that the ADAM-9 adhesive domain plays a role in regulating the motility of cells by interaction with beta1 integrins and modulates MMP synthesis. 
 Overexpression of integrin-β1 in leiomyoma promotes cell spreading and proliferation. Uterine leiomyoma, the most common tumors found in the women of the reproductive age, may cause abnormal uterine bleeding and be life threatening. Compared with myometrium, leiomyoma contains excessive extracellular matrix (ECM). However, the pathological roles of ECM in the development of leiomyoma remain largely unknown. Integrins are the major adhesion molecules on cell surface to interact with ECM. The interactions of ECM with integrins regulate cell adhesion and initiate signals for cell growth, differentiation, and   migratio  n.The aim of this study was to investigate the expression and functional role of integrin-β1 in leiomyoma pathogenesis.Levels of integrin-β1 protein were determined by Western blotting in paired normal and leiomyomal tissues (n = 15). Knockdown of integrin-β1 and inhibition of ECM-integrin interaction by disintegrin were used to evaluate the impact of integrin-β1 in cell adhesion, spreading, and proliferation.Levels of integrin-β1 were significantly up-regulated in leiomyomal cells compared with their normal counterparts. Knockdown of integrin-β1 did not affect cell adhesion on fibronectin or laminin matrix but significantly inhibits cell spreading ability. Consistent with this notion, the phosphorylation of focal adhesion kinase and the recruitment of paxillin to the focal contact were decreased in integrin-β1 knockdown cells, which attenuates contraction force. The inability of cell spreading leads to inhibition of cyclin D1 expression and impedes cell cycle progression. More importantly, disruption of ECM-integrin interaction by the small protein, disintegrin inhibited cyclin D1 expression and cell proliferation.These data demonstrate that integrin-β1 is a critical ligand to enhance cell-ECM contact force and thus promotes cell proliferation. Disruption of ECM-integrin-β1 signaling may serve as an option to inhibit the progression of leiomyoma. 
 Heparanase released from mesenchymal stem cells activates integrin beta1/HIF-2alpha/Flk-1 signaling and promotes endothelial cell   migration   and angiogenesis. Heparanase plays important roles in tumor angiogenesis. Our previous study demonstrated that hypoxic preconditioning (HPC) enhanced the angiogenic and therapeutic effects of mesenchymal stem cells (MSCs), effects that were paralleled by enhanced heparanase expression. This study was designed to elucidate the role of heparanase in the improved therapeutic properties of HPC-MSCs and to explore underlying mechanisms using an ischemic rat hind limb model. MSCs transfected with heparanase (MSC(hpa) ) or empty vector (MSC(null) ) were delivered by intramuscular injections to ischemic hind limbs. Hind limbs that received MSC(hpa) recovered blood flow more rapidly at 7 days and acquired higher capillary density at 14 days compared with MSC(null) . Conditioned medium from MSC(hpa) increased endothelial cell   migration   and promoted greater tube formation relative to that from the MSC(null) groups. Vascular endothelial growth factor receptor 2 (VEGFR2, Flk-1) and its downstream signaling pathway (p38MAPK/HSP27) were significantly increased in human umbilical vein endothelial cells (HUVECs) after treatment with MSC(hpa) conditioned medium. Each of these responses was decreased by cocultured with MSC(hpa-KD) conditioned medium. MSC(hpa) conditioned medium activated hypoxia-inducible factor-2α (HIF-2α) and increased in parallel the transcript level of Flk-1 as determined by chromatin immunoprecipitation-PCR and luciferase assays. Analyses of integrin expression revealed an important role for integrin β1 in the regulation of HIF-2α. All angiogenic effects of MSC(hpa) conditioned medium were abolished by knockdown of integrin β1, HIF-2α, and Flk-1 in HUVECs with selective shRNAs. These findings identify heparanse as a key regulator of angiogenesis by MSCs. We propose a novel pathway wherein heparanse sequentially activates integrin β1, HIF-2α, Flk-1, and p38MAPK/HSP27 with corresponding enhancement of angiogenesis. 
 Characterization of key mechanisms in trans  migration   and invasion of mesenchymal stem cells. For successful systemic stem cell therapy, mesenchymal stem cells (MSCs) must transmigrate across the endothelium and invade their target tissue. To date, most of the underlying mechanisms of trans  migration   and invasion remain to be elucidated. Improving our knowledge on these core processes might elevate the efficiency of stem cell therapy. Our aim was therefore to characterize key mechanisms involved in trans  migration   and invasion of MSCs. Co-cultivation experiments infer that MSCs integrate into the endothelial monolayer. However, the time course of adhesion, integration and trans  migration   depends on the endothelial phenotype and is most effective in venous vessels of the myocardium. Thus, a variable capacity for trans  migration   exists within the vasculature. Additionally, three-dimensional systems reveal that MSCs penetrate the endothelium and invade the surrounding tissue via plasmic podia. Furthermore, trans  migration   not only requires the interaction of vascular cell adhesion molecule-1 (VCAM-1) and very late antigen-4 (VLA-4) as verified by blocking experiments, but also triggers the clustering of beta1 integrins. In addition, in situ zymographies infer the activation of gelatinases at sites of MSC invasion into myocardial tissue. As evidenced by ELISA, MSCs secrete matrix metalloproteinase (MMP)-2 but not MMP-9. Finally, media containing additional cytokines accelerate the trans  migration  . Concluding, key players involved in trans  migration   and invasion of MSCs are the endothelial phenotype, VCAM-1/VLA-4, beta1 integrins, MMP-2 secretion and cytokines. 
 C1GALT1 promotes invasive phenotypes of hepatocellular carcinoma cells by modulating integrin β1 glycosylation and activity. Cancer cell invasion and metastasis are the primary causes of treatment failure and death in hepatocellular carcinoma (HCC). We previously reported that core 1 β1,3-galactosyltransferase (C1GALT1) is frequently overexpressed in HCC tumors and its expression is associated with advanced tumor stage, metastasis, and poor survival. However, the underlying mechanisms of C1GALT1 in HCC malignancy remain unclear. In this study, we found that overexpression of C1GALT1 enhanced HCC cell adhesion to extracellular matrix (ECM) proteins  , migrati  on, and invasion, whereas RNAi-mediated knockdown of C1GALT1 suppressed these phenotypes. The promoting effect of C1GALT1 on the metastasis of HCC cells was demonstrated in a mouse xenograft model. Mechanistic investigations showed that the C1GALT1-enhanced phenotypic changes in HCC cells were significantly suppressed by anti-integrin β1 blocking antibody. Moreover, C1GALT1 was able to modify O-glycans on integrin β1 and regulate integrin β1 activity as well as its downstream signaling. These results suggest that C1GALT1 could enhance HCC invasiveness through integrin β1 and provide novel insights into the roles of O-glycosylation in HCC metastasis. 
 Expression of integrin alpha6beta1 enhances tumorigenesis in glioma cells. The integrin alpha6beta1 and its main ligand laminin-111 are overexpressed in glioblastoma, as compared with normal brain tissue, suggesting they may be involved in glioblastoma malignancy. To address this question, we stably expressed the alpha6 integrin subunit in the U87 cell line via retroviral-mediated gene transfer. We show that cell surface expression of the alpha6beta1 integrin led to dramatic changes in tumor U87 cell behavior, both in vitro and in vivo. Nude mice receiving either subcutaneous or intracerebral inoculation of alpha6beta1-expressing cells developed substantially more voluminous tumors than mice injected with control cells. The difference in tumor growth was associated with a marked increase in vascularization in response to alpha6beta1 integrin expression and may also be related to changes in the balance between cell proliferation and survival. Indeed, expression of alpha6beta1 enhanced proliferation and decreased apoptosis of U87 cells both in the tumor and in vitro. Additionally, we demonstrate that alpha6beta1 is implicated in glioblastoma cell   migration   and invasion and that laminin-111 might mediate dissemination of alpha6beta1-positive cells in vivo. Our results highlight for the first time the considerable role of the integrin alpha6beta1 in glioma progression. 
 HIV-1-Tat protein promotes chemotaxis and invasive behavior by monocytes. Monocytes are susceptible to HIV infection and to activation by a regulatory gene product of the HIV genome, HIV-Tat. Recently, we have demonstrated that treatment with HIV-Tat up-regulates monocyte adhesion to the endothelium and increases metalloproteinase production. in the present study, we have examined the ability of the HIV-Tat protein to alter the migratory and invasive behavior of monocytes. Monocytes pretreated for 24 h with 10 ng/ml HIV-Tat exhibited enhanced migratory behavior compared with untreated monocytes in chemotaxis assays, both in the absence of a chemoattractant as well as in response to FMLP. in addition, HIV-Tat itself induced the   migration   of both untreated and HIV-Tat pretreated monocytes. Checkerboard analysis showed that monocytes migrated in response to an HIV-Tat concentration gradient, thus confirming the chemotactic characteristics of the HIV-Tat protein. Pretreatment of monocytes with 10 ng/ml HIV-Tat for 24 h also increased their ability to invade reconstituted extracellular membrane (Matrigel)-coated filters by 5-fold in the absence of chemoattractant. The presence of FMLP or HIV-Tat further enhanced invasion by both untreated and HIV-Tat-pretreated monocytes by more than 10-fold. Monocyte invasion was partially inhibited by the inclusion of anti-beta integrin Ab or tissue inhibitor of metalloproteinase (TIMP). Thus, for the first time, we present evidence that HIV-Tat can enhance the chemotactic and invasive behaviors of monocytes and propose an active role for HIV-Tat in the recruitment of monocytes into extravascular tissues, a process which may contribute to the destruction of tissues and cellular architecture often seen in patients with acquired immunodeficiency syndrome. 
 Id1-induced inhibition of p53 facilitates endothelial cell   migration   and tube formation by regulating the expression of beta1-integrin. The Id1 protein is critical for endothelial cell angiogenesis, and this function is particularly relevant to cancer development, cardiovascular disease, and wound healing. We hypothesized that Id1 enhanced   migration   and tubulogenesis by controlling the expression and function of p53. In this study, we examined cell   migration   following Id1 overexpression and silencing endothelial cells. The results showed that overexpression of Id1 enhanced cell   migration   and increased beta1-integrin expression, but inhibition of beta1-integrin blocked motility even in clones overexpressing Id1, suggesting that Id1 regulated motility through beta1-integrin. Further analysis revealed that p53, whose expression and distribution is regulated by Id1, was critical for cell   migration  , and may be involved in regulating the expression of beta1-integrin. Inhibiting p53 function using PFT-α, a functional inhibitor of p53, increased the expression of beta1-integrin and promoted cell   migratio  n even in Id1-silencing endothelial cells, demonstrating that the Id1 knockdowns induced inhibition of endothelial cell   migratio  n and the expression of beta1-integrin were controlled by p53. In addition, Id1-p53 pathway regulated the cytoskeleton formation and tubulogenesis. These results demonstrate that Id1-induced beta1-integrin expression in endothelial cells and the function of Id1 in cell   migratio  n and tubulogenesis are dependent on p53. 
 Gangliosides and beta1-integrin are required for caveolae and membrane domains. Caveolae are plasma membrane domains involved in the uptake of certain pathogens and toxins. Internalization of some cell surface integrins occurs via caveolae suggesting caveolae may play a crucial role in modulating integrin-mediated adhesion and cell   migration  . Here we demonstrate a critical role for gangliosides (sialo-glycosphingolipids) in regulating caveolar endocytosis in human skin fibroblasts. Pretreatment of cells with endoglycoceramidase (cleaves glycosphingolipids) or sialidase (modifies cell surface gangliosides and glycoproteins) selectively inhibited caveolar endocytosis by >70%, inhibited the formation of plasma membrane domains enriched in sphingolipids and cholesterol ('lipid rafts'), reduced caveolae and caveolin-1 at the plasma membrane by approximately 80%, and blunted activation of beta1-integrin, a protein required for caveolar endocytosis in these cells. These effects could be reversed by a brief incubation with gangliosides (but not with asialo-gangliosides or other sphingolipids) at 10 degrees C, suggesting that sialo-lipids are critical in supporting caveolar endocytosis. Endoglycoceramidase treatment also caused a redistribution of focal adhesion kinase, paxillin, talin, and PIP Kinase Igamma away from focal adhesions. The effects of sialidase or endoglycoceramidase on membrane domains and the distribution of caveolin-1 could be recapitulated by beta1-integrin knockdown. These results suggest that both gangliosides and beta1-integrin are required for maintenance of caveolae and plasma membrane domains. 
 SERPINA5 inhibits tumor cell   migration   by modulating the fibronectin-integrin β1 signaling pathway in hepatocellular carcinoma. In our previous study, we identified 1241 loci with somatic copy number alterations in human hepatocellular carcinoma (HCC) using Affymetrix SNP 6.0 arrays, and a putative cancer gene SERPINA5 was uncovered in a novel chromosomal region with recurrent copy number loss at 14q31.1-32.13. The SERPINA5 was reported to be deregulated in renal, breast, prostate and ovarian cancers. However, the roles of SERPINA5 in cancer remain greatly elusive. In this study, we found that the DNA dosage and expression level of the SERPINA5 gene were significantly decreased in HCC by quantitative real-time PCR. Notably, the expression levels of SERPINA5 negatively correlated with malignant progression of HCC. The SERPINA5 gene was further observed to reduce in vitro and in vivo metastatic potential of HCC cells. Moreover, secreted SERPINA5 protein also could inhibit the metastatic ability of HCC cells. Finally, we discovered that one of the mechanisms explaining SERPINA5 inhibition of HCC metastasis is through direct interaction with fibronectin and disruption of the fibronectin-integrin signaling pathway. These findings highlight an important role of SERPINA5 in the regulation of migratory and metastatic potentials of HCC and suggest a potential application of SERPINA5 in cancer treatment. 
 The homeobox transcription factor Hox D3 promotes integrin alpha5beta1 expression and function during angiogenesis. Neovascularization promotes wound healing, tumor growth, and arthritis. Endothelial cell   migration   and survival during neovascularization are regulated by adhesion proteins, including integrin alpha5beta1. Integrin alpha5beta1 is poorly expressed on normal quiescent blood vessels, but its expression is induced on tumor blood vessels and in response to angiogenic factors such as basic fibroblast growth factor, interleukin-8, tumor necrosis factor-alpha, and the angiomatrix protein Del-1. We show here that alpha5beta1 expression, and hence function, during angiogenesis is regulated by the transcription factor Hox D3, a homeobox gene that also controls the expression of endothelial cell integrin alphavbeta3 and urokinase-type plasminogen activator. Hox D3 expression in endothelial cells enhances integrin alpha5 protein and message expression, whereas Hox D3 antisense inhibits its expression. Hox D3 promotes alpha5 expression during angiogenesis in vivo, whereas inhibition of alpha5 expression by Hox D3 antisense suppresses angiogenesis. Hox D3 binds directly to the promoters of the integrin alpha5 and beta3 subunits, inducing subunit expression. As Hox D3, integrin alphavbeta3, and integrin alpha5beta1 are expressed on tumor blood vessels but not on normal quiescent vessels, these studies suggest that Hox D3 coordinately regulates the expression of integrin alpha5beta1 and integrin alphavbeta3 during angiogenesis in vivo. These studies also suggest that Hox D3 inhibition could be a useful approach to inhibit tumor angiogenesis. 
 Signaling through urokinase and urokinase receptor in lung cancer cells requires interactions with beta1 integrins. The urokinase receptor (uPAR) is upregulated upon tumor cell invasion and correlates with poor lung cancer survival. Although a cis-interaction with integrins has been ascribed to uPAR, whether this interaction alone is critical to urokinase (uPA)- and uPAR-dependent signaling and tumor promotion is unclear. Here we report the functional consequences of point mutations of uPAR (H249A-D262A) that eliminate beta1 integrin interactions but maintain uPA binding, vitronectin attachment and association with alphaV integrins, caveolin and epidermal growth factor receptor. Disruption of uPAR interactions with beta1 integrins recapitulated previously reported findings with beta1-integrin-derived peptides that attenuated matrix-dependent ERK activation, MMP expression and in vitro   migration   by human lung adenocarcinoma cell lines. The uPAR mutant cells acquired enhanced capacity to adhere to vitronectin via uPAR-alphaVbeta5-integrin, rather than through the uPAR-alpha3beta1-integrin complex and they were unable to initiate uPA signaling to activate ERK, Akt or Stat1. In an orthotopic lung cancer model, uPAR mutant cells exhibited reduced tumor size compared with cells expressing wild-type uPAR. Taken together, the results indicate that uPAR-beta1-integrin interactions are essential to signals induced by integrin matrix ligands or uPA that support lung cancer cell invasion in vitro and progression in vivo. 
 The secreted protein ANGPTL2 promotes metastasis of osteosarcoma cells through integrin α5β1, p38 MAPK, and matrix metalloproteinases. The tumor microenvironment can enhance the invasive capacity of tumor cells. We showed that expression of angiopoietin-like protein 2 (ANGPTL2) in osteosarcoma (OS) cell lines increased and the methylation of its promoter decreased with time when grown as xenografts in mice compared with culture. Compared with cells grown in normal culture conditions, the expression of genes encoding DNA demethylation-related enzymes increased in tumor cells implanted into mice or grown in hypoxic, serum-starved culture conditions. ANGPTL2 expression in OS cell lines correlated with increased tumor metastasis and decreased animal survival by promoting tumor cell intravasation mediated by the integrin α5β1, p38 mitogen-activated protein kinase, and matrix metalloproteinases. The tolloid-like 1 (TLL1) protease cleaved ANGPTL2 into fragments in vitro that did not enhance tumor progression when overexpressed in xenografts. Expression of TLL1 was weak in OS patient tumors, suggesting that ANGPTL2 may not be efficiently cleaved upon secretion from OS cells. These findings demonstrate that preventing ANGPTL2 signaling stimulated by the tumor microenvironment could inhibit tumor c  ell migra  tion and metastasis. 
 Alpha3beta1 integrin in epidermis promotes wound angiogenesis and keratinocyte-to-endothelial-cell crosstalk through the induction of MRP3. During cutaneous wound healing, epidermal keratinocytes play essential roles in the secretion of factors that promote angiogenesis. However, specific cues in the wound microenvironment that trigger the production of pro-angiogenic factors by keratinocytes, and the cellular receptors that mediate this response, remain unclear. In this study, we exploited a model of conditional integrin knockout to demonstrate impaired wound angiogenesis in mice that lack alpha3beta1 integrin in epidermis. In addition, we used genetic and shRNA approaches to determine that alpha3beta1-integrin deficiency in keratinocytes leads to reduced mRNA and protein expression of the pro-angiogenic factor mitogen-regulated protein 3 (MRP3; also known as PRL2C4), and to demonstrate that this regulation provides a mechanism of keratinocyte-to-endothelial-cell crosstalk that promotes endothelial-cell   migration  . Finally, we showed that the impaired wound angiogenesis in epidermis-specific alpha3-integrin-knockout mice is correlated with reduced expression of MRP3 in wounded epidermis. These findings identify a novel role for alpha3beta1 integrin in promoting wound angiogenesis through a mechanism of crosstalk from epidermal to endothelial cells, and they implicate MRP3 in this integrin-dependent crosstalk. Such a mechanism represents a novel paradigm for integrin-mediated regulation of wound angiogenesis that extends beyond traditional roles for integrins in cell adhesion and   migration  . 
 β-1,4-Galactosyltransferase III suppresses β1 integrin-mediated invasive phenotypes and negatively correlates with metastasis in colorectal cancer. Metastasis often occurs in colorectal cancer (CRC) patients and is the main difficulty in cancer treatment. The upregulation of poly-N-acetyllactosamine-related glycosylation is found in CRC patients and is associated with progression and metastasis in cancer. β-1,4-Galactosyltransferase III (B4GALT3) is an enzyme responsible for poly-N-acetyllactosamine synthesis, and therefore, we investigated its expression in CRC patients. We found that B4GALT3 negatively correlated with poorly differentiated histology (P < 0.001), advanced stages (P = 0.0052), regional lymph node metastasis (P = 0.0018) and distant metastasis (P = 0.0463) in CRC patients. B4GALT3 overexpression in CRC cells suppressed ce  ll migrat  ion, invasion and adhesion, whereas B4GALT3 knockdown enhanced malignant cell phenotypes. The β1 integrin-blocking antibody reversed the B4GALT3-mediated increase in cell invasion. B4GALT3 expression altered glycosylation on the N-glycan of β1 integrin probably through changes in poly-N-acetyllactosamine expression. Furthermore, more activated β1 integrin along with the activation of its downstream signaling transduction were found in B4GALT3 knockdown cells, whereas overexpression of B4GALT3 suppressed the expression of active β1 integrin and inhibited its downstream signaling. Our results suggest that B4GALT3 is negatively associated with CRC metastasis and suppresses cell invasiveness through inhibiting activation of β1 integrin. 
 Immunohistochemical assessment of fibronectin and tenascin and their integrin receptors alpha5beta1 and alpha9beta1 in gastric and colorectal cancers with lymph node and liver metastases. The aim of the current study was to assess immunohistochemically and compare the level of expression of tenascin (TN) and fibronectin (FN) and their integrin receptors alpha9beta1 and alpha5beta1 in the primary colorectal and gastric tumors, and in corresponding lymph node and liver metastases from 53 patients. We detected similar high deposition of the studied ECM proteins and their receptors in the stroma of primary tumors and in liver metastases and a lower deposition in lymph node metastases. Cytoplasmic immune reaction for FN and TN was also seen in the tumor cells. A pronounced co-localization of immune deposits for FN and TN and their receptors was found in the stroma of the center and the invasion front (IF) (p<0.0001). A significant decrease of FN immune signal was observed in the IF in primary tumors and liver metastases (p<0.0001). The levels of immunolabeling of FN and TN correlated with the differentiation grade of primary tumors (p<0.0001). In conclusion, we may say that there is heterogeneous deposition of TN, FN and their integrin receptors in the different areas of primary colorectal and gastric tumors and of their metastases. These findings imply that the studied proteins may be involved in cell processes such as growth, adhesion,   migration   and apoptosis. 
 Effects of integrin α5β1 on the proliferation an  d migrati  on of human aortic vascular smooth muscle cells. Integrin (ITG) α5β1 is a dominant fibronectin receptor that is abundantly expressed on the surface of vascular smooth muscle cells (VSMCs). However, the association between integrin α5β1 and the proliferatio  n and mig  ration of VSMCs has yet to be elucidated. The aim of the present study was to characterize the roles of ITGα5 and ITGβ1 in the proliferat  ion and m  igration of VSMCs, and to determine the effects of ITGα5β1 on integrin-linked kinase (ILK) and focal adhesion kinase (FAK) mRNA expression. Lentiviral expression vectors as well as RNA interference vectors of ITGα5 and ITGβ1 were successfully constructed and transfected into VSMCs to obtain ITGα5‑ and ITGβ1‑overexpressing or -silenced cells, respectively. Cell cycle distribution,   prolifera  tion and migration were analyzed in the transfected VSMCs in order to clarify the roles of ITGβ1 and ITGα5 in th  e prolife  ration and migration of VSMCs. ITGβ1 was markedly associated with t  he prolif  eration and migration of VSMCs, and FAK was shown to be involved in the signaling pathways of ITGβ1. ITGα5 did not exert any effects on VSMCs. The results of the present study may provide a possible therapeutic target for the prevention and treatment of early vascular disease associated with VSMCs. 
 HGF accelerates wound healing by promoting the dedifferentiation of epidermal cells through β1-integrin/ILK pathway. Skin wound healing is a critical and complex biological process after trauma. This process is activated by signaling pathways of both epithelial and nonepithelial cells, which release a myriad of different cytokines and growth factors. Hepatocyte growth factor (HGF) is a cytokine known to play multiple roles during the various stages of wound healing. This study evaluated the benefits of HGF on reepithelialization during wound healing and investigated its mechanisms of action. Gross and histological results showed that HGF significantly accelerated reepithelialization in diabetic (DB) rats. HGF increased the expressions of the cell adhesion molecules β1-integrin and the cytoskeleton remodeling protein integrin-linked kinase (ILK) in epidermal cells in vivo and in vitro. Silencing of ILK gene expression by RNA interference reduced expression of β1-integrin, ILK, and c-met in epidermal cells, concomitantly decreasing the proliferation a  nd migrat  ion ability of epidermal cells. β1-Integrin can be an important maker of poorly differentiated epidermal cells. Therefore, these data demonstrate that epidermal cells become poorly differentiated state and regained some characteristics of epidermal stem cells under the role of HGF after wound. Taken together, the results provide evidence that HGF can accelerate reepithelialization in skin wound healing by dedifferentiation of epidermal cells in a manner related to the β1-integrin/ILK pathway. 
 A novel intracellular fibulin-1D variant binds to the cytoplasmic domain of integrin beta 1 subunit. Fibulin-1 is a member of a growing family of proteins that includes eight members and is involved in cellular functions such as adhesion,   migration   and differentiation. Fibulin-1 has also been implicated in embryonic development of the heart and neural crest-derived structures. It is an integral part of the extracellular matrix (ECM) and has been shown to bind to a multitude of ECM proteins. However, fibulin-1 was first identified as a protein purified from placental extracts that binds to the cytoplasmic domain of integrin β1. Human fibulin-1 is alternatively spliced into four different isoforms namely A-D. These isoforms share a common N-terminus sequence that contains a secretion sequence but differ in their carboxy-terminal fibulin-1 module. In this report we identify a new splice variant of fibulin-1 that differs from all other fibulin-1 variants in the N-terminus sequence and has a similar carboxy-terminus sequence as fibulin-1D. This variant that we named fibulin-1D prime (fibulin-1D') lacks a secretion sequence and the anaphlatoxin region of fibulin-1 variants. The protein has an apparent molecular weight of 70.5kDa. Herein we show that fibulin-1D' binds to the intracellular domain of integrin β1 as well as to integrin α5β1. The protein was localized intracellularly in CHO cells transfected with a pEF4 plasmid containing full-length coding sequence of fibulin-1D'. We also localized the protein in human placenta. We propose that the fibulin-1D' variant might play a role in early embryo development as well as in modulating integrin β1 functions including adhesion and motility. 
 Laminin-binding integrins induce Dll4 expression and Notch signaling in endothelial cells. Integrins play a crucial role in controlling endothelial cell proliferation and   migration   during angiogenesis. The Delta-like 4 (Dll4)/Notch pathway establishes an adequate ratio between stalk and tip cell populations by restricting tip cell formation through "lateral inhibition" in response to a vascular endothelial growth factor gradient. Because angiogenesis requires a tight coordination of these cellular processes, we hypothesized that adhesion, vascular endothelial growth factor, and Notch signaling pathways are interconnected.This study was aimed at characterizing the cross-talk between integrin and Notch signaling in endothelial cells.Adhesion of primary human endothelial cells to laminin-111 triggers Dll4 expression, leading to subsequent Notch pathway activation. SiRNA-mediated knockdown of α2β1 and α6β1 integrins abolishes Dll4 induction, which discloses a selective integrin signaling acting upstream of Notch pathway. The increase in Foxc2 transcription, triggered by α2β1 binding to laminin, is required but not sufficient per se for Dll4 expression. Furthermore, vascular endothelial growth factor stimulates laminin γ1 deposition, which leads to integrin signaling and Dll4 induction. Interestingly, loss of integrins α2 or α6 mimics the effects of Dll4 silencing and induces excessive network branching in an in vitro sprouting angiogenesis assay on three-dimensional matrigel.We show that, in endothelial cells, ligation of α2β1 and α6β1 integrins induces the Notch pathway, and we disclose a novel role of basement membrane proteins in the processes controlling tip vs stalk cell selection. 
 Lebectin and lebecetin, two C-type lectins from snake venom, inhibit alpha5beta1 and alphaV-containing integrins. Integrins are essential protagonists in the complex multistep process of cancer progression and metastasis. We recently reported that lebectin, a novel C-type lectin from Macrovipera lebetina venom, displays an anti-integrin activity. In this study, we extend this observation to lebecetin, a second C-type lectin isolated from the same venom and previously reported as a potent inhibitor of platelet aggregation. Both venom lectins appear to exert their effect on cell adhesion,   migration  , invasion and proliferation by inhibiting alpha5beta1 and alphav-containing integrins. Moreover, the inhibition of alpha5beta1 and alphav integrins is likely due to the binding of venom peptides, as both lebectin and lebecetin co-immunoprecipitate with these integrins. Lebectin and lebecetin are thus the first examples of venom C-type lectins inhibiting an integrin other than the collagen receptor alpha2beta1. 
 Ganglioside GM2 mediates   migration   of tumor cells by interacting with integrin and modulating the downstream signaling pathway. The definitive role of ganglioside GM2 in mediating tumor-induced growth and progression is still unknown. Here we report a novel role of ganglioside GM2 in mediating tumor cell   migration   and uncovered its mechanism. Data shows differential expression levels of GM2-synthase as well as GM2 in different human cancer cells. siRNA mediated knockdown of GM2-synthase in CCF52, A549 and SK-RC-26B cells resulted in significant inhibition of tumor cell   migration   as well as invasion in vitro without affecting cellular proliferation. Over-expression of GM2-synthase in low-GM2 expressing SK-RC-45 cells resulted in a consequent increase in   migration   thus confirming the potential role GM2 and its downstream partners play in tumor cell   migration   and motility. Further, treatment of SK-RC-45 cells with exogenous GM2 resulted in a dramatic increase in migratory and invasive capacity with no change in proliferative capacity, thereby confirming the role of GM2 in tumorigenesis specifically by mediating tumor   migration   and invasion. Gene expression profiling of GM2-synthase silenced cells revealed altered expression of several genes involved in cell   migration   primarily those controlling the integrin mediated signaling. GM2-synthase knockdown resulted in decreased phosphorylation of FAK, Src as well as Erk, while over-expression and/or exogenous GM2 treatment caused increased FAK and Erk phosphorylation respectively. Again, GM2 mediated invasion and Erk phosphorylation is blocked in integrin knockdown SK-RC-45 cells, thus confirming that GM2 mediated   migration   and phosphorylation of Erk is integrin dependent. Finally, confocal microscopy suggested co-localization while co-immunoprecipitation and surface plasmon resonance (SPR) confirmed direct interaction of membrane bound ganglioside, GM2 with the integrin receptor. 
 Urinary-type plasminogen activator receptor/alpha 3 beta 1 integrin signaling, altered gene expression, and oral tumor progression. Oral squamous cell carcinoma (OSCC) has 50% 5-year survival rate, highlighting our limited understanding of the molecular events that contribute to disease progression. Microarray analyses of primary oral tumors have identified urinary-type plasminogen activator (uPA) and its receptor (uPAR) as key genes associated with human OSCC progression. The uPAR functions as both a proteinase receptor and an integrin ligand, modifying proteolysis,   migration  , integrin signaling, and cellular transcription. In the current study, uPAR expression levels were modified in OSCC cells followed by analysis of tumor growth in an in vivo orthotopic xenograft model and by transcriptional profiling. Overexpression of uPAR resulted in more infiltrative and less differentiated tumors, with ill-defined borders, cytologic atypia, and enhanced vascularity. Analysis of serial sections of both murine experimental tumors and microarrayed human OSCC showed a statistically significant association between uPAR and alpha(3) integrin colocalization in areas exhibiting extracellular signal-regulated kinase phosphorylation, suggesting that uPAR/alpha(3) integrin interaction potentiates extracellular signal-regulated kinase signaling in vivo. This is supported by cDNA microarray analysis, which showed differential expression of 148 genes (113 upregulated and 35 downregulated). Validation of gene expression changes in human OSCC using immunohistochemistry and quantitative real-time PCR showed increased growth factors, proteinases/inhibitors, and matrix components in uPAR-overexpressing tumors. Together, these results support a model wherein increased uPAR expression promotes alpha(3)beta(1) integrin association, resulting in increased mitogen-activated protein kinase signaling and transcriptional activation, leading to the formation of more aggressive tongue tumors. This combined approach has efficacy to identify additional biomarkers and/or prognostic indicators associated with aggressive human OSCC. 
 VEGF-induced endothelial cell   migration   requires urokinase receptor (uPAR)-dependent integrin redistribution. Vascular endothelial growth factor (VEGF)-initiated angiogenesis requires coordinated proteolytic degradation of extracellular matrix provided by the urokinase plasminogen activator/urokinase receptor (uPA/uPAR) system and regulation of cell   migration   provided by integrin-matrix interaction. In this study, we investigated the mechanisms underlying the uPAR-dependent modulation of VEGF-induced endothelial   migration  .We used flow cytometry to quantify integrins at the cell surface. Stimulation of human and murine endothelial cells with VEGF resulted in internalization of α5β1-integrins. Micropatterning and immunocytochemistry revealed co-clustering of uPAR and α5β1-integrins and retrieval via clathrin-coated vesicles. It was also contingent on receptors of the low-density lipoprotein receptor (LDL-R) family. VEGF-induced integrin redistribution was inhibited by elimination of uPAR from the endothelial cell surface or by inhibitory peptides that block the uPAR-integrin interaction. Under these conditions, the migratory response of endothelial cells upon VEGF stimulation was impaired both in vitro and in vivo.The observations indicate that uPAR is an essential component of the network through which VEGF controls endothelial c  ell migra  tion. uPAR is a bottleneck through which the VEGF-induced signal must be funnelled for both focused proteolytic activity at the leading edge and for redistribution of integrins. 
 Vesicle-associated membrane protein 2 mediates trafficking of alpha5beta1 integrin to the plasma membrane. Integrins are major receptors for cell adhesion to the extracellular matrix (ECM). As transmembrane proteins, the levels of integrins at the plasma membrane or the cell surface are ultimately determined by the balance between two vesicle trafficking events: endocytosis of integrins at the plasma membrane and exocytosis of the vesicles that transport integrins. Here, we report that vesicle-associated membrane protein 2 (VAMP2), a SNARE protein that mediates vesicle fusion with the plasma membrane, is involved in the trafficking of alpha5beta1 integrin. VAMP2 was present on vesicles containing endocytosed beta1 integrin. Small interfering RNA (siRNA) silencing of VAMP2 markedly reduced cell surface alpha5beta1 and inhibited cell adhesion and chemotactic   migration   to fibronectin, the ECM ligand of alpha5beta1, without altering cell surface expression of alpha2beta1 integrin or alpha3beta1 integrin. By contrast, silencing of VAMP8, another SNARE protein, had no effect on cell surface expression of the integrins or cell adhesion to fibronectin. In addition, VAMP2-mediated trafficking is involved in cell adhesion to collagen but not to laminin. Consistent with disruption of integrin functions in cell proliferation and survival, VAMP2 silencing diminished proliferation and triggered apoptosis. Collectively, these data indicate that VAMP2 mediates the trafficking of alpha5beta1 integrin to the plasma membrane and VAMP2-dependent integrin trafficking is critical in cell adhesion,   migration   and survival. 
 Insulin-like growth factor I controls a mutually exclusive association of RACK1 with protein phosphatase 2A and beta1 integrin to promote cell   migration  . The WD repeat scaffolding protein RACK1 can mediate integration of the insulin-like growth factor I receptor (IGF-IR) and integrin signaling in transformed cells. To address the mechanism of RACK1 function, we searched for regulatory proteins that associate with RACK1 in an IGF-I-dependent manner. The serine threonine phosphatase protein phosphatase 2A (PP2A) was found associated with RACK1 in serum-starved cells, and it dissociated immediately upon stimulation with IGF-I. This dissociation of PP2A from RACK1 and an IGF-I-mediated decrease in cellular PP2A activity did not occur in cells expressing either the serine 1248 or tyrosine 1250/1251 mutants of the IGF-IR that do not interact with RACK1. Recombinant RACK1 could bind to PP2A in vitro and restore phosphatase activity to PP2A from IGF-I-stimulated cells. Ligation of integrins with fibronectin or Matrigel was sufficient to facilitate IGF-I-mediated dissociation of PP2A from RACK1 and also to recruit beta1 integrin as PP2A dissociated. By using TAT-fused N-terminal and C-terminal deletion mutants of RACK1, we determined that both PP2A and beta1 integrin interact in the C terminus of RACK1 within WD repeats 4 to 7. This suggests that integrin ligation displaces PP2A from RACK1. MCF-7 cells overexpressing RACK1 exhibited enhanced motility, which could be reversed by the PP2A inhibitor okadaic acid. Small interfering RNA-mediated suppression of RACK1 also decreased the migratory capacity of DU145 cells. Taken together, our findings indicate that RACK1 enhances IGF-I-mediated cell   migration   through its ability to exclusively associate with either beta1 integrin or PP2A in a complex at the IGF-IR. 
 Thymidine phosphorylase and 2-deoxyribose stimulate human endothelial cell   migration   by specific activation of the integrins alpha 5 beta 1 and alpha V beta 3. Thymidine phosphorylase is an angiogenic factor that is frequently overexpressed in solid tumors, in rheumatoid arthritis, and in response to inflammatory cytokines. Our previous studies showed that cells expressing thymidine phosphorylase stimulated endothelial cell   migration   in vitro. This was a consequence of the intracellular metabolism of thymidine by thymidine phosphorylase and subsequent extracellular release of 2-deoxyribose. The mechanisms by which 2-deoxyribose might mediate thymidine phosphorylase-induced cell   migration   in vitro, however, are obscure. Here we show that both thymidine phosphorylase and 2-deoxyribose stimulated the formation of focal adhesions and the tyrosine 397 phosphorylation of focal adhesion kinase in human umbilical vein endothelial cells. Although similar actions occurred upon treatment with the angiogenic factor vascular endothelial growth factor (VEGF), thymidine phosphorylase differed from VEGF in that its effect on endothelial cell   migration   was blocked by antibodies to either integrin alpha 5 beta 1 or alpha v beta 3, whereas VEGF-induced endothelial cell   migration   was only blocked by the alpha v beta 3 antibody. Further, thymidine phosphorylase and 2-deoxyribose, but not VEGF, increased the association of both focal adhesion kinase and the focal adhesion-associated protein vinculin with integrin alpha 5 beta 1 and, in intact cells, increased the co-localization of focal adhesion kinase with alpha 5 beta 1. Thymidine phosphorylase and 2-deoxyribose-induced focal adhesion kinase phosphorylation was blocked by the antibodies to alpha 5 beta 1 and alpha v beta 3, directly linking the   migration   and signaling components of thymidine phosphorylase and 2-deoxyribose action. Cell surface expression of alpha 5 beta 1 was also increased by thymidine phosphorylase and 2-deoxyribose. These experiments are the first to demonstrate a direct effect of thymidine phosphorylase and 2-deoxyribose on signaling pathways associated with endothelial cell   migration  . 
 The collagen-binding integrin α2β1 is a novel interaction partner of the Trimeresurus flavoviridis venom protein flavocetin-A. Many snake venoms are known for their antithrombotic activity. They contain components that specifically target different platelet-activating receptors such as the collagen-binding integrin α2β1 and the von Willebrand factor receptor GPIb. In a search for an α2β1 integrin-blocking component from the venom of the habu snake (Trimeresurus flavoviridis), we employed two independent purification protocols. First, we used the integrin α2A domain, a major collagen-binding domain, as bait for affinity purification of an α2β1 integrin-binding toxin from the crude venom. Second, in parallel, we used classical protein separation protocols and tested for α2β1 integrin-inhibiting capabilities by ELISA. Using both approaches, we identified flavocetin-A as an inhibitor of α2β1 integrin. Hitherto, flavocetin-A has been reported as a GPIb inhibitor. However, flavocetin-A inhibited collagen-induced platelet aggregation even after GPIb was blocked with other inhibitors. Moreover, flavocetin-A antagonized α2β1 integrin-mediate  d adhesio  n and migration of HT1080 human fibrosarcoma cells, which lack any GPIb, on collagen. Protein chemical analyses proved that flavocetin-A binds to α2β1 integrin and its α2A domain with high affinity and in a cooperative manner, which most likely is due to its quaternary structure. Kinetic measurements confirmed the formation of a strong complex between integrin and flavocetin-A, which dissociates very slowly. This study proves that flavocetin-A, which has long been known as a GPIb inhibitor, efficiently targets α2β1 integrin and thus blocks collagen-induced platelet activation. Moreover, our findings suggest that the separation of GPIb- and α2β1 integrin-blocking members within the C-type lectin-related protein family is less strict than previously assumed. 
 Insulin-like growth factor binding protein 1 stimulates cell   migration   and binds to the alpha 5 beta 1 integrin by means of its Arg-Gly-Asp sequence. Insulin-like growth factor (IGF)-binding protein 1 (IGFBP-1) contains an Arg-Gly-Asp (RGD) integrin recognition sequence. In vitro mutagenesis was used to alter this RGD sequence to Trp-Gly-Asp (WGD).   Migration   of Chinese hamster ovary (CHO) cells expressing the wild-type protein was more than 3-fold greater in 48 hr compared with cells expressing the WGD mutant form of IGFBP-1. Similarly, wild-type IGFBP-1 added to the media of control CHO cells stimulated   migration   2-fold compared with the WGD protein. A synthetic RGD-containing peptide, when added to the medium with wild-type IGFBP-1, blocked the effect of IGFBP-1 on cell   migration  . The addition of IGF-I to the culture medium had no effect on the   migration   of cells expressing IGFBP-1 or vector alone. Affinity chromatography of 125I-labeled CHO cell membrane proteins, using IGFBP-1 coupled to agarose, identified the alpha 5 beta 1 integrin (fibronectin receptor) as the only cell surface molecule capable of binding IGFBP-1 in an RGD-dependent manner. Furthermore, wild-type IGFBP-1, but not the WGD mutant form, could be coprecipitated from CHO cells with an antibody directed against the alpha 5 integrin subunit. These studies demonstrate that IGFBP-1 stimulates CHO cell   migration   and binds to the alpha 5 beta 1 integrin receptor, both by an RGD-dependent mechanism. The effect of IGFBP-1 on   migration   is independent of IGF-I and is probably mediated through the alpha 5 beta 1 integrin. 
 Effect of KAI1/CD82 on the beta1 integrin maturation in highly migratory carcinoma cells. The KAI1/CD82 protein has been documented as the tumor metastasis suppressor in many types of human cancers. KAI1/CD82 regulates cell motility and invasiveness; however, the mechanism by which this occurs remains to be fully established. Several studies have shown that KAI1/CD82 modulates integrin-dependent signaling. It was suggested that KAI1/CD82 might function to attenuate the beta1 integrin function of inducing cellular   migration  . A wound-healing and modified Boyden chamber assays were performed to investigate the mechanism of the KAI1/CD82-mediated inhibition of cell   migration  . It was found that the migratory ability of H1299/CD82 was inhibited. The immunoblotting and biotinylation assays revealed that H1299/CD82 showed significantly decreased expression of the mature form of beta1, which was functional at the cell surface. It was confirmed that KAI1/CD82 regulates the maturation of the beta1 integrin using CD82-specific si-RNA. These results support a model in which KAI1/CD82 attenuates the maturation of the beta1 integrin precursor and thereby suppresses cell   migration  . 
 Differential induction of gelatinase B (MMP-9) and gelatinase A (MMP-2) in T lymphocytes upon alpha(4)beta(1)-mediated adhesion to VCAM-1 and the CS-1 peptide of fibronectin. Integrin alpha(4)beta(1) on the surface of T lymphocytes interacts with vascular cell adhesion molecule-1 (VCAM-1) and fibronectin during   migration   of lymphocytes from the blood to sites of inflammation. Migrating lymphocytes actively modify their environment through a number of mechanisms including proteolysis of the extracellular matrix by matrix metalloproteinases (MMP) synthesized by the cells. In this study, expression of MMP upon alpha(4)beta(1)-mediated adhesion of leukocytes to two major ligands, the IIICS-1 domain of fibronectin and VCAM-1, has been examined. Adhesion of T lymphoblastoid Jurkat cells to the CS-1 peptide induced expression of mRNA for two MMPs, gelatinase A (MMP-2) and gelatinase B (MMP-9). As evaluated by relative RT-PCR and Northern blot analyses, the level of mRNA was upregulated about 4- to 5-fold for both MMPs compared to control cells maintained in suspension. With time, both enzymes were detected in conditioned media and inside the cells, and their identities were verified by Western blotting and gelatin zymography. Adhesion of Jurkat cells to the second major alpha(4)beta(1) ligand, VCAM-1, upregulated mRNA for MMP-2 (3.5-fold) and failed to induce expression of mRNA for MMP-9. Accordingly, only MMP-2 protein was detected in conditioned media of cells adherent to VCAM-1. Occupancy of alpha(4)beta(1) on the surface of suspended cells with soluble CS-1 peptide or VCAM-1 did not upregulate synthesis and release of MMPs. A similar pattern of induction of MMPs after adhesion to CS-1 and VCAM-1 was observed in T lymphocytes isolated from human blood. These results demonstrate that adhesion of T lymphocytes through alpha(4)beta(1) to different ligands, which bind to similar or overlapping sites in the integrin, induces intracellular events leading to distinct patterns of MMPs biosynthesis. 
 The NF-κB subunit RelB regulates the   migratio  n and invasion abilities and the radio-sensitivity of prostate cancer cells. NF-κB subunits play important roles in carcinogenesis of a variety of human malignancies and response to cancer therapy; however, the contribution of an individual subunit has not been thoroughly defined. Constitutive activation of the canonical NF-κB subunit is a critical event in prostate carcinogenesis. Recent findings point out that RelB, which contributes to the non-canonical NF-κB activity, functions importantly in the prostate cancer progression. Here, we investigated systemically the functional roles of RelB in prostate cancer and examine its significance as a therapeutic target. Targeting RelB using short hairpin RNA approach in androgen-independent DU145 prostate cancer cells interfered with various biological behaviors of cells. We observed that RelB knockdown inhibited prostate cancer cell grow  th, migra  tion, and invasion, and enhanced proteasome inhibitor sensitivity. The altered expression of anti-apoptotic gene Bcl-2 played critical roles in regulating both spontaneous and radiation-induced apoptosis in the presence of RelB knockdown. For the first time, we showed that RelB knockdown significantly attenuated   the migra  tion and invasion of DU145 prostate cancer cells, due to the reduction of integrin β-1. Collectively, we provided evidence that RelB functioned as an oncogene in prostate cancer. Developing a RelB-targeted therapeutic intervention, is valuable in treating advanced, metastatic prostate cancer. 
 Cyclooxygenase-2 enhances alpha2beta1 integrin expression and cell   migration   via EP1 dependent signaling pathway in human chondrosarcoma cells. Cyclooxygenase (COX)-2, the inducible isoform of prostaglandin (PG) synthase, has been implicated in tumor metastasis. Interaction of COX-2 with its specific EP receptors on the surface of cancer cells has been reported to induce cancer invasion. However, the effects of COX-2 on   migration   activity in human chondrosarcoma cells are mostly unknown. In this study, we examined whether COX-2 and EP interaction are involved in metastasis of human chondrosarcoma.We found that over-expression of COX-2 or exogenous PGE2 increased the   migration   of human chondrosarcoma cells. We also found that human chondrosarcoma tissues and chondrosarcoma cell lines had significant expression of the COX-2 which was higher than that in normal cartilage. By using pharmacological inhibitors or activators or genetic inhibition by the EP receptors, we discovered that the EP1 receptor but not other PGE receptors is involved in PGE2-mediated cell   migration   and alpha2beta1 integrin expression. Furthermore, we found that human chondrosarcoma tissues expressed a higher level of EP1 receptor than normal cartilage. PGE2-mediated   migration   and integrin up-regulation were attenuated by phospholipase C (PLC), protein kinase C (PKC) and c-Src inhibitor. Activation of the PLCbeta, PKCalpha, c-Src and NF-kappaB signaling pathway after PGE2 treatment was demonstrated, and PGE2-induced expression of integrin and   migration   activity were inhibited by the specific inhibitor, siRNA and mutants of PLC, PKC, c-Src and NF-kappaB cascades.Our results indicated that PGE2 enhances the   migration   of chondrosarcoma cells by increasing alpha2beta1 integrin expression through the EP1/PLC/PKCalpha/c-Src/NF-kappaB signal transduction pathway. 
 N-acetylglucosaminyltransferase V negatively regulates integrin α5β1-mediated monocyte adhesion and tra  nsmigrati  on through vascular endothelium. Changes in the expression of glycosyltransferases that branch N-linked glycans are associated with many physiological and pathological events, such as cell adhesion  , migrati  on, proliferation and tumor cell malignancy. Here, the altered levels of N-acetylglucosaminyltransferase V (GnT-V) and its product β(1,6)-linked GlcNAc in monocytes were observed during inflammation. The effects of GnT-V and aberrant N-linked β(1,6) branching on monocyte adhesion through vascular endothelium and t  ransmigra  tion were investigated. During IFN-γ-induced inflammation, adhesion and transendothe  lial migr  ation of THP-1 monocytes were enhanced, and the levels of GnT-V and β(1,6)-linked GlcNAc in THP-1 monocytes were significantly decreased. Expression of the GnT-V shRNA vector in THP-1 cells reversed the abnormal IFN-γ-induced characteristics, indicating direct involvement of N-glycosylation in these biological effects. The enhanced adhesion and transendot  helial mi  gration were significantly inhibited by functional blockade with antibodies against integrin α5 or β1 in IFN-γ-induced and GnT-V knockdown THP-1 cells, demonstrating the involvement of integrin α5β1 in the monocyte-endothelium interaction. However, IFN-γ treatment and GnT-V knockdown in THP-1 cells lowered expression of N-linked β(1,6) branching on integrin α5 and β1, without affecting the total protein expression of the subunits. Decreased GnT-V expression caused marked enchancement of integrin-induced phosphorylation of focal adhesion kinase (FAK). The augmented FAK-mediated ERK phosphorylation and activation were observed in IFN-γ-induced THP-1 cells. Furthermore, ERK inhibitor pre-treatment nearly abrogated the highly elevated IFN-γ-induced monocyte   adhesion   and transmigration, concomitant with reversal of the decrease in GnT-V and β(1,6) branching. Our results demonstrate for the first time that decreased GnT-V activity due to inflammatory cytokine induction in human monocytes resulted in enchancement of integrin α5β1-dependent monocyte-vascular endotheli  um adhesi  on and transmigration. Consequently, the activation of integrin caused elevation of FAK phosphorylation. These effects promoted FAK-mediated downstream signaling, including the ERK pathway, and indicate that GnT-V may be a potential therapeutic target for vascular inflammatory conditions. 
 Vascular endothelial growth factor receptor-1 is deposited in the extracellular matrix by endothelial cells and is a ligand for the alpha 5 beta 1 integrin. Vascular endothelial growth factor receptor-1 (VEGFR-1) is a tyrosine kinase receptor for several growth factors of the VEGF family. Endothelial cells express a membrane-spanning form of VEGFR-1 and secrete a soluble variant of the receptor comprising only the extracellular region. The role of this variant has not yet been completely defined. In this study, we report that the secreted VEGFR-1 is present within the extracellular matrix deposited by endothelial cells in culture, suggesting a possible involvement in endothelial cell adhesion and   migration  . In adhesion assays, VEGFR-1 extracellular region specifically promoted endothelial cell attachment. VEGFR-1-mediated cell adhesion was divalent cation-dependent, and inhibited by antibodies directed against the alpha 5 beta 1 integrin. Moreover, VEGFR-1 promoted endothelial cell   migration  , and this effect was inhibited by anti-alpha 5 beta 1 antibodies. Direct binding of VEGFR-1 to the alpha 5 beta 1 integrin was also detected. Finally, binding to VEGFR-1 initiated endothelial cell spreading. Altogether these results indicate that the soluble VEGFR-1 secreted by endothelial cells becomes a matrix-associated protein that is able to interact with the alpha 5 beta 1 integrin, suggesting a new role of VEGFR-1 in angiogenesis, in addition to growth factor binding. 
 Urokinase-type plasminogen activator receptor interaction with β1 integrin is required for platelet-derived growth factor-AB-induced human mesenchymal stem/stromal cell   migratio  n. Mesenchymal stem cells (MSC) are well described for their role in tissue regeneration following injury. Migratory properties of endogenous or administrated MSC are critical for tissue repair processes. Platelet-derived growth factor (PDGF) is a chemotactic growth factor that elicits mesenchymal cell   migratio  n. However, it is yet to be elucidated if signaling pathways other than direct activation of PDGF receptor (PDGF-R) are involved in PDGF-induced cell   migratio  n.Knocking down and co-immunoprecipitation approaches were used to evaluate urokinase-type plasminogen activator receptor (uPAR) requirement and its interactions with proteins involved in   migratio  n mechanisms, in human MSC induced to migrate under PDGF-AB effect.We demonstrated that uPAR activation and its association with β1-integrin are required for PDGF-AB-induce  d migrati  on. This phenomenon takes place in MSC derived from bone marrow and from adipose tissue.We showed that PDGF-AB downstream signaling requires other effector molecules in MSC such as the uPA/uPAR system and β1 integrin signaling pathway known for their role   in migrat  ion. These findings provide new insights in molecular mechanisms of PDGF-AB-induc  ed migrat  ion of human MSC that may be relevant to control MSC function and tissue remodeling after injury. 
 Melanoma cells produce multiple laminin isoforms and strongly migrate on α5 laminin(s) via several integrin receptors. Melanoma cells express and interact with laminins (LMs) and other basement membrane components during invasion and metastasis. In the present study we have investigated the production and   migratio  n-promoting activity of laminin isoforms in melanoma. Immunohistochemistry of melanoma specimens and immunoprecipitation/western blotting of melanoma cell lines indicated expression of laminin-111/121, laminin-211, laminin-411/421, and laminin-511/521. Laminin-332 was not detected. In functional assays, laminin-111, laminin-332, and laminin-511, but not laminin-211 and laminin-411, strongly promoted haptotactic cell   migratio  n either constitutively or following stimulation with insulin-like growth factors. Both placenta and recombinant laminin-511 preparations were highly active, and the isolated recombinant IVa domain of LMα5 also promoted cel  l migrati  on. Function-blocking antibodies in cel  l migrati  on assays revealed α6β1 integrin as the major receptor for laminin-111, and both α3β1 and α6β1 integrins for laminin-332 and laminin-511. In contrast, isolated LMα5 IVa domain-promoted melan  oma cell   migration was largely mediated via αVβ3 integrin and inhibited by RGD peptides. Given the ubiquitous expression of α5 laminins in melanoma cells and in melanoma-target tissues/anatomical structures, as well as   the stro  ng migration-promoting activity of these laminin isoforms, the α5 laminins emerge as putative primary extracellular matrix mediators of melanoma invasion and metastasis via α3β1 and other integrin receptors. 
 Potentiation of cell invasion and matrix metalloproteinase production by alpha3beta1 integrin-mediated adhesion of gastric carcinoma cells to laminin-5. We previously reported that the adhesion of gastric carcinoma cells to the peritoneum mediated by the alpha3beta1 integrin-laminin interaction is a key step in the initial process of peritoneal metastatic dissemination. Carcinoma cells subsequently invade through the intercellular gaps of mesothelial linings. In this study, we examined the role of the interaction of carcinoma cells with laminin-5, which is a major component of submesothelial basement membranes and serves as a high-affinity ligand for alpha3beta1 integrin, in carcinoma cell invasion. Human gastric carcinoma cell lines (MKN1, GT3TKB, and NUGC-4) adhered in an alpha3beta1 integrin-dependent manner to the extracellular matrix deposited by peritoneal mesothelial cells. An in vitro invasion assay using the Boyden chamber system revealed that MKN1 cell   migration   through the membranes increased when the membranes were coated with matrices produced by mesothelial cells or with laminin-5-containing Matrigel as compared to Matrigel alone. The cell   migration   promoted by laminin-5-containing Matrigel was inhibited by the presence of anti-alpha3 integrin antibody. When MKN1 cells were cultured in a laminin-5-coated plate, these cells were promoted to produce matrix metalloproteinase (MMP)-9, as assessed by gelatin zymography, enzyme-linked immunosorbent assay, and reverse transcription-polymerase chain reaction. These results suggest that the production of MMP-9 by MKN1 cells was potentiated by the alpha3beta1 integrin-laminin-5 interaction, which facilitated their invasion via degradation of the matrix. 
 Expression of beta 1B integrin isoform in CHO cells results in a dominant negative effect on cell adhesion and motility. The integrin subunit beta 1B, a beta 1 isoform with a unique sequence at the cytoplasmic domain, forms heterodimers with integrin alpha chains and binds fibronectin, but it does not localize to focal adhesion sites (Balzac, F., A. Belkin, V. Koteliansky, Y. Balabanow, F. Altruda, L. Silengo, and G. Tarone. 1993. J. Cell Biol. 121:171-178). Here we analyze the functional properties of human beta 1B by expressing it in hamster CHO cells. When stimulated by specific antibodies, beta 1B does not trigger tyrosine phosphorylation of a 125-kD cytosolic protein, an intracellular signalling pathway that is activated both by the endogenous hamster or the transfected human beta 1A. Moreover, expression of beta 1B results in reduced spreading on fibronectin and laminin, but not on vitronectin. Expression of beta 1B also results in severe reduction of cell motility in the Boyden chamber assay. Reduced cell spreading and motility could not be accounted for by preferential association of beta 1B with a given integrin alpha subunit. These data, together with our previous results, indicate that beta 1B interferes with beta 1A function when expressed in CHO cells resulting in a dominant negative effect on cell adhesion and   migration  . 
 The cAMP-Epac-Rap1 pathway regulates cell spreading and cell adhesion to laminin-5 through the alpha3beta1 integrin but not the alpha6beta4 integrin. Laminin-5 is an important constituent of the basal lamina. The receptors for laminin-5, the integrins alpha3beta1 and alpha6beta4, have been associated with epithelial wound   migration   and carcinoma invasion. The signal transduction mechanisms that regulate these integrins are not well understood. We report here that the small GTPase Rap1 regulates the adhesion of a number of cell lines to various extracellular matrix proteins including laminin-5. cAMP also mediates cell adhesion and spreading on laminin-5, a process that is independent of protein kinase A but rather dependent on Epac1, a cAMP-dependent exchange factor for Rap. Interestingly, although both alpha3beta1 and alpha6beta4 mediate adhesion to laminin-5, only alpha3beta1-dependent adhesion is dependent on Rap1. These results provide evidence for a function of the cAMP-Epac-Rap1 pathway in cell adhesion and spreading on different extracellular matrix proteins. They also define different roles for the laminin-binding integrins in regulated cell adhesion and subsequent cell spreading. 
 Tyrosine 302 in RACK1 is essential for insulin-like growth factor-I-mediated competitive binding of PP2A and beta1 integrin and for tumor cell proliferation and   migration  . Insulin-like growth factor (IGF)-I regulates a mutually exclusive interaction of PP2A and beta1 integrin with the WD repeat scaffolding protein RACK1. This interaction is required for the integration of IGF-I receptor (IGF-IR) and adhesion signaling. Here we investigated the nature of the binding site for PP2A and beta1 integrin in RACK1. A WD7 deletion mutant of RACK1 did not associate with PP2A but retained some interaction with beta1 integrin, whereas a WD6/WD7 mutant lost the ability to bind to both PP2A and beta1 integrin. Using immobilized peptide arrays representing the entire RACK1 protein, we identified a common cluster of amino acids (FAGY) at positions 299-302 within WD7 of RACK1 which were essential for binding of both PP2A and beta1 integrin to RACK1. PP2A showed a higher level of association with a peptide in which Tyr-302 was phosphorylated compared with an unphosphorylated peptide, whereas beta1 integrin binding was not affected by phosphorylation. RACK1 mutants in which either the FAGY cluster or Tyr-302 were mutated to AAAF, or Phe, respectively, did not interact with either PP2A or beta1 integrin. These mutants were unable to rescue the decrease in PP2A activity caused by suppression of RACK1 in MCF-7 cells with small interfering RNA. MCF-7 cells and R+ (IGF-IR-overexpressing fibroblasts) expressing these mutants exhibited decreased proliferation and   migration  , whereas R- cells (IGF-IR null fibroblasts) were unaffected. Taken together, the data demonstrate that Tyr-302 in RACK1 is required for interaction with PP2A and beta1 integrin, for regulation of PP2A activity, and for IGF-I-mediated cell   migration   and proliferation. 
 Site-directed perturbation of protein kinase C- integrin interaction blocks carcinoma cell chemotaxis. Polarized cell movement is an essential requisite for cancer metastasis; thus, interference with the tumor cell motility machinery would significantly modify its metastatic behavior. Protein kinase C alpha (PKC alpha) has been implicated in the promotion of a migratory cell phenotype. We report that the phorbol ester-induced cell polarization and directional motility in breast carcinoma cells is determined by a 12-amino-acid motif (amino acids 313 to 325) within the PKC alpha V3 hinge domain. This motif is also required for a direct association between PKC alpha and beta 1 integrin. Efficient binding of beta 1 integrin to PKC alpha requires the presence of both NPXY motifs (Cyto-2 and Cyto-3) in the integrin distal cytoplasmic domains. A cell-permeant inhibitor based on the PKC-binding sequence of beta 1 integrin was shown to block both PKC alpha-driven and epidermal growth factor (EGF)-induced chemotaxis. When introduced as a minigene by retroviral transduction into human breast carcinoma cells, this inhibitor caused a striking reduction in chemotaxis towards an EGF gradient. Taken together, these findings identify a direct link between PKC alpha and beta 1 integrin that is critical for directed tumor cell   migration  . Importantly, our findings outline a new concept as to how carcinoma cell chemotaxis is enhanced and provide a conceptual basis for interfering with tumor cell dissemination. 
 Integrins mediate adherence and   migration   of T lymphocytes on human peritoneal mesothelial cells. We previously showed that a local immune response largely composed of type 1 T cells correlated with a favorable outcome of the peritonitis associated with peritoneal dialysis. To clarify how these subsets are recruited to the peritoneal cavity during inflammation, we measured integrin-mediated interactions between the T cells and human peritoneal mesothelial cells. Direct microscopy showed that lipopolysaccharide or peritoneal dialysis effluent stimulated the adherence of T cells to mesothelial cells, a process mediated by the integrins alpha6beta1 and alpha4beta1. Further, the   migration   of Th1 cell across human mesothelial cell monolayers grown on transwell surfaces was reduced by anti-alpha6beta1 integrin antibody while that of Th2 cell was inhibited by an anti-alpha4 integrin antibody. Pretreatment with either lipopolysaccharide or rapid response peritoneal dialysis effluent stimulated T cell   migration   and this was significantly decreased by the alpha6beta1 compared to the alpha4 antibody. These results suggest that integrins may play an important role in mediating selective T cell subset adhesion and   migration   across human peritoneal mesothelial cell monolayers and differential integrin expression and selective T cell subset recruitment during peritonitis may affect outcome. 
 Interferon-gamma inhibits T84 epithelial cell   migration   by redirecting transcytosis of beta1 integrin from the migrating leading edge. Intestinal inflammation is associated with epithelial damage and formation of mucosal wounds. Epithelial cells   migration   is required for wound closure. In inflammatory status, migrating epithelial cells are exposed to proinflammatory cytokines such as IFN-gamma. However, influence of such cytokines on intestinal epithelial wound closure remains unknown. The present study was designed to investigate the effect of IFN-gamma on   migration   of model T84 intestinal epithelial cells and recovery of epithelial wounds. IFN-gamma significantly inhibited rate of T84 cell   migration   and closure of epithelial wounds. This effect was accompanied by the formation of large aberrant lamellipodia at the leading edge as well as significant decrease in the number of beta(1) integrin containing focal adhesions. IFN-gamma exposure increased endocytosis of beta(1) integrin and shifted its accumulation from early/recycling endosomes at the leading edge to a yet unidentified compartment at the cell base. This redirection in beta(1) integrin transcytosis was inhibited by depolymerization of microtubules with nocodazole and was unaffected by stabilization of microtubules with docetaxel. These results suggest that IFN-gamma attenuates epithelial wound closure by microtubule-dependent redirection of beta(1) integrin transcytosis from the leading edge of migrating cells thereby inhibiting adequate turnover of focal adhesion complexes and cell   migration  . 
 A functionally relevant conformational epitope on the CD9 tetraspanin depends on the association with activated beta1 integrin. Tetraspanins associate on the cell membrane with several transmembrane proteins, including members of the integrin superfamily. The tetraspanin CD9 has been implicated in cell motility, metastasis, and sperm-egg fusion. In this study we characterize the first CD9 conformation-dependent epitope (detected by monoclonal antibody (mAb) PAINS-13) whose expression depends on changes in the activation state of associated beta(1) integrins. MAb PAINS-13 precipitates CD9 under conditions that preserve the association of this tetraspanin with integrins, but not under conditions that disrupt these interactions. Induction of activation of beta(1) integrins by temperature, divalent cation Mn(2+), or mAb TS2/16 correlated with enhanced expression of the PAINS-13 epitope on a variety of cells. Through the use of different K562 myeloid leukemia transfectant cells expressing specific members of the beta(1) integrin subfamily we show that the expression of the PAINS-13 epitope depends on CD9 association with alpha(6)beta(1) integrin. The mAb PAINS-13 reactivity has been mapped to the CD9 region comprising residues 112-154 in the NH(2) half of the large extracellular loop. Also, we show that the CD9 conformation recognized by mAb PAINS-13 is functionally relevant in beta(1) integrin-mediated cellular processes including wound healing   migration  , tubular morphogenesis, cell adhesion and spreading and in signal transduction involving phosphatidylinositol 3-kinase activation. 
 Disruption of focal adhesions by integrin cytoplasmic domain-associated protein-1 alpha. Regulation of integrin affinity and clustering plays a key role in the control of cell adhesion and   migration  . The protein ICAP-1 alpha (integrin cytoplasmic domain-associated protein-1 alpha) binds to the cytoplasmic domain of the beta(1A) integrin and controls cell spreading on fibronectin. Here, we demonstrate that, despite its ability to interact with beta(1A) integrin, ICAP-1 alpha is not recruited in focal adhesions, whereas it is colocalized with the integrin at the ruffling edges of the cells. ICAP-1 alpha induced a rapid disruption of focal adhesions, which may result from the ability of ICAP-1 alpha to inhibit the association of beta(1A) integrin with talin, which is crucial for the assembly of these structures. ICAP-1 alpha-mediated dispersion of beta(1A) integrins is not observed with beta(1D) integrins that do not bind ICAP. This strongly suggests that ICAP-1 alpha action depends on a direct interaction between ICAP-1 alpha and the cytoplasmic domain of the beta(1) chains. Altogether, these results suggest that ICAP-1 alpha plays a key role in cell adhesion by acting as a negative regulator of beta(1) integrin avidity. 
 Inhibition of IL-8-mediated MAPK activation in human neutrophils by beta1 integrin ligands. Chemokine and integrin receptors must work in concert when circulating leukocytes mobilize toward a site of tissue inflammation or infection. In a previous study, we reported that ligation of the alpha5beta1 integrin with a 120-kDa cell-binding fibronectin fragment (120-kDa FN) in suspensions of human polymorphonuclear leukocytes (PMNLs) inhibited chemotaxis toward the chemokine called interleukin-8 (IL-8). Binding of chemokines to their receptors on leukocytes leads to the activation of heterotrimeric G proteins that initiate multiple signaling cascades, including p38 and p42/p44 mitogen-activated protein kinase (MAPK) pathways. In the present study, we examine the potential interaction of beta1 integrin ligation on chemokine-mediated MAPK signaling in human PMNLs. We demonstrate that blockade of the p42/p44 MAPK signaling pathway by the inhibitor PD98059 suppresses IL-8-mediated PMNL chemotaxis. Furthermore, when PMNLs are pretreated with 120-kDa FN or an activating antibody to beta1 integrins (TS2/16), IL-8-mediated phosphorylation of p42/p44 MAPK is also inhibited. In contrast, pretreating PMNL with a specific ligand (laminin-1) for the alpha6beta1 integrin does not suppress IL-8-mediated phosphorylation of p42/p44 MAPK. These observations demonstrate a desensitization of IL-8-mediated p42/p44 MAPK signaling in response to ligation of the alpha5beta1 integrin in PMNL. Also, they suggest an interplay between integrin and chemokine signaling during PMNL   migration   through the extracellular matrix. 
 Type I phosphatidylinositol phosphate kinase beta regulates focal adhesion disassembly by promoting beta1 integrin endocytosis. Cell   migration   requires the regulated disassembly of focal adhesions, but the underlying mechanisms remain poorly defined. We have previously shown that focal adhesion disassembly requires the dynamin 2- and clathrin-dependent endocytosis of ligand-activated beta1 integrins. Here, we identify type I phosphatidylinositol phosphate kinase beta (PIPKIbeta), an enzyme that generates phosphatidylinositol-4,5-bisphosphate (PI4,5P(2)), as a key regulator of this process. We found that knockdown of PIPKIbeta by RNA interference blocks the internalization of active beta1 integrins and impairs focal adhesion turnover and cell   migration  . These defects are caused by the failure to target the endocytic machinery, including clathrin adaptors and dynamin 2, to focal adhesion sites. As a consequence, depletion of PIPKIbeta blocks clathrin assembly at adhesion plaques and prevents complex formation between dynamin 2 and focal adhesion kinase (FAK), a critical step in focal adhesion turnover. Together, our findings identify PIPKIbeta as a novel regulator of focal adhesion disassembly and suggest that PIPKIbeta spatially regulates integrin endocytosis at adhesion sites to control cell   migration  . 
 Purification and characterization of human laminin-8. Laminin-8 stimulates cell adhesion and   migration   through alpha3beta1 and alpha6beta1 integrins. Recently identified laminin isoforms containing the alpha4 chain have been shown to be expressed in the basement membrane of restricted organs such as heart, skeletal muscle, and blood vessels, especially those in embryos. We screened 38 human cell lines for the expression of the laminin alpha4 chain by reverse transcriptase-polymerase chain reaction and found that T98G glioblastoma cells express only alpha4, but not other alpha chains. Laminin-8, an isoform containing the alpha4 and beta1 chains, was purified from conditioned medium of T98G cells by gel filtration and immunoaffinity chromatography using a monoclonal antibody against laminin beta1 chain. The purified laminin isoform was composed of disulfide-linked 230-, 220-, and 200-kDa subunits, which immunoblot analysis identified as the beta1, gamma1, and alpha4 chains. Purified laminin-8 had cell adhesive activity comparable to laminin-1 but significantly weaker than laminin-5 and laminin-10/11. T98G cells adhering to laminin-8 became more elongated than those adhering to other laminin isoforms and extended multiple pseudopods. Cell adhesion to laminin-8 was abolished by an antibody against the integrin beta1 subunit or a combination of antibodies against the integrin alpha3 and alpha6 subunits, but not by either anti-alpha3 or anti-alpha6 antibody alone, suggesting that both alpha3beta1 and alpha6beta1 integrins serve as adhesion receptors for laminin-8. Consistent with these observations, K562 erythroleukemic cells transfected with either integrin alpha3 or alpha6 cDNA were capable of adhering to laminin-8 when beta1 integrins were stimulated by the beta1-activating antibody 8A2. Despite its moderate cell adhesive activity, laminin-8 was significantly potent in promoting cell   migration   when compared with other laminin isoforms and fibronectin. Cell   migration   on laminin-8 was completely inhibited by a combination of antibodies against alpha3 and alpha6 integrins, and substantially inhibited by anti-alpha3 antibody alone, suggesting that laminin-8-mediated cell   migration   is predominantly mediated by alpha3beta1 integrin. Given its potency to stimulate cell   migration   and preferential localization to the basement membrane of capillaries and embryonic tissues, laminin-8 may play a role in processes requiring enhanced cell   migration   during development, wound healing, and angiogenesis. 
 Syndecan-1 modulates β-integrin-dependent and interleukin-6-dependent functions in breast cancer cell adhesion,   migratio  n, and resistance to irradiation. Syndecan-1 is a cell surface heparan sulfate proteoglycan with various biological functions relevant to tumor progression and inflammation, including cell-cell adhesion, cell-matrix interaction, and cytokine signaling driving cell proliferation and motility. Syndecan-1 is a prognostic factor in breast cancer, and has a predicitive value for neodadjuvant chemotherapy. It is still poorly understood how syndecan-1 integrates matrix-dependent and cytokine-dependent signaling processes in the tumor microenvironment. Here, we evaluated the potential role of syndecan-1 in modulating matrix-dependent breast cancer cell   migratio  n in the presence of interleukin-6, and its potential involvement in resistance to irradiation in vitro. MDA-MB-231 breast cancer cells were transiently transfected with syndecan-1 small interfering RNA or control reagents, and this was followed by stimulation with interleukin-6 or irradiation. Cellular responses were monitored by adhesion  , migrati  on and colony formation assays, as well as analysis of cell signaling. Syndecan-1 depletion increased cell adhesion to fibronectin. Increase  d migrati  on on fibronectin was significantly suppressed by interleukin-6, and GRGDSP peptides inhibited both adhesion an  d migrati  on. Interleukin-6-induced activation of focal adhesion kinase and reduction of constitutive nuclear factor kappaB signaling were decreased in syndecan-1-deficient cells. Focal adhesion kinase hyperactivation in syndecan-1-depleted cells was associated with dramatically reduced radiation sensitivity. We conclude that loss of syndecan-1 leads to enhanced activation of β1 -integrins and focal adhesion kinase, thus increasing breast cancer cell adhesio  n, migrat  ion, and resistance to irradiation. Syndecan-1 deficiency also attenuates the modulatory effect of the inflammatory microenvironment constituent interleukin-6 on cancer ce  ll migrat  ion. 
 The structure of the talin/integrin complex at a lipid bilayer: an NMR and MD simulation study. Integrins are cell surface receptors crucial for cell   migration   and adhesion. They are activated by interactions of the talin head domain with the membrane surface and the integrin β cytoplasmic tail. Here, we use coarse-grained molecular dynamic simulations and nuclear magnetic resonance spectroscopy to elucidate the membrane-binding surfaces of the talin head (F2-F3) domain. In particular, we show that mutations in the four basic residues (K258E, K274E, R276E, and K280E) in the F2 binding surface reduce the affinity of the F2-F3 for the membrane and modify its orientation relative to the bilayer. Our results highlight the key role of anionic lipids in talin/membrane interactions. Simulation of the F2-F3 in complex with the α/β transmembrane dimer reveals information for its orientation relative to the membrane. Our studies suggest that the perturbed orientation of talin relative to the membrane in the F2 mutant would be expected to in turn perturb talin/integrin interactions. 
 Osteoblasts-derived BMP-2 enhances the motility of prostate cancer cells via activation of integrins. Bone metastases are common complications of prostate cancer cells. The bone morphogenetic protein-2 (BMP-2) is constitutively secreted by osteoblasts and plays a key role in bone formation. Integrins are the major adhesive molecules in mammalian cells, and has been associated with cancer cells metastasis to bone. The aim of this study was to investigate whether osteoblast-derived BMP-2 is associated with prostate cancer metastasis.Cancer cells   migration   activity was examined using the Transwell assay. The ERK and AKT phosphorylation was examined by using Western blot method. The siRNA was used to inhibit the expression of BMP-2. The cell surface expression of integrins was examined by using flow cytometry. A transient transfection protocol was used to examine NF-kappaB activity.We found that osteoblast conditioned medium (OBCM) increased the   migration   and cell surface expression of beta1 or beta 3 integrin in human prostate cancer cells. beta1 or beta 3 integrin monoclonal antibodies or siRNA against beta1 or beta 3 integrin inhibited the OBCM-induced increase the   migration   of prostate cancer cells. BMP-2 siRNA specifically reduced the OBCM-induced   migration   and integrins upregulation. BMP-2 siRNA also suppressed the OBCM-induced ERK, AKT and NF-kappaB activation.This study suggest that the osteoblast-derived BMP-2 act through Akt and ERK, which in turn activates IKK alpha/beta and NF-kappaB, resulting in the activations of beta1 and beta 3 integrins and contributing the   migration   of prostate cancer cells. 
 β1 integrin signaling maintains human epithelial progenitor cell survival in situ and controls proliferation, apoptosis and   migratio  n of their progeny. β1 integrin regulates multiple epithelial cell functions by connecting cells with the extracellular matrix (ECM). While β1 integrin-mediated signaling in murine epithelial stem cells is well-studied, its role in human adult epithelial progenitor cells (ePCs) in situ remains to be defined. Using microdissected, organ-cultured human scalp hair follicles (HFs) as a clinically relevant model for studying human ePCs within their natural topobiological habitat, β1 integrin-mediated signaling in ePC biology was explored by β1 integrin siRNA silencing, specific β1 integrin-binding antibodies and pharmacological inhibition of integrin-linked kinase (ILK), a key component of the integrin-induced signaling cascade. β1 integrin knock down reduced keratin 15 (K15) expression as well as the proliferation of outer root sheath keratinocytes (ORSKs). Embedding of HF epithelium into an ECM rich in β1 integrin ligands that mimic the HF mesenchyme significantly enhanced proliferat  ion and m  igration of ORSKs, while K15 and CD200 gene and protein expression were inhibited. Employing ECM-embedded β1 integrin-activating or -inhibiting antibodies allowed to identify functionally distinct human ePC subpopulations in different compartments of the HF epithelium. The β1 integrin-inhibitory antibody reduced β1 integrin expression in situ and selectively enhanced proliferation of bulge ePCs, while the β1 integrin-stimulating antibody decreased hair matrix keratinocyte apoptosis and enhanced transferrin receptor (CD71) immunoreactivity, a marker of transit amplifying cells, but did not affect bulge ePC proliferation. That the putative ILK inhibitor QLT0267 significantly r  educed OR  SK migration and proliferation and induced massive ORSK apoptosis suggests a key role for ILK in mediating the ß1 integrin effects. Taken together, these findings demonstrate that ePCs in human HFs require β1 integrin-mediated signaling for survival,   adhesion,   and migration, and that different human HF ePC subpopulations differ in their response to β1 integrin signaling. These insights may be exploited for cell-based regenerative medicine strategies that employ human HF-derived ePCs. 
 Recognition of the neural chemoattractant Netrin-1 by integrins alpha6beta4 and alpha3beta1 regulates epithelial cell adhesion and   migration  . Netrins, axon guidance cues in the CNS, have also been detected in epithelial tissues. In this study, using the embryonic pancreas as a model system, we show that Netrin-1 is expressed in a discrete population of epithelial cells, localizes to basal membranes, and specifically associates with elements of the extracellular matrix. We demonstrate that alpha6beta4 integrin mediates pancreatic epithelial cell adhesion to Netrin-1, whereas recruitment of alpha6beta4 and alpha3beta1 regulate the   migration   of CK19+/PDX1+ putative pancreatic progenitors on Netrin-1. These results provide evidence for the activation of epithelial cell adhesion and   migration   by a neural chemoattractant, and identify Netrin-1/integrin interactions as adhesive/guidance cues for epithelial cells. 
 The prion protein inhibits monocytic cell   migration   by stimulating β1 integrin adhesion and uropod formation. The broad tissue distribution and evolutionary conservation of the glycosylphosphatidylinositol (GPI)-anchored prion protein (PrP, also known as PRNP) suggests that it plays a role in cellular homeostasis. Given that integrin adhesion determines cell behavior, the proposed role of PrP in cell adhesion might underlie the various in vitro and in vivo effects associated with PrP loss-of-function, including the immune phenotypes described in PrP(-/-) mice. Here, we investigated the role of PrP in the adhesion and (transendothelial)   migratio  n of human (pro)monocytes. We found that PrP regulates β1-integrin-mediated adhesion of monocytes. Additionally, PrP controls the cell morphology and migratory behavior of monocytes: PrP-silenced cells show deficient uropod formation on immobilized VCAM and display bleb-like protrusions on the endothelium. Our data further show that PrP regulates ligand-induced integrin activation. Finally, we found that PrP controls the activation of several proteins involved in cell adhesion an  d migrati  on, including RhoA and its effector cofilin, as well as proteins of the ERM family. We propose that PrP modulates β1 integrin adhesion a  nd migrat  ion of monocytes through RhoA-induced actin remodeling mediated by cofilin, and through the regulation of ERM-mediated membrane-cytoskeleton linkage. 
 Schwann Cells Increase Prostate and Pancreatic Tumor Cell Invasion Using Laminin Binding A6 Integrin. Human pancreatic and prostate cancers metastasize along nerve axons during perineural invasion. The extracellular matrix laminin class of proteins is an abundant component of both myelinated and non-myelinated nerves. Analysis of human pancreatic and prostate tissue revealed both perineural and endoneural invasion with Schwann cells surrounded or disrupted by tumor, respectively. Tumor and nerve cell co-culture conditions were used to determine if myelinating or non-myelinating Schwann cell (S16 and S16Y, respectively) phenotype was equally likely to promote integrin-dependent cancer cell invasion and   migration   on laminin. Conditioned medium from S16 cells increased tumor cell (DU145, PC3, and CFPAC1) invasion into laminin approximately 1.3-2.0 fold compared to fetal bovine serum (FBS) treated cells. Integrin function (e.g., ITGA6p formation) increased up to 1.5 fold in prostate (DU145, PC3, RWPE-1) and pancreatic (CFPAC1) cells, and invasion was dependent on ITGA6p formation and ITGB1 as determined by function-blocking antibodies. In contrast, conditioned medium isolated from S16Y cells (non-myelinating phenotype) decreased constitutive levels of ITGA6p in the tumor cells by 50% compared to untreated cells and decreased ITGA6p formation 3.0 fold compared to S16 treated cells. Flow cytometry and western blot analysis revealed loss of ITGA6p formation as reversible and independent of overall loss of ITGA6 expression. These results suggest that the myelinating phenotype of Schwann cells within the tumor microenvironment increased integrin-dependent tumor invasion on laminin. 
 AsialoGM1 and integrin alpha2beta1 mediate prostate cancer progression. The most lethal aspect of cancer is the metastatic spread of primary tumors to distant sites. Any mechanism revealed is a target for therapy. In our previous studies, we reported that the invasive activity of the bone metastatic C4-2B prostate cancer cells could be ascribed to the reorganization of the alpha2beta1 integrin receptor and the alpha2 subunit-mediated association and activation of downstream signaling towards the activation of MMPs. In the present study, we demonstrate that expression of asialoGM1 in C4-2B cells correlates with cancer progression by influencing adhesion,   migration   and invasion, via reorganization of asialoGM1 and colocalization with integrin alpha2beta1. These observations reveal an uncharacterized complex of asialoGM1 with the integrin alpha2beta1 receptor promoting cancer metastatic potential through the previously identified integrin-mediated signaling pathway. The present findings promote further understanding of mechanisms by which glycosphingolipids modulate malignant properties and the results obtained here propose novel directions for future study. 
 Endorepellin causes endothelial cell disassembly of actin cytoskeleton and focal adhesions through alpha2beta1 integrin. Endorepellin, the COOH-terminal domain of the heparan sulfate proteoglycan perlecan, inhibits several aspects of angiogenesis. We provide evidence for a novel biological axis that links a soluble fragment of perlecan protein core to the major cell surface receptor for collagen I, alpha2beta1 integrin, and provide an initial investigation of the intracellular signaling events that lead to endorepellin antiangiogenic activity. The interaction between endorepellin and alpha2beta1 integrin triggers a unique signaling pathway that causes an increase in the second messenger cAMP; activation of two proximal kinases, protein kinase A and focal adhesion kinase; transient activation of p38 mitogen-activated protein kinase and heat shock protein 27, followed by a rapid down-regulation of the latter two proteins; and ultimately disassembly of actin stress fibers and focal adhesions. The end result is a profound block of endothelial cell   migration   and angiogenesis. Because perlecan is present in both endothelial and smooth muscle cell basement membranes, proteolytic activity during the initial stages of angiogenesis could liberate antiangiogenic fragments from blood vessels' walls, including endorepellin. 
 Introduction of bisecting GlcNAc into integrin alpha5beta1 reduces ligand binding and down-regulates cell adhesion and cell   migration  . The enzyme beta1,4-N-acetylglucosaminyltransferase III (GnT-III) catalyzes the addition of a bisecting GlcNAc residue to glycoproteins, resulting in a modulation in biological function. Our previous studies showed that the transfection of the GnT-III gene into B16 melanoma cells results in a suppression of invasive ability and lung colonization. The suppression has been postulated to be due to an increased level of E-cadherin expression on the cell surface, which in turn leads to the up-regulation of cell-cell adhesion. In this study, we report on the effects of overexpression of GnT-III on cell-matrix adhesion. The overexpression of GnT-III, but not that of an enzymatic inactive GnT-III (D323A), inhibits cell spreading and   migration   on fibronectin, a specific ligand for integrin alpha(5)beta(1), and the focal adhesion kinase phosphorylation. E(4)-PHA lectin blot analyses showed that the levels of bisecting GlcNAc structures on the integrin alpha(5) subunit as well as alpha(2) and alpha(3) subunits immunoprecipitated from GnT-III transfectants were substantially increased. In addition, the affinity of the binding of integrin alpha(5)beta(1) to fibronectin was significantly reduced by the introduction of the bisecting GlcNAc, to the alpha(5) subunit. These findings suggest that the modification of N-glycan of integrin by GnT-III inhibits its ligand binding ability, subsequently leading to the down-regulation of integrin-mediated signaling. 
 Annexin A2 regulates β1 integrin internalization and intestinal epithelial cell   migratio  n. The gastrointestinal epithelium functions as an important barrier that separates luminal contents from the underlying tissue compartment and is vital in maintaining mucosal homeostasis. Mucosal wounds in inflammatory disorders compromise the critical epithelial barrier. In response to injury, intestinal epithelial cells (IECs) rapidly migrate to reseal wounds. We have previously observed that a membrane-associated, actin binding protein, annexin A2 (AnxA2), is up-regulated in migrating IECs and plays an important role in promoting wound closure. To identify the mechanisms by which AnxA2 promotes IEC movement and wound closure, we used a loss of function approach. AnxA2-specific shRNA was utilized to generate IECs with stable down-regulation of AnxA2. Loss of AnxA2 inhibited IEC   migratio  n while promoting enhanced cell-matrix adhesion. These functional effects were associated with increased levels of β1 integrin protein, which is reported to play an important role in mediating the cell-matrix adhesive properties of epithelial cells. Because cel  l migrati  on requires dynamic turnover of integrin-based adhesions, we tested whether AnxA2 modulates internalization of cell surface β1 integrin required for forward cell movement. Indeed, pulse-chase biotinylation experiments in IECs lacking AnxA2 demonstrated a significant increase in cell surface β1 integrin that was accompanied by decreased β1 integrin internalization and degradation. These findings support an important role of AnxA2 in controlling dynamics of β1 integrin at the cell surface that in turn is required for the active turnover of cell-matrix associations,   cell mig  ration, and wound closure. 
 The lymphangiogenic vascular endothelial growth factors VEGF-C and -D are ligands for the integrin alpha9beta1. Mice homozygous for a null mutation of the integrin alpha9 subunit die 6-12 days after birth from bilateral chylothoraces suggesting an underlying defect in lymphatic development. However, until now the mechanisms by which the integrin alpha9beta1 modulates lymphangiogenesis have not been described. In this study we show that adhesion to and   migration   on the lymphangiogenic vascular endothelial growth factors (VEGF-C and -D) are alpha9beta1-dependent. Mouse embryonic fibroblasts and human colon carcinoma cells (SW-480) transfected to express alpha9beta1 adhered and/or migrated on both growth factors in a concentration-dependent fashion, and both adhesion and   migration   were abrogated by anti-alpha9beta1 function-blocking antibody. In SW-480 cells, which lack cognate receptors for VEGF-C and -D, both growth factors induced alpha9beta1-dependent Erk and paxillin phosphorylation. Human microvascular endothelial cells, which express both alpha9beta1 and VEGF-R3, also adhered to and migrated on both growth factors, and both responses were blocked by anti-alpha9beta1 antibody. Furthermore, in a solid phase binding assay recombinant VEGF-C and -D bound to purified alpha9beta1 integrin in a dose- and cation-dependent fashion showing that VEGF-C and VEGF-D are ligands for the integrin alpha9beta1. The interaction between alpha9beta1 and VEGF-C and/or -D may begin to explain the abnormal lymphatic phenotype of the alpha9 knock-out mice. 
 Identification of a novel integrin alpha 6 beta 1 binding site in the angiogenic inducer CCN1 (CYR61). The angiogenic inducer CCN1 (cysteine-rich 61, CYR61), a secreted matricellular protein of the CCN family, is a ligand of multiple integrins, including alpha 6 beta 1. Previous studies have shown that CCN1 interaction with integrin alpha 6 beta 1 mediates adhesion of fibroblasts, endothelial cells, and smooth muscle cells, as well as   migration   of smooth muscle cells. Recently, we have reported that CCN1-induced tubule formation of unactivated endothelial cells is also mediated through integrin alpha 6 beta 1. In this study, we demonstrate that human skin fibroblasts adhere specifically to the T1 sequence (GQKCIVQTTSWSQCSKS) within domain III of CCN1, and this process is blocked by anti-alpha 6 and anti-beta 1 monoclonal antibodies. Alanine substitution mutagenesis of the T1 sequence further defines the sequence TTSWSQCSKS as the critical determinant for mediating alpha 6 beta 1-dependent adhesion. Soluble T1 peptide specifically inhibits fibroblast adhesion to CCN1 in a dose-dependent manner. Furthermore, T1 also inhibits cell adhesion to other alpha 6 beta 1 ligands, including CCN2 (CTGF), CCN3 (NOV), and laminin, but not to ligands of other integrins. In addition, T1 specifically inhibits alpha 6 beta 1-dependent tubule formation of unactivated endothelial cells in a CCN1-containing collagen gel matrix. To confirm that T1 binds integrin alpha 6 beta 1 directly, we perform affinity chromatography and show that integrin alpha 6 beta 1 is isolated from an octylglucoside extract of fibroblasts on T1-coupled Affi-gel. Taken together, these findings define the T1 sequence in CCN1 as a novel binding motif for integrin alpha 6 beta 1, providing the basis for the development of peptide mimetics to examine the functional role of alpha 6 beta 1 in angiogenesis. 
 Regulation of Integrin β 1 recycling to lipid rafts by Rab1a to promote cell   migratio  n. Rab1a is a member of the Rab family of small GTPases with a well characterized function in the regulation of vesicle trafficking from the endoplasmic reticulum to the Golgi apparatus and within Golgi compartments. The integrin family heterodimeric transmembrane proteins serve as major receptors for extracellular matrix proteins, which play essential roles in cell adhesion and   migratio  n. Although effects on intracellular trafficking of integrins or other key cargos by Rab1a could influence cell   migratio  n, the regulatory mechanisms linking Rab1a to cell   migratio  n are not well understood. Here, we report identification of Rab1a as a novel regulator of cell   migratio  n using an unbiased RNAi screen targeting GTPases. Inhibition of Rab1a reduced integrin-mediated cell adhesion and spreading on fibronectins, reduced integrin β1 localization to lipid rafts, and decreased recycling of integrin β1 to the plasma membrane. Analysis of Rab1a effector molecules showed that p115 mediated Rab1a regulation of integrin recycling and lipid raft localization in ce  ll migrat  ion. Taken together, these results suggest a novel function for Rab1a in the regulation of ce  ll migrat  ion through controlling integrin β1 recycling and localization to lipid rafts via a specific downstream effector pathway. 
 Beta ig-h3 induces keratinocyte differentiation via modulation of involucrin and transglutaminase expression through the integrin alpha3beta1 and the phosphatidylinositol 3-kinase/Akt signaling pathway. Beta ig-h3 is an extracellular matrix protein whose expression is highly induced by transforming growth factor (TGF)-beta1. Whereas beta ig-h3 is known to mediate keratinocyte adhesion and   migration  , its effects on keratinocyte differentiation remain unclear. In the present study, it was demonstrated that expression of both beta ig-h3 and TGF-beta1 was enhanced during keratinocyte differentiation and that expression of the former was strongly induced by that of the latter. This study also asked whether changes in beta-h3 expression would affect keratinocyte differentiation. Indeed, down-regulation of beta ig-h3 by transfection with antisense beta ig-h3 cDNA constructs effectively inhibited keratinocyte differentiation by decreasing the promoter activities and thus expression of involucrin and transglutaminase. The result was an approximately 2-fold increase in mitotic capacity of the cells. Conversely, overexpression of beta ig-h3, either by transfection with beta ig-h3 expression plasmids or by exposure to recombinant beta ig-h3, enhanced keratinocyte differentiation by inhibiting cell proliferation and concomitantly increasing involucrin and transglutaminase expression. Recombinant beta ig-h3 also promoted keratinocyte adhesion through interaction with integrin alpha3beta1. Changes in beta ig-h3 expression did not affect intracellular calcium levels. Subsequent analysis revealed not only induction of Akt phosphorylation by recombinant beta ig-h3 but also blockage of Akt phosphorylation by LY294002, an inhibitor of phosphatidylinositol 3-kinase. Taken together, these findings indicate that enhanced beta ig-h3, induced by enhanced TGF-beta during keratinocyte differentiation, provoked cell differentiation by enhancing involucrin and transglutaminase expression through the integrin alpha3beta1 and phosphatidylinositol 3-kinase/Akt signaling pathway. Lastly, it was observed that beta ig-h3-mediated keratinocyte differentiation was caused by promotion of cell adhesion and not by calcium regulation. 
 Laminin-derived peptide AG73 regulates   migration  , invasion, and protease activity of human oral squamous cell carcinoma cells through syndecan-1 and beta1 integrin. Squamous cell carcinoma is a prevalent head and neck tumor with high mortality. We studied the role played by laminin alpha1 chain peptide AG73 on   migration  , invasion, and protease activity of cells (OSCC) from human oral squamous cell carcinoma. Immunohistochemistry and immunofluorescence analyzed expression of laminin alpha1 chain and MMP9 in oral squamous cells carcinoma in vivo and in vitro. Migratory activity of AG73-treated OSCC cells was investigated by monolayer wound assays and in chemotaxis chambers. AG73-induced invasion was assessed in Boyden chambers. Invasion depends on MMPs. Conditioned media from cells grown on AG73 was subjected to zymography. We searched for AG73 receptors related to these activities in OSCC cells. Immunofluorescence analyzed AG73-induced colocalization of syndecan-1 and beta1 integrin. Cells had these receptors silenced by siRNA, followed by treatment with AG73 and analysis of   migration  , invasion, and protease activity. Oral squamous cell carcinoma expresses laminin alpha1 chain and MMP9. OSCC cells treated with AG73 showed increased   migration  , invasion, and protease activity. AG73 induced colocalization of syndecan-1 and beta1 integrin. Knockdown of these receptors decreased AG73-dependent   migration  , invasion, and protease activity. Syndecan-1 and beta1 integrin signaling downstream of AG73 regulate   migration  , invasion, and MMP production by OSCC cells. 
 Laminin-332 and α3β1 integrin-supporte  d migrati  on of bronchial epithelial cells is modulated by fibronectin. The repair of the bronchiolar epithelium damaged by cell-mediated, physical, or chemical insult requires epithelial cel  l migrati  on over a provisional matrix composed of complexes of extracellular matrix molecules, including fibronectin and laminin. These matrix molecules suppor  t migrati  on and enhance cell adhesion. When cells adhere too tightly to their matrix they fail to move; but if they adhere too little, they are unable to develop the traction force necessary for motility. Thus, we investigated the relative contributions of laminin and fibronectin to bronchiolar cell adhesion an  d migrati  on using the immortalized bronchial lung epithelial cell line (BEP2D) and normal human bronchial epithelial (NHBE) cells, both of which assemble a laminin α3β3γ2 (LM332)/fibronectin-rich matrix. Intriguingly, BEP2D and NHBE cells migrate significantly faster on an LM332-rich matrix than on fibronectin. Moreover, addition of fibronectin to LM332 matrix suppresses motility of both cell types. Finally, fibronectin enhances the adhesion of both BEP2D and NHBE cells to LM332-coated surfaces. These results suggest that fibronectin fine tunes LM332-medi  ated migr  ation by boosting bronchiolar cell adhesion to substrate. We suggest that, during epithelial wound healing of the injured airway, fibronectin plays an important adhesive role for laminin-driven epithelial cell motility by promoting a stable cellular interaction with the provisional matrix. 
 Small GTPase Rab21 regulates cell adhesion and controls endosomal traffic of beta1-integrins. Dynamic turnover of integrin cell adhesion molecules to and from the cell surface is central to cell   migration  . We report for the first time an association between integrins and Rab proteins, which are small GTPases involved in the traffic of endocytotic vesicles. Rab21 (and Rab5) associate with the cytoplasmic domains of alpha-integrin chains, and their expression influences the endo/exocytic traffic of integrins. This function of Rab21 is dependent on its GTP/GDP cycle and proper membrane targeting. Knock down of Rab21 impairs integrin-mediated cell adhesion and motility, whereas its overexpression stimulates cell   migration   and cancer cell adhesion to collagen and human bone. Finally, overexpression of Rab21 fails to induce cell adhesion via an integrin point mutant deficient in Rab21 association. These data provide mechanistic insight into how integrins are targeted to intracellular compartments and how their traffic regulates cell adhesion. 
 Disialoganglioside GD3-synthase over expression inhibits survival and angiogenesis of pancreatic cancer cells through cell cycle arrest at S-phase and disruption of integrin-β1-mediated anchorage. Gangliosides play important roles in the development, differentiation and proliferation of mammalian cells. They bind to other cell membrane components through their terminal sialic acids. Different gangliosides influence cellular functions based on the positions and linkages of sialic acids. Expression of gangliosides mainly depends on the status of sialic acid-modulatory enzymes, such as different types of sialyltransferases and sialidases. One such sialyltransferase, disialoganglioside GD3 synthase, is specifically responsible for the production of GD3. Pancreatic ductal adenocarcinoma, making up more than 90% of pancreatic cancers, is a fatal malignancy with poor prognosis. Despite higher sialylation status, the disialoganglioside GD3 level is very low in this cancer. However, the exact status and function of this disialoganglioside is still unknown. Here, we intended to study the intracellular mechanism of disialoganglioside GD3-induced apoptosis and its correlation with the adhesion and angiogenic pathways in pancreatic cancer. We demonstrated that disialoganglioside GD3 synthase-transfected cells showed enhanced apoptosis and it caused the arrest of these cells in the S-phase of the cell cycle. Integrins, a family of transmembrane proteins play important role in cell-cell recognition, invasion, adhesion and   migratio  n. disialoganglioside GD3 co-localised with integrin-β1 and thereby inhibited it's downstream signalling in transfected cells. Transfected cells exhibited inhibition of cell adhesion with extracellular matrix proteins. Enhanced GD3 expression down regulated angiogenesis-regulatory proteins and inhibited epidermal growth factor/vascular endothelial growth factor-driven angiogenic cell growth in these cells. Taken together, our study provides support for the GD3-induced cell cycle arrest, disruption of integrin-β1-mediated anchorage, inhibition of angiogenesis and thereby induced apoptosis in pancreatic cancer cells. 
 Integrin β1 is critical for gastrin-releasing peptide receptor-mediated neuroblastoma cell   migratio  n and invasion. Gastrin-releasing peptide (GRP) and its receptor, GRP-R, are critically involved in neuroblastoma tumorigenesis; however, the molecular mechanisms and signaling pathways that are responsible for GRP/GRP-R-induced cell   migratio  n and invasion remain unclear. In this study, we sought to determine the cell signals involved in GRP/GRP-R-mediated neuroblastoma cell   migratio  n and invasion.Human neuroblastoma cell lines SK-N-SH, LAN-1, and IMR-32 were used for our study. Transwell   migratio  n and invasion assays were performed after GRP (10(-7) M) stimulation. The cDNA GEArray Microarray kit was used to determine GRP-R-induced gene expression changes. Protein and membrane expression of integrin subunits were confirmed by Western blotting and flow cytometry analysis. siRNA transfection was performed using Lipofectamine 2000. For scratch assay, a confluent monolayer of cells in 6-well plates were wounded with micropipette tip and observed microscopically at 24 to 72 h.GRP increased neuroblastoma cell   migratio  n and expressions of MMP-2 whereas the TIMP-1 level decreased. GRP-R overexpression stimulated SK-N-SH cell   migratio  n and upregulated integrin α2, α3, and β1 protein as well as mRNA expression. Targeted silencing of integrin β1 inhibited   cell migr  ation.GRP/GRP-R signaling contributes to neuroblastoma   cell migr  ation and invasion. Moreover, the integrin ß1 subunit critically regulates GRP-R-mediated neuroblastoma   cell mig  ration and invasion. 
 Reduced GM1 ganglioside in CFTR-deficient human airway cells results in decreased β1-integrin signaling and delayed wound repair. Loss of cystic fibrosis transmembrane conductance regulator (CFTR) function reduces chloride secretion and increases sodium uptake, but it is not clear why CFTR mutation also results in progressive lung inflammation and infection. We previously demonstrated that CFTR-silenced airway cells migrate more slowly during wound repair than CFTR-expressing controls. In addition, CFTR-deficient cells and mouse models have been reported to have altered sphingolipid levels. Here, we investigated the hypothesis that reduced   migratio  n in CFTR-deficient airway epithelial cells results from altered sphingolipid composition. We used cell lines derived from a human airway epithelial cell line (Calu-3) stably transfected with CFTR short hairpin RNA (CFTR-silenced) or nontargeting short hairpin RNA (controls). Cell   migratio  n was measured by electric cell substrate impedance sensing (ECIS). Lipid analyses, addition of exogenous glycosphingolipids, and immunoblotting were performed. We found that levels of the glycosphingolipid, GM1 ganglioside, were ~60% lower in CFTR-silenced cells than in controls. CFTR-silenced cells exhibited reduced levels of activated β1-integrin, phosphorylated tyrosine 576 of focal adhesion kinase (pFAK), and phosphorylation of Crk-associated substrate (pCAS). Addition of GM1 (but not GM3) ganglioside to CFTR-silenced cells restored activated β1-integrin, pFAK, and pCAS to near control levels and partially restored (~40%) ce  ll migrat  ion. Our results suggest that decreased GM1 in CFTR-silenced cells depresses β1-integrin signaling, which contributes to the delayed wound repair observed in these cells. These findings have implications for the pathology of cystic fibrosis, where altered sphingolipid levels in airway epithelial cells could result in a diminished capacity for wound repair after injury. 
 Endoglin mediates fibronectin/α5β1 integrin and TGF-β pathway crosstalk in endothelial cells. Both the transforming growth factor β (TGF-β) and integrin signalling pathways have well-established roles in angiogenesis. However, how these pathways integrate to regulate angiogenesis is unknown. Here, we show that the extracellular matrix component, fibronectin, and its cellular receptor, α5β1 integrin, specifically increase TGF-β1- and BMP-9-induced Smad1/5/8 phosphorylation via the TGF-β superfamily receptors endoglin and activin-like kinase-1 (ALK1). Fibronectin and α5β1 integrin increase Smad1/5/8 signalling by promoting endoglin/ALK1 cell surface complex formation. In a reciprocal manner, TGF-β1 activates α5β1 integrin and downstream signalling to focal adhesion kinase (FAK) in an endoglin-dependent manner. α5β1 integrin and endoglin form a complex on the cell surface and co-internalize, with their internalization regulating α5β1 integrin activation and signalling. Functionally, endoglin-mediated fibronectin/α5β1 integrin and TGF-β pathway crosstalk alter the responses of endothelial cells to TGF-β1, switching TGF-β1 from a promo  ter to a   suppressor of migration, inhibiting TGF-β1-mediated apoptosis to promote capillary stability, and partially mediating developmental angiogenesis in vivo. These studies provide a novel mechanism for the regulation of TGF-β superfamily signalling and endothelial function through crosstalk with integrin signalling pathways. 
 NG2 proteoglycan promotes endothelial cell motility and angiogenesis via engagement of galectin-3 and alpha3beta1 integrin. The NG2 proteoglycan is expressed by microvascular pericytes in newly formed blood vessels. We have used in vitro and in vivo models to investigate the role of NG2 in cross-talk between pericytes and endothelial cells (EC). Binding of soluble NG2 to the EC surface induces cell motility and multicellular network formation in vitro and stimulates corneal angiogenesis in vivo. Biochemical data demonstrate the involvement of both galectin-3 and alpha3beta1 integrin in the EC response to NG2 and show that NG2, galectin-3, and alpha3beta1 form a complex on the cell surface. Transmembrane signaling via alpha3beta1 is responsible for EC motility and morphogenesis in this system. Galectin-3-dependent oligomerization may potentiate NG2-mediated activation of alpha3beta1. In conjunction with recent studies demonstrating the early involvement of pericytes in angiogenesis, these data suggest that pericyte-derived NG2 is an important factor in promoting EC   migration   and morphogenesis during the early stages of neovascularization. 
 Vascular endothelial growth factor-induced   migration   of multiple myeloma cells is associated with beta 1 integrin- and phosphatidylinositol 3-kinase-dependent PKC alpha activation. In multiple myeloma (MM),   migration   is necessary for the homing of tumor cells to bone marrow (BM), for expansion within the BM microenvironment, and for egress into the peripheral blood. In the present study we characterize the role of vascular endothelial growth factor (VEGF) and beta(1) integrin (CD29) in MM cell   migration  . We show that protein kinase C (PKC) alpha is translocated to the plasma membrane and activated by adhesion of MM cells to fibronectin and VEGF. We identify beta(1) integrin modulating VEGF-triggered MM cell   migration   on fibronectin. We show that transient enhancement of MM cell adhesion to fibronectin triggered by VEGF is dependent on the activity of both PKC and beta(1) integrin. Moreover, we demonstrate that PKC alpha is constitutively associated with beta(1) integrin. These data are consistent with PKC alpha-dependent exocytosis of activated beta(1) integrin to the plasma membrane, where its increased surface expression mediates binding to fibronectin; conversely, catalytically active PKC alpha-driven internalization of beta(1) integrin results in MM cell de-adhesion. We show that the regulatory subunit of phosphatidylinositol (PI) 3-kinase (p85) is constitutively associated with FMS-like tyrosine kinase-1 (Flt-1). VEGF stimulates activation of PI 3-kinase, and both MM cell adhesion and   migration   are PI 3-kinase-dependent. Moreover, both VEGF-induced PI 3-kinase activation and beta(1) integrin-mediated binding to fibronectin are required for the recruitment and activation of PKC alpha. Time-lapse phase contrast video microscopy (TLVM) studies confirm the importance of these signaling components in VEGF-triggered MM cell   migration   on fibronectin. 
 Insulin-like growth factor I controls adhesion strength mediated by alpha5beta1 integrins in motile carcinoma cells. One of the intriguing questions regarding cell motility concerns the mechanism that makes stationary cells move. Here, we provide the first physical evidence that the onset of breast cancer cell motility in response to insulin-like growth factor I (IGF-I) correlates with lowering of adhesion strength from 2.52 +/- 0.20 to 1.52 +/- 0.13 microdynes/microm2 in cells attached to fibronectin via alpha5beta1 integrin. The adhesion strength depends on the dose of IGF-I and time of IGF-I treatment. Weakening of cell-matrix adhesion is blocked significantly (p < 0.01) by the catalytically inactive IGF-I receptor (IGF-IR) and the phosphoinositide 3-kinase (PI-3 kinase) inhibitor LY-294002, but it is unaffected by mitogen-activated protein kinase kinase inhibitor UO-126 and Src kinase inhibitor PP2. Sustained blockade of Rho-associated kinase (ROCK) with Y-27632 down-regulates adhesion strength in stationary, but not in IGF-I-treated, cells. Jasplakinolide, a drug that prevents actin filament disassembly, counteracts the effect of IGF-I on integrin-mediated cell adhesion. In the absence of growth factor signaling, ROCK supports a strong adhesion via alpha5beta1 integrin, whereas activation of the IGF-IR kinase reduces cell-matrix adhesion through a PI-3K-dependent, but ROCK-independent, mechanism. We propose that disassembly of the actin filaments via PI-3 kinase pathway contributes to weakening of adhesion strength and induction of cell movement. Understanding how cell adhesion and   migration   are coordinated has an important application in cancer research, developmental biology, and tissue bioengineering. 
 The endothelin-integrin axis is involved in macrophage-induced breast cancer cell chemotactic interactions with endothelial cells. Elevated macrophage infiltration in tumor tissues is associated with breast cancer metastasis. Cancer cell   migration  /invasion toward angiogenic microvasculature is a key step in metastatic spread. We therefore studied how macrophages stimulated breast cancer cell interactions with endothelial cells. Macrophages produced cytokines, such as interleukin-8 and tumor necrosis factor-α, to stimulate endothelin (ET) and ET receptor (ETR) expression in breast cancer cells and human umbilical vascular endothelial cells (HUVECs). ET-1 was induced to a greater extent from HUVECs than from breast cancer cells, resulting in a density difference that facilitated cancer cell chemotaxis toward HUVECs. Macrophages also stimulated breast cancer cell adhesion to HUVECs and transendothelial   migratio  n, which were repressed by ET-1 antibody or ETR inhibitors. The ET axis induced integrins, such as αV and β1, and their counterligands, such as intercellular adhesion molecule-2 and P-selectin, in breast cancer cells and HUVECs, and antibodies against these integrins efficiently suppressed macrophage-stimulated breast cancer cell interactions with HUVECs. ET-1 induced Ets-like kinase-1 (Elk-1), signal transducer and activator of transcription-3 (STAT-3), and nuclear factor-κB (NF-κB) phosphorylation in breast cancer cells. The use of inhibitors to prevent their phosphorylation or ectopic overexpression of dominant-negative IκBα perturbed ET-1-induced integrin αV and integrin β1 expression. The physical associations of these three transcriptional factors with the gene promoters of the two integrins were furthermore evidenced by a chromatin immunoprecipitation assay. Finally, our mouse orthotopic tumor model revealed an ET axis-mediated lung metastasis of macrophage-stimulated breast cancer cells, suggesting that the ET axis was involved in macrophage-enhanced breast cancer cell endothelial interactions. 
 Exosomes derived from human macrophages suppress endothelial cell   migration   by controlling integrin trafficking. Integrin trafficking, including internalization, recycling, and lysosomal degradation, is crucial for the regulation of cellular functions. Exosomes, nano-sized extracellular vesicles, are believed to play important roles in intercellular communications. This study demonstrates that exosomes released from human macrophages negatively regulate endothelial cell   migration   through control of integrin trafficking. Macrophage-derived exosomes promote internalization of integrin β1 in primary HUVECs. The internalized integrin β1 persistently accumulates in the perinuclear region and is not recycled back to the plasma membrane. Experimental results indicate that macrophage-derived exosomes stimulate trafficking of internalized integrin β1 to lysosomal compartments with a corresponding decrease in the integrin destined for recycling endosomes, resulting in proteolytic degradation of the integrin. Moreover, ubiquitination of HUVEC integrin β1 is enhanced by the exosomes, and exosome-mediated integrin degradation is blocked by bafilomycin A, a lysosomal degradation inhibitor. Macrophage-derived exosomes were also shown to effectively suppress collagen-induced activation of the mitogen-activated protein kinase/extracellular signal-regulated kinase signaling pathway and HU  VEC migra  tion, which are both dependent on integrin β1. These observations provide new insight into the functional significance of exosomes in the regulation of integrin trafficking. 
 Granulocyte colony-stimulating factor (G-CSF) upregulates β1 integrin and increases   migratio  n of human trophoblast Swan 71 cells via PI3K and MAPK activation. Multiple cytokines and growth factors expressed at the fetal-maternal interface are involved in the regulation of trophoblast functions and placental growth, but the role of G-CSF has not been completely established. Based on our previous study showing that G-CSF increases the activity of matrix metalloproteinase-2 and the release of vascular endothelial growth factor in Swan 71 human trophoblast cells, in this work we explore the possible contribution of G-CSF to cell   migratio  n and the G-CSF-triggered signaling pathway. We found that G-CSF induced morphological changes on actin cytoskeleton consistent with a migratory cell phenotype. G-CSF also up-regulated the expression levels of β1 integrin and promoted Swan 71 cel  l migrati  on. By using selective pharmacological inhibitors and dominant negative mutants we showed that PI3K, Erk 1/2 and p38 pathways are required for promoting Swan 71 cell motility. It was also demonstrated that PI3K behaved as an upstream regulator of Erk 1/2 and p38 MAPK. In addition, the increase of β1 integrin expression was dependent on PI3K activation. In conclusion, our results indicate that G-CSF stimulates β1 integrin expression and Swan 71 c  ell migra  tion by activating PI3K and MAPK signaling pathways, suggesting that G-CSF should be considered as an additional regulatory factor that contributes to a successful embryo implantation and to the placenta development. 
 Integrin-dependent protein tyrosine phosphorylation is a key regulatory event in collagen-IV-mediated adhesion and proliferation of human lung tumor cell line, Calu-1. The clinical phenomenon of lung cancer metastasis to specific target organs is believed to be a direct interaction between tumor cells and extracellular matrix components. During invasion, tumor cells attach to the basement membrane protein, collagen type IV, degrade it, migrate through blood vessels, and adhere to extracellular matrix proteins.Four nonsmall-cell lung cancer cells were tested for adhesion, proliferation,   migration   and morphologic alterations on collagen type IV matrix by immunoprecipitation, Western blotting, phase contrast and time lapse video microscopy.Collagen type IV promoted Calu-1 cell adhesion (< 15 minutes) and motility (< 6 hours) without any significant effect on proliferation. beta(1)-integrin is essential for collagen type IV adhesion and 8 to 10 fold higher expression of beta1-integrin on the surface of Calu-1 cells was identified. A protein tyrosine phosphatase inhibitor, peroxyvanadate, caused 50% inhibition in the adhesion process within 5 minutes but exposure to 60 micromol/L genistein for 72 hours, a protein tyrosine kinase inhibitor, drastically inhibits Calu-1 cell proliferation (> 70%). We examined the influence of beta1-integrin, peroxyvanadate and genistein on the spreading morphogenesis of Calu-1 cells on Collagen type IV. Use of either 1 microg of anti beta1-integrin inhibitory antibody (P5D2), 100 micromol/L peroxyvanadate or 60 micromol/L genistein had dramatic influence on the spreading of Calu-1 cells. Finally, Focal adhesion kinase was identified as a phosphoprotein target.We have characterized an in vitro model of matrix-specific binding of a lung cancer cell line, Calu-1 to Coll IV. Calu-1 cells use primarily a beta1-integrin mediated intracellular tyrosine phosphorylation phenomenon as the key regulatory mechanism for its binding advantage to Coll IV matrix. 
 Eotaxin activates T cells to chemotaxis and adhesion only if induced to express CCR3 by IL-2 together with IL-4. The trans  migration   and adherence of T lymphocytes through microvascular endothelium are essential events for their recruitment into inflammatory sites. In the present study, we investigated the expression of CC chemokine receptor CCR3 on T lymphocytes and the capacities of the CC chemokine eotaxin to induce chemotaxis and adhesion in T lymphocytes. We have observed a novel phenomenon that IL-2 and IL-4 induce the expression of CCR3 on T lymphocytes. We also report that CC chemokine eotaxin is a potent chemoattractant for IL-2- and IL-4-stimulated T lymphocytes, but not for freshly isolated T lymphocytes. Eotaxin attracts T lymphocytes via CCR3, documented by the fact that anti-CCR3 mAb blocks eotaxin-mediated T lymphocyte chemotaxis. In combination with IL-2 and IL-4, eotaxin enhances the expression of adhesion molecules such as ICAM-1 and several integrins (CD29, CD49a, and CD49b) on T lymphocytes and thus promotes adhesion and aggregation of T lymphocytes. The eotaxin-induced T lymphocyte adhesion could be selectively blocked by a specific cAMP-dependent protein kinase inhibitor, H-89, indicating that eotaxin activates T lymphocytes via a special cAMP-signaling pathway. Our new findings all point toward the fact that eotaxin, in association with the Th1-derived cytokine IL-2 and the Th2-derived cytokine IL-4, is an important T lymphocyte activator, stimulating the directional   migration  , adhesion, accumulation, and recruitment of T lymphocytes, and paralleled the accumulation of eosinophils and basophils during the process of certain types of inflammation such as allergy. 
 Twist induces epithelial-mesenchymal transition and cell motility in breast cancer via ITGB1-FAK/ILK signaling axis and its associated downstream network. Twist, a highly conserved basic Helix-Loop-Helix transcription factor, functions as a major regulator of epithelial-mesenchymal transition (EMT) and tumor metastasis. In different cell models, signaling pathways such as TGF-β, MAPK/ERK, WNT, AKT, JAK/STAT, Notch, and P53 have also been shown to play key roles in the EMT process, yet little is known about the signaling pathways regulated by Twist in tumor cells. Using iTRAQ-labeling combined with 2D LC-MS/MS analysis, we identified 194 proteins with significant changes of expression in MCF10A-Twist cells. These proteins reportedly play roles in EMT, cell junction organization, cell adhesion, and cell   migratio  n and invasion. ECM-receptor interaction, MAPK, PI3K/AKT, P53 and WNT signaling were found to be aberrantly activated in MCF10A-Twist cells. Ingenuity Pathways Analysis showed that integrin β1 (ITGB1) acts as a core regulator in linking integrin-linked kinase (ILK), Focal-adhesion kinase (FAK), MAPK/ERK, PI3K/AKT, and WNT signaling. Increased Twist and ITGB1 are associated with breast tumor progression. Twist transcriptionally regulates ITGB1 expression. Over-expression of ITGB1 or Twist in MCF10A led to EMT, activation of FAK/ILK, MAPK/ERK, PI3K/AKT, and WNT signaling. Knockdown of Twist or ITGB1 in BT549 and Hs578T cells decreased activity of FAK, ILK, and their downstream signaling, thus specifically impeding EMT and cell invasion. Knocking down ILK or inhibiting FAK, MAPK/ERK, or PI3K/AKT signaling also suppressed Twist-driven EMT and cell invasion. Thus, the Twist-ITGB1-FAK/ILK pathway and their downstream signaling network dictate the Twist-induced EMT process in human mammary epithelial cells and breast cancer cells. 
 The GTPase Rap1 regulates phorbol 12-myristate 13-acetate-stimulated but not ligand-induced beta 1 integrin-dependent leukocyte adhesion. Leukocyte   migration   from bloodstream to tissue requires rapid, coordinated regulation of integrin-dependent adhesion and de-adhesion. In a previous study we demonstrated that inhibition of protein geranylgeranylation inhibited phorbol ester-stimulated avidity modulation of beta(1) integrin in several leukocyte cell lines. Both RhoA and Rap1 require post-translational modification by geranylgeranylation for full function. In this report we identify Rap1, not RhoA, as a critical geranylgeranylated protein mediating phorbol ester-stimulated beta(1) and beta(2) integrin-dependent adhesion of Jurkat cells. Overexpression of the Rap1-specific GTPase-activating protein, SPA-1, or inactivated form of Rap1 (N17Rap1) blocked phorbol ester-stimulated adhesion of Jurkat cells to fibronectin (alpha(4)beta(1)) and ICAM-1 (alpha(L)beta(2)). With high concentrations of fibronectin as ligand, Jurkat cells adhered spontaneously without phorbol ester stimulation. Unlike the phorbol ester-stimulated adhesion, adhesion induced by high density ligand was not dependent upon Rap1 activation or actin cytoskeleton reorganization. Thus, the "inside-out" adhesion signal induced by phorbol ester and the "outside-in" signal induced by high density ligand involve different pathways. 
 Lumican induces human corneal epithelial cell   migration   and integrin expression via ERK 1/2 signaling. Lumican is a major proteoglycans of the human cornea. Lumican knock-out mice have been shown to lose corneal transparency and to display delayed wound healing. The purpose of this study was to define the role of lumican in corneal epithelial cell   migration  . Over-expression of lumican in human corneal epithelial (HCE-T) cells increased both cell   migration   and proliferation, and increased levels of integrins alpha2 and beta1. ERK 1/2 was also activated in lumican over-expressed cells. When we treated HCE-T cells with the ERK-specific inhibitor U0126, cell   migration   and the expression of integrin beta1 were completely blocked. These data provide evidence that lumican stimulates cell   migration   in the corneal epithelium by activating ERK 1/2, and point to a novel signaling pathway implicated in corneal epithelial cell   migration  . 
 Ubiquitination of alpha 5 beta 1 integrin controls fibroblast   migration   through lysosomal degradation of fibronectin-integrin complexes. Cell   migration   requires endocytosis and recycling of integrins, but it is not known whether degradation of these membrane proteins is involved. Here we demonstrate that in migrating cells, a fraction of the endocytosed fibronectin receptor, alpha 5 beta 1 integrin, is sorted into multivesicular endosomes together with fibronectin and degraded in lysosomes. This sorting requires fibronectin-induced ubiquitination of the alpha 5 subunit, and the activity of the endosomal sorting complex required for transport (ESCRT) machinery, which interacts with alpha 5 beta 1 integrin. Importantly, we demonstrate that both alpha 5 ubiquitination and ESCRT functions are required for proper   migration   of fibroblasts. We propose that ligand-mediated degradation of alpha 5 beta 1 integrin via the ESCRT pathway is required in order to prevent endosomal accumulation of ligand-bound integrins that might otherwise form nonproductive adhesion sites. Fibronectin and alpha 5 beta 1 integrin therefore are trafficked to lysosomes in a similar way to growth factors and their receptors. 
 MicroRNA- 130b suppresses   migration   and invasion of colorectal cancer cells through downregulation of integrin β1 [corrected]. MicroRNA 130b (miR-130b) is significantly dysregulated in various human tumor types. In this study, using a microarray assay, we characterized the upregulation of miR-130b expression in colorectal cancer (CRC) specimens. However, there is limited knowledge about the roles of aberrant miR-130b expression in CRC. Our studies in CRC cells demonstrated that miR-130b significantly decreases cell   migratio  n and invasion, but it has no evidently effects on cell proliferation and apoptosis. In the overexpression miR-130b CRC cells and the CRC specimens, we observed a decreased level of integrin β1 protein, which is considered as a key molecule involved in cell motility. The targeting of the 3'-UTR region of integrin β1 gene by miR-130b was revealed using a luciferase reporter assay. The regulation of integrin β1 by miR-130b was further shown using the miR-130b mimics and the inhibitor of miR-130b. The impaired motility of the miR-130b overexpression cells is recovered partly by the expression of integrin β1 lacking the 3'-UTR. Additionally, the knockdown of integrin β1 also gives rise to a decrease in   cell mig  ration and invasion, which is similar to the impeded motility due to overexpression of miR-130b in CRC cells. Furthermore, the inverse expressions of miR-130b and integrin β1 were observed in CRC specimens. In summary, these data demonstrate that miR-130b downregulates its target-integrin β1, leading to the i  mpaired m  igration and invasion of CRC cells. 
 ECRG2 regulates cell   migration  /invasion through urokinase-type plasmin activator receptor (uPAR)/beta1 integrin pathway. ECRG2 is a novel gene that shows sequence similarity to KAZAL-type serine protease inhibitor. We have previously demonstrated that ECRG2 inhibits   migration  /invasion of lung cancer PG cells. However, the mechanism by which ECRG2 performs these activities is a compelling question. Urokinase-type plasmin activator (uPA) binding to uPAR induces   migration  /invasion through multiple interactors including integrins. In this study, we found that ECRG2 binds specifically to the kringle domain of uPA. Moreover, we demonstrated that ECRG2 forms a complex with uPA.uPAR, that such a complex modifies the dynamical association of uPAR with beta1 integrins, and that disruption inhibits Src/MAP (mitogen-activated protein) kinase pathway, resulting in suppression of cell   migration  /invasion in an in vitro Matrigel   migration  /invasion assay. Conversely, depletion of ECRG2 markedly enhanced the association of uPAR with beta1 integrins, elevated basal Src/MAP kinase activation, and stimulated HT1080, MDA-MB-231, and MCF-7 cell   migration  /invasion. Together, our results provide evidence that ECRG2 is involved in the regulation of   migration  /invasion through uPA/uPAR/beta1 integrins/Src/MAP kinase pathway and may represent a novel therapeutic target for cancer. 
 Integrin-mediated functional polarization of Caco-2 cells through E-cadherin--actin complexes. Enterocyte differentiation is a dynamic process during which reinforcement of cell-cell adhesion favours   migration   along the crypt-to-villus axis. Functional polarization of Caco-2 cells, the most commonly used model to study intestinal differentiation, is assessed by dome formation and tightness of the monolayer and is under the control of the extracellular matrix (ECM). Furthermore, our biochemical and confocal microscopy data demonstrate that the ECM dramatically reinforces E-cadherin targeting to the upper lateral membrane, formation of the apical actin cytoskeleton and its colocalization with E-cadherin in functional complexes. In our model, these effects were produced by native laminin-5-enriched ECM as well as by type IV collagen or laminin 2, which suggests a common pathway of induction through integrin receptors. Indeed, these effects were antagonized by blocking anti-beta1- and anti-alpha6-integrin antibodies and directly induced by a stimulating anti-beta1-integrin antibody. These results demonstrate that integrin-dependent cell to ECM adhesion reinforces E-cadherin-dependent cell-cell adhesion in Caco-2 cells and further support the notion that enterocyte differentiation is supported by a molecular crosstalk between the two adhesion systems of the cell. 
 Crucial role of fibroblasts in regulating epidermal morphogenesis. Epidermis reconstructed on de-epidermized dermis (DED) was used to investigate whether fibroblasts can substitute growth factors needed for generation of a fully differentiated epidermis. For this purpose, a centrifugal seeding method was developed to reproducibly incorporate different fibroblast numbers into DED. Using (immuno)histochemical techniques, we could demonstrate that in the absence of fibroblasts the formed epidermis consisted only of two to three viable cell layers with a very thin stratum corneum layer. However, in the presence of fibroblasts keratinocyte proliferation and   migration   was stimulated and epidermal morphology markedly improved. The stimulatory effect of fibroblasts showed a biphasic character: keratinocyte proliferation increased in the initial phase but decreased in later stages of cell culture. After 3 weeks culture at the air-liquid interface, the proliferation index decreased irrespective of the number of fibroblasts present within the dermal matrix to levels observed also in native epidermis. Keratin 10 was localized in all viable suprabasal cell layers irrespective of the absence or presence of fibroblasts. Keratin 6 was downregulated with increasing numbers of fibroblasts, and keratins 16 and 17 were absent in fibroblast-populated matrices. The expression of involucrin or transglutaminase 1 showed a similar pattern as for the keratins. Irrespective of the number of fibroblasts incorporated into DED, the expression of alpha(3), alpha(6), beta(1), and beta(4) integrin subunits was upregulated. In fibroblast-free DED matrices normalization of epidermal differentiation was only achieved when the culture medium was supplemented by keratinocyte growth factor. The results of this study indicate that normalization of epidermal differentiation can be achieved using a non-contractile dermal matrix populated with fibroblasts. 
 Lubrol-RAFTs in melanoma cells: a molecular platform for tumor-promoting ephrin-B2-integrin-beta1 interaction. Ephrins control cell motility and matrix adhesion. These functions play a pivotal role in cancer progression, for example, in malignant melanomas. We have previously shown that the ephrin-B2-tumor-promoting action is partly mediated by integrin-beta1 interaction. However, the subcellular prerequisites for molecular interaction like molecular proximity and co-compartmentalization have not been elucidated yet. Specific cholesterol-rich microdomains, termed lipid rafts (RAFTs), are known to be essential for functional ephrin-B2 signalling and integrin-mediated effects. Therefore, we addressed the question whether RAFT co-compartmentalization of both molecules could provide the molecular platform for their tumor-promoting interaction. In this study, we show that overexpressed ephrin-B2 is not only compartmentalized to classical Triton X-100 RAFTs in B16 melanoma cells, but also to the recently defined Lubrol-RAFTs. Interestingly, in the melanoma cells investigated, integrin-beta1 is also preferentially detected in such Lubrol-RAFTs. Accordingly, the presence of ephrin-B2 and integrin-beta1 in RAFTs and their function in cell   migration   and matrix attachment are highly sensitive to RAFT disruption by cholesterol depletion. Confocal fluorescence microscopy analyses also support the concept of a close molecular proximity and functional interplay of ephrin-B2 and integrin-beta1 in the plasma membrane. We conclude that Lubrol-RAFTs probably represent the platform for tumor-promoting ephrin-B2-integrin-beta1 interaction, which could become an interesting target for future antitumoral therapies. 
   Migration   of renal carcinoma cells is dependent on protein kinase Cdelta via beta1 integrin and focal adhesion kinase.   Migration   and adhesion of tumor cells are essential prerequisites for the formation of metastases in malignant diseases. Protein kinase C (PKC) has been shown to regulate cell   migration  , adhesion and proliferation. In order to identify a connection between PKC isoforms and tumor progression in renal cell carcinoma (RCC), the influence of PKC isoforms on cell   migration  , adhesion and proliferation and possible influences of the activity of integrins and focal adhesion kinase (FAK) were analyzed in RCC cells. The experiments were performed in the RCC cell line CCF-RC1 after pre-incubation of the cells with the PKC inhibitors GF109203X, GO6976, RO31-8220 and rottlerin. Cell   migration   and adhesion were assessed through chemotaxis analysis and adhesion to an endothelial monolayer, respectively. Cell proliferation was analysed by a BrdU incorporation assay. The expression and activity of beta1 integrins and FAK were analysed by Western blot analysis. GF109203X reduced cell   migration   to 69%, the activity of beta1 integrins to 63% and FAK expression to 82% compared to untreated cells. Rottlerin reduced cell   migration   in a concentration-dependent manner to 36%, cell proliferation to 81%, expression and activity of beta1 integrins to 72 and 79%, and expression and activity of FAK to 56 and 76% of untreated cells, respectively. RO31-8220 also reduced the expression and activity of beta1 integrins as well as the expression of FAK to 84, 66 and 66% of untreated cells, respectively. GO6976 reduced the expression of FAK to 60% of untreated cells. Cell   migration   was only slightly reduced by GO6976 to 84% of untreated cells, and cell adhesion remained uninfluenced. These findings show a critical role of PKCdelta in the regulation of tumor cell   migration  , which seems to be caused by affecting the expression and activity of beta1 integrins and FAK. These results can provide a basis for new strategies in preventing metastases of renal cell carcinoma. 
 Gene expression profiling combined with functional analysis identify integrin beta1 (ITGB1) as a potential prognosis biomarker in triple negative breast cancer. Triple negative breast cancer (TNBC) accounts for approximately 15-20% of all types of breast cancer, and treatment is still limited. This type of breast cancer shows a high risk of recurrence, visceral metastasis, a worse prognosis, and shorter distant metastasis-free survival. Several studies have been reported that genetics factors are associated with breast cancer disease progression and patients' survival. In this study, we combined Taiwanese microarray data from the GEO database and The Cancer Genome Atlas (TCGA) database to study the role of Integrin Beta1 (ITGB1) in TNBC. Two triple negative breast cancer cell lines (MDA-MB-231; MDA-MB-468) were used to validate the functions of ITGB1. We found that a higher ITGB1 gene expression level was associated to lower survival. Silencing of ITGB1 inhibited TNBC cell   migration  , invasion and store-operated calcium influx. Our study provided a potential candidate biomarker for breast cancer cells   migration  , invasion and TNBC patients' survival. 
 Tiam1 is recruited to β1-integrin complexes by 14-3-3ζ where it mediates integrin-induced Rac1 activation and motility. 14-3-3 is an adaptor protein that localizes to the leading edge of spreading cells, returning to the cytoplasm as spreading ceases. Previously, we showed that integrin-induced Rac1 activation and spreading were inhibited by sequestration of 14-3-3ζ and restored by its overexpression. Here, we determined whether 14-3-3 mediates integrin signaling by localizing a guanine nucleotide exchange factor (GEF) to Rac1-activating integrin complexes. We showed that GST-14-3-3ζ recruited the Rac1-GEF, Tiam1, from cell lysates through Tiam1 residues 1-182 (N(1-182) Tiam1). The physiological relevance of this interaction was examined in serum-starved Hela cells plated on fibronectin. Both Tiam1 and N(1-182) Tiam1 were recruited to 14-3-3-containing β1-integrin complexes, as shown by co-localization and co-immunoprecipitation. Integrin-induced Rac1 activation was inhibited when Tiam1 was depleted with siRNA or by overexpression of catalytically inactive N(1-182) Tiam1, which was incorporated into 14-3-3/β1-integrin complexes and inhibited spreading in a manner that was overcome by constitutively active Rac1. Integrin-induced Rac1 activation, spreading  , and mig  ration were also inhibited by overexpression of 14-3-3ζ S58D, which was unable to recruit Tiam1 from lysates, co-immunoprecipitate with Tiam1, or mediate its incorporation into β1-integrin complexes. Taken together, these findings suggest a previously unrecognized mechanism of integrin-induced Rac1 activation in which 14-3-3 dimers localize Tiam1 to integrin complexes, where it mediates integrin-dependent Rac1 activation, thus initiating motility-inducing pathways. Moreover, since Tiam1 is recruited to other sites of localized Rac1 activation through its PH-CC-EX domain, the present findings show that a mechanism involving its N-terminal 182 residues is utilized to recruit Tiam1 to motility-inducing integrin complexes. 
 Prognostic role of integrin β1, E-cadherin, and rac1 expression in small cell lung cancer. Integrin β(1) mediates cellular adhesion to the extracellular matrix (ECM) and is correlated with highly invasive and metastatic behavior in small cell lung cancer (SCLC). E-cadherin (ECAD) is a calcium-dependent cell-cell adhesion receptor that restricts invasion of cells and reduces metastasis. Rac1 is involved in the regulation of the actin cytoskeleton, adhesion  , migrati  on, invasion, and tumor metastasis. The aim of this study was to examine integrin β(1) , ECAD and rac1 expression in SCLC and to analyze the prognostic value of these markers in patients with SCLC. We analyzed integrin β(1) , ECAD, and rac1 expression in 112 SCLC tissues by immunohistochemical staining. Correlative analyses between integrin β(1) , ECAD, and rac1 expression and cliniopathological factors were performed. A total of 65 patients had extensive disease (ED) (58%), and 47 had limited disease (LD) (42%). The median follow-up duration was 61 months (range: 14-117 months), and the median progression free survival (PFS) and overall survival (OS) were 6.1 months (range: 4.8-7.4 months) and 9.7 months (range: 8.1-11.3 months), respectively. The expression of integrin β(1) , ECAD, and rac1 protein was observed in 64, 73, and 99 of SCLC tissues, respectively. The correlative analyses between integrin β(1) , ECAD, or rac1 expression and various clinical parameters did not show any statistical significance. However, the ECAD expression was associated with OS in the entire cohort. In contrast, the expression of integrin β(1) and rac1 was not associated with PFS or OS. In a subgroup analysis, patients with less than two metastasis had significantly longer OS (p = 0.047) if their tumors expressed integrin β(1) compared to those without integrin β(1) expression. In addition, OS was longer for patients with ECAD positive tumors compared to those whose tumors did not express ECAD in males (p = 0.032) and patients who never smoked (p < 0.001). Multivariate analysis showed that LD (p = 0.004), overall response rate (p = 0.003), and expression of ECAD (p = 0.015) were the independent good prognostic factors for OS. LD (p = 0.024), overall response rate (p < 0.001), and less than two metastasis (p = 0.003) were prognostic factors for longer PFS. These results suggest that ECAD expression may be useful as a prognostic indicator in patients with SCLC. 
 Characterization of the oligosaccharide component of alpha3beta1 integrin from human bladder carcinoma cell line T24 and its role in adhesion and   migration  . Malignant transformation is highly associated with altered expression of cell surface N-linked oligosaccharides. These changes concern integrins, a family of cell surface glycoproteins involved in the attachment and   migration   of cells on various extracellular matrix proteins. The integrin alpha3beta1 is particularly interesting because of its role in   migration   and invasion of several types of metastatic tumours. In this study, alpha3beta1 from human bladder T24 carcinoma cells was purified and treated with peptide N-glycosidase F. Then the N-glycans of the alpha3 and beta1 subunits were characterized using matrix-assisted laser desorption ionization mass spectrometry (MALDI MS). In alpha3beta1 integrin the presence of high-mannose, hybrid and predominantly complex type N-oligosaccharides was shown. Unlike to normal epithelium cells, in both subunits of alpha3beta1 integrin from cancer cells, the sialylated tetraantennary complex type glycan Hex7HexNAc6FucSia4 was present. In a direct ligand binding assay, desialylated alpha3beta1 integrin exhibited significantly higher fibronectin-binding capability than untreated integrin, providing evidence that sialic acids play a direct role in ligand-receptor interaction. Moreover, alpha3beta1 integrin was shown to take part in T24 cell   migration   on fibronectin: anti-alpha3 antibodies induced ca 30% inhibition of wound closure. Treatment of T24 cells with swainsonine reduced the rate of bladder carcinoma cell   migration   by 16%, indicating the role of beta1,6 branched complex type glycans in this process. Our data show that alpha3beta1 integrin function may be altered by glycosylation, that both subunits contribute to these changes, and that glycosylation may be considered a newly found mechanism in the regulation of integrin function. 
 Plakophilin 2 affects cell   migration   by modulating focal adhesion dynamics and integrin protein expression. Plakophilin 2 (PKP2), a desmosome component, modulates the activity and localization of the small GTPase RhoA at sites of cell-cell contact. PKP2 regulates cortical actin rearrangement during junction formation, and its loss is accompanied by an increase in actin stress fibers. We hypothesized that PKP2 may regulate focal adhesion dynamics and cell   migration  . Here we show that PKP2-deficient cells bind efficiently to the extracellular matrix, but upon spreading display total cell areas ≈ 30% smaller than control cells. Focal adhesions in PKP2-deficient cells are ≈ 2 × larger and more stable than in control cells, and vinculin displays an increased time for fluorescence recovery after photobleaching. Furthermore, β4 and β1 integrin protein and mRNA expression is elevated in PKP2-silenced cells. Normal focal adhesion phenotypes can be restored in PKP2-null cells by dampening the RhoA pathway or silencing β1 integrin. However, integrin expression levels are not restored by RhoA signaling inhibition. These data uncover a potential role for PKP2 upstream of β1 integrin and RhoA in integrating cell-cell and cell-substrate contact signaling in basal keratinocytes necessary for the morphogenesis, homeostasis, and reepithelialization of the stratified epidermis. 
 Role of UDP-N-acetylglucosamine2-epimerase/N-acetylmannosamine kinase (GNE) in β1-integrin-mediated cell adhesion. Hereditary inclusion body myopathy (GNE myopathy) is a neuromuscular disorder due to mutation in key sialic acid biosynthetic enzyme, GNE. The pathomechanism of the disease is poorly understood as GNE is involved in other cellular functions beside sialic acid synthesis. In the present study, a HEK293 cell-based model system has been established where GNE is either knocked down or over-expressed along with pathologically relevant GNE mutants (D176V and V572L). The subcellular distribution of recombinant GNE and its mutant showed differential localization in the cell. The effect of mutation on GNE function was investigated by studying hyposialylation of cell membrane receptor, β1-integrin. Hyposialylated β1-integrin localized to internal vesicles that was restored upon supplementation with sialic acid. Fibronectin stimulation caus  ed migrat  ion of hyposialylated β1-integrin to the cell membrane and co-localization with focal adhesion kinase (FAK) leading to increased focal adhesion formation. This further activated FAK and Src, downstream signaling molecules and led to increased cell adhesion. This is the first report to show that mutation in GNE affects β1-integrin-mediated cell adhesion process in GNE mutant cells. 
 CD151 promotes α3β1 integrin-dependent organization of carcinoma cell junctions and restrains collective cell invasion. Integrins function in collectiv  e migrati  on both as major receptors for extracellular matrix and by crosstalk to adherens junctions. Despite extensive research, important questions remain about how integrin signaling mechanisms are integrated into collectiv  e migrati  on programs. Tetraspanins form cell surface complexes with a subset of integrins and thus are good candidates for regulating the balance of integrin functional inputs into cell-matrix and cell-cell interactions. For example, tetraspanin CD151 directly associates with α3β1 integrin in carcinoma cells and promotes rapid α3β1-dependent single cell motility, but CD151 also promotes organized adherens junctions and restrains collective carcinoma   cell mig  ration on 2D substrates. However, the individual roles of CD151s integrin partners in CD151s pro-junction activity in carcinoma cells were not well understood. Here we find that CD151 promotes organized carcinoma cell junctions via α3β1 integrin, by a mechanism that requires the a3b1 ligand, laminin-332. Loss of CD151 promotes collective 3D invasion and growth in vitro and in vivo, and the enhanced invasion of CD151-silenced cells is α3 integrin dependent, suggesting that CD151 can regulate the balance between α3β1s pro-junction and pro-migratory activities in collective invasion. An analysis of human cancer cases revealed that changes in CD151 expression can be linked to either better or worse clinical outcomes depending on context, including potentially divergent roles for CD151 in different subsets of breast cancer cases. Thus, the role of the CD151-α3β1 complex in carcinoma progression is context dependent, and may depend on the mode of tumor cell invasion. 
 Transient down-regulation of beta1 integrin subtypes on kidney carcinoma cells is induced by mechanical contact with endothelial cell membranes. Adhesion molecules of the integrin beta1 family are thought to be involved in the malignant progression renal cell carcinoma (RCC). Still, it is not clear how they contribute to this process. Since the hematogenous phase of tumour dissemination is the rate-limiting step in the metastatic process, we explored beta1 integrin alterations on several RCC cell lines (A498, Caki1, KTC26) before and after contacting vascular endothelium in a tumour-endothelium (HUVEC) co-culture assay. Notably, alpha2, alpha3 and alpha5 integrins became down-regulated immediately after the tumour cells attached to HUVEC, followed by re-expression shortly thereafter. Integrin down-regulation on RCC cells was caused by direct contact with endothelial cells, since the isolated endothelial membrane fragments but not the cell culture supernatant contributed to the observed effects. Integrin loss was accompanied by a reduced focal adhesion kinase (FAK) expression, FAK activity and diminished binding of tumour cells to matrix proteins. Furthermore, intracellular signalling proteins RCC cells were altered in the presence of HUVEC membrane fragments, in particular 14-3-3 epsilon, ERK2, PKCdelta, PKCepsilon and RACK1, which are involved in regulating tumour cell motility. We, therefore, speculate that contact of RCC cells with the vascular endothelium converts integrin-dependent adhesion to integrin-independent cell movement. The process of dynamic integrin regulation may be an important part in tumour cell   migration   strategy, switching the cells from being adhesive to becoming motile and invasive. 
 Human mast cells adhere to and migrate on epithelial and vascular basement membrane laminins LM-332 and LM-511 via alpha3beta1 integrin. Mast cells (MCs) are multifunctional effectors of the immune system that are distributed in many tissues, often in close association with the basement membrane of blood vessels, epithelium and nerves. Laminins (LMs), a family of large alphabetagamma heterotrimeric proteins, are major components of basement membrane that strongly promote cell adhesion and   migration  . In this study, we investigated the role of LM isoforms and their integrin receptors in human MC biology in vitro. In functional assays, alpha3-(LM-332) and alpha5-(LM-511) LMs, but not alpha1-(LM-111), alpha2-(LM-211), or alpha4-(LM-411) LMs, readily promoted adhesion and   migration   of cultured MCs. These activities were strongly enhanced by various stimuli. alpha3-LM was also able to costimulate IL-8 production. Among LM-binding integrins, MCs expressed alpha(3)beta(1), but not alpha(6)beta(1), alpha(7)beta(1), or alpha(6)beta(4), integrins. Blocking Abs to alpha(3)beta(1) integrin caused inhibition of both cell adhesion and   migration   on alpha3- and alpha5-LMs. Immunohistochemical studies on skin showed that MCs colocalized with epithelial and vascular basement membranes that expressed alpha3- and alpha5-LMs and that MCs expressed alpha(3) integrin but not alpha(6) integrin(s). These results demonstrate a role for alpha3- and alpha5-LMs and their alpha(3)beta(1) integrin receptor in MC biology. This may explain the intimate structural and functional interactions that MCs have with specific basement membranes. 
 Complementary DNA arrays identify CD63 tetraspanin and alpha3 integrin chain as differentially expressed in low and high metastatic human colon carcinoma cells. Malignant tumor cell invasion is determinant for metastasis to occur. E2 and C5 colon carcinoma cells that were derived from the parental Lovo line and that differ experimentally in spontaneous metastatic ability have been monitored for gene expression by cDNA arrays. Among genes found differentially expressed, the CD63 tetraspanin, not previously recognized in colon cancer progression, and the alpha3 integrin chain were both up-regulated in low metastatic E2 cells and were analyzed for their functional role using adhesion,   migration  , and invasion assays. Cell surface expression of CD63 and alpha3 integrin was about 2-fold higher in E2 than in C5 cells and confocal microscopy showed that CD63 and alpha3 integrin colocalized evenly on C5 cells whereas they concentrated at elongated tips of the low-metastatic more substrate-adhesive E2 cells. Antibody-interference experiments identified laminin-5 (LN-5) as a ligand interacting with the alpha3beta1/CD63 complex. Substrate-immobilized anti-CD63 antibodies enhanced tumor cell   migration   and invasion and induced prominent cell surface protrusions that were repressed by the PI3-kinase LY294002 inhibitor. Our results suggest that changes in the expression of surface CD63 and alpha3beta1 integrin interacting with LN-5 could affect migratory signals and the progression of the metastatic disease. 
 Transforming growth factor-β1 induces epithelial-mesenchymal transition and integrin α3β1-mediated ce  ll migrat  ion of HSC-4 human squamous cell carcinoma cells through Slug. We investigated whether transforming growth factor (TGF)-β1 promoted epithelial-mesenchymal transition (EMT)   and migra  tion of human oral squamous cell carcinoma (hOSCC) cells. Among 6 hOSCC cell lines investigated, Smad2 phosphorylation and TGF-β target genes expression were most clearly upregulated following TGF-β1 stimulation in HSC-4 cells, indicating that HSC-4 cells were the most responsive to TGF-β1. In addition, the expression levels of the mesenchymal markers N-cadherin and vimentin were most clearly induced in HSC-4 cells among the hOSCC cell lines by TGF-β1 stimulation. Interestingly, E-cadherin and β-catenin at the cell surface were internalized in HSC-4 cells stimulated with TGF-β1. In addition, the expression levels of the EMT-related transcription factor Slug was significantly upregulated on TGF-β1 stimulation. Moreover, the downregulation of Slug by RNA interference clearly inhibited the TGF-β1-induced expression of mesenchymal mar  ker and t  he migration of HSC-4 cells. Proteomics analysis also revealed that the expression levels of integrin α3β1-targeted proteins were upregulated in TGF-β1-stimulated HSC-4 cells. Neutral antibodies against integrin α3 and β1, as well as a focal adhesion kinase (FAK) inhibitor, clearly suppressed TGF  -β1-indu  ced cell migration. These results suggest that the EMT and integrin α3β1/F  AK pathwa  y-mediated migration of TGF-β1-stimulated HSC-4 hOSCC cells is positively controlled by Slug. 
 Ganglioside GM3 blocks the activation of epidermal growth factor receptor induced by integrin at specific tyrosine sites. The epidermal growth factor receptor (EGFR) can be activated by both direct ligand binding and cross-talk with other molecules, such as integrins. This integrin-mediated cross-talk with growth factor receptors participates in regulating cell proliferation, survival,   migration  , and invasion. Previous studies have shown that ligand-dependent EGFR activation is inhibited by GM3, the predominant ganglioside of epithelial cells, but the effect of GM3 on ligand-independent, integrin-EGFR cross-talk is unknown. Using a squamous carcinoma cell line we show that endogenous accumulation of GM3 disrupts the ligand-independent association of the integrin beta1 subunit with EGFR and results in inhibition of cell proliferation. Consistently, endogenous depletion of GM3 markedly increases the association of EGFR with tyrosine-phosphorylated integrin beta1 and promotes cell proliferation. The ligand-independent stimulation of EGFR does not require focal adhesion kinase phosphorylation or cytoskeletal rearrangement. Stimulation of EGFR and mitogen-activated protein kinase signaling by GM3 depletion involves the phosphorylation of EGFR at tyrosine residues 845, 1068, and 1148 but not 1086 or 1173. The specific blockade of phosphorylation at Tyr-845 with Src family kinase inhibition and at Tyr-1148 with phosphatidylinositol 3-kinase inhibition suggests that GM3 inhibits integrin-induced, ligand-independent EGFR phosphorylation (cross-talk) through suppression of Src family kinase and phosphatidylinositol 3-kinase signaling. 
 Alpha1beta1 integrin is crucial for accumulation of epidermal T cells and the development of psoriasis. Psoriasis is a common T cell-mediated autoimmune inflammatory disease. We show that blocking the interaction of alpha1beta1 integrin (VLA-1) with collagen prevented accumulation of epidermal T cells and immunopathology of psoriasis. Alpha1beta1 integrin, a major collagen-binding surface receptor, was exclusively expressed by epidermal but not dermal T cells. Alpha1beta1-positive T cells showed characteristic surface markers of effector memory cells and contained high levels of interferon-gamma but not interleukin-4. Blockade of alpha1beta1 inhibited   migration   of T cells into the epidermis in a clinically relevant xenotransplantation model. This was paralleled by a complete inhibition of psoriasis development, comparable to that caused by tumor necrosis factor-alpha blockers. These results define a crucial role for alpha1beta1 in controlling the accumulation of epidermal type 1 polarized effector memory T cells in a common human immunopathology and provide the basis for new strategies in psoriasis treatment focusing on T cell-extracellular matrix interactions. 
 The alpha 4 beta 1 fibronectin ligands CS-1, Hep II, and RGD induce different intracellular events in B lymphoid cells. Comparison with the effects of the endothelial ligand VCAM-1. The lymphocyte integrin alpha 4 beta 1 is the receptor for the Hep II domain and CS-1 site in fibronectin (Fn) as well as for VCAM-1. We recently showed that upon activation with anti-beta 1 mAb TS2/16, alpha 4 beta 1 also recognizes the RGD Fn sequence. To determine the physiological role of these multiple interactions, we have now studied some intracellular events induced by "resting" and activated alpha 4 beta 1 binding to its different ligands. Analyses of actin and tubulin reorganization upon adhesion of B lymphoid cells to Fn fragments or VCAM-1 showed that VCAM-1, a 38 kDa fragment (Hep II+CS-1), and the CS-1 synthetic peptide induced formation of transient cytoplasmic projections; however, cells attached to a 58 kDa (Hep II) or 80 kDa (RGD) fragments remained rounded. Using transfilter assays, we showed that VCAM-1, 38 kDa and CS-1 also induced dose-dependent B cell   migration   mediated by alpha 4 beta 1. Furthermore, these three ligands, but not the 80 kDa fragment or a synthetic peptide (H1) containing a sequence from Hep II shown to bind alpha 4 beta 1, induced tyrosine phosphorylation of a 110 kDa protein. Activation of alpha 4 beta 1 with TS2/16 inhibited the cytoplasmic protrusions and cell   migration   but did not affect the pattern of phosphorylation. Our results indicate that the various alpha 4 beta 1 ligands induce different cellular responses. Most importantly they show that alpha 4 beta 1 interaction with CS-1 is sufficient to trigger intracellular events in B cells. Furthermore, they suggest a regulation by the activation form of the receptor as well as by the ligand in events involving lymphocyte adhesion and   migration  . 
 SCAI acts as a suppressor of cancer cell invasion through the transcriptional control of beta1-integrin. Gene expression reprogramming governs cellular processes such as proliferation, differentiation and cell   migration   through the complex and tightly regulated control of transcriptional cofactors that exist in multiprotein complexes. Here we describe SCAI (suppressor of cancer cell invasion), a novel and highly conserved protein that regulates invasive cell   migration   through three-dimensional matrices. SCAI acts on the RhoA-Dia1 signal transduction pathway and localizes in the nucleus, where it binds and inhibits the myocardin-related transcription factor MAL by forming a ternary complex with serum response factor (SRF). Genome-wide expression analysis surprisingly reveals that one of the strongest upregulated genes after suppression of SCAI is beta1-integrin. Decreased levels of SCAI are tightly correlated with increased invasive cell   migration  , and SCAI is downregulated in several human tumours. Functional analysis of the beta1-integrin gene strongly argues that SCAI is a novel transcriptional cofactor that controls gene expression downstream of Dia1 to dictate changes in cell invasive behaviour. 
 miRNA-29c suppresses lung cancer cell adhesion to extracellular matrix and metastasis by targeting integrin β1 and matrix metalloproteinase2 (MMP2). Our pilot study using miRNA arrays found that miRNA-29c (miR-29c) is differentially expressed in the paired low-metastatic lung cancer cell line 95C compared to the high-metastatic lung cancer cell line 95D. Bioinformatics analysis shows that integrin β1 and matrix metalloproteinase 2 (MMP2) could be important target genes of miR-29c. Therefore, we hypothesized that miR-29c suppresses lung cancer cell adhesion to extracellular matrix (ECM) and metastasis by targeting integrin β1 and MMP2. The gain-of-function studies that raised miR-29c expression in 95D cells by using its mimics showed reductions in cell proliferation, adhesion to ECM, invasion a  nd migrat  ion. In contrasts, loss-of-function studies that reduced miR-29c by using its inhibitor in 95C cells promoted proliferation, adhesion to ECM, invasion a  nd migrat  ion. Furthermore, the dual-luciferase reporter assay demonstrated that miR-29c inhibited the expression of the luciferase gene containing the 3'-UTRs of integrin β1 and MMP2 mRNA. Western blotting indicated that miR-29c downregulated the expression of integrin β1 and MMP2 at the protein level. Gelatin zymography analysis further confirmed that miR-29c decreased MMP2 enzyme activity. Nude mice with xenograft models of lung cancer cells confirmed that miR-29c inhibited lung cancer metastasis in vivo, including bone and liver metastasis. Taken together, our results demonstrate that miR-29c serves as a tumor metastasis suppressor, which suppresses lung cancer cell adhesion to ECM and metastasis by directly inhibiting integrin β1 and MMP2 expression and by further reducing MMP2 enzyme activity. The results show that miR-29c may be a novel therapeutic candidate target to slow lung cancer metastasis. 
 Regulation of human beta-cell adhesion, motility, and insulin secretion by collagen IV and its receptor alpha1beta1. Collagens have been shown to influence the survival and function of cultured beta-cells; however, the utilization and function of individual collagen receptors in beta-cells is largely unknown. The integrin superfamily contains up to five collagen receptors, but we have determined that alpha(1)beta(1) is the primary receptor utilized by both fetal and adult beta-cells. Cultured beta-cells adhered to and migrated on collagen type IV (Col-IV), and these responses were mediated almost exclusively by alpha(1)beta(1). The   migration   of cultured beta-cells to Col-IV significantly exceeded that to other matrix components suggesting that this substrate is of unique importance for beta-cell motility. The interaction of alpha(1)beta(1) with Col-IV also resulted in significant insulin secretion at basal glucose concentrations. A subset of beta-cells in developing islets was confirmed to express alpha(1)beta(1), and this expression co-localized with Col-IV in the basal membranes of juxtaposed endothelial cells. Our findings indicate that alpha(1)beta(1) and Col-IV contribute to beta-cell functions known to be important for islet morphogenesis and glucose homeostasis. 
 Alpha4beta1 integrin and 190-kDa CD44v constitute a cell surface docking complex for gelatinase B/MMP-9 in chronic leukemic but not in normal B cells. As B-cell chronic lymphocytic leukemia (B-CLL) progresses, malignant cells extravasate and infiltrate lymphoid tissues. Several molecules, including gelatinase B/MMP-9, contribute to these processes. Although mainly a secreted protease, some MMP-9 is present at the B-CLL cell surface and the function, mode of anchoring, and interactions of this MMP-9 are unknown. Here we show that anti-MMP-9 antibodies immunoprecipitated a 190-kDa CD44v isoform and alpha4beta1 integrin from B-CLL cells, but not from normal B cells. Function-blocking antibodies to alpha4beta1 or CD44, or transfection with specific siRNAs, decreased cell-associated proMMP-9 and increased the secreted form. B-CLL cells attached to and bound proMMP-9 and active MMP-9, and this was inhibited by blocking the expression or function of alpha4beta1 or CD44. The MMP-9 hemopexin domain was critical in these interactions. alpha4beta1 and 190-kDa CD44v (but not CD44H) formed a complex at the cell surface, since they both coimmunoprecipitated with anti-alpha4, anti-beta1, or anti-CD44 antibodies. Immunofluorescence analyses confirmed that alpha4beta1 and CD44v colocalized with MMP-9. Binding of proMMP-9 inhibited B-CLL cell   migration  , and this required MMP-9 proteolytic activity. Thus, we have identified alpha4beta1 and CD44v as a novel proMMP-9 cell surface docking complex and show that cell-associated MMP-9 may regulate B-CLL cell   migration   and arrest. 
 Quantitative Glycoproteomic Analysis Identifies Platelet-Induced Increase of Monocyte Adhesion via the Up-Regulation of Very Late Antigen 5. Physiological stimuli, such as thrombin, or pathological stimuli, such as lysophosphatidic acid (LPA), activate platelets circulating in blood. Once activated, platelets bind to monocytes via P-selectin-PSGL-1 interactions but also release the stored contents of their granules. These platelet releasates, in addition to direct platelet binding, activate monocytes and facilitate their recruitment to atherosclerotic sites. Consequently, understanding the changes platelet releasates induce in monocyte membrane proteins is critical. We studied the glyco-proteome changes of THP-1 monocytic cells affected by LPA- or thrombin-induced platelet releasates. We employed lectin affinity chromatography combined with filter aided sample preparation to achieve high glyco- and membrane protein and protein sequence coverage. Using stable isotope labeling by amino acids in cell culture, we quantified 1715 proteins, including 852 membrane and 500 glycoproteins, identifying the up-regulation of multiple proteins involved in monocyte extracellular matrix binding and transendothelial   migration  . Flow cytometry indicated expression changes of integrin α5, integrin β1, PECAM-1, and PSGL-1. The observed increase in monocyte adhesion to fibronectin was determined to be mediated by the up-regulation of very late antigen 5 via a P-selectin-PSGL-1 independent mechanism. This novel aspect could be validated on CD14+ human primary monocytes, highlighting the benefits of the improved enrichment method regarding high membrane protein coverage and reliable quantification. 
 In vivo cleaved CDCP1 promotes early tumor dissemination via complexing with activated β1 integrin and induction of FAK/PI3K/Akt motility signaling. Specific cleavage of the transmembrane molecule, CUB domain-containing protein-1 (CDCP1), by plasmin-like serine proteases induces outside-in signal transduction that facilitates early stages of spontaneous metastasis leading to tumor cell intravasation, namely cell escape from the primary tumor, stromal invasion and transendothelial   migratio  n. We identified active β1 integrin as a biochemical and functional partner of the membrane-retained 70-kDa CDCP1 fragment, newly generated from its full-length 135-kDa precursor though proteolytic cleavage by serine proteases. Both in cell cultures and in live animals, active β1 integrin complexed preferentially with functionally activated, phosphorylated 70-kDa CDCP1. Complexing of β1 integrin the 70-kDa with CDCP1 fragment induced intracellular phosphorylation signaling, involving focal adhesion kinase-1 (FAK) and PI3 kinase (PI3K)-dependent Akt activation. Thus, inhibition of FAK/PI3K activities by specific inhibitors as well as short-hairpin RNA downregulation of β1 integrin significantly reduced FAK/Akt phosphorylation under conditions where CDCP1 was processed by serine proteases, indicating that FAK/PI3K/Akt pathway operates downstream of cleaved CDCP1 complexed with β1 integrin. Furthermore, this complex-dependent signaling correlated positively with high levels of tumor cell intravasation and dissemination. Correspondingly, abrogation in vivo of CDCP1 cleavage either by unique cleavage-blocking monoclonal antibody 10-D7 or by inhibition of proteolytic activity of plasmin-like serine proteases with aprotinin prevented β1 integrin/CDCP1 complexing and downstream FAK/Akt signaling concomitant with significant reduction of stromal invasion and spontaneous metastasis. Therefore, β1 integrin appears to serve as a motility-regulating partner mediating cross-talk between proteolytically cleaved, membrane-retained CDCP1 and members of FAK/PI3K/Akt pathway. This CDCP1 cleavage-induced signaling cascade constitutes a unique mechanism, independent of extracellular matrix remodeling, whereby a proteolytically cleaved CDCP1 regulates in vivo locomotion and metastasis of tumor cells through β1 integrin partnering. Our findings indicate that CDCP1 cleavage, occurring at the apex of a β1 integrin/FAK/PI3K/Akt signaling cascade, may represent a therapeutic target for CDCP1-positive cancers. 
 Identification of a novel interaction between integrin beta1 and 14-3-3beta. Integrins are cell surface receptors for extracellular matrix, which play important roles in a variety of biological processes. 14-3-3 proteins are a highly conserved family of cytoplasmic proteins that associate with several intracellular signaling molecules in regulation of various cellular functions. Here, we report identification of an interaction between the integrin beta1 cytoplasmic domain and 14-3-3beta by using the yeast two-hybrid screen. Like several other proteins, the integrin beta1 cytoplasmic domain associated with 14-3-3beta by a non-phosphoserine mechanism. The 14-3-3beta/integrin beta1 interaction was confirmed by in vitro binding assays as well as co-precipitation in vivo. Furthermore, we found that 14-3-3beta co-localized with integrin beta1 during the early stage of cell spreading on fibronectin, suggesting a potential role of the 14-3-3beta/integrin beta1 interaction in the regulation of cell adhesion. Using tetracycline-regulated expression system, we showed that overexpression of 14-3-3beta stimulated cell spreading and   migration   on fibronectin but not on poly-L-lysine. However, the induced expression of 14-3-3beta did not affect tyrosine phosphorylation of FAK or its substrates, p130(cas) and paxillin, suggesting that 14-3-3beta regulated integrin-mediated cell spreading and   migration   by FAK-independent mechanisms. Taken together, these results identify an interaction between integrin and 14-3-3 proteins and suggest a potentially novel cellular function for 14-3-3 proteins in the regulation of integrin-mediated cell adhesion and signaling events. 
 miR-134 suppresses the   migration   and invasion of non‑small cell lung cancer by targeting ITGB1. Most cancer-related deaths are caused by the development of metastatic disease. Thus, investigation of the underlying mechanisms of metastasis is urgent to design more effective targeted drugs and to treat metastatic disease more effectively. MicroRNAs (miRNAs) have emerged as potential targets for cancer treatment. In the present study, we aimed to identify the roles of miR-134 in non-small cell lung cancer (NSCLC) cel  l migrati  on and invasion. We demonstrated that overexpression of miR-134 inhibite  d migrati  on and invasion of A549 and H1299 cells. Further mechanistic investigations revealed that miR-134 inhibited epithelial-to-mesenchymal transition (EMT) evidenced by upregulation of E-cadherin expression and downregulation of vimentin expression. Using luciferase assays, we identified integrin β1 (ITGB1) as a direct target of miR-134. Performing RNAi and rescue experiments, we confirmed that miR-134 exerted its migratory and invasive suppressive role partly by downregulating ITGB1. Finally, an in vivo experiment also, to some extent, suggested that miR-134 may function as a suppressor of metastasis. Taken together, our findings suggest that miR-134 suppre  sses migr  ation and invasion of NSCLC by targeting ITGB1. 
 OSP/claudin-11 forms a complex with a novel member of the tetraspanin super family and beta1 integrin and regulates proliferation and   migration   of oligodendrocytes. Oligodendrocyte-specific protein (OSP)/claudin-11 is a major component of central nervous system myelin and forms tight junctions (TJs) within myelin sheaths. TJs are essential for forming a paracellular barrier and have been implicated in the regulation of growth and differentiation via signal transduction pathways. We have identified an OSP/claudin-11-associated protein (OAP)1, using a yeast two-hybrid screen. OAP-1 is a novel member of the tetraspanin superfamily, and it is widely expressed in several cell types, including oligodendrocytes. OAP-1, OSP/claudin-11, and beta1 integrin form a complex as indicated by coimmunoprecipitation and confocal immunocytochemistry. Overexpression of OSP/claudin-11 or OAP-1 induced proliferation in an oligodendrocyte cell line. Anti-OAP-1, anti-OSP/claudin-11, and anti-beta1 integrin antibodies inhibited   migration   of primary oligodendrocytes, and   migration   was impaired in OSP/claudin-11-deficient primary oligodendrocytes. These data suggest a role for OSP/claudin-11, OAP-1, and beta1 integrin complex in regulating proliferation and   migration   of oligodendrocytes, a process essential for normal myelination and repair. 
 Monocyte-derived dendritic cells and monocytes migrate to HIV-Tat RGD and basic peptides. Extracellular Tat released from HIV-1-infected cells is a mitogenic and motogenic factor for endothelial and Kaposi's sarcoma (KS)-derived cells and is angiogenic in vivo. Here we show for the first time that Tat induces   migration   of human dendritic cells in a concentration-dependent manner and that the Arg-Gly-Asp (RGD) and basic Tat peptides contribute to dendritic and monocyte cell   migration  . In vivo, Tat stimulates invasion of macrophages into a matrigel sponge.Monocyte and dendritic cell chemotaxis was assessed using the Boyden chamber assay.Tat induced   migration   of monocyte-derived dendritic cells at the same levels as the N-formyl-Met-Leu-Phe peptide, and of monocytes at levels comparable to RANTES. Peptide mapping of the chemotactic activity of Tat showed that the RGD domain, which has been shown to support integrin-mediated cell   migration  , and the basic domain which binds and activates the tyrosine kinase receptor KDR on endothelial cells, both had activity. Antibody-blocking experiments indicate that responses to the RGD domain was inhibited by beta1 and alpha vbeta3 integrin blocking antibodies. Combination of the Tat RGD and basic peptides did not show additive effects; however, Tat co-operated with macrophage-chemotactic protein or RANTES in inducing monocyte   migration  .Our results show that Tat can act as a chemoattractant for dendritic cells, and that both the RGD and basic domains are involved in this response. These same domains attract monocytes. The alpha vbeta3 and beta1 integrins are equally involved in Tat-induced monocyte   migration  , while the alpha vbeta3 integrin largely mediates the dendritic cell response to Tat. 
 NG2 proteoglycan promotes tumor vascularization via integrin-dependent effects on pericyte function. The NG2 proteoglycan stimulates the proliferation and   migration   of various immature cell types, including pericytes. However, the role of NG2 in mediating pericyte/endothelial cell interaction has been less clear. In this study, we show that pericyte-specific NG2 ablation causes several structural deficits in blood vessels in intracranial B16F10 melanomas, including decreased pericyte ensheathment of endothelial cells, diminished formation of endothelial junctions, and reduced assembly of the vascular basal lamina. These deficits result in decreased tumor vessel patency, increased vessel leakiness, and increased intratumoral hypoxia. NG2-dependent mechanisms of pericyte interaction with endothelial cells are further explored in pericyte/endothelial cell co-cultures. siRNA-mediated NG2 knockdown in pericytes leads to reduced formation of pericyte/endothelial networks, reduced formation of ZO-1 positive endothelial cell junctions, and increased permeability of endothelial cell monolayers. We also show that NG2 knockdown results in loss of β1 integrin activation in endothelial cells, revealing a mechanism for NG2-dependent cross talk between pericytes and endothelial cells. 
 Alpha 4 beta 1 integrin/VCAM-1 interaction activates alpha L beta 2 integrin-mediated adhesion to ICAM-1 in human T cells. Modulation of integrin affinity and/or avidity provides a regulatory mechanism by which leukocyte adhesion to endothelium is strengthened or weakened at different stages of e  migration  . In this study, we demonstrate that binding of high-affinity alpha 4 beta 1 integrins to VCAM-1 strengthens alpha L beta 2 integrin-mediated adhesion. The strength of adhesion of Jurkat cells, a human leukemia T cell line, or MnCl2-treated peripheral blood T cells to immobilized chimeric human VCAM-1/Fc, ICAM-1/Fc, or both was quantified using parallel plate flow chamber leukocyte detachment assays in which shear stress was increased incrementally (0.5-30 dynes/cm2). The strength of adhesion to VCAM-1 plus ICAM-1, or to a 40-kDa fragment of fibronectin containing the CS-1 exon plus ICAM-1, was greater than the sum of adhesion to each molecule alone. Treatment of Jurkat or blood T cells with soluble cross-linked VCAM-1/Fc or HP2/1, a mAb to alpha 4, significantly increased adhesion to ICAM-1. These treatments induced clustering of alpha L beta 2 integrins, but not the high-affinity beta 2 integrin epitope recognized by mAb 24. Up-regulated adhesion to ICAM-1 was abolished by cytochalasin D, an inhibitor of cytoskeletal rearrangement. Taken together, our data suggest that the binding of VCAM-1 or fibronectin to alpha 4 beta 1 integrins initiates a signaling pathway that increases beta 2 integrin avidity but not affinity. A role for the cytoskeleton is implicated in this process. 
 Down-regulation of ARNT promotes cancer metastasis by activating the fibronectin/integrin β1/FAK axis. The aryl hydrocarbon receptor nuclear translocator (ARNT) is broadly involved in regulating tumorigenesis by inducing genes that are involved in tumor growth and angiogenesis. Tumorigenesis usually involves normoxic conditions. However, the role of ARNT in tumor metastasis during normoxia remains unclear. Here, we demonstrate that ARNT protein levels were decreased in late-stage human colorectal cancer using immunohistochemical analysis. Down-regulation of ARNT protein promoted cancer cell   migratio  n and invasion, which was mediated by activation of the fibronectin/integrin β1/FAK signaling axis. In addition, the enhancement o  f migrati  on and invasion in ANRT knockdown cells was blocked when ARNT was restored in the cells. In xenografts in severe combined immunodeficiency mice, tumor growth was significantly inhibited in the ARNT-knockdown condition. However, the tail-vein injection animal model revealed that the depletion of ARNT-induced metastatic lung colonies was further enhanced when ARNT expression was recovered post-injection. Interestingly, chemotherapeutic drugs inhibited ARNT expression and promoted the invasion of residual tumor cells. These results suggest that ARNT may play a positive role during tumor growth (either in early-stage tumor growth or in organ metastases), but plays a negative role in tumo  r migrati  on and invasion. Therefore, the efficiency of ARNT-targeted therapy during different cancer stages should be carefully evaluated. 
 Persistent cell   migration   and adhesion rely on retrograde transport of β(1) integrin. Integrins have key functions in cell adhesion and   migratio  n. How integrins are dynamically relocalized to the leading edge in highly polarized migratory cells has remained unexplored. Here, we demonstrate that β1 integrin (known as PAT-3 in Caenorhabditis elegans), but not β3, is transported from the plasma membrane to the trans-Golgi network, to be resecreted in a polarized manner. This retrograde trafficking is restricted to the non-ligand-bound conformation of β1 integrin. Retrograde trafficking inhibition abrogates several β1-integrin-specific functions such as cell adhesion in early embryonic development of mice, and persisten  t cell mi  gration in the developing posterior gonad arm of C. elegans. Our results establish a paradigm according to which retrograde trafficking, and not endosomal recycling, is the key driver for β1 integrin function in highly polarized cells. These data more generally suggest that the retrograde route is used to relocalize plasma membrane machinery from previous sites of function to the leading edge of migratory cells. 
 Alphavbeta integrins play an essential role in BMP-2 induction of osteoblast differentiation. Both integrins and BMP-2 exert similar effects on osteoblasts. We examined the relationship between the alphav-containing integrins (alphavbeta) and BMP-2 in osteoblast function. BMP-2 stimulates alphavbeta expression. BMP-2 receptors co-localize/overlap with alphavbeta integrins, and the intact function of alphavbeta is essential in BMP-2 activity.Bone morphogenetic protein (BMP)-2 not only induces osteoblast differentiation and bone matrix mineralization, but also stimulates osteoblast   migration   on and adhesion to bone matrix proteins. The alphavbeta- and beta1- (alphabeta1) containing integrins mediate osteoblast interaction with many bone matrix proteins and play important roles in osteoblast adhesion,   migration  , and differentiation. Because alphavbeta integrins and BMP-2 share common effects on osteoblasts, we analyzed their relationship in osteoblast function.The effects of BMP-2 on integrin expression were determined by surface labeling/immunoprecipitation and cell adhesion to matrix proteins. Confocal analysis of the immunostained cells and co-immunoprecipitation of cell extracts were used to study the spatial relationship between integrins and BMP-2 receptors. A function-blocking anti-alphavbeta integrin antibody (L230) was employed to investigate the roles of alphavbeta integrins in BMP-2 function.Human osteoblasts (HOBs) express alphabeta1, alphavbeta3, alphavbeta5, alphavbeta6, and alphavbeta8 integrins at focal adhesion sites. BMP-2 increases the levels of these integrins on osteoblast surface and enhances HOB adhesion to osteopontin and vitronectin. Immunoprecipitation and immunostaining analyses show that BMP-2 receptors co-localize or overlap with alphavbeta and alphabeta1 integrins. Incubation of HOBs with L230 abolishes the antiproliferative effect of BMP-2 and reduces the capacity of BMP-2 to stimulate alkaline phosphatase activity and the expression of osteocalcin, osteopontin, and bone sialoprotein. Furthermore, L230 prevents BMP-2 induction of matrix mineralization. Although BMP-2 retains its receptor-binding capability in the presence of L230, BMP-2 stimulation of Smad signaling is abolished by L230.BMP-2 upregulates the expression of alphavbeta integrins, and these integrins, in turn, play a critical role in BMP-2 function in osteoblasts. 
 The anchoring protein RACK1 links protein kinase Cepsilon to integrin beta chains. Requirements for adhesion and motility. Integrin affinity is modulated by intracellular signaling cascades, in a process known as "inside-out" signaling, leading to changes in cell adhesion and motility. Protein kinase C (PKC) plays a critical role in integrin-mediated events; however, the mechanism that links PKC to integrins remains unclear. Here, we report that PKCepsilon positively regulates integrin-dependent adhesion, spreading, and motility of human glioma cells. PKCepsilon activation was associated with increased focal adhesion and lamellipodia formation as well as clustering of select integrins, and it is required for phorbol 12-myristate 13-acetate-induced adhesion and motility. We provide novel evidence that the scaffolding protein RACK1 mediates the interaction between integrin beta chain and activated PKCepsilon. Both depletion of RACK1 by antisense strategy and overexpression of a truncated form of RACK1 which lacks the integrin binding region resulted in decreased PKCepsilon-induced adhesion and   migration  , suggesting that RACK1 links PKCepsilon to integrin beta chains. Altogether, these results provide a novel mechanistic link between PKC activation and integrin-mediated adhesion and motility. 
 HMGB-1 induces cell motility and α5β1 integrin expression in human chondrosarcoma cells. Chondrosarcoma is a type of highly malignant tumor with a potent capacity to invade locally and cause distant metastasis. High mobility group box chromosomal protein 1 (HMGB)-1 is a widely studied, ubiquitous nuclear protein that is present in eukaryotic cells, and plays a crucial role in inflammatory response. However, the effects of HMGB-1 on human chondrosarcoma cells are largely unknown. In this study, we found that HMGB-1 increased th  e migrati  on and the expression of α5β1 integrin in human chondrosarcoma cells. Transfection of cells with receptor for advanced glycation end products (RAGE) receptor siRNA reduced HMGB-1-induced c  ell migra  tion and integrin expression. Activations of phosphatidylinositol 3-kinase (PI3K), Akt, and AP-1 pathways after HMGB-1 treatment were demonstrated, and HMGB-1-induced expression of integrin   and migra  tion activity was inhibited by the specific inhibitor and mutant of PI3K, Akt, and AP-1 cascades. Taken together, our results indicated that HMGB-1 enhances   the migra  tion of chondrosarcoma cells by increasing α5β1 integrin expression through the RAGE receptor/PI3K/Akt/c-Jun/AP-1 signal transduction pathway. 
 Integrin cytoplasmic domain-associated protein 1alpha (ICAP-1alpha ) interacts directly with the metastasis suppressor nm23-H2, and both proteins are targeted to newly formed cell adhesion sites upon integrin engagement. Cell adhesion-dependent signaling implicates cytoplasmic proteins interacting with the intracellular tails of integrins. Among those, the integrin cytoplasmic domain-associated protein 1alpha (ICAP-1alpha) has been shown to interact specifically with the beta(1) integrin cytoplasmic domain. Although it is likely that this protein plays an important role in controlling cell adhesion and   migration  , little is known about its actual function. To search for potential ICAP-1alpha-binding proteins, we used a yeast two-hybrid screen and identified the human metastatic suppressor protein nm23-H2 as a new partner of ICAP-1alpha. This direct interaction was confirmed in vitro, using purified recombinant ICAP-1alpha and nm23-H2, and by co-immunoprecipitation from CHO cell lysates over-expressing ICAP-1alpha. The physiological relevance of this interaction is provided by confocal fluorescence microscopy, which shows that ICAP-1alpha and nm23-H2 are co-localized in lamellipodia during the early stages of cell spreading. These adhesion sites are enriched in occupied beta(1) integrins and precede the formation of focal adhesions devoid of ICAP-1alpha and nm23-H2, indicating the dynamic segregation of components of matrix adhesions. This peripheral staining of ICAP-1alpha and nm23-H2 is only observed in cells spreading on fibronectin and collagen and is absent in cells spreading on poly-l-lysine, vitronectin, or laminin. This is consistent with the fact that targeting of both ICAP-1alpha and nm23-H2 to the cell periphery is dependent on beta(1) integrin engagement rather than being a consequence of cell adhesion. This finding represents the first evidence that the tumor suppressor nm23-H2 could act on beta(1) integrin-mediated cell adhesion by interacting with one of the integrin partners, ICAP-1alpha. 
 Vav1 and Rac control chemokine-promoted T lymphocyte adhesion mediated by the integrin alpha4beta1. The chemokine CXCL12 promotes T lymphocyte adhesion mediated by the integrin alpha4beta1. CXCL12 activates the GTPase Rac, as well as Vav1, a guanine-nucleotide exchange factor for Rac, concomitant with up-regulation of alpha4beta1-dependent adhesion. Inhibition of CXCL12-promoted Rac and Vav1 activation by transfection of dominant negative Rac or Vav1 forms, or by transfection of their siRNA, remarkably impaired the increase in T lymphocyte attachment to alpha4beta1 ligands in response to this chemokine. Importantly, inhibition of Vav1 expression by RNA interference resulted in a blockade of Rac activation in response to CXCL12. Adhesions in flow chambers and soluble binding assays using these transfectants indicated that initial ligand binding and adhesion strengthening mediated by alpha4beta1 were dependent on Vav1 and Rac activation by CXCL12. Finally, CXCL12-promoted T-cell transendothelial   migration   involving alpha4beta1-mediated adhesion was notably inhibited by expression of dominant negative Vav1 and Rac. These results indicate that activation of Vav1-Rac signaling pathway by CXCL12 represents an important inside-out event controlling efficient up-regulation of alpha4beta1-dependent T lymphocyte adhesion. 
 Epigenetic regulation of galectin-3 expression by β1 integrins promotes cell adhesion and   migratio  n. Introduction of the integrin β1- but not the β3-subunit in GE11 cells induces an epithelial-to-mesenchymal-transition (EMT)-like phenomenon that is characterized by the loss of cell-cell contacts, cell scattering, increased ce  ll migrat  ion and RhoA activity, and fibronectin fibrillogenesis. Because galactose-binding lectins (galectins) have been implicated in these phenomena, we investigated whether galectins are involved in the β1-induced phenotype. We examined 9 galectins and, intriguingly, found that the expression of galectin-3 (Gal-3) is specifically induced by β1 but not by β3. Using β1-β3 chimeric integrins, we show that the induction of Gal-3 expression requires the hypervariable region in the extracellular domain of β1, but not its cytoplasmic tail. Furthermore, Gal-3 expression does not depend on RhoA signaling, serum factors, or any of the major signal transduction pathways involving protein kinase C (PKC), p38 mitogen-activated protein kinase (p38MAPK), extracellular signal-regulated kinase-1/-2 (ERK-1/2), phosphatidylinositol-3-OH kinase (PI3-K), or Src kinases. Instead, Gal-3 expression is controlled in an epigenetic manner. Whereas DNA methylation of the Lgals3 promoter maintains Gal-3 silencing in GE11 cells, expression of β1 causes its demethylation, leading to transcriptional activation of the Lgals3 gene. In turn, Gal-3 expression enhances β1 integrin-mediated cell adhesion to fibronectin (FN) and laminin (LN), as we  ll as cel  l migration. Gal-3 also promotes β1-mediated cell adhesion to LN and Collagen-1 (Col)-1 in cells that endogenously express Gal-3 and β1 integrins. In conclusion, we identify a functional feedback-loop between β1 integrins and Gal-3 that involves the epigenetic induction of Gal-3 expression during integrin-induced EMT and cell scattering. 
 Proteomic analysis of α4β1 integrin adhesion complexes reveals α-subunit-dependent protein recruitment. Integrin adhesion receptors mediate cell-cell and cell-extracellular matrix interactions, which control cell morphology a  nd migrat  ion, differentiation, and tissue integrity. Integrins recruit multimolecular adhesion complexes to their cytoplasmic domains, which provide structural and mechanosensitive signaling connections between the extracellular and intracellular milieux. The different functions of specific integrin heterodimers, such as α4β1 and α5β1, have been attributed to distinct signal transduction mechanisms that are initiated by selective recruitment of adhesion complex components to integrin cytoplasmic tails. Here, we report the isolation of ligand-induced adhesion complexes associated with wild-type α4β1 integrin, an activated α4β1 variant in the absence of the α cytoplasmic domain (X4C0), and a chimeric α4β1 variant with α5 leg and cytoplasmic domains (α4Pα5L), and the cataloguing of their proteomes by MS. Using hierarchical clustering and interaction network analyses, we detail the differential recruitment of proteins and highlight enrichment patterns of proteins to distinct adhesion complexes. We identify previously unreported components of integrin adhesion complexes and observe receptor-specific enrichment of molecules with previously report  ed links   to cell migration and cell signaling processes. Furthermore, we demonstrate colocalization of MYO18A with active integrin in migrating cells. These datasets provide a resource for future studies of integrin receptor-specific signaling events. 
 Decorin regulates endothelial cell motility on collagen I through activation of insulin-like growth factor I receptor and modulation of alpha2beta1 integrin activity. The proteoglycan decorin is expressed by sprouting but not quiescent endothelial cells, and angiogenesis is dysregulated in its absence. Previously, we have shown that decorin core protein can bind to and activate insulin-like growth factor-I receptor (IGF-IR) in endothelial cells. In this study, we show that decorin promotes alpha2beta1 integrin-dependent endothelial cell adhesion and   migration   on fibrillar collagen type I. We provide evidence that decorin modulates cell-matrix interaction in this context by stimulating cytoskeletal and focal adhesion reorganization through activation of the IGF-IR and the small GTPase Rac. Further, the glycosaminoglycan moiety of decorin interacts with alpha2beta1, but not alpha1beta1 integrin, at a site distinct from the collagen I-binding A-domain, to allosterically modulate collagen I-binding activity of the integrin. We propose that induction of decorin expression in angiogenic, as opposed to quiescent, endothelial cells promotes a motile phenotype in an interstitial collagen I-rich environment by both signaling through IGF-IR and influencing alpha2beta1 integrin activity. 
 Structural basis of blocking integrin activation and deactivation for anti-inflammation. Integrins mediate leukocyte accumulation to the sites of inflammation, thereby enhancing their potential as an important therapeutic target for inflammatory disorders. Integrin activation triggered by inflammatory mediators or signaling pathway is a key step to initiate leukocyte   migration   to inflamed tissues; however, an appropriately regulated integrin deactivation is indispensable for maintaining productive leukocyte   migration  . While typical integrin antagonists that block integrin activation target the initiation of leukocyte   migration  , a novel class of experimental compounds has been designed to block integrin deactivation, thereby perturbing the progression of cell   migration  . Current review discusses the mechanisms by which integrin is activated and subsequently deactivated by focusing on its structure-function relationship. 
 Insulin-like growth factor-1 induces adhesion and   migration   in human multiple myeloma cells via activation of beta1-integrin and phosphatidylinositol 3'-kinase/AKT signaling. Insulin-like growth factor-1 (IGF-I) is a growth and survival factor in human multiple myeloma (MM) cells. Here we examine the effect of IGF-I on MM cell adhesion and   migration  , and define the role of beta1 integrin in these processes. IGF-I increases adhesion of MM.1S and OPM6 MM cells to fibronectin (FN) in a time- and dose-dependent manner, as a consequence of IGF-IR activation. Conversely, blocking anti-beta1 integrin monoclonal antibody, RGD peptide, and cytochalasin D inhibit IGF-I-induced cell adhesion to FN. IGF-I rapidly and transiently induces association of IGF-IR and beta1 integrin, with phosphorylation of IGF-IR, IRS-1, and p85(PI3-K). IGF-I also triggers phosphorylation of AKT and ERK significantly. Both IGF-IR and beta1 integrin colocalize to lipid rafts on the plasma membrane after IGF-I stimulation. In addition, IGF-I triggers polymerization of F-actin, induces phosphorylation of p125(FAK) and paxillin, and enhances beta1 integrin interaction with these focal adhesion proteins. Importantly, using pharmacological inhibitors of phosphatidylinositol 3'-kinase (PI3-K) (LY294002 and wortmannin) and extracellular signal-regulated kinase (PD98059), we demonstrate that IGF-I-induced MM cell adhesion to FN is achieved only when PI3-K/AKT is activated. IGF-I induces a 1.7-2.2 (MM.1S) and 2-2.5-fold (OPM6) increase in   migration  , whereas blocking anti-IGF-I and anti-beta1 integrin monoclonal antibodies, PI3-K inhibitors, as well as cytochalasin D abrogate IGF-I-induced MM cell trans  migration  . Finally, IGF-I induces adhesion of CD138+ patient MM cells. Therefore, these studies suggest a role for IGF-I in trafficking and localization of MM cells in the bone marrow microenvironment. Moreover, they define the functional association of IGF-IR and beta1 integrin in mediating MM cell homing, providing the preclinical rationale for novel treatment strategies targeting IGF-I/IGF-IR in MM. 
 Mantle cell lymphoma cells express high levels of CXCR4, CXCR5, and VLA-4 (CD49d): importance for interactions with the stromal microenvironment and specific targeting. Mantle cell lymphoma (MCL) is characterized by an early, widespread dissemination and residual disease after conventional treatment, but the mechanisms responsible for lymphoma cell motility and drug resistance are largely unknown. There is growing evidence suggesting that chemokine receptors and adhesion molecules are critical for malignant B-cell trafficking and homing to supportive tissue microenvironments, where they receive survival and drug resistance signals. Therefore, we examined chemokine receptor and adhesion molecule expression and function in MCL cells and their importance for   migration   and adhesion to marrow stromal cells (MSCs). We found that MCL cells display high levels of functional CXCR4 and CXCR5 chemokine receptors and VLA-4 adhesion molecules. We also report that MCL cells adhere and spontaneously migrate beneath MSCs in a CXCR4- and VLA-4-dependent fashion (pseudoemperipolesis). Moreover, we demonstrate that MSCs confer drug resistance to MCL cells, particularly to MCL cells that migrate beneath MSC. To target MCL-MSC interactions, we tested Plerixafor, a CXCR4 antagonist, and natalizumab, a VLA-4 antibody. Both agents blocked functional responses to the respective ligands and inhibited adhesive interactions between MCL cells and MSCs. These findings provide a rationale to further investigate the therapeutic potential of these drugs in MCL. 
 Clinical and functional significance of α9β1 integrin expression in breast cancer: a novel cell-surface marker of the basal phenotype that promotes tumour cell invasion. Integrin α9β1 is a receptor for ECM proteins, including Tenascin-C and the EDA domain of fibronectin, and has been shown to transduce TGFβ signalling. This study has examined the expression pattern of α9β1 in 141 frozen breast carcinoma samples and related expression to prognostic indices, molecular subtype and patient outcome. Effects of α9β1 on tum  our cell   migration and invasion were assessed using blocking antibody and gene transduction approaches. Integrin α9β1 localized to myoepithelial cells in normal ducts and acini, a pattern maintained in DCIS. A subset (17%) of invasive carcinomas exhibited tumour cell expression of α9β1, which related significantly to the basal-like phenotype, as defined by either CK5/6 or CK14 expression. Tumour expression of α9β1 showed a significant association with reduced overall patient survival (p < 0.0001; HR 5.94, 95%CI 3.26-10.82) and with reduced distant-metastasis-free survival (p < 0.0001; HR 6.37, CI 3.51-11.58). A series of breast cancer cell lines was screened for α9β1 with the highly invasive basal-like GI-101 cell line expressing signific  ant level  s. Both migration and invasion of this line were reduced significantly in the presence of α9-blocking antibody and following α9-knockdown with siRNA. Conversely, migratory and invasive behaviour of α9-negative MCF7 cells and α9-low MDA MB468 cells was enhanced significantly by over-expression of α9. Thus, α9β1 acts as a novel marker of the basal-like breast cancer subtype and expression is associated with reduced survival, while its ability to pro  mote brea  st cancer cell migration and invasion suggests that it contributes to the aggressive clinical behaviour of this tumour subtype. 
 Interleukin-8 (CXCL8) stimulates trophoblast cell   migration   and invasion by increasing levels of matrix metalloproteinase (MMP)2 and MMP9 and integrins alpha5 and beta1. Interleukin-8 (IL8/CXCL8) is present in decidua and trophoblast, which also expresses the IL8 receptors, CXCR1 and CXCR2. IL8 was shown to stimulate trophoblast   migration  . Matrix metalloproteinase (MMP)2, MMP9, and integrins alpha(5)beta(1) and alpha(1)beta(1) were found to play important roles in trophoblast invasion. We hypothesized that IL8 would increase this cell   migration   and invasion by HTR-8/SVneo cells through the activity of MMPs and integrins. Isolated first trimester of pregnancy cytotrophoblast (CT) and HTR-8/SVneo cell line were used.   Migration   was studied by monolayer wounding test, and invasion by Matrigel invasion test. The effects of IL8 on MMPs and integrin subunit expression were determined in HTR-8/SVneo cells by gelatin zymography and western blot respectively. The results that were obtained showed that exogenous IL8 stimulated HTR-8/SVneo cell   migration   and invasion. MMP2 and MMP9 levels were stimulated to 182% (P<0.01) and 134% (P<0.01) respectively. Integrin alpha(5) expression was increased to 119% (P<0.05) and integrin beta(1) expression to 173% (P<0.001) of the control values. The data that were obtained show for the first time the sensitivity of the HTR-8/SVneo cells, in addition to isolated first trimester CT, to IL8. Exogenous IL8/CXCL8 increased trophoblast cell   migration   and invasion, which may be partly attributable to stimulation of MMP2 and MMP9 levels and an increase in integrins. HTR-8/SVneo cell viability and proliferation were also increased. 
 Cross-talk of integrin alpha3beta1 and tissue factor in cell   migration  . In cancer and angiogenesis, coagulation-independent roles of tissue factor (TF) in cell   migration   are incompletely understood. Immobilized anti-TF extracellular domain antibodies induce cell spreading, but this phenomenon is epitope specific and is not induced by anti-TF 5G9. Spreading on anti-TF is beta1 integrin-dependent, indicating functional interactions of the TF extracellular domain 5G9 epitope (a presumed integrin-binding site) and integrins. Recombinant TF extracellular domain supports adhesion of cells expressing alphavbeta3 or certain beta1 integrin heterodimers (alpha3beta1, alpha4beta1, alpha5beta1, alpha6beta1, alpha9beta1) and adhesion is blocked by specific anti-integrin antibodies or mutations in the integrin ligand-binding site. Although several studies have linked TF to cell   migration  , we here demonstrate that TF specifically regulates alpha3beta1-dependent   migration   on laminin 5. Expression of TF suppresses alpha3beta1-dependent   migration  , but only when the TF cytoplasmic domain is not phosphorylated. Suppression of   migration   can be reversed by 5G9, presumably by disrupting integrin interaction, or by the protease ligand VIIa, known to induce PAR-2-dependent phosphorylation of TF. In both cases, release of alpha3beta1 inhibition is prevented by mutation of critical phosphorylation sites in the TF cytoplasmic domain. Thus, TF influences integrin-mediated   migration   through cooperative intra- and extracellular interactions and phosphorylation regulates TF's function in cell motility. 
 Alpha2beta1 integrin signalling enhances cyclooxygenase-2 expression in intestinal epithelial cells. Inflammatory bowel diseases (IBD) are linked to an increased risk of developing colon cancer, by inflammatory mediators and alterations to the extracellular matrix (ECM). The events induced by inflammatory mediators lead to dysregulated activation and induction of inflammatory genes such as cyclooxygenase-2 (COX-2). COX-2 is involved in the conversion of arachidonic acid to biologically active prostanoids and is highly upregulated in colon cancer. Since inflammation-induced changes to the extracellular matrix could affect integrin activities, we here investigated the effect of integrin signalling on the level of COX-2 expression in the non-transformed intestinal epithelial cell lines, Int 407 and IEC-6. Adhesion of these cells to a collagen I- or IV-coated surface, increased surface expression of alpha2beta1 integrin. Activation of integrins with collagen caused an increased cox-2 promoter activity, with a subsequent increase in COX-2 expression. The signalling cascade leading to this increased expression and promoter activity of cox-2, involves PKCalpha, the small GTPase Ras and NFkappaB but not Erk1/2 or Src activity. The integrin-induced increase in cellular COX-2 activity is responsible for an elevated generation of reactive oxygen species (ROS) and increased cell   migration  . This signalling pathway suggests a mechanism whereby inflammation-induced modulations of the ECM, can promote cancer transformation in the intestinal epithelial cells. 
 [Effect of calcium on the activity and expression of integrin beta1 promoter in HaCaT cells and cell   migration  ]. To study the effect of calcium on the activity and protein expression of integrin beta1 promoter in human immortal keratinocyte colony HaCaT cell and cell   migration  .(1) HaCaT cells were cultured in vitro (12-slot plate) and divided into 5 groups according to the random number table, with 18 slots in each group: reporter plasmid pGL3 promoter (positive control group, PC), pGL3 empty vector (negative control group, NC), pGL3-1756 bp (total length promoter group, TL), pGL3-1442 bp (distal promoter group, D), and pGL3-261 bp (proximal promoter group, P) was respectively used to transfect HaCaT cells in non-serum RPMI 1640 culture medium with 0.00, 0.03, 0.09, 0.30, 0.80, or 1.20 mmol/L calcium (3 slots in each group with each concentration). Luciferase activity was detected with dual-luciferase reporter assay system 24 hours after transfection. (2) HaCaT cells steadily transfected with small interfering RNA-integrin beta1 vector (steadily transfected in brief) constructed in our laboratory were normally cultured and divided into 6 parts according to the random number table. And then they were treated with former 6 different concentrations of calcium, with 3 samples for each concentration. Expression level of integrin beta1 protein was determined with Western blot. (3) Normal and steadily transfected HaCaT cells were cultured in 6-slot plate, 18 slots for each kind of cells. They were cultured with former 6 kinds of calcium culture media (divided according to the random number table, with 3 slots of cells for each concentration) for 12 hours after scratch test. Cell   migration   rate was observed and determined. (4) Data were processed with one-way analysis of variance and independent samples t test.(1) The luciferase activity of cells in TL group increased from 0.16+/-0.09 to 0.39+/-0.09 and 0.35+/-0.05 (with t value respectively 3.143, 3.140, P values all below 0.05) as calcium concentration increasing from 0.00 mmol/L to 0.09 and 0.30 mmol/L, and it decreased as calcium concentration increased to 0.80 and 1.20 mmol/L. The change pattern of luciferase activity of cells along with calcium concentration in D group was similar to that in TL group, but its activity (0.56+/-0.32, 0.64+/-0.06) at the concentration of 0.09, 0.30 mmol/L was respectively higher than that in TL group (with t value respectively 0.887, 6.122, P values all below 0.05). There was no obvious influence of calcium in either concentration on the luciferase activity of cells in P group. (2) The expression amount of integrin beta1 of steadily transfected HaCaT cells cultured with 0.03, 0.09, 0.30, 0.80, 1.20 mmol/L calcium (0.58+/-0.09, 1.40+/-0.29, 1.41+/-0.09, 0.99+/-0.10, 1.16+/-0.15) were all increased as compared with that cultured with 0.00 mmol/L calcium (0.53+/-0.10, with t value respectively 0.687, 4.880, 11.210, 5.578, 6.199, P values all below 0.05). (3)   Migration   speed of normal HaCaT cells cultured with 0.09, 0.30 mmol/L calcium increased obviously as compared with that cultured with 0.00 mmol/L calcium, and it slowed down when cultured with 0.80, 1.20 mmol/L calcium. There was no obvious difference of   migration   rate among steadily transfected HaCaT cells treated with different concentration of calcium.Distal promoter region of integrin beta1 plays a vital role in regulating integrin beta1 transcription in human epidermal cells. And calcium regulates activity, protein expression of integrin beta1 promoter and cell   migration  . 
 Integrin α9β1 promotes malignant tumor growth and metastasis by potentiating epithelial-mesenchymal transition. The integrin α9β1 binds a number of extracellular matrix components to mediate cell adhesi  on, migra  tion and tissue invasion. Although expressed in a variety of normal human cells including endothelium, it is also expressed in cancer cells. We have previously shown that α9β1 binds VEGF-A to facilitate angiogenesis, an important component of the tumor microenvironment. As α9β1 induces accelerated canc  er cell m  igration, we wished to determine what role it played in cancer growth and metastasis. In this study, we show that α9β1 expression induces molecular changes consistent with epithelial-mesenchymal transition. In addition, we found that α9β1 forms a tri-partite protein complex with β-catenin and E-cadherin, which dissociates following integrin activation and subsequent src and β-catenin phosphorylation. These findings were consistent in cells in which: α9β1 was exogenously over-expressed, or when its expression was suppressed in cancer cells endogenously expressing α9β1. These in vitro results are biologically significant as α9β1-expressing cancer cells induce greater tumor growth and metastases in mice as compared to the cells without α9β1 expression or when integrin expression is suppressed. Furthermore, integrin α9β1 is expressed in primary human small cell lung cancer and patients having a high expression of α9β1 demonstrated significantly worse long-term survival compared with patients with low α9β1 expression. These findings highlight a novel mechanism of integrin α9β1 function in human cancer. 
 Proteomic analysis of integrin-associated complexes identifies RCC2 as a dual regulator of Rac1 and Arf6. The binding of integrin adhesion receptors to their extracellular matrix ligands controls cell morphology, movement, survival, and differentiation in various developmental, homeostatic, and disease processes. Here, we report a methodology to isolate complexes associated with integrin adhesion receptors, which, like other receptor-associated signaling complexes, have been refractory to proteomic analysis. Quantitative, comparative analyses of the proteomes of two receptor-ligand pairs, alpha(4)beta(1)-vascular cell adhesion molecule-1 and alpha(5)beta(1)-fibronectin, defined both core and receptor-specific components. Regulator of chromosome condensation-2 (RCC2) was detected in the alpha(5)beta(1)-fibronectin signaling network at an intersection between the Rac1 and adenosine 5'-diphosphate ribosylation factor 6 (Arf6) subnetworks. RCC2 knockdown enhanced fibronectin-induced activation of both Rac1 and Arf6 and accelerated cell spreading, suggesting that RCC2 limits the signaling required for membrane protrusion and delivery. Dysregulation of Rac1 and Arf6 function by RCC2 knockdown also abolished persistent   migration   along fibronectin fibers, indicating a functional role for RCC2 in directional cell movement. This proteomics workflow now opens the way to further dissection and systems-level analyses of adhesion signaling. 
 The Arp2/3 activator WASH regulates α5β1-integrin-mediated invasiv  e migrati  on. The actin cytoskeleton provides scaffolding and physical force to effect fundamental processes such as motility, cytokinesis and vesicle trafficking. The Arp2/3 complex nucleates actin structures and contributes to endocytic vesicle invagination and trafficking away from the plasma membrane. Internalisation and directed recycling of integrins are major driving forces for invasive cell motility and potentially for cancer metastasis. Here, we describe a direct requirement for WASH and Arp2/3-mediated actin polymerisation on the endosomal membrane system for α5β1 integrin recycling. WASH regulates the trafficking of endosomal α5β1 integrin to the plasma membrane and is fundamental for integrin-driven cell morphology changes and integrin-mediated cancer cell invasion. Thus, we implicate WASH and Arp2/3-driven actin nucleation in receptor recycling leading to invasive motility. 
 The CXC-chemokine CXCL4 interacts with integrins implicated in angiogenesis. The human CXC-chemokine CXCL4 is a potent inhibitor of tumor-induced angiogenesis. Considering that CXCL4 is sequestered in platelet alpha-granules and released following platelet activation in the vicinity of vessel wall injury, we tested the hypothesis that CXCL4 might function as a ligand for integrins. Integrins are a family of adhesion receptors that play a crucial role in angiogenesis by regulating early angiogenic processes, such as endothelial cell adhesion and   migration  . Here, we show that CXCL4 interacts with alphavbeta3 on the surface of alphavbeta3-CHO. More importantly, human umbilical vein endothelial cells adhere to immobilized CXCL4 through alphavbeta3 integrin, and also through other integrins, such as alphavbeta5 and alpha5beta1. We further demonstrate that CXCL4-integrin interaction is of functional significance in vitro, since immobilized CXCL4 supported endothelial cell spreading and   migration   in an integrin-dependent manner. Soluble CXCL4, in turn, inhibits integrin-dependent endothelial cell adhesion and   migration  . As a whole, our study identifies integrins as novel receptors for CXCL4 that may contribute to its antiangiogenic effect. 
 DNAzyme as an efficient tool to modulate invasiveness of human carcinoma cells. In this study we evaluated efficiency of DNAzymes to modulate motility of cancer cells, an important factor in the progression and metastasis of cancers. For this purpose we targeted β1 integrins that are predominant adhesive receptors in various carcinoma cell lines (CX1.1, HT29, LOVO, LS180, PC-3). To evaluate invasiveness of cancer cells, we used a transwell   migratio  n assay that allowed analyzing chemotactic   migratio  n of colon carcinoma cell lines across an ECM-coated membrane. Their adhesive properties were also characterized by the analysis of adhesion to fibronectin, laminin and collagen. In addition, the expression of major integrin subunits, selected intact β1 integrins, and other adhesive receptors (ICAM, E-selectin, uPAR) was analyzed by flow cytometry. Inhibition of β1 integrin expression by DNAzyme to β1 mRNA almost abolished the invasiveness of the CX1.1, HT29, LS180, LOVO and PC-3 cells in vitro. These data show that DNAzymes to β1 integrin subunit can be used to inhibit invasiveness of carcinoma cells. 
 Inhibition of β1 integrin and IL-3Rβ common subunit interaction hinders tumour angiogenesis. Integrin/cytokine receptor interaction provides permissive signals leading to neoangiogenesis, and integrins are crucial for differentiation of endothelial progenitor cells (EPCs). It is known that the inflammatory interleukin-3 (IL-3), released in the tumoral microenvironment, contributes to both angiogenesis and vasculogenic processes. Herein, we generated IL-3 receptor beta common (IL-3Rβc) extracellular domain-derived fusion proteins (Fc) to elucidate the molecular mechanisms regulating these processes. Three different Fc were generated, containing the entire extracellular domain of IL-3Rβc (Fc1.4), a fragment corresponding to domains 1-3 (Fc1.3) and a fragment corresponding to domain 4 (Fc4), respectively. The ability of the fusion proteins to interfere with IL-3Rβc/β1 integrin interaction was assessed on endothelial cells (ECs), EPCs and murine-derived ECs. Pull-down experiments showed that Fc1.4 and Fc4 fusion proteins specifically interacted with β1 integrin. Fc4 and Fc1.4 fragments prevented IL-3-mediated EPC expansion, arterial morphogenesis and tumour-deri  ved EC mi  gration, without affecting cell adhesion. Fc4 in vivo inhibited the IL-3-mediated vasculogenic process, as well as inflammatory and tumour vascular growth. In conclusion, these data identify the β1 integrin-interacting domain in the juxta-membrane IL-3Rβc extracellular domain, and provide the rational for targeting this interaction to impair vascular growth. 
 VLA4 Gene Polymorphism and Susceptibility to Multiple Sclerosis in Slovaks. Multiple sclerosis (MS) is an inflammatory autoimmune disease occurring in genetically sensitive individuals. As   migration   of immune cells into the CNS is facilitated by the Very Late Antigen 4 (VLA-4) integrin molecule, the VLA4 gene may be considered as a plausible candidate genetic risk factor for susceptibility to MS. Therefore, the objective of our study was to investigate the association between two genetic polymorphisms located in the VLA4 gene and the risk of multiple sclerosis. One hundred seventeen MS patients and 165 control subjects from Slovakia were genotyped for VLA4 gene SNP polymorphisms at positions 269 (C/A) and 3061 (A/G). The same study cohorts were also genotyped for the rs3135388 polymorphism tagging the HLA-DRB1*15:01 allele, which is a known genetic factor associated with susceptibility to develop MS in many populations. Our findings show for the first time that the rs3135388 polymorphism is a strong risk factor for MS in the Slovak population. Investigation of the VLA4 gene polymorphisms revealed a significantly higher frequency of the 3061AG genotype in MS patients compared to the controls (P ≤ 0.05). We suggest that the 3061AG polymorphic variant is an independent genetic risk factor for MS development in our population as it was significantly associated with this disease. The association was also confirmed after applying multivariate logistic-regression analysis adjusted for gender, age and HLA-DRB1*15:01 positivity as possible influencing factors. 
 Tumor necrosis factor-induced neutrophil adhesion occurs via sphingosine kinase-1-dependent activation of endothelial {alpha}5{beta}1 integrin. Leukocyte recruitment plays a major role in the immune response to infectious pathogens, as well as during inflammatory and autoimmune disorders. The process of leukocyte extravasation from the blood requires a complex cascade of adhesive events between the leukocytes and the endothelium, including initial leukocyte rolling, adhesion, and finally transendothelial   migration  . Current research in this area aims to identify the key leukocyte subsets that initiate a given disease and to identify the trafficking molecule(s) that will most specifically inhibit those cells. Herein we demonstrate that tumor necrosis factor (TNF)alpha activates the integrin alpha(5)beta(1) without altering total expression levels of beta(1) integrin on human umbilical vein endothelial cells. Moreover, our studies suggest that TNFalpha-induced beta(1) activation is dependent on sphingosine kinase-1, but independent of the sphingosine-1-phosphate family of G protein-coupled receptors. We also show, using a parallel plate flow chamber assay, that neutrophil adhesion to TNFalpha-activated endothelium can be attenuated by blocking alpha(5)beta(1) or its ligand angiopoietin-2. These observations add new complexities that broaden the accepted concept of cellular trafficking with neutrophil adhesion to TNFalpha activated endothelial cells being sphingosine kinase-1, alpha(5)beta(1), and angiopoietin-2 dependent. Moreover, this work supports the notion that sphingosine kinase-1 may be the single target required for an effective broad spectrum approach to combat inflammation and immune disorders. 
 Association of the calpain/calpastatin network with subcellular organelles. The calcium-activated cysteine protease calpain is intimately involved in modulating cell adhesion and   migration  . The two ubiquitous isoforms of this protease, calpain I and II, are considered to be cytosolic proteins that can translocate to both focal complexes/adhesions or the plasma membrane. Using confocal microscopy and isopycnic density centrifugation, the results demonstrate that calpain I and II, the 30kDa regulatory subunit, and calpastatin associate with the endoplasmic reticulum and Golgi apparatus. Confocal microscopy reveals that calpain II colocalizes with the subcellular proteins calnexin and Rab6 in cells bound to laminin. To further verify this association, cell lysates prepared from laminin stimulated and unstimulated cells were subjected to isopycnic density centrifugation. The results reveal an increased association of calpain I, II, calpastatin, and the 30kDa regulatory subunit with the endoplasmic reticulum and Golgi apparatus as evidenced by their position in the gradient relative to calnexin, Rab6, caveolin, and beta1 integrin after laminin stimulation. This correlates with the accumulation of inducible calpain activity at the endoplasmic reticulum-Golgi apparatus interface. Further experiments established that calpain II colocalizes with phosphatidylinositol 4,5-bisphosphate. Finally, calpain II associates with membrane lipid rafts. These results provide new insights into how the calpain/calpastatin network is spatially and temporally regulated in cells binding to the extracellular matrix. 
 Exogenous SPARC suppresses proliferation and   migration   of prostate cancer by interacting with integrin β1. The matricellular protein secreted protein acidic and rich in cysteine (SPARC) plays an important role on tumor metastasis and progression in several cancers. However, the roles of SPARC in prostate cancer (PCa) remain unclear.To identify SPARC protein in prostate tissue, immunohistochemical analysis of SPARC was conducted using human prostate tissue microarray. To detect SPARC expression in prostate cancer (LNCaP, DU145, and PC-3) and stromal cells, RT-PCR, western blot analysis, and ELISA was conducted. To reveal the function of exogenous SPARC in PCa cells, AKT phosphorylation was confirmed by western blot analysis after coculture with stromal cells. Proliferation and   migratio  n of PCa cells were examined by addition of SPARC. The interaction between SPARC and integrin β1 was confirmed by western blot analysis after immunoprecipitation.SPARC protein was expressed well in normal tissue compared with PCa tissue. ELISA showed high secreted SPARC protein in normal prostate-derived stromal cell (PrSC) compared with PCa-derived stromal cell (PCaSC) and PCa. PCa cells cocultured with PrSC showed reduced AKT phosphorylation more than with PCaSC. PCa cells cocultured with PrSC whose SPARC was knocked-down restored AKT phosphorylation. Moreover, PCa cells treated with SPARC led to reduced AKT phosphorylation. Immunoprecipitation with SPARC revealed interaction of SPARC and integrin β1 in PCa cells. Inhibited proliferation a  nd migrat  ion of PCa cells by SPARC was restored by integrin β1 neutralizing antibody.Reduced SPARC secretion from stromal cells might affect PCa progression mediating through limiting AKT phosphorylation after interaction with integrin β1. 
 Activation of Epac stimulates integrin-dependent homing of progenitor cells. Cell therapy is a novel promising option for treatment of ischemic diseases. Administered endothelial progenitor cells (EPCs) are recruited to ischemic regions and improve neovascularization. However, the number of cells that home to ischemic tissues is restricted. The GTPase Rap1 plays an important role in the regulation of adhesion and chemotaxis. We investigated whether pharmacologic activation of Epac1, a nucleotide exchange protein for Rap1, which is directly activated by cAMP, can improve the adhesive and migratory capacity of distinct progenitor cell populations. Stimulation of Epac by a cAMP-analog increased Rap1 activity and stimulated the adhesion of human EPCs, CD34(+) hematopoietic progenitor cells, and mesenchymal stem cells (MSCs). Specifically, short-term stimulation with a specific Epac activator increased the beta2-integrin-dependent adhesion of EPCs to endothelial cell monolayers, and of EPC and CD34(+) cells to ICAM-1. Furthermore, the Epac activator enhanced the beta1-integrin-dependent adhesion of EPCs and MSCs to the matrix protein fibronectin. In addition, Epac1 activation induced the beta1- and beta2-integrin-dependent   migration   of EPCs on fibronectin and fibrinogen. Interestingly, activation of Epac rapidly increased lateral mobility of beta1- and beta2-integrins, thereby inducing integrin polarization, and stimulated beta1-integrin affinity, whereas the beta2-integrin affinity was not increased. Furthermore, prestimulation of EPCs with the Epac activator increased homing to ischemic muscles and neovascularization-promoting capacity of intravenously injected EPCs in the model of hind limb ischemia. These data demonstrate that activation of Epac1 increases integrin activity and integrin-dependent homing functions of progenitor cells and enhances their in vivo therapeutic potential. These results may provide a platform for the development of novel therapeutic approaches to improve progenitor cell homing. 
 Interplay between β1-integrin and Rho signaling regulates differential scattering and motility of pancreatic cancer cells by snail and Slug proteins. The Snail family of transcription factors has been implicated in pancreatic cancer progression. We recently showed that Snail (Snai1) promotes membrane-type 1 matrix metalloproteinase (MT1-MMP)- and ERK1/2-dependent scattering of pancreatic cancer cells in three-dimensional type I collagen. In this study, we examine the role of Slug (Snai2) in regulating pancreatic cancer cell scattering in three-dimensional type I collagen. Although Slug increased MT1-MMP expression and ERK1/2 activity, Slug-expressing cells failed to scatter in three-dimensional collagen. Moreover, in contrast to Snail-expressing cells, Slug-expressing cells did not demonstrate increased collagen I binding, collagen I-driven motility, or α2β1-integrin expression. Significantly, inhibiting β1-integrin function decrea  sed migra  tion and scattering of Snail-expressing cells in three-dimensional collagen. As Rho GTPases have been implicated in invasion   and migra  tion, we also analyzed the contribution of Rac1 and Rho signaling to the different  ial migra  tion and scattering of pancreatic cancer cells. Snail-indu  ced migra  tion and scattering were attenuated by Rac1 inhibition. In contrast, inhibiting Rho-associated kinase ROCK1/2 increa  sed migra  tion and scattering of Slug-expressing cells in three-dimensional collagen and thus phenocopied the effects of Snail in pancreatic cancer cells. Additionally, the increa  sed migra  tion and scattering in three-dimensional collagen of Slug-expressing cells following ROCK1/2 inhibition was dependent on β1-integrin function. Overall, these results demonstrate differential effects of Snail and Slug in pancreatic cancer and identify the interplay between Rho signaling and β1-integrin that functions to regulate the differential scatterin  g and mig  ration of Snail- and Slug-expressing pancreatic cancer cells. 
 Integrin expression regulates neuroblastoma attachment and   migration  . Neuroblastoma (NBL) is the most common malignant disease of infancy, and children with bone metastasis have a mortality rate greater than 90%. Two major classes of proteins, integrins and growth factors, regulate the metastatic process. We have previously shown that tumorigenic NBL cells express higher levels of the type I insulin-like growth factor receptor (IGF-IR) and that beta1 integrin expression is inversely proportional to tumorigenic potential in NBL. In the current study, we analyze the effect of beta1 integrin and IGF-IR on NBL cell attachment and   migration  . Nontumorigenic S-cells express high levels of beta1 integrin, whereas tumorigenic N-cells express little beta1 integrin. Alterations in beta1 integrin are due to regulation at the protein level, as translation is decreased in N-type cells. Moreover, inhibition of protein synthesis shows that beta1 integrin is degraded more slowly in S-type cells (SHEP) than in N-type cells (SH-SY5Y and IMR32). Inhibition of alpha5beta1 integrin prevents SHEP (but not SH-SY5Y or IMR32) cell attachment to fibronectin and increases SHEP cell   migration  . Increases in IGF-IR decrease beta1 integrin expression, and enhance SHEP cell   migration  , potentially through increased expression of alphavbeta3. These data suggest that specific classes of integrins in concert with IGF-IR regulate NBL attachment and   migration  . 
 The chemokine receptor 7 regulates cell adhesion and   migration   via beta1 integrin in metastatic squamous cell carcinoma of the head and neck.   Migration   and adhesion of tumor cells are essential prerequisites for the formation of metastases in malignant diseases. Chemokine receptor 7 (CCR7) has been shown to regulate integrin which can then facilitate adhesion of cancer cells to and/or   migration   through the extracellular matrix (ECM). In order to identify the connection between CCR7 and beta1 integrin, and the influence on cell adhesion and   migration   in metastatic squamous cell carcinoma of the head and neck (SCCHN). We use adhesion assays,   migration   assay, immunofluorescence staining, western blotting, and immunohistochemical analysis to find whether beta1 integrin can be activated by CCL19 (CCR7's ligand) and its role in SCCHN. The experiments were performed in the metastatic SCCHN cell line PCI-37B after pre-incubation of the cells with CCL19 and beta1 integrin inhibitors RGD-peptide. Our results demonstrate that CCR7 favours PCI-37B cell adhesion and   migration  , and induces reorganization of the actin cytoskeleton and up-expression of beta1 integrin protein. beta1 integrin inhibitor RGD-peptide can block all these effects. Taken together, our data indicate that CCR7 regulate cell adhesion and   migration   via beta1 integrin in metastatic SCCHN, and these results can provide a basis for new strategies in preventing metastases of SCCHN. 
 Sphingosine-1-phosphate activates chemokine-promoted myeloma cell adhesion and   migration   involving α4β1 integrin function. Myeloma cell adhesion dependent on α4β1 integrin is crucial for the progression of multiple myeloma (MM). The α4β1-dependent myeloma cell adhesion is up-regulated by the chemokine CXCL12, and pharmacological blockade of the CXCL12 receptor CXCR4 leads to defective myeloma cell homing to bone marrow (BM). Sphingosine-1-phosphate (S1P) regulates immune cell trafficking upon binding to G-protein-coupled receptors. Here we show that myeloma cells express S1P1, a receptor for S1P. We found that S1P up-regulated the α4β1-mediated myeloma cell adhesion and transendo  thelial m  igration stimulated by CXCL12. S1P promoted generation of high-affinity α4β1 that efficiently bound the α4β1 ligand VCAM-1, a finding that was associated with S1P-triggered increase in talin-β1 integrin association. Furthermore, S1P cooperated with CXCL12 for enhancement of α4β1-dependent adhesion strengthening and spreading. CXCL12 and S1P activated the DOCK2-Rac1 pathway, which was required for stimulation of myeloma cell adhesion involving α4β1. Moreover, in vivo analyses indicated that S1P contributes to optimizing the interactions of MM cells with the BM microvasculture and for their lodging inside the bone marrow. The regulation of α4β1-depe  ndent adh  esion and migration of myeloma cells by CXCL12-S1P combined activities might have important consequences for myeloma disease progression. 
 Identification of nucleolin as a lipid-raft-dependent β1-integrin-interacting protein in A375 cell   migratio  n. Lipid rafts are related to cell surface receptor function. Integrin is a major surface receptor protein in cell adhesion and   migratio  n on the extracellular matrix (ECM). Here, we showed that lipid rafts played a critical role in human melanoma A375 cell spreading and   migratio  n on fibronectin; an important component of the ECM that interacts with β1 integrin. We found that the disruption of lipid rafts did not markedly inhibit the expression and activation of β1 integrin. By coimmunoprecipitation and mass spectrometry, we investigated the influence of lipid rafts on the β1 integrin complex and identified nucleolin as a potential lipid-raft-dependent β1-integrin-interacting protein. Upon confirmation of the interaction between β1 integrin and nucleolin, further studies revealed that nucleolin colocalized with β1 integrin in lipid rafts and raft disruption interrupted their association. In addition, knockdown of nucleolin markedly attenuated A375 cell spreadi  ng and mi  gration on fibronectin. Taken together, we demonstrated that nucleolin is a critical lipid-raft-dependent β1-integrin-interacting protein in A375 cell spread  ing and m  igration on fibronectin. 
 The Tat protein of human immunodeficiency virus type-1 promotes vascular cell growth and locomotion by engaging the alpha5beta1 and alphavbeta3 integrins and by mobilizing sequestered basic fibroblast growth factor. The Tat protein of human immunodeficiency virus type-1 (HIV-1) has been shown to be released during acute infection of T cells by HIV-1 and to promote angiogenesis and Kaposi's sarcoma (KS) development in infected individuals. In this study, we investigated the molecular mechanisms responsible for the angiogenic effects of Tat. The results shown herein indicate that two different Tat domains cooperate to induce these effects by different pathways. The arginine-glycine-aspartic acid (RGD) sequence present at the carboxyterminal of Tat mediates vascular cell   migration   and invasion by binding to the alpha5beta1 and alphavbeta3 integrins. This interaction also provides endothelial cells with the adhesion signal they require to grow in response to mitogens. At the same time, the Tat basic sequence retrieves into a soluble form extracellular basic fibroblast growth factor (bFGF) bound to heparan sulfate proteoglycans by competing for heparin-binding sites. This soluble bFGF mediates Tat-induced vascular cell growth. These effects resemble those of extracellular matrix proteins, suggesting that Tat enhances angiogenesis and promotes KS progression by a molecular mimicry of these molecules. 
 TIMP-1 modulates chemotaxis of human neural stem cells through CD63 and integrin signalling. We recently reported that hNSCs (human neural stem cells) have the interesting characteristic of   migration   towards an intracranial glioma. However, the molecules and mechanisms responsible for tumour tropism are unclear. In the present study, we used microarray and proteomics analyses to identify a novel chemoattractant molecule, TIMP-1 (tissue inhibitor of metalloproteinase-1), secreted from human brain tumour tissues. We demonstrate that TIMP-1 significantly enhances hNSC adhesion and   migration   in a cell culture system. These effects were critically dependent on CD63, as shRNA-mediated ablation of CD63 expression attenuated the response. TIMP-1 significantly increased the number of FAs (focal adhesions) and cytoskeletal reorganization for cell   migration   in hNSCs, whereas knockdown of CD63 resulted in decreased hNSC spreading, FAs and   migration  , even after TIMP-1 treatment. In addition, TIMP-1 binding to CD63 activated β1 integrin-mediated signalling through Akt and FAK phosphorylation, leading to pattern changes in distribution of vinculin and F-actin (filamentous actin). Furthermore, inactivation of β1 integrin by use of a blocking antibody or inhibition of PI3K (phosphoinositide 3-kinase) signalling impaired th  e migrati  on of hNSCs towards TIMP-1. Collectively, our results underline TIMP-1 as a novel and effective key regulator of CD63 and β1 integrin-mediated signalling, which regulates hNSC adhesion a  nd migrat  ion. 
 Lipid raft regulates the initial spreading of melanoma A375 cells by modulating β1 integrin clustering. Cell adhesion and spreading require integrins-mediated cell-extracellular matrix interaction. Integrins function through binding to extracellular matrix and subsequent clustering to initiate focal adhesion formation and actin cytoskeleton rearrangement. Lipid raft, a liquid ordered plasma membrane microdomain, has been reported to play major roles in membrane motility by regulating cell surface receptor function. Here, we identified that lipid raft integrity was required for β1 integrin-mediated initial spreading of melanoma A375 cells on fibronectin. We found that lipid raft disruption with methyl-β-cyclodextrin led to the inability of focal adhesion formation and actin cytoskeleton rearrangement by preventing β1 integrin clustering. Furthermore, we explored the possible mechanism by which lipid raft regulates β1 integrin clustering and demonstrated that intact lipid raft could recruit and modify some adaptor proteins, such as talin, α-actinin, vinculin, paxillin and FAK. Lipid raft could regulate the location of these proteins in lipid raft fractions and facilitate their binding to β1 integrin, which may be crucial for β1 integrin clustering. We also showed that lipid raft disruption impaired A3  75 cell m  igration in both transwell and wound healing models. Together, these findings provide a new insight for the relationship between lipid raft and the regulation of integrins. 
 Expression of estrogen receptor beta increases integrin alpha1 and integrin beta1 levels and enhances adhesion of breast cancer cells. Estrogen effects on mammary gland development and differentiation are mediated by two receptors (ERalpha and ERbeta). Estrogen-bound ERalpha induces proliferation of mammary epithelial and cancer cells, while ERbeta is important for maintenance of the differentiated epithelium and inhibits proliferation in different cell systems. In addition, the normal breast contains higher ERbeta levels compared to the early stage breast cancers, suggesting that loss of ERbeta could be important in cancer development. Analysis of ERbeta-/- mice has consistently revealed reduced expression of cell adhesion proteins. As such, ERbeta is a candidate modulator of epithelial homeostasis and metastasis. Consequently, the aim of this study was to analyze estrogenic effects on adhesion of breast cancer cells expressing ERalpha and ERbeta. As ERbeta is widely found in breast cancer but not in cell lines, we used ERalpha positive T47-D and MCF-7 human breast cancer cells to generate cells with inducible ERbeta expression. Furthermore, the colon cancer cell lines SW480 and HT-29 were also used. Integrin alpha1 mRNA and protein levels increased following ERbeta expression. Integrin beta1-the unique partner for integrin alpha1-increased only at the protein level. ERbeta expression enhanced the formation of vinculin containing focal complexes and actin filaments, indicating a more adhesive potential. This was confirmed by adhesion assays where ERbeta increased adhesion to different extracellular matrix proteins, mostly laminin. In addition, ERbeta expression was associated to less cell   migration  . These results indicate that ERbeta affects integrin expression and clustering and consequently modulates adhesion and   migration   of breast cancer cells. 
 αv integrin processing interferes with the cross-talk between αvβ5/β6 and α2β1 integrins. Previous studies have reported that cross-talk between integrins may be an important regulator of integrin-ligand binding and subsequent signalling events that control a variety of cell functions in many tissues. We previously demonstrated that αvβ5/β6 integrin represses α2β1-depe  ndent cel  l migration. The αv subunits undergo an endoproteolytic cleavage by protein convertases, whose role in tumoral invasion has remained controversial.Inhibition of convertases by the convertase inhibitor α1-PDX (α1-antitrypsin Portland variant), leading to the cell-surface expression of an uncleaved form of the αv integrin, s  timulated   cell migration toward type I collagen. Under convertase inhibition, α2β1 engagement led to enhanced phosphorylation of both FAK (focal adhesion kinase) and MAPK (mitogen-activated protein kinase). This outside-in signalling stimulation was associated with increased levels of activated β1 integrin located in larger than usual focal-adhesion stru  ctures an  d a cell migration that was independent of the PI3K (phosphoinositide 3-kinase)/Akt (also called protein kinase B) pathway.The   increase   in cell migration observed upon convertases inhibition appears to be due to the up-regulation of β1 integrins and to their location in larger focal-adhesion structures. The endoproteolytic cleavage of αv subunits is necessary for αvβ5/β6 integrin to control α2β1 function and could thus play an essential r  ole in co  lon cancer cell migration. 
 The intermediate filament protein vimentin binds specifically to a recombinant integrin alpha2/beta1 cytoplasmic tail complex and co-localizes with native alpha2/beta1 in endothelial cell focal adhesions. Integrin receptors are crucial players in cell adhesion and   migration  . Identification and characterization of cellular proteins that interact with their short alpha and beta cytoplasmic tails will help to elucidate the molecular mechanisms by which integrins mediate bi-directional signaling across the plasma membrane. Integrin alpha2beta1 is a major collagen receptor but to date, only few proteins have been shown to interact with the alpha2 cytoplasmic tail or with the alpha2beta1 complex. In order to identify novel binding partners of a alpha2beta1cytoplasmic domain complex, we have generated recombinant GST-fusion proteins, incorporating the leucine zipper heterodimerization cassettes of Jun and Fos. To ascertain proper functionality of the recombinant proteins, interaction with natural binding partners was tested. GST-alpha2 and GST-Jun alpha2 bound His-tagged calreticulin while GST-beta1 and GST-Fos beta1 proteins bound talin. In screening assays for novel binding partners, the immobilized GST-Jun alpha2/GST-Fos beta1 heterodimeric complex, but not the single subunits, interacted specifically with endothelial cell-derived vimentin. Vimentin, an abundant intermediate filament protein, has previously been shown to co-localize with alphavbeta3-positive focal contacts. Here, we provide evidence that this interaction also occurs with alpha2beta1-enriched focal adhesions and we further show that this association is lost after prolonged adhesion of endothelial cells to collagen. 
 Lipid rafts mediate internalization of beta1-integrin in migrating intestinal epithelial cells. Intestinal mucosal inflammation is associated with epithelial wounds that rapidly reseal by   migration   of intestinal epithelial cells (IECs). Cell   migration   involves cycles of cell-matrix adhesion/deadhesion that is mediated by dynamic turnover (assembly and disassembly) of integrin-based focal adhesions. Integrin endocytosis appears to be critical for deadhesion of motile cells. However, mechanisms of integrin internalization during remodeling of focal adhesions of migrating IECs are not understood. This study was designed to define the endocytic pathway that mediates internalization of beta(1)-integrin in migrating model IECs. We observed that, in SK-CO15 and T84 colonic epithelial cells, beta(1)-integrin is internalized in a dynamin-dependent manner. Pharmacological inhibition of clathrin-mediated endocytosis or macropinocytosis and small-interfering RNA (siRNA)-mediated knock down of clathrin did not prevent beta(1)-integrin internalization. However, beta(1)-integrin internalization was inhibited following cholesterol extraction and after overexpression of lipid raft protein, caveolin-1. Furthermore, internalized beta(1)-integrin colocalized with the lipid rafts marker cholera toxin, and siRNA-mediated knockdown of caveolin-1 and flotillin-1/2 increased beta(1)-integrin endocytosis. Our data suggest that, in migrating IEC, beta(1)-integrin is internalized via a dynamin-dependent lipid raft-mediated pathway. Such endocytosis is likely to be important for disassembly of integrin-based cell-matrix adhesions and therefore in regulating IEC   migration   and wound closure. 
 Glycogen synthase kinase 3β inhibition prevents monocyte   migratio  n across brain endothelial cells via Rac1-GTPase suppression and down-regulation of active integrin conformation. Glycogen synthase kinase (GSK) 3β has been identified as a regulator of immune responses. We demonstrated previously that GSK3β inhibition in human brain microvascular endothelial cells (BMVECs) reduced monocyte adhesi  on/migrat  ion across BMVEC monolayers. Herein, we tested the idea that GSK3β inhibition in monocytes can diminish their ability to engage the brain endothelium and migrate across the blood-brain barrier. Pretreatment of primary monocytes with GSK3β inhibitors resulted in a decrease in adhesion (60%)   and migr  ation (85%), with similar results in U937 monocytic cells. Monocyte-BMVEC interactions resulted in diminished barrier integrity that was reversed by GSK3β suppression in monocytic cells. Because integrins mediate monocyte rolling/adhesion, we detected the active conformational form of very late antigen 4 after stimulation with a peptide mimicking monocyte engagement by vascular cell adhesion molecule-1. Peptide stimulation resulted in a 14- to 20-fold up-regulation of the active form of integrin in monocytes that was suppressed by GSK3β inhibitors (40% to 60%). Because small GTPases, such as Rac1, control leukocyte movement, we measured active Rac1 after monocyte activation with relevant stimuli. Stimulation enhanced the level of active Rac1 that was diminished by GSK3β inhibitors. Monocytes treated with GSK3β inhibitors showed increased levels of inhibitory sites of the actin-binding protein, cofilin, and vasodilator-stimulated phosphoprotein-regulating conformational changes of integrins. These results indicate that GSK3β inhibition in monocytes affects active integrin expression, cytoskeleton rearrangement, and adhesion via suppression of Rac1-diminishing inflammatory leukocyte responses. 
 CD9 is expressed in extravillous trophoblasts in association with integrin alpha3 and integrin alpha5. The CD9 molecule is a 24-27 kDa cell surface glycoprotein, which may be related to Schwann cell   migration   and adhesion. In this study, we examined the expression of CD9 in human extravillous trophoblasts, which invade into the endometrium during implantation and placentation. CD9 was detected immunohistochemically on the extravillous trophoblasts in the cell columns of first trimester placentae, but not on villous trophoblasts. In the second and third trimester, CD9 was highly expressed on the extravillous trophoblasts in the basal plate of placentae, and in the chorion laeve in the fetal membrane of term placentae. The molecular mass of CD9 in the chorion laeve was shown to be 27 kDa by Western blotting. The mRNA of CD9 was also detected in the chorion laeve by reverse transcription-polymerase chain reaction (RT-PCR). Proteins were purified from chorion laeve by affinity chromatography with anti-integrin alpha3 and alpha5 monoclonal antibodies and Western blotting, revealed that CD9 was associated with both integrins. These findings indicate that CD9 is a differentiation-related molecule present in the extravillous trophoblasts. Since it is associated with integrin alpha5 which has been proposed to regulate trophoblast invasion, CD9 may be implicated in trophoblast invasion at the feto-maternal interface. 
 Alphav-integrin utilization in human beta-cell adhesion, spreading, and motility. The role of individual integrins in human beta-cell development and function is largely unknown. This study describes the contribution of alpha(v)-integrins to human beta-cell adhesion, spreading, and motility. Developmental differences in alpha(v)-integrin utilization are addressed by comparing the responses of adult and fetal beta-cells, and vitronectin is used as a substrate based on its unique pattern of expression in the developing pancreas. Fetal and adult beta-cells attached equally to vitronectin and integrin alpha(v)beta(5) was found to support the adhesion of both mature and immature beta-cell populations. Fetal beta-cells were also observed to spread and migrate on vitronectin, and integrin alpha(v)beta(1) was found to be essential for these responses. In contrast to their fetal counterparts, adult beta-cells failed to either spread or migrate and this deficit was associated with a marked down-regulation of alpha(v)beta(1) expression in adult islet preparations. The integrin alpha(v)beta(3) was not found to support significant beta-cell attachment or   migration  . Based on our findings, we conclude that integrins alpha(v)beta(5) and alpha(v)beta(1) are important mediators of human beta-cell adhesion and motility, respectively. By supporting fetal beta-cell   migration  , alpha(v)beta(1) could play an important role in early motile processes required for islet neogenesis. 
 Prognostic value of β1 integrin expression in colorectal liver metastases. Integrins are cell surface adhesion molecules (CAM) that regulate via intercellular and cell-matrix signaling various cellular processes including wound healing, cell differentiation, division, growth,   migratio  n and metastatic dissemination. Although a correlation between carcinogenesis and changes in integrin expression, especially β1 integrin, has been reported, its role in colorectal liver metastases remains unclear. This study aimed to evaluate the expression of β1 integrin in colorectal liver metastases and to correlate the pattern of expression with clinicopathological features and to investigate the putative role of β1 integrin expression on survival of these patients.Formalin-fixed, paraffin-embedded (FFPE) tumor samples of 81 patients who were operated because of colorectal liver metastases without any neoadjuvant therapy were obtained and stained with hematoxylin and eosin (H & E). An immunohistochemical examination was performed using Dako, Peroxidase/DAB kit and a primary monoclonal β1 integrin (CD29, fibronectin receptor subunit beta; ab3167, Abcam plc). β1 integrin expression was evaluated according to the immunoreactive score of Remmele and Stegner and was related with clinicopathological features of prognostic significance and with disease-free and overall survival as well. Statistical analysis was performed using SPSS version 21.0.β1 integrin was overexpressed in tumor cells in 37 (48%) patients and in stromal cell in 27 (33%) patients. The β1 expression was not statistically correlated with clinicopathological features of the primary tumors but it was statistically correlated (p=0.03) with the histological grading of liver metastases. Kaplan-Meier survival analysis showed that there is a tendency but no statistically significant correlation in disease-free and overall survival.Considering that expression of β1 integrin in colorectal liver metastases remains controversial, specially its relation with survival of patients, we showed that the β1 expression represents a reliable prognostic factor regarding the grading of liver metastases of CRC and our findings imply that β1 integrin expression profiles may have further potential in identifying the stage of colorectal liver metastases and being a marker of prognosis in these patients. 
 Cholecystokinin-2 receptor modulates cell adhesion through beta 1-integrin in human pancreatic cancer cells. Several lines of evidence suggest that gastrin and the CCK-2 receptor (CCK2R) could contribute to pancreatic carcinogenesis by modulating processes such as proliferation, cell adhesion or   migration  . In the current study, we used a 'cancer gene array' and identified beta1-integrin subunit as a new gastrin-regulated gene in human pancreatic cancer cells. We also demonstrated that Src family kinases and the phosphatidylinositol-3-kinase (PI-3-kinase) pathway play a crucial role in the expression of beta1-integrin induced by gastrin. Our results also showed that gastrin modulates cell-substrate adhesion via beta1-integrin. Indeed, using blocking anti-beta1-integrin monoclonal antibodies, we completely reversed the increase in cell-substrate adhesion induced by gastrin. In addition, we observed that in response to gastrin, beta1-integrin is tyrosine phosphorylated by Src family kinases and associates with paxillin, a scaffold protein involved in focal adhesion and integrin signalling. This mechanism might be involved in gastrin-induced cell adhesion. Moreover, we showed in vivo that targeted CCK2R expression in the pancreas of Elas-CCK2 mice leads to the overexpression of beta1-integrin. This process may contribute to pancreatic tumour development observed in these transgenic animals. 
 The integrin alpha9beta1 mediates adhesion to activated endothelial cells and transendothelial neutrophil   migration   through interaction with vascular cell adhesion molecule-1. The integrin alpha9beta1 has been shown to be widely expressed on smooth muscle and epithelial cells, and to mediate adhesion to the extracellular matrix proteins osteopontin and tenascin-C. We have found that the peptide sequence this integrin recognizes in tenascin-C is highly homologous to the sequence recognized by the closely related integrin alpha4beta1, in the inducible endothelial ligand, vascular cell adhesion mole-cule-1 (VCAM-1). We therefore sought to determine whether alpha9beta1 also recognizes VCAM-1, and whether any such interaction would be biologically significant. In this report, we demonstrate that alpha9beta1 mediates stable cell adhesion to recombinant VCAM-1 and to VCAM-1 induced on human umbilical vein endothelial cells by tumor necrosis factor-alpha. Furthermore, we show that alpha9beta1 is highly and selectively expressed on neutrophils and is critical for neutrophil   migration   on VCAM-1 and tenascin-C. Finally, alpha9beta1 and alpha4 integrins contribute to neutrophil chemotaxis across activated endothelial monolayers. These observations suggest a possible role for alpha9beta1/VCAM-1 interactions in extravasation of neutrophils at sites of acute inflammation. 
 Profiling of the tetraspanin CD151 web and conspiracy of CD151/integrin β1 complex in the progression of hepatocellular carcinoma. Tetraspanin CD151 has been implicated in metastasis through forming complexes with different molecular partners. In this study, we mapped tetraspanin web proteins centered on CD151, in order to explore the role of CD151 complexes in the progression of hepatocellular carcinoma (HCC). Immunoprecipitation was used to isolate tetraspanin complexes from HCCLM3 cells using a CD151 antibody, and associated proteins were identified by mass spectrometry. The interaction of CD151 and its molecular partners, and their roles in invasiveness and metastasis of HCC cells were assayed through disruption of the CD151 network. Finally, the clinical implication of CD151 complexes in HCC patients was also examined. In this study, we identified 58 proteins, characterized the tetraspanin CD151 web, and chose integrin β1 as a main partner to further investigate. When the CD151/integrin β1 complex in HCC cells was disrupte  d, migrat  ion, invasiveness, secretion of matrix metalloproteinase 9, and metastasis were markedly influenced. However, both CD151 and integrin β1 expression were untouched. HCC patients with high expression of CD151/integrin β1 complex had the poorest prognosis of the whole cohort of patients. Together, our data show that CD151 acts as an important player in the progression of HCC in an integrin β1-dependent manner. 
  GeneRIFs  
 High expression of ITGB1 is associated with   migration   and invasion of nonsmall cell lung cancer. 
 The altered expression of anti-apoptotic gene Bcl-2 played critical roles in regulating both spontaneous and radiation-induced apoptosis in the presence of RelB knockdown. For the first time, we showed that RelB knockdown significantly attenuated the   migration   and invasion of DU145 prostate cancer cells, due to the reduction of integrin b-1 
 analysis of a beta1 integrin-dependent pattern of keratinocytes   migration   under static stretch in an in vitro co-culture model 
 The aim of the present study was to characterize the roles of ITGalpha5 and ITGbeta1 in the proliferation and   migration   of vascular smooth muscle cells. 
 Schwann cells in the tumor-nerve environment affect tumor cell   migration   and invasion through regulation of the laminin receptor A6B1 and its variant A6pB1. 
 These results indicate that the effects of GMFG on monocyte   migration   and adhesion probably occur through preventing ubiquitin-mediated proteasome degradation of alpha5beta1-integrin and facilitating effective beta1-integrin recycling back to the plasma membrane. 
 Insulin-like growth factor-independent insulin-like growth factor binding protein 3 promotes cell   migration   and lymph node metastasis of oral squamous cell carcinoma cells via integrin beta1 signaling. 
 GM2 mediats tumor cell   migration   by interacting with ITGB1 leading to downstream signaling and the phosphorylation of ERK MAP kinases. 
 G-CSF stimulates beta1 integrin expression and Swan 71 cell   migration   by activating PI3K and MAPK signaling pathways. 
 Human uPAR activation and its association with beta1-integrin are required for PDGF-AB-induced   migration  . 
 We propose that PrP modulates beta1 integrin adhesion and   migration   of monocytes through RhoA-induced actin remodeling mediated by cofilin, and through the regulation of ERM-mediated membrane-cytoskeleton linkage. 
 Suggest alpha5beta1/Arp2/Arp3/FHOD3 pathway reprograms the actin cytoskeleton to promote invasive   migration   and local invasion in vivo. 
 Data suggest expression of B4GALT3 in placenta is up-regulated in third trimester; overexpression of B4GALT3 suppresses trophoblast   migration   and enhances glycosylation of ITGB1; stability of ITGB1 may regulate trophoblast invasion in late pregnancy. 
 This study suggested that PGE2 upregulates beta1-integrin expression and cell   migration   in HCC cells by activating the PKC/NF-kappaB signaling pathway 
 PIK3R3, ITGB1, ITGAL, and ITGA6, were involved in the regulation of actin cytoskeleton that link to triple-negative breast cancer   migration  . 
 angiopoietin-2 Gln362 mediates   migration   of tumor and endothelial cells through association with alpha5beta1 integrin 
 Low integrin beta 1 is associated with   migration   and invasion of colorectal cancer. 
 SERPINA5 inhibits tumor cell   migration   by modulating the fibronectin-integrin beta1 signaling pathway in hepatocellular carcinoma. 
 It plays a role in regulation of leukocyte   migration   to a site of inflammation. 
 Data show that nucleolin is a critical lipid-raft-dependent beta1-integrin-interacting protein in A375 melanoma cell spreading and   migration   on fibronectin. 
 Data indicate that macrophage-derived exosomes regulate integrin beta1-dependent   migration   of endothelial cells. 
 Data suggest that TIMP1 (tissue inhibitor of metalloproteinase-1) acts as chemoattractant for neural stem cells (NSC); TIMP1 enhances NSC adhesion,   migration  , and cytoskeletal reorganization; these effects are dependent on CD29/CD63 (granulophysin). 
 Autophagy regulates cell   migration  , a central mechanism in cell development, angiogenesis, and tumor progression, by mitigating the cell surface expression of beta1 integrins. 
 Suggest that fibronectin fine tunes LM332-mediated   migration   by boosting bronchiolar cell adhesion to substrate via integrin alpha3beta1 integrin. 
 integrin ss1 subunit critically regulates GRP-R-mediated neuroblastoma cell   migration   and invasion 
 Integrin beta1 interacts with SPARC in prostate cancer cells and suppresses cell proliferation and   migration  . 
 Data indicate that Slug siRNA suppressed the TGF-beta1-induced integrin alpha3beta1-mediated cell   migration   ability of squamous cell carcinoma HSC-4 cells. 
 an important role of AnxA2 in controlling dynamics of beta1 integrin at the cell surface that in turn is required for the active turnover of cell-matrix associations, cell   migration  , and wound closure. 
 Sdc-1 silencing promotes increased MDA-MB-231 cell   migration   via a beta1-integrin-dependent mechanism. 
 Gal-3 expression enhances beta1 integrin-mediated cell adhesion and cell   migration  . 
 The regulation of alpha4beta1-dependent adhesion and   migration   of myeloma cells by CXCL12-S1P combined activities might have important consequences for myeloma disease progression. 
 Glycogen synthase kinase 3beta inhibition prevents monocyte   migration   across brain endothelial cells via Rac1-GTPase suppression and down-regulation of active integrin conformation. 
 Endoglin-mediated fibronectin/alpha5beta1 integrin and TGF-beta1 pathway crosstalk switched the respone to TGF-beta1 from a promoter to a suppressor of   migration  , inhibiting TGF-beta1-mediated apoptosis, and partially mediating developmental angiogenesis in vivo. 
 expression of alpha9beta1 in breast cancer; alpha9beta1 acts as novel marker of basal-like breast cancer subtype; expression associated with reduced survival; its ability to promote cell   migration   and invasion suggests it contributes to aggressiveness of this subtype 
 decreased GnT-V activity due to inflammatory cytokine induction in human monocytes resulted in enchancement of integrin alpha5beta1-dependent monocyte-vascular endothelium adhesion and trans  migration   
 HMGB-1 enhances the   migration   of chondrosarcoma cells by increasing alpha5beta1 integrin expression through the RAGE receptor/PI3K/Akt/c-Jun/AP-1 signal transduction pathway. 
 uPAR is an essential component of the network through which VEGF controls integrin redistribution and endothelial cell   migration  . 
 Results demonstrate differential effects of Snail and Slug in pancreatic cancer and identify the interplay between Rho signaling and beta1-integrin that regulates the scattering and   migration   of Snail- and Slug-expressing pancreatic cancer cells. 
 Fibronectin induces endothelial cell   migration   through beta1 integrin and Src-dependent phosphorylation of fibroblast growth factor receptor-1 at tyrosines 653/654 and 766. 
 WASH and Arp2/3 regulates integrin alpha5beta1-mediated invasive cell   migration  . 
 The results showed that overexpression of Id1 enhanced cell   migration   and increased beta1-integrin expression 
 ACAP4 protein cooperates with Grb2 protein to orchestrate epidermal growth factor-stimulated integrin beta1 recycling in cell   migration   
 Integrin alpha5beta1 might involves the abnormal proliferation and   migration   of A549 cells through mediating ERK signal transduction pathway. 
 MSC   migration   is controlled by CD73 & CD29, which in turn are regulated by mechanical stimulation of cells 
 IGF-I enhances the   migration   of chondrosarcoma cells by increasing alpha5beta1 integrin expression through the IGF-I receptor/PI3K/Akt/NF-kappaB signal transduction pathway. 
 Mesenchymal stem cell   migration   is regulated by fibronectin through alpha5beta1-integrin-mediated activation of PDGFR-beta and potentiation of PDGF-BB signals. 
 The extra nuclear AR/FlnA/integrin beta 1 complex is the key by which androgen activates signaling leading to cell   migration  . Assembly of this ternary complex may control organ development and prostate cancer metastasis. 
 Data show that interactions between beta1 integrin and ErbB4 occur in a spatiotemporally-regulated manner, and such interaction contributes to the integrin-dependent cell   migration  . 
 Lewis y can influence the biological behavior of a tumor cell as an important composition of integrins alpha5 and beta1 by some signal pathway, such as promoting cell adhesion and   migration  . 
 the three-amino acid sequence, EYP, encoded by exon 3 of VEGF-A is essential for binding of VEGF to integrin alpha9beta1 and induces adhesion and   migration   of endothelial and cancer cells 
 the CCR7/CCL21 signaling pathway leading to T lymphocyte   migration   on fibronectin is a beta1 integrin-dependent pathway involving transient ERK1/2 phosphorylation, which is modulated by PLCgamma1 
 The beta1 integrin-interacting domain in the extracellular domain of IL-3Rbeta was identified. A fragment of a protein corresponding to domain 4 of IL-3Rbeta prevented IL-3-mediated arterial morphogenesis and endothelial cell   migration  . 
 our data indicate that CCR7 regulate cell adhesion and   migration   via beta1 integrin in metastatic squamous cell carcinoma of the head and neck 
 Calcium regulates integrin beta1 activity and expression and cell   migration   of HaCaT cells. 
 Results identify PIPKIbeta as a novel regulator of focal adhesion disassembly and suggest that PIPKIbeta spatially regulates beta1 integrin endocytosis at adhesion sites to control cell   migration  . 
 Results suggest a novel function for Rab1a in the regulation of cell   migration   through controlling integrin beta1 recycling and localization to lipid rafts via a specific downstream effector pathway. 
 up-regulation of alpha9beta1 expression among all beta1 integrins during neutrophil activation and transendothelial   migration   and a synergy between alpha9beta1 and beta2 integrins in stabilizing neutrophil adhesion to endothelium under flow conditions 
 Interleukin-8 (CXCL8) stimulates trophoblast cell   migration   and invasion by increasing levels of integrins alpha5 and beta1. 
 Laminin-derived peptide AG73 regulates   migration  , invasion, and protease activity of human oral squamous cell carcinoma cells through syndecan-1 and beta1 integrin. 
 Prostaglandin E2 enhances the   migration   of chondrosarcoma cells by increasing alpha2beta1 integrin expression through the EP1/PLC/PKCalpha/c-Src/NF-kappaB signal transduction pathway. 
 SEMA3A suppression of tumor cell   migration   is dependent on alpha2beta1, the expression of which is stimulated in breast tumor cells by an autocrine SEMA3A pathway. 
 although alpha9beta1 integrin can induce and localise to focal adhesions in a high activation state, its intermediate activity state normally supports cell adhesion consistent with   migration   
 Gal-3 promotes epithelial cell   migration   by cross-linking MGAT5-modified complex N-glycans on alpha3beta1 integrin. 
 VAMP2 mediates the trafficking of alpha5beta1 integrin to the plasma membrane and VAMP2-dependent integrin trafficking is critical in cell adhesion,   migration   and survival. 
 ECRG2 is involved in the regulation of cell   migration  /invasion through uPA/uPAR/beta1 integrin pathway. 
 ERbeta affects integrin expression and clustering and consequently modulates adhesion and   migration   of breast cancer cells. 
 In intestinal crypt cells, integrin alpha8beta1 is closely involved in the regulation of adhesion,   migration   and cell proliferation via a predominant RhoA/ROCK-dependent mechanism. 
 JAM-A dimerization facilitates formation of a complex with Afadin and PDZ-GEF2 that activates Rap1A, which regulates beta1 integrin levels and cell   migration  . 
 Suggest that the specific interaction of MCT4 with beta(1)-integrin may regulate cell   migration   through modulation of focal adhesions. 
 Tyr-302 in RACK1 is required for interaction with PP2A and beta1 integrin, for regulation of PP2A activity, and for IGF-I-mediated cell   migration   and proliferation 
 Osteoblast-derived BMP-2 act through Akt and ERK, which in turn activates IKK alpha/beta and NF-kappaB, resulting in the activations of beta1 and beta 3 integrins and contributing the   migration   of prostate cancer cells. 
 Integrin alpha2beta1 may promote   migration   and invasion of neuroblastoma cells. 
 key players involved in trans  migration   and invasion of MSCs are the endothelial phenotype, VCAM-1/VLA-4, beta1 integrins, MMP-2 secretion and cytokines. 
 Show a critical role of PKCdelta in the regulation of renal carcinoma cell   migration  , which seems to be caused by affecting the expression and activity of beta1 integrins and focal adhesion kinase. 
 Lumican induces cell   migration   by activating ERK 1/2 signaling and ERK 1/2 stimulates expression of integrin beta1. 
 TGF-beta2 promoted human lens epithelial cell adhesion and   migration   in vitro. Integrin beta1 and integrin-mediated signaling are necessary for TGF-beta2-promoted adhesion and   migration   in human lens epithelial cells. 
 The process of dynamic integrin regulation may be an important part in tumour cell   migration   strategy, switching the cells from being adhesive to becoming motile and invasive. 
 that the expression of beta(1C) integrin, a very efficient inhibitor of cell proliferation, may be modulated by the maternal microenvironment and may play a fundamental role in mediating trophoblast outgrowth and   migration   during pregnancy. 
 Increases in IGF-IR decrease beta1 integrin expression, and enhance cell   migration   in neuroblastoma cells. 
 KSHV gB can activate VEGFR-3 on the microvascular endothelium and modulate endothelial cell   migration   and proliferation via an interaction between the alpha3beta1 integrin and the VEGFR-3 receptor 
 IFN-gamma attenuates epithelial wound closure by microtubule-dependent redirection of beta(1) integrin transcytosis from the leading edge of migrating cells thereby inhibiting adequate turnover of focal adhesion complexes and cell   migration  . 
 The integrin receptors of tenascin (TN) and fibronectin (FN) may be involved in cell processes such as growth, adhesion,   migration   and apoptosis. 
 support a model in which KAI1/CD82 attenuates the maturation of the beta1 integrin precursor and thereby suppresses cell   migration   
 RACK1 enhances IGF-I-mediated cell   migration   through its ability to exclusively associate with either beta1 integrin or PP2A in a complex at the IGF-IR. 
 thymidine phosphorylase and 2-deoxyribose-induced focal adhesion kinase phosphorylation was blocked by the antibodies to integrins alpha 5 beta 1 and alpha v beta 3, directly linking their   migration   and signaling components 
 alpha(v)beta(1) supports fetal beta-cell   migration   and could play an important role in early motile processes required for islet neogenesis 
 There is a desensitization of IL-8-mediated p42/p44 MAPK signaling in response to ligation of the alpha5beta1 integrin in PMNL. There may be an interplay between integrin and chemokine signaling during PMNL   migration   through the extracellular matrix. 
 beta 1 integrin has roles in cell adhesion and   migration   in development and metastasis [review] 
 Data demonstrate that alpha6beta4 integrin mediates pancreatic epithelial cell adhesion to Netrin-1, whereas recruitment of alpha6beta4 and alpha3beta1 regulate the   migration   of putative pancreatic progenitors on Netrin-1. 
  ECM  PubMed  
 Filamin A-beta1 integrin complex tunes epithelial cell response to matrix tension. The physical properties of the extracellular matrix (  ECM  ) regulate the behavior of several cell types; yet, mechanisms by which cells recognize and respond to changes in these properties are not clear. For example, breast epithelial cells undergo ductal morphogenesis only when cultured in a compliant collagen matrix, but not when the tension of the matrix is increased by loading collagen gels or by increasing collagen density. We report that the actin-binding protein filamin A (FLNa) is necessary for cells to contract collagen gels, and pull on collagen fibrils, which leads to collagen remodeling and morphogenesis in compliant, low-density gels. In stiffer, high-density gels, cells are not able to contract and remodel the matrix, and morphogenesis does not occur. However, increased FLNa-beta1 integrin interactions rescue gel contraction and remodeling in high-density gels, resulting in branching morphogenesis. These results suggest morphogenesis can be "tuned" by the balance between cell-generated contractility and opposing matrix stiffness. Our findings support a role for FLNa-beta1 integrin as a mechanosensitive complex that bidirectionally senses the tension of the matrix and, in turn, regulates cellular contractility and response to this matrix tension. 
 Filamin A-beta1 integrin complex tunes epithelial cell response to matrix tension. The physical properties of the extracellular matrix (  ECM  ) regulate the behavior of several cell types; yet, mechanisms by which cells recognize and respond to changes in these properties are not clear. For example, breast epithelial cells undergo ductal morphogenesis only when cultured in a compliant collagen matrix, but not when the tension of the matrix is increased by loading collagen gels or by increasing collagen density. We report that the actin-binding protein filamin A (FLNa) is necessary for cells to contract collagen gels, and pull on collagen fibrils, which leads to collagen remodeling and morphogenesis in compliant, low-density gels. In stiffer, high-density gels, cells are not able to contract and remodel the matrix, and morphogenesis does not occur. However, increased FLNa-beta1 integrin interactions rescue gel contraction and remodeling in high-density gels, resulting in branching morphogenesis. These results suggest morphogenesis can be "tuned" by the balance between cell-generated contractility and opposing matrix stiffness. Our findings support a role for FLNa-beta1 integrin as a mechanosensitive complex that bidirectionally senses the tension of the matrix and, in turn, regulates cellular contractility and response to this matrix tension. 
 Alpha5beta1 integrin-fibronectin interactions specify liquid to solid phase transition of 3D cellular aggregates. Tissue organization during embryonic development and wound healing depends on the ability of cells on the one hand to exchange adhesive bonds during active rearrangement and on the other to become fixed in place as tissue homeostasis is reached. Cells achieve these contradictory tasks by regulating either cell-cell adhesive bonds, mediated by cadherins, or cell-extracellular matrix (  ECM  ) connections, regulated by integrins. Integrin alpha5beta1 and soluble fibronectin (sFN) are key players in cell-  ECM   force generation and in   ECM   polymerization. Here, we explore the interplay between integrin alpha5beta1 and sFN and its influence on tissue mechanical properties and cell sorting behavior.We generated a series of cell lines varying in alpha5beta1 receptor density. We then systematically explored the effects of different sFN concentrations on aggregate biomechanical properties using tissue surface tensiometry. We found previously unreported complex behaviors including the observation that interactions between fibronectin and integrin alpha5beta1 generates biphasic tissue cohesion profiles. Specifically, we show that at constant sFn concentration, aggregate cohesion increases linearly as alpha5beta1 receptor density is increased from low to moderate levels, producing a transition from viscoelastic-liquid to pseudo viscoelastic-solid behavior. However, further increase in receptor density causes an abrupt drop in tissue cohesion and a transition back to viscoelastic-liquid properties. We propose that this may be due to depletion of sFn below a critical value in the aggregate microenvironment at high alpha5beta1 levels. We also show that differential expression of alpha5beta1 integrin can promote phase-separation between cells.The interplay between alpha5-integrin and sFn contributes significantly to tissue cohesion and, depending on their level of expression, can mediate a shift from liquid to elastic behavior. This interplay represents a tunable level of control between integrins and the   ECM   that can influence tissue cohesion and other mechanical properties, which may translate to the specification of tissue structure and function. These studies provide insights into important biological processes such as embryonic development, wound healing, and for tissue engineering applications. 
 Caveolin-1-dependent beta1 integrin endocytosis is a critical regulator of fibronectin turnover. beta1 integrins are major cell surface receptors for fibronectin. Some integrins, including beta1 integrins, are known to undergo constitutive endocytosis and recycling. Integrin endocytosis/recycling has been implicated in the regulation of cell migration. However, the mechanisms by which integrin endocytosis/recycling regulates cell migration, and other biological consequences of integrin trafficking are not completely understood. We previously showed that turnover of extracellular matrix (  ECM  ) fibronectin occurs via receptor-mediated endocytosis. Here, we investigate the biological relevance of beta1 integrin endocytosis to fibronectin matrix turnover. First, we demonstrate that beta1 integrins, including alpha5beta1 play an important role in endocytosis and turnover of matrix fibronectin. Second, we show that caveolin-1 constitutively regulates endocytosis of alpha5beta1 integrins, and that alpha5beta1 integrin endocytosis can occur in the absence of fibronectin and fibronectin matrix. We also show that downregulation of caveolin-1 expression by siRNA results in marked reduction of beta1 integrin and fibronectin endocytosis. Hence, caveolin-1-dependent beta1 integrin and fibronectin endocytosis plays a critical role in fibronectin matrix turnover, and may contribute to abnormal   ECM   remodeling that occurs in fibrotic disorders. 
 Focal adhesion kinase modulates cell adhesion strengthening via integrin activation. Focal adhesion kinase (FAK) is an essential nonreceptor tyrosine kinase regulating cell migration, adhesive signaling, and mechanosensing. Using FAK-null cells expressing FAK under an inducible promoter, we demonstrate that FAK regulates the time-dependent generation of adhesive forces. During the early stages of adhesion, FAK expression in FAK-null cells enhances integrin activation to promote integrin binding and, hence, the adhesion strengthening rate. Importantly, FAK expression regulated integrin activation, and talin was required for the FAK-dependent effects. A role for FAK in integrin activation was confirmed in human fibroblasts with knocked-down FAK expression. The FAK autophosphorylation Y397 site was required for the enhancements in adhesion strengthening and integrin-binding responses. This work demonstrates a novel role for FAK in integrin activation and the time-dependent generation of cell-  ECM   forces. 
 β1-Integrin via NF-κB signaling is essential for acquisition of invasiveness in a model of radiation treated in situ breast cancer. Ductal carcinoma in situ (DCIS) is characterized by non-invasive cancerous cell growth within the breast ducts. Although radiotherapy is commonly used in the treatment of DCIS, the effect and molecular mechanism of ionizing radiation (IR) on DCIS are not well understood, and invasive recurrence following radiotherapy remains a significant clinical problem. This study investigated the effects of IR on a clinically relevant model of Akt-driven DCIS and identified possible molecular mechanisms underlying invasive progression in surviving cells.We measured the level of phosphorylated-Akt (p-Akt) in a cohort of human DCIS specimens by immunohistochemistry (IHC) and correlated it with recurrence risk. To model human DCIS, we used Akt overexpressing human mammary epithelial cells (MCF10A-Akt) which, in three-dimensional laminin-rich extracellular matrix (  lrE  CM) and in vivo, form organotypic DCIS-like lesions with lumina expanded by pleiomorphic cells contained within an intact basement membrane. In a population of cells that survived significant IR doses in three-dimensional   lrE  CM, a malignant phenotype emerged creating a model for invasive recurrence.P-Akt was up-regulated in clinical DCIS specimens and was associated with recurrent disease. MCF10A-Akt cells that formed DCIS-like structures in three-dimensional   lrE  CM showed significant apoptosis after IR, preferentially in the luminal compartment. Strikingly, when cells that survived IR were repropagated in three-dimensional   lrE  CM, a malignant phenotype emerged, characterized by invasive activity, up-regulation of fibronectin, α5β1-integrin, matrix metalloproteinase-9 (MMP-9) and loss of E-cadherin. In addition, IR induced nuclear translocation and binding of nuclear factor-kappa B (NF-κB) to the β1-integrin promoter region, associated with up-regulation of α5β1-integrins. Inhibition of NF-κB or β1-integrin signaling abrogated emergence of the invasive activity.P-Akt is up-regulated in some human DCIS lesions and is possibly associated with recurrence. MCF10A-Akt cells form organotypic DCIS-like lesions in three-dime  nsi  onal lrECM and in vivo, and are a plausible model for some forms of human DCIS. A population of Akt-driven DCIS-like spheroids that survive IR progresses to an invasive phenotype in three-dime  nsi  onal lrECM mediated by β1-integrin and NF-κB signaling. 
 Mesenchymal stem cell migration is regulated by fibronectin through α5β1-integrin-mediated activation of PDGFR-β and potentiation of growth factor signals. Cell migration during vascular remodelling is regulated by crosstalk between growth factor receptors and integrin receptors, which together coordinate cytoskeletal and motogenic changes. Here, we report extracellular matri  x (  ECM)-directed crosstalk between platelet-derived growth factor receptor (PDGFR)-β and α5β1-integrin, which controls the migration of mesenchymal stem (stromal) cells (MSCs). Cell adhesion to fibronectin induced α5β1-integrin-dependent phosphorylation of PDGFR-β in the absence of growth factor stimulation. Phosphorylated PDGFR-β co-immunoprecipitated with α5-integrin and colocalised with α5β1-integrin in the transient tidemarks of focal adhesions. Adhesion to fibronectin also strongly potentiated PDGF-BB-induced PDGFR-β phosphorylation and focal adhesion kinase (FAK) activity, in an α5β1-integrin-dependent manner. PDGFR-β-induced phosphoinositide 3-kinase (PI3K) and Akt activity, actin reorganisation and cell migration were all regulated by fibronectin and α5β1-integrin. This synergistic relationship between α5β1-integrin and PDGFR-β is a fundamental determinant of cell migration. Thus, fibronectin-rich matrices can prime PDGFR-β to recruit mesenchymal cells at sites of vascular remodelling. 
 The role of extracellular matrix, integrins, and cytoskeleton in mechanotransduction of centrifugal loading. The study was aimed to investigate the role of the "extracellular matrix (  ECM  )-integrins-cytoskeleton" signal pathway in mechanotransduction of centrifugal loading. MG-63 osteoblasts were exposed to centrifugal loading at 209xg for 10 min. Uncentrifuged cells and centrifuged cells that have been trypsinized and suspended in liquors were designed as control. The changes in F-actin and alpha-actin cytoskeleton, gene transcription of   ECM   components, and integrins expression were analyzed by LSCM, Real-Time RT-PCR and FCM, respectively. A temporary and fast reversible change was observed in F-actin and alpha-actin cytoskeleton. And the change was paralleled with the fast autoregulation in gene transcription of   ECM   components of fibronection, osteopontin and Collagen I, and integrins expression of both alpha2 and beta1 subunits. The result suggested that cytoskeleton was a possible mechanical sensor to centrifugal stimuli, and the cytoskeleton regulation to centrifugal loading was in an   ECM  -dependent and integrin-mediated manner. 
 α3β1 integrins regulate CD151 complex assembly and membrane dynamics in carcinoma cells within 3D environments. Integrins are extracellular matrix   (E  CM) receptors that are key players in the regulation of tumour cell invasion. The laminin-binding integrin α3β1 has previously been shown to regulate adhesion and migration of carcinoma cells in part through co-operative signalling with the tetraspanin family of transmembrane proteins. However, the spatial and temporal regulation of crosstalk between these families of transmembrane proteins in intact cells remains poorly understood. Here we have used fluorescence resonance energy transfer (FRET) to demonstrate for the first time that α3β1 and the tetraspanin CD151 directly associate at the front and retracting rear of polarised migrating breast carcinoma cells in both two-dimentional (2D) and three-dimentional (3D)matrices. Furthermore, localised α3β1-CD151 binding correlates with lower CD151 homodimerisation in cells migrating on laminin or within matrigel. Loss of α3β1 integrin leads to increased CD151 homodimer formation, increased activation of Rho GTPase, loss of cell polarity and decreased invas  ion   in 3D ECM. As a result, α3-silenced cells show decreased actin-based membrane protrusion and retraction in both 2D and 3D environments. These data demonstrate that associations between α3β1 and CD151 occur dynamically within discrete subcellular compartments and act to establish local GTPase signalling to promote tumour cell invasion. These novel findings shed light on the complex crosstalk and switching between receptor complexes in response to different extracellular cues during cell invasion in 3D environments. 
 Cyclic stretch-induced TGF-β1 and fibronectin expression is mediated by β1-integrin through c-Src- and STAT3-dependent pathways in renal epithelial cells. Extracellular matrix   (E  CM) proteins, including fibronectin, may contribute to the early development and progression of renal interstitial fibrosis associated with chronic renal disease. Recent studies showed that β1-integrin is associated with the development of renal fibrosis in a murine model of unilateral ureteral obstruction (UUO). However, the molecular events responsible for β1-integrin-mediated signaling, following UUO, have yet to be determined. In this study, we investigated the mechanism by which mechanical stretch, an in vitro model for chronic obstructive nephropathy, regulates fibronectin and transforming growth factor-β1 (TGF-β1) expression in cultured human proximal tubular epithelium (HK-2) cells. Mechanical stretch upregulated fibronectin and TGF-β1 expression and activated signal transducer and transcription factor 3 (STAT3) in a time-dependent manner. Stretch-induced fibronectin and TGF-β1 were suppressed by a STAT3 inhibitor, S3I-201, and by small interfering RNA (siRNA) targeting human STAT3 (STAT3 siRNA). Similarly, fibronectin and TGF-β1 expression and STAT3 activation induced by mechanical stretch were suppressed by the Src family kinase inhibitor PP2 and by transfection of HK-2 cells with a dominant-negative mutant of c-Src (DN-Src), whereas PP3, an inactive analog of PP2, had no significant effect. Furthermore, mechanical stretch resulted in increased β1-integrin mRNA and protein levels in HK-2 cells. Furthermore, neutralizing antibody against β1-integrin and silencing of β1-integrin expression with siRNAs resulted in decreased c-Src and STAT3 activation and TGF-β1 and fibronectin expression evoked by mechanical stretch. This work demonstrates, for the first time, a role for β1-integrin in stretch-induced renal fibrosis through the activation of c-Src and STAT3 signaling pathways. 
 AMPA receptors promote perivascular glioma invasion via beta1 integrin-dependent adhesion to the extracellular matrix. High-grade gliomas release excitotoxic concentrations of glutamate, which has been shown to enhance tumor proliferation and migration. alpha-Amino-3-hydroxy-5-methylisoxazole-4-propionic acid (AMPA) glutamate receptors are abundantly expressed at the invading edge of glioblastoma specimens, suggesting they may play an important biologic role in tumor invasion. In this study, we examined potential mechanisms by which AMPA receptor (AMPAR) expression and stimulation promote glioma cell migration and invasion. Overexpression of GluR1, the most abundant AMPAR subunit in gliomas, positively correlated with glioma cell adhesion to type I and type IV collagen, which was decreased in cells with knockdown of GluR1 and with blocking antibodies to beta1 integrin. Furthermore, stimulation of the AMPAR led to detachment of cells from the extracellular matrix (  ECM  ). Immunoprecipitation studies showed that GluR1 associated with the actin cytoskeleton-linked protein band 4.1B (brain type), which may serve as a link between GluR1 and integrins. Overexpression of GluR1 correlated with increased cell-surface expression of beta1 integrin, increased phosphorylation of focal adhesion kinase (FAK-Y397), and enhanced numbers of focal adhesion (FA) complexes. Cells overexpressing GluR1 had increased colocalization of actin and paxillin at FAs and, in several glioma cell lines, significantly increased invasion in an in vitro Matrigel transwell assay. Likewise, in an intracranial xenograft model, overexpression of GluR1 led to perivascular and subependymal glioma cell invasion similar to patterns of tumor dissemination described in human glioblastoma. Together, these results suggest that AMPARs may link signals from the   ECM   to sites of FA, where signal integration promotes tumor invasion. 
 Decreased expression of alpha3 and beta1 integrin subunits is responsible for differentiation-associated changes in cells behavior in terminally differentiated human oral keratinocytes. Primary normal human oral keratinocytes (NHOKs) terminally differentiate in serial subculture. To investigate whether this subculture-induced differentiation of NHOKs affects integrin expression and cell-matrix interaction, we studied the expression levels of integrin subunits and cellular response to the extracellular matrix (  ECM  ) proteins in NHOKs at different population doublings. The phosphorylation statuses of focal adhesion kinase (FAK), extracellular signal regulated kinase (ERK), p38, and c-Jun amino-terminal kinase (JNK) were also determined in NHOK cells cultured on   ECM   proteins, to evaluate the functions of integrins with respect to cellular responses to   ECM   proteins. The expression levels of alpha3 and beta1 integrin subunits progressively decreased in NHOKs undergoing terminal differentiation. The ability of NHOKs to spread upon laminin and type I collagen significantly decreased in terminally differentiated oral keratinocytes. Keratinocyte migration was significantly increased on type I collagen for terminally differentiated NHOKs. Similar results were seen following preincubation of rapidly proliferating NHOKs with function-blocking antibodies to alpha3 or beta1 integrin subunit. In contrast, fibronectin had no effect on cellular responses in NHOKs, which were almost negligible in the expression levels of alpha5 integrin subunits. The extent of FAK phosphorylation in terminally differentiated NHOKs was notably lower than that of rapidly proliferating cells, but was enhanced in terminally differentiated cells that were cultured on type I collagen. Our results indicate that decreased expression of alpha3 and beta1 integrin subunits is responsible for differentiation-associated changes in cells behavior in terminally differentiated oral keratinocytes. Our data also show that the abrogation of the alpha5beta1 integrin function caused by omitting alpha5 subunit is linked to the loss of a cell-fibronectin interaction in human oral keratinocytes. 
   ECM   regulates MT1-MMP localization with beta1 or alphavbeta3 integrins at distinct cell compartments modulating its internalization and activity on human endothelial cells. Regulation of membrane-type 1 matrix metalloproteinase (MT1-MMP) by different extracellular matrices (  ECM  s) on human endothelial cells (ECs) has been investigated. First, MT1-MMP is found at the intercellular contacts of confluent ECs grown on beta1 integrin-dependent matrix such as type 1 collagen (COL I), fibronectin (FN), or fibrinogen (FG), but not on gelatin (GEL) or vitronectin (VN). The novel localization of MT1-MMP at cell-cell contacts is assessed by confocal videomicroscopy of MT1-MMP-GFP-transfected ECs. Moreover, MT1-MMP colocalizes with beta1 integrins at the intercellular contacts, whereas it is preferentially found with alphavbeta3 integrin at motility-associated structures on migrating ECs. In addition, clustered integrins recruit MT1-MMP and neutralizing anti-beta1 or anti-alphav integrin mAb displace MT1-MMP from its specific sites, pointing to a biochemical association that is finally demonstrated by coimmunoprecipitation assays. On the other hand, COL I, FN, or FG up-regulate cell surface MT1-MMP on confluent ECs by an impairment of its internalization, whereas expression and internalization are not modified on GEL or VN. In addition, MT1-MMP activity is diminished in confluent ECs on COL I, FN, or FG. Finally, MT1-MMP participates and cooperates with beta1 and alphavbeta3 integrins in the migration of ECs on different   ECM  . These data show a novel mechanism by which   ECM   regulates MT1-MMP association with beta1 or alphavbeta3 integrins at distinct cellular compartments, thus modulating its internalization, activity, and function on human ECs. 
 The integrin expression profile modulates orientation and dynamics of force transmission at cell-matrix adhesions. Integrin adhesion receptors connect the extracellular matrix (  ECM  ) to the cytoskeleton and serve as bidirectional mechanotransducers. During development, angiogenesis, wound healing and cancer progression, the relative abundance of fibronectin receptors, including integrins α5β1 and αvβ3, changes, thus altering the integrin composition of cell-matrix adhesions. Here, we show that enhanced αvβ3 expression can fully compensate for loss of α5β1 and other β1 integrins to support outside-in and inside-out force transmission. α5β1 and αvβ3 each mediate actin cytoskeletal remodeling in response to stiffening or cyclic stret  chi  ng of the ECM. Likewise, α5β1 and αvβ3 support cellular traction forces of comparable magnitudes and similarly increase these force  s i  n response to ECM stiffening. However, cells using αvβ3 respond to lower stiffness ranges, reorganize their actin cytoskeleton more substantially in response to stretch, and show more randomly oriented traction forces. Centripetal traction force orientation requires long stress fibers that are formed through the action of Rho kinase (ROCK) and myosin II, and that are supported by α5β1. Thus, altering the relative abundance of fibronectin-binding integrins in cell-matrix adhesions affects the spatiotemporal organization of force transmission. 
 Reduced podocyte expression of alpha3beta1 integrins and podocyte depletion in patients with primary focal segmental glomerulosclerosis and chronic PAN-treated rats. Integrins attach cells to extracellular matrix (  ECM  ) and mediate signals from   ECM   to cells or from cells to   ECM  . They regulate cell functions, including adhesion, migration, cell cycle regulation, and differentiation. Podocytes may detach from the glomerular basement membrane (GBM) and be excreted in the urine, and proteinuria is found in patients with primary focal segmental glomerulosclerosis (FSGS); both may be associated with loss of alpha3beta1integrins. In this study, we have examined the podocyte number in patients with primary FSGS and normal controls, and the alpha3- and beta1-integrin subunits expression of podocytes in patients with primary FSGS and chronic puromycin aminonucleoside (PAN)-treated rats by the morphometric, immunoperoxidase histochemical, and immunoelectron microscopic examination. We also measured their expression serially in rats that received repeated PAN injection. The results showed that the podocyte number was significantly decreased in patients with primary FSGS than in normal control (P < 0.05). The immunostaining score showed that both alpha3- and beta1-integrin subunits on podocytes in patients with primary FSGS were significantly lower than in normal controls (both P < 0.01). The number of immuno-gold particles of alpha3- and beta1-integrins at the effaced foot process area of patients with primary FSGS were also significantly decreased than that of normal controls (both P < 0.05). The immunostaining score of both alpha3- and beta1-integrin subunits was negatively correlated with the degree of glomerular sclerosing score and the amount of daily protein loss, and they were positively correlated with the number of podocytes. Chronic 12-week PAN-treated rats showed similar findings with decreased immunostaining expression and immuno-gold particles of alpha3-integrin on podocytes than in normal control (both P < 0.05). The chronic PAN-treated rats also showed a trend toward gradually decreased immunostaining expression of alpha3-integrin subunit on podocyte during the progress from normal to FSGS state. These studies indicate that podocyte expression of alpha3- and beta1-integrin subunits is significantly reduced in humans with primary FSGS and chronic PAN-treated rats, before the morphological changes of FSGS are observed. The decreased podocyte expression of alpha3beta1 integrins is closely related with podocyte depletion, glomerular sclerosis, and daily protein loss in patients with primary FSGS. 
 Sphingosine-1-phosphate induces a PDGFR-dependent cell detachment via inhibiting beta1 integrin in HEK293 cells. Several different types of interactions between sphingosine-1-phosphate (S1P) receptors and platelet-derived growth factor receptor (PDGFR) have been revealed recently. In this work, we used HEK293 cells to further investigate the potential crosstalk. Interestingly, we observed that S1P specifically induced a PDGFR-dependent cell detachment in HEK293 cells, which could be inhibited by AG1296, a specific inhibitor for PDGFR. EGFR on the other hand, did not have any effect on cell detachment. The detachment was extracellular matrix (  ECM  ) protein specific, suggesting the involvement of specific integrin molecules. When beta(1) integrin was engaged into an active state, S1P-induced cell detachment was blocked, suggesting that S1P induced an inside-out inhibitory effect on beta(1) integrin. G(i) protein and ERK activation were required for the cell detachment induced by S1P, suggesting an endogenous receptor for S1P is likely to be involved. 
 Immediate early response protein 2 regulates hepatocellular carcinoma cell adhesion and motility via integrin β1-mediated signaling pathway. Human immediate early response 2 (IER2) has been reported to function as a potential transcriptional factor or transcriptional co‑activator and seems to play a pivotal role in tumor cell motility and metastasis, however, its role and underlying mechanisms in hepatocellular carcinoma (HCC) remain unknown. Herein, we demonstrated that overexpression of IER2 in HCC cells increased cell adhesion to fibronectin, migration and invasion, whereas knockdown of IER2 displayed the opposite effects. In agreement with this phenotype, IER2 expression was positively correlated with the metastatic potential and integrin β1 (ITGB1) expression in HCC cell lines. Moreover, we demonstrated a critical role for IER2 in regulation of HCC cell‑extracellu  lar   matrix (ECM) adhesion and motility by the transcriptionally promoted ITGB1. Furthermore, we showed that ITGB1‑focal adhesion kinase (FAK)‑Src‑paxillin signal pathway activated by IER2 may contrib  ute   to the HCC cell‑ECM adhesion and motility. These results demonstrated that IER2 promoted HCC cell adhesion and motility probably by directly increasing ITGB1 expression and subsequently activating the ITGB1‑FAK‑Src‑paxillin signal pathway. 
 α5β1 integrin recycling promotes Arp2/3-independent cancer cell invasion via the formin FHOD3. Invasive migration in 3D extracellular matrix   (E  CM) is crucial to cancer metastasis, yet little is known of the molecular mechanisms that drive reorganization of the cytoskeleton as cancer cells disseminate in vivo. 2D Rac-driven lamellipodial migration is well understood, but how these features apply to 3D migration is not clear. We find that lamellipodia-like protrusions and retrograde actin flow are indeed observed in cells moving in 3  D E  CM. However, Rab-coupling protein (RCP)-driven endocytic recycling of α5β1 integrin enhances invasive migration of cancer cells into fibronectin-rich   3D   ECM, driven by RhoA and filopodial spike-based protrusions, not lamellipodia. Furthermore, we show that actin spike protrusions are Arp2/3-independent. Dynamic actin spike assembly in cells invading in vitro and in vivo is regulated by Formin homology-2 domain containing 3 (FHOD3), which is activated by RhoA/ROCK, establishing a novel mechanism through which the RCP-α5β1 pathway reprograms the actin cytoskeleton to promote invasive migration and local invasion in vivo. 
 Overexpression of the integrin beta(1A) subunit and the beta(1A) cytoplasmic domain modifies the beta-adrenergic regulation of the cardiac L-type Ca(2+)current. Integrins are a family of cell-surface receptors that link the extracellular matrix (  ECM  ) to the cellular cytoskeleton. The goal of this study was to determine the importance of the integrin beta(1) subunit in regulating cardiac L-type Ca(2+) channel function. Neonatal rat ventricular myocytes were cultured on collagen membranes and infected with adenovirus expressing either the human beta(1A) integrin (Adbeta(1A)) or a chimeric protein consisting of the cytoplasmic tail domain of the beta(1A) integrin and the extracellular/transmembrane domain of the interleukin-2 receptor (AdTAC-beta(1)). Expression of the free beta(1) integrin tail (TAC-beta(1)), but not the full-length beta(1A) integrin, altered cell morphology and disrupted normal cell adhesion. When compared with myocytes infected with control virus, neither Adbeta(1A) nor AdTAC-beta(1) infection produced any significant change in the current vs. voltage relationship of the whole-cell Ca(2+) current (I(Ca)) or the kinetics of I(Ca) decay. Expression of TAC-beta(1), but not beta(1A), induced a negative shift in the Ca(2+) channel steady-state inactivation curve. Application of the beta-adrenergic receptor agonist isoproterenol produced over a 90% increase in I(Ca) in control cells, but caused only an 18% increase in myocytes overexpressing the full-length beta(1A) integrin. In addition, beta-adrenergic stimulation resulted in a 5-10-fold increase in intracellular cAMP levels in control cells, but produced no significant response in Adbeta(1A)-infected cells. In contrast, expression of TAC-beta(1) was associated with an augmentation in the Ca(2+) channel response to isoproterenol (160% increase) and the Ca(2+) channel agonist BayK8644. Thus, integrin/  ECM   interactions may be critical in the regulation of I(Ca) 
 A mitochondrial protein, Bit1, mediates apoptosis regulated by integrins and Groucho/TLE corepressors. A delicate balance of signals regulates cell survival. One set of these signals is derived from integrin-mediated cell adhesion to the extracellular matrix (  ECM  ). Loss of cell attachment to the   ECM   causes apoptosis, a process known as anoikis. In searching for proteins involved in cell adhesion-dependent regulation of anoikis, we identified Bit1, a mitochondrial protein that is released into the cytoplasm during apoptosis. Cytoplasmic Bit1 forms a complex with AES, a small Groucho/transducin-like enhancer of split (TLE) protein, and induces cell death with characteristics of caspase-independent apoptosis. Cell attachment to fibronectin counteracts the apoptotic effect of Bit1 and AES. Increasing Bit1 expression enhances anoikis, while suppressing the expression reduces it. Thus, we have elucidated an integrin-controlled pathway that is, at least in part, responsible for the cell survival effects of cell-  ECM   interactions. 
 High-level β1-integrin expression in a subpopulation of highly tumorigenic oral cancer cells. The β1 integrin (CD29) is a putative marker for cancerous epithelial stem cells. Cancer stem cells are essential to drive tumor growth, recurrence, and metastasis. We investigated the role of β1-integrin expression in the development of malignant phenotypes of oral squamous cell carcinoma (OSCC).Immunostaining was used to analyze the expression levels of β1 integrins in different types of cell colonies and tumor spheres. The results of cell viability and migration assays with and without siRNA knockdown of β1-integrin expression were compared. Cells expressing β1 integrins were evaluated for their tumorigenicity in mice. The expression of β1 integrins in human specimens of oral cancers at different clinical stages was semiquantified based on immunohistochemical staining of the β1-integrin protein.The expression level of β1 integrins in Meng-1 oral epidermoid carcinoma   ce  lls (OECM-1) cells was significantly higher in holoclonal colonies and tumor spheres compared to control cells. The knockdown of β1-integrin expre  ssi  on in OECM-1 cells reduced cell proliferation, migration, and tumor sphere formation. Beta-1 integrin (+) cells were more tumorigenic in the mouse xenograft model than β1 integrin (-) cells. In the human specimens, the expression level of the β1-integrin protein positively correlated with the clinical stage.The expression of β1   int  egrin in OECM-1 cells is involved in the development of malignant phenotypes of OSCC.Inhibitors for β1-integrin signaling may be suitable to become target-specific therapies for OSCC. 
 Spatiotemporally-regulated interaction between β1 integrin and ErbB4 that is involved in fibronectin-dependent cell migration. Integrins are widely expressed cell surface molecules that mediate cell attachment to extracellular matrix   (EC  M) proteins. They also interact with molecules on their own membranes, and these cis-interactions play a crucial role in integrin-dependent cellular responses. We herein analysed what molecules interact with β1 integrin during biological events induced by cell attachment to differen  t E  CM proteins, using a recently established reaction, the enzyme-mediated activation of radical sources (EMARS). The interactions between β1 integrin and receptor tyrosine kinases including EGFR and ErbB4 reached a peak at 2 h after seeding HeLa S3 cells onto t  he   ECM proteins. The peak of phosphorylation of ErbB4 (at 2 h after seeding the cells onto fibronectin) coincided with the peak of the interaction with β1 integrin, while that of EGFR (at 1 day) did not. Accompanying with these findings, suppression of cell migration by a pharmacological inhibitor of the ErbB family receptors, PD168393 and an anti-ErbB4 neutralizing antibody, 12D8 was observed at 2 h after seeding. Taken together, it is deduced that interactions between β1 integrin and ErbB4 occur in a spatiotemporally-regulated manner, and such interaction contributes to the integrin-dependent cell migration. 
 Homeobox D1 regulates angiogenic functions of endothelial cells via integrin β1 expression. Homeobox (HOX) family genes, major transcription factors for embryonic development, have been also implicated in vascular development and angiogenesis, particularly with regulation of genes involved in cell-cell or cell-extracellular matrix   (EC  M) interactions. However, the cellular and molecular functions of HOXD1 in endothelial cells (ECs) are yet to be explored. We here report that HOXD1 is prominently expressed in human ECs and regulates angiogenic activities. Knockdown of HOXD1 in ECs resulted in significant inhibition of migration and adhesion as well as tube like structure formation. These effects were correlated with the reduced expression of integrin β1 (ITGB1), an important signaling component of angiogenesis. Consistently, ITGB1 promoter activity was decreased by HOXD1 knockdown in ECs. Furthermore, we identified the putative HOXD1-binding sites in the promoter region of ITGB1. Together, these findings suggest that HOXD1 plays a significant role in EC functions by regulating the expression of ITGB1. 
 Tissue-type plasminogen activator promotes murine myofibroblast activation through LDL receptor-related protein 1-mediated integrin signaling. The activation of interstitial fibroblasts to become alpha-SMA-positive myofibroblasts is an essential step in the evolution of chronic kidney fibrosis, as myofibroblasts are responsible for the production and deposition of the   ECM   components that are a hallmark of the disease. Here we describe a signaling pathway that leads to this activation. Tissue-type plasminogen activator (tPA) promoted TGF-beta1-mediated alpha-SMA and type I collagen expression in rat kidney interstitial fibroblasts. This fibrogenic effect was independent of its protease activity but required its membrane receptor, the LDL receptor-related protein 1 (LRP-1). In rat kidney fibroblasts, tPA induced rapid LRP-1 tyrosine phosphorylation and enhanced beta1 integrin recruitment by facilitating the LRP-1/beta1 integrin complex formation. Blockade or knockdown of beta1 integrin abolished type I collagen and alpha-SMA expression. Furthermore, inhibition of the integrin-linked kinase (ILK), a downstream effector of beta1 integrin, or disruption of beta1 integrin/ILK engagement, abrogated the tPA action, whereas ectopic expression of ILK mimicked tPA in promoting myofibroblast activation. In murine renal interstitium after obstructive injury, tPA and alpha-SMA colocalized with LRP-1, and tPA deficiency reduced LRP-1/beta1 integrin interaction and myofibroblast activation. These findings show that tPA induces LRP-1 tyrosine phosphorylation, which in turn facilitates the LRP-1-mediated recruitment of beta1 integrin and downstream ILK signaling, thereby leading to myofibroblast activation. This study implicates tPA as a fibrogenic cytokine that promotes the progression of kidney fibrosis. 
 The interaction between β1 integrins and ERK1/2 in osteogenic differentiation of human mesenchymal stem cells under fluid shear stress modelled by a perfusion system. Fluid shear stress (FSS) is an important biomechanical factor regulating the osteogenic differentiation of human mesenchymal stem cells (hMSCs) and is therefore widely used in bone tissue engineering. However, the mechanotransduction of FSS in hMSCs remains largely unknown. As β1 integrins are considered to be important mechanoreceptors in other cells, we suspect that β1 integrins should also be important for hMSCs to sense the stimulation of FSS. We used a perfusion culture system to produce FSS loading on hMSCs seeded in PLGA three-dimensional (3D) scaffolds and investigated the roles of β1 integrins, FAK and ERK1/2 in FSS-induced osteogenic differentiation of hMSCs. Our results showed that FSS not only markedly increased ALP activity and the expression of ALP, OCN, Runx2 and COLIα genes but also significantly enhanced the phosphorylation of ERK1/2, Runx2 and FAK. FSS-induced activation of ERK1/2 and FAK was inhibited by blockade of the connection between β1 integrin  s a  nd ECM with RGDS peptide and integrins β1 monoclonal antibody. Our study also found that FSS could upregulate the expression level of β1 integrins and that this upregulation could be abolished by PD98059. Further investigation indicated that FSS-activated ERK1/2 led to the phosphorylation of IκBα and NFκB p65. The activation of NFκB p65 resulted in the upregulation of β1 integrin expression. Therefore, it could be inferred that β1 integrins should sense the stimulation of FSS and thus activate ERK1/2 through activating of FAK, and FSS-activated ERK1/2 feedback to upregulate the expression of β1 integrins through activating NFκB. 
 A conserved interaction between beta1 integrin/PAT-3 and Nck-interacting kinase/MIG-15 that mediates commissural axon navigation in C. elegans. Integrins are heterodimeric (alphabeta) transmembrane receptors for extracellular matrix (  ECM  ) ligands. Through interactions with molecular partners at cell junctions, they provide a connection between the   ECM   and the cytoskeleton and regulate many aspects of cell behavior. A number of integrin-associated molecules have been identified; however, in many cases, their function and role in the animal remain to be clarified.We have identified the Nck-interacting kinase (NIK), a member of the STE20/germinal center kinase (GCK) family, as a partner for the beta1A integrin cytoplasmic domain. We find that NIK is expressed in the nervous system and other tissues in mouse embryos and colocalizes with actin and beta1 integrin in cellular protrusions in transfected cells. To demonstrate the functional significance of this interaction, we used Caenorhabditis elegans, since it has only one beta (PAT-3) integrin chain, two alpha (INA-1 and PAT-2) integrin chains, and a well-conserved NIK ortholog (MIG-15). Using three methods, we show that reducing mig-15 activity results in premature branching of commissures. A significant aggravation of this defect is observed when mig-15 activity is compromised in a weak ina-1 background. Neuronal-specific RNA interference against mig-15 or pat-3 leads to similar axonal defects, thus showing that both mig-15 and pat-3 act cell autonomously in neurons. Finally, we show a genetic interaction between mig-15, ina-1, and genes that encode Rac GTPases.Using several models, we provide the first evidence that the kinase NIK and integrins interact in vitro and in vivo. This interaction is required for proper axonal navigation in C. elegans. 
 The MSP receptor regulates alpha6beta4 and alpha3beta1 integrins via 14-3-3 proteins in keratinocyte migration. Growth factors, integrins, and the extracellular matrix (  ECM  ) are known to play key roles in epidermal wound healing, although the interplay between these proteins is not fully understood. We show that growth factor macrophage stimulating protein (MSP)- and its receptor Ron-mediated PI3K activation in keratinocytes induces phosphorylation of both Ron and alpha6beta4 integrin at specific 14-3-3 binding sites. Consequently, a Ron/alpha6beta4 complex formed via 14-3-3 binding displaces alpha6beta4 from its location at hemidesmosomes (structures supporting cell adhesion) and relocalizes it to lamellipodia. Concomitant activation of alpha3beta1 and keratinocyte spreading/migration on laminin-5 occurs. Further, MSP-dependent beta4 tyrosine phosphorylation evokes p38 and NF-kappaB signaling required for keratinocyte wound closure. Based on these results, we propose a mechanism based on MSP-Ron-dependent phosphorylation and 14-3-3 association, whereby the function of alpha6beta4 switches from a mechanical adhesive device into a signaling component, and might be critically involved in human epidermal wound healing. 
 Integrins protect cardiomyocytes from ischemia/reperfusion injury. Ischemic damage is recognized to cause cardiomyocyte (CM) death and myocardial dysfunction, but the role of cell-matrix interactions and integrins in this process has not been extensively studied. Expression of α7β1D integrin, the dominant integrin in normal adult CMs, increases during ischemia/reperfusion (I/R), while deficiency of β1 integrins increases ischemic damage. We hypothesized that the forced overexpression of integrins on the CM would offer protection from I/R injury. Tg mice with CM-specific overexpression of integrin α7β1D exposed to I/R had a substantial reduction in infarct size compared with that of α5β1D-overexpressing mice and WT littermate controls. Using isolated CMs, we found that α7β1D preserved mitochondrial membrane potential during hypoxia/reoxygenation (H/R) injury via inhibition of mitochondrial Ca2+ overload but did not alter H/R effects on oxidative stress. Therefore, we assessed Ca2+ handling proteins in the CM and found that β1D integrin colocalized with ryanodine receptor 2 (RyR2) in CM T-tubules, complexed with RyR2 in human and rat heart, and specifically bound to RyR2 amino acids 165-175. Integrins stabilized the RyR2 interdomain interaction, and this stabilization required integrin receptor bindi  ng   to its ECM ligand. These data suggest that α7β1D integrin modifies Ca2+ regulatory pathways and offers a means to protect the myocardium from ischemic injury. 
 The involvement of integrin β1 signaling in the migration and myofibroblastic differentiation of skin fibroblasts on anisotropic collagen-containing nanofibers. Utilization of nanofibrous matrices for skin wound repair holds great promise due to their morphological and dimensional similarity to native extracellular matrix   (EC  M). It becomes highly desired to understand how various nanofibrous matrices regulate skin cell behaviors and intracellular signaling pathways, important to tuning the functionality of tissue-engineered skin grafts and affecting the wound healing process. In this study, the phenotypic expressions of normal human dermal fibroblasts (NHDFs) on collagen-containing nanofibrous matrices with either isotropic (i.e., fibers collected randomly with no alignment) or anisotropic (i.e., fibers collected with alignment) fiber organizations were studied by immunostaining, migration assay and molecular analyses. Results showed that both nanofibrous matrices supported the attachment and growth of NHDFs similarly, while showing different cell morphology with distinct variation in focal adhesion formation and distribution. Anisotropic nanofibers significantly triggered the integrin β1 signaling pathway in NHDFs as evidenced by an increase of active integrin β1 (130 kD mature form) and phosphorylation of focal adhesion kinase (FAK) at Tyr-397. Anisotropic matrices also promoted the migration of NHDFs along the fibers, while neutralization of the integrin β1 activity abolished this promotion. Moreover, the fibroblast-to-myofibroblast differentiation was greatly enhanced for the NHDFs cultured on anisotropic nanofibrous matrices over a period of 48 h. Inhibition of cellular integrin β1 activity by neutralizing antibody eliminated this enhancement. These findings suggest the important role of integrin β1 signaling pathway in regulating the nanofiber-induced fibroblast phenotypic alteration and providing insightful understanding of the possible application of collagen-containing nanofibrous matrices for skin regeneration. 
 Integrin alpha8beta1 regulates adhesion, migration and proliferation of human intestinal crypt cells via a predominant RhoA/ROCK-dependent mechanism. Integrins are transmembrane alphabeta heterodimer receptors that function as structural and functional bridges between the cytoskeleton and   ECM   (extracellular matrix) molecules. The RGD (arginine-glycine-aspartate tripeptide motif)-dependent integrin alpha8beta1 has been shown to be involved in various cell functions in neuronal and mesenchymal-derived cell types. Its role in epithelial cells remains unknown.Integrin alpha8beta1 was found to be expressed in the crypt cell population of the human intestine but was absent from differentiating and mature epithelial cells of the villus. The function of alpha8beta1 in epithelial crypt cells was investigated at the cellular level using normal HIECs (human intestinal epithelial cells). Specific knockdown of alpha8 subunit expression using an shRNA (small-hairpin RNA) approach showed that alpha8beta1 plays important roles in RGD-dependent cell adhesion, migration and proliferation via a RhoA/ROCK (Rho-associated kinase)-dependent mechanism as demonstrated by active RhoA quantification and pharmacological inhibition of ROCK. Moreover, loss of alpha8beta1, through RhoA/ROCK, impairs FA (focal adhesion) complex integrity as demonstrated by faulty vinculin recruitment.Integrin alpha8beta1 is expressed in epithelial cells. In intestinal crypt cells, alpha8beta1 is closely involved in the regulation of adhesion, migration and cell proliferation via a predominant RhoA/ROCK-dependent mechanism. These results suggest an important role for this integrin in intestinal crypt cell homoeostasis. 
 Mechanisms involved in alpha6beta1-integrin-mediated Ca(2+) signalling. Contact of Jurkat T-lymphocytes with the extracellular matrix (  ECM  ) protein laminin resulted in long-lasting alpha6beta1-integrin-mediated Ca(2+) signalling. Both Ca(2+) release from thapsigargin-sensitive Ca(2+) stores and capacitative Ca(2+) entry via Ca(2+) channels sensitive to SKF 96365 constitute important parts of this process. Inhibition of alpha6beta1-integrin-mediated Ca(2+) signalling by (1) the src kinase inhibitor PP2, (2) the PLC inhibitor U73122, and (3) the cyclic adenosine diphosphoribose (cADPR) antagonist 7-deaza-8-Br-cADPR indicate the involvement of src tyrosine kinases and the Ca(2+)-releasing second messengers D-myo-inositol 1,4,5-trisphosphate (InsP3) and cADPR. 
 Integrin endosomal signalling suppresses anoikis. Integrin-containing focal adhesions transmit extracellular signals across the plasma membrane to modulate cell adhesion, signalling and survival. Although integrins are known to undergo continuous endo/exocytic traffic, the potential impact of endocytic traffic on integrin-induced signals is unknown. Here, we demonstrate that integrin signalling is not restricted to cell-  ECM   adhesions and identify an endosomal signalling platform that supports integrin signalling away from the plasma membrane. We show that active focal adhesion kinase (FAK), an established marker of integrin-  ECM   downstream signalling, localizes with active integrins on endosomes. Integrin endocytosis positively regulates adhesion-induced FAK activation, which is early endosome antigen-1 and small GTPase Rab21 dependent. FAK binds directly to purified endosomes and becomes activated on them, suggesting a role for endocytosis in enhancing distinct integrin downstream signalling events. Finally, endosomal integrin signalling contributes to cancer-related processes such as anoikis resistance, anchorage independence and metastasis. 
 PCTK1 regulates integrin-dependent spindle orientation via protein kinase A regulatory subunit KAP0 and myosin X. Integrin-dependent cell-extracellular matrix (  ECM  ) adhesion is a determinant of spindle orientation. However, the signaling pathways that couple integrins to spindle orientation remain elusive. Here, we show that PCTAIRE-1 kinase (PCTK1), a member of the cyclin-dependent kinases (CDKs) whose function is poorly characterized, plays an essential role in this process. PCTK1 regulates spindle orientation in a kinase-dependent manner. Phosphoproteomic analysis together with an RNA interference screen revealed that PCTK1 regulates spindle orientation through phosphorylation of Ser83 on KAP0, a regulatory subunit of protein kinase A (PKA). This phosphorylation is dispensable for KAP0 dimerization and for PKA binding but is necessary for its interaction with myosin X, a regulator of spindle orientation. KAP0 binds to the FERM domain of myosin X and enhances the association of myosin X-FERM with β1 integrin. This interaction between myosin X-FERM and β1 integrin appeared to be crucial for spindle orientation control. We propose that PCTK1-KAP0-myosin X-β1 integrin is a functional module providing a link betwe  en   ECM and the actin cytoskeleton in t  he   ECM-dependent control of spindle orientation. 
 Stimulation of beta 1 integrin induces tyrosine phosphorylation of vascular endothelial growth factor receptor-3 and modulates cell migration. Interactions between integrins and tyrosine kinase receptors can modulate a variety of cell functions. We observed a cooperative interaction between the beta(1) integrin and vascular endothelial growth factor receptor-3 (VEGFR-3 or Flt4) that appeared to be required for cell migration. By using VEGFR-3-transfected 293 cells (293/VEGFR-3) or primary dermal microvascular endothelial cells (DMEC), we found that stimulation with either soluble or immobilized extracellular matrix (  ECM  ) proteins, collagen or fibronectin (FN), resulted in the increased tyrosine phosphorylation of VEGFR-3 in the absence of a cognate ligand. This increased tyrosine phosphorylation of VEGFR-3 was diminished by pretreatment with a blocking antibody against the beta(1) integrin. Cross-linking with anti-beta(1) integrin antibody induced a similar degree of tyrosine phosphorylation of VEGFR-3. Stimulation with collagen or FN induced an association between beta(1) integrin and VEGFR-3 in both 293/VEGFR-3 and primary DMEC cells. Collagen or FN-induced tyrosine phosphorylation of VEGFR-3 was inhibited by treatment with cytochalasin D, an inhibitor of actin polymerization. Collagen or FN was able to induce the migration of 293/VEGFR-3 or DMEC cells to a limited extent. However, migration was dramatically enhanced when a gradient of the cognate ligand, VEGF-D, was added. VEGF-D failed to induce cell migration in the absence of   ECM   proteins. Introducing a mutation at the kinase domain of VEGFR-3 or treatment with blocking antibody against either VEGFR-3 or beta(1) integrin inhibited cell migration induced by   ECM   and VEGF-D, indicating that signals from both beta(1) integrin and VEGFR-3 are required for this cell function. 
 Extracellular matrix regulates endothelial functions through interaction of VEGFR-3 and integrin alpha5beta1. Endothelium extracellular matrix (  ECM  ) interactions can provide distinct spatial and molecular signals which control cellular proliferation, migration, and differentiation. Here, we investigated the role of fibronectin (FN), a major   ECM   protein, on the functions of lymphatic endothelial cells (LEC). We observed that FN, the ligand for integrin alpha5beta1, selectively promoted the growth of LEC as compared with vitronectin (VN) in the presence of the ligand for vascular endothelial growth factor receptor 3 [VEGFR-3 (VEGF-C156S)]. Upon investigating the mechanisms whereby   ECM   components regulate VEGFR-3 signaling, we found that FN transactivated VEGFR-3 and significantly enhanced the phosphorylation of VEGFR-3 induced by VEGF-C156S as compared to VN. An enhanced association of the integrin subunit alpha5 or beta1 with VEGFR-3, after stimulation with VEGF-C156S, was observed by co-immunoprecipitation. While blockade of integrin alpha5beta1 inhibited the VEGF-C156S-induced phosphorylation of VEGFR-3, no similar effect was obtained by blocking integrin alphavbeta3. FN also protected the endothelial cells from serum deprivation-induced apoptosis. Moreover, while the specific PI3 kinase inhibitor, LY294002, abolished this FN-mediated cell survival, the MAPK kinase inhibitor, PD98059, had no significant effect. Furthermore, a dominant-negative mutant of VEGFR-3 (G857R) reduced VEGF-C156S or FN-mediated cell survival, as well as the activities of PI3 kinase/Akt. Our results indicate that integrin alpha5beta1 participates in the activation of both VEGFR-3 and its downstream PI3 kinase/Akt signaling pathway, which is essential for FN-mediated lymphatic endothelial cell survival and proliferation. 
 Integrin-mediated inhibition of interleukin-8 secretion from human neutrophils by collagen type I. The function of neutrophils in the inflammatory response is modulated by contact with   ECM   proteins. We have now investigated the effect of collagen type I on secretion of the cytokine IL-8 by human neutrophils in vitro. Collagen type I inhibited the secretion of IL-8 from neutrophils maintained under basal conditions or stimulated with fMLF. This effect was accompanied by down-regulation of IL-8 mRNA, and it appeared to be specific to collagen type I among   ECM   proteins, in that it was not observed with fibronectin or laminin. The inhibitory effect of collagen type I on IL-8 secretion was dependent on collagen concentration and cell density. It was also abolished in the presence of antibodies to integrin alpha2beta1 but was not affected by antibodies to integrin alpha5beta1 or beta4. Our results thus suggest that collagen type I inhibits the secretion of IL-8 by human neutrophils in a selective manner and that this effect is mediated by the interaction of collagen with integrin alpha2beta1. 
 Epigallocatechin-3-gallate (EGCG) downregulates EGF-induced MMP-9 in breast cancer cells: involvement of integrin receptor α5β1 in the process. Epidermal growth factor receptor (EGFR/ErbB1) is a transmembrane protein with tyrosine kinase activity activated mainly by ligand, EGF. Matrix metalloproteinases (MMPs) are a family of proteinases that catalyses the destruction o  f E  CM, among which MMP-9 has important role in tumor cell invasion. Secretion of MMP-9 is stimulated by a variety of factors, EGFR being significant. Epigallocatechin-3-gallate (EGCG) is a major polyphenol of green tea that inhibits cell proliferation and invasion. Here, we study the effect of EGFR alone and in collaboration with fibronectin on the status of MMP-9 in human breast cancer cell MDA-MB-231 and its molecular mechanism; study the role of EGCG on the induced MMP-9; and elucidate the signaling molecules involved in the process.We performed zymography, immunoblots, real-time RT-PCR, cell adhesion assay, siRNA studies, and electrophoretic mobility shift assay to demonstrate the findings.EGF induces MMP-9 activity and expression; FAK, PI3 K, and ERK are mainly involved in the process. EGF also causes the transactivation of MMP-9 gene by increasing the DNA binding activity of the transcription factors. EGCG downregulates EGF-induced MMP-9 expression by inhibiting the involved regulatory kinases. EGF collaborates with fibronectin to create a synergistic response, and EGCG inhibits the synergistic response in MDA-MB-231.The study demonstrates the requirement of cross talk between cell matrix adhesion molecules and growth factor receptors to improve biological responses and shows FAK/ERK as the pivotal point of this convergence in human breast carcinoma cell line MDA-MB-231. We also establish EGCG as the potential anti-tumor agent in human breast carcinoma. 
 Interleukin-1alpha enhances integrin alpha(6)beta(1) expression and metastatic capability of human pancreatic cancer. To investigate the mechanisms of metastasis formation in metastatic human pancreatic cancer, we examined the enhancement in integrin expression, and adherence to and invasiveness into extracellular matrix (  ECM  ) proteins of human pancreatic cancer cells after exposure to interleukin (IL)-1alpha.Expression of IL-1 receptor type I (IL-1RI) and alterations in integrin subunits by IL-1alpha were examined by flow-cytometric analysis and by cellular enzyme-linked immunosorbent assay in four human pancreatic cancer cell lines (BxPC-3, PaCa-2, PANC-1, and SW1990), respectively. In addition, assays of cancer cell adhesion and invasion to   ECM   proteins were performed to investigate whether increased integrin expression affected the adhesive and invasive interaction between cancer cells and the putative integrin   ECM   ligands. Furthermore, immunohistochemistry was used to assess integrins and IL-1R1 expression in pancreatic tissues.In metastatic cancer cells, expression of the alpha(6) subunit was enhanced by IL-1alpha treatment. While metastatic cancer cells exhibited preferential adherence to and invasion into laminin, these properties were enhanced by IL-1alpha. The alpha(6) subunit and IL-1RI were strongly expressed in pancreatic tissues from pancreatic cancer patients with liver metastasis.In pancreatic cancer, IL-1alpha enhanced alpha(6)beta(1)-integrin expression, probably via increased IL-1RI levels. Our results indicated that alpha(6)beta(1)-integrin and IL-1RI expression may play important roles in metastasis formation. 
 MARVELD1 regulates integrin β1-mediated cell adhesion and actin organization via inhibiting its pre-mRNA processing. Cell adhesion on an extracellular matrix   (EC  M) participates in cell motility, invasion, cell signal transduction and gene expression. Many nuclear proteins regulate cell  -EC  M adhesion through managing the transcription of cell adhesion-related genes. Here, we identified MARVEL [MAL (The myelin and lymphocyte protein) and related proteins for vesicle trafficking and membrane link] domain containing 1 (MARVELD1) that could suppress cell spreading and complicate actin organization. Over-expression of MARVELD1 in NIH3T3 cells decreased the expression level of integrin β1 and vinculin, and further led to dephosphorylation of focal adhesion kinase (FAK) at Tyr 397. We also found that MARVELD1 partially colocalized with serine/arginine-rich splicing factor 2 (SC35) and interacted with nuclear cap binding protein subunit 2 (CBP20). Finally, we demonstrated that pre-mRNA processing of integrin β1 was affected by MARVELD1. Taken together, our studies demonstrate that MARVELD1 plays a role in pre-mRNA processing of integrin β1, and thereby regulates cell adhesion and cell motility. These studies provide a novel regulatory mechanism of c  ell  -ECM adhesion by nuclear protein in cells. 
 Comparative proteomic analysis of supportive and unsupportive extracellular matrix substrates for human embryonic stem cell maintenance. Human embryonic stem cells (hESCs) are pluripotent cells that have indefinite replicative potential and the ability to differentiate into derivatives of all three germ layers. hESCs are conventionally grown on mitotically inactivated mouse embryonic fibroblasts (MEFs) or feeder cells of human origin. In addition, feeder-free culture systems can be used to support hESCs, in which the adhesive substrate plays a key role in the regulation of stem cell self-renewal or differentiation. Extracellular matrix (  ECM  ) components define the microenvironment of the niche for many types of stem cells, but their role in the maintenance of hESCs remains poorly understood. We used a proteomic approach to characterize in detail the composition and interaction networks of   ECM  s that support the growth of self-renewing hESCs. Whereas many   ECM   components were produced by supportive and unsupportive MEF and human placental stromal fibroblast feeder cells, some proteins were only expressed in supportive   ECM  , suggestive of a role in the maintenance of pluripotency. We show that identified candidate molecules can support attachment and self-renewal of hESCs alone (fibrillin-1) or in combination with fibronectin (perlecan, fibulin-2), in the absence of feeder cells. Together, these data highlight the importance of specific   ECM   interactions in the regulation of hESC phenotype and provide a resource for future studies of hESC self-renewal. 
 Overexpression of integrin-β1 in leiomyoma promotes cell spreading and proliferation. Uterine leiomyoma, the most common tumors found in the women of the reproductive age, may cause abnormal uterine bleeding and be life threatening. Compared with myometrium, leiomyoma contains excessive extracellular matrix   (EC  M). However, the pathological roles of   EC  M in the development of leiomyoma remain largely unknown. Integrins are the major adhesion molecules on cell surface to interact with   EC  M. The interactions of   EC  M with integrins regulate cell adhesion and initiate signals for cell growth, differentiation, and migration.The aim of this study was to investigate the expression and functional role of integrin-β1 in leiomyoma pathogenesis.Levels of integrin-β1 protein were determined by Western blotting in paired normal and leiomyomal tissues (n = 15). Knockdown of integrin-β1 and inhibition   of   ECM-integrin interaction by disintegrin were used to evaluate the impact of integrin-β1 in cell adhesion, spreading, and proliferation.Levels of integrin-β1 were significantly up-regulated in leiomyomal cells compared with their normal counterparts. Knockdown of integrin-β1 did not affect cell adhesion on fibronectin or laminin matrix but significantly inhibits cell spreading ability. Consistent with this notion, the phosphorylation of focal adhesion kinase and the recruitment of paxillin to the focal contact were decreased in integrin-β1 knockdown cells, which attenuates contraction force. The inability of cell spreading leads to inhibition of cyclin D1 expression and impedes cell cycle progression. More importantly, disrup  tio  n of ECM-integrin interaction by the small protein, disintegrin inhibited cyclin D1 expression and cell proliferation.These data demonstrate that integrin-β1 is a critical ligand to enha  nce   cell-ECM contact force and thus promotes cell proliferation. Disru  pti  on of ECM-integrin-β1 signaling may serve as an option to inhibit the progression of leiomyoma. 
 Increased integrin alpha5beta1 heterodimer formation and reduced c-Jun expression are involved in integrin beta1 overexpression-mediated cell growth arrest. Integrins, heterodimers of alpha and beta subunits, are a family of cell surface molecules mediating cell-cell and cell-extracellular matrix interaction. The largest subgroup is formed by the beta(1) subunit containing integrins which consist of 12 members with different ligand-binding properties. We previously reported that overexpressed integrin beta(1) subunit in the hepatocellular carcinoma cell line SMMC-7721 imposed a growth inhibitory effect through the upregulation of p21(cip1) and p27(kip1). In this study, we confirmed the growth inhibitory effect of beta(1) subunit overexpression in different cancer cell lines. The upregulated CDK inhibitors induced by beta(1) integrin overexpression were essential for this integrin-mediated growth arrest. Reduced c-Jun level after integrin beta(1) overexpression plays an important role in the transcriptional activation of p21 through the Sp1 sites. Solely overexpressed beta(1) subunit could induce the expression of diverse alpha subunit in different cell lines, among which alpha(5) subunit was found to be correlated with integrin beta(1)-mediated growth arrest. Relative lack of   ECM  -integrin interaction might be a reason for integrin beta(1) overexpression-mediated growth arrest. These results helped us understand more about the mechanisms that integrins regulate cell growth. 
 C1GALT1 promotes invasive phenotypes of hepatocellular carcinoma cells by modulating integrin β1 glycosylation and activity. Cancer cell invasion and metastasis are the primary causes of treatment failure and death in hepatocellular carcinoma (HCC). We previously reported that core 1 β1,3-galactosyltransferase (C1GALT1) is frequently overexpressed in HCC tumors and its expression is associated with advanced tumor stage, metastasis, and poor survival. However, the underlying mechanisms of C1GALT1 in HCC malignancy remain unclear. In this study, we found that overexpression of C1GALT1 enhanced HCC cell adhesion to extracellular matrix   (E  CM) proteins, migration, and invasion, whereas RNAi-mediated knockdown of C1GALT1 suppressed these phenotypes. The promoting effect of C1GALT1 on the metastasis of HCC cells was demonstrated in a mouse xenograft model. Mechanistic investigations showed that the C1GALT1-enhanced phenotypic changes in HCC cells were significantly suppressed by anti-integrin β1 blocking antibody. Moreover, C1GALT1 was able to modify O-glycans on integrin β1 and regulate integrin β1 activity as well as its downstream signaling. These results suggest that C1GALT1 could enhance HCC invasiveness through integrin β1 and provide novel insights into the roles of O-glycosylation in HCC metastasis. 
 Alpha-toxin interferes with integrin-mediated adhesion and internalization of Staphylococcus aureus by epithelial cells. Staphylococcus aureus is an important human and animal pathogen. During infection, this bacterium is able to attach to and enter host cells by using its cell surface-associated factors to bind to the host's extracellular matrix (  ECM  ) proteins. In this study, we determined that a protein exported by S. aureus, alpha-toxin, can interfere with the integrin-mediated adhesion and internalization of S. aureus by human lung epithelial cells (A549). The downregulation of alpha-toxin production significantly increased bacterial adhesion and invasion into the epithelial cells. In contrast, bacterial adhesion and invasion was inhibited by both overproduction of alpha-toxin and the addition of alpha-toxin to the culture medium. Moreover, our results showed that the quantitative effects on invasion closely parallel those of adherence. This suggests that the effect on invasion is probably secondary to, and a consequence of, the reduced adherence caused by alpha-toxin exposure. Specifically, we demonstrated that alpha-toxin interacts with the hosts'   ECM   protein's receptor, beta1-integrin, which indicates that beta1-integrin may be a potential receptor of alpha-toxin on epithelial cells. Taken together, our results indicate that exported alpha-toxin inhibits the adhesion and internalization of S. aureus by interfering with integrin-mediated pathogen-host cell interactions. 
 Effect of dexamethasone on human osteoblasts in culture: involvement of β1 integrin and integrin-linked kinase. Adhesive interactions play a critical role in cell biology, influencing vital processes from proliferation to cell death. Integrins regulate cell  -EC  M (extracellular matrix) adhesion and must associate with phosphorylating proteins such as ILK (integrin-linked kinase). Dysregulation of ILK expression is associated with anchorage-independent growth, cell survival and inhibition of apoptosis. Glucocorticoids influence differentiation and adhesion of osteoblasts and can affect bone protein synthesis. The objective of this study was to analyse the effect of DEX (dexamethasone) on the biology of osteoblasts, together with its influence on the expression of ILK and β1 integrin. For this, primary cultures of human osteoblasts were exposed to DEX at 10-9 M (physiological dose) and 10-6 M (pharmacological dose) for 24 and 48 h. Cell viability, apoptosis and cell adhesion were analysed, as well as protein expression of β1 integrin and ILK. It was observed that cell viability and adhesion were reduced in the cultures evaluated. In comparison with the control cultures, there was slightly less apoptosis in the cultures exposed to the physiological dose and considerably more apoptosis in those exposed to the pharmacological dose. In all treated cultures, protein expression of ILK was slightly higher than in the control cultures, whereas that of β1 integrin was significantly lower. Both proteins under study were co-localized at the cell periphery in all cultures. Our results suggest that DEX causes osteoblast anoikis, probably due to decreased β1 integrin expression, which might have had a direct influence upon ILK, reducing its activation and preventing it from playing its characteristic anti-apoptotic role. 
 Phosphatidylinositol mannoside from Mycobacterium tuberculosis binds alpha5beta1 integrin (VLA-5) on CD4+ T cells and induces adhesion to fibronectin. The pathological hallmark of the host response to Mycobacterium tuberculosis is the granuloma where T cells and macrophages interact with the extracellular matrix (  ECM  ) to control the infection. Recruitment and retention of T cells within inflamed tissues depend on adhesion to the   ECM  . T cells use integrins to adhere to the   ECM  , and fibronectin (FN) is one of its major components. We have found that the major M. tuberculosis cell wall glycolipid, phosphatidylinositol mannoside (PIM), induces homotypic adhesion of human CD4+ T cells and T cell adhesion to immobilized FN. Treatment with EDTA and cytochalasin D prevented PIM-induced T cell adhesion. PIM-induced T cell adhesion to FN was blocked with mAbs against alpha5 integrin chain and with RGD-containing peptides. Alpha5beta1 (VLA-5) is one of two major FN receptors on T cells. PIM was found to bind directly to purified human VLA-5. Thus, PIM interacts directly with VLA-5 on CD4+ T lymphocytes, inducing activation of the integrin, and promoting adhesion to the   ECM   glycoprotein, FN. This is the first report of direct binding of a M. tuberculosis molecule to a receptor on human T cells resulting in a change in CD4+ T cell function. 
 Immunohistochemical assessment of fibronectin and tenascin and their integrin receptors alpha5beta1 and alpha9beta1 in gastric and colorectal cancers with lymph node and liver metastases. The aim of the current study was to assess immunohistochemically and compare the level of expression of tenascin (TN) and fibronectin (FN) and their integrin receptors alpha9beta1 and alpha5beta1 in the primary colorectal and gastric tumors, and in corresponding lymph node and liver metastases from 53 patients. We detected similar high deposition of the studied   ECM   proteins and their receptors in the stroma of primary tumors and in liver metastases and a lower deposition in lymph node metastases. Cytoplasmic immune reaction for FN and TN was also seen in the tumor cells. A pronounced co-localization of immune deposits for FN and TN and their receptors was found in the stroma of the center and the invasion front (IF) (p<0.0001). A significant decrease of FN immune signal was observed in the IF in primary tumors and liver metastases (p<0.0001). The levels of immunolabeling of FN and TN correlated with the differentiation grade of primary tumors (p<0.0001). In conclusion, we may say that there is heterogeneous deposition of TN, FN and their integrin receptors in the different areas of primary colorectal and gastric tumors and of their metastases. These findings imply that the studied proteins may be involved in cell processes such as growth, adhesion, migration and apoptosis. 
 A novel intracellular fibulin-1D variant binds to the cytoplasmic domain of integrin beta 1 subunit. Fibulin-1 is a member of a growing family of proteins that includes eight members and is involved in cellular functions such as adhesion, migration and differentiation. Fibulin-1 has also been implicated in embryonic development of the heart and neural crest-derived structures. It is an integral part of the extracellular matrix (  ECM  ) and has been shown to bind to a multitude of   ECM   proteins. However, fibulin-1 was first identified as a protein purified from placental extracts that binds to the cytoplasmic domain of integrin β1. Human fibulin-1 is alternatively spliced into four different isoforms namely A-D. These isoforms share a common N-terminus sequence that contains a secretion sequence but differ in their carboxy-terminal fibulin-1 module. In this report we identify a new splice variant of fibulin-1 that differs from all other fibulin-1 variants in the N-terminus sequence and has a similar carboxy-terminus sequence as fibulin-1D. This variant that we named fibulin-1D prime (fibulin-1D') lacks a secretion sequence and the anaphlatoxin region of fibulin-1 variants. The protein has an apparent molecular weight of 70.5kDa. Herein we show that fibulin-1D' binds to the intracellular domain of integrin β1 as well as to integrin α5β1. The protein was localized intracellularly in CHO cells transfected with a pEF4 plasmid containing full-length coding sequence of fibulin-1D'. We also localized the protein in human placenta. We propose that the fibulin-1D' variant might play a role in early embryo development as well as in modulating integrin β1 functions including adhesion and motility. 
 Vesicle-associated membrane protein 2 mediates trafficking of alpha5beta1 integrin to the plasma membrane. Integrins are major receptors for cell adhesion to the extracellular matrix (  ECM  ). As transmembrane proteins, the levels of integrins at the plasma membrane or the cell surface are ultimately determined by the balance between two vesicle trafficking events: endocytosis of integrins at the plasma membrane and exocytosis of the vesicles that transport integrins. Here, we report that vesicle-associated membrane protein 2 (VAMP2), a SNARE protein that mediates vesicle fusion with the plasma membrane, is involved in the trafficking of alpha5beta1 integrin. VAMP2 was present on vesicles containing endocytosed beta1 integrin. Small interfering RNA (siRNA) silencing of VAMP2 markedly reduced cell surface alpha5beta1 and inhibited cell adhesion and chemotactic migration to fibronectin, the   ECM   ligand of alpha5beta1, without altering cell surface expression of alpha2beta1 integrin or alpha3beta1 integrin. By contrast, silencing of VAMP8, another SNARE protein, had no effect on cell surface expression of the integrins or cell adhesion to fibronectin. In addition, VAMP2-mediated trafficking is involved in cell adhesion to collagen but not to laminin. Consistent with disruption of integrin functions in cell proliferation and survival, VAMP2 silencing diminished proliferation and triggered apoptosis. Collectively, these data indicate that VAMP2 mediates the trafficking of alpha5beta1 integrin to the plasma membrane and VAMP2-dependent integrin trafficking is critical in cell adhesion, migration and survival. 
 Molecular proximity of seprase and the urokinase-type plasminogen activator receptor on malignant melanoma cell membranes: dependence on beta1 integrins and the cytoskeleton. Previous studies have shown that several proteolytic enzymes are associated with membrane protrusions at the leading edge of migrating tumor cells. In this study we demonstrate that seprase and the urokinase plasminogen activator receptor (uPAR), co-localize in the plasma membrane of LOX malignant melanoma cells. Cells were labeled with fluorochrome-conjugated monoclonal antibodies (mAb) directed against seprase and uPAR. Proximity between these two molecules was detected with resonance energy transfer (RET) imaging, single-cell emission spectrophotometry, and single-cell excitation spectrophotometry. Significant RET signals were detected on LOX cells when adherent to uncoated and extracellular matrix (  ECM  )-coated surfaces. This indicates that seprase and uPAR are within approximately 7 nm in the plasma membrane of LOX cells. When LOX cells adhered to a 3D extracellular-like matrix, seprase-uPAR complexes were found to be associated with invadopodia. Further microscopy experiments demonstrated gelatinolytic activity, a functional attribute of seprase, in association with seprase-uPAR membrane domains. Formation of seprase-uPAR membrane complexes is dependent upon both the cytoskeleton and integrins. Specifically, the involvement of beta(1)-integrins was demonstrated by the inhibition of RET by an inhibitory anti-beta(1)-integrin mAb. Based on these findings, we speculate that formation of heterogeneous lytic domains in the invading membranes of LOX cells increases the efficiency of directed pericellular proteolysis. 
 ß1 integrin binding phosphorylates ezrin at T567 to activate a lipid raft signalsome driving invadopodia activity and invasion. Extracellular matrix   (EC  M) degradation is a critical process in tumor cell invasion and requires matrix degrading protrusions called invadopodia. The Na(+)/H(+) exchanger (NHE1) has recently been shown to be fundamental in the regulation of invadopodia actin cytoskeleton dynamics and activity. However, the structural link between the invadopodia cytoskeleton and NHE1 is still unknown. A candidate could be ezrin, a linker between the NHE1 and the actin cytoskeleton known to play a pivotal role in invasion and metastasis. However, the mechanistic basis for its role remains unknown. Here, we demonstrate that ezrin phosphorylated at T567 is highly overexpressed in the membrane of human breast tumors and positively associated with invasive growth and HER2 overexpression. Further, in the metastatic cell line, MDA-MB-231, p-ezrin was almost exclusively expressed in invadopodia lipid rafts where it co-localized in a functional complex with NHE1, EGFR, ß1-integrin and phosphorylated-NHERF1. Manipulation by mutation of ezrins T567 phosphorylation state and/or PIP2 binding capacity or of NHE1s binding to ezrin or PIP2 demonstrated that p-ezrin expression and binding to PIP2 are required for invadopodia-mediate  d E  CM degradation and invasion and identified NHE1 as the membrane protein that p-ezrin regulates to induce invadopodia formation and activity. 
 Colorectal Cancer Metastases Settle in the Hepatic Microenvironment Through α5β1 Integrin. Colorectal cancer (CRC) metastasis dissemination to secondary sites represents the critical point for the patient's survival. The microenvironment is crucial to cancer progression, influencing tumour cell behaviour by modulating the expression and activation of molecules such as integrins, the cell-extracellular matrix interacting proteins participating in different steps of the tumour metastatic process. In this work, we investigated the role of α5β1 integrin and how the microenvironment influences this adhesion molecule, in a model of colon cancer progression to the liver. The culture medium conditioned by the IHH hepatic cell line, and the extracellular matr  ix   (ECM) proteins, modulate the activation of α5β1 integrin in the colon cancer cell line HCT-116, and drives FAK phosphorylation during the process of cell adhesion to fibronectin, one of the main components of   liv  er ECM. In these conditions, α5β1 modulates the expression/activity of another integrin, α2β1, involved in the cell adhesion to collagen I. These results suggest that α5β1 integrin holds a leading role in HCT-116 colorectal cancer cells adhe  sio  n to the ECM through the modulation of the intracellular focal adhesion kinase FAK and the α2β1 integrin activity. The driving role of the tumour microenvironment on CRC dissemination, here detected, and described, strengthens and adds new value to the concept that α5β1 integrin can be an appropriate and relevant therapeutic target for the control of CRC metastases. 
 Metastasis suppressor KAI1/CD82 attenuates the matrix adhesion of human prostate cancer cells by suppressing fibronectin expression and β1 integrin activation. KAI1/CD82, a tetraspanin membrane protein functions as a metastasis suppressor in many types of human cancers and has been shown to regulate cell adhesion properties. In the present study, we investigated the underlying mechanism of KAI1/CD82-mediated changes in cell adhesion to the extracellular matrix using human prostate cancer cells. We found that high KAI1/CD82 expression attenuated short-term cell adhesion to uncoated- or fibronectin-coated plates. Moreover, high KAI1/CD82 expression generated an extracellular environment unfavorable for cell adhesion as compared to low KAI1/CD82 expression, suggesting KAI1/CD82-dependent regulation of extracellular matrix   (EC  M) molecule(s) expression and/or secretion. Among   EC  M components examined, fibronectin exhibited decreased expression and secretion in high KAI1/CD82-expressing cells. Furthermore, high KAI1/CD82 expression interfered with the activation of β (1) integrin at the cell surface while total β (1) integrin levels remained unchanged, concomitant with reduced formation of focal adhesion complex and decreased bundling of actin filaments. Finally, high KAI1/CD82 expression significantly retarded cell motility in a scratch wound assay. Taken together, our results strongly suggest that KAI1/CD82 attenuates the activation of β (1) integrin, and thereby down-regulates outside-in signaling of β (1) integrin, leading to the reduction of focal adhesion formation and fibronectin expression/secretion, which subsequently interferes with cell adhesion properties and motility. 
 Human trophoblast cell adhesion to extracellular matrix protein, entactin. Trophoblast interaction with endometrial extracellular matrix (  ECM  ) is crucial during human embryo implantation and placentation. Entactin, a ubiquitous basement membrane glycoprotein, plays a central role in   ECM   assembly, cell attachment, and chemotaxis. The present study was conducted to examine the possible role of entactin in promoting human trophoblast adhesion.Using an extended life span first trimester trophoblast cell line HTR-8/SVneo (HTR) and a cell adhesion assay, we measured the adherence of human first trimester trophoblasts to recombinant entactin and its domains. Also, we used flow cytometry and indirect immunofluorescence to detect the presence of integrins that may be involved in human trophoblast-entactin interaction; these methods were used to analyze HTR cells, as well as tissue sections and freshly isolated human trophoblasts from first trimester and term placenta.We found that first trimester trophoblast cells were highly adherent to entactin and its E and G2 domains but not to G1 or G3 domains. Using indirect immunofluorescence and flow cytometry, we found that both beta 1 and beta 3 integrin subunits were expressed on the surface of HTR trophoblast cells adhering to entactin; in contrast, beta 2 and beta 4 integrin subunits were not detected. In addition, we found that alpha v beta 3 was expressed on freshly isolated villous cytotrophoblasts and cytotrophoblast and syncytiotrophoblasts in tissue sections from term placenta. The beta 3 integrin subunit was expressed in cytotrophoblasts and syncytiotrophoblasts in villi of first trimester placental tissue sections.Recombinant entactin promotes human trophoblast cell adhesion through both its E and G2 domains and these specific adhesive interactions may be mediated by beta 1 and/or beta 3 class integrins. 
 Characterization of integrin engagement during defined human embryonic stem cell culture. Human embryonic stem (hES) cells are pluripotent, capable of differentiating into any cell type of the body, and therefore have the ability to provide insights into mechanisms of human development and disease, as well as to provide a potentially unlimited supply of cells for cell-based therapy and diagnostics. Knowledge of the adhesion receptors that hES cells employ to engage extracellular matrix (  ECM  ) proteins is of basic biological interest and can enhance the design of cell culture and implantation systems to enable these biomedical applications. Although hES cells express a variety of cell surface receptors, little is known about which integrins are involved during subculture and passage. Matrigel is broadly used as a cell adhesive matrix for hES cell culture. Here, we sought to identify which integrins hES cells exploit for adhesion to Matrigel-coated surfaces in defined medium conditions. Using RT-PCR, flow cytometry, and fluorescence immunochemistry, we found that numerous integrins were expressed by H1 hES cells; however, antibody blocking assays indicated that only alpha(v)beta(3), alpha(6), beta(1), and alpha(2)beta(1) played a significant role in the initial adhesion of the hES cells to Matrigel in defined medium conditions. We subsequently identified a cohort of synthetic peptides that, when adsorbed to the culture surface, promoted H1 hES cell attachment and proliferation, as well as maintained a pluripotent phenotype. Peptides designed to engage with alpha(v)beta(3), alpha(6), beta(1), and alpha(2)beta(1) integrins and syndecan-1 were tested both individually and in various combinations. A combination of two integrin-engaging peptides (AG-10, C-16) and one syndecan-engaging peptide (AG-73) was sufficient to promote hES cell adhesion, maintenance, and proliferation. We propose that a specific integrin "fingerprint" is necessary for maintenance of hES cell self-renewal, and synthetic culture systems must capture this engagement profile for hES cells to remain undifferentiated.-Meng, Y., Eshghi, S., Li, Y. J., Schmidt, R., Schaffer, D. V., Healy, K. E. Characterization of integrin engagement during defined human embryonic stem cell culture. 
 β1 integrin signaling maintains human epithelial progenitor cell survival in situ and controls proliferation, apoptosis and migration of their progeny. β1 integrin regulates multiple epithelial cell functions by connecting cells with the extracellular matrix   (E  CM). While β1 integrin-mediated signaling in murine epithelial stem cells is well-studied, its role in human adult epithelial progenitor cells (ePCs) in situ remains to be defined. Using microdissected, organ-cultured human scalp hair follicles (HFs) as a clinically relevant model for studying human ePCs within their natural topobiological habitat, β1 integrin-mediated signaling in ePC biology was explored by β1 integrin siRNA silencing, specific β1 integrin-binding antibodies and pharmacological inhibition of integrin-linked kinase (ILK), a key component of the integrin-induced signaling cascade. β1 integrin knock down reduced keratin 15 (K15) expression as well as the proliferation of outer root sheath keratinocytes (ORSKs). Embedding of HF epithelium i  nto   an ECM rich in β1 integrin ligands that mimic the HF mesenchyme significantly enhanced proliferation and migration of ORSKs, while K15 and CD200 gene and protein expression were inhibited. Em  plo  ying ECM-embedded β1 integrin-activating or -inhibiting antibodies allowed to identify functionally distinct human ePC subpopulations in different compartments of the HF epithelium. The β1 integrin-inhibitory antibody reduced β1 integrin expression in situ and selectively enhanced proliferation of bulge ePCs, while the β1 integrin-stimulating antibody decreased hair matrix keratinocyte apoptosis and enhanced transferrin receptor (CD71) immunoreactivity, a marker of transit amplifying cells, but did not affect bulge ePC proliferation. That the putative ILK inhibitor QLT0267 significantly reduced ORSK migration and proliferation and induced massive ORSK apoptosis suggests a key role for ILK in mediating the ß1 integrin effects. Taken together, these findings demonstrate that ePCs in human HFs require β1 integrin-mediated signaling for survival, adhesion, and migration, and that different human HF ePC subpopulations differ in their response to β1 integrin signaling. These insights may be exploited for cell-based regenerative medicine strategies that employ human HF-derived ePCs. 
 Integrins mediate adhesion of medulloblastoma cells to tenascin and activate pathways associated with survival and proliferation. Medulloblastoma spreads by leptomeningeal dissemination rather than by infiltration that characterizes other CNS tumors, eg, gliomas. This study represents an initial attempt to identify both the molecules that mediate medulloblastoma adhesion to leptomeninges and the pathways that are key to survival and proliferation of tumor following adhesion. As a first step in molecule identification, we produced adhesion of D283 medulloblastoma cells to the extracellular matrix (  ECM  ) of H4 glioma cells in vitro. Within this context, D283 cells preferentially expressed the alpha9 and beta1 integrin subunits; antibody and disintegrin blockade of alpha9 and beta1 binding eliminated the adhesion. The H4   ECM   was enriched in tenascin, a binding partner for the alpha9beta1 integrin heterodimer. Purified tenascin-C supported D283 cell adhesion. The adhesion was blocked by antibodies to alpha9 and beta1 integrin. In vivo data were similar; immunohistochemistry of primary human medulloblastomas with leptomeningeal extension demonstrated increased expression of alpha9 and beta1 integrins as well as tenascin at the interface of brain and leptomeningeal tumor. These data suggest that tumor-cell expressions of alpha9 and beta1 integrins in combination with extracellular tenascin are necessary for medulloblastoma adhesion to the leptomeninges. As a first step in the identification of pathways that mediate survival and proliferation of tumor following adhesion, we demonstrated that adhesion to H4   ECM   was associated with survival and proliferation of D283 cells as well as activation of the MAPK pathway in a growth factor deficient environment. Antibody blockade of alpha9 and beta1 integrin binding that eliminated adhesion also eliminated the in vitro survival benefit. These data suggest that adhesion of medulloblastoma to the meninges is necessary for the survival and proliferation of these tumor cells at the secondary site. 
 Additive effect of recombinant Mycobacterium tuberculosis ESAT-6 protein and ESAT-6/CFP-10 fusion protein in adhesion of macrophages through fibronectin receptors. Tuberculous granulomas are the sites of interaction between the T cells, macrophages, and extracellular matrix (  ECM  ) to control the infection caused by Mycobacterium tuberculosis (M. tuberculosis). A predominant role of RD-1-encoded secretory proteins, early secreted antigenic target-6 (ESAT-6), and culture filtrate protein-10 (CFP-10) in the formation of granulomas has recently been emphasized. However, the precise molecular events that induce the formation of these granulomatous structures are yet to be elucidated. Macrophages use integrins to adhere to fibronectin (FN) as a major component of the   ECM  . The major goal of this study was to investigate whether recombinant M. tuberculosis antigens can modulate integrin-mediated macrophage adhesion.Differentiated THP-1 cell line was stimulated with recombinant ESAT-6, CFP-10, and ESAT-6/CFP-10 proteins and evaluated for alterations in the expression levels of α5β1 and α4β1 by semiquantitative real-time polymerase chain reaction. The role of these recombinant antigens in the cytoskeleton rearrangement was determined by adhesion assay and immunofluorescent microscopy.Our data showed that ESAT-6 and ESAT-6/CFP-10 fusion proteins could induce adhesion of macrophages to FN through α4β1 integrin. An increased expression level of α4β1 integrin in comparison with α5β1 integrin in differentiated THP-1 cells was also observed. Results of immunofluorescence studies showed that recombinant proteins-treated THP-1 cells form well-organized stress fibers and focal contacts containing vinculin compared with untreated THP-1 cells.Increased expression level of α4β1 in differentiated THP-1 cells could suggest the important role of α4β1 integrin in adhesion and focal contact formation of macrophages exposed to M. tuberculosis antigens. 
 The role of integrin-β/FAK in cyclic mechanical stimulation in MG-63 cells. This study aims to explore the function of Integrin-β/FAK in the mechanical signal transduction and the connection with downstream ERK signal pathways.Human osteosarcoma MG63 cell lines were used in this study. The effects of mechanical strain on the Integrin-β₁ expression, FAK and ERK signal pathway in Human osteosarcoma MG63 cells were detected using RT-PCR and Western-blotting methods. The localization of FAK in Human osteosarcoma MG63 cells were determined using immunofluorescent method. The interaction between Integrin-β₁ and FAK were detected by using co-immunoprecipitation method.The expression of Integrin-β₁ shows a notable bimodel distribution, mechanical strain stimulation can promote Integrin-β₁ expression and the phosphorylation of FAK and ERK, mechanical strain activated FAK and ERK mediated by Integrin-β₁.Integrin-β₁ may play an important role in osteoblast proliferation differentiation process, it might feel external strain   sti  mulation through ECM composition and makes FAK phosphated through the interaction with FAK, thus causing a series of activation of signal molecules. Finally it reduces MAPK (ERK) activation and cellular responses to finish mechanical signal transduction. 
 Integrin beta1 mediates hepatocellular carcinoma cells chemotaxis to laminin. Chemotaxis is an important step during the invasion of carcinoma cells. And integrins are most important receptors mediating interaction between cells and extracellular matrix (  ECM  ). This study was designed to study integrin beta1 mediating chemotaxis of hepatocellular carcinoma (HCC) cells to laminin(LN).A micropipette technique was adopted to investigate the effect of blockade of integrin beta1 on pseudopod protrusion of HCC cells in response to LN stimulation. Chemotactic pseudopod protrusion of a HCC cell was evaluated using a dual-pipette set-up, in which two pipettes filled with LN solution were positioned in close contact with the same cell, and pseudopod protrusion into each pipette was viewed dynamically and recorded with a tape recorder. The lengths of pseudopods were measured and plotted against time to obtain a pseudopod growth curve. The integrin beta1 subunit on the surfaces of HCC cells were analyzed by flow cytometry.In dual pipette chemotaxis experiment, when the two pipettes were filled with LN(50 microg/ml, 200 microg/ml), pseudopods extended from the HCC cell into each of the pipettes nearly symmetrically, ie, with nearly identical maximum pseudopod length and similar pseudopod growth curves. Upon addition of anti-CD29 (20 microg/ml) to one of the pipettes, pseudopod protrusion was blocked nearly completely while protrusion into the opposite pipette became more evidently, with a larger maximum length. Expression of integrin beta1 was up to 95.78% to cells chosen in the experiment.Integrin beta1 subunit was an important constituent receptor subunit for mediating chemotactic pseudopod protrusion of HCC cell to LN. 
 An SH3 domain-containing GTPase-activating protein for Rho and Cdc42 associates with focal adhesion kinase. The integrin family of cell surface receptors mediates cell adhesion to components of the extracellular matrix (  ECM  ). Integrin engagement with the   ECM   initiates signaling cascades that regulate the organization of the actin-cytoskeleton and changes in gene expression. The Rho subfamily of Ras-related low-molecular-weight GTP-binding proteins and several protein tyrosine kinases have been implicated in mediating various aspects of integrin-dependent alterations in cell homeostasis. Focal adhesion kinase (FAK or pp125FAK) is one of the tyrosine kinases predicted to be a critical component of integrin signaling. To elucidate the mechanisms by which FAK participates in integrin-mediated signaling, we have used expression cloning to identify cDNAs that encode potential FAK-binding proteins. We report here the identification of a cDNA that encodes a new member of the GTPase-activating protein (GAP) family of GTPase regulators. This GAP, termed Graf (for GTPase regulator associated with FAK), binds to the C-terminal domain of FAK in an SH3 domain-dependent manner and preferentially stimulates the GTPase activity of the GTP-binding proteins RhoA and Cdc42. Subcellular localization studies using Graf-transfected chicken embryo cells indicates that Graf colocalizes with actin stress fibers, cortical actin structures, and focal adhesions. Graf mRNA is expressed in a variety of avian tissues and is particularly abundant in embryonic brain and liver. Graf represents the first example of a regulator of the Rho family of small GTP-binding proteins that exhibits binding to a protein tyrosine kinase. We suggest that Graf may function to mediate cross talk between the tyrosine kinases such as FAK and the Rho family GTPase that control steps in integrin-initiated signaling events. 
 Twist induces epithelial-mesenchymal transition and cell motility in breast cancer via ITGB1-FAK/ILK signaling axis and its associated downstream network. Twist, a highly conserved basic Helix-Loop-Helix transcription factor, functions as a major regulator of epithelial-mesenchymal transition (EMT) and tumor metastasis. In different cell models, signaling pathways such as TGF-β, MAPK/ERK, WNT, AKT, JAK/STAT, Notch, and P53 have also been shown to play key roles in the EMT process, yet little is known about the signaling pathways regulated by Twist in tumor cells. Using iTRAQ-labeling combined with 2D LC-MS/MS analysis, we identified 194 proteins with significant changes of expression in MCF10A-Twist cells. These proteins reportedly play roles in EMT, cell junction organization, cell adhesion, and cell migration and invasion.   EC  M-receptor interaction, MAPK, PI3K/AKT, P53 and WNT signaling were found to be aberrantly activated in MCF10A-Twist cells. Ingenuity Pathways Analysis showed that integrin β1 (ITGB1) acts as a core regulator in linking integrin-linked kinase (ILK), Focal-adhesion kinase (FAK), MAPK/ERK, PI3K/AKT, and WNT signaling. Increased Twist and ITGB1 are associated with breast tumor progression. Twist transcriptionally regulates ITGB1 expression. Over-expression of ITGB1 or Twist in MCF10A led to EMT, activation of FAK/ILK, MAPK/ERK, PI3K/AKT, and WNT signaling. Knockdown of Twist or ITGB1 in BT549 and Hs578T cells decreased activity of FAK, ILK, and their downstream signaling, thus specifically impeding EMT and cell invasion. Knocking down ILK or inhibiting FAK, MAPK/ERK, or PI3K/AKT signaling also suppressed Twist-driven EMT and cell invasion. Thus, the Twist-ITGB1-FAK/ILK pathway and their downstream signaling network dictate the Twist-induced EMT process in human mammary epithelial cells and breast cancer cells. 
 Integrin-mediated functional polarization of Caco-2 cells through E-cadherin--actin complexes. Enterocyte differentiation is a dynamic process during which reinforcement of cell-cell adhesion favours migration along the crypt-to-villus axis. Functional polarization of Caco-2 cells, the most commonly used model to study intestinal differentiation, is assessed by dome formation and tightness of the monolayer and is under the control of the extracellular matrix (  ECM  ). Furthermore, our biochemical and confocal microscopy data demonstrate that the   ECM   dramatically reinforces E-cadherin targeting to the upper lateral membrane, formation of the apical actin cytoskeleton and its colocalization with E-cadherin in functional complexes. In our model, these effects were produced by native laminin-5-enriched   ECM   as well as by type IV collagen or laminin 2, which suggests a common pathway of induction through integrin receptors. Indeed, these effects were antagonized by blocking anti-beta1- and anti-alpha6-integrin antibodies and directly induced by a stimulating anti-beta1-integrin antibody. These results demonstrate that integrin-dependent cell to   ECM   adhesion reinforces E-cadherin-dependent cell-cell adhesion in Caco-2 cells and further support the notion that enterocyte differentiation is supported by a molecular crosstalk between the two adhesion systems of the cell. 
 Prognostic role of integrin β1, E-cadherin, and rac1 expression in small cell lung cancer. Integrin β(1) mediates cellular adhesion to the extracellular matrix   (E  CM) and is correlated with highly invasive and metastatic behavior in small cell lung cancer (SCLC). E-cadherin (ECAD) is a calcium-dependent cell-cell adhesion receptor that restricts invasion of cells and reduces metastasis. Rac1 is involved in the regulation of the actin cytoskeleton, adhesion, migration, invasion, and tumor metastasis. The aim of this study was to examine integrin β(1) , ECAD and rac1 expression in SCLC and to analyze the prognostic value of these markers in patients with SCLC. We analyzed integrin β(1) , ECAD, and rac1 expression in 112 SCLC tissues by immunohistochemical staining. Correlative analyses between integrin β(1) , ECAD, and rac1 expression and cliniopathological factors were performed. A total of 65 patients had extensive disease (ED) (58%), and 47 had limited disease (LD) (42%). The median follow-up duration was 61 months (range: 14-117 months), and the median progression free survival (PFS) and overall survival (OS) were 6.1 months (range: 4.8-7.4 months) and 9.7 months (range: 8.1-11.3 months), respectively. The expression of integrin β(1) , ECAD, and rac1 protein was observed in 64, 73, and 99 of SCLC tissues, respectively. The correlative analyses between integrin β(1) , ECAD, or rac1 expression and various clinical parameters did not show any statistical significance. However, the ECAD expression was associated with OS in the entire cohort. In contrast, the expression of integrin β(1) and rac1 was not associated with PFS or OS. In a subgroup analysis, patients with less than two metastasis had significantly longer OS (p = 0.047) if their tumors expressed integrin β(1) compared to those without integrin β(1) expression. In addition, OS was longer for patients with ECAD positive tumors compared to those whose tumors did not express ECAD in males (p = 0.032) and patients who never smoked (p < 0.001). Multivariate analysis showed that LD (p = 0.004), overall response rate (p = 0.003), and expression of ECAD (p = 0.015) were the independent good prognostic factors for OS. LD (p = 0.024), overall response rate (p < 0.001), and less than two metastasis (p = 0.003) were prognostic factors for longer PFS. These results suggest that ECAD expression may be useful as a prognostic indicator in patients with SCLC. 
 T-cadherin enhances cell-matrix adhesiveness by regulating beta1 integrin trafficking in cutaneous squamous carcinoma cells. T-cadherin is a glycosyl-phosphatidylinositol (GPI) anchored cadherin molecule. We previously reported that T-cadherin is normally expressed on the basal keratinocytes of the epidermis and is down-regulated in cutaneous squamous cell carcinoma (SCC). We found that expression of T-cadherin in cutaneous squamous carcinoma cells regulated level of surface beta1 integrin, which functioned as extracellular matrix (  ECM  ) receptor. Involvement of T-cadherin in beta1 integrin trafficking was studied using three different stable cell lines with cytomegalovirus (CMV)-driven over-expression, tetracycline (Tet)-inducible expression and RNAi-mediated suppressed expression of T-cadherin. Pulse-chase analysis using a cholesterol-depleting reagent and a tyrosine kinase inhibitor showed that beta1 integrin mainly internalized via caveolae. Over-expression of T-cadherin suppressed the internalization of both beta1 integrin and cholera toxin (CTX), a marker of caveolae-mediated endocytosis. By Western blot analysis of tyrosine-kinase target molecules, we demonstrated a reduced level of EGF receptor (EGFR)-phosphorylation in T-cadherin over-expressing cells. In addition, studies using EGF and EGFR specific inhibitors revealed that EGFR activation stimulated beta1 integrin internalization. Taking these results together, T-cadherin may modulate cell-matrix adhesion in basal keratinocytes as well as invasive potency in SCC by regulating surface level of beta1 integrin. 
 miRNA-29c suppresses lung cancer cell adhesion to extracellular matrix and metastasis by targeting integrin β1 and matrix metalloproteinase2 (MMP2). Our pilot study using miRNA arrays found that miRNA-29c (miR-29c) is differentially expressed in the paired low-metastatic lung cancer cell line 95C compared to the high-metastatic lung cancer cell line 95D. Bioinformatics analysis shows that integrin β1 and matrix metalloproteinase 2 (MMP2) could be important target genes of miR-29c. Therefore, we hypothesized that miR-29c suppresses lung cancer cell adhesion to extracellular matrix   (E  CM) and metastasis by targeting integrin β1 and MMP2. The gain-of-function studies that raised miR-29c expression in 95D cells by using its mimics showed reductions in cell proliferation, adhesion   to   ECM, invasion and migration. In contrasts, loss-of-function studies that reduced miR-29c by using its inhibitor in 95C cells promoted proliferation, adhesion   to   ECM, invasion and migration. Furthermore, the dual-luciferase reporter assay demonstrated that miR-29c inhibited the expression of the luciferase gene containing the 3'-UTRs of integrin β1 and MMP2 mRNA. Western blotting indicated that miR-29c downregulated the expression of integrin β1 and MMP2 at the protein level. Gelatin zymography analysis further confirmed that miR-29c decreased MMP2 enzyme activity. Nude mice with xenograft models of lung cancer cells confirmed that miR-29c inhibited lung cancer metastasis in vivo, including bone and liver metastasis. Taken together, our results demonstrate that miR-29c serves as a tumor metastasis suppressor, which suppresses lung cancer cell adhesio  n t  o ECM and metastasis by directly inhibiting integrin β1 and MMP2 expression and by further reducing MMP2 enzyme activity. The results show that miR-29c may be a novel therapeutic candidate target to slow lung cancer metastasis. 
 Mechanism of beta 1-integrin-mediated hepatoma cell growth involves p27 and S-phase kinase-associated protein 2. Although cooperative interactions between growth factors and integrins, cell surface receptors for extracellular matrices (  ECM  ), have been reported, little is known about the interaction between hepatocyte growth factor (HGF) and integrin in hepatoma cells. We investigated the effects and mechanisms of integrin on the proliferation of hepatoma cells regulated by HGF. Human HepG2 hepatoma cells stably transfected with beta 1-integrin were treated with HGF and compared with parental and mock-transfected control cells. Cell proliferation and expression of cyclin-dependent kinase (Cdk) inhibitors and S-phase kinase-associated protein 2 (Skp2), were investigated. HGF dose-dependently suppressed the proliferation of parental and mock-transfected HepG2 cells. However, cells overexpressing beta 1-integrin exhibited increased proliferation in response to HGF. Although HGF increased p27 and decreased Skp2 expression in the parental and mock-transfected cells, the p27 and Skp2 levels in cells overexpressing beta 1-integrin were not altered by HGF. Interestingly, HepG2 cells overexpressing beta 1-integrin showed increased Skp2 expression. Furthermore, HGF did not reduce the proliferation of HepG2 cells transfected with antisense p27 or sense Skp2. Thus, HGF suppresses HepG2 cell proliferation by directly increasing p27 expression and indirectly decreasing Skp2 expression, and beta 1-integrin modulates the responsiveness of hepatoma cells to HGF via a p27-dependent manner by increasing Skp2. In conclusion, these results strongly suggest that integrin-mediated signals from the   ECM   can modulate growth factor-mediated signals in hepatoma cells, and may contribute to the growth of hepatocellular carcinomas. 
 Multiple beta 1 integrins mediate enhancement of human airway smooth muscle cytokine secretion by fibronectin and type I collagen. Altered airway smooth muscle (ASM) function and enrichment of the extracellular matrix (  ECM  ) with interstitial collagen and fibronectin are major pathological features of airway remodeling in asthma. We have previously shown that these   ECM   components confer enhanced ASM proliferation in vitro, but their action on its newly characterized secretory function is unknown. Here, we examined the effects of fibronectin and collagen types I, III, and V on IL-1beta-dependent secretory responses of human ASM cells, and characterized the involvement of specific integrins. Cytokine production (eotaxin, RANTES, and GM-CSF) was evaluated by ELISA, RT-PCR, and flow cytometry. Function-blocking integrin mAbs and RGD (Arg-Gly-Asp)-blocking peptides were used to identify integrin involvement. IL-1beta-dependent release of eotaxin, RANTES, and GM-CSF was enhanced by fibronectin and by fibrillar and monomeric type I collagen, with similar changes in mRNA abundance. Collagen types III and V had no effect on eotaxin or RANTES release but did modulate GM-CSF. Analogous changes in intracellular cytokine accumulation were found, but in <25% of the total ASM cell population. Function-blocking Ab and RGD peptide studies revealed that alpha2beta1, alpha5beta1, alphavbeta1, and alphavbeta3 integrins were required for up-regulation of IL-1beta-dependent ASM secretory responses by fibronectin, while alpha2beta1 was an important transducer for type I collagen. Thus, fibronectin and type I collagen enhance IL-1beta-dependent ASM secretory responses through a beta1 integrin-dependent mechanism. Enhancement of cytokine release from ASM by these   ECM   components may contribute to airway wall inflammation and remodeling in asthma. 
 Mixed extracellular matrix ligands synergistically modulate integrin adhesion and signaling. Cell adhesion to extracellular matrix (  ECM  ) components through cell-surface integrin receptors is essential to the formation, maintenance and repair of numerous tissues, and therefore represents a central theme in the design of bioactive materials that successfully interface with the body. While the adhesive responses associated with a single ligand have been extensively analyzed, the effects of multiple integrin subtypes binding to multivalent   ECM   signals remain poorly understood. In the present study, we generated a high throughput platform of non-adhesive surfaces presenting well-defined, independent densities of two integrin-specific engineered ligands for the type I collagen (COL-I) receptor alpha(2)beta(1) and the fibronectin (FN) receptor alpha(5)beta(1) to evaluate the effects of integrin cross-talk on adhesive responses. Engineered surfaces displayed ligand density-dependent adhesive effects, and mixed ligand surfaces significantly enhanced cell adhesion strength and focal adhesion assembly compared to single FN and COL-I ligand surfaces. Moreover, surfaces presenting mixed COL-I/FN ligands synergistically enhanced FAK activation compared to the single ligand substrates. The enhanced adhesive activities of the mixed ligand surfaces also promoted elevated proliferation rates. Our results demonstrate interplay between multivalent   ECM   ligands in adhesive responses and downstream cellular signaling. 
 Regulation of endodermal differentiation of human embryonic stem cells through integrin-  ECM   interactions. Many cellular responses during development are regulated by interactions between integrin receptors and extracellular matrix proteins (  ECM  Ps). Although the majority of recent studies in human embryonic stem cell (hESC) differentiation have focused on the role of growth factors, such as FGF, TGFβ, and WNT, relatively little is known about the role of   EC  MP-integrin signaling in this process. Moreover, current strategies to direct hESC differentiation into various lineages are inefficient and have yet to produce functionally mature cells in vitro. This suggests that additional factors, such as   EC  MPs, are required for the efficient differentiation of hESCs. Using a high-throughput multifactorial cellular array technology, we investigated the effect of hundreds of   EC  MP combinations and concentrations on differentiation of several hPSC lines to definitive endoderm (DE), an early embryonic cell population fated to give rise to internal organs such as the lung, liver, pancreas, stomach, and intestine. From this screen we identified fibronectin (FN) and vitronectin (VTN) as   EC  MP components that promoted DE differentiation. Analysis of integrin expression revealed that differentiation toward DE led to an increase in FN-binding integrin α5 (ITGA5) and VTN-binding integrin αV (ITGAV). Conditional short hairpin RNA-mediated knockdown of ITGA5 and ITGAV disrupted hESC differentiation toward DE. Finally, fluorescence-based cell sorting for ITGA5 and ITGAV significantly enriched cells with gene expression signatures associated with DE, demonstrating that these cell surface proteins permit isolation and enrichment of DE from hESCs. These data provide evidence that FN and VTN promote endoderm differentiation of hESCs through interaction with ITGA5 and ITGAV, and th  at   ECMP-integrin interactions are required for hESC differentiation into functionally mature cells. 
 Clinical and functional significance of α9β1 integrin expression in breast cancer: a novel cell-surface marker of the basal phenotype that promotes tumour cell invasion. Integrin α9β1 is a receptor   for   ECM proteins, including Tenascin-C and the EDA domain of fibronectin, and has been shown to transduce TGFβ signalling. This study has examined the expression pattern of α9β1 in 141 frozen breast carcinoma samples and related expression to prognostic indices, molecular subtype and patient outcome. Effects of α9β1 on tumour cell migration and invasion were assessed using blocking antibody and gene transduction approaches. Integrin α9β1 localized to myoepithelial cells in normal ducts and acini, a pattern maintained in DCIS. A subset (17%) of invasive carcinomas exhibited tumour cell expression of α9β1, which related significantly to the basal-like phenotype, as defined by either CK5/6 or CK14 expression. Tumour expression of α9β1 showed a significant association with reduced overall patient survival (p < 0.0001; HR 5.94, 95%CI 3.26-10.82) and with reduced distant-metastasis-free survival (p < 0.0001; HR 6.37, CI 3.51-11.58). A series of breast cancer cell lines was screened for α9β1 with the highly invasive basal-like GI-101 cell line expressing significant levels. Both migration and invasion of this line were reduced significantly in the presence of α9-blocking antibody and following α9-knockdown with siRNA. Conversely, migratory and invasive behaviour of α9-negative MCF7 cells and α9-low MDA MB468 cells was enhanced significantly by over-expression of α9. Thus, α9β1 acts as a novel marker of the basal-like breast cancer subtype and expression is associated with reduced survival, while its ability to promote breast cancer cell migration and invasion suggests that it contributes to the aggressive clinical behaviour of this tumour subtype. 
 Alpha2beta1 integrin signalling enhances cyclooxygenase-2 expression in intestinal epithelial cells. Inflammatory bowel diseases (IBD) are linked to an increased risk of developing colon cancer, by inflammatory mediators and alterations to the extracellular matrix (  ECM  ). The events induced by inflammatory mediators lead to dysregulated activation and induction of inflammatory genes such as cyclooxygenase-2 (COX-2). COX-2 is involved in the conversion of arachidonic acid to biologically active prostanoids and is highly upregulated in colon cancer. Since inflammation-induced changes to the extracellular matrix could affect integrin activities, we here investigated the effect of integrin signalling on the level of COX-2 expression in the non-transformed intestinal epithelial cell lines, Int 407 and IEC-6. Adhesion of these cells to a collagen I- or IV-coated surface, increased surface expression of alpha2beta1 integrin. Activation of integrins with collagen caused an increased cox-2 promoter activity, with a subsequent increase in COX-2 expression. The signalling cascade leading to this increased expression and promoter activity of cox-2, involves PKCalpha, the small GTPase Ras and NFkappaB but not Erk1/2 or Src activity. The integrin-induced increase in cellular COX-2 activity is responsible for an elevated generation of reactive oxygen species (ROS) and increased cell migration. This signalling pathway suggests a mechanism whereby inflammation-induced modulations of the   ECM  , can promote cancer transformation in the intestinal epithelial cells. 
 NF-κB regulates radioresistance mediated by β1-integrin in three-dimensional culture of breast cancer cells. β1-integrin induction enhances breast cancer cell survival after exposure to ionizing radiation (IR), but the mechanisms of this effect remain unclear. Although NF-κB initiates prosurvival signaling pathways post-IR, the molecular function of NF-κB with other key elements in radioresistance, particularly with respect to extracellular matrix-induced signaling, is not known. We discovered a typical NF-κB-binding site in the β1-integrin promoter region, indicating a possible regulatory role for NF-κB. Using three-dimensional laminin-rich extracellular matri  x (  3D lrECM) culture, we show that NF-κB is required for β1-integrin transactivation in T4-2 breast cancer cells post-IR. Inhibition of NF-κB reduced clonogenic survival and induced apoptosis and cytostasis in formed tumor colonies. In addition, T4-2 tumors with inhibition of NF-κB activity exhibit decreased growth in athymic mice, which was further reduced by IR with downregulated β1-integrin expression. Direct interactions between β1-integrin and NF-κB p65 were induced in nonmalignant breast epithelial cells, but not in malignant cells, indicating context-specific regulation. As β1-integrin also activates NF-κB, our findings reveal a novel forward feedback pathway that could be targeted to enhance therapy. 
 DNAzyme as an efficient tool to modulate invasiveness of human carcinoma cells. In this study we evaluated efficiency of DNAzymes to modulate motility of cancer cells, an important factor in the progression and metastasis of cancers. For this purpose we targeted β1 integrins that are predominant adhesive receptors in various carcinoma cell lines (CX1.1, HT29, LOVO, LS180, PC-3). To evaluate invasiveness of cancer cells, we used a transwell migration assay that allowed analyzing chemotactic migration of colon carcinoma cell lines across an   EC  M-coated membrane. Their adhesive properties were also characterized by the analysis of adhesion to fibronectin, laminin and collagen. In addition, the expression of major integrin subunits, selected intact β1 integrins, and other adhesive receptors (ICAM, E-selectin, uPAR) was analyzed by flow cytometry. Inhibition of β1 integrin expression by DNAzyme to β1 mRNA almost abolished the invasiveness of the CX1.1, HT29, LS180, LOVO and PC-3 cells in vitro. These data show that DNAzymes to β1 integrin subunit can be used to inhibit invasiveness of carcinoma cells. 
 Beta 1-integrin protects hepatoma cells from chemotherapy induced apoptosis via a mitogen-activated protein kinase dependent pathway. beta 1-integrin modulates cellular phenotype by mediating signals from the extracellular matrix (  ECM  ). Although overexpression of integrin molecules in hepatocellular carcinoma (HCC) has been reported, the role of overexpressed beta 1-integrin in the disease process of HCC is not fully understood. The authors investigated the effects of beta 1-integrin on apoptosis in hepatoma cells.Human hepatoma cell lines HepG2, Huh7, and HLE were stably transfected with full-length beta 1-integrin. Cells underwent apoptosis induced by chemotherapeutic reagents, including cis-platinum (II)-diammine dichloride, etoposide, and docetaxel. Cell survival and intracellular signaling pathways dependent on beta 1-integrin-mediated apoptosis effects were analyzed by treating cells with PD98059 (ERK inhibitor), SB203580 (p38MAP kinase inhibitor), wortmannin (phosphatidyl inositol-3-kinase inhibitor), and herbimycin A (tyrosine kinase inhibitor).All three hepatoma cell lines overexpressing beta 1-integrin were protected from apoptosis induced by chemotherapeutic reagents, whereas parental or mock transfected cells were not. Treatment with PD98059 or SB203580 abolished the protective effect on apoptosis in cells overexpressing beta 1-integrin. Neither herbimycin nor wortmannin blocked the protective effects of beta 1-integrin overexpression.These data suggest that overexpression of beta 1-integrin confers resistance to apoptosis in hepatoma cells via a MAP kinase dependent pathway. beta1-integrin mediated signaling from the   ECM   in HCC cells may contribute to chemotherapy resistance. 
 The chemokine receptor 7 regulates cell adhesion and migration via beta1 integrin in metastatic squamous cell carcinoma of the head and neck. Migration and adhesion of tumor cells are essential prerequisites for the formation of metastases in malignant diseases. Chemokine receptor 7 (CCR7) has been shown to regulate integrin which can then facilitate adhesion of cancer cells to and/or migration through the extracellular matrix (  ECM  ). In order to identify the connection between CCR7 and beta1 integrin, and the influence on cell adhesion and migration in metastatic squamous cell carcinoma of the head and neck (SCCHN). We use adhesion assays, migration assay, immunofluorescence staining, western blotting, and immunohistochemical analysis to find whether beta1 integrin can be activated by CCL19 (CCR7's ligand) and its role in SCCHN. The experiments were performed in the metastatic SCCHN cell line PCI-37B after pre-incubation of the cells with CCL19 and beta1 integrin inhibitors RGD-peptide. Our results demonstrate that CCR7 favours PCI-37B cell adhesion and migration, and induces reorganization of the actin cytoskeleton and up-expression of beta1 integrin protein. beta1 integrin inhibitor RGD-peptide can block all these effects. Taken together, our data indicate that CCR7 regulate cell adhesion and migration via beta1 integrin in metastatic SCCHN, and these results can provide a basis for new strategies in preventing metastases of SCCHN. 
 Identification of nucleolin as a lipid-raft-dependent β1-integrin-interacting protein in A375 cell migration. Lipid rafts are related to cell surface receptor function. Integrin is a major surface receptor protein in cell adhesion and migration on the extracellular matrix   (EC  M). Here, we showed that lipid rafts played a critical role in human melanoma A375 cell spreading and migration on fibronectin; an important component of the   EC  M that interacts with β1 integrin. We found that the disruption of lipid rafts did not markedly inhibit the expression and activation of β1 integrin. By coimmunoprecipitation and mass spectrometry, we investigated the influence of lipid rafts on the β1 integrin complex and identified nucleolin as a potential lipid-raft-dependent β1-integrin-interacting protein. Upon confirmation of the interaction between β1 integrin and nucleolin, further studies revealed that nucleolin colocalized with β1 integrin in lipid rafts and raft disruption interrupted their association. In addition, knockdown of nucleolin markedly attenuated A375 cell spreading and migration on fibronectin. Taken together, we demonstrated that nucleolin is a critical lipid-raft-dependent β1-integrin-interacting protein in A375 cell spreading and migration on fibronectin. 
 N-ethylmaleimide-sensitive factor attachment protein α (αSNAP) regulates matrix adhesion and integrin processing in human epithelial cells. Integrin-based adhesion to the extracellular matrix   (E  CM) plays critical roles in controlling differentiation, survival, and motility of epithelial cells. Cells attach to th  e E  CM via dynamic structures called focal adhesions (FA). FA undergo constant remodeling mediated by vesicle trafficking and fusion. A soluble N-ethylmaleimide-sensitive factor (NSF) attachment protein α (αSNAP) is an essential mediator of membrane fusion; however, its roles in regulat  ing   ECM adhesion and cell motility remain unexplored. In this study, we found that siRNA-mediated knockdown of αSNAP induced detachment of intestinal epithelial cells, whereas overexpression of αSNAP incr  eas  ed ECM adhesion and inhibited cell invasion. Loss of αSNAP impaired Golgi-dependent glycosylation and trafficking of β1 integrin and decreased phosphorylation of focal adhesion kinase (FAK) and paxillin resulting in FA disassembly. These effects of αSNAP depl  eti  on on ECM adhesion were independent of apoptosis and NSF. In agreement with our previous reports that Golgi fragmentation mediates cellular effects of αSNAP knockdown, we found that either pharmacologic or genetic disruption of the Golgi recapitulated all the effects of αSNAP de  ple  tion on ECM adhesion. Furthermore, our data implicates β1 integrin, FAK, and paxillin in mediating the observed pro-adhesive effects of αSNAP. These results reveal novel roles for αSNAP   in   regulating ECM adhesion and motility of epithelial cells. 
 Three-dimensional collagen represses cyclin E1 via β1 integrin in invasive breast cancer cells. The behavior of breast epithelial cells is influenced by their microenvironment which includes stromal cells and extracellular matrix   (EC  M). During cancer progression, the tissue microenvironment fails to control proliferation and differentiation, resulting in uncontrolled growth and invasion. Upon invasion, the   EC  M encountered by breast cancer cells changes from primarily laminin and collagen IV to primarily collagen I. We show here that culturing invasive breast cancer cells in 3-dimensional (3D) collagen I inhibits proliferation through direct regulation of cyclin E1, a G(1)/S regulator that is overexpressed in breast cancer. When the breast cancer cell line MDA-MB-231 was cultured within 3D collagen I gels, the G(1)/S transition was inhibited as compared to cells cultured on conventional 2D collagen or plastic dishes. Cells in 3D collagen downregulated cyclin E1 protein and mRNA, with no change in cyclin D1 level. Cyclin D1 was primarily cytoplasmic in 3D cultures, and this was accompanied by decreased phosphorylation of Rb, a nuclear target for both cyclin E1- and cyclin D1-associated kinases. Positive regulators of cyclin E1 expression, the transcription factor c-Myc and cold-inducible RNA binding protein (CIRP), were decreased in 3D collagen cultures, while the collagen I receptor β1 integrin was greatly increased. Inhibition of β1 integrin function rescued proliferation and cyclin E1 expression as well as c-Myc expression and Rb phosphorylation, but cyclin D1 remained cytoplasmic. We conclude that cyclin E1 is repressed independent of effects on cyclin D1 in a 3D collagen environment and dependent on β1 integrin interaction with collagen I, reducing proliferation of invasive breast cancer cells. 
 Impact of integrin-matrix interaction and signaling on insulin gene expression and the mesenchymal transition of human beta-cells. A critical shortage of donor pancreata currently prevents the development of a universal cell-based therapy for type I diabetes. The ex vivo expansion of insulin-producing beta-cells offers a potential solution but is problematic due to the inherent tendency of these cells to transition into mesenchymal-like cells that are devoid of function. Here, we demonstrate for the first time that exposure to elements of the extracellular matrix (  ECM  ) directly potentiates the mesenchymal transition of cultured fetal beta-cells and causes associated declines in insulin gene expression. Individual   ECM   constituents varied in their ability to induce such responses, with collagen-IV (C-IV) and fibronectin inducing strong responses, whereas laminin-1 had no significant effect. Mesenchymal transition and concomitant losses in insulin gene expression observed on C-IV were found to be dependent on beta(1)-integrin ligation and were augmented in the presence of hepatocyte growth factor. Importantly, selective inhibition of c-Src, c-Jun N-terminal kinase (JNK), and extracellular signal-regulated kinase (ERK) prior to exposure to C-IV prevented mesenchymal transition and effectively preserved insulin expression. Fetal beta-cells undergoing mesenchymal transition were found to acquire alpha(1)beta(1) expression, and ligation of this integrin then promotes declines in insulin gene expression and a marked increase in beta-cell motility. Inhibition of Src-, ERK-, or JNK-dependent signaling combined with the selective regulation of matrix exposure may ultimately facilitate the development of more effective beta-cell expansion protocols. 
  GeneRIFs  
 expression of beta1 integrin in O  ECM  -1 cells is involved in the development of malignant phenotypes of OSCC 
 PCTK1-KAP0-myosin X-integrin beta 1 is a functional module providing a link between   ECM   and the actin cytoskeleton in the cell-extracellular matrix (  ECM  )-dependent control of spindle orientation. 
 miR-29c serves as a tumor metastasis suppressor, which suppresses lung cancer cell adhesion to   ECM   and metastasis by directly inhibiting integrin beta1 and MMP2 expression 
 data implicates beta1 integrin, FAK, and paxillin in mediating the observed pro-adhesive effects of alphaSNAP. These results reveal novel roles for alphaSNAP in regulating   ECM   adhesion and motility of epithelial cells. 
 Pancreatic beta cell mesenchymal transition and decline in insulin gene expression are induced by elements of the   ECM  , by b1-integrin ligation, and by signaling via Src, JNK, and ERK. 
 Data show a novel mechanism by which   ECM   regulates membrane-type 1 matrix metalloproteinase (MT1-MMP) association with beta1 or alphavbeta3 integrins, thus modulating its internalization, activity, and function on human endothelial cells. 
  [Ee]xtracellular [Mm]atrix  Process  
   extracellular matrix   organization 
  PubMed  
 Epidermal growth factor (EGF) regulates α5β1 integrin activation state in human cancer cell lines through the p90RSK-dependent phosphorylation of filamin A. Regulation of integrin activation has important implications for tumor cell invasion and metastasis.EGF activates ERK/p90RSK and Rho/Rho kinase signaling in A431 and DiFi colon cancer cells, leading to phosphorylation of filamin A (FLNa) and inactivation of the α5β1 integrin receptor.EGF promotes α5β1 inactivation through the p90RSK-dependent phosphorylation of FLNa.We have identified a novel EGF-dependent mechanism controlling the α5β1 integrin activation state. Cell adhesion, motility, and invasion are regulated by the ligand-binding activity of integrin receptors, transmembrane proteins that bind   to the extracellula  r matrix. Integrins whose conformation allows for ligand binding and appropriate functional activity are said to be in an active state. Integrin activation and subsequent ligand binding are dynamically regulated by the association of cytoplasmic proteins with integrin intracellular domains. In this study, we evaluated the role of EGF in the regulation of the activation state of the α5β1 integrin receptor for fibronectin. The addition of EGF to either A431 squamous carcinoma cells or DiFi colon cancer cells resulted in loss of α5β1-dependent adhesion to fibronectin but no loss of integrin from the cell surface. EGF activated the EGF receptor/ERK/p90RSK and Rho/Rho kinase signaling pathways. Blocking either pathway inhibited EGF-mediated loss of adhesion, suggesting that they work in parallel to regulate integrin function. EGF treatment also resulted in phosphorylation of filamin A (FLNa), which binds and inactivates β1 integrins. EGF-mediated FLNa phosphorylation was completely blocked by an inhibitor of p90RSK and partially attenuated by an inhibitor of Rho kinase, suggesting that both pathways converge on FLNa to regulate integrin function. A431 clonal cell lines expressing non-phosphorylated dominant-negative FLNa were resistant to the inhibitory effects of EGF on integrin function, whereas clonal cell lines overexpressing wild-type FLNa were more sensitive to the inhibitory effect of EGF. These data suggest that EGF-dependent inactivation of α5β1 integrin is regulated through FLNa phosphorylation and cellular contractility. 
 Different splice variants of filamin-B affect myogenesis, subcellular distribution, and determine binding to integrin [beta] subunits. Integrins connect the   extracellular matrix   with the cell interior, and transduce signals through interactions of their cytoplasmic tails with cytoskeletal and signaling proteins. Using the yeast two-hybrid system, we isolated a novel splice variant (filamin-Bvar-1) of the filamentous actin cross-linking protein, filamin-B, that interacts with the cytoplasmic domain of the integrin beta1A and beta1D subunits. RT-PCR analysis showed weak, but wide, expression of filamin-Bvar-1 and a similar splice variant of filamin-A (filamin-Avar-1) in human tissues. Furthermore, alternative splice variants of filamin-B and filamin-C, from which the flexible hinge-1 region is deleted (DeltaH1), were induced during in vitro differentiation of C2C12 mouse myoblasts. We show that both filamin-Avar-1 and filamin-Bvar-1 bind more strongly than their wild-type isoforms to different integrin beta subunits. The mere presence of the high-affinity binding site for beta1A is not sufficient for targeting the filamin-Bvar-1 construct to focal contacts. Interestingly, the simultaneous deletion of the H1 region is required for the localization of filamin-B at the tips of actin stress fibers. When expressed in C2C12 cells, filamin-Bvar-1(DeltaH1) accelerates their differentiation into myotubes. Furthermore, filamin-B variants lacking the H1 region induce the formation of thinner myotubes than those in cells containing variants with this region. These findings suggest that specific combinations of filamin mRNA splicing events modulate the organization of the actin cytoskeleton and the binding affinity for integrins. 
 Filamin A regulates cell spreading and survival via beta1 integrins. Cell spreading and exploration of topographically complex substrates require tightly-regulated interactions between   extracellular matrix   receptors and the cytoskeleton, but the molecular determinants of these interactions are not defined. We examined whether the actin-binding proteins cortactin, vinculin and filamin A are involved in the formation of the earliest extensions of cells spreading over collagen or poly-L-lysine-coated smooth and beaded substrates. Spreading of human gingival fibroblasts was substantially reduced on beaded or poly-L-lysine-coated substrates. Filamin A, vinculin and cortactin were found in cell extensions on smooth collagen. HEK-293 cells also spread rapidly on smooth collagen and formed numerous cell extensions enriched with filamin A. Knockdown of filamin A in HEK-293 cells by short hairpin RNA reduced spreading and the number of cell extensions. Blocking beta1 integrin function significantly reduced cell spreading and localization of filamin A to cell extensions. Conversely, filamin A-knockdown reduced beta1 integrin-collagen binding as measured by 12G10 antibody, suggesting co-dependence between filamin A and beta1 integrin functions. TUNEL staining showed higher percentages of apoptosis after filamin A-knockdown in spreading cells. Chelation of [Ca2+]i with BAPTA/AM reduced spreading of wild-type and filamin A-knockdown cells, however wild-type cells showed recruitment of filamin A to the subcortex, indicating independent roles of filamin A and [Ca2+]i in cell spreading. We conclude that filamin A integrates with beta1 integrins to mediate cell spreading and prevent apoptosis. 
 Filamin A-beta1 integrin complex tunes epithelial cell response to matrix tension. The physical properties of the   extracellular matrix   (ECM) regulate the behavior of several cell types; yet, mechanisms by which cells recognize and respond to changes in these properties are not clear. For example, breast epithelial cells undergo ductal morphogenesis only when cultured in a compliant collagen matrix, but not when the tension of the matrix is increased by loading collagen gels or by increasing collagen density. We report that the actin-binding protein filamin A (FLNa) is necessary for cells to contract collagen gels, and pull on collagen fibrils, which leads to collagen remodeling and morphogenesis in compliant, low-density gels. In stiffer, high-density gels, cells are not able to contract and remodel the matrix, and morphogenesis does not occur. However, increased FLNa-beta1 integrin interactions rescue gel contraction and remodeling in high-density gels, resulting in branching morphogenesis. These results suggest morphogenesis can be "tuned" by the balance between cell-generated contractility and opposing matrix stiffness. Our findings support a role for FLNa-beta1 integrin as a mechanosensitive complex that bidirectionally senses the tension of the matrix and, in turn, regulates cellular contractility and response to this matrix tension. 
 Epidermal growth factor (EGF) regulates α5β1 integrin activation state in human cancer cell lines through the p90RSK-dependent phosphorylation of filamin A. Regulation of integrin activation has important implications for tumor cell invasion and metastasis.EGF activates ERK/p90RSK and Rho/Rho kinase signaling in A431 and DiFi colon cancer cells, leading to phosphorylation of filamin A (FLNa) and inactivation of the α5β1 integrin receptor.EGF promotes α5β1 inactivation through the p90RSK-dependent phosphorylation of FLNa.We have identified a novel EGF-dependent mechanism controlling the α5β1 integrin activation state. Cell adhesion, motility, and invasion are regulated by the ligand-binding activity of integrin receptors, transmembrane proteins that bind   to the extracellula  r matrix. Integrins whose conformation allows for ligand binding and appropriate functional activity are said to be in an active state. Integrin activation and subsequent ligand binding are dynamically regulated by the association of cytoplasmic proteins with integrin intracellular domains. In this study, we evaluated the role of EGF in the regulation of the activation state of the α5β1 integrin receptor for fibronectin. The addition of EGF to either A431 squamous carcinoma cells or DiFi colon cancer cells resulted in loss of α5β1-dependent adhesion to fibronectin but no loss of integrin from the cell surface. EGF activated the EGF receptor/ERK/p90RSK and Rho/Rho kinase signaling pathways. Blocking either pathway inhibited EGF-mediated loss of adhesion, suggesting that they work in parallel to regulate integrin function. EGF treatment also resulted in phosphorylation of filamin A (FLNa), which binds and inactivates β1 integrins. EGF-mediated FLNa phosphorylation was completely blocked by an inhibitor of p90RSK and partially attenuated by an inhibitor of Rho kinase, suggesting that both pathways converge on FLNa to regulate integrin function. A431 clonal cell lines expressing non-phosphorylated dominant-negative FLNa were resistant to the inhibitory effects of EGF on integrin function, whereas clonal cell lines overexpressing wild-type FLNa were more sensitive to the inhibitory effect of EGF. These data suggest that EGF-dependent inactivation of α5β1 integrin is regulated through FLNa phosphorylation and cellular contractility. 
 Filamin A-beta1 integrin complex tunes epithelial cell response to matrix tension. The physical properties of the   extracellular matrix   (ECM) regulate the behavior of several cell types; yet, mechanisms by which cells recognize and respond to changes in these properties are not clear. For example, breast epithelial cells undergo ductal morphogenesis only when cultured in a compliant collagen matrix, but not when the tension of the matrix is increased by loading collagen gels or by increasing collagen density. We report that the actin-binding protein filamin A (FLNa) is necessary for cells to contract collagen gels, and pull on collagen fibrils, which leads to collagen remodeling and morphogenesis in compliant, low-density gels. In stiffer, high-density gels, cells are not able to contract and remodel the matrix, and morphogenesis does not occur. However, increased FLNa-beta1 integrin interactions rescue gel contraction and remodeling in high-density gels, resulting in branching morphogenesis. These results suggest morphogenesis can be "tuned" by the balance between cell-generated contractility and opposing matrix stiffness. Our findings support a role for FLNa-beta1 integrin as a mechanosensitive complex that bidirectionally senses the tension of the matrix and, in turn, regulates cellular contractility and response to this matrix tension. 
 Alpha5beta1 integrin-fibronectin interactions specify liquid to solid phase transition of 3D cellular aggregates. Tissue organization during embryonic development and wound healing depends on the ability of cells on the one hand to exchange adhesive bonds during active rearrangement and on the other to become fixed in place as tissue homeostasis is reached. Cells achieve these contradictory tasks by regulating either cell-cell adhesive bonds, mediated by cadherins, or cell-  extracellular matrix   (ECM) connections, regulated by integrins. Integrin alpha5beta1 and soluble fibronectin (sFN) are key players in cell-ECM force generation and in ECM polymerization. Here, we explore the interplay between integrin alpha5beta1 and sFN and its influence on tissue mechanical properties and cell sorting behavior.We generated a series of cell lines varying in alpha5beta1 receptor density. We then systematically explored the effects of different sFN concentrations on aggregate biomechanical properties using tissue surface tensiometry. We found previously unreported complex behaviors including the observation that interactions between fibronectin and integrin alpha5beta1 generates biphasic tissue cohesion profiles. Specifically, we show that at constant sFn concentration, aggregate cohesion increases linearly as alpha5beta1 receptor density is increased from low to moderate levels, producing a transition from viscoelastic-liquid to pseudo viscoelastic-solid behavior. However, further increase in receptor density causes an abrupt drop in tissue cohesion and a transition back to viscoelastic-liquid properties. We propose that this may be due to depletion of sFn below a critical value in the aggregate microenvironment at high alpha5beta1 levels. We also show that differential expression of alpha5beta1 integrin can promote phase-separation between cells.The interplay between alpha5-integrin and sFn contributes significantly to tissue cohesion and, depending on their level of expression, can mediate a shift from liquid to elastic behavior. This interplay represents a tunable level of control between integrins and the ECM that can influence tissue cohesion and other mechanical properties, which may translate to the specification of tissue structure and function. These studies provide insights into important biological processes such as embryonic development, wound healing, and for tissue engineering applications. 
 Caveolin-1-dependent beta1 integrin endocytosis is a critical regulator of fibronectin turnover. beta1 integrins are major cell surface receptors for fibronectin. Some integrins, including beta1 integrins, are known to undergo constitutive endocytosis and recycling. Integrin endocytosis/recycling has been implicated in the regulation of cell migration. However, the mechanisms by which integrin endocytosis/recycling regulates cell migration, and other biological consequences of integrin trafficking are not completely understood. We previously showed that turnover of   extracellular matrix   (ECM) fibronectin occurs via receptor-mediated endocytosis. Here, we investigate the biological relevance of beta1 integrin endocytosis to fibronectin matrix turnover. First, we demonstrate that beta1 integrins, including alpha5beta1 play an important role in endocytosis and turnover of matrix fibronectin. Second, we show that caveolin-1 constitutively regulates endocytosis of alpha5beta1 integrins, and that alpha5beta1 integrin endocytosis can occur in the absence of fibronectin and fibronectin matrix. We also show that downregulation of caveolin-1 expression by siRNA results in marked reduction of beta1 integrin and fibronectin endocytosis. Hence, caveolin-1-dependent beta1 integrin and fibronectin endocytosis plays a critical role in fibronectin matrix turnover, and may contribute to abnormal ECM remodeling that occurs in fibrotic disorders. 
 Ablation of beta1 integrin in mammary epithelium reveals a key role for integrin in glandular morphogenesis and differentiation. Integrin-mediated adhesion regulates the development and function of a range of tissues; however, little is known about its role in glandular epithelium. To assess the contribution of beta1 integrin, we conditionally deleted its gene in luminal epithelia during different stages of mouse mammary gland development and in cultured primary mammary epithelia. Loss of beta1 integrin in vivo resulted in impaired alveologenesis and lactation. Cultured beta1 integrin-null cells displayed abnormal focal adhesion function and signal transduction and could not form or maintain polarized acini. In vivo, epithelial cells became detached from the   extracellular matrix   but remained associated with each other and did not undergo overt apoptosis. beta1 integrin-null mammary epithelial cells did not differentiate in response to prolactin stimulation because of defective Stat5 activation. In mice where beta1 integrin was deleted after the initiation of differentiation, fewer defects in alveolar morphology occurred, yet major deficiencies were also observed in milk protein and milk fat production and Stat5 activation, indicating a permissive role for beta1 integrins in prolactin signaling. This study demonstrates that beta1 integrin is critical for the alveolar morphogenesis of a glandular epithelium and for maintenance of its differentiated function. Moreover, it provides genetic evidence for the cooperation between integrin and cytokine signaling pathways. 
 Differential expression profiles between α-dystroglycan and integrin β1 in ameloblastoma: two possible perlecan signalling pathways for cellular growth and differentiation. Intercellular deposition of perlecan, a  n extracellular matr  ix molecule, results in characteristic stellate reticulum-like structures in ameloblastomas. The aims of this study were to elucidate which types of perlecan receptors function within any particular type of tissue architecture of ameloblastoma.Protein and gene expression profiles for α-dystroglycan and integrin β1 were examined comparatively with those of their ligands in ameloblastoma using surgical specimens and cells in primary culture. In the follicular-type tumour cell foci, α-dystroglycan was localized uniformly over the stellate reticulum-like cells, while integrin β1 was restricted mainly to peripheral cells facing the stroma with the interface of the basement membrane, which was also rich in perlecan. In the plexiform-type, mRNA and protein signals for α-dystroglycan were enhanced in the periphery of tumour cell foci, especially in their invading fronts. Integrin β1 was also immunolocalized in the basal cell zone, which was considered to be the proliferation centre of ameloblastoma cells. Furthermore, biosynthesis of α-dystroglycan and integrin β1 by ameloblastoma cells was confirmed in vitro using immunofluorescence and reverse transcriptase-polymerase chain reaction.Ameloblastoma cells proliferate and are differentiated by capturing perlecan differentially with α-dystroglycan and integrin β1, respectively. 
 Mechanically activated integrin switch controls alpha5beta1 function. The cytoskeleton, integrin-mediated adhesion, and substrate stiffness control a common set of cell functions required for development and homeostasis that are often deranged in cancer. The connection between these mechanical elements and chemical signaling processes is not known. Here, we show that alpha(5)beta(1) integrin switches between relaxed and tensioned states in response to myosin II-generated cytoskeletal force. Force combines with   extracellular matrix   stiffness to generate tension that triggers the integrin switch. This switch directly controls the alpha(5)beta(1)-fibronectin bond strength through engaging the synergy site in fibronectin and is required to generate signals through phosphorylation of focal adhesion kinase. In the context of tissues, this integrin switch connects cytoskeleton and   extracellular matrix   mechanics to adhesion-dependent motility and signaling pathways. 
 β1-Integrin via NF-κB signaling is essential for acquisition of invasiveness in a model of radiation treated in situ breast cancer. Ductal carcinoma in situ (DCIS) is characterized by non-invasive cancerous cell growth within the breast ducts. Although radiotherapy is commonly used in the treatment of DCIS, the effect and molecular mechanism of ionizing radiation (IR) on DCIS are not well understood, and invasive recurrence following radiotherapy remains a significant clinical problem. This study investigated the effects of IR on a clinically relevant model of Akt-driven DCIS and identified possible molecular mechanisms underlying invasive progression in surviving cells.We measured the level of phosphorylated-Akt (p-Akt) in a cohort of human DCIS specimens by immunohistochemistry (IHC) and correlated it with recurrence risk. To model human DCIS, we used Akt overexpressing human mammary epithelial cells (MCF10A-Akt) which, in three-dimensional laminin-ric  h extracellular matr  ix (lrECM) and in vivo, form organotypic DCIS-like lesions with lumina expanded by pleiomorphic cells contained within an intact basement membrane. In a population of cells that survived significant IR doses in three-dimensional lrECM, a malignant phenotype emerged creating a model for invasive recurrence.P-Akt was up-regulated in clinical DCIS specimens and was associated with recurrent disease. MCF10A-Akt cells that formed DCIS-like structures in three-dimensional lrECM showed significant apoptosis after IR, preferentially in the luminal compartment. Strikingly, when cells that survived IR were repropagated in three-dimensional lrECM, a malignant phenotype emerged, characterized by invasive activity, up-regulation of fibronectin, α5β1-integrin, matrix metalloproteinase-9 (MMP-9) and loss of E-cadherin. In addition, IR induced nuclear translocation and binding of nuclear factor-kappa B (NF-κB) to the β1-integrin promoter region, associated with up-regulation of α5β1-integrins. Inhibition of NF-κB or β1-integrin signaling abrogated emergence of the invasive activity.P-Akt is up-regulated in some human DCIS lesions and is possibly associated with recurrence. MCF10A-Akt cells form organotypic DCIS-like lesions in three-dimensional lrECM and in vivo, and are a plausible model for some forms of human DCIS. A population of Akt-driven DCIS-like spheroids that survive IR progresses to an invasive phenotype in three-dimensional lrECM mediated by β1-integrin and NF-κB signaling. 
 Mesenchymal stem cell migration is regulated by fibronectin through α5β1-integrin-mediated activation of PDGFR-β and potentiation of growth factor signals. Cell migration during vascular remodelling is regulated by crosstalk between growth factor receptors and integrin receptors, which together coordinate cytoskeletal and motogenic changes. Here, we repo  rt extracellular mat  rix (ECM)-directed crosstalk between platelet-derived growth factor receptor (PDGFR)-β and α5β1-integrin, which controls the migration of mesenchymal stem (stromal) cells (MSCs). Cell adhesion to fibronectin induced α5β1-integrin-dependent phosphorylation of PDGFR-β in the absence of growth factor stimulation. Phosphorylated PDGFR-β co-immunoprecipitated with α5-integrin and colocalised with α5β1-integrin in the transient tidemarks of focal adhesions. Adhesion to fibronectin also strongly potentiated PDGF-BB-induced PDGFR-β phosphorylation and focal adhesion kinase (FAK) activity, in an α5β1-integrin-dependent manner. PDGFR-β-induced phosphoinositide 3-kinase (PI3K) and Akt activity, actin reorganisation and cell migration were all regulated by fibronectin and α5β1-integrin. This synergistic relationship between α5β1-integrin and PDGFR-β is a fundamental determinant of cell migration. Thus, fibronectin-rich matrices can prime PDGFR-β to recruit mesenchymal cells at sites of vascular remodelling. 
 The integrin receptor alpha 8 beta 1 mediates interactions of embryonic chick motor and sensory neurons with tenascin-C. This paper identifies a neuronal receptor for tenascin-C (tenascin/cytotactin), an   extracellular matrix   protein that has previously been detected in developing sensory and motor neuron pathways and has been shown to regulate cell migration in the developing CNS. Antibodies specific for each subunit of the integrin alpha 8 beta 1 are used to demonstrate that alpha 8 beta 1 mediates neurite outgrowth of embryonic sensory and motor neurons on this   extracellular matrix   protein. In addition, expression of alpha 8 in K562 cells results in surface expression of alpha 8 beta 1 heterodimers that are shown to promote attachment of this cell line to tenascin. The major domain in tenascin that mediates neurite outgrowth is shown to be localized to fibronectin type III repeats 6-8. 
 beta1 and beta2 integrins activate different signalling pathways in monocytes. Integrin-mediated signals play an important but poorly understood role in regulating many leucocyte functions. In monocytes and macrophages, integrins of the beta2 subfamily are involved in cell-cell interactions that are important for migration of the cells through the endothelium and also for phagocytosis. On the other hand, in the same cells, beta1 integrin-mediated adhesion to   extracellular matrix   proteins results in a strong induction of immediate early genes that are important in inflammation. To investigate the signalling pathways from these two types of integrin in monocytic cells, THP-1 cells were selectively stimulated via beta1 or beta2 integrins by cross-linking each type of receptor with specific monoclonal antibodies or their natural ligands. The involvement of extracellular signal-regulated kinase (ERK), Syk and phosphoinositide 3-kinase (PI-3K) was then analysed. Nuclear factor kappaB (NF-kappaB) activation was also detected in THP-1 cells transiently transfected with an NF-kappaB-driven luciferase reporter gene. We found that binding of both types of integrin to their natural ligands activated ERK in a Syk- and PI-3K-dependent manner. Yet, cross-linking of integrins by anti-beta1 antibodies caused activation of ERK while that by anti-beta2 antibodies did not. Also both types of integrin activated NF-kappaB. However, PI-3K was required for beta1 integrin-, but not beta2 integrin-, mediated NF-kappaB activation. In addition, inhibition of PI-3K with wortmannin and LY294002 blocked beta1 integrin-mediated NF-kappaB activation, but did not affect that mediated by beta2 integrin. These data suggest that distinct integrins activate different signalling pathways in monocytic cells. 
 Alpha1 and beta1 integrins enhance the homing and differentiation of cultured prostate cancer stem cells. CD133(+) prostate cancer stem cells (PCSCs) have recently been identified in human prostate cancer tissues. The present study reports the integrin profile of prostate cancer progenitor cells and the role of alpha(1) and beta(1) integrins in the homing and differentiation of PCSCs in vitro. PCSCs were isolated from the tissue specimens of patients with prostate cancer and the expression of surface integrins and adhesion patterns were determined. Our analysis of the expression of surface integrins and their adhesion patterns of prostate cancer stem cells derived from prostate cancer tissues revealed that the levels of beta(1) and alpha(2)beta(1) integrins were significantly higher (P < 0.05) than those of the other integrins. By contrast, peripheral blood-derived CD133(+) cells from prostate cancer patients showed a high level of expression (P < 0.01) of alpha(2)beta(1), alpha(v)beta(3), alpha(v)beta(5), beta(1) and alpha(1) integrins and a minimal expression of alpha(4)beta(1) integrins. Moreover, CD133(+) cells derived from both prostate cancer tissues and peripheral blood exhibited an increased degree of attachment to   extracellular matrix   proteins (P < 0.001) and a high expression level of alpha(2)beta(1) integrin. In vitro experiments using blocking antibodies indicated that alpha(1) and beta(1) integrins have a role in the homing and differentiation of PCSCs. This is the first report to suggest the importance of integrins in mediating the homing and differentiation of PCSCs. 
 Real-time analysis of very late antigen-4 affinity modulation by shear. Shear promotes endothelial recruitment of leukocytes, cell activation, and transmigration. Mechanical stress on cells caused by shear can induce a rapid integrin conformational change and activation, followed by an increase in binding to the   extracellular matrix  . The molecular mechanism of increased avidity is unknown. We have shown previously that the affinity of the alpha(4)beta(1) integrin, very late antigen-4 (VLA-4), measured with an LDV-containing small molecule, varies with cellular avidity, measured from cell disaggregation rates. In this study, we measured in real time affinity changes of VLA-4 in response to shear. The resulting affinity was comparable with the state mediated by receptor signaling and corresponded in time with intracellular Ca(2+) responses. Ca(2+) ionophores and N,N'-[1,2-ethanediyl-bis(oxy-2,1-phenylene)]bis[N-[2-[(acetyloxy)methoxy]-2-oxoethyl]]-, bis[(acetyloxy)methyl]ester demonstrate that the affinity regulation of VLA-4 in the presence of shear was related to Ca(2+) signaling. Pertussis toxin treatment implicates G(i) in an unknown pathway that connects shear, Ca(2+) elevation, VLA-4 affinity, and cell avidity. 
 Adhesion of cultured human kidney mesangial cells to native entactin: role of integrin receptors. Entactin is an   extracellular matrix   glycoprotein which binds to laminin and is found in most renal basement membranes and in the glomerular mesangial matrix. In the present study, we have characterized specific integrin receptors on cultured human mesangial cells (CHMC) responsible for adhesion to native entactin. The integrin receptors alpha 2 beta 1, alpha 3 beta 1, alpha 5 beta 1, alpha v beta 3, alpha v beta 5, and alpha 6 complexed with either beta 1 or beta 4 could be immune precipitated from detergent extracts of metabolically labeled CHMC. Adhesion assays with inhibitory anti integrin monoclonal antibodies (mab) demonstrated that CHMC use both alpha v beta 3 and a beta 1-containing integrin to bind surfaces coated with native entactin. Optimal binding of CHMC to native entactin required the participation of cations. Using wild type and mutant recombinant entactin fragments, the binding site for the alpha v beta 3 receptor was localized to the RGD sequence on the rod or E domain of entactin. CHMC adhesion to mutant full length recombinant entactin ligands lacking the E domain RGD sequence confirmed the presence of ligand binding site(s) for beta 1 integrin receptor(s). Differences in CHMC binding characteristics to recombinant and full length entactin compared to native bovine basement membrane entactin were observed. This suggests that tertiary molecular structure may contribute to entactin ligand binding properties. Primary amino acid residue sequences and tertiary structure of entactin may play roles in forming functional cell attachment sites in native basement membrane entactin. 
 Integrins as receptors for laminins. Laminins are a family of trimeric glycoproteins present in the   extracellular matrix   and the major constituents of basement membranes. Integrins are alpha beta transmembrane receptors that play critical roles in both cell-matrix and cell-cell adhesion. Several members of the integrin family, including alpha 1 beta 1, alpha 2 beta 1, alpha 3 beta 1, alpha 6 beta 1, alpha 7 beta 1 and alpha 6 beta 4 heterodimers serve as laminin receptors on a variety of cell types. This review summarizes recent advances in understanding the involvement of individual integrins in cell interactions with laminins and the roles of laminin-binding integrins in adhesion-mediated events in vertebrates, including embryonic development, cell migration and tumor cell invasiveness, cell proliferation and differentiation, as well as basement membrane assembly. We discuss the regulation of integrin function via alternative splicing of cytoplasmic domains of alpha and beta subunits of the integrin receptors for laminins and present examples of functional collaboration between laminin-binding integrins and non-integrin laminin receptors. Advances in our understanding of the laminin-binding integrins continue to demonstrate the essential roles these receptors play in maintaining cell polarity and tissue architecture. 
 Activation of integrin alpha5beta1 delays apoptosis of Ntera2 neuronal cells. Integrins are dynamic membrane proteins that mediate adhesion of cells to the   extracellular matrix  . Integrins initiate signal transduction, alone and cooperatively with growth factor receptors, and regulate many aspects of cell behavior. We report here that alpha5beta1-mediated adhesion of Ntera2 neuronal cells to fibronectin decreased apoptosis in response to serum withdrawal. Adhesion induced phosphorylation of FAK, and strongly increased the AKT phosphorylation induced by growth factors, demonstrating for the first time in neuronal cells that integrin-mediated adhesion and growth factors cooperate to regulate AKT activity. Integrins exist on cells in different activation states, and cell survival on fibronectin was enhanced by the antibody 12G10, that modulates the conformation of beta1 in favor of its active form. The antibody 12G10 specifically delayed loss of phosphorylation of AKT on serine 473, and GSK-3beta on serine 9, induced by serum withdrawal, suggesting that these kinases are critical sensors of integrin activation on neuronal cells. 
 Nicotinamide phosphoribosyltransferase can affect metastatic activity and cell adhesive functions by regulating integrins in breast cancer. NAD(+) metabolism is an essential regulator of cellular redox reactions, energy pathways, and a substrate provider for NAD(+) consuming enzymes. We recently demonstrated that enhancement of NAD(+)/NADH levels in breast cancer cells with impaired mitochondrial NADH dehydrogenase activity, through augmentation of complex I or by supplementing tumor cell nutrients with NAD(+) precursors, inhibits tumorigenicity and metastasis. To more fully understand how aberrantly low NAD(+) levels promote tumor cell dissemination, we here asked whether inhibition of NAD(+) salvage pathway activity by reduction in nicotinamide phosphoribosyltransferase (NAMPT) expression can impact metastasis and tumor cell adhesive functions. We show that knockdown of NAMPT, the enzyme catalyzing the rate-limiting step of the NAD(+) salvage pathway, enhances metastatic aggressiveness in human breast cancer cells and involves modulation of integrin expression and function. Reduction in NAMPT expression is associated with upregulation of select adhesion receptors, particularly αvβ3 and β1 integrins, and results in increased breast cancer cell attachment   to extracellular mat  rix proteins, a key function in tumor cell dissemination. Interestingly, NAMPT downregulation prompts expression of integrin αvβ3 in a high affinity conformation, known to promote tumor cell adhesive interactions during hematogenous metastasis. NAMPT has been selected as a therapeutic target for cancer therapy based on the essential functions of this enzyme in NAD(+) metabolism, cellular redox, DNA repair and energy pathways. Notably, our results indicate that incomplete inhibition of NAMPT, which impedes NAD(+) metabolism but does not kill a tumor cell can alter its phenotype to be more aggressive and metastatic. This phenomenon could promote cancer recurrence, even if NAMPT inhibition initially reduces tumor growth. 
 The role of   extracellular matrix  , integrins, and cytoskeleton in mechanotransduction of centrifugal loading. The study was aimed to investigate the role of the "  extracellular matrix   (ECM)-integrins-cytoskeleton" signal pathway in mechanotransduction of centrifugal loading. MG-63 osteoblasts were exposed to centrifugal loading at 209xg for 10 min. Uncentrifuged cells and centrifuged cells that have been trypsinized and suspended in liquors were designed as control. The changes in F-actin and alpha-actin cytoskeleton, gene transcription of ECM components, and integrins expression were analyzed by LSCM, Real-Time RT-PCR and FCM, respectively. A temporary and fast reversible change was observed in F-actin and alpha-actin cytoskeleton. And the change was paralleled with the fast autoregulation in gene transcription of ECM components of fibronection, osteopontin and Collagen I, and integrins expression of both alpha2 and beta1 subunits. The result suggested that cytoskeleton was a possible mechanical sensor to centrifugal stimuli, and the cytoskeleton regulation to centrifugal loading was in an ECM-dependent and integrin-mediated manner. 
 α3β1 integrins regulate CD151 complex assembly and membrane dynamics in carcinoma cells within 3D environments. Integrins ar  e extracellular matr  ix (ECM) receptors that are key players in the regulation of tumour cell invasion. The laminin-binding integrin α3β1 has previously been shown to regulate adhesion and migration of carcinoma cells in part through co-operative signalling with the tetraspanin family of transmembrane proteins. However, the spatial and temporal regulation of crosstalk between these families of transmembrane proteins in intact cells remains poorly understood. Here we have used fluorescence resonance energy transfer (FRET) to demonstrate for the first time that α3β1 and the tetraspanin CD151 directly associate at the front and retracting rear of polarised migrating breast carcinoma cells in both two-dimentional (2D) and three-dimentional (3D)matrices. Furthermore, localised α3β1-CD151 binding correlates with lower CD151 homodimerisation in cells migrating on laminin or within matrigel. Loss of α3β1 integrin leads to increased CD151 homodimer formation, increased activation of Rho GTPase, loss of cell polarity and decreased invasion in 3D ECM. As a result, α3-silenced cells show decreased actin-based membrane protrusion and retraction in both 2D and 3D environments. These data demonstrate that associations between α3β1 and CD151 occur dynamically within discrete subcellular compartments and act to establish local GTPase signalling to promote tumour cell invasion. These novel findings shed light on the complex crosstalk and switching between receptor complexes in response to different extracellular cues during cell invasion in 3D environments. 
 Cyclic stretch-induced TGF-β1 and fibronectin expression is mediated by β1-integrin through c-Src- and STAT3-dependent pathways in renal epithelial cells  . Extracellular matr  ix (ECM) proteins, including fibronectin, may contribute to the early development and progression of renal interstitial fibrosis associated with chronic renal disease. Recent studies showed that β1-integrin is associated with the development of renal fibrosis in a murine model of unilateral ureteral obstruction (UUO). However, the molecular events responsible for β1-integrin-mediated signaling, following UUO, have yet to be determined. In this study, we investigated the mechanism by which mechanical stretch, an in vitro model for chronic obstructive nephropathy, regulates fibronectin and transforming growth factor-β1 (TGF-β1) expression in cultured human proximal tubular epithelium (HK-2) cells. Mechanical stretch upregulated fibronectin and TGF-β1 expression and activated signal transducer and transcription factor 3 (STAT3) in a time-dependent manner. Stretch-induced fibronectin and TGF-β1 were suppressed by a STAT3 inhibitor, S3I-201, and by small interfering RNA (siRNA) targeting human STAT3 (STAT3 siRNA). Similarly, fibronectin and TGF-β1 expression and STAT3 activation induced by mechanical stretch were suppressed by the Src family kinase inhibitor PP2 and by transfection of HK-2 cells with a dominant-negative mutant of c-Src (DN-Src), whereas PP3, an inactive analog of PP2, had no significant effect. Furthermore, mechanical stretch resulted in increased β1-integrin mRNA and protein levels in HK-2 cells. Furthermore, neutralizing antibody against β1-integrin and silencing of β1-integrin expression with siRNAs resulted in decreased c-Src and STAT3 activation and TGF-β1 and fibronectin expression evoked by mechanical stretch. This work demonstrates, for the first time, a role for β1-integrin in stretch-induced renal fibrosis through the activation of c-Src and STAT3 signaling pathways. 
 β1 integrin regulates MMP-10 dependant tubulogenesis in human lymphatic endothelial cells. Lymphatic vessel growth requires extensive remodeling of the   extracellular matri  x, a process hypothesized to be related to the expression and function of the matrix metalloproteinases. We used a protein based screening strategy to demonstrate increased matrix matalloproteinase-10 expression in human lymphatic endothelial cells undergoing collagen I induced tubulogenesis. Knock-down experiments showed that matrix metalloproteinase-10 regulated lymphatic endothelial cell tubulogenesis. β1 integrin signaling via the ERK/MAPK pathway increased matrix metalloproteinase-10 mRNA and protein expression in human lymphatic endothelial cells. These findings demonstrate a novel mechanism by which β1 integrin regulates matrix metalloproteinase-10 expression during lymphatic vessel remodeling. 
 TRPV4 channels mediate cyclic strain-induced endothelial cell reorientation through integrin-to-integrin signaling. Cyclic mechanical strain produced by pulsatile blood flow regulates the orientation of endothelial cells lining blood vessels and influences critical processes such as angiogenesis. Mechanical stimulation of stretch-activated calcium channels is known to mediate this reorientation response; however, the molecular basis remains unknown. Here, we show that cyclically stretching capillary endothelial cells adherent to flexible   extracellular matrix   substrates activates mechanosensitive TRPV4 (transient receptor potential vanilloid 4) ion channels that, in turn, stimulate phosphatidylinositol 3-kinase-dependent activation and binding of additional beta1 integrin receptors, which promotes cytoskeletal remodeling and cell reorientation. Inhibition of integrin activation using blocking antibodies and knock down of TRPV4 channels using specific small interfering RNA suppress strain-induced capillary cell reorientation. Thus, mechanical forces that physically deform   extracellular matrix   may guide capillary cell reorientation through a strain-dependent "integrin-to-integrin" signaling mechanism mediated by force-induced activation of mechanically gated TRPV4 ion channels on the cell surface. 
 Interaction of monocarboxylate transporter 4 with beta1-integrin and its role in cell migration. Monocarboxylate transporter (MCT) 4 is a heteromeric proton-coupled lactate transporter that is noncovalently linked to the   extracellular matrix   metalloproteinase inducer CD147 and is typically expressed in glycolytic tissues. There is increasing evidence to suggest that ion transporters are part of macromolecular complexes involved in regulating beta(1)-integrin adhesion and cell movement. In the present study we examined whether MCTs play a role in cell migration through their interaction with beta(1)-integrin. Using reciprocal coimmunoprecipitation assays, we found that beta(1)-integrin selectively associated with MCT4 in ARPE-19 and MDCK cells, two epithelial cell lines that express both MCT1 and MCT4. In polarized monolayers of ARPE-19 cells, MCT4 and beta(1)-integrin colocalized to the basolateral membrane, while both proteins were found in the leading edge lamellapodia of migrating cells. In scratch-wound assays, MCT4 knockdown slowed migration and increased focal adhesion size. In contrast, silencing MCT1 did not alter the rate of cell migration or focal adhesion size. Taken together, our findings suggest that the specific interaction of MCT4 with beta(1)-integrin may regulate cell migration through modulation of focal adhesions. 
 Fibulin-1 suppression of fibronectin-regulated cell adhesion and motility. Fibulin-1 is an   extracellular matrix   protein often associated with fibronectin (FN) in vivo. In this study, the ability of fibulin-1 to modulate adhesion, spreading and motility-promoting activities of FN was investigated. Fibulin-1 was found to have pronounced inhibitory effects on the cell attachment and spreading promoted by FN. Fibulin-1 was also found to inhibit the motility of a variety of cell types on FN substrata. For example, the FN-dependent haptotactic motility of breast carcinoma (MDA MB231) cells, epidermal carcinoma (A431), melanoma (A375 SM), rat pulmonary aortic smooth muscle cells (PAC1) and Chinese hamster ovary (CHO) cells was inhibited by the presence of fibulin-1 bound to FN-coated Boyden chamber membranes. Cells transfected to overproduce fibulin-1 displayed reduced velocity, distance of movement and persistence time on FN substrata. Similarly, the incorporation of fibulin-1 into FN-containing type I collagen gels inhibited the invasion of endocardial cushion mesenchymal cells migrating from cultured embryonic heart explants. By contrast, incorporation of fibulin-1 into collagen gels lacking FN had no effect on the migration of endocardial cushion cells. These results suggest that the motility-suppressive effects of fibulin-1 might be FN specific. Furthermore, such effects are cell-type specific, in that the migration of gingival fibroblasts and endothelial cells on FN substrata is not responsive to fibulin-1. Additional studies found that the mechanism for the motility-suppressive effects of fibulin-1 does not involve perturbations of interactions between alpha5beta1 or alpha4 integrins, or heparan sulfate proteoglycans with FN. However, fibulin-1 was found to inhibit extracellular signal regulated kinase (ERK) activation and to suppress phosphorylation of myosin heavy chain. This ability to influence signal transduction cascades that modulate the actin-myosin motor complex might be the basis for the effects of fibulin-1 on adhesion and motility. 
 AMPA receptors promote perivascular glioma invasion via beta1 integrin-dependent adhesion to the   extracellular matrix  . High-grade gliomas release excitotoxic concentrations of glutamate, which has been shown to enhance tumor proliferation and migration. alpha-Amino-3-hydroxy-5-methylisoxazole-4-propionic acid (AMPA) glutamate receptors are abundantly expressed at the invading edge of glioblastoma specimens, suggesting they may play an important biologic role in tumor invasion. In this study, we examined potential mechanisms by which AMPA receptor (AMPAR) expression and stimulation promote glioma cell migration and invasion. Overexpression of GluR1, the most abundant AMPAR subunit in gliomas, positively correlated with glioma cell adhesion to type I and type IV collagen, which was decreased in cells with knockdown of GluR1 and with blocking antibodies to beta1 integrin. Furthermore, stimulation of the AMPAR led to detachment of cells from the   extracellular matrix   (ECM). Immunoprecipitation studies showed that GluR1 associated with the actin cytoskeleton-linked protein band 4.1B (brain type), which may serve as a link between GluR1 and integrins. Overexpression of GluR1 correlated with increased cell-surface expression of beta1 integrin, increased phosphorylation of focal adhesion kinase (FAK-Y397), and enhanced numbers of focal adhesion (FA) complexes. Cells overexpressing GluR1 had increased colocalization of actin and paxillin at FAs and, in several glioma cell lines, significantly increased invasion in an in vitro Matrigel transwell assay. Likewise, in an intracranial xenograft model, overexpression of GluR1 led to perivascular and subependymal glioma cell invasion similar to patterns of tumor dissemination described in human glioblastoma. Together, these results suggest that AMPARs may link signals from the ECM to sites of FA, where signal integration promotes tumor invasion. 
 Decreased expression of alpha3 and beta1 integrin subunits is responsible for differentiation-associated changes in cells behavior in terminally differentiated human oral keratinocytes. Primary normal human oral keratinocytes (NHOKs) terminally differentiate in serial subculture. To investigate whether this subculture-induced differentiation of NHOKs affects integrin expression and cell-matrix interaction, we studied the expression levels of integrin subunits and cellular response to the   extracellular matrix   (ECM) proteins in NHOKs at different population doublings. The phosphorylation statuses of focal adhesion kinase (FAK), extracellular signal regulated kinase (ERK), p38, and c-Jun amino-terminal kinase (JNK) were also determined in NHOK cells cultured on ECM proteins, to evaluate the functions of integrins with respect to cellular responses to ECM proteins. The expression levels of alpha3 and beta1 integrin subunits progressively decreased in NHOKs undergoing terminal differentiation. The ability of NHOKs to spread upon laminin and type I collagen significantly decreased in terminally differentiated oral keratinocytes. Keratinocyte migration was significantly increased on type I collagen for terminally differentiated NHOKs. Similar results were seen following preincubation of rapidly proliferating NHOKs with function-blocking antibodies to alpha3 or beta1 integrin subunit. In contrast, fibronectin had no effect on cellular responses in NHOKs, which were almost negligible in the expression levels of alpha5 integrin subunits. The extent of FAK phosphorylation in terminally differentiated NHOKs was notably lower than that of rapidly proliferating cells, but was enhanced in terminally differentiated cells that were cultured on type I collagen. Our results indicate that decreased expression of alpha3 and beta1 integrin subunits is responsible for differentiation-associated changes in cells behavior in terminally differentiated oral keratinocytes. Our data also show that the abrogation of the alpha5beta1 integrin function caused by omitting alpha5 subunit is linked to the loss of a cell-fibronectin interaction in human oral keratinocytes. 
 Conformation, localization, and integrin binding of talin depend on its interaction with phosphoinositides. Talin is a structural component of focal adhesion sites and is thought to be engaged in multiple protein interactions at the cytoplasmic face of cell/matrix contacts. Talin is a major link between integrin and the actin cytoskeleton and was shown to play an important role in focal adhesion assembly. Consistent with the view that talin must be activated at these sites, we found that phosphatidylinositol 4-monophosphate and phosphatidylinositol 4,5-bisphosphate (PI4,5P(2)) bound to talin in cells in suspension or at early stages of adhesion, respectively. When phosphoinositides were associated with phospholipid bilayer, talin/phosphoinositide association was restricted to PI4,5P(2). This association led to a conformational change of the protein. Moreover, the interaction between integrin and talin was greatly enhanced by PI4,5P(2)-induced talin activation. Finally, sequestration of PI4,5P(2) by a specific pleckstrin homology domain confirms that PI4,5P(2) is necessary for proper membrane localization of talin and that this localization is essential for the maintenance of focal adhesions. Our results support a model in which PI4,5P(2) exposes the integrin-binding site on talin. We propose that PI4,5P(2)-dependent signaling modulates assembly of focal adhesions by regulating integrin-talin complexes. These results demonstrate that activation of the integrin-binding activity of talin requires not only integrin engagement to the   extracellular matrix   but also the binding of PI4,5P(2) to talin, suggesting a possible role of lipid metabolism in organizing the sequential assembly of focal adhesion components. 
 Rab25 associates with alpha5beta1 integrin to promote invasive migration in 3D microenvironments. Here, we report a direct interaction between the beta1 integrin cytoplasmic tail and Rab25, a GTPase that has been linked to tumor aggressiveness and metastasis. Rab25 promotes a mode of migration on 3D matrices that is characterized by the extension of long pseudopodia, and the association of the GTPase with alpha5beta1 promotes localization of vesicles that deliver integrin to the plasma membrane at pseudopodial tips as well as the retention of a pool of cycling alpha5beta1 at the cell front. Furthermore, Rab25-driven tumor-cell invasion into a 3D   extracellular matrix   environment is strongly dependent on ligation of fibronectin by alpha5beta1 integrin and the capacity of Rab25 to interact with beta1 integrin. These data indicate that Rab25 contributes to tumor progression by directing the localization of integrin-recycling vesicles and thereby enhancing the ability of tumor cells to invade the   extracellular matrix  . 
 Alpha9beta1 integrin in melanoma cells can signal different adhesion states for migration and anchorage. Cell surface integrins are the primary receptors for cell migration on   extracellular matrix  , and exist in several activation states regulated in part by ectodomain conformation. The alpha9 integrin subunit, which pairs only with beta1, has specific roles in the immune system and may regulate cell migration. Melanoma cells express abundant alpha9beta1 integrin, and its role in cell migration was assessed. Ligands derived from Tenascin-C and ADAM12 supported alpha9beta1 integrin-mediated cell attachment and GTP-Rac dependent migration, but not focal adhesion formation. Manganese ions induced alpha9beta1 integrin- and Rho kinase-dependent focal adhesion and stress fibre formation, suggesting that the activation status of alpha9beta1 integrin was altered. The effect of manganese ions in promoting focal adhesion formation was reproduced by beta1 integrin activating antibody. The alpha9beta1 integrin translocated to focal adhesions, where active beta1 integrin was also detected by conformation-specific antibodies. Focal adhesion assembly was commensurate with reduced cell migration. Endogenous alpha9beta1 integrin-mediated adhesion was sensitive to the PP1 chemical inhibitor and an inhibitor of endosomal vesicle recycling, but not inhibitors of protein kinase C or the small GTPase Rho. Our results demonstrated that although alpha9beta1 integrin can induce and localise to focal adhesions in a high activation state, its intermediate activity state normally supports cell adhesion consistent with migration. 
 Beta 1 integrin predicts survival in breast cancer: a clinicopathological and immunohistochemical study. The main focus of several studies concerned with cancer progression and metastasis is to analyze the mechanisms that allow cancer cells to interact and quickly adapt with their environment. Integrins, a family of transmembrane glycoproteins, play a major role in invasive and metastatic processes. Integrins are involved in cell adhesion in both cell-  extracellular matrix   and cell-cell interactions, and particularly, β1 integrin is involved in proliferation and differentiation of cells in the development of epithelial tissues. This work aimed to investigate the putative role of β1 integrin expression on survival and metastasis in patients with breast invasive ductal carcinoma (IDC). In addition, we compared the expression of β1 integrin in patients with ductal carcinoma in situ (DCIS).Through tissue microarray (TMA) slides containing 225 samples of IDC and 67 samples of DCIS, β1 integrin expression was related with several immunohistochemical markers and clinicopathologic features of prognostic significance.β1 integrin was overexpressed in 32.8% of IDC. In IDC, β1 integrin was related with HER-2 (p = 0.019) and VEGF (p = 0.011) expression and it had a significant relationship with metastasis and death (p = 0.001 and p = 0.05, respectively). Kaplan-Meier survival analysis showed that the overexpression of this protein is very significant (p = 0.002) in specific survival (number of months between diagnosis and death caused by the disease). There were no correlation between IDC and DCIS (p = 0.559) regarding β1 integrin expression.Considering that the expression of β1 integrin in breast cancer remains controversial, specially its relation with survival of patients, our findings provide further evidence that β1 integrin can be a marker of poor prognosis in breast cancer.The virtual slide(s) for this article can be found here: http://www.diagnosticpathology.diagnomx.eu/vs/6652215267393871. 
 Core3 O-glycan synthase suppresses tumor formation and metastasis of prostate carcinoma PC3 and LNCaP cells through down-regulation of alpha2beta1 integrin complex. Although there are numerous reports of carbohydrates enriched in cancer cells, very few studies have addressed the functions of carbohydrates present in normal cells that decrease in cancer cells. It has been reported that core3 O-glycans are synthesized in normal gastrointestinal cells but are down-regulated in cancer cells. To determine the roles of core3 O-glycans, we transfected PC3 and LNCaP prostate cancer cells with beta3-N-acetylglucosaminyltransferase-6 (core3 synthase) required to synthesize core3 O-glycans. Both engineered cell lines exhibited reduced migration and invasion through   extracellular matrix   components compared with mock-transfected cells. Moreover we found that alpha2beta1 integrin acquired core3 O-glycans in cells expressing core3 synthase with decreased maturation of beta1 integrin, leading to decreased levels of the alpha2beta1 integrin complex, decreased activation of focal adhesion kinase, and reduced lamellipodia formation. Upon inoculation into the prostate of nude mice, PC3 cells expressing core3 O-glycans produced much smaller tumors without metastasis to the surrounding lymph nodes in contrast to robust tumor formation and metastasis seen in mock-transfected PC3 cells. Similarly LNCaP cells expressing core3 O-glycans barely produced subcutaneous tumors in contrast to robust tumor formation by mock-transfected LNCaP cells. These findings indicate that addition of core3 O-glycans to beta1 and alpha2 integrin subunits in prostate cancer cells suppresses tumor formation and tumor metastasis. 
 The integrin expression profile modulates orientation and dynamics of force transmission at cell-matrix adhesions. Integrin adhesion receptors connect the   extracellular matrix   (ECM) to the cytoskeleton and serve as bidirectional mechanotransducers. During development, angiogenesis, wound healing and cancer progression, the relative abundance of fibronectin receptors, including integrins α5β1 and αvβ3, changes, thus altering the integrin composition of cell-matrix adhesions. Here, we show that enhanced αvβ3 expression can fully compensate for loss of α5β1 and other β1 integrins to support outside-in and inside-out force transmission. α5β1 and αvβ3 each mediate actin cytoskeletal remodeling in response to stiffening or cyclic stretching of the ECM. Likewise, α5β1 and αvβ3 support cellular traction forces of comparable magnitudes and similarly increase these forces in response to ECM stiffening. However, cells using αvβ3 respond to lower stiffness ranges, reorganize their actin cytoskeleton more substantially in response to stretch, and show more randomly oriented traction forces. Centripetal traction force orientation requires long stress fibers that are formed through the action of Rho kinase (ROCK) and myosin II, and that are supported by α5β1. Thus, altering the relative abundance of fibronectin-binding integrins in cell-matrix adhesions affects the spatiotemporal organization of force transmission. 
 Regulation of cell adhesion and anchorage-dependent growth by a new beta 1-integrin-linked protein kinase. The interaction of cells with the   extracellular matrix   regulates cell shape, motility, growth, survival, differentiation and gene expression, through integrin-mediated signal transduction. We used a two-hybrid screen to isolate genes encoding proteins that interact with the beta 1-integrin cytoplasmic domain. The most frequently isolated complementary DNA encoded a new, 59K serine/threonine protein kinase, containing four ankyrin-like repeats. We report here that this integrin-linked kinase (ILK) phosphorylated a beta 1-integrin cytoplasmic domain peptide in vitro and coimmunoprecipitated with beta 1 in lysates of mammalian cells. Endogenous ILK kinase activity was reduced in response to fibronectin. Overexpression of p59ILK disrupted epithelial cell architecture and inhibited adhesion to integrin substrates, while inducing anchorage-independent growth. We propose that ILK is a receptor-proximal protein kinase regulating integrin-mediated signal transduction. 
 Conformational regulation of the fibronectin binding and alpha 3beta 1 integrin-mediated adhesive activities of thrombospondin-1. The recognition of   extracellular matrix   components can be regulated by conformational changes that alter the activity of cell surface integrins. We now demonstrate that conformational regulation of the matrix glycoprotein thrombospondin-1 (TSP1) can also modulate its binding to an integrin receptor. F18 1G8 is a conformation-sensitive TSP1 antibody that binds weakly to soluble TSP1 in the presence of divalent cations. However, binding of the antibody to melanoma cells was strongly stimulated by adding exogenous TSP1 in the presence of calcium, suggesting that TSP1 undergoes a conformational change following its binding to the cell surface. This conformation was not induced by known cell surface TSP1 receptors, whereas binding of F18 was stimulated when TSP1 bound to fibronectin but not to heparin or fibrinogen. Conversely, binding of F18 to TSP1 enhanced TSP1 binding to fibronectin. Exogenous fibronectin also stimulated TSP1-dependent binding of F18 to melanoma cells. Binding of the fibronectin-TSP1 complex to melanoma cells was mediated by alpha4beta1 and alpha5beta1 integrins. Furthermore, binding to F18 or fibronectin strongly enhanced the adhesive activity of immobilized TSP1 for some cell types. This enhancement of adhesion was mediated by alpha3beta1 integrin and required that the alpha3beta1 integrin be in an active state. Fibronectin also enhanced TSP1 binding to purified alpha3beta1 integrin. Therefore, both fibronectin and the F18 antibody induce conformational changes in TSP1 that enhance the ability of TSP1 to be recognized by alpha3beta1 integrin. The conformational and functional regulation of TSP1 activity by fibronectin represents a novel mechanism for extracellular signal transduction. 
 Targeting of β1 integrins impairs DNA repair for radiosensitization of head and neck cancer cells. β1 Integrin-mediated cel  l-extracellular matr  ix interactions allow cancer cell survival and confer therapy resistance. It was shown that inhibition of β1 integrins sensitizes cells to radiotherapy. Here, we examined the impact of β1 integrin targeting on the repair of radiation-induced DNA double-strand breaks (DSBs). β1 Integrin inhibition was accomplished using the monoclonal antibody AIIB2 and experiments were performed in three-dimensional cell cultures and tumor xenografts of human head and neck squamous cell carcinoma (HNSCC) cell lines. AIIB2, X-ray irradiation, small interfering RNA-mediated knockdown and Olaparib treatment were performed and residual DSB number, protein and gene expression, non-homologous end joining (NHEJ) activity as well as clonogenic survival were determined. β1 Integrin targeting impaired repair of radiogenic DSB (γH2AX/53BP1, pDNA-PKcs T2609 foci) in vitro and in vivo and reduced the protein expression of Ku70, Rad50 and Nbs1. Further, we identified Ku70, Ku80 and DNA-PKcs but not poly(ADP-ribose) polymerase (PARP)-1 to reside in the β1 integrin pathway. Intriguingly, combined inhibition of β1 integrin and PARP using Olaparib was significantly more effective than either treatment alone in non-irradiated and irradiated HNSCC cells. Here, we support β1 integrins as potential cancer targets and highlight a regulatory role for β1 integrins in the repair of radiogenic DNA damage via classical NHEJ. Further, the data suggest combined targeting of β1 integrin and PARP as promising approach for radiosensitization of HNSCC. 
 RACK1, an insulin-like growth factor I (IGF-I) receptor-interacting protein, modulates IGF-I-dependent integrin signaling and promotes cell spreading and contact with   extracellular matrix  . The insulin-like growth factor I (IGF-I) receptor (IGF-IR) is known to regulate a variety of cellular processes including cell proliferation, cell survival, cell differentiation, and cell transformation. IRS-1 and Shc, substrates of the IGF-IR, are known to mediate IGF-IR signaling pathways such as those of mitogen-activated protein kinase (MAPK) and phosphatidylinositol 3-kinase (PI3K), which are believed to play important roles in some of the IGF-IR-dependent biological functions. We used the cytoplasmic domain of IGF-IR in a yeast two-hybrid interaction trap to identify IGF-IR-interacting molecules that may potentially mediate IGF-IR-regulated functions. We identified RACK1, a WD repeat family member and a Gbeta homologue, and demonstrated that RACK1 interacts with the IGF-IR but not with the closely related insulin receptor (IR). In several types of mammalian cells, RACK1 interacted with IGF-IR, protein kinase C, and beta1 integrin in response to IGF-I and phorbol 12-myristate 13-acetate stimulation. Whereas most of RACK1 resides in the cytoskeletal compartment of the cytoplasm, transformation of fibroblasts and epithelial cells by v-Src, oncogenic IR or oncogenic IGF-IR, but not by Ros or Ras, resulted in a significantly increased association of RACK1 with the membrane. We examined the role of RACK1 in IGF-IR-mediated functions by stably overexpressing RACK1 in NIH 3T3 cells that expressed an elevated level of IGF-IR. RACK1 overexpression resulted in reduced IGF-I-induced cell growth in both anchorage-dependent and anchorage-independent conditions. Overexpression of RACK1 also led to enhanced cell spreading, increased stress fibers, and increased focal adhesions, which were accompanied by increased tyrosine phosphorylation of focal adhesion kinase and paxillin. While IGF-I-induced activation of IRS-1, Shc, PI3K, and MAPK pathways was unaffected, IGF-I-inducible beta1 integrin-associated kinase activity and association of Crk with p130(CAS) were significantly inhibited by RACK1 overexpression. In RACK1-overexpressing cells, delayed cell cycle progression in G(1) or G(1)/S was correlated with retinoblastoma protein hypophophorylation, increased levels of p21(Cip1/WAF1) and p27(Kip1), and reduced IGF-I-inducible Cdk2 activity. Reduction of RACK1 protein expression by antisense oligonucleotides prevented cell spreading and suppressed IGF-I-dependent monolayer growth. Our data suggest that RACK1 is a novel IGF-IR signaling molecule that functions as a positive mediator of cell spreading and contact with   extracellular matrix  , possibly through a novel IGF-IR signaling pathway involving integrin and focal adhesion signaling molecules. 
 beta1-Integrins determine the dendritic morphology which enhances DC-SIGN-mediated particle capture by dendritic cells. The morphology of antigen-presenting dendritic cells (DCs) is characterized by the presence of numerous long dendrites. The formation of these processes is shown to require the interaction between the beta1-integrin (CD29) on the surface of the DCs and fibronectin in the   extracellular matrix  . This interaction occurs at focal contacts formed at the tips of dendrites, which contain high concentrations of the beta1-integrins, actin and the cytoskeletal proteins vinculin, paxillin and talin. Dendrites contain an extensive microtubule (MT) network, and are retracted in the presence of the MT inhibitor colchicine, suggesting that MTs are essential for dendrite stability. The dendritic morphology is shown to contribute directly to an enhanced ability to capture dendritic cell specific ICAM-3 grabbing nonintegrin (DC-SIGN)-coated beads. Time-lapse photography demonstrates that dendrites are highly dynamic structures, with cells extending and retracting multiple dendrites in different directions over a 3-h period. This motility increases the area scanned by an individual DC by over 2-fold. The unusual combination of a dendritic morphology and high motility is likely to play a major role in the efficient function of DCs as sentinels of the immune system. 
 Integrin expression during reverse remodeling in the myocardium of heart failure patients. The main anchoring proteins of myocardial cells with each other and with the   extracellular matrix   are integrins present in the membranes of myocardial cells. These integrins are important for maintaining the architecture of the myocardial tissue and the mechanotransduction in the heart. Heart failure leads to various alterations in the myocardium, such as changes in morphology, and in expression of mRNAs, miRNAs, and proteins. Left ventricular assist device (LVAD) support in heart failure patients has been described to induce reverse remodeling of the myocardium and thus to (some degree of) reversal of the aforementioned alterations. In this study, we evaluated whether changes in expression of integrins α-1, -3, -5, -6, -7, -10, -11 and β-1, -3, -5 and -6 play a role during reverse remodeling.Three-step immunoperoxidase staining procedures were applied on frozen heart tissue sections to locate the various integrins tested. Integrin mRNA expression was established by standard Q-PCR procedures.It was shown that mRNA expression of several integrins changes significantly during LVAD support, however without subsequent changes in immunohistochemical detectable quantities. Various integrins showed different locations within the myocardium.LVAD-induced reversed remodeling did not result in significant integrin protein expression, although changes in integrin mRNA expression suggested an adaptation to unloading. 
 A human integrin beta 1 subunit with a unique cytoplasmic domain generated by alternative mRNA processing. The integrin subunit (beta 1) is common to a group of plasma membrane glycoprotein heterodimers that include the fibronectin, laminin and collagen receptors. These receptors span the plasma membrane, providing a transmembrane linkage between the   extracellular matrix   and the cytoskeleton. Here, we describe a variant of the human beta 1 differing from the previously described beta 1 in the cytoplasmic domain. The variant beta 1 transcript (beta 13'v) is present in different cell types and is synthesized at lower levels compared to the beta 1 mRNA. The cytoplasmic domain of the beta 13'v is characterized by a unique 12-amino acid C-terminal sequence. A Tyr residue present in this region, and known to be phosphorylated in the beta 1, is no longer part of a consensus sequence for phosphorylation by Tyr kinases. The integrin cytoplasmic domain anchors actin fibrils to the plasma membrane by interacting with cytoskeletal proteins such as talin and fibulin. The integrin beta 13'v with the variant cytoplasmic domain is likely to mediate a new type of membrane-cytoskeleton interaction during cell-cell and cell-matrix adhesion. Analysis of genomic clones showed that the new sequences of the variant mRNA are identical to an intron located between the last two exons of the beta 1 gene, indicating that the alternative message is generated either by premature transcription termination or by lack of splicing at this site. 
 Tetraspanins CD81 and CD82 facilitate α4β1-mediated adhesion of human erythroblasts to vascular cell adhesion molecule-1. The proliferation and terminal differentiation of erythroid progenitors occurs in human bone marrow within erythroblastic islands, specialised structures consisting of a central macrophage surrounded by developing erythroid cells. Many cell-cell and cell-matrix adhesive interactions maintain and regulate the co-ordinated daily production of reticulocytes. Erythroid cells express only one integrin, α4β1, throughout differentiation, and its interactions with both macrophage Vascular Cell Adhesion Molecule-1 and w  ith extracellular ma  trix fibronectin are critical for erythropoiesis. We observed that proerythroblasts expressed a broad tetraspanin phenotype, and investigated whether any tetraspanin could modulate integrin function. A specific association between α4β1 and CD81, CD82 and CD151 was demonstrated by confocal microscopy and co-immune precipitation. We observed that antibodies to CD81 and CD82 augmented adhesion of proerythroblasts to Vascular Cell Adhesion Molecule-1 but not to the fibronectin spliceoforms FnIII12-IIICS-15 and FnIII12-15. In contrast, different anti-CD151 antibodies augmented or inhibited adhesion of proerythroblasts to Vascular Cell Adhesion Molecule-1 and the fibronectin spliceoform FnIII12-IIICS-15 but not to FnIII12-15. These results strongly suggest that tetraspanins have a functional role in terminal erythropoiesis by modulating interactions of erythroblast α4β1 with both macropha  ges and extracellula  r matrix. 
 Integrin-β5 and zyxin mediate formation of ventral stress fibers in response to transforming growth factor β. Cell adhesion to th  e extracellular matr  ix is an essential element of various biological processes. TGF-β cytokines regulate the matrix components and cell-matrix adhesions. The present study investigates the molecular organization of TGF-β-induced matrix adhesions. The study demonstrates that in various mouse and human epithelial cells TGF-β induces cellular structures containing 2 matrix adhesions bridged by a stretch of actin fibers. These structures are similar to ventral stress fibers (VSFs). Suppression of integrin-β5 by RNA interference reduces VSFs in majority of cells (> 75%), while overexpression of integrin-β5 fragments revealed a critical role of a distinct sequence in the cytoplasmic domain of integrin-β5 in the VSF structures. In addition, the integrity of actin fibers and Src kinase activity contribute to integrin-β5-mediated signaling and VSF formation. TGF-β-Smad signaling upregulates actin-regulatory proteins, such as caldesmon, zyxin, and zyxin-binding protein Csrp1 in mouse and human epithelial cells. Suppression of zyxin markedly inhibits formation of VSFs in response to TGF-β and integrin-β5. Zyxin is localized at actin fibers and matrix adhesions of VSFs and might bridge integrin-β5-mediated adhesions to actin fibers. These findings provide a platform for defining the molecular mechanism regulating the organization and activities of VSFs in response to TGF-β. 
 Reduced podocyte expression of alpha3beta1 integrins and podocyte depletion in patients with primary focal segmental glomerulosclerosis and chronic PAN-treated rats. Integrins attach cells to   extracellular matrix   (ECM) and mediate signals from ECM to cells or from cells to ECM. They regulate cell functions, including adhesion, migration, cell cycle regulation, and differentiation. Podocytes may detach from the glomerular basement membrane (GBM) and be excreted in the urine, and proteinuria is found in patients with primary focal segmental glomerulosclerosis (FSGS); both may be associated with loss of alpha3beta1integrins. In this study, we have examined the podocyte number in patients with primary FSGS and normal controls, and the alpha3- and beta1-integrin subunits expression of podocytes in patients with primary FSGS and chronic puromycin aminonucleoside (PAN)-treated rats by the morphometric, immunoperoxidase histochemical, and immunoelectron microscopic examination. We also measured their expression serially in rats that received repeated PAN injection. The results showed that the podocyte number was significantly decreased in patients with primary FSGS than in normal control (P < 0.05). The immunostaining score showed that both alpha3- and beta1-integrin subunits on podocytes in patients with primary FSGS were significantly lower than in normal controls (both P < 0.01). The number of immuno-gold particles of alpha3- and beta1-integrins at the effaced foot process area of patients with primary FSGS were also significantly decreased than that of normal controls (both P < 0.05). The immunostaining score of both alpha3- and beta1-integrin subunits was negatively correlated with the degree of glomerular sclerosing score and the amount of daily protein loss, and they were positively correlated with the number of podocytes. Chronic 12-week PAN-treated rats showed similar findings with decreased immunostaining expression and immuno-gold particles of alpha3-integrin on podocytes than in normal control (both P < 0.05). The chronic PAN-treated rats also showed a trend toward gradually decreased immunostaining expression of alpha3-integrin subunit on podocyte during the progress from normal to FSGS state. These studies indicate that podocyte expression of alpha3- and beta1-integrin subunits is significantly reduced in humans with primary FSGS and chronic PAN-treated rats, before the morphological changes of FSGS are observed. The decreased podocyte expression of alpha3beta1 integrins is closely related with podocyte depletion, glomerular sclerosis, and daily protein loss in patients with primary FSGS. 
 Sphingosine-1-phosphate induces a PDGFR-dependent cell detachment via inhibiting beta1 integrin in HEK293 cells. Several different types of interactions between sphingosine-1-phosphate (S1P) receptors and platelet-derived growth factor receptor (PDGFR) have been revealed recently. In this work, we used HEK293 cells to further investigate the potential crosstalk. Interestingly, we observed that S1P specifically induced a PDGFR-dependent cell detachment in HEK293 cells, which could be inhibited by AG1296, a specific inhibitor for PDGFR. EGFR on the other hand, did not have any effect on cell detachment. The detachment was   extracellular matrix   (ECM) protein specific, suggesting the involvement of specific integrin molecules. When beta(1) integrin was engaged into an active state, S1P-induced cell detachment was blocked, suggesting that S1P induced an inside-out inhibitory effect on beta(1) integrin. G(i) protein and ERK activation were required for the cell detachment induced by S1P, suggesting an endogenous receptor for S1P is likely to be involved. 
 β1 integrin  -extracellular matri  x interactions are essential for maintaining exocrine pancreas architecture and function. Integrin receptors are responsible for integrating   extracellular matri  x signals inside the cell. The most prominent integrin receptor, β1 integrin, has a role in cell function, survival and differentiation. Recently, we demonstrated a profound in vivo role of β1 integrin expression in the pancreas on glucose homeostasis and islet function. Here, we extend these results by examining the role of β1 integrin in exocrine pancreatic structure and function. Adult C57Bl/6 mice hemizygous for a collagen type Iα2 (Col1a2) promoter-controlled tamoxifen-inducible Cre recombinase gene and homozygous for loxP-β1 integrin were injected with tamoxifen or corn oil to generate mice deleted or not for β1 integrin. Pancreata derived from these male mice were analyzed by quantitative reverse transcriptase-polymerase chain reaction, western blot and immunofluorescence. Our results showed that β1 integrin-deficient mice displayed a significant decrease in pancreas weight with a significant reduction of amylase, regenerating islet-derived protein II and carboxypeptidase-A expression (P<0.05-0.01). Compared with control pancreata, β1 integrin-deficient pancreata showed reduced mRNA expre  ssion of extracellul  ar matrix (collagen type Iα2, fibronectin and laminin) genes (P<0.05), detached acini clusters and lost focal adhesion structure. Moreover, β1 integrin-deficient pancreatic acinar cells displayed decreased proliferation (P<0.05) and increased apoptosis (P<0.001). Apoptosis was reduced to that of controls when isolated exocrine clusters were cultured in media supplem  ented with extracell  ular matrix proteins. Taken together, these results implicate β1 integrin as an essential component for maintaining exocrine pancreatic structure and function. 
 Immediate early response protein 2 regulates hepatocellular carcinoma cell adhesion and motility via integrin β1-mediated signaling pathway. Human immediate early response 2 (IER2) has been reported to function as a potential transcriptional factor or transcriptional co‑activator and seems to play a pivotal role in tumor cell motility and metastasis, however, its role and underlying mechanisms in hepatocellular carcinoma (HCC) remain unknown. Herein, we demonstrated that overexpression of IER2 in HCC cells increased cell adhesion to fibronectin, migration and invasion, whereas knockdown of IER2 displayed the opposite effects. In agreement with this phenotype, IER2 expression was positively correlated with the metastatic potential and integrin β1 (ITGB1) expression in HCC cell lines. Moreover, we demonstrated a critical role for IER2 in regulation of   HCC cell‑extracel  lular matrix (ECM) adhesion and motility by the transcriptionally promoted ITGB1. Furthermore, we showed that ITGB1‑focal adhesion kinase (FAK)‑Src‑paxillin signal pathway activated by IER2 may contribute to the HCC cell‑ECM adhesion and motility. These results demonstrated that IER2 promoted HCC cell adhesion and motility probably by directly increasing ITGB1 expression and subsequently activating the ITGB1‑FAK‑Src‑paxillin signal pathway. 
 Cross-linking CD98 promotes integrin-like signaling and anchorage-independent growth. CD98, an early marker of T-cell activation, is an important regulator of integrin-mediated adhesion events. Previous studies suggest that CD98 is coupled to both cellular activation and transformation and is involved in the pathogenesis of viral infection, inflammatory disease, and cancer. Understanding of the molecular mechanisms underlying CD98 activity may have far-reaching practical applications in the development of novel therapeutic strategies in these disease states. Using small cell lung cancer cell lines, which are nonadherent, nonpolarized, and highly express CD98, we show that, in vitro, under physiological conditions, CD98 is constitutively associated with beta1 integrins regardless of activation status. Cross-linking CD98 with the monoclonal antibody 4F2 stimulated phosphatidylinositol (PI) 3-kinase, PI(3,4,5)P(3), and protein kinase B in the absence of integrin ligation or   extracellular matrix   engagement. Furthermore, cross-linking CD98 promoted anchorage-independent growth. Using fibroblasts derived from beta1 integrin null stem cells (GD25), wild-type GD25beta1, or GD25 cells expressing a mutation preventing beta1 integrin-dependent FAK phosphorylation, we demonstrate that a functional beta1 integrin is required for CD98 signaling. We propose that by cross-linking CD98, it acts as a "molecular facilitator" in the plasma membrane, clustering beta1 integrins to form high-density complexes. This results in integrin activation, integrin-like signaling, and anchorage-independent growth. Activation of PI 3-kinase may, in part, explain cellular transformation seen on overexpressing CD98. These results may provide a paradigm for events involved in such diverse processes as inflammation and viral-induced cell fusion. 
 Epithelial-mesenchymal transition in cervical cancer: correlation with tumor progression, epidermal growth factor receptor overexpression, and snail up-regulation. Acquisition of epithelial-mesenchymal transition (EMT) by primary carcinoma cells is associated with disrupted epithelial integrity, local invasion, and ultimately metastasis. Little is known about the existence and function of EMT in cervical cancer. This study aims to investigate the regulation of EMT in cervical squamous cell carcinoma.We investigated the molecular events of EMT in surgical specimens, which present the progression of cervical carcinoma. Two cervical cancer cell lines and the primary culture of normal cervical epithelia were used to study the regulatory mechanisms of EMT.The chronic epidermal growth factor (EGF) treatment induces the elongation of cell shape, increases cell scattering, and enhances cell invasion. EGF treatment down-regulates E-cadherin and up-regulates vimentin in cervical cancer cells. These characteristics are consistent with the morphologic changes, molecular events, and functional significance of EMT. EGF receptor (EGFR) signaling inactivates glycogen synthase kinase-3beta, which results in the nuclear accumulation of up-regulated Snail and then leads to EMT program. alpha(5)beta(1) integrin signaling and   extracellular matrix   fibronectin can modulate EGF-induced EMT. Importantly, the immunofluorescent stainings of surgical specimens indicate that cervical carcinoma progression is accompanied by EGFR overexpression, which is in parallel with decreased E-cadherin and increased vimentin. Up-regulation and nuclear accumulation of Snail correlate with EMT program in tumor tissues.EGF cooperates with alpha(5)beta(1) integrin signaling to induce EMT in cervical cancer cells via up-regulated Snail. Blockade of EGFR activity or expression may provide a potential target for the treatment of cervical cancer progression. 
 Trop-2 inhibits prostate cancer cell adhesion to fibronectin through the β1 integrin-RACK1 axis. Trop-2 is a transmembrane glycoprotein upregulated in several human carcinomas, including prostate cancer (PrCa). Trop-2 has been suggested to regulate cell-cell adhesion, given its high homology with the other member of the Trop family, Trop-1/EpCAM, and its ability to bind the tight junction proteins claudin-1 and claudin-7. However, a role for Trop-2 in cell adhesion to the   extracellular matri  x has never been postulated. Here, we show for the first time that Trop-2 expression in PrCa cells correlates with their aggressiveness. Using either shRNA-mediated silencing of Trop-2 in cells that endogenously express it, or ectopic expression of Trop-2 in cells that do not express it, we show that Trop-2 inhibits PrCa cell adhesion to fibronectin (FN). In contrast, expression of another transmembrane receptor, α(v) β(5) integrin, does not affect cell adhesion to this ligand. We find that Trop-2 does not modulate either protein or activation levels of the prominent FN receptors, β(1) integrins, but acts through increasing β(1) association with the adaptor molecule RACK1 and redistribution of RACK1 to the cell membrane. As a result of Trop-2 expression, we also observe activation of Src and FAK, known to occur upon β(1) -RACK1 interaction. These enhanced Src and FAK activities are not mediated by changes in either the activity of IGF-IR, which is known to bind RACK1, or IGF-IR's ability to associate with β(1) integrins. In summary, our data demonstrate that the transmembrane receptor Trop-2 is a regulator of PrCa cell adhesion to FN through activation of the β(1) integrin-RACK1-FAK-Src signaling axis. 
 α5β1 integrin recycling promotes Arp2/3-independent cancer cell invasion via the formin FHOD3. Invasive migration in 3  D extracellular matr  ix (ECM) is crucial to cancer metastasis, yet little is known of the molecular mechanisms that drive reorganization of the cytoskeleton as cancer cells disseminate in vivo. 2D Rac-driven lamellipodial migration is well understood, but how these features apply to 3D migration is not clear. We find that lamellipodia-like protrusions and retrograde actin flow are indeed observed in cells moving in 3D ECM. However, Rab-coupling protein (RCP)-driven endocytic recycling of α5β1 integrin enhances invasive migration of cancer cells into fibronectin-rich 3D ECM, driven by RhoA and filopodial spike-based protrusions, not lamellipodia. Furthermore, we show that actin spike protrusions are Arp2/3-independent. Dynamic actin spike assembly in cells invading in vitro and in vivo is regulated by Formin homology-2 domain containing 3 (FHOD3), which is activated by RhoA/ROCK, establishing a novel mechanism through which the RCP-α5β1 pathway reprograms the actin cytoskeleton to promote invasive migration and local invasion in vivo. 
 Overexpression of the integrin beta(1A) subunit and the beta(1A) cytoplasmic domain modifies the beta-adrenergic regulation of the cardiac L-type Ca(2+)current. Integrins are a family of cell-surface receptors that link the   extracellular matrix   (ECM) to the cellular cytoskeleton. The goal of this study was to determine the importance of the integrin beta(1) subunit in regulating cardiac L-type Ca(2+) channel function. Neonatal rat ventricular myocytes were cultured on collagen membranes and infected with adenovirus expressing either the human beta(1A) integrin (Adbeta(1A)) or a chimeric protein consisting of the cytoplasmic tail domain of the beta(1A) integrin and the extracellular/transmembrane domain of the interleukin-2 receptor (AdTAC-beta(1)). Expression of the free beta(1) integrin tail (TAC-beta(1)), but not the full-length beta(1A) integrin, altered cell morphology and disrupted normal cell adhesion. When compared with myocytes infected with control virus, neither Adbeta(1A) nor AdTAC-beta(1) infection produced any significant change in the current vs. voltage relationship of the whole-cell Ca(2+) current (I(Ca)) or the kinetics of I(Ca) decay. Expression of TAC-beta(1), but not beta(1A), induced a negative shift in the Ca(2+) channel steady-state inactivation curve. Application of the beta-adrenergic receptor agonist isoproterenol produced over a 90% increase in I(Ca) in control cells, but caused only an 18% increase in myocytes overexpressing the full-length beta(1A) integrin. In addition, beta-adrenergic stimulation resulted in a 5-10-fold increase in intracellular cAMP levels in control cells, but produced no significant response in Adbeta(1A)-infected cells. In contrast, expression of TAC-beta(1) was associated with an augmentation in the Ca(2+) channel response to isoproterenol (160% increase) and the Ca(2+) channel agonist BayK8644. Thus, integrin/ECM interactions may be critical in the regulation of I(Ca) 
 CXCR4 chemokine receptor engagement modifies integrin dependent adhesion of renal carcinoma cells. The mechanisms leading to renal cell carcinoma (RCC) metastasis are incompletely understood. Although evidence shows that the chemokine receptor CXCR4 and its ligand CXCL12 may regulate tumor dissemination, their role in RCC is not clearly defined. We examined CXCR4 expression and functionality on RCC cell lines, and explored CXCL12-triggered tumor adhesion to human endothelium (HUVEC) or   extracellular matrix   proteins. Functional CXCR4 was expressed on A498 tumor cells, enabling them to migrate towards a CXCL12 gradient. CXCR4 engagement by CXCL12 induced elevated cell adhesion to HUVEC, to immobilized fibronectin, laminin or collagen. Anti-CXCR4 antibodies or CXCR4 knock down by siRNA applied prior to CXCL12 stimulation impaired CXCL12-triggered tumor adhesion. However, blocking CXCR4 subsequent to CXCL12 stimulation did not. This pointed to an indirect control of tumor cell adhesion by CXCR4. In fact, CXCR4 engagement by CXCL12 also induced alterations of receptors of the integrin family, notably alpha3, alpha5, beta1 and beta3 subunits, and blocking beta1 integrins with a function-blocking antibody prevented CXCL12-induced A498 adhesion. Focal adhesion kinase (total and activated) and integrin-linked kinase significantly increased in CXCL12-treated A498 cells, accompanied by a distinct up-regulation of ERK1/2, JNK and p38 phosphorylation. Therefore, CXCR4 may be crucial in controlling adhesion of A498 cells via cross talking with integrin receptors. These data show that CXCR4 receptors contribute to RCC dissemination and may provide a novel link between CXCR4 chemokine receptor expression and integrin triggered RCC adhesion to the vascular wall and subendothelial matrix components. 
 Hypoxia activates beta(1)-integrin via ERK 1/2 and p38 MAP kinase in human vascular smooth muscle cells. Hypoxia plays an important role in vascular remodeling and directly affects vascular smooth muscle cell (VSMC) functions. VSMC adhesion participates in changes of vascular structure; however, little is known about VSMC adhesion under hypoxic conditions. It was the aim of the present study to investigate the effects of hypoxia on adhesion mechanisms in human VSMCs. Compared to normoxic cells, hypoxia (1% O(2), 24h) significantly increased adhesion of VSMCs to collagen I by 30.2% and fibronectin by 58.0%. This effect was completely inhibited in the presence of the pharmacological ERK 1/2 mitogen-activated protein kinase (MAPK) pathway inhibitor PD98059 (30 microM) or the p38 MAPK inhibitor SB203580 (1 microM). Basal adhesion of normoxic cells was not affected by pretreatment with PD98059 and SB203580. Hypoxia induced a time-dependent activation of ERK 1/2 and p38 MAPK activation in human VSMCs, which were completely abolished by PD98059 or SB203580, respectively. Since adhesion of VSMCs to fibronectin and collagen I involves beta(1)-integrin receptors, we used a blocking antibody against beta(1)-integrin (P5D2) to examine potential effects of hypoxia on beta(1)-integrins. P5D2 significantly reduced VSMC adhesion to fibronectin and collagen I in normoxia and hypoxia in a comparable manner; however, beta(1)-integrin protein or mRNA levels were not affected by hypoxia. As evidenced by flow cytometry, hypoxia induced a activation of beta(1)-integrins by exposing an conformationally sensitive epitope on the beta(1)-subunit. These results demonstrate that hypoxia enhances adhesion of VSMC on   extracellular matrix   proteins by activating beta(1)-integrin. 
 Chemical modulation of VLA integrin affinity in human breast cancer cells. The fact that disruption of integrin-  extracellular matrix   contacts leads to cell death, has converted cell adhesion into a potential target for the control of invasive cancer. In this work, we studied the functional consequences of the interference with the activity of the very late activation antigen (VLA) family of integrins in human breast cancer cell lines of distinct malignancy. The alpha2beta1-mediated adhesion reduced the entry of highly malignant, hormone-independent breast cancer cells into apoptosis. Adhesion of breast cancer cells through the VLA integrins alpha2beta1 and alpha5beta1 was significantly reduced by an apoptosis-inducing natural triterpenoid, dehydrothyrsiferol (DT), when studied on low amounts of   extracellular matrix  . This effect was dose-dependent, not related to cell toxicity and not shared with apoptosis-inducing standard chemotherapeutics, such as doxorubicin and taxol. The compound did not affect either the cell surface expression level of VLA integrins or cell distribution of vinculin and actin during cell spreading. In addition, neither phosphorylation of the focal adhesion kinase pp125FAK on Tyr397 nor the protein kinase B (Akt/PKB) on Ser473 was significantly altered by DT. The integrin activation level, assessed by binding of soluble collagen to the alpha2beta1 integrin, was reduced upon cell treatment with DT. Importantly, the TS2/16, an anti-beta1 activating monoclonal antibody was able to rescue DT-treated cells from apoptosis. Since the activation state of integrins is increasingly recognized as an essential factor in metastasis formation, findings presented herein reveal that the chemical regulation of integrin affinity may be a potential therapeutic strategy in cancer therapy. 
 Modulation of the transforming growth factor-beta1-induced Smad phosphorylation by the   extracellular matrix   receptor beta1-integrin. Integrins, heterodimeric receptors for the   extracellular matrix  , are known to modulate transforming growth factor-beta1 (TGF-beta1)-mediated cell behavior. However, the interplay between beta1-integrin and Smad signaling, regulated by TGF-beta1, is not clearly understood. This study focuses on the alterations of the regulatory Smads (R-Smads) by TGF-beta1 in beta1-integrin-transfected HepG2 cells. The phosphorylation at the C-terminal site of R-Smads by TGF-beta1 was impaired in the beta1-integrin-transfected cells. However, the R-Smads were constitutively phosphorylated at the linker region in a MAP kinase-dependent manner. Furthermore, the expression of a mutant Smad3, that lacks the phosphorylation sites in the linker region, restored the TGF-beta1-induced Smad transcriptional activity. These results suggest that beta1-integrin impairs the TGF-beta1-mediated signals through the altered phosphorylation of the R-Smads. 
 The integrin cytoplasmic domain-associated protein ICAP-1 binds and regulates Rho family GTPases during cell spreading. Using two-hybrid screening, we isolated the integrin cytoplasmic domain-associated protein (ICAP-1), an interactor for the COOH terminal region of the beta1A integrin cytoplasmic domain. To investigate the role of ICAP-1 in integrin-mediated adhesive function, we expressed the full-length molecule in NIH3T3 cells. ICAP-1 expression strongly prevents NIH3T3 cell spreading on   extracellular matrix  . This inhibition is transient and can be counteracted by coexpression of a constitutively activated mutant of Cdc42, suggesting that ICAP-1 acts upstream of this GTPase. In addition, we found that ICAP-1 binds both to Cdc42 and Rac1 in vitro, and its expression markedly inhibits activation of these GTPases during integrin-mediated cell adhesion to fibronectin as detected by PAK binding assay. In the attempt to define the molecular mechanism of this inhibition, we show that ICAP-1 reduces both the intrinsic and the exchange factor-induced dissociation of GDP from Cdc42; moreover, purified ICAP-1 displaces this GTPase from cellular membranes. Together, these data show for the first time that ICAP-1 regulates Rho family GTPases during integrin-mediated cell matrix adhesion, acting as guanine dissociation inhibitor. 
 Fibronectin induces endothelial cell migration through β1 integrin and Src-dependent phosphorylation of fibroblast growth factor receptor-1 at tyrosines 653/654 and 766. The   extracellular matri  x microenvironment regulates cell phenotype and function. One mechanism by which this is achieved is the transactivation of receptor tyrosine kinases by specific matrix molecules. Here, we demonstrate that the provisional matrix protein, fibronectin (FN), activates fibroblast growth factor (FGF) receptor-1 (FGFR1) independent of FGF ligand in liver endothelial cells. FN activation of FGFR1 requires β1 integrin, as evidenced by neutralizing antibody and siRNA-based studies. Complementary genetic and pharmacologic approaches identify that the non-receptor tyrosine kinase Src is required for FN transactivation of FGFR1. Whereas FGF ligand-induced phosphorylation of FGFR1 preferentially activates ERK, FN-induced phosphorylation of FGFR1 preferentially activates AKT, indicating differential downstream signaling of FGFR1 in response to alternate stimuli. Mutation analysis of known tyrosine residues of FGFR1 reveals that tyrosine 653/654 and 766 residues are required for FN-FGFR1 activation of AKT and chemotaxis. Thus, our study mechanistically dissects a new signaling pathway by which FN achieves endothelial cell chemotaxis, demonstrates how differential phosphorylation profiles of FGFR1 can achieve alternate downstream signals, and, more broadly, highlights the diversity of mechanisms by which th  e extracellular matr  ix microenvironment regulates cell behavior through transactivation of receptor tyrosine kinases. 
 beta1-integrin-mediated signaling essentially contributes to cell survival after radiation-induced genotoxic injury. Integrin-mediated adhesion to   extracellular matrix   proteins confers resistance to radiation- or drug-induced genotoxic injury. To analyse the underlying mechanisms specific for beta1-integrins, wild-type beta1A-integrin-expressing GD25beta1A cells were compared to GD25beta1B cells, which express signaling-incompetent beta1B variants. Cells grown on fibronectin, collagen-III, beta1-integrin-IgG or poly-l-lysine were exposed to 0-6 Gy X-rays in presence or depletion of growth factors and phosphatidylinositol-3 kinase (PI3K) inhibitors (LY294002, wortmannin). In order to test the relevance of these findings in tumor cells, human A-172 glioma cells were examined under the same conditions after siRNA-mediated silencing of beta1-integrins. We found that beta1A-integrin-mediated adhesion to fibronectin, collagen-III or beta1-IgG was essential for cell survival after radiation-induced genotoxic injury. Mediated by PI3K, pro-survival beta1A-integrin/Akt signaling was critically involved in this process. Additionally, the beta1-integrin downstream targets p130Cas and paxillin-impaired survival-regulating PI3K-dependent JNK. In A-172 glioma cells, beta1-integrin knockdown and PI3K inhibition confirmed the central role of beta1-integrins in Akt- and p130Cas/paxillin-mediated prosurvival signaling. These findings suggest beta1-integrins as critical regulators of cell survival after radiation-induced genotoxic injury. Elucidation of the molecular circuitry of prosurvival beta1-integrin-mediated signaling in tumor cells may promote the development of innovative molecular-targeted therapeutic antitumor strategies. 
 A mitochondrial protein, Bit1, mediates apoptosis regulated by integrins and Groucho/TLE corepressors. A delicate balance of signals regulates cell survival. One set of these signals is derived from integrin-mediated cell adhesion to the   extracellular matrix   (ECM). Loss of cell attachment to the ECM causes apoptosis, a process known as anoikis. In searching for proteins involved in cell adhesion-dependent regulation of anoikis, we identified Bit1, a mitochondrial protein that is released into the cytoplasm during apoptosis. Cytoplasmic Bit1 forms a complex with AES, a small Groucho/transducin-like enhancer of split (TLE) protein, and induces cell death with characteristics of caspase-independent apoptosis. Cell attachment to fibronectin counteracts the apoptotic effect of Bit1 and AES. Increasing Bit1 expression enhances anoikis, while suppressing the expression reduces it. Thus, we have elucidated an integrin-controlled pathway that is, at least in part, responsible for the cell survival effects of cell-ECM interactions. 
 β1 integrin regulates Arg to promote invadopodial maturation and matrix degradation. β1 integrin has been shown to promote metastasis in a number of tumor models, including breast, ovarian, pancreatic, and skin cancer; however, the mechanism by which it does so is poorly understood. Invasive membrane protrusions called invadopodia are believed to facilitat  e extracellular matr  ix degradation and intravasation during metastasis. Previous work showed that β1 integrin localizes to invadopodia, but its role in regulating invadopodial function has not been well characterized. We find that β1 integrin is required for the formation of mature, degradation-competent invadopodia in both two- and three-dimensional matrices but is dispensable for invadopodium precursor formation in metastatic human breast cancer cells. β1 integrin is activated during invadopodium precursor maturation, and forced β1 integrin activation enhances the rate of invadopodial matrix proteolysis. Furthermore, β1 integrin interacts with the tyrosine kinase Arg and stimulates Arg-dependent phosphorylation of cortactin on tyrosine 421. Silencing β1 integrin with small interfering RNA completely abrogates Arg-dependent cortactin phosphorylation and cofilin-dependent barbed-end formation at invadopodia, leading to a significant decrease in the number and stability of mature invadopodia. These results describe a fundamental role for β1 integrin in controlling actin polymerization-dependent invadopodial maturation and matrix degradation in metastatic tumor cells. 
 Spatiotemporally-regulated interaction between β1 integrin and ErbB4 that is involved in fibronectin-dependent cell migration. Integrins are widely expressed cell surface molecules that mediate cell attachment to   extracellular matri  x (ECM) proteins. They also interact with molecules on their own membranes, and these cis-interactions play a crucial role in integrin-dependent cellular responses. We herein analysed what molecules interact with β1 integrin during biological events induced by cell attachment to different ECM proteins, using a recently established reaction, the enzyme-mediated activation of radical sources (EMARS). The interactions between β1 integrin and receptor tyrosine kinases including EGFR and ErbB4 reached a peak at 2 h after seeding HeLa S3 cells onto the ECM proteins. The peak of phosphorylation of ErbB4 (at 2 h after seeding the cells onto fibronectin) coincided with the peak of the interaction with β1 integrin, while that of EGFR (at 1 day) did not. Accompanying with these findings, suppression of cell migration by a pharmacological inhibitor of the ErbB family receptors, PD168393 and an anti-ErbB4 neutralizing antibody, 12D8 was observed at 2 h after seeding. Taken together, it is deduced that interactions between β1 integrin and ErbB4 occur in a spatiotemporally-regulated manner, and such interaction contributes to the integrin-dependent cell migration. 
 beta 1 Integrin-dependent cell adhesion to EMILIN-1 is mediated by the gC1q domain. EMILIN-1 (Elastin Microfibril Interface Located ProteIN), the prototype of the EMILIN family, consists of a cysteine-rich domain (EMI domain) at the N terminus, an extended region with a high potential coiled-coil structure, a short collagenous stalk, and a self-interacting globular gC1q-l domain. EMILIN-1 is an adhesive   extracellular matrix   constituent associated with elastic fibers, detected also in the proximity of cell surfaces. To localize the cell attachment site(s), monoclonal antibodies (mAbs) against EMILIN-1 or the gC1q-1 domain were used to inhibit cell attachment to EMILIN-1. Thus, one mAb mapping to the gC1q-1 domain caused complete inhibition of cell attachment. EMILIN-1 and gC1q-1 displayed a comparable dose-dependent ability to promote cell adhesion. Adhesion kinetics was similar to that of fibronectin (FN), reaching the maximum level of attachment at 20 min, but in the absence of cations adhesion was negligible. The relative adhesion strength to detach 50% of the cells was similar for EMILIN-1 and gC1q-1 (250-270 x g) but lower than that for FN (>500). Cell adhesion to EMILIN-1 or gC1q-1 was completely blocked by a function-blocking beta(1) integrin subunit mAb. In contrast, adhesion to the complement C1q component was totally unaffected. Among the various function-blocking mAbs against the alpha integrin subunits only the anti-alpha(4) fully abrogated cell adhesion to gC1q-1 and up to 70% to EMILIN-1. Furthermore, only K562 cells transfected with the alpha(4) integrin chain, but not wild type K562, were able to adhere to EMILIN-1 and were specifically inhibited by anti-alpha(4) function-blocking mAb. Finally, cells attached to EMILIN-1 or gC1q-1, compared with cells plated on FN or vitronectin, which appeared well spread out on the substrate with prominent stress fibers and focal contacts, were much smaller with wide ruffles and a different organization status of the actin cytoskeleton along the cell periphery. This pattern was in accord with the ability of EMILIN-1 to promote cell movement. 
 Homeobox D1 regulates angiogenic functions of endothelial cells via integrin β1 expression. Homeobox (HOX) family genes, major transcription factors for embryonic development, have been also implicated in vascular development and angiogenesis, particularly with regulation of genes involved in cell-cell or cell  -extracellular matri  x (ECM) interactions. However, the cellular and molecular functions of HOXD1 in endothelial cells (ECs) are yet to be explored. We here report that HOXD1 is prominently expressed in human ECs and regulates angiogenic activities. Knockdown of HOXD1 in ECs resulted in significant inhibition of migration and adhesion as well as tube like structure formation. These effects were correlated with the reduced expression of integrin β1 (ITGB1), an important signaling component of angiogenesis. Consistently, ITGB1 promoter activity was decreased by HOXD1 knockdown in ECs. Furthermore, we identified the putative HOXD1-binding sites in the promoter region of ITGB1. Together, these findings suggest that HOXD1 plays a significant role in EC functions by regulating the expression of ITGB1. 
 The laminin binding integrin alpha6beta1 in prostate cancer perineural invasion. Metastasizing prostate tumor cells invade along nerves innervating the encapsulated human prostate gland in a process known as perineural invasion. The   extracellular matrix   laminin class of proteins line the neural route and tumor cells escaping from the gland express the laminin binding integrin alpha6beta1 as a prominent cell surface receptor. Integrin alpha6beta1 promotes aggressive disease and supports prostate tumor cell metastasis to bone. Laminins and their integrin receptors are necessary for the development and maintenance of the peripheral nervous system, indicating the potential role for integrin receptors in directing prostate tumor cell invasion on nerves during perineural invasion. 
 Matrix metalloproteinase-2 cleavage of the β1 integrin ectodomain facilitates colon cancer cell motility. Cancer cell invasion is a key element in metastasis that requires integrins for adhesion/de-adhesion, as well as matrix metalloproteinases (MMPs) for focalized proteolysis. Herein we show that MMP-2 is up-regulated in resected colorectal tumors and degrades β1 integrins with the release of fragments containing the β1 I-domain. The β1 cleavage pattern is similar to that produced by digestion of α5β1 and α2β1 with MMP-2. Two such fragments, at 25 and 75 kDa, were identified after immunoprecipitation, with monoclonal antibody BD610468 reacting with the NH(2)-terminal I-like ectodomain followed by SDS-PAGE and microsequencing using electrospray (ISI-Q-TOF-Micromass) spectrometry. Cleavage of the β1 integrin can be abolished by inhibition of MMP-2 activity; it can be induced by up-regulation of MMP-2 expression, as exemplified by HT29 colon cancer cells transfected with pCMV6-XL5-MMP-2. Co-immunoprecipitation studies of colon cancer cells showed that the β1 integrin subunit is associated with MMP-2. The MMP-2-mediated shedding of the I-like domain from β1 integrins resulted in decreased adhesion of colon cancer cells to collagen and fibronectin, thus abolishing their receptivity. Furthermore, such cells showed enhanced motility as evaluated by a "wound healing-like" assay and time-lapse microscopy, indicating their increased invasiveness. Altogether, our data demonstrate that MMP-2 amplifies the motility of colon cancer cells, not only by dig  esting the extracell  ular matrix components in the vicinity of cancer cells but also by inactivating their major β1 integrin receptors. 
 Alpha 3 beta 1 integrin promotes keratinocyte cell survival through activation of a MEK/ERK signaling pathway. Inadequate or inappropriate adhesion of epithelial cells to   extracellular matrix   leads to a form of apoptosis known as anoikis. During various tissue remodelling events, such as wound healing or carcinoma invasion, changes in the physical properties, and/or composition of the   extracellular matrix  , can lead to anoikis of epithelial cells that lack appropriate receptor-matrix interactions. Laminin-5 is the major ligand for keratinocyte adhesion in the epidermis, and it also promotes keratinocyte survival in vivo and in vitro. Integrins alpha 3 beta 1 and alpha 6 beta 4 are the major receptors for laminin-5; however, specific roles for these integrins in keratinocyte survival have not been determined. In the current study, we exploited keratinocyte cell lines derived from wild-type or alpha 3 integrin knockout mice to reveal a critical role for alpha 3 beta 1 in protecting keratinocytes from apoptosis upon serum withdrawal. We show that alpha 3 beta 1-mediated adhesion to laminin-5   extracellular matrix   inhibits proteolytic activation of caspase-3 and TUNEL-staining, both hallmarks of apoptosis. We also show that alpha 3 beta1-mediated adhesion activates focal adhesion kinase (FAK) and extracellular signal-regulated kinase (ERK), and that inhibition of either FAK or ERK signaling leads to apoptosis of keratinocytes attached to laminin-5. alpha 6 beta 4-mediated adhesion to laminin-5 only partially protects cells from apoptosis in the absence of alpha 3 beta 1, and alpha 6 beta 4 is not necessary for cell survival in the presence of alpha 3 beta 1. These results suggest that alpha 3 beta 1 is necessary and sufficient for maximal keratinocyte survival on laminin-5. We propose a model to address the potential importance of alpha 3 beta 1-mediated survival for migrating keratinocytes at the leading edge of a cutaneous wound. 
 Contact with fibrillar collagen inhibits melanoma cell proliferation by up-regulating p27KIP1. It is known that the   extracellular matrix   regulates normal cell proliferation, and it is assumed that anchorage-independent malignant cells escape this regulatory function. Here we demonstrate that human M24met melanoma cells remain responsive to growth regulatory signals that result from contact with type I collagen and that the effect on proliferation depends on the physical structure of the collagen. On polymerized fibrillar collagen, M24met cells are growth arrested at the G(1)/S checkpoint and maintain high levels of p27(KIP1) mRNA and protein. In contrast, on nonfibrillar (denatured) collagen, the cells enter the cell cycle, and p27(KIP1) is down-regulated. These growth regulatory effects involve contact between type I collagen and the collagen-binding integrin alpha(2)beta(1), which appears restricted in the presence of fibrillar collagen. Thus melanoma cells remain sensitive to negative growth regulatory signals originating from fibrillar collagen, and the proteolytic degradation of fibrils is a mechanism allowing tumor cells to escape these restrictive signals. 
 IpaA targets beta1 integrins and rho to promote actin cytoskeleton rearrangements necessary for Shigella entry. Shigella invasion into the colonic epithelium involves many steps including the formation of large membrane protrusions by the epithelial cells that facilitate bacterial engulfment. IpaA, a Shigella protein secreted into target cells upon cell contact induces a loss of actin stress fibers in cells and promotes the reorganization of actin at the site of entry. The mechanism for this is not known but is thought to involve recruitment of the focal adhesion protein vinculin to IpaA. Here we have examined the mechanism for the effects of IpaA on the actin cytoskeleton. We show that IpaA-induced loss of actin stress fibers and cell rounding do not require vinculin expression or an intact vinculin binding site on IpaA. Rather, we find that cells expressing IpaA exhibited elevated Rho activity and increased myosin light chain phosphorylation. In addition, IpaA decreases integrin affinity for   extracellular matrix   ligands by interfering with talin recruitment to the integrin cytoplasmic tail. The combination of these two effects, namely weakened adhesion and increased contractility, account for the loss of actin stress fibers and cell rounding observed in cells exposed to IpaA. 
 Mechanical control of cAMP signaling through integrins is mediated by the heterotrimeric Galphas protein. Mechanical stresses that are preferentially transmitted across the cell surface via transmembrane integrin receptors activate gene transcription by triggering production of intracellular chemical second messengers, such as cAMP. Here we show that the sensitivity of the cAMP signaling pathway to mechanical stresses transferred across beta1 integrins is mediated by force-dependent activation of the heterotrimeric G protein subunit Galphas within focal adhesions at the site of stress application. Galphas is recruited to focal adhesions that form within minutes following clustering of beta1 integrins induced by cell binding to magnetic microbeads coated with activating integrin ligands, and beta1 integrin and Galphas co-precipitate when analyzed biochemically. Stress application to activated beta1 integrins using magnetic twisting cytometry increases Galphas recruitment and activates these large G proteins within focal adhesions, as measured by binding of biotinylated azido-anilido-GTP, whereas application of similar stresses to inactivated integrins or control histocompatibility antigens has little effect. This response is relevant physiologically as application of mechanical strain to cells bound to flexible   extracellular matrix  -coated substrates induce translocation of phospho-CREB to the nucleus, which can be attenuated by inhibiting Galphas activity, either using the inhibitor melittin or suppressing its expression using siRNA. Although integrins are not typical G protein-coupled receptors, these results show that integrins focus mechanical stresses locally on heterotrimeric G proteins within focal adhesions at the site of force application, and transduce mechanical stimuli into an intracellular cAMP signaling response by activating Galphas at these membrane signaling sites. 
 Perlecan domain V is neuroprotective and proangiogenic following ischemic stroke in rodents. Stroke is the leading cause of long-term disability and the third leading cause of death in the United States. While most research thus far has focused on acute stroke treatment and neuroprotection, the exploitation of endogenous brain self-repair mechanisms may also yield therapeutic strategies. Here, we describe a distinct type of stroke treatment, the naturally occurring   extracellular matrix   fragment of perlecan, domain V, which we found had neuroprotective properties and enhanced post-stroke angiogenesis, a key component of brain repair, in rodent models of stroke. In both rat and mouse models, Western blot analysis revealed elevated levels of perlecan domain V. When systemically administered 24 hours after stroke, domain V was well tolerated, reached infarct and peri-infarct brain vasculature, and restored stroke-affected motor function to baseline pre-stroke levels in these multiple stroke models in both mice and rats. Post-stroke domain V administration increased VEGF levels via a mechanism involving brain endothelial cell α5β1 integrin, and the subsequent neuroprotective and angiogenic actions of domain V were in turn mediated via VEGFR. These results suggest that perlecan domain V represents a promising approach for stroke treatment. 
 New mechanistic insights of integrin β1 in breast cancer bone colonization. Bone metastasis is a frequent and life-threatening complication of breast cancer. The molecular mechanisms supporting the establishment of breast cancer cells in the skeleton are still not fully understood, which may be attributed to the lack of suitable models that interrogate interactions between human breast cancer cells and the bone microenvironment. Although it is well-known that integrins mediate adhesion of malignant cells to bone   extracellular matri  x, their role during bone colonization remains unclear. Here, the role of β1 integrins in bone colonization was investigated using tissue-engineered humanized in vitro and in vivo bone models. In vitro, bone-metastatic breast cancer cells with suppressed integrin β1 expression showed reduced attachment, spreading, and migration within human bone matrix compared to control cells. Cell proliferation in vitro was not affected by β1 integrin knockdown, yet tumor growth in vivo within humanized bone microenvironments was significantly inhibited upon β1 integrin suppression, as revealed by quantitative in/ex vivo fluorescence imaging and histological analysis. Tumor cells invaded bone marrow spaces in the humanized bone and formed osteolytic lesions; osteoclastic bone resorption was, however, not reduced by β1 integrin knockdown. Taken together, we demonstrate that β1 integrins have a pivotal role in bone colonization using unique tissue-engineered humanized bone models. 
 Pro-angiogenic activities of CYR61 (CCN1) mediated through integrins alphavbeta3 and alpha6beta1 in human umbilical vein endothelial cells. CYR61 (CCN1) is an   extracellular matrix  -associated protein of the CCN family, which also includes CTGF (CCN2), NOV (CCN3), WISP-1 (CCN4), WISP-2 (CCN5), and WISP-3 (CCN6). Purified CYR61 induces neovascularization in corneal implants, and Cyr61-null mice suffer embryonic death due to vascular defects, thus establishing that CYR61 is an important regulator of angiogenesis. Aberrant expression of Cyr61 is associated with breast cancer, wound healing, and vascular diseases such as atherosclerosis and restenosis. In culture, CYR61 functions through integrin-mediated pathways to promote cell adhesion, migration, and proliferation. Here we show that CYR61 can also promote cell survival and tubule formation in human umbilical vein endothelial cells. Furthermore, we have dissected the integrin receptor requirements of CYR61 with respect to its pro-angiogenic activities. Thus, CYR61-induced cell adhesion and tubule formation occur through interaction with integrin alpha(6)beta(1) in early passage endothelial cells in which integrins have not been activated. By contrast, in endothelial cells in which integrins are activated by phorbol ester or vascular endothelial growth factor, CYR61-promoted cell adhesion, migration, survival, growth factor-induced mitogenesis, and endothelial tubule formation are all mediated through integrin alpha(v)beta(3). These findings indicate that CYR61 is an activation-dependent ligand of integrin alpha(v)beta(3) and an activation-independent ligand of integrin alpha(6)beta(1) and that these integrins differentially mediate the pro-angiogenic activities of CYR61. These findings help to define the mechanisms by which CYR61 acts as an angiogenic regulator, provide a molecular interpretation for the loss of vascular integrity and increased apoptosis of vascular cells in Cyr61-null mice, and underscore the importance of CYR61 in the development and homeostasis of the vascular system. 
 Histamine effects on endothelial cell fibronectin interaction studied by atomic force microscopy. Atomic force microscopy was used to investigate the cellular response to histamine, one of the major inflammatory mediators that cause endothelial hyperpermeability and vascular leakage. AFM probes were labeled with fibronectin and used to measure binding strength between alpha5beta1 integrin and fibronectin by quantifying the force required to break single fibronectin-integrin bonds. The cytoskeletal changes, binding probability, and adhesion force before and after histamine treatment on endothelial cells were monitored. Cell topography measurements indicated that histamine induces cell shrinkage. Local cell stiffness and binding probability increased twofold after histamine treatment. The force necessary to rupture single alpha5beta1-fibronectin bond increased from 34.0 +/- 0.5 pN in control cells to 39 +/- 1 pN after histamine treatment. Experiments were also conducted to confirm the specificity of the alpha5beta1-fibronectin interaction. In the presence of soluble GRGDdSP the probability of adhesion events decreased >50% whereas the adhesion force between alpha5beta1 and fibronectin remained unchanged. These data indicate that   extracellular matrix  -integrin interactions play an important role in the endothelial cell response to changes of external chemical mediators. These changes can be recorded as direct measurements on live endothelial cells by using atomic force microscopy. 
 Cdc42 promotes transendothelial migration of cancer cells through β1 integrin. Cancer cells interact with endothelial cells during the process of metastatic spreading. Here, we use a small interfering RNA screen targeting Rho GTPases in cancer cells to identify Cdc42 as a critical regulator of cancer cell-endothelial cell interactions and transendothelial migration. We find that Cdc42 regulates β1 integrin expression at the transcriptional level via the transcription factor serum response factor (SRF). β1 integrin is the main target for Cdc42-mediating interaction of cancer cells with endothelial cells and the underlyi  ng extracellular mat  rix, as exogenous β1 integrin expression was sufficient to rescue the Cdc42-silencing phenotype. We show that Cdc42 was required in vivo for cancer cell spreading and protrusion extension along blood vessels and retention in the lungs. Interestingly, transient Cdc42 depletion was sufficient to decrease experimental lung metastases, which suggests that its role in endothelial attachment is important for metastasis. By identifying β1 integrin as a transcriptional target of Cdc42, our results provide new insight into Cdc42 function. 
 Regulation of melanoma cell migration and invasion by laminin-5 and alpha3beta1 integrin (VLA-3). We evaluated the role of soluble factors produced from epidermal cells in melanoma cell motility by using the Boyden chamber chemoinvasion system. The migration of two melanoma cell lines, A375 and Mewo, was potentiated by conditioned media of A431 epidermoid cells in a concentration-dependent manner. The enhancement of A375 melanoma cell motility induced by the conditioned medium was blocked by antibodies against either alpha3 or beta1 integrin subunit. The motility-stimulating activity was recovered in the same fraction as the alpha3 integrin-dependent adhesion-promoting activity in a high-molecular-weight (>200 kDa) fraction on Superose 12 gel chromatography, and adsorbed with an anti-laminin-5 antibody. Purified laminin-5 was capable of potentiating melanoma cell migration as measured in either the chemotaxis assay with a soluble form of laminin-5 or the haptotaxis assay with membranes coated with a mixture of laminin-5 and Matrigel. Furthermore, immobilized laminin-5 induced A375 melanoma cells to secrete matrix metalloproteinase-9 (type IV collagenase) into the culture medium. These results strongly suggest that the interaction of laminin-5 produced in the epidermis with alpha3beta1 integrin on melanoma cells is involved in cell migration, invasion, and degradation of   extracellular matrix   proteins. 
 A novel fibronectin receptor with an unexpected subunit composition (alpha v beta 1). The integrins are a family of heterodimeric cell surface receptors for   extracellular matrix   molecules. An analysis of integrin subunits expressed by a number of cell lines identified a novel heterodimer. The alpha subunit of this integrin was immunologically and electrophoretically indistinguishable from the vitronectin receptor alpha subunit (alpha v) and the beta subunit was indistinguishable from beta 1. Affinity chromatography experiments and cell adhesion assays indicated that this receptor complex is a new fibronectin receptor. Its unexpected subunit composition demonstrates the importance of the beta subunit in determining the ligand specificity of integrins and suggests that the current integrin classification scheme needs revision. 
 A conserved interaction between beta1 integrin/PAT-3 and Nck-interacting kinase/MIG-15 that mediates commissural axon navigation in C. elegans. Integrins are heterodimeric (alphabeta) transmembrane receptors for   extracellular matrix   (ECM) ligands. Through interactions with molecular partners at cell junctions, they provide a connection between the ECM and the cytoskeleton and regulate many aspects of cell behavior. A number of integrin-associated molecules have been identified; however, in many cases, their function and role in the animal remain to be clarified.We have identified the Nck-interacting kinase (NIK), a member of the STE20/germinal center kinase (GCK) family, as a partner for the beta1A integrin cytoplasmic domain. We find that NIK is expressed in the nervous system and other tissues in mouse embryos and colocalizes with actin and beta1 integrin in cellular protrusions in transfected cells. To demonstrate the functional significance of this interaction, we used Caenorhabditis elegans, since it has only one beta (PAT-3) integrin chain, two alpha (INA-1 and PAT-2) integrin chains, and a well-conserved NIK ortholog (MIG-15). Using three methods, we show that reducing mig-15 activity results in premature branching of commissures. A significant aggravation of this defect is observed when mig-15 activity is compromised in a weak ina-1 background. Neuronal-specific RNA interference against mig-15 or pat-3 leads to similar axonal defects, thus showing that both mig-15 and pat-3 act cell autonomously in neurons. Finally, we show a genetic interaction between mig-15, ina-1, and genes that encode Rac GTPases.Using several models, we provide the first evidence that the kinase NIK and integrins interact in vitro and in vivo. This interaction is required for proper axonal navigation in C. elegans. 
 Galectin-8 binding to integrins inhibits cell adhesion and induces apoptosis. The interaction of cells with the   extracellular matrix   regulates cell adhesion, motility, growth, survival and differentiation through integrin-mediated signal transduction. Here we demonstrate that galectin-8, a secreted mammalian (beta)-galactoside binding protein, inhibits adhesion of human carcinoma (1299) cells to plates coated with integrin ligands, and induces cell apoptosis. Pretreatment of the cells with Mn(2+), which increases the affinity of integrins for their ligands, abolished the inhibitory effects of galectin-8. The inhibitory effects of galectin-8 were specific and were not mimicked by plant lectins or other galectins (galectin-1 and galectin-3). In accordance with its anti-adhesive effects, transfection of galectin-8 cDNA into 1299 cells significantly reduced (by 75%) colony formation, when compared to the number of colonies formed by cells transfected with an empty vector. Affinity chromatography over immobilized galectin-8 indicated that few membrane proteins interacted with galectin-8 in a sugar-dependent manner. Microsequencing and western immunoblotting revealed that (alpha)(3)(beta)(1 )integrin derived from 1299 as well as other cells (e.g. HeLa and human endothelial cells) is a major galectin-8 binding-protein. Furthermore, immunoprecipitation and immunohistochemical studies suggested that endogenous galectin-8, secreted from 1299 cells, forms complexes with (alpha)(3)(beta)(1) integrins expressed on the surface of 1299 cells. Galectin-8 also interacts with other members of the integrin family, like (alpha)(6)(beta)(1 )integrins. In contrast, galectin-8 only minimally interacts with (alpha)(4 )or (beta)(3 )integrins. We propose that galectin-8 is an integrin binding-protein that interacts to a different extent with several, but not all members of the integrin family. Binding of galectin-8 modulates integrin interactions with the   extracellular matrix   and thus regulates cell adhesion and cell survival. 
 ACAP4 protein cooperates with Grb2 protein to orchestrate epidermal growth factor-stimulated integrin β1 recycling in cell migration. ARF6 GTPase is an important regulator of membrane trafficking and actin-based cytoskeleton dynamics active at the leading edge of migrating cells. The integrin family heterodimeric transmembrane proteins serve as major receptors for   extracellular matri  x proteins, which play essential roles in cell adhesion and migration. Our recent proteomic analyses of ARF6 effectors have identified a novel ARF6 GTPase-activating protein, ACAP4, essential for EGF-induced cell migration. However, molecular mechanisms underlying ACAP4-mediated cell migration have remained elusive. Here, we show that ACAP4 regulates integrin β1 dynamics during EGF-stimulated cell migration by interaction with Grb2. Our biochemical study shows that EGF stimulation induces phosphorylation of tyrosine 733, which enables ACAP4 to bind Grb2. This interaction of ACAP4 with Grb2 regulates integrin β1 recycling to the plasma membrane. Importantly, knockdown of ACAP4 by siRNA or overexpression of ACAP4 decreased recycling of integrin β1 to the plasma membrane and reduced integrin-mediated cell migration. Taken together, these results suggest a novel function for ACAP4 in the regulation of cell migration through controlling integrin β1 dynamics. 
 The MSP receptor regulates alpha6beta4 and alpha3beta1 integrins via 14-3-3 proteins in keratinocyte migration. Growth factors, integrins, and the   extracellular matrix   (ECM) are known to play key roles in epidermal wound healing, although the interplay between these proteins is not fully understood. We show that growth factor macrophage stimulating protein (MSP)- and its receptor Ron-mediated PI3K activation in keratinocytes induces phosphorylation of both Ron and alpha6beta4 integrin at specific 14-3-3 binding sites. Consequently, a Ron/alpha6beta4 complex formed via 14-3-3 binding displaces alpha6beta4 from its location at hemidesmosomes (structures supporting cell adhesion) and relocalizes it to lamellipodia. Concomitant activation of alpha3beta1 and keratinocyte spreading/migration on laminin-5 occurs. Further, MSP-dependent beta4 tyrosine phosphorylation evokes p38 and NF-kappaB signaling required for keratinocyte wound closure. Based on these results, we propose a mechanism based on MSP-Ron-dependent phosphorylation and 14-3-3 association, whereby the function of alpha6beta4 switches from a mechanical adhesive device into a signaling component, and might be critically involved in human epidermal wound healing. 
 The involvement of integrin β1 signaling in the migration and myofibroblastic differentiation of skin fibroblasts on anisotropic collagen-containing nanofibers. Utilization of nanofibrous matrices for skin wound repair holds great promise due to their morphological and dimensional similarity to native   extracellular matri  x (ECM). It becomes highly desired to understand how various nanofibrous matrices regulate skin cell behaviors and intracellular signaling pathways, important to tuning the functionality of tissue-engineered skin grafts and affecting the wound healing process. In this study, the phenotypic expressions of normal human dermal fibroblasts (NHDFs) on collagen-containing nanofibrous matrices with either isotropic (i.e., fibers collected randomly with no alignment) or anisotropic (i.e., fibers collected with alignment) fiber organizations were studied by immunostaining, migration assay and molecular analyses. Results showed that both nanofibrous matrices supported the attachment and growth of NHDFs similarly, while showing different cell morphology with distinct variation in focal adhesion formation and distribution. Anisotropic nanofibers significantly triggered the integrin β1 signaling pathway in NHDFs as evidenced by an increase of active integrin β1 (130 kD mature form) and phosphorylation of focal adhesion kinase (FAK) at Tyr-397. Anisotropic matrices also promoted the migration of NHDFs along the fibers, while neutralization of the integrin β1 activity abolished this promotion. Moreover, the fibroblast-to-myofibroblast differentiation was greatly enhanced for the NHDFs cultured on anisotropic nanofibrous matrices over a period of 48 h. Inhibition of cellular integrin β1 activity by neutralizing antibody eliminated this enhancement. These findings suggest the important role of integrin β1 signaling pathway in regulating the nanofiber-induced fibroblast phenotypic alteration and providing insightful understanding of the possible application of collagen-containing nanofibrous matrices for skin regeneration. 
 Integrin alpha8beta1 regulates adhesion, migration and proliferation of human intestinal crypt cells via a predominant RhoA/ROCK-dependent mechanism. Integrins are transmembrane alphabeta heterodimer receptors that function as structural and functional bridges between the cytoskeleton and ECM (  extracellular matrix  ) molecules. The RGD (arginine-glycine-aspartate tripeptide motif)-dependent integrin alpha8beta1 has been shown to be involved in various cell functions in neuronal and mesenchymal-derived cell types. Its role in epithelial cells remains unknown.Integrin alpha8beta1 was found to be expressed in the crypt cell population of the human intestine but was absent from differentiating and mature epithelial cells of the villus. The function of alpha8beta1 in epithelial crypt cells was investigated at the cellular level using normal HIECs (human intestinal epithelial cells). Specific knockdown of alpha8 subunit expression using an shRNA (small-hairpin RNA) approach showed that alpha8beta1 plays important roles in RGD-dependent cell adhesion, migration and proliferation via a RhoA/ROCK (Rho-associated kinase)-dependent mechanism as demonstrated by active RhoA quantification and pharmacological inhibition of ROCK. Moreover, loss of alpha8beta1, through RhoA/ROCK, impairs FA (focal adhesion) complex integrity as demonstrated by faulty vinculin recruitment.Integrin alpha8beta1 is expressed in epithelial cells. In intestinal crypt cells, alpha8beta1 is closely involved in the regulation of adhesion, migration and cell proliferation via a predominant RhoA/ROCK-dependent mechanism. These results suggest an important role for this integrin in intestinal crypt cell homoeostasis. 
 Mechanisms involved in alpha6beta1-integrin-mediated Ca(2+) signalling. Contact of Jurkat T-lymphocytes with the   extracellular matrix   (ECM) protein laminin resulted in long-lasting alpha6beta1-integrin-mediated Ca(2+) signalling. Both Ca(2+) release from thapsigargin-sensitive Ca(2+) stores and capacitative Ca(2+) entry via Ca(2+) channels sensitive to SKF 96365 constitute important parts of this process. Inhibition of alpha6beta1-integrin-mediated Ca(2+) signalling by (1) the src kinase inhibitor PP2, (2) the PLC inhibitor U73122, and (3) the cyclic adenosine diphosphoribose (cADPR) antagonist 7-deaza-8-Br-cADPR indicate the involvement of src tyrosine kinases and the Ca(2+)-releasing second messengers D-myo-inositol 1,4,5-trisphosphate (InsP3) and cADPR. 
 Alpha5beta1 integrin engagement increases large conductance, Ca2+-activated K+ channel current and Ca2+ sensitivity through c-src-mediated channel phosphorylation. Large conductance, calcium-activated K(+) (BK) channels are important regulators of cell excitability and recognized targets of intracellular kinases. BK channel modulation by tyrosine kinases, including focal adhesion kinase and c-src, suggests their potential involvement in integrin signaling. Recently, we found that fibronectin, an endogenous alpha5beta1 integrin ligand, enhances BK channel current through both Ca(2+)- and phosphorylation-dependent mechanisms in vascular smooth muscle. Here, we show that macroscopic currents from HEK 293 cells expressing murine BK channel alpha-subunits (mSlo) are acutely potentiated following alpha5beta1 integrin activation. The effect occurs in a Ca(2+)-dependent manner, 1-3 min after integrin engagement. After integrin activation, normalized conductance-voltage relations for mSlo are left-shifted at free Ca(2+) concentrations >or=1 microm. Overexpression of human c-src with mSlo, in the absence of integrin activation, leads to similar shifts in mSlo Ca(2+) sensitivity, whereas overexpression of catalytically inactive c-src blocks integrin-induced potentiation. However, neither integrin activation nor c-src overexpression potentiates current in BK channels containing a point mutation at Tyr-766. Biochemical tests confirmed the critical importance of residue Tyr-766 in integrin-induced channel phosphorylation. Thus, BK channel activity is enhanced by alpha5beta1 integrin activation, likely through an intracellular signaling pathway involving c-src phosphorylation of the channel alpha-subunit at Tyr-766. The net result is increased current amplitude, enhanced Ca(2+) sensitivity, and rate of activation of the BK channel, which would collectively promote smooth muscle hyperpolarization in response to integrin-  extracellular matrix   interactions. 
 Autophagy modulates cell migration and β1 integrin membrane recycling. Cell migration is dependent on a series of integrated cellular events including the membrane recycling of the   extracellular matri  x receptor integrins. In this paper, we investigate the role of autophagy in regulating cell migration. In a wound-healing assay, we observed that autophagy was reduced in cells at the leading edge than in cells located rearward. These differences in autophagy were correlated with the robustness of MTOR activity. The spatial difference in the accumulation of autophagic structures was not detected in rapamycin-treated cells, which had less migration capacity than untreated cells. In contrast, the knockdown of the autophagic protein ATG7 stimulated cell migration of HeLa cells. Accordingly, atg3(-/-) and atg5(-/-) MEFs have greater cell migration properties than their wild-type counterparts. Stimulation of autophagy increased the co-localization of β1 integrin-containing vesicles with LC3-stained autophagic vacuoles. Moreover, inhibition of autophagy slowed down the lysosomal degradation of internalized β1 integrins and promoted its membrane recycling. From these findings, we conclude that autophagy regulates cell migration, a central mechanism in cell development, angiogenesis, and tumor progression, by mitigating the cell surface expression of β1 integrins. 
 PCTK1 regulates integrin-dependent spindle orientation via protein kinase A regulatory subunit KAP0 and myosin X. Integrin-dependent cell-  extracellular matrix   (ECM) adhesion is a determinant of spindle orientation. However, the signaling pathways that couple integrins to spindle orientation remain elusive. Here, we show that PCTAIRE-1 kinase (PCTK1), a member of the cyclin-dependent kinases (CDKs) whose function is poorly characterized, plays an essential role in this process. PCTK1 regulates spindle orientation in a kinase-dependent manner. Phosphoproteomic analysis together with an RNA interference screen revealed that PCTK1 regulates spindle orientation through phosphorylation of Ser83 on KAP0, a regulatory subunit of protein kinase A (PKA). This phosphorylation is dispensable for KAP0 dimerization and for PKA binding but is necessary for its interaction with myosin X, a regulator of spindle orientation. KAP0 binds to the FERM domain of myosin X and enhances the association of myosin X-FERM with β1 integrin. This interaction between myosin X-FERM and β1 integrin appeared to be crucial for spindle orientation control. We propose that PCTK1-KAP0-myosin X-β1 integrin is a functional module providing a link between ECM and the actin cytoskeleton in the ECM-dependent control of spindle orientation. 
 N-acetylglucosaminyltransferase V regulates extravillous trophoblast invasion through glycosylation of alpha5beta1 integrin. For successful human placentation, invasion of trophoblast cells into the uterus and its associated vasculature is essential, and the regulation of this process is controlled by many factors at the fetal-maternal interface. N-acetylglucosaminyltransferase V (GnT-V) is a key enzyme that catalyzes beta1, 6-N-acetylglucosamine (beta1-6GlcNAc) branching on asparagine-linked oligosaccharides of cell proteins. GnT-V and its product, beta1-6GlcNAc, are known to regulate cellular transformation and correlate with the metastatic potential of various cancer cells. The aim of the present study was to determine whether extravillous trophoblast (EVT) expressed this molecule and examine the role of GnT-V in the regulation of human trophoblast invasion. Immunohistochemistry showed that GnT-V was strongly expressed within the cytoplasm of EVT in the anchoring villi; this expression was down-regulated in EVTs invading the decidua. Suppression of beta1-6GlcNAc glycosylation by swainsonine enhanced the migratory potential and invasive capability of both primary EVTs and the EVT cell line, HTR-8/SVneo. Down-regulation of GnT-V expression by small interfering RNA in the choriocarcinoma cell line Jar consistently enhanced the migration and invasive capacity of these cells and elevated cellular adhesion to   extracellular matrix   proteins, such as fibronectin and collagen type I/IV. The extent of beta1-6 branching of alpha5beta1 integrin was significantly reduced in small interfering GnT-V-transfected Jar cells compared with mock transfectants, although the expression of alpha1, alpha5, alpha6, and beta1 integrin on the cell surface was not changed. These results suggest that GnT-V is expressed in human EVT and is involved in regulating trophoblast invasion through modifications of the oligosaccharide chains of alpha5beta1 integrin. 
 Stimulation of beta 1 integrin induces tyrosine phosphorylation of vascular endothelial growth factor receptor-3 and modulates cell migration. Interactions between integrins and tyrosine kinase receptors can modulate a variety of cell functions. We observed a cooperative interaction between the beta(1) integrin and vascular endothelial growth factor receptor-3 (VEGFR-3 or Flt4) that appeared to be required for cell migration. By using VEGFR-3-transfected 293 cells (293/VEGFR-3) or primary dermal microvascular endothelial cells (DMEC), we found that stimulation with either soluble or immobilized   extracellular matrix   (ECM) proteins, collagen or fibronectin (FN), resulted in the increased tyrosine phosphorylation of VEGFR-3 in the absence of a cognate ligand. This increased tyrosine phosphorylation of VEGFR-3 was diminished by pretreatment with a blocking antibody against the beta(1) integrin. Cross-linking with anti-beta(1) integrin antibody induced a similar degree of tyrosine phosphorylation of VEGFR-3. Stimulation with collagen or FN induced an association between beta(1) integrin and VEGFR-3 in both 293/VEGFR-3 and primary DMEC cells. Collagen or FN-induced tyrosine phosphorylation of VEGFR-3 was inhibited by treatment with cytochalasin D, an inhibitor of actin polymerization. Collagen or FN was able to induce the migration of 293/VEGFR-3 or DMEC cells to a limited extent. However, migration was dramatically enhanced when a gradient of the cognate ligand, VEGF-D, was added. VEGF-D failed to induce cell migration in the absence of ECM proteins. Introducing a mutation at the kinase domain of VEGFR-3 or treatment with blocking antibody against either VEGFR-3 or beta(1) integrin inhibited cell migration induced by ECM and VEGF-D, indicating that signals from both beta(1) integrin and VEGFR-3 are required for this cell function. 
 Protein kinase C epsilon mediates angiotensin II-induced activation of beta1-integrins in cardiac fibroblasts. Angiotensin II (AII) promotes cardiac fibrosis by increased   extracellular matrix   production and enhanced interaction of matrix proteins with their cellular receptors, including integrins. AII and other growth factors augment beta(1)-integrin-dependent adhesion and spreading by "inside-out signaling" without affecting the total number of integrin receptors. "Inside-out signaling" involves specific signaling pathways, including protein kinase C (PKC), leading to activation of beta1-integrins. In the present study we investigated the mechanisms involved in AII-increased adhesion of adult rat cardiac fibroblasts (CFBs), obtained from Sprague-Dawley rats, to collagen I mediated by beta1-integrin.Treatment of CFBs with AII induced a concentration-dependent increase in adhesion to collagen I (2.2-fold, p<0.01) within 3-6 h of treatment. This effect was mediated by beta1-integrin via the angiotensin AT1 receptor and was significantly reduced by protein kinase C inhibition. AII significantly induced phosphorylation of PKC epsilon (PKCepsilon), which is involved in beta1-integrin-dependent adhesion and motility, and phosphorylation of the cytoplasmatic tail of beta1-integrin at threonine 788/789, required for adhesion. Phosphorylation of beta1-integrin and an increase in adhesion was also induced by l-alpha-phosphatidylinositol-3,4,5-triphosphate (l-alpha-PIP3), an activator of endogenous PKCepsilon. AII failed to increase adhesion in myofibroblasts obtained from PKCepsilon (-/-) mice, but not in cells obtained from control mice. Co-immunoprecipitation and double immunofluorescence demonstrated that AII induced a close association of PKCepsilon with beta1-integrin in CFBs.The present study demonstrates that AII increased beta1-integrin-dependent adhesion to collagen I in cardiac fibroblasts by inside-out signaling via PKCepsilon and phosphorylation of the cytoplasmatic tail of the beta1-integrin. 
 Ionizing radiation induces up-regulation of functional beta1-integrin in human lung tumour cell lines in vitro. Cell-matrix interactions are in part mediated through the beta1-integrin pathway regulating cell survival, proliferation, adhesion and migration. This study was performed to elucidate alterations of expression of the beta1-integrin and its co-localized protein kinase, integrin-linked kinase (ILK), after exposure to ionizing radiation in two lung carcinoma cell lines in the presence or absence of different beta1-integrin-dependent matrix proteins.Exponentially growing A549 and SKMES1 cells grown on fibronectin, laminin, BSA or plastic were exposed to 2 Gy or 6 Gy. Besides colony formation assays (0.5-8 Gy) and immediate plating experiments, flow cytometry (for beta1-integrin) and immunoblotting (for beta1-integrin and ILK) were carried out to analyze the protein expression. The localization of both proteins plus filamentous (f-) actin was further examined by immunofluorescence staining and laser confocal scanning microscopy. Functionality of the beta1 receptor subunit after irradiation was investigated in adhesion assays.A549 and SKMES1 cells grown on fibronectin or laminin demonstrated a significant increase in cell survival after irradiation compared to cells grown on BSA or plastic. Immediate plating of cells after irradiation on fibronectin did not show an improved survival. Flow cytometric and Western blot data showed a dose- and matrix-dependent induction of beta1-integrin and ILK expression after irradiation within 48 h. Adhesion to fibronectin or laminin compared to BSA or plastic was increased by 10-fold after irradiation, demonstrating these specific cell surface receptors to be functional. The staining of beta1-integrin and ILK in A549 cells confirmed the radiation-induced up-regulation of both proteins. Additionally, beta1-integrin and ILK co-localized with accumulated actin fibers at the cytoplasmic face of the cell membrane at confined areas.Ionizing radiation strongly induced the expression of functional beta1-integrin and ILK in the two lung cancer cell lines, A549 and SKMES1, dependent on different matrices used. Additionally, the subcellular localization of both proteins was altered by irradiation, and the individual cellular radiosensitivity was reduced in the presence of an   extracellular matrix  . On the one hand, this may result in aggravated therapeutic approaches and on the other hand, cells could adhere more strongly in their environment by the increase in functional surface receptor density preventing metastasis. Concerning intravascular located tumour cells, beta1-integrin up-regulation might enable these cells to adhere to the endothelium, which represents a prerequisite for metastatic disease. Identification of such mechanisms will provide considerable insights into the understanding of tumorigenicity and metastatic phenotypes, possibly leading to new, optimized radiochemotherapeutic regimens. 
 Functional live-cell imaging demonstrates that beta1-integrin promotes type IV collagen degradation by breast and prostate cancer cells. The ability of tumor cells to adhere to, migrate on, and remodel extracellular matrices is mediated by cell surface receptors such as beta1-integrins. Here we conducted functional live-cell imaging in real time to investigate the effects of modulating beta1-integrin expression and function on proteolytic remodeling of the   extracellular matrix  . Human breast and prostate cancer cells were grown on reconstituted basement membrane containing a quenched fluorescent form of collagen IV. Generation of cleavage products and the resulting increases in fluorescence were imaged and quantified. Decreases in the expression and activity of beta1-integrin reduced digestion of quenched fluorescent-collagen IV by the breast and prostate cancer cells and correspondingly their invasion through and migration on reconstituted basement membrane. Decreased   extracellular matrix   degradation also was associated with changes in the constituents of proteolytic pathways: decreases in secretion of the cysteine protease cathepsin B, the matrix metalloproteinase (MMP)-13, and tissue inhibitors of metalloproteinases (TIMP)-1 and 2; a decrease in expression of MMP-14 or membrane type 1 MMP; and an increase in secretion of TIMP-3. This is the first study to demonstrate through functional live-cell imaging that downregulation of beta1-integrin expression and function reduces proteolysis of collagen IV by breast and prostate cancer cells. 
   Extracellular matrix   regulates endothelial functions through interaction of VEGFR-3 and integrin alpha5beta1. Endothelium   extracellular matrix   (ECM) interactions can provide distinct spatial and molecular signals which control cellular proliferation, migration, and differentiation. Here, we investigated the role of fibronectin (FN), a major ECM protein, on the functions of lymphatic endothelial cells (LEC). We observed that FN, the ligand for integrin alpha5beta1, selectively promoted the growth of LEC as compared with vitronectin (VN) in the presence of the ligand for vascular endothelial growth factor receptor 3 [VEGFR-3 (VEGF-C156S)]. Upon investigating the mechanisms whereby ECM components regulate VEGFR-3 signaling, we found that FN transactivated VEGFR-3 and significantly enhanced the phosphorylation of VEGFR-3 induced by VEGF-C156S as compared to VN. An enhanced association of the integrin subunit alpha5 or beta1 with VEGFR-3, after stimulation with VEGF-C156S, was observed by co-immunoprecipitation. While blockade of integrin alpha5beta1 inhibited the VEGF-C156S-induced phosphorylation of VEGFR-3, no similar effect was obtained by blocking integrin alphavbeta3. FN also protected the endothelial cells from serum deprivation-induced apoptosis. Moreover, while the specific PI3 kinase inhibitor, LY294002, abolished this FN-mediated cell survival, the MAPK kinase inhibitor, PD98059, had no significant effect. Furthermore, a dominant-negative mutant of VEGFR-3 (G857R) reduced VEGF-C156S or FN-mediated cell survival, as well as the activities of PI3 kinase/Akt. Our results indicate that integrin alpha5beta1 participates in the activation of both VEGFR-3 and its downstream PI3 kinase/Akt signaling pathway, which is essential for FN-mediated lymphatic endothelial cell survival and proliferation. 
 The muscle integrin binding protein (MIBP) interacts with alpha7beta1 integrin and regulates cell adhesion and laminin matrix deposition. Integrins are alphabeta transmembrane receptors that function in key cellular processes, including cell adhesion, differentiation, and   extracellular matrix   deposition through interactions with extracellular, membrane, and cytoplasmic proteins. We previously identified and cloned a muscle beta1 integrin cytoplasmic binding protein termed MIBP and found that the expression level of MIBP is critical in the decision-making process of terminal myogenic differentiation. We report here that MIBP interacts with the alpha7beta1 integrin but not the alpha5beta1 integrin in C2C12 myoblasts, suggesting an important role of integrin alpha chains in the regulation of the beta1-MIBP interaction. Furthermore, consistent with its selective binding activity toward the alpha7beta1 laminin receptor, we have found that overexpression of MIBP in C2C12 myoblasts resulted in a significant reduction of cell adhesion to laminin and inhibition of laminin matrix deposition. By contrast, neither cell adhesion to fibronectin nor fibronectin matrix deposition was significantly altered in cells overexpressing MIBP. Finally, we show that both the protein level and tyrosine phosphorylation of paxillin, a key signaling molecule involved in the cellular control of myogenic differentiation, are increased by MIBP. These results suggest that MIBP functions in the control of myogenic differentiation by regulating alpha7beta1 integrin-mediated cell interactions with laminin matrix and intracellular signaling through paxillin. 
 C-terminal COOH of integrin β1 is necessary for β1 association with the kindlin-2 adapter protein. Protein-protein interactions are driving forces in cellular processes. As a prime example, transmembrane integrins lin  k extracellular matr  ix and intracellular proteins, resulting in bidirectional signaling that regulates cell migration, proliferation, differentiation, and survival. Here we provide the first evidence that interaction between the integrin β1 cytoplasmic tail and kindlin-2, a member of a family of adapters implicated in human disease pathogenesis, is mainly governed by the β1 C-terminal carboxylate moiety and is required for laterality organ development in zebrafish. Affinity measurements indicate that this unusual protein-protein interaction mode is coordinated by a putative carboxylate-binding motif in the kindlin-2 FERM subdomain F3. Contrary to the C terminus of proteins that engage PDZ domains, the C-terminal three residues of β1, per se, do not contribute to kindlin-2 binding or to laterality organ development. Thus, by employing zebrafish as an in situ physiological tool to correlate protein structure and function, we have discovered an unexpected association chemistry between an integrin and a key adapter involved in integrin signaling. 
 Interleukin-1alpha enhances integrin alpha(6)beta(1) expression and metastatic capability of human pancreatic cancer. To investigate the mechanisms of metastasis formation in metastatic human pancreatic cancer, we examined the enhancement in integrin expression, and adherence to and invasiveness into   extracellular matrix   (ECM) proteins of human pancreatic cancer cells after exposure to interleukin (IL)-1alpha.Expression of IL-1 receptor type I (IL-1RI) and alterations in integrin subunits by IL-1alpha were examined by flow-cytometric analysis and by cellular enzyme-linked immunosorbent assay in four human pancreatic cancer cell lines (BxPC-3, PaCa-2, PANC-1, and SW1990), respectively. In addition, assays of cancer cell adhesion and invasion to ECM proteins were performed to investigate whether increased integrin expression affected the adhesive and invasive interaction between cancer cells and the putative integrin ECM ligands. Furthermore, immunohistochemistry was used to assess integrins and IL-1R1 expression in pancreatic tissues.In metastatic cancer cells, expression of the alpha(6) subunit was enhanced by IL-1alpha treatment. While metastatic cancer cells exhibited preferential adherence to and invasion into laminin, these properties were enhanced by IL-1alpha. The alpha(6) subunit and IL-1RI were strongly expressed in pancreatic tissues from pancreatic cancer patients with liver metastasis.In pancreatic cancer, IL-1alpha enhanced alpha(6)beta(1)-integrin expression, probably via increased IL-1RI levels. Our results indicated that alpha(6)beta(1)-integrin and IL-1RI expression may play important roles in metastasis formation. 
 MARVELD1 regulates integrin β1-mediated cell adhesion and actin organization via inhibiting its pre-mRNA processing. Cell adhesion on an   extracellular matri  x (ECM) participates in cell motility, invasion, cell signal transduction and gene expression. Many nuclear proteins regulate cell-ECM adhesion through managing the transcription of cell adhesion-related genes. Here, we identified MARVEL [MAL (The myelin and lymphocyte protein) and related proteins for vesicle trafficking and membrane link] domain containing 1 (MARVELD1) that could suppress cell spreading and complicate actin organization. Over-expression of MARVELD1 in NIH3T3 cells decreased the expression level of integrin β1 and vinculin, and further led to dephosphorylation of focal adhesion kinase (FAK) at Tyr 397. We also found that MARVELD1 partially colocalized with serine/arginine-rich splicing factor 2 (SC35) and interacted with nuclear cap binding protein subunit 2 (CBP20). Finally, we demonstrated that pre-mRNA processing of integrin β1 was affected by MARVELD1. Taken together, our studies demonstrate that MARVELD1 plays a role in pre-mRNA processing of integrin β1, and thereby regulates cell adhesion and cell motility. These studies provide a novel regulatory mechanism of cell-ECM adhesion by nuclear protein in cells. 
 Comparative proteomic analysis of supportive and unsupportive   extracellular matrix   substrates for human embryonic stem cell maintenance. Human embryonic stem cells (hESCs) are pluripotent cells that have indefinite replicative potential and the ability to differentiate into derivatives of all three germ layers. hESCs are conventionally grown on mitotically inactivated mouse embryonic fibroblasts (MEFs) or feeder cells of human origin. In addition, feeder-free culture systems can be used to support hESCs, in which the adhesive substrate plays a key role in the regulation of stem cell self-renewal or differentiation.   Extracellular matrix   (ECM) components define the microenvironment of the niche for many types of stem cells, but their role in the maintenance of hESCs remains poorly understood. We used a proteomic approach to characterize in detail the composition and interaction networks of ECMs that support the growth of self-renewing hESCs. Whereas many ECM components were produced by supportive and unsupportive MEF and human placental stromal fibroblast feeder cells, some proteins were only expressed in supportive ECM, suggestive of a role in the maintenance of pluripotency. We show that identified candidate molecules can support attachment and self-renewal of hESCs alone (fibrillin-1) or in combination with fibronectin (perlecan, fibulin-2), in the absence of feeder cells. Together, these data highlight the importance of specific ECM interactions in the regulation of hESC phenotype and provide a resource for future studies of hESC self-renewal. 
 Overexpression of integrin-β1 in leiomyoma promotes cell spreading and proliferation. Uterine leiomyoma, the most common tumors found in the women of the reproductive age, may cause abnormal uterine bleeding and be life threatening. Compared with myometrium, leiomyoma contains excessive   extracellular matri  x (ECM). However, the pathological roles of ECM in the development of leiomyoma remain largely unknown. Integrins are the major adhesion molecules on cell surface to interact with ECM. The interactions of ECM with integrins regulate cell adhesion and initiate signals for cell growth, differentiation, and migration.The aim of this study was to investigate the expression and functional role of integrin-β1 in leiomyoma pathogenesis.Levels of integrin-β1 protein were determined by Western blotting in paired normal and leiomyomal tissues (n = 15). Knockdown of integrin-β1 and inhibition of ECM-integrin interaction by disintegrin were used to evaluate the impact of integrin-β1 in cell adhesion, spreading, and proliferation.Levels of integrin-β1 were significantly up-regulated in leiomyomal cells compared with their normal counterparts. Knockdown of integrin-β1 did not affect cell adhesion on fibronectin or laminin matrix but significantly inhibits cell spreading ability. Consistent with this notion, the phosphorylation of focal adhesion kinase and the recruitment of paxillin to the focal contact were decreased in integrin-β1 knockdown cells, which attenuates contraction force. The inability of cell spreading leads to inhibition of cyclin D1 expression and impedes cell cycle progression. More importantly, disruption of ECM-integrin interaction by the small protein, disintegrin inhibited cyclin D1 expression and cell proliferation.These data demonstrate that integrin-β1 is a critical ligand to enhance cell-ECM contact force and thus promotes cell proliferation. Disruption of ECM-integrin-β1 signaling may serve as an option to inhibit the progression of leiomyoma. 
 Alpha2beta1-integrin signaling by itself controls G1/S transition in a human adenocarcinoma cell line (Caco-2): implication of NADPH oxidase-dependent production of ROS. In this work, we report that type IV collagen, mainly via alpha2beta1-integrin ligation, was able to induce cyclin expression and G1/S transition in a colic adenocarcinoma cell line (Caco-2) cultured without soluble growth factors or fetal bovine serum. This process involved Erk 1/2 activation and the production of reactive oxygen species (ROS) by a membrane-bound NADPH oxidase. Data presented here show that NADPH oxidase-dependent production of ROS increased following alpha2beta1-integrin ligation with type IV collagen or with a specific monoclonal antibody (Gi9 mAb). NADPH oxidase activation and, therefore, the production of ROS were shown to be involved in the increase of alpha2beta1-integrin plasma membrane expression, p38 MAPK phosphorylation, cyclin expression, and G1/S transition. We thus identified in this work a new integrin-signaling pathway in colon tumor cells involved in cell cycle regulation by the   extracellular matrix  . 
 Increased integrin alpha5beta1 heterodimer formation and reduced c-Jun expression are involved in integrin beta1 overexpression-mediated cell growth arrest. Integrins, heterodimers of alpha and beta subunits, are a family of cell surface molecules mediating cell-cell and cell-  extracellular matrix   interaction. The largest subgroup is formed by the beta(1) subunit containing integrins which consist of 12 members with different ligand-binding properties. We previously reported that overexpressed integrin beta(1) subunit in the hepatocellular carcinoma cell line SMMC-7721 imposed a growth inhibitory effect through the upregulation of p21(cip1) and p27(kip1). In this study, we confirmed the growth inhibitory effect of beta(1) subunit overexpression in different cancer cell lines. The upregulated CDK inhibitors induced by beta(1) integrin overexpression were essential for this integrin-mediated growth arrest. Reduced c-Jun level after integrin beta(1) overexpression plays an important role in the transcriptional activation of p21 through the Sp1 sites. Solely overexpressed beta(1) subunit could induce the expression of diverse alpha subunit in different cell lines, among which alpha(5) subunit was found to be correlated with integrin beta(1)-mediated growth arrest. Relative lack of ECM-integrin interaction might be a reason for integrin beta(1) overexpression-mediated growth arrest. These results helped us understand more about the mechanisms that integrins regulate cell growth. 
 C1GALT1 promotes invasive phenotypes of hepatocellular carcinoma cells by modulating integrin β1 glycosylation and activity. Cancer cell invasion and metastasis are the primary causes of treatment failure and death in hepatocellular carcinoma (HCC). We previously reported that core 1 β1,3-galactosyltransferase (C1GALT1) is frequently overexpressed in HCC tumors and its expression is associated with advanced tumor stage, metastasis, and poor survival. However, the underlying mechanisms of C1GALT1 in HCC malignancy remain unclear. In this study, we found that overexpression of C1GALT1 enhanced HCC cell adhesion t  o extracellular matr  ix (ECM) proteins, migration, and invasion, whereas RNAi-mediated knockdown of C1GALT1 suppressed these phenotypes. The promoting effect of C1GALT1 on the metastasis of HCC cells was demonstrated in a mouse xenograft model. Mechanistic investigations showed that the C1GALT1-enhanced phenotypic changes in HCC cells were significantly suppressed by anti-integrin β1 blocking antibody. Moreover, C1GALT1 was able to modify O-glycans on integrin β1 and regulate integrin β1 activity as well as its downstream signaling. These results suggest that C1GALT1 could enhance HCC invasiveness through integrin β1 and provide novel insights into the roles of O-glycosylation in HCC metastasis. 
 Alpha-toxin interferes with integrin-mediated adhesion and internalization of Staphylococcus aureus by epithelial cells. Staphylococcus aureus is an important human and animal pathogen. During infection, this bacterium is able to attach to and enter host cells by using its cell surface-associated factors to bind to the host's   extracellular matrix   (ECM) proteins. In this study, we determined that a protein exported by S. aureus, alpha-toxin, can interfere with the integrin-mediated adhesion and internalization of S. aureus by human lung epithelial cells (A549). The downregulation of alpha-toxin production significantly increased bacterial adhesion and invasion into the epithelial cells. In contrast, bacterial adhesion and invasion was inhibited by both overproduction of alpha-toxin and the addition of alpha-toxin to the culture medium. Moreover, our results showed that the quantitative effects on invasion closely parallel those of adherence. This suggests that the effect on invasion is probably secondary to, and a consequence of, the reduced adherence caused by alpha-toxin exposure. Specifically, we demonstrated that alpha-toxin interacts with the hosts' ECM protein's receptor, beta1-integrin, which indicates that beta1-integrin may be a potential receptor of alpha-toxin on epithelial cells. Taken together, our results indicate that exported alpha-toxin inhibits the adhesion and internalization of S. aureus by interfering with integrin-mediated pathogen-host cell interactions. 
 Differential regulation of actin stress fiber assembly and proplatelet formation by alpha2beta1 integrin and GPVI in human megakaryocytes. The actin cytoskeleton plays a major role in platelet function. In contrast, its precise role in the function of megakaryocytes (MKs) is less understood but may be important for a chemoattractive response and an efficient proplatelet formation. In the marrow microenvironment, mature MKs are in contact with the   extracellular matrix  , including fibrillar collagen type I. MKs express alpha2beta1 integrin and the immunoglobulin superfamily member glycoprotein VI (GPVI), the main receptors for collagen. Using function-blocking antibodies or specific ligands, we investigated in primary human MKs how alpha2beta1 integrin and GPVI regulate stress fiber formation, the primary actin structures needed for cell contraction. Stress fiber assembly requires synergistic activation of the MAPK/Erk1/2 pathway and the small guanosine triphosphatase Rho via its effector, Rho-associated coiled-coil kinase (ROCK). alpha2beta1 integrin is crucial for stress fiber formation, whereas GPVI triggers rapid and sustained activation of the Erk1/2 pathway. Strikingly, after a longer adhesion time, proplatelet formation was significantly inhibited by the engagement of alpha2beta1 integrin, not by GPVI, likely through the Rho/ROCK pathway. Thus, proplatelet formation in human MKs could be tightly regulated by differential interactions with their collagen receptors. We propose that this interaction with collagen prevents proplatelet formation within the marrow. 
 Effect of dexamethasone on human osteoblasts in culture: involvement of β1 integrin and integrin-linked kinase. Adhesive interactions play a critical role in cell biology, influencing vital processes from proliferation to cell death. Integrins regulate cell-ECM   (extracellular matri  x) adhesion and must associate with phosphorylating proteins such as ILK (integrin-linked kinase). Dysregulation of ILK expression is associated with anchorage-independent growth, cell survival and inhibition of apoptosis. Glucocorticoids influence differentiation and adhesion of osteoblasts and can affect bone protein synthesis. The objective of this study was to analyse the effect of DEX (dexamethasone) on the biology of osteoblasts, together with its influence on the expression of ILK and β1 integrin. For this, primary cultures of human osteoblasts were exposed to DEX at 10-9 M (physiological dose) and 10-6 M (pharmacological dose) for 24 and 48 h. Cell viability, apoptosis and cell adhesion were analysed, as well as protein expression of β1 integrin and ILK. It was observed that cell viability and adhesion were reduced in the cultures evaluated. In comparison with the control cultures, there was slightly less apoptosis in the cultures exposed to the physiological dose and considerably more apoptosis in those exposed to the pharmacological dose. In all treated cultures, protein expression of ILK was slightly higher than in the control cultures, whereas that of β1 integrin was significantly lower. Both proteins under study were co-localized at the cell periphery in all cultures. Our results suggest that DEX causes osteoblast anoikis, probably due to decreased β1 integrin expression, which might have had a direct influence upon ILK, reducing its activation and preventing it from playing its characteristic anti-apoptotic role. 
 Adhesion of ST6Gal I-mediated human colon cancer cells to fibronectin contributes to cell survival by integrin beta1-mediated paxillin and AKT activation. We have recently demonstrated that ionizing radiation (IR) of cells increased the expression of beta-galactoside alpha-(2,6)-sialyltransferase (ST6Gal I) and the level of glycoprotein sialylation, especially for the key adhesion molecule integrin beta1. In addition, ST6Gal I-mediated sialylation of integrin beta1 contributed to cell adhesion-mediated radioresistance in colon cancer cells. In this study, we examined IR-induced cell adhesion to the   extracellular matrix   and evaluated the role of integrin beta1-associated downstream signaling molecules, such as paxillin and AKT. IR exposure and ST6Gal I overexpression increased adhesion of SW480 colon cancer cells to fibronectin and contributed to cell survival through the activation of paxillin and AKT. In contrast, knockdown of ST6Gal I or paxillin reduced the level of radiation-induced cell adhesion and increased the level of cell death. These results suggest that integrin beta1 sialylation may play a critical role in promoting adhesion of cancer cells by integrin beta1-mediated paxillin and AKT activation. 
 Phosphatidylinositol mannoside from Mycobacterium tuberculosis binds alpha5beta1 integrin (VLA-5) on CD4+ T cells and induces adhesion to fibronectin. The pathological hallmark of the host response to Mycobacterium tuberculosis is the granuloma where T cells and macrophages interact with the   extracellular matrix   (ECM) to control the infection. Recruitment and retention of T cells within inflamed tissues depend on adhesion to the ECM. T cells use integrins to adhere to the ECM, and fibronectin (FN) is one of its major components. We have found that the major M. tuberculosis cell wall glycolipid, phosphatidylinositol mannoside (PIM), induces homotypic adhesion of human CD4+ T cells and T cell adhesion to immobilized FN. Treatment with EDTA and cytochalasin D prevented PIM-induced T cell adhesion. PIM-induced T cell adhesion to FN was blocked with mAbs against alpha5 integrin chain and with RGD-containing peptides. Alpha5beta1 (VLA-5) is one of two major FN receptors on T cells. PIM was found to bind directly to purified human VLA-5. Thus, PIM interacts directly with VLA-5 on CD4+ T lymphocytes, inducing activation of the integrin, and promoting adhesion to the ECM glycoprotein, FN. This is the first report of direct binding of a M. tuberculosis molecule to a receptor on human T cells resulting in a change in CD4+ T cell function. 
 The signaling network of transforming growth factor beta1, protein kinase Cdelta, and integrin underlies the spreading and invasiveness of gastric carcinoma cells. Integrin-mediated cell adhesion and spreading enables cells to respond to extracellular stimuli for cellular functions. Using a gastric carcinoma cell line that is usually round in adhesion, we explored the mechanisms underlying the cell spreading process, separate from adhesion, and the biological consequences of the process. The cells exhibited spreading behavior through the collaboration of integrin-  extracellular matrix   interaction with a Smad-mediated transforming growth factor beta1 (TGFbeta1) pathway that is mediated by protein kinase Cdelta (PKCdelta). TGFbeta1 treatment of the cells replated on   extracellular matrix   caused the expression and phosphorylation of PKCdelta, which is required for expression and activation of integrins. Increased expression of integrins alpha2 and alpha3 correlated with the spreading, functioning in activation of focal adhesion molecules. Smad3, but not Smad2, overexpression enhanced the TGFbeta1 effects. Furthermore, TGFbeta1 treatment and PKCdelta activity were required for increased motility on fibronectin and invasion through matrigel, indicating their correlation with the spreading behavior. Altogether, this study clearly evidenced that the signaling network, involving the Smad-dependent TGFbeta pathway, PKCdelta expression and phosphorylation, and integrin expression and activation, regulates cell spreading, motility, and invasion of the SNU16mAd gastric carcinoma cell variant. 
 A novel intracellular fibulin-1D variant binds to the cytoplasmic domain of integrin beta 1 subunit. Fibulin-1 is a member of a growing family of proteins that includes eight members and is involved in cellular functions such as adhesion, migration and differentiation. Fibulin-1 has also been implicated in embryonic development of the heart and neural crest-derived structures. It is an integral part of the   extracellular matrix   (ECM) and has been shown to bind to a multitude of ECM proteins. However, fibulin-1 was first identified as a protein purified from placental extracts that binds to the cytoplasmic domain of integrin β1. Human fibulin-1 is alternatively spliced into four different isoforms namely A-D. These isoforms share a common N-terminus sequence that contains a secretion sequence but differ in their carboxy-terminal fibulin-1 module. In this report we identify a new splice variant of fibulin-1 that differs from all other fibulin-1 variants in the N-terminus sequence and has a similar carboxy-terminus sequence as fibulin-1D. This variant that we named fibulin-1D prime (fibulin-1D') lacks a secretion sequence and the anaphlatoxin region of fibulin-1 variants. The protein has an apparent molecular weight of 70.5kDa. Herein we show that fibulin-1D' binds to the intracellular domain of integrin β1 as well as to integrin α5β1. The protein was localized intracellularly in CHO cells transfected with a pEF4 plasmid containing full-length coding sequence of fibulin-1D'. We also localized the protein in human placenta. We propose that the fibulin-1D' variant might play a role in early embryo development as well as in modulating integrin β1 functions including adhesion and motility. 
 VEGF-induced endothelial cell migration requires urokinase receptor (uPAR)-dependent integrin redistribution. Vascular endothelial growth factor (VEGF)-initiated angiogenesis requires coordinated proteolytic degradation of   extracellular matrix   provided by the urokinase plasminogen activator/urokinase receptor (uPA/uPAR) system and regulation of cell migration provided by integrin-matrix interaction. In this study, we investigated the mechanisms underlying the uPAR-dependent modulation of VEGF-induced endothelial migration.We used flow cytometry to quantify integrins at the cell surface. Stimulation of human and murine endothelial cells with VEGF resulted in internalization of α5β1-integrins. Micropatterning and immunocytochemistry revealed co-clustering of uPAR and α5β1-integrins and retrieval via clathrin-coated vesicles. It was also contingent on receptors of the low-density lipoprotein receptor (LDL-R) family. VEGF-induced integrin redistribution was inhibited by elimination of uPAR from the endothelial cell surface or by inhibitory peptides that block the uPAR-integrin interaction. Under these conditions, the migratory response of endothelial cells upon VEGF stimulation was impaired both in vitro and in vivo.The observations indicate that uPAR is an essential component of the network through which VEGF controls endothelial cell migration. uPAR is a bottleneck through which the VEGF-induced signal must be funnelled for both focused proteolytic activity at the leading edge and for redistribution of integrins. 
 Vesicle-associated membrane protein 2 mediates trafficking of alpha5beta1 integrin to the plasma membrane. Integrins are major receptors for cell adhesion to the   extracellular matrix   (ECM). As transmembrane proteins, the levels of integrins at the plasma membrane or the cell surface are ultimately determined by the balance between two vesicle trafficking events: endocytosis of integrins at the plasma membrane and exocytosis of the vesicles that transport integrins. Here, we report that vesicle-associated membrane protein 2 (VAMP2), a SNARE protein that mediates vesicle fusion with the plasma membrane, is involved in the trafficking of alpha5beta1 integrin. VAMP2 was present on vesicles containing endocytosed beta1 integrin. Small interfering RNA (siRNA) silencing of VAMP2 markedly reduced cell surface alpha5beta1 and inhibited cell adhesion and chemotactic migration to fibronectin, the ECM ligand of alpha5beta1, without altering cell surface expression of alpha2beta1 integrin or alpha3beta1 integrin. By contrast, silencing of VAMP8, another SNARE protein, had no effect on cell surface expression of the integrins or cell adhesion to fibronectin. In addition, VAMP2-mediated trafficking is involved in cell adhesion to collagen but not to laminin. Consistent with disruption of integrin functions in cell proliferation and survival, VAMP2 silencing diminished proliferation and triggered apoptosis. Collectively, these data indicate that VAMP2 mediates the trafficking of alpha5beta1 integrin to the plasma membrane and VAMP2-dependent integrin trafficking is critical in cell adhesion, migration and survival. 
 Molecular proximity of seprase and the urokinase-type plasminogen activator receptor on malignant melanoma cell membranes: dependence on beta1 integrins and the cytoskeleton. Previous studies have shown that several proteolytic enzymes are associated with membrane protrusions at the leading edge of migrating tumor cells. In this study we demonstrate that seprase and the urokinase plasminogen activator receptor (uPAR), co-localize in the plasma membrane of LOX malignant melanoma cells. Cells were labeled with fluorochrome-conjugated monoclonal antibodies (mAb) directed against seprase and uPAR. Proximity between these two molecules was detected with resonance energy transfer (RET) imaging, single-cell emission spectrophotometry, and single-cell excitation spectrophotometry. Significant RET signals were detected on LOX cells when adherent to uncoated and   extracellular matrix   (ECM)-coated surfaces. This indicates that seprase and uPAR are within approximately 7 nm in the plasma membrane of LOX cells. When LOX cells adhered to a 3D extracellular-like matrix, seprase-uPAR complexes were found to be associated with invadopodia. Further microscopy experiments demonstrated gelatinolytic activity, a functional attribute of seprase, in association with seprase-uPAR membrane domains. Formation of seprase-uPAR membrane complexes is dependent upon both the cytoskeleton and integrins. Specifically, the involvement of beta(1)-integrins was demonstrated by the inhibition of RET by an inhibitory anti-beta(1)-integrin mAb. Based on these findings, we speculate that formation of heterogeneous lytic domains in the invading membranes of LOX cells increases the efficiency of directed pericellular proteolysis. 
 Tetraspanin CD9 is associated with very late-acting integrins in human vascular smooth muscle cells and modulates collagen matrix reorganization. CD9, a member of the tetraspanin family, and very late-acting (VLA) integrins are known to associate and form functional units on the surface of several cell types. We studied the changes in expression of CD9 and beta1-integrins (CD29, VLA) in human vascular smooth muscle cells (VSMCs) under in vitro culture conditions mimicking proliferative vascular diseases. We also investigated possible interactions between CD9 and VLA integrins in VSMCs. We found that CD9 is highly expressed in VSMCs and is subject to modulation, depending on the proliferative/contractile state of the cells. In the contractile phenotype, the levels of CD9, CD81, another tetraspanin, and CD29 are approximately 50% of those found in the proliferative phenotype. Coimmunoprecipitation experiments showed physical association between CD9 and CD29. CD9 was mainly associated with alpha2 and alpha3-integrins (CD49b and c) and also with alpha5-integrin to a weaker extent. Functionally, the addition of anti-CD9 monoclonal antibodies (MoAbs) doubled the extent of collagen gel contraction mediated by VSMCs, a model for the reorganization of the extracellular collagen matrix occurring in the vessel wall. Anti-CD29 MoAbs inhibited gel contraction, but anti-CD9 MoAbs counteracted this inhibitory effect of anti-CD29 MoAbs. Transfection of human CD9 into Chinese hamster ovary cells more than doubled the extent of Chinese hamster ovary cell-mediated collagen gel contraction (130% stimulation), confirming a role for CD9 in   extracellular matrix   reorganization. Thus, CD9 seems to be involved in the modulation of VLA integrin-mediated collagen matrix reorganization by VSMCs. These findings suggest that high CD9 expression is associated with a proliferative state of VSMCs. The role of CD9 could be to modulate the function of VLA integrins on the surface of VSMCs. 
 ß1 integrin binding phosphorylates ezrin at T567 to activate a lipid raft signalsome driving invadopodia activity and invasion.   Extracellular matri  x (ECM) degradation is a critical process in tumor cell invasion and requires matrix degrading protrusions called invadopodia. The Na(+)/H(+) exchanger (NHE1) has recently been shown to be fundamental in the regulation of invadopodia actin cytoskeleton dynamics and activity. However, the structural link between the invadopodia cytoskeleton and NHE1 is still unknown. A candidate could be ezrin, a linker between the NHE1 and the actin cytoskeleton known to play a pivotal role in invasion and metastasis. However, the mechanistic basis for its role remains unknown. Here, we demonstrate that ezrin phosphorylated at T567 is highly overexpressed in the membrane of human breast tumors and positively associated with invasive growth and HER2 overexpression. Further, in the metastatic cell line, MDA-MB-231, p-ezrin was almost exclusively expressed in invadopodia lipid rafts where it co-localized in a functional complex with NHE1, EGFR, ß1-integrin and phosphorylated-NHERF1. Manipulation by mutation of ezrins T567 phosphorylation state and/or PIP2 binding capacity or of NHE1s binding to ezrin or PIP2 demonstrated that p-ezrin expression and binding to PIP2 are required for invadopodia-mediated ECM degradation and invasion and identified NHE1 as the membrane protein that p-ezrin regulates to induce invadopodia formation and activity. 
 The androgen receptor induces integrin α6β1 to promote prostate tumor cell survival via NF-κB and Bcl-xL Independently of PI3K signaling. Recent studies indicate that androgen receptor (AR) signaling is critical for prostate cancer cell survival, even in castration-resistant disease wherein AR continues to function independently of exogenous androgens. Integrin-mediated adhesion to t  he extracellular mat  rix is also important for prostate cell survival. AR-positive prostate cancer cells express primarily integrin α6β1 and adhere to a laminin-rich matrix. In this study, we show that active nuclear-localized AR protects prostate cancer cells from death induced by phosphoinositide 3-kinase (PI3K) inhibition when cells adhere to laminin. Resistance to PI3K inhibition is mediated directly by an AR-dependent increase in integrin α6β1 mRNA transcription and protein expression. Subsequent signaling by integrin α6β1 in AR-expressing cells increased NF-κB activation and Bcl-xL expression. Blocking AR, integrin α6, NF-κB, or Bcl-xL concurrent with inhibition of PI3K was sufficient and necessary to trigger death of laminin-adherent AR-expressing cells. Taken together, these results define a novel integrin-dependent survival pathway in prostate cancer cells that is regulated by AR, independent of and parallel to the PI3K pathway. Our findings suggest that combined targeting of both the AR/α6β1 and PI3K pathways may effectively trigger prostate cancer cell death, enhancing the potential therapeutic value of PI3K inhibitors being evaluated in this setting. 
 Colorectal Cancer Metastases Settle in the Hepatic Microenvironment Through α5β1 Integrin. Colorectal cancer (CRC) metastasis dissemination to secondary sites represents the critical point for the patient's survival. The microenvironment is crucial to cancer progression, influencing tumour cell behaviour by modulating the expression and activation of molecules such as integrins, the cel  l-extracellular matr  ix interacting proteins participating in different steps of the tumour metastatic process. In this work, we investigated the role of α5β1 integrin and how the microenvironment influences this adhesion molecule, in a model of colon cancer progression to the liver. The culture medium conditioned by the IHH hepatic cell line, and   the extracellular ma  trix (ECM) proteins, modulate the activation of α5β1 integrin in the colon cancer cell line HCT-116, and drives FAK phosphorylation during the process of cell adhesion to fibronectin, one of the main components of liver ECM. In these conditions, α5β1 modulates the expression/activity of another integrin, α2β1, involved in the cell adhesion to collagen I. These results suggest that α5β1 integrin holds a leading role in HCT-116 colorectal cancer cells adhesion to the ECM through the modulation of the intracellular focal adhesion kinase FAK and the α2β1 integrin activity. The driving role of the tumour microenvironment on CRC dissemination, here detected, and described, strengthens and adds new value to the concept that α5β1 integrin can be an appropriate and relevant therapeutic target for the control of CRC metastases. 
 Differential induction of gelatinase B (MMP-9) and gelatinase A (MMP-2) in T lymphocytes upon alpha(4)beta(1)-mediated adhesion to VCAM-1 and the CS-1 peptide of fibronectin. Integrin alpha(4)beta(1) on the surface of T lymphocytes interacts with vascular cell adhesion molecule-1 (VCAM-1) and fibronectin during migration of lymphocytes from the blood to sites of inflammation. Migrating lymphocytes actively modify their environment through a number of mechanisms including proteolysis of the   extracellular matrix   by matrix metalloproteinases (MMP) synthesized by the cells. In this study, expression of MMP upon alpha(4)beta(1)-mediated adhesion of leukocytes to two major ligands, the IIICS-1 domain of fibronectin and VCAM-1, has been examined. Adhesion of T lymphoblastoid Jurkat cells to the CS-1 peptide induced expression of mRNA for two MMPs, gelatinase A (MMP-2) and gelatinase B (MMP-9). As evaluated by relative RT-PCR and Northern blot analyses, the level of mRNA was upregulated about 4- to 5-fold for both MMPs compared to control cells maintained in suspension. With time, both enzymes were detected in conditioned media and inside the cells, and their identities were verified by Western blotting and gelatin zymography. Adhesion of Jurkat cells to the second major alpha(4)beta(1) ligand, VCAM-1, upregulated mRNA for MMP-2 (3.5-fold) and failed to induce expression of mRNA for MMP-9. Accordingly, only MMP-2 protein was detected in conditioned media of cells adherent to VCAM-1. Occupancy of alpha(4)beta(1) on the surface of suspended cells with soluble CS-1 peptide or VCAM-1 did not upregulate synthesis and release of MMPs. A similar pattern of induction of MMPs after adhesion to CS-1 and VCAM-1 was observed in T lymphocytes isolated from human blood. These results demonstrate that adhesion of T lymphocytes through alpha(4)beta(1) to different ligands, which bind to similar or overlapping sites in the integrin, induces intracellular events leading to distinct patterns of MMPs biosynthesis. 
 Adhesion and protease activity in cell lines from human salivary gland tumors are regulated by the laminin-derived peptide AG73, syndecan-1 and beta1 integrin. We studied the induction of protease activity by the laminin alpha1-derived peptide AG73 in cells from adenoid cystic carcinoma (CAC2) and myoepithelioma (M1), respectively a malignant and a benign salivary gland tumors. Laminin alpha1 chain and MMP9 were immunolocalized in adenoid cystic carcinoma and myoepithelioma in vivo and in vitro. Cells grown inside AG73-enriched laminin-111 exhibited large spaces in the   extracellular matrix  , suggestive of remodeling. The broad spectrum MMP inhibitor GM6001 decreased spaces induced by AG73 in CAC2 and M1 cells. This result strongly suggests that AG73-mediated matrix remodeling involves matrix metalloproteinases. CAC2 and M1 cells cultured on AG73 showed a dose-dependent increase of MMP9 secretion, as detected by zymography. Furthermore, siRNA silencing of MMP9 decreased remodeling in 3D cultures. We searched for AG73 receptors regulating MMP9 activity in our cell lines. CAC2 and M1 cells grown on AG73 exhibited colocalization of syndecan-1 and beta1 integrin. siRNA knockdown of syndecan-1 expression in these cells resulted in decreased adhesion to AG73 and reduced protease and remodeling activity. We investigated syndecan-1 co-receptors in both cell lines. Silencing beta1 integrin inhibited adhesion to AG73, matrix remodeling and protease activity. Double-knockdown experiments were carried out to further explore syndecan-1 and beta1 integrin cooperation. CAC2 cells transfected with both syndecan-1 and beta1 integrin siRNA oligos showed significant decrease in adhesion to AG73. Simultaneous silencing of receptors also induced a decrease in protease activity. Our results suggest that syndecan-1 and beta1 integrin signaling downstream of AG73 regulate adhesion and MMP production by CAC2 and M1 cells. 
 Vascular endothelial growth factor receptor-1 is deposited in the   extracellular matrix   by endothelial cells and is a ligand for the alpha 5 beta 1 integrin. Vascular endothelial growth factor receptor-1 (VEGFR-1) is a tyrosine kinase receptor for several growth factors of the VEGF family. Endothelial cells express a membrane-spanning form of VEGFR-1 and secrete a soluble variant of the receptor comprising only the extracellular region. The role of this variant has not yet been completely defined. In this study, we report that the secreted VEGFR-1 is present within the   extracellular matrix   deposited by endothelial cells in culture, suggesting a possible involvement in endothelial cell adhesion and migration. In adhesion assays, VEGFR-1 extracellular region specifically promoted endothelial cell attachment. VEGFR-1-mediated cell adhesion was divalent cation-dependent, and inhibited by antibodies directed against the alpha 5 beta 1 integrin. Moreover, VEGFR-1 promoted endothelial cell migration, and this effect was inhibited by anti-alpha 5 beta 1 antibodies. Direct binding of VEGFR-1 to the alpha 5 beta 1 integrin was also detected. Finally, binding to VEGFR-1 initiated endothelial cell spreading. Altogether these results indicate that the soluble VEGFR-1 secreted by endothelial cells becomes a matrix-associated protein that is able to interact with the alpha 5 beta 1 integrin, suggesting a new role of VEGFR-1 in angiogenesis, in addition to growth factor binding. 
 Mac-2 binding protein is a cell-adhesive protein of the   extracellular matrix   which self-assembles into ring-like structures and binds beta1 integrins, collagens and fibronectin. Human Mac-2 binding protein (M2BP) was prepared in recombinant form from the culture medium of 293 kidney cells and consisted of a 92 kDa subunit. The protein was obtained in a native state as indicated by CD spectroscopy, demonstrating alpha-helical and beta-type structure, and by protease resistance and immunological analysis. It was highly modified by N- and O-glycosylation but not by glycosaminoglycans. Ultracentrifugation showed non-covalent association into oligomers with molar masses of 1000-1500 kDa. Electron microscopy showed ring-like shapes with diameters of 30-40 nm. M2BP bound in solid-phase assays to collagens IV, V and VI, fibronectin and nidogen, but not to fibrillar collagens I and III or other basement membrane proteins. The protein also mediated adhesion of cell lines at comparable strength with laminin. Adhesion to M2BP was inhibited by antibodies to integrin beta1 subunits but not to alpha2 and alpha6 subunits, RGD peptide or lactose. This distinguishes cell adhesion of M2BP from that of laminin and excludes involvement of lactose-binding galectin-3. Immunological assays demonstrated variable secretion by cultured human cells of M2BP, which was detected in the   extracellular matrix   of several mouse tissues. 
 Melanoma cells produce multiple laminin isoforms and strongly migrate on α5 laminin(s) via several integrin receptors. Melanoma cells express and interact with laminins (LMs) and other basement membrane components during invasion and metastasis. In the present study we have investigated the production and migration-promoting activity of laminin isoforms in melanoma. Immunohistochemistry of melanoma specimens and immunoprecipitation/western blotting of melanoma cell lines indicated expression of laminin-111/121, laminin-211, laminin-411/421, and laminin-511/521. Laminin-332 was not detected. In functional assays, laminin-111, laminin-332, and laminin-511, but not laminin-211 and laminin-411, strongly promoted haptotactic cell migration either constitutively or following stimulation with insulin-like growth factors. Both placenta and recombinant laminin-511 preparations were highly active, and the isolated recombinant IVa domain of LMα5 also promoted cell migration. Function-blocking antibodies in cell migration assays revealed α6β1 integrin as the major receptor for laminin-111, and both α3β1 and α6β1 integrins for laminin-332 and laminin-511. In contrast, isolated LMα5 IVa domain-promoted melanoma cell migration was largely mediated via αVβ3 integrin and inhibited by RGD peptides. Given the ubiquitous expression of α5 laminins in melanoma cells and in melanoma-target tissues/anatomical structures, as well as the strong migration-promoting activity of these laminin isoforms, the α5 laminins emerge as puta  tive primary extrace  llular matrix mediators of melanoma invasion and metastasis via α3β1 and other integrin receptors. 
 A lethal perinatal cardiac phenotype resulting from altered integrin function in cardiomyocytes. Integrins are heterodimeric receptors that couple the   extracellular matrix   to intracellular signaling pathways and the cyoskeleton. Integrins are strain transducers and candidates for modulators or effectors of cardiac hypertrophy.To begin to probe this function, we have transgenically expressed a chimeric protein that alters integrin function in cardiomyocytes. The transgene (Tac-beta(1D)) consists of the biologically inert extracellular and transmembrane domain of the interleukin-2 receptor alpha subunit (Tac) fused to the cytoplasmic tail of the human beta(1D) integrin driven by the cardiac alpha-myosin heavy chain promoter. Transgene expression results in a severe, usually fatal, perinatal cardiac phenotype, characterized by initial electrocardiographic abnormalities followed by extensive myocyte loss, macrophage infiltration, and replacement fibrosis.Expression of Tac-beta(1D) resulted in displacement of endogenous beta(1D) integrin from Z-lines and T-tubules, decreased expression of endogenous beta(1D), and disrupted the fibronectin pericellular matrix. These results are consistent with an essential role for beta(1) integrins in maintenance of cardiomyocyte viability and interaction with   extracellular matrix  .The appearance of conduction abnormalities before morphologic changes suggests that integrins are important in the development or maintenance of the conducting system of the heart. 
 Fas-mediated apoptosis is regulated by the   extracellular matrix   protein CCN1 (CYR61) in vitro and in vivo. Although Fas ligand (FasL) is primarily expressed by lymphoid cells, its receptor Fas (CD95/Apo-1) is broadly expressed in numerous nonlymphoid tissues and can mediate apoptosis of parenchymal cells upon injury and infiltration of inflammatory cells. Here we show that CCN1 (CYR61) and CCN2 (CTGF), matricellular proteins upregulated at sites of inflammation and wound repair, synergize with FasL to induce apoptosis by elevating cellular levels of reactive oxygen species (ROS). CCN1 acts through engagement of integrin alpha(6)beta(1) and cell surface heparan sulfate proteoglycans, leading to ROS-dependent hyperactivation of p38 mitogen-activated protein kinase in the presence of FasL to enhance mitochondrial cytochrome c release. We show that CCN1 activates neutral sphingomyelinase, which functions as a key source of CCN1-induced ROS critical for synergism with FasL. Furthermore, Fas-dependent hepatic apoptosis induced by an agonistic monoclonal anti-Fas antibody or intragastric administration of alcohol is severely blunted in knock-in mice expressing an apoptosis-defective Ccn1 allele. These results demonstrate that CCN1 is a physiologic regulator of Fas-mediated apoptosis and that the   extracellular matrix   microenvironment can modulate Fas-dependent apoptosis through CCN1 expression. 
 Potentiation of cell invasion and matrix metalloproteinase production by alpha3beta1 integrin-mediated adhesion of gastric carcinoma cells to laminin-5. We previously reported that the adhesion of gastric carcinoma cells to the peritoneum mediated by the alpha3beta1 integrin-laminin interaction is a key step in the initial process of peritoneal metastatic dissemination. Carcinoma cells subsequently invade through the intercellular gaps of mesothelial linings. In this study, we examined the role of the interaction of carcinoma cells with laminin-5, which is a major component of submesothelial basement membranes and serves as a high-affinity ligand for alpha3beta1 integrin, in carcinoma cell invasion. Human gastric carcinoma cell lines (MKN1, GT3TKB, and NUGC-4) adhered in an alpha3beta1 integrin-dependent manner to the   extracellular matrix   deposited by peritoneal mesothelial cells. An in vitro invasion assay using the Boyden chamber system revealed that MKN1 cell migration through the membranes increased when the membranes were coated with matrices produced by mesothelial cells or with laminin-5-containing Matrigel as compared to Matrigel alone. The cell migration promoted by laminin-5-containing Matrigel was inhibited by the presence of anti-alpha3 integrin antibody. When MKN1 cells were cultured in a laminin-5-coated plate, these cells were promoted to produce matrix metalloproteinase (MMP)-9, as assessed by gelatin zymography, enzyme-linked immunosorbent assay, and reverse transcription-polymerase chain reaction. These results suggest that the production of MMP-9 by MKN1 cells was potentiated by the alpha3beta1 integrin-laminin-5 interaction, which facilitated their invasion via degradation of the matrix. 
 Metastasis suppressor KAI1/CD82 attenuates the matrix adhesion of human prostate cancer cells by suppressing fibronectin expression and β1 integrin activation. KAI1/CD82, a tetraspanin membrane protein functions as a metastasis suppressor in many types of human cancers and has been shown to regulate cell adhesion properties. In the present study, we investigated the underlying mechanism of KAI1/CD82-mediated changes in cell adhesion to the   extracellular matri  x using human prostate cancer cells. We found that high KAI1/CD82 expression attenuated short-term cell adhesion to uncoated- or fibronectin-coated plates. Moreover, high KAI1/CD82 expression generated an extracellular environment unfavorable for cell adhesion as compared to low KAI1/CD82 expression, suggesting KAI1/CD82-dependent regulation of   extracellular matri  x (ECM) molecule(s) expression and/or secretion. Among ECM components examined, fibronectin exhibited decreased expression and secretion in high KAI1/CD82-expressing cells. Furthermore, high KAI1/CD82 expression interfered with the activation of β (1) integrin at the cell surface while total β (1) integrin levels remained unchanged, concomitant with reduced formation of focal adhesion complex and decreased bundling of actin filaments. Finally, high KAI1/CD82 expression significantly retarded cell motility in a scratch wound assay. Taken together, our results strongly suggest that KAI1/CD82 attenuates the activation of β (1) integrin, and thereby down-regulates outside-in signaling of β (1) integrin, leading to the reduction of focal adhesion formation and fibronectin expression/secretion, which subsequently interferes with cell adhesion properties and motility. 
 The cAMP-Epac-Rap1 pathway regulates cell spreading and cell adhesion to laminin-5 through the alpha3beta1 integrin but not the alpha6beta4 integrin. Laminin-5 is an important constituent of the basal lamina. The receptors for laminin-5, the integrins alpha3beta1 and alpha6beta4, have been associated with epithelial wound migration and carcinoma invasion. The signal transduction mechanisms that regulate these integrins are not well understood. We report here that the small GTPase Rap1 regulates the adhesion of a number of cell lines to various   extracellular matrix   proteins including laminin-5. cAMP also mediates cell adhesion and spreading on laminin-5, a process that is independent of protein kinase A but rather dependent on Epac1, a cAMP-dependent exchange factor for Rap. Interestingly, although both alpha3beta1 and alpha6beta4 mediate adhesion to laminin-5, only alpha3beta1-dependent adhesion is dependent on Rap1. These results provide evidence for a function of the cAMP-Epac-Rap1 pathway in cell adhesion and spreading on different   extracellular matrix   proteins. They also define different roles for the laminin-binding integrins in regulated cell adhesion and subsequent cell spreading. 
 Human trophoblast cell adhesion to   extracellular matrix   protein, entactin. Trophoblast interaction with endometrial   extracellular matrix   (ECM) is crucial during human embryo implantation and placentation. Entactin, a ubiquitous basement membrane glycoprotein, plays a central role in ECM assembly, cell attachment, and chemotaxis. The present study was conducted to examine the possible role of entactin in promoting human trophoblast adhesion.Using an extended life span first trimester trophoblast cell line HTR-8/SVneo (HTR) and a cell adhesion assay, we measured the adherence of human first trimester trophoblasts to recombinant entactin and its domains. Also, we used flow cytometry and indirect immunofluorescence to detect the presence of integrins that may be involved in human trophoblast-entactin interaction; these methods were used to analyze HTR cells, as well as tissue sections and freshly isolated human trophoblasts from first trimester and term placenta.We found that first trimester trophoblast cells were highly adherent to entactin and its E and G2 domains but not to G1 or G3 domains. Using indirect immunofluorescence and flow cytometry, we found that both beta 1 and beta 3 integrin subunits were expressed on the surface of HTR trophoblast cells adhering to entactin; in contrast, beta 2 and beta 4 integrin subunits were not detected. In addition, we found that alpha v beta 3 was expressed on freshly isolated villous cytotrophoblasts and cytotrophoblast and syncytiotrophoblasts in tissue sections from term placenta. The beta 3 integrin subunit was expressed in cytotrophoblasts and syncytiotrophoblasts in villi of first trimester placental tissue sections.Recombinant entactin promotes human trophoblast cell adhesion through both its E and G2 domains and these specific adhesive interactions may be mediated by beta 1 and/or beta 3 class integrins. 
 Activation signal transduction by beta1 integrin in T cells from patients with systemic lupus erythematosus. Beta 1 integrin is a representative adhesion molecule for cell-cell and cell-  extracellular matrix   interactions, and it provides costimulatory signals to T cells. However, the relevance of beta1 integrin to T cell activation in systemic lupus erythematosus (SLE) remains unclear. We undertook this study to perform a quantitative and functional analysis of beta1 integrin-mediated signaling to T cells in patients with SLE.Expression of cell surface molecules was assessed by flow cytometric analysis. Engagement of beta1 integrins was performed by crosslinking using a specific monoclonal antibody. To assess tyrosine kinases in beta1 integrin-mediated signaling, the cells were transfected with a wild-type (WT) focal adhesion kinase (FAK), a dominant-negative truncation of the FAK, or a WT PTEN expression plasmid via nucleofection.Beta 1 integrin expression was significantly up-regulated on peripheral blood T cells from patients with active SLE, particularly those with the complication of World Health Organization class IV nephritis, whereas CD28 was significantly decreased in patients with active SLE compared with normal individuals. Beta 1 integrin expression closely correlated with serum hypocomplementemia. Engagement of beta1 integrin on T cells from patients with active SLE, but not on those from normal individuals, induced cell proliferation as well as CD40L expression on T cells. Up-regulation of CD40L expression and T cell proliferation, induced by beta1 integrin stimulation, were completely inhibited by transfection of the dominant-negative truncations of FAK or WT PTEN.These results suggest that engagement of beta1 integrins on SLE T cells could induce FAK-mediated signaling and subsequent CD40L expression and proliferation. Thus, the beta1 integrin signaling cascade might serve to enhance autoreactive T cell activation. 
 Inhibition of IL-8-mediated MAPK activation in human neutrophils by beta1 integrin ligands. Chemokine and integrin receptors must work in concert when circulating leukocytes mobilize toward a site of tissue inflammation or infection. In a previous study, we reported that ligation of the alpha5beta1 integrin with a 120-kDa cell-binding fibronectin fragment (120-kDa FN) in suspensions of human polymorphonuclear leukocytes (PMNLs) inhibited chemotaxis toward the chemokine called interleukin-8 (IL-8). Binding of chemokines to their receptors on leukocytes leads to the activation of heterotrimeric G proteins that initiate multiple signaling cascades, including p38 and p42/p44 mitogen-activated protein kinase (MAPK) pathways. In the present study, we examine the potential interaction of beta1 integrin ligation on chemokine-mediated MAPK signaling in human PMNLs. We demonstrate that blockade of the p42/p44 MAPK signaling pathway by the inhibitor PD98059 suppresses IL-8-mediated PMNL chemotaxis. Furthermore, when PMNLs are pretreated with 120-kDa FN or an activating antibody to beta1 integrins (TS2/16), IL-8-mediated phosphorylation of p42/p44 MAPK is also inhibited. In contrast, pretreating PMNL with a specific ligand (laminin-1) for the alpha6beta1 integrin does not suppress IL-8-mediated phosphorylation of p42/p44 MAPK. These observations demonstrate a desensitization of IL-8-mediated p42/p44 MAPK signaling in response to ligation of the alpha5beta1 integrin in PMNL. Also, they suggest an interplay between integrin and chemokine signaling during PMNL migration through the   extracellular matrix  . 
 Molecular basis of the recognition of nephronectin by integrin alpha8beta1. Integrin alpha8beta1 interacts with a variety of Arg-Gly-Asp (RGD)-containing ligands in the   extracellular matrix  . Here, we examined the binding activities of alpha8beta1 integrin toward a panel of RGD-containing ligands. Integrin alpha8beta1 bound specifically to nephronectin with an apparent dissociation constant of 0.28 +/- 0.01 nm, but showed only marginal affinities for fibronectin and other RGD-containing ligands. The high-affinity binding to alpha8beta1 integrin was fully reproduced with a recombinant nephronectin fragment derived from the RGD-containing central "linker" segment. A series of deletion mutants of the recombinant fragment identified the LFEIFEIER sequence on the C-terminal side of the RGD motif as an auxiliary site required for high-affinity binding to alpha8beta1 integrin. Alanine scanning mutagenesis within the LFEIFEIER sequence defined the EIE sequence as a critical motif ensuring the high-affinity integrin-ligand interaction. Although a synthetic LFEIFEIER peptide failed to inhibit the binding of alpha8beta1 integrin to nephronectin, a longer peptide containing both the RGD motif and the LFEIFEIER sequence was strongly inhibitory, and was approximately 2,000-fold more potent than a peptide containing only the RGD motif. Furthermore, trans-complementation assays using recombinant fragments containing either the RGD motif or LFEIFEIER sequence revealed a clear synergism in the binding to alpha8beta1 integrin. Taken together, these results indicate that the specific high-affinity binding of nephronectin to alpha8beta1 integrin is achieved by bipartite interaction of the integrin with the RGD motif and LFEIFEIER sequence, with the latter serving as a synergy site that greatly potentiates the RGD-driven integrin-ligand interaction but has only marginal activity to secure the interaction by itself. 
 Characterization of integrin engagement during defined human embryonic stem cell culture. Human embryonic stem (hES) cells are pluripotent, capable of differentiating into any cell type of the body, and therefore have the ability to provide insights into mechanisms of human development and disease, as well as to provide a potentially unlimited supply of cells for cell-based therapy and diagnostics. Knowledge of the adhesion receptors that hES cells employ to engage   extracellular matrix   (ECM) proteins is of basic biological interest and can enhance the design of cell culture and implantation systems to enable these biomedical applications. Although hES cells express a variety of cell surface receptors, little is known about which integrins are involved during subculture and passage. Matrigel is broadly used as a cell adhesive matrix for hES cell culture. Here, we sought to identify which integrins hES cells exploit for adhesion to Matrigel-coated surfaces in defined medium conditions. Using RT-PCR, flow cytometry, and fluorescence immunochemistry, we found that numerous integrins were expressed by H1 hES cells; however, antibody blocking assays indicated that only alpha(v)beta(3), alpha(6), beta(1), and alpha(2)beta(1) played a significant role in the initial adhesion of the hES cells to Matrigel in defined medium conditions. We subsequently identified a cohort of synthetic peptides that, when adsorbed to the culture surface, promoted H1 hES cell attachment and proliferation, as well as maintained a pluripotent phenotype. Peptides designed to engage with alpha(v)beta(3), alpha(6), beta(1), and alpha(2)beta(1) integrins and syndecan-1 were tested both individually and in various combinations. A combination of two integrin-engaging peptides (AG-10, C-16) and one syndecan-engaging peptide (AG-73) was sufficient to promote hES cell adhesion, maintenance, and proliferation. We propose that a specific integrin "fingerprint" is necessary for maintenance of hES cell self-renewal, and synthetic culture systems must capture this engagement profile for hES cells to remain undifferentiated.-Meng, Y., Eshghi, S., Li, Y. J., Schmidt, R., Schaffer, D. V., Healy, K. E. Characterization of integrin engagement during defined human embryonic stem cell culture. 
 Targeting transforming growth factor (TGF)-betaRI inhibits activation of beta1 integrin and blocks vascular invasion in hepatocellular carcinoma. Vascular invasion is one of the major negative prognostic factors in patients with hepatocellular carcinoma (HCC), leading to cancer recurrence. To invade, HCC cells must penetrate the vessel wall, consisting of endothelial cells and   extracellular matrix   components, including fibronectin and fibrinogen. Employing invasive and noninvasive HCC cells, we studied the mechanism underlying vascular invasion. We show that HCC cells invade blood vessels via alpha5beta1, that is equally expressed in invasive and noninvasive cells. However, in the former, the intracytoplasmic tail of beta1 integrin is constitutively phosphorylated at threonine 788-789 and the extracellular part is conformationally activated. In noninvasive cells, beta1 integrin is not activated. Transforming growth factor (TGF)-beta1 specifically phosphorylates beta1 integrin (threonine 788-789) via Smad-2 and Smad-3, causing a conformational change of the extracellular component with an inside-out mechanism. This leads noninvasive HCC cells to behave like invasive cells. A selective TGF-betaRI inhibitor inhibits phosphorylation of the beta1 integrin intracytoplasmic tail, and blocks invasion of HCC cells, both constitutively invasive and with acquired invasive properties. In human HCC tissues with microvascular invasion, phospho-beta1 integrin was detected as well as TGF-beta1, p-Smad-2, and E-cadherin.TGF-beta1 promotes vascular invasion by activating beta1 integrin. This suggests a rationale for targeting TGF-betaRI in future clinical trials. 
 Mapping the ligand-binding pocket of integrin alpha5beta1 using a gain-of-function approach. Integrin alpha5beta1 is a key receptor for the   extracellular matrix   protein fibronectin. Antagonists of human integrin alpha5beta1 have therapeutic potential as anti-angiogenic agents in cancer and diseases of the eye. However, the structure of the integrin is unsolved and the atomic basis of fibronectin and antagonist binding by integrin alpha5beta1 is poorly understood. In the present study, we demonstrate that zebrafish alpha5beta1 integrins do not interact with human fibronectin or the human alpha5beta1 antagonists JSM6427 and cyclic peptide CRRETAWAC. Zebrafish alpha5beta1 integrins do bind zebrafish fibronectin-1, and mutagenesis of residues on the upper surface and side of the zebrafish alpha5 subunit beta-propeller domain shows that these residues are important for the recognition of the Arg-Gly-Asp (RGD) motif and the synergy sequence [Pro-His-Ser-Arg-Asn (PHSRN)] in fibronectin. Using a gain-of-function analysis involving swapping regions of the zebrafish integrin alpha5 subunit with the corresponding regions of human alpha5 we show that blades 1-4 of the beta-propeller are required for human fibronectin recognition, suggesting that fibronectin binding involves a broad interface on the side and upper face of the beta-propeller domain. We find that the loop connecting blades 2 and 3 of the beta-propeller, the D3-A3 loop, contains residues critical for antagonist recognition, with a minor role played by residues in neighbouring loops. A new homology model of human integrin alpha5beta1 supports an important function for D3-A3 loop residues Trp157 and Ala158 in the binding of antagonists. These results will aid the development of reagents that block integrin alpha5beta1 functions in vivo. 
 Fibronectin-tissue transglutaminase matrix rescues RGD-impaired cell adhesion through syndecan-4 and beta1 integrin co-signaling. Heterotropic association of tissue transglutaminase (TG2) with   extracellular matrix  -associated fibronectin (FN) can restore the adhesion of fibroblasts when the integrin-mediated direct binding to FN is impaired using RGD-containing peptide. We demonstrate that the compensatory effect of the TG-FN complex in the presence of RGD-containing peptides is mediated by TG2 binding to the heparan sulfate chains of the syndecan-4 cell surface receptor. This binding mediates activation of protein kinase Calpha (PKCalpha) and its subsequent interaction with beta(1) integrin since disruption of PKCalpha binding to beta(1) integrins with a cell-permeant competitive peptide inhibits cell adhesion and the associated actin stress fiber formation. Cell signaling by this process leads to the activation of focal adhesion kinase and ERK1/2 mitogen-activated protein kinases. Fibroblasts deficient in Raf-1 do not respond fully to the TG-FN complex unless either the full-length kinase competent Raf-1 or the kinase-inactive domain of Raf-1 is reintroduced, indicating the involvement of the Raf-1 protein in the signaling mechanism. We propose a model for a novel RGD-independent cell adhesion process that could be important during tissue injury and/or remodeling whereby TG-FN binding to syndecan-4 activates PKCalpha leading to its association with beta(1) integrin, reinforcement of actin-stress fiber organization, and MAPK pathway activation. 
 β1 integrin signaling maintains human epithelial progenitor cell survival in situ and controls proliferation, apoptosis and migration of their progeny. β1 integrin regulates multiple epithelial cell functions by connecting cells with th  e extracellular matr  ix (ECM). While β1 integrin-mediated signaling in murine epithelial stem cells is well-studied, its role in human adult epithelial progenitor cells (ePCs) in situ remains to be defined. Using microdissected, organ-cultured human scalp hair follicles (HFs) as a clinically relevant model for studying human ePCs within their natural topobiological habitat, β1 integrin-mediated signaling in ePC biology was explored by β1 integrin siRNA silencing, specific β1 integrin-binding antibodies and pharmacological inhibition of integrin-linked kinase (ILK), a key component of the integrin-induced signaling cascade. β1 integrin knock down reduced keratin 15 (K15) expression as well as the proliferation of outer root sheath keratinocytes (ORSKs). Embedding of HF epithelium into an ECM rich in β1 integrin ligands that mimic the HF mesenchyme significantly enhanced proliferation and migration of ORSKs, while K15 and CD200 gene and protein expression were inhibited. Employing ECM-embedded β1 integrin-activating or -inhibiting antibodies allowed to identify functionally distinct human ePC subpopulations in different compartments of the HF epithelium. The β1 integrin-inhibitory antibody reduced β1 integrin expression in situ and selectively enhanced proliferation of bulge ePCs, while the β1 integrin-stimulating antibody decreased hair matrix keratinocyte apoptosis and enhanced transferrin receptor (CD71) immunoreactivity, a marker of transit amplifying cells, but did not affect bulge ePC proliferation. That the putative ILK inhibitor QLT0267 significantly reduced ORSK migration and proliferation and induced massive ORSK apoptosis suggests a key role for ILK in mediating the ß1 integrin effects. Taken together, these findings demonstrate that ePCs in human HFs require β1 integrin-mediated signaling for survival, adhesion, and migration, and that different human HF ePC subpopulations differ in their response to β1 integrin signaling. These insights may be exploited for cell-based regenerative medicine strategies that employ human HF-derived ePCs. 
 Integrins mediate adhesion of medulloblastoma cells to tenascin and activate pathways associated with survival and proliferation. Medulloblastoma spreads by leptomeningeal dissemination rather than by infiltration that characterizes other CNS tumors, eg, gliomas. This study represents an initial attempt to identify both the molecules that mediate medulloblastoma adhesion to leptomeninges and the pathways that are key to survival and proliferation of tumor following adhesion. As a first step in molecule identification, we produced adhesion of D283 medulloblastoma cells to the   extracellular matrix   (ECM) of H4 glioma cells in vitro. Within this context, D283 cells preferentially expressed the alpha9 and beta1 integrin subunits; antibody and disintegrin blockade of alpha9 and beta1 binding eliminated the adhesion. The H4 ECM was enriched in tenascin, a binding partner for the alpha9beta1 integrin heterodimer. Purified tenascin-C supported D283 cell adhesion. The adhesion was blocked by antibodies to alpha9 and beta1 integrin. In vivo data were similar; immunohistochemistry of primary human medulloblastomas with leptomeningeal extension demonstrated increased expression of alpha9 and beta1 integrins as well as tenascin at the interface of brain and leptomeningeal tumor. These data suggest that tumor-cell expressions of alpha9 and beta1 integrins in combination with extracellular tenascin are necessary for medulloblastoma adhesion to the leptomeninges. As a first step in the identification of pathways that mediate survival and proliferation of tumor following adhesion, we demonstrated that adhesion to H4 ECM was associated with survival and proliferation of D283 cells as well as activation of the MAPK pathway in a growth factor deficient environment. Antibody blockade of alpha9 and beta1 integrin binding that eliminated adhesion also eliminated the in vitro survival benefit. These data suggest that adhesion of medulloblastoma to the meninges is necessary for the survival and proliferation of these tumor cells at the secondary site. 
 Recognition of the neural chemoattractant Netrin-1 by integrins alpha6beta4 and alpha3beta1 regulates epithelial cell adhesion and migration. Netrins, axon guidance cues in the CNS, have also been detected in epithelial tissues. In this study, using the embryonic pancreas as a model system, we show that Netrin-1 is expressed in a discrete population of epithelial cells, localizes to basal membranes, and specifically associates with elements of the   extracellular matrix  . We demonstrate that alpha6beta4 integrin mediates pancreatic epithelial cell adhesion to Netrin-1, whereas recruitment of alpha6beta4 and alpha3beta1 regulate the migration of CK19+/PDX1+ putative pancreatic progenitors on Netrin-1. These results provide evidence for the activation of epithelial cell adhesion and migration by a neural chemoattractant, and identify Netrin-1/integrin interactions as adhesive/guidance cues for epithelial cells. 
 Schwann Cells Increase Prostate and Pancreatic Tumor Cell Invasion Using Laminin Binding A6 Integrin. Human pancreatic and prostate cancers metastasize along nerve axons during perineural invasion. The   extracellular matrix   laminin class of proteins is an abundant component of both myelinated and non-myelinated nerves. Analysis of human pancreatic and prostate tissue revealed both perineural and endoneural invasion with Schwann cells surrounded or disrupted by tumor, respectively. Tumor and nerve cell co-culture conditions were used to determine if myelinating or non-myelinating Schwann cell (S16 and S16Y, respectively) phenotype was equally likely to promote integrin-dependent cancer cell invasion and migration on laminin. Conditioned medium from S16 cells increased tumor cell (DU145, PC3, and CFPAC1) invasion into laminin approximately 1.3-2.0 fold compared to fetal bovine serum (FBS) treated cells. Integrin function (e.g., ITGA6p formation) increased up to 1.5 fold in prostate (DU145, PC3, RWPE-1) and pancreatic (CFPAC1) cells, and invasion was dependent on ITGA6p formation and ITGB1 as determined by function-blocking antibodies. In contrast, conditioned medium isolated from S16Y cells (non-myelinating phenotype) decreased constitutive levels of ITGA6p in the tumor cells by 50% compared to untreated cells and decreased ITGA6p formation 3.0 fold compared to S16 treated cells. Flow cytometry and western blot analysis revealed loss of ITGA6p formation as reversible and independent of overall loss of ITGA6 expression. These results suggest that the myelinating phenotype of Schwann cells within the tumor microenvironment increased integrin-dependent tumor invasion on laminin. 
 Characterization of plasminogen as an adhesive ligand for integrins alphaMbeta2 (Mac-1) and alpha5beta1 (VLA-5). Plasminogen (Pg) has been implicated in many biologic processes involving extracellular proteolysis. We investigated whether Pg, by virtue of its capacity to be deposited within the   extracellular matrix  , can serve as a ligand for cell surface integrins. We report here that Pg supports cell adhesion by engaging integrins alphaMbeta2 and alpha5beta1. The immobilized Glu-Pg, but not its derivatives with the N-terminal peptide lacking, plasmin and Lys-Pg, supported efficient adhesion that was abolished by anti-alphaMbeta2 and anti-alpha5beta1 integrin-specific monoclonal antibodies (mAbs). In addition, lysine binding sites of Glu-Pg contributed to cell adhesion inasmuch as tranexamic acid and epsilon-aminocaproic acid inhibited cell adhesion. The involvement of alphaMbeta2 and alpha5)beta1 in adhesion to Glu-Pg was demonstrable with blood neutrophils, U937 monocytoid cells, and genetically engineered alphaMbeta2-transfected human embryonic kidney (HEK) 293 cells. In alphaMbeta2, the alphaMI-domain is the binding site for Glu-Pg because the "I-less" form of alphaMbeta2 did not support cell adhesion and the recombinant alphaMI-domain bound Glu-Pg directly. In comparison with cell adhesion, the binding of soluble Glu-Pg to cells and the concomitant generation of plasmin activity was inhibited by anti-alpha5beta1 but not by anti-alphaMbeta2. These findings identify Glu-Pg as an adhesive ligand for integrins alphaMbeta2 and alpha5beta1 and suggest that alpha5beta1 may participate in the binding of soluble Glu-Pg and assist in its activation. 
 Regulation of endothelial cell cytoskeletal reorganization by a secreted frizzled-related protein-1 and frizzled 4- and frizzled 7-dependent pathway: role in neovessel formation. Consistent with findings of Wnt pathway members involved in vascular cells, a role for Wnt/Frizzled signaling has recently emerged in vascular cell development. Among the few Wnt family members implicated in vessel formation in adult, Wnt7b and Frizzled 4 have been shown as involved in vessel formation in the lung and in the retina, respectively. Our previous work has shown a role for secreted Frizzled-related protein-1 (sFRP-1), a proposed Wnt signaling inhibitor, in neovascularization after an ischemic event and demonstrated its role as a potent angiogenic factor. However the mechanisms involved have not been investigated. Here, we show that sFRP-1 treatment increases endothelial cell spreading on   extracellular matrix   as revealed by actin stress fiber reorganization in an integrin-dependent manner. We demonstrate that sFRP-1 can interact with Wnt receptors Frizzled 4 and 7 on endothelial cells to transduce downstream to cellular machineries requiring Rac-1 activity in cooperation with GSK-3beta. sFRP-1 overexpression in endothelium specifically reversed the inactivation of GSK-3 beta and increased neovascularization in ischemia-induced angiogenesis in mouse hindlimb. This study illustrates a regulated pathway by sFRP-1 involving GSK-3beta and Rac-1 in endothelial cell cytoskeletal reorganization and in neovessel formation. 
 Regulation of Integrin β 1 recycling to lipid rafts by Rab1a to promote cell migration. Rab1a is a member of the Rab family of small GTPases with a well characterized function in the regulation of vesicle trafficking from the endoplasmic reticulum to the Golgi apparatus and within Golgi compartments. The integrin family heterodimeric transmembrane proteins serve as major receptors for   extracellular matri  x proteins, which play essential roles in cell adhesion and migration. Although effects on intracellular trafficking of integrins or other key cargos by Rab1a could influence cell migration, the regulatory mechanisms linking Rab1a to cell migration are not well understood. Here, we report identification of Rab1a as a novel regulator of cell migration using an unbiased RNAi screen targeting GTPases. Inhibition of Rab1a reduced integrin-mediated cell adhesion and spreading on fibronectins, reduced integrin β1 localization to lipid rafts, and decreased recycling of integrin β1 to the plasma membrane. Analysis of Rab1a effector molecules showed that p115 mediated Rab1a regulation of integrin recycling and lipid raft localization in cell migration. Taken together, these results suggest a novel function for Rab1a in the regulation of cell migration through controlling integrin β1 recycling and localization to lipid rafts via a specific downstream effector pathway. 
 Additive effect of recombinant Mycobacterium tuberculosis ESAT-6 protein and ESAT-6/CFP-10 fusion protein in adhesion of macrophages through fibronectin receptors. Tuberculous granulomas are the sites of interaction between the T cells, macrophages, and   extracellular matrix   (ECM) to control the infection caused by Mycobacterium tuberculosis (M. tuberculosis). A predominant role of RD-1-encoded secretory proteins, early secreted antigenic target-6 (ESAT-6), and culture filtrate protein-10 (CFP-10) in the formation of granulomas has recently been emphasized. However, the precise molecular events that induce the formation of these granulomatous structures are yet to be elucidated. Macrophages use integrins to adhere to fibronectin (FN) as a major component of the ECM. The major goal of this study was to investigate whether recombinant M. tuberculosis antigens can modulate integrin-mediated macrophage adhesion.Differentiated THP-1 cell line was stimulated with recombinant ESAT-6, CFP-10, and ESAT-6/CFP-10 proteins and evaluated for alterations in the expression levels of α5β1 and α4β1 by semiquantitative real-time polymerase chain reaction. The role of these recombinant antigens in the cytoskeleton rearrangement was determined by adhesion assay and immunofluorescent microscopy.Our data showed that ESAT-6 and ESAT-6/CFP-10 fusion proteins could induce adhesion of macrophages to FN through α4β1 integrin. An increased expression level of α4β1 integrin in comparison with α5β1 integrin in differentiated THP-1 cells was also observed. Results of immunofluorescence studies showed that recombinant proteins-treated THP-1 cells form well-organized stress fibers and focal contacts containing vinculin compared with untreated THP-1 cells.Increased expression level of α4β1 in differentiated THP-1 cells could suggest the important role of α4β1 integrin in adhesion and focal contact formation of macrophages exposed to M. tuberculosis antigens. 
 Beta ig-h3 induces keratinocyte differentiation via modulation of involucrin and transglutaminase expression through the integrin alpha3beta1 and the phosphatidylinositol 3-kinase/Akt signaling pathway. Beta ig-h3 is an   extracellular matrix   protein whose expression is highly induced by transforming growth factor (TGF)-beta1. Whereas beta ig-h3 is known to mediate keratinocyte adhesion and migration, its effects on keratinocyte differentiation remain unclear. In the present study, it was demonstrated that expression of both beta ig-h3 and TGF-beta1 was enhanced during keratinocyte differentiation and that expression of the former was strongly induced by that of the latter. This study also asked whether changes in beta-h3 expression would affect keratinocyte differentiation. Indeed, down-regulation of beta ig-h3 by transfection with antisense beta ig-h3 cDNA constructs effectively inhibited keratinocyte differentiation by decreasing the promoter activities and thus expression of involucrin and transglutaminase. The result was an approximately 2-fold increase in mitotic capacity of the cells. Conversely, overexpression of beta ig-h3, either by transfection with beta ig-h3 expression plasmids or by exposure to recombinant beta ig-h3, enhanced keratinocyte differentiation by inhibiting cell proliferation and concomitantly increasing involucrin and transglutaminase expression. Recombinant beta ig-h3 also promoted keratinocyte adhesion through interaction with integrin alpha3beta1. Changes in beta ig-h3 expression did not affect intracellular calcium levels. Subsequent analysis revealed not only induction of Akt phosphorylation by recombinant beta ig-h3 but also blockage of Akt phosphorylation by LY294002, an inhibitor of phosphatidylinositol 3-kinase. Taken together, these findings indicate that enhanced beta ig-h3, induced by enhanced TGF-beta during keratinocyte differentiation, provoked cell differentiation by enhancing involucrin and transglutaminase expression through the integrin alpha3beta1 and phosphatidylinositol 3-kinase/Akt signaling pathway. Lastly, it was observed that beta ig-h3-mediated keratinocyte differentiation was caused by promotion of cell adhesion and not by calcium regulation. 
 Increased expression of tenascin-C-binding epithelial integrins in human bullous keratopathy corneas. We previously found an abnormal deposition of an   extracellular matrix   glycoprotein, tenascin-C (TN-C), in human corneas with pseudophakic/aphakic bullous keratopathy (PBK/ABK). In this work, we studied cellular TN-C receptors in normal and PBK/ABK corneas. Cryostat sections of normal and PBK/ABK corneas were stained by immuno-fluorescence for TN-C receptors: alpha2, alpha8, alpha9, alphaVbeta3, beta1, and beta6 integrins, and annexin II. Beta6 integrin mRNA levels were assessed by semiquantitative reverse transcription-polymerase chain reaction (RT-PCR) using beta2-microglobulin gene to normalize the samples. In PBK/ABK compared to normal corneas, relatively minor changes were observed for alpha2 and beta1 integrins, and for annexin II. Alpha8, alpha9, and beta6 subunits of TN-C receptors, alpha8beta1 alpha9beta1, and alphaVbeta6, respectively, were absent from normal central corneas but were found in the central epithelium of PBK/ABK corneas. Beta6 integrin showed the most significant accumulation. It correlated best with the expression of TN-C rather than with the expression of other alphaVbeta6 ligands, fibronectin, and vitronectin. RT-PCR analysis also showed elevated levels of beta6 mRNA in PBK/ABK compared to normal corneas. Therefore, accumulation of TN-C in PBK/ABK corneas was accompanied by an increased expression of its three binding integrins, especially alphaVbeta6 in the corneal epithelium. The interaction of tenascin-C with these integrins may contribute to the fibrotic process that occurs in PBK/ABK corneas. 
 Laminin-332 and α3β1 integrin-supported migration of bronchial epithelial cells is modulated by fibronectin. The repair of the bronchiolar epithelium damaged by cell-mediated, physical, or chemical insult requires epithelial cell migration over a provisional matrix composed of complexes o  f extracellular matr  ix molecules, including fibronectin and laminin. These matrix molecules support migration and enhance cell adhesion. When cells adhere too tightly to their matrix they fail to move; but if they adhere too little, they are unable to develop the traction force necessary for motility. Thus, we investigated the relative contributions of laminin and fibronectin to bronchiolar cell adhesion and migration using the immortalized bronchial lung epithelial cell line (BEP2D) and normal human bronchial epithelial (NHBE) cells, both of which assemble a laminin α3β3γ2 (LM332)/fibronectin-rich matrix. Intriguingly, BEP2D and NHBE cells migrate significantly faster on an LM332-rich matrix than on fibronectin. Moreover, addition of fibronectin to LM332 matrix suppresses motility of both cell types. Finally, fibronectin enhances the adhesion of both BEP2D and NHBE cells to LM332-coated surfaces. These results suggest that fibronectin fine tunes LM332-mediated migration by boosting bronchiolar cell adhesion to substrate. We suggest that, during epithelial wound healing of the injured airway, fibronectin plays an important adhesive role for laminin-driven epithelial cell motility by promoting a stable cellular interaction with the provisional matrix. 
 Disialoganglioside GD3-synthase over expression inhibits survival and angiogenesis of pancreatic cancer cells through cell cycle arrest at S-phase and disruption of integrin-β1-mediated anchorage. Gangliosides play important roles in the development, differentiation and proliferation of mammalian cells. They bind to other cell membrane components through their terminal sialic acids. Different gangliosides influence cellular functions based on the positions and linkages of sialic acids. Expression of gangliosides mainly depends on the status of sialic acid-modulatory enzymes, such as different types of sialyltransferases and sialidases. One such sialyltransferase, disialoganglioside GD3 synthase, is specifically responsible for the production of GD3. Pancreatic ductal adenocarcinoma, making up more than 90% of pancreatic cancers, is a fatal malignancy with poor prognosis. Despite higher sialylation status, the disialoganglioside GD3 level is very low in this cancer. However, the exact status and function of this disialoganglioside is still unknown. Here, we intended to study the intracellular mechanism of disialoganglioside GD3-induced apoptosis and its correlation with the adhesion and angiogenic pathways in pancreatic cancer. We demonstrated that disialoganglioside GD3 synthase-transfected cells showed enhanced apoptosis and it caused the arrest of these cells in the S-phase of the cell cycle. Integrins, a family of transmembrane proteins play important role in cell-cell recognition, invasion, adhesion and migration. disialoganglioside GD3 co-localised with integrin-β1 and thereby inhibited it's downstream signalling in transfected cells. Transfected cells exhibited inhibition of cell adhesion wit  h extracellular matr  ix proteins. Enhanced GD3 expression down regulated angiogenesis-regulatory proteins and inhibited epidermal growth factor/vascular endothelial growth factor-driven angiogenic cell growth in these cells. Taken together, our study provides support for the GD3-induced cell cycle arrest, disruption of integrin-β1-mediated anchorage, inhibition of angiogenesis and thereby induced apoptosis in pancreatic cancer cells. 
 Endoglin mediates fibronectin/α5β1 integrin and TGF-β pathway crosstalk in endothelial cells. Both the transforming growth factor β (TGF-β) and integrin signalling pathways have well-established roles in angiogenesis. However, how these pathways integrate to regulate angiogenesis is unknown. Here, we show that   the extracellular m  atrix component, fibronectin, and its cellular receptor, α5β1 integrin, specifically increase TGF-β1- and BMP-9-induced Smad1/5/8 phosphorylation via the TGF-β superfamily receptors endoglin and activin-like kinase-1 (ALK1). Fibronectin and α5β1 integrin increase Smad1/5/8 signalling by promoting endoglin/ALK1 cell surface complex formation. In a reciprocal manner, TGF-β1 activates α5β1 integrin and downstream signalling to focal adhesion kinase (FAK) in an endoglin-dependent manner. α5β1 integrin and endoglin form a complex on the cell surface and co-internalize, with their internalization regulating α5β1 integrin activation and signalling. Functionally, endoglin-mediated fibronectin/α5β1 integrin and TGF-β pathway crosstalk alter the responses of endothelial cells to TGF-β1, switching TGF-β1 from a promoter to a suppressor of migration, inhibiting TGF-β1-mediated apoptosis to promote capillary stability, and partially mediating developmental angiogenesis in vivo. These studies provide a novel mechanism for the regulation of TGF-β superfamily signalling and endothelial function through crosstalk with integrin signalling pathways. 
 Contributions of the integrin β1 tail to cell adhesive forces. Integrin receptors connect the   extracellular matri  x to the cell cytoskeleton to provide essential forces and signals. To examine the contributions of the β1 integrin cytoplasmic tail to adhesive forces, we generated cell lines expressing wild-type and tail mutant β1 integrins in β1-null fibroblasts. Deletion of β1 significantly reduced cell spreading, focal adhesion assembly, and adhesive forces, and expression of human β1 (hβ1) integrin in these cells restored adhesive functions. Cells expressing a truncated tail mutant had impaired spreading, fewer and smaller focal adhesions, reduced integrin binding to fibronectin, and lower adhesion strength and traction forces compared to hβ1-expressing cells. All these metrics were equivalent to those for β1-null cells, demonstrating that the β1 tail is essential to these adhesive functions. Expression of the constitutively-active D759A hβ1 mutant restored many of these adhesive functions in β1-null cells, although with important differences when compared to wild-type β1. Even though there were no differences in integrin-fibronectin binding and adhesion strength between hβ1- and hβ1-D759A-expressing cells, hβ1-D759A-expressing cells assembled more but smaller adhesions than hβ1-expressing cells. Importantly, hβ1-D759A-expressing cells generated lower traction forces compared to hβ1-expressing cells. These differences between hβ1- and hβ1-D759A-expressing cells suggest that regulation of integrin activation is important for fine-tuning cell spreading, focal adhesion assembly, and traction force generation. 
 Direct interactions with the integrin β1 cytoplasmic tail activate the Abl2/Arg kinase. Integrins are heterodimeric α/  β extracellular mat  rix adhesion receptors that couple physically to the actin cytoskeleton and regulate kinase signaling pathways to control cytoskeletal remodeling and adhesion complex formation and disassembly. β1 integrins signal through the Abl2/Arg (Abl-related gene) nonreceptor tyrosine kinase to control fibroblast cell motility, neuronal dendrite morphogenesis and stability, and cancer cell invasiveness, but the molecular mechanisms by which integrin β1 activates Arg are unknown. We report here that the Arg kinase domain interacts directly with a lysine-rich membrane-proximal segment in the integrin β1 cytoplasmic tail, that Arg phosphorylates the membrane-proximal Tyr-783 in the β1 tail, and that the Arg Src homology domain then engages this phosphorylated region in the tail. We show that these interactions mediate direct binding between integrin β1 and Arg in vitro and in cells and activate Arg kinase activity. These findings provide a model for understanding how β1-containing integrins interact with and activate Abl family kinases. 
 Integrin beta1 mediates hepatocellular carcinoma cells chemotaxis to laminin. Chemotaxis is an important step during the invasion of carcinoma cells. And integrins are most important receptors mediating interaction between cells and   extracellular matrix   (ECM). This study was designed to study integrin beta1 mediating chemotaxis of hepatocellular carcinoma (HCC) cells to laminin(LN).A micropipette technique was adopted to investigate the effect of blockade of integrin beta1 on pseudopod protrusion of HCC cells in response to LN stimulation. Chemotactic pseudopod protrusion of a HCC cell was evaluated using a dual-pipette set-up, in which two pipettes filled with LN solution were positioned in close contact with the same cell, and pseudopod protrusion into each pipette was viewed dynamically and recorded with a tape recorder. The lengths of pseudopods were measured and plotted against time to obtain a pseudopod growth curve. The integrin beta1 subunit on the surfaces of HCC cells were analyzed by flow cytometry.In dual pipette chemotaxis experiment, when the two pipettes were filled with LN(50 microg/ml, 200 microg/ml), pseudopods extended from the HCC cell into each of the pipettes nearly symmetrically, ie, with nearly identical maximum pseudopod length and similar pseudopod growth curves. Upon addition of anti-CD29 (20 microg/ml) to one of the pipettes, pseudopod protrusion was blocked nearly completely while protrusion into the opposite pipette became more evidently, with a larger maximum length. Expression of integrin beta1 was up to 95.78% to cells chosen in the experiment.Integrin beta1 subunit was an important constituent receptor subunit for mediating chemotactic pseudopod protrusion of HCC cell to LN. 
 Integrin-dependent protein tyrosine phosphorylation is a key regulatory event in collagen-IV-mediated adhesion and proliferation of human lung tumor cell line, Calu-1. The clinical phenomenon of lung cancer metastasis to specific target organs is believed to be a direct interaction between tumor cells and   extracellular matrix   components. During invasion, tumor cells attach to the basement membrane protein, collagen type IV, degrade it, migrate through blood vessels, and adhere to   extracellular matrix   proteins.Four nonsmall-cell lung cancer cells were tested for adhesion, proliferation, migration and morphologic alterations on collagen type IV matrix by immunoprecipitation, Western blotting, phase contrast and time lapse video microscopy.Collagen type IV promoted Calu-1 cell adhesion (< 15 minutes) and motility (< 6 hours) without any significant effect on proliferation. beta(1)-integrin is essential for collagen type IV adhesion and 8 to 10 fold higher expression of beta1-integrin on the surface of Calu-1 cells was identified. A protein tyrosine phosphatase inhibitor, peroxyvanadate, caused 50% inhibition in the adhesion process within 5 minutes but exposure to 60 micromol/L genistein for 72 hours, a protein tyrosine kinase inhibitor, drastically inhibits Calu-1 cell proliferation (> 70%). We examined the influence of beta1-integrin, peroxyvanadate and genistein on the spreading morphogenesis of Calu-1 cells on Collagen type IV. Use of either 1 microg of anti beta1-integrin inhibitory antibody (P5D2), 100 micromol/L peroxyvanadate or 60 micromol/L genistein had dramatic influence on the spreading of Calu-1 cells. Finally, Focal adhesion kinase was identified as a phosphoprotein target.We have characterized an in vitro model of matrix-specific binding of a lung cancer cell line, Calu-1 to Coll IV. Calu-1 cells use primarily a beta1-integrin mediated intracellular tyrosine phosphorylation phenomenon as the key regulatory mechanism for its binding advantage to Coll IV matrix. 
 An SH3 domain-containing GTPase-activating protein for Rho and Cdc42 associates with focal adhesion kinase. The integrin family of cell surface receptors mediates cell adhesion to components of the   extracellular matrix   (ECM). Integrin engagement with the ECM initiates signaling cascades that regulate the organization of the actin-cytoskeleton and changes in gene expression. The Rho subfamily of Ras-related low-molecular-weight GTP-binding proteins and several protein tyrosine kinases have been implicated in mediating various aspects of integrin-dependent alterations in cell homeostasis. Focal adhesion kinase (FAK or pp125FAK) is one of the tyrosine kinases predicted to be a critical component of integrin signaling. To elucidate the mechanisms by which FAK participates in integrin-mediated signaling, we have used expression cloning to identify cDNAs that encode potential FAK-binding proteins. We report here the identification of a cDNA that encodes a new member of the GTPase-activating protein (GAP) family of GTPase regulators. This GAP, termed Graf (for GTPase regulator associated with FAK), binds to the C-terminal domain of FAK in an SH3 domain-dependent manner and preferentially stimulates the GTPase activity of the GTP-binding proteins RhoA and Cdc42. Subcellular localization studies using Graf-transfected chicken embryo cells indicates that Graf colocalizes with actin stress fibers, cortical actin structures, and focal adhesions. Graf mRNA is expressed in a variety of avian tissues and is particularly abundant in embryonic brain and liver. Graf represents the first example of a regulator of the Rho family of small GTP-binding proteins that exhibits binding to a protein tyrosine kinase. We suggest that Graf may function to mediate cross talk between the tyrosine kinases such as FAK and the Rho family GTPase that control steps in integrin-initiated signaling events. 
 Sequence and tissue distribution of the human integrin alpha 8 subunit: a beta 1-associated alpha subunit expressed in smooth muscle cells. Integrins are a major family of cell adhesion molecules involved in cell-cell and cell-  extracellular matrix   interactions. Each integrin is a heterodimeric glycoprotein composed of an alpha and a beta subunit. We now report the cDNA sequence and distribution of a new human integrin alpha subunit. This sequence is 78% identical to the previously reported chicken alpha 8 integrin sequence. Thus, we have designated this subunit as human alpha 8. By northern blot analysis, an alpha 8 probe detects two mRNA species of approximately 6.5 and 4.0 kb in neuroglioma H4 cells. An anti-alpha 8 polyclonal antibody precipitates a protein complex containing the beta 1 subunit associated with the putative alpha 8 subunit, which has an apparent molecular mass of 180 kDa (non-reduced) or 155 kDa and 25 kDa (reduced). Immunohistochemistry with anti-alpha 8 polyclonal antibody in adult rat tissues shows prominent staining in vascular and visceral smooth muscle. In addition, the antibody strongly stained kidney mesangial cells and a cell type in the alveolar wall of the lungs, most likely corresponding to alveolar myofibroblasts. These results suggest that in adult mammalian tissues, alpha 8 is predominantly expressed in smooth muscle and smooth muscle-like contractile cells. 
 IFATS collection: Combinatorial peptides identify alpha5beta1 integrin as a receptor for the matricellular protein SPARC on adipose stromal cells. The biological features of adipose stromal (stem) cells (ASC), which serve as progenitors for differentiated cells of white adipose tissue (WAT), are still largely undefined. In an initiative to identify functional ASC surface receptors, we screened a combinatorial library for peptide ligands binding to patient-derived ASC. We demonstrate that both primary and cultured human and mouse stromal cells express a conserved receptor targeted by peptides found to mimic SPARC, a matricellular protein that is required for normal WAT development. A signaling receptor for SPARC has not as yet been determined. By using the SPARC-mimicking peptides CMLAGWIPC (termed hPep) and CWLGEWLGC (termed mPep), isolated by panning on human and mouse cells, respectively, we identified the alpha5beta1 integrin complex as a candidate receptor for SPARC. On the basis of these results, we evaluated ASC responses to SPARC or SPARC-mimicking peptide exposure. Our results suggest that extracellular SPARC binds to alpha5beta1 integrin at sites of focal adhesions, an interaction disrupting firm attachment of ASC to   extracellular matrix  . We propose that SPARC-mediated mobilization of ASC through its effect on alpha5beta1 integrin complex provides a functional basis for the regulation of WAT body composition by SPARC. We also show that alpha5beta1 integrin is a potential target for ASC-selective intracellular delivery of bioactive peptides and gene therapy vectors directed by the SPARC-mimicking peptides. Disclosure of potential conflicts of interest is found at the end of this article. 
 Integrin-mediated functional polarization of Caco-2 cells through E-cadherin--actin complexes. Enterocyte differentiation is a dynamic process during which reinforcement of cell-cell adhesion favours migration along the crypt-to-villus axis. Functional polarization of Caco-2 cells, the most commonly used model to study intestinal differentiation, is assessed by dome formation and tightness of the monolayer and is under the control of the   extracellular matrix   (ECM). Furthermore, our biochemical and confocal microscopy data demonstrate that the ECM dramatically reinforces E-cadherin targeting to the upper lateral membrane, formation of the apical actin cytoskeleton and its colocalization with E-cadherin in functional complexes. In our model, these effects were produced by native laminin-5-enriched ECM as well as by type IV collagen or laminin 2, which suggests a common pathway of induction through integrin receptors. Indeed, these effects were antagonized by blocking anti-beta1- and anti-alpha6-integrin antibodies and directly induced by a stimulating anti-beta1-integrin antibody. These results demonstrate that integrin-dependent cell to ECM adhesion reinforces E-cadherin-dependent cell-cell adhesion in Caco-2 cells and further support the notion that enterocyte differentiation is supported by a molecular crosstalk between the two adhesion systems of the cell. 
 Prognostic role of integrin β1, E-cadherin, and rac1 expression in small cell lung cancer. Integrin β(1) mediates cellular adhesion to th  e extracellular matr  ix (ECM) and is correlated with highly invasive and metastatic behavior in small cell lung cancer (SCLC). E-cadherin (ECAD) is a calcium-dependent cell-cell adhesion receptor that restricts invasion of cells and reduces metastasis. Rac1 is involved in the regulation of the actin cytoskeleton, adhesion, migration, invasion, and tumor metastasis. The aim of this study was to examine integrin β(1) , ECAD and rac1 expression in SCLC and to analyze the prognostic value of these markers in patients with SCLC. We analyzed integrin β(1) , ECAD, and rac1 expression in 112 SCLC tissues by immunohistochemical staining. Correlative analyses between integrin β(1) , ECAD, and rac1 expression and cliniopathological factors were performed. A total of 65 patients had extensive disease (ED) (58%), and 47 had limited disease (LD) (42%). The median follow-up duration was 61 months (range: 14-117 months), and the median progression free survival (PFS) and overall survival (OS) were 6.1 months (range: 4.8-7.4 months) and 9.7 months (range: 8.1-11.3 months), respectively. The expression of integrin β(1) , ECAD, and rac1 protein was observed in 64, 73, and 99 of SCLC tissues, respectively. The correlative analyses between integrin β(1) , ECAD, or rac1 expression and various clinical parameters did not show any statistical significance. However, the ECAD expression was associated with OS in the entire cohort. In contrast, the expression of integrin β(1) and rac1 was not associated with PFS or OS. In a subgroup analysis, patients with less than two metastasis had significantly longer OS (p = 0.047) if their tumors expressed integrin β(1) compared to those without integrin β(1) expression. In addition, OS was longer for patients with ECAD positive tumors compared to those whose tumors did not express ECAD in males (p = 0.032) and patients who never smoked (p < 0.001). Multivariate analysis showed that LD (p = 0.004), overall response rate (p = 0.003), and expression of ECAD (p = 0.015) were the independent good prognostic factors for OS. LD (p = 0.024), overall response rate (p < 0.001), and less than two metastasis (p = 0.003) were prognostic factors for longer PFS. These results suggest that ECAD expression may be useful as a prognostic indicator in patients with SCLC. 
 Roles of integrins in human induced pluripotent stem cell growth on Matrigel and vitronectin. Human induced pluripotent stem cells (iPSCs) hold promise as a source of adult-derived, patient-specific pluripotent cells for use in cell-based regenerative therapies. However, current methods of cell culture are tedious and expensive, and the mechanisms underlying cell proliferation are not understood. In this study, we investigated expression and function of iPSC integrin   extracellular matrix   receptors to better understand the molecular mechanisms of cell adhesion, survival, and proliferation. We show that iPSC lines generated using Oct-3/4, Sox-2, Nanog, and Lin-28 express a repertoire of integrins similar to that of hESCs, with prominent expression of subunits alpha5, alpha6, alphav, beta1, and beta5. Integrin function was investigated in iPSCs cultured without feeder layers on Matrigel or vitronectin, in comparison to human embryonic stem cells. beta1 integrins were required for adhesion and proliferation on Matrigel, as shown by immunological blockade experiments. On vitronectin, the integrin alphavbeta5 was required for initial attachment, but inhibition of both alphavbeta5 and beta1 was required to significantly decrease iPSC proliferation. Furthermore, iPSCs cultured on vitronectin for 9 passages retained normal karyotype, pluripotency marker expression, and capacity to differentiate in vitro. These studies suggest that vitronectin, or derivatives thereof, might substitute for Matrigel in a more defined system for iPSC culture. 
 MicroRNA-124 suppresses oral squamous cell carcinoma motility by targeting ITGB1. Alterations in the levels of molecules which interact with the   extracellular matrix  , such as integrins, are associated with invasion of oral squamous cell carcinomas (OSCC). The molecular mechanisms underlying dysregulation of integrin expression in OSCC, however, remain unclear. Here, we show that microRNA-124, a small non-coding RNA down-regulated in OSCC, is able to downregulate expression of integrin beta-1 (ITGB1) by interacting with its 3' untranslated region. Over-expression of miR-124 attenuates endogenous ITGB1 expression and reduces the adherence and motility of OSCC cells, suggesting disruption of miR-124-mediated repression of ITGB1 may be a key factor in OSCC progression. 
 Phosphoinositide-3-OH kinase-dependent regulation of glycogen synthase kinase 3 and protein kinase B/AKT by the integrin-linked kinase. Integrin-linked kinase (ILK) is an ankyrin-repeat containing serine-threonine protein kinase capable of interacting with the cytoplasmic domains of integrin beta1, beta2, and beta3 subunits. Overexpression of ILK in epithelial cells disrupts cell-  extracellular matrix   as well as cell-cell interactions, suppresses suspension-induced apoptosis (also called Anoikis), and stimulates anchorage-independent cell cycle progression. In addition, ILK induces nuclear translocation of beta-catenin, where the latter associates with a T cell factor/lymphocyte enhancer-binding factor 1 (TCF/LEF-1) to form an activated transcription factor. We now demonstrate that ILK activity is rapidly, but transiently, stimulated upon attachment of cells to fibronectin, as well as by insulin, in a phosphoinositide-3-OH kinase [Pi(3)K]-dependent manner. Furthermore, phosphatidylinositol(3,4,5)trisphosphate specifically stimulates the activity of ILK in vitro, and in addition, membrane targetted constitutively active Pi(3)K activates ILK in vivo. We also demonstrate here that ILK is an upstream effector of the Pi(3)K-dependent regulation of both protein kinase B (PKB/AKT) and glycogen synthase kinase 3 (GSK-3). Specifically, ILK can directly phosphorylate GSK-3 in vitro and when stably, or transiently, overexpressed in cells can inhibit GSK-3 activity, whereas the overexpression of kinase-deficient ILK enhances GSK-3 activity. In addition, kinase-active ILK can phosphorylate PKB/AKT on serine-473, whereas kinase-deficient ILK severely inhibits endogenous phosphorylation of PKB/AKT on serine-473, demonstrating that ILK is involved in agonist stimulated, Pi(3)K-dependent, PKB/AKT activation. ILK is thus a receptor-proximal effector for the Pi(3)K-dependent,   extracellular matrix   and growth factor mediated, activation of PKB/AKT, and inhibition of GSK-3. 
 Characterization of the oligosaccharide component of alpha3beta1 integrin from human bladder carcinoma cell line T24 and its role in adhesion and migration. Malignant transformation is highly associated with altered expression of cell surface N-linked oligosaccharides. These changes concern integrins, a family of cell surface glycoproteins involved in the attachment and migration of cells on various   extracellular matrix   proteins. The integrin alpha3beta1 is particularly interesting because of its role in migration and invasion of several types of metastatic tumours. In this study, alpha3beta1 from human bladder T24 carcinoma cells was purified and treated with peptide N-glycosidase F. Then the N-glycans of the alpha3 and beta1 subunits were characterized using matrix-assisted laser desorption ionization mass spectrometry (MALDI MS). In alpha3beta1 integrin the presence of high-mannose, hybrid and predominantly complex type N-oligosaccharides was shown. Unlike to normal epithelium cells, in both subunits of alpha3beta1 integrin from cancer cells, the sialylated tetraantennary complex type glycan Hex7HexNAc6FucSia4 was present. In a direct ligand binding assay, desialylated alpha3beta1 integrin exhibited significantly higher fibronectin-binding capability than untreated integrin, providing evidence that sialic acids play a direct role in ligand-receptor interaction. Moreover, alpha3beta1 integrin was shown to take part in T24 cell migration on fibronectin: anti-alpha3 antibodies induced ca 30% inhibition of wound closure. Treatment of T24 cells with swainsonine reduced the rate of bladder carcinoma cell migration by 16%, indicating the role of beta1,6 branched complex type glycans in this process. Our data show that alpha3beta1 integrin function may be altered by glycosylation, that both subunits contribute to these changes, and that glycosylation may be considered a newly found mechanism in the regulation of integrin function. 
 Plakophilin 2 affects cell migration by modulating focal adhesion dynamics and integrin protein expression. Plakophilin 2 (PKP2), a desmosome component, modulates the activity and localization of the small GTPase RhoA at sites of cell-cell contact. PKP2 regulates cortical actin rearrangement during junction formation, and its loss is accompanied by an increase in actin stress fibers. We hypothesized that PKP2 may regulate focal adhesion dynamics and cell migration. Here we show that PKP2-deficient cells bind efficiently to the   extracellular matrix  , but upon spreading display total cell areas ≈ 30% smaller than control cells. Focal adhesions in PKP2-deficient cells are ≈ 2 × larger and more stable than in control cells, and vinculin displays an increased time for fluorescence recovery after photobleaching. Furthermore, β4 and β1 integrin protein and mRNA expression is elevated in PKP2-silenced cells. Normal focal adhesion phenotypes can be restored in PKP2-null cells by dampening the RhoA pathway or silencing β1 integrin. However, integrin expression levels are not restored by RhoA signaling inhibition. These data uncover a potential role for PKP2 upstream of β1 integrin and RhoA in integrating cell-cell and cell-substrate contact signaling in basal keratinocytes necessary for the morphogenesis, homeostasis, and reepithelialization of the stratified epidermis. 
 Transforming growth factor beta 1 increases fibronectin deposition through integrin receptor alpha 5 beta 1 on human airway smooth muscle. Integrin receptors signal to and from the   extracellular matrix  . Altered expression of the integrin receptors, such as the fibronectin receptor alpha(5)beta(1), might be implicated in   extracellular matrix   accumulation in airway remodeling in asthma.We examined the effect of TGF-beta stimulation on integrin alpha(5)beta(1) expression and the role of alpha(5)beta(1) in fibronectin deposition and proliferation.Integrin subunit alpha(5) and beta(1) expression in airway smooth muscle (ASM) from subjects with and without asthma was examined by means of PCR and flow cytometry. The effect of blocking alpha(5)beta(1) receptor on ASM proliferation to FBS was assessed by using the 3-(4,5-dimethylthiazol-2-yl)-2,5-diphenyltetrazolium assay. Cells were stimulated with TGF-beta in the presence or absence of extracellular signal-regulated kinase, phosphoinositide-3 kinase, or p38 inhibitors and antibodies to the alpha(5) and beta(1) subunits. The effect of blocking alpha(5)beta(1) receptor on fibronectin deposition was assessed by means of immunocytochemistry.Proliferation of ASM cells from asthmatic and nonasthmatic subjects was inhibited by blocking the fibronectin receptor subunit alpha(5)beta(1). TGF-beta-induced alpha(5)beta(1) was extracellular signal-regulated kinase dependent but not phosphoinositide-3 kinase or p38 dependent. Blockade of the alpha(5)beta(1) receptor inhibited TGF-beta-induced fibronectin matrix deposition.Through its increased expression by the profibrotic stimulus TGF-beta, integrin alpha(5)beta(1) might be important in regulating fibronectin deposition. 
 Crystal structure of α5β1 integrin ectodomain: atomic details of the fibronectin receptor. Integrin α5β1 is a major cellular receptor for   the extracellular ma  trix protein fibronectin and plays a fundamental role during mammalian development. A crystal structure of the α5β1 integrin headpiece fragment bound by an allosteric inhibitory antibody was determined at a 2.9-Å resolution both in the absence and presence of a ligand peptide containing the Arg-Gly-Asp (RGD) sequence. The antibody-bound β1 chain accommodated the RGD ligand with very limited structural changes, which may represent the initial step of cell adhesion mediated by nonactivated integrins. Furthermore, a molecular dynamics simulation pointed to an important role for Ca(2+) in the conformational coupling between the ligand-binding site and the rest of the molecule. The RGD-binding pocket is situated at the center of a trenchlike exposed surface on the top face of α5β1 devoid of glycosylation sites. The structure also enabled the precise prediction of the acceptor residue for the auxiliary synergy site of fibronectin on the α5 subunit, which was experimentally confirmed by mutagenesis and kinetic binding assays. 
 CD151 promotes α3β1 integrin-dependent organization of carcinoma cell junctions and restrains collective cell invasion. Integrins function in collective migration both as major receptors fo  r extracellular matr  ix and by crosstalk to adherens junctions. Despite extensive research, important questions remain about how integrin signaling mechanisms are integrated into collective migration programs. Tetraspanins form cell surface complexes with a subset of integrins and thus are good candidates for regulating the balance of integrin functional inputs into cell-matrix and cell-cell interactions. For example, tetraspanin CD151 directly associates with α3β1 integrin in carcinoma cells and promotes rapid α3β1-dependent single cell motility, but CD151 also promotes organized adherens junctions and restrains collective carcinoma cell migration on 2D substrates. However, the individual roles of CD151s integrin partners in CD151s pro-junction activity in carcinoma cells were not well understood. Here we find that CD151 promotes organized carcinoma cell junctions via α3β1 integrin, by a mechanism that requires the a3b1 ligand, laminin-332. Loss of CD151 promotes collective 3D invasion and growth in vitro and in vivo, and the enhanced invasion of CD151-silenced cells is α3 integrin dependent, suggesting that CD151 can regulate the balance between α3β1s pro-junction and pro-migratory activities in collective invasion. An analysis of human cancer cases revealed that changes in CD151 expression can be linked to either better or worse clinical outcomes depending on context, including potentially divergent roles for CD151 in different subsets of breast cancer cases. Thus, the role of the CD151-α3β1 complex in carcinoma progression is context dependent, and may depend on the mode of tumor cell invasion. 
 T-cadherin enhances cell-matrix adhesiveness by regulating beta1 integrin trafficking in cutaneous squamous carcinoma cells. T-cadherin is a glycosyl-phosphatidylinositol (GPI) anchored cadherin molecule. We previously reported that T-cadherin is normally expressed on the basal keratinocytes of the epidermis and is down-regulated in cutaneous squamous cell carcinoma (SCC). We found that expression of T-cadherin in cutaneous squamous carcinoma cells regulated level of surface beta1 integrin, which functioned as   extracellular matrix   (ECM) receptor. Involvement of T-cadherin in beta1 integrin trafficking was studied using three different stable cell lines with cytomegalovirus (CMV)-driven over-expression, tetracycline (Tet)-inducible expression and RNAi-mediated suppressed expression of T-cadherin. Pulse-chase analysis using a cholesterol-depleting reagent and a tyrosine kinase inhibitor showed that beta1 integrin mainly internalized via caveolae. Over-expression of T-cadherin suppressed the internalization of both beta1 integrin and cholera toxin (CTX), a marker of caveolae-mediated endocytosis. By Western blot analysis of tyrosine-kinase target molecules, we demonstrated a reduced level of EGF receptor (EGFR)-phosphorylation in T-cadherin over-expressing cells. In addition, studies using EGF and EGFR specific inhibitors revealed that EGFR activation stimulated beta1 integrin internalization. Taking these results together, T-cadherin may modulate cell-matrix adhesion in basal keratinocytes as well as invasive potency in SCC by regulating surface level of beta1 integrin. 
 BB0172, a Borrelia burgdorferi outer membrane protein that binds integrin α3β1. Lyme disease is a multisystemic disorder caused by Borrelia burgdorferi infection. Upon infection, some B. burgdorferi genes are upregulated, including members of the microbial surface components recognizing adhesive matrix molecule (MSCRAMM) protein family, which facilitate B. burgdorferi adherence t  o extracellular matr  ix components of the host. Comparative genome analysis has revealed a new family of B. burgdorferi proteins containing the von Willebrand factor A (vWFA) domain. In the present study, we characterized the expression and membrane association of the vWFA domain-containing protein BB0172 by using in vitro transcription/translation systems in the presence of microsomal membranes and with detergent phase separation assays. Our results showed evidence of BB0172 localization in the outer membrane, the orientation of the vWFA domain to the extracellular environment, and its function as a metal ion-dependent integrin-binding protein. This is the first report of a borrelial adhesin with a metal ion-dependent adhesion site (MIDAS) motif that is similar to those observed in eukaryotic integrins and has a similar function. 
 Cyclic mechanical reinforcement of integrin-ligand interactions. Cells regulate adhesion in response to internally generated and externally applied forces. Integrins connect the   extracellular matrix   to the cytoskeleton and provide cells with mechanical anchorages and signaling platforms. Here we show that cyclic forces applied to a fibronectin-integrin α5β1 bond switch the bond from a short-lived state with 1 s lifetime to a long-lived state with 100 s lifetime. We term this phenomenon "cyclic mechanical reinforcement," as the bond strength remembers the history of force application and accumulates over repeated cycles, but does not require force to be sustained. Cyclic mechanical reinforcement strengthens the fibronectin-integrin α5β1 bond through the RGD binding site of the ligand with the synergy binding site greatly facilitating the process. A flexible integrin hybrid domain is also important for cyclic mechanical reinforcement. Our results reveal a mechanical regulation of receptor-ligand interactions and identify a molecular mechanism for cell adhesion strengthening by cyclic forces. 
 Perlecan proteolysis induces an alpha2beta1 integrin- and Src family kinase-dependent anti-apoptotic pathway in fibroblasts in the absence of focal adhesion kinase activation. Dysregulation of apoptosis in endothelial cells (EC) and fibroblasts contributes to fibrosis. We have shown previously that apoptosis of EC triggers the proteolysis of   extracellular matrix   components and the release of a C-terminal fragment of perlecan, which in turn inhibits apoptosis of fibroblasts. Here we have defined the receptors and pathways implicated in this anti-apoptotic response in fibroblasts. Neutralizing alpha2beta1 integrin activity in fibroblasts exposed to either medium conditioned by apoptotic EC (SSC) or a recombinant perlecan C-terminal fragment (LG3) prevented resistance to apoptosis and is associated with decreased levels of Akt phosphorylation. Co-incubation of fibroblasts for 24 h with SSC or LG3 in the presence of PP2 (AG1879), a biochemical inhibitor of Src family kinases (SFKs) and focal adhesion kinase, showed a significantly decreased anti-apoptotic response. However, focal adhesion kinase gene silencing with RNA interference did not inhibit the anti-apoptotic response in fibroblasts. Src phosphorylation was increased in fibroblasts exposed to SSC, and transfection of fibroblasts with constitutively active Src mutants induced an anti-apoptotic response that was not further increased by SSC. Also, Src(-/-)Fyn(-/-) fibroblasts failed to mount an anti-apoptotic response in presence of SSC for 24 h but developed a complete anti-apoptotic response when exposed to SSC for 7 days. These results suggest that   extracellular matrix   fragments produced by apoptotic EC initiate a state of resistance to apoptosis in fibroblasts via an alpha2beta1 integrin/SFK (Src and Fyn)/phosphatidylinositol 3-kinase (PI3K)-dependent pathway. In the long term, additional SFK members are recruited for sustaining the anti-apoptotic response, which could play crucial roles in abnormal fibrogenic healing. 
 Alpha1beta1 integrin is crucial for accumulation of epidermal T cells and the development of psoriasis. Psoriasis is a common T cell-mediated autoimmune inflammatory disease. We show that blocking the interaction of alpha1beta1 integrin (VLA-1) with collagen prevented accumulation of epidermal T cells and immunopathology of psoriasis. Alpha1beta1 integrin, a major collagen-binding surface receptor, was exclusively expressed by epidermal but not dermal T cells. Alpha1beta1-positive T cells showed characteristic surface markers of effector memory cells and contained high levels of interferon-gamma but not interleukin-4. Blockade of alpha1beta1 inhibited migration of T cells into the epidermis in a clinically relevant xenotransplantation model. This was paralleled by a complete inhibition of psoriasis development, comparable to that caused by tumor necrosis factor-alpha blockers. These results define a crucial role for alpha1beta1 in controlling the accumulation of epidermal type 1 polarized effector memory T cells in a common human immunopathology and provide the basis for new strategies in psoriasis treatment focusing on T cell-  extracellular matrix   interactions. 
 miRNA-29c suppresses lung cancer cell adhesion to   extracellular matrix   and metastasis by targeting integrin β1 and matrix metalloproteinase2 (MMP2). Our pilot study using miRNA arrays found that miRNA-29c (miR-29c) is differentially expressed in the paired low-metastatic lung cancer cell line 95C compared to the high-metastatic lung cancer cell line 95D. Bioinformatics analysis shows that integrin β1 and matrix metalloproteinase 2 (MMP2) could be important target genes of miR-29c. Therefore, we hypothesized that miR-29c suppresses lung cancer cell adhesion t  o extracellular matr  ix (ECM) and metastasis by targeting integrin β1 and MMP2. The gain-of-function studies that raised miR-29c expression in 95D cells by using its mimics showed reductions in cell proliferation, adhesion to ECM, invasion and migration. In contrasts, loss-of-function studies that reduced miR-29c by using its inhibitor in 95C cells promoted proliferation, adhesion to ECM, invasion and migration. Furthermore, the dual-luciferase reporter assay demonstrated that miR-29c inhibited the expression of the luciferase gene containing the 3'-UTRs of integrin β1 and MMP2 mRNA. Western blotting indicated that miR-29c downregulated the expression of integrin β1 and MMP2 at the protein level. Gelatin zymography analysis further confirmed that miR-29c decreased MMP2 enzyme activity. Nude mice with xenograft models of lung cancer cells confirmed that miR-29c inhibited lung cancer metastasis in vivo, including bone and liver metastasis. Taken together, our results demonstrate that miR-29c serves as a tumor metastasis suppressor, which suppresses lung cancer cell adhesion to ECM and metastasis by directly inhibiting integrin β1 and MMP2 expression and by further reducing MMP2 enzyme activity. The results show that miR-29c may be a novel therapeutic candidate target to slow lung cancer metastasis. 
 Integrin β1 mediates epithelial growth factor-induced invasion in human ovarian cancer cells. Integrins function as cell  -extracellular matri  x adhesion proteins and have been implicated in tumor progression. In ovarian tumors, elevated integrin β1 expression correlates with high clinical stage and poor patient survival. In this study, we report that EGF treatment up-regulated integrin β1 mRNA and protein levels in ovarian cancer cells. Moreover, pharmacological inhibition of MEK totally abolished EGF-induced integrin β1 up-regulation and cell invasion suggesting that MAPK/ERK signaling is required for EGF-induced integrin β1 up-regulation and cell invasion. Furthermore, we found that knockdown of integrin β1 expression reduced the intrinsic invasiveness of ovarian cancer cells and the EGF-induced cell invasion. Finally, we found that overexpression of integrin β1 was sufficient to promote ovarian cancer cell invasion. This study demonstrates that integrin β1 mediates EGF-induced cell invasion in ovarian cancer. 
 Beta 1D integrin displaces the beta 1A isoform in striated muscles: localization at junctional structures and signaling potential in nonmuscle cells. The cytoplasmic domains of integrins provide attachment of these   extracellular matrix   receptors to the cytoskeleton and play a critical role in integrin-mediated signal transduction. In this report we describe the identification, expression, localization, and initial functional characterization of a novel form of beta 1 integrin, termed beta 1D. This isoform contains a unique alternatively spliced cytoplasmic domain of 50 amino acids, with the last 24 amino acids encoded by an additional exon. Of these 24 amino acids, 11 are conserved when compared to the beta 1A isoform, but 13 are unique (Zhidkova, N. I., A. M. Belkin, and R. Mayne. 1995. Biochem. Biophys. Res. Commun. 214:279-285; van der Flier, A., I. Kuikman, C. Baudoin, R, van der Neuf, and A. Sonnenberg. 1995. FEBS Lett. 369:340-344). Using an anti-peptide antibody against the beta 1D integrin subunit, we demonstrated that the beta 1D isoform is synthesized only in skeletal and cardiac muscles, while very low amounts of beta 1A were detected by immunoblot in striated muscles. Whereas beta 1A could not be detected in adult skeletal muscle fibers and cardiomyocytes by immunofluorescence, beta 1D was localized to the sarcolemma of both cell types. In skeletal muscle, beta 1D was concentrated in costameres, myotendinous, and neuromuscular junctions. In cardiac muscle this beta 1 isoform was found in costamers and intercalated discs. beta 1D was associated with alpha 7A and alpha 7B in adult skeletal muscle. In cardiomyocytes of adult heart, alpha 7B was the major partner for the beta 1D isoform. beta 1D could not be detected in proliferating C2C12 myoblasts, but it appeared immediately after myoblast fusion and its amount continued to rise during myotube growth and maturation. In contrast, expression of the beta 1A isoform was downregulated during myodifferentiation in culture and it was completely displaced by beta 1D in mature differentiated myotubes. We also analyzed some functional properties of the beta 1D integrin subunit. Expression of human beta 1D in CHO cells led to its localization at focal adhesions. Clustering of this integrin isoform on the cell surface stimulated tyrosine phosphorylation of pp125FAK (focal adhesion kinase) and caused transient activation of mitogen-activated protein (MAP) kinases. These data indicate that beta 1D and beta 1A integrin isoforms are functionally similar with regard to integrin-mediated signaling. 
 Quantitative Glycoproteomic Analysis Identifies Platelet-Induced Increase of Monocyte Adhesion via the Up-Regulation of Very Late Antigen 5. Physiological stimuli, such as thrombin, or pathological stimuli, such as lysophosphatidic acid (LPA), activate platelets circulating in blood. Once activated, platelets bind to monocytes via P-selectin-PSGL-1 interactions but also release the stored contents of their granules. These platelet releasates, in addition to direct platelet binding, activate monocytes and facilitate their recruitment to atherosclerotic sites. Consequently, understanding the changes platelet releasates induce in monocyte membrane proteins is critical. We studied the glyco-proteome changes of THP-1 monocytic cells affected by LPA- or thrombin-induced platelet releasates. We employed lectin affinity chromatography combined with filter aided sample preparation to achieve high glyco- and membrane protein and protein sequence coverage. Using stable isotope labeling by amino acids in cell culture, we quantified 1715 proteins, including 852 membrane and 500 glycoproteins, identifying the up-regulation of multiple proteins involved in monocyte   extracellular matrix   binding and transendothelial migration. Flow cytometry indicated expression changes of integrin α5, integrin β1, PECAM-1, and PSGL-1. The observed increase in monocyte adhesion to fibronectin was determined to be mediated by the up-regulation of very late antigen 5 via a P-selectin-PSGL-1 independent mechanism. This novel aspect could be validated on CD14+ human primary monocytes, highlighting the benefits of the improved enrichment method regarding high membrane protein coverage and reliable quantification. 
 Eukaryotic translation initiation factor 4E binding protein 1 (4EBP-1) function is suppressed by Src and protein phosphatase 2A (PP2A) on   extracellular matrix  . Human lung fibroblasts utilize integrins to attach and proliferate on type I collagen. β1 integrin is the major integrin subunit for this attachment. Integrins coordinate cellular responses to cell-cell and cell  -extracellular matri  x interactions that regulate a variety of biological processes. Although β1 integrin-mediated signaling pathways in lung fibroblasts have been studied, a detailed molecular mechanism regulating translational control of gene expression by 4EBP-1 is not understood. 4EBP-1 inhibits cap-dependent translation by binding to the eIF4E translation initiation factor. We found that when lung fibroblasts attach to collagen via β1 integrin, high Src activity suppresses 4EBP-1 expression via PP2A, and the decrease of 4EBP-1 is due to protein degradation. The inhibition of Src activity dramatically increases PP2A and 4EBP-1 expression. Furthermore ectopic expression of PP2A, or PP2A silencing using PP2A siRNA confirmed that 4EBP-1 is regulated by PP2A. In addition, we found that 4EBP-1 inhibition by fibroblast attachment to collagen increases cap-dependent translation. Our study showed that when lung fibroblasts are attached to collagen matrix, the β1 integrin/Src/PP2A-mediated 4EBP-1 regulatory pathway is activated. We suggest that β1 integrin-mediated signaling pathway may be a crucial event in regulating fibroblast translational control machinery on collagen matrix. 
 In vivo cleaved CDCP1 promotes early tumor dissemination via complexing with activated β1 integrin and induction of FAK/PI3K/Akt motility signaling. Specific cleavage of the transmembrane molecule, CUB domain-containing protein-1 (CDCP1), by plasmin-like serine proteases induces outside-in signal transduction that facilitates early stages of spontaneous metastasis leading to tumor cell intravasation, namely cell escape from the primary tumor, stromal invasion and transendothelial migration. We identified active β1 integrin as a biochemical and functional partner of the membrane-retained 70-kDa CDCP1 fragment, newly generated from its full-length 135-kDa precursor though proteolytic cleavage by serine proteases. Both in cell cultures and in live animals, active β1 integrin complexed preferentially with functionally activated, phosphorylated 70-kDa CDCP1. Complexing of β1 integrin the 70-kDa with CDCP1 fragment induced intracellular phosphorylation signaling, involving focal adhesion kinase-1 (FAK) and PI3 kinase (PI3K)-dependent Akt activation. Thus, inhibition of FAK/PI3K activities by specific inhibitors as well as short-hairpin RNA downregulation of β1 integrin significantly reduced FAK/Akt phosphorylation under conditions where CDCP1 was processed by serine proteases, indicating that FAK/PI3K/Akt pathway operates downstream of cleaved CDCP1 complexed with β1 integrin. Furthermore, this complex-dependent signaling correlated positively with high levels of tumor cell intravasation and dissemination. Correspondingly, abrogation in vivo of CDCP1 cleavage either by unique cleavage-blocking monoclonal antibody 10-D7 or by inhibition of proteolytic activity of plasmin-like serine proteases with aprotinin prevented β1 integrin/CDCP1 complexing and downstream FAK/Akt signaling concomitant with significant reduction of stromal invasion and spontaneous metastasis. Therefore, β1 integrin appears to serve as a motility-regulating partner mediating cross-talk between proteolytically cleaved, membrane-retained CDCP1 and members of FAK/PI3K/Akt pathway. This CDCP1 cleavage-induced signaling cascade constitutes a unique mechanism, indepen  dent of extracellula  r matrix remodeling, whereby a proteolytically cleaved CDCP1 regulates in vivo locomotion and metastasis of tumor cells through β1 integrin partnering. Our findings indicate that CDCP1 cleavage, occurring at the apex of a β1 integrin/FAK/PI3K/Akt signaling cascade, may represent a therapeutic target for CDCP1-positive cancers. 
 Structural diversity in integrin/talin interactions. The adhesion of integrins to the   extracellular matrix   is regulated by binding of the cytoskeletal protein talin to the cytoplasmic tail of the β-integrin subunit. Structural studies of this interaction have hitherto largely focused on the β3-integrin, one member of the large and diverse integrin family. Here, we employ NMR to probe interactions and dynamics, revealing marked structural diversity in the contacts between β1A, β1D, and β3 tails and the Talin1 and Talin2 isoforms. Coupled with analysis of recent structures of talin/β tail complexes, these studies elucidate the thermodynamic determinants of this heterogeneity and explain why the Talin2/β1D isoforms, which are co-localized in striated muscle, form an unusually tight interaction. We also show that talin/integrin affinity can be enhanced 1000-fold by deleting two residues in the β tail. Together, these studies illustrate how the integrin/talin interaction has been fine-tuned to meet varying biological requirements. 
 Identification of a novel interaction between integrin beta1 and 14-3-3beta. Integrins are cell surface receptors for   extracellular matrix  , which play important roles in a variety of biological processes. 14-3-3 proteins are a highly conserved family of cytoplasmic proteins that associate with several intracellular signaling molecules in regulation of various cellular functions. Here, we report identification of an interaction between the integrin beta1 cytoplasmic domain and 14-3-3beta by using the yeast two-hybrid screen. Like several other proteins, the integrin beta1 cytoplasmic domain associated with 14-3-3beta by a non-phosphoserine mechanism. The 14-3-3beta/integrin beta1 interaction was confirmed by in vitro binding assays as well as co-precipitation in vivo. Furthermore, we found that 14-3-3beta co-localized with integrin beta1 during the early stage of cell spreading on fibronectin, suggesting a potential role of the 14-3-3beta/integrin beta1 interaction in the regulation of cell adhesion. Using tetracycline-regulated expression system, we showed that overexpression of 14-3-3beta stimulated cell spreading and migration on fibronectin but not on poly-L-lysine. However, the induced expression of 14-3-3beta did not affect tyrosine phosphorylation of FAK or its substrates, p130(cas) and paxillin, suggesting that 14-3-3beta regulated integrin-mediated cell spreading and migration by FAK-independent mechanisms. Taken together, these results identify an interaction between integrin and 14-3-3 proteins and suggest a potentially novel cellular function for 14-3-3 proteins in the regulation of integrin-mediated cell adhesion and signaling events. 
 Fibulin-5 regulates keloid-derived fibroblast-like cells through integrin beta-1. Keloid scar is pathological tissue that appears after skin injury, and that is more aggressive than hypertrophic scars. Keloid scars are characterized by increased proliferation of fibroblast-like cells (FLCs) and the accumulation of   extracellular matrix  , mainly collagen. Fibulin-5, a glycoprotein secreted by many cell types, is a component of the   extracellular matrix  . We investigated the effect of fibulin-5 on the adhesion and proliferation of FLCs derived from keloid scars and the role of integrin beta-1 in these activities.Fibroblast-like cells were isolated from six keloid scars and cultured on plates coated with fibulin-5 or with gelatin. Cells were incubated for 72-96 h to examine proliferation rates and incubated for 240 min, with washings at 20, 40, 60, 90, 120, 180 min, to assess adhesion rates. To examine the role of integrin beta-1, the anti-human integrin beta-1 (CD29) antibody was added to the culture medium.Fibroblast-like cells from keloids cultured on a fibulin-5-coated surface showed a significantly reduced proliferation rate and a delayed adhesion rate, compared to cells cultured on gelatin-coated dishes. Adherence of these cells to fibulin-5 pre-coated wells was significantly reduced in the presence of anti-human integrin beta-1 (CD29) antibodies. Our current findings are similar to previously observed reduced proliferation in vascular smooth muscle cells overexpressing fibulin-5. We did not test the effects of fibulin-5 on normal fibroblasts.This study demonstrates the pivotal role of the extracellular protein, fibulin-5, on the adhesion and proliferation of human keloid-derived cells, through binding to integrin beta-1. 
 The alpha(2)beta(1) and alpha(3)beta(1) integrins do not mediate attachment of endometrial cells to peritoneal mesothelium. To evaluate the possible role of mesothelial alpha(2)beta(1) and alpha(3)beta(1) integrins in the attachment of endometrial stromal cells (ESCs) and endometrial epithelial cells (EECs).In vitro study.University medical center.Women of reproductive age (n = 26).Mesothelial cells were grown on collagen IV. Endometrial stromal cells and EECs were plated on mesothelial cells for 1 hour. Before plating, mesothelial cells or endometrial cells were incubated with antibodies to alpha2, alpha3, and beta1 integrin subunits. The effect of these antibodies on ESC and EEC binding to collagen IV and collagen I was also examined. The expression of collagen I, collagen IV, fibronectin, and laminin by cultured ESCs and EECs was examined.The anti-integrin antibodies had no effect on endometrial binding to mesothelium. The beta1 integrin antibody decreased binding of ESCs and EECs to the collagen matrices. In culture, ESCs and EECs expressed collagen I, collagen IV, fibronectin, and laminin to varying degrees.The initial adhesion of ESCs and EECs to mesothelium is not mediated by beta1 integrins. In contrast, ESC and EEC attachment to collagen IV and collagen I, which are present in the submesothelial   extracellular matrix  , is mediated by beta1 integrins. 
 Integrin β1 regulates leiomyoma cytoskeletal integrity and growth. Uterine leiomyomas are characterized by an excessive   extracellular matri  x, increased mechanical stress, and increased active RhoA. Previously, we observed that mechanical signaling was attenuated in leiomyoma, but the mechanisms responsible remain unclear. Integrins, especially integrin β1, are transmembrane adhesion receptors that coupl  e extracellular matr  ix stresses to the intracellular cytoskeleton to influence cell proliferation and differentiation. Here we characterized integrin and laminin to signaling in leiomyoma cells. We observed a 2.25±0.32 fold increased expression of integrin β1 in leiomyoma cells, compared to myometrial cells. Antibody-mediated inhibition of integrin β1 led to significant growth inhibition in leiomyoma cells and a loss of cytoskeletal integrity. Specifically, polymerization of actin filaments and formation of focal adhesions were reduced by inhibition of integrin β1. Inhibition of integrin β1 in leiomyoma cells led to 0.81±0.02 fold decrease in active RhoA, and resembled levels found in serum-starved cells. Likewise, inhibition of integrin β1 was accompanied by a decrease in phospho-ERK. Compared to myometrial cells, leiomyoma cells demonstrated increased expression of integrin α6 subunit to laminin receptor (1.91±0.11 fold), and increased expression of laminin 5α (1.52±0.02), laminin 5β (3.06±0.92), and laminin 5γ (1.66±0.06). Of note, leiomyoma cells grown on laminin matrix appear to realign themselves. Taken together, the findings reveal that the attenuated mechanical signaling in leiomyoma cells is accompanied by an increased expression and a dependence on integrin β1 signaling in leiomyoma cells, compared to myometrial cells. 
 Syndecan- and integrin-binding peptides synergistically accelerate cell adhesion. Integrins and syndecans mediate cell adhesion to   extracellular matrix   and their synergistic cooperation is implicated in cell adhesion processes. We previously identified two active peptides, AG73 and EF1, from the laminin alpha1 chain LG4 module, that promote cell attachment through syndecan- and alpha2beta1 integrin-binding, respectively. Here, we examined time-dependent cell attachment on the mixed peptides AG73/EF1. The AG73/EF1 promoted stronger and more rapid cell attachment, spreading, FAK phosphorylation that reached a maximum at 20 min than that on AG73 (40 min) or EF1 (90 min) supplied singly. Thus, the syndecan- and alpha2beta1 integrin-binding peptides synergistically affect cells and accelerate cell adhesion. 
 Multiple beta 1 integrins mediate enhancement of human airway smooth muscle cytokine secretion by fibronectin and type I collagen. Altered airway smooth muscle (ASM) function and enrichment of the   extracellular matrix   (ECM) with interstitial collagen and fibronectin are major pathological features of airway remodeling in asthma. We have previously shown that these ECM components confer enhanced ASM proliferation in vitro, but their action on its newly characterized secretory function is unknown. Here, we examined the effects of fibronectin and collagen types I, III, and V on IL-1beta-dependent secretory responses of human ASM cells, and characterized the involvement of specific integrins. Cytokine production (eotaxin, RANTES, and GM-CSF) was evaluated by ELISA, RT-PCR, and flow cytometry. Function-blocking integrin mAbs and RGD (Arg-Gly-Asp)-blocking peptides were used to identify integrin involvement. IL-1beta-dependent release of eotaxin, RANTES, and GM-CSF was enhanced by fibronectin and by fibrillar and monomeric type I collagen, with similar changes in mRNA abundance. Collagen types III and V had no effect on eotaxin or RANTES release but did modulate GM-CSF. Analogous changes in intracellular cytokine accumulation were found, but in <25% of the total ASM cell population. Function-blocking Ab and RGD peptide studies revealed that alpha2beta1, alpha5beta1, alphavbeta1, and alphavbeta3 integrins were required for up-regulation of IL-1beta-dependent ASM secretory responses by fibronectin, while alpha2beta1 was an important transducer for type I collagen. Thus, fibronectin and type I collagen enhance IL-1beta-dependent ASM secretory responses through a beta1 integrin-dependent mechanism. Enhancement of cytokine release from ASM by these ECM components may contribute to airway wall inflammation and remodeling in asthma. 
 Distinct structural requirements for binding of the integrins alphavbeta6, alphavbeta3, alphavbeta5, alpha5beta1 and alpha9beta1 to osteopontin. The   extracellular matrix   protein, osteopontin, is a ligand for several members of the integrin family, including alpha5beta1, alphavbeta3, alphavbeta5 and alpha9beta1. Osteopontin is a substrate for a number of extracellular proteases, including thrombin and the metalloproteases MMP-3 and MMP-7, which cleave osteopontin at sites close to or within the mapped integrin binding sites. Using affinity chromatography and cell adhesion assays, we now identify the integrin alphavbeta6 as an additional osteopontin receptor. Utilizing a series of recombinant forms of osteopontin, we compared the structural requirements for alphavbeta6 binding with those for the 4 other osteopontin-binding integrins. Like alpha5beta1, alphavbeta3 and alphavbeta5 (but not alpha9beta1), alphavbeta6 binds to the RGD site in osteopontin, since RGD peptide or mutation of this site to RAA completely inhibits alphavbeta6-mediated cell adhesion. For both alpha9beta1 and alpha5beta1, the N-terminal fragment generated by thrombin cleavage is a much better ligand than full length osteopontin, whereas thrombin-cleavage does not appear to be required for optimal adhesion to alphavbeta3, alphavbeta5 or alphavbeta6. A recombinant fragment predicted to be generated by MMP cleavage no longer supported alpha5beta1 or alpha9beta1-mediated adhesion, but adhesion mediated by alphavbeta5 or alphavbeta6 was unaffected. Finally, adhesion of alphavbeta5 or alphavbeta6 was inhibited by mutation of two aspartic acid residues upstream of the RGD site, whereas adhesion mediated by alphavbeta3, alpha5beta1 or alpha9beta1 was unaffected by these mutations. These results suggest that the hierarchy of integrin interactions with osteopontin can undergo complex regulation at least in part through the action of extracellular proteases. 
 Proteomic analysis of α4β1 integrin adhesion complexes reveals α-subunit-dependent protein recruitment. Integrin adhesion receptors mediate cell-cell and ce  ll-extracellular mat  rix interactions, which control cell morphology and migration, differentiation, and tissue integrity. Integrins recruit multimolecular adhesion complexes to their cytoplasmic domains, which provide structural and mechanosensitive signaling connections between the extracellular and intracellular milieux. The different functions of specific integrin heterodimers, such as α4β1 and α5β1, have been attributed to distinct signal transduction mechanisms that are initiated by selective recruitment of adhesion complex components to integrin cytoplasmic tails. Here, we report the isolation of ligand-induced adhesion complexes associated with wild-type α4β1 integrin, an activated α4β1 variant in the absence of the α cytoplasmic domain (X4C0), and a chimeric α4β1 variant with α5 leg and cytoplasmic domains (α4Pα5L), and the cataloguing of their proteomes by MS. Using hierarchical clustering and interaction network analyses, we detail the differential recruitment of proteins and highlight enrichment patterns of proteins to distinct adhesion complexes. We identify previously unreported components of integrin adhesion complexes and observe receptor-specific enrichment of molecules with previously reported links to cell migration and cell signaling processes. Furthermore, we demonstrate colocalization of MYO18A with active integrin in migrating cells. These datasets provide a resource for future studies of integrin receptor-specific signaling events. 
 Mixed   extracellular matrix   ligands synergistically modulate integrin adhesion and signaling. Cell adhesion to   extracellular matrix   (ECM) components through cell-surface integrin receptors is essential to the formation, maintenance and repair of numerous tissues, and therefore represents a central theme in the design of bioactive materials that successfully interface with the body. While the adhesive responses associated with a single ligand have been extensively analyzed, the effects of multiple integrin subtypes binding to multivalent ECM signals remain poorly understood. In the present study, we generated a high throughput platform of non-adhesive surfaces presenting well-defined, independent densities of two integrin-specific engineered ligands for the type I collagen (COL-I) receptor alpha(2)beta(1) and the fibronectin (FN) receptor alpha(5)beta(1) to evaluate the effects of integrin cross-talk on adhesive responses. Engineered surfaces displayed ligand density-dependent adhesive effects, and mixed ligand surfaces significantly enhanced cell adhesion strength and focal adhesion assembly compared to single FN and COL-I ligand surfaces. Moreover, surfaces presenting mixed COL-I/FN ligands synergistically enhanced FAK activation compared to the single ligand substrates. The enhanced adhesive activities of the mixed ligand surfaces also promoted elevated proliferation rates. Our results demonstrate interplay between multivalent ECM ligands in adhesive responses and downstream cellular signaling. 
 Regulation of endodermal differentiation of human embryonic stem cells through integrin-ECM interactions. Many cellular responses during development are regulated by interactions between integrin receptors and   extracellular matrix   proteins (ECMPs). Although the majority of recent studies in human embryonic stem cell (hESC) differentiation have focused on the role of growth factors, such as FGF, TGFβ, and WNT, relatively little is known about the role of ECMP-integrin signaling in this process. Moreover, current strategies to direct hESC differentiation into various lineages are inefficient and have yet to produce functionally mature cells in vitro. This suggests that additional factors, such as ECMPs, are required for the efficient differentiation of hESCs. Using a high-throughput multifactorial cellular array technology, we investigated the effect of hundreds of ECMP combinations and concentrations on differentiation of several hPSC lines to definitive endoderm (DE), an early embryonic cell population fated to give rise to internal organs such as the lung, liver, pancreas, stomach, and intestine. From this screen we identified fibronectin (FN) and vitronectin (VTN) as ECMP components that promoted DE differentiation. Analysis of integrin expression revealed that differentiation toward DE led to an increase in FN-binding integrin α5 (ITGA5) and VTN-binding integrin αV (ITGAV). Conditional short hairpin RNA-mediated knockdown of ITGA5 and ITGAV disrupted hESC differentiation toward DE. Finally, fluorescence-based cell sorting for ITGA5 and ITGAV significantly enriched cells with gene expression signatures associated with DE, demonstrating that these cell surface proteins permit isolation and enrichment of DE from hESCs. These data provide evidence that FN and VTN promote endoderm differentiation of hESCs through interaction with ITGA5 and ITGAV, and that ECMP-integrin interactions are required for hESC differentiation into functionally mature cells. 
 Mouse AMACO, a kidney and skin basement membrane associated molecule that mediates RGD-dependent cell attachment. The VWA domain-containing   extracellular matrix   protein AMACO has not been extensively characterized and its function remains unknown. It has been proposed as a potential cancer marker and carries a rare O-glucosylation and O-fucosylation on its first EGF-like domain. AMACO is a basement membrane associated protein, however its exact localization has not been determined. Here we show by immunogold electron microscopy of mouse kidney and skin that AMACO does not occur within the basement membrane but rather subjacent to the basement membrane at its stromal surface. In skin, AMACO often colocalizes with triple-helical domains of collagen VII containing anchoring fibrils as they emerge from the basal lamina. However, the immunogold patterns for AMACO and the C-terminal end of collagen VII show discrete differences, indicating that AMACO and collagen VII do not colocalize at anchoring plaques. In contrast, the localization pattern of AMACO partially overlaps with that for collagen XVIII. In addition, mouse AMACO was shown to support beta1 integrin-mediated adhesion of a keratinocyte-like cell line, HaCaT, and a fibroblast cell line, Wi26, in an RGD-dependent manner, most likely using an RGD-motif near the C-terminus of AMACO. However, the loss of cell adhesion to the C-terminal part of the human AMACO, due to the unique absence of an RGD sequence in the human protein, suggests that cell adhesion is not AMACO's major function. 
 Alpha2beta1 integrin signalling enhances cyclooxygenase-2 expression in intestinal epithelial cells. Inflammatory bowel diseases (IBD) are linked to an increased risk of developing colon cancer, by inflammatory mediators and alterations to the   extracellular matrix   (ECM). The events induced by inflammatory mediators lead to dysregulated activation and induction of inflammatory genes such as cyclooxygenase-2 (COX-2). COX-2 is involved in the conversion of arachidonic acid to biologically active prostanoids and is highly upregulated in colon cancer. Since inflammation-induced changes to the   extracellular matrix   could affect integrin activities, we here investigated the effect of integrin signalling on the level of COX-2 expression in the non-transformed intestinal epithelial cell lines, Int 407 and IEC-6. Adhesion of these cells to a collagen I- or IV-coated surface, increased surface expression of alpha2beta1 integrin. Activation of integrins with collagen caused an increased cox-2 promoter activity, with a subsequent increase in COX-2 expression. The signalling cascade leading to this increased expression and promoter activity of cox-2, involves PKCalpha, the small GTPase Ras and NFkappaB but not Erk1/2 or Src activity. The integrin-induced increase in cellular COX-2 activity is responsible for an elevated generation of reactive oxygen species (ROS) and increased cell migration. This signalling pathway suggests a mechanism whereby inflammation-induced modulations of the ECM, can promote cancer transformation in the intestinal epithelial cells. 
 Integrin α9β1 promotes malignant tumor growth and metastasis by potentiating epithelial-mesenchymal transition. The integrin α9β1 binds a number   of extracellular ma  trix components to mediate cell adhesion, migration and tissue invasion. Although expressed in a variety of normal human cells including endothelium, it is also expressed in cancer cells. We have previously shown that α9β1 binds VEGF-A to facilitate angiogenesis, an important component of the tumor microenvironment. As α9β1 induces accelerated cancer cell migration, we wished to determine what role it played in cancer growth and metastasis. In this study, we show that α9β1 expression induces molecular changes consistent with epithelial-mesenchymal transition. In addition, we found that α9β1 forms a tri-partite protein complex with β-catenin and E-cadherin, which dissociates following integrin activation and subsequent src and β-catenin phosphorylation. These findings were consistent in cells in which: α9β1 was exogenously over-expressed, or when its expression was suppressed in cancer cells endogenously expressing α9β1. These in vitro results are biologically significant as α9β1-expressing cancer cells induce greater tumor growth and metastases in mice as compared to the cells without α9β1 expression or when integrin expression is suppressed. Furthermore, integrin α9β1 is expressed in primary human small cell lung cancer and patients having a high expression of α9β1 demonstrated significantly worse long-term survival compared with patients with low α9β1 expression. These findings highlight a novel mechanism of integrin α9β1 function in human cancer. 
 Proteomic analysis of integrin-associated complexes identifies RCC2 as a dual regulator of Rac1 and Arf6. The binding of integrin adhesion receptors to their   extracellular matrix   ligands controls cell morphology, movement, survival, and differentiation in various developmental, homeostatic, and disease processes. Here, we report a methodology to isolate complexes associated with integrin adhesion receptors, which, like other receptor-associated signaling complexes, have been refractory to proteomic analysis. Quantitative, comparative analyses of the proteomes of two receptor-ligand pairs, alpha(4)beta(1)-vascular cell adhesion molecule-1 and alpha(5)beta(1)-fibronectin, defined both core and receptor-specific components. Regulator of chromosome condensation-2 (RCC2) was detected in the alpha(5)beta(1)-fibronectin signaling network at an intersection between the Rac1 and adenosine 5'-diphosphate ribosylation factor 6 (Arf6) subnetworks. RCC2 knockdown enhanced fibronectin-induced activation of both Rac1 and Arf6 and accelerated cell spreading, suggesting that RCC2 limits the signaling required for membrane protrusion and delivery. Dysregulation of Rac1 and Arf6 function by RCC2 knockdown also abolished persistent migration along fibronectin fibers, indicating a functional role for RCC2 in directional cell movement. This proteomics workflow now opens the way to further dissection and systems-level analyses of adhesion signaling. 
 CD44 engagement promotes matrix-derived survival through the CD44-SRC-integrin axis in lipid rafts. CD44 is present in detergent-resistant, cholesterol-rich microdomains, called lipid rafts, in many types of cells. However, the functional significance of CD44 in lipid rafts is still unknown. We have previously demonstrated that osteopontin-mediated engagement of CD44 spliced variant isoforms promotes an   extracellular matrix  -derived survival signal through integrin activation. By using a series of CD44 mutants and pharmacological inhibitors selectively targeted to various cellular pathways, we show in this study that engagement of CD44 induces lipid raft coalescence to facilitate a CD44-Src-integrin signaling axis in lipid rafts, leading to increased matrix-derived survival. Palmitoylation of the membrane-proximal cysteine residues and carboxyl-terminal linkage to the actin cytoskeleton both contribute to raft targeting of CD44. The enrichment of integrin beta1 in lipid rafts is tightly coupled to CD44 ligation-elicited lipid raft reorganization and associated with temporally delayed endocytosis. Through the interaction with the CD44 carboxyl-terminal ankyrin domain, Src is cotranslocated to lipid rafts, where it induces integrin activation via an inside-out mechanism. Collectively, this study demonstrates an important role of the dynamic raft reorganization induced by CD44 clustering in eliciting the matrix-derived survival signal. 
 NF-κB regulates radioresistance mediated by β1-integrin in three-dimensional culture of breast cancer cells. β1-integrin induction enhances breast cancer cell survival after exposure to ionizing radiation (IR), but the mechanisms of this effect remain unclear. Although NF-κB initiates prosurvival signaling pathways post-IR, the molecular function of NF-κB with other key elements in radioresistance, particularly with respec  t to extracellular m  atrix-induced signaling, is not known. We discovered a typical NF-κB-binding site in the β1-integrin promoter region, indicating a possible regulatory role for NF-κB. Using three-dimensional lamin  in-rich extracellula  r matrix (3D lrECM) culture, we show that NF-κB is required for β1-integrin transactivation in T4-2 breast cancer cells post-IR. Inhibition of NF-κB reduced clonogenic survival and induced apoptosis and cytostasis in formed tumor colonies. In addition, T4-2 tumors with inhibition of NF-κB activity exhibit decreased growth in athymic mice, which was further reduced by IR with downregulated β1-integrin expression. Direct interactions between β1-integrin and NF-κB p65 were induced in nonmalignant breast epithelial cells, but not in malignant cells, indicating context-specific regulation. As β1-integrin also activates NF-κB, our findings reveal a novel forward feedback pathway that could be targeted to enhance therapy. 
 An activating beta1 integrin mutation increases the conversion of benign to malignant skin tumors. Identifying the physiologic relevance of cancer-associated genetic polymorphisms is a major challenge. Several changes in the coding sequence of beta integrin subunits have now been described in human tumors. One of these, T188Ibeta1, was identified as a heterozygous mutation in a poorly differentiated squamous cell carcinoma (SCC) and shown to activate   extracellular matrix   adhesion and inhibit keratinocyte differentiation in vitro. To study its contribution to tumor development, we overexpressed the mutant or wild-type (WT) human beta1 subunit in the basal layer of mouse epidermis using the keratin 14 promoter. The transgenic integrins were expressed at the cell surface and were functional, with the T188Ibeta1 subunit promoting cell spreading to a greater extent than WTbeta1. Epidermal proliferation and differentiation were unaffected and no expansion of the stem cell compartment was detected. During chemical carcinogenesis, both transgenes increased papilloma formation, but only the T188Ibeta1 transgene stimulated the conversion of papillomas to SCCs. Papillomas bearing the mutation showed increased Erk activity and reduced differentiation. SCCs expressing T188Ibeta1 were less well-differentiated than those expressing WTbeta1. These observations establish that the expression of a genetic variant in the I-like domain of beta1 integrins does not affect normal epidermal homeostasis, but increases tumor susceptibility and influences tumor type. 
 A regulated interaction between alpha5beta1 integrin and osteopontin. The   extracellular matrix   protein osteopontin (OPN) interacts with a number of integrins, namely alphavbeta1, alphavbeta3, alphavbeta5, alpha9beta1, alpha8beta1, and alpha4beta1. We have investigated the interaction of alpha5beta1 integrin with OPN using K562 cells, which only express alpha5beta1. alpha5beta1 is in a low activation state in this cell line, but can be stimulated to a higher activation state by the phorbol ester TPA. Treating K562 wild-type cells (K562-WT) with TPA stimulated an interaction between alpha5beta1 and OPN. No interaction was seen in the absence of TPA. alpha5beta1 selectively interacted with a GST fusion protein of the N-terminal fragment of OPN (aa17-168), which is generated in vivo by thrombin cleavage of OPN. Expression of the alpha4 integrin in K562 cells (K562-alpha4beta1) stimulated alpha5beta1-dependent binding to aa17-168 in the absence of TPA, suggesting that alpha4beta1 activates alpha5beta1 in K562 cells. Adhesion via alpha5beta1 is mediated by the Arg-Gly-Asp (RGD) motif of OPN, as mutating this sequence to Arg-Ala-Asp (RAD) blocked binding of both cell types. These data demonstrate that thrombin cleavage regulates the adhesive properties of OPN and that alpha5beta1 integrin can interact with thrombin-cleaved osteopontin when in a high activation state. 
 Association of the calpain/calpastatin network with subcellular organelles. The calcium-activated cysteine protease calpain is intimately involved in modulating cell adhesion and migration. The two ubiquitous isoforms of this protease, calpain I and II, are considered to be cytosolic proteins that can translocate to both focal complexes/adhesions or the plasma membrane. Using confocal microscopy and isopycnic density centrifugation, the results demonstrate that calpain I and II, the 30kDa regulatory subunit, and calpastatin associate with the endoplasmic reticulum and Golgi apparatus. Confocal microscopy reveals that calpain II colocalizes with the subcellular proteins calnexin and Rab6 in cells bound to laminin. To further verify this association, cell lysates prepared from laminin stimulated and unstimulated cells were subjected to isopycnic density centrifugation. The results reveal an increased association of calpain I, II, calpastatin, and the 30kDa regulatory subunit with the endoplasmic reticulum and Golgi apparatus as evidenced by their position in the gradient relative to calnexin, Rab6, caveolin, and beta1 integrin after laminin stimulation. This correlates with the accumulation of inducible calpain activity at the endoplasmic reticulum-Golgi apparatus interface. Further experiments established that calpain II colocalizes with phosphatidylinositol 4,5-bisphosphate. Finally, calpain II associates with membrane lipid rafts. These results provide new insights into how the calpain/calpastatin network is spatially and temporally regulated in cells binding to the   extracellular matrix  . 
 Fibrillar collagen type I stimulation of apolipoprotein B secretion in Caco-2 cells is mediated by beta1 integrin. Caco-2 cells spontaneously differentiate into enterocyte-like cells and secrete apolipoprotein B (apoB) lipoproteins. We evaluated the effect of different   extracellular matrix   proteins on lipoprotein secretion by these cells. Caco-2 cells grown on human amnion connective tissue (HACT) secreted twice as much apoB as control cells on Transwells, but secreted similar amounts of apoA1. Cells cultured on fibrillar collagen type I secreted increased amounts of apoB similar to the cells cultured on HACT, but cells cultured on non-fibrillar collagen type I, type IV collagen or laminin-1 did not. The increased secretion was nullified by a function inhibiting anti-integrin beta1 monoclonal antibody. Therefore, interactions between type I collagen and beta1 integrins augment apoB secretion by Caco-2 cells. Cells on HACT formed a more uniform columnar epithelium with lipid droplets polarized to the basolateral membrane. We also studied the effect of   extracellular matrix   proteins on transepithelial resistance (TER) of differentiated Caco-2 cells. TER in cells cultured on HACT was similar to that on Transwells, but cells on laminin-1 and collagen IV exhibited higher TER. Thus, various   extracellular matrix   proteins regulate apoB secretion and TER differently. This new observation that   extracellular matrix   proteins can enhance apoB secretion in Caco-2 cells could be useful to explore the modulation of lipid transport by these proteins. 
 Beta 1-integrin protects hepatoma cells from chemotherapy induced apoptosis via a mitogen-activated protein kinase dependent pathway. beta 1-integrin modulates cellular phenotype by mediating signals from the   extracellular matrix   (ECM). Although overexpression of integrin molecules in hepatocellular carcinoma (HCC) has been reported, the role of overexpressed beta 1-integrin in the disease process of HCC is not fully understood. The authors investigated the effects of beta 1-integrin on apoptosis in hepatoma cells.Human hepatoma cell lines HepG2, Huh7, and HLE were stably transfected with full-length beta 1-integrin. Cells underwent apoptosis induced by chemotherapeutic reagents, including cis-platinum (II)-diammine dichloride, etoposide, and docetaxel. Cell survival and intracellular signaling pathways dependent on beta 1-integrin-mediated apoptosis effects were analyzed by treating cells with PD98059 (ERK inhibitor), SB203580 (p38MAP kinase inhibitor), wortmannin (phosphatidyl inositol-3-kinase inhibitor), and herbimycin A (tyrosine kinase inhibitor).All three hepatoma cell lines overexpressing beta 1-integrin were protected from apoptosis induced by chemotherapeutic reagents, whereas parental or mock transfected cells were not. Treatment with PD98059 or SB203580 abolished the protective effect on apoptosis in cells overexpressing beta 1-integrin. Neither herbimycin nor wortmannin blocked the protective effects of beta 1-integrin overexpression.These data suggest that overexpression of beta 1-integrin confers resistance to apoptosis in hepatoma cells via a MAP kinase dependent pathway. beta1-integrin mediated signaling from the ECM in HCC cells may contribute to chemotherapy resistance. 
 Endothelial expression of TNF receptor-1 generates a proapoptotic signal inhibited by integrin α6β1 in glioblastoma. Activation of TNF receptor 1 (TNF-R1) can generate signals that promote either apoptosis or survival. In this study, we show that these signals can be determined by the character of th  e extracellular matr  ix in the tumor microenvironment. Specifically, through studies of glioblastoma, we showed that TNFα stimulation induced apoptosis of primary brain endothelial cells (EC) attached to collagen or fibronectin (which engage integrins α2β1/α3β1 and α5β1, respectively), but did not induce apoptosis of ECs attached to laminin (which engages integrins α6β1 and α3β1). TNF-R1 expression was significantly higher in ECs in glioblastoma (GBM) tumors compared with ECs in normal brain specimens. TNFα was also expressed in GBM tumor-associated ECs, which was associated with longer patient survival. ECs plated on anti-integrin α2 or α3 antibody were susceptible to TNFα-induced apoptosis, whereas those plated on anti-integrin α6 antibody were not. Moreover, the ECs plated on laminin, but not collagen, expressed cellular FLICE inhibitory protein (cFLIP) and TNFα stimulation of laminin-attached cells in which cFLIP had been downregulated resulted in the induction of apoptosis. In contrast, attachment to laminin did not induce cFLIP expression in GBM tumor stem cells. Together, our findings indicate that the laminin receptor integrin α6β1 promotes the survival of brain ECs by inhibiting prodeath signaling by TNF-R1, in part by inducing cFLIP expression. 
 The chemokine receptor 7 regulates cell adhesion and migration via beta1 integrin in metastatic squamous cell carcinoma of the head and neck. Migration and adhesion of tumor cells are essential prerequisites for the formation of metastases in malignant diseases. Chemokine receptor 7 (CCR7) has been shown to regulate integrin which can then facilitate adhesion of cancer cells to and/or migration through the   extracellular matrix   (ECM). In order to identify the connection between CCR7 and beta1 integrin, and the influence on cell adhesion and migration in metastatic squamous cell carcinoma of the head and neck (SCCHN). We use adhesion assays, migration assay, immunofluorescence staining, western blotting, and immunohistochemical analysis to find whether beta1 integrin can be activated by CCL19 (CCR7's ligand) and its role in SCCHN. The experiments were performed in the metastatic SCCHN cell line PCI-37B after pre-incubation of the cells with CCL19 and beta1 integrin inhibitors RGD-peptide. Our results demonstrate that CCR7 favours PCI-37B cell adhesion and migration, and induces reorganization of the actin cytoskeleton and up-expression of beta1 integrin protein. beta1 integrin inhibitor RGD-peptide can block all these effects. Taken together, our data indicate that CCR7 regulate cell adhesion and migration via beta1 integrin in metastatic SCCHN, and these results can provide a basis for new strategies in preventing metastases of SCCHN. 
 Role for beta1 integrin and its associated alpha3, alpha5, and alpha6 subunits in development of the human fetal pancreas. The integrin receptors play a major role in tissue morphogenesis and homeostasis by regulating cell interactions with   extracellular matrix   proteins. We have examined the expression pattern of integrin subunits in the human fetal pancreas (8-20 weeks fetal age) and the relevance of beta1 integrin function for insulin gene expression and islet cell survival. Its subunits alpha3, alpha5, and alpha6 beta1 integrins are expressed in ductal cells at 8 weeks, before glucagon- and insulin-immunoreactive cells bud off; their levels gradually increase in both ductal cells and islet clusters up to 20 weeks. Colocalization of alpha3, alpha5 and alpha6 beta1 integrins with endocrine cell markers was frequently observed in 8- to 20-week fetal pancreatic cells. When the beta1 integrin receptor was functionally blocked in cultured islet-epithelial clusters with a beta1 immunoneutralizing antibody or following transient beta1 integrin small interfering RNA treatment, there was inhibition of cell adhesion to extracellular matrices, decreased expression of insulin, and increased cell apoptosis. These data offer evidence for dynamic and cell-specific changes in integrin expression during human pancreatic islet neogenesis. They also provide an initial insight into a molecular basis for cell-matrix interactions during islet development and suggest that beta1 integrin plays a vital role in regulating islet cell adhesion, gene expression, and survival. 
 Identification of nucleolin as a lipid-raft-dependent β1-integrin-interacting protein in A375 cell migration. Lipid rafts are related to cell surface receptor function. Integrin is a major surface receptor protein in cell adhesion and migration on the   extracellular matri  x (ECM). Here, we showed that lipid rafts played a critical role in human melanoma A375 cell spreading and migration on fibronectin; an important component of the ECM that interacts with β1 integrin. We found that the disruption of lipid rafts did not markedly inhibit the expression and activation of β1 integrin. By coimmunoprecipitation and mass spectrometry, we investigated the influence of lipid rafts on the β1 integrin complex and identified nucleolin as a potential lipid-raft-dependent β1-integrin-interacting protein. Upon confirmation of the interaction between β1 integrin and nucleolin, further studies revealed that nucleolin colocalized with β1 integrin in lipid rafts and raft disruption interrupted their association. In addition, knockdown of nucleolin markedly attenuated A375 cell spreading and migration on fibronectin. Taken together, we demonstrated that nucleolin is a critical lipid-raft-dependent β1-integrin-interacting protein in A375 cell spreading and migration on fibronectin. 
 Integrin β1 mediates vaccinia virus entry through activation of PI3K/Akt signaling. Vaccinia virus has a broad range of infectivity in many cell lines and animals. Although it is known that the vaccinia mature virus binds to cell surface glycosaminoglycans and   extracellular matri  x proteins, whether additional cellular receptors are required for virus entry remains unclear. Our previous studies showed that the vaccinia mature virus enters through lipid rafts, suggesting the involvement of raft-associated cellular proteins. Here we demonstrate that one lipid raft-associated protein, integrin β1, is important for vaccinia mature virus entry into HeLa cells. Vaccinia virus associates with integrin β1 in lipid rafts on the cell surface, and the knockdown of integrin β1 in HeLa cells reduces vaccinia mature virus entry. Additionally, vaccinia mature virus infection is reduced in a mouse cell line, GD25, that is deficient in integrin β1 expression. Vaccinia mature virus infection triggers the activation of phosphatidylinositol 3-kinase (PI3K)/Akt signaling, and the treatment of cells with inhibitors to block P13K activation reduces virus entry in an integrin β1-dependent manner, suggesting that integrin β1-mediates PI3K/Akt activation induced by vaccinia virus and that this signaling pathway is essential for virus endocytosis. The inhibition of integrin β1-mediated cell adhesion results in a reduction of vaccinia virus entry and the disruption of focal adhesion and PI3K/Akt activation. In summary, our results show that the binding of vaccinia mature virus to cells mimics the outside-in activation process of integrin functions to facilitate vaccinia virus entry into HeLa cells. 
 N-ethylmaleimide-sensitive factor attachment protein α (αSNAP) regulates matrix adhesion and integrin processing in human epithelial cells. Integrin-based adhesion to th  e extracellular matr  ix (ECM) plays critical roles in controlling differentiation, survival, and motility of epithelial cells. Cells attach to the ECM via dynamic structures called focal adhesions (FA). FA undergo constant remodeling mediated by vesicle trafficking and fusion. A soluble N-ethylmaleimide-sensitive factor (NSF) attachment protein α (αSNAP) is an essential mediator of membrane fusion; however, its roles in regulating ECM adhesion and cell motility remain unexplored. In this study, we found that siRNA-mediated knockdown of αSNAP induced detachment of intestinal epithelial cells, whereas overexpression of αSNAP increased ECM adhesion and inhibited cell invasion. Loss of αSNAP impaired Golgi-dependent glycosylation and trafficking of β1 integrin and decreased phosphorylation of focal adhesion kinase (FAK) and paxillin resulting in FA disassembly. These effects of αSNAP depletion on ECM adhesion were independent of apoptosis and NSF. In agreement with our previous reports that Golgi fragmentation mediates cellular effects of αSNAP knockdown, we found that either pharmacologic or genetic disruption of the Golgi recapitulated all the effects of αSNAP depletion on ECM adhesion. Furthermore, our data implicates β1 integrin, FAK, and paxillin in mediating the observed pro-adhesive effects of αSNAP. These results reveal novel roles for αSNAP in regulating ECM adhesion and motility of epithelial cells. 
 Three-dimensional collagen represses cyclin E1 via β1 integrin in invasive breast cancer cells. The behavior of breast epithelial cells is influenced by their microenvironment which includes stromal cells and   extracellular matri  x (ECM). During cancer progression, the tissue microenvironment fails to control proliferation and differentiation, resulting in uncontrolled growth and invasion. Upon invasion, the ECM encountered by breast cancer cells changes from primarily laminin and collagen IV to primarily collagen I. We show here that culturing invasive breast cancer cells in 3-dimensional (3D) collagen I inhibits proliferation through direct regulation of cyclin E1, a G(1)/S regulator that is overexpressed in breast cancer. When the breast cancer cell line MDA-MB-231 was cultured within 3D collagen I gels, the G(1)/S transition was inhibited as compared to cells cultured on conventional 2D collagen or plastic dishes. Cells in 3D collagen downregulated cyclin E1 protein and mRNA, with no change in cyclin D1 level. Cyclin D1 was primarily cytoplasmic in 3D cultures, and this was accompanied by decreased phosphorylation of Rb, a nuclear target for both cyclin E1- and cyclin D1-associated kinases. Positive regulators of cyclin E1 expression, the transcription factor c-Myc and cold-inducible RNA binding protein (CIRP), were decreased in 3D collagen cultures, while the collagen I receptor β1 integrin was greatly increased. Inhibition of β1 integrin function rescued proliferation and cyclin E1 expression as well as c-Myc expression and Rb phosphorylation, but cyclin D1 remained cytoplasmic. We conclude that cyclin E1 is repressed independent of effects on cyclin D1 in a 3D collagen environment and dependent on β1 integrin interaction with collagen I, reducing proliferation of invasive breast cancer cells. 
 Conformational regulation of alpha 4 beta 1-integrin affinity by reducing agents. "Inside-out" signaling is independent of and additive to reduction-regulated integrin activation. The alpha(4)beta(1)-integrin (very late antigen-4 (VLA-4), CD49d/CD29) is an adhesion receptor involved in the interaction of lymphocytes, dendritic cells, and stem cells with the   extracellular matrix   and endothelial cells. This and other integrins have the ability to regulate their affinity for ligands through a process termed "inside-out" signaling that affects cell adhesion avidity. Several mechanisms are known to regulate integrin affinity and conformation: conformational changes induced by separation of the C-terminal tails, divalent ions, and reducing agents. Recently, we described a fluorescent LDV-containing small molecule that was used to monitor VLA-4 affinity changes in live cells (Chigaev, A., Blenc, A. M., Braaten, J. V., Kumaraswamy, N., Kepley, C. L., Andrews, R. P., Oliver, J. M., Edwards, B. S., Prossnitz, E. R., Larson, R. S., and Sklar, L. A. (2001) J. Biol. Chem. 276, 48670-48678). Using the same molecule, we also developed a fluorescence resonance energy transfer-based assay to probe the "switchblade-like" opening of VLA-4 upon activation. Here, we investigated the effect of reducing agents on the affinity and conformational state of the VLA-4 integrin simultaneously with cell activation initiated by inside-out signaling through G protein-coupled receptors or Mn(2+) in live cells in real time. We found that reducing agents (dithiothreitol and 2,3-dimercapto-1-propanesulfonic acid) induced multiple states of high affinity of VLA-4, where the affinity change was accompanied by an extension of the integrin molecule. Bacitracin, an inhibitor of the reductive function of the plasma membrane, diminished the effect of dithiothreitol, but had no effect on inside-out signaling. Based on this result and differences in the kinetics of integrin activation, we conclude that conformational activation of VLA-4 by inside-out signaling is independent of and additive to reduction-regulated integrin activation. 
 The Tat protein of human immunodeficiency virus type-1 promotes vascular cell growth and locomotion by engaging the alpha5beta1 and alphavbeta3 integrins and by mobilizing sequestered basic fibroblast growth factor. The Tat protein of human immunodeficiency virus type-1 (HIV-1) has been shown to be released during acute infection of T cells by HIV-1 and to promote angiogenesis and Kaposi's sarcoma (KS) development in infected individuals. In this study, we investigated the molecular mechanisms responsible for the angiogenic effects of Tat. The results shown herein indicate that two different Tat domains cooperate to induce these effects by different pathways. The arginine-glycine-aspartic acid (RGD) sequence present at the carboxyterminal of Tat mediates vascular cell migration and invasion by binding to the alpha5beta1 and alphavbeta3 integrins. This interaction also provides endothelial cells with the adhesion signal they require to grow in response to mitogens. At the same time, the Tat basic sequence retrieves into a soluble form extracellular basic fibroblast growth factor (bFGF) bound to heparan sulfate proteoglycans by competing for heparin-binding sites. This soluble bFGF mediates Tat-induced vascular cell growth. These effects resemble those of   extracellular matrix   proteins, suggesting that Tat enhances angiogenesis and promotes KS progression by a molecular mimicry of these molecules. 
 Skin equivalent tensional force alters keloid fibroblast behavior and phenotype. Skin tension may influence keloid scar behavior, development, and spreading, e.g., butterfly-shaped keloid disease in the sternum. Here, we developed a three-dimensional (3D) in vitro model to mimic in vivo tension and evaluate keloid fibroblast (KF) behavior and   extracellular matrix   synthesis under tension. In vivo skin tension measured in volunteers (n = 4) using 3D image photogrammetry enabled prediction of actual force (35 mN). A novel cell force monitor applied tension in a fibroblast-populated 3D collagen lattice replicating the in vivo force. The effect of tension on keloid (n = 10) fibroblast (KF) and normal skin (n = 10) fibroblasts (NF) at set time points (6, 12, and 24 hours) was measured in Hsp27, PAI-2, and α2β1 integrin, tension-related genes demonstrating significant (p < 0.05) time-dependent regulation of these genes in NF vs. KF with and without tension. KF showed higher (p < 0.05) proliferation post-tension. Knockdown of all three genes in 24 and 48 hours with and without tension showed significant down-regulation in NF vs. KF. Additionally, we show significant (p < 0.05) modi  fication of the expr  ession of extracellular matrix-related genes post-tension following down-regulation of Hsp27, PAI-2, or α2β1 integrin. Finally, we demonstrate significant alteration in NF compared with KF morphology following knockdown. In conclusion, this study shows induction of tension-related genes expression following mechano-regulation in KFs, with potential relevance to its development and therapy. 
 Lipid raft regulates the initial spreading of melanoma A375 cells by modulating β1 integrin clustering. Cell adhesion and spreading require integrins-mediated cell  -extracellular matri  x interaction. Integrins function through binding to   extracellular matri  x and subsequent clustering to initiate focal adhesion formation and actin cytoskeleton rearrangement. Lipid raft, a liquid ordered plasma membrane microdomain, has been reported to play major roles in membrane motility by regulating cell surface receptor function. Here, we identified that lipid raft integrity was required for β1 integrin-mediated initial spreading of melanoma A375 cells on fibronectin. We found that lipid raft disruption with methyl-β-cyclodextrin led to the inability of focal adhesion formation and actin cytoskeleton rearrangement by preventing β1 integrin clustering. Furthermore, we explored the possible mechanism by which lipid raft regulates β1 integrin clustering and demonstrated that intact lipid raft could recruit and modify some adaptor proteins, such as talin, α-actinin, vinculin, paxillin and FAK. Lipid raft could regulate the location of these proteins in lipid raft fractions and facilitate their binding to β1 integrin, which may be crucial for β1 integrin clustering. We also showed that lipid raft disruption impaired A375 cell migration in both transwell and wound healing models. Together, these findings provide a new insight for the relationship between lipid raft and the regulation of integrins. 
 Expression of estrogen receptor beta increases integrin alpha1 and integrin beta1 levels and enhances adhesion of breast cancer cells. Estrogen effects on mammary gland development and differentiation are mediated by two receptors (ERalpha and ERbeta). Estrogen-bound ERalpha induces proliferation of mammary epithelial and cancer cells, while ERbeta is important for maintenance of the differentiated epithelium and inhibits proliferation in different cell systems. In addition, the normal breast contains higher ERbeta levels compared to the early stage breast cancers, suggesting that loss of ERbeta could be important in cancer development. Analysis of ERbeta-/- mice has consistently revealed reduced expression of cell adhesion proteins. As such, ERbeta is a candidate modulator of epithelial homeostasis and metastasis. Consequently, the aim of this study was to analyze estrogenic effects on adhesion of breast cancer cells expressing ERalpha and ERbeta. As ERbeta is widely found in breast cancer but not in cell lines, we used ERalpha positive T47-D and MCF-7 human breast cancer cells to generate cells with inducible ERbeta expression. Furthermore, the colon cancer cell lines SW480 and HT-29 were also used. Integrin alpha1 mRNA and protein levels increased following ERbeta expression. Integrin beta1-the unique partner for integrin alpha1-increased only at the protein level. ERbeta expression enhanced the formation of vinculin containing focal complexes and actin filaments, indicating a more adhesive potential. This was confirmed by adhesion assays where ERbeta increased adhesion to different   extracellular matrix   proteins, mostly laminin. In addition, ERbeta expression was associated to less cell migration. These results indicate that ERbeta affects integrin expression and clustering and consequently modulates adhesion and migration of breast cancer cells. 
 IFN-gamma induces endothelial cells to proliferate and to invade the   extracellular matrix   in response to the HIV-1 Tat protein: implications for AIDS-Kaposi's sarcoma pathogenesis. Previous studies indicated that the Tat protein of HIV functions as a progression factor in Kaposi's sarcoma (KS), an angioproliferative disease common and aggressive in HIV-1-infected individuals (AIDS-KS). In particular, Tat that is released by infected cells stimulates the growth and invasion of spindle cells of endothelial origin derived from KS lesions (KS cells). Other work suggested that inflammatory cytokines may act as initiating factors in KS since they induce normal endothelial cells to acquire the same phenotype and functional features of KS cells, including the responsiveness to Tat. In this study, we show that among the inflammatory cytokines increased in AIDS-KS lesions, IFN-gamma alone is sufficient to induce endothelial cells to proliferate and to invade the   extracellular matrix   in response to Tat. This is because IFN-gamma up-regulates the expression and activity of the receptors for Tat identified as the integrins alpha5beta1 and alpha(v)beta3. These results suggest that, by triggering Tat effects, IFN-gamma plays a major role in AIDS-KS pathogenesis. 
 Impact of integrin-matrix interaction and signaling on insulin gene expression and the mesenchymal transition of human beta-cells. A critical shortage of donor pancreata currently prevents the development of a universal cell-based therapy for type I diabetes. The ex vivo expansion of insulin-producing beta-cells offers a potential solution but is problematic due to the inherent tendency of these cells to transition into mesenchymal-like cells that are devoid of function. Here, we demonstrate for the first time that exposure to elements of the   extracellular matrix   (ECM) directly potentiates the mesenchymal transition of cultured fetal beta-cells and causes associated declines in insulin gene expression. Individual ECM constituents varied in their ability to induce such responses, with collagen-IV (C-IV) and fibronectin inducing strong responses, whereas laminin-1 had no significant effect. Mesenchymal transition and concomitant losses in insulin gene expression observed on C-IV were found to be dependent on beta(1)-integrin ligation and were augmented in the presence of hepatocyte growth factor. Importantly, selective inhibition of c-Src, c-Jun N-terminal kinase (JNK), and extracellular signal-regulated kinase (ERK) prior to exposure to C-IV prevented mesenchymal transition and effectively preserved insulin expression. Fetal beta-cells undergoing mesenchymal transition were found to acquire alpha(1)beta(1) expression, and ligation of this integrin then promotes declines in insulin gene expression and a marked increase in beta-cell motility. Inhibition of Src-, ERK-, or JNK-dependent signaling combined with the selective regulation of matrix exposure may ultimately facilitate the development of more effective beta-cell expansion protocols. 
 RGD-independent binding of integrin alpha9beta1 to the ADAM-12 and -15 disintegrin domains mediates cell-cell interaction. ADAMs (a disintegrin and metalloproteases) mediate several important processes (e.g. tumor necrosis factor-alpha release, fertilization, and myoblast fusion). The ADAM disintegrin domains generally lack RGD motifs, and their receptors are virtually unknown. Here we show that integrin alpha(9)beta(1) specifically interacts with the recombinant ADAMs-12 and -15 disintegrin domains in an RGD-independent manner. We also show that interaction between ADAM-12 or -15 and alpha(9)beta(1) supports cell-cell interaction. Interestingly, the cation requirement and integrin activation status required for alpha(9)beta(1)/ADAM-mediated cell adhesion and cell-cell interaction is similar to those required for known integrin-  extracellular matrix   interaction. These results are quite different from recent reports that ADAM-2/alpha(6)beta(1) interaction during sperm/egg fusion requires an integrin activation status distinct from that for   extracellular matrix   interaction. These results suggest that alpha(9)beta(1) may be a major receptor for ADAMs that lack RGD motifs, and that, considering a wide distribution of ADAMs and alpha(9)beta(1), this interaction may be of potential biological and pathological significance. 
 The integrin alpha9beta1 mediates adhesion to activated endothelial cells and transendothelial neutrophil migration through interaction with vascular cell adhesion molecule-1. The integrin alpha9beta1 has been shown to be widely expressed on smooth muscle and epithelial cells, and to mediate adhesion to the   extracellular matrix   proteins osteopontin and tenascin-C. We have found that the peptide sequence this integrin recognizes in tenascin-C is highly homologous to the sequence recognized by the closely related integrin alpha4beta1, in the inducible endothelial ligand, vascular cell adhesion mole-cule-1 (VCAM-1). We therefore sought to determine whether alpha9beta1 also recognizes VCAM-1, and whether any such interaction would be biologically significant. In this report, we demonstrate that alpha9beta1 mediates stable cell adhesion to recombinant VCAM-1 and to VCAM-1 induced on human umbilical vein endothelial cells by tumor necrosis factor-alpha. Furthermore, we show that alpha9beta1 is highly and selectively expressed on neutrophils and is critical for neutrophil migration on VCAM-1 and tenascin-C. Finally, alpha9beta1 and alpha4 integrins contribute to neutrophil chemotaxis across activated endothelial monolayers. These observations suggest a possible role for alpha9beta1/VCAM-1 interactions in extravasation of neutrophils at sites of acute inflammation. 
  GeneRIFs  
 alpha5beta1 and alphavbeta3 each mediate actin cytoskeletal remodeling in response to stiffening or cyclic stretching of the   extracellular matrix  . 
 PCTK1-KAP0-myosin X-integrin beta 1 is a functional module providing a link between ECM and the actin cytoskeleton in the cell-  extracellular matrix   (ECM)-dependent control of spindle orientation. 
 Data suggest that ITGB1 is critical ligand to enhance cell-  extracellular matrix   contact force and thus promotes cell proliferation; ITGB1 knockdown affects leiomyomal cell spreading but not adhesion; ITGB1 may function in progression of leiomyoma. 
 Examined the role of beta1 integrin in exocrine pancreatic structure and function. Compared with control pancreata, beta1 integrin-deficient pancreata showed reduced mRNA expression of   extracellular matrix   (collagen type Ialpha2, fibronectin and laminin) genes. 
 Beta1 integrin is the main target for Cdc42-mediating interaction of cancer cells with endothelial cells and the underlying   extracellular matrix  , as exogenous beta1 integrin expression was sufficient to rescue the Cdc42-silencing phenotype. 
 MMP-2 amplifies the motility of colon cancer cells, not only by digesting the   extracellular matrix   components in the vicinity of cancer cells but also by inactivating their major beta1 integrin receptors. 
 SIRPalpha facilitates cross-talk between the prolactin receptor (PRLr) and beta1 integrin and provides a basis for   extracellular matrix   regulation of PRLr action in breast cancer. 
 Data suggest that the alpha5 laminins emerge as putative primary   extracellular matrix   mediators of melanoma invasion and metastasis via alpha3/alpha6beta1 and other integrin receptors. 
 This work demonstrates a novel role for FAK in integrin activation and the time-dependent generation of cell-  extracellular matrix   forces. 
 The result suggested that cytoskeleton was a possible mechanical sensor to centrifugal stimuli, and the cytoskeleton regulation to centrifugal loading was in an   extracellular matrix  -dependent and integrin-mediated manner. 
   extracellular matrix   fragments produced by apoptotic EC initiate a state of resistance to apoptosis in fibroblasts via an alpha2beta1 integrin/SFK (Src and Fyn)/phosphatidylinositol 3-kinase (PI3K)-dependent pathway 
 initial adhesion of endometrial cells to mesothelium is not mediated by beta1 integrins but attachment to collagen IV and collagen I, which are present in the submesothelial   extracellular matrix  , is mediated by beta1 integrins 
 results are consistent with an essential role for beta(1) integrins in maintenance of cardiomyocyte viability and interaction with   extracellular matrix   
 These results demonstrate that integrin-dependent cell to   extracellular matrix   adhesion reinforces E-cadherin-dependent cell-cell adhesion in Caco-2 cells. 
 There is a desensitization of IL-8-mediated p42/p44 MAPK signaling in response to ligation of the alpha5beta1 integrin in PMNL. There may be an interplay between integrin and chemokine signaling during PMNL migration through the   extracellular matrix  . 
 The formation of dendrites is shown to require the interaction between the beta1-integrin (CD29) on the surface of the DCs and fibronectin in the   extracellular matrix  . 
  [Ii]nvasive  PubMed  
 Pro-prion binds filamin A, facilitating its interaction with integrin beta1, and contributes to melanomagenesis. Filamin A (FLNA) is an integrator of cell mechanics and signaling. The spreading and migration observed in FLNA sufficient A7 melanoma cells but not in the parental FLNA deficient M2 cells have been attributed to FLNA. In A7 and M2 cells, the normal prion (PrP) exists as pro-PrP, retaining its glycosylphosphatidyl-inositol (GPI) anchor peptide signal sequence (GPI-PSS). The GPI-PSS of PrP has a FLNA binding motif and binds FLNA. Reducing PrP expression in A7 cells alters the spatial distribution of FLNA and organization of actin and diminishes cell spreading and migration. Integrin β1 also binds FLNA. In A7 cells, FLNA, PrP, and integrin β1 exist as two independent, yet functionally linked, complexes; they are FLNA with PrP or FLNA with integrin β1. Reducing PrP expression in A7 cells decreases the amount of integrin β1 bound to FLNA. A PrP GPI-PSS synthetic peptide that crosses the cell membrane inhibits A7 cell spreading and migration. Thus, in A7 cells FLNA does not act alone; the binding of pro-PrP enhances association between FLNA and integrin β1, which then promotes cell spreading and migration. Pro-PrP is detected in melanoma in situ but not in melanoc  yte. Inv  asive melanoma has more pro-PrP. The binding of pro-PrP to FLNA, therefore, contributes to melanomagenesis. 
 Androgen-induced cell migration: role of androgen receptor/filamin A association. Androgen receptor (AR) controls male morphogenesis, gametogenesis and prostate growth as well as development of prostate cancer. These findings support a role for AR in cell migration and   invasive  ness. However, the molecular mechanism involved in AR-mediated cell migration still remains elusive.Mouse embryo NIH3T3 fibroblasts and highly metastatic human fibrosarcoma HT1080 cells harbor low levels of transcriptionally incompetent AR. We now report that, through extra nuclear action, AR triggers migration of both cell types upon stimulation with physiological concentrations of the androgen R1881. We analyzed the initial events leading to androgen-induced cell migration and observed that challenging NIH3T3 cells with 10 nM R1881 rapidly induces interaction of AR with filamin A (FlnA) at cytoskeleton. AR/FlnA complex recruits integrin beta 1, thus activating its dependent cascade. Silencing of AR, FlnA and integrin beta 1 shows that this ternary complex controls focal adhesion kinase (FAK), paxillin and Rac, thereby driving cell migration. FAK-null fibroblasts migrate poorly and Rac inhibition by EHT impairs motility of androgen-treated NIH3T3 cells. Interestingly, FAK and Rac activation by androgens are independent of each other. Findings in human fibrosarcoma HT1080 cells strengthen the role of Rac in androgen signaling. The Rac inhibitor significantly impairs androgen-induced migration in these cells. A mutant AR, deleted of the sequence interacting with FlnA, fails to mediate FAK activation and paxillin tyrosine phosphorylation in androgen-stimulated cells, further reinforcing the role of AR/FlnA interaction in androgen-mediated motility.The present report, for the first time, indicates that the extra nuclear AR/FlnA/integrin beta 1 complex is the key by which androgen activates signaling leading to cell migration. Assembly of this ternary complex may control organ development and prostate cancer metastasis. 
 Androgen-induced cell migration: role of androgen receptor/filamin A association. Androgen receptor (AR) controls male morphogenesis, gametogenesis and prostate growth as well as development of prostate cancer. These findings support a role for AR in cell migration and   invasive  ness. However, the molecular mechanism involved in AR-mediated cell migration still remains elusive.Mouse embryo NIH3T3 fibroblasts and highly metastatic human fibrosarcoma HT1080 cells harbor low levels of transcriptionally incompetent AR. We now report that, through extra nuclear action, AR triggers migration of both cell types upon stimulation with physiological concentrations of the androgen R1881. We analyzed the initial events leading to androgen-induced cell migration and observed that challenging NIH3T3 cells with 10 nM R1881 rapidly induces interaction of AR with filamin A (FlnA) at cytoskeleton. AR/FlnA complex recruits integrin beta 1, thus activating its dependent cascade. Silencing of AR, FlnA and integrin beta 1 shows that this ternary complex controls focal adhesion kinase (FAK), paxillin and Rac, thereby driving cell migration. FAK-null fibroblasts migrate poorly and Rac inhibition by EHT impairs motility of androgen-treated NIH3T3 cells. Interestingly, FAK and Rac activation by androgens are independent of each other. Findings in human fibrosarcoma HT1080 cells strengthen the role of Rac in androgen signaling. The Rac inhibitor significantly impairs androgen-induced migration in these cells. A mutant AR, deleted of the sequence interacting with FlnA, fails to mediate FAK activation and paxillin tyrosine phosphorylation in androgen-stimulated cells, further reinforcing the role of AR/FlnA interaction in androgen-mediated motility.The present report, for the first time, indicates that the extra nuclear AR/FlnA/integrin beta 1 complex is the key by which androgen activates signaling leading to cell migration. Assembly of this ternary complex may control organ development and prostate cancer metastasis. 
 Pro-prion binds filamin A, facilitating its interaction with integrin beta1, and contributes to melanomagenesis. Filamin A (FLNA) is an integrator of cell mechanics and signaling. The spreading and migration observed in FLNA sufficient A7 melanoma cells but not in the parental FLNA deficient M2 cells have been attributed to FLNA. In A7 and M2 cells, the normal prion (PrP) exists as pro-PrP, retaining its glycosylphosphatidyl-inositol (GPI) anchor peptide signal sequence (GPI-PSS). The GPI-PSS of PrP has a FLNA binding motif and binds FLNA. Reducing PrP expression in A7 cells alters the spatial distribution of FLNA and organization of actin and diminishes cell spreading and migration. Integrin β1 also binds FLNA. In A7 cells, FLNA, PrP, and integrin β1 exist as two independent, yet functionally linked, complexes; they are FLNA with PrP or FLNA with integrin β1. Reducing PrP expression in A7 cells decreases the amount of integrin β1 bound to FLNA. A PrP GPI-PSS synthetic peptide that crosses the cell membrane inhibits A7 cell spreading and migration. Thus, in A7 cells FLNA does not act alone; the binding of pro-PrP enhances association between FLNA and integrin β1, which then promotes cell spreading and migration. Pro-PrP is detected in melanoma in situ but not in melanoc  yte. Inv  asive melanoma has more pro-PrP. The binding of pro-PrP to FLNA, therefore, contributes to melanomagenesis. 
 A tumor-associated beta 1 integrin mutation that abrogates epithelial differentiation control. SCC4 human keratinocytes are derived from a squamous cell carcinoma of the tongue and undergo very little spontaneous differentiation. Introduction of a wild-type beta 1 integrin subunit into SCC4 cells stimulates differentiation, suggesting either that the cells have a defect in the integrin signaling pathways that control differentiation or that the beta1 subunit itself is defective. Here we describe a heterozygous mutation in the SCC4 beta 1 subunit. The mutation, T188I, maps to the I-like domain. It results in constitutive activation of ligand binding, irrespective of the partner alpha subunit, in solid phase assays with recombinant protein and in living cells. The mutation promotes cell spreading, but not proliferation, motility, or   invasive  ness. It results in sustained activation of Erk MAPK independent of cell spreading. When introduced into SCC4 keratinocytes, the wild-type beta1 integrin stimulates differentiation, whereas the mutant is inactive. Activation of beta 1 integrins in normal keratinocytes also suppresses differentiation. These results establish, for the first time, mutation as a mechanism by which integrins can contribute to neoplasia, because the degree of differentiation in epithelial cancers is inversely correlated with prognosis. They also provide new insights into how integrins regulate keratinocyte differentiation. 
 CD151-α3β1 integrin complexes are prognostic markers of glioblastoma and cooperate with EGFR to drive tumor cell motility and invasion. Glioblastoma, one of the most aggressive forms of brain cancer, is featured by high tumor cell motility an  d invasi  veness, which not only fuel tumor infiltration, but also enable escape from surgical or other clinical interventions. Thus, better understanding of how these malignant traits are controlled will be key to the discovery of novel biomarkers and therapies against this deadly disease. Tetraspanin CD151 and its associated α3β1 integrin have been implicated in facilitating tumor progression across multiple cancer types. How these adhesion molecules are involved in the progression of glioblastoma, however, remains largely unclear. Here, we examined an in-house tissue microarray-based cohort of 96 patient biopsies and TCGA dataset to evaluate the clinical significance of CD151 and α3β1 integrin. Functional and signaling analyses were also conducted to understand how these molecules promote the aggressiveness of glioblastoma at molecular and cellular levels. Results from our analyses showed that CD151 and α3 integrin were significantly elevated in glioblastomas at both protein and mRNA levels, and exhibited strong inverse correlation with patient survival (p < 0.006). These adhesion molecules also formed tight protein complexes and synergized with EGF/EGFR to accelerate tumor cell motility and invasion. Furthermore, disruption of such complexes enhanced the survival of tumor-bearing mice in a xenograft model, and impaired activation of FAK and small GTPases. Also, knockdown- or pharmacological agent-based attenuation of EGFR, FAK or Graf (ARHGAP26)/small GTPase-mediated pathways markedly mitigated the aggressiveness of glioblastoma cells. Collectively, our findings provide clinical, molecular and cellular evidence of CD151-α3β1 integrin complexes as promising prognostic biomarkers and therapeutic targets for glioblastoma. 
 Functional relevance of urinary-type plasminogen activator receptor-alpha3beta1 integrin association in proteinase regulatory pathways. Squamous cell carcinoma of the oral cavity is characterized by persistent, disorganized expression of integrin alpha3beta1 and enhanced production of urinary-type plasminogen activator (uPA) and its receptor (uPAR) relative to normal oral mucosa. Because multivalent aggregation of alpha3beta1 integrin up-regulates uPA and induces a dramatic co-clustering of uPAR, we explored the hypothesis that lateral ligation of alpha3beta1 integrin by uPAR contributes to uPA regulation in oral mucosal cells. To investigate mechanisms by which uPAR/alpha3beta1 binding enhances uPA expression, integrin-dependent signal activation was assessed. Both Src and ERK1/2 were phosphorylated in response to integrin aggregation, and blocking Src kinase activity completely abrogated ERK1/2 activation and uPA induction, whereas inhibition of epidermal growth factor receptor tyrosine kinase activity did not alter uPA expression. Proteinase up-regulation occurred at the transcriptional level and mutation of the AP1 (-1967) site in the uPA promoter blocked the uPAR/integrin-mediated transcriptional activation. Because uPAR is redistributed to clustered alpha3beta1 integrins, the requirement for uPAR/alpha3beta1 interaction in uPA regulation was assessed. Clustering of alpha3beta1 in the presence of a peptide (alpha325) that disrupts uPAR/alpha3beta1 integrin binding prevented uPA induction. Depletion of cell surface uPAR using small interfering RNA also blocked uPA induction following integrin alpha3beta1 clustering. These results were confirmed using a genetic strategy in which alpha3 null epithelial cells reconstituted with wild type alpha3 integrin, but not a mutant alpha3 unable to bind uPAR, induced uPA expression upon integrin clustering, confirming the critical role of uPAR in integrin-regulated proteinase expression. Disruption of uPAR/alpha3beta1 binding using peptide alpha325 or small interfering RNA blocked filopodia formation and matrix invasion, indicating that this interaction stimulates   invasive   behavior. Together these data support a model wherein matrix-induced clustering ofalpha3beta1 integrin promotes uPAR/alpha3beta1 interaction, thereby potentiating cellular signal transduction pathways culminating in activation of uPA expression and enhanced uPA-dependent   invasive   behavior. 
 The diacylglycerol kinase α/atypical PKC/β1 integrin pathway in SDF-1α mammary carcino  ma invas  iveness. Diacylglycerol kinase α (DGKα), by phosphorylating diacylglycerol into phosphatidic acid, provides a key signal driving cell migration and matrix invasion. We previously demonstrated that in epithelial cells activation of DGKα activity promotes cytoskeletal remodeling and matrix invasion by recruiting atypical PKC at ruffling sites and by promoting RCP-mediated recycling of α5β1 integrin to the tip of pseudopods. In here we investigate the signaling pathway by which DGKα mediates SDF-1α-induced matrix invasion of M  DA-MB-23  1 invasive breast carcinoma cells. Indeed we showed that, following SDF-1α stimulation, DGKα is activated and localized at cell protrusion, thus promoting their elongation and mediating SDF-1α induced MMP-9 metalloproteinase secretion and matrix invasion. Phosphatidic acid generated by DGKα promotes localization at cell protrusions of atypical PKCs which play an essential role downstream of DGKα by promoting Rac-mediated protrusion elongation and localized recruitment of β1 integrin and MMP-9. We finally demonstrate that activation of DGKα, atypical PKCs signaling and β1 integrin are all essenti  al for M  DA-MB-231 invasiveness. These data indicates the existence of a SDF-1α induced DGKα - atypical PKC - β1 integrin signaling pathway, which is essential for matrix invasion of carcinoma cells. 
 β1-Integrin via NF-κB signaling is essential for acquisition o  f invasi  veness in a model of radiation treated in situ breast cancer. Ductal carcinoma in situ (DCIS) is characterized by no  n-invasi  ve cancerous cell growth within the breast ducts. Although radiotherapy is commonly used in the treatment of DCIS, the effect and molecular mechanism of ionizing radiation (IR) on DCIS are not well understood, an  d invasi  ve recurrence following radiotherapy remains a significant clinical problem. This study investigated the effects of IR on a clinically relevant model of Akt-driven DCIS and identified possible molecular mechanisms underlyin  g invasi  ve progression in surviving cells.We measured the level of phosphorylated-Akt (p-Akt) in a cohort of human DCIS specimens by immunohistochemistry (IHC) and correlated it with recurrence risk. To model human DCIS, we used Akt overexpressing human mammary epithelial cells (MCF10A-Akt) which, in three-dimensional laminin-rich extracellular matrix (lrECM) and in vivo, form organotypic DCIS-like lesions with lumina expanded by pleiomorphic cells contained within an intact basement membrane. In a population of cells that survived significant IR doses in three-dimensional lrECM, a malignant phenotype emerged creating a model fo  r invasi  ve recurrence.P-Akt was up-regulated in clinical DCIS specimens and was associated with recurrent disease. MCF10A-Akt cells that formed DCIS-like structures in three-dimensional lrECM showed significant apoptosis after IR, preferentially in the luminal compartment. Strikingly, when cells that survived IR were repropagated in three-dimensional lrECM, a malignant phenotype emerged, characterized b  y invasi  ve activity, up-regulation of fibronectin, α5β1-integrin, matrix metalloproteinase-9 (MMP-9) and loss of E-cadherin. In addition, IR induced nuclear translocation and binding of nuclear factor-kappa B (NF-κB) to the β1-integrin promoter region, associated with up-regulation of α5β1-integrins. Inhibition of NF-κB or β1-integrin signaling abrogated emergen  ce of th  e invasive activity.P-Akt is up-regulated in some human DCIS lesions and is possibly associated with recurrence. MCF10A-Akt cells form organotypic DCIS-like lesions in three-dimensional lrECM and in vivo, and are a plausible model for some forms of human DCIS. A population of Akt-driven DCIS-like spheroids that survive IR progres  ses to a  n invasive phenotype in three-dimensional lrECM mediated by β1-integrin and NF-κB signaling. 
 Changes in adhesive and migratory characteristics of hepatocellular carcinoma (HCC) cells induced by expression of alpha3beta1 integrin. The   invasive   and metastatic potentials of hepatocellular carcinoma are positively correlated with the expression level of alpha3beta1 integrin, a high-affinity adhesion receptor for laminin isoforms including laminin-5. In this study, we investigated changes in the adhesive and   invasive   behaviors of human HCC HepG2 cells after transfection with cDNA for alpha3 integrin in order to elucidate the direct involvement of this integrin in these cellular processes. We introduced cDNA for splice variants of alpha3 integrin (alpha3A and alpha3B) into the cells, and selected two transfectant clones (HepG2-3A and HepG2-3B), which express the alpha3A and alpha3B integrins, respectively. Both transfectant cells adhered almost equally to laminin-5-coated plates in an alpha3 integrin-dependent manner, indicating that transfected alpha3Abeta1 and alpha3Bbeta1 integrins were functionally active in these cells. The migratory and   invasive   potentials of the transfectant cells were assessed by scratch wound assay and in vitro chemoinvasion assay. The results demonstrated that the migration of HepG2-3A and HepG2-3B cells but not of mock transfectant (HepG2-M) cells was stimulated on the plates coated with laminin-5. Furthermore, HepG2-3A and HepG2-3B cells were found to be more   invasive   into laminin-5-containing matrices than were HepG2-M cells. These results strongly suggest that enhanced expression of alpha3beta1 integrin on HCC cells is directly involved in their malignant phenotypes such as invasion and metastasis. 
 β-1,4-Galactosyltransferase III enhances   invasiv  e phenotypes via β1-integrin and predicts poor prognosis in neuroblastoma. Neuroblastoma (NB) is a neural crest-derived tumor that commonly occurs in childhood. β-1,4-Galactosyltransferase III (B4GALT3) is highly expressed in human fetal brain and is responsible for the generation of poly-N-acetyllactosamine, which plays a critical role in tumor progression. We therefore investigated the expression and role of B4GALT3 in NB.We examined B4GALT3 expression in tumor specimens from 101 NB patients by immunohistochemistry and analyzed the correlation between B4GALT3 expression and clinicopathologic factors or survival. The functional role of B4GALT3 expression was investigated by overexpression or knockdown of B4GALT3 in NB cells for in vitro and in vivo studies.We found that B4GALT3 expression correlated with advanced clinical stages (P = 0.040), unfavorable Shimada histology (P < 0.001), and lower survival rate (P < 0.001). Multivariate analysis showed that B4GALT3 expression is an independent prognostic factor for poor survival of NB patients. B4GALT3 overexpression increased migration, invasion, and tumor growth of NB cells, whereas B4GALT3 knockdown suppressed the malignant phenotypes of NB cells. Mechanistic investigation showed that B4GALT3-enhanced migration and invasion were significantly suppressed by β1-integrin blocking antibody. Furthermore, B4GALT3 overexpression increased lactosamine glycans on β1-integrin, increased expression of mature β1-integrin via delayed degradation, and enhanced phosphorylation of focal adhesion kinase. Conversely, these properties were decreased by knockdown of B4GALT3 in NB cells.Our findings suggest that B4GALT3 predicts an unfavorable prognosis for NB and may reg  ulate in  vasive phenotypes through modulating glycosylation, degradation, and signaling of β1-integrin in NB cells. 
 Integrins as receptors for laminins. Laminins are a family of trimeric glycoproteins present in the extracellular matrix and the major constituents of basement membranes. Integrins are alpha beta transmembrane receptors that play critical roles in both cell-matrix and cell-cell adhesion. Several members of the integrin family, including alpha 1 beta 1, alpha 2 beta 1, alpha 3 beta 1, alpha 6 beta 1, alpha 7 beta 1 and alpha 6 beta 4 heterodimers serve as laminin receptors on a variety of cell types. This review summarizes recent advances in understanding the involvement of individual integrins in cell interactions with laminins and the roles of laminin-binding integrins in adhesion-mediated events in vertebrates, including embryonic development, cell migration and tumor cell   invasive  ness, cell proliferation and differentiation, as well as basement membrane assembly. We discuss the regulation of integrin function via alternative splicing of cytoplasmic domains of alpha and beta subunits of the integrin receptors for laminins and present examples of functional collaboration between laminin-binding integrins and non-integrin laminin receptors. Advances in our understanding of the laminin-binding integrins continue to demonstrate the essential roles these receptors play in maintaining cell polarity and tissue architecture. 
 Neuropilin-1 interacts with integrin beta1 and modulates pancreatic cancer cell growth, survival and invasion. Neuropilin-1 (Np-1) is a coreceptor for vascular endothelial growth factor-A (VEGF-A), and both are expressed at high levels in pancreatic ductal adenocarcinomas (PDACs). While VEGF-A has been implicated in tumor angiogenesis, the role of Np-1 in PDAC is less clearly defined. Accordingly, PANC-1 pancreatic cancer cells, which express relatively high levels of Np-1, were transfected with the Np-1 antisense cDNA. By comparison with sham transfected cells, Np-1 antisense expressing clones (Np-1AS) exhibited decreased anchorage independent growth, adhesion and   invasive  ness, and prolonged doubling times. Np-1AS were also more sensitive to the pro-apoptotic actions of ActD, as evidenced by PARP cleavage, caspase 9 activation and annexin V staining. ActD decreased Bcl-xL and STAT5 levels in the antisense expressing cells, but not in sham-transfected cells, and did not alter STAT3, Bcl-2, phospho-AKT, AKT, Bad, Bax or Bak levels. Immunoprecipitation followed by immunoblotting revealed that Np-1 associated with integrin beta1 and integrin beta1 blockade attenuated adhesion. However, Np-AS expressing clones exhibited enhanced tyrosine phosphorylated focal adhesion kinase. Thus, Np-1 confers a growth and survival advantage to PANC-1 cells, and interacts with integrin beta1 to coordinate signaling events that promote cell adherence and   invasive  ness. 
 Aberrant expression of E-cadherin and integrin β-1 in trophoblasts is associated with malignant gestational trophoblastic diseases. To investigate the association between malignant gestational trophoblastic diseases and aberrant local expression of E-cadherin and integrin β-1 in the context of identifying a method to predict and diagnose malignant gestational trophoblastic diseases at an early stage.We used immunohistochemistry to assess E-cadherin and integrin β-1 expression profiles in normal chorionic villus, hydatidiform mole, a  nd invas  ive mole.E-cadherin expression gradually decreased from normal tissue to malignant tissue. Staining for E-cadherin was positive in 90% of normal trophoblastic villus in early pregnancy, 80% in benign hydatidiform mole, and 50%   in invas  ive mole. Notabl  y, invas  ive mole had significantly lower E-cadherin expression than benign mole (P < 0.05). However, there was no significant difference in staining between benign and malignant moles (15% vs 10%, P > 0.05). Integrin β-1 expression was identical in benign hydatidiform mole and malignant mole (90% and 90%), but this value was significantly higher than that in normal trophoblastic villus (40%, both P < 0.05). Moreover, malignant mole staining was significantly higher than in the benign mole (30% vs 15%, P < 0.05).Aberrant expression of E-cadherin and integrin β-1 is closely related to malignant transformation and hydatidiform mole development. Changes in the expression of E-cadherin and integrin β-1 are informative and may improve the early diagnosis of mali  gnant/in  vasive moles. 
 Targeting of integrin beta1 and kinesin 2alpha by microRNA 183. MicroRNA 183 (miR-183) has been reported to inhibit tumor   invasive  ness and is believed to be involved in the development and function of ciliated neurosensory organs. We have recently found that expression of miR-183 increased after the induction of cellular senescence by exposure to H(2)O(2). To gain insight into the biological roles of miR-183 we investigated two potential novel targets: integrin beta1 (ITGB1) and kinesin 2alpha (KIF2A). miR-183 significantly decreased the expression of ITGB1 and KIF2A measured by Western blot. Targeting of the 3'-untranslated region (3'-UTR) of ITGB1 and KIF2A by miR-183 was confirmed by luciferase assay. Transfection with miR-183 led to a significant decrease in cell invasion and migration capacities of HeLa cells that could be rescued by expression of ITGB1 lacking the 3'-UTR. Although miR-183 had no effects on cell adhesion in HeLa cells, it significantly decreased adhesion to laminin, gelatin, and collagen type I in normal human diploid fibroblasts and human trabecular meshwork cells. These effects were also rescued by expression of ITGB1 lacking the 3'-UTR. Targeting of KIF2A by miR-183 resulted in some increase in the formation of cells with monopolar spindles in HeLa cells but not in human diploid fibroblast or human trabecular meshwork cells. The regulation of ITGB1 expression by miR-183 provides a new mechanism for the anti-metastatic role of miR-183 and suggests that this miRNA could influence the development and function in neurosensory organs, and contribute to functional alterations associated with cellular senescence in human diploid fibroblasts and human trabecular meshwork cells. 
 Staged stromal extracellular 3D matrices differentially regulate breast cancer cell responses through PI3K and beta1-integrins. Interactions between cancer cells and stroma are critical for growth and   invasive  ness of epithelial tumors. The biochemical mechanisms behind tumor-stromal interactions leading to increased   invasive  ness and metastasis are mostly unknown. The goal of this study was to analyze the direct effects of staged stroma-derived extracellular matrices on breast cancer cell behavior.Early and late three-dimensional matrices were produced by NIH-3T3 and tumor-associated murine fibroblasts, respectively. After removing fibroblasts, extracted matrices were re-cultured with breast epithelial cells of assorted characteristics: MCF-10A (non-tumorigenic), MCF-7 (tumorigenic, non-  invasive  ), and MDA-MB-231 (tumorigenic,   invasive  ). Effects prompted by staged matrices on epithelial cell's growth, morphology and invasion were determined. Also, matrix-induced velocity, directionality and relative track orientation of   invasive   cells were assessed in the presence or absence of inhibitors of phosphoinositide-3 kinase (PI3K) and/or beta-1 integrin.We observed that assorted breast epithelial cells reacted differently to two-dimensional vs. staged, control (early) and tumor-associated (late), three-dimensional matrices. MCF-10A had a proliferative advantage on two-dimensional substrates while MCF-7 and MDA-MB-231 showed no difference. MCF-10A and MCF-7 formed morphologically distinguishable aggregates within three-dimensional matrices, while MDA-MB-231 exhibited increased spindle-shape morphologies and directional movements within three-dimensional matrices. Furthermore, MDA-MB-231 acquired a pattern of parallel oriented organization within tumor-associated, but not control matrices. Moreover, tumor-associated matrices induced PI3K and beta1-integrin dependent Akt/PKB activity in MDA-MB-231 cells. Interestingly, beta1-integrin (but not PI3K) regulated tumor-associated matrix-induced mesenchymal invasion which, when inhibited, resulted in a change of   invasive   strategy rather than impeding invasion altogether.We propose that both cells and matrices are important to promote effective breast cancer cell invasion through three-dimensional matrices and that beta1-integrin inhibition is not necessarily sufficient to block tumor-matrix induced breast cancer cell invasion. Additionally, we believe that characterizing stroma staging (e.g., early vs. late or tumor-associated) might be beneficial for predicting matrix-induced cancer cell responses in order to facilitate the selection of therapies. 
 Association of CD98, integrin β1, integrin β3 and Fak with the progression and liver metastases of colorectal cancer. CD98-mediated β1 and β3 integrins activation can induce Fak phosphorylation which eventually promotes cell survival, proliferation, and migration. We evaluated the expression of CD98, integrin β1, integrin β3 and Fak in 45 cases of matched colorectal cancer (CRC) and liver metastases as well as 35 cases of CRC without liver metastases. There was a gradual increase of the expression of CD98, integrin β1, integrin β3 and Fak as tumor progressed from normal colon to carcinoma to budding tumor cells   at the   invasive front and to liver metastases. The expression of CD98 and integrin β1 in CRC with liver metastases was significantly higher than that in CRC without liver metastases. Furthermore, for those liver metastases with desmoplastic growth pattern, expression of CD98, integrin β1, integrin β3 and Fak at the metastases center was as strong as that at the metastases periphery. For those liver metastases with pushing or replacement growth patterns, more intense expression of these markers was found at the metastases center than the periphery. Overexpression of CD98, integrin β1, integrin β3 and Fak is associated with the progression and liver metastases of CRC. Overexpression of these markers in liver metastases requires direct contact between tumor cells and the stroma. 
 Rab25 associates with alpha5beta1 integrin to promote   invasive   migration in 3D microenvironments. Here, we report a direct interaction between the beta1 integrin cytoplasmic tail and Rab25, a GTPase that has been linked to tumor aggressiveness and metastasis. Rab25 promotes a mode of migration on 3D matrices that is characterized by the extension of long pseudopodia, and the association of the GTPase with alpha5beta1 promotes localization of vesicles that deliver integrin to the plasma membrane at pseudopodial tips as well as the retention of a pool of cycling alpha5beta1 at the cell front. Furthermore, Rab25-driven tumor-cell invasion into a 3D extracellular matrix environment is strongly dependent on ligation of fibronectin by alpha5beta1 integrin and the capacity of Rab25 to interact with beta1 integrin. These data indicate that Rab25 contributes to tumor progression by directing the localization of integrin-recycling vesicles and thereby enhancing the ability of tumor cells to invade the extracellular matrix. 
 Beta 1 integrin predicts survival in breast cancer: a clinicopathological and immunohistochemical study. The main focus of several studies concerned with cancer progression and metastasis is to analyze the mechanisms that allow cancer cells to interact and quickly adapt with their environment. Integrins, a family of transmembrane glycoproteins, play a major role in   invasive   and metastatic processes. Integrins are involved in cell adhesion in both cell-extracellular matrix and cell-cell interactions, and particularly, β1 integrin is involved in proliferation and differentiation of cells in the development of epithelial tissues. This work aimed to investigate the putative role of β1 integrin expression on survival and metastasis in patients with breas  t invasi  ve ductal carcinoma (IDC). In addition, we compared the expression of β1 integrin in patients with ductal carcinoma in situ (DCIS).Through tissue microarray (TMA) slides containing 225 samples of IDC and 67 samples of DCIS, β1 integrin expression was related with several immunohistochemical markers and clinicopathologic features of prognostic significance.β1 integrin was overexpressed in 32.8% of IDC. In IDC, β1 integrin was related with HER-2 (p = 0.019) and VEGF (p = 0.011) expression and it had a significant relationship with metastasis and death (p = 0.001 and p = 0.05, respectively). Kaplan-Meier survival analysis showed that the overexpression of this protein is very significant (p = 0.002) in specific survival (number of months between diagnosis and death caused by the disease). There were no correlation between IDC and DCIS (p = 0.559) regarding β1 integrin expression.Considering that the expression of β1 integrin in breast cancer remains controversial, specially its relation with survival of patients, our findings provide further evidence that β1 integrin can be a marker of poor prognosis in breast cancer.The virtual slide(s) for this article can be found here: http://www.diagnosticpathology.diagnomx.eu/vs/6652215267393871. 
 Involvement of intercellular adhesion molecule-1 and beta1 integrin in the internalization process to human endothelial cells of group B Streptococcus clinical isolates. The mechanism by which group B Streptococcus (GBS) interacts with human cells and disrupts physiological processes is an intriguing area of investigation and continues to unfold. The aim of this study was to investigate the adherence and intracellular viability within endothelial ECV304 cells of GBS serotypes Ia, III and V isolates from patients and asymptomatic carriers. The GBS isolates from patients (GBS-Ia 90222-urine, GBS-III 90356-liquor and GBS-V 90186-blood strains) exhibited a more efficient adherence and survival mechanisms to endothelial cells than those from asymptomatic carriers (GBS-Ia 85147-oropharynx, GBS-III 80340 and GBS-V 88641-vagina strains), independent of bacterial serotypes. Treatment of endothelial ECV304 cells with EDTA demonstrated that Ca2+-dependent molecules modulated the adherence and internalization process of GBS-Ia and III to ECV304 cells. SDS-PAGE analysis of samples of biotinylated ECV304 extracts treated with GBS clinical isolates (urine 90222-Ia, liquor 90356-III and blood 90186-V strains) revealed fragments ranging from approximately 61 to approximately 179 kDa. Results of immunoassays with ECV304 membrane proteins showed that ICAM-1 molecules interacted only with GBS-III liquor 90356 strain while beta1 integrin interacted with GBS-III liquor 90356 and GBS-V blood 90186   invasive   strains. Thus, the interaction between ICAM-1 and beta1-integrin seems an additional means by which GBS exploits host endothelial cells during infection. These findings add to the current understanding of the roles played by multiple receptor-ligand systems in the uptake and pathogenesis of GBS infection. 
 A novel protease-docking function of integrin at invadopodia. Invadopodia are membrane extensions of aggressive tumor cells that function in the activation of membrane-bound proteases occurring during tumor cell invasion. We explore a novel and provocative activity of integrins in docking proteases to sites of invasion, termed invadopodia. In the absence of collagen, alpha(3)beta(1) integrin and the gelatinolytic enzyme, seprase, exist as nonassociating membrane proteins. Type I collagen substratum induces the association of alpha(3)beta(1) integrin with seprase as a complex on invadopodia. The results show that alpha(3)beta(1) integrin is a docking protein for seprase to form functional invadopodia. In addition, alpha(5)beta(1) integrin may participate in the adhesion process necessary for invadopodial formation. Thus, alpha(3)beta(1) and alpha(5)beta(1) integrins play major organizational roles in the adhesion and formation of invadopodia, promoting   invasive   cell behavior. 
 β1 integrin controls EGFR signaling and tumorigenic properties of lung cancer cells. Lung cancer is the leading cause of cancer death worldwide. The epidermal growth factor receptor (EGFR) represents the main target for non-small cell lung cancer (NSCLC) therapy, as its overexpression or constitutive activation contributes to malignancy and correlates with poor prognosis. Our previous work demonstrated that in epithelial cells β1 integrin is required for propagating EGFR signaling from the plasma membrane to the nucleus. In this study, we silenced β1 integrin in human NSCLC A549 cells. The β1 integrin-silenced cells show a defective activation of the EGFR signaling cascade, leading to decreased in vitro proliferation, enhanced sensitivity to cisplatin and Gefitinib, impaired migration   and inva  sive behavior. Inhibitory effects on tumor growth and on the EGFR pathway were also observed in in vivo experiments. Moreover, β1 integrin silencing increases the amount of EGFR on the cell surface, suggesting that β1 integrin is required for efficient constitutive EGFR turnover at the cell membrane. Although the rate of EGF internalization and recycling is not affected in silenced cells, EGFR signaling is recovered only by expression of the Rab-coupling protein RCP, indicating that β1 integrin sustains the endocytic machinery required for EGFR signaling. Overall, these results show that β1 integrin is an essential regulator of EGFR signaling and tumorigenic properties of lung cancer cells, and that its silencing might represent an adjuvant approach to anti-EGFR therapy. 
 Integrin-mediated invasion of Staphylococcus aureus into human cells requires Src family protein-tyrosine kinases. Staphylococcus aureus, a common cause of nosocomial infections, is able to invade eukaryotic cells by indirectly engaging beta1 integrin-containing host receptors, whereas non-pathogenic Staphylococcus carnosus is not   invasive  . Here, we identify intracellular signals involved in integrin-initiated internalization of S. aureus. In particular, the host cell actin cytoskeleton and Src family protein-tyrosine kinases (PTKs) are essential to mediate S. aureus invasion. Src PTKs are activated in response to pathogenic S. aureus, but not S. carnosus. In addition, pharmacological and genetic interference with Src PTK function reduces bacterial internalization. Importantly, Src PTK-deficient cells are resistant to S. aureus invasion, demonstrating the essentiality of host Src PTKs in integrin-mediated uptake of this pathogen. 
 Expression of laminin-5 and integrins in actinic cheilitis and superficially   invasive   squamous cell carcinomas of the lip. The progression of carcinogenesis entails the detachment of cells, invasion and migration of neoplastic cells. Alterations in epithelial adhesion and basement membrane proteins might mediate the early stages of carcinogenesis. This study investigated the expression of adhesion molecules and the basement membrane protein laminin-5 in actinic cheilitis (AC) and incipient squamous cell carcinoma of the lower lip to understand early photocarcinogenesis. Ln-5γ2 chain as well as α3, β1 subunits of α3β1 heterodimer and β4 subunit of integrin α6β4 were evaluated by immunohistochemistry in 16 cases of AC and 16 cases of superf  icially   invasive squamous cell carcinoma (SISCC). Most AC cases showed reduced expression of β1, β4 and α3 integrins, and SISCCs lacked β1, β4 and α3 int  egrins i  n the invasive front. AC cases were negative for the Ln-5γ2 chain. Five cases of SISCC (31%) showed heterogeneous Ln-5γ2 chain ex  pression   in the invasive front of the tumor. Integrin β1, β4 and α3 expression is lost during the early stages of lip carcinogenesis. Expressio  n of Ln-  5γ2 in the invasive front in cases and its correlation with tumor progression suggest that it mediates the acquisition of the migrating and invading epithelial cell phenotype. 
 α5β1 integrin recycling promotes Arp2/3-independent cancer cell invasion via the formin FHOD3  . Invasi  ve migration in 3D extracellular matrix (ECM) is crucial to cancer metastasis, yet little is known of the molecular mechanisms that drive reorganization of the cytoskeleton as cancer cells disseminate in vivo. 2D Rac-driven lamellipodial migration is well understood, but how these features apply to 3D migration is not clear. We find that lamellipodia-like protrusions and retrograde actin flow are indeed observed in cells moving in 3D ECM. However, Rab-coupling protein (RCP)-driven endocytic recycling of α5β1 integrin enhan  ces inva  sive migration of cancer cells into fibronectin-rich 3D ECM, driven by RhoA and filopodial spike-based protrusions, not lamellipodia. Furthermore, we show that actin spike protrusions are Arp2/3-independent. Dynamic actin spike assembly in cells invading in vitro and in vivo is regulated by Formin homology-2 domain containing 3 (FHOD3), which is activated by RhoA/ROCK, establishing a novel mechanism through which the RCP-α5β1 pathway reprograms the actin cytoskeleton to pr  omote in  vasive migration and local invasion in vivo. 
 Effects and mechanism of integrin-β1 gene expression inhibited by shRNA in invasion of pancreatic carcinoma PANC-1 cells. To investigate the effects of integrin-β1 gene expression inhibited by shRNA on invasion of pancreatic carcinoma PANC-1 cells in vitro.The eukaryotic expression plasmid of short hairpin RNA (shRNA) targeting integrin-β1 gene (integrin-β1-shRNA) was constructed and transfected into PANC-1 cells. The expressions of integrin-β1 mRNA and protein were detected by real-time quantitative polymerase chain reaction (PCR) and western blot assay, respectively.   The inv  asive ability of PANC-1 cells was observed with a transwell cell culture chamber and the expressions of MMP-2 and MMP-9 were assayed.Compared to the untransfected group, recombinant expression plasmid integrin-β1-shRNA resulted in reduction of integrin-β1 mRNA and protein by 78.58%±7.24% and 92.88%±3.18%, respectively and the average number of invading PANC-1 cells were decreased from 52±5 to 21±4 (p<0.01) and the expressions of MMP-2 and MMP-9 were inhibited.Recombinant expression plasmid integrin-β1- shRNA can inhibit the expression of integrin-β1 gene and suppress the invasion of PANC-1 cells in vitro significantly. 
 The kinase LMTK3 promotes invasion in breast cancer through GRB2-mediated induction of integrin β₁. Lemur tyrosine kinase 3 (LMTK3) is associated with cell proliferation and endocrine resistance in breast cancer. We found that, in cultured breast cancer cell lines, LMTK3 promotes the development of a metastatic phenotype by inducing the expression of genes encoding integrin subunit  s. Invas  ive behavior in various breast cancer cell lines positively correlated with the abundance of LMTK3. Overexpression of LMTK3 in a breast cancer cell line with low endogenous LMTK3 abundance promoted actin cytoskeleton remodeling, focal adhesion formation, and adhesion to collagen and fibronectin in culture. Using SILAC (stable isotope labeling by amino acids in cell culture) proteomic analysis, we found that LMTK3 increased the abundance of integrin subunits α5 and β1, encoded by ITGA5 and ITGB1. This effect depended on the CDC42 Rho family guanosine triphosphatase, which was in turn activated by the interaction between LMTK3 and growth factor receptor-bound protein 2 (GRB2), an adaptor protein that mediates receptor tyrosine kinase-induced activation of RAS and downstream signaling. Knockdown of GRB2 suppressed LMTK3-induced CDC42 activation, blocked ITGA5 and ITGB1 expression promoted by the transcription factor serum response factor (SRF), and red  uced inv  asive activity. Furthermore, abundance of LMTK3 positively correlated with that of the integrin β1 subunit in breast cancer patient's tumors. Our findings suggest a role for LMTK3 in promoting integrin activity during breast cancer progression and metastasis. 
 Chemical modulation of VLA integrin affinity in human breast cancer cells. The fact that disruption of integrin-extracellular matrix contacts leads to cell death, has converted cell adhesion into a potential target for the control of   invasive   cancer. In this work, we studied the functional consequences of the interference with the activity of the very late activation antigen (VLA) family of integrins in human breast cancer cell lines of distinct malignancy. The alpha2beta1-mediated adhesion reduced the entry of highly malignant, hormone-independent breast cancer cells into apoptosis. Adhesion of breast cancer cells through the VLA integrins alpha2beta1 and alpha5beta1 was significantly reduced by an apoptosis-inducing natural triterpenoid, dehydrothyrsiferol (DT), when studied on low amounts of extracellular matrix. This effect was dose-dependent, not related to cell toxicity and not shared with apoptosis-inducing standard chemotherapeutics, such as doxorubicin and taxol. The compound did not affect either the cell surface expression level of VLA integrins or cell distribution of vinculin and actin during cell spreading. In addition, neither phosphorylation of the focal adhesion kinase pp125FAK on Tyr397 nor the protein kinase B (Akt/PKB) on Ser473 was significantly altered by DT. The integrin activation level, assessed by binding of soluble collagen to the alpha2beta1 integrin, was reduced upon cell treatment with DT. Importantly, the TS2/16, an anti-beta1 activating monoclonal antibody was able to rescue DT-treated cells from apoptosis. Since the activation state of integrins is increasingly recognized as an essential factor in metastasis formation, findings presented herein reveal that the chemical regulation of integrin affinity may be a potential therapeutic strategy in cancer therapy. 
 Neisseria meningitidis adhesin NadA targets beta1 integrins: functional similarity to Yersinia invasin. Meningococci are facultative-pathogenic bacteria endowed with a set of adhesins allowing colonization of the human upper respiratory tract, leading to fulminant meningitis and septicemia. The Neisseria adhesin NadA was identified in about 50% of N. meningitidis isolates and is closely related to the Yersinia adhesin YadA, the prototype of the oligomeric coiled-coil adhesin (Oca) family. NadA is known to be involved in cell adhesion, invasion, and induction of proinflammatory cytokines. Because of the enormous diversity of neisserial cell adhesins the analysis of the specific contribution of NadA in meningococcal host interactions is limited. Therefore, we used a non-  invasive   Y. enterocolitica mutant as carrier to study the role of NadA in host cell interaction. NadA was shown to be efficiently produced and localized in its oligomeric form on the bacterial surface of Y. enterocolitica. Additionally, NadA mediated a β1 integrin-dependent adherence with subsequent internalization of yersiniae by a β1 integrin-positive cell line. Using recombinant NadA(24-210) protein and human and murine β1 integrin-expressing cell lines we could demonstrate the role of the β1 integrin subunit as putative receptor for NadA. Subsequent inhibition assays revealed specific interaction of NadA(24-210) with the human β1 integrin subunit. Cumulatively, these results indicate that Y. enterocolitica is a suitable toolbox system for analysis of the adhesive properties of NadA, revealing strong evidence that β1 integrins are important receptors for NadA. Thus, this study demonstrated for the first time a direct interaction between the Oca-family member NadA and human β1 integrins. 
 Autocrine semaphorin3A stimulates alpha2 beta1 integrin expression/function in breast tumor cells. The axon repulsion factor semaphorin3A (SEMA3A) and its receptor neuropilin-1 (NP-1) are expressed in breast tumor cells, and function as suppressors of tumor cell migration. Based on the knowledge that both SEMA3A and the alpha2beta1 integrin suppress breast tumor cell migration, we studied the impact of SEMA3A signaling on alpha2beta1 integrin expression/function. The incubation of breast tumor cells with SEMA3A increased alpha2 and beta1 integrin levels, and stimulated tumor cell adhesion to the alpha2beta1-binding matrix protein collagen I. Conversely, reducing SEMA3A expression in breast tumor cells decreased alpha2beta1 levels and collagen adhesion. The ability of SEMA3A to increase tumor cell adhesion to collagen was dependent on both the SEMA3A receptor NP-1 and the glycogen synthase kinase-3. The incubation of breast tumor cells with SEMA3A disrupted the actin cytoskeleton, and reduced both tumor cell migratory and   invasive   behavior. Importantly, using an alpha2beta1-neutralizing antibody, we demonstrated that SEMA3A suppression of tumor cell migration is dependent on alpha2beta1. Our studies indicate that expression of the alpha2beta1 integrin, a suppressor of metastatic breast tumor growth, is stimulated in breast tumor cells by an autocrine SEMA3A pathway. 
 CD151 amplifies signaling by integrin α6β1 to PI3K and induces the epithelial-mesenchymal transition in HCC cells. Overexpression of CD151 is associated with poor prognosis for hepatocellular carcinoma (HCC), yet its role in pathogenesis is not known.We analyzed the expression of the integrin subunit α6 by quantitative, real-time polymerase chain reaction and immunoblot analyses of 120 HCC tissue samples; its clinical significance was investigated using tissue microarray (TMAs) analysis of samples from 335 patients with HCC. Immunoprecipitation was used to assess the relationship between α6 and CD151. The molecular effects of high expression levels of α6 and CD151 in HCC cells were determined using RNA interference and pharmacologic approaches.Overexpression of α6 correlated with poor prognosis of patients with HCC; α6 formed a complex with endogenous CD151 in HCC cells. In cells that expressed high levels of α6 and CD151, laminin-5 promoted cell spreading by inducing the epithelial-mesenchymal transition (EMT); this effect was not observed in cells that expressed high levels of only α6 or CD151. Cells that expressed high levels of α6 and CD151 underwent the EMT in response to laminin-5, through hyperactivation of phosphatidylinositol-3-kinase (PI3K), primarily induced via the PI3K-protein kinase B (Akt)-Snail-phosphatase and tensin homolog feedback pathway. The EMT was reversed by PI3K inhibitors and antibodies against CD151 or α6 in vitro, and was delayed by specific interference with CD151 and α6 in vivo.High expression levels of CD151 and   α6 pro  mote invasiveness of HCC cells. Either of these proteins, or PI3K signaling, might be targets for therapeutics for subgroups of patients with HCC. 
 β1 integrin regulates Arg to promote invadopodial maturation and matrix degradation. β1 integrin has been shown to promote metastasis in a number of tumor models, including breast, ovarian, pancreatic, and skin cancer; however, the mechanism by which it does so is poorly understood  . Invasi  ve membrane protrusions called invadopodia are believed to facilitate extracellular matrix degradation and intravasation during metastasis. Previous work showed that β1 integrin localizes to invadopodia, but its role in regulating invadopodial function has not been well characterized. We find that β1 integrin is required for the formation of mature, degradation-competent invadopodia in both two- and three-dimensional matrices but is dispensable for invadopodium precursor formation in metastatic human breast cancer cells. β1 integrin is activated during invadopodium precursor maturation, and forced β1 integrin activation enhances the rate of invadopodial matrix proteolysis. Furthermore, β1 integrin interacts with the tyrosine kinase Arg and stimulates Arg-dependent phosphorylation of cortactin on tyrosine 421. Silencing β1 integrin with small interfering RNA completely abrogates Arg-dependent cortactin phosphorylation and cofilin-dependent barbed-end formation at invadopodia, leading to a significant decrease in the number and stability of mature invadopodia. These results describe a fundamental role for β1 integrin in controlling actin polymerization-dependent invadopodial maturation and matrix degradation in metastatic tumor cells. 
 Matrix metalloproteinase-2 cleavage of the β1 integrin ectodomain facilitates colon cancer cell motility. Cancer cell invasion is a key element in metastasis that requires integrins for adhesion/de-adhesion, as well as matrix metalloproteinases (MMPs) for focalized proteolysis. Herein we show that MMP-2 is up-regulated in resected colorectal tumors and degrades β1 integrins with the release of fragments containing the β1 I-domain. The β1 cleavage pattern is similar to that produced by digestion of α5β1 and α2β1 with MMP-2. Two such fragments, at 25 and 75 kDa, were identified after immunoprecipitation, with monoclonal antibody BD610468 reacting with the NH(2)-terminal I-like ectodomain followed by SDS-PAGE and microsequencing using electrospray (ISI-Q-TOF-Micromass) spectrometry. Cleavage of the β1 integrin can be abolished by inhibition of MMP-2 activity; it can be induced by up-regulation of MMP-2 expression, as exemplified by HT29 colon cancer cells transfected with pCMV6-XL5-MMP-2. Co-immunoprecipitation studies of colon cancer cells showed that the β1 integrin subunit is associated with MMP-2. The MMP-2-mediated shedding of the I-like domain from β1 integrins resulted in decreased adhesion of colon cancer cells to collagen and fibronectin, thus abolishing their receptivity. Furthermore, such cells showed enhanced motility as evaluated by a "wound healing-like" assay and time-lapse microscopy, indicating their   increas  ed invasiveness. Altogether, our data demonstrate that MMP-2 amplifies the motility of colon cancer cells, not only by digesting the extracellular matrix components in the vicinity of cancer cells but also by inactivating their major β1 integrin receptors. 
 Integrin alpha5beta1 controls invasion of human breast carcinoma cells by direct and indirect modulation of MMP-2 collagenase activity. Integrins control a variety of signal transduction pathways central to cell survival, proliferation, and differentiation and their functions and expression levels are altered in many types of cancer. Although alpha5beta1 is one of the most studied integrins in cancer, its functions in different aspects of this disease have not been completely elucidated. In particular, controversial data exist on its role in tumor invasion and metastasis. In order to establish mechanisms underlying involvement of alpha5beta1 integrin in invasion, we depleted its expression in MCF-7Dox human breast carcinoma cells via siRNA. We demonstrated that concomitant to alpha5beta1 integrin depletion, was a sharp decrease in MMP-2 collagenase expression and inhibition of the   invasive  ness of these cells in vitro. Similar reduction of invasion potential was observed upon siRNA-mediated silencing of the MMP-2 gene. Down-regulation of alpha5beta1 integrin was accompanied by a substantial decrease in the amounts of active (phosphorylated) forms of Akt, Erk1/2 kinases and c-Jun oncoprotein. Moreover, in MCF-7Dox cells, blocking the activity of above kinases by specific inhibitors strongly reduced expression of MMP-2 and c-Jun, and suppressed invasion of the cells in vitro. Similar results were observed upon siRNA-mediated silencing of c-Jun expression. Co-immunoprecipitation experiments demonstrated that alpha5beta1 integrin interacts with MMP-2 collagenase on the surface of MCF-7Dox breast carcinoma and SKMel-147 human melanoma cells. Our data suggest that alpha5beta1 integrin controls invasion of the studied cells via regulation of MMP-2 collagenase expression which can occur either through signaling pathways involving PI-3K, Akt, and Erk protein kinases and the c-Jun or via direct recruitment of MMP-2 to the cell surface. 
 Formin-like 2 Promotes β1-Integrin Trafficking and   Invasiv  e Motility Downstream of PKCα. Regulated turnover of integrin receptors is essential for cell adhesion and migration. Pathways selectively regulating β1-integrin recycling are implicated in cancer invasion and metastasis, yet proteins required for the internalization of this p  ro-invas  ive integrin remain to be identified. Here, we uncover formin-like 2 (FMNL2) as a critical regulator of β1-integrin internalization downstream of protein kinase C (PKC). PKCα associates with and phosphorylates FMNL2 at S1072 within its Diaphanous autoregulatory region, leading to the release of formin autoinhibition. Phosphorylation of FMNL2 triggers its rapid relocation and promotes its interaction with the cytoplasmic tails of the α-integrin subunits for β1-integrin endocytosis. FMNL2 drives β1-integrin internalizat  ion and   invasive motility in a phosphorylation-dependent manner, while a FMNL2 mutant defective in actin assembly interferes with β1-integrin endocytosis and cancer cell invasion. Our data establish a role for FMNL2 in the regulation of β1-integrin and provide a mechanistic understanding of the function of FMNL2   in cance  r invasiveness. 
 A specific microdomain ("glycosynapse 3") controls phenotypic conversion and reversion of bladder cancer cells through GM3-mediated interaction of alpha3beta1 integrin with CD9. Cell motility is highly dependent on the organization and function of microdomains composed of integrin, proteolipid/tetraspanin CD9, and ganglioside (Ono, M., Handa, K., Sonnino, S., Withers, D. A., Nagai, H., and Hakomori, S. (2001) Biochemistry 40, 6414-6421; Kawakami, Y., Kawakami, K., Steelant, W. F. A., Ono, M., Baek, R. C., Handa, K., Withers, D. A., and Hakomori, S. (2002) J. Biol. Chem. 277, 34349-34358), later termed "glycosynapse 3" (Hakomori, S., and Handa, K. (2002) FEBS Lett. 531, 88-92, 2002). Human bladder cancer cell lines KK47 (non  invasive   and nonmetastatic) and YTS1 (highly   invasive   and metastatic), both derived from transitional bladder epithelia, are very similar in terms of integrin composition and levels of tetraspanin CD9. Tetraspanin CD82 is absent in both. The major difference is in the level of ganglioside GM3, which is several times higher in KK47 than in YTS1. We now report that the GM3 level reflects glycosynapse function as follows: (i) a stronger interaction of integrin alpha3 with CD9 in KK47 than in YTS1; (ii) conversion of benign, low motility KK47 to   invasive  , high motility cells by depletion of GM3 by P4 (D-threo-1-phenyl-2-palmitoylamino-3-pyrrolidino-1-propanol) treatment or by knockdown of CD9 by the RNA interference method; (iii) reversion of high motility YTS1 to low motility phenotype like that of KK47 by exogenous GM3 addition, whereby the alpha3-to-CD9 interaction was enhanced; (iv) low GM3 level activated c-Src in YTS1 or in P4-treated KK47, and high GM3 level by exogenous addition caused Csk translocation into glycosynapse, with subsequent inhibition of c-Src activation; (v) inhibition of c-Src by "PP2" in YTS1 greatly reduced cell motility. Thus, GM3 in glycosynapse 3 plays a dual role in defining glycosynapse 3 function. One is by modulating the interaction of alpha3 with CD9; the other is by activating or inhibiting the c-Src activity, possibly through Csk translocation. High GM3 level decreases tumor cell motility/  invasive  ness, whereas low GM3 level enhances tumor cell motility/  invasive  ness. Oncogenic transformation and its reversion can be explained through the difference in glycosynapse organization. 
 Laminin isoforms and their integrin receptors in glioma cell migration and   invasive  ness: Evidence for a role of alpha5-laminin(s) and alpha3beta1 integrin. Glioma cell infiltration of brain tissue often occurs along the basement membrane (BM) of blood vessels. In the present study we have investigated the role of laminins, major structural components of BMs and strong promoters of cell migration. Immunohistochemical studies of glioma tumor tissue demonstrated expression of alpha2-, alpha3-, alpha4- and alpha5-, but not alpha1-, laminins by the tumor vasculature. In functional assays, alpha3 (Lm-332/laminin-5)- and alpha5 (Lm-511/laminin-10)-laminins strongly promoted migration of all glioma cell lines tested. alpha1-Laminin (Lm-111/laminin-1) displayed lower activity, whereas alpha2 (Lm-211/laminin-2)- and alpha4 (Lm-411/laminin-8)-laminins were practically inactive. Global integrin phenotyping identified alpha3beta1 as the most abundant integrin in all the glioma cell lines, and this laminin-binding integrin exclusively or largely mediate the cell migration. Moreover, pretreatment of U251 glioma cells with blocking antibodies to alpha3beta1 integrin followed by intracerebral injection into nude mice inhibited invasion of the tumor cells into the brain tissue. The cell lines secreted Lm-211, Lm-411 and Lm-511, at different ratios. The results indicate that glioma cells secrete alpha2-, alpha4- and alpha5-laminins and that alpha3- and alpha5-laminins, found in brain vasculature, selectively promote glioma cell migration. They identify alpha3beta1 as the predominant integrin and laminin receptor in glioma cells, and as a brain invasion-mediating integrin. 
 Overexpression of AKT2/protein kinase Bbeta leads to up-regulation of beta1 integrins, increased invasion, and metastasis of human breast and ovarian cancer cells. To determine how AKT2 might contribute to tumor cell progression, a full-length, wild-type, human AKT2/protein kinase B (PKB)beta cDNA was transfected into a panel of eight human breast and ovarian cancer cells. AKT2 transfectants demonstrated increased adhesion and invasion through collagen IV because of up-regulation of beta1 integrins. In addition, AKT2 cells were more metastatic than control cells in vivo. Increased invasion by AKT2 was blocked by preincubation with an anti-beta1 integrin function blocking antibody, exposure to wortmannin, and by expression of phosphatase and tensin homologue tumor suppressor (PTEN). Confocal microscopy performed on transfected human breast cancer cells showed that unlike AKT1, AKT2 protein predominantly localized adjacent to the collagen IV matrix during cellular attachment. Overexpression of AKT2, but not AKT1 or AKT3, was sufficient to duplicate the   invasive   effects of phosphoinositide 3-OH kinase (PI3-K) transfected in breast cancer cells. Furthermore, expression of kinase dead AKT2(181 amino acid methionine [M]), and not kinase dead AKT1(179M) or AKT3(177M), was capable of blocking invasion induced by either human epidermal growth factor receptor-2 (HER-2) overexpression or by activation of PI3-K. Taken together, these data indicate that AKT2 mediates PI3-K-dependent effects on adhesion, motility, invasion, and metastasis in vivo. 
 Metastatic properties of prostate cancer cells are controlled by VEGF. Mechanisms of metastasis, the major complication of prostate cancer, are poorly understood. In this study, we define molecular mechanisms that may contribute to the highly   invasive   potential of prostate cancer cells. Vascular endothelial growth factor (VEGF), its receptors (VEGFRs), and alpha5beta1 integrin were expressed by prostate cancer cells in vitro and by prostate tumors in vivo, and their expression was elevated at sites of bone metastasis compared to original prostate tumor. VEGF, through interaction with its receptors, regulated adhesive and migratory properties of the cancer cells. Specifically, the highly metastatic prostate cancer cell subline LNCaP-C4-2 showed a decreased adhesive but an enhanced migratory response to fibronectin, a ligand for alpha5beta1 integrin, compared to its nonmetastatic counterpart. A similar pattern was also observed when bone sialoprotein was used as a ligand in migration assays. Increased migration of metastatic prostate cancer cells to fibronectin and bone sialoprotein was regulated by VEGF via VEGFR-2. Tumor suppressor PTEN was involved in control of VEGF/VEGFR-2 stimulated prostate cancer cell adhesion as well as proliferation. 
 N-acetylglucosaminyltransferase V regulates extravillous trophoblast invasion through glycosylation of alpha5beta1 integrin. For successful human placentation, invasion of trophoblast cells into the uterus and its associated vasculature is essential, and the regulation of this process is controlled by many factors at the fetal-maternal interface. N-acetylglucosaminyltransferase V (GnT-V) is a key enzyme that catalyzes beta1, 6-N-acetylglucosamine (beta1-6GlcNAc) branching on asparagine-linked oligosaccharides of cell proteins. GnT-V and its product, beta1-6GlcNAc, are known to regulate cellular transformation and correlate with the metastatic potential of various cancer cells. The aim of the present study was to determine whether extravillous trophoblast (EVT) expressed this molecule and examine the role of GnT-V in the regulation of human trophoblast invasion. Immunohistochemistry showed that GnT-V was strongly expressed within the cytoplasm of EVT in the anchoring villi; this expression was down-regulated in EVTs invading the decidua. Suppression of beta1-6GlcNAc glycosylation by swainsonine enhanced the migratory potential and   invasive   capability of both primary EVTs and the EVT cell line, HTR-8/SVneo. Down-regulation of GnT-V expression by small interfering RNA in the choriocarcinoma cell line Jar consistently enhanced the migration and   invasive   capacity of these cells and elevated cellular adhesion to extracellular matrix proteins, such as fibronectin and collagen type I/IV. The extent of beta1-6 branching of alpha5beta1 integrin was significantly reduced in small interfering GnT-V-transfected Jar cells compared with mock transfectants, although the expression of alpha1, alpha5, alpha6, and beta1 integrin on the cell surface was not changed. These results suggest that GnT-V is expressed in human EVT and is involved in regulating trophoblast invasion through modifications of the oligosaccharide chains of alpha5beta1 integrin. 
 Regulation of urokinase receptor function and pericellular proteolysis by the integrin alpha(5)beta(1). Interactions between the uPA receptor (uPAR) and various integrins, including alpha(5)beta(1), are known to modulate integrin-dependent cell adhesion, and we have shown that the integrin-associated tetraspanin protein CD82 down-regulates uPAR-dependent plasminogen activation by affecting alpha(5)beta(1) cellular localisation. Here we have investigated whether overexpression of alpha(5)beta(1) directly affects uPAR-dependent pericellular proteolysis. CHO cells overexpressing alpha(5)beta(1) were found to activate plasminogen at a rate up to 18-fold faster than B2CHO cells which are alpha(5)-deficient. This effect was dependent on the activation state of alpha(5)beta(1), as it was maximal in the presence of Mn(2+). To determine the role of uPAR-alpha(5)beta(1) interactions in this effect, we determined the adhesion of these cells to immobilised soluble uPAR (suPAR). Neither cell-type was found to adhere to suPAR, but both cell types were found to adhere to an anti-uPAR monoclonal antibody in a uPAR- and integrin-dependent manner. This adhesion was 10-fold greater in the absence of alpha(5)beta(1), possibly implicating the involvement of non-alpha(5)-integrins. Soluble forms of the various components were used to investigate the molecular basis of these effects, but no direct interactions could be demonstrated between alpha(5)beta(1) and either uPAR, uPA or uPA-uPAR complex. This suggests that assembly of these components on the plasma membrane is required to influence uPAR function, increasing uPAR-dependent pericellular proteolysis and decreasing uPAR-dependent cell adhesion. These interactions may be modified by other integrins, suggesting a complex interplay between uPAR and integrins on the cell surface with the potential to regulate   invasive   cell migration. 
 Down-regulation of epithelial cadherin is required to initiate metastatic outgrowth of breast cancer. Reduced epithelial cadherin (E-cad) is a hallmark of   invasive   carcinomas that have acquired epithelial-mesenchymal transition (EMT) phenotypes. Here we show that down-regulated E-cad expression induced by transforming growth factor-β (TGF-β) and EMT preceded breast cancer outgrowth in three-dimensional (3D) organotypic assays and in the lungs of mice. Pharmacological inhibitors against focal adhesion kinase prevented metastatic outgrowth of newly seeded organoids, but not that of their fully established counterparts. Interrogating the D2-HAN (hyperplastic alveolar nodule) model of breast cancer dormancy and metastasis showed that dormant D2.OR cells produced branched organoid morphologies in 3D-cultures, and expressed robust quantities of E-cad that was uncoupled from regulation by TGF-β. In contrast, metastatic D2.A1 organoids were spherical and wholly lacked E-cad expression. Interestingly, D2.A1 cells engineered to re-express E-cad formed branched organoids, down-regulated β1 integrin expression, and failed to undergo metastatic outgrowth. The tumor-suppressing function of E-cad was inactivated by increased microenvironmental rigidity, and was not recapitulated by expression of an E-cad mutant lacking its extracellular domain. Twist expression, but not that of Snail, reinitiated metastatic outgrowth in dormant D2.OR cells. Our findings show that EMT and its down-regulated expression of E-cad circumvent breast cancer dormancy in part by facilitating β1 integrin expression necessary for metastatic outgrowth. 
 Interleukin-1alpha enhances integrin alpha(6)beta(1) expression and metastatic capability of human pancreatic cancer. To investigate the mechanisms of metastasis formation in metastatic human pancreatic cancer, we examined the enhancement in integrin expression, and adherence to and   invasive  ness into extracellular matrix (ECM) proteins of human pancreatic cancer cells after exposure to interleukin (IL)-1alpha.Expression of IL-1 receptor type I (IL-1RI) and alterations in integrin subunits by IL-1alpha were examined by flow-cytometric analysis and by cellular enzyme-linked immunosorbent assay in four human pancreatic cancer cell lines (BxPC-3, PaCa-2, PANC-1, and SW1990), respectively. In addition, assays of cancer cell adhesion and invasion to ECM proteins were performed to investigate whether increased integrin expression affected the adhesive and   invasive   interaction between cancer cells and the putative integrin ECM ligands. Furthermore, immunohistochemistry was used to assess integrins and IL-1R1 expression in pancreatic tissues.In metastatic cancer cells, expression of the alpha(6) subunit was enhanced by IL-1alpha treatment. While metastatic cancer cells exhibited preferential adherence to and invasion into laminin, these properties were enhanced by IL-1alpha. The alpha(6) subunit and IL-1RI were strongly expressed in pancreatic tissues from pancreatic cancer patients with liver metastasis.In pancreatic cancer, IL-1alpha enhanced alpha(6)beta(1)-integrin expression, probably via increased IL-1RI levels. Our results indicated that alpha(6)beta(1)-integrin and IL-1RI expression may play important roles in metastasis formation. 
 CXCL12/CXCR4 activation by cancer-associated fibroblasts promotes integrin β1 clustering and   invasiv  eness in gastric cancer. Cancer-associated fibroblasts (CAFs) are reportedly involved in invasion and metastasis in several types of cancer, including gastric cancer (GC), through the stimulation of CXCL12/CXCR4 signaling. However, the mechanisms underlying these tumor-promoting effects are not well understood, which limits the potential to develop therapeutic targets against CAF-mediated CXCL12/CXCR4 signaling. CXCL12 expression was analyzed in resected GC tissues from 110 patients by immunohistochemistry (IHC). We established primary cultures of normal fibroblasts (NFs) and CAFs from the GC tissues and examined the functional differences between these primary fibroblasts using co-culture assays with GC cell lines. We evaluated the efficacy of a CXCR4 antagonist (AMD3100) and a FAK inhibitor (PF-573,228) on the   invasiv  e ability of GC cells. High CXCL12 expression levels were significantly associated with larger tumor size, increased tumor depth, lymphatic invasion and poor prognosis in GC. CXCL12/CXCR4 activation by CAFs mediated integrin β1 clustering at the cell surface and promoted th  e invasi  ve ability of GC cells. Notably, AMD3100 was more efficient than PF-573,228 at inhibiting GC cell invasion through the suppression of integrin β1/FAK signaling. These results suggest that CXCL12 derived from CAFs promotes GC cell invasion by enhancing the clustering of integrin β1 in GC cells, resulting in GC progression. Taken together, the inhibition of CXCL12/CXCR4 signaling in GC cells may be a promising therapeutic strategy against GC cell invasion. 
 Activation of integrin β1 mediates the increased malignant potential of ovarian cancer cells exerted by inflammatory cytokines. Epithelial ovarian cancer (EOC) is a highly lethal gynecological malignancy since it could not be discovered until at late stage. Identifying the molecular phenotype alteration during the development and progression of ovarian cancer is an urgent demand for the targeted intervention therapy. Recently, inflammation and Integrin beta 1(ITGB1), a subunit of heterodimeric transmembrane receptors family, had been pointed out to be involved in promoting ovarian tumorigenesis and cancer progression, respectively. However, the relationship between ITGB1 and the inflammatory mediators in ovarian cancer progression remains obscure. In the present study, ITGB1 was observed to be frequently upregulated in ovarian cancer, overexpression of ITGB1 led to a more   invasiv  e and mesenchymal phenotype. Furthermore, our results also provided evidence concerning the role of inflammatory cytokines (IL-6, TGF-β1 and SDF-1) in ITGB1 expression as well as in the malignant potential of ovarian cancer cells. Consistently, sh-RNA mediated knocking down of ITGB1 evidently reduced tumor growth and peritoneal dissemination in in vivo Nod-scid SKOV3 orthotopic xenograft mice. Overall, the present data suggested that ITGB1 upregulation was involved in the regulation of tumorigenesis and disease exacerbation exerted by inflammatory cytokines as IL-6, TGF-β1 and SDF-1, and suggested that targeting ITGB1 and the underlying inflammatory modulator was an attractive strategy for therapeutic intervention during ovarian carcinogenesis. 
 C1GALT1 promotes   invasive   phenotypes of hepatocellular carcinoma cells by modulating integrin β1 glycosylation and activity. Cancer cell invasion and metastasis are the primary causes of treatment failure and death in hepatocellular carcinoma (HCC). We previously reported that core 1 β1,3-galactosyltransferase (C1GALT1) is frequently overexpressed in HCC tumors and its expression is associated with advanced tumor stage, metastasis, and poor survival. However, the underlying mechanisms of C1GALT1 in HCC malignancy remain unclear. In this study, we found that overexpression of C1GALT1 enhanced HCC cell adhesion to extracellular matrix (ECM) proteins, migration, and invasion, whereas RNAi-mediated knockdown of C1GALT1 suppressed these phenotypes. The promoting effect of C1GALT1 on the metastasis of HCC cells was demonstrated in a mouse xenograft model. Mechanistic investigations showed that the C1GALT1-enhanced phenotypic changes in HCC cells were significantly suppressed by anti-integrin β1 blocking antibody. Moreover, C1GALT1 was able to modify O-glycans on integrin β1 and regulate integrin β1 activity as well as its downstream signaling. These results suggest that C1GALT1 could enhance   HCC inv  asiveness through integrin β1 and provide novel insights into the roles of O-glycosylation in HCC metastasis. 
 HIV-1-Tat protein promotes chemotaxis and   invasive   behavior by monocytes. Monocytes are susceptible to HIV infection and to activation by a regulatory gene product of the HIV genome, HIV-Tat. Recently, we have demonstrated that treatment with HIV-Tat up-regulates monocyte adhesion to the endothelium and increases metalloproteinase production. in the present study, we have examined the ability of the HIV-Tat protein to alter the migratory and   invasive   behavior of monocytes. Monocytes pretreated for 24 h with 10 ng/ml HIV-Tat exhibited enhanced migratory behavior compared with untreated monocytes in chemotaxis assays, both in the absence of a chemoattractant as well as in response to FMLP. in addition, HIV-Tat itself induced the migration of both untreated and HIV-Tat pretreated monocytes. Checkerboard analysis showed that monocytes migrated in response to an HIV-Tat concentration gradient, thus confirming the chemotactic characteristics of the HIV-Tat protein. Pretreatment of monocytes with 10 ng/ml HIV-Tat for 24 h also increased their ability to invade reconstituted extracellular membrane (Matrigel)-coated filters by 5-fold in the absence of chemoattractant. The presence of FMLP or HIV-Tat further enhanced invasion by both untreated and HIV-Tat-pretreated monocytes by more than 10-fold. Monocyte invasion was partially inhibited by the inclusion of anti-beta integrin Ab or tissue inhibitor of metalloproteinase (TIMP). Thus, for the first time, we present evidence that HIV-Tat can enhance the chemotactic and   invasive   behaviors of monocytes and propose an active role for HIV-Tat in the recruitment of monocytes into extravascular tissues, a process which may contribute to the destruction of tissues and cellular architecture often seen in patients with acquired immunodeficiency syndrome. 
 The secreted protein ANGPTL2 promotes metastasis of osteosarcoma cells through integrin α5β1, p38 MAPK, and matrix metalloproteinases. The tumor microenvironment can enhance th  e invasi  ve capacity of tumor cells. We showed that expression of angiopoietin-like protein 2 (ANGPTL2) in osteosarcoma (OS) cell lines increased and the methylation of its promoter decreased with time when grown as xenografts in mice compared with culture. Compared with cells grown in normal culture conditions, the expression of genes encoding DNA demethylation-related enzymes increased in tumor cells implanted into mice or grown in hypoxic, serum-starved culture conditions. ANGPTL2 expression in OS cell lines correlated with increased tumor metastasis and decreased animal survival by promoting tumor cell intravasation mediated by the integrin α5β1, p38 mitogen-activated protein kinase, and matrix metalloproteinases. The tolloid-like 1 (TLL1) protease cleaved ANGPTL2 into fragments in vitro that did not enhance tumor progression when overexpressed in xenografts. Expression of TLL1 was weak in OS patient tumors, suggesting that ANGPTL2 may not be efficiently cleaved upon secretion from OS cells. These findings demonstrate that preventing ANGPTL2 signaling stimulated by the tumor microenvironment could inhibit tumor cell migration and metastasis. 
 β-1,4-Galactosyltransferase III suppresses β1 integrin-mediate  d invasi  ve phenotypes and negatively correlates with metastasis in colorectal cancer. Metastasis often occurs in colorectal cancer (CRC) patients and is the main difficulty in cancer treatment. The upregulation of poly-N-acetyllactosamine-related glycosylation is found in CRC patients and is associated with progression and metastasis in cancer. β-1,4-Galactosyltransferase III (B4GALT3) is an enzyme responsible for poly-N-acetyllactosamine synthesis, and therefore, we investigated its expression in CRC patients. We found that B4GALT3 negatively correlated with poorly differentiated histology (P < 0.001), advanced stages (P = 0.0052), regional lymph node metastasis (P = 0.0018) and distant metastasis (P = 0.0463) in CRC patients. B4GALT3 overexpression in CRC cells suppressed cell migration, invasion and adhesion, whereas B4GALT3 knockdown enhanced malignant cell phenotypes. The β1 integrin-blocking antibody reversed the B4GALT3-mediated increase in cell invasion. B4GALT3 expression altered glycosylation on the N-glycan of β1 integrin probably through changes in poly-N-acetyllactosamine expression. Furthermore, more activated β1 integrin along with the activation of its downstream signaling transduction were found in B4GALT3 knockdown cells, whereas overexpression of B4GALT3 suppressed the expression of active β1 integrin and inhibited its downstream signaling. Our results suggest that B4GALT3 is negatively associated with CRC metastasis and suppresse  s cell i  nvasiveness through inhibiting activation of β1 integrin. 
 The signaling network of transforming growth factor beta1, protein kinase Cdelta, and integrin underlies the spreading and   invasive  ness of gastric carcinoma cells. Integrin-mediated cell adhesion and spreading enables cells to respond to extracellular stimuli for cellular functions. Using a gastric carcinoma cell line that is usually round in adhesion, we explored the mechanisms underlying the cell spreading process, separate from adhesion, and the biological consequences of the process. The cells exhibited spreading behavior through the collaboration of integrin-extracellular matrix interaction with a Smad-mediated transforming growth factor beta1 (TGFbeta1) pathway that is mediated by protein kinase Cdelta (PKCdelta). TGFbeta1 treatment of the cells replated on extracellular matrix caused the expression and phosphorylation of PKCdelta, which is required for expression and activation of integrins. Increased expression of integrins alpha2 and alpha3 correlated with the spreading, functioning in activation of focal adhesion molecules. Smad3, but not Smad2, overexpression enhanced the TGFbeta1 effects. Furthermore, TGFbeta1 treatment and PKCdelta activity were required for increased motility on fibronectin and invasion through matrigel, indicating their correlation with the spreading behavior. Altogether, this study clearly evidenced that the signaling network, involving the Smad-dependent TGFbeta pathway, PKCdelta expression and phosphorylation, and integrin expression and activation, regulates cell spreading, motility, and invasion of the SNU16mAd gastric carcinoma cell variant. 
 Expression of Toll-like receptor 4 and beta 1 integrin in breast cancer. Toll-like receptor (TLR) 4 signaling pathway has been shown to support tumor cell growth in vitro and in vivo. Its stimulation on breast cancer cell lines induces β1 integrin and promotes tumor   invasiv  eness. However, its role in predicting clinical behavior of tumor is not yet clarified. Therefore, we investigated TLR4 and β1 integrin expression on 133 primary breast cancer samples by immunohistochemistry and correlated it with overall survival and disease-free survival of patients as well as with clinicopathological characteristics of the tumor. We found higher β1 integrin expression   in invas  ive lobular cancer in comparison with other tumor types. No significant association of TLR4 and β1 integrin expression with overall survival or disease-free survival was seen. Therefore, we conclude that expression of these markers is of biological interest but appears to be of little additional use as predictive clinical marker. 
 Ganglioside GM2 mediates migration of tumor cells by interacting with integrin and modulating the downstream signaling pathway. The definitive role of ganglioside GM2 in mediating tumor-induced growth and progression is still unknown. Here we report a novel role of ganglioside GM2 in mediating tumor cell migration and uncovered its mechanism. Data shows differential expression levels of GM2-synthase as well as GM2 in different human cancer cells. siRNA mediated knockdown of GM2-synthase in CCF52, A549 and SK-RC-26B cells resulted in significant inhibition of tumor cell migration as well as invasion in vitro without affecting cellular proliferation. Over-expression of GM2-synthase in low-GM2 expressing SK-RC-45 cells resulted in a consequent increase in migration thus confirming the potential role GM2 and its downstream partners play in tumor cell migration and motility. Further, treatment of SK-RC-45 cells with exogenous GM2 resulted in a dramatic increase in migratory and   invasive   capacity with no change in proliferative capacity, thereby confirming the role of GM2 in tumorigenesis specifically by mediating tumor migration and invasion. Gene expression profiling of GM2-synthase silenced cells revealed altered expression of several genes involved in cell migration primarily those controlling the integrin mediated signaling. GM2-synthase knockdown resulted in decreased phosphorylation of FAK, Src as well as Erk, while over-expression and/or exogenous GM2 treatment caused increased FAK and Erk phosphorylation respectively. Again, GM2 mediated invasion and Erk phosphorylation is blocked in integrin knockdown SK-RC-45 cells, thus confirming that GM2 mediated migration and phosphorylation of Erk is integrin dependent. Finally, confocal microscopy suggested co-localization while co-immunoprecipitation and surface plasmon resonance (SPR) confirmed direct interaction of membrane bound ganglioside, GM2 with the integrin receptor. 
 Expression of beta1-integrins and N-cadherin in bladder cancer and melanoma cell lines. Changes in the expression of integrins and cadherins might contribute to the progression, invasion and metastasis of transitional cell cancer of the bladder and of melanomas. The expression of alpha5 (P < 0.001), alpha2 and beta1 (P < 0.05 - P < 0.001) integrin subunits in melanoma cells from noncutaneous metastatic sites (WM9, A375) were significantly increased as compared to cutaneous primary tumor (WM35) and metastatic (WM239) cell lines. These differences might be ascribed to the   invasive   character of melanoma cells and their metastasis to the noncutaneous locations. The significantly heterogeneous expression of beta1 integrin subunit in two malignant bladder cancer cell lines (T24 and Hu456) and nonsignificant differences in the expression of alpha2, alpha3, and alpha5 subunits between malignant and non-malignant human bladder cell lines do not allow an unanimous conclusion on the role of these intergrin subunits in the progression of transitional cancer of bladder. The adhesion molecule, expressed in all studied melanoma and bladder cell lines, that reacted with anti-Pan cadherin monoclonal antibodies was identified as N-cadherin except in the HCV29 non-malignant ureter cell line. However, neither this nor any other bladder or melanoma cell line expressed E-cadherin. The obtained results imply that the replacement of E-cadherin by N-cadherin accompanied by a simultaneous increase in expression of alpha2, alpha3 and alpha5 integrin subunits clearly indicates an increase of   invasive  ness of melanoma and, to a lesser extent, of transitional cell cancer of bladder. High expression of N-cadherin and alpha5 integrin subunit seems to be associated with the most   invasive   melanoma phenotype. 
 ß1 integrin binding phosphorylates ezrin at T567 to activate a lipid raft signalsome driving invadopodia activity and invasion. Extracellular matrix (ECM) degradation is a critical process in tumor cell invasion and requires matrix degrading protrusions called invadopodia. The Na(+)/H(+) exchanger (NHE1) has recently been shown to be fundamental in the regulation of invadopodia actin cytoskeleton dynamics and activity. However, the structural link between the invadopodia cytoskeleton and NHE1 is still unknown. A candidate could be ezrin, a linker between the NHE1 and the actin cytoskeleton known to play a pivotal role in invasion and metastasis. However, the mechanistic basis for its role remains unknown. Here, we demonstrate that ezrin phosphorylated at T567 is highly overexpressed in the membrane of human breast tumors and positively associated with   invasiv  e growth and HER2 overexpression. Further, in the metastatic cell line, MDA-MB-231, p-ezrin was almost exclusively expressed in invadopodia lipid rafts where it co-localized in a functional complex with NHE1, EGFR, ß1-integrin and phosphorylated-NHERF1. Manipulation by mutation of ezrins T567 phosphorylation state and/or PIP2 binding capacity or of NHE1s binding to ezrin or PIP2 demonstrated that p-ezrin expression and binding to PIP2 are required for invadopodia-mediated ECM degradation and invasion and identified NHE1 as the membrane protein that p-ezrin regulates to induce invadopodia formation and activity. 
 hTERT mediates gastric cancer metastasis partially through the indirect targeting of ITGB1 by microRNA-29a. Human telomerase reverse transcriptase (hTERT) plays a key role in tumor invasion and metastasis, but the mechanism of its involvement in these processes is not clear. The purpose of this study is to investigate the possible molecular mechanism of hTERT in the promotion of gastric cancer (GC) metastasis. We found that the up-regulation of hTERT in gastric cancer cells could inhibit the expression of miR-29a and enhance the expression of Integrin β1 (ITGB1). In addition, the   invasiv  e capacity of gastric cancer cells was also highly increased after hTERT overexpression. Our study also found that the restoration of miR-29a suppressed the expression of ITGB1 and inhibited GC cell metastasis both in vitro and in vivo. Taken together, our results suggested that hTERT may promote GC metastasis through the hTERT-miR-29a-ITGB1 regulatory pathway. 
 Effect of KAI1/CD82 on the beta1 integrin maturation in highly migratory carcinoma cells. The KAI1/CD82 protein has been documented as the tumor metastasis suppressor in many types of human cancers. KAI1/CD82 regulates cell motility and   invasive  ness; however, the mechanism by which this occurs remains to be fully established. Several studies have shown that KAI1/CD82 modulates integrin-dependent signaling. It was suggested that KAI1/CD82 might function to attenuate the beta1 integrin function of inducing cellular migration. A wound-healing and modified Boyden chamber assays were performed to investigate the mechanism of the KAI1/CD82-mediated inhibition of cell migration. It was found that the migratory ability of H1299/CD82 was inhibited. The immunoblotting and biotinylation assays revealed that H1299/CD82 showed significantly decreased expression of the mature form of beta1, which was functional at the cell surface. It was confirmed that KAI1/CD82 regulates the maturation of the beta1 integrin using CD82-specific si-RNA. These results support a model in which KAI1/CD82 attenuates the maturation of the beta1 integrin precursor and thereby suppresses cell migration. 
 The expression of integrinbeta1 and FAK in pituitary adenomas and their correlation with   invasive  ness. The expression and possible role of integrin-focal adhesion kinase signal pathway in   invasive   pituitary adenomas were explored. Forty-nine human pituitary adenomas were detected for the expression of integrinbeta1 (INTbeta1) and focal adhesion kinase (FAK) by immunohistochemistry, and their correlation with the   invasive  ness of pituitary adenomas as well as between themselves was analyzed. The results showed that INTbeta1 was expressed in 46 cases (93.9%) and FAK in 36 cases (73.5%), respectively, and their expression levels were highly correlated with tumor   invasive  ness, but not with the tumor types. It was suggested that the integrin-focal adhesion kinase signal pathway plays a role in the   invasive  ness of pituitary adenomas. 
 SPARC promotes cathepsin B-mediated melanoma   invasive  ness through a collagen I/α2β1 integrin axis. In melanoma, the extracellular protein SPARC (secreted protein acidic and rich in cysteine) is related to tumor progression. Some of the evidence that links SPARC to melanoma progression indicates that SPARC may be involved in the acquisition of mesenchymal traits that favor metastatic dissemination. However, no molecular pathways that link extracellular SPARC to a mesenchymal phenotype have been described. In this study, global protein expression analysis of the melanoma secretome following enforced downregulation of SPARC expression led us to elucidate a new molecular mechanism by which SPARC promotes cathepsin B-mediated melanom  a invasi  veness using collagen I and α2β1 integrins as mediators. Interestingly, we also found that the transforming growth factor (TGF)-β1 contribution to cathepsin B-mediated invasion is highly SPARC dependent. In addition, induction of the E-cadherin to N-cadherin switch by SPARC enabled melanoma cells to transmigrate across an endothelial layer through a mechanism independent to that of enhancing invasion. Finally, SPARC also enhanced the extracellular expression of other proteins involved in epithelial-mesenchymal transformation, such as family with sequence similarity 3, member C/interleukin-like EMT-inducer. Our findings demonstrate a previously unreported molecular pathway for SPARC activity on invasion and support an active role of SPARC in the mesenchymal transformation that contributes to melanoma dissemination. 
 Targeting transforming growth factor (TGF)-betaRI inhibits activation of beta1 integrin and blocks vascular invasion in hepatocellular carcinoma. Vascular invasion is one of the major negative prognostic factors in patients with hepatocellular carcinoma (HCC), leading to cancer recurrence. To invade, HCC cells must penetrate the vessel wall, consisting of endothelial cells and extracellular matrix components, including fibronectin and fibrinogen. Employing   invasive   and non  invasive   HCC cells, we studied the mechanism underlying vascular invasion. We show that HCC cells invade blood vessels via alpha5beta1, that is equally expressed in   invasive   and non  invasive   cells. However, in the former, the intracytoplasmic tail of beta1 integrin is constitutively phosphorylated at threonine 788-789 and the extracellular part is conformationally activated. In non  invasive   cells, beta1 integrin is not activated. Transforming growth factor (TGF)-beta1 specifically phosphorylates beta1 integrin (threonine 788-789) via Smad-2 and Smad-3, causing a conformational change of the extracellular component with an inside-out mechanism. This leads non  invasive   HCC cells to behave like   invasive   cells. A selective TGF-betaRI inhibitor inhibits phosphorylation of the beta1 integrin intracytoplasmic tail, and blocks invasion of HCC cells, both constitutively   invasive   and with acquired   invasive   properties. In human HCC tissues with microvascular invasion, phospho-beta1 integrin was detected as well as TGF-beta1, p-Smad-2, and E-cadherin.TGF-beta1 promotes vascular invasion by activating beta1 integrin. This suggests a rationale for targeting TGF-betaRI in future clinical trials. 
 AsialoGM1 and integrin alpha2beta1 mediate prostate cancer progression. The most lethal aspect of cancer is the metastatic spread of primary tumors to distant sites. Any mechanism revealed is a target for therapy. In our previous studies, we reported that the   invasive   activity of the bone metastatic C4-2B prostate cancer cells could be ascribed to the reorganization of the alpha2beta1 integrin receptor and the alpha2 subunit-mediated association and activation of downstream signaling towards the activation of MMPs. In the present study, we demonstrate that expression of asialoGM1 in C4-2B cells correlates with cancer progression by influencing adhesion, migration and invasion, via reorganization of asialoGM1 and colocalization with integrin alpha2beta1. These observations reveal an uncharacterized complex of asialoGM1 with the integrin alpha2beta1 receptor promoting cancer metastatic potential through the previously identified integrin-mediated signaling pathway. The present findings promote further understanding of mechanisms by which glycosphingolipids modulate malignant properties and the results obtained here propose novel directions for future study. 
 Introduction of bisecting GlcNAc into integrin alpha5beta1 reduces ligand binding and down-regulates cell adhesion and cell migration. The enzyme beta1,4-N-acetylglucosaminyltransferase III (GnT-III) catalyzes the addition of a bisecting GlcNAc residue to glycoproteins, resulting in a modulation in biological function. Our previous studies showed that the transfection of the GnT-III gene into B16 melanoma cells results in a suppression of   invasive   ability and lung colonization. The suppression has been postulated to be due to an increased level of E-cadherin expression on the cell surface, which in turn leads to the up-regulation of cell-cell adhesion. In this study, we report on the effects of overexpression of GnT-III on cell-matrix adhesion. The overexpression of GnT-III, but not that of an enzymatic inactive GnT-III (D323A), inhibits cell spreading and migration on fibronectin, a specific ligand for integrin alpha(5)beta(1), and the focal adhesion kinase phosphorylation. E(4)-PHA lectin blot analyses showed that the levels of bisecting GlcNAc structures on the integrin alpha(5) subunit as well as alpha(2) and alpha(3) subunits immunoprecipitated from GnT-III transfectants were substantially increased. In addition, the affinity of the binding of integrin alpha(5)beta(1) to fibronectin was significantly reduced by the introduction of the bisecting GlcNAc, to the alpha(5) subunit. These findings suggest that the modification of N-glycan of integrin by GnT-III inhibits its ligand binding ability, subsequently leading to the down-regulation of integrin-mediated signaling. 
 Rab5c promotes AMAP1-PRKD2 complex formation to enhance β1 integrin recycling in EGF-induced cancer invasion. Epidermal growth factor receptor (EGFR) signaling is one of the crucial factors in breast cancer malignancy. Breast cancer cells often overexpress Arf6 and its effector, AMAP1/ASAP1/DDEF1; in these cells, EGFR signaling may activate the Arf6 pathway to induce invasion and metastasis. Active recycling of some integrins is crucial for invasion and metastasis. Here, we show that the Arf6-AMAP1 pathway links to the machinery that recycles β1 integrins, such as α3β1, to promote cell invasion upon EGFR stimulation. We found that AMAP1 had the ability to bind directly to PRKD2 and hence to make a complex with the cytoplasmic tail of the β1 subunit. Moreover, GTP-Rab5c also bound to AMAP1, and activation of Rab5c by EGFR signaling was necessary to promote the intracellular association of AMAP1 and PRKD2. Our results suggest a novel mechanism by which EGFR signaling promotes   the inv  asiveness of some breast cancer cells via integrin recycling. 
 Integrin expression levels correlate with invasion, metastasis and prognosis of oral squamous cell carcinoma. The present study evaluated the relationship between alpha 3, alpha 6A, and beta 1 integrin expression in cancer cells at the   invasive   front of oral squamous cell carcinoma (OSCC) and survival rates, as well as the clinical and pathological characteristics. Sections of 100 specimens of primary OSCC were immunostained to assess alpha 3, alpha 6A, and beta 1 integrin expression in cancer cells at the   invasive   front. OSCC patients with higher expression levels of alpha 3, alpha 6A, and beta 1 integrin had significantly better prognosis than those with lower expression levels (median survival at low vs. high expression levels: alpha 3, 37.1 months vs. 55.7 months; alpha 6A , 38.3 months vs. 47.9 months; and beta 1, 26.1 months vs. 46.1 months) (P < 0.05). In addition, beta 1 integrin expression showed the highest correlation with clinical and pathological characteristics. This study concludes that alpha 3, alpha 6A, and beta 1 integrin expression in cancer cells at the   invasive   front are related to the mode of invasion and prognosis in OSCC. 
 Loss of miR-200b promotes invasion via activating the Kindlin-2/integrin β1/AKT pathway in esophageal squamous cell carcinoma: An E-cadherin-independent mechanism. Our previous studies have shown that loss of miR-200b enhances the   invasiv  eness of esophageal squamous cell carcinoma (ESCC) cells. However, whether the miR-200-ZEB1/2-E-cadherin regulatory cascade, a master regulator of epithelial-to-mesenchymal transition (EMT), is involved in the regulation of ESCC invasion remains elusive. Here, we show that miR-200b represses ESCC cell invasion in vivo without altering the expression of E-cadherin and vimentin, two surrogate markers of EMT. However, an inverse correlation was observed between the expression levels of miR-200b and ZEB1/2 in both ESCC cell lines (n = 7, P < 0.05) and ESCC tumor samples (n = 88, P < 0.05). Methylation of E-cadherin gene was found to block the regulation of E-cadherin by the miR-200b-ZEB1/2 axis, indicating that an E-cadherin-independent mechanism can mediate the biological function of miR-200b in ESCC. We revealed that miR-200b suppresses the integrin β1-AKT pathway via targeting Kindlin-2 to mitigate ESCC cel  l invasi  veness. In two independent cohorts of ESCC samples (n = 20 and n = 53, respectively), Kindlin-2 expression positively correlated with the activation status of both the integrin signaling pathway and the PI3K-AKT signaling pathway (both P < 0.01). These data highlight that suppression of the Kindlin-2-integrin β1-AKT regulatory axis is an alternative mechanism underlying the tumor suppressor function of miR-200b in ESCC. 
 Direct interactions with the integrin β1 cytoplasmic tail activate the Abl2/Arg kinase. Integrins are heterodimeric α/β extracellular matrix adhesion receptors that couple physically to the actin cytoskeleton and regulate kinase signaling pathways to control cytoskeletal remodeling and adhesion complex formation and disassembly. β1 integrins signal through the Abl2/Arg (Abl-related gene) nonreceptor tyrosine kinase to control fibroblast cell motility, neuronal dendrite morphogenesis and stability, and cancer c  ell inva  siveness, but the molecular mechanisms by which integrin β1 activates Arg are unknown. We report here that the Arg kinase domain interacts directly with a lysine-rich membrane-proximal segment in the integrin β1 cytoplasmic tail, that Arg phosphorylates the membrane-proximal Tyr-783 in the β1 tail, and that the Arg Src homology domain then engages this phosphorylated region in the tail. We show that these interactions mediate direct binding between integrin β1 and Arg in vitro and in cells and activate Arg kinase activity. These findings provide a model for understanding how β1-containing integrins interact with and activate Abl family kinases. 
 Influence of exogenous tissue factor on estrogen receptor alpha expression in breast cancer cells: involvement of beta1-integrin, PAR2, and mitogen-activated protein kinase activation. Increased expression of tissue factor (TF) has been associated with   invasive   forms of breast cancer. Conversely, the loss of estrogen receptor alpha (ERalpha) is associated with increased cell   invasive  ness. We have examined the influence of exogenous truncated recombinant TF (rTF) on ERalpha expression and cell   invasive  ness and investigated the mechanism of rTF signaling. The influence of rTF on ERalpha expression in MCF-7 and T47D cell lines was investigated using reverse transcription-PCR and ELISA. Cell invasion was measured using Boyden chamber-based invasion assays. Additionally, the interaction of fluorescein-labeled rTF with the surface of MCF-7 cells and particularly with beta(1)-integrin was examined. Treatment of cells with rTF resulted in the down-regulation of ERalpha mRNA and protein over 24 h, which required beta(1)-integrin and involved the mitogen-activated protein kinase pathway but did not require PAR2 activation. The addition of rTF reduced estradiol-mediated cell proliferation as well as increased cell   invasive  ness requiring both PAR2 and beta(1)-integrin activation. Fluorescein-labeled rTF was shown to bind to the surface of MCF-7 cells within 5 min and peaked at 15 min. The bound rTF colocalized with cellular beta(1)-integrin and was disrupted in the presence of excess unlabeled rTF and an anti-beta(1) polyclonal antibody. Finally, affinity purification of beta(1)-integrin using rTF-conjugated agarose showed a requirement for the presence of divalent cations but not factor VIIa. The results indicate that rTF is capable of down-regulating ERalpha expression in breast cancer cells, resulting in decreases in estrogen-mediated cell proliferation and increased   invasive  ness. Furthermore, the mechanisms by which rTF induces these changes involve both PAR2 and beta(1)-integrin. 
 Prognostic role of integrin β1, E-cadherin, and rac1 expression in small cell lung cancer. Integrin β(1) mediates cellular adhesion to the extracellular matrix (ECM) and is correlated with highl  y invasi  ve and metastatic behavior in small cell lung cancer (SCLC). E-cadherin (ECAD) is a calcium-dependent cell-cell adhesion receptor that restricts invasion of cells and reduces metastasis. Rac1 is involved in the regulation of the actin cytoskeleton, adhesion, migration, invasion, and tumor metastasis. The aim of this study was to examine integrin β(1) , ECAD and rac1 expression in SCLC and to analyze the prognostic value of these markers in patients with SCLC. We analyzed integrin β(1) , ECAD, and rac1 expression in 112 SCLC tissues by immunohistochemical staining. Correlative analyses between integrin β(1) , ECAD, and rac1 expression and cliniopathological factors were performed. A total of 65 patients had extensive disease (ED) (58%), and 47 had limited disease (LD) (42%). The median follow-up duration was 61 months (range: 14-117 months), and the median progression free survival (PFS) and overall survival (OS) were 6.1 months (range: 4.8-7.4 months) and 9.7 months (range: 8.1-11.3 months), respectively. The expression of integrin β(1) , ECAD, and rac1 protein was observed in 64, 73, and 99 of SCLC tissues, respectively. The correlative analyses between integrin β(1) , ECAD, or rac1 expression and various clinical parameters did not show any statistical significance. However, the ECAD expression was associated with OS in the entire cohort. In contrast, the expression of integrin β(1) and rac1 was not associated with PFS or OS. In a subgroup analysis, patients with less than two metastasis had significantly longer OS (p = 0.047) if their tumors expressed integrin β(1) compared to those without integrin β(1) expression. In addition, OS was longer for patients with ECAD positive tumors compared to those whose tumors did not express ECAD in males (p = 0.032) and patients who never smoked (p < 0.001). Multivariate analysis showed that LD (p = 0.004), overall response rate (p = 0.003), and expression of ECAD (p = 0.015) were the independent good prognostic factors for OS. LD (p = 0.024), overall response rate (p < 0.001), and less than two metastasis (p = 0.003) were prognostic factors for longer PFS. These results suggest that ECAD expression may be useful as a prognostic indicator in patients with SCLC. 
 T-cadherin enhances cell-matrix adhesiveness by regulating beta1 integrin trafficking in cutaneous squamous carcinoma cells. T-cadherin is a glycosyl-phosphatidylinositol (GPI) anchored cadherin molecule. We previously reported that T-cadherin is normally expressed on the basal keratinocytes of the epidermis and is down-regulated in cutaneous squamous cell carcinoma (SCC). We found that expression of T-cadherin in cutaneous squamous carcinoma cells regulated level of surface beta1 integrin, which functioned as extracellular matrix (ECM) receptor. Involvement of T-cadherin in beta1 integrin trafficking was studied using three different stable cell lines with cytomegalovirus (CMV)-driven over-expression, tetracycline (Tet)-inducible expression and RNAi-mediated suppressed expression of T-cadherin. Pulse-chase analysis using a cholesterol-depleting reagent and a tyrosine kinase inhibitor showed that beta1 integrin mainly internalized via caveolae. Over-expression of T-cadherin suppressed the internalization of both beta1 integrin and cholera toxin (CTX), a marker of caveolae-mediated endocytosis. By Western blot analysis of tyrosine-kinase target molecules, we demonstrated a reduced level of EGF receptor (EGFR)-phosphorylation in T-cadherin over-expressing cells. In addition, studies using EGF and EGFR specific inhibitors revealed that EGFR activation stimulated beta1 integrin internalization. Taking these results together, T-cadherin may modulate cell-matrix adhesion in basal keratinocytes as well as   invasive   potency in SCC by regulating surface level of beta1 integrin. 
 Transient down-regulation of beta1 integrin subtypes on kidney carcinoma cells is induced by mechanical contact with endothelial cell membranes. Adhesion molecules of the integrin beta1 family are thought to be involved in the malignant progression renal cell carcinoma (RCC). Still, it is not clear how they contribute to this process. Since the hematogenous phase of tumour dissemination is the rate-limiting step in the metastatic process, we explored beta1 integrin alterations on several RCC cell lines (A498, Caki1, KTC26) before and after contacting vascular endothelium in a tumour-endothelium (HUVEC) co-culture assay. Notably, alpha2, alpha3 and alpha5 integrins became down-regulated immediately after the tumour cells attached to HUVEC, followed by re-expression shortly thereafter. Integrin down-regulation on RCC cells was caused by direct contact with endothelial cells, since the isolated endothelial membrane fragments but not the cell culture supernatant contributed to the observed effects. Integrin loss was accompanied by a reduced focal adhesion kinase (FAK) expression, FAK activity and diminished binding of tumour cells to matrix proteins. Furthermore, intracellular signalling proteins RCC cells were altered in the presence of HUVEC membrane fragments, in particular 14-3-3 epsilon, ERK2, PKCdelta, PKCepsilon and RACK1, which are involved in regulating tumour cell motility. We, therefore, speculate that contact of RCC cells with the vascular endothelium converts integrin-dependent adhesion to integrin-independent cell movement. The process of dynamic integrin regulation may be an important part in tumour cell migration strategy, switching the cells from being adhesive to becoming motile and   invasive  . 
 SCAI acts as a suppressor of cancer cell invasion through the transcriptional control of beta1-integrin. Gene expression reprogramming governs cellular processes such as proliferation, differentiation and cell migration through the complex and tightly regulated control of transcriptional cofactors that exist in multiprotein complexes. Here we describe SCAI (suppressor of cancer cell invasion), a novel and highly conserved protein that regulates   invasive   cell migration through three-dimensional matrices. SCAI acts on the RhoA-Dia1 signal transduction pathway and localizes in the nucleus, where it binds and inhibits the myocardin-related transcription factor MAL by forming a ternary complex with serum response factor (SRF). Genome-wide expression analysis surprisingly reveals that one of the strongest upregulated genes after suppression of SCAI is beta1-integrin. Decreased levels of SCAI are tightly correlated with increased   invasive   cell migration, and SCAI is downregulated in several human tumours. Functional analysis of the beta1-integrin gene strongly argues that SCAI is a novel transcriptional cofactor that controls gene expression downstream of Dia1 to dictate changes in cell   invasive   behaviour. 
 Integrin β1 mediates epithelial growth factor-induced invasion in human ovarian cancer cells. Integrins function as cell-extracellular matrix adhesion proteins and have been implicated in tumor progression. In ovarian tumors, elevated integrin β1 expression correlates with high clinical stage and poor patient survival. In this study, we report that EGF treatment up-regulated integrin β1 mRNA and protein levels in ovarian cancer cells. Moreover, pharmacological inhibition of MEK totally abolished EGF-induced integrin β1 up-regulation and cell invasion suggesting that MAPK/ERK signaling is required for EGF-induced integrin β1 up-regulation and cell invasion. Furthermore, we found that knockdown of integrin β1 expression reduced the intr  insic in  vasiveness of ovarian cancer cells and the EGF-induced cell invasion. Finally, we found that overexpression of integrin β1 was sufficient to promote ovarian cancer cell invasion. This study demonstrates that integrin β1 mediates EGF-induced cell invasion in ovarian cancer. 
 The role of beta1 integrin subfamily in anchorage-dependent apoptosis of breast carcinoma cells differing in multidrug resistance. Integrin expression was investigated in MCF-7 human breast adenocarcinoma line and in the MCF-7Dox line, which was selected from MCF-7 by a resistance to multiple antitumor drugs (MDR). We have shown that acquisition of MDR was accompanied by a drastically reduced expression of some integrins of the beta1-subfamily (alpha2beta1, alpha3beta1, alpha6beta1) and of alpha vbeta5 intergin in the adenocarcinoma cells. In contrast, expression of alpha5beta1 integrin was markedly increased in the MDR cells. Along with multiple antitumor drug resistance, MCF-7Dox cells demonstrate elevated resistance to anchorage-dependent apoptosis (anoikis) and enhanced in vitro   invasive   activity. To elucidate the implication of beta1-integrins in the above phenotypic modifications, the effect of beta1-integrin signaling was assayed. Stimulation of beta1-mediated signaling was accomplished by treating of the cells with antibodies to the beta1-subunit common for members of the beta1-subfamily. These data show that activation of beta1-integrin signaling markedly upregulated anoikis of the adenocarcinoma cells. 
 miR-134 suppresses the migration and invasion of non‑small cell lung cancer by targeting ITGB1. Most cancer-related deaths are caused by the development of metastatic disease. Thus, investigation of the underlying mechanisms of metastasis is urgent to design more effective targeted drugs and to treat metastatic disease more effectively. MicroRNAs (miRNAs) have emerged as potential targets for cancer treatment. In the present study, we aimed to identify the roles of miR-134 in non-small cell lung cancer (NSCLC) cell migration and invasion. We demonstrated that overexpression of miR-134 inhibited migration and invasion of A549 and H1299 cells. Further mechanistic investigations revealed that miR-134 inhibited epithelial-to-mesenchymal transition (EMT) evidenced by upregulation of E-cadherin expression and downregulation of vimentin expression. Using luciferase assays, we identified integrin β1 (ITGB1) as a direct target of miR-134. Performing RNAi and rescue experiments, we confirmed that miR-134 exerted its migratory   and inva  sive suppressive role partly by downregulating ITGB1. Finally, an in vivo experiment also, to some extent, suggested that miR-134 may function as a suppressor of metastasis. Taken together, our findings suggest that miR-134 suppresses migration and invasion of NSCLC by targeting ITGB1. 
 Transcriptional regulation of the beta1C integrin splice variant in human prostate adenocarcinoma. Members of the integrin family of cell adhesion receptors influence several important aspects of cancer cell behaviour, including motility and   invasive  ness, cell growth and cell survival. beta1C integrin, an alternatively spliced variant of the human beta1 integrin, has been shown to inhibit cell proliferation. We have previously demonstrated that beta1C integrin mRNA and protein are present in normal prostate and are down-regulated in prostate adenocarcinoma. To explore some of the molecular mechanisms regulating beta1C integrin gene expression, we have analysed the transcriptional activity of the beta1 integrin gene in neoplastic and normal human prostate tissue. Run-on analysis demonstrates that the transcription rate of the beta1 integrin gene is significantly reduced in prostate cancer specimens compared to normal prostate, thus accounting for the reduction in mRNA levels of the beta1 integrin variants. Moreover, the decrease in transcriptional activity of the beta1 integrin gene directly correlates to the reduction of beta1C integrin steady-state mRNA levels (r=0.78). 
 The cytoplasmic extension of the integrin β6 subunit regulates epithelial-to-mesenchymal transition. Prognosis for oral cancer patients has not improved in over 60 years due to invasion and recurrence. To understand the   invasiv  e behavior of this tumor, we evaluated the role of the αvβ6 integri  n. Invas  ive oral SCC cells express the αvβ6 integrin, which contains an 11-amino-acid extension on its β-subunit unique to the integrin family. We determined that this β6 cytoplasmic extension regulates the composition of the intermediate filament network and the organization of signaling structures called focal contacts. The auto-phosphorylation of FAK, which is localized to focal contacts, was also regulated by the β6-cytoplasmic tail, as were the transcription factors Notch and STAT3. Lastly, we also determined that activation of MAPK required the full-length β6 integrin. Together these results indicate that the signaling critical to epithelial-to-mesenchymal transition (EMT) is regulated by the β6 integrin cytoplasmic domain. 
 Evaluation of candidate stromal epithelial cross-talk genes identifies association between risk of serous ovarian cancer and TERT, a cancer susceptibility "hot-spot". We hypothesized that variants in genes expressed as a consequence of interactions between ovarian cancer cells and the host micro-environment could contribute to cancer susceptibility. We therefore used a two-stage approach to evaluate common single nucleotide polymorphisms (SNPs) in 173 genes involved in stromal epithelial interactions in the Ovarian Cancer Association Consortium (OCAC). In the discovery stage, cases with epithelial ovarian cancer (n=675) and controls (n=1,162) were genotyped at 1,536 SNPs using an Illumina GoldenGate assay. Based on Positive Predictive Value estimates, three SNPs-PODXL rs1013368, ITGA6 rs13027811, and MMP3 rs522616-were selected for replication using TaqMan genotyping in up to 3,059 serous   invasive   cases and 8,905 controls from 16 OCAC case-control studies. An additional 18 SNPs with Pper-allele<0.05 in the discovery stage were selected for replication in a subset of five OCAC studies (n=1,233 serous   invasive   cases; n=3,364 controls). The discovery stage associations in PODXL, ITGA6, and MMP3 were attenuated in the larger replication set (adj. Pper-allele>or=0.5). However genotypes at TERT rs7726159 were associated with ovarian cancer risk in the smaller, five-study replication study (Pper-allele=0.03). Combined analysis of the discovery and replication sets for this TERT SNP showed an increased risk of serous ovarian cancer among non-Hispanic whites [adj. ORper-allele 1.14 (1.04-1.24) p=0.003]. Our study adds to the growing evidence that, like the 8q24 locus, the telomerase reverse transcriptase locus at 5p15.33, is a general cancer susceptibility locus. 
 Clinical and functional significance of α9β1 integrin expression in breast cancer: a novel cell-surface marker of the basal phenotype that promotes tumour cell invasion. Integrin α9β1 is a receptor for ECM proteins, including Tenascin-C and the EDA domain of fibronectin, and has been shown to transduce TGFβ signalling. This study has examined the expression pattern of α9β1 in 141 frozen breast carcinoma samples and related expression to prognostic indices, molecular subtype and patient outcome. Effects of α9β1 on tumour cell migration and invasion were assessed using blocking antibody and gene transduction approaches. Integrin α9β1 localized to myoepithelial cells in normal ducts and acini, a pattern maintained in DCIS. A subse  t (17%)   of invasive carcinomas exhibited tumour cell expression of α9β1, which related significantly to the basal-like phenotype, as defined by either CK5/6 or CK14 expression. Tumour expression of α9β1 showed a significant association with reduced overall patient survival (p < 0.0001; HR 5.94, 95%CI 3.26-10.82) and with reduced distant-metastasis-free survival (p < 0.0001; HR 6.37, CI 3.51-11.58). A series of breast cancer cell lines was screened for α9β1   with th  e highly invasive basal-like GI-101 cell line expressing significant levels. Both migration and invasion of this line were reduced significantly in the presence of α9-blocking antibody and following α9-knockdown with siRNA. Convers  ely, mig  ratory and invasive behaviour of α9-negative MCF7 cells and α9-low MDA MB468 cells was enhanced significantly by over-expression of α9. Thus, α9β1 acts as a novel marker of the basal-like breast cancer subtype and expression is associated with reduced survival, while its ability to promote breast cancer cell migration and invasion suggests that it contributes to the aggressive clinical behaviour of this tumour subtype. 
 Lung cancer cells that survive ionizing radiation show increased integrin α2β1- and EGFR-dependen  t invasi  veness. Ionizing radiation (IR)-enhanced tumo  r invasi  veness is emerging as a contributor to the limited benefit of radiotherapy; however, its mechanism is still unclear. We previously showed that subcloned lung adenocarcinoma A549 cells (P cells), which survived 10 Gy IR (IR cells), acquired hig  h invasi  veness in vitro. Here, we tried to identify the mechanism by which IR cells increase thei  r invasi  veness by examining altered gene expression and signaling pathways in IR cells compared with those in P cells. To simulate the microenvironment in vivo, cells were embedded in a three-dimensional (3D) collagen type I gel, in which the IR cells were elongated, while the P cells were spherical. The integrin expression pattern was surveyed, and expression levels of the integrin α2 and β1 subunits were significantly elevated in IR cells. Knockdown of α2 expression or functional blockade of integrin α2β1 resulted in a round morphology of IR cells, and abrogated their invasion in the collagen matrix, suggesting the molecule's essential role in cell spread and invasion in 3D collagen. Epidermal growth factor receptor (EGFR) also presented enhanced expression and activation in IR cells. Treatment with EGFR tyrosine kinase inhibitor, PD168393, decreased the ratio of elongated cells an  d cell i  nvasiveness. Signaling molecules, including extracellular signal-regulated kinase-1/2 (Erk1/2) and Akt, exhibited higher activation in IR cells. Inhibition of Akt activation by treating with phosphoinositide 3-kinase (PI3K) inhibitor LY294002 decreased IR cell invasion, whereas inhibition of Erk1/2 activation by mitogen-activated protein kinase kinase (MEK) inhibitor U0126 did not. Our results show that integrin α2β1 and EGFR cooperatively promot  e higher   invasiveness of IR-survived lung cancer cells, mediated in part by the PI3K/Akt signaling pathway, and might serve as alternative targets in combination with radiotherapy. 
 The Arp2/3 activator WASH regulates α5β1-integrin-mediate  d invasi  ve migration. The actin cytoskeleton provides scaffolding and physical force to effect fundamental processes such as motility, cytokinesis and vesicle trafficking. The Arp2/3 complex nucleates actin structures and contributes to endocytic vesicle invagination and trafficking away from the plasma membrane. Internalisation and directed recycling of integrins are major driving forces fo  r invasi  ve cell motility and potentially for cancer metastasis. Here, we describe a direct requirement for WASH and Arp2/3-mediated actin polymerisation on the endosomal membrane system for α5β1 integrin recycling. WASH regulates the trafficking of endosomal α5β1 integrin to the plasma membrane and is fundamental for integrin-driven cell morphology changes and integrin-mediated cancer cell invasion. Thus, we implicate WASH and Arp2/3-driven actin nucleation in receptor recycling leadi  ng to in  vasive motility. 
 DNAzyme as an efficient tool to modulate   invasive  ness of human carcinoma cells. In this study we evaluated efficiency of DNAzymes to modulate motility of cancer cells, an important factor in the progression and metastasis of cancers. For this purpose we targeted β1 integrins that are predominant adhesive receptors in various carcinoma cell lines (CX1.1, HT29, LOVO, LS180, PC-3). To evaluate   invasiv  eness of cancer cells, we used a transwell migration assay that allowed analyzing chemotactic migration of colon carcinoma cell lines across an ECM-coated membrane. Their adhesive properties were also characterized by the analysis of adhesion to fibronectin, laminin and collagen. In addition, the expression of major integrin subunits, selected intact β1 integrins, and other adhesive receptors (ICAM, E-selectin, uPAR) was analyzed by flow cytometry. Inhibition of β1 integrin expression by DNAzyme to β1 mRNA almost abolished   the inva  siveness of the CX1.1, HT29, LS180, LOVO and PC-3 cells in vitro. These data show that DNAzymes to β1 integrin subunit can be used to inh  ibit inv  asiveness of carcinoma cells. 
 CD147-targeting siRNA inhibits cell-matrix adhesion of human malignant melanoma cells by phosphorylating focal adhesion kinase. CD147/basigin, highly expressed on the surface of malignant tumor cells including malignant melanoma (MM) cells, plays a critical role in the   invasive  ness and metastasis of MM. Metastasis is an orchestrated process comprised of multiple steps including adhesion and invasion. Integrin, a major adhesion molecule, co-localizes with CD147/basigin on the cell surface. Using the human MM cell line A375 that highly expresses CD147/basigin, we investigated whether CD147/basigin is involved in adhesion in association with integrin. CD147/basigin was knocked-down using siRNA targeting CD147 to elucidate the role of CD147/basigin. Cell adhesion was evaluated by adhesion assay on matrix-coated plates. The localization of integrin was inspected under a confocal microscope and the expression and phosphorylation of focal adhesion kinase (FAK), a downstream kinase of integrin, were examined by western blot analysis. Silencing of CD147/basigin in A375 cells by siRNA induced the phosphorylation of FAK at Y397. Integrin identified on the surface of parental cells was distributed in a speckled fashion in the cytoplasm of CD147 knockdown cells, resulting in morphological changes from a round to a polygonal shape with pseudopodial protrusions. Silencing of CD147/basigin in A375 cells clearly weakened their adhesiveness to collagen I and IV. Our results suggest that CD147/basigin regulates the adhesion of MM cells to extracellular matrices and of integrin β1 signaling via the phosphorylation of FAK. 
 The DSCs-expressed CD82 controls the   invasive  ness of trophoblast cells via integrinbeta1/MAPK/MAPK3/1 signaling pathway in human first-trimester pregnancy. CD82 is recognized as a wide-spectrum tumor metastasis suppressor that inhibits cancer cell motility and   invasive  ness. At the human maternal-fetal interface, the decidua is believed to effectively limit the inappropriate invasion of trophoblasts. Here we have found the transcription and translation of CD82 in decidual stromal cells (DSCs), whereas trophoblast cells do not express CD82. The in-cell Western analysis reveals attenuation of CD82 translation in DSCs by human chorionic gonadotropin (hCG), but not by estrogen or progesterone. It is demonstrated that silencing of CD82 by RNA interference increases integrinbeta1, decreases TIMP1 expression in DSCs, and promotes the invasion of the first-trimester human trophoblasts in the coculture. Moreover, U0126, or anti-integrinbeta1 neutralizing antibody, reverses the decreased TIMP1 expression and the increased   invasive  ness of trophoblast cells, and the antibody also inhibits the MAPK3/1 phosphorylation induced by CD82 silence. After transfection with CD82, the   invasive   index of BeWo cells decreases significantly with TIMP1 increase. The results above indicate that the DSCs-expressed CD82 up-regulates the expression of TIMP1 in an autocrine manner and inhibits the   invasive  ness of human first-trimester trophoblast cells partly through the integrinbeta1/MAPK/MAPK3/1 signaling pathway. Furthermore, we have found that the mRNA and protein level of CD82 in decidua of the miscarriage is significantly higher than that of the normal early pregnancy, which implies that the abnormal higher CD82 expression in decidua restricts appropriate invasion of trophoblasts that leads to early pregnancy wastage. 
 Three-dimensional collagen represses cyclin E1 via β1 integrin in   invasiv  e breast cancer cells. The behavior of breast epithelial cells is influenced by their microenvironment which includes stromal cells and extracellular matrix (ECM). During cancer progression, the tissue microenvironment fails to control proliferation and differentiation, resulting in uncontrolled growth and invasion. Upon invasion, the ECM encountered by breast cancer cells changes from primarily laminin and collagen IV to primarily collagen I. We show here that culturing   invasiv  e breast cancer cells in 3-dimensional (3D) collagen I inhibits proliferation through direct regulation of cyclin E1, a G(1)/S regulator that is overexpressed in breast cancer. When the breast cancer cell line MDA-MB-231 was cultured within 3D collagen I gels, the G(1)/S transition was inhibited as compared to cells cultured on conventional 2D collagen or plastic dishes. Cells in 3D collagen downregulated cyclin E1 protein and mRNA, with no change in cyclin D1 level. Cyclin D1 was primarily cytoplasmic in 3D cultures, and this was accompanied by decreased phosphorylation of Rb, a nuclear target for both cyclin E1- and cyclin D1-associated kinases. Positive regulators of cyclin E1 expression, the transcription factor c-Myc and cold-inducible RNA binding protein (CIRP), were decreased in 3D collagen cultures, while the collagen I receptor β1 integrin was greatly increased. Inhibition of β1 integrin function rescued proliferation and cyclin E1 expression as well as c-Myc expression and Rb phosphorylation, but cyclin D1 remained cytoplasmic. We conclude that cyclin E1 is repressed independent of effects on cyclin D1 in a 3D collagen environment and dependent on β1 integrin interaction with collagen I, reducing proliferation   of inva  sive breast cancer cells. 
 The NLRP3 Inflammasome Is a Pathogen Sensor for   Invasive   Entamoeba histolytica via Activation of α5β1 Integrin at the Macrophage-Amebae Intercellular Junction. Entamoeba histolytica (Eh) is an extracellular protozoan parasite of humans that invades the colon to cause life-threatening intestinal and extra-intestinal amebiasis. Colonized Eh is asymptomatic, however, when trophozoites adhere to host cells there is a considerable inflammatory response that is critical in the pathogenesis of amebiasis. The host and/or parasite factors that trigger the inflammatory response to invading Eh are not well understood. We recently identified that Eh adherence to macrophages induces inflammasome activation and in the present study we sought to determine the molecular events upon contact that coordinates this response. Here we report that Eh contact-dependent activation of α5β1 integrin is critical for activation of the NLRP3 inflammasome. Eh-macrophage contact triggered recruitment of α5β1 integrin and NLRP3 into the intercellular junction, where α5β1 integrin underwent activation by an integrin-binding cysteine protease on the parasite surface, termed EhCP5. As a result of its activation, α5β1 integrin induced ATP release into the extracellular space through opening of pannexin-1 channels that signalled through P2X7 receptors to deliver a critical co-stimulatory signal that activated the NLRP3 inflammasome. Both the cysteine protease activity and integrin-binding domain of EhCP5 were required to trigger α5β1 integrin that led to ATP release and NLRP3 inflammasome activation. These findings reveal engagement of α5β1 integrin across the parasite-host junction is a key regulatory step that initiates robust inflammatory responses to Eh. We propose that α5β1 integrin distinguishes Eh direct contact and functions with NLRP3 as pathogeni  city sen  sor for invasive Eh infection. 
 Ameloblastoma and adenomatoid odontogenic tumor: the role of alpha2beta1, alpha3beta1, and alpha5beta1 integrins in local   invasive  ness and architectural characteristics. Ameloblastoma is an odontogenic neoplasm characterized by local   invasive  ness and a tendency toward recurrence, whereas adenomatoid odontogenic tumor (AOT) is an indolent neoplasm. The objective of the present study was to immunohistochemically analyze the role of alpha2beta1, alpha3beta1, and alpha5beta1 integrins in the cellular events and cell-matrix interactions that occur in these tumors and their consequent repercussions on the architectural arrangement and biologic behavior of these lesions. Paraffin-embedded specimens from 30 ameloblastomas (20 solid and 10 unicystic tumors) and 12 AOTs were submitted to immunohistochemistry using the catalyzed signal amplification system. A difference in the pattern of integrin expression was observed between the various histologic types of ameloblastoma. No significant difference in labeling intensity was observed between unicystic and solid ameloblastomas, but comparison between ameloblastomas and AOT showed a significantly stronger expression of alpha5beta1 integrin in the former (P < .05). Our findings suggest an important role of the integrins studied in the architectural characteristics of ameloblastomas and AOTs and a possible participation of alpha5beta1 integrin in the mechanism of local invasion of ameloblastomas. 
 Profiling of the tetraspanin CD151 web and conspiracy of CD151/integrin β1 complex in the progression of hepatocellular carcinoma. Tetraspanin CD151 has been implicated in metastasis through forming complexes with different molecular partners. In this study, we mapped tetraspanin web proteins centered on CD151, in order to explore the role of CD151 complexes in the progression of hepatocellular carcinoma (HCC). Immunoprecipitation was used to isolate tetraspanin complexes from HCCLM3 cells using a CD151 antibody, and associated proteins were identified by mass spectrometry. The interaction of CD151 and its molecular partners, and their roles in   invasiv  eness and metastasis of HCC cells were assayed through disruption of the CD151 network. Finally, the clinical implication of CD151 complexes in HCC patients was also examined. In this study, we identified 58 proteins, characterized the tetraspanin CD151 web, and chose integrin β1 as a main partner to further investigate. When the CD151/integrin β1 complex in HCC cells was disrupted, migratio  n, invas  iveness, secretion of matrix metalloproteinase 9, and metastasis were markedly influenced. However, both CD151 and integrin β1 expression were untouched. HCC patients with high expression of CD151/integrin β1 complex had the poorest prognosis of the whole cohort of patients. Together, our data show that CD151 acts as an important player in the progression of HCC in an integrin β1-dependent manner. 
 [Expression of integrin-beta1 in oral leukoplakia and early   invasive   carcinoma and its relationship with cell proliferation]. To investigate the expression and significance of integrin-beta1 in oral leukoplakia and early   invasive   carcinoma.The expression of integrin-beta1 and Ki-67 was examined in 12 normal oral mucosa, 10 simple hyperplasia, 24 epithelial dysplasia and 14 early   invasive   carcinoma by immunohistochemistry.In normal and simple hyperplasia epithelium, integrin-beta1 was mostly expressed in the basal cell membrane, and Ki-67 in the nuclei of basal and the parabasal layers. In dysplasia epithelium and early   invasive   carcinoma, integrin-beta1 showed membrane staining and Ki-67 showed nuclear staining in dysplastic basal cells, sprinkle cells and SCC cells. Integrin-beta1 and Ki-67 were overexpressed in 7 and 10 of 24 dysplasia cases and in 8 of 14 early   invasive   carcinoma cases respectively. There was a significant difference in integrin-beta1 and Ki-67 expression among the four groups (chi2 = 10.651, P = 0.014; chi2 = 14.831, P = 0.002), in integrin-beta1 expression among normal oral mucosa, simple hyperplasia and early   invasive   carcinoma (P = 0.008, P = 0.013) and in Ki-67 expression among normal oral mucosa and dysplasia, early   invasive   carcinoma (P = 0.026, P = 0.001), and among simple hyperplasia and early   invasive   carcinoma (P = 0.005). A significant correlation between integrin-beta1 and Ki-67 (R = 0.442, P < 0.01) was found.Integrin-beta1 showed increased staining in dysplasia epithelium cells and SCC cells, and may correlate to the proliferation of the dysplasia cells. 
  GeneRIFs  
 CXCL12/CXCR4 activation by cancer-associated fibroblasts mediated integrin beta1 clustering at the cell surface and promoted the   invasive   ability of gastric cancer cells 
 Suggest alpha5beta1/Arp2/Arp3/FHOD3 pathway reprograms the actin cytoskeleton to promote   invasive   migration and local invasion in vivo. 
 These data establish a role for FMNL2 in the regulation of beta1-integrin and provide a mechanistic understanding of the function of FMNL2 in cancer   invasive  ness. 
 C1GALT1 promotes   invasive   phenotypes of hepatocellular carcinoma cells by modulating integrin beta1 glycosylation and activity. 
 beta1-Integrin via NF-kappaB signaling is essential for acquisition of   invasive  ness in a model of radiation treated in situ breast cancer. 
 beta-1,4-Galactosyltransferase III suppresses beta1 integrin-mediated   invasive   phenotypes and negatively correlates with metastasis in colorectal cancer. 
 Integrin alpha2 beta1 and EGFR cooperatively promote higher   invasive  ness of ionizing radiation - survived lung cancer cells, mediated in part by the PI3K/Akt signaling pathway, and might serve as alternative targets in combination with radiotherapy. 
 beta-1,4-Galactosyltransferase III enhances   invasive   phenotypes via beta1-integrin and predicts poor prognosis in neuroblastoma. 
 Most actinic cheilitis cases showed reduced expression of integrin beta1 and superficially   invasive   squamous cell carcinoma lacked intergrin beta1 in the   invasive   front. 
 High integrin beta 1 is associated with   invasive   lobular cancer in comparison with other breast tumor types. 
 WASH and Arp2/3 regulates integrin alpha5beta1-mediated   invasive   cell migration. 
 Analysis of the data led to the elucidation of a new molecular mechanism by which SPARC promotes cathepsin B-mediated melanoma   invasive  ness using collagen I and alpha2beta1 integrins as mediators. 
 High expression levels of CD151 and alpha6 promote   invasive  ness of HCC cells. Either of these proteins, or PI3K signaling, might be targets for therapeutics for subgroups of patients with HCC. 
 Inhibition of beta1 integrin expression by DNAzyme to beta1 mRNA almost abolished the   invasive  ness of the CX1.1, HT29, LS180, LOVO and PC-3 cells in vitro. 
 The integrin beta1-focal adhesion kinase signal pathway plays a role in the   invasive  ness of pituitary adenomas. 
 DSCs-expressed CD82 up-regulates the expression of TIMP1 in an autocrine manner and inhibits the   invasive  ness of human first-trimester trophoblast cells partly through the integrinbeta1/MAPK/MAPK3/1 signaling pathway 
 alpha 3, alpha 6A, and beta 1 integrin expression in cancer cells at the   invasive   front are related to the mode of invasion and prognosis in oral squamous cell carcinoma 
 Np-1 confers a growth and survival advantage to pancreas cancer cells, and interacts with integrin beta1 to coordinate signaling events that promote cell adherence and   invasive  ness. 
 The process of dynamic integrin regulation may be an important part in tumour cell migration strategy, switching the cells from being adhesive to becoming motile and   invasive  . 
 T-cadherin may modulate cell-matrix adhesion in basal keratinocytes as well as   invasive   potency in squamous cell carcinoma by regulating surface level of beta1 integrin 
 ICAM-1 molecules interacted only with group B Streptococcus(GBS)-III liquor 90356 strain while beta1 integrin interacted with GBS-III liquor 90356 and GBS-V blood 90186   invasive   strains 
 This study supports matrix-induced clustering of alpha3beta1 integrin promotes uPAR/alpha3beta1 interaction; potentiating cellular signal transduction pathways culminating in activation of uPA expression and enhanced uPA-dependent   invasive   behavior. 
  [Mm]etastasize  PubMed  
 Insulin-like growth factor-independent insulin-like growth factor binding protein 3 promotes cell migration and lymph node metastasis of oral squamous cell carcinoma cells by requirement of integrin β1. Frequent metastasis to the cervical lymph nodes leads to poor survival of patients with oral squamous cell carcinoma (OSCC). To understand the underlying mechanisms of lymph node metastasis, two sublines were successfully isolated from cervical lymph nodes of nude mice through in vivo selection, and identified as originating from poorly metastatic parental cells. These two sublines specifically   metastasiz  ed to cervical lymph nodes in 83% of mice, whereas OEC-M1 cells did not   metastasiz  e after injection into the oral cavity. After gene expression analysis, we identified insulin-like growth factor binding protein 3 (IGFBP3) as one of the significantly up-regulated genes in the sublines in comparison with their parental cells. Consistently, meta-analysis of the public microarray datasets and IGFBP3 immunohistochemical analysis revealed increased both levels of IGFBP3 mRNA and protein in human OSCC tissues when compared to normal oral or adjacent nontumorous tissues. Interestingly, the up-regulated IGFBP3 mRNA expression was significantly associated with OSCC patients with lymph node metastasis. IGFBP3 knockdown in the sublines impaired and ectopic IGFBP3 expression in the parental cells promoted migration, transendothelial migration and lymph node metastasis of orthotopic transplantation. Additionally, ectopic expression of IGFBP3 with an IGF-binding defect sustained the IGFBP3-enhanced biological functions. Results indicated that IGFBP3 regulates metastasis-related functions of OSCC cells through an IGF-independent mechanism. Furthermore, exogenous IGFBP3 was sufficient to induce cell motility and extracellular signal-regulated kinase (ERK) activation. The silencing of integrin β1 was able to impair exogenous IGFBP3-mediated migration and ERK phosphorylation, suggesting a critical role of integrin β1 in IGFBP3-enchanced functions. 
 Talin1 phosphorylation activates β1 integrins: a novel mechanism to promote prostate cancer bone metastasis. Talins are adaptor proteins that regulate focal adhesion signaling by conjugating integrins to the cytoskeleton. Talins directly bind integrins and are essential for integrin activation. We previously showed that β1 integrins are activated in metastatic prostate cancer (PCa) cells, increasing PCa metastasis to lymph nodes and bone. However, how β1 integrins are activated in PCa cells is unknown. In this study, we identified a novel mechanism of β1 integrin activation. Using knockdown experiments, we first demonstrated that talin1, but not talin2, is important in β1 integrin activation. We next showed that talin1 S425 phosphorylation, but not total talin1 expression, correlates with metastatic potential of PCa cells. Expressing a non-phosphorylatable mutant, talin1(S425A), in talin1-silenced PC3-MM2 and C4-2B4 PCa cells, decreased activation of β1 integrins, integrin-mediated adhesion, motility and increased the sensitivity of the cells to anoikis. In contrast, reexpression of the phosphorylation-mimicking mutant talin1(S425D) led to increased β1 integrin activation and generated biologic effects opposite to talin1(S425A) expression. In the highly metastatic PC3-MM2 cells, expression of a non-phosphorylatable mutant, talin1(S425A), in talin1-silenced PC3-MM2 cells, abolished their ability to colonize in the bone following intracardiac injection, while reexpression of phosphorylation-mimicking mutant talin1(S425D) restored their abil  ity to meta  stasize to bone. Immunohistochemical staining demonstrated that talin S425 phosphorylation is significantly increased in human bone metastases when compared with normal tissues, primary tumors or lymph node metastases. We further showed that p35 expression, an activator of Cdk5, and Cdk5 activity were increased in metastatic tumor cells, and that Cdk5 kinase activity is responsible for talin1 phosphorylation and subsequent β1 integrin activation. Together, our study reveals Cdk5-mediated phosphorylation of talin1 leading to β1 integrin activation is a novel mechanism that increases metastatic potential of PCa cells. 
 Schwann Cells Increase Prostate and Pancreatic Tumor Cell Invasion Using Laminin Binding A6 Integrin. Human pancreatic and prostate cancers   metastasize   along nerve axons during perineural invasion. The extracellular matrix laminin class of proteins is an abundant component of both myelinated and non-myelinated nerves. Analysis of human pancreatic and prostate tissue revealed both perineural and endoneural invasion with Schwann cells surrounded or disrupted by tumor, respectively. Tumor and nerve cell co-culture conditions were used to determine if myelinating or non-myelinating Schwann cell (S16 and S16Y, respectively) phenotype was equally likely to promote integrin-dependent cancer cell invasion and migration on laminin. Conditioned medium from S16 cells increased tumor cell (DU145, PC3, and CFPAC1) invasion into laminin approximately 1.3-2.0 fold compared to fetal bovine serum (FBS) treated cells. Integrin function (e.g., ITGA6p formation) increased up to 1.5 fold in prostate (DU145, PC3, RWPE-1) and pancreatic (CFPAC1) cells, and invasion was dependent on ITGA6p formation and ITGB1 as determined by function-blocking antibodies. In contrast, conditioned medium isolated from S16Y cells (non-myelinating phenotype) decreased constitutive levels of ITGA6p in the tumor cells by 50% compared to untreated cells and decreased ITGA6p formation 3.0 fold compared to S16 treated cells. Flow cytometry and western blot analysis revealed loss of ITGA6p formation as reversible and independent of overall loss of ITGA6 expression. These results suggest that the myelinating phenotype of Schwann cells within the tumor microenvironment increased integrin-dependent tumor invasion on laminin. 
  [Mm]etastasis  PubMed  
 Epidermal growth factor (EGF) regulates α5β1 integrin activation state in human cancer cell lines through the p90RSK-dependent phosphorylation of filamin A. Regulation of integrin activation has important implications for tumor cell invasion an  d metastas  is.EGF activates ERK/p90RSK and Rho/Rho kinase signaling in A431 and DiFi colon cancer cells, leading to phosphorylation of filamin A (FLNa) and inactivation of the α5β1 integrin receptor.EGF promotes α5β1 inactivation through the p90RSK-dependent phosphorylation of FLNa.We have identified a novel EGF-dependent mechanism controlling the α5β1 integrin activation state. Cell adhesion, motility, and invasion are regulated by the ligand-binding activity of integrin receptors, transmembrane proteins that bind to the extracellular matrix. Integrins whose conformation allows for ligand binding and appropriate functional activity are said to be in an active state. Integrin activation and subsequent ligand binding are dynamically regulated by the association of cytoplasmic proteins with integrin intracellular domains. In this study, we evaluated the role of EGF in the regulation of the activation state of the α5β1 integrin receptor for fibronectin. The addition of EGF to either A431 squamous carcinoma cells or DiFi colon cancer cells resulted in loss of α5β1-dependent adhesion to fibronectin but no loss of integrin from the cell surface. EGF activated the EGF receptor/ERK/p90RSK and Rho/Rho kinase signaling pathways. Blocking either pathway inhibited EGF-mediated loss of adhesion, suggesting that they work in parallel to regulate integrin function. EGF treatment also resulted in phosphorylation of filamin A (FLNa), which binds and inactivates β1 integrins. EGF-mediated FLNa phosphorylation was completely blocked by an inhibitor of p90RSK and partially attenuated by an inhibitor of Rho kinase, suggesting that both pathways converge on FLNa to regulate integrin function. A431 clonal cell lines expressing non-phosphorylated dominant-negative FLNa were resistant to the inhibitory effects of EGF on integrin function, whereas clonal cell lines overexpressing wild-type FLNa were more sensitive to the inhibitory effect of EGF. These data suggest that EGF-dependent inactivation of α5β1 integrin is regulated through FLNa phosphorylation and cellular contractility. 
 Androgen-induced cell migration: role of androgen receptor/filamin A association. Androgen receptor (AR) controls male morphogenesis, gametogenesis and prostate growth as well as development of prostate cancer. These findings support a role for AR in cell migration and invasiveness. However, the molecular mechanism involved in AR-mediated cell migration still remains elusive.Mouse embryo NIH3T3 fibroblasts and highly metastatic human fibrosarcoma HT1080 cells harbor low levels of transcriptionally incompetent AR. We now report that, through extra nuclear action, AR triggers migration of both cell types upon stimulation with physiological concentrations of the androgen R1881. We analyzed the initial events leading to androgen-induced cell migration and observed that challenging NIH3T3 cells with 10 nM R1881 rapidly induces interaction of AR with filamin A (FlnA) at cytoskeleton. AR/FlnA complex recruits integrin beta 1, thus activating its dependent cascade. Silencing of AR, FlnA and integrin beta 1 shows that this ternary complex controls focal adhesion kinase (FAK), paxillin and Rac, thereby driving cell migration. FAK-null fibroblasts migrate poorly and Rac inhibition by EHT impairs motility of androgen-treated NIH3T3 cells. Interestingly, FAK and Rac activation by androgens are independent of each other. Findings in human fibrosarcoma HT1080 cells strengthen the role of Rac in androgen signaling. The Rac inhibitor significantly impairs androgen-induced migration in these cells. A mutant AR, deleted of the sequence interacting with FlnA, fails to mediate FAK activation and paxillin tyrosine phosphorylation in androgen-stimulated cells, further reinforcing the role of AR/FlnA interaction in androgen-mediated motility.The present report, for the first time, indicates that the extra nuclear AR/FlnA/integrin beta 1 complex is the key by which androgen activates signaling leading to cell migration. Assembly of this ternary complex may control organ development and prostate cancer   metastasis  . 
 Epidermal growth factor (EGF) regulates α5β1 integrin activation state in human cancer cell lines through the p90RSK-dependent phosphorylation of filamin A. Regulation of integrin activation has important implications for tumor cell invasion an  d metastas  is.EGF activates ERK/p90RSK and Rho/Rho kinase signaling in A431 and DiFi colon cancer cells, leading to phosphorylation of filamin A (FLNa) and inactivation of the α5β1 integrin receptor.EGF promotes α5β1 inactivation through the p90RSK-dependent phosphorylation of FLNa.We have identified a novel EGF-dependent mechanism controlling the α5β1 integrin activation state. Cell adhesion, motility, and invasion are regulated by the ligand-binding activity of integrin receptors, transmembrane proteins that bind to the extracellular matrix. Integrins whose conformation allows for ligand binding and appropriate functional activity are said to be in an active state. Integrin activation and subsequent ligand binding are dynamically regulated by the association of cytoplasmic proteins with integrin intracellular domains. In this study, we evaluated the role of EGF in the regulation of the activation state of the α5β1 integrin receptor for fibronectin. The addition of EGF to either A431 squamous carcinoma cells or DiFi colon cancer cells resulted in loss of α5β1-dependent adhesion to fibronectin but no loss of integrin from the cell surface. EGF activated the EGF receptor/ERK/p90RSK and Rho/Rho kinase signaling pathways. Blocking either pathway inhibited EGF-mediated loss of adhesion, suggesting that they work in parallel to regulate integrin function. EGF treatment also resulted in phosphorylation of filamin A (FLNa), which binds and inactivates β1 integrins. EGF-mediated FLNa phosphorylation was completely blocked by an inhibitor of p90RSK and partially attenuated by an inhibitor of Rho kinase, suggesting that both pathways converge on FLNa to regulate integrin function. A431 clonal cell lines expressing non-phosphorylated dominant-negative FLNa were resistant to the inhibitory effects of EGF on integrin function, whereas clonal cell lines overexpressing wild-type FLNa were more sensitive to the inhibitory effect of EGF. These data suggest that EGF-dependent inactivation of α5β1 integrin is regulated through FLNa phosphorylation and cellular contractility. 
 Androgen-induced cell migration: role of androgen receptor/filamin A association. Androgen receptor (AR) controls male morphogenesis, gametogenesis and prostate growth as well as development of prostate cancer. These findings support a role for AR in cell migration and invasiveness. However, the molecular mechanism involved in AR-mediated cell migration still remains elusive.Mouse embryo NIH3T3 fibroblasts and highly metastatic human fibrosarcoma HT1080 cells harbor low levels of transcriptionally incompetent AR. We now report that, through extra nuclear action, AR triggers migration of both cell types upon stimulation with physiological concentrations of the androgen R1881. We analyzed the initial events leading to androgen-induced cell migration and observed that challenging NIH3T3 cells with 10 nM R1881 rapidly induces interaction of AR with filamin A (FlnA) at cytoskeleton. AR/FlnA complex recruits integrin beta 1, thus activating its dependent cascade. Silencing of AR, FlnA and integrin beta 1 shows that this ternary complex controls focal adhesion kinase (FAK), paxillin and Rac, thereby driving cell migration. FAK-null fibroblasts migrate poorly and Rac inhibition by EHT impairs motility of androgen-treated NIH3T3 cells. Interestingly, FAK and Rac activation by androgens are independent of each other. Findings in human fibrosarcoma HT1080 cells strengthen the role of Rac in androgen signaling. The Rac inhibitor significantly impairs androgen-induced migration in these cells. A mutant AR, deleted of the sequence interacting with FlnA, fails to mediate FAK activation and paxillin tyrosine phosphorylation in androgen-stimulated cells, further reinforcing the role of AR/FlnA interaction in androgen-mediated motility.The present report, for the first time, indicates that the extra nuclear AR/FlnA/integrin beta 1 complex is the key by which androgen activates signaling leading to cell migration. Assembly of this ternary complex may control organ development and prostate cancer   metastasis  . 
 The efficacy evaluation of cryosurgery in pancreatic cancer patients with the expression of CD44v6, integrin-β1, CA199, and CEA. Increased expression of cell adhesion molecule CD44v6, integrin-β1, carbohydrate antigen 199 (CA199), and carcinoembryonic antigen (CEA) are closely associated with the progression an  d metastas  is of numerous cancers. In this study, peripheral blood mononuclear cell (PBMC) and serum samples were collected from 37 pancreatic cancer patients and 12 healthy people. A novel triplex TaqMan real-time reverse transcription polymerase chain reaction assay was used to measure the expression levels of CD44v6 and integrin-β1 gene in PBMCs, while chemiluminescence and enzyme-linked immunosorbent assay were used to measure the levels of CA199 and CEA expression in serum. The results showed that both the levels of CD44v6 and integrin-β1 expression had significant correlation with clinical stage, lymph node, and li  ver metast  asis of pancreatic cancer (P < 0.05). Age, tumor size, tumor differentiation, clinical stage, lymph nodes, and li  ver metast  asis were significantly associated with the levels of CA199 and CEA expression (P < 0.05). The levels of CD44v6, integrin-β1, CA199, and CEA expression in the patients prior cryosurgery and chemotherapy were significantly higher than those in the control group (P < 0.05), whereas no significant difference was found between the patients 1 month post cryosurgery and control group (P > 0.05). The expression levels of CD44v6, integrin-β1, CA199, and CEA in the patients 1 month post cryosurgery were significantly lower than those in the patients prior cryosurgery (P < 0.05). Interestingly, no significant difference was found for the CD44v6, integrin-β1, CA199, and CEA levels between the patients prior and post-chemotherapy (P > 0.05). The higher expression of CD44v6, integrin-β1, CA199, and CEA are closely related to the progress  ion and me  tastasis of pancreatic cancer and may play a important role in the curative evaluation of cryosurgery of pancreatic cancer. 
 Changes in adhesive and migratory characteristics of hepatocellular carcinoma (HCC) cells induced by expression of alpha3beta1 integrin. The invasive and metastatic potentials of hepatocellular carcinoma are positively correlated with the expression level of alpha3beta1 integrin, a high-affinity adhesion receptor for laminin isoforms including laminin-5. In this study, we investigated changes in the adhesive and invasive behaviors of human HCC HepG2 cells after transfection with cDNA for alpha3 integrin in order to elucidate the direct involvement of this integrin in these cellular processes. We introduced cDNA for splice variants of alpha3 integrin (alpha3A and alpha3B) into the cells, and selected two transfectant clones (HepG2-3A and HepG2-3B), which express the alpha3A and alpha3B integrins, respectively. Both transfectant cells adhered almost equally to laminin-5-coated plates in an alpha3 integrin-dependent manner, indicating that transfected alpha3Abeta1 and alpha3Bbeta1 integrins were functionally active in these cells. The migratory and invasive potentials of the transfectant cells were assessed by scratch wound assay and in vitro chemoinvasion assay. The results demonstrated that the migration of HepG2-3A and HepG2-3B cells but not of mock transfectant (HepG2-M) cells was stimulated on the plates coated with laminin-5. Furthermore, HepG2-3A and HepG2-3B cells were found to be more invasive into laminin-5-containing matrices than were HepG2-M cells. These results strongly suggest that enhanced expression of alpha3beta1 integrin on HCC cells is directly involved in their malignant phenotypes such as invasion and   metastasis  . 
 βig-h3 promotes human osteosarcoma cells   metastasi  s by interacting with integrin α2β1 and activating PI3K signaling pathway. Osteosarcoma, the most common primary bone tumor in children and young adolescents, is characterized by local invasion and dista  nt metasta  sis. But the detailed mechanisms of osteosarco  ma metasta  sis are not well known. In the present study, we found that βig-h3 promotes metastatic potential of human osteosarcoma cells in vitro and in vivo. Furthermore, βig-h3 co-localized with integrin α2β1 in osteosarcoma cells. But βig-h3 did not change integrin α2β1 expression in Saos-2 cells. Interaction of βig-h3 with integrin α2  β1 mediat  es metastasis of human osteosarcoma cells. The second FAS1 domain of βig-h3 but not the first FAS1 domain, the third FAS1 domain or the fourth FAS1 domain mediates human osteo  sarcoma ce  lls metastasis, which is the α2β1 integrin-interacting domain. We further demonstrated that PI3K/AKT signaling pathway is involved in βig-h3-induced human os  teosarcoma   cells metastasis process. Together, these results reveal βi  g-h3 enhan  ces the metastasis potentials of human osteosarcoma cells via integrin α2β1-mediated PI3K/AKT signal pathways. The discovery of βig-h3-mediated pathway helps us to understand the mechanism o  f human os  teosarcoma metastasis and provides evidence for the possibility that βig-h3 can be a potential therapeutic target for osteosarcoma treatment. 
 Interleukin-1alpha enhances the aggressive behavior of pancreatic cancer cells by regulating the alpha6beta1-integrin and urokinase plasminogen activator receptor expression. In human pancreatic cancer progression, the alpha6beta1-integrin is expressed on cancer cell surface during invasion and   metastasis   formation. In this study, we investigated whether interleukin (IL)-1alpha induces the alterations of integrin subunits and urokinase plasminogen activator/urokinase plasminogen activator receptor (uPA/uPAR) expression in pancreatic cancer cells. We hypothesize that the alterations of integrin subunits and uPA/uPAR expression make an important role in signaling pathways responsible for biological behavior of pancreatic cancer cells.IL-1alpha upregulated the expression of alpha6 and beta1 integrins without any alterations of alpha5 and alphav integrins expression. IL-1alpha also induced enhancement in the expression of uPA/uPAR in pancreatic cancer cells. IL-1alpha enhanced the proliferation, adhesion, and migration in pancreatic cancer cells, and IL-1alpha-induced alterations of uPA/uPAR expression correlated with the increased the migration of pancreatic cancer cells. Upregulation of alpha6 integrin subunit and uPA/uPAR correlated with the activation of Ras and downstream extracellular signal-regulated kinase (ERK) pathways. IL-1alpha-induced activation of Ras and downstream ERK can be inhibited by using inhibitory antibodies against alpha6 and beta1 integrin and uPAR, consistent with the inhibition of proliferation, adhesion and migration of pancreatic cancer cells. Immunohistochemical analysis demonstrated a significant association between strong expressions of alpha6 integrin with uPAR in pancreatic cancer specimens. Furthermore, the strong expression of alpha6 integrin and uPAR was found to be independent prognosticator in pancreatic cancer patients.Based on these findings, we conclude that IL-1alpha can induce selective upregulation of alpha6beta1-integrin and uPA/uPAR in pancreatic cancer cells and these changes may modulate the aggressive functions of pancreatic cancer. 
 Extracellular membrane-proximal domain of HAb18G/CD147 binds to metal ion-dependent adhesion site (MIDAS) motif of integrin β1 to modulate malignant properties of hepatoma cells. Several lines of evidence suggest that HAb18G/CD147 interacts with the integrin variants α3β1 and α6β1. However, the mechanism of the interaction remains largely unknown. In this study, mammalian protein-protein interaction trap (MAPPIT), a mammalian two-hybrid method, was used to study the CD147-integrin β1 subunit interaction. CD147 in human hepatocellular carcinoma (HCC) cells was interfered with by small hairpin RNA. Nude mouse xenograft model and metastatic model of HCC were used to detect the role of CD147 in carcinogenesi  s and meta  stasis. We found that the extracellular membrane-proximal domain of HAb18G/CD147 (I-type domain) binds at the metal ion-dependent adhesion site in the βA domain of the integrin β1 subunit, and Asp(179) in the I-type domain of HAb18G/CD147 plays an important role in the interaction. The levels of the proteins that act downstream of integrin, including focal adhesion kinase (FAK) and phospho-FAK, were decreased, and the cytoskeletal structures of HCC cells were rearranged bearing the HAb18G/CD147 deletion. Simultaneously, the migration and invasion capacities, secretion of matrix metalloproteinases, colony formation rate in vitro, and tumor growth and metastatic potential in vivo were decreased. These results indicate that the interaction of HAb18G/CD147 extracellular I-type domain with the integrin β1 metal ion-dependent adhesion site motif activates the downstream FAK signaling pathway, subsequently enhancing the malignant properties of HCC cells. 
 Novel focal adhesion protein kindlin-2 promotes the invasion of gastric cancer cells through phosphorylation of integrin β1 and β3. We have found that the expression of the novel focal adhesion protein kindlin-2 had a significant positive correlation with poor survival in gastric cancer. However, the mechanism by which kindlin-2 acts in gastric cancer warrants further evaluation.Kindlin-2 mRNA expression in gastric cancer cell lines was measured by realtime RT-PCR under normal and hypoxic conditions. Cell proliferation, apoptosis, cell cycle, tumor adhesion, cell invasion ability, and phosphorylation of integrin β1 and β3 proteins were measured to assess the influence of kindlin-2 on the malignant behavior of gastric cancer cells.Kindlin-2 mRNA expression was highest in the dist  ant metast  asis gastric cancer cell line Hs-746T. Cell proliferation, adhesion with endothelium and collagen IV, invasion rate, and angiogenesis genes expression, as well as phosphorylation of integrin β1 and β3 in Hs-746T, were decreased significantly after kindlin-2 downregulation, but there was no change in apoptosis and cell cycle.Kindlin-2 might promote the invasion of gastric cancer cells through enhancing proliferation and adhesion by the phosphorylation of integrin β1 and β3. 
 Nicotinamide phosphoribosyltransferase can affect metastatic activity and cell adhesive functions by regulating integrins in breast cancer. NAD(+) metabolism is an essential regulator of cellular redox reactions, energy pathways, and a substrate provider for NAD(+) consuming enzymes. We recently demonstrated that enhancement of NAD(+)/NADH levels in breast cancer cells with impaired mitochondrial NADH dehydrogenase activity, through augmentation of complex I or by supplementing tumor cell nutrients with NAD(+) precursors, inhibits tumorigenicity and   metastasis  . To more fully understand how aberrantly low NAD(+) levels promote tumor cell dissemination, we here asked whether inhibition of NAD(+) salvage pathway activity by reduction in nicotinamide phosphoribosyltransferase (NAMPT) expression can impact   metastasis   and tumor cell adhesive functions. We show that knockdown of NAMPT, the enzyme catalyzing the rate-limiting step of the NAD(+) salvage pathway, enhances metastatic aggressiveness in human breast cancer cells and involves modulation of integrin expression and function. Reduction in NAMPT expression is associated with upregulation of select adhesion receptors, particularly αvβ3 and β1 integrins, and results in increased breast cancer cell attachment to extracellular matrix proteins, a key function in tumor cell dissemination. Interestingly, NAMPT downregulation prompts expression of integrin αvβ3 in a high affinity conformation, known to promote tumor cell adhesive interactions during hematoge  nous metas  tasis. NAMPT has been selected as a therapeutic target for cancer therapy based on the essential functions of this enzyme in NAD(+) metabolism, cellular redox, DNA repair and energy pathways. Notably, our results indicate that incomplete inhibition of NAMPT, which impedes NAD(+) metabolism but does not kill a tumor cell can alter its phenotype to be more aggressive and metastatic. This phenomenon could promote cancer recurrence, even if NAMPT inhibition initially reduces tumor growth. 
 Thrombin induces tumor invasion through the induction and association of matrix metalloproteinase-9 and beta1-integrin on the cell surface. The procoagulatory serine protease, thrombin, is known to induce invasion and   metastasis   in various cancers, but the mechanisms by which it promotes tumorigenesis are poorly understood. Because the 92-kDa gelatinase (MMP-9) is a known mediator of tumor cell invasion, we sought to determine whether and how thrombin regulates MMP-9. The thrombin receptor, PAR-1, and MMP-9 are expressed in osteosarcomas, as determined by immunohistochemistry. Stimulation of U2-OS osteosarcoma cells with thrombin and a thrombin receptor-activating peptide induced pro-MMP-9 secretion as well as cell surface-associated pro-MMP-9 expression and proteolytic activity. This was paralleled by an increase in MMP-9 mRNA and MMP-9 promoter activity. Thrombin-induced invasion of U2-OS cells through Matrigel was mediated by the phosphatidylinositol 3-kinase signaling pathway and could be inhibited with an MMP-9 antibody. The stimulation of MMP-9 by thrombin was paralleled by an increase in beta1-integrin mRNA and beta1-integrin expression on the cell surface, which was also mediated by phosphatidylinositol 3-kinase and was required for invasion. Thrombin activation induced and co-localized both beta1-integrin and pro-MMP-9 on the cell membrane, as evidenced by co-immunoprecipitation, confocal microscopy, and a protein binding assay. The thrombin-mediated association of these two proteins, as well as thrombin-mediated invasion of U2-OS cells, could be blocked with a cyclic peptide and with an antibody preventing binding of the MMP-9 hemopexin domain to beta1-integrin. These results suggest that thrombin induces expression and association of beta1-integrin with MMP-9 and that the cell surface localization of the protease by the integrin promotes tumor cell invasion. 
 Overexpression of malignancy-associated laminins and laminin receptors by angiotropic human melanoma cells in a chick chorioallantoic membrane model. As distinct from intravascular/lymphatic dissemination, extravascular migratory   metastasis   (EVMM) has been described as a potential additional mechanism of melanoma spread in which tumor cells migrate along the external surfaces of vessels. Angiotropic melanoma cells are linked to the endothelium by a matrix containing laminin. In addition, it has been shown that C16 laminin-derived peptide increases extravascular migration of human green fluorescent protein (GFP) melanoma cells along vessels in a chicken chorioallantoic membrane model (CAM). In this study, we have tested the hypothesis that expression levels of some genes related to lamimin and   metastasis   are differentially expressed in vascularized angiotropic melanoma areas vs. avascular melanoma areas from the same tumor.C8161 human melanoma cells in a shell-less chick CAM assay were used to study EVMM associated with the presence of vascularized angiotropic melanoma areas. For both high-quality histomorphology and RNA preservation in paraffin-embedded tissue, we used a methanol-based fixative coupled with microwave-assisted rapid tissue processing as previously described. Using laser capture microdissection, angiotropic melanoma areas as well as avascular areas were microdissected. Using quantitative real time polymerase chain reaction (QRT-PCR), six genes have been studied: LAMC2 (laminin gamma2 chain), LAMA4 (laminin alpha4 chain), ITGB1 (integrin beta1), ITGB3 (integrin beta3), RSPA (ribosomal protein), and MMP2 (matrix metallopeptidase 2). QRT-PCR data were normalized to human GAPDH housekeeping gene and values were compared against Human Total RNA. Final results were expressed as percentage of expression.All tumors demonstrated a similar pattern, i.e. EVMM of angiotropic melanoma cells. The microdissected histopathological sections presented both angiotropic areas and avascular areas. All genes were overexpressed in angiotropic melanoma areas vs. avascular melanoma areas, especially LAMC2, LAMA4 and ITGB3 (respectively, 165.18, 208.86, and 483.69%).This study shows that several genes related to laminin are overexpressed in angiotropic melanoma areas vs. avascular melanoma areas. Since extravascular migration of melanoma cells along vessels has been demonstrated in the CAM model, taken together these results suggests that some laminins and laminin receptors may play a role in extravascular migratory   metastasis  . This model may represent a promising strategy to analyze differential gene expression in EVMM. 
 Upregulation of Eps8 in oral squamous cell carcinoma promotes cell migration and invasion through integrin-dependent Rac1 activation. Oral squamous cell carcinoma (OSCC) is a lethal disease and early death usually occurs as a result of local invasion and regional lymph node metastases. Current treatment regimens are, to a certain degree, inadequate, with a 5-year mortality rate of around 50% and novel therapeutic targets are urgently required. Using expression microarrays, we identified the eps8 gene as being overexpressed in OSCC cell lines relative to normal oral keratinocytes, and confirmed these findings using RT-PCR and western blotting. In human tissues, we found that Eps8 was upregulated in OSCC (32% of primary tumors) compared with normal oral mucosa, and that expression correlated significantly with lymph node   metastasis   (P=0.032), suggesting a disease-promoting effect. Using OSCC cell lines, we assessed the functional role of Eps8 in tumor cells. Although suppression of Eps8 produced no effect on cell proliferation, both cell spreading and migration were markedly inhibited. The latter cell functions may be modulated through the small GTP-ase, Rac1 and we used pull-down assays to investigate the role of Eps8 in Rac1 signaling. We found that alphavbeta6- and alpha5beta1-integrin-dependent activation of Rac1 was mediated through Eps8. Knockdown of either Eps8 or Rac1, inhibited integrin-dependent cell migration similarly and transient expression of constitutively active Rac1 restored migration of cells in which Eps8 expression had been suppressed. We also showed that knockdown of Eps8 inhibited tumor cell invasion in an organotypic model of OSCC. These data suggest that Eps8 and Rac1 are part of an integrated signaling pathway modulating integrin-dependent tumour cell motility and identify Eps8 as a possible therapeutic target. 
 IGF-I enhances α5β1 integrin expression and cell motility in human chondrosarcoma cells. Chondrosarcoma is a type of highly malignant tumor with a potent capacity to invade locally and cause distan  t metastas  is. Chondrosarcoma shows a predilection fo  r metastas  is to the lungs. Integrins are the major adhesive molecules in mammalian cells and have been associated wit  h metastas  is of cancer cells. Insulin-like growth factor-I (IGF)-I plays an important role in regulating cell growth, proliferation, survival, and metabolism. However, the effects of IGF-I in migration and integrin expression in chondrosarcoma cells are largely unknown. In this study, we found that IGF-I increased the migration and the expression of α5β1 integrin in human chondrosarcoma cells. Pretreatment of cells with IGF-I receptor antibody reduced IGF-I-induced cell migration and integrin expression. Activations of phosphatidylinositol 3-kinase (PI3K), Akt, and nuclear factor-κB (NF-κB) pathways after IGF-I treatment were demonstrated, and IGF-I-induced expression of integrin and migration activity was inhibited by the specific inhibitor and mutant of PI3K, Akt, and NF-κB cascades. Taken together, our results indicated that IGF-I enhances the migration of chondrosarcoma cells by increasing α5β1 integrin expression through the IGF-I receptor/PI3K/Akt/NF-κB signal transduction pathway. 
 Trop-2 promotes prostate cancer   metastasis   by modulating β(1) integrin functions. The molecular mechanisms underlying metastatic dissemination are still not completely understood. We have recently shown that β(1) integrin-dependent cell adhesion to fibronectin and signaling is affected by a transmembrane molecule, Trop-2, which is frequently upregulated in human carcinomas. Here, we report that Trop-2 promotes metastatic dissemination of prostate cancer cells in vivo and is abundantly expressed i  n metastas  is from human prostate cancer. We also show here that Trop-2 promotes prostate cancer cell migration on fibronectin, a phenomenon dependent on β(1) integrins. Mechanistically, we demonstrate that Trop-2 and the α(5)β(1) integrin associate through their extracellular domains, causing relocalization of α(5)β(1) and the β(1)-associated molecule talin from focal adhesions to the leading edges. Trop-2 effect is specific as this molecule does not modulate migration on vitronectin, does not associate with the major vitronectin receptor, α(v)β(3) integrin, and does not affect localization of α(v)β(3) integrin as well as vinculin in focal adhesions. We show that Trop-2 enhances directional prostate cancer cell migration, through modulation of Rac1 GTPase activity. Finally, we show that Trop-2 induces activation of PAK4, a kinase that has been reported to mediate cancer cell migration. In conclusion, we provide the first evidence that β(1) integrin-dependent migratory and metastatic competence of prostate cancer cells is enhanced by Trop-2. 
 Staged stromal extracellular 3D matrices differentially regulate breast cancer cell responses through PI3K and beta1-integrins. Interactions between cancer cells and stroma are critical for growth and invasiveness of epithelial tumors. The biochemical mechanisms behind tumor-stromal interactions leading to increased invasiveness and   metastasis   are mostly unknown. The goal of this study was to analyze the direct effects of staged stroma-derived extracellular matrices on breast cancer cell behavior.Early and late three-dimensional matrices were produced by NIH-3T3 and tumor-associated murine fibroblasts, respectively. After removing fibroblasts, extracted matrices were re-cultured with breast epithelial cells of assorted characteristics: MCF-10A (non-tumorigenic), MCF-7 (tumorigenic, non-invasive), and MDA-MB-231 (tumorigenic, invasive). Effects prompted by staged matrices on epithelial cell's growth, morphology and invasion were determined. Also, matrix-induced velocity, directionality and relative track orientation of invasive cells were assessed in the presence or absence of inhibitors of phosphoinositide-3 kinase (PI3K) and/or beta-1 integrin.We observed that assorted breast epithelial cells reacted differently to two-dimensional vs. staged, control (early) and tumor-associated (late), three-dimensional matrices. MCF-10A had a proliferative advantage on two-dimensional substrates while MCF-7 and MDA-MB-231 showed no difference. MCF-10A and MCF-7 formed morphologically distinguishable aggregates within three-dimensional matrices, while MDA-MB-231 exhibited increased spindle-shape morphologies and directional movements within three-dimensional matrices. Furthermore, MDA-MB-231 acquired a pattern of parallel oriented organization within tumor-associated, but not control matrices. Moreover, tumor-associated matrices induced PI3K and beta1-integrin dependent Akt/PKB activity in MDA-MB-231 cells. Interestingly, beta1-integrin (but not PI3K) regulated tumor-associated matrix-induced mesenchymal invasion which, when inhibited, resulted in a change of invasive strategy rather than impeding invasion altogether.We propose that both cells and matrices are important to promote effective breast cancer cell invasion through three-dimensional matrices and that beta1-integrin inhibition is not necessarily sufficient to block tumor-matrix induced breast cancer cell invasion. Additionally, we believe that characterizing stroma staging (e.g., early vs. late or tumor-associated) might be beneficial for predicting matrix-induced cancer cell responses in order to facilitate the selection of therapies. 
 Expression and correlation of Lewis y antigen and integrins α5 and β1 in ovarian serous and mucinous carcinoma. This study investigates the expression and the clinical significance of Lewis y and integrins α5 and β1 in serous and mucinous ovarian tumors and then evaluates the association between them.Lewis y and integrin α5 and β1 expression are detected on tissues from malignant, borderline, and benign ovarian serous and mucinous tumors and normal tissues. Their expression and relationship are assessed in paraffin sections using immunohistochemistry and double-labeling immunofluorescence method.Lewis y was mainly expressed in ovarian serous and mucinous cancers (88.33%); its positive rate was obviously higher than rates in the borderline (60.00%, P < 0.05) and benign ovarian tumors (35.00%, P < 0.01) and normal ovarian tissues (0, P < 0.01) and was not associated with clinicopathological characteristics. Integrins α5 (85.00%) and β1 (81.67%) were also mainly expressed in ovarian serous and mucinous cancers; their positive rates were all obviously higher than those in benign ovarian tumors (60.00% and 55.00%, respectively; all P < 0.05) and normal tissues (40.00% and 30.00%, respectively; all P < 0.01). Increased expression of integrins α5 and β1 correlated with higher clinical stage (P < 0.05) but were not associated with histological types, differentiation degree, and   lymphatic   metastasis (P > 0.05). The expression intensity of Lewis y and integrins α5 and β1 was significant with clinical stage and differentiation degree (all P < 0.05) in ovarian cancer; positive significant correlation between Lewis y antigen and integrins α5 and β1 was observed in serous and mucinous ovarian cancer tissues.A close correlation between Lewis y, integrins α5 and β1, and ovarian cancer was observed. Lewis y can influence the biological behavior of a tumor cell as an important composition of integrins α5 and β1 by some signal pathway, such as promoting cell adhesion and migration, and this study provides theoretical evidence of ovarian cancer biological treatment. 
 Up-regulation of TGM2 with ITGB1 and SDC4 is important in the development and   metastasis   of renal cell carcinoma. Tissue transglutaminase (TGM2) up-regulation is involved in the progression and dissemination of carcinomas through β1 integrin (ITGB1) association. Given that TGM2 interaction with syndecan-4 (SDC4) on the cell surface is important in the activation of ITGB1 and integrin-mediated survival signaling, we investigated the roles of TGM2, ITGB1, and SDC4 in the development and   metastasi  s of renal cell carcinoma (RCC).Expression levels of TGM2, ITGB1, and SDC4 mRNA were analyzed in primary tumor samples (n = 95) and their healthy counterparts in addition to control and RCC epithelial cell lines. TGM2 catalytic activity in 60 randomly selected patient samples was measured by enzyme-linked sorbent plate assay.TGM2 expression ratio showed a significant 2.9-fold decrease in 67 (70.5%) of the primary RCC tumors (P <0.0001) independent of clinical covariates, including tumor node   metastasi  s (TNM) staging and histopathologic grading. For the remaining 28 (29.5%) tumors, a 1.95-fold increase was recorded in the TGM2 expression levels, which also showed a significant increase in ITGB1 and SDC4 expression levels in 82.6% of the overexpression cases (P <0.001). Up-regulation of TGM2 along with ITGB1 and SCD4 was associated with   metastasi  s and a marked decrease in tumor necrosis. Consistently, RCC cell lines exhibited higher levels of TGM2 expression compared with the control epithelial cell line with a significant up-regulation of ITGB1 and SCD4 recorded for the metastatic lines.Our findings suggest that TGM2 up-regulation along with ITGB1 and SDC4 plays an important role in the development of RCC tumors and advanced RCC with   metastasi  s. 
 Rab25 associates with alpha5beta1 integrin to promote invasive migration in 3D microenvironments. Here, we report a direct interaction between the beta1 integrin cytoplasmic tail and Rab25, a GTPase that has been linked to tumor aggressiveness and   metastasis  . Rab25 promotes a mode of migration on 3D matrices that is characterized by the extension of long pseudopodia, and the association of the GTPase with alpha5beta1 promotes localization of vesicles that deliver integrin to the plasma membrane at pseudopodial tips as well as the retention of a pool of cycling alpha5beta1 at the cell front. Furthermore, Rab25-driven tumor-cell invasion into a 3D extracellular matrix environment is strongly dependent on ligation of fibronectin by alpha5beta1 integrin and the capacity of Rab25 to interact with beta1 integrin. These data indicate that Rab25 contributes to tumor progression by directing the localization of integrin-recycling vesicles and thereby enhancing the ability of tumor cells to invade the extracellular matrix. 
 Ligand-independent activation of c-Met by fibronectin and α(5)β(1)-integrin regulates ovarian cancer invasion an  d metastas  is. The role of the fibronectin receptor, α(5)β(1)-integrin, as an adhesion receptor and in angiogenesis is well established. However, its role in cancer cell invasion   and metast  asis is less clear. We describe a novel mechanism by which fibronectin regulates ovarian cancer cell signaling and promo  tes metast  asis. Fibronectin binding to α(5)β(1)-integrin led to a direct association of α(5)-integrin with the receptor tyrosine kinase, c-Met, activating it in a hepatocyte growth factor/scatter factor (HGF/SF) independent manner. Subsequently, c-Met associated with Src, and activated Src and focal adhesion kinase (FAK). Inhibition of α(5)β(1)-integrin decreased the phosphorylation of c-Met, FAK and Src, both in vitro and in vivo. Independent activation of c-Met by its native ligand, HGF/SF, or overexpression of a constitutively active FAK in HeyA8 cells could overcome the effect of α(5)β(1)-integrin inhibition on tumor cell invasion, indicating that α(5)β(1)-integrin is upstream of c-Met, Src and FAK. Inhibition of α(5)β(1)-integrin on cancer cells in two xenograft models of   ovarian ca  ncer metastasis resulted in a significant decrease of tumor burden, which was independent of the effect of α(5)β(1)-integrin on angiogenesis. These data suggest that fibronectin promotes ovarian can  cer invasi  on and metastasis through an α(5)β(1)-integrin/c-Met/FAK/Src-dependent signaling pathway, transducing signals through c-Met in an HGF/SF-independent manner. 
 Beta 1 integrin predicts survival in breast cancer: a clinicopathological and immunohistochemical study. The main focus of several studies concerned with cancer progression and   metastasis   is to analyze the mechanisms that allow cancer cells to interact and quickly adapt with their environment. Integrins, a family of transmembrane glycoproteins, play a major role in invasive and metastatic processes. Integrins are involved in cell adhesion in both cell-extracellular matrix and cell-cell interactions, and particularly, β1 integrin is involved in proliferation and differentiation of cells in the development of epithelial tissues. This work aimed to investigate the putative role of β1 integrin expression on survival an  d metastas  is in patients with breast invasive ductal carcinoma (IDC). In addition, we compared the expression of β1 integrin in patients with ductal carcinoma in situ (DCIS).Through tissue microarray (TMA) slides containing 225 samples of IDC and 67 samples of DCIS, β1 integrin expression was related with several immunohistochemical markers and clinicopathologic features of prognostic significance.β1 integrin was overexpressed in 32.8% of IDC. In IDC, β1 integrin was related with HER-2 (p = 0.019) and VEGF (p = 0.011) expression and it had a significant rela  tionship w  ith metastasis and death (p = 0.001 and p = 0.05, respectively). Kaplan-Meier survival analysis showed that the overexpression of this protein is very significant (p = 0.002) in specific survival (number of months between diagnosis and death caused by the disease). There were no correlation between IDC and DCIS (p = 0.559) regarding β1 integrin expression.Considering that the expression of β1 integrin in breast cancer remains controversial, specially its relation with survival of patients, our findings provide further evidence that β1 integrin can be a marker of poor prognosis in breast cancer.The virtual slide(s) for this article can be found here: http://www.diagnosticpathology.diagnomx.eu/vs/6652215267393871. 
 Core3 O-glycan synthase suppresses tumor formation and   metastasis   of prostate carcinoma PC3 and LNCaP cells through down-regulation of alpha2beta1 integrin complex. Although there are numerous reports of carbohydrates enriched in cancer cells, very few studies have addressed the functions of carbohydrates present in normal cells that decrease in cancer cells. It has been reported that core3 O-glycans are synthesized in normal gastrointestinal cells but are down-regulated in cancer cells. To determine the roles of core3 O-glycans, we transfected PC3 and LNCaP prostate cancer cells with beta3-N-acetylglucosaminyltransferase-6 (core3 synthase) required to synthesize core3 O-glycans. Both engineered cell lines exhibited reduced migration and invasion through extracellular matrix components compared with mock-transfected cells. Moreover we found that alpha2beta1 integrin acquired core3 O-glycans in cells expressing core3 synthase with decreased maturation of beta1 integrin, leading to decreased levels of the alpha2beta1 integrin complex, decreased activation of focal adhesion kinase, and reduced lamellipodia formation. Upon inoculation into the prostate of nude mice, PC3 cells expressing core3 O-glycans produced much smaller tumors without   metastasis   to the surrounding lymph nodes in contrast to robust tumor formation and   metastasis   seen in mock-transfected PC3 cells. Similarly LNCaP cells expressing core3 O-glycans barely produced subcutaneous tumors in contrast to robust tumor formation by mock-transfected LNCaP cells. These findings indicate that addition of core3 O-glycans to beta1 and alpha2 integrin subunits in prostate cancer cells suppresses tumor formation and tumor   metastasis  . 
 [The expression of integrin beta 1 in normal hepatic tissues, hepatic cirrhosis tissues and hepatocellular carcinoma]. To investigate the expression of integrin beta 1 in hepatic cirrhosis (HC) and hepatocellular carcinoma (HCC).The expression of integrin beta 1 in HCC, HC and normal liver tissues was detected by reverse transcriptase-polymerase chain reaction (RT-PCR) and laser scanning confocal microscopy (LSCM). The association between the integrin beta 1 expression and clinical pathological features were analyzed.(1) The levels of integrin beta 1 mRNA and protein in the HCC (1.30+/-0.24, 90.50+/-33.50) and HC (1.58+/-0.31, 123.10+/-38.90) were much higher than that in the normal hepatic tissue (0.37+/-0.08, 11.90+/-6.00) (P less than 0.05). (2) The expression of integrin beta 1 was associated with HC (r = 0.692), Edmondson pathologic grade (F = 13.618), encapsulation (F = 17.857) and   metastasis   (F = 38.857) (P less than 0.01).Integrin beta 1 may play an important role in the development of hepatic fibrosis, hepatic cirrhosis and hepatocellular carcinoma. 
 Tumor cell cycle arrest induced by shear stress: Roles of integrins and Smad. Interstitial flow in and around tumor tissue affects the mechanical microenvironment to modulate tumor cell growth and   metastasis  . We investigated the roles of flow-induced shear stress in modulating cell cycle distribution in four tumor cell lines and the underlying mechanisms. In all four cell lines, incubation under static conditions for 24 or 48 h led to G(0)/G(1) arrest; in contrast, shear stress (12 dynes/cm(2)) induced G(2)/M arrest. The molecular basis of the shear effect was analyzed, and the presentation on molecular mechanism is focused on human MG63 osteosarcoma cells. Shear stress induced increased expressions of cyclin B1 and p21(CIP1) and decreased expressions of cyclins A, D1, and E, cyclin-dependent protein kinases (Cdk)-1, -2, -4, and -6, and p27(KIP1) as well as a decrease in Cdk1 activity. Using specific antibodies and small interfering RNA, we found that the shear-induced G(2)/M arrest and corresponding changes in G(2)/M regulatory protein expression and activity were mediated by alpha(v)beta(3) and beta(1) integrins through bone morphogenetic protein receptor type IA-specific Smad1 and Smad5. Shear stress also down-regulated runt-related transcription factor 2 (Runx2) binding activity and osteocalcin and alkaline phosphatase expressions in MG63 cells; these responses were mediated by alpha(v)beta(3) and beta(1) integrins through Smad5. Our findings provide insights into the mechanism by which shear stress induces G(2)/M arrest in tumor cells and inhibits cell differentiation and demonstrate the importance of mechanical microenvironment in modulating molecular signaling, gene expression, cell cycle, and functions in tumor cells. 
 Cleavage of ST6Gal I by radiation-induced BACE1 inhibits golgi-anchored ST6Gal I-mediated sialylation of integrin β1 and migration in colon cancer cells. Previously, we found that β-galactoside α2,6-sialyltransferase (ST6Gal I), an enzyme that adds sialic acids to N-linked oligosaccharides of glycoproteins and is frequently overexpressed in cancer cells, is up-regulated by ionizing radiation (IR) and cleaved to a form possessing catalytic activity comparable to that of the Golgi-localized enzyme. Moreover, this soluble form is secreted into the culture media. Induction of ST6Gal I significantly increased the migration of colon cancer cells via sialylation of integrin β1. Here, we further investigated the mechanisms underlying ST6Gal I cleavage, solubilization and release from cells, and addressed its functions, focusing primarily on cancer cell migration.We performed immunoblotting and lectin affinity assay to analyze the expression of ST6 Gal I and level of sialylated integrin β1. After ionizing radiation, migration of cells was measured by in vitro migration assay. α2, 6 sialylation level of cell surface was analyzed by flow cytometry. Cell culture media were concentrated and then analyzed for soluble ST6Gal I levels using an α2, 6 sialyltransferase sandwich ELISA.We found that ST6Gal I was cleaved by BACE1 (β-site amyloid precursor protein-cleaving enzyme), which was specifically overexpressed in response to IR. The soluble form of ST6Gal I, which also has sialyltransferase enzymatic activity, was cleaved from the Golgi membrane and then released into the culture media. Both non-cleaved and cleaved forms of ST6Gal I significantly increased colon cancer cell migration in a sialylation-dependent manner. The pro-migratory effect of the non-cleaved form of ST6Gal I was dependent on integrin β1 sialylation, whereas that of the cleaved form of ST6Gal I was not, suggesting that other intracellular sialylated molecules apart from cell surface molecules such as integrin β1 might be involved in mediating the pro-migratory effects of the soluble form of ST6Gal I. Moreover, production of soluble form ST6Gal I by BACE 1 inhibited integrin β1 sialylation and migration by Golgi-anchored form of ST6Gal I.Our results suggest that soluble ST6Gal I, possibly in cooperation with the Golgi-bound form, may participate in cancer progr  ession and   metastasis prior to being secreted from cancer cells. 
 Silencing profilin-1 inhibits gastric cancer progression via integrin β1/focal adhesion kinase pathway modulation. To investigate the role of profilin-1 (PFN1) in gastric cancer and the underlying mechanisms.Immunohistochemical analysis, quantitative real-time polymerase chain reaction (qRT-PCR) and Western blot were performed to detect PFN1 expression in clinical gastric carcinoma and adjacent tissues, and the association of PFN1 expression with patient clinicopathological characteristics was analyzed. PFN1 was knocked down to investigate the role of this protein in cell proliferation and   metastasi  s in the SGC-7901 cell line. To explore the underlying mechanisms, the expression of integrin β1 and the activity of focal adhesion kinase (FAK) and the downstream proteins extracellular-regulated kinase (ERK)1/2, P38 mitogen-activated protein kinase (MAPK), phosphatidylinositol 3-kinase (PI3K), AKT and mammalian target of rapamycin (mTOR) were measured through Western blot or qRT-PCR analysis. Fibronectin (FN), a ligand of integrin β1, was used to verify the correlation between alterations in the integrin β1/FAK pathway and changes in tumor cell aggressiveness upon PFN1 perturbation.Immunohistochemical, Western blot and qRT-PCR analyses revealed that PFN1 expression was higher at both the protein and mRNA levels in gastric carcinoma tissues compared with the adjacent tissues. In addition, high PFN1 expression (53/75, 70.4%) was correlated with tumor infiltration, lymph n  ode metast  asis and TNM stage in gastric cancer, but not with gender, age, location, tumor size, or histological differentiation. In vitro experiments showed that PFN1 knockdown inhibited the proliferation of SGC-7901 cells through the induction G0/G1 arrest. Silencing PFN1 inhibited cell migration and invasion and down-regulated the expression of matrix metalloproteinase (MMP)-2 and MMP9. Moreover, silencing PFN1 reduced the expression of integrin β1 at the protein level and inhibited the activity of FAK, and the downstream effectors ERK1/2, P38MAPK, PI3K, AKT and mTOR. FN-promoted cell proliferation   and metas  tasis via the integrin β1/FAK pathway was ameliorated by PFN1 silencing.These findings suggest that PFN1 plays a critical role in gastric carcinoma progression, and these effects are likely mediated through the integrin β1/FAK pathway. 
 The interaction affinity between vascular cell adhesion molecule-1 (VCAM-1) and very late antigen-4 (VLA-4) analyzed by quantitative FRET. Very late antigen-4 (VLA-4), a member of integrin superfamily, interacts with its major counter ligand vascular cell adhesion molecule-1 (VCAM-1) and plays an important role in leukocyte adhesion to vascular endothelium and immunological synapse formation. However, irregular expressions of these proteins may also lead to several autoimmune diseases and   metastasis   cancer. Thus, quantifying the interaction affinity of the VCAM-1/VLA-4 interaction is of fundamental importance in further understanding the nature of this interaction and drug discovery. In this study, we report an 'in solution' steady state organic fluorophore based quantitative fluorescence resonance energy transfer (FRET) assay to quantify this interaction in terms of the dissociation constant (Kd). We have used, in our FRET assay, the Alexa Fluor 488-VLA-4 conjugate as the donor, and Alexa Fluor 546-VCAM-1 as the acceptor. From the FRET signal analysis, Kd of this interaction was determined to be 41.82 ± 2.36 nM. To further confirm our estimation, we have employed surface plasmon resonance (SPR) technique to obtain Kd = 39.60 ± 1.78 nM, which is in good agreement with the result obtained by FRET. This is the first reported work which applies organic fluorophore based 'in solution' simple quantitative FRET assay to obtain the dissociation constant of the VCAM-1/VLA-4 interaction, and is also the first quantification of this interaction. Moreover, the value of Kd can serve as an indicator of abnormal protein-protein interactions; hence, this assay can potentially be further developed into a drug screening platform of VLA-4/VCAM-1 as well as other protein-ligand interactions. 
 Identification of integrin β1 as a prognostic biomarker for human lung adenocarcinoma using 2D-LC-MS/MS combined with iTRAQ technology. To discover novel lung adenocarcinoma (AdC) biomarkers, isobaric tags for relative and absolute quantitation (iTRAQ)-tagging combined with 2D-LC-MS/MS analysis was used to identify differentially expressed plasma membrane proteins in lung AdC and paired paraneoplastic normal lung tissues (PNLTs) adjacent to tumors. In this study, significant caveolin-1 downregulation and integrin β1 upregulation was observed in primary lung AdC vs. PNLT. As there has been no report on the association of integrin β1 with lung AdC, immunohistochemical staining was performed to detect the expression of integrin β1 in an independent set of archival tissue specimens including 42 cases of PLNT, 46 cases of without lymph n  ode metast  asis primary AdC (non-LNM AdC) and 62 cases of LNM AdC; the correlation of their expression levels with clinicopathological characteristics and clinical outcomes were evaluated. Based on the data, upregulation of integrin β1 was significantly correlated with advanced clinical stage and lymph   node metas  tasis. Integrin β1 overexpression was significantly associated with advanced clinical stage (P<0.05), lymph   node meta  stasis (P<0.05), increased relapse rate (P<0.05) and decreased overall survival (P<0.05) in AdCs. Cox regression analysis indicated that integrin β1 overexpression is an independent prognostic factor. The data suggest that integrin β1 is a potential biomarker for LNM and prognosis of AdC and integrin β1 upregulation may play an important role in the pathogenesis of AdC. 
 Expression of CD44v6 and integrin-β1 for the prognosis evaluation of pancreatic cancer patients after cryosurgery. Many previous studies demonstrated that cell adhesion molecules CD44v6 and integrin-β1 had been extensively investigated as potential prognostic markers of various cancers. However, data in PC are scarce.We now investigate CD44v6 and integrin-β1 mRNA expression in PBMC by a triplex real-time RT-PCR assay and protein expression in plasma by ELISA. All specimens were collected from 54 PC patients who received the treatment of cryosurgery as well as 20 healthy individuals (control).The mRNA and protein expression levels of CD44v6 and integrin-β1 in patients were significantly increased compared with control group (P<0.05). The high CD44v6 mRNA and protein expression were significantly correlated with clinical stage, tumor differentiation, LNM, li  ver metast  asis and decreased median DFS (P<0.05), while the high integrin-β1 mRNA and protein expression were significantly correlated with clinical stage, LNM, l  iver metas  tasis and decreased median DFS (P<0.05). Clinical stage, LNM, l  iver metas  tasis, CD44v6 mRNA and protein expression were the independent predictors of survival in PC patients (P<0.05). Moreover, CD44v6 and integrin-β1 mRNA and protein expression levels were significantly decreased in patients in 3 months after cryosurgery (P<0.05). No significant difference was found in CD44v6 mRNA and protein expression between patients in 3 months after cryosurgery and control group (P>0.05).CD44v6 and integrin-β1 mRNA and protein expression in blood may serve as biomarkers for the developme  nt and met  astasis of PC, and as prognostic indicators for PC. They may become useful predictors in assessing outcome of PC patients after cryosurgery.The virtual slides for this article can be found here: http://www.diagnosticpathology.diagnomx.eu/vs/4035308681009006. 
 The   metastasis  -promoting phosphatase PRL-3 shows activity toward phosphoinositides. Phosphatase of regenerating liver 3 (PRL-3) is suggested as a biomarker and therapeutic target in several cancers. It has a well-established causative role in cancer   metastasis  . However, little is known about its natural substrates, pathways, and biological functions, and only a few protein substrates have been suggested so far. To improve our understanding of the substrate specificity and molecular determinants of PRL-3 activity, the wild-type (WT) protein, two supposedly catalytically inactive mutants D72A and C104S, and the reported hyperactive mutant A111S were tested in vitro for substrate specificity and activity toward phosphopeptides and phosphoinositides (PIPs), their structural stability, and their ability to promote cell migration using stable HEK293 cell lines. We discovered that WT PRL-3 does not dephosphorylate the tested phosphopeptides in vitro. However, as shown by two complementary biochemical assays, PRL-3 is active toward the phosphoinositide PI(4,5)P(2). Our experimental results substantiated by molecular docking studies suggest that PRL-3 is a phosphatidylinositol 5-phosphatase. The C104S variant was shown to be not only catalytically inactive but also structurally destabilized and unable to promote cell migration, whereas WT PRL-3 promotes cell migration. The D72A mutant is structurally stable and does not dephosphorylate the unnatural substrate 3-O-methylfluorescein phosphate (OMFP). However, we observed residual in vitro activity of D72A against PI(4,5)P(2), and in accordance with this, it exhibits the same cellular phenotype as WT PRL-3. Our analysis of the A111S variant shows that the hyperactivity toward the unnatural OMFP substrate is not apparent in dephosphorylation assays with phosphoinositides: the mutant is completely inactive against PIPs. We observed significant structural destabilization of this variant. The cellular phenotype of this mutant equals that of the catalytically inactive C104S mutant. These results provide a possible explanation for the absence of the conserved Ser of the PTP catalytic motif in the PRL family. The correlation of the phosphatase activity toward PI(4,5)P(2) with the observed phenotypes for WT PRL-3 and the mutants suggests a link between the PI(4,5)P(2) dephosphorylation by PRL-3 and its role in cell migration. 
 Immediate early response protein 2 regulates hepatocellular carcinoma cell adhesion and motility via integrin β1-mediated signaling pathway. Human immediate early response 2 (IER2) has been reported to function as a potential transcriptional factor or transcriptional co‑activator and seems to play a pivotal role in tumor cell motilit  y and meta  stasis, however, its role and underlying mechanisms in hepatocellular carcinoma (HCC) remain unknown. Herein, we demonstrated that overexpression of IER2 in HCC cells increased cell adhesion to fibronectin, migration and invasion, whereas knockdown of IER2 displayed the opposite effects. In agreement with this phenotype, IER2 expression was positively correlated with the metastatic potential and integrin β1 (ITGB1) expression in HCC cell lines. Moreover, we demonstrated a critical role for IER2 in regulation of HCC cell‑extracellular matrix (ECM) adhesion and motility by the transcriptionally promoted ITGB1. Furthermore, we showed that ITGB1‑focal adhesion kinase (FAK)‑Src‑paxillin signal pathway activated by IER2 may contribute to the HCC cell‑ECM adhesion and motility. These results demonstrated that IER2 promoted HCC cell adhesion and motility probably by directly increasing ITGB1 expression and subsequently activating the ITGB1‑FAK‑Src‑paxillin signal pathway. 
 Epithelial-mesenchymal transition in cervical cancer: correlation with tumor progression, epidermal growth factor receptor overexpression, and snail up-regulation. Acquisition of epithelial-mesenchymal transition (EMT) by primary carcinoma cells is associated with disrupted epithelial integrity, local invasion, and ultimately   metastasis  . Little is known about the existence and function of EMT in cervical cancer. This study aims to investigate the regulation of EMT in cervical squamous cell carcinoma.We investigated the molecular events of EMT in surgical specimens, which present the progression of cervical carcinoma. Two cervical cancer cell lines and the primary culture of normal cervical epithelia were used to study the regulatory mechanisms of EMT.The chronic epidermal growth factor (EGF) treatment induces the elongation of cell shape, increases cell scattering, and enhances cell invasion. EGF treatment down-regulates E-cadherin and up-regulates vimentin in cervical cancer cells. These characteristics are consistent with the morphologic changes, molecular events, and functional significance of EMT. EGF receptor (EGFR) signaling inactivates glycogen synthase kinase-3beta, which results in the nuclear accumulation of up-regulated Snail and then leads to EMT program. alpha(5)beta(1) integrin signaling and extracellular matrix fibronectin can modulate EGF-induced EMT. Importantly, the immunofluorescent stainings of surgical specimens indicate that cervical carcinoma progression is accompanied by EGFR overexpression, which is in parallel with decreased E-cadherin and increased vimentin. Up-regulation and nuclear accumulation of Snail correlate with EMT program in tumor tissues.EGF cooperates with alpha(5)beta(1) integrin signaling to induce EMT in cervical cancer cells via up-regulated Snail. Blockade of EGFR activity or expression may provide a potential target for the treatment of cervical cancer progression. 
 Stromal cell-derived factor-1/CXC receptor 4 and β1 integrin interaction regulates urokinase-type plasminogen activator expression in human colorectal cancer cells. The stromal cell-derived factor-1 (SDF-1)/CXC receptor 4 (CXCR4) axis has been shown to play a role in colorectal cancer progression. In addition, the protease urokinase-type plasminogen activator (uPA) is an important factor in tumor cell invasion and   metastasi  s. However, the mechanism by which SDF-1 mediates uPA expression in human colorectal cancer cells remains unknown. We investigated the molecular mechanism governing the interaction between SDF-1 stimulation and uPA expression in three human colon cancer cell lines (DLD-1, SW48, and COLO 205). We found that SDF-1 stimulation led to an increase in the expression and secretion of uPA in these cells. Experiments involving specific inhibitors and small interfering RNA demonstrated that the activation of p38 mitogen-activated protein kinase (MAPK) and phosphatidylinositol 3-kinase (PI3K)/Akt pathways are critical for SDF-1-induced uPA expression. Analysis of transcription factor binding using ELISA and chromatin immunoprecipitation assays revealed that SDF-1 increased Sp1- and AP-1-DNA-binding activities in DLD-1 cells. Inhibition of Sp1 and AP-1 activation blocked the SDF-1-induced expression and activity of the uPA promoter. The effect of SDF-1 on DLD-1 signaling and uPA expression was mediated by the CXCR4/β1 integrin axis. In summary, our findings elucidate the mechanisms of SDF-1/CXCR4 downstream signaling and provide insights into the function of SDF-1 in colon cancer cells. 
 α5β1 integrin recycling promotes Arp2/3-independent cancer cell invasion via the formin FHOD3. Invasive migration in 3D extracellular matrix (ECM) is crucial to cance  r metastas  is, yet little is known of the molecular mechanisms that drive reorganization of the cytoskeleton as cancer cells disseminate in vivo. 2D Rac-driven lamellipodial migration is well understood, but how these features apply to 3D migration is not clear. We find that lamellipodia-like protrusions and retrograde actin flow are indeed observed in cells moving in 3D ECM. However, Rab-coupling protein (RCP)-driven endocytic recycling of α5β1 integrin enhances invasive migration of cancer cells into fibronectin-rich 3D ECM, driven by RhoA and filopodial spike-based protrusions, not lamellipodia. Furthermore, we show that actin spike protrusions are Arp2/3-independent. Dynamic actin spike assembly in cells invading in vitro and in vivo is regulated by Formin homology-2 domain containing 3 (FHOD3), which is activated by RhoA/ROCK, establishing a novel mechanism through which the RCP-α5β1 pathway reprograms the actin cytoskeleton to promote invasive migration and local invasion in vivo. 
 The kinase LMTK3 promotes invasion in breast cancer through GRB2-mediated induction of integrin β₁. Lemur tyrosine kinase 3 (LMTK3) is associated with cell proliferation and endocrine resistance in breast cancer. We found that, in cultured breast cancer cell lines, LMTK3 promotes the development of a metastatic phenotype by inducing the expression of genes encoding integrin subunits. Invasive behavior in various breast cancer cell lines positively correlated with the abundance of LMTK3. Overexpression of LMTK3 in a breast cancer cell line with low endogenous LMTK3 abundance promoted actin cytoskeleton remodeling, focal adhesion formation, and adhesion to collagen and fibronectin in culture. Using SILAC (stable isotope labeling by amino acids in cell culture) proteomic analysis, we found that LMTK3 increased the abundance of integrin subunits α5 and β1, encoded by ITGA5 and ITGB1. This effect depended on the CDC42 Rho family guanosine triphosphatase, which was in turn activated by the interaction between LMTK3 and growth factor receptor-bound protein 2 (GRB2), an adaptor protein that mediates receptor tyrosine kinase-induced activation of RAS and downstream signaling. Knockdown of GRB2 suppressed LMTK3-induced CDC42 activation, blocked ITGA5 and ITGB1 expression promoted by the transcription factor serum response factor (SRF), and reduced invasive activity. Furthermore, abundance of LMTK3 positively correlated with that of the integrin β1 subunit in breast cancer patient's tumors. Our findings suggest a role for LMTK3 in promoting integrin activity during breast cancer progressio  n and meta  stasis. 
 CXCR4 chemokine receptor engagement modifies integrin dependent adhesion of renal carcinoma cells. The mechanisms leading to renal cell carcinoma (RCC)   metastasis   are incompletely understood. Although evidence shows that the chemokine receptor CXCR4 and its ligand CXCL12 may regulate tumor dissemination, their role in RCC is not clearly defined. We examined CXCR4 expression and functionality on RCC cell lines, and explored CXCL12-triggered tumor adhesion to human endothelium (HUVEC) or extracellular matrix proteins. Functional CXCR4 was expressed on A498 tumor cells, enabling them to migrate towards a CXCL12 gradient. CXCR4 engagement by CXCL12 induced elevated cell adhesion to HUVEC, to immobilized fibronectin, laminin or collagen. Anti-CXCR4 antibodies or CXCR4 knock down by siRNA applied prior to CXCL12 stimulation impaired CXCL12-triggered tumor adhesion. However, blocking CXCR4 subsequent to CXCL12 stimulation did not. This pointed to an indirect control of tumor cell adhesion by CXCR4. In fact, CXCR4 engagement by CXCL12 also induced alterations of receptors of the integrin family, notably alpha3, alpha5, beta1 and beta3 subunits, and blocking beta1 integrins with a function-blocking antibody prevented CXCL12-induced A498 adhesion. Focal adhesion kinase (total and activated) and integrin-linked kinase significantly increased in CXCL12-treated A498 cells, accompanied by a distinct up-regulation of ERK1/2, JNK and p38 phosphorylation. Therefore, CXCR4 may be crucial in controlling adhesion of A498 cells via cross talking with integrin receptors. These data show that CXCR4 receptors contribute to RCC dissemination and may provide a novel link between CXCR4 chemokine receptor expression and integrin triggered RCC adhesion to the vascular wall and subendothelial matrix components. 
 Angiopoietin-like 4 protein elevates the prosurvival intracellular O2(-):H2O2 ratio and confers anoikis resistance to tumors. Cancer is a leading cause of death worldwide. Tumor cells exploit various signaling pathways to promote their growth and   metastasis  . To our knowledge, the role of angiopoietin-like 4 protein (ANGPTL4) in cancer remains undefined. Here, we found that elevated ANGPTL4 expression is widespread in tumors, and its suppression impairs tumor growth associated with enhanced apoptosis. Tumor-derived ANGPTL4 interacts with integrins to stimulate NADPH oxidase-dependent production of O(2)(-). A high ratio of O(2)(-):H(2)O(2) oxidizes/activates Src, triggering the PI3K/PKBα and ERK prosurvival pathways to confer anoikis resistance, thus promoting tumor growth. ANGPTL4 deficiency results in diminished O(2)(-) production and a reduced O(2)(-):H(2)O(2) ratio, creating a cellular environment conducive to apoptosis. ANGPTL4 is an important redox player in cancer and a potential therapeutic target. 
 Chemical modulation of VLA integrin affinity in human breast cancer cells. The fact that disruption of integrin-extracellular matrix contacts leads to cell death, has converted cell adhesion into a potential target for the control of invasive cancer. In this work, we studied the functional consequences of the interference with the activity of the very late activation antigen (VLA) family of integrins in human breast cancer cell lines of distinct malignancy. The alpha2beta1-mediated adhesion reduced the entry of highly malignant, hormone-independent breast cancer cells into apoptosis. Adhesion of breast cancer cells through the VLA integrins alpha2beta1 and alpha5beta1 was significantly reduced by an apoptosis-inducing natural triterpenoid, dehydrothyrsiferol (DT), when studied on low amounts of extracellular matrix. This effect was dose-dependent, not related to cell toxicity and not shared with apoptosis-inducing standard chemotherapeutics, such as doxorubicin and taxol. The compound did not affect either the cell surface expression level of VLA integrins or cell distribution of vinculin and actin during cell spreading. In addition, neither phosphorylation of the focal adhesion kinase pp125FAK on Tyr397 nor the protein kinase B (Akt/PKB) on Ser473 was significantly altered by DT. The integrin activation level, assessed by binding of soluble collagen to the alpha2beta1 integrin, was reduced upon cell treatment with DT. Importantly, the TS2/16, an anti-beta1 activating monoclonal antibody was able to rescue DT-treated cells from apoptosis. Since the activation state of integrins is increasingly recognized as an essential factor in   metastasis   formation, findings presented herein reveal that the chemical regulation of integrin affinity may be a potential therapeutic strategy in cancer therapy. 
 High affinity interaction of integrin alpha4beta1 (VLA-4) and vascular cell adhesion molecule 1 (VCAM-1) enhances migration of human melanoma cells across activated endothelial cell layers. The capacity of tumor cells to form metastatic foci correlates with their ability to interact with and migrate through endothelial cell layers. This process involves multiple adhesive interactions between tumor cells and the endothelium. Only little is known about the molecular nature of these interactions during extravasation of tumor cells. In human melanoma cells, the integrin alphavbeta3 is involved in transendothelial migration and its expression correlates with   metastasis  . However, many human melanoma cells do not express beta3 integrins. Therefore, it remained unclear how these cells undergo transendothelial migration. In this study we show that human melanoma cells with different metastatic potency, which do not express beta2 or beta3 integrins, express the VCAM-1 receptor alpha4beta1. VCAM-1 is up-regulated on activated endothelial cells and is known to promote transendothelial migration of leukocytes. Interestingly, despite comparable cell surface levels of alpha4beta1, only the highly metastatic melanoma cell lines MV3 and BLM, but not the low metastatic cell lines IF6 and 530, bind VCAM-1 with high affinity without further stimulation, and are therefore able to adhere to and migrate on isolated VCAM-1. Moreover, we demonstrate that function-blocking antibodies against the integrin alpha4beta1, as well as siRNA-mediated knock-down of the alpha4 subunit in these highly metastatic human melanoma cells reduce their transendothelial migration. These data imply that only high affinity interactions between the integrin alpha4beta1 on melanoma cells and VCAM-1 on activated endothelial cells may enhance the metastatic capacity of human beta2/beta3-negative melanoma cells. 
 Insulin-like growth factor-independent insulin-like growth factor binding protein 3 promotes cell migration and lymph node   metastasis   of oral squamous cell carcinoma cells by requirement of integrin β1. Frequent   metastasi  s to the cervical lymph nodes leads to poor survival of patients with oral squamous cell carcinoma (OSCC). To understand the underlying mechanisms of lymph node   metastasi  s, two sublines were successfully isolated from cervical lymph nodes of nude mice through in vivo selection, and identified as originating from poorly metastatic parental cells. These two sublines specifically metastasized to cervical lymph nodes in 83% of mice, whereas OEC-M1 cells did not metastasize after injection into the oral cavity. After gene expression analysis, we identified insulin-like growth factor binding protein 3 (IGFBP3) as one of the significantly up-regulated genes in the sublines in comparison with their parental cells. Consistently, meta-analysis of the public microarray datasets and IGFBP3 immunohistochemical analysis revealed increased both levels of IGFBP3 mRNA and protein in human OSCC tissues when compared to normal oral or adjacent nontumorous tissues. Interestingly, the up-regulated IGFBP3 mRNA expression was significantly associated with OSCC patients with lymph node   metastasi  s. IGFBP3 knockdown in the sublines impaired and ectopic IGFBP3 expression in the parental cells promoted migration, transendothelial migration and lymph node   metastasi  s of orthotopic transplantation. Additionally, ectopic expression of IGFBP3 with an IGF-binding defect sustained the IGFBP3-enhanced biological functions. Results indicated that IGFBP3 regulates   metastasi  s-related functions of OSCC cells through an IGF-independent mechanism. Furthermore, exogenous IGFBP3 was sufficient to induce cell motility and extracellular signal-regulated kinase (ERK) activation. The silencing of integrin β1 was able to impair exogenous IGFBP3-mediated migration and ERK phosphorylation, suggesting a critical role of integrin β1 in IGFBP3-enchanced functions. 
 PRL-3 promotes the motility, invasion, and   metastasis   of LoVo colon cancer cells through PRL-3-integrin beta1-ERK1/2 and-MMP2 signaling. Phosphatase of regenerating liver-3 (PRL-3) plays a causative role in tumor   metastasis  , but the underlying mechanisms are not well understood. In our previous study, we observed that PRL-3 could decrease tyrosine phosphorylation of integrin beta1 and enhance activation of ERK1/2 in HEK293 cells. Herein we aim to explore the association of PRL-3 with integrin beta1 signaling and its functional implications in motility, invasion, and   metastasis   of colon cancer cell LoVo.Transwell chamber assay and nude mouse model were used to study motility and invasion, and metastsis of LoVo colon cancer cells, respectively. Knockdown of integrin beta1 by siRNA or lentivirus were detected with Western blot and RT-PCR. The effect of PRL-3 on integrin beta1, ERK1/2, and MMPs that mediate motility, invasion, and   metastasis   were measured by Western blot, immunofluorencence, co-immunoprecipitation and zymographic assays.We demonstrated that PRL-3 associated with integrin beta1 and its expression was positively correlated with ERK1/2 phosphorylation in colon cancer tissues. Depletion of integrin beta1 with siRNA, not only abrogated the activation of ERK1/2 stimulated by PRL-3, but also abolished PRL-3-induced motility and invasion of LoVo cells in vitro. Similarly, inhibition of ERK1/2 phosphorylation with U0126 or MMP activity with GM6001 also impaired PRL-3-induced invasion. In addition, PRL-3 promoted gelatinolytic activity of MMP2, and this stimulation correlated with decreased TIMP2 expression. Moreover, PRL-3-stimulated lung   metastasis   of LoVo cells in a nude mouse model was inhibited when integrin beta1 expression was interfered with shRNA.Our results suggest that PRL-3's roles in motility, invasion, and   metastasis   in colon cancer are critically controlled by the integrin beta1-ERK1/2-MMP2 signaling. 
 Genome-wide screening identified that miR-134 acts as a   metastasis   suppressor by targeting integrin β1 in hepatocellular carcinoma. MicroRNAs (miRNAs) are small, single-stranded, non-coding RNAs that play pivotal roles in human cancer development and progression, such as tumor   metastasi  s. Here, we identified the miRNAs that regulate hepatocellular carcinoma (HCC) cell migration by a high-throughput screening method using the classical wound-healing assay with time-lapse video microscopy and validation with a transwell migration assay. Eleven miRNAs (miR-134, -146b-3p, -188-3p, -525-3p, -661, -767-5p, -891a, -891b, -1244, -1247 and miR-1471) were found to promote or inhibit HCC cell migration. Further investigation revealed that miR-134 suppressed the invasion and   metastasi  s of HCC cells in vitro and in vivo, and integrin beta 1 (ITGB1) was a direct and functional target gene of miR-134. Moreover, miR-134 inhibited the phosphorylation of focal adhesion kinase (FAK) and the activation of RhoA downstream of the ITGB1 pathway, thereby decreasing stress fiber formation and cell adhesion in HCC cells. In conclusion, we demonstrated that miR-134 is a novel   metastasi  s suppressor in HCC and could be a potential therapeutic target for the treatment of HCC. 
 High-level β1-integrin expression in a subpopulation of highly tumorigenic oral cancer cells. The β1 integrin (CD29) is a putative marker for cancerous epithelial stem cells. Cancer stem cells are essential to drive tumor growth, recurrence, an  d metastas  is. We investigated the role of β1-integrin expression in the development of malignant phenotypes of oral squamous cell carcinoma (OSCC).Immunostaining was used to analyze the expression levels of β1 integrins in different types of cell colonies and tumor spheres. The results of cell viability and migration assays with and without siRNA knockdown of β1-integrin expression were compared. Cells expressing β1 integrins were evaluated for their tumorigenicity in mice. The expression of β1 integrins in human specimens of oral cancers at different clinical stages was semiquantified based on immunohistochemical staining of the β1-integrin protein.The expression level of β1 integrins in Meng-1 oral epidermoid carcinoma cells (OECM-1) cells was significantly higher in holoclonal colonies and tumor spheres compared to control cells. The knockdown of β1-integrin expression in OECM-1 cells reduced cell proliferation, migration, and tumor sphere formation. Beta-1 integrin (+) cells were more tumorigenic in the mouse xenograft model than β1 integrin (-) cells. In the human specimens, the expression level of the β1-integrin protein positively correlated with the clinical stage.The expression of β1 integrin in OECM-1 cells is involved in the development of malignant phenotypes of OSCC.Inhibitors for β1-integrin signaling may be suitable to become target-specific therapies for OSCC. 
 β1 integrin regulates Arg to promote invadopodial maturation and matrix degradation. β1 integrin has been shown to promot  e metastas  is in a number of tumor models, including breast, ovarian, pancreatic, and skin cancer; however, the mechanism by which it does so is poorly understood. Invasive membrane protrusions called invadopodia are believed to facilitate extracellular matrix degradation and intravasation durin  g metastas  is. Previous work showed that β1 integrin localizes to invadopodia, but its role in regulating invadopodial function has not been well characterized. We find that β1 integrin is required for the formation of mature, degradation-competent invadopodia in both two- and three-dimensional matrices but is dispensable for invadopodium precursor formation in metastatic human breast cancer cells. β1 integrin is activated during invadopodium precursor maturation, and forced β1 integrin activation enhances the rate of invadopodial matrix proteolysis. Furthermore, β1 integrin interacts with the tyrosine kinase Arg and stimulates Arg-dependent phosphorylation of cortactin on tyrosine 421. Silencing β1 integrin with small interfering RNA completely abrogates Arg-dependent cortactin phosphorylation and cofilin-dependent barbed-end formation at invadopodia, leading to a significant decrease in the number and stability of mature invadopodia. These results describe a fundamental role for β1 integrin in controlling actin polymerization-dependent invadopodial maturation and matrix degradation in metastatic tumor cells. 
 The laminin binding integrin alpha6beta1 in prostate cancer perineural invasion. Metastasizing prostate tumor cells invade along nerves innervating the encapsulated human prostate gland in a process known as perineural invasion. The extracellular matrix laminin class of proteins line the neural route and tumor cells escaping from the gland express the laminin binding integrin alpha6beta1 as a prominent cell surface receptor. Integrin alpha6beta1 promotes aggressive disease and supports prostate tumor cell   metastasis   to bone. Laminins and their integrin receptors are necessary for the development and maintenance of the peripheral nervous system, indicating the potential role for integrin receptors in directing prostate tumor cell invasion on nerves during perineural invasion. 
 miR-199a-5p regulates β1 integrin through Ets-1 to suppress invasion in breast cancer. Increasing evidence has revealed that miR-199a-5p is actively involved in tumor invasion and   metastasi  s as well as in the decline of breast cancer tissues. In this research, overexpression of miR-199a-5p weakened motility and invasion of breast cancer cells MCF-7 and MDA-MB-231. Upregulation of Ets-1 increased breast cancer cell invasion, but the mechanism by which miR-199a-5p modulates activation of Ets-1 in breast cancer was not clarified. We investigated the relationship between miR-199a-5p and Ets-1 on the basis of 158 primary breast cancer case specimens, and the results showed that Ets-1 expression was inversely correlated with endogenous miR-199a-5p. Overexpression of miR-199a-5p reduced the mRNA and protein levels of Ets-1 in MCF-7 and MDA-MB-231 cells, whereas anti-miR-199a-5p elevated Ets-1. siRNA-mediated Ets-1 knockdown phenocopied the inhibition invasion of miR-199a-5p in vitro. Moreover, luciferase reporter assay revealed that miR-199a-5p directly targeted 3'-UTR of Ets-1 mRNA. This research revealed that miR-199a-5p could descend the levels of β1 integrin by targeting 3'-UTR of Ets-1 to alleviate the invasion of breast cancer via FAK/Src/Akt/mTOR signaling pathway. Our results provide insight into the regulation of β1 integrin through miR-199a-5p-mediated Ets-1 silence and will help in designing new therapeutic strategies to inhibit signal pathways induced by miR-199a-5p in breast cancer invasion. 
 Expression of E-selectin, integrin beta1 and immunoglobulin superfamily member in human gastric carcinoma cells and its clinicopathologic significance. To study the expression levels of E- selectin, integrin beta1 and immunoglobulin superfamily member-intercellular adhesion molecule-1 (ICAM-1) in human gastric carcinoma cells, and to explore the relationship between these three kinds of cell adhesion molecules and gastric carcinoma.The serum contents of E-selectin, integrin beta1 and ICAM-1 were detected by enzyme-linked immunosorbent assay (ELISA), in 47 healthy individuals (control group) and in 57 patients with gastric carcinoma (gastric carcinoma group) respectively prior to operation and 7 d after operation.The serum E-selectin, ECAM-1 and integrin beta1 were found to be expressed in both control and gastric carcinoma groups. However, they were highly expressed in patients with gastric carcinoma patients before operation or with unresectable tumours. The expression levels of ICAM-1 and integrin beta1 were significantly higher in gastric carcinoma patients than in controls (P < 0.01). A comparison of the E-selectin levels between the two groups showed statistically insignificant difference (P = 0.64). In addition, the expression levels were all decreased substantially in the postoperative patients subjected to radical resection of the tumours, indicating that the high level expressions of these compounds might be the important factor for predicting the prognosis of these patients.Serum E-selectin, ICAM-1 and integrin beta1 expression levels are probably related to the   metastasis   and relapse of gastric cancer. 
 Matrix metalloproteinase-2 cleavage of the β1 integrin ectodomain facilitates colon cancer cell motility. Cancer cell invasion is a key element in   metastasi  s that requires integrins for adhesion/de-adhesion, as well as matrix metalloproteinases (MMPs) for focalized proteolysis. Herein we show that MMP-2 is up-regulated in resected colorectal tumors and degrades β1 integrins with the release of fragments containing the β1 I-domain. The β1 cleavage pattern is similar to that produced by digestion of α5β1 and α2β1 with MMP-2. Two such fragments, at 25 and 75 kDa, were identified after immunoprecipitation, with monoclonal antibody BD610468 reacting with the NH(2)-terminal I-like ectodomain followed by SDS-PAGE and microsequencing using electrospray (ISI-Q-TOF-Micromass) spectrometry. Cleavage of the β1 integrin can be abolished by inhibition of MMP-2 activity; it can be induced by up-regulation of MMP-2 expression, as exemplified by HT29 colon cancer cells transfected with pCMV6-XL5-MMP-2. Co-immunoprecipitation studies of colon cancer cells showed that the β1 integrin subunit is associated with MMP-2. The MMP-2-mediated shedding of the I-like domain from β1 integrins resulted in decreased adhesion of colon cancer cells to collagen and fibronectin, thus abolishing their receptivity. Furthermore, such cells showed enhanced motility as evaluated by a "wound healing-like" assay and time-lapse microscopy, indicating their increased invasiveness. Altogether, our data demonstrate that MMP-2 amplifies the motility of colon cancer cells, not only by digesting the extracellular matrix components in the vicinity of cancer cells but also by inactivating their major β1 integrin receptors. 
 Integrin alpha5beta1 controls invasion of human breast carcinoma cells by direct and indirect modulation of MMP-2 collagenase activity. Integrins control a variety of signal transduction pathways central to cell survival, proliferation, and differentiation and their functions and expression levels are altered in many types of cancer. Although alpha5beta1 is one of the most studied integrins in cancer, its functions in different aspects of this disease have not been completely elucidated. In particular, controversial data exist on its role in tumor invasion and   metastasis  . In order to establish mechanisms underlying involvement of alpha5beta1 integrin in invasion, we depleted its expression in MCF-7Dox human breast carcinoma cells via siRNA. We demonstrated that concomitant to alpha5beta1 integrin depletion, was a sharp decrease in MMP-2 collagenase expression and inhibition of the invasiveness of these cells in vitro. Similar reduction of invasion potential was observed upon siRNA-mediated silencing of the MMP-2 gene. Down-regulation of alpha5beta1 integrin was accompanied by a substantial decrease in the amounts of active (phosphorylated) forms of Akt, Erk1/2 kinases and c-Jun oncoprotein. Moreover, in MCF-7Dox cells, blocking the activity of above kinases by specific inhibitors strongly reduced expression of MMP-2 and c-Jun, and suppressed invasion of the cells in vitro. Similar results were observed upon siRNA-mediated silencing of c-Jun expression. Co-immunoprecipitation experiments demonstrated that alpha5beta1 integrin interacts with MMP-2 collagenase on the surface of MCF-7Dox breast carcinoma and SKMel-147 human melanoma cells. Our data suggest that alpha5beta1 integrin controls invasion of the studied cells via regulation of MMP-2 collagenase expression which can occur either through signaling pathways involving PI-3K, Akt, and Erk protein kinases and the c-Jun or via direct recruitment of MMP-2 to the cell surface. 
 Formin-like 2 Promotes β1-Integrin Trafficking and Invasive Motility Downstream of PKCα. Regulated turnover of integrin receptors is essential for cell adhesion and migration. Pathways selectively regulating β1-integrin recycling are implicated in cancer invasion a  nd metasta  sis, yet proteins required for the internalization of this pro-invasive integrin remain to be identified. Here, we uncover formin-like 2 (FMNL2) as a critical regulator of β1-integrin internalization downstream of protein kinase C (PKC). PKCα associates with and phosphorylates FMNL2 at S1072 within its Diaphanous autoregulatory region, leading to the release of formin autoinhibition. Phosphorylation of FMNL2 triggers its rapid relocation and promotes its interaction with the cytoplasmic tails of the α-integrin subunits for β1-integrin endocytosis. FMNL2 drives β1-integrin internalization and invasive motility in a phosphorylation-dependent manner, while a FMNL2 mutant defective in actin assembly interferes with β1-integrin endocytosis and cancer cell invasion. Our data establish a role for FMNL2 in the regulation of β1-integrin and provide a mechanistic understanding of the function of FMNL2 in cancer invasiveness. 
 Functional role of endothelial adhesion molecules in the early stages of brain   metastasis  . Cellular adhesion molecules (CAMs), which are normally associated with leukocyte trafficking, have also been shown to play an essential role in tumor   metastasis   to non-CNS sites. However, the role played by CAMs in brain   metastasis   is largely unexplored. It is known that leukocyte recruitment to the brain is very atypical and that mechanisms of disease in peripheral tissues cannot be extrapolated to the brain. Here, we have established the spatiotemporal expression of 12 key CAMs in the initial phases of tumor seeding in 2 different models of brain   metastasis  .BALB/c or SCID mice were injected intracardially (10(5) cells/100 μL phosphate-buffered saline with either 4T1-GFP or MDA231BR-GFP cells, respectively (n = 4-6/group), and expression of the CAMs was determined by immunohistochemistry and immunofluorescence colocalisation.Endothelial expression of E-selectin, VCAM-1, ALCAM, ICAM-1, VLA-4, and β4 integrin was markedly increased early in tumor seeding. At the same time, the natural ligands to these adhesion molecules were highly expressed on the metastatic tumor cells both in vitro and in vivo. Two of these ligands showed particularly high tumor cell expression (ALCAM and VLA-4), and consequently their functional role in tumor seeding was determined. Antibody neutralization of either ALCAM or VLA-4 significantly reduced tumor seeding within the brain (>60% decrease in tumor number/mm(2) brain; P < .05-0.01).These findings suggest that ALCAM/ALCAM and VLA-4/VCAM-1 interactions play an important functional role in the early stages o  f metastas  is seeding in the brain. Moreover, this work identifies a specific subset of ligand-receptor interactions that may yield new therapeutic and diagnostic targets for brai  n metastas  is. 
 The pivotal role of integrin β1 in   metastasi  s of head and neck squamous cell carcinoma. This study aimed to understand the prognostic value of integrin β1 expression in head and neck squamous cell carcinoma (HNSCC) and the mechanism underlying its association with metastatic HNSCC.Archival HNSCC tissues including 99 nonmetastatic primary tumors and 101 metastatic primary tumors were examined for the association of integrin β1 expression wi  th metasta  sis and disease prognosis by appropriate statistical methods. Fluorescence-activated cell sorting was used to separate the integrin β1(high/+) cell population from the integrin β1(low/-) population in HNSCC cell lines. These two populations and integrin β1 shRNA knockdown HNSCC cells were examined for the effect of integrin β1 on invasion in vitro and on lymph node and lung metastases in a xenograft mouse model. Expression and activation of matrix metalloproteinases (MMP) were examined by zymography.Statistical analysis showed that integrin β1 expression was significantly higher in the metastatic primary tumors than in the nonmetastatic tumors (42.6% vs. 24.8%, P < 0.0001 and P < 0.0001 by univariate and multivariate analyses, respectively). In patients with lym  ph node me  tastasis, integrin β1 expression was inversely correlated with overall survival (P = 0.035). The integrin β1 knockdown or integrin β1(low/-) HNSCC cells showed a significant reduction in lymph node and lung metastases in vivo (P < 0.001 and P < 0.05, respectively). Significantly reduced Matrigel invasion capability was also found in integrin β1 knockdown or integrin β1(low/-) HNSCC cells (P < 0.01). Finally, zymography results showed integrin β1-affected HNSCC invasion by regulating MMP-2 activation.These findings indicate that integrin β1 has a major impact on HNSCC prognosis through its   regulatio  n of metastasis. 
 New mechanistic insights of integrin β1 in breast cancer bone colonization. Bone   metastasi  s is a frequent and life-threatening complication of breast cancer. The molecular mechanisms supporting the establishment of breast cancer cells in the skeleton are still not fully understood, which may be attributed to the lack of suitable models that interrogate interactions between human breast cancer cells and the bone microenvironment. Although it is well-known that integrins mediate adhesion of malignant cells to bone extracellular matrix, their role during bone colonization remains unclear. Here, the role of β1 integrins in bone colonization was investigated using tissue-engineered humanized in vitro and in vivo bone models. In vitro, bone-metastatic breast cancer cells with suppressed integrin β1 expression showed reduced attachment, spreading, and migration within human bone matrix compared to control cells. Cell proliferation in vitro was not affected by β1 integrin knockdown, yet tumor growth in vivo within humanized bone microenvironments was significantly inhibited upon β1 integrin suppression, as revealed by quantitative in/ex vivo fluorescence imaging and histological analysis. Tumor cells invaded bone marrow spaces in the humanized bone and formed osteolytic lesions; osteoclastic bone resorption was, however, not reduced by β1 integrin knockdown. Taken together, we demonstrate that β1 integrins have a pivotal role in bone colonization using unique tissue-engineered humanized bone models. 
 Cancer stem-like cells enriched with CD29 and CD44 markers exhibit molecular characteristics with epithelial-mesenchymal transition in squamous cell carcinoma. Increasing evidences have indicated that only a phenotypic subset of cancer cells, termed as the cancer stem cells (CSCs), is capable of initiating tumor growth and provide a reservoir of cells that cause tumor recurrence after therapy. Epithelial-mesenchymal transition (EMT), a cell type change from an epithelial cobblestone phenotype to an elongated fibroblastic phenotype, plays a critical role not only in tumor   metastasis   but also in tumor recurrence and contributes to drug resistance. Accumulating evidence has shown that cells with an EMT phenotype are rich sources for CSCs, suggesting a biological link between EMT and CSCs; thus study on the link will help understand the cellular and molecular mechanisms of tumor   metastasis   and drug resistance. CD29 is involved in EMT through cross-talk with cadherins and CD44 has been reported as a successful used marker for CSCs. Here, we try to address whether combination of CD29 and CD44 could be used to identify cancer stem-like cells undergoing EMT in squamous cell carcinoma (SCC) and compare the molecular differences between CD29high/CD44high and CD29low/CD44low cells in SCC. Expression pattern of CD29 and CD44 was analyzed in tissues of skin SCC and cultured A431 cells by immunostaining. Subtype cells of CD29high/CD44high and CD29low/CD44low A431 were sorted by fluorescence-activated cell sorting and proliferating abilities were assayed by cell counting, colony forming and tumorigenicity in NOD/SCID mice. Finally, to probe more deeply into the molecular differences between CD29high/CD44high and CD29low/CD44low A431 cells, gene microarray analysis was applied to compare gene expression profiling. Staining of CD29 and CD44 showed similar heterogeneous expression pattern with positive cells located in the invasion front of SCC tissue as well as in cultured A431 cells. Sorted CD29high/CD44high A431 cells had higher proliferating ability in vitro and in NOD/SCID mice as compared with CD29low/CD44low cells. Gene profiling identified differentiated gene expressions between CD29high/CD44high and CD29low/CD44low A431 cells. These genes are involved in cell cycle, cell malignant transformation,   metastasis  , drug resistance and EMT, implying that CD29high/CD44high cells have properties of CSCs and EMT. Our present results demonstrated heterogeneous gene expression patterns and different biological behavior in SCC. Combination of CD29 and CD44 can be used as markers to enrich CSCs in human SCC. Moreover, CD29high/CD44high cells exhibit molecular characteristics of EMT, suggesting that CSC-associated pathways were involved in EMT. Studies on correlation of CSCs and the cells undergoing EMT may explain some aspects of tumor progression and drug resistance. 
 Cdc42 promotes transendothelial migration of cancer cells through β1 integrin. Cancer cells interact with endothelial cells during the process of metastatic spreading. Here, we use a small interfering RNA screen targeting Rho GTPases in cancer cells to identify Cdc42 as a critical regulator of cancer cell-endothelial cell interactions and transendothelial migration. We find that Cdc42 regulates β1 integrin expression at the transcriptional level via the transcription factor serum response factor (SRF). β1 integrin is the main target for Cdc42-mediating interaction of cancer cells with endothelial cells and the underlying extracellular matrix, as exogenous β1 integrin expression was sufficient to rescue the Cdc42-silencing phenotype. We show that Cdc42 was required in vivo for cancer cell spreading and protrusion extension along blood vessels and retention in the lungs. Interestingly, transient Cdc42 depletion was sufficient to decrease experimental lung metastases, which suggests that its role in endothelial attachment is important   for metast  asis. By identifying β1 integrin as a transcriptional target of Cdc42, our results provide new insight into Cdc42 function. 
 Overexpression of AKT2/protein kinase Bbeta leads to up-regulation of beta1 integrins, increased invasion, and   metastasis   of human breast and ovarian cancer cells. To determine how AKT2 might contribute to tumor cell progression, a full-length, wild-type, human AKT2/protein kinase B (PKB)beta cDNA was transfected into a panel of eight human breast and ovarian cancer cells. AKT2 transfectants demonstrated increased adhesion and invasion through collagen IV because of up-regulation of beta1 integrins. In addition, AKT2 cells were more metastatic than control cells in vivo. Increased invasion by AKT2 was blocked by preincubation with an anti-beta1 integrin function blocking antibody, exposure to wortmannin, and by expression of phosphatase and tensin homologue tumor suppressor (PTEN). Confocal microscopy performed on transfected human breast cancer cells showed that unlike AKT1, AKT2 protein predominantly localized adjacent to the collagen IV matrix during cellular attachment. Overexpression of AKT2, but not AKT1 or AKT3, was sufficient to duplicate the invasive effects of phosphoinositide 3-OH kinase (PI3-K) transfected in breast cancer cells. Furthermore, expression of kinase dead AKT2(181 amino acid methionine [M]), and not kinase dead AKT1(179M) or AKT3(177M), was capable of blocking invasion induced by either human epidermal growth factor receptor-2 (HER-2) overexpression or by activation of PI3-K. Taken together, these data indicate that AKT2 mediates PI3-K-dependent effects on adhesion, motility, invasion, and   metastasis   in vivo. 
 Integrin endosomal signalling suppresses anoikis. Integrin-containing focal adhesions transmit extracellular signals across the plasma membrane to modulate cell adhesion, signalling and survival. Although integrins are known to undergo continuous endo/exocytic traffic, the potential impact of endocytic traffic on integrin-induced signals is unknown. Here, we demonstrate that integrin signalling is not restricted to cell-ECM adhesions and identify an endosomal signalling platform that supports integrin signalling away from the plasma membrane. We show that active focal adhesion kinase (FAK), an established marker of integrin-ECM downstream signalling, localizes with active integrins on endosomes. Integrin endocytosis positively regulates adhesion-induced FAK activation, which is early endosome antigen-1 and small GTPase Rab21 dependent. FAK binds directly to purified endosomes and becomes activated on them, suggesting a role for endocytosis in enhancing distinct integrin downstream signalling events. Finally, endosomal integrin signalling contributes to cancer-related processes such as anoikis resistance, anchorage independence and   metastasis  . 
 Integrative analyses identify osteopontin, LAMB3 and ITGB1 as critical pro-metastatic genes for lung cancer. To explore the key regulatory genes associated with lung cancer in order to reduce its occurrence and progress through silencing these key genes.To identify the key regulatory genes involved in lung cancer, we performed a combination of gene array and bioinformatics analyses to compare gene transcription profiles in 3 monoclonal cell strains with high, medium or low metastatic abilities, which were separated from the SPC-A-1sci and SPC-A-1 cell lines by limiting dilution monoclone assay. We then analyzed those genes' biological activities by knocking down their expression in SPC-A-1sci cells using siRNA and lenti-viral shRNA vectors, followed by determinations of the invasion and migration capabilities of the resulting cell lines in vitro as well as their potential for inducing occurrence and   metastasis   of lung cancer in vivo. To examine the clinical relevance of these findings, we analyzed the expression levels of the identified genes in human lung cancer tissues (n = 135) and matched adjacent normal tissues by immunohistochemical (IHC) staining.Three monoclonal cell strains characterized with high, medium or low metastatic abilities were successfully selected. Gene array and bioinformatics analyses implied that osteopontin, LAMB3 and ITGB1 were key genes involved in lung cancer. Knockdown of these genes suppressed human lung cancer cell invasion   and metast  asis in vitro and in vivo. Clinical sample analyses indicated that osteopontin, LAMB3 and ITGB1 protein expression levels were higher in lung cancer patients, compared to non-cancerous adjacent tissues, and correlated with lympha  tic metast  asis.We confirmed that osteopontin, LAMB3 and ITGB1 played important roles in the occurrence   and metast  asis of lung cancer, thus provided important clues to understanding the molecular mechanism   of metast  asis and contributing to the therapeutic treatment of lung cancer. 
 Metastatic properties of prostate cancer cells are controlled by VEGF. Mechanisms of   metastasis  , the major complication of prostate cancer, are poorly understood. In this study, we define molecular mechanisms that may contribute to the highly invasive potential of prostate cancer cells. Vascular endothelial growth factor (VEGF), its receptors (VEGFRs), and alpha5beta1 integrin were expressed by prostate cancer cells in vitro and by prostate tumors in vivo, and their expression was elevated at sites of bone   metastasis   compared to original prostate tumor. VEGF, through interaction with its receptors, regulated adhesive and migratory properties of the cancer cells. Specifically, the highly metastatic prostate cancer cell subline LNCaP-C4-2 showed a decreased adhesive but an enhanced migratory response to fibronectin, a ligand for alpha5beta1 integrin, compared to its nonmetastatic counterpart. A similar pattern was also observed when bone sialoprotein was used as a ligand in migration assays. Increased migration of metastatic prostate cancer cells to fibronectin and bone sialoprotein was regulated by VEGF via VEGFR-2. Tumor suppressor PTEN was involved in control of VEGF/VEGFR-2 stimulated prostate cancer cell adhesion as well as proliferation. 
 Integrin alpha4beta1 signaling is required for lymphangiogenesis and tumor   metastasis  . Recent studies have shown that lymphangiogenesis or the growth of lymphatic vessels at the periphery of tumors promotes tumor   metastasis   to lymph nodes. We show here that the fibronectin-binding integrin alpha4beta1 and its ligand fibronectin are novel functional markers of proliferative lymphatic endothelium. Tumors and lymphangiogenic growth factors, such as vascular endothelial growth factor-C (VEGF-C) and VEGF-A, induce lymphatic vessel expression of integrin alpha4beta1. Integrin alpha4beta1 then promotes growth factor and tumor-induced lymphangiogenesis, as genetic loss of integrin alpha4beta1 expression in Tie2Cre+ alpha4(loxp/loxp) mice or genetic loss of alpha4 signaling in alpha4Y991A knock-in mice blocks growth factor and tumor-induced lymphangiogenesis, as well as tumor   metastasis   to lymph nodes. In addition, antagonists of integrin alpha4beta1 suppress lymphangiogenesis and tumor   metastasis  . Our studies show that integrin alpha4beta1 and the signals it transduces regulate the adhesion, migration, invasion, and survival of proliferating lymphatic endothelial cells. As suppression of alpha4beta1 expression, signal transduction, or function in tumor lymphatic endothelium not only inhibits tumor lymphangiogenesis but also prevents metastatic disease, these results show that integrin alpha4beta1-mediated tumor lymphangiogenesis promotes   metastasis   and is a useful target for the suppression of metastatic disease. 
 Increased expression of α5β1-integrin is a prognostic marker for patients with gastric cancer. The study was to evaluate the association of expression level of α5β1-integrin with clinicopathologic features and prognosis in gastric cancer (GC).The expression of α5β1-integrin in normal gastric mucosa and GC tissue was detected with immunohistochemistry. The level of α5 and β1 mRNA in GC tissues and non-neoplastic tissues was evaluated in 48 paired cases by quantitative real-time polymerase chain reaction (qRT-PCR). Survival analysis by the Kaplan-Meier method was performed to assess prognostic significance.The α5β1-integrin expression was detected in 68.3 % (127/186) GC samples, and there was a significant difference on their positive expression rate between GC tissue and normal gastric mucosa (P < 0.001). The positive expression rate of α5β1-integrin in patients with poor histologic differentiation (P = 0.001),   lymph nod  e metastasis (P < 0.001), and recurrence (P < 0.001) group was heightened. Using Kaplan-Meier analysis, a comparison of survival curves of low versus high expresser of α5β1-integrin revealed a highly significant difference in human GC tissue (P = 0.002), which suggested that overexpression of α5β1-integrin is associated with a worse prognosis. Multivariate analyses showed that α5β1-integrin expression was independent risk factor predicting overall survival [Hazard ratio (HR) 1.594, 95 % confidence interval (CI) 1.236-2.408, P = 0.006] and disease-free survival [HR 3.952, 95 % CI 1.676-9.861, P = 0.003] in GC.The α5β1-integrin promotes angiogenesis and associa  tes with l  ymph node metastasis, vascular invasion and poor prognosis of GC. The current study shows that α5β1-integrin may be an independent prognostic factor for GC patients. 
 Tumor cell alpha3beta1 integrin and vascular laminin-5 mediate pulmonary arrest and   metastasis  . Arrest of circulating tumor cells in distant organs is required for hematogenous   metastasis  , but the tumor cell surface molecules responsible have not been identified. Here, we show that the tumor cell alpha3beta1 integrin makes an important contribution to arrest in the lung and to early colony formation. These analyses indicated that pulmonary arrest does not occur merely due to size restriction, and raised the question of how the tumor cell alpha3beta1 integrin contacts its best-defined ligand, laminin (LN)-5, a basement membrane (BM) component. Further analyses revealed that LN-5 is available to the tumor cell in preexisting patches of exposed BM in the pulmonary vasculature. The early arrest of tumor cells in the pulmonary vasculature through interaction of alpha3beta1 integrin with LN-5 in exposed BM provides both a molecular and a structural basis for cell arrest during pulmonary   metastasis  . 
 Ionizing radiation induces up-regulation of functional beta1-integrin in human lung tumour cell lines in vitro. Cell-matrix interactions are in part mediated through the beta1-integrin pathway regulating cell survival, proliferation, adhesion and migration. This study was performed to elucidate alterations of expression of the beta1-integrin and its co-localized protein kinase, integrin-linked kinase (ILK), after exposure to ionizing radiation in two lung carcinoma cell lines in the presence or absence of different beta1-integrin-dependent matrix proteins.Exponentially growing A549 and SKMES1 cells grown on fibronectin, laminin, BSA or plastic were exposed to 2 Gy or 6 Gy. Besides colony formation assays (0.5-8 Gy) and immediate plating experiments, flow cytometry (for beta1-integrin) and immunoblotting (for beta1-integrin and ILK) were carried out to analyze the protein expression. The localization of both proteins plus filamentous (f-) actin was further examined by immunofluorescence staining and laser confocal scanning microscopy. Functionality of the beta1 receptor subunit after irradiation was investigated in adhesion assays.A549 and SKMES1 cells grown on fibronectin or laminin demonstrated a significant increase in cell survival after irradiation compared to cells grown on BSA or plastic. Immediate plating of cells after irradiation on fibronectin did not show an improved survival. Flow cytometric and Western blot data showed a dose- and matrix-dependent induction of beta1-integrin and ILK expression after irradiation within 48 h. Adhesion to fibronectin or laminin compared to BSA or plastic was increased by 10-fold after irradiation, demonstrating these specific cell surface receptors to be functional. The staining of beta1-integrin and ILK in A549 cells confirmed the radiation-induced up-regulation of both proteins. Additionally, beta1-integrin and ILK co-localized with accumulated actin fibers at the cytoplasmic face of the cell membrane at confined areas.Ionizing radiation strongly induced the expression of functional beta1-integrin and ILK in the two lung cancer cell lines, A549 and SKMES1, dependent on different matrices used. Additionally, the subcellular localization of both proteins was altered by irradiation, and the individual cellular radiosensitivity was reduced in the presence of an extracellular matrix. On the one hand, this may result in aggravated therapeutic approaches and on the other hand, cells could adhere more strongly in their environment by the increase in functional surface receptor density preventing   metastasis  . Concerning intravascular located tumour cells, beta1-integrin up-regulation might enable these cells to adhere to the endothelium, which represents a prerequisite for metastatic disease. Identification of such mechanisms will provide considerable insights into the understanding of tumorigenicity and metastatic phenotypes, possibly leading to new, optimized radiochemotherapeutic regimens. 
 HPW-RX40 restores anoikis sensitivity of human breast cancer cells by inhibiting integrin/FAK signaling. Anoikis is defined as apoptosis, which is induced by inappropriate cell-matrix interactions. Cancer cells with anoikis resistance tend to undergo   metastasis  , and this phenomenon has been reported to be associated with integrin and FAK activity. HPW-RX40 is a derivative of 3,4-methylenedioxy-β-nitrostyrene, which is known to prevent platelet aggregation by inhibition of integrin. In the present study, we investigated the effect of HPW-RX40 on an anoikis-resistant human breast cancer cell line MDA-MB-231. HPW-RX40 inhibited cell aggregation and induced cell death in suspending MDA-MB-231 cells, but had only little effect on the monolayer growth of adherent cells. Analysis of caspase activation and poly (ADP-ribose) polymerase (PARP) cleavage confirmed anoikis in HPW-RX40-treated suspending cancer cells. HPW-RX40 also affected the Bcl-2 family proteins in detached cancer cells. Furthermore, HPW-RX40 inhibited detachment-induced activation of FAK and the downstream phosphorylation of Src and paxillin, but did not affect this pathway in adherent cancer cells. We also found that the expression and activation of β1 integrin in MDA-MB-231 cells were reduced by HPW-RX40. The combination of HPW-RX40 with an EGFR inhibitor led to enhanced anoikis and inhibition of the FAK pathway in breast cancer cells. Taken together, our results suggest that HPW-RX40 restores the anoikis sensitivity in the metastatic breast cancer cells by inhibiting integrin and subsequent FAK activation, and reveal a potential strategy for prevention of tumo  r metastas  is. 
 Down-regulation of epithelial cadherin is required to initiate metastatic outgrowth of breast cancer. Reduced epithelial cadherin (E-cad) is a hallmark of invasive carcinomas that have acquired epithelial-mesenchymal transition (EMT) phenotypes. Here we show that down-regulated E-cad expression induced by transforming growth factor-β (TGF-β) and EMT preceded breast cancer outgrowth in three-dimensional (3D) organotypic assays and in the lungs of mice. Pharmacological inhibitors against focal adhesion kinase prevented metastatic outgrowth of newly seeded organoids, but not that of their fully established counterparts. Interrogating the D2-HAN (hyperplastic alveolar nodule) model of breast cancer dormancy an  d metastas  is showed that dormant D2.OR cells produced branched organoid morphologies in 3D-cultures, and expressed robust quantities of E-cad that was uncoupled from regulation by TGF-β. In contrast, metastatic D2.A1 organoids were spherical and wholly lacked E-cad expression. Interestingly, D2.A1 cells engineered to re-express E-cad formed branched organoids, down-regulated β1 integrin expression, and failed to undergo metastatic outgrowth. The tumor-suppressing function of E-cad was inactivated by increased microenvironmental rigidity, and was not recapitulated by expression of an E-cad mutant lacking its extracellular domain. Twist expression, but not that of Snail, reinitiated metastatic outgrowth in dormant D2.OR cells. Our findings show that EMT and its down-regulated expression of E-cad circumvent breast cancer dormancy in part by facilitating β1 integrin expression necessary for metastatic outgrowth. 
 [Effect of Integrin α5β1-mediated ERK signal pathway on proliferation and migration of A549 cells]. Recent studies have shown that integrin α5β1 as a core in the integrin family plays an important role   in metast  asis, invasion and poor difference of non-small cell lung cancer. In this study, A549 cells were cultured and treated with integrin α5β1 small interfering RNA (siRNA) and extracelluar signal-regulated protein kinase (ERK) inhibitor PD98059 to investigate the effect of integrin α5β1 on proliferation and migration of A549 cells and explore its signal transduction mechanism.A549 cells were divided into four groups: Untransfection, Lipofectamine, Integrin α5β1 siRNA-transfected group and PD98059 group. The protein expression levels of integrin α5β1 were detected by Western blot analysis and the expression levels of integrin α5β1 mRNA was measured by reverse transcription-polymerase chain reaction (RT-PCR). The protein expression level of ERK1/2, MMP-9 and caspase-3 were measured by Western blot analysis. The proliferation and apoptosis of A549 cells were measured by MTT assay and Annexin-V FITC PI double staining.Integrin α5β1 siRNA could inhibit the phosphorylated ratio of ERK by down-regulate the expression of ERK 1/2 proteins. In addition, integrin α5β1 siRNA or PD98059 could inhibit the proliferation of A549 cells, induce the apoptosis of A549 cells, up-regulate the expression of caspase-3 and down-regulate the expression of MMP-9.Integrin α5β1 might involves the abnormal proliferation and migration of A549 cells through mediating ERK signal transduction pathway. 
 Interleukin-1alpha enhances integrin alpha(6)beta(1) expression and metastatic capability of human pancreatic cancer. To investigate the mechanisms of   metastasis   formation in metastatic human pancreatic cancer, we examined the enhancement in integrin expression, and adherence to and invasiveness into extracellular matrix (ECM) proteins of human pancreatic cancer cells after exposure to interleukin (IL)-1alpha.Expression of IL-1 receptor type I (IL-1RI) and alterations in integrin subunits by IL-1alpha were examined by flow-cytometric analysis and by cellular enzyme-linked immunosorbent assay in four human pancreatic cancer cell lines (BxPC-3, PaCa-2, PANC-1, and SW1990), respectively. In addition, assays of cancer cell adhesion and invasion to ECM proteins were performed to investigate whether increased integrin expression affected the adhesive and invasive interaction between cancer cells and the putative integrin ECM ligands. Furthermore, immunohistochemistry was used to assess integrins and IL-1R1 expression in pancreatic tissues.In metastatic cancer cells, expression of the alpha(6) subunit was enhanced by IL-1alpha treatment. While metastatic cancer cells exhibited preferential adherence to and invasion into laminin, these properties were enhanced by IL-1alpha. The alpha(6) subunit and IL-1RI were strongly expressed in pancreatic tissues from pancreatic cancer patients with liver   metastasis  .In pancreatic cancer, IL-1alpha enhanced alpha(6)beta(1)-integrin expression, probably via increased IL-1RI levels. Our results indicated that alpha(6)beta(1)-integrin and IL-1RI expression may play important roles in   metastasis   formation. 
 CXCL12/CXCR4 activation by cancer-associated fibroblasts promotes integrin β1 clustering and invasiveness in gastric cancer. Cancer-associated fibroblasts (CAFs) are reportedly involved in invasion and   metastasi  s in several types of cancer, including gastric cancer (GC), through the stimulation of CXCL12/CXCR4 signaling. However, the mechanisms underlying these tumor-promoting effects are not well understood, which limits the potential to develop therapeutic targets against CAF-mediated CXCL12/CXCR4 signaling. CXCL12 expression was analyzed in resected GC tissues from 110 patients by immunohistochemistry (IHC). We established primary cultures of normal fibroblasts (NFs) and CAFs from the GC tissues and examined the functional differences between these primary fibroblasts using co-culture assays with GC cell lines. We evaluated the efficacy of a CXCR4 antagonist (AMD3100) and a FAK inhibitor (PF-573,228) on the invasive ability of GC cells. High CXCL12 expression levels were significantly associated with larger tumor size, increased tumor depth, lymphatic invasion and poor prognosis in GC. CXCL12/CXCR4 activation by CAFs mediated integrin β1 clustering at the cell surface and promoted the invasive ability of GC cells. Notably, AMD3100 was more efficient than PF-573,228 at inhibiting GC cell invasion through the suppression of integrin β1/FAK signaling. These results suggest that CXCL12 derived from CAFs promotes GC cell invasion by enhancing the clustering of integrin β1 in GC cells, resulting in GC progression. Taken together, the inhibition of CXCL12/CXCR4 signaling in GC cells may be a promising therapeutic strategy against GC cell invasion. 
 Amphiregulin enhances alpha6beta1 integrin expression and cell motility in human chondrosarcoma cells through Ras/Raf/MEK/ERK/AP-1 pathway. Chondrosarcoma is a malignant tumor that produces cartilage matrix. The most lethal aspect is its metastatic property. We demonstrated that amphiregulin (AR) is significantly upregulated in highly aggressive cells. AR silencing markedly suppressed cell migration. Exogenous AR markedly increased cell migration by transactivation of α6β1 integrin expression. A neutralizing α6β1 integrin antibody can abolish AR-induced cell motility. Knockdown of AR inhib  its metast  asis of cells to the lung in vivo. Furthermore, elevated AR expression is positively correlated with α6β1 integrin levels and higher grades in patients. These findings can potentially serve as biomarker and therapeutic approach for controlling chondrosa  rcoma meta  stasis. 
 [Expression of E-cadherin and beta1-integrin mRNA in endometrial cancer]. The metastatic ability of tumors is characteristic for malignant neoplasms and constitutes the main cause of therapeutics failures.   Metastasis   formation involves the sequence of processes such as proteolytic degradation of the basement membrane, migration, intravasation, extravasation, proliferation and angiogenesis. Cadherins and integrins are groups of proteins directly involved in these processes. In the present study we analyzed the mRNA expression of CDH1 and ITGB1 genes by real-time polymerase chain reaction (RT-PCR). The study included 106 endometrial carcinomas. CDH1 and ITGB1 mRNA expression was found in all of the studied samples. Generally the CDH1 and ITGB1 mRNA expression was significantly higher in well-differentiated rather than poorly differentiated tumors.The mRNA expression levels of CDH1 and ITGB1 in series of 83 samples of endometrial carcinoma were studied by real time RT-PCR method. Statistical analysis of the obtained results was performed.CDH1 and ITGB1 gene expression was observed in all examined tissues and was correlated with cancer malignancy (G). In high grade malignant tumors (G1), CDH1 and ITGB1 gene expression was the highest, in G2 and G3 tumors the expression of both genes was gradually lowering. Moreover the statistically significant correlation between CDH1 and ITGB1 gene expression was observed. (Spearman test, r=0.29, p<O.01).CDH1 and ITGB1 mRNA expression seems to be an important marker of cancer progression and metastases in endometrial malignant tumors. 
 C1GALT1 promotes invasive phenotypes of hepatocellular carcinoma cells by modulating integrin β1 glycosylation and activity. Cancer cell invasion and   metastasi  s are the primary causes of treatment failure and death in hepatocellular carcinoma (HCC). We previously reported that core 1 β1,3-galactosyltransferase (C1GALT1) is frequently overexpressed in HCC tumors and its expression is associated with advanced tumor stage  , metastas  is, and poor survival. However, the underlying mechanisms of C1GALT1 in HCC malignancy remain unclear. In this study, we found that overexpression of C1GALT1 enhanced HCC cell adhesion to extracellular matrix (ECM) proteins, migration, and invasion, whereas RNAi-mediated knockdown of C1GALT1 suppressed these phenotypes. The promoting effect of C1GALT1 on th  e metastas  is of HCC cells was demonstrated in a mouse xenograft model. Mechanistic investigations showed that the C1GALT1-enhanced phenotypic changes in HCC cells were significantly suppressed by anti-integrin β1 blocking antibody. Moreover, C1GALT1 was able to modify O-glycans on integrin β1 and regulate integrin β1 activity as well as its downstream signaling. These results suggest that C1GALT1 could enhance HCC invasiveness through integrin β1 and provide novel insights into the roles of O-glycosylation i  n HCC meta  stasis. 
 Integrin alpha2beta 1 (α2β1) promotes prostate cancer skeleta  l metastas  is. Men who die of prostate cancer (PCa) do so because of systemic metastases, the most frequent of which are within the skeleton. Recent data suggest that the colonization of the skeleton is mediated in part by collagen type I, the most abundant protein within the bone. We have shown that enhanced collagen I binding through increased expression of integrin α2β1 stimulated in vitro invasion and promoted the growth of PCa cells within the bone. Accordingly, we sought to determine whether α2β1 integrin is a potential mediator of ske  letal meta  stasis. To examine whether α2β1 integrin media  tes PCa me  tastasis, α2 integrin was over-expressed in low-tumorigenic LNCaP PCa cells or selectively knocked-down in highly metastatic LNCaPcol PCa cells. We document that the over-expression of α2 cDNA stimulated whereas α2 shRNA inhibited the ability of transduced cells to bind to or migrate towards collagen in vitro. Correspondingly, α2 integrin knock-down reduced the tumor burden of intra-osseous tumors compared to control-transduced cells. To investigate the clinical significance of α2β1 expression in PCa, α2β1 protein was measured in prostatic tissues and in soft tissue and bone metastases. The data demonstrate that α2β1 protein was elevated in PCa skeletal metastases compared to either PCa primary lesions or soft tissue metastases suggesting that α2β1 contribut  es to the   selective metastasis to the bone. Taken together, these data support that α2β1 integrin is nee  ded for th  e efficient metastasis of PCa cells to the skeleton. 
 Significance of the expression of integrin β1, VEGF and MVD in hypopharyngeal squamous cell carcinoma. This study aimed to determine the expression of integrin β1 and vascular endothelial growth factor (VEGF) and microvascular density (MVD) by CD105 staining in hypopharyngeal squamous cell carcinoma to determine their association with clinicopathologic characteristics, and to determine their role and the effects of their interactions in the development and progression of hypopharyngeal squamous cell carcinomas. The expression of integrin β1 and VEGF and MVD in hypopharyngeal squamous cell carcinomas and normal hypopharyngeal tissues were evaluated using immunohistochemistry. The Image-Pro Plus software was used to determine the mean optical density of the immunohistochemical images. Integrin β1 expression was significantly higher in hypopharyngeal squamous cell carcinoma tissues (78.00%) than in normal hypopharyngeal tissues (35.00%; P = 0.001) and significantly differed across pathologic grades and different T stages, and regarding the presence of cervical lymph n  ode metast  asis (P < 0.05). VEGF expression was significantly higher in hypopharyngeal squamous cell carcinoma tissues (74.00%) than in normal hypopharyngeal tissues (30.00%; P = 0.002), VEGF overexpression differed significantly across different pathologic grades and different T stages, and regarding the presence of cervical lymph n  ode metast  asis (P < 0.05). The MVD count was significantly higher in hypopharyngeal squamous cell carcinoma tissues (37.10 ± 5.95) than in normal hypopharyngeal tissues (8.70 ± 3.34; P = 0.000). MVD differed significantly across different pathologic grades and different T stages, and regarding the presence of cervical lymph   node meta  stasis (P < 0.05). The expression of integrin β1 and VEGF and the MVD count exhibited no significant differences in terms of age, gender, history of smoking, and clinical stages (P > 0.05). VEGF expression was positively associated with the MVD count of hypopharyngeal squamous cell carcinomas (r = 0.582, P = 0.000); however, integrin β1 was not associated with VEGF or MVD (P > 0.05). Integrin β1 and VEGF are overexpressed and MVD increased in hypopharyngeal squamous cell carcinomas. VEGF is positively correlated with MVD. 
 Talin1 phosphorylation activates β1 integrins: a novel mechanism to promote prostate cancer bone   metastasi  s. Talins are adaptor proteins that regulate focal adhesion signaling by conjugating integrins to the cytoskeleton. Talins directly bind integrins and are essential for integrin activation. We previously showed that β1 integrins are activated in metastatic prostate cancer (PCa) cells, increasing PC  a metastas  is to lymph nodes and bone. However, how β1 integrins are activated in PCa cells is unknown. In this study, we identified a novel mechanism of β1 integrin activation. Using knockdown experiments, we first demonstrated that talin1, but not talin2, is important in β1 integrin activation. We next showed that talin1 S425 phosphorylation, but not total talin1 expression, correlates with metastatic potential of PCa cells. Expressing a non-phosphorylatable mutant, talin1(S425A), in talin1-silenced PC3-MM2 and C4-2B4 PCa cells, decreased activation of β1 integrins, integrin-mediated adhesion, motility and increased the sensitivity of the cells to anoikis. In contrast, reexpression of the phosphorylation-mimicking mutant talin1(S425D) led to increased β1 integrin activation and generated biologic effects opposite to talin1(S425A) expression. In the highly metastatic PC3-MM2 cells, expression of a non-phosphorylatable mutant, talin1(S425A), in talin1-silenced PC3-MM2 cells, abolished their ability to colonize in the bone following intracardiac injection, while reexpression of phosphorylation-mimicking mutant talin1(S425D) restored their ability to metastasize to bone. Immunohistochemical staining demonstrated that talin S425 phosphorylation is significantly increased in human bone metastases when compared with normal tissues, primary tumors or lymph node metastases. We further showed that p35 expression, an activator of Cdk5, and Cdk5 activity were increased in metastatic tumor cells, and that Cdk5 kinase activity is responsible for talin1 phosphorylation and subsequent β1 integrin activation. Together, our study reveals Cdk5-mediated phosphorylation of talin1 leading to β1 integrin activation is a novel mechanism that increases metastatic potential of PCa cells. 
 SERPINA5 inhibits tumor cell migration by modulating the fibronectin-integrin β1 signaling pathway in hepatocellular carcinoma. In our previous study, we identified 1241 loci with somatic copy number alterations in human hepatocellular carcinoma (HCC) using Affymetrix SNP 6.0 arrays, and a putative cancer gene SERPINA5 was uncovered in a novel chromosomal region with recurrent copy number loss at 14q31.1-32.13. The SERPINA5 was reported to be deregulated in renal, breast, prostate and ovarian cancers. However, the roles of SERPINA5 in cancer remain greatly elusive. In this study, we found that the DNA dosage and expression level of the SERPINA5 gene were significantly decreased in HCC by quantitative real-time PCR. Notably, the expression levels of SERPINA5 negatively correlated with malignant progression of HCC. The SERPINA5 gene was further observed to reduce in vitro and in vivo metastatic potential of HCC cells. Moreover, secreted SERPINA5 protein also could inhibit the metastatic ability of HCC cells. Finally, we discovered that one of the mechanisms explaining SERPINA5 inhibition of HCC   metastasi  s is through direct interaction with fibronectin and disruption of the fibronectin-integrin signaling pathway. These findings highlight an important role of SERPINA5 in the regulation of migratory and metastatic potentials of HCC and suggest a potential application of SERPINA5 in cancer treatment. 
 [The expression and significance of integrin beta1 and focal adhesion kinase and its clinical value in laryngeal carcinoma]. To study the expressions and clinical significance of integrin beta1 and focal adhesion kinase (FAK) in laryngeal carcinoma (LSCC).The mRNA and protein levels of integrin beta1 and focal adhesion kinase and the surrounding tissue of laryngeal carcinoma in 48 specimens and 20 specimens of vocal cord polyp were detected by reverse transcription-polymerase chain reaction (RT-PCR) and immunohistochemistry SABC method.The mRNA levels and positive rates of integrin beta1 and FAK protein were significantly higher in laryngeal carcinoma than that in the surrounding tissue and vocal cord polyp (P < 0.05). The expression levels of integrin beta1 and FAK were significantly higher in the group with cervical lymph node   metastasis   than those without (P < 0.05), and they were significantly higher in the tissue of stage of T3 and T4 than those of T1 and T2 (P < 0.05). But pathological grades was not significantly related with the expression levels of integrin beta1 or FAK (P > 0.05).The expression levels of integrin beta1 and FAK were increased in LSCC, and they may contribute significantly to invasion and   metastasis   of LSCC. 
 The secreted protein ANGPTL2 promotes   metastasis   of osteosarcoma cells through integrin α5β1, p38 MAPK, and matrix metalloproteinases. The tumor microenvironment can enhance the invasive capacity of tumor cells. We showed that expression of angiopoietin-like protein 2 (ANGPTL2) in osteosarcoma (OS) cell lines increased and the methylation of its promoter decreased with time when grown as xenografts in mice compared with culture. Compared with cells grown in normal culture conditions, the expression of genes encoding DNA demethylation-related enzymes increased in tumor cells implanted into mice or grown in hypoxic, serum-starved culture conditions. ANGPTL2 expression in OS cell lines correlated with increased tumo  r metastas  is and decreased animal survival by promoting tumor cell intravasation mediated by the integrin α5β1, p38 mitogen-activated protein kinase, and matrix metalloproteinases. The tolloid-like 1 (TLL1) protease cleaved ANGPTL2 into fragments in vitro that did not enhance tumor progression when overexpressed in xenografts. Expression of TLL1 was weak in OS patient tumors, suggesting that ANGPTL2 may not be efficiently cleaved upon secretion from OS cells. These findings demonstrate that preventing ANGPTL2 signaling stimulated by the tumor microenvironment could inhibit tumor cell migration   and metast  asis. 
 β-1,4-Galactosyltransferase III suppresses β1 integrin-mediated invasive phenotypes and negatively correlates wit  h metastas  is in colorectal cancer  . Metastas  is often occurs in colorectal cancer (CRC) patients and is the main difficulty in cancer treatment. The upregulation of poly-N-acetyllactosamine-related glycosylation is found in CRC patients and is associated with progression an  d metastas  is in cancer. β-1,4-Galactosyltransferase III (B4GALT3) is an enzyme responsible for poly-N-acetyllactosamine synthesis, and therefore, we investigated its expression in CRC patients. We found that B4GALT3 negatively correlated with poorly differentiated histology (P < 0.001), advanced stages (P = 0.0052), regional lymph no  de metasta  sis (P = 0.0018) and dista  nt metasta  sis (P = 0.0463) in CRC patients. B4GALT3 overexpression in CRC cells suppressed cell migration, invasion and adhesion, whereas B4GALT3 knockdown enhanced malignant cell phenotypes. The β1 integrin-blocking antibody reversed the B4GALT3-mediated increase in cell invasion. B4GALT3 expression altered glycosylation on the N-glycan of β1 integrin probably through changes in poly-N-acetyllactosamine expression. Furthermore, more activated β1 integrin along with the activation of its downstream signaling transduction were found in B4GALT3 knockdown cells, whereas overexpression of B4GALT3 suppressed the expression of active β1 integrin and inhibited its downstream signaling. Our results suggest that B4GALT3 is negatively associated wi  th CRC met  astasis and suppresses cell invasiveness through inhibiting activation of β1 integrin. 
 Lebectin and lebecetin, two C-type lectins from snake venom, inhibit alpha5beta1 and alphaV-containing integrins. Integrins are essential protagonists in the complex multistep process of cancer progression and   metastasis  . We recently reported that lebectin, a novel C-type lectin from Macrovipera lebetina venom, displays an anti-integrin activity. In this study, we extend this observation to lebecetin, a second C-type lectin isolated from the same venom and previously reported as a potent inhibitor of platelet aggregation. Both venom lectins appear to exert their effect on cell adhesion, migration, invasion and proliferation by inhibiting alpha5beta1 and alphav-containing integrins. Moreover, the inhibition of alpha5beta1 and alphav integrins is likely due to the binding of venom peptides, as both lebectin and lebecetin co-immunoprecipitate with these integrins. Lebectin and lebecetin are thus the first examples of venom C-type lectins inhibiting an integrin other than the collagen receptor alpha2beta1. 
 Calreticulin activates β1 integrin via fucosylation by fucosyltransferase 1 in J82 human bladder cancer cells. Fucosylation regulates various pathological events in cells. We reported that different levels of CRT (calreticulin) affect the cell adhesion and   metastasi  s of bladder cancer. However, the precise mechanism of tumour   metastasi  s regulated by CRT remains unclear. Using a DNA array, we identified FUT1 (fucosyltransferase 1) as a gene regulated by CRT expression levels. CRT regulated cell adhesion through α1,2-linked fucosylation of β1 integrin and this modification was catalysed by FUT1. To clarify the roles for FUT1 in bladder cancer, we transfected the human FUT1 gene into CRT-RNAi stable cell lines. FUT1 overexpression in CRT-RNAi cells resulted in increased levels of β1 integrin fucosylation and rescued cell adhesion to type-I collagen. Treatment with UEA-1 (Ulex europaeus agglutinin-1), a lectin that recognizes FUT1-modified glycosylation structures, did not affect cell adhesion. In contrast, a FUT1-specific fucosidase diminished the activation of β1 integrin. These results indicated that α1,2-fucosylation of β1 integrin was not involved in integrin-collagen interaction, but promoted β1 integrin activation. Moreover, we demonstrated that CRT regulated FUT1 mRNA degradation at the 3'-UTR. In conclusion, the results of the present study suggest that CRT stabilized FUT1 mRNA, thereby leading to an increase in fucosylation of β1 integrin. Furthermore, increased fucosylation levels activate β1 integrin, rather than directly modifying the integrin-binding sites. 
 The α₂β₁ integrin   is a meta  stasis suppressor in mouse models and human cancer. Integrins regulate cell-cell and cell-matrix adhesion and thereby play critical roles in tumor progressio  n and meta  stasis. Although work in preclinical models suggests that β1 integrins may sti  mulate met  astasis of a number of cancers, expression of the β1 subunit alone has not been shown to be a useful prognostic indicator in human cancer patients. Here we have demonstrated that the α2β1 integrin s  uppresses   metastasis in a clinically relevant spontaneous mouse model of breast cancer. These data are consistent with previous studies indicating high expression of α2β1 integrin in normal breast epithelium and loss of α2β1 in poorly differentiated breast cancer. They are also consistent with our systematic analysis of microarray databases of human breast and prostate cancer, which revealed that decreased expression of the gene encoding α2 integrin, but not genes encoding α1, α3, or β1 integrin, was predictive of metastatic dissemination and decreased survival. The predictive value of α2 expression persisted within both good-risk and poor-risk cohorts defined by estrogen receptor and lymph node status. Thus, the α2β1 integrin functionally i  nhibits br  east tumor metastasis, and α2 expression may serve as an important biomarker of metastatic potential and patient survival. 
 Expression of beta1-integrins and N-cadherin in bladder cancer and melanoma cell lines. Changes in the expression of integrins and cadherins might contribute to the progression, invasion and   metastasis   of transitional cell cancer of the bladder and of melanomas. The expression of alpha5 (P < 0.001), alpha2 and beta1 (P < 0.05 - P < 0.001) integrin subunits in melanoma cells from noncutaneous metastatic sites (WM9, A375) were significantly increased as compared to cutaneous primary tumor (WM35) and metastatic (WM239) cell lines. These differences might be ascribed to the invasive character of melanoma cells and their   metastasis   to the noncutaneous locations. The significantly heterogeneous expression of beta1 integrin subunit in two malignant bladder cancer cell lines (T24 and Hu456) and nonsignificant differences in the expression of alpha2, alpha3, and alpha5 subunits between malignant and non-malignant human bladder cell lines do not allow an unanimous conclusion on the role of these intergrin subunits in the progression of transitional cancer of bladder. The adhesion molecule, expressed in all studied melanoma and bladder cell lines, that reacted with anti-Pan cadherin monoclonal antibodies was identified as N-cadherin except in the HCV29 non-malignant ureter cell line. However, neither this nor any other bladder or melanoma cell line expressed E-cadherin. The obtained results imply that the replacement of E-cadherin by N-cadherin accompanied by a simultaneous increase in expression of alpha2, alpha3 and alpha5 integrin subunits clearly indicates an increase of invasiveness of melanoma and, to a lesser extent, of transitional cell cancer of bladder. High expression of N-cadherin and alpha5 integrin subunit seems to be associated with the most invasive melanoma phenotype. 
 ß1 integrin binding phosphorylates ezrin at T567 to activate a lipid raft signalsome driving invadopodia activity and invasion. Extracellular matrix (ECM) degradation is a critical process in tumor cell invasion and requires matrix degrading protrusions called invadopodia. The Na(+)/H(+) exchanger (NHE1) has recently been shown to be fundamental in the regulation of invadopodia actin cytoskeleton dynamics and activity. However, the structural link between the invadopodia cytoskeleton and NHE1 is still unknown. A candidate could be ezrin, a linker between the NHE1 and the actin cytoskeleton known to play a pivotal role in invasion and   metastasi  s. However, the mechanistic basis for its role remains unknown. Here, we demonstrate that ezrin phosphorylated at T567 is highly overexpressed in the membrane of human breast tumors and positively associated with invasive growth and HER2 overexpression. Further, in the metastatic cell line, MDA-MB-231, p-ezrin was almost exclusively expressed in invadopodia lipid rafts where it co-localized in a functional complex with NHE1, EGFR, ß1-integrin and phosphorylated-NHERF1. Manipulation by mutation of ezrins T567 phosphorylation state and/or PIP2 binding capacity or of NHE1s binding to ezrin or PIP2 demonstrated that p-ezrin expression and binding to PIP2 are required for invadopodia-mediated ECM degradation and invasion and identified NHE1 as the membrane protein that p-ezrin regulates to induce invadopodia formation and activity. 
 hTERT mediates gastric cancer   metastasis   partially through the indirect targeting of ITGB1 by microRNA-29a. Human telomerase reverse transcriptase (hTERT) plays a key role in tumor invasion and   metastasis  , but the mechanism of its involvement in these processes is not clear. The purpose of this study is to investigate the possible molecular mechanism of hTERT in the promotion of gastric cancer (GC)   metastasis  . We found that the up-regulation of hTERT in gastric cancer cells could inhibit the expression of miR-29a and enhance the expression of Integrin β1 (ITGB1). In addition, the invasive capacity of gastric cancer cells was also highly increased after hTERT overexpression. Our study also found that the restoration of miR-29a suppressed the expression of ITGB1 and inhibited GC cell   metastasi  s both in vitro and in vivo. Taken together, our results suggested that hTERT may promote GC   metastasi  s through the hTERT-miR-29a-ITGB1 regulatory pathway. 
 Effect of KAI1/CD82 on the beta1 integrin maturation in highly migratory carcinoma cells. The KAI1/CD82 protein has been documented as the tumor   metastasis   suppressor in many types of human cancers. KAI1/CD82 regulates cell motility and invasiveness; however, the mechanism by which this occurs remains to be fully established. Several studies have shown that KAI1/CD82 modulates integrin-dependent signaling. It was suggested that KAI1/CD82 might function to attenuate the beta1 integrin function of inducing cellular migration. A wound-healing and modified Boyden chamber assays were performed to investigate the mechanism of the KAI1/CD82-mediated inhibition of cell migration. It was found that the migratory ability of H1299/CD82 was inhibited. The immunoblotting and biotinylation assays revealed that H1299/CD82 showed significantly decreased expression of the mature form of beta1, which was functional at the cell surface. It was confirmed that KAI1/CD82 regulates the maturation of the beta1 integrin using CD82-specific si-RNA. These results support a model in which KAI1/CD82 attenuates the maturation of the beta1 integrin precursor and thereby suppresses cell migration. 
 Colorectal Cancer Metastases Settle in the Hepatic Microenvironment Through α5β1 Integrin. Colorectal cancer (CRC  ) metastas  is dissemination to secondary sites represents the critical point for the patient's survival. The microenvironment is crucial to cancer progression, influencing tumour cell behaviour by modulating the expression and activation of molecules such as integrins, the cell-extracellular matrix interacting proteins participating in different steps of the tumour metastatic process. In this work, we investigated the role of α5β1 integrin and how the microenvironment influences this adhesion molecule, in a model of colon cancer progression to the liver. The culture medium conditioned by the IHH hepatic cell line, and the extracellular matrix (ECM) proteins, modulate the activation of α5β1 integrin in the colon cancer cell line HCT-116, and drives FAK phosphorylation during the process of cell adhesion to fibronectin, one of the main components of liver ECM. In these conditions, α5β1 modulates the expression/activity of another integrin, α2β1, involved in the cell adhesion to collagen I. These results suggest that α5β1 integrin holds a leading role in HCT-116 colorectal cancer cells adhesion to the ECM through the modulation of the intracellular focal adhesion kinase FAK and the α2β1 integrin activity. The driving role of the tumour microenvironment on CRC dissemination, here detected, and described, strengthens and adds new value to the concept that α5β1 integrin can be an appropriate and relevant therapeutic target for the control of CRC metastases. 
 Cyclooxygenase-2 enhances alpha2beta1 integrin expression and cell migration via EP1 dependent signaling pathway in human chondrosarcoma cells. Cyclooxygenase (COX)-2, the inducible isoform of prostaglandin (PG) synthase, has been implicated in tumor   metastasis  . Interaction of COX-2 with its specific EP receptors on the surface of cancer cells has been reported to induce cancer invasion. However, the effects of COX-2 on migration activity in human chondrosarcoma cells are mostly unknown. In this study, we examined whether COX-2 and EP interaction are involved in   metastasis   of human chondrosarcoma.We found that over-expression of COX-2 or exogenous PGE2 increased the migration of human chondrosarcoma cells. We also found that human chondrosarcoma tissues and chondrosarcoma cell lines had significant expression of the COX-2 which was higher than that in normal cartilage. By using pharmacological inhibitors or activators or genetic inhibition by the EP receptors, we discovered that the EP1 receptor but not other PGE receptors is involved in PGE2-mediated cell migration and alpha2beta1 integrin expression. Furthermore, we found that human chondrosarcoma tissues expressed a higher level of EP1 receptor than normal cartilage. PGE2-mediated migration and integrin up-regulation were attenuated by phospholipase C (PLC), protein kinase C (PKC) and c-Src inhibitor. Activation of the PLCbeta, PKCalpha, c-Src and NF-kappaB signaling pathway after PGE2 treatment was demonstrated, and PGE2-induced expression of integrin and migration activity were inhibited by the specific inhibitor, siRNA and mutants of PLC, PKC, c-Src and NF-kappaB cascades.Our results indicated that PGE2 enhances the migration of chondrosarcoma cells by increasing alpha2beta1 integrin expression through the EP1/PLC/PKCalpha/c-Src/NF-kappaB signal transduction pathway. 
 Melanoma cells produce multiple laminin isoforms and strongly migrate on α5 laminin(s) via several integrin receptors. Melanoma cells express and interact with laminins (LMs) and other basement membrane components during invasion and   metastasi  s. In the present study we have investigated the production and migration-promoting activity of laminin isoforms in melanoma. Immunohistochemistry of melanoma specimens and immunoprecipitation/western blotting of melanoma cell lines indicated expression of laminin-111/121, laminin-211, laminin-411/421, and laminin-511/521. Laminin-332 was not detected. In functional assays, laminin-111, laminin-332, and laminin-511, but not laminin-211 and laminin-411, strongly promoted haptotactic cell migration either constitutively or following stimulation with insulin-like growth factors. Both placenta and recombinant laminin-511 preparations were highly active, and the isolated recombinant IVa domain of LMα5 also promoted cell migration. Function-blocking antibodies in cell migration assays revealed α6β1 integrin as the major receptor for laminin-111, and both α3β1 and α6β1 integrins for laminin-332 and laminin-511. In contrast, isolated LMα5 IVa domain-promoted melanoma cell migration was largely mediated via αVβ3 integrin and inhibited by RGD peptides. Given the ubiquitous expression of α5 laminins in melanoma cells and in melanoma-target tissues/anatomical structures, as well as the strong migration-promoting activity of these laminin isoforms, the α5 laminins emerge as putative primary extracellular matrix mediators of melanoma   invasion a  nd metastasis via α3β1 and other integrin receptors. 
   Metastasis   suppressor KAI1/CD82 attenuates the matrix adhesion of human prostate cancer cells by suppressing fibronectin expression and β1 integrin activation. KAI1/CD82, a tetraspanin membrane protein functions as a   metastasi  s suppressor in many types of human cancers and has been shown to regulate cell adhesion properties. In the present study, we investigated the underlying mechanism of KAI1/CD82-mediated changes in cell adhesion to the extracellular matrix using human prostate cancer cells. We found that high KAI1/CD82 expression attenuated short-term cell adhesion to uncoated- or fibronectin-coated plates. Moreover, high KAI1/CD82 expression generated an extracellular environment unfavorable for cell adhesion as compared to low KAI1/CD82 expression, suggesting KAI1/CD82-dependent regulation of extracellular matrix (ECM) molecule(s) expression and/or secretion. Among ECM components examined, fibronectin exhibited decreased expression and secretion in high KAI1/CD82-expressing cells. Furthermore, high KAI1/CD82 expression interfered with the activation of β (1) integrin at the cell surface while total β (1) integrin levels remained unchanged, concomitant with reduced formation of focal adhesion complex and decreased bundling of actin filaments. Finally, high KAI1/CD82 expression significantly retarded cell motility in a scratch wound assay. Taken together, our results strongly suggest that KAI1/CD82 attenuates the activation of β (1) integrin, and thereby down-regulates outside-in signaling of β (1) integrin, leading to the reduction of focal adhesion formation and fibronectin expression/secretion, which subsequently interferes with cell adhesion properties and motility. 
 Site-directed perturbation of protein kinase C- integrin interaction blocks carcinoma cell chemotaxis. Polarized cell movement is an essential requisite for cancer   metastasis  ; thus, interference with the tumor cell motility machinery would significantly modify its metastatic behavior. Protein kinase C alpha (PKC alpha) has been implicated in the promotion of a migratory cell phenotype. We report that the phorbol ester-induced cell polarization and directional motility in breast carcinoma cells is determined by a 12-amino-acid motif (amino acids 313 to 325) within the PKC alpha V3 hinge domain. This motif is also required for a direct association between PKC alpha and beta 1 integrin. Efficient binding of beta 1 integrin to PKC alpha requires the presence of both NPXY motifs (Cyto-2 and Cyto-3) in the integrin distal cytoplasmic domains. A cell-permeant inhibitor based on the PKC-binding sequence of beta 1 integrin was shown to block both PKC alpha-driven and epidermal growth factor (EGF)-induced chemotaxis. When introduced as a minigene by retroviral transduction into human breast carcinoma cells, this inhibitor caused a striking reduction in chemotaxis towards an EGF gradient. Taken together, these findings identify a direct link between PKC alpha and beta 1 integrin that is critical for directed tumor cell migration. Importantly, our findings outline a new concept as to how carcinoma cell chemotaxis is enhanced and provide a conceptual basis for interfering with tumor cell dissemination. 
 A functionally relevant conformational epitope on the CD9 tetraspanin depends on the association with activated beta1 integrin. Tetraspanins associate on the cell membrane with several transmembrane proteins, including members of the integrin superfamily. The tetraspanin CD9 has been implicated in cell motility,   metastasis  , and sperm-egg fusion. In this study we characterize the first CD9 conformation-dependent epitope (detected by monoclonal antibody (mAb) PAINS-13) whose expression depends on changes in the activation state of associated beta(1) integrins. MAb PAINS-13 precipitates CD9 under conditions that preserve the association of this tetraspanin with integrins, but not under conditions that disrupt these interactions. Induction of activation of beta(1) integrins by temperature, divalent cation Mn(2+), or mAb TS2/16 correlated with enhanced expression of the PAINS-13 epitope on a variety of cells. Through the use of different K562 myeloid leukemia transfectant cells expressing specific members of the beta(1) integrin subfamily we show that the expression of the PAINS-13 epitope depends on CD9 association with alpha(6)beta(1) integrin. The mAb PAINS-13 reactivity has been mapped to the CD9 region comprising residues 112-154 in the NH(2) half of the large extracellular loop. Also, we show that the CD9 conformation recognized by mAb PAINS-13 is functionally relevant in beta(1) integrin-mediated cellular processes including wound healing migration, tubular morphogenesis, cell adhesion and spreading and in signal transduction involving phosphatidylinositol 3-kinase activation. 
 Osteoblasts-derived BMP-2 enhances the motility of prostate cancer cells via activation of integrins. Bone metastases are common complications of prostate cancer cells. The bone morphogenetic protein-2 (BMP-2) is constitutively secreted by osteoblasts and plays a key role in bone formation. Integrins are the major adhesive molecules in mammalian cells, and has been associated with cancer cells   metastasis   to bone. The aim of this study was to investigate whether osteoblast-derived BMP-2 is associated with prostate cancer   metastasis  .Cancer cells migration activity was examined using the Transwell assay. The ERK and AKT phosphorylation was examined by using Western blot method. The siRNA was used to inhibit the expression of BMP-2. The cell surface expression of integrins was examined by using flow cytometry. A transient transfection protocol was used to examine NF-kappaB activity.We found that osteoblast conditioned medium (OBCM) increased the migration and cell surface expression of beta1 or beta 3 integrin in human prostate cancer cells. beta1 or beta 3 integrin monoclonal antibodies or siRNA against beta1 or beta 3 integrin inhibited the OBCM-induced increase the migration of prostate cancer cells. BMP-2 siRNA specifically reduced the OBCM-induced migration and integrins upregulation. BMP-2 siRNA also suppressed the OBCM-induced ERK, AKT and NF-kappaB activation.This study suggest that the osteoblast-derived BMP-2 act through Akt and ERK, which in turn activates IKK alpha/beta and NF-kappaB, resulting in the activations of beta1 and beta 3 integrins and contributing the migration of prostate cancer cells. 
 Rab5c promotes AMAP1-PRKD2 complex formation to enhance β1 integrin recycling in EGF-induced cancer invasion. Epidermal growth factor receptor (EGFR) signaling is one of the crucial factors in breast cancer malignancy. Breast cancer cells often overexpress Arf6 and its effector, AMAP1/ASAP1/DDEF1; in these cells, EGFR signaling may activate the Arf6 pathway to induce invasion and   metastasi  s. Active recycling of some integrins is crucial for invasion and   metastasi  s. Here, we show that the Arf6-AMAP1 pathway links to the machinery that recycles β1 integrins, such as α3β1, to promote cell invasion upon EGFR stimulation. We found that AMAP1 had the ability to bind directly to PRKD2 and hence to make a complex with the cytoplasmic tail of the β1 subunit. Moreover, GTP-Rab5c also bound to AMAP1, and activation of Rab5c by EGFR signaling was necessary to promote the intracellular association of AMAP1 and PRKD2. Our results suggest a novel mechanism by which EGFR signaling promotes the invasiveness of some breast cancer cells via integrin recycling. 
 Integrin expression levels correlate with invasion,   metastasis   and prognosis of oral squamous cell carcinoma. The present study evaluated the relationship between alpha 3, alpha 6A, and beta 1 integrin expression in cancer cells at the invasive front of oral squamous cell carcinoma (OSCC) and survival rates, as well as the clinical and pathological characteristics. Sections of 100 specimens of primary OSCC were immunostained to assess alpha 3, alpha 6A, and beta 1 integrin expression in cancer cells at the invasive front. OSCC patients with higher expression levels of alpha 3, alpha 6A, and beta 1 integrin had significantly better prognosis than those with lower expression levels (median survival at low vs. high expression levels: alpha 3, 37.1 months vs. 55.7 months; alpha 6A , 38.3 months vs. 47.9 months; and beta 1, 26.1 months vs. 46.1 months) (P < 0.05). In addition, beta 1 integrin expression showed the highest correlation with clinical and pathological characteristics. This study concludes that alpha 3, alpha 6A, and beta 1 integrin expression in cancer cells at the invasive front are related to the mode of invasion and prognosis in OSCC. 
 miR-17 inhibits ovarian cancer cell peritoneal   metastasis   by targeting ITGA5 and ITGB1. An essential step in the peritoneal spread of ovarian cancer is the adhesion and implantation of tumor cells to the mesothelium layer. Integrin α5 and β1 have been reported to mediate the initial adhesion process and to correlate with disease survival in ovarian cancer. However, the molecular mechanism of integrin α5β1 dysregulation in tumorigenesi  s and meta  stasis remained enigmatic. In the present study, using the US NCI60 database, we identified miR-17 as a candidate regulator targeting both integrin α5 and β1. The level of miR-17 was evidently inversely correlated with that of α5 and β1 in ovarian cancer cell lines. Specifically, miR-17 bound directly to the 3' untranslated region (3'UTR) of α5 and β1 and suppressed their expression. Forced expression of miR-17 led to markedly diminished adhesion and invasion of ovarian cancer cells in vitro, and notably reduced metastatic nodules inside the peritoneal cavity in in vivo SKOV3 xenografts model. Moreover, ectopic expression of miR-17 in ovarian cancer cells resulted in repressed ILK phosphorylation as well as decreased production of active matrix metalloproteinase-2 (MMP-2). Our results indicated that miR-17 hampered ovarian cancer peritoneal propagation by targeting integrin α5 and β1. These findings supported the utility of miR-17/α5β1 to be considered as valuable marker for metastatic potential of ovarian cancer cells, or a therapeutic target in ovarian cancer treatment. 
 Differential modulation of human melanoma cell metalloproteinase expression by alpha2beta1 integrin and CD44 triple-helical ligands derived from type IV collagen. Tumor cell binding to components of the basement membrane is well known to trigger intracellular signaling pathways. Signaling ultimately results in the modulation of gene expression, facilitating   metastasis  . Type IV collagen is the major structural component of the basement membrane and is known to be a polyvalent ligand, possessing sequences bound by the alpha1beta1, alpha2beta1, and alpha3beta1 integrins, as well as cell surface proteoglycan receptors, such as CD44/chondroitin sulfate proteoglycan (CSPG). The role of alpha2beta1 integrin and CD44/CSPG receptor binding on human melanoma cell activation has been evaluated herein using triple-helical peptide ligands incorporating the alpha1(IV)382-393 and alpha1(IV)1263-1277 sequences, respectively. Gene expression and protein production of matrix metalloproteinases-1 (MMP-1), -2, -3, -13, and -14 were modulated with the alpha2beta1-specific sequence, whereas the CD44-specific sequence yielded significant stimulation of MMP-8 and lower levels of modulation of MMP-1, -2, -13, and -14. Analysis of enzyme activity confirmed different melanoma cell proteolytic potentials based on engagement of either the alpha2beta1 integrin or CD44/CSPG. These results are indicative of specific activation events that tumor cells undergo upon binding to select regions of basement membrane collagen. Based on the present study, triple-helical peptide ligands provide a general approach for monitoring the regulation of proteolysis in cellular systems. 
 The endothelin-integrin axis is involved in macrophage-induced breast cancer cell chemotactic interactions with endothelial cells. Elevated macrophage infiltration in tumor tissues is associated with breast cancer   metastasis  . Cancer cell migration/invasion toward angiogenic microvasculature is a key step in metastatic spread. We therefore studied how macrophages stimulated breast cancer cell interactions with endothelial cells. Macrophages produced cytokines, such as interleukin-8 and tumor necrosis factor-α, to stimulate endothelin (ET) and ET receptor (ETR) expression in breast cancer cells and human umbilical vascular endothelial cells (HUVECs). ET-1 was induced to a greater extent from HUVECs than from breast cancer cells, resulting in a density difference that facilitated cancer cell chemotaxis toward HUVECs. Macrophages also stimulated breast cancer cell adhesion to HUVECs and transendothelial migration, which were repressed by ET-1 antibody or ETR inhibitors. The ET axis induced integrins, such as αV and β1, and their counterligands, such as intercellular adhesion molecule-2 and P-selectin, in breast cancer cells and HUVECs, and antibodies against these integrins efficiently suppressed macrophage-stimulated breast cancer cell interactions with HUVECs. ET-1 induced Ets-like kinase-1 (Elk-1), signal transducer and activator of transcription-3 (STAT-3), and nuclear factor-κB (NF-κB) phosphorylation in breast cancer cells. The use of inhibitors to prevent their phosphorylation or ectopic overexpression of dominant-negative IκBα perturbed ET-1-induced integrin αV and integrin β1 expression. The physical associations of these three transcriptional factors with the gene promoters of the two integrins were furthermore evidenced by a chromatin immunoprecipitation assay. Finally, our mouse orthotopic tumor model revealed an ET axis-media  ted lung m  etastasis of macrophage-stimulated breast cancer cells, suggesting that the ET axis was involved in macrophage-enhanced breast cancer cell endothelial interactions. 
 Overexpression of HAb18G/CD147 promotes invasion and   metastasis   via alpha3beta1 integrin mediated FAK-paxillin and FAK-PI3K-Ca2+ pathways. Mechanism of HAb18G/CD147 underlying the   metastasis   process of human hepatoma cells has not been determined. In the present study, we found that integrin alpha3beta1 colocalizes with HAb18G/CD147 in human 7721 hepatoma cells. The enhancing effect of HAb18G/CD147 on adhesion, invasion capacities and matrix metalloproteinases (MMPs) secretion was decreased by integrin alpha3beta1 antibodies (p<0.01). The expressions of integrin downstream molecules including focal adhesion kinase (FAK), phospho-FAK (p-FAK), paxillin, and phospho-paxillin (p-paxillin) were increased in human hepatoma cells overexpressing HAb18G/CD147. Deletion of HAb18G/CD147 reduces the quantity of focal adhesions and rearranges cytoskeleton. Wortmannin and LY294002, specific phosphatidylinositol kinase (PI3K) inhibitors, reversed the effect of HAb18G/CD147 on the regulation of intracellular Ca(2+) mobilization, significantly reducing cell adhesion, invasion and MMPs secretion potential (p<0.01). Together, these results suggest that HAb18G/CD147 enhances the invasion and metastatic potentials of human hepatoma cells via integrin alpha3beta1-mediated FAK-paxillin and FAKPI3K-Ca(2+) signal pathways. 
 Integrin-dependent protein tyrosine phosphorylation is a key regulatory event in collagen-IV-mediated adhesion and proliferation of human lung tumor cell line, Calu-1. The clinical phenomenon of lung cancer   metastasis   to specific target organs is believed to be a direct interaction between tumor cells and extracellular matrix components. During invasion, tumor cells attach to the basement membrane protein, collagen type IV, degrade it, migrate through blood vessels, and adhere to extracellular matrix proteins.Four nonsmall-cell lung cancer cells were tested for adhesion, proliferation, migration and morphologic alterations on collagen type IV matrix by immunoprecipitation, Western blotting, phase contrast and time lapse video microscopy.Collagen type IV promoted Calu-1 cell adhesion (< 15 minutes) and motility (< 6 hours) without any significant effect on proliferation. beta(1)-integrin is essential for collagen type IV adhesion and 8 to 10 fold higher expression of beta1-integrin on the surface of Calu-1 cells was identified. A protein tyrosine phosphatase inhibitor, peroxyvanadate, caused 50% inhibition in the adhesion process within 5 minutes but exposure to 60 micromol/L genistein for 72 hours, a protein tyrosine kinase inhibitor, drastically inhibits Calu-1 cell proliferation (> 70%). We examined the influence of beta1-integrin, peroxyvanadate and genistein on the spreading morphogenesis of Calu-1 cells on Collagen type IV. Use of either 1 microg of anti beta1-integrin inhibitory antibody (P5D2), 100 micromol/L peroxyvanadate or 60 micromol/L genistein had dramatic influence on the spreading of Calu-1 cells. Finally, Focal adhesion kinase was identified as a phosphoprotein target.We have characterized an in vitro model of matrix-specific binding of a lung cancer cell line, Calu-1 to Coll IV. Calu-1 cells use primarily a beta1-integrin mediated intracellular tyrosine phosphorylation phenomenon as the key regulatory mechanism for its binding advantage to Coll IV matrix. 
 Twist induces epithelial-mesenchymal transition and cell motility in breast cancer via ITGB1-FAK/ILK signaling axis and its associated downstream network. Twist, a highly conserved basic Helix-Loop-Helix transcription factor, functions as a major regulator of epithelial-mesenchymal transition (EMT) and tumor   metastasis  . In different cell models, signaling pathways such as TGF-β, MAPK/ERK, WNT, AKT, JAK/STAT, Notch, and P53 have also been shown to play key roles in the EMT process, yet little is known about the signaling pathways regulated by Twist in tumor cells. Using iTRAQ-labeling combined with 2D LC-MS/MS analysis, we identified 194 proteins with significant changes of expression in MCF10A-Twist cells. These proteins reportedly play roles in EMT, cell junction organization, cell adhesion, and cell migration and invasion. ECM-receptor interaction, MAPK, PI3K/AKT, P53 and WNT signaling were found to be aberrantly activated in MCF10A-Twist cells. Ingenuity Pathways Analysis showed that integrin β1 (ITGB1) acts as a core regulator in linking integrin-linked kinase (ILK), Focal-adhesion kinase (FAK), MAPK/ERK, PI3K/AKT, and WNT signaling. Increased Twist and ITGB1 are associated with breast tumor progression. Twist transcriptionally regulates ITGB1 expression. Over-expression of ITGB1 or Twist in MCF10A led to EMT, activation of FAK/ILK, MAPK/ERK, PI3K/AKT, and WNT signaling. Knockdown of Twist or ITGB1 in BT549 and Hs578T cells decreased activity of FAK, ILK, and their downstream signaling, thus specifically impeding EMT and cell invasion. Knocking down ILK or inhibiting FAK, MAPK/ERK, or PI3K/AKT signaling also suppressed Twist-driven EMT and cell invasion. Thus, the Twist-ITGB1-FAK/ILK pathway and their downstream signaling network dictate the Twist-induced EMT process in human mammary epithelial cells and breast cancer cells. 
 Expression of integrin β1 and its significance in squamous cell carcinoma of the cervix. The aim of the present study was to examine the expression of integrin β1 in squamous cell carcinoma (SCC) of the cervix and its association with the clinicopathological features of patients. The expression of integrin β1 in 87 SCC cervical tissues and 32 normal cervical tissues was detected using an enzyme-linked immunosorbent assay, western blot analysis and the immunohistochemical streptavidin-peroxidase method. Integrin β1 expression was greater in SCC cervical tissues compared with that in normal cervical tissues (P<0.05), and its mean expression level in the SCC cervical tissues was also markedly higher compared with that in the normal cervical tissues (P<0.05). In terms of the association between the expression of integrin β1 with clinicopathological features, patients with stage IIA SCC had higher integrin β1 positive rates compared with patients with stage I SCC (P<0.05). The integrin β1 positive rates in SCC tissues with histological grade 3 were also significantly higher than that in the SCC tissues with histological grade 1 (P<0.05). Furthermore, patients with cervical SCC with lymp  h node met  astasis showed increased integrin β1 positive expression compared with those without lym  ph node me  tastasis (P<0.05). In conclusion, the expression of integrin β1 protein in cervical SCC tissues was significantly higher than that in the normal cervical tissues, and it increased with the clinical stage and the degree of malignancy. 
 Inhibition of peritoneal   metastasis   of human gastric cancer cells by dextran sulphate through the reduction in HIF-1α and ITGβ1 expression. The aim of the present study was to investigate the effects of dextran sulphate (DS) on HIF-1α and integrin β1 (ITGβ1) expression in human gastric cancer cells, the correlation between HIF-1α and ITGβ1 expression and the influence of DS on the peri  toneal met  astasis of human gastric cancer cells. In in vitro experiments, BGC-823 cells in the experimental and control groups were administered DS and PBS, respectively, and exposed to hypoxic conditions for different periods. Immunocytochemistry, western blot and RT-PCR analyses were used to evaluate HIF-1α and ITGβ1 expression levels. In in vivo experiments, an animal model was established by injecting BGC-823 cells into nude mice. The experimental and control groups received DS and PBS injections, respectively. The mice were euthanized at different times, and the number of tumor nodules in the celiac implantation was recorded. Immunohistochemistry, RT-PCR and western blot analyses were used to detect HIF-1α and ITGβ1 expression in the tumor nodules of the greater omentum. The in vitro and in vivo results revealed that HIF-1α and ITGβ1 expression levels in the experimental group were significantly lower than those in the control group (P<0.05), and the expression levels of these factors were positively correlated with each other. The number of tumor nodules in the in vivo experiments was notably less in the experimental group than that noted in the control group (P<0.01). In conclusion, DS may act through inhibition of HIF-1α expression, which decreased ITGβ1 expression, consequently   reducing t  umor metastasis. 
 Gene expression profiling combined with functional analysis identify integrin beta1 (ITGB1) as a potential prognosis biomarker in triple negative breast cancer. Triple negative breast cancer (TNBC) accounts for approximately 15-20% of all types of breast cancer, and treatment is still limited. This type of breast cancer shows a high risk of recurrence, visceral   metastasis  , a worse prognosis, and shorter distant   metastasis  -free survival. Several studies have been reported that genetics factors are associated with breast cancer disease progression and patients' survival. In this study, we combined Taiwanese microarray data from the GEO database and The Cancer Genome Atlas (TCGA) database to study the role of Integrin Beta1 (ITGB1) in TNBC. Two triple negative breast cancer cell lines (MDA-MB-231; MDA-MB-468) were used to validate the functions of ITGB1. We found that a higher ITGB1 gene expression level was associated to lower survival. Silencing of ITGB1 inhibited TNBC cell migration, invasion and store-operated calcium influx. Our study provided a potential candidate biomarker for breast cancer cells migration, invasion and TNBC patients' survival. 
 Prognostic role of integrin β1, E-cadherin, and rac1 expression in small cell lung cancer. Integrin β(1) mediates cellular adhesion to the extracellular matrix (ECM) and is correlated with highly invasive and metastatic behavior in small cell lung cancer (SCLC). E-cadherin (ECAD) is a calcium-dependent cell-cell adhesion receptor that restricts invasion of cells and reduce  s metastas  is. Rac1 is involved in the regulation of the actin cytoskeleton, adhesion, migration, invasion, and tumo  r metastas  is. The aim of this study was to examine integrin β(1) , ECAD and rac1 expression in SCLC and to analyze the prognostic value of these markers in patients with SCLC. We analyzed integrin β(1) , ECAD, and rac1 expression in 112 SCLC tissues by immunohistochemical staining. Correlative analyses between integrin β(1) , ECAD, and rac1 expression and cliniopathological factors were performed. A total of 65 patients had extensive disease (ED) (58%), and 47 had limited disease (LD) (42%). The median follow-up duration was 61 months (range: 14-117 months), and the median progression free survival (PFS) and overall survival (OS) were 6.1 months (range: 4.8-7.4 months) and 9.7 months (range: 8.1-11.3 months), respectively. The expression of integrin β(1) , ECAD, and rac1 protein was observed in 64, 73, and 99 of SCLC tissues, respectively. The correlative analyses between integrin β(1) , ECAD, or rac1 expression and various clinical parameters did not show any statistical significance. However, the ECAD expression was associated with OS in the entire cohort. In contrast, the expression of integrin β(1) and rac1 was not associated with PFS or OS. In a subgroup analysis, patients with less t  han two me  tastasis had significantly longer OS (p = 0.047) if their tumors expressed integrin β(1) compared to those without integrin β(1) expression. In addition, OS was longer for patients with ECAD positive tumors compared to those whose tumors did not express ECAD in males (p = 0.032) and patients who never smoked (p < 0.001). Multivariate analysis showed that LD (p = 0.004), overall response rate (p = 0.003), and expression of ECAD (p = 0.015) were the independent good prognostic factors for OS. LD (p = 0.024), overall response rate (p < 0.001), and less   than two   metastasis (p = 0.003) were prognostic factors for longer PFS. These results suggest that ECAD expression may be useful as a prognostic indicator in patients with SCLC. 
 Complementary DNA arrays identify CD63 tetraspanin and alpha3 integrin chain as differentially expressed in low and high metastatic human colon carcinoma cells. Malignant tumor cell invasion is determinant for   metastasis   to occur. E2 and C5 colon carcinoma cells that were derived from the parental Lovo line and that differ experimentally in spontaneous metastatic ability have been monitored for gene expression by cDNA arrays. Among genes found differentially expressed, the CD63 tetraspanin, not previously recognized in colon cancer progression, and the alpha3 integrin chain were both up-regulated in low metastatic E2 cells and were analyzed for their functional role using adhesion, migration, and invasion assays. Cell surface expression of CD63 and alpha3 integrin was about 2-fold higher in E2 than in C5 cells and confocal microscopy showed that CD63 and alpha3 integrin colocalized evenly on C5 cells whereas they concentrated at elongated tips of the low-metastatic more substrate-adhesive E2 cells. Antibody-interference experiments identified laminin-5 (LN-5) as a ligand interacting with the alpha3beta1/CD63 complex. Substrate-immobilized anti-CD63 antibodies enhanced tumor cell migration and invasion and induced prominent cell surface protrusions that were repressed by the PI3-kinase LY294002 inhibitor. Our results suggest that changes in the expression of surface CD63 and alpha3beta1 integrin interacting with LN-5 could affect migratory signals and the progression of the metastatic disease. 
 miRNA-29c suppresses lung cancer cell adhesion to extracellular matrix and   metastasis   by targeting integrin β1 and matrix metalloproteinase2 (MMP2). Our pilot study using miRNA arrays found that miRNA-29c (miR-29c) is differentially expressed in the paired low-metastatic lung cancer cell line 95C compared to the high-metastatic lung cancer cell line 95D. Bioinformatics analysis shows that integrin β1 and matrix metalloproteinase 2 (MMP2) could be important target genes of miR-29c. Therefore, we hypothesized that miR-29c suppresses lung cancer cell adhesion to extracellular matrix (ECM) an  d metastas  is by targeting integrin β1 and MMP2. The gain-of-function studies that raised miR-29c expression in 95D cells by using its mimics showed reductions in cell proliferation, adhesion to ECM, invasion and migration. In contrasts, loss-of-function studies that reduced miR-29c by using its inhibitor in 95C cells promoted proliferation, adhesion to ECM, invasion and migration. Furthermore, the dual-luciferase reporter assay demonstrated that miR-29c inhibited the expression of the luciferase gene containing the 3'-UTRs of integrin β1 and MMP2 mRNA. Western blotting indicated that miR-29c downregulated the expression of integrin β1 and MMP2 at the protein level. Gelatin zymography analysis further confirmed that miR-29c decreased MMP2 enzyme activity. Nude mice with xenograft models of lung cancer cells confirmed that miR-29c inhibited lung ca  ncer metas  tasis in vivo, including bone and l  iver metas  tasis. Taken together, our results demonstrate that miR-29c serves as a t  umor metas  tasis suppressor, which suppresses lung cancer cell adhesion to ECM   and metas  tasis by directly inhibiting integrin β1 and MMP2 expression and by further reducing MMP2 enzyme activity. The results show that miR-29c may be a novel therapeutic candidate target to slow lung c  ancer meta  stasis. 
 [Expression and significance of integrin beta1 and integrin-linked kinase in laryngeal carcinoma]. To study the expressions and clinical significance of integrin beta1 and integrin-linked kinase(ILK) in laryngeal carcinoma (LSCC).The mRNA and protein levels of integrinbeta1 and integrin-linked kinase in 43 specimens of laryngeal carcinoma, its surrounding tissue and 18 specimens of vocal cord polyp were detected by reverse transcription-polymerase chain reaction (RT-PCR) and immunohistochemistry SABC method.The mRNA levels of integrin beta1 and ILK protein were significantly higher in laryngeal carcinoma than that in its surrounding tissue and vocal cord polyp (P < 0.05). There was significant correlation between the levels of integrin beta1 expression and the status of cervical lymph node, either between the level of ILK expression and cervical lymph node status (P < 0.05). There was significant correlation between the levels of integrin beta1 expression and T-category, either between the level of ILK expression and T-category (P < 0.05). But pathological grades was not significantly related with the level of integrin beta1 or ILK expression (P > 0.05).The expressions of integrin beta1 and ILK are increased in LSCC. Integrin beta1 and ILK may contribute significantly to invasion and   metastasis   of LSCC. 
 In vivo cleaved CDCP1 promotes early tumor dissemination via complexing with activated β1 integrin and induction of FAK/PI3K/Akt motility signaling. Specific cleavage of the transmembrane molecule, CUB domain-containing protein-1 (CDCP1), by plasmin-like serine proteases induces outside-in signal transduction that facilitates early stages of spontaneous   metastasi  s leading to tumor cell intravasation, namely cell escape from the primary tumor, stromal invasion and transendothelial migration. We identified active β1 integrin as a biochemical and functional partner of the membrane-retained 70-kDa CDCP1 fragment, newly generated from its full-length 135-kDa precursor though proteolytic cleavage by serine proteases. Both in cell cultures and in live animals, active β1 integrin complexed preferentially with functionally activated, phosphorylated 70-kDa CDCP1. Complexing of β1 integrin the 70-kDa with CDCP1 fragment induced intracellular phosphorylation signaling, involving focal adhesion kinase-1 (FAK) and PI3 kinase (PI3K)-dependent Akt activation. Thus, inhibition of FAK/PI3K activities by specific inhibitors as well as short-hairpin RNA downregulation of β1 integrin significantly reduced FAK/Akt phosphorylation under conditions where CDCP1 was processed by serine proteases, indicating that FAK/PI3K/Akt pathway operates downstream of cleaved CDCP1 complexed with β1 integrin. Furthermore, this complex-dependent signaling correlated positively with high levels of tumor cell intravasation and dissemination. Correspondingly, abrogation in vivo of CDCP1 cleavage either by unique cleavage-blocking monoclonal antibody 10-D7 or by inhibition of proteolytic activity of plasmin-like serine proteases with aprotinin prevented β1 integrin/CDCP1 complexing and downstream FAK/Akt signaling concomitant with significant reduction of stromal invasion and spont  aneous met  astasis. Therefore, β1 integrin appears to serve as a motility-regulating partner mediating cross-talk between proteolytically cleaved, membrane-retained CDCP1 and members of FAK/PI3K/Akt pathway. This CDCP1 cleavage-induced signaling cascade constitutes a unique mechanism, independent of extracellular matrix remodeling, whereby a proteolytically cleaved CDCP1 regulates in vivo locomot  ion and me  tastasis of tumor cells through β1 integrin partnering. Our findings indicate that CDCP1 cleavage, occurring at the apex of a β1 integrin/FAK/PI3K/Akt signaling cascade, may represent a therapeutic target for CDCP1-positive cancers. 
 IL-18 regulates melanoma VLA-4 integrin activation through a Hierarchized sequence of inflammatory factors. Very late antigen-4 (VLA-4) is frequently overexpressed on melanoma cells contributing to inflammation-dependent   metastasis  . Melanoma cell adhesion to endothelium via VLA-4-vascular cell adhesion molecule-1 (VCAM-1) interaction was used to study VLA-4 activation during melanoma cell response to inflammation. Cooperation among major inflammatory mediators was analyzed in melanoma cells exposed to single inflammatory factors in the presence of inhibitors for other assayed mediators. A stepwise cascade of hierarchized molecules heterogeneously made and used during melanoma response to IL-18, induced hydrogen peroxide (H2O2), in turn activating VLA-4 and melanoma cell adhesion to endothelium. The cascade involved prostaglandin E2 (PGE2) production from melanoma induced by IL-18-dependent tumor necrosis factor-α (TNFα); next, PGE2-induced IL-1β via vascular endothelial growth factor (VEGF) secretion, which in turn induced VLA-4 activation via cyclooxygenase 2-dependent H2O2. This sequence operated in IL-18R/VLA-4/VEGF-expressing murine (B16) and human (A375 and 883) melanomas, but not in those without this phenotype. Separation of active VLA-4-expressing B16 melanoma cells through immobilized VCAM-1 verified their higher IL-18R/TNFR1/VEGFR2 expression and metastatic growth than inactive VLA-4-expressing cells. However, cooperation among melanoma cell sub-populations with heterogeneous cytokine receptor levels may occur through VLA-4-stimulating factors, leading to intratumoral amplification of metastatic potential. Therefore, expression of the VLA-4-stimulating factor sequence may help to predict melanoma prometastatic risk, and offers therapeutic targets for metastatic melanoma deactivation through VLA-4 activation blockade. 
 miR-134 suppresses the migration and invasion of non‑small cell lung cancer by targeting ITGB1. Most cancer-related deaths are caused by the development of metastatic disease. Thus, investigation of the underlying mechanisms o  f metastas  is is urgent to design more effective targeted drugs and to treat metastatic disease more effectively. MicroRNAs (miRNAs) have emerged as potential targets for cancer treatment. In the present study, we aimed to identify the roles of miR-134 in non-small cell lung cancer (NSCLC) cell migration and invasion. We demonstrated that overexpression of miR-134 inhibited migration and invasion of A549 and H1299 cells. Further mechanistic investigations revealed that miR-134 inhibited epithelial-to-mesenchymal transition (EMT) evidenced by upregulation of E-cadherin expression and downregulation of vimentin expression. Using luciferase assays, we identified integrin β1 (ITGB1) as a direct target of miR-134. Performing RNAi and rescue experiments, we confirmed that miR-134 exerted its migratory and invasive suppressive role partly by downregulating ITGB1. Finally, an in vivo experiment also, to some extent, suggested that miR-134 may function as a suppresso  r of metas  tasis. Taken together, our findings suggest that miR-134 suppresses migration and invasion of NSCLC by targeting ITGB1. 
 Functional polymorphisms of ITGB1 are associated with clinical outcome of Chinese patients with resected colorectal cancer. Integrin β1 (ITGB1) has been recognized to play a major role in tumor growth, invasion and   metastasi  s. However, effects of single-nucleotide polymorphisms (SNPs) in ITGB1 gene on the prognosis of patients with colorectal cancer (CRC) have not been reported.A total of 372 patients with resected colorectal adenocarcinoma were enrolled in our study. Three functional SNPs (rs2230395, rs1187075 and rs1187076) in ITGB1 were selected and genotyped using the Sequenom iPLEX genotyping system.We identified two SNPs (rs2230395 and rs1187075) in ITGB1 gene to be significantly associated with CRC overall survival (OS). Compared with the homozygous wild-type (AA) and heterozygous variant (AC), rs2230395 homozygous variant (CC) conferred a 1.55-fold (95 % CI 1.00-2.41, P = 0.049) increased risk of death. Similar result was obtained for homozygous variant (AA) in rs1187075 with a 1.62-fold (95 % CI 1.08-2.42, P = 0.020). In stratified analysis, this association in rs2230395 remained to be significant in patients receiving chemotherapy, but not in those without chemotherapy. We further evaluated the effects of chemotherapy on CRC survival in subgroups stratified by rs2230395 and rs1187075 genotypes. We found that chemotherapy resulted in a significantly better OS in patients with the homozygous wild-type (WW) or heterozygous variant (WV) genotype in both rs2230395 and rs1187075 when compared with patients with homozygous variant (VV) genotype.Our data suggest that ITGB1 SNPs might be a prognostic biomarker for CRC patients, especially in those receiving chemotherapy. Our findings warrant validation in larger independent populations. 
 Down-regulation of ARNT promotes cancer   metastasis   by activating the fibronectin/integrin β1/FAK axis. The aryl hydrocarbon receptor nuclear translocator (ARNT) is broadly involved in regulating tumorigenesis by inducing genes that are involved in tumor growth and angiogenesis. Tumorigenesis usually involves normoxic conditions. However, the role of ARNT in tumor   metastasi  s during normoxia remains unclear. Here, we demonstrate that ARNT protein levels were decreased in late-stage human colorectal cancer using immunohistochemical analysis. Down-regulation of ARNT protein promoted cancer cell migration and invasion, which was mediated by activation of the fibronectin/integrin β1/FAK signaling axis. In addition, the enhancement of migration and invasion in ANRT knockdown cells was blocked when ARNT was restored in the cells. In xenografts in severe combined immunodeficiency mice, tumor growth was significantly inhibited in the ARNT-knockdown condition. However, the tail-vein injection animal model revealed that the depletion of ARNT-induced metastatic lung colonies was further enhanced when ARNT expression was recovered post-injection. Interestingly, chemotherapeutic drugs inhibited ARNT expression and promoted the invasion of residual tumor cells. These results suggest that ARNT may play a positive role during tumor growth (either in early-stage tumor growth or in organ metastases), but plays a negative role in tumor migration and invasion. Therefore, the efficiency of ARNT-targeted therapy during different cancer stages should be carefully evaluated. 
 Integrin VLA-4 enhances sialyl-Lewisx/a-negative melanoma adhesion to and extravasation through the endothelium under low flow conditions. During their passage through the circulatory system, tumor cells undergo extensive interactions with various host cells including endothelial cells. The capacity of tumor cells to form   metastasis   is related to their ability to interact with and extravasate through endothelial cell layers, which involves multiple adhesive interactions between tumor cells and endothelium (EC). Thus it is essential to identify the adhesive receptors on the endothelial and melanoma surface that mediate those specific adhesive interactions. P-selectin and E-selectin have been reported as adhesion molecules that mediate the cell-cell interaction of endothelial cells and melanoma cells. However, not all melanoma cells express ligands for selectins. In this study, we elucidated the molecular constituents involved in the endothelial adhesion and extravasation of sialyl-Lewis(x/a)-negative melanoma cell lines under flow in the presence and absence of polymorphonuclear neutrophils (PMNs). Results show the interactions of alpha(4)beta(1) (VLA-4) on sialyl-Lewis(x/a)-negative melanoma cells and vascular adhesion molecule (VCAM-1) on inflamed EC supported melanoma adhesion to and subsequent extravasation through the EC in low shear flow. These findings provide clear evidence for a direct role of the VLA-4/VCAM-1 pathway in melanoma cell adhesion to and extravasation through the vascular endothelium in a shear flow. PMNs facilitated melanoma cell extravasation under both low and high shear conditions via the involvement of distinct molecular mechanisms. In the low shear regime, beta(2)-integrins were sufficient to enhance melanoma cell extravasation, whereas in the high shear regime, selectin ligands and beta(2)-integrins on PMNs were necessary for facilitating the melanoma extravasation process. 
 LPS-induced TLR4 signaling in human colorectal cancer cells increases beta1 integrin-mediated cell adhesion and liver   metastasis  . Infectious complications resulting from resection of colorectal cancer (CRC) elevates the risk of cancer recurrence and   metastasis  , but the reason for this risk relationship is unknown. Defining the mechanisms responsible may offer opportunities to improve outcomes in a majority of patients whose tumors are resected as part of their therapy. The complex formed between Toll receptor TLR4 and myeloid differentiation factor MD2 defines a major cell surface receptor for lipopolysaccharide (LPS), a gram-negative bacterial antigen that has been implicated in infectious complications after CRC resection. As the TLR4/MD2 complex is expressed on CRC cells, we hypothesized that LPS may promote liver   metastasis   in CRC by stimulating TLR4 signaling. In support of this hypothesis, we report here that LPS enhances liver   metastasis   of human CRC cells that express TLR4/MD2 after intrasplenic graft of immunocompromised nude mice. Compared with TLR4 nonexpressing, nonmetastatic CRC cells, we observed increased in vitro adherence to different extracellular matrices and human umbilical vein endothelial cells (HUVEC). Furthermore, we observed an increased likelihood of in vivo capture within hepatic sinusoids after LPS treatment. No differences were apparent in phosphorylation of p38 and MAPK isoforms, but in metastatic CRC cells expressing surface TLR4 treatment with LPS increased Ser473 phosphorylation of AKT kinase. We showed that enhanced adherence elicited by LPS in these cells could be blocked at three different levels, using Eritoran (TLR4 small molecule antagonist), PI-103 (PI3K inhibitor), or anti-β1 integrin blocking antibodies. Taken together, the results indicate that stimulation of the TLR4/MD2 complex by LPS activates PI3K/AKT signaling and promotes downstream β1 integrin function, thereby increasing the adhesiveness and metastatic capacity of CRC cells. Our findings suggest that inhibiting LPS-induced TLR4 signaling could improve therapeutic outcomes by preventing cance  r metastas  is during the perioperative period of CRC resection. 
 Expression and prognostic significance of CEACAM6, ITGB1, and CYR61 in peripheral blood of patients with gastric cancer. We examined CEACAM6, ITGB1, and cyr61 concentrations from patients with gastric cancers (GCs) to assess their clinical application for diagnosing and monitoring diseases.Real-time reverse transcription-polymerase chain reaction was used to detect the expressions of CEA, CEACAM6, ITGB1, IGF1R, CK20, cyr61, and S100A4 in peripheral blood karyocyte from 82 patients with GC, 24 patients with recurrence, and 37 healthy volunteers. Receiver operating characteristics (ROC) curves were constructed.There were significant association between these CEACAM6, ITGB1, and cyr61 and TNM Stages and distant   metastasis  . The AUC of CEACAM6 was 0.884 ± 0.044 (P = 0.0001), the AUC of cyr61 was 0.833 ± 0.047 (P = 0.0001), and the AUC of ITGB1 was 0.838 ± 0.042 (P = 0.0001) by differentiating preoperative GC patients from healthy volunteers from ROC curve analysis. The AUC of CEACAM6 was 0.761 ± 0.066 (P = 0.001), the AUC of CYR61 was 0.762 ± 0.063 (P = 0.001), and the AUC of ITGB1 was 0.824 ± 0.051 (P = 0.0001), by differentiating recurrence of GC from healthy volunteers from ROC curve analysis.The method of detecting the expression of CEACAM6, ITGB1, and CYR61 in peripheral blood of GC patients was more sensitive than CEA, IGF1R, CK20, and S100A4 for the early diagnos  is of meta  stasis and recurrence. 
 HMGB-1 induces cell motility and α5β1 integrin expression in human chondrosarcoma cells. Chondrosarcoma is a type of highly malignant tumor with a potent capacity to invade locally and cause distan  t metastas  is. High mobility group box chromosomal protein 1 (HMGB)-1 is a widely studied, ubiquitous nuclear protein that is present in eukaryotic cells, and plays a crucial role in inflammatory response. However, the effects of HMGB-1 on human chondrosarcoma cells are largely unknown. In this study, we found that HMGB-1 increased the migration and the expression of α5β1 integrin in human chondrosarcoma cells. Transfection of cells with receptor for advanced glycation end products (RAGE) receptor siRNA reduced HMGB-1-induced cell migration and integrin expression. Activations of phosphatidylinositol 3-kinase (PI3K), Akt, and AP-1 pathways after HMGB-1 treatment were demonstrated, and HMGB-1-induced expression of integrin and migration activity was inhibited by the specific inhibitor and mutant of PI3K, Akt, and AP-1 cascades. Taken together, our results indicated that HMGB-1 enhances the migration of chondrosarcoma cells by increasing α5β1 integrin expression through the RAGE receptor/PI3K/Akt/c-Jun/AP-1 signal transduction pathway. 
 Integrin cytoplasmic domain-associated protein 1alpha (ICAP-1alpha ) interacts directly with the   metastasis   suppressor nm23-H2, and both proteins are targeted to newly formed cell adhesion sites upon integrin engagement. Cell adhesion-dependent signaling implicates cytoplasmic proteins interacting with the intracellular tails of integrins. Among those, the integrin cytoplasmic domain-associated protein 1alpha (ICAP-1alpha) has been shown to interact specifically with the beta(1) integrin cytoplasmic domain. Although it is likely that this protein plays an important role in controlling cell adhesion and migration, little is known about its actual function. To search for potential ICAP-1alpha-binding proteins, we used a yeast two-hybrid screen and identified the human metastatic suppressor protein nm23-H2 as a new partner of ICAP-1alpha. This direct interaction was confirmed in vitro, using purified recombinant ICAP-1alpha and nm23-H2, and by co-immunoprecipitation from CHO cell lysates over-expressing ICAP-1alpha. The physiological relevance of this interaction is provided by confocal fluorescence microscopy, which shows that ICAP-1alpha and nm23-H2 are co-localized in lamellipodia during the early stages of cell spreading. These adhesion sites are enriched in occupied beta(1) integrins and precede the formation of focal adhesions devoid of ICAP-1alpha and nm23-H2, indicating the dynamic segregation of components of matrix adhesions. This peripheral staining of ICAP-1alpha and nm23-H2 is only observed in cells spreading on fibronectin and collagen and is absent in cells spreading on poly-l-lysine, vitronectin, or laminin. This is consistent with the fact that targeting of both ICAP-1alpha and nm23-H2 to the cell periphery is dependent on beta(1) integrin engagement rather than being a consequence of cell adhesion. This finding represents the first evidence that the tumor suppressor nm23-H2 could act on beta(1) integrin-mediated cell adhesion by interacting with one of the integrin partners, ICAP-1alpha. 
 Adhesion of osteosarcoma cells to the 70-kDa core region of thrombospondin-1 is mediated by the alpha 4 beta 1 integrin. Thrombospondin-1 (TSP-1) is an extracellular glycoprotein that is involved in a variety of physiological processes such as tumor cell adhesion, invasion, and   metastasis  . It has been hypothesized that TSP-1 provides an adhesive matrix for osteosarcoma cells. Here we present data showing that TSP-1 can promote cell substrate adhesion to U2OS and SAOS cells through the alpha 4 beta 1 integrin. The dose-dependent adhesion to TSP-1 was inhibited by anti-integrin antibodies directed against the alpha 4 or beta 1 subunit, but not by control antibodies against other integrins. To localize the potential alpha 4 beta 1-binding site within the TSP-1 molecule, the protein was subjected to limited proteolysis with chymotrypsin in the absence of calcium. The stable 70-kDa core fragment produced under these conditions promoted alpha 4 beta 1-dependent osteosarcoma cell adhesion in a manner similar to that of the intact protein. Moreover adhesion experiments with neutralizing antibodies revealed that the adhesion was totally dependent on the alpha 4 beta 1 interaction. Further blocking experiments with potential inhibitory peptides revealed that the alpha 4 beta 1-mediated adhesion was not influenced by peptides containing the RGD sequence. Attachment to the 70-kDa fragment was strongly inhibited by the CS-1 peptide, which represents the most active recognition domain for alpha 4 beta 1 integrin in fibronectin. The present data provide evidence that TSP-1 contains an alpha 4 beta 1 integrin-binding site within the 70-kDa core region. 
 Expression of focal adhesion kinase and alpha5 and beta1 integrins in carcinomas and its clinical significance. To detect the expression pattern of FAK (focal adhesion kinase) and integrin alpha5 and beta1 subunits in different kinds of cancerous tissues and to study their correlation with clinicopathological data including tumor type, grade and lymph node status.Using an immunohistochemical technique, we examined the expression of FAK and integrin and subunits in cancerous and noncancerous tissues obtained from 75 patients with gastric carcinomas, 21 colorectal carcinomas, 16 hepatocellular carcinomas, 20 uterocervical carcinomas, and 20 breast carcinomas.The staining of FAK was stronger in cancerous than in noncancerous areas. Enhanced expression of FAKwas detected in poor-differentiated carcinoma of the stomach and colorectum. Tumors with lymph node metastases had more FAK protein than those without metastases. In addition, the deeper the extent of tumor infiltration, the higher the FAK expression. The expression of integrin alpha5 and beta1 subunits was lower in cancerous areas than in noncancerous areas, but it was higher in well-differentiated cancerous tissues than in poor differentiated tissues. The relationship between the expression of integrin alpha5 and beta1 subunits and infiltration or   metastasis   was not significant. Cancerous tissues with stronger FAK expression (++ or +++) also had a higher expression of integrin alpha5 and beta1 subunits in the tumor and its unaffected margins.FAK is a better marker for carcinogenesis and the progression of cancer than integrin alpha5 and beta1 subunit, and it may be not only a transformation-linked enzyme but also a progression-linked enzyme. 
 Clinical and functional significance of α9β1 integrin expression in breast cancer: a novel cell-surface marker of the basal phenotype that promotes tumour cell invasion. Integrin α9β1 is a receptor for ECM proteins, including Tenascin-C and the EDA domain of fibronectin, and has been shown to transduce TGFβ signalling. This study has examined the expression pattern of α9β1 in 141 frozen breast carcinoma samples and related expression to prognostic indices, molecular subtype and patient outcome. Effects of α9β1 on tumour cell migration and invasion were assessed using blocking antibody and gene transduction approaches. Integrin α9β1 localized to myoepithelial cells in normal ducts and acini, a pattern maintained in DCIS. A subset (17%) of invasive carcinomas exhibited tumour cell expression of α9β1, which related significantly to the basal-like phenotype, as defined by either CK5/6 or CK14 expression. Tumour expression of α9β1 showed a significant association with reduced overall patient survival (p < 0.0001; HR 5.94, 95%CI 3.26-10.82) and with r  educed dis  tant-metastasis-free survival (p < 0.0001; HR 6.37, CI 3.51-11.58). A series of breast cancer cell lines was screened for α9β1 with the highly invasive basal-like GI-101 cell line expressing significant levels. Both migration and invasion of this line were reduced significantly in the presence of α9-blocking antibody and following α9-knockdown with siRNA. Conversely, migratory and invasive behaviour of α9-negative MCF7 cells and α9-low MDA MB468 cells was enhanced significantly by over-expression of α9. Thus, α9β1 acts as a novel marker of the basal-like breast cancer subtype and expression is associated with reduced survival, while its ability to promote breast cancer cell migration and invasion suggests that it contributes to the aggressive clinical behaviour of this tumour subtype. 
 Timp1 interacts with beta-1 integrin and CD63 along melanoma genesis and confers anoikis resistance by activating PI3-K signaling pathway independently of Akt phosphorylation. Anoikis resistance is one of the abilities acquired along tumor progression. This characteristic is associated with   metastasis   development, since tumorigenic cells must survive independently of cell-matrix interactions in this process. In our laboratory, it was developed a murine melanocyte malignant transformation model associated with a sustained stressful condition. After subjecting melan-a melanocytes to 1, 2, 3 and 4 cycles of anchorage impediment, anoikis resistant cells were established and named 1C, 2C, 3C and 4C, respectively. These cells showed altered morphology and PMA independent cell growth, but were not tumorigenic, corresponding to pre-malignant cells. After limiting dilution of 4C pre-malignant cells, melanoma cell lines with different characteristics were obtained. Previous data from our group showed that increased Timp1 expression correlated with anoikis-resistant phenotype. Timp1 was shown to confer anchorage-independent growth capability to melan-a melanocytes and render melanoma cells more aggressive when injected into mice. However, the mechanisms involved in anoikis regulation by Timp1 in tumorigenic cells are not clear yet.The β1-integrin and Timp1 expression were evaluated by Western blotting and CD63 protein expression by flow cytometry using specific antibodies. To analyze the interaction among Timp1, CD63 and β1-integrin, immunoprecipitation assays were performed, anoikis resistance capability was evaluated in the presence or not of the PI3-K inhibitors, Wortmannin and LY294002. Relative expression of TIMP1 and CD63 in human metastatic melanoma cells was analyzed by real time PCR.Differential association among Timp1, CD63 and β1-integrins was observed in melan-a melanocytes, 4C pre-malignant melanocytes and 4C11- and 4C11+ melanoma cells. Timp1 present in conditioned medium of melanoma cells rendered melan-a melanocytes anoikis-resistant through PI3-K signaling pathway independently of Akt activation. In human melanoma cell lines, in which TIMP1 and beta-1 integrin were also found to be interacting, TIMP1 and CD63 levels together was shown to correlate significantly with colony formation capacity.Our results show that Timp1 is assembled in a supramolecular complex containing CD63 and β1-integrins along melanoma genesis and confers anoikis resistance by activating PI3-K signaling pathway, independently of Akt phosphorylation. In addition, our data point TIMP1, mainly together with CD63, as a potential biomarker of melanoma. 
 Integrin α9β1 promotes malignant tumor growth an  d metastas  is by potentiating epithelial-mesenchymal transition. The integrin α9β1 binds a number of extracellular matrix components to mediate cell adhesion, migration and tissue invasion. Although expressed in a variety of normal human cells including endothelium, it is also expressed in cancer cells. We have previously shown that α9β1 binds VEGF-A to facilitate angiogenesis, an important component of the tumor microenvironment. As α9β1 induces accelerated cancer cell migration, we wished to determine what role it played in cancer gro  wth and me  tastasis. In this study, we show that α9β1 expression induces molecular changes consistent with epithelial-mesenchymal transition. In addition, we found that α9β1 forms a tri-partite protein complex with β-catenin and E-cadherin, which dissociates following integrin activation and subsequent src and β-catenin phosphorylation. These findings were consistent in cells in which: α9β1 was exogenously over-expressed, or when its expression was suppressed in cancer cells endogenously expressing α9β1. These in vitro results are biologically significant as α9β1-expressing cancer cells induce greater tumor growth and metastases in mice as compared to the cells without α9β1 expression or when integrin expression is suppressed. Furthermore, integrin α9β1 is expressed in primary human small cell lung cancer and patients having a high expression of α9β1 demonstrated significantly worse long-term survival compared with patients with low α9β1 expression. These findings highlight a novel mechanism of integrin α9β1 function in human cancer. 
 The Arp2/3 activator WASH regulates α5β1-integrin-mediated invasive migration. The actin cytoskeleton provides scaffolding and physical force to effect fundamental processes such as motility, cytokinesis and vesicle trafficking. The Arp2/3 complex nucleates actin structures and contributes to endocytic vesicle invagination and trafficking away from the plasma membrane. Internalisation and directed recycling of integrins are major driving forces for invasive cell motility and potentially for cance  r metastas  is. Here, we describe a direct requirement for WASH and Arp2/3-mediated actin polymerisation on the endosomal membrane system for α5β1 integrin recycling. WASH regulates the trafficking of endosomal α5β1 integrin to the plasma membrane and is fundamental for integrin-driven cell morphology changes and integrin-mediated cancer cell invasion. Thus, we implicate WASH and Arp2/3-driven actin nucleation in receptor recycling leading to invasive motility. 
 DNAzyme as an efficient tool to modulate invasiveness of human carcinoma cells. In this study we evaluated efficiency of DNAzymes to modulate motility of cancer cells, an important factor in the progression and   metastasis   of cancers. For this purpose we targeted β1 integrins that are predominant adhesive receptors in various carcinoma cell lines (CX1.1, HT29, LOVO, LS180, PC-3). To evaluate invasiveness of cancer cells, we used a transwell migration assay that allowed analyzing chemotactic migration of colon carcinoma cell lines across an ECM-coated membrane. Their adhesive properties were also characterized by the analysis of adhesion to fibronectin, laminin and collagen. In addition, the expression of major integrin subunits, selected intact β1 integrins, and other adhesive receptors (ICAM, E-selectin, uPAR) was analyzed by flow cytometry. Inhibition of β1 integrin expression by DNAzyme to β1 mRNA almost abolished the invasiveness of the CX1.1, HT29, LS180, LOVO and PC-3 cells in vitro. These data show that DNAzymes to β1 integrin subunit can be used to inhibit invasiveness of carcinoma cells. 
 [Effects of laparoscopy-assisted radical gastrectomy on the expression of intercellular adhesion molecule 1 and integrin beta(1) in peritoneal mesothelial cells and its significance]. To investigate the changes of the expression of intercellular adhesion molecule-1 (ICAM-1) and integrin beta(1) in peritoneal mesothelial cells during laparoscopy-assisted radical gastrectomy(LARG) and to explore the possible effects of LARG on the peritoneal   metastasis  .From April to August 2008, LARG was performed for 26 patients with gastric cancer (laparoscopy group), while 20 cases underwent open radical gastrectomy(open group). Peritoneum of right upper belly was collected at 3 operation time points(the beginning, 2 hours, 4 hours). The expressions of ICAM-1 and integrin beta(1) in peritoneal mesothelial cells at 3 time points were detected by immunohistochemistry.With the operation prolonging, the expression of ICAM-1 and integrin beta(1) was increased gradually in both LARG and open groups. The expression of integrin beta(1) in two groups was obviously increased at 4-hour time point as compared to the beginning(P<0.05). Besides, there were no significant differences of these two adhesion molecules among the three operation time points between two groups(P>0.05).Compared with open surgery, LARG is not associated with a greater effect on the expression of ICAM-1 and integrin beta(1) in peritoneal mesothelial cells, and may not promote peritoneal   metastasis   of gastric cancer through increasing the expression of adhesion molecule in peritoneal mesothelial cells. 
 AlphaV- and beta1-integrin subunits are commonly expressed in malignant effusions from ovarian carcinoma patients. The objective was to study expression of alphav- and beta1-integrin subunits in effusions, primary tumors, and solid metastases of ovarian carcinoma patients, as well as to evaluate its potential association with previously studied   metastasis  -associated molecules and clinicopathologic parameters.Sections from 121 malignant effusions and 30 corresponding primary and metastatic lesions were evaluated for protein expression of the alphav- and beta1-integrin subunits using immunohistochemistry (IHC). A subset of effusions was additionally studied using immunoblotting (IB) and flow cytometry (FCM). mRNA in situ hybridization (ISH) was performed in 58 effusions and 30 biopsies.Protein expression of alphav- and beta1-integrin subunits was detected in carcinoma cells in 116/121 (96%) and 113/121 (93%) effusions, respectively. alphav protein expression was limited to carcinoma cells. IB and FCM confirmed IHC results. mRNA for alphav- and beta1-integrin subunits was detected in carcinoma cells in 37/58 (64%) and 33/58 (57%) effusions, respectively. Both protein and mRNA expression were higher in peritoneal effusions, significantly for alphav mRNA (P = 0.042) and beta1 protein (P = 0.023). beta1 protein expression in effusions was more frequently detected in better-differentiated tumors (P = 0.006). alphav-integrin subunit expression correlated with that of the previously studied matrix metalloproteinase-9 (MMP-9) (P = 0.006) and the MMP inducer EMMPRIN (P = 0.001). Expression of beta1-integrin subunit showed an association with that of EMMPRIN (P = 0.029), basic fibroblast growth factor (P < 0.001), and the MMP inhibitor TIMP-2 (P = 0.025). In carcinoma cells of solid lesions, alphav protein was uniformly present, while beta1 expression was limited to 15/30 (50%) specimens. As in effusions, protein expression of alphav subunit was cancer-specific, while beta1 protein was detected also in stromal fibroblasts and endothelial cells.The alphav- and beta1-integrin subunits are frequently expressed in ovarian carcinoma cells in effusions, and the alphav-integrin subunit is a powerful diagnostic marker for carcinoma cells. The reduced expression of the beta1-integrin subunit in solid lesions may be attributed to the role of other subunits at these stages, such as the beta3 subunit as part of the alphavbeta3-vitronectin receptor. The high expression of integrin subunits with a role of binding mesothelium, invasion, and angiogenesis in carcinoma cells in both peritoneal and pleural effusions suggests that cells at both sites have metastatic potential. 
 Exogenous SPARC suppresses proliferation and migration of prostate cancer by interacting with integrin β1. The matricellular protein secreted protein acidic and rich in cysteine (SPARC) plays an important role on tumor   metastasi  s and progression in several cancers. However, the roles of SPARC in prostate cancer (PCa) remain unclear.To identify SPARC protein in prostate tissue, immunohistochemical analysis of SPARC was conducted using human prostate tissue microarray. To detect SPARC expression in prostate cancer (LNCaP, DU145, and PC-3) and stromal cells, RT-PCR, western blot analysis, and ELISA was conducted. To reveal the function of exogenous SPARC in PCa cells, AKT phosphorylation was confirmed by western blot analysis after coculture with stromal cells. Proliferation and migration of PCa cells were examined by addition of SPARC. The interaction between SPARC and integrin β1 was confirmed by western blot analysis after immunoprecipitation.SPARC protein was expressed well in normal tissue compared with PCa tissue. ELISA showed high secreted SPARC protein in normal prostate-derived stromal cell (PrSC) compared with PCa-derived stromal cell (PCaSC) and PCa. PCa cells cocultured with PrSC showed reduced AKT phosphorylation more than with PCaSC. PCa cells cocultured with PrSC whose SPARC was knocked-down restored AKT phosphorylation. Moreover, PCa cells treated with SPARC led to reduced AKT phosphorylation. Immunoprecipitation with SPARC revealed interaction of SPARC and integrin β1 in PCa cells. Inhibited proliferation and migration of PCa cells by SPARC was restored by integrin β1 neutralizing antibody.Reduced SPARC secretion from stromal cells might affect PCa progression mediating through limiting AKT phosphorylation after interaction with integrin β1. 
 Integrin expression regulates neuroblastoma attachment and migration. Neuroblastoma (NBL) is the most common malignant disease of infancy, and children with bone   metastasis   have a mortality rate greater than 90%. Two major classes of proteins, integrins and growth factors, regulate the metastatic process. We have previously shown that tumorigenic NBL cells express higher levels of the type I insulin-like growth factor receptor (IGF-IR) and that beta1 integrin expression is inversely proportional to tumorigenic potential in NBL. In the current study, we analyze the effect of beta1 integrin and IGF-IR on NBL cell attachment and migration. Nontumorigenic S-cells express high levels of beta1 integrin, whereas tumorigenic N-cells express little beta1 integrin. Alterations in beta1 integrin are due to regulation at the protein level, as translation is decreased in N-type cells. Moreover, inhibition of protein synthesis shows that beta1 integrin is degraded more slowly in S-type cells (SHEP) than in N-type cells (SH-SY5Y and IMR32). Inhibition of alpha5beta1 integrin prevents SHEP (but not SH-SY5Y or IMR32) cell attachment to fibronectin and increases SHEP cell migration. Increases in IGF-IR decrease beta1 integrin expression, and enhance SHEP cell migration, potentially through increased expression of alphavbeta3. These data suggest that specific classes of integrins in concert with IGF-IR regulate NBL attachment and migration. 
 CD147-targeting siRNA inhibits cell-matrix adhesion of human malignant melanoma cells by phosphorylating focal adhesion kinase. CD147/basigin, highly expressed on the surface of malignant tumor cells including malignant melanoma (MM) cells, plays a critical role in the invasiveness and   metastasis   of MM.   Metastasis   is an orchestrated process comprised of multiple steps including adhesion and invasion. Integrin, a major adhesion molecule, co-localizes with CD147/basigin on the cell surface. Using the human MM cell line A375 that highly expresses CD147/basigin, we investigated whether CD147/basigin is involved in adhesion in association with integrin. CD147/basigin was knocked-down using siRNA targeting CD147 to elucidate the role of CD147/basigin. Cell adhesion was evaluated by adhesion assay on matrix-coated plates. The localization of integrin was inspected under a confocal microscope and the expression and phosphorylation of focal adhesion kinase (FAK), a downstream kinase of integrin, were examined by western blot analysis. Silencing of CD147/basigin in A375 cells by siRNA induced the phosphorylation of FAK at Y397. Integrin identified on the surface of parental cells was distributed in a speckled fashion in the cytoplasm of CD147 knockdown cells, resulting in morphological changes from a round to a polygonal shape with pseudopodial protrusions. Silencing of CD147/basigin in A375 cells clearly weakened their adhesiveness to collagen I and IV. Our results suggest that CD147/basigin regulates the adhesion of MM cells to extracellular matrices and of integrin β1 signaling via the phosphorylation of FAK. 
 The CD9/CD81 tetraspanin complex and tetraspanin CD151 regulate α3β1 integrin-dependent tumor cell behaviors by overlapping but distinct mechanisms. Integrin α3β1 potently promotes cell motility on its ligands, laminin-332 and laminin-511, and this may help to explain why α3β1 has repeatedly been linked to breast carcinoma progressio  n and meta  stasis. The pro-migratory functions of α3β1 depend strongly on lateral interactions with cell surface tetraspanin proteins. Tetraspanin CD151 interacts directly with the α3 integrin subunit and links α3β1 integrin to other tetraspanins, including CD9 and CD81. Loss of CD151 disrupts α3β1 association with other tetraspanins and impairs α3β1-dependent motility. However, the extent to which tetraspanins other than CD151 are required for specific α3β1 functions is unclear. To begin to clarify which aspects of α3β1 function require which tetraspanins, we created breast carcinoma cells depleted of both CD9 and CD81 by RNA interference. Silencing both of these closely related tetraspanins was required to uncover their contributions to α3β1 function. We then directly compared our CD9/CD81-silenced cells to CD151-silenced cells. Both CD9/CD81-silenced cells and CD151-silenced cells showed delayed α3β1-dependent cell spreading on laminin-332. Surprisingly, however, once fully spread, CD9/CD81-silenced cells, but not CD151-silenced cells, displayed impaired α3β1-dependent directed motility and altered front-rear cell morphology. Also unexpectedly, the CD9/CD81 complex, but not CD151, was required to promote α3β1 association with PKCα in breast carcinoma cells, and a PKC inhibitor mimicked aspects of the CD9/CD81-silenced cell motility defect. Our data reveal overlapping, but surprisingly distinct contributions of specific tetraspanins to α3β1 integrin function. Importantly, some of CD9/CD81's α3β1 regulatory functions may not require CD9/CD81 to be physically linked to α3β1 by CD151. 
 The DSCs-expressed CD82 controls the invasiveness of trophoblast cells via integrinbeta1/MAPK/MAPK3/1 signaling pathway in human first-trimester pregnancy. CD82 is recognized as a wide-spectrum tumor   metastasis   suppressor that inhibits cancer cell motility and invasiveness. At the human maternal-fetal interface, the decidua is believed to effectively limit the inappropriate invasion of trophoblasts. Here we have found the transcription and translation of CD82 in decidual stromal cells (DSCs), whereas trophoblast cells do not express CD82. The in-cell Western analysis reveals attenuation of CD82 translation in DSCs by human chorionic gonadotropin (hCG), but not by estrogen or progesterone. It is demonstrated that silencing of CD82 by RNA interference increases integrinbeta1, decreases TIMP1 expression in DSCs, and promotes the invasion of the first-trimester human trophoblasts in the coculture. Moreover, U0126, or anti-integrinbeta1 neutralizing antibody, reverses the decreased TIMP1 expression and the increased invasiveness of trophoblast cells, and the antibody also inhibits the MAPK3/1 phosphorylation induced by CD82 silence. After transfection with CD82, the invasive index of BeWo cells decreases significantly with TIMP1 increase. The results above indicate that the DSCs-expressed CD82 up-regulates the expression of TIMP1 in an autocrine manner and inhibits the invasiveness of human first-trimester trophoblast cells partly through the integrinbeta1/MAPK/MAPK3/1 signaling pathway. Furthermore, we have found that the mRNA and protein level of CD82 in decidua of the miscarriage is significantly higher than that of the normal early pregnancy, which implies that the abnormal higher CD82 expression in decidua restricts appropriate invasion of trophoblasts that leads to early pregnancy wastage. 
 Expression of estrogen receptor beta increases integrin alpha1 and integrin beta1 levels and enhances adhesion of breast cancer cells. Estrogen effects on mammary gland development and differentiation are mediated by two receptors (ERalpha and ERbeta). Estrogen-bound ERalpha induces proliferation of mammary epithelial and cancer cells, while ERbeta is important for maintenance of the differentiated epithelium and inhibits proliferation in different cell systems. In addition, the normal breast contains higher ERbeta levels compared to the early stage breast cancers, suggesting that loss of ERbeta could be important in cancer development. Analysis of ERbeta-/- mice has consistently revealed reduced expression of cell adhesion proteins. As such, ERbeta is a candidate modulator of epithelial homeostasis and   metastasis  . Consequently, the aim of this study was to analyze estrogenic effects on adhesion of breast cancer cells expressing ERalpha and ERbeta. As ERbeta is widely found in breast cancer but not in cell lines, we used ERalpha positive T47-D and MCF-7 human breast cancer cells to generate cells with inducible ERbeta expression. Furthermore, the colon cancer cell lines SW480 and HT-29 were also used. Integrin alpha1 mRNA and protein levels increased following ERbeta expression. Integrin beta1-the unique partner for integrin alpha1-increased only at the protein level. ERbeta expression enhanced the formation of vinculin containing focal complexes and actin filaments, indicating a more adhesive potential. This was confirmed by adhesion assays where ERbeta increased adhesion to different extracellular matrix proteins, mostly laminin. In addition, ERbeta expression was associated to less cell migration. These results indicate that ERbeta affects integrin expression and clustering and consequently modulates adhesion and migration of breast cancer cells. 
 Profiling of the tetraspanin CD151 web and conspiracy of CD151/integrin β1 complex in the progression of hepatocellular carcinoma. Tetraspanin CD151 has been implicated in   metastasi  s through forming complexes with different molecular partners. In this study, we mapped tetraspanin web proteins centered on CD151, in order to explore the role of CD151 complexes in the progression of hepatocellular carcinoma (HCC). Immunoprecipitation was used to isolate tetraspanin complexes from HCCLM3 cells using a CD151 antibody, and associated proteins were identified by mass spectrometry. The interaction of CD151 and its molecular partners, and their roles in invasiveness and   metastasi  s of HCC cells were assayed through disruption of the CD151 network. Finally, the clinical implication of CD151 complexes in HCC patients was also examined. In this study, we identified 58 proteins, characterized the tetraspanin CD151 web, and chose integrin β1 as a main partner to further investigate. When the CD151/integrin β1 complex in HCC cells was disrupted, migration, invasiveness, secretion of matrix metalloproteinase 9, a  nd metasta  sis were markedly influenced. However, both CD151 and integrin β1 expression were untouched. HCC patients with high expression of CD151/integrin β1 complex had the poorest prognosis of the whole cohort of patients. Together, our data show that CD151 acts as an important player in the progression of HCC in an integrin β1-dependent manner. 
  GeneRIFs  
 TERT may promote gastric cancer   metastasis   through the TERT-miR-29a-ITGB1 regulatory pathway. 
 Insulin-like growth factor-independent insulin-like growth factor binding protein 3 promotes cell migration and lymph node   metastasis   of oral squamous cell carcinoma cells via integrin beta1 signaling. 
 Down-regulation of ARNT promotes colorectal cancer   metastasis   by activating the fibronectin/integrin beta1/FAK axis. 
 These results reveal betaig-h3 enhances the   metastasis   potentials of human osteosarcoma cells via integrin alpha2beta1-mediated PI3K/AKT signal pathways. 
 The alpha5beta1-integrin promotes angiogenesis and associates with lymph node   metastasis  , vascular invasion and poor prognosis of gastric cancer 
 TGM2 up-regulation along with ITGB1 and SDC4 plays an important role in the development of RCC tumors and advanced RCC with   metastasis   
 Data indicate that miR-134 suppressed the invasion and   metastasis   of hepatocellular carcinoma cells in vitro and in vivo, and integrin beta 1 (ITGB1) was a direct and functional target gene of miR-134. 
 High VLA-4 expression is associated with brain   metastasis  . 
 ANGPTL2 expression in OS cell lines correlated with increased tumor   metastasis   and decreased animal survival by promoting tumor cell intravasation mediated by the integrin alpha5beta1, p38 mitogen-activated protein kinase, and matrix metalloproteinases 
 beta-1,4-Galactosyltransferase III suppresses beta1 integrin-mediated invasive phenotypes and negatively correlates with   metastasis   in colorectal cancer. 
 CD44v6 and integrin-beta1 mRNA and protein expression in blood may serve as biomarkers for the development and   metastasis   of pancreatic cancer 
 miR-29c serves as a tumor   metastasis   suppressor, which suppresses lung cancer cell adhesion to ECM and   metastasis   by directly inhibiting integrin beta1 and MMP2 expression 
 The data suggest that integrin beta1 is a potential biomarker for lymph node   metastasis   and prognosis of lung adenocarcinoma and integrin beta1 upregulation may play an important role in the pathogenesis of lung adenocarcinoma. 
 osteopontin, LAMB3 and ITGB1 played important roles in the occurrence and   metastasis   of lung cancer, thus provided important clues to understanding the molecular mechanism of   metastasis  . 
 data demonstrate that alpha2beta1 protein was elevated in PCa skeletal metastases compared to either PCa primary lesions or soft tissue metastases suggesting that alpha2beta1 contributes to the selective   metastasis   to the bone. 
 These findings indicate that integrin beta1 has a major impact on HNSCC prognosis through its regulation of   metastasis  . 
 The higher expression of CD44v6, integrin-beta1, CA199, and CEA are closely related to the progression and   metastasis   of pancreatic cancer. 
   Metastasis   suppressor KAI1/CD82 attenuates the matrix adhesion of human prostate cancer cells by suppressing fibronectin expression and beta1 integrin activation. 
 The extra nuclear AR/FlnA/integrin beta 1 complex is the key by which androgen activates signaling leading to cell migration. Assembly of this ternary complex may control organ development and prostate cancer   metastasis  . 
 fibronectin promotes ovarian cancer invasion and   metastasis   through an alpha(5)beta(1)-integrin/c-Met/FAK/Src-dependent signaling pathway. 
 Data suggest that the alpha5 laminins emerge as putative primary extracellular matrix mediators of melanoma invasion and   metastasis   via alpha3/alpha6beta1 and other integrin receptors. 
 alpha2beta1 integrin functionally inhibits breast tumor   metastasis  , and alpha2 expression may serve as an important biomarker of metastatic potential and patient survival 
 alpha4beta1-mediated tumor lymphangiogenesis promotes   metastasis   
 results suggest that PRL-3's roles in motility, invasion, and   metastasis   in colon cancer are critically controlled by the integrin beta1-ERK1/2-MMP2 signaling 
 These results strongly suggest that enhanced expression of alpha3beta1 integrin on hepatocellular carcinoma cells is directly involved in their malignant phenotypes such as invasion and   metastasis  . 
 Overexpression of AKT2/protein kinase Bbeta leads to up-regulation, increased invasion, and   metastasis   of human breast and ovarian cancer cells. 
 Serum E-selectin, ICAM-1 and integrin beta1 expression levels are probably related to the   metastasis   and relapse of gastric cancer. 
 Data suggest that the early arrest of tumor cells in the pulmonary vasculature through interaction of alpha3beta1 integrin with laminin-5 in exposed basement membrane provides a basis for cell arrest during pulmonary   metastasis  . 
 The integrin, beta 1 was expressed by prostate cancer cells in vitro and by prostate tumors in vivo, and their expression was elevated at sites of bone   metastasis   compared to original prostate tumor. 
 beta 1 integrin has roles in cell adhesion and migration in development and   metastasis   [review] 
  [Cc]ell [Ii]nvasion  PubMed  
 Epidermal growth factor (EGF) regulates α5β1 integrin activation state in human cancer cell lines through the p90RSK-dependent phosphorylation of filamin A. Regulation of integrin activation has important implications for tumo  r cell invasi  on and metastasis.EGF activates ERK/p90RSK and Rho/Rho kinase signaling in A431 and DiFi colon cancer cells, leading to phosphorylation of filamin A (FLNa) and inactivation of the α5β1 integrin receptor.EGF promotes α5β1 inactivation through the p90RSK-dependent phosphorylation of FLNa.We have identified a novel EGF-dependent mechanism controlling the α5β1 integrin activation state. Cell adhesion, motility, and invasion are regulated by the ligand-binding activity of integrin receptors, transmembrane proteins that bind to the extracellular matrix. Integrins whose conformation allows for ligand binding and appropriate functional activity are said to be in an active state. Integrin activation and subsequent ligand binding are dynamically regulated by the association of cytoplasmic proteins with integrin intracellular domains. In this study, we evaluated the role of EGF in the regulation of the activation state of the α5β1 integrin receptor for fibronectin. The addition of EGF to either A431 squamous carcinoma cells or DiFi colon cancer cells resulted in loss of α5β1-dependent adhesion to fibronectin but no loss of integrin from the cell surface. EGF activated the EGF receptor/ERK/p90RSK and Rho/Rho kinase signaling pathways. Blocking either pathway inhibited EGF-mediated loss of adhesion, suggesting that they work in parallel to regulate integrin function. EGF treatment also resulted in phosphorylation of filamin A (FLNa), which binds and inactivates β1 integrins. EGF-mediated FLNa phosphorylation was completely blocked by an inhibitor of p90RSK and partially attenuated by an inhibitor of Rho kinase, suggesting that both pathways converge on FLNa to regulate integrin function. A431 clonal cell lines expressing non-phosphorylated dominant-negative FLNa were resistant to the inhibitory effects of EGF on integrin function, whereas clonal cell lines overexpressing wild-type FLNa were more sensitive to the inhibitory effect of EGF. These data suggest that EGF-dependent inactivation of α5β1 integrin is regulated through FLNa phosphorylation and cellular contractility. 
 Epidermal growth factor (EGF) regulates α5β1 integrin activation state in human cancer cell lines through the p90RSK-dependent phosphorylation of filamin A. Regulation of integrin activation has important implications for tumo  r cell invasi  on and metastasis.EGF activates ERK/p90RSK and Rho/Rho kinase signaling in A431 and DiFi colon cancer cells, leading to phosphorylation of filamin A (FLNa) and inactivation of the α5β1 integrin receptor.EGF promotes α5β1 inactivation through the p90RSK-dependent phosphorylation of FLNa.We have identified a novel EGF-dependent mechanism controlling the α5β1 integrin activation state. Cell adhesion, motility, and invasion are regulated by the ligand-binding activity of integrin receptors, transmembrane proteins that bind to the extracellular matrix. Integrins whose conformation allows for ligand binding and appropriate functional activity are said to be in an active state. Integrin activation and subsequent ligand binding are dynamically regulated by the association of cytoplasmic proteins with integrin intracellular domains. In this study, we evaluated the role of EGF in the regulation of the activation state of the α5β1 integrin receptor for fibronectin. The addition of EGF to either A431 squamous carcinoma cells or DiFi colon cancer cells resulted in loss of α5β1-dependent adhesion to fibronectin but no loss of integrin from the cell surface. EGF activated the EGF receptor/ERK/p90RSK and Rho/Rho kinase signaling pathways. Blocking either pathway inhibited EGF-mediated loss of adhesion, suggesting that they work in parallel to regulate integrin function. EGF treatment also resulted in phosphorylation of filamin A (FLNa), which binds and inactivates β1 integrins. EGF-mediated FLNa phosphorylation was completely blocked by an inhibitor of p90RSK and partially attenuated by an inhibitor of Rho kinase, suggesting that both pathways converge on FLNa to regulate integrin function. A431 clonal cell lines expressing non-phosphorylated dominant-negative FLNa were resistant to the inhibitory effects of EGF on integrin function, whereas clonal cell lines overexpressing wild-type FLNa were more sensitive to the inhibitory effect of EGF. These data suggest that EGF-dependent inactivation of α5β1 integrin is regulated through FLNa phosphorylation and cellular contractility. 
 Mammary-derived growth inhibitor (MDGI) interacts with integrin α-subunits and suppresses integrin activity and invasion. The majority of mortality associated with cancer is due to formation of metastases from the primary tumor. Adhesion mediated by different integrin heterodimers has an important role during cell migration and invasion. Protein interactions with the β1-integrin cytoplasmic tail are known to influence integrin affinity for extracellular ligands, but regulating binding partners for the α-subunit cytoplasmic tails have remained elusive. In this study, we show that mammary-derived growth inhibitor (MDGI) (also known as FABP-3 or H-FABP) binds directly to the cytoplasmic tail of integrin α-subunits and its expression inhibits integrin activity. In breast cancer cell lines, MDGI expression correlates with suppression of the active conformation of integrins. This results in reduced integrin adhesion to type I collagen and fibronectin and inhibition of cell migration and invasion. In tissue microarray of 1331 breast cancer patients, patients with MDGI-positive tumors had more favorable 10-year distant disease-free survival compared with patients with MDGI-negative tumors. Our data indicate that MDGI is a novel interacting partner for integrin α-subunits, and its expression modulates integrin activity and suppre  sses cell inv  asion in breast cancer patients. Retained MDGI expression is associated with favorable prognosis. 
 Relative roles of GM1 ganglioside, N-acylneuraminic acids, and α2β1 integrin in mediating rotavirus infection. N-acetyl- and N-glycolylneuraminic acids (Sia) and α2β1 integrin are frequently used by rotaviruses as cellular receptors through recognition by virion spike protein VP4. The VP4 subunit VP8*, derived from Wa rotavirus, binds the internal N-acetylneuraminic acid on ganglioside GM1. Wa infection is increased by enhanced internal Sia access following terminal Sia removal from main glycan chains with sialidase. The GM1 ligand cholera toxin B (CTB) reduces Wa infectivity. Here, we found sialidase treatment increased cellular GM1 availability and the infectivity of several other human (including RV-3) and animal rotaviruses, typically rendering them susceptible to methyl α-d-N-acetylneuraminide treatment, but did not alter α2β1 usage. CTB reduced the infectivity of these viruses. Aceramido-GM1 inhibited Wa and RV-3 infectivity in untreated and sialidase-treated cells, and GM1 supplementation increased their infectivity, demonstrating the importance of GM1 for infection. Wa recognition of α2β1 and internal Sia were at least partially independent. Rotavirus usage of GM1 was mapped to VP4 using virus reassortants, and RV-3 VP8* bound aceramido-GM1 by saturation transfer difference nuclear magnetic resonance (STD NMR). Most rotaviruses recognizing terminal Sia did not use GM1, including RRV. RRV VP8* interacted minimally with aceramido-GM1 by STD NMR. Unusually, TFR-41 rotavirus infectivity depended upon terminal Sia and GM1. Competition of CTB, Sia, and/or aceramido-GM1 with cell binding by VP8* from representative rotaviruses showed that rotavirus Sia and GM1 preferences resulted from VP8*-cell binding. Our major finding is that infection by human rotaviruses of commonly occurring VP4 serotypes involves VP8* binding to cell surface GM1 glycan, typically including the internal N-acetylneuraminic acid.Rotaviruses, the major cause of severe infantile gastroenteritis, recognize cell surface receptors through virus spike protein VP4. Several animal rotaviruses are known to bind sialic acids at the termini of main carbohydrate chains. Conversely, only a single human rotavirus is known to bind sialic acid. Interestingly, VP4 of this rotavirus bound to sialic acid that forms a branch on the main carbohydrate chain of the GM1 ganglioside. Here, we use several techniques to demonstrate that other human rotaviruses exhibit similar GM1 usage properties. Furthermore, binding by VP4 to cell surface GM1, involving branched sialic acid recognition, is shown to facilitate infection. In contrast, most animal rotaviruses that bind terminal sialic acids did not utilize GM1 for VP4 cell binding or infection. These studies support a significant role for GM1 in mediat  ing host cell   invasion by human rotaviruses. 
 Novel focal adhesion protein kindlin-2 promotes the invasion of gastric cancer cells through phosphorylation of integrin β1 and β3. We have found that the expression of the novel focal adhesion protein kindlin-2 had a significant positive correlation with poor survival in gastric cancer. However, the mechanism by which kindlin-2 acts in gastric cancer warrants further evaluation.Kindlin-2 mRNA expression in gastric cancer cell lines was measured by realtime RT-PCR under normal and hypoxic conditions. Cell proliferation, apoptosis, cell cycle, tumor adhesion  , cell invasi  on ability, and phosphorylation of integrin β1 and β3 proteins were measured to assess the influence of kindlin-2 on the malignant behavior of gastric cancer cells.Kindlin-2 mRNA expression was highest in the distant metastasis gastric cancer cell line Hs-746T. Cell proliferation, adhesion with endothelium and collagen IV, invasion rate, and angiogenesis genes expression, as well as phosphorylation of integrin β1 and β3 in Hs-746T, were decreased significantly after kindlin-2 downregulation, but there was no change in apoptosis and cell cycle.Kindlin-2 might promote the invasion of gastric cancer cells through enhancing proliferation and adhesion by the phosphorylation of integrin β1 and β3. 
 Thrombin induces tumor invasion through the induction and association of matrix metalloproteinase-9 and beta1-integrin on the cell surface. The procoagulatory serine protease, thrombin, is known to induce invasion and metastasis in various cancers, but the mechanisms by which it promotes tumorigenesis are poorly understood. Because the 92-kDa gelatinase (MMP-9) is a known mediator of tumor   cell invasion  , we sought to determine whether and how thrombin regulates MMP-9. The thrombin receptor, PAR-1, and MMP-9 are expressed in osteosarcomas, as determined by immunohistochemistry. Stimulation of U2-OS osteosarcoma cells with thrombin and a thrombin receptor-activating peptide induced pro-MMP-9 secretion as well as cell surface-associated pro-MMP-9 expression and proteolytic activity. This was paralleled by an increase in MMP-9 mRNA and MMP-9 promoter activity. Thrombin-induced invasion of U2-OS cells through Matrigel was mediated by the phosphatidylinositol 3-kinase signaling pathway and could be inhibited with an MMP-9 antibody. The stimulation of MMP-9 by thrombin was paralleled by an increase in beta1-integrin mRNA and beta1-integrin expression on the cell surface, which was also mediated by phosphatidylinositol 3-kinase and was required for invasion. Thrombin activation induced and co-localized both beta1-integrin and pro-MMP-9 on the cell membrane, as evidenced by co-immunoprecipitation, confocal microscopy, and a protein binding assay. The thrombin-mediated association of these two proteins, as well as thrombin-mediated invasion of U2-OS cells, could be blocked with a cyclic peptide and with an antibody preventing binding of the MMP-9 hemopexin domain to beta1-integrin. These results suggest that thrombin induces expression and association of beta1-integrin with MMP-9 and that the cell surface localization of the protease by the integrin promotes tumor   cell invasion  . 
 Upregulation of Eps8 in oral squamous cell carcinoma promotes cell migration and invasion through integrin-dependent Rac1 activation. Oral squamous cell carcinoma (OSCC) is a lethal disease and early death usually occurs as a result of local invasion and regional lymph node metastases. Current treatment regimens are, to a certain degree, inadequate, with a 5-year mortality rate of around 50% and novel therapeutic targets are urgently required. Using expression microarrays, we identified the eps8 gene as being overexpressed in OSCC cell lines relative to normal oral keratinocytes, and confirmed these findings using RT-PCR and western blotting. In human tissues, we found that Eps8 was upregulated in OSCC (32% of primary tumors) compared with normal oral mucosa, and that expression correlated significantly with lymph node metastasis (P=0.032), suggesting a disease-promoting effect. Using OSCC cell lines, we assessed the functional role of Eps8 in tumor cells. Although suppression of Eps8 produced no effect on cell proliferation, both cell spreading and migration were markedly inhibited. The latter cell functions may be modulated through the small GTP-ase, Rac1 and we used pull-down assays to investigate the role of Eps8 in Rac1 signaling. We found that alphavbeta6- and alpha5beta1-integrin-dependent activation of Rac1 was mediated through Eps8. Knockdown of either Eps8 or Rac1, inhibited integrin-dependent cell migration similarly and transient expression of constitutively active Rac1 restored migration of cells in which Eps8 expression had been suppressed. We also showed that knockdown of Eps8 inhibited tumor   cell invasion   in an organotypic model of OSCC. These data suggest that Eps8 and Rac1 are part of an integrated signaling pathway modulating integrin-dependent tumour cell motility and identify Eps8 as a possible therapeutic target. 
 [Effect of integrin alpha2beta1 on invasion and migration of neuroblastoma cells]. To study the effect of integrin alpha2beta1 on invasion and migration of SK-N-SH neuroblastoma cells.Neuroblastoma SK-N-SH cell line was cultured in the modified eagle's medium. The effects of monoclonal antibodies to integrin alpha2 and integrin beta1 on migration and invasion were measured by inclined test and polycarbonate filters incorporated in modified Transwell chambers respectively. The migration and invasion cells were stained with Gimsa staining and counted under a 200 multiplied microscope. The blocking rate of migration and invasion of cells was calculated.The number of migrated SK-N-SH cells in the anti-alpha2 and anti-beta1 treatment groups (50.9+/-10.5 and 54.3+/-9.0 respectively) was significantly less than that in the control group without monoclonal antibody treatment (98.1+/-7.4) (P<0.01), with a blocking rate of cell migration of 48.1% and 44.5% respectively. The invasion to matrigel of SK-N-SH cells exposed monoclonal antibodies to integrin alpha2 and integrin beta1 was significantly blocked compared with the control SK-N-SH cells, with the number of invasion cells in the anti-alpha2 and anti-beta1 treatment groups of 25.3 +/- 4.4 and 18.8 +/- 3.9 respectively vs 41.5 +/- 4.8 in the control group (P<0.01). The blocking rate of   cell invasion   in the anti-alpha2 and anti-beta1 treatment groups was 39.0% and 54.7% respectively.Integrin alpha2beta1 may promote migration and invasion of neuroblastoma cells. 
 α3β1 integrins regulate CD151 complex assembly and membrane dynamics in carcinoma cells within 3D environments. Integrins are extracellular matrix (ECM) receptors that are key players in the regulation of tumou  r cell invasi  on. The laminin-binding integrin α3β1 has previously been shown to regulate adhesion and migration of carcinoma cells in part through co-operative signalling with the tetraspanin family of transmembrane proteins. However, the spatial and temporal regulation of crosstalk between these families of transmembrane proteins in intact cells remains poorly understood. Here we have used fluorescence resonance energy transfer (FRET) to demonstrate for the first time that α3β1 and the tetraspanin CD151 directly associate at the front and retracting rear of polarised migrating breast carcinoma cells in both two-dimentional (2D) and three-dimentional (3D)matrices. Furthermore, localised α3β1-CD151 binding correlates with lower CD151 homodimerisation in cells migrating on laminin or within matrigel. Loss of α3β1 integrin leads to increased CD151 homodimer formation, increased activation of Rho GTPase, loss of cell polarity and decreased invasion in 3D ECM. As a result, α3-silenced cells show decreased actin-based membrane protrusion and retraction in both 2D and 3D environments. These data demonstrate that associations between α3β1 and CD151 occur dynamically within discrete subcellular compartments and act to establish local GTPase signalling to pr  omote tumour   cell invasion. These novel findings shed light on the complex crosstalk and switching between receptor complexes in response to different extracellular   cues during   cell invasion in 3D environments. 
 AMPA receptors promote perivascular glioma invasion via beta1 integrin-dependent adhesion to the extracellular matrix. High-grade gliomas release excitotoxic concentrations of glutamate, which has been shown to enhance tumor proliferation and migration. alpha-Amino-3-hydroxy-5-methylisoxazole-4-propionic acid (AMPA) glutamate receptors are abundantly expressed at the invading edge of glioblastoma specimens, suggesting they may play an important biologic role in tumor invasion. In this study, we examined potential mechanisms by which AMPA receptor (AMPAR) expression and stimulation promote glioma cell migration and invasion. Overexpression of GluR1, the most abundant AMPAR subunit in gliomas, positively correlated with glioma cell adhesion to type I and type IV collagen, which was decreased in cells with knockdown of GluR1 and with blocking antibodies to beta1 integrin. Furthermore, stimulation of the AMPAR led to detachment of cells from the extracellular matrix (ECM). Immunoprecipitation studies showed that GluR1 associated with the actin cytoskeleton-linked protein band 4.1B (brain type), which may serve as a link between GluR1 and integrins. Overexpression of GluR1 correlated with increased cell-surface expression of beta1 integrin, increased phosphorylation of focal adhesion kinase (FAK-Y397), and enhanced numbers of focal adhesion (FA) complexes. Cells overexpressing GluR1 had increased colocalization of actin and paxillin at FAs and, in several glioma cell lines, significantly increased invasion in an in vitro Matrigel transwell assay. Likewise, in an intracranial xenograft model, overexpression of GluR1 led to perivascular and subependymal glioma   cell invasion   similar to patterns of tumor dissemination described in human glioblastoma. Together, these results suggest that AMPARs may link signals from the ECM to sites of FA, where signal integration promotes tumor invasion. 
 Prostaglandin E2 stimulates β1-integrin expression in hepatocellular carcinoma through the EP1 receptor/PKC/NF-κB pathway. Prostaglandin E2 (PGE2) has been implicated i  n cell invasi  on in hepatocellular carcinoma (HCC), via increased β1-integrin expression and cell migration; however, the mechanism remains unclear. PGE2 exerts its effects via four subtypes of the E prostanoid receptor (EP receptor 1-4). The present study investigated the effect of EP1 receptor activation on β1-integrin expression and cell migration in HCC. Cell migration increased by 60% in cells treated with 17-PT-PGE2 (EP1 agonist), which was suppressed by pretreatment with a β1-integrin polyclonal antibody. PGE2 increased β1-integrin expression by approximately 2-fold. EP1 receptor transfection or treatment with 17-PT-PGE2 mimicked the effect of PGE2 treatment. EP1 siRNA blocked PGE2-mediated β1-integrin expression. 17-PT-PGE2 treatment induced PKC and NF-κB activation; PKC and NF-κB inhibitors suppressed 17-PT-PGE2-mediated β1-integrin expression. FoxC2, a β1-integrin transcription factor, was also upregulated by 17-PT-PGE2. NF-κB inhibitor suppressed 17-PT-PGE2-mediated FoxC2 upregulation. Immunohistochemistry showed p65, FoxC2, EP1 receptor and β1-integrin were all highly expressed in the HCC cases. This study suggested that PGE2 upregulates β1-integrin expression and cell migration in HCC cells by activating the PKC/NF-κB signaling pathway. Targeting PGE2/EP1/PKC/NF-κB/FoxC2/β1-integrin pathway may represent a new therapeutic strategy for the prevention and treatment of this cancer. 
 Targeting of integrin beta1 and kinesin 2alpha by microRNA 183. MicroRNA 183 (miR-183) has been reported to inhibit tumor invasiveness and is believed to be involved in the development and function of ciliated neurosensory organs. We have recently found that expression of miR-183 increased after the induction of cellular senescence by exposure to H(2)O(2). To gain insight into the biological roles of miR-183 we investigated two potential novel targets: integrin beta1 (ITGB1) and kinesin 2alpha (KIF2A). miR-183 significantly decreased the expression of ITGB1 and KIF2A measured by Western blot. Targeting of the 3'-untranslated region (3'-UTR) of ITGB1 and KIF2A by miR-183 was confirmed by luciferase assay. Transfection with miR-183 led to a significant decrease in   cell invasion   and migration capacities of HeLa cells that could be rescued by expression of ITGB1 lacking the 3'-UTR. Although miR-183 had no effects on cell adhesion in HeLa cells, it significantly decreased adhesion to laminin, gelatin, and collagen type I in normal human diploid fibroblasts and human trabecular meshwork cells. These effects were also rescued by expression of ITGB1 lacking the 3'-UTR. Targeting of KIF2A by miR-183 resulted in some increase in the formation of cells with monopolar spindles in HeLa cells but not in human diploid fibroblast or human trabecular meshwork cells. The regulation of ITGB1 expression by miR-183 provides a new mechanism for the anti-metastatic role of miR-183 and suggests that this miRNA could influence the development and function in neurosensory organs, and contribute to functional alterations associated with cellular senescence in human diploid fibroblasts and human trabecular meshwork cells. 
 β-1,4-Galactosyltransferase III suppresses extravillous trophoblast invasion through modifying β1-integrin glycosylation. Glycosylation controls diverse protein functions and regulates various cellular phenotypes. Trophoblast invasion is essential for normal placental development. However, the role of glycosylation in human placenta throughout pregnancy is still unclear. The β-1,4-galactosyltransferase III (B4GALT3) has been found to regulate canc  er cell invas  ion. We therefore investigated the expression of B4GALT3 in placenta and its roles in trophoblast.B4GALT3 protein expression was examined by quantitative Western blotting analysis in human placentas. For identification of B4GALT3-positive cells in normal human placenta, immunohistochemistry and immunofluorescence methods were used. To investigate effects of B4GALT3 on extravillous trophoblast (EVT)-like cell and primary EVT cells, we analyzed cell growth, adhesion, migration, and invasion in mock and B4GALT3-transfected cell.B4GALT3 expression significantly increased in third trimester human placenta. Immunostaining revealed that B4GALT3 expressed in placental villous cytotrophoblast, syncytiotrophoblast, and a subpopulation of EVT cells throughout pregnancy. Interestingly, we found increases in the expression level and percentage of B4GALT3-positive cells in third trimester EVT, but not in syncytiotrophoblasts and cytotrophoblasts of placental villi. Overexpression of B4GALT3 in HTR8/SVneo cells and primary trophoblast cells significantly suppressed cell migration. In addition, B4GALT3 suppress  ed cell invas  ion, and enhanced cell adhesion to laminin in HTR8/SVneo cells. Notably, we found that B4GALT3 modified glycans on β1-integrin, suppressed focal adhesion kinase (FAK) signaling, and enhanced β1-integrin degradation.We propose that B4GALT3-mediated glycosylation change not only enhances β1-integrin binding to laminin, but also attenuates β1-integrin stability. Our findings suggest that B4GALT3 is a critical regulator for suppressing EVT invasion in the late stages of pregnancy. 
 Staged stromal extracellular 3D matrices differentially regulate breast cancer cell responses through PI3K and beta1-integrins. Interactions between cancer cells and stroma are critical for growth and invasiveness of epithelial tumors. The biochemical mechanisms behind tumor-stromal interactions leading to increased invasiveness and metastasis are mostly unknown. The goal of this study was to analyze the direct effects of staged stroma-derived extracellular matrices on breast cancer cell behavior.Early and late three-dimensional matrices were produced by NIH-3T3 and tumor-associated murine fibroblasts, respectively. After removing fibroblasts, extracted matrices were re-cultured with breast epithelial cells of assorted characteristics: MCF-10A (non-tumorigenic), MCF-7 (tumorigenic, non-invasive), and MDA-MB-231 (tumorigenic, invasive). Effects prompted by staged matrices on epithelial cell's growth, morphology and invasion were determined. Also, matrix-induced velocity, directionality and relative track orientation of invasive cells were assessed in the presence or absence of inhibitors of phosphoinositide-3 kinase (PI3K) and/or beta-1 integrin.We observed that assorted breast epithelial cells reacted differently to two-dimensional vs. staged, control (early) and tumor-associated (late), three-dimensional matrices. MCF-10A had a proliferative advantage on two-dimensional substrates while MCF-7 and MDA-MB-231 showed no difference. MCF-10A and MCF-7 formed morphologically distinguishable aggregates within three-dimensional matrices, while MDA-MB-231 exhibited increased spindle-shape morphologies and directional movements within three-dimensional matrices. Furthermore, MDA-MB-231 acquired a pattern of parallel oriented organization within tumor-associated, but not control matrices. Moreover, tumor-associated matrices induced PI3K and beta1-integrin dependent Akt/PKB activity in MDA-MB-231 cells. Interestingly, beta1-integrin (but not PI3K) regulated tumor-associated matrix-induced mesenchymal invasion which, when inhibited, resulted in a change of invasive strategy rather than impeding invasion altogether.We propose that both cells and matrices are important to promote effective breast cancer   cell invasion   through three-dimensional matrices and that beta1-integrin inhibition is not necessarily sufficient to block tumor-matrix induced breast cancer   cell invasion  . Additionally, we believe that characterizing stroma staging (e.g., early vs. late or tumor-associated) might be beneficial for predicting matrix-induced cancer cell responses in order to facilitate the selection of therapies. 
 Rab25 associates with alpha5beta1 integrin to promote invasive migration in 3D microenvironments. Here, we report a direct interaction between the beta1 integrin cytoplasmic tail and Rab25, a GTPase that has been linked to tumor aggressiveness and metastasis. Rab25 promotes a mode of migration on 3D matrices that is characterized by the extension of long pseudopodia, and the association of the GTPase with alpha5beta1 promotes localization of vesicles that deliver integrin to the plasma membrane at pseudopodial tips as well as the retention of a pool of cycling alpha5beta1 at the cell front. Furthermore, Rab25-driven tumor-  cell invasion   into a 3D extracellular matrix environment is strongly dependent on ligation of fibronectin by alpha5beta1 integrin and the capacity of Rab25 to interact with beta1 integrin. These data indicate that Rab25 contributes to tumor progression by directing the localization of integrin-recycling vesicles and thereby enhancing the ability of tumor cells to invade the extracellular matrix. 
 Ligand-independent activation of c-Met by fibronectin and α(5)β(1)-integrin regulates ovarian cancer invasion and metastasis. The role of the fibronectin receptor, α(5)β(1)-integrin, as an adhesion receptor and in angiogenesis is well established. However, its role in can  cer cell inva  sion and metastasis is less clear. We describe a novel mechanism by which fibronectin regulates ovarian cancer cell signaling and promotes metastasis. Fibronectin binding to α(5)β(1)-integrin led to a direct association of α(5)-integrin with the receptor tyrosine kinase, c-Met, activating it in a hepatocyte growth factor/scatter factor (HGF/SF) independent manner. Subsequently, c-Met associated with Src, and activated Src and focal adhesion kinase (FAK). Inhibition of α(5)β(1)-integrin decreased the phosphorylation of c-Met, FAK and Src, both in vitro and in vivo. Independent activation of c-Met by its native ligand, HGF/SF, or overexpression of a constitutively active FAK in HeyA8 cells could overcome the effect of α(5)β(1)-integrin inhibitio  n on tumor ce  ll invasion, indicating that α(5)β(1)-integrin is upstream of c-Met, Src and FAK. Inhibition of α(5)β(1)-integrin on cancer cells in two xenograft models of ovarian cancer metastasis resulted in a significant decrease of tumor burden, which was independent of the effect of α(5)β(1)-integrin on angiogenesis. These data suggest that fibronectin promotes ovarian cancer invasion and metastasis through an α(5)β(1)-integrin/c-Met/FAK/Src-dependent signaling pathway, transducing signals through c-Met in an HGF/SF-independent manner. 
 A novel protease-docking function of integrin at invadopodia. Invadopodia are membrane extensions of aggressive tumor cells that function in the activation of membrane-bound proteases occurring during tumor   cell invasion  . We explore a novel and provocative activity of integrins in docking proteases to sites of invasion, termed invadopodia. In the absence of collagen, alpha(3)beta(1) integrin and the gelatinolytic enzyme, seprase, exist as nonassociating membrane proteins. Type I collagen substratum induces the association of alpha(3)beta(1) integrin with seprase as a complex on invadopodia. The results show that alpha(3)beta(1) integrin is a docking protein for seprase to form functional invadopodia. In addition, alpha(5)beta(1) integrin may participate in the adhesion process necessary for invadopodial formation. Thus, alpha(3)beta(1) and alpha(5)beta(1) integrins play major organizational roles in the adhesion and formation of invadopodia, promoting invasive cell behavior. 
 Epithelial-mesenchymal transition in cervical cancer: correlation with tumor progression, epidermal growth factor receptor overexpression, and snail up-regulation. Acquisition of epithelial-mesenchymal transition (EMT) by primary carcinoma cells is associated with disrupted epithelial integrity, local invasion, and ultimately metastasis. Little is known about the existence and function of EMT in cervical cancer. This study aims to investigate the regulation of EMT in cervical squamous cell carcinoma.We investigated the molecular events of EMT in surgical specimens, which present the progression of cervical carcinoma. Two cervical cancer cell lines and the primary culture of normal cervical epithelia were used to study the regulatory mechanisms of EMT.The chronic epidermal growth factor (EGF) treatment induces the elongation of cell shape, increases cell scattering, and enhances   cell invasion  . EGF treatment down-regulates E-cadherin and up-regulates vimentin in cervical cancer cells. These characteristics are consistent with the morphologic changes, molecular events, and functional significance of EMT. EGF receptor (EGFR) signaling inactivates glycogen synthase kinase-3beta, which results in the nuclear accumulation of up-regulated Snail and then leads to EMT program. alpha(5)beta(1) integrin signaling and extracellular matrix fibronectin can modulate EGF-induced EMT. Importantly, the immunofluorescent stainings of surgical specimens indicate that cervical carcinoma progression is accompanied by EGFR overexpression, which is in parallel with decreased E-cadherin and increased vimentin. Up-regulation and nuclear accumulation of Snail correlate with EMT program in tumor tissues.EGF cooperates with alpha(5)beta(1) integrin signaling to induce EMT in cervical cancer cells via up-regulated Snail. Blockade of EGFR activity or expression may provide a potential target for the treatment of cervical cancer progression. 
 Stromal cell-derived factor-1/CXC receptor 4 and β1 integrin interaction regulates urokinase-type plasminogen activator expression in human colorectal cancer cells. The stromal cell-derived factor-1 (SDF-1)/CXC receptor 4 (CXCR4) axis has been shown to play a role in colorectal cancer progression. In addition, the protease urokinase-type plasminogen activator (uPA) is an important factor in tumor   cell invasio  n and metastasis. However, the mechanism by which SDF-1 mediates uPA expression in human colorectal cancer cells remains unknown. We investigated the molecular mechanism governing the interaction between SDF-1 stimulation and uPA expression in three human colon cancer cell lines (DLD-1, SW48, and COLO 205). We found that SDF-1 stimulation led to an increase in the expression and secretion of uPA in these cells. Experiments involving specific inhibitors and small interfering RNA demonstrated that the activation of p38 mitogen-activated protein kinase (MAPK) and phosphatidylinositol 3-kinase (PI3K)/Akt pathways are critical for SDF-1-induced uPA expression. Analysis of transcription factor binding using ELISA and chromatin immunoprecipitation assays revealed that SDF-1 increased Sp1- and AP-1-DNA-binding activities in DLD-1 cells. Inhibition of Sp1 and AP-1 activation blocked the SDF-1-induced expression and activity of the uPA promoter. The effect of SDF-1 on DLD-1 signaling and uPA expression was mediated by the CXCR4/β1 integrin axis. In summary, our findings elucidate the mechanisms of SDF-1/CXCR4 downstream signaling and provide insights into the function of SDF-1 in colon cancer cells. 
 α5β1 integrin recycling promotes Arp2/3-independent cance  r cell invasi  on via the formin FHOD3. Invasive migration in 3D extracellular matrix (ECM) is crucial to cancer metastasis, yet little is known of the molecular mechanisms that drive reorganization of the cytoskeleton as cancer cells disseminate in vivo. 2D Rac-driven lamellipodial migration is well understood, but how these features apply to 3D migration is not clear. We find that lamellipodia-like protrusions and retrograde actin flow are indeed observed in cells moving in 3D ECM. However, Rab-coupling protein (RCP)-driven endocytic recycling of α5β1 integrin enhances invasive migration of cancer cells into fibronectin-rich 3D ECM, driven by RhoA and filopodial spike-based protrusions, not lamellipodia. Furthermore, we show that actin spike protrusions are Arp2/3-independent. Dynamic actin spike assembly in cells invading in vitro and in vivo is regulated by Formin homology-2 domain containing 3 (FHOD3), which is activated by RhoA/ROCK, establishing a novel mechanism through which the RCP-α5β1 pathway reprograms the actin cytoskeleton to promote invasive migration and local invasion in vivo. 
 Vascular endothelial growth factor A (VEGF-A) induces endothelial and cancer cell migration through direct binding to integrin {alpha}9{beta}1: identification of a specific {alpha}9{beta}1 binding site. Integrin α9β1 mediates accelerated cell adhesion and migration through interactions with a number of diverse extracellular ligands. We have shown previously that it directly binds the vascular endothelial growth factors (VEGF) A, C, and D and contributes to VEGF-induced angiogenesis and lymphangiogenesis. Until now, the α9β1 binding site in VEGF has not been identified. Here, we report that the three-amino acid sequence, EYP, encoded by exon 3 of VEGF-A is essential for binding of VEGF to integrin α9β1 and induces adhesion and migration of endothelial and cancer cells. EYP is specific for α9β1 binding and neither requires nor activates VEGFR-2, the cognate receptor for VEGF-A. Following binding to EYP, integrin α9β1 transduces cell migration through direct activation of the integrin signaling intermediates Src and focal adhesion kinase. This interaction is biologically important because it mediates in vitro endothelial cell tube formation, wound healing, a  nd cancer cel  l invasion. These novel findings identify EYP as a potential site for directed pharmacotherapy. 
 The laminin binding integrin alpha6beta1 in prostate cancer perineural invasion. Metastasizing prostate tumor cells invade along nerves innervating the encapsulated human prostate gland in a process known as perineural invasion. The extracellular matrix laminin class of proteins line the neural route and tumor cells escaping from the gland express the laminin binding integrin alpha6beta1 as a prominent cell surface receptor. Integrin alpha6beta1 promotes aggressive disease and supports prostate tumor cell metastasis to bone. Laminins and their integrin receptors are necessary for the development and maintenance of the peripheral nervous system, indicating the potential role for integrin receptors in directing prostate tumor   cell invasion   on nerves during perineural invasion. 
 miR-199a-5p regulates β1 integrin through Ets-1 to suppress invasion in breast cancer. Increasing evidence has revealed that miR-199a-5p is actively involved in tumor invasion and metastasis as well as in the decline of breast cancer tissues. In this research, overexpression of miR-199a-5p weakened motility and invasion of breast cancer cells MCF-7 and MDA-MB-231. Upregulation of Ets-1 increased breast cancer   cell invasio  n, but the mechanism by which miR-199a-5p modulates activation of Ets-1 in breast cancer was not clarified. We investigated the relationship between miR-199a-5p and Ets-1 on the basis of 158 primary breast cancer case specimens, and the results showed that Ets-1 expression was inversely correlated with endogenous miR-199a-5p. Overexpression of miR-199a-5p reduced the mRNA and protein levels of Ets-1 in MCF-7 and MDA-MB-231 cells, whereas anti-miR-199a-5p elevated Ets-1. siRNA-mediated Ets-1 knockdown phenocopied the inhibition invasion of miR-199a-5p in vitro. Moreover, luciferase reporter assay revealed that miR-199a-5p directly targeted 3'-UTR of Ets-1 mRNA. This research revealed that miR-199a-5p could descend the levels of β1 integrin by targeting 3'-UTR of Ets-1 to alleviate the invasion of breast cancer via FAK/Src/Akt/mTOR signaling pathway. Our results provide insight into the regulation of β1 integrin through miR-199a-5p-mediated Ets-1 silence and will help in designing new therapeutic strategies to inhibit signal pathways induced by miR-199a-5p in breast cancer invasion. 
 Matrix metalloproteinase-2 cleavage of the β1 integrin ectodomain facilitates colon cancer cell motility. Cancer   cell invasio  n is a key element in metastasis that requires integrins for adhesion/de-adhesion, as well as matrix metalloproteinases (MMPs) for focalized proteolysis. Herein we show that MMP-2 is up-regulated in resected colorectal tumors and degrades β1 integrins with the release of fragments containing the β1 I-domain. The β1 cleavage pattern is similar to that produced by digestion of α5β1 and α2β1 with MMP-2. Two such fragments, at 25 and 75 kDa, were identified after immunoprecipitation, with monoclonal antibody BD610468 reacting with the NH(2)-terminal I-like ectodomain followed by SDS-PAGE and microsequencing using electrospray (ISI-Q-TOF-Micromass) spectrometry. Cleavage of the β1 integrin can be abolished by inhibition of MMP-2 activity; it can be induced by up-regulation of MMP-2 expression, as exemplified by HT29 colon cancer cells transfected with pCMV6-XL5-MMP-2. Co-immunoprecipitation studies of colon cancer cells showed that the β1 integrin subunit is associated with MMP-2. The MMP-2-mediated shedding of the I-like domain from β1 integrins resulted in decreased adhesion of colon cancer cells to collagen and fibronectin, thus abolishing their receptivity. Furthermore, such cells showed enhanced motility as evaluated by a "wound healing-like" assay and time-lapse microscopy, indicating their increased invasiveness. Altogether, our data demonstrate that MMP-2 amplifies the motility of colon cancer cells, not only by digesting the extracellular matrix components in the vicinity of cancer cells but also by inactivating their major β1 integrin receptors. 
 Formin-like 2 Promotes β1-Integrin Trafficking and Invasive Motility Downstream of PKCα. Regulated turnover of integrin receptors is essential for cell adhesion and migration. Pathways selectively regulating β1-integrin recycling are implicated in cancer invasion and metastasis, yet proteins required for the internalization of this pro-invasive integrin remain to be identified. Here, we uncover formin-like 2 (FMNL2) as a critical regulator of β1-integrin internalization downstream of protein kinase C (PKC). PKCα associates with and phosphorylates FMNL2 at S1072 within its Diaphanous autoregulatory region, leading to the release of formin autoinhibition. Phosphorylation of FMNL2 triggers its rapid relocation and promotes its interaction with the cytoplasmic tails of the α-integrin subunits for β1-integrin endocytosis. FMNL2 drives β1-integrin internalization and invasive motility in a phosphorylation-dependent manner, while a FMNL2 mutant defective in actin assembly interferes with β1-integrin endocytosis an  d cancer cell   invasion. Our data establish a role for FMNL2 in the regulation of β1-integrin and provide a mechanistic understanding of the function of FMNL2 in cancer invasiveness. 
 Identification of regulatory elements of human alpha 6 integrin subunit gene. The integrin alpha 6 subunit associates with either the beta 1 or beta 4 subunit to form receptors for laminin, a major component of the basement membrane. Here, we characterized basal promoter of the human integrin alpha 6 subunit gene. The transcription start site, mapped by primer extension analysis, was 208 bp upstream of the translation start site. The promoter region lacked canonical TATA and GC boxes, but did contain a TATA-like sequence (GATAAA) 23 bp upstream of the major transcription start site. Consensus binding sites for Sp1 and the NF-kappa B complex were also present in the promoter region. A putative glucocorticoid/progesterone receptor responsive element (GRE/PRE), together with the Ap1 and c-myc binding sites located around 350-360 bp upstream of the transcription start site, represented positive regulatory sequences. Our current study showed a molecular model by which progesterone promotes tumor   cell invasion   through the basement membrane by up-regulating the laminin receptor. 
 Integrative analyses identify osteopontin, LAMB3 and ITGB1 as critical pro-metastatic genes for lung cancer. To explore the key regulatory genes associated with lung cancer in order to reduce its occurrence and progress through silencing these key genes.To identify the key regulatory genes involved in lung cancer, we performed a combination of gene array and bioinformatics analyses to compare gene transcription profiles in 3 monoclonal cell strains with high, medium or low metastatic abilities, which were separated from the SPC-A-1sci and SPC-A-1 cell lines by limiting dilution monoclone assay. We then analyzed those genes' biological activities by knocking down their expression in SPC-A-1sci cells using siRNA and lenti-viral shRNA vectors, followed by determinations of the invasion and migration capabilities of the resulting cell lines in vitro as well as their potential for inducing occurrence and metastasis of lung cancer in vivo. To examine the clinical relevance of these findings, we analyzed the expression levels of the identified genes in human lung cancer tissues (n = 135) and matched adjacent normal tissues by immunohistochemical (IHC) staining.Three monoclonal cell strains characterized with high, medium or low metastatic abilities were successfully selected. Gene array and bioinformatics analyses implied that osteopontin, LAMB3 and ITGB1 were key genes involved in lung cancer. Knockdown of these genes suppressed human lung can  cer cell inva  sion and metastasis in vitro and in vivo. Clinical sample analyses indicated that osteopontin, LAMB3 and ITGB1 protein expression levels were higher in lung cancer patients, compared to non-cancerous adjacent tissues, and correlated with lymphatic metastasis.We confirmed that osteopontin, LAMB3 and ITGB1 played important roles in the occurrence and metastasis of lung cancer, thus provided important clues to understanding the molecular mechanism of metastasis and contributing to the therapeutic treatment of lung cancer. 
 A functional genetic screen reveals new regulators of β1-integrin activity. β1 integrins constitute a large group of widely distributed adhesion receptors, which regulate the ability of cells to interact with their surroundings. This regulation of the expression and activity of integrins is crucial for tissue homeostasis and development and contributes to inflammation and cancer. We report an RNA interference screen to uncover genes involved in the regulation of β1-integrin activity using cell spot microarray technology in cancer cell lines. Altogether, ten cancer and two normal cell lines were used to identify regulators of β1 integrin activity. Cell biological analysis of the identified β1-integrin regulatory genes revealed that modulation of integrin activity can influ  ence cell inv  asion in a three-dimensional matrix. We demonstrate with loss-of-function and rescue experiments that CD9 activates and MMP8 inactivates β1 integrins and that both proteins associate with β1 integrins in cells. Furthermore, CD9 and MMP8 regulate cancer cell extravasation in vivo. Our discovery of new regulators of β1-integrin activity highlight the complexity of integrin activity regulation and provide a set of new genes involved in regulation of integrin function. 
 Epigallocatechin-3-gallate (EGCG) downregulates EGF-induced MMP-9 in breast cancer cells: involvement of integrin receptor α5β1 in the process. Epidermal growth factor receptor (EGFR/ErbB1) is a transmembrane protein with tyrosine kinase activity activated mainly by ligand, EGF. Matrix metalloproteinases (MMPs) are a family of proteinases that catalyses the destruction of ECM, among which MMP-9 has important role in tumo  r cell invasi  on. Secretion of MMP-9 is stimulated by a variety of factors, EGFR being significant. Epigallocatechin-3-gallate (EGCG) is a major polyphenol of green tea that inhibits cell proliferation and invasion. Here, we study the effect of EGFR alone and in collaboration with fibronectin on the status of MMP-9 in human breast cancer cell MDA-MB-231 and its molecular mechanism; study the role of EGCG on the induced MMP-9; and elucidate the signaling molecules involved in the process.We performed zymography, immunoblots, real-time RT-PCR, cell adhesion assay, siRNA studies, and electrophoretic mobility shift assay to demonstrate the findings.EGF induces MMP-9 activity and expression; FAK, PI3 K, and ERK are mainly involved in the process. EGF also causes the transactivation of MMP-9 gene by increasing the DNA binding activity of the transcription factors. EGCG downregulates EGF-induced MMP-9 expression by inhibiting the involved regulatory kinases. EGF collaborates with fibronectin to create a synergistic response, and EGCG inhibits the synergistic response in MDA-MB-231.The study demonstrates the requirement of cross talk between cell matrix adhesion molecules and growth factor receptors to improve biological responses and shows FAK/ERK as the pivotal point of this convergence in human breast carcinoma cell line MDA-MB-231. We also establish EGCG as the potential anti-tumor agent in human breast carcinoma. 
 CXCL12/CXCR4 activation by cancer-associated fibroblasts promotes integrin β1 clustering and invasiveness in gastric cancer. Cancer-associated fibroblasts (CAFs) are reportedly involved in invasion and metastasis in several types of cancer, including gastric cancer (GC), through the stimulation of CXCL12/CXCR4 signaling. However, the mechanisms underlying these tumor-promoting effects are not well understood, which limits the potential to develop therapeutic targets against CAF-mediated CXCL12/CXCR4 signaling. CXCL12 expression was analyzed in resected GC tissues from 110 patients by immunohistochemistry (IHC). We established primary cultures of normal fibroblasts (NFs) and CAFs from the GC tissues and examined the functional differences between these primary fibroblasts using co-culture assays with GC cell lines. We evaluated the efficacy of a CXCR4 antagonist (AMD3100) and a FAK inhibitor (PF-573,228) on the invasive ability of GC cells. High CXCL12 expression levels were significantly associated with larger tumor size, increased tumor depth, lymphatic invasion and poor prognosis in GC. CXCL12/CXCR4 activation by CAFs mediated integrin β1 clustering at the cell surface and promoted the invasive ability of GC cells. Notably, AMD3100 was more efficient than PF-573,228 at inhibiting G  C cell invasi  on through the suppression of integrin β1/FAK signaling. These results suggest that CXCL12 derived from CAFs promotes   GC cell invas  ion by enhancing the clustering of integrin β1 in GC cells, resulting in GC progression. Taken together, the inhibition of CXCL12/CXCR4 signaling in GC cells may be a promising therapeutic strategy against   GC cell inva  sion. 
 Integrin-mediated host   cell invasion   by type 1-piliated uropathogenic Escherichia coli. Uropathogenic Escherichia coli (UPEC), the primary causative agent of urinary tract infections, typically express filamentous adhesive organelles called type 1 pili that mediate both bacterial attachment to and invasion of bladder urothelial cells. Several host proteins have previously been identified as receptors for type 1 pili, but none have been conclusively shown to promote UPEC entry into host bladder cells. Using overlay assays with FimH, the purified type 1 pilus adhesin, and mass spectroscopy, we have identified beta1 and alpha3 integrins as key host receptors for UPEC. FimH recognizes N-linked oligosaccharides on these receptors, which are expressed throughout the urothelium. In a bladder cell culture system, beta1 and alpha3 integrin receptors co-localize with invading type 1-piliated bacteria and F-actin. FimH-mediated bacterial invasion of host bladder cells is inhibited by beta1 and alpha3 integrin-specific antibodies and by disruption of the beta1 integrin gene in the GD25 fibroblast cell line. Phosphorylation site mutations within the cytoplasmic tail of beta1 integrin that alter integrin signaling also variably affect UPEC entry into host cells, by either attenuating or boosting invasion frequencies. Furthermore, focal adhesion and Src family kinases, which propagate integrin-linked signaling and downstream cytoskeletal rearrangements, are shown to be required for FimH-dependent bacterial invasion of target host cells. Cumulatively, these results indicate that beta1 and alpha3 integrins are functionally important receptors for type 1 pili-expressing bacteria within the urinary tract and possibly at other sites within the host. 
 C1GALT1 promotes invasive phenotypes of hepatocellular carcinoma cells by modulating integrin β1 glycosylation and activity. Cancer   cell invasio  n and metastasis are the primary causes of treatment failure and death in hepatocellular carcinoma (HCC). We previously reported that core 1 β1,3-galactosyltransferase (C1GALT1) is frequently overexpressed in HCC tumors and its expression is associated with advanced tumor stage, metastasis, and poor survival. However, the underlying mechanisms of C1GALT1 in HCC malignancy remain unclear. In this study, we found that overexpression of C1GALT1 enhanced HCC cell adhesion to extracellular matrix (ECM) proteins, migration, and invasion, whereas RNAi-mediated knockdown of C1GALT1 suppressed these phenotypes. The promoting effect of C1GALT1 on the metastasis of HCC cells was demonstrated in a mouse xenograft model. Mechanistic investigations showed that the C1GALT1-enhanced phenotypic changes in HCC cells were significantly suppressed by anti-integrin β1 blocking antibody. Moreover, C1GALT1 was able to modify O-glycans on integrin β1 and regulate integrin β1 activity as well as its downstream signaling. These results suggest that C1GALT1 could enhance HCC invasiveness through integrin β1 and provide novel insights into the roles of O-glycosylation in HCC metastasis. 
 Galectin-1 is implicated in the protein kinase C epsilon/vimentin-controlled trafficking of integrin-beta1 in glioblastoma cells. Cell motility and resistance to apoptosis characterize glioblastoma (GBM) growth and malignancy. In our current work we report that galectin-1, a homodimeric adhesion molecule and carbohydrate-binding protein with affinity for beta-galactosides, is linked with cell surface expression of integrin beta1 and the process of integrin trafficking. Using immunofluorescence, depletion of galectin-1 through both stable knockdown and transient-targeted small interfering RNA (siRNA) treatment induces an intracellular accumulation of integrin-beta1 coincident with a diminution of integrin-beta1 at points of cellular adhesion at the cell membrane. Galectin-1 depletion does not alter the gene expression level of integrin-beta1. Transient galectin-1 depletion effectuates as well the perinuclear accumulation of protein kinase C epsilon (PKCepsilon) and the intermediate filament vimentin, both of which have been shown to mediate integrin recycling in motile cells. Our results argue for the involvement of galectin-1 in the PKCepsilon/vimentin-controlled trafficking of integrin-beta1. The understanding of molecular mediators such as galectin-1 and the pathways through which they drive the   cell invasion   so descriptive of GBM is anticipated to reveal potential therapeutic targets that promote glioma malignancy. 
 Signaling through urokinase and urokinase receptor in lung cancer cells requires interactions with beta1 integrins. The urokinase receptor (uPAR) is upregulated upon tumor   cell invasion   and correlates with poor lung cancer survival. Although a cis-interaction with integrins has been ascribed to uPAR, whether this interaction alone is critical to urokinase (uPA)- and uPAR-dependent signaling and tumor promotion is unclear. Here we report the functional consequences of point mutations of uPAR (H249A-D262A) that eliminate beta1 integrin interactions but maintain uPA binding, vitronectin attachment and association with alphaV integrins, caveolin and epidermal growth factor receptor. Disruption of uPAR interactions with beta1 integrins recapitulated previously reported findings with beta1-integrin-derived peptides that attenuated matrix-dependent ERK activation, MMP expression and in vitro migration by human lung adenocarcinoma cell lines. The uPAR mutant cells acquired enhanced capacity to adhere to vitronectin via uPAR-alphaVbeta5-integrin, rather than through the uPAR-alpha3beta1-integrin complex and they were unable to initiate uPA signaling to activate ERK, Akt or Stat1. In an orthotopic lung cancer model, uPAR mutant cells exhibited reduced tumor size compared with cells expressing wild-type uPAR. Taken together, the results indicate that uPAR-beta1-integrin interactions are essential to signals induced by integrin matrix ligands or uPA that support lung cancer   cell invasion   in vitro and progression in vivo. 
 β-1,4-Galactosyltransferase III suppresses β1 integrin-mediated invasive phenotypes and negatively correlates with metastasis in colorectal cancer. Metastasis often occurs in colorectal cancer (CRC) patients and is the main difficulty in cancer treatment. The upregulation of poly-N-acetyllactosamine-related glycosylation is found in CRC patients and is associated with progression and metastasis in cancer. β-1,4-Galactosyltransferase III (B4GALT3) is an enzyme responsible for poly-N-acetyllactosamine synthesis, and therefore, we investigated its expression in CRC patients. We found that B4GALT3 negatively correlated with poorly differentiated histology (P < 0.001), advanced stages (P = 0.0052), regional lymph node metastasis (P = 0.0018) and distant metastasis (P = 0.0463) in CRC patients. B4GALT3 overexpression in CRC cells suppressed cell migration, invasion and adhesion, whereas B4GALT3 knockdown enhanced malignant cell phenotypes. The β1 integrin-blocking antibody reversed the B4GALT3-mediated increase   in cell inva  sion. B4GALT3 expression altered glycosylation on the N-glycan of β1 integrin probably through changes in poly-N-acetyllactosamine expression. Furthermore, more activated β1 integrin along with the activation of its downstream signaling transduction were found in B4GALT3 knockdown cells, whereas overexpression of B4GALT3 suppressed the expression of active β1 integrin and inhibited its downstream signaling. Our results suggest that B4GALT3 is negatively associated with CRC metastasis and suppresses cell invasiveness through inhibiting activation of β1 integrin. 
 ß1 integrin binding phosphorylates ezrin at T567 to activate a lipid raft signalsome driving invadopodia activity and invasion. Extracellular matrix (ECM) degradation is a critical process in tumor   cell invasio  n and requires matrix degrading protrusions called invadopodia. The Na(+)/H(+) exchanger (NHE1) has recently been shown to be fundamental in the regulation of invadopodia actin cytoskeleton dynamics and activity. However, the structural link between the invadopodia cytoskeleton and NHE1 is still unknown. A candidate could be ezrin, a linker between the NHE1 and the actin cytoskeleton known to play a pivotal role in invasion and metastasis. However, the mechanistic basis for its role remains unknown. Here, we demonstrate that ezrin phosphorylated at T567 is highly overexpressed in the membrane of human breast tumors and positively associated with invasive growth and HER2 overexpression. Further, in the metastatic cell line, MDA-MB-231, p-ezrin was almost exclusively expressed in invadopodia lipid rafts where it co-localized in a functional complex with NHE1, EGFR, ß1-integrin and phosphorylated-NHERF1. Manipulation by mutation of ezrins T567 phosphorylation state and/or PIP2 binding capacity or of NHE1s binding to ezrin or PIP2 demonstrated that p-ezrin expression and binding to PIP2 are required for invadopodia-mediated ECM degradation and invasion and identified NHE1 as the membrane protein that p-ezrin regulates to induce invadopodia formation and activity. 
 Potentiation of   cell invasion   and matrix metalloproteinase production by alpha3beta1 integrin-mediated adhesion of gastric carcinoma cells to laminin-5. We previously reported that the adhesion of gastric carcinoma cells to the peritoneum mediated by the alpha3beta1 integrin-laminin interaction is a key step in the initial process of peritoneal metastatic dissemination. Carcinoma cells subsequently invade through the intercellular gaps of mesothelial linings. In this study, we examined the role of the interaction of carcinoma cells with laminin-5, which is a major component of submesothelial basement membranes and serves as a high-affinity ligand for alpha3beta1 integrin, in carcinoma   cell invasion  . Human gastric carcinoma cell lines (MKN1, GT3TKB, and NUGC-4) adhered in an alpha3beta1 integrin-dependent manner to the extracellular matrix deposited by peritoneal mesothelial cells. An in vitro invasion assay using the Boyden chamber system revealed that MKN1 cell migration through the membranes increased when the membranes were coated with matrices produced by mesothelial cells or with laminin-5-containing Matrigel as compared to Matrigel alone. The cell migration promoted by laminin-5-containing Matrigel was inhibited by the presence of anti-alpha3 integrin antibody. When MKN1 cells were cultured in a laminin-5-coated plate, these cells were promoted to produce matrix metalloproteinase (MMP)-9, as assessed by gelatin zymography, enzyme-linked immunosorbent assay, and reverse transcription-polymerase chain reaction. These results suggest that the production of MMP-9 by MKN1 cells was potentiated by the alpha3beta1 integrin-laminin-5 interaction, which facilitated their invasion via degradation of the matrix. 
 Schwann Cells Increase Prostate and Pancreatic Tumor   Cell Invasion   Using Laminin Binding A6 Integrin. Human pancreatic and prostate cancers metastasize along nerve axons during perineural invasion. The extracellular matrix laminin class of proteins is an abundant component of both myelinated and non-myelinated nerves. Analysis of human pancreatic and prostate tissue revealed both perineural and endoneural invasion with Schwann cells surrounded or disrupted by tumor, respectively. Tumor and nerve cell co-culture conditions were used to determine if myelinating or non-myelinating Schwann cell (S16 and S16Y, respectively) phenotype was equally likely to promote integrin-dependent cancer   cell invasion   and migration on laminin. Conditioned medium from S16 cells increased tumor cell (DU145, PC3, and CFPAC1) invasion into laminin approximately 1.3-2.0 fold compared to fetal bovine serum (FBS) treated cells. Integrin function (e.g., ITGA6p formation) increased up to 1.5 fold in prostate (DU145, PC3, RWPE-1) and pancreatic (CFPAC1) cells, and invasion was dependent on ITGA6p formation and ITGB1 as determined by function-blocking antibodies. In contrast, conditioned medium isolated from S16Y cells (non-myelinating phenotype) decreased constitutive levels of ITGA6p in the tumor cells by 50% compared to untreated cells and decreased ITGA6p formation 3.0 fold compared to S16 treated cells. Flow cytometry and western blot analysis revealed loss of ITGA6p formation as reversible and independent of overall loss of ITGA6 expression. These results suggest that the myelinating phenotype of Schwann cells within the tumor microenvironment increased integrin-dependent tumor invasion on laminin. 
 Rab5c promotes AMAP1-PRKD2 complex formation to enhance β1 integrin recycling in EGF-induced cancer invasion. Epidermal growth factor receptor (EGFR) signaling is one of the crucial factors in breast cancer malignancy. Breast cancer cells often overexpress Arf6 and its effector, AMAP1/ASAP1/DDEF1; in these cells, EGFR signaling may activate the Arf6 pathway to induce invasion and metastasis. Active recycling of some integrins is crucial for invasion and metastasis. Here, we show that the Arf6-AMAP1 pathway links to the machinery that recycles β1 integrins, such as α3β1, to prom  ote cell inva  sion upon EGFR stimulation. We found that AMAP1 had the ability to bind directly to PRKD2 and hence to make a complex with the cytoplasmic tail of the β1 subunit. Moreover, GTP-Rab5c also bound to AMAP1, and activation of Rab5c by EGFR signaling was necessary to promote the intracellular association of AMAP1 and PRKD2. Our results suggest a novel mechanism by which EGFR signaling promotes the invasiveness of some breast cancer cells via integrin recycling. 
 The interaction of HAb18G/CD147 with integrin alpha6beta1 and its implications for the invasion potential of human hepatoma cells. HAb18G/CD147 plays pivotal roles in invasion by hepatoma cells, but the underlying mechanism remains unclear. Our previous study demonstrated that overexpression of HAb18G/CD147 promotes invasion by interacting with integrin alpha3beta1. However, it has never been investigated whether alpha3beta1 is solely responsible for this process or if other integrin family members also interact with HAb18G/CD147 in human hepatoma cells.Human SMMC-7721 and FHCC98 cells were cultured and transfected with siRNA fragments against HAb18G/CD147. The expression levels of HAb18G/CD147 and integrin alpha6beta1 were determined by immunofluorescent double-staining and confocal imaging analysis. Co-immunoprecipitation and Western blot analyses were performed to examine the native conformations of HAb18G/CD147 and integrin alpha6beta1. Invasion potential was evaluated with an invasion assay and gelatin zymography.We found that integrin alpha6beta1 co-localizes and interacts with HAb18G/CD147 in human hepatoma cells. The enhancing effects of HAb18G/CD147 on invasion capacity and secretion of matrix metalloproteinases (MMPs) were partially blocked by integrin alpha6beta1 antibodies (P < 0.01). Wortmannin, a specific phosphatidylinositol kinase (PI3K) inhibitor that reverses the effect of HAb18G/CD147 on the regulation of intracellular Ca2+ mobilization, significantly reduced   cell invasion   potential and secretion of MMPs in human hepatoma cells (P < 0.05). Importantly, no additive effect between Wortmannin and alpha6beta1 antibodies was observed, indicating that alpha6beta1 and PI3K transmit the signal in an upstream-downstream relationship.These results suggest that alpha6beta1 interacts with HAb18G/CD147 to mediate tumor invasion and metastatic processes through the PI3K pathway. 
 Loss of miR-200b promotes invasion via activating the Kindlin-2/integrin β1/AKT pathway in esophageal squamous cell carcinoma: An E-cadherin-independent mechanism. Our previous studies have shown that loss of miR-200b enhances the invasiveness of esophageal squamous cell carcinoma (ESCC) cells. However, whether the miR-200-ZEB1/2-E-cadherin regulatory cascade, a master regulator of epithelial-to-mesenchymal transition (EMT), is involved in the regulation of ESCC invasion remains elusive. Here, we show that miR-200b represses ESCC   cell invasio  n in vivo without altering the expression of E-cadherin and vimentin, two surrogate markers of EMT. However, an inverse correlation was observed between the expression levels of miR-200b and ZEB1/2 in both ESCC cell lines (n = 7, P < 0.05) and ESCC tumor samples (n = 88, P < 0.05). Methylation of E-cadherin gene was found to block the regulation of E-cadherin by the miR-200b-ZEB1/2 axis, indicating that an E-cadherin-independent mechanism can mediate the biological function of miR-200b in ESCC. We revealed that miR-200b suppresses the integrin β1-AKT pathway via targeting Kindlin-2 to mitigate ESCC cell invasiveness. In two independent cohorts of ESCC samples (n = 20 and n = 53, respectively), Kindlin-2 expression positively correlated with the activation status of both the integrin signaling pathway and the PI3K-AKT signaling pathway (both P < 0.01). These data highlight that suppression of the Kindlin-2-integrin β1-AKT regulatory axis is an alternative mechanism underlying the tumor suppressor function of miR-200b in ESCC. 
 E-cadherin loss promotes the initiation of squamous cell carcinoma invasion through modulation of integrin-mediated adhesion. Much remains to be learned about how cell-cell and cell-matrix interactions are coordinated to influence the earliest development of neoplasia. We used novel 3D human tissue reconstructs that mimic premalignant disease in normal epidermis, to directly investigate how loss of E-cadherin function directs conversion to malignant disease. We used a genetically tagged variant of Ha-Ras-transformed human keratinocytes (II-4) expressing dominant-interfering E-cadherin fusion protein (H-2k(d)-Ecad). These cells were admixed with normal human keratinocytes and tumor cell fate was monitored in 3D reconstructed epidermis upon transplantation to immunodeficient mice. Tumor initiation was suppressed in tissues harboring control- and mock-infected II-4 cells that lost contact with the stromal interface. By contrast, H-2k(d)-Ecad-expressing cells persisted at this interface, thus enabling incipient tumor   cell invasion   upon in vivo transplantation. Loss of intercellular adhesion was linked to elevated cell surface expression of alpha2, alpha3 and beta1 integrins and increased adhesion to laminin-1 and Types I and IV collagen that was blocked with beta1-integrin antibodies, suggesting that invasion was linked to initial II-4 cell attachment at the stromal interface. Collectively, these results outline a novel aspect to loss of E-cadherin function that is linked to the mutually interdependent regulation of cell-cell and cell-matrix adhesion and has significant consequences for the conversion of premalignancy to cancer. 
 Twist induces epithelial-mesenchymal transition and cell motility in breast cancer via ITGB1-FAK/ILK signaling axis and its associated downstream network. Twist, a highly conserved basic Helix-Loop-Helix transcription factor, functions as a major regulator of epithelial-mesenchymal transition (EMT) and tumor metastasis. In different cell models, signaling pathways such as TGF-β, MAPK/ERK, WNT, AKT, JAK/STAT, Notch, and P53 have also been shown to play key roles in the EMT process, yet little is known about the signaling pathways regulated by Twist in tumor cells. Using iTRAQ-labeling combined with 2D LC-MS/MS analysis, we identified 194 proteins with significant changes of expression in MCF10A-Twist cells. These proteins reportedly play roles in EMT, cell junction organization, cell adhesion, and cell migration and invasion. ECM-receptor interaction, MAPK, PI3K/AKT, P53 and WNT signaling were found to be aberrantly activated in MCF10A-Twist cells. Ingenuity Pathways Analysis showed that integrin β1 (ITGB1) acts as a core regulator in linking integrin-linked kinase (ILK), Focal-adhesion kinase (FAK), MAPK/ERK, PI3K/AKT, and WNT signaling. Increased Twist and ITGB1 are associated with breast tumor progression. Twist transcriptionally regulates ITGB1 expression. Over-expression of ITGB1 or Twist in MCF10A led to EMT, activation of FAK/ILK, MAPK/ERK, PI3K/AKT, and WNT signaling. Knockdown of Twist or ITGB1 in BT549 and Hs578T cells decreased activity of FAK, ILK, and their downstream signaling, thus specifically impeding EMT an  d cell invasi  on. Knocking down ILK or inhibiting FAK, MAPK/ERK, or PI3K/AKT signaling also suppressed Twist-driven EMT an  d cell invasi  on. Thus, the Twist-ITGB1-FAK/ILK pathway and their downstream signaling network dictate the Twist-induced EMT process in human mammary epithelial cells and breast cancer cells. 
 Influence of exogenous tissue factor on estrogen receptor alpha expression in breast cancer cells: involvement of beta1-integrin, PAR2, and mitogen-activated protein kinase activation. Increased expression of tissue factor (TF) has been associated with invasive forms of breast cancer. Conversely, the loss of estrogen receptor alpha (ERalpha) is associated with increased cell invasiveness. We have examined the influence of exogenous truncated recombinant TF (rTF) on ERalpha expression and cell invasiveness and investigated the mechanism of rTF signaling. The influence of rTF on ERalpha expression in MCF-7 and T47D cell lines was investigated using reverse transcription-PCR and ELISA.   Cell invasion   was measured using Boyden chamber-based invasion assays. Additionally, the interaction of fluorescein-labeled rTF with the surface of MCF-7 cells and particularly with beta(1)-integrin was examined. Treatment of cells with rTF resulted in the down-regulation of ERalpha mRNA and protein over 24 h, which required beta(1)-integrin and involved the mitogen-activated protein kinase pathway but did not require PAR2 activation. The addition of rTF reduced estradiol-mediated cell proliferation as well as increased cell invasiveness requiring both PAR2 and beta(1)-integrin activation. Fluorescein-labeled rTF was shown to bind to the surface of MCF-7 cells within 5 min and peaked at 15 min. The bound rTF colocalized with cellular beta(1)-integrin and was disrupted in the presence of excess unlabeled rTF and an anti-beta(1) polyclonal antibody. Finally, affinity purification of beta(1)-integrin using rTF-conjugated agarose showed a requirement for the presence of divalent cations but not factor VIIa. The results indicate that rTF is capable of down-regulating ERalpha expression in breast cancer cells, resulting in decreases in estrogen-mediated cell proliferation and increased invasiveness. Furthermore, the mechanisms by which rTF induces these changes involve both PAR2 and beta(1)-integrin. 
 Host cell kinases, α5 and β1 integrins, and Rac1 signalling on the microtubule cytoskeleton are important for non-typable Haemophilus influenzae invasion of respiratory epithelial cells. Non-typable Haemophilus influenzae (NTHi) is a common commensal of the human nasopharynx, but causes opportunistic infection when the respiratory tract is compromised by infection or disease. The ability of NTHi to invade epithelial cells has been described, but the underlying molecular mechanisms are poorly characterized. We previously determined that NTHi promotes phosphorylation of the serine-threonine kinase Akt in A549 human lung epithelial cells, and that Akt phosphorylation and NTH  i cell invasi  on are prevented by inhibition of phosphoinositide 3-kinase (PI3K). Because PI3K-Akt signalling is associated with several host cell networks, the purpose of the current study was to identify eukaryotic molecules important for NTHi epithelial invasion. We found that inhibition of Akt activity reduced NTHi internalization; differently, bacterial entry was increased by phospholipase Cγ1 inhibition but was not affected by protein kinase inhibition. We also found that α5 and β1 integrins, and the tyrosine kinases focal adhesion kinase and Src, are important for NTHi   A549 cell inv  asion. NTHi internalization was shown to be favoured by activation of Rac1 guanosine triphosphatase (GTPase), together with the guanine nucleotide exchange factor Vav2 and the effector Pak1. Also, Pak1 might be associated with inactivation of the microtubule destabilizing agent Op18/stathmin, to facilitate microtubule polymerization and NTHi entry. Conversely, inhibition of RhoA GTPase and its effector ROCK increased the number of internalized bacteria. Src and Rac1 were found to be important for NTHi-triggered Akt phosphorylation. An increase in host cyclic AMP reduced bacterial entry, which was linked to protein kinase A. These findings suggest that NTHi finely manipulates host signalling molecules to invade respiratory epithelial cells. 
 CD151 promotes α3β1 integrin-dependent organization of carcinoma cell junctions and restrains collectiv  e cell invasi  on. Integrins function in collective migration both as major receptors for extracellular matrix and by crosstalk to adherens junctions. Despite extensive research, important questions remain about how integrin signaling mechanisms are integrated into collective migration programs. Tetraspanins form cell surface complexes with a subset of integrins and thus are good candidates for regulating the balance of integrin functional inputs into cell-matrix and cell-cell interactions. For example, tetraspanin CD151 directly associates with α3β1 integrin in carcinoma cells and promotes rapid α3β1-dependent single cell motility, but CD151 also promotes organized adherens junctions and restrains collective carcinoma cell migration on 2D substrates. However, the individual roles of CD151s integrin partners in CD151s pro-junction activity in carcinoma cells were not well understood. Here we find that CD151 promotes organized carcinoma cell junctions via α3β1 integrin, by a mechanism that requires the a3b1 ligand, laminin-332. Loss of CD151 promotes collective 3D invasion and growth in vitro and in vivo, and the enhanced invasion of CD151-silenced cells is α3 integrin dependent, suggesting that CD151 can regulate the balance between α3β1s pro-junction and pro-migratory activities in collective invasion. An analysis of human cancer cases revealed that changes in CD151 expression can be linked to either better or worse clinical outcomes depending on context, including potentially divergent roles for CD151 in different subsets of breast cancer cases. Thus, the role of the CD151-α3β1 complex in carcinoma progression is context dependent, and may depend on the m  ode of tumor   cell invasion. 
 Complementary DNA arrays identify CD63 tetraspanin and alpha3 integrin chain as differentially expressed in low and high metastatic human colon carcinoma cells. Malignant tumor   cell invasion   is determinant for metastasis to occur. E2 and C5 colon carcinoma cells that were derived from the parental Lovo line and that differ experimentally in spontaneous metastatic ability have been monitored for gene expression by cDNA arrays. Among genes found differentially expressed, the CD63 tetraspanin, not previously recognized in colon cancer progression, and the alpha3 integrin chain were both up-regulated in low metastatic E2 cells and were analyzed for their functional role using adhesion, migration, and invasion assays. Cell surface expression of CD63 and alpha3 integrin was about 2-fold higher in E2 than in C5 cells and confocal microscopy showed that CD63 and alpha3 integrin colocalized evenly on C5 cells whereas they concentrated at elongated tips of the low-metastatic more substrate-adhesive E2 cells. Antibody-interference experiments identified laminin-5 (LN-5) as a ligand interacting with the alpha3beta1/CD63 complex. Substrate-immobilized anti-CD63 antibodies enhanced tumor cell migration and invasion and induced prominent cell surface protrusions that were repressed by the PI3-kinase LY294002 inhibitor. Our results suggest that changes in the expression of surface CD63 and alpha3beta1 integrin interacting with LN-5 could affect migratory signals and the progression of the metastatic disease. 
 SCAI acts as a suppressor of cancer   cell invasion   through the transcriptional control of beta1-integrin. Gene expression reprogramming governs cellular processes such as proliferation, differentiation and cell migration through the complex and tightly regulated control of transcriptional cofactors that exist in multiprotein complexes. Here we describe SCAI (suppressor of cancer   cell invasion  ), a novel and highly conserved protein that regulates invasive cell migration through three-dimensional matrices. SCAI acts on the RhoA-Dia1 signal transduction pathway and localizes in the nucleus, where it binds and inhibits the myocardin-related transcription factor MAL by forming a ternary complex with serum response factor (SRF). Genome-wide expression analysis surprisingly reveals that one of the strongest upregulated genes after suppression of SCAI is beta1-integrin. Decreased levels of SCAI are tightly correlated with increased invasive cell migration, and SCAI is downregulated in several human tumours. Functional analysis of the beta1-integrin gene strongly argues that SCAI is a novel transcriptional cofactor that controls gene expression downstream of Dia1 to dictate changes in cell invasive behaviour. 
 Integrin β1 mediates epithelial growth factor-induced invasion in human ovarian cancer cells. Integrins function as cell-extracellular matrix adhesion proteins and have been implicated in tumor progression. In ovarian tumors, elevated integrin β1 expression correlates with high clinical stage and poor patient survival. In this study, we report that EGF treatment up-regulated integrin β1 mRNA and protein levels in ovarian cancer cells. Moreover, pharmacological inhibition of MEK totally abolished EGF-induced integrin β1 up-regulation   and cell inva  sion suggesting that MAPK/ERK signaling is required for EGF-induced integrin β1 up-regulation   and cell inv  asion. Furthermore, we found that knockdown of integrin β1 expression reduced the intrinsic invasiveness of ovarian cancer cells and the EGF-in  duced cell in  vasion. Finally, we found that overexpression of integrin β1 was sufficient to promote ovarian   cancer cell i  nvasion. This study demonstrates that integrin β1 mediates EGF-  induced cell   invasion in ovarian cancer. 
 Clinical and functional significance of α9β1 integrin expression in breast cancer: a novel cell-surface marker of the basal phenotype that promotes tumou  r cell invasi  on. Integrin α9β1 is a receptor for ECM proteins, including Tenascin-C and the EDA domain of fibronectin, and has been shown to transduce TGFβ signalling. This study has examined the expression pattern of α9β1 in 141 frozen breast carcinoma samples and related expression to prognostic indices, molecular subtype and patient outcome. Effects of α9β1 on tumour cell migration and invasion were assessed using blocking antibody and gene transduction approaches. Integrin α9β1 localized to myoepithelial cells in normal ducts and acini, a pattern maintained in DCIS. A subset (17%) of invasive carcinomas exhibited tumour cell expression of α9β1, which related significantly to the basal-like phenotype, as defined by either CK5/6 or CK14 expression. Tumour expression of α9β1 showed a significant association with reduced overall patient survival (p < 0.0001; HR 5.94, 95%CI 3.26-10.82) and with reduced distant-metastasis-free survival (p < 0.0001; HR 6.37, CI 3.51-11.58). A series of breast cancer cell lines was screened for α9β1 with the highly invasive basal-like GI-101 cell line expressing significant levels. Both migration and invasion of this line were reduced significantly in the presence of α9-blocking antibody and following α9-knockdown with siRNA. Conversely, migratory and invasive behaviour of α9-negative MCF7 cells and α9-low MDA MB468 cells was enhanced significantly by over-expression of α9. Thus, α9β1 acts as a novel marker of the basal-like breast cancer subtype and expression is associated with reduced survival, while its ability to promote breast cancer cell migration and invasion suggests that it contributes to the aggressive clinical behaviour of this tumour subtype. 
 Lung cancer cells that survive ionizing radiation show increased integrin α2β1- and EGFR-dependent invasiveness. Ionizing radiation (IR)-enhanced tumor invasiveness is emerging as a contributor to the limited benefit of radiotherapy; however, its mechanism is still unclear. We previously showed that subcloned lung adenocarcinoma A549 cells (P cells), which survived 10 Gy IR (IR cells), acquired high invasiveness in vitro. Here, we tried to identify the mechanism by which IR cells increase their invasiveness by examining altered gene expression and signaling pathways in IR cells compared with those in P cells. To simulate the microenvironment in vivo, cells were embedded in a three-dimensional (3D) collagen type I gel, in which the IR cells were elongated, while the P cells were spherical. The integrin expression pattern was surveyed, and expression levels of the integrin α2 and β1 subunits were significantly elevated in IR cells. Knockdown of α2 expression or functional blockade of integrin α2β1 resulted in a round morphology of IR cells, and abrogated their invasion in the collagen matrix, suggesting the molecule's essential role in cell spread and invasion in 3D collagen. Epidermal growth factor receptor (EGFR) also presented enhanced expression and activation in IR cells. Treatment with EGFR tyrosine kinase inhibitor, PD168393, decreased the ratio of elongated cells and cell invasiveness. Signaling molecules, including extracellular signal-regulated kinase-1/2 (Erk1/2) and Akt, exhibited higher activation in IR cells. Inhibition of Akt activation by treating with phosphoinositide 3-kinase (PI3K) inhibitor LY294002 decrea  sed IR cell i  nvasion, whereas inhibition of Erk1/2 activation by mitogen-activated protein kinase kinase (MEK) inhibitor U0126 did not. Our results show that integrin α2β1 and EGFR cooperatively promote higher invasiveness of IR-survived lung cancer cells, mediated in part by the PI3K/Akt signaling pathway, and might serve as alternative targets in combination with radiotherapy. 
 The Arp2/3 activator WASH regulates α5β1-integrin-mediated invasive migration. The actin cytoskeleton provides scaffolding and physical force to effect fundamental processes such as motility, cytokinesis and vesicle trafficking. The Arp2/3 complex nucleates actin structures and contributes to endocytic vesicle invagination and trafficking away from the plasma membrane. Internalisation and directed recycling of integrins are major driving forces for invasive cell motility and potentially for cancer metastasis. Here, we describe a direct requirement for WASH and Arp2/3-mediated actin polymerisation on the endosomal membrane system for α5β1 integrin recycling. WASH regulates the trafficking of endosomal α5β1 integrin to the plasma membrane and is fundamental for integrin-driven cell morphology changes and integrin-mediated c  ancer cell in  vasion. Thus, we implicate WASH and Arp2/3-driven actin nucleation in receptor recycling leading to invasive motility. 
 N-ethylmaleimide-sensitive factor attachment protein α (αSNAP) regulates matrix adhesion and integrin processing in human epithelial cells. Integrin-based adhesion to the extracellular matrix (ECM) plays critical roles in controlling differentiation, survival, and motility of epithelial cells. Cells attach to the ECM via dynamic structures called focal adhesions (FA). FA undergo constant remodeling mediated by vesicle trafficking and fusion. A soluble N-ethylmaleimide-sensitive factor (NSF) attachment protein α (αSNAP) is an essential mediator of membrane fusion; however, its roles in regulating ECM adhesion and cell motility remain unexplored. In this study, we found that siRNA-mediated knockdown of αSNAP induced detachment of intestinal epithelial cells, whereas overexpression of αSNAP increased ECM adhesion and inhi  bited cell in  vasion. Loss of αSNAP impaired Golgi-dependent glycosylation and trafficking of β1 integrin and decreased phosphorylation of focal adhesion kinase (FAK) and paxillin resulting in FA disassembly. These effects of αSNAP depletion on ECM adhesion were independent of apoptosis and NSF. In agreement with our previous reports that Golgi fragmentation mediates cellular effects of αSNAP knockdown, we found that either pharmacologic or genetic disruption of the Golgi recapitulated all the effects of αSNAP depletion on ECM adhesion. Furthermore, our data implicates β1 integrin, FAK, and paxillin in mediating the observed pro-adhesive effects of αSNAP. These results reveal novel roles for αSNAP in regulating ECM adhesion and motility of epithelial cells. 
 The group B streptococcal alpha C protein binds alpha1beta1-integrin through a novel KTD motif that promotes internalization of GBS within human epithelial cells. Group B Streptococcus (GBS) is the leading cause of bacterial pneumonia, sepsis and meningitis among neonates and a cause of morbidity among pregnant women and immunocompromised adults. GBS epithelial   cell invasion   is associated with expression of alpha C protein (ACP). Loss of ACP expression results in a decrease in GBS internalization and translocation across human cervical epithelial cells (ME180). Soluble ACP and its 170 amino acid N-terminal region (NtACP), but not the repeat protein RR', bind to ME180 cells and reduce internalization of wild-type GBS to levels obtained with an ACP-deficient isogenic mutant. In the current study, ACP colocalized with alpha(1)beta(1)-integrin, resulting in integrin clustering as determined by laser scanning confocal microscopy. NtACP contains two structural domains, D1 and D2. D1 is structurally similar to fibronectin's integrin-binding region (FnIII10). D1's (KT)D146 motif is structurally similar to the FnIII10 (RG)D1495 integrin-binding motif, suggesting that ACP binds alpha(1)beta(1)-integrin via the D1 domain. The (KT)D146A mutation within soluble NtACP reduced its ability to bind alpha(1)beta(1)-integrin and inhibit GBS internalization within ME180 cells. Thus ACP binding to human epithelial cell integrins appears to contribute to GBS internalization within epithelial cells. 
 The Chlamydia trachomatis Ctad1 invasin exploits the human integrin β1 receptor for host cell entry. Infection of human cells by the obligate intracellular bacterium Chlamydia trachomatis requires adhesion and internalization of the infectious elementary body (EB). This highly complex process is poorly understood. Here, we characterize Ctad1 (CT017) as a new adhesin and invasin from C. trachomatis serovar E. Recombinant Ctad1 (rCtad1) binds to human cells via two bacterial SH3 domains located in its N-terminal half. Pre-incubation of host cells with rCtad1 reduces subsequent adhesion and infectivity of bacteria. Interestingly, protein-coated latex beads revealed Ctad1 being an invasin. rCtad1 interacts with the integrin β1 subunit on human epithelial cells, and induces clustering of integrins at EB attachment sites. Receptor activation induces ERK1/2 phosphorylation. Accordingly, rCtad1 binding to integrin β1-negative cells is significantly impaired, as is the chlamydial infection. Thus interaction of C. trachomatis Ctad1 with integrin β1 mediates EB adhesion and induces signaling processes that promote h  ost-cell inva  sion. 
  GeneRIFs  
 interaction of C. trachomatis Ctad1 with integrin beta1 mediates EB adhesion and induces signaling processes that promote host-  cell invasion  . 
 the role of the CD151-alpha3beta1 complex in carcinoma progression is context dependent, and may depend on the mode of tumor   cell invasion  . 
 This study demonstrates that integrin beta1 mediates EGF-induced   cell invasion   in ovarian cancer. 
 Laminins and their integrin receptors are necessary for the development and maintenance of the peripheral nervous system, indicating the potential role for integrin receptors in directing prostate tumor   cell invasion   on nerves during perineural invasion. 
 results suggest that thrombin induces expression and association of beta1-integrin with MMP-9 and that the cell surface localization of the protease by the integrin promotes tumor   cell invasion   
   
  
 PDIA6 
 
  NCBI Gene  
  GeneRIF  
  Pubmed  
 
 
  Search Term  Rank  Fields  
  [Mm]igration  6  PubMed(2)  
  [Ee]xtracellular [Mm]atrix  8  PubMed(1)  
  [Ii]nvasive  10  PubMed(1)  
  [Mm]etastasis  12  PubMed(2)  
 
 
 hide/show details for PDIA6 
  [Mm]igration  PubMed  
 Restoration of miR-127-3p and miR-376a-3p counteracts the neoplastic phenotype of giant cell tumor of bone derived stromal cells by targeting COA1, GLE1 and PDIA6. Although generally benign, giant cell tumors of bone (GCTB) display an aggressive behavior associated with significant bone destruction and lung metastasis in rare cases. This and the very high recurrence rate observed after surgical resection ranging from 20 to 55% necessitates the development of more effective treatment strategies. To identify valuable therapeutic targets, we screened a previously identified microRNA signature consisting of 23 microRNAs predominantly down-regulated in GCTB. We preselected eight candidate microRNAs and analyzed the impact of their restored expression on the neoplastic phenotype of GCTB stromal cells (GCTSC). A consistent and significant inhibition of cell proliferation,   migration  , colony formation and spheroid formation could be induced by transfection of primary GCTSC cell lines with miR-127-3p and miR-376a-3p, respectively. Genome wide expression analysis of miR-127-3p and miR-376a-3p transfected cells revealed four novel target genes for each microRNA. Luciferase reporter assays demonstrated direct interactions of miR-127-3p with COA1 and direct interaction of miR-376a-3p with GLE1 and PDIA6, suggesting a pivotal role of these genes in the molecular etiology of GTCB. Interestingly, both microRNAs are located within a chromosomal region frequently silenced in GCTB and many other types of cancers, indicating that these microRNAs and their target genes are valuable therapeutic targets for the treatment of GCTB and possibly other tumor entities. 
 Galectin-9 binding to cell surface protein disulfide isomerase regulates the redox environment to enhance T-cell   migration   and HIV entry. Interaction of cell surface glycoproteins with endogenous lectins on the cell surface regulates formation and maintenance of plasma membrane domains, clusters signaling complexes, and controls the residency time of glycoproteins on the plasma membrane. Galectin-9 is a soluble, secreted lectin that binds to glycoprotein receptors to form galectin-glycoprotein lattices on the cell surface. Whereas galectin-9 binding to specific glycoprotein receptors induces death of CD4 Th1 cells, CD4 Th2 cells are resistant to galectin-9 death due to alternative glycosylation. On Th2 cells, galectin-9 binds cell surface protein disulfide isomerase (PDI), increasing retention of PDI on the cell surface and altering the redox status at the plasma membrane. Cell surface PDI regulates integrin function on platelets and also enhances susceptibility of T cells to infection with HIV. We find that galectin-9 binding to PDI on Th2 cells results in increased cell   migration   through extracellular matrix via β3 integrins, identifying a unique mechanism to regulate T-cell   migratio  n. In addition, galectin-9 binding to PDI on T cells potentiates infection with HIV. We identify a mechanism for regulating cell surface redox status via a galectin-glycoprotein lattice, to regulate distinct T-cell functions. 
  [Ee]xtracellular [Mm]atrix  PubMed  
 Galectin-9 binding to cell surface protein disulfide isomerase regulates the redox environment to enhance T-cell migration and HIV entry. Interaction of cell surface glycoproteins with endogenous lectins on the cell surface regulates formation and maintenance of plasma membrane domains, clusters signaling complexes, and controls the residency time of glycoproteins on the plasma membrane. Galectin-9 is a soluble, secreted lectin that binds to glycoprotein receptors to form galectin-glycoprotein lattices on the cell surface. Whereas galectin-9 binding to specific glycoprotein receptors induces death of CD4 Th1 cells, CD4 Th2 cells are resistant to galectin-9 death due to alternative glycosylation. On Th2 cells, galectin-9 binds cell surface protein disulfide isomerase (PDI), increasing retention of PDI on the cell surface and altering the redox status at the plasma membrane. Cell surface PDI regulates integrin function on platelets and also enhances susceptibility of T cells to infection with HIV. We find that galectin-9 binding to PDI on Th2 cells results in increased cell migration through   extracellular matrix   via β3 integrins, identifying a unique mechanism to regulate T-cell migration. In addition, galectin-9 binding to PDI on T cells potentiates infection with HIV. We identify a mechanism for regulating cell surface redox status via a galectin-glycoprotein lattice, to regulate distinct T-cell functions. 
  [Ii]nvasive  PubMed  
 PDIA3 and PDIA6 gene expression as an aggressiveness marker in primary ductal breast cancer. Changes in the expression of the protein disulfide isomerase genes PDIA3 and PDIA6 may increase endoplasmic reticulum stress, leading to cellular instability and neoplasia. We evaluated the expression of PDIA3 and PDIA6 in   invasive   ductal carcinomas. Using reverse transcription-quantitative polymerase chain reaction, we compared the mRNA expression level in 45 samples of   invasive   ductal carcinoma with that in normal breast samples. Increased expression of the PDIA3 gene in carcinomas (P = 0.0009) was observed. In addition, PDIA3 expression was increased in tumors with lymph node metastasis (P = 0.009) and with grade III (P < 0.02). The PDIA6 gene showed higher expression levels in the presence of lymph node metastasis (U = 99.00, P = 0.0476) and lower expression for negative hormone receptors status (P = 0.0351). Our results suggest that alterations in PDIA3/6 expression levels may be involved in the breast carcinogenic process and should be further investigated as a marker of aggressiveness. 
  [Mm]etastasis  PubMed  
 Restoration of miR-127-3p and miR-376a-3p counteracts the neoplastic phenotype of giant cell tumor of bone derived stromal cells by targeting COA1, GLE1 and PDIA6. Although generally benign, giant cell tumors of bone (GCTB) display an aggressive behavior associated with significant bone destruction and lung   metastasis   in rare cases. This and the very high recurrence rate observed after surgical resection ranging from 20 to 55% necessitates the development of more effective treatment strategies. To identify valuable therapeutic targets, we screened a previously identified microRNA signature consisting of 23 microRNAs predominantly down-regulated in GCTB. We preselected eight candidate microRNAs and analyzed the impact of their restored expression on the neoplastic phenotype of GCTB stromal cells (GCTSC). A consistent and significant inhibition of cell proliferation, migration, colony formation and spheroid formation could be induced by transfection of primary GCTSC cell lines with miR-127-3p and miR-376a-3p, respectively. Genome wide expression analysis of miR-127-3p and miR-376a-3p transfected cells revealed four novel target genes for each microRNA. Luciferase reporter assays demonstrated direct interactions of miR-127-3p with COA1 and direct interaction of miR-376a-3p with GLE1 and PDIA6, suggesting a pivotal role of these genes in the molecular etiology of GTCB. Interestingly, both microRNAs are located within a chromosomal region frequently silenced in GCTB and many other types of cancers, indicating that these microRNAs and their target genes are valuable therapeutic targets for the treatment of GCTB and possibly other tumor entities. 
 PDIA3 and PDIA6 gene expression as an aggressiveness marker in primary ductal breast cancer. Changes in the expression of the protein disulfide isomerase genes PDIA3 and PDIA6 may increase endoplasmic reticulum stress, leading to cellular instability and neoplasia. We evaluated the expression of PDIA3 and PDIA6 in invasive ductal carcinomas. Using reverse transcription-quantitative polymerase chain reaction, we compared the mRNA expression level in 45 samples of invasive ductal carcinoma with that in normal breast samples. Increased expression of the PDIA3 gene in carcinomas (P = 0.0009) was observed. In addition, PDIA3 expression was increased in tumors with lymph node   metastasis   (P = 0.009) and with grade III (P < 0.02). The PDIA6 gene showed higher expression levels in the presence of lymph node   metastasis   (U = 99.00, P = 0.0476) and lower expression for negative hormone receptors status (P = 0.0351). Our results suggest that alterations in PDIA3/6 expression levels may be involved in the breast carcinogenic process and should be further investigated as a marker of aggressiveness. 
   
  
 PDCD6IP 
 
  NCBI Gene  
  GeneRIF  
  Pubmed  
 
 
  Search Term  Rank  Fields  
  [Ff]ilopodia  1  PubMed(1)  
  [Mm]igration  6  PubMed(1)  
  [Ee]xtracellular [Mm]atrix  8  PubMed(2)  
  [Mm]etastasis  12  PubMed(1); GeneRIFs(1)  
 
 
 hide/show details for PDCD6IP 
  [Ff]ilopodia  PubMed  
 Identification of Rab GTPase-activating protein-like protein (RabGAPLP) as a novel Alix/AIP1-interacting protein. Alix/AIP1 is a multifunctional adaptor protein involved in endocytosis, cell adhesion, and cell death. By yeast two-hybrid screening we identified a novel Alix/AIP1-interacting protein named Rab GTPase-activating protein-like protein (RabGAPLP). Interaction between Alix and RabGAPLP was confirmed by pull-down assays using fusion proteins of either glutathione-S-transferase (GST) or chitin-binding domain (CBD) and lysates of cultured mammalian cells expressing the respective proteins. Partial colocalization of FLAG-tagged RabGAPLP and green fluorescent protein (GFP)-fused Alix was observed at cell edges and   filopodia  -like structures by fluorescence confocal laser scanning microscopic analysis. The identity of RabGAPLP to merlin-associated protein (MAP), one of the interacting partners of neurofibromatosis type 2 (NF2) tumor suppressor gene product (merlin), implies cross-talk of membrane traffic and cell adhesion. 
  [Mm]igration  PubMed  
 Analysis of the myosin-II-responsive focal adhesion proteome reveals a role for β-Pix in negative regulation of focal adhesion maturation. Focal adhesions undergo myosin-II-mediated maturation wherein they grow and change composition to modulate integrin signalling for cell   migratio  n, growth and differentiation. To determine how focal adhesion composition is affected by myosin II activity, we performed proteomic analysis of isolated focal adhesions and compared protein abundance in focal adhesions from cells with and without myosin II inhibition. We identified 905 focal adhesion proteins, 459 of which changed in abundance with myosin II inhibition, defining the myosin-II-responsive focal adhesion proteome. The abundance of 73% of the proteins in the myosin-II-responsive focal adhesion proteome was enhanced by contractility, including proteins involved in Rho-mediated focal adhesion maturation and endocytosis- and calpain-dependent focal adhesion disassembly. During myosin II inhibition, 27% of proteins in the myosin-II-responsive focal adhesion proteome, including proteins involved in Rac-mediated lamellipodial protrusion, were enriched in focal adhesions, establishing that focal adhesion protein recruitment is also negatively regulated by contractility. We focused on the Rac guanine nucleotide exchange factor β-Pix, documenting its role in the negative regulation of focal adhesion maturation and the promotion of lamellipodial protrusion and focal adhesion turnover to drive cel  l migrati  on. 
  [Ee]xtracellular [Mm]atrix  PubMed  
 Proteomic analysis of human parotid gland exosomes by multidimensional protein identification technology (MudPIT). Human ductal saliva contributes over a thousand unique proteins to whole oral fluids. The mechanism by which most of these proteins are secreted by salivary glands remains to be determined. The present study used a mass spectrometry-based, shotgun proteomics approach to explore the possibility that a subset of the proteins found in saliva are derived from exosomes, membrane-bound vesicles of endosomal origin within multivesicular endosomes. Using MudPIT (multidimensional protein identification technology) mass spectrometry, we catalogued 491 proteins in the exosome fraction of human parotid saliva. Many of these proteins were previously observed in ductal saliva from parotid glands (265 proteins). Furthermore, 72 of the proteins in parotid exosomes overlap with those previously identified as urinary exosome proteins, proteins which are also frequently associated with exosomes from other tissues and cell types. Gene Ontology (GO) and KEGG pathway analyses found that cytosolic proteins comprise the largest category of proteins in parotid exosomes (43%), involved in such processes as phosphatidylinositol signaling system, calcium signaling pathway, inositol metabolism, protein export, and signal transduction, among others; whereas the integral plasma membrane proteins and associated/peripheral plasma membrane proteins (26%) were associated with   extracellular matrix  -receptor interaction, epithelial cell signaling, T-cell and B-cell receptor signaling, cytokine receptor interaction, and antigen processing and presentation, among other biological functions. In addition, these putative saliva exosomal proteins were linked to specific diseases (e.g., neurodegenerative disorders, prion disease, cancers, type I and II diabetes). Consequently, parotid glands secrete exosomes that reflect the metabolic and functional status of the gland and may also carry informative protein markers useful in the diagnosis and treatment of systemic diseases. 
 Isolation of novel and known genes from a human fetal cochlear cDNA library using subtractive hybridization and differential screening. We used a combination of subtractive hybridization and differential screening strategies to identify genes that may function normally in hearing and, when mutated, result in deafness. A human fetal cochlear (membranous labyrinth) cDNA library was subtracted against total human fetal brain RNAs by an avidin-biotin-based procedure to enrich for cochlear transcripts. Subtracted cochlear clones were differentially screened with 32P-labeled total cochlear and total brain cDNA probes. Sequence analysis of clones that hybridized more intensely with cochlear than with brain cDNA probes revealed some previously characterized genes, including mitochondrial sequences, collagen type I alpha-2 (COL1A2), collagen type II alpha-1 (COL2A1), collagen type III alpha-1 (COL3A1), spermidine/spermine N1-acetyltransferase (SAT), osteonectin (SPARC), and peripheral myelin protein 22 (PMP22). Also identified were clones that are potential novel cochlear genes. Northern blots of cochlear and brain RNAs probed with COL1A2, COL2A1, COL3A1, SAT, SPARC, PMP22, and a novel sequence, designated Coch-5B2, confirm results of the subtractive procedure by showing preferential cochlear expression. A number of these genes serve structural or regulatory functions in   extracellular matrix   or neural conduction; defects in some of these genes are associated with disorders involving hearing loss. Partial sequence analysis of Coch-5B2 reveals a von Willebrand factor type A-like domain in this cDNA. To assess the cochlear specificity of Coch-5B2, a Northern blot panel of 14 human fetal tissue RNAs was probed with Coch-5B2, showing differential expression of this novel gene in the cochlea. 
  [Mm]etastasis  PubMed  
 AIP1 Expression in Tumor Niche Suppresses Tumor Progression and   Metastasis  . Studies from tumor cells suggest that tumor-suppressor AIP1 inhibits epithelial-mesenchymal transition (EMT). However, the role of AIP1 in the tumor microenvironment has not been examined. We show that a global or vascular endothelial cell (EC)-specific deletion of the AIP1 gene in mice augments tumor growth and   metastasis   in melanoma and breast cancer models. AIP1-deficient vascular environment not only enhances tumor neovascularization and increases premetastatic niche formation, but also secretes tumor EMT-promoting factors. These effects from AIP1 loss are associated with increased VEGFR2 signaling in the vascular EC and could be abrogated by systemic administration of VEGFR2 kinase inhibitors. Mechanistically, AIP1 blocks VEGFR2-dependent signaling by directly binding to the phosphotyrosine residues within the activation loop of VEGFR2. Our data reveal that AIP1, by inhibiting VEGFR2-dependent signaling in tumor niche, suppresses tumor EMT switch, tumor angiogenesis, and tumor premetastatic niche formation to limit tumor growth and   metastasis  . 
  GeneRIFs  
 Our data reveal that AIP1, by inhibiting VEGFR2-dependent signaling in tumor niche, suppresses tumor EMT switch, tumor angiogenesis, and tumor premetastatic niche formation to limit tumor growth and   metastasis  . 
   
  
 PLS3 
 
  NCBI Gene  
  GeneRIF  
  Pubmed  
 
 
  Search Term  Rank  Fields  
  [Mm]igration  6  PubMed(3); GeneRIFs(2)  
  [Ii]nvasive  10  PubMed(2)  
  [Mm]etastasis  12  PubMed(3); GeneRIFs(1)  
  [Cc]ell [Ii]nvasion  13  PubMed(2)  
 
 
 hide/show details for PLS3 
  [Mm]igration  PubMed  
 T-plastin expression downstream to the calcineurin/NFAT pathway is involved in keratinocyte   migration  . Cutaneous wound healing requires keratinocyte proliferation,   migration   and differentiation to restore the barrier function of the skin. The calcineurin/nuclear factor of activated-T-cell (NFAT) signaling pathway has been recently shown to be involved in keratinocyte growth, differentiation and   migration  . It is induced by an increased intracellular calcium rate and its inhibition results in decreased capacities of keratinocytes to migrate. Nevertheless, the link between calcineurin activation and keratinocyte   migration   remains unknown. Recently, Orai1, a pore subunit of a store-operated calcium channel that favors calcium influx, was shown to play a critical role to control proliferation and   migration   of basal keratinocytes. Of interest, the actin-bundling T-plastin is crucial in cell motility through cross-linking to actin filament and its synthesis was shown to be induced by calcium influx and regulated by the calcineurin/NFAT pathway in tumor Sezary cells. We investigated herein the role of the calcineurin/NFAT pathway-dependent T-plastin in keratinocyte   migration  , by quantifying T-plastin expression in keratinocytes and by analyzing their   migration   under calcineurin inhibition or knockdown of NFAT2 or T-plastin. We did confirm the role of the calcineurin/NFAT pathway in keratinocyte   migration   as shown by their decreased capacities to migrate after FK506 treatment or siNFAT2 transfection in both scratching and Boyden assays. The expression of NFAT2 and T-plastin in keratinocytes was decreased under FK506 treatment, suggesting that T-plastin plays a role in keratinocyte   migration   downstream to the calcineurin/NFAT pathway. Accordingly, siRNA knockdown of T-plastin expression also decreased their   migration   capacities. Actin lamellipodia formation as well as FAK and β6-integrin expression were also significantly decreased after treatment with FK506 or siRNA, reinforcing that NFAT2-dependent T-plastin expression plays a role in keratinocyte   migratio  n. These results indicate that T-plastin might be considered as a major actor in the mechanisms underlying calcineurin/NFAT-dependent keratinocyte   migratio  n and may explain wound-healing defects observed in patients under calcineurin inhibitor long-term treatment. 
 Plastin polymorphisms predict gender- and stage-specific colon cancer recurrence after adjuvant chemotherapy. Tumor recurrence after curative resection remains a major problem in patients with locally advanced colorectal cancer treated with adjuvant chemotherapy. Genetic single-nucleotide polymorphisms (SNP) may serve as useful molecular markers to predict clinical outcomes in these patients and identify targets for future drug development. Recent in vitro and in vivo studies have demonstrated that the plastin genes PLS3 and LCP1 are overexpressed in colon cancer cells and play an important role in tumor cell invasion, adhesion, and   migration  . Hence, we hypothesized that functional genetic variations of plastin may have direct effects on the progression and prognosis of locally advanced colorectal cancer. We tested whether functional tagging polymorphisms of PLS3 and LCP1 predict time to tumor recurrence (TTR) in 732 patients (training set, 234; validation set, 498) with stage II/III colorectal cancer. The PLS3 rs11342 and LCP1 rs4941543 polymorphisms were associated with a significantly increased risk for recurrence in the training set. PLS3 rs6643869 showed a consistent association with TTR in the training and validation set, when stratified by gender and tumor location. Female patients with the PLS3 rs6643869 AA genotype had the shortest median TTR compared with those with any G allele in the training set [1.7 vs. 9.4 years; HR, 2.84; 95% confidence interval (CI), 1.32-6.1; P = 0.005] and validation set (3.3 vs. 13.7 years; HR, 2.07; 95% CI, 1.09-3.91; P = 0.021). Our findings suggest that several SNPs of the PLS3 and LCP1 genes could serve as gender- and/or stage-specific molecular predictors of tumor recurrence in stage II/III patients with colorectal cancer as well as potential therapeutic targets. 
 Inducible expression and pathophysiologic functions of T-plastin in cutaneous T-cell lymphoma. A molecular feature of Sézary syndrome (SS) is the abnormal expression of T-plastin by malignant T cells. Herein, we investigated the molecular mechanisms involved in T-plastin synthesis and the functions of this actin-binding protein, with a special interest in chemoresistance and   migratio  n. We confirm the specific expression of T-plastin in peripheral blood lymphocytes (PBLs) from SS patients and its total absence in PBLs from patients with mycosis fungoides, inflammatory cutaneous or hematologic diseases, and from healthy volunteers. Only 3 of 4 SS patients did constitutively express T-plastin. To assess whether T-plastin expression was inducible, T-plastin-negative PBLs were stimulated by phorbol 12-myristate 13-acetate and ionomycin. Our results demonstrate that T-plastin synthesis was induced in negative PBLs from SS patients, other studied patients, and healthy volunteers. Both constitutive and calcium-induced T-plastin expression was down-regulated by calcineurin inhibitors and involved nuclear factor of activated T cells transcription pathway. Constitutive T-plastin expression in SS was associated with resistance to etoposide-induced apoptosis and cell   migratio  n toward chemokines (TARC/CCL17, IP-10). In conclusion, T-plastin is a marker restricted to malignant lymphocytes from SS patients and plays a role for cell survival and   migratio  n. This opens new strategies for the treatment of SS advanced stages. 
  GeneRIFs  
 T-plastin expression downstream to the calcineurin/NFAT pathway is involved in keratinocyte   migration  . 
 T-plastin is a marker restricted to malignant lymphocytes from Sezary syndrome patients and plays a role for cell survival and   migration  . 
  [Ii]nvasive  PubMed  
 Changes in T-plastin expression with human trophoblast differentiation. During the first trimester of pregnancy, the human placenta is an actively dividing and highly   invasive   tumour-like tissue, while near term, it represents a fully developed, non-  invasive   unit. In order to understand the molecular basis of this marked difference in the placental phenotypes, an approach based on a differential display-reverse transcription-polymerase chain reaction (DD-RT-PCR) was adopted to analyse changes in gene expression, using total RNA isolated from first-trimester and term placental villi. Using this approach, T-plastin was initially identified as being differentially expressed in the human first-trimester placenta. T-plastin is an actin-bundling protein and is known to be highly expressed in actively dividing cells and up-regulated in several carcinomas. Using a homogenous population of cytotrophoblasts and syncytiotrophoblasts isolated from human placentae, the present authors demonstrate the differential expression of T-plastin in cytotrophoblasts compared with the terminally differentiated syncytiotrophoblasts. The down-regulation of T-plastin expression is further demonstrated in human trophoblastic BeWo cells induced to differentiate using transforming growth factor (TGF)beta1, a growth factor known for its anti-proliferative and anti-  invasive   response in placental cells. These studies suggest that expression of T-plastin in the placental context may indeed be associated with the enhanced replicative potential of placental trophoblasts. 
 Aberrant expression of plastin-3 via copy number gain induces the epithelial-mesenchymal transition in circulating colorectal cancer cells. Plastin-3 (PLS3) is a novel marker for circulating tumor cells (CTCs) in colorectal cancer (CRC). We sought to investigate the mechanisms mediating the aberrant expression of PLS3, the role of PLS3 in the epithelial-mesenchymal transition (EMT), and its association with the acquisition of   invasive   and metastatic abilities in human CRC.The expression levels of PLS3 messenger RNA in the tumor drainage venous blood (TDB) were examined in 177 CRC cases, and the associations between PLS3 expression and Xq23 copy numbers were analyzed in 132 CRC samples. We then established a stable PLS3-expressing CRC cell line and assessed the role of PLS3 in the EMT.In clinical CRC cases, high expression of PLS3 in CTCs of TDB as well as peripheral blood was established as an independent prognostic factor of overall survival (p < 0.001), and the copy number gain of Xq23, which is the locus of the PLS3 gene, was significantly related to PLS3 overexpression. PLS3 induced the EMT via transforming growth factor (TGF)-β signaling and resulted in the acquisition   of invas  ive ability in CRC cells.The aberrant expression of PLS3 was associated with copy number gain in CTCs from primary tumors and was involved in the regulation of the EMT, contributing to a poor prognosis in CRC patients. 
  [Mm]etastasis  PubMed  
 Plastin3 is a novel marker for circulating tumor cells undergoing the epithelial-mesenchymal transition and is associated with colorectal cancer prognosis. Circulating tumor cells (CTC) in blood have attracted attention both as potential seeds for   metastasis   and as biomarkers. However, most CTC detection systems might miss epithelial-mesenchymal transition (EMT)-induced metastatic cells because detection is based on epithelial markers. First, to discover novel markers capable of detecting CTCs in which EMT has not been repressed, microarray analysis of 132 colorectal cancers (CRC) from Japanese patients was conducted, and 2,969 genes were detected that were overexpressed relative to normal colon mucosa. From the detected genes, we selected those that were overexpressed CRC with distant   metastasis  . Then, we analyzed the CRC   metastasis  -specific genes (n = 22) to determine whether they were expressed in normal circulation. As a result, PLS3 was discovered as a CTC marker that was expressed in metastatic CRC cells but not in normal circulation. Using fluorescent immunocytochemistry, we validated that PLS3 was expressed in EMT-induced CTC in peripheral blood from patients with CRC with distant   metastasis  . PLS3-expressing cells were detected in the peripheral blood of approximately one-third of an independent set of 711 Japanese patients with CRC. Multivariate analysis showed that PLS3-positive CTC was independently associated with prognosis in the training set (n = 381) and the validation set [n = 330; HR = 2.17; 95% confidence interval (CI) = 1.38-3.40 and HR = 3.92; 95% CI = 2.27-6.85]. The association between PLS3-positive CTC and prognosis was particularly strong in patients with Dukes B (HR = 4.07; 95% CI = 1.50-11.57) and Dukes C (HR = 2.57; 95% CI = 1.42-4.63). PLS3 is a novel marker for metastatic CRC cells, and it possesses significant prognostic value. 
 A common gene variant in PLS3 predicts colon cancer recurrence in women. Recent evidence suggests that PLS3 (T-Plastin), an important member of the actin filamentous network, significantly influences cell invasion and   metastasis  . Germline polymorphisms within the PLS3 gene may impact the gene's function, resulting in inter-individual differences in tumor recurrence capacity. In the present study, we investigated the association of germline polymorphisms in PLS3 to predict time to recurrence (TTR) in patients with stage II and III colon cancer. A total of 264 patients with histologically confirmed colon cancer were included in this retrospective study. Germline DNA was genotyped for rs871773 C>T, rs757124 C>G, rs1557770 G>T, rs6643869 G>A, and rs2522188 C>T in the PLS3 gene by 5'-exonuclease (TaqMan™) technology. As the PLS3 gene is located on the X chromosome, a gender-specific statistical analysis was performed. In univariate analysis, the minor allele of PLS3 rs871773 C>T was significantly associated with decreased TTR in women (hazard ratio (HR) = 5.02; 95 % confidence interval (CI) = 1.251-20.114; p = 0.023) and remained significantly associated in multivariate analysis (HR = 6.165; 95 % CI = 1.538-24.716; p = 0.010). Female patients carrying the C/T genotype in PLS3 rs871773 showed a median TTR of 69 months. In contrast, female patients with homozygous C/C had a median TTR of 112 months. There were no significant associations between PLS3 rs871773 C>T and TTR in male and between the other polymorphisms and TTR in male or female colon cancer patients. In conclusion, we identified a common gene variant in PLS3 as an independent prognostic marker in female patients with stage II and III colon cancer. Larger prospective trials are warranted to confirm these findings. 
 Expression of the PLS3 Gene in Circulating Cells in Patients with Colorectal Cancer. Circulating tumor cells (CTC) are cells in circulating blood that have the antigen and gene features of tumor cells of a specific type. Since they can be potentially used in diagnostics and monitoring of treatment of many tumors, they have been attracting attention of researchers worldwide. Plastin-3 (PL S3) is one of such markers of CTC. The aim of the study was to assess expression of PL S3 in CTC in patients with colorectal cancer, to conduct a statistical analysis and to demonstrate a link between expression of PL S3 and progress of the disease, level of CEA and Ca19-9 markers, gender and age of the patients.A group of 85 patients of the Department of General and Colorectal Surgery of the Medical University in Łódź were enrolled in this study. Circulating tumor cells were isolated from whole blood of patients with colorectal cancer and an analysis of PL S3 gene expression in CTC was conducted. The next step was to conduct a statistical analysis and to demonstrate a link between expression of PL S3 in patients' CTC and progress of the disease, level of CEA and Ca 19-9 markers, gender and age of the patients.PL S3 is a marker which can be potentially used in prediction and monitoring of colorectal cancer. A link between expression of PL S3 in CTC of patients with colorectal cancer a  nd metasta  sis to lymph nodes has been demonstrated. It may be of key importance how PL S3 could impact the qualification to supplementary cancer treatment in patients with stage II colorectal cancer. A link between expression of PL S3 gene in CTC and gender requires further in-depth studies. It is beyond doubt that PL S3 must be further investigated to determine its role in diagnostics, prediction, treatment and monitoring of treatment of colorectal cancer. 
  GeneRIFs  
 Overexpression of PLS3 is associated with epithelial-mesenchymal transition and is associated with   metastasis   in colorectal cancer 
  [Cc]ell [Ii]nvasion  PubMed  
 A common gene variant in PLS3 predicts colon cancer recurrence in women. Recent evidence suggests that PLS3 (T-Plastin), an important member of the actin filamentous network, significantly influences   cell invasion   and metastasis. Germline polymorphisms within the PLS3 gene may impact the gene's function, resulting in inter-individual differences in tumor recurrence capacity. In the present study, we investigated the association of germline polymorphisms in PLS3 to predict time to recurrence (TTR) in patients with stage II and III colon cancer. A total of 264 patients with histologically confirmed colon cancer were included in this retrospective study. Germline DNA was genotyped for rs871773 C>T, rs757124 C>G, rs1557770 G>T, rs6643869 G>A, and rs2522188 C>T in the PLS3 gene by 5'-exonuclease (TaqMan™) technology. As the PLS3 gene is located on the X chromosome, a gender-specific statistical analysis was performed. In univariate analysis, the minor allele of PLS3 rs871773 C>T was significantly associated with decreased TTR in women (hazard ratio (HR) = 5.02; 95 % confidence interval (CI) = 1.251-20.114; p = 0.023) and remained significantly associated in multivariate analysis (HR = 6.165; 95 % CI = 1.538-24.716; p = 0.010). Female patients carrying the C/T genotype in PLS3 rs871773 showed a median TTR of 69 months. In contrast, female patients with homozygous C/C had a median TTR of 112 months. There were no significant associations between PLS3 rs871773 C>T and TTR in male and between the other polymorphisms and TTR in male or female colon cancer patients. In conclusion, we identified a common gene variant in PLS3 as an independent prognostic marker in female patients with stage II and III colon cancer. Larger prospective trials are warranted to confirm these findings. 
 Plastin polymorphisms predict gender- and stage-specific colon cancer recurrence after adjuvant chemotherapy. Tumor recurrence after curative resection remains a major problem in patients with locally advanced colorectal cancer treated with adjuvant chemotherapy. Genetic single-nucleotide polymorphisms (SNP) may serve as useful molecular markers to predict clinical outcomes in these patients and identify targets for future drug development. Recent in vitro and in vivo studies have demonstrated that the plastin genes PLS3 and LCP1 are overexpressed in colon cancer cells and play an important role in tumor   cell invasion  , adhesion, and migration. Hence, we hypothesized that functional genetic variations of plastin may have direct effects on the progression and prognosis of locally advanced colorectal cancer. We tested whether functional tagging polymorphisms of PLS3 and LCP1 predict time to tumor recurrence (TTR) in 732 patients (training set, 234; validation set, 498) with stage II/III colorectal cancer. The PLS3 rs11342 and LCP1 rs4941543 polymorphisms were associated with a significantly increased risk for recurrence in the training set. PLS3 rs6643869 showed a consistent association with TTR in the training and validation set, when stratified by gender and tumor location. Female patients with the PLS3 rs6643869 AA genotype had the shortest median TTR compared with those with any G allele in the training set [1.7 vs. 9.4 years; HR, 2.84; 95% confidence interval (CI), 1.32-6.1; P = 0.005] and validation set (3.3 vs. 13.7 years; HR, 2.07; 95% CI, 1.09-3.91; P = 0.021). Our findings suggest that several SNPs of the PLS3 and LCP1 genes could serve as gender- and/or stage-specific molecular predictors of tumor recurrence in stage II/III patients with colorectal cancer as well as potential therapeutic targets. 
   
  
 WDR1 
 
  NCBI Gene  
  GeneRIF  
  Pubmed  
 
 
  Search Term  Rank  Fields  
  [Pp]odosome(s)?  5  Component(1)  
  [Mm]igration  6  Process(1); PubMed(1); GeneRIFs(1)  
 
 
 hide/show details for WDR1 
  [Pp]odosome(s)?  Component  
   podosome   
  [Mm]igration  Process  
 neutrophil   migration   
  PubMed  
 Critical roles of actin-interacting protein 1 in cytokinesis and chemotactic   migration   of mammalian cells. Cofilin regulates actin filament dynamics by stimulating actin filament disassembly and plays a critical role in cytokinesis and chemotactic   migration  . Aip1 (actin-interacting protein 1), also called WDR1 (WD-repeat protein 1), is a highly conserved WD-repeat protein in eukaryotes and promotes cofilin-mediated actin filament disassembly in vitro; however, little is known about the mechanisms by which Aip1 functions in cytokinesis and cell   migration   in mammalian cells. In the present study, we investigated the roles of Aip1 in cytokinesis and chemotactic   migration   of human cells by silencing the expression of Aip1 using siRNA (small interfering RNA). Knockdown of Aip1 in HeLa cells increased the percentage of multinucleate cells; this effect was reversed by expression of an active form of cofilin. In Aip1-knockdown cells, the cleavage furrow ingressed normally from anaphase to early telophase; however, an excessive accumulation of actin filaments was observed on the contractile ring in late telophase. These results suggest that Aip1 plays a crucial role in the completion of cytokinesis by promoting cofilin-mediated actin filament disassembly in telophase. We have also shown that Aip1 knockdown significantly suppressed chemokine-induced chemotactic   migration   of Jurkat T-lymphoma cells, and this was blocked by expression of an active form of cofilin. Whereas control cells mostly formed a single lamellipodium in response to chemokine stimulation, Aip1 knockdown cells abnormally exhibited multiple protrusions around the cells before and after cell stimulation. This indicates that Aip1 plays an important role in directional cell   migration   by restricting the stimulus-induced membrane protrusion to one direction via promoting cofilin activity. 
  GeneRIFs  
 Data indicates that Aip1 plays an important role in directional cell   migration   by restricting the stimulus-induced membrane protrusion to one direction via promoting cofilin activity. 
   
  
 ECT2 
 
  NCBI Gene  
  GeneRIF  
  Pubmed  
 
 
  Search Term  Rank  Fields  
  [Ii]nvadopodia  3  PubMed(1); GeneRIFs(1)  
  [Mm]igration  6  PubMed(4); GeneRIFs(1)  
  [Ii]nvasive  10  PubMed(5)  
  [Mm]etastasis  12  PubMed(7); GeneRIFs(1)  
 
 
 hide/show details for ECT2 
  [Ii]nvadopodia  PubMed  
 Localization of the   invadopodia  -related proteins actinin-1 and cortactin to matrix-contact-side cytoplasm of cancer cells in surgically resected lung adenocarcinomas. Actin-associated proteins at cell-matrix-contact sites form   invadopodia   in cancer cells and participate in migration, matrix degradation and invasion. We investigated an alteration of subcellular localization of   invadopodia  -related actin-associated proteins, actinin-1 and cortactin, in lung adenocarcinomas, its clinical significance, and its possible regulatory factors.  Invadopodia  -related proteins, actinin-1 and cortactin, were immunohistochemically examined in 90 cases of lung adenocarcinomas. Expression of   invadopodia  -associated proteins and their possible regulators in lung adenocarcinomas were examined by real-time RT-PCR, database search, and immunohistochemistry.Actinin-1 and cortactin showed matrix-contact-side localization in adenocarcinoma cells, but rarely in normal bronchiolar epithelial cells, alveolar cells, or precursor lesion atypical adenomatous hyperplasia cells. Immunoelectron-microscopic examination of adenocarcinoma cells revealed actinin-1 localization to matrix-contact-side cytoplasm with cytoplasmic protrusions. Matrix-contact-side localization of actinin-1 and cortactin was correlated with tumor stages, lymph node metastasis, vascular permeation, and loss of basement membrane. The tumor-specific survival rate was worse for the group in which matrix-contact-side localization of cortactin was high than for the low group. mRNA of the Rho guanine exchange factor epithelial cell transforming sequence-2 (Ect2) tended to be overexpressed in lung adenocarcinomas and cytoplasmic expression of Ect2 tended to be correlated with matrix-contact-side localization of actinin-1.Matrix-contact-side localization of   invadopodia  -related proteins in the lung adenocarcinoma cells were correlated with invasion, metastasis, and poor prognosis. Ect2 was a possible regulator of matrix-contact-side localization of   invadopodia  -related proteins. 
  GeneRIFs  
 Ect2 was a possible regulator of matrix-contact-side localization of   invadopodia  -related proteins. 
  [Mm]igration  PubMed  
 ECT2 regulates the Rho/ERK signalling axis to promote early recurrence in human hepatocellular carcinoma. Early recurrence is the major obstacle for improving the outcome of patients with hepatocellular carcinoma (HCC). Therefore, identifying key molecules contributing to early HCC recurrence can enable the development of novel therapeutic strategies for the clinical management of HCC. Epithelial cell transforming sequence 2 (ECT2) has been implicated in human cancers, but its function in HCC is largely unknown.ECT2 expression was studied by microarrays, immunoblotting and immunohistochemistry in human HCC samples. siRNA- and lentiviral vector-mediated knockdown were employed to decipher the molecular functions of ECT2.The upregulation of ECT2 is significantly associated with early recurrent HCC disease and poor survival. Knockdown of ECT2 markedly suppressed Rho GTPases activities, enhanced apoptosis, attenuated oncogenicity and reduced the metastatic ability of HCC cells. Moreover, knockdown of ECT2 or Rho also suppressed ERK activation, while the silencing of Rho or ERK led to a marked reduction in cell   migration  . Stable knockdown of ECT2 in vivo resulted in significant retardation of tumour growth and the suppression of ERK activation. High expression of ECT2 correlates with high ERK phosphorylation and poor survival of HCC patients. Furthermore, ECT2 enhances the expression and stability of RACGAP1, accelerating ECT2-mediated Rho activation to promote metastasis.ECT2 is closely associated with the activation of the Rho/ERK signalling axis to promote early HCC recurrence. In addition, ECT2 can crosstalk with RACGAP1 to catalyse the GTP exchange involved in Rho signalling to further regulate tumour initiation and metastasis. 
 ECT2 and RASAL2 mediate mesenchymal-amoeboid transition in human astrocytoma cells. Malignant astrocytomas are highly invasive brain tumors. The Rho family of cytoskeletal GTPases are key regulators of astrocytoma   migration   and invasion; expression of the guanine nucleotide exchange factor ECT2 is elevated in primary astrocytomas and predicts both survival and malignancy. Mice bearing orthotopically implanted astrocytoma cells with diminished ECT2 levels following ECT2 knockdown exhibit longer survival. Although ECT2 is normally expressed in the nucleus, we show that ECT2 is aberrantly localized to the cytoplasm in both astrocytoma cell lines and primary human astrocytomas, and colocalizes with RAC1 and CDC42 at the leading edge of migrating astrocytoma cells. Inhibition of ECT2 expression by RNA interference resulted in decreased RAC1 and CDC42 activity, but no change in RHO activity, suggesting that ECT2 is capable of activating these pro-migratory Rho family members. ECT2 overexpression in astrocytoma cells resulted in a transition to an amoeboid phenotype that was abolished with the ROCK inhibitor, Y-27632. Cytoplasmic fractionation of astrocytoma cells followed by ECT2 immunoprecipitation and mass spectrometry were used to identify protein-binding partners that modulate the activity of ECT2 toward RAC1 and RHO/ROCK. We identified RASAL2 as an ECT2-interacting protein that regulates RHO activity in astrocytoma cells. RASAL2 knockdown leads to a conversion to an amoeboid phenotype. Our studies reveal that ECT2 has a novel role in mesenchymal-amoeboid transition in human astrocytoma cells. 
 The guanine nucleotide exchange factors trio, Ect2, and Vav3 mediate the invasive behavior of glioblastoma. Malignant gliomas are characterized by their ability to invade normal brain tissue. We have previously shown that the small GTPase Rac1 plays a role in both   migration   and invasion in gliomas. Here, we aim to identify Rac-activating guanine nucleotide exchange factors (GEFs) that mediate glioblastoma invasiveness. Using a brain tumor expression database, we identified three GEFs, Trio, Ect2, and Vav3, that are expressed at higher levels in glioblastoma versus low-grade glioma. The expression of these GEFs is also associated with poor patient survival. Quantitative real-time polymerase chain reaction and immunohistochemical analyses on an independent set of tumors confirmed that these GEFs are overexpressed in glioblastoma as compared with either nonneoplastic brain or low-grade gliomas. In addition, depletion of Trio, Ect2, and Vav3 by siRNA oligonucleotides suppresses glioblastoma cell   migration   and invasion. Depletion of either Ect2 or Trio also reduces the rate of cell proliferation. These results suggest that targeting GEFs may present novel strategies for anti-invasive therapy for malignant gliomas. 
 Localization of the invadopodia-related proteins actinin-1 and cortactin to matrix-contact-side cytoplasm of cancer cells in surgically resected lung adenocarcinomas. Actin-associated proteins at cell-matrix-contact sites form invadopodia in cancer cells and participate in   migration  , matrix degradation and invasion. We investigated an alteration of subcellular localization of invadopodia-related actin-associated proteins, actinin-1 and cortactin, in lung adenocarcinomas, its clinical significance, and its possible regulatory factors.Invadopodia-related proteins, actinin-1 and cortactin, were immunohistochemically examined in 90 cases of lung adenocarcinomas. Expression of invadopodia-associated proteins and their possible regulators in lung adenocarcinomas were examined by real-time RT-PCR, database search, and immunohistochemistry.Actinin-1 and cortactin showed matrix-contact-side localization in adenocarcinoma cells, but rarely in normal bronchiolar epithelial cells, alveolar cells, or precursor lesion atypical adenomatous hyperplasia cells. Immunoelectron-microscopic examination of adenocarcinoma cells revealed actinin-1 localization to matrix-contact-side cytoplasm with cytoplasmic protrusions. Matrix-contact-side localization of actinin-1 and cortactin was correlated with tumor stages, lymph node metastasis, vascular permeation, and loss of basement membrane. The tumor-specific survival rate was worse for the group in which matrix-contact-side localization of cortactin was high than for the low group. mRNA of the Rho guanine exchange factor epithelial cell transforming sequence-2 (Ect2) tended to be overexpressed in lung adenocarcinomas and cytoplasmic expression of Ect2 tended to be correlated with matrix-contact-side localization of actinin-1.Matrix-contact-side localization of invadopodia-related proteins in the lung adenocarcinoma cells were correlated with invasion, metastasis, and poor prognosis. Ect2 was a possible regulator of matrix-contact-side localization of invadopodia-related proteins. 
  GeneRIFs  
 Data show that Trio, Ect2, and Vav3 are expressed at higher levels in glioblastoma versus low-grade glioma, and are involved in tumor cell   migration   and invasion. 
  [Ii]nvasive  PubMed  
 Involvement of epithelial cell transforming sequence-2 oncoantigen in lung and esophageal cancer progression. This study aims to isolate potential molecular targets for diagnosis, treatment, and/or prevention of lung and esophageal carcinomas.We screened for genes that were frequently overexpressed in the tumors through gene expression profile analyses of 101 lung cancers and 19 esophageal squamous cell carcinomas (ESCC) by cDNA microarray consisting of 27,648 genes or expressed sequence tags. In this process, we identified epithelial cell transforming sequence 2 (ECT2) as a candidate. Tumor tissue microarray was applied to examine the expression of ECT2 protein in 242 archived non-small-cell lung cancers (NSCLC) and 240 ESCC specimens and to investigate its prognostic value. A role of ECT2 in lung and esophageal cancer cell growth and/or survival was examined by small interfering RNA experiments. Cellular   invasive   activity of ECT2 in mammalian cells was examined using Matrigel assays.Northern blot and immunohistochemical analyses detected expression of ECT2 only in testis among 23 normal tissues. Immunohistochemical staining showed that a high level of ECT2 expression was associated with poor prognosis for patients with NSCLC (P = 0.0004) as well as ESCC (P = 0.0088). Multivariate analysis indicated it to be an independent prognostic factor for NSCLC (P = 0.0005). Knockdown of ECT2 expression by small interfering RNAs effectively suppressed lung and esophageal cancer cell growth. In addition, induction of exogenous expression of ECT2 in mammalian cells promoted cellular   invasive   activity.ECT2 cancer-testis antigen is likely to be a prognostic biomarker in clinic and a potential therapeutic target for the development of anticancer drugs and cancer vaccines for lung and esophageal cancers. 
 ECT2 amplification and overexpression as a new prognostic biomarker for early-stage lung adenocarcinoma. Genetic abnormality in early-stage lung adenocarcinoma was examined to search for new prognostic biomarkers. Six in situ lung adenocarcinomas and nine small but   invasive   adenocarcinomas were examined by array-comparative genomic hybridization, and candidate genes of interest were screened. To examine gene abnormalities, 83 cases of various types of lung carcinoma were examined by quantitative real-time genomic PCR and immunohistochemistry. The results were then verified using another set of early-stage adenocarcinomas. Array-comparative genomic hybridization indicated frequent amplification at chromosome 3q26. Of the seven genes located in this region, we focused on the epithelial cell transforming sequence 2 (ECT2) oncogene, as ECT2 amplification was detected only in   invasive   adenocarcinoma, and not in in situ carcinoma. Quantitative PCR and immunohistochemistry analyses also detected overexpression of ECT2 in   invasive   adenocarcinoma, and this was correlated with both the Ki-67 labeling index and mitotic index. In addition, it was associated with disease-free survival and overall survival of patients with lung adenocarcinoma. These results were verified using another set of early-stage adenocarcinomas resected at another hospital. Abnormality of the ECT2 gene occurs at a relatively early stage of lung adenocarcinogenesis and would be applicable as a new biomarker for prognostication of patients with lung adenocarcinoma. 
 ECT2 and RASAL2 mediate mesenchymal-amoeboid transition in human astrocytoma cells. Malignant astrocytomas are highly   invasive   brain tumors. The Rho family of cytoskeletal GTPases are key regulators of astrocytoma migration and invasion; expression of the guanine nucleotide exchange factor ECT2 is elevated in primary astrocytomas and predicts both survival and malignancy. Mice bearing orthotopically implanted astrocytoma cells with diminished ECT2 levels following ECT2 knockdown exhibit longer survival. Although ECT2 is normally expressed in the nucleus, we show that ECT2 is aberrantly localized to the cytoplasm in both astrocytoma cell lines and primary human astrocytomas, and colocalizes with RAC1 and CDC42 at the leading edge of migrating astrocytoma cells. Inhibition of ECT2 expression by RNA interference resulted in decreased RAC1 and CDC42 activity, but no change in RHO activity, suggesting that ECT2 is capable of activating these pro-migratory Rho family members. ECT2 overexpression in astrocytoma cells resulted in a transition to an amoeboid phenotype that was abolished with the ROCK inhibitor, Y-27632. Cytoplasmic fractionation of astrocytoma cells followed by ECT2 immunoprecipitation and mass spectrometry were used to identify protein-binding partners that modulate the activity of ECT2 toward RAC1 and RHO/ROCK. We identified RASAL2 as an ECT2-interacting protein that regulates RHO activity in astrocytoma cells. RASAL2 knockdown leads to a conversion to an amoeboid phenotype. Our studies reveal that ECT2 has a novel role in mesenchymal-amoeboid transition in human astrocytoma cells. 
 The guanine nucleotide exchange factors trio, Ect2, and Vav3 mediate the   invasive   behavior of glioblastoma. Malignant gliomas are characterized by their ability to invade normal brain tissue. We have previously shown that the small GTPase Rac1 plays a role in both migration and invasion in gliomas. Here, we aim to identify Rac-activating guanine nucleotide exchange factors (GEFs) that mediate glioblastoma   invasive  ness. Using a brain tumor expression database, we identified three GEFs, Trio, Ect2, and Vav3, that are expressed at higher levels in glioblastoma versus low-grade glioma. The expression of these GEFs is also associated with poor patient survival. Quantitative real-time polymerase chain reaction and immunohistochemical analyses on an independent set of tumors confirmed that these GEFs are overexpressed in glioblastoma as compared with either nonneoplastic brain or low-grade gliomas. In addition, depletion of Trio, Ect2, and Vav3 by siRNA oligonucleotides suppresses glioblastoma cell migration and invasion. Depletion of either Ect2 or Trio also reduces the rate of cell proliferation. These results suggest that targeting GEFs may present novel strategies for anti-  invasive   therapy for malignant gliomas. 
 Epithelial cell transformation sequence 2 is a potential biomarker of unfavorable survival in human gliomas. High-grade primary gliomas are   invasive   and have poor outcome. The identification of biomarkers predictive of outcome in patients with gliomas is crucial for clinical follow-up. Epithelial cell transformation sequence 2 (ECT2) modulates cancer invasion, progression, metastasis and cell cycle regulation. However, its role in determining the clinical outcome of human gliomas warrants further elucidation.This study hypothesized that ECT2 is over-expressed in human gliomas. We analysis de-linked data (GDS1815/219787_s_at/ECT2) in primary high-grade glioma, and exclude 23 sheets of data without detailed information. An additional database (GDS1962/234992_x_at/ECT2) was also included to evaluation ECT2 gene expression in each pathologic grading.Analysis of the Gene Expression Omnibus (GEO) profile showed that ECT2 mRNA expression level was higher in WHO grade IV (n = 81) than in grade II (n = 7, P = 0.0126) gliomas and non-tumor controls (n = 23; P = 1.65 Χ 10⁻⁸). Kaplan-Meier analysis showed unfavorable survival in patients with high ECT2 mRNA levels (n = 10) than in those with low ECT2 expression (n = 67) (median survival, 106 vs. 46 weeks, P < 0.0001, by log-rank test, Hazard ratio: 0.07850, 95% CI: 0.02402-0.2565).ECT2 expression is positively correlated with WHO pathologic grading and unfavorable survival, suggesting that ECT2 may be a potential therapeutic candidate in human gliomas. 
  [Mm]etastasis  PubMed  
 ECT2 regulates the Rho/ERK signalling axis to promote early recurrence in human hepatocellular carcinoma. Early recurrence is the major obstacle for improving the outcome of patients with hepatocellular carcinoma (HCC). Therefore, identifying key molecules contributing to early HCC recurrence can enable the development of novel therapeutic strategies for the clinical management of HCC. Epithelial cell transforming sequence 2 (ECT2) has been implicated in human cancers, but its function in HCC is largely unknown.ECT2 expression was studied by microarrays, immunoblotting and immunohistochemistry in human HCC samples. siRNA- and lentiviral vector-mediated knockdown were employed to decipher the molecular functions of ECT2.The upregulation of ECT2 is significantly associated with early recurrent HCC disease and poor survival. Knockdown of ECT2 markedly suppressed Rho GTPases activities, enhanced apoptosis, attenuated oncogenicity and reduced the metastatic ability of HCC cells. Moreover, knockdown of ECT2 or Rho also suppressed ERK activation, while the silencing of Rho or ERK led to a marked reduction in cell migration. Stable knockdown of ECT2 in vivo resulted in significant retardation of tumour growth and the suppression of ERK activation. High expression of ECT2 correlates with high ERK phosphorylation and poor survival of HCC patients. Furthermore, ECT2 enhances the expression and stability of RACGAP1, accelerating ECT2-mediated Rho activation to promote   metastasis  .ECT2 is closely associated with the activation of the Rho/ERK signalling axis to promote early HCC recurrence. In addition, ECT2 can crosstalk with RACGAP1 to catalyse the GTP exchange involved in Rho signalling to further regulate tumour initiation and   metastasis  . 
 Up-regulation of ECT2 is associated with poor prognosis in gastric cancer patients. The aim of this study was to investigate the expression of ECT2 in gastric cancer and its clinical significance.We investigated the differentially expressed genes between gastric cancer tissues and normal gastric mucosa by cDNA microarray, and then we found ECT2 was up-regulated in gastric cancer. What is more, we verified ECT2 expression level by quantitative real-time reverse transcription-polymerase chain reaction (qRT-PCR) and measured its protein level by immunohistochemistry (IHC). qRT-PCR analysis indicated ECT2 was significantly up-regulated in gastric cancer and Immunohistochemistry confirmed the percentage of ECT2-positive specimens was significantly higher in gastric carcinoma than in non-tumor tissues. Up-regulation of ECT2 is associated with the degree of histological differentiation (P = 0.007), invasion depth (P = 0.047), lymph node   metastasis   (P = 0.016), distant   metastasis   (P = 0.021) and TNM stage (P = 0.016), patients with up-regulated ECT2 had a lower overall survival rate (P = 0.000). Cox regression analysis revealed that up-regulation of ECT2 is an independent prognostic factor in gastric cancer patients (P = 0.012).Up-regulation of ECT2 might contribute to the progression of gastric carcinogenesis and may be a useful prognostic indicator in gastric cancer. 
 Elevated levels of epithelial cell transforming sequence 2 predicts poor prognosis for prostate cancer. Epithelial cell transforming sequence 2 (Ect2) was originally reported as an oncogene that is involved in several types of human cancers. However, little is known about its expression and function in prostate cancer. Immunohistochemical staining for Ect2 was performed on a human tissue microarray. The staining intensity was analyzed in association with clinical pathological parameters such as Gleason score, pathological grade, clinical stage, tumor invasion, lymph node and distant   metastasis  . Furthermore, we repeated such analysis and investigated the prognostic value of Ect2 using the TCGA (The Cancer Genome Atlas) Dataset. Our immunohistochemical results showed that the expression levels of Ect2 protein were enhanced in human prostate cancer tissues. There existed positive correlations between the expression levels of Ect2 and several clinicopathological parameters, including advanced clinical stage, enhanced tumor invasion and lymph node   metastasis  . Similarly, we found that the expression levels of Ect2 were positively related to Gleason score, tumor invasion, lymph node   metastasis   and high distant   metastasis   in the TCGA Dataset. Kaplan-Meier analysis revealed that lower levels of Ect2 mRNA predicted higher overall survivals and biochemical recurrence (BCR)-free survivals in all patients or non-metastatic patients. Multivariate analysis by Cox regression showed that the expression of Ect2 could be an independent prognostic marker of poor BCR-free survivals. Therefore, levels of Ect2 may serve as a novel marker for the diagnosis or prognosis of prostate cancer. 
 Epithelial cell transformation sequence 2 is a potential biomarker of unfavorable survival in human gliomas. High-grade primary gliomas are invasive and have poor outcome. The identification of biomarkers predictive of outcome in patients with gliomas is crucial for clinical follow-up. Epithelial cell transformation sequence 2 (ECT2) modulates cancer invasion, progression,   metastasis   and cell cycle regulation. However, its role in determining the clinical outcome of human gliomas warrants further elucidation.This study hypothesized that ECT2 is over-expressed in human gliomas. We analysis de-linked data (GDS1815/219787_s_at/ECT2) in primary high-grade glioma, and exclude 23 sheets of data without detailed information. An additional database (GDS1962/234992_x_at/ECT2) was also included to evaluation ECT2 gene expression in each pathologic grading.Analysis of the Gene Expression Omnibus (GEO) profile showed that ECT2 mRNA expression level was higher in WHO grade IV (n = 81) than in grade II (n = 7, P = 0.0126) gliomas and non-tumor controls (n = 23; P = 1.65 Χ 10⁻⁸). Kaplan-Meier analysis showed unfavorable survival in patients with high ECT2 mRNA levels (n = 10) than in those with low ECT2 expression (n = 67) (median survival, 106 vs. 46 weeks, P < 0.0001, by log-rank test, Hazard ratio: 0.07850, 95% CI: 0.02402-0.2565).ECT2 expression is positively correlated with WHO pathologic grading and unfavorable survival, suggesting that ECT2 may be a potential therapeutic candidate in human gliomas. 
 Clinical significance of ECT2 expression in tissue and serum of gastric cancer patients. The ECT2 (epithelial cell transforming sequence 2) oncogene acted as a guanine nucleotide exchange factor for RhoGTPases, and regulates cytokinesis; thus, it may play a role in the pathogenesis of gastric cancer. In this study, we investigated the expression ECT2 gene in tissues and serum of gastric cancer patients to explore its clinical significance. ECT2 mRNA expression levels in tissues and serum were examined by RT-PCR, and ECT2 protein expression in tissue was evaluated by Western blot, and was further validated by immunohistochemistry and enzyme-linked immunosorbent assay at serum level. ECT2 level was significantly increased in the GC tissues and serum compared to normal control. ECT2 expression was positively correlated with the histologic differentiation, stages of TNM, and lymph node   metastasis   in GC (P < 0.05). Our results suggest that ECT2 plays an important role during GC progression and it may become a new diagnostic marker and therapeutic molecular target for management of GC. 
 Prognostic value of microRNA-223/epithelial cell transforming sequence 2 signaling in patients with osteosarcoma. MicroRNA-223 (miR-223) has been demonstrated to be implicated in cell proliferation and cell cycle progression of osteosarcoma cell lines by regulating its target gene epithelial cell transforming sequence 2 (ECT2). However, the clinical significance of the deregulation of the miR-223/Ect2 axis in human osteosarcoma has not been fully elucidated. To address this problem, we firstly showed that the expression levels of miR-223 and Ect2 messenger RNA were, respectively, down-regulated and up-regulated in osteosarcoma tissues compared with those in noncancerous bone tissues significantly (both P < .001), according to the results of quantitative real-time reverse transcription-polymerase chain reaction. Notably, miR-223 down-regulation was negatively correlated with Ect2 messenger RNA up-regulation in osteosarcoma tissues (r = -0.68, P = .01). Then, the combined low miR-223 expression and high Ect2 expression (miR-223-low/Ect2-high) was significantly associated with high tumor grade (P = .01), poor response to chemotherapy (P = .01), positive   metastasis   (P < .001), and recurrence (P < .001) of osteosarcomas. Moreover, patients with miR-223-low/Ect2-high expression had the shortest overall survival (P < .001) and disease-free survival (P < .001) compared with patients in the other 3 groups (miR-223-low/Ect2-low, miR-223-high/Ect2-high, and miR-223-high/Ect2-low). Furthermore, the multivariate analysis identified miR-223/Ect2 expression and the status of   metastasis   as independent prognostic factors for overall survival and disease-free survival. In conclusion, our data offer convincing evidence that the deregulation of miR-223 and its target gene ECT2 may be associated with the aggressive tumor progression of human osteosarcoma. Of note, the combined miR-223 down-regulation and Ect2 up-regulation may be a possible marker of poor prognosis in this malignancy. 
 Localization of the invadopodia-related proteins actinin-1 and cortactin to matrix-contact-side cytoplasm of cancer cells in surgically resected lung adenocarcinomas. Actin-associated proteins at cell-matrix-contact sites form invadopodia in cancer cells and participate in migration, matrix degradation and invasion. We investigated an alteration of subcellular localization of invadopodia-related actin-associated proteins, actinin-1 and cortactin, in lung adenocarcinomas, its clinical significance, and its possible regulatory factors.Invadopodia-related proteins, actinin-1 and cortactin, were immunohistochemically examined in 90 cases of lung adenocarcinomas. Expression of invadopodia-associated proteins and their possible regulators in lung adenocarcinomas were examined by real-time RT-PCR, database search, and immunohistochemistry.Actinin-1 and cortactin showed matrix-contact-side localization in adenocarcinoma cells, but rarely in normal bronchiolar epithelial cells, alveolar cells, or precursor lesion atypical adenomatous hyperplasia cells. Immunoelectron-microscopic examination of adenocarcinoma cells revealed actinin-1 localization to matrix-contact-side cytoplasm with cytoplasmic protrusions. Matrix-contact-side localization of actinin-1 and cortactin was correlated with tumor stages, lymph node   metastasis  , vascular permeation, and loss of basement membrane. The tumor-specific survival rate was worse for the group in which matrix-contact-side localization of cortactin was high than for the low group. mRNA of the Rho guanine exchange factor epithelial cell transforming sequence-2 (Ect2) tended to be overexpressed in lung adenocarcinomas and cytoplasmic expression of Ect2 tended to be correlated with matrix-contact-side localization of actinin-1.Matrix-contact-side localization of invadopodia-related proteins in the lung adenocarcinoma cells were correlated with invasion,   metastasis  , and poor prognosis. Ect2 was a possible regulator of matrix-contact-side localization of invadopodia-related proteins. 
  GeneRIFs  
 ECT2 can crosstalk with RACGAP1 to catalyse the GTP exchange involved in Rho signalling to further regulate tumour initiation and   metastasis  . 
   
  
 MAPK14 
 
  NCBI Gene  
  GeneRIF  
  Pubmed  
 
 
  Search Term  Rank  Fields  
  [Mm]igration  6  Process(1); PubMed(79); GeneRIFs(30)  
  ECM  7  PubMed(5)  
  [Ee]xtracellular [Mm]atrix  8  PubMed(21); GeneRIFs(3)  
  [Ii]nvasive  10  PubMed(33); GeneRIFs(3)  
  [Mm]etastasize  11  PubMed(2)  
  [Mm]etastasis  12  PubMed(42); GeneRIFs(5)  
  [Cc]ell [Ii]nvasion  13  PubMed(14); GeneRIFs(4)  
 
 
 hide/show details for MAPK14 
  [Mm]igration  Process  
 positive regulation of blood vessel endothelial cell   migration   
  PubMed  
 The secreted protein ANGPTL2 promotes metastasis of osteosarcoma cells through integrin α5β1, p38 MAPK, and matrix metalloproteinases. The tumor microenvironment can enhance the invasive capacity of tumor cells. We showed that expression of angiopoietin-like protein 2 (ANGPTL2) in osteosarcoma (OS) cell lines increased and the methylation of its promoter decreased with time when grown as xenografts in mice compared with culture. Compared with cells grown in normal culture conditions, the expression of genes encoding DNA demethylation-related enzymes increased in tumor cells implanted into mice or grown in hypoxic, serum-starved culture conditions. ANGPTL2 expression in OS cell lines correlated with increased tumor metastasis and decreased animal survival by promoting tumor cell intravasation mediated by the integrin α5β1, p38 mitogen-activated protein kinase, and matrix metalloproteinases. The tolloid-like 1 (TLL1) protease cleaved ANGPTL2 into fragments in vitro that did not enhance tumor progression when overexpressed in xenografts. Expression of TLL1 was weak in OS patient tumors, suggesting that ANGPTL2 may not be efficiently cleaved upon secretion from OS cells. These findings demonstrate that preventing ANGPTL2 signaling stimulated by the tumor microenvironment could inhibit tumor c  ell migra  tion and metastasis. 
 The secreted protein ANGPTL2 promotes metastasis of osteosarcoma cells through integrin α5β1, p38 MAPK, and matrix metalloproteinases. The tumor microenvironment can enhance the invasive capacity of tumor cells. We showed that expression of angiopoietin-like protein 2 (ANGPTL2) in osteosarcoma (OS) cell lines increased and the methylation of its promoter decreased with time when grown as xenografts in mice compared with culture. Compared with cells grown in normal culture conditions, the expression of genes encoding DNA demethylation-related enzymes increased in tumor cells implanted into mice or grown in hypoxic, serum-starved culture conditions. ANGPTL2 expression in OS cell lines correlated with increased tumor metastasis and decreased animal survival by promoting tumor cell intravasation mediated by the integrin α5β1, p38 mitogen-activated protein kinase, and matrix metalloproteinases. The tolloid-like 1 (TLL1) protease cleaved ANGPTL2 into fragments in vitro that did not enhance tumor progression when overexpressed in xenografts. Expression of TLL1 was weak in OS patient tumors, suggesting that ANGPTL2 may not be efficiently cleaved upon secretion from OS cells. These findings demonstrate that preventing ANGPTL2 signaling stimulated by the tumor microenvironment could inhibit tumor c  ell migra  tion and metastasis. 
 Hypoxia-induced Bcl-2 expression in endothelial cells via p38 MAPK pathway. Angiogenesis and apoptosis are reciprocal processes in endothelial cells. Bcl-2, an anti-apoptotic protein, has been found to have angiogenic activities. The purpose of this study was to determine the role of Bcl-2 in hypoxia-induced angiogenesis in endothelial cells and to investigate the underlying mechanisms. Human aortic endothelial cells (HAECs) were exposed to hypoxia followed by reoxygenation. Myocardial ischemia and reperfusion mouse model was used and Bcl-2 expression was assessed. Bcl-2 expression increased in a time-dependent manner in response to hypoxia from 2 to 72h. Peak expression occurred at 12h (3- to 4-fold, p<0.05). p38 inhibitor (SB203580) blocked hypoxia-induced Bcl-2 expression, whereas PKC, ERK1/2 and PI3K inhibitors did not. Knockdown of Bcl-2 resulted in decreased HAECs' proliferation and   migration  . Over-expression of Bcl-2 increased HAECs' tubule formation, whereas knockdown of Bcl-2 inhibited this process. In this model of myocardial ischemia and reperfusion, Bcl-2 expression was increased and was associated with increased p38 MAPK activation. Our results showed that hypoxia induces Bcl-2 expression in HAECs via p38 MAPK pathway. 
 Interleukin-17A stimulates   migration   of periodontal ligament fibroblasts via p38 MAPK/NF-κB -dependent MMP-1 expression. Interleukin-17 (IL-17) is a cytokine secreted predominantly by Th17 cells. Although IL-17 is primarily associated with the induction of tissue inflammation, the other biological functions of IL-17, including its wound-healing functions, have yet to be thoroughly explored. Fibroblast proliferation and   migratio  n play essential roles in periodontal wound-healing responses. In this study, we report that IL-17A can increase the   migratio  n and expression of matrix metalloproteinase (MMP)-1 in human periodontal ligament (PDL) fibroblasts but has no effect on PDL fibroblast proliferation. IL-17A-induced MMP-1 expression led to cell   migratio  n, which was attenuated by pre-treatment with IL-17 receptor neutralizing antibody and small interfering RNA (siRNA) for MMP-1. The IL-17A-induced cell   migratio  n was also attenuated by its tissue inhibitor of matrix metalloproteinase (TIMP)-1. In addition, a p38 mitogen-activated protein kinase (MAPK) inhibitor (SB203580) inhibited IL-17A-induced increase of the   migratio  n and MMP-1 upregulation of PDL fibroblasts. The involvement of p38 MAPK in IL-17A-induced MMP-1 expression and cell   migratio  n was further confirmed by transfection of p38α siRNA. A nuclear factor kappaB (NF-κB) inhibitor (pyrrolidine dithiocarbamate) also suppressed the ce  ll migrat  ion and MMP-1 expression enhanced by IL-17A. Moreover, transfection with p38α siRNA inhibited IL-17A-induced NF-κB nuclear translocation as well as NF-κB binding activity. Our results suggest that IL-17A enhance  s the mig  ration of PDL fibroblasts by increasing MMP-1 expression through the IL-17 receptor, p38 MAPK, and NF-κB signal transduction pathways. 
 IL-1beta-stimulated activation of ERK1/2 and p38alpha MAPK mediates the transcriptional up-regulation of IL-6, IL-8 and GRO-alpha in HeLa cells. Epithelial cells represent the first line of defense against infection. Here we have studied the production of inflammatory mediators induced by IL-1beta in the HeLa epithelial cell line. We found that GRO-alpha, IL-6 and IL-8 were the only three inflammatory mediators elevated out of 36 tested. Specific inhibition of p38alpha MAP kinase or preventing the activation of ERK1/ERK2 partially reduced the production of these substances, while the combined blockade of both pathways almost abolished secretion. The suppression of these signaling pathways mainly reduced transcription of the genes encoding GRO-alpha, IL-6 and IL-8, rather than affecting mRNA stability, translation or secretion. The production of these three inflammatory mediators was shown to account for the ability of the HeLa cell culture medium to stimulate the   migration   of monocytes/macrophages, suggesting a key role for p38 MAPK and ERK1/ERK2 in orchestrating the epithelial cell response to infection. 
 The p38 mitogen-activated protein kinases modulate endothelial cell survival and tissue repair. This study is designed to investigate the role of p38 MAPK in modulating human pulmonary artery endothelial cells (HPAECs) survival and tissue repair functions.HPAECs (passage 8-12) were used for all experiments. Cells were treated with IL-1β (0.5 or 2 ng/ml) or p38 inhibitor (SB203580 or SB220025, 5 μM each). Cells were also transfected with 50 nM siRNAs. Cell length was measured using ImageJ software. Collagen gel contraction and wound close assay were performed to evaluate tissue repair functions.IL-1β activated p38 MAPK and induced morphologic change of HPAECs. The p38 inhibitors further augmented IL-1β-induced cell morphologic change, prevented cell death, and augmented collagen gel contraction. Suppression of p38α, γ, or δ, but not p38β resulted in cell morphologic alteration, and suppressing any one of p38 isoforms by siRNAs increased cell survival. Suppression of p38α or δ augmented gel contraction. While p38α suppression st  imulated   cell migration, suppressing the rest of three isoforms   inhibit   cell migration. Nuclear factor p65-siRNA blocked IL-1β-induced cell morphologic change, but did not affect p38 inhibitor-induced change.These findings suggest that p38 MAPK may negatively modulate tissue repair functions of endothelial cells via p65 independent pathway. 
 Mammary gland specific expression of Brk/PTK6 promotes delayed involution and tumor formation associated with activation of p38 MAPK. Protein tyrosine kinases (PTKs) are frequently overexpressed and/or activated in human malignancies, and regulate cancer cell proliferation, cellular survival, and   migration  . As such, they have become promising molecular targets for new therapies. The non-receptor PTK termed breast tumor kinase (Brk/PTK6) is overexpressed in approximately 86% of human breast tumors. The role of Brk in breast pathology is unclear.We expressed a WAP-driven Brk/PTK6 transgene in FVB/n mice, and analyzed mammary glands from wild-type (wt) and transgenic mice after forced weaning. Western blotting and immunohistochemistry (IHC) studies were conducted to visualize markers of mammary gland involution, cell proliferation and apoptosis, as well as Brk, STAT3, and activated p38 mitogen-activated protein kinase (MAPK) in mammary tissues and tumors from WAP-Brk mice. Human (HMEC) or mouse (HC11) mammary epithelial cells were stably or transiently transfected with Brk cDNA to assay p38 MAPK signaling and cell survival in suspension or in response to chemotherapeutic agents.Brk-transgenic dams exhibited delayed mammary gland involution and aged mice developed infrequent tumors with reduced latency relative to wt mice. Consistent with delayed involution, mammary glands of transgenic animals displayed decreased STAT3 phosphorylation, a marker of early-stage involution. Notably, p38 MAPK, a pro-survival signaling mediator downstream of Brk, was activated in mammary glands of Brk transgenic relative to wt mice. Brk-dependent signaling to p38 MAPK was recapitulated by Brk overexpression in the HC11 murine mammary epithelial cell (MEC) line and human MEC, while Brk knock-down in breast cancer cells blocked EGF-stimulated p38 signaling. Additionally, human or mouse MECs expressing Brk exhibited increased anchorage-independent survival and resistance to doxorubicin. Finally, breast tumor biopsies were subjected to IHC analysis for co-expression of Brk and phospho-p38 MAPK; ductal and lobular carcinomas expressing Brk were significantly more likely to express elevated phospho-p38 MAPK.These studies illustrate that forced expression of Brk/PTK6 in non-transformed mammary epithelial cells mediates p38 MAPK phosphorylation and promotes increased cellular survival, delayed involution, and latent tumor formation. Brk expression in human breast tumors may contribute to progression by inducing p38-driven pro-survival signaling pathways. 
 Triggering of DC   migration   by dengue virus stimulation of COX-2-dependent signaling cascades in vitro highlights the significance of these cascades beyond inflammation. A term "bone-breaking fever" is used in Chinese medicine to describe the symptoms of patients infected with dengue virus (DV). We examined the significance of the COX-prostaglandin pathway in human DC infected by DV. We show that DV infection induced the expression of COX-2 and the production of prostaglandin E2 (PGE2) in DC, and stimulated the DNA binding of NF-kappaB and the kinase activity of both IkappaBalpha kinase (IKK) alpha and beta. DV infection also activated MAPK and AP-1 signaling. Both IkappaBalpha kinase-NF-kappaB and MAPK-AP-1 were upstream of COX-2 activation. Our investigation into the significance of COX-2-PGE2 pathway also revealed that DV infection enhances DC   migration   by inducing CC chemokine receptor 7 (CCR7) expression, and that blocking COX-2 or MAPK activity suppresses DV-induced DC   migration  . Our data also suggest that PGE2 can induce CCR7 expression on DC and that antagonists of the PGE2 receptors EP2 and EP4 suppress DV-induced DC   migration  . We further show that the increased CCR7 expression was observed in both DV-infected and bystander DC, suggesting the presence of secondary effects in inducing CCR7 expression. Collectively, this study reveals not only the pathways involved in COX-2 synthesis in DV-infected DC but also the autocrine action of PGE2 on the   migration   of DV-infected DC. 
 Activation of p38 has opposing effects on the proliferation and   migration   of endothelial cells. Pathological conditions such as hypertension and hyperglycemia as well as abrasions following balloon angioplasty all lead to endothelial dysfunction that impacts disease morbidity. These conditions are associated with the elaboration of a variety of cytokines and increases in p38 activity in endothelial cells. However, the relationship between enhanced p38 activity and endothelial cell function remains poorly understood. To investigate the effect of enhanced p38 MAPK activity on endothelial cell function, we expressed an activated mutant of MEK6 (MEK6E), an upstream regulator of p38. Expression of MEK6E activated p38 and resulted in phosphorylation of its downstream substrate, heat shock protein 27 (Hsp27). Activation of p38 was not sufficient to induce apoptosis; however, it did induce p38-dependent cell cycle arrest. MEK6E expression was sufficient to inhibit ERK phosphorylation triggered by growth factors and integrin engagement. MAPK phosphatase-1 (MKP-1) expression was increased upon p38 activation, and expression of a "substrate-trapping" MKP-1 was sufficient to restore ERK activity. Activation of p38 was sufficient to induce cell   migration  , which was accompanied by alterations in actin architecture characterized by enhanced lamellipodia. Co-expression of a mutant form of Hsp27, lacking all three phosphorylation sites, reversed MEK6E-induced cell   migration   and altered the cytoskeletal changes induced by p38 activation. Collectively, these results suggest that cellular decisions regarding   migration   and proliferation are influenced by p38 activity and that prolonged activation of p38 may result in an anti-angiogenic phenotype that contributes to endothelial dysfunction. 
 COX-2 promotes breast cancer cell radioresistance via p38/MAPK-mediated cellular anti-apoptosis and invasiveness. Radioresistance is one of the major barriers to improve the survival rate of breast cancer patients. Cyclooxygenase 2 (COX-2) is usually overexpressed in highly invasive and metastatic breast cancer, which may indicate an association with breast cancer radioresistance. The function role of COX-2 was investigated by using a radioresistant breast cancer cell line MDA-MB-231/RR10 and its parental cell line MDA-MB-231 cells before or after COX-2 was silenced by a specific small hairpin RNA (shRNA). The cell proliferation,   migration  , invasion, colony formation, and apoptosis were measured by CCK-8, scratch-wound, transwell, clone formation assay, and flow cytometry. Protein and mRNA expression were analyzed by Western blot and quantitative reverse transcriptase-polymerase chain reaction. COX-2 is upregulated in MDA-MB-231/RR10 cells compared with in MDA-MB-231 cells, and silencing of COX-2 expression by shRNA in MDA-MB-231/RR10 cells decreases the expression of Bcl-2 and Bcl-XL, but increases the proapoptotic protein BAK, leading to the increased apoptosis following treatment with γ-irradiation in comparison with those in control cells. Silencing of COX-2 also increases the expression of β-catenin and E-cadherin, two anti-invasion proteins, resulting in reduced cel  l migrati  on and invasion tested by transwell chambers and wound-healing assays. Further study demonstrated that COX-2-induced radioresistance is negatively regulated through the phosphorylation of p38 at Tyr182, and that the phosphorylation of p38 induced by TNF-alpha reduces the expression of Bcl-2, BCL-XL, but increases β-catenin and E-cadherin, leading to the decreased invasiveness of cells. Our data suggest that COX-2, p38, Bcl-2, Bcl-XL, β-catenin, and E-cadherin may be considered as potential therapeutic targets against radioresistant breast cancer. 
 NSAID inhibition of prostate cancer cell   migration   is mediated by Nag-1 Induction via the p38 MAPK-p75(NTR) pathway. The nonsteroidal anti-inflammatory drugs (NSAID) R-flurbiprofen and ibuprofen have been shown to induce expression of p75(NTR) (neurotrophin receptor) in prostate cancer cell lines. p75(NTR), a tumor necrosis factor receptor superfamily member, is a proapoptotic protein that functions as a tumor suppressor in the human prostate. Expression of p75(NTR) is lost as prostate cancer progresses and is minimal in several metastatic prostate cancer cell lines. NSAIDs induce p75(NTR) through activation of the p38 mitogen-activated protein kinase (MAPK) pathway, with a concomitant decrease in cell survival. Here, we show that treatment with R-flurbiprofen and ibuprofen induces expression of the NSAID-activated gene-1 (Nag-1) protein, a divergent member of the TGF beta (TGF-β) family, in PC-3 cells. Using the selective pharmacologic inhibitor of p38 MAPK, SB202190, and p38 MAPK-specific siRNA (small interfering RNA), we show that Nag-1 induction following NSAID treatment is mediated by the p38 MAPK pathway. p75(NTR)-specific siRNA pretreatment shows that Nag-1 induction by NSAIDs is downstream of p75(NTR) induction. Decreased survival of NSAID-treated cells is rescued by p75(NTR)-specific siRNA but not by Nag-1 siRNA. Transwell chamber and in vitro wound healing assays demonstrate decreased cell   migratio  n upon NSAID treatment. Pretreatment of PC-3 cells with p75(NTR) and Nag-1-specific siRNA shows that NSAID inhibition of cell   migratio  n is mediated by Nag-1 and p75(NTR). These results demonstrate a role for Nag-1 in NSAID inhibition of cell   migratio  n, but not survival. 
 Activation of synovial cell p38 MAP kinase by macrophage   migration   inhibitory factor. Macrophage   migration   inhibitory factor (MIF) is a proinflammatory cytokine important in animal models of rheumatoid arthritis (RA). We investigated the utilization by MIF of mitogen activated protein (MAP) kinase signalling pathways in the stimulation of fibroblast-like synoviocytes (FLS), cyclooxygenase-2 (COX-2), prostaglandin E(2) (PGE(2)), and interleukin 6 (IL-6) and IL-8 expression.Cultured human RA FLS were treated with recombinant MIF. Activation of MAPK was measured by Western blotting and blocked using specific inhibitors. The expression of COX-2, PGE(2), IL-6, and IL-8 were measured using flow cytometry, ELISA, and real-time polymerase chain reaction.MIF induced the phosphorylation of FLS p38 and extracellular-signal regulated kinase (ERK) MAP kinase. MIF significantly induced COX-2 and IL-6 protein and mRNA expression as well as PGE(2) and IL-8 production. Antagonism of p38 MAP kinase inhibited MIF induction of COX-2, PGE(2), and IL-6. In contrast, antagonism of ERK had no effect on COX-2, PGE(2), or IL-6. Neither antagonist inhibited MIF-induced IL-8.MIF activates RA FLS COX-2 and IL-6 expression via p38 MAP kinase activation and induces IL-8 via p38 and ERK MAP kinase-independent pathways. 
 H-Ras-specific activation of Rac-MKK3/6-p38 pathway: its critical role in invasion and   migration   of breast epithelial cells. Human tumors frequently exhibit constitutively activated Ras signaling, which contributes to the malignant phenotype. Mounting evidence suggests unique roles of the Ras family members, H-Ras, N-Ras and K-Ras, in normal and pathological conditions. In an effort to dissect distinct Ras isoform-specific functions in malignant phenotypic changes, we previously established H-Ras- and N-Ras-activated MCF10A human breast epithelial cell lines. Using these, we showed that p38 kinase is a key signaling molecule differentially regulated between H-Ras and N-Ras, leading to H-Ras-specific induction of invasive and migrative phenotypes. The present study is to further investigate H-Ras- and N-Ras-mediated signaling pathways and to unveil how these pathways are integrated for regulation of invasive/migrative phenotypic conversion of human breast epithelial cells. Here we report that the Rac-MAPK kinase (MKK)3/6-p38 pathway is a unique signaling pathway activated by H-Ras, leading to the invasive/migrative phenotype. In contrast, Raf-MEK-ERK and phosphatidylinositol 3-kinase-Akt pathways, which are fundamental to proliferation and differentiation, are activated by both H-Ras and N-Ras. A significant role for p38 in cell invasion is further supported by the observation that p38 activation by MKK6 transfection is sufficient to induce invasive and migrative phenotypes in MCF10A cells. Activation of the MKK6-p38 pathway results in a marked induction of matrix metalloproteinase (MMP)-2, whereas it had little effect on MMP-9, suggesting MMP-2 up-regulation by MKK6-p38 pathway as a key step for H-Ras-induced invasion and   migration  . We also provide evidence for cross-talk among the Rac, Raf, and phosphatidylinositol 3-kinase pathways critical for regulation of MMP-2 and MMP-9 expression and invasive phenotype. Taken together, the present study elucidated the role of the Rac-MKK3/6-p38 pathway leading to H-Ras-specific induction of malignant progression in breast epithelial cells, providing implications for developing therapeutic strategies for mammary carcinoma to target Ras downstream signaling molecules required for malignant cancer cell behavior but less critical for normal cell functions. 
 Acacetin inhibits the invasion and   migration   of human non-small cell lung cancer A549 cells by suppressing the p38α MAPK signaling pathway. Lung cancer is one of the most common malignancies in the world and its metastasis is the major cause of death in cancer patients. Acacetin (5,7-dihydroxy-4'-methoxyflavone), a flavonoid compound, has anti-peroxidative and anti-inflammatory effects. The effect of acacetin on invasion and   migratio  n in human NSCLC A549 cells was investigated. First, the result demonstrated acacetin could exhibit an inhibitory effect on the abilities of the adhesion, morphology/actin cytoskeleton arrangement, invasion, and   migratio  n by cell-matrix adhesion assay, immunofluorescence assay, Boyden chamber assay, and wound-healing assay. Molecular data showed that the effect of acacetin in A549 cells might be mediated via sustained inactivation of the phosphorylation of mixed-lineage protein kinase 3 (MLK3), mitogen-activated protein kinase kinases 3/6 (MKK3/6), and p38α MAPK signal involved in the downregulation of the expressions of matrix metalloproteinase-2 (MMP-2), matrix metalloproteinase-9 (MMP-9), and urokinase-type plasminogen activator (u-PA). Next, acacetin significantly decreased in the phosphorylation and degradation of inhibitor of kappaBα (IκBα), and the nuclear levels of nuclear factor kappa B (NF-κB), c-Fos, and c-Jun. Also, the treatment with acacetin to A549 cells also leads to a concentration-dependent inhibition on the binding abilities of NF-κB and activator protein-1 (AP-1). Furthermore, the treatment of specific inhibitor for p38 MAPK (SB203580) to A549 cells could cause reduced activities of MMP-2/9 and u-PA. In addition, acacetin significantly decreased the levels of phospho-p38α MAPK, MMP-2/9, and u-PA in p38α-cDNA-transfected cells concomitantly with a marked reduction on cell inva  sion and   migration. Our results revealed   the anti-  migration and anti-invasion effects of acacetin, which may act as a promising therapeutic agent for the treatment of lung cancer. 
 Interleukin-8 induces the endothelial cell   migration   through the Rac 1/RhoA-p38MAPK pathway. Endothelial cell   migration   is essential for tumor angiogenesis, and interleukin-8 (IL-8) has been shown to play an important role in tumor growth, angiogenesis, and metastasis. The objective of this study was to investigate the molecular mechanism of IL-8 induced endothelial cell   migration   in vitro.Fluorescence microscope was used to study the distribution of cytoskeleton. The expression of Rac1 and RhoA protein was detected by western blotting. After endothelial cells were transfected by lipofectamine 2000 reagent, the Transwell chamber motility assay was applied to observe the   migration   of endothelial cells induced by IL-8. The active p38MAPK (mitogen-activated protein kinase) was evaluated by the p38MAPK activation assay.We demonstrated that IL-8 activated cell   migration   can be impaired by p38MAPK inhibitor, suggesting the participation of p38MAPK in the cell   migration  . Our results indicated that p38MAPK signaling is required for membrane ruffles, lamellipodia extensions, and actin stress fibers formation induced by IL-8. Furthermore, p38MAPK inhibitor led to increased Rac1 and RhoA expression in IL-8 treated EA.hy926 cells. In addition, IL-8 induced p38MAPK activation was suppressed by dominant-negative mutant for Rac1 and RhoA.Our study demonstrates that IL-8-Rac1/RhoA-p38MAPK signaling pathway plays a vital role in the IL-8-induced endothelial cell   migration  , and it provides new insight into the molecular mechanisms by which IL-8 contributes to tumor angiogenesis and metastasis. 
 p38 MAPK inhibits breast cancer metastasis through regulation of stromal expansion. p38 MAPK signaling controls cell growth, proliferation and the cell cycle under stress conditions. However, the function of p38 activation in tumor metastasis is still not well understood. We report that p38 activation in breast cancer cells inhibits tumor metastasis but does not substantially modulate primary tumor growth. Stable p38 knockdown in breast cancer cells suppressed NF-κB p65 activation, inhibiting miR-365 expression and resulting in increased IL-6 secretion. The inhibitory effect of p38 signaling on metastasis was mediated by suppression of mesenchymal stem cell (MSC)   migratio  n to the primary tumor and sites of metastasis, where MSCs can differentiate into cancer-associated fibroblasts to promote tumor metastasis. The   migratio  n of MSCs to these sites relies on CXCR4-SDF1 signaling in the tumor microenvironment. Analysis of human primary and metastatic breast cancer tumors showed that p38 activation was inversely associated with IL-6 and vimentin expression. This study suggests that combination analysis of p38 MAPK and IL-6 signaling in patients with breast cancer may improve prognosis and treatment of metastatic breast cancer. 
 Close encounters: regulation of vertebrate skeletal myogenesis by cell-cell contact. Cells of the vertebrate skeletal muscle lineage develop in a highly ordered process that includes specification,   migration   and differentiation into multinucleated myofibers. The changes in gene expression and cell morphology that occur during myogenic differentiation must be coordinated with each other in a spatiotemporal fashion; one way that this might occur is through regulation of these processes by cell-cell adhesion and resultant signaling. The past several years have witnessed the identification of molecules that are likely to be mediators of the promyogenic effects of cell-cell contact and some of the mechanisms by which they work. These include: the community factor, embryonic fibroblast growth factor (eFGF); classical cadherins, which mediate both adhesion and signaling; and cadherin-associated immunoglobulin superfamily members such as CDO, BOC and neogenin. Genetic evidence for the promyogenic roles of some of these factors is emerging. In other cases, potential compensatory or redundant functions necessitate future construction of double or triple mutants. Mechanistic studies in vitro indicate that specific cadherins and immunoglobulin superfamily proteins exert some of their effects in an interdependent fashion by signaling from a multiprotein complex found at sites of cell-cell contact. 
 Norepinephrine stimulates pancreatic cancer cell proliferation,   migration   and invasion via β-adrenergic receptor-dependent activation of P38/MAPK pathway. There is growing evidence that chronic stress is associated with cancer pathogenesis and progression, but the mechanisms involved in this association are poorly understood. This study aims to examine the effect of stress hormone norepinephrine (NE) on proliferation,   migratio  n and invasion of human pancreatic cancer cells and the molecular pathway involved.PANC-1 cells were examined for the expression of β-Adrenergic receptor (β-AR) subtypes, β1-AR, β2-AR by RT-PCR and western blotting, respectively. The PANC-1 cells proliferation, cell cycle distribution,   cell migr  ation and invasion were assessed by CCK-8, flow cytometry, scratch wound healing and transwell Matrigel, respectively.PANC- 1 cells expressed both β1-AR and β2-AR protein. Exposure of PANC-1 cells to increasing concentrations of NE stimulated cell proliferation in a dose-dependent fashion. In addition, NE increased S-phase population and decreased G1 and G2-phase population of PANC- 1 cells. Furthermore, NE significantly en  hanced mi  gration and invasion of PANC-1 cells. More strikingly, we observed that NE elevated P38/MAPK phosphorylation (Phos-p38) level. These stimulatory effects were completely abolished by β-AR antagonist propranolol (PRO) or P38/MAPK inhibitor SB203580.NE could stimulate pancreatic cancer cell prolife  ration, m  igration and invasion through a β-AR-dependent activation of P38/MAPK pathway involved. 
 Deacetylated GM3 promotes uPAR-associated membrane molecular complex to activate p38 MAPK in metastatic melanoma. GM3, the simplest ganglioside, regulates cell proliferation,   migration  , and invasion by influencing cell signaling at the membrane level. Although the classic N-acetylated form of GM3 (NeuAcLacCer) is commonly expressed and has been well studied, deacetylated GM3 (NeuNH2LacCer, d-GM3) has been poorly investigated, despite its presence in metastatic tumors but not in noninvasive melanomas or benign nevi. We have recently found that d-GM3 stimulates cell   migration   and invasion by activating urokinase plasminogen activator receptor (uPAR) signaling to augment matrix metalloproteinase-2 (MMP-2) function. However, the mechanisms by which d-GM3/uPAR increase MMP-2 expression and activation are not clear. By modifying the expression of d-GM3 genetically and biochemically, we found that decreasing d-GM3 expression inhibits, whereas overexpressing d-GM3 stimulates, p38 mitogen-activated protein kinase (MAPK) activity to influence MMP-2 expression and activation. p38 MAPK (p38) activation requires the formation of a membrane complex that contains uPAR, caveolin-1, and integrin α5β1 in membrane lipid rafts. In addition, knocking down or inhibiting focal adhesion kinase (FAK), phosphoinositide 3-kinase (PI3K), or Src kinase significantly reduces d-GM3-induced p38 phosphorylation and activation. Taken together, these results suggest that d-GM3 enhances the metastatic phenotype by activating p38 signaling through uPAR/integrin signaling with FAK, PI3K, and Src kinase as intermediates. Elucidation of the mechanisms by which d-GM3, a newly discovered, potential biomarker of metastatic melanomas, promotes cell metastasis will help us to understand the function of d-GM3 in metastatic melanomas and may lead to novel GM3-based cancer therapies. 
 Polycyclic aromatic hydrocarbon (PAH)-mediated upregulation of hepatic microRNA-181 family promotes cancer cell   migration   by targeting MAPK phosphatase-5, regulating the activation of p38 MAPK. Growing evidence indicates that changes in microRNA (miRNA) expression in cancer induced by chemical carcinogens play an important role in cancer development and progression by regulating related genes. However, the mechanisms underlying miRNA involvement in hepatocarcinogenesis induced by polycyclic aromatic hydrocarbons (PAHs) remain unclear. Thus, the identification of aberrant miRNA expression during PAH-induced cancer cell   migration   will lead to a better understanding of the substantial role of miRNAs in cancer progression. In the present study, miRNA expression profiling showed significant upregulation of miR-181a, -181b, and -181d in human hepatocellular carcinoma cells (HepG2 line) exposed to benzo[a]anthracene (BA) and benzo[k]fluoranthene (BF). MAPK phosphatase-5 (MKP-5), a validated miR-181 target that deactivates MAPKs, was markedly suppressed while phosphorylation of p38 MAPK was increased after BA and BF exposure. The   migration   of HepG2 cells, observed using the scratch wound-healing assay, also increased in a dose-dependent manner. Depletion of miR-181 family members by miRNA inhibitors enhanced the expression of MKP-5 and suppressed the phosphorylation of p38 MAPK. Furthermore, the depletion of the miR-181 family inhibited cancer cell   migration  . Based on these results, we conclude that the miR-181 family plays a critical role in PAH-induced hepatocarcinogenesis by targeting MKP-5, resulting in the regulation of p38 MAPK activation. 
 MKK3 and -6-dependent activation of p38alpha MAP kinase is required for cytoskeletal changes in pulmonary microvascular endothelial cells induced by ICAM-1 ligation. Previous studies demonstrated that neutrophil adherence induces ICAM-1-dependent cytoskeletal changes in TNF-alpha-treated pulmonary microvascular endothelial cells that are prevented by a pharmacological inhibitor of p38 MAP kinase. This study determined whether neutrophil adherence induces activation of p38 MAP kinase in endothelial cells, the subcellular localization of phosphorylated p38, which MAP kinase kinases lead to p38 activation, which p38 isoform is activated, and what the downstream targets may be. Confocal microscopy showed that neutrophil adhesion for 2 or 6 min induced an increase in phosphorylated p38 in endothelial cells that was punctate and concentrated in the central region of the endothelial cells. Studies using small interfering RNA (siRNA) to inhibit the protein expression of MAP kinase kinase 3 and 6, either singly or in combination, showed that both MAP kinase kinases were required for p38 phosphorylation. Studies using an antisense oligonucleotide to p38alpha demonstrated that inhibition of the protein expression of p38alpha 1) inhibited activation of p38 MAP kinase without affecting the protein expression of p38beta; 2) prevented phosphorylation of heat shock protein 27, an actin binding protein that may induce actin polymerization upon phosphorylation; 3) attenuated cytoskeletal changes; and 4) attenuated neutrophil   migration   to the EC borders. Thus MAP kinase kinase3- and 6-dependent activation of the alpha-isoform of p38 MAP kinase is required for the cytoskeletal changes induced by neutrophil adherence and influences subsequent neutrophil   migration   toward endothelial cell junctions. 
 SASH1 is a scaffold molecule in endothelial TLR4 signaling. Recognition of microbial products by TLRs is critical for mediating innate immune responses to invading pathogens. In this study, we identify a novel scaffold protein in TLR4 signaling called SAM and SH3 domain containing protein 1 (SASH1). Sash1 is expressed across all microvascular beds and functions as a scaffold molecule to independently bind TRAF6, TAK1, IκB kinase α, and IκB kinase β. This interaction fosters ubiquitination of TRAF6 and TAK1 and promotes LPS-induced NF-κB, JNK, and p38 activation, culminating in increased production of proinflammatory cytokines and increased LPS-induced endothe  lial migr  ation. Our findings suggest that SASH1 acts to assemble a signaling complex downstream of TLR4 to activate early endothelial responses to receptor activation. 
 Interleukin-32 induces the differentiation of monocytes into macrophage-like cells. After e  migration   from the bone marrow to the peripheral blood, monocytes enter tissues and differentiate into macrophages, the prototype scavenger of the immune system. By ingesting and killing microorganisms and removing cellular debris, macrophages also process antigens as a first step in mounting a specific immune response. IL-32 is a cytokine inducing proinflammatory cytokines and chemokines via p38-MAPK and NF-kappaB. In the present study, we demonstrate that IL-32 induces differentiation of human blood monocytes as well as THP-1 leukemic cells into macrophage-like cells with functional phagocytic activity for live bacteria. Muramyl dipepide (MDP), the ligand for the intracellular nuclear oligomerization domain (NOD) 2 receptor, has no effect on differentiation alone but augments the monocyte-to-macrophage differentiation by IL-32. Unexpectedly, IL-32 reversed GM-CSF/IL-4-induced dendritic cell differentiation to macrophage-like cells. Whereas the induction of TNFalpha, IL-1beta, and IL-6 by IL-32 is mediated by p38-MAPK, IL-32-induced monocyte-to-macrophage differentiation is mediated through nonapoptotic, caspase-3-dependent mechanisms. Thus, IL-32 not only contributes to host responses through the induction of proinflammatory cytokines but also directly affects specific immunity by differentiating monocytes into macrophage-like cells. 
 Blood-derived human osteoclast resorption activity is impaired by Hyaluronan-CD44 engagement via a p38-dependent mechanism. The control of bone resorption is crucial in osteolytic diseases. Once attached to bone, osteoclasts (OCs) initiate the resorption process through the activation of a complex cascade of morphological and biochemical changes. Hyaluronan (HA), an extracellular glycosaminoglycan long non-branching polysaccharide, is expressed in bone matrices. Here we demonstrate that HA counter-balances the erosion activity of human mature OCs by significantly reducing their degradative potential. HA treatment of fully differentiated OCs derived from human peripheral blood monocytes inhibited   migration   on collagen as well as bone resorption. HA-mediated effects were primarily due to TRAcP, MMP-9, and cathepsin K down-regulation and to the increased levels of TIMP-1, a natural MMP-9 inhibitor. Binding of HA to mature OCs was entirely mediated by CD44: function-blocking anti-CD44 antibodies fully abrogated HA effects, and the engagement of HA receptor caused a rapid de-phosphorylation of Ser325 in the CD44 cytoplasmic tail. The inhibitory action by HA was associated with a transient up-phosphorylation of Pyk2, a novel persistent phosphorylation of p38 and the down-regulation of NFATc1 transcription factor. Our results provide a direct evidence for the involvement of CD44 in the HA-dependent regulation of OC activity and suggest a signaling pathway that could be unique in OC function inhibition. 
 Suppression of phorbol-12-myristate-13-acetate-induced tumor cell invasion by apigenin via the inhibition of p38 mitogen-activated protein kinase-dependent matrix metalloproteinase-9 expression. Apigenin has special interest for the development of chemopreventive agents against cancer because it is a widely distributed plant flavonoid that has antitumor properties. In this study, we investigated the apigenin effects on the protease-mediated invasiveness in human metastatic cancer cell lines Caski, SK-Hep1, and MDA-231. We found that apigenin markedly inhibits the phorbol-12-myristate-13-acetate (PMA)-induced increase in MMP-9 expression and activity in several cancer cell lines. These effects of apigenin are dose-dependent and correlate with the suppression of MMP-9 mRNA expression levels. PMA caused about a 5-fold induction in MMP-9 promoter activity, which was also suppressed by apigenin treatment in Caski cells. We found that apigenin could inhibit PMA-induced phosphorylation of p38 mitogen-activated protein kinase (p38 MAPK), which was involved in the down-regulation of the expression of matrix metalloproteinase-9 (MMP-9) at mRNA levels. Furthermore, the treatment of inhibitors specific for p38 MAPK (SB203580) to Caski cells caused the reduction of MMP-9 expression. Restoration of p38 expression partly increased PMA-induced MMP-9 secretion blocked by apigenin treatment in Caski cells. These results showed apigenin might inhibit the invasion and   migration   abilities of Caski cells by reducing the MMP-9 expression through suppressing the p38 MAPK signaling pathway. These findings indicate that apigenin might be a useful strategy for controlling metastasis and the invasiveness of tumors. 
 Tissue factor/activated factor VIIa induces matrix metalloproteinase-7 expression through activation of c-Fos via ERK1/2 and p38 MAPK signaling pathways in human colon cancer cell. Increased expression of tissue factor (TF) is associated with tumor invasion and metastasis in human colorectal cancer. We have previously observed that TF/FVIIa upregulates matrix metalloproteinase-7 (MMP-7) expression at the transcriptional level in colon cancer cells. MMP-7 overexpression is believed to play an important role in tumor invasion and metastasis. The aim of this study is to elucidate the molecular mechanisms by which TF/FVIIa induced MMP-7 expression and cell invasion in vitro.Reverse transcription polymerase chain reaction, Western blot, luciferase assay, and chromatin immunoprecipitation (ChIP) were used to determine the potential mechanism and signaling pathways by which TF/FVIIa induced MMP-7 expression and cell invasion in LoVo cells. Small interfering RNA (siRNA) and cell invasion assay was used to examine whether blocking c-Fos expression could abolish FVIIa-mediated upregulation of MMP-7 and cell invasion in vitro.The results showed that FVIIa induced the upregulation of MMP-7 both at the mRNA and protein levels in a time- and dose-dependent manner and increased the invasive behavior of LoVo cells. FVIIa enhanced the promoter activity of MMP-7, and the activator protein-1 (AP-1) binding site was responsible for the activation. Site mutation of the AP-1 binding site in the promoter almost completely abolished FVIIa-mediated response. Furthermore, ChIP assay confirmed that FVIIa promoted the direct binding of c-Fos with the MMP-7 promoter in vivo. FVIIa also induced the expression and nuclear accumulation of the AP-1 subunit c-Fos. siRNA-mediated knockdown of c-Fos eliminated FVIIa-stimulated MMP-7 expression and cell   migration   in vitro. In addition, selective mitogen-activated protein kinase (MAPK) kinase (MEK1/2) inhibitor (PD98059) and p38 MAPK inhibitor SB203580 suppressed MMP-7 upregulation induced by FVIIa.Our data suggest that a novel TF/FVIIa/MAPK/c-Fos/MMP-7 axis plays an important role in modulating the invasion of colon cancer cells and blockage of this pathway holds promise to treat colon cancer metastasis. 
 Galectin-1 promotes lung cancer progression and chemoresistance by upregulating p38 MAPK, ERK, and cyclooxygenase-2. This study is aimed at investigating the role and novel molecular mechanisms of galectin-1 in lung cancer progression.The role of galectin-1 in lung cancer progression was evaluated both in vitro and in vivo by short hairpin RNA (shRNA)-mediated knockdown of galectin-1 in lung adenocarcinoma cell lines. To explore novel molecular mechanisms underlying galectin-1-mediated tumor progression, we analyzed gene expression profiles and signaling pathways using reverse transcription PCR and Western blotting. A tissue microarray containing samples from patients with lung cancer was used to examine the expression of galectin-1 in lung cancer.We found overexpression of galectin-1 in non-small cell lung cancer (NSCLC) cell lines. Suppression of endogenous galectin-1 in lung adenocarcinoma resulted in reduction of the cell   migration  , invasion, and anchorage-independent growth in vitro and tumor growth in mice. In particular, COX-2 was downregulated in galectin-1-knockdown cells. The decreased tumor invasion and anchorage-independent growth abilities were rescued after reexpression of COX-2 in galectin-1-knockdown cells. Furthermore, we found that TGF-β1 promoted COX-2 expression through galectin-1 interaction with Ras and subsequent activation of p38 mitogen-activated protein kinase (MAPK), extracellular signal-regulated kinase (ERK), and NF-κB pathway. Galectin-1 knockdown sensitized lung cancer cells to platinum-based chemotherapy (cisplatin). In addition, galectin-1 and COX-2 expression was correlated with the progression of lung adenocarcinoma, and high clinical relevance of both proteins was evidenced (n = 47).p38 MAPK, ERK, and COX-2 activation are novel mediators for the galectin-1-promoted tumor progression and chemoresistance in lung cancer. Galectin-1 may be an innovative target for combined modality therapy for lung cancer. 
 Hyaluronan oligosaccharides induce matrix metalloproteinase 13 via transcriptional activation of NFkappaB and p38 MAP kinase in articular chondrocytes. Hyaluronan exerts a variety of biological effects on cells including changes in cell   migration  , proliferation, and matrix metabolism. However, the signaling pathways associated with the action of hyaluronan on cells have not been clearly defined. In some cells, signaling is induced by the loss of cell-hyaluronan interactions. The goal of this study was to use hyaluronan oligosaccharides as a molecular tool to explore the effects of changes in cell-hyaluronan interactions and determine the underlying molecular events that become activated. In this study, hyaluronan oligosaccharides induced the loss of extracellular matrix proteoglycan and collagen from cultured slices of normal adult human articular cartilage. This loss was coincident with an increased expression of matrix metalloproteinase (MMP)-13. MMP-13 expression was also induced in articular chondrocytes by hyaluronan (HA) hexasaccharides but not by HA tetrasaccharides nor high molecular weight hyaluronan. MMP-13 promoter-reporter constructs in CD44-null COS-7 cells revealed that both CD44-dependent and CD44-independent events mediate the induction of MMP-13 by hyaluronan oligosaccharides. Electromobility gel shift assays demonstrated the activation of chondrocyte NFkappaB by hyaluronan oligosaccharides. NFkappaB activation was also documented in C-28/I2 immortalized human chondrocytes by luciferase promoter assays and phosphorylation of IKK-alpha/beta. The link between activation of NFkappaB and MMP-13 induction by HA oligosaccharides was further confirmed through the use of the NFkappaB inhibitor helenalin. Inhibition of MAP kinases also demonstrated the involvement of p38 MAP kinase in the hyaluronan oligosaccharide induction of MMP-13. Our findings suggest that hyaluronan-CD44 interactions affect matrix metabolism via activation of NFkappaB and p38 MAP kinase. 
 Carbonic anhydrase XII promotes invasion and   migration   ability of MDA-MB-231 breast cancer cells through the p38 MAPK signaling pathway. Carbonic anhydrase (CA) XII, an extracellular enzyme involved in the regulation of the microenvironment acidity and tumor malignant phenotype, was originally identified as a protein overexpressed in some types of cancers, including breast cancer. However, the cellular function and mechanism of CAXII remained unclear. In this study, the effects of CAXII expression on invasion and   migration   of breast cancer cells was investigated. Gene knockdown of CAXII in the human breast cancer cell line MDA-MB-231 resulted in decreased invasion and   migration   by interfering with the p38 MAPK pathway. CAXII knockdown also decreased the expression of matrix metalloproteinase (MMP)-2, MMP-9, and urokinase-type plasminogen activator (u-PA), but increased tissue inhibitor of metalloproteinases (TIMP)-2 and plasminogen activator inhibitor (PAI)-1 expression. Furthermore, decreased invasive and   migration   ability of CAXII-knockdown cells were restored by an overexpression of CAXII. Results also showed that CAXII knockdown may decrease anchorage-independent growth and cell growth by inhibiting CDK6 and cyclin D1 expression. Furthermore, the impact of CAXII knockdown on invasion,   migration   and cell growth was further evidenced by effects on tumor size and metastasis of MDA-MB-231 cells in vivo. Taken together, these data suggested that CAXII may affect the capability of invasion and   migration   of MDA-MB-231 cells, which may be mediated through the p38 MAPK pathway. 
 CARMA3 regulates the invasion,   migration  , and apoptosis of non-small cell lung cancer cells by activating NF-кB and suppressing the P38 MAPK signaling pathway. In our previous study, CARMA3 overexpression in lung cancer cells promoted cell proliferation and invasion; however, the mechanism underlying the role of CARMA3 in cancer cell invasion remained unclear. In the present study, knockdown of CARMA3 in A549 and H1299 cells suppressed cell invasion and   migratio  n, and downregulated matrix metalloprotease 9 expression at the protein and mRNA levels, as shown by Western blotting and real-time PCR. CARMA3 knockdown increased cell apoptosis, as shown by flow cytometry, increased the mRNA and protein expression levels of Bax and Caspase3, and downregulated Bcl-2 in A549 and H1299 cells. Phosphorylated P38 levels increased and NF-кB activation decreased following knockdown of CARMA3. SB203580, a P38 MAPK inhibitor, activated NF-кB, increased ce  ll migrat  ion, and inhibited cell apoptosis after knockdown of CARMA3 compared to knockdown of CARMA3 without SB203580. These findings indicate that CARMA3 may suppress the activation of the P38 MAPK signaling pathway to regulate invasio  n, migrat  ion and apoptosis of lung cancer cells by activating NF-кB (P65) in the nucleus. 
 Suppression of urokinase plasminogen activator receptor inhibits proliferation and   migration   of pancreatic adenocarcinoma cells via regulation of ERK/p38 signaling. Pancreatic ductal adenocarcinoma (PDAC) expresses high levels of urokinase-type plasminogen activator (uPA), its receptor (uPAR) and plasminogen activator inhibitor (PAI)-2, which may play an important role in PDAC progression. The overexpression of uPAR predicted short survival in PDAC patients. In this study, two different PDAC cell lines were used to examine the effect of small interfering (si) RNAs to uPAR, uPA and PAI-2 on proliferation, apoptosis,   migration   and MAP kinase activation. In both PDAC cell lines, siRNA to uPAR significantly inhibited cell proliferation and   migration   and stimulated apoptosis, to a greater extent than uPA siRNA. When either PDAC cell line was treated with uPAR siRNA, the level of phosphorylated ERK (p-ERK) decreased substantially, whereas phosphorylated p38 (p-p38) increased when compared to non-silencing control, uPA siRNA or PAI-2 siRNA treatment. This resulted in enhancement of the p-p38/p-ERK ratio which favors cancer cell arrest. Interestingly, uPAR protein expression was suppressed by p-ERK inhibition and stimulated with p-p38 inhibition, suggesting the presence of a positive feedback loop between uPAR and ERK. In summary, our data indicate that, of the uPA system, uPAR exerts the strongest effects on PDAC cells, by acting through the ERK signaling pathway via a positive feedback loop. Disruption of this loop with uPAR siRNA or inhibitor of p-ERK, inhibits PDAC proliferation and   migration   and promotes apoptosis. These findings suggest that uPAR strongly contributes to PDAC progression and may be considered as a potential anti-pancreatic cancer target. 
 T-cadherin protects endothelial cells from oxidative stress-induced apoptosis. In vascular tissue, T-cadherin (T-cad) is up-regulated in vivo under disease conditions associated with oxidative stress and concomitant cell   migration  , proliferation and apoptosis/survival. Using cultures of human umbilical vein endothelial cells (HUVEC), we examined whether there is a functional relationship between oxidative stress, T-cad expression, and cell survival status. Culture of HUVEC under conditions of oxidative stress (e.g., serum deprivation, inclusion of H2O2) resulted in increased T-cad expression. Oxidative stress-induced increases in T-cad were inhibited by the free radical-scavenging antioxidant, N-acetylcysteine, and the flavin-containing oxidase inhibitor, diphenyleneiodonium. Thus reactive oxygen species (ROS) contribute to stress-induced elevation of T-cad in HUVEC. Compared with control cells, HUVEC overexpressing T-cad (T-cad+-HUVEC) had higher phosphorylation levels for phosphatidylinositol 3-kinase (PI3K) target Akt and mTOR target p70(S6K) (survival pathway regulators), but lower levels for p38MAPK (death pathway regulator). T-cad+-HUVEC exposed to stress (serum-deprivation, TNF-alpha, actinomycin D, staurosporine) exhibited reduced caspase activation together with increased cell survival. Protection against stress-induced apoptosis in T-cad+-HUVEC was abrogated by either PI3K-inhibitor wortmannin or mTOR-inhibitor rapamycin. We conclude that T-cad overexpression in HUVEC protects against stress-induced apoptosis through activation of the PI3K/Akt/mTOR survival signal pathway and concomitant suppression of the p38 MAPK proapoptotic pathway. ROS-induced changes in T-cad expression may play an important role in controlling tissue cellularity during vascular remodeling. 
 miR-20a represses endothelial cell   migration   by targeting MKK3 and inhibiting p38 MAP kinase activation in response to VEGF. Endothelial cell   migration   induced in response to vascular endothelial growth factor (VEGF) is a crucial step of angiogenesis and it depends on the activation of the p38 MAP-kinase pathway downstream of VEGFR2. In this study, we investigated the role of microRNAs (miRNAs) in regulating these processes. We found that the VEGF-induced p38 activation and cell   migration   are modulated by overexpression of Argonaute 2, a key protein in the functioning of miRNAs. Thereafter, we found that miR-20a expression is increased by VEGF and that its ectopic expression inhibits VEGF-induced actin remodeling and cell   migration  . Moreover, the expression of miR-20a impairs the formation of branched capillaries in a tissue-engineered model of angiogenesis. In addition, the lentivirus-mediated expression of miR-20a precursor (pmiR-20a) is associated with a decrease in the VEGF-induced activation of p38. In contrast, these processes are increased by inhibiting miR-20a with a specific antagomir. Interestingly, miR-20a does not modulate VEGFR2 or p38 protein expression level. miR-20a does not affect either the expression of other known actors of the p38 MAP kinase pathway except MKK3. Indeed, by using quantitative PCR and Western Blot analysis, we found that pmiR-20a decreases the expression of MKK3 and we obtained evidence indicating that miR-20a specifically binds to the 3'UTR region of MKK3 mRNA. In accordance, the VEGF-induced activation of p38 and cell   migration   are impaired when the MKK3 expression is knocked down by siRNA. We conclude that miR-20a acts in a feedback loop to repress the expression of MKK3 and to negatively regulate the p38 pathway-mediated VEGF-induced endothelial cell   migration   and angiogenesis. 
 p38 and ERK1/2 coordinate cellular   migration   and proliferation in epithelial wound healing: evidence of cross-talk activation between MAP kinase cascades. One important action of growth factors is their participation in tissue repair; however, the signaling pathways involved are poorly understood. In a model of corneal wound healing, we found that two paracrine growth factors, hepatocyte growth factor (HGF) and keratinocyte growth factor (KGF), induced rapid and marked activation and prompt nuclear accumulation of phospho-p38 (p-p38) and -ERK1/2 (p-ERK1/2), but not of JNK (p-JNK1/2), in corneal epithelial cells. Interruption of p38 and ERK1/2 signaling pathways by pretreatment with inhibitors SB203580 and PD98059 and subsequent stimulation with HGF or KGF abolished the activation and nuclear localization. Inhibition of either one of these mitogen-activated protein kinases, p38 or ERK1/2, induced a robust cross-activation of the other. In immunofluorescence studies of wounded cornea, p-p38, unlike p-ERK1/2, was immediately detectable in epithelium after injury. Inhibition of p38 by SB203580 blocked   migration   of epithelial cells almost completely. In contrast, PD98059 seemed to slightly increase the   migration  , through concomitant activation of p38. Unlike ERK1/2, p38 did not significantly contribute to proliferation of epithelial cells. Inhibition of either the ERK1/2 or p38 pathway resulted in delayed corneal epithelial wound healing. Interruption of both signaling cascades additively inhibited the wound-healing process. These findings demonstrate that both p38 and ERK1/2 coordinate the dynamics of wound healing: while growth factor-stimulated p38 induces epithelial   migration  , ERK1/2 activation induces proliferation. The cross-talk between these two signal cascades and the selective action of p38 in   migration   appear to be important to corneal wound healing, and possibly wound healing in general, and may offer novel drug targets for tissue repair. 
 LPS-induced MCP-1 expression in human microvascular endothelial cells is mediated by the tyrosine kinase, Pyk2 via the p38 MAPK/NF-kappaB-dependent pathway. Bacterial endotoxin (lipopolysaccharide or LPS) has potent pro-inflammatory properties and acts on many cell types including endothelial cells. Secretion of the CC chemokine, MCP-1 (CCL2) by LPS-activated endothelial cells contributes substantially to the pathogenesis of sepsis. However, the mechanism involved in LPS-induced MCP-1 production in endothelial cells is not well understood. Using human microvascular endothelial cells (HMVEC), we analyzed the involvement of the non-receptor tyrosine kinase, Pyk2, in LPS-mediated MCP-1 production. There was a marked activation of the non-receptor tyrosine kinase, Pyk2, in response to LPS. Inhibition of Pyk2 activity using a pharmacological inhibitor, Tyrphostin A9 significantly attenuated LPS-induced Pyk2 tyrosine phosphorylation, p38 MAP kinase (MAPK) activation, NF-kappaB activation, and MCP-1 expression. Furthermore, specific inactivation of Pyk2 activity by transducing microvascular endothelial cells with catalytically inactive Pyk2 mutant (AAV-Pyk2MT) or Pyk2-specific siRNA significantly blocked LPS-induced MCP-1 production. The supernatants of these LPS-stimulated cells with attenuated Pyk2 activity demonstrated decreased trans-endothelial monocyte   migration   in comparison to LPS-treated controls, thus confirming the inhibition of functional MCP-1 production. In summary, our data suggest a critical role for the Pyk2 mediated pathway involving p38 MAP kinase and NF-kappaB in LPS-induced MCP-1 production in human microvascular endothelial cells. 
 Crucial implication of protein kinase C (PKC)-delta, PKC-zeta, ERK-1/2, and p38 MAPK in   migration   of human asthmatic eosinophils. Asthma is associated with an eosinophil infiltration into the bronchial mucosa. 5-Oxo-6,8,11,14(E,Z,Z,Z)-eicosatetraenoic acid (5-oxo-ETE), a potent eosinophil chemotactic factor, activates cell motility, adherence, and proteolysis, notably, by promoting CD11b expression, matrix metalloproteinase (MMP)-9 secretion, and plasmin generation. We investigated the intracellular signaling pathways implicated in these various steps by using different, selective inhibitors. Human eosinophil   migration   through a reconstituted basement membrane in response to 5-oxo-ETE was greatly inhibited (>or=72%) by the protein kinase C (PKC)-delta, PKC-zeta, ERK-1/2, and p38 inhibitors. Our findings indicate that PKC-delta mediates cell motility, CD11b expression, and MMP-9 granule release. PKC-zeta is also largely involved in eosinophil   migration  , although its specific targets remain undefined. ERK-1/2 and p38 modulate CD11b expression; ERK-1/2 is also involved in long-term MMP-9 secretion and p38 in the plasmin activation system. We demonstrated the crucial implication of PKC-delta, PKC-zeta, ERK-1/2, and p38 in human blood eosinophil   migration   through extracellular matrix components. Targeting specific pathways may have therapeutic potential for the treatment of allergic airway inflammation. 
 An involvement of SR-B1 mediated p38 MAPK signaling pathway in serum amyloid A-induced angiogenesis in rheumatoid arthritis. Serum amyloid A (SAA) has been reported high expression in autoimmune diseases, such as rheumatoid arthritis (RA). However, detailed molecular mechanisms induced by SAA in the pathogenesis of RA are still unclear. Herein, we focused on the role of SAA-SR-B1 mediated p38 MAPK signaling pathway in the process of RA angiogenesis. Our results showed that both SAA and SR-B1 predominantly localized to vascular endothelial cells, lining and sublining layers in RA synovium. In a series of in vitro experiments with human umbilical vein endothelial cells (HUVECs), SAA induced the endothelial cells (ECs) proliferation,   migration   and tube formation. However, blockage of SR-B1 and p38 MAPK inhibited SAA-induced cells proliferation,   migration   and tube formation. In conclusion, our data showed a possible molecular mechanism for SAA-SR-B1 induced angiogenesis events via p38 MAPK signaling pathway. 
 Interleukin-8 secretion by fibroblasts induced by low density lipoproteins is p38 MAPK-dependent and leads to cell spreading and wound closure. We have previously reported (Dobreva, I., Waeber, G., Mooser, V., James, R. W., and Widmann, C. (2003) J. Lipid Res. 44, 2382-2390) that low density lipoproteins (LDLs) induce activation of the p38 MAPK pathway, resulting in fibroblast spreading and lamellipodia formation. Here, we show that LDL-stimulated fibroblast spreading and wound sealing are due to secretion of a soluble factor. Using an antibody-based human protein array, interleukin-8 (IL-8) was identified as the main cytokine whose concentration was increased in supernatants from LDL-stimulated cells. Incubation of supernatants from LDL-treated cells with an anti-IL-8 blocking antibody completely abolished their ability to induce cell spreading and mediate wound closure. In addition, fibroblasts treated with recombinant IL-8 spread to the same extent as cells incubated with LDL or supernatants from LDL-treated cells. The ability of LDL and IL-8 to induce fibroblast spreading was mediated by the IL-8 receptor type II (CXCR-2). Furthermore, LDL-induced IL-8 production and subsequent wound closure required the activation of the p38 MAPK pathway, because both processes were abrogated by a specific p38 inhibitor. Therefore, the capacity of LDLs to induce fibroblast spreading and accelerate wound closure relies on their ability to stimulate IL-8 secretion in a p38 MAPK-dependent manner. Regulation of fibroblast shape and   migration   by lipoproteins may be relevant to atherosclerosis that is characterized by increased LDL cholesterol levels, IL-8 production, and extensive remodeling of the vessel wall. 
 Involvement of connexin 43 in angiotensin II-induced   migration   and proliferation of saphenous vein smooth muscle cells via the MAPK-AP-1 signaling pathway. Proliferation and   migration   of vascular smooth muscle cells (VSMCs) lead to intimal thickening and influence the long-term patency of venous graft post coronary arterial bypass graft. There is increasing evidence that connexins are involved in the development of intimal hyperplasia and restenosis. We assessed connexin 43 (Cx43) expression and its role in angiotensin II-induced proliferation and   migration   of smooth muscle cells and the signal pathways involved in human saphenous vein bypass conduits. Angiotensin II significantly increased gap junctional intercellular communication and induced the expression of Cx43 in human saphenous vein SMCs in a dose- and time-dependent manner through angiotensin II type 1 receptor. The effect of angiotensin II was blocked by siRNA of ERK 1/2, p38 MAPK and JNK, respectively. Overexpression of Cx43 markedly increased the proliferation of saphenous vein SMCs. However, siRNA for Cx43 inhibited angiotensin II-induced proliferation, cyclin E expression and   migration   of human saphenous vein SMCs. In dual-luciferase reporter assay, angiotensin II markedly activated AP-1 transcription factor, which was significantly attenuated by a dominant-negative AP-1 (A-Fos) with subsequent inhibition of angiotensin II-induced transcriptional expression of Cx43. These data demonstrate the role of Cx43 in the proliferation and   migration   of human saphenous vein SMCs and angiotensin II-induced Cx43 expression via mitogen-activated protein kinases (MAPK)-AP-1 signaling pathway. 
 Analysis of protein-protein interactions in cross-talk pathways reveals CRKL protein as a novel prognostic marker in hepatocellular carcinoma. Deciphering the network of signaling pathways in cancer via protein-protein interactions (PPIs) at the cellular level is a promising approach but remains incomplete. We used an in situ proximity ligation assay to identify and quantify 67 endogenous PPIs among 21 interlinked pathways in two hepatocellular carcinoma (HCC) cells, Huh7 (minimally migratory cells) and Mahlavu (highly migratory cells). We then applied a differential network biology analysis and determined that the novel interaction, CRKL-FLT1, has a high centrality ranking, and the expression of this interaction is strongly correlated with the migratory ability of HCC and other cancer cell lines. Knockdown of CRKL and FLT1 in HCC cells leads to a decrease in cell   migration   via ERK signaling and the epithelial-mesenchymal transition process. Our immunohistochemical analysis shows high expression levels of the CRKL and CRKL-FLT1 pair that strongly correlate with reduced disease-free and overall survival in HCC patient samples, and a multivariate analysis further established CRKL and the CRKL-FLT1 as novel prognosis markers. This study demonstrated that functional exploration of a disease network with interlinked pathways via PPIs can be used to discover novel biomarkers. 
 ErbB2-enhanced invasiveness of H-Ras MCF10A breast cells requires MMP-13 and uPA upregulation via p38 MAPK signaling. Overexpression of ErbB2 has been frequently found in mammary carcinoma. We have previously shown that the aberrant activation of H-Ras induces human breast cell invasion and   migration  . The present study was aimed at investigating the effect of ErbB2 overexpression on H-Ras-induced breast cell invasion and to elucidate the underlying mechanisms. Herein, we show that overexpression of ErbB2 promotes invasive and migratory abilities of H-Ras-activated MCF10A human breast epithelial cells through upregulation of matrix metalloproteinase (MMP)-13 and urokinase-type plasminogen activator (uPA). We also demonstrate that the p38 MAPK is an important signaling molecule in the ErbB2-induced upregulation of MMP-13 and uPA and invasion/  migration   of H-Ras MCF10A cells overexpressing ErbB2. The present study elucidating the molecular mechanism underlying ErbB2-induced promotion of H-Ras MCF10A cell invasion may provide invaluable information for understanding breast cancer progression and establishing therapeutic interventions for breast cancer. 
 The growth-promoting effect of KGF on limbal epithelial cells is mediated by upregulation of DeltaNp63alpha through the p38 pathway. Corneal epithelial stem cells are thought to reside in the limbus, the transition zoon between cornea and conjunctiva. Keratinocyte growth factor (KGF) and hepatocyte growth factor (HGF) are two paracrine factors that regulate the proliferation,   migration   and differentiation of the limbal epithelial cells; however, the underlying mechanisms are still poorly understood. In an ex vivo limbal explant culture, we found that KGF is a more potent growth stimulator for the epithelial outgrowth than HGF. Immunofluorescence studies of the epithelial outgrowth from cells treated with HGF or KGF showed similar expression patterns of keratin-3 and keratin-14. Interestingly, p63 was highly expressed in KGF-treated limbal epithelial sheets but not in those treated with HGF. Kinase inhibitor studies showed that induction of DeltaNp63alpha expression by KGF is mediated via the p38 pathway. The effect of KGF on limbal epithelial outgrowth was significantly reduced when endogenous DeltaNp63alpha was suppressed, suggesting that KGF-induced limbal epithelial outgrowth is dependent on the expression of DeltaNp63alpha. Our findings strongly suggest that limbal keratocytes regulate limbal epithelial cell growth and differentiation through a KGF paracrine loop, with DeltaNp63alpha expression as one of the downstream targets. 
 IL-1β-induced activation of p38 promotes metastasis in gastric adenocarcinoma via upregulation of AP-1/c-fos, MMP2 and MMP9. Interleukin-1β (IL-1β) has been implicated in the progression of gastric adenocarcinoma (GA); however, the molecular mechanisms of action of IL-1β in GA are poorly characterized. P38 and JNK are the major MAPK family members that regulate IL-1β signaling pathways. Here, we investigated the role of both p38 and JNK in IL-1β-induced GA   cell mig  ration, invasion and metastatic potential.The effects of IL-1β-induced p38 and JNK activation in GA cells were determined using in vitro Tra  nswell mi  gration and invasion assays of MKN-45 and AGS cells, or an in vivo metastasis assay in nude mice. The IL-1β-induced p38 signaling pathway was further characterized in GA cells. Activation of the IL-1β/p38 signaling pathway was also assessed in human primary GA tissues by immunohistochemistry.IL-1β-induced activation of p38 increase  d GA cell   migration and invasion in vitro and promoted the metastatic potential of GA cells in vivo; these effects were attenuated by p38 siRNA or the p38 inhibitor SB202190. MMP2 or MMP9 siRNAs and the MMP2/9 inhibitor BiPS also inhibited IL-1β-induc  ed GA cel  l migration and invasion in vitro. IL-1β-induced p38 activation significantly increased MMP2 and MMP9 mRNA and protein expression and activity. Luciferase reporter assays demonstrated that the activator protein-1 (AP-1) and the AP-1 binding sites of the MMP9 promoter (-670/MMP9) were activated by IL-1β-induced p38 activation. Phospho-p38 was significantly upregulated in human GA tissues (compared to matched non-neoplastic tissues), and significantly associated with lymph node metastasis, and invasion beyond the serosa. Expression of phospho-p38 significantly correlated with IL-1β, MMP2, MMP9, and c-fos expression in both human GA tissues and GA cell metastases in the lungs of nude mice. IL-1β was also capable of activating JNK in GA cells, but activation of JNK was not associate  d with GA   cell migration and invasion. Therefore, IL-  1β-induc  ed the migration and invasion in GA cells were regulated by p38, but not by JNK.IL-1β-induced p38 activation and the IL-1β/p38/AP-1(c-fos)/MMP2 & MMP9 pathway play an important role in metastasis in GA; this pathway may provide a novel therapeutic target for GA. 
 Vascular endothelial growth factor induces heat shock protein (HSP) 27 serine 82 phosphorylation and endothelial tubulogenesis via protein kinase D and independent of p38 kinase. Proteomic analysis identified HSP27 phosphorylation as a major change in protein phosphorylation stimulated by Vascular Endothelial Growth Factor (VEGF) in Human Umbilical Vein Endothelial Cells (HUVEC). VEGF-induced HSP27 phosphorylation at serines 15, 78 and 82, but whereas HSP27 phosphorylation induced by H2O2 and TNFalpha was completely blocked by the p38 kinase inhibitor, SB203580, VEGF-stimulated serine 82 phosphorylation was resistant to SB203580 and small interfering(si)RNA-mediated knockdown of p38 kinase and MAPKAPK2. The PKC inhibitor, GF109203X, partially reduced VEGF-induced HSP27 serine 82 phosphorylation, and SB203580 plus GF109203X abolished phosphorylation. VEGF activated Protein Kinase D (PKD) via PKC, and siRNAs targeted to PKD1 and PKD2 inhibited VEGF-induced HSP27 serine 82 phosphorylation. Furthermore recombinant PKD selectively phosphorylated HSP27 at serine 82 in vitro, and PKD2 activated by VEGF in HUVECs also phosphorylated HSP27 selectively at this site. Knockdown of HSP27 and PKDs markedly inhibited VEGF-induced HUVEC   migration   and tubulogenesis, whereas inhibition of the p38 kinase pathway using either SB203580 or siRNAs against p38alpha or MAPKAPK2, had no significant effect on the chemotactic response to VEGF. These findings identify a novel pathway for VEGF-induced HSP27 serine 82 phosphorylation via PKC-mediated PKD activation and direct phosphorylation of HSP27 by PKD, and show that PKDs and HSP27 play major roles in the angiogenic response to VEGF. 
 Collagen XVII participates in keratinocyte adhesion to collagen IV, and in p38MAPK-dependent   migration   and cell signaling. Collagen XVII (COL17) participates in keratinocyte adhesion and possibly   migration  , as COL17 defects disrupt keratinocyte-basal lamina adhesion and underlie the disease non-Herlitz junctional epidermolysis bullosa. Using small interference RNA (siRNA) to knock down COL17 expression in HaCaT cells, we assessed cell characteristics, including adhesion,   migration  , and signaling. Control and siRNA-transfected keratinocytes showed no difference in adhesion on plastic dishes after incubation for 8 hours in serum-free keratinocyte-growth medium; however, when grown on collagen IV alone or BD matrigel (containing collagen IV and laminin isoforms), COL17-deficient cells showed significantly reduced adhesion compared with controls (P<0.01), and mitogen-activated protein kinase (MAPK)/ERK kinase (MEK)1/2 and MAPK showed reduced phosphorylation. Furthermore, COL17-deficient HaCaT cells plated on plastic exhibited reduced motility that was p38MAPK-dependent (after addition of the p38MAPK inhibitor SB203580). Together, these results suggest that COL17 has significantly wider signaling roles than were previously thought, including the involvement of COL17 in keratinocyte adhesion to collagen IV, in p38MAPK-dependent cell   migration  , and multiple cell signaling events pertaining to MEK1/2 phosphorylation. 
 hNUDC promotes the cell proliferation and differentiation in a leukemic cell line via activation of the thrombopoietin receptor (Mpl). We have recently identified a human homolog of a fungal nuclear   migration   protein (hNUDC) that binds specifically with the extracellular domain of thrombopoietin receptor (Mpl). Preliminary studies with human CD34(+) cells cultured in serum-free medium and normal mice showed that hNUDC appears to act as a cytokine, triggering many of the same responses as thrombopoietin (TPO). More intriguingly, recent data gained using a NIH 3T3 system have demonstrated that hNUDC exerts its biological activities through activation of Mpl. In this study, we further compared the biological functions of hNUDC with TPO in an EPO-dependent UT-7 cell line that was engineered to express the thrombopoietin receptor (Mpl). These Mpl-expressing cells following stimulation by either hNUDC or TPO exhibited overlapping patterns of megakaryocytic proliferation and differentiation, manifested by cell morphological change, polyploidy and expression of CD41(+). Similar with TPO, hNUDC induced a sustained activation of the extracellular signal-regulated protein kinases-1 and -2 (ERK1/2) as well as p38 mitogen-activated kinase (p38 MAPK) pathways and these activations were inhibited in the presence of PD98059 or SB203580. Further evidence is provided that PD98059 or SB203580 inhibited hNUDC- or TPO-induced cell proliferation and differentiation, suggesting that ERK1/2 and p38 MAPK pathways are necessary in megakaryocyte development. 
 microRNA-130a Promotes Human Keratinocyte Viability and   Migration   and Inhibits Apoptosis Through Direct Regulation of STK40-Mediated NF-κB Pathway and Indirect Regulation of SOX9-Meditated JNK/MAPK Pathway: A Potential Role in Psoriasis. Psoriasis is a chronic inflammatory skin disorder. The aim of this study was to determine a potential role of microRNA (miR)-130a in psoriasis, and underlying mechanism. Expression levels of miR-130a in psoriasis specimens and normal skin tissues were analyzed. MiR-130a mimic, inhibitor, miR-control, small interfering RNA (siRNA) specific serine/threonine kinase 40 (STK40), or sex-determining region Y chromosome-box 9 (SOX9) were transfected to human keratinocyte HaCaT cells, respectively. After transfection, the cell viability, apoptosis, and   migratio  n were determined. Luciferase reporter assay, quantitative reverse transcription-polymerase chain reaction, and western blot were performed to explore whether STK40 was a target of miR-130a. The effects of aberrant expressions of miR-130a, STK40, or SOX9 on key proteins of NF-κB and c-Jun N-terminal kinase (JNK)/mitogen-activated protein kinase (MAPK) pathway were assessed. The miR-130a levels were significantly higher in patients with psoriasis compared to the healthy controls (p < 0.01). Overexpressing miR-130a strikingly promoted HaCaT cell viabilit  y and mig  ration and inhibited apoptosis (p < 0.01 or p < 0.05). We confirmed that STK40 was a direct target of miR-130a, and STK40 was involved in miR-130a-induced cell functions. Overexpressing miR-130a significantly upregulated NF-κB p65, SOX9, p-c-Jun, p-JNK, and p-p38MAPK proteins and silencing miR-130a downregulated them. In addition, silencing STK40 alleviated the effects of anti-miR-130a on SOX9 expression. Furthermore, silencing SOX9 also decreased levels of p-c-Jun, p-JNK, and p-p38MAPK proteins. MiR-130a regulates human keratinocyte Ha  CaT viabi  lity, migration and apoptosis might be by direct regulation of STK40-mediated NF-κB pathway and by indirect regulation of SOX9-mediated downstream JNK/MAPK signaling pathway. 
 p38 and a p38-interacting protein are critical for downregulation of E-cadherin during mouse gastrulation. During vertebrate gastrulation, an epithelial to mesenchymal transition (EMT) is necessary for   migration   of mesoderm from the primitive streak. We demonstrate that p38 MAP kinase and a p38-interacting protein (p38IP) are critically required for downregulation of E-cadherin during gastrulation. In an ENU-mutagenesis screen we identified the droopy eye (drey) mutation, which affects splicing of p38IP. p38IP(drey) mutant embryos display incompletely penetrant defects in neural tube closure, eye development, and gastrulation. A stronger allele (p38IP(RRK)) exhibits gastrulation defects in which mesoderm   migration   is defective due to deficiency in E-cadherin protein downregulation in the primitive streak. We show that p38IP binds directly to p38 and is required for p38 activation in vivo. Moreover, both p38 and p38IP are required for E-cadherin downregulation during gastrulation. Finally, p38 regulates E-cadherin protein expression downstream from NCK-interacting kinase (NIK) and independently of the regulation of transcription by Fibroblast Growth Factor (Fgf) signaling and Snail. 
 Comprehensive analysis of phosphorylation sites in Tensin1 reveals regulation by p38MAPK. Tensin1 is the archetype of a family of focal adhesion proteins. Tensin1 has a phosphotyrosine binding domain that binds the cytoplasmic tail of β-integrin, a Src homology 2 domain that binds focal adhesion kinase, p130Cas, and the RhoGAP called deleted in liver cancer-1, a phosphatase and tensin homology domain that binds protein phosphatase-1α and other regions that bind F-actin. The association between tensin1 and these partners affects cell polarization  , migrati  on, and invasion. In this study we analyzed the phosphorylation of human S-tag-tensin1 expressed in HEK293 cells by mass spectrometry. Peptides covering >90% of the sequence initially revealed 50 phosphorylated serine/phosphorylated threonine (pSer/pThr) but no phosphorylated tyrosine (pTyr) sites. Addition of peroxyvanadate to cells to inhibit protein tyrosine phosphatases exposed 10 pTyr sites and addition of calyculin A to cells to inhibit protein phosphatases type 1 and 2A gave a total of 62 pSer/pThr sites. We also characterized two sites modified by O-linked N-acetylglucosamine. Tensin1 F302A, which does not bind protein phosphatase-1, showed > twofold enhanced phosphorylation of seven sites. The majority of pSer/pThr have adjacent proline (Pro) residues and we show endogenous p38 mitogen activated protein kinase (MAPK) associated with and phosphorylated tensin1 in an in vitro kinase assay. Recombinant p38α MAPK also phosphorylated S-tag-tensin1, resulting in decreased binding with deleted in liver cancer-1. Activation of p38 MAPK in cells by sorbitol-induced hyperosmotic stress increased phosphorylation of S-tag-tensin1, which reduced binding to deleted in liver cancer-1 and increased binding to endogenous pTyr proteins, including p130Cas and focal adhesion kinase. These data demonstrate that tensin1 is extensively phosphorylated on Ser/Thr residues in cells and phosphorylation by p38 MAPK regulates the specificity of the tensin1 Src homology 2 domain for binding to different proteins. Tensin1 provides a hub for connecting signaling pathways involving p38 MAP kinase, tyrosine kinases and RhoGTPases. 
 p38 kinase-mediated transactivation of the epidermal growth factor receptor is required for dedifferentiation of renal epithelial cells after oxidant injury. Renal proximal tubular cell (RPTC) dedifferentiation is thought to be a prerequisite for regenerative proliferation and   migration   after renal injury. However, the specific mediators and the mechanisms that regulate RPTC dedifferentiation have not been elucidated. Because epidermal growth factor (EGF) receptor activity is required for recovery from acute renal failure, we examined the role of the EGF receptor in dedifferentiation and the mechanisms of EGF receptor transactivation in primary cultures of RPTCs after oxidant injury. Exposure of confluent RPTCs to H2O2 resulted in 40% cell death, and surviving RPTCs acquired a dedifferentiated phenotype (e.g. elongated morphology and vimetin expression). The EGF receptor, p38, Src, and MKK3 were activated after oxidant injury and inhibition of the EGF receptor or p38 with specific inhibitors (AG1478 and SB203580, respectively) blocked RPTC dedifferentiation. Treatment with SB203580 or adenoviral overexpression of dominant negative p38alpha or its upstream activator, MKK3, inhibited EGF receptor phosphorylation induced by oxidant injury, whereas AG1478 had no effect on p38 phosphorylation. Inhibition of Src with 4-amino-5-(4-methylphenyl)-7-(t-butyl)pyrazolo[3,4-d]-pyrimidine (PP1) blocked MKK3 and p38 activation, and inhibition of MKK3 blocked p38 activation. In addition, inactivation of Src, MKK3, p38, or the EGF receptor blocked tyrosine phosphorylation of beta-catenin, a key signaling intermediate that is involved in the epithelial-mesenchymal transition and vimentin expression. These results reveal that p38 mediates EGF receptor activation after oxidant injury; that Src activates MMK3, which, in turn, activates p38; and that the EGF receptor signaling pathway plays a critical role in RPTC dedifferentiation. 
 p38MAPK inhibition: a new combined approach to reduce neuroblastoma resistance under etoposide treatment. Neuroblastoma (NB) is the second most common solid pediatric tumor and is characterized by clinical and biological heterogeneity, and stage-IV of the disease represents 50% of all cases. Considering the limited success of present chemotherapy treatment, it has become necessary to find new and effective therapies. In this context, our approach consists of identifying and targeting key molecular pathways associated with NB chemoresistance. This study has been carried out on three stage-IV NB cell lines with different status of MYCN amplification. Cells were exposed to a standard chemotherapy agent, namely etoposide, either alone or in combination with particular drugs, which target intracellular signaling pathways. Etoposide alone induced a concentration-dependent reduction of cell viability and, at very high doses, totally counteracted cell tumorigenicity and neurosphere formation. In addition, etoposide activated p38 mitogen-activated protein kinase (MAPK), AKT and c-Jun N-terminal kinase. Pre-treatment with SB203580, a p38MAPK inhibitor, dramatically sensibilized NB cells to etoposide, strongly reducing the dosage needed to inhibit tumorigenicity and neurosphere formation. Importantly, SB203580-etoposide cotreatment also reduced cell   migration   and invasion by affecting cyclooxygenase-2, intercellular adhesion molecule-1, C-X-C chemokine receptor-4 and matrix metalloprotease-9. Collectively, our results suggest that p38MAPK inhibition, in combination with standard chemotherapy, could represent an effective strategy to counteract NB resistance in stage-IV patients. 
 VCAM-1 upregulation via PKCdelta-p38 kinase-linked cascade mediates the TNF-alpha-induced leukocyte adhesion and e  migration   in the lung airway epithelium. Vascular cell adhesion molecule (VCAM)-1 plays a central role in the recruitment of inflammatory cells, and its expression is rapidly induced by proinflammatory cytokines such as TNF-alpha. In the present study, we show that pretreatment with rottlerin, a specific inhibitor of protein kinase C (PKC)-delta, or transient transfection with antisense PKCdelta oligonucleotides significantly inhibits TNF-alpha-induced expression of VCAM-1, but not of intercellular adhesion molecule (ICAM)-1 in human lung epithelium A549 cells. In addition, TNF-alpha was shown to induce the expression of VCAM-1 in a p38 kinase-dependent manner; also, TNF-alpha-induced p38 kinase activation was blocked by inhibition of PKCdelta, suggesting that p38 kinase is apparently situated downstream of PKCdelta in the TNF-alpha-signaling pathway to VCAM-1 expression. Notably, inhibition of the PKCdelta-p38 kinase cascade also attenuated the TNF-alpha-induced adhesion of neutrophils to lung epithelium and the trafficking of leukocytes across the epithelium into the airway lumen in vivo. Together, these findings indicate that signaling via PKCdelta-p38 kinase-linked cascade specifically induces expression of VCAM-1 in lung epithelium in response to TNF-alpha and that this effect is both functionally and clinically significant. 
 Suppression of ATAD2 inhibits hepatocellular carcinoma progression through activation of p53- and p38-mediated apoptotic signaling. The ATPase family, AAA domain containing 2 (ATAD2) is highly expressed in multiple cancers. We aim to understand the clinical and biological significance of ATAD2 over-expression in hepatocellular carcinoma (HCC), as a means to validate it as a therapeutic target in HCC. We demonstrated that ATAD2 was over-expressed in HCC patients, where high ATAD2 levels were significantly correlated with aggressive phenotypes such as high AFP levels, advanced tumor stages, and vascular invasion. Using RNA interference, suppression of ATAD2 in HCC cell lines decreased cell viability,   migration  , and invasion, and induced apoptosis in vitro. Furthermore, we identified p53 and p38 as key proteins that mediate apoptosis induced by ATAD2 suppression. In HCC cells, we demonstrated that ATAD2 directly interacted with MKK3/6, which prevented p38 activation and therefore inhibited p38-mediated apoptosis. In vivo, suppression of ATAD2 impaired the growth of HepG2 and Hep3B subcutaneous xenografts, accompanied by enhanced apoptosis and p-p53 and p-p38 levels. Our results validate that ATAD2 is an important negative regulator of apoptosis, and that neutralizing its activity has promising anti-tumor effects in HCC cells. 
 Naringin inhibits the invasion and   migration   of human glioblastoma cell via downregulation of MMP-2 and MMP-9 expression and inactivation of p38 signaling pathway. Gliomas are the most common and malignant primary brain tumors. They are associated with a poor prognosis despite the availability of multiple therapeutic options. Naringin, a common dietary flavonoid abundantly present in fruits and vegetables, is believed to possess strong anti-proliferative and anti-cancer properties. However, there are no reports describing its effects on the invasion and   migration   of glioblastoma cell lines. Our results showed that the treatment of U251 glioma cell lines with different concentrations of naringin inhibited the invasion and   migration   of these cells. In addition, we revealed a decrease in the levels of matrix metalloproteinases (MMP-2) and (MMP-9) expression as well as proteinase activity in U251 glioma cells. In contrast, the expression of tissue inhibitor of metalloproteinases (TIMP-1) and (TIMP-2) was increased. Furthermore, naringin treatment decreased significantly the phosphorylated level of p38. Combined treatment with a p38 inhibitor (SB203580) resulted in the synergistic reduction of MMP-2 and MMP-9 expressions correlated with an increase of TIMP-1 and TIMP-2 expressions and the anti-invasive properties. However, p38 chemical activator (anisomycin) could block these effects produced by naringin, suggesting a direct downregulation of the p38 signaling pathway. These data suggest that naringin may have therapeutic potential for controlling invasiveness of malignant gliomas by inhibiting of p38 signal transduction pathways. 
 Glial cell-derived neurotrophic factor (GDNF) promotes low-grade Hs683 glioma cell   migration   through JNK, ERK-1/2 and p38 MAPK signaling pathways. Invasion of tumor cells is the primary cause of therapeutic failure in the treatment of malignant gliomas. In an attempt to investigate the properties of the malignant progression of glioma cells, we examined the correlation between cell   migration   and glial cell-derived neurotrophic factor (GDNF) secretion of two glioma cell lines which differ in their invasive phenotypes. Here, we show that the high-grade C6 cells are more migrative and secrete more GDNF than the low-grade Hs683 cells. GDNF signaling is more highly activated in C6 cells than in Hs683 cells. Treatment of the Hs683 cells with GDNF significantly increased   migration   comparable to the C6 cells, revealing the autocrine and/or paracrine effect of GDNF on promotion of the glioma cell   migration  . We then examined the involvement of mitogen-activated protein kinases (MAPKs) including c-Jun N-terminal protein kinase (JNK), extracellular signal-regulated kinases (ERKs) and p38 MAPK in Hs683 cell   migration   induced by GDNF. A prominent activation of JNK, ERKs and p38 MAPK was observed in the GDNF-treated cells. Functional studies showed that the activation of these MAPKs was critical for Hs683 cell   migration   induced by GDNF. Our findings revealing molecular mechanisms for the promoting effect of GDNF on glioma cell   migration   may provide an insight into a better understanding to the malignant progression of human gliomas. 
   Migration   induced by epidermal and hepatocyte growth factors in oral squamous carcinoma cells in vitro: role of MEK/ERK, p38 and PI-3 kinase/Akt. Cell   migration   is a necessary part of malignant invasiveness. Oral squamous cell carcinomas (OSCC) have a great tendency for local invasive growth. We have investigated signalling pathways involved in cell   migration   induced by epidermal growth factor (EGF) and hepatocyte growth factor (HGF) in OSCC cells and examined the effects of various experimental and clinically approved anti-tumour signal inhibitors on the migratory activity.  Migration   was studied in three human OSCC cell lines, using a scratch wound assay in vitro and time-lapse cinematography. Specific phosphorylation of signalling proteins was assessed by Western blotting.In the E10 cell line, EGF and HGF induced phosphorylation of EGF receptor (EGFR) and Met, respectively, phosphorylation of ERK1/2, p38 and Akt, and dose-dependent activation of cell   migration  . Addition of the EGFR-specific inhibitors cetuximab (antibody) or gefitinib (tyrosine kinase blocker) abolished cell   migration   elicited by EGF. Similarly, a Met kinase inhibitor (SU11274) blocked HGF-induced cell   migration  . Furthermore, when three cell lines were treated with blockers of the MEK/ERK, p38 or the PI-3 kinase/Akt pathways, the migratory response to both EGF and HGF was inhibited, but to varying degrees. Notably, in E10 and D12 cells, HGF-induced   migration   was particularly sensitive to PI-3 K-inhibition, while in C12 cells, both HGF- and EGF-induced   migration   were highly sensitive to p38-blockade.The results demonstrate that the MEK/ERK, p38 and PI-3 kinase pathways are all involved in mediating the increased   migration   in OSCC cell lines induced by EGF and HGF, but their relative importance and the effects of specific signal inhibitors differ. 
 Downregulation of LAPTM5 suppresses cell proliferation and viability inducing cell cycle arrest at G0/G1 phase of bladder cancer cells. Our transcriptome analysis revealed in bladder cancer (BCa) tissues a significant induction of lysosomal-associated multispanning membrane protein 5 (LAPTM5), a lysosomal membrane protein preferentially expressing in immune cells and hematopoietic cells. Transportation of LAPTM5 from Golgi to lysosome could be inhibited by deficiency of Nedd4, a key member of E3 ubiquitin ligase family overexpressing in invasive BCa and promoting its progression. Therefore, we hypothesize that LAPTM5 may be closely correlated with BCa tumorigenesis. In human BCa tissues, we observed that LAPTM5 was significantly induced at both mRNA and protein levels, which is consistent with our microarray result. Furthermore, we established a BCa cell model with downregulated LAPTM5, revealing a significantly delayed growth rate in the BCa cells with knockdown of LAPTM5. Moreover, cell cycle arrest at G0/G1 phase was triggered by decreased LAPTM5 as well, which could lead to delayed BCa cell growth. In contrast, no significant alteration of apoptosis in the BCa cells with downregulated LAPTM5 was noticed. Analysis of the changes of   migratio  n and invasion, showed significant reduced LAPTM5 suppressed cell metastasis. Furthermore, proteins involved in epithelial-mesenchymal transition (EMT) were strongly altered, which plays a central role in metastasis. In addition, phosphorylated ERK1/2 and p38, key members of mitogen-activated protein kinase (MAPK) family regulating BCa tumorigenesis, were strongly decreased. Taken together, our results suggested that decreased LAPTM5 inhibited proliferation and viability, as well as induced G0/G1 cell cycle arrest possibly via deactivation of ERK1/2 and p38 in BCa cells. 
 p38 MAP kinase is necessary for melanoma-mediated regulation of VE-cadherin disassembly. Vascular endothelial (VE)-cadherin is localized to the endothelial borders and the adherens junctions, which are regulated by changes in mitogen-activated protein (MAP) kinases, GTPases, and intracellular calcium. We previously showed that melanoma cells induce VE-cadherin disassembly through contact with human umbilical vein endothelial cells in coculture. However, the exact mechanism by which melanoma cells signal endothelial cells to induce VE-cadherin junction disassembly is not well understood. In this study, VE-cadherin junction disassembly was further examined under fluorescence microscopy. We found that melanoma-induced VE-cadherin junction disassembly and upregulation of p38 MAP kinase in endothelial cells is regulated by both soluble factors from melanomas, particularly interleukin (IL)-8, IL-6, and IL-1beta, and through vascular cell adhesion molecule-1. Neutralizing melanoma-secreted soluble factors reduced endothelial gap formation. Endothelial cells transfected with MAP kinase kinase 6, a direct activator of p38 MAP kinase, increased VE-cadherin-mediated gap formation, facilitating melanoma transendothelial   migration  . In contrast, endothelial cells transfected with small-interfering RNA against p38 MAP kinase expression largely prevented melanoma transendothelial   migration   in Boyden chamber experiments. These findings indicate that p38 MAP kinase proteins regulate VE-cadherin junction disassembly, facilitating melanoma   migration   across endothelial cells. 
 Promotion of cancer cell   migration  : an insulin-like growth factor (IGF)-independent action of IGF-binding protein-6. A family of six high affinity IGF-binding proteins (IGFBPs 1-6) plays an important role in modulating IGF activities. Recent studies suggest that some IGFBPs may have IGF-independent effects, including induction of apoptosis and modulation of cell   migration  . However, very little is known about possible IGF-independent actions of IGFBP-6. We have generated a non-IGF-binding IGFBP-6 mutant by substituting Ala for four amino acid residues (Pro(93)/Leu(94)/Leu(97)/Leu(98)) in its N-domain IGF-binding site. A >10,000-fold loss of binding affinity for IGF-I and IGF-II was observed using charcoal solution binding assay, BIAcore biosensor, and ligand blotting. Wild-type and mutant IGFBP-6, as well as IGF-II, induced cell   migration   in RD rhabdomyosarcoma and LIM 1215 colon cancer cells. Cell   migration   was mediated by the C-domain of IGFBP-6. Transient p38 phosphorylation was observed in RD cells after treatment with IGFBP-6, whereas no change was seen in phospho-ERK1/2 levels. Phospho-JNK was not detected. IGFBP-6-induced cell   migration   was inhibited by SB203580, an inhibitor of p38 MAPK, and PD98059, an inhibitor of ERK1/2 MAPK activation. In contrast, SP600125, a JNK MAPK inhibitor, had no effect on   migration  . Knockdown of p38 MAPK using short interfering RNA blocked IGFBP-6-induced   migration   of RD cells. These results indicate that p38 MAPK is involved in IGFBP-6-induced IGF-independent RD cell   migration  . 
 Tenascin-W is found in malignant mammary tumors, promotes alpha8 integrin-dependent motility and requires p38MAPK activity for BMP-2 and TNF-alpha induced expression in vitro. Tenascins represent a family of extracellular matrix glycoproteins with distinctive expression patterns. Here we have analyzed the most recently described member, tenascin-W, in breast cancer. Mammary tumors isolated from transgenic mice expressing hormone-induced oncogenes reveal tenascin-W in the stroma around lesions with a high likelihood of metastasis. The presence of tenascin-W was correlated with the expression of its putative receptor, alpha8 integrin. HC11 cells derived from normal mammary epithelium do not express alpha8 integrin and fail to cross tenascin-W-coated filters. However, 4T1 mammary carcinoma cells do express alpha8 integrin and their   migration   is stimulated by tenascin-W. The expression of tenascin-W is induced by BMP-2 but not by TGF-beta1, though the latter is a potent inducer of tenascin-C. The expression of tenascin-W is dependent on p38MAPK and JNK signaling pathways. Since preinflammatory cytokines also act through p38MAPK and JNK signaling pathways, the possible role of TNF-alpha in tenascin-W expression was also examined. TNF-alpha induced the expression of both tenascin-W and tenascin-C, and this induction was p38MAPK- and cyclooxygenase-dependent. Our results show that tenascin-W may be a useful diagnostic marker for breast malignancies, and that the induction of tenascin-W in the tumor stroma may contribute to the invasive behavior of tumor cells. 
 CCL27 expression is regulated by both p38 MAPK and IKKβ signalling pathways. The skin-specific chemokine CCL27 is believed to play a pivotal role in establishing the inflammatory infiltrate characteristic for common inflammatory skin diseases. Through binding to the chemokine receptor 10 (CCR10), CCL27 mediates inflammation by promoting lymphocyte   migratio  n into the skin. Little is known about the regulation of CCL27 gene expression. The purpose of our study was to investigate the regulation of the IL-1β-induced CCL27 gene expression in normal human keratinocytes (NHEK). Preincubation of NHEK with the inhibitory κB (IκB) kinase (IKK) inhibitor, SC-514, or the p38 mitogen-activated protein kinase (MAPK) inhibitor, SB202190, revealed a profound reduction in both CCL27 mRNA and CCL27 protein expression indicating the significance of these pathways in the regulation of CCL27 expression. Furthermore, the impact of inhibitors of mitogen- and stress-activated kinase 1 (MSK1) or the mitogen-activated protein kinase-interacting kinases (Mnk1+2), downstream kinases of p38 MAPK, on IL-1β-induced CCL27 expression in NHEK were investigated. We identified seven NF-κB binding elements upstream from the CCL27 gene start codon using electrophoretic mobility shift assay (EMSA). Supershift analyses demonstrated the involvement of the p50/p65 NF-κB heterodimer. We conclude that IL-1β-induced CCL27 gene expression in NHEK is regulated through the p38 MAPK/MSK1/Mnk1+2 as well as the IKKβ/NF-κB signalling pathways. 
 Flt-1 in colorectal cancer cells is required for the tumor invasive effect of placental growth factor through a p38-MMP9 pathway. Placenta growth factor (PlGF), a dimeric glycoprotein with 53% homology to VEGF, binds to VEGF receptor-1 (Flt-1), but not to VEGF receptor-2 (Flk-1), and may function by modulating VEGF activity. We previously have showed that PlGF displays prognostic value in colorectal cancer (CRC) but the mechanism remains elucidated.Overexpression of PlGF increased the invasive/  migration   ability and decreased apoptosis in CRC cells showing Flt-1 expression. Increased   migration   was associated with increasing MMP9 via p38 MAPK activation. Tumors grew faster, larger; with higher vascularity from PlGF over-expression cells in xenograft assay. In two independent human CRC tissue cohorts, PlGF, MMP9, and Flt-1 expressions were higher in the advanced than the localized disease group. PlGF expression correlated with MMP9, and Flt-1 expression. CRC patients with high PlGF and high Flt-1 expression in tissue had poor prognosis.PlGF/Flt-1 signaling plays an important role in CRC progression, blocking PlGF/Flt-1 signaling maybe an alternative therapy for CRC. 
 Lysophosphatidic acid induces prostate cancer PC3 cell   migration   via activation of LPA(1), p42 and p38alpha. Prostate cancer cell   migration   is an essential event both in the progression of prostate cancer and in the steps leading to metastasis. We report here that lysophosphatidic acid (LPA), a potent bioactive phospholipid, induces prostate cancer PC3 cell   migration   via the activation of the LPA(1) receptor, which is linked to a PTX-sensitive activation mechanism of the mitogen-activated protein kinases (MAPK). Our results demonstrate that parallel activation of ERK1/2 and p38, but not JNK, is responsible for LPA-stimulated PC3 cell   migration  . Furthermore, using small interfering RNA (siRNA) technology, and overexpressing dominant-negative mutants of p38 MAPK isotypes of alpha, beta, gamma and delta, we have identified that the activation of ERK2 (p42) and p38alpha, but not of ERK1 and the other isoforms of p38 MAPK, is required for LPA-induced   migration  . Our study provides the first evidence for a functional role of p42 and p38alpha in LPA-induced mammalian cell   migration  , and also demonstrates, for the first time, that the receptor LPA(1) mediates prostate cancer cell   migration  . The results of the present study suggest that LPA, the receptor LPA(1), ERK2 and p38alpha are important regulators for prostate cancer cell invasion and thus could play a significant role in the development of metastasis. 
 Bidirectional regulation of neutrophil   migration   by mitogen-activated protein kinases. To kill invading bacteria, neutrophils must interpret spatial cues, migrate and reach target sites. Although the initiation of chemotactic   migration   has been extensively studied, little is known about its termination. Here we found that two mitogen-activated protein kinases (MAPKs) had opposing roles in neutrophil trafficking. The extracellular signal-regulated kinase Erk potentiated activity of the G protein-coupled receptor kinase GRK2 and inhibited neutrophil   migration  , whereas the MAPK p38 acted as a noncanonical GRK that phosphorylated the formyl peptide receptor FPR1 and facilitated neutrophil   migration   by blocking GRK2 function. Therefore, the dynamic balance between Erk and p38 controlled neutrophil 'stop' and 'go' activity, which ensured that neutrophils reached their final destination as the first line of host defense. 
 TNF-alpha promotes a stop signal that inhibits neutrophil polarization and   migration   via a p38 MAPK pathway. Neutrophils are a major component of the inflammatory response in patients with asthma and other inflammatory conditions. Proinflammatory cytokines, such as tumor necrosis factor alpha (TNF-alpha), are increased in the airway of patients with severe asthma and have been implicated in the recruitment of neutrophils into areas of inflammation. Here, we show that TNF-alpha induces a stop signal that promotes firm neutrophil adhesion and inhibits neutrophil polarization and chemotaxis to chemoattractants including interleukin-8 and C5a. TNF-alpha treatment of neutrophils plated on a fibrinogen-coated surface promotes firm neutrophil adhesion and the formation of vinculin-containing focal complexes. TNF-alpha induces a more than tenfold increase in p38 mitogen-activated protein kinase (MAPK) but not extracellular signal-regulated kinase phosphorylation. Inhibition of p38 MAPK in neutrophils treated with TNF-alpha causes neutrophil polarization and motility. These findings suggest that TNF-alpha initiates a stop signal through a p38 MAPK pathway, which may promote the retention of neutrophils in inflammatory sites. Together, our data suggest that inhibition of p38 MAPK may be an attractive target to limit inflammatory responses that are mediated by TNF-alpha. 
 Epidermal growth factor-stimulated extravillous cytotrophoblast motility is mediated by the activation of PI3-K, Akt and both p38 and p42/44 mitogen-activated protein kinases. Trophoblast invasion is a temporally and spatially regulated scheme of events that can dictate pregnancy outcome. Evidence suggests that the potent mitogen epidermal growth factor (EGF) regulates cytotrophoblast (CTB) differentiation and invasion during early pregnancy.In the present study, the first trimester extravillous CTB cell line SGHPL-4 was used to investigate the signalling pathways involved in the motile component of EGF-mediated CTB   migration  /invasion. EGF induced the phosphorylation of the phosphatidylinositol 3-kinase (PI3-K)-dependent proteins, Akt and GSK-3beta as well as both p42/44 MAPK and p38 mitogen-activated protein kinases (MAPK). EGF-stimulated motility was significantly reduced following the inhibition of PI3-K (P < 0.001), Akt (P < 0.01) and both p42/44 MAPK (P < 0.001) and p38 MAPKs (P < 0.001) but not the inhibition of GSK-3beta. Further analysis indicated that the p38 MAPK inhibitor SB 203580 inhibited EGF-stimulated phosphorylation of Akt on serine 473, which may be responsible for the effect SB 203580 has on CTB motility. Although Akt activation leads to GSK-3beta phosphorylation and the subsequent expression of beta-catenin, activation of this pathway by 1-azakenpaullone was insufficient to stimulate the motile phenotype.We demonstrate a role for PI3-K, p42/44 MAPK and p38 MAPK in the stimulation of CTB cell motility by EGF, however activation of beta-catenin alone was insufficient to stimulate cell motility. 
 Lysophosphatidic acid enhances human hepatocellular carcinoma cell   migration  , invasion and adhesion through P38 MAPK pathway. Lysophosphatidic acid (LPA) has diverse biological activities implicated in tumor progression, including increasing cell   migration  , invasion and metastasis. However, the underlying mechanisms for LPA-induced human hepatocellular carcinoma (HCC)   migration   and invasion are poorly understood.We sought to determine the promoting effect of LPA on HCC   migration  , invasion and adhesion by transwell and matrix adhesion assays, respectively. Levels of matrix metalloproteinase, MMP-2 and MMP- 9 from cells were assayed by ELISA and p38 mitogenactivated protein kinase (MAPK) signaling was determined by western blot analysis.In the present study, LPA was found to increase HCC cell   migration  , invasion and adhesion. In addition, LPA increased MMP-9 expression level and induced activation of the p38 mitogen- activated protein kinase (MAPK) signaling. Furthermore, pharmacological inhibition of p38 MAPK signal pathway with SB203580 significantly attenuated LPA-induced HCC cell   migration  , invasion, and adhesion and abrogated LPA-induced MMP-9 expression and p38 MAPK phosphorylation.We demonstrated a mechanism that LPA can activate p38 MAPK signaling, which is required for LPA-induced HCC cell   migration  , invasion, adhesion and MMP-9 expression, providing a novel biomarker and potential therapeutic target for HCC. 
 Oncogenic K-Ras down-regulates Rac1 and RhoA activity and enhances   migration   and invasion of pancreatic carcinoma cells through activation of p38. Activating mutations in the K-ras gene are genetic alterations frequently found in human carcinomas, particularly in pancreatic adenocarcinomas. Mutation of the K-ras gene is thought to be an early and important event in pancreatic tumor initiation, but the precise role of the mutant K-Ras proteins in neoplastic progression is still unknown. In the present study, we have characterized the influence of oncogenic K-Ras on the phenotype and on the signal transduction of epitheloid PANC-1 pancreatic carcinoma cells by generating PANC-1 cell clones, which stably express EGFP(enhanced green fluorescent protein)-K-Ras (V12). EGFP-K-Ras (V12)-expressing cells exhibited a more fibroblastoid cellular phenotype with irregular cell shape and disorganized cytokeratin filaments. Moreover, these cells showed a marked enhancement of their migratory and invasive properties. Stable expression of EGFP-K-Ras (V12) down-regulated the activity of Rac1 and RhoA, resulting in reduced subcortical actin filaments and stress fibers, which might contribute to the epithelial dedifferentiation. Characterization of the activity of mitogen-activated protein kinases revealed that EGFP-K-Ras (V12) enhanced the activity of p38, but did not affect the activities of the Raf/MEK/ERK cascade and JNK. While inhibition of either MEK or JNK activity had no effect on EGFP-K-Ras (V12)-induced   migration  , inhibition of p38 activity markedly reduced EGFP-K-Ras (V12)-induced   migration  . Collectively, the results suggest that oncogenic K-Ras enhances the malignant phenotype and identify the mitogen-activated protein kinase p38 as a target to inhibit oncogenic K-Ras-induced pancreatic tumor cell   migration  . 
 Eosinophil   migration   induced by mast cell chymase is mediated by extracellular signal-regulated kinase pathway. Mast cell chymase is known to induce eosinophil   migration   in vivo and in vitro. In the present study, we investigated possible involvement of mitogen-activated protein (MAP) kinases; extracellular signal-regulated kinase (ERK), c-Jun amino-terminal kinase (JNK), and p38, in the chymase-induced eosinophil   migration  . Human chymase induced a rapid phosphorylation of ERK1/2 and p38 in human eosinophilic leukemia EoL-1 cells, while no phosphorylation was detected in JNK. The chymase-induced phosphorylation of ERK and p38 was inhibited by pertussis toxin. Similar results were obtained in the experiments using mouse chymase and eosinophils. U0126 (the inhibitor for MAP/ERK kinase) suppressed chymase-induced   migration   of EoL-1 cells and mouse eosinophils. However, SB203580 (p38 inhibitor) and SP600125 (JNK inhibitor) showed little effect on the   migration  . It is suggested therefore that chymase activates ERK and p38 probably through G-protein-coupled receptor, and that ERK but not p38 cascade may have a crucial role in chymase-induced   migration   of eosinophils. 
 Resveratrol inhibits hyperglycemia-driven ROS-induced invasion and   migration   of pancreatic cancer cells via suppression of the ERK and p38 MAPK signaling pathways. Increasing evidence suggests that there is a strong relationship between diabetes mellitus (DM) and pancreatic cancer. Our previous study revealed that hyperglycemia could enhance the invasive and migratory activities of pancreatic cancer cells. Resveratrol, a natural polyphenolic phytoalexin, has many biological and pharmaceutical properties, including antioxidant and anti-tumorigenic capabilities. The aim of the present study was to evaluate whether resveratrol affects hyperglycemia-induced reactive oxygen species (ROS) production as well as the invasion and   migration   of pancreatic cancer and its underlying mechanisms. Human pancreatic cancer Panc-1 cells were exposed to high glucose condition with or without resveratrol, N-acetylcysteine (NAC, a scavenger of free radicals), PD 98059 (an ERK inhibitor) or SB 203580 (a p38 MAPK inhibitor). The intracellular ROS and hydrogen peroxide (H2O2) were determined using 2,7-dichlorodihydrofluorecein diacetate and H2O2 assay. MTT, wound healing assay and transwell matrigel invasion assay were used to detect the proliferation,   migration   and invasion potential of cancer cells. The expressions of uPA, E-cadherin and Glut-1 were examined using QT-PCR and western blot analysis at mRNA and protein levels. The activation of p-ERK, p-p38 and p-NF-κB were measured by western blot analysis. The results of the present study showed that resveratrol could significantly decrease high glucose-induced production of ROS and H2O2 in Panc-1 cells. Resveratrol was also able to inhibit high glucose-induced proliferation,   migratio  n and invasion of pancreatic cancer cells. High glucose-modulated expression of uPA, E-cadherin and Glut-1 were inhibited by resveratrol. In addition, high glucose-induced activation of ERK and p38 MAPK signaling pathways as well as the transcription factor NF-κB could also be suppressed by resveratrol. Furthermore, resveratrol was able to suppress H2O2-induce  d migrati  on and invasion abilities of pancreatic cancer cells. Taken together, these data indicate that resveratrol plays an important role in suppressing hyperglycemia-driven ROS-induced pancreatic cancer progression by inhibiting the ERK and p38 MAPK signaling pathways, providing evidence that resveratrol might be a potential candidate for chemoprevention of pancreatic cancer. 
 Genetic evidence for critical roles of P38α protein in regulating mast cell differentiation and chemotaxis through distinct mechanisms. Mast cells mediate a range of immune responses. However, the mechanisms that contribute to their development remain poorly understood. Here, using a P38α conditional knockout system, we provide evidence to suggest that P38α plays critical roles in regulating mast cell differentiation a  nd migrat  ion via distinct mechanisms. Induced deletion of P38α in bone marrow cells retards the maturation of mast cells in part by inhibiting the activation of cAMP response element-binding protein and expression of microphthalmia-associated transcription factor, which encourages the generation of basophils over mast cells. In fully differentiated mast cells, absence of P38α inhibits stem cell factor-induced activation of Akt and ERK, which is associated with reduced chemotaxis. In vivo, conditional deletion of P38α results in reduced numbers of mast cells in certain tissues and a failure to reconstitute these cells in W(sh) mice transplanted with P38α(-/-) Lin(-)c-kit(+)Sca-1(+) (LKS(+)) cells. Our findings suggest that P38α plays a dual role in mast cell development by regulating IL-3-induced differentiation of mast cell progenitor cells as well as by regulating stem cell factor-  induced m  igration of fully differentiated mast cells. 
 IVIG inhibits TNF-α-induced MMP9 expression and activity in monocytes by suppressing NF-κB and P38 MAPK activation. Matrix metalloproteinase-9 (MMP9) has been involved in inflammatory and pathologic processes of coronary artery lesions (CAL) in Kawasaki disease (KD). Intravenous immunoglobulin (IVIG), a traditional treatment for Kawasaki disease, could decrease the expressions of MMP9. The purpose of this study was to investigate the protective effect of IVIG in chemotacti  c migrati  on of monocyte and the regulation of MMP9 induced by tumor necrosis factor-α (TNF-α) in U937s. Studies were carried out with real time polymerase chain reaction (RT-PCR), zymographic, Western blotting and immunofluorescence. U93  7s' migra  tion was enhanced by TNF-α stimulation, while was inhibited by IVIG pretreatment. MMP9 expression and activity in U937s were also significantly enhanced by TNF-α and inhibited by IVIVG pretreatment. During inflammatory stimulus, nuclear factor kappa B (NF-κB) and P38 Mitogenactivated protein kinase (P38 MAPK) pathways play a significant role in regulating MMP9 gene expression. TNF-α induced nuclear translocation of NF-κB and P38 MAPK activation in U937s were inhibited significantly by IVIG. Furthermore, we clarified that nuclear NF-κB and P38 MAPK pathways play pivotal roles in regulati  ng U937s'   migration and MMP9 expressions using PDTC and SB203580, which were specific inhibitors of NF-κB and p38 MAPK pathways. IVIG displays striking biological effects, notably promotin  g monocyt  e migration. These effects involve the NF-κB and p38 pathways, and increased MMP9 activity. It might be a crucial mechanism of IVIG reducing the occurrence of CAL that IVIG inhibited monocytes expressing MMP9 and decreased   chemotact  ic migration of monocyte. 
 Fibroblast growth factor 18 promotes proliferation and   migration   of H460 cells via the ERK and p38 signaling pathways. Recently, fibroblast growth factor 18 (FGF18) expression was reported to be upregulated in colon cancer and ovarian cancer, and increased expression of FGF18 mRNA and protein is associated with tumor progression and poor overall survival in patients; however, its role in lung cancer remains to be explored. In the present study, the effect and underlying molecular mechanisms of FGF18 on H460 cells were investigated. Cell proliferation and cell cycle alterations were detected using a 3-(4, 5-dimethylthiazol-2-yl)-2, 5-diphenyltetrazolium bromide assay and flow cytometry. A wound healing assay was conducted to detect cell   migration  . Reverse transcription-quantitative polymerase chain reaction and western blotting were performed to measure extracellular signal-regulated kinase (ERK), p38 and matrix metalloproteinase 26 (MMP26) expression. Knockdown of FGF18 using short interfering RNA (siRNA-FGF18) suppressed H460 cell proliferation, inhibited cell   migratio  n via the downregulation of MMP26 levels, with siRNA-FGF18 additionally inhibiting the ERK and p38 signaling pathway. The present study indicates that FGF18 serves an essential role in the growth and   migratio  n of non-small cell lung cancer (NSCLC) cells by regulating the ERK, p38 signaling pathways and MMP26 protein levels, suggesting that FGF18 may be a potential molecular drug target for the treatment NSCLC. 
 HB-EGF synthesis and release induced by cholesterol depletion of human epidermal keratinocytes is controlled by extracellular ATP and involves both p38 and ERK1/2 signaling pathways. The heparin-binding EGF-like growth factor (HB-EGF) is an autocrine/paracrine keratinocyte growth factor, which binds to the epidermal growth factor (EGF) receptor family and plays a critical role during the re-epithelialization of cutaneous wound by stimulating the keratinocytes proliferation and   migration  . In this study, cellular stressing condition in autocrine cultures of human keratinocytes was induced by cholesterol depletion using methyl-beta-cyclodextrin (MβCD). MβCD treatment induces the expression and the release of HB-EGF. By analysis of the culture media, large amounts of cellular ATP were measured particularly after 1 h of MβCD treatment. To investigate whether ATP contributes to the expression of HB-EGF, the nonhydrolyzable ATP analogue, ATP-γ-S, was used to mimic the extracellular ATP released. We report that keratinocytes stimulated with ATP-γ-S induce HB-EGF expression and activate EGFR and ERK1/2. Using an antagonist of P2 purinergic receptors, we demonstrate that HB-EGF synthesis induced by lipid rafts disruption is dependent on ATP interaction with P2 purinergic receptors. Moreover, our data suggest that both MAPKs p38 and ERK1/2 are involved together or independently in the regulation of HB-EGF gene expression. These findings provide new insight into the signaling pathway by which HB-EGF is expressed after lipid rafts disruption. In summary, after lipid raft disruption, keratinocytes release large amount of extracellular ATP. ATP induces HB-EGF synthesis and release by interacting with the P2 purinergic receptor and through p38 and ERK1/2 signaling in response to a challenging environment. A release of ATP acts as an early stress response in keratinocytes. 
 Up-regulation of galectin-9 induces cell   migration   in human dendritic cells infected with dengue virus. Galectin-9 (Gal-9) exerts immunosuppressive effects by inducing apoptosis in T cells that produce interferon-γ and interleukin (IL)-17. However, Gal-9 can be pro-inflammatory in lipopolysaccharide-stimulated monocytes. Using microarray analysis, we observed that Gal-9 was up-regulated in human dendritic cells (DCs) after dengue virus (DV) infection. The investigation into the immunomodulatory effects and mechanisms of Gal-9 in DCs exposed to DV revealed that DV infection specifically increased mRNA and protein levels of Gal-9 but not those of Gal-1 or Gal-3. Blocking p38, but not c-Jun N-terminal kinase or extracellular signal-regulated kinase (ERK), inhibited DV-induced expression of Gal-9. Reduction in Gal-9 by small interference RNA treatment suppressed DV-stimulated   migratio  n of DCs towards the chemoattractants CCL19 and CCL21. In addition, DV-induced IL-12p40 production was reduced after knockdown of Gal-9 in DCs. Furthermore, Gal-9 deficiency suppressed DV-induced activation of nuclear factor-κB. Inhibition of DV-induced D  C migrati  on under conditions of Gal-9 deficiency was mediated through suppressing ERK activation but not by regulating the expression of CCR7, the receptor for CCL19 and CCL21. Both the reduction in IL-12 production and the suppression of ERK activity might account for the inhibition of DV-induced D  C migrati  on after knockdown of Gal-9. In summary, this study reveals the roles of Gal-9 in DV-induce  d migrati  on of DCs. The findings indicate that Gal-9 might be a therapeutic target for preventing immunopathogenesis induced by DV infection. 
 Silibinin inhibits the invasion and   migration   of renal carcinoma 786-O cells in vitro, inhibits the growth of xenografts in vivo and enhances chemosensitivity to 5-fluorouracil and paclitaxel. Silibinin is a flavonoid antioxidant that is widely used for its anti-hepatotoxic properties. It exerts a dose-dependent inhibition on the invasion and   migration   of 786-O renal cell carcinoma (RCC) cells in the absence of cytotoxicity. 786-O cells were treated with silibinin at various concentrations, up to 50 µM, for a defined period and then subjected to gelatin zymography, casein zymography, and Western blot to investigate the impacts of silibinin on metalloproteinase (MMP) -2, -9, urokinase plasminogen activator (u-PA), and MAPK pathway signaling proteins, respectively. The results showed that silibinin decreased MMP-2, MMP-9, u-PA, p-p38, and p-Erk1/2 expressions in a concentration-dependent manner. The reduced expressions of MMP-2 and u-PA, as well as inhibition of cell invasion were obtained in the cultures pre-treated with PD98059 (Erk1/2 inhibitor) and SB203580 (p38 inhibitor). An in vivo anti-tumor study with a nude mice xenograft model by a subcutaneous inoculation of 786-O cells demonstrated small solid tumors after eight days following cell inoculation. There was a 70.1% reduction in tumor volume and 69.7% reduction in tumor weight by silibinin feeding on day 44, compared to those of controls. Moreover, combination treatment with silibinin and 5-fluorouracil, paclitaxel, vinblastine, or RAD-001 enhanced the chemosensitivity of 5-fluorouracil and paclitaxel. In conclusion, silibinin inhibits the invasion a  nd migrat  ion of 786-O cells in vitro, inhibits the growth of xenografts in vivo, and enhances chemosensitivity to 5-fluorouracil and paclitaxel. © 2011 Wiley-Liss, Inc. 
 p38 MAPK and ERK activation by 9-cis-retinoic acid induces chemokine receptors CCR1 and CCR2 expression in human monocytic THP-1 cells. 9-cis-Retinoic acid (9CRA) plays an important role in the immune response; this includes cytokine production and cell   migration  . We have previously demonstrated that 9CRA increases expression of chemokine receptors CCR1 and CCR2 in human monocytes. To better understand how 9CRA induces CCR1 and CCR2 expression, we examined the contribution of signaling proteins in human monocytic THP-1 cells. The mRNA and surface protein up-regulation of CCR1 and CCR2 in 9CRA-stimulated cells were weakly blocked by the pretreatment of SB202190, a p38 MAPK inhibitor, and PD98059, an upstream ERK inhibitor. Activation of p38 MAPK and ERK1/2 was induced in both a time and dose-dependent manner after 9CRA stimulation. Both p38 MAPK and ERK1/2 phosphorylation peaked at 2 h after a 100 nM 9CRA treatment. 9CRA increased calcium influx and chemotactic activity in response to CCR1-dependent chemokines, Lkn-1/CCL15, MIP-1alpha/CCL3, and RANTES/CCL5, and the CCR2-specific chemokine, MCP-1/CCL2. Both SB202190 and PD98059 pretreatment diminished the increased calcium mobilization and chemotactic ability due to 9CRA. SB202190 inhibited the expression and functional activities of CCR1 and CCR2 more effectively than did PD98059. Therefore, our results demonstrate that 9CRA transduces the signal through p38 MAPK and ERK1/2 for CCR1 and CCR2 up-regulation, and may regulate the pro-inflammatory process through the p38 MAPK and ERK-dependent signaling pathways. 
 Targeted inhibition of p38alpha MAPK suppresses tumor-associated endothelial cell   migration   in response to hypericin-based photodynamic therapy. Photodynamic therapy (PDT) is an established anticancer modality and hypericin is a promising photosensitizer for the treatment of bladder tumors. We show that exposure of bladder cancer cells to hypericin PDT leads to a rapid rise in the cytosolic calcium concentration which is followed by the generation of arachidonic acid by phospholipase A2 (PLA2). PLA2 inhibition significantly protects cells from the PDT-induced intrinsic apoptosis and attenuates the activation of p38 MAPK, a survival signal mediating the up-regulation of cyclooxygenase-2 that converts arachidonic acid into prostanoids. Importantly, inhibition of p38alpha MAPK blocks the release of vascular endothelial growth factor and suppresses tumor-promoted endothelial cell   migration  , a key step in angiogenesis. Hence, targeted inhibition of p38alpha MAPK could be therapeutically beneficial to PDT, since it would prevent COX-2 expression, the inducible release of growth and angiogenic factors by the cancer cells, and cause an increase in the levels of free arachidonic acid, which promotes apoptosis. 
 p38 Mitogen-activated protein kinase stabilizes mRNAs that contain cyclooxygenase-2 and tumor necrosis factor AU-rich elements by inhibiting deadenylation. AU-rich elements (AREs) in 3'-untranslated regions of mRNAs confer instability. They target mRNAs for rapid deadenylation and degradation and may enhance decapping. The p38 MAPK pathway stabilizes many otherwise unstable ARE-containing mRNAs encoding proteins involved in inflammation; however, the mRNA decay step(s) regulated by the signaling pathway are unknown. To investigate whether it regulates deadenylation or the decay of the mRNA body, we used a tetracycline-regulated beta-globin mRNA reporter system to transcribe pulses of mRNA of uniform length. We measured on Northern gels the   migration   of reporter mRNAs isolated from cells transfected only with reporter plasmid or co-transfected with an active mutant of MAPK kinase-6, and treated either with or without the p38 MAPK inhibitor SB 203580. Differences in   migration   were shown by RNase H mapping with oligo(dT) to be due to poly(A) shortening. Insertion of an ARE into the beta-globin reporter mRNA promoted rapid deadenylation and decay of hypo-adenylated reporter mRNA. p38 MAPK activation inhibited the deadenylation of reporter mRNAs containing either the cyclooxygenase-2 or tumor necrosis factor AREs. The regulation of deadenylation by p38 MAPK was found to be specific because deadenylation of the beta-globin reporter mRNA either lacking an ARE or containing the c-Myc 3'-untranslated region (which is not p38 MAPK-responsive) was unaffected by p38 MAPK. It was concluded that the p38 MAPK pathway predominantly regulates deadenylation, rather than decay of the mRNA body, and this provides an explanation for why p38 MAPK regulates mRNA stability in some situations and translation in others. 
  GeneRIFs  
 p38 serves an essential role in the growth and   migration   of non-small cell lung cancer cells. 
 IVIG inhibits TNF-alpha-induced MMP9 expression and activity in monocytes by suppressing NF-kappaB and P38 MAPK activation, preventing monocyte   migration  . 
 Findings indicate that CARMA3 may suppress the activation of the P38 MAPK signaling pathway to regulate invasion,   migration   and apoptosis of lung cancer cells by activating NF-small ka, CyrillicB (P65) in the nucleus. 
 Suggest that IL-17A enhances the   migration   of PDL fibroblasts by increasing MMP-1 expression through the IL-17 receptor, p38 MAPK, and NF-kappaB signal transduction pathways. 
 Depletion of miR-181 family members by miRNA inhibitors enhanced the expression of MKP-5 and suppressed the phosphorylation of p38 MAPK after exposure to PAHs, which promotes cancer cell   migration  . 
 miR-20a acts in a feedback loop to repress the expression of MKK3 and to negatively regulate the p38 pathway-mediated VEGF-induced endothelial cell   migration   and angiogenesis 
 EGF and HGF induced cell   migration   in human oral squamous carcinoma cell lines was dependent on the MEK ERK, the p38 and the PI-3 kinase pathways for both growth factors. 
 P38alpha plays a dual role in mast cell development by regulating IL-3-induced differentiation of mast cell progenitor cells as well as by regulating stem cell factor-induced   migration   of fully differentiated mast cells. 
 IL-8-Rac1/RhoA-p38MAPK signaling pathway plays a vital role in the IL-8-induced endothelial cell   migration  . 
 Lysophosphatidic acid activates p38 MAPK signaling in hepatocellular carcinoma promoting cell   migration  , invasion and adhesion. 
 Norepinephrine could stimulate pancreatic cancer cell proliferation,   migration   and invasion through a beta-adrenergic receptor-dependent activation of P38/MAPK pathway. 
 The MAPK p38 acted as a noncanonical G protein-coupled receptor kinase that phosphorylated the formyl peptide receptor FPR1 and facilitated neutrophil   migration   by blocking the G protein-coupled receptor kinase GRK2 function. 
 Acacetin inhibits the invasion and   migration   of human non-small cell lung cancer A549 cells by suppressing the p38alpha MAPK signaling pathway. 
 Apigenin might inhibit the invasion and   migration   abilities of Caski cells by reducing the MMP-9 expression through suppressing the p38 MAPK signaling pathway. 
 Carbonic anhydrase XII may affect the capability of invasion and   migration   of MDA-MB-231 cells, which may be mediated through the p38 MAPK pathway. 
 These findings indicate that p38 MAP kinase proteins regulate VE-cadherin junction disassembly, facilitating melanoma   migration   across endothelial cells. 
 Data revealed that DV infection enhances DC   migration   by inducing CCR7 expression, and that blocking COX-2 or MAPK activity suppresses DV-induced DC   migration  . 
 Collagen XVII participates in keratinocyte adhesion to collagen IV, and in p38MAPK-dependent   migration   and cell signaling. 
 siRNA to uPAR significantly inhibited cell proliferation and   migration   and stimulated apoptosis, to a greater extent than uPA siRNA. 
 crucial implication of PKC-delta, PKC-zeta, ERK-1/2, and p38 in human blood eosinophil   migration   through extracellular matrix components 
 These data demonstrate the role of Cx43 in the proliferation and   migration   of human saphenous vein smooth muscle cells and angiotensin II-induced Cx43 expression via mitogen-activated protein kinases (MAPK)-AP-1 signaling pathway. 
 The production of GRO-alpha, IL-6 and IL-8 was shown to account for the ability of the HeLa cell culture medium to stimulate the   migration   of monocytes/macrophages, suggesting a key role for p38 MAPK and ERK1/ERK2. 
 p38 MAPK is involved in IGFBP-6-induced IGF-independent rhabdomyosarcoma cell   migration   
 The results suggest that oncogenic K-Ras enhances the malignant phenotype and identify the mitogen-activated protein kinase p38 as a target to inhibit oncogenic K-Ras-induced pancreatic tumor cell   migration  . 
 Results show that activation of c-Jun N-terminal protein kinase, ERKs 1 and 2, and p38 MAP kinase is critical for Hs683 glioma cell   migration   induced by GDNF. 
 p38 and ERK1/2 have roles in coordinating cellular   migration   and proliferation in epithelial wound healing 
 MAP kinase kinase3- and 6-dependent activation of the alpha-isoform of p38 MAP kinase is required for the cytoskeletal changes induced by neutrophil adherence and influences subsequent neutrophil   migration   toward endothelial cell junctions 
 mast cell chymase activates ERK and p38 probably through G-protein-coupled receptor, and the ERK but not p38 cascade may have a crucial role in chymase-induced   migration   of eosinophils 
 Importantly, inhibition of p38alpha MAPK blocks the release of vascular endothelial growth factor and suppresses tumor-promoted endothelial cell   migration  , a key step in angiogenesis. 
 H-Ras-specific activation of Rac-MKK3/6-p38 pathway has a role in invasion and   migration   of breast epithelial cells 
  ECM  PubMed  
 TGF-β1, in association with the increased expression of connective tissue growth factor, induce the hypertrophy of the ligamentum flavum through the p38 MAPK pathway. Hypertrophy of the ligamentum flavum (LF) is one of the key pathomechanisms of lumbar spinal stenosis (LSS). Transforming growth factor (TGF)-β1 is abundantly expressed in hypertrophied degenerative LF tissues from LSS. However, the molecular mechanisms underling the association between TGF-β1 and LF hypertrophy have not yet been fully elucidated. In this study, we investigated the important role of the mitogen-activated protein kinase (MAPK) pathway in the pathogenesis of LSS by analyzing the expression of connective tissue growth factor (CTGF) and extracellular   mat  rix (ECM) components (collagen I and collagen III) in TGF-β1-treated LF cells. Cell growth assay revealed that TGF-β1, in association with CTGF, enhanced the the proliferation of LF cells, and we found that TGF-β1 also elevated CTGF expression and subsequently enhanced the mRNA expression of collagen I and collagen III. The increased mRNA expression levels of CTGF, collagen I and collagen III were abolished by p38 inhibitors. Both immunofluorescence imaging and western blot analysis of p38 and p-p38 revealed the increased expression and phosphorylation of p38. Silencing the expression of p38 by siRNA in LF cells decreased the protein expression of p38, p-p38 and CTGF, as well as the mRNA expression of CTGF, collagen I and collagen III. Taken together, our findings indicate that TGF-β1, in association with the increased expression of CTGF, contribute to t  he   homeostasis of the ECM and to the hypertrophy of LF through the p38 MAPK pathway. 
 Role of Smad2/3 and p38 MAP kinase in TGF-β1-induced epithelial-mesenchymal transition of pulmonary epithelial cells. Idiopathic pulmonary fibrosis is characterized by myofibroblast accumulation, extracellular matrix   (EC  M) remodeling, and excessive collagen deposition.   EC  M-producing myofibroblasts may originate from epithelial cells through epithelial to mesenchymal transition (EMT). TGF-β1 is an inducer of EMT in pulmonary epithelial cells in vitro and in vivo, though the mechanisms are unclear. We hypothesized that TGF-β1 induced EMT through Smad-dependent and -independent processes. To test this hypothesis, we studied the roles and mechanisms of TGF-β1-induced Smad and p38 mitogen-activated protein kinase (MAPK) signaling in EMT-related changes in pulmonary epithelial cells. Exposure of pulmonary epithelial 1HAEo(-) cells to TGF-β1 resulted in morphological and molecular changes of EMT over a 96-h period; loss of cell-cell contact, cell elongation, down-regulation of E-cadherin, up-regulation of fibronectin, and up-regulation of collagen I. Both Smad2/3 and p38 MAPK signaling pathways were activated by TGF-β1. However, neither Smad2/3 nor p38 MAPK were required for the down-regulation of E-cadherin, yet p38 MAPK was associated with fibronectin up-regulation. Both Smad2/3 and p38 MAPK had a role in regulation of TGF-β1-induced collagen expression. Furthermore, these data demonstrate that Smads and p38 MAPK differentially regulate EMT-related changes in pulmonary epithelial cells. 
 Sustained expression of NADPH oxidase 4 by p38 MAPK-Akt signaling potentiates radiation-induced differentiation of lung fibroblasts. Radiation-induced fibrosis (RIF) is a long-term adverse effect of curative radiotherapy; however, the distinct molecular mechanisms of RIF in neighboring normal tissue are not fully understood. We investigated the mechanisms underlying radiation-induced fibroblast differentiation into myofibroblasts. Lung fibroblasts produced reactive oxygen species (ROS) immediately after irradiation, the level of which remained increased for 24 h. The NADPH oxidase inhibitor, diphenyleneiodonium (DPI), suppressed ROS production and significantly decreased the radiation-induced expression of alpha-smooth muscle actin (alpha-SMA) and fibronectin (FN). The mRNA and protein expression of Nox4 was increased by radiation, and siRNA knockdown of Nox4 reduced alpha-SMA and FN levels. Increased phosphorylation of p38MAPK, Erk, and PI3k/Akt was observed after irradiation. Inhibitors of p38 MAPK and Akt, but not of Erk, reduced radiation-induced fibroblast differentiation and Nox4 expression. Notably, DPI partially decreased phosphorylation of p38MAPK and Akt, suggesting that p38MAPK, Akt, and Nox4 may cooperate in a positive feedback loop. Nox4 expression was also increased during bleomycin-induced fibroblast differentiation, and downregulation of Nox4 reduced alpha-SMA levels and extracellular matrix (  ECM  ) accumulation. These results demonstrate that interfering Nox4 activation can be a potential strategy to disrupt fibrotic process. 
 Cathepsins B, K, and L are regulated by a defined collagen type II peptide via activation of classical protein kinase C and p38 MAP kinase in articular chondrocytes. Degradation of the extracellular matrix (  ECM  ) is a prominent feature in osteoarthritis (OA), which is mainly because of the imbalance between anabolic and catabolic processes in chondrocytes resulting in cartilage and bone destruction. Various proteases act in concert to degrade matrix components, e.g. type II collagen, MMPs, ADAMTS, and cathepsins. Protease-generated collagen fragments may foster the destructive process. However, the signaling pathways associated with the action of collagen fragments on chondrocytes have not been clearly defined. The present data demonstrate that the N-terminal telopeptide of collagen type II enhances expression of cathepsins B, K, and L in articular chondrocytes at mRNA, protein, and activity levels, mediated at least in part through extracellular calcium. We also demonstrate that the induction is associated with the activation of protein kinase C and p38 MAP kinase. 
 Increased phosphorylation and activation of mitogen-activated protein kinase p38 in scleroderma fibroblasts. Transforming growth factor-beta (TGF-beta) stimulates the transcription of the alpha2(I) collagen gene. The dermal fibroblast activation in systemic sclerosis (SSc) may be a result of stimulation by autocrine TGF-beta. In this study, we investigated whether p38 mitogen-activated protein kinase (MAPK) is involved in TGF-beta-induced transcriptional activation of the human alpha2(I) collagen gene in normal dermal fibroblasts and in upregulated extracellular matrix (  ECM  ) expression in SSc fibroblasts. Type I collagen expression induced by TGF-beta was suppressed by the specific p38 MAPK inhibitors SB203580 or SB202190 in normal fibroblasts. TGF-beta induced phosphorylation and activation of p38 MAPK in normal dermal fibroblasts. Transient transfection of dominant-negative mutant p38 MAPK into normal fibroblasts abolished TGF-beta-induced promoter activity of the human alpha2(I) collagen gene in normal fibroblasts. Moreover, constitutive phosphorylation and activation of p38 MAPK was demonstrated in SSc fibroblasts, and the inhibition of p38 MAPK using specific p38 MAPK inhibitors or dominant-negative mutant p38 MAPK abolished the upregulated expression of type I collagen or fibronectin in SSc fibroblasts. These results strongly suggest the contribution of p38 MAPK signaling to the TGF-beta-mediated regulation of the human alpha2(I) collagen gene in normal dermal fibroblasts and constitutive upregulated expression of type I collagen and fibronectin in SSc fibroblasts. 
  [Ee]xtracellular [Mm]atrix  PubMed  
 Hypoxia activates beta(1)-integrin via ERK 1/2 and p38 MAP kinase in human vascular smooth muscle cells. Hypoxia plays an important role in vascular remodeling and directly affects vascular smooth muscle cell (VSMC) functions. VSMC adhesion participates in changes of vascular structure; however, little is known about VSMC adhesion under hypoxic conditions. It was the aim of the present study to investigate the effects of hypoxia on adhesion mechanisms in human VSMCs. Compared to normoxic cells, hypoxia (1% O(2), 24h) significantly increased adhesion of VSMCs to collagen I by 30.2% and fibronectin by 58.0%. This effect was completely inhibited in the presence of the pharmacological ERK 1/2 mitogen-activated protein kinase (MAPK) pathway inhibitor PD98059 (30 microM) or the p38 MAPK inhibitor SB203580 (1 microM). Basal adhesion of normoxic cells was not affected by pretreatment with PD98059 and SB203580. Hypoxia induced a time-dependent activation of ERK 1/2 and p38 MAPK activation in human VSMCs, which were completely abolished by PD98059 or SB203580, respectively. Since adhesion of VSMCs to fibronectin and collagen I involves beta(1)-integrin receptors, we used a blocking antibody against beta(1)-integrin (P5D2) to examine potential effects of hypoxia on beta(1)-integrins. P5D2 significantly reduced VSMC adhesion to fibronectin and collagen I in normoxia and hypoxia in a comparable manner; however, beta(1)-integrin protein or mRNA levels were not affected by hypoxia. As evidenced by flow cytometry, hypoxia induced a activation of beta(1)-integrins by exposing an conformationally sensitive epitope on the beta(1)-subunit. These results demonstrate that hypoxia enhances adhesion of VSMC on   extracellular matrix   proteins by activating beta(1)-integrin. 
 Hypoxia activates beta(1)-integrin via ERK 1/2 and p38 MAP kinase in human vascular smooth muscle cells. Hypoxia plays an important role in vascular remodeling and directly affects vascular smooth muscle cell (VSMC) functions. VSMC adhesion participates in changes of vascular structure; however, little is known about VSMC adhesion under hypoxic conditions. It was the aim of the present study to investigate the effects of hypoxia on adhesion mechanisms in human VSMCs. Compared to normoxic cells, hypoxia (1% O(2), 24h) significantly increased adhesion of VSMCs to collagen I by 30.2% and fibronectin by 58.0%. This effect was completely inhibited in the presence of the pharmacological ERK 1/2 mitogen-activated protein kinase (MAPK) pathway inhibitor PD98059 (30 microM) or the p38 MAPK inhibitor SB203580 (1 microM). Basal adhesion of normoxic cells was not affected by pretreatment with PD98059 and SB203580. Hypoxia induced a time-dependent activation of ERK 1/2 and p38 MAPK activation in human VSMCs, which were completely abolished by PD98059 or SB203580, respectively. Since adhesion of VSMCs to fibronectin and collagen I involves beta(1)-integrin receptors, we used a blocking antibody against beta(1)-integrin (P5D2) to examine potential effects of hypoxia on beta(1)-integrins. P5D2 significantly reduced VSMC adhesion to fibronectin and collagen I in normoxia and hypoxia in a comparable manner; however, beta(1)-integrin protein or mRNA levels were not affected by hypoxia. As evidenced by flow cytometry, hypoxia induced a activation of beta(1)-integrins by exposing an conformationally sensitive epitope on the beta(1)-subunit. These results demonstrate that hypoxia enhances adhesion of VSMC on   extracellular matrix   proteins by activating beta(1)-integrin. 
 TGF-β1, in association with the increased expression of connective tissue growth factor, induce the hypertrophy of the ligamentum flavum through the p38 MAPK pathway. Hypertrophy of the ligamentum flavum (LF) is one of the key pathomechanisms of lumbar spinal stenosis (LSS). Transforming growth factor (TGF)-β1 is abundantly expressed in hypertrophied degenerative LF tissues from LSS. However, the molecular mechanisms underling the association between TGF-β1 and LF hypertrophy have not yet been fully elucidated. In this study, we investigated the important role of the mitogen-activated protein kinase (MAPK) pathway in the pathogenesis of LSS by analyzing the expression of connective tissue growth factor (CT  GF) and extracellula  r matrix (ECM) components (collagen I and collagen III) in TGF-β1-treated LF cells. Cell growth assay revealed that TGF-β1, in association with CTGF, enhanced the the proliferation of LF cells, and we found that TGF-β1 also elevated CTGF expression and subsequently enhanced the mRNA expression of collagen I and collagen III. The increased mRNA expression levels of CTGF, collagen I and collagen III were abolished by p38 inhibitors. Both immunofluorescence imaging and western blot analysis of p38 and p-p38 revealed the increased expression and phosphorylation of p38. Silencing the expression of p38 by siRNA in LF cells decreased the protein expression of p38, p-p38 and CTGF, as well as the mRNA expression of CTGF, collagen I and collagen III. Taken together, our findings indicate that TGF-β1, in association with the increased expression of CTGF, contribute to the homeostasis of the ECM and to the hypertrophy of LF through the p38 MAPK pathway. 
 Role of Smad2/3 and p38 MAP kinase in TGF-β1-induced epithelial-mesenchymal transition of pulmonary epithelial cells. Idiopathic pulmonary fibrosis is characterized by myofibroblast accumulation,   extracellular matri  x (ECM) remodeling, and excessive collagen deposition. ECM-producing myofibroblasts may originate from epithelial cells through epithelial to mesenchymal transition (EMT). TGF-β1 is an inducer of EMT in pulmonary epithelial cells in vitro and in vivo, though the mechanisms are unclear. We hypothesized that TGF-β1 induced EMT through Smad-dependent and -independent processes. To test this hypothesis, we studied the roles and mechanisms of TGF-β1-induced Smad and p38 mitogen-activated protein kinase (MAPK) signaling in EMT-related changes in pulmonary epithelial cells. Exposure of pulmonary epithelial 1HAEo(-) cells to TGF-β1 resulted in morphological and molecular changes of EMT over a 96-h period; loss of cell-cell contact, cell elongation, down-regulation of E-cadherin, up-regulation of fibronectin, and up-regulation of collagen I. Both Smad2/3 and p38 MAPK signaling pathways were activated by TGF-β1. However, neither Smad2/3 nor p38 MAPK were required for the down-regulation of E-cadherin, yet p38 MAPK was associated with fibronectin up-regulation. Both Smad2/3 and p38 MAPK had a role in regulation of TGF-β1-induced collagen expression. Furthermore, these data demonstrate that Smads and p38 MAPK differentially regulate EMT-related changes in pulmonary epithelial cells. 
 Sustained expression of NADPH oxidase 4 by p38 MAPK-Akt signaling potentiates radiation-induced differentiation of lung fibroblasts. Radiation-induced fibrosis (RIF) is a long-term adverse effect of curative radiotherapy; however, the distinct molecular mechanisms of RIF in neighboring normal tissue are not fully understood. We investigated the mechanisms underlying radiation-induced fibroblast differentiation into myofibroblasts. Lung fibroblasts produced reactive oxygen species (ROS) immediately after irradiation, the level of which remained increased for 24 h. The NADPH oxidase inhibitor, diphenyleneiodonium (DPI), suppressed ROS production and significantly decreased the radiation-induced expression of alpha-smooth muscle actin (alpha-SMA) and fibronectin (FN). The mRNA and protein expression of Nox4 was increased by radiation, and siRNA knockdown of Nox4 reduced alpha-SMA and FN levels. Increased phosphorylation of p38MAPK, Erk, and PI3k/Akt was observed after irradiation. Inhibitors of p38 MAPK and Akt, but not of Erk, reduced radiation-induced fibroblast differentiation and Nox4 expression. Notably, DPI partially decreased phosphorylation of p38MAPK and Akt, suggesting that p38MAPK, Akt, and Nox4 may cooperate in a positive feedback loop. Nox4 expression was also increased during bleomycin-induced fibroblast differentiation, and downregulation of Nox4 reduced alpha-SMA levels and   extracellular matrix   (ECM) accumulation. These results demonstrate that interfering Nox4 activation can be a potential strategy to disrupt fibrotic process. 
 Hyaluronan oligosaccharides induce matrix metalloproteinase 13 via transcriptional activation of NFkappaB and p38 MAP kinase in articular chondrocytes. Hyaluronan exerts a variety of biological effects on cells including changes in cell migration, proliferation, and matrix metabolism. However, the signaling pathways associated with the action of hyaluronan on cells have not been clearly defined. In some cells, signaling is induced by the loss of cell-hyaluronan interactions. The goal of this study was to use hyaluronan oligosaccharides as a molecular tool to explore the effects of changes in cell-hyaluronan interactions and determine the underlying molecular events that become activated. In this study, hyaluronan oligosaccharides induced the loss of   extracellular matrix   proteoglycan and collagen from cultured slices of normal adult human articular cartilage. This loss was coincident with an increased expression of matrix metalloproteinase (MMP)-13. MMP-13 expression was also induced in articular chondrocytes by hyaluronan (HA) hexasaccharides but not by HA tetrasaccharides nor high molecular weight hyaluronan. MMP-13 promoter-reporter constructs in CD44-null COS-7 cells revealed that both CD44-dependent and CD44-independent events mediate the induction of MMP-13 by hyaluronan oligosaccharides. Electromobility gel shift assays demonstrated the activation of chondrocyte NFkappaB by hyaluronan oligosaccharides. NFkappaB activation was also documented in C-28/I2 immortalized human chondrocytes by luciferase promoter assays and phosphorylation of IKK-alpha/beta. The link between activation of NFkappaB and MMP-13 induction by HA oligosaccharides was further confirmed through the use of the NFkappaB inhibitor helenalin. Inhibition of MAP kinases also demonstrated the involvement of p38 MAP kinase in the hyaluronan oligosaccharide induction of MMP-13. Our findings suggest that hyaluronan-CD44 interactions affect matrix metabolism via activation of NFkappaB and p38 MAP kinase. 
 p38 MAPK mediates fibrogenic signal through Smad3 phosphorylation in rat myofibroblasts. Hepatic stellate cells (HSCs) spontaneously transdifferentiate into myofibroblast (MFB)-phenotype on plastic dishes. This response recapitulates the features of activation in vivo. Transforming growth factor beta (TGF-beta) plays a prominent role in stimulating liver fibrogenesis by MFBs. In quiescent HSCs, TGF-beta signaling involves TGF-beta type I receptor (TbetaRI)-mediated phosphorylation of serine residues within the conserved SSXS motif at the C-terminus of Smad2 and Smad3. The middle linker regions of Smad2 and Smad3 also are phosphorylated by mitogen-activated protein kinase (MAPK). This study elucidates the change of Smad3-mediated signals during the transdifferentiation process. By using antibodies highly specific to the phosphorylated C-terminal region and the phosphorylated linker region of Smad3, we found that TGF-beta-dependent Smad3 phosphorylation at the C-terminal region decreased, but that the phosphorylation at the linker region increased in the process of transdifferentiation. TGF-beta activated the p38 MAPK pathway, further leading to Smad3 phosphorylation at the linker region in the cultured MFBs, irrespective of Smad2. The phosphorylation promoted hetero-complex formation and nuclear translocation of Smad3 and Smad4. Once combined with TbetaRI-phosphorylated Smad2, the Smad3 and Smad4 complex bound to plasminogen activator inhibitor-type I promoter could enhance the transcription. In addition, Smad3 phosphorylation mediated by the activated TbetaRI was impaired severely in MFBs during chronic liver injury, whereas Smad3 phosphorylation at the linker region was remarkably induced by p38 MAPK pathway. In conclusion, p38 MAPK-dependent Smad3 phosphorylation promoted   extracellular matrix   production in MFBs both in vitro and in vivo. 
 Stromal cell-derived factor-1 (SDF-1) enhances cells invasion by αvβ6 integrin-mediated signaling in ovarian cancer. Ovarian carcinoma is a common gynecological malignancy and a great threat to health as a result of metastasis. The chemokine stromal-derived factor (SDF-1) plays multiple roles in tumor pathogenesis. However, the precise molecular mechanism underlying SDF-1-induced ovarian cancer cell invasion is still undefined. αvβ6 integrin is an important factor in tumor progression. Therefore, we speculate that SDF-1-enhanced ovarian cancer cell invasion is related to αvβ6 integrin-mediated signaling. After culturing with SDF-1, an obvious time- and dose-dependent increase in αvβ6 integrin was demonstrated. Furthermore, CXC receptor 4 (CXCR4) was responsible for SDF-1-induced αvβ6 integrin expression. Simultaneously, SDF-1 was found to dramaticall  y enhance extracellu  lar matrix degradation via urokinase-type plasminogen activator (uPA) expression and cell invasion by αvβ6 integrin expression; these reinforce failed to be increased when pretreatment was performed with the CXCR4 inhibitor AMD3100 or anti-αvβ6 integrin antibody, respectively. In addition, αvβ6 integrin induced the phosphorylation of p38 MAPK and PI3 K/Akt, contributing to the up-regulation of uPA, as treatment with the specific inhibitor for p38 mitogen-activated protein kinases (MAPK) (SB203580) or phosphatidylinositol 3-kinase (PI3 K)/Akt (LY294002) strikingly abrogated uPA expression. Taken together, these results demonstrated that SDF-1 enhanced ovarian cancer cell invasion through αvβ6 integrin-mediated uPA expression via the p38 MAPK and PI3 K/Akt pathway. Consequently, our findings will provide a new explanation about how SDF-1 aggravates the pathogenesis of ovarian cancer. 
 Cathepsins B, K, and L are regulated by a defined collagen type II peptide via activation of classical protein kinase C and p38 MAP kinase in articular chondrocytes. Degradation of the   extracellular matrix   (ECM) is a prominent feature in osteoarthritis (OA), which is mainly because of the imbalance between anabolic and catabolic processes in chondrocytes resulting in cartilage and bone destruction. Various proteases act in concert to degrade matrix components, e.g. type II collagen, MMPs, ADAMTS, and cathepsins. Protease-generated collagen fragments may foster the destructive process. However, the signaling pathways associated with the action of collagen fragments on chondrocytes have not been clearly defined. The present data demonstrate that the N-terminal telopeptide of collagen type II enhances expression of cathepsins B, K, and L in articular chondrocytes at mRNA, protein, and activity levels, mediated at least in part through extracellular calcium. We also demonstrate that the induction is associated with the activation of protein kinase C and p38 MAP kinase. 
 Crucial implication of protein kinase C (PKC)-delta, PKC-zeta, ERK-1/2, and p38 MAPK in migration of human asthmatic eosinophils. Asthma is associated with an eosinophil infiltration into the bronchial mucosa. 5-Oxo-6,8,11,14(E,Z,Z,Z)-eicosatetraenoic acid (5-oxo-ETE), a potent eosinophil chemotactic factor, activates cell motility, adherence, and proteolysis, notably, by promoting CD11b expression, matrix metalloproteinase (MMP)-9 secretion, and plasmin generation. We investigated the intracellular signaling pathways implicated in these various steps by using different, selective inhibitors. Human eosinophil migration through a reconstituted basement membrane in response to 5-oxo-ETE was greatly inhibited (>or=72%) by the protein kinase C (PKC)-delta, PKC-zeta, ERK-1/2, and p38 inhibitors. Our findings indicate that PKC-delta mediates cell motility, CD11b expression, and MMP-9 granule release. PKC-zeta is also largely involved in eosinophil migration, although its specific targets remain undefined. ERK-1/2 and p38 modulate CD11b expression; ERK-1/2 is also involved in long-term MMP-9 secretion and p38 in the plasmin activation system. We demonstrated the crucial implication of PKC-delta, PKC-zeta, ERK-1/2, and p38 in human blood eosinophil migration through   extracellular matrix   components. Targeting specific pathways may have therapeutic potential for the treatment of allergic airway inflammation. 
 Fluid shear stress regulates metalloproteinase-1 and 2 in human periodontal ligament cells: involvement of extracellular signal-regulated kinase (ERK) and P38 signaling pathways. Matrix metalloproteinase (MMP)-1, 2, with their endogenous inhibitors, tissue inhibitor of metalloproteinase (TIMP)-1, 2 are critical for   extracellular matrix   remodeling in human periodontal ligament (PDL) and their expression are sensitive to mechanical stresses. Shear stress as the main type of mechanical stress in tooth movement is involved in matrix turnover. However, how shear stress regulates MMPs and TIMPs system is still unclear. In this study, we investigated the effect of fluid shear stress on expression of MMP-1, 2 and TIMP-1, 2 in human PDL cells and the possible roles of mitogen-activated protein kinases in this process. Three levels of fluid shear stresses (6, 9 and 12 dyn/cm(2)) were loaded on PDL cells for 2, 4, 8 and 12h. The results indicated that fluid shear stress rearranged cytoskeleton in PDL cells. Fluid shear stress increased expression of MMP-1, 2, TIMP-1 and suppressed TIMP-2 expression. MAP kinases including extracellular signal-regulated kinase (ERK), c-Jun N-terminal kinase (JNK) and p38 were activated rapidly by fluid shear stress. The ERK inhibitor blocked fluid shear stress induced MMP-1 expression and P38 inhibitor reduced fluid shear stress stimulated MMP-2 expression. Our study suggested that fluid shear stress involved in PDL remodeling via regulating MMP-1, 2 and TIMP-1, 2 expression. ERK regulated fluid shear stress induced MMP-1 expression and P38 play a role in fluid shear stress induced MMP-2 upregulation. 
 Microarray analysis of Dupuytren's disease cells: the profibrogenic role of the TGF-β inducible p38 MAPK pathway. Dupuytren's disease (DD) is a nodular palmar fibromatosis that causes irreversible permanent contracture of fingers and results in the loss of hand function. Surgery still remains the only available solution for DD patients but cannot permanently cure the disease nor reduce high recurrence rates. With this rationale, we designed a study aimed at an improved understanding of the molecular mechanisms underlying DD. Our major focus was an analysis of the global gene expression profile and signalling pathways in DD cells with the aim of identifying novel biomarkers and/or therapeutic targets.Primary cells were cultured from surgically removed diseased and healthy tissue. Microarray expression analysis (HG-U133A array, Affymetrix) and qPCR was performed with total RNA isolated from primary DD cells. Mechanistic studies involving inhibition of p38 phosphorylation were performed on normal human fibroblasts' and primary DD cells' in vitro models. Expression of stem cell markers in primary fibroblasts/myofibroblasts was assessed as well.We identified 3 p38MAPK signalling pathway regulatory genes, THBS1, GADD45α and NUAK1, all involved in cellular proliferation and production of th  e extracellular matr  ix proteins. Inhibition of the p38MAPK signalling pathway induced down-regulation of myofibroblast markers, α-smooth muscle actin and palladin. A stem-cell like subpopulation positive for CD90 marker was identified among primary DD cells.The study reveals involvement of the p38 MAPK pathway as a possible signalling cascade in the pathogenesis of Dupuytren's disease. Moreover, a particular stem cell-like CD90(+) subpopulation was identified that might contribute to DD development. 
 p38 mitogen-activated protein kinase-dependent activation of protein phosphatases 1 and 2A inhibits MEK1 and MEK2 activity and collagenase 1 (MMP-1) gene expression. Degradation of collagenous   extracellular matrix   by collagenase 1 (also known as matrix metalloproteinase 1 [MMP-1]) plays a role in the pathogenesis of various destructive disorders, such as rheumatoid arthritis, chronic ulcers, and tumor invasion and metastasis. Here, we have investigated the role of distinct mitogen-activated protein kinase (MAPK) pathways in the regulation of MMP-1 gene expression. The activation of the extracellular signal-regulated kinase 1 (ERK1)/ERK2 (designated ERK1,2) pathway by oncogenic Ras, constitutively active Raf-1, or phorbol ester resulted in potent stimulation of MMP-1 promoter activity and mRNA expression. In contrast, activation of stress-activated c-Jun N-terminal kinase and p38 pathways by expression of constitutively active mutants of Rac, transforming growth factor beta-activated kinase 1 (TAK1), MAPK kinase 3 (MKK3), or MKK6 or by treatment with arsenite or anisomycin did not alone markedly enhance MMP-1 promoter activity. Constitutively active MKK6 augmented Raf-1-mediated activation of the MMP-1 promoter, whereas active mutants of TAK1 and MKK3b potently inhibited the stimulatory effect of Raf-1. Activation of p38 MAPK by arsenite also potently abrogated stimulation of MMP-1 gene expression by constitutively active Ras and Raf-1 and by phorbol ester. Specific activation of p38alpha by adenovirus-delivered constitutively active MKK3b resulted in potent inhibition of the activity of ERK1,2 and its upstream activator MEK1,2. Furthermore, arsenite prevented phorbol ester-induced phosphorylation of ERK1,2 kinase-MEK1,2, and this effect was dependent on p38-mediated activation of protein phosphatase 1 (PP1) and PP2A. These results provide evidence that activation of signaling cascade MKK3-MKK3b-->p38alpha blocks the ERK1,2 pathway at the level of MEK1,2 via PP1-PP2A and inhibits the activation of MMP-1 gene expression. 
 Transforming growth factor beta1 induces alphavbeta3 integrin expression in human lung fibroblasts via a beta3 integrin-, c-Src-, and p38 MAPK-dependent pathway. In response to transforming growth factor beta1 (TGFbeta) stimulation, fibroblasts modify their integrin repertoire and adhesive capabilities to certain   extracellular matrix   proteins. Although TGFbeta has been shown to increase the expression of specific alphav integrins, the mechanisms underlying this are unknown. In this study we demonstrate that TGFbeta1 increased both beta3 integrin subunit mRNA and protein levels as well as surface expression of alphavbeta3 in human lung fibroblasts. TGFbeta1-induced alphavbeta3 expression was strongly adhesion-dependent and associated with increased focal adhesion kinase and c-Src kinase phosphorylation. Inhibition of beta3 integrin activation by the Arg-Gly-Asp tripeptide motif-specific disintegrin echistatin or alphavbeta3 blocking antibody prevented the increase in beta3 but not beta5 integrin expression. In addition, echistatin inhibited TGFbeta1-induced p38 MAPK but not Smad3 activation. Furthermore, inhibition of the Src family kinases, but not focal adhesion kinase, completely abrogated TGFbeta1-induced expression of alphavbeta3 and p38 MAPK phosphorylation but not beta5 integrin expression and Smad3 activation. The TGFbeta1-induced alphavbeta3 expression was blocked by pharmacologic and genetic inhibition of p38 MAPK- but not Smad2/3-, Sp1-, ERK-, phosphatidylinositol 3-kinase, and NF-kappaB-dependent pathways. Our results demonstrate that TGFbeta1 induces alphavbeta3 integrin expression via a beta3 integrin-, c-Src-, and p38 MAPK-dependent pathway. These data identify a novel mechanism for TGFbeta1 signaling in human lung fibroblasts by which they may contribute to normal and pathological wound healing. 
 Transcriptional profiling defines the roles of ERK and p38 kinases in epidermal keratinocytes. Epidermal keratinocytes respond to extracellular influences by activating cytoplasmic signal transduction pathways that change gene expression. Using pathway-specific transcriptional profiling, we identified the genes regulated by two such pathways, p38 and ERK. These pathways are at the fulcrum of epidermal differentiation, proliferative and inflammatory skin diseases. We used SB203580 and PD98059 as specific inhibitors and Affymetrix Hu133Av2 microarrays, to identify the genes regulated after 1, 4, 24, and 48 h and compared them to genes regulated by JNK. Unexpectedly, inhibition of MAPK pathways is compensated by activation of the NFkappaB pathway and suppression of the DUSP enzymes. Both pathways promote epidermal differentiation; however, there is a surprising disconnect between the expression of steroid synthesis enzymes and differentiation markers. The p38 pathway induces the expression of   extracellular matrix   and proliferation-associated genes, while suppressing microtubule-associated genes. The ERK pathway induces nuclear envelope and mRNA splicing proteins, while suppressing steroid synthesis and mitochondrial energy production enzymes. Transcription factors SRY, c-FOS, and N-Myc are the principal targets of the p38 pathway, Elk-1 SAP1 and HLH2 of ERK, while FREAC-4, ARNT and USF are shared. The results suggest a list of targets potentially useful in therapeutic interventions in cutaneous diseases and wound healing. 
 Aberrant integrin activation induces p38 MAPK phosphorylation resulting in suppressed Fas-mediated apoptosis in T cells: implications for rheumatoid arthritis. Delayed Fas-mediated apoptosis in T cells is associated with inflammatory diseases including rheumatoid arthritis (RA). CD3(+) T cells in RA synovia expressed high amounts of phospho-p38 MAPK. Exposure to RA synovial fluid or soluble collagen, a degradation product of   extracellular matrix   abundant in RA synovium, induced the phosphorylation of p38 MAPK in Jurkat T cells accompanied by resistance against Fas-mediated apoptosis. Blocking beta1 integrin by antibody diminished this effect. In addition, ectopic expression of auto-activated beta1 integrin variant in T cells profoundly induced the phosphorylation of p38 MAPK. Suppression of p38 MAPK sensitized T cells to Fas-mediated apoptosis and increased caspase-8 and caspase-3 cleavage. A physical interaction of p38 MAPK and caspase-8 was demonstrated by using confocal microscopic imaging and co-immunoprecipitation assay. RA synovial fluid markedly increased the formation of phospho-p38 MAPK/caspase-8 complex in Jurkat T cells. In conclusion, abnormal activation of p38 MAPK to prevent Fas-mediated apoptosis may represent a common survival mechanism of RA synovial T cells contributing to the persistent inflammation of affected synovium. 
 Tenascin-W is found in malignant mammary tumors, promotes alpha8 integrin-dependent motility and requires p38MAPK activity for BMP-2 and TNF-alpha induced expression in vitro. Tenascins represent a family of   extracellular matrix   glycoproteins with distinctive expression patterns. Here we have analyzed the most recently described member, tenascin-W, in breast cancer. Mammary tumors isolated from transgenic mice expressing hormone-induced oncogenes reveal tenascin-W in the stroma around lesions with a high likelihood of metastasis. The presence of tenascin-W was correlated with the expression of its putative receptor, alpha8 integrin. HC11 cells derived from normal mammary epithelium do not express alpha8 integrin and fail to cross tenascin-W-coated filters. However, 4T1 mammary carcinoma cells do express alpha8 integrin and their migration is stimulated by tenascin-W. The expression of tenascin-W is induced by BMP-2 but not by TGF-beta1, though the latter is a potent inducer of tenascin-C. The expression of tenascin-W is dependent on p38MAPK and JNK signaling pathways. Since preinflammatory cytokines also act through p38MAPK and JNK signaling pathways, the possible role of TNF-alpha in tenascin-W expression was also examined. TNF-alpha induced the expression of both tenascin-W and tenascin-C, and this induction was p38MAPK- and cyclooxygenase-dependent. Our results show that tenascin-W may be a useful diagnostic marker for breast malignancies, and that the induction of tenascin-W in the tumor stroma may contribute to the invasive behavior of tumor cells. 
 Arachidonic acid stimulates cell adhesion through a novel p38 MAPK-RhoA signaling pathway that involves heat shock protein 27. Rho GTPases are critical components of cellular signal transduction pathways. Both hyperactivity and overexpression of these proteins have been observed in human cancers and have been implicated as important factors in metastasis. We previously showed that dietary n-6 fatty acids increase cancer cell adhesion to   extracellular matrix   proteins, such as type IV collagen. Here we report that in MDA-MB-435 human melanoma cells, arachidonic acid activates RhoA, and inhibition of RhoA signaling with either C3 exoenzyme or dominant negative Rho blocked arachidonic acid-induced cell adhesion. Inhibition of the Rho kinase (ROCK) with either small molecule inhibitors or ROCK II-specific small interfering RNA (siRNA) blocked the fatty acid-induced adhesion. However, unlike other systems, inhibition of ROCK did not block the activation of p38 mitogen-activated protein kinase (MAPK); instead, Rho activation depended on p38 MAPK activity and the presence of heat shock protein 27 (HSP27), which is phosphorylated downstream of p38 after arachidonic acid treatment. HSP27 associated with p115RhoGEF in fatty acid-treated cells, and this association was blocked when p38 was inhibited. Furthermore, siRNA knockdown of HSP27 blocked the fatty acid-stimulated Rho activity. Expression of dominant negative p115-RhoGEF or p115RhoGEF-specific siRNA inhibited both RhoA activation and adhesion on type IV collagen, whereas a constitutively active p115RhoGEF restored the arachidonic acid stimulation in cells in which the p38 MAPK had been inhibited. These data suggest that n-6 dietary fatty acids stimulate a set of interactions that regulates cell adhesion through RhoA and ROCK II via a p38 MAPK-dependent association of HSP27 and p115RhoGEF. 
 Increased phosphorylation and activation of mitogen-activated protein kinase p38 in scleroderma fibroblasts. Transforming growth factor-beta (TGF-beta) stimulates the transcription of the alpha2(I) collagen gene. The dermal fibroblast activation in systemic sclerosis (SSc) may be a result of stimulation by autocrine TGF-beta. In this study, we investigated whether p38 mitogen-activated protein kinase (MAPK) is involved in TGF-beta-induced transcriptional activation of the human alpha2(I) collagen gene in normal dermal fibroblasts and in upregulated   extracellular matrix   (ECM) expression in SSc fibroblasts. Type I collagen expression induced by TGF-beta was suppressed by the specific p38 MAPK inhibitors SB203580 or SB202190 in normal fibroblasts. TGF-beta induced phosphorylation and activation of p38 MAPK in normal dermal fibroblasts. Transient transfection of dominant-negative mutant p38 MAPK into normal fibroblasts abolished TGF-beta-induced promoter activity of the human alpha2(I) collagen gene in normal fibroblasts. Moreover, constitutive phosphorylation and activation of p38 MAPK was demonstrated in SSc fibroblasts, and the inhibition of p38 MAPK using specific p38 MAPK inhibitors or dominant-negative mutant p38 MAPK abolished the upregulated expression of type I collagen or fibronectin in SSc fibroblasts. These results strongly suggest the contribution of p38 MAPK signaling to the TGF-beta-mediated regulation of the human alpha2(I) collagen gene in normal dermal fibroblasts and constitutive upregulated expression of type I collagen and fibronectin in SSc fibroblasts. 
 Iron deficiency activates pro-inflammatory signaling in macrophages and foam cells via the p38 MAPK-NF-κB pathway. Major bleeding in patients with acute coronary syndrome (ACS) increases the risk of recurrent ACS and mortality. However, the mechanism involved is poorly understood. Bleeding induces iron deficiency. Iron deficiency enhances inflammation in other diseases. Thus, in this paper, the particular effect of iron deficiency on atherosclerotic plaque destabilization, especially the pro-inflammatory role of iron deficiency in atheroma and the mechanism involved were investigated  .Extracellular matri  x metalloproteinase inducer (EMMPRIN) and matrix metalloproteinase-9 (MMP-9) mRNA levels were investigated by RT-PCR. EMMPRIN and MMP-9 protein levels, nuclear factor (NF)-κB-p65 protein levels, peroxisome proliferator-activated receptor γ (PPARγ) protein levels, and mitogen-activated protein kinase (MAPK) phosphorylation were determined by western blotting. MMP-9 enzymatic activity was assayed by gelatin zymography.Iron deficiency enhanced EMMPRIN, MMP-9 production, and MMP-9 enzymatic activity in THP-1 derived macrophages and foam cells. Iron deficiency elicited the activation of NF-κB and p38 MAPK. By using the p38 inhibitor and NF-κB inhibitor, the study established that EMMPRIN and MMP-9 inductions by iron deficiency required the consecutive upstream activation of p38 MAPK and NF-κB. This pro-inflammatory action was not prevented by PPARγ agonist. Meanwhile, iron deficiency did not modulate PPARγ expression. Retinoid X receptor agonist suppressed the effects of iron deficiency on EMMPRIN , MMP-9, and NF-κB, but not on MAPK activation.Iron deficiency enhances atheroma inflammation through p38 MAPK-NF-κB-EMMPRIN/MMP-9 pathway. Our findings provide a potential mechanism for the association of major bleeding with recurrent ACS and mortality in patients with ACS. 
 Phosphorylation of c-Fos by members of the p38 MAPK family. Role in the AP-1 response to UV light. Exposure to sources of UV radiation, such as sunlight, induces a number of cellular alterations that are highly dependent on its ability to affect gene expression. Among them, the rapid activation of genes coding for two subfamilies of proto-oncoproteins, Fos and Jun, which constitute the AP-1 transcription factor, plays a key role in the subsequent regulation of expression of genes involved in DNA repair, cell proliferation, cell cycle arrest, death by apoptosis, and tissue and   extracellular matrix   remodeling proteases. Besides being regulated at the transcriptional level, Jun and Fos transcriptional activities are also regulated by phosphorylation as a result of the activation of intracellular signaling cascades. In this regard, the phosphorylation of c-Jun by UV-induced JNK has been readily documented, whereas a role for Fos proteins in UV-mediated responses and the identification of Fos-activating kinases has remained elusive. Here we identify p38 MAPKs as proteins that can associate with c-Fos and phosphorylate its transactivation domain both in vitro and in vivo. This phosphorylation is transduced into changes in its transcriptional ability as p38-activated c-Fos enhances AP1-driven gene expression. Our findings indicate that as a consequence of the activation of stress pathways induced by UV light, endogenous c-Fos becomes a substrate of p38 MAPKs and, for the first time, provide evidence that support a critical role for p38 MAPKs in mediating stress-induced c-Fos phosphorylation and gene transcription activation. Using a specific pharmacological inhibitor for p38alpha and -beta, we found that most likely these two isoforms mediate UV-induced c-Fos phosphorylation in vivo. Thus, these newly described pathways act concomitantly with the activation of c-Jun by JNK/MAPKs, thereby contributing to the complexity of AP1-driven gene transcription regulation. 
  GeneRIFs  
 TGF-beta1, in association with the increased expression of CTGF, contribute to the homeostasis of the   extracellular matrix   and to the hypertrophy of ligamentum flavum through the p38 MAPK pathway. 
 crucial implication of PKC-delta, PKC-zeta, ERK-1/2, and p38 in human blood eosinophil migration through   extracellular matrix   components 
 The p38 pathway induces the expression of   extracellular matrix   and proliferation-associated genes, while suppressing microtubule-associated genes. 
  [Ii]nvasive  PubMed  
 The secreted protein ANGPTL2 promotes metastasis of osteosarcoma cells through integrin α5β1, p38 MAPK, and matrix metalloproteinases. The tumor microenvironment can enhance th  e invasi  ve capacity of tumor cells. We showed that expression of angiopoietin-like protein 2 (ANGPTL2) in osteosarcoma (OS) cell lines increased and the methylation of its promoter decreased with time when grown as xenografts in mice compared with culture. Compared with cells grown in normal culture conditions, the expression of genes encoding DNA demethylation-related enzymes increased in tumor cells implanted into mice or grown in hypoxic, serum-starved culture conditions. ANGPTL2 expression in OS cell lines correlated with increased tumor metastasis and decreased animal survival by promoting tumor cell intravasation mediated by the integrin α5β1, p38 mitogen-activated protein kinase, and matrix metalloproteinases. The tolloid-like 1 (TLL1) protease cleaved ANGPTL2 into fragments in vitro that did not enhance tumor progression when overexpressed in xenografts. Expression of TLL1 was weak in OS patient tumors, suggesting that ANGPTL2 may not be efficiently cleaved upon secretion from OS cells. These findings demonstrate that preventing ANGPTL2 signaling stimulated by the tumor microenvironment could inhibit tumor cell migration and metastasis. 
 The secreted protein ANGPTL2 promotes metastasis of osteosarcoma cells through integrin α5β1, p38 MAPK, and matrix metalloproteinases. The tumor microenvironment can enhance th  e invasi  ve capacity of tumor cells. We showed that expression of angiopoietin-like protein 2 (ANGPTL2) in osteosarcoma (OS) cell lines increased and the methylation of its promoter decreased with time when grown as xenografts in mice compared with culture. Compared with cells grown in normal culture conditions, the expression of genes encoding DNA demethylation-related enzymes increased in tumor cells implanted into mice or grown in hypoxic, serum-starved culture conditions. ANGPTL2 expression in OS cell lines correlated with increased tumor metastasis and decreased animal survival by promoting tumor cell intravasation mediated by the integrin α5β1, p38 mitogen-activated protein kinase, and matrix metalloproteinases. The tolloid-like 1 (TLL1) protease cleaved ANGPTL2 into fragments in vitro that did not enhance tumor progression when overexpressed in xenografts. Expression of TLL1 was weak in OS patient tumors, suggesting that ANGPTL2 may not be efficiently cleaved upon secretion from OS cells. These findings demonstrate that preventing ANGPTL2 signaling stimulated by the tumor microenvironment could inhibit tumor cell migration and metastasis. 
 Interaction of Neisseria meningitidis with human brain microvascular endothelial cells: role of MAP- and tyrosine kinases in invasion and inflammatory cytokine release. Neisseria meningitidis traversal across the blood-cerebrospinal fluid barrier is an essential step in the pathogenesis of bacterial meningitis. We have previously shown that invasion of human brain microvascular endothelial cells (HBMEC) by meningococci is mediated by bacterial outer membrane protein Opc that binds fibronectin, thereby anchoring the bacterium to the integrin alpha 5 beta 1-receptor on the endothelial cell surface. However, subsequent signal transduction mechanisms essential for or regulated by N. meningitidis adhesion and invasion, or HBMEC responses to N. meningitidis are unknown. In this report we investigated the role of c-Jun N-terminal kinases 1 and 2 (JNK1 and JNK2), p38 mitogen-activated (MAP) kinase and protein tyrosine kinases in endothelial-N. meningitidis interaction. Binding of meningococci to HBMEC phosphorylated and activated JNK1 and JNK2 and p38 MAPK as well as their direct substrates c-Jun and MAP kinase activated kinase-2 (MAPKAPK-2), respectively. Non-  invasive   meningococcal strains lacking opc gene (opc mutants and sequence type 11 complex meningococci) still activated p38 MAPK, however, failed to activate JNK. Inhibition of JNK1 and JNK2 significantly reduced internalization of N. meningitidis by HBMEC without affecting its adherence. Blocking the endothelial integrin alpha 5 beta 1 also decreased N. meningitidis-induced JNK activation in HBMEC. These findings indicate the crucial role of JNK signalling pathway in N. meningitidis invasion in HBMEC. In contrast, p38 MAPK pathway was important for the control of interleukin-6 (IL-6) and IL-8 release by HBMEC. Genistein, a protein tyrosine kinase inhibitor, decreased both invasion of N. meningitidis into HBMEC and IL-6 and IL-8 release, indicating that protein tyrosine kinases, which link signals from integrins to intracellular signalling pathways are essential for both bacterial internalization and cytokine secretion by HBMEC. 
 Protein kinase A gating of a pseudopodial-located RhoA/ROCK/p38/NHE1 signal module regulates invasion in breast cancer cell lines. Metastasis results from a sequence of selective events often involving interactions with elements of the tumor-specific physiological microenvironment. The low-serum component of this microenvironment confers increased motility and invasion in breast cancer cells by activating the Na+/H+ exchanger isoform 1 (NHE1). The present study was undertaken to characterize the signal transduction mechanisms underlying this serum deprivation-dependent activation of both the NHE1 and the concomitant   invasive   characteristics such as leading edge pseudopodia development and penetration of matrigel in breast cancer cell lines representing different stages of metastatic progression. Using pharmacological and genetic manipulation together with transport and kinase activity assays, we observe that the activation of the NHE1 and subsequent invasion by serum deprivation in metastatic human breast cells is coordinated by a sequential RhoA/p160ROCK/p38MAPK signaling pathway gated by direct protein kinase A phosphorylation and inhibition of RhoA. Fluorescence resonance energy transfer imaging of RhoA activity and immunofluorescence analysis of phospho-RhoA and NHE1 show that serum deprivation dynamically remodels the cell, forming long, leading edge pseudopodia and that this signal module is preferentially compartmentalized in these leading edge pseudopodia, suggesting a tight topographic relation of the signaling module to an invasion-specific cell structure. 
 COX-2 promotes breast cancer cell radioresistance via p38/MAPK-mediated cellular anti-apoptosis and   invasive  ness. Radioresistance is one of the major barriers to improve the survival rate of breast cancer patients. Cyclooxygenase 2 (COX-2) is usually overexpressed in highly   invasive   and metastatic breast cancer, which may indicate an association with breast cancer radioresistance. The function role of COX-2 was investigated by using a radioresistant breast cancer cell line MDA-MB-231/RR10 and its parental cell line MDA-MB-231 cells before or after COX-2 was silenced by a specific small hairpin RNA (shRNA). The cell proliferation, migration, invasion, colony formation, and apoptosis were measured by CCK-8, scratch-wound, transwell, clone formation assay, and flow cytometry. Protein and mRNA expression were analyzed by Western blot and quantitative reverse transcriptase-polymerase chain reaction. COX-2 is upregulated in MDA-MB-231/RR10 cells compared with in MDA-MB-231 cells, and silencing of COX-2 expression by shRNA in MDA-MB-231/RR10 cells decreases the expression of Bcl-2 and Bcl-XL, but increases the proapoptotic protein BAK, leading to the increased apoptosis following treatment with γ-irradiation in comparison with those in control cells. Silencing of COX-2 also increases the expression of β-catenin and E-cadherin, two anti-invasion proteins, resulting in reduced cell migration and invasion tested by transwell chambers and wound-healing assays. Further study demonstrated that COX-2-induced radioresistance is negatively regulated through the phosphorylation of p38 at Tyr182, and that the phosphorylation of p38 induced by TNF-alpha reduces the expression of Bcl-2, BCL-XL, but increases β-catenin and E-cadherin, leading to the decreas  ed invas  iveness of cells. Our data suggest that COX-2, p38, Bcl-2, Bcl-XL, β-catenin, and E-cadherin may be considered as potential therapeutic targets against radioresistant breast cancer. 
 TAB1beta (transforming growth factor-beta-activated protein kinase 1-binding protein 1beta ), a novel splicing variant of TAB1 that interacts with p38alpha but not TAK1. The mitogen-activated protein kinases (MAPKs) play an important role in a variety of biological processes. Activation of MAPKs is mediated by phosphorylation on specific regulatory tyrosine and threonine sites. We have recently found that activation of p38alpha MAPK can be carried out not only by its upstream MAPK kinases (MKKs) but also by p38alpha autophosphorylation. p38alpha autoactivation requires an interaction of p38alpha with TAB1 (transforming growth factor-beta-activated protein kinase 1-binding protein 1). The autoactivation mechanism of p38alpha has been found to be important in cellular responses to a number of physiologically relevant stimuli. Here, we report the characterization of a splicing variant of TAB1, TAB1beta. TAB1 and TAB1beta share the first 10 exons. The 11th and 12th exons of TAB1 were spliced out in TAB1beta, and an extra exon, termed exon beta, downstream of exons 11 and 12 in the genome was used as the last exon in TAB1beta. The mRNA of TAB1beta was expressed in all cell lines examined. The TAB1beta mRNA encodes a protein with an identical sequence to TAB1 except the C-terminal 69 amino acids were replaced with an unrelated 27-amino acid sequence. Similar to TAB1, TAB1beta interacts with p38alpha but not other MAPKs and stimulates p38alpha autoactivation. Different from TAB1, TAB1beta does not bind or activate TAK1. Inhibition of TAB1beta expression with RNA interference in MDA231 breast cancer cells resulted in the reduction of basal activity of p38alpha and   invasive  ness of MDA231 cells, suggesting that TauAlphaBeta1beta is involved in regulating p38alpha activity in physiological conditions. 
 TLR4 ligand/H₂O₂ enhances TGF-β1 signaling to induce metastatic potential of   non-inv  asive breast cancer cells by activating non-Smad pathways. TGF-β1 has the potential to activate multiple signaling pathways required for inducing metastatic potential of tumor cells. However, TGF-β1 was inefficient in inducing metastatic potential of ma  ny non-i  nvasive human tumor cells. Here we report that the enhancement of TGF-β1 signaling is required for inducing metastatic potential   of non-  invasive breast cancer cells. TGF-β1 alone could not efficiently induce the sustained activation of Smad and non-Smad pathway  s in non  -invasive breast cancer cells. TLR4 ligand (LPS) and H₂O₂ cooperated with TGF-β1 to enhance the sustained activation of non-Smad pathways, including p38MAPK, ERK, JNK, PI3K, and NF-κB. The activation of MAPK and PI3K pathways resulted in a positive feed-back effect on TGF-β1 signaling by down-regulating Nm23-H1 expression and up-regulating the expression of TβRI and TβRII, favoring further activation of multiple signaling pathways. Moreover, the enhanced TGF-β1 signaling induced higher expression of SNAI2, which also promoted TβRII expression. Therefore, the sustained activation levels of both Smad and non-Smad pathways were gradually increased after prolonged stimulation with TGF-β1/H₂O₂/LPS. Consistent with the activation pattern of   signali  ng pathways, the invasive capacity and a  noikis-r  esistance of non-invasive breast cancer cells were gradually increased after prolonged stimulation with TGF-β1/H₂O₂/LPS. The metastatic potential induced by TGF-β1/H₂O₂/LPS was sufficient for tumor cells to extravasate and form metastatic foci in an experimental metastasis model in nude mice. The findings in this study suggested that the enhanced signaling is required for inducing higher metastatic capacity of tumor cells, and that targeting one of stimuli or signaling pathways might be potential approach in comprehensive strategy for tumor therapy. 
 H-Ras-specific activation of Rac-MKK3/6-p38 pathway: its critical role in invasion and migration of breast epithelial cells. Human tumors frequently exhibit constitutively activated Ras signaling, which contributes to the malignant phenotype. Mounting evidence suggests unique roles of the Ras family members, H-Ras, N-Ras and K-Ras, in normal and pathological conditions. In an effort to dissect distinct Ras isoform-specific functions in malignant phenotypic changes, we previously established H-Ras- and N-Ras-activated MCF10A human breast epithelial cell lines. Using these, we showed that p38 kinase is a key signaling molecule differentially regulated between H-Ras and N-Ras, leading to H-Ras-specific induction of   invasive   and migrative phenotypes. The present study is to further investigate H-Ras- and N-Ras-mediated signaling pathways and to unveil how these pathways are integrated for regulation of   invasive  /migrative phenotypic conversion of human breast epithelial cells. Here we report that the Rac-MAPK kinase (MKK)3/6-p38 pathway is a unique signaling pathway activated by H-Ras, leading to the   invasive  /migrative phenotype. In contrast, Raf-MEK-ERK and phosphatidylinositol 3-kinase-Akt pathways, which are fundamental to proliferation and differentiation, are activated by both H-Ras and N-Ras. A significant role for p38 in cell invasion is further supported by the observation that p38 activation by MKK6 transfection is sufficient to induce   invasive   and migrative phenotypes in MCF10A cells. Activation of the MKK6-p38 pathway results in a marked induction of matrix metalloproteinase (MMP)-2, whereas it had little effect on MMP-9, suggesting MMP-2 up-regulation by MKK6-p38 pathway as a key step for H-Ras-induced invasion and migration. We also provide evidence for cross-talk among the Rac, Raf, and phosphatidylinositol 3-kinase pathways critical for regulation of MMP-2 and MMP-9 expression and   invasive   phenotype. Taken together, the present study elucidated the role of the Rac-MKK3/6-p38 pathway leading to H-Ras-specific induction of malignant progression in breast epithelial cells, providing implications for developing therapeutic strategies for mammary carcinoma to target Ras downstream signaling molecules required for malignant cancer cell behavior but less critical for normal cell functions. 
 Estrogen receptorβ2 regulates interlukin-12 receptorβ2 expression via p38 mitogen-activated protein kinase signaling and inhibits non-small-cell lung cancer proliferation and invasion. Lung cancer is one of the most common types of cancer and is the leading cause of cancer-related mortality worldwide. Estrogens are known to be involved in the development and progression of non-small-cell lung cancer (NSCLC). These effects are initially mediated through binding of estrogen to estrogen receptors (ERs), in particular ERβ2. Our preliminary studies demonstrated that ERβ2 and interleukin-12 receptorβ2 (IL-12Rβ2) expression are correlated in NSCLC. The present study investigated the expression of these proteins in NSCLC cells and how changes in their expression affected cell proliferation and invasion. In addition, it aimed to explore whether p38 mitogen-activated protein kinase (p38MAPK) is involved in the regulation of IL-12Rβ2 expression by ERβ2. An immunocytochemical array was used to observe the distribution of ERβ2 and IL-12Rβ2. Co-immuoprecipitation was employed to observe the interaction between p38MAPK and IL-12Rβ2, by varying the expression of ERβ2 and p38MAPK. Western-blot analysis and reverse transcription-polymerase chain reaction assays were used to investigate the mechanism underlying ERβ2 regulation of IL-12Rβ2 expression. 3-(4,5-dimethylthiazol-2-yl)-2,5-diphenyltetrazolium bromide, scratch wound healing and Transwell assays were used to investigate the impact of ERβ2 on   prolifer  ative, invasive and migratory abilities of NSCLC cells. ERβ2 was predominantly found in the cytoplasm and nucleus, whilst IL-12Rβ2 was largely confined to the cytoplasm, although a degree of expression was observed in the nucleus. Compared with normal bronchial epithelial cells, IL-12Rβ2 and ERβ2 were overexpressed in the NSCLC cell groups. Coimmuoprecipitation demonstrated an interaction between p38MAPK and IL-12Rβ2. ERβ2 appeared to upregulate IL-12Rβ2 expression and inhibition of p38MAPK attenuated this effect. ERβ2 and IL-12Rβ2 expression inhibited the proliferation, metastasis and invasion of NSCLC cell lines, but knockout of IL-12Rβ2, even in the presence of ERβ2, led to an increase in NSC  LC cell   proliferation and invasiveness. In conclusion, to the best of our knowledge this study is the first to demonstrate that IL-12Rβ2 may be important in the mechanisms underlying ERβ2 inhibition of NSCLC development, and that this interaction may be mediated via p38MAPK. 
 Deacetylated GM3 promotes uPAR-associated membrane molecular complex to activate p38 MAPK in metastatic melanoma. GM3, the simplest ganglioside, regulates cell proliferation, migration, and invasion by influencing cell signaling at the membrane level. Although the classic N-acetylated form of GM3 (NeuAcLacCer) is commonly expressed and has been well studied, deacetylated GM3 (NeuNH2LacCer, d-GM3) has been poorly investigated, despite its presence in metastatic tumors but not in non  invasive   melanomas or benign nevi. We have recently found that d-GM3 stimulates cell migration and invasion by activating urokinase plasminogen activator receptor (uPAR) signaling to augment matrix metalloproteinase-2 (MMP-2) function. However, the mechanisms by which d-GM3/uPAR increase MMP-2 expression and activation are not clear. By modifying the expression of d-GM3 genetically and biochemically, we found that decreasing d-GM3 expression inhibits, whereas overexpressing d-GM3 stimulates, p38 mitogen-activated protein kinase (MAPK) activity to influence MMP-2 expression and activation. p38 MAPK (p38) activation requires the formation of a membrane complex that contains uPAR, caveolin-1, and integrin α5β1 in membrane lipid rafts. In addition, knocking down or inhibiting focal adhesion kinase (FAK), phosphoinositide 3-kinase (PI3K), or Src kinase significantly reduces d-GM3-induced p38 phosphorylation and activation. Taken together, these results suggest that d-GM3 enhances the metastatic phenotype by activating p38 signaling through uPAR/integrin signaling with FAK, PI3K, and Src kinase as intermediates. Elucidation of the mechanisms by which d-GM3, a newly discovered, potential biomarker of metastatic melanomas, promotes cell metastasis will help us to understand the function of d-GM3 in metastatic melanomas and may lead to novel GM3-based cancer therapies. 
 The MLK-related kinase (MRK) is a novel RhoC effector that mediates lysophosphatidic acid (LPA)-stimulated tumor cell invasion. The small GTPase RhoC is overexpressed in many   invasive   tumors and is essential for metastasis. Despite its high structural homology to RhoA, RhoC appears to perform functions that are different from those controlled by RhoA. The identity of the signaling components that are differentially regulated by these two GTPases is only beginning to emerge. Here, we show that the MAP3K protein MRK directly binds to the GTP-bound forms of both RhoA and RhoC in vitro. However, siRNA-mediated depletion of MRK in cells phenocopies depletion of RhoC, rather than that of RhoA. MRK depletion, like that of RhoC, inhibits LPA-stimulated cell invasion, while depletion of RhoA increases invasion. We also show that active MRK enhances LPA-stimulated invasion, further supporting a role for MRK in the regulation of invasion. Depletion of either RhoC or MRK causes sustained myosin light chain phosphorylation after LPA stimulation. In addition, activation of MRK causes a reduction in myosin light chain phosphorylation. In contrast, as expected, depletion of RhoA inhibits myosin light chain phosphorylation. We also present evidence that both RhoC and MRK are required for LPA-induced stimulation of the p38 and ERK MAP kinases. In conclusion, we have identified MRK as a novel RhoC effector that controls LPA-stimulated cell invasion at least in part by regulating myosin dynamics, ERK and p38. 
 Expression of extracellular signal-regulated kinase1/2 and p38 mitogen-activated protein kinase in the   invasive   trophoblasts at the human placental bed. Mitogen-activated protein kinases (MAP kinases) participate in signal transduction pathways that control embryogenesis, cell differentiation, cell proliferation and cell death. The roles of extracellular signal-regulated kinase1/2 (ERK1/2) and p38 MAP kinase in the differentiation and invasion of human trophoblasts have been studied. However, the in vivo expression and activation of ERK1/2 and p38 at the placental bed have not been elucidated.The study group consisted of placental bed biopsy tissues obtained from the pregnancies without preeclampsia (n=24) and with preeclampsia (n=8) between 31 and 40 weeks of gestation. We evaluated the expressions and phosphorylations of ERK1/2 and p38 MAP kinase in the   invasive   trophoblasts in the placental bed tissues using immunohistochemistry.p38 and phospho-p38 MAP kinase were not detected in   invasive   trophoblasts in cases or controls. ERK1/2 and phospho-ERK1/2 were positive in   invasive   trophoblasts albeit with variable staining. Phosphorylation of ERK1/2 was significantly less frequent in   invasive   trophoblasts in placental bed biopsies from women with preeclampsia compared with normotensive controls.These findings suggest that preeclampsia is associated with decreased activation of ERK1/2 in   invasive   trophoblasts in vivo. 
 Suppression of phorbol-12-myristate-13-acetate-induced tumor cell invasion by apigenin via the inhibition of p38 mitogen-activated protein kinase-dependent matrix metalloproteinase-9 expression. Apigenin has special interest for the development of chemopreventive agents against cancer because it is a widely distributed plant flavonoid that has antitumor properties. In this study, we investigated the apigenin effects on the protease-mediated   invasive  ness in human metastatic cancer cell lines Caski, SK-Hep1, and MDA-231. We found that apigenin markedly inhibits the phorbol-12-myristate-13-acetate (PMA)-induced increase in MMP-9 expression and activity in several cancer cell lines. These effects of apigenin are dose-dependent and correlate with the suppression of MMP-9 mRNA expression levels. PMA caused about a 5-fold induction in MMP-9 promoter activity, which was also suppressed by apigenin treatment in Caski cells. We found that apigenin could inhibit PMA-induced phosphorylation of p38 mitogen-activated protein kinase (p38 MAPK), which was involved in the down-regulation of the expression of matrix metalloproteinase-9 (MMP-9) at mRNA levels. Furthermore, the treatment of inhibitors specific for p38 MAPK (SB203580) to Caski cells caused the reduction of MMP-9 expression. Restoration of p38 expression partly increased PMA-induced MMP-9 secretion blocked by apigenin treatment in Caski cells. These results showed apigenin might inhibit the invasion and migration abilities of Caski cells by reducing the MMP-9 expression through suppressing the p38 MAPK signaling pathway. These findings indicate that apigenin might be a useful strategy for controlling metastasis and the   invasive  ness of tumors. 
 Tissue factor/activated factor VIIa induces matrix metalloproteinase-7 expression through activation of c-Fos via ERK1/2 and p38 MAPK signaling pathways in human colon cancer cell. Increased expression of tissue factor (TF) is associated with tumor invasion and metastasis in human colorectal cancer. We have previously observed that TF/FVIIa upregulates matrix metalloproteinase-7 (MMP-7) expression at the transcriptional level in colon cancer cells. MMP-7 overexpression is believed to play an important role in tumor invasion and metastasis. The aim of this study is to elucidate the molecular mechanisms by which TF/FVIIa induced MMP-7 expression and cell invasion in vitro.Reverse transcription polymerase chain reaction, Western blot, luciferase assay, and chromatin immunoprecipitation (ChIP) were used to determine the potential mechanism and signaling pathways by which TF/FVIIa induced MMP-7 expression and cell invasion in LoVo cells. Small interfering RNA (siRNA) and cell invasion assay was used to examine whether blocking c-Fos expression could abolish FVIIa-mediated upregulation of MMP-7 and cell invasion in vitro.The results showed that FVIIa induced the upregulation of MMP-7 both at the mRNA and protein levels in a time- and dose-dependent manner and increased the   invasive   behavior of LoVo cells. FVIIa enhanced the promoter activity of MMP-7, and the activator protein-1 (AP-1) binding site was responsible for the activation. Site mutation of the AP-1 binding site in the promoter almost completely abolished FVIIa-mediated response. Furthermore, ChIP assay confirmed that FVIIa promoted the direct binding of c-Fos with the MMP-7 promoter in vivo. FVIIa also induced the expression and nuclear accumulation of the AP-1 subunit c-Fos. siRNA-mediated knockdown of c-Fos eliminated FVIIa-stimulated MMP-7 expression and cell migration in vitro. In addition, selective mitogen-activated protein kinase (MAPK) kinase (MEK1/2) inhibitor (PD98059) and p38 MAPK inhibitor SB203580 suppressed MMP-7 upregulation induced by FVIIa.Our data suggest that a novel TF/FVIIa/MAPK/c-Fos/MMP-7 axis plays an important role in modulating the invasion of colon cancer cells and blockage of this pathway holds promise to treat colon cancer metastasis. 
 Aspergillus fumigatus-induced interleukin-8 synthesis by respiratory epithelial cells is controlled by the phosphatidylinositol 3-kinase, p38 MAPK, and ERK1/2 pathways and not by the toll-like receptor-MyD88 pathway. Previous studies have established that phagocytes are key cells of the pulmonary innate immune defense against A. fumigatus, an opportunistic fungus responsible of   invasive   pulmonary aspergillosis. Macrophages detect A. fumigatus via Toll-like receptors 2 and 4 (TLR2 and -4) and respond by the MyD88-NF-kappaB-dependent synthesis of inflammatory mediators. In the present study, we demonstrate that respiratory epithelial cells also sense A. fumigatus and participate in the host defense. Thus, the interaction of respiratory epithelial cells with germinating but not resting conidia of A. fumigatus results in interleukin (IL)-8 synthesis that is controlled by phosphatidylinositol 3-kinase, p38 MAPK, and ERK1/2. Using MyD88-dominant negative transfected cells, we also show that IL-8 production is not dependent on the TLR-MyD88 pathway, although the MyD88 pathway is activated by A. fumigatus and leads to NF-kappaB activation. Thus, our results provide evidence for the existence of two independent signaling pathways activated in respiratory epithelial cells by A. fumigatus, one that is MyD88-dependent and another that is My88-independent and involved in IL-8 synthesis. 
 Carbonic anhydrase XII promotes invasion and migration ability of MDA-MB-231 breast cancer cells through the p38 MAPK signaling pathway. Carbonic anhydrase (CA) XII, an extracellular enzyme involved in the regulation of the microenvironment acidity and tumor malignant phenotype, was originally identified as a protein overexpressed in some types of cancers, including breast cancer. However, the cellular function and mechanism of CAXII remained unclear. In this study, the effects of CAXII expression on invasion and migration of breast cancer cells was investigated. Gene knockdown of CAXII in the human breast cancer cell line MDA-MB-231 resulted in decreased invasion and migration by interfering with the p38 MAPK pathway. CAXII knockdown also decreased the expression of matrix metalloproteinase (MMP)-2, MMP-9, and urokinase-type plasminogen activator (u-PA), but increased tissue inhibitor of metalloproteinases (TIMP)-2 and plasminogen activator inhibitor (PAI)-1 expression. Furthermore, decreased   invasive   and migration ability of CAXII-knockdown cells were restored by an overexpression of CAXII. Results also showed that CAXII knockdown may decrease anchorage-independent growth and cell growth by inhibiting CDK6 and cyclin D1 expression. Furthermore, the impact of CAXII knockdown on invasion, migration and cell growth was further evidenced by effects on tumor size and metastasis of MDA-MB-231 cells in vivo. Taken together, these data suggested that CAXII may affect the capability of invasion and migration of MDA-MB-231 cells, which may be mediated through the p38 MAPK pathway. 
 Expression and activation of P38 MAP kinase in   invasive   ductal breast cancers: correlation with expression of the estrogen receptor, HER2 and downstream signaling phosphorylated proteins. MAP kinase signaling proteins have major implications in the molecular oncogenesis of breast cancers and have been extensively investigated as putative targets for therapy. This study reports the investigation of the expression of P38 MAPK and its phosphorylated form (p-P38 MAPK) in clinical specimens of   invasive   breast carcinomas and their correlation with estrogen receptor (ER) and HER2 expression, as well as MAPK and PI3 kinase-AKT pathway signaling phosphorylated proteins. Expression levels of P38 MAPK and p-P38 MAPK as well as p-AKT, p-GSK3β, p-S6 kinase, p-MEK1 and p-ERK1/2 were quantitatively assessed using multiplex bead immunoassay in frozen specimens from 45   invasiv  e ductal breast cancers. Twenty-nine specimens were ER+, 15 were HER2+ and 10 were triple‑negative breast cancers (TNBCs). P38 MAPK was found to be expressed in all tumor specimens and was significantly (P=0.002) overexpressed in ER+ tumors. P38 MAPK expression was lower in TNBCs than in all of the other tumors. The median expression of p-P38 MAPK was also higher in ER+ tumors while lower in the TNBCs. HER2 status had no effect on P38 MAPK and p-P38 MAPK expression. No variation in the phosphorylation rate of P38 MAPK was observed in relation with ER, HER2 or TNBC status. Significantly higher (P=0.0048) expression of p-AKT was observed in HER2+ tumors. No significant difference in p-MEK1, p-GSK3β and p-S6K expression was found in any other comparisons based on ER and HER2 expression subtypes. Investigation of the expression of multiple phosphorylated signaling proteins can be used for personalized targeted therapy.   In inva  sive breast cancer, the overexpression of P38 MAPK may serve as a biomarker for the evaluation of P38 MAPK inhibitors. 
 Piperine inhibits IL-1β-induced IL-6 expression by suppressing p38 MAPK and STAT3 activation in gastric cancer cells. Piperine, a kind of natural alkaloid found in peppers, has been reported to exhibit anti-oxidative and anti-tumor activities, both in vitro and in vivo. Interleukin-6 (IL-6) is an important cytokine that activates the signal transduction, promotes tumor cell metastasis, and induces malignancy, including in gastric cancer. However, the effects of piperine on IL-6 expression in gastric cancer cells have not yet been well defined. In this study, we investigated the effects of piperine on the IL-6 expression, and examined the underlying signaling pathways via RT-PCR, promoter studies and Western blotting in human gastric cancer TMK-1 cells. Our results showed that piperine inhibited interleukin-1β (IL-1β)-induced IL-6 expression in a dose-dependent manner. In addition, piperine also inhibited IL-6 promoter activity. Experiments with mitogen-activated protein kinase (MAPK) inhibitors and dominant negative mutant p38 MAPK indicated that p38 MAPK was essential for IL-6 expression in the TMK-1 cells. Additionally, signal transducer and activator of transcription 3 (STAT3) was also involved in the IL-1β-induced IL-6 expression in gastric cancer cells. Piperine inhibited IL-1β-induced p38 MAPK and STAT3 activation and, in turn, blocked the IL-1β-induced IL-6 expression. Furthermore, gastric cancer cells pretreated with IL-1β showed markedly en  hanced i  nvasiveness, which was partially abrogated by treatment with IL-6 siRNA, piperine, and inhibitors of p38 MAPK and STAT3. These results suggest that piperine may exert at least part of its anti-cancer effect by controlling IL-6 expression through the suppression of p38 MAPK and STAT3. 
 ErbB2-enhanced   invasive  ness of H-Ras MCF10A breast cells requires MMP-13 and uPA upregulation via p38 MAPK signaling. Overexpression of ErbB2 has been frequently found in mammary carcinoma. We have previously shown that the aberrant activation of H-Ras induces human breast cell invasion and migration. The present study was aimed at investigating the effect of ErbB2 overexpression on H-Ras-induced breast cell invasion and to elucidate the underlying mechanisms. Herein, we show that overexpression of ErbB2 promotes   invasive   and migratory abilities of H-Ras-activated MCF10A human breast epithelial cells through upregulation of matrix metalloproteinase (MMP)-13 and urokinase-type plasminogen activator (uPA). We also demonstrate that the p38 MAPK is an important signaling molecule in the ErbB2-induced upregulation of MMP-13 and uPA and invasion/migration of H-Ras MCF10A cells overexpressing ErbB2. The present study elucidating the molecular mechanism underlying ErbB2-induced promotion of H-Ras MCF10A cell invasion may provide invaluable information for understanding breast cancer progression and establishing therapeutic interventions for breast cancer. 
 Naringin inhibits the invasion and migration of human glioblastoma cell via downregulation of MMP-2 and MMP-9 expression and inactivation of p38 signaling pathway. Gliomas are the most common and malignant primary brain tumors. They are associated with a poor prognosis despite the availability of multiple therapeutic options. Naringin, a common dietary flavonoid abundantly present in fruits and vegetables, is believed to possess strong anti-proliferative and anti-cancer properties. However, there are no reports describing its effects on the invasion and migration of glioblastoma cell lines. Our results showed that the treatment of U251 glioma cell lines with different concentrations of naringin inhibited the invasion and migration of these cells. In addition, we revealed a decrease in the levels of matrix metalloproteinases (MMP-2) and (MMP-9) expression as well as proteinase activity in U251 glioma cells. In contrast, the expression of tissue inhibitor of metalloproteinases (TIMP-1) and (TIMP-2) was increased. Furthermore, naringin treatment decreased significantly the phosphorylated level of p38. Combined treatment with a p38 inhibitor (SB203580) resulted in the synergistic reduction of MMP-2 and MMP-9 expressions correlated with an increase of TIMP-1 and TIMP-2 expressions and the anti-  invasive   properties. However, p38 chemical activator (anisomycin) could block these effects produced by naringin, suggesting a direct downregulation of the p38 signaling pathway. These data suggest that naringin may have therapeutic potential for controlling   invasive  ness of malignant gliomas by inhibiting of p38 signal transduction pathways. 
 Glial cell-derived neurotrophic factor (GDNF) promotes low-grade Hs683 glioma cell migration through JNK, ERK-1/2 and p38 MAPK signaling pathways. Invasion of tumor cells is the primary cause of therapeutic failure in the treatment of malignant gliomas. In an attempt to investigate the properties of the malignant progression of glioma cells, we examined the correlation between cell migration and glial cell-derived neurotrophic factor (GDNF) secretion of two glioma cell lines which differ in their   invasive   phenotypes. Here, we show that the high-grade C6 cells are more migrative and secrete more GDNF than the low-grade Hs683 cells. GDNF signaling is more highly activated in C6 cells than in Hs683 cells. Treatment of the Hs683 cells with GDNF significantly increased migration comparable to the C6 cells, revealing the autocrine and/or paracrine effect of GDNF on promotion of the glioma cell migration. We then examined the involvement of mitogen-activated protein kinases (MAPKs) including c-Jun N-terminal protein kinase (JNK), extracellular signal-regulated kinases (ERKs) and p38 MAPK in Hs683 cell migration induced by GDNF. A prominent activation of JNK, ERKs and p38 MAPK was observed in the GDNF-treated cells. Functional studies showed that the activation of these MAPKs was critical for Hs683 cell migration induced by GDNF. Our findings revealing molecular mechanisms for the promoting effect of GDNF on glioma cell migration may provide an insight into a better understanding to the malignant progression of human gliomas. 
 Migration induced by epidermal and hepatocyte growth factors in oral squamous carcinoma cells in vitro: role of MEK/ERK, p38 and PI-3 kinase/Akt. Cell migration is a necessary part of malignant   invasive  ness. Oral squamous cell carcinomas (OSCC) have a great tendency for local   invasive   growth. We have investigated signalling pathways involved in cell migration induced by epidermal growth factor (EGF) and hepatocyte growth factor (HGF) in OSCC cells and examined the effects of various experimental and clinically approved anti-tumour signal inhibitors on the migratory activity.Migration was studied in three human OSCC cell lines, using a scratch wound assay in vitro and time-lapse cinematography. Specific phosphorylation of signalling proteins was assessed by Western blotting.In the E10 cell line, EGF and HGF induced phosphorylation of EGF receptor (EGFR) and Met, respectively, phosphorylation of ERK1/2, p38 and Akt, and dose-dependent activation of cell migration. Addition of the EGFR-specific inhibitors cetuximab (antibody) or gefitinib (tyrosine kinase blocker) abolished cell migration elicited by EGF. Similarly, a Met kinase inhibitor (SU11274) blocked HGF-induced cell migration. Furthermore, when three cell lines were treated with blockers of the MEK/ERK, p38 or the PI-3 kinase/Akt pathways, the migratory response to both EGF and HGF was inhibited, but to varying degrees. Notably, in E10 and D12 cells, HGF-induced migration was particularly sensitive to PI-3 K-inhibition, while in C12 cells, both HGF- and EGF-induced migration were highly sensitive to p38-blockade.The results demonstrate that the MEK/ERK, p38 and PI-3 kinase pathways are all involved in mediating the increased migration in OSCC cell lines induced by EGF and HGF, but their relative importance and the effects of specific signal inhibitors differ. 
 Downregulation of LAPTM5 suppresses cell proliferation and viability inducing cell cycle arrest at G0/G1 phase of bladder cancer cells. Our transcriptome analysis revealed in bladder cancer (BCa) tissues a significant induction of lysosomal-associated multispanning membrane protein 5 (LAPTM5), a lysosomal membrane protein preferentially expressing in immune cells and hematopoietic cells. Transportation of LAPTM5 from Golgi to lysosome could be inhibited by deficiency of Nedd4, a key member of E3 ubiquitin ligase family overexpressing in   invasiv  e BCa and promoting its progression. Therefore, we hypothesize that LAPTM5 may be closely correlated with BCa tumorigenesis. In human BCa tissues, we observed that LAPTM5 was significantly induced at both mRNA and protein levels, which is consistent with our microarray result. Furthermore, we established a BCa cell model with downregulated LAPTM5, revealing a significantly delayed growth rate in the BCa cells with knockdown of LAPTM5. Moreover, cell cycle arrest at G0/G1 phase was triggered by decreased LAPTM5 as well, which could lead to delayed BCa cell growth. In contrast, no significant alteration of apoptosis in the BCa cells with downregulated LAPTM5 was noticed. Analysis of the changes of migration and invasion, showed significant reduced LAPTM5 suppressed cell metastasis. Furthermore, proteins involved in epithelial-mesenchymal transition (EMT) were strongly altered, which plays a central role in metastasis. In addition, phosphorylated ERK1/2 and p38, key members of mitogen-activated protein kinase (MAPK) family regulating BCa tumorigenesis, were strongly decreased. Taken together, our results suggested that decreased LAPTM5 inhibited proliferation and viability, as well as induced G0/G1 cell cycle arrest possibly via deactivation of ERK1/2 and p38 in BCa cells. 
 Gelsolin promotes cell growth and invasion through the upregulation of p-AKT and p-P38 pathway in osteosarcoma. The gelsolin (GSN) has been involved in the regulation of tumor formation and development, but the biological role of GSN in the pathogenesis of osteosarcoma (OS) has not been well characterized. In this study, we show that high expression of GSN was observed in OS tissues and correlated with tumor size, advanced Enneking stage, and poor patient prognosis. Knockdown of GSN significantly inhibited cell proliferation and   invasive  ness in the OS cell lines. Furthermore, stable overexpression of GSN in OS cells resulted in a significant increase in cell growth and motility with the downregulation of p-AKT and p-P38 pathway. Finally, overexpression of GSN promoted growth of OS tumors in SCID mice. Taken together, these results provided the first evidence that GSN might contribute to OS cancer development and could be an attractive therapeutic target for patients with OS. 
 Tenascin-W is found in malignant mammary tumors, promotes alpha8 integrin-dependent motility and requires p38MAPK activity for BMP-2 and TNF-alpha induced expression in vitro. Tenascins represent a family of extracellular matrix glycoproteins with distinctive expression patterns. Here we have analyzed the most recently described member, tenascin-W, in breast cancer. Mammary tumors isolated from transgenic mice expressing hormone-induced oncogenes reveal tenascin-W in the stroma around lesions with a high likelihood of metastasis. The presence of tenascin-W was correlated with the expression of its putative receptor, alpha8 integrin. HC11 cells derived from normal mammary epithelium do not express alpha8 integrin and fail to cross tenascin-W-coated filters. However, 4T1 mammary carcinoma cells do express alpha8 integrin and their migration is stimulated by tenascin-W. The expression of tenascin-W is induced by BMP-2 but not by TGF-beta1, though the latter is a potent inducer of tenascin-C. The expression of tenascin-W is dependent on p38MAPK and JNK signaling pathways. Since preinflammatory cytokines also act through p38MAPK and JNK signaling pathways, the possible role of TNF-alpha in tenascin-W expression was also examined. TNF-alpha induced the expression of both tenascin-W and tenascin-C, and this induction was p38MAPK- and cyclooxygenase-dependent. Our results show that tenascin-W may be a useful diagnostic marker for breast malignancies, and that the induction of tenascin-W in the tumor stroma may contribute to the   invasive   behavior of tumor cells. 
 Inhibition of p38 MAPK signaling augments skin tumorigenesis via NOX2 driven ROS generation. p38 mitogen-activated protein kinases (MAPKs) respond to a wide range of extracellular stimuli. While the inhibition of p38 signaling is implicated in the impaired capacity to repair ultraviolet (UV)-induced DNA damage-a primary risk factor for human skin cancers-its mechanism of action in skin carcinogenesis remains unclear, as both anti-proliferative and survival functions have been previously described. In this study, we utilized cultured keratinocytes, murine tumorigenesis models, and human cutaneous squamous cell carcinoma (SCC) specimens to assess the effect of p38 in this regard. UV irradiation of normal human keratinocytes increased the expression of all four p38 isoforms (α/β/γ/δ); whereas irradiation of p53-deficient A431 keratinocytes derived from a human SCC selectively decreased p38α, without affecting other isoforms. p38α levels are decreased in the majority of human cutaneous SCCs assessed by tissue microarray, suggesting a tumor-suppressive effect of p38α in SCC pathogenesis. Genetic and pharmacological inhibition of p38α and in A431 cells increased cell proliferation, which was in turn associated with increases in NAPDH oxidase (NOX2) activity as well as intracellular reactive oxygen species (ROS). These changes led to e  nhanced   invasiveness of A431 cells as assessed by the matrigel invasion assay. Chronic treatment of p53-/-/SKH-1 mice with the p38 inhibitor SB203580 accelerated UV-induced SCC carcinogenesis and increased the expression of NOX2. NOX2 knockdown suppressed the augmented growth of A431 xenografts treated with SB203580. These findings indicate that in the absence of p53, p38α deficiency drives SCC growth and progression that is associated with enhanced NOX2 expression and ROS formation. 
 Flt-1 in colorectal cancer cells is required for the tumor   invasive   effect of placental growth factor through a p38-MMP9 pathway. Placenta growth factor (PlGF), a dimeric glycoprotein with 53% homology to VEGF, binds to VEGF receptor-1 (Flt-1), but not to VEGF receptor-2 (Flk-1), and may function by modulating VEGF activity. We previously have showed that PlGF displays prognostic value in colorectal cancer (CRC) but the mechanism remains elucidated.Overexpression of PlGF increased the   invasive  /migration ability and decreased apoptosis in CRC cells showing Flt-1 expression. Increased migration was associated with increasing MMP9 via p38 MAPK activation. Tumors grew faster, larger; with higher vascularity from PlGF over-expression cells in xenograft assay. In two independent human CRC tissue cohorts, PlGF, MMP9, and Flt-1 expressions were higher in the advanced than the localized disease group. PlGF expression correlated with MMP9, and Flt-1 expression. CRC patients with high PlGF and high Flt-1 expression in tissue had poor prognosis.PlGF/Flt-1 signaling plays an important role in CRC progression, blocking PlGF/Flt-1 signaling maybe an alternative therapy for CRC. 
 The NHERF1 PDZ2 domain regulates PKA-RhoA-p38-mediated NHE1 activation and invasion in breast tumor cells. Understanding the signal transduction systems governing invasion is fundamental for the design of therapeutic strategies against metastasis. Na(+)/H(+) exchanger regulatory factor (NHERF1) is a postsynaptic density 95/disc-large/zona occludens (PDZ) domain-containing protein that recruits membrane receptors/transporters and cytoplasmic signaling proteins into functional complexes. NHERF1 expression is altered in breast cancer, but its effective role in mammary carcinogenesis remains undefined. We report here that NHERF1 overexpression in human breast tumor biopsies is associated with metastatic progression, poor prognosis, and hypoxia-inducible factor-1alpha expression. In cultured tumor cells, hypoxia and serum deprivation increase NHERF1 expression, promote the formation of leading-edge pseudopodia, and redistribute NHERF1 to these pseudopodia. This pseudopodial localization of NHERF1 was verified in breast biopsies and in three-dimensional Matrigel culture. Furthermore, serum deprivation and hypoxia stimulate the Na(+)/H(+) exchanger, invasion, and activate a protein kinase A (PKA)-gated RhoA/p38 invasion signal module. Significantly, NHERF1 overexpression was sufficient to induce these morphological and functional changes, and it potentiated their induction by serum deprivation. Functional experiments with truncated and binding groove-mutated PDZ domain constructs demonstrated that NHERF1 regulates these processes through its PDZ2 domain. We conclude that NHERF1 overexpression enhances the   invasive   phenotype in breast cancer cells, both alone and in synergy with exposure to the tumor microenvironment, via the coordination of PKA-gated RhoA/p38 signaling. 
 MAP-ing glioma invasion: mitogen-activated protein kinase kinase 3 and p38 drive glioma invasion and progression and predict patient survival. Although astrocytic brain tumors do not metastasize systemically, during tumorigenesis glioma cells adopt an   invasive   phenotype that is poorly targeted by conventional therapies; hence, glioma patients die of recurrence from the locally   invasive   tumor population. Our work is aimed at identifying and validating novel therapeutic targets and biomarkers in   invasive   human gliomas. Transcriptomes of   invasive   glioma cells relative to stationary cognates were produced from a three-dimensional spheroid in vitro invasion assay by laser capture microdissection and whole human genome expression microarrays. Qualitative differential expression of candidate invasion genes was confirmed by quantitative reverse transcription-PCR, clinically by immunohistochemistry on tissue microarray, by immunoblotting on surgical specimens, and on two independent gene expression data sets of glial tumors. Cell-based assays and ex vivo brain slice invasion studies were used for functional validation. We identify mitogen-activated protein kinase (MAPK) kinase 3 (MKK3) as a key activator of p38 MAPK in glioma; MKK3 activation is strongly correlated with p38 activation in vitro and in vivo. We further report that these members of the MAPK family are strong promoters of tumor invasion, progression, and poor patient survival. Inhibition of either candidate leads to significantly reduced glioma   invasive  ness in vitro. Consistent with the concept of synthetic lethality, we show that inhibition of invasion by interference with these genes greatly sensitizes arrested glioma cells to cytotoxic therapies. Our findings therefore argue that interference with MKK3 signaling through a novel treatment combination of p38 inhibitor plus temozolomide heightens the vulnerability of glioma to chemotherapy. 
 Peroxiredoxin 1 promotes pancreatic cancer cell invasion by modulating p38 MAPK activity. The aim of this study was to investigate the role of peroxiredoxin 1 (Prdx1) in the   invasive  ness of pancreatic ductal adenocarcinoma (PDAC) cells.Immunohistochemistry was used to determine overexpression of Prdx1 in human PDAC tissues. Immunoprecipitation and immunocytochemistry were used to determine the interaction and intracellular distribution of Prdx1 and a member of the mitogen-activated protein kinase (MAPK) family protein, p38 MAPK, in PDAC cells. Finally, immunocytochemistry and Matrigel invasion assay were used to examine the effects of Prdx1 and p38 MAPK on the formation of cell protrusions and PDAC cell invasion.Prdx1 is overexpressed in human PDAC tissues. Peroxiredoxin 1 interacts with active forms of p38 MAPK, and complexes of Prdx1 and phosphorylated p38 MAPK localize at the leading edges of migrating PDAC cells. Suppression of Prdx1 decreases active p38 MAPK localized in cell protrusions and inhibits the   invasive  ness of PDAC cells. Consequently, suppression of Prdx1 inhibits membrane ruffling and protrusions. The p38 MAPK inhibitor SB203580 also decreases the formation of membrane protrusions and inhibits   invasive  ness.Prdx1 associates with the formation of membrane protrusions through modulation of the activity of p38 MAPK, which in turn promotes PDAC cell invasion. 
 Oncogenic K-Ras down-regulates Rac1 and RhoA activity and enhances migration and invasion of pancreatic carcinoma cells through activation of p38. Activating mutations in the K-ras gene are genetic alterations frequently found in human carcinomas, particularly in pancreatic adenocarcinomas. Mutation of the K-ras gene is thought to be an early and important event in pancreatic tumor initiation, but the precise role of the mutant K-Ras proteins in neoplastic progression is still unknown. In the present study, we have characterized the influence of oncogenic K-Ras on the phenotype and on the signal transduction of epitheloid PANC-1 pancreatic carcinoma cells by generating PANC-1 cell clones, which stably express EGFP(enhanced green fluorescent protein)-K-Ras (V12). EGFP-K-Ras (V12)-expressing cells exhibited a more fibroblastoid cellular phenotype with irregular cell shape and disorganized cytokeratin filaments. Moreover, these cells showed a marked enhancement of their migratory and   invasive   properties. Stable expression of EGFP-K-Ras (V12) down-regulated the activity of Rac1 and RhoA, resulting in reduced subcortical actin filaments and stress fibers, which might contribute to the epithelial dedifferentiation. Characterization of the activity of mitogen-activated protein kinases revealed that EGFP-K-Ras (V12) enhanced the activity of p38, but did not affect the activities of the Raf/MEK/ERK cascade and JNK. While inhibition of either MEK or JNK activity had no effect on EGFP-K-Ras (V12)-induced migration, inhibition of p38 activity markedly reduced EGFP-K-Ras (V12)-induced migration. Collectively, the results suggest that oncogenic K-Ras enhances the malignant phenotype and identify the mitogen-activated protein kinase p38 as a target to inhibit oncogenic K-Ras-induced pancreatic tumor cell migration. 
 Resveratrol inhibits hyperglycemia-driven ROS-induced invasion and migration of pancreatic cancer cells via suppression of the ERK and p38 MAPK signaling pathways. Increasing evidence suggests that there is a strong relationship between diabetes mellitus (DM) and pancreatic cancer. Our previous study revealed that hyperglycemia could enhance the   invasive   and migratory activities of pancreatic cancer cells. Resveratrol, a natural polyphenolic phytoalexin, has many biological and pharmaceutical properties, including antioxidant and anti-tumorigenic capabilities. The aim of the present study was to evaluate whether resveratrol affects hyperglycemia-induced reactive oxygen species (ROS) production as well as the invasion and migration of pancreatic cancer and its underlying mechanisms. Human pancreatic cancer Panc-1 cells were exposed to high glucose condition with or without resveratrol, N-acetylcysteine (NAC, a scavenger of free radicals), PD 98059 (an ERK inhibitor) or SB 203580 (a p38 MAPK inhibitor). The intracellular ROS and hydrogen peroxide (H2O2) were determined using 2,7-dichlorodihydrofluorecein diacetate and H2O2 assay. MTT, wound healing assay and transwell matrigel invasion assay were used to detect the proliferation, migration and invasion potential of cancer cells. The expressions of uPA, E-cadherin and Glut-1 were examined using QT-PCR and western blot analysis at mRNA and protein levels. The activation of p-ERK, p-p38 and p-NF-κB were measured by western blot analysis. The results of the present study showed that resveratrol could significantly decrease high glucose-induced production of ROS and H2O2 in Panc-1 cells. Resveratrol was also able to inhibit high glucose-induced proliferation, migration and invasion of pancreatic cancer cells. High glucose-modulated expression of uPA, E-cadherin and Glut-1 were inhibited by resveratrol. In addition, high glucose-induced activation of ERK and p38 MAPK signaling pathways as well as the transcription factor NF-κB could also be suppressed by resveratrol. Furthermore, resveratrol was able to suppress H2O2-induced migration and invasion abilities of pancreatic cancer cells. Taken together, these data indicate that resveratrol plays an important role in suppressing hyperglycemia-driven ROS-induced pancreatic cancer progression by inhibiting the ERK and p38 MAPK signaling pathways, providing evidence that resveratrol might be a potential candidate for chemoprevention of pancreatic cancer. 
 Phosphorylation of the homeotic tumor suppressor Cdx2 mediates its ubiquitin-dependent proteasome degradation. The Caudal-related homeodomain transcription factor Cdx2 plays a key role in intestinal cell fate determination. Reduction of Cdx2 expression is a feature of many human colon carcinomas and inactivation of one cdx2 allele facilitates the development of   invasive   adenocarcinoma in the murine colon. Here, we investigated the post-translational regulation of Cdx2. We showed that various forms of Cdx2 coexist in the intestine and colon cancer cell lines, some of them being phosphorylated forms. We found that cyclin-dependent kinase 2 phosphorylated Cdx2 in vitro and in vivo. Using site-specific mutagenesis, we identified serine 281 as a new key residue for Cdx2 phosphorylation. Intriguingly, serine 281 belongs to a conserved motif of four evenly spaced serines (the 4S motif) similar to the one controlling beta-catenin degradation by the proteasome pathway. A nonphosphorylated mutant Cdx2 lacking the 4S motif (4S>A) exhibited reduced polyubiquitination upon proteasome inhibition and increased stability compared to wild-type Cdx2. In addition, we found that this mutant was less efficient to suppress colony formation than wild-type Cdx2. Thus, our data highlight a novel post-translational mechanism controlling Cdx2 degradation via phosphorylation and polyubiquitination, which may be of importance for intestinal development and cancer. 
  GeneRIFs  
 Flt-1 in colorectal cancer cells is required for the tumor   invasive   effect of placental growth factor through a p38-MMP9 pathway 
 p38 and phospho-p38 MAP kinase were not detected in   invasive   trophoblasts in preeclampsia cases or controls. 
 NHERF1 overexpression enhances the   invasive   phenotype in breast cancer cells, both alone and in synergy with exposure to the tumor microenvironment, via the coordination of PKA-gated RhoA/p38 signaling. 
  [Mm]etastasize  PubMed  
 MAP-ing glioma invasion: mitogen-activated protein kinase kinase 3 and p38 drive glioma invasion and progression and predict patient survival. Although astrocytic brain tumors do not   metastasize   systemically, during tumorigenesis glioma cells adopt an invasive phenotype that is poorly targeted by conventional therapies; hence, glioma patients die of recurrence from the locally invasive tumor population. Our work is aimed at identifying and validating novel therapeutic targets and biomarkers in invasive human gliomas. Transcriptomes of invasive glioma cells relative to stationary cognates were produced from a three-dimensional spheroid in vitro invasion assay by laser capture microdissection and whole human genome expression microarrays. Qualitative differential expression of candidate invasion genes was confirmed by quantitative reverse transcription-PCR, clinically by immunohistochemistry on tissue microarray, by immunoblotting on surgical specimens, and on two independent gene expression data sets of glial tumors. Cell-based assays and ex vivo brain slice invasion studies were used for functional validation. We identify mitogen-activated protein kinase (MAPK) kinase 3 (MKK3) as a key activator of p38 MAPK in glioma; MKK3 activation is strongly correlated with p38 activation in vitro and in vivo. We further report that these members of the MAPK family are strong promoters of tumor invasion, progression, and poor patient survival. Inhibition of either candidate leads to significantly reduced glioma invasiveness in vitro. Consistent with the concept of synthetic lethality, we show that inhibition of invasion by interference with these genes greatly sensitizes arrested glioma cells to cytotoxic therapies. Our findings therefore argue that interference with MKK3 signaling through a novel treatment combination of p38 inhibitor plus temozolomide heightens the vulnerability of glioma to chemotherapy. 
 Crosstalk between p38 and Smad3 through TGF-β1 in JEG-3 choriocarcinoma cells. Choriocarcinoma is a highly malignant trophoblastic tumor related to pregnancy that often occurs with a complete hydatidiform mole. It grows quickly and can also widely   metastasiz  e to other organs or tissues through both the venous and lymphatic systems. The transforming growth factor-β1 (TGF-β1) belongs to a growth factor superfamily and has been suggested to play a critical role in regulating the genesis and development of choriocarcinoma through a variety of Smad-independent pathways, including the p38 MAPK pathway. Previous studies indicated that TGF-β can activate the p38 MAPK pathway. In this study, we investigated Smad and p38 MAPK signaling in JEG-3 choriocarcinoma cells using p38 MAPK inhibitor and TGF-β receptor inhibitor. Immunofluorescence and western blot assays were used to detect the proteins in Smad and p38 MAPK pathways. Our data demonstrated that TGF-β can activate Smad3 and induce Smad3 translocation into the nucleus in JEG-3 cells. Blockade of the TGF-β pathway significantly reduced the expression levels of p38 and phospho-p38. p38 MAPK inhibitors (SB 203580) can attenuate TGF-β1-induced Smad3 expression and suppress the activation of smad3. These findings indicate crosstalk between p38 and smad3 through TGF-β1 in choriocarcinoma cells. 
  [Mm]etastasis  PubMed  
 The secreted protein ANGPTL2 promotes   metastasis   of osteosarcoma cells through integrin α5β1, p38 MAPK, and matrix metalloproteinases. The tumor microenvironment can enhance the invasive capacity of tumor cells. We showed that expression of angiopoietin-like protein 2 (ANGPTL2) in osteosarcoma (OS) cell lines increased and the methylation of its promoter decreased with time when grown as xenografts in mice compared with culture. Compared with cells grown in normal culture conditions, the expression of genes encoding DNA demethylation-related enzymes increased in tumor cells implanted into mice or grown in hypoxic, serum-starved culture conditions. ANGPTL2 expression in OS cell lines correlated with increased tumo  r metastas  is and decreased animal survival by promoting tumor cell intravasation mediated by the integrin α5β1, p38 mitogen-activated protein kinase, and matrix metalloproteinases. The tolloid-like 1 (TLL1) protease cleaved ANGPTL2 into fragments in vitro that did not enhance tumor progression when overexpressed in xenografts. Expression of TLL1 was weak in OS patient tumors, suggesting that ANGPTL2 may not be efficiently cleaved upon secretion from OS cells. These findings demonstrate that preventing ANGPTL2 signaling stimulated by the tumor microenvironment could inhibit tumor cell migration   and metast  asis. 
 The secreted protein ANGPTL2 promotes   metastasis   of osteosarcoma cells through integrin α5β1, p38 MAPK, and matrix metalloproteinases. The tumor microenvironment can enhance the invasive capacity of tumor cells. We showed that expression of angiopoietin-like protein 2 (ANGPTL2) in osteosarcoma (OS) cell lines increased and the methylation of its promoter decreased with time when grown as xenografts in mice compared with culture. Compared with cells grown in normal culture conditions, the expression of genes encoding DNA demethylation-related enzymes increased in tumor cells implanted into mice or grown in hypoxic, serum-starved culture conditions. ANGPTL2 expression in OS cell lines correlated with increased tumo  r metastas  is and decreased animal survival by promoting tumor cell intravasation mediated by the integrin α5β1, p38 mitogen-activated protein kinase, and matrix metalloproteinases. The tolloid-like 1 (TLL1) protease cleaved ANGPTL2 into fragments in vitro that did not enhance tumor progression when overexpressed in xenografts. Expression of TLL1 was weak in OS patient tumors, suggesting that ANGPTL2 may not be efficiently cleaved upon secretion from OS cells. These findings demonstrate that preventing ANGPTL2 signaling stimulated by the tumor microenvironment could inhibit tumor cell migration   and metast  asis. 
 [Correlation of p38 mitogen-activated protein kinase signal transduction pathway to uPA expression in breast cancer]. p38 mitogen-activated protein kinase (p38MAPK) signal transduction pathway regulates the expression of various   metastasis  -related genes. Urokinase-type plasminogen activator (uPA) plays an important role in cancer invasion and   metastasis  . This study was to explore the correlation of p38MAPK signal transduction pathway to uPA expression in breast cancer.SP immunohistochemistry was used to test the expression of p-p38, p-Akt, p-Erk, and uPA in 60 specimens of breast cancer. Western blot was used to detect the protein levels of p-p38 and uPA in breast cancer MDA-MB-231 and MCF-7 cells and the protein level of uPA in MDA-MB-231 cells after blocking p38MAPK signal transduction pathway by SB203580, a specific inhibitor of p38MAPK. The correlations of p-p38, p-Akt, p-Erk, and uPA expression to the clinicopathologic characteristics of breast cancer, and the correlations of p-p38, p-Akt, and p-Erk expression to uPA expression were analyzed. The mechanism of p38MAPK signal transduction pathway regulating the protein expression of uPA in breast cancer cells was investigated. The correlation of p-p38 and uPA expression to prognosis of breast cancer was analyzed.The positive rates of p-p38, p-Akt, p-Erk, and uPA proteins in breast cancer tissues were 56.7%, 95.0%, 93.3%, and 60.0%, respectively. The expression of p-p38 was positively correlated to the expression of uPA (r=0.316, P<0.05), while the expression of p-Akt and p-Erk was not related to uPA expression. The expression of p-p38 and uPA was correlated to lymph node   metastasis   and TNM stage (P<0.05), but not to patients' age and tumor size (P>0.05). The expression of p-Akt and p-Erk were correlated to lymph node   metastasis   (P<0.05), but not to TNM stage, patients' age, and tumor size (P>0.05). The protein levels of p-p38 and uPA in MDA-MB-231 cells were higher than that in MCF-7 cells. SB203580 concentration-dependently inhibited p38MAPK pathway and induced uPA protein expression. The expression of p-p38 and uPA was negatively correlated to prognosis of breast cancer (log-rank=4.98, 5.40, P=0.026, 0.020).p38MAPK signal transduction pathway might improve breast cancer progression by up-regulating uPA expression, and might be an important route in invasion and   metastasis   of breast cancer. p-p38 and uPA might help to evaluate prognosis of breast cancer. 
 Mechanical regulation of cancer cell apoptosis and autophagy: roles of bone morphogenetic protein receptor, Smad1/5, and p38 MAPK. Mechanical forces induced by interstitial fluid flow in and surrounding tissues and by blood/lymphatic flow in vessels may modulate cancer cell invasion and   metastasis   and anticancer drug delivery. Our previous study demonstrated that laminar flow-induced shear stress induces G2/M arrest in tumor cells. However, whether shear stress modulates final cell fate remains unclear. In this study, we investigated the role of flow-induced shear stress in modulating the survival of four human tumor cell lines, i.e., Hep3B hepatocarcinoma cells, MG63 osteosarcoma cells, SCC25 oral squamous carcinoma cells, and A549 carcinomic alveolar basal epithelial cells. Laminar shear stress (LSS) ranging from 0.5 to 12dyn/cm(2) induced death of these four tumor cell lines. In contrast to LSS at 0.5dyn/cm(2), oscillatory shear stress (OSS) at 0.5±4dyn/cm(2) cannot induce cancer cell death. Both LSS and OSS had no effect on human normal hepatocyte, lung epithelial, and endothelial cells. Application of LSS to these four cell lines increased the percentage of cells stained positively for annexin V-FITC, with up-regulations of cleaved caspase-8, -9, and -3, and PARP. In addition, LSS also induced Hep3B cell autophagy, as detected by acidic vesicular organelle formation, LC3B transformation, and p62/SQSTM1 degradation. By transfecting with small interfering RNA, we found that the shear-induced apoptosis and autophagy are mediated by bone morphogenetic protein receptor type (BMPR)-IB, BMPR-specific Smad1 and Smad5, and p38 mitogen-activated protein kinase in Hep3B cells. Our findings provide insights into the molecular mechanisms by which shear stress induces apoptosis and autophagy in tumor cells. 
 Protein kinase A gating of a pseudopodial-located RhoA/ROCK/p38/NHE1 signal module regulates invasion in breast cancer cell lines.   Metastasis   results from a sequence of selective events often involving interactions with elements of the tumor-specific physiological microenvironment. The low-serum component of this microenvironment confers increased motility and invasion in breast cancer cells by activating the Na+/H+ exchanger isoform 1 (NHE1). The present study was undertaken to characterize the signal transduction mechanisms underlying this serum deprivation-dependent activation of both the NHE1 and the concomitant invasive characteristics such as leading edge pseudopodia development and penetration of matrigel in breast cancer cell lines representing different stages of metastatic progression. Using pharmacological and genetic manipulation together with transport and kinase activity assays, we observe that the activation of the NHE1 and subsequent invasion by serum deprivation in metastatic human breast cells is coordinated by a sequential RhoA/p160ROCK/p38MAPK signaling pathway gated by direct protein kinase A phosphorylation and inhibition of RhoA. Fluorescence resonance energy transfer imaging of RhoA activity and immunofluorescence analysis of phospho-RhoA and NHE1 show that serum deprivation dynamically remodels the cell, forming long, leading edge pseudopodia and that this signal module is preferentially compartmentalized in these leading edge pseudopodia, suggesting a tight topographic relation of the signaling module to an invasion-specific cell structure. 
 Phosphorylated p38, a negative prognostic biomarker, complements TNM staging prognostication in colorectal cancer. Phosphorylated p38 (p-p38) played a pivotal role in the regulation of disease progression and correlated with tumor prognosis. Here, we characterized the prognostic effect of p-p38 in colorectal cancer (CRC). Three hundred and sixteen CRC patients in stages I-III were recruited in this study. P-p38 expression was semi-quantitatively evaluated using tissue microarrays and immunohistochemistry staining. Overall survival (OS), disease-free survival (DFS), local failure-free survival (LFFS), and distant   metastasis  -free survival (DMFS) of patient subgroups, segregated by p-p38 expression level and clinical stage, were compared using Kaplan-Meier analysis. We found that p-p38 was overexpressed in 48.1 % (152/316) CRC tissues, whereas low or deficiently expressed in normal adjacent epithelia. Overexpression of p-p38 predicted poor OS (P < 0.001), DFS (P = 0.002), LFFS (P = 0.016), and DMFS (P = 0.025) in CRC. Importantly, patient subgroups in the early stage (stages I + II) and with low p-p38 had similar OS, PFS, LFFS, and DMFS probabilities to that of stage I, whereas those with high p-p38 were similar to stage III disease. In addition, for stage III disease, the subgroup with low p-p38 had a similar survival probability to that of stage I, whereas the subgroup with high p-p38 had the worst survival. Multivariate Cox analysis confirmed that p-p38 was indeed a significantly independent factor for death, recurrence, and distant metastases in CRC. Our results demonstrated that p-p38 was a negative independent prognostic factor for CRC. Complementing TNM staging with p-p38 might refine the risk definition more accurately for a subset of patients. 
 TLR4 ligand/H₂O₂ enhances TGF-β1 signaling to induce metastatic potential of non-invasive breast cancer cells by activating non-Smad pathways. TGF-β1 has the potential to activate multiple signaling pathways required for inducing metastatic potential of tumor cells. However, TGF-β1 was inefficient in inducing metastatic potential of many non-invasive human tumor cells. Here we report that the enhancement of TGF-β1 signaling is required for inducing metastatic potential of non-invasive breast cancer cells. TGF-β1 alone could not efficiently induce the sustained activation of Smad and non-Smad pathways in non-invasive breast cancer cells. TLR4 ligand (LPS) and H₂O₂ cooperated with TGF-β1 to enhance the sustained activation of non-Smad pathways, including p38MAPK, ERK, JNK, PI3K, and NF-κB. The activation of MAPK and PI3K pathways resulted in a positive feed-back effect on TGF-β1 signaling by down-regulating Nm23-H1 expression and up-regulating the expression of TβRI and TβRII, favoring further activation of multiple signaling pathways. Moreover, the enhanced TGF-β1 signaling induced higher expression of SNAI2, which also promoted TβRII expression. Therefore, the sustained activation levels of both Smad and non-Smad pathways were gradually increased after prolonged stimulation with TGF-β1/H₂O₂/LPS. Consistent with the activation pattern of signaling pathways, the invasive capacity and anoikis-resistance of non-invasive breast cancer cells were gradually increased after prolonged stimulation with TGF-β1/H₂O₂/LPS. The metastatic potential induced by TGF-β1/H₂O₂/LPS was sufficient for tumor cells to extravasate and form   metastatic   foci in an experimental metastasis model in nude mice. The findings in this study suggested that the enhanced signaling is required for inducing higher metastatic capacity of tumor cells, and that targeting one of stimuli or signaling pathways might be potential approach in comprehensive strategy for tumor therapy. 
 Acacetin inhibits the invasion and migration of human non-small cell lung cancer A549 cells by suppressing the p38α MAPK signaling pathway. Lung cancer is one of the most common malignancies in the world and its   metastasi  s is the major cause of death in cancer patients. Acacetin (5,7-dihydroxy-4'-methoxyflavone), a flavonoid compound, has anti-peroxidative and anti-inflammatory effects. The effect of acacetin on invasion and migration in human NSCLC A549 cells was investigated. First, the result demonstrated acacetin could exhibit an inhibitory effect on the abilities of the adhesion, morphology/actin cytoskeleton arrangement, invasion, and migration by cell-matrix adhesion assay, immunofluorescence assay, Boyden chamber assay, and wound-healing assay. Molecular data showed that the effect of acacetin in A549 cells might be mediated via sustained inactivation of the phosphorylation of mixed-lineage protein kinase 3 (MLK3), mitogen-activated protein kinase kinases 3/6 (MKK3/6), and p38α MAPK signal involved in the downregulation of the expressions of matrix metalloproteinase-2 (MMP-2), matrix metalloproteinase-9 (MMP-9), and urokinase-type plasminogen activator (u-PA). Next, acacetin significantly decreased in the phosphorylation and degradation of inhibitor of kappaBα (IκBα), and the nuclear levels of nuclear factor kappa B (NF-κB), c-Fos, and c-Jun. Also, the treatment with acacetin to A549 cells also leads to a concentration-dependent inhibition on the binding abilities of NF-κB and activator protein-1 (AP-1). Furthermore, the treatment of specific inhibitor for p38 MAPK (SB203580) to A549 cells could cause reduced activities of MMP-2/9 and u-PA. In addition, acacetin significantly decreased the levels of phospho-p38α MAPK, MMP-2/9, and u-PA in p38α-cDNA-transfected cells concomitantly with a marked reduction on cell invasion and migration. Our results revealed the anti-migration and anti-invasion effects of acacetin, which may act as a promising therapeutic agent for the treatment of lung cancer. 
 Estrogen receptorβ2 regulates interlukin-12 receptorβ2 expression via p38 mitogen-activated protein kinase signaling and inhibits non-small-cell lung cancer proliferation and invasion. Lung cancer is one of the most common types of cancer and is the leading cause of cancer-related mortality worldwide. Estrogens are known to be involved in the development and progression of non-small-cell lung cancer (NSCLC). These effects are initially mediated through binding of estrogen to estrogen receptors (ERs), in particular ERβ2. Our preliminary studies demonstrated that ERβ2 and interleukin-12 receptorβ2 (IL-12Rβ2) expression are correlated in NSCLC. The present study investigated the expression of these proteins in NSCLC cells and how changes in their expression affected cell proliferation and invasion. In addition, it aimed to explore whether p38 mitogen-activated protein kinase (p38MAPK) is involved in the regulation of IL-12Rβ2 expression by ERβ2. An immunocytochemical array was used to observe the distribution of ERβ2 and IL-12Rβ2. Co-immuoprecipitation was employed to observe the interaction between p38MAPK and IL-12Rβ2, by varying the expression of ERβ2 and p38MAPK. Western-blot analysis and reverse transcription-polymerase chain reaction assays were used to investigate the mechanism underlying ERβ2 regulation of IL-12Rβ2 expression. 3-(4,5-dimethylthiazol-2-yl)-2,5-diphenyltetrazolium bromide, scratch wound healing and Transwell assays were used to investigate the impact of ERβ2 on proliferative, invasive and migratory abilities of NSCLC cells. ERβ2 was predominantly found in the cytoplasm and nucleus, whilst IL-12Rβ2 was largely confined to the cytoplasm, although a degree of expression was observed in the nucleus. Compared with normal bronchial epithelial cells, IL-12Rβ2 and ERβ2 were overexpressed in the NSCLC cell groups. Coimmuoprecipitation demonstrated an interaction between p38MAPK and IL-12Rβ2. ERβ2 appeared to upregulate IL-12Rβ2 expression and inhibition of p38MAPK attenuated this effect. ERβ2 and IL-12Rβ2 expression inhib  ited the p  roliferation, metastasis and invasion of NSCLC cell lines, but knockout of IL-12Rβ2, even in the presence of ERβ2, led to an increase in NSCLC cell proliferation and invasiveness. In conclusion, to the best of our knowledge this study is the first to demonstrate that IL-12Rβ2 may be important in the mechanisms underlying ERβ2 inhibition of NSCLC development, and that this interaction may be mediated via p38MAPK. 
 LY2228820 dimesylate, a selective inhibitor of p38 mitogen-activated protein kinase, reduces angiogenic endothelial cord formation in vitro and in vivo. LY2228820 dimesylate is a highly selective small molecule inhibitor of p38α and p38β mitogen-activated protein kinases (MAPKs) that is currently under clinical investigation for human malignancies. p38 MAPK is implicated in a wide range of biological processes, in particular those that support tumorigenesis. One such process, angiogenesis, is required for tumor growth an  d metastas  is, and many new cancer therapies are therefore directed against the tumor vasculature. Using an in vitro co-culture endothelial cord formation assay, a surrogate of angiogenesis, we investigated the role of p38 MAPK in growth factor- and tumor-driven angiogenesis using LY2228820 dimesylate treatment and by shRNA gene knockdown. p38 MAPK was activated in endothelial cells upon growth factor stimulation, with inhibition by LY2228820 dimesylate treatment causing a significant decrease in VEGF-, bFGF-, EGF-, and IL-6-induced endothelial cord formation and an even more dramatic decrease in tumor-driven cord formation. In addition to involvement in downstream cytokine signaling, p38 MAPK was important for VEGF, bFGF, EGF, IL-6, and other proangiogenic cytokine secretion in stromal and tumor cells. LY2228820 dimesylate results were substantiated using p38α MAPK-specific shRNA and shRNA against the downstream p38 MAPK effectors MAPKAPK-2 and HSP27. Using in vivo models of functional neoangiogenesis, LY2228820 dimesylate treatment reduced hemoglobin content in a plug assay and decreased VEGF-A-stimulated vascularization in a mouse ear model. Thus, p38α MAPK is implicated in tumor angiogenesis through direct tumoral effects and through reduction of proangiogenic cytokine secretion via the microenvironment. 
 Interleukin-8 induces the endothelial cell migration through the Rac 1/RhoA-p38MAPK pathway. Endothelial cell migration is essential for tumor angiogenesis, and interleukin-8 (IL-8) has been shown to play an important role in tumor growth, angiogenesis, and   metastasis  . The objective of this study was to investigate the molecular mechanism of IL-8 induced endothelial cell migration in vitro.Fluorescence microscope was used to study the distribution of cytoskeleton. The expression of Rac1 and RhoA protein was detected by western blotting. After endothelial cells were transfected by lipofectamine 2000 reagent, the Transwell chamber motility assay was applied to observe the migration of endothelial cells induced by IL-8. The active p38MAPK (mitogen-activated protein kinase) was evaluated by the p38MAPK activation assay.We demonstrated that IL-8 activated cell migration can be impaired by p38MAPK inhibitor, suggesting the participation of p38MAPK in the cell migration. Our results indicated that p38MAPK signaling is required for membrane ruffles, lamellipodia extensions, and actin stress fibers formation induced by IL-8. Furthermore, p38MAPK inhibitor led to increased Rac1 and RhoA expression in IL-8 treated EA.hy926 cells. In addition, IL-8 induced p38MAPK activation was suppressed by dominant-negative mutant for Rac1 and RhoA.Our study demonstrates that IL-8-Rac1/RhoA-p38MAPK signaling pathway plays a vital role in the IL-8-induced endothelial cell migration, and it provides new insight into the molecular mechanisms by which IL-8 contributes to tumor angiogenesis and   metastasis  . 
 Kiss-1 suppresses MMP-9 expression by activating p38 MAP kinase in human stomach cancer. Kiss-1 has been identified as a putative   metastasis   suppressor gene in various human malignancies. However, there is little information about its possible role in gastric carcinoma. In this study, we determined whether the Kiss-1 gene negatively regulates MMP-9 expression. cDNA microarray technology was used to identify the genes associated with   metastasis   by hepatocyte growth factor (HGF) in the gastric cancer cell lines, NUGC-3 and MKN-28. The levels of Kiss-1 RNA and protein were confirmed to be upregulated in HGF-treated gastric cancer cells. HGF induced Kiss-1 and MMP-9 production in a dose-dependent manner. In order to investigate roles of HGF signaling in tumor progression and   metastasis  , we measured effects of a specific MEK1 inhibitor (PD 098059) and a p38 kinase inhibitor (SB 203580) on HGF-mediated cell proliferation and MMP-9. Pretreatment with PD 098059 reduced MMP-9 and HGF-mediated cell proliferation, but increased Kiss-I expression. In contrast, SB 203580 pretreatment enhanced MMP-9 and cell prolifera-tion, but decreased Kiss-1 expression. Cotreatment of PD098059 and SB203580 increased the p38 phosphorylation stimulated by HGF. These results suggest that the HGF-mediated Kiss-1 overexpression is regulated mainly by the p38 activation and, furthermore, the activation of ERK might affect HGF-mediated Kiss-1 expression indirectly by the regulation of p38 kinase. Consistent with this result, p38 phosphorylation was strongly repressed by the knock-down of Kiss-1. Downregulation of Kiss-1 using Kiss-1 shRNA also increased in vitro cell invasion. In conclusion, Kiss-1 suppresses MMP-9 expression by activating the p38 MAP kinase signaling pathway. 
 p38 MAPK inhibits breast cancer   metastasis   through regulation of stromal expansion. p38 MAPK signaling controls cell growth, proliferation and the cell cycle under stress conditions. However, the function of p38 activation in tumor   metastasis   is still not well understood. We report that p38 activation in breast cancer cells inhibits tumor   metastasis   but does not substantially modulate primary tumor growth. Stable p38 knockdown in breast cancer cells suppressed NF-κB p65 activation, inhibiting miR-365 expression and resulting in increased IL-6 secretion. The inhibitory effect of p38 signaling on   metastasi  s was mediated by suppression of mesenchymal stem cell (MSC) migration to the primary tumor and sites of   metastasi  s, where MSCs can differentiate into cancer-associated fibroblasts to promote tumor   metastasi  s. The migration of MSCs to these sites relies on CXCR4-SDF1 signaling in the tumor microenvironment. Analysis of human primary and metastatic breast cancer tumors showed that p38 activation was inversely associated with IL-6 and vimentin expression. This study suggests that combination analysis of p38 MAPK and IL-6 signaling in patients with breast cancer may improve prognosis and treatment of metastatic breast cancer. 
 Deacetylated GM3 promotes uPAR-associated membrane molecular complex to activate p38 MAPK in metastatic melanoma. GM3, the simplest ganglioside, regulates cell proliferation, migration, and invasion by influencing cell signaling at the membrane level. Although the classic N-acetylated form of GM3 (NeuAcLacCer) is commonly expressed and has been well studied, deacetylated GM3 (NeuNH2LacCer, d-GM3) has been poorly investigated, despite its presence in metastatic tumors but not in noninvasive melanomas or benign nevi. We have recently found that d-GM3 stimulates cell migration and invasion by activating urokinase plasminogen activator receptor (uPAR) signaling to augment matrix metalloproteinase-2 (MMP-2) function. However, the mechanisms by which d-GM3/uPAR increase MMP-2 expression and activation are not clear. By modifying the expression of d-GM3 genetically and biochemically, we found that decreasing d-GM3 expression inhibits, whereas overexpressing d-GM3 stimulates, p38 mitogen-activated protein kinase (MAPK) activity to influence MMP-2 expression and activation. p38 MAPK (p38) activation requires the formation of a membrane complex that contains uPAR, caveolin-1, and integrin α5β1 in membrane lipid rafts. In addition, knocking down or inhibiting focal adhesion kinase (FAK), phosphoinositide 3-kinase (PI3K), or Src kinase significantly reduces d-GM3-induced p38 phosphorylation and activation. Taken together, these results suggest that d-GM3 enhances the metastatic phenotype by activating p38 signaling through uPAR/integrin signaling with FAK, PI3K, and Src kinase as intermediates. Elucidation of the mechanisms by which d-GM3, a newly discovered, potential biomarker of metastatic melanomas, promotes cel  l metastas  is will help us to understand the function of d-GM3 in metastatic melanomas and may lead to novel GM3-based cancer therapies. 
 The MLK-related kinase (MRK) is a novel RhoC effector that mediates lysophosphatidic acid (LPA)-stimulated tumor cell invasion. The small GTPase RhoC is overexpressed in many invasive tumors and is essential for   metastasis  . Despite its high structural homology to RhoA, RhoC appears to perform functions that are different from those controlled by RhoA. The identity of the signaling components that are differentially regulated by these two GTPases is only beginning to emerge. Here, we show that the MAP3K protein MRK directly binds to the GTP-bound forms of both RhoA and RhoC in vitro. However, siRNA-mediated depletion of MRK in cells phenocopies depletion of RhoC, rather than that of RhoA. MRK depletion, like that of RhoC, inhibits LPA-stimulated cell invasion, while depletion of RhoA increases invasion. We also show that active MRK enhances LPA-stimulated invasion, further supporting a role for MRK in the regulation of invasion. Depletion of either RhoC or MRK causes sustained myosin light chain phosphorylation after LPA stimulation. In addition, activation of MRK causes a reduction in myosin light chain phosphorylation. In contrast, as expected, depletion of RhoA inhibits myosin light chain phosphorylation. We also present evidence that both RhoC and MRK are required for LPA-induced stimulation of the p38 and ERK MAP kinases. In conclusion, we have identified MRK as a novel RhoC effector that controls LPA-stimulated cell invasion at least in part by regulating myosin dynamics, ERK and p38. 
 Characterization of LY2228820 dimesylate, a potent and selective inhibitor of p38 MAPK with antitumor activity. p38α mitogen-activated protein kinase (MAPK) is activated in cancer cells in response to environmental factors, oncogenic stress, radiation, and chemotherapy. p38α MAPK phosphorylates a number of substrates, including MAPKAP-K2 (MK2), and regulates the production of cytokines in the tumor microenvironment, such as TNF-α, interleukin-1β (IL-1β), IL-6, and CXCL8 (IL-8). p38α MAPK is highly expressed in human cancers and may play a role in tumor growth, inva  sion, meta  stasis, and drug resistance. LY2228820 dimesylate (hereafter LY2228820), a trisubstituted imidazole derivative, is a potent and selective, ATP-competitive inhibitor of the α- and β-isoforms of p38 MAPK in vitro (IC(50) = 5.3 and 3.2 nmol/L, respectively). In cell-based assays, LY2228820 potently and selectively inhibited phosphorylation of MK2 (Thr334) in anisomycin-stimulated HeLa cells (at 9.8 nmol/L by Western blot analysis) and anisomycin-induced mouse RAW264.7 macrophages (IC(50) = 35.3 nmol/L) with no changes in phosphorylation of p38α MAPK, JNK, ERK1/2, c-Jun, ATF2, or c-Myc ≤ 10 μmol/L. LY2228820 also reduced TNF-α secretion by lipopolysaccharide/IFN-γ-stimulated macrophages (IC(50) = 6.3 nmol/L). In mice transplanted with B16-F10 melanoma, tumor phospho-MK2 (p-MK2) was inhibited by LY2228820 in a dose-dependent manner [threshold effective dose (TED)(70) = 11.2 mg/kg]. Significant target inhibition (>40% reduction in p-MK2) was maintained for 4 to 8 hours following a single 10 mg/kg oral dose. LY2228820 produced significant tumor growth delay in multiple in vivo cancer models (melanoma, non-small cell lung cancer, ovarian, glioma, myeloma, breast). In summary, LY2228820 is a p38 MAPK inhibitor, which has been optimized for potency, selectivity, drug-like properties (such as oral bioavailability), and efficacy in animal models of human cancer. 
 Expression and clinical significance of the nin one binding protein and p38 MAPK in prostate carcinoma. Prostate carcinoma is a major cause of morbidity and mortality. The MAPK Signaling Pathway plays an important role in multiple tumors, including prostate carcinoma. MAPK signaling is mediated by ERK1/2, JNK and p38 MAPK, which are important in the control of cell proliferation, differentiation and apoptosis. However, relatively little is known about the regulatory mechanism of p38 MAPK in prostate cancers. NOB1 is among the most novel topic in MAPK studies currently. Recent studies found its vital role in tumor   metastasis   in glioblastoma proliferation, however, its expression profile and its prognostic value in prostate carcinoma have not been investigated.To determine the relationship between NOB1 and p38 MAPK expressions, a population-based study was conducted for immunohistochemical staining analysis of tumor tissues, in matched malignant and nonmalignant prostatectomy samples from 132 PCa patients. Moreover, Western blot analysis and NOB1 interference studies of prostate cancer cell lines. To evaluate the diagnostic and prognostic between NOB1 and p38 MAPK in prostate cancer (PCa) tissue after radical prostatectomy, the hypothesis that prostate cancers with NOB1 expression have distinct clinical, prognostic and molecular attributes was tested.Among 132 prostate cancers, NOB1 expression was detected in 117 (88.7%) tumors by immunohistochemistry. NOB1 and p38 MAPK expression had significant positive correlation with carcinogenesis, tumor progression and patient survival. Immunohistochemically, NOB1 expression in prostate cancer was independently associated with p38 MAPK activation (P=0.0002). Furthermore, p38 MAPK expression was completely suppressed by NOB1 interference in the prostate cancer cell lines DU-145 and PC-3.NOB1 expression status was closely correlated with important histopathologic characteristics and the recurrence and   metastasis   of prostate carcinomas. These data support a potential link between NOB1 and p38 MAPK, and suggest that NOB1 may identify a subset of prostate cancer patients with a poor prognosis. This study proved that NOB1 in PCa tissue can be used, in combination with traditional clinicopathological factors, as promising diagnostic and prognostic tools. 
 Suppression of phorbol-12-myristate-13-acetate-induced tumor cell invasion by apigenin via the inhibition of p38 mitogen-activated protein kinase-dependent matrix metalloproteinase-9 expression. Apigenin has special interest for the development of chemopreventive agents against cancer because it is a widely distributed plant flavonoid that has antitumor properties. In this study, we investigated the apigenin effects on the protease-mediated invasiveness in human metastatic cancer cell lines Caski, SK-Hep1, and MDA-231. We found that apigenin markedly inhibits the phorbol-12-myristate-13-acetate (PMA)-induced increase in MMP-9 expression and activity in several cancer cell lines. These effects of apigenin are dose-dependent and correlate with the suppression of MMP-9 mRNA expression levels. PMA caused about a 5-fold induction in MMP-9 promoter activity, which was also suppressed by apigenin treatment in Caski cells. We found that apigenin could inhibit PMA-induced phosphorylation of p38 mitogen-activated protein kinase (p38 MAPK), which was involved in the down-regulation of the expression of matrix metalloproteinase-9 (MMP-9) at mRNA levels. Furthermore, the treatment of inhibitors specific for p38 MAPK (SB203580) to Caski cells caused the reduction of MMP-9 expression. Restoration of p38 expression partly increased PMA-induced MMP-9 secretion blocked by apigenin treatment in Caski cells. These results showed apigenin might inhibit the invasion and migration abilities of Caski cells by reducing the MMP-9 expression through suppressing the p38 MAPK signaling pathway. These findings indicate that apigenin might be a useful strategy for controlling   metastasis   and the invasiveness of tumors. 
 Tissue factor/activated factor VIIa induces matrix metalloproteinase-7 expression through activation of c-Fos via ERK1/2 and p38 MAPK signaling pathways in human colon cancer cell. Increased expression of tissue factor (TF) is associated with tumor invasion and   metastasis   in human colorectal cancer. We have previously observed that TF/FVIIa upregulates matrix metalloproteinase-7 (MMP-7) expression at the transcriptional level in colon cancer cells. MMP-7 overexpression is believed to play an important role in tumor invasion and   metastasis  . The aim of this study is to elucidate the molecular mechanisms by which TF/FVIIa induced MMP-7 expression and cell invasion in vitro.Reverse transcription polymerase chain reaction, Western blot, luciferase assay, and chromatin immunoprecipitation (ChIP) were used to determine the potential mechanism and signaling pathways by which TF/FVIIa induced MMP-7 expression and cell invasion in LoVo cells. Small interfering RNA (siRNA) and cell invasion assay was used to examine whether blocking c-Fos expression could abolish FVIIa-mediated upregulation of MMP-7 and cell invasion in vitro.The results showed that FVIIa induced the upregulation of MMP-7 both at the mRNA and protein levels in a time- and dose-dependent manner and increased the invasive behavior of LoVo cells. FVIIa enhanced the promoter activity of MMP-7, and the activator protein-1 (AP-1) binding site was responsible for the activation. Site mutation of the AP-1 binding site in the promoter almost completely abolished FVIIa-mediated response. Furthermore, ChIP assay confirmed that FVIIa promoted the direct binding of c-Fos with the MMP-7 promoter in vivo. FVIIa also induced the expression and nuclear accumulation of the AP-1 subunit c-Fos. siRNA-mediated knockdown of c-Fos eliminated FVIIa-stimulated MMP-7 expression and cell migration in vitro. In addition, selective mitogen-activated protein kinase (MAPK) kinase (MEK1/2) inhibitor (PD98059) and p38 MAPK inhibitor SB203580 suppressed MMP-7 upregulation induced by FVIIa.Our data suggest that a novel TF/FVIIa/MAPK/c-Fos/MMP-7 axis plays an important role in modulating the invasion of colon cancer cells and blockage of this pathway holds promise to treat colon cancer   metastasis  . 
 Carbonic anhydrase XII promotes invasion and migration ability of MDA-MB-231 breast cancer cells through the p38 MAPK signaling pathway. Carbonic anhydrase (CA) XII, an extracellular enzyme involved in the regulation of the microenvironment acidity and tumor malignant phenotype, was originally identified as a protein overexpressed in some types of cancers, including breast cancer. However, the cellular function and mechanism of CAXII remained unclear. In this study, the effects of CAXII expression on invasion and migration of breast cancer cells was investigated. Gene knockdown of CAXII in the human breast cancer cell line MDA-MB-231 resulted in decreased invasion and migration by interfering with the p38 MAPK pathway. CAXII knockdown also decreased the expression of matrix metalloproteinase (MMP)-2, MMP-9, and urokinase-type plasminogen activator (u-PA), but increased tissue inhibitor of metalloproteinases (TIMP)-2 and plasminogen activator inhibitor (PAI)-1 expression. Furthermore, decreased invasive and migration ability of CAXII-knockdown cells were restored by an overexpression of CAXII. Results also showed that CAXII knockdown may decrease anchorage-independent growth and cell growth by inhibiting CDK6 and cyclin D1 expression. Furthermore, the impact of CAXII knockdown on invasion, migration and cell growth was further evidenced by effects on tumor size and   metastasis   of MDA-MB-231 cells in vivo. Taken together, these data suggested that CAXII may affect the capability of invasion and migration of MDA-MB-231 cells, which may be mediated through the p38 MAPK pathway. 
 Stromal cell-derived factor-1 (SDF-1) enhances cells invasion by αvβ6 integrin-mediated signaling in ovarian cancer. Ovarian carcinoma is a common gynecological malignancy and a great threat to health as a result o  f metastas  is. The chemokine stromal-derived factor (SDF-1) plays multiple roles in tumor pathogenesis. However, the precise molecular mechanism underlying SDF-1-induced ovarian cancer cell invasion is still undefined. αvβ6 integrin is an important factor in tumor progression. Therefore, we speculate that SDF-1-enhanced ovarian cancer cell invasion is related to αvβ6 integrin-mediated signaling. After culturing with SDF-1, an obvious time- and dose-dependent increase in αvβ6 integrin was demonstrated. Furthermore, CXC receptor 4 (CXCR4) was responsible for SDF-1-induced αvβ6 integrin expression. Simultaneously, SDF-1 was found to dramatically enhance extracellular matrix degradation via urokinase-type plasminogen activator (uPA) expression and cell invasion by αvβ6 integrin expression; these reinforce failed to be increased when pretreatment was performed with the CXCR4 inhibitor AMD3100 or anti-αvβ6 integrin antibody, respectively. In addition, αvβ6 integrin induced the phosphorylation of p38 MAPK and PI3 K/Akt, contributing to the up-regulation of uPA, as treatment with the specific inhibitor for p38 mitogen-activated protein kinases (MAPK) (SB203580) or phosphatidylinositol 3-kinase (PI3 K)/Akt (LY294002) strikingly abrogated uPA expression. Taken together, these results demonstrated that SDF-1 enhanced ovarian cancer cell invasion through αvβ6 integrin-mediated uPA expression via the p38 MAPK and PI3 K/Akt pathway. Consequently, our findings will provide a new explanation about how SDF-1 aggravates the pathogenesis of ovarian cancer. 
 [Expression of p38MAPK in salivary adenoid cystic carcinoma and their significance]. To investigate the expression of p38 mitogen-activated protein kinase (p38MAPK) in salivary adenoid cystic carcinoma (SACC) tissues and their clinicopathologic significance.The protein and mRNA expressions of p38MAPK were examined by immunohistochemistry and in situ hybridization, respectively, in 52 cases of SACC and in 11 normal salivary gland tissues adjacent to the tumors on a tissue microarray platform.The positive rate of p38MAPK protein expression in the paracancerous normal salivary gland tissues and that in SACC were 36.4% and 96.2%, respectively, showing a significant statistical difference (P < 0.01). The protein expression of p38MAPK was positively correlated with the lymph node   metastasis   and nerve involvement (P < 0.05). The positive rates of p38MAPK mRNA in the paracancerous tissues and in the SACC tissues were 45.5% and 94.2%, respectively, with a significant statistical difference (P < 0.01). The mRNA expression of p38MAPK was positively correlated with the lymph node   metastasis   and nerve involvement (P < 0.05). In the SACC, there was a notable positive correlation between the p38MAPK protein and mRNA expressions (r = 0.409, P < 0.01).The expression of p38MAPK is up-regulated in salivary adenoid cystic carcinoma. p38MARK may be involved in the tumorigenesis, development and   metastasis   of this cancer. 
 Piperine inhibits IL-1β-induced IL-6 expression by suppressing p38 MAPK and STAT3 activation in gastric cancer cells. Piperine, a kind of natural alkaloid found in peppers, has been reported to exhibit anti-oxidative and anti-tumor activities, both in vitro and in vivo. Interleukin-6 (IL-6) is an important cytokine that activates the signal transduction, promotes tumor cell   metastasi  s, and induces malignancy, including in gastric cancer. However, the effects of piperine on IL-6 expression in gastric cancer cells have not yet been well defined. In this study, we investigated the effects of piperine on the IL-6 expression, and examined the underlying signaling pathways via RT-PCR, promoter studies and Western blotting in human gastric cancer TMK-1 cells. Our results showed that piperine inhibited interleukin-1β (IL-1β)-induced IL-6 expression in a dose-dependent manner. In addition, piperine also inhibited IL-6 promoter activity. Experiments with mitogen-activated protein kinase (MAPK) inhibitors and dominant negative mutant p38 MAPK indicated that p38 MAPK was essential for IL-6 expression in the TMK-1 cells. Additionally, signal transducer and activator of transcription 3 (STAT3) was also involved in the IL-1β-induced IL-6 expression in gastric cancer cells. Piperine inhibited IL-1β-induced p38 MAPK and STAT3 activation and, in turn, blocked the IL-1β-induced IL-6 expression. Furthermore, gastric cancer cells pretreated with IL-1β showed markedly enhanced invasiveness, which was partially abrogated by treatment with IL-6 siRNA, piperine, and inhibitors of p38 MAPK and STAT3. These results suggest that piperine may exert at least part of its anti-cancer effect by controlling IL-6 expression through the suppression of p38 MAPK and STAT3. 
 IL-1β-induced activation of p38 promotes   metastasi  s in gastric adenocarcinoma via upregulation of AP-1/c-fos, MMP2 and MMP9. Interleukin-1β (IL-1β) has been implicated in the progression of gastric adenocarcinoma (GA); however, the molecular mechanisms of action of IL-1β in GA are poorly characterized. P38 and JNK are the major MAPK family members that regulate IL-1β signaling pathways. Here, we investigated the role of both p38 and JNK in IL-1β-induced GA cell migration, invasion and metastatic potential.The effects of IL-1β-induced p38 and JNK activation in GA cells were determined using in vitro Transwell migration and invasion assays of MKN-45 and AGS cells, or an i  n vivo met  astasis assay in nude mice. The IL-1β-induced p38 signaling pathway was further characterized in GA cells. Activation of the IL-1β/p38 signaling pathway was also assessed in human primary GA tissues by immunohistochemistry.IL-1β-induced activation of p38 increased GA cell migration and invasion in vitro and promoted the metastatic potential of GA cells in vivo; these effects were attenuated by p38 siRNA or the p38 inhibitor SB202190. MMP2 or MMP9 siRNAs and the MMP2/9 inhibitor BiPS also inhibited IL-1β-induced GA cell migration and invasion in vitro. IL-1β-induced p38 activation significantly increased MMP2 and MMP9 mRNA and protein expression and activity. Luciferase reporter assays demonstrated that the activator protein-1 (AP-1) and the AP-1 binding sites of the MMP9 promoter (-670/MMP9) were activated by IL-1β-induced p38 activation. Phospho-p38 was significantly upregulated in human GA tissues (compared to matched non-neoplastic tissues), and significantly associated wit  h lymph no  de metastasis, and invasion beyond the serosa. Expression of phospho-p38 significantly correlated with IL-1β, MMP2, MMP9, and c-fos expression in both human GA tissues and GA cell metastases in the lungs of nude mice. IL-1β was also capable of activating JNK in GA cells, but activation of JNK was not associated with GA cell migration and invasion. Therefore, IL-1β-induced the migration and invasion in GA cells were regulated by p38, but not by JNK.IL-1β-induced p38 activation and the IL-1β/p38/AP-1(c-fos)/MMP2 & MMP9 pathway play an   important   role in metastasis in GA; this pathway may provide a novel therapeutic target for GA. 
 Induction of matrix metalloproteinase-9 by galectin-7 through p38 MAPK signaling in HeLa human cervical epithelial adenocarcinoma cells. The expression of matrix metalloproteinase-9 (MMP-9) has been reported in various cancers. Its expression is associated with tumorigenesis and tumor   metastasis  . Studies have shown that galectin-7, a beta-galactoside-binding animal lectin, is involved in various processes including the suppression of tumor growth. However, a recent study reported that the development of thymic lymphoma is accelerated by galectin-7 expression. In this report, we demonstrate that the expression of MMP-9 was increased by galectin-7 in human cervix epithelial cells (HeLa). When the galectin-7 gene was transfected to HeLa cells with the ECFP vector, the expression of MMP-9 mRNA increased, as compared to non-transfected cells. As a result, MMP-9 protein levels also increased, as indicated by Western blot and gelatin zymography. In addition, galectin-7-transfected cells exhibited increased invasion in the matrigel invasion system. Expression of MMP-9 is involved in several signaling pathways by various stimulation factors. Therefore, we investigated how the signaling pathway in galectin-7-transfected cells differs from that of non-transfected cells. Upon transfection of galectin-7, p38 MAPK was activated and SB203580, a chemical inhibitor of p38 MAPK, reversed the effects of galectin-7. These results indicate that galectin-7 increases the expression of MMP-9 through the p38 MAPK signaling pathway. 
 Dysregulation of the cell survival/anti-apoptotic NF-kappaB pathway by the novel humanized BM-ca anti-CD20 mAb: implication in chemosensitization. Treatment of patients with relapsed or refractory low grade follicular B-NHL lymphoma with rituximab (chimeric anti-CD20 mAb) has resulted in approximately 50% response rate. The mechanism underlying the failure of rituximab to affect the remaining 50% of the patients is not clear, though their tumors express CD20. The in vivo effector functions of rituximab include ADCC, CDC and seldom apoptosis. In addition, we have reported that rituximab signals the cells and inhibits several intracellular cell survival pathways that are responsible for the immuno and chemo-sensitizing effects of rituximab on resistant B-NHL cell lines. The objective of this study was to develop novel and fully humanized anti-CD20 monoclonal antibodies with enhanced effector functions and molecular signaling that may potentiate their therapeutic efficacy. Novel humanized anti-CD20 monoclonal antibodies were derived from a chimerized form of murine anti-CD20 1K11791, shown to exert a more potent ADCC, CDC and apoptotic activities compared to rituximab. A representative humanized monoclonal antibody, BM-ca was used to examine its biological effect and molecular signaling using Ramos B-NHL cell line as a model. The studies were also performed in parallel with rituximab treatment for comparison. Ramos cells were treated with various concentrations of BM-ca monoclonal antibody. Inhibition of cell proliferation was observed in a concentration-dependent manner, suggesting cell signal perturbations must have occurred. Compared to untreated cells, treatment with BM-ca inhibited both the constitutively activated NF-kappaB and p38 MAPK pathways, as assessed by inhibition of both phospho-p65 and phospho-IkappaBalpha and phospho-p38, respectively, but not the unphosphorylated forms. BM-ca significantly induced the expression of the   metastasis   suppressor and immune surveillance cancer gene product, Raf-1 kinase inhibitor protein (RKIP). These alterations resulted in inhibition of anti-apoptotic gene products and sensitized Ramos cells to apoptosis by CDDP. In comparison with rituximab, BM-ca showed qualitative and quantitative differences in the above analyses. These findings demonstrate that BM-ca triggers CD20 expressing B-NHL cells resulting in a significant alteration of several gene products that regulate cell growth and chemoresistance. 
 p38 mitogen-activated protein kinase-dependent activation of protein phosphatases 1 and 2A inhibits MEK1 and MEK2 activity and collagenase 1 (MMP-1) gene expression. Degradation of collagenous extracellular matrix by collagenase 1 (also known as matrix metalloproteinase 1 [MMP-1]) plays a role in the pathogenesis of various destructive disorders, such as rheumatoid arthritis, chronic ulcers, and tumor invasion and   metastasis  . Here, we have investigated the role of distinct mitogen-activated protein kinase (MAPK) pathways in the regulation of MMP-1 gene expression. The activation of the extracellular signal-regulated kinase 1 (ERK1)/ERK2 (designated ERK1,2) pathway by oncogenic Ras, constitutively active Raf-1, or phorbol ester resulted in potent stimulation of MMP-1 promoter activity and mRNA expression. In contrast, activation of stress-activated c-Jun N-terminal kinase and p38 pathways by expression of constitutively active mutants of Rac, transforming growth factor beta-activated kinase 1 (TAK1), MAPK kinase 3 (MKK3), or MKK6 or by treatment with arsenite or anisomycin did not alone markedly enhance MMP-1 promoter activity. Constitutively active MKK6 augmented Raf-1-mediated activation of the MMP-1 promoter, whereas active mutants of TAK1 and MKK3b potently inhibited the stimulatory effect of Raf-1. Activation of p38 MAPK by arsenite also potently abrogated stimulation of MMP-1 gene expression by constitutively active Ras and Raf-1 and by phorbol ester. Specific activation of p38alpha by adenovirus-delivered constitutively active MKK3b resulted in potent inhibition of the activity of ERK1,2 and its upstream activator MEK1,2. Furthermore, arsenite prevented phorbol ester-induced phosphorylation of ERK1,2 kinase-MEK1,2, and this effect was dependent on p38-mediated activation of protein phosphatase 1 (PP1) and PP2A. These results provide evidence that activation of signaling cascade MKK3-MKK3b-->p38alpha blocks the ERK1,2 pathway at the level of MEK1,2 via PP1-PP2A and inhibits the activation of MMP-1 gene expression. 
 Stress-activated kinase pathway alteration is a frequent event in bladder cancer. The stress-activated MAP kinases (SAPK) signaling pathways play a critical role in the cellular response to toxins and physical stress, mediate inflammation, and modulate carcinogenesis and tumor   metastasis  . The stress-activated MAP kinases (MAPK) c-Jun N-terminal kinase (JNK) and p38 are activated upon phosphorylation by a widely expressed and conserved family of upstream MAP kinase kinases (MAP2K). Signaling mediated by p38 and JNK has well-established importance in cancer, yet the contribution of this pathway in urothelial bladder cancer is not understood. This study evaluated stress-activated MAP kinase pathway expression in cell lines derived from human urothelial carcinomas.Total protein lysates from a panel of human urothelial bladder cancer cell lines (RT4, T24, UMUC-3, J82, 5637, 253J, and 253J-BV) were analyzed by immunoblotting for the JNK and p38 MAPKs, as well as MKK3, MKK4, MKK6, and MKK7. Quantitative real time PCR was utilized to determine mRNA expression levels of the MAP2Ks. Stress stimuli (sorbitol, hydrogen peroxide, and UV irradiation) were used to active p38, which was measured by phospho-antibody.Although protein levels were variable, all cell lines expressed p38 and JNK. On the other hand, with the exception of the well-differentiated cell line RT4, each cell line had a reduction or absence of expression of one or more MAP2K. 253J and 253J-BV exhibited no expression of MKK6, even when an excess of protein was queried. mRNA levels indicated that both transcriptional and post-transcriptional mechanisms are involved in the regulation of MAP2Ks. Decreased MAP2K expression correlated with decreased ability to activate p38 in response to stress stimuli.Aberrant MAP2K protein expression indicates that altered cellular signal transduction mediated via JNK and p38 may be common in bladder cancer. Down-regulation of MAP2Ks likely occurs at both the transcriptional and post-transcriptional levels. Consistent with the known function of p38 and JNK in apoptosis, defects in normal pathway function caused by decreased expression of upstream MAP2Ks may provide a survival advantage to bladder cancer cells. Further investigations should focus on identifying a functional role for these pathways in bladder cancer development. 
 The status of phosphorylated p38 in esophageal squamous cell carcinoma. The p38 mitogen-activated protein kinase (MAPK) is a member of the MAPK family, which is initially found to be activated by stress stimuli, proinflammatory cytokines, and growth factors. However, its role in the pathogenesis of esophageal squamous cell carcinoma (ESCC) is largely unkown, so we investigate the role of phosphorylated p38 (p-p38) MAPK in ESCC. First of all, in vitro cell line ECa109, SB203580 as selective inhibitor of p38, can suppress the growth of esophageal cancer cell in a dose- and time-dependent way, suggesting that ECa109 cell growth and proliferation was closely associated with p-p38. Using western-blot analysis of fresh 16 paired surgically resected ESCC and matched non-tumor adjacent tissues (NAT), we showed that p-p38 was significantly expressed higher in NAT compared to ESCC. Moreover, expressions of p-p38 were further confirmed by 162 paired of formalin-fixed paraffin-embedded (FFPE) ESCC and NAT by immunohistochemistry, the same trend result was obtained through statistical analysis that there was increased expression of p-p38 in NAT as compared with ESCC (P < 0.01), and expression of p-p38 was not significantly associated with lymph node  s metastas  is (P > 0.05) and ESCC differentiation degree (P > 0.05). Taken together, all the results we obtained demonstrated that p-p38 plays a key role in the malignant transformation of ESCC. 
 Computational identification of a p38SAPK-regulated transcription factor network required for tumor cell quiescence. The stress-activated kinase p38 plays key roles in tumor suppression and induction of tumor cell dormancy. However, the mechanisms behind these functions remain poorly understood. Using computational tools, we identified a transcription factor (TF) network regulated by p38alpha/beta and required for human squamous carcinoma cell quiescence in vivo. We found that p38 transcriptionally regulates a core network of 46 genes that includes 16 TFs. Activation of p38 induced the expression of the TFs p53 and BHLHB3, while inhibiting c-Jun and FoxM1 expression. Furthermore, induction of p53 by p38 was dependent on c-Jun down-regulation. Accordingly, RNAi down-regulation of BHLHB3 or p53 interrupted tumor cell quiescence, while down-regulation of c-Jun or FoxM1 or overexpression of BHLHB3 in malignant cells mimicked the onset of quiescence. Our results identify components of the regulatory mechanisms driving p38-induced cancer cell quiescence. These may regulate dormancy of residual disease that usually precedes the onset of   metastasis   in many cancers. 
 CCL20/CCR6 promotes cell proliferation and   metastasis   in laryngeal cancer by activating p38 pathway. Chemokine and its receptors play important roles in laryngeal cancer development and progression. CCR6 is the receptor of CCL20 chemokine, but its function in laryngeal cancer is not known. The aim of this study is to explore the roles of CCR6 and its regulation mechanism in laryngeal cancer. We found CCR6 expression was higher in laryngeal cancer tissues compared with their normal controls, so did in laryngeal cancer cells. Cellular function indicated that down-regulation of CCR6 in laryngeal cancer cells could suppress cell proliferation and   metastasis  . Further research showed that CCR6 could activate p38, which was related with the changes of microRNA (miRNA) profile in laryngeal cancer cells. We also found that CCR6 was positively associated with miR-20a-5p, miR-489 and negatively related to miR-29-3p, miR-632 and miR-1276 in laryngeal cancer tissues. 
 Downregulation of LAPTM5 suppresses cell proliferation and viability inducing cell cycle arrest at G0/G1 phase of bladder cancer cells. Our transcriptome analysis revealed in bladder cancer (BCa) tissues a significant induction of lysosomal-associated multispanning membrane protein 5 (LAPTM5), a lysosomal membrane protein preferentially expressing in immune cells and hematopoietic cells. Transportation of LAPTM5 from Golgi to lysosome could be inhibited by deficiency of Nedd4, a key member of E3 ubiquitin ligase family overexpressing in invasive BCa and promoting its progression. Therefore, we hypothesize that LAPTM5 may be closely correlated with BCa tumorigenesis. In human BCa tissues, we observed that LAPTM5 was significantly induced at both mRNA and protein levels, which is consistent with our microarray result. Furthermore, we established a BCa cell model with downregulated LAPTM5, revealing a significantly delayed growth rate in the BCa cells with knockdown of LAPTM5. Moreover, cell cycle arrest at G0/G1 phase was triggered by decreased LAPTM5 as well, which could lead to delayed BCa cell growth. In contrast, no significant alteration of apoptosis in the BCa cells with downregulated LAPTM5 was noticed. Analysis of the changes of migration and invasion, showed significant reduced LAPTM5 suppressed cell   metastasi  s. Furthermore, proteins involved in epithelial-mesenchymal transition (EMT) were strongly altered, which plays a central role in   metastasi  s. In addition, phosphorylated ERK1/2 and p38, key members of mitogen-activated protein kinase (MAPK) family regulating BCa tumorigenesis, were strongly decreased. Taken together, our results suggested that decreased LAPTM5 inhibited proliferation and viability, as well as induced G0/G1 cell cycle arrest possibly via deactivation of ERK1/2 and p38 in BCa cells. 
 Tenascin-W is found in malignant mammary tumors, promotes alpha8 integrin-dependent motility and requires p38MAPK activity for BMP-2 and TNF-alpha induced expression in vitro. Tenascins represent a family of extracellular matrix glycoproteins with distinctive expression patterns. Here we have analyzed the most recently described member, tenascin-W, in breast cancer. Mammary tumors isolated from transgenic mice expressing hormone-induced oncogenes reveal tenascin-W in the stroma around lesions with a high likelihood of   metastasis  . The presence of tenascin-W was correlated with the expression of its putative receptor, alpha8 integrin. HC11 cells derived from normal mammary epithelium do not express alpha8 integrin and fail to cross tenascin-W-coated filters. However, 4T1 mammary carcinoma cells do express alpha8 integrin and their migration is stimulated by tenascin-W. The expression of tenascin-W is induced by BMP-2 but not by TGF-beta1, though the latter is a potent inducer of tenascin-C. The expression of tenascin-W is dependent on p38MAPK and JNK signaling pathways. Since preinflammatory cytokines also act through p38MAPK and JNK signaling pathways, the possible role of TNF-alpha in tenascin-W expression was also examined. TNF-alpha induced the expression of both tenascin-W and tenascin-C, and this induction was p38MAPK- and cyclooxygenase-dependent. Our results show that tenascin-W may be a useful diagnostic marker for breast malignancies, and that the induction of tenascin-W in the tumor stroma may contribute to the invasive behavior of tumor cells. 
 Interaction of Snail and p38 mitogen-activated protein kinase results in shorter overall survival of ovarian cancer patients. Epithelial ovarian cancer is a highly metastatic disease and the leading cause of death among cancer of the female genital tract. Abnormal epidermal growth factor receptor (EGFR) signalling has been shown to be involved in epithelial-mesenchymal transition (EMT), an early step during   metastasis  . Additionally, over-expression of the E-cadherin repressor Snail, a key regulator of EMT, has previously been found to be associated with unfavourable prognostic features. Thus, the aim of our study was to elucidate the role of EGFR-dependent signalling pathways for Snail expression in ovarian cancer. For this purpose, we analysed 25 formalin-fixed and paraffin-embedded (FFPE) primary tumours and their corresponding metastases for the expression of 25 signalling pathway molecules by reverse phase protein arrays. We found a significant correlation of Snail with EGFR((Tyr1086)) and p38 MAPK((Thr180/Tyr182)) in primary ovarian carcinoma and with EGFR((Tyr1086)) in their corresponding   metastasis  . Additionally, we showed that high expression levels of Snail in primary tumours combined with high expression levels of the phosphorylated p38 MAPK((Thr180/Tyr182)) in   metastasis   lead to an increased risk for death in ovarian carcinoma patients. Thus, for future combinatorial cancer therapy, drug combinations that best target the deregulated protein network in each individual patient should be selected. 
 Arachidonic acid stimulates cell adhesion through a novel p38 MAPK-RhoA signaling pathway that involves heat shock protein 27. Rho GTPases are critical components of cellular signal transduction pathways. Both hyperactivity and overexpression of these proteins have been observed in human cancers and have been implicated as important factors in   metastasis  . We previously showed that dietary n-6 fatty acids increase cancer cell adhesion to extracellular matrix proteins, such as type IV collagen. Here we report that in MDA-MB-435 human melanoma cells, arachidonic acid activates RhoA, and inhibition of RhoA signaling with either C3 exoenzyme or dominant negative Rho blocked arachidonic acid-induced cell adhesion. Inhibition of the Rho kinase (ROCK) with either small molecule inhibitors or ROCK II-specific small interfering RNA (siRNA) blocked the fatty acid-induced adhesion. However, unlike other systems, inhibition of ROCK did not block the activation of p38 mitogen-activated protein kinase (MAPK); instead, Rho activation depended on p38 MAPK activity and the presence of heat shock protein 27 (HSP27), which is phosphorylated downstream of p38 after arachidonic acid treatment. HSP27 associated with p115RhoGEF in fatty acid-treated cells, and this association was blocked when p38 was inhibited. Furthermore, siRNA knockdown of HSP27 blocked the fatty acid-stimulated Rho activity. Expression of dominant negative p115-RhoGEF or p115RhoGEF-specific siRNA inhibited both RhoA activation and adhesion on type IV collagen, whereas a constitutively active p115RhoGEF restored the arachidonic acid stimulation in cells in which the p38 MAPK had been inhibited. These data suggest that n-6 dietary fatty acids stimulate a set of interactions that regulates cell adhesion through RhoA and ROCK II via a p38 MAPK-dependent association of HSP27 and p115RhoGEF. 
 Lysophosphatidic acid induces prostate cancer PC3 cell migration via activation of LPA(1), p42 and p38alpha. Prostate cancer cell migration is an essential event both in the progression of prostate cancer and in the steps leading to   metastasis  . We report here that lysophosphatidic acid (LPA), a potent bioactive phospholipid, induces prostate cancer PC3 cell migration via the activation of the LPA(1) receptor, which is linked to a PTX-sensitive activation mechanism of the mitogen-activated protein kinases (MAPK). Our results demonstrate that parallel activation of ERK1/2 and p38, but not JNK, is responsible for LPA-stimulated PC3 cell migration. Furthermore, using small interfering RNA (siRNA) technology, and overexpressing dominant-negative mutants of p38 MAPK isotypes of alpha, beta, gamma and delta, we have identified that the activation of ERK2 (p42) and p38alpha, but not of ERK1 and the other isoforms of p38 MAPK, is required for LPA-induced migration. Our study provides the first evidence for a functional role of p42 and p38alpha in LPA-induced mammalian cell migration, and also demonstrates, for the first time, that the receptor LPA(1) mediates prostate cancer cell migration. The results of the present study suggest that LPA, the receptor LPA(1), ERK2 and p38alpha are important regulators for prostate cancer cell invasion and thus could play a significant role in the development of   metastasis  . 
 The NHERF1 PDZ2 domain regulates PKA-RhoA-p38-mediated NHE1 activation and invasion in breast tumor cells. Understanding the signal transduction systems governing invasion is fundamental for the design of therapeutic strategies against   metastasis  . Na(+)/H(+) exchanger regulatory factor (NHERF1) is a postsynaptic density 95/disc-large/zona occludens (PDZ) domain-containing protein that recruits membrane receptors/transporters and cytoplasmic signaling proteins into functional complexes. NHERF1 expression is altered in breast cancer, but its effective role in mammary carcinogenesis remains undefined. We report here that NHERF1 overexpression in human breast tumor biopsies is associated with metastatic progression, poor prognosis, and hypoxia-inducible factor-1alpha expression. In cultured tumor cells, hypoxia and serum deprivation increase NHERF1 expression, promote the formation of leading-edge pseudopodia, and redistribute NHERF1 to these pseudopodia. This pseudopodial localization of NHERF1 was verified in breast biopsies and in three-dimensional Matrigel culture. Furthermore, serum deprivation and hypoxia stimulate the Na(+)/H(+) exchanger, invasion, and activate a protein kinase A (PKA)-gated RhoA/p38 invasion signal module. Significantly, NHERF1 overexpression was sufficient to induce these morphological and functional changes, and it potentiated their induction by serum deprivation. Functional experiments with truncated and binding groove-mutated PDZ domain constructs demonstrated that NHERF1 regulates these processes through its PDZ2 domain. We conclude that NHERF1 overexpression enhances the invasive phenotype in breast cancer cells, both alone and in synergy with exposure to the tumor microenvironment, via the coordination of PKA-gated RhoA/p38 signaling. 
 Involvement of p38alpha mitogen-activated protein kinase in lung   metastasis   of tumor cells. To study the role of p38 mitogen-activated protein kinase (p38) activity during the process of   metastasis  , p38alpha(+/-) mice were subjected to an in vivo   metastasis   assay. The number of lung colonies of tumor cells intravenously injected in p38alpha(+/-) mice was markedly decreased compared with that in wild-type (WT) mice. On the other hand, the time-dependent increase in tumor volume after subcutaneous tumor cells transplantation was comparable between WT and p38alpha(+/-) mice. Platelets of p38alpha(+/-) mice were poorly bound to tumor cells in vitro and in vivo compared with those of WT mice. E- and P-selectin mRNAs were markedly induced in the lung after intravenous injection of tumor cells. However, the induction of these selectin mRNAs in p38alpha(+/-) mice was weaker than that in WT mice. Furthermore, the resting expression levels of E-selectin in lung endothelial cells and P-selectin in platelets of p38alpha(+/-) mice were suppressed compared with those of WT mice. The number of tumor cells attached on lung endothelial cells of p38alpha(+/-) mice was significantly reduced compared with that of WT mice. The transmigrating activity of tumor cells through lung endothelial cells of p38alpha(+/-) mice was similar to that of WT mice. These results suggest that p38alpha plays an important role in extravasation of tumor cells, possibly through regulating the formation of tumor-platelet aggregates and their interaction with the endothelium involved in a step of hematogenous   metastasis  . 
 Lysophosphatidic acid enhances human hepatocellular carcinoma cell migration, invasion and adhesion through P38 MAPK pathway. Lysophosphatidic acid (LPA) has diverse biological activities implicated in tumor progression, including increasing cell migration, invasion and   metastasis  . However, the underlying mechanisms for LPA-induced human hepatocellular carcinoma (HCC) migration and invasion are poorly understood.We sought to determine the promoting effect of LPA on HCC migration, invasion and adhesion by transwell and matrix adhesion assays, respectively. Levels of matrix metalloproteinase, MMP-2 and MMP- 9 from cells were assayed by ELISA and p38 mitogenactivated protein kinase (MAPK) signaling was determined by western blot analysis.In the present study, LPA was found to increase HCC cell migration, invasion and adhesion. In addition, LPA increased MMP-9 expression level and induced activation of the p38 mitogen- activated protein kinase (MAPK) signaling. Furthermore, pharmacological inhibition of p38 MAPK signal pathway with SB203580 significantly attenuated LPA-induced HCC cell migration, invasion, and adhesion and abrogated LPA-induced MMP-9 expression and p38 MAPK phosphorylation.We demonstrated a mechanism that LPA can activate p38 MAPK signaling, which is required for LPA-induced HCC cell migration, invasion, adhesion and MMP-9 expression, providing a novel biomarker and potential therapeutic target for HCC. 
 Activation of mitogen-activated protein kinases is required for alpha1-adrenergic agonist-induced cell scattering in transfected HepG2 cells. Activation of alpha1B-adrenergic receptors ((alpha1B)AR) by phenylephrine (PE) induces scattering of HepG2 cells stably transfected with the (alpha1B)AR (TFG2 cells). Scattering was also observed after stimulation of TFG2 cells with phorbol myristate acetate (PMA) but not with hepatocyte growth factor/scatter factor, epidermal growth factor, or insulin. PMA but not phenylephrine rapidly activated PKCalpha in TFG2 cells, and the highly selective PKC inhibitor bisindolylmaleimide (GFX) completely abolished PMA-induced but not PE-induced scattering. PE rapidly activated p44/42 mitogen-activated protein kinase (MAPK), p38 MAPK, c-Jun N-terminal kinase (JNK), and AP1 (c-fos/c-jun). Selective blockade of p42/44 MAPK activity by PD98059 or by transfection of a MEK1 dominant negative adenovirus significantly inhibited the PE-induced scattering of TFG2 cells. Selective inhibition of p38 MAPK by SB203850 or SB202190 also blocked PE-induced scattering, whereas treatment of TFG2 cells with the PI3 kinase inhibitors LY294002 or wortmannin did not inhibit PE-induced scattering. Blocking JNK activation with a dominant negative mutant of JNK or blocking AP1 activation with a dominant negative mutant of c-jun (TAM67) significantly inhibited PE-induced cell scattering. These data indicate that PE-induced scattering of TFG2 cells is mediated by complex mechanisms, including activation of p42/44 MAPK, p38 MAPK, and JNK. Cell spreading has been reported to play important roles in wound repair, tumor invasion, and   metastasis  . Therefore, catecholamines acting via the (alpha1)AR may modulate these physiological and pathological processes. 
 MAPK phosphatase-1 contributes to trichostatin A inhibition of cyclooxygenase-2 expression in human umbilical vascular endothelial cells exposed to lipopolysaccharide. Histone deacetylase (HDAC) inhibitors have emerged as a new class of antitumor agents because they were demonstrated to induce cell cycle arrest, promote cell apoptosis, and inhibit   metastasis  . Recently, HDAC inhibitors were also shown to exhibit pronounced anti-inflammatory properties. However, the underlying mechanism contributing to the suppression of inflammatory responses by HDAC inhibitors remains to be fully defined. In the present study, we explored the actions of trichostatin A (TSA), a potent HDAC inhibitor, on lipopolysaccharide (LPS)-induced cyclooxygenase (COX)-2 expression in human umbilical vascular endothelial cells (HUVECs).HUVECs were exposed to LPS in the absence or presence of TSA. COX-2 expression and signaling molecules (JNK, p38MAPK and c-jun) activated by LPS were assessed.The LPS-induced cox-2 messenger RNA and protein were markedly suppressed by TSA. TSA inhibited JNK and p38MAPK phosphorylation in cells exposed to LPS. Treatment of cells with a JNK signaling inhibitor (JNK inhibitor II) or a p38MAPK inhibitor (p38MAPK inhibitor III) markedly inhibited LPS-induced COX-2 expression. TSA suppression of JNK and p38MAPK phosphorylation and subsequent COX-2 expression were restored by selective inhibition of MKP-1 using MKP-1 siRNA. In addition, TSA caused an increase in MKP-1 phosphatase activity in HUVECs. In conclusion, TSA may cause MKP-1 activation to dephosphorylate JNK and p38MAPK, leading to the downregulation of COX-2 in HUVECs stimulated by LPS, a proinflammatory stimulus.MKP-1 contributes to TSA's protective actions in HUVECs exposed to LPS. The present study also supports the therapeutic value of TSA in treating inflammatory vascular diseases. 
 IL-6 induces neuroendocrine dedifferentiation and cell proliferation in non-small cell lung cancer cells. Interleukin-6 (IL-6) has been identified as an important growth regulator of lung cancer cells. Elevation of serum levels of IL-6 has been found in a subpopulation of lung cancer patients, but rarely in patients with benign lung diseases. Approximately 15% of non-small cell lung cancer (NSCLC) tumors exhibit neuroendocrine (NE) properties (NSCLC-NE) and have been suggested to have the biological characteristics similar to small cell lung cancer (SCLC) with early   metastasis   and initial responsiveness to chemotherapy. We recently showed that IL-6 promotes cell proliferation and downregulates the expression of neuron-specific enolase (NSE, one of the major NE markers) in NSCLC-NE cells. In this study, we show that IL-6 stimulates a transient increase of tyrosine phosphorylation of STAT3 in a dose-dependent fashion. Inhibition of STAT3 signaling pathway by either AG-490 (JAK2-specific inhibitor) or overexpression of STAT3Y705F (a dominant-negative STAT3) reverses NSE expression in IL-6- treated NSCLC-NE cells. In addition, IL-6 induces phosphorylation and activation of p38 MAPK. SB-203580, a p38 MAPK-specific inhibitor, inhibits IL-6-induced p38 MAPK phosphorylating activity and suppresses IL-6-stimulated cell proliferation. Together, our results indicate that STAT3 signaling pathway is involved in IL-6-induced NE differentiation and that p38 MAPK is associated with IL-6-stimulated growth regulation in NSCLC-NE cells. These data suggest that both kinase pathways play critical roles in the pathogenesis of NSCLC-NE malignancies, providing new molecular targets for future therapeutic approaches. 
  GeneRIFs  
 This study evaluated the role of CCL20 and CCR6 in the regulation of laryngeal neoplasms; it showed that these proteins acted on proliferation and   metastasis   via the p38 pathway and multiple microRNAs. 
 ANGPTL2 expression in OS cell lines correlated with increased tumor   metastasis   and decreased animal survival by promoting tumor cell intravasation mediated by the integrin alpha5beta1, p38 mitogen-activated protein kinase, and matrix metalloproteinases 
 the IL-1beta/p38/AP-1(c-fos)/MMP2 & MMP9 pathway play an important role in   metastasis   in gastric adenocarcinoma 
 The results of the present study suggest that lysophosphatidic acid (LPA), the receptor LPA(1), ERK2 and p38alpha are important regulators for prostate cancer cell invasion and thus could play a significant role in the development of   metastasis  . 
 p38alpha plays an important role in extravasation of tumor cells, possibly through regulating the formation of tumor-platelet aggregates and their interaction with the endothelium involved in a step of hematogenous   metastasis   
  [Cc]ell [Ii]nvasion  PubMed  
 Mechanical regulation of cancer cell apoptosis and autophagy: roles of bone morphogenetic protein receptor, Smad1/5, and p38 MAPK. Mechanical forces induced by interstitial fluid flow in and surrounding tissues and by blood/lymphatic flow in vessels may modulate cancer   cell invasion   and metastasis and anticancer drug delivery. Our previous study demonstrated that laminar flow-induced shear stress induces G2/M arrest in tumor cells. However, whether shear stress modulates final cell fate remains unclear. In this study, we investigated the role of flow-induced shear stress in modulating the survival of four human tumor cell lines, i.e., Hep3B hepatocarcinoma cells, MG63 osteosarcoma cells, SCC25 oral squamous carcinoma cells, and A549 carcinomic alveolar basal epithelial cells. Laminar shear stress (LSS) ranging from 0.5 to 12dyn/cm(2) induced death of these four tumor cell lines. In contrast to LSS at 0.5dyn/cm(2), oscillatory shear stress (OSS) at 0.5±4dyn/cm(2) cannot induce cancer cell death. Both LSS and OSS had no effect on human normal hepatocyte, lung epithelial, and endothelial cells. Application of LSS to these four cell lines increased the percentage of cells stained positively for annexin V-FITC, with up-regulations of cleaved caspase-8, -9, and -3, and PARP. In addition, LSS also induced Hep3B cell autophagy, as detected by acidic vesicular organelle formation, LC3B transformation, and p62/SQSTM1 degradation. By transfecting with small interfering RNA, we found that the shear-induced apoptosis and autophagy are mediated by bone morphogenetic protein receptor type (BMPR)-IB, BMPR-specific Smad1 and Smad5, and p38 mitogen-activated protein kinase in Hep3B cells. Our findings provide insights into the molecular mechanisms by which shear stress induces apoptosis and autophagy in tumor cells. 
 H-Ras-specific activation of Rac-MKK3/6-p38 pathway: its critical role in invasion and migration of breast epithelial cells. Human tumors frequently exhibit constitutively activated Ras signaling, which contributes to the malignant phenotype. Mounting evidence suggests unique roles of the Ras family members, H-Ras, N-Ras and K-Ras, in normal and pathological conditions. In an effort to dissect distinct Ras isoform-specific functions in malignant phenotypic changes, we previously established H-Ras- and N-Ras-activated MCF10A human breast epithelial cell lines. Using these, we showed that p38 kinase is a key signaling molecule differentially regulated between H-Ras and N-Ras, leading to H-Ras-specific induction of invasive and migrative phenotypes. The present study is to further investigate H-Ras- and N-Ras-mediated signaling pathways and to unveil how these pathways are integrated for regulation of invasive/migrative phenotypic conversion of human breast epithelial cells. Here we report that the Rac-MAPK kinase (MKK)3/6-p38 pathway is a unique signaling pathway activated by H-Ras, leading to the invasive/migrative phenotype. In contrast, Raf-MEK-ERK and phosphatidylinositol 3-kinase-Akt pathways, which are fundamental to proliferation and differentiation, are activated by both H-Ras and N-Ras. A significant role for p38 in   cell invasion   is further supported by the observation that p38 activation by MKK6 transfection is sufficient to induce invasive and migrative phenotypes in MCF10A cells. Activation of the MKK6-p38 pathway results in a marked induction of matrix metalloproteinase (MMP)-2, whereas it had little effect on MMP-9, suggesting MMP-2 up-regulation by MKK6-p38 pathway as a key step for H-Ras-induced invasion and migration. We also provide evidence for cross-talk among the Rac, Raf, and phosphatidylinositol 3-kinase pathways critical for regulation of MMP-2 and MMP-9 expression and invasive phenotype. Taken together, the present study elucidated the role of the Rac-MKK3/6-p38 pathway leading to H-Ras-specific induction of malignant progression in breast epithelial cells, providing implications for developing therapeutic strategies for mammary carcinoma to target Ras downstream signaling molecules required for malignant cancer cell behavior but less critical for normal cell functions. 
 Acacetin inhibits the invasion and migration of human non-small cell lung cancer A549 cells by suppressing the p38α MAPK signaling pathway. Lung cancer is one of the most common malignancies in the world and its metastasis is the major cause of death in cancer patients. Acacetin (5,7-dihydroxy-4'-methoxyflavone), a flavonoid compound, has anti-peroxidative and anti-inflammatory effects. The effect of acacetin on invasion and migration in human NSCLC A549 cells was investigated. First, the result demonstrated acacetin could exhibit an inhibitory effect on the abilities of the adhesion, morphology/actin cytoskeleton arrangement, invasion, and migration by cell-matrix adhesion assay, immunofluorescence assay, Boyden chamber assay, and wound-healing assay. Molecular data showed that the effect of acacetin in A549 cells might be mediated via sustained inactivation of the phosphorylation of mixed-lineage protein kinase 3 (MLK3), mitogen-activated protein kinase kinases 3/6 (MKK3/6), and p38α MAPK signal involved in the downregulation of the expressions of matrix metalloproteinase-2 (MMP-2), matrix metalloproteinase-9 (MMP-9), and urokinase-type plasminogen activator (u-PA). Next, acacetin significantly decreased in the phosphorylation and degradation of inhibitor of kappaBα (IκBα), and the nuclear levels of nuclear factor kappa B (NF-κB), c-Fos, and c-Jun. Also, the treatment with acacetin to A549 cells also leads to a concentration-dependent inhibition on the binding abilities of NF-κB and activator protein-1 (AP-1). Furthermore, the treatment of specific inhibitor for p38 MAPK (SB203580) to A549 cells could cause reduced activities of MMP-2/9 and u-PA. In addition, acacetin significantly decreased the levels of phospho-p38α MAPK, MMP-2/9, and u-PA in p38α-cDNA-transfected cells concomitantly with a marked redu  ction on cell   invasion and migration. Our results revealed the anti-migration and anti-invasion effects of acacetin, which may act as a promising therapeutic agent for the treatment of lung cancer. 
 Kiss-1 suppresses MMP-9 expression by activating p38 MAP kinase in human stomach cancer. Kiss-1 has been identified as a putative metastasis suppressor gene in various human malignancies. However, there is little information about its possible role in gastric carcinoma. In this study, we determined whether the Kiss-1 gene negatively regulates MMP-9 expression. cDNA microarray technology was used to identify the genes associated with metastasis by hepatocyte growth factor (HGF) in the gastric cancer cell lines, NUGC-3 and MKN-28. The levels of Kiss-1 RNA and protein were confirmed to be upregulated in HGF-treated gastric cancer cells. HGF induced Kiss-1 and MMP-9 production in a dose-dependent manner. In order to investigate roles of HGF signaling in tumor progression and metastasis, we measured effects of a specific MEK1 inhibitor (PD 098059) and a p38 kinase inhibitor (SB 203580) on HGF-mediated cell proliferation and MMP-9. Pretreatment with PD 098059 reduced MMP-9 and HGF-mediated cell proliferation, but increased Kiss-I expression. In contrast, SB 203580 pretreatment enhanced MMP-9 and cell prolifera-tion, but decreased Kiss-1 expression. Cotreatment of PD098059 and SB203580 increased the p38 phosphorylation stimulated by HGF. These results suggest that the HGF-mediated Kiss-1 overexpression is regulated mainly by the p38 activation and, furthermore, the activation of ERK might affect HGF-mediated Kiss-1 expression indirectly by the regulation of p38 kinase. Consistent with this result, p38 phosphorylation was strongly repressed by the knock-down of Kiss-1. Downregulation of Kiss-1 using Kiss-1 shRNA also increased in vitro   cell invasion  . In conclusion, Kiss-1 suppresses MMP-9 expression by activating the p38 MAP kinase signaling pathway. 
 The MLK-related kinase (MRK) is a novel RhoC effector that mediates lysophosphatidic acid (LPA)-stimulated tumor   cell invasion  . The small GTPase RhoC is overexpressed in many invasive tumors and is essential for metastasis. Despite its high structural homology to RhoA, RhoC appears to perform functions that are different from those controlled by RhoA. The identity of the signaling components that are differentially regulated by these two GTPases is only beginning to emerge. Here, we show that the MAP3K protein MRK directly binds to the GTP-bound forms of both RhoA and RhoC in vitro. However, siRNA-mediated depletion of MRK in cells phenocopies depletion of RhoC, rather than that of RhoA. MRK depletion, like that of RhoC, inhibits LPA-stimulated   cell invasion  , while depletion of RhoA increases invasion. We also show that active MRK enhances LPA-stimulated invasion, further supporting a role for MRK in the regulation of invasion. Depletion of either RhoC or MRK causes sustained myosin light chain phosphorylation after LPA stimulation. In addition, activation of MRK causes a reduction in myosin light chain phosphorylation. In contrast, as expected, depletion of RhoA inhibits myosin light chain phosphorylation. We also present evidence that both RhoC and MRK are required for LPA-induced stimulation of the p38 and ERK MAP kinases. In conclusion, we have identified MRK as a novel RhoC effector that controls LPA-stimulated   cell invasion   at least in part by regulating myosin dynamics, ERK and p38. 
 Suppression of phorbol-12-myristate-13-acetate-induced tumor   cell invasion   by apigenin via the inhibition of p38 mitogen-activated protein kinase-dependent matrix metalloproteinase-9 expression. Apigenin has special interest for the development of chemopreventive agents against cancer because it is a widely distributed plant flavonoid that has antitumor properties. In this study, we investigated the apigenin effects on the protease-mediated invasiveness in human metastatic cancer cell lines Caski, SK-Hep1, and MDA-231. We found that apigenin markedly inhibits the phorbol-12-myristate-13-acetate (PMA)-induced increase in MMP-9 expression and activity in several cancer cell lines. These effects of apigenin are dose-dependent and correlate with the suppression of MMP-9 mRNA expression levels. PMA caused about a 5-fold induction in MMP-9 promoter activity, which was also suppressed by apigenin treatment in Caski cells. We found that apigenin could inhibit PMA-induced phosphorylation of p38 mitogen-activated protein kinase (p38 MAPK), which was involved in the down-regulation of the expression of matrix metalloproteinase-9 (MMP-9) at mRNA levels. Furthermore, the treatment of inhibitors specific for p38 MAPK (SB203580) to Caski cells caused the reduction of MMP-9 expression. Restoration of p38 expression partly increased PMA-induced MMP-9 secretion blocked by apigenin treatment in Caski cells. These results showed apigenin might inhibit the invasion and migration abilities of Caski cells by reducing the MMP-9 expression through suppressing the p38 MAPK signaling pathway. These findings indicate that apigenin might be a useful strategy for controlling metastasis and the invasiveness of tumors. 
 Tissue factor/activated factor VIIa induces matrix metalloproteinase-7 expression through activation of c-Fos via ERK1/2 and p38 MAPK signaling pathways in human colon cancer cell. Increased expression of tissue factor (TF) is associated with tumor invasion and metastasis in human colorectal cancer. We have previously observed that TF/FVIIa upregulates matrix metalloproteinase-7 (MMP-7) expression at the transcriptional level in colon cancer cells. MMP-7 overexpression is believed to play an important role in tumor invasion and metastasis. The aim of this study is to elucidate the molecular mechanisms by which TF/FVIIa induced MMP-7 expression and   cell invasion   in vitro.Reverse transcription polymerase chain reaction, Western blot, luciferase assay, and chromatin immunoprecipitation (ChIP) were used to determine the potential mechanism and signaling pathways by which TF/FVIIa induced MMP-7 expression and   cell invasion   in LoVo cells. Small interfering RNA (siRNA) and   cell invasion   assay was used to examine whether blocking c-Fos expression could abolish FVIIa-mediated upregulation of MMP-7 and   cell invasion   in vitro.The results showed that FVIIa induced the upregulation of MMP-7 both at the mRNA and protein levels in a time- and dose-dependent manner and increased the invasive behavior of LoVo cells. FVIIa enhanced the promoter activity of MMP-7, and the activator protein-1 (AP-1) binding site was responsible for the activation. Site mutation of the AP-1 binding site in the promoter almost completely abolished FVIIa-mediated response. Furthermore, ChIP assay confirmed that FVIIa promoted the direct binding of c-Fos with the MMP-7 promoter in vivo. FVIIa also induced the expression and nuclear accumulation of the AP-1 subunit c-Fos. siRNA-mediated knockdown of c-Fos eliminated FVIIa-stimulated MMP-7 expression and cell migration in vitro. In addition, selective mitogen-activated protein kinase (MAPK) kinase (MEK1/2) inhibitor (PD98059) and p38 MAPK inhibitor SB203580 suppressed MMP-7 upregulation induced by FVIIa.Our data suggest that a novel TF/FVIIa/MAPK/c-Fos/MMP-7 axis plays an important role in modulating the invasion of colon cancer cells and blockage of this pathway holds promise to treat colon cancer metastasis. 
 Caveolin-1 acts as a tumor suppressor by down-regulating epidermal growth factor receptor-mitogen-activated protein kinase signaling pathway in pancreatic carcinoma cell lines. To investigate the effect of caveolin-1 (cav1) in pancreatic carcinoma panc1 cell growth in vitro and in vivo.Caveolin-1 gene was transferred into panc1 cells, and stably overexpressed cav1 clones were established. Proliferation and anchorage-independent growth capacity in vitro were detected by 3-[4,5-dimethylthiazol-2-yl]-2,5-diphenyltetrazolium bromide and colony formation assays in soft agar. Flow cytometry was used to analyze cell cycle and apoptosis. The invasion ability was measured by Transwell invasion assay. Activities of signal molecules in epidermal growth factor receptor-mitogen-activated protein kinase (EGFR-MAPK) signal pathway were determined by Western blots. Tumor growth in vivo was evaluated by tumorigenesis assay in nude mice.Stably overexpressing cav1 cells exhibited slower growth and reduced the capacity of anchorage-independent growth. Overexpression of cav1 reduced   cell invasion   capacity and promoted cell apoptosis. The activities of EGFR-MAPK signal pathway were also inhibited significantly by overexpression of cav1, in addition, overexpression of cav1 in panc1 cells reduced tumor formation in vivo.The cav1 may act as a candidate tumor suppressor gene in human panceatic carcinoma, and this effect may be related with the inhibition of EGFR-MAPK signal cascade. 
 CARMA3 regulates the invasion, migration, and apoptosis of non-small cell lung cancer cells by activating NF-кB and suppressing the P38 MAPK signaling pathway. In our previous study, CARMA3 overexpression in lung cancer cells promoted cell proliferation and invasion; however, the mechanism underlying the role of CARMA3 in cancer   cell invasio  n remained unclear. In the present study, knockdown of CARMA3 in A549 and H1299 cells suppressed   cell invasio  n and migration, and downregulated matrix metalloprotease 9 expression at the protein and mRNA levels, as shown by Western blotting and real-time PCR. CARMA3 knockdown increased cell apoptosis, as shown by flow cytometry, increased the mRNA and protein expression levels of Bax and Caspase3, and downregulated Bcl-2 in A549 and H1299 cells. Phosphorylated P38 levels increased and NF-кB activation decreased following knockdown of CARMA3. SB203580, a P38 MAPK inhibitor, activated NF-кB, increased cell migration, and inhibited cell apoptosis after knockdown of CARMA3 compared to knockdown of CARMA3 without SB203580. These findings indicate that CARMA3 may suppress the activation of the P38 MAPK signaling pathway to regulate invasion, migration and apoptosis of lung cancer cells by activating NF-кB (P65) in the nucleus. 
 Stromal cell-derived factor-1 (SDF-1) enhances cells invasion by αvβ6 integrin-mediated signaling in ovarian cancer. Ovarian carcinoma is a common gynecological malignancy and a great threat to health as a result of metastasis. The chemokine stromal-derived factor (SDF-1) plays multiple roles in tumor pathogenesis. However, the precise molecular mechanism underlying SDF-1-induced ovarian cance  r cell invasi  on is still undefined. αvβ6 integrin is an important factor in tumor progression. Therefore, we speculate that SDF-1-enhanced ovarian can  cer cell inva  sion is related to αvβ6 integrin-mediated signaling. After culturing with SDF-1, an obvious time- and dose-dependent increase in αvβ6 integrin was demonstrated. Furthermore, CXC receptor 4 (CXCR4) was responsible for SDF-1-induced αvβ6 integrin expression. Simultaneously, SDF-1 was found to dramatically enhance extracellular matrix degradation via urokinase-type plasminogen activator (uPA) expre  ssion and cel  l invasion by αvβ6 integrin expression; these reinforce failed to be increased when pretreatment was performed with the CXCR4 inhibitor AMD3100 or anti-αvβ6 integrin antibody, respectively. In addition, αvβ6 integrin induced the phosphorylation of p38 MAPK and PI3 K/Akt, contributing to the up-regulation of uPA, as treatment with the specific inhibitor for p38 mitogen-activated protein kinases (MAPK) (SB203580) or phosphatidylinositol 3-kinase (PI3 K)/Akt (LY294002) strikingly abrogated uPA expression. Taken together, these results demonstrated that SDF-1 enhanced   ovarian canc  er cell invasion through αvβ6 integrin-mediated uPA expression via the p38 MAPK and PI3 K/Akt pathway. Consequently, our findings will provide a new explanation about how SDF-1 aggravates the pathogenesis of ovarian cancer. 
 ErbB2-enhanced invasiveness of H-Ras MCF10A breast cells requires MMP-13 and uPA upregulation via p38 MAPK signaling. Overexpression of ErbB2 has been frequently found in mammary carcinoma. We have previously shown that the aberrant activation of H-Ras induces human breast   cell invasion   and migration. The present study was aimed at investigating the effect of ErbB2 overexpression on H-Ras-induced breast   cell invasion   and to elucidate the underlying mechanisms. Herein, we show that overexpression of ErbB2 promotes invasive and migratory abilities of H-Ras-activated MCF10A human breast epithelial cells through upregulation of matrix metalloproteinase (MMP)-13 and urokinase-type plasminogen activator (uPA). We also demonstrate that the p38 MAPK is an important signaling molecule in the ErbB2-induced upregulation of MMP-13 and uPA and invasion/migration of H-Ras MCF10A cells overexpressing ErbB2. The present study elucidating the molecular mechanism underlying ErbB2-induced promotion of H-Ras MCF10A   cell invasion   may provide invaluable information for understanding breast cancer progression and establishing therapeutic interventions for breast cancer. 
 Lysophosphatidic acid induces prostate cancer PC3 cell migration via activation of LPA(1), p42 and p38alpha. Prostate cancer cell migration is an essential event both in the progression of prostate cancer and in the steps leading to metastasis. We report here that lysophosphatidic acid (LPA), a potent bioactive phospholipid, induces prostate cancer PC3 cell migration via the activation of the LPA(1) receptor, which is linked to a PTX-sensitive activation mechanism of the mitogen-activated protein kinases (MAPK). Our results demonstrate that parallel activation of ERK1/2 and p38, but not JNK, is responsible for LPA-stimulated PC3 cell migration. Furthermore, using small interfering RNA (siRNA) technology, and overexpressing dominant-negative mutants of p38 MAPK isotypes of alpha, beta, gamma and delta, we have identified that the activation of ERK2 (p42) and p38alpha, but not of ERK1 and the other isoforms of p38 MAPK, is required for LPA-induced migration. Our study provides the first evidence for a functional role of p42 and p38alpha in LPA-induced mammalian cell migration, and also demonstrates, for the first time, that the receptor LPA(1) mediates prostate cancer cell migration. The results of the present study suggest that LPA, the receptor LPA(1), ERK2 and p38alpha are important regulators for prostate cancer   cell invasion   and thus could play a significant role in the development of metastasis. 
 Peroxiredoxin 1 promotes pancreatic cancer   cell invasion   by modulating p38 MAPK activity. The aim of this study was to investigate the role of peroxiredoxin 1 (Prdx1) in the invasiveness of pancreatic ductal adenocarcinoma (PDAC) cells.Immunohistochemistry was used to determine overexpression of Prdx1 in human PDAC tissues. Immunoprecipitation and immunocytochemistry were used to determine the interaction and intracellular distribution of Prdx1 and a member of the mitogen-activated protein kinase (MAPK) family protein, p38 MAPK, in PDAC cells. Finally, immunocytochemistry and Matrigel invasion assay were used to examine the effects of Prdx1 and p38 MAPK on the formation of cell protrusions and PDAC   cell invasion  .Prdx1 is overexpressed in human PDAC tissues. Peroxiredoxin 1 interacts with active forms of p38 MAPK, and complexes of Prdx1 and phosphorylated p38 MAPK localize at the leading edges of migrating PDAC cells. Suppression of Prdx1 decreases active p38 MAPK localized in cell protrusions and inhibits the invasiveness of PDAC cells. Consequently, suppression of Prdx1 inhibits membrane ruffling and protrusions. The p38 MAPK inhibitor SB203580 also decreases the formation of membrane protrusions and inhibits invasiveness.Prdx1 associates with the formation of membrane protrusions through modulation of the activity of p38 MAPK, which in turn promotes PDAC   cell invasion  . 
 Silibinin inhibits the invasion and migration of renal carcinoma 786-O cells in vitro, inhibits the growth of xenografts in vivo and enhances chemosensitivity to 5-fluorouracil and paclitaxel. Silibinin is a flavonoid antioxidant that is widely used for its anti-hepatotoxic properties. It exerts a dose-dependent inhibition on the invasion and migration of 786-O renal cell carcinoma (RCC) cells in the absence of cytotoxicity. 786-O cells were treated with silibinin at various concentrations, up to 50 µM, for a defined period and then subjected to gelatin zymography, casein zymography, and Western blot to investigate the impacts of silibinin on metalloproteinase (MMP) -2, -9, urokinase plasminogen activator (u-PA), and MAPK pathway signaling proteins, respectively. The results showed that silibinin decreased MMP-2, MMP-9, u-PA, p-p38, and p-Erk1/2 expressions in a concentration-dependent manner. The reduced expressions of MMP-2 and u-PA, as well as inhibition   of cell invas  ion were obtained in the cultures pre-treated with PD98059 (Erk1/2 inhibitor) and SB203580 (p38 inhibitor). An in vivo anti-tumor study with a nude mice xenograft model by a subcutaneous inoculation of 786-O cells demonstrated small solid tumors after eight days following cell inoculation. There was a 70.1% reduction in tumor volume and 69.7% reduction in tumor weight by silibinin feeding on day 44, compared to those of controls. Moreover, combination treatment with silibinin and 5-fluorouracil, paclitaxel, vinblastine, or RAD-001 enhanced the chemosensitivity of 5-fluorouracil and paclitaxel. In conclusion, silibinin inhibits the invasion and migration of 786-O cells in vitro, inhibits the growth of xenografts in vivo, and enhances chemosensitivity to 5-fluorouracil and paclitaxel. © 2011 Wiley-Liss, Inc. 
  GeneRIFs  
 Prdx1 associates with the formation of membrane protrusions through modulation of the activity of p38 MAPK, which in turn promotes pancreatic ductal adenocarcinoma   cell invasion  . 
 Results show that stromal cell-derived factor-1 (SDF-1) enhanced ovarian cancer   cell invasion   through alphavbeta6 integrin-mediated urokinase-type plasminogen activator (uPA) expression via the p38 MAPK and PI3 K/Akt pathway. 
 MRK is a novel RhoC effector that controls LPA-stimulated   cell invasion   at least in part by regulating myosin dynamics, ERK and p38 
 The results of the present study suggest that lysophosphatidic acid (LPA), the receptor LPA(1), ERK2 and p38alpha are important regulators for prostate cancer   cell invasion   and thus could play a significant role in the development of metastasis. 
   
  
 FERMT2 
 
  NCBI Gene  
  GeneRIF  
  Pubmed  
 
 
  Search Term  Rank  Fields  
  [Ii]nvadopodia  3  PubMed(1)  
  [Mm]igration  6  PubMed(10)  
  ECM  7  PubMed(3)  
  [Ee]xtracellular [Mm]atrix  8  PubMed(7)  
  [Ii]nvasive  10  PubMed(3); GeneRIFs(2)  
  [Mm]etastasis  12  PubMed(5); GeneRIFs(1)  
  [Cc]ell [Ii]nvasion  13  PubMed(4)  
 
 
 hide/show details for FERMT2 
  [Ii]nvadopodia  PubMed  
 Dense fibrillar collagen is a potent inducer of   invadopodia   via a specific signaling network. Cell interactions with the extracellular matrix (ECM) can regulate multiple cellular activities and the matrix itself in dynamic, bidirectional processes. One such process is local proteolytic modification of the ECM.   Invadopodia   of tumor cells are actin-rich proteolytic protrusions that locally degrade matrix molecules and mediate invasion. We report that a novel high-density fibrillar collagen (HDFC) matrix is a potent inducer of   invadopodia  , both in carcinoma cell lines and in primary human fibroblasts. In carcinoma cells, HDFC matrix induced formation of   invadopodia   via a specific integrin signaling pathway that did not require growth factors or even altered gene and protein expression. In contrast, phosphoproteomics identified major changes in a complex phosphosignaling network with kindlin2 serine phosphorylation as a key regulatory element. This kindlin2-dependent signal transduction network was required for efficient induction of   invadopodia   on dense fibrillar collagen and for local degradation of collagen. This novel phosphosignaling mechanism regulates cell surface   invadopodia   via kindlin2 for local proteolytic remodeling of the ECM. 
  [Mm]igration  PubMed  
 Analysis of the myosin-II-responsive focal adhesion proteome reveals a role for β-Pix in negative regulation of focal adhesion maturation. Focal adhesions undergo myosin-II-mediated maturation wherein they grow and change composition to modulate integrin signalling for cell   migratio  n, growth and differentiation. To determine how focal adhesion composition is affected by myosin II activity, we performed proteomic analysis of isolated focal adhesions and compared protein abundance in focal adhesions from cells with and without myosin II inhibition. We identified 905 focal adhesion proteins, 459 of which changed in abundance with myosin II inhibition, defining the myosin-II-responsive focal adhesion proteome. The abundance of 73% of the proteins in the myosin-II-responsive focal adhesion proteome was enhanced by contractility, including proteins involved in Rho-mediated focal adhesion maturation and endocytosis- and calpain-dependent focal adhesion disassembly. During myosin II inhibition, 27% of proteins in the myosin-II-responsive focal adhesion proteome, including proteins involved in Rac-mediated lamellipodial protrusion, were enriched in focal adhesions, establishing that focal adhesion protein recruitment is also negatively regulated by contractility. We focused on the Rac guanine nucleotide exchange factor β-Pix, documenting its role in the negative regulation of focal adhesion maturation and the promotion of lamellipodial protrusion and focal adhesion turnover to drive cel  l migrati  on. 
 C-terminal COOH of integrin β1 is necessary for β1 association with the kindlin-2 adapter protein. Protein-protein interactions are driving forces in cellular processes. As a prime example, transmembrane integrins link extracellular matrix and intracellular proteins, resulting in bidirectional signaling that regulates cel  l migrati  on, proliferation, differentiation, and survival. Here we provide the first evidence that interaction between the integrin β1 cytoplasmic tail and kindlin-2, a member of a family of adapters implicated in human disease pathogenesis, is mainly governed by the β1 C-terminal carboxylate moiety and is required for laterality organ development in zebrafish. Affinity measurements indicate that this unusual protein-protein interaction mode is coordinated by a putative carboxylate-binding motif in the kindlin-2 FERM subdomain F3. Contrary to the C terminus of proteins that engage PDZ domains, the C-terminal three residues of β1, per se, do not contribute to kindlin-2 binding or to laterality organ development. Thus, by employing zebrafish as an in situ physiological tool to correlate protein structure and function, we have discovered an unexpected association chemistry between an integrin and a key adapter involved in integrin signaling. 
 C-terminal COOH of integrin β1 is necessary for β1 association with the kindlin-2 adapter protein. Protein-protein interactions are driving forces in cellular processes. As a prime example, transmembrane integrins link extracellular matrix and intracellular proteins, resulting in bidirectional signaling that regulates cel  l migrati  on, proliferation, differentiation, and survival. Here we provide the first evidence that interaction between the integrin β1 cytoplasmic tail and kindlin-2, a member of a family of adapters implicated in human disease pathogenesis, is mainly governed by the β1 C-terminal carboxylate moiety and is required for laterality organ development in zebrafish. Affinity measurements indicate that this unusual protein-protein interaction mode is coordinated by a putative carboxylate-binding motif in the kindlin-2 FERM subdomain F3. Contrary to the C terminus of proteins that engage PDZ domains, the C-terminal three residues of β1, per se, do not contribute to kindlin-2 binding or to laterality organ development. Thus, by employing zebrafish as an in situ physiological tool to correlate protein structure and function, we have discovered an unexpected association chemistry between an integrin and a key adapter involved in integrin signaling. 
 Kindlin-2 phosphorylation by Src at Y193 enhances Src activity and is involved in Migfilin recruitment to the focal adhesions. Kindlin-2 regulates external to internal cell signaling by interaction with integrins in a process that involves the tyrosine kinase, Src. However, the underlying mechanisms remain elusive. Here we report that Src binds to and phosphorylates Kindlin-2 at Y193. Reciprocally, Kindlin-2-Y193 phosphorylation activates and maintains Src kinase activity. Kindlin-2-Y193 phosphorylation is also involved in its binding capacity with Migfilin and the recruitment of Migfilin to the focal adhesions. Functionally, we demonstrate that Kindlin-2-Y193 phosphorylation regulates Kindlin-2-mediated cell spreading and   migration  . These findings suggest that Src, Kindlin-2 and Migfilin together constitute a positive feedback loop that controls Src activity and regulates integrin-mediated cellular functions. 
 Opposite role of Kindlin-1 and Kindlin-2 in lung cancers. Lung cancer is highly heterogenous and is composed of various subtypes that are in diverse differential stages. The newly identified integrin-interacting proteins Kindlin-1 and Kindlin-2 are the activators of transmembrane receptor integrins that play important roles in cancer progression. In this report we present the expression profiles of Kindlin-1 and Kindlin-2 in lung cancers using patient specimens and established their correlation with lung cancer progression. We found that Kindlin-1 was expressed in epithelia-derived non-small-cell lung cancer, especially in squamous cell lung cancer but expressed at low levels in poorly differentiated large cell lung cancer. However, Kindlin-2 was highly expressed in large cell lung cancer. Both Kindlin-1 and Kindlin-2 were found not expressed or expressed at very low levels in neuroendocrine-derived small cell lung cancer. Importantly, the Kindlin-1 expression level was positively correlated with the differentiation of squamous cell lung cancer. Surprisingly, we found that the very homologous Kindlin family proteins, Kindlin-1 and Kindlin-2, displayed counteracting functional roles in lung cancer cells. Ectopic expression of Kindlin-1 in non-small-cell lung cancer cells inhibited in vitro cell   migration   and in vivo tumor growth, while Kindlin-2 promoted these functions. Mechanistically, Kindlin-1 prohibited epithelail to mesenchymal transition in non-small-cell lung cancer cells, while Kindlin-2 enhanced epithelail to mesenchymal transition in these cells. Taken together, we demonstrated that Kindlin-1 and Kindlin-2 differentially regulate lung cancer cell progression. Further, the expression levels of Kindlin-1 might be potentially used as a marker for lung cancer differentiation and targeting Kindlin-2 might block the invasive growth of large cell lung cancer. 
 Role of kindlin-2 in fibroblast functions: implications for wound healing. Kindlins are a protein family that regulates cell-matrix interactions. Although kindlin-2 is known to be essential for the early developmental processes, its role in the homeostasis of adult tissues has remained elusive. In this study, we used new domain-specific antibodies and small interfering RNA technology to uncover physiological functions of kindlin-2 in human dermal fibroblasts in vitro and in adult skin in situ. In primary dermal fibroblasts, kindlin-2 is essential for the integrin clustering and activation, and it regulates cell adhesion and directed   migration   by guiding formation and maturation of focal adhesions (FAs) and organization of the cytoskeleton. These functions are linked to the transmission of mechanical cues between cells and their microenvironment, typically in processes wherein fibroblasts differentiate to myofibroblasts under mechanical tension. In concert, kindlin-2 was shown to be required for the stabilization and maturation of FAs and stress fibers in activated fibroblasts and myofibroblasts. These findings implicated that in physiological wound closure and tissue repair, the prediction was validated by strong upregulation of kindlin-2 in contractile myofibroblasts during middle stages of wound healing in human skin. Taken together, the data reveal a physiological role for kindlin-2 in skin fibroblasts under normal steady-state conditions and during tissue regeneration. 
 Kindlin-2 is expressed in malignant mesothelioma and is required for tumor cell adhesion and   migration  . Kindlin-2 is a novel integrin-interacting focal adhesion protein that belongs to the Kindlin family. Focal adhesion proteins control cytoskeleton dynamics and promote cancer cell growth, survival,   migration   and metastasis. Little is known, however, about expression of Kindlin-2 in association with human cancer. We now reveal high Kindlin-2 expression in malignant mesothelioma (MM) cell lines using an affinity-purified anti-Kindlin-2 antibody. Furthermore, we show by immunohistochemistry that Kindlin-2 is highly expressed in 92 of 102 (90%) MMs with epitheliod; sarcomatoid, biphasic and poorly differentiated morphologies. In addition, Kindlin-2 expression correlates to cell proliferation, suggesting a role for Kindlin-2 in tumor growth. We also detect increased expression of Kindlin-2 at the invasion front of tumors concurrent with increased expression of integrin-linked kinase, a Kindlin-binding protein. Besides the high expression of Kindlin-2 in pleural MMs, pleural metastases of lung adenocarcinoma also express large amounts of Kindlin-2, but not Kindlin-1. Notably, in vitro, when endogenous Kindlin-2 was knocked down with RNAi in MM cells, this impaired cell spreading, adhesion and   migration  . Overall, our study suggests that heightened expression of Kindlin-2 might contribute to tumor progression in MM. 
 Kindlin-1 and -2 have overlapping functions in epithelial cells implications for phenotype modification. Kindlins are a novel family of intracellular adaptor proteins in integrin-containing focal adhesions. Kindlin-1 and -2 are expressed in the skin, but whether and how they cooperate in adult epithelial cells have remained elusive. We uncovered the overlapping roles of kindlin-1 and -2 in maintaining epithelial integrity and show that the phenotype of kindlin-1-deficient cells can be modulated by regulating kindlin-2 gene expression and vice versa. The experimental evidence is provided by use of human keratinocyte cell lines that express both kindlins, just kindlin-1 or kindlin-2, or none of them. Double deficiency of kindlin-1 and -2 had significant negative effects on focal adhesion formation and actin cytoskeleton organization, cell adhesion, survival, directional   migration  , and activation of β(1) integrin, whereas deficiency of one kindlin only showed variable perturbation of these functions. Cell motility and formation of cell-cell contacts were particularly affected by lack of kindlin-2. These results predict that kindlin-1 and -2 can functionally compensate for each other, at least in part. The high physiologic and pathologic significance of the compensation was emphasized by the discovery of environmental regulation of kindlin-2 expression. UV-B irradiation induced loss of kindlin-2 in keratinocytes. This first example of environmental regulation of kindlin expression has implications for phenotype modulation in Kindler syndrome, a skin disorder caused by kindlin-1 deficiency. 
 Molecular basis of kindlin-2 binding to integrin-linked kinase pseudokinase for regulating cell adhesion. Integrin-linked kinase (ILK) is a distinct intracellular adaptor essential for integrin-mediated cell-extracellular matrix adhesion, cell spreading, and   migration  . Acting as a major docking platform in focal adhesions, ILK engages many proteins to dynamically link integrins with the cytoskeleton, but the underlying mechanism remains elusive. Here, we have characterized the interaction of ILK with kindlin-2, a key regulator for integrin bidirectional signaling. We show that human kindlin-2 binds to human ILK with high affinity. Using systematic mapping approaches, we have identified a major ILK binding site involving a 20-residue fragment (residues 339-358) in kindlin-2. NMR-based analysis reveals a helical conformation of this fragment that utilizes its leucine-rich surface to recognize the ILK pseudokinase domain in a mode that is distinct from another ILK pseudokinase domain binding protein, α-parvin. Structure-based mutational experiments further demonstrate that the kindlin-2 binding to ILK is crucial for the kindlin-2 localization to focal adhesions and cell spreading (integrin outside-in signaling) but dispensable for the kindlin-2-mediated integrin activation (integrin inside-out signaling). These data define a specific mode of the kindlin-2/ILK interaction with mechanistic implications as to how it spatiotemporally mediates integrin signaling and cell adhesion. 
 Kindlin-2 (Mig-2): a co-activator of beta3 integrins. Integrin activation is essential for dynamically linking the extracellular environment and cytoskeletal/signaling networks. Activation is controlled by integrins' short cytoplasmic tails (CTs). It is widely accepted that the head domain of talin (talin-H) can mediate integrin activation by binding to two sites in integrin beta's CT; in integrin beta(3) this is an NPLY(747) motif and the membrane-proximal region. Here, we show that the C-terminal region of integrin beta(3) CT, composed of a conserved TS(752)T region and NITY(759) motif, supports integrin activation by binding to a cytosolic binding partner, kindlin-2, a widely distributed PTB domain protein. Co-transfection of kindlin-2 with talin-H results in a synergistic enhancement of integrin alpha(IIb)beta(3) activation. Furthermore, siRNA knockdown of endogenous kindlin-2 impairs talin-induced alpha(IIb)beta(3) activation in transfected CHO cells and blunts alpha(v)beta(3)-mediated adhesion and   migration   of endothelial cells. Our results thus identify kindlin-2 as a novel regulator of integrin activation; it functions as a coactivator. 
  ECM  PubMed  
 Dense fibrillar collagen is a potent inducer of invadopodia via a specific signaling network. Cell interactions with the extracellular matrix (  ECM  ) can regulate multiple cellular activities and the matrix itself in dynamic, bidirectional processes. One such process is local proteolytic modification of the   ECM  . Invadopodia of tumor cells are actin-rich proteolytic protrusions that locally degrade matrix molecules and mediate invasion. We report that a novel high-density fibrillar collagen (HDFC) matrix is a potent inducer of invadopodia, both in carcinoma cell lines and in primary human fibroblasts. In carcinoma cells, HDFC matrix induced formation of invadopodia via a specific integrin signaling pathway that did not require growth factors or even altered gene and protein expression. In contrast, phosphoproteomics identified major changes in a complex phosphosignaling network with kindlin2 serine phosphorylation as a key regulatory element. This kindlin2-dependent signal transduction network was required for efficient induction of invadopodia on dense fibrillar collagen and for local degradation of collagen. This novel phosphosignaling mechanism regulates cell surface invadopodia via kindlin2 for local proteolytic remodeling of the   ECM  . 
 Loss of kindlin-1, a human homolog of the Caenorhabditis elegans actin-extracellular-matrix linker protein UNC-112, causes Kindler syndrome. Kindler syndrome is an autosomal recessive disorder characterized by neonatal blistering, sun sensitivity, atrophy, abnormal pigmentation, and fragility of the skin. Linkage and homozygosity analysis in an isolated Panamanian cohort and in additional inbred families mapped the gene to 20p12.3. Loss-of-function mutations were identified in the FLJ20116 gene (renamed "KIND1" [encoding kindlin-1]). Kindlin-1 is a human homolog of the Caenorhabditis elegans protein UNC-112, a membrane-associated structural/signaling protein that has been implicated in linking the actin cytoskeleton to the extracellular matrix (  ECM  ). Thus, Kindler syndrome is, to our knowledge, the first skin fragility disorder caused by a defect in actin-  ECM   linkage, rather than keratin-  ECM   linkage. 
 The novel focal adhesion gene kindlin-2 promotes the invasion of gastric cancer cells mediated by tumor-associated macrophages. Kindlin-2 is a novel focal adhesion gene mediating the cell-extracellular matrix (  ECM  ) adhesion. Tumor-associated macrophages (TAMs) play an important role in linking chronic inflammation to cancer progression. Both kindlin-2 and TAMs have been found to promote the invasion of gastric cancer cells in our previous studies. However, the correlation between kindlin-2 and TAMs remains unclear. Real-time RT-PCR was used to investigate kindlin-2 expression in the AGS, NCI and Hs-746T gastric cancer cell lines co-cultured with TAMs under normal or hypoxic conditions. IL8, IL10, IL11, IL17b, IL18, IL22 and IL24 expressions were measured by real-time RT-PCR in the gastric cancer lines with varying levels of kindlin-2 expression, as well as after downregulation of kindlin-2 mRNA expression by the siRNA method. We found that kindlin-2 was upregulated in all three gastric cancer cell lines when co-cultured with TAMs under normal conditions. Under hypoxic conditions, the induction of kindlin-2 expression induced by macrophages was significantly downregulated in the Hs-746T cell line. IL8, IL11, IL17b, IL22 and IL24 expression was significantly higher in gastric cell lines with high kindlin-2 expression. Downregulation of kindlin-2 mRNA decreased IL10, IL11, IL17b, IL22 and IL24 expression but IL8 and IL18 expression was upregulated. Therefore, the novel focal adhesion gene kindlin-2 may play an important role in promoting the invasion of gastric cancer cells mediated by TAMs through regulating interleukin expression. 
  [Ee]xtracellular [Mm]atrix  PubMed  
 Migfilin and Mig-2 link focal adhesions to filamin and the actin cytoskeleton and function in cell shape modulation. Cell-  extracellular matrix   adhesion is an important determinant of cell morphology. We show here that migfilin, a LIM-containing protein, localizes to cell-matrix adhesions, associates with actin filaments, and is essential for cell shape modulation. Migfilin interacts with the cell-matrix adhesion protein Mig-2 (mitogen inducible gene-2), a mammalian homolog of UNC-112, and the actin binding protein filamin through its C- and N-terminal domains, respectively. Loss of Mig-2 or migfilin impairs cell shape modulation. Mig-2 recruits migfilin to cell-matrix adhesions, while the interaction with filamin mediates the association of migfilin with actin filaments. Migfilin therefore functions as an important scaffold at cell-matrix adhesions. Together, Mig-2, migfilin and filamin define a connection between cell matrix adhesions and the actin cytoskeleton and participate in the orchestration of actin assembly and cell shape modulation. 
 C-terminal COOH of integrin β1 is necessary for β1 association with the kindlin-2 adapter protein. Protein-protein interactions are driving forces in cellular processes. As a prime example, transmembrane integrins lin  k extracellular matr  ix and intracellular proteins, resulting in bidirectional signaling that regulates cell migration, proliferation, differentiation, and survival. Here we provide the first evidence that interaction between the integrin β1 cytoplasmic tail and kindlin-2, a member of a family of adapters implicated in human disease pathogenesis, is mainly governed by the β1 C-terminal carboxylate moiety and is required for laterality organ development in zebrafish. Affinity measurements indicate that this unusual protein-protein interaction mode is coordinated by a putative carboxylate-binding motif in the kindlin-2 FERM subdomain F3. Contrary to the C terminus of proteins that engage PDZ domains, the C-terminal three residues of β1, per se, do not contribute to kindlin-2 binding or to laterality organ development. Thus, by employing zebrafish as an in situ physiological tool to correlate protein structure and function, we have discovered an unexpected association chemistry between an integrin and a key adapter involved in integrin signaling. 
 C-terminal COOH of integrin β1 is necessary for β1 association with the kindlin-2 adapter protein. Protein-protein interactions are driving forces in cellular processes. As a prime example, transmembrane integrins lin  k extracellular matr  ix and intracellular proteins, resulting in bidirectional signaling that regulates cell migration, proliferation, differentiation, and survival. Here we provide the first evidence that interaction between the integrin β1 cytoplasmic tail and kindlin-2, a member of a family of adapters implicated in human disease pathogenesis, is mainly governed by the β1 C-terminal carboxylate moiety and is required for laterality organ development in zebrafish. Affinity measurements indicate that this unusual protein-protein interaction mode is coordinated by a putative carboxylate-binding motif in the kindlin-2 FERM subdomain F3. Contrary to the C terminus of proteins that engage PDZ domains, the C-terminal three residues of β1, per se, do not contribute to kindlin-2 binding or to laterality organ development. Thus, by employing zebrafish as an in situ physiological tool to correlate protein structure and function, we have discovered an unexpected association chemistry between an integrin and a key adapter involved in integrin signaling. 
 Dense fibrillar collagen is a potent inducer of invadopodia via a specific signaling network. Cell interactions with the   extracellular matrix   (ECM) can regulate multiple cellular activities and the matrix itself in dynamic, bidirectional processes. One such process is local proteolytic modification of the ECM. Invadopodia of tumor cells are actin-rich proteolytic protrusions that locally degrade matrix molecules and mediate invasion. We report that a novel high-density fibrillar collagen (HDFC) matrix is a potent inducer of invadopodia, both in carcinoma cell lines and in primary human fibroblasts. In carcinoma cells, HDFC matrix induced formation of invadopodia via a specific integrin signaling pathway that did not require growth factors or even altered gene and protein expression. In contrast, phosphoproteomics identified major changes in a complex phosphosignaling network with kindlin2 serine phosphorylation as a key regulatory element. This kindlin2-dependent signal transduction network was required for efficient induction of invadopodia on dense fibrillar collagen and for local degradation of collagen. This novel phosphosignaling mechanism regulates cell surface invadopodia via kindlin2 for local proteolytic remodeling of the ECM. 
 Loss of kindlin-1, a human homolog of the Caenorhabditis elegans actin-extracellular-matrix linker protein UNC-112, causes Kindler syndrome. Kindler syndrome is an autosomal recessive disorder characterized by neonatal blistering, sun sensitivity, atrophy, abnormal pigmentation, and fragility of the skin. Linkage and homozygosity analysis in an isolated Panamanian cohort and in additional inbred families mapped the gene to 20p12.3. Loss-of-function mutations were identified in the FLJ20116 gene (renamed "KIND1" [encoding kindlin-1]). Kindlin-1 is a human homolog of the Caenorhabditis elegans protein UNC-112, a membrane-associated structural/signaling protein that has been implicated in linking the actin cytoskeleton to the   extracellular matrix   (ECM). Thus, Kindler syndrome is, to our knowledge, the first skin fragility disorder caused by a defect in actin-ECM linkage, rather than keratin-ECM linkage. 
 The novel focal adhesion gene kindlin-2 promotes the invasion of gastric cancer cells mediated by tumor-associated macrophages. Kindlin-2 is a novel focal adhesion gene mediating the cell-  extracellular matrix   (ECM) adhesion. Tumor-associated macrophages (TAMs) play an important role in linking chronic inflammation to cancer progression. Both kindlin-2 and TAMs have been found to promote the invasion of gastric cancer cells in our previous studies. However, the correlation between kindlin-2 and TAMs remains unclear. Real-time RT-PCR was used to investigate kindlin-2 expression in the AGS, NCI and Hs-746T gastric cancer cell lines co-cultured with TAMs under normal or hypoxic conditions. IL8, IL10, IL11, IL17b, IL18, IL22 and IL24 expressions were measured by real-time RT-PCR in the gastric cancer lines with varying levels of kindlin-2 expression, as well as after downregulation of kindlin-2 mRNA expression by the siRNA method. We found that kindlin-2 was upregulated in all three gastric cancer cell lines when co-cultured with TAMs under normal conditions. Under hypoxic conditions, the induction of kindlin-2 expression induced by macrophages was significantly downregulated in the Hs-746T cell line. IL8, IL11, IL17b, IL22 and IL24 expression was significantly higher in gastric cell lines with high kindlin-2 expression. Downregulation of kindlin-2 mRNA decreased IL10, IL11, IL17b, IL22 and IL24 expression but IL8 and IL18 expression was upregulated. Therefore, the novel focal adhesion gene kindlin-2 may play an important role in promoting the invasion of gastric cancer cells mediated by TAMs through regulating interleukin expression. 
 Molecular basis of kindlin-2 binding to integrin-linked kinase pseudokinase for regulating cell adhesion. Integrin-linked kinase (ILK) is a distinct intracellular adaptor essential for integrin-mediated cell-  extracellular matrix   adhesion, cell spreading, and migration. Acting as a major docking platform in focal adhesions, ILK engages many proteins to dynamically link integrins with the cytoskeleton, but the underlying mechanism remains elusive. Here, we have characterized the interaction of ILK with kindlin-2, a key regulator for integrin bidirectional signaling. We show that human kindlin-2 binds to human ILK with high affinity. Using systematic mapping approaches, we have identified a major ILK binding site involving a 20-residue fragment (residues 339-358) in kindlin-2. NMR-based analysis reveals a helical conformation of this fragment that utilizes its leucine-rich surface to recognize the ILK pseudokinase domain in a mode that is distinct from another ILK pseudokinase domain binding protein, α-parvin. Structure-based mutational experiments further demonstrate that the kindlin-2 binding to ILK is crucial for the kindlin-2 localization to focal adhesions and cell spreading (integrin outside-in signaling) but dispensable for the kindlin-2-mediated integrin activation (integrin inside-out signaling). These data define a specific mode of the kindlin-2/ILK interaction with mechanistic implications as to how it spatiotemporally mediates integrin signaling and cell adhesion. 
  [Ii]nvasive  PubMed  
 Opposite role of Kindlin-1 and Kindlin-2 in lung cancers. Lung cancer is highly heterogenous and is composed of various subtypes that are in diverse differential stages. The newly identified integrin-interacting proteins Kindlin-1 and Kindlin-2 are the activators of transmembrane receptor integrins that play important roles in cancer progression. In this report we present the expression profiles of Kindlin-1 and Kindlin-2 in lung cancers using patient specimens and established their correlation with lung cancer progression. We found that Kindlin-1 was expressed in epithelia-derived non-small-cell lung cancer, especially in squamous cell lung cancer but expressed at low levels in poorly differentiated large cell lung cancer. However, Kindlin-2 was highly expressed in large cell lung cancer. Both Kindlin-1 and Kindlin-2 were found not expressed or expressed at very low levels in neuroendocrine-derived small cell lung cancer. Importantly, the Kindlin-1 expression level was positively correlated with the differentiation of squamous cell lung cancer. Surprisingly, we found that the very homologous Kindlin family proteins, Kindlin-1 and Kindlin-2, displayed counteracting functional roles in lung cancer cells. Ectopic expression of Kindlin-1 in non-small-cell lung cancer cells inhibited in vitro cell migration and in vivo tumor growth, while Kindlin-2 promoted these functions. Mechanistically, Kindlin-1 prohibited epithelail to mesenchymal transition in non-small-cell lung cancer cells, while Kindlin-2 enhanced epithelail to mesenchymal transition in these cells. Taken together, we demonstrated that Kindlin-1 and Kindlin-2 differentially regulate lung cancer cell progression. Further, the expression levels of Kindlin-1 might be potentially used as a marker for lung cancer differentiation and targeting Kindlin-2 might block the   invasive   growth of large cell lung cancer. 
 Kindlin-2 expression in arsenite- and cadmium-transformed bladder cancer cell lines and in archival specimens of human bladder cancer. To confirm a microarray study that suggested that Kindlin-2 might play a role in the development and progression of bladder cancer. There has been no previous examination of Kindlin-2 expression in human bladder cancer.A combination of real-time polymerase chain reaction, Western analysis, and immunohistochemistry was used to characterize Kindlin-2 expression in arsenite (As(+3))- and cadmium (Cd(+2))-transformed human cell lines, their tumor transplants in immunocompromised mice, and in archival specimens of human bladder and bladder cancer.The results show that the Kindlin-2 expression patterns in the cell lines were not duplicated in the tumor tissues. However, it was shown that Kindlin-2 was expressed in the stromal element of all the transplanted tumors and archival specimens of human bladder cancer. It was also shown that a small number of high-grade   invasive   urothelial cancers have focal expression of Kindlin-2 in the tumor cells.Kindlin-2 is expressed in the stromal component of most, if not all, human bladder cancers. Kindlin-2 is not expressed in normal urothelium. Kindlin-2 is expressed in a small subset of high-grade   invasive   bladder cancers and may have potential as a prognostic marker for tumor progression. 
 Kindlin-2 promotes   invasive  ness of prostate cancer cells via NF-κB-dependent upregulation of matrix metalloproteinases.   Invasiv  e progression is the major lethal cause of prostate cancer. In this study, we aimed to investigate the role of kindlin-2, an integrin-binding focal adhesion protein, in the regulation of   invasiv  eness of prostate cancer. We found that downregulation of kindlin-2 using small interfering RNA (siRNA) technology significantly inhibited the invasion of PC-3 and DU-145 prostate cancer cells in a Matrigel Transwell assay. Conversely, overexpression of kindlin-2 promoted the   invasiv  eness of prostate cancer cells. Kindlin-2 overexpression was found to activate nuclear factor (NF)-κB-dependent signaling and upregulate the expression of matrix metalloproteinase-9 (MMP-9) and MMP-2, whereas kindlin-2 silencing led to opposing effects on the expression of NF-κB and MMPs. Most importantly, kindlin-2-induc  ed invas  iveness was almost completely abolished by pretreatment with pyrrolidine dithiocarbamate (an inhibitor of NF-κB signaling) or co-transfection with MMP-9 or MMP-2 siRNA. Taken together, our data indicate that kindlin-2 promotes   the inva  siveness of prostate cancer cells largely through NF-κB-dependent upregulation of MMP-9 and MMP-2. Further studies are warranted to evaluate the significance of kindlin-2 as a therapeutic target for metastatic prostate cancer. 
  GeneRIFs  
 data indicate that kindlin-2 promotes the   invasive  ness of prostate cancer cells largely through NF-kappaB-dependent upregulation of MMP-9 and MMP-2 
 Kindlin-2 is expressed in a small subset of high-grade   invasive   bladder cancers and may have a role in tumor progression 
  [Mm]etastasis  PubMed  
 Novel focal adhesion protein kindlin-2 promotes the invasion of gastric cancer cells through phosphorylation of integrin β1 and β3. We have found that the expression of the novel focal adhesion protein kindlin-2 had a significant positive correlation with poor survival in gastric cancer. However, the mechanism by which kindlin-2 acts in gastric cancer warrants further evaluation.Kindlin-2 mRNA expression in gastric cancer cell lines was measured by realtime RT-PCR under normal and hypoxic conditions. Cell proliferation, apoptosis, cell cycle, tumor adhesion, cell invasion ability, and phosphorylation of integrin β1 and β3 proteins were measured to assess the influence of kindlin-2 on the malignant behavior of gastric cancer cells.Kindlin-2 mRNA expression was highest in the dist  ant metast  asis gastric cancer cell line Hs-746T. Cell proliferation, adhesion with endothelium and collagen IV, invasion rate, and angiogenesis genes expression, as well as phosphorylation of integrin β1 and β3 in Hs-746T, were decreased significantly after kindlin-2 downregulation, but there was no change in apoptosis and cell cycle.Kindlin-2 might promote the invasion of gastric cancer cells through enhancing proliferation and adhesion by the phosphorylation of integrin β1 and β3. 
 Novel focal adhesion protein kindlin-2 promotes the invasion of gastric cancer cells through phosphorylation of integrin β1 and β3. We have found that the expression of the novel focal adhesion protein kindlin-2 had a significant positive correlation with poor survival in gastric cancer. However, the mechanism by which kindlin-2 acts in gastric cancer warrants further evaluation.Kindlin-2 mRNA expression in gastric cancer cell lines was measured by realtime RT-PCR under normal and hypoxic conditions. Cell proliferation, apoptosis, cell cycle, tumor adhesion, cell invasion ability, and phosphorylation of integrin β1 and β3 proteins were measured to assess the influence of kindlin-2 on the malignant behavior of gastric cancer cells.Kindlin-2 mRNA expression was highest in the dist  ant metast  asis gastric cancer cell line Hs-746T. Cell proliferation, adhesion with endothelium and collagen IV, invasion rate, and angiogenesis genes expression, as well as phosphorylation of integrin β1 and β3 in Hs-746T, were decreased significantly after kindlin-2 downregulation, but there was no change in apoptosis and cell cycle.Kindlin-2 might promote the invasion of gastric cancer cells through enhancing proliferation and adhesion by the phosphorylation of integrin β1 and β3. 
 Kindlin-2: a novel adhesion protein related to tumor invasion, lymph node   metastasis  , and patient outcome in gastric cancer. Kindlin-2 has been confirmed as an essential element of bidirectional integrin signaling. In recent years, the relationship between Kindlin-2 expression and cancers has been a focus of interest. However, the relationship between Kindlin-2 expression in gastric cancer and tumor invasion,   metastasis  , and the outcome of patients have not been studied.Kindlin-2 expression at protein and RNA levels were detected by Western blot and real-time polymerase chain reaction in 40 pairs of gastric cancer samples. In addition, the correlations between Kindlin-2 expression and clinicopathologic factors as well as the prognosis of the patients were analyzed. Multivariate Cox regression was used to study the effect of Kindlin-2 expression on overall and progression-free survival.We found that Kindlin-2 was up-regulated both at RNA (P = .027) and protein levels (P = .014) in gastric cancer tissues. Tumor samples with high Kindlin-2 expression (Kindlin-2/β-actin:tumor tissue/paraneoplastic tissue, ≥2) was observed in 55% of the patients. Moreover, Kindlin-2 expression had a significant positive correlation with tumor stromal invasion (P = .014), lymph no  de metasta  sis (P = .007), and TNM stage (P = .014). Patients with high Kindlin-2 expression had significantly poorer overall survival (P = .012) and progression-free survival (P = .012). High Kindlin-2 expression was an independent risk factor of progression-free survival (hazard ratio, 5.2; 95% confidence interval, 1.1-3.3; P = .032).Kindlin-2 may play an important role in the development of gastric cancer and it is a potential factor that could be used to evaluate the outcome of gastric cancer. Kindlin-2 may shed new light on evaluating the prognosis and targeted therapy of gastric cancer. 
 Kindlin-2 expression in peritumoral stroma is associated with poor prognosis in pancreatic ductal adenocarcinoma. Kindlin-2 is a novel focal adhesion protein reported to be expressed in breast, lung, and gastric cancers. This study aimed to investigate the significance of kindlin-2 expression in pancreatic ductal adenocarcinomas (PDACs).We performed immunohistochemical analysis on kindlin-2 on PDAC samples from 95 patients. We investigated the association between kindlin-2 expression and clinicopathological parameters of PDAC and the survival time of patients with PDAC who underwent pancreatectomy.Kindlin-2 was highly expressed in the peritumoral stroma of PDACs. Stromal kindlin-2 expression was related to nodal   metastasis   (P = 0.03). Univariate analysis showed that patients with positive kindlin-2 expression had significantly shorter survival times than those with negative kindlin-2 expression (P = 0.01). In addition, multivariate analysis revealed that kindlin-2 expression was an independent factor of poor prognosis in patients with PDAC after R0 resection (RR = 2.15; P = 0.04).Kindlin-2 expression in stromal components is significantly associated with poor prognosis of patients with PDAC, suggesting that kindlin-2 is a prognostic marker for patients with PDAC. 
 Kindlin-2 is expressed in malignant mesothelioma and is required for tumor cell adhesion and migration. Kindlin-2 is a novel integrin-interacting focal adhesion protein that belongs to the Kindlin family. Focal adhesion proteins control cytoskeleton dynamics and promote cancer cell growth, survival, migration and   metastasis  . Little is known, however, about expression of Kindlin-2 in association with human cancer. We now reveal high Kindlin-2 expression in malignant mesothelioma (MM) cell lines using an affinity-purified anti-Kindlin-2 antibody. Furthermore, we show by immunohistochemistry that Kindlin-2 is highly expressed in 92 of 102 (90%) MMs with epitheliod; sarcomatoid, biphasic and poorly differentiated morphologies. In addition, Kindlin-2 expression correlates to cell proliferation, suggesting a role for Kindlin-2 in tumor growth. We also detect increased expression of Kindlin-2 at the invasion front of tumors concurrent with increased expression of integrin-linked kinase, a Kindlin-binding protein. Besides the high expression of Kindlin-2 in pleural MMs, pleural metastases of lung adenocarcinoma also express large amounts of Kindlin-2, but not Kindlin-1. Notably, in vitro, when endogenous Kindlin-2 was knocked down with RNAi in MM cells, this impaired cell spreading, adhesion and migration. Overall, our study suggests that heightened expression of Kindlin-2 might contribute to tumor progression in MM. 
  GeneRIFs  
 Kindlin-2 was highly expressed in the peritumoral stroma of pancreatic ductal adenocarcinomas. Stromal kindlin-2 expression was related to nodal   metastasis   (P = 0.03). 
  [Cc]ell [Ii]nvasion  PubMed  
 Novel focal adhesion protein kindlin-2 promotes the invasion of gastric cancer cells through phosphorylation of integrin β1 and β3. We have found that the expression of the novel focal adhesion protein kindlin-2 had a significant positive correlation with poor survival in gastric cancer. However, the mechanism by which kindlin-2 acts in gastric cancer warrants further evaluation.Kindlin-2 mRNA expression in gastric cancer cell lines was measured by realtime RT-PCR under normal and hypoxic conditions. Cell proliferation, apoptosis, cell cycle, tumor adhesion  , cell invasi  on ability, and phosphorylation of integrin β1 and β3 proteins were measured to assess the influence of kindlin-2 on the malignant behavior of gastric cancer cells.Kindlin-2 mRNA expression was highest in the distant metastasis gastric cancer cell line Hs-746T. Cell proliferation, adhesion with endothelium and collagen IV, invasion rate, and angiogenesis genes expression, as well as phosphorylation of integrin β1 and β3 in Hs-746T, were decreased significantly after kindlin-2 downregulation, but there was no change in apoptosis and cell cycle.Kindlin-2 might promote the invasion of gastric cancer cells through enhancing proliferation and adhesion by the phosphorylation of integrin β1 and β3. 
 Novel focal adhesion protein kindlin-2 promotes the invasion of gastric cancer cells through phosphorylation of integrin β1 and β3. We have found that the expression of the novel focal adhesion protein kindlin-2 had a significant positive correlation with poor survival in gastric cancer. However, the mechanism by which kindlin-2 acts in gastric cancer warrants further evaluation.Kindlin-2 mRNA expression in gastric cancer cell lines was measured by realtime RT-PCR under normal and hypoxic conditions. Cell proliferation, apoptosis, cell cycle, tumor adhesion  , cell invasi  on ability, and phosphorylation of integrin β1 and β3 proteins were measured to assess the influence of kindlin-2 on the malignant behavior of gastric cancer cells.Kindlin-2 mRNA expression was highest in the distant metastasis gastric cancer cell line Hs-746T. Cell proliferation, adhesion with endothelium and collagen IV, invasion rate, and angiogenesis genes expression, as well as phosphorylation of integrin β1 and β3 in Hs-746T, were decreased significantly after kindlin-2 downregulation, but there was no change in apoptosis and cell cycle.Kindlin-2 might promote the invasion of gastric cancer cells through enhancing proliferation and adhesion by the phosphorylation of integrin β1 and β3. 
 Kindlin 2 promotes breast cancer invasion via epigenetic silencing of the microRNA200 gene family. Kindlin 2, as a focal adhesion protein, controls integrin activation and regulates Wnt signaling in an integrin-binding independent manner. However, the association of Kindlin 2 with cancer-related microRNAs is unknown. Here, we report that Kindlin 2 markedly downregulates the expression of miR-200 family by inducing CpG island hypermethylation. Mechanistically, Kindlin 2 forms a complex with DNMT3A in the cell nucleus and the two proteins co-occupy the promoter of miRNA-200b. Functionally, repression of miR-200b is required for Kindlin 2-induced breast cancer   cell invasion   and tumor formation. Our data indicate that Kindlin 2 plays a novel role in epigenetic repression of miR-200 family, a mechanism that promotes breast cancer invasion. 
 Kindlin 2 forms a transcriptional complex with β-catenin and TCF4 to enhance Wnt signalling. Kindlin 2, as a focal adhesion protein, controls integrin activation. However, the association of Kindlin 2 with cancer-related signalling pathways is unknown. Here we identified a new direct interaction between Kindlin 2 and the active β-catenin. Importantly, Kindlin 2 forms a tripartite complex with β-catenin and TCF4. Mechanistically, Kindlin 2 selectively strengthens the occupancy of β-catenin on the Wnt target gene Axin2 and enhances Axin2 gene expression. Functionally, the β-catenin-Axin2-Snail cascade is required for Kindlin 2-induced tu  mour cell inv  asion. Our data indicate that Kindlin 2 is a new regulator of Wnt signalling, providing a mechanistic insight into the role of Kindlin 2 in cancer progression. 
   
  
 HSPA5 
 
  NCBI Gene  
  GeneRIF  
  Pubmed  
 
 
  Search Term  Rank  Fields  
  [Mm]igration  6  Process(1); PubMed(23); GeneRIFs(8)  
  ECM  7  PubMed(3); GeneRIFs(2)  
  [Ee]xtracellular [Mm]atrix  8  PubMed(6); Component(1); GeneRIFs(1)  
  [Ii]nvasive  10  PubMed(16); GeneRIFs(3)  
  [Mm]etastasize  11  PubMed(2)  
  [Mm]etastasis  12  PubMed(40); GeneRIFs(10)  
  [Cc]ell [Ii]nvasion  13  PubMed(8)  
 
 
 hide/show details for HSPA5 
  [Mm]igration  Process  
 positive regulation of cell   migration   
  PubMed  
 Analysis of the myosin-II-responsive focal adhesion proteome reveals a role for β-Pix in negative regulation of focal adhesion maturation. Focal adhesions undergo myosin-II-mediated maturation wherein they grow and change composition to modulate integrin signalling for cell   migratio  n, growth and differentiation. To determine how focal adhesion composition is affected by myosin II activity, we performed proteomic analysis of isolated focal adhesions and compared protein abundance in focal adhesions from cells with and without myosin II inhibition. We identified 905 focal adhesion proteins, 459 of which changed in abundance with myosin II inhibition, defining the myosin-II-responsive focal adhesion proteome. The abundance of 73% of the proteins in the myosin-II-responsive focal adhesion proteome was enhanced by contractility, including proteins involved in Rho-mediated focal adhesion maturation and endocytosis- and calpain-dependent focal adhesion disassembly. During myosin II inhibition, 27% of proteins in the myosin-II-responsive focal adhesion proteome, including proteins involved in Rac-mediated lamellipodial protrusion, were enriched in focal adhesions, establishing that focal adhesion protein recruitment is also negatively regulated by contractility. We focused on the Rac guanine nucleotide exchange factor β-Pix, documenting its role in the negative regulation of focal adhesion maturation and the promotion of lamellipodial protrusion and focal adhesion turnover to drive cel  l migrati  on. 
 Unraveling the role of KIAA1199, a novel endoplasmic reticulum protein, in cancer cell   migration  . Cell   migration   is a critical determinant of cancer metastasis, and a better understanding of the genes involved will lead to the identification of novel targets aimed at preventing cancer dissemination. KIAA1199 has been shown to be upregulated in human cancers, yet its role in cancer progression was hitherto unknown.Clinical relevance was assessed by examining KIAA1199 expression in human cancer specimens. In vitro and in vivo studies were employed to determine the function of KIAA1199 in cancer progression. Cellular localization of KIAA1199 was microscopically determined. SNAP-tag pull-down assays were used to identify binding partner(s) of KIAA1199. Calcium levels were evaluated using spectrofluorometric and fluorescence resonance energy transfer analyses. Signaling pathways were dissected by Western blotting. Student t test was used to assess differences. All statistical tests were two-sided.KIAA1199 was upregulated in invasive breast cancer specimens and inversely associated with patient survival rate. Silencing of KIAA1199 in MDA-MB-435 cancer cells resulted in a mesenchymal-to-epithelial transition that reduced cell migratory ability in vitro (75% reduction; P < .001) and decreased metastasis in vivo (80% reduction; P < .001). Gain-of-function assays further demonstrated the role of KIAA1199 in cell   migration  . KIAA1199-enhanced cell   migration   required endoplasmic reticulum (ER) localization, where it forms a stable complex with the chaperone binding immunoglobulin protein (BiP). A novel ER-retention motif within KIAA1199 that is required for its ER localization, BiP interaction, and enhanced cell   migration   was identified. Mechanistically, KIAA1199 was found to mediate ER calcium leakage, and the resultant increase in cytosolic calcium ultimately led to protein kinase C alpha activation and cell   migration  .KIAA1199 serves as a novel cell   migration  -promoting gene and plays a critical role in maintaining cancer mesenchymal status. 
 A critical role for GRP78/BiP in the tumor microenvironment for neovascularization during tumor growth and metastasis. Glucose-regulated protein 78 (GRP78)/BiP is a multifunctional protein which plays a major role in endoplasmic reticulum (ER) protein processing, protein quality control, maintaining ER homeostasis, and controlling cell signaling and viability. Previously, using a transgene-induced mammary tumor model, we showed that Grp78 heterozygosity impeded cancer growth through suppression of tumor cell proliferation and promotion of apoptosis and the Grp78(+/-) mice exhibited dramatic reduction (70%) in the microvessel density (MVD) of the endogenous mammary tumors, while having no effect on the MVD of normal organs. This observation suggests that GRP78 may critically regulate the function of the host vasculature within the tumor microenvironment. In this article, we interrogated the role of GRP78 in the tumor microenvironment. In mouse tumor models in which wild-type (WT), syngeneic mammary tumor cells were injected into the host, we showed that Grp78(+/-) mice suppressed tumor growth and angiogenesis during the early phase but not during the late phase of tumor growth. Growth of metastatic lesions of WT, syngeneic melanoma cells in the Grp78(+/-) mice was potently suppressed. We created conditional heterozygous knockout of GRP78 in the host endothelial cells and showed severe reduction of tumor angiogenesis and metastatic growth, with minimal effect on normal tissue MVD. Furthermore, knockdown of GRP78 expression in immortalized human endothelial cells showed that GRP78 is a critical mediator of angiogenesis by regulating cell proliferation, survival, and   migration  . Our findings suggest that concomitant use of current chemotherapeutic agents and novel therapies against GRP78 may offer a powerful dual approach to arrest cancer initiation, progression, and metastasis. 
 Grp78 Is Critical for Amelogenin-Induced Cell   Migration   in a Multipotent Clonal Human Periodontal Ligament Cell Line. Periodontal ligament stem cells (PDLSCs) are known to play a pivotal role in regenerating the periodontium. Amelogenin, which belongs to a family of extracellular matrix (ECM) proteins, is a potential bioactive molecule for periodontal regenerative therapy. However, its downstream target molecules and/or signaling patterns are still unknown. Our recent proteomic study identified glucose-regulated protein 78 (Grp78) as a new amelogenin-binding protein. In this study, we demonstrate, for the first time, the cellular responses induced by the biological interaction between amelogenin and Grp78 in the human undifferentiated PDL cell line 1-17, which possesses the most typical characteristics of PDLSCs. Confocal co-localization experiments revealed the internalization of recombinant amelogenin (rM180) via binding to cell surface Grp78, and the endocytosis was inhibited by the silencing of Grp78 in 1-17 cells. Microarray analysis indicated that rM180 and Grp78 regulate the expression profiles of cell   migration  -associated genes in 1-17 cells. Moreover, Grp78 overexpression enhanced rM180-induced cell   migration   and adhesion without affecting cell proliferation, while silencing of Grp78 diminished these activities. Finally, binding of rM180 to Grp78 promoted the formation of lamellipodia, and the simultaneous activation of Rac1 was also demonstrated by NSC23766, a widely accepted Rac1 inhibitor. These results suggest that Grp78 is essential for enhancing amelogenin-induced   migration   in 1-17 cells. The biological interaction of amelogenin with Grp78 offers significant therapeutic potential for understanding the biological components and specific functions involved in the signal transduction of amelogenin-induced periodontal tissue regeneration. 
 Mild endoplasmic reticulum stress promotes retinal neovascularization via induction of BiP/GRP78. Endoplasmic reticulum (ER) stress occurs as a result of accumulation of unfolded or misfolded proteins in the ER and is involved in the mechanisms of various diseases, such as cancer and neurodegeneration. The goal of the present study was to clarify the relationship between ER stress and pathological neovascularization in the retina. Proliferation and   migration   of human retinal microvascular endothelial cells (HRMEC) were assessed in the presence of ER stress inducers, such as tunicamycin and thapsigargin. The expression of ER chaperone immunoglobulin heavy-chain binding protein (BiP), known as Grp78, was evaluated by real time RT-PCR, immunostaining, and Western blotting. Tunicamycin or thapsigargin was injected into the intravitreal body of oxygen-induced retinopathy (OIR) model mice at postnatal day 14 (P14) and retinal neovascularization was quantified at P17. The expression and localization of BiP in the retina was also evaluated in the OIR model. Exposure to tunicamycin and thapsigargin increased the proliferation and   migration   of HRMEC. Tunicamycin enhanced the expression of BiP in HRMEC at both the mRNA level and at the protein level on the cell surface, and increased the formation of a BiP/T-cadherin immunocomplex. In OIR model mice, retinal neovascularization was accelerated by treatments with ER stress inducers. BiP was particularly observed in the pathological vasculature and retinal microvascular endothelial cells, and the increase of BiP expression was correlated with retinal neovascularization. In conclusion, ER stress may contribute to the formation of abnormal vasculature in the retina via BiP complexation with T-cadherin, which then promotes endothelial cell proliferation and   migration  . 
 Glucose-regulated protein 78 contributes to the proliferation and tumorigenesis of human colorectal carcinoma via AKT and ERK pathways. Glucose-regulated protein 78 (GRP78), a molecular chaperon in the endoplasmic reticulum (ER), is overexpressed in a variety of tumor types and plays a critical role in cancer cell proliferation,   migratio  n, invasion and drug resistance. However, the mechanisms underlying the role of GRP78 in tumor carcinogenesis remain largely unknown. In the present study, we found that GRP78 knockdown in colorectal carcinoma (CRC) cells significantly inhibited cell proliferation, colony formation and tumorigenesis in vitro and in vivo. The proliferation inhibition of CRC cells by GRP78 knockdown was associated with an S phase arrest, a reduced G1/S transition, and a downregulation of phosphorylation of AKT and ERK1/2, key cell cycle regulatory proteins. In addition, GRP78 knockdown enhanced the apoptosis induced by 5-fluorouracil (5-FU) in CRC cells. Taken together, our results indicate that GRP78 plays an important role in the development and progression of CRC and may have therapeutic potential for CRC patients. 
 Molecular chaperones as a common set of proteins that regulate the invasion phenotype of head and neck cancer. The goal of this study was to establish a common set of molecules that regulate cell invasion in head and neck cancer (HNC).Five invasive sublines derived from HNC cell lines were established using the Matrigel selection method. Proteomic technology, MetaCore algorithm, and reverse transcriptase-PCR methods were used to search for molecules that contribute to the invasion phenotype. Cellular functional analyses and clinical association studies were applied to examine the significance of the molecules.Fifty-two proteins were identified in more than two of the four independent proteomic experiments, including 10 (19%) molecular chaperones. Seven chaperones were confirmed to be differentially expressed in five sublines, Hsp90α, Hsp90β, Hsp90-B1/Gp96, Hsp70-A5/Grp78, and HYOU1, that upregulate, whereas Hsp60 and glucosidase-α neutral AB (GANAB) downregulate. Four molecules were further investigated. In all cell lines, knockdown of Hsp60 or GANAB and silencing of Gp96 or Grp78 considerably enhanced or reduced ce  ll migrat  ion and invasion, respectively. Clinical association studies consistently revealed that low levels of Hsp60 or GANAB and high levels of Gp96 or Grp78 are significantly associated with advanced cancer (P < 0.001 to P = 0.047, respectively, for the four molecules) and poor survival (P < 0.001 to P = 0.025, respectively, for the four molecules).Our study defined molecular chaperones as a common set of proteins that regulate the invasion phenotype of HNC. Loss of the tumor suppression function of Hsp60 or GANAB and acquisition of the oncogenic function of Gp96 or Grp78 contribute to aggressive cancers. These molecules may serve as prognostic markers and targets for cancer drug development. 
 Cell-surface GRP78 facilitates colorectal cancer cell   migration   and invasion. Glucose regulated protein 78 (GRP78) is predominantly located in the endoplasmic reticulum as a molecular chaperone. It has also been found on the membranes of some cancer cells, acting as a receptor for a wide variety of ligands. However, its presence on colorectal cancer (CRC) cell surface and its role in CRC metastatic progression remain elusive. Here we reported that GRP78 was predominantly present in the form of clustering aggregates on CRC cell surfaces, and its surface abundance was strongly correlated with CRC differentiation stage. Interestingly, we observed that cell-surface GRP78 had an interaction with the ECM adhesion molecule β1-integrin and was involved in cell-matrix adhesion through regulation of focal adhesion kinase (FAK). Moreover, the present data also implicated that surface GRP78 promoted the cell invasion process, and this effect was at least partly mediated through its association with uPA-uPAR protease system. Together, our data suggests that surface GRP78 promotes CRC cell   migratio  n and invasion by regulating cell-matrix adhesion and ECM degradation, which is independent of its signaling receptor function. 
 Glucose-regulated protein 78 mediates hormone-independent prostate cancer progression and metastasis through maspin and COX-2 expression. Glucose-regulated protein 78 (GRP78) plays an essential role in embryonic development and in the progression and therapeutic resistance of many cancers. However, little is known about the function of GRP78 in hormone-independent prostate cancer. Here, we found that the expression levels of GRP78 were higher in PC-3 cells than in DU-145 cells. When the expression of GRP78 was silenced using a GRP78-specific small interfering RNA in PC-3 cells, the growth rate and adhesive ability were reduced. Cell   migration   was dramatically decreased in GRP78-depleted cells. Dissection of the involved signal pathways revealed that maspin expression was upregulated after silencing GRP78 expression. The upregulation of maspin and downregulation of COX-2 may cause the decrease in cell proliferation and   migration   observed after silencing GRP78 expression. Silencing GRP78 expression may suppress the proliferation, adhesion, and   migration   of prostate cancer cells via maspin and COX-2 regulation. 
 Glucose-regulated protein 78 regulates multiple malignant phenotypes in head and neck cancer and may serve as a molecular target of therapeutic intervention. Glucose-regulated protein 78 (Grp78) is an endoplasmic reticulum chaperone protein and is overexpressed in various cancers. However, it is unclear how significance of this molecule play an active role contributing to the oncogenic effect of head and neck cancer (HNC). To investigate the potential function of Grp78, six HNC cell lines were used. We found that Grp78 is highly expressed in all six cell lines and many of the proteins were localized in the periphery regions, implying other function of this molecule aside from endoplasmic reticulum stress response. Knockdown of Grp78 by small interfering RNA significantly reduced cell growth and colony formation to 53% to 12% compared with that of controls in all six HNC cell lines. Using in vitro wound healing and Matrigel invasion assays, we found that cell   migration   and invasive ability were also inhibited to 23% to 2% in all these cell lines tested. In vivo xenograft studies showed that administration of Grp78-small interfering RNA plasmid into HNC xenografts significantly inhibited both tumor growth in situ (>60% inhibition at day 34) and liver metastasis (>90% inhibition at day 20). Our study showed that Grp78 actively regulates multiple malignant phenotypes, including cell growth,   migration  , and invasion. Because knockdown Grp78 expression succeeds in the reduction of tumor growth and metastatic potential, this molecule may serve as a molecular target of therapeutic intervention for HNC. 
 Deacetylation of HSPA5 by HDAC6 leads to GP78-mediated HSPA5 ubiquitination at K447 and suppresses metastasis of breast cancer. Heat-shock protein 5 (HSPA5) is a marker for poor prognosis in breast cancer patients and has an important role in cancer progression, including promoting drug resistance and metastasis. In this study, we identify that the specific lysine residue 447 (K447) of HSPA5 could be modified with polyubiquitin for subsequent degradation through the ubiquitin proteasomal system, leading to the suppression of cell   migration   and invasion of breast cancer. We further found that GP78, an E3 ubiquitin ligase, interacted with the C-terminal region of HSPA5 and mediated HSPA5 ubiquitination and degradation. Knock down of GP78 significantly increased the expression of HSPA5 and enhanced   migration  /invasive ability of breast cancer cells. Knock down of histone deacetylase-6 (HDAC6) increased the acetylation of HSPA5 at lysine residues 353 (K353) and reduced GP78-mediated ubiquitination of HSPA5 at K447 and then increased cell   migration  /invasion. In addition, we demonstrate that E3 ubiquitin ligase GP78 preferentially binds to deacetylated HSPA5. Notably, the expression levels of GP78 inversely correlated with HSPA5 levels in breast cancer patients. Patients with low GP78 expression significantly correlated with invasiveness of breast cancer, advanced tumor stages and poor clinical outcome. Taken together, our results provide new mechanistic insights into the understanding that deacetylation of HSPA5 by HDAC6 facilitates GP78-mediated HSPA5 ubiquitination and suggest that post-translational regulation of HSPA5 protein is critical for HSPA5-mediated metastatic properties of breast cancer. 
 GRP78 mediates cell growth and invasiveness in endometrial cancer. Recent studies have indicated that endoplasmic reticulum stress, the unfolded protein response activation and altered GRP78 expression can play an important role in a variety of tumors development and progression. Very recently we reported for the first time that GRP78 is increased in endometrial tumors. However, whether GRP78 could play a role in the growth and/or invasiveness of endometrial cancer cells is still unknown. Here we report that the silencing of GRP78 expression affects both cell growth and invasiveness of Ishikawa and AN3CA cells, analyzed by the (3-(4,5-dimethylthiazol-2-yl)-2,5-diphenyl tetrazolium bromide) and transwell   migration   assay, respectively. At variance with Ishikawa cells, AN3CA cells showed, besides an endoplasmic reticulum, also a plasma membrane GRP78 localization, evidenced by both immunofluorescence and cell membrane biotinylation experiments. Intriguingly, flow cytometry experiments showed that the treatment with a specific antibody targeting GRP78 C-terminal domain caused apoptosis in AN3CA but not in Ishikawa cells. Induction of apoptosis in AN3CA cells was not mediated by the p53 pathway activation but was rather associated to reduced AKT phosphorylation. Interestingly, immunofluorescence analysis evidenced that endometrioid adenocarcinoma tissues displayed, similarly to AN3CA cells, also a GRP78 plasma membrane localization. These data suggest that GRP78 and its plasma membrane localization, might play a role in endometrial cancer development and progression and might constitute a novel target for the treatment of endometrial cancer. 
 Survivin-mediated cancer cell   migration   through GRP78 and epithelial-mesenchymal transition (EMT) marker expression in Mahlavu cells. Survivin has multiple functions during the progression of cancer. However, the role of survivin in the progression and metastasis of hepatocellular carcinoma (HCC) remains unknown.Survivin expression in HCC cells (Mahlavu and Hep3B) was assessed using reverse transcription real-time PCR and Western blot analyses. In addition, survivin expression in HCC cells was manipulated using small interfering RNA (siRNA) or overexpression and proliferation and transwell   migration   assays were performed to monitor the effect of manipulated survivin expression on the growth rate and migratory ability of the transfected cells.Among the HCC cell lines tested, we found high endogenous expression of survivin mRNA and protein in Mahlavu cells. After silencing survivin expression in Mahlavu cells, there was a dramatic decrease in the cell growth rate and an increase in the metastatic potential of the cells. Overexpression of survivin in Hep3B cells suppressed the ability of the cell to migrate. The mechanism of enhanced cell   migration   caused by decreased survivin expression is mediated through the downregulation of glucose-regulated protein 78 (GRP78) and the upregulation of the epithelial-mesenchymal transition (EMT) marker, vimentin.Survivin may mediate metastasis in HCC. The knockdown of survivin expression may enhance cancer metastasis through the downregulation of GRP78 and upregulation of vimentin expression. 
 Activation of GRP78 on endothelial cell membranes by an ADAM15-derived peptide induces angiogenesis. Impaired angiogenesis is one of the features of ischemic diseases. We have previously identified, by screening a phage display peptide library, a peptide that induces angiogenesis in endothelial cells under hypoxic conditions by binding the cell's membrane heat shock protein GRP78. Protein data base search identified 4 amino acids (HWRR) of that synthetic peptide present on the ADAM15 metalloprotease domain, a protein considered to be involved in neovascularization. Three peptides were synthesized according to the ADAM15 sequence placing HWRR at different positions. Peptide ADoPep1 exhibited significant angiogenic properties under hypoxic conditions as determined by cell proliferation,   migration   and tube formation. In a mouse hind limb ischemia model, a single injection of the peptide restored blood perfusion. The identified peptide was found to activate GRP78 on endothelial cell membrane and siRNA directed against the GRP78 mRNA interfered with induction of angiogenesis by the peptide. The peptide binding induced a decrease in heat shock protein GRP78 that is overexpressed under hypoxic conditions. The mechanism of peptide-induced angiogenic activity involves inhibition of apoptosis as well as increased Akt phosphorylation and ERK 1/2 activation. The peptide did not induce VEGF receptor-2 protein synthesis and phosphorylation, suggesting a VEGF-independent mechanism of angiogenesis. 
 Knockdown of glucose-regulated protein 78/binding immunoglobulin heavy chain protein expression by asymmetric small interfering RNA induces apoptosis in prostate cancer cells and attenuates migratory capability. Glucose-regulated protein 78 [GRP78, also termed binding immunoglobulin heavy chain protein (Bip)] may be involved in cancer progression and metastasis. However, to date there has been minimal investigation into its potential role in human prostate cancer cells. Recent studies have demonstrated that asymmetric small interfering RNA (asiRNA)-mediated gene silencing is more effective and longer-lasting than conventional symmetric siRNA. Thus, the current study aimed to investigate the effects of GRP78-specific asiRNA on human prostate cancer cells. A series of asiRNAs was synthesized and their efficiency in silencing GRP78 expression in PC-3 human prostate cancer cells was evaluated. The effects of knockdown using asiRNAs were compared to those of knockdown using symmetric siRNAs. The effect of GRP78 silencing on PC-3 cell apoptosis and   migration  , and the possible mechanisms governing these biological processes were examined. Compared with the symmetric siRNA, transfection with the 15 base pair asiRNA (asiGRP78-3) resulted in greater downregulation of GRP78 expression. GRP78 depletion in PC-3 cells resulted in increased apoptosis and decreased   migration   of these cells. Experiments investigating the underlying mechanisms of these effects revealed that knockdown of GRP78 attenuated protein kinase B activation and decreased the expression of pro-caspase 9, pro-caspase 3 and vimentin. In conclusion, knockdown of GRP78/Bip expression with asymmetric siRNA led to increased prostate cancer cell apoptosis and reduced cellular   migration  . 
 Glucose-regulated protein 78 (GRP78) regulates colon cancer metastasis through EMT biomarkers and the NRF-2/HO-1 pathway. Glucose-regulated protein 78 (GRP78) is a key chaperone and stress response protein. Previous studies have demonstrated that high GRP78 expression may be correlated with cancer progression and therapeutic response. However, the role of GRP78 in the metastasis of colon cancer is unclear. In this study, we used small interfering RNA (siRNA) to knock down GRP78 expression in colon cancer cells (HT-29 and DLD-1 cells). In wound-healing   migration   assays, we found that GRP78-knockdown (GRP78KD) cells showed better wound-healing ability than control cells. We also found that GRP78KD cells displayed a better migratory ability than control cells in   migration   and invasion assays. As we further dissected the underlying molecular mechanism, we found that silencing GRP78 may cause an increase in vimentin expression and a decrease in the E-cadherin level, which was correlated with the increase in migratory ability. In addition, we found that GRP78KD may activate the NRF-2/HO-1 pathway, and this activation was also correlated with the increase in cell invasiveness. Furthermore, we examined GRP78 expression in a tissue array and found that the GRP78 expression in metastatic adenocarcinoma in lymph nodes tended to be weaker than that in primary colonic adenocarcinoma. In conclusion, a low level of GRP78 may cause an increase in metastasis ability in colon cancer cells by altering E-cadherin and vimentin expression and activating the NRF-2/HO-1 signaling pathway. Our study demonstrates that low expression of GRP78 may correlate with a high risk of metastasis in colon cancer. 
 E1A-mediated inhibition of HSPA5 suppresses cell   migration   and invasion in triple-negative breast cancer. Triple-negative breast cancer (TNBC) is defined by reduced expression of the estrogen receptor, progesterone receptor, and HER2. TNBC is an especially aggressive group of breast cancers with poor prognosis. There are currently no validated molecular targets to effectively treat this disease. Thus, it is necessary to identify effective molecular targets and therapeutic strategies for TNBC patients.The expression of HSPA5 in patients with breast cancer was examined by immunohistochemistry. The association of HSPA5 expression with tumor grade and metastatic events in TNBC patients was analyzed using the Oncomine database. The knockdown and overexpression of HSPA5 protein were performed to investigate the effects on E1A-suppressed cell   migratio  n/invasion of TNBC using in vitro transwell assays and tumor growth/experimental metastasis studies in animal models.The expression of HSPA5 was positively correlated with high-grade tumors, metastatic events, and poor overall survival in breast cancer patients with TNBC. E1A-inhibited HSPA5 expression suppressed cell   migratio  n/invasive ability of TNBC cell lines. Moreover, E1A significantly abolished lung metastases from breast cancer cells by inhibiting HSPA5 expression in a xenograft tumor model.The overexpression of HSPA5 is critical for high-risk metastasis of breast cancer and TNBC. The results of our study suggest that HSPA5 may be a crucial mediator of E1A-suppressed metastatic ability of breast cancer cells. Thus, E1A may be a potential target for diagnosis and individualized treatment in clinical practice. 
 Cripto/GRP78 modulation of the TGF-β pathway in development and oncogenesis. Cripto is a small, GPI-anchored signaling protein that regulates cellular survival, proliferation, differentiation and   migratio  n during normal developmental processes and tumorigenesis. Cripto functions as an obligatory co-receptor for the TGF-β ligands Nodal, GDF1 and GDF3 but attenuates signaling of others such as activin-A, activin-B and TGF-β1. Soluble, secreted forms of Cripto also activate Src, ras/raf/MAPK and PI3K/Akt pathways via a mechanism that remains largely obscure. This review describes the biological roles and signaling mechanisms of Cripto, highlighting our identification of the 78 kDa glucose regulated protein (GRP78) as a cell surface receptor/co-factor required for Cripto signaling via both TGF-β and Src/MAPK/PI3K pathways. We discuss emerging evidence indicating that Cripto/GRP78 signaling regulates normal somatic stem cells and their tumorigenic counterparts. 
 Silencing of glucose-regulated protein 78 (GRP78) enhances cell   migration   through the upregulation of vimentin in hepatocellular carcinoma cells. Glucose-regulated protein 78 (GRP78) plays an important role in embryonic development and cancer progression. However, there is little information regarding the regulation of GRP78 in hepatocellular carcinoma (HCC) metastasis.We used RNA silencing and cDNA expression vectors to manipulate target gene expression in HCC cells. The transwell   migration   assay and xCelligence biosensor system were applied to determine the proliferatory and migratory ability of the HCC cells.In this study, we found that GRP78 silencing enhanced cell   migration   in both HepJ5 and Mahlavu cells. Overexpressed GRP78 in skHep1 cells suppressed the migratory ability. In the insight mechanism dissection for GRP78-mediated cancer   migration  , we found that downregulation of GRP78 caused the increase of vimentin expression on HCC cells. Suppressed vimentin expression also decreased the migratory ability on HCC, indicating that vimentin expression levels modulated the cell migratory ability.We found that silencing GRP78 in HCC cells may enhance cell   migration   through the increase of vimentin expression. 
 Cell Surface GRP78 Accelerated Breast Cancer Cell Proliferation and   Migration   by Activating STAT3. High levels of cell surface glucose regulated protein 78 (sGRP78) have been implicated in cancer growth, survival, metastasis, and chemotherapy resistance. However, the underlying mechanism remains largely unknown. Here we report that the level of sGRP78 expression in human breast tumors gradually increases during cancer progression. Overexpression of GRP78 significantly enhanced its membrane distribution in human MCF-7 breast cancer cells, but had no effect on endoplasmic reticulum (ER) stress. High levels of sGRP78 facilitated cell proliferation and   migration  , as well as suppressed cell apoptosis. Neutralization of sGRP78 by a specific antibody against GRP78 alleviated sGRP78-induced cell growth and   migration  . Importantly, high phosphorylation levels of the signal transducer and activator of transcription 3 (STAT3) were found in human breast tumors that express sGRP78 and MCF-7 cells infected with adenovirus encoding human GRP78. Pretreatment with a GRP78 antibody suppressed STAT3 phosphorylation. Furthermore, genetic and pharmacological inhibition of STAT3 reversed the impacts of GRP78 on cell proliferation, apoptosis, and   migration  . These findings indicate that STAT3 mediates sGRP78-promoted breast cancer cell growth and   migration  . 
 Elevated GRP78 expression is associated with poor prognosis in patients with pancreatic cancer. Glucose-regulated protein 78 (GRP78) is a member of the heat-shock protein 70 family. We evaluated the expression of GRP78 using tissue microarray-based immunohistochemistry in tumor tissues and adjacent nontumor tissues from 180 pancreatic ductal adenocarcinoma (PDAC) patients. The associations between the expression levels of GRP78, clinicopathological factors, and overall survival were evaluated. The results showed that the expression of GRP78 was significantly higher in PDAC cells than in normal pancreatic duct cells within adjacent nontumor tissues (p < 0.05). The increased expression of GRP78 in the tumor tissues was significantly correlated with a higher T-stage (p < 0.05) and a shorter overall survival (OS, p < 0.05). In an in vitro study, the regulation of GRP78 in the PDAC cell lines affected the pro  liferatio  n, migration, and invasion of PDAC cells through the regulation of CyclinD1, cyclin-dependent kinase (CDK) 4, CDK6, phospho-signal transducer, activator of transcription 3 (p-STAT3), janus kinase 2 (JAK2), ras homolog gene family member A (RhoA), Rho-associated kinase 1 (ROCK1), and sterile alpha motif domain containing protein 4 (Smad4). The present data suggest that GRP78 plays a crucial role in the pro  liferatio  n, migration, and invasion of pancreatic cancer cells and may be a suitable prognostic marker in PDAC. 
 Japanese encephalitis virus co-opts the ER-stress response protein GRP78 for viral infectivity. The serum-free medium from Japanese encephalitis virus (JEV) infected Baby Hamster Kidney-21 (BHK-21) cell cultures was analyzed by liquid chromatography tandem mass spectrometry (LC-MS) to identify host proteins that were secreted upon viral infection. Five proteins were identified, including the molecular chaperones Hsp90, GRP78, and Hsp70. The functional role of GRP78 in the JEV life cycle was then investigated. Co-  migration   of GRP78 with JEV particles in sucrose density gradients was observed and co-localization of viral E protein with GRP78 was detected by immunofluorescence analysis in vivo. Knockdown of GRP78 expression by siRNA did not effect viral RNA replication, but did impair mature viral production. Mature viruses that do not co-fractionate with GPR78 displayed a significant decrease in viral infectivity. Our results support the hypothesis that JEV co-opts host cell GPR78 for use in viral maturation and in subsequent cellular infections. 
 Glucose regulated protein 78 promotes cell invasion via regulation of uPA production and secretion in colon cancer cells. Glucose regulated protein 78 (GRP78) is frequently highly expressed in tumor cells, contributing to the acquisition of several phenotypic cancer hallmarks. GRP78 expression is also positively correlated with tumor metastasis, and promotes hepatocellular carcinoma cell invasion via increasing cell motility, however, other mechanisms involving the prometastatic roles of GRP78 remain to be elucidated. Here we report that forced GRP78 expression promotes colon cancer cell   migration   and invasion through upregulating MMP-2, MMP-9 and especially uPA production. These effects of GRP78 are mediated by enhancing the activation of β-catenin signaling. Interestingly, we identify that GRP78 interacts with uPA both in the cells and in the culture medium, suggesting that GRP78 protein is likely to directly facilitate uPA secretion via protein-protein interaction. Taken together, our findings demonstrate for the first time that besides stimulation of cell motility, GRP78 can act by increasing proteases production to promote tumor cell invasion. 
  GeneRIFs  
 The present data suggest that GRP78 plays a crucial role in the proliferation,   migration  , and invasion of pancreatic cancer cells and may be a suitable prognostic marker in pancreatic ductal adenocarcinoma. 
 STAT3 mediates GRP78-promoted breast cancer cell growth and   migration  . 
 knockdown of GRP78/Bip expression with asymmetric siRNA led to increased prostate cancer cell apoptosis and reduced cellular   migration  . 
 Silencing GRP78 expression may suppress the proliferation, adhesion, and   migration   of prostate cancer cells via maspin and COX-2 regulation. 
 Data suggests that surface GRP78 promotes CRC cell   migration   and invasion by regulating cell-matrix adhesion and ECM degradation, which is independent of its signaling receptor function. 
 silencing GRP78 in hepatocellular carcinoma cells may enhance cell   migration   through the increase of vimentin expression. 
 Knockdown of GRP78 expression in immortalized human endothelial cells showed that GRP78 is a critical mediator of angiogenesis by regulating cell proliferation, survival, and   migration  . 
 Grp78 actively regulates multiple malignant phenotypes, including cell growth,   migration  , and invasion. 
  ECM  PubMed  
 Grp78 Is Critical for Amelogenin-Induced Cell Migration in a Multipotent Clonal Human Periodontal Ligament Cell Line. Periodontal ligament stem cells (PDLSCs) are known to play a pivotal role in regenerating the periodontium. Amelogenin, which belongs to a family of extracellular matrix (  ECM  ) proteins, is a potential bioactive molecule for periodontal regenerative therapy. However, its downstream target molecules and/or signaling patterns are still unknown. Our recent proteomic study identified glucose-regulated protein 78 (Grp78) as a new amelogenin-binding protein. In this study, we demonstrate, for the first time, the cellular responses induced by the biological interaction between amelogenin and Grp78 in the human undifferentiated PDL cell line 1-17, which possesses the most typical characteristics of PDLSCs. Confocal co-localization experiments revealed the internalization of recombinant amelogenin (rM180) via binding to cell surface Grp78, and the endocytosis was inhibited by the silencing of Grp78 in 1-17 cells. Microarray analysis indicated that rM180 and Grp78 regulate the expression profiles of cell migration-associated genes in 1-17 cells. Moreover, Grp78 overexpression enhanced rM180-induced cell migration and adhesion without affecting cell proliferation, while silencing of Grp78 diminished these activities. Finally, binding of rM180 to Grp78 promoted the formation of lamellipodia, and the simultaneous activation of Rac1 was also demonstrated by NSC23766, a widely accepted Rac1 inhibitor. These results suggest that Grp78 is essential for enhancing amelogenin-induced migration in 1-17 cells. The biological interaction of amelogenin with Grp78 offers significant therapeutic potential for understanding the biological components and specific functions involved in the signal transduction of amelogenin-induced periodontal tissue regeneration. 
 Cell-surface GRP78 facilitates colorectal cancer cell migration and invasion. Glucose regulated protein 78 (GRP78) is predominantly located in the endoplasmic reticulum as a molecular chaperone. It has also been found on the membranes of some cancer cells, acting as a receptor for a wide variety of ligands. However, its presence on colorectal cancer (CRC) cell surface and its role in CRC metastatic progression remain elusive. Here we reported that GRP78 was predominantly present in the form of clustering aggregates on CRC cell surfaces, and its surface abundance was strongly correlated with CRC differentiation stage. Interestingly, we observed that cell-surface GRP78 had an interaction with the   ECM   adhesion molecule β1-integrin and was involved in cell-matrix adhesion through regulation of focal adhesion kinase (FAK). Moreover, the present data also implicated that surface GRP78 promoted the cell invasion process, and this effect was at least partly mediated through its association with uPA-uPAR protease system. Together, our data suggests that surface GRP78 promotes CRC cell migration and invasion by regulating cell-matrix adhesion and   EC  M degradation, which is independent of its signaling receptor function. 
 Knockdown of glucose-regulated protein 78 decreases the invasion, metalloproteinase expression and   ECM   degradation in hepatocellular carcinoma cells. We have reported previously that overexpression of glucose-regulated protein 78 (GRP78) promotes the invasion of hepatocellular carcinoma. However, whether GRP78 knockdown affects the extracellular matrix degradation has not been elucidated. Here we are going to determine whether GRP78 knockdown affect the   ECM   degradation and the role of MMP-2 and MMP-9 in these process in hepatocellular carcinoma cells.Human hepatocellular carcinoma cell line SMMC7721 and HepG2 were cultured in DMEM supplemented with 10% FBS, RT-PCR and western blot were used to detect the endogenous expression of GRP78, MMP-2, MMP-9 and TIMP-2 in SMMC7721 and HepG2. GRP78 shRNAs were transfected using lipofection2000. Transwell assay and wound healing assay were used to analyze the invasion of each transfectant. Gelatin zymography and FITC-gelatin degradation assay were employed to investigate the capabilities of   ECM   degradation of each transfectant. MTT assay was used to determine the proliferation status. Western blot was employed to detect the expression of matrix metalloproteinase 2(MMP-2), MMP-9, MMP-14, and tissue inhibitor of metalloproteinases 2(TIMP-2), focal adhesion kinase (FAK), ERK1/2, JNK and Src.According to the expression levels of GRP78, MMP-2, MMP-9, MMP-14 and TIMP-1 in hepatocellular carcinoma cell lines SMMC7721 and hepG2, we used SMMC7721 as the in vitro invasion model for further functional analysis. Using this model, we found that GRP78 knockdown decreased the invasion of tumor cells, and this inhibitory effect was independent of cell proliferation. In hepatocellular carcinoma cells, Grp78 knockdown inhibited   ECM   degradation and the decreased activity and expression of MMP-2, but not MMP-9 contributed largely to this impact. Further analysis revealed that the decreased activity and expression of MMP-2 is mediated by JNK.Knockdown of GRP78 decreases   ECM   degradation, and downregulates the expression and activity of MMP-2 and TIMP-2. These results further demonstrate that GRP78 is a potential target for inhibiting the invasion of hepatocellular carcinoma cells. 
  GeneRIFs  
 Data suggests that surface GRP78 promotes CRC cell migration and invasion by regulating cell-matrix adhesion and   ECM   degradation, which is independent of its signaling receptor function. 
 Knockdown of GRP78 decreases   ECM   degradation, and downregulates the expression and activity of MMP-2 and TIMP-2. 
  [Ee]xtracellular [Mm]atrix  PubMed  
 Proteomic analysis of human parotid gland exosomes by multidimensional protein identification technology (MudPIT). Human ductal saliva contributes over a thousand unique proteins to whole oral fluids. The mechanism by which most of these proteins are secreted by salivary glands remains to be determined. The present study used a mass spectrometry-based, shotgun proteomics approach to explore the possibility that a subset of the proteins found in saliva are derived from exosomes, membrane-bound vesicles of endosomal origin within multivesicular endosomes. Using MudPIT (multidimensional protein identification technology) mass spectrometry, we catalogued 491 proteins in the exosome fraction of human parotid saliva. Many of these proteins were previously observed in ductal saliva from parotid glands (265 proteins). Furthermore, 72 of the proteins in parotid exosomes overlap with those previously identified as urinary exosome proteins, proteins which are also frequently associated with exosomes from other tissues and cell types. Gene Ontology (GO) and KEGG pathway analyses found that cytosolic proteins comprise the largest category of proteins in parotid exosomes (43%), involved in such processes as phosphatidylinositol signaling system, calcium signaling pathway, inositol metabolism, protein export, and signal transduction, among others; whereas the integral plasma membrane proteins and associated/peripheral plasma membrane proteins (26%) were associated with   extracellular matrix  -receptor interaction, epithelial cell signaling, T-cell and B-cell receptor signaling, cytokine receptor interaction, and antigen processing and presentation, among other biological functions. In addition, these putative saliva exosomal proteins were linked to specific diseases (e.g., neurodegenerative disorders, prion disease, cancers, type I and II diabetes). Consequently, parotid glands secrete exosomes that reflect the metabolic and functional status of the gland and may also carry informative protein markers useful in the diagnosis and treatment of systemic diseases. 
 SILAC-based proteomics of human primary endothelial cell morphogenesis unveils tumor angiogenic markers. Proteomics has been successfully used for cell culture on dishes, but more complex cellular systems have proven to be challenging and so far poorly approached with proteomics. Because of the complexity of the angiogenic program, we still do not have a complete understanding of the molecular mechanisms involved in this process, and there have been no in depth quantitative proteomic studies. Plating endothelial cells on matrigel recapitulates aspects of vessel growth, and here we investigate this mechanism by using a spike-in SILAC quantitative proteomic approach. By comparing proteomic changes in primary human endothelial cells morphogenesis on matrigel to general adhesion mechanisms in cells spreading on culture dish, we pinpoint pathways and proteins modulated by endothelial cells. The cell-  extracellular matrix   adhesion proteome depends on the adhesion substrate, and a detailed proteomic profile of the   extracellular matrix   secreted by endothelial cells identified CLEC14A as a matrix component, which binds to MMRN2. We verify deregulated levels of these proteins during tumor angiogenesis in models of multistage carcinogenesis. This is the most in depth quantitative proteomic study of endothelial cell morphogenesis, which shows the potential of applying high accuracy quantitative proteomics to in vitro models of vessel growth to shed new light on mechanisms that accompany pathological angiogenesis. The mass spectrometry proteomics data have been deposited to the ProteomeXchange Consortium with the data set identifier PXD000359. 
 Grp78 Is Critical for Amelogenin-Induced Cell Migration in a Multipotent Clonal Human Periodontal Ligament Cell Line. Periodontal ligament stem cells (PDLSCs) are known to play a pivotal role in regenerating the periodontium. Amelogenin, which belongs to a family of   extracellular matrix   (ECM) proteins, is a potential bioactive molecule for periodontal regenerative therapy. However, its downstream target molecules and/or signaling patterns are still unknown. Our recent proteomic study identified glucose-regulated protein 78 (Grp78) as a new amelogenin-binding protein. In this study, we demonstrate, for the first time, the cellular responses induced by the biological interaction between amelogenin and Grp78 in the human undifferentiated PDL cell line 1-17, which possesses the most typical characteristics of PDLSCs. Confocal co-localization experiments revealed the internalization of recombinant amelogenin (rM180) via binding to cell surface Grp78, and the endocytosis was inhibited by the silencing of Grp78 in 1-17 cells. Microarray analysis indicated that rM180 and Grp78 regulate the expression profiles of cell migration-associated genes in 1-17 cells. Moreover, Grp78 overexpression enhanced rM180-induced cell migration and adhesion without affecting cell proliferation, while silencing of Grp78 diminished these activities. Finally, binding of rM180 to Grp78 promoted the formation of lamellipodia, and the simultaneous activation of Rac1 was also demonstrated by NSC23766, a widely accepted Rac1 inhibitor. These results suggest that Grp78 is essential for enhancing amelogenin-induced migration in 1-17 cells. The biological interaction of amelogenin with Grp78 offers significant therapeutic potential for understanding the biological components and specific functions involved in the signal transduction of amelogenin-induced periodontal tissue regeneration. 
 Human telomerase reverse transcriptase and glucose-regulated protein 78 increase the life span of articular chondrocytes and their repair potential. Like all mammalian cells, normal adult chondrocytes have a limited replicative life span, which decreases with age. To facilitate the therapeutic use of chondrocytes from older donors, a method is needed to prolong their life span.We transfected chondrocytes with hTERT or GRP78 and cultured them in a 3-dimensional atelocollagen honeycomb-shaped scaffold with a membrane seal. Then, we measured the amount of nuclear DNA and glycosaminoglycans (GAGs) and the expression level of type II collagen as markers of cell proliferation and   extracellular matrix   formation, respectively, in these cultures. In addition, we allografted this tissue-engineered cartilage into osteochondral defects in old rabbits to assess their repair activity in vivo.Our results showed different degrees of differentiation in terms of GAG content between chondrocytes from old and young rabbits. Chondrocytes that were cotransfected with hTERT and GRP78 showed higher cellular proliferation and expression of type II collagen than those of nontransfected chondrocytes, regardless of the age of the cartilage donor. In addition, the in vitro growth rates of hTERT- or GRP78-transfected chondrocytes were higher than those of nontransfected chondrocytes, regardless of donor age. In vivo, the tissue-engineered cartilage implants exhibited strong repairing activity, maintained a chondrocyte-specific phenotype, and produced   extracellular matrix   components.Focal gene delivery to aged articular chondrocytes exhibited strong repairing activity and may be therapeutically useful for articular cartilage regeneration. 
 Expression and distribution of grp-78/bip in mineralizing tissues and mesenchymal cells. Glucose-regulated protein 78 (GRP-78) is one of the many endoplasmic reticulum chaperone proteins that have been shown to possess multifunctional roles. We have previously demonstrated that GRP-78 functions as a receptor for dentin matrix protein 1 (DMP1) and is required for DMP1-mediated calcium release; that it is a secreted protein and can bind to type I collagen and DMP1 extracellularly and aid in the nucleation of calcium phosphate. We provide evidence in this study that tyrosine phosphorylation is required for DMP1/GRP-78-mediated calcium release in mesenchymal cells. We further demonstrate that GRP-78 is localized in the nucleus of mesenchymal cells and that the cell surface GRP-78 is not associated with the G-protein Gαq in mesenchymal cells. Results from this study show that during development of mineralized tissues, increased expression of GRP-78 can be observed in condensing cartilage and mesenchymal cells of the alveolar bone, endochondral bone and dental pulp. Additionally, we show that GRP-78 is present in the mineralizing matrices of teeth, bone and in the   extracellular matri  x of differentiating human marrow stromal cells and dental pulp stem cells. Collectively, our observations provide a new perspective on GRP-78 with respect to mineralized matrix formation. 
 Knockdown of glucose-regulated protein 78 decreases the invasion, metalloproteinase expression and ECM degradation in hepatocellular carcinoma cells. We have reported previously that overexpression of glucose-regulated protein 78 (GRP78) promotes the invasion of hepatocellular carcinoma. However, whether GRP78 knockdown affects the   extracellular matrix   degradation has not been elucidated. Here we are going to determine whether GRP78 knockdown affect the ECM degradation and the role of MMP-2 and MMP-9 in these process in hepatocellular carcinoma cells.Human hepatocellular carcinoma cell line SMMC7721 and HepG2 were cultured in DMEM supplemented with 10% FBS, RT-PCR and western blot were used to detect the endogenous expression of GRP78, MMP-2, MMP-9 and TIMP-2 in SMMC7721 and HepG2. GRP78 shRNAs were transfected using lipofection2000. Transwell assay and wound healing assay were used to analyze the invasion of each transfectant. Gelatin zymography and FITC-gelatin degradation assay were employed to investigate the capabilities of ECM degradation of each transfectant. MTT assay was used to determine the proliferation status. Western blot was employed to detect the expression of matrix metalloproteinase 2(MMP-2), MMP-9, MMP-14, and tissue inhibitor of metalloproteinases 2(TIMP-2), focal adhesion kinase (FAK), ERK1/2, JNK and Src.According to the expression levels of GRP78, MMP-2, MMP-9, MMP-14 and TIMP-1 in hepatocellular carcinoma cell lines SMMC7721 and hepG2, we used SMMC7721 as the in vitro invasion model for further functional analysis. Using this model, we found that GRP78 knockdown decreased the invasion of tumor cells, and this inhibitory effect was independent of cell proliferation. In hepatocellular carcinoma cells, Grp78 knockdown inhibited ECM degradation and the decreased activity and expression of MMP-2, but not MMP-9 contributed largely to this impact. Further analysis revealed that the decreased activity and expression of MMP-2 is mediated by JNK.Knockdown of GRP78 decreases ECM degradation, and downregulates the expression and activity of MMP-2 and TIMP-2. These results further demonstrate that GRP78 is a potential target for inhibiting the invasion of hepatocellular carcinoma cells. 
  Component  
   extracellular matrix   
  GeneRIFs  
 GRP-78 is present in the mineralizing matrices of teeth, bone and in the   extracellular matrix   of differentiating human marrow stromal cells and dental pulp stem cells. 
  [Ii]nvasive  PubMed  
 GRP78 as a marker of pre-eclampsia: an exploratory study. Although the exact mechanisms that lead to shallow invasion or defective trophoblastic differentiation in pre-eclampsia are still unknown, it is widely admitted that the etiology of pre-eclampsia is a defect in trophoblast invasion of the uterine spiral arteries. We have previously observed that the status of a chaperone protein, glucose regulated protein 78 (GRP78) is associated with the   invasive   properties of cytotrophoblastic cells; we therefore hypothesized that circulating GRP78 could serve as a diagnostic tool in pre-eclampsia. In a prospective case-control study, we quantified GRP78 autoantibodies, complexes of GRP78 with autoantibodies and GRP78 (C-term fragment, N-term fragment and full-length GRP78) by ELISA. Plasma from women diagnosed with pre-eclampsia (n = 16), from women during the first trimester of pregnancy who subsequently developed pre-eclampsia (n = 10) and from healthy pregnant women (controls, n = 58 at term, n = 26 at first trimester) were analysed and compared. We observed no significant difference between pre-eclamptic and healthy pregnant women for autoantibodies-GRP78 complexes or total GRP78 at both first trimester and at delivery. In contrast, the ratio of C-terminal GRP78 over full length GRP78 was significantly different in plasma of pre-eclamptic patients as compared with controls both during first trimester (P < 0.004) and at term (P < 0.0001). Our findings suggest that circulating C-terminal GRP78 reflect the   invasive   properties of cells, and could be used as a predictive marker for pre-eclampsia early in pregnancy. 
 Unraveling the role of KIAA1199, a novel endoplasmic reticulum protein, in cancer cell migration. Cell migration is a critical determinant of cancer metastasis, and a better understanding of the genes involved will lead to the identification of novel targets aimed at preventing cancer dissemination. KIAA1199 has been shown to be upregulated in human cancers, yet its role in cancer progression was hitherto unknown.Clinical relevance was assessed by examining KIAA1199 expression in human cancer specimens. In vitro and in vivo studies were employed to determine the function of KIAA1199 in cancer progression. Cellular localization of KIAA1199 was microscopically determined. SNAP-tag pull-down assays were used to identify binding partner(s) of KIAA1199. Calcium levels were evaluated using spectrofluorometric and fluorescence resonance energy transfer analyses. Signaling pathways were dissected by Western blotting. Student t test was used to assess differences. All statistical tests were two-sided.KIAA1199 was upregulated in   invasive   breast cancer specimens and inversely associated with patient survival rate. Silencing of KIAA1199 in MDA-MB-435 cancer cells resulted in a mesenchymal-to-epithelial transition that reduced cell migratory ability in vitro (75% reduction; P < .001) and decreased metastasis in vivo (80% reduction; P < .001). Gain-of-function assays further demonstrated the role of KIAA1199 in cell migration. KIAA1199-enhanced cell migration required endoplasmic reticulum (ER) localization, where it forms a stable complex with the chaperone binding immunoglobulin protein (BiP). A novel ER-retention motif within KIAA1199 that is required for its ER localization, BiP interaction, and enhanced cell migration was identified. Mechanistically, KIAA1199 was found to mediate ER calcium leakage, and the resultant increase in cytosolic calcium ultimately led to protein kinase C alpha activation and cell migration.KIAA1199 serves as a novel cell migration-promoting gene and plays a critical role in maintaining cancer mesenchymal status. 
 Role of GRP78 in promoting therapeutic-resistant breast cancer. Over 230,000 new cases of   invasive   breast cancer are diagnosed annually within the USA. Recurrent breast cancer remains a mostly incurable disease with drug resistance, tumor latency and distant metastases driving breast tumor recurrence and morbidity. Understanding drug resistance is a critical component of combating breast cancer. Recently, the protein chaperone GRP78 and the unfolded protein response were implicated as drivers of drug resistance. Preclinical studies show inhibiting GRP78 can reverse drug resistance. Furthermore, drugs developed to target GRP78 show clinical promise in several ongoing clinical trials. 
 Patterns of GRP78 and MTJ1 expression in primary cutaneous malignant melanoma. Cell surface expression of glucose-regulated protein 78 (GRP78) occurs in several types of cancer; however, its role in the behavior of primary cutaneous melanoma is not well studied. The association of cell surface GRP78 with other proteins such as MTJ1 stimulates cell proliferation. In this study, we characterized the pattern of expression of GRP78 and MTJ1 in   invasive   primary cutaneous melanomas and analyzed the relationships between the pattern of expression and various clinicopathological parameters. We found two patterns of GRP78 expression in   invasive   primary cutaneous melanoma. One pattern showed a gradual fading of protein expression from superficial to deeper levels within the same tumor. The second pattern of expression showed a similar fading with an abrupt regaining of expression at the deep   invasive   edge of the melanoma. These two distinct patterns of GRP78 expression correlated with both patient survival and depth of tumor invasion. A moderate MTJ1 expression was found to be associated with decreased patient survival; however, no significant associations were observed between patterns of GRP78 and MTJ1 expression. Our study (1) describes two distinct patterns of GRP78 in   invasive   primary cutaneous melanoma, (2) inversely correlates regain of GRP78 expression with patient survival, and (3) suggests a modifying effect of MTJ1 on GRP78 in enhancing tumor aggressiveness. 
 Overexpression of GRP78 is associated with malignant transformation in epithelial ovarian tumors. Glucose-regulated protein 78 (GRP78) is one of the best-characterized endoplasmic reticulum chaperone proteins. The aim of this study was to explore the potential implications of GRP78 in the development of epithelial ovarian carcinomas (EOCs) and the possible clinical usefulness of GRP78 expression as a prognostic biomarker for EOCs.A total of 133 women were enrolled. This pilot study consisted of 96 cases of EOCs and 37 cases of benign ovarian tumors as controls. The expression of GRP78 protein was studied using tissue microarray technology and immunohistochemistry.Of the 96   invasive   carcinomas analyzed, 25 cases (26.0%) showed moderate GRP78 expression and 65 cases (67.7%) showed strong GRP78 expression. Conversely, 17 (45.9%) of the 37 patients with benign ovarian tumors showed no or weak GRP78 staining. Our results demonstrated that GRP78 expression was significantly higher in EOCs than in benign ovarian tumors (P<0.001). However, no statistically significant association was found between GRP78 expression and survival.The present study suggests that GRP78 plays a significant role in malignant transformation of epithelial ovarian tumors and may represent an important biomarker for EOCs. 
 Molecular chaperones as a common set of proteins that regulate the invasion phenotype of head and neck cancer. The goal of this study was to establish a common set of molecules that regulate cell invasion in head and neck cancer (HNC).Five   invasive   sublines derived from HNC cell lines were established using the Matrigel selection method. Proteomic technology, MetaCore algorithm, and reverse transcriptase-PCR methods were used to search for molecules that contribute to the invasion phenotype. Cellular functional analyses and clinical association studies were applied to examine the significance of the molecules.Fifty-two proteins were identified in more than two of the four independent proteomic experiments, including 10 (19%) molecular chaperones. Seven chaperones were confirmed to be differentially expressed in five sublines, Hsp90α, Hsp90β, Hsp90-B1/Gp96, Hsp70-A5/Grp78, and HYOU1, that upregulate, whereas Hsp60 and glucosidase-α neutral AB (GANAB) downregulate. Four molecules were further investigated. In all cell lines, knockdown of Hsp60 or GANAB and silencing of Gp96 or Grp78 considerably enhanced or reduced cell migration and invasion, respectively. Clinical association studies consistently revealed that low levels of Hsp60 or GANAB and high levels of Gp96 or Grp78 are significantly associated with advanced cancer (P < 0.001 to P = 0.047, respectively, for the four molecules) and poor survival (P < 0.001 to P = 0.025, respectively, for the four molecules).Our study defined molecular chaperones as a common set of proteins that regulate the invasion phenotype of HNC. Loss of the tumor suppression function of Hsp60 or GANAB and acquisition of the oncogenic function of Gp96 or Grp78 contribute to aggressive cancers. These molecules may serve as prognostic markers and targets for cancer drug development. 
 Differential influence of anticancer treatments and angiogenesis on the seric titer of autoantibody used as tumor and metastasis biomarker. Early detection of tumor-specific autoantibodies (auto-Abs) has the potential to be used for cancer screening and diagnosis. Whether auto-Ab may be useful to track metastatic progression or response to treatment is, however, largely unknown. To address these issues, the serological proteome was analyzed in an   invasive   but treatment-responsive mouse tumor model. Among 40 serum-reactive proteins identified by multiplex analysis, we chose to focus on glucose-regulated protein 78 (GRP78), a chaperone protein involved in the endoplasmic reticulum stress response. We first validated GRP78 as a protein overexpressed and mislocalized in tumor cells. We then documented that an increase in GRP78 auto-Ab titer preceded the detection of a palpable tumor mass, correlated with metastatic progression, and was influenced by the onset of tumor neovascularization. We also found that chemotherapy and radiotherapy, both leading to inhibition of tumor growth, oppositely influenced the anti-GRP78 immune response. Whereas radiation increased the concentration of GRP78 auto-Ab by three-fold, the auto-Ab titer was reduced in response to bolus or metronomic administration of cyclophosphamide. Finally, we established a decrease in auto-Ab-producing B lymphocytes in response to chemotherapy and the overexpression of GRP78 together with a strong immunoglobulin response in irradiated tumors. In conclusion, we identified GRP78 auto-Ab as an early marker of tumor and metastatic progressions. However, the multiple influences of anticancer treatments on the humoral immune system calls for caution when exploiting such auto-Ab as markers of the tumor response. 
 Glucose-regulated protein 78 regulates multiple malignant phenotypes in head and neck cancer and may serve as a molecular target of therapeutic intervention. Glucose-regulated protein 78 (Grp78) is an endoplasmic reticulum chaperone protein and is overexpressed in various cancers. However, it is unclear how significance of this molecule play an active role contributing to the oncogenic effect of head and neck cancer (HNC). To investigate the potential function of Grp78, six HNC cell lines were used. We found that Grp78 is highly expressed in all six cell lines and many of the proteins were localized in the periphery regions, implying other function of this molecule aside from endoplasmic reticulum stress response. Knockdown of Grp78 by small interfering RNA significantly reduced cell growth and colony formation to 53% to 12% compared with that of controls in all six HNC cell lines. Using in vitro wound healing and Matrigel invasion assays, we found that cell migration and   invasive   ability were also inhibited to 23% to 2% in all these cell lines tested. In vivo xenograft studies showed that administration of Grp78-small interfering RNA plasmid into HNC xenografts significantly inhibited both tumor growth in situ (>60% inhibition at day 34) and liver metastasis (>90% inhibition at day 20). Our study showed that Grp78 actively regulates multiple malignant phenotypes, including cell growth, migration, and invasion. Because knockdown Grp78 expression succeeds in the reduction of tumor growth and metastatic potential, this molecule may serve as a molecular target of therapeutic intervention for HNC. 
 Interleukin (IL)11 mediates protein secretion and modification in human extravillous trophoblasts. Human trophoblast invasion and differentiation are essential for a successful pregnancy outcome. Dysregulation of these processes can lead to placental pathologies such as pre-eclampsia. The molecular mechanisms; however, are poorly understood. Interleukin (IL)11--a cytokine that regulates endometrial epithelial cell adhesion, trophoblast motility and invasion during implantation--may be involved in some of these processes.The effect of IL11 on protein expression was investigated in trophoblastic HTR8/SVneo cells and primary extravillous trophoblasts (EVTs) purified from first- trimester placentas. Two-dimension (2D)-differential in-gel electrophoresis analyses revealed that 731 spots were significantly differentially regulated by IL11 in HTR8/SVneo cells: seven spots were analyzed by liquid chromatography-tandem mass spectrometry and 14 unique proteins identified. Protein disulfide isomerase family A, member 3 (PDIA3; endoplasmic reticulum p57) and glucose-regulated protein 78 (GRP78) were further validated to be regulated by IL11 in HTR8/SVneo and primary EVT. One dimension western blot analysis confirmed that PDIA3 was down-regulated in EVT. 2D western blot analysis revealed that GRP78 was post-translationally modified following IL11 treatment. Moreover, IL11 stimulated the secretion of GRP78 in EVT.Data suggest that IL11, possibly via signal transducers and activators of transcription 3 signaling pathway, regulates PDIA3 protein expression and modification/secretion of GRP78. This is the first study to identify PDIA3 and GRP78 as IL11 targets in   invasive   trophoblasts and identifies a possible mechanism by which IL11 regulates trophoblast function. 
 Deacetylation of HSPA5 by HDAC6 leads to GP78-mediated HSPA5 ubiquitination at K447 and suppresses metastasis of breast cancer. Heat-shock protein 5 (HSPA5) is a marker for poor prognosis in breast cancer patients and has an important role in cancer progression, including promoting drug resistance and metastasis. In this study, we identify that the specific lysine residue 447 (K447) of HSPA5 could be modified with polyubiquitin for subsequent degradation through the ubiquitin proteasomal system, leading to the suppression of cell migration and invasion of breast cancer. We further found that GP78, an E3 ubiquitin ligase, interacted with the C-terminal region of HSPA5 and mediated HSPA5 ubiquitination and degradation. Knock down of GP78 significantly increased the expression of HSPA5 and enhanced migration/  invasive   ability of breast cancer cells. Knock down of histone deacetylase-6 (HDAC6) increased the acetylation of HSPA5 at lysine residues 353 (K353) and reduced GP78-mediated ubiquitination of HSPA5 at K447 and then increased cell migration/invasion. In addition, we demonstrate that E3 ubiquitin ligase GP78 preferentially binds to deacetylated HSPA5. Notably, the expression levels of GP78 inversely correlated with HSPA5 levels in breast cancer patients. Patients with low GP78 expression significantly correlated with   invasive  ness of breast cancer, advanced tumor stages and poor clinical outcome. Taken together, our results provide new mechanistic insights into the understanding that deacetylation of HSPA5 by HDAC6 facilitates GP78-mediated HSPA5 ubiquitination and suggest that post-translational regulation of HSPA5 protein is critical for HSPA5-mediated metastatic properties of breast cancer. 
 GRP78 mediates cell growth and   invasive  ness in endometrial cancer. Recent studies have indicated that endoplasmic reticulum stress, the unfolded protein response activation and altered GRP78 expression can play an important role in a variety of tumors development and progression. Very recently we reported for the first time that GRP78 is increased in endometrial tumors. However, whether GRP78 could play a role in the growth and/or   invasive  ness of endometrial cancer cells is still unknown. Here we report that the silencing of GRP78 expression affects both cell growth and   invasive  ness of Ishikawa and AN3CA cells, analyzed by the (3-(4,5-dimethylthiazol-2-yl)-2,5-diphenyl tetrazolium bromide) and transwell migration assay, respectively. At variance with Ishikawa cells, AN3CA cells showed, besides an endoplasmic reticulum, also a plasma membrane GRP78 localization, evidenced by both immunofluorescence and cell membrane biotinylation experiments. Intriguingly, flow cytometry experiments showed that the treatment with a specific antibody targeting GRP78 C-terminal domain caused apoptosis in AN3CA but not in Ishikawa cells. Induction of apoptosis in AN3CA cells was not mediated by the p53 pathway activation but was rather associated to reduced AKT phosphorylation. Interestingly, immunofluorescence analysis evidenced that endometrioid adenocarcinoma tissues displayed, similarly to AN3CA cells, also a GRP78 plasma membrane localization. These data suggest that GRP78 and its plasma membrane localization, might play a role in endometrial cancer development and progression and might constitute a novel target for the treatment of endometrial cancer. 
 Glucose-regulated protein 78 (GRP78) regulates colon cancer metastasis through EMT biomarkers and the NRF-2/HO-1 pathway. Glucose-regulated protein 78 (GRP78) is a key chaperone and stress response protein. Previous studies have demonstrated that high GRP78 expression may be correlated with cancer progression and therapeutic response. However, the role of GRP78 in the metastasis of colon cancer is unclear. In this study, we used small interfering RNA (siRNA) to knock down GRP78 expression in colon cancer cells (HT-29 and DLD-1 cells). In wound-healing migration assays, we found that GRP78-knockdown (GRP78KD) cells showed better wound-healing ability than control cells. We also found that GRP78KD cells displayed a better migratory ability than control cells in migration and invasion assays. As we further dissected the underlying molecular mechanism, we found that silencing GRP78 may cause an increase in vimentin expression and a decrease in the E-cadherin level, which was correlated with the increase in migratory ability. In addition, we found that GRP78KD may activate the NRF-2/HO-1 pathway, and this activation was also correlated with the increase in cell   invasive  ness. Furthermore, we examined GRP78 expression in a tissue array and found that the GRP78 expression in metastatic adenocarcinoma in lymph nodes tended to be weaker than that in primary colonic adenocarcinoma. In conclusion, a low level of GRP78 may cause an increase in metastasis ability in colon cancer cells by altering E-cadherin and vimentin expression and activating the NRF-2/HO-1 signaling pathway. Our study demonstrates that low expression of GRP78 may correlate with a high risk of metastasis in colon cancer. 
 Overexpression of GRP78 in complete hydatidiform moles. Hydatidiform moles, subdivided into partial moles (PM) and complete moles (CM), are abnormal pregnancies with a disturbed   invasive   behavior. We had previously shown that MMP-2 and p53 proteins are overexpressed in CM versus PM, and that in primary cytotrophoblasts p53 protein is stabilized by complexing to the 78kDa glucose-regulated protein (GRP78) which is involved in cytotrophoblasts invasion process. The present study aims to compare the transcript expression profile of p53, MMP-2 and GRP78 in hydatidiform moles.A retrospective study was performed by RT-qPCR and immunostaining on paraffin-embedded tissues of 19 PM, 16 CM and 16 control (CTRL) samples of gestational age 8-12 weeks.Expression of MMP-2 transcript was significantly overexpressed in CM compared to CTRL samples (p=0.031). In contrast, expression of p53 transcript was similar among the samples. This suggests a regulation of p53 in CM at the protein level. GRP78 cDNA was significantly overexpressed in CM compared to CTRL (p=0.021) and to PM (p=0.011). At the protein level, immunostaining of GRP78 was on average stronger in CM than PM samples.Collectively, present data suggest that in CM, p53 is normally expressed at the mRNA level but probably complexes at the protein level with the overexpressed GRP78, leading to accumulation of p53 protein. Moreover, since GRP78 and MMP-2 are increased in CM and known to play key roles in invasion, our results suggest that GRP78 and MMP-2 should be investigated as prognostic markers of hydatidiform moles. 
 E1A-mediated inhibition of HSPA5 suppresses cell migration and invasion in triple-negative breast cancer. Triple-negative breast cancer (TNBC) is defined by reduced expression of the estrogen receptor, progesterone receptor, and HER2. TNBC is an especially aggressive group of breast cancers with poor prognosis. There are currently no validated molecular targets to effectively treat this disease. Thus, it is necessary to identify effective molecular targets and therapeutic strategies for TNBC patients.The expression of HSPA5 in patients with breast cancer was examined by immunohistochemistry. The association of HSPA5 expression with tumor grade and metastatic events in TNBC patients was analyzed using the Oncomine database. The knockdown and overexpression of HSPA5 protein were performed to investigate the effects on E1A-suppressed cell migration/invasion of TNBC using in vitro transwell assays and tumor growth/experimental metastasis studies in animal models.The expression of HSPA5 was positively correlated with high-grade tumors, metastatic events, and poor overall survival in breast cancer patients with TNBC. E1A-inhibited HSPA5 expression suppressed cell migration  /invasiv  e ability of TNBC cell lines. Moreover, E1A significantly abolished lung metastases from breast cancer cells by inhibiting HSPA5 expression in a xenograft tumor model.The overexpression of HSPA5 is critical for high-risk metastasis of breast cancer and TNBC. The results of our study suggest that HSPA5 may be a crucial mediator of E1A-suppressed metastatic ability of breast cancer cells. Thus, E1A may be a potential target for diagnosis and individualized treatment in clinical practice. 
 E6^E7, a novel splice isoform protein of human papillomavirus 16, stabilizes viral E6 and E7 oncoproteins via HSP90 and GRP78. Transcripts of human papillomavirus 16 (HPV16) E6 and E7 oncogenes undergo alternative RNA splicing to produce multiple splice isoforms. However, the importance of these splice isoforms is poorly understood. Here we report a critical role of E6^E7, a novel isoform containing the 41 N-terminal amino acid (aa) residues of E6 and the 38 C-terminal aa residues of E7, in the regulation of E6 and E7 stability. Through mass spectrometric analysis, we identified that HSP90 and GRP78, which are frequently upregulated in cervical cancer tissues, are two E6^E7-interacting proteins responsible for the stability and function of E6^E7, E6, and E7. Although GRP78 and HSP90 do not bind each other, GRP78, but not HSP90, interacts with E6 and E7. E6^E7 protein, in addition to self-binding, interacts with E6 and E7 in the presence of GRP78 and HSP90, leading to the stabilization of E6 and E7 by prolonging the half-life of each protein. Knocking down E6^E7 expression in HPV16-positive CaSki cells by a splice junction-specific small interfering RNA (siRNA) destabilizes E6 and E7 and prevents cell growth. The same is true for the cells with a GRP78 knockdown or in the presence of an HSP90 inhibitor. Moreover, mapping and alignment analyses for splicing elements in 36 alpha-HPVs (α-HPVs) suggest the possible expression of E6^E7 mostly by other oncogenic or possibly oncogenic α-HPVs (HPV18, -30, -31, -39, -42, -45, -56, -59, -70, and -73). HPV18 E6^E7 is detectable in HPV18-positive HeLa cells and HPV18-infected raft tissues. All together, our data indicate that viral E6^E7 and cellular GRP78 or HSP90 might be novel targets for cervical cancer therapy.HPV16 is the most prevalent HPV genotype, being responsible for 60%   of inva  sive cervical cancer cases worldwide. What makes HPV16 so potent in the development of cervical cancer remains a mystery. We discovered in this study that, besides producing two well-known oncoproteins, E6 and E7, seen in other high-risk HPVs, HPV16 produces E6^E7, a novel splice isoform of E6 and E7. E6^E7, in addition to self-interacting, binds cellular chaperone proteins, HSP90 and GRP78, and viral E6 and E7 to increase the steady-state levels and half-lives of viral oncoproteins, leading to cell proliferation. The splicing cis elements in the regulation of HPV16 E6^E7 production are highly conserved in 11 oncogenic or possibly oncogenic HPVs, and we confirmed the production of HPV18 E6^E7 in HPV18-infected cells. This study provides new insight into the mechanism of splicing, the interplay between different products of the polycistronic viral message, and the role of the host chaperones as they function. 
 The endoplasmic reticulum stress markers GRP78 and CHOP predict disease-free survival and responsiveness to chemotherapy in breast cancer. Glucose-regulated protein (GRP) 78 and C/-EBP homologous protein (CHOP) are commonly used as markers of endoplasmic reticulum (ER) stress. As an ER chaperone, GRP78 functions as a potent anti-apoptotic factor and confers drug resistance, whereas CHOP is a key initiating factor of ER stress-related cell death. We aimed at investigating the predictive values of GRP78 and CHOP in breast cancer patients who underwent adjuvant chemotherapy. An immunohistochemistry screen for GRP78 and CHOP was performed using a tissue microarray containing 250 tumors from female patients diagnosed with   invasive   ductal breast carcinoma at the Fudan University Shanghai Cancer Center. The staining results were scored semi-quantitatively, and a prediction model was constructed to verify the hypothesis. In this retrospective cohort study, CHOP correlated with prolonged disease-free survival (HR = 0.385, 95 % CI 0.215-0.688; P = 0.001), whereas GRP78 showed an opposite association (HR = 4.573; 95 % CI 2.291-9.128; P < 0.001). Moreover, in a GRP78-positive subset, CHOP overexpression correlated with a lower risk of recurrence. In the receiver operating characteristic analysis, the prediction capability of the predictive model combining the above two markers surpassed that of the traditional model (P = 0.0085 for the area under the curve comparison). Within the anthracycline-treatment subgroup, the combined GRP78 and CHOP exhibited similar predictive significance. Cumulatively, our findings suggest a tight association between ER stress markers and clinical outcomes for patients with breast cancer. 
  GeneRIFs  
 PDIA3 and GRP78 are IL11 targets in   invasive   trophoblasts 
 Our study a modifying effect of MTJ1 on GRP78 in enhancing tumor aggressiveness in   invasive   primary cutaneous melanoma 
 Findings suggest that circulating C-terminal GRP78 reflect the   invasive   properties of cells, and could be used as a predictive marker for pre-eclampsia early in pregnancy. 
  [Mm]etastasize  PubMed  
 Colon cancer cells expressing cell surface GRP78 as a marker for reduced tumorigenicity. The glucose regulated heat shock protein 78 (GRP78) is a central regulator of ER (endoplasmic reticulum) stress due to its pro-survival property. Up regulated GRP78 expression in tumor cells has been correlated with aggressive malignancies whereas some reports have predicted an improved prognosis. Over-expression of GRP78 in the ER promotes its localization to the cell surface on several cell types including tumor cells.In order to elucidate whether GRP78 receptor positive and negative tumor cells manifest different properties in colorectal cancer, we first artificially separated GRP78 positive and negative sub-populations from HM7 and HCT116 cell lines using anti GRP78 antibody coated magnetic beads.Only GRP78 negative cells were highly proliferative, induced significant growth in tumor size in nude mice and   metastasize  d to the liver in a human metastatic colorectal carcinoma model in mice. In contrast, GRP78 positive cells manifested reduced proliferation, colony formation, tumor growth and liver metastases. The reduced tumorigenicity of GRP78 positive subpopulation was abrogated by silencing GRP78 expression using siRNA oligomers. In our efforts to induce cell surface GRP78, we subjected the cells to doxorubicin and taxol that increased significantly the percent of GRP78 positive population. Cells pre-incubated with doxorubicin exhibited reduced proliferation and tumor growth in mice.This study demonstrates the significance of cell surface GRP78 in colon cancer, which may be used as a marker for reduced tumorigenicity. 
 Expression patterns of Trk-A, Trk-B, GRP78, and p75NRT in olfactory neuroblastoma. Olfactory neuroblastoma is an uncommon neuroectodermal tumor of the sinonasal tract. It represents 2% to 3% of sinonasal neoplasms. Most olfactory neuroblastoma behave locally aggressive with 30% recurrence rates. A subset   metastasize  s to lymph nodes and/or distant sites. Grading of olfactory neuroblastoma involves a combination of factors with low-grade tumors having better survival than high-grade tumors. The grade does not always predict prognosis, however, as metastases can be seen in all grades of olfactory neuroblastoma. Trk-A, Trk-B, and p75NRT are neurotrophin receptors associated with numerous solid malignancies, particularly pediatric neuroblastoma. GRP78 is an endoplasmic reticulum protein, associated with differentiation of neuroblastic cells. Trk-A, p75NRT, and GRP78 overexpression are favorable prognostic factors in pediatric neuroblastoma, whereas Trk-B is associated with a poorer prognosis in these tumors. Olfactory neuroblastoma is clinically distinct from pediatric neuroblastoma but shares some histological features. Trk-A and p75NRT have been demonstrated in olfactory neuroblastoma previously. Trk-B and GRP78 have not been investigated in olfactory neuroblastoma. None of these markers have been correlated with grade or outcome in olfactory neuroblastoma. To investigate the role of Trk-A, Trk-B, p75NRT, and GRP78, a series of 20 olfactory neuroblastomas was stained with these antibodies. Trk-A and Trk-B stained most cases of olfactory neuroblastoma (90% and 85%). GRP78 stained most cases (90%), although weakly. P75NRT demonstrated focal membranous staining in a sustentacular pattern (60%). None of these markers correlated with Hyams grade. None of these markers definitively correlated with patient outcome. Neurotrophin receptors do not appear to have a prognostic role; however, Trk's may play an oncogenic role in olfactory neuroblastoma. 
  [Mm]etastasis  PubMed  
 Proteomics analysis of the interactome of N-myc downstream regulated gene 1 and its interactions with the androgen response program in prostate cancer cells. NDRG1 is known to play important roles in both androgen-induced cell differentiation and inhibition of prostate cancer   metastasis  . However, the proteins associated with NDRG1 function are not fully enumerated. Using coimmunoprecipitation and mass spectrometry analysis, we identified 58 proteins that interact with NDRG1 in prostate cancer cells. These proteins include nuclear proteins, adhesion molecules, endoplasmic reticulum (ER) chaperons, proteasome subunits, and signaling proteins. Integration of our data with protein-protein interaction data from the Human Proteome Reference Database allowed us to build a comprehensive interactome map of NDRG1. This interactome map consists of several modules such as a nuclear module and a cell membrane module; these modules explain the reported versatile functions of NDRG1. We also determined that serine 330 and threonine 366 of NDRG1 were phosphorylated and demonstrated that the phosphorylation of NDRG1 was prominently mediated by protein kinase A (PKA). Further, we showed that NDRG1 directly binds to beta-catenin and E-cadherin. However, the phosphorylation of NDRG1 did not interrupt the binding of NDRG1 to E-cadherin and beta-catenin. Finally, we showed that the inhibition of NDRG1 expression by RNA interference decreased the ER inducible chaperon GRP94 expression, directly proving that NDRG1 is involved in the ER stress response. Intriguingly, we observed that many members of the NDRG1 interactome are androgen-regulated and that the NDRG1 interactome links to the androgen response network through common interactions with beta-catenin and heat shock protein 90. Therefore we overlaid the transcriptomic expression changes in the NDRG1 interactome in response to androgen treatment and built a dual dynamic picture of the NDRG1 interactome in response to androgen. This interactome map provides the first road map for understanding the functions of NDRG1 in cells and its roles in human diseases, such as prostate cancer, which can progress from androgen-dependent curable stages to androgen-independent incurable stages. 
 Unraveling the role of KIAA1199, a novel endoplasmic reticulum protein, in cancer cell migration. Cell migration is a critical determinant of cancer   metastasis  , and a better understanding of the genes involved will lead to the identification of novel targets aimed at preventing cancer dissemination. KIAA1199 has been shown to be upregulated in human cancers, yet its role in cancer progression was hitherto unknown.Clinical relevance was assessed by examining KIAA1199 expression in human cancer specimens. In vitro and in vivo studies were employed to determine the function of KIAA1199 in cancer progression. Cellular localization of KIAA1199 was microscopically determined. SNAP-tag pull-down assays were used to identify binding partner(s) of KIAA1199. Calcium levels were evaluated using spectrofluorometric and fluorescence resonance energy transfer analyses. Signaling pathways were dissected by Western blotting. Student t test was used to assess differences. All statistical tests were two-sided.KIAA1199 was upregulated in invasive breast cancer specimens and inversely associated with patient survival rate. Silencing of KIAA1199 in MDA-MB-435 cancer cells resulted in a mesenchymal-to-epithelial transition that reduced cell migratory ability in vitro (75% reduction; P < .001) and decreased   metastasis   in vivo (80% reduction; P < .001). Gain-of-function assays further demonstrated the role of KIAA1199 in cell migration. KIAA1199-enhanced cell migration required endoplasmic reticulum (ER) localization, where it forms a stable complex with the chaperone binding immunoglobulin protein (BiP). A novel ER-retention motif within KIAA1199 that is required for its ER localization, BiP interaction, and enhanced cell migration was identified. Mechanistically, KIAA1199 was found to mediate ER calcium leakage, and the resultant increase in cytosolic calcium ultimately led to protein kinase C alpha activation and cell migration.KIAA1199 serves as a novel cell migration-promoting gene and plays a critical role in maintaining cancer mesenchymal status. 
 A critical role for GRP78/BiP in the tumor microenvironment for neovascularization during tumor growth and   metastasis  . Glucose-regulated protein 78 (GRP78)/BiP is a multifunctional protein which plays a major role in endoplasmic reticulum (ER) protein processing, protein quality control, maintaining ER homeostasis, and controlling cell signaling and viability. Previously, using a transgene-induced mammary tumor model, we showed that Grp78 heterozygosity impeded cancer growth through suppression of tumor cell proliferation and promotion of apoptosis and the Grp78(+/-) mice exhibited dramatic reduction (70%) in the microvessel density (MVD) of the endogenous mammary tumors, while having no effect on the MVD of normal organs. This observation suggests that GRP78 may critically regulate the function of the host vasculature within the tumor microenvironment. In this article, we interrogated the role of GRP78 in the tumor microenvironment. In mouse tumor models in which wild-type (WT), syngeneic mammary tumor cells were injected into the host, we showed that Grp78(+/-) mice suppressed tumor growth and angiogenesis during the early phase but not during the late phase of tumor growth. Growth of metastatic lesions of WT, syngeneic melanoma cells in the Grp78(+/-) mice was potently suppressed. We created conditional heterozygous knockout of GRP78 in the host endothelial cells and showed severe reduction of tumor angiogenesis and metastatic growth, with minimal effect on normal tissue MVD. Furthermore, knockdown of GRP78 expression in immortalized human endothelial cells showed that GRP78 is a critical mediator of angiogenesis by regulating cell proliferation, survival, and migration. Our findings suggest that concomitant use of current chemotherapeutic agents and novel therapies against GRP78 may offer a powerful dual approach to arrest cancer initiation, progression, and   metastasis  . 
 Inhibition of established micrometastases by targeted drug delivery via cell surface-associated GRP78. The major cause of morbidity in breast cancer is development of metastatic disease, for which few effective therapies exist. Because tumor cell dissemination is often an early event in breast cancer progression and can occur before diagnosis, new therapies need to focus on targeting established metastatic disease in secondary organs. We report an effective therapy based on targeting cell surface-localized glucose-regulated protein 78 (GRP78). GRP78 is expressed normally in the endoplasmic reticulum, but many tumors and disseminated tumor cells are subjected to environmental stresses and exhibit elevated levels of GRP78, some of which are localized at the plasma membrane.Here, we show that matched primary tumors and metastases from patients who died from advanced breast cancer also express high levels of GRP78. We used a peptidomimetic targeting strategy that uses a known GRP78-binding peptide fused to a proapoptotic moiety [designated bone   metastasis   targeting peptide 78 (BMTP78)] and show that it can selectively kill breast cancer cells that express surface-localized GRP78. Furthermore, in preclinical   metastasis   models, we show that administration of BMTP78 can inhibit primary tumor growth as well as prolong overall survival by reducing the extent of outgrowth of established lung and bone micrometastases.The data presented here provide strong evidence that it is possible to induce cell death in established micrometastases by peptide-mediated targeting of cell surface-localized GRP in advanced breast cancers. The significance to patients with advanced breast cancer of a therapy that can reduce established metastatic disease should not be underestimated. 
 Overexpression of GRP78 and GRP94 are markers for aggressive behavior and poor prognosis in gastric carcinomas. Glucose-related proteins (GRPs) are ubiquitously expressed in endoplasmic reticulum and able to assist in protein folding and assembly; consequently, they are considered as molecular chaperones. GRP78 and GRP94 expression was induced by glucose starvation and up-regulated in the malignancies. To clarify the roles of both molecules in tumorigenesis and progression of gastric carcinomas, immunohistochemistry was used on tissue microarray containing gastric carcinomas, adenomas, and nonneoplastic mucosa using the antibodies against GRP78 and GRP94, with a comparison of their expression with clinicopathological parameters of carcinomas. Gastric carcinoma cell lines (MKN28, AGS, MKN45, KATO-III, and HGC-27) were studied for both proteins by immunohistochemistry and Western blot. There was more expression of both proteins in gastric carcinoma and adenoma than in nonneoplastic mucosas (P < .05). All gastric carcinoma cell lines showed their expression at different levels. They were positively correlated with tumor size, depth of invasion, lymphatic and venous invasion, lymph node   metastasis  , and Union Internationale Contre le Cancer staging (P < .05), with positive relationship between both proteins (P < .05). Univariate analysis indicated the postsurgical cumulative survival rate of patients with positive GRP78 or GRP94 expression to be lower than that in those without GRP78 or GRP94 expression (P < .05), but the close link disappeared if stratified according to depth of invasion (P > .05). Multivariate analysis showed that age, depth of invasion, lymphatic invasion, lymph node   metastasis  , Union Internationale Contre le Cancer staging, and Lauren classification (P < .05), but not GRP78 and GRP94 expression, were independent prognostic factors for carcinomas (P > .05). Up-regulated expression of GRP78 and GRP94 was possibly involved in pathogenesis, growth, invasion, and   metastasis   of gastric carcinomas. They were considered objective and effective markers for the aggressive behavior and poor prognosis in gastric carcinomas. 
 Glucose-regulated protein 78 silencing down-regulates vascular endothelial growth factor/vascular endothelial growth factor receptor 2 pathway to suppress human colon cancer tumor growth. Up to 20% of colorectal cancer (CRC) is diagnosed with distant   metastasis  . The combination of chemotherapy with anti-vascular endothelial growth factor (VEGF) antibody can improve patient survival. Glucose-regulated protein 78 (GRP78) has an important role in cancer progression, but little is known about its role in VEGF production in CRC. The aim of this study was to explore the mechanism of GRP78 in two human colon cancer cell lines.We first checked the expression of GRP78 in human normal and colon cancer tissues and two colon cancer cell lines. Glucose-regulated protein 78 was knocked down using GRP78 small interfering RNA (siRNA) in HT29 and DLD-1 cells. We examined knockdown cells by the cell growth kinetics in vitro and tumor growth rate in vivo, respectively. We also investigated the effect of GRP78 siRNA on the expression of hypoxia inducible factor (HIF-1α), VEGF, and VEGF receptor 2 (VEGFR2).Compared with their adjacent normal tissue, we detected high expression levels of GRP78 of surgically removed colon cancer tissues. Using GRP78 siRNA, we reduced the expression of GRP78 in HT29 and DLD-1 cells. The GRP78 knockdown cells had a lower proliferation rate with fewer colony-forming units in vitro and produced smaller tumors in vivo. In dissecting the mechanism underlying the reduced cell growth, we found that the down-regulation of GRP78 decreased the production of HIF-1α, VEGF, and VEGFR2 and suppressed angiogenesis.Silencing GRP78 not only inhibits tumor, but also decreases the expression of VEGF and VEGFR2. Collectively, therapy targeting for GRP78 may inhibit the formation of colon cancer tumors via the HIF-1α/VEGF/VEGFR2 pathway. 
 The role of c-Src in the invasion and   metastasis   of hepatocellular carcinoma cells induced by association of cell surface GRP78 with activated α2M. Emerging data have suggested that cell surface GRP78 is a multifunctional receptor and has been linked to proliferative and antiapoptotic signaling cascades. Activated α2-macroglobin (α2M*) is a natural circulating ligand of cell surface GRP78. Association of cell surface GRP78 with α2M* is involved in the regulation of cell proliferation, survival and apoptosis in human cancers.The invasion   and metast  asis of HCC cells were examined using transwell and wound healing assay; Cell surface expression of GRP78 was detected by in cell western assay. Translocation of GRP78 from cytosol to cell surface was observed by transfection of GRP78-EGFP plus TRIRC-WGA staining. The levels of Src, phosphor-Src, FAK, phospho-FAK, EGFR, phospho-EGFR, phospho-Cortactin, phospho-Paxillin were determined by western blot. Cell surface expression of GRP78 in HCC tissue samples was observed by immunofluorescence. The distribution of Paxillin and Cortactin in HCC cells was also observed by immunofluorescence. The interaction between GRP78 and Src were detected by far-western blot, co-immunoprecipitation and GST pulldown. GRP78 mRNA was detected by RT-PCR.In the current study, we showed that association of cell surface GRP78 with α2M* stimulated the invasion   and metas  tasis of HCC. Cell surface GRP78 could interact directly with c-Src, promoted the phosphorylation of c-Src at Y416. Inhibition of the tyrosine kinase activity of c-Src with PP2 reverted the stimulatory effect caused by association of cell surface GRP78 with α2M*. Moreover, association of cell surface GRP78 with α2M* facilitates the interaction between EGFR and c-Src and consequently phosphorylated EGFR at Y1101 and Y845, promoting the invasi  on and met  astasis of HCCs. However, inhibition of the tyrosine kinase of c-Src do not affect the interaction between EGFR and Src.c-Src plays a critical role in the invasi  on and met  astasis of HCC induced by association of cell surface GRP78 with α2M*. Cell surface GRP78 directly binds and phosphorylates c-Src. As a consequence, c-Src phosphorylated EGFR, promoting the invas  ion and me  tastasis of HCCs. 
 Polymorphisms of glucose-regulated protein 78 and risk of colorectal cancer: a case-control study in southwest China. Glucose-regulated protein 78 (GRP78), an endoplasmic reticulum chaperone, up-regulation serves as an efficient mechanism to promote malignant transformation of colorectal cancer (CRC) and protect CRC cells against apoptosis. Recently, the analysis of GRP78 polymorphisms has already determined that GRP78 rs391957 polymorphism could predict clinical outcome in CRC patients. Thus, we tested whether GRP78 polymorphisms are related to the risk of CRC. In this study, we detected two GRP78 polymorphisms (rs391957 (C>T) and rs430397 (G>A)) in 414 CRC cases and 502 hospital-based cancer-free healthy controls in Southwest China using a polymerase chain reaction-restriction fragment length polymorphism technique. Compared with the CC genotype, carriers of CT and TT genotypes of rs391957 polymorphism had higher risks of CRC (odds ratio (OR) = 1.39, 95% confidence interval (CI) = 1.06-1.83 for CT genotype and OR = 2.10, 95% CI = 1.06-4.14 for TT genotype, respectively). In CRC cases, the variant T allele was significantly associated with tumor invasion stage (P = 0.030), but not with st  atus of ly  mph nodes metastasis (P = 0.052). Compared with the GG genotype, carriers of GA and AA genotypes of rs430397 polymorphism had higher risks of CRC (OR = 1.63, 95% CI = 1.23-2.15 for GA genotype and OR = 2.92, 95% CI = 1.23-6.94 for AA genotype, respectively). The rs430397 polymorphism was not associated with the clinicopathological characteristics of CRC. These data provide the first evidence that GRP78 rs391957 and rs430397 polymorphisms could serve as markers to predict the risk of CRC. 
 Differential influence of anticancer treatments and angiogenesis on the seric titer of autoantibody used as tumor and   metastasis   biomarker. Early detection of tumor-specific autoantibodies (auto-Abs) has the potential to be used for cancer screening and diagnosis. Whether auto-Ab may be useful to track metastatic progression or response to treatment is, however, largely unknown. To address these issues, the serological proteome was analyzed in an invasive but treatment-responsive mouse tumor model. Among 40 serum-reactive proteins identified by multiplex analysis, we chose to focus on glucose-regulated protein 78 (GRP78), a chaperone protein involved in the endoplasmic reticulum stress response. We first validated GRP78 as a protein overexpressed and mislocalized in tumor cells. We then documented that an increase in GRP78 auto-Ab titer preceded the detection of a palpable tumor mass, correlated with metastatic progression, and was influenced by the onset of tumor neovascularization. We also found that chemotherapy and radiotherapy, both leading to inhibition of tumor growth, oppositely influenced the anti-GRP78 immune response. Whereas radiation increased the concentration of GRP78 auto-Ab by three-fold, the auto-Ab titer was reduced in response to bolus or metronomic administration of cyclophosphamide. Finally, we established a decrease in auto-Ab-producing B lymphocytes in response to chemotherapy and the overexpression of GRP78 together with a strong immunoglobulin response in irradiated tumors. In conclusion, we identified GRP78 auto-Ab as an early marker of tumor and metastatic progressions. However, the multiple influences of anticancer treatments on the humoral immune system calls for caution when exploiting such auto-Ab as markers of the tumor response. 
 Glucose-regulated protein 78 mediates hormone-independent prostate cancer progression and   metastasis   through maspin and COX-2 expression. Glucose-regulated protein 78 (GRP78) plays an essential role in embryonic development and in the progression and therapeutic resistance of many cancers. However, little is known about the function of GRP78 in hormone-independent prostate cancer. Here, we found that the expression levels of GRP78 were higher in PC-3 cells than in DU-145 cells. When the expression of GRP78 was silenced using a GRP78-specific small interfering RNA in PC-3 cells, the growth rate and adhesive ability were reduced. Cell migration was dramatically decreased in GRP78-depleted cells. Dissection of the involved signal pathways revealed that maspin expression was upregulated after silencing GRP78 expression. The upregulation of maspin and downregulation of COX-2 may cause the decrease in cell proliferation and migration observed after silencing GRP78 expression. Silencing GRP78 expression may suppress the proliferation, adhesion, and migration of prostate cancer cells via maspin and COX-2 regulation. 
 Glucose-regulated protein 78 regulates multiple malignant phenotypes in head and neck cancer and may serve as a molecular target of therapeutic intervention. Glucose-regulated protein 78 (Grp78) is an endoplasmic reticulum chaperone protein and is overexpressed in various cancers. However, it is unclear how significance of this molecule play an active role contributing to the oncogenic effect of head and neck cancer (HNC). To investigate the potential function of Grp78, six HNC cell lines were used. We found that Grp78 is highly expressed in all six cell lines and many of the proteins were localized in the periphery regions, implying other function of this molecule aside from endoplasmic reticulum stress response. Knockdown of Grp78 by small interfering RNA significantly reduced cell growth and colony formation to 53% to 12% compared with that of controls in all six HNC cell lines. Using in vitro wound healing and Matrigel invasion assays, we found that cell migration and invasive ability were also inhibited to 23% to 2% in all these cell lines tested. In vivo xenograft studies showed that administration of Grp78-small interfering RNA plasmid into HNC xenografts significantly inhibited both tumor growth in situ (>60% inhibition at day 34) and liver   metastasis   (>90% inhibition at day 20). Our study showed that Grp78 actively regulates multiple malignant phenotypes, including cell growth, migration, and invasion. Because knockdown Grp78 expression succeeds in the reduction of tumor growth and metastatic potential, this molecule may serve as a molecular target of therapeutic intervention for HNC. 
 Deacetylation of HSPA5 by HDAC6 leads to GP78-mediated HSPA5 ubiquitination at K447 and suppresses   metastasis   of breast cancer. Heat-shock protein 5 (HSPA5) is a marker for poor prognosis in breast cancer patients and has an important role in cancer progression, including promoting drug resistance and   metastasis  . In this study, we identify that the specific lysine residue 447 (K447) of HSPA5 could be modified with polyubiquitin for subsequent degradation through the ubiquitin proteasomal system, leading to the suppression of cell migration and invasion of breast cancer. We further found that GP78, an E3 ubiquitin ligase, interacted with the C-terminal region of HSPA5 and mediated HSPA5 ubiquitination and degradation. Knock down of GP78 significantly increased the expression of HSPA5 and enhanced migration/invasive ability of breast cancer cells. Knock down of histone deacetylase-6 (HDAC6) increased the acetylation of HSPA5 at lysine residues 353 (K353) and reduced GP78-mediated ubiquitination of HSPA5 at K447 and then increased cell migration/invasion. In addition, we demonstrate that E3 ubiquitin ligase GP78 preferentially binds to deacetylated HSPA5. Notably, the expression levels of GP78 inversely correlated with HSPA5 levels in breast cancer patients. Patients with low GP78 expression significantly correlated with invasiveness of breast cancer, advanced tumor stages and poor clinical outcome. Taken together, our results provide new mechanistic insights into the understanding that deacetylation of HSPA5 by HDAC6 facilitates GP78-mediated HSPA5 ubiquitination and suggest that post-translational regulation of HSPA5 protein is critical for HSPA5-mediated metastatic properties of breast cancer. 
 Glucose-regulated protein 78 mediates the anticancer efficacy of shikonin in hormone-refractory prostate cancer cells. Glucose-regulated protein 78 (GRP78) is a key modulator of prostate cancer progression and therapeutic resistance. Prostate cancer is a worldwide health problem, and therapeutic resistance is a critical obstacle for the treatment of hormone-refractory prostate cancer patients. Shikonin inhibits prostate cancer proliferation and   metastasis  . However, the role of GRP78 in the cytotoxic effect of shikonin in prostate cancer cells remains unclear. GRP78 expression was abolished using small interfering RNA (siRNA), and the anticancer effects of shikonin were assessed using MTT assays, the XCELLigence biosensor, flow cytometric cell cycle analysis, and Annexin V-PI apoptotic assays. PC-3 cells expressed more GRP78 than DU-145 cells, and the MTT assays revealed that DU-145 cells were more sensitive to shikonin than PC-3 cells. GRP78 knockdown (GRP78KD) PC-3 cells were more sensitive to shikonin treatment than scrambled siRNA control cells. Based on cell cycle analysis and AnnexinV-PI apoptotic assays, apoptosis dramatically increased in GRP78KD cells compared with the control PC-3 in response to shikonin. Finally, in response to shikonin treatment, Mcl-1 and Bcl-2 levels increased in the scrambled control cells treated with shikonin, whereas Bcl-2 decreased and Mcl-1 slightly increased in the GRP78KD PC-3 cells. The levels of Bax and Bad did not change in the scrambled control or GRP78KD cells after shikonin treatment. These results are consistent with the increased sensitivity to shikonin after knockdown of GRP78. GRP78 expression may determine the therapeutic efficacy of shikonin against prostate cancer cells. 
 Expressions of GRP78 and Bax associate with differentiation,   metastasis  , and apoptosis in non-small cell lung cancer. The purpose of this study was to detect the expressions of GRP78 and Bax in human non-small cell lung cancer (NSCLC) tissues, to analyze their correlations with carcinogenesis and the development of NSCLC, and to investigate the relationship of GRP78 expression to   metastasis   and apoptosis in the NSCLC cell line HCC827. The positive expression rates of GRP78 and Bax in NSCLC lung tissues were 59.7% and 34.7% by RT-PCR, respectively. The mRNA and protein expression levels of GRP78 in NSCLC tissues were significantly higher than that in the relatively normal surrounding lung tissues (p < 0.05); the lesser the degree of tumor differentiation was, the higher the mRNA and protein expression levels of GRP78 were (p < 0.05). The mRNA and protein expression levels of GRP78 from patients in advanced pathological stages (III-IV) were significantly higher than the corresponding levels in patients in early pathological stages (I-II) (p < 0.05); the mRNA and protein expression levels of GRP78 in patients with positive lymph node   metastasis   were significantly higher than those in patients with negative lymph node   metastasis   (p < 0.05). The mRNA and protein expression levels of Bax in the above cases showed the opposite trend of the mRNA and protein expression levels of GRP78. However, the mRNA and protein expression levels of both GRP78 and Bax were independent of the patient’s sex, the patient’s age, the tumor size and the histological type (adenocarcinoma or squamous cell carcinoma) of NSCLC (p > 0.05). The mRNA expression level of GRP78 and the mRNA expression level of Bax in human NSCLC tissues were negatively correlated (r = -0.353, p = 0.002). After transfection of GRP78 siRNA in HCC827 cells, the GRP78 protein expression level was significantly decreased (p < 0.01), while the Bax protein expression level was significantly increased (p < 0.01); the number of cells that passed through the Transwell chamber was significantly less in the non-transfected control group compared to the transfected control group (p < 0.01). The number of apoptotic cells was significantly greater in the non-transfected control group compared to the transfected control group (p < 0.01). The expression levels of GRP78 and Bax were related to the carcinogenesis, development   and metast  asis of NSCLC. GRP78 expression with siRNA interference in the human NSCLC cell line HCC827 can red  uce metast  asis and promote apoptosis in HCC827 cells. 
 Co-silencing of Birc5 (survivin) and Hspa5 (Grp78) induces apoptosis in hepatoma cells more efficiently than single gene interference. Birc5 (previously known as survivin) is a cancer-specific protein. Due to the upregulation of its expression in various human malignancies and its key role in apoptosis, proliferation and angiogenesis, Birc5 has attracted attention as a target for anticancer therapies. In this study, when Birc5 was silenced in HepG2 cells, 29.7±3.3% cells underwent apoptosis as expected. It was found that the expression levels of glucose-regulated protein 78 (Hspa5, previously known as Grp78) was increased by almost 3-fold in Birc5-silenced HepG2 cells. Hspa5, a master regulator of the anti-apoptotic unfolded protein response signalling network, can also promote tumor proliferation, survival and   metastasi  s. Hence, we hypothesized that the co-silencing of Birc5 and Hspa5 may exert a stronger apoptosis-inducing effect than single gene interference. To verify this, the expression levels of Birc5 and Hspa5 in human hepatocellular carcinoma tissues were determined. Immunohistochemical staining showed that the expression of Birc5 and Hspa5 was elevated in 28 out of 31 samples. Additionally, plasmid-based siRNA against Birc5 and/or Hspa5 were constructed and transfected into the human hepatocellular liver carcinoma cell line, HepG2. Compared with the HepG2 cells, in which Birc5 or Hspa5 were silenced alone, only 44.2±3.4% of the co-silenced cells proliferated, and 40.3±3.7% co-silenced cells underwent apoptosis (p<0.05). Furthermore, tumor formation from inoculated subcutaneous co-silenced cells in nude mice was inhibited significantly. The current study suggests that Birc5 and Hspa5 could be important survival factors for hepatoma carcinoma cells and that the simultaneous knockdown of Birc5 and Hspa5 is more effective in inducing apoptosis in HepG2 cells than the knockdown of Birc5 or Hspa5 alone. The co-silencing of Birc5 and Hspa5 could be warranted for cancer therapy. 
 Survivin-mediated cancer cell migration through GRP78 and epithelial-mesenchymal transition (EMT) marker expression in Mahlavu cells. Survivin has multiple functions during the progression of cancer. However, the role of survivin in the progression and   metastasis   of hepatocellular carcinoma (HCC) remains unknown.Survivin expression in HCC cells (Mahlavu and Hep3B) was assessed using reverse transcription real-time PCR and Western blot analyses. In addition, survivin expression in HCC cells was manipulated using small interfering RNA (siRNA) or overexpression and proliferation and transwell migration assays were performed to monitor the effect of manipulated survivin expression on the growth rate and migratory ability of the transfected cells.Among the HCC cell lines tested, we found high endogenous expression of survivin mRNA and protein in Mahlavu cells. After silencing survivin expression in Mahlavu cells, there was a dramatic decrease in the cell growth rate and an increase in the metastatic potential of the cells. Overexpression of survivin in Hep3B cells suppressed the ability of the cell to migrate. The mechanism of enhanced cell migration caused by decreased survivin expression is mediated through the downregulation of glucose-regulated protein 78 (GRP78) and the upregulation of the epithelial-mesenchymal transition (EMT) marker, vimentin.Survivin may mediate   metastasis   in HCC. The knockdown of survivin expression may enhance cancer   metastasis   through the downregulation of GRP78 and upregulation of vimentin expression. 
 Transcriptional repression of the prosurvival endoplasmic reticulum chaperone GRP78/BIP by E2F1. The endoplasmic reticulum chaperone GRP78/BIP plays a central role in the prosurvival machinery, and its enhanced expression has been implicated in drug resistance, carcinogenesis, and   metastasis  . E2F1, as part of an antitumor safeguard mechanism, promotes apoptosis regardless of functional p53. Using cells that are defective in p53, we show that E2F1 represses GRP78/BIP at the transcriptional level, and this requires its DNA binding domain. Analysis of human GRP78/BIP promoter reporter constructs revealed that the region between -371 and -109 of the proximal promoter contains major E2F1-responsive elements. Toward understanding the underlying mechanism of this regulation, we performed chromatin immunoprecipitation and gel shift assays, demonstrating that E2F1 directly binds to GC-rich regions in the distal GC-box and endoplasmic reticulum stress response element -126 by interfering with the binding of positive regulatory proteins Sp1 and TFII-I of the ER stress response element-binding factor complex. We further show that TFII-I, which is required for optimal stress induction of GRP78/BIP, is suppressed by E2F1 on the protein level. Finally, our studies suggest a molecular link between the inhibition of GRP78/BIP and E2F1-mediated chemosensitization of tumor cells, underscoring its relevance for cancer treatment. Together, the data provide a new mechanism for the incompletely understood tumor suppressor function of E2F1. 
 De-acetylation and degradation of HSPA5 is critical for E1A   metastasis   suppression in breast cancer cells. Elevated expression of heat shock protein 5 (HSPA5) promotes drug resistance and   metastasis   and is a marker of poor prognosis in breast cancer patients. Adenovirus type 5 E1A gene therapy has demonstrated antitumor efficacy but the mechanisms of   metastasis  -inhibition are unclear. Here, we report that E1A interacts with p300 histone acetyltransferase (HAT) and blocks p300-mediated HSPA5 acetylation at K353, which in turn promotes HSPA5 ubiquitination by GP78 (E3 ubiquitin ligase) and subsequent proteasome-mediated degradation. Our findings point out the Ying-Yang regulation of two different post-translational modifications (ubiquitination and acetylation) of HSPA5 in tumor   metastasis  . 
 Expression of glucose regulated protein 78 (GRP78) determines colorectal cancer response to chemotherapy. Colorectal cancer (CRC) is an important health problem all over the world. A great improvement in the screening and early detection of CRC has been achieved. However, a new molecular prognostic marker is largely required. Glucose regulated protein 78-kDa (GRP78) is the central regulator of the endoplasmic reticulum (ER) and has an important role in the proliferation, differentiation and resistance to chemotherapy in cancer cells.The aim of the present study was to investigate the impact of elevated level of GRP78 on CRC prognosis and chemosensitivity.Sixty eight CRC tissue samples were collected and protein expression of GRP78 was evaluated using immunohistochemistry. The clinicopathological factors of the patients were correlated with GRP78 level.GRP78 expression increased with the progression from early to advanced CRC stages. In addition, GRP78 level was increased with the progression from early T1-2 to late T3-4 tumor localization (p< 0.05). Moreover, a significant association was found between GRP78 expression and response to chemotherapy (p< 0.05). Association between GRP78 expression and patient's clinical characteristics including lymph node involvement and   metastasis   was not significant.Results suggest the possibility to use GRP78 as a biomarker for progression of CRC and its chemosensitivity to therapy. 
 Knockdown of glucose-regulated protein 78/binding immunoglobulin heavy chain protein expression by asymmetric small interfering RNA induces apoptosis in prostate cancer cells and attenuates migratory capability. Glucose-regulated protein 78 [GRP78, also termed binding immunoglobulin heavy chain protein (Bip)] may be involved in cancer progression and   metastasis  . However, to date there has been minimal investigation into its potential role in human prostate cancer cells. Recent studies have demonstrated that asymmetric small interfering RNA (asiRNA)-mediated gene silencing is more effective and longer-lasting than conventional symmetric siRNA. Thus, the current study aimed to investigate the effects of GRP78-specific asiRNA on human prostate cancer cells. A series of asiRNAs was synthesized and their efficiency in silencing GRP78 expression in PC-3 human prostate cancer cells was evaluated. The effects of knockdown using asiRNAs were compared to those of knockdown using symmetric siRNAs. The effect of GRP78 silencing on PC-3 cell apoptosis and migration, and the possible mechanisms governing these biological processes were examined. Compared with the symmetric siRNA, transfection with the 15 base pair asiRNA (asiGRP78-3) resulted in greater downregulation of GRP78 expression. GRP78 depletion in PC-3 cells resulted in increased apoptosis and decreased migration of these cells. Experiments investigating the underlying mechanisms of these effects revealed that knockdown of GRP78 attenuated protein kinase B activation and decreased the expression of pro-caspase 9, pro-caspase 3 and vimentin. In conclusion, knockdown of GRP78/Bip expression with asymmetric siRNA led to increased prostate cancer cell apoptosis and reduced cellular migration. 
 Glucose-regulated protein 78 (GRP78) regulates colon cancer   metastasis   through EMT biomarkers and the NRF-2/HO-1 pathway. Glucose-regulated protein 78 (GRP78) is a key chaperone and stress response protein. Previous studies have demonstrated that high GRP78 expression may be correlated with cancer progression and therapeutic response. However, the role of GRP78 in the   metastasis   of colon cancer is unclear. In this study, we used small interfering RNA (siRNA) to knock down GRP78 expression in colon cancer cells (HT-29 and DLD-1 cells). In wound-healing migration assays, we found that GRP78-knockdown (GRP78KD) cells showed better wound-healing ability than control cells. We also found that GRP78KD cells displayed a better migratory ability than control cells in migration and invasion assays. As we further dissected the underlying molecular mechanism, we found that silencing GRP78 may cause an increase in vimentin expression and a decrease in the E-cadherin level, which was correlated with the increase in migratory ability. In addition, we found that GRP78KD may activate the NRF-2/HO-1 pathway, and this activation was also correlated with the increase in cell invasiveness. Furthermore, we examined GRP78 expression in a tissue array and found that the GRP78 expression in metastatic adenocarcinoma in lymph nodes tended to be weaker than that in primary colonic adenocarcinoma. In conclusion, a low level of GRP78 may cause an increase in   metastasis   ability in colon cancer cells by altering E-cadherin and vimentin expression and activating the NRF-2/HO-1 signaling pathway. Our study demonstrates that low expression of GRP78 may correlate with a high risk of   metastasis   in colon cancer. 
 Overexpressed GRP78 affects EMT and cell-matrix adhesion via autocrine TGF-β/Smad2/3 signaling. Glucose-regulated protein of 78kD (GRP78) is a multifunctional protein belonging to the heat shock protein 70 family. Overexpression of GRP78 triggered by environmental and physiological stresses is positively correlated with the occurrence and progression of various tumors, but the molecular mechanisms have not been well established. The present study indicated that overexpression of GRP78 in colon cancer cells could promote cell-matrix adhesion through the upregulation of fibronectin, integrin-β1 and phosphorylated FAK. Meanwhile, it resulted in a visible epithelial-mesenchymal transition in DLD1 cells, and the Snail-2 played the key role during the process. More importantly, the data indicated that GRP78 overexpression facilitated the expression and secretion of TGF-β1, which further activated the downstream Smad2/3 signaling module to effectuate the cell-matrix adhesion and epithelial-mesenchymal transition. Taken together, this study provides a novel molecular mechanism involving in the effects of GRP78 on colon canc  er metasta  sis. 
 Glucose-regulated protein GRP78 is up-regulated in prostate cancer and correlates with recurrence and survival. Chemotherapy resistance is a significant contributor to treatment failure and death in men with hormone-refractory prostate cancer. One unexplored mechanism for drug resistance is the induction of stress response proteins referred to as the glucose-regulated proteins (GRPs). We sought to determine the level of expression of GRP78, the best characterized GRP in lymph node-positive prostate cancer. Archived, paraffin-embedded, radical prostatectomy specimens were obtained from 153 patients with lymph node-positive prostate cancer (stage D1). The level of GRP78 expression was determined by immunohistochemistry. We assessed the expression and specificity of GRP78 immunoreactivity in benign prostatic tissue, prostate cancer, and lymph node   metastasis  . We correlated the intensity of immunopositivity with prostate cancer recurrence and survival. Whereas immunohistochemical staining demonstrated that all prostate tissue was immunoreactive for GRP78, the intensity of expression was markedly higher in the primary tumor compared with areas of benign epithelium. GRP78 expression was also evident in lymph node metastases although less intensely than in the primary tumor. Patients with strong GRP78 immunoreactivity in the primary tumor are at higher risk for clinical recurrence (relative risk = 2.0, P = .019) and death (relative risk = 1.8, P = .024) than patients with weak GRP78 expression. This finding confirms that GRP78 protein expression is significantly higher in prostate cancer than in benign prostatic tissue. The intensity of expression is significantly associated with survival and clinical recurrence. GRP78 has considerable potential not only as a prognostic indicator but also as a potential therapeutic target. 
 Reversed expression of GRIM-1 and GRP78 in human non-small cell lung cancer. Gene associated with retinoid and interferon-induced mortality 1 (GRIM-1) acts as a tumor growth suppressor via apoptosis induction. However, GRIM-1 expression in human non-small cell lung cancer (NSCLC) and its potential interaction with another apoptosis-associated protein-glucose-regulated protein 78 (GRP78)-are as yet unknown. Using 40 surgical specimens, we showed significantly lower expression of GRIM-1 in NSCLC at both protein and messenger RNA (mRNA) levels compared with that in normal tissues (P < .01 and P < .001, respectively). Interestingly, these tumors tended to express higher basal amounts of GRP78 protein and mRNA (P < .05 and P < .001, respectively). Similarly, in the NSCLC tissues, weaker staining for GRIM-1 (main intensity + to ++) but stronger staining for GRP78 (main intensity +++ to ++++) was observed. Correlation analysis showed that protein and mRNA expression or the percentage of cells immunoreactive for GRIM-1 was negatively correlated with that of GRP78 (r = -0.279, r = -0.326, or r = -0.571, respectively). Coimmunoprecipitation and transient transfection revealed that GRIM-1 interacted with GRP78 and suppressed GRP78 protein expression. In addition, there was no correlation between GRIM-1 expression and clinical characteristics, whereas GRP78 expression was significantly correlated with tumor-nodes-  metastasis   (TNM) stage (stage 3 + 4 versus stage 1 + 2). In conclusion, the expression of GRIM-1 and GRP78 was negatively correlated in human NSCLC tissues, and the down-regulation of GRP78 by GRIM-1 provides a possible mechanism for their interaction. This study suggests a novel potential molecular pathway inactivated during the development of NSCLC. 
 New prognosis biomarkers identified by dynamic proteomic analysis of colorectal cancer. The initiation, promotion and progression of human cancer are complex, polygenic, multi-factored processes. Through systematic proteomic analysis, different stages of CRC (colorectal cancer) biopsies were examined, and 199 differentially expressed proteins were detected between TNM (the tumor, nodes, and   metastasis  ) stages I-IV and normal tissue (One-Way Analysis of Variance, ANOVA; p≤ 0.05). Instead of looking for biomarkers to distinguish CRC from normal or identify metastatic tumors, we focused on the variation tendency of CRC carcinogenesis and the dynamic expression patterns of proteins among the different stages. Som (self-organizing map clustering) analysis revealed eight unique expression patterns and that the cancer-related proteins were dynamically expressed, and their expression levels changed continuously throughout tumorigenesis. Molecular evidence emerged much earlier than visible, clinical or histological changes, which shows the potential prospect of building molecular staging. Proteins identified by MALDI-TOF MS (Matrix-Assisted Laser Desorption/Ionization Time of Flight Mass Spectrometry) were mainly involved in energy metabolism, acetylation and signaling pathways. Validation experiments using immunoblotting and immunohistochemistry (IHC) agreed with the 2D-DIGE (two-dimensional difference in gel electrophoresis) data. After survival classifier and LOOCV (leave-one-out cross-validation) analyses, the new prognostic biomarkers (78 kDa Glucose-Regulated Protein precursor (GRP78), Fructose-bisphosphate Aldolase A (ALDOA), Carbonic Anhydrase I (CA1) and Peptidyl-prolyl cis-trans isomerase A or Cyclophilin A (PPIA)) provided good survival prediction for TNM stage I-IV patients. The new biomarkers derived from the dynamic patterns of these proteins' expression provide is a good supplementary method for determining prognosis for CRC, especially for the TNM stage III and IV patients. 
 Overexpression of GRP78 and GRP94 is involved in colorectal carcinogenesis. Glucose-related proteins (GRPs) are ubiquitously expressed in the endoplasmic reticulum and assist in protein folding and assembly, consequently considered to be molecular chaperones. GRP78 and GRP94 expression was induced by glucose starvation and up-regulated in samples taken from several different malignant tissues. To clarify the roles of both molecules in tumorigenesis and progression of colorectal carcinomas, immunohistochemistry (IHC) was performed on tissue microarrays containing colorectal carcinomas, adenomas and the non-neoplastic mucosa (NNM) using antibodies against GRP78 and GRP94. Their expression was correlated with the clinicopathological parameters of carcinomas. Both proteins were also studied in colorectal carcinoma cell lines (DLD-1, HCT-15, SW480 and WiDr) by IHC and Western blot. There was a gradually increased GRP78 expression from colorectal NNMs, carcinomas, to low-grade and high-grade adenomas (P<0.05), while up-regulated GRP94 expression from NNM, low-grade adenoma, high-grade adenoma, to carcinoma (P<0.05). The expression was similar in all the carcinoma cell lines. GRP78 expression was negatively correlated with lymphatic invasion or low GRP94 expression of the carcinomas (P<0.05), while there was no correlation of GRP94 expression with other parameters of carcinomas (P>0.05). Multivariate analysis showed that venous invasion, lymph node   metastasis   and UICC staging (P<0.05), but not age, sex, tumor size, differentiation, depth of invasion, lymphatic invasion, GRP78 and GRP94 expression (P>0.05), were independent prognostic factors for carcinomas. It is suggested that up-regulated expression of GRP78 and GRP94 could possibly be involved in the pathogenesis of colorectal carcinomas. 
 E1A-mediated inhibition of HSPA5 suppresses cell migration and invasion in triple-negative breast cancer. Triple-negative breast cancer (TNBC) is defined by reduced expression of the estrogen receptor, progesterone receptor, and HER2. TNBC is an especially aggressive group of breast cancers with poor prognosis. There are currently no validated molecular targets to effectively treat this disease. Thus, it is necessary to identify effective molecular targets and therapeutic strategies for TNBC patients.The expression of HSPA5 in patients with breast cancer was examined by immunohistochemistry. The association of HSPA5 expression with tumor grade and metastatic events in TNBC patients was analyzed using the Oncomine database. The knockdown and overexpression of HSPA5 protein were performed to investigate the effects on E1A-suppressed cell migration/invasion of TNBC using in vitro transwell assays and tumor growth/experimental   metastasi  s studies in animal models.The expression of HSPA5 was positively correlated with high-grade tumors, metastatic events, and poor overall survival in breast cancer patients with TNBC. E1A-inhibited HSPA5 expression suppressed cell migration/invasive ability of TNBC cell lines. Moreover, E1A significantly abolished lung metastases from breast cancer cells by inhibiting HSPA5 expression in a xenograft tumor model.The overexpression of HSPA5 is critical for high-risk   metastasi  s of breast cancer and TNBC. The results of our study suggest that HSPA5 may be a crucial mediator of E1A-suppressed metastatic ability of breast cancer cells. Thus, E1A may be a potential target for diagnosis and individualized treatment in clinical practice. 
 Grp78 promotes the invasion of hepatocellular carcinoma. Glucose regulated protein 78 (Grp78) is involved in the invasion and   metastasis   in many human cancers including gastric cancer, breast cancer, prostate cancer. But the role of Grp78 in the invasion of human hepatocellular carcinoma has not been reported. In this article, we examined if Grp78 was associated with the invasion of hepatocellular carcinoma and explored the possible underlying mechanism.The Grp78 and FAK expression levels in 44 patients with hepatocellular carcinoma were examined using immunohistochemistry. Grp78 overexpressing SMMC7721 cells were established by pcDNA3.1 (+)-Grp78 transfection and screened by G418. Grp78 and FAK levels in Grp78 overexpressing cells were down-regulated by siRNA transfection. The invasion status of tumor cells was evaluated by transwell assay in vitro, and chick embryo   metastasis   model in vivo. Cell spreading was determined by cell spreading assay, and quantitatively measured by Orisis software HUG. Grp78, pY397 FAK, pY576/577 FAK and FAK levels were detected by western blot. RhoA activity was detected by GST pulldown assay. The distribution of actin cytoskeleton was observed by fluorescent staining.Grp78 expression levels in 44 patients with hepatocellular carcinoma were negatively correlated with tumor grading, and positively correlated with portal invasion and intra-hepatic invasion. Overexpression of Grp78 in SMMC7721 cells promoted the invasion of cancer cells in vitro and in vivo, and this increase in tumor cell invasion was blocked by Grp78 siRNA knockdown. Our results also revealed that overexpression of Grp78 in SMMC7721 cells accelerated the process of cell spreading and promoted lamellipodia formation. Further analysis showed that overexpression of Grp78 in SMMC7721 cells increased pY397 and pY576/577 levels of FAK. Grp78 siRNA knockdown decreased FAK activation and activity. Our results also revealed that Grp78 overexpression in SMMC7721 cells decreased RhoA-GTP level, and Grp78 siRNA knockdown rescued RhoA-GTP level in Grp78 overexpressing cells, indicating Grp78 inhibited RhoA activity in hepatocellular carcinoma cells. Furthermore, overexpression of Grp78 in SMMC7721 cells increased phospho-p190RhoGAP level. FAK siRNA knockdown in Grp78 overexpressing cells reversed phospho-p190RhoGAP level. These data suggested that Grp78 inhibited RhoA activity by up-regulated phospho-p190RhoGAP level and Grp78 mediated p190RhoGAP phosphorylation is FAK dependent.Grp78 promoted the invasion of hepatocellular carcinoma both in vitro and in vivo. Overexpression of Grp78 in hepatocellular carcinoma cells enhanced the activation and activity of FAK which negatively regulated Rock kinase activity by promoting the phosphorylation of p190RhoGAP. 
 Silencing of glucose-regulated protein 78 (GRP78) enhances cell migration through the upregulation of vimentin in hepatocellular carcinoma cells. Glucose-regulated protein 78 (GRP78) plays an important role in embryonic development and cancer progression. However, there is little information regarding the regulation of GRP78 in hepatocellular carcinoma (HCC)   metastasis  .We used RNA silencing and cDNA expression vectors to manipulate target gene expression in HCC cells. The transwell migration assay and xCelligence biosensor system were applied to determine the proliferatory and migratory ability of the HCC cells.In this study, we found that GRP78 silencing enhanced cell migration in both HepJ5 and Mahlavu cells. Overexpressed GRP78 in skHep1 cells suppressed the migratory ability. In the insight mechanism dissection for GRP78-mediated cancer migration, we found that downregulation of GRP78 caused the increase of vimentin expression on HCC cells. Suppressed vimentin expression also decreased the migratory ability on HCC, indicating that vimentin expression levels modulated the cell migratory ability.We found that silencing GRP78 in HCC cells may enhance cell migration through the increase of vimentin expression. 
 GRP78 promotes the invasion of pancreatic cancer cells by FAK and JNK. The major characteristics of pancreatic cancer are its excessive local invasion and early systemic dissemination. The glucose-regulated protein is over-expressed in many human cancers including pancreatic cancer and correlated with invasion and   metastasis   in many cancers. To investigate the effect of Grp78 on the invasion of pancreatic cancer, we used western blot and Transwell assay. We found Grp78 is expressed at lower levels in capan-2 and higher expressed in MiaPaCa-2 cells, and Grp78 expression levels were correlated with the invasion potentials of tumor cells. Then,we increased the expression of Grp78 in capan-2 cells and decreased the expression of Grp78 in MiaPaCa-2 cells. We found that over-expression of Grp78 caused significant increase in the expression of TIMP-1, TIMP-2, MMP-14, MMP-2, and MMP-9 in Capan-2 cells. Consistently, knockdown of Grp78 decreased the expression of them in MiaPaCa-2 cells. Gelatin zymography showed Grp78 over-expression stimulated the activities of MMP-2 and MMP-9, while GRP78 knockdown reduced the activities of MMP-2 and MMP-9. Cytoskeleton staining showed that knockdown of Grp78 caused a marked increase in cytoskeleton F-actin stress fibers in MiaPaCa-2 cells. Consistently, GRP78 knockdown hyperactivated RhoA and inhibited significantly Rac activity. Grp78 over-expression decreases the RhoA and stimulated Rac activity. We also found that Grp78 modulated FAK and JNK signaling pathways. Over-expression of GRP78 in Capan-2 activated FAK and JNK. Finally, we demonstrated that knockdown of FAK by shRNA in combination with blockade of JNK signaling pathway with SP600125 completely inhibited GRP78-induced cancer cell invasion. GRP78 is involved in the regulation of pancreatic cancer invasion. FAK and JNK are the key downstream effectors of GRP78. 
 Cell Surface GRP78 Accelerated Breast Cancer Cell Proliferation and Migration by Activating STAT3. High levels of cell surface glucose regulated protein 78 (sGRP78) have been implicated in cancer growth, survival,   metastasis  , and chemotherapy resistance. However, the underlying mechanism remains largely unknown. Here we report that the level of sGRP78 expression in human breast tumors gradually increases during cancer progression. Overexpression of GRP78 significantly enhanced its membrane distribution in human MCF-7 breast cancer cells, but had no effect on endoplasmic reticulum (ER) stress. High levels of sGRP78 facilitated cell proliferation and migration, as well as suppressed cell apoptosis. Neutralization of sGRP78 by a specific antibody against GRP78 alleviated sGRP78-induced cell growth and migration. Importantly, high phosphorylation levels of the signal transducer and activator of transcription 3 (STAT3) were found in human breast tumors that express sGRP78 and MCF-7 cells infected with adenovirus encoding human GRP78. Pretreatment with a GRP78 antibody suppressed STAT3 phosphorylation. Furthermore, genetic and pharmacological inhibition of STAT3 reversed the impacts of GRP78 on cell proliferation, apoptosis, and migration. These findings indicate that STAT3 mediates sGRP78-promoted breast cancer cell growth and migration. 
 Monoclonal antibody against cell surface GRP78 as a novel agent in suppressing PI3K/AKT signaling, tumor growth, and   metastasis  . The ER chaperone GRP78 translocates to the surface of tumor cells and promotes survival,   metastasis  , and resistance to therapy. An oncogenic function of cell surface GRP78 has been attributed to the activation of the phosphoinositide 3-kinase (PI3K) pathway. We intend to use a novel anti-GRP78 monoclonal antibody (MAb159) to attenuate PI3K signaling and inhibit tumor growth and   metastasis  .MAb159 was characterized biochemically. Antitumor activity was tested in cancer cell culture, tumor xenograft models, tumor   metastasis   models, and spontaneous tumor models. Cancer cells and tumor tissues were analyzed for PI3K activity. MAb159 was humanized and validated for diagnostic and therapeutic application.MAb159 specifically recognized surface GRP78, triggered GRP78 endocytosis, and localized to tumors but not to normal organs in vivo. MAb159 inhibited tumor cell proliferation and enhanced tumor cell death both in vitro and in vivo. In MAb159-treated tumors, PI3K signaling was inhibited without compensatory MAPK pathway activation. Furthermore, MAb159 halted or reversed tumor progression in the spontaneous PTEN-loss-driven prostate and leukemia tumor models, and inhibited tumor growth and   metastasis   in xenograft models. Humanized MAb159, which retains high affinity, tumor specific localization, and the antitumor activity, was nontoxic in mice, and had desirable pharmacokinetics.GRP78-specific antibody MAb159 modulates the PI3K pathway and inhibits tumor growth and   metastasis  . Humanized MAb159 will enter human trials shortly. 
 Proteomic profiling of hepatocellular carcinoma in Chinese cohort reveals heat-shock proteins (Hsp27, Hsp70, GRP78) up-regulation and their associated prognostic values. To facilitate the identification of candidate molecular biomarkers that are linked to the pathogenesis of hepatocellular carcinoma (HCC), we investigated protein-expression profiles of 146 tissue specimens including 67 pairs of tumors and adjacent non-tumors resected from HCC patients as well as 12 normal livers by 2-DE. Among the 1800 spots displayed in the liver proteome, a total of 90 protein species were found to be significantly different between the three groups (P < 0.05). Three of the top candidate markers up-regulated in HCC, with high receiver operating characteristic (ROC) curves, were identified by MS/MS analysis and belonged to the chaperone members: heat-shock protein (Hsp)27, Hsp70 and glucose-regulated protein (GRP)78. Over-expression of these chaperone proteins in HCC tissues was confirmed by Western blotting and immunohistochemistry. In correlation with clinico-pathological parameters, expression of Hsp27 was linked to alpha-fetoprotein level (P = 0.007) whereas up-regulation of GRP78 was associated with tumor venous infiltration (P = 0.035). No significant association of Hsp70 with any pathologic features was observed. The present HCC proteome analysis revealed that in response to the stressful cancerous microenvironment, tumor cells strived to increase the expression of chaperone proteins for cyto-protective function and to enhance tumor growth and   metastasis  . 
 Glucose-regulated protein 78 and heparanase expression in oral squamous cell carcinoma: correlations and prognostic significance. The aim of the present study was to investigate the expression of glucose-related protein 78 (GRP78) and heparanase (HPA) in oral squamous cell carcinoma (OSCC) and their relationship with clinicopathological parameters and potential implications for survival.A total of 46 patients with OSCC and 10 normal individuals were recruited for the study. GRP78 and HPA expression were determined in the lesion tissues using immunohistochemical analysis. The correlation between GRP78 and HPA was assessed using the Spearman correlation analysis. The associations of GRP78 and HPA with clinicopathological characteristics and survival were examined using the x2-test, Kaplan-Meier, or Cox regression.Patients with OSCC showed a statistically significant higher prevalence of GRP78 and HPA expression than normal oral tissues. GRP78 and HPA expression was positively correlated with size, TNM stage, histological grade, lymphatic   metastasis  , and distant   metastasis   in OSCC patients. GRP78 expression was also positively correlated with HPA expression. Positive GRP78 and HPA expression was inversely correlated with survival in OSCC patients.HPA expression was found to be positively correlated with GRP78 expression. GRP78 and HPA are biomarkers that may have the potential to guide the treatment of oral cancer patients. 
 Decreased GRP78 protein expression is a potential prognostic marker of oral squamous cell carcinoma in Taiwan. Oral squamous cell carcinoma (OSCC) is an aggressive tumor and its occurrence in Taiwan is closely related to chronic smoking, alcohol consumption, and especially to betel quid chewing. It became the fourth most common malignant tumor of Taiwanese men in 2006. Unfortunately, there are few biomarkers for diagnosis and treatment of this disease.To find potential markers, two domestic cell lines (OC2 and OCSL) derived from different grades of OSCC were established and their proteins were compared by global proteomic analysis. The expression differences of GRP78 protein in these two cell lines and clinical samples from OSCC patients were verified.Of the 11 candidate proteins expressed differentially in both cell lines, six [heat shock protein 90 kDa beta member 1 (94 kDa glucose-regulated protein; GRP94), protein disulfide-isomerase precursor, vimentin, tubulin beta-2C chain, 78 kDa glucose-regulated protein precursor (GRP78), and annexin A2] were increased in OC2 cells (low-grade OSCC), and five (heat shock protein 90-beta, annexin A1, stress-induced phosphoprotein 1, elongation factor-2, and integrin alpha-3 precursor) were increased in OCSL cells (high-grade OSCC). Some of these proteins have been previously associated with malignant tumors, but no previous association of GRP78 with OSCC has been reported. GRP78 protein expression in these two OSCC cell lines was confirmed by Western blotting. Immunohistochemical staining of clinical samples from OSCC patients revealed that decreased GRP78 protein expression was significantly correlated with advance tumor stage (p < 0.001) and neck lymph node   metastasis   (p = 0.001).GRP78 protein is a possible biomarker of oral cancer in Taiwan. 
 Glucose regulated protein 78 promotes cell invasion via regulation of uPA production and secretion in colon cancer cells. Glucose regulated protein 78 (GRP78) is frequently highly expressed in tumor cells, contributing to the acquisition of several phenotypic cancer hallmarks. GRP78 expression is also positively correlated with tumor   metastasis  , and promotes hepatocellular carcinoma cell invasion via increasing cell motility, however, other mechanisms involving the prometastatic roles of GRP78 remain to be elucidated. Here we report that forced GRP78 expression promotes colon cancer cell migration and invasion through upregulating MMP-2, MMP-9 and especially uPA production. These effects of GRP78 are mediated by enhancing the activation of β-catenin signaling. Interestingly, we identify that GRP78 interacts with uPA both in the cells and in the culture medium, suggesting that GRP78 protein is likely to directly facilitate uPA secretion via protein-protein interaction. Taken together, our findings demonstrate for the first time that besides stimulation of cell motility, GRP78 can act by increasing proteases production to promote tumor cell invasion. 
 Expression of GRP78 predicts taxane-based therapeutic resistance and recurrence of human gastric cancer. Cancer cells adapt to chronic stress in the tumor microenvironment by inducing the expression of glucose-regulated protein 78 (GRP78), a major endoplasmic reticulum chaperone with Ca(2+)-binding and antiapoptotic properties. The effect in and potential role of its expression in progression of and prognosis for gastric cancer (GC) are unclear. In the present study, we investigated the clinical value of GRP78 expression in judgment of the severity of and prognosis for GC in a retrospective cohort study of 160 patients who underwent D2 radical gastrectomy and adjuvant chemotherapy. GRP78 expression was detected using immunohistochemistry. The relationships of GRP78 expression with age, sex, differentiation, invasion depth, disease stage, lymph node   metastasis  , and time to recurrence (TTR) were analyzed. The GRP78 expression was higher in tumors from patients with deep tumor infiltration, with poor differentiation, at late disease stages, and with lymph node   metastasis   than that in tumors from patients without. Also, GRP78 positivity was associated with short TTR (hazard ratio [HR], 1.75; 95% confidence interval [CI], 1.07-4.85; P=0.041). Subgroup analysis revealed that the HR in the GRP78-high group increased significantly in patients who did not receive taxane-containing regimens (HR, 2.21; 95% CI, 1.23-7.36; P=0.038). In contrast, in the patients who received taxane-based chemotherapy, the association between GRP78 positivity and increased risk of recurrence was not statistically significant (HR, 1.16; 95% CI, 0.81-2.98; P=0.111). In the patients with GRP78 expression, those who underwent taxane-containing chemotherapy had longer median TTRs than did those who did not undergo this treatment (P=0.017). Downregulation of GRP78 expression markedly inhibited proliferation of the GC cells at the G1 phase, whereas GRP78 overexpression promoted cell-cycle progression. These findings suggest that GRP78 overexpression promotes GC cells proliferation and is an independent indicator of poor prognosis for GC. 
 High Expression of GRP78 Promotes Invasion and Metastases in Patients with Esophageal Squamous Cell Carcinoma. Esophageal squamous cell carcinoma (ESCC) has a poor prognosis due to its high frequency of   metastasis   and invasion. Recent studies have suggested glucose-regulated protein 78KD (GRP78) may play important roles in progression and development of malignant tumors. However, the mechanisms of invasion and   metastasis   of ESCC in relation to GRP78 still remain obscure.The aim of this study was to investigate the effect of GRP78 on invasion and   metastasis   of ESCC and to explore its potential mechanism.GRP78 expression levels in ESCC tissues were examined by immunohistochemistry. RT-PCR and western blot were used to test the relative expression of GRP78 in non-metastatic and high-metastatic ESCC cells. In vitro and in vivo studies were both performed to investigate the role of GRP78 in invasion and   metastasis   of ESCC cells. The expression of   metastasis  -related proteins was examined by western blot in GRP78-depleted cells.The expression of GRP78 is correlated with invasion,   metastasis   and poor prognosis in ESCC patients. GRP78 expression was significantly higher in highly metastatic cells compared with ESCC non-metastatic cells. In addition, down-regulation of GRP78 significantly inhibited the metastatic potential of ESCC cells in both in vitro and in vivo studies. The expression of MMP-2 and MMP-9 were down-regulated in GRP78-depleted ESCC cells.The present study demonstrated that GRP78 plays important roles in invasion and   metastasis   of ESCC, indicating that GRP78 might be used as a potential prognostic and therapeutic marker in patients with ESCC by modulating the expression of MMP-2 and MMP-9. 
 Mitochondrial ATAD3A combines with GRP78 to regulate the WASF3   metastasis  -promoting protein. AAA domain containing 3A (ATAD3A) is an integral mitochondrial membrane protein with unknown function, although we now show that high-level expression is associated with poor survival in breast cancer patients. Using a mass spectrometry approach we have demonstrated that ATAD3A interacts with the WASF3   metastasis  -promoting protein. Knockdown of ATAD3A leads to decreased WASF3 protein levels in breast and colon cancer cells. Silencing ATAD3A also results in loss of both cell anchorage-independent growth and invasion and suppression of tumor growth and   metastasis   in vivo using immuno-compromised mice. HSP70 is responsible for stabilizing WASF3 in the cytoplasm, but inactivation of HSP70 does not lead to the loss of WASF3 stability at the mitochondrial membrane, where presumably it is protected through its interaction with ATAD3A. In response to endoplasmic reticulum (ER) stress, increases in the GRP78 protein level leads to increased WASF3 protein levels. We also show that ATAD3A was present in a WASF3-GRP78 complex, and suppression of GRP78 led to destabilization of WASF3 at the mitochondrial membrane, which was ATAD3A dependent. Furthermore, ATAD3A-mediated suppression of CDH1/E-cadherin occurs through its regulation of GRP78-mediated WASF3 stability. Proteolysis experiments using isolated mitochondria demonstrates the presence of the N-terminal end of WASF3 within the mitochondria, which is the interaction site with the N-terminal end of ATAD3A. It appears, therefore, that stabilization of WASF3 function occurs through its interaction with ATAD3A and GRP78, which may provide a bridge between the ER and mitochondria, allowing communication between the two organelles. These findings also suggest that pharmacologic inhibition of ATAD3A could be an effective therapeutic strategy to treat human cancer. 
 MDA-9 and GRP78 as potential diagnostic biomarkers for early detection of melanoma   metastasis  . Metastatic melanoma, the primary cause of skin cancer-related death, warrants new diagnostic and therapeutic approaches that target the regulatory machinery at molecular level. The heterogeneity and complexity of melanoma result in the difficulty to find biomarkers and targets for early detection and treatment. Here, we investigated   metastasis  -associated proteins by comparing the proteomic profiles of primary cutaneous melanomas to their matched lymph node metastases, which minimizes heterogeneity among samples from different patients. Results of two-dimensional gel electrophoresis (2-DE) followed by proteomic analysis revealed eight differentially expressed proteins. Among them, seven proteins (α-enolase, cofilin-1, LDH, m-β-actin, Nm23, GRP78, and MDA-9) showed increased and one (annexin A2) showed decreased expression in metastatic lymph node tissues than in primary melanomas. MDA-9 and GRP78 were the most highly expressed proteins in lymph node metastases, which was validated by immunohistochemical staining. Moreover, exosomes from serum samples of metastatic melanoma patients contained higher levels of MDA-9 and GRP78 than those of patients without metastases, indicating the potential of MDA-9 and GRP78 to be biomarkers for early detection o  f metastas  is. Further, small interfering RNA (siRNA)-mediated knockdown confirmed a functional role for MDA-9 and GRP78 to promote cell invasion in the A375 cells. Finally, we showed that GRP78 co-localized with MDA-9 in 293T cells. Taken together, our findings support MDA-9, co-expressed with GRP78, as a melanoma protein associated with lymph nod  e metastas  is. Investigating how MDA-9 and GRP78 interact to contribute to melanom  a metastas  is and disease progression could reveal new potential avenues of targeted therapy and/or useful biomarkers for diagnosis and prognosis. 
  GeneRIFs  
 Cell surface GRP78 directly binds and phosphorylates c-Src. As a consequence, c-Src phosphorylated EGFR, promoting the invasion and   metastasis   of HCCs. 
 The present study demonstrated that GRP78 plays important roles in invasion and   metastasis   of esophageal squamous cell carcinoma. 
 Elevated expression of heat shock protein 5 promotes drug resistance and   metastasis   in breast cancer. 
 MDA-9, co-expressed with GRP78, as a melanoma protein associated with lymph node   metastasis  . Investigating how MDA-9 and GRP78 interact to contribute to melanoma   metastasis   and disease progression could reveal new potential avenues of targeted therapy 
 A low level of GRP78 may cause an increase in   metastasis  . 
 GRP78-specific antibody MAb159 modulates the PI3K pathway and inhibits tumor growth and   metastasis   
 The expression levels of GRP78 and Bax were related to the carcinogenesis, development and   metastasis   of non-small cell lung cancer. 
 The knockdown of survivin expression may enhance cancer   metastasis   through the downregulation of GRP78 and upregulation of vimentin expression 
 immunohistochemical staining of clinical samples from oral squamous cell carcinoma patients revealed that decreased GRP78 protein expression was significantly correlated with advance tumor stage (p < 0.001) and neck lymph node   metastasis   (p = 0.001) 
 Up-regulated expression of GRP78 and GRP94 was possibly involved in pathogenesis, growth, invasion, and   metastasis   of gastric carcinomas. 
  [Cc]ell [Ii]nvasion  PubMed  
 Molecular chaperones as a common set of proteins that regulate the invasion phenotype of head and neck cancer. The goal of this study was to establish a common set of molecules that regulate   cell invasion   in head and neck cancer (HNC).Five invasive sublines derived from HNC cell lines were established using the Matrigel selection method. Proteomic technology, MetaCore algorithm, and reverse transcriptase-PCR methods were used to search for molecules that contribute to the invasion phenotype. Cellular functional analyses and clinical association studies were applied to examine the significance of the molecules.Fifty-two proteins were identified in more than two of the four independent proteomic experiments, including 10 (19%) molecular chaperones. Seven chaperones were confirmed to be differentially expressed in five sublines, Hsp90α, Hsp90β, Hsp90-B1/Gp96, Hsp70-A5/Grp78, and HYOU1, that upregulate, whereas Hsp60 and glucosidase-α neutral AB (GANAB) downregulate. Four molecules were further investigated. In all cell lines, knockdown of Hsp60 or GANAB and silencing of Gp96 or Grp78 considerably enhanced or reduced cell migration and invasion, respectively. Clinical association studies consistently revealed that low levels of Hsp60 or GANAB and high levels of Gp96 or Grp78 are significantly associated with advanced cancer (P < 0.001 to P = 0.047, respectively, for the four molecules) and poor survival (P < 0.001 to P = 0.025, respectively, for the four molecules).Our study defined molecular chaperones as a common set of proteins that regulate the invasion phenotype of HNC. Loss of the tumor suppression function of Hsp60 or GANAB and acquisition of the oncogenic function of Gp96 or Grp78 contribute to aggressive cancers. These molecules may serve as prognostic markers and targets for cancer drug development. 
 Cell-surface GRP78 facilitates colorectal cancer cell migration and invasion. Glucose regulated protein 78 (GRP78) is predominantly located in the endoplasmic reticulum as a molecular chaperone. It has also been found on the membranes of some cancer cells, acting as a receptor for a wide variety of ligands. However, its presence on colorectal cancer (CRC) cell surface and its role in CRC metastatic progression remain elusive. Here we reported that GRP78 was predominantly present in the form of clustering aggregates on CRC cell surfaces, and its surface abundance was strongly correlated with CRC differentiation stage. Interestingly, we observed that cell-surface GRP78 had an interaction with the ECM adhesion molecule β1-integrin and was involved in cell-matrix adhesion through regulation of focal adhesion kinase (FAK). Moreover, the present data also implicated that surface GRP78 promoted the   cell invasio  n process, and this effect was at least partly mediated through its association with uPA-uPAR protease system. Together, our data suggests that surface GRP78 promotes CRC cell migration and invasion by regulating cell-matrix adhesion and ECM degradation, which is independent of its signaling receptor function. 
 Glucose-regulated protein 78 demonstrates antiviral effects but is more suitable for hepatocellular carcinoma prevention in hepatitis B. Hepatitis B virus (HBV) is the leading cause of liver cirrhosis and hepatocellular carcinoma in Asia and Africa. Existing antivirals cannot cure HBV or eliminate risk of hepatocellular carcinoma. Glucose-regulated protein 78 (GRP78) can inhibit HBV replication, but promote virion secretion and hepatocellular cancer   cell invasion  . For these reasons, the overall effect of GRP78 on HBV production and whether to utilize the HBV replication-inhibitory effect of GRP78 up-regulation or the hepatocellular cancer   cell invasion  -inhibitory effect of its down-regulation were further investigated in order to improve the efficacy of current antiviral therapy.GRP78 regulations in HepG2.2.15 cells were conducted by transfections of expressing vector and small interfering RNA, respectively. The changes in HBV replication, hepatitis B e antigen (HBeAg) synthesis and hepatoma cell motility were monitored.GRP78 overall decreased HBV production due to its HBV replication-inhibitory effect time-dependently overwhelming virion secretion-promoting effect in HepG2.2.15 cells. Unlike the parental cells (HepG2), HepG2.2.15 cells demonstrated decreased expressions of the major genes in the interferon-β1-dependent pathway. Moreover, the expressions of these genes were not affected by GRP78 regulations. However, GRP78 was found to inhibit HBeAg secretion and to increase the retro-transportation of capsid assembly-interfering HBeAg precursor from the endoplasmic reticulum into the cytosol where new viral nucleocapsids formed. Furthermore, GRP78 overexpression promoted wound healing process (the motility) of HepG2.2.15 cells. In contrast, GRP78 knockdown enhanced HBV replication and HBeAg secretion, but they were abolished by entecavir and furin inhibitor, respectively.GRP78 mainly demonstrates anti-HBV effects, reducing HBV production and HBeAg secretion. With due regard to the hepatocellular cancer invasion risk of the overexpression and the rectifiability of the unpleasant effects of the knockdown, GRP78 down-regulation may be more suitable to serve as an additive strategy to cover the hepatocellular cancer prevention shortage of current antiviral therapy in the future. 
 The cell surface GRP78 facilitates the invasion of hepatocellular carcinoma cells. Invasion is a major characteristic of hepatocellular carcinoma and one of the main causes of refractory to treatment. We have previously reported that GRP78 promotes the invasion of hepatocellular carcinoma although the mechanism underlying this change remains uncertain. In this paper, we explored the role of the cell surface GRP78 in the regulation of cancer   cell invasion   in hepatocellular carcinoma cells. We found that neutralization of the endogenous cell surface GRP78 with the anti-GRP78 antibody inhibited the adhesion and invasion in hepatocellular carcinoma cell lines Mahlavu and SMMC7721. However, forced expression of the cell surface GRP78 facilitated the adhesion and invasion in SMMC7721. We further demonstrated that inhibition of the endogenous cell surface GRP78 specifically inhibited the secretion and activity of MMP-2 but did not affect the secretion and activity of MMP-9. We also found that inhibition of the cell surface GRP78 increased E-Cadherin expression and decreased N-Cadherin level. On the contrary, forced expression of the cell surface GRP78 increased N-Cadherin expression and decreased E-Cadherin level, suggesting that the cell surface GRP78 plays critical role in the regulation of EMT process. These findings suggest that the cell surface GRP78 plays a stimulatory role in the invasion process and may be a potential anti-invasion target for the treatment of hepatocellular carcinoma. 
 Grp78 promotes the invasion of hepatocellular carcinoma. Glucose regulated protein 78 (Grp78) is involved in the invasion and metastasis in many human cancers including gastric cancer, breast cancer, prostate cancer. But the role of Grp78 in the invasion of human hepatocellular carcinoma has not been reported. In this article, we examined if Grp78 was associated with the invasion of hepatocellular carcinoma and explored the possible underlying mechanism.The Grp78 and FAK expression levels in 44 patients with hepatocellular carcinoma were examined using immunohistochemistry. Grp78 overexpressing SMMC7721 cells were established by pcDNA3.1 (+)-Grp78 transfection and screened by G418. Grp78 and FAK levels in Grp78 overexpressing cells were down-regulated by siRNA transfection. The invasion status of tumor cells was evaluated by transwell assay in vitro, and chick embryo metastasis model in vivo. Cell spreading was determined by cell spreading assay, and quantitatively measured by Orisis software HUG. Grp78, pY397 FAK, pY576/577 FAK and FAK levels were detected by western blot. RhoA activity was detected by GST pulldown assay. The distribution of actin cytoskeleton was observed by fluorescent staining.Grp78 expression levels in 44 patients with hepatocellular carcinoma were negatively correlated with tumor grading, and positively correlated with portal invasion and intra-hepatic invasion. Overexpression of Grp78 in SMMC7721 cells promoted the invasion of cancer cells in vitro and in vivo, and this increase in tumor   cell invasion   was blocked by Grp78 siRNA knockdown. Our results also revealed that overexpression of Grp78 in SMMC7721 cells accelerated the process of cell spreading and promoted lamellipodia formation. Further analysis showed that overexpression of Grp78 in SMMC7721 cells increased pY397 and pY576/577 levels of FAK. Grp78 siRNA knockdown decreased FAK activation and activity. Our results also revealed that Grp78 overexpression in SMMC7721 cells decreased RhoA-GTP level, and Grp78 siRNA knockdown rescued RhoA-GTP level in Grp78 overexpressing cells, indicating Grp78 inhibited RhoA activity in hepatocellular carcinoma cells. Furthermore, overexpression of Grp78 in SMMC7721 cells increased phospho-p190RhoGAP level. FAK siRNA knockdown in Grp78 overexpressing cells reversed phospho-p190RhoGAP level. These data suggested that Grp78 inhibited RhoA activity by up-regulated phospho-p190RhoGAP level and Grp78 mediated p190RhoGAP phosphorylation is FAK dependent.Grp78 promoted the invasion of hepatocellular carcinoma both in vitro and in vivo. Overexpression of Grp78 in hepatocellular carcinoma cells enhanced the activation and activity of FAK which negatively regulated Rock kinase activity by promoting the phosphorylation of p190RhoGAP. 
 GRP78 promotes the invasion of pancreatic cancer cells by FAK and JNK. The major characteristics of pancreatic cancer are its excessive local invasion and early systemic dissemination. The glucose-regulated protein is over-expressed in many human cancers including pancreatic cancer and correlated with invasion and metastasis in many cancers. To investigate the effect of Grp78 on the invasion of pancreatic cancer, we used western blot and Transwell assay. We found Grp78 is expressed at lower levels in capan-2 and higher expressed in MiaPaCa-2 cells, and Grp78 expression levels were correlated with the invasion potentials of tumor cells. Then,we increased the expression of Grp78 in capan-2 cells and decreased the expression of Grp78 in MiaPaCa-2 cells. We found that over-expression of Grp78 caused significant increase in the expression of TIMP-1, TIMP-2, MMP-14, MMP-2, and MMP-9 in Capan-2 cells. Consistently, knockdown of Grp78 decreased the expression of them in MiaPaCa-2 cells. Gelatin zymography showed Grp78 over-expression stimulated the activities of MMP-2 and MMP-9, while GRP78 knockdown reduced the activities of MMP-2 and MMP-9. Cytoskeleton staining showed that knockdown of Grp78 caused a marked increase in cytoskeleton F-actin stress fibers in MiaPaCa-2 cells. Consistently, GRP78 knockdown hyperactivated RhoA and inhibited significantly Rac activity. Grp78 over-expression decreases the RhoA and stimulated Rac activity. We also found that Grp78 modulated FAK and JNK signaling pathways. Over-expression of GRP78 in Capan-2 activated FAK and JNK. Finally, we demonstrated that knockdown of FAK by shRNA in combination with blockade of JNK signaling pathway with SP600125 completely inhibited GRP78-induced cancer   cell invasion  . GRP78 is involved in the regulation of pancreatic cancer invasion. FAK and JNK are the key downstream effectors of GRP78. 
 Glucose regulated protein 78 promotes   cell invasion   via regulation of uPA production and secretion in colon cancer cells. Glucose regulated protein 78 (GRP78) is frequently highly expressed in tumor cells, contributing to the acquisition of several phenotypic cancer hallmarks. GRP78 expression is also positively correlated with tumor metastasis, and promotes hepatocellular carcinoma   cell invasion   via increasing cell motility, however, other mechanisms involving the prometastatic roles of GRP78 remain to be elucidated. Here we report that forced GRP78 expression promotes colon cancer cell migration and invasion through upregulating MMP-2, MMP-9 and especially uPA production. These effects of GRP78 are mediated by enhancing the activation of β-catenin signaling. Interestingly, we identify that GRP78 interacts with uPA both in the cells and in the culture medium, suggesting that GRP78 protein is likely to directly facilitate uPA secretion via protein-protein interaction. Taken together, our findings demonstrate for the first time that besides stimulation of cell motility, GRP78 can act by increasing proteases production to promote tumor   cell invasio  n. 
 MDA-9 and GRP78 as potential diagnostic biomarkers for early detection of melanoma metastasis. Metastatic melanoma, the primary cause of skin cancer-related death, warrants new diagnostic and therapeutic approaches that target the regulatory machinery at molecular level. The heterogeneity and complexity of melanoma result in the difficulty to find biomarkers and targets for early detection and treatment. Here, we investigated metastasis-associated proteins by comparing the proteomic profiles of primary cutaneous melanomas to their matched lymph node metastases, which minimizes heterogeneity among samples from different patients. Results of two-dimensional gel electrophoresis (2-DE) followed by proteomic analysis revealed eight differentially expressed proteins. Among them, seven proteins (α-enolase, cofilin-1, LDH, m-β-actin, Nm23, GRP78, and MDA-9) showed increased and one (annexin A2) showed decreased expression in metastatic lymph node tissues than in primary melanomas. MDA-9 and GRP78 were the most highly expressed proteins in lymph node metastases, which was validated by immunohistochemical staining. Moreover, exosomes from serum samples of metastatic melanoma patients contained higher levels of MDA-9 and GRP78 than those of patients without metastases, indicating the potential of MDA-9 and GRP78 to be biomarkers for early detection of metastasis. Further, small interfering RNA (siRNA)-mediated knockdown confirmed a functional role for MDA-9 and GRP78 to promot  e cell invasi  on in the A375 cells. Finally, we showed that GRP78 co-localized with MDA-9 in 293T cells. Taken together, our findings support MDA-9, co-expressed with GRP78, as a melanoma protein associated with lymph node metastasis. Investigating how MDA-9 and GRP78 interact to contribute to melanoma metastasis and disease progression could reveal new potential avenues of targeted therapy and/or useful biomarkers for diagnosis and prognosis. 
   
  
 GLCE 
 
  NCBI Gene  
  GeneRIF  
  Pubmed  
 
 
  Search Term  Rank  Fields  
  [Ee]xtracellular [Mm]atrix  8  PubMed(1)  
  [Mm]etastasis  12  PubMed(1)  
 
 
 hide/show details for GLCE 
  [Ee]xtracellular [Mm]atrix  PubMed  
 The human D-glucuronyl C5-epimerase gene is transcriptionally activated through the beta-catenin-TCF4 pathway. Heparan sulphate (HS) is a ubiquitous constituent of the   extracellular matrix   that is required for the biological activity of circulating soluble and insoluble extracellular ligands. GLCE (D-glucuronyl C5-epimerase), an enzyme responsible for the epimerization of D-glucuronic acid into L-iduronic acid of HS, endows the nascent polysaccharide chain with the ability to bind to growth factors and cytokines. In order to examine the mechanism of regulation of GLCE expression, the functional organization of the human GLCE gene promoter has been investigated. Studies utilizing stepwise deleted and site-directed mutagenized promoter constructs have shed light on the functional relevance of two cis-acting binding elements for the beta-catenin-TCF4 complex (where TCF4 stands for T-cell factor 4) that are located in the enhancer region of the promoter. The ability of the putative binding sequences to bind the beta-catenin-TCF4 complex has been confirmed through electrophoretic mobility-shift and supershift analyses. We have found that, in a set of human colon carcinoma cell lines, the expression of GLCE correlates with the degree of activation of the beta-catenin-TCF4 transactivation complex. Furthermore, the ectopic expression of beta-catenin-TCF4 in cells that constitutively express low levels of the transactivation complex produces a significant increase of GLCE transcript level and, at the same time, enhances the rate of D-glucuronic acid epimerization in HS. The data obtained are consistent with the idea that the beta-catenin-TCF4 transactivation pathway plays a major role in modulating GLCE expression, thus contributing to the regulation of HS biosynthesis and its structural organization. 
  [Mm]etastasis  PubMed  
 D-glucuronyl C5-epimerase cell type specifically affects angiogenesis pathway in different prostate cancer cells. D-glucuronyl C5-epimerase (GLCE) is involved in breast and lung carcinogenesis as a potential tumor suppressor gene, acting through inhibition of tumor angiogenesis and invasion/  metastasis   pathways. However, in prostate tumors, increased GLCE expression is associated with advanced disease, suggesting versatile effects of GLCE in different cancers. To investigate further the potential cancer-promoting effect of GLCE in prostate cancer, GLCE was ectopically re-expressed in morphologically different LNCaP and PC3 prostate cancer cells. Transcriptional profiles of normal PNT2 prostate cells, LNCaP, PC3 and DU145 prostate cancer cells, and GLCE-expressing LNCaP and PC3 cells were determined. Comparative analysis revealed the genes whose expression was changed in prostate cancer cells compared with normal PNT2 cells, and those differently expressed between the cancer cell lines (ACTA2, IL6, SERPINE1, TAGLN, SEMA3A, and CDH2). GLCE re-expression influenced mainly angiogenesis-involved genes (ANGPT1, SERPINE1, IGF1, PDGFB, TNF, IL8, TEK, IFNA1, and IFNB1) but in a cell type-specific manner (from basic deregulation of angiogenesis in LNCaP cells to significant activation in PC3 cells). Invasion/  metastasis   pathway was also affected (MMP1, MMP2, MMP9, S100A4, ITGA1, ITGB3, ERBB2, and FAS). The obtained results suggest activation of angiogenesis as a main molecular mechanism of pro-oncogenic effect of GLCE in prostate cancer. GLCE up-regulation plus expression pattern of a panel of six genes, discriminating morphologically different prostate cancer cell sub-types, is suggested as a potential marker of aggressive prostate cancer. 
   
  
 UGCG 
 
  NCBI Gene  
  GeneRIF  
  Pubmed  
 
 
  Search Term  Rank  Fields  
  [Mm]igration  6  PubMed(1)  
  [Ii]nvasive  10  PubMed(2)  
  [Mm]etastasis  12  PubMed(4); GeneRIFs(1)  
 
 
 hide/show details for UGCG 
  [Mm]igration  PubMed  
 Dysregulated sphingolipid metabolism in endometriosis. In endometriosis, the establishment and subsistence of ectopic lesions outside the endometrium suggest an altered cellular state for pathological hyperplasia. Sphingolipids are bioactive compounds, and their biosynthesis and metabolism modulate a range of cellular processes including proliferation,   migration   and apoptosis. We demonstrate that aberrations in sphingolipid metabolism occur in women with endometriosis.Targeted mass spectrometry on >120 sphingolipids were measured in the sera (n = 62), peritoneal fluid (n = 63), and endometrial tissue (n = 14) of women with and without endometriosis. Quantitative RT-PCR and immunohistochemistry were performed on endometrial tissues determine the expression levels of sphingolipid enzymes.Sphingolipidomics identified the in vivo accumulation of numerous sphingolipids, including the functionally antagonistic glucosylceramides and ceramides in the serum and PF of women with endometriosis. We found upregulation of specific sphingolipid enzymes, namely sphingomyelin synthase 1 (SMS1), sphingomyelinase 3 (SMPD3), and glucosylceramide synthase (GCS) in the endometrium of endometriotic women with corresponding increased GlcCer, decreased sphingomyelin levels, and decreased apoptosis in the endometrium.Our sphingolipidomics approach provided evidence of altered sphingolipid metabolism flux in serum, peritoneal fluid, and endometrial tissue in women with endometriosis. The results provide new information on how sphingolipids and eutopic endometrium may contribute to the pathophysiology of endometriosis. The results also have implications for the use of sphingolipids as potential biomarkers. 
  [Ii]nvasive  PubMed  
 Expression of glucosylceramide synthase in   invasive   ductal breast cancer may be correlated with high estrogen receptor status and low HER-2 status. Breast cancer is one of the most common causes of cancer-related deaths in women worldwide. Studies on glucosylceramide synthase (GCS) activity suggest that this enzyme has a role in the development of multidrug resistance in many cancer cells. However, few studies have shown the expression of GCS in   invasive   ductal breast cancer and breast intraductal proliferative lesions.In total, 196 samples from patients with   invasive   ductal breast cancer and 61 samples of breast intraductal proliferative lesions were collected. Immunohistochemical analyses were conducted to determine the expression of GCS and other related proteins.Expression of GCS was high in estrogen receptor (ER)-positive and HER-2 negative samples. In contrast, the expression of GCS in   invasive   ductal cancer was significantly lower than that in intraductal proliferative lesions.Our data demonstrates a correlation between the expression of the GCS protein and ER-positive/HER-2 negative breast cancer. Furthermore, in contrast to previous reports, the expression of GCS protein was shown to be much higher in ductal carcinoma in-situ than that in   invasive   ductal cancer.The virtual slide(s) for this article can be found here: http://www.diagnosticpathology.diagnomx.eu/vs/1559854430111589. 
 Doxorubicin influences the expression of glucosylceramide synthase in   invasive   ductal breast cancer. Glucosylceramide synthase (GCS) is one enzyme that provides a major route for ceramide clearance. Recent evidence has indicated an important role for GCS in multidrug resistance (MDR) tumors. Doxorubicin (DOX)can modulate the expression of GCS in leukemia and ovary cell lines. However, few studies have investigated their relationship in breast cancer;We collected 84 excision biopsies from patients with   invasive   ductal breast cancer of whom 33 patients had undergone preoperative chemotherapy. Immunohistochemistry was used to analyze the expression of GCS protein and significantly showed that the expression of GCS was higher in the samples from patients treated with preoperative chemotherapy(p = 0.018). In order to investigate the underlying mechanism, breast cancer cell lines were cultured with different concentrations of DOX, and mRNA and protein levels of GCS were then detected;DOX significantly upregulated the expression of GCS at both the mRNA and protein level in ERα-positive MCF-7 cells.We then block down the Sp1 site of GCS promoter, which inhibited the DOX-mediated increase in GCS expression; and after Erα was inhibited in MCF-7 cells, the up-regulation of GCS by DOX also been inhibited.In conclusion, our data demonstrated the novel finding that DOX could modulate the expression of GCS through the Sp1 site of GCS promoter in ERα-positive breast cancer cells. 
  [Mm]etastasis  PubMed  
 Expression and significance of glucosylceramide synthase in colorectal carcinoma tissues. To investigate the impression of glucosylceramide synthase (GCS) in colorectal carcinoma tissues.Immunohistochemical method was used to detect the expression of GCS in 188 cases of colorectal carcinoma tissues and 80 cases of non-cancerous colorectal tissues. The expression of GCS and the relation with age, gender, tumor location, Tumor stage, differentiation, lymph node   metastasis   were analyzed. Thus, we further discussed whether to accept neoadjuvant chemotherapy can affect the expression of GCS.GCS was overexpressed in colorectal carcinoma tissues compared with non-cancerous colorectal tissues; the expression of GCS was associated with lymph node   metastasis  , but not associated with age, gender, tumor location, differentiation and Tumor stage. What's more, the positive expression rate of GCS in patients who receiving neoadjuvant chemotherapy was higher than that in untreated patients.GCS was upregulated in colorectal carcinoma tissues. It might not only play an important role in controlling the generation and progression of colorectal carcinoma, but also be a factor of MDR. 
 Glucosylceramide synthase, a factor in modulating drug resistance, is overexpressed in metastatic breast carcinoma. Drug resistance causes treatment failure in approximately 50% of breast cancer patients with chemotherapy. Overexpression of glucosylceramide synthase (GCS) confers drug resistance in cancer cells, and suppression of GCS sensitizes cancers to chemotherapy in preclinical studies. Thus, GCS becomes a potential target to reverse drug resistance; however, little is known about GCS expression levels in normal tissues and whether GCS overexpression is associated with metastatic cancers. Herewith, we report our studies in GCS expression levels and breast cancer from patients. GCS levels were analyzed using cancer profiling arrays, breast cancer histo-arrays and quantitative RT-PCR in tumor tissues. We found that breast (18 exp. index) and other hormone-dependent organs (testis, cervix, ovary, prostate) displayed the lowest levels of GCS mRNA, whereas liver (52 exp. index) and other organs (kidney, bladder, stomach) displayed the highest levels of GCS. GCS mRNA levels were significantly elevated in tumors of breast, cervix, rectum and small intestine, as compared to each paired normal tissue. In mammary tissue, GCS overexpression was detected in breast cancers with   metastasis  , but not in benign fibroadenoma or primary tumors. GCS overexpression was coincident with HER2 expression (γ2=0.84) in ER-negative breast adenocarcinoma. In tumor specimens, GCS mRNA was elevated by 4-fold and significantly associated with stage III (5/7), lymph node-positive (7/8) and estrogen receptor-positive breast cancers (7/9). GCS expression was significantly and selectively elevated in breast cancer, in particular in metastatic disease. GCS overexpression was highly associated with ER-positive and HER2-positive breast cancer with   metastasi  s. Although a small study, these data suggest that GCS may be a prognostic indicator and potential target for the treatment of chemotherapy-refractory breast cancer. 
 Upregulation of glucosylceramide synthase protein in papillary thyroid carcinoma. Glucosylceramide synthase (GCS) can reduce ceramide levels and help cells escape ceramide-induced apoptosis, thus leading to multidrug resistance (MDR). However, its expression and clinical significance in thyroid neoplasms still remain unclear. We aimed to elucidate the expression of GCS and explore its correlation with the clinicopathological characteristics in papillary thyroid carcinomas (PTCs).We retrospectively investigated GCS protein expression level in tissue specimens obtained from 108 consecutive PTC patients by immunohistochemistry and Western blotting.GCS was weakly positive or negative in normal follicular cells, but it was frequently overexpressed in PTC cells. GCS overexpression was associated with primary tumor size, local infiltration, lymph node   metastasis  , and local recurrence, but not associated with gender, age, pathological variants, tumor multifocality, tumor stage or distant   metastasis  . Western blotting also showed that GCS protein levels were much higher in PTCs' tissues than in normal thyroid tissues.GCS was upregulated in PTCs and might be an independent factor affecting prognosis. 
 Up-regulation of glucosylceramide synthase in urinary bladder neoplasms. To investigate the relationships between the clinicopathologic features and the expression of GCS in bladder cancer.Using immunohistochemistry and Western blotting method, 75 bladder cancer specimens were tested for expression of GCS. The correlation of GCS with clinicopathologic features of the patients was analyzed in combination with clinical data. Statistics analyses were done with SPSS 13.0 software, χ(2) test, Fisher's exact test, Kaplan-Meier method, Log-rank test.High and low level expression of GCS explored by immunohistochemistry were 61.3 (46/75) and 39.6 (29/75), respectively. The high expression group (n = 46) showed a significant correlation with high histologic grade (P = 0.021) and tended to show (P = 0.045) that up-expression of GCS was positive related to BNs with lymph node   metastasi  s among the various clinicopathologic characteristics. The overall 5-year survival and disease-free survival rates were 39.5% and 18.4%, respectively. Mean overall survival time was 60.3 months for the low expression group and 45.1 months for the high expression group. Mean disease-free survival was 36.2 months for the low-expression group and 27.3 months for the high-expression group.Our study suggested that up-regulation of GCS might make an aggressive choice of surgical therapy. A high expression of GCS seemed to be an indicator of poor prognosis. 
  GeneRIFs  
 GCS overexpression was highly associated with ER-positive and HER2-positive breast cancer with   metastasis  . 
   
  
 GPSM2 
 
  NCBI Gene  
  GeneRIF  
  Pubmed  
 
 
  Search Term  Rank  Fields  
  [Mm]igration  6  PubMed(3); GeneRIFs(1)  
  ECM  7  PubMed(1)  
  [Ee]xtracellular [Mm]atrix  8  PubMed(1)  
  [Ii]nvasive  10  PubMed(1)  
  [Mm]etastasis  12  PubMed(1)  
 
 
 hide/show details for GPSM2 
  [Mm]igration  PubMed  
 LGN Directs Interphase Endothelial Cell Behavior via the Microtubule Network. Angiogenic sprouts require coordination of endothelial cell (EC) behaviors as they extend and branch. Microtubules influence behaviors such as cell   migration   and cell-cell interactions via regulated growth and shrinkage. Here we investigated the role of the mitotic polarity protein LGN in EC behaviors and sprouting angiogenesis. Surprisingly, reduced levels of LGN did not affect oriented division of EC within a sprout, but knockdown perturbed overall sprouting. At the cell level, LGN knockdown compromised cell-cell adhesion and   migration  . EC with reduced LGN levels also showed enhanced growth and stabilization of microtubules that correlated with perturbed   migration  . These results fit a model whereby LGN influences interphase microtubule dynamics in endothelial cells to regulate   migration  , cell adhesion, and sprout extension, and reveal a novel non-mitotic role for LGN in sprouting angiogenesis. 
 The GPSM2/LGN GoLoco motifs are essential for hearing. The planar cell polarity (PCP) pathway is responsible for polarizing and orienting cochlear hair cells during development through movement of a primary cilium, the kinocilium. GPSM2/LGN, a mitotic spindle-orienting protein associated with deafness in humans, is a PCP effector involved in kinocilium   migration  . Here, we link human and mouse truncating mutations in the GPSM2/LGN gene, both leading to hearing loss. The human variant, p.(Trp326*), was identified by targeted genomic enrichment of genes associated with deafness, followed by massively parallel sequencing. Lgn (ΔC) mice, with a targeted deletion truncating the C-terminal GoLoco motifs, are profoundly deaf and show misorientation of the hair bundle and severe malformations in stereocilia shape that deteriorates over time. Full-length protein levels are greatly reduced in mutant mice, with upregulated mRNA levels. The truncated Lgn (ΔC) allele is translated in vitro, suggesting that mutant mice may have partially functioning Lgn. Gαi and aPKC, known to function in the same pathway as Lgn, are dependent on Lgn for proper localization. The polarization of core PCP proteins is not affected in Lgn mutants; however, Lgn and Gαi are misoriented in a PCP mutant, supporting the role of Lgn as a PCP effector. The kinocilium, previously shown to be dependent on Lgn for robust localization, is essential for proper localization of Lgn, as well as Gαi and aPKC, suggesting that cilium function plays a role in positioning of apical proteins. Taken together, our data provide a mechanism for the loss of hearing found in human patients with GPSM2/LGN variants. 
 High expression of G-protein signaling modulator 2 in hepatocellular carcinoma facilitates tumor growth and metastasis by activating the PI3K/AKT signaling pathway. The aim of this study was to investigate the role of G-protein signaling modulator 2 in the carcinogenesis and progression of hepatocellular carcinoma. We previously showed that G-protein signaling modulator 2 was upregulated in hepatitis B virus-related hepatocellular carcinoma tissues through a hierarchical clustering analysis. With this study, we first assessed the expression pattern of G-protein signaling modulator 2 in hepatocellular carcinoma specimens and adjacent noncancerous tissues; clinical data were analyzed, along survival times, utilizing the Kaplan-Meier method. Moreover, the functions of G-protein signaling modulator 2 were examined using small-interfering RNAs in vitro. The results showed that G-protein signaling modulator 2 was clearly overexpressed in hepatocellular carcinoma tissues and cell lines and that the G-protein signaling modulator 2 expression level was related to tumor size and hepatitis B virus infection. Furthermore, G-protein signaling modulator 2 knockdown studies suggested that G-protein signaling modulator 2 accelerates cell growth, cell cycle,   migration  , and invasion and inhibits apoptosis, acting as an oncogene in hepatocellular carcinoma. Western blotting indicated that silencing of G-protein signaling modulator 2 in HepG2 and SMMC-7721 cells increased the expression levels of Bax, caspase-3, and E-cadherin, while notably suppressing the cyclin-dependent kinase 4, cyclin-dependent kinase 6, CyclinD1, Snail1, Vimentin, and matrix metallopeptidase 9 expression levels, compared with that in the control groups. In addition, we found that G-protein signaling modulator 2 can affect the expression of key proteins involved in protein kinase B activation. In conclusion, high expression of G-protein signaling modulator 2 was involved in the pathological processes of hepatocellular carcinoma through activation of the phosphatidylinositol 3-kinase/protein kinase B signaling pathway, which may provide an attractive potential diagnostic biomarker and therapeutic target for treatment of hepatocellular carcinoma. 
  GeneRIFs  
 results fit a model whereby LGN influences interphase microtubule dynamics in endothelial cells to regulate   migration  , cell adhesion, and sprout extension, and reveal a novel non-mitotic role for LGN in sprouting angiogenesis 
  ECM  PubMed  
 Par1b links lumen polarity with LGN-NuMA positioning for distinct epithelial cell division phenotypes. Columnar epithelia establish their luminal domains and their mitotic spindles parallel to the basal surface and undergo symmetric cell divisions in which the cleavage furrow bisects the apical domain. Hepatocyte lumina interrupt the lateral domain of neighboring cells perpendicular to two basal domains and their cleavage furrow rarely bifurcates the luminal domains. We determine that the serine/threonine kinase Par1b defines lumen position in concert with the position of the astral microtubule anchoring complex LGN-NuMA to yield the distinct epithelial division phenotypes. Par1b signaling via the extracellular matrix (  ECM  ) in polarizing cells determined RhoA/Rho-kinase activity at cell-cell contact sites. Columnar MDCK and Par1b-depleted hepatocytic HepG2 cells featured high RhoA activity that correlated with robust LGN-NuMA recruitment to the metaphase cortex, spindle alignment with the substratum, and columnar organization. Reduced RhoA activity at the metaphase cortex in HepG2 cells and Par1b-overexpressing MDCK cells correlated with a single or no LGN-NuMA crescent, tilted spindles, and the development of lateral lumen polarity. 
  [Ee]xtracellular [Mm]atrix  PubMed  
 Par1b links lumen polarity with LGN-NuMA positioning for distinct epithelial cell division phenotypes. Columnar epithelia establish their luminal domains and their mitotic spindles parallel to the basal surface and undergo symmetric cell divisions in which the cleavage furrow bisects the apical domain. Hepatocyte lumina interrupt the lateral domain of neighboring cells perpendicular to two basal domains and their cleavage furrow rarely bifurcates the luminal domains. We determine that the serine/threonine kinase Par1b defines lumen position in concert with the position of the astral microtubule anchoring complex LGN-NuMA to yield the distinct epithelial division phenotypes. Par1b signaling via the   extracellular matrix   (ECM) in polarizing cells determined RhoA/Rho-kinase activity at cell-cell contact sites. Columnar MDCK and Par1b-depleted hepatocytic HepG2 cells featured high RhoA activity that correlated with robust LGN-NuMA recruitment to the metaphase cortex, spindle alignment with the substratum, and columnar organization. Reduced RhoA activity at the metaphase cortex in HepG2 cells and Par1b-overexpressing MDCK cells correlated with a single or no LGN-NuMA crescent, tilted spindles, and the development of lateral lumen polarity. 
  [Ii]nvasive  PubMed  
 Centrosome-related genes, genetic variation, and risk of breast cancer. Centrosome amplification has been detected in premalignant lesions and in situ tumors in the breast and in over 70% of   invasive   breast tumors, and has been associated with aneuploidy and tumor development. Based on these observations, the contribution of commonly inherited genetic variation in candidate genes related to centrosome structure and function to breast cancer risk was evaluated in an association study. Seven-hundred and 82 single nucleotide polymorphisms (SNPs) from 101 centrosomal genes were analyzed in 798 breast cancer cases and 843 controls from the Mayo Clinic Breast Cancer Study to assess the association between these SNPs (both individually and combined) and risk of breast cancer in this population. Eleven SNPs out of 782 from six genes displayed associations with breast cancer risk (P < 0.01). Haplotypes in five genes also displayed significant associations with risk. A two SNP combination of rs10145182 in NIN and rs2134808 in the TUBG1 locus (P-interaction = 0.00001), suggested SNPs in mediators of microtubule nucleation from the centrosome contribute to breast cancer. Evaluation of the simultaneous significance of all SNPs in the centrosome pathway suggested that the centrosome pathway is highly enriched (P = 4.76 × 10(-50)) for SNPs that are associated with breast cancer risk. Collections of weakly associated genetic variants in the centrosome pathway, rather than individual highly significantly associated SNPs, may account for a putative role for the centrosome pathway in predisposition to breast cancer. 
  [Mm]etastasis  PubMed  
 High expression of G-protein signaling modulator 2 in hepatocellular carcinoma facilitates tumor growth and   metastasis   by activating the PI3K/AKT signaling pathway. The aim of this study was to investigate the role of G-protein signaling modulator 2 in the carcinogenesis and progression of hepatocellular carcinoma. We previously showed that G-protein signaling modulator 2 was upregulated in hepatitis B virus-related hepatocellular carcinoma tissues through a hierarchical clustering analysis. With this study, we first assessed the expression pattern of G-protein signaling modulator 2 in hepatocellular carcinoma specimens and adjacent noncancerous tissues; clinical data were analyzed, along survival times, utilizing the Kaplan-Meier method. Moreover, the functions of G-protein signaling modulator 2 were examined using small-interfering RNAs in vitro. The results showed that G-protein signaling modulator 2 was clearly overexpressed in hepatocellular carcinoma tissues and cell lines and that the G-protein signaling modulator 2 expression level was related to tumor size and hepatitis B virus infection. Furthermore, G-protein signaling modulator 2 knockdown studies suggested that G-protein signaling modulator 2 accelerates cell growth, cell cycle, migration, and invasion and inhibits apoptosis, acting as an oncogene in hepatocellular carcinoma. Western blotting indicated that silencing of G-protein signaling modulator 2 in HepG2 and SMMC-7721 cells increased the expression levels of Bax, caspase-3, and E-cadherin, while notably suppressing the cyclin-dependent kinase 4, cyclin-dependent kinase 6, CyclinD1, Snail1, Vimentin, and matrix metallopeptidase 9 expression levels, compared with that in the control groups. In addition, we found that G-protein signaling modulator 2 can affect the expression of key proteins involved in protein kinase B activation. In conclusion, high expression of G-protein signaling modulator 2 was involved in the pathological processes of hepatocellular carcinoma through activation of the phosphatidylinositol 3-kinase/protein kinase B signaling pathway, which may provide an attractive potential diagnostic biomarker and therapeutic target for treatment of hepatocellular carcinoma. 
   
  
 PIK3CD 
 
  NCBI Gene  
  GeneRIF  
  Pubmed  
 
 
  Search Term  Rank  Fields  
  [Mm]igration  6  Process(1); PubMed(12); GeneRIFs(4)  
  [Ee]xtracellular [Mm]atrix  8  PubMed(1)  
  [Ii]nvasive  10  PubMed(3)  
  [Mm]etastasis  12  PubMed(1)  
 
 
 hide/show details for PIK3CD 
  [Mm]igration  Process  
 positive regulation of cell   migration   
  PubMed  
 Phosphoinositide 3-kinase δ regulates   migratio  n and invasion of synoviocytes in rheumatoid arthritis. Cartilage destruction mediated by invasive fibroblast-like synoviocytes (FLS) plays a central role in pathogenesis of rheumatoid arthritis (RA). Increased cell   migratio  n and degradation of extracellular matrix are fundamental to these processes. The class I PI3Ks control cell survival, proliferation, and   migratio  n, which might be involved in cartilage damage in RA. PI3Kδ isoform was recently identified as a key regulator of FLS growth and survival, suggesting that it could contribute to synoviocyte aggressive behavior. Therefore, we assessed the role of PI3Kδ in RA synoviocy  te migrat  ion and invasion. We observed that PI3Kδ inhibition or small interfering RNA knockdown decreased platelet-derived growth factor (PDGF)-media  ted migra  tion and invasion of FLS. We then showed that PI3Kδ regulates the organization of actin cytoskeleton and lamellipodium formation during PDGF stimulation. To gain insight into molecular mechanisms, we examined the effect of PI3Kδ inhibition on Rac1/PAK, FAK, and JNK activation. Our studies suggest that Rac1/PAK is key target of PDGF-mediated PI3Kδ signaling, whereas FAK and JNK are not involved. Thus, PI3Kδ contributes to multiple aspects of the pathogenic FLS behavior in RA. These observations, together with previous findings that PI3Kδ regulates FLS growth and survival, suggest that PI3Kδ inhibition could be chondroprotective in RA by modulating synoviocyt  e growth,   migration, and invasion. 
 miR-30a suppresses cell   migration   and invasion through downregulation of PIK3CD in colorectal carcinoma. MicroRNAs (miRNAs) play key roles in tumor metastasis. The aim of this study was to determine the regulation and function of miR-30a in colorectal carcinoma (CRC) metastasis.The expression of miR-30a was detected in CRC cell lines and samples by qRT-PCR. The anti-metastatic effect of miR-30a was determined by both in vitro and in vivo assays. A luciferase reporter assay was performed to determine target association between miR-30a and phosphoinositide 3-kinase catalytic subunit delta (PIK3CD).miR-30a was significantly downregulated in highly metastatic CRC cell lines and metastatic tissues. Overexpression of miR-30a suppressed CRC cell   migration   and invasion in vitro and liver metastasis in vivo, whereas miR-30a deletion dramatically promoted cell   migration   and invasion. Further studies revealed that PIK3CD is a direct target of miR-30a as miR-30a bounds directly to the 3'-UTR of PIK3CD, subsequently reducing its expression. Similar to the restoring miR-30a expression, PIK3CD downregulation inhibited cell   migration   and invasion, whereas PIK3CD overexpression rescued the suppressive effect of miR-30a. Moreover, significant downregulation of miR-30a in metastatic CRC tissues was found to be inversely correlated with PIK3CD expression. Mechanistic studies revealed that miR-30a down-regulated the expression of key components of the Akt/mTOR pathway, whereas PIK3CD overexpression reversed this negative effect.Our findings indicate that miR-30a might function as a metastasis suppressor in CRC. miR-30a may be a potential therapeutic target to block CRC metastasis. 
 Phosphoinositide 3-kinase delta (PI3Kδ) in leukocyte signaling and function. PI3Kδ is a lipid kinase of the PI3K class IA family involved in early signaling events of leukocytes responding to a wide variety of stimuli. The leukocyte specificity of PI3Kδ is defined by its expression, whereas its signaling function is via the production of phosphoinositide 3,4,5-triphosphates at the proximity of activated receptors for recruiting other signaling molecules. The importance of PI3Kδ in B cell development and function is most apparent, and its role in other leukocyte cell types can be easily demonstrated as well. PI3Kδ participates in the development, activation   and migr  ation of T cells and NK cells. The role of PI3Kδ in myeloid cell activities, such as inflammation driven cell infiltration, neutrophil oxidative burst, immune complex mediated macrophage activation, as well as mast cell maturation and degranulation, has been well illustrated in various studies. As a result of the broad effects of PI3Kδ in leukocyte functions, the disruption of PI3Kδ expression or activity leads to decreased inflammatory and immune responses in vivo. The protective role of PI3Kδ inactivation in animal models of arthritis, asthma or obstructive respiratory diseases has been demonstrated. These findings suggest the potential efficacy achievable with PI3Kδ inhibitors in the treatment of autoimmune and respiratory diseases. 
 MiR-125b inhibits anaplastic thyroid cancer cell   migration   and invasion by targeting PIK3CD. Emerging evidences indicate that dysregulated microRNAs are implicated in the process of tumorigenesis and progression. The miRNA-125b (miR-125b) is downregulated and identified as tumor supressor in various cancers including thyroid cancer. However, the role and mechanism of miR-125b in anaplastic thyroid cancer (ATC)   migration   and invasion remain unknown. In the present study, the expression levels of miR-125b were downregulated and the expression levels of phosphoinositide 3-kinase catalytic subunit delta (PIK3CD) were upregulated in ATC tissues and cell lines. Moreover, miR-125b expression was negatively related to PIK3CD expression in ATC tissues. A computational search and luciferase assay identified PIK3CD as a direct target of miR-125b in ATC and PIK3CD expression was downregulated by miR-125b in ATC cells. In terms of function, miR-125b repressed   migration   and invasion of ATC cells, whereas PIK3CD overexpression reversed this effect. Furthermore, we showed that exogenous miR-125b decreased the PI3K, phospho-Akt, and phospho-mTOR expression in ATC cells. In conclusion, these results indicated that miR-125b suppressed ATC cell   migration   and invasion by targeting PIK3CD expression, and suggested novel potential therapeutic targets for the treatment of ATC. 
 Phosphoinositide 3-kinase p110 delta regulates natural antibody production, marginal zone and B-1 B cell function, and autoantibody responses. B-1 and marginal zone (MZ) B cells produce natural Abs, make Ab responses to microbial pathogens, and contribute to autoimmunity. Although the delta isoform of the PI3K p110 catalytic subunit is essential for development of these innate-like B cells, its role in the localization, activation, and function of normal B-1 and MZ B cells is not known. Using IC87114, a highly selective inhibitor of p110delta enzymatic activity, we show that p110delta is important for murine B-1 and MZ B cells to respond to BCR clustering, the TLR ligands LPS and CpG DNA, and the chemoattractants CXCL13 and sphingosine 1-phosphate. In these innate-like B cells, p110delta activity mediates BCR-, TLR- and chemoattractant-induced activation of the Akt prosurvival kinase, chemoattractant-induced   migration  , and TLR-induced proliferation. Moreover, we found that TLR-stimulated Ab responses by B-1 and MZ B cells, as well as the localization of MZ B cells in the spleen, depend on p110delta activity. Finally, we show that the in vivo production of natural Abs requires p110delta and that p110delta inhibitors can reduce in vivo autoantibody responses. Thus, targeting p110delta may be a novel approach for regulating innate-like B cells and for treating Ab-mediated autoimmune diseases. 
 MiR-125b inhibits cell biological progression of Ewing's sarcoma by suppressing the PI3K/Akt signalling pathway. Increasing evidence has suggested the close relationship between microRNAs (miRNAs) dysregulation and the carcinogenesis of Ewing's sarcoma (ES), among of which miR-125b has been reported to be decreased in ES tissues recently. Strikingly, ectopic expression of miR-125b could suppress cell proliferation of ES cell line A673, suggesting the tumor suppressor role of miR-125b in ES. However, the other accurate mechanistic functions and relative molecule mechanisms are largely unknown.Herein, we completed a series of experiments to investigate the role of miR-125b in Ewing's sarcoma. We restored the expression of miR-125b in ES cell line A673 through transfection with miR-125b mimics. To further understand the role of miR-125b in ES, we detected the effects of miR-125b on the cell proliferation,   migration   and invasion, cell cycle as well as cell apoptosis.We found that restored expression of miR-125b in ES cell line A673 inhibited cell proliferation,   migration   and invasion, arrested cell cycle progression, and induced cell apoptosis. Moreover, bioinformatic prediction suggested the oncogene, phosphoinositide-3-kinase catalytic subunit delta (PIK3CD), was a target gene of miR-125b in ES cells. Further quantitative RT-PCR and western blot assays identified over-expression of miR-125b suppressed the expression of PIK3CD mRNA and protein. PIK3CD participates in regulating the PI3K signaling pathway, which has been reported to play an important role in the development of ES. Suppression of PIK3CD down-regulated the expression of phospho-AKT and phospho-mTOR proteins and inhibited the biologic progression of A673 cells.Collectively, these data suggest that miR-125b functions as a tumor suppressor by targeting the PI3K/Akt/mTOR signaling pathway, and may provide potential therapy strategy for ES patients by targeting miRNA expression. 
 The p110delta isoform of PI 3-kinase negatively controls RhoA and PTEN. Inactivation of PI 3-kinase (PI3K) signalling is critical for tumour suppression by PTEN. This is thought to be a unidirectional relationship in which PTEN degrades the lipids produced by PI3K, thus controlling cell proliferation, survival and   migration  . We now show that this relationship is in fact bidirectional, whereby PI3K reciprocally controls PTEN. We report that the p110delta PI3K negatively regulates PTEN, through a pathway involving inhibition of RhoA. Inactivation of p110delta in macrophages led to reduced Akt and Rac1 activation, but paradoxically to increased RhoA and PTEN activity. Partial inactivation of p190RhoGAP and a reduced binding of cytoplasmic RhoA to the cyclin-dependent kinase inhibitor p27 both contributed to the increased RhoA-GTP levels upon p110delta inactivation. Pharmacological inhibition of ROCK, a downstream effector kinase of RhoA, restored all signalling and functional defects of p110delta inactivation, including Akt phosphorylation, chemotaxis and proliferation. This work identifies the RhoA/ROCK pathway as a major target of p110delta-mediated PI3K signalling, and establishes for the first time that PI3K controls itself, via a feedback loop involving PTEN. 
 HIV-1 Tat promotes monocyte chemoattractant protein-1 secretion followed by trans  migration   of monocytes. The mechanism whereby HIV-infected cells transit from the bloodstream into tissues is not well defined. This phenomenon was addressed by studying the effects of HIV-1 Tat, a protein secreted by infected cells, on human lung microvascular endothelial cells (HMVEC-Ls). It was found that monocyte chemoattractant protein-1 (MCP-1) was released from HMVEC-Ls in a dose- and time-dependent manner after Tat treatment. MCP-1 is a potent beta-chemokine that recruits monocytes and T cells and promotes cell adhesion and trans  migration   across an endothelial monolayer. It was also observed that MCP-1 and the culture medium from Tat-treated HMVEC-Ls were chemotactic for CD14(+) monocytes from human peripheral blood and for THP-1, a promonocytic cell line used as a model system. To characterize the signaling pathways underlying the observed induction of MCP-1, HMVEC-Ls were treated with 2 different protein kinase inhibitors: PD98059, a MAP kinase inhibitor, and GF109203X, a protein kinase C (PKC) inhibitor. MCP-1 release was significantly reduced when PKC was inhibited, and slightly decreased when PI3 kinase was blocked; no effect on MCP-1 release was observed on MAP kinase inhibition. Similarly, trans  migration   of THP-1 cells was significantly impaired by the PKC inhibitor, but not by the other tested inhibitors. These data indicate that the HIV-1 Tat protein may act as a protocytokine by causing the release of MCP-1 from the endothelial monolayer, and thereby facilitating monocyte trans  migration   into tissues via a PKC signaling pathway. 
 Essential role of phosphoinositide 3-kinase delta in neutrophil directional movement. Neutrophil chemotaxis is a critical component of the innate immune response. Neutrophils can sense an extremely shallow gradient of chemoattractants and produce relatively robust chemotactic behavior. This directional   migration   requires cell polarization with actin polymerization occurring predominantly in the leading edge. Synthesis of phosphatidylinositol (3,4,5) trisphosphate (PIP3) by phosphoinositide 3-kinase (PI3K) contributes to asymmetric F-actin synthesis and cell polarization during neutrophil chemotaxis. To determine the contribution of the hemopoietic cell-restricted PI3K delta in neutrophil chemotaxis, we have developed a potent and selective PI3K delta inhibitor, IC87114. IC87114 inhibited polarized morphology of neutrophils, fMLP-stimulated PIP3 production and chemotaxis. Tracking analysis of IC87114-treated neutrophils indicated that PI3K delta activity was required for the directional component of chemotaxis, but not for random movement. Inhibition of PI3K delta, however, did not block F-actin synthesis or neutrophil adhesion. These results demonstrate that PI3K delta can play a selective role in the amplification of PIP3 levels that lead to neutrophil polarization and directional   migration  . 
 Expression of functional sphingosine-1 phosphate receptor-1 is reduced by B cell receptor signaling and increased by inhibition of PI3 kinase δ but not SYK or BTK in chronic lymphocytic leukemia cells. BCR signaling pathway inhibitors such as ibrutinib, idelalisib, and fostamatinib (respective inhibitors of Bruton's tyrosine kinase, PI3Kδ, and spleen tyrosine kinase) represent a significant therapeutic advance in B cell malignancies, including chronic lymphocytic leukemia (CLL). These drugs are distinctive in increasing blood lymphocytes while simultaneously shrinking enlarged lymph nodes, suggesting anatomical redistribution of CLL cells from lymph nodes into the blood. However, the mechanisms underlying this phenomenon are incompletely understood. In this study, we showed that the egress receptor, sphingosine-1-phosphate (S1P) receptor 1 (S1PR1), was expressed at low levels in normal germinal centers and CLL lymph nodes in vivo but became upregulated on normal B cells and, to a variable and lesser extent, CLL cells following in vitro incubation in S1P-free medium. Spontaneous recovery of S1PR1 expression on normal B and CLL cells was prevented by BCR cross-linking, whereas treatment of CLL cells with idelalisib increased S1PR1 expression an  d migrati  on toward S1P, the greatest increase occurring in cases with unmutated IgH V region genes. Intriguingly, ibrutinib and fostamatinib had no effect on S1PR1 expression or function. Conversely, chemokine-induce  d migrati  on, which requires integrin activation and is essential for the entry of lymphocytes into lymph nodes as well as their retention, was blocked by ibrutinib and fostamatinib, but not idelalisib. In summary, our results suggest that different BCR signaling inhibitors redistribute CLL cells from lymph nodes into the blood through distinct mechanisms: idelalisib actively promotes egress by upregulating S1PR1, whereas fostamatinib and ibrutinib may reduce CLL cell entry and retention by suppressing chemokine-induced integrin activation. 
 The catalytic phosphoinositol 3-kinase isoform p110δ is required for glioma cell   migratio  n and invasion. Glioblastoma multiforme (GBM) is a highly invasive and aggressive primary brain tumour in which loss of phosphatase and tensin homologue deleted on chromosome 10 (PTEN), a negative regulator of PI3K signalling, is a common feature. PTEN/PI3K/Akt signalling is involved in the regulation of proliferation, apoptosis and cell   migratio  n. Deregulation of PI3K signalling is considered an essential driver in gliomagenesis. However, the role of different PI3K isoforms in glioma is still largely unclear. Here we show that the catalytic PI3K isoform p110δ is consistently expressed at a high level in various glioma cell lines. We used small interfering RNA to selectively deplete p110δ and to determine its tumourigenic roles in PTEN-deficient cells. Interestingly, knockdown of p110δ decreased the c  ell migra  tion and invasion ability of all GBM cell lines tested. Mechanistically, p110δ knockdown reduced the protein levels of focal adhesion kinase and cell division cycle 42, key regulators of cell  ular migr  ation. In contrast, pharmacologic inhibition of p110δ by IC87114 or CAL-101 also clearly impaired glioma   cell mig  ration but had no obvious effect on the invasion capacity thus pinpointing to possible kinase-dependent and -independent roles of p110δ in glioma pathology. In summary, our data provide novel evidence that in glioma cells p110δ is a key regulator of cell movement and thus may contribute to the highly invasive phenotype of GBM. Isoform specific targeting of PI3Kδ may be beneficial in the treatment of glioblastoma multiforme by specifically inhibiting tum  our cell   migration capacity. 
 Targeted inhibition of dominant PI3-kinase catalytic isoforms increase expression of stem cell genes in glioblastoma cancer stem cell models. Cancer stem cells (CSC) exhibit therapy resistance and drive self-renewal of the tumour, making cancer stem cells an important target for therapy. The PI3K signalling pathway has been the focus of considerable research effort, including in glioblastoma (GBM), a cancer that is notoriously resistant to conventional therapy. Different isoforms of the catalytic sub-unit have been associated with proliferation,   migration   and differentiation in stem cells and cancer stem cells. Blocking these processes in CSC would improve patient outcome. We examined the effect of isoform specific PI3K inhibitors in two models of GBM CSC, an established GBM stem cell line 08/04 and a neurosphere formation model. We identified the dominant catalytic PI3K isoform for each model, and inhibition of the dominant isoform blocked AKT phosphorylation, as did pan-PI3K/mTOR inhibition. Analysis of SOX2, OCT4 and MSI1 expression revealed that inhibition of the dominant p110 subunit increased expression of cancer stem cell genes, while pan-PI3K/mTOR inhibition caused a similar, though not identical, increase in cancer stem cell gene expression. This suggested that PI3K inhibition enhanced, rather than blocked, CSC activity. Careful analysis of the response to specific isoform inhibition will be necessary before specific subunit inhibitors can be successfully deployed against GBM CSC. 
  GeneRIFs  
 PI3K isoforms p110a, p110b and p110g were selectively inhibited and effects on proliferation and   migration   assessed. Inhibition increased transcription of cancer stem cell genes in both models, suggesting de-differentiation in response to blockade of the PI3K/AKT/mTOR signalling axis. 
 miR-125b suppressed anaplastic thyroid cancer cell   migration   and invasion by targeting PIK3CD expression. 
 Isoform specific targeting of PI3Kdelta may be beneficial in the treatment of glioblastoma multiforme by specifically inhibiting tumour cell   migration   capacity. 
 PI3K delta can play a selective role in the amplification of phosphatidylinositol (3,4,5) trisphosphate (PIP3) levels that lead to neutrophil polarization and regulate directional   migration  , but not random locomotion. 
  [Ee]xtracellular [Mm]atrix  PubMed  
 Phosphoinositide 3-kinase δ regulates migration and invasion of synoviocytes in rheumatoid arthritis. Cartilage destruction mediated by invasive fibroblast-like synoviocytes (FLS) plays a central role in pathogenesis of rheumatoid arthritis (RA). Increased cell migration and degradation of   extracellular matri  x are fundamental to these processes. The class I PI3Ks control cell survival, proliferation, and migration, which might be involved in cartilage damage in RA. PI3Kδ isoform was recently identified as a key regulator of FLS growth and survival, suggesting that it could contribute to synoviocyte aggressive behavior. Therefore, we assessed the role of PI3Kδ in RA synoviocyte migration and invasion. We observed that PI3Kδ inhibition or small interfering RNA knockdown decreased platelet-derived growth factor (PDGF)-mediated migration and invasion of FLS. We then showed that PI3Kδ regulates the organization of actin cytoskeleton and lamellipodium formation during PDGF stimulation. To gain insight into molecular mechanisms, we examined the effect of PI3Kδ inhibition on Rac1/PAK, FAK, and JNK activation. Our studies suggest that Rac1/PAK is key target of PDGF-mediated PI3Kδ signaling, whereas FAK and JNK are not involved. Thus, PI3Kδ contributes to multiple aspects of the pathogenic FLS behavior in RA. These observations, together with previous findings that PI3Kδ regulates FLS growth and survival, suggest that PI3Kδ inhibition could be chondroprotective in RA by modulating synoviocyte growth, migration, and invasion. 
  [Ii]nvasive  PubMed  
 Phosphoinositide 3-kinase δ regulates migration and invasion of synoviocytes in rheumatoid arthritis. Cartilage destruction mediated by   invasiv  e fibroblast-like synoviocytes (FLS) plays a central role in pathogenesis of rheumatoid arthritis (RA). Increased cell migration and degradation of extracellular matrix are fundamental to these processes. The class I PI3Ks control cell survival, proliferation, and migration, which might be involved in cartilage damage in RA. PI3Kδ isoform was recently identified as a key regulator of FLS growth and survival, suggesting that it could contribute to synoviocyte aggressive behavior. Therefore, we assessed the role of PI3Kδ in RA synoviocyte migration and invasion. We observed that PI3Kδ inhibition or small interfering RNA knockdown decreased platelet-derived growth factor (PDGF)-mediated migration and invasion of FLS. We then showed that PI3Kδ regulates the organization of actin cytoskeleton and lamellipodium formation during PDGF stimulation. To gain insight into molecular mechanisms, we examined the effect of PI3Kδ inhibition on Rac1/PAK, FAK, and JNK activation. Our studies suggest that Rac1/PAK is key target of PDGF-mediated PI3Kδ signaling, whereas FAK and JNK are not involved. Thus, PI3Kδ contributes to multiple aspects of the pathogenic FLS behavior in RA. These observations, together with previous findings that PI3Kδ regulates FLS growth and survival, suggest that PI3Kδ inhibition could be chondroprotective in RA by modulating synoviocyte growth, migration, and invasion. 
 p37δ is a new isoform of PI3K p110δ that increases cell proliferation and is overexpressed in tumors. The phosphatidylinositol 3-kinases (PI3Ks) regulate cell growth, proliferation and survival, and are frequently affected in human cancer. PI3K is composed of a catalytic subunit, p110, and a regulatory subunit, p85. The PI3K catalytic subunit p110δ is encoded by PIK3CD and contains p85- and RAS-binding domains, and a kinase domain. Here we present an alternatively spliced PIK3CD transcript encoding a previously unknown protein, p37δ, and demonstrate that this protein is expressed in human ovarian and colorectal tumors. p37δ retains the p85-binding domain and a fraction of the RAS-binding domain, lacks the catalytic domain, and has a unique carboxyl-terminal region. In contrast to p110δ, which stabilizes p85, p37δ promoted p85 sequestering. Despite the truncated RAS-binding domain, p37δ bound to RAS and we found a strong positive correlation between the protein levels of p37δ and RAS. Overexpressing p37δ, but not p110δ, increased the prolife  ration a  nd invasive properties of HEK-293 cells and mouse embryonic fibroblasts. Cells overexpressing p37δ showed a quicker phosphorylation response of AKT and ERK1/2 following serum stimulation. Ubiquitous expression of human p37δ in the fruit fly increased body size, DNA content and phosphorylated ERK1/2 levels. Thus, p37δ appears to be a new tumor-specific isoform of p110δ with growth-promoting properties. 
 The catalytic phosphoinositol 3-kinase isoform p110δ is required for glioma cell migration and invasion. Glioblastoma multiforme (GBM) is a highly   invasiv  e and aggressive primary brain tumour in which loss of phosphatase and tensin homologue deleted on chromosome 10 (PTEN), a negative regulator of PI3K signalling, is a common feature. PTEN/PI3K/Akt signalling is involved in the regulation of proliferation, apoptosis and cell migration. Deregulation of PI3K signalling is considered an essential driver in gliomagenesis. However, the role of different PI3K isoforms in glioma is still largely unclear. Here we show that the catalytic PI3K isoform p110δ is consistently expressed at a high level in various glioma cell lines. We used small interfering RNA to selectively deplete p110δ and to determine its tumourigenic roles in PTEN-deficient cells. Interestingly, knockdown of p110δ decreased the cell migration and invasion ability of all GBM cell lines tested. Mechanistically, p110δ knockdown reduced the protein levels of focal adhesion kinase and cell division cycle 42, key regulators of cellular migration. In contrast, pharmacologic inhibition of p110δ by IC87114 or CAL-101 also clearly impaired glioma cell migration but had no obvious effect on the invasion capacity thus pinpointing to possible kinase-dependent and -independent roles of p110δ in glioma pathology. In summary, our data provide novel evidence that in glioma cells p110δ is a key regulator of cell movement and thus may contribute to the   highly   invasive phenotype of GBM. Isoform specific targeting of PI3Kδ may be beneficial in the treatment of glioblastoma multiforme by specifically inhibiting tumour cell migration capacity. 
  [Mm]etastasis  PubMed  
 miR-30a suppresses cell migration and invasion through downregulation of PIK3CD in colorectal carcinoma. MicroRNAs (miRNAs) play key roles in tumor   metastasis  . The aim of this study was to determine the regulation and function of miR-30a in colorectal carcinoma (CRC)   metastasis  .The expression of miR-30a was detected in CRC cell lines and samples by qRT-PCR. The anti-metastatic effect of miR-30a was determined by both in vitro and in vivo assays. A luciferase reporter assay was performed to determine target association between miR-30a and phosphoinositide 3-kinase catalytic subunit delta (PIK3CD).miR-30a was significantly downregulated in highly metastatic CRC cell lines and metastatic tissues. Overexpression of miR-30a suppressed CRC cell migration and invasion in vitro and liver   metastasis   in vivo, whereas miR-30a deletion dramatically promoted cell migration and invasion. Further studies revealed that PIK3CD is a direct target of miR-30a as miR-30a bounds directly to the 3'-UTR of PIK3CD, subsequently reducing its expression. Similar to the restoring miR-30a expression, PIK3CD downregulation inhibited cell migration and invasion, whereas PIK3CD overexpression rescued the suppressive effect of miR-30a. Moreover, significant downregulation of miR-30a in metastatic CRC tissues was found to be inversely correlated with PIK3CD expression. Mechanistic studies revealed that miR-30a down-regulated the expression of key components of the Akt/mTOR pathway, whereas PIK3CD overexpression reversed this negative effect.Our findings indicate that miR-30a might function as a   metastasis   suppressor in CRC. miR-30a may be a potential therapeutic target to block CRC   metastasis  . 
   
  
 VTA1 
 
  NCBI Gene  
  GeneRIF  
  Pubmed  
 
 
  Search Term  Rank  Fields  
  [Ee]xtracellular [Mm]atrix  8  PubMed(1)  
 
 
 hide/show details for VTA1 
  [Ee]xtracellular [Mm]atrix  PubMed  
 Proteomic analysis of human parotid gland exosomes by multidimensional protein identification technology (MudPIT). Human ductal saliva contributes over a thousand unique proteins to whole oral fluids. The mechanism by which most of these proteins are secreted by salivary glands remains to be determined. The present study used a mass spectrometry-based, shotgun proteomics approach to explore the possibility that a subset of the proteins found in saliva are derived from exosomes, membrane-bound vesicles of endosomal origin within multivesicular endosomes. Using MudPIT (multidimensional protein identification technology) mass spectrometry, we catalogued 491 proteins in the exosome fraction of human parotid saliva. Many of these proteins were previously observed in ductal saliva from parotid glands (265 proteins). Furthermore, 72 of the proteins in parotid exosomes overlap with those previously identified as urinary exosome proteins, proteins which are also frequently associated with exosomes from other tissues and cell types. Gene Ontology (GO) and KEGG pathway analyses found that cytosolic proteins comprise the largest category of proteins in parotid exosomes (43%), involved in such processes as phosphatidylinositol signaling system, calcium signaling pathway, inositol metabolism, protein export, and signal transduction, among others; whereas the integral plasma membrane proteins and associated/peripheral plasma membrane proteins (26%) were associated with   extracellular matrix  -receptor interaction, epithelial cell signaling, T-cell and B-cell receptor signaling, cytokine receptor interaction, and antigen processing and presentation, among other biological functions. In addition, these putative saliva exosomal proteins were linked to specific diseases (e.g., neurodegenerative disorders, prion disease, cancers, type I and II diabetes). Consequently, parotid glands secrete exosomes that reflect the metabolic and functional status of the gland and may also carry informative protein markers useful in the diagnosis and treatment of systemic diseases. 
   
  
 TLN2 
 
  NCBI Gene  
  GeneRIF  
  Pubmed  
 
 
  Search Term  Rank  Fields  
  [Mm]igration  6  PubMed(3); Summary(1)  
  [Ee]xtracellular [Mm]atrix  8  PubMed(5)  
  [Ii]nvasive  10  GeneRIFs(1)  
  [Mm]etastasis  12  PubMed(1)  
 
 
 hide/show details for TLN2 
  [Mm]igration  PubMed  
 Analysis of the myosin-II-responsive focal adhesion proteome reveals a role for β-Pix in negative regulation of focal adhesion maturation. Focal adhesions undergo myosin-II-mediated maturation wherein they grow and change composition to modulate integrin signalling for cell   migratio  n, growth and differentiation. To determine how focal adhesion composition is affected by myosin II activity, we performed proteomic analysis of isolated focal adhesions and compared protein abundance in focal adhesions from cells with and without myosin II inhibition. We identified 905 focal adhesion proteins, 459 of which changed in abundance with myosin II inhibition, defining the myosin-II-responsive focal adhesion proteome. The abundance of 73% of the proteins in the myosin-II-responsive focal adhesion proteome was enhanced by contractility, including proteins involved in Rho-mediated focal adhesion maturation and endocytosis- and calpain-dependent focal adhesion disassembly. During myosin II inhibition, 27% of proteins in the myosin-II-responsive focal adhesion proteome, including proteins involved in Rac-mediated lamellipodial protrusion, were enriched in focal adhesions, establishing that focal adhesion protein recruitment is also negatively regulated by contractility. We focused on the Rac guanine nucleotide exchange factor β-Pix, documenting its role in the negative regulation of focal adhesion maturation and the promotion of lamellipodial protrusion and focal adhesion turnover to drive cel  l migrati  on. 
 Cytoskeletal proteins talin and vinculin in integrin-mediated adhesion. The cytoskeletal proteins talin and vinculin form part of a macromolecular complex on the cytoplasmic face of integrin-mediated cellular junctions with the extracellular matrix. Recent genetic, biochemical and structural data show that talin is essential for the assembly of such junctions, whereas vinculin appears to be important in regulating adhesion dynamics and cell   migration  . 
 Both Talin-1 and Talin-2 correlate with malignancy potential of the human hepatocellular carcinoma MHCC-97 L cell. Talin-1 (TLN-1) and TLN-2 are implicated in many cellular processes, but their roles in hepatocellular carcinoma (HCC) remain unclear. This study aimed to assess cell cycle distribution, anoikis, invasion and   migration   in human HCC MHCC-97 L cells.MHCC-97 L cells, which highly express TLN-1, were transduced with TLN-1 shRNA (experimental group) or scramble shRNA (negative control group); non-transduced MHCC-97 L cells were used as blank controls. TLN-1 and TLN-2 mRNA and protein levels were detected by real-time RT-PCR and western blot, respectively. Then, cell cycle distribution and anoikis were assessed by flow cytometry. In addition,   migration   and invasion abilities were assessed using Transwell and cell scratch assays. Finally, a xenograft nude mouse model was established to further assess cell tumorigenicity.Compared with the blank and negative control groups, TLN-1/2 mRNA and protein levels were significantly reduced in the experiment group. TLN-1/2 knockdown cells showed significantly more cells in the G0/G1 phase (79.24%) in comparison with both blank (65.36%) and negative (62.69%) control groups; conversely, less cells were found in G2/M and S phases in the experimental group compared with controls. Moreover, anoikis was enhanced (P < 0.05), while invasion and   migration   abilities were reduced (P < 0.05) in TLN-1/2 knockdown cells compared with controls. TLN-1/2 knockdown inhibited MHCC-97 L cell   migration   (Percentage of wound healing area: experimental group: 32.6 ± 0.7% vs. negative controls: 50.1 ± 0.6% and blank controls: 53.6 ± 0.6%, both P < 0.01). Finally, the tumors obtained with TLN-1/2 knockdown cells were smaller (P < 0.05) compared with controls.Both TLN-1 and TLN-2 levels correlate with tumorigenicity in human HCC, indicating that these molecules constitute important molecular targets for the diagnosis and/or treatment of HCC. 
  Summary  
 This gene encodes a protein related to talin 1, a cytoskeletal protein that plays a significant role in the assembly of actin filaments and in spreading and   migration   of various cell types, including fibroblasts and osteoclasts. This protein has a different pattern of expression compared to talin 1 but, like talin 1, is thought to associate with unique transmembrane receptors to form novel linkages between extracellular matrices and the actin cytoskeleton. [provided by RefSeq, Jul 2008] 
  [Ee]xtracellular [Mm]atrix  PubMed  
 Structural diversity in integrin/talin interactions. The adhesion of integrins to the   extracellular matrix   is regulated by binding of the cytoskeletal protein talin to the cytoplasmic tail of the β-integrin subunit. Structural studies of this interaction have hitherto largely focused on the β3-integrin, one member of the large and diverse integrin family. Here, we employ NMR to probe interactions and dynamics, revealing marked structural diversity in the contacts between β1A, β1D, and β3 tails and the Talin1 and Talin2 isoforms. Coupled with analysis of recent structures of talin/β tail complexes, these studies elucidate the thermodynamic determinants of this heterogeneity and explain why the Talin2/β1D isoforms, which are co-localized in striated muscle, form an unusually tight interaction. We also show that talin/integrin affinity can be enhanced 1000-fold by deleting two residues in the β tail. Together, these studies illustrate how the integrin/talin interaction has been fine-tuned to meet varying biological requirements. 
 Structural diversity in integrin/talin interactions. The adhesion of integrins to the   extracellular matrix   is regulated by binding of the cytoskeletal protein talin to the cytoplasmic tail of the β-integrin subunit. Structural studies of this interaction have hitherto largely focused on the β3-integrin, one member of the large and diverse integrin family. Here, we employ NMR to probe interactions and dynamics, revealing marked structural diversity in the contacts between β1A, β1D, and β3 tails and the Talin1 and Talin2 isoforms. Coupled with analysis of recent structures of talin/β tail complexes, these studies elucidate the thermodynamic determinants of this heterogeneity and explain why the Talin2/β1D isoforms, which are co-localized in striated muscle, form an unusually tight interaction. We also show that talin/integrin affinity can be enhanced 1000-fold by deleting two residues in the β tail. Together, these studies illustrate how the integrin/talin interaction has been fine-tuned to meet varying biological requirements. 
 Cytoskeletal proteins talin and vinculin in integrin-mediated adhesion. The cytoskeletal proteins talin and vinculin form part of a macromolecular complex on the cytoplasmic face of integrin-mediated cellular junctions with the   extracellular matrix  . Recent genetic, biochemical and structural data show that talin is essential for the assembly of such junctions, whereas vinculin appears to be important in regulating adhesion dynamics and cell migration. 
 Integrin-mediated cell adhesion: the cytoskeletal connection. Members of the integrin family of cell adhesion molecules play a pivotal role in the interaction between animal cells and the   extracellular matrix  . This article reviews the evidence (i) that the integrin beta-subunit cytoplasmic domain is important in the localization of integrins to focal adhesions, and for integrin-mediated cell adhesion/spreading; and (ii) that the integrin beta-subunit can be linked to F-actin via the actin-binding proteins talin, alpha-actinin and filamin. Talin has two or more actin-binding sites, and three binding sites for the cytoskeletal protein vinculin. Because vinculin can also bind F-actin, it may cross-link talin and actin, thereby stabilizing the interaction. In addition, vinculin contains a binding site for VASP (vasodilator-stimulated phospho-protein), a protein which may serve to recruit a profilin/G-actin complex to talin, which has actin-nucleating activity. Evidence that talin, vinculin and alpha-actinin are important in the assembly of focal adhesions, obtained using antisense technology and protein microinjection, is reviewed. To analyse the role of talin in focal adhesions, we have disrupted both copies of the talin gene in mouse embryonic stem (ES) cells. Undifferentiated talin (-/-) ES cell mutants are unable to assemble focal adhesions when plated on fibronectin, whereas vinculin (-/-) ES cells are able to do so. Finally, the role of small GTP-binding proteins in the assembly of focal adhesions is discussed, along with our recent studies using streptolysin-O-permeabilized Swiss 3T3 cells which suggest that the GTP-binding protein ADP-ribosylation factor-1 (ARF-1) is important in targeting the protein paxillin to focal adhesions. 
 Type I gamma phosphatidylinositol phosphate kinase targets and regulates focal adhesions. The ability of cells to form cell contacts, adhere to the   extracellular matrix  , change morphology, and migrate is essential for development, wound healing, metastasis, cell survival and the immune response. These events depend on the binding of integrin to the   extracellular matrix  , and assembly of focal adhesions, which are complexes comprising scaffolding and signalling proteins organized by adhesion to the   extracellular matrix  . Phosphatidylinositol-4,5-bisphosphate (PtdIns(4,5)P(2)) regulates interactions between these proteins, including the interaction of vinculin with actin and talin. The binding of talin to beta-integrin is strengthened by PtdIns(4,5)P(2), suggesting that the basis of focal adhesion assembly is regulated by this lipid mediator. Here we show that the type I phosphatidylinositol phosphate kinase isoform-gamma 661 (PIPKI gamma 661), an enzyme that makes PtdIns(4,5)P(2), is targeted to focal adhesions by an association with talin. PIPKI gamma 661 is tyrosine phosphorylated by focal adhesion associated kinase signalling, increasing both the activity of phosphatidylinositol phosphate kinase and its association with talin. This defines a mechanism for spatial generation of PtdIns(4,5)P(2) at focal adhesions. 
  [Ii]nvasive  GeneRIFs  
 Data show that SCGB3A1 was down-regulated in   invasive   compared with DCIS, whereas talin 2 (TLN2) and PTGS1 were up-regulated in   invasive   compared with DCIS. 
  [Mm]etastasis  PubMed  
 Type I gamma phosphatidylinositol phosphate kinase targets and regulates focal adhesions. The ability of cells to form cell contacts, adhere to the extracellular matrix, change morphology, and migrate is essential for development, wound healing,   metastasis  , cell survival and the immune response. These events depend on the binding of integrin to the extracellular matrix, and assembly of focal adhesions, which are complexes comprising scaffolding and signalling proteins organized by adhesion to the extracellular matrix. Phosphatidylinositol-4,5-bisphosphate (PtdIns(4,5)P(2)) regulates interactions between these proteins, including the interaction of vinculin with actin and talin. The binding of talin to beta-integrin is strengthened by PtdIns(4,5)P(2), suggesting that the basis of focal adhesion assembly is regulated by this lipid mediator. Here we show that the type I phosphatidylinositol phosphate kinase isoform-gamma 661 (PIPKI gamma 661), an enzyme that makes PtdIns(4,5)P(2), is targeted to focal adhesions by an association with talin. PIPKI gamma 661 is tyrosine phosphorylated by focal adhesion associated kinase signalling, increasing both the activity of phosphatidylinositol phosphate kinase and its association with talin. This defines a mechanism for spatial generation of PtdIns(4,5)P(2) at focal adhesions. 
   
  
 BMPR1B 
 
  NCBI Gene  
  GeneRIF  
  Pubmed  
 
 
  Search Term  Rank  Fields  
  [Mm]igration  6  PubMed(3)  
  [Ii]nvasive  10  PubMed(1)  
  [Mm]etastasis  12  PubMed(7)  
  [Cc]ell [Ii]nvasion  13  PubMed(2)  
 
 
 hide/show details for BMPR1B 
  [Mm]igration  PubMed  
 The protein kinase LKB1 negatively regulates bone morphogenetic protein receptor signaling. The protein kinase LKB1 regulates cell metabolism and growth and is implicated in intestinal and lung cancer. Bone morphogenetic protein (BMP) signaling regulates cell differentiation during development and tissue homeostasis. We demonstrate that LKB1 physically interacts with BMP type I receptors and requires Smad7 to promote downregulation of the receptor. Accordingly, LKB1 suppresses BMP-induced osteoblast differentiation and affects BMP signaling in Drosophila wing longitudinal vein morphogenesis. LKB1 protein expression and Smad1 phosphorylation analysis in a cohort of non-small cell lung cancer patients demonstrated a negative correlation predominantly in a subset enriched in adenocarcinomas. Lung cancer patient data analysis indicated strong correlation between LKB1 loss-of-function mutations and high BMP2 expression, and these two events further correlated with expression of a gene subset functionally linked to apoptosis and   migration  . This new mechanism of BMP receptor regulation by LKB1 has ramifications in physiological organogenesis and disease. 
 BMPR1B up-regulation via a miRNA binding site variation defines endometriosis susceptibility and CA125 levels. Bone morphogenetic protein receptor I B (BMPR1B) is a transmembrane receptor mediating TGF-β signal transduction. Recent studies indicate a tumor suppressor role for BMPR1B in ovarian cancer. Polymorphism at BMPR1B 3'UTR within the miR-125b binding site alters its binding affinity toward the miRNA, which may result in insufficient post-transcriptional repression.Single-nucleotide polymorphisms rs1970801, rs1434536, and rs11097457 near the miR-125b binding site in BMPR1B were genotyped by Taqman assay on 193 endometriosis patients and 202 healthy controls. BMPR1B and CA125 levels in ectopic endometrial tissues were evaluated by quantitative PCR and immunohistochemistry. Luciferase reporter assay was utilized to verify regulatory roles of BMPR1B 3'UTR with allelic variants of rs1434536 in a cell line model. Cell proliferation and   migratio  n were recorded, while expression of BMPR1B, CA125, glucocorticoid receptor (GCCR) and IL-1β were measured by quantitative PCR in endometrial cells transfected with wild-type or mutated miR-125b.This study found two endometriosis-associated SNPs, rs1434536 (P = 0.010) and rs1970801 (P = 0.0087), located within and next to a miR-125b binding site on BMPR1B. Interestingly, patients with homozygous variant alleles at rs1434536 showed significantly lower serum CA125 levels. Immunohistochemistry staining further confirmed inverse correlation between BMPR1B and CA125 levels in three rs1434536 genotypes. Cell assays demonstrated the variant allele of rs1434536 up-regulating BMPR1B at both mRNA and protein levels, which negatively correlated with CA125 and IL-1β levels. Disruption of the binding between miR-125b and BMPR1B hampered abnormal cell proliferation.SNPs of BMPR1B within and next to the miR-125b binding site manifested strong correlation with endometriosis development in a Taiwanese cohort. Disrupting the binding of miR-125b toward BMPR1B would increase protein expression, diminishing abnormal cell proliferation as well as serum and cellular CA125 levels. Genetic variation at the miR-125b binding site may play functional roles to protect against endometriosis progression. 
 SB-431542 is a potent and specific inhibitor of transforming growth factor-beta superfamily type I activin receptor-like kinase (ALK) receptors ALK4, ALK5, and ALK7. Small molecule inhibitors have proven extremely useful for investigating signal transduction pathways and have the potential for development into therapeutics for inhibiting signal transduction pathways whose activities contribute to human diseases. Transforming growth factor beta (TGF-beta) is a member of a large family of pleiotropic cytokines that are involved in many biological processes, including growth control, differentiation,   migration  , cell survival, adhesion, and specification of developmental fate, in both normal and diseased states. TGF-beta superfamily members signal through a receptor complex comprising a type II and type I receptor, both serine/threonine kinases. Here, we characterize a small molecule inhibitor (SB-431542) that was identified as an inhibitor of activin receptor-like kinase (ALK)5 (the TGF-beta type I receptor). We demonstrate that it inhibits ALK5 and also the activin type I receptor ALK4 and the nodal type I receptor ALK7, which are very highly related to ALK5 in their kinase domains. It has no effect on the other, more divergent ALK family members that recognize bone morphogenetic proteins (BMPs). Consistent with this, we demonstrate that SB-431542 is a selective inhibitor of endogenous activin and TGF-beta signaling but has no effect on BMP signaling. To demonstrate the specificity of SB-431542, we tested its effect on several other signal transduction pathways whose activities depend on the concerted activation of multiple kinases. SB-431542 has no effect on components of the ERK, JNK, or p38 MAP kinase pathways or on components of the signaling pathways activated in response to serum. 
  [Ii]nvasive  PubMed  
 Low expression of BMPRIB indicates poor prognosis of breast cancer and is insensitive to taxane-anthracycline chemotherapy. Bone morphogenetic protein receptor type IB (BMPRIB) is one osteogenesis factor, which function in breast cancer has been rarely explored until recently. In the clinical study presented here, involving a cohort of 368   invasive   ductal carcinoma (IDC) patients, we identified that patients with low expression of BMPRIB exhibited poor prognosis, especially in the luminal B subtype. We also provided the first piece of evidence that low level of BMPRIB was a promoting factor for breast cancer patients to develop bone metastasis, but not lung, liver or brain. The first of its kind, we reported that patients with high expression of BMPRIB exhibited favorable prognosis by a retrospective analysis consisting of 168 patients treated with TE (taxane and anthracycline) regimens. And the patients with high expression of BMPRIB were more sensitive to TE regimens in the detection of 32 paired pre-neoadjuvant and post-neoadjuvant specimens. Overall, our study concluded that low expression of BMPRIB indicated poor prognosis of breast cancer and was insensitive to taxane-anthracycline chemotherapy. Our findings also lay a foundation to help clinicians improve identification of patients for TE regimens by BMPRIB in the era of precision medicine. 
  [Mm]etastasis  PubMed  
 Mechanical regulation of cancer cell apoptosis and autophagy: roles of bone morphogenetic protein receptor, Smad1/5, and p38 MAPK. Mechanical forces induced by interstitial fluid flow in and surrounding tissues and by blood/lymphatic flow in vessels may modulate cancer cell invasion and   metastasis   and anticancer drug delivery. Our previous study demonstrated that laminar flow-induced shear stress induces G2/M arrest in tumor cells. However, whether shear stress modulates final cell fate remains unclear. In this study, we investigated the role of flow-induced shear stress in modulating the survival of four human tumor cell lines, i.e., Hep3B hepatocarcinoma cells, MG63 osteosarcoma cells, SCC25 oral squamous carcinoma cells, and A549 carcinomic alveolar basal epithelial cells. Laminar shear stress (LSS) ranging from 0.5 to 12dyn/cm(2) induced death of these four tumor cell lines. In contrast to LSS at 0.5dyn/cm(2), oscillatory shear stress (OSS) at 0.5±4dyn/cm(2) cannot induce cancer cell death. Both LSS and OSS had no effect on human normal hepatocyte, lung epithelial, and endothelial cells. Application of LSS to these four cell lines increased the percentage of cells stained positively for annexin V-FITC, with up-regulations of cleaved caspase-8, -9, and -3, and PARP. In addition, LSS also induced Hep3B cell autophagy, as detected by acidic vesicular organelle formation, LC3B transformation, and p62/SQSTM1 degradation. By transfecting with small interfering RNA, we found that the shear-induced apoptosis and autophagy are mediated by bone morphogenetic protein receptor type (BMPR)-IB, BMPR-specific Smad1 and Smad5, and p38 mitogen-activated protein kinase in Hep3B cells. Our findings provide insights into the molecular mechanisms by which shear stress induces apoptosis and autophagy in tumor cells. 
 Mechanical regulation of cancer cell apoptosis and autophagy: roles of bone morphogenetic protein receptor, Smad1/5, and p38 MAPK. Mechanical forces induced by interstitial fluid flow in and surrounding tissues and by blood/lymphatic flow in vessels may modulate cancer cell invasion and   metastasis   and anticancer drug delivery. Our previous study demonstrated that laminar flow-induced shear stress induces G2/M arrest in tumor cells. However, whether shear stress modulates final cell fate remains unclear. In this study, we investigated the role of flow-induced shear stress in modulating the survival of four human tumor cell lines, i.e., Hep3B hepatocarcinoma cells, MG63 osteosarcoma cells, SCC25 oral squamous carcinoma cells, and A549 carcinomic alveolar basal epithelial cells. Laminar shear stress (LSS) ranging from 0.5 to 12dyn/cm(2) induced death of these four tumor cell lines. In contrast to LSS at 0.5dyn/cm(2), oscillatory shear stress (OSS) at 0.5±4dyn/cm(2) cannot induce cancer cell death. Both LSS and OSS had no effect on human normal hepatocyte, lung epithelial, and endothelial cells. Application of LSS to these four cell lines increased the percentage of cells stained positively for annexin V-FITC, with up-regulations of cleaved caspase-8, -9, and -3, and PARP. In addition, LSS also induced Hep3B cell autophagy, as detected by acidic vesicular organelle formation, LC3B transformation, and p62/SQSTM1 degradation. By transfecting with small interfering RNA, we found that the shear-induced apoptosis and autophagy are mediated by bone morphogenetic protein receptor type (BMPR)-IB, BMPR-specific Smad1 and Smad5, and p38 mitogen-activated protein kinase in Hep3B cells. Our findings provide insights into the molecular mechanisms by which shear stress induces apoptosis and autophagy in tumor cells. 
 Hepatocyte growth factor up-regulates the expression of the bone morphogenetic protein (BMP) receptors, BMPR-IB and BMPR-II, in human prostate cancer cells. Hepatocyte growth factor (HGF) plays multiple roles in cancer, by acting as a motility, invasion and angiogenesis stimulating factor, which promotes   metastasis   and tumour growth. Bone morphogenetic proteins (BMPs) are members of the TGF-beta superfamily. The effects of BMPs are mediated by two subgroups of receptors, type I and type II. Recent studies have shown that some BMPs, via their signaling pathways, affect the growth of prostate cancer cells. BMPR-IB and BMPR-II have been reported to be expressed at low levels in prostate cancer. However, little is known about the crosstalk between HGF and BMP pathways. In this study, prostate cancer cells (PC-3 and DU-145) were exposed to HGF at different concentrations (1-75 ng/ml) for 18 h, or were treated with HGF at 40 ng/ml over various time periods (up to 24 h). The effect of HGF on BMP receptor expression was further investigated in a nude mouse PC-3 xenograft model. Mice were treated with either HGF, the HGF antagonist NK4, or a combination of both. The expression of BMPR-IB and BMPR-II mRNA was up-regulated by HGF, as shown by both conventional PCR and quantitative PCR. An elevation of BMPR-IB and BMPR-II at the protein level was confirmed by both Western blot analysis and immunocytochemical staining. In a murine prostate tumour model, infusion of recombinant HGF resulted in an increase in the levels of both BMPR-IB and BMPR-II transcript in prostate tumours. Concomitant delivery of NK4, an HGF antagonist, prevented this effect. In conclusion, HGF up-regulates the expression of the bone morphogenetic protein receptors, BMPR-IB and BMPR-II, in prostate cancer cells, both in vitro and in vivo. This may have important implications in the development of bone   metastasis   in prostate cancer. 
 Reduced expression of BMPR-IB correlates with poor prognosis and increased proliferation of breast cancer cells. Breast cancer is the leading female cancer in the UK. Recent work has shown that Bone Morphogenetic Proteins (BMPs) and their receptors may be involved in the progression of breast cancer. The aim of the current study is to identify the role of BMPR-IB, one of these receptors, in breast cancer.Expression of BMPR-IB was examined in a cohort of breast tissue samples. The transcript level was determined using quantitative real time-PCR and protein levels were assessed with immunohistochemical staining (IHC). Constructed ribozyme transgenes were used to knock-down BMPR-IB in MDA-MB-231 cells, and the effect this had on in vitro cell growth was examined.Decreased staining of BMPR-IB was seen in most of the breast tumour samples examined compared to normal breast tissues. Q-PCR analysis revealed lower expression levels of BMPR-IB transcript in patient samples with a predicted poor prognosis (Nottingham prognosis index (NPI) >5.4) (8.2+/-17, p=0.03) compared to samples with a good prognosis (NPI <3.4) (469+/-244). Higher BMPR-IB expression was seen in samples from disease free patients compared to those with poorer prognosis; including patients with   metastasis  , local recurrence and breast cancer deaths, (p=0.026). Furthermore, down-regulation of BMPR-IB in MDA-MB-231cells led to a promotion of in vitro cell growth.This study shows that decreased expression of BMPR-IB correlates with poor prognosis in breast cancer patients and leads to increased cell proliferation of breast cancer cells in vitro. This suggests that BMPR-IB mediates an inhibitory effect on breast cancer cells. 
 Low expression of BMPRIB indicates poor prognosis of breast cancer and is insensitive to taxane-anthracycline chemotherapy. Bone morphogenetic protein receptor type IB (BMPRIB) is one osteogenesis factor, which function in breast cancer has been rarely explored until recently. In the clinical study presented here, involving a cohort of 368 invasive ductal carcinoma (IDC) patients, we identified that patients with low expression of BMPRIB exhibited poor prognosis, especially in the luminal B subtype. We also provided the first piece of evidence that low level of BMPRIB was a promoting factor for breast cancer patients to develop bone   metastasis  , but not lung, liver or brain. The first of its kind, we reported that patients with high expression of BMPRIB exhibited favorable prognosis by a retrospective analysis consisting of 168 patients treated with TE (taxane and anthracycline) regimens. And the patients with high expression of BMPRIB were more sensitive to TE regimens in the detection of 32 paired pre-neoadjuvant and post-neoadjuvant specimens. Overall, our study concluded that low expression of BMPRIB indicated poor prognosis of breast cancer and was insensitive to taxane-anthracycline chemotherapy. Our findings also lay a foundation to help clinicians improve identification of patients for TE regimens by BMPRIB in the era of precision medicine. 
 Cloning of human bone morphogenetic protein type IB receptor (BMPR-IB) and its expression in prostate cancer in comparison with other BMPRs. Bone   metastasis   is a common event in prostate cancer, and it is known that some of the bone morphogenetic proteins (BMPs) are expressed in prostate cancer cells, while no study on the expression of their receptors, BMPRs, has been reported. Here we report cloning and sequence analysis of the human BMPR-IB cDNA. We also analysed the expression of transcripts of three types of the BMPR genes in human tissues and prostate cancer cell lines. The BMPR-IB mRNA was present in various organs, but the highest level was found in the prostate. Moreover, the amount of BMPR-IB mRNA was significantly low in prostate cancer tissues after androgen withdrawal and was also low in prostate cancer cell lines. RT-PCR analysis showed that the BMPR-IB message was upregulated by androgen stimulation in the LNCaP cell line which expresses the androgen receptor. By contrast, the mRNA levels of BMPR-IA and BMPR-II were not significantly different among non-cancerous and cancerous prostate tissues. It was also suggested that human BMPR-IA and BMPR-IB might have different biological functions in the prostate, although their sequences were 85.3% identical in the serine-threonine kinase domain. 
 Expression of bone morphogenetic protein receptors type-IA, -IB and -II correlates with tumor grade in human prostate cancer tissues. Bone morphogenetic proteins (BMPs) are potential regulators of prostate cancer cell growth and   metastasis   that signal through an interaction with BMP membrane receptors (BMPRs) type I and type II. In the present study, Western blot and immunohistochemical analysis of BMPRs were carried out in benign and malignant human prostate tissues to explain the loss of BMP response in human prostate cancer cells. The results demonstrated that the benign prostate specimens expressed high levels of all three BMPRs. In normal prostate, BMPRs were localized predominantly to epithelial cells. Among prostate cancer specimens, well-differentiated cancers were positive for the expression of BMPR-II, BMPR-IA, and BMPR-IB, for the most part. In contrast, only 1 of 10 poorly differentiated prostate cancer cases was positive for each of the three BMPRs (P < 0.005 for all three receptors). Taken together, these results indicate that human prostate cancer cells frequently exhibit loss of expression of BMPRs and suggest that loss of BMPRs may play an important role during the progression of prostate cancer. 
  [Cc]ell [Ii]nvasion  PubMed  
 Mechanical regulation of cancer cell apoptosis and autophagy: roles of bone morphogenetic protein receptor, Smad1/5, and p38 MAPK. Mechanical forces induced by interstitial fluid flow in and surrounding tissues and by blood/lymphatic flow in vessels may modulate cancer   cell invasion   and metastasis and anticancer drug delivery. Our previous study demonstrated that laminar flow-induced shear stress induces G2/M arrest in tumor cells. However, whether shear stress modulates final cell fate remains unclear. In this study, we investigated the role of flow-induced shear stress in modulating the survival of four human tumor cell lines, i.e., Hep3B hepatocarcinoma cells, MG63 osteosarcoma cells, SCC25 oral squamous carcinoma cells, and A549 carcinomic alveolar basal epithelial cells. Laminar shear stress (LSS) ranging from 0.5 to 12dyn/cm(2) induced death of these four tumor cell lines. In contrast to LSS at 0.5dyn/cm(2), oscillatory shear stress (OSS) at 0.5±4dyn/cm(2) cannot induce cancer cell death. Both LSS and OSS had no effect on human normal hepatocyte, lung epithelial, and endothelial cells. Application of LSS to these four cell lines increased the percentage of cells stained positively for annexin V-FITC, with up-regulations of cleaved caspase-8, -9, and -3, and PARP. In addition, LSS also induced Hep3B cell autophagy, as detected by acidic vesicular organelle formation, LC3B transformation, and p62/SQSTM1 degradation. By transfecting with small interfering RNA, we found that the shear-induced apoptosis and autophagy are mediated by bone morphogenetic protein receptor type (BMPR)-IB, BMPR-specific Smad1 and Smad5, and p38 mitogen-activated protein kinase in Hep3B cells. Our findings provide insights into the molecular mechanisms by which shear stress induces apoptosis and autophagy in tumor cells. 
 Mechanical regulation of cancer cell apoptosis and autophagy: roles of bone morphogenetic protein receptor, Smad1/5, and p38 MAPK. Mechanical forces induced by interstitial fluid flow in and surrounding tissues and by blood/lymphatic flow in vessels may modulate cancer   cell invasion   and metastasis and anticancer drug delivery. Our previous study demonstrated that laminar flow-induced shear stress induces G2/M arrest in tumor cells. However, whether shear stress modulates final cell fate remains unclear. In this study, we investigated the role of flow-induced shear stress in modulating the survival of four human tumor cell lines, i.e., Hep3B hepatocarcinoma cells, MG63 osteosarcoma cells, SCC25 oral squamous carcinoma cells, and A549 carcinomic alveolar basal epithelial cells. Laminar shear stress (LSS) ranging from 0.5 to 12dyn/cm(2) induced death of these four tumor cell lines. In contrast to LSS at 0.5dyn/cm(2), oscillatory shear stress (OSS) at 0.5±4dyn/cm(2) cannot induce cancer cell death. Both LSS and OSS had no effect on human normal hepatocyte, lung epithelial, and endothelial cells. Application of LSS to these four cell lines increased the percentage of cells stained positively for annexin V-FITC, with up-regulations of cleaved caspase-8, -9, and -3, and PARP. In addition, LSS also induced Hep3B cell autophagy, as detected by acidic vesicular organelle formation, LC3B transformation, and p62/SQSTM1 degradation. By transfecting with small interfering RNA, we found that the shear-induced apoptosis and autophagy are mediated by bone morphogenetic protein receptor type (BMPR)-IB, BMPR-specific Smad1 and Smad5, and p38 mitogen-activated protein kinase in Hep3B cells. Our findings provide insights into the molecular mechanisms by which shear stress induces apoptosis and autophagy in tumor cells. 
   
  
 LIMS1 
 
  NCBI Gene  
  GeneRIF  
  Pubmed  
 
 
  Search Term  Rank  Fields  
  [Mm]igration  6  PubMed(13); GeneRIFs(1)  
  ECM  7  PubMed(5)  
  [Ee]xtracellular [Mm]atrix  8  PubMed(12)  
  [Ii]nvasive  10  PubMed(6); GeneRIFs(1)  
  [Mm]etastasis  12  PubMed(5); GeneRIFs(2)  
 
 
 hide/show details for LIMS1 
  [Mm]igration  PubMed  
 Analysis of the myosin-II-responsive focal adhesion proteome reveals a role for β-Pix in negative regulation of focal adhesion maturation. Focal adhesions undergo myosin-II-mediated maturation wherein they grow and change composition to modulate integrin signalling for cell   migratio  n, growth and differentiation. To determine how focal adhesion composition is affected by myosin II activity, we performed proteomic analysis of isolated focal adhesions and compared protein abundance in focal adhesions from cells with and without myosin II inhibition. We identified 905 focal adhesion proteins, 459 of which changed in abundance with myosin II inhibition, defining the myosin-II-responsive focal adhesion proteome. The abundance of 73% of the proteins in the myosin-II-responsive focal adhesion proteome was enhanced by contractility, including proteins involved in Rho-mediated focal adhesion maturation and endocytosis- and calpain-dependent focal adhesion disassembly. During myosin II inhibition, 27% of proteins in the myosin-II-responsive focal adhesion proteome, including proteins involved in Rac-mediated lamellipodial protrusion, were enriched in focal adhesions, establishing that focal adhesion protein recruitment is also negatively regulated by contractility. We focused on the Rac guanine nucleotide exchange factor β-Pix, documenting its role in the negative regulation of focal adhesion maturation and the promotion of lamellipodial protrusion and focal adhesion turnover to drive cel  l migrati  on. 
 Structure of an ultraweak protein-protein complex and its crucial role in regulation of cell morphology and motility. Weak protein-protein interactions (PPIs) (K(D) > 10(-6) M) are critical determinants of many biological processes. However, in contrast to a large growing number of well-characterized, strong PPIs, the weak PPIs, especially those with K(D) > 10(-4) M, are poorly explored. Genome wide, there exist few 3D structures of weak PPIs with K(D) > 10(-4) M, and none with K(D) > 10(-3) M. Here, we report the NMR structure of an extremely weak focal adhesion complex (K(D) approximately 3 x 10(-3) M) between Nck-2 SH3 domain and PINCH-1 LIM4 domain. The structure exhibits a remarkably small and polar interface with distinct binding modes for both SH3 and LIM domains. Such an interface suggests a transient Nck-2/PINCH-1 association process that may trigger rapid focal adhesion turnover during integrin signaling. Genetic rescue experiments demonstrate that this interface is indeed involved in mediating cell shape change and   migration  . Together, the data provide a molecular basis for an ultraweak PPI in regulating focal adhesion dynamics during integrin signaling. 
 Thymosin beta4 activates integrin-linked kinase and promotes cardiac cell   migration  , survival and cardiac repair. Heart disease is a leading cause of death in newborn children and in adults. Efforts to promote cardiac repair through the use of stem cells hold promise but typically involve isolation and introduction of progenitor cells. Here, we show that the G-actin sequestering peptide thymosin beta4 promotes myocardial and endothelial cell   migration   in the embryonic heart and retains this property in postnatal cardiomyocytes. Survival of embryonic and postnatal cardiomyocytes in culture was also enhanced by thymosin beta4. We found that thymosin beta4 formed a functional complex with PINCH and integrin-linked kinase (ILK), resulting in activation of the survival kinase Akt (also known as protein kinase B). After coronary artery ligation in mice, thymosin beta4 treatment resulted in upregulation of ILK and Akt activity in the heart, enhanced early myocyte survival and improved cardiac function. These findings suggest that thymosin beta4 promotes cardiomyocyte   migration  , survival and repair and the pathway it regulates may be a new therapeutic target in the setting of acute myocardial damage. 
 Identification of PINCH in Schwann cells and DRG neurons: shuttling and signaling after nerve injury. Particularly interesting new cysteine-histidine rich protein (PINCH) is a double zinc finger domain (LIM)-only adapter protein that functions to recruit the integrin-linked kinase (ILK) to sites of integrin activation. Genetic studies have shown that PINCH and ILK are required for integrin signaling. Since integrin activation is associated with Schwann cell   migration  , neurite outgrowth and regeneration, this study examined PINCH in the normal peripheral nervous system and after chronic constriction injury (CCI) in adult Sprague-Dawley rats. Immunohistochemistry identified PINCH immunoreactivity in cell bodies of dorsal root ganglia (DRG) neurons, axons, satellite cells, and Schwann cells. PINCH immunostaining was localized to the membrane of uninjured DRG cell bodies consistent with its localization at a site of integrin activation. In contrast, 5 days following CCI, PINCH immunostaining was diffuse throughout the DRG cell cytoplasm. Confocal microscopy of primary and transformed Schwann cells localized PINCH in cytoplasmic, perinuclear and nuclear areas. Examination of the PINCH sequence revealed a putative leucine-rich nuclear export signal (NES) and an overlapping basic nuclear localization signal (NLS). To demonstrate nuclear export of PINCH, rabbit anti-PINCH IgG was microinjected into Schwann cell nuclei and allowed to combine with PINCH contained within the nucleus. Immunofluorescence showed that the PINCH and anti-PINCH IgG complex rapidly translocated to the cytoplasm. Treatment with leptomycin B caused nuclear accumulation of PINCH, indicating that the CRM1 pathway mediates nuclear export of PINCH. ILK activity in Schwann cells was enhanced by platelet-derived growth factor (PDGF) and tumor necrosis factor alpha. PINCH immunoprecipitates from PDGF- and TNFalpha-stimulated Schwann cells contained several high-molecular-weight threonine-phosphorylated proteins. Taken together, these results indicate that PINCH is an abundant shuttling/signaling protein in Schwann cells and DRG neurons. 
 Particularly interesting cysteine- and histidine-rich protein in cardiac development and remodeling. Integrin-mediated cell-extracellular matrix interaction plays key roles in tissue morphogenesis and integrity. The Lin11-Isl-1-Mec-3 (LIM) domain-only particularly interesting cysteine- and histidine-rich (PINCH) protein functions as an adaptor essential for the assembly and function of the focal adhesion complex that links integrin signaling to the cytoskeleton and other intracellular signaling pathways and regulates diverse cellular processes such as cell adhesion,   migration  , growth, differentiation, and survival. Recent biochemical and genetic studies have greatly advanced our knowledge surrounding the molecular interactions and functions of each component of the focal adhesion complex and revealed a requirement for PINCH in early embryogenesis, in morphogenesis of the neural crest and cardiac outflow, and in myocardial growth and remodeling. In this review article, we will provide an overview of the current knowledge of the molecular interactions of PINCH with other components of focal adhesions, highlighting recent discoveries of the in vivo role of PINCH and discuss its potential implication for human heart disease. 
 PINCH1 is transcriptional regulator in podocytes that interacts with WT1 and represses podocalyxin expression. PINCH1, an adaptor protein containing five LIM domains, plays an important role in regulating the integrin-mediated cell adhesion,   migration   and epithelial-mesenchymal transition. PINCH1 is induced in the fibrotic kidney after injury, and it primarily localizes at the sites of focal adhesion. Whether it can translocate to the nucleus and directly participate in gene regulation is completely unknown.Using cultured glomerular podocytes as a model system, we show that PINCH1 expression was induced by TGF-β1, a fibrogenic cytokine that promotes podocyte dysfunction. Interestingly, increased PINCH1 not only localized at the sites of focal adhesions, but also underwent nuclear translocation after TGF-β1 stimulation. This nuclear translocation of PINCH1 was apparently dependent on the putative nuclear export/localization signals (NES/NLS) at its C-terminus, as deletion or site-directed mutations abolished its nuclear shuttling. Co-immunoprecipitation and pull-down experiments revealed that PINCH1 interacted with Wilms tumor 1 protein (WT1), a nuclear transcription factor that is essential for regulating podocyte-specific gene expression in adult kidney. Interaction of PINCH1 and WT1 was mediated by the LIM1 domain of PINCH1 and C-terminal zinc-finger domain of WT1, which led to the suppression of the WT1-mediated podocalyxin expression in podocytes. PINCH1 also repressed podocalyxin gene transcription in a promoter-luciferase reporter assay.These results indicate that PINCH1 can shuttle into the nucleus from cytoplasm in podocytes, wherein it interacts with WT1 and suppresses podocyte-specific gene expression. Our studies reveal a previously unrecognized, novel function of PINCH1, in which it acts as a transcriptional regulator through controlling specific gene expression. 
 The structural basis of integrin-linked kinase-PINCH interactions. The heterotrimeric complex between integrin-linked kinase (ILK), PINCH, and parvin is an essential signaling platform, serving as a convergence point for integrin and growth-factor signaling and regulating cell adhesion, spreading, and   migration  . We report a 1.6-A crystal structure of the ILK ankyrin repeat domain bound to the PINCH1 LIM1 domain, revealing the molecular basis of ILK-PINCH interactions and providing a structural description of this region of ILK. This structure identifies 5 ankyrin repeats in ILK, explains previous deletion mutagenesis data, permits identification of ILK and PINCH1 point mutations that disrupt the interaction, shows how zincs are coordinated by PINCH1 LIM1, and suggests that conformational flexibility and twisting between the 2 zinc fingers within the LIM1 domain may be important for ILK binding. These data provide an atomic-resolution description of a key interaction in the ILK-PINCH-parvin scaffolding complex. 
 Signaling via PINCH: Functions, binding partners and implications in human diseases. Particularly interesting new cysteine-histidine-rich protein (PINCH) is a LIM-domain-only adaptor that plays important roles in cytoskeletal organization and extracellular matrix adhesion,   migration  , proliferation and survival. Mammalian cells have two functional PINCH proteins, PINCH1 and PINCH2. PINCH not only binds to Nck2 and engages in the signaling of growth factor receptors, but also forms a ternary complex with ILK and parvin (IPP complex). Normally, the IPP complex locates to focal adhesions participating in the signaling of integrins and mediating the interaction of cytoskeleton and extracellular matrix (ECM). Accumulative evidence indicates that abnormalities in PINCH signaling are involved in the pathogenesis of important diseases, such as cancers, renal diseases, cardiomyopathy, and HIV. Therefore, clarifying the functions of PINCH and its interactions with key factors is important for better understanding of signaling events both in health and disease. 
 Molecular dissection of PINCH-1 reveals a mechanism of coupling and uncoupling of cell shape modulation and survival. How cells couple and uncouple regulation of cellular processes such as shape change and survival is an important question in molecular cell biology. PINCH-1, a widely expressed protein consisting of five LIM domains and a C-terminal tail, is an essential focal adhesion protein with multiple functions including regulation of the integrin-linked kinase (ILK) level, cell shape, and survival signaling. We show here that the LIM1-mediated interaction with ILK regulates all these three processes. By contrast, the LIM4-mediated interaction with Nck-2, which regulates cell morphology and   migration  , is not required for the control of the ILK level and survival. Remarkably, a short 15-residue tail C-terminal to LIM5 is required for both cell shape modulation and survival, albeit it is not required for the control of the ILK level. The C-terminal tail not only regulates PINCH-1 localization to focal adhesions but also functions after it localizes there. These findings suggest that PINCH-1 functions as a molecular platform for coupling and uncoupling diverse cellular processes via overlapping but yet distinct domain interactions. 
 Prognostic significance of PINCH signalling in human pancreatic ductal adenocarcinoma. Prognostic markers for pancreatic ductal adenocarcinoma (PDA) have failed to accurately predict patient prognosis. Recently, interest has developed in the accuracy of integrin-associated PINCH protein expression in human cancers as a predictive marker of tumour status. The goal of this study was to define the expression of PINCH protein in PDA.Human PDA samples and orthotopic tumours from a murine model were analysed by immunohistochemistry for PINCH expression. In the animal model, PINCH expression was compared between primary and metastatic tumours. In the human samples, PINCH expression was correlated with stage, nodal involvement, margin status and overall survival.In the murine model, there was greater PINCH expression in metastatic tumours than in primary tumours. In the human PDA samples, greater staining for PINCH in the tumour cells was correlated with higher T status. Additionally, high PINCH expression in the stroma was associated with decreased overall survival.Findings of increased PINCH protein in more advanced stages of human PDA, as well as in metastatic tumours in the animal model, support the hypothesis that PINCH is an important controller of cell survival and   migration  . Additionally, the importance of the differential expression of PINCH in the human tumour and stroma warrants further evaluation. 
 Structural basis of focal adhesion localization of LIM-only adaptor PINCH by integrin-linked kinase. The LIM-only adaptor PINCH (the particularly interesting cysteine- and histidine-rich protein) plays a pivotal role in the assembly of focal adhesions (FAs), supramolecular complexes that transmit mechanical and biochemical information between extracellular matrix and actin cytoskeleton, regulating diverse cell adhesive processes such as cell   migration  , cell spreading, and survival. A key step for the PINCH function is its localization to FAs, which depends critically on the tight binding of PINCH to integrin-linked kinase (ILK). Here we report the solution NMR structure of the core ILK.PINCH complex (28 kDa, K(D) approximately 68 nm) involving the N-terminal ankyrin repeat domain (ARD) of ILK and the first LIM domain (LIM1) of PINCH. We show that the ILK ARD exhibits five sequentially stacked ankyrin repeat units, which provide a large concave surface to grip the two contiguous zinc fingers of the PINCH LIM1. The highly electrostatic interface is evolutionally conserved but differs drastically from those of known ARD and LIM bound to other types of protein domains. Consistently mutation of a hot spot in LIM1, which is not conserved in other LIM domains, disrupted the PINCH binding to ILK and abolished the PINCH targeting to FAs. These data provide atomic insight into a novel modular recognition and demonstrate how PINCH is specifically recruited by ILK to mediate the FA assembly and cell-extracellular matrix communication. 
 The Rsu-1-PINCH1-ILK complex is regulated by Ras activation in tumor cells. The link between Ras transformation and enhanced cell   migration   due to altered integrin signaling is well established in tumorigenesis, however there remain gaps in our understanding of its mechanism. The Ras suppressor, Rsu-1, has recently been linked to the IPP (integrin-linked kinase {ILK}, PINCH-1/LIMS1, parvin) focal adhesion complex based on its interaction with the LIM 5 domain of PINCH1. Defining the role of the Rsu1-PINCH1-ILK-parvin complex in tumorigenesis is important because both ILK and PINCH1 are elevated in certain tumors while ectopic expression of Rsu-1 blocks tumorigenesis. Our studies previously identified an alternatively spliced isoform of Rsu-1 in high-grade gliomas. We report here the detection of a truncated (p29) Rsu-1 protein, which correlates with the presence of the alternatively spliced Rsu-1 RNA. This RNA and the respective protein were detected in human tumor cell lines that contain high levels of activated Ras, and inhibitor studies demonstrate that the Mek-ERK pathway regulates expression of this truncated Rsu-1 product. We also show that Rsu-1 co-localizes with ILK at focal contacts and co-immunoprecipitates with the ILK-PINCH1 complex in non-transformed cells, but following Ras transformation the association of Rsu-1 with the PINCH1-ILK complex is greatly reduced. Using a human breast cancer cell line, our in vitro studies demonstrate that the depletion of Rsu-1 full-length protein enhances cell   migration   coincident with an increase in Rac-GTP while the depletion of the p29 Rsu-1 truncated protein inhibits   migration  . These findings indicate that Rsu-1 may inhibit cell   migration   by stabilizing the IPP adhesion complex and that Ras activation perturbs this inhibitory function by modulating both Rsu-1 splicing and association of full-length Rsu-1 with IPP. Hence, our findings demonstrate that Rsu-1 links the Ras pathway with the IPP complex and the perturbations of cell attachment-dependent signaling that occur in the malignant process. 
 Pinch-1 was up-regulated in leukemia BMSC and its possible effect. Pinch-1, a widely expressed focal adhesion protein, has been demonstrated to be up-regulated in multiple solid tumor-associated stromal cells, particularly at invasive edges. It was supposed that Pinch-1 was intimately associated with development and progression of tumors. The expression of Pinch-1 in hematopoietic microenvironment in patients with leukemia remains unclear. This study focused on the expression of Pinch-1 in bone marrow stromal cells (BMSCs) from leukemia patients and its possible effect. BMSC was isolated and cultured from bone marrow in leukemia patients and normal healthy donors. RT-PCR and Western blot analysis were performed to determine Pinch-1 mRNA and protein level in BMSC, respectively. Lentiviral vector containing Pinch-1 siRNA was constructed, and the recombinant lentivirus particle was packaged in 293 cells. Effectiveness of Pinch-1 siRNA was determined by Western blot. The proliferation, apoptosis and motility of leukemia BMSC subjected to Pinch-1 knockdown using siRNA were tested by flow cytometry, TUNEL assay and Transwell system, respectively. Pinch-1 mRNA and protein were significantly up-regulated in ALL and AML BMSC compared to normal BMSC (p<0.01). Although there was no difference in Pinch-1 mRNA between ALL and AML BMSC, cellular levels of Pinch-1 protein in ALL BMSC were significantly higher than that in AML BMSC (p<0.01). Overexpressed Pinch-1 was significantly reduced in leukemia BMSC transfected with Pinch-1 siRNA evidenced by Western blot. Flow cytometry analysis showed that the percentage of cells in S+G2 phases in leukemia BMSC transfected with Pinch-1 siRNA was significantly lower than control (p<0.01). The percentage of apoptotic cells in leukemia BMSC transfected with Pinch-1 siRNA was 19.8±1.0%, significantly higher than controls (p<0.01). The number of leukemia BMSC transfected with Pinch-1 siRNA that migrated to the lower chamber after culturing for 24 h was 8.4±1.1 per field, significantly lower than controls (p<0.01). Pinch-1 mRNA and protein in leukemia BMSC were up-regulated drastically compared with BMSC from healthy donors. Leukemia BMSC displayed hypoproliferation, decrease  d migrati  on and increased apoptosis after transfecting Pinch-1 siRNA. 
  GeneRIFs  
 Findings of increased PINCH protein in more advanced stages of human pancreatic ductal adenocarcinoma, as well as in metastatic tumours in the animal model, support the hypothesis that PINCH is an important controller of cell survival and   migration  . 
  ECM  PubMed  
 The roles of two distinct regions of PINCH-1 in the regulation of cell attachment and spreading. Cells attach to the extracellular matrix (  ECM  ) through integrins to form focal adhesion complexes, and this process is followed by the extension of lamellipodia to enable cell spreading. PINCH-1, an adaptor protein essential for the regulation of cell-  ECM   adhesion, consists of five tandem LIM domains and a small C-terminal region. PINCH-1 is known to interact with integrin-linked kinase (ILK) and Ras suppressor protein 1 (Rsu-1); however, the precise mechanism by which this complex regulates cell-  ECM   adhesion is not fully understood. We report here that the LIM1 domain of PINCH-1, which associates with ILK to stabilize the expression of this protein, is sufficient for cell attachment but not for cell spreading. In contrast, the C-terminal region of PINCH-1, which binds to Rsu-1, plays a pivotal role in cell spreading but not in cell attachment. We also show that PINCH-1 associates with Rsu-1 to activate Rac1 and that Rac1 activation is necessary for cell spreading. Thus, these data reveal how specific domains of PINCH-1 direct two independent pathways: one utilizing ILK to allow cell attachment, and the other recruiting Rsu-1 to activate Rac1 in order to promote cell spreading. 
 Ras suppressor-1 promotes apoptosis in breast cancer cells by inhibiting PINCH-1 and activating p53-upregulated-modulator of apoptosis (PUMA); verification from metastatic breast cancer human samples. Metastasis, responsible for most deaths from breast cancer (BC), is a multistep process leading to cancer cell spread. Extracellular matrix (  ECM  )-related adhesion and apoptosis resistance play pivotal role in metastasis. Ras suppressor-1 (RSU-1) localizes to cell-  ECM   adhesions and binds to pro-survival adhesion protein PINCH-1. Little is known about the role of RSU-1 in BC. In the present study, we investigated the role of RSU-1 in BC metastasis using two BC cell lines that differ in terms of their metastatic potential and a set of 32 human BC samples from patients with or without lymph node metastasis. We show that RSU-1 is upregulated in the aggressive MDA-MB-231 cells compared to MCF-7 and that its silencing by siRNA leads to upregulation of PINCH-1, induction of proliferation and reduction of apoptosis through downregulation of the pro-apoptotic gene p53-upregulated-modulator-of-apoptosis (PUMA). Our findings in the cell lines were further validated in the human BC tissues where normal adjacent tissues were used as controls. We demonstrate for the first time, that RSU-1 expression is upregulated in metastatic BC samples and downregulated in non-metastatic while it is negatively correlated with PINCH-1 and positively correlated with PUMA expression, suggesting that a pro-apoptotic mechanism is in place in metastatic BC samples and identifying RSU-1 as a potentially interesting molecule that needs to be evaluated further as a novel BC metastasis biomarker. 
 Signaling via PINCH: Functions, binding partners and implications in human diseases. Particularly interesting new cysteine-histidine-rich protein (PINCH) is a LIM-domain-only adaptor that plays important roles in cytoskeletal organization and extracellular matrix adhesion, migration, proliferation and survival. Mammalian cells have two functional PINCH proteins, PINCH1 and PINCH2. PINCH not only binds to Nck2 and engages in the signaling of growth factor receptors, but also forms a ternary complex with ILK and parvin (IPP complex). Normally, the IPP complex locates to focal adhesions participating in the signaling of integrins and mediating the interaction of cytoskeleton and extracellular matrix (  ECM  ). Accumulative evidence indicates that abnormalities in PINCH signaling are involved in the pathogenesis of important diseases, such as cancers, renal diseases, cardiomyopathy, and HIV. Therefore, clarifying the functions of PINCH and its interactions with key factors is important for better understanding of signaling events both in health and disease. 
 PINCH mRNA overexpression in colorectal carcinomas correlated with VEGF and FAS mRNA expression. Particularly interesting new cysteine-histidine-rich protein (PINCH) was found to be up-regulated in the stroma of colorectal carcinomas (CRCs) in our previous studies and was involved in angiogenesis through activation of fibroblasts in extracellular matrix (  ECM  ) in response to tumors. Here, we examined PINCH mRNA expression in colorectal cancer and investigated its relationship with the clinicopathological features and proliferation cell nuclear antigen (PCNA), vascular endothelial growth factor (VEGF) and FAS.The primary cancer tissues, adjacent noncancerous tissues and the proximal and distant margins of normal mucosa were collected from 81 colorectal cancer patients during surgery. PINCH, PCNA, VEGF and FAS mRNA expression was examined by reverse transcriptional PCR (RT-PCR).PINCH mRNA expression was significantly increased in primary tumors compared with that in adjacent noncancerous tissues, and the proximal and distant margins of normal mucosa (p<0.0001). Expression of PINCH mRNA in colon cancer tended to be higher than expression in rectal cancer (p=0.051). Tumors which had infiltrated through the wall of the colorectum trended to have higher PINCH mRNA expression (p=0.073). PINCH mRNA expression in primary tumors was positively related to the expression of PCNA mRNA (r=0.534, p=0.010), VEGF mRNA (r=0.431, p=0.022) and FAS mRNA (r=0.542, p=0.012).PINCH mRNA was overexpressed in colorectal cancer and associated with PCNA mRNA, VEGF mRNA and FAS mRNA expression. PINCH mRNA was involved in the development of colorectal cancer and might play a role in the epithelial mesenchymal transition in the rectum differently than in the colon, through the adenomatous polyposis coli (APC)/catenin pathway. 
 Targeting of the EGFR/β1 integrin connecting proteins PINCH1 and Nck2 radiosensitizes three-dimensional SCC cell cultures. Epidermal growth factor receptor (EGFR) signaling plays an important role in tumor cell resistance to therapy. In addition to ligand binding, mutual and cooperative interactions of EGFR with integrin cell adhesion receptors critically influence proper downstream signaling through a number of bridging adapter proteins. In the present study, we analyzed the role of two of these adapter proteins, called PINCH1 and Nck2, for cellular radioresistance in combination with EGFR-targeting using the monoclonal antibody cetuximab. siRNA-mediated knockdown of PINCH1 or Nck2 resulted in enhanced radiosensitivity of 3D grown human squamous cell carcinoma cell lines FaDu (head and neck) and A431 (epidermis) comparable with effects seen after cetuximab treatment. Combination of knockdown and cetuximab did not result in additive nor synergistic effects regarding clonogenic radiation survival. Modifications in MAPK, Akt and FAK phosphorylation occurred upon cetuximab treatment as well as PINCH1 or Nck2 depletion. We further found this tumor cell radiosensitization to be due to attenuated repair of DNA double strand breaks and altered Rad50 and Nbs1 expression but without changes in other DNA repair proteins such as ATM, DNA-PK and Mre11. Our data suggest that the adaptor proteins PINCH1 and Nck2 critically contribute to cellular radioresistance and proper EGFR signaling in 3D l  rEC  M grown human squamous cell carcinoma cells. Further investigations are warranted to identify the intracellular signaling network controlled by EGFR, PINCH1 and Nck2. 
  [Ee]xtracellular [Mm]atrix  PubMed  
 Integrin-linked kinase is localized to cell-matrix focal adhesions but not cell-cell adhesion sites and the focal adhesion localization of integrin-linked kinase is regulated by the PINCH-binding ANK repeats. Integrin-linked kinase (ILK) is a ubiquitously expressed protein serine/threonine kinase that has been implicated in integrin-, growth factor- and Wnt-signaling pathways. In this study, we show that ILK is a constituent of cell-matrix focal adhesions. ILK was recruited to focal adhesions in all types of cells examined upon adhesion to a variety of   extracellular matrix   proteins. By contrast, ILK was absent in E-cadherin-mediated cell-cell adherens junctions. In previous studies, we have identified PINCH, a protein consisting of five LIM domains, as an ILK binding protein. We demonstrate in this study that the ILK-PINCH interaction requires the N-terminal-most ANK repeat (ANK1) of ILK and one (the C-terminal) of the two zinc-binding modules within the LIM1 domain of PINCH. The ILK ANK repeats domain, which is capable of interacting with PINCH in vitro, could also form a complex with PINCH in vivo. However, the efficiency of the complex formation or the stability of the complex was markedly reduced in the absence of the C-terminal domain of ILK. The PINCH binding defective ANK1 deletion ILK mutant, unlike the wild-type ILK, was unable to localize and cluster in focal adhesions, suggesting that the interaction with PINCH is necessary for focal adhesion localization and clustering of ILK. The N-terminal ANK repeats domain, however, is not sufficient for mediating focal adhesion localization of ILK, as an ILK mutant containing the ANK repeats domain but lacking the C-terminal integrin binding site failed to localize in focal adhesions. These results suggest that focal adhesions are a major subcellular compartment where ILK functions in intracellular signal transduction, and provide important evidence for a critical role of PINCH and integrins in regulating ILK cellular function. 
 The roles of two distinct regions of PINCH-1 in the regulation of cell attachment and spreading. Cells attach to the   extracellular matrix   (ECM) through integrins to form focal adhesion complexes, and this process is followed by the extension of lamellipodia to enable cell spreading. PINCH-1, an adaptor protein essential for the regulation of cell-ECM adhesion, consists of five tandem LIM domains and a small C-terminal region. PINCH-1 is known to interact with integrin-linked kinase (ILK) and Ras suppressor protein 1 (Rsu-1); however, the precise mechanism by which this complex regulates cell-ECM adhesion is not fully understood. We report here that the LIM1 domain of PINCH-1, which associates with ILK to stabilize the expression of this protein, is sufficient for cell attachment but not for cell spreading. In contrast, the C-terminal region of PINCH-1, which binds to Rsu-1, plays a pivotal role in cell spreading but not in cell attachment. We also show that PINCH-1 associates with Rsu-1 to activate Rac1 and that Rac1 activation is necessary for cell spreading. Thus, these data reveal how specific domains of PINCH-1 direct two independent pathways: one utilizing ILK to allow cell attachment, and the other recruiting Rsu-1 to activate Rac1 in order to promote cell spreading. 
 Ras suppressor-1 promotes apoptosis in breast cancer cells by inhibiting PINCH-1 and activating p53-upregulated-modulator of apoptosis (PUMA); verification from metastatic breast cancer human samples. Metastasis, responsible for most deaths from breast cancer (BC), is a multistep process leading to cancer cell spread.   Extracellular matrix   (ECM)-related adhesion and apoptosis resistance play pivotal role in metastasis. Ras suppressor-1 (RSU-1) localizes to cell-ECM adhesions and binds to pro-survival adhesion protein PINCH-1. Little is known about the role of RSU-1 in BC. In the present study, we investigated the role of RSU-1 in BC metastasis using two BC cell lines that differ in terms of their metastatic potential and a set of 32 human BC samples from patients with or without lymph node metastasis. We show that RSU-1 is upregulated in the aggressive MDA-MB-231 cells compared to MCF-7 and that its silencing by siRNA leads to upregulation of PINCH-1, induction of proliferation and reduction of apoptosis through downregulation of the pro-apoptotic gene p53-upregulated-modulator-of-apoptosis (PUMA). Our findings in the cell lines were further validated in the human BC tissues where normal adjacent tissues were used as controls. We demonstrate for the first time, that RSU-1 expression is upregulated in metastatic BC samples and downregulated in non-metastatic while it is negatively correlated with PINCH-1 and positively correlated with PUMA expression, suggesting that a pro-apoptotic mechanism is in place in metastatic BC samples and identifying RSU-1 as a potentially interesting molecule that needs to be evaluated further as a novel BC metastasis biomarker. 
 Novel expression of PINCH in the central nervous system and its potential as a biomarker for human immunodeficiency virus-associated neurodegeneration. Particularly interesting cysteine histidine-rich (PINCH) protein functions as a shuttling protein in Schwann cells after peripheral nerve damage, during repair and remodeling, and in maintaining neuronal polarity. However, the presence of PINCH in the human CNS during disease has not been addressed. Because HIV-associated damage to cells of the CNS involves dysregulation of neuronal signaling and white matter damage, we hypothesized that PINCH may play a role in neuropathological processes during the course of HIV infection. To determine the expression of PINCH in the CNS, brain, and cerebrospinal fluid (CSF) obtained at autopsy from HIV patients with no CNS alterations, HIV encephalitic (HIVE) patients, and HIV-negative individuals with no CNS alterations were examined for PINCH immunoreactivity. Our results show that PINCH is expressed robustly in the brains and CSF of HIV patients, but is nearly undetectable in HIV-negative individuals. However, HIVE patients' CSF contained significantly less PINCH than HIV patients with no CNS alterations. PINCH immunolabeling was significantly more intense in the white matter than in the grey matter and was associated exclusively with neuronal cell bodies or processes, or with the   extracellular matrix  . Given the recently discovered importance of PINCH in maintaining neuronal fitness, our observations that PINCH is robustly expressed in the CNS of HIV patients suggests an important role for PINCH in HIV-associated neurodegenerative processes. Understanding mechanisms by which PINCH functions during HIV-associated CNS alterations will provide new insight into potential treatments to limit neurological alterations in HIV. 
 PINCH-1 regulates the ERK-Bim pathway and contributes to apoptosis resistance in cancer cells. Resistance to apoptosis is a hallmark of cancer cells. We report here that PINCH-1, a cytoplasmic component of cell-  extracellular matrix   adhesions, is required for protection of multiple types of cancer cells from apoptosis. Furthermore, using HT-1080 fibrosarcoma cells as a model system, we have investigated the signaling pathway through which PINCH-1 contributes to apoptosis resistance. Loss of PINCH-1 markedly increases the level of Bim and promotes Bim translocation to mitochondria, resulting in activation of the intrinsic apoptosis pathway. Depletion of Bim completely blocked apoptosis induced by the loss of PINCH-1. Thus, PINCH-1 contributes to apoptosis resistance through suppression of Bim. Mechanistically, PINCH-1 suppresses Bim not only transcriptionally but also post-transcriptionally. PINCH-1 promotes activating phosphorylation of Src family kinase and ERK1/2. Consistent with this, ERK1/2-mediated Ser(69) phosphorylation of Bim, a key signal for turnover of Bim, is suppressed by the removal of PINCH-1. Our results demonstrate a strong dependence of multiple types of apoptosis-resistant cancer cells on PINCH-1 and provide new insights into the molecular mechanism by which cancer cells are protected from apoptosis. 
 Particularly interesting cysteine- and histidine-rich protein in cardiac development and remodeling. Integrin-mediated cell-  extracellular matrix   interaction plays key roles in tissue morphogenesis and integrity. The Lin11-Isl-1-Mec-3 (LIM) domain-only particularly interesting cysteine- and histidine-rich (PINCH) protein functions as an adaptor essential for the assembly and function of the focal adhesion complex that links integrin signaling to the cytoskeleton and other intracellular signaling pathways and regulates diverse cellular processes such as cell adhesion, migration, growth, differentiation, and survival. Recent biochemical and genetic studies have greatly advanced our knowledge surrounding the molecular interactions and functions of each component of the focal adhesion complex and revealed a requirement for PINCH in early embryogenesis, in morphogenesis of the neural crest and cardiac outflow, and in myocardial growth and remodeling. In this review article, we will provide an overview of the current knowledge of the molecular interactions of PINCH with other components of focal adhesions, highlighting recent discoveries of the in vivo role of PINCH and discuss its potential implication for human heart disease. 
 Signaling via PINCH: Functions, binding partners and implications in human diseases. Particularly interesting new cysteine-histidine-rich protein (PINCH) is a LIM-domain-only adaptor that plays important roles in cytoskeletal organization and   extracellular matrix   adhesion, migration, proliferation and survival. Mammalian cells have two functional PINCH proteins, PINCH1 and PINCH2. PINCH not only binds to Nck2 and engages in the signaling of growth factor receptors, but also forms a ternary complex with ILK and parvin (IPP complex). Normally, the IPP complex locates to focal adhesions participating in the signaling of integrins and mediating the interaction of cytoskeleton and   extracellular matrix   (ECM). Accumulative evidence indicates that abnormalities in PINCH signaling are involved in the pathogenesis of important diseases, such as cancers, renal diseases, cardiomyopathy, and HIV. Therefore, clarifying the functions of PINCH and its interactions with key factors is important for better understanding of signaling events both in health and disease. 
 PINCH mRNA overexpression in colorectal carcinomas correlated with VEGF and FAS mRNA expression. Particularly interesting new cysteine-histidine-rich protein (PINCH) was found to be up-regulated in the stroma of colorectal carcinomas (CRCs) in our previous studies and was involved in angiogenesis through activation of fibroblasts in   extracellular matrix   (ECM) in response to tumors. Here, we examined PINCH mRNA expression in colorectal cancer and investigated its relationship with the clinicopathological features and proliferation cell nuclear antigen (PCNA), vascular endothelial growth factor (VEGF) and FAS.The primary cancer tissues, adjacent noncancerous tissues and the proximal and distant margins of normal mucosa were collected from 81 colorectal cancer patients during surgery. PINCH, PCNA, VEGF and FAS mRNA expression was examined by reverse transcriptional PCR (RT-PCR).PINCH mRNA expression was significantly increased in primary tumors compared with that in adjacent noncancerous tissues, and the proximal and distant margins of normal mucosa (p<0.0001). Expression of PINCH mRNA in colon cancer tended to be higher than expression in rectal cancer (p=0.051). Tumors which had infiltrated through the wall of the colorectum trended to have higher PINCH mRNA expression (p=0.073). PINCH mRNA expression in primary tumors was positively related to the expression of PCNA mRNA (r=0.534, p=0.010), VEGF mRNA (r=0.431, p=0.022) and FAS mRNA (r=0.542, p=0.012).PINCH mRNA was overexpressed in colorectal cancer and associated with PCNA mRNA, VEGF mRNA and FAS mRNA expression. PINCH mRNA was involved in the development of colorectal cancer and might play a role in the epithelial mesenchymal transition in the rectum differently than in the colon, through the adenomatous polyposis coli (APC)/catenin pathway. 
 Assembly of the PINCH-ILK-CH-ILKBP complex precedes and is essential for localization of each component to cell-matrix adhesion sites. PINCH, integrin-linked kinase (ILK) and calponin homology-containing ILK-binding protein (CH-ILKBP) form a ternary complex that plays crucial roles at cell-  extracellular matrix   adhesion sites. To understand the mechanism underlying the complex formation and recruitment to cell-adhesion sites we have undertaken a combined structural, mutational and cell biological analysis. Three-dimensional structure-based point mutations identified specific PINCH and ILK sites that mediate the complex formation. Analyses of the binding defective point mutants revealed that the assembly of the PINCH-ILK-CH-ILKBP complex is essential for their localization to cell-  extracellular matrix   adhesion sites. The formation of the PINCH-ILK-CH-ILKBP complex precedes integrin-mediated cell adhesion and spreading. Furthermore, inhibition of protein kinase C, but not that of actin polymerization, inhibited the PINCH-ILK-CH-ILKBP complex formation, suggesting that the PINCH-ILK-CH-ILKBP complex likely serves as a downstream effector of protein kinase C in the cellular control of focal adhesion assembly. Finally, we provide evidence that the formation of the PINCH-ILK-CH-ILKBP complex, while necessary, is not sufficient for ILK localization to cell-  extracellular matrix   adhesion sites. These results provide new insights into the molecular mechanism underlying the assembly and regulation of cell-matrix adhesion structures. 
 The role of the focal adhesion protein PINCH1 for the radiosensitivity of adhesion and suspension cell cultures. Focal adhesion (FA) signaling mediated by adhesion to   extracellular matrix   and growth factor receptors contributes to the regulation of the cellular stress response to external stimuli. Critical to focal adhesion assembly and signaling is the adapter protein PINCH1. To evaluate whether the prosurvival function of PINCH1 in radiation cell survival depends on cell adhesion, we examined PINCH1(fl/fl) and PINCH1(-/-) mouse embryonic fibroblasts and human cancer cell lines. Here, we found that the enhanced cellular radiosensitivity mediated by PINCH1 depletion observed under adhesion conditions is conserved when cells are irradiated under suspension conditions. This unsuspected finding could not be explained by the observed modification of adhesion and growth factor associated signaling involving FAK, Paxillin, p130(CAS), Src, AKT, GSK3β and ERK1/2 under suspension and serum withdrawal relative to adhesion conditions with serum. Our data suggest that the adapter protein PINCH1 critically participates in the regulation of the cellular radiosensitivity of normal and malignant cells similarly under adhesion and suspension conditions. 
 Structural basis of focal adhesion localization of LIM-only adaptor PINCH by integrin-linked kinase. The LIM-only adaptor PINCH (the particularly interesting cysteine- and histidine-rich protein) plays a pivotal role in the assembly of focal adhesions (FAs), supramolecular complexes that transmit mechanical and biochemical information between   extracellular matrix   and actin cytoskeleton, regulating diverse cell adhesive processes such as cell migration, cell spreading, and survival. A key step for the PINCH function is its localization to FAs, which depends critically on the tight binding of PINCH to integrin-linked kinase (ILK). Here we report the solution NMR structure of the core ILK.PINCH complex (28 kDa, K(D) approximately 68 nm) involving the N-terminal ankyrin repeat domain (ARD) of ILK and the first LIM domain (LIM1) of PINCH. We show that the ILK ARD exhibits five sequentially stacked ankyrin repeat units, which provide a large concave surface to grip the two contiguous zinc fingers of the PINCH LIM1. The highly electrostatic interface is evolutionally conserved but differs drastically from those of known ARD and LIM bound to other types of protein domains. Consistently mutation of a hot spot in LIM1, which is not conserved in other LIM domains, disrupted the PINCH binding to ILK and abolished the PINCH targeting to FAs. These data provide atomic insight into a novel modular recognition and demonstrate how PINCH is specifically recruited by ILK to mediate the FA assembly and cell-  extracellular matrix   communication. 
 Structural and functional insights into PINCH LIM4 domain-mediated integrin signaling. PINCH is an adaptor protein found in focal adhesions, large cellular complexes that link   extracellular matrix   to the actin cytoskeleton. PINCH, which contains an array of five LIM domains, has been implicated as a platform for multiple protein-protein interactions that mediate integrin signaling within focal adhesions. We had previously characterized the LIM1 domain of PINCH, which functions in focal adhesions by binding specifically to integrin-linked kinase. Using NMR spectroscopy, we show here that the PINCH LIM4 domain, while maintaining the conserved LIM scaffold, recognizes the third SH3 domain of another adaptor protein, Nck2 (also called Nckbeta or Grb4), in a manner distinct from that of the LIM1 domain. Point mutation of LIM residues in the SH3-binding interface disrupted LIM-SH3 interaction and substantially impaired localization of PINCH to focal adhesions. These data provide novel structural insight into LIM domain-mediated protein-protein recognition and demonstrate that the PINCH-Nck2 interaction is an important component of the focal adhesion assembly during integrin signaling. 
  [Ii]nvasive  PubMed  
 PINCH protein expression in normal endometrium, atypical endometrial hyperplasia and endometrioid endometrial carcinoma. Particularly interesting new cysteine-histidine rich protein (PINCH), as an adapter protein of the LIM family for signal transduction in the integrin and growth factor pathway, is upregulated in the stroma of several common types of cancers and involved in promoting tumor progression. In the present study, we examined PINCH expression in normal endometrium, atypical endometrial hyperplasia and endometrioid carcinoma, and further studied the relationships of PINCH expression with clinicopathological variables in cancer patients.PINCH expression was examined by immunohistochemistry in 23 normal endometrial samples, 18 atypical endometrial hyperplasias and 48 endometrioid endometrial carcinomas.The PINCH expression in the stroma of cancer (71%) was significantly increased compared to either normal endometrium (17%, p < 0.0001) or atypical hyperplasia (39%, p = 0.017), along with 9 cancers that had stronger PINCH expressions at the   invasive   margin of the cancers compared to the inner cancers. PINCH expression in cancer was higher in the patients with hypertension (p = 0.041) and estrogen exposure time >30 years (p = 0.021). On the other hand, PINCH expression was not related to menopausal status, gravid status, blood sugar/lipid, family background of cancer, histological grade, myometrial invasion, cervical involvement, lymph nodal metastases, growth pattern, estrogen and progestogen receptors (p > 0.05). conclusion: The results suggest that PINCH seems to play a role, presently unknown, in the tumorigenesis and development of endometrial cancer that merits further study. 
 Impact of PINCH expression on survival in colorectal cancer patients. The adaptor protein PINCH is overexpressed in the stroma of several types of cancer, and is an independent prognostic marker in colorectal cancer. In this study we further investigate the relationship of PINCH and survival regarding the response to chemotherapy in colorectal cancer.Paraffin-embedded tissue sections from 251 primary adenocarcinomas, 149 samples of adjacent normal mucosa, 57 samples of distant normal mucosa and 75 lymph node metastases were used for immunohistochemical staining. Stromal staining for PINCH increased from normal mucosa to primary tumour to metastasis. Strong staining in adjacent normal mucosa was related to worse survival independently of sex, age, tumour location, differentiation and stage (p = 0.044, HR, 1.60, 95% CI, 1.01-2.52). PINCH staining at the   invasive   margin tended to be related to survival (p = 0.051). In poorly differentiated tumours PINCH staining at the   invasive   margin was related to survival independently of sex, age and stage (p = 0.013, HR, 1.90, 95% CI, 1.14-3.16), while in better differentiated tumours it was not. In patients with weak staining, adjuvant chemotherapy was related to survival (p = 0.010, 0.013 and 0.013 in entire tumour area,   invasive   margin and inner tumour area, respectively), but not in patients with strong staining. However, in the multivariate analysis no such relationship was seen.PINCH staining in normal adjacent mucosa was related to survival. Further, PINCH staining at the tumour   invasive   margin was related to survival in poorly differentiated tumours but not in better differentiated tumours, indicating that the impact of PINCH on prognosis was dependent on differentiation status. 
 Up-regulation of PINCH in the stroma of oral squamous cell carcinoma predicts nodal metastasis. Particularly interesting new cysteine-histidine rich protein (PINCH), an adapter protein involved in integrin and growth factor signalling, is up-regulated in the stroma of colorectal, breast, prostate, lung and skin cancer. Strong stromal immunostaining for PINCH is an independent prognostic indicator for reduced survival in colorectal cancer, suggesting that PINCH is involved in the signalling that promotes tumour progression. Since no study on PINCH has been carried out in oral squamous cell carcinoma (OSCC), this study aimed to determine PINCH expression in OSCC and its clinicopathological significance. PINCH protein expression was examined by immunohistochemistry in 20 normal oral mucosa and in 57 OSCC specimens. The frequency of strong PINCH immunostaining was higher in tumour-associated stroma of OSCC (37%) as compared to normal oral mucosa (10%) (p=0.02). Strong PINCH stromal immunostaining predicted nodal metastasis: 19/26 (73%) OSCC cases with nodal metastasis had strong PINCH immunostaining compared to 9/31 (29%) cases without nodal metastasis (p=0.02). The PINCH expression in OSCC was more intense in stroma at the   invasive   edge than in intratumoural stroma. In conclusion, the up-regulation of PINCH protein in stroma may be involved in promoting invasion and metastasis in OSCC. 
 PINCH expression and its clinicopathological significance in gastric adenocarcinoma. Particularly interesting new cysteine-histidine rich protein (PINCH) is an important component of the local adhesion complexes and upregulated in several types of malignancies, and involved in the incidence and development of tumours. PINCH expression is also independently correlated with poorer survival in patients with colorectal cancer. However, there is no study of PINCH in gastric cancer, therefore, the aim of this project was to investigate PINCH expression and its clinicopathological significance in gastric adenocarcinoma.PINCH expression was immunohistochemically examined in normal gastric mucous (n=30) and gastric adenocarcinoma (n=73), from gastric cancer patients.PINCH expression in the associated-stroma of gastric cancers was heterogeneous, and its positive rate (75%) was higher than that of normal gastric mucosa (43%, <formula>X^{2}</formula> =9.711, p=0.002). The stronger staining was observed at the   invasive   edge of tumour when compared to the inner area of tumour. The rate of positive PINCH (88%) in the cases with lymph node metastasis was higher than that (52%) in the cases without metastasis (<formula>X^{2}</formula>=11.151, p=0.001). PINCH expression was not correlated with patients' gender, age, tumour size, differentiation and invasion depth (p> 0.05). COMCLUSION: PINCH protein might play an important role in the tumourigenesis and metastasis of gastric adenocarcinoma. 
 PINCH expression and its significance in esophageal squamous cell carcinoma. Particularly interesting new cysteine-histidine rich protein (PINCH), as a newly discovered protein of LIM family members, may play a role in signal transduction of integrin and growth factor, and involved in the incidence and development of tumors. PINCH protein is overexpressed in tumor-associated stroma of several types of tumors. However, there is no study of the PINCH in esophageal cancer, therefore we investigated PINCH expression in esophageal squamous cell carcinomas and its clinicopathological significance in the patients. PINCH expression was immunohistochemically examined in 20 normal esophageal samples and 64 esophageal squamous cell carcinomas. The results showed that PINCH expression in the stroma of cancers was heterogeneous, and its positive rate (56%) was higher than that of normal esophageal mucosa (5%, p<0.0001). The stronger staining was observed at the   invasive   edge of tumor when compared to the inner area of tumor. The rate of positive PINCH (90%) in the cases with lymph node metastasis was higher than that (41%) in the cases without metastasis (p<0.0001). PINCH expression was not correlated with patients' gender, age, tumor location, size and differentiation (p>0.05). The results suggest that PINCH protein may be a marker of tumor associated-stroma involving tumor development, and predicting the ability of invasion and metastasis of esophageal squamous cell carcinoma. 
 Pinch-1 was up-regulated in leukemia BMSC and its possible effect. Pinch-1, a widely expressed focal adhesion protein, has been demonstrated to be up-regulated in multiple solid tumor-associated stromal cells, particularly at   invasive   edges. It was supposed that Pinch-1 was intimately associated with development and progression of tumors. The expression of Pinch-1 in hematopoietic microenvironment in patients with leukemia remains unclear. This study focused on the expression of Pinch-1 in bone marrow stromal cells (BMSCs) from leukemia patients and its possible effect. BMSC was isolated and cultured from bone marrow in leukemia patients and normal healthy donors. RT-PCR and Western blot analysis were performed to determine Pinch-1 mRNA and protein level in BMSC, respectively. Lentiviral vector containing Pinch-1 siRNA was constructed, and the recombinant lentivirus particle was packaged in 293 cells. Effectiveness of Pinch-1 siRNA was determined by Western blot. The proliferation, apoptosis and motility of leukemia BMSC subjected to Pinch-1 knockdown using siRNA were tested by flow cytometry, TUNEL assay and Transwell system, respectively. Pinch-1 mRNA and protein were significantly up-regulated in ALL and AML BMSC compared to normal BMSC (p<0.01). Although there was no difference in Pinch-1 mRNA between ALL and AML BMSC, cellular levels of Pinch-1 protein in ALL BMSC were significantly higher than that in AML BMSC (p<0.01). Overexpressed Pinch-1 was significantly reduced in leukemia BMSC transfected with Pinch-1 siRNA evidenced by Western blot. Flow cytometry analysis showed that the percentage of cells in S+G2 phases in leukemia BMSC transfected with Pinch-1 siRNA was significantly lower than control (p<0.01). The percentage of apoptotic cells in leukemia BMSC transfected with Pinch-1 siRNA was 19.8±1.0%, significantly higher than controls (p<0.01). The number of leukemia BMSC transfected with Pinch-1 siRNA that migrated to the lower chamber after culturing for 24 h was 8.4±1.1 per field, significantly lower than controls (p<0.01). Pinch-1 mRNA and protein in leukemia BMSC were up-regulated drastically compared with BMSC from healthy donors. Leukemia BMSC displayed hypoproliferation, decreased migration and increased apoptosis after transfecting Pinch-1 siRNA. 
  GeneRIFs  
 PINCH staining at the tumour   invasive   margin was related to survival in poorly differentiated tumours but not in better differentiated tumours, indicating that the impact of PINCH on prognosis was dependent on differentiation status. 
  [Mm]etastasis  PubMed  
 Impact of PINCH expression on survival in colorectal cancer patients. The adaptor protein PINCH is overexpressed in the stroma of several types of cancer, and is an independent prognostic marker in colorectal cancer. In this study we further investigate the relationship of PINCH and survival regarding the response to chemotherapy in colorectal cancer.Paraffin-embedded tissue sections from 251 primary adenocarcinomas, 149 samples of adjacent normal mucosa, 57 samples of distant normal mucosa and 75 lymph node metastases were used for immunohistochemical staining. Stromal staining for PINCH increased from normal mucosa to primary tumour to   metastasis  . Strong staining in adjacent normal mucosa was related to worse survival independently of sex, age, tumour location, differentiation and stage (p = 0.044, HR, 1.60, 95% CI, 1.01-2.52). PINCH staining at the invasive margin tended to be related to survival (p = 0.051). In poorly differentiated tumours PINCH staining at the invasive margin was related to survival independently of sex, age and stage (p = 0.013, HR, 1.90, 95% CI, 1.14-3.16), while in better differentiated tumours it was not. In patients with weak staining, adjuvant chemotherapy was related to survival (p = 0.010, 0.013 and 0.013 in entire tumour area, invasive margin and inner tumour area, respectively), but not in patients with strong staining. However, in the multivariate analysis no such relationship was seen.PINCH staining in normal adjacent mucosa was related to survival. Further, PINCH staining at the tumour invasive margin was related to survival in poorly differentiated tumours but not in better differentiated tumours, indicating that the impact of PINCH on prognosis was dependent on differentiation status. 
 Ras suppressor-1 promotes apoptosis in breast cancer cells by inhibiting PINCH-1 and activating p53-upregulated-modulator of apoptosis (PUMA); verification from metastatic breast cancer human samples.   Metastasis  , responsible for most deaths from breast cancer (BC), is a multistep process leading to cancer cell spread. Extracellular matrix (ECM)-related adhesion and apoptosis resistance play pivotal role in   metastasis  . Ras suppressor-1 (RSU-1) localizes to cell-ECM adhesions and binds to pro-survival adhesion protein PINCH-1. Little is known about the role of RSU-1 in BC. In the present study, we investigated the role of RSU-1 in BC   metastasis   using two BC cell lines that differ in terms of their metastatic potential and a set of 32 human BC samples from patients with or without lymph node   metastasis  . We show that RSU-1 is upregulated in the aggressive MDA-MB-231 cells compared to MCF-7 and that its silencing by siRNA leads to upregulation of PINCH-1, induction of proliferation and reduction of apoptosis through downregulation of the pro-apoptotic gene p53-upregulated-modulator-of-apoptosis (PUMA). Our findings in the cell lines were further validated in the human BC tissues where normal adjacent tissues were used as controls. We demonstrate for the first time, that RSU-1 expression is upregulated in metastatic BC samples and downregulated in non-metastatic while it is negatively correlated with PINCH-1 and positively correlated with PUMA expression, suggesting that a pro-apoptotic mechanism is in place in metastatic BC samples and identifying RSU-1 as a potentially interesting molecule that needs to be evaluated further as a novel BC   metastasis   biomarker. 
 Up-regulation of PINCH in the stroma of oral squamous cell carcinoma predicts nodal   metastasis  . Particularly interesting new cysteine-histidine rich protein (PINCH), an adapter protein involved in integrin and growth factor signalling, is up-regulated in the stroma of colorectal, breast, prostate, lung and skin cancer. Strong stromal immunostaining for PINCH is an independent prognostic indicator for reduced survival in colorectal cancer, suggesting that PINCH is involved in the signalling that promotes tumour progression. Since no study on PINCH has been carried out in oral squamous cell carcinoma (OSCC), this study aimed to determine PINCH expression in OSCC and its clinicopathological significance. PINCH protein expression was examined by immunohistochemistry in 20 normal oral mucosa and in 57 OSCC specimens. The frequency of strong PINCH immunostaining was higher in tumour-associated stroma of OSCC (37%) as compared to normal oral mucosa (10%) (p=0.02). Strong PINCH stromal immunostaining predicted nodal   metastasis  : 19/26 (73%) OSCC cases with nodal   metastasis   had strong PINCH immunostaining compared to 9/31 (29%) cases without nodal   metastasis   (p=0.02). The PINCH expression in OSCC was more intense in stroma at the invasive edge than in intratumoural stroma. In conclusion, the up-regulation of PINCH protein in stroma may be involved in promoting invasion and   metastasis   in OSCC. 
 PINCH expression and its clinicopathological significance in gastric adenocarcinoma. Particularly interesting new cysteine-histidine rich protein (PINCH) is an important component of the local adhesion complexes and upregulated in several types of malignancies, and involved in the incidence and development of tumours. PINCH expression is also independently correlated with poorer survival in patients with colorectal cancer. However, there is no study of PINCH in gastric cancer, therefore, the aim of this project was to investigate PINCH expression and its clinicopathological significance in gastric adenocarcinoma.PINCH expression was immunohistochemically examined in normal gastric mucous (n=30) and gastric adenocarcinoma (n=73), from gastric cancer patients.PINCH expression in the associated-stroma of gastric cancers was heterogeneous, and its positive rate (75%) was higher than that of normal gastric mucosa (43%, <formula>X^{2}</formula> =9.711, p=0.002). The stronger staining was observed at the invasive edge of tumour when compared to the inner area of tumour. The rate of positive PINCH (88%) in the cases with lymph node   metastasis   was higher than that (52%) in the cases without   metastasis   (<formula>X^{2}</formula>=11.151, p=0.001). PINCH expression was not correlated with patients' gender, age, tumour size, differentiation and invasion depth (p> 0.05). COMCLUSION: PINCH protein might play an important role in the tumourigenesis and   metastasis   of gastric adenocarcinoma. 
 PINCH expression and its significance in esophageal squamous cell carcinoma. Particularly interesting new cysteine-histidine rich protein (PINCH), as a newly discovered protein of LIM family members, may play a role in signal transduction of integrin and growth factor, and involved in the incidence and development of tumors. PINCH protein is overexpressed in tumor-associated stroma of several types of tumors. However, there is no study of the PINCH in esophageal cancer, therefore we investigated PINCH expression in esophageal squamous cell carcinomas and its clinicopathological significance in the patients. PINCH expression was immunohistochemically examined in 20 normal esophageal samples and 64 esophageal squamous cell carcinomas. The results showed that PINCH expression in the stroma of cancers was heterogeneous, and its positive rate (56%) was higher than that of normal esophageal mucosa (5%, p<0.0001). The stronger staining was observed at the invasive edge of tumor when compared to the inner area of tumor. The rate of positive PINCH (90%) in the cases with lymph node   metastasis   was higher than that (41%) in the cases without   metastasis   (p<0.0001). PINCH expression was not correlated with patients' gender, age, tumor location, size and differentiation (p>0.05). The results suggest that PINCH protein may be a marker of tumor associated-stroma involving tumor development, and predicting the ability of invasion and   metastasis   of esophageal squamous cell carcinoma. 
  GeneRIFs  
 PINCH protein might play an important role in the tumourigenesis and   metastasis   of gastric adenocarcinoma. 
 Up-regulation of PINCH protein in stroma may be involved in promoting invasion and   metastasis   in oral squamous cell cacinoma. 
   
  
 KPNA6 
 
  NCBI Gene  
  GeneRIF  
  Pubmed  
 
 
  Search Term  Rank  Fields  
  [Mm]igration  6  PubMed(1)  
 
 
 hide/show details for KPNA6 
  [Mm]igration  PubMed  
 Role of the karyopherin pathway in human immunodeficiency virus type 1 nuclear import. The interaction of the human immunodeficiency virus type 1 (HIV-1) nucleoprotein complex with the cell nuclear import machinery is necessary for viral replication in macrophages and for the establishment of infection in quiescent T lymphocytes. The karyophilic properties of two viral proteins, matrix (MA) and Vpr, are keys to this process. Here, we show that an early step of HIV-1 nuclear import is the recognition of the MA nuclear localization signal (NLS) by Rch1, a member of the karyopherin-alpha family. Furthermore, we demonstrate that an N-terminally truncated form of Rch1 which binds MA but fails to localize to the nucleus efficiently blocks MA- but not Vpr-mediated HIV-1 nuclear import. Correspondingly, NLS peptide inhibits the nuclear   migration   of MA but not that of Vpr and prevents the infection of terminally differentiated macrophages by vpr-defective virus but not wild-type virus. These results are consistent with a model in which Rch1 or another member of the karyopherin-alpha family, through the recognition of the MA NLS, participates in docking the HIV-1 nucleoprotein complex at the nuclear pore. In addition, our data suggest that Vpr governs HIV-1 nuclear import through a distinct pathway. 
   
  
 GBF1 
 
  NCBI Gene  
  GeneRIF  
  Pubmed  
 
 
  Search Term  Rank  Fields  
  [Ii]nvasive  10  PubMed(1)  
  [Mm]etastasis  12  PubMed(1)  
 
 
 hide/show details for GBF1 
  [Ii]nvasive  PubMed  
 GEP100 links epidermal growth factor receptor signalling to Arf6 activation to induce breast cancer invasion. Epidermal growth factor (EGF) receptor (EGFR) signalling is implicated in tumour invasion and metastasis. However, whether there are EGFR signalling pathways specifically used for tumour invasion still remains elusive. Overexpression of Arf6 and its effector, AMAP1, correlates with and is crucial for the   invasive   phenotypes of different breast cancer cells. Here we identify the mechanism by which Arf6 is activated to induce tumour invasion. We found that GEP100/BRAG2, a guanine nucleotide exchanging factor (GEF) for Arf6, is responsible for the   invasive   activity of MDA-MB-231 breast cancer cells, whereas the other ArfGEFs are not. GEP100, through its pleckstrin homology domain, bound directly to Tyr1068/1086-phosphorylated EGFR to activate Arf6. Overexpression of GEP100, together with Arf6, caused non-  invasive   MCF7 cells to become   invasive  , which was dependent on EGF stimulation. Moreover, GEP100 knockdown blocked tumour metastasis. GEP100 was expressed in 70% of primary breast ductal carcinomas, and was preferentially co-expressed with EGFR in the malignant cases. Our results indicate that GEP100 links EGFR signalling to Arf6 activation to induce   invasive   activities of some breast cancer cells, and hence may contribute to their metastasis and malignancy. 
  [Mm]etastasis  PubMed  
 GEP100 links epidermal growth factor receptor signalling to Arf6 activation to induce breast cancer invasion. Epidermal growth factor (EGF) receptor (EGFR) signalling is implicated in tumour invasion and   metastasis  . However, whether there are EGFR signalling pathways specifically used for tumour invasion still remains elusive. Overexpression of Arf6 and its effector, AMAP1, correlates with and is crucial for the invasive phenotypes of different breast cancer cells. Here we identify the mechanism by which Arf6 is activated to induce tumour invasion. We found that GEP100/BRAG2, a guanine nucleotide exchanging factor (GEF) for Arf6, is responsible for the invasive activity of MDA-MB-231 breast cancer cells, whereas the other ArfGEFs are not. GEP100, through its pleckstrin homology domain, bound directly to Tyr1068/1086-phosphorylated EGFR to activate Arf6. Overexpression of GEP100, together with Arf6, caused non-invasive MCF7 cells to become invasive, which was dependent on EGF stimulation. Moreover, GEP100 knockdown blocked tumour   metastasis  . GEP100 was expressed in 70% of primary breast ductal carcinomas, and was preferentially co-expressed with EGFR in the malignant cases. Our results indicate that GEP100 links EGFR signalling to Arf6 activation to induce invasive activities of some breast cancer cells, and hence may contribute to their   metastasis   and malignancy. 
   
  
 DOCK3 
 
  NCBI Gene  
  GeneRIF  
  Pubmed  
 
 
  Search Term  Rank  Fields  
  [Mm]igration  6  PubMed(1); GeneRIFs(1)  
  [Mm]etastasis  12  PubMed(2)  
 
 
 hide/show details for DOCK3 
  [Mm]igration  PubMed  
 Inhibition of RAC1-GEF DOCK3 by miR-512-3p contributes to suppression of metastasis in non-small cell lung cancer. MicroRNAs are a class of small non-coding RNAs regulating gene expression. In this study, we demonstrated that retinoic acid (RA) treatment increases the expression of miR-512-3p. Overexpression of miR-512-3p inhibited cell adhesion,   migration  , and invasion in non-small cell lung cancer (NSCLC) cell lines A549 and H1299. miR-512-3p inhibitor partially reversed these effects in H1299 cells stably expressing miR-512. We identified DOCK3, a RAC1-GEF (guanine nucleotide exchange factor), as a target gene of miR-512-3p. Overexpression of miR-512-3p led to the decrease of DOCK3 protein but not its mRNA. Knockdown of DOCK3 resulted in similar effects on adhesion,   migration  , and invasion as observed of miR-512-3p overexpression. Active RAC1 pull-down assay indicated that overexpression of miR-512-3p could decrease the activity of RAC1 with a higher efficiency than that of DOCK3 knockdown. Furthermore, expression of miR-512-3p was suppressed in most NSCLC patient tumor samples compared to its paired normal controls, suggesting that miR-512-3p might play a crucial role in lung cancer development. In conclusion, our results supported that miR-512-3p could inhibit tumor cell adhesion,   migration  , and invasion by regulating the RAC1 activity via DOCK3 in NSCLC A549 and H1299 cell lines. 
  GeneRIFs  
 Results supported that miR-512-3p could inhibit tumor cell adhesion,   migration  , and invasion by regulating the RAC1 activity via DOCK3 in NSCLC A549 and H1299 cell lines. 
  [Mm]etastasis  PubMed  
 Inhibition of RAC1-GEF DOCK3 by miR-512-3p contributes to suppression of   metastasis   in non-small cell lung cancer. MicroRNAs are a class of small non-coding RNAs regulating gene expression. In this study, we demonstrated that retinoic acid (RA) treatment increases the expression of miR-512-3p. Overexpression of miR-512-3p inhibited cell adhesion, migration, and invasion in non-small cell lung cancer (NSCLC) cell lines A549 and H1299. miR-512-3p inhibitor partially reversed these effects in H1299 cells stably expressing miR-512. We identified DOCK3, a RAC1-GEF (guanine nucleotide exchange factor), as a target gene of miR-512-3p. Overexpression of miR-512-3p led to the decrease of DOCK3 protein but not its mRNA. Knockdown of DOCK3 resulted in similar effects on adhesion, migration, and invasion as observed of miR-512-3p overexpression. Active RAC1 pull-down assay indicated that overexpression of miR-512-3p could decrease the activity of RAC1 with a higher efficiency than that of DOCK3 knockdown. Furthermore, expression of miR-512-3p was suppressed in most NSCLC patient tumor samples compared to its paired normal controls, suggesting that miR-512-3p might play a crucial role in lung cancer development. In conclusion, our results supported that miR-512-3p could inhibit tumor cell adhesion, migration, and invasion by regulating the RAC1 activity via DOCK3 in NSCLC A549 and H1299 cell lines. 
 Rac activation and inactivation control plasticity of tumor cell movement. Tumor cells exhibit two different modes of individual cell movement. Mesenchymal-type movement is characterized by an elongated cellular morphology and requires extracellular proteolysis. In amoeboid movement, cells have a rounded morphology, are less dependent on proteases, and require high Rho-kinase signaling to drive elevated levels of actomyosin contractility. These two modes of cell movement are interconvertible. We show that mesenchymal-type movement in melanoma cells is driven by activation of the GTPase Rac through a complex containing NEDD9, a recently identified melanoma   metastasis   gene, and DOCK3, a Rac guanine nucleotide exchange factor. Rac signals through WAVE2 to direct mesenchymal movement and suppress amoeboid movement through decreasing actomyosin contractility. Conversely, in amoeboid movement, Rho-kinase signaling activates a Rac GAP, ARHGAP22, that suppresses mesenchymal movement by inactivating Rac. We demonstrate tight interplay between Rho and Rac in determining different modes of tumor cell movement, revealing how tumor cells switch between different modes of movement. 
   
  
 LLGL1 
 
  NCBI Gene  
  GeneRIF  
  Pubmed  
 
 
  Search Term  Rank  Fields  
  [Mm]igration  6  PubMed(4)  
  [Ii]nvasive  10  PubMed(1)  
  [Mm]etastasis  12  PubMed(1); GeneRIFs(1)  
 
 
 hide/show details for LLGL1 
  [Mm]igration  PubMed  
 Aberrant splicing of Hugl-1 is associated with hepatocellular carcinoma progression. Lethal giant larvae functions as a cell polarity regulator and a tumor suppressor in Drosophila. Its evolutionary conservation implies a tumor suppressor role for its human homologue, Hugl-1. The aims of this study were to characterize Hugl-1 and to determine the clinical significance of Hugl-1 alterations in hepatocellular carcinoma (HCC).Sequence alterations of Hugl-1 from 80 HCC specimens and 5 HCC cell lines were characterized by reverse transcription-PCR and sequence analysis. Western blot was used for determining Hugl-1 expression. The biological activities of Hugl-1 and its aberrant variants were examined by 3-(4,5-dimethylthiazol-2-yl)-2,5-diphenyltetrazolium bromide assay, wound healing assay, Boyden chamber assay, and tumorigenicity assay.In 32.5% (26 of 80) of the specimens and 20.0% (one of five) of HCC cell lines, 23 unique aberrant Hugl-1 transcripts were identified, most of which resulted from skipping part of and/or entire exon or insertion of intron sequences. The majority of these aberrant Hugl-1 transcripts encoded truncated proteins lacking one or more conserved WD-40 repeat motifs. Two truncated Hugl-1 proteins were found exclusively in HCC tissues. Aberrant Hugl-1 transcripts (78.3%, 20 of 23) had a short "direct repeat" sequence flanking their deleted regions. The abnormal Hugl-1 was significantly correlated with poor differentiation and large tumor size of HCC. Overexpression of two representative HCC-derived aberrant Hugl-1 variants promoted HCC cell   migration  , invasion, and tumorigenicity in nude mice.We provide the first evidence that Hugl-1 mRNA is frequently mutated by aberrant splicing exclusively in HCC, which may be involved in HCC progression. 
 Reduced Expression of Hugl 1 Contributes to the Progression of Lung Squamous Cell Carcinoma. Cell polarity and cell-cell adhesion play a critical role in the regulation of normal tissue architecture and function. Disruption of cell adhesion and cell polarity is often associated with neoplastic tumors. Loss of apical-basal polarity in epithelial cells is one of the hallmarks of aggressive and invasive cancers. Several polarity proteins including atypical protein kinase C (aPKC), Par 6, Par 3, and Lethal giant larvae (Lgl, the human homologues of which are called Hugl 1 and Hugl 2) are localized at the leading edge of migrating cells, and play critical roles during directional   migration  . Herein, we investigated the expression of aPKC, Par 6, Par 3, Hugl 1, and Hugl 2 in lung squamous cell carcinoma (SqCC). An inverse correlation was observed between the expression of Hugl 1 and lung SqCC progression. Results of immunohistochemistry and real-time RT-PCR analyses showed that reduced expression of Hugl 1 predicts poor survival in lung SqCC patients. The expression of Hugl 1 was inversely correlated with both overall survival rate and tumor stage. On the other hand, no associations were observed between the expressions of Hugl 2, Par 6, and Par 3 and lung SqCC progression. These findings indicate that the reduced expression of Hugl 1 could be considered as a poor prognostic factor in human lung cancers. 
 Reduced expression of Hugl-1, the human homologue of Drosophila tumour suppressor gene lgl, contributes to progression of colorectal cancer. The human gene, human giant larvae (Hugl-1/Llg1/Lgl1) has significant homology to the Drosophila tumour suppressor gene lethal(2)giant larvae (lgl). The lgl gene codes for a cortical cytoskeleton protein, Lgl, that binds Myosin II and is involved in maintaining cell polarity and epithelial integrity. The human protein, Hugl-1 contains several conserved functional domains found in Lgl, suggesting that these proteins may have closely related functions. Whether loss of Hugl expression plays a role in human tumorigenesis has so far not been extensively investigated. Thus, we evaluated tumour tissues from 94 patients undergoing surgery for colorectal cancer (CRC) for loss of Hugl-1 transcription and compared our findings with the clinical data from each of these patients. We found that Hugl-1 was lost in 75% of tumour samples and these losses were associated with advanced stage and particularly with lymph node metastases. Reduced Hugl-1 expression during the adenoma-carcinoma sequence occurring as early as in colorectal adenomas was detected by both immunohistochemical and reverse transcription-polymerase chain reaction analysis. Functional assays with ecdysone-inducible cell lines revealed that Hugl-1 expression increased cell adhesion and decreased cell   migration  . Our studies thus indicate that downregulation of Hugl-1 contributes to CRC progression. 
 Expression of Hugl-1 is strongly reduced in malignant melanoma. The human gene Hugl-1 (Llgl/Lgl1) has significant homology to the Drosophila tumor suppressor gene lethal(2)giant larvae (lgl). The lgl gene codes for a cortical cytoskeleton protein, Lgl, that is involved in maintaining cell polarity and epithelial integrity. We speculate that Hugl-1 might play a role in epithelial-mesenchymal transition (EMT) and that loss of Hugl-1 expression plays a role in the development or progression of malignant melanoma. Thus, we evaluated melanoma cell lines and tissue samples of malignant melanoma for loss of Hugl-1 transcription. We found that Hugl-1 was downregulated or lost in all cell lines and in most of the tumor samples analysed, and that these losses were associated with advanced stage of the disease. Reduced Hugl-1 expression occurred as early as in primary tumors detected by both immunohistochemical and reverse transcription-polymerase chain reaction (RT-PCR) analysis. Functional assays with stable Hugl-1-transfected cell lines revealed that Hugl-1 expression increased cell adhesion and decreased cell   migration  . Further, downregulation of MMP2 and MMP14 (MT1-MMP) and re-expression of E-cadherin was found in the Hugl-1-expressing cell clones supporting a role of Hugl-1 in EMT. Our studies thus indicate that loss of Hugl-1 expression contributes to melanoma progression. 
  [Ii]nvasive  PubMed  
 Reduced Expression of Hugl 1 Contributes to the Progression of Lung Squamous Cell Carcinoma. Cell polarity and cell-cell adhesion play a critical role in the regulation of normal tissue architecture and function. Disruption of cell adhesion and cell polarity is often associated with neoplastic tumors. Loss of apical-basal polarity in epithelial cells is one of the hallmarks of aggressive and   invasive   cancers. Several polarity proteins including atypical protein kinase C (aPKC), Par 6, Par 3, and Lethal giant larvae (Lgl, the human homologues of which are called Hugl 1 and Hugl 2) are localized at the leading edge of migrating cells, and play critical roles during directional migration. Herein, we investigated the expression of aPKC, Par 6, Par 3, Hugl 1, and Hugl 2 in lung squamous cell carcinoma (SqCC). An inverse correlation was observed between the expression of Hugl 1 and lung SqCC progression. Results of immunohistochemistry and real-time RT-PCR analyses showed that reduced expression of Hugl 1 predicts poor survival in lung SqCC patients. The expression of Hugl 1 was inversely correlated with both overall survival rate and tumor stage. On the other hand, no associations were observed between the expressions of Hugl 2, Par 6, and Par 3 and lung SqCC progression. These findings indicate that the reduced expression of Hugl 1 could be considered as a poor prognostic factor in human lung cancers. 
  [Mm]etastasis  PubMed  
 Loss of Hugl-1 expression associates with lymph node   metastasis   in endometrial cancer. Mutation of neoplastic tumor suppressor genes, scribble, discs large, and lethal giant larvae (lgl), causes disruption of cell polarity and overproliferation of Drosophila epithelial cells and neuroblasts. Reduced expression of human homologue of lgl, Hugl-1, has been reported to be involved in development and progression of human colon cancer and malignant melanoma. To explore the association between Hugl-1 expression and clinical character in endometrial cancer, we examined the expression of Hugl-1 in primary endometrial cancer tissues. The expression of Hugl-1 mRNA in 86 primary endometrial cancer tissues was examined using semiquantitative reverse transcription polymerase chain reaction (RT-PCR). All samples were categorized into two groups: Hugl-1 positive and Hugl-1 negative. Clinical data of each group were analyzed by Fisher's exact probability test and survival rates of each group were compared by Kaplan-Meier method and Log-rank test. Loss of Hugl-1 expression had correlation with the higher incidence of lymph node   metastasis  , but not to the patient's age at onset, distant   metastasis  , clinical stage, lymph or venous vessel invasion, or histopathological grade of differentiation. The Hugl-1-positive group had poorer prognosis compared with the Hugl-1-negative group. These results indicate that loss of Hugl-1 expression in endometrial cancer may contribute to lymph node   metastasis   and it can be a factor of poor prognosis. 
  GeneRIFs  
 loss of Hugl-1 expression in endometrial cancer may contribute to lymph node   metastasis   and it can be a factor of poor prognosis. 
   
  
 COPA 
 
  NCBI Gene  
  GeneRIF  
  Pubmed  
 
 
  Search Term  Rank  Fields  
  [Ee]xtracellular [Mm]atrix  8  PubMed(1)  
  [Ii]nvasive  10  PubMed(1)  
  [Mm]etastasis  12  PubMed(1)  
 
 
 hide/show details for COPA 
  [Ee]xtracellular [Mm]atrix  PubMed  
 Proteomic analysis of human parotid gland exosomes by multidimensional protein identification technology (MudPIT). Human ductal saliva contributes over a thousand unique proteins to whole oral fluids. The mechanism by which most of these proteins are secreted by salivary glands remains to be determined. The present study used a mass spectrometry-based, shotgun proteomics approach to explore the possibility that a subset of the proteins found in saliva are derived from exosomes, membrane-bound vesicles of endosomal origin within multivesicular endosomes. Using MudPIT (multidimensional protein identification technology) mass spectrometry, we catalogued 491 proteins in the exosome fraction of human parotid saliva. Many of these proteins were previously observed in ductal saliva from parotid glands (265 proteins). Furthermore, 72 of the proteins in parotid exosomes overlap with those previously identified as urinary exosome proteins, proteins which are also frequently associated with exosomes from other tissues and cell types. Gene Ontology (GO) and KEGG pathway analyses found that cytosolic proteins comprise the largest category of proteins in parotid exosomes (43%), involved in such processes as phosphatidylinositol signaling system, calcium signaling pathway, inositol metabolism, protein export, and signal transduction, among others; whereas the integral plasma membrane proteins and associated/peripheral plasma membrane proteins (26%) were associated with   extracellular matrix  -receptor interaction, epithelial cell signaling, T-cell and B-cell receptor signaling, cytokine receptor interaction, and antigen processing and presentation, among other biological functions. In addition, these putative saliva exosomal proteins were linked to specific diseases (e.g., neurodegenerative disorders, prion disease, cancers, type I and II diabetes). Consequently, parotid glands secrete exosomes that reflect the metabolic and functional status of the gland and may also carry informative protein markers useful in the diagnosis and treatment of systemic diseases. 
  [Ii]nvasive  PubMed  
 COPA and SLC4A4 are required for cellular entry of arginine-rich peptides. Cell-penetrating peptides (CPPs) have gained attention as promising tools to enable the delivery of various molecules in a non-  invasive   manner. Among the CPPs, TAT and poly-arginine have been extensively utilized in numerous studies for the delivery of functional proteins, peptides, and macromolecules to analyze cellular signaling. However, the molecular mechanisms of cellular entry remain largely unknown. Here, we applied siRNA library screening to identify the regulatory genes for the cellular entry of poly-arginine peptide based on microscopic observation of the entry of fluorescent peptides in siRNA-treated cells. In this screening, we identified the cell membrane gene SLC4A4 and the trafficking regulator gene COPA, which also plays an important role in early endosome maturation. These results demonstrated that cellular entry of poly-arginine requires at least two different steps, probably binding on the cell surface and endosomal entry. The identification of genes for cellular entry of poly-arginine provides insights into its mechanisms and should further aid in the development of highly efficient cell-penetrating peptides. 
  [Mm]etastasis  PubMed  
 Positional mapping for amplified DNA sequences on 1q21-q22 in hepatocellular carcinoma indicates candidate genes over-expression. Comparative genomic hybridization analysis on hepatocellular carcinoma (HCC) indicated frequent gains of 1q and an amplicon at 1q21-q22. Current cytogenetic evidences confer much importance on 1q21-q22, where a role in drug resistance, tumor   metastasis   and shorter patient survival had been implicated.Using positional mapping by interphase cytogenetics, we investigated the amplicon 1q21-q22 in five HCC cases. Three amplification maxima represented by yeast artificial chromosomes (YACs) 955E11, 876B11 and 945D5 that mapped to regions 1q21.1, 1q21.2 and 1q22, respectively, were indicated. We further investigated candidate genes expression in the mapped YACs by quantitative reverse-transcription-polymerase chain reaction. A panel of genes encoding protein transcripts involved in apoptosis, cell cycle progression, calcium binding and jumping translocation was studied.Among ten HCC cases with the amplicon 1q21-q22 examined, we found a significant gene expression level of JTB, SHC1, CCT3 and COPA in the tumors than the paired adjacent non-malignant liver tissues (P< or =0.04).Our interphase findings on 1q21-q22 pinpointed three affected loci between D1S305 and D1S2369. Up-regulation of candidate genes identified within these over-represented regions may represent targets in the progression of HCC and may carry prognostic significance. 
   
  
 EIF4EBP2 
 
  NCBI Gene  
  GeneRIF  
  Pubmed  
 
 
  Search Term  Rank  Fields  
  [Ii]nvasive  10  PubMed(2)  
 
 
 hide/show details for EIF4EBP2 
  [Ii]nvasive  PubMed  
 Evaluation of candidate stromal epithelial cross-talk genes identifies association between risk of serous ovarian cancer and TERT, a cancer susceptibility "hot-spot". We hypothesized that variants in genes expressed as a consequence of interactions between ovarian cancer cells and the host micro-environment could contribute to cancer susceptibility. We therefore used a two-stage approach to evaluate common single nucleotide polymorphisms (SNPs) in 173 genes involved in stromal epithelial interactions in the Ovarian Cancer Association Consortium (OCAC). In the discovery stage, cases with epithelial ovarian cancer (n=675) and controls (n=1,162) were genotyped at 1,536 SNPs using an Illumina GoldenGate assay. Based on Positive Predictive Value estimates, three SNPs-PODXL rs1013368, ITGA6 rs13027811, and MMP3 rs522616-were selected for replication using TaqMan genotyping in up to 3,059 serous   invasive   cases and 8,905 controls from 16 OCAC case-control studies. An additional 18 SNPs with Pper-allele<0.05 in the discovery stage were selected for replication in a subset of five OCAC studies (n=1,233 serous   invasive   cases; n=3,364 controls). The discovery stage associations in PODXL, ITGA6, and MMP3 were attenuated in the larger replication set (adj. Pper-allele>or=0.5). However genotypes at TERT rs7726159 were associated with ovarian cancer risk in the smaller, five-study replication study (Pper-allele=0.03). Combined analysis of the discovery and replication sets for this TERT SNP showed an increased risk of serous ovarian cancer among non-Hispanic whites [adj. ORper-allele 1.14 (1.04-1.24) p=0.003]. Our study adds to the growing evidence that, like the 8q24 locus, the telomerase reverse transcriptase locus at 5p15.33, is a general cancer susceptibility locus. 
 Evaluation of candidate stromal epithelial cross-talk genes identifies association between risk of serous ovarian cancer and TERT, a cancer susceptibility "hot-spot". We hypothesized that variants in genes expressed as a consequence of interactions between ovarian cancer cells and the host micro-environment could contribute to cancer susceptibility. We therefore used a two-stage approach to evaluate common single nucleotide polymorphisms (SNPs) in 173 genes involved in stromal epithelial interactions in the Ovarian Cancer Association Consortium (OCAC). In the discovery stage, cases with epithelial ovarian cancer (n=675) and controls (n=1,162) were genotyped at 1,536 SNPs using an Illumina GoldenGate assay. Based on Positive Predictive Value estimates, three SNPs-PODXL rs1013368, ITGA6 rs13027811, and MMP3 rs522616-were selected for replication using TaqMan genotyping in up to 3,059 serous   invasive   cases and 8,905 controls from 16 OCAC case-control studies. An additional 18 SNPs with Pper-allele<0.05 in the discovery stage were selected for replication in a subset of five OCAC studies (n=1,233 serous   invasive   cases; n=3,364 controls). The discovery stage associations in PODXL, ITGA6, and MMP3 were attenuated in the larger replication set (adj. Pper-allele>or=0.5). However genotypes at TERT rs7726159 were associated with ovarian cancer risk in the smaller, five-study replication study (Pper-allele=0.03). Combined analysis of the discovery and replication sets for this TERT SNP showed an increased risk of serous ovarian cancer among non-Hispanic whites [adj. ORper-allele 1.14 (1.04-1.24) p=0.003]. Our study adds to the growing evidence that, like the 8q24 locus, the telomerase reverse transcriptase locus at 5p15.33, is a general cancer susceptibility locus. 
   
  
 PNLIP 
 
  NCBI Gene  
  GeneRIF  
  Pubmed  
 
 
  Search Term  Rank  Fields  
  [Ii]nvasive  10  PubMed(1)  
 
 
 hide/show details for PNLIP 
  [Ii]nvasive  PubMed  
 Abnormal serum pancreatic enzymes, but not pancreatitis, are associated with an increased risk of malignancy in patients with intraductal papillary mucinous neoplasms. Pancreatitis is associated with intraductal papillary mucinous neoplasm (IPMN). This association is in part due to inflammation from pancreatic ductal obstruction. Although the correlation between pancreatitis and the malignant potential of IPMN is unclear, the 2012 International Consensus Guidelines (ICG) consider pancreatitis a "worrisome feature." We hypothesized that serum pancreatic enzymes, markers of inflammation, are a better predictor of malignancy than pancreatitis in patients with IPMN.Between 1992 and 2012, 364 patients underwent resection for IPMN at a single university hospital. In the past decade, serum amylase and lipase were collected prospectively as an inflammatory marker in 203 patients with IPMN at initial surveillance and "cyst clinic" visits. The latest serum pancreatic enzyme values within 3 months preoperatively were studied. Pancreatitis was defined according to the 2012 revision of the Atlanta Consensus.Of the 203 eligible patients, there were 76 with pancreatitis. Pancreatitis was not associated with an increased rate of malignancy (P = .51)   or invas  iveness (P = .08). Serum pancreatic enzymes categorically outside of normal range (high or low) were also not associated with malignanc  y or inv  asiveness. In contrast, as a continuous variable, the higher the serum pancreatic enzymes were, the greater the rat  e of inv  asive IPMN. Of the 127 remaining patients without pancreatitis, serum pancreatic enzymes outside of normal range (low and high) were each associated with a greater rate of malignancy (P < .0001 and P = .0009, respectively). Serum pancreatic enzyme levels above normal range (high) were associated with a greater   rate of   invasiveness (P = .02).In patients with IPMN without a history of pancreatitis, serum pancreatic enzymes outside of the normal range are associated with a greater risk of malignancy. In patients with a history of pancreatitis, there is a positive correlation between the levels of serum pancreatic enzymes and the p  resence   of invasive IPMN. These data suggest serum pancreatic enzymes may be useful markers in stratification of pancreatic cancer risk in patients with IPMN. 
   
  
 SERAC1 
 
  NCBI Gene  
  GeneRIF  
  Pubmed  
 
 
  Search Term  Rank  Fields  
  [Ee]xtracellular [Mm]atrix  8  Process(1); Component(1)  
 
 
 hide/show details for SERAC1 
  [Ee]xtracellular [Mm]atrix  Process  
   extracellular matrix   organization 
  Component  
 proteinaceous   extracellular matrix   
   
  
 CHRD 
 
  NCBI Gene  
  GeneRIF  
  Pubmed  
 
 
  Search Term  Rank  Fields  
  [Mm]igration  6  Process(1)  
  [Ii]nvasive  10  PubMed(1)  
 
 
 hide/show details for CHRD 
  [Mm]igration  Process  
 negative regulation of cell   migration   
  [Ii]nvasive  PubMed  
 Chordin is underexpressed in ovarian tumors and reduces tumor cell motility. Ovarian cancers mostly derive from the monolayer epithelium that covers the ovary. There are currently very few molecular clues to the etiology of this cancer. Bone morphogenetic proteins (BMPs) are required for follicular development and female fertility and are expressed in the ovarian surface epithelium (OSE). We previously reported the expression of human chordin (CHRD), a BMP extracellular regulator, in the ovary. Here we show that CHRD is underexpressed in epithelium ovary cancer and epithelial cancer cell lines as compared with normal tissues and OSE, respectively. Besides, we detected BMP expression in all ovarian cell lines analyzed. To determine the functional relevance of the absence of CHRD mRNA in tumors and cancer cell lines, we studied the effects of CHRD on two cancer cell lines, BG1 and PEO14. Migratory and   invasive   properties were greatly reduced, whereas cell adhesion to the support was enhanced. In addition, we detected chordin (Chrd) expression in OSE of rat ovaries in a pattern similar to that of BMP4. Altogether, these results suggest that CHRD could participate in regulating BMP activity in normal OSE physiology, and that its mis-expression in OSE may facilitate cancer incidence and/or progression. 
   
  
 VCL 
 
  NCBI Gene  
  GeneRIF  
  Pubmed  
 
 
  Search Term  Rank  Fields  
  [Pp]odosome(s)?  5  PubMed(1); Component(1)  
  [Mm]igration  6  Process(1); PubMed(21); GeneRIFs(1)  
  ECM  7  PubMed(3)  
  [Ee]xtracellular [Mm]atrix  8  PubMed(19); GeneRIFs(1)  
  [Mm]etastasize  11  PubMed(1)  
  [Mm]etastasis  12  PubMed(2)  
  [Cc]ell [Ii]nvasion  13  PubMed(2)  
 
 
 hide/show details for VCL 
  [Pp]odosome(s)?  PubMed  
 Phosphatidylinositol 3,4,5-trisphosphate directs association of Src homology 2-containing signaling proteins with gelsolin.   Podosomes   are adhesion structures in osteoclasts and are structurally related to focal adhesions mediating cell motility during bone resorption. Here we show that gelsolin coprecipitates some of the focal adhesion-associated proteins such as c-Src, phosphoinositide 3-kinase (PI3K), p130(Cas), focal adhesion kinase, integrin alpha(v)beta(3), vinculin, talin, and paxillin. These proteins were inducibly tyrosine-phosphorylated in response to integrin activation by osteopontin. Previous studies have defined unique biochemical properties of gelsolin related to phosphatidylinositol 3,4,5-trisphosphate in osteoclast   podosomes  , and here we demonstrate phosphatidylinositol 3,4,5-trisphosphate/gelsolin function in mediating organization of the   podosome   signaling complex. Overlay and GST pull-down assays demonstrated strong phosphatidylinositol 3,4,5-trisphosphate-PI3K interactions based on the Src homology 2 domains of PI3K. Furthermore, lipid extraction of lysates from activated osteoclasts eliminated interaction between gelsolin, c-Src, PI3K, and focal adhesion kinase despite equal amounts of gelsolin in both the lipid-extracted and unextracted experiment. The cytoplasmic protein tyrosine phosphatase (PTP)-proline-glutamic acid-serine-threonine amino acid sequences (PEST) was also found to be associated with gelsolin in osteoclast   podosomes   and with stimulation of alpha(v)beta(3)-regulated phosphorylation of PTP-PEST. We conclude that gelsolin plays a key role in recruitment of signaling proteins to the plasma membrane through phospholipid-protein interactions and by regulation of their phosphorylation status through its association with PTP-PEST. Because both gelsolin deficiency and PI3K inhibition impair bone resorption, we conclude that phosphatidylinositol 3,4,5-trisphosphate-based protein interactions are critical for osteoclast function. 
  Component  
   podosome   
  [Mm]igration  Process  
 negative regulation of cell   migration   
  PubMed  
 Cytoskeletal proteins talin and vinculin in integrin-mediated adhesion. The cytoskeletal proteins talin and vinculin form part of a macromolecular complex on the cytoplasmic face of integrin-mediated cellular junctions with the extracellular matrix. Recent genetic, biochemical and structural data show that talin is essential for the assembly of such junctions, whereas vinculin appears to be important in regulating adhesion dynamics and cell   migration  . 
 Analysis of the myosin-II-responsive focal adhesion proteome reveals a role for β-Pix in negative regulation of focal adhesion maturation. Focal adhesions undergo myosin-II-mediated maturation wherein they grow and change composition to modulate integrin signalling for cell   migratio  n, growth and differentiation. To determine how focal adhesion composition is affected by myosin II activity, we performed proteomic analysis of isolated focal adhesions and compared protein abundance in focal adhesions from cells with and without myosin II inhibition. We identified 905 focal adhesion proteins, 459 of which changed in abundance with myosin II inhibition, defining the myosin-II-responsive focal adhesion proteome. The abundance of 73% of the proteins in the myosin-II-responsive focal adhesion proteome was enhanced by contractility, including proteins involved in Rho-mediated focal adhesion maturation and endocytosis- and calpain-dependent focal adhesion disassembly. During myosin II inhibition, 27% of proteins in the myosin-II-responsive focal adhesion proteome, including proteins involved in Rac-mediated lamellipodial protrusion, were enriched in focal adhesions, establishing that focal adhesion protein recruitment is also negatively regulated by contractility. We focused on the Rac guanine nucleotide exchange factor β-Pix, documenting its role in the negative regulation of focal adhesion maturation and the promotion of lamellipodial protrusion and focal adhesion turnover to drive cel  l migrati  on. 
 Loss of vinculin and membrane-bound β-catenin promotes metastasis and predicts poor prognosis in colorectal cancer. Loss of cell-cell adhesion is important for the development of cancer invasion and metastasis. Vinculin, a key adhesion-related protein, can affect metastasis and prognosis in several tumours. Here, we determined the biological roles of vinculin in the metastasis of colorectal cancer (CRC) and evaluated its clinical significance as a potential disease biomarker.The expression level of vinculin in CRC cell lines and tissues was measured using Real-Time PCR and western blotting. Moreover, vinculin function was analysed using Transwell assays and in vivo metastasis assays in gain- and loss-of-function experiments. Furthermore, the impact of vinculin together with membrane-bound β-catenin on the prognosis of 228 CRC patients was investigated by immunohistochemistry. Additionally, the expression of epithelial-mesenchymal transition (EMT) indicators was verified by immunohistochemistry in CRC tissues obtained from these patients.Vinculin expression was found to be significantly downregulated in highly metastatic CRC cell lines and metastatic tissues. Both in vitro and in vivo experiments showed that vinculin suppressed invasion  , migrati  on and metastasis in CRC cells and that this suppression could be attenuated by silencing β-catenin. Moreover, the expression of vinculin and membrane-bound β-catenin were positively correlated in CRC tissues, and lack of vinculin expression emerged as an independent prognostic factor in patients with CRC. Finally, the loss of vinculin and membrane-bound β-catenin was associated with node metastasis, organ metastasis and expression of EMT indicators.Our results suggest that vinculin may play specific roles in the EMT and metastasis of CRC and that loss of vinculin could be used as a prognostic factor for CRC. 
 Mechanisms and Functions of Vinculin Interactions with Phospholipids at Cell Adhesion Sites. The cytoskeletal protein vinculin is a major regulator of cell adhesion and attaches to the cell surface by binding to specific phospholipids. Structural, biochemical, and biological studies provided much insight into how vinculin binds to membranes, what components it recognizes, and how lipid binding is regulated. Here we discuss the roles and mechanisms of phospholipids in regulating the structure and function of vinculin and of its muscle-specific metavinculin splice variant. A full appreciation of these processes is necessary for understanding how vinculin regulates cell motility,   migration  , and wound healing, and for understanding of its role in cancer and cardiovascular diseases. 
 KIAA1797/FOCAD encodes a novel focal adhesion protein with tumour suppressor function in gliomas. In a strategy to identify novel genes involved in glioma pathogenesis by molecular characterization of chromosomal translocation breakpoints, we identified the KIAA1797 gene, encoding a protein with an as yet undefined function, to be disrupted by a 7;9 translocation in a primary glioblastoma culture. Array-based comparative genomic hybridization detected deletions involving KIAA1797 in around half of glioblastoma cell lines and glioblastomas investigated. Quantification of messenger RNA levels in human tissues demonstrated highest KIAA1797 expression in brain, reduced levels in all glioblastoma cell lines and most glioblastomas and similar levels in glial and neuronal cells by analysis of different hippocampal regions from murine brain. Antibodies against KIAA1797 were generated and showed similar protein levels in cortex and subcortical white matter of human brain, while levels were significantly reduced in glioblastomas with KIAA1797 deletion. By immunofluorescence of astrocytoma cells, KIAA1797 co-localized with vinculin in focal adhesions. Physical interaction between KIAA1797 and vinculin was demonstrated via co-immunoprecipitation. Functional in vitro assays demonstrated a significant decrease in colony formation,   migration   and invasion capacity of LN18 and U87MG glioma cells carrying a homozygous KIAA1797 deletion ectopically expressing KIAA1797 compared with mock-transduced cells. In an in vivo orthotopic xenograft mouse model, U87MG tumour lesions expressing KIAA1797 had a significantly reduced volume compared to tumours not expressing KIAA1797. In summary, the frequently deleted KIAA1797 gene encodes a novel focal adhesion complex protein with tumour suppressor function in gliomas, which we name 'focadhesin'. Since KIAA1797 genetic variation has been implicated in Alzheimer's disease, our data are also relevant for neurodegeneration. 
 Vinculin controls focal adhesion formation by direct interactions with talin and actin. Focal adhesions (FAs) regulate cell   migration  . Vinculin, with its many potential binding partners, can interconnect signals in FAs. Despite the well-characterized structure of vinculin, the molecular mechanisms underlying its action have remained unclear. Here, using vinculin mutants, we separate the vinculin head and tail regions into distinct functional domains. We show that the vinculin head regulates integrin dynamics and clustering and the tail regulates the link to the mechanotransduction force machinery. The expression of vinculin constructs with unmasked binding sites in the head and tail regions induces dramatic FA growth, which is mediated by their direct interaction with talin. This interaction leads to clustering of activated integrin and an increase in integrin residency time in FAs. Surprisingly, paxillin recruitment, induced by active vinculin constructs, occurs independently of its potential binding site in the vinculin tail. The vinculin tail, however, is responsible for the functional link of FAs to the actin cytoskeleton. We propose a new model that explains how vinculin orchestrates FAs. 
 Actomyosin-dependent formation of the mechanosensitive talin-vinculin complex reinforces actin anchoring. The force generated by the actomyosin cytoskeleton controls focal adhesion dynamics during cell   migration  . This process is thought to involve the mechanical unfolding of talin to expose cryptic vinculin-binding sites. However, the ability of the actomyosin cytoskeleton to directly control the formation of a talin-vinculin complex and the resulting activity of the complex are not known. Here we develop a microscopy assay with pure proteins in which the self-assembly of actomyosin cables controls the association of vinculin to a talin-micropatterned surface in a reversible manner. Quantifications indicate that talin refolding is limited by vinculin dissociation and modulated by the actomyosin network stability. Finally, we show that the activation of vinculin by stretched talin induces a positive feedback that reinforces the actin-talin-vinculin association. This in vitro reconstitution reveals the mechanism by which a key molecular switch senses and controls the connection between adhesion complexes and the actomyosin cytoskeleton. 
 Focal adhesion kinase-dependent regulation of adhesive forces involves vinculin recruitment to focal adhesions. FAK (focal adhesion kinase), an essential non-receptor tyrosine kinase, plays pivotal roles in migratory responses, adhesive signalling and mechanotransduction. FAK-dependent regulation of cell   migration   involves focal adhesion turnover dynamics as well as actin cytoskeleton polymerization and lamellipodia protrusion. Whereas roles for FAK in migratory and mechanosensing responses have been established, the contribution of FAK to the generation of adhesive forces is not well understood.Using FAK-null cells expressing wild-type and mutant FAK under an inducible tetracycline promoter, we analysed the role of FAK in the generation of steady-state adhesive forces using micropatterned substrates and a hydrodynamic adhesion assay. FAK expression reduced steady-state strength by 30% compared with FAK-null cells. FAK expression reduced VCL (vinculin) localization to focal adhesions by 35% independently of changes in integrin binding and localization of talin and paxillin. RNAi (RNA interference) knock-down of VCL abrogated the FAK-dependent differences in adhesive forces. FAK-dependent changes in VCL localization and adhesive forces were confirmed in human primary fibroblasts with FAK knocked down by RNAi. The autophosphorylation Tyr-397 and kinase domain Tyr-576/Tyr-577 sites were differentially required for FAK-mediated adhesive responses.We demonstrate that FAK reduces steady-state adhesion strength by modulating VCL recruitment to focal adhesions. These findings provide insights into the role of FAK in mechanical interactions between a cell and the extracellular matrix. 
 Lipid binding promotes oligomerization and focal adhesion activity of vinculin. Adherens junctions (AJs) and focal adhesion (FA) complexes are necessary for cell   migration   and morphogenesis, and for the development, growth, and survival of all metazoans. Vinculin is an essential regulator of both AJs and FAs, where it provides links to the actin cytoskeleton. Phosphatidylinositol 4,5-bisphosphate (PIP2) affects the functions of many targets, including vinculin. Here we report the crystal structure of vinculin in complex with PIP2, which revealed that PIP2 binding alters vinculin structure to direct higher-order oligomerization and suggests that PIP2 and F-actin binding to vinculin are mutually permissive. Forced expression of PIP2-binding-deficient mutants of vinculin in vinculin-null mouse embryonic fibroblasts revealed that PIP2 binding is necessary for maintaining optimal FAs, for organization of actin stress fibers, and for cell   migration   and spreading. Finally, photobleaching experiments indicated that PIP2 binding is required for the control of vinculin dynamics and turnover in FAs. Thus, through oligomerization, PIP2 directs a transient vinculin sequestration at FAs that is necessary for proper FA function. 
 CAP interacts with cytoskeletal proteins and regulates adhesion-mediated ERK activation and motility. CAP/Ponsin belongs to the SoHo family of adaptor molecules that includes ArgBP2 and Vinexin. These proteins possess an N-terminal sorbin homology (SoHo) domain and three C-terminal SH3 domains that bind to diverse signaling molecules involved in a variety of cellular processes. Here, we show that CAP binds to the cytoskeletal proteins paxillin and vinculin. CAP localizes to cell-extracellular matrix (ECM) adhesion sites, and this process requires binding to vinculin. Overexpression of CAP induces the aggregation of paxillin, vinculin and actin at cell-ECM adhesion sites. Moreover, CAP inhibits adhesion-dependent processes such as cell spreading and focal adhesion turnover, whereas a CAP mutant that is unable to localize to cell-ECM adhesion sites is incapable of exerting these effects. Finally, depletion of CAP by siRNA-mediated knockdown leads to enhanced cell spreading,   migration   and the activation of the PAK/MEK/ERK pathway in REF52 cells. Taken together, these results indicate that CAP is a cytoskeletal adaptor protein involved in modulating adhesion-mediated signaling events that lead to cell   migration  . 
 Molecular mechanism of vinculin activation and nanoscale spatial organization in focal adhesions. Focal adhesions (FAs) link the extracellular matrix to the actin cytoskeleton to mediate cell adhesion,   migration  , mechanosensing and signalling. FAs have conserved nanoscale protein organization, suggesting that the position of proteins within FAs regulates their activity and function. Vinculin binds different FA proteins to mediate distinct cellular functions, but how vinculin's interactions are spatiotemporally organized within FAs is unknown. Using interferometric photoactivation localization super-resolution microscopy to assay vinculin nanoscale localization and a FRET biosensor to assay vinculin conformation, we found that upward repositioning within the FA during FA maturation facilitates vinculin activation and mechanical reinforcement of FAs. Inactive vinculin localizes to the lower integrin signalling layer in FAs by binding to phospho-paxillin. Talin binding activates vinculin and targets active vinculin higher in FAs where vinculin can engage retrograde actin flow. Thus, specific protein interactions are spatially segregated within FAs at the nanoscale to regulate vinculin activation and function. 
 Vinculin negatively regulates transcription of MT1-MMP through MEK/ERK pathway. Vinculin regulates a variety of cellular functions partly through stabilization of tumor suppressor PTEN. In order to study the role of vinculin in tumor progression other than PTEN stabilization, vinculin was knocked down in PTEN-deficient squamous cell carcinoma HSC-4 cells. Knockdown of vinculin induced phenotypical change by reducing cell-cell and cell-extracellular matrix adhesions, and enhanced MT1-MMP expression at transcription level and subsequent cell   migration  . Up-regulation of MT1-MMP transcription by vinculin knockdown was abrogated by ERK inhibition. These results suggest that vinculin negatively regulates malignant phenotype of tumor cells including MT1-MMP transcription through MEK/ERK pathway. 
 Direct evidence of vinculin tail-lipid membrane interaction in beta-sheet conformation. The focal adhesion protein vinculin (1066 residues) plays an important role in cell adhesion and   migration  . The interaction between vinculin and lipid membranes is necessary to ensure these processes. There are three putative lipid-membrane interaction sites located at the vinculin tail domain two that form amphipathic alpha-helices (residues 935-978 and 1020-1040) and one that remains unstructured (residues 1052-1066) during crystallization. In this work, the structural and biochemical properties of the last 21 residues of the vinculin tail domain were investigated. Differential scanning calorimetry was performed in the presence of lipid vesicles consisting of dimyristoyl-L-alpha-phosphatidylcholine and dimyristoyl-L-alpha-phosphatidylglycerol at various molar ratios. The results demonstrate that this peptide inserts into lipid vesicle membranes. Examining the secondary structure of this peptide by molecular dynamics simulations and circular dichroism spectroscopy, we show that it adopts an antiparallel beta sheet backbone geometry that could ensure the association with lipid vesicles. 
 The metavinculin tail domain directs constitutive interactions with raver1 and vinculin RNA. Vinculin is a key regulator of the actin cytoskeleton attachment to the cell membrane at cellular adhesion sites, which is crucial for processes such as cell motility and   migration  , development, survival, and wound healing. Vinculin loss results in embryonic lethality, cardiovascular diseases, and cancer. Its tail domain, Vt, is crucial for vinculin activation and focal adhesion turnover and binds to the actin cytoskeleton and acidic phospholipids upon which it unfurls. The RNA binding protein raver1 regulates the assembly of focal adhesions transcriptionally by binding to vinculin. The muscle-specific splice form, metavinculin, is characterized by a 68-residue insert in the tail domain (MVt) and correlates with hereditary idiopathic dilated cardiomyopathy. Here, we report that metavinculin can bind to raver1 in its inactive state. Our crystal structure explains this permissivity, where an extended coil unique to MVt is unfurled in the MVtΔ954:raver1 complex structure. Our binding assays show that raver1 forms a ternary complex with MVt and vinculin mRNA. These findings suggest that the metavinculin:raver1:RNA complex is constitutively recruited to adhesion complexes. 
 Serum- and glucocorticoid-dependent kinase-1-induced cell   migration   is dependent on vinculin and regulated by the membrane androgen receptor. The serum- and glucocorticoid-dependent kinases 1-3 (SGK1-3) are downstream effectors of phosphatidylinositol 3-kinases, implicated in various cell responses including colon cancer tumorigenesis in mice. Here, we investigated the role of SGK1 in the regulation of cell motility. Using Caco-2 colon tumor and HEK293 embryonic kidney cells, we report that transfection with the constitutively active SGK1 mutant (SGK1-SD) significantly enhanced cell motility. The cell-adhesion protein vinculin was effectively dephosphorylated in SGK1-SD-transfected cells. Treatment of the cells with phosphatase inhibitors restored vinculin phosphorylation and inhibited cell   migration  , indicating a significant role for vinculin phosphorylation in SGK1-induced motility. SGK1-SD-enhanced cell motility was inhibited by activation of membrane androgen-binding sites (mAR) via testosterone-conjugates in both cell lines, whereas intracellular androgen receptor (iAR)-silencing and flutamide treatment revealed that these effects were clearly independent of the interaction of SGK1 with the classical androgen receptors (iAR). More importantly, mAR activation restored vinculin phosphorylation in SGK1-SD-transfected cells, whereas silencing of vinculin fully reversed the mAR-induced inhibition of the migratory capacity, implying that this protein is directly involved in cell motility regulation by SGK1 and mAR. This study indicates for the first time that SGK1 regulates cell   migration   via vinculin dephosphorylation, a mechanism that is controlled by mAR function. 
 Integrin β4 and vinculin contained in exosomes are potential markers for progression of prostate cancer associated with taxane-resistance. Treatment with taxanes for castration-resistant prostate cancer often leads to the development of resistance. It has been recently demonstrated that exosomes present in the body fluids contain proteins and RNAs in the cells from which they are derived and could serve as a diagnostic marker for various diseases. In the present study, we aimed to identify proteins contained in exosomes that could be markers for progression and taxane-resistance of prostate cancer. Exosomes were isolated by differential centrifugation from the culture medium of taxane-resistant human prostate cancer PC-3 cells (PC-3R) and their parental PC-3 cells. Isolated exosomes were subjected to iTRAQ-based quantitative proteomic analysis. Exosomes were also isolated from the culture medium by using anti-CD9 antibody-conjugated magnetic beads. Protein expression was knocked down by siRNA transfection followed by analysis of the silencing effects. Proteomic analysis showed that integrin β4 (ITGB4) and vinculin (VCL) were upregulated in exosomes derived from PC-3R cells compared to PC-3 cells. The elevation of ITGB4 and VCL was confirmed in exosomes captured by anti-CD9 antibody from the culture medium of PC-3R cells. Silencing of ITGB4 and VCL expression did not affect proliferation and taxane-resistance of PC-3R cells, but ITGB4 knockdown attenuated both cel  l migrati  on and invasion and VCL knockdown reduced invasion. Our results suggest that ITGB4 and VCL in exosomes could be useful markers for progression of prostate cancer associated with taxane-resistance, providing the basis for development of an exosome-based diagnostic system. 
 Becoming stable and strong: the interplay between vinculin exchange dynamics and adhesion strength during adhesion site maturation. The coordinated formation and release of focal adhesions is necessary for cell attachment and   migration  . According to current models, these processes are caused by temporal variations in protein composition. Protein incorporation into focal adhesions is believed to be controlled by phosphorylation. Here, we tested the exchange dynamics of GFP-vinculin as marker protein of focal adhesions using the method of Fluorescence Recovery After Photobleaching. The relevance of the phosphorylation state of the protein, the age of focal adhesions and the acting force were investigated. For stable focal adhesions of stationary keratinocytes, we determined an exchangeable vinculin fraction of 52% and a recovery halftime of 57 s. Nascent focal adhesions of moving cells contained a fraction of exchanging vinculin of 70% with a recovery halftime of 36 s. Upon maturation, mean saturation values and recovery halftimes decreased to levels of 49% and 42 s, respectively. Additionally, the fraction of stably incorporated vinculin increased with cell forces and decreased with vinculin phosphorylation within these sites. Experiments on a nonphosphorylatable vinculin mutant construct at phosphorylation site tyr1065 confirmed the direct interplay between phosphorylation and exchange dynamics of adhesion proteins during adhesion site maturation. 
 Investigating lasp-2 in cell adhesion: new binding partners and roles in motility. Focal adhesions are intricate protein complexes that facilitate cell attachment,   migration  , and cellular communication. Lasp-2 (LIM-nebulette), a member of the nebulin family of actin-binding proteins, is a newly identified component of these complexes. To gain further insights into the functional role of lasp-2, we identified two additional binding partners of lasp-2: the integral focal adhesion proteins vinculin and paxillin. Of interest, the interaction of lasp-2 with its binding partners vinculin and paxillin is significantly reduced in the presence of lasp-1, another nebulin family member. The presence of lasp-2 appears to enhance the interaction of vinculin and paxillin with each other; however, as with the interaction of lasp-2 with vinculin or paxillin, this effect is greatly diminished in the presence of excess lasp-1. This suggests that the interplay between lasp-2 and lasp-1 could be an adhesion regulatory mechanism. Lasp-2's potential role in metastasis is revealed, as overexpression of lasp-2 in either SW620 or PC-3B1 cells-metastatic cancer cell lines-increases cell   migration   but impedes cell invasion, suggesting that the enhanced interaction of vinculin and paxillin may functionally destabilize focal adhesion composition. Taken together, these data suggest that lasp-2 has an important role in coordinating and regulating the composition and dynamics of focal adhesions. 
 Effects of hypergravity on the cell shape and on the organization of cytoskeleton and extracelluar matrix molecules of in vitro human dermal fibroblasts. In vitro human dermal fibroblasts were submitted to normal gravity (1 g) or to chronic hypergravity ranging from 2 to 20 g for 8 days. Changes only appeared above 15 g. The majority of 20 g-subjected cells showed fine filipods in the shape of a star whereas most control cells had rounded shapes and spread by forming lamellipodia. Indirect immunofluorescence staining of vinculin, alpha-actinin and actin stress fibers showed changes of the arrangement anchoring points of stress fibers under hypergravity. Tubulin staining showed that the centrosomal material generally located above the nucleus in control cells had migrated to the nucleus side in 20 g-exposed cells. After 8 d of culture under 20 g hypergravity the thickness of fibronectin network seemed to be increased and bundles of fibrils appeared linking ordered arrays of fibers. The fibrils of collagen I formed better delimited and thicker bundles of fibers. We may assume that 20 g hypergravity can induce changes in fibroblast cell shape,   migration   way, and anchorage leading to a reorganization of extracellular matrix without concomitant change of cell proliferation. 
 Recruitment of the Arp2/3 complex to vinculin: coupling membrane protrusion to matrix adhesion. Cell   migration   involves many steps, including membrane protrusion and the development of new adhesions. Here we have investigated whether there is a link between actin polymerization and integrin engagement. In response to signals that trigger membrane protrusion, the actin-related protein (Arp)2/3 complex transiently binds to vinculin, an integrin-associated protein. The interaction is regulated, requiring phosphatidylinositol-4,5-bisphosphate and Rac1 activation, and is sufficient to recruit the Arp2/3 complex to new sites of integrin aggregation. Binding of the Arp2/3 complex to vinculin is direct and does not depend on the ability of vinculin to associate with actin. We have mapped the binding site for the Arp2/3 complex to the hinge region of vinculin, and a point mutation in this region selectively blocks binding to the Arp2/3 complex. Compared with WT vinculin, expression of this mutant in vinculin-null cells results in diminished lamellipodial protrusion and spreading on fibronectin. The recruitment of the Arp2/3 complex to vinculin may be one mechanism through which actin polymerization and membrane protrusion are coupled to integrin-mediated adhesion. 
 Developmental regulation of focal contact protein expression in human melanocytes. Focal contacts are transmembrane links between the extracellular matrix and the actin cytoskeleton that play a critical role in directed cell   migration  , adhesion, and normal growth. Several different component proteins of the focal contact show developmentally dependent changes in expression, suggesting that this is an important mechanism by which focal contact formation is controlled during embryogenesis. In this report we examine the expression of focal contact-associated proteins in human fetal and neonatal melanocytes using Western blotting. We show that expression of paxillin, a 69-kDa vinculin binding protein, is fourfold higher in neonatal melanocytes than in fetal melanocytes. Further, we show that talin, a high molecular weight structural protein that links integrins to the actin cytoskeleton, is proteolytically cleaved in fetal, but not in neonatal melanocytes. Immunofluorescence microscopy of cells grown on fibronectin confirmed the presence of paxillin, talin, and vinculin at the ends of actin stress fibers at presumptive focal contacts in melanocytes. Adhesion experiments to extracellular matrix ligands revealed significant differences in adhesion of fetal and neonatal melanocytes to fibronectin. The developmentally specific changes in focal contact protein expression observed suggest that this may be an important mechanism by which focal contact assembly is controlled in human melanocytes during development. 
  GeneRIFs  
 Data show that SGK1 regulates cell   migration   via vinculin dephosphorylation, a mechanism that is controlled by membrane androgen receptor function. 
  ECM  PubMed  
 CAP interacts with cytoskeletal proteins and regulates adhesion-mediated ERK activation and motility. CAP/Ponsin belongs to the SoHo family of adaptor molecules that includes ArgBP2 and Vinexin. These proteins possess an N-terminal sorbin homology (SoHo) domain and three C-terminal SH3 domains that bind to diverse signaling molecules involved in a variety of cellular processes. Here, we show that CAP binds to the cytoskeletal proteins paxillin and vinculin. CAP localizes to cell-extracellular matrix (  ECM  ) adhesion sites, and this process requires binding to vinculin. Overexpression of CAP induces the aggregation of paxillin, vinculin and actin at cell-  ECM   adhesion sites. Moreover, CAP inhibits adhesion-dependent processes such as cell spreading and focal adhesion turnover, whereas a CAP mutant that is unable to localize to cell-  ECM   adhesion sites is incapable of exerting these effects. Finally, depletion of CAP by siRNA-mediated knockdown leads to enhanced cell spreading, migration and the activation of the PAK/MEK/ERK pathway in REF52 cells. Taken together, these results indicate that CAP is a cytoskeletal adaptor protein involved in modulating adhesion-mediated signaling events that lead to cell migration. 
 Actomyosin-generated tension controls the molecular kinetics of focal adhesions. Focal adhesions (FAs) have key roles in the interaction of cells with the extracellular matrix (  ECM  ) and in adhesion-mediated signaling. These dynamic, multi-protein structures sense the   ECM   both chemically and physically, and respond to external and internal forces by changing their size and signaling activity. However, this mechanosensitivity is still poorly understood at the molecular level. Here, we present direct evidence that actomyosin contractility regulates the molecular kinetics of FAs. We show that the molecular turnover of proteins within FAs is primarily regulated by their dissociation rate constant (k(off)), which is sensitive to changes in forces applied to the FA. We measured the early changes in k(off) values for three FA proteins (vinculin, paxillin and zyxin) upon inhibition of actomyosin-generated forces using two methods - high temporal resolution FRAP and direct measurement of FA protein dissociation in permeabilized cells. When myosin II contractility was inhibited, the k(off) values for all three proteins changed rapidly, in a highly protein-specific manner: dissociation of vinculin from FAs was facilitated, whereas dissociation of paxillin and zyxin was attenuated. We hypothesize that these early kinetic changes initiate FA disassembly by affecting the molecular turnover of FAs and altering their composition. 
 Vinculin tension distributions of individual stress fibers within cell-matrix adhesions. Actomyosin stress fibers (SFs) enable cells to exert traction on planar extracellular matrices (  ECM  s) by tensing focal adhesions (FAs) at the cell-  ECM   interface. Although it is widely appreciated that the spatial and temporal distribution of these tensile forces play key roles in polarity, motility, fate choice, and other defining cell behaviors, virtually nothing is known about how an individual SF quantitatively contributes to tensile loads borne by specific molecules within associated FAs. We address this key open question by using femtosecond laser ablation to sever single SFs in cells while tracking tension across vinculin using a molecular optical sensor. We show that disruption of a single SF reduces tension across vinculin in FAs located throughout the cell, with enriched vinculin tension reduction in FAs oriented parallel to the targeted SF. Remarkably, however, some subpopulations of FAs exhibit enhanced vinculin tension upon SF irradiation and undergo dramatic, unexpected transitions between tension-enhanced and tension-reduced states. These changes depend strongly on the location of the severed SF, consistent with our earlier finding that different SF pools are regulated by distinct myosin activators. We critically discuss the extent to which these measurements can be interpreted in terms of whole-FA tension and traction and propose a model that relates SF tension to adhesive loads and cell shape stability. These studies represent the most direct and high-resolution intracellular measurements of SF contributions to tension on specific FA proteins to date and offer a new paradigm for investigating regulation of adhesive complexes by cytoskeletal force. 
  [Ee]xtracellular [Mm]atrix  PubMed  
 Cytoskeletal proteins talin and vinculin in integrin-mediated adhesion. The cytoskeletal proteins talin and vinculin form part of a macromolecular complex on the cytoplasmic face of integrin-mediated cellular junctions with the   extracellular matrix  . Recent genetic, biochemical and structural data show that talin is essential for the assembly of such junctions, whereas vinculin appears to be important in regulating adhesion dynamics and cell migration. 
 Integrin-mediated cell adhesion: the cytoskeletal connection. Members of the integrin family of cell adhesion molecules play a pivotal role in the interaction between animal cells and the   extracellular matrix  . This article reviews the evidence (i) that the integrin beta-subunit cytoplasmic domain is important in the localization of integrins to focal adhesions, and for integrin-mediated cell adhesion/spreading; and (ii) that the integrin beta-subunit can be linked to F-actin via the actin-binding proteins talin, alpha-actinin and filamin. Talin has two or more actin-binding sites, and three binding sites for the cytoskeletal protein vinculin. Because vinculin can also bind F-actin, it may cross-link talin and actin, thereby stabilizing the interaction. In addition, vinculin contains a binding site for VASP (vasodilator-stimulated phospho-protein), a protein which may serve to recruit a profilin/G-actin complex to talin, which has actin-nucleating activity. Evidence that talin, vinculin and alpha-actinin are important in the assembly of focal adhesions, obtained using antisense technology and protein microinjection, is reviewed. To analyse the role of talin in focal adhesions, we have disrupted both copies of the talin gene in mouse embryonic stem (ES) cells. Undifferentiated talin (-/-) ES cell mutants are unable to assemble focal adhesions when plated on fibronectin, whereas vinculin (-/-) ES cells are able to do so. Finally, the role of small GTP-binding proteins in the assembly of focal adhesions is discussed, along with our recent studies using streptolysin-O-permeabilized Swiss 3T3 cells which suggest that the GTP-binding protein ADP-ribosylation factor-1 (ARF-1) is important in targeting the protein paxillin to focal adhesions. 
 A helix replacement mechanism directs metavinculin functions. Cells require distinct adhesion complexes to form contacts with their neighbors or the   extracellular matrix  , and vinculin links these complexes to the actin cytoskeleton. Metavinculin, an isoform of vinculin that harbors a unique 68-residue insert in its tail domain, has distinct actin bundling and oligomerization properties and plays essential roles in muscle development and homeostasis. Moreover, patients with sporadic or familial mutations in the metavinculin-specific insert invariably develop fatal cardiomyopathies. Here we report the high resolution crystal structure of the metavinculin tail domain, as well as the crystal structures of full-length human native metavinculin (1,134 residues) and of the full-length cardiomyopathy-associated DeltaLeu954 metavinculin deletion mutant. These structures reveal that an alpha-helix (H1') and extended coil of the metavinculin insert replace alpha-helix H1 and its preceding extended coil found in the N-terminal region of the vinculin tail domain to form a new five-helix bundle tail domain. Further, biochemical analyses demonstrate that this helix replacement directs the distinct actin bundling and oligomerization properties of metavinculin. Finally, the cardiomyopathy associated DeltaLeu954 and Arg975Trp metavinculin mutants reside on the replaced extended coil and the H1' alpha-helix, respectively. Thus, a helix replacement mechanism directs metavinculin's unique functions. 
 Focal adhesion kinase-dependent regulation of adhesive forces involves vinculin recruitment to focal adhesions. FAK (focal adhesion kinase), an essential non-receptor tyrosine kinase, plays pivotal roles in migratory responses, adhesive signalling and mechanotransduction. FAK-dependent regulation of cell migration involves focal adhesion turnover dynamics as well as actin cytoskeleton polymerization and lamellipodia protrusion. Whereas roles for FAK in migratory and mechanosensing responses have been established, the contribution of FAK to the generation of adhesive forces is not well understood.Using FAK-null cells expressing wild-type and mutant FAK under an inducible tetracycline promoter, we analysed the role of FAK in the generation of steady-state adhesive forces using micropatterned substrates and a hydrodynamic adhesion assay. FAK expression reduced steady-state strength by 30% compared with FAK-null cells. FAK expression reduced VCL (vinculin) localization to focal adhesions by 35% independently of changes in integrin binding and localization of talin and paxillin. RNAi (RNA interference) knock-down of VCL abrogated the FAK-dependent differences in adhesive forces. FAK-dependent changes in VCL localization and adhesive forces were confirmed in human primary fibroblasts with FAK knocked down by RNAi. The autophosphorylation Tyr-397 and kinase domain Tyr-576/Tyr-577 sites were differentially required for FAK-mediated adhesive responses.We demonstrate that FAK reduces steady-state adhesion strength by modulating VCL recruitment to focal adhesions. These findings provide insights into the role of FAK in mechanical interactions between a cell and the   extracellular matrix  . 
 Vinculin--a dynamic regulator of cell adhesion. The ability of cells to tightly adhere to one another and to the   extracellular matrix   is fundamentally important in numerous biological processes, including embryogenesis, wound healing and maintenance of tissue integrity. Vinculin, a protein localized at the cytoplasmic face of cell-matrix and cell-cell adhesions, is required for strong cell adhesion. Two new crystal structures reveal that vinculin exhibits a high degree of structural plasticity upon ligand binding that might promote rapid changes in cell adhesion. 
 Evaluation of sarcoglycans, vinculin-talin-integrin system and filamin2 in alpha- and gamma-sarcoglycanopathy: an immunohistochemical study. The sarcoglycan subcomplex (SGC) is a well-known system of interaction between   extracellular matrix   and sarcolemma-associated cytoskeleton in skeletal and cardiac muscle. The SGC is included in the DGC made up of sarcoplasmic subcomplex and a dystroglycan subcomplex. Recent developments in molecular genetics have demonstrated that the mutation of each single sarcoglycan gene, causes a series of recessive autosomal muscular dystrophies, dystrophin-positive, called sarcoglycanopathies or limb girdle muscular dystrophies. Our recent studies have demonstrated that costameres are a proteic machinery made up of DGC and vinculin-talin-integrin system, also revealing the colocalization of sarcoglycans and integrins in adult human skeletal muscle. These results may support the hypothesis of the existence of a bidirectional signalling between sarcoglycans and integrins in cultured L6 myocytes. The hypothesis of bidirectional signalling between sarcoglycans and integrins could be supported by the identification of a skeletal and cardiac muscle filamin2 as a gamma-sarcoglycan, delta-sarcoglycan and, hypothetically, beta1 integrin interacting protein. Our results, acquired with an immunofluorescence study on adult human skeletal muscle affected by LGMD type 2D and 2C, showed that in LGMD2D: a) alpha-sarcoglycan staining is severely reduced; b) the beta-gamma-delta-sarcoglycan subunit and all tested integrins staining are clearly detectable; c) filamin2 is normal and shows a costameric distribution. In LGMD2C: a) alpha-sarcoglycan staining is preserved; b) the beta-gamma-delta-sarcoglycan subunit staining is severely reduced; c) the alpha7B-integrin is slightly reduced and beta1D-integrin is severely reduced; d) filamin2 is severely reduced. Other tested proteins of the two systems show a normal staining pattern in both sarcoglycanopathies. Our study seems to confirm, for the first time on adult human skeletal muscle of subjects affected by LGMDs, the hypo-theses of: a) the existence, in mouse myotubes in culture, of two distinct subunits in sarcoglycans subcomplex; b) the presence of a bidirectional signalling between sarcoglycans and integrins, previously demonstrated on rat cultured L6 myocytes; c) the interaction of FLN2 with both sarcoglycans and integrins. These results may stimulate the search of yet unidentified common interactors of both fiber-  extracellular matrix   interaction systems. 
 Distribution of cytoskeletal proteins, integrins, leukocyte adhesion molecules and   extracellular matrix   proteins in plastic-embedded human and rat kidneys. To study the distribution of cytoskeletal proteins (actin, alpha-actinin, vinculin, beta-tubulin, keratin, vimentin, desmin), adhesion molecules for cell-matrix interations (very later antigens [VLA1-61, beta1, beta2 [CD18], vitronectin receptor [alphavbeta3], CD 11b), leukocyte adhesion molecules (ICAM-1) and   extracellular matrix   proteins (collagen IV, fibronectin, laminin, vitronectin) in human and rat kidneys by using a superior processing and immunohistochemical staining technique.Human and rat kidneys were fixed in 2% paraformaldehyde, dehydrated in acetone and processed in a new, low-toxic glycol, methacrylate mixture, especially developed for immunohistochemistry. Both the glomeruli and the interstitial areas were carefully examined and scored semiquantitatively.Immunostained plastic sections showed excellent morphology combined with remarkably well preserved antigenicity.The above mentioned provides an excellent tool for the accurate localization of a wide variety of antigens at the light microscopic level. 
 CAP interacts with cytoskeletal proteins and regulates adhesion-mediated ERK activation and motility. CAP/Ponsin belongs to the SoHo family of adaptor molecules that includes ArgBP2 and Vinexin. These proteins possess an N-terminal sorbin homology (SoHo) domain and three C-terminal SH3 domains that bind to diverse signaling molecules involved in a variety of cellular processes. Here, we show that CAP binds to the cytoskeletal proteins paxillin and vinculin. CAP localizes to cell-  extracellular matrix   (ECM) adhesion sites, and this process requires binding to vinculin. Overexpression of CAP induces the aggregation of paxillin, vinculin and actin at cell-ECM adhesion sites. Moreover, CAP inhibits adhesion-dependent processes such as cell spreading and focal adhesion turnover, whereas a CAP mutant that is unable to localize to cell-ECM adhesion sites is incapable of exerting these effects. Finally, depletion of CAP by siRNA-mediated knockdown leads to enhanced cell spreading, migration and the activation of the PAK/MEK/ERK pathway in REF52 cells. Taken together, these results indicate that CAP is a cytoskeletal adaptor protein involved in modulating adhesion-mediated signaling events that lead to cell migration. 
 Force-dependent vinculin binding to talin in live cells: a crucial step in anchoring the actin cytoskeleton to focal adhesions. Mechanical forces play a pivotal role in the regulation of focal adhesions (FAs) where the actin cytoskeleton is anchored to the   extracellular matrix   through integrin and a variety of linker proteins including talin and vinculin. The localization of vinculin at FAs depends on mechanical forces. While in vitro studies have demonstrated the force-induced increase in vinculin binding to talin, it remains unclear whether such a mechanism exists at FAs in vivo. In this study, using fibroblasts cultured on elastic silicone substrata, we have examined the role of forces in modulating talin-vinculin binding at FAs. Stretching the substrata caused vinculin accumulation at talin-containing FAs, and this accumulation was abrogated by expressing the talin-binding domain of vinculin (domain D1, which inhibits endogenous vinculin from binding to talin). These results indicate that mechanical forces loaded to FAs facilitate vinculin binding to talin at FAs. In cell-protruding regions, the actin network moved backward over talin-containing FAs in domain D1-expressing cells while it was anchored to FAs in control cells, suggesting that the force-dependent vinculin binding to talin is crucial for anchoring the actin cytoskeleton to FAs in living cells. 
 Dystrophin-glycoprotein complex and vinculin-talin-integrin system in human adult cardiac muscle. Costameres were identified, for the first time, in skeletal and cardiac muscle, as regions associated with the sarcolemma, consisting of densely clustered patches of vinculin; they have many characteristics common to the cell-  extracellular matrix  -type of adherens junctions. Costameres are considered 'proteic machinery' and they appear to comprise two protein complexes, the dystrophin-glycoprotein complex (DGC) and the vinculin-talin-integrin system. In comparison to skeletal muscle, few studies have focused on cardiac muscle regarding these two complexes, and study is generally relative to dystrophin or to cardiac diseases, such as cardiomyopathies. However, insufficient data are available on these proteins in healthy human cardiomyocytes. For this reason, we performed an immunohistochemical study using human cardiac muscle fibers, in order to define the real distribution and the spatial relationship between the proteins in these two complexes. Our data showed a real costameric distribution of DGC and of the vinculin-talin-integrin system; all tested proteins were present in T-tubule and in intercalated disks. Moreover, our data demonstrated that all tested proteins of DGC colocalized with each other, as all tested components of the vinculin-talin-integrin system, and that all tested proteins of DGC colocalized with all tested proteins of the vinculin-talin-integrin system. Finally, all tested proteins of the two complexes were localized in the region of the sarcolemma over the I band, in 100% of our observations. The present study, for the first time, analyzed the majority of proteins of DGC and of the vinculin-talin-integrin system in cardiac muscle fibers, and it confirmed that DGC and the vinculin-talin-integrin system have a role in the transduction of mechanical force to the   extracellular matrix  . Finally it attributed a key role in the regulation of action potential duration to cardiac myocytes. 
 Molecular mechanism of vinculin activation and nanoscale spatial organization in focal adhesions. Focal adhesions (FAs) link the   extracellular matrix   to the actin cytoskeleton to mediate cell adhesion, migration, mechanosensing and signalling. FAs have conserved nanoscale protein organization, suggesting that the position of proteins within FAs regulates their activity and function. Vinculin binds different FA proteins to mediate distinct cellular functions, but how vinculin's interactions are spatiotemporally organized within FAs is unknown. Using interferometric photoactivation localization super-resolution microscopy to assay vinculin nanoscale localization and a FRET biosensor to assay vinculin conformation, we found that upward repositioning within the FA during FA maturation facilitates vinculin activation and mechanical reinforcement of FAs. Inactive vinculin localizes to the lower integrin signalling layer in FAs by binding to phospho-paxillin. Talin binding activates vinculin and targets active vinculin higher in FAs where vinculin can engage retrograde actin flow. Thus, specific protein interactions are spatially segregated within FAs at the nanoscale to regulate vinculin activation and function. 
 Vinculin negatively regulates transcription of MT1-MMP through MEK/ERK pathway. Vinculin regulates a variety of cellular functions partly through stabilization of tumor suppressor PTEN. In order to study the role of vinculin in tumor progression other than PTEN stabilization, vinculin was knocked down in PTEN-deficient squamous cell carcinoma HSC-4 cells. Knockdown of vinculin induced phenotypical change by reducing cell-cell and cell-  extracellular matrix   adhesions, and enhanced MT1-MMP expression at transcription level and subsequent cell migration. Up-regulation of MT1-MMP transcription by vinculin knockdown was abrogated by ERK inhibition. These results suggest that vinculin negatively regulates malignant phenotype of tumor cells including MT1-MMP transcription through MEK/ERK pathway. 
 Primary sequence of paxillin contains putative SH2 and SH3 domain binding motifs and multiple LIM domains: identification of a vinculin and pp125Fak-binding region. Paxillin is a cytoskeletal protein involved in actin-membrane attachment at sites of cell adhesion to the   extracellular matrix  . Extensive tyrosine phosphorylation of this protein occurs during integrin-mediated cell adhesion, embryonic development, fibroblast transformation and following stimulation of cells by mitogens that operate through the family of seven membrane-spanning G-protein-coupled receptors. Paxillin binds in vitro to the focal adhesion protein vinculin as well as to the SH3 domain of c-src and, when tyrosine phosphorylated, to the SH2 domain of v-crk. Here, we report the complementary DNA, and derived amino acid sequence, that codes for approximately 90% of the paxillin protein. We have identified a region in the amino-terminal half of the protein that supports the binding of both vinculin and the focal adhesion tyrosine kinase, pp125Fak. Although there is no significant overall homology with other identified proteins, the carboxyl third of paxillin contains one LIM domain and three LIM-like sequences. The LIM motif is common to a number of transcription factors and to two other focal adhesion proteins, zyxin and cysteine-rich protein. In addition to several potential tyrosine phosphorylation sites there are five tyrosine-containing sequences that conform to SH2-binding motifs. The protein also contains a short proline-rich region indicative of a SH3-binding domain. Taken together, these data suggest that paxillin is a unique cytoskeletal protein capable of interaction with a variety of intracellular signalling, and structural, molecules important in growth control and the regulation of cytoskeletal organization.(ABSTRACT TRUNCATED AT 250 WORDS) 
 Actomyosin-generated tension controls the molecular kinetics of focal adhesions. Focal adhesions (FAs) have key roles in the interaction of cells with the   extracellular matrix   (ECM) and in adhesion-mediated signaling. These dynamic, multi-protein structures sense the ECM both chemically and physically, and respond to external and internal forces by changing their size and signaling activity. However, this mechanosensitivity is still poorly understood at the molecular level. Here, we present direct evidence that actomyosin contractility regulates the molecular kinetics of FAs. We show that the molecular turnover of proteins within FAs is primarily regulated by their dissociation rate constant (k(off)), which is sensitive to changes in forces applied to the FA. We measured the early changes in k(off) values for three FA proteins (vinculin, paxillin and zyxin) upon inhibition of actomyosin-generated forces using two methods - high temporal resolution FRAP and direct measurement of FA protein dissociation in permeabilized cells. When myosin II contractility was inhibited, the k(off) values for all three proteins changed rapidly, in a highly protein-specific manner: dissociation of vinculin from FAs was facilitated, whereas dissociation of paxillin and zyxin was attenuated. We hypothesize that these early kinetic changes initiate FA disassembly by affecting the molecular turnover of FAs and altering their composition. 
 WAVE2 regulates high-affinity integrin binding by recruiting vinculin and talin to the immunological synapse. T-cell-receptor (TCR)-mediated integrin activation is required for T-cell-antigen-presenting cell conjugation and adhesion to   extracellular matrix   components. While it has been demonstrated that the actin cytoskeleton and its regulators play an essential role in this process, no mechanism has been established which directly links TCR-induced actin polymerization to the activation of integrins. Here, we demonstrate that TCR stimulation results in WAVE2-ARP2/3-dependent F-actin nucleation and the formation of a complex containing WAVE2, ARP2/3, vinculin, and talin. The verprolin-connecting-acidic (VCA) domain of WAVE2 mediates the formation of the ARP2/3-vinculin-talin signaling complex and talin recruitment to the immunological synapse (IS). Interestingly, although vinculin is not required for F-actin or integrin accumulation at the IS, it is required for the recruitment of talin. In addition, RNA interference of either WAVE2 or vinculin inhibits activation-dependent induction of high-affinity integrin binding to VCAM-1. Overall, these findings demonstrate a mechanism in which signals from the TCR produce WAVE2-ARP2/3-mediated de novo actin polymerization, leading to integrin clustering and high-affinity binding through the recruitment of vinculin and talin. 
 Molecular interactions of N-RAP, a nebulin-related protein of striated muscle myotendon junctions and intercalated disks. N-RAP is a recently discovered muscle-specific protein that is concentrated at the myotendon junctions in skeletal muscle and at the intercalated disks in cardiac muscle. The C-terminal half of N-RAP contains a region with sequence homology to nebulin, while a LIM domain is found at its N-terminus. N-RAP is hypothesized to perform an anchoring function, linking the terminal actin filaments of myofibrils to protein complexes located beneath the sarcolemma. We used a solid-phase assay to screen myofibrillar and junctional proteins for binding to several recombinant fragments of N-RAP, including the nebulin-like super repeat region (N-RAP-SR), the N-terminal half including the LIM domain (N-RAP-NH), and the region of N-RAP between the super repeat region and the LIM domain (N-RAP-IB). Actin is the only myofibrillar protein tested that exhibits specific binding to N-RAP, with high-affinity binding to N-RAP super repeats, and 10-fold weaker binding to N-RAP-IB. In contrast, myosin, isolated myosin heads, tropomyosin, and troponin exhibited no specific interaction with N-RAP domains. A recombinant fragment corresponding to the C-terminal one-fourth of vinculin also binds specifically to N-RAP super repeats, while no specific N-RAP binding activity was observed for other regions of the vinculin molecule. Finally, talin binds with high affinity to the LIM domain of N-RAP. These results support our hypothesis that N-RAP is part of a complex of proteins that anchors the terminal actin filaments of the myofibril to the membrane, and functions in transmitting tension from the myofibrils to the   extracellular matrix  . 
 The C-terminal tail domain of metavinculin, vinculin's splice variant, severs actin filaments. Vinculin and its splice variant, metavinculin (MV), are key elements of multiple protein assemblies linking the   extracellular matrix   to the actin cytoskeleton. Vinculin is expressed ubiquitously, whereas MV is mainly expressed in smooth and cardiac muscle tissue. The only difference in amino acid sequence between the isoforms is a 68-residue insert in the C-terminal tail domain of MV (MVt). Although the functional role of this insert remains elusive, its importance is exemplified by point mutations that are associated with dilated and hypertrophic cardiomyopathy. In vinculin, the actin binding site resides in the tail domain. In this paper, we show that MVt binds actin filaments similarly to the vinculin tail domain. Unlike its splice variant, MVt did not bundle actin filaments. Instead, MVt promoted severing of actin filaments, most efficiently at substoichiometric concentrations. This surprising and seemingly contradictory alteration of vinculin function by the 68-residue insert may be essential for modulating compliance of vinculin-induced actin bundles when exposed to rapidly increasing external forces. 
 Effects of hypergravity on the cell shape and on the organization of cytoskeleton and extracelluar matrix molecules of in vitro human dermal fibroblasts. In vitro human dermal fibroblasts were submitted to normal gravity (1 g) or to chronic hypergravity ranging from 2 to 20 g for 8 days. Changes only appeared above 15 g. The majority of 20 g-subjected cells showed fine filipods in the shape of a star whereas most control cells had rounded shapes and spread by forming lamellipodia. Indirect immunofluorescence staining of vinculin, alpha-actinin and actin stress fibers showed changes of the arrangement anchoring points of stress fibers under hypergravity. Tubulin staining showed that the centrosomal material generally located above the nucleus in control cells had migrated to the nucleus side in 20 g-exposed cells. After 8 d of culture under 20 g hypergravity the thickness of fibronectin network seemed to be increased and bundles of fibrils appeared linking ordered arrays of fibers. The fibrils of collagen I formed better delimited and thicker bundles of fibers. We may assume that 20 g hypergravity can induce changes in fibroblast cell shape, migration way, and anchorage leading to a reorganization of   extracellular matrix   without concomitant change of cell proliferation. 
 Developmental regulation of focal contact protein expression in human melanocytes. Focal contacts are transmembrane links between the   extracellular matrix   and the actin cytoskeleton that play a critical role in directed cell migration, adhesion, and normal growth. Several different component proteins of the focal contact show developmentally dependent changes in expression, suggesting that this is an important mechanism by which focal contact formation is controlled during embryogenesis. In this report we examine the expression of focal contact-associated proteins in human fetal and neonatal melanocytes using Western blotting. We show that expression of paxillin, a 69-kDa vinculin binding protein, is fourfold higher in neonatal melanocytes than in fetal melanocytes. Further, we show that talin, a high molecular weight structural protein that links integrins to the actin cytoskeleton, is proteolytically cleaved in fetal, but not in neonatal melanocytes. Immunofluorescence microscopy of cells grown on fibronectin confirmed the presence of paxillin, talin, and vinculin at the ends of actin stress fibers at presumptive focal contacts in melanocytes. Adhesion experiments to   extracellular matrix   ligands revealed significant differences in adhesion of fetal and neonatal melanocytes to fibronectin. The developmentally specific changes in focal contact protein expression observed suggest that this may be an important mechanism by which focal contact assembly is controlled in human melanocytes during development. 
  GeneRIFs  
 the vinculin-talin-integrin system have a role in the transduction of mechanical force to the   extracellular matrix   
  [Mm]etastasize  PubMed  
 Malignant pheochromocytomas/paragangliomas harbor mutations in transport and cell adhesion genes. One out of ten patients with pheochromocytoma (PCC) and paraganglioma (PGL) develop malignant disease. Today there are no reliable pathological methods to predict malignancy at the time of diagnosis. Tumors harboring mutations in the succinate dehydrogenase subunit B (SDHB) gene often   metastasize   but the sequential genetic events resulting in malignant progression are not fully understood. The aim of this study was to identify somatic mutations that contribute to the malignant transformation of PCC/PGL. We performed pair-wise (tumor-normal) whole-exome sequencing to analyze the somatic mutational landscape in five malignant and four benign primary PCC/sympathetic PGL (sPGL), including two biological replicates from each specimen. In total, 225 unique somatic mutations were identified in 215 genes, with an average mutation rate of 0.54 mutations/megabase. Malignant tumors had a significantly higher number of mutations compared to benign tumors (p < 0.001). Three novel genes were identified as recurrently mutated; MYCN, MYO5B and VCL, and mutations in these genes were exclusively found in malignant sPGL tumors. Mutations in the MYO5B gene could be verified in two publicly available data sets. A gene ontology analysis of mutated genes showed enrichment of cellular functions related to cytoskeletal protein binding, myosin complex and motor activity, many of which had functions in Rab and Rac/Rho GTPase pathways. In conclusion, we have identified recurrent mutations in genes related to intracellular transport and cell adhesion, and we have confirmed MYO5B to be recurrently mutated in PCC/PGL cases with malignant potential. Our study suggests that deregulated Rab and Rac/Rho pathways may be important in PCC/PGL tumorigenesis. 
  [Mm]etastasis  PubMed  
 Loss of vinculin and membrane-bound β-catenin promotes   metastasi  s and predicts poor prognosis in colorectal cancer. Loss of cell-cell adhesion is important for the development of cancer invasion and   metastasi  s. Vinculin, a key adhesion-related protein, can affect   metastasi  s and prognosis in several tumours. Here, we determined the biological roles of vinculin in the   metastasi  s of colorectal cancer (CRC) and evaluated its clinical significance as a potential disease biomarker.The expression level of vinculin in CRC cell lines and tissues was measured using Real-Time PCR and western blotting. Moreover, vinculin function was analysed using Transwell assays and in vivo   metastasi  s assays in gain- and loss-of-function experiments. Furthermore, the impact of vinculin together with membrane-bound β-catenin on the prognosis of 228 CRC patients was investigated by immunohistochemistry. Additionally, the expression of epithelial-mesenchymal transition (EMT) indicators was verified by immunohistochemistry in CRC tissues obtained from these patients.Vinculin expression was found to be significantly downregulated in highly metastatic CRC cell lines and metastatic tissues. Both in vitro and in vivo experiments showed that vinculin suppressed invasion, migration an  d metastas  is in CRC cells and that this suppression could be attenuated by silencing β-catenin. Moreover, the expression of vinculin and membrane-bound β-catenin were positively correlated in CRC tissues, and lack of vinculin expression emerged as an independent prognostic factor in patients with CRC. Finally, the loss of vinculin and membrane-bound β-catenin was associated with   node metas  tasis, o  rgan metas  tasis and expression of EMT indicators.Our results suggest that vinculin may play specific roles in the EMT   and metas  tasis of CRC and that loss of vinculin could be used as a prognostic factor for CRC. 
 Investigating lasp-2 in cell adhesion: new binding partners and roles in motility. Focal adhesions are intricate protein complexes that facilitate cell attachment, migration, and cellular communication. Lasp-2 (LIM-nebulette), a member of the nebulin family of actin-binding proteins, is a newly identified component of these complexes. To gain further insights into the functional role of lasp-2, we identified two additional binding partners of lasp-2: the integral focal adhesion proteins vinculin and paxillin. Of interest, the interaction of lasp-2 with its binding partners vinculin and paxillin is significantly reduced in the presence of lasp-1, another nebulin family member. The presence of lasp-2 appears to enhance the interaction of vinculin and paxillin with each other; however, as with the interaction of lasp-2 with vinculin or paxillin, this effect is greatly diminished in the presence of excess lasp-1. This suggests that the interplay between lasp-2 and lasp-1 could be an adhesion regulatory mechanism. Lasp-2's potential role in   metastasis   is revealed, as overexpression of lasp-2 in either SW620 or PC-3B1 cells-metastatic cancer cell lines-increases cell migration but impedes cell invasion, suggesting that the enhanced interaction of vinculin and paxillin may functionally destabilize focal adhesion composition. Taken together, these data suggest that lasp-2 has an important role in coordinating and regulating the composition and dynamics of focal adhesions. 
  [Cc]ell [Ii]nvasion  PubMed  
 Novel vinculin binding site of the IpaA invasin of Shigella. Internalization of Shigella into host epithelial cells, where the bacteria replicates and spreads to neighboring cells, requires a type 3 secretion system (T3SS) effector coined IpaA. IpaA binds directly to and activates the cytoskeletal protein vinculin after injection in the host cell cytosol, and this was previously thought to be directed by two amphipathic α-helical vinculin-binding sites (VBS) found in the C-terminal tail domain of IpaA. Here, we report a third VBS, IpaA-VBS3, that is located N-terminal to the other two VBSs of IpaA and show that one IpaA molecule can bind up to three vinculin molecules. Biochemical in vitro Shigella invasion assays and the 1.6 Å crystal structure of the vinculin·IpaA-VBS3 complex showed that IpaA-VBS3 is functionally redundant with the other two IpaA-VBSs   in cell invas  ion and in activating the latent F-actin binding functions of vinculin. Multiple VBSs in IpaA are reminiscent of talin, which harbors 11 VBSs. However, most of the talin VBSs have low affinity and are buried in helix bundles, whereas all three of the VBSs of IpaA are high affinity, readily available, and in close proximity to each other in the IpaA structure. Although deletion of IpaA-VBS3 has no detectable effects on Shigella invasion of epithelial cells, deletion of all three VBSs impaired bacterial invasion to levels found in an ipaA null mutant strain. Thus, IpaA-directed mimicry of talin in activating vinculin occurs through three high affinity VBSs that are essential for Shigella pathogenesis. 
 Investigating lasp-2 in cell adhesion: new binding partners and roles in motility. Focal adhesions are intricate protein complexes that facilitate cell attachment, migration, and cellular communication. Lasp-2 (LIM-nebulette), a member of the nebulin family of actin-binding proteins, is a newly identified component of these complexes. To gain further insights into the functional role of lasp-2, we identified two additional binding partners of lasp-2: the integral focal adhesion proteins vinculin and paxillin. Of interest, the interaction of lasp-2 with its binding partners vinculin and paxillin is significantly reduced in the presence of lasp-1, another nebulin family member. The presence of lasp-2 appears to enhance the interaction of vinculin and paxillin with each other; however, as with the interaction of lasp-2 with vinculin or paxillin, this effect is greatly diminished in the presence of excess lasp-1. This suggests that the interplay between lasp-2 and lasp-1 could be an adhesion regulatory mechanism. Lasp-2's potential role in metastasis is revealed, as overexpression of lasp-2 in either SW620 or PC-3B1 cells-metastatic cancer cell lines-increases cell migration but impedes   cell invasion  , suggesting that the enhanced interaction of vinculin and paxillin may functionally destabilize focal adhesion composition. Taken together, these data suggest that lasp-2 has an important role in coordinating and regulating the composition and dynamics of focal adhesions. 
   
  
 SLC26A11 
 
  NCBI Gene  
  GeneRIF  
  Pubmed  
 
 
  Search Term  Rank  Fields  
  [Mm]igration  6  PubMed(1)  
 
 
 hide/show details for SLC26A11 
  [Mm]igration  PubMed  
 Molecular and functional characterization of SLC26A11, a sodium-independent sulfate transporter from high endothelial venules. Lymphocyte e  migration   from the blood into most secondary lymphoid organs and chronically inflamed tissues occurs at the level of high endothelial venules (HEV). A unique characteristic of HEV endothelial cells (HEVEC) is their capacity to incorporate large amounts of sulfate into sialomucin-type counter-receptors for the lymphocyte homing receptor L-selectin. We have previously shown that sulfate uptake into HEVEC is mediated by two distinct functional classes of sulfate transporters: Na+-coupled transporters and sulfate/anion exchangers. Here, we report the molecular characterization from human HEVEC of SLC26A11, a novel member of the SLC26 sulfate/anion exchanger family. Functional expression studies in COS-7 and Sf9 insect cells revealed that SLC26A11 is targeted to the cell membrane and exhibits Na+-independent sulfate transport activity, sensitive to the anion exchanger inhibitor 4,4'-diisothiocyanostilbene-2,2'-disulfonic acid (DIDS). Northern blot analysis showed the highest SLC26A11 transcript levels in placenta, kidney, and brain. The SLC26A11 gene mapped to human chromosome 17q25, very close to the hereditary hearing loss diseases loci DFNA20, DFNA26, and USH1G. RT-PCR analysis of SLC26 sulfate transporters in human HEVEC revealed coexpression of SLC26A11 with SLC26A2/DTDST and lack of SLC26A1/SAT1, SLC26A3/DRA, and SLC26A8/TAT1. Together, our results indicate that SLC26A11 is a novel Na+-independent sulfate transporter that may cooperate with SLC26A2 to mediate DIDS-sensitive sulfate uptake into HEVEC. 
   
  
 MYL6 
 
  NCBI Gene  
  GeneRIF  
  Pubmed  
 
 
  Search Term  Rank  Fields  
  [Mm]igration  6  PubMed(1)  
  [Ee]xtracellular [Mm]atrix  8  PubMed(1); Component(1)  
  [Mm]etastasis  12  PubMed(1)  
 
 
 hide/show details for MYL6 
  [Mm]igration  PubMed  
 RAC3 is a pro-migratory co-activator of ERα. Estrogen receptor alpha (ERα) is a ligand-dependent nuclear receptor that is important in breast cancer genesis, behavior and response to hormone-based therapies. A T7 phage display screen against full-length human ERα, coupled with genome-wide exon arrays, was used to identify RAC3 as a putative ERα co-regulator. RAC3 is a Rho family small GTPase that is associated with cytoskeletal rearrangement. We demonstrate a novel role for nuclear RAC3 as an ERα transcriptional activator, with prognostic implications for metastatic disease. Through in vitro and cell-based studies, RAC3 was shown to exist in a GTP-bound state and act as a ligand specific ERα co-activator of E2-induced transcription. Overexpression of RAC3 induced pro-growth and pro-migratory genes that resulted in incr  eased mig  ration of ERα-positive breast cancer cells. Chemical inhibition and genetic knockdown of RAC3 antagonized E2-induced cell proliferation  , cell mi  gration and ERα mediated gene expression, indicating that RAC3 is necessary for full ERα transcriptional activity. In agreement with the molecular and cellular data, RAC3 overexpression in ERα-positive breast cancers correlated with a significant decrease in recurrence free survival and a significant increase in the odds ratio of metastasis. In conclusion, RAC3 is a novel ERα co-activator that pro  motes cel  l migration and has prognostic value for ERα-positive breast cancer metastasis. RAC3 may also be a useful therapeutic target for ERα-positive breast cancers. 
  [Ee]xtracellular [Mm]atrix  PubMed  
 SILAC-based proteomics of human primary endothelial cell morphogenesis unveils tumor angiogenic markers. Proteomics has been successfully used for cell culture on dishes, but more complex cellular systems have proven to be challenging and so far poorly approached with proteomics. Because of the complexity of the angiogenic program, we still do not have a complete understanding of the molecular mechanisms involved in this process, and there have been no in depth quantitative proteomic studies. Plating endothelial cells on matrigel recapitulates aspects of vessel growth, and here we investigate this mechanism by using a spike-in SILAC quantitative proteomic approach. By comparing proteomic changes in primary human endothelial cells morphogenesis on matrigel to general adhesion mechanisms in cells spreading on culture dish, we pinpoint pathways and proteins modulated by endothelial cells. The cell-  extracellular matrix   adhesion proteome depends on the adhesion substrate, and a detailed proteomic profile of the   extracellular matrix   secreted by endothelial cells identified CLEC14A as a matrix component, which binds to MMRN2. We verify deregulated levels of these proteins during tumor angiogenesis in models of multistage carcinogenesis. This is the most in depth quantitative proteomic study of endothelial cell morphogenesis, which shows the potential of applying high accuracy quantitative proteomics to in vitro models of vessel growth to shed new light on mechanisms that accompany pathological angiogenesis. The mass spectrometry proteomics data have been deposited to the ProteomeXchange Consortium with the data set identifier PXD000359. 
  Component  
   extracellular matrix   
  [Mm]etastasis  PubMed  
 RAC3 is a pro-migratory co-activator of ERα. Estrogen receptor alpha (ERα) is a ligand-dependent nuclear receptor that is important in breast cancer genesis, behavior and response to hormone-based therapies. A T7 phage display screen against full-length human ERα, coupled with genome-wide exon arrays, was used to identify RAC3 as a putative ERα co-regulator. RAC3 is a Rho family small GTPase that is associated with cytoskeletal rearrangement. We demonstrate a novel role for nuclear RAC3 as an ERα transcriptional activator, with prognostic implications for metastatic disease. Through in vitro and cell-based studies, RAC3 was shown to exist in a GTP-bound state and act as a ligand specific ERα co-activator of E2-induced transcription. Overexpression of RAC3 induced pro-growth and pro-migratory genes that resulted in increased migration of ERα-positive breast cancer cells. Chemical inhibition and genetic knockdown of RAC3 antagonized E2-induced cell proliferation, cell migration and ERα mediated gene expression, indicating that RAC3 is necessary for full ERα transcriptional activity. In agreement with the molecular and cellular data, RAC3 overexpression in ERα-positive breast cancers correlated with a significant decrease in recurrence free survival and a significant increase in the odds   ratio of   metastasis. In conclusion, RAC3 is a novel ERα co-activator that promotes cell migration and has prognostic value for ERα-positive br  east cance  r metastasis. RAC3 may also be a useful therapeutic target for ERα-positive breast cancers. 
   
  
 GMFG 
 
  NCBI Gene  
  GeneRIF  
  Pubmed  
 
 
  Search Term  Rank  Fields  
  [Mm]igration  6  PubMed(5); GeneRIFs(2)  
  [Ee]xtracellular [Mm]atrix  8  PubMed(1)  
  [Mm]etastasis  12  PubMed(1); GeneRIFs(1)  
 
 
 hide/show details for GMFG 
  [Mm]igration  PubMed  
 Glia Maturation Factor-γ Regulates Monocyte   Migratio  n through Modulation of β1-Integrin. Monocyt  e migrati  on requires the dynamic redistribution of integrins through a regulated endo-exocytosis cycle, but the complex molecular mechanisms underlying this process have not been fully elucidated. Glia maturation factor-γ (GMFG), a novel regulator of the Arp2/3 complex, has been shown to regulate direction  al migrat  ion of neutrophils and T-lymphocytes. In this study, we explored the important role of GMFG in monocyte chemotaxis, adhesion, and β1-integrin turnover. We found that knockdown of GMFG in monocytes resulted in impaired chemotac  tic migra  tion toward formyl-Met-Leu-Phe (fMLP) and stromal cell-derived factor 1α (SDF-1α) as well as decreased α5β1-integrin-mediated chemoattractant-stimulated adhesion. These GMFG knockdown impaired effects could be reversed by cotransfection of GFP-tagged full-length GMFG. GMFG knockdown cells reduced the cell surface and total protein levels of α5β1-integrin and increased its degradation. Importantly, we demonstrate that GMFG mediates the ubiquitination of β1-integrin through knockdown or overexpression of GMFG. Moreover, GMFG knockdown retarded the efficient recycling of β1-integrin back to the plasma membrane following normal endocytosis of α5β1-integrin, suggesting that the involvement of GMFG in maintaining α5β1-integrin stability may occur in part by preventing ubiquitin-mediated degradation and promoting β1-integrin recycling. Furthermore, we observed that GMFG interacted with syntaxin 4 (STX4) and syntaxin-binding protein 4 (STXBP4); however, only knockdown of STXBP4, but not STX4,   reduced m  onocyte migration and decreased β1-integrin cell surface expression. Knockdown of STXBP4 also substantially inhibited β1-integrin recycling in human monocytes. These results indicate that the effects o  f GMFG on   monocyte migration and adhesion probably occur through preventing ubiquitin-mediated proteasome degradation of α5β1-integrin and facilitating effective β1-integrin recycling back to the plasma membrane. 
 Glia Maturation Factor-γ Regulates Monocyte   Migratio  n through Modulation of β1-Integrin. Monocyt  e migrati  on requires the dynamic redistribution of integrins through a regulated endo-exocytosis cycle, but the complex molecular mechanisms underlying this process have not been fully elucidated. Glia maturation factor-γ (GMFG), a novel regulator of the Arp2/3 complex, has been shown to regulate direction  al migrat  ion of neutrophils and T-lymphocytes. In this study, we explored the important role of GMFG in monocyte chemotaxis, adhesion, and β1-integrin turnover. We found that knockdown of GMFG in monocytes resulted in impaired chemotac  tic migra  tion toward formyl-Met-Leu-Phe (fMLP) and stromal cell-derived factor 1α (SDF-1α) as well as decreased α5β1-integrin-mediated chemoattractant-stimulated adhesion. These GMFG knockdown impaired effects could be reversed by cotransfection of GFP-tagged full-length GMFG. GMFG knockdown cells reduced the cell surface and total protein levels of α5β1-integrin and increased its degradation. Importantly, we demonstrate that GMFG mediates the ubiquitination of β1-integrin through knockdown or overexpression of GMFG. Moreover, GMFG knockdown retarded the efficient recycling of β1-integrin back to the plasma membrane following normal endocytosis of α5β1-integrin, suggesting that the involvement of GMFG in maintaining α5β1-integrin stability may occur in part by preventing ubiquitin-mediated degradation and promoting β1-integrin recycling. Furthermore, we observed that GMFG interacted with syntaxin 4 (STX4) and syntaxin-binding protein 4 (STXBP4); however, only knockdown of STXBP4, but not STX4,   reduced m  onocyte migration and decreased β1-integrin cell surface expression. Knockdown of STXBP4 also substantially inhibited β1-integrin recycling in human monocytes. These results indicate that the effects o  f GMFG on   monocyte migration and adhesion probably occur through preventing ubiquitin-mediated proteasome degradation of α5β1-integrin and facilitating effective β1-integrin recycling back to the plasma membrane. 
 Expression of glia maturation factor γ is associated with colorectal cancer metastasis and its downregulation suppresses colorectal cancer cell   migratio  n and invasion in vitro. Glia maturation factor γ (GMFG) functions to reorganize the actin cytoskeleton and appears to play a causative role in ce  ll migrat  ion and adherence. The present study assessed GMFG expression in colorectal cancer cells and tissue specimens and then explored the role of GMFG in colorectal cancer progression in vitro. GMFG protein was highly expressed in colorectal cancer tissues and a metastatic colon cancer cell line. Knockdown of GMFG expression using GMFG siRNA or anti-GMFG antibody decreased the capacity of colon cancer LoVo c  ell migra  tion and invasion in vitro, while recombinant GMFG treatment induced LoVo   cell migr  ation. Furthermore, GMFG knockdown also decreased expression of MMP2 protein and reversed epithelial-mesenchymal transition (EMT) phenotypes in LoVo cells. Co-culture of LoVo cells with human umbilical vein endothelial cells (HUVECs) and exogenous GMFG treatment promoted LoVo   cell migr  ation and invasion. The data from the present study indicate that GMFG should be further evaluated as a biomarker for detection of colorectal cancer metastasis and that the targeting of GMFG expression or function could be a novel strategy in the future control of colorectal cancer. 
 Glia maturation factor gamma regulates the   migration   and adherence of human T lymphocytes. Lymphocyte   migration   and chemotaxis are essential for effective immune surveillance. A critical aspect of   migration   is cell polarization and the extension of pseudopodia in the direction of movement. However, our knowledge of the underlying molecular mechanisms responsible for these events is incomplete. Proteomic analysis of the isolated leading edges of CXCL12 stimulated human T cell lines was used to identify glia maturation factor gamma (GMFG) as a component of the pseudopodia. This protein is predominantly expressed in hematopoietic cells and it has been shown to regulate cytoskeletal branching. The present studies were undertaken to examine the role of GMFG in lymphocyte   migration  .Microscopic analysis of migrating T-cells demonstrated that GMFG was distributed along the axis of movement with enrichment in the leading edge and behind the nucleus of these cells. Inhibition of GMFG expression in T cell lines and IL-2 dependent human peripheral blood T cells with shRNAmir reduced cellular basal and chemokine induced   migration   responses. The failure of the cells with reduced GMFG to migrate was associated with an apparent inability to detach from the substrates that they were moving on. It was also noted that these cells had an increased adherence to extracellular matrix proteins such as fibronectin. These changes in adherence were associated with altered patterns of β1 integrin expression and increased levels of activated integrins as detected with the activation specific antibody HUTS4. GMFG loss was also shown to increase the expression of the β2 integrin LFA-1 and to increase the adhesion of these cells to ICAM-1.The present studies demonstrate that GMFG is a component of human T cell pseudopodia required fo  r migrati  on. The reduction i  n migrati  on and increased adherence properties associated with inhibition of GMFG expression suggest that GMFG activity influences the regulation of integrin mediated adhesion. 
 Glia maturation factor-γ negatively modulates TLR4 signaling by facilitating TLR4 endocytic trafficking in macrophages. TLR4 signaling must be tightly regulated to provide both effective immune protection and avoid inflammation-induced pathology. Thus, the mechanisms that negatively regulate the TLR4-triggered inflammatory response are of particular importance. Glia maturation factor-γ (GMFG), a novel actin depolymerization factor/cofilin superfamily protein that is expressed in inflammatory cells, has been implicated in mediating neutrophil and T cel  l migrati  on, but its function in macrophage immune response remains unclear. In the current study, the role of GMFG in the LPS-induced TLR4-signaling pathway was investigated in THP-1 macrophages and human primary macrophages. LPS stimulation of macrophages decreased GMFG mRNA and protein expression. We show that GMFG negatively regulates LPS-induced activation of NF-κB-, MAPK-, and IRF3-signaling pathways and subsequent production of proinflammatory cytokines and type I IFN in human macrophages. We found that endogenous GMFG localized within early and late endosomes. GMFG knockdown delayed LPS-induced TLR4 internalization and caused prolonged TLR4 retention at the early endosome, suggesting that TLR4 transport from early to late endosomes is interrupted, which may contribute to enhanced LPS-induced TLR4 signaling. Taken together, our findings suggest that GMFG functions as a negative regulator of TLR4 signaling by facilitating TLR4 endocytic trafficking in macrophages. 
  GeneRIFs  
 These results indicate that the effects of GMFG on monocyte   migration   and adhesion probably occur through preventing ubiquitin-mediated proteasome degradation of alpha5beta1-integrin and facilitating effective beta1-integrin recycling back to the plasma membrane. 
 regulates the   migration   and adherence of human T lymphocytes 
  [Ee]xtracellular [Mm]atrix  PubMed  
 Glia maturation factor gamma regulates the migration and adherence of human T lymphocytes. Lymphocyte migration and chemotaxis are essential for effective immune surveillance. A critical aspect of migration is cell polarization and the extension of pseudopodia in the direction of movement. However, our knowledge of the underlying molecular mechanisms responsible for these events is incomplete. Proteomic analysis of the isolated leading edges of CXCL12 stimulated human T cell lines was used to identify glia maturation factor gamma (GMFG) as a component of the pseudopodia. This protein is predominantly expressed in hematopoietic cells and it has been shown to regulate cytoskeletal branching. The present studies were undertaken to examine the role of GMFG in lymphocyte migration.Microscopic analysis of migrating T-cells demonstrated that GMFG was distributed along the axis of movement with enrichment in the leading edge and behind the nucleus of these cells. Inhibition of GMFG expression in T cell lines and IL-2 dependent human peripheral blood T cells with shRNAmir reduced cellular basal and chemokine induced migration responses. The failure of the cells with reduced GMFG to migrate was associated with an apparent inability to detach from the substrates that they were moving on. It was also noted that these cells had an increased adherence to   extracellular matrix   proteins such as fibronectin. These changes in adherence were associated with altered patterns of β1 integrin expression and increased levels of activated integrins as detected with the activation specific antibody HUTS4. GMFG loss was also shown to increase the expression of the β2 integrin LFA-1 and to increase the adhesion of these cells to ICAM-1.The present studies demonstrate that GMFG is a component of human T cell pseudopodia required for migration. The reduction in migration and increased adherence properties associated with inhibition of GMFG expression suggest that GMFG activity influences the regulation of integrin mediated adhesion. 
  [Mm]etastasis  PubMed  
 Expression of glia maturation factor γ is associated with colorectal cancer   metastasi  s and its downregulation suppresses colorectal cancer cell migration and invasion in vitro. Glia maturation factor γ (GMFG) functions to reorganize the actin cytoskeleton and appears to play a causative role in cell migration and adherence. The present study assessed GMFG expression in colorectal cancer cells and tissue specimens and then explored the role of GMFG in colorectal cancer progression in vitro. GMFG protein was highly expressed in colorectal cancer tissues and a metastatic colon cancer cell line. Knockdown of GMFG expression using GMFG siRNA or anti-GMFG antibody decreased the capacity of colon cancer LoVo cell migration and invasion in vitro, while recombinant GMFG treatment induced LoVo cell migration. Furthermore, GMFG knockdown also decreased expression of MMP2 protein and reversed epithelial-mesenchymal transition (EMT) phenotypes in LoVo cells. Co-culture of LoVo cells with human umbilical vein endothelial cells (HUVECs) and exogenous GMFG treatment promoted LoVo cell migration and invasion. The data from the present study indicate that GMFG should be further evaluated as a biomarker for detection of colorectal ca  ncer metas  tasis and that the targeting of GMFG expression or function could be a novel strategy in the future control of colorectal cancer. 
  GeneRIFs  
 High GMF-GAMMA expression is associated with colorectal cancer   metastasis  . 
   
  
 ABCC3 
 
  NCBI Gene  
  GeneRIF  
  Pubmed  
 
 
  Search Term  Rank  Fields  
  [Mm]igration  6  PubMed(1)  
  [Ii]nvasive  10  PubMed(1)  
  [Mm]etastasis  12  PubMed(3)  
 
 
 hide/show details for ABCC3 
  [Mm]igration  PubMed  
 ABCC multidrug transporters in childhood neuroblastoma: clinical and biological effects independent of cytotoxic drug efflux. Although the prognostic value of the ATP-binding cassette, subfamily C (ABCC) transporters in childhood neuroblastoma is usually attributed to their role in cytotoxic drug efflux, certain observations have suggested that these multidrug transporters might contribute to the malignant phenotype independent of cytotoxic drug efflux.A v-myc myelocytomatosis viral related oncogene, neuroblastoma derived (MYCN)-driven transgenic mouse neuroblastoma model was crossed with an Abcc1-deficient mouse strain (658 hMYCN(1/-), 205 hMYCN(+/1) mice) or, alternatively, treated with the ABCC1 inhibitor, Reversan (n = 20). ABCC genes were suppressed using short interfering RNA or overexpressed by stable transfection in neuroblastoma cell lines BE(2)-C, SH-EP, and SH-SY5Y, which were then assessed for wound closure ability, clonogenic capacity, morphological differentiation, and cell growth. Real-time quantitative polymerase chain reaction was used to examine the clinical significance of ABCC family gene expression in a large prospectively accrued cohort of patients (n = 209) with primary neuroblastomas. Kaplan-Meier survival analysis and Cox regression were used to test for associations with event-free and overall survival. Except where noted, all statistical tests were two-sided.Inhibition of ABCC1 statistically significantly inhibited neuroblastoma development in hMYCN transgenic mice (mean age for palpable tumor: treated mice, 47.2 days; control mice, 41.9 days; hazard ratio [HR] = 9.3, 95% confidence interval [CI] = 2.65 to 32; P < .001). Suppression of ABCC1 in vitro inhibited wound closure (P < .001) and clonogenicity (P = .006); suppression of ABCC4 enhanced morphological differentiation (P < .001) and inhibited cell growth (P < .001). Analysis of 209 neuroblastoma patient tumors revealed that, in contrast with ABCC1 and ABCC4, low rather than high ABCC3 expression was associated with reduced event-free survival (HR of recurrence or death = 2.4, 95% CI = 1.4 to 4.2; P = .001), with 23 of 53 patients with low ABCC3 expression experiencing recurrence or death compared with 31 of 155 patients with high ABCC3. Moreover, overexpression of ABCC3 in vitro inhibited neuroblastoma cell   migration   (P < .001) and clonogenicity (P = .03). The combined expression of ABCC1, ABCC3, and ABCC4 was associated with patients having an adverse event, such that of the 12 patients with the "poor prognosis" expression pattern, 10 experienced recurrence or death (HR of recurrence or death = 12.3, 95% CI = 6 to 27; P < .001).ABCC transporters can affect neuroblastoma biology independently of their role in chemotherapeutic drug efflux, enhancing their potential as targets for therapeutic intervention. 
  [Ii]nvasive  PubMed  
 Expression profile of early lung adenocarcinoma: identification of MRP3 as a molecular marker for early progression. Early lung adenocarcinoma is well-recognized as a small-sized non-  invasive   adenocarcinoma or localized non-mucinous bronchioloalveolar carcinoma (LNMBAC); however, the molecular events associated with these early lesions are not clear. To determine the genes involved in tumorigenesis at the early stage of lung adenocarcinoma, we compared the mRNA expression profiles of LNMBAC and normal lungs with an oligonucleotide array. Immunohistochemical analyses were performed to confirm the expression of detected genes. We identified 183 differentially expressed genes, of which 15 were up-regulated and 168 down-regulated. Among them, most up-regulated genes, such as AQP3 and Claudin-4, were expressed in both adenocarcinoma cells and type II alveolar pneumocytes, corresponding to the histological similarity between these cell types. However, multidrug resistant protein 3 (MRP3) was only expressed on tumour cell membranes and not in type II alveolar pneumocytes, as confirmed by immunohistochemistry. Moreover, the number of MRP3-positive cells significantly increased from AAH (the precursor lesion of lung adenocarcinoma) to LNMBAC. We conclude that MRP3 could be a novel molecular marker for LNMBAC, whose expression increases during the early progression of tumourigenesis. 
  [Mm]etastasis  PubMed  
 Overexpression of ABCC3 promotes cell proliferation, drug resistance, and aerobic glycolysis and is associated with poor prognosis in urinary bladder cancer patients. Human urinary bladder cancer (UBC) is the one of the most common malignancies worldwide and occurs at a higher frequency in male individuals. ATP-binding cassette, subfamily C, member 3 (ABCC3), a member of the ABC transporter family, is highly expressed in tumor cells, where it actively effluxes a broad spectrum of metabolites. However, the expression and role of ABCC3 in human UBC remains unclear. Our study aimed to identify the expression status of ABCC3 in UBC cases and investigate the biological effects on UBC in cells. We found that both mRNA and protein levels of ABCC3 were significantly higher in UBC tissues than normal tissues. Immunochemistry evaluation of ABCC3 expression in 122 UBC clinical specimens showed that high expression of ABCC3 had a positive correlation with UBC tumor size, advanced tumor node   metastasis   stage, and malignant histology. Moreover, high ABCC3 expression was linked to poor overall survival in UBC. ABCC3 effects on cell proliferation and drug resistance were measured by colony formation and methylthiazolyldiphenyl-tetrazolium bromide (MTT) assays. ABCC3-knockdown cells showed a significant decrease in cell growth and drug resistance. RNA interference of ABCC3 also caused downregulation of lactate dehydrogenase A (LDHA), which positively correlated with ABCC3 expression in UBC specimens. In addition, cancer cell glycolytic ability was decreased upon ABCC3 knockdown. The activity of LDHA was also abrogated in ABCC3-deficient UBC cells, and the blockade of LDHA increased UBC cells sensitivity to Cis-diamine dichloroplatinum (CDDP). In summary, our study suggests ABCC3 is an important oncoprotein involved in glycolysis and drug resistance. These data also indicates that ABCC3 could be a potential prognostic marker and promising therapeutic target in UBC. 
 A role for calcium in the regulation of ATP-binding cassette, sub-family C, member 3 (ABCC3) gene expression in a model of epidermal growth factor-mediated breast cancer epithelial-mesenchymal transition. Epithelial-mesenchymal transition (EMT), a process implicated in cancer   metastasis  , is associated with the transcriptional regulation of members of the ATP-binding cassette superfamily of efflux pumps, and drug resistance in breast cancer cells. Epidermal growth factor (EGF)-induced EMT in MDA-MB-468 breast cancer cells is calcium signal dependent. In this study induction of EMT was shown to result in the transcriptional up-regulation of ATP-binding cassette, subfamily C, member 3 (ABCC3), a member of the ABC transporter superfamily, which has a recognized role in multidrug resistance. Buffering of cytosolic free calcium inhibited EGF-mediated ABCC3 increases, indicating a calcium-dependent mode of regulation. Silencing of TRPM7 (an ion channel involved in EMT associated vimentin induction) did not inhibit ABCC3 up-regulation. Silencing of the store operated calcium entry (SOCE) pathway components ORAI1 and STIM1 also did not alter ABCC3 induction by EGF. However, the calcium permeable ion channel transient receptor potential cation channel, subfamily C, member 1 (TRPC1) appears to contribute to the regulation of both basal and EGF-induced ABCC3 mRNA. Improved understanding of the relationship between calcium signaling, EMT and the regulation of genes important in therapeutic resistance may help identify novel therapeutic targets for breast cancer. 
 Modulation of MDR1 and MRP3 gene expression in lung cancer cells after paclitaxel and carboplatin exposure. Carboplatin-paclitaxel is a reference regimen in the treatment of locally advanced or disseminated non-small cell lung cancer (NSCLC). This paper discusses the multidrug resistance developed with this drug combination, which is one of the major obstacles to successful treatment. In order to understand and overcome the drug resistance pattern of NSCLC after carboplatin plus paclitaxel exposure, levels of mRNA expression of multidrug resistance 1 (MDR1) and multidrug resistance-associated protein 3 (MRP3) were investigated in primary NSCLC cell lines (A-549 and A-427) and a   metastasis  -derived NSCLC cell line (NODO). Our results showed that exposure of the three NSCLC lines to plasma concentrations of paclitaxel (5 μM) produced an increase in MDR1 expression, while MRP3 showed no alteration in expression. By contrast, the same cells exposed to carboplatin plasma concentrations (30 μM) showed overexpression of MRP3. In these cells, MDR1 showed no expression changes. Interestingly, the combination of both paclitaxel and carboplatin caused increased expression of the MDR1 drug resistance gene rather than the individual treatments. These results suggest that carboplatin and paclitaxel may induce drug resistance mediated by MDR1 and MRP3, which may be enhanced by the simultaneous use of both drugs. 
   
  
 STRAP 
 
  NCBI Gene  
  GeneRIF  
  Pubmed  
 
 
  Search Term  Rank  Fields  
  [Mm]igration  6  Process(1); PubMed(1)  
  [Mm]etastasis  12  PubMed(1)  
  [Cc]ell [Ii]nvasion  13  PubMed(1)  
 
 
 hide/show details for STRAP 
  [Mm]igration  Process  
 negative regulation of epithelial cell   migration   
  PubMed  
 MAWBP and MAWD inhibit proliferation and invasion in gastric cancer. To investigate role of putative mitogen-activated protein kinase activator with WD40 repeats (MAWD)/MAWD binding protein (MAWBP) in gastric cancer (GC).MAWBP and MAWD mRNA expression level was examined by real-time reverse transcriptase-polymerase chain reaction and semi-quantitative polymerase chain reaction in six GC cell lines. Western blotting was used to examine the protein expression levels. We developed GC cells that stably overexpressed MAWBP and MAWD, and downregulated expression by RNA interference assay. Proliferation and   migration   of these GC cells were analyzed by 3-(4,5-dimethyl-2-thiazolyl)-2,5-diphenyl tetrazolium bromide (MTT), soft agar, tumorigenicity,   migration   and transwell assays. The effect of expression of MAWBP and MAWD on transforming growth factor (TGF)-β1-induced epithelial-mesenchymal transition (EMT) was examined by transfection of MAWBP and MAWD into GC cells. We detected the levels of EMT markers E-cadherin, N-cadherin and Snail in GC cells overexpressing MAWBP and MAWD by Western blotting. The effect of MAWBP and MAWD on TGF-β signal was detected by analysis of phosphorylation level and nuclear translocation of Smad3 using Western blotting and immunofluorescence.Among the GC cell lines, expression of endogenous MAWBP and MAWD was lowest in SGC7901 cells and highest in BGC823 cells. MAWBP and MAWD were stably overexpressed in SGC7901 cells and knocked down in BGC823 cells. MAWBP and MAWD inhibited GC cell proliferation in vitro and in vivo. MTT assay showed that overexpression of MAWBP and MAWD suppressed growth of SGC7901 cells (P < 0.001), while knockdown of these genes promoted growth of BGC823 cells (P < 0.001). Soft agar colony formation experiments showed that overexpression of MAWBP and MAWD alone or together reduced colony formation compared with vector group in SGC7901 (86.25 ± 8.43, 12.75 ± 4.49, 30 ± 6.41 vs 336.75 ± 22.55, P < 0.001), and knocked-down MAWBP and MAWD demonstrated opposite effects (131.25 ± 16.54, 88.75 ± 11.12, 341.75 ± 22.23 vs 30.25 ± 8.07, P < 0.001). Tumorigenicity experiments revealed that overexpressed MAWBP and MAWD inhibited GC cell proliferation in vivo (P < 0.001). MAWBP and MAWD also inhibited GC cell invasion. Transwell assay showed that the number of traverse cells of MAWBP, MAWD and coexpression group were more than that in vector group (84 ± 16.57, 98.33 ± 9.8, 29 ± 16.39 vs 298 ± 11.86, P < 0.001). Coexpression of MAWBP and MAWD significantly decreased the cells traversing the matrix membrane. Conversely, knocked-down MAWBP and MAWD correspondingly promoted invasion of GC cells (100.67 ± 14.57, 72.66 ± 8.51, 330.67 ± 20.55 vs 27 ± 11.53, P < 0.001). More importantly, coexpression of MAWBP and MAWD promoted EMT. Cells that coexpressed MAWBP and MAWD displayed a pebble-like shape and tight cell-cell adhesion, while vector cells showed a classical mesenchymal phenotype. Western blotting showed that expression of E-cadherin was increased, and expression of N-cadherin and Snail was decreased when cells coexpressed MAWBP and MAWD and were treated with TGF-β1. Nuclear translocation of p-Smad3 was reduced by attenuating its phosphorylation.Coexpression of MAWBP and MAWD inhibited EMT, and EMT-aided malignant cell progression was suppressed. 
  [Mm]etastasis  PubMed  
 NM23-H1 tumor suppressor physically interacts with serine-threonine kinase receptor-associated protein, a transforming growth factor-beta (TGF-beta) receptor-interacting protein, and negatively regulates TGF-beta signaling. NM23-H1 is a member of the NM23/NDP kinase gene family and a putative   metastasis   suppressor. Previously, a screen for NM23-H1-interacting proteins that could potentially modulate its activity identified serine-threonine kinase receptor-associated protein (STRAP), a transforming growth factor (TGF)-beta receptor-interacting protein. Through the use of cysteine to serine amino acid substitution mutants of NM23-H1 (C4S, C109S, and C145S) and STRAP (C152S, C270S, and C152S/C270S), we demonstrated that the association between these two proteins is dependent on Cys(145) of NM23-H1 and Cys(152) and Cys(270) of STRAP but did not appear to involve Cys(4) and Cys(109) of NM23-H1, suggesting that a disulfide linkage involving Cys(145) of NM23-H1 and Cys(152) or Cys(270) of STRAP mediates complex formation. The interaction was dependent on the presence of dithiothreitol or beta-mercaptoethanol but not H(2)O(2). Ectopic expression of wild-type NM23-H1, but not NM23-H1(C145S), negatively regulated TGF-beta signaling in a dose-dependent manner, enhanced stable association between the TGF-beta receptor and Smad7, and prevented nuclear translocation of Smad3. Similarly, wild-type NM23-H1 inhibited TGF-beta-induced apoptosis and growth inhibition, whereas NM23-H1(C145S) had no effect. Knockdown of NM23-H1 by small interfering RNA stimulated TGF-beta signaling. Coexpression of wild-type STRAP, but not STRAP(C152S/C270S), significantly stimulated NM23-H1-induced growth of HaCaT cells. These results suggest that the direct interaction of NM23-H1 and STRAP is important for the regulation of TGF-beta-dependent biological activity as well as NM23-H1 activity. 
  [Cc]ell [Ii]nvasion  PubMed  
 MAWBP and MAWD inhibit proliferation and invasion in gastric cancer. To investigate role of putative mitogen-activated protein kinase activator with WD40 repeats (MAWD)/MAWD binding protein (MAWBP) in gastric cancer (GC).MAWBP and MAWD mRNA expression level was examined by real-time reverse transcriptase-polymerase chain reaction and semi-quantitative polymerase chain reaction in six GC cell lines. Western blotting was used to examine the protein expression levels. We developed GC cells that stably overexpressed MAWBP and MAWD, and downregulated expression by RNA interference assay. Proliferation and migration of these GC cells were analyzed by 3-(4,5-dimethyl-2-thiazolyl)-2,5-diphenyl tetrazolium bromide (MTT), soft agar, tumorigenicity, migration and transwell assays. The effect of expression of MAWBP and MAWD on transforming growth factor (TGF)-β1-induced epithelial-mesenchymal transition (EMT) was examined by transfection of MAWBP and MAWD into GC cells. We detected the levels of EMT markers E-cadherin, N-cadherin and Snail in GC cells overexpressing MAWBP and MAWD by Western blotting. The effect of MAWBP and MAWD on TGF-β signal was detected by analysis of phosphorylation level and nuclear translocation of Smad3 using Western blotting and immunofluorescence.Among the GC cell lines, expression of endogenous MAWBP and MAWD was lowest in SGC7901 cells and highest in BGC823 cells. MAWBP and MAWD were stably overexpressed in SGC7901 cells and knocked down in BGC823 cells. MAWBP and MAWD inhibited GC cell proliferation in vitro and in vivo. MTT assay showed that overexpression of MAWBP and MAWD suppressed growth of SGC7901 cells (P < 0.001), while knockdown of these genes promoted growth of BGC823 cells (P < 0.001). Soft agar colony formation experiments showed that overexpression of MAWBP and MAWD alone or together reduced colony formation compared with vector group in SGC7901 (86.25 ± 8.43, 12.75 ± 4.49, 30 ± 6.41 vs 336.75 ± 22.55, P < 0.001), and knocked-down MAWBP and MAWD demonstrated opposite effects (131.25 ± 16.54, 88.75 ± 11.12, 341.75 ± 22.23 vs 30.25 ± 8.07, P < 0.001). Tumorigenicity experiments revealed that overexpressed MAWBP and MAWD inhibited GC cell proliferation in vivo (P < 0.001). MAWBP and MAWD also inh  ibited GC cel  l invasion. Transwell assay showed that the number of traverse cells of MAWBP, MAWD and coexpression group were more than that in vector group (84 ± 16.57, 98.33 ± 9.8, 29 ± 16.39 vs 298 ± 11.86, P < 0.001). Coexpression of MAWBP and MAWD significantly decreased the cells traversing the matrix membrane. Conversely, knocked-down MAWBP and MAWD correspondingly promoted invasion of GC cells (100.67 ± 14.57, 72.66 ± 8.51, 330.67 ± 20.55 vs 27 ± 11.53, P < 0.001). More importantly, coexpression of MAWBP and MAWD promoted EMT. Cells that coexpressed MAWBP and MAWD displayed a pebble-like shape and tight cell-cell adhesion, while vector cells showed a classical mesenchymal phenotype. Western blotting showed that expression of E-cadherin was increased, and expression of N-cadherin and Snail was decreased when cells coexpressed MAWBP and MAWD and were treated with TGF-β1. Nuclear translocation of p-Smad3 was reduced by attenuating its phosphorylation.Coexpression of MAWBP and MAWD inhibited EMT, and EMT-aided malignant cell progression was suppressed. 
   
  
 FMNL1 
 
  NCBI Gene  
  GeneRIF  
  Pubmed  
 
 
  Search Term  Rank  Fields  
  [Pp]odosome(s)?  5  PubMed(1); GeneRIFs(1)  
  [Mm]igration  6  Process(1); PubMed(5); GeneRIFs(1)  
  [Mm]etastasis  12  PubMed(1)  
 
 
 hide/show details for FMNL1 
  [Pp]odosome(s)?  PubMed  
 The formin FRL1 (FMNL1) is an essential component of macrophage   podosomes  .   Podosomes   are highly dynamic actin-rich adhesion structures in cells of myeloid lineage and some transformed cells. Unlike transformed mesenchymal cell types,   podosomes   are the sole adhesion structure in macrophage and thus mediate all contact with adhesion substrate, including movement through complex tissues for immune surveillance. The existence of   podosomes   in inflammatory macrophages and transformed cell types suggest an important role in tissue invasion. The proteome, assembly, and maintenance of   podosomes   are emerging, but remain incompletely defined. Previously, we reported a formin homology sequence and actin assembly activity in association with macrophage beta-3 integrin. In this study we demonstrate by quantitative reverse transcriptase polymerase chain reaction and Western blotting that the formin FRL1 is specifically upregulated during monocyte differentiation to macrophages. We show that the formin FRL1 localizes to the actin-rich cores of primary macrophage   podosomes  . FRL1 co-precipitates with beta-3 integrin and both fixed and live cell fluorescence microscopy show that endogenous and overexpressed FRL1 selectively localize to macrophage   podosomes  . Targeted disruption of FRL1 by siRNA results in reduced cell adhesion and disruption of   podosome   dynamics. Our data suggest that FRL1 is responsible for modifying actin at the macrophage   podosome   and may be involved in actin cytoskeleton dynamics during adhesion and migration within tissues. 
  GeneRIFs  
 Our data suggest that FRL1 is responsible for modifying actin at the macrophage   podosome   and may be involved in actin cytoskeleton dynamics during adhesion and migration within tissues. 
  [Mm]igration  Process  
 substrate-dependent cell   migration   
  PubMed  
 Identification and characterization of human FMNL1, FMNL2 and FMNL3 genes in silico. FMNL (NM_005892.2) is a 5'-truncated partial cDNA encoding a Formin-homology protein related to DAAM1, DAAM2, DIAPH1 and DIAPH2. Here, we identified three members of FMNL gene family in the human genome by using bioinformatics. FMNL1 gene, corresponding to 5'-truncated KW-13 and FMNL cDNAs, was located within reference genomic contig NT_010748.9 (nucleotide position 100576-125849, forward orientation). FMNL2 gene, corresponding to KIAA1902 and FHOD2 cDNAs, was located within NT_005151.10 (nucleotide position 122465-436828, forward orientation). FMNL3 gene, corresponding to 5'-truncated DKFZp762B245 and KIAA2014 cDNAs, was located within NT_026397.10 (nucleotide position 209769-279037, reverse orientation). FMNL1, FMNL2 and FMNL3 genes encode A and B isoforms with the C-terminal divergence due to alternative splicing (cassette splicing of exon 26). FMNL1A (1100 aa), FMNL1B (1114 aa), FMNL2A (1087 aa), FMNL2B (1093 aa), FMNL3A (1028 aa) and FMNL3B (1027 aa) consist of FDD, FH1 and FH2 domains. Total amino-acid identity were as follows: FMNL1A vs. FMNL2A, 59.3%; FMNL1A vs. FMNL3A, 56.1%; FMNL2A vs. FMNL3A, 68.6%. FMNL1 gene was mapped to human chromosome 17q21. FMNL2 gene was linked to FNBP3/HYPA gene on chromosome 2q23.3, while FMNL3 gene was linked to FNBP3L/HYPC gene on chromosome 12q13. FMNL1 mRNA was expressed in natural killer cells, Burkitt lymphoma, pancreatic cancer, prostate cancer, and lung large cell carcinoma, FMNL2 mRNA in several normal tissues, diffuse-type gastric cancer, breast cancer, chondrosarcoma, melanoma, and glioblastoma, and FMNL3 mRNA in gastric cancer. FMNL1, FMNL2 and FMNL3 might be implicated in polarity control, invasion,   migration  , or metastasis through regulation of the Rho-related signaling pathway. 
 Identification and characterization of a set of conserved and new regulators of cytoskeletal organization, cell morphology and   migration  . Cell   migration   is essential during development and in human disease progression including cancer. Most cell   migration   studies concentrate on known or predicted components of   migration   pathways.Here we use data from a genome-wide RNAi morphology screen in Drosophila melanogaster cells together with bioinformatics to identify 26 new regulators of morphology and cytoskeletal organization in human cells. These include genes previously implicated in a wide range of functions, from mental retardation, Down syndrome and Huntington's disease to RNA and DNA-binding genes. We classify these genes into seven groups according to phenotype and identify those that affect cell   migration  . We further characterize a subset of seven genes, FAM40A, FAM40B, ARC, FMNL3, FNBP3/FBP11, LIMD1 and ZRANB1, each of which has a different effect on cell shape, actin filament distribution and cell   migration  . Interestingly, in several instances closely related isoforms with a single Drosophila homologue have distinct phenotypes. For example, FAM40B depletion induces cell elongation and tail retraction defects, whereas FAM40A depletion reduces cell spreading.Our results identify multiple regulators of cell   migration   and cytoskeletal signalling that are highly conserved between Drosophila and humans, and show that closely related paralogues can have very different functions in these processes. 
 FMNL1 promotes proliferation and   migration   of leukemia cells. The human FMNL1 is expressed predominantly in hematopoietic cells and has been described previously as overexpressed in hematopoietic malignancies. However, it is not known whether FMNL1 contributes to leukemogenesis. Here, we investigate the FMNL1 function using two different human leukemia models: Namalwa and K562 cell lines. FMNL1 depletion reduced cell proliferation and colony formation in both leukemic cell types, as well as a decrease in the tumor growth of FMNL1-depleted Namalwa cell xenografts. In addition, there was a decrease in   migration   and in TEM in FMNL1-depleted Namalwa cells. FMNL1 endogenously associates with Rac1, and FMNL1 silencing resulted in an increased Rac1 activity. The reduced   migration   observed in FMNL1-depleted cells was restored by inhibiting Rac activity. Our results indicate that FMNL1 stimulates leukemia cell proliferation as well as   migration  . This suggests that FMNL1 contributes to leukemogenesis and could act in part through Rac1 regulation. 
 FRL, a novel formin-related protein, binds to Rac and regulates cell motility and survival of macrophages. We have isolated a cDNA, frl (formin-related gene in leukocytes), a novel mammalian member of the formin gene family. The frl cDNA encodes a 160-kDa protein, FRL, that possesses FH1, FH2, and FH3 domains that are well conserved among other Formin-related proteins. An FRL protein is mainly localized in the cytosol and is highly expressed in spleen, lymph node, and bone marrow cells. Formin-related genes and proteins have been reported to play crucial roles in morphogenesis, cell polarity, and cytokinesis through interaction with Rho family small GTPases. FRL binds to Rac at its N-terminal region including the FH3 domain and associates with profilin at the FH1 domain. In a macrophage cell line, P388D1, overexpression of a truncated form of FRL containing only the FH3 domain (FH3-FRL) strongly inhibited cell adhesion to fibronectin and   migration   upon stimulation with a chemokine. Moreover, expression of the truncated FH3-FRL protein resulted in apoptotic cell death of P388D1 cells, suggesting that the truncated FH3-FRL protein may interfere with signals of FRL. Overexpression in the P388D1 cells of full-length FRL or of the truncated protein containing the FH3 and FH1 domains, with simultaneous expression of the truncated FH3-FRL protein, blocked apoptotic cell death and inhibition of cell adhesion and   migration  . These results suggest that FRL may play a role in the control of reorganization of the actin cytoskeleton in association with Rac and also in the regulation of the signal for cell survival. 
 The formin FRL1 (FMNL1) is an essential component of macrophage podosomes. Podosomes are highly dynamic actin-rich adhesion structures in cells of myeloid lineage and some transformed cells. Unlike transformed mesenchymal cell types, podosomes are the sole adhesion structure in macrophage and thus mediate all contact with adhesion substrate, including movement through complex tissues for immune surveillance. The existence of podosomes in inflammatory macrophages and transformed cell types suggest an important role in tissue invasion. The proteome, assembly, and maintenance of podosomes are emerging, but remain incompletely defined. Previously, we reported a formin homology sequence and actin assembly activity in association with macrophage beta-3 integrin. In this study we demonstrate by quantitative reverse transcriptase polymerase chain reaction and Western blotting that the formin FRL1 is specifically upregulated during monocyte differentiation to macrophages. We show that the formin FRL1 localizes to the actin-rich cores of primary macrophage podosomes. FRL1 co-precipitates with beta-3 integrin and both fixed and live cell fluorescence microscopy show that endogenous and overexpressed FRL1 selectively localize to macrophage podosomes. Targeted disruption of FRL1 by siRNA results in reduced cell adhesion and disruption of podosome dynamics. Our data suggest that FRL1 is responsible for modifying actin at the macrophage podosome and may be involved in actin cytoskeleton dynamics during adhesion and   migration   within tissues. 
  GeneRIFs  
 Our data suggest that FRL1 is responsible for modifying actin at the macrophage podosome and may be involved in actin cytoskeleton dynamics during adhesion and   migration   within tissues. 
  [Mm]etastasis  PubMed  
 Identification and characterization of human FMNL1, FMNL2 and FMNL3 genes in silico. FMNL (NM_005892.2) is a 5'-truncated partial cDNA encoding a Formin-homology protein related to DAAM1, DAAM2, DIAPH1 and DIAPH2. Here, we identified three members of FMNL gene family in the human genome by using bioinformatics. FMNL1 gene, corresponding to 5'-truncated KW-13 and FMNL cDNAs, was located within reference genomic contig NT_010748.9 (nucleotide position 100576-125849, forward orientation). FMNL2 gene, corresponding to KIAA1902 and FHOD2 cDNAs, was located within NT_005151.10 (nucleotide position 122465-436828, forward orientation). FMNL3 gene, corresponding to 5'-truncated DKFZp762B245 and KIAA2014 cDNAs, was located within NT_026397.10 (nucleotide position 209769-279037, reverse orientation). FMNL1, FMNL2 and FMNL3 genes encode A and B isoforms with the C-terminal divergence due to alternative splicing (cassette splicing of exon 26). FMNL1A (1100 aa), FMNL1B (1114 aa), FMNL2A (1087 aa), FMNL2B (1093 aa), FMNL3A (1028 aa) and FMNL3B (1027 aa) consist of FDD, FH1 and FH2 domains. Total amino-acid identity were as follows: FMNL1A vs. FMNL2A, 59.3%; FMNL1A vs. FMNL3A, 56.1%; FMNL2A vs. FMNL3A, 68.6%. FMNL1 gene was mapped to human chromosome 17q21. FMNL2 gene was linked to FNBP3/HYPA gene on chromosome 2q23.3, while FMNL3 gene was linked to FNBP3L/HYPC gene on chromosome 12q13. FMNL1 mRNA was expressed in natural killer cells, Burkitt lymphoma, pancreatic cancer, prostate cancer, and lung large cell carcinoma, FMNL2 mRNA in several normal tissues, diffuse-type gastric cancer, breast cancer, chondrosarcoma, melanoma, and glioblastoma, and FMNL3 mRNA in gastric cancer. FMNL1, FMNL2 and FMNL3 might be implicated in polarity control, invasion, migration, or   metastasis   through regulation of the Rho-related signaling pathway. 
   
  
 MYL12A 
 
  NCBI Gene  
  GeneRIF  
  Pubmed  
 
 
  Search Term  Rank  Fields  
  [Mm]igration  6  PubMed(1)  
  [Cc]ell [Ii]nvasion  13  PubMed(1)  
 
 
 hide/show details for MYL12A 
  [Mm]igration  PubMed  
 Chelerythrine perturbs lamellar actomyosin filaments by selective inhibition of myotonic dystrophy kinase-related Cdc42-binding kinase. Cell movement requires forces generated by non-muscle myosin II (NM II) for coordinated protrusion and retraction. The Cdc42/Rac effector MRCK regulates a specific actomyosin network in the lamella essential for cell protrusion and   migration  . Together with the Rho effector ROK required for cell rear retraction, they cooperatively regulate cell motility and tumour cell invasion. Despite the increasing importance of ROK inhibitors for both experimental and clinical purposes, there is a lack of specific inhibitors for other related kinases such as MRCK. Here, we report the identification of chelerythrine chloride as a specific MRCK inhibitor. Its ability to block cellular activity of MRCK resulted in the specific loss of NM II-associated MLC phosphorylation in the lamella, and the consequential suppression of cell   migration  . 
  [Cc]ell [Ii]nvasion  PubMed  
 Chelerythrine perturbs lamellar actomyosin filaments by selective inhibition of myotonic dystrophy kinase-related Cdc42-binding kinase. Cell movement requires forces generated by non-muscle myosin II (NM II) for coordinated protrusion and retraction. The Cdc42/Rac effector MRCK regulates a specific actomyosin network in the lamella essential for cell protrusion and migration. Together with the Rho effector ROK required for cell rear retraction, they cooperatively regulate cell motility and tumour   cell invasion  . Despite the increasing importance of ROK inhibitors for both experimental and clinical purposes, there is a lack of specific inhibitors for other related kinases such as MRCK. Here, we report the identification of chelerythrine chloride as a specific MRCK inhibitor. Its ability to block cellular activity of MRCK resulted in the specific loss of NM II-associated MLC phosphorylation in the lamella, and the consequential suppression of cell migration. 
   
  
 MYL9 
 
  NCBI Gene  
  GeneRIF  
  Pubmed  
 
 
  Search Term  Rank  Fields  
  [Ff]ilopodia  1  PubMed(1)  
  [Mm]igration  6  PubMed(4); GeneRIFs(3)  
  [Mm]etastasis  12  PubMed(4)  
 
 
 hide/show details for MYL9 
  [Ff]ilopodia  PubMed  
 MAL/SRF complex is involved in platelet formation and megakaryocyte migration by regulating MYL9 (MLC2) and MMP9. Megakaryoblastic leukemia 1 (MAL) is a transcriptional coactivator of serum response factor (SRF). In acute megakaryoblastic leukemia, the MAL gene is translocated and fused with the gene encoding one twenty-two (OTT). Herein, we show that MAL expression increases during the late differentiation steps of neonate and adult human megakaryopoiesis and localized into the nucleus after Rho GTPase activation by adhesion on collagen I or convulxin. MAL knockdown in megakaryocyte progenitors reduced the percentage of cells forming   filopodia  , lamellipodia, and stress fibers after adhesion on the same substrates, and reduced proplatelet formation. MAL repression led to dysmorphic megakaryocytes with disorganized demarcation membranes and alpha granules heterogeneously scattered in the cytoplasm. Gene expression profiling revealed a marked decrease in metalloproteinase 9 (MMP-9) and MYL9 expression after MAL inhibition. Luciferase assays in HEK293T cells and chromatin immunoprecipitation in primary megakaryocytes showed that the MAL/SRF complex directly regulates MYL9 and MMP9 in vitro. Megakaryocyte migration in response to stromal cell-derived factor 1, through Matrigel was considerably decreased after MAL knockdown, implicating MMP9 in migration. Finally, the use of a shRNA to decrease MYL9 expression showed that MYL9 was involved in proplatelet formation. MAL/SRF complex is thus involved in platelet formation and megakaryocyte migration by regulating MYL9 and MMP9. 
  [Mm]igration  PubMed  
 Myocardin-related transcription factor A is up-regulated by 17β-estradiol and promotes   migratio  n of MCF-7 breast cancer cells via transactivation of MYL9 and CYR61. Many lines of evidence have suggested that estrogen plays important roles not only in the initiation and proliferation of breast cancer, but also in cancer metastasis. However, the mechanistic basis of the latter events is poorly understood. In addition, recent studies have suggested that myocardin-related transcription factor A (MRTF-A) might be related to cancer metastasis. However, as reports are contradictory, certain of its roles still remain confusing. In the present study, we showed that excessive 17β-estradiol could promote th  e migrati  on of MCF-7 breast cancer cells and up-regulate the expression of MRTF-A, myosin regulatory light chain 9 (MYL9), and cysteine-rich angiogenic inducer 61 (CYR61). Overexpression of MRTF-A significantly promoted th  e migrati  on of MCF-7 cells through its transactivation effects on MYL9 and CYR61 genes, while RNA interference-mediated knockdown of MRTF-A strongly inhibited transcription and expression of the target genes and reduced th  e migrati  on ability of MCF-7 cells. These results provided novel evidence supporting the metastasis-promoting functions of MRTF-A, and implied that MRTF-A might be a switch for the estrogen pathway to change its proliferation-promoting roles int  o migrati  on-stimulating roles in breast cancer. 
 MAL/SRF complex is involved in platelet formation and megakaryocyte   migration   by regulating MYL9 (MLC2) and MMP9. Megakaryoblastic leukemia 1 (MAL) is a transcriptional coactivator of serum response factor (SRF). In acute megakaryoblastic leukemia, the MAL gene is translocated and fused with the gene encoding one twenty-two (OTT). Herein, we show that MAL expression increases during the late differentiation steps of neonate and adult human megakaryopoiesis and localized into the nucleus after Rho GTPase activation by adhesion on collagen I or convulxin. MAL knockdown in megakaryocyte progenitors reduced the percentage of cells forming filopodia, lamellipodia, and stress fibers after adhesion on the same substrates, and reduced proplatelet formation. MAL repression led to dysmorphic megakaryocytes with disorganized demarcation membranes and alpha granules heterogeneously scattered in the cytoplasm. Gene expression profiling revealed a marked decrease in metalloproteinase 9 (MMP-9) and MYL9 expression after MAL inhibition. Luciferase assays in HEK293T cells and chromatin immunoprecipitation in primary megakaryocytes showed that the MAL/SRF complex directly regulates MYL9 and MMP9 in vitro. Megakaryocyte   migration   in response to stromal cell-derived factor 1, through Matrigel was considerably decreased after MAL knockdown, implicating MMP9 in   migration  . Finally, the use of a shRNA to decrease MYL9 expression showed that MYL9 was involved in proplatelet formation. MAL/SRF complex is thus involved in platelet formation and megakaryocyte   migration   by regulating MYL9 and MMP9. 
 Nogo-B regulates   migration   and contraction of airway smooth muscle cells by decreasing ARPC 2/3 and increasing MYL-9 expression. Abnormal proliferation, apoptosis,   migration   and contraction of airway smooth muscle (ASM) cells in airway remodeling in asthma are basically excessive repair responses to a network of inflammatory mediators such as PDGF, but the mechanisms of such responses remain unclear. Nogo-B, a member of the reticulum family 4(RTN4), is known to play a key role in arteriogenesis and tissue repair. Further studies are needed to elucidate the role of Nogo-B in airway smooth muscle abnormalities.A mouse model of chronic asthma was established by repeated OVA inhalation and subjected to Nogo-B expression analysis using immunohistochemistry and Western Blotting. Then, primary human bronchial smooth muscle cells (HBSMCs) were cultured in vitro and a siRNA interference was performed to knockdown the expression of Nogo-B in the cells. The effects of Nogo-B inhibition on PDGF-induced HBSMCs proliferation,   migration   and contraction were evaluated. Finally, a proteomic analysis was conducted to unveil the underlying mechanisms responsible for the function of Nogo-B.Total Nogo-B expression was approximately 3.08-fold lower in chronic asthmatic mice compared to naïve mice, which was obvious in the smooth muscle layer of the airways. Interference of Nogo-B expression by siRNA resulted nearly 96% reduction in mRNA in cultured HBSMCs. In addition, knockdown of Nogo-B using specific siRNA significantly decreased PDGF-induced   migratio  n of HBSMCs by 2.3-fold, and increased the cellular contraction by 16% compared to negative controls, but had limited effects on PDGF-induced proliferation. Furthermore, using proteomic analysis, we demonstrate that the expression of actin related protein 2/3 complex subunit 5 (ARPC 2/3) decreased and, myosin regulatory light chain 9 isoform a (MYL-9) increased after Nogo-B knockdown.These data define a novel role for Nogo-B in airway remodeling in chronic asthma. Endogenous Nogo-B, which may exert its effects through ARPC 2/3 and MYL-9, is necessary for the   migratio  n and contraction of airway smooth muscle cells. 
 Histone methyltransferase SMYD3 promotes MRTF-A-mediated transactivation of MYL9 and   migration   of MCF-7 breast cancer cells. Myocardin-related transcription factor-A (MRTF-A) is a Rho signal-responsive transcriptional coactivator of serum response factor (SRF). Recent studies indicated that MRTF-A might be an important regulator of mammary gland and be involved in cancer metastasis. However, the roles of histone modification in the MRTF-A-dependent signal pathway and tumor   migration   are still not very clear. Here, we report that histone methylation is required for the MRTF-A-mediated upregulation of myosin regulatory light chain 9 (MYL9), an important cytoskeletal component which is implicated in cell   migration  . Furthermore, we demonstrate that SET and MYND domain containing protein 3 (SMYD3), a hitone methyltransferase (HMT) associated with carcinogenesis, might be the one which is responsible for the histone methylation occurred in the MRTF-A-mediated- transactivation of MYL9 and   migration   of breast cancer cells. Overexpression of SMYD3 promotes MRTF-A-mediated upregulation of MYL9 and   migration   of MCF-7 breast cancer cells, while contrary results were observed when the endogenous MRTF-A and SMYD3 were suppressed with specific siRNAs. In addition, the mutation analysis suggested that this cooperative transactivation is mainly mediated via the proximal binding element of MRTF-A in the promoter of MYL9, and the HMT activity of SMYD3 is required as well. Our findings reveal a new mechanism by which MRTF-A and SMYD3 functions in transcriptional regulation and cell   migration  , and provide a better understanding for metastasis of breast cancer. 
  GeneRIFs  
 Overexpression of MRTF-A significantly promoted the   migration   of MCF-7 cells through its transactivation effects on MYL9 and CYR61 genes 
 Overexpression of SMYD3 promotes MRTF-A-mediated upregulation of MYL9 and   migration   of MCF-7 breast cancer cells 
 Endogenous Nogo-B, which may exert its effects through ARPC 2/3 and MYL-9, is necessary for the   migration   and contraction of airway smooth muscle cells. 
  [Mm]etastasis  PubMed  
 Myocardin-related transcription factor A is up-regulated by 17β-estradiol and promotes migration of MCF-7 breast cancer cells via transactivation of MYL9 and CYR61. Many lines of evidence have suggested that estrogen plays important roles not only in the initiation and proliferation of breast cancer, but also in cancer   metastasi  s. However, the mechanistic basis of the latter events is poorly understood. In addition, recent studies have suggested that myocardin-related transcription factor A (MRTF-A) might be related to cancer   metastasi  s. However, as reports are contradictory, certain of its roles still remain confusing. In the present study, we showed that excessive 17β-estradiol could promote the migration of MCF-7 breast cancer cells and up-regulate the expression of MRTF-A, myosin regulatory light chain 9 (MYL9), and cysteine-rich angiogenic inducer 61 (CYR61). Overexpression of MRTF-A significantly promoted the migration of MCF-7 cells through its transactivation effects on MYL9 and CYR61 genes, while RNA interference-mediated knockdown of MRTF-A strongly inhibited transcription and expression of the target genes and reduced the migration ability of MCF-7 cells. These results provided novel evidence supporting th  e metastas  is-promoting functions of MRTF-A, and implied that MRTF-A might be a switch for the estrogen pathway to change its proliferation-promoting roles into migration-stimulating roles in breast cancer. 
 MYLK and MYL9 expression in non-small cell lung cancer identified by bioinformatics analysis of public expression data. Gene expression microarrays are widely used to investigate molecular targets in cancers, including lung cancer. In this study, we analyzed online non-small cell lung cancer (NSCLC) microarray databases, to screen the key genes and pathways related to NSCLC by bioinformatics analyses. And then, the expression levels of two selected genes in the down-regulated co-pathways, myosin light chain kinase (MYLK) and myosin regulatory light chain 9 (MYL9), were determined in tumor, paired paraneoplastic, and normal lung tissues. First, gene set enrichment analysis and meta-analysis were conducted to identify key genes and pathways that contribute to NSCLC carcinogenesis. Second, using the total RNA and protein extracted from lung cancer tissues (n = 240), adjacent non-cancer tissues (n = 240), and normal lung tissues (n = 300), we examined the MYLK and MYL9 expression levels by quantitative real-time PCR and Western blot. Finally, we explored the correlations between mRNA and protein expressions of these two genes and the clinicopathological parameters of NSCLC. Fifteen up-regulated and nine down-regulated co-pathways were observed. A number of differentially expressed genes (CALM1, THBS1, CSF3, BMP2, IL6ST, MYLK, ROCK2, IL3RA, MYL9, PPP2CA, CSF2RB, CNAQ, GRIA2, IL10RA, IL10RB, IL11RA, LIFR, PLCB4, and RAC3) were identified (P < 0.01) in the down-regulated co-pathways. The expression levels of MYLK and MYL9, which act downstream of the vascular smooth muscle contraction signal pathway and focal adhesion pathway, were significantly lower in cancer tissue than those in the paraneoplastic and normal tissues (P < 0.05). Moreover, the expression levels of these two genes in stages III and IV NSCLC were significantly increased, when compared to stages I and II, and expressions levels in N  SCLC with   lymphatic metastasis were higher than tha  t without   lymphatic metastasis (P < 0.05). Additionally, significant lower expression levels of the two genes were found in smokers than in nonsmokers (P < 0.05). In contrast, gender, differentiated degrees, and pathohistological type appeared to have no impact on these gene expressions (P > 0.05). These findings suggested that low MYLK and MYL9 expressions might be associated with the development of NSCLC. These gene  s may be a  lso relevant to NSCLC metastasis. Future investigations with large sample sizes needed to verify these findings. 
 Decreased expression of myosin light chain MYL9 in stroma predicts malignant progression and poor biochemical recurrence-free survival in prostate cancer. The aim of this study was to investigate the associations of myosin light chain (MYL9) downregulation with tumor progression and prognosis in patients with prostate cancer (PCa). MYL9 protein expression in human PCa and non-cancerous prostate tissues was detected by Western blot and immunohistochemistry analyses, which was validated by microarray-based Taylor data at mRNA level. Then, the associations of MYL9 expression with clinicopathological features and clinical outcome of PCa patients were statistically analyzed. Both Western blot and immunohistochemistry analyses found that MYL9 expression was significantly decreased (both P < 0.001) in PCa tissues compared with those in non-cancerous prostate tissues. In addition, MYL9 was mainly expressed in the cytoplasm of stromal cells of prostate tissues, and the decreased expression of MYL9 in PCa tissues was significantly correlated with the older age of patients (P = 0.011), the higher Gleason score (P < 0.001), the advanced pathological stage (P = 0.002), the presence of   metastasis   (P < 0.001) and PSA failure (P = 0.001). Furthermore, both univariate and multivariate analyses showed that the downregulation of MYL9 was an independent predictor of shorter overall survival (P = 0.026 and P = 0.009, respectively) and biochemical recurrence-free survival (P = 0.001 and P = 0.002, respectively). Our data strongly confirmed for the first time that the decreased expression of MYL9 may play an important role in tumor progression of PCa. More importantly, the downregulation of MYL9 may efficiently predict both overall and biochemical recurrence-free survivals in PCa patients. 
 Histone methyltransferase SMYD3 promotes MRTF-A-mediated transactivation of MYL9 and migration of MCF-7 breast cancer cells. Myocardin-related transcription factor-A (MRTF-A) is a Rho signal-responsive transcriptional coactivator of serum response factor (SRF). Recent studies indicated that MRTF-A might be an important regulator of mammary gland and be involved in cancer   metastasis  . However, the roles of histone modification in the MRTF-A-dependent signal pathway and tumor migration are still not very clear. Here, we report that histone methylation is required for the MRTF-A-mediated upregulation of myosin regulatory light chain 9 (MYL9), an important cytoskeletal component which is implicated in cell migration. Furthermore, we demonstrate that SET and MYND domain containing protein 3 (SMYD3), a hitone methyltransferase (HMT) associated with carcinogenesis, might be the one which is responsible for the histone methylation occurred in the MRTF-A-mediated- transactivation of MYL9 and migration of breast cancer cells. Overexpression of SMYD3 promotes MRTF-A-mediated upregulation of MYL9 and migration of MCF-7 breast cancer cells, while contrary results were observed when the endogenous MRTF-A and SMYD3 were suppressed with specific siRNAs. In addition, the mutation analysis suggested that this cooperative transactivation is mainly mediated via the proximal binding element of MRTF-A in the promoter of MYL9, and the HMT activity of SMYD3 is required as well. Our findings reveal a new mechanism by which MRTF-A and SMYD3 functions in transcriptional regulation and cell migration, and provide a better understanding for   metastasis   of breast cancer. 
   
  
 IST1 
 
  NCBI Gene  
  GeneRIF  
  Pubmed  
 
 
  Search Term  Rank  Fields  
  [Ee]xtracellular [Mm]atrix  8  PubMed(1)  
  [Ii]nvasive  10  PubMed(3)  
 
 
 hide/show details for IST1 
  [Ee]xtracellular [Mm]atrix  PubMed  
 Proteomic analysis of human parotid gland exosomes by multidimensional protein identification technology (MudPIT). Human ductal saliva contributes over a thousand unique proteins to whole oral fluids. The mechanism by which most of these proteins are secreted by salivary glands remains to be determined. The present study used a mass spectrometry-based, shotgun proteomics approach to explore the possibility that a subset of the proteins found in saliva are derived from exosomes, membrane-bound vesicles of endosomal origin within multivesicular endosomes. Using MudPIT (multidimensional protein identification technology) mass spectrometry, we catalogued 491 proteins in the exosome fraction of human parotid saliva. Many of these proteins were previously observed in ductal saliva from parotid glands (265 proteins). Furthermore, 72 of the proteins in parotid exosomes overlap with those previously identified as urinary exosome proteins, proteins which are also frequently associated with exosomes from other tissues and cell types. Gene Ontology (GO) and KEGG pathway analyses found that cytosolic proteins comprise the largest category of proteins in parotid exosomes (43%), involved in such processes as phosphatidylinositol signaling system, calcium signaling pathway, inositol metabolism, protein export, and signal transduction, among others; whereas the integral plasma membrane proteins and associated/peripheral plasma membrane proteins (26%) were associated with   extracellular matrix  -receptor interaction, epithelial cell signaling, T-cell and B-cell receptor signaling, cytokine receptor interaction, and antigen processing and presentation, among other biological functions. In addition, these putative saliva exosomal proteins were linked to specific diseases (e.g., neurodegenerative disorders, prion disease, cancers, type I and II diabetes). Consequently, parotid glands secrete exosomes that reflect the metabolic and functional status of the gland and may also carry informative protein markers useful in the diagnosis and treatment of systemic diseases. 
  [Ii]nvasive  PubMed  
 Proteomic identification of exosomal LRG1: a potential urinary biomarker for detecting NSCLC. In the present research, we aimed to screen for non-small cell lung cancer (NSCLC)-related proteins in urinary exosomes by comparing urinary exosomes proteome of normal controls and NSCLC patients. Urinary exosomes were isolated by ultracentrifugation and identified by electron microscopy. Exosomal proteins were separated by 1-D SDS-PAGE and the differentially expressed bands between healthy controls and NSCLC patients ranging in size from 35 to 45 kD were cut from the gel. After tryptic digestion, 18 proteins were identified by nano-HPLC-chip-MS/MS. The differential expression of leucine-rich α-2-glycoprotein (LRG1) was further validated in urinary exosomes by Western blot and in lung tissue by immunohistochemistry. The LRG1 was found to be expressed at higher levels in urinary exosomes and lung tissue of NSCLC patients. These results suggested that LRG1 may be a candidate biomarker for n  on-invas  ive diagnosis of NSCLC in urine. 
 Influence of whole arm loss of chromosome 16q on gene expression patterns in oestrogen receptor-positive,   invasive   breast cancer. A whole chromosome arm loss of 16q belongs to the most frequent and earliest chromosomal alterations in   invasive   and in situ breast cancers of all common subtypes. Besides E-cadherin, several putative tumour suppressor genes residing on 16q in breast cancer have been investigated. However, the significance of these findings has remained unclear. Thus, other mechanisms leading to gene loss of function (eg haploinsufficiency, or distortion of multiple regulative subnetworks) remain to be tested as a hypothesis. To define the effect on gene expression of whole-arm loss of chromosome 16q in   invasive   breast cancer, we performed global gene expression analysis on a series of 18 genetically extensively characterized   invasive   ductal breast carcinomas and verified the results by quantitative real-time PCR (qRT-PCR). The distribution of the differential genes across the genome and their expression status was studied. A second approach by qRT-PCR in an independent series of 30 breast carcinomas helped to narrow down the observed effect. Whole-arm chromosome 16q losses, irrespective of other chromosomal changes, are associated with decreased expression of a number of candidate genes located on 16q (eg CDA08, CGI-128, SNTB2, NQO1, SF3B3, KIAA0174, ATBF1, GABARAPL2, KARS, GCSH, MBTPS1 and ZDHHC7) in breast carcinomas with a low degree of genetic instability. qRT-PCR provided evidence to suggest that the expression of these genes was reduced in a gene dosage-dependent manner. The differential expression of the candidate genes according to the chromosomal 16q-status vanished in genetically advanced breast cancer cases and changed ER status. These results corroborate previous reports about the importance of whole-arm loss of chromosome 16q in breast carcinogenesis and give evidence for the first time that haploinsufficiency, in the sense of a gene dosage effect, might be an important contributing factor in the early steps of breast carcinogenesis. 
 Overexpression of OLC1 promotes tumorigenesis of human esophageal squamous cell carcinoma. OLC1 was recently identified to be a potential oncogene. However, the role of OLC1 in human esophageal cell carcinoma (ESCC) is unknown. The aim of this study was therefore to evaluate the expression of OLC1 in human ESCC from normal, premalignant, and malignant lesions, and to clarify the mechanisms by which OLC1 contributes to the progression of ESCC.Two hundred and fourteen paired ESCC specimens, and an independent set from 28 ESCC patients, were used to analyze the correlation between OLC1 expression and the pathological characteristics of tumors using immunohistochemistry. Stable OLC1-overexpressing and OLC1-interfering esophageal cancer cells were established and a series of experimental methods were used to investigate the biological functions and mechanisms of action of OLC1.We showed that OLC1 was overexpressed in 145 of 214 (67.8%) of human ESCC specimens, compared with in only 59 of 214 (27.57%) paired adjacent normal tissues (P<0.001). OLC1 overexpression occurred at a rate of 35% (10/28) at the stage of mild/moderate dysplasia, but was significantly upregulated to 66% (22/33) at the stages of severe dysplasia and in situ carcinoma, while 71% positive staining (22/28) was observed in   invasive   carcinoma tissues compared with normal tissues (P<0.05). We also provided evidence that OLC1 abnormalities significantly altered the cell proliferation and apoptosis induced by cytotoxic agents. OLC1 overexpression suppressed apoptosis, and was associated with attenuated caspase-3 activation and increased Bcl-2 stability.Our study provides strong evidence suggesting OLC1 abnormalities may contribute to the development of human ESCC and have some important clinical significance. 
   
  
 RSU1 
 
  NCBI Gene  
  GeneRIF  
  Pubmed  
 
 
  Search Term  Rank  Fields  
  [Mm]igration  6  PubMed(3)  
  ECM  7  PubMed(4)  
  [Ee]xtracellular [Mm]atrix  8  PubMed(4)  
  [Ii]nvasive  10  PubMed(1)  
  [Mm]etastasis  12  PubMed(2)  
 
 
 hide/show details for RSU1 
  [Mm]igration  PubMed  
 The Rsu-1-PINCH1-ILK complex is regulated by Ras activation in tumor cells. The link between Ras transformation and enhanced cell   migration   due to altered integrin signaling is well established in tumorigenesis, however there remain gaps in our understanding of its mechanism. The Ras suppressor, Rsu-1, has recently been linked to the IPP (integrin-linked kinase {ILK}, PINCH-1/LIMS1, parvin) focal adhesion complex based on its interaction with the LIM 5 domain of PINCH1. Defining the role of the Rsu1-PINCH1-ILK-parvin complex in tumorigenesis is important because both ILK and PINCH1 are elevated in certain tumors while ectopic expression of Rsu-1 blocks tumorigenesis. Our studies previously identified an alternatively spliced isoform of Rsu-1 in high-grade gliomas. We report here the detection of a truncated (p29) Rsu-1 protein, which correlates with the presence of the alternatively spliced Rsu-1 RNA. This RNA and the respective protein were detected in human tumor cell lines that contain high levels of activated Ras, and inhibitor studies demonstrate that the Mek-ERK pathway regulates expression of this truncated Rsu-1 product. We also show that Rsu-1 co-localizes with ILK at focal contacts and co-immunoprecipitates with the ILK-PINCH1 complex in non-transformed cells, but following Ras transformation the association of Rsu-1 with the PINCH1-ILK complex is greatly reduced. Using a human breast cancer cell line, our in vitro studies demonstrate that the depletion of Rsu-1 full-length protein enhances cell   migration   coincident with an increase in Rac-GTP while the depletion of the p29 Rsu-1 truncated protein inhibits   migration  . These findings indicate that Rsu-1 may inhibit cell   migration   by stabilizing the IPP adhesion complex and that Ras activation perturbs this inhibitory function by modulating both Rsu-1 splicing and association of full-length Rsu-1 with IPP. Hence, our findings demonstrate that Rsu-1 links the Ras pathway with the IPP complex and the perturbations of cell attachment-dependent signaling that occur in the malignant process. 
 The Rsu-1-PINCH1-ILK complex is regulated by Ras activation in tumor cells. The link between Ras transformation and enhanced cell   migration   due to altered integrin signaling is well established in tumorigenesis, however there remain gaps in our understanding of its mechanism. The Ras suppressor, Rsu-1, has recently been linked to the IPP (integrin-linked kinase {ILK}, PINCH-1/LIMS1, parvin) focal adhesion complex based on its interaction with the LIM 5 domain of PINCH1. Defining the role of the Rsu1-PINCH1-ILK-parvin complex in tumorigenesis is important because both ILK and PINCH1 are elevated in certain tumors while ectopic expression of Rsu-1 blocks tumorigenesis. Our studies previously identified an alternatively spliced isoform of Rsu-1 in high-grade gliomas. We report here the detection of a truncated (p29) Rsu-1 protein, which correlates with the presence of the alternatively spliced Rsu-1 RNA. This RNA and the respective protein were detected in human tumor cell lines that contain high levels of activated Ras, and inhibitor studies demonstrate that the Mek-ERK pathway regulates expression of this truncated Rsu-1 product. We also show that Rsu-1 co-localizes with ILK at focal contacts and co-immunoprecipitates with the ILK-PINCH1 complex in non-transformed cells, but following Ras transformation the association of Rsu-1 with the PINCH1-ILK complex is greatly reduced. Using a human breast cancer cell line, our in vitro studies demonstrate that the depletion of Rsu-1 full-length protein enhances cell   migration   coincident with an increase in Rac-GTP while the depletion of the p29 Rsu-1 truncated protein inhibits   migration  . These findings indicate that Rsu-1 may inhibit cell   migration   by stabilizing the IPP adhesion complex and that Ras activation perturbs this inhibitory function by modulating both Rsu-1 splicing and association of full-length Rsu-1 with IPP. Hence, our findings demonstrate that Rsu-1 links the Ras pathway with the IPP complex and the perturbations of cell attachment-dependent signaling that occur in the malignant process. 
 Rsu1 contributes to cell adhesion and spreading in MCF10A cells via effects on P38 map kinase signaling. The ILK, PINCH, Parvin (IPP) complex regulates adhesion and   migration   via binding of ILK to β1 integrin and α-parvin thus linking focal adhesions to actin cytoskeleton. ILK also binds the adaptor protein PINCH which connects signaling proteins including Rsu1 to the complex. A recent study of Rsu1 and PINCH1 in non-transformed MCF10A human mammary epithelial cells revealed that the siRNA-mediated depletion of either Rsu1 or PINCH1 decreased the number of focal adhesions (FAs) and altered the distribution and localization of FA proteins. This correlated with reduced adhesion, failure to spread or migrate in response to EGF and a loss of actin stress fibers and caveolae. The depletion of Rsu1 caused significant reduction in PINCH1 implying that Rsu1 may function in part by regulating levels of PINCH1. However, Rsu1, but not PINCH1, was required for EGF-induced activation of p38 Map kinase and ATF2 phosphorylation, suggesting a Rsu1 function independent from the IPP complex. Reconstitution of Rsu1-depleted cells with a Rsu1 mutant (N92D) that does not bind to PINCH1 failed to restore FAs o  r migrati  on but did promote IPP-independent spreading and constitutive as well as EGF-induced p38 activation. In this commentary we discuss p38 activity in adhesion and how Rsu1 expression may be linked to Map kinase kinase (MKK) activation and detachment-induced stress kinase signaling. 
  ECM  PubMed  
 The roles of two distinct regions of PINCH-1 in the regulation of cell attachment and spreading. Cells attach to the extracellular matrix (  ECM  ) through integrins to form focal adhesion complexes, and this process is followed by the extension of lamellipodia to enable cell spreading. PINCH-1, an adaptor protein essential for the regulation of cell-  ECM   adhesion, consists of five tandem LIM domains and a small C-terminal region. PINCH-1 is known to interact with integrin-linked kinase (ILK) and Ras suppressor protein 1 (Rsu-1); however, the precise mechanism by which this complex regulates cell-  ECM   adhesion is not fully understood. We report here that the LIM1 domain of PINCH-1, which associates with ILK to stabilize the expression of this protein, is sufficient for cell attachment but not for cell spreading. In contrast, the C-terminal region of PINCH-1, which binds to Rsu-1, plays a pivotal role in cell spreading but not in cell attachment. We also show that PINCH-1 associates with Rsu-1 to activate Rac1 and that Rac1 activation is necessary for cell spreading. Thus, these data reveal how specific domains of PINCH-1 direct two independent pathways: one utilizing ILK to allow cell attachment, and the other recruiting Rsu-1 to activate Rac1 in order to promote cell spreading. 
 Ras suppressor-1 promotes apoptosis in breast cancer cells by inhibiting PINCH-1 and activating p53-upregulated-modulator of apoptosis (PUMA); verification from metastatic breast cancer human samples. Metastasis, responsible for most deaths from breast cancer (BC), is a multistep process leading to cancer cell spread. Extracellular matrix (  ECM  )-related adhesion and apoptosis resistance play pivotal role in metastasis. Ras suppressor-1 (RSU-1) localizes to cell-  ECM   adhesions and binds to pro-survival adhesion protein PINCH-1. Little is known about the role of RSU-1 in BC. In the present study, we investigated the role of RSU-1 in BC metastasis using two BC cell lines that differ in terms of their metastatic potential and a set of 32 human BC samples from patients with or without lymph node metastasis. We show that RSU-1 is upregulated in the aggressive MDA-MB-231 cells compared to MCF-7 and that its silencing by siRNA leads to upregulation of PINCH-1, induction of proliferation and reduction of apoptosis through downregulation of the pro-apoptotic gene p53-upregulated-modulator-of-apoptosis (PUMA). Our findings in the cell lines were further validated in the human BC tissues where normal adjacent tissues were used as controls. We demonstrate for the first time, that RSU-1 expression is upregulated in metastatic BC samples and downregulated in non-metastatic while it is negatively correlated with PINCH-1 and positively correlated with PUMA expression, suggesting that a pro-apoptotic mechanism is in place in metastatic BC samples and identifying RSU-1 as a potentially interesting molecule that needs to be evaluated further as a novel BC metastasis biomarker. 
 Ras suppressor-1 promotes apoptosis in breast cancer cells by inhibiting PINCH-1 and activating p53-upregulated-modulator of apoptosis (PUMA); verification from metastatic breast cancer human samples. Metastasis, responsible for most deaths from breast cancer (BC), is a multistep process leading to cancer cell spread. Extracellular matrix (  ECM  )-related adhesion and apoptosis resistance play pivotal role in metastasis. Ras suppressor-1 (RSU-1) localizes to cell-  ECM   adhesions and binds to pro-survival adhesion protein PINCH-1. Little is known about the role of RSU-1 in BC. In the present study, we investigated the role of RSU-1 in BC metastasis using two BC cell lines that differ in terms of their metastatic potential and a set of 32 human BC samples from patients with or without lymph node metastasis. We show that RSU-1 is upregulated in the aggressive MDA-MB-231 cells compared to MCF-7 and that its silencing by siRNA leads to upregulation of PINCH-1, induction of proliferation and reduction of apoptosis through downregulation of the pro-apoptotic gene p53-upregulated-modulator-of-apoptosis (PUMA). Our findings in the cell lines were further validated in the human BC tissues where normal adjacent tissues were used as controls. We demonstrate for the first time, that RSU-1 expression is upregulated in metastatic BC samples and downregulated in non-metastatic while it is negatively correlated with PINCH-1 and positively correlated with PUMA expression, suggesting that a pro-apoptotic mechanism is in place in metastatic BC samples and identifying RSU-1 as a potentially interesting molecule that needs to be evaluated further as a novel BC metastasis biomarker. 
 Elimination of Ras Suppressor-1 from hepatocellular carcinoma cells hinders their in vitro metastatic properties. Extracellular matrix (  ECM  ) is of great significance for homeostasis in the liver. In fact, one of the stages leading to hepatocellular carcinoma (HCC) includes accumulation of excess   ECM  . Ras Suppressor-1 (RSU-1) is localized in the cell-  ECM   adhesions but its role in HCC is unexplored.We investigated the expression and role of RSU-1 in two HCC cell lines that differ in aggressiveness; non-invasive Alexander cells and highly invasive HepG2 cells.Our results showed that RSU-1 expression is elevated in HepG2 cells both at the mRNA and protein level, while its silencing leads to increased cell proliferation in both cell lines. Interestingly, RSU-1 depletion from highly invasive HepG2 cells reduces cell adhesion and invasion.This is the first study to provide in vitro evidence for the involvement of RSU-1 in HCC cell invasive behavior. 
  [Ee]xtracellular [Mm]atrix  PubMed  
 The roles of two distinct regions of PINCH-1 in the regulation of cell attachment and spreading. Cells attach to the   extracellular matrix   (ECM) through integrins to form focal adhesion complexes, and this process is followed by the extension of lamellipodia to enable cell spreading. PINCH-1, an adaptor protein essential for the regulation of cell-ECM adhesion, consists of five tandem LIM domains and a small C-terminal region. PINCH-1 is known to interact with integrin-linked kinase (ILK) and Ras suppressor protein 1 (Rsu-1); however, the precise mechanism by which this complex regulates cell-ECM adhesion is not fully understood. We report here that the LIM1 domain of PINCH-1, which associates with ILK to stabilize the expression of this protein, is sufficient for cell attachment but not for cell spreading. In contrast, the C-terminal region of PINCH-1, which binds to Rsu-1, plays a pivotal role in cell spreading but not in cell attachment. We also show that PINCH-1 associates with Rsu-1 to activate Rac1 and that Rac1 activation is necessary for cell spreading. Thus, these data reveal how specific domains of PINCH-1 direct two independent pathways: one utilizing ILK to allow cell attachment, and the other recruiting Rsu-1 to activate Rac1 in order to promote cell spreading. 
 Ras suppressor-1 promotes apoptosis in breast cancer cells by inhibiting PINCH-1 and activating p53-upregulated-modulator of apoptosis (PUMA); verification from metastatic breast cancer human samples. Metastasis, responsible for most deaths from breast cancer (BC), is a multistep process leading to cancer cell spread.   Extracellular matrix   (ECM)-related adhesion and apoptosis resistance play pivotal role in metastasis. Ras suppressor-1 (RSU-1) localizes to cell-ECM adhesions and binds to pro-survival adhesion protein PINCH-1. Little is known about the role of RSU-1 in BC. In the present study, we investigated the role of RSU-1 in BC metastasis using two BC cell lines that differ in terms of their metastatic potential and a set of 32 human BC samples from patients with or without lymph node metastasis. We show that RSU-1 is upregulated in the aggressive MDA-MB-231 cells compared to MCF-7 and that its silencing by siRNA leads to upregulation of PINCH-1, induction of proliferation and reduction of apoptosis through downregulation of the pro-apoptotic gene p53-upregulated-modulator-of-apoptosis (PUMA). Our findings in the cell lines were further validated in the human BC tissues where normal adjacent tissues were used as controls. We demonstrate for the first time, that RSU-1 expression is upregulated in metastatic BC samples and downregulated in non-metastatic while it is negatively correlated with PINCH-1 and positively correlated with PUMA expression, suggesting that a pro-apoptotic mechanism is in place in metastatic BC samples and identifying RSU-1 as a potentially interesting molecule that needs to be evaluated further as a novel BC metastasis biomarker. 
 Ras suppressor-1 promotes apoptosis in breast cancer cells by inhibiting PINCH-1 and activating p53-upregulated-modulator of apoptosis (PUMA); verification from metastatic breast cancer human samples. Metastasis, responsible for most deaths from breast cancer (BC), is a multistep process leading to cancer cell spread.   Extracellular matrix   (ECM)-related adhesion and apoptosis resistance play pivotal role in metastasis. Ras suppressor-1 (RSU-1) localizes to cell-ECM adhesions and binds to pro-survival adhesion protein PINCH-1. Little is known about the role of RSU-1 in BC. In the present study, we investigated the role of RSU-1 in BC metastasis using two BC cell lines that differ in terms of their metastatic potential and a set of 32 human BC samples from patients with or without lymph node metastasis. We show that RSU-1 is upregulated in the aggressive MDA-MB-231 cells compared to MCF-7 and that its silencing by siRNA leads to upregulation of PINCH-1, induction of proliferation and reduction of apoptosis through downregulation of the pro-apoptotic gene p53-upregulated-modulator-of-apoptosis (PUMA). Our findings in the cell lines were further validated in the human BC tissues where normal adjacent tissues were used as controls. We demonstrate for the first time, that RSU-1 expression is upregulated in metastatic BC samples and downregulated in non-metastatic while it is negatively correlated with PINCH-1 and positively correlated with PUMA expression, suggesting that a pro-apoptotic mechanism is in place in metastatic BC samples and identifying RSU-1 as a potentially interesting molecule that needs to be evaluated further as a novel BC metastasis biomarker. 
 Elimination of Ras Suppressor-1 from hepatocellular carcinoma cells hinders their in vitro metastatic properties.   Extracellular matrix   (ECM) is of great significance for homeostasis in the liver. In fact, one of the stages leading to hepatocellular carcinoma (HCC) includes accumulation of excess ECM. Ras Suppressor-1 (RSU-1) is localized in the cell-ECM adhesions but its role in HCC is unexplored.We investigated the expression and role of RSU-1 in two HCC cell lines that differ in aggressiveness; non-invasive Alexander cells and highly invasive HepG2 cells.Our results showed that RSU-1 expression is elevated in HepG2 cells both at the mRNA and protein level, while its silencing leads to increased cell proliferation in both cell lines. Interestingly, RSU-1 depletion from highly invasive HepG2 cells reduces cell adhesion and invasion.This is the first study to provide in vitro evidence for the involvement of RSU-1 in HCC cell invasive behavior. 
  [Ii]nvasive  PubMed  
 Elimination of Ras Suppressor-1 from hepatocellular carcinoma cells hinders their in vitro metastatic properties. Extracellular matrix (ECM) is of great significance for homeostasis in the liver. In fact, one of the stages leading to hepatocellular carcinoma (HCC) includes accumulation of excess ECM. Ras Suppressor-1 (RSU-1) is localized in the cell-ECM adhesions but its role in HCC is unexplored.We investigated the expression and role of RSU-1 in two HCC cell lines that differ in aggressiveness; non-  invasive   Alexander cells and highly   invasive   HepG2 cells.Our results showed that RSU-1 expression is elevated in HepG2 cells both at the mRNA and protein level, while its silencing leads to increased cell proliferation in both cell lines. Interestingly, RSU-1 depletion from highly   invasive   HepG2 cells reduces cell adhesion and invasion.This is the first study to provide in vitro evidence for the involvement of RSU-1 in HCC cell   invasive   behavior. 
  [Mm]etastasis  PubMed  
 Ras suppressor-1 promotes apoptosis in breast cancer cells by inhibiting PINCH-1 and activating p53-upregulated-modulator of apoptosis (PUMA); verification from metastatic breast cancer human samples.   Metastasis  , responsible for most deaths from breast cancer (BC), is a multistep process leading to cancer cell spread. Extracellular matrix (ECM)-related adhesion and apoptosis resistance play pivotal role in   metastasis  . Ras suppressor-1 (RSU-1) localizes to cell-ECM adhesions and binds to pro-survival adhesion protein PINCH-1. Little is known about the role of RSU-1 in BC. In the present study, we investigated the role of RSU-1 in BC   metastasis   using two BC cell lines that differ in terms of their metastatic potential and a set of 32 human BC samples from patients with or without lymph node   metastasis  . We show that RSU-1 is upregulated in the aggressive MDA-MB-231 cells compared to MCF-7 and that its silencing by siRNA leads to upregulation of PINCH-1, induction of proliferation and reduction of apoptosis through downregulation of the pro-apoptotic gene p53-upregulated-modulator-of-apoptosis (PUMA). Our findings in the cell lines were further validated in the human BC tissues where normal adjacent tissues were used as controls. We demonstrate for the first time, that RSU-1 expression is upregulated in metastatic BC samples and downregulated in non-metastatic while it is negatively correlated with PINCH-1 and positively correlated with PUMA expression, suggesting that a pro-apoptotic mechanism is in place in metastatic BC samples and identifying RSU-1 as a potentially interesting molecule that needs to be evaluated further as a novel BC   metastasis   biomarker. 
 Ras suppressor-1 promotes apoptosis in breast cancer cells by inhibiting PINCH-1 and activating p53-upregulated-modulator of apoptosis (PUMA); verification from metastatic breast cancer human samples.   Metastasis  , responsible for most deaths from breast cancer (BC), is a multistep process leading to cancer cell spread. Extracellular matrix (ECM)-related adhesion and apoptosis resistance play pivotal role in   metastasis  . Ras suppressor-1 (RSU-1) localizes to cell-ECM adhesions and binds to pro-survival adhesion protein PINCH-1. Little is known about the role of RSU-1 in BC. In the present study, we investigated the role of RSU-1 in BC   metastasis   using two BC cell lines that differ in terms of their metastatic potential and a set of 32 human BC samples from patients with or without lymph node   metastasis  . We show that RSU-1 is upregulated in the aggressive MDA-MB-231 cells compared to MCF-7 and that its silencing by siRNA leads to upregulation of PINCH-1, induction of proliferation and reduction of apoptosis through downregulation of the pro-apoptotic gene p53-upregulated-modulator-of-apoptosis (PUMA). Our findings in the cell lines were further validated in the human BC tissues where normal adjacent tissues were used as controls. We demonstrate for the first time, that RSU-1 expression is upregulated in metastatic BC samples and downregulated in non-metastatic while it is negatively correlated with PINCH-1 and positively correlated with PUMA expression, suggesting that a pro-apoptotic mechanism is in place in metastatic BC samples and identifying RSU-1 as a potentially interesting molecule that needs to be evaluated further as a novel BC   metastasis   biomarker. 
   
  
 HSP90B1 
 
  NCBI Gene  
  GeneRIF  
  Pubmed  
 
 
  Search Term  Rank  Fields  
  [Ff]ilopodia  1  PubMed(1); GeneRIFs(1)  
  [Pp]odosome(s)?  5  PubMed(1)  
  [Mm]igration  6  PubMed(7); GeneRIFs(1)  
  ECM  7  PubMed(1)  
  [Ee]xtracellular [Mm]atrix  8  PubMed(3); Component(1)  
  [Ii]nvasive  10  PubMed(8); GeneRIFs(2)  
  [Mm]etastasize  11  PubMed(1)  
  [Mm]etastasis  12  PubMed(19); GeneRIFs(4)  
  [Cc]ell [Ii]nvasion  13  PubMed(4); GeneRIFs(1)  
 
 
 hide/show details for HSP90B1 
  [Ff]ilopodia  PubMed  
 Endoplasmic Reticulum-resident Heat Shock Protein 90 (HSP90) Isoform Glucose-regulated Protein 94 (GRP94) Regulates Cell Polarity and Cancer Cell Migration by Affecting Intracellular Transport. Heat shock protein 90 (HSP90) is a molecular chaperone that is up-regulated in cancer and is required for the folding of numerous signaling proteins. Consequently, HSP90 represents an ideal target for the development of new anti-cancer agents. The human HSP90 isoform, glucose-regulated protein 94 (GRP94), resides in the endoplasmic reticulum and regulates secretory pathways, integrins, and Toll-like receptors, which contribute to regulating immunity and metastasis. However, the cellular function of GRP94 remains underinvestigated. We report that GRP94 knockdown cells are defective in intracellular transport and, consequently, negatively impact the trafficking of F-actin toward the cellular cortex, integrin α2 and integrin αL toward the cell membrane an  d filopod  ia, and secretory vesicles containing the HSP90α-AHA1-survivin complex toward the leading edge. As a result, GRP94 knockdown cells form a multipolar spindle instead of bipolar morphology and consequently manifest a defect in cell migration and adhesion. 
  GeneRIFs  
 GRP94 knockdown cells are defective in intracellular transport and, consequently, negatively impact the trafficking of F-actin toward the cellular cortex, integrin alpha2 and integrin alphaL toward the cell membrane and   filopodia   
  [Pp]odosome(s)?  PubMed  
 Stable complexes formed by Grp94 with human IgG promoting angiogenic differentiation of HUVECs by a cytokine-like mechanism. To explore the molecular mechanisms by which complexes of Grp94 with IgG, purified from the plasma of diabetic subjects, could drive an inflammatory risk in vascular cells, native Grp94 was co-incubated with human, non-immune IgG to obtain the formation of complexes that were then tested on human umbilical vein endothelial cells (HUVECs). Co-incubation of Grp94 with IgG led to the formation of stable, SDS-resistant complexes that displayed effects partly similar and partly significantly different from those of Grp94 alone. Both Grp94 alone and with IgG stimulated the cell growth and promoted angiogenesis by a mechanism of autocrine/paracrine activation of the expression of heat shock protein (HSP)90 and HSP70. However, the most striking alterations in the cell cytoskeleton, characterized by dramatic rearrangement of actin and increased formation of   podosomes  , were induced by Grp94 with IgG, and were mediated by the enhanced expression of HSP90. At variance with Grp94 alone, Grp94 with IgG promoted the angiogenic differentiation by activating a signaling pathway apparently independent of the intense stimulation of the ERK1/2 pathway that was instead more directly involved in mediating the proliferative effects on HUVECs. Results show unprecedented cytokine-like effects of Grp94 and a so far undisclosed capacity to bind irreversibly IgG, forming complexes that, with respect to Grp94 alone, display a more intense angiogenic transforming capacity that may predict an increased inflammatory risk in vascular cells in vivo. 
  [Mm]igration  PubMed  
 Analysis of the myosin-II-responsive focal adhesion proteome reveals a role for β-Pix in negative regulation of focal adhesion maturation. Focal adhesions undergo myosin-II-mediated maturation wherein they grow and change composition to modulate integrin signalling for cell   migratio  n, growth and differentiation. To determine how focal adhesion composition is affected by myosin II activity, we performed proteomic analysis of isolated focal adhesions and compared protein abundance in focal adhesions from cells with and without myosin II inhibition. We identified 905 focal adhesion proteins, 459 of which changed in abundance with myosin II inhibition, defining the myosin-II-responsive focal adhesion proteome. The abundance of 73% of the proteins in the myosin-II-responsive focal adhesion proteome was enhanced by contractility, including proteins involved in Rho-mediated focal adhesion maturation and endocytosis- and calpain-dependent focal adhesion disassembly. During myosin II inhibition, 27% of proteins in the myosin-II-responsive focal adhesion proteome, including proteins involved in Rac-mediated lamellipodial protrusion, were enriched in focal adhesions, establishing that focal adhesion protein recruitment is also negatively regulated by contractility. We focused on the Rac guanine nucleotide exchange factor β-Pix, documenting its role in the negative regulation of focal adhesion maturation and the promotion of lamellipodial protrusion and focal adhesion turnover to drive cel  l migrati  on. 
 Molecular chaperones as a common set of proteins that regulate the invasion phenotype of head and neck cancer. The goal of this study was to establish a common set of molecules that regulate cell invasion in head and neck cancer (HNC).Five invasive sublines derived from HNC cell lines were established using the Matrigel selection method. Proteomic technology, MetaCore algorithm, and reverse transcriptase-PCR methods were used to search for molecules that contribute to the invasion phenotype. Cellular functional analyses and clinical association studies were applied to examine the significance of the molecules.Fifty-two proteins were identified in more than two of the four independent proteomic experiments, including 10 (19%) molecular chaperones. Seven chaperones were confirmed to be differentially expressed in five sublines, Hsp90α, Hsp90β, Hsp90-B1/Gp96, Hsp70-A5/Grp78, and HYOU1, that upregulate, whereas Hsp60 and glucosidase-α neutral AB (GANAB) downregulate. Four molecules were further investigated. In all cell lines, knockdown of Hsp60 or GANAB and silencing of Gp96 or Grp78 considerably enhanced or reduced ce  ll migrat  ion and invasion, respectively. Clinical association studies consistently revealed that low levels of Hsp60 or GANAB and high levels of Gp96 or Grp78 are significantly associated with advanced cancer (P < 0.001 to P = 0.047, respectively, for the four molecules) and poor survival (P < 0.001 to P = 0.025, respectively, for the four molecules).Our study defined molecular chaperones as a common set of proteins that regulate the invasion phenotype of HNC. Loss of the tumor suppression function of Hsp60 or GANAB and acquisition of the oncogenic function of Gp96 or Grp78 contribute to aggressive cancers. These molecules may serve as prognostic markers and targets for cancer drug development. 
 Molecular chaperones as a common set of proteins that regulate the invasion phenotype of head and neck cancer. The goal of this study was to establish a common set of molecules that regulate cell invasion in head and neck cancer (HNC).Five invasive sublines derived from HNC cell lines were established using the Matrigel selection method. Proteomic technology, MetaCore algorithm, and reverse transcriptase-PCR methods were used to search for molecules that contribute to the invasion phenotype. Cellular functional analyses and clinical association studies were applied to examine the significance of the molecules.Fifty-two proteins were identified in more than two of the four independent proteomic experiments, including 10 (19%) molecular chaperones. Seven chaperones were confirmed to be differentially expressed in five sublines, Hsp90α, Hsp90β, Hsp90-B1/Gp96, Hsp70-A5/Grp78, and HYOU1, that upregulate, whereas Hsp60 and glucosidase-α neutral AB (GANAB) downregulate. Four molecules were further investigated. In all cell lines, knockdown of Hsp60 or GANAB and silencing of Gp96 or Grp78 considerably enhanced or reduced ce  ll migrat  ion and invasion, respectively. Clinical association studies consistently revealed that low levels of Hsp60 or GANAB and high levels of Gp96 or Grp78 are significantly associated with advanced cancer (P < 0.001 to P = 0.047, respectively, for the four molecules) and poor survival (P < 0.001 to P = 0.025, respectively, for the four molecules).Our study defined molecular chaperones as a common set of proteins that regulate the invasion phenotype of HNC. Loss of the tumor suppression function of Hsp60 or GANAB and acquisition of the oncogenic function of Gp96 or Grp78 contribute to aggressive cancers. These molecules may serve as prognostic markers and targets for cancer drug development. 
 Glucose-regulated protein 94 is a novel glioma biomarker and promotes the aggressiveness of glioma via Wnt/β-catenin signaling pathway. Malignant glioma is the most common type of primary brain tumor and represents one of the most aggressive and lethal human cancer types. Glioma recurrence is a common event; however, the relevant molecular mechanisms in this setting are not well-understood. In this study, we investigated glucose-regulated protein 94 (GRP94) expressions in human glioma and aimed to determine the roles of GRP94 expression affects cell proliferation, invasion, and regulatory signaling in human glioma U87 cells. Our results showed that GRP94 was overexpressed at both mRNA and protein levels in high-grade glioblastoma as compared with normal brain tissues. High GRP94 levels also predict shorter overall survival of glioma patients. RNAi-mediated silencing of GRP94 suppressed cellular proliferation, colony formation ability in glioma cells. Depletion of GRP94 also inhibited cell   migratio  n and invasion ability in glioma cell. Furthermore, gene microarray analysis revealed that GRP94 depletion caused the dysregulation of critical pathway, Wnt/β-catenin signaling pathway. We next demonstrated GRP94 regulates Wnt/β-catenin signaling pathway to promote the proliferation of glioblastoma cells. Conclusion, our findings establish GRP94 as progression markers and druggable targets in glioblastoma, relating their oncogenic effects to activation of the Wnt/β-catenin signaling pathway. 
 Glucose-regulated protein 94 mediates metastasis by CCT8 and the JNK pathway in hepatocellular carcinoma. Hepatocellular carcinoma (HCC) is one of the leading causes of cancer death worldwide. Cancer metastasis is a major obstacle in clinical cancer therapy. The mechanisms underlying the metastasis of HCC remain unclear. Glucose-regulated protein 94 (GRP94) is a key protein involved in mediating cancer progression, and it is highly expressed in HCC specimens. However, the role of GRP94 in cancer metastasis is unclear. A specific short hairpin RNA (shRNA) was employed to knock down GRP94 gene expression in HCC cell lines. Wound-healing   migration  , transwell   migration  , and invasion assays were performed to determine the   migration   and invasive ability of HCC cells. We demonstrated that silencing GRP94 inhibited HCC cell wound healing,   migration  , and invasion. Furthermore, our findings indicated that GRP94 knockdown might attenuate HCC cell metastasis by inhibiting CCT8/c-Jun/EMT signaling. Our study indicated that silencing GRP94 significantly reduced the   migration   and invasion abilities of HCC cells. Moreover, depleting GRP94 inhibited cell   migration   and invasion by downregulating CCT8/c-Jun signaling. Thus, our data suggest that the GRP94/CCT8/c-Jun/EMT signaling cascade might be a new therapeutic target for HCC. 
 Endoplasmic Reticulum-resident Heat Shock Protein 90 (HSP90) Isoform Glucose-regulated Protein 94 (GRP94) Regulates Cell Polarity and Cancer Cell   Migration   by Affecting Intracellular Transport. Heat shock protein 90 (HSP90) is a molecular chaperone that is up-regulated in cancer and is required for the folding of numerous signaling proteins. Consequently, HSP90 represents an ideal target for the development of new anti-cancer agents. The human HSP90 isoform, glucose-regulated protein 94 (GRP94), resides in the endoplasmic reticulum and regulates secretory pathways, integrins, and Toll-like receptors, which contribute to regulating immunity and metastasis. However, the cellular function of GRP94 remains underinvestigated. We report that GRP94 knockdown cells are defective in intracellular transport and, consequently, negatively impact the trafficking of F-actin toward the cellular cortex, integrin α2 and integrin αL toward the cell membrane and filopodia, and secretory vesicles containing the HSP90α-AHA1-survivin complex toward the leading edge. As a result, GRP94 knockdown cells form a multipolar spindle instead of bipolar morphology and consequently manifest a defect in ce  ll migrat  ion and adhesion. 
 Overexpression of GRP94 in breast cancer cells resistant to oxidative stress promotes high levels of cancer cell proliferation and   migration  : implications for tumor recurrence. Targeting the altered redox status of cancer cells is emerging as an interesting approach to potentiate chemotherapy. However, to maximize the effectiveness of this strategy and define the correct chemotherapeutic associations, it is important to understand the biological consequences of chronically exposing cancer cells to reactive oxygen species (ROS). Using an H(2)O(2)-generating system, we selected a ROS-resistant MCF-7 breast cancer cell line, namely Resox cells. By exploring different survival pathways that are usually induced during oxidative stress, we identified a constitutive overexpression of the endoplasmic reticulum chaperone, GRP94, in these cells, whereas levels of its cytoplasmic homolog HSP90, or GRP78, were not modified. This overexpression was not mediated by constitutive unfolded protein response (UPR) activation. The increase in GRP94 is tightly linked to an increase in cell proliferation and   migration   capacities, as shown by GRP94-silencing experiments. Interestingly, we also observed that GRP94 silencing inhibits   migration   and proliferation of the highly aggressive MDA-MB-231 cells. By immunohistochemistry, we showed that GRP94 expression was higher in recurrent human breast cancers than in their paired primary neoplasias. Similar to the situation in the Resox cells, this increase was not associated with an increase in UPR activation in recurrent tumors. In conclusion, this study suggests that GRP94 overexpression may be a hallmark of aggressiveness and recurrence in breast cancers. 
  GeneRIFs  
 higher expression in recurrent breast cancer promotes cancer cell proliferation and   migration   
  ECM  PubMed  
 Comparative proteomic analysis of supportive and unsupportive extracellular matrix substrates for human embryonic stem cell maintenance. Human embryonic stem cells (hESCs) are pluripotent cells that have indefinite replicative potential and the ability to differentiate into derivatives of all three germ layers. hESCs are conventionally grown on mitotically inactivated mouse embryonic fibroblasts (MEFs) or feeder cells of human origin. In addition, feeder-free culture systems can be used to support hESCs, in which the adhesive substrate plays a key role in the regulation of stem cell self-renewal or differentiation. Extracellular matrix (  ECM  ) components define the microenvironment of the niche for many types of stem cells, but their role in the maintenance of hESCs remains poorly understood. We used a proteomic approach to characterize in detail the composition and interaction networks of   ECM  s that support the growth of self-renewing hESCs. Whereas many   ECM   components were produced by supportive and unsupportive MEF and human placental stromal fibroblast feeder cells, some proteins were only expressed in supportive   ECM  , suggestive of a role in the maintenance of pluripotency. We show that identified candidate molecules can support attachment and self-renewal of hESCs alone (fibrillin-1) or in combination with fibronectin (perlecan, fibulin-2), in the absence of feeder cells. Together, these data highlight the importance of specific   ECM   interactions in the regulation of hESC phenotype and provide a resource for future studies of hESC self-renewal. 
  [Ee]xtracellular [Mm]atrix  PubMed  
 SILAC-based proteomics of human primary endothelial cell morphogenesis unveils tumor angiogenic markers. Proteomics has been successfully used for cell culture on dishes, but more complex cellular systems have proven to be challenging and so far poorly approached with proteomics. Because of the complexity of the angiogenic program, we still do not have a complete understanding of the molecular mechanisms involved in this process, and there have been no in depth quantitative proteomic studies. Plating endothelial cells on matrigel recapitulates aspects of vessel growth, and here we investigate this mechanism by using a spike-in SILAC quantitative proteomic approach. By comparing proteomic changes in primary human endothelial cells morphogenesis on matrigel to general adhesion mechanisms in cells spreading on culture dish, we pinpoint pathways and proteins modulated by endothelial cells. The cell-  extracellular matrix   adhesion proteome depends on the adhesion substrate, and a detailed proteomic profile of the   extracellular matrix   secreted by endothelial cells identified CLEC14A as a matrix component, which binds to MMRN2. We verify deregulated levels of these proteins during tumor angiogenesis in models of multistage carcinogenesis. This is the most in depth quantitative proteomic study of endothelial cell morphogenesis, which shows the potential of applying high accuracy quantitative proteomics to in vitro models of vessel growth to shed new light on mechanisms that accompany pathological angiogenesis. The mass spectrometry proteomics data have been deposited to the ProteomeXchange Consortium with the data set identifier PXD000359. 
 Proteomic analysis of human parotid gland exosomes by multidimensional protein identification technology (MudPIT). Human ductal saliva contributes over a thousand unique proteins to whole oral fluids. The mechanism by which most of these proteins are secreted by salivary glands remains to be determined. The present study used a mass spectrometry-based, shotgun proteomics approach to explore the possibility that a subset of the proteins found in saliva are derived from exosomes, membrane-bound vesicles of endosomal origin within multivesicular endosomes. Using MudPIT (multidimensional protein identification technology) mass spectrometry, we catalogued 491 proteins in the exosome fraction of human parotid saliva. Many of these proteins were previously observed in ductal saliva from parotid glands (265 proteins). Furthermore, 72 of the proteins in parotid exosomes overlap with those previously identified as urinary exosome proteins, proteins which are also frequently associated with exosomes from other tissues and cell types. Gene Ontology (GO) and KEGG pathway analyses found that cytosolic proteins comprise the largest category of proteins in parotid exosomes (43%), involved in such processes as phosphatidylinositol signaling system, calcium signaling pathway, inositol metabolism, protein export, and signal transduction, among others; whereas the integral plasma membrane proteins and associated/peripheral plasma membrane proteins (26%) were associated with   extracellular matrix  -receptor interaction, epithelial cell signaling, T-cell and B-cell receptor signaling, cytokine receptor interaction, and antigen processing and presentation, among other biological functions. In addition, these putative saliva exosomal proteins were linked to specific diseases (e.g., neurodegenerative disorders, prion disease, cancers, type I and II diabetes). Consequently, parotid glands secrete exosomes that reflect the metabolic and functional status of the gland and may also carry informative protein markers useful in the diagnosis and treatment of systemic diseases. 
 Comparative proteomic analysis of supportive and unsupportive   extracellular matrix   substrates for human embryonic stem cell maintenance. Human embryonic stem cells (hESCs) are pluripotent cells that have indefinite replicative potential and the ability to differentiate into derivatives of all three germ layers. hESCs are conventionally grown on mitotically inactivated mouse embryonic fibroblasts (MEFs) or feeder cells of human origin. In addition, feeder-free culture systems can be used to support hESCs, in which the adhesive substrate plays a key role in the regulation of stem cell self-renewal or differentiation.   Extracellular matrix   (ECM) components define the microenvironment of the niche for many types of stem cells, but their role in the maintenance of hESCs remains poorly understood. We used a proteomic approach to characterize in detail the composition and interaction networks of ECMs that support the growth of self-renewing hESCs. Whereas many ECM components were produced by supportive and unsupportive MEF and human placental stromal fibroblast feeder cells, some proteins were only expressed in supportive ECM, suggestive of a role in the maintenance of pluripotency. We show that identified candidate molecules can support attachment and self-renewal of hESCs alone (fibrillin-1) or in combination with fibronectin (perlecan, fibulin-2), in the absence of feeder cells. Together, these data highlight the importance of specific ECM interactions in the regulation of hESC phenotype and provide a resource for future studies of hESC self-renewal. 
  Component  
   extracellular matrix   
  [Ii]nvasive  PubMed  
 Molecular chaperones as a common set of proteins that regulate the invasion phenotype of head and neck cancer. The goal of this study was to establish a common set of molecules that regulate cell invasion in head and neck cancer (HNC).Five   invasive   sublines derived from HNC cell lines were established using the Matrigel selection method. Proteomic technology, MetaCore algorithm, and reverse transcriptase-PCR methods were used to search for molecules that contribute to the invasion phenotype. Cellular functional analyses and clinical association studies were applied to examine the significance of the molecules.Fifty-two proteins were identified in more than two of the four independent proteomic experiments, including 10 (19%) molecular chaperones. Seven chaperones were confirmed to be differentially expressed in five sublines, Hsp90α, Hsp90β, Hsp90-B1/Gp96, Hsp70-A5/Grp78, and HYOU1, that upregulate, whereas Hsp60 and glucosidase-α neutral AB (GANAB) downregulate. Four molecules were further investigated. In all cell lines, knockdown of Hsp60 or GANAB and silencing of Gp96 or Grp78 considerably enhanced or reduced cell migration and invasion, respectively. Clinical association studies consistently revealed that low levels of Hsp60 or GANAB and high levels of Gp96 or Grp78 are significantly associated with advanced cancer (P < 0.001 to P = 0.047, respectively, for the four molecules) and poor survival (P < 0.001 to P = 0.025, respectively, for the four molecules).Our study defined molecular chaperones as a common set of proteins that regulate the invasion phenotype of HNC. Loss of the tumor suppression function of Hsp60 or GANAB and acquisition of the oncogenic function of Gp96 or Grp78 contribute to aggressive cancers. These molecules may serve as prognostic markers and targets for cancer drug development. 
 Molecular chaperones as a common set of proteins that regulate the invasion phenotype of head and neck cancer. The goal of this study was to establish a common set of molecules that regulate cell invasion in head and neck cancer (HNC).Five   invasive   sublines derived from HNC cell lines were established using the Matrigel selection method. Proteomic technology, MetaCore algorithm, and reverse transcriptase-PCR methods were used to search for molecules that contribute to the invasion phenotype. Cellular functional analyses and clinical association studies were applied to examine the significance of the molecules.Fifty-two proteins were identified in more than two of the four independent proteomic experiments, including 10 (19%) molecular chaperones. Seven chaperones were confirmed to be differentially expressed in five sublines, Hsp90α, Hsp90β, Hsp90-B1/Gp96, Hsp70-A5/Grp78, and HYOU1, that upregulate, whereas Hsp60 and glucosidase-α neutral AB (GANAB) downregulate. Four molecules were further investigated. In all cell lines, knockdown of Hsp60 or GANAB and silencing of Gp96 or Grp78 considerably enhanced or reduced cell migration and invasion, respectively. Clinical association studies consistently revealed that low levels of Hsp60 or GANAB and high levels of Gp96 or Grp78 are significantly associated with advanced cancer (P < 0.001 to P = 0.047, respectively, for the four molecules) and poor survival (P < 0.001 to P = 0.025, respectively, for the four molecules).Our study defined molecular chaperones as a common set of proteins that regulate the invasion phenotype of HNC. Loss of the tumor suppression function of Hsp60 or GANAB and acquisition of the oncogenic function of Gp96 or Grp78 contribute to aggressive cancers. These molecules may serve as prognostic markers and targets for cancer drug development. 
 Dendritic cells, T-cell infiltration, and Grp94 expression in cholangiocellular carcinoma. Although dendritic cells (DCs) play an important role in tumor immunity, there have been no reports on their role in cholangiocellular carcinoma (CCC). In 26 formalin-fixed, paraffin-embedded tissue sections from patients with CCC, cells positive for CD83 (a marker of mature DCs), CD1a (a marker of immature DCs), and CD8 and CD4 (T cell markers) were counted, and expression of glucose-regulated protein (grp) 94, which is considered to participate in the maturation of DCs, was evaluated by immunohistochemistry and Western blot analysis to study the relationship between their expression and patients' disease outcome. The number of CD83-positive DCs at the   invasive   margin of CCCs correlated significantly with the number of CD8-positive or CD4-positive T cells in the cancerous region and was significantly higher in grp94-positive cancer than in grp94-negative cancer (P = 0.0006). CD83-positive patients (positive cells in   invasive   margin > 12.4/field) had both a significantly lower incidence of lymph node metastasis (23.1% vs 69.2%; P = 0.0206) and a better outcome than CD83-negative patients (P <0.001). We conclude that mature DCs are distributed predominantly at the   invasive   margin of cancers, and a significantly higher number of mature DCs at the   invasive   margin are observed in patients with grp94-positive cancer cells. Mature DCs may enhance CD8- and CD4-positive cell infiltration into cancers and improve prognosis in patients with CCC, due in part to abatement of lymph node metastasis. 
 Glucose-regulated protein 94 mediates metastasis by CCT8 and the JNK pathway in hepatocellular carcinoma. Hepatocellular carcinoma (HCC) is one of the leading causes of cancer death worldwide. Cancer metastasis is a major obstacle in clinical cancer therapy. The mechanisms underlying the metastasis of HCC remain unclear. Glucose-regulated protein 94 (GRP94) is a key protein involved in mediating cancer progression, and it is highly expressed in HCC specimens. However, the role of GRP94 in cancer metastasis is unclear. A specific short hairpin RNA (shRNA) was employed to knock down GRP94 gene expression in HCC cell lines. Wound-healing migration, transwell migration, and invasion assays were performed to determine the migration and   invasive   ability of HCC cells. We demonstrated that silencing GRP94 inhibited HCC cell wound healing, migration, and invasion. Furthermore, our findings indicated that GRP94 knockdown might attenuate HCC cell metastasis by inhibiting CCT8/c-Jun/EMT signaling. Our study indicated that silencing GRP94 significantly reduced the migration and invasion abilities of HCC cells. Moreover, depleting GRP94 inhibited cell migration and invasion by downregulating CCT8/c-Jun signaling. Thus, our data suggest that the GRP94/CCT8/c-Jun/EMT signaling cascade might be a new therapeutic target for HCC. 
 Gp96 and its different expression in breast carcinomas. The aim of our work was to determine the expression of glycoprotein 96 (gp96; glucose-regulated protein 94 - GRP94) in 69 samples of breast carcinoma. Enzyme immunohistochemical method was chosen for the detection of gp96 protein and its expression was compared in breast cancer cells versus normal breast cells. We found higher expression of gp96 protein in breast carcinoma cells and low or no expression in normal breast cells. Furthermore, we demonstrate first time, that ductal   invasive   breast carcinomas showed higher expression of gp96 than lobular   invasive   breast carcinomas. 
 The endoplasmic reticulum stress response chaperone: Gp96, a host receptor for Crohn disease-associated adherent-  invasive   Escherichia coli. Ileal lesions of Crohn disease (CD) patients are abnormally colonized by adherent-  invasive   Escherichia coli (AIEC) producing outer membrane vesicles (OMVs) that contribute to the bacterial invasion process. In addition, endoplasmic stress was recently reported to have a key role in CD patients after the discovery, in CD patients, of several single nucleotide polymorphisms within gene encoding key component of the ER stress response and of increased expression of ER-localised stress response proteins. We recently demonstrated that the ER-stress response chaperone Gp96 is strongly expressed on the apical surface of ileal epithelial cells in CD patients and acts as a host cell receptor for OMVs, promoting AIEC invasion. In this addendum, we provide a synopsis on current data concerning AIEC virulence, summarize our recent findings and also discuss, in more detail, unresolved issues and the potential implications of our findings.  
 Identification of NTN4, TRA1, and STC2 as prognostic markers in breast cancer in a screen for signal sequence encoding proteins. In a previous screen using a signal-trap library, we identified a number of secreted proteins up-regulated in primary tumor cells isolated from   invasive   breast cancers. The purpose of this study was to assess the expression of these genes in human   invasive   breast tumors and to determine the significance of their expression for prognosis in breast cancer.A tissue microarray containing 245   invasive   breast tumors from women treated with curative surgery followed by anthracycline-based chemotherapy and hormone therapy for the estrogen receptor (ER)-positive tumors was screened by in situ hybridization with probes against thrombospondin 3 (TSP3), insulin-like growth factor binding protein 7 (IGFBP7), tumor rejection antigen 1 (TRA1), stanniocalcin 2 (STC2), and netrin 4 (NTN4). Correlations between categorical variables were done using the chi(2) test and Fisher's exact test. Cumulative survival probabilities were calculated using the Kaplan-Meier method and multivariate survival analysis was done with Cox hazard model. A series of breast cancers were also stained with NTN4 antibodies.All five genes examined were expressed in   invasive   breast tumor cells. NTN4 protein expression was also confirmed by immunohistochemistry. Together, these data validate the design and screening of the signal-trap library. Univariate survival analysis revealed that expressions of TRA1, STC2, and NTN4 are correlated with longer disease-free survival and that TRA1 and NTN4 are associated with longer overall survival. Multivariate analysis showed that NTN4 is an independent prognostic factor of overall survival.This article describes the identification of three secreted proteins, NTN4, TRA1, and STC2, as potential novel prognostic markers in breast cancer. 
 GP96 is over-expressed in oral cavity cancer and is a poor prognostic indicator for patients receiving radiotherapy. Oral cavity cancers (ORC) are the most common cancers, and standard treatment is radical surgery with postoperative radiotherapy. However, locoregional failure remains a major problem, indicating radioresistance an important issue. Our previous work has shown that GP96 contributed to radioresistance in nasopharyngeal and oral cancer cell lines. In this study, we determined clinical significance of GP96 in ORC by evaluation of GP96 expression and its association with disease prognosis in patients receiving radiotherapyTotal of 79 ORC patients (77 males, median age: 48 years old) receiving radical surgery and postoperative radiotherapy between Oct 1999 and Dec 2004 were enrolled. Patients in pathological stages II, III and IV were 16.5%, 16.5% and 67%, respectively. For each patient, a pair of carcinoma tissue and grossly adjacent normal mucosa was obtained. GP96-expression was examined by western blot analysis, and the association with clinicopathological status was determined.Three-year locoregional control (LRC), distant metastasis-free survival (DMFS), disease-specific survival (DSS) and overall survival (OS) rates were 69%, 79%, 63% and 57%, respectively. We found that 55 patients (70%) displayed GP96-overexpression in the tumor tissue, which correlated with a higher pN stage (p = 0.020) and tumor depth (> 10 mm) (p = 0.045). Nodal extracapsular spreading (ECS) and GP96-overexpression predicted adverse LRC (p = 0.049 and p = 0.008). When stratified by nodal ECS, the adverse impact of GP96 remained significant in three-year LRC (p = 0.004). In multivariate analysis, GP96-overexpression was also an independent predictor of LRC, DSS and OS (p = 0.018, p = 0.011 and p = 0.012).GP96 may play roles in radioresistance which attributes to tumor   invasive  ness in oral cancer patients receiving radiotherapy. GP96 may serve as a novel prognostic marker of radiotherapy. However, further independent studies are required to validate our findings in a larger series. 
  GeneRIFs  
 GP96 may play roles in radioresistance which attributes to tumor   invasive  ness in oral cancer patients receiving radiotherapy 
 The endoplasmic reticulum stress response chaperone Gp96 is strongly expressed on the apical surface of ileal epithelial cells in Crohn disease patients, promoting adherent-  invasive   Escherichia coli invasion. 
  [Mm]etastasize  PubMed  
 FN14 and GRP94 expression are prognostic/predictive biomarkers of brain metastasis outcome that open up new therapeutic strategies. Brain metastasis is a devastating problem in patients with breast, lung and melanoma tumors. GRP94 and FN14 are predictive biomarkers over-expressed in primary breast carcinomas that   metastasize  d in brain. To further validate these brain metastasis biomarkers, we performed a multicenter study including 318 patients with breast carcinomas. Among these patients, there were 138 patients with metastasis, of whom 84 had brain metastasis. The likelihood of developing brain metastasis increased by 5.24-fold (95%CI 2.83-9.71) and 2.55- (95%CI 1.52-4.3) in the presence of FN14 and GRP94, respectively. Moreover, FN14 was more sensitive than ErbB2 (38.27 vs. 24.68) with similar specificity (89.43 vs. 89.55) to predict brain metastasis and had identical prognostic value than triple negative patients (p < 0.0001). Furthermore, we used GRP94 and FN14 pathways and GUILD, a network-based disease-gene prioritization program, to pinpoint the genes likely to be therapeutic targets, which resulted in FN14 as the main modulator and thalidomide as the best scored drug. The treatment of mice with brain metastasis improves survival decreasing reactive astrocytes and angiogenesis, and down-regulate FN14 and its ligand TWEAK. In conclusion our results indicate that FN14 and GRP94 are prediction/prognosis markers which open up new possibilities for preventing/treating brain metastasis. 
  [Mm]etastasis  PubMed  
 Overexpression of GRP78 and GRP94 is involved in colorectal carcinogenesis. Glucose-related proteins (GRPs) are ubiquitously expressed in the endoplasmic reticulum and assist in protein folding and assembly, consequently considered to be molecular chaperones. GRP78 and GRP94 expression was induced by glucose starvation and up-regulated in samples taken from several different malignant tissues. To clarify the roles of both molecules in tumorigenesis and progression of colorectal carcinomas, immunohistochemistry (IHC) was performed on tissue microarrays containing colorectal carcinomas, adenomas and the non-neoplastic mucosa (NNM) using antibodies against GRP78 and GRP94. Their expression was correlated with the clinicopathological parameters of carcinomas. Both proteins were also studied in colorectal carcinoma cell lines (DLD-1, HCT-15, SW480 and WiDr) by IHC and Western blot. There was a gradually increased GRP78 expression from colorectal NNMs, carcinomas, to low-grade and high-grade adenomas (P<0.05), while up-regulated GRP94 expression from NNM, low-grade adenoma, high-grade adenoma, to carcinoma (P<0.05). The expression was similar in all the carcinoma cell lines. GRP78 expression was negatively correlated with lymphatic invasion or low GRP94 expression of the carcinomas (P<0.05), while there was no correlation of GRP94 expression with other parameters of carcinomas (P>0.05). Multivariate analysis showed that venous invasion, lymph node   metastasis   and UICC staging (P<0.05), but not age, sex, tumor size, differentiation, depth of invasion, lymphatic invasion, GRP78 and GRP94 expression (P>0.05), were independent prognostic factors for carcinomas. It is suggested that up-regulated expression of GRP78 and GRP94 could possibly be involved in the pathogenesis of colorectal carcinomas. 
 Overexpression of GRP78 and GRP94 are markers for aggressive behavior and poor prognosis in gastric carcinomas. Glucose-related proteins (GRPs) are ubiquitously expressed in endoplasmic reticulum and able to assist in protein folding and assembly; consequently, they are considered as molecular chaperones. GRP78 and GRP94 expression was induced by glucose starvation and up-regulated in the malignancies. To clarify the roles of both molecules in tumorigenesis and progression of gastric carcinomas, immunohistochemistry was used on tissue microarray containing gastric carcinomas, adenomas, and nonneoplastic mucosa using the antibodies against GRP78 and GRP94, with a comparison of their expression with clinicopathological parameters of carcinomas. Gastric carcinoma cell lines (MKN28, AGS, MKN45, KATO-III, and HGC-27) were studied for both proteins by immunohistochemistry and Western blot. There was more expression of both proteins in gastric carcinoma and adenoma than in nonneoplastic mucosas (P < .05). All gastric carcinoma cell lines showed their expression at different levels. They were positively correlated with tumor size, depth of invasion, lymphatic and venous invasion, lymph node   metastasis  , and Union Internationale Contre le Cancer staging (P < .05), with positive relationship between both proteins (P < .05). Univariate analysis indicated the postsurgical cumulative survival rate of patients with positive GRP78 or GRP94 expression to be lower than that in those without GRP78 or GRP94 expression (P < .05), but the close link disappeared if stratified according to depth of invasion (P > .05). Multivariate analysis showed that age, depth of invasion, lymphatic invasion, lymph node   metastasis  , Union Internationale Contre le Cancer staging, and Lauren classification (P < .05), but not GRP78 and GRP94 expression, were independent prognostic factors for carcinomas (P > .05). Up-regulated expression of GRP78 and GRP94 was possibly involved in pathogenesis, growth, invasion, and   metastasis   of gastric carcinomas. They were considered objective and effective markers for the aggressive behavior and poor prognosis in gastric carcinomas. 
 Overexpression of GRP78 and GRP94 is involved in colorectal carcinogenesis. Glucose-related proteins (GRPs) are ubiquitously expressed in the endoplasmic reticulum and assist in protein folding and assembly, consequently considered to be molecular chaperones. GRP78 and GRP94 expression was induced by glucose starvation and up-regulated in samples taken from several different malignant tissues. To clarify the roles of both molecules in tumorigenesis and progression of colorectal carcinomas, immunohistochemistry (IHC) was performed on tissue microarrays containing colorectal carcinomas, adenomas and the non-neoplastic mucosa (NNM) using antibodies against GRP78 and GRP94. Their expression was correlated with the clinicopathological parameters of carcinomas. Both proteins were also studied in colorectal carcinoma cell lines (DLD-1, HCT-15, SW480 and WiDr) by IHC and Western blot. There was a gradually increased GRP78 expression from colorectal NNMs, carcinomas, to low-grade and high-grade adenomas (P<0.05), while up-regulated GRP94 expression from NNM, low-grade adenoma, high-grade adenoma, to carcinoma (P<0.05). The expression was similar in all the carcinoma cell lines. GRP78 expression was negatively correlated with lymphatic invasion or low GRP94 expression of the carcinomas (P<0.05), while there was no correlation of GRP94 expression with other parameters of carcinomas (P>0.05). Multivariate analysis showed that venous invasion, lymph node   metastasis   and UICC staging (P<0.05), but not age, sex, tumor size, differentiation, depth of invasion, lymphatic invasion, GRP78 and GRP94 expression (P>0.05), were independent prognostic factors for carcinomas. It is suggested that up-regulated expression of GRP78 and GRP94 could possibly be involved in the pathogenesis of colorectal carcinomas. 
 Overexpression of GRP78 and GRP94 are markers for aggressive behavior and poor prognosis in gastric carcinomas. Glucose-related proteins (GRPs) are ubiquitously expressed in endoplasmic reticulum and able to assist in protein folding and assembly; consequently, they are considered as molecular chaperones. GRP78 and GRP94 expression was induced by glucose starvation and up-regulated in the malignancies. To clarify the roles of both molecules in tumorigenesis and progression of gastric carcinomas, immunohistochemistry was used on tissue microarray containing gastric carcinomas, adenomas, and nonneoplastic mucosa using the antibodies against GRP78 and GRP94, with a comparison of their expression with clinicopathological parameters of carcinomas. Gastric carcinoma cell lines (MKN28, AGS, MKN45, KATO-III, and HGC-27) were studied for both proteins by immunohistochemistry and Western blot. There was more expression of both proteins in gastric carcinoma and adenoma than in nonneoplastic mucosas (P < .05). All gastric carcinoma cell lines showed their expression at different levels. They were positively correlated with tumor size, depth of invasion, lymphatic and venous invasion, lymph node   metastasis  , and Union Internationale Contre le Cancer staging (P < .05), with positive relationship between both proteins (P < .05). Univariate analysis indicated the postsurgical cumulative survival rate of patients with positive GRP78 or GRP94 expression to be lower than that in those without GRP78 or GRP94 expression (P < .05), but the close link disappeared if stratified according to depth of invasion (P > .05). Multivariate analysis showed that age, depth of invasion, lymphatic invasion, lymph node   metastasis  , Union Internationale Contre le Cancer staging, and Lauren classification (P < .05), but not GRP78 and GRP94 expression, were independent prognostic factors for carcinomas (P > .05). Up-regulated expression of GRP78 and GRP94 was possibly involved in pathogenesis, growth, invasion, and   metastasis   of gastric carcinomas. They were considered objective and effective markers for the aggressive behavior and poor prognosis in gastric carcinomas. 
 Decreased adhesiveness, resistance to anoikis and suppression of GRP94 are integral to the survival of circulating tumor cells in prostate cancer. The presence of circulating tumor cells (CTC) is common in prostate cancer patients, however until recently their clinical significance was unknown. The CTC stage is essential for the formation of distant metastases, and their continuing presence after radical prostatectomy has been shown to predict recurrent or latent disease. Despite their mechanistic and prognostic importance, due both to their scarcity and difficulties in their isolation, little is known about the characteristics that enable their production and survival. The aim of this study was to investigate the molecular mechanisms underlying the survival of CTC cells. A novel CTC cell line from the bloodstream of an orthotopic mouse model of castration-resistant prostate cancer was established and compared with the primary tumor using attachment assays, detachment culture, Western blot, flow cytometry and 2D gel electrophoresis. Decreased adhesiveness and expression of adhesion molecules E-cadherin, beta4-integrin and gamma-catenin, together with resistance to detachment and drug-induced apoptosis and upregulation of Bcl-2 were integral to the development of CTC and their survival. Using proteomic studies, we observed that the GRP94 glycoprotein was suppressed in CTC. GRP94 was also shown to be suppressed in a tissue microarray study of 79 prostate cancer patients, indicating its possible role in prostate cancer progression. Overall, this study suggests molecular alterations accounting for the release and survival of CTC, which may be used as drug targets for either anti-metastatic therapy or the suppression of latent disease. We also indicate the novel involvement of GRP94 suppression in prostate cancer   metastasis  . 
 Dendritic cells, T-cell infiltration, and Grp94 expression in cholangiocellular carcinoma. Although dendritic cells (DCs) play an important role in tumor immunity, there have been no reports on their role in cholangiocellular carcinoma (CCC). In 26 formalin-fixed, paraffin-embedded tissue sections from patients with CCC, cells positive for CD83 (a marker of mature DCs), CD1a (a marker of immature DCs), and CD8 and CD4 (T cell markers) were counted, and expression of glucose-regulated protein (grp) 94, which is considered to participate in the maturation of DCs, was evaluated by immunohistochemistry and Western blot analysis to study the relationship between their expression and patients' disease outcome. The number of CD83-positive DCs at the invasive margin of CCCs correlated significantly with the number of CD8-positive or CD4-positive T cells in the cancerous region and was significantly higher in grp94-positive cancer than in grp94-negative cancer (P = 0.0006). CD83-positive patients (positive cells in invasive margin > 12.4/field) had both a significantly lower incidence of lymph node   metastasis   (23.1% vs 69.2%; P = 0.0206) and a better outcome than CD83-negative patients (P <0.001). We conclude that mature DCs are distributed predominantly at the invasive margin of cancers, and a significantly higher number of mature DCs at the invasive margin are observed in patients with grp94-positive cancer cells. Mature DCs may enhance CD8- and CD4-positive cell infiltration into cancers and improve prognosis in patients with CCC, due in part to abatement of lymph node   metastasis  . 
 Eight proteins play critical roles in RCC with bone   metastasis   via mitochondrial dysfunction. Most kidney cancers are renal cell carcinomas (RCC). RCC lacks early warning signs and 70 % of patients with RCC develop metastases. Among them, 50 % of patients having skeletal metastases developed a dismal survival of less than 10 % at 5 years. Therefore, exploring the key driving proteins and pathways involved in RCC bone   metastasis   could benefit patients' therapy and prolong their survival. We examined the difference between the OS-RC-2 cells and the OS-RC-2-BM5 cells (subpopulation from OS-RC-2) of RCC with proteomics. Then we employed Western-blot, immunohistochemistry and the clinical database (oncomine) to screen and verify the key proteins and then we analyzed the functions and the related pathways of selected key proteins with system biology approaches. Our proteomic data revealed 26 significant changed spots (fold change <0.5 and >1.9, P < 0.05) between two cells. The Western blotting results validated for these identified spots were consistent with the proteomics'. From the public clinical database, 23 out of 26 proteins were connected with RCC metastases and 9 out of 23 with survival time directly (P < 0.05). Finally, only 8 out of 9 proteins had significantly positive results in tissues of RCC patients with bone   metastasis   compared with primary tumor (P < 0.05). System biology analyzing results showed these eight proteins mainly distributed in oxidative phosphorylation which indicates that mitochondria dysfunction played the critical role to regulate cells   metastasis  . Our article used a variety of experimental techniques to find eight proteins which abnormally regulated mitochondrial function to achieve a successful induction for RCC   metastasis   to bone. 
 Proteomic analyses reveal high expression of decorin and endoplasmin (HSP90B1) are associated with breast cancer   metastasis   and decreased survival. Breast cancer is the most common malignancy among women worldwide in terms of incidence and mortality. About 10% of North American women will be diagnosed with breast cancer during their lifetime and 20% of those will die of the disease. Breast cancer is a heterogeneous disease and biomarkers able to correctly classify patients into prognostic groups are needed to better tailor treatment options and improve outcomes. One powerful method used for biomarker discovery is sample screening with mass spectrometry, as it allows direct comparison of protein expression between normal and pathological states. The purpose of this study was to use a systematic and objective method to identify biomarkers with possible prognostic value in breast cancer patients, particularly in identifying cases most likely to have lymph node   metastasis   and to validate their prognostic ability using breast cancer tissue microarrays.Differential proteomic analyses were employed to identify candidate biomarkers in primary breast cancer patients. These analyses identified decorin (DCN) and endoplasmin (HSP90B1) which play important roles regulating the tumour microenvironment and in pathways related to tumorigenesis. This study indicates that high expression of Decorin is associated with lymph node   metastasis   (p<0.001), higher number of positive lymph nodes (p<0.0001) and worse overall survival (p = 0.01). High expression of HSP90B1 is associated with dist  ant metast  asis (p<0.0001) and decreased overall survival (p<0.0001) these patients also appear to benefit significantly from hormonal treatment.Using quantitative proteomic profiling of primary breast cancers, two new promising prognostic and predictive markers were found to identify patients with worse survival. In addition HSP90B1 appears to identify a group of patients with dist  ant metast  asis with otherwise good prognostic features. 
 Glucose-regulated protein 94 mediates   metastasis   by CCT8 and the JNK pathway in hepatocellular carcinoma. Hepatocellular carcinoma (HCC) is one of the leading causes of cancer death worldwide. Cancer   metastasis   is a major obstacle in clinical cancer therapy. The mechanisms underlying the   metastasis   of HCC remain unclear. Glucose-regulated protein 94 (GRP94) is a key protein involved in mediating cancer progression, and it is highly expressed in HCC specimens. However, the role of GRP94 in cancer   metastasis   is unclear. A specific short hairpin RNA (shRNA) was employed to knock down GRP94 gene expression in HCC cell lines. Wound-healing migration, transwell migration, and invasion assays were performed to determine the migration and invasive ability of HCC cells. We demonstrated that silencing GRP94 inhibited HCC cell wound healing, migration, and invasion. Furthermore, our findings indicated that GRP94 knockdown might attenuate HCC cell   metastasis   by inhibiting CCT8/c-Jun/EMT signaling. Our study indicated that silencing GRP94 significantly reduced the migration and invasion abilities of HCC cells. Moreover, depleting GRP94 inhibited cell migration and invasion by downregulating CCT8/c-Jun signaling. Thus, our data suggest that the GRP94/CCT8/c-Jun/EMT signaling cascade might be a new therapeutic target for HCC. 
 Immunolocalisation of heat shock protein 72 and glycoprotein 96 in colonic adenocarcinoma. Heat shock protein 72 (HSP72) and glycoprotein 96 (gp96) are highly expressed in cancer tissues. Recent studies indicate the possible roles of HSP72 and gp96 in the development and progression of colonic carcinomas, but detailed information is still ambiguous. In this study, we investigated the correlation between clinical pathology and immunolocalisation of HSP72 and gp96 in human colonic carcinoma. The distribution of HSP72 and gp96 was studied in 160 human colonic carcinomas, with or without   metastasis  , as well as in mucous membranes adjacent to cancers by means of immunohistochemistry. HSP72 immunoreactivity was detected in 145 of 160 primary tumours (90.6%) and in 44 of 160 mucous membranes adjacent to cancers (27.5%). Gp96 was detected in 81.3% colonic carcinomas and in 13.8% mucous membranes adjacent to cancer. Immunolocalisation of HSP72 and gp96 was mainly cytoplasmic. HSP72 and gp96 immunolabelling was significantly higher in colonic carcinomas with   metastasis   than in those without   metastasis   (P<0.05). The results indicate a significant correlation between the immunopositivity of HSP72 and gp96 and the progression of colonic carcinomas. Immunolabelling of HSP72 and gp96 may be useful as diagnostic or prognostic markers in colonic carcinoma. 
 Correlation between clinicopathology and expression of heat shock protein 72 and glycoprotein 96 in human esophageal squamous cell carcinoma. Heat shock protein 72 (HSP72) and glycoprotein 96 (gp96) are highly expressed in cancer tissues. Recent studies indicate the possible roles of HSP72 and gp96 in the development and progression of gastrointestinal carcinomas but detailed information is still ambiguous. We investigated the correlation between clinicopathology and expression of HSP72 and gp96 in human esophageal squamous cell carcinoma. The expression of HSP72 and gp96 was studied in 120 human esophageal squamous cell carcinomas with or without   metastasis   as well as in mucous membrane adjacent to cancers by way of immunohistochemistry. HSP72 immunoreactivities were detected in 112 of 120 primary tumors (93.3%) and in 30 of 120 mucous membranes adjacent to cancers (25.0%). Gp96 detected in esophageal squamous cell carcinoma and inmucous membrane adjacent to cancer was 85.0% and 20.0%, respectively. Both HSP72 and gp96 stained in cytoplasm. HSP72 and gp96 expression in esophageal squamous cell carcinomas with   metastasis   was significantly higher than those with non  metastasis   (P < .05). The results indicate that there exists a significant correlation between the expression of HSP72 and gp96 and the progression of esophageal squamous cell carcinomas. HSP72 and gp96 expression were significantly associated with the presence of tumor infiltration, lymph node, and remote   metastasis  . 
 Expression of endoplasmic reticulum stress proteins is a candidate marker of brain   metastasis   in both ErbB-2+ and ErbB-2- primary breast tumors. The increasing incidence of breast cancer brain   metastasis   in patients with otherwise well-controlled systemic cancer is a key challenge in cancer research. It is necessary to understand the properties of brain-tropic tumor cells to identify patients at risk for brain   metastasis  . Here we attempt to identify functional phenotypes that might enhance brain   metastasis  . To obtain an accurate classification of brain   metastasis   proteins, we mapped organ-specific brain   metastasis   gene expression signatures onto an experimental protein-protein interaction network based on brain metastatic cells. Thirty-seven proteins were differentially expressed between brain metastases and non-brain metastases. Analysis of metastatic tissues, the use of bioinformatic approaches, and the characterization of protein expression in tumors with or without   metastasis   identified candidate markers. A multivariate analysis based on stepwise logistic regression revealed GRP94, FN14, and inhibin as the best combination to discriminate between brain and non-brain metastases (ROC AUC = 0.85, 95% CI = 0.73 to 0.96 for the combination of the three proteins). These markers substantially improve the discrimination of brain   metastasis   compared with ErbB-2 alone (AUC = 0.76, 95% CI = 0.60 to 0.93). Furthermore, GRP94 was a better negative marker (LR = 0.16) than ErbB-2 (LR = 0.42). We conclude that, in breast carcinomas, certain proteins associated with the endoplasmic reticulum stress phenotype are candidate markers of brain   metastasis  . 
 HSP72 and gp96 in gastroenterological cancers. Heat shock protein 72 (HSP72) and glycoprotein 96 (gp96) are highly expressed in cancer tissues. Recent studies indicate the possible roles of HSP72 and gp96 in the development and progression of gastrointestinal carcinomas but detailed mechanisms are still ambiguous. Human esophageal cancer, gastric cancer, colon cancer and liver cancer are common gastrointestinal malignant carcinomas in the world. The studies indicated that there existed a significant correlation between the expression of HSP72, gp96 and the development and progression of digestive carcinomas. HSP72 and gp96 expression were significantly associated with the presence of tumor infiltration, lymph node and remote   metastasis  . Interestingly, studies have found that HSP72 chaperoned alpha-fetoprotein (AFP), HBx in hepatocellular carcinoma, and CD44 in colonic carcinomas. The further researches demonstrated that HSP72-AFP or gp96-AFP recombined vaccine could elicit specific anti-tumor immunity. The high-level expression of HSP72 and gp96 may be not only used as diagnostic or prognostic markers for gastrointestinal carcinomas but also as better immunotherapeutic vaccines in the cancers. 
 Endoplasmic Reticulum-resident Heat Shock Protein 90 (HSP90) Isoform Glucose-regulated Protein 94 (GRP94) Regulates Cell Polarity and Cancer Cell Migration by Affecting Intracellular Transport. Heat shock protein 90 (HSP90) is a molecular chaperone that is up-regulated in cancer and is required for the folding of numerous signaling proteins. Consequently, HSP90 represents an ideal target for the development of new anti-cancer agents. The human HSP90 isoform, glucose-regulated protein 94 (GRP94), resides in the endoplasmic reticulum and regulates secretory pathways, integrins, and Toll-like receptors, which contribute to regulating immunity and   metastasis  . However, the cellular function of GRP94 remains underinvestigated. We report that GRP94 knockdown cells are defective in intracellular transport and, consequently, negatively impact the trafficking of F-actin toward the cellular cortex, integrin α2 and integrin αL toward the cell membrane and filopodia, and secretory vesicles containing the HSP90α-AHA1-survivin complex toward the leading edge. As a result, GRP94 knockdown cells form a multipolar spindle instead of bipolar morphology and consequently manifest a defect in cell migration and adhesion. 
 FN14 and GRP94 expression are prognostic/predictive biomarkers of brain   metastasis   outcome that open up new therapeutic strategies. Brain   metastasis   is a devastating problem in patients with breast, lung and melanoma tumors. GRP94 and FN14 are predictive biomarkers over-expressed in primary breast carcinomas that metastasized in brain. To further validate these brain   metastasis   biomarkers, we performed a multicenter study including 318 patients with breast carcinomas. Among these patients, there were 138 patients with   metastasis  , of whom 84 had brain   metastasis  . The likelihood of developing brain   metastasis   increased by 5.24-fold (95%CI 2.83-9.71) and 2.55- (95%CI 1.52-4.3) in the presence of FN14 and GRP94, respectively. Moreover, FN14 was more sensitive than ErbB2 (38.27 vs. 24.68) with similar specificity (89.43 vs. 89.55) to predict brain   metastasis   and had identical prognostic value than triple negative patients (p < 0.0001). Furthermore, we used GRP94 and FN14 pathways and GUILD, a network-based disease-gene prioritization program, to pinpoint the genes likely to be therapeutic targets, which resulted in FN14 as the main modulator and thalidomide as the best scored drug. The treatment of mice with brain   metastasis   improves survival decreasing reactive astrocytes and angiogenesis, and down-regulate FN14 and its ligand TWEAK. In conclusion our results indicate that FN14 and GRP94 are prediction/prognosis markers which open up new possibilities for preventing/treating brain   metastasis  . 
 Involvement of ER-α36 in the malignant growth of gastric carcinoma cells is associated with GRP94 overexpression. This study aimed to examine the involvement of glucose-regulated protein 94 (GRP94) in oestrogen receptor-α36 (ER-α36)-mediated oestrogen signalling in gastric cancer development.A total of 130 formalin-fixed and paraffin-embedded gastric tumour samples with corresponding normal gastric and tumour-adjacent tissues were used. High levels of GRP94 expression (2+ or 3+) were observed in 109 of 130 gastric carcinomas (83.85%) by immunohistochemistry, and in 13 of 18 tumour specimens (72.22%) with Western blot analysis. GRP94 expression was correlated positively with gender, tumour stage, lymph no  de metasta  sis and ER-α36 expression (P < 0.05). Oestrogen treatment up-regulated both GRP94 and ER-α36 expression in gastric cancer SGC7901 cells. In addition, steady state levels of GRP94 protein were decreased in established gastric cancer SGC7901 cells with knocked-down levels of ER-α36 expression and in xenograft tumours formed by these cells. Forced expression of recombinant ER-α36 in SGC7901 cells, however, up-regulated the levels of GRP94 expression.Glucose-regulated protein 94 is a downstream effector of ER-α36-mediated oestrogen signalling, and may be involved in ER-α36 function during gastric carcinogenesis. 
 [Expression and significance of gp96 and immune-related gene CTLA-4, CD8 in lung cancer tissues]. It has been proven that gp96 plays an important role in specific cytotoxic immune response which is involved in anti-tumor effect in the body. The aim of this study is to investigate the biological significance of heat shock protein gp96 and immune-related gene CTLA-4, CD8 expressions in lung cancer tissues of different progressive stages.We used Envision immunohistochemistry method to detect the levels of expression of gp96, CTLA-4, CD8 in tissue microarray, which contained 89 primary lung cancer tissues, 12 lymph node   metastasis   lung cancer tissues, 12 precancerous lesions and 10 normal lung tissues, and analyzed the relationship between their expressions and clinicopathological parameters.(1) The positive rate of gp96 in primary lung cancer was remarkably higher than that in precancerous lesion and normal lung tissue (P<0.05). The positive rate of CTLA-4 in primary lung cancer tissue and precancerous lesion was significantly higher than that in normal lung tissue (P<0.05). The positive rate of CD8 in primary lung cancer tissue was significantly higher than that in normal lung tissue (P<0.05). The positive rate of gp96 in CD8-positive lymphocytes in the high expression group was less than that in the low group (P<0.05). (2) The positive rate of gp96 was closely related to sex, differentiation and clinical stage (P<0.05), but not to age, gross type, histological type and lymph node   metastasis   (P>0.05). The positive rate of CTLA-4 was closely related to age and differentiation (P<0.05), but not to sex, gross type, histological type, clinical stage and lymph node   metastasis   (P>0.05). CD8 expression was related to clinical stage (P<0.05), but not to sex, age, gross type, histological type, differentiation and lymph node   metastasis   (P>0.05). The positive rates of gp96, CTLA-4 were higher than that of CD8 in squamous cell carcinoma and SCLC, respectively. (3) There was positive correlation between gp96 and CTLA-4; there was negative correlation between gp96 and CD8, and there was negative correlation between CD8 and CTLA-4 (P<0.05).Gene expression of PD-L1 on lung cancer cell line can decrease the cytolytic effect of CTL on target cells. 
 HSP90B1 overexpression predicts poor prognosis in NSCLC patients. Non-small cell lung cancer (NSCLC) accounts for 85 % of lung cancer-related mortality worldwide. The heat shock protein 90B1 (HSP90B1) and DNA damage-inducible transcript 3 (DDIT3) are endoplasmic reticulum stress-related proteins that are associated with many malignancies. However, the roles of two proteins on NSCLC remain uncovered. To investigate the correlation between the expressions of HSP90B1 and DDIT3 and clinicopathological parameters of NSCLC as well as the significance of prognosis in NSCLC, a total of 143 NSCLC tissue samples and 45 control tissues samples were assessed. NSCLC patients were followed up from the day of surgery and ended by March 2014. The expressions of HSP90B1 and DDIT3 proteins were detected in all paraffin-embedded biopsy samples by immunochemistry. The HSP90B1 was highly expressed (65.2 %) in the 143 NSCLC patients, and its high expression was correlated with clinical stages (P = 0.001) and lymph n  ode metast  asis (P = 0.016). Similarly, DDIT3 was highly expressed in 43 (30.1 %) of 143 NSCLC patients, but only correlated with lymp  h node met  astasis. Furthermore, Log-rank test suggested that high HSP90B1 expression may predict shorter survival (overall survival (OS)) and disease-free survival (DFS) for NSCLC patients. Cox model multivariate analyses indicated that HSP90B1 overexpression was an independent poor prognostic factor for both of OS and DFS. Therefore, HSP90B1 and DDIT3 may the potential biomarker to predict the NSCLC clinicopathological progress. Meanwhile, high HSP90B1 expression means poor prognosis, and HSP90B1 can be a promising prognosis factor for NSCLC. 
 GP96 is over-expressed in oral cavity cancer and is a poor prognostic indicator for patients receiving radiotherapy. Oral cavity cancers (ORC) are the most common cancers, and standard treatment is radical surgery with postoperative radiotherapy. However, locoregional failure remains a major problem, indicating radioresistance an important issue. Our previous work has shown that GP96 contributed to radioresistance in nasopharyngeal and oral cancer cell lines. In this study, we determined clinical significance of GP96 in ORC by evaluation of GP96 expression and its association with disease prognosis in patients receiving radiotherapyTotal of 79 ORC patients (77 males, median age: 48 years old) receiving radical surgery and postoperative radiotherapy between Oct 1999 and Dec 2004 were enrolled. Patients in pathological stages II, III and IV were 16.5%, 16.5% and 67%, respectively. For each patient, a pair of carcinoma tissue and grossly adjacent normal mucosa was obtained. GP96-expression was examined by western blot analysis, and the association with clinicopathological status was determined.Three-year locoregional control (LRC), distant   metastasis  -free survival (DMFS), disease-specific survival (DSS) and overall survival (OS) rates were 69%, 79%, 63% and 57%, respectively. We found that 55 patients (70%) displayed GP96-overexpression in the tumor tissue, which correlated with a higher pN stage (p = 0.020) and tumor depth (> 10 mm) (p = 0.045). Nodal extracapsular spreading (ECS) and GP96-overexpression predicted adverse LRC (p = 0.049 and p = 0.008). When stratified by nodal ECS, the adverse impact of GP96 remained significant in three-year LRC (p = 0.004). In multivariate analysis, GP96-overexpression was also an independent predictor of LRC, DSS and OS (p = 0.018, p = 0.011 and p = 0.012).GP96 may play roles in radioresistance which attributes to tumor invasiveness in oral cancer patients receiving radiotherapy. GP96 may serve as a novel prognostic marker of radiotherapy. However, further independent studies are required to validate our findings in a larger series. 
  GeneRIFs  
 Results indicate that FN14 and GRP94 are prediction/prognosis markers which open up new possibilities for preventing/treating brain   metastasis   in breast cancer patients. 
 High HSP90B1 expression is associated with bone   metastasis   in renal cell carcinoma. 
 the novel involvement of GRP94 suppression in prostate cancer   metastasis  . 
 Up-regulated expression of GRP78 and GRP94 was possibly involved in pathogenesis, growth, invasion, and   metastasis   of gastric carcinomas. 
  [Cc]ell [Ii]nvasion  PubMed  
 Molecular chaperones as a common set of proteins that regulate the invasion phenotype of head and neck cancer. The goal of this study was to establish a common set of molecules that regulate   cell invasion   in head and neck cancer (HNC).Five invasive sublines derived from HNC cell lines were established using the Matrigel selection method. Proteomic technology, MetaCore algorithm, and reverse transcriptase-PCR methods were used to search for molecules that contribute to the invasion phenotype. Cellular functional analyses and clinical association studies were applied to examine the significance of the molecules.Fifty-two proteins were identified in more than two of the four independent proteomic experiments, including 10 (19%) molecular chaperones. Seven chaperones were confirmed to be differentially expressed in five sublines, Hsp90α, Hsp90β, Hsp90-B1/Gp96, Hsp70-A5/Grp78, and HYOU1, that upregulate, whereas Hsp60 and glucosidase-α neutral AB (GANAB) downregulate. Four molecules were further investigated. In all cell lines, knockdown of Hsp60 or GANAB and silencing of Gp96 or Grp78 considerably enhanced or reduced cell migration and invasion, respectively. Clinical association studies consistently revealed that low levels of Hsp60 or GANAB and high levels of Gp96 or Grp78 are significantly associated with advanced cancer (P < 0.001 to P = 0.047, respectively, for the four molecules) and poor survival (P < 0.001 to P = 0.025, respectively, for the four molecules).Our study defined molecular chaperones as a common set of proteins that regulate the invasion phenotype of HNC. Loss of the tumor suppression function of Hsp60 or GANAB and acquisition of the oncogenic function of Gp96 or Grp78 contribute to aggressive cancers. These molecules may serve as prognostic markers and targets for cancer drug development. 
 Molecular chaperones as a common set of proteins that regulate the invasion phenotype of head and neck cancer. The goal of this study was to establish a common set of molecules that regulate   cell invasion   in head and neck cancer (HNC).Five invasive sublines derived from HNC cell lines were established using the Matrigel selection method. Proteomic technology, MetaCore algorithm, and reverse transcriptase-PCR methods were used to search for molecules that contribute to the invasion phenotype. Cellular functional analyses and clinical association studies were applied to examine the significance of the molecules.Fifty-two proteins were identified in more than two of the four independent proteomic experiments, including 10 (19%) molecular chaperones. Seven chaperones were confirmed to be differentially expressed in five sublines, Hsp90α, Hsp90β, Hsp90-B1/Gp96, Hsp70-A5/Grp78, and HYOU1, that upregulate, whereas Hsp60 and glucosidase-α neutral AB (GANAB) downregulate. Four molecules were further investigated. In all cell lines, knockdown of Hsp60 or GANAB and silencing of Gp96 or Grp78 considerably enhanced or reduced cell migration and invasion, respectively. Clinical association studies consistently revealed that low levels of Hsp60 or GANAB and high levels of Gp96 or Grp78 are significantly associated with advanced cancer (P < 0.001 to P = 0.047, respectively, for the four molecules) and poor survival (P < 0.001 to P = 0.025, respectively, for the four molecules).Our study defined molecular chaperones as a common set of proteins that regulate the invasion phenotype of HNC. Loss of the tumor suppression function of Hsp60 or GANAB and acquisition of the oncogenic function of Gp96 or Grp78 contribute to aggressive cancers. These molecules may serve as prognostic markers and targets for cancer drug development. 
 Listeria monocytogenes triggers the cell surface expression of Gp96 protein and interacts with its N terminus to support cellular infection. Listeria monocytogenes is an intracellular food-borne pathogen causing listeriosis in humans. This bacterium deploys an arsenal of virulence factors that act in concert to promote cellular infection. Bacterial surface proteins are of primary importance in the process of host   cell invasion  . They interact with host cellular receptors, inducing/modulating specific cellular responses. We previously identified Vip, a Listeria surface protein covalently attached to the bacterial cell wall acting as a key virulence factor. We have shown that Vip interacts with Gp96 localized at the surface of host cells during invasion and that this interaction is critical for a successful infection in vivo. To better understand the importance of Vip-Gp96 interaction during infection, we aimed to characterize this interaction at the molecular level. Here we demonstrate that, during infection, L. monocytogenes triggers the cellular redistribution of Gp96, inducing its exposure at the cell surface. Upon infection, Gp96 N-terminal domain is exposed to the extracellular milieu in L2071 fibroblasts and interacts with Vip expressed by Listeria. We identified Gp96 (Asp(1)-Leu(170)) as sufficient to interact with Vip; however, we also showed that the region Tyr(179)-Leu(390) of Gp96 is important for the interaction. Our findings unravel the Listeria-induced surface expression of Gp96 and the topology of its insertion on the plasma membrane and improve our knowledge on the Vip-Gp96 interaction during Listeria infection. 
 Gp96 is a receptor for a novel Listeria monocytogenes virulence factor, Vip, a surface protein. By comparative genomics, we have identified a gene of the intracellular pathogen Listeria monocytogenes that encodes an LPXTG surface protein absent from nonpathogenic Listeria species. This gene, vip, is positively regulated by PrfA, the transcriptional activator of the major Listeria virulence factors. Vip is anchored to the Listeria cell wall by sortase A and is required for entry into some mammalian cells. Using a ligand overlay approach, we identified a cellular receptor for Vip, the endoplasmic reticulum (ER) resident chaperone Gp96 recently shown to interact with TLRs. The Vip-Gp96 interaction is critical for bacterial entry into some cells. Comparative infection studies using oral and intravenous inoculation of nontransgenic and transgenic mice expressing human E-cadherin demonstrated a role for Vip in Listeria virulence, not only at the intestine level but also in late stages of the infectious process. Vip thus appears as a new virulence factor exploiting Gp96 as a receptor for   cell invasion   and/or signalling events that may interfere with the host immune response in the course of the infection. 
  GeneRIFs  
 Gp96 is exploited by Listeria monocytogenes as a receptor for   cell invasion   or signalling events that may interfere with the host immune response in the course of Listeria infection. 
   
  
 GMPPB 
 
  NCBI Gene  
  GeneRIF  
  Pubmed  
 
 
  Search Term  Rank  Fields  
  [Ee]xtracellular [Mm]atrix  8  PubMed(1)  
 
 
 hide/show details for GMPPB 
  [Ee]xtracellular [Mm]atrix  PubMed  
 Congenital muscular dystrophy and generalized epilepsy caused by GMPPB mutations. The alpha-dystroglycanopathies are genetically heterogeneous muscular dystrophies that result from hypoglycosylation of alpha-dystroglycan (α-DG). Alpha-dystroglycan is an essential link between the   extracellular matri  x and the muscle fiber sarcolemma, and proper glycosylation is critical for its ability to bind to ligands in the   extracellular matri  x. We sought to identify the genetic basis of alpha-dystroglycanopathy in a family wherein the affected individuals presented with congenital muscular dystrophy, brain abnormalities and generalized epilepsy. We performed whole exome sequencing and identified compound heterozygous GMPPB mutations in the affected children. GMPPB is an enzyme in the glycosylation pathway, and GMPPB mutations were recently linked to eight cases of alpha-dystroglycanopathy with a range of symptoms. We identified a novel mutation in GMPPB (p.I219T) as well as a previously published mutation (p.R287Q). Thus, our work further confirms a role for GMPPB defects in alpha-dystroglycanopathy, and suggests that glycosylation may play a role in the neuronal membrane channels or networks involved in the physiology of generalized epilepsy syndromes. This article is part of a Special Issue entitled RNA Metabolism 2013. 
   
  
 AGBL4 
 
  NCBI Gene  
  GeneRIF  
  Pubmed  
 
 
  Search Term  Rank  Fields  
  [Mm]etastasis  12  PubMed(1)  
 
 
 hide/show details for AGBL4 
  [Mm]etastasis  PubMed  
 Clinical Relevance of Plasma DNA Methylation in Colorectal Cancer Patients Identified by Using a Genome-Wide High-Resolution Array. DNA methylation is a potential tumor marker for several cancers, including colorectal cancer (CRC), because of its heritable and stable characteristics.Using a high-resolution, genome-wide approach, we epigenotyped >450,000 CpG sites in tumor and adjacent non-tumor tissues from 23 microsatellite instability (MSI)/microsatellite stability (MSS) CRC cases. Using matrix-assisted laser desorption ionization-time of flight mass spectrometry, the methylation status of five frequently hypermethylated genes were confirmed in 75 independent CRC series and 353 CRC patients with available plasma.Compared with non-tumor tissues, 13 MSI tumors had 34,836 (7 %) aberrant methylation sites, 87 % of which were hypermethylated. In contrast, only 9,806 (2 %) differentially methylated sites were identified in ten MSS cases (62 % hypermethylated). In both MSI and MSS, 228 promoter-associated CpG islands were hypermethylated, with AGBL4, ZNF625, MDFI, TWIST1, and FLI1 being most frequently hypermethylated. In an independent set of 35 MSI and 40 MSS cases, the methylation status of these five genes significantly differed between tumor and adjacent non-tumor tissues. Of 353 CRC patients, 230 (65.2 %), 232 (65.7 %), and 247 (70.0 %) had AGBL4, FLI1, and TWIST1 promoter hypermethylation in circulating cell-free DNA, respectively. In patients without   metastasis  , the sensitivity of any two or three hypermethylation markers was 52.8-57.8 and 27.9-38.9 %, respectively. The sensitivity of any two or three markers was significantly high in patients with stage IV disease (73.0 and 55.6 %, respectively). The prognostic value of these epimarkers was inconclusive.DNA methylation patterns differed in CRC subtypes. The identified hypermethylation markers in CRC patients may have good sensitivity in different CRC stages. 
   
  
 ROCK2 
 
  NCBI Gene  
  GeneRIF  
  Pubmed  
 
 
  Search Term  Rank  Fields  
  [Ff]ilopodia  1  PubMed(1)  
  [Ii]nvadopodia  3  PubMed(1); GeneRIFs(1)  
  [Mm]igration  6  Process(1); PubMed(29); GeneRIFs(6)  
  ECM  7  PubMed(3)  
  [Ee]xtracellular [Mm]atrix  8  PubMed(3); GeneRIFs(2)  
  [Ii]nvasive  10  PubMed(11)  
  [Mm]etastasis  12  PubMed(19); GeneRIFs(4)  
  [Cc]ell [Ii]nvasion  13  PubMed(6); GeneRIFs(1)  
 
 
 hide/show details for ROCK2 
  [Ff]ilopodia  PubMed  
 Rho-kinase 2 is frequently overexpressed in hepatocellular carcinoma and involved in tumor invasion. Deregulation of Rho family small guanosine triphosphatases has been implicated in human carcinogenesis. Rho-kinases are downstream effectors of Rho guanosine triphosphatases in the regulation of cytoskeletal reorganization and cell motility. However, their functions in human cancers remain elusive. In this study, we aimed to investigate the role of Rho-kinases in hepatocellular carcinoma (HCC) tumor progression and invasion. We first examined the expression of the two Rho-kinases (ROCK1 and ROCK2) in human HCC, and found that ROCK2 was frequently overexpressed in primary HCCs (22/41 [53.66%]). Clinico-pathological analysis revealed that overexpression of ROCK2 was significantly associated with the presence of tumor microsatellite formation (P = 0.005), suggesting that deregulation of ROCK2 may contribute to the intrahepatic metastasis of HCC. Consistently, we demonstrated that stable overexpression of ROCK2 significantly enhanced cell motility and invasiveness in HCC cells. Conversely, stable knockdown of ROCK2 by short hairpin RNA approach remarkably reduced HCC cell migration and invasion. Moreover, orthotopic liver xenograft models provided further support that stable knockdown of ROCK2 suppressed HCC invasion in vivo. Stable knockdown of ROCK2 in HCC cells significantly inhibited Golgi reorientation, myosin phosphatase phosphorylation, and formations of stress fibers,   filopodia  , and lamellipodia; these molecular and cellular events are crucial for cell motility and cancer invasion.Our results indicate that ROCK2 was overexpressed in human HCCs, and this overexpression was associated with a more aggressive biological behavior. Our findings also demonstrate that ROCK2 played a significant role in regulating cytoskeletal events and contributed to the invasion of HCC. 
  [Ii]nvadopodia  PubMed  
 Matrix rigidity differentially regulates   invadopodia   activity through ROCK1 and ROCK2. ROCK activity increases due to ECM rigidity in the tumor microenvironment and promotes a malignant phenotype via actomyosin contractility. Invasive migration is facilitated by actin-rich adhesive protrusions known as   invadopodia   that degrade the ECM.   Invadopodia   activity is dependent on matrix rigidity and contractile forces suggesting that mechanical factors may regulate these subcellular structures through ROCK-dependent actomyosin contractility. However, emerging evidence indicates that the ROCK1 and ROCK2 isoforms perform different functions in cells suggesting that alternative mechanisms may potentially regulate rigidity-dependent   invadopodia   activity. In this study, we found that matrix rigidity drives ROCK signaling in cancer cells but that ROCK1 and ROCK2 differentially regulate   invadopodia   activity through separate signaling pathways via contractile (NM II) and non-contractile (LIMK) mechanisms. These data suggest that the mechanical rigidity of the tumor microenvironment may drive ROCK signaling through distinct pathways to enhance the invasive migration required for cancer progression and metastasis. 
  GeneRIFs  
 ROCK1 and ROCK2 differentially regulate   invadopodia   activity through separate signaling pathways. 
  [Mm]igration  Process  
 positive regulation of endothelial cell   migration   
  PubMed  
 Interplay between β1-integrin and Rho signaling regulates differential scattering and motility of pancreatic cancer cells by snail and Slug proteins. The Snail family of transcription factors has been implicated in pancreatic cancer progression. We recently showed that Snail (Snai1) promotes membrane-type 1 matrix metalloproteinase (MT1-MMP)- and ERK1/2-dependent scattering of pancreatic cancer cells in three-dimensional type I collagen. In this study, we examine the role of Slug (Snai2) in regulating pancreatic cancer cell scattering in three-dimensional type I collagen. Although Slug increased MT1-MMP expression and ERK1/2 activity, Slug-expressing cells failed to scatter in three-dimensional collagen. Moreover, in contrast to Snail-expressing cells, Slug-expressing cells did not demonstrate increased collagen I binding, collagen I-driven motility, or α2β1-integrin expression. Significantly, inhibiting β1-integrin function decrea  sed migra  tion and scattering of Snail-expressing cells in three-dimensional collagen. As Rho GTPases have been implicated in invasion   and migra  tion, we also analyzed the contribution of Rac1 and Rho signaling to the different  ial migra  tion and scattering of pancreatic cancer cells. Snail-indu  ced migra  tion and scattering were attenuated by Rac1 inhibition. In contrast, inhibiting Rho-associated kinase ROCK1/2 increa  sed migra  tion and scattering of Slug-expressing cells in three-dimensional collagen and thus phenocopied the effects of Snail in pancreatic cancer cells. Additionally, the increa  sed migra  tion and scattering in three-dimensional collagen of Slug-expressing cells following ROCK1/2 inhibition was dependent on β1-integrin function. Overall, these results demonstrate differential effects of Snail and Slug in pancreatic cancer and identify the interplay between Rho signaling and β1-integrin that functions to regulate the differential scatterin  g and mig  ration of Snail- and Slug-expressing pancreatic cancer cells. 
 Interplay between β1-integrin and Rho signaling regulates differential scattering and motility of pancreatic cancer cells by snail and Slug proteins. The Snail family of transcription factors has been implicated in pancreatic cancer progression. We recently showed that Snail (Snai1) promotes membrane-type 1 matrix metalloproteinase (MT1-MMP)- and ERK1/2-dependent scattering of pancreatic cancer cells in three-dimensional type I collagen. In this study, we examine the role of Slug (Snai2) in regulating pancreatic cancer cell scattering in three-dimensional type I collagen. Although Slug increased MT1-MMP expression and ERK1/2 activity, Slug-expressing cells failed to scatter in three-dimensional collagen. Moreover, in contrast to Snail-expressing cells, Slug-expressing cells did not demonstrate increased collagen I binding, collagen I-driven motility, or α2β1-integrin expression. Significantly, inhibiting β1-integrin function decrea  sed migra  tion and scattering of Snail-expressing cells in three-dimensional collagen. As Rho GTPases have been implicated in invasion   and migra  tion, we also analyzed the contribution of Rac1 and Rho signaling to the different  ial migra  tion and scattering of pancreatic cancer cells. Snail-indu  ced migra  tion and scattering were attenuated by Rac1 inhibition. In contrast, inhibiting Rho-associated kinase ROCK1/2 increa  sed migra  tion and scattering of Slug-expressing cells in three-dimensional collagen and thus phenocopied the effects of Snail in pancreatic cancer cells. Additionally, the increa  sed migra  tion and scattering in three-dimensional collagen of Slug-expressing cells following ROCK1/2 inhibition was dependent on β1-integrin function. Overall, these results demonstrate differential effects of Snail and Slug in pancreatic cancer and identify the interplay between Rho signaling and β1-integrin that functions to regulate the differential scatterin  g and mig  ration of Snail- and Slug-expressing pancreatic cancer cells. 
 Rho-kinase 2 is frequently overexpressed in hepatocellular carcinoma and involved in tumor invasion. Deregulation of Rho family small guanosine triphosphatases has been implicated in human carcinogenesis. Rho-kinases are downstream effectors of Rho guanosine triphosphatases in the regulation of cytoskeletal reorganization and cell motility. However, their functions in human cancers remain elusive. In this study, we aimed to investigate the role of Rho-kinases in hepatocellular carcinoma (HCC) tumor progression and invasion. We first examined the expression of the two Rho-kinases (ROCK1 and ROCK2) in human HCC, and found that ROCK2 was frequently overexpressed in primary HCCs (22/41 [53.66%]). Clinico-pathological analysis revealed that overexpression of ROCK2 was significantly associated with the presence of tumor microsatellite formation (P = 0.005), suggesting that deregulation of ROCK2 may contribute to the intrahepatic metastasis of HCC. Consistently, we demonstrated that stable overexpression of ROCK2 significantly enhanced cell motility and invasiveness in HCC cells. Conversely, stable knockdown of ROCK2 by short hairpin RNA approach remarkably reduced HCC cell   migration   and invasion. Moreover, orthotopic liver xenograft models provided further support that stable knockdown of ROCK2 suppressed HCC invasion in vivo. Stable knockdown of ROCK2 in HCC cells significantly inhibited Golgi reorientation, myosin phosphatase phosphorylation, and formations of stress fibers, filopodia, and lamellipodia; these molecular and cellular events are crucial for cell motility and cancer invasion.Our results indicate that ROCK2 was overexpressed in human HCCs, and this overexpression was associated with a more aggressive biological behavior. Our findings also demonstrate that ROCK2 played a significant role in regulating cytoskeletal events and contributed to the invasion of HCC. 
 MiR-200b/200c/429 subfamily negatively regulates Rho/ROCK signaling pathway to suppress hepatocellular carcinoma metastasis. MiR-200 family is an important regulator of epithelial-mesenchymal transition and has been implicated in human carcinogenesis. However, their expression and functions in human cancers remain controversial. In the work presented here, we showed that miR-200 family members were frequently down-regulated in hepatocellular carcinoma (HCC). Although all five members of miR-200 family inhibited ZEB1/2 expression in HCC cell lines, we showed that overexpression only of the miR-200b/200c/429 subfamily, but not the miR-200a/141 subfamily, resulted in impeded HCC cell   migration  . Further investigations led to the identification of RhoA and ROCK2 as specific down-stream targets of the miR-200b/200c/429 subfamily. We demonstrated that the miR-200b/200c/429 subfamily inhibited HCC cell   migration   through modulating Rho/ROCK mediated cell cytoskeletal reorganization and cell-substratum adhesion. Re-expression of miR-200b significantly suppressed lung metastasis of HCC cells in an orthotopic liver implantation model in vivo. In conclusion, our findings identified the miR-200b/200c/429 subfamily as metastasis suppressor microRNAs in human HCC and highlighted the functional discrepancy among miR-200 family members. 
 Opposing signaling of ROCK1 and ROCK2 determines the switching of substrate specificity and the mode of   migration   of glioblastoma cells. Despite current advances in therapy, the prognosis of patients with glioblastoma has not improved sufficiently in recent decades. This is due mainly to the highly invasive capacity of glioma cells. Little is known about the mechanisms underlying this particular characteristic. While the Rho-kinase (ROCK)-dependent signaling pathways involved in glioma   migration   have yet to be determined, they show promise as one of the candidates in targeted glioblastoma therapy. There are two ROCK isoforms: ROCK1, which is upregulated in glioblastoma tissue compared to normal brain tissue, and ROCK2, which is also expressed in normal brain tissue. Blockage of both of these ROCK isoforms with pharmacologic inhibitors regulates the   migration   process. We examined the activities of ROCK1 and ROCK2 using knockdown cell lines and the newly developed stripe assay. Selective knockdown of either ROCK1 or ROCK2 exerted antidromic effects on glioma   migration  : while ROCK1 deletion altered the substrate-dependent   migration  , deletion of ROCK2 did not. Furthermore, ROCK1 knockdown reduced cell proliferation, whereas ROCK2 knockdown enhanced it. Along the signaling pathways, key regulators of the ROCK pathway are differentially affected by ROCK1 and ROCK2. These data suggest that the balanced activation of ROCKs is responsible for the substrate-specific   migration   and the proliferation of glioblastoma cells. 
 Rock2 promotes the invasion and metastasis of hepatocellular carcinoma by modifying MMP2 ubiquitination and degradation. Rho-associated coiled-coil-containing protein kinase 2 (Rock2) is a downstream effector of Rho that plays an important role in the tumorigenesis and progression of hepatocellular carcinoma (HCC). Matrix metalloproteinase 2 (MMP2) is a master regulator of tumor metastasis. In this study, we investigated the collections of Rock2 and MMP2 in HCCs and determined the potential role and molecular mechanism of Rock2 in MMP2-mediated invasiveness and metastasis. We found that Rock2 and MMP2 were markedly overexpressed in HCCs compared with the corresponding adjacent tissues, where a positive correlation in their expression was found. The knockdown of Rock2 significantly decreased MMP2 expression and inhibited the invasion and metastasis of HCC in vitro and in vivo. Additionally, the upregulation of MMP2 rescued the decreased   migration   and invasion induced by the knockdown of Rock2, whereas the knockdown of MMP2 decreased Rock2-enhanced HCC   migration   and invasion. Mechanistically, Rock2 stabilized MMP2 by preventing its ubiquitination and degradation. Together, our results link two drivers of invasion and metastasis in HCC and identify a novel pathway for MMP2 control. 
 CD99 suppresses osteosarcoma cell   migration   through inhibition of ROCK2 activity. CD99, a transmembrane protein encoded by MIC2 gene is involved in multiple cellular events including cell adhesion and   migration  , apoptosis, cell differentiation and regulation of protein trafficking either in physiological or pathological conditions. In osteosarcoma, CD99 is expressed at low levels and functions as a tumour suppressor. The full-length protein (CD99wt) and the short-form harbouring a deletion in the intracytoplasmic domain (CD99sh) have been associated with distinct functional outcomes with respect to tumour malignancy. In this study, we especially evaluated modulation of cell-cell contacts, reorganisation of the actin cytoskeleton and modulation of signalling pathways by comparing osteosarcoma cells characterised by different metastasis capabilities and CD99 expression, to identify molecular mechanisms responsible for metastasis. Our data indicate that forced expression of CD99wt induces recruitment of N-cadherin and β-catenin to adherens junctions. In addition, transfection of CD99wt inhibits the expression of several molecules crucial to the remodelling of the actin cytoskeleton, such as ACTR2, ARPC1A, Rho-associated, coiled-coil containing protein kinase 2 (ROCK2) as well as ezrin, an ezrin/radixin/moesin family member that has been clearly associated with tumour progression and metastatic spread in osteosarcoma. Functional studies point to ROCK2 as a crucial intracellular mediator regulating osteosarcoma   migratio  n. By maintaining c-Src in an inactive conformation, CD99wt inhibits ROCK2 signalling and this leads to ezrin decrease at cell membrane while N-cadherin and β-catenin translocate to the plasma membrane and function as main molecular bridges for actin cytoskeleton. Taken together, we propose that the re-expression of CD99wt, which is generally present in osteoblasts but lost in osteosarcoma, through inhibition of c-Src and ROCK2 activity, manages to increase contact strength and reactivate sto  p-migrati  on signals that counteract the otherwise dominant promigratory action of ezrin in osteosarcoma cells. 
 Matrix rigidity differentially regulates invadopodia activity through ROCK1 and ROCK2. ROCK activity increases due to ECM rigidity in the tumor microenvironment and promotes a malignant phenotype via actomyosin contractility. Invasive   migration   is facilitated by actin-rich adhesive protrusions known as invadopodia that degrade the ECM. Invadopodia activity is dependent on matrix rigidity and contractile forces suggesting that mechanical factors may regulate these subcellular structures through ROCK-dependent actomyosin contractility. However, emerging evidence indicates that the ROCK1 and ROCK2 isoforms perform different functions in cells suggesting that alternative mechanisms may potentially regulate rigidity-dependent invadopodia activity. In this study, we found that matrix rigidity drives ROCK signaling in cancer cells but that ROCK1 and ROCK2 differentially regulate invadopodia activity through separate signaling pathways via contractile (NM II) and non-contractile (LIMK) mechanisms. These data suggest that the mechanical rigidity of the tumor microenvironment may drive ROCK signaling through distinct pathways to enhance the invasive   migration   required for cancer progression and metastasis. 
 Rho kinase inhibitors block melanoma cell   migration   and inhibit metastasis. There is an urgent need to identify new therapeutic opportunities for metastatic melanoma. Fragment-based screening has led to the discovery of orally available, ATP-competitive AKT kinase inhibitors, AT13148 and CCT129254. These compounds also inhibit the Rho-kinases ROCK 1 and ROCK 2 and we show they potently inhibit ROCK activity in melanoma cells in culture and in vivo. Treatment of melanoma cells with CCT129254 or AT13148 dramatically reduces cell invasion, impairing both "amoeboid-like" and mesenchymal-like modes of invasion in culture. Intravital imaging shows that CCT129254 or AT13148 treatment reduces the motility of melanoma cells in vivo. CCT129254 inhibits melanoma metastasis when administered 2 days after orthotopic intradermal injection of the cells, or when treatment starts after metastases have arisen. Mechanistically, our data suggest that inhibition of ROCK reduces the ability of melanoma cells to efficiently colonize the lungs. These results suggest that these novel inhibitors of ROCK may be beneficial in the treatment of metastasis. 
 Phosphorylation and mRNA splicing of collapsin response mediator protein-2 determine inhibition of rho-associated protein kinase (ROCK) II function in carcinoma cell   migration   and invasion. The Rho-associated protein kinases (ROCK I and II) are central regulators of important cellular processes such as   migration   and invasion downstream of the GTP-Rho. Recently, we reported collapsin response mediator protein (CRMP)-2 as an endogenous ROCK II inhibitor. To reveal how the CRMP-2-ROCK II interaction is controlled, we further mapped the ROCK II interaction site of CRMP-2 and examined whether phosphorylation states of CRMP-2 affected the interaction. Here, we show that an N-terminal fragment of the long CRMP-2 splice variant (CRMP-2L) alone binds ROCK II and inhibits colon carcinoma cell   migration   and invasion. Furthermore, the interaction of CRMP-2 and ROCK II is partially regulated by glycogen synthase kinase (GSK)-3 phosphorylation of CRMP-2, downstream of PI3K. Inhibition of PI3K reduced interaction of CRMP-2 with ROCK II, an effect rescued by simultaneous inhibition of GSK3. Inhibition of PI3K also reduced colocalization of ROCK II and CRMP-2 at the cell periphery in human breast carcinoma cells. Mimicking GSK3 phosphorylation of CRMP-2 significantly reduced CRMP-2 binding of recombinant full-length and catalytic domain of ROCK II. These data implicate GSK3 in the regulation of ROCK II-CRMP-2 interactions. Using phosphorylation-mimetic and -resistant CRMP-2L constructs, it was revealed that phosphorylation of CRMP-2L negatively regulates its inhibitory function in ROCK-dependent haptotactic cell   migration  , as well as invasion of human colon carcinoma cells. Collectively, the presented data show that CRMP-2-dependent regulation of ROCK II activity is mediated through interaction of the CRMP-2L N terminus with the ROCK II catalytic domain as well as by GSK3-dependent phosphorylation of CRMP-2. 
 Inhibition of Rho-dependent kinases ROCK I/II activates VEGF-driven retinal neovascularization and sprouting angiogenesis. Vascular endothelial growth factor (VEGF) is an endothelial-specific growth factor that activates the small GTPase RhoA. While the role of RhoA for VEGF-driven endothelial   migration   and angiogenesis has been studied in detail, the function of its target proteins, the Rho-dependent kinases ROCK I and II, are controversially discussed. Using the mouse model of oxygen-induced proliferative retinopathy, ROCK I/II inhibition by H-1152 resulted in increased angiogenesis. This enhanced angiogenesis, however, was completely blocked by the VEGF-receptor antagonist PTK787/ZK222584. Loss-of-function experiments in endothelial cells revealed that inhibition of ROCK I/II using the pharmacological inhibitor H-1152 and ROCK I/II-specific small-interfering RNAs resulted in a rise of VEGF-driven sprouting angiogenesis. These functional data were biochemically substantiated by showing an enhanced VEGF-receptor kinase insert domain receptor phosphorylation and extracellular signal-regulated kinase 1/2 activation after inhibition of ROCK I/II. Thus our data identify that the inhibition of Rho-dependent kinases ROCK I/II activates angiogenesis both, in vitro and in vivo. 
 [Ouabain induces Rho-dependent rock activation and membrane blebbing incultured endothelial cells]. Small G protein Rho and its most studied effectors, ROCK I and ROCK II, are involved in several cellular fuctions including smooth muscle and non-muscle cell contractions, cell   migration   and apoptosis. Activation of ROCK I by caspase-3 and activation of ROCK II by granzyme B are essential for membrane blebbing in the execution phase of apoptosis. In contrast, it has been demonstrated that Rho signaling is critical for blebbing developed after serum removal. As it was shown by us previously, ouabain induces membrane blebbing and proteolitic cleavage of ROCK I and ROCK II via caspases in human umbilical endothelial cells. However, caspase inhibitors do not prevent ouabain-induced blebs. Ouabain induces concentration-dependent cell death and membrane blebbing in endothelial cells. The aim of this study was to identify the possible role of Rho in ouabain-induced membrane blebbing. Pretreatment of endothelial cells with a Rho inhibitor CT04 did not inhibit the ouabain-induced cell death but prevented the development ofbleb formation. These results indicate that bleb formation is dependent on Rho activity in ouabain-induced cell death in HUVECs. Taken together, these results suggest that the mechanism of membrane bleb formation might be different depending on cell type and cell death-stimuli. 
 ROCK inhibitor Y-27632 inhibits the growth,   migration  , and invasion of Tca8113 and CAL-27 cells in tongue squamous cell carcinoma. The objective of this study is to determine the effects of Rho-associated coiled-coil containing protein kinase (ROCK) inhibitor Y-27632 on the growth, invasion, and   migration   of Tca8113 and CAL-27 cells in tongue squamous cell carcinoma (TSCC). The methods of the study are as follows: After being routinely cultured for 24 h, Tca8113 and CAL-27 cells were treated with Y-27632 solution. The morphological change of Y-27632-treated cells was observed under an optical microscope and an inverted microscope; MTT assay was performed to measure the optical density (OD) of cells and calculate cell growth inhibition rate; the change of apoptosis was detected by AnnexinV-FITC/PI assay; cell invasion and   migratio  n were measured by Transwell assay. The results were as follows: (1) With increasing concentration of Y-27632, cell morphology changed and cell apoptosis appeared; (2) MTT assay showed that inhibition effect of Y-27632 on Tca8113 and CAL-27 cells was enhanced with increasing concentrations and time (all P < 0.01); (3) Apoptosis showed that, compared with controls, the number of apoptosis cells in experimental groups was significantly increased (all P < 0.01). Apoptosis rate was elevated with increasing concentrations of Y-27632; (4) Transwell assay showed, after a treatment with Y-27632, the number of migrated and invaded Tca8113 and CAL-27 cells in each group was statistically different (all P < 0.01); compared with controls, the number of migrated cell in groups treated with Y-27632 was decreased and less Tca8113 and CAL-27 cells in experimental groups passed through polycarbonate membrane (all P < 0.05). The study concludes that Y-27632 can inhibit the growt  h, invasi  on, and migration of Tca8113 and CAL-27 cells, suggesting that Y-27632 may be therapeutically useful in TSCC. 
 Rocks: multifunctional kinases in cell behaviour. ROCKs, or Rho kinases, are serine/threonine kinases that are involved in many aspects of cell motility, from smooth-muscle contraction to cell   migration   and neurite outgrowth. Recent experiments have defined new functions of ROCKs in cells, including centrosome positioning and cell-size regulation, which might contribute to various physiological and pathological states. 
 Caffeine inhibits   migration   in glioma cells through the ROCK-FAK pathway. Glioma is the most malignant brain tumor that has the ability to migrate and invade the CNS. In this study, we investigated the signaling mechanism of caffeine on the   migration   of glioma cells.The effect of caffeine on cell   migration   was evaluated using Transwell and wound healing assays. The expression of the focal adhesion complex as it related to cell   migration   was assayed using Western blotting and immunostaining.Caffeine decreased the   migration   of rat C6 and human U87MG glioma cells and down-regulated the expression of phosphorylated focal adhesion kinase (p-FAK) and p-paxillin. Caffeine also decreased p-FAK staining at the edge of glioma cells and disassembled actin stress fibers. Additionally, caffeine elevated expression of phosphorylated myosin light chain (p-MLC), an effect that could be blocked by Y27632, a rho-associated protein kinase (ROCK) inhibitor, but not myosin light chain kinase inhibitor, ML-7. Y27632 also inhibited the caffeine-reduced expression of p-FAK and p-paxillin as well as cell   migration  .Caffeine decreased the   migration   of glioma cell through the ROCK-focal adhesion complex pathway; this mechanism may be useful as part of clinical therapy in the future. 
 Abnormal activation of Ras/Raf/MAPK and RhoA/ROCKII signalling pathways in eutopic endometrial stromal cells of patients with endometriosis. BACKGROUND Enhanced proliferation and survival of eutopic endometrial cells from patients with endometriosis compared with healthy women is associated with abnormal activation of extra-cellular signal-regulated kinases 1 and 2 (ERK1/2). Given the role of Ras/Raf/mitogen-activated protein kinase (MAPK) and RhoA/ROCKII signalling pathways in the regulation of cell proliferation and   migration  , we analysed their possible roles in endometriosis. METHODS Primary eutopic endometrial stromal cells of patients with endometriosis (Eu-hESC, n= 16) and endometriosis-free controls (Co-hESC, n= 14) were harvested and subjected to proliferation and   migration   assays as well as kinase activity assays and immunoblot analysis of proteins from the Ras/Raf/MAPK and RhoA/ROCKII signalling pathways. Effects of ROCKII (Y-27632) and MAPK (U0126) inhibitors or siRNA knockdown of ROCKII, Raf-1 and B-Raf were analysed. RESULTS The proliferation rate of Eu-hESC was 54% higher than Co-hESC. Eu-hESC also displayed a 75% higher   migration   rate than Co-hESC. Eu-hESC displayed higher levels of ERK phosphorylation (83%) and p27 expression (61%) and lower levels of Raf-1 protein (47%) compared with controls. In addition to an inhibitory effect on cell proliferation, ROCKII knockdown led to significant down-regulation of cyclinD1 and p27 but did not affect ERK phosphorylation. Down-regulation of Raf-1 by siRNA was dispensable for cell proliferation control but led to an increase in ROCKII activity and a decrease in cell   migration  . B-Raf was shown to act as a regulator of hESC proliferation by modulating cellular ERK1/2 activity and cyclinD1 levels. Eu-hESC displayed 2.4-fold higher B-Raf activity compared with Co-hESC and therefore exhibit abnormally activated Ras/Raf/MAPK signalling. CONCLUSIONS We show that the same molecular mechanisms operate in Co- and Eu-hESC. The differences in cell proliferation and   migration   between both cell types are likely due to increased activation of Ras/Raf/MAPK and RhoA/ROCKII signalling pathways in cells from endometriosis patients. 
 Differential regulation of adhesion complex turnover by ROCK1 and ROCK2. ROCK1 and ROCK2 are serine/threonine kinases that function downstream of the small GTP-binding protein RhoA. Rho signalling via ROCK regulates a number of cellular functions including organisation of the actin cytoskeleton, cell adhesion and cell   migration  .In this study we use RNAi to specifically knockdown ROCK1 and ROCK2 and analyse their role in assembly of adhesion complexes in human epidermal keratinocytes. We observe that loss of ROCK1 inhibits signalling via focal adhesion kinase resulting in a failure of immature adhesion complexes to form mature stable focal adhesions. In contrast, loss of ROCK2 expression results in a significant reduction in adhesion complex turnover leading to formation of large, stable focal adhesions. Interestingly, loss of either ROCK1 or ROCK2 expression significantly impairs cell   migration   indicating both ROCK isoforms are required for normal keratinocyte   migration  .ROCK1 and ROCK2 have distinct and separate roles in adhesion complex assembly and turnover in human epidermal keratinocytes. 
 Vascular endothelial growth factor C promotes cervical cancer metastasis via up-regulation and activation of RhoA/ROCK-2/moesin cascade. The elevated expression of vascular endothelial growth factor C (VEGF-C) is correlated with clinical cervical cancer metastasis and patient survival, which is interpreted by VEGF-C functions to stimulate angiogenesis and lymphatic genesis. However, the direct impact of VEGF-C on cervical cancer cell motility remains largely unknown.In this study, we investigated the effects of VEGF-C on actin cytoskeleton remodeling and on cervical cancer cell   migration   and invasion and how the actin-regulatory protein, moesin regulated these effects through RhoA/ROCK-2 signaling pathway.On cervical carcinoma cell line SiHa cells, exposure of VEGF-C triggered remodeling of the actin cytoskeleton and the formation of membrane ruffles, which was required for cell movement. VEGF-C significantly enhanced SiHa cells horizontal   migration   and three-dimensional invasion into matrices. These actions were dependent on increased expression and phosphorylation of the actin-regulatory protein moesin and specific moesin siRNA severely impaired VEGF-C stimulated-cell   migration  . The extracellular small GTPase RhoA/ROCK-2 cascade mediated the increased moesin expression and phosphorylation, which was discovered by the use of Y-27632, a specific inhibitor of Rho kinase and by transfected constitutively active, dominant-negative RhoA as well as ROCK-2 SiRNA. Furthermore, in the surgical cervical specimen from the patients with FIGO stage at cervical intra-epithelial neoplasia and I-II cervical squamous cell carcinoma, the expression levels of moesin were found to be significantly correlated with tumor malignancy and metastasis.These results implied that VEGF-C promoted cervical cancer metastasis by upregulation and activation of moesin protein through RhoA/ROCK-2 pathway. Our findings offer new insight into the role of VEGF-C on cervical cancer progression and may provide potential targets for cervical cancer therapy. 
 Rho kinases regulate corneal epithelial wound healing. We have previously shown that Rho small GTPase is required for modulating both cell   migration   and proliferation through cytoskeleton reorganization and focal adhesion formation in response to wounding. In the present study, we investigated the role of Rho kinases (ROCKs), major effectors of Rho GTPase, in mediating corneal epithelial wound healing. Both ROCK 1 and 2 were expressed and activated in THCE cells, an SV40-immortalized human corneal epithelial cell (HCEC) line, in response to wounding, lysophosphatidic acid, and heparin-binding EGF-like growth factor (HB-EGF) stimulations. The ROCK inhibitor Y-27632 efficiently antagonized ROCK activities without affecting Rho activation in wounded HCECs. Y-27632 promoted basal and HB-EGF-enhanced scratch wound healing and enhanced cell   migration   and adhesion to matrices, while retarded HB-EGF induced cell proliferation. E-cadherin- and beta-catenin-mediated cell-cell junction and actin cytoskeleton organization were disrupted by Y-27632. Y-27632 impaired the formation and maintenance of tight junction barriers indicated by decreased trans-epithelial resistance and disrupted occludin staining. We conclude that ROCK activities enhance cell proliferation, promote epithelial differentiation, but negatively modulate cell   migration   and cell adhesion and therefore play a role in regulating corneal epithelial wound healing. 
 Is there any association between childhood cardiac septal defects and ROCK2 gene polymorphism? Rho/Rho-kinase pathway plays a critical role in the regulation of cellular functions such as proliferation and   migration  . One of the possible theories of the development of ventricular septal defects is cell   migration   disorder. The aim of this study was to analyze the genotype distributions and allele frequencies for the ROCK2 gene Thr431Asn polymorphisms in the development of cardiac septal defects in a Turkish population. In this case-control study, 300 patients with cardiac defects (150 patients with ventricular and 150 patients with atrial septal defects) and control group (150 healthy control subjects) were investigated. A single-nucleotide polymorphism in ROCK2 gene Thr431Asn was analyzed by real-time PCR using a Light-Cycler. Neither genotype distributions nor the allele frequencies for the Thr431Asn polymorphism showed a significant difference between the groups. These results suggest that there is no association of the ROCK2 gene Thr431Asn polymorphism with the development of cardiac septal defects in pediatric patients. 
 Tubers from patients with tuberous sclerosis complex are characterized by changes in microtubule biology through ROCK2 signalling. Most patients with tuberous sclerosis complex (TSC) develop cortical tubers that cause severe neurological disabilities. It has been suggested that defects in neuronal differentiation and/or   migration   underlie the appearance of tubers. However, the precise molecular alterations remain largely unknown. Here, by combining cytological and immunohistochemical analyses of tubers from nine TSC patients (four of them diagnosed with TSC2 germline mutations), we show that alteration of microtubule biology through ROCK2 signalling contributes to TSC neuropathology. All tubers showed a larger number of binucleated neurons than expected relative to control cortex. An excess of normal and altered cytokinetic figures was also commonly observed. Analysis of centrosomal markers suggested increased microtubule nucleation capacity, which was supported by the analysis of an expression dataset from cortical tubers and control cortex, and subsequently linked to under-expression of Rho-associated coiled-coil containing kinase 2 (ROCK2). Thus, augmented microtubule nucleation capacity was observed in mouse embryonic fibroblasts and human fibroblasts deficient in the Tsc2/TSC2 gene product, tuberin. Consistent with ROCK2 under-expression, microtubule acetylation was found to be increased with tuberin deficiency; this alteration was abrogated by rapamycin treatment and mimicked by HDAC6 inhibition. Together, the results of this study support the hypothesis that loss of TSC2 expression can alter microtubule organization and dynamics, which, in turn, deregulate cell division and potentially impair neuronal differentiation. 
 RhoC and ROCKs regulate cancer cell interactions with endothelial cells. RhoC is a member of the Rho GTPase family that is implicated in cancer progression by stimulating cancer cell invasiveness. Here we report that RhoC regulates the interaction of cancer cells with vascular endothelial cells (ECs), a crucial step in the metastatic process. RhoC depletion by RNAi reduces PC3 prostate cancer cell adhesion to ECs, intercalation between ECs as well as transendothelial   migration   in vitro. Depletion of the kinases ROCK1 and ROCK2, two known RhoC downstream effectors, similarly decreases cancer interaction with ECs. RhoC also regulates the extension of protrusions made by cancer cells on vascular ECs in vivo. Transient RhoC depletion is sufficient to reduce both early PC3 cell retention in the lungs and experimental metastasis formation in vivo. Our results indicate RhoC plays a central role in cancer cell interaction with vascular ECs, which is a critical event for cancer progression. 
 Testosterone promotes vascular endothelial cell   migration   via upregulation of ROCK-2/moesin cascade. Cross-sectional studies have demonstrated a reverse relationship between serum level of testosterone (T) and the incidence rate of cardiovascular disease in men, indicating that T exerts beneficial effects in cardiovascular system. However, the endothelial effects of T are poorly understood. Actin remodeling is essential for endothelial cell movement and vascular repair and this process is controlled by the actin-binding protein moesin. In the present study, we studied the effects of T on actin remodeling, moesin expression and phosphorylation, as well as cell   migration   in cultured human umbilical endothelial cells (hUVECs). We found that T provoked the formation of cortical actin complexes and membrane protrusions in endothelial cells. Treatment with T induced dose- and time-dependent increase of moesin expression and phosphorylation, which was inhibited by the addition of androgen receptor antagonist hydroxyflutamide (HF). Moreover, T enhanced ROCK-2 activity. The ROCK-2 inhibitor Y27632 or the transfection of ROCK-2 siRNA largely inhibited T-induced moesin expression and phosphorylation, indicating that ROCK-2 pathway is crucial for these effects. T promoted endothelial cell   migration  , which was inhibited by the addition of HF or Y27632. In conclusion, T induces actin cytoskeleton remodeling by regulating moesin expression and activation, resulting in enhanced endothelial cell   migration  . Our work adds new insights into endothelial mechanisms of T, which is relevant for its vascular actions. 
 Crucial role of ROCK2 in vascular smooth muscle cells for hypoxia-induced pulmonary hypertension in mice. Rho/Rho-kinase (ROCK) pathway in vascular smooth muscle cells (VSMCs) plays an important role in the pathogenesis of cardiovascular diseases, including pulmonary arterial hypertension (PAH). Rho-kinase has 2 isoforms, ROCK1 and ROCK2, with different functions in different cells; ROCK1 for circulating inflammatory cells and ROCK2 for the vasculature. In the present study, we aimed to examine whether ROCK2 in VSMC is involved in the pathogenesis of PAH.In patients with PAH, the expression of ROCK2 was increased in pulmonary arterial media and primary pulmonary arterial smooth muscle cells when compared with controls. To investigate the role of ROCK2 in VSMC, we generated VSMC-specific heterozygous ROCK2-deficient (ROCK2(+/-)) mice and VSMC-specific ROCK2-overexpressing transgenic (ROCK2-Tg) mice. The extent of hypoxia-induced pulmonary hypertension was reduced in ROCK2(+/-) mice and was enhanced in ROCK2-Tg mice compared with respective littermates. The protein expression of ROCK activity and phosphorylated extracellular signal-regulated kinase and the number of Ki67-positive proliferating cells in the lung were reduced in ROCK2(+/-) mice and were increased in ROCK2-Tg mice compared with respective littermates. In cultured mouse aortic VSMC,   migration   and proliferation activities were reduced in ROCK2(+/-) mice, and   migration   activity was increased in ROCK2-Tg mice compared with respective littermates. In addition, in primary pulmonary arterial smooth muscle cells from a patient with PAH, ROCK2 was required for   migration   and proliferation through ROCK and extracellular signal-regulated kinase activation.ROCK2 in VSMC contributes to the pathogenesis of PAH. 
 Phosphorylation of adducin by Rho-kinase plays a crucial role in cell motility. Adducin is a membrane skeletal protein that binds to actin filaments (F-actin) and thereby promotes the association of spectrin with F-actin to form a spectrin-actin meshwork beneath plasma membranes such as ruffling membranes. Rho-associated kinase (Rho- kinase), which is activated by the small guanosine triphosphatase Rho, phosphorylates alpha-adducin and thereby enhances the F-actin-binding activity of alpha-adducin in vitro. Here we identified the sites of phosphorylation of alpha-adducin by Rho-kinase as Thr445 and Thr480. We prepared antibody that specifically recognized alpha-adducin phosphorylated at Thr445, and found by use of this antibody that Rho-kinase phosphorylated alpha-adducin at Thr445 in COS7 cells in a Rho-dependent manner. Phosphorylated alpha-adducin accumulated in the membrane ruffling area of Madin-Darby canine kidney (MDCK) epithelial cells and the leading edge of scattering cells during the action of tetradecanoylphorbol-13-acetate (TPA) or hepatocyte growth factor (HGF). The microinjection of Botulinum C3 ADP-ribosyl-transferase, dominant negative Rho-kinase, or alpha-adducinT445A,T480A (substitution of Thr445 and Thr480 by Ala) inhibited the TPA-induced membrane ruffling in MDCK cells and wound-induced   migration   in NRK49F cells. alpha-AdducinT445D,T480D (substitution of Thr445 and Thr480 by Asp), but not alpha-adducinT445A,T480A, counteracted the inhibitory effect of the dominant negative Rho-kinase on the TPA-induced membrane ruffling in MDCK cells. Taken together, these results indicate that Rho-kinase phosphorylates alpha-adducin downstream of Rho in vivo, and that the phosphorylation of adducin by Rho-kinase plays a crucial role in the regulation of membrane ruffling and cell motility. 
 The microRNA miR-139 suppresses metastasis and progression of hepatocellular carcinoma by down-regulating Rho-kinase 2. We investigated mechanisms of hepatocellular carcinoma (HCC) metastasis and identified an antimetastatic microRNA (miRNA), miR-139, that is down-regulated in human HCC samples.Effects of stable and transient expression of miRNA-139 and its inhibitors were studied in the human HCC cell lines SMMC-7721 and BEL7402; cells were analyzed for   migration   and invasion. Liver samples from patients with metastatic HCC were analyzed for levels of miRNA-139; data were compared with survival data using the Kaplan-Meier method and compared between groups by the log-rank test. Tumor formation and metastasis from human HCC MHCC97L cells that did or did not express miR-139 were analyzed in mice.Down-regulation of miR-139 in HCC was associated significantly with poor prognosis of patients and features of metastatic tumors, including venous invasion, microsatellite formation, absence of tumor encapsulation, and reduced differentiation. miR-139 expression was reduced in metastatic HCC tumors compared with primary tumors. Overexpression of miR-139 in HCC cells significantly reduced cell   migration   and invasion in vitro and the incidence and severity of lung metastasis from orthotopic liver tumors in mice. miR-139 interacted with the 3' untranslated region of Rho-kinase 2 (ROCK2) and reduced its expression in HCC cells. Levels of miR-139 were correlated inversely with ROCK2 protein in human HCC samples. Overexpression of miR-139 did not inhibit HCC cell motility when ROCK2 was knocked down.The microRNA miR-139 interacts with ROCK2 and reduces its expression in HCC cells. Down-regulation of miR-139 increased the invasive abilities of HCC cells in vitro and HCC metastasis in vivo. Expression of miR-139 is reduced in human metastatic HCC samples and correlates with prognosis. 
 Downregulation of the Rho GTPase signaling pathway is involved in the microRNA-138-mediated inhibition of cell   migration   and invasion in tongue squamous cell carcinoma. Tumor metastasis is the dominant cause of death in cancer patients, including patients with oral tongue squamous cell carcinoma (TSCC). Previously, we reported that reduced miR-138 level is correlated with enhanced metastatic potential in TSCC cells. Here, we demonstrate that miR-138 suppresses TSCC cell   migration   and invasion by regulating 2 key genes in the Rho GTPase signaling pathway: RhoC and ROCK2. Direct targeting of miR-138 to specific sequences located in the 3'-untranslated regions of both RhoC and ROCK2 mRNAs was confirmed using luciferase reporter gene assays. Ectopic transfection of miR-138 reduced the expression of both RhoC and ROCK2 in TSCC cells. These reduced expressions, in consequence, led to the reorganization of the stress fibers and the subsequent cell morphology change to a round bleb-like shape as well as the suppression of cell   migration   and invasion. In contrast, knockdown of miR-138 in TSCC cells enhanced the expression of RhoC and ROCK2, which resulted in an altered, elongated cell morphology, enhanced cell stress fiber formation and accelerated cell   migration   and invasion. Taken together, our results suggest that miR-138 plays an important role in TSCC cell   migration   and invasion by concurrently targeting RhoC and ROCK2, and miR-138 may serve as a novel therapeutic target for TSCC patients at risk of metastatic disease. 
 Overexpression of ROCK1 and ROCK2 inhibits human laryngeal squamous cell carcinoma. Rho-associated coiled-coil containing protein kinase (ROCK) over-expression has been implicated in the progression of many tumor types. The aim of this study was to explore the roles of ROCK1 and ROCK2 in human laryngeal squamous cell carcinoma (LSCC). ROCK1 and ROCK2 expression levels were examined in 50 cases of human LSCC samples by immunohistochemistry. Effects of ROCK1 and ROCK2 on LSCC cell proliferation and motility were investigated in the presence of the ROCK inhibitor Y-27632. The results showed that ROCK1 expression was positively correlated with tumor size and lymph node metastasis (P < 0.05); ROCK2 positively correlated with tumor size (P < 0.05). Inhibition of ROCK1 and ROCK2 by Y-27632 significantly inhibits proliferation,   migration  , and invasion of LSCC cells. Our data indicate that expression of ROCK1 and ROCK2 are closely associated with tumor growth and lymph node metastasis of LSCC. Thus, these two ROCK isoforms may be useful as molecular makers for LSCC diagnosis and may be useful therapeutic targets as well. 
 Phosphorylated caveolin-1 regulates Rho/ROCK-dependent focal adhesion dynamics and tumor cell   migration   and invasion. Rho/ROCK signaling and caveolin-1 (Cav1) are implicated in tumor cell   migration   and metastasis; however, the underlying molecular mechanisms remain poorly defined. Cav1 was found here to be an independent predictor of decreased survival in breast and rectal cancer and significantly associated with the presence of distant metastasis for colon cancer patients. Rho/ROCK signaling promotes tumor cell   migration   by regulating focal adhesion (FA) dynamics through tyrosine (Y14) phosphorylation of Cav1. Phosphorylated Cav1 is localized to protrusive domains of tumor cells and Cav1 tyrosine phosphorylation is dependent on Src kinase and Rho/ROCK signaling. Increased levels of phosphorylated Cav1 were associated with elevated GTP-RhoA levels in metastatic tumor cells of various tissue origins. Stable expression and knockdown studies of Cav1 in tumor cells showed that phosphorylated Cav1 expression stimulates Rho activation, stabilizes FAK association with FAs, and promotes cell   migration   and invasion in a ROCK-dependent and Src-dependent manner. Tyrosine-phosphorylated Cav1, therefore, functions as an effector of Rho/ROCK signaling in the regulation of FA turnover and, thereby, tumor cell   migration   and invasion. These studies define a feedback loop between Rho/ROCK, Src, and phosphorylated Cav1 in tumor cell protrusions, identifying a novel function for Cav1 in tumor metastasis that may contribute to the poor prognosis of some Cav1-expressing tumors. 
  GeneRIFs  
 Results demonstrated that the miR-200b/200c/429 subfamily inhibited HCC cell   migration   through modulating Rho/ROCK mediated cell cytoskeletal reorganization and cell-substratum adhesion. 
 upregulation of MMP2 rescued the decreased   migration   and invasion induced by the knockdown of Rock2, whereas the knockdown of MMP2 decreased Rock2-enhanced hepatocellular carcinoma   migration   and invasion 
 Selective knockdown of ROCK2 exerted antidromic effects on glioma   migration  . 
 Results demonstrate differential effects of Snail and Slug in pancreatic cancer and identify the interplay between Rho signaling and beta1-integrin that regulates the scattering and   migration   of Snail- and Slug-expressing pancreatic cancer cells. 
 results suggest that miR-138 plays an important role in TSCC cell   migration   and invasion by concurrently targeting RhoC and ROCK2, and miR-138 may serve as a novel therapeutic target for TSCC patients at risk of metastatic disease 
 Rock1 and 2 are activated in response to wounding. ROCK activities enhance cell proliferation and epithelial differentiation, but negatively modulate cell   migration   and adhesion and therefore play a role in regulating corneal epithelial wound healing. 
  ECM  PubMed  
 Substrate Stiffness Influences Doxorubicin-Induced p53 Activation via ROCK2 Expression. The physical properties of the extracellular matrix (  ECM  ), such as stiffness, are involved in the determination of the characteristics of cancer cells, including chemotherapy sensitivity. Resistance to chemotherapy is often linked to dysfunction of tumor suppressor p53; however, it remains elusive whether the   ECM   microenvironment interferes with p53 activation in cancer cells. Here, we show that, in MCF-7 breast cancer cells, extracellular stiffness influences p53 activation induced by the antitumor drug doxorubicin. Cell growth inhibition by doxorubicin was increased in response to   ECM   rigidity in a p53-dependent manner. The expression of Rho-associated coiled coil-containing protein kinase (ROCK) 2, which induces the activation of myosin II, was significantly higher when cells were cultured on stiffer   ECM   substrates. Knockdown of ROCK2 expression or pharmacological inhibition of ROCK decreased doxorubicin-induced p53 activation. Our results suggest that a soft   ECM   causes downregulation of ROCK2 expression, which drives resistance to chemotherapy by repressing p53 activation. 
 Matrix rigidity differentially regulates invadopodia activity through ROCK1 and ROCK2. ROCK activity increases due to   ECM   rigidity in the tumor microenvironment and promotes a malignant phenotype via actomyosin contractility. Invasive migration is facilitated by actin-rich adhesive protrusions known as invadopodia that degrade the   ECM  . Invadopodia activity is dependent on matrix rigidity and contractile forces suggesting that mechanical factors may regulate these subcellular structures through ROCK-dependent actomyosin contractility. However, emerging evidence indicates that the ROCK1 and ROCK2 isoforms perform different functions in cells suggesting that alternative mechanisms may potentially regulate rigidity-dependent invadopodia activity. In this study, we found that matrix rigidity drives ROCK signaling in cancer cells but that ROCK1 and ROCK2 differentially regulate invadopodia activity through separate signaling pathways via contractile (NM II) and non-contractile (LIMK) mechanisms. These data suggest that the mechanical rigidity of the tumor microenvironment may drive ROCK signaling through distinct pathways to enhance the invasive migration required for cancer progression and metastasis. 
 Rho-associated kinases are crucial for myofibroblast differentiation and production of extracellular matrix in scleroderma fibroblasts. Rho-associated kinases (Rock) are the major cellular mediators of Rho GTPases and play an important role in the organization of the actin cytoskeleton. Inhibitors of Rock are currently being evaluated for the treatment of pulmonary arterial hypertension. This study was undertaken to analyze the role of Rock in the activation of fibroblasts in systemic sclerosis (SSc).Rock signaling was inhibited using chemical inhibitors and small interfering RNA. The expression of extracellular matrix (  ECM  ) proteins and alpha-smooth muscle actin was analyzed by real-time polymerase chain reaction, Western blotting, and SirCol assay. Metabolic activity was quantified by MTT assay. Cell viability was assessed by staining with annexin V and propidium iodide. The role of MAP kinases was investigated using selective inhibitors and Western blotting.Inhibition of Rock strongly reduced the synthesis of the major   ECM   proteins at the messenger RNA level as well as the protein level. Counterregulatory changes in the expression of tissue inhibitors of metalloproteinases and matrix metalloproteinases were not observed. Inhibition of Rock prevented myofibroblast differentiation. Transforming growth factor beta activated ERK in a Rock-dependent manner, and ERK mediated in part the stimulatory effects of Rock on myofibroblast differentiation. Toxic adverse effects of the inhibition of Rock were not observed.Our findings demonstrate that Rock potently stimulates the differentiation of resting fibroblasts into myofibroblasts and the production of   ECM   at biologically relevant concentrations without cell toxicity. These findings, along with the beneficial effects of Rock inhibition on vascular disease, indicate that inhibition of Rock might be an interesting novel therapeutic approach for the treatment of SSc. 
  [Ee]xtracellular [Mm]atrix  PubMed  
 Substrate Stiffness Influences Doxorubicin-Induced p53 Activation via ROCK2 Expression. The physical properties of the   extracellular matrix   (ECM), such as stiffness, are involved in the determination of the characteristics of cancer cells, including chemotherapy sensitivity. Resistance to chemotherapy is often linked to dysfunction of tumor suppressor p53; however, it remains elusive whether the ECM microenvironment interferes with p53 activation in cancer cells. Here, we show that, in MCF-7 breast cancer cells, extracellular stiffness influences p53 activation induced by the antitumor drug doxorubicin. Cell growth inhibition by doxorubicin was increased in response to ECM rigidity in a p53-dependent manner. The expression of Rho-associated coiled coil-containing protein kinase (ROCK) 2, which induces the activation of myosin II, was significantly higher when cells were cultured on stiffer ECM substrates. Knockdown of ROCK2 expression or pharmacological inhibition of ROCK decreased doxorubicin-induced p53 activation. Our results suggest that a soft ECM causes downregulation of ROCK2 expression, which drives resistance to chemotherapy by repressing p53 activation. 
 ROCK2 associates with lectin-like oxidized LDL receptor-1 and mediates oxidized LDL-induced IL-8 production. Oxidatively modified low-density lipoprotein (OxLDL) is a contributing factor of endothelial dysfunction, an early cellular event during atherogenesis. In endothelial cells, OxLDL has been shown to stimulate proinflammatory responses, increase lipid accumulation, and induce the expression of adhesion and   extracellular matrix   degrading molecules. The primary receptor for OxLDL on endothelial cells has been identified as a member of the scavenger receptor family called lectin-like OxLDL receptor-1 (LOX-1). A number of studies on LOX-1 have implicated its role in multiple cardiovascular diseases including atherosclerosis. To better understand the molecular mechanisms underlying the role of LOX-1 in endothelial cells, we identified interacting proteins in an affinity-purified LOX-1 receptor complex from human aortic endothelial HAECT cells by mass spectrometry. Two molecules involved in Rho signaling pathway, ARHGEF1 and ROCK2, were identified, and their associations with LOX-1 were confirmed in reciprocal immunoprecipitation studies. Particularly, ROCK2 was found to dynamically associate with LOX-1 in the presence of OxLDL. In addition, OxLDL treatment stimulated ROCK2 catalytic activity, and ROCK2 inhibition attenuated NF-kappaB activation and IL-8 production resulting from OxLDL activation of LOX-1. In summary, a functional proteomics approach has enabled us to identify novel LOX-1 interactors that potentially contribute to the cellular and signaling functions of LOX-1. 
 Rho-associated kinases are crucial for myofibroblast differentiation and production of   extracellular matrix   in scleroderma fibroblasts. Rho-associated kinases (Rock) are the major cellular mediators of Rho GTPases and play an important role in the organization of the actin cytoskeleton. Inhibitors of Rock are currently being evaluated for the treatment of pulmonary arterial hypertension. This study was undertaken to analyze the role of Rock in the activation of fibroblasts in systemic sclerosis (SSc).Rock signaling was inhibited using chemical inhibitors and small interfering RNA. The expression of   extracellular matrix   (ECM) proteins and alpha-smooth muscle actin was analyzed by real-time polymerase chain reaction, Western blotting, and SirCol assay. Metabolic activity was quantified by MTT assay. Cell viability was assessed by staining with annexin V and propidium iodide. The role of MAP kinases was investigated using selective inhibitors and Western blotting.Inhibition of Rock strongly reduced the synthesis of the major ECM proteins at the messenger RNA level as well as the protein level. Counterregulatory changes in the expression of tissue inhibitors of metalloproteinases and matrix metalloproteinases were not observed. Inhibition of Rock prevented myofibroblast differentiation. Transforming growth factor beta activated ERK in a Rock-dependent manner, and ERK mediated in part the stimulatory effects of Rock on myofibroblast differentiation. Toxic adverse effects of the inhibition of Rock were not observed.Our findings demonstrate that Rock potently stimulates the differentiation of resting fibroblasts into myofibroblasts and the production of ECM at biologically relevant concentrations without cell toxicity. These findings, along with the beneficial effects of Rock inhibition on vascular disease, indicate that inhibition of Rock might be an interesting novel therapeutic approach for the treatment of SSc. 
  GeneRIFs  
 a soft   extracellular matrix   causes downregulation of ROCK2 expression, which drives resistance to chemotherapy by repressing p53 activation. 
 Rock potently stimulates the differentiation of resting fibroblasts into myofibroblasts and the production of   extracellular matrix   in scleroderma fibroblasts. 
  [Ii]nvasive  PubMed  
 Rho-kinase 2 is frequently overexpressed in hepatocellular carcinoma and involved in tumor invasion. Deregulation of Rho family small guanosine triphosphatases has been implicated in human carcinogenesis. Rho-kinases are downstream effectors of Rho guanosine triphosphatases in the regulation of cytoskeletal reorganization and cell motility. However, their functions in human cancers remain elusive. In this study, we aimed to investigate the role of Rho-kinases in hepatocellular carcinoma (HCC) tumor progression and invasion. We first examined the expression of the two Rho-kinases (ROCK1 and ROCK2) in human HCC, and found that ROCK2 was frequently overexpressed in primary HCCs (22/41 [53.66%]). Clinico-pathological analysis revealed that overexpression of ROCK2 was significantly associated with the presence of tumor microsatellite formation (P = 0.005), suggesting that deregulation of ROCK2 may contribute to the intrahepatic metastasis of HCC. Consistently, we demonstrated that stable overexpression of ROCK2 significantly enhanced cell motility and   invasive  ness in HCC cells. Conversely, stable knockdown of ROCK2 by short hairpin RNA approach remarkably reduced HCC cell migration and invasion. Moreover, orthotopic liver xenograft models provided further support that stable knockdown of ROCK2 suppressed HCC invasion in vivo. Stable knockdown of ROCK2 in HCC cells significantly inhibited Golgi reorientation, myosin phosphatase phosphorylation, and formations of stress fibers, filopodia, and lamellipodia; these molecular and cellular events are crucial for cell motility and cancer invasion.Our results indicate that ROCK2 was overexpressed in human HCCs, and this overexpression was associated with a more aggressive biological behavior. Our findings also demonstrate that ROCK2 played a significant role in regulating cytoskeletal events and contributed to the invasion of HCC. 
 Opposing signaling of ROCK1 and ROCK2 determines the switching of substrate specificity and the mode of migration of glioblastoma cells. Despite current advances in therapy, the prognosis of patients with glioblastoma has not improved sufficiently in recent decades. This is due mainly to the highly   invasive   capacity of glioma cells. Little is known about the mechanisms underlying this particular characteristic. While the Rho-kinase (ROCK)-dependent signaling pathways involved in glioma migration have yet to be determined, they show promise as one of the candidates in targeted glioblastoma therapy. There are two ROCK isoforms: ROCK1, which is upregulated in glioblastoma tissue compared to normal brain tissue, and ROCK2, which is also expressed in normal brain tissue. Blockage of both of these ROCK isoforms with pharmacologic inhibitors regulates the migration process. We examined the activities of ROCK1 and ROCK2 using knockdown cell lines and the newly developed stripe assay. Selective knockdown of either ROCK1 or ROCK2 exerted antidromic effects on glioma migration: while ROCK1 deletion altered the substrate-dependent migration, deletion of ROCK2 did not. Furthermore, ROCK1 knockdown reduced cell proliferation, whereas ROCK2 knockdown enhanced it. Along the signaling pathways, key regulators of the ROCK pathway are differentially affected by ROCK1 and ROCK2. These data suggest that the balanced activation of ROCKs is responsible for the substrate-specific migration and the proliferation of glioblastoma cells. 
 Rock2 promotes the invasion and metastasis of hepatocellular carcinoma by modifying MMP2 ubiquitination and degradation. Rho-associated coiled-coil-containing protein kinase 2 (Rock2) is a downstream effector of Rho that plays an important role in the tumorigenesis and progression of hepatocellular carcinoma (HCC). Matrix metalloproteinase 2 (MMP2) is a master regulator of tumor metastasis. In this study, we investigated the collections of Rock2 and MMP2 in HCCs and determined the potential role and molecular mechanism of Rock2 in MMP2-mediated   invasive  ness and metastasis. We found that Rock2 and MMP2 were markedly overexpressed in HCCs compared with the corresponding adjacent tissues, where a positive correlation in their expression was found. The knockdown of Rock2 significantly decreased MMP2 expression and inhibited the invasion and metastasis of HCC in vitro and in vivo. Additionally, the upregulation of MMP2 rescued the decreased migration and invasion induced by the knockdown of Rock2, whereas the knockdown of MMP2 decreased Rock2-enhanced HCC migration and invasion. Mechanistically, Rock2 stabilized MMP2 by preventing its ubiquitination and degradation. Together, our results link two drivers of invasion and metastasis in HCC and identify a novel pathway for MMP2 control. 
 Matrix rigidity differentially regulates invadopodia activity through ROCK1 and ROCK2. ROCK activity increases due to ECM rigidity in the tumor microenvironment and promotes a malignant phenotype via actomyosin contractility.   Invasive   migration is facilitated by actin-rich adhesive protrusions known as invadopodia that degrade the ECM. Invadopodia activity is dependent on matrix rigidity and contractile forces suggesting that mechanical factors may regulate these subcellular structures through ROCK-dependent actomyosin contractility. However, emerging evidence indicates that the ROCK1 and ROCK2 isoforms perform different functions in cells suggesting that alternative mechanisms may potentially regulate rigidity-dependent invadopodia activity. In this study, we found that matrix rigidity drives ROCK signaling in cancer cells but that ROCK1 and ROCK2 differentially regulate invadopodia activity through separate signaling pathways via contractile (NM II) and non-contractile (LIMK) mechanisms. These data suggest that the mechanical rigidity of the tumor microenvironment may drive ROCK signaling through distinct pathways to enhance the   invasive   migration required for cancer progression and metastasis. 
 Low Expression of miR-448 Induces EMT and Promotes Invasion by Regulating ROCK2 in Hepatocellular Carcinoma. miR-448 has been reported to exhibit abnormal expression in hepatocellular carcinoma (HCC), however, the essential role of miR-448 in HCC progression is still unclear.real-time PCR was used to detect the expression of miRNAs and candidate genes in HCC samples (n=117). miR-448 mimics and inhibitor were tansfected in human HCC cells. The transwell assay was used to examine the cell   invasive   ability. The regulation mechanism was confirmed by luciferase reporter assay. The markers of EMT were detected by using Western blot.miR-448 was decreased in HCC samples and associated with HCC development. Inhibition of miR-448 significantly promoted cell invasion, while the effect of miR-448 up-regulation was reverse. miR-448 could regulate ROCK2 in hepatocellular carcinoma. Knockdown of ROCK2 expression partially reversed the effect of miR-448 inhibitor. Abnormal expression of miR-448 could regulate the markers of epithelial-mesenchymal transition (EMT).miR-448 may contribute to the progression of HCC via regulating ROCK2 expression. 
 Inhibition of TMEM45A suppresses proliferation, induces cell cycle arrest and reduces cell invasion in human ovarian cancer cells. The association of TMEM45A with various cancers has been recently reported. However, the biological function of TMEM45A in ovarian cancer remains unclear. The present study aimed to elucidate the role of TMEM45A in regulating the biological behavior of ovarian cancer cells. We compared the expression of TMEM45A between ovarian cancer tissues and normal tissues based on RNA-sequencing data of the ovarian cancer cohort from The Cancer Genome Atlas (TCGA) project and our real-time PCR data from 25 pairs of ovarian cancer and their matched non-cancerous tissue samples. The expression of TMEM45A was then suppressed in two ovarian cancer cell lines, HO-8910 and A2780, by RNA interference. Cell proliferation, cell cycle distribution, adhesion and   invasive   ability were then detected using the Cell Counting Kit-8 assay (CCK-8), propidium iodide (PI) staining, and cell adhesion and Transwell assays, respectively. In addition, the mRNA and protein levels of transforming growth factor-β (TGF-β1 and TGF-β2), Ras homolog family member A (RhoA) and Rho-associated kinase 2 (ROCK2) were detected with real-time PCR and western blotting, respectively. TCGA data and our real-time PCR results demonstrated the overexpression of TMEM45A in ovarian cancer. Silencing of TMEM45A significantly inhibited cell proliferation and significantly increased the cell population in the G1 phase. Moreover, knockdown of TMEM45A also inhibited cell adhesion as well as cell invasion. More importantly, suppression of TMEM45A notably downregulated the expression of TGF-β1, TGF-β2, RhoA and ROCK2. In conclusion, TMEM45A may function as an oncogene for ovarian cancer, and inhibition of TMEM45A may be a therapeutic strategy for ovarian cancer. 
 RhoC and ROCKs regulate cancer cell interactions with endothelial cells. RhoC is a member of the Rho GTPase family that is implicated in cancer progression by stimulating cancer cell   invasive  ness. Here we report that RhoC regulates the interaction of cancer cells with vascular endothelial cells (ECs), a crucial step in the metastatic process. RhoC depletion by RNAi reduces PC3 prostate cancer cell adhesion to ECs, intercalation between ECs as well as transendothelial migration in vitro. Depletion of the kinases ROCK1 and ROCK2, two known RhoC downstream effectors, similarly decreases cancer interaction with ECs. RhoC also regulates the extension of protrusions made by cancer cells on vascular ECs in vivo. Transient RhoC depletion is sufficient to reduce both early PC3 cell retention in the lungs and experimental metastasis formation in vivo. Our results indicate RhoC plays a central role in cancer cell interaction with vascular ECs, which is a critical event for cancer progression. 
 ROCK1 and ROCK2 are required for non-small cell lung cancer anchorage-independent growth and invasion. Evidence is emerging that the closely related ROCK1 and ROCK2 serine/threonine kinases support the   invasive   and metastatic growth of a spectrum of human cancer types. Therefore, inhibitors of ROCK are under preclinical development. However, a key step in their development involves the identification of genetic biomarkers that will predict ROCK inhibitor antitumor activity. One identified mechanism for ROCK activation in cancer involves the loss of function of the DLC1 tumor suppressor gene, which encodes a GTPase activating protein (RhoGAP) for the RhoA and RhoC small GTPases. DLC-1 loss may lead to hyperactivation of RhoA/C and its downstream effectors, the ROCK kinases. We therefore determined whether loss of DLC-1 protein expression identifies non-small cell lung carcinoma (NSCLC) cell lines whose growth and invasion phenotypes are sensitive to ROCK inhibition. We identified and characterized a novel small molecule pharmacologic inhibitor of ROCK and additionally applied genetic approaches to impair ROCK1 and/or ROCK2 activity, and we determined that although NSCLC anchorage-dependent growth was ROCK-independent, both anchorage-independent growth and Matrigel invasion were ROCK-dependent. However, loss of DLC-1 expression did not correlate with ROCK activation or with OXA-06 sensitivity. Unexpectedly, suppression of ROCK1 or ROCK2 expression alone was sufficient to impair anchorage-independent growth, supporting their nonoverlapping roles in oncogenesis. Mechanistically, the block in anchorage-independent growth was associated with accumulation of cells in the G(0)-G(1) phase of the cell cycle, but not increased anoikis. We conclude that ROCK may be a useful therapeutic target for NSCLC. 
 Immunohistochemical evaluation of ROCK activation in   invasive   breast cancer. Two isoforms of Rho-associated coiled-coil kinase (ROCK), ROCKI and ROCKII, play an important role in many cellular processes. Despite the accumulating evidence showing that ROCK could be a potential cancer therapeutic target, the relevant tumor types to ROCK activation are not well clarified. The aim of this study was to evaluate the ROCK activation status in different tumor types of breast cancer.We evaluated the immunoreactivities of phosphorylation-specific antibodies of ROCKI and ROCKII to inform their kinase activation in 275 of breast carcinoma tissues, including 56 of carcinoma in situ, 116 of   invasive   carcinoma, and 103 of   invasive   carcinoma with metastasis. ROCKII activation signal detected in nucleus was significantly correlated with tumor metastasis, while ROCKI and cytosolic ROCKII activation signals made no significant difference in that metastasis. Furthermore, nuclear ROCKII activation signal was associated with poor clinical outcome and correlated with late tumor stage, low expression of estrogen receptor (ER) and progesterone receptor (PR), overexpression of human epidermal growth factor receptor 2 (HER2) and high Ki67 labeling index.Nuclear ROCKII activation signal might contribute to the tumor metastasis in breast cancer. Differences in ROCK activation that underlie the phenotypes of breast cancer could enhance our understanding for the use of ROCK inhibitors in cancer therapy. 
 The expression and prognostic value of ROCK I and ROCK II and their role in human breast cancer. The role and expression of ROCKI and ROCKII in human breast cancer was investigated and the effect on clinical outcome assessed. ROCK knockdown cells (MDA-MB-231DeltaROCKI and MDA-MB-231DeltaROCKII were tested for their in vitro   invasive  ness, motility and in vivo tumour growth. Samples of fresh frozen breast tumour tissue (n=113) and normal background tissue (n=30) were processed for immunohistochemical and quantitative RT-PCR analyses. MDA-MB-231DeltaROCKI and MDA-MB-231DeltaROCKII cells showed significantly decreased   invasive  ness compared with control cells (mean +/- SEM 4.33+/-0.84 MDA-MB-231DeltaROCKI vs. 18.4+/-1.4 control, p<0.001; 6.8+/-1.2 MDA-MB-231DeltaROCKII vs. 18.4+/-1.4 control, p<0.001). Similarly, both exhibited reduced motility compared with control cells (p<0.001) and lost their response to HGF. Importantly, no significant difference existed between knockdown and control cells in in vivo tumour growth. ROCKI was significantly higher in human mammary tumours than normal background tissue (2.9+/-1.1 vs. 0.29+/-0.13, p=0.023), although expression of ROCKII was fairly consistent in both (2050+/-646 vs. 2303+/-2079). ROCKI expression was greater in patients who died from breast cancer than in patients who remained disease free (11.6+/-7.1 vs. 1.95+/-0.95). Higher levels of ROCKI were associated with increased grade (0.95+/-0.73 grade-1; 2.11+/-1.72 grade-2; and 4.06+/-1.99 grade-3). Levels of ROCKI, but not ROCKII, were significantly correlated with overall survival of patients (p=0.004, Univariate analysis, median follow-up 120 month). These results show that ROCKI and possibly ROCKII are key factors in regulation of motility/invasion of breast cancer cells. This, together with significant correlation between ROCKI and poor outcome in clinical breast cancer, indicates that it is a potential target in human breast cancer. 
 The microRNA miR-139 suppresses metastasis and progression of hepatocellular carcinoma by down-regulating Rho-kinase 2. We investigated mechanisms of hepatocellular carcinoma (HCC) metastasis and identified an antimetastatic microRNA (miRNA), miR-139, that is down-regulated in human HCC samples.Effects of stable and transient expression of miRNA-139 and its inhibitors were studied in the human HCC cell lines SMMC-7721 and BEL7402; cells were analyzed for migration and invasion. Liver samples from patients with metastatic HCC were analyzed for levels of miRNA-139; data were compared with survival data using the Kaplan-Meier method and compared between groups by the log-rank test. Tumor formation and metastasis from human HCC MHCC97L cells that did or did not express miR-139 were analyzed in mice.Down-regulation of miR-139 in HCC was associated significantly with poor prognosis of patients and features of metastatic tumors, including venous invasion, microsatellite formation, absence of tumor encapsulation, and reduced differentiation. miR-139 expression was reduced in metastatic HCC tumors compared with primary tumors. Overexpression of miR-139 in HCC cells significantly reduced cell migration and invasion in vitro and the incidence and severity of lung metastasis from orthotopic liver tumors in mice. miR-139 interacted with the 3' untranslated region of Rho-kinase 2 (ROCK2) and reduced its expression in HCC cells. Levels of miR-139 were correlated inversely with ROCK2 protein in human HCC samples. Overexpression of miR-139 did not inhibit HCC cell motility when ROCK2 was knocked down.The microRNA miR-139 interacts with ROCK2 and reduces its expression in HCC cells. Down-regulation of miR-139 increased the   invasive   abilities of HCC cells in vitro and HCC metastasis in vivo. Expression of miR-139 is reduced in human metastatic HCC samples and correlates with prognosis. 
  [Mm]etastasis  PubMed  
 Rho-kinase 2 is frequently overexpressed in hepatocellular carcinoma and involved in tumor invasion. Deregulation of Rho family small guanosine triphosphatases has been implicated in human carcinogenesis. Rho-kinases are downstream effectors of Rho guanosine triphosphatases in the regulation of cytoskeletal reorganization and cell motility. However, their functions in human cancers remain elusive. In this study, we aimed to investigate the role of Rho-kinases in hepatocellular carcinoma (HCC) tumor progression and invasion. We first examined the expression of the two Rho-kinases (ROCK1 and ROCK2) in human HCC, and found that ROCK2 was frequently overexpressed in primary HCCs (22/41 [53.66%]). Clinico-pathological analysis revealed that overexpression of ROCK2 was significantly associated with the presence of tumor microsatellite formation (P = 0.005), suggesting that deregulation of ROCK2 may contribute to the intrahepatic   metastasis   of HCC. Consistently, we demonstrated that stable overexpression of ROCK2 significantly enhanced cell motility and invasiveness in HCC cells. Conversely, stable knockdown of ROCK2 by short hairpin RNA approach remarkably reduced HCC cell migration and invasion. Moreover, orthotopic liver xenograft models provided further support that stable knockdown of ROCK2 suppressed HCC invasion in vivo. Stable knockdown of ROCK2 in HCC cells significantly inhibited Golgi reorientation, myosin phosphatase phosphorylation, and formations of stress fibers, filopodia, and lamellipodia; these molecular and cellular events are crucial for cell motility and cancer invasion.Our results indicate that ROCK2 was overexpressed in human HCCs, and this overexpression was associated with a more aggressive biological behavior. Our findings also demonstrate that ROCK2 played a significant role in regulating cytoskeletal events and contributed to the invasion of HCC. 
 MiR-200b/200c/429 subfamily negatively regulates Rho/ROCK signaling pathway to suppress hepatocellular carcinoma   metastasis  . MiR-200 family is an important regulator of epithelial-mesenchymal transition and has been implicated in human carcinogenesis. However, their expression and functions in human cancers remain controversial. In the work presented here, we showed that miR-200 family members were frequently down-regulated in hepatocellular carcinoma (HCC). Although all five members of miR-200 family inhibited ZEB1/2 expression in HCC cell lines, we showed that overexpression only of the miR-200b/200c/429 subfamily, but not the miR-200a/141 subfamily, resulted in impeded HCC cell migration. Further investigations led to the identification of RhoA and ROCK2 as specific down-stream targets of the miR-200b/200c/429 subfamily. We demonstrated that the miR-200b/200c/429 subfamily inhibited HCC cell migration through modulating Rho/ROCK mediated cell cytoskeletal reorganization and cell-substratum adhesion. Re-expression of miR-200b significantly suppressed lung   metastasis   of HCC cells in an orthotopic liver implantation model in vivo. In conclusion, our findings identified the miR-200b/200c/429 subfamily as   metastasis   suppressor microRNAs in human HCC and highlighted the functional discrepancy among miR-200 family members. 
 Rock2 promotes the invasion and   metastasis   of hepatocellular carcinoma by modifying MMP2 ubiquitination and degradation. Rho-associated coiled-coil-containing protein kinase 2 (Rock2) is a downstream effector of Rho that plays an important role in the tumorigenesis and progression of hepatocellular carcinoma (HCC). Matrix metalloproteinase 2 (MMP2) is a master regulator of tumor   metastasis  . In this study, we investigated the collections of Rock2 and MMP2 in HCCs and determined the potential role and molecular mechanism of Rock2 in MMP2-mediated invasiveness and   metastasis  . We found that Rock2 and MMP2 were markedly overexpressed in HCCs compared with the corresponding adjacent tissues, where a positive correlation in their expression was found. The knockdown of Rock2 significantly decreased MMP2 expression and inhibited the invasion and   metastasis   of HCC in vitro and in vivo. Additionally, the upregulation of MMP2 rescued the decreased migration and invasion induced by the knockdown of Rock2, whereas the knockdown of MMP2 decreased Rock2-enhanced HCC migration and invasion. Mechanistically, Rock2 stabilized MMP2 by preventing its ubiquitination and degradation. Together, our results link two drivers of invasion and   metastasis   in HCC and identify a novel pathway for MMP2 control. 
 CD99 suppresses osteosarcoma cell migration through inhibition of ROCK2 activity. CD99, a transmembrane protein encoded by MIC2 gene is involved in multiple cellular events including cell adhesion and migration, apoptosis, cell differentiation and regulation of protein trafficking either in physiological or pathological conditions. In osteosarcoma, CD99 is expressed at low levels and functions as a tumour suppressor. The full-length protein (CD99wt) and the short-form harbouring a deletion in the intracytoplasmic domain (CD99sh) have been associated with distinct functional outcomes with respect to tumour malignancy. In this study, we especially evaluated modulation of cell-cell contacts, reorganisation of the actin cytoskeleton and modulation of signalling pathways by comparing osteosarcoma cells characterised by different   metastasis   capabilities and CD99 expression, to identify molecular mechanisms responsible for   metastasis  . Our data indicate that forced expression of CD99wt induces recruitment of N-cadherin and β-catenin to adherens junctions. In addition, transfection of CD99wt inhibits the expression of several molecules crucial to the remodelling of the actin cytoskeleton, such as ACTR2, ARPC1A, Rho-associated, coiled-coil containing protein kinase 2 (ROCK2) as well as ezrin, an ezrin/radixin/moesin family member that has been clearly associated with tumour progression and metastatic spread in osteosarcoma. Functional studies point to ROCK2 as a crucial intracellular mediator regulating osteosarcoma migration. By maintaining c-Src in an inactive conformation, CD99wt inhibits ROCK2 signalling and this leads to ezrin decrease at cell membrane while N-cadherin and β-catenin translocate to the plasma membrane and function as main molecular bridges for actin cytoskeleton. Taken together, we propose that the re-expression of CD99wt, which is generally present in osteoblasts but lost in osteosarcoma, through inhibition of c-Src and ROCK2 activity, manages to increase contact strength and reactivate stop-migration signals that counteract the otherwise dominant promigratory action of ezrin in osteosarcoma cells. 
 Matrix rigidity differentially regulates invadopodia activity through ROCK1 and ROCK2. ROCK activity increases due to ECM rigidity in the tumor microenvironment and promotes a malignant phenotype via actomyosin contractility. Invasive migration is facilitated by actin-rich adhesive protrusions known as invadopodia that degrade the ECM. Invadopodia activity is dependent on matrix rigidity and contractile forces suggesting that mechanical factors may regulate these subcellular structures through ROCK-dependent actomyosin contractility. However, emerging evidence indicates that the ROCK1 and ROCK2 isoforms perform different functions in cells suggesting that alternative mechanisms may potentially regulate rigidity-dependent invadopodia activity. In this study, we found that matrix rigidity drives ROCK signaling in cancer cells but that ROCK1 and ROCK2 differentially regulate invadopodia activity through separate signaling pathways via contractile (NM II) and non-contractile (LIMK) mechanisms. These data suggest that the mechanical rigidity of the tumor microenvironment may drive ROCK signaling through distinct pathways to enhance the invasive migration required for cancer progression and   metastasis  . 
 Rho kinase inhibitors block melanoma cell migration and inhibit   metastasis  . There is an urgent need to identify new therapeutic opportunities for metastatic melanoma. Fragment-based screening has led to the discovery of orally available, ATP-competitive AKT kinase inhibitors, AT13148 and CCT129254. These compounds also inhibit the Rho-kinases ROCK 1 and ROCK 2 and we show they potently inhibit ROCK activity in melanoma cells in culture and in vivo. Treatment of melanoma cells with CCT129254 or AT13148 dramatically reduces cell invasion, impairing both "amoeboid-like" and mesenchymal-like modes of invasion in culture. Intravital imaging shows that CCT129254 or AT13148 treatment reduces the motility of melanoma cells in vivo. CCT129254 inhibits melanoma   metastasis   when administered 2 days after orthotopic intradermal injection of the cells, or when treatment starts after metastases have arisen. Mechanistically, our data suggest that inhibition of ROCK reduces the ability of melanoma cells to efficiently colonize the lungs. These results suggest that these novel inhibitors of ROCK may be beneficial in the treatment of   metastasis  . 
 Protein kinase C activation disrupts epithelial apical junctions via ROCK-II dependent stimulation of actomyosin contractility. Disruption of epithelial cell-cell adhesions represents an early and important stage in tumor   metastasis  . This process can be modeled in vitro by exposing cells to chemical tumor promoters, phorbol esters and octylindolactam-V (OI-V), known to activate protein kinase C (PKC). However, molecular events mediating PKC-dependent disruption of epithelial cell-cell contact remain poorly understood. In the present study we investigate mechanisms by which PKC activation induces disassembly of tight junctions (TJs) and adherens junctions (AJs) in a model pancreatic epithelium.Exposure of HPAF-II human pancreatic adenocarcinoma cell monolayers to either OI-V or 12-O-tetradecanoylphorbol-13-acetate caused rapid disruption and internalization of AJs and TJs. Activity of classical PKC isoenzymes was responsible for the loss of cell-cell contacts which was accompanied by cell rounding, phosphorylation and relocalization of the F-actin motor nonmuscle myosin (NM) II. The OI-V-induced disruption of AJs and TJs was prevented by either pharmacological inhibition of NM II with blebbistatin or by siRNA-mediated downregulation of NM IIA. Furthermore, AJ/TJ disassembly was attenuated by inhibition of Rho-associated kinase (ROCK) II, but was insensitive to blockage of MLCK, calmodulin, ERK1/2, caspases and RhoA GTPase.Our data suggest that stimulation of PKC disrupts epithelial apical junctions via ROCK-II dependent activation of NM II, which increases contractility of perijunctional actin filaments. This mechanism is likely to be important for cancer cell dissociation and tumor   metastasis  . 
 Expression of Wnt11 and Rock2 protein with clinical characteristics of esophageal squamous cell carcinoma in Kazakh and Han patients. Esophageal squamous cell carcinoma (ESCC) is one of the most malignancies with a very poor outcome in China. Wnt11 and Rock2, new identified proteins highly associated with   metastasis   of many cancers, which were never reported in esophageal squamous cell carcinoma (ESCC). Here we measured the expression levels of Wnt11 and Rock2 in tissues from 265 patients with ESCC. Immunohistochemical staining was employed to detect the correlation of Wnt11 and Rock2 expression with clinicopathological features.The expression of Wnt11 and Rock2 was detected by immunohistochemistry in esophageal squamous cell carcinomas and normal esophageal tissues. A chi-square test was used to assess the statistical significance of the correlations between Wnt11, Rock2 expression and different clinicopathological parameters, respectively.The high-expression of Wnt11 and Rock2 was observed in ESCCs. Seventy-five cases of ESCC (51.7%) showed a positive expression of Wnt11, which indicated a significant association with the AJCC stage (P=0.007). Ninety-eight cases of ESCC (65.5%) showed a positive expression of Rock2, which indicated a significant association with ethnic background. There were no close correlations between Rock2 expression and gender, tumor location, AJCC stage, lymph node   metastasis  . Specifically, the expression of Rock2 was significantly different between Hans and Kazaks ethnicities (P=0.000). In Kaplan-Meier curve analysis, no significant correlation was observed between the expression of Wnt11, Rock-2 and the poor prognosis of ESCCs.Our finding suggests that the over-expression of Rock2 may play an important role in the carcinogenesis and progression, and may become a new underlying molecular marker in the diagnosis and treatment in ESCC. 
 Vascular endothelial growth factor C promotes cervical cancer   metastasis   via up-regulation and activation of RhoA/ROCK-2/moesin cascade. The elevated expression of vascular endothelial growth factor C (VEGF-C) is correlated with clinical cervical cancer   metastasis   and patient survival, which is interpreted by VEGF-C functions to stimulate angiogenesis and lymphatic genesis. However, the direct impact of VEGF-C on cervical cancer cell motility remains largely unknown.In this study, we investigated the effects of VEGF-C on actin cytoskeleton remodeling and on cervical cancer cell migration and invasion and how the actin-regulatory protein, moesin regulated these effects through RhoA/ROCK-2 signaling pathway.On cervical carcinoma cell line SiHa cells, exposure of VEGF-C triggered remodeling of the actin cytoskeleton and the formation of membrane ruffles, which was required for cell movement. VEGF-C significantly enhanced SiHa cells horizontal migration and three-dimensional invasion into matrices. These actions were dependent on increased expression and phosphorylation of the actin-regulatory protein moesin and specific moesin siRNA severely impaired VEGF-C stimulated-cell migration. The extracellular small GTPase RhoA/ROCK-2 cascade mediated the increased moesin expression and phosphorylation, which was discovered by the use of Y-27632, a specific inhibitor of Rho kinase and by transfected constitutively active, dominant-negative RhoA as well as ROCK-2 SiRNA. Furthermore, in the surgical cervical specimen from the patients with FIGO stage at cervical intra-epithelial neoplasia and I-II cervical squamous cell carcinoma, the expression levels of moesin were found to be significantly correlated with tumor malignancy and   metastasis  .These results implied that VEGF-C promoted cervical cancer   metastasis   by upregulation and activation of moesin protein through RhoA/ROCK-2 pathway. Our findings offer new insight into the role of VEGF-C on cervical cancer progression and may provide potential targets for cervical cancer therapy. 
 MiR-126 suppresses colon cancer cell proliferation and invasion via inhibiting RhoA/ROCK signaling pathway. Recent data strongly suggests the profound role of miRNAs in cancer progression. Here, we showed miR-126 expression was much lower in HCT116, SW620 and HT-29 colon cancer cells with highly metastatic potential and miR-126 downregulation was more frequent in colorectal cancers with   metastasis  . Restored miR-126 expression inhibited HT-29 cell growth, cell-cycle progression and invasion. Mechanically, microarray results combined with bioinformatic and experimental analysis demonstrated miR-126 exerted cancer suppressor role via inhibiting RhoA/ROCK signaling pathway. These results suggest miR-126 function as a potential tumor suppressor in colon cancer progression and miR-126/RhoA/ROCK may be a novel candidate for developing rational therapeutic strategies. 
 RhoC and ROCKs regulate cancer cell interactions with endothelial cells. RhoC is a member of the Rho GTPase family that is implicated in cancer progression by stimulating cancer cell invasiveness. Here we report that RhoC regulates the interaction of cancer cells with vascular endothelial cells (ECs), a crucial step in the metastatic process. RhoC depletion by RNAi reduces PC3 prostate cancer cell adhesion to ECs, intercalation between ECs as well as transendothelial migration in vitro. Depletion of the kinases ROCK1 and ROCK2, two known RhoC downstream effectors, similarly decreases cancer interaction with ECs. RhoC also regulates the extension of protrusions made by cancer cells on vascular ECs in vivo. Transient RhoC depletion is sufficient to reduce both early PC3 cell retention in the lungs and experimental   metastasis   formation in vivo. Our results indicate RhoC plays a central role in cancer cell interaction with vascular ECs, which is a critical event for cancer progression. 
 Immunohistochemical evaluation of ROCK activation in invasive breast cancer. Two isoforms of Rho-associated coiled-coil kinase (ROCK), ROCKI and ROCKII, play an important role in many cellular processes. Despite the accumulating evidence showing that ROCK could be a potential cancer therapeutic target, the relevant tumor types to ROCK activation are not well clarified. The aim of this study was to evaluate the ROCK activation status in different tumor types of breast cancer.We evaluated the immunoreactivities of phosphorylation-specific antibodies of ROCKI and ROCKII to inform their kinase activation in 275 of breast carcinoma tissues, including 56 of carcinoma in situ, 116 of invasive carcinoma, and 103 of invasive carcinoma with   metastasis  . ROCKII activation signal detected in nucleus was significantly correlated with tumor   metastasis  , while ROCKI and cytosolic ROCKII activation signals made no significant difference in that   metastasis  . Furthermore, nuclear ROCKII activation signal was associated with poor clinical outcome and correlated with late tumor stage, low expression of estrogen receptor (ER) and progesterone receptor (PR), overexpression of human epidermal growth factor receptor 2 (HER2) and high Ki67 labeling index.Nuclear ROCKII activation signal might contribute to the tumor   metastasis   in breast cancer. Differences in ROCK activation that underlie the phenotypes of breast cancer could enhance our understanding for the use of ROCK inhibitors in cancer therapy. 
 MicroRNA-144 suppresses osteosarcoma growth and   metastasis   by targeting ROCK1 and ROCK2. Osteosarcoma (OS) is the most common primary tumor of bone. MicroRNAs (miRNAs) are a class of endogenously expressed small non-coding RNAs that are strongly implicated in cancerous processes. However, our current understanding of the biological role of miRNAs in OS remains incomplete. In the present study, miR-144 was markedly downregulated in OS cell lines and clinical specimens. Low-level expression of miR-144 was significantly associated with distant   metastasis   and poor prognosis. Functional studies demonstrated that ectopic expression of miR-144 suppresses tumor cell proliferation and   metastasis   in vitro as well as in vivo. Furthermore, we identified Rho-associated kinases 1 and 2 (ROCK1 and ROCK2) as direct targets for miR-144 binding, resulting in suppression of their expression. Exogenous expression of ROCK1 or ROCK2 in 143B-miR-144 cells partially restored miR-144-inhibited cell proliferation and invasion. In clinical OS specimens, ROCK1 and ROCK2 levels were elevated, relative to that in paired normal bone tissues, and inversely correlated with miR-144 expression. Taken together, miR-144 suppresses OS progression by directly downregulating ROCK1 and ROCK2 expression, and may be a promising therapeutic target for OS. 
 Rock2 stabilizes β-catenin to promote tumor invasion and   metastasi  s in colorectal cancer. Rho-associated coiled-coil-containing protein kinase 2 (Rock2) is an effector for the small GTPase Rho and plays an important role in tumor progression and   metastasi  s. However, the effect of Rock2 in colorectal cancer (CRC) still remains unclear. In this study, we found that Rock2 expression was markedly increased in clinical CRC tissues compared with adjacent non-cancerous tissues. High expression of Rock2 was correlated with tumor   metastasi  s and poor prognosis in CRC. In addition, the knockdown of Rock2 suppressed the invasion and   metastasi  s of CRC cells both in vitro and in vivo. Furthermore, we found that the β-catenin/TCF4 pathway contributed to the effects of Rock2 in CRC cells, and Rock2 stabilized β-catenin by preventing its ubiquitination and degradation. Taken together, this novel pathway for β-catenin control plays a biologically relevant role i  n CRC meta  stasis. 
 [Relationship between ROCK2 expression and clinicopathologic characteristics of esophageal squamous cell carcinoma]. To investigate the expression and relationship of Rho-associated protein kinase 2 (ROCK2) and clinical characteristics in esophageal squamous cell carcinoma (ESCC).Immunohistochemistry was performed to assay the expression of ROCK2 in tumor tissues and adjacent normal epithelium from 118 ESCC patients in tissue microarray. The relationship between ROCK2 level and clinicopathologic profiles such as age, gender, location, smoking, differentiation degree, T stage, lymph node   metastasis   and TNM stage were analyzed.The ROCK2 expression was up-regulated in 54 of 118 (45.76%) ESCC tissues. The up-regulated expression of ROCK2 was observed in 55.74% (34/61) ESCC tissues of patients under 61 years old. And it was significantly higher than that in 35.09% (20/57) of patients over 61 years old (χ(2) = 5.062, P = 0.024). In addition, the rate of up-regulation of ROCK2 was significantly higher in high-grade differentiation group (58.70%, 27/46) than that in moderate-grade and low-grade differentiation group (37.50%, 27/72) (χ(2) = 5.080, P = 0.024).The up-regulated expression of ROCK2 is correlated with patient age and differentiation grade of ESCC. 
 The microRNA miR-139 suppresses   metastasis   and progression of hepatocellular carcinoma by down-regulating Rho-kinase 2. We investigated mechanisms of hepatocellular carcinoma (HCC)   metastasis   and identified an antimetastatic microRNA (miRNA), miR-139, that is down-regulated in human HCC samples.Effects of stable and transient expression of miRNA-139 and its inhibitors were studied in the human HCC cell lines SMMC-7721 and BEL7402; cells were analyzed for migration and invasion. Liver samples from patients with metastatic HCC were analyzed for levels of miRNA-139; data were compared with survival data using the Kaplan-Meier method and compared between groups by the log-rank test. Tumor formation and   metastasis   from human HCC MHCC97L cells that did or did not express miR-139 were analyzed in mice.Down-regulation of miR-139 in HCC was associated significantly with poor prognosis of patients and features of metastatic tumors, including venous invasion, microsatellite formation, absence of tumor encapsulation, and reduced differentiation. miR-139 expression was reduced in metastatic HCC tumors compared with primary tumors. Overexpression of miR-139 in HCC cells significantly reduced cell migration and invasion in vitro and the incidence and severity of lung   metastasis   from orthotopic liver tumors in mice. miR-139 interacted with the 3' untranslated region of Rho-kinase 2 (ROCK2) and reduced its expression in HCC cells. Levels of miR-139 were correlated inversely with ROCK2 protein in human HCC samples. Overexpression of miR-139 did not inhibit HCC cell motility when ROCK2 was knocked down.The microRNA miR-139 interacts with ROCK2 and reduces its expression in HCC cells. Down-regulation of miR-139 increased the invasive abilities of HCC cells in vitro and HCC   metastasis   in vivo. Expression of miR-139 is reduced in human metastatic HCC samples and correlates with prognosis. 
 Downregulation of the Rho GTPase signaling pathway is involved in the microRNA-138-mediated inhibition of cell migration and invasion in tongue squamous cell carcinoma. Tumor   metastasis   is the dominant cause of death in cancer patients, including patients with oral tongue squamous cell carcinoma (TSCC). Previously, we reported that reduced miR-138 level is correlated with enhanced metastatic potential in TSCC cells. Here, we demonstrate that miR-138 suppresses TSCC cell migration and invasion by regulating 2 key genes in the Rho GTPase signaling pathway: RhoC and ROCK2. Direct targeting of miR-138 to specific sequences located in the 3'-untranslated regions of both RhoC and ROCK2 mRNAs was confirmed using luciferase reporter gene assays. Ectopic transfection of miR-138 reduced the expression of both RhoC and ROCK2 in TSCC cells. These reduced expressions, in consequence, led to the reorganization of the stress fibers and the subsequent cell morphology change to a round bleb-like shape as well as the suppression of cell migration and invasion. In contrast, knockdown of miR-138 in TSCC cells enhanced the expression of RhoC and ROCK2, which resulted in an altered, elongated cell morphology, enhanced cell stress fiber formation and accelerated cell migration and invasion. Taken together, our results suggest that miR-138 plays an important role in TSCC cell migration and invasion by concurrently targeting RhoC and ROCK2, and miR-138 may serve as a novel therapeutic target for TSCC patients at risk of metastatic disease. 
 Overexpression of ROCK1 and ROCK2 inhibits human laryngeal squamous cell carcinoma. Rho-associated coiled-coil containing protein kinase (ROCK) over-expression has been implicated in the progression of many tumor types. The aim of this study was to explore the roles of ROCK1 and ROCK2 in human laryngeal squamous cell carcinoma (LSCC). ROCK1 and ROCK2 expression levels were examined in 50 cases of human LSCC samples by immunohistochemistry. Effects of ROCK1 and ROCK2 on LSCC cell proliferation and motility were investigated in the presence of the ROCK inhibitor Y-27632. The results showed that ROCK1 expression was positively correlated with tumor size and lymph node   metastasis   (P < 0.05); ROCK2 positively correlated with tumor size (P < 0.05). Inhibition of ROCK1 and ROCK2 by Y-27632 significantly inhibits proliferation, migration, and invasion of LSCC cells. Our data indicate that expression of ROCK1 and ROCK2 are closely associated with tumor growth and lymph node   metastasis   of LSCC. Thus, these two ROCK isoforms may be useful as molecular makers for LSCC diagnosis and may be useful therapeutic targets as well. 
 Phosphorylated caveolin-1 regulates Rho/ROCK-dependent focal adhesion dynamics and tumor cell migration and invasion. Rho/ROCK signaling and caveolin-1 (Cav1) are implicated in tumor cell migration and   metastasis  ; however, the underlying molecular mechanisms remain poorly defined. Cav1 was found here to be an independent predictor of decreased survival in breast and rectal cancer and significantly associated with the presence of distant   metastasis   for colon cancer patients. Rho/ROCK signaling promotes tumor cell migration by regulating focal adhesion (FA) dynamics through tyrosine (Y14) phosphorylation of Cav1. Phosphorylated Cav1 is localized to protrusive domains of tumor cells and Cav1 tyrosine phosphorylation is dependent on Src kinase and Rho/ROCK signaling. Increased levels of phosphorylated Cav1 were associated with elevated GTP-RhoA levels in metastatic tumor cells of various tissue origins. Stable expression and knockdown studies of Cav1 in tumor cells showed that phosphorylated Cav1 expression stimulates Rho activation, stabilizes FAK association with FAs, and promotes cell migration and invasion in a ROCK-dependent and Src-dependent manner. Tyrosine-phosphorylated Cav1, therefore, functions as an effector of Rho/ROCK signaling in the regulation of FA turnover and, thereby, tumor cell migration and invasion. These studies define a feedback loop between Rho/ROCK, Src, and phosphorylated Cav1 in tumor cell protrusions, identifying a novel function for Cav1 in tumor   metastasis   that may contribute to the poor prognosis of some Cav1-expressing tumors. 
  GeneRIFs  
 Study shows that nuclear ROCKII activation signal might contribute to the tumor   metastasis   in breast cancer. 
 Our data indicate that expression of ROCK1 and ROCK2 are closely associated with tumor growth and lymph node   metastasis   of laryngeal squamous cell carcinoma 
 Results suggest that VEGF-C promoted cervical cancer   metastasis   by upregulation and activation of moesin protein through RhoA/ROCK-2 pathway. 
 a feedback loop between Rho/ROCK, Src, and phosphorylated Cav1 in tumor cell protrusions, identifying a novel function for Cav1 in tumor   metastasis   that may contribute to the poor prognosis of some Cav1-expressing tumors. 
  [Cc]ell [Ii]nvasion  PubMed  
 Low Expression of miR-448 Induces EMT and Promotes Invasion by Regulating ROCK2 in Hepatocellular Carcinoma. miR-448 has been reported to exhibit abnormal expression in hepatocellular carcinoma (HCC), however, the essential role of miR-448 in HCC progression is still unclear.real-time PCR was used to detect the expression of miRNAs and candidate genes in HCC samples (n=117). miR-448 mimics and inhibitor were tansfected in human HCC cells. The transwell assay was used to examine the cell invasive ability. The regulation mechanism was confirmed by luciferase reporter assay. The markers of EMT were detected by using Western blot.miR-448 was decreased in HCC samples and associated with HCC development. Inhibition of miR-448 significantly promoted   cell invasion  , while the effect of miR-448 up-regulation was reverse. miR-448 could regulate ROCK2 in hepatocellular carcinoma. Knockdown of ROCK2 expression partially reversed the effect of miR-448 inhibitor. Abnormal expression of miR-448 could regulate the markers of epithelial-mesenchymal transition (EMT).miR-448 may contribute to the progression of HCC via regulating ROCK2 expression. 
 Rho kinase inhibitors block melanoma cell migration and inhibit metastasis. There is an urgent need to identify new therapeutic opportunities for metastatic melanoma. Fragment-based screening has led to the discovery of orally available, ATP-competitive AKT kinase inhibitors, AT13148 and CCT129254. These compounds also inhibit the Rho-kinases ROCK 1 and ROCK 2 and we show they potently inhibit ROCK activity in melanoma cells in culture and in vivo. Treatment of melanoma cells with CCT129254 or AT13148 dramatically reduces   cell invasion  , impairing both "amoeboid-like" and mesenchymal-like modes of invasion in culture. Intravital imaging shows that CCT129254 or AT13148 treatment reduces the motility of melanoma cells in vivo. CCT129254 inhibits melanoma metastasis when administered 2 days after orthotopic intradermal injection of the cells, or when treatment starts after metastases have arisen. Mechanistically, our data suggest that inhibition of ROCK reduces the ability of melanoma cells to efficiently colonize the lungs. These results suggest that these novel inhibitors of ROCK may be beneficial in the treatment of metastasis. 
 Expression of Wnt11 and Rock2 in esophageal squamous cell carcinoma by activation of the WNT/PCP pathway and its clinical significance. The purpose of this study was to investigate the relation between expression of Wnt11, Rho-associated protein kinase 2 (Rock2), and its clinical characteristics in esophageal squamous cell carcinoma (ESCC). Expression of Wnt11 and Rock2 protein was examined by using immunohistochemistry that contained 260 paraffin-embedded specimens of ESCC and its adjacent normal tissues; expression of Wnt11 and Rock2 protein was verified by Western-blotting that contained 20 specimens of ESCC and its adjacent normal tissues. The positive rates of Wnt11 protein in normal esophageal epithelium tissue was 29.8% and in esophageal carcinomas tissue was 31.9%; there was no significant difference between the two groups(P>0.05); The positive rates of Rock2 protein in normal esophageal epithelium tissue was 12.3% and in esophageal carcinomas tissues was 56.5%, there was a significant difference between the two groups (p<0.05). The expression of Rock2 protein was significantly related with the invasion of vascular and there was no significantly difference between the expression of Rock2 protein and ESCC patients' tumor location, differentiation, T stage, and lymph node metastases. The abnormal expression of Rock2 protein may promote tumor   cell invasion  . 
 Autocrine motility factor receptor signaling pathway promotes   cell invasion   via activation of ROCK-2 in esophageal squamous cell cancer cells. Aberrant overexpression of autocrine motility factor and its receptor (AMFR) is observed in many cancers but not in esophageal squamous cell cancer (ESCC). In this study, upregulated rho-associated protein kinase 2, p-cofilin and intracellular adhesion molecule-1, and downregulated E-cadherin were found in ESCC cells transfected with the plasmid pcDNA 3.1-AMFR-C, while opposite results were observed in ESCC cells transfected with siRNA against AMFR. Additionally, an elevated invasion of ESCC cells by AMFR was reversed by rho-associated protein kinase 2 inhibitor Y-27632, suggesting that AMFR pathway promotes invasion of ESCC cells and may be a potential target for ESCC therapy. 
 Inhibition of TMEM45A suppresses proliferation, induces cell cycle arrest and reduces   cell invasion   in human ovarian cancer cells. The association of TMEM45A with various cancers has been recently reported. However, the biological function of TMEM45A in ovarian cancer remains unclear. The present study aimed to elucidate the role of TMEM45A in regulating the biological behavior of ovarian cancer cells. We compared the expression of TMEM45A between ovarian cancer tissues and normal tissues based on RNA-sequencing data of the ovarian cancer cohort from The Cancer Genome Atlas (TCGA) project and our real-time PCR data from 25 pairs of ovarian cancer and their matched non-cancerous tissue samples. The expression of TMEM45A was then suppressed in two ovarian cancer cell lines, HO-8910 and A2780, by RNA interference. Cell proliferation, cell cycle distribution, adhesion and invasive ability were then detected using the Cell Counting Kit-8 assay (CCK-8), propidium iodide (PI) staining, and cell adhesion and Transwell assays, respectively. In addition, the mRNA and protein levels of transforming growth factor-β (TGF-β1 and TGF-β2), Ras homolog family member A (RhoA) and Rho-associated kinase 2 (ROCK2) were detected with real-time PCR and western blotting, respectively. TCGA data and our real-time PCR results demonstrated the overexpression of TMEM45A in ovarian cancer. Silencing of TMEM45A significantly inhibited cell proliferation and significantly increased the cell population in the G1 phase. Moreover, knockdown of TMEM45A also inhibited cell adhesion as well   as cell invas  ion. More importantly, suppression of TMEM45A notably downregulated the expression of TGF-β1, TGF-β2, RhoA and ROCK2. In conclusion, TMEM45A may function as an oncogene for ovarian cancer, and inhibition of TMEM45A may be a therapeutic strategy for ovarian cancer. 
 ROCK inhibitor Y-27632 inhibits the growth, migration, and invasion of Tca8113 and CAL-27 cells in tongue squamous cell carcinoma. The objective of this study is to determine the effects of Rho-associated coiled-coil containing protein kinase (ROCK) inhibitor Y-27632 on the growth, invasion, and migration of Tca8113 and CAL-27 cells in tongue squamous cell carcinoma (TSCC). The methods of the study are as follows: After being routinely cultured for 24 h, Tca8113 and CAL-27 cells were treated with Y-27632 solution. The morphological change of Y-27632-treated cells was observed under an optical microscope and an inverted microscope; MTT assay was performed to measure the optical density (OD) of cells and calculate cell growth inhibition rate; the change of apoptosis was detected by AnnexinV-FITC/PI assay;   cell invasio  n and migration were measured by Transwell assay. The results were as follows: (1) With increasing concentration of Y-27632, cell morphology changed and cell apoptosis appeared; (2) MTT assay showed that inhibition effect of Y-27632 on Tca8113 and CAL-27 cells was enhanced with increasing concentrations and time (all P < 0.01); (3) Apoptosis showed that, compared with controls, the number of apoptosis cells in experimental groups was significantly increased (all P < 0.01). Apoptosis rate was elevated with increasing concentrations of Y-27632; (4) Transwell assay showed, after a treatment with Y-27632, the number of migrated and invaded Tca8113 and CAL-27 cells in each group was statistically different (all P < 0.01); compared with controls, the number of migrated cell in groups treated with Y-27632 was decreased and less Tca8113 and CAL-27 cells in experimental groups passed through polycarbonate membrane (all P < 0.05). The study concludes that Y-27632 can inhibit the growth, invasion, and migration of Tca8113 and CAL-27 cells, suggesting that Y-27632 may be therapeutically useful in TSCC. 
  GeneRIFs  
 knockdown of TMEM45A also inhibited cell adhesion as well as   cell invasion  . More importantly, suppression of TMEM45A notably downregulated the expression of TGF-b1, TGF-b2, RhoA and ROCK2. 
   
  
 IQSEC1 
 
  NCBI Gene  
  GeneRIF  
  Pubmed  
 
 
  Search Term  Rank  Fields  
  [Ff]ilopodia  1  PubMed(1)  
  [Mm]igration  6  PubMed(7); GeneRIFs(2)  
  [Ee]xtracellular [Mm]atrix  8  PubMed(1); GeneRIFs(1)  
  [Ii]nvasive  10  PubMed(6); GeneRIFs(1)  
  [Mm]etastasis  12  PubMed(8); GeneRIFs(3)  
  [Cc]ell [Ii]nvasion  13  PubMed(3); GeneRIFs(1)  
 
 
 hide/show details for IQSEC1 
  [Ff]ilopodia  PubMed  
 Phosphatidylinositol-4-phosphate 5-kinase and GEP100/Brag2 protein mediate antiangiogenic signaling by semaphorin 3E-plexin-D1 through Arf6 protein. The semaphorins are a family of secreted or membrane-bound proteins that are known to guide axons in the developing nervous system. Genetic evidence revealed that a class III semaphorin, semaphorin 3E (Sema3E), and its receptor Plexin-D1 also control the vascular patterning during development. At the molecular level, we have recently shown that Sema3E acts on Plexin-D1 expressed in endothelial cells, thus initiating a novel antiangiogenic signaling pathway that results in the retraction of   filopodia   in endothelial tip cells. Sema3E induces the rapid disassembly of integrin-mediated adhesive structures, thereby inhibiting endothelial cell adhesion to the extracellular matrix. This process requires the activation of small GTPase Arf6 (ADP-ribosylation factor 6), which regulates intracellular trafficking of β1 integrin. However, the molecular mechanisms by which Sema3E-Plexin-D1 activates Arf6 remained to be identified. Here we show that GEP100 (guanine nucleotide exchange protein 100)/Brag2, a guanine nucleotide exchange factor for Arf6, mediates Sema3E-induced Arf6 activation in endothelial cells. We provide evidence that upon activation by Sema3E, Plexin-D1 recruits phosphatidylinositol-4-phosphate 5-kinase, and its enzymatic lipid product, phosphatidylinositol 4,5-bisphosphate, binds to the pleckstrin homology domain of GEP100. Phosphatidylinositol 4,5-bisphosphate binding to GEP100 enhances its guanine nucleotide exchange factor activity toward Arf6, thus resulting in the disassembly of integrin-mediated focal adhesions and endothelial cell collapse. Our present study reveals a novel phospholipid-regulated antiangiogenic signaling pathway whereby Sema3E activates Arf6 through Plexin-D1 and consequently controls integrin-mediated endothelial cell attachment to the extracellular matrix and migration. 
  [Mm]igration  PubMed  
 Brag2 differentially regulates β1- and β3-integrin-dependent adhesion in endothelial cells and is involved in developmental and pathological angiogenesis. β1-Integrins are essential for angiogenesis. The mechanisms regulating integrin function in endothelial cells (EC) and their contribution to angiogenesis remain elusive. Brag2 is a guanine nucleotide exchange factor for the small Arf-GTPases Arf5 and Arf6. The role of Brag2 in EC and angiogenesis and the underlying molecular mechanisms remain unclear. siRNA-mediated Brag2-silencing reduced EC angiogenic sprouting a  nd migrat  ion. Brag2-siRNA transfection differentially affected α5β1- and αVβ3-integrin function: specifically, Brag2-silencing increased focal/fibrillar adhesions and adhesion on β1-integrin ligands (fibronectin and collagen), while reducing the adhesion on the αVβ3-integrin ligand, vitronectin. Consistent with these results, Brag2-silencing enhanced surface expression of α5β1-integrin, while reducing surface expression of αVβ3-integrin. Mechanistically, Brag2-mediated αVβ3-integrin-recycling and β1-integrin endocytosis and specifically of the active/matrix-bound α5β1-integrin present in fibrillar/focal adhesions (FA), suggesting that Brag2 contributes to the disassembly of FA via β1-integrin endocytosis. Arf5 and Arf6 are promoting downstream of Brag2 angiogenic sprouting, β1-integrin endocytosis and the regulation of FA. In vivo silencing of the Brag2-orthologues in zebrafish embryos using morpholinos perturbed vascular development. Furthermore, in vivo intravitreal injection of plasmids containing Brag2-shRNA reduced pathological ischemia-induced retinal and choroidal neovascularization. These data reveal that Brag2 is essential for developmental and pathological angiogenesis by promoting EC sprouting through regulation of adhesion by mediating β1-integrin internalization and link for the first time the process of β1-integrin endocytosis with angiogenesis. 
 Brag2 differentially regulates β1- and β3-integrin-dependent adhesion in endothelial cells and is involved in developmental and pathological angiogenesis. β1-Integrins are essential for angiogenesis. The mechanisms regulating integrin function in endothelial cells (EC) and their contribution to angiogenesis remain elusive. Brag2 is a guanine nucleotide exchange factor for the small Arf-GTPases Arf5 and Arf6. The role of Brag2 in EC and angiogenesis and the underlying molecular mechanisms remain unclear. siRNA-mediated Brag2-silencing reduced EC angiogenic sprouting a  nd migrat  ion. Brag2-siRNA transfection differentially affected α5β1- and αVβ3-integrin function: specifically, Brag2-silencing increased focal/fibrillar adhesions and adhesion on β1-integrin ligands (fibronectin and collagen), while reducing the adhesion on the αVβ3-integrin ligand, vitronectin. Consistent with these results, Brag2-silencing enhanced surface expression of α5β1-integrin, while reducing surface expression of αVβ3-integrin. Mechanistically, Brag2-mediated αVβ3-integrin-recycling and β1-integrin endocytosis and specifically of the active/matrix-bound α5β1-integrin present in fibrillar/focal adhesions (FA), suggesting that Brag2 contributes to the disassembly of FA via β1-integrin endocytosis. Arf5 and Arf6 are promoting downstream of Brag2 angiogenic sprouting, β1-integrin endocytosis and the regulation of FA. In vivo silencing of the Brag2-orthologues in zebrafish embryos using morpholinos perturbed vascular development. Furthermore, in vivo intravitreal injection of plasmids containing Brag2-shRNA reduced pathological ischemia-induced retinal and choroidal neovascularization. These data reveal that Brag2 is essential for developmental and pathological angiogenesis by promoting EC sprouting through regulation of adhesion by mediating β1-integrin internalization and link for the first time the process of β1-integrin endocytosis with angiogenesis. 
 GEP100/BRAG2: activator of ADP-ribosylation factor 6 for regulation of cell adhesion and actin cytoskeleton via E-cadherin and alpha-catenin. GEP(100) (p100) was identified as an ADP-ribosylation factor (ARF) guanine nucleotide-exchange protein (GEP) that partially colocalized with ARF6 in the cell periphery. p100 preferentially accelerated guanosine 5[gamma-thio]triphosphate (GTPgammaS) binding by ARF6, which participates in protein trafficking near the plasma membrane, including receptor recycling, cell adhesion, and cell   migration  . Here we report that yeast two-hybrid screening of a human fetal brain cDNA library using p100 as bait revealed specific interaction with alpha-catenin, which is known as a regulator of adherens junctions and actin cytoskeleton remodeling. Interaction of p100 with alpha-catenin was confirmed by coimmunoprecipitation of the endogenous proteins from human HepG2 or CaSki cells, although colocalization was difficult to demonstrate microscopically. alpha-Catenin enhanced GTPgammaS binding by ARF6 in vitro in the presence of p100. Depletion of p100 by small interfering RNA (siRNA) treatment in HepG2 cells resulted in E-cadherin content 3-fold that in control cells and blocked hepatocyte growth factor-induced redistribution of E-cadherin, consistent with a known role of ARF6 in this process. F-actin was markedly decreased in normal rat kidney (NRK) cells overexpressing wild-type p100, but not its GEP-inactive mutants, also consistent with the conclusion that p100 has an important role in the activation of ARF6 for its functions in both E-cadherin recycling and actin remodeling. 
 Down-regulation of GEP100 causes increase in E-cadherin levels and inhibits pancreatic cancer cell invasion. Invasion and metastasis are major reasons for pancreatic cancer death and identifying signaling molecules that are specifically used in tumor invasion is of great significance. The purpose of this study was to elucidate the role of GEP100 in pancreatic cancer cell invasion and metastasis and the corresponding molecular mechanism.Stable cell lines with GEP100 knocked-down were established by transfecting GEP100 shRNA vector into PaTu8988 cells and selected by puromycin. qRT-PCR and Western blot were performed to detect gene expression. Matrigel-invasion assay was used to detect cancer cell invasion in vitro. Liver metastasis in vivo was determined by splenic injection of indicated cell lines followed by spleen resection. Immunofluorescence study was used to detect the intracellular localization of E-cadherin.We found that the expression level of GEP100 protein was closely related to the invasive ability of a panel of 6 different human pancreatic cancer cell lines. Down-regulation of GEP100 in PaTu8988 cells significantly decreased invasive activity by Matrigel invasion assay, without affecting   migration  , invasion and viability. The inhibited invasive activity was rescued by over-expression of GEP100 cDNA. In vivo study showed that liver metastasis was significantly decreased in the PaTu8988 cells with GEP100 stably knocked-down. In addition, an epithelial-like morphological change, mimicking a mesenchymal to epithelial transition (MET) was induced by GEP100 down-regulation. The expression of E-cadherin protein was increased 2-3 folds accompanied by its redistribution to the cell-cell contacts, while no obvious changes were observed for E-cadherin mRNA. Unexpectedly, the mRNA of Slug was increased by GEP100 knock-down.These findings provided important evidence that GEP100 plays a significant role in pancreatic cancer invasion through regulating the expression of E-cadherin and the process of MET, indicating the possibility of it becoming a potential therapeutic target against pancreatic cancer. 
 GEP100/Arf6 is required for epidermal growth factor-induced ERK/Rac1 signaling and cell   migration   in human hepatoma HepG2 cells. Epidermal growth factor (EGF) signaling is implicated in the invasion and metastasis of hepatoma cells. However, the signaling pathways for EGF-induced motility of hepatoma cells remain undefined.We found that EGF dose-dependently stimulated the   migration   of human hepatoma cells HepG2, with the maximal effect at 10 ng/mL. Additionally, EGF increased Arf6 activity, and ectopic expression of Arf6 T27N, a dominant negative Arf6 mutant, largely abolish EGF-induced cell   migration  . Blocking GEP100 with GEP100 siRNA or GEP100-△PH, a pleckstrin homology (PH) domain deletion mutant of GEP100, blocked EGF-induced Arf6 activity and cel  l migrati  on. EGF also increased ERK and Rac1 activity. Ectopic expression GEP100 siRNA, GEP100-△PH, or Arf6-T27N suppressed EGF-induced ERK and Rac1 activity. Furthermore, blocking ERK signaling with its inhibitor U0126 remarkably inhibited both EGF-induced Rac1 activation as well as c  ell migra  tion, and ectopic expression of inactive mutant form of Rac1 (Rac1-T17N) also largely abolished EGF-induced c  ell migra  tion.Taken together, this study highlights the function of the PH domain of GEP100 and its regulated Arf6/ERK/Rac1 signaling cascade in EGF-induced hepatoma c  ell migra  tion. These findings could provide a rationale for designing new therapy based on inhibition of hepatoma metastasis. 
 Phosphatidylinositol-4-phosphate 5-kinase and GEP100/Brag2 protein mediate antiangiogenic signaling by semaphorin 3E-plexin-D1 through Arf6 protein. The semaphorins are a family of secreted or membrane-bound proteins that are known to guide axons in the developing nervous system. Genetic evidence revealed that a class III semaphorin, semaphorin 3E (Sema3E), and its receptor Plexin-D1 also control the vascular patterning during development. At the molecular level, we have recently shown that Sema3E acts on Plexin-D1 expressed in endothelial cells, thus initiating a novel antiangiogenic signaling pathway that results in the retraction of filopodia in endothelial tip cells. Sema3E induces the rapid disassembly of integrin-mediated adhesive structures, thereby inhibiting endothelial cell adhesion to the extracellular matrix. This process requires the activation of small GTPase Arf6 (ADP-ribosylation factor 6), which regulates intracellular trafficking of β1 integrin. However, the molecular mechanisms by which Sema3E-Plexin-D1 activates Arf6 remained to be identified. Here we show that GEP100 (guanine nucleotide exchange protein 100)/Brag2, a guanine nucleotide exchange factor for Arf6, mediates Sema3E-induced Arf6 activation in endothelial cells. We provide evidence that upon activation by Sema3E, Plexin-D1 recruits phosphatidylinositol-4-phosphate 5-kinase, and its enzymatic lipid product, phosphatidylinositol 4,5-bisphosphate, binds to the pleckstrin homology domain of GEP100. Phosphatidylinositol 4,5-bisphosphate binding to GEP100 enhances its guanine nucleotide exchange factor activity toward Arf6, thus resulting in the disassembly of integrin-mediated focal adhesions and endothelial cell collapse. Our present study reveals a novel phospholipid-regulated antiangiogenic signaling pathway whereby Sema3E activates Arf6 through Plexin-D1 and consequently controls integrin-mediated endothelial cell attachment to the extracellular matrix and   migratio  n. 
 GEP100-Arf6-AMAP1-cortactin pathway frequently used in cancer invasion is activated by VEGFR2 to promote angiogenesis. Angiogenesis and cancer invasiveness greatly contribute to cancer malignancy.Arf6 and its effector, AMAP1, are frequently overexpressed in breast cancer, and constitute a central pathway to induce the invasion and metastasis. In this pathway, Arf6 is activated by EGFR via GEP100. Arf6 is highly expressed also in human umbilical vein endothelial cells (HUVECs) and is implicated in angiogenesis. Here, we found that HUVECs also highly express AMAP1, and that vascular endothelial growth factor receptor-2 (VEGFR2) recruits GEP100 to activate Arf6. AMAP1 functions by binding to cortactin in cancer invasion and metastasis. We demonstrate that the same GEP100-Arf6-AMAP1-cortactin pathway is essential for angiogenesis activities, including cell   migration   and tubular formation, as well as for the enhancement of cell permeability and VE-cadherin endocytosis of VEGF-stimulated HUVECs. Components of this pathway are highly expressed in pathologic angiogenesis, and blocking of this pathway effectively inhibits VEGF- or tumor-induced angiogenesis and choroidal neovascularization. The GEP100-Arf6-AMAP1-cortactin pathway, activated by receptor tyrosine kinases, appears to be common in angiogenesis and cancer invasion and metastasis, and provides their new therapeutic targets. 
  GeneRIFs  
 GEP100/Arf6 is required for epidermal growth factor-induced ERK/Rac1 signaling and cell   migration   in human hepatoma HepG2 cells. 
 a novel phospholipid-regulated antiangiogenic signaling pathway whereby Sema3E activates Arf6 through Plexin-D1 and consequently controls integrin-mediated endothelial cell attachment to the extracellular matrix and   migration  . 
  [Ee]xtracellular [Mm]atrix  PubMed  
 Phosphatidylinositol-4-phosphate 5-kinase and GEP100/Brag2 protein mediate antiangiogenic signaling by semaphorin 3E-plexin-D1 through Arf6 protein. The semaphorins are a family of secreted or membrane-bound proteins that are known to guide axons in the developing nervous system. Genetic evidence revealed that a class III semaphorin, semaphorin 3E (Sema3E), and its receptor Plexin-D1 also control the vascular patterning during development. At the molecular level, we have recently shown that Sema3E acts on Plexin-D1 expressed in endothelial cells, thus initiating a novel antiangiogenic signaling pathway that results in the retraction of filopodia in endothelial tip cells. Sema3E induces the rapid disassembly of integrin-mediated adhesive structures, thereby inhibiting endothelial cell adhesion to the   extracellular matrix  . This process requires the activation of small GTPase Arf6 (ADP-ribosylation factor 6), which regulates intracellular trafficking of β1 integrin. However, the molecular mechanisms by which Sema3E-Plexin-D1 activates Arf6 remained to be identified. Here we show that GEP100 (guanine nucleotide exchange protein 100)/Brag2, a guanine nucleotide exchange factor for Arf6, mediates Sema3E-induced Arf6 activation in endothelial cells. We provide evidence that upon activation by Sema3E, Plexin-D1 recruits phosphatidylinositol-4-phosphate 5-kinase, and its enzymatic lipid product, phosphatidylinositol 4,5-bisphosphate, binds to the pleckstrin homology domain of GEP100. Phosphatidylinositol 4,5-bisphosphate binding to GEP100 enhances its guanine nucleotide exchange factor activity toward Arf6, thus resulting in the disassembly of integrin-mediated focal adhesions and endothelial cell collapse. Our present study reveals a novel phospholipid-regulated antiangiogenic signaling pathway whereby Sema3E activates Arf6 through Plexin-D1 and consequently controls integrin-mediated endothelial cell attachment to the   extracellular matri  x and migration. 
  GeneRIFs  
 a novel phospholipid-regulated antiangiogenic signaling pathway whereby Sema3E activates Arf6 through Plexin-D1 and consequently controls integrin-mediated endothelial cell attachment to the   extracellular matrix   and migration. 
  [Ii]nvasive  PubMed  
 GEP100 links epidermal growth factor receptor signalling to Arf6 activation to induce breast cancer invasion. Epidermal growth factor (EGF) receptor (EGFR) signalling is implicated in tumour invasion and metastasis. However, whether there are EGFR signalling pathways specifically used for tumour invasion still remains elusive. Overexpression of Arf6 and its effector, AMAP1, correlates with and is crucial for the   invasive   phenotypes of different breast cancer cells. Here we identify the mechanism by which Arf6 is activated to induce tumour invasion. We found that GEP100/BRAG2, a guanine nucleotide exchanging factor (GEF) for Arf6, is responsible for the   invasive   activity of MDA-MB-231 breast cancer cells, whereas the other ArfGEFs are not. GEP100, through its pleckstrin homology domain, bound directly to Tyr1068/1086-phosphorylated EGFR to activate Arf6. Overexpression of GEP100, together with Arf6, caused non-  invasive   MCF7 cells to become   invasive  , which was dependent on EGF stimulation. Moreover, GEP100 knockdown blocked tumour metastasis. GEP100 was expressed in 70% of primary breast ductal carcinomas, and was preferentially co-expressed with EGFR in the malignant cases. Our results indicate that GEP100 links EGFR signalling to Arf6 activation to induce   invasive   activities of some breast cancer cells, and hence may contribute to their metastasis and malignancy. 
 GEP100 links epidermal growth factor receptor signalling to Arf6 activation to induce breast cancer invasion. Epidermal growth factor (EGF) receptor (EGFR) signalling is implicated in tumour invasion and metastasis. However, whether there are EGFR signalling pathways specifically used for tumour invasion still remains elusive. Overexpression of Arf6 and its effector, AMAP1, correlates with and is crucial for the   invasive   phenotypes of different breast cancer cells. Here we identify the mechanism by which Arf6 is activated to induce tumour invasion. We found that GEP100/BRAG2, a guanine nucleotide exchanging factor (GEF) for Arf6, is responsible for the   invasive   activity of MDA-MB-231 breast cancer cells, whereas the other ArfGEFs are not. GEP100, through its pleckstrin homology domain, bound directly to Tyr1068/1086-phosphorylated EGFR to activate Arf6. Overexpression of GEP100, together with Arf6, caused non-  invasive   MCF7 cells to become   invasive  , which was dependent on EGF stimulation. Moreover, GEP100 knockdown blocked tumour metastasis. GEP100 was expressed in 70% of primary breast ductal carcinomas, and was preferentially co-expressed with EGFR in the malignant cases. Our results indicate that GEP100 links EGFR signalling to Arf6 activation to induce   invasive   activities of some breast cancer cells, and hence may contribute to their metastasis and malignancy. 
 Epidermal growth factor receptor-GEP100-Arf6 axis affects the prognosis of lung adenocarcinoma. The overexpression of Arf6 and GEP100 is responsible for the   invasive   activity that is crucial for the activation of the epidermal growth factor receptor (EGFR) signaling pathways in human cancer. However, whether or not the expression of the EGFR-GEP100-Arf6 axis can be used as a biomarker for the prognosis of lung cancer has yet to be fully determined. Tumor specimens were collected from 182 patients who underwent a complete resection for lung adenocarcinoma. We analyzed phospho-EGFR (p-EGFR), GEP100, and Arf6 expression levels in the primary tumor by immunohistochemical analysis. The expression of p-EGFR, GEP100, and Arf6 was observed in 65 (35.7%), 95 (52.2%), and 20 (11.0%) patients, respectively. Significant associations between p-EGFR and GEP100 expression and vessel invasion were identified. The expression of these individual molecules was not associated with any statistically significant differences in survival. However, triple positive expression of p-EGFR, GEP100, and Arf6 was significantly associated with an increased risk of death based on the multivariate analysis. The EGFR-GEP100-Arf6 axis affected the prognosis of patients with primary lung adenocarcinoma. The combination of p-EGFR, GEP100, and Arf6 staining can predict the prognosis of patients after surgery. 
 Down-regulation of GEP100 causes increase in E-cadherin levels and inhibits pancreatic cancer cell invasion. Invasion and metastasis are major reasons for pancreatic cancer death and identifying signaling molecules that are specifically used in tumor invasion is of great significance. The purpose of this study was to elucidate the role of GEP100 in pancreatic cancer cell invasion and metastasis and the corresponding molecular mechanism.Stable cell lines with GEP100 knocked-down were established by transfecting GEP100 shRNA vector into PaTu8988 cells and selected by puromycin. qRT-PCR and Western blot were performed to detect gene expression. Matrigel-invasion assay was used to detect cancer cell invasion in vitro. Liver metastasis in vivo was determined by splenic injection of indicated cell lines followed by spleen resection. Immunofluorescence study was used to detect the intracellular localization of E-cadherin.We found that the expression level of GEP100 protein was closely related to the   invasive   ability of a panel of 6 different human pancreatic cancer cell lines. Down-regulation of GEP100 in PaTu8988 cells significantly decreased   invasive   activity by Matrigel invasion assay, without affecting migration, invasion and viability. The inhibited   invasive   activity was rescued by over-expression of GEP100 cDNA. In vivo study showed that liver metastasis was significantly decreased in the PaTu8988 cells with GEP100 stably knocked-down. In addition, an epithelial-like morphological change, mimicking a mesenchymal to epithelial transition (MET) was induced by GEP100 down-regulation. The expression of E-cadherin protein was increased 2-3 folds accompanied by its redistribution to the cell-cell contacts, while no obvious changes were observed for E-cadherin mRNA. Unexpectedly, the mRNA of Slug was increased by GEP100 knock-down.These findings provided important evidence that GEP100 plays a significant role in pancreatic cancer invasion through regulating the expression of E-cadherin and the process of MET, indicating the possibility of it becoming a potential therapeutic target against pancreatic cancer. 
 Engagement of overexpressed Her2 with GEP100 induces autonomous   invasive   activities and provides a biomarker for metastases of lung adenocarcinoma. Overexpression of Her2/ErbB2/Neu in cancer is often correlated with recurrent distant metastasis, although the mechanism still remains largely elusive. We have previously shown that EGFR, when tyrosine-phosphorylated, binds to GEP100/BRAG2 to activate Arf6, which induces cancer invasion and metastasis. We now show that overexpressed Her2 in lung adenocarcinoma cells also employs GEP100. Like EGFR-GEP100 binding, this association is primarily mediated by the pleckstrin homology (PH) domain of GEP100 and Tyr1139/Tyr1196 of Her2. Tyr1139/Tyr1196 are autonomously phosphorylated, when Her2 is overexpressed. Accordingly,   invasive   activities mediated by the Her2-GEP100 pathway are not dependent on external factors. Blocking Her2-GEP100 binding, as well as its signaling pathway all inhibit cancer   invasive   activities. Moreover, our clinical study indicates that co-overexpression of Her2 with GEP100 in primary lung adenocarcinomas of patients is correlated with the presence of their node-metastasis with a statistical significance. Since the GEP100 PH domain interacts with both Her2 and EGFR, targeting this domain may provide novel cancer therapeutics. 
 GEP100-Arf6-AMAP1-cortactin pathway frequently used in cancer invasion is activated by VEGFR2 to promote angiogenesis. Angiogenesis and cancer   invasive  ness greatly contribute to cancer malignancy.Arf6 and its effector, AMAP1, are frequently overexpressed in breast cancer, and constitute a central pathway to induce the invasion and metastasis. In this pathway, Arf6 is activated by EGFR via GEP100. Arf6 is highly expressed also in human umbilical vein endothelial cells (HUVECs) and is implicated in angiogenesis. Here, we found that HUVECs also highly express AMAP1, and that vascular endothelial growth factor receptor-2 (VEGFR2) recruits GEP100 to activate Arf6. AMAP1 functions by binding to cortactin in cancer invasion and metastasis. We demonstrate that the same GEP100-Arf6-AMAP1-cortactin pathway is essential for angiogenesis activities, including cell migration and tubular formation, as well as for the enhancement of cell permeability and VE-cadherin endocytosis of VEGF-stimulated HUVECs. Components of this pathway are highly expressed in pathologic angiogenesis, and blocking of this pathway effectively inhibits VEGF- or tumor-induced angiogenesis and choroidal neovascularization. The GEP100-Arf6-AMAP1-cortactin pathway, activated by receptor tyrosine kinases, appears to be common in angiogenesis and cancer invasion and metastasis, and provides their new therapeutic targets. 
  GeneRIFs  
 Results indicate that GEP100 links EGFR signalling to Arf6 activation to induce   invasive   activities of some breast cancer cells, and hence may contribute to their metastasis and malignancy. 
  [Mm]etastasis  PubMed  
 GEP100 links epidermal growth factor receptor signalling to Arf6 activation to induce breast cancer invasion. Epidermal growth factor (EGF) receptor (EGFR) signalling is implicated in tumour invasion and   metastasis  . However, whether there are EGFR signalling pathways specifically used for tumour invasion still remains elusive. Overexpression of Arf6 and its effector, AMAP1, correlates with and is crucial for the invasive phenotypes of different breast cancer cells. Here we identify the mechanism by which Arf6 is activated to induce tumour invasion. We found that GEP100/BRAG2, a guanine nucleotide exchanging factor (GEF) for Arf6, is responsible for the invasive activity of MDA-MB-231 breast cancer cells, whereas the other ArfGEFs are not. GEP100, through its pleckstrin homology domain, bound directly to Tyr1068/1086-phosphorylated EGFR to activate Arf6. Overexpression of GEP100, together with Arf6, caused non-invasive MCF7 cells to become invasive, which was dependent on EGF stimulation. Moreover, GEP100 knockdown blocked tumour   metastasis  . GEP100 was expressed in 70% of primary breast ductal carcinomas, and was preferentially co-expressed with EGFR in the malignant cases. Our results indicate that GEP100 links EGFR signalling to Arf6 activation to induce invasive activities of some breast cancer cells, and hence may contribute to their   metastasis   and malignancy. 
 GEP100 links epidermal growth factor receptor signalling to Arf6 activation to induce breast cancer invasion. Epidermal growth factor (EGF) receptor (EGFR) signalling is implicated in tumour invasion and   metastasis  . However, whether there are EGFR signalling pathways specifically used for tumour invasion still remains elusive. Overexpression of Arf6 and its effector, AMAP1, correlates with and is crucial for the invasive phenotypes of different breast cancer cells. Here we identify the mechanism by which Arf6 is activated to induce tumour invasion. We found that GEP100/BRAG2, a guanine nucleotide exchanging factor (GEF) for Arf6, is responsible for the invasive activity of MDA-MB-231 breast cancer cells, whereas the other ArfGEFs are not. GEP100, through its pleckstrin homology domain, bound directly to Tyr1068/1086-phosphorylated EGFR to activate Arf6. Overexpression of GEP100, together with Arf6, caused non-invasive MCF7 cells to become invasive, which was dependent on EGF stimulation. Moreover, GEP100 knockdown blocked tumour   metastasis  . GEP100 was expressed in 70% of primary breast ductal carcinomas, and was preferentially co-expressed with EGFR in the malignant cases. Our results indicate that GEP100 links EGFR signalling to Arf6 activation to induce invasive activities of some breast cancer cells, and hence may contribute to their   metastasis   and malignancy. 
 Down-regulation of GEP100 causes increase in E-cadherin levels and inhibits pancreatic cancer cell invasion. Invasion and   metastasis   are major reasons for pancreatic cancer death and identifying signaling molecules that are specifically used in tumor invasion is of great significance. The purpose of this study was to elucidate the role of GEP100 in pancreatic cancer cell invasion and   metastasis   and the corresponding molecular mechanism.Stable cell lines with GEP100 knocked-down were established by transfecting GEP100 shRNA vector into PaTu8988 cells and selected by puromycin. qRT-PCR and Western blot were performed to detect gene expression. Matrigel-invasion assay was used to detect cancer cell invasion in vitro. Liver   metastasis   in vivo was determined by splenic injection of indicated cell lines followed by spleen resection. Immunofluorescence study was used to detect the intracellular localization of E-cadherin.We found that the expression level of GEP100 protein was closely related to the invasive ability of a panel of 6 different human pancreatic cancer cell lines. Down-regulation of GEP100 in PaTu8988 cells significantly decreased invasive activity by Matrigel invasion assay, without affecting migration, invasion and viability. The inhibited invasive activity was rescued by over-expression of GEP100 cDNA. In vivo study showed that liver   metastasis   was significantly decreased in the PaTu8988 cells with GEP100 stably knocked-down. In addition, an epithelial-like morphological change, mimicking a mesenchymal to epithelial transition (MET) was induced by GEP100 down-regulation. The expression of E-cadherin protein was increased 2-3 folds accompanied by its redistribution to the cell-cell contacts, while no obvious changes were observed for E-cadherin mRNA. Unexpectedly, the mRNA of Slug was increased by GEP100 knock-down.These findings provided important evidence that GEP100 plays a significant role in pancreatic cancer invasion through regulating the expression of E-cadherin and the process of MET, indicating the possibility of it becoming a potential therapeutic target against pancreatic cancer. 
 GEP100/Arf6 is required for epidermal growth factor-induced ERK/Rac1 signaling and cell migration in human hepatoma HepG2 cells. Epidermal growth factor (EGF) signaling is implicated in the invasion and   metastasis   of hepatoma cells. However, the signaling pathways for EGF-induced motility of hepatoma cells remain undefined.We found that EGF dose-dependently stimulated the migration of human hepatoma cells HepG2, with the maximal effect at 10 ng/mL. Additionally, EGF increased Arf6 activity, and ectopic expression of Arf6 T27N, a dominant negative Arf6 mutant, largely abolish EGF-induced cell migration. Blocking GEP100 with GEP100 siRNA or GEP100-△PH, a pleckstrin homology (PH) domain deletion mutant of GEP100, blocked EGF-induced Arf6 activity and cell migration. EGF also increased ERK and Rac1 activity. Ectopic expression GEP100 siRNA, GEP100-△PH, or Arf6-T27N suppressed EGF-induced ERK and Rac1 activity. Furthermore, blocking ERK signaling with its inhibitor U0126 remarkably inhibited both EGF-induced Rac1 activation as well as cell migration, and ectopic expression of inactive mutant form of Rac1 (Rac1-T17N) also largely abolished EGF-induced cell migration.Taken together, this study highlights the function of the PH domain of GEP100 and its regulated Arf6/ERK/Rac1 signaling cascade in EGF-induced hepatoma cell migration. These findings could provide a rationale for designing new therapy based on inhibition of hepat  oma metast  asis. 
 GEP100 regulates epidermal growth factor-induced MDA-MB-231 breast cancer cell invasion through the activation of Arf6/ERK/uPAR signaling pathway. GEP100, a guanine nucleotide exchanging factor (GEF) for Arf6, plays a pivotal role in promoting breast cancer cell invasion both in vitro and in vivo. However, the precise mechanism for GEP100-mediated cell invasion is still poorly understood. In this study, we found that down-regulation of endogenous GEP100 in MDA-MB-231 cells significantly inhibited EGF-induced cell invasion, which was rescued by over-expression of ectopic GEP100. EGF increased Arf6 activity, ERK phosphorylation, and uPAR expression in a time dependent manner. Additionally, blocking Arf6 with Arf6 siRNA largely abolished EGF-induced cell invasion. GEP100 siRNA or Arf6 siRNA suppressed EGF-induced ERK activity and uPAR expression. Furthermore, blocking ERK signaling with U0126, a specific inhibitor for MEK, markedly inhibited EGF-induced uPAR expression and consequently cell invasion. Inhibition of uPAR expression by uPAR siRNA also significantly abolished EGF-induced cell invasion. Taken together, this study illustrates that GEP100 regulates an Arf6/ERK/uPAR signaling cascade in EGF-induced breast cancer cell invasion. These findings could provide a rationale for designing new therapies based on inhibition of breast cancer   metastasis  . 
 Co-overexpression of GEP100 and AMAP1 proteins correlates with rapid local recurrence after breast conservative therapy. A major problem of current cancer research and therapy is prediction of tumor recurrence after initial treatment, rather than the simple biological characterization of the malignancy and proliferative properties of tumors. Breast conservation therapy (BCT) is a well-approved, standard treatment for patients with early stages of breast cancer, which consists of lumpectomy and whole-breast irradiation. In spite of extensive studies, only 'age' and 'Ki-67 positivity' have been identified to be well correlated with local recurrence after BCT. An Arf6 pathway, activated by GEP100 under receptor tyrosine kinases (RTKs) and employs AMAP1 as its effector, is crucial for invasion and   metastasis   of some breast cancer cells. This pathway activates β1 integrins and perturbs E-cadherin-based adhesions, hence appears to be integral for epithelial-mesenchymal transdifferentiation (EMT). We here show that expression of the Arf6 pathway components statistically correlates with rapid local recurrence after BCT. We retrospectively analyzed four hundred seventy-nine patients who received BCT in Hokkaido University Hospital, and found 20 patients had local recurrence. We then analyzed pathological samples of patients who experienced local recurrence by use of Kaplan-Meier analysis, Stepwise regression analysis and the t-test, coupled with immunostaining, and found that co-overexpression of GEP100 and AMAP1 correlates with rapidity of the local recurrence. Their margin-status, node-positivity, and estrogen receptor (ER)- or progesterone receptor (PgR)-positivity did not correlated with the rapidity. This study is the first to show that expression of a certain set of proteins correlates with the rapidity of local recurrence. Our results are useful not only for prediction, but highlight the possibility of developing novel strategies to block local recurrence. We also discuss why mRNAs encoding these proteins have not been identified to correlate with local recurrence by previous conventional gene expression profiling analyses. 
 Engagement of overexpressed Her2 with GEP100 induces autonomous invasive activities and provides a biomarker for metastases of lung adenocarcinoma. Overexpression of Her2/ErbB2/Neu in cancer is often correlated with recurrent distant   metastasis  , although the mechanism still remains largely elusive. We have previously shown that EGFR, when tyrosine-phosphorylated, binds to GEP100/BRAG2 to activate Arf6, which induces cancer invasion and   metastasis  . We now show that overexpressed Her2 in lung adenocarcinoma cells also employs GEP100. Like EGFR-GEP100 binding, this association is primarily mediated by the pleckstrin homology (PH) domain of GEP100 and Tyr1139/Tyr1196 of Her2. Tyr1139/Tyr1196 are autonomously phosphorylated, when Her2 is overexpressed. Accordingly, invasive activities mediated by the Her2-GEP100 pathway are not dependent on external factors. Blocking Her2-GEP100 binding, as well as its signaling pathway all inhibit cancer invasive activities. Moreover, our clinical study indicates that co-overexpression of Her2 with GEP100 in primary lung adenocarcinomas of patients is correlated with the presence of their node-  metastasis   with a statistical significance. Since the GEP100 PH domain interacts with both Her2 and EGFR, targeting this domain may provide novel cancer therapeutics. 
 GEP100-Arf6-AMAP1-cortactin pathway frequently used in cancer invasion is activated by VEGFR2 to promote angiogenesis. Angiogenesis and cancer invasiveness greatly contribute to cancer malignancy.Arf6 and its effector, AMAP1, are frequently overexpressed in breast cancer, and constitute a central pathway to induce the invasion and   metastasis  . In this pathway, Arf6 is activated by EGFR via GEP100. Arf6 is highly expressed also in human umbilical vein endothelial cells (HUVECs) and is implicated in angiogenesis. Here, we found that HUVECs also highly express AMAP1, and that vascular endothelial growth factor receptor-2 (VEGFR2) recruits GEP100 to activate Arf6. AMAP1 functions by binding to cortactin in cancer invasion and   metastasis  . We demonstrate that the same GEP100-Arf6-AMAP1-cortactin pathway is essential for angiogenesis activities, including cell migration and tubular formation, as well as for the enhancement of cell permeability and VE-cadherin endocytosis of VEGF-stimulated HUVECs. Components of this pathway are highly expressed in pathologic angiogenesis, and blocking of this pathway effectively inhibits VEGF- or tumor-induced angiogenesis and choroidal neovascularization. The GEP100-Arf6-AMAP1-cortactin pathway, activated by receptor tyrosine kinases, appears to be common in angiogenesis and cancer invasion and   metastasis  , and provides their new therapeutic targets. 
  GeneRIFs  
 Data suggest that GEP100-Arf6-AMAP1-cortactin pathway, activated by VEGFR2, appears to be common in angiogenesis and cancer invasion and   metastasis  , and provides their new therapeutic targets. 
 Cinical study indicates that co-overexpression of Her2 with GEP100 in primary lung adenocarcinomas of patients is correlated with the presence of their node-  metastasis   with a statistical significance. 
 Results indicate that GEP100 links EGFR signalling to Arf6 activation to induce invasive activities of some breast cancer cells, and hence may contribute to their   metastasis   and malignancy. 
  [Cc]ell [Ii]nvasion  PubMed  
 Down-regulation of GEP100 causes increase in E-cadherin levels and inhibits pancreatic cancer   cell invasion  . Invasion and metastasis are major reasons for pancreatic cancer death and identifying signaling molecules that are specifically used in tumor invasion is of great significance. The purpose of this study was to elucidate the role of GEP100 in pancreatic cancer   cell invasion   and metastasis and the corresponding molecular mechanism.Stable cell lines with GEP100 knocked-down were established by transfecting GEP100 shRNA vector into PaTu8988 cells and selected by puromycin. qRT-PCR and Western blot were performed to detect gene expression. Matrigel-invasion assay was used to detect cancer   cell invasion   in vitro. Liver metastasis in vivo was determined by splenic injection of indicated cell lines followed by spleen resection. Immunofluorescence study was used to detect the intracellular localization of E-cadherin.We found that the expression level of GEP100 protein was closely related to the invasive ability of a panel of 6 different human pancreatic cancer cell lines. Down-regulation of GEP100 in PaTu8988 cells significantly decreased invasive activity by Matrigel invasion assay, without affecting migration, invasion and viability. The inhibited invasive activity was rescued by over-expression of GEP100 cDNA. In vivo study showed that liver metastasis was significantly decreased in the PaTu8988 cells with GEP100 stably knocked-down. In addition, an epithelial-like morphological change, mimicking a mesenchymal to epithelial transition (MET) was induced by GEP100 down-regulation. The expression of E-cadherin protein was increased 2-3 folds accompanied by its redistribution to the cell-cell contacts, while no obvious changes were observed for E-cadherin mRNA. Unexpectedly, the mRNA of Slug was increased by GEP100 knock-down.These findings provided important evidence that GEP100 plays a significant role in pancreatic cancer invasion through regulating the expression of E-cadherin and the process of MET, indicating the possibility of it becoming a potential therapeutic target against pancreatic cancer. 
 The pleckstrin homology (PH) domain of the Arf exchange factor Brag2 is an allosteric binding site. Brag2, a Sec7 domain (sec7d)-containing guanine nucleotide exchange factor, regulates cell adhesion and tumor   cell invasion  . Brag2 catalyzes nucleotide exchange, converting Arf·GDP to Arf·GTP. Brag2 contains a pleckstrin homology (PH) domain, and its nucleotide exchange activity is stimulated by phosphatidylinositol 4,5-bisphosphate (PIP(2)). Here we determined kinetic parameters for Brag2 and examined the basis for regulation by phosphoinositides. Using myristoylated Arf1·GDP as a substrate, the k(cat) was 1.8 ± 0.1/s as determined by single turnover kinetics, and the K(m) was 0.20 ± 0.07 μm as determined by substrate saturation kinetics. PIP(2) decreased the K(m) and increased the k(cat) of the reaction. The effect of PIP(2) required the PH domain of Brag2 and the N terminus of Arf and was largely independent of Arf myristoylation. Structural analysis indicated that the linker between the sec7d and the PH domain in Brag2 may directly contact Arf. In support, we found that a Brag2 fragment containing the sec7d and the linker was more active than sec7d alone. We conclude that Brag2 is allosterically regulated by PIP(2) binding to the PH domain and that activity depends on the interdomain linker. Thus, the PH domain and the interdomain linker of Brag2 may be targets for selectively regulating the activity of Brag2. 
 GEP100 regulates epidermal growth factor-induced MDA-MB-231 breast cancer   cell invasion   through the activation of Arf6/ERK/uPAR signaling pathway. GEP100, a guanine nucleotide exchanging factor (GEF) for Arf6, plays a pivotal role in promoting breast cancer   cell invasion   both in vitro and in vivo. However, the precise mechanism for GEP100-mediated   cell invasion   is still poorly understood. In this study, we found that down-regulation of endogenous GEP100 in MDA-MB-231 cells significantly inhibited EGF-induced   cell invasion  , which was rescued by over-expression of ectopic GEP100. EGF increased Arf6 activity, ERK phosphorylation, and uPAR expression in a time dependent manner. Additionally, blocking Arf6 with Arf6 siRNA largely abolished EGF-induced   cell invasion  . GEP100 siRNA or Arf6 siRNA suppressed EGF-induced ERK activity and uPAR expression. Furthermore, blocking ERK signaling with U0126, a specific inhibitor for MEK, markedly inhibited EGF-induced uPAR expression and consequently   cell invasion  . Inhibition of uPAR expression by uPAR siRNA also significantly abolished EGF-induced   cell invasion  . Taken together, this study illustrates that GEP100 regulates an Arf6/ERK/uPAR signaling cascade in EGF-induced breast cancer   cell invasion  . These findings could provide a rationale for designing new therapies based on inhibition of breast cancer metastasis. 
  GeneRIFs  
 GEP100 regulates an Arf6/ERK/uPAR signaling cascade in EGF-induced breast cancer   cell invasion  . 
   
  
 DNAJC3 
 
  NCBI Gene  
  GeneRIF  
  Pubmed  
 
 
  Search Term  Rank  Fields  
  [Ee]xtracellular [Mm]atrix  8  PubMed(1)  
 
 
 hide/show details for DNAJC3 
  [Ee]xtracellular [Mm]atrix  PubMed  
 Proteomic analysis of human parotid gland exosomes by multidimensional protein identification technology (MudPIT). Human ductal saliva contributes over a thousand unique proteins to whole oral fluids. The mechanism by which most of these proteins are secreted by salivary glands remains to be determined. The present study used a mass spectrometry-based, shotgun proteomics approach to explore the possibility that a subset of the proteins found in saliva are derived from exosomes, membrane-bound vesicles of endosomal origin within multivesicular endosomes. Using MudPIT (multidimensional protein identification technology) mass spectrometry, we catalogued 491 proteins in the exosome fraction of human parotid saliva. Many of these proteins were previously observed in ductal saliva from parotid glands (265 proteins). Furthermore, 72 of the proteins in parotid exosomes overlap with those previously identified as urinary exosome proteins, proteins which are also frequently associated with exosomes from other tissues and cell types. Gene Ontology (GO) and KEGG pathway analyses found that cytosolic proteins comprise the largest category of proteins in parotid exosomes (43%), involved in such processes as phosphatidylinositol signaling system, calcium signaling pathway, inositol metabolism, protein export, and signal transduction, among others; whereas the integral plasma membrane proteins and associated/peripheral plasma membrane proteins (26%) were associated with   extracellular matrix  -receptor interaction, epithelial cell signaling, T-cell and B-cell receptor signaling, cytokine receptor interaction, and antigen processing and presentation, among other biological functions. In addition, these putative saliva exosomal proteins were linked to specific diseases (e.g., neurodegenerative disorders, prion disease, cancers, type I and II diabetes). Consequently, parotid glands secrete exosomes that reflect the metabolic and functional status of the gland and may also carry informative protein markers useful in the diagnosis and treatment of systemic diseases. 
   
  
 SIL1 
 
  NCBI Gene  
  GeneRIF  
  Pubmed  
 
 
  Search Term  Rank  Fields  
  [Mm]igration  6  PubMed(1); GeneRIFs(1)  
 
 
 hide/show details for SIL1 
  [Mm]igration  PubMed  
 SIL1, a causative cochaperone gene of Marinesco-Söjgren syndrome, plays an essential role in establishing the architecture of the developing cerebral cortex. Marinesco-Sjögren syndrome (MSS) is a rare autosomal recessively inherited disorder with mental retardation (MR). Recently, mutations in the SIL1 gene, encoding a co-chaperone which regulates the chaperone HSPA5, were identified as a major cause of MSS. We here examined the pathophysiological significance of SIL1 mutations in abnormal corticogenesis of MSS. SIL1-silencing caused neurona  l migrati  on delay during corticogenesis ex vivo. While RNAi-resistant SIL1 rescued the defects, three MSS-causing SIL1 mutants tested did not. These mutants had lower affinities to HSPA5 in vitro, and SIL1-HSPA5 interaction as well as HSPA5 function was found to be crucial for neurona  l migrati  on ex vivo. Furthermore time-lapse imaging revealed morphological disorganization associated with abnorma  l migrati  on of SIL1-deficient neurons. These results suggest that the mutations prevent SIL1 from interacting with and regulating HSPA5, leading to abnormal neuronal morphology an  d migrati  on. Consistent with this, when SIL1 was silenced in cortical neurons in one hemisphere, axonal growth in the contralateral hemisphere was delayed. Taken together, abnormal neurona  l migrati  on and interhemispheric axon development may contribute to MR in MSS. 
  GeneRIFs  
 The mutations prevent SIL1 from interacting with and regulating HSPA5, leading to abnormal neuronal morphology and   migration  . 
   
  
 PABPN1 
 
  NCBI Gene  
  GeneRIF  
  Pubmed  
 
 
  Search Term  Rank  Fields  
  [Ii]nvasive  10  PubMed(1)  
 
 
 hide/show details for PABPN1 
  [Ii]nvasive  PubMed  
 Oculopharyngeal muscular dystrophy: phenotypic and genotypic studies in a UK population. Oculopharyngeal muscular dystrophy (OPMD) is an autosomal dominant disorder of late onset that commonly presents with ptosis and dysphagia. The genetic basis of the condition has been identified recently as a stable trinucleotide repeat expansion in exon 1 of the poly(A) binding protein 2 gene (PABP2), in which (GCG)(6) is the normal repeat length. The prevalence of OPMD is greatest in patients of French-Canadian origin. It is not clear if expansion repeat length is a reliable test in other populations. In this study, we analysed the phenotypic and genotypic characteristics of 31 patients with OPMD in the UK. Ptosis was the first reported symptom in two-thirds of the patients, and half of the subjects studied had evidence of ophthalmoplegia. All but one family had a pathological expansion in the PABP2 gene, ranging from (GCG)(8) to (GCG)(13). In contrast to the French-Canadian population, (GCG)(10) was almost as common as (GCG)(9), evidence against a strong founder effect in the UK population. There was a weak association between repeat length and age of disease onset. Patients with longer repeat lengths, such as (GCG)(13), developed severe limb weakness early in the disease. We were unable to detect the (GCG)(7) polymorphism in over 200 normal controls, suggesting that the frequency of this expansion is lower than that found in the French-Canadian population. One family was negative for the expansion. Affected members presented with the classical features of OPMD, namely ptosis, dysphagia and cytoplasmic inclusions on muscle biopsy, although with some atypical features, such as early age of onset, high serum levels of creatine kinase and a profound ophthalmoplegia. This family is an example of a GCG expansion-negative oculopharyngeal syndrome requiring further genetic investigation. We conclude that PABP2 analysis is a reliable non-  invasive   diagnostic test for OPMD in the UK population. 
   
  
 CAPZA1 
 
  NCBI Gene  
  GeneRIF  
  Pubmed  
 
 
  Search Term  Rank  Fields  
  [Mm]igration  6  PubMed(2); GeneRIFs(1)  
  [Mm]etastasis  12  PubMed(2)  
 
 
 hide/show details for CAPZA1 
  [Mm]igration  PubMed  
 Prognostic value of CAPZA1 overexpression in gastric cancer. F-actin capping protein α1 subunit (CAPZA1) was previously identified in a proteomic analysis of human gastric cancer clinical specimens and selected for further study. The association between CAPZA1 overexpression, detected by immunohistochemistry, and clinicopathological features including survival were evaluated. In vitro gain-of-function and loss-of-function approaches were utilized to assess the function of CPAZA1 in malignancy. Univariate analysis revealed that poorly differentiated disease, according to the World Health Organization (WHO) classification, advanced T stage, positive lymph nodes, high TNM stage, D2 lymph node dissection, adjuvant chemotherapy and CAPZA1 underexpression were significantly associated with cancer-related death (p<0.05); however, only high TNM stage remained significantly associated by multivariate analysis (p<0.01). CAPZA1 overexpression was associated with well differentiated histology, smaller tumor size, lower T stage, absence of lymph node metastasis, lower TNM stage, lower recurrence rate and longer survival time, compared to CAPZA1 underexpression. In vitro, forced expression of CAPZA1 caused a significant decrease in gastric cancer cell   migratio  n and invasion, whereas CAPZA1 depletion had the opposite effect. The present study suggests that CAPZA1 could be a marker of good prognosis in gastric cancer and shows that CAPZA1 is associated with decreased cancer cell   migratio  n and invasion. 
 Mammalian CARMIL inhibits actin filament capping by capping protein. Actin polymerization in cells occurs via filament elongation at the barbed end. Proteins that cap the barbed end terminate this elongation. Heterodimeric capping protein (CP) is an abundant and ubiquitous protein that caps the barbed end. We find that the mouse homolog of the adaptor protein CARMIL (mCARMIL) binds CP with high affinity and decreases its affinity for the barbed end. Addition of mCARMIL to cell extracts increases the rate and extent of Arp2/3 or spectrin-actin seed-induced polymerization. In cells, GFP-mCARMIL concentrates in lamellipodia and increases the fraction of cells with large lamellipodia. Decreasing mCARMIL levels by siRNA transfection lowers the F-actin level and slows cell   migration   through a mechanism that includes decreased lamellipodia protrusion. This phenotype is reversed by full-length mCARMIL but not mCARMIL lacking the domain that binds CP. Thus, mCARMIL is a key regulator of CP and has profound effects on cell behavior. 
  GeneRIFs  
 The present study suggests that CAPZA1 could be a marker of good prognosis in gastric cancer and shows that CAPZA1 is associated with decreased cancer cell   migration   and invasion. 
  [Mm]etastasis  PubMed  
 Differential expression patterns of capping protein, protein phosphatase 1, and casein kinase 1 may serve as diagnostic markers for malignant melanoma. Early and accurate diagnosis of malignant melanoma is critical for patient survival. However, currently used diagnostic markers are insufficiently specific, which limits their utility. We aimed to identify molecular markers that are more specific to malignant melanoma, thereby aiding in melanoma diagnosis and treatment. A PCR-based suppression subtractive hybridization was used to identify capping protein Z-line α1, protein phosphatase 1 catalytic subunit β isoform (PP1CB), and casein kinase 1 α1 (CSNK1A1) as being differentially expressed between melanoma cells and normal melanocytes. Quantitative reverse transcription-PCR and western blot analysis confirmed that these genes were overexpressed in melanoma cells. In addition, immunohistochemical assays revealed that the expression of PP1CB and CSNK1A1 was significantly greater in human melanoma specimens than nevi (P<0.0001). Combined application of PP1CB and CSNK1A showed high sensitivity and specificity for melanoma. Thus, our data suggest that PP1CB and CSNK1A1 are potential biomarkers for distinguishing malignant melanoma from other melanocytic lesions. In addition, because capping protein Z-line α1, PP1CB, and CSNK1A1 are involved in cell motility, which underlies invasion   and metast  asis of human cancer; they may be novel targets for antimetastatic therapies as well. 
 Prognostic value of CAPZA1 overexpression in gastric cancer. F-actin capping protein α1 subunit (CAPZA1) was previously identified in a proteomic analysis of human gastric cancer clinical specimens and selected for further study. The association between CAPZA1 overexpression, detected by immunohistochemistry, and clinicopathological features including survival were evaluated. In vitro gain-of-function and loss-of-function approaches were utilized to assess the function of CPAZA1 in malignancy. Univariate analysis revealed that poorly differentiated disease, according to the World Health Organization (WHO) classification, advanced T stage, positive lymph nodes, high TNM stage, D2 lymph node dissection, adjuvant chemotherapy and CAPZA1 underexpression were significantly associated with cancer-related death (p<0.05); however, only high TNM stage remained significantly associated by multivariate analysis (p<0.01). CAPZA1 overexpression was associated with well differentiated histology, smaller tumor size, lower T stage, absence of lymph node   metastasi  s, lower TNM stage, lower recurrence rate and longer survival time, compared to CAPZA1 underexpression. In vitro, forced expression of CAPZA1 caused a significant decrease in gastric cancer cell migration and invasion, whereas CAPZA1 depletion had the opposite effect. The present study suggests that CAPZA1 could be a marker of good prognosis in gastric cancer and shows that CAPZA1 is associated with decreased cancer cell migration and invasion. 
   
  
 CAPZA2 
 
  NCBI Gene  
  GeneRIF  
  Pubmed  
 
 
  Search Term  Rank  Fields  
  [Mm]igration  6  PubMed(1)  
 
 
 hide/show details for CAPZA2 
  [Mm]igration  PubMed  
 Mammalian CARMIL inhibits actin filament capping by capping protein. Actin polymerization in cells occurs via filament elongation at the barbed end. Proteins that cap the barbed end terminate this elongation. Heterodimeric capping protein (CP) is an abundant and ubiquitous protein that caps the barbed end. We find that the mouse homolog of the adaptor protein CARMIL (mCARMIL) binds CP with high affinity and decreases its affinity for the barbed end. Addition of mCARMIL to cell extracts increases the rate and extent of Arp2/3 or spectrin-actin seed-induced polymerization. In cells, GFP-mCARMIL concentrates in lamellipodia and increases the fraction of cells with large lamellipodia. Decreasing mCARMIL levels by siRNA transfection lowers the F-actin level and slows cell   migration   through a mechanism that includes decreased lamellipodia protrusion. This phenotype is reversed by full-length mCARMIL but not mCARMIL lacking the domain that binds CP. Thus, mCARMIL is a key regulator of CP and has profound effects on cell behavior. 
   
  
 HMGCR 
 
  NCBI Gene  
  GeneRIF  
  Pubmed  
 
 
  Search Term  Rank  Fields  
  [Mm]igration  6  PubMed(5); GeneRIFs(2)  
  [Ii]nvasive  10  PubMed(3)  
  [Mm]etastasis  12  PubMed(2)  
 
 
 hide/show details for HMGCR 
  [Mm]igration  PubMed  
 Artemisinic acid inhibits melanogenesis through downregulation of C/EBP α-dependent expression of HMG-CoA reductase gene. Cholesterol is associated with the regulation of melanogenesis which is the major physiological defense against solar irradiation. The present study was designed to determine the effects of artemisinic acid on melanogenesis and its mechanisms of action in human epidermal melanocytes. In this study, we found that artemisinic acid inhibited melanin content. The mRNA levels of microphthalmia-associated transcription factor (MITF) and its downstream genes tyrosinase, tyrosinase-related protein (TRP)-1, and TRP-2 were reduced by artemisinic acid treatment. Additionally, the mRNA levels of melanogenesis-related genes (c-KIT, stem cell factor (SCF), and macrophage   migratio  n inhibitory factor (MIF)) were down-regulated by artemisinic acid. Furthermore, cAMP production and protein kinase A (PKA) activity were suppressed by artemisinic acid. Moreover, attempts to elucidate a possible mechanism underlying the artemisinic acid-mediated effects revealed that artemisinic acid regulated melanogenesis by inhibiting cholesterol synthesis through downregulation of the hydroxymethylglutaryl CoA (HMG CoA) reductase gene, which was mediated through reduced expression of the CCAAT/enhancer-binding protein (C/EBP) α gene. Taken together, these findings indicate that the inhibition of melanogenesis by artemisinic acid occurs through reduced expression of the HMG CoA reductase gene, which is mediated by C/EBP α inhibition and suggest that artemisinic acid may be useful as a hyperpigmentation inhibitor. 
 HMGCR is necessary for the tumorigenecity of esophageal squamous cell carcinoma and is regulated by Myc. Hydroxymethylglutaryl coenzyme A reductase (HMGCR), the rate-limiting enzyme of mevalonate pathway, has been involved in the tumorigenesis of several tumor types. Our previous study has showed that statin, the inhibitor of HMGCR, inhibited the tumorigenecity of esophageal squamous cell carcinoma (ESCC) in vitro and in vivo. However, the function of HMGCR in the carcinogenesis of ESCC cells remains unknown. In this study, we have observed the up-regulation of HMGCR in ESCC tissues compared with the paired normal tissues. Over-expression of HMGCR in ESCC cells promoted cell growth and   migration  , while knockdown of the expression of HMGCR inhibited the growth,   migration   and colony formation of ESCC cells in vitro and in vivo. Furthermore, we found that oncogene Myc positively regulated the expression of HMGCR. Taken together, our study revealed the pivotal function of HMGCR and mevalonate pathway in the progression of ESCC and supported the clinical application of statin. 
 HMGCR positively regulated the growth and   migration   of glioblastoma cells. The metabolic program of cancer cells is significant different from the normal cells, which makes it possible to develop novel strategies targeting cancer cells. Mevalonate pathway and its rate-limiting enzyme HMG-CoA reductase (HMGCR) have shown important roles in the progression of several cancer types. However, their roles in glioblastoma cells remain unknown. In this study, up-regulation of HMGCR in the clinical glioblastoma samples was observed. Forced expression of HMGCR promoted the growth and   migration   of U251 and U373 cells, while knocking down the expression of HMGCR inhibited the growth,   migration   and metastasis of glioblastoma cells. Molecular mechanism studies revealed that HMGCR positively regulated the expression of TAZ, an important mediator of Hippo pathway, and the downstream target gene connective tissue growth factor (CTGF), suggesting HMGCR might activate Hippo pathway in glioblastoma cells. Taken together, our study demonstrated the oncogenic roles of HMGCR in glioblastoma cells and HMGCR might be a promising therapeutic target. 
 HMGCR is up-regulated in gastric cancer and promotes the growth and   migration   of the cancer cells. Alteration of metabolic profile is one of the hallmarks of cancer cells. Statin, the inhibitors for synthesis of cholesterol, has shown anti-cancer effects on the gastric cancer cells. However, the functions of its target, HMGCR, in the progression of gastric cancer remain unknown. In the present study, we investigated the expression profile and the biological functions of HMGCR in gastric cancer. It was found that the expression of HMGCR was increased in gastric cancer tissues. Over-expression of HMGCR promoted the growth and   migration   of gastric cancer cells, while knocking down the expression of HMGCR inhibited the growth,   migration   and tumorigenesis of gastric cancer cells. In the further molecular mechanism study, HMGCR was shown to activate Hedgehog/Gli1 signaling and promoted the expression of Gli1 target genes. Taken together, this study demonstrated the tumor-promoting effects of HMGCR in gastric cancer and suggested HMGCR as a promising therapeutic target. 
 HMG-CoA reductase guides migrating primordial germ cells. The enzyme 3-hydroxy-3-methylglutaryl coenzyme A (HMG-CoA) reductase is best known for catalysing a rate-limiting step in cholesterol biosynthesis, but it also participates in the production of a wide variety of other compounds. Some clinical benefits attributed to inhibitors of HMG-CoA reductase are now thought to be independent of any serum cholesterol-lowering effect. Here we describe a new cholesterol-independent role for HMG-CoA reductase, in regulating a developmental process: primordial germ cell   migration  . We show that in Drosophila this enzyme is highly expressed in the somatic gonad and that it is necessary for primordial germ cells to migrate to this tissue. Misexpression of HMG-CoA reductase is sufficient to attract primordial germ cells to tissues other than the gonadal mesoderm. We conclude that the regulated expression of HMG-CoA reductase has a critical developmental function in providing spatial information to guide migrating primordial germ cells. 
  GeneRIFs  
 Gene expression profile and the biological functions of HMGCR in gastric cancer were studied. It was found that the expression of HMGCR was increased in gastric cancer tissues. Over-expression of HMGCR promoted the growth and   migration   of gastric cancer cells, while knocking down the expression of HMGCR inhibited the growth,   migration   and tumorigenesis of gastric cancer cells. 
 Over-expression of HMGCR in esophageal squamous cell carcinoma (ESCC) cells promoted cell growth and   migration  . 
  [Ii]nvasive  PubMed  
 Genetic susceptibility to distinct bladder cancer subphenotypes. Clinical, pathologic, and molecular evidence indicate that bladder cancer is heterogeneous with pathologic/molecular features that define distinct subphenotypes with different prognoses. It is conceivable that specific patterns of genetic susceptibility are associated with particular subphenotypes.To examine evidence for the contribution of germline genetic variation to bladder cancer heterogeneity.The Spanish Bladder Cancer/EPICURO Study is a case-control study based in 18 hospitals located in five areas in Spain. Cases were patients with a newly diagnosed, histologically confirmed, urothelial cell carcinoma of the bladder from 1998 to 2001. Case diagnoses were reviewed and uniformly classified by pathologists following the World Health Organisation/International Society of Urological Pathology 1999 criteria. Controls were hospital-matched patients (n=1149).A total of 1526 candidate variants in 423 candidate genes were analysed. Three distinct subphenotypes were defined according to stage and grade: low-grade nonmuscle   invasive   (n=586), high-grade nonmuscle   invasive   (n=219), and muscle   invasive   (n=246). The association between each variant and subphenotype was assessed by polytomous risk models adjusting for potential confounders. Heterogeneity in genetic susceptibility among subphenotypes was also tested.Two established bladder cancer susceptibility genotypes, NAT2 slow-acetylation and GSTM1-null, exhibited similar associations among the subphenotypes, as did VEGF-rs25648, which was previously identified in our study. Other variants conferred risks for specific tumour subphenotypes such as PMS2-rs6463524 and CD4-rs3213427 (respective heterogeneity p values of 0.006 and 0.004), which were associated with muscle-  invasive   tumours (per-allele odds ratios [95% confidence interval] of 0.56 [0.41-0.77] and 0.71 [0.57-0.88], respectively) but not with non-muscle-  invasive   tumours. Heterogeneity p values were not robust in multiple testing according to their false-discovery rate.These exploratory analyses suggest that genetic susceptibility loci might be related to the molecular/pathologic diversity of bladder cancer. Validation through large-scale replication studies and the study of additional genes and single nucleotide polymorphisms are required. 
 Genetic susceptibility to distinct bladder cancer subphenotypes. Clinical, pathologic, and molecular evidence indicate that bladder cancer is heterogeneous with pathologic/molecular features that define distinct subphenotypes with different prognoses. It is conceivable that specific patterns of genetic susceptibility are associated with particular subphenotypes.To examine evidence for the contribution of germline genetic variation to bladder cancer heterogeneity.The Spanish Bladder Cancer/EPICURO Study is a case-control study based in 18 hospitals located in five areas in Spain. Cases were patients with a newly diagnosed, histologically confirmed, urothelial cell carcinoma of the bladder from 1998 to 2001. Case diagnoses were reviewed and uniformly classified by pathologists following the World Health Organisation/International Society of Urological Pathology 1999 criteria. Controls were hospital-matched patients (n=1149).A total of 1526 candidate variants in 423 candidate genes were analysed. Three distinct subphenotypes were defined according to stage and grade: low-grade nonmuscle   invasive   (n=586), high-grade nonmuscle   invasive   (n=219), and muscle   invasive   (n=246). The association between each variant and subphenotype was assessed by polytomous risk models adjusting for potential confounders. Heterogeneity in genetic susceptibility among subphenotypes was also tested.Two established bladder cancer susceptibility genotypes, NAT2 slow-acetylation and GSTM1-null, exhibited similar associations among the subphenotypes, as did VEGF-rs25648, which was previously identified in our study. Other variants conferred risks for specific tumour subphenotypes such as PMS2-rs6463524 and CD4-rs3213427 (respective heterogeneity p values of 0.006 and 0.004), which were associated with muscle-  invasive   tumours (per-allele odds ratios [95% confidence interval] of 0.56 [0.41-0.77] and 0.71 [0.57-0.88], respectively) but not with non-muscle-  invasive   tumours. Heterogeneity p values were not robust in multiple testing according to their false-discovery rate.These exploratory analyses suggest that genetic susceptibility loci might be related to the molecular/pathologic diversity of bladder cancer. Validation through large-scale replication studies and the study of additional genes and single nucleotide polymorphisms are required. 
 The target for statins, HMG-CoA reductase, is expressed in ductal carcinoma-in situ and may predict patient response to radiotherapy. Patients with ductal carcinoma-in-situ (DCIS) are currently not prescribed adjuvant systemic treatment after surgery and radiotherapy. Prediction of DCIS patients who would benefit from radiotherapy is warranted. Statins have been suggested to exert radio-sensitizing effects. The target for cholesterol-lowering statins is HMG-CoA reductase (HMGCR), the rate-limiting enzyme in the mevalonate pathway. The aim of this study was to examine HMGCR expression in DCIS and study its treatment predictive value.A population-based cohort including 458 women diagnosed with primary DCIS between 1986 and 2004 were followed until November 2011 to study long-term survival. Tumor tissue microarrays were constructed, and immunohistochemical analyses were performed to detect cytoplasmic protein expression of HMGCR. The association between DCIS HMGCR expression and   invasive   breast cancer recurrence-free survival (RFSinv) and overall survival (OS) was analyzed by Kaplan-Meier curves, log rank test, and Cox proportional hazard analysis.HMGCR was strongly expressed in 24 % of the assessed DCIS samples, moderately expressed in 46 %, and weakly expressed in 23 %; no expression was detected in 7 % of the samples. During the follow-up time (median 13.8 years), 61 patients were diagnosed with an   invasive   breast cancer recurrence, and 80 patients died. A crude analysis showed no survival benefit from radiotherapy. However, patients with strong HMGCR expression showed an improved RFSinv (log rank, p = 0.03) and OS (log rank, p = 0.04) after radiotherapy. No statistically significant interaction was observed for HMGCR and radiotherapy (RFSinv p = 0.69 and OS p = 0.29).This study demonstrates HMGCR expression in DCIS and suggests HMGCR as a predictive marker of response to postoperative radiotherapy in DCIS, although the test for interaction was nonsignificant. Future DCIS studies addressing the potential of statin treatment targeting HMGCR are warranted. 
  [Mm]etastasis  PubMed  
 HMG-CoA reductase expression in primary colorectal cancer correlates with favourable clinicopathological characteristics and an improved clinical outcome. An association between tumor-specific HMG-CoA reductase (HMGCR) expression and good prognosis has previously been demonstrated in breast and ovarian cancer. In this study, the expression, clinicopathological correlates and prognostic value of HMGCR expression in colorectal cancer was examined.Immunohistochemical expression of HMGCR was assessed in tissue microarrays with primary tumours from 557 incident cases of colorectal cancer in the Malmö Diet and Cancer Study. Pearson's Chi Square test was applied to explore the associations between HMGCR expression and clinicopathological factors and other investigative biomarkers. Kaplan Meier analysis and Cox proportional hazards modeling were used to assess the relationship between HMGCR expression and cancer-specific survival (CSS) according to negative vs positive HMGCR expression. A total number of 535 (96.0%) tumours were suitable for analysis, of which 61 (11.4%) were HMGCR negative. Positive cytoplasmic HMGCR expression was associated with distant   metastasi  s-free disease at diagnosis (p = 0.002), lack of vascular invasion (p = 0.043), microsatellite-instability (p = 0.033), expression of cyclin D1 (p = <0.001) and p21 (p = <0.001). Positive HMGCR expression was significantly associated with a prolonged CSS in unadjusted Cox regression analysis in the entire cohort (HR = 1.79; 95% CI 1.20-2.66) and in Stage III-IV disease (HR = 1.71; 95% CI 1.09-2.68), but not after adjustment for established clinicopathological parameters.Findings from this prospective cohort study demonstrate that HMGCR is differentially expressed in colorectal cancer and that positive expression is associated with favourable tumour characteristics and a prolonged survival in unadjusted analysis. The utility of HMGCR as a predictor of response to neoadjuvant or adjuvant statin treatment in colorectal cancer merits further study.The virtual slides for this article can be found here: http://www.diagnosticpathology.diagnomx.eu/vs/2115647072103464. 
 HMGCR positively regulated the growth and migration of glioblastoma cells. The metabolic program of cancer cells is significant different from the normal cells, which makes it possible to develop novel strategies targeting cancer cells. Mevalonate pathway and its rate-limiting enzyme HMG-CoA reductase (HMGCR) have shown important roles in the progression of several cancer types. However, their roles in glioblastoma cells remain unknown. In this study, up-regulation of HMGCR in the clinical glioblastoma samples was observed. Forced expression of HMGCR promoted the growth and migration of U251 and U373 cells, while knocking down the expression of HMGCR inhibited the growth, migration and   metastasis   of glioblastoma cells. Molecular mechanism studies revealed that HMGCR positively regulated the expression of TAZ, an important mediator of Hippo pathway, and the downstream target gene connective tissue growth factor (CTGF), suggesting HMGCR might activate Hippo pathway in glioblastoma cells. Taken together, our study demonstrated the oncogenic roles of HMGCR in glioblastoma cells and HMGCR might be a promising therapeutic target. 
   
  
 CAP1 
 
  NCBI Gene  
  GeneRIF  
  Pubmed  
 
 
  Search Term  Rank  Fields  
  [Mm]igration  6  Process(1); PubMed(7)  
  [Ee]xtracellular [Mm]atrix  8  PubMed(2)  
  [Ii]nvasive  10  PubMed(1)  
  [Mm]etastasis  12  PubMed(6); GeneRIFs(3)  
 
 
 hide/show details for CAP1 
  [Mm]igration  Process  
 ameboidal-type cell   migration   
  PubMed  
 Analysis of the myosin-II-responsive focal adhesion proteome reveals a role for β-Pix in negative regulation of focal adhesion maturation. Focal adhesions undergo myosin-II-mediated maturation wherein they grow and change composition to modulate integrin signalling for cell   migratio  n, growth and differentiation. To determine how focal adhesion composition is affected by myosin II activity, we performed proteomic analysis of isolated focal adhesions and compared protein abundance in focal adhesions from cells with and without myosin II inhibition. We identified 905 focal adhesion proteins, 459 of which changed in abundance with myosin II inhibition, defining the myosin-II-responsive focal adhesion proteome. The abundance of 73% of the proteins in the myosin-II-responsive focal adhesion proteome was enhanced by contractility, including proteins involved in Rho-mediated focal adhesion maturation and endocytosis- and calpain-dependent focal adhesion disassembly. During myosin II inhibition, 27% of proteins in the myosin-II-responsive focal adhesion proteome, including proteins involved in Rac-mediated lamellipodial protrusion, were enriched in focal adhesions, establishing that focal adhesion protein recruitment is also negatively regulated by contractility. We focused on the Rac guanine nucleotide exchange factor β-Pix, documenting its role in the negative regulation of focal adhesion maturation and the promotion of lamellipodial protrusion and focal adhesion turnover to drive cel  l migrati  on. 
 Overexpression of CAP1 and its significance in tumor cell proliferation,   migration   and invasion in glioma. Adenylate cyclase-associated protein 1 (CAP1), a protein related to the regulation of actin filaments and the Ras/cAMP pathway, is associated with tumor progression. Nevertheless, the expression level and effects of CAP1 in regards to glioma have not been reported. In the present study, we examined the expression of CAP1 in glioma and tumor adjacent normal brain tissues by tissue microarray and immunohistochemistry. Our results showed that CAP1 was overexpressed in glioma tissues in comparison with that noted in the tumor adjacent normal brain tissues and increased staining of CAP1 was found to be correlated with WHO stage. In addition, we discovered that knockdown of CAP1 by specific RNA interference markedly inhibited cell growth and caused downregulation of the proliferation markers, PCNA and cyclin A. We further demonstrated that knockdown of CAP1 inhibited cell metastatic abilities by downregulating N-cadherin and vimentin and upregulating E-cadherin. These findings revealed that CAP1 expression is markedly increased in human glioma and that downregulation of CAP1 in tumors may serve as a treatment for glioma patients. 
 Downregulated expression of the cyclase-associated protein 1 (CAP1) reduces   migration   in esophageal squamous cell carcinoma. Overexpression of cyclase-associated proteins has been associated with poor prognosis in several human cancers. Cyclase-associated protein 1 is a member of the cyclase-associated proteins which contributes to tumor progression. The aim of the present study was to examine the expression of cyclase-associated protein 1 and to elucidate its clinicopathologic signiﬁcance in a larger series of esophageal squamous cell carcinoma.Immunohistochemical and western blot analyses were performed in esophageal squamous cell carcinoma tissues. Survival analyses were performed by using the Kaplan-Meier method. The role of cyclase-associated protein 1 i  n migrati  on was studied in esophageal squamous cell carcinoma cell lines of TE1 through knocking down cyclase-associated protein 1 with siRNA and overexpression of cyclase-associated protein 1. The regulation of cyclase-associated protein 1 o  n migrati  on was determined by transwell and wound-healing assays.Immunohistochemical analysis showed that cyclase-associated protein 1 expression was negatively associated with E-cadherin and signiﬁcantly associated with lymph node metastases. Survival analysis revealed that cyclase-associated protein 1 overexpression was signiﬁcantly associated with overall survival (P = 0.011). Knock down of cyclase-associated protein 1 in TE1 cells resulted in decreased vimentin and F-actin levels and the capabilit  y for mig  ration. In addition, overexpression of cyclase-associated protein 1 promote  d the mig  ration of TE1 cells.These ﬁndings suggest that cyclase-associated protein 1 is involved in the metastasis of esophageal squamous cell carcinoma, and that elevated levels of cyclase-associated protein 1 expression may indicate a poor prognosis for patients with esophageal squamous cell carcinoma. 
 High expression of adenylate cyclase-associated protein 1 accelerates the proliferation,   migration   and invasion of neural glioma cells. Adenylate cyclase-associated protein 1 (CAP1), a conserved member of cyclase-associated proteins was reported to be associated with the proliferation,   migration   or invasion of the tumors of pancreas, breast and liver, and was involved in astrocyte proliferation after acute Traumatic Brain Injury (TBI). In this study, we sought to investigate the character of CAP1 in the pathological process of human glioma by detecting human glioma specimens and cell lines. 43 of 100 specimens showed high expression of CAP1 via immunohistochemistry. With statistics analysis, we found out the expression level of CAP1 was correlated with the WHO grades of human glioma and was great positively related to Ki-67 (p<0.01). In vitro, silencing CAP1 in U251 and U87MG, the glioma cell lines with the relatively higher expression of CAP1, induced the proliferation of the cells significantly retarded,   migration   and invasion as well. Obviously, our results indicated that CAP1 participated in the molecular pathological process of glioma indeed, and in a certain sense, CAP1 might be a potential and promising molecular target for glioma diagnosis and therapies in the future. 
 Upregulated expression of CAP1 is associated with tumor   migration   and metastasis in hepatocellular carcinoma. Hepatocellular carcinoma (HCC) is one of the most common cancers that exhibits high incidences of intrahepatic metastasis and tumor recurrence. Adenylate cyclase-associated protein 1 (CAP1), a protein involved in the regulation of actin filaments, was recently reported to play a role in cell motility and the pathology of pancreatic cancer. In this study, we examined a potential role of CAP1 in HCC progression, and found that CAP1 was overexpressed in HCC specimens compared with adjacent noncancerous liver tissues by Western blot analysis and real-time PCR assay. Further, immunohistochemical analysis in 107 HCC specimens revealed that overexpression of CAP1 was closely correlated only with tumor metastasis, but not with other clinicopathologic parameters. Univariate and multivariate survival analyses showed that CAP1 could be an independent prognostic factor for patients' survival. In addition, immunofluorescent assay demonstrated that CAP1 was colocalized with actin in the leading edge of lamellipodium in HCC cells. Importantly, knocking-down the expression of CAP1 using small interfering RNA (siRNA) targeting CAP1 led to impaired   migration   of HCC cells. Collectively, our results indicated that upregulated expression of CAP1 might contribute heavily to the metastasis of HCC. 
 Overexpression of adenylate cyclase-associated protein 1 is associated with metastasis of lung cancer. Lung cancer ranks first in both prevalence and mortality rates among all types of cancer. Metastasis is the main cause of treatment failure. Biomarkers are critical to early diagnosis and prediction and monitoring of progressive lesions. Several biomarkers have been identified for lung cancer but none have been routinely used clinically. The present study assessed the diagnostic and prognostic value of cyclase-associated protein 1 (CAP1) for lung cancer. CAP1 mRNA abundance and protein content were determined by real-time PCR and western blot analysis and/or immunostaining in biopsy specimens (24 neoplastic and 6 non-neoplastic) freshly collected at surgical lung resection, in 82 pathologically banked lung cancer specimens and in cultured non-invasive (95-C) and invasive (95-D) lung cancer cells. Multivariate regression analysis was performed to correlate immunoreactive CAP1 signal with cancer type and stage. In vitro cell   migration   was performed to determine the effect of RNA interference-mediated CAP1 gene silencing on invasiveness of 95-D cells. These analyses collectively demonstrated that: i) both CAP1 mRNA abundance and protein content were significantly higher in neoplastic compared to non-neoplastic specimens and in metastatic compared to non-metastatic specimens but not different between adenocarcinoma and squamous cell carcinoma; ii) immunoreactive CAP1 signal was significantly stronger in metastatic specimens and 95-D cells compared to non-metastatic specimens and 95-C cells; and iii) RNA interference-mediated CAP1 gene silencing adequately attenuated the invasive capacity of 95-D cells in vitro. These findings suggest that overexpression of CAP1 in lung cancer cells, particularly at the metastatic stage, may have significant clinical implications as a diagnostic/prognostic factor for lung cancer. 
 Knocking down the expression of adenylate cyclase-associated protein 1 inhibits the proliferation and   migration   of breast cancer cells. Adenylate cyclase-associated protein 1 (CAP1) is a conserved protein that was found to be up-regulated in breast cancer and related to the   migration   of breast cancer. We verified its roles in breast cancer specimens and cell lines. In our results, 71 of 100 specimens of breast cancer showed high levels of CAP1 by immunohistochemistry. Associated with statistical analysis, we saw that CAP1 was related to the grade of breast cancer. In MDA-MB-231, the expression of CAP1 was the highest and by knocking down the expression of CAP1 in MDA-MB-231, its ability for proliferating and migrating apparently decreased and induced changes in morphology, which were related to the arrangement of F-actin. Therefore, CAP1 might be a potential molecular targeted therapy for surgery and immune treatment. 
  [Ee]xtracellular [Mm]atrix  PubMed  
 Proteomic analysis of human parotid gland exosomes by multidimensional protein identification technology (MudPIT). Human ductal saliva contributes over a thousand unique proteins to whole oral fluids. The mechanism by which most of these proteins are secreted by salivary glands remains to be determined. The present study used a mass spectrometry-based, shotgun proteomics approach to explore the possibility that a subset of the proteins found in saliva are derived from exosomes, membrane-bound vesicles of endosomal origin within multivesicular endosomes. Using MudPIT (multidimensional protein identification technology) mass spectrometry, we catalogued 491 proteins in the exosome fraction of human parotid saliva. Many of these proteins were previously observed in ductal saliva from parotid glands (265 proteins). Furthermore, 72 of the proteins in parotid exosomes overlap with those previously identified as urinary exosome proteins, proteins which are also frequently associated with exosomes from other tissues and cell types. Gene Ontology (GO) and KEGG pathway analyses found that cytosolic proteins comprise the largest category of proteins in parotid exosomes (43%), involved in such processes as phosphatidylinositol signaling system, calcium signaling pathway, inositol metabolism, protein export, and signal transduction, among others; whereas the integral plasma membrane proteins and associated/peripheral plasma membrane proteins (26%) were associated with   extracellular matrix  -receptor interaction, epithelial cell signaling, T-cell and B-cell receptor signaling, cytokine receptor interaction, and antigen processing and presentation, among other biological functions. In addition, these putative saliva exosomal proteins were linked to specific diseases (e.g., neurodegenerative disorders, prion disease, cancers, type I and II diabetes). Consequently, parotid glands secrete exosomes that reflect the metabolic and functional status of the gland and may also carry informative protein markers useful in the diagnosis and treatment of systemic diseases. 
 The identification of myocilin-associated proteins in the human trabecular meshwork. Myocilin forms high molecular weight complexes in vivo presumably due to interaction with itself and other myocilin binding proteins. To identify myocilin interacting proteins, yeast 2-hybrid analysis was performed on >1x10(6) human trabecular meshwork cDNA clones. Coimmunoprecipitation and Far Western analysis were also performed on cell lysates obtained from fresh human trabecular meshworks or cultured human monolayer trabecular cell lines. Among the different methods, 46 candidate myocilin-associated proteins were identified, including molecules associated with the   extracellular matrix  , cytoskeleton, signaling, and metabolism. The most consistent interaction was myocilin-myocilin binding. Yeast-2 hybrid and Far Western analysis also found an association between myocilin and glyceraldehyde-3-phosphate dehydrogenase (GAPDH). None of the other candidate myocilin interacting proteins were identified in more than one method. Characterization of these potential interacting proteins may help to better understand the function of myocilin in the trabecular meshwork and aqueous outflow pathway. 
  [Ii]nvasive  PubMed  
 Overexpression of adenylate cyclase-associated protein 1 is associated with metastasis of lung cancer. Lung cancer ranks first in both prevalence and mortality rates among all types of cancer. Metastasis is the main cause of treatment failure. Biomarkers are critical to early diagnosis and prediction and monitoring of progressive lesions. Several biomarkers have been identified for lung cancer but none have been routinely used clinically. The present study assessed the diagnostic and prognostic value of cyclase-associated protein 1 (CAP1) for lung cancer. CAP1 mRNA abundance and protein content were determined by real-time PCR and western blot analysis and/or immunostaining in biopsy specimens (24 neoplastic and 6 non-neoplastic) freshly collected at surgical lung resection, in 82 pathologically banked lung cancer specimens and in cultured non-  invasive   (95-C) and   invasive   (95-D) lung cancer cells. Multivariate regression analysis was performed to correlate immunoreactive CAP1 signal with cancer type and stage. In vitro cell migration was performed to determine the effect of RNA interference-mediated CAP1 gene silencing on   invasive  ness of 95-D cells. These analyses collectively demonstrated that: i) both CAP1 mRNA abundance and protein content were significantly higher in neoplastic compared to non-neoplastic specimens and in metastatic compared to non-metastatic specimens but not different between adenocarcinoma and squamous cell carcinoma; ii) immunoreactive CAP1 signal was significantly stronger in metastatic specimens and 95-D cells compared to non-metastatic specimens and 95-C cells; and iii) RNA interference-mediated CAP1 gene silencing adequately attenuated the   invasive   capacity of 95-D cells in vitro. These findings suggest that overexpression of CAP1 in lung cancer cells, particularly at the metastatic stage, may have significant clinical implications as a diagnostic/prognostic factor for lung cancer. 
  [Mm]etastasis  PubMed  
 Downregulated expression of the cyclase-associated protein 1 (CAP1) reduces migration in esophageal squamous cell carcinoma. Overexpression of cyclase-associated proteins has been associated with poor prognosis in several human cancers. Cyclase-associated protein 1 is a member of the cyclase-associated proteins which contributes to tumor progression. The aim of the present study was to examine the expression of cyclase-associated protein 1 and to elucidate its clinicopathologic signiﬁcance in a larger series of esophageal squamous cell carcinoma.Immunohistochemical and western blot analyses were performed in esophageal squamous cell carcinoma tissues. Survival analyses were performed by using the Kaplan-Meier method. The role of cyclase-associated protein 1 in migration was studied in esophageal squamous cell carcinoma cell lines of TE1 through knocking down cyclase-associated protein 1 with siRNA and overexpression of cyclase-associated protein 1. The regulation of cyclase-associated protein 1 on migration was determined by transwell and wound-healing assays.Immunohistochemical analysis showed that cyclase-associated protein 1 expression was negatively associated with E-cadherin and signiﬁcantly associated with lymph node metastases. Survival analysis revealed that cyclase-associated protein 1 overexpression was signiﬁcantly associated with overall survival (P = 0.011). Knock down of cyclase-associated protein 1 in TE1 cells resulted in decreased vimentin and F-actin levels and the capability for migration. In addition, overexpression of cyclase-associated protein 1 promoted the migration of TE1 cells.These ﬁndings suggest that cyclase-associated protein 1 is involved   in the me  tastasis of esophageal squamous cell carcinoma, and that elevated levels of cyclase-associated protein 1 expression may indicate a poor prognosis for patients with esophageal squamous cell carcinoma. 
 Upregulated expression of CAP1 is associated with tumor migration and   metastasis   in hepatocellular carcinoma. Hepatocellular carcinoma (HCC) is one of the most common cancers that exhibits high incidences of intrahepatic   metastasis   and tumor recurrence. Adenylate cyclase-associated protein 1 (CAP1), a protein involved in the regulation of actin filaments, was recently reported to play a role in cell motility and the pathology of pancreatic cancer. In this study, we examined a potential role of CAP1 in HCC progression, and found that CAP1 was overexpressed in HCC specimens compared with adjacent noncancerous liver tissues by Western blot analysis and real-time PCR assay. Further, immunohistochemical analysis in 107 HCC specimens revealed that overexpression of CAP1 was closely correlated only with tumor   metastasis  , but not with other clinicopathologic parameters. Univariate and multivariate survival analyses showed that CAP1 could be an independent prognostic factor for patients' survival. In addition, immunofluorescent assay demonstrated that CAP1 was colocalized with actin in the leading edge of lamellipodium in HCC cells. Importantly, knocking-down the expression of CAP1 using small interfering RNA (siRNA) targeting CAP1 led to impaired migration of HCC cells. Collectively, our results indicated that upregulated expression of CAP1 might contribute heavily to the   metastasis   of HCC. 
 Adenylate cyclase-associated protein 1 overexpressed in pancreatic cancers is involved in cancer cell motility. Pancreatic cancer has the worst prognosis among cancers due to the difficulty of early diagnosis and its aggressive behavior. To characterize the aggressiveness of pancreatic cancers on gene expression, pancreatic cancer xenografts transplanted into severe combined immunodeficient mice served as a panel for gene-expression profiling. As a result of profiling, the adenylate cyclase-associated protein 1 (CAP1) gene was shown to be overexpressed in all of the xenografts. The expression of CAP1 protein in all 73 cases of pancreatic cancer was recognized by immunohistochemical analyses. The ratio of CAP1-positive tumor cells in clinical specimens was correlated with the presence of lymph node   metastasis   and neural invasion, and also with the poor prognosis of patients. Immunocytochemical analyses in pancreatic cancer cells demonstrated that CAP1 colocalized to the leading edge of lamellipodia with actin. Knockdown of CAP1 by RNA interference resulted in the reduction of lamellipodium formation, motility, and invasion of pancreatic cancer cells. This is the first report demonstrating the overexpression of CAP1 in pancreatic cancers and suggesting the involvement of CAP1 in the aggressive behavior of pancreatic cancer cells. 
 Overexpression of adenylate cyclase-associated protein 1 is associated with   metastasis   of lung cancer. Lung cancer ranks first in both prevalence and mortality rates among all types of cancer.   Metastasis   is the main cause of treatment failure. Biomarkers are critical to early diagnosis and prediction and monitoring of progressive lesions. Several biomarkers have been identified for lung cancer but none have been routinely used clinically. The present study assessed the diagnostic and prognostic value of cyclase-associated protein 1 (CAP1) for lung cancer. CAP1 mRNA abundance and protein content were determined by real-time PCR and western blot analysis and/or immunostaining in biopsy specimens (24 neoplastic and 6 non-neoplastic) freshly collected at surgical lung resection, in 82 pathologically banked lung cancer specimens and in cultured non-invasive (95-C) and invasive (95-D) lung cancer cells. Multivariate regression analysis was performed to correlate immunoreactive CAP1 signal with cancer type and stage. In vitro cell migration was performed to determine the effect of RNA interference-mediated CAP1 gene silencing on invasiveness of 95-D cells. These analyses collectively demonstrated that: i) both CAP1 mRNA abundance and protein content were significantly higher in neoplastic compared to non-neoplastic specimens and in metastatic compared to non-metastatic specimens but not different between adenocarcinoma and squamous cell carcinoma; ii) immunoreactive CAP1 signal was significantly stronger in metastatic specimens and 95-D cells compared to non-metastatic specimens and 95-C cells; and iii) RNA interference-mediated CAP1 gene silencing adequately attenuated the invasive capacity of 95-D cells in vitro. These findings suggest that overexpression of CAP1 in lung cancer cells, particularly at the metastatic stage, may have significant clinical implications as a diagnostic/prognostic factor for lung cancer. 
 High expression and prognostic role of CAP1 and CtBP2 in breast carcinoma: associated with E-cadherin and cell proliferation. Overexpression of C-terminal binding protein-2 (CtBP2) has been noted to correlate with cancer   metastasis   in several human cancers including breast cancer. The aim of this study was to examine the effect of cyclase-associated protein 1 (CAP1) overexpression on CtBP2 expression and related mechanism in the   metastasis   of breast cancer. Immunohistochemical analysis was performed in 100 human breast carcinoma samples, and the data were correlated with clinicopathologic features. Furthermore, Western blot analysis was performed for CAP1 and CtBP2 in breast carcinoma samples and cell lines to evaluate their protein levels and molecular interaction. We found that the expression of CAP1 was positively related to CtBP2 expression (P<0.01); moreover, CAP1 expression was significantly correlated with histologic grade (P<0.01) and negatively related to E-cadherin expression (P<0.01). Meanwhile, CtBP2 expression obtained similar results. Kaplan-Meier survival analysis showed that overexpression of CAP1 and CtBP2 exhibited a significant correlation with poor prognosis in human breast cancer (P<0.01). While in vitro, we employed siRNA technique to knockdown CAP1 and CtBP2 expressions and observed their effects on MDA-MB-231 cells growth. CtBP2 depletion by siRNA-inhibited cell proliferation, resulted in increased E-cadherin levels. Moreover, knockdown of CAP1 resulted in decreased CtBP2 and increased E-cadherin expression. On the basis of these results, we suggested that CAP1's oncogenic abilities appear to be triggered at least in part by the modulation of CtBP2 and E-cadherin, which might serve as a future target for breast cancer. 
 Overexpression of adenylate cyclase-associated protein 1 may predict brain   metastasis   in non-small cell lung cancer. This study was designed to establish a biomarker risk model for predicting brain   metastasis   (BM) in non-small cell lung cancer (NSCLC). The model comprises 120 cases of NSCLC that were treated and followed up for 4 years. The patients were divided into the BM (n=50) and non-BM (other visceral   metastasis   and those without recurrence) (n=70) groups. Immunohistochemical and western blot analyses were performed in metastatic tissues of NSCLC. Multivariate regression analysis was performed to correlate the immunoreactive cyclase-associated protein 1 (CAP1) signal with BM. Survival analyses were performed by using the Kaplan-Meier method. CAP1 protein content and immunoreactivity were significantly increased in BM specimens compared to other-metastatic specimens. The survival analysis revealed that CAP1 overexpression was significantly associated with survival (P<0.05). The ROC test suggested that the area under the curve was 73.33% (P<0.001; 95% CI, 63.5-83.2%). When P=0.466, the sensitivity and specificity reached 79.5 and 67.1%, respectively. These findings suggested that CAP1 is involved in the BM of NSCLC, and that elevated levels of CAP1 expression may indicate a poor prognosis for patients with BM. The CAP1 molecular model may be useful in the prediction of the risk of BM in NSCLC. 
  GeneRIFs  
 CAP1 is involved in the brain   metastasis   in non-small cell lung cancer.CAP1 overexpression is associated with poor prognosis in non-small cell lung cancer patients. 
 results indicate that upregulated expression of CAP1 might contribute heavily to the   metastasis   of hepatocellular carcinoma. 
 Findings suggest that CAP1 (cyclase-associated protein 1) is involved in the   metastasis   of esophageal squamous cell carcinoma, and increased expression may indicate a poor prognosis. 
   
  
 ARPC1A 
 
  NCBI Gene  
  GeneRIF  
  Pubmed  
 
 
  Search Term  Rank  Fields  
  [Mm]igration  6  PubMed(1); GeneRIFs(1)  
  [Mm]etastasis  12  PubMed(1)  
 
 
 hide/show details for ARPC1A 
  [Mm]igration  PubMed  
 Characterization of the 7q21-q22 amplicon identifies ARPC1A, a subunit of the Arp2/3 complex, as a regulator of cell   migration   and invasion in pancreatic cancer. Pancreatic cancer is a highly aggressive malignancy and one of the leading causes of cancer deaths, mainly due to the lack of methods for early diagnosis and the lack of effective therapies. Recent CGH microarray studies have revealed several regions that are recurrently amplified in pancreatic cancer; these are thus likely to contain genes that contribute to cancer pathogenesis and thereby could serve as novel diagnostic and therapeutic targets. Here, we performed a detailed characterization of the 7q21-q22 amplicon in pancreatic cancer to identify putative amplification target genes. Fluorescence in situ hybridization analyses in 16 pancreatic cancer cell lines and 29 primary pancreatic tumors revealed an increased copy number in approximately 25% of cases in both sample groups, and the cell line data also allowed us to identify a 0.77 Mb amplicon core region containing ten transcripts. Gene expression analyses by qRT-PCR highlighted the ARPC1A gene as having the statistically most significant correlation between amplification and elevated expression (P = 0.004). Silencing of ARPC1A by RNA interference in AsPC-1 cells having high level amplification and expression resulted in a slight decrease in cell proliferation, but a massive reduction in cell   migration   and invasion. ARPC1A codes for the p41 subunit of the Arp2/3 protein complex, which is a key player in actin polymerization and thus regulates cell mobility. Taken together, our data implicate ARPC1A as a novel target for the 7q21-q22 amplification and a regulator of cell   migration   and invasion in pancreatic cancer, thus making it an interesting target for antimetastasis therapy. 
  GeneRIFs  
 data implicate ARPC1A as a novel target for the 7q21-q22 amplification and a regulator of cell   migration   and invasion in pancreatic cancer 
  [Mm]etastasis  PubMed  
 Characterization of the 7q21-q22 amplicon identifies ARPC1A, a subunit of the Arp2/3 complex, as a regulator of cell migration and invasion in pancreatic cancer. Pancreatic cancer is a highly aggressive malignancy and one of the leading causes of cancer deaths, mainly due to the lack of methods for early diagnosis and the lack of effective therapies. Recent CGH microarray studies have revealed several regions that are recurrently amplified in pancreatic cancer; these are thus likely to contain genes that contribute to cancer pathogenesis and thereby could serve as novel diagnostic and therapeutic targets. Here, we performed a detailed characterization of the 7q21-q22 amplicon in pancreatic cancer to identify putative amplification target genes. Fluorescence in situ hybridization analyses in 16 pancreatic cancer cell lines and 29 primary pancreatic tumors revealed an increased copy number in approximately 25% of cases in both sample groups, and the cell line data also allowed us to identify a 0.77 Mb amplicon core region containing ten transcripts. Gene expression analyses by qRT-PCR highlighted the ARPC1A gene as having the statistically most significant correlation between amplification and elevated expression (P = 0.004). Silencing of ARPC1A by RNA interference in AsPC-1 cells having high level amplification and expression resulted in a slight decrease in cell proliferation, but a massive reduction in cell migration and invasion. ARPC1A codes for the p41 subunit of the Arp2/3 protein complex, which is a key player in actin polymerization and thus regulates cell mobility. Taken together, our data implicate ARPC1A as a novel target for the 7q21-q22 amplification and a regulator of cell migration and invasion in pancreatic cancer, thus making it an interesting target for anti  metastasis   therapy. 
   
  
 HGS 
 
  NCBI Gene  
  GeneRIF  
  Pubmed  
 
 
  Search Term  Rank  Fields  
  [Mm]igration  6  PubMed(1)  
  [Mm]etastasis  12  PubMed(2)  
 
 
 hide/show details for HGS 
  [Mm]igration  PubMed  
 Targeting the hepatocyte growth factor/c-Met signaling pathway in renal cell carcinoma. The product of a proto-oncogene, the c-Met protein is a transmembrane receptor tyrosine kinase. Its only known ligand, hepatocyte growth factor/scatter factor, regulates cell growth, motility,   migration  , invasion, proliferation, and angiogenesis. Dysregulation of c-Met and hepatocyte growth factor have been observed in both clear cell and non-clear cell renal cell carcinomas (RCCs), although only papillary RCCs harbor activating mutations in the MET gene. In clear cell RCC, there is evidence of a direct link between loss of von Hippel-Lindau and up-regulation of c-Met. As in other cancers, high expression of c-Met correlates with worse outcomes in RCC. In vitro and in vivo preclinical RCC models demonstrate cancer control with small molecule and antibodies against c-Met. Given these findings, the c-Met pathway is a logical therapeutic target in RCC, and several agents are in clinical testing with early signs of efficacy. 
  [Mm]etastasis  PubMed  
 Inhibition of tumor growth and   metastasis   by depletion of vesicular sorting protein Hrs: its regulatory role on E-cadherin and beta-catenin. Abnormally high signals from receptor tyrosine kinases (RTK) are associated with carcinogenesis, and impaired deactivation of RTKs may also be a mechanism in cancer. Hepatocyte growth factor-regulated tyrosine kinase substrate (Hrs) is one of the master regulators that sort activated receptors toward lysosomes and shut down their signals. Hrs contains a ubiquitin-interacting motif and is involved in the endosomal sorting of monoubiquitinated membrane proteins, such as growth factor receptor and E-cadherin. Here, we investigated the role of Hrs in determining the malignancy of cancer cells and discovered that the targeted disruption of Hrs by small interfering RNA effectively attenuated the proliferation, anchorage-independent growth, tumorigenesis, and metastatic potential of HeLa cells in vitro and in vivo. The restoration of Hrs expression increased cell proliferation and anchorage-independent growth in a mouse embryonic fibroblast line established from a Hrs knockout mouse. Further analysis revealed that Hrs depletion was associated with the up-regulation of E-cadherin and reduced beta-catenin signaling. The aberrant accumulation of E-cadherin most likely resulted from impaired E-cadherin degradation in lysosomes. These results suggest that Hrs may play a critical role in determining the malignancy of cancer cells by regulating the degradation of E-cadherin. 
 Hepatocyte growth factor-regulated tyrosine kinase substrate (HGS) and guanylate kinase 1 (GUK1) are differentially expressed in GH-secreting adenomas. Pituitary tumors, adenomas in their vast majority, represent around 10-15% of the intracranial neoplasms. Pituitary carcinomas are exceedingly rare. Clinically, these neoplasms cause hormonal dysfunctions, and mass effect symptoms as headache and visual disorders in the case of macroadenomas. Pituitary tumorigenesis is still poorly understood. In order to investigate the expression of cancer-related genes in pituitary tumors, we employed a human cancer cDNA macroarray membrane with 1176 well-characterized human genes related to cancer and tumor biology. We were able to identify several differentially expressed genes, among them hepatocyte growth factor-regulated tyrosine kinase substrate (HGS) and guanylate kinase 1 (GUK1) which were over expressed in a pool of clinically nonfunctioning pituitary adenomas, compared with a spinal cord   metastasis   of a nonfunctioning pituitary carcinoma. HGS and GUK1 mRNA expression were chosen to be validated by quantitative RT-qPCR, however, only GUK1 had the differential expression confirmed between the adenomas and the   metastasis   of a pituitary carcinoma. We have also investigated HGS and GUK1 mRNA expressions in a series of 46 pituitary adenomas (18 nonfunctioning, 12 GH-secreting, nine PRL-secreting, and seven ACTH-secreting adenomas). HGS and GUK1 were significantly over expressed in GH-secreting adenomas, compared with ACTH-secreting adenomas and nonfunctioning tumors, and with PRL-secreting adenomas, respectively. We have shown that these genes, involved in tumorigenesis in other tissues, are as well over expressed in the pituitary tumors, however, their role in the oncogenesis of these tumors need to be further investigated. 
   
  
 ANO10 
 
  NCBI Gene  
  GeneRIF  
  Pubmed  
 
 
  Search Term  Rank  Fields  
  [Mm]igration  6  PubMed(1)  
 
 
 hide/show details for ANO10 
  [Mm]igration  PubMed  
 A Coding Variant of ANO10, Affecting Volume Regulation of Macrophages, Is Associated with Borrelia Seropositivity. In a first genome-wide association study (GWAS) approach to anti-Borrelia seropositivity, we identified two significant single nucleotide polymorphisms (SNPs) (rs17850869, P = 4.17E-09; rs41289586, P = 7.18E-08). Both markers, located on chromosomes 16 and 3, respectively, are within or close to genes previously connected to spinocerebellar ataxia. The risk SNP rs41289586 represents a missense variant (R263H) of anoctamin 10 (ANO10), a member of a protein family encoding Cl(-) channels and phospholipid scramblases. ANO10 augments volume-regulated Cl(-) currents (IHypo) in Xenopus oocytes, HEK293 cells, lymphocytes and macrophages and controls volume regulation by enhancing regulatory volume decrease (RVD). ANO10 supports   migration   of macrophages and phagocytosis of spirochetes. The R263H variant is inhibitory on IHypo, RVD and intracellular Ca(2+) signals, which may delay spirochete clearance, thereby sensitizing adaptive immunity. Our data demonstrate for the first time that ANO10 has a central role in innate immune defense against Borrelia infection. 
   
  
 CYTH1 
 
  NCBI Gene  
  GeneRIF  
  Pubmed  
 
 
  Search Term  Rank  Fields  
  [Mm]igration  6  PubMed(2); GeneRIFs(2)  
  [Ee]xtracellular [Mm]atrix  8  PubMed(1); GeneRIFs(1)  
 
 
 hide/show details for CYTH1 
  [Mm]igration  PubMed  
 Cytohesin-1 regulates human blood neutrophil adhesion to endothelial cells through β2 integrin activation. Cytohesin-1 is a guanine nucleotide exchange factor for ADP ribosylation factor 6 (Arf6) in human blood neutrophils and differentiated PLB-985 neutrophil-like cells. Cytohesin-1 regulates adhesion and the transendothelial   migratio  n of monocytes, dendritic cells and T lymphocytes through activation of the β2 integrin LFA-1. In this study we investigated the role of cytohesin-1 in neutrophil and neutrophil-like cell adhesion to HUVECs, immobilized ICAM-1, and the α4β1 and α5β1 integrin extracellular matrix ligand fibronectin. We show that cytohesin-1 knockdown or inhibition with secinH3 inhibits fMLF-mediated cell adhesion to HUVECs and immobilized ICAM-1, whereas cytohesin-1 over-expression has the opposing effect. Binding of PLB-985 cells to HUVECs correlated with expression of the high-affinity β2 integrin epitope recognized by mAb24. Adhesion to HUVECs was inhibited by soluble ICAM-1, anti-ICAM-1, anti-CD11a and anti-CD18, but not anti-CD11b, blocking antibodies. We also demonstrate that cytohesin-1 knockdown promotes fMLF-mediated cell adhesion to fibronectin whereas cytohesin-1 over-expression has the opposing effect. Crosstalk between β1 and β2 integrins also exists since inhibition of β1 integrin functions with blocking antibodies enhanced adhesion of PLB-985 over-expressing cytohesin-1 to ICAM-1. We suggest that cytohesin-1 is a key regulator of neutrophil adhesion to endothelial cells and to components of extracellular matrix, which may influe  nce cell   emigration through its dual opposing effect on β2 and β1 integrin activation. 
 Cytohesin-1 controls the activation of RhoA and modulates integrin-dependent adhesion and   migration   of dendritic cells. Adhesion and motility of mammalian leukocytes are essential requirements for innate and adaptive immune defense mechanisms. We show here that the guanine nucleotide exchange factor cytohesin-1, which had previously been demonstrated to be an important component of beta-2 integrin activation in lymphocytes, regulates the activation of the small GTPase RhoA in primary dendritic cells (DCs). Cytohesin-1 and RhoA are both required for the induction of chemokine-dependent conformational changes of the integrin beta-2 subunit of DCs during adhesion under physiological flow conditions. Furthermore, use of RNAi in murine bone marrow DCs (BM-DCs) revealed that interference with cytohesin-1 signaling impairs   migration   of wild-type dendritic cells in complex 3D environments and in vivo. This phenotype was not observed in the complete absence of integrins. We thus demonstrate an essential role of cytohesin-1/RhoA during ameboid   migration   in the presence of integrins and further suggest that DCs without integrins switch to a different   migration   mode. 
  GeneRIFs  
 findings suggest that cytohesin-1 is a key regulator of neutrophil adhesion to endothelial cells and to components of extracellular matrix, which may influence cell e  migration   through its dual opposing effect on beta2 and beta1 integrin activation 
 demonstrate an essential role of cytohesin-1/RhoA during ameboid   migration   in the presence of integrins 
  [Ee]xtracellular [Mm]atrix  PubMed  
 Cytohesin-1 regulates human blood neutrophil adhesion to endothelial cells through β2 integrin activation. Cytohesin-1 is a guanine nucleotide exchange factor for ADP ribosylation factor 6 (Arf6) in human blood neutrophils and differentiated PLB-985 neutrophil-like cells. Cytohesin-1 regulates adhesion and the transendothelial migration of monocytes, dendritic cells and T lymphocytes through activation of the β2 integrin LFA-1. In this study we investigated the role of cytohesin-1 in neutrophil and neutrophil-like cell adhesion to HUVECs, immobilized ICAM-1, and the α4β1 and α5β1 int  egrin extracellular   matrix ligand fibronectin. We show that cytohesin-1 knockdown or inhibition with secinH3 inhibits fMLF-mediated cell adhesion to HUVECs and immobilized ICAM-1, whereas cytohesin-1 over-expression has the opposing effect. Binding of PLB-985 cells to HUVECs correlated with expression of the high-affinity β2 integrin epitope recognized by mAb24. Adhesion to HUVECs was inhibited by soluble ICAM-1, anti-ICAM-1, anti-CD11a and anti-CD18, but not anti-CD11b, blocking antibodies. We also demonstrate that cytohesin-1 knockdown promotes fMLF-mediated cell adhesion to fibronectin whereas cytohesin-1 over-expression has the opposing effect. Crosstalk between β1 and β2 integrins also exists since inhibition of β1 integrin functions with blocking antibodies enhanced adhesion of PLB-985 over-expressing cytohesin-1 to ICAM-1. We suggest that cytohesin-1 is a key regulator of neutrophil adhesion to endothelial cells and to comp  onents of extracellu  lar matrix, which may influence cell emigration through its dual opposing effect on β2 and β1 integrin activation. 
  GeneRIFs  
 findings suggest that cytohesin-1 is a key regulator of neutrophil adhesion to endothelial cells and to components of   extracellular matrix  , which may influence cell emigration through its dual opposing effect on beta2 and beta1 integrin activation 
   
  
 RAB6A 
 
  NCBI Gene  
  GeneRIF  
  Pubmed  
 
 
  Search Term  Rank  Fields  
  ECM  7  PubMed(1)  
  [Ee]xtracellular [Mm]atrix  8  PubMed(1)  
 
 
 hide/show details for RAB6A 
  ECM  PubMed  
 CLASPs link focal-adhesion-associated microtubule capture to localized exocytosis and adhesion site turnover. Turnover of integrin-based focal adhesions (FAs) with the extracellular matrix (  ECM  ) is essential for coordinated cell movement. In collectively migrating human keratinocytes, FAs assemble near the leading edge, grow and mature as a result of contractile forces and disassemble underneath the advancing cell body. We report that clustering of microtubule-associated CLASP1 and CLASP2 proteins around FAs temporally correlates with FA turnover. CLASPs and LL5β (also known as PHLDB2), which recruits CLASPs to FAs, facilitate FA disassembly. CLASPs are further required for FA-associated   EC  M degradation, and matrix metalloprotease inhibition slows FA disassembly similarly to CLASP or PHLDB2 (LL5β) depletion. Finally, CLASP-mediated microtubule tethering at FAs establishes an FA-directed transport pathway for delivery, docking and localized fusion of exocytic vesicles near FAs. We propose that CLASPs couple microtubule organization, vesicle transport and cell interactions with th  e E  CM, establishing a local secretion pathway that facilitates FA turnover by severing cell-matrix connections. 
  [Ee]xtracellular [Mm]atrix  PubMed  
 CLASPs link focal-adhesion-associated microtubule capture to localized exocytosis and adhesion site turnover. Turnover of integrin-based focal adhesions (FAs) with the   extracellular matrix   (ECM) is essential for coordinated cell movement. In collectively migrating human keratinocytes, FAs assemble near the leading edge, grow and mature as a result of contractile forces and disassemble underneath the advancing cell body. We report that clustering of microtubule-associated CLASP1 and CLASP2 proteins around FAs temporally correlates with FA turnover. CLASPs and LL5β (also known as PHLDB2), which recruits CLASPs to FAs, facilitate FA disassembly. CLASPs are further required for FA-associated ECM degradation, and matrix metalloprotease inhibition slows FA disassembly similarly to CLASP or PHLDB2 (LL5β) depletion. Finally, CLASP-mediated microtubule tethering at FAs establishes an FA-directed transport pathway for delivery, docking and localized fusion of exocytic vesicles near FAs. We propose that CLASPs couple microtubule organization, vesicle transport and cell interactions with the ECM, establishing a local secretion pathway that facilitates FA turnover by severing cell-matrix connections. 
   
  
 SHC1 
 
  NCBI Gene  
  GeneRIF  
  Pubmed  
 
 
  Search Term  Rank  Fields  
  [Mm]igration  6  Process(1); PubMed(19); GeneRIFs(5)  
  ECM  7  PubMed(1)  
  [Ee]xtracellular [Mm]atrix  8  PubMed(3); GeneRIFs(1)  
  [Ii]nvasive  10  PubMed(4); GeneRIFs(1)  
  [Mm]etastasis  12  PubMed(12); GeneRIFs(1)  
 
 
 hide/show details for SHC1 
  [Mm]igration  Process  
 leukocyte   migration   
  PubMed  
 Analysis of protein-protein interactions in cross-talk pathways reveals CRKL protein as a novel prognostic marker in hepatocellular carcinoma. Deciphering the network of signaling pathways in cancer via protein-protein interactions (PPIs) at the cellular level is a promising approach but remains incomplete. We used an in situ proximity ligation assay to identify and quantify 67 endogenous PPIs among 21 interlinked pathways in two hepatocellular carcinoma (HCC) cells, Huh7 (minimally migratory cells) and Mahlavu (highly migratory cells). We then applied a differential network biology analysis and determined that the novel interaction, CRKL-FLT1, has a high centrality ranking, and the expression of this interaction is strongly correlated with the migratory ability of HCC and other cancer cell lines. Knockdown of CRKL and FLT1 in HCC cells leads to a decrease in cell   migration   via ERK signaling and the epithelial-mesenchymal transition process. Our immunohistochemical analysis shows high expression levels of the CRKL and CRKL-FLT1 pair that strongly correlate with reduced disease-free and overall survival in HCC patient samples, and a multivariate analysis further established CRKL and the CRKL-FLT1 as novel prognosis markers. This study demonstrated that functional exploration of a disease network with interlinked pathways via PPIs can be used to discover novel biomarkers. 
 TNFα signals via p66(Shc) to induce E-Selectin, promote leukocyte tran  smigratio  n and enhance permeability in human endothelial cells. Endothelial cells participate in inflammatory events leading to atherogenesis by regulating endothelial cell permeability via the expression of VE-Cadherin and β-catenin and leukocyte recruitment via the expression of E-Selectins and other adhesion molecules. The protein p66(Shc) acts as a sensor/inducer of oxidative stress and may promote vascular dysfunction. The objective of this study was to investigate the role of p66(Shc) in tumor necrosis factor TNFα-induced E-Selectin expression and function in human umbilical vein endothelial cells (HUVEC). Exposure of HUVEC to 50 ng/ml TNFα resulted in increased leukocyte t  ransmigra  tion through the endothelial monolayer and E-Selectin expression, in association with augmented phosphorylation of both p66(Shc) on Ser(36) and the stress kinase c-Jun NH2-terminal protein kinase (JNK)-1/2, and higher intracellular reactive oxygen species (ROS) levels. Overexpression of p66(Shc) in HUVEC resulted in enhanced p66(Shc) phosphorylation on Ser(36), increased ROS and E-Selectin levels, and amplified endothelial cell permeability and leukocyte t  ransmigra  tion through the HUVEC monolayer. Conversely, overexpression of a phosphorylation-defective p66(Shc) protein, in which Ser(36) was replaced by Ala, did not augment ROS and E-Selectin levels, nor modify cell permeability or leukocyte t  ransmigra  tion beyond those found in wild-type cells. Moreover, siRNA-mediated silencing of p66(Shc) resulted in marked reduction of E-Selectin expression and leukocyte t  ransmigra  tion. In conclusion, p66(Shc) acts as a novel intermediate in the TNFα pathway mediating endothelial dysfunction, and its action requires JNK-dependent phosphorylation of p66(Shc) on Ser(36). 
 G protein-coupled chemoattractant receptors regulate Lyn tyrosine kinase.Shc adapter protein signaling complexes. Receptors for chemoattractants that direct the   migration   of phagocytic leukocytes to sites of injury/infection also modulate many other leukocyte functions that are critical to the inflammatory response. These chemoattractant receptors, members of the G protein-coupled heptahelical receptor family, have been classically linked to cell activation via phospholipase C, calcium, and protein kinase C. We show here that activation of the N-formyl peptide chemoattractant receptor stimulates an additional protein kinase C-independent pathway through the Src-related tyrosine kinase, Lyn, in human neutrophils. We demonstrate that activation of Lyn is associated with binding to the Shc adapter protein, which becomes phosphorylated on tyrosine residues. This interaction appears to be mediated via the Shc SH2 domain. Complexes of phosphorylated Lyn and Shc with phosphatidylinositol 3-kinase are rapidly formed in stimulated neutrophils, correlating with phosphatidylinositol 3,4,5-trisphosphate [corrected] formation and cell activation. This signaling pathway involving a Src-related kinase and the Shc adapter protein provides a potential mechanism linking chemoattractant receptors to downstream events involving Rac activation and NADPH oxidase. Regulation of Shc by G protein-coupled receptors may also allow these receptors to modulate the activity of the Ras/mitogen-activated protein kinase cascade. 
   Migration   of renal tumor cells depends on dephosphorylation of Shc by PTEN. The tumor suppressor PTEN is a phosphatase using FAK and Shc as direct substrates, and Akt as a key effector via PIP3. PTEN regulates cell   migration   and may influence metastases. We quantified PTEN in 135 clear cell renal cell carcinomas (ccRCC) by Western blot analysis and found statistically significant lower PTEN expression in patients who died, usually caused by metastases, within 5 years after surgery, compared to those surviving this time period. In athymic mice, PTEN transfected 786-O cells were injected into the tail vein and metastatic load of the lungs was quantified. We observed a strongly reduced metastatic load after PTEN transfection. For analyses of the PTEN activities, transfections with mutated PTEN genes were performed, leading to loss of lipid phosphatase activity and/or protein phosphatase activity, and of the C-terminal tail. Cell   migration   was analyzed in a Boyden chamber and phosphorylation of PTEN downstream targets Akt, FAK and Shc by Western blotting. 786-O cells transfected with the functional PTEN gene showed profoundly diminished   migration  . Transfection with a mutated PTEN isoform leading to loss of protein phosphatase activity, but not of lipid phosphatase activity, abolished this effect. Shc but not FAK seems to mediate this effect. These results show a critical role of PTEN in metastasis of RCC, depending on protein phosphatase activity via Shc. This new insight opens an alley of additional approaches complementing current cancer therapy and metastasis prediction in RCC. 
 Insulin-like growth factor-I stimulates Shc-dependent phosphatidylinositol 3-kinase activation via Grb2-associated p85 in vascular smooth muscle cells. Insulin-like growth factor-I (IGF-I) stimulates vascular smooth muscle cell proliferation and   migration   by activating both MAPK and phosphatidylinositol 3-kinase (PI3K). Vascular smooth muscle cells (VSMCs) maintained in 25 mm glucose sustain MAPK activation via increased Shc phosphorylation and Grb2 association resulting in an enhanced mitogenic response compared with cells grown in 5 mm glucose. PI3K plays a major role in IGF-I-stimulated VSMC   migration  , and hyperglycemia augments this response. In contrast to MAPK activation the role of Shc in modulating PI3K in response to IGF-I has not been determined. In this study we show that impaired Shc association with Grb2 results in decreased Grb2-p85 association, SHPS-1-p85 recruitment, and PI3K activation in response to IGF-I. Exposure of VSMCs to cell-permeable peptides, which contained polyproline sequences from p85 proposed to mediate Grb2 association, resulted in inhibition of Grb2-p85 binding and AKT phosphorylation. Transfected cells that expressed p85 mutant that had specific prolines mutated to alanines resulted in less Grb2-p85 association, and a Grb2 mutant (W36A/W193A) that attenuated p85 binding showed decreased association of p85 with SHPS-1, PI3K activation, AKT phosphorylation, cell proliferation, and   migration   in response to IGF-I. Cellular exposure to 25 mm glucose, which is required for Shc phosphorylation in response to IGF-I, resulted in enhanced Grb2 binding to p85, activation of PI3K activity, and increased AKT phosphorylation as compared with cells exposed to 5 mm glucose. We conclude that in VSMCs exposed to hyperglycemia, IGF-I stimulation of Shc facilitates the transfer of Grb2 to p85 resulting in enhanced PI3K activation and AKT phosphorylation leading to enhanced cell proliferation and   migration  . 
 The adaptor protein Shc integrates growth factor and ECM signaling during postnatal angiogenesis. Angiogenesis requires integration of cues from growth factors, extracellular matrix (ECM) proteins, and their receptors in endothelial cells. In the present study, we show that the adaptor protein Shc is required for angiogenesis in zebrafish, mice, and cell-culture models. Shc knockdown zebrafish embryos show defects in intersegmental vessel sprouting in the trunk. Shc flox/flox; Tie2-Cre mice display reduced angiogenesis in the retinal neovascularization model and in response to VEGF in the Matrigel plug assay in vivo. Functional studies reveal a model in which Shc is required for integrin-mediated spreading and   migration   specifically on fibronectin, as well as endothelial cell survival in response to VEGF. Mechanistically, Shc is required for activation of the Akt pathway downstream of both integrin and VEGF signaling, as well as for integration of signals from these 2 receptors when cells are grown on fibronectin. Therefore, we have identified a unique mechanism in which signals from 2 critical angiogenic signaling axes, integrins and VEGFR-2, converge at Shc to regulate postnatal angiogenesis. 
 Adaptor ShcA protein binds tyrosine kinase Tie2 receptor and regulates   migration   and sprouting but not survival of endothelial cells. Angiopoietin-1 can promote   migration  , sprouting, and survival of endothelial cells through activation of different signaling pathways triggered by the Tie2 tyrosine kinase receptor. ShcA adapter proteins are targets of activated tyrosine kinases and are implicated in the transmission of activation signals to the Ras/mitogen-activated protein kinase pathway. Here we report the identification of an interaction between the adapter protein ShcA and the cytoplasmic domain of Tie2 through in vitro co-immunoprecipitation analysis. Stimulation of endogenous Tie2 in endothelial cells with its ligand angiopoietin-1 increased its association with ShcA and phosphorylation of the adapter protein. The interaction requires the SH2 domain of ShcA and the tyrosine phosphorylation of Tie2 as shown by pull-down experiments. Furthermore, Tyr-1101 of Tie2 was identified as the primary binding site for the SH2 domain of ShcA. Overexpression of a dominant-negative form of ShcA affects angiopoietin-1-induced chemotaxis and sprouting, although it has no effect on survival of endothelial cells. Furthermore, this mutant partially reduces the tyrosine phosphorylation of the regulatory p85 subunit of phosphatidylinositol 3-kinase. Together, our results identified a novel interaction between Tie2 with the adapter molecule ShcA and suggested that this interaction may play a role in the regulation of   migration   and three-dimensional organization of endothelial cells induced by angiopoietin-1. 
 EphB1 recruits c-Src and p52Shc to activate MAPK/ERK and promote chemotaxis. Eph receptors and their ligands (ephrins) play an important role in axonal guidance, topographic mapping, and angiogenesis. The signaling pathways mediating these activities are starting to emerge and are highly cell- and receptor-type specific. Here we demonstrate that activated EphB1 recruits the adaptor proteins Grb2 and p52Shc and promotes p52Shc and c-Src tyrosine phosphorylation as well as MAPK/extracellular signal-regulated kinase (ERK) activation. EphB1-mediated increase of cell   migration   was abrogated by the MEK inhibitor PD98059 and Src inhibitor PP2. In contrast, cell adhesion, which we previously showed to be c-jun NH2-terminal kinase (JNK) dependent, was unaffected by ERK1/2 and Src inhibition. Expression of dominant-negative c-Src significantly reduced EphB1-dependent ERK1/2 activation and chemotaxis. Site-directed mutagenesis experiments demonstrate that tyrosines 600 and 778 of EphB1 are required for its interaction with c-Src and p52Shc. Furthermore, phosphorylation of p52Shc by c-Src is essential for its recruitment to EphB1 signaling complexes through its phosphotyrosine binding domain. Together these findings highlight a new aspect of EphB1 signaling, whereby the concerted action of c-Src and p52Shc activates MAPK/ERK and regulates events involved in cell motility. 
 Distinct phosphotyrosine-dependent functions of the ShcA adaptor protein are required for transforming growth factor β (TGFβ)-induced breast cancer cel  l migrati  on, invasion, and metastasis. The ErbB2 and TGFβ signaling pathways cooperate to promote the migratory, invasive, and metastatic behavior of breast cancer cells. We previously demonstrated that ShcA is necessary for these synergistic interactions. Through a structure/function approach, we now show that the phosphotyrosine-binding, but not the Src homology 2, domain of ShcA is required for TGFβ-indu  ced migra  tion and invasion of ErbB2-expressing breast cancer cells. We further demonstrate that the tyrosine phosphorylation sites within ShcA (Tyr(239)/Tyr(240) and Tyr(313)) transduce distinct and non-redundant signals that promote these TGFβ-mediated effects. We demonstrate that Grb2 is required specifically downstream of Tyr(313), whereas the Tyr(239)/Tyr(240) phosphorylation sites require the Crk adaptor proteins to augment TGFβ-in  duced mig  ration and invasion. Furthermore, ShcA Tyr(313) phosphorylation enhances tumor cell survival, and ShcA Tyr(239)/Tyr(240) signaling promotes endothelial cell recruitment into ErbB2-expressing breast tumors in vivo, whereas all three ShcA tyrosine residues are required for efficient breast cancer metastasis to the lungs. Our data uncover a novel ShcA-dependent signaling axis downstream of TGFβ and ErbB2 that requires both the Grb2 and Crk adaptor proteins to increase the migratory and invasive properties of breast cancer cells. In addition, signaling downstream of specific ShcA tyrosine residues facilitates the survival, vascularization, and metastatic spread of breast tumors. 
 The motogenic and mitogenic responses to HGF are amplified by the Shc adaptor protein. The receptor of Hepatocyte Growth Factor-Scatter Factor (HGF) is a tyrosine kinase which regulates cell motility and growth. After ligand-induced tyrosine phosphorylation, the HGF receptor associates with the Shc adaptor, via the SH2 domain. Site-directed mutagenesis of the HGF receptor indicates that phosphotyrosines Y1349VHV and Y1356VNV can work as docking sites for Shc. The Kd of this interaction, measured in real time using synthetic phosphopeptides and recombinant Shc on a BIAcore biosensor, is 150 nm for both sites. After stimulation of the HGF receptor, Shc is phosphorylated on Y317VNV, generating an high affinity binding site for Grb2 (Kd = 15 nM). This duplicates the high affinity binding site for Grb2 present on the HGF receptor (Y1356VNV). Thus HGF stimulation can trigger the Ras pathway by recruiting Grb2 both directly through the receptor, and indirectly, through Shc. Overexpression of wild-type Shc, but not of the Y317-->F mutant, enhances cell   migration   and growth in response to HGF. These data show that Shc is a relevant substrate of the HGF receptor, and works as an 'amplifier' of the motogenic as well as of the mitogenic response. 
 Novel role of p66Shc in ROS-dependent VEGF signaling and angiogenesis in endothelial cells. p66Shc, a longevity adaptor protein, is demonstrated as a key regulator of reactive oxygen species (ROS) metabolism involved in aging and cardiovascular diseases. Vascular endothelial growth factor (VEGF) stimulates endothelial cell (EC)   migration   and proliferation primarily through the VEGF receptor-2 (VEGFR2). We have shown that ROS derived from Rac1-dependent NADPH oxidase are involved in VEGFR2 autophosphorylation and angiogenic-related responses in ECs. However, a role of p66Shc in VEGF signaling and physiological responses in ECs is unknown. Here we show that VEGF promotes p66Shc phosphorylation at Ser36 through the JNK/ERK or PKC pathway as well as Rac1 binding to a nonphosphorylated form of p66Shc in ECs. Depletion of endogenous p66Shc with short interfering RNA inhibits VEGF-induced Rac1 activity and ROS production. Fractionation of caveolin-enriched lipid raft demonstrates that p66Shc plays a critical role in VEGFR2 phosphorylation in caveolae/lipid rafts as well as downstream p38MAP kinase activation. This in turn stimulates VEGF-induced EC   migration  , proliferation, and capillary-like tube formation. These studies uncover a novel role of p66Shc as a positive regulator for ROS-dependent VEGFR2 signaling linked to angiogenesis in ECs and suggest p66Shc as a potential therapeutic target for various angiogenesis-dependent diseases. 
 Copine-III interacts with ErbB2 and promotes tumor cell   migration  . ErbB2 amplification and overexpression in breast cancer correlates with aggressive disease and poor prognosis. To find novel ErbB2-interacting proteins, we used stable isotope labeling of amino acids in cell culture followed by peptide affinity pull-downs and identified specific binders using relative quantification by mass spectrometry. Copine-III, a member of a Ca(2+)-dependent phospholipid-binding protein family, was identified as binding to phosphorylated Tyr1248 of ErbB2. In breast cancer cells, Copine-III requires Ca(2+) for binding to the plasma membrane, where it interacts with ErbB2 upon receptor stimulation, an interaction that is dependent on receptor activity. Copine-III also binds receptor of activated C kinase 1 and colocalizes with phosphorylated focal adhesion kinase at the leading edge of migrating cells. Importantly, knockdown of Copine-III in T47D breast cancer cells causes a decrease in Src kinase activation and ErbB2-dependent wound healing. Our data suggest that Copine-III is a novel player in the regulation of ErbB2-dependent cancer cell motility. In primary breast tumors, high CPNE3 RNA levels significantly correlate with ERBB2 amplification. Moreover, in an in situ tissue microarray analysis, we detected differential protein expression of Copine-III in normal versus breast, prostate and ovarian tumors, suggesting a more general role for Copine-III in carcinogenesis. 
 Mutant p53 disrupts role of ShcA protein in balancing Smad protein-dependent and -independent signaling activity of transforming growth factor-β (TGF-β). Biomarkers are lacking for identifying the switch of transforming growth factor-β (TGF-β) from tumor-suppressing to tumor-promoting. Mutated p53 (mp53) has been suggested to switch TGF-β to a tumor promoter. However, we found that mp53 does not always promote the oncogenic role of TGF-β. Here, we show that endogenous mp53 knockdown enhanced   cell mig  ration and phosphorylation of ERK in DU145 prostate cancer cells. Furthermore, ectopic expression of mp53 in p53-null PC-3 prostate cancer cells enhanced Smad-dependent signaling but inhibited TGF-β-induce  d cell mi  gration by down-regulating activated ERK. Reactivation of ERK by the expression of its activator, MEK-1, restored TGF-β-induc  ed cell m  igration. Because TGF-β is known to activate the MAPK/ERK pathway through direct phosphorylation of the adaptor protein ShcA and MAPK/ERK signaling is pivotal to tumor progression, we investigated whether ShcA contributed to mp53-induced ERK inhibition and the conversion of the role of TGF-β during carcinogenesis. We found that mp53 expression led to a decrease of phosphorylated p52ShcA/ERK levels and an increase of phosphorylated Smad levels in a panel of mp53-expressing cancer cell lines and in mammary glands and tumors from mp53 knock-in mice. By manipulating ShcA levels to regulate ERK and Smad signaling in human untransformed and cancer cell lines, we showed that the role of TGF-β in regulating anchorage-dependent and -independent   growth an  d migration can be shifted between growth suppr  ession an  d migration promotion. Thus, our results for the first time suggest that mp53 disrupts the role of ShcA in balancing the Smad-dependent and -independent signaling activity of TGF-β and that ShcA/ERK signaling is a major pathway regulating the tumor-promoting activity of TGF-β. 
 The adaptor proteins p66Shc and Grb2 regulate the activation of the GTPases ARF1 and ARF6 in invasive breast cancer cells. Signals downstream of growth factor receptors play an important role in mammary carcinogenesis. Recently, we demonstrated that the small GTPases ARF1 and ARF6 were shown to be activated downstream of the epidermal growth factor receptor (EGFR) and act as a key regulator of growth,   migration  , and invasion of breast cancer cells. However, the mechanism via which the EGFR recruits and activates ARF1 and ARF6 to transmit signals has yet to be fully elucidated. Here, we identify adaptor proteins Grb2 and p66Shc as important regulators mediating ARF activation. We demonstrate that ARF1 can be found in complex with Grb2 and p66Shc upon EGF stimulation of the basal-like breast cancer MDA-MB-231 cell line. However, we report that these two adaptors regulate ARF1 activation differently, with Grb2 promoting ARF1 activation and p66Shc blocking this response. Furthermore, we show that Grb2 is essential for the recruitment of ARF1 to the EGFR, whereas p66Shc hindered ARF1 receptor recruitment. We demonstrate that the negative regulatory role of p66Shc stemmed from its ability to block the recruitment of Grb2/ARF1 to the EGFR. Conversely, p66Shc potentiates ARF6 activation as well as the recruitment of this ARF isoform to the EGFR. Interestingly, we demonstrate that Grb2 is also required for the activation and receptor recruitment of ARF6. Additionally, we show an important role for p66Shc in modulating ARF activation, cell growth, and   migration   in HER2-positive breast cancer cells. Together, our results highlight a central role for adaptor proteins p66Shc and Grb2 in the regulation of ARF1 and ARF6 activation in invasive breast cancer cells. 
 CD9-mediated activation of the p46 Shc isoform leads to apoptosis in cancer cells. CD9, a member of the tetraspanin family, has been shown to be involved in a range of cellular activities, including   migration  , proliferation and adhesion, but the molecular mechanisms by which it mediates such events is unclear. Here, we found that anti-CD9 monoclonal antibody ALB6 inhibited cell proliferation, reduced cell viability and induced not only morphological changes specific to apoptosis but also molecular changes, as evidenced by TUNEL and annexin-V staining. For the possible mechanism of ALB6-induced apoptosis, ALB6 activated the c-Jun NH2-terminal kinase/stress-activated protein kinase (JNK/SAPK) and p38 mitogen-activated-protein kinase (MAPK) within 5-15 minutes, as well as caspase-3 within 24-48 hours. It is noteworthy that ALB6 induced tyrosine phosphorylation of the p46 Shc isoform specifically and that the overexpression of its dominant-negative form completely suppressed the ALB6-induced activation of JNK/SAPK, p38 MAPK and caspase-3, resulting in the inhibition of apoptotic cell death. These results suggest that CD9 might regulate apoptosis through the specialized signals in human cancer cell lines. 
 Focal adhesion kinase enhances signaling through the Shc/extracellular signal-regulated kinase pathway in anaplastic astrocytoma tumor biopsy samples. Focal adhesion kinase (FAK) is a nonreceptor tyrosine kinase that on activation generates signals that can modulate crucial cell functions, including cell proliferation,   migration  , and survival. In vitro, overexpression of FAK has been shown to promote cell proliferation by signaling through the Ras/mitogen-activated protein kinase cascade in several cell types. We have shown previously that overexpression of exogenous FAK lacking alternative splicing in malignant astrocytoma clones injected intracerebrally into SCID mouse brains promotes tumor cell proliferation. Here, we show that in anaplastic astrocytoma biopsy samples, FAK is expressed as an unspliced variant and migrates with a faster mobility similar to that observed in embryonic brain. Compared with nonneoplastic adult brain biopsies, the levels of FAK protein are elevated as are its levels of activation as assessed by autophosphorylation and overall tyrosine phosphorylation. The activity of Src kinase in these tumors is also elevated, as well as the activity of Src kinase associated with FAK; the latter may result in enhanced Src kinase phosphorylation of FAK. Phosphorylated Shc is associated with FAK in the anaplastic astrocytoma biopsy samples and in astrocytoma cells overexpressing FAK in vitro but not in nonneoplastic brain biopsy samples. Elevated extracellular signal-regulated kinase-2 activation and elevated expression of cyclins D and E are also found in anaplastic astrocytoma biopsy samples. These data provide evidence that the increased FAK activity in these tumors contributes to phosphorylation of Shc and likely to the promotion of Ras activity, extracellular signal-regulated kinase-2 activation, and cell proliferation in vivo. 
 ShcA Protects against Epithelial-Mesenchymal Transition through Compartmentalized Inhibition of TGF-β-Induced Smad Activation. Epithelial-mesenchymal transition (EMT) is a normal cell differentiation event during development and contributes pathologically to carcinoma and fibrosis progression. EMT often associates with increased transforming growth factor-β (TGF-β) signaling, and TGF-β drives EMT, in part through Smad-mediated reprogramming of gene expression. TGF-β also activates the Erk MAPK pathway through recruitment and Tyr phosphorylation of the adaptor protein ShcA by the activated TGF-β type I receptor. We found that ShcA protects the epithelial integrity of nontransformed cells against EMT by repressing TGF-β-induced, Smad-mediated gene expression. p52ShcA competed with Smad3 for TGF-β receptor binding, and down-regulation of ShcA expression enhanced autocrine TGF-β/Smad signaling and target gene expression, whereas increased p52ShcA expression resulted in decreased Smad3 binding to the TGF-β receptor, decreased Smad3 activation, and increased Erk MAPK and Akt signaling. Furthermore, p52ShcA sequestered TGF-β receptor complexes to caveolin-associated membrane compartments, and reducing ShcA expression enhanced the receptor localization in clathrin-associated membrane compartments that enable Smad activation. Consequently, silencing ShcA expression induced EMT, with incr  eased cel  l migration, invasion, and dissemination, and increased stem cell generation and mammosphere formation, dependent upon autocrine TGF-β signaling. These findings position ShcA as a determinant of the epithelial phenotype by repressing TGF-β-induced Smad activation through differential partitioning of receptor complexes at the cell surface. 
 Indirect recruitment of the signalling adaptor Shc to the fibroblast growth factor receptor 2 (FGFR2). The adaptor protein Shc (Src homology and collagen-containing protein) plays an important role in the activation of signalling pathways downstream of RTKs (receptor tyrosine kinases) regulating diverse cellular functions, such as differentiation, adhesion,   migration   and mitogenesis. Despite being phosphorylated downstream of members of the FGFR (fibroblast growth factor receptor) family, a direct interaction of Shc with this receptor family has not been described to date. Various studies have suggested potential binding sites for the Shc PTB domain (phosphotyrosine-binding domain) and/or the SH2 (Src homology 2) domain on FGFR1, but no interaction of full-length Shc with these sites has been reported in vivo. In the present study, we investigated the importance of the SH2 domain and the PTB domain in recruitment of Shc to FGFR2(IIIc) to characterize the interaction of these two proteins. Confocal microscopy revealed extensive co-localization of Shc with FGFR2. The PTB domain was identified as the critical component of Shc which mediates membrane localization. Results from FLIM (fluorescence lifetime imaging microscopy) revealed that the interaction between Shc and FGFR2 is indirect, suggesting that the adaptor protein forms part of a signalling complex containing the receptor. We identified the non-RTK Src as a protein which potentially mediates the formation of such a ternary complex. Although an interaction between Src and Shc has been described previously, in the present study we implicate the Shc SH2 domain as a novel mediator of this association. The recruitment of Shc to FGFR2 via an indirect mechanism provides new insight into the regulation of protein assembly and activation of various signalling pathways downstream of this RTK. 
 Activation of the EphA2 tyrosine kinase stimulates the MAP/ERK kinase signaling cascade. Intracellular signaling by receptor tyrosine kinases regulates many different aspects of cell behavior. Recent studies in our laboratory and others have demonstrated that the EphA2 receptor tyrosine kinase critically regulates tumor cell growth,   migration   and invasiveness. Although the cellular consequences of EphA2 signaling have been the focus of recent attention, the biochemical changes that are triggered by ligand-mediated activation of EphA2 remain largely unknown. Herein, we demonstrate that ligand stimulation of EphA2 promotes the nucleus translocation and phosphorylation of ERK kinases, followed by an increase in nuclear induction of the Elk-1 transcription factor. Ligand-mediated activation allows EphA2 to form a molecular complex with the SHC and GRB2 adaptor proteins. Specifically, we demonstrate that tyrosine phosphorylated EphA2 interacts with the PTB and SH2 domains of SHC. We also show that the interaction of EphA2 with GRB2 is indirect and mediated by SHC and that this complex is necessary for EphA2-mediated activation of ERK kinases. These studies provide a novel mechanism to demonstrate how EphA2 can convey information from the cell exterior to the nucleus. 
  GeneRIFs  
 TNFalpha signals via p66(Shc) to induce E-Selectin, promote leukocyte trans  migration   and enhance permeability in human endothelial cells. 
 Distinct phosphotyrosine-dependent functions of the ShcA adaptor protein are required for transforming growth factor beta (TGFbeta)-induced breast cancer cell   migration  , invasion, and metastasis 
   Migration   of renal tumor cells depends on dephosphorylation of Shc by PTEN. 
 in VSMCs exposed to hyperglycemia, IGF-I stimulation of Shc facilitates the transfer of Grb2 to p85 resulting in enhanced PI3K activation and AKT phosphorylation leading to enhanced cell proliferation and   migration   
 there is a novel interaction between Tie2 with the adapter molecule ShcA that may play a role in the regulation of   migration   and three-dimensional organization of endothelial cells induced by angiopoietin-1 
  ECM  PubMed  
 The adaptor protein Shc integrates growth factor and   ECM   signaling during postnatal angiogenesis. Angiogenesis requires integration of cues from growth factors, extracellular matrix (  ECM  ) proteins, and their receptors in endothelial cells. In the present study, we show that the adaptor protein Shc is required for angiogenesis in zebrafish, mice, and cell-culture models. Shc knockdown zebrafish embryos show defects in intersegmental vessel sprouting in the trunk. Shc flox/flox; Tie2-Cre mice display reduced angiogenesis in the retinal neovascularization model and in response to VEGF in the Matrigel plug assay in vivo. Functional studies reveal a model in which Shc is required for integrin-mediated spreading and migration specifically on fibronectin, as well as endothelial cell survival in response to VEGF. Mechanistically, Shc is required for activation of the Akt pathway downstream of both integrin and VEGF signaling, as well as for integration of signals from these 2 receptors when cells are grown on fibronectin. Therefore, we have identified a unique mechanism in which signals from 2 critical angiogenic signaling axes, integrins and VEGFR-2, converge at Shc to regulate postnatal angiogenesis. 
  [Ee]xtracellular [Mm]atrix  PubMed  
 The adaptor protein Shc integrates growth factor and ECM signaling during postnatal angiogenesis. Angiogenesis requires integration of cues from growth factors,   extracellular matrix   (ECM) proteins, and their receptors in endothelial cells. In the present study, we show that the adaptor protein Shc is required for angiogenesis in zebrafish, mice, and cell-culture models. Shc knockdown zebrafish embryos show defects in intersegmental vessel sprouting in the trunk. Shc flox/flox; Tie2-Cre mice display reduced angiogenesis in the retinal neovascularization model and in response to VEGF in the Matrigel plug assay in vivo. Functional studies reveal a model in which Shc is required for integrin-mediated spreading and migration specifically on fibronectin, as well as endothelial cell survival in response to VEGF. Mechanistically, Shc is required for activation of the Akt pathway downstream of both integrin and VEGF signaling, as well as for integration of signals from these 2 receptors when cells are grown on fibronectin. Therefore, we have identified a unique mechanism in which signals from 2 critical angiogenic signaling axes, integrins and VEGFR-2, converge at Shc to regulate postnatal angiogenesis. 
 Outside-in integrin signal transduction. Alpha IIb beta 3-(GP IIb IIIa) tyrosine phosphorylation induced by platelet aggregation. alpha IIb beta 3-(GP IIb IIIa) is the most abundant integrin expressed on platelets and plays a critical role in platelet aggregation and normal hemostasis. In response to platelet stimulation by agonists such as thrombin, alpha IIb beta 3 becomes a receptor for the adhesive proteins fibrinogen, von Willebrand factor, vitronectin, and fibronectin. Binding of   extracellular matrix   ligands allows the integrin to transmit a signal to the inside of the cell, but the exact mechanisms whereby integrins transduce these signals remains unclear. In this paper we demonstrate that the beta 3 subunit of alpha IIb beta 3 was phosphorylated on tyrosine residues in response to thrombin-induced platelet aggregation. However, tyrosine phosphorylation was not observed when platelets were stimulated by thrombin in the presence of an inhibitor of aggregation. Phosphotyrosine was only detected when platelets were solubilized under protein-denaturing conditions. A peptide corresponding to residues 740-762 of the beta 3 cytoplasmic domain was capable of binding the signaling proteins SHC and GRB2. GRB2 binding occurred only when both tyrosine residues (Tyr-747 and Tyr-759) were phosphorylated. SHC binding also occurred to a peptide monophosphorylated at Tyr-759. The data suggest that tyrosine phosphorylation of an integrin beta subunit may be important in initiating outside-in signaling cascades by inducing association of signaling components directly with the integrin. 
 The discoidin domain receptor tyrosine kinases are activated by collagen. Two mammalian receptor tyrosine kinases (DDR1 and DDR2) have extracellular domains closely related to a D. discoideum lectin, discoidin, required for cell aggregation. Here, we show that the mammalian DDR receptors bind and are activated by specific types of collagen. Stimulation of DDR receptor tyrosine kinase activity requires the native triple-helical structure of collagen and occurs over an extended period of time. Collagen activation of DDR1 induces phosphorylation of a docking site for the Shc phosphotyrosine binding domain, whose presence is controlled by alternative splicing. Activation of DDR2 by collagen results in the up-regulation of matrix metalloproteinase-1 expression. These results suggest that the discoidin-related DDR tyrosine kinases are novel collagen receptors with the potential to control cellular responses to the   extracellular matrix  . 
  GeneRIFs  
 regulates the alternative splicing of XAF1 in   extracellular matrix  -detachment induced autophagy to coordinate with the anoikic cell death 
  [Ii]nvasive  PubMed  
 Distinct phosphotyrosine-dependent functions of the ShcA adaptor protein are required for transforming growth factor β (TGFβ)-induced breast cancer cell migration, invasion, and metastasis. The ErbB2 and TGFβ signaling pathways cooperate to promote the migrator  y, invas  ive, and metastatic behavior of breast cancer cells. We previously demonstrated that ShcA is necessary for these synergistic interactions. Through a structure/function approach, we now show that the phosphotyrosine-binding, but not the Src homology 2, domain of ShcA is required for TGFβ-induced migration and invasion of ErbB2-expressing breast cancer cells. We further demonstrate that the tyrosine phosphorylation sites within ShcA (Tyr(239)/Tyr(240) and Tyr(313)) transduce distinct and non-redundant signals that promote these TGFβ-mediated effects. We demonstrate that Grb2 is required specifically downstream of Tyr(313), whereas the Tyr(239)/Tyr(240) phosphorylation sites require the Crk adaptor proteins to augment TGFβ-induced migration and invasion. Furthermore, ShcA Tyr(313) phosphorylation enhances tumor cell survival, and ShcA Tyr(239)/Tyr(240) signaling promotes endothelial cell recruitment into ErbB2-expressing breast tumors in vivo, whereas all three ShcA tyrosine residues are required for efficient breast cancer metastasis to the lungs. Our data uncover a novel ShcA-dependent signaling axis downstream of TGFβ and ErbB2 that requires both the Grb2 and Crk adaptor proteins to increase the migrato  ry and i  nvasive properties of breast cancer cells. In addition, signaling downstream of specific ShcA tyrosine residues facilitates the survival, vascularization, and metastatic spread of breast tumors. 
 The adaptor proteins p66Shc and Grb2 regulate the activation of the GTPases ARF1 and ARF6 in   invasive   breast cancer cells. Signals downstream of growth factor receptors play an important role in mammary carcinogenesis. Recently, we demonstrated that the small GTPases ARF1 and ARF6 were shown to be activated downstream of the epidermal growth factor receptor (EGFR) and act as a key regulator of growth, migration, and invasion of breast cancer cells. However, the mechanism via which the EGFR recruits and activates ARF1 and ARF6 to transmit signals has yet to be fully elucidated. Here, we identify adaptor proteins Grb2 and p66Shc as important regulators mediating ARF activation. We demonstrate that ARF1 can be found in complex with Grb2 and p66Shc upon EGF stimulation of the basal-like breast cancer MDA-MB-231 cell line. However, we report that these two adaptors regulate ARF1 activation differently, with Grb2 promoting ARF1 activation and p66Shc blocking this response. Furthermore, we show that Grb2 is essential for the recruitment of ARF1 to the EGFR, whereas p66Shc hindered ARF1 receptor recruitment. We demonstrate that the negative regulatory role of p66Shc stemmed from its ability to block the recruitment of Grb2/ARF1 to the EGFR. Conversely, p66Shc potentiates ARF6 activation as well as the recruitment of this ARF isoform to the EGFR. Interestingly, we demonstrate that Grb2 is also required for the activation and receptor recruitment of ARF6. Additionally, we show an important role for p66Shc in modulating ARF activation, cell growth, and migration in HER2-positive breast cancer cells. Together, our results highlight a central role for adaptor proteins p66Shc and Grb2 in the regulation of ARF1 and ARF6 activation in   invasive   breast cancer cells. 
 Activation of the EphA2 tyrosine kinase stimulates the MAP/ERK kinase signaling cascade. Intracellular signaling by receptor tyrosine kinases regulates many different aspects of cell behavior. Recent studies in our laboratory and others have demonstrated that the EphA2 receptor tyrosine kinase critically regulates tumor cell growth, migration and   invasive  ness. Although the cellular consequences of EphA2 signaling have been the focus of recent attention, the biochemical changes that are triggered by ligand-mediated activation of EphA2 remain largely unknown. Herein, we demonstrate that ligand stimulation of EphA2 promotes the nucleus translocation and phosphorylation of ERK kinases, followed by an increase in nuclear induction of the Elk-1 transcription factor. Ligand-mediated activation allows EphA2 to form a molecular complex with the SHC and GRB2 adaptor proteins. Specifically, we demonstrate that tyrosine phosphorylated EphA2 interacts with the PTB and SH2 domains of SHC. We also show that the interaction of EphA2 with GRB2 is indirect and mediated by SHC and that this complex is necessary for EphA2-mediated activation of ERK kinases. These studies provide a novel mechanism to demonstrate how EphA2 can convey information from the cell exterior to the nucleus. 
 HER2 kinase domain mutation results in constitutive phosphorylation and activation of HER2 and EGFR and resistance to EGFR tyrosine kinase inhibitors. HER2/Neu gene mutations have been identified in lung cancer. Expression of a HER2 mutant containing a G776(YVMA) insertion in exon 20 was more potent than wild-type HER2 in associating with and activating signal transducers, phosphorylating EGFR, and inducing survival,   invasive  ness, and tumorigenicity. HER2(YVMA) transphosphorylated kinase-dead EGFR(K721R) and EGFR(WT) in the presence of EGFR tyrosine kinase inhibitors (TKIs). Knockdown of mutant HER2 in H1781 lung cancer cells increased apoptosis and restored sensitivity to EGFR TKIs. The HER2 inhibitors lapatinib, trastuzumab, and CI-1033 inhibited growth of H1781 cells and cells expressing exogenous HER2(YVMA). These data suggest that (1) HER2(YVMA) activates cellular substrates more potently than HER2(WT); and (2) cancer cells expressing this mutation remain sensitive to HER2-targeted therapies but insensitive to EGFR TKIs. 
  GeneRIFs  
 a central role for adaptor proteins p66Shc and Grb2 in the regulation of ARF1 and ARF6 activation in   invasive   breast cancer cells. 
  [Mm]etastasis  PubMed  
 Positional mapping for amplified DNA sequences on 1q21-q22 in hepatocellular carcinoma indicates candidate genes over-expression. Comparative genomic hybridization analysis on hepatocellular carcinoma (HCC) indicated frequent gains of 1q and an amplicon at 1q21-q22. Current cytogenetic evidences confer much importance on 1q21-q22, where a role in drug resistance, tumor   metastasis   and shorter patient survival had been implicated.Using positional mapping by interphase cytogenetics, we investigated the amplicon 1q21-q22 in five HCC cases. Three amplification maxima represented by yeast artificial chromosomes (YACs) 955E11, 876B11 and 945D5 that mapped to regions 1q21.1, 1q21.2 and 1q22, respectively, were indicated. We further investigated candidate genes expression in the mapped YACs by quantitative reverse-transcription-polymerase chain reaction. A panel of genes encoding protein transcripts involved in apoptosis, cell cycle progression, calcium binding and jumping translocation was studied.Among ten HCC cases with the amplicon 1q21-q22 examined, we found a significant gene expression level of JTB, SHC1, CCT3 and COPA in the tumors than the paired adjacent non-malignant liver tissues (P< or =0.04).Our interphase findings on 1q21-q22 pinpointed three affected loci between D1S305 and D1S2369. Up-regulation of candidate genes identified within these over-represented regions may represent targets in the progression of HCC and may carry prognostic significance. 
 Positional mapping for amplified DNA sequences on 1q21-q22 in hepatocellular carcinoma indicates candidate genes over-expression. Comparative genomic hybridization analysis on hepatocellular carcinoma (HCC) indicated frequent gains of 1q and an amplicon at 1q21-q22. Current cytogenetic evidences confer much importance on 1q21-q22, where a role in drug resistance, tumor   metastasis   and shorter patient survival had been implicated.Using positional mapping by interphase cytogenetics, we investigated the amplicon 1q21-q22 in five HCC cases. Three amplification maxima represented by yeast artificial chromosomes (YACs) 955E11, 876B11 and 945D5 that mapped to regions 1q21.1, 1q21.2 and 1q22, respectively, were indicated. We further investigated candidate genes expression in the mapped YACs by quantitative reverse-transcription-polymerase chain reaction. A panel of genes encoding protein transcripts involved in apoptosis, cell cycle progression, calcium binding and jumping translocation was studied.Among ten HCC cases with the amplicon 1q21-q22 examined, we found a significant gene expression level of JTB, SHC1, CCT3 and COPA in the tumors than the paired adjacent non-malignant liver tissues (P< or =0.04).Our interphase findings on 1q21-q22 pinpointed three affected loci between D1S305 and D1S2369. Up-regulation of candidate genes identified within these over-represented regions may represent targets in the progression of HCC and may carry prognostic significance. 
 Feedback loop between p66(Shc) and Nrf2 promotes lung cancer progression. p66(Shc), one of the SHC1 gene encoding proteins, promotes cell death and reports cell anchorage status, mediating anoikis in vitro and functioning as a   metastasis   suppressor in vivo. However, very little is known about p66(Shc) gene regulation in cancer cells. Here, we show that methylation of a specific CpG site in the early post-transcriptional region correlates with p66(Shc) repression in clinical human lung cancer samples and cancer cell lines. We also find that the stress related transcription factor Nrf2 associates with p66(Shc) gene promoter in the methylated region, and promotes p66(Shc) transcription. However, p66(Shc) induction by Nrf2 requires demethylation of the Nrf2 binding site in p66(Shc) promoter. Knock-down of p66(Shc) leads to a positive feedback upregulation of Nrf2 expression and accordingly, Nrf2 is found to be highly expressed in tumors with low p66(Shc) expression. Further, Nrf2 expression level positively correlates with tumor grade of patients. Thus, we propose that epigenetic repression of p66(Shc) in cancer cells might be a key factor leading to Nrf2 upregulation, increased cell survival, and tumor progression. 
 p66Shc--a longevity redox protein in human prostate cancer progression and   metastasis   : p66Shc in cancer progression and   metastasis  . p66Shc, a 66 kDa proto-oncogene Src homologous-collagen homologue (Shc) adaptor protein, is classically known in mediating receptor tyrosine kinase signaling and recently identified as a sensor to oxidative stress-induced apoptosis and as a longevity protein in mammals. The expression of p66Shc is decreased in mice and increased in human fibroblasts upon aging and in aging-related diseases, including prostate cancer. p66Shc protein level correlates with the proliferation of several carcinoma cells and can be regulated by steroid hormones. Recent advances point that p66Shc protein plays a role in mediating cross-talk between steroid hormones and redox signals by serving as a common convergence point in signaling pathways on cell proliferation and apoptosis. This article first reviews the unique function of p66Shc protein in regulating oxidative stress-induced apoptosis. Subsequently, we discuss its novel role in androgen-regulated prostate cancer cell proliferation and   metastasis   and the mechanism by which it mediates androgen action via the redox signaling pathway. The data together indicate that p66Shc might be a useful biomarker for the prognosis of prostate cancer and serve as an effective target for its cancer treatment. 
 Migration of renal tumor cells depends on dephosphorylation of Shc by PTEN. The tumor suppressor PTEN is a phosphatase using FAK and Shc as direct substrates, and Akt as a key effector via PIP3. PTEN regulates cell migration and may influence metastases. We quantified PTEN in 135 clear cell renal cell carcinomas (ccRCC) by Western blot analysis and found statistically significant lower PTEN expression in patients who died, usually caused by metastases, within 5 years after surgery, compared to those surviving this time period. In athymic mice, PTEN transfected 786-O cells were injected into the tail vein and metastatic load of the lungs was quantified. We observed a strongly reduced metastatic load after PTEN transfection. For analyses of the PTEN activities, transfections with mutated PTEN genes were performed, leading to loss of lipid phosphatase activity and/or protein phosphatase activity, and of the C-terminal tail. Cell migration was analyzed in a Boyden chamber and phosphorylation of PTEN downstream targets Akt, FAK and Shc by Western blotting. 786-O cells transfected with the functional PTEN gene showed profoundly diminished migration. Transfection with a mutated PTEN isoform leading to loss of protein phosphatase activity, but not of lipid phosphatase activity, abolished this effect. Shc but not FAK seems to mediate this effect. These results show a critical role of PTEN in   metastasis   of RCC, depending on protein phosphatase activity via Shc. This new insight opens an alley of additional approaches complementing current cancer therapy and   metastasis   prediction in RCC. 
 Use of signal specific receptor tyrosine kinase oncoproteins reveals that pathways downstream from Grb2 or Shc are sufficient for cell transformation and   metastasis  . Many human cancers have been associated with the deregulation of receptor tyrosine kinases (RTK). However, the individual contribution of receptor-associated signaling proteins in cellular transformation and   metastasis   is poorly understood. To examine the role of RTK activated signal transduction pathways to processes involved in cell transformation, we have exploited the oncogenic derivative of the Met RTK (Tpr-Met). Unlike other RTKs, twin tyrosine residues in the carboxy-terminal tail of the Met oncoprotein and receptor are required for all biological and transforming activities, and a mutant lacking these tyrosines is catalytically active but non transforming. Using this mutant we have inserted oligonucleotide cassettes, each encoding a binding site for a specific signaling protein derived from other RTKs. We have generated variant forms of the Tpr-Met oncoprotein with the ability to bind individually to the p85 subunit of PI3'K, PLCgamma, or to the Grb2 or Shc adaptor proteins. Variants that recruit the Shc or Grb2 adaptor proteins generated foci of morphologically transformed fibroblast cells and induced anchorage-independent growth, scattering of epithelial cells and experimental   metastasis  . In contrast, variants that bind and activate PI3'K or PLCgamma failed to generate readily detectable foci. Although cell lines expressing the PI3'K variant grew in soft-agar, these cells were non metastatic. Using this unique RTK oncoprotein model, we have established that Grb2 or Shc dependent signaling pathways are sufficient for cell transformation and metastatic spread. 
 The expression of p66Shc protein in benign, premalignant, and malignant gastrointestinal lesions. ROS produced from Oxidative stress have long been recognized to be involved in carcinogenesis. p66Shc generates H(2)O(2) by oxidizing cytochrome c, and its expression has been reported to be elevated in several tumors. However, the expression of p66Shc in gastric cancer has not been reported, and its role in colorectal cancer has not been well elucidated. This study investigated p66Shc expression in benign, premalignant, and malignant gastric and colorectal lesions. p66Shc expression in 146 gastric tumors, 136 colorectal tumors, 45 gastric hyperplastic polyps, 33 gastric low-grade intraepithelial neoplasias, 41 gastric high-grade intraepithelial neoplasias, 42 colorectal hyperplastic polyps, 21 colorectal low-grade intraepithelial neoplasias, 38 colorectal high-grade intraepithelial neoplasias, and 30 normal gastric and colorectal tissues was measured by immunohistochemistry. Most normal gastric and colorectal tissues exhibited low or no p66Shc expression (93.4 %), while most gastric and colorectal tumors exhibited moderate to high p66Shc expression (78.1 %-80.9 %). The p66Shc expression in normal gastric and colorectal tissues were significantly lower than that in the low-grade intraepithelial neoplasias (p < 0.05), high-grade intraepithelial neoplasias (p < 0.01), and gastric adenocarcinomas (p < 0.01 or <0.001). No differences in p66Shc expression were observed in gastric and colorectal hyperplastic polyps compared to the normal tissues. No statistically significant differences in p66Shc expression were observed between patients with different disease stages, different tumor grades, and with or without   lymph nod  e metastasis in gastric and colorectal cancers. In conclusion, p66Shc may be involved in the carcinogenesis of gastric and colorectal cancers and could be a marker for the diagnosis of gastric and colorectal cancers. 
 Downregulated adaptor protein p66(Shc) mitigates autophagy process by low nutrient and enhances apoptotic resistance in human lung adenocarcinoma A549 cells. Macroautophagy or autophagy is a lysosome-dependent process in which enzymatic degradation and recycling of cytosolic components occur in stressful contexts. The mechanisms underlying the signaling from starvation to the regulation of autophagy are not fully understood. We previously showed that the Src family member p66(Shc) (focal adhesion-associated 66 kDa isoform of the Src homology and collagen) promotes anoikis and suppresses tumor   metastasis   via k-Ras-dependent control of proliferation and survival. However, the role of p66(Shc) in low-nutrient-induced autophagy-related pathways remains elusive. In this work, human lung adenocarcinoma A549 cells were used to further investigate the biological effects of p66(Shc) on autophagy and apoptotic resistance. Here, we show that deficiency of p66(Shc) mitigates the low-nutrient-induced autophagy process in the levels of microtubule-associated protein 1A light chain protein 3B (LC3B) conversion, in the number of autophagic vacuoles and in p62/sequestosome 1 protein degradation. However, autophagy-related protein Beclin 1 was not significantly changed during low-nutrient treatment. Furthermore, we found that prolonged phosphorylation of extracellular signaling-regulated kinase (Erk)1/2, but not phosphorylation of Akt is significantly sustained when p66(Shc) expression is inhibited by shRNA. In addition, cleavage of caspase 7 and poly(ADP-ribose) polymerase, but not caspase 6 and 9 are retarded with this effect compared to the shRNA control cells. Together, these findings suggest the possibility that p66(Shc) plays a pivotal role in coordinately regulating autophagy process and apoptotic resistance in A549 cells under nutrient-limited conditions. 
 Distinct phosphotyrosine-dependent functions of the ShcA adaptor protein are required for transforming growth factor β (TGFβ)-induced breast cancer cell migration, invasion, an  d metastas  is. The ErbB2 and TGFβ signaling pathways cooperate to promote the migratory, invasive, and metastatic behavior of breast cancer cells. We previously demonstrated that ShcA is necessary for these synergistic interactions. Through a structure/function approach, we now show that the phosphotyrosine-binding, but not the Src homology 2, domain of ShcA is required for TGFβ-induced migration and invasion of ErbB2-expressing breast cancer cells. We further demonstrate that the tyrosine phosphorylation sites within ShcA (Tyr(239)/Tyr(240) and Tyr(313)) transduce distinct and non-redundant signals that promote these TGFβ-mediated effects. We demonstrate that Grb2 is required specifically downstream of Tyr(313), whereas the Tyr(239)/Tyr(240) phosphorylation sites require the Crk adaptor proteins to augment TGFβ-induced migration and invasion. Furthermore, ShcA Tyr(313) phosphorylation enhances tumor cell survival, and ShcA Tyr(239)/Tyr(240) signaling promotes endothelial cell recruitment into ErbB2-expressing breast tumors in vivo, whereas all three ShcA tyrosine residues are required for efficient breast c  ancer meta  stasis to the lungs. Our data uncover a novel ShcA-dependent signaling axis downstream of TGFβ and ErbB2 that requires both the Grb2 and Crk adaptor proteins to increase the migratory and invasive properties of breast cancer cells. In addition, signaling downstream of specific ShcA tyrosine residues facilitates the survival, vascularization, and metastatic spread of breast tumors. 
 p66(Shc) restrains Ras hyperactivation and suppresses metastatic behavior. Normal tissue cells survive and proliferate only while anchored to solid substrate. Conversely, transformed cells both survive and proliferate following detachment, having lost attachment context through unclear mechanisms. p66(Shc) is a focal adhesion-associated protein that reports cell attachment through a RhoA-dependent mechanosensory test. We find that human small cell lung cancer (SCLC) cells and mouse Lewis lung carcinoma (LLC), which display aggressive metastatic behavior, lack both p66(Shc) and retinoblastoma (pRB) and bypass anoikis. Re-expression of p66(Shc) in these cells restores anoikis and provides striking protection from   metastasis   by LLC cells in vivo. Notably, knockdown of p66(Shc) in normal epithelial cells leads to unrestrained Ras activation, preventing anoikis through downstream suppression of RhoA but blocking proliferation in a pRB-dependent manner, thus mimicking oncogenic Ras. Conversely, LLC and SCLC cells display constitutive Ras activation necessary to bypass anoikis, which is reversed by re-expression of p66(Shc). p66(Shc) therefore coordinates Ras-dependent control of proliferation and anchorage sensation, which can be defeated in the evolution of highly metastatic tumors by combined loss of both p66(Shc) and pRB. 
 The SRC-associated protein CUB Domain-Containing Protein-1 regulates adhesion and motility. Multiple SRC-family kinases (SFKs) are commonly activated in carcinoma and appear to have a role in   metastasis   through incompletely understood mechanisms. Recent studies have shown that CDCP1 (CUB (complement C1r/C1s, Uegf, Bmp1) Domain-Containing Protein-1) is a transmembrane protein and an SRC substrate potentially involved in   metastasis  . Here we show that increased SFK and CDCP1 tyrosine phosphorylation is, surprisingly, associated with a decrease in FAK phosphorylation. This appears to be true in human tumors as shown by our correlation analysis of a mass spectrometric data set of affinity-purified phosphotyrosine peptides obtained from normal and cancer lung tissue samples. Induction of tyrosine phosphorylation of CDCP1 in cell culture, including by a mAb that binds to its extracellular domain, promoted changes in SFK and FAK tyrosine phosphorylation, as well as in PKC(TM), a protein known to associate with CDCP1, and these changes are accompanied by increases in adhesion and motility. Thus, signaling events that accompany the CDCP1 tyrosine phosphorylation observed in cell lines and human lung tumors may explain how the CDCP1/SFK complex regulates motility and adhesion. 
 Detachment-Based Equilibrium of Anoikic Cell Death and Autophagic Cell Survival Through Adaptor Protein p66(Shc). Anoikis (detachment-induced cell death) confers a tumor-suppressive function in metastatic cancer cells. Autophagy, a conserved self-degradative process, enhances the anoikis resistance of detached cancer cells by maintaining cellular homeostasis. However, the mechanism of regulating cell fate-decision by balancing anoikis and autophagy has been poorly understood. Our previous studies have shown that the adaptor protein p66(Shc) mediates anoikis through RhoA activation and inhibits tumor   metastasis   in vivo. We also found that p66(Shc) depletion mitigates nutrient-deprivation-induced autophagy. These findings suggest p66(Shc) may coordinately regulate these two processes. To verify this hypothesis, we investigated the effect of p66(Shc) on the cell death of detached lung cancer cells, and measured autophagy markers and autophagic flux. Results showed that p66(Shc) depletion significantly inhibited anoikis, and reduced the formation of LC3B-II and the degradation of Sequestosome 1 (SQSTM1, p62) in detachment-induced cells. Using monodansylcadaverine (MDC)-LysoTracker double staining and monomeric Cherry (mCherry)-GFP-LC3 assay, we found that the autophagic flux was also mitigated by p66(Shc) depletion. In addition, p66(Shc) knockdown increased the formation of full-length X-linked inhibitor of apoptosis (XIAP)-associated factor 1 (XAF1), which enhances anoikis sensitivity. In conclusion, p66(Shc) plays an essential role in detachment-based equilibrium of anoikic cell death and autophagic cell survival. 
  GeneRIFs  
 Distinct phosphotyrosine-dependent functions of the ShcA adaptor protein are required for transforming growth factor beta (TGFbeta)-induced breast cancer cell migration, invasion, and   metastasis   
   
  
 MSN 
 
  NCBI Gene  
  GeneRIF  
  Pubmed  
 
 
  Search Term  Rank  Fields  
  [Ff]ilopodia  1  PubMed(9); GeneRIFs(1); Summary(1)  
  [Ff]ilopodium  2  Component(1)  
  [Ii]nvadopodia  3  PubMed(1); GeneRIFs(1)  
  [Ii]nvadopodium  4  PubMed(1)  
  [Pp]odosome(s)?  5  Process(1); PubMed(1); GeneRIFs(1)  
  [Mm]igration  6  Process(3); PubMed(20); GeneRIFs(2)  
  [Ee]xtracellular [Mm]atrix  8  PubMed(8); GeneRIFs(1)  
  [Ii]nvasive  10  PubMed(7)  
  [Mm]etastasis  12  PubMed(16); GeneRIFs(6)  
  [Cc]ell [Ii]nvasion  13  PubMed(3); GeneRIFs(1)  
 
 
 hide/show details for MSN 
  [Ff]ilopodia  PubMed  
 Phosphorylation of threonine 558 in the carboxyl-terminal actin-binding domain of moesin by thrombin activation of human platelets. The phosphorylation and localization of the membrane-linking protein moesin was analyzed during early activation of platelets with thrombin. Activated platelets elaborate   filopodia   and spread to assume flat pancake-like shapes, and moesin is localized in   filopodia   and cell body. In resting platelets, approximately 25% of moesin molecules are phosphorylated as shown by metabolic labeling with 32P(i) and by isoelectric focusing. Within seconds after exposure to thrombin, phosphorylation increases, reaching a maximum of 35% labeled molecules by 1 min, followed by a decrease to a new basal level within 5 min. This modification affects a single residue, Thr558, which is located within or close to a binding site for F-actin. Rapid shifts (0-100%) in the number of phosphorylated molecules are observed in the presence of inhibitors of serine/threonine kinases and phosphatases. Inhibitors affecting tyrosine phosphorylation also modulate phosphorylation at this site suggesting that the enzymes involved in the modification of Thr558 are regulated by tyrosine phosphorylation. Platelets respond to both extremes of modification by forming extremely long   filopodia   and the absence of spreading on glass. Completely phosphorylated moesin is concentrated together with F-actin in the center of the cell. The rapid modification of moesin at or near its actin-binding domain suggests a model for regulated membrane-cytoskeleton interaction during cell activation. 
 Protein phosphatase 2C inactivates F-actin binding of human platelet moesin. During activation of platelets by thrombin phosphorylation of Thr(558) in the C-terminal domain of the membrane-F-actin linking protein moesin increases transiently, and this correlates with protrusion of   filopodia  l structures. Calyculin A enhances phosphorylation of moesin by inhibition of phosphatases. To measure this moesin-specific activity, a nonradioactive enzyme-linked immunosorbent assay method was developed with the synthetic peptide Cys-Lys(555)-Tyr-Lys-Thr(P)-Leu-Arg(560) coupled to bovine serum albumin as the substrate and moesin phosphorylation state-specific polyclonal antibodies for the detection and quantitation of dephosphorylation. Calyculin A-sensitive and -insensitive protein-threonine phosphatase activities were detected in platelet lysates and separated by DEAE-cellulose chromatography. The calyculin A-sensitive enzyme was identified as a type 1 protein phosphatase. The calyculin A-insensitive enzyme activity was purified to homogeneity by phenyl- Sepharose, protamine-, and phosphonic acid peptide-agarose chromatography and characterized biochemically and immunologically as a 53-kDa protein(s) and a type 2C protein phosphatase (PP2C). Phosphorylation of Thr(558) is necessary for F-actin binding of moesin in vitro. The purified enzyme, as well as bacterially made PP2Calpha and PP2Cbeta, efficiently dephosphorylate(s) highly purified platelet phospho-moesin. This reverses the activating effect of phosphorylation, and moesin no longer co-sediments with actin filaments. In vivo, regulation of these phosphatase activities are likely to influence dynamic interactions between the actin cytoskeleton and membrane constituents linked to moesin. 
 Structure and localization on the X chromosome of the gene coding for the human   filopodia  l protein moesin (MSN). Moesin is a member of a recently discovered family of closely related proteins that includes ezrin, radixin, and merlin. It is widely expressed in different tissues and cells and has been localized to   filopodia   and other membranous protrusions that are important for cell-cell recognition and signaling and cell movement. Here, we have localized the coding gene (MSN) to Xq11.2-q12 by Southern and Western blot analyses of Chinese hamster x human somatic cell hybrids and by fluorescence chromosomal in situ hybridization. Moesin-like sequences were identified on chromosomes 5 and 6. The murine Msn locus was mapped to the X chromosome as well by studying a rodent x mouse hybrid panel. The structure of the human moesin gene has been determined. The 12 exons are distributed over > 30 kb, and the exon/intron junctions demarcate individual highly conserved domains. Primer extension analysis revealed two major start transcription sites, 184 and 133 bp upstream of the initiation codon. The 5'-flanking region is GC-rich, lacks a TATA box, and contains four SP1 and one AP1 binding sites. 
 Moesin regulates the motility of oral cancer cells via MT1-MMP and E-cadherin/p120-catenin adhesion complex. The present study aimed to clarify the role of Moesin in oral squamous cell carcinoma (OSCC) progression, especially in regulation of cell motility.Immunohistochemistry and western blotting were used to investigate the expression of Moesin, E-cadherin, p120-catenin and MT1-MMP in normal epithelia, dysplasia and OSCCs. Then, Moesin was knockdown by siRNA in OSCC cell lines, WSU-HN6 and CAL27, and the biological role of Moesin in cell adhesion and motility was evaluated by transwell system, cell spreading and aggregation assays. The interactions between Moesin, MT1-MMP and E-cadherin/p120-catenin complex were determined by co-immunoprecipitation and immunofluorescence.Moesin expression was found decreased in the membrane and increased in cytoplasm during the malignant transformation of oral epithelia, and cytoplasmic overexpression of Moesin correlated with nodal metastasis and poor prognosis of OSCCs. Furthermore, Moesin-silencing induced an increased cell-cell adhesion but decreased invasiveness, which was subsequently demonstrated might due to Moesin-mediated E-cadherin and p120-catenin interaction. Meantime, Moesin-silencing significantly down-regulated MT1-MMP expression, accompanied by reduced cell motility and impaired   filopodia   formation, which was also observed when MT1-MMP knockdown by RNAi or tissue inhibitor (TIMP2), indicating the involvement of MT1-MMP in Moesin-mediated cell motility. Finally, the relationship between Moesin, E-cadherin and MT1-MMP was confirmed in OSCC tissue samples.Taken together, our results indicate Moesin may regulate cell motility through its interactions with MT1-MMP and E-cadherin/p120-catenin adhesion complex and cytoplasmic expression of Moesin correlates with nodal metastasis and poor prognosis of OSCCs, indicating Moesin may be a potential candidate for targeted gene therapy for OSCCs. 
 Subcellular localization of moesin in dynamic   filopodia  , retraction fibers, and other structures involved in substrate exploration, attachment, and cell-cell contacts. Moesin, a member of the talin-4.1 superfamily, is a linking protein of the submembraneous cytoskeleton. It is expressed in variable amounts in cells of different phenotypes such as macrophages, lymphocytes, fibroblastic, endothelial, epithelial, and neuronal cell lines. In this report we show that moesin is not randomly distributed throughout the cortical cytoskeleton, but rather that it is concentrated in specialized microdomains. It is localized in the intracellular core of microextensions known as   filopodia  , microvilli, microspikes, and retraction fibers. This subcellular distribution follows closely the dynamic changes in cell shape that take place when cells attach, spread, and move spontaneously or in response to extracellular signals. This suggests a similar function for moesin in diverse cell types related to the dynamic restructuring of domains of the plasma membrane and underlying membrane skeleton. Support for this comes from studies on PC-12 cells, which respond to NGF by extending neurites and moesin is redistributed from a diffuse localization to growth cone   filopodia  . In fibroblastic (NIH3T3) or macrophage (RAW264.7) cell lines, moesin is found in   filopodia   appearing at random on the cell surface soon after the cells are placed in culture, begin to attach, and spread. In polarized epithelial cells (LLC-PK1), moesin is associated with peripheral   filopodia   and apical microvilli. The cellular microextensions containing moesin are devoid of microtubules, focal contact proteins such as vinculin, and cortical cytoskeletal elements such as protein 4.1, but they do contain varying amounts of actin microfilaments. This localization of moesin in microextensions is not influenced by cytochalasin B. Treatment of cells with phorbolester (PMA) causes rapid cell spreading, disappearance of   filopodia   and retraction fibers, and moesin does not accumulate in the actin-rich lamellae that form at the cellular edges. After removal of PMA, cells retract and moesin again becomes concentrated in   filopodia   and retraction fibers. These studies support the hypothesis that   filopodia  , retraction fibers, and other microextensions of the plasma membrane are unique cellular microdomains with characteristic submembraneous components. Moesin could be involved in the dynamic restructuring of such microdomains by regulating binding interactions between the plasma membrane and the actin cytoskeleton. 
 Tyrosine Phosphorylation of Moesin in Arachidonic Acid-Stimulated Human Platelets. Moesin, a member of the ezrin/radixin/moesin (ERM) family of cytoskeletal proteins, has been implicated in dynamic membrane-based processes such as the formation and stabilization of   filopodia  . Ezrin is known to be a substrate of tyrosine kinases in activated T cells and epithelial growth factor-stimulated A431 cells. For the closely related 77-kD protein moesin, which shares 72% identity with ezrin on the basis of their amino acid sequences, a reversible phosphorylation on tyrosine residues has not yet been described. Because our scanning electron microscopy studies revealed the appearance of multiple, up to 3 µm long   filopodi  a on the surface of activated human platelets, we investigated the participation of moesin in dynamic shape changes on platelet stimulation with arachidonic acid. Antimoesin immunoprecipitates obtained under denaturing conditions from lysates of resting platelets contained only low amounts of tyrosine-phosphorylated moesin. In lysates of arachidonic acid-stimulated platelets, the level of tyrosine phosphorylation was significantly increased. This activation-dependent phosphorylation of moesin was verified by probing antiphosphotyrosine immunoprecipitates from unstimulated and stimulated platelets with antimoesin antibodies. Tyrosine-phosphorylated moesin was detectable only in the presence of the tyrosine phosphatase inhibitor vanadate, suggesting that a coordinated balance between kinase and phosphatase activities controls the steady-state level of moesin phosphorylation. 
 Phosphorylation of ezrin/radixin/moesin proteins by LRRK2 promotes the rearrangement of actin cytoskeleton in neuronal morphogenesis. Leucine-rich repeat kinase 2 (LRRK2) functions as a putative protein kinase of ezrin, radixin, and moesin (ERM) family proteins. A Parkinson's disease-related G2019S substitution in the kinase domain of LRRK2 further enhances the phosphorylation of ERM proteins. The phosphorylated ERM (pERM) proteins are restricted to the   filopodia   of growing neurites in which they tether filamentous actin (F-actin) to the cytoplasmic membrane and regulate the dynamics of   filopodia   protrusion. Here, we show that, in cultured neurons derived from LRRK2 G2019S transgenic mice, the number of pERM-positive and F-actin-enriched   filopodia   was significantly increased, and this correlates with the retardation of neurite outgrowth. Conversely, deletion of LRRK2, which lowered the pERM and F-actin contents in   filopodia  , promoted neurite outgrowth. Furthermore, inhibition of ERM phosphorylation or actin polymerization rescued the G2019S-dependent neuronal growth defects. These data support a model in which the G2019S mutation of LRRK2 causes a gain-of-function effect that perturbs the homeostasis of pERM and F-actin in sprouting neurites critical for neuronal morphogenesis. 
 NHE-RF, a regulatory cofactor for Na(+)-H+ exchange, is a common interactor for merlin and ERM (MERM) proteins. We have identified the human homologue of a regulatory cofactor of Na(+)-H+ exchanger (NHE-RF) as a novel interactor for merlin, the neurofibromatosis 2 tumor suppressor protein. NHE-RF mediates protein kinase A regulation of Na(+)-H+ exchanger NHE3 to which it is thought to bind via one of its two PDZ domains. The carboxyl-terminal region of NHE-RF, downstream of the PDZ domains, interacts with the amino-terminal protein 4.1 domain-containing segment of merlin in yeast two-hybrid assays. This interaction also occurs in affinity binding assays with full-length NHE-RF expressed in COS-7 cells. NHE-RF binds to the related ERM proteins, moesin and radixin. We have localized human NHE-RF to actin-rich structures such as membrane ruffles, microvilli, and   filopodia   in HeLa and COS-7 cells, where it co-localizes with merlin and moesin. These findings suggest that hNHE-RF and its binding partners may participate in a larger complex (one component of which might be a Na(+)-H+ exchanger) that could be crucial for the actin filament assembly activated by the ERM proteins and for the tumor suppressor function of merlin. 
 Platelet moesin interacts with PECAM-1 (CD31). Platelet activation results information of   filopodia   and cell spreading by extension of lamellae. Moesin is a member of the ezrin/radixin/moesin (ERM) family of proteins, which localize in cell extensions like   filopodia   and function as cross-linkers between the actin cytoskeleton and the plasma membrane. Here we investigated whether the adhesion molecule PECAM-1 (CD31) is a membrane-binding partner for moesin in platelets. Our data show that moesin co-immunoprecipitated with PECAM-1 in lysates from thrombin-stimulated, but not resting platelets. Furthermore, PECAM-1 co-localized with moesin at the cell periphery and in   filopodia   of glass-activated platelets. Our observations suggest that moesin may play a role in platelet adhesion, linking PECAM-1 with the actin cytoskeleton. 
  GeneRIFs  
 PECAM-1 co-localized with moesin at the cell periphery and in   filopodia   of glass-activated platelets; moesin may play a role in platelet adhesion, linking PECAM-1 with the actin cytoskeleton 
  Summary  
 Moesin (for membrane-organizing extension spike protein) is a member of the ERM family which includes ezrin and radixin. ERM proteins appear to function as cross-linkers between plasma membranes and actin-based cytoskeletons. Moesin is localized to   filopodia   and other membranous protrusions that are important for cell-cell recognition and signaling and for cell movement. [provided by RefSeq, Jul 2008] 
  [Ff]ilopodium  Component  
   filopodium   
  [Ii]nvadopodia  PubMed  
 Talin regulates moesin-NHE-1 recruitment to   invadopodia   and promotes mammary tumor metastasis.   Invadopodia   are actin-rich protrusions that degrade the extracellular matrix and are required for stromal invasion, intravasation, and metastasis. The role of the focal adhesion protein talin in regulating these structures is not known. Here, we demonstrate that talin is required for   invadopodia  l matrix degradation and three-dimensional extracellular matrix invasion in metastatic breast cancer cells. The sodium/hydrogen exchanger 1 (NHE-1) is linked to the cytoskeleton by ezrin/radixin/moesin family proteins and is known to regulate invadopodium-mediated matrix degradation. We show that the talin C terminus binds directly to the moesin band 4.1 ERM (FERM) domain to recruit a moesin-NHE-1 complex to   invadopodia  . Silencing talin resulted in a decrease in cytosolic pH at   invadopodia   and blocked cofilin-dependent actin polymerization, leading to impaired invadopodium stability and matrix degradation. Furthermore, talin is required for mammary tumor cell motility, intravasation, and spontaneous lung metastasis in vivo. Thus, our findings provide a novel understanding of how intracellular pH is regulated and a molecular mechanism by which talin enhances tumor cell invasion and metastasis. 
  GeneRIFs  
 Talin has a role in regulating moesin-NHE-1 recruitment to   invadopodia   in breast cancer 
  [Ii]nvadopodium  PubMed  
 Talin regulates moesin-NHE-1 recruitment to invadopodia and promotes mammary tumor metastasis. Invadopodia are actin-rich protrusions that degrade the extracellular matrix and are required for stromal invasion, intravasation, and metastasis. The role of the focal adhesion protein talin in regulating these structures is not known. Here, we demonstrate that talin is required for invadopodial matrix degradation and three-dimensional extracellular matrix invasion in metastatic breast cancer cells. The sodium/hydrogen exchanger 1 (NHE-1) is linked to the cytoskeleton by ezrin/radixin/moesin family proteins and is known to regulate   invadopodium  -mediated matrix degradation. We show that the talin C terminus binds directly to the moesin band 4.1 ERM (FERM) domain to recruit a moesin-NHE-1 complex to invadopodia. Silencing talin resulted in a decrease in cytosolic pH at invadopodia and blocked cofilin-dependent actin polymerization, leading to impaired   invadopodium   stability and matrix degradation. Furthermore, talin is required for mammary tumor cell motility, intravasation, and spontaneous lung metastasis in vivo. Thus, our findings provide a novel understanding of how intracellular pH is regulated and a molecular mechanism by which talin enhances tumor cell invasion and metastasis. 
  [Pp]odosome(s)?  Process  
 positive regulation of   podosome   assembly 
  PubMed  
 Phosphorylation of moesin by Jun N-terminal kinase is important for   podosome   rosette formation in Src-transformed fibroblasts.   Podosomes   are actin-based membrane protrusions that facilitate extracellular matrix degradation and motility of invasive cells.   Podosomes   can self-organize into large rosette-like structures in Src-transformed fibroblasts, osteoclasts and some highly invasive cancer cells. However, the mechanism of this assembly remains obscure. In this study, we show that the suppression of Jun N-terminal kinase (JNK) by the JNK inhibitor SP600125 or short-hairpin RNA inhibited   podosome   rosette formation in SrcY527F-transformed NIH3T3 fibroblasts. In addition, SrcY527F was less able to induce   podosome   rosettes in JNK1-null or JNK2-null mouse embryo fibroblasts than in wild-type counterparts. The kinase activity of JNK was essential for promoting   podosome   rosette formation but not for its localization to   podosome   rosettes. Moesin, a member of the ERM (ezrin, radixin and moesin) protein family, was identified as a substrate of JNK. We show that the phosphorylation of moesin at Thr558 by JNK was important for   podosome   rosette formation in SrcY527F-transformed NIH3T3 fibroblasts. Taken together, our results unveil a novel role of JNK in   podosome   rosette formation through the phosphorylation of moesin. 
  GeneRIFs  
 Phosphorylation of moesin at Thr558 by JNK is important for   podosome   rosette formation. 
  [Mm]igration  Process  
 T cell   migration   
 leukocyte   migration   
 regulation of lymphocyte   migration   
  PubMed  
 Analysis of the myosin-II-responsive focal adhesion proteome reveals a role for β-Pix in negative regulation of focal adhesion maturation. Focal adhesions undergo myosin-II-mediated maturation wherein they grow and change composition to modulate integrin signalling for cell   migratio  n, growth and differentiation. To determine how focal adhesion composition is affected by myosin II activity, we performed proteomic analysis of isolated focal adhesions and compared protein abundance in focal adhesions from cells with and without myosin II inhibition. We identified 905 focal adhesion proteins, 459 of which changed in abundance with myosin II inhibition, defining the myosin-II-responsive focal adhesion proteome. The abundance of 73% of the proteins in the myosin-II-responsive focal adhesion proteome was enhanced by contractility, including proteins involved in Rho-mediated focal adhesion maturation and endocytosis- and calpain-dependent focal adhesion disassembly. During myosin II inhibition, 27% of proteins in the myosin-II-responsive focal adhesion proteome, including proteins involved in Rac-mediated lamellipodial protrusion, were enriched in focal adhesions, establishing that focal adhesion protein recruitment is also negatively regulated by contractility. We focused on the Rac guanine nucleotide exchange factor β-Pix, documenting its role in the negative regulation of focal adhesion maturation and the promotion of lamellipodial protrusion and focal adhesion turnover to drive cel  l migrati  on. 
 Vascular endothelial growth factor C promotes cervical cancer metastasis via up-regulation and activation of RhoA/ROCK-2/moesin cascade. The elevated expression of vascular endothelial growth factor C (VEGF-C) is correlated with clinical cervical cancer metastasis and patient survival, which is interpreted by VEGF-C functions to stimulate angiogenesis and lymphatic genesis. However, the direct impact of VEGF-C on cervical cancer cell motility remains largely unknown.In this study, we investigated the effects of VEGF-C on actin cytoskeleton remodeling and on cervical cancer cell   migration   and invasion and how the actin-regulatory protein, moesin regulated these effects through RhoA/ROCK-2 signaling pathway.On cervical carcinoma cell line SiHa cells, exposure of VEGF-C triggered remodeling of the actin cytoskeleton and the formation of membrane ruffles, which was required for cell movement. VEGF-C significantly enhanced SiHa cells horizontal   migration   and three-dimensional invasion into matrices. These actions were dependent on increased expression and phosphorylation of the actin-regulatory protein moesin and specific moesin siRNA severely impaired VEGF-C stimulated-cell   migration  . The extracellular small GTPase RhoA/ROCK-2 cascade mediated the increased moesin expression and phosphorylation, which was discovered by the use of Y-27632, a specific inhibitor of Rho kinase and by transfected constitutively active, dominant-negative RhoA as well as ROCK-2 SiRNA. Furthermore, in the surgical cervical specimen from the patients with FIGO stage at cervical intra-epithelial neoplasia and I-II cervical squamous cell carcinoma, the expression levels of moesin were found to be significantly correlated with tumor malignancy and metastasis.These results implied that VEGF-C promoted cervical cancer metastasis by upregulation and activation of moesin protein through RhoA/ROCK-2 pathway. Our findings offer new insight into the role of VEGF-C on cervical cancer progression and may provide potential targets for cervical cancer therapy. 
 Testosterone promotes vascular endothelial cell   migration   via upregulation of ROCK-2/moesin cascade. Cross-sectional studies have demonstrated a reverse relationship between serum level of testosterone (T) and the incidence rate of cardiovascular disease in men, indicating that T exerts beneficial effects in cardiovascular system. However, the endothelial effects of T are poorly understood. Actin remodeling is essential for endothelial cell movement and vascular repair and this process is controlled by the actin-binding protein moesin. In the present study, we studied the effects of T on actin remodeling, moesin expression and phosphorylation, as well as cell   migration   in cultured human umbilical endothelial cells (hUVECs). We found that T provoked the formation of cortical actin complexes and membrane protrusions in endothelial cells. Treatment with T induced dose- and time-dependent increase of moesin expression and phosphorylation, which was inhibited by the addition of androgen receptor antagonist hydroxyflutamide (HF). Moreover, T enhanced ROCK-2 activity. The ROCK-2 inhibitor Y27632 or the transfection of ROCK-2 siRNA largely inhibited T-induced moesin expression and phosphorylation, indicating that ROCK-2 pathway is crucial for these effects. T promoted endothelial cell   migration  , which was inhibited by the addition of HF or Y27632. In conclusion, T induces actin cytoskeleton remodeling by regulating moesin expression and activation, resulting in enhanced endothelial cell   migration  . Our work adds new insights into endothelial mechanisms of T, which is relevant for its vascular actions. 
 Testosterone promotes vascular endothelial cell   migration   via upregulation of ROCK-2/moesin cascade. Cross-sectional studies have demonstrated a reverse relationship between serum level of testosterone (T) and the incidence rate of cardiovascular disease in men, indicating that T exerts beneficial effects in cardiovascular system. However, the endothelial effects of T are poorly understood. Actin remodeling is essential for endothelial cell movement and vascular repair and this process is controlled by the actin-binding protein moesin. In the present study, we studied the effects of T on actin remodeling, moesin expression and phosphorylation, as well as cell   migration   in cultured human umbilical endothelial cells (hUVECs). We found that T provoked the formation of cortical actin complexes and membrane protrusions in endothelial cells. Treatment with T induced dose- and time-dependent increase of moesin expression and phosphorylation, which was inhibited by the addition of androgen receptor antagonist hydroxyflutamide (HF). Moreover, T enhanced ROCK-2 activity. The ROCK-2 inhibitor Y27632 or the transfection of ROCK-2 siRNA largely inhibited T-induced moesin expression and phosphorylation, indicating that ROCK-2 pathway is crucial for these effects. T promoted endothelial cell   migration  , which was inhibited by the addition of HF or Y27632. In conclusion, T induces actin cytoskeleton remodeling by regulating moesin expression and activation, resulting in enhanced endothelial cell   migration  . Our work adds new insights into endothelial mechanisms of T, which is relevant for its vascular actions. 
 Vascular endothelial growth factor C promotes cervical cancer metastasis via up-regulation and activation of RhoA/ROCK-2/moesin cascade. The elevated expression of vascular endothelial growth factor C (VEGF-C) is correlated with clinical cervical cancer metastasis and patient survival, which is interpreted by VEGF-C functions to stimulate angiogenesis and lymphatic genesis. However, the direct impact of VEGF-C on cervical cancer cell motility remains largely unknown.In this study, we investigated the effects of VEGF-C on actin cytoskeleton remodeling and on cervical cancer cell   migration   and invasion and how the actin-regulatory protein, moesin regulated these effects through RhoA/ROCK-2 signaling pathway.On cervical carcinoma cell line SiHa cells, exposure of VEGF-C triggered remodeling of the actin cytoskeleton and the formation of membrane ruffles, which was required for cell movement. VEGF-C significantly enhanced SiHa cells horizontal   migration   and three-dimensional invasion into matrices. These actions were dependent on increased expression and phosphorylation of the actin-regulatory protein moesin and specific moesin siRNA severely impaired VEGF-C stimulated-cell   migration  . The extracellular small GTPase RhoA/ROCK-2 cascade mediated the increased moesin expression and phosphorylation, which was discovered by the use of Y-27632, a specific inhibitor of Rho kinase and by transfected constitutively active, dominant-negative RhoA as well as ROCK-2 SiRNA. Furthermore, in the surgical cervical specimen from the patients with FIGO stage at cervical intra-epithelial neoplasia and I-II cervical squamous cell carcinoma, the expression levels of moesin were found to be significantly correlated with tumor malignancy and metastasis.These results implied that VEGF-C promoted cervical cancer metastasis by upregulation and activation of moesin protein through RhoA/ROCK-2 pathway. Our findings offer new insight into the role of VEGF-C on cervical cancer progression and may provide potential targets for cervical cancer therapy. 
 Moesin orchestrates cortical polarity of melanoma tumour cells to initiate 3D invasion. Tumour cell dissemination through corporal fluids (blood, lymph and body cavity fluids) is a distinctive feature of the metastatic process. Tumour cell transition from fluid to adhesive conditions involves an early polarization event and major rearrangements of the submembrane cytoskeleton that remain poorly understood. As regulation of cortical actin-membrane binding might be important in this process, we investigated the role of ezrin and moesin, which are key crosslinking proteins of the ERM (ezrin, radixin, moesin) family. We used short interfering RNA (siRNA) to show that moesin is crucial for invasion by melanoma cells in 3D matrices and in early lung colonization. Using live imaging, we show that following initial adhesion to the endothelium or 3D matrices, moesin is redistributed away from the region of adhesion, thereby generating a polarized cortex: a stable cortical actin dome enriched in moesin and an invasive membrane domain full of blebs. Using Lifeact-GFP, a 17-amino-acid peptide that binds F-actin, we show the initial symmetry breaking of cortical actin cytoskeleton during early attachment of round cells. We also demonstrated that ezrin and moesin are differentially distributed during initial invasion of 3D matrices, and, specifically, that moesin controls adhesion-dependent activation of Rho and subsequent myosin II contractility. Our results reveal that polarized moesin plays a role in orienting Rho activation, myosin II contractility, and cortical actin stability, which is crucial for driving directional vertical   migration   instead of superficial spreading on the fluid-to-solid tissue interface. We propose that this mechanism of cortical polarization could sustain extravasation of fluid-borne tumour cells during the process of metastasis. 
 A novel serine-rich motif in the intercellular adhesion molecule 3 is critical for its ezrin/radixin/moesin-directed subcellular targeting. Intercellular adhesion molecule 3 (ICAM-3) is a leukocyte-specific receptor involved in primary immune responses. We have investigated the interaction between ICAM-3 and ezrin/radixin/moesin (ERM) proteins and its role in LFA-1-induced cell-cell interactions and membrane positioning of ICAM-3 in polarized migrating lymphocytes. Protein-protein binding assays demonstrated a phosphatidylinositol 4,5-bisphosphate-induced association between ICAM-3 and the amino-terminal domain of ERM proteins. This interaction was not essential for the binding of ICAM-3 to LFA-1. Dynamic fluorescence videomicroscopy studies of cells demonstrated that moesin and ICAM-3 coordinately redistribute on the plasma membrane during lymphocyte   migration  . Furthermore, overexpression of the amino-terminal domain of moesin, which lacks the consensus moesin actin-binding site, caused the subcellular mislocalization of ICAM-3. A CD4 chimerical protein containing the cytoplasmic tail of ICAM-3 was targeted to the trailing edge. Point mutation of Ser(487), Ser(489), and Ser(496) to alanine in the juxtamembrane region of ICAM-3 significantly impaired both ERM binding and polarization of ICAM-3. ERM-directed polarization of ICAM-3 was also impaired by phosphorylation-like mutation of Ser(487) and Ser(489), but not of Ser(496). Our results underscore the key role of specific serine residues within the cytoplasmic region of ICAM-3 for its ERM-directed positioning at the trailing edge of motile lymphocytes. 
 Regulation of NK cell trafficking by CD81. NK cells, a heterogeneous sub-population of lymphocytes, are critically involved in the regulation of both innate and adaptive immune responses in humans. Besides their participation in the control of tumors and viral infections, they also regulate inflammatory processes, mediating both beneficial and detrimental effects. To effectively fulfil their role in immune surveillance, proper trafficking of NK cells is essential. However, the mechanisms and factors governing NK cell recruitment are only poorly dissected. Here, we describe the functional role of tetraspanins, a family of evolutionary conserved cell-surface proteins, in modulating   migration   and trans  migration   of human NK cells. We demonstrate expression of various tetraspanins on NK cells. Furthermore, we show that stimulation of the NK cell-expressed tetraspanin CD81 induces phosphorylation of ezrin/radixin/moesin proteins and leads to NK cell polarization thereby facilitating NK cell   migration   toward various chemokines/cytokines. Finally, we provide evidence for a role of CD81 in promoting adhesion of NK cells to components of the extracellular matrix, a prerequisite for extravasation of lymphocytes in inflamed tissues. Thus, our data suggest that the tetraspanin CD81 is importantly involved in the regulation of NK cell recruitment. 
 Dynamic interaction of VCAM-1 and ICAM-1 with moesin and ezrin in a novel endothelial docking structure for adherent leukocytes. Ezrin, radixin, and moesin (ERM) regulate cortical morphogenesis and cell adhesion by connecting membrane adhesion receptors to the actin-based cytoskeleton. We have studied the interaction of moesin and ezrin with the vascular cell adhesion molecule (VCAM)-1 during leukocyte adhesion and transendothelial   migration   (TEM). VCAM-1 interacted directly with moesin and ezrin in vitro, and all of these molecules colocalized at the apical surface of endothelium. Dynamic assessment of this interaction in living cells showed that both VCAM-1 and moesin were involved in lymphoblast adhesion and spreading on the endothelium, whereas only moesin participated in TEM, following the same distribution pattern as ICAM-1. During leukocyte adhesion in static or under flow conditions, VCAM-1, ICAM-1, and activated moesin and ezrin clustered in an endothelial actin-rich docking structure that anchored and partially embraced the leukocyte containing other cytoskeletal components such as alpha-actinin, vinculin, and VASP. Phosphoinositides and the Rho/p160 ROCK pathway, which participate in the activation of ERM proteins, were involved in the generation and maintenance of the anchoring structure. These results provide the first characterization of an endothelial docking structure that plays a key role in the firm adhesion of leukocytes to the endothelium during inflammation. 
 Role of moesin in hyaluronan induced cell   migration   in glioblastoma multiforme. A major barrier to effective treatment of glioblastoma multiforme (GBM) is the invasion of glioma cells into the brain parenchyma rendering local therapies such as surgery and radiation therapy ineffective. GBM patients with such highly invasive and infiltrative tumors have poor prognosis with a median survival time of only about a year. However, the mechanisms leading to increased cell   migration  , invasion and diffused behavior of glioma cells are still poorly understood.In the current study, we applied quantitative proteomics for the identification of differentially expressed proteins in GBMs as compared to non-malignant brain tissues.Our study led to the identification of 23 proteins showing overexpression in GBM; these include membrane proteins, moesin and CD44. The results were verified using Western blotting and immunohistochemistry in independent set of GBM and non-malignant brain tissues. Both GBM tissues and glioma cell lines (U87 / U373) demonstrated membranous expression of moesin and CD44, as revealed by immunohistochemistry and immunofluorescence, respectively. Notably, glioma cells transfected with moesin siRNA displayed reduced   migration   and invasion on treatment with hyaluronan (HA), an important component of the extracellular matrix in GBM. CD44, a transmembrane glycoprotein, acts as a major receptor for hyaluronan (HA). Using co-immunoprecipitation assays, we further demonstrated that moesin interacts with CD44 in glioma cells only after treatment with HA; this implicates a novel role of moesin in HA-CD44 signaling in gliomas.Our results suggest that development of inhibitors which interfere with CD44-moesin interactions may open a new avenue in the future to mitigate cellular   migration   in gliomas. 
 Phosphorylated ERM is responsible for increased T cell polarization, adhesion, and   migration   in patients with systemic lupus erythematosus. Systemic lupus erythematosus (SLE) is an autoimmune/inflammatory disease characterized by autoantibody production and abnormal T cells that infiltrate tissues through not well-known mechanisms. We report that SLE T lymphocytes display increased levels of CD44, ezrin, radixin, and moesin (ERM) phosphorylation, stronger actin polymerization, higher polar cap formation, and enhanced adhesion and chemotactic   migration   compared with T cells from patients with rheumatoid arthritis and normal individuals. Silencing of CD44 by CD44 small interfering RNA in SLE T cells inhibited significantly their ability to adhere and migrate as did treatment with Rho kinase and actin polymerization inhibitors. Forced expression of T567D-ezrin, a phosphorylation-mimic form, enhanced remarkably the adhesion and   migration   rate of normal T cells. Anti-CD3/TCR autoantibodies present in SLE sera caused increased ERM phosphorylation, adhesion, and   migration   in normal T cells. pERM and CD44 are highly expressed in T cells infiltrating in the kidneys of patients with lupus nephritis. These data prove that increased ERM phosphorylation represents a key molecular abnormality that guides T cell adhesion and   migration   in SLE patients. 
 Role of Moesin in Advanced Glycation End Products-Induced Angiogenesis of Human Umbilical Vein Endothelial Cells. Disorders of angiogenesis are related to microangiopathies during the development of diabetic vascular complications, but the effect of advanced glycation end products (AGEs) on angiogenesis and the mechanism has not been completely unveiled. We previous demonstrated that moesin belonging to the ezrin-radixin-moesin (ERM) protein family protein played a critical role in AGE-induced hyper-permeability in human umbilical vein endothelial cells (HUVECs). Here, we investigated the impact of moesin on AGE-induced HUVEC proliferation,   migration  , and tubulogenesis. Silencing of moesin decreased cell motility and tube formation but not cell proliferation. It also attenuated cellular F-actin reassembly. Further, phosphorylation of threonine at the 558 amino acid residue (Thr 558) in moesin suppressed AGE-induced HUVEC proliferation,   migration  , and tube formation, while the activating mutation of moesin at Thr 558 enhanced HUVEC angiogenesis. Further, the inhibition of either RhoA activity by adenovirus or ROCK activation with inhibitor Y27632 decreased AGE-induced moesin phosphorylation and subsequently suppressed HUVEC angiogenesis. These results indicate that the Thr 558 phosphorylation in moesin mediates endothelial angiogenesis. AGEs promoted HUVEC angiogenesis by inducing moesin phosphorylation via RhoA/ROCK pathway. 
 Radixin regulates cell   migration   and cell-cell adhesion through Rac1. The ERM proteins ezrin, radixin and moesin are adaptor proteins that link plasma membrane receptors to the actin cytoskeleton. Ezrin and moesin have been implicated in cell polarization and cell   migration  , but little is known about the involvement of radixin in these processes. Here we show that radixin is required for   migration   of PC3 prostate cancer cells, and that radixin, but not ezrin or moesin, depletion by RNA interference increases cell spread area and cell-cell adhesion mediated by adherens junctions. Radixin depletion also alters actin organization, and distribution of active phosphorylated ezrin and moesin. Similar effects were observed in MDA-MB-231 breast cancer cells. The phenotype of radixin-depleted cells is similar to that induced by constitutively active Rac1, and Rac1 is required for the radixin knockdown phenotype. Radixin depletion also increases the activity of Rac1 but not Cdc42 or RhoA. Analysis of Rac guanine nucleotide exchange factors (GEFs) suggests that radixin affects the activity of Vav GEFs. Indeed, Vav GEF depletion reverses the phenotype of radixin knockdown and reduces the effect of radixin knockdown on Rac1 activity. Our results indicate that radixin plays an important role in promoting cell   migration   by regulating Rac1-mediated epithelial polarity and formation of adherens junctions through Vav GEFs. 
 ERM stable knockdown by siRNA reduced in vitro   migration   and invasion of human SGC-7901 cells. Three closely related proteins, ezrin, radixin, and moesin (ERM), which primarily act as a linker between the plasma membrane and the cytoskeleton, are involved in many cellular functions, including regulation of actin cytoskeleton, control of cell shape, adhesion and motility, and modulation of signaling pathways. Although, ezrin is now recognized as a key component in tumor metastasis, the functional role of the radixin and moesin in tumor metastasis has not been established. In the present study, we chose highly metastatic human gastric carcinoma SGC-7901 cells, which express all of the ERM proteins as a model to examine the functional roles of these proteins in tumor metastasis. Ezrin, radixin or moesin stable knockdown SGC-7901 cell lines were established using siRNA methodology. In vitro cell   migration   and invasion studies showed that either ezrin, radixin or moesin specific deficiency in the cells caused the substantial reduction of the cell   migration   and invasion. Western blotting and immunofluorescence analysis showed that the expression of E-cadherin was also significantly increased when any member of ERM proteins was downregulated. Our results indicated that these three ERM proteins play similar roles in the SGC-7901 cell metastatic potential and their roles of upregulating the expression of E-cadherin may be important in tumor progression. 
 Anchoring of protein kinase A by ERM (ezrin-radixin-moesin) proteins is required for proper netrin signaling through DCC (deleted in colorectal cancer). Netrin-1, acting through its principal receptor DCC (deleted in colorectal cancer), serves as an axon guidance cue during neural development and also contributes to vascular morphogenesis, epithelial   migration  , and the pathogenesis of some tumors. Several lines of evidence suggest that netrin-DCC signaling can regulate and be regulated by the cAMP-dependent protein kinase, PKA, although the molecular details of this relationship are poorly understood. Specificity in PKA signaling is often achieved through differential subcellular localization of the enzyme by interaction with protein kinase A anchoring proteins (AKAPs). Here, we show that AKAP function is required for DCC-mediated activation of PKA and phosphorylation of cytoskeletal regulatory proteins of the Mena/VASP (vasodilator-stimulated phosphoprotein) family. Moreover, we show that DCC and PKA physically interact and that this association is mediated by the ezrin-radixin-moesin (ERM) family of plasma membrane-actin cytoskeleton cross-linking proteins. Silencing of ERM protein expression inhibits DCC-PKA interaction, DCC-mediated PKA activation, and phosphorylation of Mena/VASP proteins as well as growth cone morphology and neurite outgrowth. Finally, although expression of wild-type radixin partially rescued growth cone morphology and tropism toward netrin in ERM-knockdown cells, expression of an AKAP-deficient mutant of radixin did not fully rescue growth cone morphology and switched netrin tropism from attraction to repulsion. These data support a model in which ERM-mediated anchoring of PKA activity to DCC is required for proper netrin/DCC-mediated signaling. 
 Tumor suppressive microRNA-133a regulates novel targets: moesin contributes to cancer cell proliferation and invasion in head and neck squamous cell carcinoma. Recently, many studies suggest that microRNAs (miRNAs) contribute to the development, invasion and metastasis of various types of human cancers. Our recent study revealed that expression of microRNA-133a (miR-133a) was significantly reduced in head and neck squamous cell carcinoma (HNSCC) and that restoration of miR-133a inhibited cell proliferation,   migration   and invasion in HNSCC cell lines, suggesting that miR-133a function as a tumor suppressor. Genome-wide gene expression analysis of miR-133a transfectants and TargetScan database showed that moesin (MSN) was a promising candidate of miR-133a target gene. MSN is a member of the ERM (ezrin, radixin and moesin) protein family and ERM function as cross-linkers between plasma membrane and actin-based cytoskeleton. The functions of MSN in cancers are controversial in previous reports. In this study, we focused on MSN and investigated whether MSN was regulated by tumor suppressive miR-133a and contributed to HNSCC oncogenesis. Restoration of miR-133a in HNSCC cell lines (FaDu, HSC3, IMC-3 and SAS) suppressed the MSN expression both in mRNA and protein level. Silencing study of MSN in HNSCC cell lines demonstrated significant inhibitions of cell proliferation,   migration   and invasion activities in si-MSN transfectants. In clinical specimen with HNSCC, the expression level of MSN was significantly up-regulated in cancer tissues compared to adjacent non-cancerous tissues. These data suggest that MSN may function as oncogene and is regulated by tumor suppressive miR-133a. Our analysis data of novel tumor-suppressive miR-133a-mediated cancer pathways could provide new insights into the potential mechanisms of HNSCC oncogenesis. 
 Immunohistochemical assessment of ezrin and moesin in colorectal carcinoma. Ezrin and moesin are important molecules of the ERM family of proteins, which regulate cell adhesion and   migration  . The aim of this study was to evaluate the intensity and pattern of ezrin and moesin expression in colorectal carcinoma (CRC) together with correlating their expression with the clinico-pathologic features of this neoplasm. This study was carried out on 48 CRC and 10 adenoma specimens. All adenoma and 95% of CRC cases showed both ezrin and moesin expression. Ezrin was predominantly cytoplasmic in adenoma cases in comparison to membranous localization in carcinoma cases. Moesin was predominantly expressed in stroma (inflammatory cells and fibroblasts) in carcinoma (89.1%) compared with adenoma (50%). High H-score of ezrin expression was associated with adenocarcinoma type (P = .024) and was inversely correlated with mitotic count (P = .005). High H-score of moesin expression was associated with early Dukes staging of CRC (P = .016), absence of lymph node involvement (P = .022), and low number of involved lymph nodes (P = .04). The association of ezrin with favorable prognostic parameters may be due to its prominent membranous localization. The stroma of CRC could stand against invasion by expression of moesin. Ezrin and moesin are independently expressed from each other. 
 Moesin-dependent cytoskeleton remodelling is associated with an anaplastic phenotype of pancreatic cancer. Cell motility is controlled by the dynamic cytoskeleton and its related proteins, such as members of the ezrin/radixin/moesin (ERM) family, which act as signalling molecules inducing cytoskeleton remodelling. Although ERM proteins have been identified as important factors in various malignancies, functional redundancy between these proteins has hindered the dissection of their individual contribution. The aim of the present study was to analyse the functional role of moesin in pancreatic malignancies. Cancer cells of different malignant lesions of human and transgenic mice pancreata were evaluated by immunohistochemistry. For functional analysis, cell growth, adhesion and invasion assays were carried out after transient and stable knock-down of moesin expression in pancreatic cancer cells. In vivo tumourigenicity was determined using orthotopic and metastatic mouse tumour models. We now show that moesin knock-down increases   migration  , invasion and metastasis and influences extracellular matrix organization of pancreatic cancer. Moesin-regulated migratory activities of pancreatic cancer cells were in part promoted through cellular translocation of beta-catenin, and re-distribution and organization of the cytoskeleton. Analysis of human and different transgenic mouse pancreatic cancers demonstrated that moesin is a phenotypic marker for anaplastic carcinoma, suggesting that this ERM protein plays a specific role in pancreatic carcinogenesis. 
 LOK is a major ERM kinase in resting lymphocytes and regulates cytoskeletal rearrangement through ERM phosphorylation. ERM (ezrin-radixin-moesin) proteins mediate linkage of actin cytoskeleton to plasma membrane in many cells. ERM activity is regulated in part by phosphorylation at a C-terminal threonine, but the identity of ERM kinases is unknown in lymphocytes and incompletely defined in other mammalian cells. Our studies show that lymphocyte-oriented kinase (LOK) is an ERM kinase in vitro and in vivo. Mass spectrometric analysis indicates LOK is abundant at the lymphocyte plasma membrane and immunofluorescence studies show LOK enrichment at the plasma membrane near ERM. In vitro peptide specificity analyses characterize LOK as a basophilic kinase whose optimal substrate sequence resembles the ERM site, including unusual preference for tyrosine at P-2. LOK's activity on moesin peptide and protein was comparable to reported ERM kinases ROCK and PKC but unlike them LOK displayed preferential specificity for moesin compared to traditional basophilic kinase substrates. Two genetic approaches demonstrate a role for LOK in ERM phosphorylation: cell transfection with LOK kinase domain augments ERM phosphorylation and lymphocytes from LOK knockout mice have >50% reduction in ERM phosphorylation. The findings on localization and specificity argue that LOK is a direct ERM kinase. The knockout mice have normal hematopoietic cell development but notably lymphocyte   migration   and polarization in response to chemokine are enhanced. These functional alterations fit the current understanding of the role of ERM phosphorylation in regulating cortical reorganization. Thus, these studies identify a new ERM kinase of importance in lymphocytes and confirm the role of ERM phosphorylation in regulating cell shape and motility. 
 X-linked primary immunodeficiency associated with hemizygous mutations in the moesin (MSN) gene. We investigated 7 male patients (from 5 different families) presenting with profound lymphopenia, hypogammaglobulinemia, fluctuating monocytopenia and neutropenia, a poor immune response to vaccine antigens, and increased susceptibility to bacterial and varicella zoster virus infections.We sought to characterize the genetic defect involved in a new form of X-linked immunodeficiency.We performed genetic analyses and an exhaustive phenotypic and functional characterization of the lymphocyte compartment.We observed hemizygous mutations in the moesin (MSN) gene (located on the X chromosome and coding for MSN) in all 7 patients. Six of the latter had the same missense mutation, which led to an amino acid substitution (R171W) in the MSN four-point-one, ezrin, radixin, moesin domain. The seventh patient had a nonsense mutation leading to a premature stop codon mutation (R533X). The naive T-cell counts were particularly low for age, and most CD8(+) T cells expressed the senescence marker CD57. This phenotype was associated with impaired T-cell proliferation, which was rescued by expression of wild-type MSN. MSN-deficient T cells also displayed poor chemokine receptor expression, increased adhesion molecule expression, and altere  d migrati  on and adhesion capacities.Our observations establish a causal link between an ezrin-radixin-moesin protein mutation and a primary immunodeficiency that could be referred to as X-linked moesin-associated immunodeficiency. 
  GeneRIFs  
 Data show that stimulation of the NK cell-expressed tetraspanin CD81 induces phosphorylation of ezrin/radixin/moesin proteins and leads to NK cell polarization thereby facilitating NK cell   migration   toward various chemokines/cytokines. 
 increased CD44, ezrin, radixin, and moesin phosphorylation represents a key molecular abnormality that guides T cell adhesion and   migration   in SLE patients. 
  [Ee]xtracellular [Mm]atrix  PubMed  
 Proteomic analysis of human parotid gland exosomes by multidimensional protein identification technology (MudPIT). Human ductal saliva contributes over a thousand unique proteins to whole oral fluids. The mechanism by which most of these proteins are secreted by salivary glands remains to be determined. The present study used a mass spectrometry-based, shotgun proteomics approach to explore the possibility that a subset of the proteins found in saliva are derived from exosomes, membrane-bound vesicles of endosomal origin within multivesicular endosomes. Using MudPIT (multidimensional protein identification technology) mass spectrometry, we catalogued 491 proteins in the exosome fraction of human parotid saliva. Many of these proteins were previously observed in ductal saliva from parotid glands (265 proteins). Furthermore, 72 of the proteins in parotid exosomes overlap with those previously identified as urinary exosome proteins, proteins which are also frequently associated with exosomes from other tissues and cell types. Gene Ontology (GO) and KEGG pathway analyses found that cytosolic proteins comprise the largest category of proteins in parotid exosomes (43%), involved in such processes as phosphatidylinositol signaling system, calcium signaling pathway, inositol metabolism, protein export, and signal transduction, among others; whereas the integral plasma membrane proteins and associated/peripheral plasma membrane proteins (26%) were associated with   extracellular matrix  -receptor interaction, epithelial cell signaling, T-cell and B-cell receptor signaling, cytokine receptor interaction, and antigen processing and presentation, among other biological functions. In addition, these putative saliva exosomal proteins were linked to specific diseases (e.g., neurodegenerative disorders, prion disease, cancers, type I and II diabetes). Consequently, parotid glands secrete exosomes that reflect the metabolic and functional status of the gland and may also carry informative protein markers useful in the diagnosis and treatment of systemic diseases. 
 Phosphorylation of moesin by Jun N-terminal kinase is important for podosome rosette formation in Src-transformed fibroblasts. Podosomes are actin-based membrane protrusions that facilitate   extracellular matrix   degradation and motility of invasive cells. Podosomes can self-organize into large rosette-like structures in Src-transformed fibroblasts, osteoclasts and some highly invasive cancer cells. However, the mechanism of this assembly remains obscure. In this study, we show that the suppression of Jun N-terminal kinase (JNK) by the JNK inhibitor SP600125 or short-hairpin RNA inhibited podosome rosette formation in SrcY527F-transformed NIH3T3 fibroblasts. In addition, SrcY527F was less able to induce podosome rosettes in JNK1-null or JNK2-null mouse embryo fibroblasts than in wild-type counterparts. The kinase activity of JNK was essential for promoting podosome rosette formation but not for its localization to podosome rosettes. Moesin, a member of the ERM (ezrin, radixin and moesin) protein family, was identified as a substrate of JNK. We show that the phosphorylation of moesin at Thr558 by JNK was important for podosome rosette formation in SrcY527F-transformed NIH3T3 fibroblasts. Taken together, our results unveil a novel role of JNK in podosome rosette formation through the phosphorylation of moesin. 
 Regulation of NK cell trafficking by CD81. NK cells, a heterogeneous sub-population of lymphocytes, are critically involved in the regulation of both innate and adaptive immune responses in humans. Besides their participation in the control of tumors and viral infections, they also regulate inflammatory processes, mediating both beneficial and detrimental effects. To effectively fulfil their role in immune surveillance, proper trafficking of NK cells is essential. However, the mechanisms and factors governing NK cell recruitment are only poorly dissected. Here, we describe the functional role of tetraspanins, a family of evolutionary conserved cell-surface proteins, in modulating migration and transmigration of human NK cells. We demonstrate expression of various tetraspanins on NK cells. Furthermore, we show that stimulation of the NK cell-expressed tetraspanin CD81 induces phosphorylation of ezrin/radixin/moesin proteins and leads to NK cell polarization thereby facilitating NK cell migration toward various chemokines/cytokines. Finally, we provide evidence for a role of CD81 in promoting adhesion of NK cells to components of the   extracellular matrix  , a prerequisite for extravasation of lymphocytes in inflamed tissues. Thus, our data suggest that the tetraspanin CD81 is importantly involved in the regulation of NK cell recruitment. 
 Role of moesin in hyaluronan induced cell migration in glioblastoma multiforme. A major barrier to effective treatment of glioblastoma multiforme (GBM) is the invasion of glioma cells into the brain parenchyma rendering local therapies such as surgery and radiation therapy ineffective. GBM patients with such highly invasive and infiltrative tumors have poor prognosis with a median survival time of only about a year. However, the mechanisms leading to increased cell migration, invasion and diffused behavior of glioma cells are still poorly understood.In the current study, we applied quantitative proteomics for the identification of differentially expressed proteins in GBMs as compared to non-malignant brain tissues.Our study led to the identification of 23 proteins showing overexpression in GBM; these include membrane proteins, moesin and CD44. The results were verified using Western blotting and immunohistochemistry in independent set of GBM and non-malignant brain tissues. Both GBM tissues and glioma cell lines (U87 / U373) demonstrated membranous expression of moesin and CD44, as revealed by immunohistochemistry and immunofluorescence, respectively. Notably, glioma cells transfected with moesin siRNA displayed reduced migration and invasion on treatment with hyaluronan (HA), an important component of the   extracellular matrix   in GBM. CD44, a transmembrane glycoprotein, acts as a major receptor for hyaluronan (HA). Using co-immunoprecipitation assays, we further demonstrated that moesin interacts with CD44 in glioma cells only after treatment with HA; this implicates a novel role of moesin in HA-CD44 signaling in gliomas.Our results suggest that development of inhibitors which interfere with CD44-moesin interactions may open a new avenue in the future to mitigate cellular migration in gliomas. 
 Spatial regulation of cyclic AMP-Epac1 signaling in cell adhesion by ERM proteins. Epac1 is a guanine nucleotide exchange factor for the small G protein Rap and is involved in membrane-localized processes such as integrin-mediated cell adhesion and cell-cell junction formation. Cyclic AMP (cAMP) directly activates Epac1 by release of autoinhibition and in addition induces its translocation to the plasma membrane. Here, we show an additional mechanism of Epac1 recruitment, mediated by activated ezrin-radixin-moesin (ERM) proteins. Epac1 directly binds with its N-terminal 49 amino acids to ERM proteins in their open conformation. Receptor-induced activation of ERM proteins results in increased binding of Epac1 and consequently the clustered localization of Epac1 at the plasma membrane. Deletion of the N terminus of Epac1, as well as disruption of the Epac1-ERM interaction by an interfering radixin mutant or small interfering RNA (siRNA)-mediated depletion of the ERM proteins, impairs Epac1-mediated cell adhesion. We conclude that ERM proteins are involved in the spatial regulation of Epac1 and cooperate with cAMP- and Rap-mediated signaling to regulate adhesion to the   extracellular matrix  . 
 Tumor necrosis factor-alpha regulates transforming growth factor-beta-dependent epithelial-mesenchymal transition by promoting hyaluronan-CD44-moesin interaction. Aberrant epithelial-mesenchymal transition (EMT) is involved in development of fibrotic disorders and cancer invasion. Alterations of cell-  extracellular matrix   interaction also contribute to those pathological conditions. However, the functional interplay between EMT and cell-  extracellular matrix   interactions remains poorly understood. We now show that the inflammatory mediator tumor necrosis factor-alpha (TNF-alpha) induces the formation of fibrotic foci by cultured retinal pigment epithelial cells through activation of transforming growth factor-beta (TGF-beta) signaling in a manner dependent on hyaluronan-CD44-moesin interaction. TNF-alpha promoted CD44 expression and moesin phosphorylation by protein kinase C, leading to the pericellular interaction of hyaluronan and CD44. Formation of the hyaluronan-CD44-moesin complex resulted in both cell-cell dissociation and increased cellular motility through actin remodeling. Furthermore, this complex was found to be associated with TGF-beta receptor II and clathrin at actin microdomains, leading to activation of TGF-beta signaling. We established an in vivo model of TNF-alpha-induced fibrosis in the mouse eye, and such ocular fibrosis was attenuated in CD44-null mice. The production of hyaluronan and its interaction with CD44, thus, play an essential role in TNF-alpha-induced EMT and are potential therapeutic targets in fibrotic disorders. 
 Talin regulates moesin-NHE-1 recruitment to invadopodia and promotes mammary tumor metastasis. Invadopodia are actin-rich protrusions that degrade the   extracellular matrix   and are required for stromal invasion, intravasation, and metastasis. The role of the focal adhesion protein talin in regulating these structures is not known. Here, we demonstrate that talin is required for invadopodial matrix degradation and three-dimensional   extracellular matrix   invasion in metastatic breast cancer cells. The sodium/hydrogen exchanger 1 (NHE-1) is linked to the cytoskeleton by ezrin/radixin/moesin family proteins and is known to regulate invadopodium-mediated matrix degradation. We show that the talin C terminus binds directly to the moesin band 4.1 ERM (FERM) domain to recruit a moesin-NHE-1 complex to invadopodia. Silencing talin resulted in a decrease in cytosolic pH at invadopodia and blocked cofilin-dependent actin polymerization, leading to impaired invadopodium stability and matrix degradation. Furthermore, talin is required for mammary tumor cell motility, intravasation, and spontaneous lung metastasis in vivo. Thus, our findings provide a novel understanding of how intracellular pH is regulated and a molecular mechanism by which talin enhances tumor cell invasion and metastasis. 
 Moesin-dependent cytoskeleton remodelling is associated with an anaplastic phenotype of pancreatic cancer. Cell motility is controlled by the dynamic cytoskeleton and its related proteins, such as members of the ezrin/radixin/moesin (ERM) family, which act as signalling molecules inducing cytoskeleton remodelling. Although ERM proteins have been identified as important factors in various malignancies, functional redundancy between these proteins has hindered the dissection of their individual contribution. The aim of the present study was to analyse the functional role of moesin in pancreatic malignancies. Cancer cells of different malignant lesions of human and transgenic mice pancreata were evaluated by immunohistochemistry. For functional analysis, cell growth, adhesion and invasion assays were carried out after transient and stable knock-down of moesin expression in pancreatic cancer cells. In vivo tumourigenicity was determined using orthotopic and metastatic mouse tumour models. We now show that moesin knock-down increases migration, invasion and metastasis and influences   extracellular matrix   organization of pancreatic cancer. Moesin-regulated migratory activities of pancreatic cancer cells were in part promoted through cellular translocation of beta-catenin, and re-distribution and organization of the cytoskeleton. Analysis of human and different transgenic mouse pancreatic cancers demonstrated that moesin is a phenotypic marker for anaplastic carcinoma, suggesting that this ERM protein plays a specific role in pancreatic carcinogenesis. 
  GeneRIFs  
 Data suggest that ezrin-radixin-moesin proteins are involved in the spatial regulation of Epac1 and cooperate with cAMP- and Rap-mediated signaling to regulate adhesion to the   extracellular matrix  . 
  [Ii]nvasive  PubMed  
 Phosphorylation of moesin by Jun N-terminal kinase is important for podosome rosette formation in Src-transformed fibroblasts. Podosomes are actin-based membrane protrusions that facilitate extracellular matrix degradation and motility of   invasive   cells. Podosomes can self-organize into large rosette-like structures in Src-transformed fibroblasts, osteoclasts and some highly   invasive   cancer cells. However, the mechanism of this assembly remains obscure. In this study, we show that the suppression of Jun N-terminal kinase (JNK) by the JNK inhibitor SP600125 or short-hairpin RNA inhibited podosome rosette formation in SrcY527F-transformed NIH3T3 fibroblasts. In addition, SrcY527F was less able to induce podosome rosettes in JNK1-null or JNK2-null mouse embryo fibroblasts than in wild-type counterparts. The kinase activity of JNK was essential for promoting podosome rosette formation but not for its localization to podosome rosettes. Moesin, a member of the ERM (ezrin, radixin and moesin) protein family, was identified as a substrate of JNK. We show that the phosphorylation of moesin at Thr558 by JNK was important for podosome rosette formation in SrcY527F-transformed NIH3T3 fibroblasts. Taken together, our results unveil a novel role of JNK in podosome rosette formation through the phosphorylation of moesin. 
 Moesin orchestrates cortical polarity of melanoma tumour cells to initiate 3D invasion. Tumour cell dissemination through corporal fluids (blood, lymph and body cavity fluids) is a distinctive feature of the metastatic process. Tumour cell transition from fluid to adhesive conditions involves an early polarization event and major rearrangements of the submembrane cytoskeleton that remain poorly understood. As regulation of cortical actin-membrane binding might be important in this process, we investigated the role of ezrin and moesin, which are key crosslinking proteins of the ERM (ezrin, radixin, moesin) family. We used short interfering RNA (siRNA) to show that moesin is crucial for invasion by melanoma cells in 3D matrices and in early lung colonization. Using live imaging, we show that following initial adhesion to the endothelium or 3D matrices, moesin is redistributed away from the region of adhesion, thereby generating a polarized cortex: a stable cortical actin dome enriched in moesin and an   invasive   membrane domain full of blebs. Using Lifeact-GFP, a 17-amino-acid peptide that binds F-actin, we show the initial symmetry breaking of cortical actin cytoskeleton during early attachment of round cells. We also demonstrated that ezrin and moesin are differentially distributed during initial invasion of 3D matrices, and, specifically, that moesin controls adhesion-dependent activation of Rho and subsequent myosin II contractility. Our results reveal that polarized moesin plays a role in orienting Rho activation, myosin II contractility, and cortical actin stability, which is crucial for driving directional vertical migration instead of superficial spreading on the fluid-to-solid tissue interface. We propose that this mechanism of cortical polarization could sustain extravasation of fluid-borne tumour cells during the process of metastasis. 
 Role of moesin in hyaluronan induced cell migration in glioblastoma multiforme. A major barrier to effective treatment of glioblastoma multiforme (GBM) is the invasion of glioma cells into the brain parenchyma rendering local therapies such as surgery and radiation therapy ineffective. GBM patients with such highly   invasive   and infiltrative tumors have poor prognosis with a median survival time of only about a year. However, the mechanisms leading to increased cell migration, invasion and diffused behavior of glioma cells are still poorly understood.In the current study, we applied quantitative proteomics for the identification of differentially expressed proteins in GBMs as compared to non-malignant brain tissues.Our study led to the identification of 23 proteins showing overexpression in GBM; these include membrane proteins, moesin and CD44. The results were verified using Western blotting and immunohistochemistry in independent set of GBM and non-malignant brain tissues. Both GBM tissues and glioma cell lines (U87 / U373) demonstrated membranous expression of moesin and CD44, as revealed by immunohistochemistry and immunofluorescence, respectively. Notably, glioma cells transfected with moesin siRNA displayed reduced migration and invasion on treatment with hyaluronan (HA), an important component of the extracellular matrix in GBM. CD44, a transmembrane glycoprotein, acts as a major receptor for hyaluronan (HA). Using co-immunoprecipitation assays, we further demonstrated that moesin interacts with CD44 in glioma cells only after treatment with HA; this implicates a novel role of moesin in HA-CD44 signaling in gliomas.Our results suggest that development of inhibitors which interfere with CD44-moesin interactions may open a new avenue in the future to mitigate cellular migration in gliomas. 
 Moesin regulates the motility of oral cancer cells via MT1-MMP and E-cadherin/p120-catenin adhesion complex. The present study aimed to clarify the role of Moesin in oral squamous cell carcinoma (OSCC) progression, especially in regulation of cell motility.Immunohistochemistry and western blotting were used to investigate the expression of Moesin, E-cadherin, p120-catenin and MT1-MMP in normal epithelia, dysplasia and OSCCs. Then, Moesin was knockdown by siRNA in OSCC cell lines, WSU-HN6 and CAL27, and the biological role of Moesin in cell adhesion and motility was evaluated by transwell system, cell spreading and aggregation assays. The interactions between Moesin, MT1-MMP and E-cadherin/p120-catenin complex were determined by co-immunoprecipitation and immunofluorescence.Moesin expression was found decreased in the membrane and increased in cytoplasm during the malignant transformation of oral epithelia, and cytoplasmic overexpression of Moesin correlated with nodal metastasis and poor prognosis of OSCCs. Furthermore, Moesin-silencing induced an increased cell-cell adhesion but decreased   invasive  ness, which was subsequently demonstrated might due to Moesin-mediated E-cadherin and p120-catenin interaction. Meantime, Moesin-silencing significantly down-regulated MT1-MMP expression, accompanied by reduced cell motility and impaired filopodia formation, which was also observed when MT1-MMP knockdown by RNAi or tissue inhibitor (TIMP2), indicating the involvement of MT1-MMP in Moesin-mediated cell motility. Finally, the relationship between Moesin, E-cadherin and MT1-MMP was confirmed in OSCC tissue samples.Taken together, our results indicate Moesin may regulate cell motility through its interactions with MT1-MMP and E-cadherin/p120-catenin adhesion complex and cytoplasmic expression of Moesin correlates with nodal metastasis and poor prognosis of OSCCs, indicating Moesin may be a potential candidate for targeted gene therapy for OSCCs. 
 Dynamic actin remodeling during epithelial-mesenchymal transition depends on increased moesin expression. Remodeling of actin filaments is necessary for epithelial-mesenchymal transition (EMT); however, understanding of how this is regulated in real time is limited. We used an actin filament reporter and high-resolution live-cell imaging to analyze the regulated dynamics of actin filaments during transforming growth factor-β-induced EMT of mammary epithelial cells. Progressive changes in cell morphology were accompanied by reorganization of actin filaments from thin cortical bundles in epithelial cells to thick, parallel, contractile bundles that disassembled more slowly but remained dynamic in transdifferentiated cells. We show that efficient actin filament remodeling during EMT depends on increased expression of the ezrin/radixin/moesin (ERM) protein moesin. Cells suppressed for moesin expression by short hairpin RNA had fewer, thinner, and less stable actin bundles, incomplete morphological transition, and decreased   invasiv  e capacity. These cells also had less α-smooth muscle actin and phosphorylated myosin light chain in cortical patches, decreased abundance of the adhesion receptor CD44 at membrane protrusions, and attenuated autophosphorylation of focal adhesion kinase. Our findings suggest that increased moesin expression promotes EMT by regulating adhesion and contractile elements for changes in actin filament organization. We propose that the transciptional program driving EMT controls progressive remodeling of actin filament architectures. 
 Differential expression of moesin in breast cancers and its implication in epithelial-mesenchymal transition. Moesin belongs to the ERM (ezrin, radixin and moesin) family. Recent in-vitro studies have shown the possible involvement of moesin in epithelial-mesenchymal transition (EMT), but correlating in-vivo evidence is lacking.To study the biological significance of moesin, we used immunohistochemistry to investigate the in-situ expression profiles of moesin in 322 breast carcinomas of different subtypes, including 23 cases of metaplastic carcinoma (MCB) which is pathogenetically considered to involve EMT. Moesin was highly expressed in 95.7% of cases of MCB, and in 16% of cases of   invasive   ductal carcinoma (IDC), but was negative in all other subtypes of breast carcinomas. In IDCs, moesin expression correlated positively with a high histological grade (P < 0.001), basal-like phenotype (P < 0.001) and poor overall survival (P = 0.0263). Transfection of MCF7 cells with Snail, one of the key regulators of EMT, showed up-regulation of moesin at the transcriptional level. Finally, mRNA level of moesin correlated positively with Snail and EMT-related genes in a microarray data set using primary breast cancer samples.These results offer biological evidence of moesin as an EMT marker, support the association between moesin, Snail and EMT and suggest a role for moesin in breast cancer prognostication. 
 Proteomic analysis reveals overexpression of moesin and cytokeratin 17 proteins in colorectal carcinoma. The study of tumor biomarkers was gradually facilitated by the adoption of proteomic strategies due to less   invasive  ness and higher sensitivity. Colorectal cancer is one of the most commonly occurring cancers worldwide and its incidence has markedly increased in Korea. While the adoption of proteomic strategies facilitated the study of tumor biomarkers, to date, no common agreement has been derived from proteomic investigations regarding tumor markers of colorectal cancer. This study was designed to find molecules differentially expressed in colorectal cancer compared to non-tumor mucosa. Four colorectal adenocarcinoma and corresponding non-tumor tissue samples were analyzed to find previously unknown proteins via two-dimensional electrophoresis and MALDI-TOF/MS spectrometry. Western blot assays and tissue microarray (TMA) immunohistochemistry were performed to validate the identified proteins. Among the twelve up-regulated and one down-regulated proteins identified, moesin, cytokeratin (KRT) 17 and carbonic anhydrase I were validated by western blot analysis and/or immunohistochemistry. On immunohistochemistry, both moesin and KRT17 demonstrated a tendency of increased expression as pT stage advanced. Both moesin and KRT17 were not expressed in normal colorectal epithelium. These two proteins may play a role in cancer invasion and/or metastasis in colorectal carcinoma, and could be candidate biomarkers for the diagnosis and prognosis of colorectal cancer. 
  [Mm]etastasis  PubMed  
 Vascular endothelial growth factor C promotes cervical cancer   metastasis   via up-regulation and activation of RhoA/ROCK-2/moesin cascade. The elevated expression of vascular endothelial growth factor C (VEGF-C) is correlated with clinical cervical cancer   metastasis   and patient survival, which is interpreted by VEGF-C functions to stimulate angiogenesis and lymphatic genesis. However, the direct impact of VEGF-C on cervical cancer cell motility remains largely unknown.In this study, we investigated the effects of VEGF-C on actin cytoskeleton remodeling and on cervical cancer cell migration and invasion and how the actin-regulatory protein, moesin regulated these effects through RhoA/ROCK-2 signaling pathway.On cervical carcinoma cell line SiHa cells, exposure of VEGF-C triggered remodeling of the actin cytoskeleton and the formation of membrane ruffles, which was required for cell movement. VEGF-C significantly enhanced SiHa cells horizontal migration and three-dimensional invasion into matrices. These actions were dependent on increased expression and phosphorylation of the actin-regulatory protein moesin and specific moesin siRNA severely impaired VEGF-C stimulated-cell migration. The extracellular small GTPase RhoA/ROCK-2 cascade mediated the increased moesin expression and phosphorylation, which was discovered by the use of Y-27632, a specific inhibitor of Rho kinase and by transfected constitutively active, dominant-negative RhoA as well as ROCK-2 SiRNA. Furthermore, in the surgical cervical specimen from the patients with FIGO stage at cervical intra-epithelial neoplasia and I-II cervical squamous cell carcinoma, the expression levels of moesin were found to be significantly correlated with tumor malignancy and   metastasis  .These results implied that VEGF-C promoted cervical cancer   metastasis   by upregulation and activation of moesin protein through RhoA/ROCK-2 pathway. Our findings offer new insight into the role of VEGF-C on cervical cancer progression and may provide potential targets for cervical cancer therapy. 
 Vascular endothelial growth factor C promotes cervical cancer   metastasis   via up-regulation and activation of RhoA/ROCK-2/moesin cascade. The elevated expression of vascular endothelial growth factor C (VEGF-C) is correlated with clinical cervical cancer   metastasis   and patient survival, which is interpreted by VEGF-C functions to stimulate angiogenesis and lymphatic genesis. However, the direct impact of VEGF-C on cervical cancer cell motility remains largely unknown.In this study, we investigated the effects of VEGF-C on actin cytoskeleton remodeling and on cervical cancer cell migration and invasion and how the actin-regulatory protein, moesin regulated these effects through RhoA/ROCK-2 signaling pathway.On cervical carcinoma cell line SiHa cells, exposure of VEGF-C triggered remodeling of the actin cytoskeleton and the formation of membrane ruffles, which was required for cell movement. VEGF-C significantly enhanced SiHa cells horizontal migration and three-dimensional invasion into matrices. These actions were dependent on increased expression and phosphorylation of the actin-regulatory protein moesin and specific moesin siRNA severely impaired VEGF-C stimulated-cell migration. The extracellular small GTPase RhoA/ROCK-2 cascade mediated the increased moesin expression and phosphorylation, which was discovered by the use of Y-27632, a specific inhibitor of Rho kinase and by transfected constitutively active, dominant-negative RhoA as well as ROCK-2 SiRNA. Furthermore, in the surgical cervical specimen from the patients with FIGO stage at cervical intra-epithelial neoplasia and I-II cervical squamous cell carcinoma, the expression levels of moesin were found to be significantly correlated with tumor malignancy and   metastasis  .These results implied that VEGF-C promoted cervical cancer   metastasis   by upregulation and activation of moesin protein through RhoA/ROCK-2 pathway. Our findings offer new insight into the role of VEGF-C on cervical cancer progression and may provide potential targets for cervical cancer therapy. 
 Expression of ezrin and moesin related to invasion,   metastasis   and prognosis of laryngeal squamous cell carcinoma. We examined the expression of ezrin and moesin in laryngeal squamous cell carcinoma (LSCC) and their correlation with patient clinicopathological characteristics and overall survival. Immunohistochemical staining and reverse transcription-polymerase chain reaction (RT-PCR) for ezrin and moesin were applied to 60 carcinoma tissues, adjacent normal tissues, and 33 metastatic lymph nodes. Survival functions were estimated using the Kaplan-Meier method and compared by the log-rank test. RT-PCR demonstrated that the intensity ratios of ezrin and moesin to β-actin were higher in LSCC than in adjacent normal mucous membrane (P < 0.05). Furthermore, intensity ratios were higher in cervical metastatic lymph nodes than in LSCC (P < 0.05). Immunohistochemical staining showed that ezrin and moesin were well distributed in the cell membrane and cytoplasm. Expression was significantly different between LSCC and adjacent normal tissues (P < 0.05); moreover, expression in the cervical metastatic lymph nodes was higher than in LSCC (P < 0.05). Expression of ezrin and moesin was significantly related to clinical stage, T stage, and cervical lymph node   metastasi  s (P < 0.05), except that moesin showed no significant relationship with clinical stage (P > 0.05). Patients with negative ezrin and moesin expression had a significantly longer overall survival time compared to patients with moderate and intense ezrin and moesin expression (P < 0.001, P < 0.05). Ezrin and moesin expression is related to LSCC invasion and   metastasi  s, and may be important molecular markers for predicting prognosis and therapeutic targets in LSCC patients. 
 Moesin orchestrates cortical polarity of melanoma tumour cells to initiate 3D invasion. Tumour cell dissemination through corporal fluids (blood, lymph and body cavity fluids) is a distinctive feature of the metastatic process. Tumour cell transition from fluid to adhesive conditions involves an early polarization event and major rearrangements of the submembrane cytoskeleton that remain poorly understood. As regulation of cortical actin-membrane binding might be important in this process, we investigated the role of ezrin and moesin, which are key crosslinking proteins of the ERM (ezrin, radixin, moesin) family. We used short interfering RNA (siRNA) to show that moesin is crucial for invasion by melanoma cells in 3D matrices and in early lung colonization. Using live imaging, we show that following initial adhesion to the endothelium or 3D matrices, moesin is redistributed away from the region of adhesion, thereby generating a polarized cortex: a stable cortical actin dome enriched in moesin and an invasive membrane domain full of blebs. Using Lifeact-GFP, a 17-amino-acid peptide that binds F-actin, we show the initial symmetry breaking of cortical actin cytoskeleton during early attachment of round cells. We also demonstrated that ezrin and moesin are differentially distributed during initial invasion of 3D matrices, and, specifically, that moesin controls adhesion-dependent activation of Rho and subsequent myosin II contractility. Our results reveal that polarized moesin plays a role in orienting Rho activation, myosin II contractility, and cortical actin stability, which is crucial for driving directional vertical migration instead of superficial spreading on the fluid-to-solid tissue interface. We propose that this mechanism of cortical polarization could sustain extravasation of fluid-borne tumour cells during the process of   metastasis  . 
 Moesin regulates the motility of oral cancer cells via MT1-MMP and E-cadherin/p120-catenin adhesion complex. The present study aimed to clarify the role of Moesin in oral squamous cell carcinoma (OSCC) progression, especially in regulation of cell motility.Immunohistochemistry and western blotting were used to investigate the expression of Moesin, E-cadherin, p120-catenin and MT1-MMP in normal epithelia, dysplasia and OSCCs. Then, Moesin was knockdown by siRNA in OSCC cell lines, WSU-HN6 and CAL27, and the biological role of Moesin in cell adhesion and motility was evaluated by transwell system, cell spreading and aggregation assays. The interactions between Moesin, MT1-MMP and E-cadherin/p120-catenin complex were determined by co-immunoprecipitation and immunofluorescence.Moesin expression was found decreased in the membrane and increased in cytoplasm during the malignant transformation of oral epithelia, and cytoplasmic overexpression of Moesin correlated with nodal   metastasis   and poor prognosis of OSCCs. Furthermore, Moesin-silencing induced an increased cell-cell adhesion but decreased invasiveness, which was subsequently demonstrated might due to Moesin-mediated E-cadherin and p120-catenin interaction. Meantime, Moesin-silencing significantly down-regulated MT1-MMP expression, accompanied by reduced cell motility and impaired filopodia formation, which was also observed when MT1-MMP knockdown by RNAi or tissue inhibitor (TIMP2), indicating the involvement of MT1-MMP in Moesin-mediated cell motility. Finally, the relationship between Moesin, E-cadherin and MT1-MMP was confirmed in OSCC tissue samples.Taken together, our results indicate Moesin may regulate cell motility through its interactions with MT1-MMP and E-cadherin/p120-catenin adhesion complex and cytoplasmic expression of Moesin correlates with nodal   metastasis   and poor prognosis of OSCCs, indicating Moesin may be a potential candidate for targeted gene therapy for OSCCs. 
 [Study of the correlation of papillary thyroid carcinoma's invasion with Ezrin, Moesin and E-Cadherin]. To investigate the effects of Ezrin, Moesin and E-Cadherin on the invasion and   metastasis   of papillary thyroid carcinoma(PTC).The 81 paraffin-embedded specimen of PTC treated in The Second Clinical College of Harbin Medical University from 2005 to 2008 were examined immunohistochemically for Ezrin, Moesin, and E-Cadherin. Normal thyroid tissues adjacent to cancer were control. The correlation of Ezrin, Moesin, and E-Cadherin expressions and clinical features of PTC, including gender, age, tumor size, invasion, lymph node   metastasis  , and TNM stage, was analyzed.Positive staining rates of Ezrin and Moesin in PTC were significantly higher than those in control (χ(2) were 61.691 and 57.949, P < 0.01, respectively). Positive staining rate of E-Cadherin in PTC was significantly lower than that in control (χ(2) = 64.241, P < 0.01). The positive expression rates of Ezrin and Moesin in PTC increased in the subgroups with age ≥ 45 years, local invasion, lymph n  ode metast  asis and stage III-IV, with significant difference respectively (P < 0.01), while the positive expression rate of E-Cadherin in PTC significantly decreased (P < 0.01).The up-regulation expressions of Ezrin and Moesin and the down-regulation expression of E-Cadherin were associated with the invasion   and metast  asis of PTC. Combined detection of Ezrin, Moesin and E-Cadherin in PTC might served as an important predictor for the invasi  on, metast  asis and prognosis of PTC. 
 ERM stable knockdown by siRNA reduced in vitro migration and invasion of human SGC-7901 cells. Three closely related proteins, ezrin, radixin, and moesin (ERM), which primarily act as a linker between the plasma membrane and the cytoskeleton, are involved in many cellular functions, including regulation of actin cytoskeleton, control of cell shape, adhesion and motility, and modulation of signaling pathways. Although, ezrin is now recognized as a key component in tumor   metastasis  , the functional role of the radixin and moesin in tumor   metastasis   has not been established. In the present study, we chose highly metastatic human gastric carcinoma SGC-7901 cells, which express all of the ERM proteins as a model to examine the functional roles of these proteins in tumor   metastasis  . Ezrin, radixin or moesin stable knockdown SGC-7901 cell lines were established using siRNA methodology. In vitro cell migration and invasion studies showed that either ezrin, radixin or moesin specific deficiency in the cells caused the substantial reduction of the cell migration and invasion. Western blotting and immunofluorescence analysis showed that the expression of E-cadherin was also significantly increased when any member of ERM proteins was downregulated. Our results indicated that these three ERM proteins play similar roles in the SGC-7901 cell metastatic potential and their roles of upregulating the expression of E-cadherin may be important in tumor progression. 
 Proteomic profiling in pancreatic cancer with and without lymph node   metastasis  . The aim of the study was to observe different protein profiles in pancreatic cancer with and without lymph node   metastasis   (LNM), and search for novel LNM-associated proteins, which would help to understand the metastatic mechanisms and provide targets for therapeutic interventions. Cancer nests were manually miscrodissected from 8 LNM and 7 non-LNM pancreatic cancer tissues, and the protein extracts were then separated by difference gel electrophoresis (DIGE) and identified by MALDI-TOF-TOF. Four differently regulated proteins, ezrin, radixin, moesin, and c14orf166, were selected for further validation by Western blot and immunohistochemistry. In DIGE analysis, we identified 18 up-regulated proteins and 15 down-regulated proteins in LNM pancreatic cancer nests compared with non-LNM ones. Western blot and immunohistochemical analyses confirmed that radixin, moesin and c14orf166, but not ezrin, had significantly higher expression levels in LNM pancreatic cancers than in non-LNM controls. In conclusion, the specific protein profiles found in this study might provide new insights into the mechanism of lymph node   metastasis  . For the first time, c14orf166 was identified asa novel   metastasis  -associated protein, and the roles of radixin, moesin and c14orf166 in cancer   metastasis   deserve further investigations. 
 Clinical significance of cellular distribution of moesin in patients with oral squamous cell carcinoma. Moesin is a linking protein of the submembraneous cytoskeleton and plays a key role in the control of cell morphology, adhesion, and motility. The aim of the present study was to elucidate the clinical significance of expression patterns of moesin in patients with oral squamous cell carcinoma (OSCC).Immunohistochemistry for moesin monoclonal antibody was performed on 103 paraffin-embedded specimens from patients with primary OSCC, including 30 patients with locoregional lymph node   metastasis  , and in the sections from nude mice transplanted with two cell lines derived from a single human tongue cancer (SQUU-A and SQUU-B).Expression patterns of moesin in OSCCs were divided into three groups: membranous pattern; mixed pattern; and cytoplasmic pattern. These expression patterns correlated with tumor size, lymph node   metastasis  , mode of invasion, differentiation, and lymphocytic infiltration. In about two-thirds of the patients with metastatic lymph node, homogeneous cytoplasmic expression was detected in the metastatic lymph nodes. In addition, SQUU-B with high metastatic potential showed more reduced levels of membrane-bound moesin than SQUU-A with low metastatic potential. A multivariate analysis demonstrated that expression patterns of moesin can be an independent prognostic factor.Our results suggest that moesin expression contributed to discriminating between patients with the potentiality for locoregional lymph node   metastasis   and those with a better prognosis and might improve the definition of suitable therapy for each. 
 Moesin expression is a marker of basal breast carcinomas. Basal breast cancers (BBCs) have a high risk of   metastasis  , recurrence and death. Formal subtype definition relies on gene expression but can be approximated by protein expression. New markers are needed to help in the management of the basal subtype of breast cancer. In a previous transcriptional analysis of breast cell lines we found that Moesin expression was a potential basal marker. We show here that Moesin protein expression is a basal marker in breast tumors. In a tissue microarray (TMA) containing 547 sporadic breast cancers, of which 108 were profiled for gene expression, Moesin was expressed in 31% of all tumors and in 82% of the basal tumors. To confirm that Moesin expression remained associated with the basal phenotype in specific types of BBCs, we analyzed Moesin expression in 2 other TMAs containing 40 medullary breast cancers (MBCs) and 27 BRCA1-associated breast cancers (BRCA1-BCs), respectively. Moesin was strongly expressed in MBCs (87%; p = 2.4 x 10(-5)) and in BRCA1-BCs (58%; p = 1.3 x 10(-5)) as compared with non-MBCs and sporadic cases. Moesin-expressing tumors display features of BBCs, such as high proliferation rate, hormone receptors negativity, expression of putative basal/myoepithelial markers (CAV1, CD10, CK5/6, CK14, EGFR, P53, P-cadherin and SMA). Survival analysis showed a reduced specific survival and   metastasis  -free survival in Moesin-expressing tumors by log-rank test (p(SS) = 0.014 and p(MFS) = 0.014). In multivariate analysis, Moesin expression was nearly an independent prognostic marker of poor outcome as shown by Cox proportional hazard model in patients without lymph node   metastasis   (p = 0.052, HR = 2.38, CI 95[0.99-5.69]). 
 Tumor suppressive microRNA-133a regulates novel targets: moesin contributes to cancer cell proliferation and invasion in head and neck squamous cell carcinoma. Recently, many studies suggest that microRNAs (miRNAs) contribute to the development, invasion and   metastasis   of various types of human cancers. Our recent study revealed that expression of microRNA-133a (miR-133a) was significantly reduced in head and neck squamous cell carcinoma (HNSCC) and that restoration of miR-133a inhibited cell proliferation, migration and invasion in HNSCC cell lines, suggesting that miR-133a function as a tumor suppressor. Genome-wide gene expression analysis of miR-133a transfectants and TargetScan database showed that moesin (MSN) was a promising candidate of miR-133a target gene. MSN is a member of the ERM (ezrin, radixin and moesin) protein family and ERM function as cross-linkers between plasma membrane and actin-based cytoskeleton. The functions of MSN in cancers are controversial in previous reports. In this study, we focused on MSN and investigated whether MSN was regulated by tumor suppressive miR-133a and contributed to HNSCC oncogenesis. Restoration of miR-133a in HNSCC cell lines (FaDu, HSC3, IMC-3 and SAS) suppressed the MSN expression both in mRNA and protein level. Silencing study of MSN in HNSCC cell lines demonstrated significant inhibitions of cell proliferation, migration and invasion activities in si-MSN transfectants. In clinical specimen with HNSCC, the expression level of MSN was significantly up-regulated in cancer tissues compared to adjacent non-cancerous tissues. These data suggest that MSN may function as oncogene and is regulated by tumor suppressive miR-133a. Our analysis data of novel tumor-suppressive miR-133a-mediated cancer pathways could provide new insights into the potential mechanisms of HNSCC oncogenesis. 
 Talin regulates moesin-NHE-1 recruitment to invadopodia and promotes mammary tumor   metastasis  . Invadopodia are actin-rich protrusions that degrade the extracellular matrix and are required for stromal invasion, intravasation, and   metastasis  . The role of the focal adhesion protein talin in regulating these structures is not known. Here, we demonstrate that talin is required for invadopodial matrix degradation and three-dimensional extracellular matrix invasion in metastatic breast cancer cells. The sodium/hydrogen exchanger 1 (NHE-1) is linked to the cytoskeleton by ezrin/radixin/moesin family proteins and is known to regulate invadopodium-mediated matrix degradation. We show that the talin C terminus binds directly to the moesin band 4.1 ERM (FERM) domain to recruit a moesin-NHE-1 complex to invadopodia. Silencing talin resulted in a decrease in cytosolic pH at invadopodia and blocked cofilin-dependent actin polymerization, leading to impaired invadopodium stability and matrix degradation. Furthermore, talin is required for mammary tumor cell motility, intravasation, and spontaneous lung   metastasis   in vivo. Thus, our findings provide a novel understanding of how intracellular pH is regulated and a molecular mechanism by which talin enhances tumor cell invasion and   metastasis  . 
 Expression of moesin and CD44 is associated with poor prognosis in gastric adenocarcinoma. CD44 has been reported as a negative prognostic marker in gastric cancer. It interacts with moesin in epithelial-mesenchymal transition. To date, to our knowledge, there has been no clinical study dealing with the relationship between moesin and gastric adenocarcinoma. We analysed the expression of moesin and CD44 in gastric adenocarcinoma tissue, and correlations with clinicopathological factors.A retrospective analysis was made of 430 patients who had undergone gastrectomy at the Korea University Guro Hospital between 2002 and 2005 for gastric adenocarcinoma. Using tissue microarray and immunohistochemical staining, moesin expression was observed in 192 (44.7%) cases; it was associated significantly with poorly differentiated histology, invasion depth, lymph node   metastasis  , lymphatic invasion and advanced pathological TNM stage. CD44 expression was not correlated with clinicopathological features or moesin expression. Moesin expression was a strong predictor of lymph node   metastasis   in logistic regression analysis. Both moesin expression and CD44 expression were associated significantly with poor overall survival in univariate analysis. Furthermore, in multivariate analysis, moesin and CD44 were independent markers of poor prognosis, along with pathological TNM stage and older patient age.Moesin expression and CD44 expression might be useful markers of poor prognosis in gastric adenocarcinoma. 
 Moesin-dependent cytoskeleton remodelling is associated with an anaplastic phenotype of pancreatic cancer. Cell motility is controlled by the dynamic cytoskeleton and its related proteins, such as members of the ezrin/radixin/moesin (ERM) family, which act as signalling molecules inducing cytoskeleton remodelling. Although ERM proteins have been identified as important factors in various malignancies, functional redundancy between these proteins has hindered the dissection of their individual contribution. The aim of the present study was to analyse the functional role of moesin in pancreatic malignancies. Cancer cells of different malignant lesions of human and transgenic mice pancreata were evaluated by immunohistochemistry. For functional analysis, cell growth, adhesion and invasion assays were carried out after transient and stable knock-down of moesin expression in pancreatic cancer cells. In vivo tumourigenicity was determined using orthotopic and metastatic mouse tumour models. We now show that moesin knock-down increases migration, invasion and   metastasis   and influences extracellular matrix organization of pancreatic cancer. Moesin-regulated migratory activities of pancreatic cancer cells were in part promoted through cellular translocation of beta-catenin, and re-distribution and organization of the cytoskeleton. Analysis of human and different transgenic mouse pancreatic cancers demonstrated that moesin is a phenotypic marker for anaplastic carcinoma, suggesting that this ERM protein plays a specific role in pancreatic carcinogenesis. 
 Proteomic analysis reveals overexpression of moesin and cytokeratin 17 proteins in colorectal carcinoma. The study of tumor biomarkers was gradually facilitated by the adoption of proteomic strategies due to less invasiveness and higher sensitivity. Colorectal cancer is one of the most commonly occurring cancers worldwide and its incidence has markedly increased in Korea. While the adoption of proteomic strategies facilitated the study of tumor biomarkers, to date, no common agreement has been derived from proteomic investigations regarding tumor markers of colorectal cancer. This study was designed to find molecules differentially expressed in colorectal cancer compared to non-tumor mucosa. Four colorectal adenocarcinoma and corresponding non-tumor tissue samples were analyzed to find previously unknown proteins via two-dimensional electrophoresis and MALDI-TOF/MS spectrometry. Western blot assays and tissue microarray (TMA) immunohistochemistry were performed to validate the identified proteins. Among the twelve up-regulated and one down-regulated proteins identified, moesin, cytokeratin (KRT) 17 and carbonic anhydrase I were validated by western blot analysis and/or immunohistochemistry. On immunohistochemistry, both moesin and KRT17 demonstrated a tendency of increased expression as pT stage advanced. Both moesin and KRT17 were not expressed in normal colorectal epithelium. These two proteins may play a role in cancer invasion and/or   metastasi  s in colorectal carcinoma, and could be candidate biomarkers for the diagnosis and prognosis of colorectal cancer. 
 MiR-200 can repress breast cancer   metastasis   through ZEB1-independent but moesin-dependent pathways. The microRNA-200 (miR-200) family has a critical role in regulating epithelial-mesenchymal transition and cancer cell invasion through inhibition of the E-cadherin transcriptional repressors ZEB1 and ZEB2. Recent studies have indicated that the miR-200 family may exert their effects at distinct stages in the metastatic process, with an overall effect of enhancing   metastasis   in a syngeneic mouse breast cancer model. We find in a xenograft orthotopic model of breast cancer   metastasis   that ectopic expression of members of the miR-200b/200c/429, but not the miR-141/200a, functional groups limits tumour cell invasion and   metastasis  . Despite modulation of the ZEB1-E-cadherin axis, restoration of ZEB1 in miR-200b-expressing cells was not able to alter metastatic potential suggesting that other targets contribute to this process. Instead, we found that miR-200b repressed several actin-associated genes, with the knockdown of the ezrin-radixin-moesin family member moesin alone phenocopying the repression of cell invasion by miR-200b. Moesin was verified to be directly targeted by miR-200b, and restoration of moesin in miR-200b-expressing cells was sufficient to alleviate metastatic repression. In breast cancer cell lines and patient samples, the expression of moesin significantly inversely correlated with miR-200 expression, and high levels of moesin were associated with poor relapse-free survival. These findings highlight the context-dependent effects of miR-200 in breast cancer   metastasis   and demonstrate the existence of a moesin-dependent pathway, distinct from the ZEB1-E-cadherin axis, through which miR-200 can regulate tumour cell plasticity and   metastasis  . 
  GeneRIFs  
 results indicate Moesin may regulate cell motility through its interactions with MT1-MMP and E-cadherin/p120-catenin adhesion complex and cytoplasmic expression of Moesin correlates with nodal   metastasis   and poor prognosis of OSCCs 
 Ezrin and moesin expression is related to LSCC invasion and   metastasis  , and may be important molecular markers for predicting prognosis and therapeutic targets in LSCC patients 
 The up-regulation of Ezrin and Moesin and the down-regulation of E-Cadherin were associated with the invasion and   metastasis   of papillary thyroid carcinoma. 
 Results suggest that VEGF-C promoted cervical cancer   metastasis   by upregulation and activation of moesin protein through RhoA/ROCK-2 pathway. 
 c14orf166 was identified asa novel   metastasis  -associated protein, and the roles of radixin, moesin and c14orf166 in pancreatic cancer   metastasis   deserve further investigations. 
 moesin has a role in lymph node   metastasis   of oral squamous cell carcinoma 
  [Cc]ell [Ii]nvasion  PubMed  
 Intracellular sphingosine kinase 2-derived sphingosine-1-phosphate mediates epidermal growth factor-induced ezrin-radixin-moesin phosphorylation and cancer   cell invasion  . The bioactive sphingolipid sphingosine-1-phosphate (S1P) mediates cellular proliferation, mitogenesis, inflammation, and angiogenesis. These biologies are mediated through S1P binding to specific GPCRs [sphingosine-1-phosphate receptor (S1PR)1-5] and some other less well-characterized intracellular targets. Ezrin-radixin-moesin (ERM) proteins, a family of adaptor molecules linking the cortical actin cytoskeleton to the plasma membrane, are emerging as critical regulators of cancer invasion via regulation of cell morphology and motility. Recently, we identified S1P as an acute ERM activator (via phosphorylation) through its action on S1PR2. In this work, we dissect the mechanism of S1P generation downstream of epidermal growth factor (EGF) leading to ERM phosphorylation and cancer invasion. Using pharmacologic inhibitors, small interfering RNA technologies, and genetic approaches, we demonstrate that sphingosine kinase (SK)2, and not SK1, is essential and sufficient in EGF-mediated ERM phosphorylation in HeLa cells. In fact, knocking down SK2 decreased ERM activation 2.5-fold. Furthermore, we provide evidence that SK2 is necessary to mediate EGF-induced invasion. In addition, overexpressing SK2 causes a 2-fold increase in HeLa   cell invasion  . Surprisingly, and for the first time, we find that this event, although dependent on S1PR2 activation, does not generate and does not require extracellular S1P secretion, therefore introducing a potential novel model of autocrine/intracrine action of S1P that still involves its GPCRs. These results define new mechanistic insights for EGF-mediated invasion and novel actions of SK2, therefore setting the stage for novel targets in the treatment of growth factor-driven malignancies. 
 Talin regulates moesin-NHE-1 recruitment to invadopodia and promotes mammary tumor metastasis. Invadopodia are actin-rich protrusions that degrade the extracellular matrix and are required for stromal invasion, intravasation, and metastasis. The role of the focal adhesion protein talin in regulating these structures is not known. Here, we demonstrate that talin is required for invadopodial matrix degradation and three-dimensional extracellular matrix invasion in metastatic breast cancer cells. The sodium/hydrogen exchanger 1 (NHE-1) is linked to the cytoskeleton by ezrin/radixin/moesin family proteins and is known to regulate invadopodium-mediated matrix degradation. We show that the talin C terminus binds directly to the moesin band 4.1 ERM (FERM) domain to recruit a moesin-NHE-1 complex to invadopodia. Silencing talin resulted in a decrease in cytosolic pH at invadopodia and blocked cofilin-dependent actin polymerization, leading to impaired invadopodium stability and matrix degradation. Furthermore, talin is required for mammary tumor cell motility, intravasation, and spontaneous lung metastasis in vivo. Thus, our findings provide a novel understanding of how intracellular pH is regulated and a molecular mechanism by which talin enhances tumor   cell invasion   and metastasis. 
 MiR-200 can repress breast cancer metastasis through ZEB1-independent but moesin-dependent pathways. The microRNA-200 (miR-200) family has a critical role in regulating epithelial-mesenchymal transition and cancer   cell invasion   through inhibition of the E-cadherin transcriptional repressors ZEB1 and ZEB2. Recent studies have indicated that the miR-200 family may exert their effects at distinct stages in the metastatic process, with an overall effect of enhancing metastasis in a syngeneic mouse breast cancer model. We find in a xenograft orthotopic model of breast cancer metastasis that ectopic expression of members of the miR-200b/200c/429, but not the miR-141/200a, functional groups limits tumour   cell invasion   and metastasis. Despite modulation of the ZEB1-E-cadherin axis, restoration of ZEB1 in miR-200b-expressing cells was not able to alter metastatic potential suggesting that other targets contribute to this process. Instead, we found that miR-200b repressed several actin-associated genes, with the knockdown of the ezrin-radixin-moesin family member moesin alone phenocopying the repression of   cell invasion   by miR-200b. Moesin was verified to be directly targeted by miR-200b, and restoration of moesin in miR-200b-expressing cells was sufficient to alleviate metastatic repression. In breast cancer cell lines and patient samples, the expression of moesin significantly inversely correlated with miR-200 expression, and high levels of moesin were associated with poor relapse-free survival. These findings highlight the context-dependent effects of miR-200 in breast cancer metastasis and demonstrate the existence of a moesin-dependent pathway, distinct from the ZEB1-E-cadherin axis, through which miR-200 can regulate tumour cell plasticity and metastasis. 
  GeneRIFs  
 Intracellular sphingosine kinase 2-derived sphingosine-1-phosphate mediates epidermal growth factor-induced ezrin-radixin-moesin phosphorylation and cancer   cell invasion  . 
   
  
 UTP20 
 
  NCBI Gene  
  GeneRIF  
  Pubmed  
 
 
  Search Term  Rank  Fields  
  [Mm]etastasis  12  PubMed(5); Conserved Domains(1); Alternative Name(2)  
 
 
 hide/show details for UTP20 
  [Mm]etastasis  PubMed  
 Differential gene expression in mammary carcinoma cell lines: identification of DRIM, a new gene down-regulated in   metastasis  . Differential display technique was applied to a pair of cell lines derived from human breast carcinoma cell line MDA-MB 435 with metastatic and non-metastatic properties in the nude mouse system, with the objective to isolate genes involved in   metastasis  . DRIM (Down-Regulated In   Metastasis  ) was the only gene found to be differentially expressed in this system. DRIM encodes a protein comprising 2785 amino acids with significant homology to a protein in yeast and C. elegans. The protein contains a conserved positively charged tail and several HEAT repeats, designated after four functionally characterized proteins in which the repeat was detected. Most of the hydrophobic regions of DRIM can be assigned to HEAT repeats. Expression of DRIM at the RNA level was investigated in several normal tissues and tumor cell lines. 
 Mapping nucleolar localization sequences of 1A6/DRIM. Human 1A6/downregulated in   metastasis   (DRIM) is a nucleolar protein with multiple HEAT-repeat motifs (Huntington, elongation factor 3, a subunit of protein phosphatase 2A, target of rapamycin). The yeast homologue to 1A6/DRIM, Utp20, is part of the small subunit processome and functions in 18S RNA processing. In the present study, we utilized the green fluorescent protein as the fusion protein marker to investigate the sequence responsible for 1A6/DRIM accumulation in nucleolus. Deletion sequence analysis demonstrated that a single region located between amino acids 2744 and 2761 at the C-terminus of 1A6/DRIM is capable of nucleolar accumulation. Two basic amino acid clusters within this region are essential for nucleolar accumulation. The sequences required for nucleolar accumulation overlaps the putative nuclear localization signal of 1A6/DRIM. 
 Characterization of the promoter of 1A6/DRIM, a novel cancer-related gene and identification of its transcriptional activator. 1A6/DRIM (Down-regulated in   Metastasis  ) has been reported to express at a high level in the gastric cancer tissues and the premalignant lesions implicating the involvement of 1A6/DRIM in cell transformation. Although the information regarding the putative functions and distribution of the 1A6/DRIM in different tissues and cell lines has been increasing recently, its promoter and promoter-regulating factors remain unknown. In this study, the transcription initiation site of 1A6/DRIM was confirmed to be located at 147 bp upstream of the ATG codon using the primer extension analysis. The minimal promoter region of the 1A6/DRIM is located between -47 and +42 of the transcription initiation site measured by luciferase reporter assays using a set of deletion constructs. In addition, an E-box is shown to be an essential element for transcriptional regulation of 1A6/DRIM demonstrated by luciferase assay with different deletion and mutation constructs. Finally, a transcription factor, upstream stimulatory factor 2 (USF2) was found to be an activator of the 1A6/DRIM through binding to the E-box demonstrated by luciferase reporter assay, electrophoretic mobility shift assay, and chromatin immunoprecipitation (ChIP) assay. The structural analysis of the 1A6/DRIM promoter and the identification of its potential regulatory effecter may help us to understand its biological functions in regulating cancer development. 
 KIAA0649, a 1A6/DRIM-interacting protein with the oncogenic potential. We identified a 1A6/DRIM (down-regulated in   metastasis  ) interacting protein, KIAA0649 during the yeast two-hybrid screen. The interaction between KIAA0649 and 1A6/DRIM was further confirmed by GST-pull-down and co-immunoprecipitation assays. KIAA0649 was originally identified from human brain tissue. However, its biological function remains unknown. In this report, we showed that KIAA0649 mRNA is widely expressed in human multiple tissues and cell lines. We have also demonstrated that KIAA0649 has oncogenic characteristics: it enhances colony formation, allows anchorage-independent growth. Moreover, KIAA0649 exogenous expression in NIH3T3 fibroblasts caused tumor development in nude mice. 
 The interrelationship between DRIM gene expression and cytogenetic and phenotypic characteristics in human breast tumor cell lines. In order to facilitate the identification of genes involved in the metastatic phenotype we have previously developed a pair of cell lines from the human breast carcinoma cell line MDA-MB-435, which have diametrically opposite metastatic potential in athymic mice. Differential display analysis of this model previously identified a novel gene, DRIM (down regulated in   metastasis  ), the decreased expression of which correlated with metastatic capability. DRIM encodes a protein comprising 2785 amino acids with significant homology to a protein in yeast and C. elegans, but little else is currently known about its function or pattern of expression. In a detailed analysis of the DRIM gene locus we quantitatively evaluated gene dosage and the expression of DRIM transcripts in a panel of breast cell lines of known metastatic phenotype.Fluorescent in situ hybridization (FISH) analyses mapped a single DRIM gene locus to human chromosome 12q23~24, a region of conserved synteny to mouse chromosome 10. We confirmed higher expression of DRIM mRNA in the non-metastatic MDA-MB-435 clone NM2C5, relative to its metastatic counterpart M4A4, but this appeared to be due to the presence of an extra copy of the DRIM gene in the cell line's genome. The other non-metastatic cell lines in the series (T47D MCF-7, SK-BR-3 and ZR-75-1) contained either 3 or 4 chromosomal copies of DRIM gene. However, the expression level of DRIM mRNA in M4A4 was found to be 2-4 fold higher than in unrelated breast cells of non-metastatic phenotype.Whilst DRIM expression is decreased in metastatic M4A4 cells relative to its non-metastatic isogenic counterpart, neither DRIM gene dosage nor DRIM mRNA levels correlated with metastatic propensity in a series of human breast tumor cell lines examined. Collectively, these findings indicate that the expression pattern of the DRIM gene in relation to the pathogenesis of breast tumor   metastasis   is more complex than previously recognized. 
  Conserved Domains  
 pfam07539: DRIM; Down-regulated in   metastasis   
  Alternative Name  
 down regulated in   metastasis   
 down-regulated in   metastasis   protein 
   
  
 CARMIL1 
 
  NCBI Gene  
  GeneRIF  
  Pubmed  
 
 
  Search Term  Rank  Fields  
  [Ii]nvadopodia  3  PubMed(1)  
  [Mm]igration  6  Process(3); PubMed(4); GeneRIFs(1)  
  [Ee]xtracellular [Mm]atrix  8  PubMed(1)  
 
 
 hide/show details for CARMIL1 
  [Ii]nvadopodia  PubMed  
 Cell Migration and   Invadopodia   Formation Require a Membrane-binding Domain of CARMIL2. CARMILs regulate capping protein (CP), a critical determinant of actin assembly and actin-based cell motility. Vertebrates have three conserved CARMIL genes with distinct functions. In migrating cells, CARMIL2 is important for cell polarity, lamellipodial assembly, ruffling, and macropinocytosis. In cells, CARMIL2 localizes with a distinctive dual pattern to vimentin intermediate filaments and to membranes at leading edges and macropinosomes. The mechanism by which CARMIL2 localizes to membranes has not been defined. Here, we report that CARMIL2 has a conserved membrane-binding domain composed of basic and hydrophobic residues, which is necessary and sufficient for membrane localization, based on expression studies in cells and on direct binding of purified protein to lipids. Most important, we find that the membrane-binding domain is necessary for CARMIL2 to function in cells, based on rescue expression with a set of biochemically defined mutants. CARMIL1 and CARMIL3 contain similar membrane-binding domains, based on sequence analysis and on experiments, but other CPI motif proteins, such as CD2AP, do not. Based on these results, we propose a model in which the membrane-binding domain of CARMIL2 tethers this multidomain protein to the membrane, where it links dynamic vimentin filaments with regulation of actin assembly via CP. 
  [Mm]igration  Process  
 cell   migration   
 positive regulation of cell   migration   
 positive regulation of cell   migration   
  PubMed  
 Mammalian CARMIL inhibits actin filament capping by capping protein. Actin polymerization in cells occurs via filament elongation at the barbed end. Proteins that cap the barbed end terminate this elongation. Heterodimeric capping protein (CP) is an abundant and ubiquitous protein that caps the barbed end. We find that the mouse homolog of the adaptor protein CARMIL (mCARMIL) binds CP with high affinity and decreases its affinity for the barbed end. Addition of mCARMIL to cell extracts increases the rate and extent of Arp2/3 or spectrin-actin seed-induced polymerization. In cells, GFP-mCARMIL concentrates in lamellipodia and increases the fraction of cells with large lamellipodia. Decreasing mCARMIL levels by siRNA transfection lowers the F-actin level and slows cell   migration   through a mechanism that includes decreased lamellipodia protrusion. This phenotype is reversed by full-length mCARMIL but not mCARMIL lacking the domain that binds CP. Thus, mCARMIL is a key regulator of CP and has profound effects on cell behavior. 
 Distinct roles for CARMIL isoforms in cell   migration  . Molecular mechanisms for cell   migration  , especially how signaling and cytoskeletal systems are integrated, are not understood well. Here, we examined the role of CARMIL (capping protein, Arp2/3, and Myosin-I linker) family proteins in migrating cells. Vertebrates express three conserved genes for CARMIL, and we examined the functions of the two CARMIL genes expressed in migrating human cultured cells. Both isoforms, CARMIL1 and 2, were necessary for cell   migration  , but for different reasons. CARMIL1 localized to lamellipodia and macropinosomes, and loss of its function caused loss of lamellipodial actin, along with defects in protrusion, ruffling, and macropinocytosis. CARMIL1-knockdown cells showed loss of activation of Rac1, and CARMIL1 was biochemically associated with the GEF Trio. CARMIL2, in contrast, colocalized with vimentin intermediate filaments, and loss of its function caused a distinctive multipolar phenotype. Loss of CARMIL2 also caused decreased levels of myosin-IIB, which may contribute to the polarity phenotype. Expression of one CARMIL isoform was not able to rescue the knockdown phenotypes of the other. Thus, the two isoforms are both important for cell   migration  , but they have distinct functions. 
 Physiological role of the interaction between CARMIL1 and capping protein. The regulation of free barbed ends is central to the control of dynamic actin assembly and actin-based motility in cells. Capping protein (CP) is known to regulate barbed ends and control actin assembly in cells. The CARMIL family of proteins can bind and inhibit CP in vitro, but the physiological significance of the interaction of CARMIL with CP in cells is poorly understood. Mammalian cells lacking CARMIL1 have defects in lamellipodia, macropinocytosis, cell   migration  , and Rac1 activation. Here we investigate the physiological significance of the CARMIL1-CP interaction, using a point mutant with a well-defined biochemical defect. We find that the CARMIL1-CP interaction is essential for the assembly of lamellipodia, the formation of ruffles, and the process of macropinocytosis. In contrast, the interaction of CARMIL1 with CP shows little to no importance for other functions of CARMIL1, including localization of CARMIL1 to the membrane, activation of Rac1, and cell   migration  . One implication is that lamellipodia are only marginally important for cell   migration   in a wound-healing model. The results also suggest that the ability of CARMIL1 to inhibit CP in cells may be regulated. 
 Cell   Migration   and Invadopodia Formation Require a Membrane-binding Domain of CARMIL2. CARMILs regulate capping protein (CP), a critical determinant of actin assembly and actin-based cell motility. Vertebrates have three conserved CARMIL genes with distinct functions. In migrating cells, CARMIL2 is important for cell polarity, lamellipodial assembly, ruffling, and macropinocytosis. In cells, CARMIL2 localizes with a distinctive dual pattern to vimentin intermediate filaments and to membranes at leading edges and macropinosomes. The mechanism by which CARMIL2 localizes to membranes has not been defined. Here, we report that CARMIL2 has a conserved membrane-binding domain composed of basic and hydrophobic residues, which is necessary and sufficient for membrane localization, based on expression studies in cells and on direct binding of purified protein to lipids. Most important, we find that the membrane-binding domain is necessary for CARMIL2 to function in cells, based on rescue expression with a set of biochemically defined mutants. CARMIL1 and CARMIL3 contain similar membrane-binding domains, based on sequence analysis and on experiments, but other CPI motif proteins, such as CD2AP, do not. Based on these results, we propose a model in which the membrane-binding domain of CARMIL2 tethers this multidomain protein to the membrane, where it links dynamic vimentin filaments with regulation of actin assembly via CP. 
  GeneRIFs  
 The two CARMIL isoforms are both important for cell   migration  , but they have distinct functions.[CARMIL1, CARMIL2] 
  [Ee]xtracellular [Mm]atrix  PubMed  
 Proteomic analysis of human parotid gland exosomes by multidimensional protein identification technology (MudPIT). Human ductal saliva contributes over a thousand unique proteins to whole oral fluids. The mechanism by which most of these proteins are secreted by salivary glands remains to be determined. The present study used a mass spectrometry-based, shotgun proteomics approach to explore the possibility that a subset of the proteins found in saliva are derived from exosomes, membrane-bound vesicles of endosomal origin within multivesicular endosomes. Using MudPIT (multidimensional protein identification technology) mass spectrometry, we catalogued 491 proteins in the exosome fraction of human parotid saliva. Many of these proteins were previously observed in ductal saliva from parotid glands (265 proteins). Furthermore, 72 of the proteins in parotid exosomes overlap with those previously identified as urinary exosome proteins, proteins which are also frequently associated with exosomes from other tissues and cell types. Gene Ontology (GO) and KEGG pathway analyses found that cytosolic proteins comprise the largest category of proteins in parotid exosomes (43%), involved in such processes as phosphatidylinositol signaling system, calcium signaling pathway, inositol metabolism, protein export, and signal transduction, among others; whereas the integral plasma membrane proteins and associated/peripheral plasma membrane proteins (26%) were associated with   extracellular matrix  -receptor interaction, epithelial cell signaling, T-cell and B-cell receptor signaling, cytokine receptor interaction, and antigen processing and presentation, among other biological functions. In addition, these putative saliva exosomal proteins were linked to specific diseases (e.g., neurodegenerative disorders, prion disease, cancers, type I and II diabetes). Consequently, parotid glands secrete exosomes that reflect the metabolic and functional status of the gland and may also carry informative protein markers useful in the diagnosis and treatment of systemic diseases. 
   
  
 SHOC2 
 
  NCBI Gene  
  GeneRIF  
  Pubmed  
 
 
  Search Term  Rank  Fields  
  [Mm]igration  6  PubMed(2); GeneRIFs(1)  
 
 
 hide/show details for SHOC2 
  [Mm]igration  PubMed  
 An MRAS, SHOC2, and SCRIB complex coordinates ERK pathway activation with polarity and tumorigenic growth. SHOC2 is mutated in Noonan syndrome and plays a key role in the activation of the ERK-MAPK pathway, which is upregulated in the majority of human cancers. SHOC2 functions as a PP1-regulatory protein and as an effector of MRAS. Here we show that SHOC2 and MRAS form a complex with SCRIB, a polarity protein with tumor suppressor properties. SCRIB functions as a PP1-regulatory protein and antagonizes SHOC2-mediated RAF dephosphorylation through a mechanism involving competition for PP1 molecules within the same macromolecular complex. SHOC2 function is selectively required for the malignant properties of tumor cells with mutant RAS, and both MRAS and SHOC2 play a key role in polarized   migration  . We propose that MRAS, through its ability to recruit a complex with paradoxical components, coordinates ERK pathway spatiotemporal dynamics with polarity and that this complex plays a key role during tumorigenic growth. 
 soc-2 encodes a leucine-rich repeat protein implicated in fibroblast growth factor receptor signaling. Activation of fibroblast growth factor (FGF) receptors elicits diverse cellular responses including growth, mitogenesis,   migration  , and differentiation. The intracellular signaling pathways that mediate these important processes are not well understood. In Caenorhabditis elegans, suppressors of clr-1 identify genes, termed soc genes, that potentially mediate or activate signaling through the EGL-15 FGF receptor. We demonstrate that three soc genes, soc-1, soc-2, and sem-5, suppress the activity of an activated form of the EGL-15 FGF receptor, consistent with the soc genes functioning downstream of EGL-15. We show that soc-2 encodes a protein composed almost entirely of leucine-rich repeats, a domain implicated in protein-protein interactions. We identified a putative human homolog, SHOC-2, which is 54% identical to SOC-2. We find that shoc-2 maps to 10q25, shoc-2 mRNA is expressed in all tissues assayed, and SHOC-2 protein is cytoplasmically localized. Within the leucine-rich repeats of both SOC-2 and SHOC-2 are two YXNX motifs that are potential tyrosine-phosphorylated docking sites for the SEM-5/GRB2 Src homology 2 domain. However, phosphorylation of these residues is not required for SOC-2 function in vivo, and SHOC-2 is not observed to be tyrosine phosphorylated in response to FGF stimulation. We conclude that this genetic system has allowed for the identification of a conserved gene implicated in mediating FGF receptor signaling in C. elegans. 
  GeneRIFs  
 Both MRAS and SHOC2 play a key role in polarized   migration  . 
   
  
 AKT2 
 
  NCBI Gene  
  GeneRIF  
  Pubmed  
 
 
  Search Term  Rank  Fields  
  [Mm]igration  6  Process(2); PubMed(28); GeneRIFs(9)  
  ECM  7  PubMed(1)  
  [Ee]xtracellular [Mm]atrix  8  PubMed(5)  
  [Ii]nvasive  10  PubMed(10); GeneRIFs(1)  
  [Mm]etastasize  11  PubMed(1)  
  [Mm]etastasis  12  PubMed(30); GeneRIFs(6)  
  [Cc]ell [Ii]nvasion  13  PubMed(4); GeneRIFs(1)  
 
 
 hide/show details for AKT2 
  [Mm]igration  Process  
 positive regulation of cell   migration   
 regulation of cell   migration   
  PubMed  
 Opposing roles for Akt1 and Akt2 in Rac/Pak signaling and cell   migration  . The Akt/PKB isoforms have different roles in animals, with Akt2 primarily regulating metabolic signaling and Akt1 regulating growth and survival. Here we show distinct roles for Akt1 and Akt2 in mouse embryo fibroblast cell   migration   and regulation of the cytoskeleton. Akt1-deficient cells responded poorly to platelet-derived growth factor while Akt2-deficient cells had a dramatically enhanced response, resulting in a substantial increase in dorsal ruffling. Swapping domains between Akt1 and Akt2 demonstrated that the N-terminal region containing the pleckstrin homology domain and a linker region distinguishes the two isoforms, while the catalytic domains are interchangeable. Akt2 knock-out cells also migrated faster than wild-type cells, especially through extracellular matrix (ECM), while Akt1 knock-out cells migrated more slowly than wild-type cells. Consistently, Akt2 knock-out cells had elevated Pak1 and Rac activities, suggesting that Akt2 inhibits Rac and Pak1. Both Akt2 and Akt1 associated in complexes with Pak1, but only Akt2 inhibited Pak1 in kinase assays, suggesting an underlying molecular basis for the different cellular phenotypes. Together these data provide evidence for an unexpected functional link between Akt2 and Pak1 that opposes the actions of Akt1 on cell   migration  . 
 Distinct roles of AKT isoforms in regulating β1-integrin activity,   migratio  n, and invasion in prostate cancer. AKT1 and AKT2 kinases have been shown to play opposite roles in breast cancer   migratio  n and invasion. In this study, an RNA interference screen for integrin activity inhibitors identified AKT1 as an inhibitor of β1-integrin activity in prostate cancer. Validation experiments investigating all three AKT isoforms demonstrated that, unlike in breast cancer, both AKT1 and AKT2 function as negative regulators of cel  l migrati  on and invasion in PC3 prostate cancer cells. Down-regulation of AKT1 and AKT2, but not AKT3, induced activation of cell surface β1-integrins and enhanced adhesio  n, migrat  ion, and invasion. Silencing of AKT1 and AKT2 also resulted in increased focal adhesion size. Importantly, the mechanisms involved in integrin activity regulation were distinct for the two AKT isoforms. Silencing of AKT1 relieved feedback suppression of the expression and activity of several receptor tyrosine kinases, including EGFR and MET, with established cross-talk with β1-integrins. Silencing of AKT2, on the other hand, induced up-regulation of the microRNA-200 (miR-200) family, and overexpression of miR-200 was sufficient to induce integrin activity and c  ell migra  tion in PC3 cells. Taken together, these data define an inhibitory role for both AKT1 and AKT2 in prostate can  cer migra  tion and invasion and highlight the cell type-specific actions of AKT kinases in the regulation of cell motility. 
 Distinct roles of Akt1 and Akt2 in regulating cell   migration   and epithelial-mesenchymal transition. The Akt family of kinases are activated by growth factors and regulate pleiotropic cellular activities. In this study, we provide evidence for isoform-specific positive and negative roles for Akt1 and -2 in regulating growth factor-stimulated phenotypes in breast epithelial cells. Insulin-like growth factor-I receptor (IGF-IR) hyperstimulation induced hyperproliferation and antiapoptotic activities that were reversed by Akt2 down-regulation. In contrast, Akt1 down-regulation in IGF-IR-stimulated cells promoted dramatic neomorphic effects characteristic of an epithelial-mesenchymal transition (EMT) and enhanced cell   migration   induced by IGF-I or EGF stimulation. The phenotypic effects of Akt1 down-regulation were accompanied by enhanced extracellular signal-related kinase (ERK) activation, which contributed to the induction of   migration   and EMT. Interestingly, down-regulation of Akt2 suppressed the EMT-like morphological conversion induced by Akt1 down-regulation in IGF-IR-overexpressing cells and inhibited   migration   in EGF-stimulated cells. These results highlight the distinct functions of Akt isoforms in regulating growth factor-stimulated EMT and cell   migration  , as well as the importance of Akt1 in cross-regulating the ERK signaling pathway. 
 Advanced glycation end products of bovine serum albumin-induced endothelial-to-mesenchymal transition in cultured human and monkey endothelial cells via protein kinase B signaling cascades. Advanced glycation end products of BSA (AGE-BSA) participate in the pathogenesis of diabetic vascular disease. However, the role of AGE-BSA in diabetic retinopathy, especially in retinal neovascularization, remains incomplete. This study aimed to determine the contributions of AGE-BSA in the endothelial-to-mesenchymal transition (EnMT) of cultured human and monkey endothelial cell lines and the mechanism that may be related with the transition.Monkey choroid-retinal endothelial cells (RF/6A) and human umbilical vein endothelial cells (HUVEC) were cultured in Dulbecco's modified Eagle's Medium (DMEM) and Ham's F12 medium containing 200 mg/l AGE-BSA. The expression of VE-cadherin, β-catenin, vimentin, N-cadherin, and protein kinase B (AKT2) was observed by immunocytochemistry and flow cytometry. Cell motility was determined by   migratio  n assays; the endothelial function of the formatting tube was measured by tube formation assays, while the change in the polarity was measured using resistance instruments.The characteristics of EnMT included loss of endothelial markers of VE-cadherin and β-catenin, which were replaced by mesenchymal markers of vimentin and N-cadherin, enhance  d migrati  on and tube formation, and diminished polarity. AGE-BSA contributed to upregulation of the protein expression of VE-cadherin and β-catenin and downregulation of protein expression of vimentin and N-cadherin, leading to enhanc  ed migrat  ion and tube formation and diminished polarity. During this process, expression of AKT2 was upregulated.AGE-BSA can induce EnMT of cultured human and monkey endothelial cells. The signal pathway involving AKT2 may play a role in this process. 
 Roles of AKT1 and AKT2 in non-small cell lung cancer cell survival, growth, and   migration  . Although AKT ⁄ protein kinase B is constitutively active in nonsmall cell lung cancer (NSCLC) cells and is an attractive target for enhancing the cytotoxicity of therapeutic agents, the distinct roles of the AKT isoforms in NSCLC are largely unknown. In the present study, we investigated the roles of AKT1 and AKT2 in NSCLC cells using RNAi. The siRNA targeting of AKT1 or AKT2 effectively decreased protein levels of AKT1 and AKT2, respectively, in A549 and H460 cells. Cisplatin treatment of these cells increased apoptotic cell death compared with control. The siRNA-induced knockdown of AKT1 in H460 cells significantly decreased basal MEK⁄ ERK1 ⁄ 2 activity, resulting in nuclear factor-κB activation, whereas knockdown of AKT2 resulted in anti-apoptotic Bcl-2 family protein MCL-1 (MCL-1) cleavage, the collapse of mitochondrial membrane potential, cytochrome c release, and activation of the caspase cascade. Consequently, both siRNA treatments enhanced the chemosensitivity of H460 cells to cisplatin. However, neither AKT1 nor AKT2 siRNA treatment had any effect of p27 expression, and although both treatments tended to induced G₂ ⁄M phase arrest, the effect was not statistically significant. Treatment with AKT1 siRNA markedly decreased colony formation   growth an  d migration, but AKT2 siRNA had no significant effects on these parameters. These data suggest that AKT1 and AKT2 both contribute to cell survival, albeit via different mechanisms, and that the effects on cell   growth an  d migration are predominantly regulated by AKT1. These findings may aid in refining targeted strategies for the inhibition of AKT isoforms towards the sensitization of NSCLC cells to therapeutic agents. 
 Regulation of cancer cell survival,   migration  , and invasion by Twist: AKT2 comes to interplay. Metastasis, the foremost cause of mortality in cancer patients, is increasingly recognized as a coordinated biological process. The multistep process of metastasis posts difficulty in studying its mechanism and molecular basis. Recent works have shown that the basic helix-loop-helix transcriptional factor Twist and the serine/threonine kinase AKT play pivotal roles in tumor development and progression. Our recent study has shown that AKT2 is a transcriptional regulatory target of Twist and acts downstream of Twist to promote cancer cell survival,   migration  , and invasion. Functional convergence of Twist and AKT2 underscores the importance of this signaling pathway in tumor development and progression and as a potential therapeutic target. 
 miR-612 negatively regulates colorectal cancer growth and metastasis by targeting AKT2. Colorectal cancer (CRC) is one of the most common cancers worldwide, with a particularly high incidence in developed countries. Distant metastasis and recurrence are the main causes of CRC-related deaths. MicroRNAs (miRNAs) in the serum make them potential biomarkers for cancers, as reported in serum or tumor tissues from CRC patients. In this study, we found that miR-612 expression was significantly lower in CRC tissues or cells compared with peritumor tissues or normal cells, and lower in metastatic CRC specimens compared with non-metastatic specimens, whereas AKT2 exhibited opposite trend. Gain-of-function and loss-of-function assays showed that miR-612 inhibited CRC cell proliferation and   migration   in vitro by Cell Counting Kit-8 and transwell assays. Further analysis revealed that miR-612 directly suppressed AKT2, which in turn inhibited the downstream epithelial-mesenchymal transition-related signaling pathway. These results were additionally validated in vivo by tumorigenesis and liver metastasis experiments. The results of this study suggested a critical role of miR-612 in the development of CRC. 
 Function of Akt/PKB signaling to cell motility, invasion and the tumor stroma in cancer. The serine/threonine protein kinase Akt is a major signal transducer of the phosphoinositide 3-kinase (PI 3-K) pathway in all cells and tissues and plays a pivotal role in the maintenance of cellular processes including cell growth, proliferation, survival and metabolism. The frequent aberrant activation of the PI 3-K/Akt pathway in human cancer has made it an attractive therapeutic target. Numerous studies have provided a comprehensive understanding of the specific functions of Akt signaling in cancer cells as well as the surrounding tumor microenvironment and this has informed and enabled the development of therapeutic drugs to target both PI 3-K and Akt. However, recent studies have provided evidence for distinct functions of the three mammalian Akt isoforms, particularly with respect to the regulation of cell motility and metastasis of breast cancer. Here we discuss the mechanisms by which Akt signaling contributes to invasive   migration   and tumor metastasis, and highlight recent advances in our understanding of the contribution of the Akt pathway in the tumor-associated stroma. 
 MicroRNA-137 Contributes to Dampened Tumorigenesis in Human Gastric Cancer by Targeting AKT2. MiRNAs play important roles in tumorigenesis. This study focused on exploring the effects and regulation mechanism of miRNA-137 on the biological behaviors of gastric cancer. Total RNA was extracted from tissues of 100 patients with gastric cancer and from four gastric cancer cell lines. Expression of miR-137 was detected by real-time PCR from 100 patients. The effects of miR-137 overexpression on gastric cancer cells' proliferation, apoptosis,   migration   and invasion ability were investigated in vitro and in vivo. The target gene of miR-137 was predicted by Targetscan on line software, screened by dual luciferase reporter gene assay and demonstrated by western blot. As a result, the expression of miR-137 was significant reduced in gastric cancer cell line HGC-27, HGC-803, SGC-7901 and MKN-45 as well as in gastric cancer tissues compared with GES-1 cell or matched adjacent non-neoplastic tissues (p<0.001). The re-introduction of miR-137 into gastric cancer cells was able to inhibit cell proliferation,   migration   and invasion. The in vivo experiments demonstrated that the miR-137 overexpression can reduce the gastric cancer cell proliferation and metastasis. Bioinformatic and western blot analysis indicated that the miR-137 acted as tumor suppressor roles on gastric cancer cells through targeting AKT2 and further affecting the Bad and GSK-3β. In conclusion, the miR-137 which is frequently down-regulated in gastric cancer is potentially involved in gastric cancer tumorigenesis and metastasis by regulating AKT2 related signal pathways. 
 Distinct and specific roles of AKT1 and AKT2 in androgen-sensitive and androgen-independent prostate cancer cells. AKT isoforms are expressed in prostate cancer and their expression and localization have different associations with clinical characteristics. However, the distinct roles of the AKT isoforms in prostate cancer cells are largely unknown. In the present study, we demonstrate distinct roles for AKT1 and AKT2 in cell growth and   migration  . Ablation of AKT1 and AKT2 decreased the proliferation of the androgen-independent cell line PC-3, although by different mechanisms. AKT1 ablation induced loss of cell adhesion and subsequent apoptosis. AKT2 (but not AKT1) ablation promoted cell cycle arrest at G0/G1, associated with downregulation of cyclin D, CDK6 and CDK2, and upregulation and cytoplasmic-to-nuclear redistribution of p27. The increase of p27 protein levels was due to more gene transcription and an increase in protein stability. The increased stability of p27 was induced by delocalisation of Skp2 and a lower level of p27 phosphorylation at Thr187. AKT1 and AKT2 ablation inhibited and stimulated PC-3 cell   migration  , respectively. An AKT isoform-specific function could be associated with its subcellular localization. We found that AKT1 and AKT2 were mainly localised in the cytoplasm and nucleus, respectively. In androgen-sensitive cell line LNCaP, the ablation of AKT1 or AKT2 caused apoptosis but in androgen-independent LNCaP sublines, the effect of AKT1 ablation was lower; whereas no changes were observed after AKT2 ablation. Taken together, our data show that AKT1 and AKT2 have non-redundant roles in the regulation of PC-3 cell proliferation and   migration  . These could be explained by their subcellular localization and/or the specific regulation of downstream effectors. Furthermore, contribution of AKT isoforms to the progression of prostate cancer may change from an androgen-sensitive to a hormone-refractory stage. These findings may help design new targeted strategies for inhibiting AKT isoforms in prostate cancer. 
 Akt2 regulates expression of the actin-bundling protein palladin. The phosphatidylinositol 3-kinase/Akt pathway is responsible for key aspects of tumor progression, and is frequently hyperactivated in cancer. We have recently identified palladin, an actin-bundling protein that functions to control the actin cytoskeleton, as an Akt1-specific substrate that inhibits breast cancer cell   migration  . Here we have identified a role for Akt isoforms in the regulation of palladin expression. Akt2, but not Akt1, enhances palladin expression by maintaining protein stability and upregulating transcription. These data reveal that Akt signaling regulates the stability of palladin, and further supports the notion that Akt isoforms have distinct and specific roles in tumorigenesis. 
 miR-148a suppresses human renal cell carcinoma malignancy by targeting AKT2. MicroRNA-148a (miR-148a) has been reported to be deregulated in different tumor types, whereas the biological function of miR-148a in renal cell carcinoma (RCC) largely remains unexplored. In the present study we investigated the clinical significance, biological effects, and the underlying molecular mechanisms of miR-148 in RCC. Here, we showed that miR-148a was significantly downregulated in RCC tissues and cell lines. Low expression of miR-148a in RCC tissues was associated with large tumor size, advanced TNM stage, and lymph node metastasis. Functional assays revealed that overexpression of miR-148a significantly inhibited RCC cell proliferation, colony formation,   migration   and invasion in vitro and suppressed RCC xenograft tumor growth in vivo. In addition, using quantitative RT-PCR (qRT-PCR), western blot analysis and luciferase reporter assays, AKT2 was confirmed to be a direct target of miR-148a. AKT2 expression was upregulated, and was negatively correlated with miR-148a expression in RCC tissues (r=-0.641, P<0.001). Silencing of AKT2 phenotypically copied miR-148a-induced phenotypes, whereas re-expression of AKT2 reversed the suppressive effects of miR-148a in RCC cells. Further mechanistic investigations showed that miR-148a exerted its antitumor activity via inhibition of the AKT pathway in vitro and in vivo. Taken together, these findings suggest that miR-148a functions as tumor suppressor in RCC by targeting AKT2. 
 FoxD3-regulated microRNA-137 suppresses tumour growth and metastasis in human hepatocellular carcinoma by targeting AKT2. microRNAs, frequently deregulated in human cancer, have been implicated in the progression of hepatocarcinogenesis. Here, we show that microRNA (miR)-137 is significantly down-regulated in hepatocellular carcinoma (HCC). Its decreased expression is associated with vein invasion, incomplete Involucrum, and distant metastasis. Multivariate analysis suggests that miR-137 is an independent indicator for poor survival. We next show that over-expression of miR-137 suppresses cell proliferation,   migration   and invasion in vitro. Conversely, miR-137 inhibition promotes HCC cell growth. We also identify AKT2 as a key target of miR-137 in this context. Statistical data reveal a reverse correlation of AKT2 and miR-137 expression in HCC patients. Silencing of AKT2 phenotypically copied miR-137-induced phenotypes, whereas re-expression of AKT2 reversed the suppressive effects of miR-137. Further investigations showed that miR-137 exerted its anti-tumour activity via inhibiting the AKT2/mTOR pathway. Moreover, we demonstrate that FoxD3 directly binds to the promoter of miR-137 and activates its transcription. In vivo studies confirm that FoxD3-regulated miR-137 inhibited HCC growth and metastasis via targeting AKT2. Together, our findings indicate that miR-137 is a valuable biomarker for HCC prognosis and the FoxD3/miR-137/AKT2 regulatory network plays an important role in HCC progression. 
 RLN2 Is a Positive Regulator of AKT-2-Induced Gene Expression Required for Osteosarcoma Cells Invasion and Chemoresistance. The aim of the study was to determine the effect of H2 relaxin (RLN2) on invasion,   migration  , and chemosensitivity to cisplatin in human osteosarcoma U2-OS and MG-63 cells and then to investigate the effect of RLN2 on the AKT/NF-κB signaling pathway. The expression of RLN2, p-AKT (Ser473), and p-ERK1/2 (Phospho-Thr202/Tyr204) proteins was detected by western blot in OS tissues from 21 patients with pulmonary metastatic disease, and the correlation between RLN2 and p-AKT or RLN2 and p-ERK1/2 expression was investigated. RLN2 expression was inhibited by RLN2 siRNA transfection in the MG-63 cells. RLN2 was overexpressed in the U2-OS cells by treatment with recombinant relaxin. The results showed that positive relation was found between RLN2 and p-AKT expression in tissues of OS. Silencing RLN2 inhibited cell migratory and invasive ability and angiogenesis formation and increased the chemosensitivity to cisplatin in MG-63 cells. RLN2 overexpression promoted migratory and invasive ability and angiogenesis and increased the chemoresistance to cisplatin in U2-OS cells. Silencing RLN2 inhibited the activity of AKT/NF-κB signaling pathway in MG-63 cells, and vice versa. Blockage of both pathways by specific inhibitors abrogated RLN2-induced survival and invasion of OS cells, and vice versa. Our results indicated RLN2 confers to migratory and invasive ability, angiogenesis, and chemoresistance to cisplatin via modulating the AKT/NF-κB signaling pathway in vitro. 
 Arachidonic acid promotes   migration   and invasion through a PI3K/Akt-dependent pathway in MDA-MB-231 breast cancer cells. Arachidonic acid (AA) is a common dietary n-6 cis polyunsaturated fatty acid that under physiological conditions is present in an esterified form in cell membrane phospholipids, however it might be present in the extracellular microenvironment. AA and its metabolites mediate FAK activation, adhesion and   migration   in MDA-MB-231 breast cancer cells. However, it remains to be investigated whether AA promotes invasion and the signal transduction pathways involved in   migration   and invasion. Here, we demonstrate that AA induces Akt2 activation and invasion in MDA-MB-231 cells. Akt2 activation requires the activity of Src, EGFR, and PIK3, whereas   migration   and invasion require Akt, PI3K, EGFR and metalloproteinases activity. Moreover, AA also induces NFκB-DNA binding activity through a PI3K and Akt-dependent pathway. Our findings demonstrate, for the first time, that Akt/PI3K and EGFR pathways mediate   migratio  n and invasion induced by AA in MDA-MB-231 breast cancer cells. 
 Antisense and dominant-negative AKT2 cDNA inhibits glioma cell invasion. The aim of this study was to explore the potential role of AKT2 in glioma cell invasion. Therefore, dominant-negative (DN-AKT2) and antisense AKT2 constructs (AS-AKT2) were transfected into rat C6 glioma cells with elevated endogenous AKT2 expression. In situ hybridization and Western blot analysis were used to identify AKT2 expression. Spheroid culturing was used to assess cell   migration   and invasion in Matrigel from spheroids. Cell motility and invasion were also evaluated by scratch and Transwell invasion assays, respectively. The secretion of matrix metalloproteinases (MMPs), MMP2 and MMP9, was determined by gelatin zymography. AKT2 expression was inhibited in C6 cells transfected with AS-AKT2 but did not significantly change in cells transfected with DN-AKT2. The cell   migration   distance from spheroids or the number of cells migrating into the acellular space created by scratching was reduced in cells transfected with DN-AKT2 or AS-AKT2 compared to the control cells. The invasive distance of cells from the spheroids in Matrigel sandwich and the number of invading cells through the Matrigel were also decreased in the DN-AKT2- and AS-AKT2-transfected cells. Gelatin zymography showed that the production of MMP2 and MMP9 was inhibited in transfected cells. In conclusion, AKT2 plays an important role in glioma cell motility and invasion. Therapy based on AKT inhibition may complement currently available treatment to control glioma cell invasion. 
 Akt2 is required for macrophage chemotaxis. Tumor-associated macrophages play an important role in tumorigenesis and metastasis. Trafficking of macrophages to the proximity of tumors is mediated by CSF-1, a growth factor. In this study, we investigated the role of PKB/Akt in CSF-1-induced macrophage   migration  . Disruption of Akt2 expression by small interference RNA impaired chemotaxis of both THP-1 cells and mouse peritoneal macrophages. Phosphorylation of PKCzeta, an essential component in chemotaxis signaling pathway, was reduced. LIMK/Cofilin, downstream of PKCzeta, regulated cytoskeleton rearrangement during cell   migration  . Disruption of Akt2 expression inhibited CSF-1-induced LIMK/Cofilin phosphorylation, which contributed to defects in actin polymerization and chemotaxis. Furthermore, MCP-1, a chemokine, -induced macrophage chemotaxis was also impaired. Taken together, our results demonstrated that Akt2 plays an essential role in both CSF-1- and chemokine-induced chemotaxis of macrophages. 
 Small ribosomal protein subunit S7 suppresses ovarian tumorigenesis through regulation of the PI3K/AKT and MAPK pathways. Small ribosomal protein subunit S7 (RPS7) has been reported to be associated with various malignancies, but the role of RPS7 in ovarian cancer remains unclear. In this study, we found that silencing of RPS7 by a specific shRNA promoted ovarian cancer cell proliferation, accelerated cell cycle progression, and slightly reduced cell apoptosis and response to cisplatin treatment. Knockdown of RPS7 resulted in increased expression of P85α, P110α, and AKT2. Although the basal levels of ERK1/2, MEK1/2, and P38 were inconsistently altered in ovarian cancer cells, the phosphorylated forms of MEK1/2 (Ser217/221), ERK1/2 (Thr202/Tyr204), JNK1/2 (Thr183/Tyr185), and P38 (Thr180/Tyr182) were consistently reduced after RPS7 was silenced. Both the in vitro anchorage-independent colony formation and in vivo animal tumor formation capability of cells were enhanced after RPS7 was depleted. We also showed that silencing of RPS7 enhanced ovarian cancer cel  l migrati  on and invasion. In sum, our results suggest that RPS7 suppresses ovarian tumorigenesis and metastasis through PI3K/AKT and MAPK signal pathways. Thus, RPS7 may be used as a potential marker for diagnosis and treatment of ovarian cancer. 
 MiRNA-615-5p functions as a tumor suppressor in pancreatic ductal adenocarcinoma by targeting AKT2. Aberrant microRNA (miRNA) expression is associated with tumor development. This study aimed to elucidate the role of miR-615-5p in the development of pancreatic ductal adenocarcinoma (PDAC).Locked nucleic acid in situ hybridization (LNA-ISH) was performed to compare miR-615-5p expression in patients between PDAC and matched adjacent normal tissues. Effects of miR-615-5p overexpression on cell proliferation, apoptosis, colony formation,   migration  , and invasion were determined in the pancreatic cancer cell lines PANC-1 and MIA PaCa-2. Effects of miR-615-5p on AKT2 were examined by dual-luciferase reporter assay. Lentivirus expressing miR-615 was used to create stable overexpression cell lines, which were subsequently used in mouse xenograft and metastasis models to assess tumor growth, apoptosis and metastasis.miR-615-5p expression was significantly lower in PDAC than in adjacent normal tissues. Low levels of miR-615-5p were independently associated with poor prognosis (HR: 2.243, 95% CI: 1.190-4.227, P=0.013). AKT2 protein expression was inversely correlated with miR-615-5p expression (r=-0.3, P=0.003). miR-615-5p directly targeted the 3'-untranslated region of AKT2 mRNA and repressed its expression. miR-615-5p overexpression inhibited pancreatic cancer cell proliferation,   migration  , and invasion in vitro, and tumor growth and metastasis in vivo. Furthermore, miR-615-5p overexpression also induced pancreatic cancer cell apoptosis both in vitro and in vivo.These results show that miR-615-5p inhibits pancreatic cancer cell proliferation,   migration  , and invasion by targeting AKT2. The data implicate miR-615-5p in the prognosis and treatment of PDAC. 
 Different functions of AKT1 and AKT2 in molecular pathways, cell   migration   and metabolism in colon cancer cells. AKT is a central protein in many cellular pathways such as cell survival, proliferation, glucose uptake, metabolism, angiogenesis, as well as radiation and drug response. The three isoforms of AKT (AKT1, AKT2 and AKT3) are proposed to have different physiological functions, properties and expression patterns in a cell type-dependent manner. As of yet, not much is known about the influence of the different AKT isoforms in the genome and their effects in the metabolism of colorectal cancer cells. In the present study, DLD-1 isogenic AKT1, AKT2 and AKT1/2 knockout colon cancer cell lines were used as a model system in conjunction with the parental cell line in order to further elucidate the differences between the AKT isoforms and how they are involved in various cellular pathways. This was done using genome wide expression analyses, metabolic profiling and cell   migration   assays. In conclusion, downregulation of genes in the cell adhesion, extracellular matrix and Notch-pathways and upregulation of apoptosis and metastasis inhibitory genes in the p53-pathway, confirm that the knockout of both AKT1 and AKT2 will attenuate metastasis and tumor cell growth. This was verified with a reduction in   migration   rate in the AKT1 KO and AKT2 KO and most explicitly in the AKT1/2 KO. Furthermore, the knockout of AKT1, AKT2 or both, resulted in a reduction in lactate and alanine, suggesting that the metabolism of carbohydrates and glutathione was impaired. This was further verified in gene expression analyses, showing downregulation of genes involved in glucose metabolism. Additionally, both AKT1 KO and AKT2 KO demonstrated an impaired fatty acid metabolism. However, genes were upregulated in the Wnt and cell proliferation pathways, which could oppose this effect. AKT inhibition should therefore be combined with other effectors to attain the best effect. 
 Akt2 regulates metastatic potential in neuroblastoma. Activation of PI3K/AKT pathway correlates with poor prognosis in patients with neuroblastoma. Our previous studies have demonstrated that PI3K/AKT signaling is critical for the oncogenic transformations induced by gastrin-releasing peptide (GRP) and its receptor, GRP-R, in neuroblastoma. Moreover, PI3K/AKT-dependent oncogenic transformations require N-myc, an extensively studied oncogene in neuroblastoma. Whether AKT directly regulates the expression of N-myc oncogene is yet to be determined. Here, we report a novel finding that of the three AKT isoforms, AKT2 specifically regulated N-myc expression in neuroblastoma cells. We also confirmed that GRP-R is upstream of AKT2 and in turn, regulated N-myc expression via AKT2 in neuroblastoma cells. Functional assays demonstrated that attenuation of AKT2 impaired cell proliferation and anchorage-independent cell growth, and decreased the secretion of angiogenic factor VEGF in vitro. Furthermore, silencing AKT2 inhibited   migration   and invasion of neuroblastoma cells in vitro. Xenografts established by injecting AKT2 silenced human neuroblastoma cells into murine spleen expressed decreased levels of AKT2 and resulted in fewer liver metastases compared to controls in vivo. Hence, our study highlights the potential molecular mechanism(s) mediating the oncogenic role of GRP/GRP-R and demonstrates a novel role for AKT2 in neuroblastoma tumorigenesis, indicating that targeting the GRP/GRP-R/AKT2 axis may be important for developing novel therapeutics in the treatment of clinically aggressive neuroblastoma. 
 MiR-650 inhibits proliferation,   migration   and invasion of rheumatoid arthritis synovial fibroblasts by targeting AKT2. This study was aimed to investigate the effects of miR-650 on the proliferation,   migration  , invasion and apoptosis of rheumatoid arthritis synovial fibroblasts (RASFs).Synovial tissue samples were collected from 16 rheumatoid arthritis (RA) patients and 13 patients with joint trauma undergoing joint replacement surgery. The RASFs were isolated and cultured. MiR-650 and AKT2 expressions in both synovial tissues and cells were detected using the quantitative real-time PCR (qRT-PCR), western blot and immunohistochemistry (IHC). Dual luciferase reporter gene assay was employed to evaluate the effect of miR-650 on the luciferase activity of AKT2. Then RASFs were transfected with miR-650 mimics, miR-650 inhibitors and pLenti6/V5-AKT2 respectively. And changes in cellular proliferation,   migration  , invasion and apoptosis were detected through MTT assay, wound healing assay, Transwell invasion assay and flow cytometry analysis, respectively.MiR-650 was significantly down-regulated in RASFs than normal cells, whereas AKT2 was up-regulated in RASFs. Dual luciferase reporter gene assay showed that miR-650 could specifically bind to the 3'UTR of AKT2 and significantly repress the luciferase activity. MiR-650 significantly decreased the expression of AKT2. Down-regulation of miR-650 or up-regulation of AKT2 could increase proliferation,   migration  , invasion of RASFs, and decrease RASFs apoptosis. The conversed results were observed, when miR-650 was up-regulated in RASFs.MiR-650 could inhibit the proliferation,   migration   and invasion of RASFs through targeted regulation of AKT2 expression. 
 Twist transcriptionally up-regulates AKT2 in breast cancer cells leading to increased   migration  , invasion, and resistance to paclitaxel. Metastasis, the cardinal feature of malignant tumors, is an important clinical variable in patient prognosis. To understand the basis for metastasis, we systematically selected for highly invasive cells from breast cancer cell lines, MCF7 and MDA-MB-453, with moderate to low invasive ability using Boyden chamber invasion assay. The four-cycle selected invasive lines, named MCF7-I4 and MDA-MB-453-I4, respectively, displayed epithelial-mesenchymal transition (EMT) and dramatically enhanced invasive ability. EMT changes were corroborated with decreased level of E-cadherin and increased vimentin, fibronectin, and beta(1) integrin. Twist, a basic helix-loop-helix transcription factor, and AKT2, a known proto-oncogene, were found to be elevated in the invasive cells compared with the parental. Ectopic expression and knockdown of Twist by short interference RNA resulted in significant increase and reduction, respectively, of AKT2 protein and mRNA expression. Twist bound to E-box elements on AKT2 promoter and enhanced its transcriptional activity. Moreover, silencing AKT2 decreased Twist-promoted   migration  , invasion, and paclitaxel resistance. Reintroducing AKT2 largely rescued the phenotype resulted from knockdown of Twist in I4 cells, suggesting that AKT2 is a downstream target and functional mediator of Twist. Finally, we observed a 68.8% correlation of elevated Twist and AKT2 expression in late-stage breast cancers as oppose to 13% in early-stage breast cancers. Our study identifies Twist as a positive transcriptional regulator of AKT2 expression, and Twist-AKT2 signaling is involved in promoting invasive ability and survival of breast cancer cells. 
 Plasmin triggers chemotaxis of monocyte-derived dendritic cells through an Akt2-dependent pathway and promotes a T-helper type-1 response. Dendritic cells (DC) accumulate in atherosclerotic arteries where they can modulate atherogenesis. We investigated whether plasmin might alter the function of human DC.Stimulation of monocyte-derived DC with plasmin elicited a time-dependent actin polymerization and chemotaxis comparable to that triggered by the standard chemoattractant formyl-methionyl-leucyl-phenylalanine. Plasmin triggered rapid activation of Akt and mitogen-activated protein kinases, followed by phosphorylation of the regulatory myosin light chain and chemotaxis. For the chemotactic DC   migration  , the activation of Akt and p38 and extracellular signal-regulated kinase 1/2 mitogen-activated protein kinases were indispensable, as shown by pharmacological inhibitors. DC express Akt1 and Akt2, but not Akt3. However, in DC, plasmin activates exclusively Akt2 via a p38 mitogen-activated protein kinase-dependent pathway. Accordingly, knockdown of Akt2 with short-hairpin RNA, but not of Akt1, blocked the plasmin-induced extracellular signal-regulated kinase 1/2 activation and the chemotactic response. Moreover, plasmin-stimulated DC induced polarization of CD4(+) T cells toward the interferon-gamma-producing, proinflammatory Th1 phenotype. Consistent with a role for DC and adaptive immune response in atherogenesis, we demonstrate DC in human atherosclerotic vessels and show that plasmin is abundant in human atherosclerotic lesions, where it colocalizes with DC.Plasmin generation in the atherosclerotic vessel wall might contribute to accumulation of DC, activation of the adaptive immune response, and aggravation of atherosclerosis. 
 Angiopoietin-2, an angiogenic regulator, promotes initial growth and survival of breast cancer metastases to the lung through the integrin-linked kinase (ILK)-AKT-B cell lymphoma 2 (Bcl-2) pathway. The early onsets of breast cancer metastasis involve cell retention, survival, and resistant to apoptosis and subsequent growth at target vascular beds and tissues in distant organs. We previously reported that angiopoietin-2 (Ang2), an angiogenic regulator stimulates MCF-7 breast tumor metastasis from their orthotopic sites to distant organs through the α(5)β(1) integrin/integrin-linked kinase (ILK)/Akt pathway. Here, by using an experimental tumor metastasis model and in vitro studies, we further dissect the underlying mechanism by which Ang2 promotes the initial growth and survival of MCF-7 breast cancer metastasis in the lung of animals. We show that Ang2 increases cell survival and suppresses cell apoptosis through ILK-induced phosphorylation of Akt1, Akt2, and up-regulation of Bcl-2 in breast cancer cells. Inhibition of ILK, Akt1, and Akt2, and their effector Bcl-2 diminishes Ang2-stimulated breast cancer cell survival and Ang2-attenuated apoptosis in vitro, and initial survival and growth of breast cancer metastasis in the lung of animals. Additionally, siRNA knockdown of endogenous Ang2 in three human metastatic breast cancer cell lines also inhibits phosphorylation of Akt, expression of Bcl-2, and tumor cell survival  , migrati  on, and increases cell apoptosis. Since increased expression of Ang2 correlates with elevated potential of human breast cancer metastasis in clinic, our data underscore the importance that up-regulated Ang2 not only stimulates breast cancer growth and metastasis at late stages of the process, but is also critical at the initiating stages of metastases onset, thereby suggesting Ang2 as a promising therapeutic target for treating patients with metastatic breast cancer. 
 Mitochondrial DNA depletion in prostate epithelial cells promotes anoikis resistance and invasion through activation of PI3K/Akt2. Neoplastic transformation of prostate epithelium involves aberrant activation of anti-apoptotic and pro-invasive pathways triggered by multiple poorly understood genetic events. We demonstrated earlier that depletion of mitochondrial DNA (mtDNA) induces prostate cancer progression. Here, using normal prostate epithelial PNT1A cells we demonstrate that mtDNA depletion prevents detachment-induced apoptosis (anoikis) and promotes migratory capabilities onto basement membrane proteins through upregulation of p85 and p110 phosphatidylinositol 3-kinase (PI3K) subunits, which results in Akt2 activation and phosphorylation of downstream substrates GSK3beta, c-Myc, MMP-9, Mdm2, and p53. Pharmacological or genetic PI3K inhibition, siRNA-mediated Akt2 depletion, as well as mtDNA reconstitution were sufficient to restore sensitivity to anoikis and curtail cell   migration  . Moreover, Akt2 activation induced glucose transporter 1 (GLUT1) expression, glucose uptake, and lactate production, common phenotypic changes seen in neoplastic cells. In keeping with these findings, several prostate carcinoma cell lines displayed reduced mtDNA content and increased PI3K/Akt2 levels when compared to normal PNT1A cells, and Akt2 downregulation prevented their survival,   migration   and glycolytic metabolism. On a tissue microarray, we also found a statistically significant decrease in mtDNA-encoded cytochrome oxidase I in prostate carcinomas. Taken together, these results provide novel mechanistic evidence supporting the notion that mtDNA mutations may confer survival and migratory advantage to prostate cancer cells through Akt2 signaling. 
 Downregulation of uPA inhibits   migration   and PI3k/Akt signaling in glioblastoma cells. The ability of glioma cells to migrate great distances from a primary tumor mass is the primary cause of tumor recurrence. The urokinase-type plasminogen activator (uPA) is a serine protease that can initiate proteolytic cascades, which result in remodeling of extracellular matrix and basement membrane, allowing cells to move across and through these barriers. The binding between uPA and its receptor uPAR also mediates several signaling events that seem to contribute to the evolution of a migratory phenotype. In this study, we determined how the downregulation of uPA affects the signaling pathways leading to cell   migration  . Stably transfecting human glioblastoma cells with antisense uPA decreased the amount of cell-bound uPA and disrupted actin cytoskeleton formation and cell   migration  . The phosphatidylinositol 3-kinase (PI3k) and Akt signaling pathway has been suggested to mediate   migration   in various cancer cells. The antisense-uPA clones also had less phosphorylated PI3k and Akt than control cells, a finding associated with decreased cell   migration  , G2/M-phase arrest, and decreased clonogenic survival. Decreased activation of PI3k and the antiapoptotic factor Akt was not sufficient to induce apoptosis in the antisense-uPA clones, but staurosporine sensitized them to apoptosis to a greater extent than control cells. These results indicate that PI3k/Akt pathway is involved in the signaling cascade required to induce cell   migration   and that uPA has a direct role in regulating   migration  . 
 Signalling specificity in the Akt pathway in breast cancer. Aberrant activation of fundamental cellular processes, such as proliferation,   migration   and survival, underlies the development of numerous human pathophysiologies, including cancer. One of the most frequently hyperactivated pathways in cancer is the phosphoinositide 3-kinase (PI3K)/Akt signalling cascade. Three isoforms of the serine/threonine protein kinase Akt (Akt1, Akt2 and Akt3) function to regulate cell survival, growth, proliferation and metabolism. Strikingly, non-redundant and even opposing functions of Akt isoforms in the regulation of phenotypes associated with malignancy in humans have been described. However, the mechanisms by which Akt isoform-specificity is conferred are largely unknown. In the present review, we highlight recent findings that have contributed to our understanding of the complexity of Akt isoform-specific signalling and discussed potential mechanisms by which this isoform-specificity is conferred. An understanding of the mechanisms of Akt isoform-specificity has important implications for the development of isoform-specific Akt inhibitors and will be critical to finding novel targets to treat disease. 
  GeneRIFs  
 MiR-650 could inhibit the proliferation,   migration   and invasion of rheumatoid arthritis synovial fibroblasts through targeted regulation of AKT2 expression. 
 miR-615-5p inhibits pancreatic cancer cell proliferation,   migration  , and invasion by targeting AKT2 
 AA induces Akt2 activation and invasion in MDA-MB-231 cells. Akt2 activation requires the activity of Src, EGFR, and PIK3, whereas   migration   and invasion require Akt, PI3K, EGFR and metalloproteinases activity. 
 AKT1 and AKT2 have non-redundant roles in the regulation of prostate cancer cell proliferation and   migration  . 
 these data define an inhibitory role for both AKT1 and AKT2 in prostate cancer   migration   and invasion and highlight the cell type-specific actions of AKT kinases in the regulation of cell motility. 
 AKT1 and AKT2 both contribute to cell survival, albeit via different mechanisms; the effects on cell growth and   migration   are predominantly regulated by AKT1 
 The expression levels and activities of exogenous Akt1 and Akt2 are almost identical, Akt2 exerted a greater inhibitory effect on both proliferation and cell   migration  . 
 Akt1 and Akt2 have opposing roles in Rac/Pak signaling and cell   migration   
 These results indicate that PI3k/Akt pathway is involved in the signaling cascade of glioma cells required to induce cell   migration  . 
  ECM  PubMed  
 Opposing roles for Akt1 and Akt2 in Rac/Pak signaling and cell migration. The Akt/PKB isoforms have different roles in animals, with Akt2 primarily regulating metabolic signaling and Akt1 regulating growth and survival. Here we show distinct roles for Akt1 and Akt2 in mouse embryo fibroblast cell migration and regulation of the cytoskeleton. Akt1-deficient cells responded poorly to platelet-derived growth factor while Akt2-deficient cells had a dramatically enhanced response, resulting in a substantial increase in dorsal ruffling. Swapping domains between Akt1 and Akt2 demonstrated that the N-terminal region containing the pleckstrin homology domain and a linker region distinguishes the two isoforms, while the catalytic domains are interchangeable. Akt2 knock-out cells also migrated faster than wild-type cells, especially through extracellular matrix (  ECM  ), while Akt1 knock-out cells migrated more slowly than wild-type cells. Consistently, Akt2 knock-out cells had elevated Pak1 and Rac activities, suggesting that Akt2 inhibits Rac and Pak1. Both Akt2 and Akt1 associated in complexes with Pak1, but only Akt2 inhibited Pak1 in kinase assays, suggesting an underlying molecular basis for the different cellular phenotypes. Together these data provide evidence for an unexpected functional link between Akt2 and Pak1 that opposes the actions of Akt1 on cell migration. 
  [Ee]xtracellular [Mm]atrix  PubMed  
 Phosphoinositide-3-OH kinase-dependent regulation of glycogen synthase kinase 3 and protein kinase B/AKT by the integrin-linked kinase. Integrin-linked kinase (ILK) is an ankyrin-repeat containing serine-threonine protein kinase capable of interacting with the cytoplasmic domains of integrin beta1, beta2, and beta3 subunits. Overexpression of ILK in epithelial cells disrupts cell-  extracellular matrix   as well as cell-cell interactions, suppresses suspension-induced apoptosis (also called Anoikis), and stimulates anchorage-independent cell cycle progression. In addition, ILK induces nuclear translocation of beta-catenin, where the latter associates with a T cell factor/lymphocyte enhancer-binding factor 1 (TCF/LEF-1) to form an activated transcription factor. We now demonstrate that ILK activity is rapidly, but transiently, stimulated upon attachment of cells to fibronectin, as well as by insulin, in a phosphoinositide-3-OH kinase [Pi(3)K]-dependent manner. Furthermore, phosphatidylinositol(3,4,5)trisphosphate specifically stimulates the activity of ILK in vitro, and in addition, membrane targetted constitutively active Pi(3)K activates ILK in vivo. We also demonstrate here that ILK is an upstream effector of the Pi(3)K-dependent regulation of both protein kinase B (PKB/AKT) and glycogen synthase kinase 3 (GSK-3). Specifically, ILK can directly phosphorylate GSK-3 in vitro and when stably, or transiently, overexpressed in cells can inhibit GSK-3 activity, whereas the overexpression of kinase-deficient ILK enhances GSK-3 activity. In addition, kinase-active ILK can phosphorylate PKB/AKT on serine-473, whereas kinase-deficient ILK severely inhibits endogenous phosphorylation of PKB/AKT on serine-473, demonstrating that ILK is involved in agonist stimulated, Pi(3)K-dependent, PKB/AKT activation. ILK is thus a receptor-proximal effector for the Pi(3)K-dependent,   extracellular matrix   and growth factor mediated, activation of PKB/AKT, and inhibition of GSK-3. 
 Cross-linking CD98 promotes integrin-like signaling and anchorage-independent growth. CD98, an early marker of T-cell activation, is an important regulator of integrin-mediated adhesion events. Previous studies suggest that CD98 is coupled to both cellular activation and transformation and is involved in the pathogenesis of viral infection, inflammatory disease, and cancer. Understanding of the molecular mechanisms underlying CD98 activity may have far-reaching practical applications in the development of novel therapeutic strategies in these disease states. Using small cell lung cancer cell lines, which are nonadherent, nonpolarized, and highly express CD98, we show that, in vitro, under physiological conditions, CD98 is constitutively associated with beta1 integrins regardless of activation status. Cross-linking CD98 with the monoclonal antibody 4F2 stimulated phosphatidylinositol (PI) 3-kinase, PI(3,4,5)P(3), and protein kinase B in the absence of integrin ligation or   extracellular matrix   engagement. Furthermore, cross-linking CD98 promoted anchorage-independent growth. Using fibroblasts derived from beta1 integrin null stem cells (GD25), wild-type GD25beta1, or GD25 cells expressing a mutation preventing beta1 integrin-dependent FAK phosphorylation, we demonstrate that a functional beta1 integrin is required for CD98 signaling. We propose that by cross-linking CD98, it acts as a "molecular facilitator" in the plasma membrane, clustering beta1 integrins to form high-density complexes. This results in integrin activation, integrin-like signaling, and anchorage-independent growth. Activation of PI 3-kinase may, in part, explain cellular transformation seen on overexpressing CD98. These results may provide a paradigm for events involved in such diverse processes as inflammation and viral-induced cell fusion. 
 Opposing roles for Akt1 and Akt2 in Rac/Pak signaling and cell migration. The Akt/PKB isoforms have different roles in animals, with Akt2 primarily regulating metabolic signaling and Akt1 regulating growth and survival. Here we show distinct roles for Akt1 and Akt2 in mouse embryo fibroblast cell migration and regulation of the cytoskeleton. Akt1-deficient cells responded poorly to platelet-derived growth factor while Akt2-deficient cells had a dramatically enhanced response, resulting in a substantial increase in dorsal ruffling. Swapping domains between Akt1 and Akt2 demonstrated that the N-terminal region containing the pleckstrin homology domain and a linker region distinguishes the two isoforms, while the catalytic domains are interchangeable. Akt2 knock-out cells also migrated faster than wild-type cells, especially through   extracellular matrix   (ECM), while Akt1 knock-out cells migrated more slowly than wild-type cells. Consistently, Akt2 knock-out cells had elevated Pak1 and Rac activities, suggesting that Akt2 inhibits Rac and Pak1. Both Akt2 and Akt1 associated in complexes with Pak1, but only Akt2 inhibited Pak1 in kinase assays, suggesting an underlying molecular basis for the different cellular phenotypes. Together these data provide evidence for an unexpected functional link between Akt2 and Pak1 that opposes the actions of Akt1 on cell migration. 
 Different functions of AKT1 and AKT2 in molecular pathways, cell migration and metabolism in colon cancer cells. AKT is a central protein in many cellular pathways such as cell survival, proliferation, glucose uptake, metabolism, angiogenesis, as well as radiation and drug response. The three isoforms of AKT (AKT1, AKT2 and AKT3) are proposed to have different physiological functions, properties and expression patterns in a cell type-dependent manner. As of yet, not much is known about the influence of the different AKT isoforms in the genome and their effects in the metabolism of colorectal cancer cells. In the present study, DLD-1 isogenic AKT1, AKT2 and AKT1/2 knockout colon cancer cell lines were used as a model system in conjunction with the parental cell line in order to further elucidate the differences between the AKT isoforms and how they are involved in various cellular pathways. This was done using genome wide expression analyses, metabolic profiling and cell migration assays. In conclusion, downregulation of genes in the cell adhesion,   extracellular matrix   and Notch-pathways and upregulation of apoptosis and metastasis inhibitory genes in the p53-pathway, confirm that the knockout of both AKT1 and AKT2 will attenuate metastasis and tumor cell growth. This was verified with a reduction in migration rate in the AKT1 KO and AKT2 KO and most explicitly in the AKT1/2 KO. Furthermore, the knockout of AKT1, AKT2 or both, resulted in a reduction in lactate and alanine, suggesting that the metabolism of carbohydrates and glutathione was impaired. This was further verified in gene expression analyses, showing downregulation of genes involved in glucose metabolism. Additionally, both AKT1 KO and AKT2 KO demonstrated an impaired fatty acid metabolism. However, genes were upregulated in the Wnt and cell proliferation pathways, which could oppose this effect. AKT inhibition should therefore be combined with other effectors to attain the best effect. 
 Downregulation of uPA inhibits migration and PI3k/Akt signaling in glioblastoma cells. The ability of glioma cells to migrate great distances from a primary tumor mass is the primary cause of tumor recurrence. The urokinase-type plasminogen activator (uPA) is a serine protease that can initiate proteolytic cascades, which result in remodeling of   extracellular matrix   and basement membrane, allowing cells to move across and through these barriers. The binding between uPA and its receptor uPAR also mediates several signaling events that seem to contribute to the evolution of a migratory phenotype. In this study, we determined how the downregulation of uPA affects the signaling pathways leading to cell migration. Stably transfecting human glioblastoma cells with antisense uPA decreased the amount of cell-bound uPA and disrupted actin cytoskeleton formation and cell migration. The phosphatidylinositol 3-kinase (PI3k) and Akt signaling pathway has been suggested to mediate migration in various cancer cells. The antisense-uPA clones also had less phosphorylated PI3k and Akt than control cells, a finding associated with decreased cell migration, G2/M-phase arrest, and decreased clonogenic survival. Decreased activation of PI3k and the antiapoptotic factor Akt was not sufficient to induce apoptosis in the antisense-uPA clones, but staurosporine sensitized them to apoptosis to a greater extent than control cells. These results indicate that PI3k/Akt pathway is involved in the signaling cascade required to induce cell migration and that uPA has a direct role in regulating migration. 
  [Ii]nvasive  PubMed  
 Overexpression of AKT2/protein kinase Bbeta leads to up-regulation of beta1 integrins, increased invasion, and metastasis of human breast and ovarian cancer cells. To determine how AKT2 might contribute to tumor cell progression, a full-length, wild-type, human AKT2/protein kinase B (PKB)beta cDNA was transfected into a panel of eight human breast and ovarian cancer cells. AKT2 transfectants demonstrated increased adhesion and invasion through collagen IV because of up-regulation of beta1 integrins. In addition, AKT2 cells were more metastatic than control cells in vivo. Increased invasion by AKT2 was blocked by preincubation with an anti-beta1 integrin function blocking antibody, exposure to wortmannin, and by expression of phosphatase and tensin homologue tumor suppressor (PTEN). Confocal microscopy performed on transfected human breast cancer cells showed that unlike AKT1, AKT2 protein predominantly localized adjacent to the collagen IV matrix during cellular attachment. Overexpression of AKT2, but not AKT1 or AKT3, was sufficient to duplicate the   invasive   effects of phosphoinositide 3-OH kinase (PI3-K) transfected in breast cancer cells. Furthermore, expression of kinase dead AKT2(181 amino acid methionine [M]), and not kinase dead AKT1(179M) or AKT3(177M), was capable of blocking invasion induced by either human epidermal growth factor receptor-2 (HER-2) overexpression or by activation of PI3-K. Taken together, these data indicate that AKT2 mediates PI3-K-dependent effects on adhesion, motility, invasion, and metastasis in vivo. 
 Overexpression of AKT2/protein kinase Bbeta leads to up-regulation of beta1 integrins, increased invasion, and metastasis of human breast and ovarian cancer cells. To determine how AKT2 might contribute to tumor cell progression, a full-length, wild-type, human AKT2/protein kinase B (PKB)beta cDNA was transfected into a panel of eight human breast and ovarian cancer cells. AKT2 transfectants demonstrated increased adhesion and invasion through collagen IV because of up-regulation of beta1 integrins. In addition, AKT2 cells were more metastatic than control cells in vivo. Increased invasion by AKT2 was blocked by preincubation with an anti-beta1 integrin function blocking antibody, exposure to wortmannin, and by expression of phosphatase and tensin homologue tumor suppressor (PTEN). Confocal microscopy performed on transfected human breast cancer cells showed that unlike AKT1, AKT2 protein predominantly localized adjacent to the collagen IV matrix during cellular attachment. Overexpression of AKT2, but not AKT1 or AKT3, was sufficient to duplicate the   invasive   effects of phosphoinositide 3-OH kinase (PI3-K) transfected in breast cancer cells. Furthermore, expression of kinase dead AKT2(181 amino acid methionine [M]), and not kinase dead AKT1(179M) or AKT3(177M), was capable of blocking invasion induced by either human epidermal growth factor receptor-2 (HER-2) overexpression or by activation of PI3-K. Taken together, these data indicate that AKT2 mediates PI3-K-dependent effects on adhesion, motility, invasion, and metastasis in vivo. 
 MicroRNAs differentially regulated by Akt isoforms control EMT and stem cell renewal in cancer cells. Although Akt is known to play a role in human cancer, the relative contribution of its three isoforms to oncogenesis remains to be determined. We expressed each isoform individually in an Akt1(-/-)/Akt2(-/-)/Akt3(-/-) cell line. MicroRNA profiling of growth factor-stimulated cells revealed unique microRNA signatures for cells with each isoform. Among the differentially regulated microRNAs, the abundance of the miR-200 family was decreased in cells bearing Akt2. Knockdown of Akt1 in transforming growth factor-beta (TGFbeta)-treated MCF10A cells also decreased the abundance of miR-200; however, knockdown of Akt2, or of both Akt1 and Akt2, did not. Furthermore, Akt1 knockdown in MCF10A cells promoted TGFbeta-induced epithelial-mesenchymal transition (EMT) and a stem cell-like phenotype. Carcinomas developing in MMTV-cErbB2/Akt1(-/-) mice showed increased   invasive  ness because of miR-200 down-regulation. Finally, the ratio of Akt1 to Akt2 and the abundance of miR-200 and of the messenger RNA encoding E-cadherin in a set of primary and metastatic human breast cancers were consistent with the hypothesis that in many cases breast cancer metastasis may be under the control of the Akt-miR-200-E-cadherin axis. We conclude that induction of EMT is controlled by microRNAs whose abundance depends on the balance between Akt1 and Akt2 rather than on the overall activity of Akt. 
 Function of Akt/PKB signaling to cell motility, invasion and the tumor stroma in cancer. The serine/threonine protein kinase Akt is a major signal transducer of the phosphoinositide 3-kinase (PI 3-K) pathway in all cells and tissues and plays a pivotal role in the maintenance of cellular processes including cell growth, proliferation, survival and metabolism. The frequent aberrant activation of the PI 3-K/Akt pathway in human cancer has made it an attractive therapeutic target. Numerous studies have provided a comprehensive understanding of the specific functions of Akt signaling in cancer cells as well as the surrounding tumor microenvironment and this has informed and enabled the development of therapeutic drugs to target both PI 3-K and Akt. However, recent studies have provided evidence for distinct functions of the three mammalian Akt isoforms, particularly with respect to the regulation of cell motility and metastasis of breast cancer. Here we discuss the mechanisms by which Akt signaling contributes to   invasive   migration and tumor metastasis, and highlight recent advances in our understanding of the contribution of the Akt pathway in the tumor-associated stroma. 
 RLN2 Is a Positive Regulator of AKT-2-Induced Gene Expression Required for Osteosarcoma Cells Invasion and Chemoresistance. The aim of the study was to determine the effect of H2 relaxin (RLN2) on invasion, migration, and chemosensitivity to cisplatin in human osteosarcoma U2-OS and MG-63 cells and then to investigate the effect of RLN2 on the AKT/NF-κB signaling pathway. The expression of RLN2, p-AKT (Ser473), and p-ERK1/2 (Phospho-Thr202/Tyr204) proteins was detected by western blot in OS tissues from 21 patients with pulmonary metastatic disease, and the correlation between RLN2 and p-AKT or RLN2 and p-ERK1/2 expression was investigated. RLN2 expression was inhibited by RLN2 siRNA transfection in the MG-63 cells. RLN2 was overexpressed in the U2-OS cells by treatment with recombinant relaxin. The results showed that positive relation was found between RLN2 and p-AKT expression in tissues of OS. Silencing RLN2 inhibited cell migratory and   invasiv  e ability and angiogenesis formation and increased the chemosensitivity to cisplatin in MG-63 cells. RLN2 overexpression promoted migratory and   invasiv  e ability and angiogenesis and increased the chemoresistance to cisplatin in U2-OS cells. Silencing RLN2 inhibited the activity of AKT/NF-κB signaling pathway in MG-63 cells, and vice versa. Blockage of both pathways by specific inhibitors abrogated RLN2-induced survival and invasion of OS cells, and vice versa. Our results indicated RLN2 confers to migratory an  d invasi  ve ability, angiogenesis, and chemoresistance to cisplatin via modulating the AKT/NF-κB signaling pathway in vitro. 
 Antisense and dominant-negative AKT2 cDNA inhibits glioma cell invasion. The aim of this study was to explore the potential role of AKT2 in glioma cell invasion. Therefore, dominant-negative (DN-AKT2) and antisense AKT2 constructs (AS-AKT2) were transfected into rat C6 glioma cells with elevated endogenous AKT2 expression. In situ hybridization and Western blot analysis were used to identify AKT2 expression. Spheroid culturing was used to assess cell migration and invasion in Matrigel from spheroids. Cell motility and invasion were also evaluated by scratch and Transwell invasion assays, respectively. The secretion of matrix metalloproteinases (MMPs), MMP2 and MMP9, was determined by gelatin zymography. AKT2 expression was inhibited in C6 cells transfected with AS-AKT2 but did not significantly change in cells transfected with DN-AKT2. The cell migration distance from spheroids or the number of cells migrating into the acellular space created by scratching was reduced in cells transfected with DN-AKT2 or AS-AKT2 compared to the control cells. The   invasive   distance of cells from the spheroids in Matrigel sandwich and the number of invading cells through the Matrigel were also decreased in the DN-AKT2- and AS-AKT2-transfected cells. Gelatin zymography showed that the production of MMP2 and MMP9 was inhibited in transfected cells. In conclusion, AKT2 plays an important role in glioma cell motility and invasion. Therapy based on AKT inhibition may complement currently available treatment to control glioma cell invasion. 
 Twist transcriptionally up-regulates AKT2 in breast cancer cells leading to increased migration, invasion, and resistance to paclitaxel. Metastasis, the cardinal feature of malignant tumors, is an important clinical variable in patient prognosis. To understand the basis for metastasis, we systematically selected for highly   invasive   cells from breast cancer cell lines, MCF7 and MDA-MB-453, with moderate to low   invasive   ability using Boyden chamber invasion assay. The four-cycle selected   invasive   lines, named MCF7-I4 and MDA-MB-453-I4, respectively, displayed epithelial-mesenchymal transition (EMT) and dramatically enhanced   invasive   ability. EMT changes were corroborated with decreased level of E-cadherin and increased vimentin, fibronectin, and beta(1) integrin. Twist, a basic helix-loop-helix transcription factor, and AKT2, a known proto-oncogene, were found to be elevated in the   invasive   cells compared with the parental. Ectopic expression and knockdown of Twist by short interference RNA resulted in significant increase and reduction, respectively, of AKT2 protein and mRNA expression. Twist bound to E-box elements on AKT2 promoter and enhanced its transcriptional activity. Moreover, silencing AKT2 decreased Twist-promoted migration, invasion, and paclitaxel resistance. Reintroducing AKT2 largely rescued the phenotype resulted from knockdown of Twist in I4 cells, suggesting that AKT2 is a downstream target and functional mediator of Twist. Finally, we observed a 68.8% correlation of elevated Twist and AKT2 expression in late-stage breast cancers as oppose to 13% in early-stage breast cancers. Our study identifies Twist as a positive transcriptional regulator of AKT2 expression, and Twist-AKT2 signaling is involved in promoting   invasive   ability and survival of breast cancer cells. 
 Mitochondrial DNA depletion in prostate epithelial cells promotes anoikis resistance and invasion through activation of PI3K/Akt2. Neoplastic transformation of prostate epithelium involves aberrant activation of anti-apoptotic and pro-  invasive   pathways triggered by multiple poorly understood genetic events. We demonstrated earlier that depletion of mitochondrial DNA (mtDNA) induces prostate cancer progression. Here, using normal prostate epithelial PNT1A cells we demonstrate that mtDNA depletion prevents detachment-induced apoptosis (anoikis) and promotes migratory capabilities onto basement membrane proteins through upregulation of p85 and p110 phosphatidylinositol 3-kinase (PI3K) subunits, which results in Akt2 activation and phosphorylation of downstream substrates GSK3beta, c-Myc, MMP-9, Mdm2, and p53. Pharmacological or genetic PI3K inhibition, siRNA-mediated Akt2 depletion, as well as mtDNA reconstitution were sufficient to restore sensitivity to anoikis and curtail cell migration. Moreover, Akt2 activation induced glucose transporter 1 (GLUT1) expression, glucose uptake, and lactate production, common phenotypic changes seen in neoplastic cells. In keeping with these findings, several prostate carcinoma cell lines displayed reduced mtDNA content and increased PI3K/Akt2 levels when compared to normal PNT1A cells, and Akt2 downregulation prevented their survival, migration and glycolytic metabolism. On a tissue microarray, we also found a statistically significant decrease in mtDNA-encoded cytochrome oxidase I in prostate carcinomas. Taken together, these results provide novel mechanistic evidence supporting the notion that mtDNA mutations may confer survival and migratory advantage to prostate cancer cells through Akt2 signaling. 
 Amplification of AKT2 in human pancreatic cells and inhibition of AKT2 expression and tumorigenicity by antisense RNA. We previously demonstrated that the putative oncogene AKT2 is amplified and overexpressed in some human ovarian carcinomas. We have now identified amplification of AKT2 in approximately 10% of pancreatic carcinomas (2 of 18 cell lines and 1 of 10 primary tumor specimens). The two cell lines with altered AKT2 (PANC1 and ASPC1) exhibited 30-fold and 50-fold amplification of AKT2, respectively, and highly elevated levels of AKT2 RNA and protein. PANC1 cells were transfected with antisense AKT2, and several clones were established after G418 selection. The expression of AKT2 protein in these clones was greatly decreased by the antisense RNA. Furthermore, tumorigenicity in nude mice was markedly reduced in PANC1 cells expressing antisense AKT2 RNA. To examine further whether overexpression of AKT2 plays a significant role in pancreatic tumorigenesis, PANC1 cells and ASPC1 cells, as well as pancreatic carcinoma cells that do not overexpress AKT2 (COLO 357), were transfected with antisense AKT2, and their growth and   invasive  ness were characterized by a rat tracheal xenotransplant assay. ASPC1 and PANC1 cells expressing antisense AKT2 RNA remained confined to the tracheal lumen, whereas the respective parental cells invaded the tracheal wall. In contrast, no difference was seen in the growth pattern between parental and antisense-treated COLO 357 cells. These data suggest that overexpression of AKT2 contributes to the malignant phenotype of a subset of human ductal pancreatic cancers. 
 Vimentin is a novel AKT1 target mediating motility and invasion. The PI3K/AKT signaling pathway is aberrant in a wide variety of cancers. Downstream effectors of AKT are involved in survival, growth and metabolic-related pathways. In contrast, contradictory data relating to AKT effects on cell motility and invasion, crucial prometastatic processes, have been reported pointing to a potential cell type and isoform type-specific AKT-driven function. By implication, study of AKT signaling should optimally be conducted in an appropriate intracellular environment. Prognosis in soft-tissue sarcoma (STS), the aggressive malignancies of mesenchymal origin, is poor, reflecting our modest ability to control metastasis, an effort hampered by lack of insight into molecular mechanisms driving STS progression and dissemination. We examined the impact of the cancer progression-relevant AKT pathway on the mesenchymal tumor cell internal milieu. We demonstrate that AKT1 activation induces STS cell motility and   invasive  ness at least partially through a novel interaction with the intermediate filament vimentin (Vim). The binding of AKT (tail region) to Vim (head region) results in Vim Ser39 phosphorylation enhancing the ability of Vim to induce motility and invasion while protecting Vim from caspase-induced proteolysis. Moreover, vimentin phosphorylation was shown to enhance tumor and metastasis growth in vivo. Insights into this mesenchymal-related molecular mechanism may facilitate the development of critically lacking therapeutic options for these devastating malignancies. 
  GeneRIFs  
 Twist as a positive transcriptional regulator of AKT2 expression; Twist-AKT2 signaling is involved in promoting   invasive   ability and survival of breast cancer cells. 
  [Mm]etastasize  PubMed  
 Akt2 overexpression plays a critical role in the establishment of colorectal cancer metastasis. Colorectal cancer is the second leading cause of cancer-related deaths in the United States. Understanding the distinct genetic and epigenetic changes contributing to the establishment and growth of metastatic lesions is crucial for the development of novel therapeutic strategies. In a search for key regulators of colorectal cancer metastasis establishment, we have found that the serine/threonine kinase Akt2, a known proto-oncogene, is highly expressed in late-stage colorectal cancer and metastatic tumors. Suppression of Akt2 expression in highly metastatic colorectal carcinoma cells inhibits their ability to   metastasize   in an experimental liver metastasis model. Overexpression of wild-type Akt1 did not restore metastatic potential in cells with downregulated Akt2, thus suggesting non-redundant roles for the individual Akt isoforms. In contrast, Akt2 overexpression in wild-type PTEN expressing SW480 colorectal cancer cells led to the formation of micrometastases; however, loss of PTEN is required for sustained formation of overt metastasis. Finally, we found that the consequence of PTEN loss and Akt2 overexpression function synergistically to promote metastasis. These results support a role for Akt2 overexpression in metastatic colorectal cancer and establish a mechanistic link between Akt2 overexpression and PTEN mutation in metastatic tumor establishment and growth. Taken together, these data suggest that Akt family members have distinct functional roles in tumor progression and that selective targeting of the PI3K/Akt2 pathway may provide a novel treatment strategy for colorectal cancer metastasis. 
  [Mm]etastasis  PubMed  
 Overexpression of AKT2/protein kinase Bbeta leads to up-regulation of beta1 integrins, increased invasion, and   metastasis   of human breast and ovarian cancer cells. To determine how AKT2 might contribute to tumor cell progression, a full-length, wild-type, human AKT2/protein kinase B (PKB)beta cDNA was transfected into a panel of eight human breast and ovarian cancer cells. AKT2 transfectants demonstrated increased adhesion and invasion through collagen IV because of up-regulation of beta1 integrins. In addition, AKT2 cells were more metastatic than control cells in vivo. Increased invasion by AKT2 was blocked by preincubation with an anti-beta1 integrin function blocking antibody, exposure to wortmannin, and by expression of phosphatase and tensin homologue tumor suppressor (PTEN). Confocal microscopy performed on transfected human breast cancer cells showed that unlike AKT1, AKT2 protein predominantly localized adjacent to the collagen IV matrix during cellular attachment. Overexpression of AKT2, but not AKT1 or AKT3, was sufficient to duplicate the invasive effects of phosphoinositide 3-OH kinase (PI3-K) transfected in breast cancer cells. Furthermore, expression of kinase dead AKT2(181 amino acid methionine [M]), and not kinase dead AKT1(179M) or AKT3(177M), was capable of blocking invasion induced by either human epidermal growth factor receptor-2 (HER-2) overexpression or by activation of PI3-K. Taken together, these data indicate that AKT2 mediates PI3-K-dependent effects on adhesion, motility, invasion, and   metastasis   in vivo. 
 Overexpression of AKT2/protein kinase Bbeta leads to up-regulation of beta1 integrins, increased invasion, and   metastasis   of human breast and ovarian cancer cells. To determine how AKT2 might contribute to tumor cell progression, a full-length, wild-type, human AKT2/protein kinase B (PKB)beta cDNA was transfected into a panel of eight human breast and ovarian cancer cells. AKT2 transfectants demonstrated increased adhesion and invasion through collagen IV because of up-regulation of beta1 integrins. In addition, AKT2 cells were more metastatic than control cells in vivo. Increased invasion by AKT2 was blocked by preincubation with an anti-beta1 integrin function blocking antibody, exposure to wortmannin, and by expression of phosphatase and tensin homologue tumor suppressor (PTEN). Confocal microscopy performed on transfected human breast cancer cells showed that unlike AKT1, AKT2 protein predominantly localized adjacent to the collagen IV matrix during cellular attachment. Overexpression of AKT2, but not AKT1 or AKT3, was sufficient to duplicate the invasive effects of phosphoinositide 3-OH kinase (PI3-K) transfected in breast cancer cells. Furthermore, expression of kinase dead AKT2(181 amino acid methionine [M]), and not kinase dead AKT1(179M) or AKT3(177M), was capable of blocking invasion induced by either human epidermal growth factor receptor-2 (HER-2) overexpression or by activation of PI3-K. Taken together, these data indicate that AKT2 mediates PI3-K-dependent effects on adhesion, motility, invasion, and   metastasis   in vivo. 
 Astrocyte elevated gene-1 interacts with Akt isoform 2 to control glioma growth, survival, and pathogenesis. The oncogene astrocyte elevated gene-1 (AEG-1; MTDH) is highly expressed in glioblastoma multiforme (GBM) and many other types of cancer, where it activates multiple signaling pathways that drive proliferation, invasion, angiogenesis, chemoresistance, radioresistance, and   metastasis  . AEG-1 activates the Akt signaling pathway and Akt and c-Myc are positive regulators of AEG-1 transcription, generating a positive feedback loop between AEG-1 and Akt in regulating tumorigenesis. Here, we describe in GBM cells a direct interaction between an internal domain of AEG-1 and the PH domain of Akt2, a major driver in GBM. Expression and interaction of AEG-1 and Akt2 are elevated in GBM and contribute to tumor cell survival, proliferation, and invasion. Clinically, in silico gene expression and immunohistochemical analyses of patient specimens showed that AEG-1 and Akt2 expression correlated with GBM progression and reduced patient survival. AEG-1-Akt2 interaction prolonged stabilization of Akt2 phosphorylation at S474, regulating downstream signaling cascades that enable cell proliferation and survival. Disrupting AEG-1-Akt2 interaction by competitive binding of the Akt2-PH domain led to reduced cell viability and invasion. When combined with AEG-1 silencing, conditional expression of Akt2-PH markedly increased survival in an orthotopic mouse model of human GBM. Our study uncovers a novel molecular mechanism by which AEG-1 augments glioma progression and offers a rationale to block AEG-1-Akt2 signaling function as a novel GBM treatment. 
 MiR-194 deregulation contributes to colorectal carcinogenesis via targeting AKT2 pathway. Recent studies have increasingly linked microRNAs to colorectal cancer (CRC). MiR-194 has been reported deregulated in different tumor types, whereas the function of miR-194 in CRC largely remains unexplored. Here we investigated the biological effects, mechanisms and clinical significance of miR-194. Functional assay revealed that overexpression of miR-194 inhibited CRC cell viability and invasion in vitro and suppressed CRC xenograft tumor growth in vivo. Conversely, block of miR-194 in APC(Min/+) mice promoted tumor growth. Furthermore, miR-194 reduced the expression of AKT2 both in vitro and in vivo. Clinically, the expression of miR-194 gradually decreased from 20 normal colorectal mucosa (N-N) cases through 40 colorectal adenomas (CRA) cases and then to 40 CRC cases, and was negatively correlated with AKT2 and pAKT2 expression. Furthermore, expression of miR-194 in stool samples was gradually decreased from 20 healthy cases, 20 CRA cases, then to 28 CRC cases. Low expression of miR-194 in CRC tissues was associated with large tumor size (P=0.006), lymph node   metastasis   (P=0.012) and shorter survival (HR =2.349, 95% CI = 1.242 to 4.442; P=0.009). In conclusion, our data indicated that miR-194 acted as a tumor suppressor in the colorectal carcinogenesis via targeting PDK1/AKT2/XIAP pathway, and could be a significant diagnostic and prognostic biomarker for CRC. 
 Genetic inactivation of AKT1, AKT2, and PDPK1 in human colorectal cancer cells clarifies their roles in tumor growth regulation. Phosphotidylinositol-3-kinase (PI3K) signaling is altered in the majority of human cancers. To gain insight into the roles of members of this pathway in growth regulation, we inactivated AKT1, AKT2, or PDPK1 genes by targeted homologous recombination in human colon cancer cell lines. Knockout of either AKT1 or AKT2 had minimum effects on cell growth or downstream signaling. In contrast, knockout of both AKT1 and AKT2 resulted in markedly reduced proliferation in vitro when growth factors were limiting and severely affected experimental   metastasis   in mice. Unexpectedly, AKT1 and AKT2 appeared to regulate growth through FOXO proteins, but not through either GSK3beta or mTOR. In contrast, inactivation of PDPK1 affected GSK3beta and mTOR activation. These findings show that the PI3K signaling pathway is wired differently in human cancer cells than in other cell types or organisms, which has important implications for the design and testing of drugs that target this pathway. 
 miR-302b suppresses cell invasion and   metastasis   by directly targeting AKT2 in human hepatocellular carcinoma cells. MicroRNAs (miRNAs) have been shown to play essential roles in regulating the activity of human hepatocellular carcinoma (HCC) cells, thereby contributing to the suppression of invasion and   metastasis  . In this study, using gain and loss of function assays, we demonstrated that miR-302b was frequently down-regulated in clinical HCC specimens, as compared with 15 corresponding adjacent normal tissues. Overexpression of miR-302b suppressed HCC cell invasion and   metastasis  . Regulation of NF-κB and matrix metalloproteinase (MMP)-2 expression by miR-302b was mediated via AKT2 in SMMC-7721 cells. Silencing AKT2 produced effects similar to those of miR-302b overexpression, which included inhibiting SMMC-7721 cell invasion and   metastasi  s and dereasing NF-κB and MMP-2 expression. Furthermore, overexpression of AKT2 attenuated the effects of miR-302b overexpression. Taken together, our findings indicate that miR-302b inhibits SMMC-7721 cell invasion an  d metastas  is by targeting AKT2, suggesting that miR-302b might represent a potential therapeutic target for HCC intervention. 
 MicroRNAs differentially regulated by Akt isoforms control EMT and stem cell renewal in cancer cells. Although Akt is known to play a role in human cancer, the relative contribution of its three isoforms to oncogenesis remains to be determined. We expressed each isoform individually in an Akt1(-/-)/Akt2(-/-)/Akt3(-/-) cell line. MicroRNA profiling of growth factor-stimulated cells revealed unique microRNA signatures for cells with each isoform. Among the differentially regulated microRNAs, the abundance of the miR-200 family was decreased in cells bearing Akt2. Knockdown of Akt1 in transforming growth factor-beta (TGFbeta)-treated MCF10A cells also decreased the abundance of miR-200; however, knockdown of Akt2, or of both Akt1 and Akt2, did not. Furthermore, Akt1 knockdown in MCF10A cells promoted TGFbeta-induced epithelial-mesenchymal transition (EMT) and a stem cell-like phenotype. Carcinomas developing in MMTV-cErbB2/Akt1(-/-) mice showed increased invasiveness because of miR-200 down-regulation. Finally, the ratio of Akt1 to Akt2 and the abundance of miR-200 and of the messenger RNA encoding E-cadherin in a set of primary and metastatic human breast cancers were consistent with the hypothesis that in many cases breast cancer   metastasis   may be under the control of the Akt-miR-200-E-cadherin axis. We conclude that induction of EMT is controlled by microRNAs whose abundance depends on the balance between Akt1 and Akt2 rather than on the overall activity of Akt. 
 Regulation of cancer cell survival, migration, and invasion by Twist: AKT2 comes to interplay.   Metastasis  , the foremost cause of mortality in cancer patients, is increasingly recognized as a coordinated biological process. The multistep process of   metastasis   posts difficulty in studying its mechanism and molecular basis. Recent works have shown that the basic helix-loop-helix transcriptional factor Twist and the serine/threonine kinase AKT play pivotal roles in tumor development and progression. Our recent study has shown that AKT2 is a transcriptional regulatory target of Twist and acts downstream of Twist to promote cancer cell survival, migration, and invasion. Functional convergence of Twist and AKT2 underscores the importance of this signaling pathway in tumor development and progression and as a potential therapeutic target. 
 miR-612 negatively regulates colorectal cancer growth and   metastasis   by targeting AKT2. Colorectal cancer (CRC) is one of the most common cancers worldwide, with a particularly high incidence in developed countries. Distant   metastasis   and recurrence are the main causes of CRC-related deaths. MicroRNAs (miRNAs) in the serum make them potential biomarkers for cancers, as reported in serum or tumor tissues from CRC patients. In this study, we found that miR-612 expression was significantly lower in CRC tissues or cells compared with peritumor tissues or normal cells, and lower in metastatic CRC specimens compared with non-metastatic specimens, whereas AKT2 exhibited opposite trend. Gain-of-function and loss-of-function assays showed that miR-612 inhibited CRC cell proliferation and migration in vitro by Cell Counting Kit-8 and transwell assays. Further analysis revealed that miR-612 directly suppressed AKT2, which in turn inhibited the downstream epithelial-mesenchymal transition-related signaling pathway. These results were additionally validated in vivo by tumorigenesis and liver   metastasis   experiments. The results of this study suggested a critical role of miR-612 in the development of CRC. 
 Function of Akt/PKB signaling to cell motility, invasion and the tumor stroma in cancer. The serine/threonine protein kinase Akt is a major signal transducer of the phosphoinositide 3-kinase (PI 3-K) pathway in all cells and tissues and plays a pivotal role in the maintenance of cellular processes including cell growth, proliferation, survival and metabolism. The frequent aberrant activation of the PI 3-K/Akt pathway in human cancer has made it an attractive therapeutic target. Numerous studies have provided a comprehensive understanding of the specific functions of Akt signaling in cancer cells as well as the surrounding tumor microenvironment and this has informed and enabled the development of therapeutic drugs to target both PI 3-K and Akt. However, recent studies have provided evidence for distinct functions of the three mammalian Akt isoforms, particularly with respect to the regulation of cell motility and   metastasis   of breast cancer. Here we discuss the mechanisms by which Akt signaling contributes to invasive migration and tumor   metastasis  , and highlight recent advances in our understanding of the contribution of the Akt pathway in the tumor-associated stroma. 
 MicroRNA-137 Contributes to Dampened Tumorigenesis in Human Gastric Cancer by Targeting AKT2. MiRNAs play important roles in tumorigenesis. This study focused on exploring the effects and regulation mechanism of miRNA-137 on the biological behaviors of gastric cancer. Total RNA was extracted from tissues of 100 patients with gastric cancer and from four gastric cancer cell lines. Expression of miR-137 was detected by real-time PCR from 100 patients. The effects of miR-137 overexpression on gastric cancer cells' proliferation, apoptosis, migration and invasion ability were investigated in vitro and in vivo. The target gene of miR-137 was predicted by Targetscan on line software, screened by dual luciferase reporter gene assay and demonstrated by western blot. As a result, the expression of miR-137 was significant reduced in gastric cancer cell line HGC-27, HGC-803, SGC-7901 and MKN-45 as well as in gastric cancer tissues compared with GES-1 cell or matched adjacent non-neoplastic tissues (p<0.001). The re-introduction of miR-137 into gastric cancer cells was able to inhibit cell proliferation, migration and invasion. The in vivo experiments demonstrated that the miR-137 overexpression can reduce the gastric cancer cell proliferation and   metastasis  . Bioinformatic and western blot analysis indicated that the miR-137 acted as tumor suppressor roles on gastric cancer cells through targeting AKT2 and further affecting the Bad and GSK-3β. In conclusion, the miR-137 which is frequently down-regulated in gastric cancer is potentially involved in gastric cancer tumorigenesis and   metastasi  s by regulating AKT2 related signal pathways. 
 Identification of functional domains in AKT responsible for distinct roles of AKT isoforms in pressure-stimulated cancer cell adhesion. Cell adhesion is a critical step in cancer   metastasis  , activated by extracellular forces such as pressure and shear. Reducing AKT1, but not AKT2, ablates the increase in cancer cell adhesion associated with 15 mm Hg increased extracellular pressure. To identify the determinants of this AKT isoform specificity, we exchanged the pleckstrin homology (PH) domains and/or hinge regions of AKT1 and AKT2. Wild type isoforms or these chimeras were overexpressed in Caco-2 cells in the absence or presence of isoform-specific siRNA to suppress endogenous AKT1. Pressure-induced AKT translocation and phosphorylation to the membrane were compared, along with the stimulation of cell adhesion by pressure. Pressure stimulated translocation of AKT1, but not AKT2 to the plasma membrane. Among our chimeras, only the chimeric AKT2 (chimera2), in which both the AKT2 PH domain and hinge region had been replaced by those of AKT1, translocated to the membrane in response to pressure. Similarly, only chimera2 rescued the function of AKT1 in mediating pressure-stimulated adhesion after endogenous AKT1 had been reduced. Pressure also promoted phosphorylation of AKT1 but not AKT2, and expression of a nonphosphorylatable double point mutant prevented pressure-stimulated adhesion. Among the chimeras, pressure promoted only chimera2 phosphorylation. These results identify the AKT1 PH domain and hinge region as functional domains which jointly permit AKT1 translocation and phosphorylation in response to extracellular pressure and distinguish determine the specificity of AKT1 in mediating the effects of extracellular pressure on cancer cell adhesion. These may be useful targets for interventions to inhibit   metastasis  . 
 Molecular alterations in AKT and its protein activation in human lung carcinomas. To evaluate the involvement of aberrant Akt in lung carcinomas, the frequency of Akt overexpression/activation and gene gains of AKT1 and AKT2 were investigated. Immunohistochemistry in 135 cases revealed overexpression of total-Akt in 62% and phosphorylated-Akt in 44%. A statistically significant correlation between Akt activation and lymph node   metastasis   was observed. Immunoblot analyses revealed almost consistent results. Fluorescence in situ hybridization for AKT1 and AKT2 genes in 62 carcinomas exhibiting total-Akt overexpression indicated amplification of AKT1 in 6.5% of total-Akt-expressing cases (3.5% of total cases) and AKT1 gain by polysomy of chromosome 14 in 40% (high level in 8% and low level in 32%). For AKT2, amplification was observed in 6.5% of total-Akt-expressing cases (3.5% of total cases) and polysomy of chromosome 19 in 44% (high level in 16% and low level in 28%). Total-Akt overexpression and "fluorescence in situ hybridization-positive" gene gain (defined as "amplification/high level polysomy") of AKT2 were more prevalent in small cell carcinoma. However, Akt activation and fluorescence in situ hybridization-positive AKT1 gene gain were most frequent in large cell carcinoma. Fluorescence in situ hybridization-positive AKTs gene gain accompanied overexpression/activation of Akt and all fluorescence in situ hybridization-positive carcinomas harbored wild-type EGFR in disomy in this sample group examined; therefore, fluorescence in situ hybridization-positive gene gains of AKTs versus EGFR or EGFR mutation occurred in a reciprocal manner. In conclusion, amplification of AKT1 and/or AKT2 and high-level polysomy were found in 16% of total cases, and this defined subset was characterized by the overexpression/activation of Akt, reciprocal to EGFR aberrations. Thus, novel Akt-targeted therapy could be applicable for this particular group of carcinomas in which Akt may be critically involved. 
 let-7b/g silencing activates AKT signaling to promote gastric carcinogenesis. Aberrant AKT activation contributes to gastric cancer cell survival and chemotherapy resistance, however its regulation is poorly understood. microRNAs have been established to be important regulators in gastric carcinogenesis. Here, we showed the functional role and putative target of let-7b and let-7g (let-7b/g) in gastric carcinogenesis.The expression of let-7b/g in gastric cancer cell lines and primary tumors were evaluated by miRNA qRT-PCR. The putative target gene of let-7b/g was explored by TargetScan followed by further validation. Functional analyses including MTT proliferation, monolayer colony formation, cell invasion assays and in vivo study were performed in both ectopic expression and knockdown approaches.let-7b/g was found down-regulated in gastric cancer and its downregulation was associated with poor survival and correlated with lymph node   metastasis  . let-7b/g inhibited AKT2 expression by directly binding to its 3'UTR, reduced p-AKT (S473) activation and suppressed expression of the downstream effector pS6. AKT2 mRNA expression showed negative correlation with the expression of let-7b/g in primary tumors. Short interfering RNA (siRNA) mediated knockdown of AKT2 phenocopied the tumor-suppressive effects of let-7b/g. Moreover, AKT2 re-expression partly abrogated the growth-inhibitory effect of let-7b/g.In conclusion, our findings reveal decreased let-7b/g contributes to aberrant AKT activation in gastric tumorigenesis and provide a potential therapeutic strategy for gastric cancer. 
 miR-148a suppresses human renal cell carcinoma malignancy by targeting AKT2. MicroRNA-148a (miR-148a) has been reported to be deregulated in different tumor types, whereas the biological function of miR-148a in renal cell carcinoma (RCC) largely remains unexplored. In the present study we investigated the clinical significance, biological effects, and the underlying molecular mechanisms of miR-148 in RCC. Here, we showed that miR-148a was significantly downregulated in RCC tissues and cell lines. Low expression of miR-148a in RCC tissues was associated with large tumor size, advanced TNM stage, and lymph node   metastasis  . Functional assays revealed that overexpression of miR-148a significantly inhibited RCC cell proliferation, colony formation, migration and invasion in vitro and suppressed RCC xenograft tumor growth in vivo. In addition, using quantitative RT-PCR (qRT-PCR), western blot analysis and luciferase reporter assays, AKT2 was confirmed to be a direct target of miR-148a. AKT2 expression was upregulated, and was negatively correlated with miR-148a expression in RCC tissues (r=-0.641, P<0.001). Silencing of AKT2 phenotypically copied miR-148a-induced phenotypes, whereas re-expression of AKT2 reversed the suppressive effects of miR-148a in RCC cells. Further mechanistic investigations showed that miR-148a exerted its antitumor activity via inhibition of the AKT pathway in vitro and in vivo. Taken together, these findings suggest that miR-148a functions as tumor suppressor in RCC by targeting AKT2. 
 [The expression of oncogene AKT2 in laryngeal squamous cell carcinoma and its clinical significance]. To investigate the expression and clinical significance of oncogene AKT2 in laryngeal squamous cell carcinoma (LSCC).The expression of AKT2 protein was measured with immunohistochemistry (Elivision two steps) in 60 cases of LSCC tissue, 28 cases of peri-cancer tissue and 16 cases of normal laryngeal epithelium, the relationship between AKT2 expression and clinical pathologic factors in LSCC was also analyzed.The positive rates of AKT2 protein expression in LSCC and peri-cancer tissue were 56.7% (34/60), 14.3% (4/28) respectively. There was no expression of AKT2 in normal laryngeal epithelium. The positive rate of AKT2 protein expression in LSCC was significantly higher than that in peri-cancer tissue and normal laryngeal epithelium (P < 0.05, respectively). The positive rate of AKT2 was correlated with tumor site, lymph node   metastasis   and clinical stage of LSCC (P < 0.05, respectively), but wasn't correlated with patient's age, sex, and T stage (P > 0.05, respectively).AKT2 might play an important role in the tumorigenesis and development of LSCC, and it was suggested that AKT2 could be a useful marker for predicting tumorigenesis, invasion and   metastasis   potential of LSCC. 
 Akt2 overexpression plays a critical role in the establishment of colorectal cancer   metastasis  . Colorectal cancer is the second leading cause of cancer-related deaths in the United States. Understanding the distinct genetic and epigenetic changes contributing to the establishment and growth of metastatic lesions is crucial for the development of novel therapeutic strategies. In a search for key regulators of colorectal cancer   metastasis   establishment, we have found that the serine/threonine kinase Akt2, a known proto-oncogene, is highly expressed in late-stage colorectal cancer and metastatic tumors. Suppression of Akt2 expression in highly metastatic colorectal carcinoma cells inhibits their ability to metastasize in an experimental liver   metastasis   model. Overexpression of wild-type Akt1 did not restore metastatic potential in cells with downregulated Akt2, thus suggesting non-redundant roles for the individual Akt isoforms. In contrast, Akt2 overexpression in wild-type PTEN expressing SW480 colorectal cancer cells led to the formation of micrometastases; however, loss of PTEN is required for sustained formation of overt   metastasis  . Finally, we found that the consequence of PTEN loss and Akt2 overexpression function synergistically to promote   metastasis  . These results support a role for Akt2 overexpression in metastatic colorectal cancer and establish a mechanistic link between Akt2 overexpression and PTEN mutation in metastatic tumor establishment and growth. Taken together, these data suggest that Akt family members have distinct functional roles in tumor progression and that selective targeting of the PI3K/Akt2 pathway may provide a novel treatment strategy for colorectal cancer   metastasis  . 
 FoxD3-regulated microRNA-137 suppresses tumour growth and   metastasis   in human hepatocellular carcinoma by targeting AKT2. microRNAs, frequently deregulated in human cancer, have been implicated in the progression of hepatocarcinogenesis. Here, we show that microRNA (miR)-137 is significantly down-regulated in hepatocellular carcinoma (HCC). Its decreased expression is associated with vein invasion, incomplete Involucrum, and distant   metastasis  . Multivariate analysis suggests that miR-137 is an independent indicator for poor survival. We next show that over-expression of miR-137 suppresses cell proliferation, migration and invasion in vitro. Conversely, miR-137 inhibition promotes HCC cell growth. We also identify AKT2 as a key target of miR-137 in this context. Statistical data reveal a reverse correlation of AKT2 and miR-137 expression in HCC patients. Silencing of AKT2 phenotypically copied miR-137-induced phenotypes, whereas re-expression of AKT2 reversed the suppressive effects of miR-137. Further investigations showed that miR-137 exerted its anti-tumour activity via inhibiting the AKT2/mTOR pathway. Moreover, we demonstrate that FoxD3 directly binds to the promoter of miR-137 and activates its transcription. In vivo studies confirm that FoxD3-regulated miR-137 inhibited HCC growth and   metastasis   via targeting AKT2. Together, our findings indicate that miR-137 is a valuable biomarker for HCC prognosis and the FoxD3/miR-137/AKT2 regulatory network plays an important role in HCC progression. 
 Identification of AKT kinases as unfavorable prognostic factors for hepatocellular carcinoma by a combination of expression profile, interaction network analysis and clinical validation. identification of key markers that differentiate occurrence and progression of hepatocellular carcinoma (HCC) is of great significance to develop novel prognostic factors and improve therapeutic strategies. The aim of this study was to screen novel markers for HCC by combining expression profile, interaction network analysis and clinical validation.HCC significant molecules which were differentially expressed in HCC tissues were obtained from five existing HCC related databases (OncoDB.HCC, HCC.net, dbHCCvar, EHCO and Liverome). The protein-protein interaction network of HCC significant proteins was constructed and 331 candidate HCC markers were identified by calculating four topological features of the network ('Degree', 'Betweenness', 'Closeness' and 'K-coreness'). According to the enrichment analysis on Gene ontology items and KEGG pathways, these candidate HCC markers were more frequently involved in cellular protein metabolic processes, translational elongation and intracellular signaling cascade, which are associated with cancer development and   metastasis  . Among 331 candidate HCC markers, the three AKT kinase family members (AKT1-AKT3) were selected for clinical validation by immunohistochemistry analysis using 130 HCC specimens and matched adjacent non-neoplastic liver tissues. Interestingly, the upregulation of AKT1, AKT2 and AKT3 proteins were all significantly associated with tumor aggressiveness and poor prognosis in patients with HCC.this study provided an integrated analysis by combining expression profile and interaction network analysis to identify a list of biologically significant HCC related markers and pathways. Further experimental validation also indicated that AKT1, AKT2 and AKT3 proteins may all be novel unfavorable prognostic factors for patients with HCC. 
 Akt2 is required for macrophage chemotaxis. Tumor-associated macrophages play an important role in tumorigenesis and   metastasis  . Trafficking of macrophages to the proximity of tumors is mediated by CSF-1, a growth factor. In this study, we investigated the role of PKB/Akt in CSF-1-induced macrophage migration. Disruption of Akt2 expression by small interference RNA impaired chemotaxis of both THP-1 cells and mouse peritoneal macrophages. Phosphorylation of PKCzeta, an essential component in chemotaxis signaling pathway, was reduced. LIMK/Cofilin, downstream of PKCzeta, regulated cytoskeleton rearrangement during cell migration. Disruption of Akt2 expression inhibited CSF-1-induced LIMK/Cofilin phosphorylation, which contributed to defects in actin polymerization and chemotaxis. Furthermore, MCP-1, a chemokine, -induced macrophage chemotaxis was also impaired. Taken together, our results demonstrated that Akt2 plays an essential role in both CSF-1- and chemokine-induced chemotaxis of macrophages. 
 Small ribosomal protein subunit S7 suppresses ovarian tumorigenesis through regulation of the PI3K/AKT and MAPK pathways. Small ribosomal protein subunit S7 (RPS7) has been reported to be associated with various malignancies, but the role of RPS7 in ovarian cancer remains unclear. In this study, we found that silencing of RPS7 by a specific shRNA promoted ovarian cancer cell proliferation, accelerated cell cycle progression, and slightly reduced cell apoptosis and response to cisplatin treatment. Knockdown of RPS7 resulted in increased expression of P85α, P110α, and AKT2. Although the basal levels of ERK1/2, MEK1/2, and P38 were inconsistently altered in ovarian cancer cells, the phosphorylated forms of MEK1/2 (Ser217/221), ERK1/2 (Thr202/Tyr204), JNK1/2 (Thr183/Tyr185), and P38 (Thr180/Tyr182) were consistently reduced after RPS7 was silenced. Both the in vitro anchorage-independent colony formation and in vivo animal tumor formation capability of cells were enhanced after RPS7 was depleted. We also showed that silencing of RPS7 enhanced ovarian cancer cell migration and invasion. In sum, our results suggest that RPS7 suppresses ovarian tumorigenesis an  d metastas  is through PI3K/AKT and MAPK signal pathways. Thus, RPS7 may be used as a potential marker for diagnosis and treatment of ovarian cancer. 
 MiRNA-615-5p functions as a tumor suppressor in pancreatic ductal adenocarcinoma by targeting AKT2. Aberrant microRNA (miRNA) expression is associated with tumor development. This study aimed to elucidate the role of miR-615-5p in the development of pancreatic ductal adenocarcinoma (PDAC).Locked nucleic acid in situ hybridization (LNA-ISH) was performed to compare miR-615-5p expression in patients between PDAC and matched adjacent normal tissues. Effects of miR-615-5p overexpression on cell proliferation, apoptosis, colony formation, migration, and invasion were determined in the pancreatic cancer cell lines PANC-1 and MIA PaCa-2. Effects of miR-615-5p on AKT2 were examined by dual-luciferase reporter assay. Lentivirus expressing miR-615 was used to create stable overexpression cell lines, which were subsequently used in mouse xenograft and   metastasis   models to assess tumor growth, apoptosis and   metastasis  .miR-615-5p expression was significantly lower in PDAC than in adjacent normal tissues. Low levels of miR-615-5p were independently associated with poor prognosis (HR: 2.243, 95% CI: 1.190-4.227, P=0.013). AKT2 protein expression was inversely correlated with miR-615-5p expression (r=-0.3, P=0.003). miR-615-5p directly targeted the 3'-untranslated region of AKT2 mRNA and repressed its expression. miR-615-5p overexpression inhibited pancreatic cancer cell proliferation, migration, and invasion in vitro, and tumor growth and   metastasis   in vivo. Furthermore, miR-615-5p overexpression also induced pancreatic cancer cell apoptosis both in vitro and in vivo.These results show that miR-615-5p inhibits pancreatic cancer cell proliferation, migration, and invasion by targeting AKT2. The data implicate miR-615-5p in the prognosis and treatment of PDAC. 
 Different functions of AKT1 and AKT2 in molecular pathways, cell migration and metabolism in colon cancer cells. AKT is a central protein in many cellular pathways such as cell survival, proliferation, glucose uptake, metabolism, angiogenesis, as well as radiation and drug response. The three isoforms of AKT (AKT1, AKT2 and AKT3) are proposed to have different physiological functions, properties and expression patterns in a cell type-dependent manner. As of yet, not much is known about the influence of the different AKT isoforms in the genome and their effects in the metabolism of colorectal cancer cells. In the present study, DLD-1 isogenic AKT1, AKT2 and AKT1/2 knockout colon cancer cell lines were used as a model system in conjunction with the parental cell line in order to further elucidate the differences between the AKT isoforms and how they are involved in various cellular pathways. This was done using genome wide expression analyses, metabolic profiling and cell migration assays. In conclusion, downregulation of genes in the cell adhesion, extracellular matrix and Notch-pathways and upregulation of apoptosis and   metastasis   inhibitory genes in the p53-pathway, confirm that the knockout of both AKT1 and AKT2 will attenuate   metastasis   and tumor cell growth. This was verified with a reduction in migration rate in the AKT1 KO and AKT2 KO and most explicitly in the AKT1/2 KO. Furthermore, the knockout of AKT1, AKT2 or both, resulted in a reduction in lactate and alanine, suggesting that the metabolism of carbohydrates and glutathione was impaired. This was further verified in gene expression analyses, showing downregulation of genes involved in glucose metabolism. Additionally, both AKT1 KO and AKT2 KO demonstrated an impaired fatty acid metabolism. However, genes were upregulated in the Wnt and cell proliferation pathways, which could oppose this effect. AKT inhibition should therefore be combined with other effectors to attain the best effect. 
 The prognostic impact of Akt isoforms, PI3K and PTEN related to female steroid hormone receptors in soft tissue sarcomas. The PI3K/Akt pathway is involved in cellular survival pathways by inhibiting apoptotic processes and stimulating cell growth and proliferation. Its negative prognostic value has been proven in many types of cancer. In soft tissue sarcomas, the expression profiles of the PI3K/Akt pathway components are poorly defined and their significance uncertain. We aimed to investigate the prognostic impact of Akt (Akt1) phosphorylated at threonine308 and serine473, Akt2, Akt3, PI3K and PTEN, alone and in coexpression with ER and PgR in non-gastrointestinal stromal tumor soft tissue sarcomas (non-GIST STSs).Tumor samples and clinical data from 249 patients with non-GIST STS were obtained, and tissue microarrays (TMAs) were constructed. Immunohistochemistry (IHC) was used to evaluate marker expression in tumor cells.In univariate analyses, the expression levels of p-Akt Thr308 (P = 0.002), Akt2 (P = 0.008) and PI3K (P < 0.001) were significant prognostic factors. In the multivariate analysis, high PI3K expression was an independent negative prognosticator (HR = 1.5, 95% CI = 1.0-2.2, P = 0.042) in addition to advanced age, tumor depth, high malignancy grade,   metastasis   at diagnosis, surgery and positive resection margins. p-Akt Thr308 expression had strong unfavorable effect in men only (P = 0.009). In contrast, p-Akt Ser473 expression had strong unfavorable impact in women (P = 0.023). PgR-/p-Akt Ser473+ phenotype tended to have less favorable impact in women (P = 0.087), but was the most favorable one in men (P = 0.010).Expression of PI3K was significantly associated with aggressive behavior and shorter DSS in non-GIST STSs. The site of Akt phosphorylation seems to have gender-dependent impact on survival in STS patients. 
 Twist transcriptionally up-regulates AKT2 in breast cancer cells leading to increased migration, invasion, and resistance to paclitaxel.   Metastasis  , the cardinal feature of malignant tumors, is an important clinical variable in patient prognosis. To understand the basis for   metastasis  , we systematically selected for highly invasive cells from breast cancer cell lines, MCF7 and MDA-MB-453, with moderate to low invasive ability using Boyden chamber invasion assay. The four-cycle selected invasive lines, named MCF7-I4 and MDA-MB-453-I4, respectively, displayed epithelial-mesenchymal transition (EMT) and dramatically enhanced invasive ability. EMT changes were corroborated with decreased level of E-cadherin and increased vimentin, fibronectin, and beta(1) integrin. Twist, a basic helix-loop-helix transcription factor, and AKT2, a known proto-oncogene, were found to be elevated in the invasive cells compared with the parental. Ectopic expression and knockdown of Twist by short interference RNA resulted in significant increase and reduction, respectively, of AKT2 protein and mRNA expression. Twist bound to E-box elements on AKT2 promoter and enhanced its transcriptional activity. Moreover, silencing AKT2 decreased Twist-promoted migration, invasion, and paclitaxel resistance. Reintroducing AKT2 largely rescued the phenotype resulted from knockdown of Twist in I4 cells, suggesting that AKT2 is a downstream target and functional mediator of Twist. Finally, we observed a 68.8% correlation of elevated Twist and AKT2 expression in late-stage breast cancers as oppose to 13% in early-stage breast cancers. Our study identifies Twist as a positive transcriptional regulator of AKT2 expression, and Twist-AKT2 signaling is involved in promoting invasive ability and survival of breast cancer cells. 
 Angiopoietin-2, an angiogenic regulator, promotes initial growth and survival of breast cancer metastases to the lung through the integrin-linked kinase (ILK)-AKT-B cell lymphoma 2 (Bcl-2) pathway. The early onsets of breast cancer   metastasis   involve cell retention, survival, and resistant to apoptosis and subsequent growth at target vascular beds and tissues in distant organs. We previously reported that angiopoietin-2 (Ang2), an angiogenic regulator stimulates MCF-7 breast tumor   metastasis   from their orthotopic sites to distant organs through the α(5)β(1) integrin/integrin-linked kinase (ILK)/Akt pathway. Here, by using an experimental tumo  r metastas  is model and in vitro studies, we further dissect the underlying mechanism by which Ang2 promotes the initial growth and survival of MCF-7 breast cance  r metastas  is in the lung of animals. We show that Ang2 increases cell survival and suppresses cell apoptosis through ILK-induced phosphorylation of Akt1, Akt2, and up-regulation of Bcl-2 in breast cancer cells. Inhibition of ILK, Akt1, and Akt2, and their effector Bcl-2 diminishes Ang2-stimulated breast cancer cell survival and Ang2-attenuated apoptosis in vitro, and initial survival and growth of breast cance  r metastas  is in the lung of animals. Additionally, siRNA knockdown of endogenous Ang2 in three human metastatic breast cancer cell lines also inhibits phosphorylation of Akt, expression of Bcl-2, and tumor cell survival, migration, and increases cell apoptosis. Since increased expression of Ang2 correlates with elevated potential of human breast cance  r metastas  is in clinic, our data underscore the importance that up-regulated Ang2 not only stimulates breast cancer growth an  d metastas  is at late stages of the process, but is also critical at the initiating stages of metastases onset, thereby suggesting Ang2 as a promising therapeutic target for treating patients with metastatic breast cancer. 
 Akt2/ZEB2 may be a biomarker for exfoliant cells in ascitic fluid in advanced grades of serous ovarian carcinoma. Ovarian cancers present a mild clinical course when diagnosed early but an aggressive pathway when diagnosed in the peri- and postmenopausal periods. However, the predictability of tumor progression is stochastic and is difficult to predict. In the present study, we hypothesized to examine the key pathways that are dysregulated to promote epithelial-mesenchymal transition in serous ovarian carcinoma. Examination of these steps would help to identify ascitic fluid with cells poised for   metastasis   or otherwise. We focused on examining the Akt2 expression, mainly because of its report as being overamplified in the aggressive variants of ovarian cancer, as well as TGFbeta-sensitivity of Akt2 that forms the key basis for   metastasis   initiation of most kinds of carcinoma. We obtained primary ovarian carcinoma samples as well as ascitic fluid and distantly metastatic ovarian carcinoma to examine the expression of Akt2. The results of the study demonstrated that in malignant exfoliated ovarian cancer cells, Smad4 expression was tremendously increased in the nuclei, suggesting nuclear translocation of Smad, which thereafter may have activated ZEB2, and thereafter genomically affected the expression of E-cadherin, myosin II, and vimentin, key components for initiating and sustaining   metastasis  . All of these may have been stimulated by increased cellular expression of Akt2 in metastatic variants of the serous ovarian carcinoma. The reliance on Akt2 and TGF beta signaling may also potentiate the case for Akt inhibitors or small molecule inhibitors of TGFbeta signaling like doxycycline as adjunct chemotherapy in serous ovarian carcinoma, especially the metastatic variants. 
 Vimentin is a novel AKT1 target mediating motility and invasion. The PI3K/AKT signaling pathway is aberrant in a wide variety of cancers. Downstream effectors of AKT are involved in survival, growth and metabolic-related pathways. In contrast, contradictory data relating to AKT effects on cell motility and invasion, crucial prometastatic processes, have been reported pointing to a potential cell type and isoform type-specific AKT-driven function. By implication, study of AKT signaling should optimally be conducted in an appropriate intracellular environment. Prognosis in soft-tissue sarcoma (STS), the aggressive malignancies of mesenchymal origin, is poor, reflecting our modest ability to control   metastasis  , an effort hampered by lack of insight into molecular mechanisms driving STS progression and dissemination. We examined the impact of the cancer progression-relevant AKT pathway on the mesenchymal tumor cell internal milieu. We demonstrate that AKT1 activation induces STS cell motility and invasiveness at least partially through a novel interaction with the intermediate filament vimentin (Vim). The binding of AKT (tail region) to Vim (head region) results in Vim Ser39 phosphorylation enhancing the ability of Vim to induce motility and invasion while protecting Vim from caspase-induced proteolysis. Moreover, vimentin phosphorylation was shown to enhance tumor and   metastasis   growth in vivo. Insights into this mesenchymal-related molecular mechanism may facilitate the development of critically lacking therapeutic options for these devastating malignancies. 
 Reduction of Akt2 expression inhibits chemotaxis signal transduction in human breast cancer cells. Protein kinase Czeta PKCzeta mediates cancer cell chemotaxis by regulating cytoskeleton rearrangement and cell adhesion. In the research for its upstream regulator, we investigated the role of Akt2 in chemotaxis and   metastasis   of human breast cancer cells. Reduction of Akt2 expression by siRNA inhibited chemotaxis of MDA-MB-231, T47D, and MCF7 cells, three representative human breast cancer cells. Expression of a wild type Akt2 in siRNA transfected cells rescued the phenotype. EGF-induced integrin beta1 phosphorylation was dampened, consistent with defects in adhesion. Phosphorylation of LIMK and cofilin, a critical step of cofilin recycle and actin polymerization, was also impaired. Thus, Akt2 regulates both cell adhesion and cytoskeleton rearrangement during chemotaxis. Depletion of Akt2 by siRNA impaired the activation of PKCzeta while inhibition of PKCzeta did not interfere with EGF induced phosphorylation of Akt. Furthermore, EGF induced co-immunoprecipitation between PKCzeta and Akt2, but not Akt1, suggesting that a direct interaction between PKCzeta and Akt2 in chemotaxis. Protein levels of integrin beta1, LIMK, cofilin, and PKCzeta didn't alter, suggesting that Akt2 does not regulate the expression of these signaling molecules. In a Severe Combine Immunodeficiency mouse model, Akt2 depleted MDA-MB-231 cells showed a marked reduction in   metastasis   to mouse lungs, demonstrating the biological relevancy of Akt2 in cancer   metastasis   in vivo. Taken together, our results suggest that Akt2 directly mediates EGF-induced chemotactic signaling pathways through PKCzeta and its expression is critical during the extravasation of circulating cancer cells. 
 Significance of Akt activation and AKT gene increases in soft tissue tumors. To clarify the aberrations of AKT genes, their protein products and clinicopathologic significance in bone and soft tissue tumors, expression profiles of total Akt, its isoforms and activated Akt, and increases in copy number of AKT1/AKT2 genes were examined. Immunohistochemical analysis in 77 cases revealed overexpression of total Akt, Akt1, Akt2, and phosphorylated Akt in 84.4%, 67.5%, 72.7%, and 71.4%, respectively. Positive results were also observed in benign lesions but at a lower frequency. Overexpression of Akt1 was more frequent than that of Akt2 in well-differentiated liposarcoma (6/7 versus 3/7 cases) and schwannoma (4/4 versus 1/4 cases), whereas Akt2 overexpression and Akt activation were more frequent than Akt1 overexpression in malignant nerve sheath (3/4 and 4/4, respectively, versus 2/4 cases) and muscular tumors (8/9 and 8/9 versus 4/9 cases). By fluorescence in situ hybridization analysis, increase of gene copy number was observed in 13.3% for AKT1 and in 25.0% for AKT2 due to polysomy of chromosome 14 or 19, respectively, but not gene amplification. One case of schwannoma exhibited polysomy of both chromosomes 14 and 19. Akt activation was correlated with total Akt cytoplasmic localization (P = .0031) and subsequent   metastasis   (P = .0454). Moreover, AKT2 gene increase correlated with tumor size (P = .0352) and   metastasis   (P = .0344). In conclusion, in a defined subset of bone and soft tissue tumors, including benign tumors, Akt was frequently overexpressed and activated, and AKT1/2 copy number was increased. Because abnormality of Akt/AKT correlated with clinicopathologic profiles, novel therapies targeting isoform-specific Akts may be useful for these particular types of tumors. 
  GeneRIFs  
 miR-302b inhibits SMMC-7721 cell invasion and   metastasis   by targeting AKT2 
 miR-137 which is frequently down-regulated in gastric cancer is potentially involved in gastric cancer tumorigenesis and   metastasis   by regulating AKT2 related signal pathways 
 The positive rate of AKT2 protein expression in laryngeal squamous cell carcinoma was significantly higher than that in peri-cancer tissue and normal laryngeal epithelium, and was correlated with tumor site, lymph node   metastasis   and clinical stage. 
 Akt2 up-regulates beta1 integrins & promotes adhesion & invasion of breast cancer cells in vitro & also   metastasis   in vivo. It opposes Akt1. Review. 
 the consequence of PTEN loss and Akt2 overexpression function synergistically to promote   metastasis   
 data indicate that AKT2 mediates PI3-K-dependent effects on adhesion, motility, invasion, and   metastasis   in vivo 
  [Cc]ell [Ii]nvasion  PubMed  
 miR-302b suppresses   cell invasion   and metastasis by directly targeting AKT2 in human hepatocellular carcinoma cells. MicroRNAs (miRNAs) have been shown to play essential roles in regulating the activity of human hepatocellular carcinoma (HCC) cells, thereby contributing to the suppression of invasion and metastasis. In this study, using gain and loss of function assays, we demonstrated that miR-302b was frequently down-regulated in clinical HCC specimens, as compared with 15 corresponding adjacent normal tissues. Overexpression of miR-302b suppressed HCC   cell invasion   and metastasis. Regulation of NF-κB and matrix metalloproteinase (MMP)-2 expression by miR-302b was mediated via AKT2 in SMMC-7721 cells. Silencing AKT2 produced effects similar to those of miR-302b overexpression, which included inhibiting SMMC-7721   cell invasio  n and metastasis and dereasing NF-κB and MMP-2 expression. Furthermore, overexpression of AKT2 attenuated the effects of miR-302b overexpression. Taken together, our findings indicate that miR-302b inhibits SMMC-772  1 cell invasi  on and metastasis by targeting AKT2, suggesting that miR-302b might represent a potential therapeutic target for HCC intervention. 
 let-7b/g silencing activates AKT signaling to promote gastric carcinogenesis. Aberrant AKT activation contributes to gastric cancer cell survival and chemotherapy resistance, however its regulation is poorly understood. microRNAs have been established to be important regulators in gastric carcinogenesis. Here, we showed the functional role and putative target of let-7b and let-7g (let-7b/g) in gastric carcinogenesis.The expression of let-7b/g in gastric cancer cell lines and primary tumors were evaluated by miRNA qRT-PCR. The putative target gene of let-7b/g was explored by TargetScan followed by further validation. Functional analyses including MTT proliferation, monolayer colony formation,   cell invasion   assays and in vivo study were performed in both ectopic expression and knockdown approaches.let-7b/g was found down-regulated in gastric cancer and its downregulation was associated with poor survival and correlated with lymph node metastasis. let-7b/g inhibited AKT2 expression by directly binding to its 3'UTR, reduced p-AKT (S473) activation and suppressed expression of the downstream effector pS6. AKT2 mRNA expression showed negative correlation with the expression of let-7b/g in primary tumors. Short interfering RNA (siRNA) mediated knockdown of AKT2 phenocopied the tumor-suppressive effects of let-7b/g. Moreover, AKT2 re-expression partly abrogated the growth-inhibitory effect of let-7b/g.In conclusion, our findings reveal decreased let-7b/g contributes to aberrant AKT activation in gastric tumorigenesis and provide a potential therapeutic strategy for gastric cancer. 
 Antisense and dominant-negative AKT2 cDNA inhibits glioma   cell invasion  . The aim of this study was to explore the potential role of AKT2 in glioma   cell invasion  . Therefore, dominant-negative (DN-AKT2) and antisense AKT2 constructs (AS-AKT2) were transfected into rat C6 glioma cells with elevated endogenous AKT2 expression. In situ hybridization and Western blot analysis were used to identify AKT2 expression. Spheroid culturing was used to assess cell migration and invasion in Matrigel from spheroids. Cell motility and invasion were also evaluated by scratch and Transwell invasion assays, respectively. The secretion of matrix metalloproteinases (MMPs), MMP2 and MMP9, was determined by gelatin zymography. AKT2 expression was inhibited in C6 cells transfected with AS-AKT2 but did not significantly change in cells transfected with DN-AKT2. The cell migration distance from spheroids or the number of cells migrating into the acellular space created by scratching was reduced in cells transfected with DN-AKT2 or AS-AKT2 compared to the control cells. The invasive distance of cells from the spheroids in Matrigel sandwich and the number of invading cells through the Matrigel were also decreased in the DN-AKT2- and AS-AKT2-transfected cells. Gelatin zymography showed that the production of MMP2 and MMP9 was inhibited in transfected cells. In conclusion, AKT2 plays an important role in glioma cell motility and invasion. Therapy based on AKT inhibition may complement currently available treatment to control glioma   cell invasion  . 
 Akt2 and acid ceramidase cooperate to induce   cell invasion   and resistance to apoptosis. Both Akt 2 and acid ceramidase (ASAH1) are found aberrantly overexpressed in cancer cells, but whether these two enzymes cooperate to induce malignant transformation is not known. We found that in immortalized, non-transformed cells, ectopic co-expression of Akt2 and ASAH1 is significantly more effective than expression of each gene alone at inducing   cell invasion   and at conferring resistance to apoptosis. Consistent with these observations, siRNA-mediated depletion of both Akt2 and ASAH1 is much more potent than depleting each alone at inhibiting cell viability/proliferation and   cell invasion  . Furthermore, pharmacological inhibitors of Akt (TCN or MK-2206) and ASAH1 (B13) synergize to inhibit cell viability/proliferation, and combinations of these drugs are more effective than single-agent treatments at inhibiting   cell invasion  . Taken together, the results suggest that these two enzymes cooperate to induce malignant transformation and warrant further preclinical studies to evaluate the potential of combining inhibitors of Akt and ASAH1 to treat cancer. 
  GeneRIFs  
 miR-302b inhibits SMMC-7721   cell invasion   and metastasis by targeting AKT2 
   
  
 RAB7A 
 
  NCBI Gene  
  GeneRIF  
  Pubmed  
 
 
  Search Term  Rank  Fields  
  [Mm]igration  6  PubMed(2); GeneRIFs(1)  
  [Ii]nvasive  10  PubMed(2)  
  [Mm]etastasis  12  PubMed(1)  
  [Cc]ell [Ii]nvasion  13  PubMed(1)  
 
 
 hide/show details for RAB7A 
  [Mm]igration  PubMed  
 Rab GTPase regulation of VEGFR2 trafficking and signaling in endothelial cells. Vascular endothelial growth factor receptor 2 (VEGFR2) is a receptor tyrosine kinase that regulates vascular physiology. However, mechanism(s) by which VEGFR2 signaling and trafficking is coordinated are not clear. Here, we have tested endocytic Rab GTPases for regulation of VEGFR2 trafficking and signaling linked to endothelial cell   migration  .Quiescent VEGFR2 displays endosomal localization and colocalization with the Rab5a GTPase, an early endosome fusion regulator. Expression of GTP or GDP-bound Rab5a mutants block activated VEGFR2 trafficking and degradation. Manipulation of Rab7a GTPase activity associated with late endosomes using overexpression of wild-type or mutant proteins blocks activated VEGFR2 trafficking and degradation. Depletion of Rab7a decreased VEGFR2 Y1175 phosphorylation but increased p42/44 (pERK1/2) MAPK phosphorylation. Endothelial cell   migration   was increased by Rab5a depletion but decreased by Rab7a depletion.Rab5a and Rab7a regulate VEGFR2 trafficking toward early and late endosomes. Our data suggest that VEGFR2-mediated regulation of endothelial function is dependent on different but specific Rab-mediated GTP hydrolysis activity required for endosomal trafficking. 
 rab7 activity affects epidermal growth factor:epidermal growth factor receptor degradation by regulating endocytic trafficking from the late endosome. The epidermal growth factor receptor (EGFR) is a member of the receptor tyrosine kinase family. Ligand (epidermal growth factor or EGF) binding to the EGFR results in the coordinated activation and integration of biochemical signaling events to mediate cell growth,   migration  , and differentiation. One mechanism the cell utilizes to orchestrate these events is ligand-mediated endocytosis through the canonical clathrin-mediated endocytic pathway. Identification of proteins that regulate the intracellular movement of the EGF.EGFR complex is an important first step in dissecting how specificity of EGFR signaling is conferred. We examined the role of the small molecular weight guanine nucleotide-binding protein (G-protein) rab7 as a regulator of the distal stages of the endocytic pathway. Through the transient expression of activating and inactivating mutants of rab7 in HeLa cells, we have determined that rab7 activity directly correlates with the rate of radiolabeled EGF and EGFR degradation. Furthermore, when inhibitory mutants of rab7 are expressed, the internalized EGF.EGFR complex accumulates in high-density endosomes that are characteristic of the late endocytic pathway. Thus, we conclude that rab7 regulates the endocytic trafficking of the EGF.EGFR complex by regulating its lysosomal degradation. 
  GeneRIFs  
 Rab GTPase regulation of VEGFR2 trafficking and signaling in endothelial cells; Endothelial cell   migration   was increased by Rab5a depletion but decreased by Rab7a depletion 
  [Ii]nvasive  PubMed  
 Novel RING E3 ubiquitin ligases in breast cancer. Defects in ubiquitin E3 ligases are implicated in the pathogenesis of several human diseases, including cancer, because of their central role in the control of diverse signaling pathways. RING E3 ligases promote the ubiquitination of proteins that are essential to a variety of cellular events. Identification of which ubiquitin ligases specifically affect distinct cellular processes is essential to the development of targeted therapeutics for these diseases. Here we discuss two novel RING E3 ligases, BCA2 and RNF11, that are closely linked to human breast cancer. BCA2 E3 ligase is coregulated with estrogen receptor and plays a role in the regulation of epidermal growth factor receptor (EGF-R) trafficking. RNF11 is a small RING E3 ligase that affects transforming growth factorbeta and EGF-R signaling and is overexpressed in   invasive   breast cancers. These two proteins demonstrate the complexity of RING E3 ligase interactions in breast cancer and are potential targets for therapeutic interventions. 
 Supporting a role for the GTPase Rab7 in prostate cancer progression. Invasion and subsequent metastasis is the major cause of death from most cancers including prostate cancer. Herein we report on the potential tumor suppressive properties of Rab7, a GTPase that regulates trafficking of lysosomes. The movement of lysosomes to the cell surface in response to environmental cues increases the secretion of proteinases and cell invasion. We determined that Troglitazone and other members of the Thiazolidinedione family inhibit cell-surface directed lysosome trafficking and cathepsin B secretion through a Rab7-dependent mechanism. Moreover, Rab7 shRNA expressing cells were found to be more   invasive   in vitro and in vivo. Increased   invasive  ness was accompanied by elevated expression of the c-Met receptor and prolonged downstream signaling, thereby supporting a role for Rab7 as a mediator of signaling down-regulation. Taken together, these results suggested that Rab7 acts as a negative regulator of prostate tumor growth and invasion, providing further evidence for its potential as a tumor suppressor. 
  [Mm]etastasis  PubMed  
 Supporting a role for the GTPase Rab7 in prostate cancer progression. Invasion and subsequent   metastasis   is the major cause of death from most cancers including prostate cancer. Herein we report on the potential tumor suppressive properties of Rab7, a GTPase that regulates trafficking of lysosomes. The movement of lysosomes to the cell surface in response to environmental cues increases the secretion of proteinases and cell invasion. We determined that Troglitazone and other members of the Thiazolidinedione family inhibit cell-surface directed lysosome trafficking and cathepsin B secretion through a Rab7-dependent mechanism. Moreover, Rab7 shRNA expressing cells were found to be more invasive in vitro and in vivo. Increased invasiveness was accompanied by elevated expression of the c-Met receptor and prolonged downstream signaling, thereby supporting a role for Rab7 as a mediator of signaling down-regulation. Taken together, these results suggested that Rab7 acts as a negative regulator of prostate tumor growth and invasion, providing further evidence for its potential as a tumor suppressor. 
  [Cc]ell [Ii]nvasion  PubMed  
 Supporting a role for the GTPase Rab7 in prostate cancer progression. Invasion and subsequent metastasis is the major cause of death from most cancers including prostate cancer. Herein we report on the potential tumor suppressive properties of Rab7, a GTPase that regulates trafficking of lysosomes. The movement of lysosomes to the cell surface in response to environmental cues increases the secretion of proteinases and   cell invasion  . We determined that Troglitazone and other members of the Thiazolidinedione family inhibit cell-surface directed lysosome trafficking and cathepsin B secretion through a Rab7-dependent mechanism. Moreover, Rab7 shRNA expressing cells were found to be more invasive in vitro and in vivo. Increased invasiveness was accompanied by elevated expression of the c-Met receptor and prolonged downstream signaling, thereby supporting a role for Rab7 as a mediator of signaling down-regulation. Taken together, these results suggested that Rab7 acts as a negative regulator of prostate tumor growth and invasion, providing further evidence for its potential as a tumor suppressor. 
   
  
 ADAM10 
 
  NCBI Gene  
  GeneRIF  
  Pubmed  
 
 
  Search Term  Rank  Fields  
  [Mm]igration  6  Process(1); PubMed(46); GeneRIFs(19)  
  [Ee]xtracellular [Mm]atrix  8  Process(1); PubMed(7)  
  [Ii]nvasive  10  PubMed(14); GeneRIFs(2)  
  [Mm]etastasis  12  PubMed(23); GeneRIFs(6)  
  [Cc]ell [Ii]nvasion  13  PubMed(6)  
 
 
 hide/show details for ADAM10 
  [Mm]igration  Process  
 positive regulation of cell   migration   
  PubMed  
 Analysis of the myosin-II-responsive focal adhesion proteome reveals a role for β-Pix in negative regulation of focal adhesion maturation. Focal adhesions undergo myosin-II-mediated maturation wherein they grow and change composition to modulate integrin signalling for cell   migratio  n, growth and differentiation. To determine how focal adhesion composition is affected by myosin II activity, we performed proteomic analysis of isolated focal adhesions and compared protein abundance in focal adhesions from cells with and without myosin II inhibition. We identified 905 focal adhesion proteins, 459 of which changed in abundance with myosin II inhibition, defining the myosin-II-responsive focal adhesion proteome. The abundance of 73% of the proteins in the myosin-II-responsive focal adhesion proteome was enhanced by contractility, including proteins involved in Rho-mediated focal adhesion maturation and endocytosis- and calpain-dependent focal adhesion disassembly. During myosin II inhibition, 27% of proteins in the myosin-II-responsive focal adhesion proteome, including proteins involved in Rac-mediated lamellipodial protrusion, were enriched in focal adhesions, establishing that focal adhesion protein recruitment is also negatively regulated by contractility. We focused on the Rac guanine nucleotide exchange factor β-Pix, documenting its role in the negative regulation of focal adhesion maturation and the promotion of lamellipodial protrusion and focal adhesion turnover to drive cel  l migrati  on. 
 TLR4-mediated galectin-1 production triggers epithelial-mesenchymal transition in colon cancer cells through ADAM10- and ADAM17-associated lactate production. Toll-like receptor 4 (TLR4) activation is a key contributor to the carcinogenesis of colon cancer. Overexpression of galectin-1 (Gal-1) also correlates with increased invasive activity of colorectal cancer. Lactate production is a critical predictive factor of risk of metastasis, but the functional relationship between intracellular lactate and Gal-1 expression in TLR4-activated colon cancer remains unknown. In this study, we investigated the underlying mechanism and role of Gal-1 in metastasis and invasion of colorectal cancer (CRC) cells after TLR4 stimulation. Exposure to the TLR4 ligand lipopolysaccharide (LPS) increased expression of Gal-1, induced EMT-related cytokines, triggered the activation of glycolysis-related enzymes, and promoted lactate production. Gene silencing of TLR4 and Gal-1 in CRC cells inhibited lactate-mediated epithelial-mesenchymal transition (EMT) after TLR4 stimulation. Gal-1-mediated activation of a disintegrin and metalloproteinase 10 (ADAM10) and ADAM 17 increased the invasion activity and expression of mesenchymal characteristics in LPS-activated CRC cells. Conversely, inhibition of ADAM10 or ADAM17 effectively blocked the generation of lactate and the   migration   capacity of LPS-treated CRC cells. Thus, the TLR4/Gal-1 signaling pathway regulates lactate-mediated EMT processes through the activation of ADAM10 and ADAM17 in CRC cells. 
 ADAM10 controls collagen signaling and cell   migration   on collagen by shedding the ectodomain of discoidin domain receptor 1 (DDR1). Discoidin domain receptor 1 (DDR1) is a receptor tyrosine kinase that binds and transmits signals from various collagens in epithelial cells. However, how DDR1-dependent signaling is regulated has not been understood. Here we report that collagen binding induces ADAM10-dependent ectodomain shedding of DDR1. DDR1 shedding is not a result of an activation of its signaling pathway, since DDR1 mutants defective in signaling were shed in an efficient manner. DDR1 and ADAM10 were found to be in a complex on the cell surface, but shedding did not occur unless collagen bound to DDR1. Using a shedding-resistant DDR1 mutant, we found that ADAM10-dependent DDR1 shedding regulates the half-life of collagen-induced phosphorylation of the receptor. Our data also revealed that ADAM10 plays an important role in regulating DDR1-mediated cell adhesion to achieve efficient cell   migration   on collagen matrices. 
 ADAM10 overexpression in human non-small cell lung cancer correlates with cell   migration   and invasion through the activation of the Notch1 signaling pathway. A disintegrin and metalloproteinase 10 (ADAM10) was identified as a key protease in the ectodomain shedding of various substrates, such as Notch1 protein, ErbB2 and E-cadherin, which are important in the development of non-small cell lung cancer (NSCLC). The aim of this study was to investi-gate the role of ADAM10 in NSCLC metastasis.We characterized the expression of ADAM10 and Notch1 in human NSCLC tissues in vivo. Immunohistochemical analysis indicated that ADAM10 expression was significantly increased in the NSCLC tissues, particularly in the metastatic tissues. Futhermore, ADAM10 overexpression positively correlated with Notch1 expression in the NSCLC tissues. The in vitro downregulation of ADAM10 expression using ADAM10 short hairpin RNA (shRNA) reduced the   migration   and invasion of NSCLC cells. We present further evidence that ADAM10 promotes NSCLC cell   migration   and invasion via the activation of the Notch1 signaling pathway. Taken together, our results suggest that ADAM10 may serve as a potential target for the therapeutic intervention of NSCLC metastasis. The data provided in this study may aid in the further understanding of the function of ADAM10 in the progression of NSCLC and open new perspectives for the diagnosis and treatment of NSCLC. 
 ADAM10 regulates endothelial permeability and T-Cell trans  migration   by proteolysis of vascular endothelial cadherin. Vascular endothelial (VE)-cadherin is the major adhesion molecule of endothelial adherens junctions. It plays an essential role in controlling endothelial permeability, vascular integrity, leukocyte trans  migration  , and angiogenesis. Elevated levels of soluble VE-cadherin are associated with diseases like coronary atherosclerosis. Previous data showed that the extracellular domain of VE-cadherin is released by an unknown metalloprotease activity during apoptosis. In this study, we used gain-of-function analyses, inhibitor studies, and RNA interference experiments to analyze the proteolytic release of VE-cadherin in human umbilical vein endothelial cells (HUVECs). We found that VE-cadherin is specifically cleaved by the disintegrin and metalloprotease ADAM10 in its ectodomain, releasing a soluble fragment and generating a carboxyl-terminal membrane-bound stub, which is a substrate for a subsequent gamma-secretase cleavage. This ADAM10-mediated proteolysis could be induced by Ca(2+) influx and staurosporine treatment, indicating that ADAM10-mediated VE-cadherin cleavage contributes to the dissolution of adherens junctions during endothelial cell activation and apoptosis, respectively. In contrast, protein kinase C activation or inhibition did not modulate VE-cadherin processing. Increased ADAM10 expression was functionally associated with an increase in endothelial permeability. Remarkably, our data indicate that ADAM10 activity also contributes to the thrombin-induced decrease of endothelial cell-cell adhesion. Moreover, knockdown of ADAM10 in HUVECs as well as in T cells by small interfering RNA impaired T-cell trans  migration  . Taken together, our data identify ADAM10 as a novel regulator of vascular permeability and demonstrate a hitherto unknown function of ADAM10 in the regulation of VE-cadherin-dependent endothelial cell functions and leukocyte transendothelial   migration  . 
 ADAM 10 is over expressed in oral squamous cell carcinoma and contributes to invasive behaviour through a functional association with αvβ6 integrin. A disintegrin and metalloprotease (ADAM) proteins are upregulated in cancer and can interact with integrin receptors. We investigated whether such interactions may have functional significance in oral squamous cell carcinoma (OSCC). ADAM 10 expression was increased in OSCC tissue and cell lines compared to normal oral mucosa. Silencing of ADAM 10 reduce  d migrati  on and invasion specifically in OSCC cells over-expressing αvβ6 integrin. This may result from ADAM 10-induced up-regulation of MMPs. We conclude ADAM 10 may influence OSCC invasion by functionally interacting with αvβ6 integrin which we have previously shown is over expressed in OSCC. 
 Human mesenchymal stem cells induce E-cadherin degradation in breast carcinoma spheroids by activating ADAM10. Mesenchymal stem cells (MSCs) have been shown to communicate with tumor cells. We analyzed the effect of human MSCs (hMSCs) on breast cancer cells in three-dimensional cultures. By using GFP expression and immunohistochemistry, we show that hMSCs invade 3D breast cancer cell aggregates. hMSCs caused breast cancer spheroids to become disorganized which was accompanied by a disruption of cell-cell adhesion, E-cadherin cleavage, and nuclear translocation of E-cadherin, but not by epithelial/mesenchymal transition or by an increase in ERK1/2 activity. In addition, hMSCs enhanced the motility of breast cancer cells. Inhibition of ADAM10 (a disintegrin and metalloprotease 10), known to cleave E-cadherin, prevented both hMSC-mediated E-cadherin cleavage and enhanced   migration  . Our data suggest that hMSCs interfere with cell-cell adhesion and enhance   migration   of breast cancer cells by activating ADAM10. 
 TspanC8 tetraspanins regulate ADAM10/Kuzbanian trafficking and promote Notch activation in flies and mammals. The metalloprotease ADAM10/Kuzbanian catalyzes the ligand-dependent ectodomain shedding of Notch receptors and activates Notch. Here, we show that the human tetraspanins of the evolutionary conserved TspanC8 subfamily (Tspan5, Tspan10, Tspan14, Tspan15, Tspan17, and Tspan33) directly interact with ADAM10, regulate its exit from the endoplasmic reticulum, and that four of them regulate ADAM10 surface expression levels. In an independent RNAi screen in Drosophila, two TspanC8 genes were identified as Notch regulators. Functional analysis of the three Drosophila TspanC8 genes (Tsp3A, Tsp86D, and Tsp26D) indicated that these genes act redundantly to promote Notch signaling. During oogenesis, TspanC8 genes were up-regulated in border cells and regulated Kuzbanian distribution, Notch activity, and cell   migration  . Furthermore, the human TspanC8 tetraspanins Tspan5 and Tspan14 positively regulated ligand-induced ADAM10-dependent Notch1 signaling. We conclude that TspanC8 tetraspanins have a conserved function in the regulation of ADAM10 trafficking and activity, thereby positively regulating Notch receptor activation. 
 Notch1 Autoactivation via Transcriptional Regulation of Furin, Which Sustains Notch1 Signaling by Processing Notch1-Activating Proteases ADAM10 and Membrane Type 1 Matrix Metalloproteinase. Notch1 is an evolutionarily conserved transmembrane receptor involved in melanoma growth. Notch1 is first cleaved by furin in the Golgi apparatus to produce the biologically active heterodimer. Following ligand binding, Notch1 is cleaved at the cell membrane by proteases such as ADAM10 and -17 and membrane type 1 matrix metalloproteinase (MT1-MMP), the latter of which we recently identified as a novel protease involved in Notch1 processing. The final cleavage is γ-secretase dependent and releases the active Notch intracellular domain (NIC). We now demonstrate that Notch1 directly regulates furin expression. Aside from activating Notch1, furin cleaves and activates several proteases, including MT1-MMP, ADAM10, and ADAM17. By chromatin immunoprecipitation and a reporter assay, we demonstrate that Notch1 binds at position -1236 of the furin promoter and drives furin expression. The Notch1-dependent enhancement of furin expression increases the activities of MT1-MMP and ADAM10 but not that of ADAM17, as demonstrated by short hairpin RNA (shRNA) knockdown of furin, and promotes the cleavage of Notch1 itself. These data highlight a novel positive-feedback loop whereby Notch1-dependent furin expression can induce Notch1 signaling by increasing Notch1 processing and by potentiating the activity of the proteases responsible for Notch1 activation. This leads to Notch1 signal amplification, which can promote melanoma tumor growth and progression, as demonstrated by the inhibition of cell   migratio  n and invasion upon furin inhibition downstream of Notch1. Disruption of such feedback signaling might represent an avenue for the treatment of melanoma. 
 Knockdown of ADAM10 inhibits   migration   and invasion of fibroblast-like synoviocytes in rheumatoid arthritis. Rheumatoid arthritis (RA) is a systemic autoimmune disease with high rates of morbidity and mortality. Previous studies proposed that the A disintegrin and metalloprotease (ADAM) family is involved in the regulation of inflammation and arthritis. Thus, the present study investigated whether ADAM10 is involved in the progression of RA. The effects of ADAM10 small interfering (si)RNA on the expression levels of tumor necrosis factor (TNF)‑α, interleukin (IL)‑6, IL‑8 and chemokine (C-X-C motif) ligand 16 (CXCL16) were determined in fibroblast‑like synoviocytes (FLS). In addition, the effects of ADAM10 siRNA on cell proliferation, inva  sion and   migration in human RA‑FLS were assessed in vitro. The therapeutic efficacy and side‑effects of ADAM10 siRNA were examined in a mouse model of collagen‑induced arthritis (CIA). In vitro, ADAM10 silencing suppressed the expression of TNF‑α, IL‑6, IL‑8 and CXCL16 in lipopolysaccharide (LPS)‑stimulated human RA‑FLS. LPS‑in  duced RA�  ��FLS proliferation, migration and invasion were significantly attenuated by ADAM10 knockdown. ADAM10 silencing inhibited the secretion of vascular endothelial growth factor A (VEGF‑A) and matrix metalloproteinase (MMP)‑3 and ‑9 from LPS‑stimulated human RA‑FLS, in addition to inhibiting the phosphoinositide 3‑kinase/AKT activation in LPS‑stimulated human RA‑FLS. In vivo, treatment with siRNA against ADAM10 for three weeks reduced the arthritis score. Serum levels of VEGF‑A, MMP‑3 and MMP‑9 were also reduced in CIA mice. These observations indicate that the inhibition of ADAM10 may be a viable therapeutic target in the amelioration of disease   progressi  on in RA by attenuating FLS proliferation, migration and invasion. 
 ADAM10 mediates the cell invasion and metastasis of human esophageal squamous cell carcinoma via regulation of E-cadherin activity. A disintegrin and metalloprotease 10 (ADAM10) is involved in the tumorigenesis, invasion and metastasis of several types of solid tumors. However, the potential role of ADAM10 in human esophageal squamous cell carcinoma (ESCC) is not yet well understood. The present study showed that ADAM10 was overexpressed in human ESCC tissues in vivo, and positively associated with depth of tumor invasion, lymph node metastasis and TNM stage, contributing to tumor carcinogenesis, invasion and metastasis. Additionally, ADAM10 was overexpressed in 3 types of ESCC cell lines in vitro, as compared to that in normal esophageal epithelial cells (NEECs); and moreover, ESCC cells with high ADAM10 expression obtained enhanced invasion and   migration   ability. Subsequently, ADAM10 silencing by small interfering (si) RNA in ESCC cell line, EC-1, reduced cell invasion,   migration   and proliferation in vitro. Finally, ADAM10 negatively regulated E-cadherin in ESCC in vivo and in vitro. In conclusion, active ADAM10 promotes the carcinogenesis, invasion, metastasis and proliferation of ESCC and controls invasion and metastasis at least in part through the shedding of E-cadherin activity, which makes it a potential biomarker and a useful therapeutic target for ESCC. 
 Effects of ADAM10 upregulation on progression,   migration  , and prognosis of nasopharyngeal carcinoma. A disintegrin and metalloprotease 10 (ADAM10) is a typical member of the ADAMs family, which has been reported to be upregulated in various types of cancers and contribute to cancer progression and metastasis. However, little is known about the role of ADAM10 in nasopharyngeal carcinoma (NPC). The purpose of this study is to explore ADAM10 expression status and its biological functions in NPC. We first examined the expression of ADAM10 in NPC tissues and cell lines by immunohistochemistry, Western blotting, PCR, and immunofluorescence analysis. We observed that ADAM10 was significantly elevated in NPC and its expression level was correlated with T classification (P = 0.044), distant metastasis (P = 0.016), TNM clinical stage (P = 0.013), and proliferation marker Ki-67 expression (P = 0.001). Patients with NPC with high expression of ADAM10 had shorter overall survival rates. In addition, knockdown of ADAM10 by RNAi was found to inhibit the CNE-2 cell proliferation and   migration  . Our findings hinted that overexpression of ADAM10 promotes the progression and   migration   of NPC, which makes it a potential therapeutic target for the treatment of tumor metastases in NPC. 
 Amyloid precursor protein regulates   migration   and metalloproteinase gene expression in prostate cancer cells. Amyloid precursor protein (APP) is a type I transmembrane protein, and one of its processed forms, β-amyloid, is considered to play a central role in the development of Alzheimer's disease. We previously showed that APP is a primary androgen-responsive gene in prostate cancer and that its increased expression is correlated with poor prognosis for patients with prostate cancer. APP has also been implicated in several human malignancies. Nevertheless, the mechanism underlying the pro-proliferative effects of APP on cancers is still not well-understood. In the present study, we explored a pathophysiological role for APP in prostate cancer cells using siRNA targeting APP (siAPP). The proliferation and   migratio  n of LNCaP and DU145 prostate cancer cells were significantly suppressed by siAPP. Differentially expressed genes in siAPP-treated cells compared to control siRNA-treated cells were identified by microarray analysis. Notably, several metalloproteinase genes, such as ADAM10 and ADAM17, and epithelial-mesenchymal transition (EMT)-related genes, such as VIM, and SNAI2, were downregulated in siAPP-treated cells as compared to control cells. The expression of these genes was upregulated in LNCaP cells stably expressing APP when compared with control cells. APP-overexpressing LNCaP cells exhibited enhanced   migratio  n in comparison to control cells. These results suggest that APP may contribute to the proliferation and   migratio  n of prostate cancer cells by modulating the expression of metalloproteinase and EMT-related genes. 
 Cell   migration   in response to the amino-terminal fragment of urokinase requires epidermal growth factor receptor activation through an ADAM-mediated mechanism. Cell   migration   is an integral component of intimal hyperplasia development and proteases are pivotal in the process. Understanding the role of urokinase signaling within the cells of vasculature remains poorly defined. The study examines the role of amino-terminal fragment (ATF) of urokinase on a pivotal cross-talk receptor, epidermal growth factor receptor (EGFR). EGFR is transactivated by both G-protein-coupled receptors and receptor tyrosine kinases and is key to many of their responses. We hypothesize that A Disintegrin and Metalloproteinase Domains (ADAM) allows the transactivation of EGFR by ATF.To determine the role of ADAM in EGFR transactivation by ATF in human vascular smooth muscle cells (VSMC) during cell   migration  .Human coronary VSMC were cultured in vitro. Assays of EGFR phosphorylation were examined in response to ATF (10 nM) in the presence and absence of the matrix metalloprotease (MMP) inhibitor GM6001, the ADAM inhibitors TAPI-0 and TAPI-1, heparin binding epidermal growth factor (HB-EGF) inhibitor, CRM197, HB-EGF inhibitory antibodies, epidermal growth factor (EGF) inhibitory antibodies, and the EGFR inhibitor AG1478. The small interference ribonucleic acid (siRNA) against EGFR and ADAM-9, ADAM-10, ADAM-12, and adenoviral delivered Gbg inhibitor, betaARK(CT) were also used.ATF produced concentration-dependent VSMC   migration   (by wound assay and Boyden chamber), which was inhibited by increasing concentrations of AG1478. ATF was shown to induce time-dependent EGFR phosphorylation, which peaked at fourfold greater than control. Pre-incubation with the Gbetagamma inhibitor betaARK(CT) inhibited EGFR activation by ATF. This migratory and EGFR response was inhibited by AG1478 in a concentration-dependent manner. Incubation with siRNA against EGFR blocked the ATF-mediated migratory and EGFR responses. EGFR phosphorylation by ATF was blocked by inhibition of MMP activity and the ligand HB-EGF. The presence of the ADAM inhibitors, TAPI-0 and TAPI-1 significantly decreased EGFR activation. EGFR phosphorylation by EGF was not interrupted by inhibition of MMP, ADAMs, or HB-EGF. Direct blockade of the EGFR prevented activation by both ATF and EGF. Incubation with siRNA to ADAM-9 and -10 significantly reduced HB-EGF release from VSMC and EGFR activation in response to ATF. The siRNA against ADAM-12 had no effect.ATF can induce transactivation of EGFR by an ADAM-mediated, HB-EGF-dependent process. Targeting a pivotal cross-talk receptor such as EGFR is an attractive molecular target to inhibit cell   migration  . 
 A disintegrin and metalloprotease-10 is correlated with disease activity and mediates monocyte   migration   and adhesion in rheumatoid arthritis. A disintegrin and metalloproteases (ADAMs) are a family of proteins that have been reported to be involved in several inflammatory conditions. We examined the secretion of ADAM-10 in biological fluids from patients with rheumatoid arthritis (RA) and the role it plays in monocyte   migration  . ADAM-10 levels were measured using enzyme-linked immunosorbent assays and immunofluorescence. To examine the role of ADAM-10 in RA synovial fluids (SFs), we studied THP-1 (human acute monocyte leukemia cell line) and monocyte chemotaxis. To determine whether ADAM-10 plays a role in cell proliferation in the RA synovium, we assayed the proliferation of ADAM-10 small interfering RNA (siRNA)-transfected RA fibroblast-like synoviocytes (FLSs). The ADAM-10 level in RA serum was significantly higher than that in normal serum and was correlated with a disease activity score of 28. ADAM-10-depleted RA SFs showed a decrease in THP-1 and monocyte migratory activity compared with that of sham-depleted controls. ADAM-10 siRNA inhibited monocyte adhesion to RA FLSs. Finally, blocking ADAM-10 secretion in RA FLSs resulted in decreased production of fractalkine/CX3CL1 and vascular endothelial cell growth factor. These data indicate that ADAM-10 plays a role in monocyte   migration   in RA and suggest that targeting ADAM-10 may provide a method of decreasing inflammation and potentially treating other inflammatory diseases. 
 Regulation of ADAM10 and ADAM17 by Sorafenib Inhibits Epithelial-to-Mesenchymal Transition in Epstein-Barr Virus-Infected Retinal Pigment Epithelial Cells. The a-disintegrin-and-metalloprotease (ADAM) family proteins are widely expressed in the different layers of the retina throughout development. The effect of ADAM proteins on the epithelial-to-mesenchymal transition (EMT) in proliferative vitreoretinopathy (PVR) or AMD is yet to be elucidated. In this study we used Epstein-Barr virus (EBV)-transformed adult retinal pigment epithelial (ARPE) cells to investigate how sorafenib, a multikinase inhibitor, modulates ADAM proteins to control EMT.Epithelial to mesenchymal transition and related mechanisms in EBV-infected ARPE cells were determined by RT-PCR, Western blot, invasion assay, ELISA assay, and gene silencing with siRNA.Mesenchymal-like ARPE/EBV cells exhibited considerably increased cellular   migration   and invasion compared with ARPE cells and produced EMT-related cytokines. Sorafenib significantly inhibited production of TGF-β1, VEGF, IL-6, IL-8, MCP-1, and TNF-α and blocked the activation o  f migrati  on-related signaling molecules, such as HIF-1α, p-STAT3, MMP2, and Ang-1. The expression of mature ADAM10, ADAM17, and cleaved Notch 1 proteins in ARPE/EBV cells was downregulated after treatment with sorafenib through the regulatory activity of nardilysin (NRD-1). Gene silencing of NRD-1 in ARPE/EBV cells attenuated secretion of EMT-related cytokines and expression of ADAM10 and 17 and upregulated epithelial markers.Sorafenib controls the mesenchymal characteristics of EBV-infected ARPE cells. Nardilysin and ADAM family proteins might be new targets for the prevention or control of EMT in retinal diseases. 
 ADAM-10 and -17 regulate endometriotic cell   migration   via concerted ligand and receptor shedding feedback on kinase signaling. A Disintegrin and Metalloproteinases (ADAMs) are the principal enzymes for shedding receptor tyrosine kinase (RTK) ectodomains and ligands from the cell surface. Multiple layers of activity regulation, feedback, and catalytic promiscuity impede our understanding of context-dependent ADAM "sheddase" function and our ability to predictably target that function in disease. This study uses combined measurement and computational modeling to examine how various growth factor environments influence sheddase activity and cell   migration   in the invasive disease of endometriosis. We find that ADAM-10 and -17 dynamically integrate numerous signaling pathways to direct cell motility. Data-driven modeling reveals that induced cell   migration   is a quantitative function of positive feedback through EGF ligand release and negative feedback through RTK shedding. Although sheddase inhibition prevents autocrine ligand shedding and resultant EGF receptor transactivation, it also leads to an accumulation of phosphorylated receptors (HER2, HER4, and MET) on the cell surface, which subsequently enhances Jnk/p38 signaling. Jnk/p38 inhibition reduces cell   migration   by blocking sheddase activity while additionally preventing the compensatory signaling from accumulated RTKs. In contrast, Mek inhibition reduces ADAM-10 and -17 activities but fails to inhibit compensatory signaling from accumulated RTKs, which actually enhances cell motility in some contexts. Thus, here we present a sheddase-based mechanism of rapidly acquired resistance to Mek inhibition through reduced RTK shedding that can be overcome with rationally directed combination inhibitor treatment. We investigate the clinical relevance of these findings using targeted proteomics of peritoneal fluid from endometriosis patients and find growth-factor-driven ADAM-10 activity and MET shedding are jointly dysregulated with disease. 
 Epidermal growth factor-regulated activation of Rac GTPase enhances CD44 cleavage by metalloproteinase disintegrin ADAM10. Invasive tumour cells, such as gliomas, frequently express EGF (epidermal growth factor) receptor at a high level and they exhibit enhanced cell   migration   in response to EGF. We reported previously that tumour cell   migration   is associated with ectodomain cleavage of CD44, the major adhesion molecule that is implicated in tumour invasion and metastasis, and that the cleavage is enhanced by ligation of CD44. In the present study, we show that EGF promotes CD44 cleavage and CD44-dependent cell   migration  . Introduction of a dominant-negative mutant of the small GTPase Rac1 or depletion of Rac1 by RNAi (RNA interference) abrogated CD44 cleavage induced by EGF. Treatment with PD98059, an inhibitor for MEK (mitogen-activated protein kinase/extracellular-signal-regulated kinase kinase), also suppressed the CD44 cleavage. Furthermore, RNAi studies showed that EGF induced ADAM10 (a disintegrin and metalloproteinase 10)-dependent CD44 cleavage and cell   migration  . These results indicate that EGF induces ADAM10-mediated CD44 cleavage through Rac1 and mitogen-activated protein kinase activation, and thereby promotes tumour cell   migration   and invasion. 
 ADAM10-mediated cleavage of L1 adhesion molecule at the cell surface and in released membrane vesicles. Cells can release membrane components in a soluble form and as membrane vesicles. L1, an important molecule for cell   migration   of neural and tumor cells, is released by membrane-proximal cleavage, and soluble L1 promotes cell   migration  . Release of L1 is enhanced by shedding inducers such as phorbol ester and pervanadate, but it is also enhanced by depletion of cellular cholesterol with methyl-beta-cyclodextrin (MCD). How such different compounds can induce shedding is presently unknown. We show here that ADAM10 is involved in L1 cleavage, which occurs at the cell surface and in the Golgi apparatus. MCD and pervanadate treatment induced the release of microvesicles containing full-length L1 and the active form of ADAM10. L1 cleavage occurred in isolated vesicles. L1-containing microvesicles could trigger haptotactic cell   migration  . Only the neural L1 form carrying the RSLE signal for clathrin-dependent endocytosis was recruited and cleaved in vesicles. Phorbol ester treatment activated L1 cleavage predominantly at the cell surface. Our results provide evidence for two pathways of L1 cleavage, based on ADAM10 localization, that can be activated differentially: 1) direct cleavage at the cell surface, and 2) release and cleavage in secretory vesicles most likely derived from the Golgi apparatus. The findings establish a novel role for ADAM10 as a vesicle-based protease. 
 Melittin modulates keratinocyte function through P2 receptor-dependent ADAM activation. Melittin, the major component of the bee venom, is an amphipathic, cationic peptide with a wide spectrum of biological properties that is being considered as an anti-inflammatory and anti-cancer agent. It modulates multiple cellular functions but the underlying mechanisms are not clearly understood. Here, we report that melittin activates disintegrin-like metalloproteases (ADAMs) and that downstream events likely contribute to the biological effects evoked by the peptide. Melittin stimulated the proteolysis of ADAM10 and ADAM17 substrates in human neutrophil granulocytes, endothelial cells and murine fibroblasts. In human HaCaT keratinocytes, melittin induced shedding of the adhesion molecule E-cadherin and release of TGF-α, which was accompanied by transactivation of the EGF receptor and ERK1/2 phosphorylation. This was followed by functional consequences such as increased keratinocyte proliferation and enhanced cell   migratio  n. Evidence is provided that ATP release and activation of purinergic P2 receptors are involved in melittin-induced ADAM activation. E-cadherin shedding and EGFR phosphorylation were dose-dependently reduced in the presence of ATPases or P2 receptor antagonists. The involvement of P2 receptors was underscored in experiments with HEK cells, which lack the P2X7 receptor and showed strikingly increased response to melittin stimulation after transfection with this receptor. Our study provides new insight into the mechanism of melittin function which should be of interest particularly in the context of its potential use as an anti-inflammatory or anti-cancer agent. 
 Human metalloprotease-disintegrin Kuzbanian regulates sympathoadrenal cell fate in development and neoplasia. The development of the sympathetic nervous system involves cell-cell interactions that regulate the fate and   migration   of progenitor neural cells. Recent evidence shows that focal membrane-bound protease activity is critical for such interactions. The Drosophila kuzbanian (kuz) gene is required in neurogenesis and encodes a highly conserved, membrane-bound metalloprotease- disintegrin closley related to theTNF-alphaconvertingenzyme (TACE). We have characterized the human and mouse kuz homologs and mapped human kuz to chromosome 15q22. During mouse embryonic development Kuz is expressed mainly in the sympathoadrenal and olfactory neural precursors. Once sympathoadrenal cells differentiate into chromaffin cells in the adult adrenal medulla, they no longer express Kuz. However, we found that tumors of sympathoadrenal origin, such as pheochromocytomas and neuroblastomas, overexpress Kuz. Further, transfection of a kuz construct lacking the protease domain, but not the full-length construct, induces neurite formation in PC12 chromaffin tumor cells. Taken together our results suggest a critical role for Kuz in regulation of sympathoadrenal cell fate. 
 Leukocytes require ADAM10 but not ADAM17 for their   migration   and inflammatory recruitment into the alveolar space. Inflammation is a key process in various diseases, characterized by leukocyte recruitment to the inflammatory site. This study investigates the role of a disintegrin and a metalloproteinase (ADAM) 10 and ADAM17 for leukocyte   migration   in vitro and in a murine model of acute pulmonary inflammation. Inhibition experiments or RNA knockdown indicated that monocytic THP-1 cells and primary human neutrophils require ADAM10 but not ADAM17 for efficient chemokine-induced cell   migration  . Signaling and adhesion events that are linked to cell   migration   such as p38 and ρ GTPase-family activation, F-actin polymerization, adhesion to fibronectin, and up-regulation of α5 integrin were also dependent on ADAM10 but not ADAM17. This was confirmed with leukocytes isolated from mice lacking either ADAM10 or ADAM17 in all hematopoietic cells (vav 1 guanine nucleotide exchange factor [Vav]-Adam10(-/-) or Vav-Adam17(-/-) mice). In lipopolysaccharide-induced acute pulmonary inflammation, alveolar recruitment of neutrophils and monocytes was transiently increased in Vav-Adam17(-/-) but steadily reduced in Vav-Adam10(-/-) mice. This deficit in alveolar leukocyte recruitment was also observed in LysM-Adam10(-/-) mice lacking ADAM10 in myeloid cells and correlated with protection against edema formation. Thus, with regard to leukocyt  e migrati  on, leukocyte-expressed ADAM10 but not ADAM17 displays proinflammatory activities and may therefore serve as a target to limit inflammatory cell recruitment. 
 Cleavage of E-cadherin by ADAM10 mediates epithelial cell sorting downstream of EphB signalling. The formation and maintenance of complex organs requires segregation of distinct cell populations into defined territories (that is, cell sorting) and the establishment of boundaries between them. Here we have investigated the mechanism by which Eph/ephrin signalling controls the compartmentalization of cells in epithelial tissues. We show that EphB/ephrin-B signalling in epithelial cells regulates the formation of E-cadherin-based adhesions. EphB receptors interact with E-cadherin and with the metalloproteinase ADAM10 at sites of adhesion and their activation induces shedding of E-cadherin by ADAM10 at interfaces with ephrin-B1-expressing cells. This process results in asymmetric localization of E-cadherin and, as a consequence, in differences in cell affinity between EphB-positive and ephrin-B-positive cells. Furthermore, genetic inhibition of ADAM10 activity in the intestine of mice results in a lack of compartmentalization of Paneth cells within the crypt stem cell niche, a defect that phenocopies that of EphB3-null mice. These results provide important insights into the regulation of cell   migration   in the intestinal epithelium and may help in the understanding of the nature of the cell sorting process in other epithelial tissues where Eph-ephrin interactions play a central role. 
 Formation of the collateral circulation is regulated by vascular endothelial growth factor-A and a disintegrin and metalloprotease family members 10 and 17. The density of native (preexisting) collaterals varies widely and is a significant determinant of variation in severity of stroke, myocardial infarction, and peripheral artery disease. However, little is known about mechanisms responsible for formation of the collateral circulation in healthy tissues.We previously found that variation in vascular endothelial growth factor (VEGF) expression causes differences in collateral density of newborn and adult mice. Herein, we sought to determine mechanisms of collaterogenesis in the embryo and the role of VEGF in this process.Pial collaterals begin forming between embryonic day 13.5 and 14.5 as sprout-like extensions from arterioles of existing cerebral artery trees. Global VEGF-A overexpressing mice (Vegf(hi/+)) formed more, and Vegf(lo/+) formed fewer, collaterals during embryogenesis, in association with differences in vascular patterning. Conditional global reduction of Vegf or Flk1 only during collaterogenesis significantly reduced collateral formation, but now without affecting vascular patterning, and the effects remained in adulthood. Endothelial-specific Vegf reduction had no effect on collaterogenesis. Endothelial-specific reduction of a disintegrin-and-metalloprotease-domain-10 (Adam10) and inhibition of γ-secretase increased collateral formation, consistent with their roles in VEGF-induced Notch1 activation and suppression of prosprouting signals. Endothelial-specific knockdown of Adam17 reduced collateral formation, consistent with its roles in endothelial cell   migratio  n and embryonic vascular stabilization, but not in activation of ligand-bound Notch1. These effects also remained in adulthood.Formation of pial collaterals occurs during a narrow developmental window via a sprouting angiogenesis-like mechanism, requires paracrine VEGF stimulation of fetal liver kinase 1-Notch signaling, and adult collateral number is dependent on embryonic collaterogenesis. 
 ADAM10 promotes pituitary adenoma cell   migration   by regulating cleavage of CD44 and L1. ADAM10 is a metalloproteinase that regulates invasiveness in many tumors. Here, we found that ADAM10 expression correlates with the invasiveness of pituitary adenomas and contributes to invasion by cleaving L1 and CD44. In high-grade pituitary adenoma patients, ADAM10 expression levels were found to be elevated compared with low-grade pituitary adenomas. In a phorbol 12-myristate 13-acetate (PMA)-stimulated pituitary adenoma cell line, AtT-20 cells, we found that the cleavage of L1 was correspondingly enhanced with the increased interaction between Src and Shc. Increases in PMA-induced L1 cleavage and the phosphorylation of residue 418 of Src (418Src) were promoted by overexpression of ADAM10. Inversely, knockdown of Adam10 suppressed PMA-induced L1 cleavage and the phosphorylation of Src, which was blocked by the Src inhibitor PP2 and the MEK inhibitor PD98059. On the other hand, calcium flux activation in AtT-20 cells resulted in increased CD44 cleavage, with reduction of the interaction between calmodulin and ADAM10. The induction of enhanced CD44 cleavage by calcium flux activation was inhibited by knockdown of Adam10. In addition, Adam10 knockdown repressed AtT-20 cell   migration  , which was reversed by CD44EXT (CD44 ectodomain cleavage). Collectively, these data indicated that ADAM10 facilitated cell   migration   through modulation of CD44 and L1 cleavage. 
 ADAM10 regulates proliferation, invasion, and chemoresistance of bladder cancer cells. A disintegrin and metalloprotease 10 (ADAM10) is upregulated in several cancers and associates with malignant cancer progression. However, its expression pattern in bladder cancer remains unexplored. In the present study, we examined ADAM10 expression in 105 bladder cancer specimens using immunohistochemistry. We found ADAM10 overexpression in 51 of 105 (48.5 %) bladder cancer specimens. ADAM10 overexpression associated with advanced tumor stage (p = 0.001) and tumor grade (p = 0.018). To explore its biological functions in bladder cancer cells, small interfering RNA (siRNA) knockdown was performed in 5,637 and T24 cell lines. Cell Counting Kit-8 (CCK8) assay and Matrigel invasion assay showed that ADAM10 depletion decreased cell prolife  ration, m  igration, and invasion. In addition, ADAM10 knockdown increased the level of cisplatin-induced apoptosis. In conclusion, ADAM10 is overexpressed in bladder cancer and regulates malignant cell growth and invasion, which makes it a candidate therapeutic target. 
 Increase of ADAM10 level in coronary artery in-stent restenosis segments in diabetic minipigs: high ADAM10 expression promoting growth and   migration   in human vascular smooth muscle cells via Notch 1 and 3. This study aimed to identify major proteins in the pathogenesis of coronary artery in-stent restenosis (ISR) in diabetic minipigs with sirolimus-eluting stenting, and to investigate the roles of key candidate molecules, particularly ADAM10, in human arterial smooth muscle cells (HASMCs).The stents were implanted in the coronary arteries of 15 diabetic and 26 non-diabetic minipigs, and angiography was repeated at six months. The intima of one vascular segment with significant ISR and one with non-ISR in diabetic minipigs were isolated and cultured in conditioned medium (CM). The CM was analyzed by LC-MS/MS to uncover proteins whose levels were significantly increased (≥ 1.5-fold) in ISR than in non-ISR tissues. After literature searching, we focused on the identified proteins, whose biological functions were most potentially related to ISR pathophysiology. Among them, ADAM10 was significantly increased in diabetic and non-diabetic ISR tissues as compared with non-ISR controls. In cell experiments, retrovirus-mediated overexpression of ADAM10 promoted growth an  d migrati  on of HASMCs. The effects of ADAM10 were more remarkable in high-glucose culture than in low-glucose culture. Using shRNA and an inhibitor of γ-secretase (GSI), we found that the influences of ADAM10 were in part mediated by Notch1 and notch 3 pathway, which up-regulated Notch downstream genes and enhanced nuclear translocation of the small intracellular component of Notch1 and Notch3.This study has identified significantly increased expression of ADAM10 in the ISR versus non-ISR segment in diabetic minipigs and implicates ADAM10 in the enhanced neointimal formation observed in diabetes after vascular injury. 
 ADAM-10 is overexpressed in rheumatoid arthritis synovial tissue and mediates angiogenesis. To examine the expression of ADAM-10 in rheumatoid arthritis (RA) synovial tissue (ST) and the role it plays in angiogenesis.ADAM-10 expression was determined using immunohistology, Western blotting, and quantitative polymerase chain reaction. In order to examine the role of ADAM-10 in angiogenesis, we performed in vitro Matrigel tube formation and chemotaxis assays using human microvascular endothelial cells (HMVECs) transfected with control or ADAM-10 small interfering RNA (siRNA). To determine whether ADAM-10 plays a role in angiogenesis in the context of RA, we performed Matrigel assays using a coculture system of HMVECs and RA synovial fibroblasts.Endothelial cells and lining cells within RA ST expressed high levels of ADAM-10 compared with cells within osteoarthritis ST and normal ST. ADAM-10 expression was significantly elevated at the protein and messenger RNA levels in HMVECs and RA synovial fibroblasts stimulated with proinflammatory mediators compared with unstimulated cells. ADAM-10 siRNA-treated HMVECs had decreased endothelial cell tube formation and   migration   compared with control siRNA-treated HMVECs. In addition, ADAM-10 siRNA-treated HMVECs from the RA synovial fibroblast coculture system had decreased endothelial cell tube formation compared with control siRNA-treated HMVECs.These data show that ADAM-10 is overexpressed in RA and suggest that ADAM-10 may play a role in RA angiogenesis. ADAM-10 may be a potential therapeutic target in inflammatory angiogenic diseases such as RA. 
 ADAM10 releases a soluble form of the GPNMB/Osteoactivin extracellular domain with angiogenic properties. Glycoprotein non-metastatic melanoma protein B (GPNMB)/Osteoactivin (OA) is a transmembrane protein expressed in approximately 40-75% of breast cancers. GPNMB/OA promotes the   migration  , invasion and metastasis of breast cancer cells; it is commonly expressed in basal/triple-negative breast tumors and is associated with shorter recurrence-free and overall survival times in patients with breast cancer. Thus, GPNMB/OA represents an attractive target for therapeutic intervention in breast cancer; however, little is known about the functions of GPNMB/OA within the primary tumor microenvironment.We have employed mouse and human breast cancer cells to investigate the effects of GPNMB/OA on tumor growth and angiogenesis. GPNMB/OA-expressing tumors display elevated endothelial recruitment and reduced apoptosis when compared to vector control-derived tumors. Primary human breast cancers characterized by high vascular density also display elevated levels of GPNMB/OA when compared to those with low vascular density. Using immunoblot and ELISA assays, we demonstrate the GPNMB/OA ectodomain is shed from the surface of breast cancer cells. Transient siRNA-mediated knockdown studies of known sheddases identified ADAM10 as the protease responsible for GPNMB/OA processing. Finally, we demonstrate that the shed extracellular domain (ECD) of GPNMB/OA can promote endothelial   migration   in vitro.GPNMB/OA expression promotes tumor growth, which is associated with enhanced endothelial recruitment. We identify ADAM10 as a sheddase capable of releasing the GPNMB/OA ectodomain from the surface of breast cancer cells, which induces endothelial cell   migration  . Thus, ectodomain shedding may serve as a novel mechanism by which GPNMB/OA promotes angiogenesis in breast cancer. 
 ADAM10 is upregulated in melanoma metastasis compared with primary melanoma. ADAM10 (a disintegrin and metalloproteinase 10) is involved in the ectodomain shedding of various substrates, including adhesion molecules such as L1 cell adhesion molecule (L1-CAM) and CD44, which are known to have important roles in the development of malignant melanoma. In our study, we characterized the expression of ADAM10 in melanoma cells in vitro and in vivo. Immunohistochemical analysis on tissue microarrays indicated that ADAM10 expression was significantly elevated in melanoma metastasis compared with primary melanomas. In vitro downregulation of ADAM10 with specific small interfering RNA (siRNA) resulted in a suppression of the anchorage-independent cell growth and reduced the   migration   of melanoma cells. In addition, overexpression of ADAM10 induced the   migration   of melanoma cells. In cell lines from melanoma patients with metastasis, ADAM10 was significantly overexpressed, and ADAM10 expression correlated with increased cell proliferation. Furthermore, we present evidence that ADAM10 is involved in the release of L1-CAM from melanoma cells. It is important that knockdown of cellular L1-CAM reduced the   migration   of melanoma cells and abrogated the chemoresistance against cisplatin. In contrast, soluble L1-CAM had no effect on melanoma cell   migration   or cell survival. Taken together, our data demonstrate that ADAM10 and L1-CAM have important roles during melanoma progression and both molecules represent attractive targets for therapeutical intervention of melanomas. 
 The disintegrin/metalloproteinase ADAM10 is essential for the establishment of the brain cortex. The metalloproteinase and major amyloid precursor protein (APP) alpha-secretase candidate ADAM10 is responsible for the shedding of proteins important for brain development, such as cadherins, ephrins, and Notch receptors. Adam10(-/-) mice die at embryonic day 9.5, due to major defects in development of somites and vasculogenesis. To investigate the function of ADAM10 in brain, we generated Adam10 conditional knock-out (cKO) mice using a Nestin-Cre promotor, limiting ADAM10 inactivation to neural progenitor cells (NPCs) and NPC-derived neurons and glial cells. The cKO mice die perinatally with a disrupted neocortex and a severely reduced ganglionic eminence, due to precocious neuronal differentiation resulting in an early depletion of progenitor cells. Premature neuronal differentiation is associated with aberrant neuronal   migration   and a disorganized laminar architecture in the neocortex. Neurospheres derived from Adam10 cKO mice have a disrupted sphere organization and segregated more neurons at the expense of astrocytes. We found that Notch-1 processing was affected, leading to downregulation of several Notch-regulated genes in Adam10 cKO brains, in accordance with the central role of ADAM10 in this signaling pathway and explaining the neurogenic phenotype. Finally, we found that alpha-secretase-mediated processing of APP was largely reduced in these neurons, demonstrating that ADAM10 represents the most important APP alpha-secretase in brain. Our study reveals that ADAM10 plays a central role in the developing brain by controlling mainly Notch-dependent pathways but likely also by reducing surface shedding of other neuronal membrane proteins including APP. 
 Effect of A disintegrin and metalloproteinase 10 gene silencing on the proliferation, invasion and   migration   of the human tongue squamous cell carcinoma cell line TCA8113. The present study aimed to investigate the effect of A disintegrin and metalloproteinase 10 (ADAM10) gene silencing on the proliferation,   migration   and invasion of the human tongue squamous cell carcinoma cell line TCA8113. RNA interference was used to knock down the expression of ADAM10 in the TCA8113 cell line and the proliferation,   migration   and invasive ability of the treated cells were observed in vitro. The expression levels of epidermal growth factor receptor (EGFR) and E-cadherin in the treated cells were determined by western blot analysis. The proliferation,   migration   and invasion abilities of cells in the ADAM10 siRNA-treated group were significantly lower than those in the control groups (P<0.05). In addition, compared with the control groups, the expression levels of EGFR and E-cadherin in the ADAM10 siRNA-treated cells were significantly decreased (P<0.05) and increased (P<0.05), respectively. These results suggested that ADAM10 is important in regulating the proliferation, invasion and   migration   of the human tongue squamous cell carcinoma cell line TCA8113 and that the mechanism may, at least in part, be associated with the upregulation of EGFR and the downregulation of E-cadherin. 
 Reciprocal effects between microRNA-140-5p and ADAM10 suppress   migration   and invasion of human tongue cancer cells. ADAM10, overexpressed in tongue squamous cell carcinoma (TSCC), has been well documented for its role in tumor progression and metastasis. In the present study, we evaluated the inhibition effect of microRNAs (miRNAs) on the TSCC and identified that miR-140-5p could directly targets ADAM10 and inhibits the invasion and   migration   of TSCC cells. LAMC1, HDAC7 and PAX6, clustered into   migration  -related genes, were validated to be direct targets of miR-140-5p, while IGF1R and PSEN1 were not responsible to the regulation. Most intriguingly, ERBB4 was upregulated by miR-140-5p even though the interaction between ERBB4 3'UTR and miR-140-5p existed simultaneously. Meanwhile, ADAM10 is involved in the "positive" regulation of ERBB4 and negative regulation of PAX6 by miR-140-5p. Taken together, our results suggest that miR-140-5p play a role in TSCC cell   migration   and invasion, and two brand new relationships between miRNA and its targets emerged: (1) ADAM10 is not just a direct target of miR-140-5p, the repressed ADAM10 also helps to enhance the effect of miR-140-5p to other target genes: ERBB4 and PAX6; (2) ERBB4 is "positively" regulated by miR-140-5p. 
 ADAM10 is overexpressed in human hepatocellular carcinoma and contributes to the proliferation, invasion and   migration   of HepG2 cells. The overexpression of A disintegrin and metalloproteinase 10 (ADAM10) has been found to be closely associated with the development and progression of various types of tumors. However, ADAM10 expression in hepatocellular carcinoma (HCC) and its significance remain largely unknown. The present study aimed to investigate the expression of ADAM10 in human HCC and the effect of ADAM10 gene silencing by siRNA on the proliferation, invasion and   migration   of HepG2 human hepatoma cells. Immunohistochemistry was performed to examine the expression of ADAM10 in human HCC tissues and in the adjacent non-cancer tissues from 30 patients with HCC. RNA interference was used to knock down ADAM10 expression in HepG2 human hepatoma cells and the proliferation and   migration   as well as the invasive ability of the treated cells were observed in vitro. The expression of ADAM10 protein in HCC tissues was significantly higher when compared to that in adjacent non-tumor tissues (P<0.05). The high expression of ADAM10 in cancer was significantly correlated with clinical outcomes (P<0.05). Silencing of ADAM10 resulted in inhibition of proliferation and   migration   as well as invasion of HepG2 human hepatoma cells (P<0.05). These studies suggest that ADAM10 plays an important role in regulating proliferation, invasion and   migration   of HepG2 cells. High expression of ADAM10 may be a valuable predictive factor for HCC prognosis, and ADAM10 is potentially an important therapeutic target for the prevention of tumor development and progression in HCC. 
 ADAM-10-mediated N-cadherin cleavage is protein kinase C-alpha dependent and promotes glioblastoma cell   migration  . MMPs (matrix metalloproteinases) and the related "a disintegrin and metalloproteinases" (ADAMs) promote tumorigenesis by cleaving extracellular matrix and protein substrates, including N-cadherin. Although N-cadherin is thought to regulate cell adhesion,   migration  , and invasion, its role has not been characterized in glioblastomas (GBMs). In this study, we investigated the expression and function of posttranslational N-cadherin cleavage in GBM cells as well as its regulation by protein kinase C (PKC). N-Cadherin cleavage occurred at a higher level in glioblastoma cells than in non-neoplastic astrocytes. Treatment with the PKC activator phorbol 12-myristate 13-acetate (PMA) increased N-cadherin cleavage, which was reduced by pharmacological inhibitors and short interfering RNA (siRNA) specific for ADAM-10 or PKC-alpha. Furthermore, treatment of GBM cells with PMA induced the translocation of ADAM-10 to the cell membrane, the site at which N-cadherin was cleaved, and this translocation was significantly reduced by the PKC-alpha inhibitor Gö6976 [12-(2-cyanoethyl)-6,7,12,13-tetrahydro-13-methyl-5-oxo-5H-indolo[2,3-a]pyrrolo[3,4-c]carbazole] or PKC-alpha short hairpin RNA. In functional studies, N-cadherin cleavage was required for GBM cell   migratio  n, as depletion of N-cadherin cleavage by N-cadherin siRNA, ADAM-10 siRNA, or a cleavage-site mutant N-cadherin, decreased GBM cell   migratio  n. Together, these results suggest that N-cadherin cleavage is regulated by a PKC-alpha-ADAM-10 cascade in GBM cells and may be involved in mediating GBM cell   migratio  n. 
 Identification of SH3 domain proteins interacting with the cytoplasmic tail of the a disintegrin and metalloprotease 10 (ADAM10). The a disintegrin and metalloproteases (ADAMs) play a pivotal role in the control of development, adhesion,   migration  , inflammation and cancer. Although numerous substrates of ADAM10 have been identified, the regulation of its surface expression and proteolytic activity is still poorly defined. One current hypothesis is that both processes are in part modulated by protein-protein interactions mediated by the intracellular portion of the protease. For related proteases, especially proline-rich regions serving as docking sites for Src homology domain 3 (SH3) domain-containing proteins proved to be important for mediating regulatory interactions. In order to identify ADAM10-binding SH3 domain proteins, we screened the All SH3 Domain Phager library comprising 305 human SH3 domains using a GST fusion protein with the intracellular region of human ADAM10 as a bait for selection. Of a total of 291 analyzed phage clones, we found 38 SH3 domains that were precipitated with the ADAM10-derived fusion protein but not with GST. We verified the binding to the cytosolic portion of ADAM10 for several candidates by co-immunoprecipitation and/or pull down analyses. Intriguingly, several of the identified proteins have been implicated in regulating surface appearance and/or proteolytic activity of related ADAMs. Thus, it seems likely that they also play a role in ADAM10 biology. 
 Silencing ADAM10 inhibits the in vitro and in vivo growth of hepatocellular carcinoma cancer cells. A disintegrin and metalloprotease 10 (ADAM10) is a transmembrane protein associated with metastasis in a number of types of cancer. Little is known, however, regarding the role of ADAM10 in hepatocellular carcinoma (HCC). The aim of the present study was to evaluate whether downregulation of ADAM10 effects HCC cell proliferation, cell cycle, cell   migration   and cell invasion. A recombinant small hairpin RNA expression vector carrying ADAM10 was constructed and then transfected into the HepG2 human HCC cell line. In vitro cell proliferation, cell cycle, cell   migration   and cell invasion, and in vivo tumor growth were determined following the downregulation of ADAM10 by RNA interference. The results revealed that downregulation of ADAM10 expression in HepG2 tumor cells using the RNA silencing approach significantly suppressed cell proliferation, cell   migration   and cell invasion in vitro, and tumor growth in vivo. Furthermore, ADAM10 silencing was able to significantly reduce constitutive phosphorylation of phosphoinositide 3-kinase (PI3K) and Akt, which implies that ADAM10 is, at least partially, involved in the activation of the PI3K/Akt signaling pathway. These results suggest that ADAM10 is important in regulating the proliferation and metastasis of HCC. Thus, ADAM10 is a promising therapeutic target for the prevention of tumor metastases in HCC. 
 Synergistic effects of co-expression plasmid‑based ADAM10-specific siRNA and GRIM-19 on hepatocellular carcinoma in vitro and in vivo. A disintegrin and metalloproteinase 10 (ADAM10) has been demonstrated to correlate with hepatocellular carcinoma (HCC) grade and clinical outcome and its potential as a target for HCC therapy has been established. Gene associated with retinoid-interferon-induced mortality 19 (GRIM-19), a signal transducer and activator of transcription 3 (Stat3)-inhibitory protein, was identified as a potential tumor suppressor associated with growth inhibition and cell apoptosis. In the present study, we investigated whether a combined treatment with ADAM10-specific siRNA and GRIM19 gene could have an enhanced anticancer effectiveness on HCC in vitro and in vivo. We developed a dual expression plasmid that co-expressed ADAM10-specific siRNA and GRIM19, to evaluate its effects on HCC growth. Our results showed that simultaneous expression of ADAM10-specific siRNA and GRIM19 (pSi-ADAM10-GRIM19) in HepG2 cancer cells significantly inhibited the proliferation  , migrati  on and invasion, and induced cell apoptosis in vitro, and it also suppressed tumor growth in a nude mouse model when compared to the controls, either ADAM10-specific siRNA or GRIM-19 alone. In summary, our data demonstrated that a combined strategy of co-expressed ADAM10-specific siRNA and GRIM19 synergistically and more effectively suppressed HCC tumor growth, and has therapeutic potential for the treatment of HCC. 
 Elevated serum CXCL16 is an independent predictor of poor survival in ovarian cancer and may reflect pro-metastatic ADAM protease activity. In certain cancers, expression of CXCL16 and its receptor CXCR6 associate with lymphocyte infiltration, possibly aiding anti-tumour immune response. In other cancers, CXCL16 and CXCR6 associate with pro-metastatic activity. In the current study, we aimed to characterise the role of CXCL16, sCXCL16, and CXCR6 in ovarian cancer (OC).CXCL16/CXCR6 expression was analysed on tissue microarray containing 306 OC patient samples. Pre-treatment serum sCXCL16 was determined in 118 patients using ELISA. In vitro, (primary) OC cells were treated with an ADAM-10/ADAM-17 inhibitor (TAPI-2) and an ADAM-10-specific inhibitor (GI254023x), whereupon CXCL16 levels were evaluated on the cell membrane (immunofluorescent analysis, western blots) and in culture supernatants (ELISA). In addition, cell   migration   was assessed using scratch assays.sCXCL16 independently predicted for poor survival (hazard ratio=2.28, 95% confidence interval=1.29-4.02, P=0.005), whereas neither CXCL16 nor CXCR6 expression correlated with survival. Further, CXCL16/CXCR6 expression and serum sCXCL16 levels did not associate with lymphocyte infiltration. In vitro inhibition of both ADAM-17 and ADAM-10, but especially the latter, decreased CXCL16 membrane shedding and strongly reduced cell   migration   of A2780 and cultured primary OC-derived malignant cells.High serum sCXCL16 is a prognostic marker for poor survival of OC patients, possibly reflecting ADAM-10 and ADAM-17 pro-metastatic activity. Therefore, serum sCXCL16 levels may be a pseudomarker that identifies patients with highly metastatic tumours. 
 A disintegrin and metalloprotease 10 is a novel mediator of vascular endothelial growth factor-induced endothelial cell function in angiogenesis and is associated with atherosclerosis. To elucidate the downstream mechanisms of vascular endothelial growth factor receptor 2 (VEGFR2), a key receptor in angiogenesis, which has been associated with atherosclerotic plaque growth and instability.By using a yeast-2-hybrid assay, we identified A Disintegrin And Metalloprotease 10 (ADAM10) as a novel binding partner of VEGFR2. ADAM10 is a metalloprotease with sheddase activity involved in cell   migration  ; however, its exact function in endothelial cells (ECs), angiogenesis, and atherosclerosis is largely unknown. For the first time to our knowledge, we show ADAM10 expression in human atherosclerotic lesions, associated with plaque progression and neovascularization. We demonstrate ADAM10 expression and activity in ECs to be induced by VEGF; also, ADAM10 mediates the ectodomain shedding of VEGFR2. Furthermore, VEGF induces ADAM10-mediated cleavage of vascular endothelium (VE)-cadherin, which could increase vascular permeability and facilitate EC   migration  . Indeed, VEGF increases vascular permeability in an ADAM10- and ADAM17-dependent way; inhibition of ADAM10 reduces EC   migration   and chemotaxis.These data provide the first evidence of ADAM10 expression in atherosclerosis and neovascularization. ADAM10 plays a functional role in VEGF-induced EC function. These data open perspectives for novel therapeutic interventions in vascular diseases. 
 Expression profile of ADAM10 and ADAM17 in allergic rhinitis. A disintegrin and metalloprotease (ADAM) is a multifunctional gene family that contributes to the homeostasis of the extracellular matrix, transduction of specific intracellular signals, organogenesis, inflammation, tissue remodeling, adhesion, and cell   migration  . ADAM17 is the best characterized sheddase, with widespread putative substrates, including various inflammatory modulators. ADAM10 is the most similar ADAM to ADAM17 in terms of both protein sequence and the structural properties of their catalytic domains. The objective of this work was to assess the expression of ADAM10 and ADAM17 in allergic rhinitis to gain insight into their respective roles.The expression of ADAM10 and ADAM17 was investigated in the nasal mucosa under allergic and nonallergic conditions. Tissue samples were evaluated by reverse-transcription polymerase chain reaction (RT-PCR) and Western blotting, and data were analyzed semiquantitatively with densitometry.The ADAM17 messenger RNA (mRNA) level was significantly (p < 0.001) lower in the allergic nasal mucosa than in the nonallergic nasal mucosa, whereas the ADAM10 mRNA level was significantly (p < 0.001) lower in the nonallergic nasal mucosa. The ADAM17 protein levels were also significantly (p < 0.001) lower in the allergic nasal mucosa, whereas the ADAM10 protein levels were lower in the nonallergic nasal mucosa (p = 0.002).Decreased expression of ADAM17 and increased expression of ADAM10 may contribute to the development of allergic rhinitis through unknown pathways. We suggest that understanding the expression profile of ADAM17 and ADAM10 might help to elucidate the mechanism of allergic rhinitis. 
 Protocadherin-12 cleavage is a regulated process mediated by ADAM10 protein: evidence of shedding up-regulation in pre-eclampsia. Protocadherins are a group of transmembrane proteins with homophilic binding activity, members of the cadherin superfamily. Apart from their role in adhesion, the cellular functions of protocadherins are essentially unknown. Protocadherin (PCDH)12 was previously identified in invasive trophoblasts and endothelial and mesangial cells in the mouse. Invalidation studies revealed that the protein was required for optimal placental development. In this article, we show that its human homolog is abundantly expressed in various trophoblast subtypes of the human placenta and at lower levels in endothelial cells. We demonstrate that PCDH12 is shed at high rates in vitro. The shedding mechanism depends on ADAM10 and results in reduced cellular adhesion in a cell   migration   assay. PCDH12 is subsequently cleaved by the γ-secretase complex, and its cytoplasmic domain is rapidly degraded by the proteasome. PCDH12 shedding is regulated by interlinked intracellular pathways, including those involving protein kinase C, PI3K, and cAMP, that either increase or inhibit cleavage. In endothelial cells, VEGF, prostaglandin E(2), or histamine regulates PCDH12 shedding. The extracellular domain of PCDH12 was also detected in human serum and urine, thus providing evidence of PCDH12 shedding in vivo. Importantly, we observed an increase in circulating PCDH12 in pregnant women who later developed a pre-eclampsia, a frequent pregnancy syndrome and a major cause of maternal and fetal morbidity and mortality. In conclusion, we speculate that, like in mice, PCDH12 may play an important role in human placental development and that proteolytic cleavage in response to external factors, such as cytokines and pathological settings, regulates its activity. 
 The role of CXCL16 and its processing metalloproteinases ADAM10 and ADAM17 in the proliferation and   migration   of human mesangial cells. In this study, we analyzed the regulation and functional role of CXCL16 in human mesangial cells (hMCs). We can show, that CXCL16 is constitutively expressed in hMCs and is further up-regulated by cytokine mix (IFNgamma, TNFalpha, and IL1beta). The constitutive release of CXCL16 from hMCs was rapidly induced by the stimulation with cytokines. We identified ADAM10 and ADAM17 as being responsible for the cytokine-induced shedding of CXCL16. Notably, targeting ADAM10 and ADAM17 in hMCs decreased the chemotaxis of T-Jurkat cells, whereas the inhibition of CXCL16 had no significant influence. This suggests that both proteases are important players in the recruitment of immune cells into the glomerulus, but other substrates than CXCL16 are involved in this process. Finally, we could show that the inhibition of CXCL16, ADAM10, and ADAM17 led to a strong reduction of cell proliferation and   migration   of hMCs. This finding could be important to develop novel diagnostic and therapeutic strategies to treat mesangial proliferative kidney diseases. 
 MicroR-140-5p suppresses tumor cell   migration   and invasion by targeting ADAM10-mediated Notch1 signaling pathway in hypopharyngeal squamous cell carcinoma. MicroRNAs (miRNAs) are small non-coding RNAs of approximately 22 nucleotides that negatively regulate gene expression at the post-transcriptional level. Downexpression of miR-140-5p was reported in some human cancers, and combined with a reduction of cell   migration   and invasion, suggesting that miR-140-5p functions as a tumor suppressor. However, little is known about the expression and function of miR-140-5p in hypopharyngeal squamous cell carcinoma (HSCC). In this research, we found that miR-140-5p was significantly downregulated in HSCC tissues and correlated to tumor classification and lymph node metastasis. Restoration of miR-140-5p suppressed the   migration   and invasion of FaDu cells, and decreased the protein expression levels of ADAM10. Furthermore, the luciferase reporter assay revealed that miR-140-5p was directly bound to ADAM10 mRNA and knockdown of ADAM10 could inhibit FaDu cell   migration   and invasion and reduced the protein expression levels of and Notch1 intracellular domain (NICD1). Of note, knockdown of Notch1 could inhibit the   migration   and invasion of FaDu cells and rescued the effect of miR-140-5p inhibitor in FaDu cells. Taken together, our study demonstrates that miR-140-5p suppresses tumor   migration   and invasion by inhibiting ADAM10-mediated Notch1 signaling pathway and suggests that miR-140-5p could have potential therapeutic applications in HSCC. 
 Evidence for an interaction of the metalloprotease-disintegrin tumour necrosis factor alpha convertase (TACE) with mitotic arrest deficient 2 (MAD2), and of the metalloprotease-disintegrin MDC9 with a novel MAD2-related protein, MAD2beta. Metalloprotease-disintegrins are a family of transmembrane glycoproteins that have a role in fertilization, sperm   migration  , myoblast fusion, neural development and ectodomain shedding. In the present study we used the yeast two-hybrid system to search for proteins that interact with the cytoplasmic domain of two metalloprotease-disintegrins, tumour necrosis factor alpha convertase (TACE; ADAM17) and MDC9 (ADAM9; meltrin gamma). We have identified mitotic arrest deficient 2 (MAD2) as a binding partner of the TACE cytoplasmic domain, and a novel MAD2-related protein, MAD2beta, as a binding partner of the MDC9 cytoplasmic domain. MAD2beta has 23% sequence identity with MAD2, which is a component of the spindle assembly (or mitotic) checkpoint mechanism. Northern blot analysis of human tissues indicates that MAD2beta mRNA is expressed ubiquitously. The interaction of the TACE and MDC9 cytoplasmic domains with their binding partners has been confirmed biochemically. The independent identification of MAD2 and MAD2beta as potential interacting partners of distinct metalloprotease-disintegrins raises the possibility of a link between metalloprotease-disintegrins and the cell cycle, or of functions for MAD2 and MAD2beta that are not related to cell cycle control. 
 ADAM10 mediates E-cadherin shedding and regulates epithelial cell-cell adhesion,   migration  , and beta-catenin translocation. E-cadherin controls a wide array of cellular behaviors, including cell-cell adhesion, differentiation, and tissue development. We show here that E-cadherin is cleaved specifically by ADAM (a disintegrin and metalloprotease) 10 in its ectodomain. Analysis of ADAM10-deficient fibroblasts, inhibitor studies, and RNA interference-mediated down-regulation of ADAM10 demonstrated that ADAM10 is responsible not only for the constitutive shedding but also for the regulated shedding of this adhesion molecule in fibroblasts and keratinocytes. ADAM10-mediated E-cadherin shedding affects epithelial cell-cell adhesion as well as cell   migration  . Furthermore, the shedding of E-cadherin by ADAM10 modulates the beta-catenin subcellular localization and downstream signaling. ADAM10 overexpression in epithelial cells increased the expression of the beta-catenin downstream gene cyclin D1 dose-dependently and enhanced cell proliferation. In ADAM10-deficient mouse embryos, the C-terminal E-cadherin fragment is not generated, and the full-length protein accumulates, highlighting the in vivo relevance for ADAM10 in E-cadherin shedding. Our data strongly suggest that this protease constitutes a major regulatory element for the multiple functions of E-cadherin under physiological as well as pathological conditions. 
  GeneRIFs  
 Knockdown of ADAM10 inhibits   migration   and invasion of fibroblast-like synoviocytes in rheumatoid arthritis 
 that miR-140-5p suppresses tumor   migration   and invasion by inhibiting ADAM10-mediated Notch1 signaling pathway 
 Our findings hinted that overexpression of ADAM10 promotes the progression and   migration   of nasopharyngeal carcinoma 
 ADAM10 plays an important role in regulating DDR1-mediated cell adhesion to achieve efficient cell   migration   on collagen matrices. 
 ADAM-10 plays a role in monocyte   migration   in rheumatoid arthritis. 
 These results suggested that ADAM10 is important in regulating the proliferation, invasion and   migration   of the human tongue squamous cell carcinoma cell line TCA8113. 
 Simultaneous expression of ADAM10-specific siRNA and GRIM19 in HepG2 cancer cells significantly inhibited the proliferation,   migration   and invasion. 
 APP regulates   migration   and ADAM10/ADAM17 gene expression in prostate cancer cells. 
 Thus, with regard to leukocyte   migration  , leukocyte-expressed ADAM10 but not ADAM17 displays proinflammatory activities and may therefore serve as a target to limit inflammatory cell recruitment. 
 Silencing of ADAM10 resulted in inhibition of proliferation and   migration   as well as invasion of HepG2 human hepatoma cells. 
 ADAM-10 and -17 regulate endometriotic cell   migration   via concerted ligand and receptor shedding feedback on kinase signaling. 
 ADAM10 promotes non-small cell lung cancer cell   migration   and invasion via the activation of the Notch1 signaling pathway 
 ADAM10 facilitated cell   migration   through modulation of CD44 and L1 cleavage. 
 hMSCs interfere with cell-cell adhesion and enhance   migration   of breast cancer cells by activating ADAM10 
 Cell   migration   in response to the amino-terminal fragment of urokinase requires epidermal growth factor receptor activation through an ADAM9/10-mediated mechanism. 
 N-cadherin cleavage is regulated by a protein kinase C-alpha-ADAM-10 cascade in glioblastoma (GBM) cells and may be involved in mediating GBM cell   migration  . 
 ADAM10 is a novel regulator of vascular permeability and demonstrates a hitherto unknown function in the regulation of VE-cadherin-dependent endothelial cell functions and leukocyte transendothelial   migration   
 ADAM10 has a role in E-cadherin shedding and epithelial cell-cell adhesion,   migration  , and beta-catenin translocation 
 EGF induces ADAM10-mediated CD44 cleavage through Rac1 and mitogen-activated protein kinase activation, and thereby promotes tumour cell   migration   and invasion. 
  [Ee]xtracellular [Mm]atrix  Process  
   extracellular matrix   disassembly 
  PubMed  
 High percentage of ADAM-10 positive melanoma cells correlates with paucity of tumor-infiltrating lymphocytes but does not predict prognosis in cutaneous melanoma patients. ADAM-10 (CDw156, CD156c, and kuzbanian) is a protein belonging to a superfamily of metalloproteases, enzymes capable of degrading the   extracellular matrix  . ADAMs have also been shown to be primarily involved in ectodomain cleavage. The aim of the study was to assess the expression and intracellular location of ADAM-10 in 104 primary skin melanomas and 16 metastatic lesions from regional lymph nodes. Also, prognostic significance of ADAM-10 expression in primary tumor cells and metastatic lesion cells was evaluated during 5-year observation. It was revealed that high expression of ADAM-10 positive cells was strictly related with lower intensity of tumor-infiltrating lymphocytes (P = 0.037), which suggests that ADAM-10 regulates immunoresponse in melanoma initiation and progression. No statistically significant correlations were found between ADAM-10 expression in primary tumor cells and nodal metastases and other histopathological parameters analyzed. Decreased immunoreactivity of ADAM-10 in cancer cells from regional lymph nodes was correlated with worse prognosis; however this correlation was statistically nonsignificant (P = 0.065). Review of the literature shows that our study is the first one ever to describe the significance of ADAM-10 expression in correlation with detailed histopathological parameters of the primary tumor and data on long-term survival of cutaneous melanoma patients. 
 ADAM10 is the constitutive functional sheddase of CD44 in human melanoma cells. CD44 proteins are cell surface receptors for hyaluronic acid (HA), a component of the   extracellular matrix   that has multiple effects on cell behavior. CD44 can be shed from the cell surface by proteolytic cleavage. The resulting soluble form can interfere with the interaction between HA and membrane-bound CD44. Soluble CD44 can abolish the cell proliferation-promoting effect of HA on melanoma cell lines, suggesting that a better understanding of the shedding process might identify ways of blocking tumor cell proliferation. ADAM10, ADAM17, and MMP14 have previously been implicated in the shedding of CD44 from various tumor cells. Using immunohistochemistry we demonstrate that ADAM10 and ADAM17 but not MMP14 are significantly expressed on melanoma cells in histological sections. In human melanoma cell lines expression of these proteases could be blocked by transfection with appropriate siRNAs. However, only blocking of ADAM10 expression led to decreased shedding of CD44. In parallel, cell proliferation was promoted. Confocal microscopy demonstrated that ADAM10 and CD44 colocalize on the cell surface. We conclude that ADAM10 is the predominant protease involved in the constitutive shedding of endogenous CD44 from melanoma cells, and that enhancement of ADAM10 activity could be an approach to decrease the proliferation of melanoma cells. 
 Upregulation of the alpha-secretase ADAM10--risk or reason for hope? A decade ago, a disintegrin and metalloproteinase 10 (ADAM10) was identified as an alpha-secretase and as a key proteinase in the processing of the amyloid precursor protein. Accordingly, the important role that it plays in Alzheimer's disease was manifested. Animal models with an overexpression of ADAM10 revealed a beneficial profile of the metalloproteinase with respect to learning and memory, plaque load and synaptogenesis. Therefore, ADAM10 presents a worthwhile target with respect to the treatment of a neurodegenerative disease such as Morbus Alzheimer. Initially, ADAM10 was suggested to be an enzyme, shaping the   extracellular matrix   by cleavage of collagen type IV, or to be a tumour necrosis factor alpha convertase. In a relatively short time, a wide variety of additional substrates (with amyloid precursor protein probably being the most prominent) has been identified and the search is still ongoing. Hence, any side effects concerning the therapeutic enhancement of ADAM10 alpha-secretase activity have to be considered. The present review summarizes our knowledge about the structure and function of ADAM10 and highlights the opportunities for enhancing the expression and/or activity of the alpha-secretase as a therapeutic target. 
 ADAM-10-mediated N-cadherin cleavage is protein kinase C-alpha dependent and promotes glioblastoma cell migration. MMPs (matrix metalloproteinases) and the related "a disintegrin and metalloproteinases" (ADAMs) promote tumorigenesis by cleaving   extracellular matrix   and protein substrates, including N-cadherin. Although N-cadherin is thought to regulate cell adhesion, migration, and invasion, its role has not been characterized in glioblastomas (GBMs). In this study, we investigated the expression and function of posttranslational N-cadherin cleavage in GBM cells as well as its regulation by protein kinase C (PKC). N-Cadherin cleavage occurred at a higher level in glioblastoma cells than in non-neoplastic astrocytes. Treatment with the PKC activator phorbol 12-myristate 13-acetate (PMA) increased N-cadherin cleavage, which was reduced by pharmacological inhibitors and short interfering RNA (siRNA) specific for ADAM-10 or PKC-alpha. Furthermore, treatment of GBM cells with PMA induced the translocation of ADAM-10 to the cell membrane, the site at which N-cadherin was cleaved, and this translocation was significantly reduced by the PKC-alpha inhibitor Gö6976 [12-(2-cyanoethyl)-6,7,12,13-tetrahydro-13-methyl-5-oxo-5H-indolo[2,3-a]pyrrolo[3,4-c]carbazole] or PKC-alpha short hairpin RNA. In functional studies, N-cadherin cleavage was required for GBM cell migration, as depletion of N-cadherin cleavage by N-cadherin siRNA, ADAM-10 siRNA, or a cleavage-site mutant N-cadherin, decreased GBM cell migration. Together, these results suggest that N-cadherin cleavage is regulated by a PKC-alpha-ADAM-10 cascade in GBM cells and may be involved in mediating GBM cell migration. 
 Expression profile of ADAM10 and ADAM17 in allergic rhinitis. A disintegrin and metalloprotease (ADAM) is a multifunctional gene family that contributes to the homeostasis of the   extracellular matrix  , transduction of specific intracellular signals, organogenesis, inflammation, tissue remodeling, adhesion, and cell migration. ADAM17 is the best characterized sheddase, with widespread putative substrates, including various inflammatory modulators. ADAM10 is the most similar ADAM to ADAM17 in terms of both protein sequence and the structural properties of their catalytic domains. The objective of this work was to assess the expression of ADAM10 and ADAM17 in allergic rhinitis to gain insight into their respective roles.The expression of ADAM10 and ADAM17 was investigated in the nasal mucosa under allergic and nonallergic conditions. Tissue samples were evaluated by reverse-transcription polymerase chain reaction (RT-PCR) and Western blotting, and data were analyzed semiquantitatively with densitometry.The ADAM17 messenger RNA (mRNA) level was significantly (p < 0.001) lower in the allergic nasal mucosa than in the nonallergic nasal mucosa, whereas the ADAM10 mRNA level was significantly (p < 0.001) lower in the nonallergic nasal mucosa. The ADAM17 protein levels were also significantly (p < 0.001) lower in the allergic nasal mucosa, whereas the ADAM10 protein levels were lower in the nonallergic nasal mucosa (p = 0.002).Decreased expression of ADAM17 and increased expression of ADAM10 may contribute to the development of allergic rhinitis through unknown pathways. We suggest that understanding the expression profile of ADAM17 and ADAM10 might help to elucidate the mechanism of allergic rhinitis. 
 Presenilin-dependent gamma-secretase activity mediates the intramembranous cleavage of CD44. CD44 is the major adhesion molecule for the   extracellular matrix   components and is implicated in a wide variety of physiological and pathological processes including the regulation of tumor cell growth and metastasis. Our previous studies have shown that CD44 undergoes sequential proteolytic cleavages in the extracellular and transmembrane domains and the cleavage product derived from CD44 intramembranous cleavage acts as a signal transduction molecule. However, the underlying mechanism of the intramembranous cleavage of CD44 remains to be elucidated. In the present study, we report for the first time that CD44 is a substrate of the presenilin (PS)-dependent gamma-secretase. We demonstrate that the intramembranous cleavage of CD44 induced by 12-O-tetradecanoylphorbol 13-acetate (TPA) treatment or mechanical scraping is blocked by gamma-secretase inhibitors in U251MG cells and that this cleavage is also inhibited in PS-deficient mouse embryonic fibroblasts. Furthermore, we showed that PS1 is redistributed to ruffling areas of the plasma membrane similarly to CD44 after TPA treatment, supporting our biochemical observation that PS1 is involved in the intramembranous cleavage of CD44. Our present findings suggest important implications for understanding CD44-dependent signal transduction and a potential role of PS/gamma-secretase activity in the functional regulation of adhesion molecules. 
 Differential expression of ADAM (a disintegrin and metalloproteinase) genes between human first trimester villous and extravillous trophoblast cells. A disintegrin and metalloproteinases (ADAMs) are members of the metzincin family of zinc-dependent metalloproteinases that play pivotal roles in the proteolytic degradation of the   extracellular matrix   for cell invasion. Few studies have investigated the ADAM subtypes that are expressed in first trimester trophoblast cells. The purpose of this study was to elucidate the differential expression profiles of ADAMs between first trimester villous trophoblast cells (VTs) and extravillous trophoblast cells (EVTs). We isolated EVTs from explanted human first trimester chorionic villi and investigated the mRNA expression levels of five members of the ADAM family (ADAMTS1, ADAMTS2, ADAM10, ADAM12, and ADAM17) using real-time PCR. Chorionic villous tips were defined as first trimester VTs. Of the differentially expressed ADAM genes between first trimester VTs and EVTs, ADAMTS1 was expressed at a significantly higher level in EVTs than in VTs. In contrast, both ADAM10 and ADAM12 were expressed at significantly higher levels in VTs than in EVTs. No differences were found in the mRNA levels of ADAMTS2 and ADAM17 between the two cell types. Moreover, we demonstrated that in VTs, the expression level of ADAM12 was significantly downregulated in the late first trimester (10-13 gestational weeks) compared to the middle first trimester (7-8 weeks). These results suggest that first trimester trophoblast cells express ADAM genes in cell type- and gestational age-dependent manners. Our data provide additional insight into the functions of ADAMs in the human placenta. 
  [Ii]nvasive  PubMed  
 TLR4-mediated galectin-1 production triggers epithelial-mesenchymal transition in colon cancer cells through ADAM10- and ADAM17-associated lactate production. Toll-like receptor 4 (TLR4) activation is a key contributor to the carcinogenesis of colon cancer. Overexpression of galectin-1 (Gal-1) also correlates with increased   invasive   activity of colorectal cancer. Lactate production is a critical predictive factor of risk of metastasis, but the functional relationship between intracellular lactate and Gal-1 expression in TLR4-activated colon cancer remains unknown. In this study, we investigated the underlying mechanism and role of Gal-1 in metastasis and invasion of colorectal cancer (CRC) cells after TLR4 stimulation. Exposure to the TLR4 ligand lipopolysaccharide (LPS) increased expression of Gal-1, induced EMT-related cytokines, triggered the activation of glycolysis-related enzymes, and promoted lactate production. Gene silencing of TLR4 and Gal-1 in CRC cells inhibited lactate-mediated epithelial-mesenchymal transition (EMT) after TLR4 stimulation. Gal-1-mediated activation of a disintegrin and metalloproteinase 10 (ADAM10) and ADAM 17 increased the invasion activity and expression of mesenchymal characteristics in LPS-activated CRC cells. Conversely, inhibition of ADAM10 or ADAM17 effectively blocked the generation of lactate and the migration capacity of LPS-treated CRC cells. Thus, the TLR4/Gal-1 signaling pathway regulates lactate-mediated EMT processes through the activation of ADAM10 and ADAM17 in CRC cells. 
 ADAM 10 is over expressed in oral squamous cell carcinoma and contributes to   invasive   behaviour through a functional association with αvβ6 integrin. A disintegrin and metalloprotease (ADAM) proteins are upregulated in cancer and can interact with integrin receptors. We investigated whether such interactions may have functional significance in oral squamous cell carcinoma (OSCC). ADAM 10 expression was increased in OSCC tissue and cell lines compared to normal oral mucosa. Silencing of ADAM 10 reduced migration and invasion specifically in OSCC cells over-expressing αvβ6 integrin. This may result from ADAM 10-induced up-regulation of MMPs. We conclude ADAM 10 may influence OSCC invasion by functionally interacting with αvβ6 integrin which we have previously shown is over expressed in OSCC. 
 ADAM10 correlates with uveal melanoma metastasis and promotes in vitro invasion. Uveal melanoma (UM) is a rare ocular tumor that may lead to deadly metastases in 50% of patients. A disintegrin and metalloproteinase (ADAM)10, ADAM17, and the HGF-receptor c-Met support   invasive  ness in different tumors. Here, we report that high ADAM10, MET, and, to a lesser extent, ADAM17 gene expression correlates with poor progression-free survival in UM patients (hazard ratio 2.7, 2.6, and 1.9, respectively). About 60% of primary UM expresses c-Met and/or ADAM10 proteins. Four UM cell lines display high levels of ADAM10 and ADAM17, which constitutively cleave c-Met, inducing the release of soluble c-Met. ADAM10/17 pharmacological inhibition or gene silencing reduces c-Met shedding, but has limited impact on surface c-Met, which is overexpressed. Importantly, ADAM10 silencing inhibits UM cell invasion driven by FCS or HGF, while ADAM17 silencing has a limited effect. Altogether our data indicate that ADAM10 has a pro-  invasive   role and may contribute to UM progression. 
 Expression of L1-CAM and ADAM10 in human colon cancer cells induces metastasis. L1-CAM, a neuronal cell adhesion receptor, is also expressed in a variety of cancer cells. Recent studies identified L1-CAM as a target gene of beta-catenin-T-cell factor (TCF) signaling expressed at the   invasive   front of human colon cancer tissue. We found that L1-CAM expression in colon cancer cells lacking L1-CAM confers metastatic capacity, and mice injected in their spleen with such cells form liver metastases. We identified ADAM10, a metalloproteinase that cleaves the L1-CAM extracellular domain, as a novel target gene of beta-catenin-TCF signaling. ADAM10 overexpression in colon cancer cells displaying endogenous L1-CAM enhanced L1-CAM cleavage and induced liver metastasis, and ADAM10 also enhanced metastasis in colon cancer cells stably transfected with L1-CAM. DNA microarray analysis of genes induced by L1-CAM in colon cancer cells identified a cluster of genes also elevated in a large set of human colon carcinoma tissue samples. Expression of these genes in normal colon epithelium was low. These results indicate that there is a gene program induced by L1-CAM in colon cancer cells that is also present in colorectal cancer tissue and suggest that L1-CAM can serve as target for colon cancer therapy. 
 ADAM-10 and -17 regulate endometriotic cell migration via concerted ligand and receptor shedding feedback on kinase signaling. A Disintegrin and Metalloproteinases (ADAMs) are the principal enzymes for shedding receptor tyrosine kinase (RTK) ectodomains and ligands from the cell surface. Multiple layers of activity regulation, feedback, and catalytic promiscuity impede our understanding of context-dependent ADAM "sheddase" function and our ability to predictably target that function in disease. This study uses combined measurement and computational modeling to examine how various growth factor environments influence sheddase activity and cell migration in the   invasive   disease of endometriosis. We find that ADAM-10 and -17 dynamically integrate numerous signaling pathways to direct cell motility. Data-driven modeling reveals that induced cell migration is a quantitative function of positive feedback through EGF ligand release and negative feedback through RTK shedding. Although sheddase inhibition prevents autocrine ligand shedding and resultant EGF receptor transactivation, it also leads to an accumulation of phosphorylated receptors (HER2, HER4, and MET) on the cell surface, which subsequently enhances Jnk/p38 signaling. Jnk/p38 inhibition reduces cell migration by blocking sheddase activity while additionally preventing the compensatory signaling from accumulated RTKs. In contrast, Mek inhibition reduces ADAM-10 and -17 activities but fails to inhibit compensatory signaling from accumulated RTKs, which actually enhances cell motility in some contexts. Thus, here we present a sheddase-based mechanism of rapidly acquired resistance to Mek inhibition through reduced RTK shedding that can be overcome with rationally directed combination inhibitor treatment. We investigate the clinical relevance of these findings using targeted proteomics of peritoneal fluid from endometriosis patients and find growth-factor-driven ADAM-10 activity and MET shedding are jointly dysregulated with disease. 
 Epidermal growth factor-regulated activation of Rac GTPase enhances CD44 cleavage by metalloproteinase disintegrin ADAM10.   Invasive   tumour cells, such as gliomas, frequently express EGF (epidermal growth factor) receptor at a high level and they exhibit enhanced cell migration in response to EGF. We reported previously that tumour cell migration is associated with ectodomain cleavage of CD44, the major adhesion molecule that is implicated in tumour invasion and metastasis, and that the cleavage is enhanced by ligation of CD44. In the present study, we show that EGF promotes CD44 cleavage and CD44-dependent cell migration. Introduction of a dominant-negative mutant of the small GTPase Rac1 or depletion of Rac1 by RNAi (RNA interference) abrogated CD44 cleavage induced by EGF. Treatment with PD98059, an inhibitor for MEK (mitogen-activated protein kinase/extracellular-signal-regulated kinase kinase), also suppressed the CD44 cleavage. Furthermore, RNAi studies showed that EGF induced ADAM10 (a disintegrin and metalloproteinase 10)-dependent CD44 cleavage and cell migration. These results indicate that EGF induces ADAM10-mediated CD44 cleavage through Rac1 and mitogen-activated protein kinase activation, and thereby promotes tumour cell migration and invasion. 
 Metastasis-associated C4.4A, a GPI-anchored protein cleaved by ADAM10 and ADAM17. Metalloproteases play a complex role in tumor progression. While the activity of some ADAM, ADAMTS and matrix metalloproteases (MMPs) seems to be protumorigenic, the activity of others seems to prevent tumor progression. The identification of the array of substrates of a given metalloprotease (degradome) seems an adequate approach to predict the effect of the inhibition of a metalloprotease in tumors. Here, we present the proteomic identification of a novel substrate for ADAM10 and -17. We used SILAC (Stable Isotope Labeling by Amino acids in Cell culture), a proteomic technique based on the differential metabolic labeling of cells in different conditions. This was applied to MCF7 cells derived from an   invasive   mammary tumor, and the same cells expressing shRNAs that knock down ADAM10 or -17. Following this approach, we have identified C4.4A as a substrate to both metalloproteases. Since C4.4A is likely involved in tumor invasion, these results indicate that the cleavage of C4.4A by ADAM10 and ADAM17 contributes to tumor progression. 
 PAX2 regulates ADAM10 expression and mediates anchorage-independent cell growth of melanoma cells. PAX transcription factors play an important role during development and carcinogenesis. In this study, we investigated PAX2 protein levels in melanocytes and melanoma cells by Western Blot and immunofluorescence analysis and characterized the role of PAX2 in the pathogenesis of melanoma. In vitro we found weak PAX2 protein expression in keratinocytes and melanocytes. Compared to melanocytes increased PAX2 protein levels were detectable in melanoma cell lines. Interestingly, in tissue sections of melanoma patients nuclear PAX2 expression strongly correlated with nuclear atypia and the degree of prominent nucleoli, indicating an association of PAX2 with a more atypical cellular phenotype. In addition, with chromatin immunoprecipitation assay, PAX2 overexpression and PAX2 siRNA we present compelling evidence that PAX2 can regulate ADAM10 expression, a metalloproteinase known to play important roles in melanoma metastasis. In human tissue samples we found co-expression of PAX2 and ADAM10 in melanocytes of benign nevi and in melanoma cells of patients with malignant melanoma. Importantly, the downregulation of PAX2 by specific siRNA inhibited the anchorage independent cell growth and decreased the migratory and   invasive   capacity of melanoma cells. Furthermore, the downregulation of PAX2 abrogated the chemoresistance of melanoma cells against cisplatin, indicating that PAX2 expression mediates cell survival and plays important roles during melanoma progression. 
 ADAM10 promotes pituitary adenoma cell migration by regulating cleavage of CD44 and L1. ADAM10 is a metalloproteinase that regulates   invasive  ness in many tumors. Here, we found that ADAM10 expression correlates with the   invasive  ness of pituitary adenomas and contributes to invasion by cleaving L1 and CD44. In high-grade pituitary adenoma patients, ADAM10 expression levels were found to be elevated compared with low-grade pituitary adenomas. In a phorbol 12-myristate 13-acetate (PMA)-stimulated pituitary adenoma cell line, AtT-20 cells, we found that the cleavage of L1 was correspondingly enhanced with the increased interaction between Src and Shc. Increases in PMA-induced L1 cleavage and the phosphorylation of residue 418 of Src (418Src) were promoted by overexpression of ADAM10. Inversely, knockdown of Adam10 suppressed PMA-induced L1 cleavage and the phosphorylation of Src, which was blocked by the Src inhibitor PP2 and the MEK inhibitor PD98059. On the other hand, calcium flux activation in AtT-20 cells resulted in increased CD44 cleavage, with reduction of the interaction between calmodulin and ADAM10. The induction of enhanced CD44 cleavage by calcium flux activation was inhibited by knockdown of Adam10. In addition, Adam10 knockdown repressed AtT-20 cell migration, which was reversed by CD44EXT (CD44 ectodomain cleavage). Collectively, these data indicated that ADAM10 facilitated cell migration through modulation of CD44 and L1 cleavage. 
 Effect of A disintegrin and metalloproteinase 10 gene silencing on the proliferation, invasion and migration of the human tongue squamous cell carcinoma cell line TCA8113. The present study aimed to investigate the effect of A disintegrin and metalloproteinase 10 (ADAM10) gene silencing on the proliferation, migration and invasion of the human tongue squamous cell carcinoma cell line TCA8113. RNA interference was used to knock down the expression of ADAM10 in the TCA8113 cell line and the proliferation, migration and   invasive   ability of the treated cells were observed in vitro. The expression levels of epidermal growth factor receptor (EGFR) and E-cadherin in the treated cells were determined by western blot analysis. The proliferation, migration and invasion abilities of cells in the ADAM10 siRNA-treated group were significantly lower than those in the control groups (P<0.05). In addition, compared with the control groups, the expression levels of EGFR and E-cadherin in the ADAM10 siRNA-treated cells were significantly decreased (P<0.05) and increased (P<0.05), respectively. These results suggested that ADAM10 is important in regulating the proliferation, invasion and migration of the human tongue squamous cell carcinoma cell line TCA8113 and that the mechanism may, at least in part, be associated with the upregulation of EGFR and the downregulation of E-cadherin. 
 ADAM10 is overexpressed in human hepatocellular carcinoma and contributes to the proliferation, invasion and migration of HepG2 cells. The overexpression of A disintegrin and metalloproteinase 10 (ADAM10) has been found to be closely associated with the development and progression of various types of tumors. However, ADAM10 expression in hepatocellular carcinoma (HCC) and its significance remain largely unknown. The present study aimed to investigate the expression of ADAM10 in human HCC and the effect of ADAM10 gene silencing by siRNA on the proliferation, invasion and migration of HepG2 human hepatoma cells. Immunohistochemistry was performed to examine the expression of ADAM10 in human HCC tissues and in the adjacent non-cancer tissues from 30 patients with HCC. RNA interference was used to knock down ADAM10 expression in HepG2 human hepatoma cells and the proliferation and migration as well as the   invasive   ability of the treated cells were observed in vitro. The expression of ADAM10 protein in HCC tissues was significantly higher when compared to that in adjacent non-tumor tissues (P<0.05). The high expression of ADAM10 in cancer was significantly correlated with clinical outcomes (P<0.05). Silencing of ADAM10 resulted in inhibition of proliferation and migration as well as invasion of HepG2 human hepatoma cells (P<0.05). These studies suggest that ADAM10 plays an important role in regulating proliferation, invasion and migration of HepG2 cells. High expression of ADAM10 may be a valuable predictive factor for HCC prognosis, and ADAM10 is potentially an important therapeutic target for the prevention of tumor development and progression in HCC. 
 The disintegrin-metalloproteinases ADAM 10, 12 and 17 are upregulated in invading peripheral tumor cells of basal cell carcinomas. Members of the a disintegrin and metalloproteinase (ADAM) family are expressed in malignant tumors and participate in the pathogenesis of cancer. However, the presence of ADAM 10, 12, 17 and their role in basal cell carcinoma (BCC) have not been described. The purpose of this study was to investigate expression of ADAM 10, 12 and 17 in BCC.Expression of ADAM 10, 12 and 17 was analyzed by immunohistochemistry in skin tissues obtained from 25 patients with different types of BCC.Immunoreactivity of ADAM 10, 12 and 17 was increased at the peripheral tumor margin compared with central areas of BCC tumor cell nests. Immunoreactivity of ADAM 10 and 12 was increased in the deep margin of invading tumor cell nests in mixed BCC. Focally increased expression of ADAM 12 was detected in squamous differentiated tumor cells of nodular BCC. In addition, immunoreactivity of ADAM 17 was increased in superficial BCC.ADAM 10, 12 and 17 showed different expression pattern in BCC histologic subtypes, indicating their different role in the BCC pathogenesis. Overexpression of ADAM 10, 12 and 17 immunoreactivity in deep invasion area of BCC indicates that these three proteases may play an important role in the locally   invasive   and highly destructive growth behavior of BCC. Additionally, we suggest that ADAM 17 may play an important role in early development of BCC. 
 Protocadherin-12 cleavage is a regulated process mediated by ADAM10 protein: evidence of shedding up-regulation in pre-eclampsia. Protocadherins are a group of transmembrane proteins with homophilic binding activity, members of the cadherin superfamily. Apart from their role in adhesion, the cellular functions of protocadherins are essentially unknown. Protocadherin (PCDH)12 was previously identified in   invasive   trophoblasts and endothelial and mesangial cells in the mouse. Invalidation studies revealed that the protein was required for optimal placental development. In this article, we show that its human homolog is abundantly expressed in various trophoblast subtypes of the human placenta and at lower levels in endothelial cells. We demonstrate that PCDH12 is shed at high rates in vitro. The shedding mechanism depends on ADAM10 and results in reduced cellular adhesion in a cell migration assay. PCDH12 is subsequently cleaved by the γ-secretase complex, and its cytoplasmic domain is rapidly degraded by the proteasome. PCDH12 shedding is regulated by interlinked intracellular pathways, including those involving protein kinase C, PI3K, and cAMP, that either increase or inhibit cleavage. In endothelial cells, VEGF, prostaglandin E(2), or histamine regulates PCDH12 shedding. The extracellular domain of PCDH12 was also detected in human serum and urine, thus providing evidence of PCDH12 shedding in vivo. Importantly, we observed an increase in circulating PCDH12 in pregnant women who later developed a pre-eclampsia, a frequent pregnancy syndrome and a major cause of maternal and fetal morbidity and mortality. In conclusion, we speculate that, like in mice, PCDH12 may play an important role in human placental development and that proteolytic cleavage in response to external factors, such as cytokines and pathological settings, regulates its activity. 
 Inhibiting adenoid cystic carcinoma cells growth and metastasis by blocking the expression of ADAM 10 using RNA interference. Adenoid cystic carcinoma is one of the most common types of salivary gland cancers. The poor long-term prognosis for patients with adenoid cystic carcinoma is mainly due to local recurrence and distant metastasis. Disintegrin and metalloprotease 10 (ADAM 10) is a transmembrane protein associated with metastasis in a number of diverse of cancers. The aim of this study was to analyze the relationship between ADAM 10 and the   invasive   and metastatic potentials as well as the proliferation capability of adenoid cystic carcinoma cells in vitro and in vivo.Immunohistochemistry and Western blot analysis were applied to detect ADAM 10 expression levels in metastatic cancer tissues, corresponding primary adenoid cystic carcinoma tissues, adenoid cystic carcinoma cell lines with high metastatic potential, and adenoid cystic carcinoma cell lines with low metastatic potential. RNA interference was used to knockdown ADAM 10 expression in adenoid cystic carcinoma cell lines with high metastatic potential. Furthermore, the   invasive   and metastatic potentials as well as the proliferation capability of the treated cells were observed in vitro and in vivo.It was observed that ADAM 10 was expressed at a significantly higher level in metastatic cancer tissues and in adenoid cystic carcinoma cell lines with high metastatic potential than in corresponding primary adenoid cystic carcinomas and adenoid cystic carcinoma cell lines with low metastatic potential. Additionally, silencing of ADAM 10 resulted in inhibition of cell growth and invasion in vitro as well as inhibition of cancer metastasis in an experimental murine model of lung metastases in vivo.These studies suggested that ADAM 10 plays an important role in regulating proliferation and metastasis of adenoid cystic carcinoma cells. ADAM 10 is potentially an important therapeutic target for the prevention of tumor metastases in adenoid cystic carcinoma. 
  GeneRIFs  
 ADAM10 has a pro-  invasive   role and may contribute to uveal melanoma progression. 
 Overexpression of ADAM 10, 12 and 17 immunoreactivity in deep invasion area of BCC indicates that these three proteases may play an important role in the locally   invasive   and highly destructive growth behavior of basal cell carcinoma 
  [Mm]etastasis  PubMed  
 TLR4-mediated galectin-1 production triggers epithelial-mesenchymal transition in colon cancer cells through ADAM10- and ADAM17-associated lactate production. Toll-like receptor 4 (TLR4) activation is a key contributor to the carcinogenesis of colon cancer. Overexpression of galectin-1 (Gal-1) also correlates with increased invasive activity of colorectal cancer. Lactate production is a critical predictive factor of risk of   metastasis  , but the functional relationship between intracellular lactate and Gal-1 expression in TLR4-activated colon cancer remains unknown. In this study, we investigated the underlying mechanism and role of Gal-1 in   metastasis   and invasion of colorectal cancer (CRC) cells after TLR4 stimulation. Exposure to the TLR4 ligand lipopolysaccharide (LPS) increased expression of Gal-1, induced EMT-related cytokines, triggered the activation of glycolysis-related enzymes, and promoted lactate production. Gene silencing of TLR4 and Gal-1 in CRC cells inhibited lactate-mediated epithelial-mesenchymal transition (EMT) after TLR4 stimulation. Gal-1-mediated activation of a disintegrin and metalloproteinase 10 (ADAM10) and ADAM 17 increased the invasion activity and expression of mesenchymal characteristics in LPS-activated CRC cells. Conversely, inhibition of ADAM10 or ADAM17 effectively blocked the generation of lactate and the migration capacity of LPS-treated CRC cells. Thus, the TLR4/Gal-1 signaling pathway regulates lactate-mediated EMT processes through the activation of ADAM10 and ADAM17 in CRC cells. 
 ADAM10 overexpression in human non-small cell lung cancer correlates with cell migration and invasion through the activation of the Notch1 signaling pathway. A disintegrin and metalloproteinase 10 (ADAM10) was identified as a key protease in the ectodomain shedding of various substrates, such as Notch1 protein, ErbB2 and E-cadherin, which are important in the development of non-small cell lung cancer (NSCLC). The aim of this study was to investi-gate the role of ADAM10 in NSCLC   metastasis  .We characterized the expression of ADAM10 and Notch1 in human NSCLC tissues in vivo. Immunohistochemical analysis indicated that ADAM10 expression was significantly increased in the NSCLC tissues, particularly in the metastatic tissues. Futhermore, ADAM10 overexpression positively correlated with Notch1 expression in the NSCLC tissues. The in vitro downregulation of ADAM10 expression using ADAM10 short hairpin RNA (shRNA) reduced the migration and invasion of NSCLC cells. We present further evidence that ADAM10 promotes NSCLC cell migration and invasion via the activation of the Notch1 signaling pathway. Taken together, our results suggest that ADAM10 may serve as a potential target for the therapeutic intervention of NSCLC   metastasis  . The data provided in this study may aid in the further understanding of the function of ADAM10 in the progression of NSCLC and open new perspectives for the diagnosis and treatment of NSCLC. 
 ADAM10 correlates with uveal melanoma   metastasis   and promotes in vitro invasion. Uveal melanoma (UM) is a rare ocular tumor that may lead to deadly metastases in 50% of patients. A disintegrin and metalloproteinase (ADAM)10, ADAM17, and the HGF-receptor c-Met support invasiveness in different tumors. Here, we report that high ADAM10, MET, and, to a lesser extent, ADAM17 gene expression correlates with poor progression-free survival in UM patients (hazard ratio 2.7, 2.6, and 1.9, respectively). About 60% of primary UM expresses c-Met and/or ADAM10 proteins. Four UM cell lines display high levels of ADAM10 and ADAM17, which constitutively cleave c-Met, inducing the release of soluble c-Met. ADAM10/17 pharmacological inhibition or gene silencing reduces c-Met shedding, but has limited impact on surface c-Met, which is overexpressed. Importantly, ADAM10 silencing inhibits UM cell invasion driven by FCS or HGF, while ADAM17 silencing has a limited effect. Altogether our data indicate that ADAM10 has a pro-invasive role and may contribute to UM progression. 
 ADAM10 mediates the cell invasion and   metastasis   of human esophageal squamous cell carcinoma via regulation of E-cadherin activity. A disintegrin and metalloprotease 10 (ADAM10) is involved in the tumorigenesis, invasion and   metastasis   of several types of solid tumors. However, the potential role of ADAM10 in human esophageal squamous cell carcinoma (ESCC) is not yet well understood. The present study showed that ADAM10 was overexpressed in human ESCC tissues in vivo, and positively associated with depth of tumor invasion, lymph node   metastasis   and TNM stage, contributing to tumor carcinogenesis, invasion and   metastasis  . Additionally, ADAM10 was overexpressed in 3 types of ESCC cell lines in vitro, as compared to that in normal esophageal epithelial cells (NEECs); and moreover, ESCC cells with high ADAM10 expression obtained enhanced invasion and migration ability. Subsequently, ADAM10 silencing by small interfering (si) RNA in ESCC cell line, EC-1, reduced cell invasion, migration and proliferation in vitro. Finally, ADAM10 negatively regulated E-cadherin in ESCC in vivo and in vitro. In conclusion, active ADAM10 promotes the carcinogenesis, invasion,   metastasis   and proliferation of ESCC and controls invasion and   metastasis   at least in part through the shedding of E-cadherin activity, which makes it a potential biomarker and a useful therapeutic target for ESCC. 
 Effects of ADAM10 upregulation on progression, migration, and prognosis of nasopharyngeal carcinoma. A disintegrin and metalloprotease 10 (ADAM10) is a typical member of the ADAMs family, which has been reported to be upregulated in various types of cancers and contribute to cancer progression and   metastasis  . However, little is known about the role of ADAM10 in nasopharyngeal carcinoma (NPC). The purpose of this study is to explore ADAM10 expression status and its biological functions in NPC. We first examined the expression of ADAM10 in NPC tissues and cell lines by immunohistochemistry, Western blotting, PCR, and immunofluorescence analysis. We observed that ADAM10 was significantly elevated in NPC and its expression level was correlated with T classification (P = 0.044), distant   metastasis   (P = 0.016), TNM clinical stage (P = 0.013), and proliferation marker Ki-67 expression (P = 0.001). Patients with NPC with high expression of ADAM10 had shorter overall survival rates. In addition, knockdown of ADAM10 by RNAi was found to inhibit the CNE-2 cell proliferation and migration. Our findings hinted that overexpression of ADAM10 promotes the progression and migration of NPC, which makes it a potential therapeutic target for the treatment of tumor metastases in NPC. 
 Expression of L1-CAM and ADAM10 in human colon cancer cells induces   metastasis  . L1-CAM, a neuronal cell adhesion receptor, is also expressed in a variety of cancer cells. Recent studies identified L1-CAM as a target gene of beta-catenin-T-cell factor (TCF) signaling expressed at the invasive front of human colon cancer tissue. We found that L1-CAM expression in colon cancer cells lacking L1-CAM confers metastatic capacity, and mice injected in their spleen with such cells form liver metastases. We identified ADAM10, a metalloproteinase that cleaves the L1-CAM extracellular domain, as a novel target gene of beta-catenin-TCF signaling. ADAM10 overexpression in colon cancer cells displaying endogenous L1-CAM enhanced L1-CAM cleavage and induced liver   metastasis  , and ADAM10 also enhanced   metastasis   in colon cancer cells stably transfected with L1-CAM. DNA microarray analysis of genes induced by L1-CAM in colon cancer cells identified a cluster of genes also elevated in a large set of human colon carcinoma tissue samples. Expression of these genes in normal colon epithelium was low. These results indicate that there is a gene program induced by L1-CAM in colon cancer cells that is also present in colorectal cancer tissue and suggest that L1-CAM can serve as target for colon cancer therapy. 
 Activation of microRNA-494-targeting Bmi1 and ADAM10 by silibinin ablates cancer stemness and predicts favourable prognostic value in head and neck squamous cell carcinomas. Tumor initiating cells (TICs) possessing cancer stemness were shown to be enriched after therapy, resulting in the relapse and   metastasis   of head and neck squamous cell carcinomas (HNC). An effective therapeutic approach suppressing the HNC-TICs would be a potential method to improve the treatments for HNC. We observed that the treatment of silibinin (SB) dose dependently down-regulated the ALDH1 activity, CD133 positivity, stemness signatures expression, self-renewal property, and chemoresistance in ALDH1+CD44+ HNC-TICs. Using miRNA-microarray and mechanistic studies, SB increased the expression of microRNA-494 (miR-494) and both Bmi1 and ADAM10 were identified as the novel targets of miR-494. Moreover, overexpression of miR-494 results in a reduction in cancer stemness. However, knockdown of miR-494 in CD44-ALDH1- non-HNC-TICs enhanced cancer stemness and oncogenicity, while co-knockdown of Bmi1 and ADAM10 effectively reversed these phenomena. Mice model showed that SB treatment by oral gavage to xenograft tumors reduced tumor growth and prolonged the survival time of tumor-bearing mice by activation of miR-494-inhibiting Bmi1/ADAM10 expression. Survival analysis indicated that a miR494highBmi1lowADAM10low phenotype predicted a favourable clinical outcome. We conclude that the inhibition of tumor aggressiveness in HNC-TICs by SB was mediated by up-regulation miR-494, suggesting that SB would be a valuable anti-cancer drug for treatment of HNC. 
 Epidermal growth factor-regulated activation of Rac GTPase enhances CD44 cleavage by metalloproteinase disintegrin ADAM10. Invasive tumour cells, such as gliomas, frequently express EGF (epidermal growth factor) receptor at a high level and they exhibit enhanced cell migration in response to EGF. We reported previously that tumour cell migration is associated with ectodomain cleavage of CD44, the major adhesion molecule that is implicated in tumour invasion and   metastasis  , and that the cleavage is enhanced by ligation of CD44. In the present study, we show that EGF promotes CD44 cleavage and CD44-dependent cell migration. Introduction of a dominant-negative mutant of the small GTPase Rac1 or depletion of Rac1 by RNAi (RNA interference) abrogated CD44 cleavage induced by EGF. Treatment with PD98059, an inhibitor for MEK (mitogen-activated protein kinase/extracellular-signal-regulated kinase kinase), also suppressed the CD44 cleavage. Furthermore, RNAi studies showed that EGF induced ADAM10 (a disintegrin and metalloproteinase 10)-dependent CD44 cleavage and cell migration. These results indicate that EGF induces ADAM10-mediated CD44 cleavage through Rac1 and mitogen-activated protein kinase activation, and thereby promotes tumour cell migration and invasion. 
   Metastasis  -associated C4.4A, a GPI-anchored protein cleaved by ADAM10 and ADAM17. Metalloproteases play a complex role in tumor progression. While the activity of some ADAM, ADAMTS and matrix metalloproteases (MMPs) seems to be protumorigenic, the activity of others seems to prevent tumor progression. The identification of the array of substrates of a given metalloprotease (degradome) seems an adequate approach to predict the effect of the inhibition of a metalloprotease in tumors. Here, we present the proteomic identification of a novel substrate for ADAM10 and -17. We used SILAC (Stable Isotope Labeling by Amino acids in Cell culture), a proteomic technique based on the differential metabolic labeling of cells in different conditions. This was applied to MCF7 cells derived from an invasive mammary tumor, and the same cells expressing shRNAs that knock down ADAM10 or -17. Following this approach, we have identified C4.4A as a substrate to both metalloproteases. Since C4.4A is likely involved in tumor invasion, these results indicate that the cleavage of C4.4A by ADAM10 and ADAM17 contributes to tumor progression. 
 Nuclear translocation and signalling of L1-CAM in human carcinoma cells requires ADAM10 and presenilin/gamma-secretase activity. L1-CAM (L1 cell-adhesion molecule), or more simply L1, plays an important role in the progression of human carcinoma. Overexpression promotes tumour-cell invasion and motility, growth in nude mice and tumour   metastasis  . It is feasible that L1-dependent signalling contributes to these effects. However, little is known about its mechanism in tumour cells. We reported previously that L1 is cleaved by ADAM (a disintegrin and metalloprotease) and that the cytoplasmic part is essential for L1 function. Here we analysed more closely the role of proteolytic cleavage in L1-mediated nuclear signalling. Using OVMz carcinoma cells and L1-transfected cells as a model, we found that ADAM10-mediated cleavage of L1 proceeds in lipid raft and non-raft domains. The cleavage product, L1-32, is further processed by PS (presenilin)/gamma-secretase to release L1-ICD, an L1 intracellular domain of 28 kDa. Overexpression of dominant-negative PS1 or use of a specific gamma-secretase inhibitor leads to an accumulation of L1-32. Fluorescence and biochemical analysis revealed a nuclear localization for L1-ICD. Moreover, inhibition of ADAM10 and/or gamma-secretase blocks nuclear translocation of L1-ICD and L1-dependent gene regulation. Overexpression of recombinant L1-ICD mediates gene regulation in a similar manner to full-length L1. Our results establish for the first time that regulated proteolytic processing by ADAM10 and PS/gamma-secretase is essential for the nuclear signalling of L1 in human carcinoma cell lines. 
 PAX2 regulates ADAM10 expression and mediates anchorage-independent cell growth of melanoma cells. PAX transcription factors play an important role during development and carcinogenesis. In this study, we investigated PAX2 protein levels in melanocytes and melanoma cells by Western Blot and immunofluorescence analysis and characterized the role of PAX2 in the pathogenesis of melanoma. In vitro we found weak PAX2 protein expression in keratinocytes and melanocytes. Compared to melanocytes increased PAX2 protein levels were detectable in melanoma cell lines. Interestingly, in tissue sections of melanoma patients nuclear PAX2 expression strongly correlated with nuclear atypia and the degree of prominent nucleoli, indicating an association of PAX2 with a more atypical cellular phenotype. In addition, with chromatin immunoprecipitation assay, PAX2 overexpression and PAX2 siRNA we present compelling evidence that PAX2 can regulate ADAM10 expression, a metalloproteinase known to play important roles in melanoma   metastasis  . In human tissue samples we found co-expression of PAX2 and ADAM10 in melanocytes of benign nevi and in melanoma cells of patients with malignant melanoma. Importantly, the downregulation of PAX2 by specific siRNA inhibited the anchorage independent cell growth and decreased the migratory and invasive capacity of melanoma cells. Furthermore, the downregulation of PAX2 abrogated the chemoresistance of melanoma cells against cisplatin, indicating that PAX2 expression mediates cell survival and plays important roles during melanoma progression. 
 A disintegrin and metalloprotease (ADAM)10 is highly expressed in hepatocellular carcinoma and is associated with tumour progression. A disintegrin and metalloprotease (ADAM)10 has been implicated in the progression of various solid tumours. Little is known, however, about its role in hepatocellular carcinoma (HCC). The aim of the present study was to evaluate the protein and transcript level expression of ADAM10 in HCC patients.Samples of HCC and adjacent noncancerous liver tissue were taken during liver resection surgery. Immunostaining was used to measure ADAM10 protein expression levels and quantitative reverse- transcription polymerase chain reaction was used to measure ADAM10 mRNA expression levels. Levels of ADAM10 were compared, and a survival analysis undertaken.In total, 98 HCC patient samples were studied. There were significant associations between protein levels of ADAM10 and tumour grade, amount of tumour differentiation, tumour size and the presence of   metastasis  . Furthermore, ADAM10 protein expression was significantly associated with shortened patient survival.ADAM10 is strongly expressed in a large proportion of HCC cases, which is in agreement with findings in other tumour entities. Expression of ADAM10 may serve as a useful molecular marker for HCC. 
 ADAM10 releases a soluble form of the GPNMB/Osteoactivin extracellular domain with angiogenic properties. Glycoprotein non-metastatic melanoma protein B (GPNMB)/Osteoactivin (OA) is a transmembrane protein expressed in approximately 40-75% of breast cancers. GPNMB/OA promotes the migration, invasion and   metastasis   of breast cancer cells; it is commonly expressed in basal/triple-negative breast tumors and is associated with shorter recurrence-free and overall survival times in patients with breast cancer. Thus, GPNMB/OA represents an attractive target for therapeutic intervention in breast cancer; however, little is known about the functions of GPNMB/OA within the primary tumor microenvironment.We have employed mouse and human breast cancer cells to investigate the effects of GPNMB/OA on tumor growth and angiogenesis. GPNMB/OA-expressing tumors display elevated endothelial recruitment and reduced apoptosis when compared to vector control-derived tumors. Primary human breast cancers characterized by high vascular density also display elevated levels of GPNMB/OA when compared to those with low vascular density. Using immunoblot and ELISA assays, we demonstrate the GPNMB/OA ectodomain is shed from the surface of breast cancer cells. Transient siRNA-mediated knockdown studies of known sheddases identified ADAM10 as the protease responsible for GPNMB/OA processing. Finally, we demonstrate that the shed extracellular domain (ECD) of GPNMB/OA can promote endothelial migration in vitro.GPNMB/OA expression promotes tumor growth, which is associated with enhanced endothelial recruitment. We identify ADAM10 as a sheddase capable of releasing the GPNMB/OA ectodomain from the surface of breast cancer cells, which induces endothelial cell migration. Thus, ectodomain shedding may serve as a novel mechanism by which GPNMB/OA promotes angiogenesis in breast cancer. 
 ADAM10 is upregulated in melanoma   metastasis   compared with primary melanoma. ADAM10 (a disintegrin and metalloproteinase 10) is involved in the ectodomain shedding of various substrates, including adhesion molecules such as L1 cell adhesion molecule (L1-CAM) and CD44, which are known to have important roles in the development of malignant melanoma. In our study, we characterized the expression of ADAM10 in melanoma cells in vitro and in vivo. Immunohistochemical analysis on tissue microarrays indicated that ADAM10 expression was significantly elevated in melanoma   metastasis   compared with primary melanomas. In vitro downregulation of ADAM10 with specific small interfering RNA (siRNA) resulted in a suppression of the anchorage-independent cell growth and reduced the migration of melanoma cells. In addition, overexpression of ADAM10 induced the migration of melanoma cells. In cell lines from melanoma patients with   metastasis  , ADAM10 was significantly overexpressed, and ADAM10 expression correlated with increased cell proliferation. Furthermore, we present evidence that ADAM10 is involved in the release of L1-CAM from melanoma cells. It is important that knockdown of cellular L1-CAM reduced the migration of melanoma cells and abrogated the chemoresistance against cisplatin. In contrast, soluble L1-CAM had no effect on melanoma cell migration or cell survival. Taken together, our data demonstrate that ADAM10 and L1-CAM have important roles during melanoma progression and both molecules represent attractive targets for therapeutical intervention of melanomas. 
 Reciprocal effects between microRNA-140-5p and ADAM10 suppress migration and invasion of human tongue cancer cells. ADAM10, overexpressed in tongue squamous cell carcinoma (TSCC), has been well documented for its role in tumor progression and   metastasis  . In the present study, we evaluated the inhibition effect of microRNAs (miRNAs) on the TSCC and identified that miR-140-5p could directly targets ADAM10 and inhibits the invasion and migration of TSCC cells. LAMC1, HDAC7 and PAX6, clustered into migration-related genes, were validated to be direct targets of miR-140-5p, while IGF1R and PSEN1 were not responsible to the regulation. Most intriguingly, ERBB4 was upregulated by miR-140-5p even though the interaction between ERBB4 3'UTR and miR-140-5p existed simultaneously. Meanwhile, ADAM10 is involved in the "positive" regulation of ERBB4 and negative regulation of PAX6 by miR-140-5p. Taken together, our results suggest that miR-140-5p play a role in TSCC cell migration and invasion, and two brand new relationships between miRNA and its targets emerged: (1) ADAM10 is not just a direct target of miR-140-5p, the repressed ADAM10 also helps to enhance the effect of miR-140-5p to other target genes: ERBB4 and PAX6; (2) ERBB4 is "positively" regulated by miR-140-5p. 
 ADAM 10 expression in primary uveal melanoma as prognostic factor for risk of   metastasis  . Uveal melanoma is the most frequent primary intraocular neoplasm in adults. Although malignant melanoma may be located at any point in the uveal tract, the choroid and ciliary body are more frequent locations than the iris. In the present study, we examined ADAM10 expression levels in primary uveal melanoma both with and without   metastasis  , and we evaluated their association with other high risk characteristics for   metastasis   in order to assess if ADAM10 can be used to predict   metastasis  . This study included a total of 52 patients, 23 men and 29 women, with uveal melanoma. A significantly high expression of ADAM-10 was seen in patients with   metastasis   (11/13, 84.6%), but not in patients without   metastasis   (15/39, 38.5%). In conclusion we found that ADAM10 expression was associated with a more rapid metastatic progression confirming its role in uveal melanoma   metastasis  . 
 ADAM 10 is associated with gastric cancer progression and prognosis of patients. The metalloproteinase domain-containing protein 10 (ADAM 10) has been implicated in the development and progression of gastric cancer.Expression of ADAM 10 and C-erbB-2 were examined immunochemically in 436 clinicopathologically characterized gastric cancer cases.Protein levels of ADAM 10 and C-erbB-2 were up-regulated in gastric cancer lesions compared with adjacent non-cancerous tissues. Positive expression of ADAM 10 correlated with age, size of tumor, location of tumor, depth of invasion, vessel invasion, lymph node, and distant   metastasis   and TNM stage, and also with expression of C-erbB-2. In stages I, II, and III, the 5-year survival rate of patients with high ADAM 10 expression was significantly lower than in patients with low expression. However, in stage IV, ADAM 10 expression did not correlate with the 5-year survival rate. Further multivariate analysis suggests that up-regulation of ADAM 10 and C-erbB-2 were independent prognostic indicators for the disease, along with depth of invasion, lymph node and distant   metastasis   and TNM stage.Expression of ADAM 10 in gastric cancer is significantly associated with lymph node and distant   metastasis  , high C-erbB-2 expression, and poor prognosis. ADAM 10 and C-erbB-2 proteins could be useful markers to predict tumor progression and prognosis. 
 Silencing ADAM10 inhibits the in vitro and in vivo growth of hepatocellular carcinoma cancer cells. A disintegrin and metalloprotease 10 (ADAM10) is a transmembrane protein associated with   metastasis   in a number of types of cancer. Little is known, however, regarding the role of ADAM10 in hepatocellular carcinoma (HCC). The aim of the present study was to evaluate whether downregulation of ADAM10 effects HCC cell proliferation, cell cycle, cell migration and cell invasion. A recombinant small hairpin RNA expression vector carrying ADAM10 was constructed and then transfected into the HepG2 human HCC cell line. In vitro cell proliferation, cell cycle, cell migration and cell invasion, and in vivo tumor growth were determined following the downregulation of ADAM10 by RNA interference. The results revealed that downregulation of ADAM10 expression in HepG2 tumor cells using the RNA silencing approach significantly suppressed cell proliferation, cell migration and cell invasion in vitro, and tumor growth in vivo. Furthermore, ADAM10 silencing was able to significantly reduce constitutive phosphorylation of phosphoinositide 3-kinase (PI3K) and Akt, which implies that ADAM10 is, at least partially, involved in the activation of the PI3K/Akt signaling pathway. These results suggest that ADAM10 is important in regulating the proliferation and   metastasis   of HCC. Thus, ADAM10 is a promising therapeutic target for the prevention of tumor metastases in HCC. 
 Nuclear translocation of ADAM-10 contributes to the pathogenesis and progression of human prostate cancer. A disintegrin and metalloproteases (ADAM) are cell membrane-anchored proteins with potential implications for the   metastasis   of human cancer cells via cell adhesion and protease activities. In prostate cancer (PC), the ADAM-10 protein showed a nuclear localization whereas in benign prostate hypertrophy (BPH) it was predominantly bound to the cell membrane. We hypothesized that the pathogenesis and progression of PC are attributable to the nuclear translocation of ADAM-10. Immunoblotting revealed that after 5alpha-dihydrotestosterone treatment, a 60-kDa active form of ADAM-10 was increased in the nuclear fraction but decreased in the cell membrane and cytoplasmic fractions of human androgen-dependent PC cells. Immunocytochemistry revealed that after 5alpha-dihydrotestosterone treatment, the ADAM-10 protein was translocated from the cell membrane to the nucleus. Coimmunoprecipitation of androgen receptor and ADAM-10 was detected in the nuclear fraction but not in the cell membrane and cytoplasmic fractions. Immunohistochemical study of 64 PC and 20 BPH samples showed that the intensity of ADAM-10 staining was significantly higher in the nuclei of PC cells than in the nuclei of BPH cells (P < 0.0001). It was also significantly lower in the cell membrane of PC cells than in the cell membrane of BPH cells (P = 0.0017). Nuclear staining intensity was significantly correlated with the clinical T-factor (P = 0.004), the Gleason score (P < 0.0001) and preoperative prostate-specific antigen levels (P = 0.0061). ADAM-10 small interfering RNA transfectants showed a significant decrease in cell growth compared to the controls. Our results suggest that in human PC, the nuclear translocation of ADAM-10 coupled with the androgen receptor is involved in tumor growth and progression. 
 Hypoxia induces escape from innate immunity in cancer cells via increased expression of ADAM10: role of nitric oxide. One key to malignant progression is the acquired ability of tumor cells to escape immune-mediated lysis. Whereas tumor hypoxia is known to play a causal role in cancer   metastasis   and resistance to therapy, the link between hypoxia and immune escape in cancer remains poorly understood. Here, we show that hypoxia induces tumor cell resistance to lysis mediated by immune effectors and that this resistance to lysis occurs via a hypoxia-inducible factor-1 (HIF-1)-dependent pathway linked to increased expression of the metalloproteinase ADAM10. This enzyme is required for the hypoxia-induced shedding of MHC class I chain-related molecule A (MICA), a ligand that triggers the cytolytic action of immune effectors, from the surface of tumor cells. Indeed, our findings show a mechanistic link between hypoxia-induced accumulation of the α-subunit of HIF-1 (HIF-1α), increased expression of ADAM10, and decreased surface MICA levels leading to tumor cell resistance to lysis mediated by innate immune effectors. Nitric oxide mimetic agents interfered with the hypoxia-induced accumulation of HIF-1α and with the hypoxia-induced upregulation of ADAM10 expression required for decreased surface MICA expression and resistance to lysis. Furthermore, treatment of tumor-bearing mice with nitroglycerin, a nitric oxide mimetic, attenuated tumor growth by a mechanism that relied upon innate immune effector cells. Together, these findings reveal a novel mechanism by which the hypoxic tumor microenvironment contributes to immune escape in cancer, lending support to potential immunotherapeutic strategies involving the use of nitric oxide mimetics. 
 Presenilin-dependent gamma-secretase activity mediates the intramembranous cleavage of CD44. CD44 is the major adhesion molecule for the extracellular matrix components and is implicated in a wide variety of physiological and pathological processes including the regulation of tumor cell growth and   metastasis  . Our previous studies have shown that CD44 undergoes sequential proteolytic cleavages in the extracellular and transmembrane domains and the cleavage product derived from CD44 intramembranous cleavage acts as a signal transduction molecule. However, the underlying mechanism of the intramembranous cleavage of CD44 remains to be elucidated. In the present study, we report for the first time that CD44 is a substrate of the presenilin (PS)-dependent gamma-secretase. We demonstrate that the intramembranous cleavage of CD44 induced by 12-O-tetradecanoylphorbol 13-acetate (TPA) treatment or mechanical scraping is blocked by gamma-secretase inhibitors in U251MG cells and that this cleavage is also inhibited in PS-deficient mouse embryonic fibroblasts. Furthermore, we showed that PS1 is redistributed to ruffling areas of the plasma membrane similarly to CD44 after TPA treatment, supporting our biochemical observation that PS1 is involved in the intramembranous cleavage of CD44. Our present findings suggest important implications for understanding CD44-dependent signal transduction and a potential role of PS/gamma-secretase activity in the functional regulation of adhesion molecules. 
 MicroR-140-5p suppresses tumor cell migration and invasion by targeting ADAM10-mediated Notch1 signaling pathway in hypopharyngeal squamous cell carcinoma. MicroRNAs (miRNAs) are small non-coding RNAs of approximately 22 nucleotides that negatively regulate gene expression at the post-transcriptional level. Downexpression of miR-140-5p was reported in some human cancers, and combined with a reduction of cell migration and invasion, suggesting that miR-140-5p functions as a tumor suppressor. However, little is known about the expression and function of miR-140-5p in hypopharyngeal squamous cell carcinoma (HSCC). In this research, we found that miR-140-5p was significantly downregulated in HSCC tissues and correlated to tumor classification and lymph node   metastasis  . Restoration of miR-140-5p suppressed the migration and invasion of FaDu cells, and decreased the protein expression levels of ADAM10. Furthermore, the luciferase reporter assay revealed that miR-140-5p was directly bound to ADAM10 mRNA and knockdown of ADAM10 could inhibit FaDu cell migration and invasion and reduced the protein expression levels of and Notch1 intracellular domain (NICD1). Of note, knockdown of Notch1 could inhibit the migration and invasion of FaDu cells and rescued the effect of miR-140-5p inhibitor in FaDu cells. Taken together, our study demonstrates that miR-140-5p suppresses tumor migration and invasion by inhibiting ADAM10-mediated Notch1 signaling pathway and suggests that miR-140-5p could have potential therapeutic applications in HSCC. 
 Inhibiting adenoid cystic carcinoma cells growth and   metastasis   by blocking the expression of ADAM 10 using RNA interference. Adenoid cystic carcinoma is one of the most common types of salivary gland cancers. The poor long-term prognosis for patients with adenoid cystic carcinoma is mainly due to local recurrence and distant   metastasis  . Disintegrin and metalloprotease 10 (ADAM 10) is a transmembrane protein associated with   metastasis   in a number of diverse of cancers. The aim of this study was to analyze the relationship between ADAM 10 and the invasive and metastatic potentials as well as the proliferation capability of adenoid cystic carcinoma cells in vitro and in vivo.Immunohistochemistry and Western blot analysis were applied to detect ADAM 10 expression levels in metastatic cancer tissues, corresponding primary adenoid cystic carcinoma tissues, adenoid cystic carcinoma cell lines with high metastatic potential, and adenoid cystic carcinoma cell lines with low metastatic potential. RNA interference was used to knockdown ADAM 10 expression in adenoid cystic carcinoma cell lines with high metastatic potential. Furthermore, the invasive and metastatic potentials as well as the proliferation capability of the treated cells were observed in vitro and in vivo.It was observed that ADAM 10 was expressed at a significantly higher level in metastatic cancer tissues and in adenoid cystic carcinoma cell lines with high metastatic potential than in corresponding primary adenoid cystic carcinomas and adenoid cystic carcinoma cell lines with low metastatic potential. Additionally, silencing of ADAM 10 resulted in inhibition of cell growth and invasion in vitro as well as inhibition of cancer   metastasis   in an experimental murine model of lung metastases in vivo.These studies suggested that ADAM 10 plays an important role in regulating proliferation and   metastasis   of adenoid cystic carcinoma cells. ADAM 10 is potentially an important therapeutic target for the prevention of tumor metastases in adenoid cystic carcinoma. 
  GeneRIFs  
 we found that ADAM10 expression was associated with a more rapid metastatic progression confirming its role in uveal melanoma   metastasis  . 
 Active ADAM10 promotes the carcinogenesis, invasion,   metastasis   and proliferation of ESCC and controls invasion and   metastasis   at least in part through the shedding of E-cadherin activity. 
 These results suggest that ADAM10 is important in regulating the proliferation and   metastasis   of HCC. Thus, ADAM10 is a promising therapeutic target for the prevention of tumor metastases in HCC. 
 PAX2 can regulate ADAM10 expression, a metalloproteinase known to play important roles in melanoma   metastasis  . 
 Expression of ADAM 10 in gastric cancer is significantly associated with lymph node and distant   metastasis  , high C-erbB-2 expression, and poor prognosis. 
 ADAM10 overexpression in colon cancer cells displaying endogenous L1-CAM enhanced L1-CAM cleavage and induced liver   metastasis  , and ADAM10 also enhanced   metastasis   in colon cancer cells stably transfected with L1-CAM. 
  [Cc]ell [Ii]nvasion  PubMed  
 ADAM10 correlates with uveal melanoma metastasis and promotes in vitro invasion. Uveal melanoma (UM) is a rare ocular tumor that may lead to deadly metastases in 50% of patients. A disintegrin and metalloproteinase (ADAM)10, ADAM17, and the HGF-receptor c-Met support invasiveness in different tumors. Here, we report that high ADAM10, MET, and, to a lesser extent, ADAM17 gene expression correlates with poor progression-free survival in UM patients (hazard ratio 2.7, 2.6, and 1.9, respectively). About 60% of primary UM expresses c-Met and/or ADAM10 proteins. Four UM cell lines display high levels of ADAM10 and ADAM17, which constitutively cleave c-Met, inducing the release of soluble c-Met. ADAM10/17 pharmacological inhibition or gene silencing reduces c-Met shedding, but has limited impact on surface c-Met, which is overexpressed. Importantly, ADAM10 silencing inhibits UM   cell invasion   driven by FCS or HGF, while ADAM17 silencing has a limited effect. Altogether our data indicate that ADAM10 has a pro-invasive role and may contribute to UM progression. 
 ADAM10 mediates the   cell invasion   and metastasis of human esophageal squamous cell carcinoma via regulation of E-cadherin activity. A disintegrin and metalloprotease 10 (ADAM10) is involved in the tumorigenesis, invasion and metastasis of several types of solid tumors. However, the potential role of ADAM10 in human esophageal squamous cell carcinoma (ESCC) is not yet well understood. The present study showed that ADAM10 was overexpressed in human ESCC tissues in vivo, and positively associated with depth of tumor invasion, lymph node metastasis and TNM stage, contributing to tumor carcinogenesis, invasion and metastasis. Additionally, ADAM10 was overexpressed in 3 types of ESCC cell lines in vitro, as compared to that in normal esophageal epithelial cells (NEECs); and moreover, ESCC cells with high ADAM10 expression obtained enhanced invasion and migration ability. Subsequently, ADAM10 silencing by small interfering (si) RNA in ESCC cell line, EC-1, reduced   cell invasion  , migration and proliferation in vitro. Finally, ADAM10 negatively regulated E-cadherin in ESCC in vivo and in vitro. In conclusion, active ADAM10 promotes the carcinogenesis, invasion, metastasis and proliferation of ESCC and controls invasion and metastasis at least in part through the shedding of E-cadherin activity, which makes it a potential biomarker and a useful therapeutic target for ESCC. 
 Self-control of HGF regulation on human trophoblast   cell invasion   via enhancing c-Met receptor shedding by ADAM10 and ADAM17. Hepatocyte growth factor (HGF)/c-Met signaling has been implicated in mammalian placental development. Integral c-Met can be released from endothelial cell membrane by proteolysis to form a soluble, truncated protein [soluble Met (sMet)], which is biochemically able to bind HGF and may disrupt HGF/c-Met signaling. By far, production of sMet in human placenta has not been reported, and the shedding mechanism remains unclear.In this study, production of sMet in healthy pregnant placenta and preeclamptic ones was compared, and the role of sMet on trophoblast   cell invasion   as well as the regulation of c-Met shedding by HGF were investigated in an immortal trophoblast cell line, B6Tert-1.Placenta productions of sMet, pro- and active forms of a disintegrin and metalloprotease 10 (ADAM10) and ADAM17 in preeclamptic patients were significantly higher than those in normal pregnant women. In B6Tert-1 cells, the HGF-induced promotion on   cell invasion   and activation of MAPK and AKT could be extensively blocked by sMet. ADAM10 and ADAM17, but not ADAM12, were explored to be sheddases of c-Met. HGF down-regulated c-Met receptor expression, whereas it up-regulated pro- and active/mature forms of ADAM10 and ADAM17 expression, which resulted in enhanced sMet production. Stimulation of H(2)O(2) caused an increase in active ADAM10, pro-ADAM17, and active ADAM17 levels and thus excessive c-Met shedding.HGF could negatively self-control its regulatory effect on trophoblast   cell invasion   via enhancing proteolysis of its receptor. Unbalancing of HGF self-control by oxidative stress may lead to impeding placentation in relevance to preeclampsia. 
 Nuclear translocation and signalling of L1-CAM in human carcinoma cells requires ADAM10 and presenilin/gamma-secretase activity. L1-CAM (L1 cell-adhesion molecule), or more simply L1, plays an important role in the progression of human carcinoma. Overexpression promotes tumour-  cell invasion   and motility, growth in nude mice and tumour metastasis. It is feasible that L1-dependent signalling contributes to these effects. However, little is known about its mechanism in tumour cells. We reported previously that L1 is cleaved by ADAM (a disintegrin and metalloprotease) and that the cytoplasmic part is essential for L1 function. Here we analysed more closely the role of proteolytic cleavage in L1-mediated nuclear signalling. Using OVMz carcinoma cells and L1-transfected cells as a model, we found that ADAM10-mediated cleavage of L1 proceeds in lipid raft and non-raft domains. The cleavage product, L1-32, is further processed by PS (presenilin)/gamma-secretase to release L1-ICD, an L1 intracellular domain of 28 kDa. Overexpression of dominant-negative PS1 or use of a specific gamma-secretase inhibitor leads to an accumulation of L1-32. Fluorescence and biochemical analysis revealed a nuclear localization for L1-ICD. Moreover, inhibition of ADAM10 and/or gamma-secretase blocks nuclear translocation of L1-ICD and L1-dependent gene regulation. Overexpression of recombinant L1-ICD mediates gene regulation in a similar manner to full-length L1. Our results establish for the first time that regulated proteolytic processing by ADAM10 and PS/gamma-secretase is essential for the nuclear signalling of L1 in human carcinoma cell lines. 
 Silencing ADAM10 inhibits the in vitro and in vivo growth of hepatocellular carcinoma cancer cells. A disintegrin and metalloprotease 10 (ADAM10) is a transmembrane protein associated with metastasis in a number of types of cancer. Little is known, however, regarding the role of ADAM10 in hepatocellular carcinoma (HCC). The aim of the present study was to evaluate whether downregulation of ADAM10 effects HCC cell proliferation, cell cycle, cell migration and   cell invasion  . A recombinant small hairpin RNA expression vector carrying ADAM10 was constructed and then transfected into the HepG2 human HCC cell line. In vitro cell proliferation, cell cycle, cell migration and   cell invasion  , and in vivo tumor growth were determined following the downregulation of ADAM10 by RNA interference. The results revealed that downregulation of ADAM10 expression in HepG2 tumor cells using the RNA silencing approach significantly suppressed cell proliferation, cell migration and   cell invasion   in vitro, and tumor growth in vivo. Furthermore, ADAM10 silencing was able to significantly reduce constitutive phosphorylation of phosphoinositide 3-kinase (PI3K) and Akt, which implies that ADAM10 is, at least partially, involved in the activation of the PI3K/Akt signaling pathway. These results suggest that ADAM10 is important in regulating the proliferation and metastasis of HCC. Thus, ADAM10 is a promising therapeutic target for the prevention of tumor metastases in HCC. 
 Differential expression of ADAM (a disintegrin and metalloproteinase) genes between human first trimester villous and extravillous trophoblast cells. A disintegrin and metalloproteinases (ADAMs) are members of the metzincin family of zinc-dependent metalloproteinases that play pivotal roles in the proteolytic degradation of the extracellular matrix for   cell invasion  . Few studies have investigated the ADAM subtypes that are expressed in first trimester trophoblast cells. The purpose of this study was to elucidate the differential expression profiles of ADAMs between first trimester villous trophoblast cells (VTs) and extravillous trophoblast cells (EVTs). We isolated EVTs from explanted human first trimester chorionic villi and investigated the mRNA expression levels of five members of the ADAM family (ADAMTS1, ADAMTS2, ADAM10, ADAM12, and ADAM17) using real-time PCR. Chorionic villous tips were defined as first trimester VTs. Of the differentially expressed ADAM genes between first trimester VTs and EVTs, ADAMTS1 was expressed at a significantly higher level in EVTs than in VTs. In contrast, both ADAM10 and ADAM12 were expressed at significantly higher levels in VTs than in EVTs. No differences were found in the mRNA levels of ADAMTS2 and ADAM17 between the two cell types. Moreover, we demonstrated that in VTs, the expression level of ADAM12 was significantly downregulated in the late first trimester (10-13 gestational weeks) compared to the middle first trimester (7-8 weeks). These results suggest that first trimester trophoblast cells express ADAM genes in cell type- and gestational age-dependent manners. Our data provide additional insight into the functions of ADAMs in the human placenta. 
   
  
 RPS6KB1 
 
  NCBI Gene  
  GeneRIF  
  Pubmed  
 
 
  Search Term  Rank  Fields  
  [Ff]ilopodia  1  PubMed(1)  
  [Ff]ilopodium  2  PubMed(1)  
  [Mm]igration  6  Process(2); PubMed(12); GeneRIFs(6)  
  [Ii]nvasive  10  PubMed(5); GeneRIFs(1)  
  [Mm]etastasize  11  PubMed(1)  
  [Mm]etastasis  12  PubMed(7); GeneRIFs(2)  
  [Cc]ell [Ii]nvasion  13  PubMed(3)  
 
 
 hide/show details for RPS6KB1 
  [Ff]ilopodia  PubMed  
 Targeting protein phosphatase 1 (PP1) to the actin cytoskeleton: the neurabin I/PP1 complex regulates cell morphology. Neurabin I, a neuronal actin-binding protein, binds protein phosphatase 1 (PP1) and p70 ribosomal S6 protein kinase (p70S6K), both proteins implicated in cytoskeletal dynamics. We expressed wild-type and mutant neurabins fused to green fluorescent protein in Cos7, HEK293, and hippocampal neurons. Biochemical and cellular studies showed that an N-terminal F-actin-binding domain dictated neurabin I localization at actin cytoskeleton and promoted disassembly of stress fibers. Deletion of the C-terminal coiled-coil and sterile alpha motif domains abolished neurabin I dimerization and induced filopodium extension. Immune complex assays showed that neurabin I recruited an active PP1 via a PP1-docking sequence,(457)KIKF(460). Mutation of the PP1-binding motif or PP1 inhibition by okadaic acid and calyculin A abolished   filopodia   and restored stress fibers in cells expressing neurabin I. In vitro and in vivo studies suggested that the actin-binding domain attenuated protein kinase A (PKA) phosphorylation of neurabin I. Modification of a major PKA site, serine-461, impaired PP1 binding. Finally, p70S6K was excluded from neurabin I/PP1 complexes and required the displacement of PP1 for recruitment to neurabin I. These studies provided new insights into the assembly and regulation of a neurabin I/PP1 complex that controls actin rearrangement to promote spine development in mammalian neurons. 
  [Ff]ilopodium  PubMed  
 Targeting protein phosphatase 1 (PP1) to the actin cytoskeleton: the neurabin I/PP1 complex regulates cell morphology. Neurabin I, a neuronal actin-binding protein, binds protein phosphatase 1 (PP1) and p70 ribosomal S6 protein kinase (p70S6K), both proteins implicated in cytoskeletal dynamics. We expressed wild-type and mutant neurabins fused to green fluorescent protein in Cos7, HEK293, and hippocampal neurons. Biochemical and cellular studies showed that an N-terminal F-actin-binding domain dictated neurabin I localization at actin cytoskeleton and promoted disassembly of stress fibers. Deletion of the C-terminal coiled-coil and sterile alpha motif domains abolished neurabin I dimerization and induced   filopodium   extension. Immune complex assays showed that neurabin I recruited an active PP1 via a PP1-docking sequence,(457)KIKF(460). Mutation of the PP1-binding motif or PP1 inhibition by okadaic acid and calyculin A abolished filopodia and restored stress fibers in cells expressing neurabin I. In vitro and in vivo studies suggested that the actin-binding domain attenuated protein kinase A (PKA) phosphorylation of neurabin I. Modification of a major PKA site, serine-461, impaired PP1 binding. Finally, p70S6K was excluded from neurabin I/PP1 complexes and required the displacement of PP1 for recruitment to neurabin I. These studies provided new insights into the assembly and regulation of a neurabin I/PP1 complex that controls actin rearrangement to promote spine development in mammalian neurons. 
  [Mm]igration  Process  
 cell   migration   
 positive regulation of smooth muscle cell   migration   
  PubMed  
 The mTOR Kinase Inhibitor INK128 Blunts   Migration   of Cultured Retinal Pigment Epithelial Cells. Retinal pigment epithelium (RPE) cell   migration   in response to disease has been reported for age-related macular degeneration, proliferative vitreoretinopathy, and proliferative diabetic retinopathy. The complex molecular process of RPE cell   migration   is regulated in part by growth factors and cytokines, and activation of the PI3/AKT/mTOR signaling pathway. Rapamycin, an allosteric mTOR inhibitor, has been shown to block only one of the primary downstream mTOR effectors, p70 S6 kinase 1, in many cell types. INK128, a selective mTOR ATP binding site competitor, blocks both p70 S6 kinase 1 and a second primary downstream effector, 4E-BP1. We performed scratch assays using differentiated ARPE-19 and primary porcine RPE cells to assess the effect of mTOR inhibition on cell   migration  . We found that INK128-mediated blocking of both p70 S6 kinase 1 and 4E-BP1 was much more effective at preventing RPE cell   migration   than rapamycin-mediated inhibition of p70 S6 kinase 1 alone. 
 Molecular crosstalk between p70S6k and MAPK cell signaling pathways. We report here for the first time that the specific MAPK kinase (MEK) inhibitor, PD-98059, completely knocked out granulocyte-macrophage colony-stimulating factor (GM-CSF)-stimulated MAPK activity but also partially inactivated the ribosomal kinase p70S6K. Since a connection between the two major signaling pathways, Ras/MEK/MAPK and PI3-K/p70S6K was suspected, experiments were designed to prove a molecular crosstalk between those. First, p70S6K protein could be co-immunoprecipitated with anti-MAPK antibodies, MAPK protein was similarly present in anti-p70S6K immunoprecipitates, indicating close spatial proximity of both signaling molecules. Second, p70S6K enzymatic activity was found in anti-MAPK immunoprecipitates and MAPK in anti-p70S6K immunoprecipitates, being the latter activity higher in samples derived from GM-CSF-treated cells. Since an upstream activator of p70S6K, phosphatidylinositol (PI)3-kinase, has been associated to cell movement in phagocytic cells, we studied a possible participation of p70S6K in chemotaxis and whether MAPK had an input. Our data show that functional chemotaxis was inhibited by rapamycin, a specific p70S6K inhibitor, as well as by PD-98059. Thus, a connection between these two kinases extends from the molecular level to cell   migration  , a key functionality in non-proliferative, mature phagocytes such as neutrophils. 
 Mammalian target of rapamycin (mTOR) and S6 kinase down-regulate phospholipase D2 basal expression and function. The mammalian target of rapamycin (mTOR) and S6 kinase (S6K) pathway is essential for cell differentiation, growth, and survival. Phospholipase D2 (PLD2) plays a key role in mTOR/S6K mitogenic signaling. However, the impact of PLD on mTOR/S6K gene expression is not known. Here we show that interleukin-8 (IL-8) increases mRNA expression levels for PLD2, mTOR, and S6K, with PLD2 preceding mTOR/S6K in time. Silencing of PLD2 gene expression abrogated IL-8-induced mTOR/S6K mRNA expression, whereas silencing of mTOR or S6K gene expression resulted in large (>3-fold and >5-fold, respectively) increased levels of PLD2 RNA, which was paralleled by increases in protein expression and lipase activity. Treatment of cells with 0.5 nm rapamycin induced a similar trend. These results suggest that, under basal conditions, PLD2 expression and concomitant activity is negatively regulated by the mTOR/S6K signaling pathway. Down-regulation of PLD2 was confirmed in differentiated HL-60 leukocytes overexpressing an mTOR-wild type, but not an mTOR kinase-dead construct. At the cellular level, overexpression of mTOR-wild type resulted in lower basal cell   migration  , which was reversed by treatment with IL-8. We propose that IL-8 reverses an mTOR/S6K-led down-regulation of PLD2 expression and enables PLD2 to fully function as a facilitator for cell   migration  . 
 miR-195 Inhibits Tumor Progression by Targeting RPS6KB1 in Human Prostate Cancer. To investigate the involvement of hsa-miRNA-195-5p (miR-195) in progression and prognosis of human prostate cancer.qRT-PCR was performed to detect miR-195 expression in both prostate cancer cell lines and clinical tissue samples. Its clinical significance was statistically analyzed. The roles of miR-195 and its candidate target gene, ribosomal protein S6 kinase, 70 kDa, polypeptide 1 (RPS6KB1) in prostate cancer progression were confirmed on the basis of both in vitro and in vivo systems.miR-195 downregulation in prostate cancer tissues was significantly associated with high Gleason score (P = 0.001), positive metastasis failure (P < 0.001), and biochemical recurrence (BCR, P < 0.001). Survival analysis identified miR-195 as an independent prognostic factor for BCR-free survival of prostate cancer patients (P = 0.022). Then, we confirmed the tumor suppressive role of miR-195 through prostate cancer cell invasion,   migration  , and apoptosis assays in vitro, along with tumor xenograft growth, angiogenesis, and invasion in vivo according to both gain-of-function and loss-of-function experiments. In addition, RPS6KB1 was identified as a novel direct target of miR-195 through proteomic expression profiling combined with bioinformatic target prediction and luciferase reporter assay. Moreover, the reexpression and knockdown of RPS6KB1 could respectively rescue and imitate the effects induced by miR-195. Importantly, RPS6KB1 expression was closely correlated with aggressive progression and poor prognosis in prostate cancer patients as opposed to miR-195. Furthermore, we identified MMP-9, VEGF, BAD, and E-cadherin as the downstream effectors of miR-195-RPS6KB1 axis.The newly identified miR-195-RPS6KB1 axis partially illustrates the molecular mechanism of prostate cancer progression and represents a novel potential therapeutic target for prostate cancer treatment. 
 p70 S6 kinase in the control of actin cytoskeleton dynamics and directed   migration   of ovarian cancer cells. Ovarian cancer is highly metastatic with a poor prognosis. The serine/threonine kinase, p70 S6 kinase (p70(S6K)), which is a downstream effector of phosphatidylinositol 3-kinase/Akt pathway, is frequently activated in ovarian cancer. Here, we show that p70(S6K) is a critical regulator of the actin cytoskeleton in the acquisition of the metastatic phenotype. This regulation is through two important activities: p70(S6K) acts as an actin filament cross-linking protein and as a Rho family GTPase-activating protein. Ectopic expression of constitutively active p70(S6K) in ovarian cancer cells induced a marked reorganization of the actin cytoskeleton and promoted directional cell   migration  . Using cosedimentation and differential sedimentation assays, p70(S6K) was found to directly bind to and cross-link actin filaments. Immunofluorescence studies showed p70(S6K) colocalized with cytochalasin D-sensitive actin at the leading edge of motile cells. The p70(S6K) did not affect the kinetics of spontaneous actin polymerization, but could stabilize actin filaments by the inhibition of cofilin-induced actin depolymerization. In addition, we showed that p70(S6K) stimulated the rapid activation of both Rac1 and Cdc42, and their downstream effector p21-activated kinase (PAK1), but not RhoA. Depletion of p70(S6K) expression or inhibition of its activity resulted in significant inhibition of actin cytoskeleton reorganization and reduced   migration  , with a concomitant reduction in Rac1, Cdc42 and PAK1 activation, confirming that the effect was p70(S6K) specific. Similarly, the actin cytoskeleton reorganization/migratory phenotype could be reversed by expression of dominant negative Rac1 and Cdc42, or inhibition of PAK1. These results reveal a new direction for understanding the oncogenic roles of p70(S6K) in tumor progression. 
 Activation of the PI3K/Akt/mTOR/p70S6K pathway is involved in S100A4-induced viability and   migration   in colorectal cancer cells. The S100 protein family member S100A4 regulates various cellular functions. Previous studies have shown that elevated expression of S100A4 is associated with progression and metastasis of colorectal cancer (CRC). However, little is known about whether and how S100A4 contributes to CRC development. In our present study, the elevated expression of S100A4 in CRC tissues compared to matched adjacent normal tissues was confirmed by immunohistochemistry, semi-quantitative RT-PCR and Western blot. Adenovirus-mediated S100A4 overexpression obviously enhanced viability and   migration   of CRC cells, which was detected by MTT assay and transwell assay, respectively. Additionally, S100A4 overexpression increased the phosphorylation levels of Akt, mTOR and p70S6K. These effects of S100A4 were abolished by treatment with either the specific PI3K/Akt inhibitor LY294002, or the specific mTOR/p70S6K inhibitor rapamycin. Furthermore, overexpression of S100A4 resulted in upregulation of VEGF and downregulation of E-cadherin, which were strongly reversed by either LY294002 or rapamycin. Altogether, our results demonstrate that activation of the PI3K/Akt/mTOR/p70S6K signaling pathway is involved in S100A4-induced viability,   migration  , upregulation of VEGF and downregulation of E-cadherin in CRC cells. 
 Activation of p70S6K induces expression of matrix metalloproteinase 9 associated with hepatocyte growth factor-mediated invasion in human ovarian cancer cells. The expression of hepatocyte growth factor (HGF) receptor, encoded by the Met oncogene, is elevated in ovarian and a variety of cancers. Here we show that human ovarian cancer cells with high Met expression were more sensitive to the cell motility and invasion effect of HGF. Met down-regulation by small interfering RNAs or K252a resulted in reduced   migration   in response to HGF. The invasive/migratory phenotype activated by HGF can be blocked by specific inhibitors of the phosphatidylinositol-3-kinase (PI3K) cascade, inhibitor of p70(S6K), and also the expression of a dominant-negative Akt, demonstrating that HGF transmits the motogenic signal through PI3K and Akt to p70(S6K). A significant role for p70(S6K) in cell invasion is further supported by the observation that expression of constitutively active forms of p70(S6K) is sufficient to induce invasive and migratory phenotypes in ovarian cancer cells. Importantly, activation of p70(S6K) stimulated expression and proteolytic activity of matrix metalloproteinase (MMP)-9 and cellular invasion, whereas it had little effect on MMP-2, suggesting for the first time that MMP-9 up-regulation by p70(S6K) as a key step for HGF-induced invasion and   migration  . These data suggest that interfering p70(S6K) may provide a novel means of controlling tumor cell invasiveness. 
 Targeting p70S6K prevented lung metastasis in a breast cancer xenograft model. Overexpression of p70S6K in breast cancer patients is associated with aggressive disease and poor prognosis. Recent studies showed that patients with breast cancer with increased p70S6K phosphorylation had poor survival and increased metastasis. The purpose of our study was to determine whether knockdown of p70S6K would inhibit cell growth, invasion, and metastasis in breast cancer. We therefore stably knocked down p70S6K expression in MDA-231, a highly metastatic breast cancer cell line, using a lentiviral short hairpin RNA (shRNA) based approach. Inhibition of p70S6K led to inhibition of cell growth,   migration  , and invasion in vitro. To determine the role of p70S6K in breast cancer tumorigenesis and metastasis, we used an MDA-231 orthotopic and metastatic animal model. In the orthotopic model, mice injected with MDA-231-p70S6K shRNA cells developed significantly smaller tumors than control mice injected with MDA-231 control shRNA cells (P < 0.01). No metastasis was observed in the p70S6K downregulated group, whereas lung metastasis was detected in all mice in the control group. To determine the role of p70S6K on growth and invasion, we tested downstream signaling targets by Western blot analysis. Knockdown of p70S6K inhibited phosphorylation of focal adhesion kinase, tissue transglutaminase 2, and cyclin D1 proteins, which promote cell growth, survival, and invasion. In addition, downregulation of p70S6K induced expression of PDCD4, a tumor-suppressor protein. In conclusion, we showed that p70S6K plays an important role in metastasis by regulating key proteins like cyclin D1, PDCD4, focal adhesion kinase, E-cadherin, beta-catenin, and tissue transglutaminase 2, which are essential for cell attachment, survival, invasion, and metastasis in breast cancer. 
 Phosphorylated osteopontin promotes   migration   of human choriocarcinoma cells via a p70 S6 kinase-dependent pathway. This study examined the role of osteopontin (OPN), a phosphorylated secreted glycoprotein, in the promotion of trophoblastic cell   migration  , an early event in the embryo implantation process. Three human choriocarcinoma cell lines, namely JAR, BeWo, and JEG-3, were treated with variants of OPN differing in the extent of phosphorylation following sequential dephosphorylation with tartrate-resistant acid phosphatase (TRAP), and their migratory response was measured. The highly phosphorylated human milk form of OPN (OPN-1) strongly triggered   migration   in all three cell lines, whereas the less phosphorylated variants, OPN-2a and OPN-2b, failed to stimulate   migration  . JAR cell   migration   in response to OPN-1 was accompanied by a rapid rearrangement of actin filaments to the cellular membrane. Using broad spectrum protein kinase profiling, we identified p70 S6 kinase as a major signal transduction pathway activated by OPN-1 during the migratory response in JAR cells. Activation was blocked completely by rapamycin and LY294002, thus demonstrating that OPN-1-stimulated   migration   occurs through mTOR and PI3K pathways, respectively. Conversely, PD98059 did not affect the activation of p70 S6 kinase by OPN-1, therefore, this response does not involve the Ras/ MAPK signaling cascade. Together, these data show that the highly phosphorylated human OPN-1 can stimulate trophoblastic cell   migration   and provides evidence for the involvement of the PI3K/mTOR/p70 S6 kinase pathway in the JAR cells response. Because both OPN and TRAP are expressed in the uterus during early pregnancy, it is conceivable that extracellular phosphatases such as TRAP may modify OPN charge state and thus modulate cell   migration  . 
 Role of PI3K and AKT specific isoforms in ovarian cancer cell   migration  , invasion and proliferation through the p70S6K1 pathway. Ovarian cancer is the leading cause of death from gynecological malignancy for women. The amplification of the PI3K catalytic subunit (p110alpha) and the lost function of PTEN are frequently detected in ovarian cancer cells. PI3K plays an important role in tumorigenesis. To specifically inhibit PI3K activity in ovarian cancer cells, we constructed small interfering RNA (siRNA) against p110alpha. The expression of p110alpha siRNA significantly decreased cell   migration  , invasion, and proliferation compared to the siSCR control cells. The expression of p110alpha siRNA induced CDK inhibitor p27(KIP1) levels, and decreased levels of cyclin D1, CDK4, and phosphorylated retinoblastoma protein. PI3K transmits the mytogenic signal through AKT. AKT has three isoforms in the cells: AKT1, AKT2 and AKT3. We found that inhibition of AKT1 is sufficient to affect cell   migration  , invasion, and proliferation. Expression of AKT1 siRNA had a similar effect as p110alpha siRNA in the cells. We showed the roles of specific PI3K and AKT isoforms in the cells, which are important to understanding the mechanism of PI3K/AKT signaling in ovarian cancer cells. Both p110alpha and AKT1 siRNA-expressing cells decreased the activation of p70S6K1. Inhibition of p70S6K1 activity by its siRNA also decreased cell   migration  , invasion, and proliferation associated with the induction of p27(KIP1) levels, and with the inhibition of cell cycle-associated proteins including cyclin D1, CDK2, and phosphorylated retinoblastoma protein. This study demonstrates the important role of the PI3K/AKT/mTOR/p70S6K1 pathway in cell proliferation,   migration  , and invasion in ovarian cancer cells by using siRNA-mediated gene silencing as a reverse genetic method. 
 Dual inhibition by S6K1 and Elf4E is essential for controlling cellular growth and invasion in bladder cancer. We investigated how dual inhibition of the molecular mechanism of mammalian target of rapamycin (mTOR) downstream of S6K1 and the eukaryotic initiation factor 4E (eIF4E) can lead to suppression of proliferation and progression of urothelial carcinoma.We characterized the molecular mechanism of the mTOR pathway in the T24 and 5637 urothelial carcinoma cell lines by interfering with different molecular components using rapamycin and short interfering (siRNA) technology (S6K1 or elF4E) and analyzed the effects on molecular activation status, cell growth, proliferation, and invasion.A high concentration of rapamycin (10 μM) blocked both S6K1 and elF4E phosphorylation and inhibited cell proliferation in T24 and 5637 cells. The inhibition of both S6K1 and elF4E phosphorylation by rapamycin reduced cell viability and proliferation more than transfection of siRNA against S6K1 or elF4E in 5637 and T24 cells. Cells silenced for S6K1 or elF4E expression exhibited significantly reduced cell   migratio  n and invasion compared with those of the control but inhibition of both S6K1 and elF4E phosphorylation by rapamycin reduced cell   migratio  n and invasion more than siRNA transfection against S6K1 or elF4E in 5637 and T24 cells.These findings suggest that both the mTOR pathway downstream of eukaryotic initiation factor 4E and S6K1 can be successfully inhibited, therefore, the recurrence of bladder cancer can be prevented by high-dose rapamycin only, suggesting that 4E-BP1 might be still under mTORC1. 
 Vascular tumors have increased p70 S6-kinase activation and are inhibited by topical rapamycin. Vascular tumors are endothelial cell neoplasms whose cellular and molecular mechanisms, leading to tumor formation, are poorly understood, and current therapies have limited efficacy with significant side effects. We have investigated mechanistic (mammalian) target of rapamycin (mTOR) signaling in benign and malignant vascular tumors, and the effects of mTOR kinase inhibitor as a potential therapy for these lesions. Human vascular tumors (infantile hemangioma and angiosarcoma) were analyzed by immunohistochemical stains and western blot for the phosphorylation of p70 S6-kinase (S6K) and S6 ribosomal protein (S6), which are activated downstream of mTOR complex-1 (mTORC1). To assess the function of S6K, tumor cells with genetic knockdown of S6K were analyzed for cell proliferation and   migration  . The effects of topical rapamycin, an mTOR inhibitor, on mTORC1 and mTOR complex-2 (mTORC2) activities, as well as on tumor growth and   migration  , were determined. Vascular tumors showed increased activation of S6K and S6. Genetic knockdown of S6K resulted in reduced tumor cell proliferation and   migration  . Rapamycin fully inhibited mTORC1 and partially inhibited mTORC2 activities, including the phosphorylation of Akt (serine 473) and PKCα, in vascular tumor cells. Rapamycin significantly reduced vascular tumor growth in vitro and in vivo. As a potential localized therapy for cutaneous vascular tumors, topically applied rapamycin effectively reduced tumor growth with limited systemic drug absorption. These findings reveal the importance of mTOR signaling pathways in benign and malignant vascular tumors. The mTOR pathway is an important therapeutic target in vascular tumors, and topical mTOR inhibitors may provide an alternative and well-tolerated therapy for the treatment of cutaneous vascular lesions. 
  GeneRIFs  
 Results suggest that blocking both the mTOR kinase downstream targets 4E-BP1 protein and p70 S6 kinase 1, but not p70 S6 kinase 1 alone, prevents the   migration   of retinal pigment epithelium (RPE) cells. 
 our results demonstrate that activation of the PI3K/Akt/mTOR/p70S6K signaling pathway is involved in S100A4-induced viability,   migration  , upregulation of VEGF and downregulation of E-cadherin in CRC cells. 
 Data indicate that the combined activity of pS6K and elf4E regulates cell proliferatioin, cell   migration   and invasion in bladder cancer cells. 
 Vascular tumors (both angiosarcomas and capillary infantile hemangiomas) exhibit increased activation of p70-S6K; knockdown of p70-S6K results in reduced tumor cell proliferation and   migration  . 
 IL-8 reverses an mTOR/S6K-led down-regulation of PLD2 expression and enables PLD2 to fully function as a facilitator for cell   migration   
 Inhibition of p70S6K1 activity by its siRNA also decreased cell   migration  , invasion, and proliferation associated with the induction of p27(KIP1) levels, and with the inhibition of cell cycle-associated proteins. 
  [Ii]nvasive  PubMed  
 Activation of p70S6K induces expression of matrix metalloproteinase 9 associated with hepatocyte growth factor-mediated invasion in human ovarian cancer cells. The expression of hepatocyte growth factor (HGF) receptor, encoded by the Met oncogene, is elevated in ovarian and a variety of cancers. Here we show that human ovarian cancer cells with high Met expression were more sensitive to the cell motility and invasion effect of HGF. Met down-regulation by small interfering RNAs or K252a resulted in reduced migration in response to HGF. The   invasive  /migratory phenotype activated by HGF can be blocked by specific inhibitors of the phosphatidylinositol-3-kinase (PI3K) cascade, inhibitor of p70(S6K), and also the expression of a dominant-negative Akt, demonstrating that HGF transmits the motogenic signal through PI3K and Akt to p70(S6K). A significant role for p70(S6K) in cell invasion is further supported by the observation that expression of constitutively active forms of p70(S6K) is sufficient to induce   invasive   and migratory phenotypes in ovarian cancer cells. Importantly, activation of p70(S6K) stimulated expression and proteolytic activity of matrix metalloproteinase (MMP)-9 and cellular invasion, whereas it had little effect on MMP-2, suggesting for the first time that MMP-9 up-regulation by p70(S6K) as a key step for HGF-induced invasion and migration. These data suggest that interfering p70(S6K) may provide a novel means of controlling tumor cell   invasive  ness. 
 Expression of phosphorylated mTOR and its regulatory protein is related to biological behaviors of ameloblastoma. The present study investigated the relationship between the expression of p-mTOR, p-4EBP1 and p-p70S6K in the cytoplasm and nucleus of ameloblastoma (AB) cells and the   invasive  ness of ABs. Immunohistochemistry was performed to detect the expression of p-mTOR, p-4EBP1 and p-p70S6K in ABs and the level of these proteins in the nucleus and cytoplasm was scored. There was ectopic expression of p-mTOR in ABs. 27 AB patients were positive for p-mTOR expression in the nucleus and 47 for p-mTOR expression in the cytoplasm. The ectopic expression of p-4EBP-1 was also noted. 23 patients (27%) were positive for p-4EBP-1 expression in the nucleus and 55 (64.7%) for p-4EBP-1 expression in the cytoplasm. The ectopic expression of p-p70S6K was also noted. Of these patients, 33 were positive for p-p70S6K expression in the nucleus (38.8%) and 45 for p-p70S6K expression in the cytoplasm (52.9%). Statistical analysis showed the expression of three proteins in the nucleus of patients with recurrent cancer was markedly higher than that in those with primary cancer. The expression of p-mTOR, p-4E-BP1 and p-p70S6K in the nucleus was related to the   invasive  ness of ABs. Multivariable analysis with Cox proportional hazards model showed the p-mTOR expression had influence on AB recurrence (OR: 6.417, 95%CI: 1.428-28.824). The possibility of AB recurrence in patients with nuclear p-mTOR expression was 6.417 folds higher than that in those with cytoplasmic p-mTOR expression. The nuclear expression of p-mTOR, p-4E-BP1 and p-p70S6K was associated with biological behaviors (  invasive  ness) of ABs, and p-mTOR was an independent predictor of AB. 
 ERBB2, TBX2, RPS6KB1, and MYC alterations in breast tissues of BRCA1 and BRCA2 mutation carriers. Breast cancer risk is greatly increased in women who carry mutations in the BRCA1 or BRCA2 genes. Because breast cancer initiation is different between BRCA1/2 mutation carriers and women who do not carry mutations, it is possible that the mechanism of breast cancer progression is also different. Histopathologic and genetic studies have supported this hypothesis. To test this hypothesis further, we utilized a large cohort of women who underwent therapeutic mastectomy (TM) and contralateral prophylactic mastectomy (PM). From this cohort, we developed case groups of women with a family history of breast cancer with BRCA1/2 deleterious mutations, with unclassified variant alterations, and with no detected mutation and matched these cases with sporadic controls from the same TM and PM cohort. Fluorescence in situ hybridization was performed on paraffin sections by use of dual-color probes for ERBB2/CEP17, MYC/CEP8, TBX2/CEP17, and RPS6KB1/CEP17. All malignant and benign lesions, including putative precursor lesions, were studied. The   invasive   cancers from deleterious mutation carriers had a higher prevalence of duplication of MYC (P = 0.006) and TBX2 (P = 0.0008) compared to controls and a lower prevalence of ERBB2 amplification (P = 0.011). Coduplication of MYC and TBX2 was common in the in situ and   invasive   lesions from the deleterious mutation carriers. The odds ratio of having a BRCA1/2 mutation is 31.4 (95% CI = 1.7-569) when MYC and TBX2 are coduplicated but ERBB2 is normal. Unclassified variant carriers/no mutation detected and sporadic controls had a similar prevalence of alterations, suggesting that hereditary patients with no deleterious mutations follow a progression pathway similar to that of sporadic cases. With the exception of one atypical ductal hyperplasia lesion, no putative precursor lesion showed any detectable alteration of the probes tested. There was no significant intratumoral heterogeneity of genetic alterations. Our data confirm that a specific pattern of genomic instability characterizes BRCA1/2-related cancers and that this pattern has implications for the biology of these cancers. Moreover, our current and previous results emphasize the interaction between phenotype and genotype in BRCA1/2-related breast cancers and that a combination of morphologic features and alterations of ERBB2, MYC, and TBX2 may better define mechanisms of tumor progression, as well as determine which patients are more likely to carry BRCA1/2 mutations. 
 p130Cas promotes   invasive  ness of three-dimensional ErbB2-transformed mammary acinar structures by enhanced activation of mTOR/p70S6K and Rac1. ErbB2 over-expression is detected in approximately 25% of   invasive   breast cancers and is strongly associated with poor patient survival. We have previously demonstrated that p130Cas adaptor is a crucial mediator of ErbB2 transformation. Here, we analysed the molecular mechanisms through which p130Cas controls ErbB2-dependent invasion in three-dimensional cultures of mammary epithelial cells. Concomitant p130Cas over-expression and ErbB2 activation enhance PI3K/Akt and Erk1/2 MAPK signalling pathways and promote invasion of mammary acini. By using pharmacological inhibitors, we demonstrate that both signalling cascades are required for the   invasive   behaviour of p130Cas over-expressing and ErbB2 activated acini. Erk1/2 MAPK and PI3K/Akt signalling triggers invasion through distinct downstream effectors involving mTOR/p70S6K and Rac1 activation, respectively. Moreover, in silico analyses indicate that p130Cas expression in ErbB2 positive human breast cancers significantly correlates with higher risk to develop distant metastasis, thus underlying the value of the p130Cas/ErbB2 synergism in regulating breast cancer invasion. In conclusion, high levels of p130Cas favour progression of ErbB2-transformed cells towards an   invasive   phenotype. 
 MEK/ERK-dependent uPAR expression is required for motility via phosphorylation of P70S6K in human hepatocarcinoma cells. Motility and   invasive  ness events require specific intracellular signaling cascade activations. In cancer liver cells, one of these mechanisms could involve the MAPK MEK/ERK cascade activation which has been shown over expressed and activated in hepatocellular carcinoma. To study whether the MEK/ERK cascade is involved in the motility of HCC, we examined the effect of MEK inhibitor and ERK2 silencing using monolayer wound-healing assays and fluoroblock invasion systems. Evidence was provided that the MAPK cascade is a key transduction pathway which controls HCC cells motility and   invasive  ness. We could disconnect proliferation to motility using mitomycin C and we established that RNAi-mediated inhibition of ERK2 led to strongly reduced cell motility. To improve our understanding, we analysed the regulation and the role of urokinase receptor (uPAR) in this process. We provided evidence that uPAR was under a MEK/ERK dependent mechanism and blocking uPAR activity using specific antagonist or inhibiting its expression by RNA interference which resulted in complete inhibition of motility. Moreover, we found in MAPK inhibited cultures and in uPAR silencing cells that p70S6K phosphorylation on residue Thr-389 was significantly reduced, whereas Ser-421/Thr-424 phosphorylation did not change. We highlighted that the FRAP/mTOR pathway did not affect motility and Thr-389 phosphorylation. Furthermore, we demonstrated that p70S6K inhibition by RNA interference completely inhibited hepatocarcinoma cell motility. Therefore, targeting uPAR and/or MEK/ERK/S6K by RNA interference could be a major therapeutic strategy for the future treatment of   invasive   hepatocarcinoma cells. 
  GeneRIFs  
 The nuclear expression of p-mTOR, p-4E-BP1 and p-p70S6K was associated with biological behaviors (  invasive  ness) of ameloblastomas, and p-mTOR was an independent predictor of them. 
  [Mm]etastasize  PubMed  
 Fisetin inhibits human melanoma cell growth through direct binding to p70S6K and mTOR: findings from 3-D melanoma skin equivalents and computational modeling. The incidence of melanoma continues to rise. Inspite of treatment advances, the prognosis remains grim once the disease has   metastasize  d, emphasizing the need to explore additional therapeutic strategies. One such approach is through the use of mechanism-based dietary intervention. We previously showed that the flavonoid fisetin inhibits melanoma cell proliferation, in vitro and in vivo. Here, we studied fisetin-mediated regulation of kinases involved in melanoma growth and progression. Time-course analysis in 3-D melanoma constructs that transitioned from radial to vertical growth showed that fisetin treatment resulted in significant decrease in melanocytic lesions in contrast to untreated controls that showed large tumor nests and invading disseminated cells. Further studies in melanoma cultures and mouse xenografts showed that fisetin-mediated growth inhibition was associated with dephosphorylation of AKT, mTOR and p70S6K proteins. In silico modeling indicated direct interaction of fisetin with mTOR and p70S6K with favorable free energy values. These findings were validated by cell-free competition assays that established binding of fisetin to p70S6K and mTOR while little affinity was detected with AKT. Kinase activity studies reflected similar trend with % inhibition observed for p70S6K and mTOR at lower doses than AKT. Our studies characterized, for the first time, the differential interactions of any botanical agent with kinases involved in melanoma growth and demonstrate that fisetin inhibits mTOR and p70S6K through direct binding while the observed inhibitory effect of fisetin on AKT is mediated indirectly, through targeting interrelated pathways. 
  [Mm]etastasis  PubMed  
 Clinically significant association between the maximum standardized uptake value on 18F-FDG PET and expression of phosphorylated Akt and S6 kinase for prediction of the biological characteristics of renal cell cancer. The relationship between the clinicopathological features and molecular changes associated with standardized uptake value (SUV) determined by Positron emission tomography (PET) with [18F] fluorodeoxyglucose (18F-FDG PET) in human renal cell carcinoma (RCC) has not been elucidated. On the other hand, overactivation of the phosphatidylinositol 3'kinase (PI3K), serine/threonine kinase Akt, and mammalian target of rapamycin (mTOR) pathway has been detected in a variety of human cancers, including RCC. So far, little is known about the relationship between the SUV and these proteins in human RCC. Thus, it is important to study the relevance of SUV with clinicopathological features in human RCCs from a molecular point of view.Seventy-seven consecutive patients with RCC who underwent nephrectomy and pretreatment determination of the maximum SUV (SUVmax) by 18F-FDG PET were analyzed. We investigated the relationship between the SUVmax, phosphorylated-Akt (Ser-473) (pAkt(Ser-473)), phosphorylated-Akt (Thr-308) (pAkt(Thr-308), and phosphorylated-S6 ribosomal protein (Ser-235/236) (pS6) protein levels in the primary tumor and various clinicopathological features.The average SUVmax of the primary tumor was 6.9 (1.5 to 40.3). A higher SUVmax was correlated with higher expression of pAkt(Ser-473), pAkt (Thr-308), and pS6 protein in the primary tumor. A higher SUVmax and increased expression of pAkt (Ser-473), pAkt (Thr-308), and pS6 of the primary tumor was associated with less tumor differentiation, a higher pT stage, regional lymph node involvement, microscopic vascular invasion, and distant   metastasis  , as well as with early relapse following radical nephrectomy in patients who had localized or locally advanced RCC without distant   metastasis   (cTanyNanyM0) and with shorter overall survival in all patients.A higher SUVmax on 18F-FDG PET is associated with elevated tumor levels of pAkt and pS6 protein and with aggressive behavior and metastatic potential of RCC, as well as with early relapse following radical nephrectomy and shorter overall survival. These findings suggest that SUVmax may be useful for predicting the biological characteristics of RCC. 
 miR-195 Inhibits Tumor Progression by Targeting RPS6KB1 in Human Prostate Cancer. To investigate the involvement of hsa-miRNA-195-5p (miR-195) in progression and prognosis of human prostate cancer.qRT-PCR was performed to detect miR-195 expression in both prostate cancer cell lines and clinical tissue samples. Its clinical significance was statistically analyzed. The roles of miR-195 and its candidate target gene, ribosomal protein S6 kinase, 70 kDa, polypeptide 1 (RPS6KB1) in prostate cancer progression were confirmed on the basis of both in vitro and in vivo systems.miR-195 downregulation in prostate cancer tissues was significantly associated with high Gleason score (P = 0.001), positive   metastasis   failure (P < 0.001), and biochemical recurrence (BCR, P < 0.001). Survival analysis identified miR-195 as an independent prognostic factor for BCR-free survival of prostate cancer patients (P = 0.022). Then, we confirmed the tumor suppressive role of miR-195 through prostate cancer cell invasion, migration, and apoptosis assays in vitro, along with tumor xenograft growth, angiogenesis, and invasion in vivo according to both gain-of-function and loss-of-function experiments. In addition, RPS6KB1 was identified as a novel direct target of miR-195 through proteomic expression profiling combined with bioinformatic target prediction and luciferase reporter assay. Moreover, the reexpression and knockdown of RPS6KB1 could respectively rescue and imitate the effects induced by miR-195. Importantly, RPS6KB1 expression was closely correlated with aggressive progression and poor prognosis in prostate cancer patients as opposed to miR-195. Furthermore, we identified MMP-9, VEGF, BAD, and E-cadherin as the downstream effectors of miR-195-RPS6KB1 axis.The newly identified miR-195-RPS6KB1 axis partially illustrates the molecular mechanism of prostate cancer progression and represents a novel potential therapeutic target for prostate cancer treatment. 
 Activation of the PI3K/Akt/mTOR/p70S6K pathway is involved in S100A4-induced viability and migration in colorectal cancer cells. The S100 protein family member S100A4 regulates various cellular functions. Previous studies have shown that elevated expression of S100A4 is associated with progression and   metastasis   of colorectal cancer (CRC). However, little is known about whether and how S100A4 contributes to CRC development. In our present study, the elevated expression of S100A4 in CRC tissues compared to matched adjacent normal tissues was confirmed by immunohistochemistry, semi-quantitative RT-PCR and Western blot. Adenovirus-mediated S100A4 overexpression obviously enhanced viability and migration of CRC cells, which was detected by MTT assay and transwell assay, respectively. Additionally, S100A4 overexpression increased the phosphorylation levels of Akt, mTOR and p70S6K. These effects of S100A4 were abolished by treatment with either the specific PI3K/Akt inhibitor LY294002, or the specific mTOR/p70S6K inhibitor rapamycin. Furthermore, overexpression of S100A4 resulted in upregulation of VEGF and downregulation of E-cadherin, which were strongly reversed by either LY294002 or rapamycin. Altogether, our results demonstrate that activation of the PI3K/Akt/mTOR/p70S6K signaling pathway is involved in S100A4-induced viability, migration, upregulation of VEGF and downregulation of E-cadherin in CRC cells. 
 Downregulation of p70S6K Enhances Cell Sensitivity to Rapamycin in Esophageal Squamous Cell Carcinoma. It has been demonstrated that mTOR/p70S6K pathway was abnormally activated in many cancers and rapamycin and its analogs can restrain tumor growth through inhibiting this pathway, but some tumors including esophageal squamous cell carcinoma (ESCC) appear to be insensitive to rapamycin in recent studies. In the present study, we explored the measures to improve the sensitivity of ESCC cells to rapamycin and identified the clinical significance of the expression of phosphorylated p70S6K (p-p70S6K). The results showed that, after downregulating the expression of p70S6K and p-p70S6K by p70S6K siRNA, the inhibitory effects of rapamycin on cell proliferation, cell cycle, and tumor growth were significantly enhanced in vitro and in vivo. Furthermore, p-p70S6K had strong positive expression in ESCC tissues and its expression was closely related to lymph node   metastasis   and the TNM staging. These results indicated that p-p70S6K may participate in the invasion and   metastasis   in the development of ESCC and downregulation of the expression of p-p70S6K could improve the sensitivity of cells to rapamycin in ESCC. 
 mTOR/p70S6K signal transduction pathway contributes to osteosarcoma progression and patients' prognosis. The mTOR/p70S6K signal transduction pathway plays a key role in the regulation of cancer cells' survival and proliferation. However, its roles in osteosarcoma, which is one of the most rapidly growing sarcomas, remain unknown. This study investigated for the first time the correlation between the mTOR/p70S6K signal transduction pathway in human osteosarcoma and patients' prognosis. The expression patterns of mTOR and p70S6K in paraffin-embedded specimens gathered from 65 patients with primary osteosarcoma were detected by the method of immunohistochemistry using antibodies against mTOR and p70S6K. Kaplan-Meier survival and Cox regression analyses were performed to evaluate the prognosis of patients. Immunostaining revealed that the mTOR/p70S6K signal transduction pathway is activated in human osteosarcoma. Additionally, positive expression of mTOR and p70S6K proteins was significantly correlated with surgical stage,   metastasis   pattern and percentage of dead cells of osteosarcoma. Moreover, in univariate analysis, surgical stage,   metastasis   pattern and percentage of dead cells, mTOR and p70S6K expression showed significant influence on overall survival (OS) and disease-free survival (DFS). In multivariate analysis, surgical stage (IIA vs. IIB/III),   metastasis   pattern (without vs. with), percentage of dead cells (≥90 vs. <90%), mTOR expression pattern (negative vs. positive) and p70S6K expression pattern (negative vs. positive) were significant for DFS and OS. Our results demonstrate the correlation of mTOR and p70S6K expression patterns with the oncological progression of osteosarcoma patients, suggesting the prognostic significance of the mTOR/p70S6K signal transduction pathway in osteosarcoma patients, which may lay a foundation for making further investigations on the mTOR/p70S6K signal transduction pathway as a potential target for osteosarcoma therapy. 
 Targeting p70S6K prevented lung   metastasis   in a breast cancer xenograft model. Overexpression of p70S6K in breast cancer patients is associated with aggressive disease and poor prognosis. Recent studies showed that patients with breast cancer with increased p70S6K phosphorylation had poor survival and increased   metastasis  . The purpose of our study was to determine whether knockdown of p70S6K would inhibit cell growth, invasion, and   metastasis   in breast cancer. We therefore stably knocked down p70S6K expression in MDA-231, a highly metastatic breast cancer cell line, using a lentiviral short hairpin RNA (shRNA) based approach. Inhibition of p70S6K led to inhibition of cell growth, migration, and invasion in vitro. To determine the role of p70S6K in breast cancer tumorigenesis and   metastasis  , we used an MDA-231 orthotopic and metastatic animal model. In the orthotopic model, mice injected with MDA-231-p70S6K shRNA cells developed significantly smaller tumors than control mice injected with MDA-231 control shRNA cells (P < 0.01). No   metastasis   was observed in the p70S6K downregulated group, whereas lung   metastasis   was detected in all mice in the control group. To determine the role of p70S6K on growth and invasion, we tested downstream signaling targets by Western blot analysis. Knockdown of p70S6K inhibited phosphorylation of focal adhesion kinase, tissue transglutaminase 2, and cyclin D1 proteins, which promote cell growth, survival, and invasion. In addition, downregulation of p70S6K induced expression of PDCD4, a tumor-suppressor protein. In conclusion, we showed that p70S6K plays an important role in   metastasis   by regulating key proteins like cyclin D1, PDCD4, focal adhesion kinase, E-cadherin, beta-catenin, and tissue transglutaminase 2, which are essential for cell attachment, survival, invasion, and   metastasis   in breast cancer. 
 p130Cas promotes invasiveness of three-dimensional ErbB2-transformed mammary acinar structures by enhanced activation of mTOR/p70S6K and Rac1. ErbB2 over-expression is detected in approximately 25% of invasive breast cancers and is strongly associated with poor patient survival. We have previously demonstrated that p130Cas adaptor is a crucial mediator of ErbB2 transformation. Here, we analysed the molecular mechanisms through which p130Cas controls ErbB2-dependent invasion in three-dimensional cultures of mammary epithelial cells. Concomitant p130Cas over-expression and ErbB2 activation enhance PI3K/Akt and Erk1/2 MAPK signalling pathways and promote invasion of mammary acini. By using pharmacological inhibitors, we demonstrate that both signalling cascades are required for the invasive behaviour of p130Cas over-expressing and ErbB2 activated acini. Erk1/2 MAPK and PI3K/Akt signalling triggers invasion through distinct downstream effectors involving mTOR/p70S6K and Rac1 activation, respectively. Moreover, in silico analyses indicate that p130Cas expression in ErbB2 positive human breast cancers significantly correlates with higher risk to develop distant   metastasis  , thus underlying the value of the p130Cas/ErbB2 synergism in regulating breast cancer invasion. In conclusion, high levels of p130Cas favour progression of ErbB2-transformed cells towards an invasive phenotype. 
  GeneRIFs  
 These results indicated that p-p70S6K may participate in the invasion and   metastasis   in the development of ESCC and downregulation of the expression of p-p70S6K could improve the sensitivity of cells to rapamycin in ESCC. 
 p70S6K plays an important role in   metastasis   by regulating key proteins which are essential for cell attachment, survival, invasion, and   metastasis   in breast cancer. 
  [Cc]ell [Ii]nvasion  PubMed  
 High expression of ErbB2 contributes to cholangiocarcinoma   cell invasion   and proliferation through AKT/p70S6K. To compare the impact of ErbB2 on   cell invasion   and proliferation in cholangiocarcinoma (CCA) cell lines.Level of endogenous ErbB2 expression in three CCA cell lines, namely HuCCA-1, KKU-100 and KKU-M213, was determined by real-time reverse-transcriptase polymerase chain reaction. Two ErbB2 inhibitory methods, a small molecule ErbB2 kinase inhibitor (AG825) and siRNA, were used to disrupt ErbB2 function in the cell lines. CCA   cell invasion  , motility and proliferation under ErbB2-disrupted conditions were detected using Transwell and 3-(4,5-dimethylthiazol-2-yl)-2,5-diphenyltetrazolium bromide assays. In addition, ErbB2 downstream effectors were investigated by Western blotting analysis.Suppression of ErbB2 activity, using a specific kinase inhibitor (AG825), reduced invasion, motility and proliferation of all three CCA cell lines. The ability of this drug to inhibit neoplastic properties (invasion, motility and proliferation) increased concomitantly with the level of ErbB2 expression. Similarly, knockdown of ErbB2 level by siRNA inhibited   cell invasion   and proliferation of KKU-M213, a high-ErbB2-expressing cell, better than those of the lower-ErbB2-expressing cells, HuCCA-1 and KKU-100. Thus, both inhibitory methods indicated that there is more ErbB2-dependency for malignancy of the high-ErbB2-expressing cell, KKU-M213, than for that of low-ErbB2-expressing ones. In addition, interrupting ErbB2 activity decreased phosphorylation of AKT and p70S6K, but not extracellular signal-regulated kinase 1/2, in the high-ErbB2-expressing CCA cell line.Our data indicated that high ErbB2 expression enhances CCA invasion, motility and proliferation via the AKT/p70S6K pathway, which suggests the possibility of targeting these molecules for CCA therapy. 
 miR-195 Inhibits Tumor Progression by Targeting RPS6KB1 in Human Prostate Cancer. To investigate the involvement of hsa-miRNA-195-5p (miR-195) in progression and prognosis of human prostate cancer.qRT-PCR was performed to detect miR-195 expression in both prostate cancer cell lines and clinical tissue samples. Its clinical significance was statistically analyzed. The roles of miR-195 and its candidate target gene, ribosomal protein S6 kinase, 70 kDa, polypeptide 1 (RPS6KB1) in prostate cancer progression were confirmed on the basis of both in vitro and in vivo systems.miR-195 downregulation in prostate cancer tissues was significantly associated with high Gleason score (P = 0.001), positive metastasis failure (P < 0.001), and biochemical recurrence (BCR, P < 0.001). Survival analysis identified miR-195 as an independent prognostic factor for BCR-free survival of prostate cancer patients (P = 0.022). Then, we confirmed the tumor suppressive role of miR-195 through prostate cancer   cell invasion  , migration, and apoptosis assays in vitro, along with tumor xenograft growth, angiogenesis, and invasion in vivo according to both gain-of-function and loss-of-function experiments. In addition, RPS6KB1 was identified as a novel direct target of miR-195 through proteomic expression profiling combined with bioinformatic target prediction and luciferase reporter assay. Moreover, the reexpression and knockdown of RPS6KB1 could respectively rescue and imitate the effects induced by miR-195. Importantly, RPS6KB1 expression was closely correlated with aggressive progression and poor prognosis in prostate cancer patients as opposed to miR-195. Furthermore, we identified MMP-9, VEGF, BAD, and E-cadherin as the downstream effectors of miR-195-RPS6KB1 axis.The newly identified miR-195-RPS6KB1 axis partially illustrates the molecular mechanism of prostate cancer progression and represents a novel potential therapeutic target for prostate cancer treatment. 
 Activation of p70S6K induces expression of matrix metalloproteinase 9 associated with hepatocyte growth factor-mediated invasion in human ovarian cancer cells. The expression of hepatocyte growth factor (HGF) receptor, encoded by the Met oncogene, is elevated in ovarian and a variety of cancers. Here we show that human ovarian cancer cells with high Met expression were more sensitive to the cell motility and invasion effect of HGF. Met down-regulation by small interfering RNAs or K252a resulted in reduced migration in response to HGF. The invasive/migratory phenotype activated by HGF can be blocked by specific inhibitors of the phosphatidylinositol-3-kinase (PI3K) cascade, inhibitor of p70(S6K), and also the expression of a dominant-negative Akt, demonstrating that HGF transmits the motogenic signal through PI3K and Akt to p70(S6K). A significant role for p70(S6K) in   cell invasion   is further supported by the observation that expression of constitutively active forms of p70(S6K) is sufficient to induce invasive and migratory phenotypes in ovarian cancer cells. Importantly, activation of p70(S6K) stimulated expression and proteolytic activity of matrix metalloproteinase (MMP)-9 and cellular invasion, whereas it had little effect on MMP-2, suggesting for the first time that MMP-9 up-regulation by p70(S6K) as a key step for HGF-induced invasion and migration. These data suggest that interfering p70(S6K) may provide a novel means of controlling tumor cell invasiveness. 
   
  
 RPS6KB2 
 
  NCBI Gene  
  GeneRIF  
  Pubmed  
 
 
  Search Term  Rank  Fields  
  [Mm]igration  6  PubMed(2)  
  [Ee]xtracellular [Mm]atrix  8  PubMed(1)  
  [Ii]nvasive  10  PubMed(1)  
  [Mm]etastasis  12  PubMed(2)  
 
 
 hide/show details for RPS6KB2 
  [Mm]igration  PubMed  
 Sustained induction of ERK, protein kinase B, and p70 S6 kinase regulates cell spreading and formation of F-actin microspikes upon ligation of integrins by galectin-8, a mammalian lectin. Galectin-8, a member of the galectin family of mammalian lectins, is a secreted protein that promotes cell adhesion and   migration   upon binding to a subset of integrins through sugar-protein interactions. Ligation of integrins by galectin-8 triggers a distinct pattern of cytoskeletal organization, including formation of F-actin-containing microspikes. This is associated with activation of integrin-mediated signaling cascades (ERK and phosphatidylinositol 3 kinase (PI3K)) that are much more robust and are of longer duration than those induced upon cell adhesion to fibronectin. Indeed, formation of microspikes is enhanced 40% in cells that overexpress protein kinase B, the downstream effector of PI3K. Inhibition of PI3K activity induced by wortmannin partially inhibits cell adhesion and spreading while largely inhibiting microspike formation in cells adherent to galectin-8. Furthermore, the inhibitory effects of wortmannin are markedly accentuated in cells overexpressing PKB or p70S6K (CHO(PKB) and CHO(p70S6K) cells), whose adhesion and spreading on galectin-8 (but not on fibronectin) is inhibited approximately 25-35% in the presence of wortmannin. The above results suggest that galectin-8 is an extracellular matrix protein that triggers a unique repertoire of integrin-mediated signals, which leads to a distinctive cytoskeletal organization and microspike formation. They further suggest that downstream effectors of PI3K, including PKB and p70 S6 kinase, in part mediate cell adhesion, spreading, and microspike formation induced by galectin-8. 
 MicroRNA-193a-3p and -5p suppress the metastasis of human non-small-cell lung cancer by downregulating the ERBB4/PIK3R3/mTOR/S6K2 signaling pathway. The metastatic cascade is a complex and multistep process with many potential barriers. Recent evidence has shown that microRNAs (miRNAs) are involved in carcinogenesis and tumor progression in non-small-cell lung cancer (NSCLC). In this study, by comparing the miRNA expression profiles of SPC-A-1sci (high metastatic) and SPC-A-1 (weakly metastatic) cells, we demonstrated that the downregulation and function of miR-193a-3p and miR-193a-5p in NSCLC metastasis and the expression of these miRNAs was suppressed in NSCLC compared with corresponding non-tumorous tissues. Decreased miR-193a-3p/5p expression was significantly associated with tumor node metastasis (TNM) and lymph node metastasis. Furthermore, functional assays showed that the overexpression of miR-193a-3p/5p inhibited NSCLC cell   migration  , invasion and epithelial-mesenchymal transition (EMT) in vitro and lung metastasis formation in vivo. In addition, we discovered that ERBB4 and S6K2 were the direct targets of miR-193a-3p and that PIK3R3 and mTOR were the direct targets of miR-193a-5p in NSCLC. We also observed that miR-193a-3p/5p could inactivate the AKT/mTOR signaling pathway. Thus, miR-193a-3p/5p functions as a tumor suppressor and has an important role in NSCLC metastasis through ERBB signaling pathway. 
  [Ee]xtracellular [Mm]atrix  PubMed  
 Sustained induction of ERK, protein kinase B, and p70 S6 kinase regulates cell spreading and formation of F-actin microspikes upon ligation of integrins by galectin-8, a mammalian lectin. Galectin-8, a member of the galectin family of mammalian lectins, is a secreted protein that promotes cell adhesion and migration upon binding to a subset of integrins through sugar-protein interactions. Ligation of integrins by galectin-8 triggers a distinct pattern of cytoskeletal organization, including formation of F-actin-containing microspikes. This is associated with activation of integrin-mediated signaling cascades (ERK and phosphatidylinositol 3 kinase (PI3K)) that are much more robust and are of longer duration than those induced upon cell adhesion to fibronectin. Indeed, formation of microspikes is enhanced 40% in cells that overexpress protein kinase B, the downstream effector of PI3K. Inhibition of PI3K activity induced by wortmannin partially inhibits cell adhesion and spreading while largely inhibiting microspike formation in cells adherent to galectin-8. Furthermore, the inhibitory effects of wortmannin are markedly accentuated in cells overexpressing PKB or p70S6K (CHO(PKB) and CHO(p70S6K) cells), whose adhesion and spreading on galectin-8 (but not on fibronectin) is inhibited approximately 25-35% in the presence of wortmannin. The above results suggest that galectin-8 is an   extracellular matrix   protein that triggers a unique repertoire of integrin-mediated signals, which leads to a distinctive cytoskeletal organization and microspike formation. They further suggest that downstream effectors of PI3K, including PKB and p70 S6 kinase, in part mediate cell adhesion, spreading, and microspike formation induced by galectin-8. 
  [Ii]nvasive  PubMed  
 Roles of phosphatidylinositol 3'-kinase and mammalian target of rapamycin/p70 ribosomal protein S6 kinase in K-Ras-mediated transformation of intestinal epithelial cells. Phosphatidylinositol 3'-kinase (PI3K) activity is required for Ras- mediated transformation of intestinal epithelial cells (IECs). The mammalian target of rapamycin (mTOR) and its downstream pathways control the translation of specific mRNAs that are required for cell proliferation and transformation. Here, we elucidated the roles of PI3K and mTOR in K-Ras-mediated transformation of IECs (IEC-6). Induction of K-Ras activated PI3K and mTOR in IECs. p70 ribosomal protein S6 kinase activity was induced by K-Ras in a PI3K- and mTOR-dependent manner. K-Ras did not significantly alter the phosphorylation of eukaryotic initiation factor 4E-binding protein 1. Treatment with either LY-294002 or rapamycin inhibited IEC proliferation and resulted in G(1) growth arrest. However, it was noted that inhibition of mTOR enhanced K-Ras-mediated morphological transformation and increased   invasive  ness of IECs in a mitogen-activated protein/extracellular signal-regulated kinase-dependent manner. Furthermore, inhibition of PI3K or mTOR impaired the growth of an array of colon cancer cells. Spindle transformation, reduced E-cadherin, and increased   invasive  ness were observed in LY-294002-treated Moser cells. Thus, our results suggest that K-Ras-mediated transformation of IECs involves activation of the PI3K/mTOR pathway. Inhibition of PI3K/mTOR activity leads to G(1) growth arrest of transformed IECs. On the other hand, inhibition of PI3K or mTOR may induce the epithelial to mesenchymal transdifferentiation of IECs under certain circumstances. 
  [Mm]etastasis  PubMed  
 MicroRNA-193a-3p and -5p suppress the   metastasis   of human non-small-cell lung cancer by downregulating the ERBB4/PIK3R3/mTOR/S6K2 signaling pathway. The metastatic cascade is a complex and multistep process with many potential barriers. Recent evidence has shown that microRNAs (miRNAs) are involved in carcinogenesis and tumor progression in non-small-cell lung cancer (NSCLC). In this study, by comparing the miRNA expression profiles of SPC-A-1sci (high metastatic) and SPC-A-1 (weakly metastatic) cells, we demonstrated that the downregulation and function of miR-193a-3p and miR-193a-5p in NSCLC   metastasis   and the expression of these miRNAs was suppressed in NSCLC compared with corresponding non-tumorous tissues. Decreased miR-193a-3p/5p expression was significantly associated with tumor node   metastasis   (TNM) and lymph node   metastasis  . Furthermore, functional assays showed that the overexpression of miR-193a-3p/5p inhibited NSCLC cell migration, invasion and epithelial-mesenchymal transition (EMT) in vitro and lung   metastasis   formation in vivo. In addition, we discovered that ERBB4 and S6K2 were the direct targets of miR-193a-3p and that PIK3R3 and mTOR were the direct targets of miR-193a-5p in NSCLC. We also observed that miR-193a-3p/5p could inactivate the AKT/mTOR signaling pathway. Thus, miR-193a-3p/5p functions as a tumor suppressor and has an important role in NSCLC   metastasis   through ERBB signaling pathway. 
 Degradation of Tiam1 by casein kinase 1 and the SCFβTrCP ubiquitin ligase controls the duration of mTOR-S6K signaling. Tiam1 (T-cell lymphoma invasion and   metastasi  s 1) is a guanine nucleotide exchange factor that specifically controls the activity of the small GTPase Rac, a key regulator of cell adhesion, proliferation, and survival. Here, we report that in response to mitogens, Tiam1 is degraded by the ubiquitin-proteasome system via the SCF(βTrCP) ubiquitin ligase. Mitogenic stimulation triggers the binding of Tiam1 to the F-box protein βTrCP via its degron sequence and subsequent Tiam1 ubiquitylation and proteasomal degradation. The proteolysis of Tiam1 is prevented by βTrCP silencing, inhibition of CK1 and MEK, or mutation of the Tiam1 degron site. Expression of a stable Tiam1 mutant that is unable to interact with βTrCP results in sustained activation of the mTOR/S6K signaling and increased apoptotic cell death. We propose that the SCF(βTrCP)-mediated degradation of Tiam1 controls the duration of the mTOR-S6K signaling pathway in response to mitogenic stimuli. 
   
  
 INPP5F 
 
  NCBI Gene  
  GeneRIF  
  Pubmed  
 
 
  Search Term  Rank  Fields  
  [Mm]igration  6  PubMed(1)  
 
 
 hide/show details for INPP5F 
  [Mm]igration  PubMed  
 Spatiotemporal control of phosphatidylinositol 4-phosphate by Sac2 regulates endocytic recycling. It is well established that the spatial- and temporal-restricted generation and turnover of phosphoinositides (PIs) by a cascade of PI-metabolizing enzymes is a key regulatory mechanism in the endocytic pathway. Here, we demonstrate that the Sac1 domain-containing protein Sac2 is a PI 4-phosphatase that specifically hydrolyzes phosphatidylinositol 4-phosphate in vitro. We further show that Sac2 colocalizes with early endosomal markers and is recruited to transferrin (Tfn)-containing vesicles during endocytic recycling. Exogenous expression of the catalytically inactive mutant Sac2C458S resulted in altered cellular distribution of Tfn receptors and delayed Tfn recycling. Furthermore, genomic ablation of Sac2 caused a similar perturbation on Tfn and integrin recycling as well as defects in cell   migration  . Structural characterization of Sac2 revealed a unique pleckstrin-like homology Sac2 domain conserved in all Sac2 orthologues. Collectively, our findings provide evidence for the tight regulation of PIs by Sac2 in the endocytic recycling pathway. 
   
  
 STAT5B 
 
  NCBI Gene  
  GeneRIF  
  Pubmed  
 
 
  Search Term  Rank  Fields  
  [Mm]igration  6  Process(1); PubMed(14); GeneRIFs(5)  
  [Ii]nvasive  10  PubMed(6); GeneRIFs(1)  
  [Mm]etastasize  11  PubMed(1)  
  [Mm]etastasis  12  PubMed(4); GeneRIFs(1)  
  [Cc]ell [Ii]nvasion  13  PubMed(2); GeneRIFs(1)  
 
 
 hide/show details for STAT5B 
  [Mm]igration  Process  
 mast cell   migration   
  PubMed  
 Glioma-derived macrophage   migration   inhibitory factor (MIF) promotes mast cell recruitment in a STAT5-dependent manner. Recently, glioma research has increased its focus on the diverse types of cells present in brain tumors. We observed previously that gliomas are associated with a profound accumulation of mast cells (MCs) and here we investigate the underlying mechanism. Gliomas express a plethora of chemoattractants. First, we demonstrated pronounced   migration   of human MCs toward conditioned medium from cultures of glioma cell lines. Subsequent cytokine array analyses of media from cells, cultured in either serum-containing or -free conditions, revealed a number of candidates which were secreted in high amounts in both cell lines. Among these, we then focused on macrophage   migration   inhibitory factor (MIF), which has been reported to be pro-inflammatory and -tumorigenic. Infiltration of MCs was attenuated by antibodies that neutralized MIF. Moreover, a positive correlation between the number of MCs and the level of MIF in a large cohort of human glioma tissue samples was observed. Further, both glioma-conditioned media and purified MIF promoted differential phosphorylation of a number of signaling molecules, including signal transducer and activator of transcription 5 (STAT5), in MCs. Inhibition of pSTAT5 signaling significantly attenuated the   migration   of MCs toward glioma cell-conditioned medium shown to contain MIF. In addition, analysis of tissue microarrays (TMAs) of high-grade gliomas revealed a direct correlation between the level of pSTAT5 in MCs and the level of MIF in the medium. In conclusion, these findings indicate the important influence of signaling cascades involving MIF and STAT5 on the recruitment of MCs to gliomas. 
 Inhibition of STAT5 induces G1 cell cycle arrest and reduces tumor cell invasion in human colorectal cancer cells. Abnormalities in the signal transducer and activator of transcription (STAT) pathway are involved in the oncogenesis of several cancers. However, the mechanism by which dysregulated STAT5 signaling contributes to the progression of human colorectal cancer (CRC) has not been elucidated. To investigate the role of STAT5 in CRC progression, we depleted STAT5 with a small interfering RNA (siRNA). Our results demonstrate that STAT5 is involved in CRC cell growth, cell cycle progression, invasion and   migration   through regulation of gene expression, such as Bcl-2, p16(ink4a), p21(waf1/cip1), p27(kip1), E-cadherin, the focal adhesion kinase (FAK), vascular endothelial growth factor (VEGF) and matrix metalloproteinases. In addition, immunohistochemical staining reveals upregulation of STAT5 during CRC tumorigenesis. Moreover, phospho-STAT5 (pSTAT5) is predominantly localized in the cytoplasm of adenomas cells and colon adenocarcinoma cells, but primarily presented in the nucleus of normal colonic epithelium cells. Thus, pSTAT5 protein is shuttled from the nucleus to the cytoplasm in the oncogenesis of CRC, suggesting that activated STAT5 may also have cytoplasmic functions. In support of this hypothesis, we found that STAT5 formed a complex with p44/42 MAPK and SAPK/JNK in CRC cells, suggesting cross talk between STAT5 signaling and the MAPK pathway in the development of human CRC. Our findings illustrate the biological significance of STAT5 signaling in CRC progression, and provide novel evidence that intervention in STAT5 signaling may have potential therapeutic value in the prevention of human colorectal cancer. 
 The combination of methylsulfonylmethane and tamoxifen inhibits the Jak2/STAT5b pathway and synergistically inhibits tumor growth and metastasis in ER-positive breast cancer xenografts. Combination therapy, which reduces the dosage intensity of the individual drugs while increasing their efficacy, is not a novel approach for the treatment of cancer. Methylsulfonylmethane (MSM) is an organic sulfur compound shown to act against tumor cells. Tamoxifen is a commercially available therapeutic agent for breast malignancies.In the current study, we analyzed the combinatorial effect of MSM and tamoxifen on the suppression of ER-positive breast cancer xenograft growth and metastasis. Additionally, we also validated the molecular targets by which the drug combination regulated tumor growth and metastasis.We observed that the combination of MSM and tamoxifen regulated cell viability and   migration   in vitro. The intragastric administration of MSM and subcutaneous implantation of tamoxifen tablets led to tumor growth suppression and inhibition of the Janus kinase 2 (Jak2)/signal transducer and activator of transcription 5b (STAT5b) pathway. Our study also assessed the regulation of signaling molecules implicated in the growth, progression, differentiation, and   migration   of cancer cells, such as Jak2, STAT5b, insulin-like growth factor-1Rβ, and their phosphorylation status.Study results indicated that this combination therapy inhibited tumor growth and metastasis. Therefore, this drug combination may have a synergistic and powerful anticancer effect against breast cancer. 
 Constitutive activation of signal transducer and activator of transcription 5 contributes to tumor growth, epithelial-mesenchymal transition, and resistance to epidermal growth factor receptor targeting. Signal transducer and activator of transcription 5 (STAT5) is activated in squamous cell carcinoma of the head and neck (SCCHN), where targeting of STAT5 inhibits tumor growth in vitro and in vivo. The role of STAT5 activation in carcinogenesis, tumor progression, and response to therapy remains incompletely understood. In this study, we investigated the effects of STAT5 activation on squamous epithelial carcinogenesis and response to therapy.The functional consequences of STAT5 activation in squamous epithelial carcinogenesis were examined using cells derived from normal (Het-1A) and transformed mucosal epithelial cells engineered to express constitutive-active mutants of STAT5.The growth rate of stable clones derived from both normal and transformed squamous epithelial cells expressing the constitutive-active STAT5 was increased. In SCCHN xenografts, tumor volumes were increased in constitutive-active STAT5 mutant cells compared with vector-transfected controls. Constitutive activation of STAT5 significantly increased cell   migration   and invasion through Matrigel, as well as the transforming efficiency of SCCHN cells in vitro, as assessed by soft agar assays. The constitutive-active STAT5 clones derived from SCCHN cells showed changes consistent with an epithelial-mesenchymal transition including decreased expression of E-cadherin and increased vimentin in comparison with control transfectants. In these cells, STAT5 activation was associated with resistance to cisplatin-mediated apoptosis and growth inhibition induced by the epidermal growth factor receptor tyrosine kinase inhibitor, erlotinib.These results suggest that constitutive STAT5 signaling enhances tumor growth, invasion, and epithelial-to-mesenchymal transition in squamous epithelial carcinogenesis and may contribute to resistance to epidermal growth factor receptor tyrosine kinase inhibitor and chemotherapy. 
 ABL-N may induce apoptosis of human prostate cancer cells through suppression of KLF5, ICAM-1 and Stat5b, and upregulation of Bax/Bcl-2 ratio: An in vitro and in vivo study. Identification of novel botanicals that can selectively induce apoptosis and arrest growth of cancer cells without producing cytotoxic effects is highly appreciable for cancer therapy. The present study aimed to investigate the possibility of acetylbritannilactone (ABL) derivative 5-(5-(ethylperoxy)pentan-2-yl)-6-methyl-3-methylene-2-oxo-2,3,3a,4,7,7a‑hexahydroben-zofuran-4-yl2-(6-methoxynaphthalen-2-yl) propanoate (ABL-N) as a therapeutic agent in human prostate cancer and potential mechanisms. Human prostate cancer cells were treated with ABL-N of different concentrations (0, 5, 10, 20, 30 and 40 µmol/l). Cell viabili  ty, migra  tion and apoptosis were determined. Activities of caspases were assayed, as well as protein expression of cancer‑related proteins KLF5, Stat5b and ICAM-1 in PC3 cells. The therapeutic effect of ABL-N was further evaluated in our tumor xenografts. ABL-N inhibited growth of prostate cancer cells in a dose-dependent manner, without obvious effect on normal human prostate epithelial PrEC cells. ABL-N administration induced apoptosis of PC3 cells in a dose-dependent manner, along with the enhanced activity of caspases and increased Bax/Bcl-2 ratio. Expression of KLF5, Stat5b and ICAM-1 was significantly downregulated in PC3 cells. Our in vivo study further confirmed that ABL-N significantly inhibited the tumor growth of PC3 cells in the xenograft mouse model. ABL-N induces apoptosis of prostate cancer cells through activation of caspases, increasing the ratio of Bax/Bcl-2, as well as suppression of KLF5, Stat5b and ICAM-1 expressions. The present study indicated that ABL-N may be a potential therapeutic drug for human prostate cancer, and our data supported further studies to explore the therapeutic potential of ABL-N in other types of human cancer. 
 A novel role for signal transducer and activator of transcription 5b (STAT5b) in beta1-integrin-mediated human breast cancer cell   migration  . Signal transducer and activator of transcription (STAT) 5b is a transcription factor involved in pro-proliferative and pro-survival signaling in a number of solid tumors, including breast cancer. The contribution of STAT5b to breast cancer cell motility has not been explored. This work aims to elucidate the role of STAT5b in breast cancer cell   migration  .STAT5b was knocked down by using siRNA in two aggressive, highly migratory breast cancer cell lines (BT-549 and MDA-MB-231), and transwell   migration   assays were performed to determine the importance of STAT5b for their   migration  . Knockdown-rescue experiments were used to validate the specificity of STAT5b knockdown and to determine which regions/functions of STAT5b are necessary for its role in   migration  . Live-cell imaging of wound healing and spreading was carried out to examine cell morphology and motility after STAT5b knockdown.Knockdown of STAT5b, but not STAT5a, inhibited   migration   of BT-549 and MDA-MB-231 breast cancer cells to serum by 60% to 80%, and inhibited   migration   equally over a range of serum concentrations (0.1% to 10% serum). Migratory inhibition upon STAT5b knockdown could be rescued by reintroduction of wild-type STAT5b, as well as Y699F- and dominant-negative STAT5b mutants, but not an SH2 domain defective R618K-STAT5b mutant. beta1- integrin-mediated   migration   of breast cancer cells to fibronectin was inhibited with STAT5b knockdown, and loss of STAT5b correlated with loss of directional   migration   and formation of multiple, highly contractile protrusions upon attachment to fibronectin.The data presented here demonstrate that STAT5b is integral to breast cancer cell   migration   and identify a novel, SH2-dependent function of STAT5b in regulating beta1-integrin-mediated   migration   of highly aggressive breast cancer cells. 
 Osteolytic prostate cancer cells induce the expression of specific cytokines in bone-forming osteoblasts through a Stat3/5-dependent mechanism. Prostate cancer primarily metastasizes to bone, and the interaction of cancer cells with bone cells results in a local activation of bone formation and/or bone resorption. Since the cellular and molecular mechanisms underlying the development of these tumor-induced osteoblastic or osteolytic lesions are still poorly understood, we have compared the effects of two prostate cancer cell lines, osteoblastic MDA-PCa-2b cells and osteolytic PC-3 cells, on bone-forming osteoblasts. Using Affymetrix Gene Chip hybridization followed by qRT-PCR confirmation we were able to identify specific genes, including Smpd3 and Dmp1, whose expression is significantly reduced upon treatment with PC-3-conditioned medium. Moreover, we observed that PC-3-conditioned medium led to a marked induction of several cytokine genes, including Cxcl5, Cxcl12 and Tnfsf11, the latter one encoding for the osteoclast differentiation factor Rankl. Likewise, when we analyzed the effects of MDA-PCa-2b- and PC-3-conditioned medium on signal transduction in osteoblasts we did not only observe opposite effects on the canonical Wnt signalling pathway, but also a specific induction of Erk and Stat phosphorylation by PC-3-conditioned medium. Most importantly, the induction of Cxcl5, Cxcl12 and Tnfsf11 in osteoblasts by PC-3-conditioned medium was abrogated by the Stat3/5 inhibitor piceatannol, whereas the selective blockade of Stat1 and Erk activation had no effect. Together with the finding, that activated Stat3 in osteoblasts was detectable in bone biopsies from patients with osteolytic metastases, our data suggest that the Stat3/5-dependent activation of cytokine expression in osteoblasts may have a significant impact on cancer cell   migration   and proliferation, but also on osteoclast activation. 
 STAT protein recruitment and activation in c-Kit deletion mutants. Stem cell factor (SCF) and its tyrosine kinase receptor, c-Kit, play a crucial role in regulating   migration   and proliferation of melanoblasts, germ cells, and hemopoietic cell progenitors by activating a number of intracellular signaling molecules. Here we report that SCF stimulation of myeloid cells or fibroblasts ectopically expressing c-Kit induces physical association with and tyrosine phosphorylation of three signal transducers and activators of transcription (STATs) as follows: STAT1alpha, STAT5A, and STAT5B. Other STAT proteins are not recruited upon SCF stimulation. Recruitment of STATs leads to their dimerization, nuclear translocation, and binding to specific promoter-responsive elements. Whereas STAT1alpha, possibly in the form of homodimers, binds to the sis-inducible DNA element, STAT5 proteins, either as STAT5A/STAT5B or STAT5/STAT1alpha heterodimers, bind to the prolactin-inducible element of the beta-casein promoter. The tyrosine kinase activity of Kit appears essential for STAT activation since a kinase-defective mutant lacking a kinase insert domain was inactive in STAT signaling. However, another mutant that lacked the carboxyl-terminal region retained STAT1alpha activation and nuclear translocation but was unable to fully activate STAT5 proteins, although it mediated their transient phosphorylation. These results indicate that different intracellular domains of c-Kit are involved in activation of the various STAT proteins. 
 Jak2-Stat5a/b Signaling Induces Epithelial-to-Mesenchymal Transition and Stem-Like Cell Properties in Prostate Cancer. Active Stat5a/b predicts early recurrence and disease-specific death in prostate cancer (PC), which both typically are caused by development of metastatic disease. Herein, we demonstrate that Stat5a/b induces epithelial-to-mesenchymal transition (EMT) of PC cells, as shown by Stat5a/b regulation of EMT marker expression (Twist1, E-cadherin, N-cadherin, vimentin, and fibronectin) in PC cell lines, xenograft tumors in vivo, and patient-derived PCs ex vivo using organ explant cultures. Jak2-Stat5a/b signaling induced functional end points of EMT as well, indicated by disruption of epithelial cell monolayers and increased   migration   and adhesion of PC cells to fibronectin. Knockdown of Twist1 suppressed Jak2-Stat5a/b-induced EMT properties of PC cells, which were rescued by re-introduction of Twist1, indicating that Twist1 mediates Stat5a/b-induced EMT in PC cells. While promoting EMT, Jak2-Stat5a/b signaling induced stem-like properties in PC cells, such as sphere formation and expression of cancer stem cell markers, including BMI1. Mechanistically, both Twist1 and BMI1 were critical for Stat5a/b induction of stem-like features, because genetic knockdown of Twist1 suppressed Stat5a/b-induced BMI1 expression and sphere formation in stem cell culture conditions, which were rescued by re-introduction of BMI1. By using human prolactin knock-in mice, we demonstrate that prolactin-Stat5a/b signaling promoted metastases formation of PC cells in vivo. In conclusion, our data support the concept that Jak2-Stat5a/b signaling promotes metastatic progression of PC by inducing EMT and stem cell properties in PC cells. 
 STAT5 regulates glioma cell invasion by pathways dependent and independent of STAT5 DNA binding. STAT5 activation in primary glioma was analyzed using antibody directed against phosphorylated STAT5 (pSTAT5), in all samples robust pSTAT5 immunostaining was detected, predominantly in the nucleus. We then used immunofluorescence, transcription factor binding assays and western blotting to study EGF-modulated STAT5 activation in the human U87 glioma cell line. EGF was found to upregulate pSTAT5 levels and enhances STAT5 DNA-binding activity. To address the role of STAT5 in glioma cell invasion, resting and EGF-treated U87 cells were treated with siRNA directed against STAT5 and the extent of glioma cell   migration   into a Matrigel matrix was monitored. EGF treatment markedly enhanced matrix invasion, and knockdown of STAT5 expression with siRNA significantly downregulated matrix invasion by both EGF-stimulated and resting glioma cells. However, transfection with a decoy oligonucleotide abolished transcriptional activation of a STAT5 target gene but failed to inhibit matrix invasion in resting U87 cells. By contrast, the decoy nucleotide abolished EGF enhancement of matrix invasion. STAT5 therefore promotes glioma cell invasion via at least two different pathways: a pathway dependent of STAT5 DNA binding, and a second pathway independent of STAT5 DNA binding. 
 Stat5 promotes metastatic behavior of human prostate cancer cells in vitro and in vivo. There are no effective therapies for disseminated prostate cancer. Constitutive activation of Stat5 in prostate cancer is associated with cancer lesions of high histological grade. We have shown that Stat5 is activated in 61% of distant metastases of clinical prostate cancer. Active Stat5 increased metastases formation of prostate cancer cells in nude mice by 11-fold in an experimental metastases assay. Active Stat5 promoted   migration   and invasion of prostate cancer cells, and induced rearrangement of the microtubule network. Active Stat5 expression was associated with decreased cell surface E-cadherin levels, while heterotypic adhesion of prostate cancer cells to endothelial cells was stimulated by active Stat5. Activation of Stat5 and Stat5-induced binding of prostate cancer cells to endothelial cells were decreased by inhibition of Src but not of Jak2. Gene expression profiling indicated that 21% of Stat5-regulated genes in prostate cancer cells were related to metastases, while 7.9% were related to proliferation and 3.9% to apoptosis. The work presented here provides the first evidence of Stat5 involvement in the induction of metastatic behavior of human prostate cancer cells in vitro and in vivo. Stat5 may provide a therapeutic target protein for disseminated prostate cancer. 
 Signal transducer and activator of transcription (STAT)-5A and STAT5B differentially regulate human mammary carcinoma cell behavior. Increased activation of signal transducer and activator of transcription (STAT)-5 has been reported in various malignancies including mammary carcinoma. However, it is only recently that potentially distinct roles of STAT5A and STAT5B in neoplasia have begun to emerge. Herein we systematically delineate the functions of STAT5A and STAT5B in human mammary carcinoma cell lines MCF-7 and T47D. Forced expression of constitutively active (CA) STAT5A enhanced both survival and anchorage-independent growth of human mammary carcinoma cells but concordantly suppressed cell motility as revealed in colony scattering, cell   migration  , and invasion assays. In contrast, forced expression of CA STAT5B exhibited lower potency than CA STAT5A in enhancing survival and anchorage-independent growth of mammary carcinoma cells and exerted no effects on cell motility. Differential expression of genes that regulate cellular survival and motility was concomitantly observed on forced expression of CA STAT5A or CA STAT5B. Small interfering RNA-mediated depletion of STAT5A significantly impaired anchorage-independent growth of human mammary carcinoma cells, whereas a smaller reduction was observed upon small interfering RNA-mediated depletion of STAT5B. Depletion of endogenous STAT5A also significantly enhanced cell motility, whereas depletion of endogenous STAT5B exhibited no effect. Xenograft studies provided data concordant with the in vitro effects of the two STAT5 isoforms. We therefore demonstrate that STAT5A and STAT5B differentially regulate behavior of human mammary carcinoma cells. 
 The 15(S)-hydroxyeicosatetraenoic acid-induced angiogenesis requires Janus kinase 2-signal transducer and activator of transcription-5B-dependent expression of interleukin-8. To understand the molecular basis underlying 15(S)-hydroxyeicosatetraenoic acid (15(S)-HETE)-induced angiogenesis, we have studied the role of the Janus kinase-signal transducer and activator of transcription (Jak-STAT) signaling. The 15(S)-HETE stimulated tyrosine phosphorylation of Jak2 in a time-dependent manner in human retinal microvascular endothelial cells (HRMVECs). Inhibition of Jak2 activation via adenovirus-mediated expression of its dominant-negative mutant attenuated 15(S)-HETE-induced HRMVEC   migration   and tube formation and Matrigel plug angiogenesis. Similarly, 15(S)-HETE activated tyrosine phosphorylation of STAT-5B in a time-dependent manner. Dominant-negative mutant-mediated interference of STAT-5B activation suppressed 15(S)-HETE-induced HRMVEC   migration   and tube formation and Matrigel plug angiogenesis. The 15(S)-HETE induced interleukin-8 (IL-8) expression in Jak2-STAT-5B-dependent manner in HRMVECs. In addition, neutralizing anti-IL-8 antibodies reduced 15(S)-HETE-induced HRMVEC   migration   and tube formation and Matrigel plug angiogenesis. Cloning and Transfac analysis of IL-8 promoter revealed the presence of 1 putative STAT-binding sequence at -476 nt, and electrophoretic mobility shift assay and chromatin immunoprecipitation analysis showed the binding of STAT-5B to this site in response to 15(S)-HETE. Mutational analysis showed that STAT binding site is essential for 15(S)-HETE-induced IL-8 promoter activity. Together, these observations suggest that 15(S)-HETE-induced angiogenesis requires Jak2-STAT-5B-dependent expression of IL-8. 
 Role of arachidonic acid metabolism in Stat5 activation induced by oleic acid in MDA-MB-231 breast cancer cells. Epidemiological studies and animal models suggest an association between high levels of dietary fat intake and an increased risk of breast cancer. In breast cancer cells, the free fatty acid oleic acid (OLA) induces proliferation,   migration  , invasion and an increase of MMP-9 secretion. However, the role of OLA on Stat5 activation and the participation of COX-2 and LOXs activity in Stat5 activation induced by OLA remain to be investigated. We demonstrate here that stimulation of MDA-MB-231 breast cancer cells with 100 μM OLA induces Stat5 phosphorylation at Tyr-694 and an increase of Stat5-DNA complex formation. The Stat5 DNA-binding activity requires COX-2, LOXs, metalloproteinases and Src activities. In addition, OLA induces cell   migratio  n through a Stat5-dependent pathway. In summary, our findings establish that OLA induces cell   migratio  n through a Stat5-dependent pathway and that Stat5 activation requires AA metabolites in MDA-MB-231 breast cancer cells. 
  GeneRIFs  
 Our study also assessed the regulation of signaling molecules implicated in the growth, progression, differentiation, and   migration   of cancer cells, such as Jak2, STAT5b, insulin-like growth factor-1Rbeta, and their phosphorylation status 
 Macrophage   migration   inhibitory factor induced accumulation of mast cells in vivo is associated with activation of STAT5. 
 Oleic acid induces cell   migration   through a Stat5-dependent pathway and Stat5 activation requires arachidonic acid metabolites in MDA-MB-231 breast cancer cells. 
 The Stat3/5-dependent activation of cytokine expression in osteoblasts may have a significant impact on cancer cell   migration   and proliferation, but also on osteoclast activation. 
 STAT5b is integral to breast cancer cell   migration  . A novel, SH2-dependent function of STAT5b regulates beta1-integrin-mediated   migration   of highly aggressive breast cancer cells. 
  [Ii]nvasive  PubMed  
 Degradation of the transcription factors NF-κB, STAT3, and STAT5 is involved in Entamoeba histolytica-induced cell death in Caco-2 colonic epithelial cells. Entamoeba histolytica is a tissue  -invasiv  e protozoan parasite causing dysentery in humans. During infection of colonic tissues, amoebic trophozoites are able to kill host cells via apoptosis or necrosis, both of which trigger IL-8-mediated acute inflammatory responses. However, the signaling pathways involved in host cell death induced by E. histolytica have not yet been fully defined. In this study, we examined whether calpain plays a role in the cleavage of pro-survival transcription factors during cell death of colonic epithelial cells, induced by live E. histolytica trophozoites. Incubation with amoebic trophozoites induced activation of m-calpain in a time- and dose-dependent manner. Moreover, incubation with amoebae resulted in marked degradation of STAT proteins (STAT3 and STAT5) and NF-κB (p65) in Caco-2 cells. However, IκB, an inhibitor of NF-κB, was not cleaved in Caco-2 cells following adherence of E. histolytica. Entamoeba-induced cleavage of STAT proteins and NF-κB was partially inhibited by pretreatment of cells with a cell-permeable calpain inhibitor, calpeptin. In contrast, E. histolytica did not induce cleavage of caspase-3 in Caco-2 cells. Furthermore, pretreatment of Caco-2 cells with a calpain inhibitor, calpeptin (but not the pan-caspase inhibitor, z-VAD-fmk) or m-calpain siRNA partially reduced Entamoeba-induced DNA fragmentation in Caco-2 cells. These results suggest that calpain plays an important role in E. histolytica-induced degradation of NF-κB and STATs in colonic epithelial cells, which ultimately accelerates cell death. 
 STAT5A/B gene locus undergoes amplification during human prostate cancer progression. The molecular mechanisms underlying progression of prostate cancer (PCa) to castrate-resistant (CR) and metastatic disease are poorly understood. Our previous mechanistic work shows that inhibition of transcription factor Stat5 by multiple alternative methods induces extensive rapid apoptotic death of Stat5-positive PCa cells in vitro and inhibits PCa xenograft tumor growth in nude mice. Furthermore, STAT5A/B induces   invasiv  e behavior of PCa cells in vitro and in vivo, suggesting involvement of STAT5A/B in PCa progression. Nuclear STAT5A/B protein levels are increased in high-grade PCas, CR PCas, and distant metastases, and high nuclear STAT5A/B expression predicts early disease recurrence and PCa-specific death in clinical PCas. Based on these findings, STAT5A/B represents a therapeutic target protein for advanced PCa. The mechanisms underlying increased Stat5 protein levels in PCa are unclear. Herein, we demonstrate amplification at the STAT5A/B gene locus in a significant fraction of clinical PCa specimens. STAT5A/B gene amplification was more frequently found in PCas of high histologic grades and in CR distant metastases. Quantitative in situ analysis revealed that STAT5A/B gene amplification was associated with increased STAT5A/B protein expression in PCa. Functional studies showed that increased STAT5A/B copy numbers conferred growth advantage in PCa cells in vitro and as xenograft tumors in vivo. The work presented herein provides the first evidence of somatic STAT5A/B gene amplification in clinical PCas. 
 Nuclear expression of STAT5 in intraductal papillary mucinous neoplasms of the pancreas. Intraductal papillary mucinous neoplasms (IPMNs) are non  invasive   lesions of the pancreas and classified as intraductal papillary mucinous adenomas (IPMAs), borderline IPMNs, and intraductal papillary mucinous carcinomas (IPMCs). Expression patterns of the specific genes alter during IPMN progression. Based on the evidence that signal transducers and activators of transcription (STAT) 5 play important roles in tumor development, we tested STAT5 expression in IPMAs, borderline IPMNs, and IPMCs by immunohistochemical method. STAT5 frequently expressed in the nuclei of tumor cells of borderline IPMNs or IPMCs but was not observed in those of IPMAs. Nuclear expression of STAT5 protein correlated to the Ki-67 labeling index of the examined IPMNs. STAT5 protein could contribute to the progression and proliferation of IPMNs. 
 Stat5 activation inhibits prolactin-induced AP-1 activity: distinct prolactin-initiated signals in tumorigenesis dependent on cell context. The essential role of prolactin (PRL) in normal mammary gland growth and differentiation has implicated this hormone in the development and progression of breast cancer. Although Stat5 is the best-characterized mediator of PRL signals, PRL also activates multiple other signals, whose roles in normal and pathologic processes are not well understood. We have shown that PRL stimulates activating protein-1 (AP-1) activity in breast cancer cells, and can cooperate with estradiol in this pathway. AP-1 modulates many processes critical for carcinogenesis, including cell proliferation, survival, transformation, invasion and angiogenesis, and is elevated in many neoplasms, including breast tumors. Here, we investigated the relationship between PRL signals to AP-1 and Stat5. We found that PRL activation of Stat5a and Stat5b, but not Stat1 or Stat3, reduced PRL signals to AP-1, without altering estradiol-induced AP-1 activity. The truncation mutant, Stat5/Delta53C, but not Stat5Y699F, was an effective inhibitor, consistent with a requirement for Stat5 dimerization and nuclear accumulation, but not its C-terminal transactivation activity. The association of Stat5 with AP-1 proteins suggests that this underlies the inhibition. Predictably, the ability of PRL to activate Stat5 and AP-1 was inversely related in mammary cell lines. Further, reduction of Stat5 protein with siRNA in T47D cells, which contain elevated Stat5, increased PRL-induced AP-1 signals, transcripts for the AP-1 target, matrix metalloproteinase-2 and associated   invasive   behavior. This study points to the importance of cell context in determining the spectrum of PRL-induced actions, which is critical for understanding the contributions of PRL to breast cancer. 
 Hemin inhibits cyclin D1 and IGF-1 expression via STAT5b under hypoxia in ERalpha-negative MDA-MB 231 breast cancer cells. Cyclin D1 and insulin-like growth factor 1 receptor (IGF-1R) are key regulators of cell proliferation that are overexpressed in most breast cancers. The purpose of the present study was to investigate the molecular mechanism by which hemin exerts its inhibitory effects on aggressive breast cancer cells. We found that hemin regulates cyclin D1 and IGF-1R proteins and insulin-like growth factor-1 gene expression through STAT5b in breast cancer cells. We confirmed that STAT5b, cyclin D1, and IGF-1R is up-regulated by hypoxia, and the increased STAT5b binds strongly to the STAT5-binding sites contained within the distal 5'-flanking region of IGF-1 gene in breast cancer cells. EMSA studies showed that STAT5 binding activity to the IGF-1 and cyclin D1 promoter was distinctly decreased by hemin in STAT5b-transfected COS-7 or MDA-MB 231 cells. IGF-1 gene expression was also decreased by hemin in mammary epithelial cells. STAT5b expression was inhibited in siRNA experiments and by hemin, leading to decreased levels of IGF-1. These results provide a basis for molecular targets in cancer treatment via the STAT5b/IGF-1 or /cyclin D1 pathway in solid tumor cells. These data indicate that hemin inhibits the cyclin D1 and IGF-1 expression via STAT5b under hypoxia in ERalpha-negative breast cancer cells. These findings are valuable toward understanding the role of hemin-induced inhibition of cyclin D1 and IGF-1 expression under hypoxia in   invasive   and metastatic breast cancer. 
 Suppression of STAT5b in pancreatic cancer cells leads to attenuated gemcitabine chemoresistance, adhesion and invasion. Pancreatic ductal adenocarcinoma (PDAC) is one of the most lethal solid malignancies, and there is an urgent need for new therapeutic strategies based on the molecular biology of PDAC. Signal transducers and activators of transcription 5 (STAT5) are known to be activated in a variety of malignancies and involved in tumor proliferation, apoptosis, and invasion, whereas the expression and biological role of STAT5b in PDAC are less clearly defined. In the present study, we examined the expression and role of STAT5b in human pancreatic cancer cell lines. Expressions of STAT5b mRNA and protein were detected in eight kinds of pancreatic cancer cells. Confocal microscopy and western blot analysis indicated that STAT5b is localized in both cytoplasm and nuclei. Immunoprecipitation analysis revealed tyrosine phosphorylation of STAT5b in pancreatic cancer cells. These results indicate that STAT5b in pancreatic cancer cells is constitutively activated. STAT5b shRNA clones in PANC-1 cells, which express relatively high levels of STAT5b, exhibited reduced chemoresistance against gemcitabine, adhesion and invasion compared to sham. On the other hand, AsPC-1 and BxPC3 cells, which express relatively low levels of STAT5b, exhibited reduced chemoresistance compared to PANC-1 cells. Moreover, STAT5b overexpression clones in AsPC-1 cells exhibited increased chemoresistance compared to sham. STAT5b shRNA clones in PANC-1 cells were more sensitive to the proapoptotic actions of gemcitabine, as evidenced by PARP and cleaved caspase-3 activation. Gemcitabine also significantly reduced Bcl-xL levels in the STAT5b shRNA-expressing cells. We also investigated the clinicopathological characteristics of STAT5b expression of PDAC. Although a significant correlation between STAT5b expression and overall survival rates was not observed, a significant correlation with main pancreatic duct invasion was observed. These findings suggest that STAT5b confers gemcitabine chemoresistance and promotes cell adherence and   invasive  ness in pancreatic cancer cells. Targeting STAT5b may lead to novel therapeutic strategies for PDAC. 
  GeneRIFs  
 STAT5b confers gemcitabine chemoresistance and promotes cell adherence and   invasive  ness in pancreatic cancer cells. 
  [Mm]etastasize  PubMed  
 Osteolytic prostate cancer cells induce the expression of specific cytokines in bone-forming osteoblasts through a Stat3/5-dependent mechanism. Prostate cancer primarily   metastasize  s to bone, and the interaction of cancer cells with bone cells results in a local activation of bone formation and/or bone resorption. Since the cellular and molecular mechanisms underlying the development of these tumor-induced osteoblastic or osteolytic lesions are still poorly understood, we have compared the effects of two prostate cancer cell lines, osteoblastic MDA-PCa-2b cells and osteolytic PC-3 cells, on bone-forming osteoblasts. Using Affymetrix Gene Chip hybridization followed by qRT-PCR confirmation we were able to identify specific genes, including Smpd3 and Dmp1, whose expression is significantly reduced upon treatment with PC-3-conditioned medium. Moreover, we observed that PC-3-conditioned medium led to a marked induction of several cytokine genes, including Cxcl5, Cxcl12 and Tnfsf11, the latter one encoding for the osteoclast differentiation factor Rankl. Likewise, when we analyzed the effects of MDA-PCa-2b- and PC-3-conditioned medium on signal transduction in osteoblasts we did not only observe opposite effects on the canonical Wnt signalling pathway, but also a specific induction of Erk and Stat phosphorylation by PC-3-conditioned medium. Most importantly, the induction of Cxcl5, Cxcl12 and Tnfsf11 in osteoblasts by PC-3-conditioned medium was abrogated by the Stat3/5 inhibitor piceatannol, whereas the selective blockade of Stat1 and Erk activation had no effect. Together with the finding, that activated Stat3 in osteoblasts was detectable in bone biopsies from patients with osteolytic metastases, our data suggest that the Stat3/5-dependent activation of cytokine expression in osteoblasts may have a significant impact on cancer cell migration and proliferation, but also on osteoclast activation. 
  [Mm]etastasis  PubMed  
 The combination of methylsulfonylmethane and tamoxifen inhibits the Jak2/STAT5b pathway and synergistically inhibits tumor growth and   metastasis   in ER-positive breast cancer xenografts. Combination therapy, which reduces the dosage intensity of the individual drugs while increasing their efficacy, is not a novel approach for the treatment of cancer. Methylsulfonylmethane (MSM) is an organic sulfur compound shown to act against tumor cells. Tamoxifen is a commercially available therapeutic agent for breast malignancies.In the current study, we analyzed the combinatorial effect of MSM and tamoxifen on the suppression of ER-positive breast cancer xenograft growth and   metastasis  . Additionally, we also validated the molecular targets by which the drug combination regulated tumor growth and   metastasis  .We observed that the combination of MSM and tamoxifen regulated cell viability and migration in vitro. The intragastric administration of MSM and subcutaneous implantation of tamoxifen tablets led to tumor growth suppression and inhibition of the Janus kinase 2 (Jak2)/signal transducer and activator of transcription 5b (STAT5b) pathway. Our study also assessed the regulation of signaling molecules implicated in the growth, progression, differentiation, and migration of cancer cells, such as Jak2, STAT5b, insulin-like growth factor-1Rβ, and their phosphorylation status.Study results indicated that this combination therapy inhibited tumor growth and   metastasi  s. Therefore, this drug combination may have a synergistic and powerful anticancer effect against breast cancer. 
 [Expression and clinical significance of COX-2, p-Stat3, and p-Stat5 in esophageal carcinoma]. Cyclooxygenase-2 (COX-2) and signal transducers and activators of transcription (STAT) are closely correlated to the genesis of tumors. This study was to investigate the expression and clinical significance of COX-2, p-Stat3 and p-Stat5 (the activated forms of Stat3 and Stat5) in various lesions of esophageal tissues, and to analyze their correlations to clinicopathologic features of esophageal squamous cell carcinoma (ESCC).The expression of COX-2, p-Stat3, and p-Stat5 in 59 specimens of ESCC, 24 specimens of squamous dysplasia, and 18 specimens of normal squamous epithelium was examined by SP immunohistochemistry. Their correlations to clinicopathologic features of ESCC were analyzed.The protein level of COX-2 was significantly higher in ESCC and squamous dysplasia than in normal squamous epithelium (2.10+/-1.77 and 1.85+/-1.24 vs. 0.83+/-0.46, P<0.05). The protein level of p-Stat3 was 0 in normal squamous epithelium, 0.76+/-0.59 in squamous dysplasia, and 2.83+/-1.27 in ESCC. The protein level of p-Stat5 was 1.98+/-0.78 in normal squamous epithelium, 3.92+/-0.41 in squamous dysplasia, and 5.02+/-0.34 in ESCC. There were significant differences among the 3 groups (P<0.05). In ESCC, COX-2 expression was correlated to lymph node   metastasis   and differentiation (P<0.05); p-Stat3 expression was correlated to tumor invasion depth (P<0.05); but p-Stat5 expression had no correlation to clinicopathologic features. COX-2 expression was positively correlated to both p-Stat3 expression and p-Stat5 expression in ESCC.The up-regulation of COX-2, p-Stat3, and p-Stat5 may be correlated to the carcinogenesis of ESCC. The activation of Stat3 is correlated to the aggressive behavior of ESCC. 
 Negative cross talk between NFAT1 and Stat5 signaling in breast cancer. The molecular mechanisms that modulate the activity of the signal transducers and activators of transcription 5 (Stat5) during the progression of breast cancer remain elusive. Here, we present evidence that the calcineurin/nuclear factor of activated T cells (NFAT) pathway negatively regulates the activation of Stat5, and vice versa in breast cancer. NFAT1 interacts with Stat5 in breast cancer cells, and their physical association is mediated by the DNA binding and transactivation domains of Stat5. Ectopically expressed NFAT1 is capable of inhibiting Stat5-dependent functions, including Stat5 transactivation, Stat5-mediated transcription of the downstream target gene expression, and binding of Stat5a to the Stat5 target promoter. By contrast, overexpression of a selective NFAT inhibitor VIVIT reversed NFAT1-mediated suppression of Stat5-dependent gene expression, whereas silencing of NFAT1 through RNA interference enhanced prolactin-induced, Stat5-mediated gene transcription, and breast cancer cell proliferation. A reciprocal inhibitory effect of Stat5 activity on NFAT1 signaling was also observed, implying these two signaling cascades antagonize each other in breast cancer. Importantly, analysis of a matched breast cancer progression tissue microarray revealed a negative correlation between levels of NFAT1 and Stat5 (pY694) during the progression of breast cancer. Taken together, these studies highlight a novel negative cross talk between the NFAT1- and Stat5-signaling cascades that may affect breast tumor formation, growth, and   metastasis  . 
 Expression of STAT5, COX-2 and PIAS3 in correlation with NSCLC histhopathological features. Signal transducers and activators of transcription (STATs), their inhibitors and cyclooxygenase-2 (COX-2) participate in transformations of many various types of cancers. The aim of the present study was to evaluate the relationship between STAT5A/B, COX-2, and PIAS3 mRNA expression and tumor staging,   metastasis   status, and histopathological subtype in 71 patients with confirmed non-small cell lung cancer (NSCLC) diagnosis. Total RNA was isolated from NSCLC tissue samples and the expression of the studied genes was assessed using TaqMan probes in real-time PCR assay. The expression levels of STAT5A, STAT5B, and COX-2 genes were increased in 69%, 79%, and 71% NSCLC samples respectively, while PIAS3 expression was decreased in the majority (69%) of the studied tissues. Statistically significant differences were observed between STAT5 isoforms (P = 0.0008), with higher expression of STAT5B. We found statistically significant positive correlation between STAT5B and COX-2 (rho = 0.045), and significant negative correlation between STAT5B and PIAS3 (rho = -0.049). The negative correlation between STAT5B and PIAS3 (rho = -0.43) was also observed in T2a+T2b tumor group. Additionally, STAT5B and COX-2 expression levels were significantly different between T1a+T1b and T2a+T2b tumors (P = 0.002 and P = 0.041, respectively), with higher expression of both genes in T2 tumor stage. PIAS3 expression was significantly lower in NSCC subtype as compared with SCC subtype (P = 0.017). Also, STAT5A and STAT5B immunoexpression was assessed, and the results indicated significantly higher protein levels in NSCLC patients as compared with controls (P = 0.048 and P = 0.034, respectively). High STAT5B immunoexpression was positively correlated with STAT5B gene expression in tumors (rho = 0.755). STAT5B protein level was also significantly higher in T2a+T2b tumors, reflecting high STAT5B gene expression in this group. There was no statistically significant association between mRNA and protein expression levels of the studied genes and patients' characteristics: age, gender, smoking. The obtained results highlight the importance of the genes STAT5B and COX-2 in lung cancer progression. 
  GeneRIFs  
 a novel negative cross talk between the NFAT1- and Stat5-signaling cascades that may affect breast tumor formation, growth, and   metastasis   
  [Cc]ell [Ii]nvasion  PubMed  
 Inhibition of STAT5 induces G1 cell cycle arrest and reduces tumor   cell invasion   in human colorectal cancer cells. Abnormalities in the signal transducer and activator of transcription (STAT) pathway are involved in the oncogenesis of several cancers. However, the mechanism by which dysregulated STAT5 signaling contributes to the progression of human colorectal cancer (CRC) has not been elucidated. To investigate the role of STAT5 in CRC progression, we depleted STAT5 with a small interfering RNA (siRNA). Our results demonstrate that STAT5 is involved in CRC cell growth, cell cycle progression, invasion and migration through regulation of gene expression, such as Bcl-2, p16(ink4a), p21(waf1/cip1), p27(kip1), E-cadherin, the focal adhesion kinase (FAK), vascular endothelial growth factor (VEGF) and matrix metalloproteinases. In addition, immunohistochemical staining reveals upregulation of STAT5 during CRC tumorigenesis. Moreover, phospho-STAT5 (pSTAT5) is predominantly localized in the cytoplasm of adenomas cells and colon adenocarcinoma cells, but primarily presented in the nucleus of normal colonic epithelium cells. Thus, pSTAT5 protein is shuttled from the nucleus to the cytoplasm in the oncogenesis of CRC, suggesting that activated STAT5 may also have cytoplasmic functions. In support of this hypothesis, we found that STAT5 formed a complex with p44/42 MAPK and SAPK/JNK in CRC cells, suggesting cross talk between STAT5 signaling and the MAPK pathway in the development of human CRC. Our findings illustrate the biological significance of STAT5 signaling in CRC progression, and provide novel evidence that intervention in STAT5 signaling may have potential therapeutic value in the prevention of human colorectal cancer. 
 STAT5 regulates glioma   cell invasion   by pathways dependent and independent of STAT5 DNA binding. STAT5 activation in primary glioma was analyzed using antibody directed against phosphorylated STAT5 (pSTAT5), in all samples robust pSTAT5 immunostaining was detected, predominantly in the nucleus. We then used immunofluorescence, transcription factor binding assays and western blotting to study EGF-modulated STAT5 activation in the human U87 glioma cell line. EGF was found to upregulate pSTAT5 levels and enhances STAT5 DNA-binding activity. To address the role of STAT5 in glioma   cell invasion  , resting and EGF-treated U87 cells were treated with siRNA directed against STAT5 and the extent of glioma cell migration into a Matrigel matrix was monitored. EGF treatment markedly enhanced matrix invasion, and knockdown of STAT5 expression with siRNA significantly downregulated matrix invasion by both EGF-stimulated and resting glioma cells. However, transfection with a decoy oligonucleotide abolished transcriptional activation of a STAT5 target gene but failed to inhibit matrix invasion in resting U87 cells. By contrast, the decoy nucleotide abolished EGF enhancement of matrix invasion. STAT5 therefore promotes glioma   cell invasion   via at least two different pathways: a pathway dependent of STAT5 DNA binding, and a second pathway independent of STAT5 DNA binding. 
  GeneRIFs  
 STAT5 regulates glioma   cell invasion   via at least two different pathways: one that is dependent of STAT5 DNA binding, and a second pathway independent of STAT5 DNA binding. 
   
  
 PGLYRP1 
 
  NCBI Gene  
  GeneRIF  
  Pubmed  
 
 
  Search Term  Rank  Fields  
  [Mm]igration  6  PubMed(1); GeneRIFs(1)  
  [Mm]etastasis  12  PubMed(1)  
 
 
 hide/show details for PGLYRP1 
  [Mm]igration  PubMed  
 A new role for PGRP-S (Tag7) in immune defense: lymphocyte   migration   is induced by a chemoattractant complex of Tag7 with Mts1. PGRP-S (Tag7) is an innate immunity protein involved in the antimicrobial defense systems, both in insects and in mammals. We have previously shown that Tag7 specifically interacts with several proteins, including Hsp70 and the calcium binding protein S100A4 (Mts1), providing a number of novel cellular functions. Here we show that Tag7-Mts1 complex causes chemotactic   migration   of lymphocytes, with NK cells being a preferred target. Cells of either innate immunity (neutrophils and monocytes) or acquired immunity (CD4(+) and CD8(+) lymphocytes) can produce this complex, which confirms the close connection between components of the 2 branches of immune response. 
  GeneRIFs  
 Tag7-Mts1 complex causes chemotactic   migration   of lymphocytes, with NK cells being a preferred target. 
  [Mm]etastasis  PubMed  
 Opposite roles of metastasin (S100A4) in two potentially tumoricidal mechanisms involving human lymphocyte protein Tag7 and Hsp70. We compare the physical and functional interactions between three widespread multifunctional proteins [metastasin (Mts1/S100A4), innate immunity-related Tag7/PGRP-S, and Hsp70] in two experimental models relevant to host-tumor relationships on humoral and cellular levels. (i) Tag7 and Hsp70 in solution or in a lymphocyte make a stable binary complex that is highly cytotoxic for some tumor cells. Here, we show that Mts1 prevents Tag7.Hsp70 assembly in solution, and an excess of Mts1 disrupts the existing Tag7.Hsp70 complex; accordingly, Tag7.Hsp70 cytotoxicity (exemplified with L929 cells) is diminished in the presence of excess Mts1. (ii) Tag7 exposed on a specialized subset of lymphokine-activated killer cells makes specific contact with Hsp70 exposed on some HLA-negative tumor cells, thus enabling FasL/Fas-mediated induction of apoptosis. Here, we show that some CD4(+)CD25(+) cells coexpose Mts1 with Tag7 and FasL, that Mts1 and Tag7 closely contact the same Hsp70 molecule on the target K562 cell (as evidenced by cross-linking), and that killing of such targets is abolished by Mts1-specific antibodies (or selective removal of Mts1-exposing lymphocytes). Thus, this phenotype active against immunoevasive cancerous cells is defined as CD4(+)CD25(+), FasL(+), Tag7(+)Mts1(+) (approximately 0.5% of total lymphocytes in culture). Remarkably, similar effectors with at least the same activity are often found in fresh donor blood samples (approximately 10(4) effectors/mL). Thus, our models suggest that interactions between the three proteins in different situations may have opposite functional outcomes as regards antitumor defense, immune escape, and   metastasis  . 
   
  
 RAC1 
 
  NCBI Gene  
  GeneRIF  
  Pubmed  
 
 
  Search Term  Rank  Fields  
  [Ff]ilopodia  1  PubMed(27); GeneRIFs(1)  
  [Ff]ilopodium  2  PubMed(2)  
  [Ii]nvadopodia  3  PubMed(2); GeneRIFs(2)  
  [Ii]nvadopodium  4  PubMed(1)  
  [Pp]odosome(s)?  5  PubMed(2)  
  [Mm]igration  6  Process(4); PubMed(264); GeneRIFs(87); Alternative Name(1)  
  ECM  7  PubMed(13)  
  [Ee]xtracellular [Mm]atrix  8  PubMed(36); Component(1)  
  [Ii]nvadosome  9  PubMed(1); GeneRIFs(1)  
  [Ii]nvasive  10  PubMed(71); GeneRIFs(11)  
  [Mm]etastasize  11  PubMed(2)  
  [Mm]etastasis  12  PubMed(100); GeneRIFs(19)  
  [Cc]ell [Ii]nvasion  13  PubMed(37); GeneRIFs(14)  
 
 
 hide/show details for RAC1 
  [Ff]ilopodia  PubMed  
 The small GTPase RalA targets filamin to induce   filopodia  . The Ras-related small GTPases Rac, Rho, Cdc42, and RalA bind filamin, an actin filament-crosslinking protein that also links membrane and other intracellular proteins to actin. Of these GTPases only RalA binds filamin in a GTP-specific manner, and GTP-RalA elicits actin-rich filopods on surfaces of Swiss 3T3 cells and recruits filamin into the   filopodia  l cytoskeleton. Either a dominant negative RalA construct or the RalA-binding domain of filamin 1 specifically block Cdc42-induced filopod formation, but a Cdc42 inhibitor does not impair RalA's effects, which, unlike Cdc42, are Rac independent. RalA does not generate   filopodia   in filamin-deficient human melanoma cells, whereas transfection of filamin 1 restores the functional response. RalA therefore is a downstream intermediate in Cdc42-mediated filopod production and uses filamin in this pathway. 
 Cross-talk between Rac1 and Cdc42 GTPases regulates formation of   filopodia   required for dengue virus type-2 entry into HMEC-1 cells. Infection with dengue virus type-2 (DENV-2) begins with virus adherence to cell surface receptors. In endothelial cells (HMEC-1), a cell model for DENV-2 infection, alpha 5 beta 3 integrin has been identified as a putative receptor for the virus. Previous work had suggested that the actin cytoskeleton of HMEC-1 cells plays an important role in virus entry and infection. In the present work, fixed and living HMEC-1 cells expressing enhanced green fluorescent protein-actin were monitored for actin reorganization after virus inoculation, utilizing fluorescence and time lapse microscopy. Cell infection and production of infective viruses were quantified using an anti-E protein antibody and by measuring the p.f.u. ml(-1). Specific drugs that antagonize actin organization and regulate actin-signalling pathways were tested in viral adhesion and infection assays, as were the expression of dominant-negative Rac1 and Cdc42 proteins. Disorganization of actin precluded infection, while microtubule depolymerization had no effect. Activation of Rac1 and Cdc42 signalling, which occurs upon virus binding, induced reorganization of actin to form   filopodia   in the cellular periphery. Formation of   filopodia   was a requirement for virus entry and further cell infection. Expression of the dominant-negative proteins Rac1 and Cdc42 confirmed the role of these GTPases in the actin reorganization that is required to form   filopodia  . In addition, inhibition of the ATPase activity of myosin II greatly decreased infection, suggesting its participation in   filopodia  l stability. We show here, for the first time, that internalization of DENV-2 into endothelial cells requires viral induction of dynamic   filopodia   regulated by Rac1 and Cdc42 cross-talk and myosin II motor activities. 
 Desmoglein 3 acting as an upstream regulator of Rho GTPases, Rac-1/Cdc42 in the regulation of actin organisation and dynamics. Desmoglein 3 (Dsg3), a member of the desmoglein sub-family, serves as an adhesion molecule in desmosomes. Our previous study showed that overexpression of human Dsg3 in several epithelial lines induces formation of membrane protrusions, a phenotype suggestive of Rho GTPase activation. Here we examined the interaction between Dsg3 and actin in detail and showed that endogenous Dsg3 colocalises and interacts with actin, particularly the junctional actin in a Rac1-dependent manner. Ablation of Rac1 activity by dominant negative Rac1 mutant (N17Rac1) or the Rac1 specific inhibitor (NSC23766) directly disrupts the interaction between Dsg3 and actin. Assembly of the junctional actin at the cell borders is accompanied with enhanced levels of Dsg3, while inhibition of Dsg3 by RNAi results in profound changes in the organisation of actin cytoskeleton. In accordance, overexpression of Dsg3 results in a remarkable increase of Rac1 and Cdc42 activities and to a lesser extent, RhoA. The enhancements in Rho GTPases are accompanied by the pronounced actin-based membrane structures such as lamellipodia and   filopodia  , enhanced rate of actin turnover and cell polarisation. Together, our results reveal an important novel function for Dsg3 in promoting actin dynamics through regulating Rac1 and Cdc42 activation in epithelial cells. 
 Serine-71 phosphorylation of Rac1/Cdc42 diminishes the pathogenic effect of Clostridium difficile toxin A. Clostridium difficile toxin A and B (TcdA/TcdB) are glucosyltransferases that glucosylate GTPases of the Rho family. The epidermal growth factor (EGF) positively modulates C. difficile toxin-induced disturbance of the intestinal barrier function by an unknown mechanism. We found that EGF-treated CaCo-2 monolayers were less susceptible to TcdA-catalysed glucosylation of Rac1 but not of RhoA, which correlated with phosphorylation of Rac1 at Ser-71. Phospho-Rac1/phospho-Cdc42 (Ser-71) still bound to the PAK-CRIB domain indicating an active state. A more detailed characterization of phospho-Rac1 was performed using the phosphomimetic mutant Rac1 S71E. Ectopic expression of Rac1 S71E induced a specific phenotype of cells showing an increase in   filopodia  l structures that were also induced by EGF. Rac1 S71E (and Cdc42 S71E) but not Rac1 S71A was at least fivefold weaker substrate for TcdA-catalysed glucosylation compared with wild type Rac1. The protective effect was checked in transfection experiments where Rac1 S71E and, to a lesser extent, Cdc42 S71E reduced the TcdA-induced cytopathic effect. Thus, Ser-71 phosphorylation of Rac1 might be interesting for modulation of microbial pathogenesis where Rho GTPases, especially Rac1 and Cdc42, are involved. In addition, this is the first description of a specific functional outcome of Rac1 phosphorylation at Ser-71. 
 Myristoylated Alanine-Rich Protein Kinase Substrate (MARCKS) Regulates Small GTPase Rac1 and Cdc42 Activity and Is a Critical Mediator of Vascular Smooth Muscle Cell Migration in Intimal Hyperplasia Formation. Transcription of the myristoylated alanine-rich C kinase substrate (MARCKS) is upregulated in animal models of intimal hyperplasia. MARCKS knockdown inhibits vascular smooth muscle cell (VSMC) migration in vitro; however, the mechanism is as yet unknown. We sought to elucidate the mechanism of MARCKS-mediated motility and determine whether MARCKS knockdown reduces intimal hyperplasia formation in vivo.MARCKS knockdown blocked platelet-derived growth factor (PDGF)-induced translocation of cortactin to the cell cortex, impaired both lamellipodia and   filopodia   formation, and attenuated motility of human coronary artery smooth muscle cells (CASMCs). Activation of the small GTPases, Rac1 and Cdc42, was prevented by MARCKS knockdown. Phosphorylation of MARCKS resulted in a transient shift of MARCKS from the plasma membrane to the cytosol. MARCKS knockdown significantly decreased membrane-associated phosphatidylinositol 4,5-bisphosphate (PIP2) levels. Cotransfection with an intact, unphosphorylated MARCKS, which has a high binding affinity for PIP2, restored membrane-associated PIP2 levels and was indispensable for activation of Rac1 and Cdc42 and, ultimately, VSMC migration. Overexpression of MARCKS in differentiated VSMCs increased membrane PIP2 abundance, Rac1 and Cdc42 activity, and cell motility. MARCKS protein was upregulated early in the development of intimal hyperplasia in the murine carotid ligation model. Decreased MARKCS expression, but not total knockdown, attenuated intimal hyperplasia formation.MARCKS upregulation increases VSMC motility by activation of Rac1 and Cdc42. These effects are mediated by MARCKS sequestering PIP2 at the plasma membrane. This study delineates a novel mechanism for MARCKS-mediated VSMC migration and supports the rational for MARCKS knockdown to prevent intimal hyperplasia. 
 Cdc42/Rac1-dependent activation of the p21-activated kinase (PAK) regulates human platelet lamellipodia spreading: implication of the cortical-actin binding protein cortactin. Platelet activation by thrombin or thrombin receptor-activating peptide (TRAP) results in extensive actin reorganization that leads to   filopodia   emission and lamellae spreading concomitantly with activation of the Rho family small G proteins, Cdc42 and Rac1. Evidence has been provided that direct binding of Cdc42-guanosine triphosphate (GTP) and Rac1-GTP to the N-terminal regulatory domain of the p21-activated kinase (PAK) stimulates PAK activation and actin reorganization. In the present study, we have investigated the relationship between shape change and PAK activation. We show that thrombin, TRAP, or monoclonal antibody (MoAb) anti-Fc(gamma)RIIA IV.3 induces an activation of Cdc42 and Rac1. The GpVI ligand, convulxin (CVX), that forces platelets to lamellae spreading efficiently activates Rac1. Thrombin, TRAP, MoAb IV.3, and CVX stimulate autophosphorylation and kinase activity of PAK. Inhibition of Cdc42 and Rac1 with clostridial toxin B inhibits PAK activation and lamellae spreading. The cortical-actin binding protein, p80/85 cortactin, is constitutively associated with PAK in resting platelets and dissociates from PAK after thrombin stimulation. Inhibition of PAK autophosphorylation by toxin B prevents the dissociation of cortactin. These results suggest that Cdc42/Rac1-dependent activation of PAK may trigger early platelet shape change, at least in part through the regulation of cortactin binding to PAK. 
 Prostaglandin E2-mediated migration of human trophoblast requires RAC1 and CDC42. The invasion of maternal decidua and uterine spiral arteries by a trophoblast subpopulation called extravillous trophoblast (EVT) is essential for the establishment of a normal placenta and an adequate blood flow toward the fetus. Derangements in these processes underlie pregnancy-related diseases like preeclampsia and intrauterine growth restriction. Many growth factors, growth factor binding proteins, and extracellular matrix components can positively or negatively regulate the proliferation, migration, and/or invasiveness of these EVT cells. RHO GTPases, including RHOA, RAC1, and CDC42, are ubiquitous proteins that control cytoskeletal changes by forming stress fibers and projecting lamellipodia and   filopodia   during cellular migration. We had previously shown that prostaglandin (PG) E(2) produced in abundance by the decidua promotes the migration of first-trimester human EVTs by increasing the intracellular concentration of calcium and activating calpain. Using our well-characterized immortalized EVT cell line, HTR-8/SVneo, as well as villus explants from first-trimester placentae, this study examined the role of RHO GTPases RAC1 and CDC42 in PGE(2)-mediated migratory responses of these cells. Though a RAC1 inhibitor, NSC23766 as well as RAC1 knockdown by siRNA decreased the migration of HTR-8/SVneo cells in a Transwell migration assay, this inhibition could not be restored by PGE(2) or 17-phenyl trinor PGE(2) (PGE receptor PTGER1 agonist) or PGE(1) Alcohol (PGE receptor PTGER4 agonist). Similar results were noted for EVT cell spreading in villus explants. Furthermore, CDC42 silencing using siRNA inhibited PGE(2)-induced migration of HTR-8/SVneo cells. Finally, the treatment of EVT cells with PGE(2), PTGER1 agonist, or PTGER4 agonist activated RAC1 and CDC42 at 10 min, suggesting that RAC1 and CDC42 play an essential role in PGE(2)-mediated migration of human EVTs. 
 Estrogen and resveratrol regulate Rac and Cdc42 signaling to the actin cytoskeleton of metastatic breast cancer cells. Estrogen and structurally related molecules play critical roles in breast cancer. We reported that resveratrol (50 microM), an estrogen-like phytosterol from grapes, acts in an antiestrogenic manner in breast cancer cells to reduce cell migration and to induce a global and sustained extension of actin structures called   filopodia  . Herein, we report that resveratrol-induced   filopodia   formation is time-dependent and concentration-dependent. In contrast to resveratrol at 50 microM, resveratrol at 5 microM acts in a manner similar to estrogen by increasing lamellipodia, as well as cell migration and invasion. Because Rho GTPases regulate the extension of actin structures, we investigated a role for Rac and Cdc42 in estrogen and resveratrol signaling. Our results demonstrate that 50 microM resveratrol decreases Rac and Cdc42 activity, whereas estrogen and 5 microM resveratrol increase Rac activity in breast cancer cells. MDA-MB-231 cells expressing dominant-negative Cdc42 or dominant-negative Rac retain   filopodia   response to 50 microM resveratrol. Lamellipodia response to 5 microM resveratrol, estrogen, or epidermal growth factor is inhibited in cells expressing dominant-negative Rac, indicating that Rac regulates estrogen and resveratrol (5 microM) signaling to the actin cytoskeleton. These results indicate that signaling to the actin cytoskeleton by low and high concentrations of resveratrol may be differentially regulated by Rac and Cdc42. 
 DEF6, a novel PH-DH-like domain protein, is an upstream activator of the Rho GTPases Rac1, Cdc42, and RhoA. In this paper, we describe the characterization of DEF6, a novel PH-DH-like protein related to SWAP-70 that functions as an upstream activator of Rho GTPases. In NIH 3T3 cells, stimulation of the PI 3-kinase signaling pathway with either H2O2 or platelet-derived growth factor (PDGF) resulted in the translocation of an overexpressed DEF6-GFP fusion protein to the cell membrane and induced the formation of   filopodia   and lamellipodia. In contrast to full-length DEF6, expression of the DH-like (DHL) domain as a GFP fusion protein potently induced actin polymerization, including stress fiber formation in COS-7 cells, in the absence of PI 3-kinase signaling, indicating that it was constitutively active. The GTP-loading of Cdc42 was strongly enhanced in NIH 3T3 cells expressing the DH domain while   filopodia   formation, membrane ruffling, and stress fiber formation could be inhibited by the co-expression of the DH domain with dominant negative mutants of either N17Rac1, N17Cdc42, or N19RhoA, respectively. This indicated that DEF6 acts upstream of the Rho GTPases resulting in the activation of the Cdc42, Rac1, and RhoA signaling pathways. In vitro, DEF6 specifically interacted with Rac1, Rac2, Cdc42, and RhoA, suggesting a direct role for DEF6 in the activation of Rho GTPases. The ability of DEF6 to both stimulate actin polymerization and bind to filamentous actin suggests a role for DEF6 in regulating cell shape, polarity, and movement. 
 Rac regulates the interaction of fascin with protein kinase C in cell migration. Fascin is an actin-bundling protein that is low or absent in normal epithelia; its upregulation correlates with poor prognosis in many human carcinomas. We have recently demonstrated in mouse xenograft models that fascin contributes to tumour development and metastasis through its dual actin-bundling and active PKC-binding activities. Rac was implicated as a regulator of fascin-dependent colon carcinoma cell migration in vitro. Here, we tested the hypothesis that Rac regulates the interaction of fascin with active PKC. The major conventional PKC in colon carcinoma cells is protein kinase Cgamma (PKCgamma). Endogenous PKCgamma, fascin and Rac1 colocalised at lamellipodial margins of migrating cells. Colocalisation of fascin and PKCgamma depended on Rac activity, and inhibition of Rac decreased PKCgamma activity in cell extracts but not in vitro. Fluorescence resonance energy transfer/fluorescence lifetime imaging microscopy uncovered that fascin and PKCgamma interact in protrusions and   filopodia   of migrating cells. Mechanistically, the interaction depended on phosphorylated fascin, active PKCgamma and active Rac, but not on active Cdc42. The activity of Rac on the fascin/PKC complex was mediated in part by Pak. Elucidation of this novel pathway for regulation of the fascin/PKCgamma complex in migrating carcinoma cells suggests novel targets for therapeutic intervention in metastasis. 
 Cell migration and signaling specificity is determined by the phosphatidylserine recognition motif of Rac1. The Rho guanosine triphosphatases (GTPases) control cell shape and motility and are frequently overexpressed during malignant growth. These proteins act as molecular switches cycling between active GTP- and inactive GDP-bound forms. Despite being membrane anchored via their isoprenylated C termini, Rho GTPases rapidly translocate between membrane and cytosolic compartments. Here, we show that the Rho GTPase Rac1 preferentially interacts with phosphatidylserine (PS)-containing bilayers through its polybasic motif (PBM). Rac1 isoprenylation contributes to membrane avidity but is not critical for PS recognition. The similar protein Cdc42 (cell division cycle 42), however, only associates with PS when prenylated. Conversely, other Rho GTPases such as Rac2, Rac3, and RhoA do not bind to PS even when they are prenylated. Cell stimulation with PS induces translocation of Rac1 toward the plasma membrane and stimulates GTP loading, membrane ruffling, and   filopodia   formation. This stimulation also promotes Cdc42 activation and phosphorylation of mitogen-activated protein kinase through Rac1/PS signaling. Consequently, the PBM specifically directs Rac1 to effect cytoskeletal rearrangement and cell migration by selective membrane phospholipid targeting. 
 Proteasomal degradation of cytotoxic necrotizing factor 1-activated rac. The cytotoxic necrotizing factor 1 (CNF1) from Escherichia coli has been shown to activate members of the Rho family by deamidation of glutamine 63. This amino acid is essential for hydrolysis of GTP, and any substitution results in a constitutively active Rho. Activation of Rho induces the formation of stress fibers,   filopodia  , and membrane ruffles due to activation of RhoA, Cdc42, and Rac, respectively. Here we show that the level of endogenous Rac decreased in CNF1-treated HEK293 and HeLa cells. The amount of mRNA remained unaffected, leaving the possibility that Rac is subject to proteolytic degradation. Treatment of cells with lactacystin, an inhibitor of the 26S proteasome, protected Rac from degradation. We have previously shown that CNF1 activates the c-Jun N-terminal kinase (JNK) only transiently in HeLa cells (M. Lerm, J. Selzer, A. Hoffmeyer, U. R. Rapp, K. Aktories, and G. Schmidt, Infect. Immun. 67:496-503, 1998). Here we show that CNF1-induced JNK activation is stabilized in the presence of lactacystin. The data indicate that Rac is degraded by a proteasome-dependent pathway in CNF1-treated cells. 
 PAK5, a new brain-specific kinase, promotes neurite outgrowth in N1E-115 cells. We have characterized a new member of the mammalian PAK family of serine/threonine kinases, PAK5, which is a novel target of the Rho GTPases Cdc42 and Rac. The kinase domain and GTPase-binding domain (GBD) of PAK5 are most closely related in sequence to those of mammalian PAK4. Outside of these domains, however, PAK5 is completely different in sequence from any known mammalian proteins. PAK5 does share considerable sequence homology with the Drosophila MBT protein (for "mushroom body tiny"), however, which is thought to play a role in development of cells in Drosophila brain. Interestingly, PAK5 is highly expressed in mammalian brain and is not expressed in most other tissues. We have found that PAK5, like Cdc42, promotes the induction of   filopodia  . In N1E-115 neuroblastoma cells, expression of PAK5 also triggered the induction of neurite-like processes, and a dominant-negative PAK5 mutant inhibited neurite outgrowth. Expression of activated PAK1 caused no noticeable changes in these cells. An activated mutant of PAK5 had an even more dramatic effect than wild-type PAK5, indicating that the morphologic changes induced by PAK5 are directly related to its kinase activity. Although PAK5 activates the JNK pathway, dominant-negative JNK did not inhibit neurite outgrowth. In contrast, the induction of neurites by PAK5 was abolished by expression of activated RhoA. Previous work has shown that Cdc42 and Rac promote neurite outgrowth by a pathway that is antagonistic to Rho. Our results suggest, therefore, that PAK5 operates downstream to Cdc42 and Rac and antagonizes Rho in the pathway, leading to neurite development. 
 A 68-kDa kinase and NADPH oxidase component p67phox are targets for Cdc42Hs and Rac1 in neutrophils. Cdc42Hs and Rac1 are members of the Ras superfamily of small molecular weight (p21) GTP binding proteins. Cdc42Hs induces   filopodia   formation in Swiss 3T3 fibroblasts while Rac1 induces membrane ruffling. Rac1 also activates superoxide production by the components (cytochrome b, p40phox, p67phox, and p47phox) of the neutrophil oxidase. To isolate target proteins involved in these signaling pathways, we have probed proteins from neutrophil cytosol immobilized on nitrocellulose with Cdc42Hs labeled with [gamma-32P]GTP. Cdc42Hs probe detected binding protein(s) of 66-68 kDa in neutrophil cytosol. Rac1 probe also detected the 66-68-kDa proteins, suggesting the possibility that p67phox may be a binding protein for both of these p21 proteins. Indeed, Cdc42Hs and Rac1 were found to bind specifically to purified recombinant p67phox but not the other oxidase components. A 68-kDa Cdc42Hs binding protein was purified from neutrophil cytosol and found to be related to the recently described p65pak kinase from brain. These results suggest that the p68 kinase and p67phox are targets for Cdc42Hs and Rac1 in neutrophils. 
 A MAPK-Driven Feedback Loop Suppresses Rac Activity to Promote RhoA-Driven Cancer Cell Invasion. Cell migration in 3D microenvironments is fundamental to development, homeostasis and the pathobiology of diseases such as cancer. Rab-coupling protein (RCP) dependent co-trafficking of α5β1 and EGFR1 promotes cancer cell invasion into fibronectin (FN) containing extracellular matrix (ECM), by potentiating EGFR1 signalling at the front of invasive cells. This promotes a switch in RhoGTPase signalling to inhibit Rac1 and activate a RhoA-ROCK-Formin homology domain-containing 3 (FHOD3) pathway and generat  e filopod  ial actin-spike protrusions which drive invasion. To further understand the signalling network that drives RCP-driven invasive migration, we generated a Boolean logical model based on existing network pathways/models, where each node can be interrogated by computational simulation. The model predicted an unanticipated feedback loop, whereby Raf/MEK/ERK signalling maintains suppression of Rac1 by inhibiting the Rac-activating Sos1-Eps8-Abi1 complex, allowing RhoA activity to predominate in invasive protrusions. MEK inhibition was sufficient to promote lamellipodia formation and oppos  e filopod  ial actin-spike formation, and led to activation of Rac and inactivation of RhoA at the leading edge of cells moving in 3D matrix. Furthermore, MEK inhibition abrogated RCP/α5β1/EGFR1-driven invasive migration. However, upon knockdown of Eps8 (to suppress the Sos1-Abi1-Eps8 complex), MEK inhibition had no effect on RhoGTPase activity and did not oppose invasive migration, suggesting that MEK-ERK signalling suppresses the Rac-activating Sos1-Abi1-Eps8 complex to maintain RhoA activity and prom  ote filop  odial actin-spike formation and invasive migration. Our study highlights the predictive potential of mathematical modelling approaches, and demonstrates that a simple intervention (MEK-inhibition) could be of therapeutic benefit in preventing invasive migration and metastasis. 
 Tiam1 and Rac1 are required for platelet-activating factor-induced endothelial junctional disassembly and increase in vascular permeability. It is known that platelet-activating factor (PAF) induces severe endothelial barrier leakiness, but the signaling mechanisms remain unclear. Here, using a wide range of biochemical and morphological approaches applied in both mouse models and cultured endothelial cells, we addressed the mechanisms of PAF-induced disruption of interendothelial junctions (IEJs) and of increased endothelial permeability. The formation of interendothelial gaps filled with   filopodia   and lamellipodia is the cellular event responsible for the disruption of endothelial barrier. We observed that PAF ligation of its receptor induced the activation of the Rho GTPase Rac1. Following PAF exposure, both Rac1 and its guanine nucleotide exchange factor Tiam1 were found associated with a membrane fraction from which they co-immunoprecipitated with PAF receptor. In the same time frame with Tiam1-Rac1 translocation, the junctional proteins ZO-1 and VE-cadherin were relocated from the IEJs, and formation of numerous interendothelial gaps was recorded. Notably, the response was independent of myosin light chain phosphorylation and thus distinct from other mediators, such as histamine and thrombin. The changes in actin status are driven by the PAF-induced localized actin polymerization as a consequence of Rac1 translocation and activation. Tiam1 was required for the activation of Rac1, actin polymerization, relocation of junctional associated proteins, and disruption of IEJs. Thus, PAF-induced IEJ disruption and increased endothelial permeability requires the activation of a Tiam1-Rac1 signaling module, suggesting a novel therapeutic target against increased vascular permeability associated with inflammatory diseases. 
 Formyl peptide receptor-1 activation enhances intestinal epithelial cell restitution through phosphatidylinositol 3-kinase-dependent activation of Rac1 and Cdc42. Inflammatory disorders of the gastrointestinal tract result in the breakdown of the intestinal epithelial barrier in the form of erosion and ulceration. To reestablish the epithelial barrier, the epithelium must efficiently migrate to reseal wounds. Numerous signaling cascades are involved in the induction and regulation of this complex process. N-formyl peptide receptors comprise a group of Gi-coupled receptors that regulate innate immune responses. Previously, we identified the expression of functional N-formyl peptide receptors in model SK-CO15 intestinal epithelial cells and observed a role for activation of these receptors in regulating cellular invasive behavior. In these studies, we performed formyl peptide receptor-1 (FPR) localization and evaluated its role in regulating intestinal epithelial cell wound closure. Immunolocalization studies using a recently developed specific monoclonal anti-FPR Ab demonstrated its localization along the lateral membrane of crypt epithelial cells in normal human colonic epithelium. In vitro studies using the classical FPR agonist fMLF showed that FPR activation significantly enhances model intestinal epithelial cell restitution and that FPR localized along actin filaments in lamellipodial and   filopodia  l extrusions. The increase in cell migration was associated with activation of PI3K, Rac1, and Cdc42. Pharmacologic inhibition of PI3K activity abrogated the fMLF-induced increase in wound closure and activation of both Rac1 and Cdc42. Inhibition of Rac1 and Cdc42 using pharmacologic inhibitors and dominant negative mutants also inhibited the fMLF-induced increase in cell migration. Taken together, theses results support a novel role for FPR stimulation in enhancing intestinal epithelial cell restitution through PI3K-dependent activation of Rac1 and Cdc42. 
 The novel synaptogenic protein Farp1 links postsynaptic cytoskeletal dynamics and transsynaptic organization. Synaptic adhesion organizes synapses, yet the signaling pathways that drive and integrate synapse development remain incompletely understood. We screened for regulators of these processes by proteomically analyzing synaptic membranes lacking the synaptogenic adhesion molecule SynCAM 1. This identified FERM, Rho/ArhGEF, and Pleckstrin domain protein 1 (Farp1) as strongly reduced in SynCAM 1 knockout mice. Farp1 regulates dendritic   filopodia  l dynamics in immature neurons, indicating roles in synapse formation. Later in development, Farp1 is postsynaptic and its 4.1 protein/ezrin/radixin/moesin (FERM) domain binds SynCAM 1, assembling a synaptic complex. Farp1 increases synapse number and modulates spine morphology, and SynCAM 1 requires Farp1 for promoting spines. In turn, SynCAM 1 loss reduces the ability of Farp1 to elevate spine density. Mechanistically, Farp1 activates the GTPase Rac1 in spines downstream of SynCAM 1 clustering, and promotes F-actin assembly. Farp1 furthermore triggers a retrograde signal regulating active zone composition via SynCAM 1. These results reveal a postsynaptic signaling pathway that engages transsynaptic interactions to coordinate synapse development. 
 Regulation of Rac1 and Cdc42 activation in thrombin- and collagen-stimulated CHRF-288-11 cells. Rac1 and Cdc42 are members of the Rho family of small GTPases and have been shown to promote the formation of lamellipodia and   filopodia   at the leading edge of motile cells and affect cell migration. In this study the authors have investigated the activation of Rac1 and Cdc42 by thrombin or collagen using the megakaryocytic cell line, CHRF-288-11. Maximal activation of Rac1 by thrombin or collagen was observed at 3 and 1 min, respectively. Similar results were obtained for the activation of Cdc42 by thrombin or collagen. The activation of Rac1 by thrombin and collagen was inhibited by the calmodulin-specific inhibitor, W7. However, W7 had no effect on the activation of Cdc42 by thrombin or collagen. The less potent calmodulin inhibitor, W5, did not have any effect on Rac1 or Cdc42 activation by thrombin or collagen. Transient over-expression of calmodulin in CHRF cells increased the basal and thrombin-mediated activation of Rac1 when compared to control but had no effect on the basal and thrombin-mediated activation of Cdc42 when compared to control. The results suggest that calmodulin regulates the activation of Rac1 in CHRF-288-11 cells in a manner similar to that in the human platelets. 
 Rac1 is essential for platelet lamellipodia formation and aggregate stability under flow. The role of Rac family proteins in platelet spreading on matrix proteins under static and flow conditions has been investigated by using Rac-deficient platelets. Murine platelets form   filopodia   and undergo limited spreading on fibrinogen independent of Rac1 and Rac2. In the presence of thrombin, marked lamellipodia formation is observed on fibrinogen, which is abrogated in the absence of Rac1. However, Rac1 is not required for thrombin-induced aggregation or elevation of F-actin levels. Formation of lamellipodia on collagen and laminin is also Rac1-dependent. Analysis of platelet adhesion dynamics on collagen under flow conditions in vitro revealed that Rac1 is required for platelet aggregate stability at arterial rates of shear, as evidenced by a dramatic increase in platelet embolization. Furthermore, studies employing intravital microscopy demonstrated that Rac1 plays a critical role in the development of stable thrombi at sites of vascular injury in vivo. Thus, our data demonstrated that Rac1 is essential for lamellipodia formation in platelets and indicated that Rac1 is required for aggregate integrity leading to thrombus formation under physiologically relevant levels of shear both in vitro and in vivo. 
 The TRE17 oncogene encodes a component of a novel effector pathway for Rho GTPases Cdc42 and Rac1 and stimulates actin remodeling. The Rho family GTPases Cdc42 and Rac1 play fundamental roles in transformation and actin remodeling. Here, we demonstrate that the TRE17 oncogene encodes a component of a novel effector pathway for these GTPases. TRE17 coprecipitated specifically with the active forms of Cdc42 and Rac1 in vivo. Furthermore, the subcellular localization of TRE17 was dramatically regulated by these GTPases and mitogens. Under serum-starved conditions, TRE17 localized predominantly to filamentous structures within the cell. Epidermal growth factor (EGF) induced relocalization of TRE17 to the plasma membrane in a Cdc42-/Rac1-dependent manner. Coexpression of activated alleles of Cdc42 or Rac1 also caused complete redistribution of TRE17 to the plasma membrane, where it partially colocalized with the GTPases in   filopodia   and ruffles, respectively. Membrane recruitment of TRE17 by EGF or the GTPases was dependent on actin polymerization. Finally, we found that a C-terminal truncation mutant of TRE17 induced the accumulation of cortical actin, mimicking the effects of activated Cdc42. Together, these results identify TRE17 as part of a novel effector complex for Cdc42 and Rac1, potentially contributing to their effects on actin remodeling. The present study provides insights into the regulation and cellular function of this previously uncharacterized oncogene. 
 The Ras-related GTPase Rac1 binds tubulin. The Ras-related Rho family are involved in controlling actin-based changes in cell morphology. Microinjection of Rac1, RhoA, and Cdc42Hs into Swiss 3T3 cells induces pinocytosis and membrane ruffling, stress fiber formation, and   filopodia   formation, respectively. To identify target proteins involved in these signaling pathways cell extracts immobilized on nitrocellulose have been probed with [gamma-32P]GTP-labeled Rac1, RhoA, and Cdc42Hs. We have identified two 55-kDa brain proteins which bind Rac1 but not RhoA or Cdc42Hs. These 55-kDa proteins were abundant, had pI values of around 5.5, and could be purified by Q-Sepharose chromatography. The characteristics on two-dimensional gel analysis suggested the proteins comprised alpha- and beta-tubulin. Indeed, beta-tubulin specific antibodies detected one of the purified 55-kDa proteins. Rac1 bound pure tubulin (purified by cycles of polymerization and depolymerization) only in the GTP-bound state. The GTPase negative Rac1 point mutants, G12V and Q61L, did not significantly affect the ability of Rac1 to interact with tubulin while the "effector-site" mutant D38A prevented interaction. These results suggest that the Rac1-tubulin interaction may play a role in Rac1 function. 
 A Rac/Cdc42-specific exchange factor, GEFT, induces cell proliferation, transformation, and migration. The Rho family of small GTPases, including Rho, Rac, and Cdc42, play essential roles in diverse cellular functions. The ability of Rho family GTPases to participate in signaling events is determined by the ratio of inactive (GDP-bound) and active (GTP-bound) forms in the cell. The activation of Rho family proteins requires the exchange of bound GDP for GTP, a process catalyzed by the Dbl family of guanine nucleotide exchange factors (GEFs). The GEFs have high affinity for the guanine nucleotide-free state of the GTPases and are thought to promote GDP release by stabilizing an intermediate transition state. In this study, we have identified and characterized a new Rac/Cdc42-specific Dbl family guanine nucleotide exchange factor, named GEFT. GEFT is highly expressed in the excitable tissues, including brain, heart, and muscle. Low or very little expression was detected in other nonexcitable tissues. GEFT has specific exchange activity for Rac and Cdc42 in our in vitro GTPase exchange assays and glutathione S-transferase-PAK pull-down assays with GTP-bound Rac1 and Cdc42. Overexpression of GEFT leads to changes in cell morphology and actin cytoskeleton re-organization, including the formation of membrane microspikes,   filopodia  , and lamilliopodia. Furthermore, expression of GEFT in NIH3T3 cells promotes foci formation, cell proliferation, and cell migration, possibly through the activation of transcriptional factors involved in cell growth and proliferation. Together, our data suggest that GEFT is a Rac/Cdc42-specific GEF protein that regulates cell morphology, cell proliferation, and transformation. 
 Kaempferol-3-O-rutinoside from Afgekia mahidoliae promotes keratinocyte migration through FAK and Rac1 activation. The restoration of the epidermal epithelium through re-epithelialization is a critical process in wound healing. Directed keratinocyte migration to the wound is required, and the retardation of this process may result in a chronic, non-healing wound. The present study contributes to research aiming to identify promising compounds that promote wound healing using a human keratinocyte model. The effects of three kaempferol glycosides from an Afgekia mahidoliae leaf extract, kaempferol-3-O-arabinoside, kaempferol-3-O-glucoside, and kaempferol-3-O-rutinoside, on keratinocyte migration were determined. Interestingly, kaempferol-3-O-rutinoside exhibited a pronounced effect on wound closure in comparison to the parental kaempferol and other glycosides. The mechanism by which kaempferol-3-O-rutinoside enhances cell migration involves the induction of   filopodia   and lamellipodia formation, increased cellular levels of phosphorylated FAK (Tyr 397) and phosphorylated Akt (Ser 473), and up-regulation of active Rac1-GTP. The data obtained in this study may support the development of this compound for use in wound healing therapies. 
 Kank attenuates actin remodeling by preventing interaction between IRSp53 and Rac1. In this study, insulin receptor substrate (IRS) p53 is identified as a binding partner for Kank, a kidney ankyrin repeat-containing protein that functions to suppress cell proliferation and regulate the actin cytoskeleton. Kank specifically inhibits the binding of IRSp53 with active Rac1 (Rac1(G12V)) but not Cdc42 (cdc42(G12V)) and thus inhibits the IRSp53-dependent development of lamellipodia without affecting the formation of   filopodia  . Knockdown (KD) of Kank by RNA interference results in increased lamellipodial development, whereas KD of both Kank and IRSp53 has little effect. Moreover, insulin-induced membrane ruffling is inhibited by overexpression of Kank. Kank also suppresses integrin-dependent cell spreading and IRSp53-induced neurite outgrowth. Our results demonstrate that Kank negatively regulates the formation of lamellipodia by inhibiting the interaction between Rac1 and IRSp53. 
 Cdc42 induces   filopodia   by promoting the formation of an IRSp53:Mena complex. The Rho GTPases Rho, Rac, and Cdc42 regulate the organization of the actin cytoskeleton by interacting with multiple, distinct downstream effector proteins. Cdc42 controls the formation of actin bundle-containing   filopodia   at the cellular periphery. The molecular mechanism for this remains as yet unclear.We report here that Cdc42 interacts with IRSp53/BAP2 alpha, an SH3 domain-containing scaffold protein, at a partial CRIB motif and that an N-terminal fragment of IRSp53 binds, via an intramolecular interaction, to the CRIB motif-containing central region. Overexpression of IRSp53 in fibroblasts leads to the formation of   filopodia  , and both this and Cdc42-induced   filopodia   are inhibited by expression of the N-terminal IRSp53 fragment. Using affinity chromatography, we have identified Mena, an Ena/VASP family member, as interacting with the SH3 domain of IRSp53. Mena and IRSp53 act synergistically to promote   filopodia   formation.We conclude that the interaction of Cdc42 with the partial CRIB motif of IRSp53 relieves an intramolecular, autoinhibitory interaction with the N terminus, allowing the recruitment of Mena to the IRSp53 SH3 domain. This IRSp53:Mena complex initiates actin filament assembly into   filopodia  . 
 CdGAP, a novel proline-rich GTPase-activating protein for Cdc42 and Rac. Cdc42 mediates several signaling pathways leading to actin reorganization, transcriptional activation, and cell cycle control. Mutational analysis of Cdc42 has revealed that actin reorganization and transcriptional activation are induced through independent signaling pathways. The Y40C effector mutant of Cdc42 no longer interacts with many of its known target proteins, such as p65(PAK) and WASP, yet this mutant can still induce   filopodia   formation. To identify Cdc42 targets involved in actin rearrangements, we have screened a yeast two-hybrid cDNA library using the Y40C mutant of Cdc42 as a bait. We report here the identification of a novel serine- and proline-rich GTPase-activating protein, CdGAP, which is active in vitro on both Cdc42 and Rac. Microinjection of CdGAP into serum-starved fibroblasts inhibits both platelet-derived growth factor-induced lamellipodia and bradykinin-induced   filopodia   mediated by Rac and Cdc42, respectively. CdGAP does not show in vitro activity toward Rho, and it has no effect on lysophosphatidic acid-induced stress fiber formation when microinjected into fibroblasts. The carboxyl terminus of CdGAP reveals potential protein kinase C phosphorylation sites and five SH3 binding motifs. Thus, CdGAP is a novel GAP that is likely to participate in Cdc42- and Rac-induced signaling pathways leading to actin reorganization. 
  GeneRIFs  
 Internalization of DENV-2 into endothelial cells requires viral induction of dynamic   filopodia   regulated by Rac1 and Cdc42 cross-talk and myosin II motor activities. 
  [Ff]ilopodium  PubMed  
 Streptococcus pneumoniae ClpL modulates adherence to A549 human lung cells through Rap1/Rac1 activation. Caseinolytic protease L (ClpL) is a member of the HSP100/Clp chaperone family, which is found mainly in Gram-positive bacteria. ClpL is highly expressed during infection for refolding of stress-induced denatured proteins, some of which are important for adherence. However, the role of ClpL in modulating pneumococcal virulence is poorly understood. Here, we show that ClpL impairs pneumococcal adherence to A549 lung cells by inducing and activating Rap1 and Rac1, thus increasing phosphorylation of cofilin (inactive form). Moreover, infection with a clpL mutant (ΔclpL) causes a greater degree of   filopodiu  m formation than D39 wild-type (WT) infection. Inhibition of Rap1 and Rac1 impairs   filopodiu  m formation and pneumococcal adherence. Therefore, ClpL can reduce pneumococcal adherence to A549 cells, likely via modulation of Rap1- and Rac1-mediated   filopodiu  m formation. These results demonstrate a potential role for ClpL in pneumococcal resistance to host cell adherence during infection. This study provides insight into further understanding the interactions between hosts and pathogens. 
 IRSp53 is an essential intermediate between Rac and WAVE in the regulation of membrane ruffling. Neural Wiskott-Aldrich syndrome protein (N-WASP) functions in several intracellular events including   filopodium   formation, vesicle transport and movement of Shigella frexneri and vaccinia virus, by stimulating rapid actin polymerization through the Arp2/3 complex. N-WASP is regulated by the direct binding of Cdc42 (refs 7, 8), which exposes the domain in N-WASP that activates the Arp2/3 complex. A WASP-related protein, WAVE/Scar, functions in Rac-induced membrane ruffling; however, Rac does not bind directly to WAVE, raising the question of how WAVE is regulated by Rac. Here we demonstrate that IRSp53, a substrate for insulin receptor with unknown function, is the 'missing link' between Rac and WAVE. Activated Rac binds to the amino terminus of IRSp53, and carboxy-terminal Src-homology-3 domain of IRSp53 binds to WAVE to form a trimolecular complex. From studies of ectopic expression, we found that IRSp53 is essential for Rac to induce membrane ruffling, probably because it recruits WAVE, which stimulates actin polymerization mediated by the Arp2/3 complex. 
  [Ii]nvadopodia  PubMed  
 A Trio-Rac1-Pak1 signalling axis drives   invadopodia   disassembly. Rho family GTPases control cell migration and participate in the regulation of cancer metastasis.   Invadopodia  , associated with invasive tumour cells, are crucial for cellular invasion and metastasis. To study Rac1 GTPase in   invadopodia   dynamics, we developed a genetically encoded, single-chain Rac1 fluorescence resonance energy (FRET) transfer biosensor. The biosensor shows Rac1 activity exclusion from the core of   invadopodia  , and higher activity when   invadopodia   disappear, suggesting that reduced Rac1 activity is necessary for their stability, and Rac1 activation is involved in disassembly. Photoactivating Rac1 at   invadopodia   confirmed this previously unknown Rac1 function. We describe here an   invadopodia   disassembly model, where a signalling axis involving TrioGEF, Rac1, Pak1, and phosphorylation of cortactin, causes   invadopodia   dissolution. This mechanism is critical for the proper turnover of invasive structures during tumour cell invasion, where a balance of proteolytic activity and locomotory protrusions must be carefully coordinated to achieve a maximally invasive phenotype. 
 Podocalyxin-like 1 promotes   invadopodia   formation and metastasis through activation of Rac1/Cdc42/cortactin signaling in breast cancer cells. Metastatic disease is the leading cause of cancer mortality. Identifying biomarkers and regulatory mechanisms is important toward developing diagnostic and therapeutic tools against metastatic cancer. In this study, we demonstrated that podocalyxin-like 1 (PODXL) is overexpressed in breast tumor cells and increased in lymph node metastatic cancer. Mechanistically, we found that the expression of PODXL was associated with cell motility and invasiveness. Suppression of PODXL in MDA-MB-231 cells reduced lamellipodia formation and focal adhesion kinase (FAK) and paxillin phosphorylation. PODXL knockdown reduced the formation of   invadopodia  , such as inhibiting the colocalization of F-actin with cortactin and suppressing phosphorylation of cortactin and neural Wiskott-Aldrich syndrome protein. Conversely, overexpression of PODXL in MCF7 cells induced F-actin/cortactin colocalization and enhanced   invadopodia   formation and activation.   Invadopodia   activity and tumor invasion in PODXL-knockdown cells are similar to that in cortactin-knockdown cells. We further found that the DTHL motif in PODXL is crucial for regulating cortactin phosphorylation and Rac1/Cdc42 activation. Inhibition of Rac1/Cdc42 impeded PODXL-mediated cortactin activation and FAK and paxillin phosphorylation. Moreover, inhibition of PODXL in MDA-MB-231 cells significantly suppressed tumor colonization in the lungs and distant metastases, similar to those in cortactin-knockdown cells. These findings show that overexpression of PODXL enhanced   invadopodia   formation and tumor metastasis by inducing Rac1/Cdc42/cortactin signaling network. 
  GeneRIFs  
 These findings show that overexpression of PODXL enhanced   invadopodia   formation and tumor metastasis by inducing Rac1/Cdc42/cortactin signaling network. 
 Describe here an   invadopodia   disassembly model, where a signalling axis involving TrioGEF, Rac1, Pak1, and phosphorylation of cortactin, causes   invadopodia   dissolution. 
  [Ii]nvadopodium  PubMed  
 Glycogen synthase kinase 3β sustains invasion of glioblastoma via the focal adhesion kinase, Rac1, and c-Jun N-terminal kinase-mediated pathway. The failure of current treatment options for glioblastoma stems from their inability to control tumor cell proliferation and invasion. Biologically targeted therapies offer great hope and one promising target is glycogen synthase kinase-3β (GSK3β), implicated in various diseases, including cancer. We previously reported that inhibition of GSK3β compromises the survival and proliferation of glioblastoma cells, induces their apoptosis, and sensitizes them to temozolomide and radiation. Here, we explore whether GSK3β also contributes to the highly invasive nature of glioblastoma. The effects of GSK3β inhibition on migration and invasion of glioblastoma cells were examined by wound-healing and Transwell assays, as well as in a mouse model of glioblastoma. We also investigated changes in cellular microarchitectures, cytoskeletal components, and proteins responsible for cell motility and invasion. Inhibition of GSK3β attenuated the migration and invasion of glioblastoma cells in vitro and that of tumor cells in a mouse model of glioblastoma. These effects were associated with suppression of the molecular axis involving focal adhesion kinase, guanine nucleotide exchange factors/Rac1 and c-Jun N-terminal kinase. Changes in cellular phenotypes responsible for cell motility and invasion were also observed, including decreased formation of lamellipod  ia and invad  opodium-like microstructures and alterations in the subcellular localization, and activity of Rac1 and F-actin. These changes coincided with decreased expression of matrix metalloproteinases. Our results confirm the potential of GSK3β as an attractive therapeutic target against glioblastoma invasion, thus highlighting a second role in this tumor type in addition to its involvement in chemo- and radioresistance. 
  [Pp]odosome(s)?  PubMed  
 ARF6 promotes the formation of Rac1 and WAVE-dependent ventral F-actin rosettes in breast cancer cells in response to epidermal growth factor. Coordination between actin cytoskeleton assembly and localized polarization of intracellular trafficking routes is crucial for cancer cell migration. ARF6 has been implicated in the endocytic recycling of surface receptors and membrane components and in actin cytoskeleton remodeling. Here we show that overexpression of an ARF6 fast-cycling mutant in MDA-MB-231 breast cancer-derived cells to mimick ARF6 hyperactivation observed in invasive breast tumors induced a striking rearrangement of the actin cytoskeleton at the ventral cell surface. This phenotype consisted in the formation of dynamic actin-based   podosome   rosette-like structures expanding outward as wave positive for F-actin and actin cytoskeleton regulatory components including cortactin, Arp2/3 and SCAR/WAVE complexes and upstream Rac1 regulator. Ventral rosette-like structures were similarly induced in MDA-MB-231 cells in response to epidermal growth factor (EGF) stimulation and to Rac1 hyperactivation. In addition, interference with ARF6 expression attenuated activation and plasma membrane targeting of Rac1 in response to EGF treatment. Our data suggest a role for ARF6 in linking EGF-receptor signaling to Rac1 recruitment and activation at the plasma membrane to promote breast cancer cell directed migration. 
 Rac1 and Rac2 regulate macrophage morphology but are not essential for migration. Rac GTPases are believed to contribute to migration in leukocytes by transducing signals from cell surface receptors to the actin and microtubule cytoskeletons. Mammals have three closely related Rac isoforms, Rac1, Rac2 and Rac3, and it is widely assumed that cell migration requires the activity of these Rac GTPases. We have previously shown that Rac1-null mouse macrophages have altered cell shape and reduced membrane ruffling but normal migration speed. Here we investigate the behaviour of macrophages lacking Rac2 (Rac2(-/-)) or Rac1 and Rac2 (Rac1/2(-/-)). Rac2(-/-) macrophages have reduced F-actin levels and lack   podosomes  , which are integrin-based adhesion sites, and their migration speed is similar to or slightly slower than wild-type macrophages, depending on the substrate. Unexpectedly, Rac1/2(-/-) macrophages, which do not express Rac1, Rac2 or Rac3, migrate at a similar speed to wild-type macrophages on a variety of substrates and perform chemotaxis normally, although their morphology and mode of migration is altered. However, Rac1(-/-) and Rac1/2(-/-) but not Rac2(-/-) macrophages are impaired in their ability to invade through Matrigel. Together, these data show that Rac1 and Rac2 have distinct roles in regulating cell morphology, migration and invasion, but are not essential for macrophage migration or chemotaxis. 
  [Mm]igration  Process  
 cerebral cortex radially oriented cell   migration   
 embryonic olfactory bulb interneuron precursor   migration   
 regulation of cell   migration   
 regulation of fibroblast   migration   
  PubMed  
 CBAP functions as a novel component in chemokine-induced ZAP70-mediated T-cell adhesion and   migration  . Activated chemokine receptor initiates inside-out signaling to transiently trigger activation of integrins, a process involving multiple components that have not been fully characterized. Here we report that GM-CSF/IL-3/IL-5 receptor common beta-chain-associated protein (CBAP) is required to optimize this inside-out signaling and activation of integrins. First, knockdown of CBAP expression in human Jurkat T cells caused attenuated CXC chemokine ligand-12 (CXCL12)-induced cell   migration   and integrin α4β1- and αLβ2-mediated cell adhesion in vitro, which could be rescued sufficiently upon expression of murine CBAP proteins. Freshly isolated CBAP-deficient primary T cells also exhibited diminution of chemotaxis toward CC chemokine ligand-21 (CCL21) and CXCL12, and these chemokines-induced T-cell adhesions in vitro. Adoptive transfer of isolated naive T cells demonstrated that CBAP deficiency significantly reduced lymph node homing ability in vivo. Final  ly, migra  tion of T cell-receptor-activated T cells induced by inflammatory chemokines was also attenuated in CBAP-deficient cells. Further analyses revealed that CBAP constitutively associated with both integrin β1 and ZAP70 and that CBAP is required for chemokine-induced initial binding of the talin-Vav1 complex to integrin β1 and to facilitate subsequent ZAP70-mediated dissociation of the talin-Vav1 complex and Vav1 phosphorylation. Within such an integrin signaling complex, CBAP likely functions as an adaptor and ultimately leads to activation of both integrin α4β1 and Rac1. Taken together, our data suggest that CBAP indeed can function as a novel signaling component within the ZAP70/Vav1/talin complex and plays an important role in regulating chemokine-promoted T-cell trafficking. 
 Interleukin-8 induces the endothelial cell   migration   through the Rac 1/RhoA-p38MAPK pathway. Endothelial cell   migration   is essential for tumor angiogenesis, and interleukin-8 (IL-8) has been shown to play an important role in tumor growth, angiogenesis, and metastasis. The objective of this study was to investigate the molecular mechanism of IL-8 induced endothelial cell   migration   in vitro.Fluorescence microscope was used to study the distribution of cytoskeleton. The expression of Rac1 and RhoA protein was detected by western blotting. After endothelial cells were transfected by lipofectamine 2000 reagent, the Transwell chamber motility assay was applied to observe the   migration   of endothelial cells induced by IL-8. The active p38MAPK (mitogen-activated protein kinase) was evaluated by the p38MAPK activation assay.We demonstrated that IL-8 activated cell   migration   can be impaired by p38MAPK inhibitor, suggesting the participation of p38MAPK in the cell   migration  . Our results indicated that p38MAPK signaling is required for membrane ruffles, lamellipodia extensions, and actin stress fibers formation induced by IL-8. Furthermore, p38MAPK inhibitor led to increased Rac1 and RhoA expression in IL-8 treated EA.hy926 cells. In addition, IL-8 induced p38MAPK activation was suppressed by dominant-negative mutant for Rac1 and RhoA.Our study demonstrates that IL-8-Rac1/RhoA-p38MAPK signaling pathway plays a vital role in the IL-8-induced endothelial cell   migration  , and it provides new insight into the molecular mechanisms by which IL-8 contributes to tumor angiogenesis and metastasis. 
 Oncogenic K-Ras down-regulates Rac1 and RhoA activity and enhances   migration   and invasion of pancreatic carcinoma cells through activation of p38. Activating mutations in the K-ras gene are genetic alterations frequently found in human carcinomas, particularly in pancreatic adenocarcinomas. Mutation of the K-ras gene is thought to be an early and important event in pancreatic tumor initiation, but the precise role of the mutant K-Ras proteins in neoplastic progression is still unknown. In the present study, we have characterized the influence of oncogenic K-Ras on the phenotype and on the signal transduction of epitheloid PANC-1 pancreatic carcinoma cells by generating PANC-1 cell clones, which stably express EGFP(enhanced green fluorescent protein)-K-Ras (V12). EGFP-K-Ras (V12)-expressing cells exhibited a more fibroblastoid cellular phenotype with irregular cell shape and disorganized cytokeratin filaments. Moreover, these cells showed a marked enhancement of their migratory and invasive properties. Stable expression of EGFP-K-Ras (V12) down-regulated the activity of Rac1 and RhoA, resulting in reduced subcortical actin filaments and stress fibers, which might contribute to the epithelial dedifferentiation. Characterization of the activity of mitogen-activated protein kinases revealed that EGFP-K-Ras (V12) enhanced the activity of p38, but did not affect the activities of the Raf/MEK/ERK cascade and JNK. While inhibition of either MEK or JNK activity had no effect on EGFP-K-Ras (V12)-induced   migration  , inhibition of p38 activity markedly reduced EGFP-K-Ras (V12)-induced   migration  . Collectively, the results suggest that oncogenic K-Ras enhances the malignant phenotype and identify the mitogen-activated protein kinase p38 as a target to inhibit oncogenic K-Ras-induced pancreatic tumor cell   migration  . 
 Knockdown of Coronin-1C disrupts Rac1 activation and impairs tumorigenic potential in hepatocellular carcinoma cells. Coronin-1C is an important F-actin binding protein which is critical for cell motility. Furthermore, the expression of this protein was found to be increased in diffuse tumors and was correlated with the degree of tumor malignancy. However, the mechanism(s) through which this protein enhances malignancy in hepatocellular carcinoma (HCC) is poorly understood. In this study, we found that Coronin-1C was overexpressed in human HCC tissues compared with the adjacent non-tumor tissues. Overexpression of Coronin-1C enhanced the cell   migration   in the human HCC cell line BEL-7402, whereas suppressed cell   migration   and proliferation were observed in Coronin-1C-knockdown BEL-7402 cells together with impaired cell polarity, disrupted cytoskeleton and decreased Rac-1 activation. Moreover, the Coronin-1C knockdown cells displayed a lower degree of malignancy by inducing smaller tumors in nude mice. Thus, we demonstrated a relationship between Coronin-1C overexpression and human HCC growth through enhancement of tumor cell proliferation and   migration  , which are correlated with Rac-1 activation. 
 Radixin regulates cell   migration   and cell-cell adhesion through Rac1. The ERM proteins ezrin, radixin and moesin are adaptor proteins that link plasma membrane receptors to the actin cytoskeleton. Ezrin and moesin have been implicated in cell polarization and cell   migration  , but little is known about the involvement of radixin in these processes. Here we show that radixin is required for   migration   of PC3 prostate cancer cells, and that radixin, but not ezrin or moesin, depletion by RNA interference increases cell spread area and cell-cell adhesion mediated by adherens junctions. Radixin depletion also alters actin organization, and distribution of active phosphorylated ezrin and moesin. Similar effects were observed in MDA-MB-231 breast cancer cells. The phenotype of radixin-depleted cells is similar to that induced by constitutively active Rac1, and Rac1 is required for the radixin knockdown phenotype. Radixin depletion also increases the activity of Rac1 but not Cdc42 or RhoA. Analysis of Rac guanine nucleotide exchange factors (GEFs) suggests that radixin affects the activity of Vav GEFs. Indeed, Vav GEF depletion reverses the phenotype of radixin knockdown and reduces the effect of radixin knockdown on Rac1 activity. Our results indicate that radixin plays an important role in promoting cell   migration   by regulating Rac1-mediated epithelial polarity and formation of adherens junctions through Vav GEFs. 
 FRL, a novel formin-related protein, binds to Rac and regulates cell motility and survival of macrophages. We have isolated a cDNA, frl (formin-related gene in leukocytes), a novel mammalian member of the formin gene family. The frl cDNA encodes a 160-kDa protein, FRL, that possesses FH1, FH2, and FH3 domains that are well conserved among other Formin-related proteins. An FRL protein is mainly localized in the cytosol and is highly expressed in spleen, lymph node, and bone marrow cells. Formin-related genes and proteins have been reported to play crucial roles in morphogenesis, cell polarity, and cytokinesis through interaction with Rho family small GTPases. FRL binds to Rac at its N-terminal region including the FH3 domain and associates with profilin at the FH1 domain. In a macrophage cell line, P388D1, overexpression of a truncated form of FRL containing only the FH3 domain (FH3-FRL) strongly inhibited cell adhesion to fibronectin and   migration   upon stimulation with a chemokine. Moreover, expression of the truncated FH3-FRL protein resulted in apoptotic cell death of P388D1 cells, suggesting that the truncated FH3-FRL protein may interfere with signals of FRL. Overexpression in the P388D1 cells of full-length FRL or of the truncated protein containing the FH3 and FH1 domains, with simultaneous expression of the truncated FH3-FRL protein, blocked apoptotic cell death and inhibition of cell adhesion and   migration  . These results suggest that FRL may play a role in the control of reorganization of the actin cytoskeleton in association with Rac and also in the regulation of the signal for cell survival. 
 Upregulation of Eps8 in oral squamous cell carcinoma promotes cell   migration   and invasion through integrin-dependent Rac1 activation. Oral squamous cell carcinoma (OSCC) is a lethal disease and early death usually occurs as a result of local invasion and regional lymph node metastases. Current treatment regimens are, to a certain degree, inadequate, with a 5-year mortality rate of around 50% and novel therapeutic targets are urgently required. Using expression microarrays, we identified the eps8 gene as being overexpressed in OSCC cell lines relative to normal oral keratinocytes, and confirmed these findings using RT-PCR and western blotting. In human tissues, we found that Eps8 was upregulated in OSCC (32% of primary tumors) compared with normal oral mucosa, and that expression correlated significantly with lymph node metastasis (P=0.032), suggesting a disease-promoting effect. Using OSCC cell lines, we assessed the functional role of Eps8 in tumor cells. Although suppression of Eps8 produced no effect on cell proliferation, both cell spreading and   migration   were markedly inhibited. The latter cell functions may be modulated through the small GTP-ase, Rac1 and we used pull-down assays to investigate the role of Eps8 in Rac1 signaling. We found that alphavbeta6- and alpha5beta1-integrin-dependent activation of Rac1 was mediated through Eps8. Knockdown of either Eps8 or Rac1, inhibited integrin-dependent cell   migration   similarly and transient expression of constitutively active Rac1 restored   migration   of cells in which Eps8 expression had been suppressed. We also showed that knockdown of Eps8 inhibited tumor cell invasion in an organotypic model of OSCC. These data suggest that Eps8 and Rac1 are part of an integrated signaling pathway modulating integrin-dependent tumour cell motility and identify Eps8 as a possible therapeutic target. 
 Proteomic analysis of integrin-associated complexes identifies RCC2 as a dual regulator of Rac1 and Arf6. The binding of integrin adhesion receptors to their extracellular matrix ligands controls cell morphology, movement, survival, and differentiation in various developmental, homeostatic, and disease processes. Here, we report a methodology to isolate complexes associated with integrin adhesion receptors, which, like other receptor-associated signaling complexes, have been refractory to proteomic analysis. Quantitative, comparative analyses of the proteomes of two receptor-ligand pairs, alpha(4)beta(1)-vascular cell adhesion molecule-1 and alpha(5)beta(1)-fibronectin, defined both core and receptor-specific components. Regulator of chromosome condensation-2 (RCC2) was detected in the alpha(5)beta(1)-fibronectin signaling network at an intersection between the Rac1 and adenosine 5'-diphosphate ribosylation factor 6 (Arf6) subnetworks. RCC2 knockdown enhanced fibronectin-induced activation of both Rac1 and Arf6 and accelerated cell spreading, suggesting that RCC2 limits the signaling required for membrane protrusion and delivery. Dysregulation of Rac1 and Arf6 function by RCC2 knockdown also abolished persistent   migration   along fibronectin fibers, indicating a functional role for RCC2 in directional cell movement. This proteomics workflow now opens the way to further dissection and systems-level analyses of adhesion signaling. 
 Role of non-genomic androgen signalling in suppressing proliferation of fibroblasts and fibrosarcoma cells. The functions of androgen receptor (AR) in stromal cells are still debated in spite of the demonstrated importance of these cells in organ development and diseases. Here, we show that physiological androgen concentration (10 nM R1881 or DHT) fails to induce DNA synthesis, while it consistently stimulates cel  l migrati  on in mesenchymal and transformed mesenchymal cells. Ten nanomolar R1881 triggers p27 Ser10 phosphorylation and its stabilization in NIH3T3 fibroblasts. Activation of Rac and its downstream effector DYRK 1B is responsible for p27 Ser10 phosphorylation and cell quiescence. Ten nanomolar androgen also inhibits transformation induced by oncogenic Ras in NIH3T3 fibroblasts. Overexpression of an AR mutant unable to interact with filamin A, use of a small peptide displacing AR/filamin A interaction, and filamin A knockdown indicate that the androgen-triggered AR/filamin A complex regulates the pathway leading to p27 Ser10 phosphorylation and cell cycle arrest. As the AR/filamin A complex is also responsible fo  r migrati  on stimulated by 10 nM androgen, our report shows that the androgen-triggered AR/filamin A complex controls, through Rac 1, the decision of cells to halt cell cycle   and migra  tion. This study reveals a new and unexpected role of androgen/AR signalling in coordinating stromal cell functions. 
 Inhibition of RAC1-GEF DOCK3 by miR-512-3p contributes to suppression of metastasis in non-small cell lung cancer. MicroRNAs are a class of small non-coding RNAs regulating gene expression. In this study, we demonstrated that retinoic acid (RA) treatment increases the expression of miR-512-3p. Overexpression of miR-512-3p inhibited cell adhesion,   migration  , and invasion in non-small cell lung cancer (NSCLC) cell lines A549 and H1299. miR-512-3p inhibitor partially reversed these effects in H1299 cells stably expressing miR-512. We identified DOCK3, a RAC1-GEF (guanine nucleotide exchange factor), as a target gene of miR-512-3p. Overexpression of miR-512-3p led to the decrease of DOCK3 protein but not its mRNA. Knockdown of DOCK3 resulted in similar effects on adhesion,   migration  , and invasion as observed of miR-512-3p overexpression. Active RAC1 pull-down assay indicated that overexpression of miR-512-3p could decrease the activity of RAC1 with a higher efficiency than that of DOCK3 knockdown. Furthermore, expression of miR-512-3p was suppressed in most NSCLC patient tumor samples compared to its paired normal controls, suggesting that miR-512-3p might play a crucial role in lung cancer development. In conclusion, our results supported that miR-512-3p could inhibit tumor cell adhesion,   migration  , and invasion by regulating the RAC1 activity via DOCK3 in NSCLC A549 and H1299 cell lines. 
 Epidermal growth factor-regulated activation of Rac GTPase enhances CD44 cleavage by metalloproteinase disintegrin ADAM10. Invasive tumour cells, such as gliomas, frequently express EGF (epidermal growth factor) receptor at a high level and they exhibit enhanced cell   migration   in response to EGF. We reported previously that tumour cell   migration   is associated with ectodomain cleavage of CD44, the major adhesion molecule that is implicated in tumour invasion and metastasis, and that the cleavage is enhanced by ligation of CD44. In the present study, we show that EGF promotes CD44 cleavage and CD44-dependent cell   migration  . Introduction of a dominant-negative mutant of the small GTPase Rac1 or depletion of Rac1 by RNAi (RNA interference) abrogated CD44 cleavage induced by EGF. Treatment with PD98059, an inhibitor for MEK (mitogen-activated protein kinase/extracellular-signal-regulated kinase kinase), also suppressed the CD44 cleavage. Furthermore, RNAi studies showed that EGF induced ADAM10 (a disintegrin and metalloproteinase 10)-dependent CD44 cleavage and cell   migration  . These results indicate that EGF induces ADAM10-mediated CD44 cleavage through Rac1 and mitogen-activated protein kinase activation, and thereby promotes tumour cell   migration   and invasion. 
 Analysis of the myosin-II-responsive focal adhesion proteome reveals a role for β-Pix in negative regulation of focal adhesion maturation. Focal adhesions undergo myosin-II-mediated maturation wherein they grow and change composition to modulate integrin signalling for cell   migratio  n, growth and differentiation. To determine how focal adhesion composition is affected by myosin II activity, we performed proteomic analysis of isolated focal adhesions and compared protein abundance in focal adhesions from cells with and without myosin II inhibition. We identified 905 focal adhesion proteins, 459 of which changed in abundance with myosin II inhibition, defining the myosin-II-responsive focal adhesion proteome. The abundance of 73% of the proteins in the myosin-II-responsive focal adhesion proteome was enhanced by contractility, including proteins involved in Rho-mediated focal adhesion maturation and endocytosis- and calpain-dependent focal adhesion disassembly. During myosin II inhibition, 27% of proteins in the myosin-II-responsive focal adhesion proteome, including proteins involved in Rac-mediated lamellipodial protrusion, were enriched in focal adhesions, establishing that focal adhesion protein recruitment is also negatively regulated by contractility. We focused on the Rac guanine nucleotide exchange factor β-Pix, documenting its role in the negative regulation of focal adhesion maturation and the promotion of lamellipodial protrusion and focal adhesion turnover to drive cel  l migrati  on. 
 Prognostic role of integrin β1, E-cadherin, and rac1 expression in small cell lung cancer. Integrin β(1) mediates cellular adhesion to the extracellular matrix (ECM) and is correlated with highly invasive and metastatic behavior in small cell lung cancer (SCLC). E-cadherin (ECAD) is a calcium-dependent cell-cell adhesion receptor that restricts invasion of cells and reduces metastasis. Rac1 is involved in the regulation of the actin cytoskeleton, adhesion  , migrati  on, invasion, and tumor metastasis. The aim of this study was to examine integrin β(1) , ECAD and rac1 expression in SCLC and to analyze the prognostic value of these markers in patients with SCLC. We analyzed integrin β(1) , ECAD, and rac1 expression in 112 SCLC tissues by immunohistochemical staining. Correlative analyses between integrin β(1) , ECAD, and rac1 expression and cliniopathological factors were performed. A total of 65 patients had extensive disease (ED) (58%), and 47 had limited disease (LD) (42%). The median follow-up duration was 61 months (range: 14-117 months), and the median progression free survival (PFS) and overall survival (OS) were 6.1 months (range: 4.8-7.4 months) and 9.7 months (range: 8.1-11.3 months), respectively. The expression of integrin β(1) , ECAD, and rac1 protein was observed in 64, 73, and 99 of SCLC tissues, respectively. The correlative analyses between integrin β(1) , ECAD, or rac1 expression and various clinical parameters did not show any statistical significance. However, the ECAD expression was associated with OS in the entire cohort. In contrast, the expression of integrin β(1) and rac1 was not associated with PFS or OS. In a subgroup analysis, patients with less than two metastasis had significantly longer OS (p = 0.047) if their tumors expressed integrin β(1) compared to those without integrin β(1) expression. In addition, OS was longer for patients with ECAD positive tumors compared to those whose tumors did not express ECAD in males (p = 0.032) and patients who never smoked (p < 0.001). Multivariate analysis showed that LD (p = 0.004), overall response rate (p = 0.003), and expression of ECAD (p = 0.015) were the independent good prognostic factors for OS. LD (p = 0.024), overall response rate (p < 0.001), and less than two metastasis (p = 0.003) were prognostic factors for longer PFS. These results suggest that ECAD expression may be useful as a prognostic indicator in patients with SCLC. 
 Opposing roles for Akt1 and Akt2 in Rac/Pak signaling and cell   migration  . The Akt/PKB isoforms have different roles in animals, with Akt2 primarily regulating metabolic signaling and Akt1 regulating growth and survival. Here we show distinct roles for Akt1 and Akt2 in mouse embryo fibroblast cell   migration   and regulation of the cytoskeleton. Akt1-deficient cells responded poorly to platelet-derived growth factor while Akt2-deficient cells had a dramatically enhanced response, resulting in a substantial increase in dorsal ruffling. Swapping domains between Akt1 and Akt2 demonstrated that the N-terminal region containing the pleckstrin homology domain and a linker region distinguishes the two isoforms, while the catalytic domains are interchangeable. Akt2 knock-out cells also migrated faster than wild-type cells, especially through extracellular matrix (ECM), while Akt1 knock-out cells migrated more slowly than wild-type cells. Consistently, Akt2 knock-out cells had elevated Pak1 and Rac activities, suggesting that Akt2 inhibits Rac and Pak1. Both Akt2 and Akt1 associated in complexes with Pak1, but only Akt2 inhibited Pak1 in kinase assays, suggesting an underlying molecular basis for the different cellular phenotypes. Together these data provide evidence for an unexpected functional link between Akt2 and Pak1 that opposes the actions of Akt1 on cell   migration  . 
 Valvular dystrophy associated filamin A mutations reveal a new role of its first repeats in small-GTPase regulation. Filamin A (FlnA) is a ubiquitous actin binding protein which anchors various transmembrane proteins to the cell cytoskeleton and provides a scaffold to many cytoplasmic signaling proteins involved in actin cytoskeleton remodeling in response to mechanical stress and cytokines stimulation. Although the vast majority of FlnA binding partners interact with the carboxy-terminal immunoglobulin like (Igl) repeats of FlnA, little is known on the role of the amino-N-terminal repeats. Here, using cardiac mitral valvular dystrophy associated FlnA-G288R and P637Q mutations located in the N-terminal Igl repeat 1 and 4 respectively as a model, we identified a new role of FlnA N-terminal repeats in small Rho-GTPases regulation. Using FlnA-deficient melanoma and HT1080 cell lines as expression systems we showed that FlnA mutations reduce cell spreading and   migration   capacities. Furthermore, we defined a signaling network in which FlnA mutations alter the balance between RhoA and Rac1 GTPases activities in favor of RhoA and provided evidences for a role of the Rac1 specific GTPase activating protein FilGAP in this process. Together our work ascribed a new role to the N-terminal repeats of FlnA in Small GTPases regulation and supports a conceptual framework for the role of FlnA mutations in cardiac valve diseases centered around signaling molecules regulating cellular actin cytoskeleton in response to mechanical stress. 
 Glycogen synthase kinase 3β inhibition prevents monocyte   migratio  n across brain endothelial cells via Rac1-GTPase suppression and down-regulation of active integrin conformation. Glycogen synthase kinase (GSK) 3β has been identified as a regulator of immune responses. We demonstrated previously that GSK3β inhibition in human brain microvascular endothelial cells (BMVECs) reduced monocyte adhesi  on/migrat  ion across BMVEC monolayers. Herein, we tested the idea that GSK3β inhibition in monocytes can diminish their ability to engage the brain endothelium and migrate across the blood-brain barrier. Pretreatment of primary monocytes with GSK3β inhibitors resulted in a decrease in adhesion (60%)   and migr  ation (85%), with similar results in U937 monocytic cells. Monocyte-BMVEC interactions resulted in diminished barrier integrity that was reversed by GSK3β suppression in monocytic cells. Because integrins mediate monocyte rolling/adhesion, we detected the active conformational form of very late antigen 4 after stimulation with a peptide mimicking monocyte engagement by vascular cell adhesion molecule-1. Peptide stimulation resulted in a 14- to 20-fold up-regulation of the active form of integrin in monocytes that was suppressed by GSK3β inhibitors (40% to 60%). Because small GTPases, such as Rac1, control leukocyte movement, we measured active Rac1 after monocyte activation with relevant stimuli. Stimulation enhanced the level of active Rac1 that was diminished by GSK3β inhibitors. Monocytes treated with GSK3β inhibitors showed increased levels of inhibitory sites of the actin-binding protein, cofilin, and vasodilator-stimulated phosphoprotein-regulating conformational changes of integrins. These results indicate that GSK3β inhibition in monocytes affects active integrin expression, cytoskeleton rearrangement, and adhesion via suppression of Rac1-diminishing inflammatory leukocyte responses. 
 Inhibition of RAC1-GEF DOCK3 by miR-512-3p contributes to suppression of metastasis in non-small cell lung cancer. MicroRNAs are a class of small non-coding RNAs regulating gene expression. In this study, we demonstrated that retinoic acid (RA) treatment increases the expression of miR-512-3p. Overexpression of miR-512-3p inhibited cell adhesion,   migration  , and invasion in non-small cell lung cancer (NSCLC) cell lines A549 and H1299. miR-512-3p inhibitor partially reversed these effects in H1299 cells stably expressing miR-512. We identified DOCK3, a RAC1-GEF (guanine nucleotide exchange factor), as a target gene of miR-512-3p. Overexpression of miR-512-3p led to the decrease of DOCK3 protein but not its mRNA. Knockdown of DOCK3 resulted in similar effects on adhesion,   migration  , and invasion as observed of miR-512-3p overexpression. Active RAC1 pull-down assay indicated that overexpression of miR-512-3p could decrease the activity of RAC1 with a higher efficiency than that of DOCK3 knockdown. Furthermore, expression of miR-512-3p was suppressed in most NSCLC patient tumor samples compared to its paired normal controls, suggesting that miR-512-3p might play a crucial role in lung cancer development. In conclusion, our results supported that miR-512-3p could inhibit tumor cell adhesion,   migration  , and invasion by regulating the RAC1 activity via DOCK3 in NSCLC A549 and H1299 cell lines. 
 Role of non-genomic androgen signalling in suppressing proliferation of fibroblasts and fibrosarcoma cells. The functions of androgen receptor (AR) in stromal cells are still debated in spite of the demonstrated importance of these cells in organ development and diseases. Here, we show that physiological androgen concentration (10 nM R1881 or DHT) fails to induce DNA synthesis, while it consistently stimulates cel  l migrati  on in mesenchymal and transformed mesenchymal cells. Ten nanomolar R1881 triggers p27 Ser10 phosphorylation and its stabilization in NIH3T3 fibroblasts. Activation of Rac and its downstream effector DYRK 1B is responsible for p27 Ser10 phosphorylation and cell quiescence. Ten nanomolar androgen also inhibits transformation induced by oncogenic Ras in NIH3T3 fibroblasts. Overexpression of an AR mutant unable to interact with filamin A, use of a small peptide displacing AR/filamin A interaction, and filamin A knockdown indicate that the androgen-triggered AR/filamin A complex regulates the pathway leading to p27 Ser10 phosphorylation and cell cycle arrest. As the AR/filamin A complex is also responsible fo  r migrati  on stimulated by 10 nM androgen, our report shows that the androgen-triggered AR/filamin A complex controls, through Rac 1, the decision of cells to halt cell cycle   and migra  tion. This study reveals a new and unexpected role of androgen/AR signalling in coordinating stromal cell functions. 
 Knockdown of Coronin-1C disrupts Rac1 activation and impairs tumorigenic potential in hepatocellular carcinoma cells. Coronin-1C is an important F-actin binding protein which is critical for cell motility. Furthermore, the expression of this protein was found to be increased in diffuse tumors and was correlated with the degree of tumor malignancy. However, the mechanism(s) through which this protein enhances malignancy in hepatocellular carcinoma (HCC) is poorly understood. In this study, we found that Coronin-1C was overexpressed in human HCC tissues compared with the adjacent non-tumor tissues. Overexpression of Coronin-1C enhanced the cell   migration   in the human HCC cell line BEL-7402, whereas suppressed cell   migration   and proliferation were observed in Coronin-1C-knockdown BEL-7402 cells together with impaired cell polarity, disrupted cytoskeleton and decreased Rac-1 activation. Moreover, the Coronin-1C knockdown cells displayed a lower degree of malignancy by inducing smaller tumors in nude mice. Thus, we demonstrated a relationship between Coronin-1C overexpression and human HCC growth through enhancement of tumor cell proliferation and   migration  , which are correlated with Rac-1 activation. 
 Valvular dystrophy associated filamin A mutations reveal a new role of its first repeats in small-GTPase regulation. Filamin A (FlnA) is a ubiquitous actin binding protein which anchors various transmembrane proteins to the cell cytoskeleton and provides a scaffold to many cytoplasmic signaling proteins involved in actin cytoskeleton remodeling in response to mechanical stress and cytokines stimulation. Although the vast majority of FlnA binding partners interact with the carboxy-terminal immunoglobulin like (Igl) repeats of FlnA, little is known on the role of the amino-N-terminal repeats. Here, using cardiac mitral valvular dystrophy associated FlnA-G288R and P637Q mutations located in the N-terminal Igl repeat 1 and 4 respectively as a model, we identified a new role of FlnA N-terminal repeats in small Rho-GTPases regulation. Using FlnA-deficient melanoma and HT1080 cell lines as expression systems we showed that FlnA mutations reduce cell spreading and   migration   capacities. Furthermore, we defined a signaling network in which FlnA mutations alter the balance between RhoA and Rac1 GTPases activities in favor of RhoA and provided evidences for a role of the Rac1 specific GTPase activating protein FilGAP in this process. Together our work ascribed a new role to the N-terminal repeats of FlnA in Small GTPases regulation and supports a conceptual framework for the role of FlnA mutations in cardiac valve diseases centered around signaling molecules regulating cellular actin cytoskeleton in response to mechanical stress. 
 Radixin regulates cell   migration   and cell-cell adhesion through Rac1. The ERM proteins ezrin, radixin and moesin are adaptor proteins that link plasma membrane receptors to the actin cytoskeleton. Ezrin and moesin have been implicated in cell polarization and cell   migration  , but little is known about the involvement of radixin in these processes. Here we show that radixin is required for   migration   of PC3 prostate cancer cells, and that radixin, but not ezrin or moesin, depletion by RNA interference increases cell spread area and cell-cell adhesion mediated by adherens junctions. Radixin depletion also alters actin organization, and distribution of active phosphorylated ezrin and moesin. Similar effects were observed in MDA-MB-231 breast cancer cells. The phenotype of radixin-depleted cells is similar to that induced by constitutively active Rac1, and Rac1 is required for the radixin knockdown phenotype. Radixin depletion also increases the activity of Rac1 but not Cdc42 or RhoA. Analysis of Rac guanine nucleotide exchange factors (GEFs) suggests that radixin affects the activity of Vav GEFs. Indeed, Vav GEF depletion reverses the phenotype of radixin knockdown and reduces the effect of radixin knockdown on Rac1 activity. Our results indicate that radixin plays an important role in promoting cell   migration   by regulating Rac1-mediated epithelial polarity and formation of adherens junctions through Vav GEFs. 
 Glycogen synthase kinase 3β inhibition prevents monocyte   migratio  n across brain endothelial cells via Rac1-GTPase suppression and down-regulation of active integrin conformation. Glycogen synthase kinase (GSK) 3β has been identified as a regulator of immune responses. We demonstrated previously that GSK3β inhibition in human brain microvascular endothelial cells (BMVECs) reduced monocyte adhesi  on/migrat  ion across BMVEC monolayers. Herein, we tested the idea that GSK3β inhibition in monocytes can diminish their ability to engage the brain endothelium and migrate across the blood-brain barrier. Pretreatment of primary monocytes with GSK3β inhibitors resulted in a decrease in adhesion (60%)   and migr  ation (85%), with similar results in U937 monocytic cells. Monocyte-BMVEC interactions resulted in diminished barrier integrity that was reversed by GSK3β suppression in monocytic cells. Because integrins mediate monocyte rolling/adhesion, we detected the active conformational form of very late antigen 4 after stimulation with a peptide mimicking monocyte engagement by vascular cell adhesion molecule-1. Peptide stimulation resulted in a 14- to 20-fold up-regulation of the active form of integrin in monocytes that was suppressed by GSK3β inhibitors (40% to 60%). Because small GTPases, such as Rac1, control leukocyte movement, we measured active Rac1 after monocyte activation with relevant stimuli. Stimulation enhanced the level of active Rac1 that was diminished by GSK3β inhibitors. Monocytes treated with GSK3β inhibitors showed increased levels of inhibitory sites of the actin-binding protein, cofilin, and vasodilator-stimulated phosphoprotein-regulating conformational changes of integrins. These results indicate that GSK3β inhibition in monocytes affects active integrin expression, cytoskeleton rearrangement, and adhesion via suppression of Rac1-diminishing inflammatory leukocyte responses. 
 Interleukin-8 induces the endothelial cell   migration   through the Rac 1/RhoA-p38MAPK pathway. Endothelial cell   migration   is essential for tumor angiogenesis, and interleukin-8 (IL-8) has been shown to play an important role in tumor growth, angiogenesis, and metastasis. The objective of this study was to investigate the molecular mechanism of IL-8 induced endothelial cell   migration   in vitro.Fluorescence microscope was used to study the distribution of cytoskeleton. The expression of Rac1 and RhoA protein was detected by western blotting. After endothelial cells were transfected by lipofectamine 2000 reagent, the Transwell chamber motility assay was applied to observe the   migration   of endothelial cells induced by IL-8. The active p38MAPK (mitogen-activated protein kinase) was evaluated by the p38MAPK activation assay.We demonstrated that IL-8 activated cell   migration   can be impaired by p38MAPK inhibitor, suggesting the participation of p38MAPK in the cell   migration  . Our results indicated that p38MAPK signaling is required for membrane ruffles, lamellipodia extensions, and actin stress fibers formation induced by IL-8. Furthermore, p38MAPK inhibitor led to increased Rac1 and RhoA expression in IL-8 treated EA.hy926 cells. In addition, IL-8 induced p38MAPK activation was suppressed by dominant-negative mutant for Rac1 and RhoA.Our study demonstrates that IL-8-Rac1/RhoA-p38MAPK signaling pathway plays a vital role in the IL-8-induced endothelial cell   migration  , and it provides new insight into the molecular mechanisms by which IL-8 contributes to tumor angiogenesis and metastasis. 
 Prognostic role of integrin β1, E-cadherin, and rac1 expression in small cell lung cancer. Integrin β(1) mediates cellular adhesion to the extracellular matrix (ECM) and is correlated with highly invasive and metastatic behavior in small cell lung cancer (SCLC). E-cadherin (ECAD) is a calcium-dependent cell-cell adhesion receptor that restricts invasion of cells and reduces metastasis. Rac1 is involved in the regulation of the actin cytoskeleton, adhesion  , migrati  on, invasion, and tumor metastasis. The aim of this study was to examine integrin β(1) , ECAD and rac1 expression in SCLC and to analyze the prognostic value of these markers in patients with SCLC. We analyzed integrin β(1) , ECAD, and rac1 expression in 112 SCLC tissues by immunohistochemical staining. Correlative analyses between integrin β(1) , ECAD, and rac1 expression and cliniopathological factors were performed. A total of 65 patients had extensive disease (ED) (58%), and 47 had limited disease (LD) (42%). The median follow-up duration was 61 months (range: 14-117 months), and the median progression free survival (PFS) and overall survival (OS) were 6.1 months (range: 4.8-7.4 months) and 9.7 months (range: 8.1-11.3 months), respectively. The expression of integrin β(1) , ECAD, and rac1 protein was observed in 64, 73, and 99 of SCLC tissues, respectively. The correlative analyses between integrin β(1) , ECAD, or rac1 expression and various clinical parameters did not show any statistical significance. However, the ECAD expression was associated with OS in the entire cohort. In contrast, the expression of integrin β(1) and rac1 was not associated with PFS or OS. In a subgroup analysis, patients with less than two metastasis had significantly longer OS (p = 0.047) if their tumors expressed integrin β(1) compared to those without integrin β(1) expression. In addition, OS was longer for patients with ECAD positive tumors compared to those whose tumors did not express ECAD in males (p = 0.032) and patients who never smoked (p < 0.001). Multivariate analysis showed that LD (p = 0.004), overall response rate (p = 0.003), and expression of ECAD (p = 0.015) were the independent good prognostic factors for OS. LD (p = 0.024), overall response rate (p < 0.001), and less than two metastasis (p = 0.003) were prognostic factors for longer PFS. These results suggest that ECAD expression may be useful as a prognostic indicator in patients with SCLC. 
 Proteomic analysis of integrin-associated complexes identifies RCC2 as a dual regulator of Rac1 and Arf6. The binding of integrin adhesion receptors to their extracellular matrix ligands controls cell morphology, movement, survival, and differentiation in various developmental, homeostatic, and disease processes. Here, we report a methodology to isolate complexes associated with integrin adhesion receptors, which, like other receptor-associated signaling complexes, have been refractory to proteomic analysis. Quantitative, comparative analyses of the proteomes of two receptor-ligand pairs, alpha(4)beta(1)-vascular cell adhesion molecule-1 and alpha(5)beta(1)-fibronectin, defined both core and receptor-specific components. Regulator of chromosome condensation-2 (RCC2) was detected in the alpha(5)beta(1)-fibronectin signaling network at an intersection between the Rac1 and adenosine 5'-diphosphate ribosylation factor 6 (Arf6) subnetworks. RCC2 knockdown enhanced fibronectin-induced activation of both Rac1 and Arf6 and accelerated cell spreading, suggesting that RCC2 limits the signaling required for membrane protrusion and delivery. Dysregulation of Rac1 and Arf6 function by RCC2 knockdown also abolished persistent   migration   along fibronectin fibers, indicating a functional role for RCC2 in directional cell movement. This proteomics workflow now opens the way to further dissection and systems-level analyses of adhesion signaling. 
 Upregulation of Eps8 in oral squamous cell carcinoma promotes cell   migration   and invasion through integrin-dependent Rac1 activation. Oral squamous cell carcinoma (OSCC) is a lethal disease and early death usually occurs as a result of local invasion and regional lymph node metastases. Current treatment regimens are, to a certain degree, inadequate, with a 5-year mortality rate of around 50% and novel therapeutic targets are urgently required. Using expression microarrays, we identified the eps8 gene as being overexpressed in OSCC cell lines relative to normal oral keratinocytes, and confirmed these findings using RT-PCR and western blotting. In human tissues, we found that Eps8 was upregulated in OSCC (32% of primary tumors) compared with normal oral mucosa, and that expression correlated significantly with lymph node metastasis (P=0.032), suggesting a disease-promoting effect. Using OSCC cell lines, we assessed the functional role of Eps8 in tumor cells. Although suppression of Eps8 produced no effect on cell proliferation, both cell spreading and   migration   were markedly inhibited. The latter cell functions may be modulated through the small GTP-ase, Rac1 and we used pull-down assays to investigate the role of Eps8 in Rac1 signaling. We found that alphavbeta6- and alpha5beta1-integrin-dependent activation of Rac1 was mediated through Eps8. Knockdown of either Eps8 or Rac1, inhibited integrin-dependent cell   migration   similarly and transient expression of constitutively active Rac1 restored   migration   of cells in which Eps8 expression had been suppressed. We also showed that knockdown of Eps8 inhibited tumor cell invasion in an organotypic model of OSCC. These data suggest that Eps8 and Rac1 are part of an integrated signaling pathway modulating integrin-dependent tumour cell motility and identify Eps8 as a possible therapeutic target. 
 Opposing roles for Akt1 and Akt2 in Rac/Pak signaling and cell   migration  . The Akt/PKB isoforms have different roles in animals, with Akt2 primarily regulating metabolic signaling and Akt1 regulating growth and survival. Here we show distinct roles for Akt1 and Akt2 in mouse embryo fibroblast cell   migration   and regulation of the cytoskeleton. Akt1-deficient cells responded poorly to platelet-derived growth factor while Akt2-deficient cells had a dramatically enhanced response, resulting in a substantial increase in dorsal ruffling. Swapping domains between Akt1 and Akt2 demonstrated that the N-terminal region containing the pleckstrin homology domain and a linker region distinguishes the two isoforms, while the catalytic domains are interchangeable. Akt2 knock-out cells also migrated faster than wild-type cells, especially through extracellular matrix (ECM), while Akt1 knock-out cells migrated more slowly than wild-type cells. Consistently, Akt2 knock-out cells had elevated Pak1 and Rac activities, suggesting that Akt2 inhibits Rac and Pak1. Both Akt2 and Akt1 associated in complexes with Pak1, but only Akt2 inhibited Pak1 in kinase assays, suggesting an underlying molecular basis for the different cellular phenotypes. Together these data provide evidence for an unexpected functional link between Akt2 and Pak1 that opposes the actions of Akt1 on cell   migration  . 
 Oncogenic K-Ras down-regulates Rac1 and RhoA activity and enhances   migration   and invasion of pancreatic carcinoma cells through activation of p38. Activating mutations in the K-ras gene are genetic alterations frequently found in human carcinomas, particularly in pancreatic adenocarcinomas. Mutation of the K-ras gene is thought to be an early and important event in pancreatic tumor initiation, but the precise role of the mutant K-Ras proteins in neoplastic progression is still unknown. In the present study, we have characterized the influence of oncogenic K-Ras on the phenotype and on the signal transduction of epitheloid PANC-1 pancreatic carcinoma cells by generating PANC-1 cell clones, which stably express EGFP(enhanced green fluorescent protein)-K-Ras (V12). EGFP-K-Ras (V12)-expressing cells exhibited a more fibroblastoid cellular phenotype with irregular cell shape and disorganized cytokeratin filaments. Moreover, these cells showed a marked enhancement of their migratory and invasive properties. Stable expression of EGFP-K-Ras (V12) down-regulated the activity of Rac1 and RhoA, resulting in reduced subcortical actin filaments and stress fibers, which might contribute to the epithelial dedifferentiation. Characterization of the activity of mitogen-activated protein kinases revealed that EGFP-K-Ras (V12) enhanced the activity of p38, but did not affect the activities of the Raf/MEK/ERK cascade and JNK. While inhibition of either MEK or JNK activity had no effect on EGFP-K-Ras (V12)-induced   migration  , inhibition of p38 activity markedly reduced EGFP-K-Ras (V12)-induced   migration  . Collectively, the results suggest that oncogenic K-Ras enhances the malignant phenotype and identify the mitogen-activated protein kinase p38 as a target to inhibit oncogenic K-Ras-induced pancreatic tumor cell   migration  . 
 Epidermal growth factor-regulated activation of Rac GTPase enhances CD44 cleavage by metalloproteinase disintegrin ADAM10. Invasive tumour cells, such as gliomas, frequently express EGF (epidermal growth factor) receptor at a high level and they exhibit enhanced cell   migration   in response to EGF. We reported previously that tumour cell   migration   is associated with ectodomain cleavage of CD44, the major adhesion molecule that is implicated in tumour invasion and metastasis, and that the cleavage is enhanced by ligation of CD44. In the present study, we show that EGF promotes CD44 cleavage and CD44-dependent cell   migration  . Introduction of a dominant-negative mutant of the small GTPase Rac1 or depletion of Rac1 by RNAi (RNA interference) abrogated CD44 cleavage induced by EGF. Treatment with PD98059, an inhibitor for MEK (mitogen-activated protein kinase/extracellular-signal-regulated kinase kinase), also suppressed the CD44 cleavage. Furthermore, RNAi studies showed that EGF induced ADAM10 (a disintegrin and metalloproteinase 10)-dependent CD44 cleavage and cell   migration  . These results indicate that EGF induces ADAM10-mediated CD44 cleavage through Rac1 and mitogen-activated protein kinase activation, and thereby promotes tumour cell   migration   and invasion. 
 beta4 integrin and epidermal growth factor coordinately regulate electric field-mediated directional   migration   via Rac1. Endogenous DC electric fields (EF) are present during embryogenesis and are generated in vivo upon wounding, providing guidance cues for directional cell   migration   (galvanotaxis) required in these processes. To understand the role of beta (beta)4 integrin in directional   migration  , the migratory paths of either primary human keratinocytes (NHK), beta4 integrin-null human keratinocytes (beta4-), or those in which beta4 integrin was reexpressed (beta4+), were tracked during exposure to EFs of physiological magnitude (100 mV/mm). Although the expression of beta4 integrin had no effect on the rate of cell movement, it was essential for directional (cathodal)   migration   in the absence of epidermal growth factor (EGF). The addition of EGF potentiated the directional response, suggesting that at least two distinct but synergistic signaling pathways coordinate galvanotaxis. Expression of either a ligand binding-defective beta4 (beta4+AD) or beta4 with a truncated cytoplasmic tail (beta4+CT) resulted in loss of directionality in the absence of EGF, whereas inhibition of Rac1 blinded the cells to the EF even in the presence of EGF. In summary, both the beta4 integrin ligand-binding and cytoplasmic domains together with EGF were required for the synergistic activation of a Rac-dependent signaling pathway that was essential for keratinocyte directional   migration   in response to a galvanotactic stimulus. 
 Vitreous-induced cytoskeletal rearrangements via the Rac1 GTPase-dependent signaling pathway in human retinal pigment epithelial cells. Proliferative vitreoretinopathy (PVR) is mainly caused by retinal pigment epithelial (RPE) cell   migration  , invasion, proliferation and transformation into fibroblast-like cells that produce the extracellular matrix (ECM). The vitreous humor is known to play an important role in PVR. An epithelial-to-mesenchymal transdifferentiation (EMT) of human RPE cells induced by 25% vitreous treatment has been linked to stimulation of the mesenchymal phenotype,   migration   and invasion. Here, we characterized the effects of the vitreous on the cell morphology and cytoskeleton in human RPE cells. The signaling pathway that mediates these effects was investigated. Serum-starved RPE cells were incubated with 25% vitreous, and the morphological changes were examined by phase-contrast microscopy. Filamentous actin (F-actin) was examined by immunofluorescence and confocal microscopy. Protein phosphorylation of AKT, ERK1/2, Smad2/3, LIM kinase (LIMK) 1 and cofilin was analyzed by Western blot analysis. Vitreous treatment induced cytoskeletal rearrangements, activated Rac1 and enhanced the phosphorylation of AKT, ERK1/2 and Smad2/3. When the cells were treated with a Rac activation-specific inhibitor, the cytoskeletal rearrangements were prevented, and the phosphorylation of Smad2/3 was blocked. Vitreous treatment also enhanced the phosphorylation of LIMK1 and cofilin and the Rac inhibitor blocked this effect. We propose that vitreous-transformed human RPE cells undergo cytoskeletal rearrangements via Rac1 GTPase-dependent pathways that modulate LIMK1 and cofilin activity. The TGFβ-like activity of the vitreous may participate in this effect. Actin polymerization causes the cytoskeletal rearrangements that lead to the plasticity of vitreous-transformed RPE cells in PVR. 
 Regulation of CXCR4 conformation by the small GTPase Rac1: implications for HIV infection. The chemokine receptor CXCR4 is a critical regulator of cell   migration   and serves as a coreceptor for HIV-1. The chemokine stromal cell derived factor-1, also known as CXCL12, binds to CXCR4 and exerts its biologic functions partly through the small guanosine triphosphate hydrolase (GTPase) Rac1 (ras-related C3 botulinum toxin substrate 1). We show in different cell types, including CD34(+) hematopoietic stem and progenitor cells, that inhibition of Rac1 causes a reversible conformational change in CXCR4, but not in the related receptors CXCR7 or CCR5. Biochemical experiments showed that Rac1 associates with CXCR4. The conformational change of CXCR4 on Rac1 inhibition blocked receptor internalization and impaired CXCL12-induced Gα(i) protein activation. Importantly, we found that the conformation adopted by CXCR4 after Rac1 inhibition prevents HIV-1 infection of both the U87-CD4-CXCR4 cell line and of primary peripheral blood mononuclear cells. In conclusion, our data show that Rac1 activity is required to maintain CXCR4 in the responsive conformation that allows receptor signaling and facilitates HIV-1 infection; this implies that Rac1 positively regulates CXCR4 function and identifies the Rac1-CXCR4 axis as a new target for preventing HIV-1 infection. 
 Rac1 signaling modulates BCL-6-mediated repression of gene transcription. Rac1 is a member of the Rho family of small GTPases that not only regulates signaling pathways involved in cell adhesion and   migration   but also regulates gene transcription. Here we show that the transcriptional repressor BCL-6 is regulated by Rac1 signaling. Transfection of active Rac1 mutants into colorectal DLD-1 cells led to increased expression of a BCL-6-controlled luciferase reporter construct. Conversely, inhibition of endogenous Rac1 activation by the Rac1 inhibitor NSC23766 decreased reporter activity. Moreover, BCL-6 lost its typical localization to nuclear dots upon activation of Rac1 and became predominantly soluble in a non-chromatin-bound cell fraction. Rac1 signaling also regulated the expression of endogenous BCL-6-regulated genes, including the p50 precursor NF-kappaB1/p105 and the cell adhesion molecule CD44. Interestingly, these effects were not stimulated by the alternative splice variant Rac1b. The mechanism of BCL-6 inhibition does not involve formation of a stable Rac1/BCL-6 complex and is independent of Rac-induced reactive oxygen species production or Jun NH(2)-terminal kinase activation. We show that PAK1 mediates inhibition downstream of Rac and can directly phosphorylate BCL-6. Together, these data provide substantial evidence that Rac1 signaling inhibits the transcriptional repressor BCL-6 in colorectal cells and reveal a novel pathway that links Rac1 signaling to the regulation of gene transcription. 
 PH domain of ELMO functions in trans to regulate Rac activation via Dock180. The members of the Dock180 superfamily of proteins are novel guanine nucleotide exchange factors (GEF) for Rho family GTPases and are linked to multiple biological processes from worms to mammals. ELMO is a critical regulator of Dock180, and the Dock180-ELMO complex functions as a bipartite GEF for Rac. We identified a mechanism wherein the PH domain of ELMO, by binding the Dock180-Rac complex in trans, stabilizes Rac in the nucleotide-free transition state. Mutagenesis studies reveal that this ELMO PH domain-dependent regulation is essential for the Dock180-ELMO complex to function in phagocytosis and cell   migration  . Genetic rescue studies in Caenorhabditis elegans using ELMO and its homolog CED-12 support the above observations in vivo. These data reveal a new mode of action of PH domains and a novel, evolutionarily conserved mechanism by which a bipartite GEF can activate Rac. 
 MicroRNA-200b suppresses arsenic-transformed cell   migration   by targeting protein kinase Cα and Wnt5b-protein kinase Cα positive feedback loop and inhibiting Rac1 activation. MicroRNA-200b (miR-200b) is a member of miR-200 family that has been found to inhibit cel  l migrati  on and cancer metastasis; however, the underlying mechanism is not well understood. We previously reported that miR-200 expression is depleted in arsenic-transformed human bronchial epithelial cells with highly migratory and invasive characteristics, whereas stably re-expressing miR-200b strongly suppresses arsenic-transformed cel  l migrati  on. This study was performed to investigate how miR-200b inhibits arsenic-transformed cel  l migrati  on. We found that protein kinase Cα (PKCα) is significantly up-regulated in arsenic-transformed cells. Combining bioinformatics analysis with PKCα 3'-untranslated region vector luciferase reporter assays, we showed that PKCα is a direct target of miR-200b. Inhibiting PKCα activity or knocking down PKCα expression drastically reduc  ed cell m  igration, phenocoping the inhibitory effect of overexpressing miR-200b. In contrast, forced expression of PKCα in miR-200b overexpressing cells impaired the inhibitory effect of miR-200b   on cell   migration. In addition, we also found a positive feedback loop between Wnt5b and PKCα in arsenic-transformed cells. Knocking down Wnt5b expression reduced phospho-PKC levels   and cell   migration; and knocking down PKCα expression decreased Wnt5b leve  l and cel  l migration. Moreover, forced expression of PKCα increased Wnt5b and phospho-PKC leve  ls and ce  ll migration. Further mechanistic studies revealed that Rac1 is highly activated in arsenic-transformed cells and stably expressing miR-200b abolishes Rac1 activation changing actin cytoskeleton organization. Manipulating PKCα or Wnt5b expression levels significantly altered the level of active Rac1. Together, these findings indicate that miR-200b suppresses arsenic-tran  sformed c  ell migration by targeting PKCα and Wnt5b-PKCα positive feedback loop and subsequently inhibiting Rac1 activation. 
 Protein kinase C-epsilon regulates sphingosine 1-phosphate-mediated   migration   of human lung endothelial cells through activation of phospholipase D2, protein kinase C-zeta, and Rac1. The signaling pathways by which sphingosine 1-phosphate (S1P) potently stimulates endothelial cell   migration   and angiogenesis are not yet fully defined. We, therefore, investigated the role of protein kinase C (PKC) isoforms, phospholipase D (PLD), and Rac in S1P-induced   migration   of human pulmonary artery endothelial cells (HPAECs). S1P-induced   migration   was sensitive to S1P(1) small interfering RNA (siRNA) and pertussis toxin, demonstrating coupling of S1P(1) to G(i). Overexpression of dominant negative (dn) PKC-epsilon or -zeta, but not PKC-alpha or -delta, blocked S1P-induced   migration  . Although S1P activated both PLD1 and PLD2, S1P-induced   migration   was attenuated by knocking down PLD2 or expressing dnPLD2 but not PLD1. Blocking PKC-epsilon, but not PKC-zeta, activity attenuated S1P-mediated PLD stimulation, demonstrating that PKC-epsilon, but not PKC-zeta, was upstream of PLD. Transfection of HPAECs with dnRac1 or Rac1 siRNA attenuated S1P-induced   migration  . Furthermore, transfection with PLD2 siRNA, infection of HPAECs with dnPKC-zeta, or treatment with myristoylated PKC-zeta peptide inhibitor abrogated S1P-induced Rac1 activation. These results establish that S1P signals through S1P(1) and G(i) to activate PKC-epsilon and, subsequently, a PLD2-PKC-zeta-Rac1 cascade. Activation of this pathway is necessary to stimulate the   migration   of lung endothelial cells, a key component of the angiogenic process. 
 Hypoxia/reoxygenation-experienced cancer cell   migration   and metastasis are regulated by Rap1- and Rac1-GTPase activation via the expression of thymosin beta-4. Signaling by small guanosine triphosphatases (GTPase), Rap1/Rac1, is one of the major pathways controlling cancer cell   migration   and tumor metastasis. Thymosin beta-4 (Tβ4), an actin-sequestering protein, has been shown to increase   migratio  n of cancer cells. Episodes of hypoxia and re-oxygenation (H/R) are an important phenomenon in tumor microenvironment (TME). We investigated whether Tβ4 could play as an intermediary to crosstalk between Rac1- and Rap1- GTPase activation under hypoxia/reoxygenation (H/R) conditions. Inhibition of Tβ4 expression using transcription activator-like effector nucleases (TALEN) significantly decreased lung metastasis of B16F10 cells. Rac1 and Rap1 activity, as well as cancer ce  ll migrat  ion, increased following induction of Tβ4 expression in normoxia- or H/R-experienced cells, but were barely detectable in Tβ4-depleted cells. Rap1-regulated Rac1 activity was decreased by a dominant negative Rap1 (Rap1N17), and increased by 8-(4-chloro-phenylthio)-2'-O-methyladenosine-3',5'-cyclic monophosphate (CPT), a Rap1 activator. In contrast, a Rac1-specific inhibitor, NSC23766, and dominant negative Rac1 (Rac1N17) enhanced Tβ4 expression and aberrant Rap1 activity. While NSC23766 and Rac1N17 incompletely inhibited tumor metastasis in vivo, and H/R-experienced cancer   cell mig  ration in vitro, more efficient attenuation of cancer   cell mig  ration was accomplished by simultaneous inactivation of Rap1 and Rac1 with Rap1N17 and Rac1N17, respectively. These data suggest that a combination therapy targeting both Rap1 and Rac1 activity may be an effective method of inhibiting tumor metastasis. 
 Aberrant methylation of PSD disturbs Rac1-mediated immune responses governing neutrophil chemotaxis and apoptosis in ulcerative colitis-associated carcinogenesis. We previously reported that the Pleckstrin and Sec7 domain-containing (PSD) gene is preferentially methylated in patients with ulcerative colitis (UC) who developed colorectal cancer (CRC), and is implicated in UC-associated carcinogenesis through its inhibition of apoptosis. This study aimed to determine the potential effect of PSD methylation on its downstream molecule, Ras-related C3 botulinum toxin substrate 1 (Rac1), which governs neutrophil chemotaxis and apoptosis signaling. PSD was knocked down in a normal human fibroblast cell line (HNDF) and a neutrophil-like cell line (HL-60). Both NHDF and HL-60 cells exhibited numerous filamentous-actin (F-actin) rich membrane extensions, resulting in the activation of Rac1; this activation was hampered by PSD silencing. Lipopolysaccharide, a reactive oxygen species (ROS) inducer, stimulated NHDF cells to release ROS and activated caspase‑3/7 in the presence of neutrophils, which was inhibited by PSD knockdown  . Migrati  on assays demonstrated that chemotaxis of HL-60 cells was affected by PSD silencing in NHDF cells. Tissue sections from 6 UC patients with CRC and 15 UC patients without CRC were examined. To verify Rac1-mediated chemotaxis in tissue sections, we evaluated the grade of neutrophil infiltration by histological assessment and assessed F-actin and PSD expression by immunohistochemistry. Neutrophil infiltration, F-actin and PSD expression were significantly decreased in specimens from UC patients with PSD methylation compared with those without. Decreased levels of F-actin expression were observed in colorectal mucosa, as well as in infiltrating cells with PSD methylation. PSD expression was preferentially inhibited in colorectal mucosa by PSD methylation, whereas PSD expression was rarely observed in infiltrating cells, regardless of PSD methylation status. These data indicate that aberrant methylation of PSD occurs in UC-associated colorectal mucosa, enabling circumvention of Rac1-mediated immune responses governing neutrophil chemotaxis and apoptosis, and thus plays a pivotal role in the mechanisms underlying UC-associated carcinogenesis. 
 Role of Rac1 and oxygen radicals in collagenase-1 expression induced by cell shape change. Integrin-mediated reorganization of cell shape leads to an altered cellular phenotype. Disruption of the actin cytoskeleton, initiated by binding of soluble antibody to alpha5beta1 integrin, led to increased expression of the collagenase-1 gene in rabbit synovial fibroblasts. Activation of the guanosine triphosphate-binding protein Rac1, which was downstream of the integrin, was necessary for this process, and expression of activated Rac1 was sufficient to increase expression of collagenase-1. Rac1 activation generated reactive oxygen species that were essential for nuclear factor kappa B-dependent transcriptional regulation of interleukin-1alpha, which, in an autocrine manner, induced collagenase-1 gene expression. Remodeling of the extracellular matrix and consequent alterations of integrin-mediated adhesion and cytoarchitecture are central to development, wound healing, inflammation, and malignant disease. The resulting activation of Rac1 may lead to altered gene regulation and alterations in cellular morphogenesis,   migration  , and invasion. 
 Increased fibroblast growth factor-inducible 14 expression levels promote glioma cell invasion via Rac1 and nuclear factor-kappaB and correlate with poor patient outcome. Glial tumors progress to malignant grades by heightened proliferation and relentless dispersion throughout the central nervous system. Understanding genetic and biochemical processes that foster these behaviors is likely to reveal specific and effective targets for therapeutic intervention. Our current report shows that the fibroblast growth factor-inducible 14 (Fn14), a member of the tumor necrosis factor (TNF) receptor superfamily, is expressed at high levels in migrating glioma cells in vitro and invading glioma cells in vivo. Forced Fn14 overexpression stimulates glioma cell   migration   and invasion, and depletion of Rac1 by small interfering RNA inhibits this cellular response. Activation of Fn14 signaling by the ligand TNF-like weak inducer of apoptosis (TWEAK) stimulates   migration   and up-regulates expression of Fn14; this TWEAK effect requires Rac1 and nuclear factor-kappaB (NF-kappaB) activity. The Fn14 promoter region contains NF-kappaB binding sites, which mediate positive feedback causing sustained overexpression of Fn14 and enduring glioma cell invasion. Furthermore, Fn14 gene expression levels increase with glioma grade and inversely correlate with patient survival. These results show that the Fn14 cascade operates as a positive feedback mechanism for elevated and sustained Fn14 expression. Such a feedback loop argues for aggressive targeting of the Fn14 axis as a unique and specific driver of glioma malignant behavior. 
 Rac1 is correlated with aggressiveness and a potential therapeutic target for gastric cancer. Rac1 is a member of the Rho GTPase family. Rac1 activity is critical in regulating cytoskeleton organization and thus, modulates a diverse spectrum of cellular functions in normal and malignant cells. The aims of the present study were to investigate the expression pattern and clinical significance of Rac1, as well as the role of Rac1 in gastric cancer tumorigenesis and metastasis. The expression of Rac1 in human gastric cancer was explored by immunohistochemistry. The correlation of Rac1 expression with the clinicopathological characteristics and the survival of patients were analyzed by Pearson's Chi-square and Kaplan-Meier analyses, respectively. Rac1 overexpression cell model was used to examine in vitro and in vivo effects of Rac1 in cell growth,   migration   and invasion. Rac1 was highly expressed in gastric cancer tissues and correlated with differentiation, local invasion, lymph node metastasis and Lauren's classification. Rac1 expression in gastric cancer predicted shorter survival. Overexpression of Rac1 in gastric cancer cells dramatically induced Rac1 activation and rendered a more aggressive phenotype such as increased cell growth and   migration  /invasion in vitro and in vivo. Inhibiting Rac1 activity by specific inhibitor abrogated the effects of Rac1 on the malignant phenotype. Our clinical findings demonstrated that Rac1 was well correlated with aggressiveness and a negative prognostic factor. In addition, our data on experimental cell models supported the fundamental role of Rac1 in gastric cancer. Given its pivotal role in gastric tumorigenesis and progression, Rac1 can serve as a promising therapeutic target for gastric cancer. 
 Rac1 mediates laminar shear stress-induced vascular endothelial cell   migration  . The   migration   of endothelial cells (ECs) plays an important role in vascular remodeling and regeneration. ECs are constantly subjected to shear stress resulting from blood flow and are able to convert mechanical stimuli into intracellular signals that affect cellular behaviors and functions. The aim of this study is to elucidate the effects of Rac1, which is the member of small G protein family, on EC   migration   under different laminar shear stress (5.56, 10.02, and 15.27 dyn/cm(2)). The cell   migration   distance under laminar shear stress increased significantly than that under the static culture condition. Especially, under relative high shear stress (15.27 dyn/cm(2)) there was a higher difference at 8 h (P<0.01) and 2 h (P<0.05) compared with static controls. RT-PCR results further showed increasing mRNA expression of Rac1 in ECs exposed to laminar shear stress than that exposed to static culture. Using plasmids encoding the wild-type (WT), an activated mutant (Q61L), and a dominant-negative mutant (T17N), plasmids encoding Rac1 were transfected into EA.hy 926 cells. The average net   migration   distance of Rac1Q61L group increased significantly, while Rac1T17N group decreased significantly in comparison with the static controls. These results indicated that Rac1 mediated shear stress-induced EC   migration  . Our findings conduce to elucidate the molecular mechanisms of EC   migration   induced by shear stress, which is expected to understand the pathophysiological basis of wound healing in health and diseases. 
 Rac1 recruits the adapter protein CMS/CD2AP to cell-cell contacts. Rac1 is a member of the Rho family of small GTPases, which regulate cell adhesion and   migration   through their control of the actin cytoskeleton. Rho-GTPases are structurally very similar, with the exception of a hypervariable domain in the C terminus. Using peptide-based pulldown assays in combination with mass spectrometry, we previously showed that the hypervariable domain in Rac1 mediates specific protein-protein interactions. Most recently, we found that the Rac1 C terminus associates to the ubiquitously expressed adapter protein CMS/CD2AP. CD2AP is critical for the formation and maintenance of a specialized cell-cell contact between kidney podocyte foot processes, the slit diaphragm. Here, CD2AP links the cell adhesion protein nephrin to the actin cytoskeleton. In addition, CMS/CD2AP binds actin-regulating proteins, such as CAPZ and cortactin, and has been implicated in the internalization of growth factor receptors. We found that CD2AP specifically interacts with the C-terminal domain of Rac1 but not with that of other Rho family members. Efficient interaction between Rac1 and CD2AP requires both the proline-rich domain and the poly-basic region in the Rac1 C terminus, and at least two of the three N-terminal SH3 domains of CD2AP. CD2AP co-localizes with Rac1 to membrane ruffles, and small interfering RNA-based experiments showed that CD2AP links Rac1 to CAPZ and cortactin. Finally, expression of constitutive active Rac1 recruits CD2AP to cell-cell contacts in epithelial cells, where we found CD2AP to participate in the control of the epithelial barrier function. These data identify CD2AP as a novel Rac1-associated adapter protein that participates in the regulation of epithelial cell-cell contact. 
 Myristoylated Alanine-Rich Protein Kinase Substrate (MARCKS) Regulates Small GTPase Rac1 and Cdc42 Activity and Is a Critical Mediator of Vascular Smooth Muscle Cell   Migration   in Intimal Hyperplasia Formation. Transcription of the myristoylated alanine-rich C kinase substrate (MARCKS) is upregulated in animal models of intimal hyperplasia. MARCKS knockdown inhibits vascular smooth muscle cell (VSMC)   migration   in vitro; however, the mechanism is as yet unknown. We sought to elucidate the mechanism of MARCKS-mediated motility and determine whether MARCKS knockdown reduces intimal hyperplasia formation in vivo.MARCKS knockdown blocked platelet-derived growth factor (PDGF)-induced translocation of cortactin to the cell cortex, impaired both lamellipodia and filopodia formation, and attenuated motility of human coronary artery smooth muscle cells (CASMCs). Activation of the small GTPases, Rac1 and Cdc42, was prevented by MARCKS knockdown. Phosphorylation of MARCKS resulted in a transient shift of MARCKS from the plasma membrane to the cytosol. MARCKS knockdown significantly decreased membrane-associated phosphatidylinositol 4,5-bisphosphate (PIP2) levels. Cotransfection with an intact, unphosphorylated MARCKS, which has a high binding affinity for PIP2, restored membrane-associated PIP2 levels and was indispensable for activation of Rac1 and Cdc42 and, ultimately, VSMC   migration  . Overexpression of MARCKS in differentiated VSMCs increased membrane PIP2 abundance, Rac1 and Cdc42 activity, and cell motility. MARCKS protein was upregulated early in the development of intimal hyperplasia in the murine carotid ligation model. Decreased MARKCS expression, but not total knockdown, attenuated intimal hyperplasia formation.MARCKS upregulation increases VSMC motility by activation of Rac1 and Cdc42. These effects are mediated by MARCKS sequestering PIP2 at the plasma membrane. This study delineates a novel mechanism for MARCKS-mediated VSMC   migration   and supports the rational for MARCKS knockdown to prevent intimal hyperplasia. 
 ARF1 controls Rac1 signaling to regulate   migration   of MDA-MB-231 invasive breast cancer cells. ADP-ribosylation factors (ARFs) are monomeric G proteins that regulate many cellular processes such as reorganization of the actin cytoskeleton. We have previously shown that ARF1 is overexpressed in highly invasive breast cancer cells and contribute to their enhanced   migration  . In this study, we propose to define the molecular mechanism by which ARF1 regulates this complex cellular response by investigating the role of this ARF GTPase on the activation process of Rac1, a Rho GTPase, associated with lamellipodia formation during cell   migration  . Here, we first show that inhibition of ARF1 or Rac1 expression markedly impacts the ability of MDA-MB-231 cells to migrate upon EGF stimulation. However, the effect of ARF1 depletion can be reversed by overexpression of the Rac1 active mutant, Rac1 Q(61)L. Depletion of ARF1 also impairs the ability of EGF stimulation to promote GTP-loading of Rac1. To further investigate the possible cross-talk between ARF1 and Rac1, we next examined whether they could form a complex. We observed that the two GTPases could directly interact independently of the nature of the nucleotide bound to them. EGF treatment however resulted in the association of Rac1 with its effector IRSp53, which was completely abrogated in ARF1 depleted cells. We present evidences that this ARF isoform is responsible for the plasma membrane targeting of both Rac1 and IRSp53, a step essential for lamellipodia formation. In conclusion, this study provides a new mechanism by which ARF1 regulates cell   migration   and identifies this GTPase as a promising pharmacological target to reduce metastasis formation in breast cancer patients. 
 Integrin α6β4 cooperates with LPA signaling to stimulate Rac through AKAP-Lbc-mediated RhoA activation. The α(6)β(4) integrin promotes carcinoma invasion through its ability to promote direc  ted migra  tion and polarization of carcinoma cells. In this study, we explore how the α(6)β(4) integrin cooperates with lysophosphatidic acid (LPA) to activate Rho and Rac small GTPases. Through the use of dominant negative Rho constructs, C3 exotransferase, and Rho kinase inhibitor, we find that Rho is critical for LPA-dependent chemotaxis and lamellae formation. However, utilization of specific Rho isoforms depends on integrin α(6)β(4) expression status. Integrin α(6)β(4)-negative MDA-MB-435 cells utilize only RhoC for motility, whereas integrin α(6)β(4)-expressing cells utilize RhoC but additionally activate and utilize RhoA for LPA-dependent cell motility and lamellae formation. Notably, the activation of RhoA by cooperative LPA and integrin α(6)β(4) signaling requires the Rho guanine nucleotide exchange factor AKAP-Lbc. We also determine that integrin α(6)β(4) cannot activate Rac1 directly but promotes LPA-mediated Rac1 activation that is dependent on RhoA activity and de novo β(1) integrin ligation. Finally, we find that the regulation of Rac1 and RhoA in response to LPA is differentially regulated by phosphodiesterases, PKA, and phosphatidylinositol 3-kinase, thus supporting their spatially distinct compartmentalization. In summary, signaling from integrin α(6)β(4) facilitates LPA-stimulated chemotaxis through preferential activation of RhoA, which, in turn, facilitates activation of Rac1. 
 Dynamin 2 potentiates invasive   migration   of pancreatic tumor cells through stabilization of the Rac1 GEF Vav1. The large GTPase Dynamin 2 (Dyn2) is markedly upregulated in pancreatic cancer, is a potent activator of metastatic   migration  , and is required for Rac1-mediated formation of lamellipodia. Here we demonstrate an unexpected mechanism of Dyn2 action in these contexts via direct binding to the Rac1 guanine nucleotide exchange factor (GEF) Vav1. Surprisingly, disruption of the Dyn2-Vav1 interaction targets Vav1 to the lysosome for degradation via an interaction with the cytoplasmic chaperone Hsc70, resulting in a dramatic reduction of Vav1 protein stability. Importantly, a specific mutation in Vav1 near its Dyn2-binding C-terminal Src homology 3 (SH3) domain prevents Hsc70 binding, resulting in a stabilization of Vav1 levels. Dyn2 binding regulates the interaction of Vav1 with Hsc70 to control the stability and subsequent activity of this oncogenic GEF. These findings elucidate how Dyn2 activates Rac1, lamellipod protrusion, and invasive cellular   migration   and provide insight into how this specific Vav is ectopically expressed in pancreatic tumors. 
 Distinct predictive performance of Rac1 and Cdc42 in cell   migration  . We propose a new computation-based approach for elucidating how signaling molecules are decoded in cell   migration  . In this approach, we performed FRET time-lapse imaging of Rac1 and Cdc42, members of Rho GTPases which are responsible for cell motility, and quantitatively identified the response functions that describe the conversion from the molecular activities to the morphological changes. Based on the identified response functions, we clarified the profiles of how the morphology spatiotemporally changes in response to local and transient activation of Rac1 and Cdc42, and found that Rac1 and Cdc42 activation triggers laterally propagating membrane protrusion. The response functions were also endowed with property of differentiator, which is beneficial for maintaining sensitivity under adaptation to the mean level of input. Using the response function, we could predict the morphological change from molecular activity, and its predictive performance provides a new quantitative measure of how much the Rho GTPases participate in the cell   migration  . Interestingly, we discovered distinct predictive performance of Rac1 and Cdc42 depending on the   migration   modes, indicating that Rac1 and Cdc42 contribute to persistent and random   migration  , respectively. Thus, our proposed predictive approach enabled us to uncover the hidden information processing rules of Rho GTPases in the cell   migration  . 
 Semaphorin 5A and plexin-B3 regulate human glioma cell motility and morphology through Rac1 and the actin cytoskeleton. Semaphorins are implicated in glioma progression, although little is known about the underlying mechanisms. We have reported plexin-B3 expression in human gliomas, which upon stimulation by Sema5A causes significant inhibition of cell   migration   and invasion. The concomitant inactivation of Rac1 is of mechanistic importance because forced expression of constitutively active Rac1 abolishes these inhibitory effects. Furthermore, Sema5A induces prominent cell collapse and ramification of processes reminiscent of astrocytic morphology, which temporally associate with extensive disassembly of actin stress fibers and disruption of focal adhesions, followed by accumulation of actin patches in protrusions. Mechanistically, Sema5A induces transient protein kinase C (PKC) phosphorylation of fascin-1, which can reduce its actin-binding/bundling activities and temporally parallels its translocation from cell body to extending processes. PKC inhibition or fascin-1 knockdown is sufficient to abrogate Sema5A-induced morphological differentiation, whereas the process is hastened by forced expression of fascin-1. Intriguingly, Sema5A induces re-expression of glial fibrillary acidic protein (GFAP), which when silenced restricts differentiation of glioma cells to bipolar instead of multipolar morphology. Therefore, we hypothesize complementary functions of fascin-1 and GFAP in the early and late phases of Sema5A-induced astrocytic differentiation of gliomas, respectively. In summary, Sema5A and plexin-B3 impede motility but promote differentiation of human gliomas. These effects are plausibly compromised in high-grade human astrocytomas in which Sema5A expression is markedly reduced, hence leading to infiltrative and anaplastic characteristics. This is evident by increased invasiveness of glioma cells when endogenous Sema5A is silenced. Therefore, Sema5A and plexin-B3 represent potential novel targets in counteracting glioma progression. 
 S1P(2) receptors mediate inhibition of glioma cell   migration   through Rho signaling pathways independent of PTEN. Sphingosine 1-phosphate (S1P) induced the inhibition of glioma cell   migration  . Here, we characterized the signaling mechanisms involved in the inhibitory action by S1P. In human GNS-3314 glioblastoma cells, the S1P-induced inhibition of cell   migration   was associated with activation of RhoA and suppression of Rac1. The inhibitory action of S1P was recovered by a small interference RNA specific to S1P(2) receptor, a carboxyl-terminal region of Galpha12 or Galpha13, an RGS domain of p115RhoGEF, and a dominant-negative mutant of RhoA. The inhibitory action of S1P through S1P(2) receptors was also observed in both U87MG glioblastoma and 1321N1 astrocytoma cells, which have no protein expression of a phosphatase and tensin homolog deleted on chromosome 10 (PTEN). These results suggest that S1P(2) receptors/G(12/13)-proteins/Rho signaling pathways mediate S1P-induced inhibition of glioma cell   migration  . However, PTEN, recently postulated as an indispensable molecule for the inhibition of cell   migration  , may not be critical for the S1P(2) receptor-mediated action in glioma cells. 
 Rab5 isoforms orchestrate a "division of labor" in the endocytic network; Rab5C modulates Rac-mediated cell motility. Rab5, the prototypical Rab GTPase and master regulator of the endocytic pathway, is encoded as three differentially expressed isoforms, Rab5A, Rab5B and Rab5C. Here, we examined the differential effects of Rab5 isoform silencing on cell motility and report that Rab5C, but neither Rab5A nor Rab5B, is selectively associated with the growth factor-activation of Rac1 and with enhanced cell motility. Initial observations revealed that silencing of Rab5C expression, but neither Rab5A nor Rab5C, led to spindle-shaped cells that displayed reduced formation of membrane ruffles. When subjected to a scratch wound assay, cells depleted of Rab5C, but not Rab5A or Rab5B, demonstrated reduced cell   migration  . U937 cells depleted of Rab5C also displayed reduced cell motility in a Transwell plate   migration   assay. To examine activation of Rac, HeLa cells stably expressing GFP-Rac1 were independently depleted of Rab5A, Rab5B or Rab5C and seeded onto coverslips imprinted with a crossbow pattern. 3-D GFP-Rac1 images of micro-patterned cells show that GFP-Rac1 was less localized to the cell periphery in the absence of Rab5C. To confirm the connection between Rab5C and Rac activation, HeLa cells depleted of Rab5 isoforms were starved and then stimulated with EGF. Rac1 pull-down assays revealed that EGF-stimulated Rac1 activity was significantly suppressed in Rab5C-suppressed cells. To determine whether events upstream of Rac activation were affected by Rab5C, we observed that EGF-stimulated Akt phosphorylation was suppressed in cells depleted of Rab5C. Finally, since spatio-temporal assembly/disassembly of adhesion complexes are essential components of cell   migration  , we examined the effect of Rab5 isoform depletion on the formation of focal adhesion complexes. Rab5C-depleted HeLa cells have significantly fewer focal adhesion foci, in accordance with the lack of persistent lamellipodial protrusions and reduced directional   migration  . We conclude that Rab5 isoforms selectively oversee the multiple signaling and trafficking events associated with the endocytic network. 
 Tiam1 interaction with the PAR complex promotes talin-mediated Rac1 activation during polarized cell   migration  . Migrating cells acquire front-rear polarity with a leading edge and a trailing tail for directional movement. The Rac exchange factor Tiam1 participates in polarized cell   migration   with the PAR complex of PAR3, PAR6, and atypical protein kinase C. However, it remains largely unknown how Tiam1 is regulated and contributes to the establishment of polarity in migrating cells. We show here that Tiam1 interacts directly with talin, which binds and activates integrins to mediate their signaling. Tiam1 accumulated at adhesions in a manner dependent on talin and the PAR complex. The interactions of talin with Tiam1 and the PAR complex were required for adhesion-induced Rac1 activation, cell spreading, and   migration   toward integrin substrates. Furthermore, Tiam1 acted with talin to regulate adhesion turnover. Thus, we propose that Tiam1, with the PAR complex, binds to integrins through talin and, together with the PAR complex, thereby regulates Rac1 activity and adhesion turnover for polarized   migration  . 
 Maspin controls mammary tumor cell   migration   through inhibiting Rac1 and Cdc42, but not the RhoA GTPase. Rac1 and Cdc42 are members of the Rho family of small GTPases that play essential roles in diverse cellular functions, including cell   migration  . The activities of these Rho family proteins are controlled by growth factor receptor activation and cell-ECM interactions. Here, we show that maspin, a well-documented tumor suppressor gene, also controls cell motility through inhibiting Rac1/Cdc42 activity. Using the GST-PAK and GST-Rho binding protein pull-down assays for GTP-bound Rac1, Cdc42, and RhoA, we showed that treatment of MDA-MB-231 tumor cells with recombinant maspin for a short time period significantly inhibited the activity of Rac1 and Cdc42, but not RhoA. The reactive site loop (RSL) within maspin protein is the functional domain involved in the inhibition. Maspin mutants with the RSL deleted or a point mutation in the RSL region lost their inhibitory activity. We further examined the ability of maspin to inhibit Rac1- and Cdc42-mediated signaling pathways and transcription factors. Treatment of MDA-MB-231 cells with maspin led to the inhibition of JNK kinase activity as assayed by immuno-kinase assays. In addition, the AP-1 transcription activity downstream of JNK kinase pathway was also reduced. Together, we have identified Rac1 and Cdc42 as the downstream targets that mediate the inhibition of mammary tumor cell   migration   by maspin. 
 Positive regulation of   migration   and invasion by vasodilator-stimulated phosphoprotein via Rac1 pathway in human breast cancer cells. This study aimed to investigate the role of the cytoskeleton-associated protein vasodilator-stimulated phosphoprotein (VASP) on the   migration   and invasion of human breast cancer cells and its relationship to Rac1 which is a member of the Rho family and has been found to be implicated in tumorigenesis, invasion and metastasis. We detected the mRNA and protein expression levels of VASP and Rac1 of the non-invasive breast cancer cell line MCF-7 as well as the invasive cell line MDA-MB-231 by RT-PCR and Western blotting. GST pull-down assay was used to examine the activity of Rac1. Accordingly, the cell invasive   migration   ability was analyzed in a wound-healing assay (2D) and transwell assays (3D   migration   and invasion). We then used VASP-siRNA to inhibit the expression of VASP in breast cancer cells in order to study the relationship between the VASP expression level and the invasive   migration   ability of breast cancer cells. Rac1-siRNA was used to decrease the expression of Rac1, and observe its effect on the VASP expression level together with the   migration   and invasion ability of MCF-7 and MDA-MB-231 cells. Our results revealed that the invasive breast cancer cell line MDA-MB-231 showed a higher Rac1 activity and VASP expression level compared with the non-invasive MCF-7. Inhibition of Rac1 or VASP by siRNA, respectively, decreased the   migration   and invasion ability of breast cancer cells and the transfection of Rac1 siRNA-mediated reduction of VASP expression at mRNA and protein levels. Collectively, our data showed that the higher expression level of VASP contributed to a higher invasive   migration   capacity of human breast cancer cells which was controlled by the Rac1 pathway. 
 Changes of cytoskeleton and cell cycle in Lovo cells via deletion of Rac1. Rac1, the better characterized Rac subfamily member, can regulate a large variety of different functions, including the organization of the actin cytoskeleton, cell   migration  , cell cycle progression, and cell survival through engagement of specific effectors. However, very little is currently known about the expression of Rac1 in colorectal cancer cells and the roles of Rac1 in the cell cycle progression and cell survival of human colorectal cancer cells.To assess the change of cytoskeleton and cell cycle in Lovo (human colorectal cancer) cell via deletion of Rac1 with RNA interference.Rac1 protein of all selected human colorectal cancer cells and in human colorectal tissue was detected by Western blotting, Rac1-shRNA was used to silence the Rac1 to reduce its expression specifically in Lovo cells.Rac1 protein was overexpressed in human colorectal cancer cells and in human colorectal tissue, RNA interference-mediated deletion of Rac1 strongly prolonged cell cycle progression and enhanced cell apoptosis of Lovo cells in vitro.depletion of Rac1 by the use of RNAi can arrest Lovo Cells in G<formula>_{0}</formula>/G<formula>_{1}</formula> and induce LoVo cells apoptosis. 
 Rab23 is overexpressed in human astrocytoma and promotes cell   migration   and invasion through regulation of Rac1. Rab23 overexpression has been implicated in several human cancers. However, its biological roles and molecular mechanism in astrocytoma have not been elucidated. The aim of this study is to explore clinical significance and biological roles of Rab23 in astrocytoma. We observed negative Rab23 staining in normal astrocytes and positive staining in 39 out of 86 (45 %) astrocytoma specimens using immunohistochemistry. The positive rate of Rab23 was higher in grades III and IV (56.5 %, 26/46) than grades I + II astrocytomas (32.5 %, 13/40, p < 0.05). Transfection of Rab23 plasmid was performed induced A172 cell proliferation, colony formation, invasio  n, and mi  gration, while Rab23 depletion with siRNA reduced these abilities of U87 cells. In addition, we found that Rab23 transfection upregulated while its depletion reduced Rac1 activity. Treatment of transfected cells with a Rac1 inhibitor decreased Rac1 activity and invasion. In conclusion, Rab23 serves as an important oncoprotein in human astrocytoma by regulating cell invasi  on and mi  gration through Rac1 activity. 
 Rac1+ cells distributed in accordance with CD 133+ cells in glioblastomas and the elevated invasiveness of CD 133+ glioma cells with higher Rac1 activity. Recent studies have suggested that cancer stem cells are one of the major causes for tumor recurrence due to their resistance to radiotherapy and chemotherapy. Although the highly invasive nature of glioblastoma (GBM) cells is also implicated in the failure of current therapies, it is not clear how glioma stem cells (GSCs) are involved in invasiveness. Rac1 activity is necessary for inducing reorganization of actin cytoskeleton and cell movement. In this study, we aimed to investigate the distribution characteristics of CD133+ cells and Rac1+ cells in GBM as well as Rac1 activity in CD133+ GBM cells, and analyze the   migration   and invasion potential of these cells.A series of 21 patients with GBM were admitted consecutively and received tumor resection in Tianjin Medical University General Hospital during the first half of the year 2011. Tissue specimens were collected both from the peripheral and the central parts for each tumor under magnetic resonance imaging (MRI) navigation guidance. Immunohistochemical staining was used to detect the CD133+ cells and Rac1+ cells distribution in GBM specimens. Double-labeling immunofluorescence was further used to analyze CD133 and Rac1 co-expression and the relationship between CD133+ cells distribution and Rac1 expression. Serum-free medium culture and magnetic sorting were used to isolate CD133+ cells from U87 cell line. Rac1 activation assay was conducted to assess the activation of Rac1 in CD133+ and CD133 - U87 cells. The   migration   and invasive ability of CD133+ and CD133 - U87 cells were determined by cell   migration   and invasion assays in vitro. Student's t-test and one-way analysis of variance (ANOVA) test were used to determine statistical significance in this study.In the central parts of GBMs, CD133+ cells were found to cluster around necrosis and occasionally cluster around the vessels under the microscope by immunohistological staining. In the peripheral parts of the tumors, CD133+ cells were lined up along the basement membrane of the vessels and myelinated nerve fibers. Rac1 expression was high and diffused in the central parts of the GBMs, and the Rac1+ cells were distributed basically in accordance with CD133+ cells both in the central and peripheral parts of GBMs. In double-labeling immunofluorescence, Rac1 was expressed in (83.14 ± 4.23)% of CD133+ cells, and CD133 and Rac1 co-expressed cells were located around the vessels in GBMs. Significantly higher amounts of Rac1-GTP were expressed in the CD133+ cells (0.378 ± 0.007), compared to CD133- cells (0.195 ± 0.004) (t = 27.81; P < 0.05). CD133+ cells had stronger ability to migrate (74.34 ± 2.40 vs. 38.72 ± 2.60, t = 42.71, P < 0.005) and invade (52.00 ± 2.28 vs. 31.26 ± 1.82, t = 30.76, P < 0.005), compared to their counterpart CD133- cells in transwel  l cell mi  gration/invasion assay.These data suggest that CD133+ GBM cells highly express Rac1 and have greater potential to migrate and invade through activated Rac1-GTP. The accordance of distribution between Rac1+ cells and CD133+ cells in GBMs implies that Rac1 might be an inhibited target to prevent invasi  on and mi  gration and to avoid malignant glioma recurrence. 
 Receptor tyrosine kinase c-Met controls the cytoskeleton from different endosomes via different pathways. Receptor tyrosine kinases (RTKs) are increasingly recognized as having the capacity to signal post-internalization. Signalling outputs and/or duration, and subsequent cellular outcome, are thought to be distinct when emanating from endosomes compared with those from the plasma membrane. Here we show, in invasive, basal-like human breast cell models, that different mechanisms are engaged by the RTK c-Met in two different endosomes to control the actin cytoskeleton via the key migratory signal output Rac1. Despite an acute activation of Rac1 from peripheral endosomes (PEs), c-Met needs to traffic to a perinuclear endosome (PNE) to sustain Rac1 signalling, trigger optimal membrane ruffling, cell   migration   and invasion. Unexpectedly, in the PNE but not in the PE, PI3K and the Rac-GEF Vav2 are required. Thus we describe a novel endosomal signalling mechanism whereby one signal output, Rac1, is stimulated through distinct pathways by the same RTK depending on which endosome it is localized to in the cell. 
 Unconventional Rac-GEF activity is mediated through the Dock180-ELMO complex. Mammalian Dock180 and ELMO proteins, and their homologues in Caenorhabditis elegans and Drosophila melanogaster, function as critical upstream regulators of Rac during development and cell   migration  . The mechanism by which Dock180 or ELMO mediates Rac activation is not understood. Here, we identify a domain within Dock180 (denoted Docker) that specifically recognizes nucleotide-free Rac and can mediate GTP loading of Rac in vitro. The Docker domain is conserved among known Dock180 family members in metazoans and in a yeast protein. In cells, binding of Dock180 to Rac alone is insufficient for GTP loading, and a Dock180 ELMO1 interaction is required. We can also detect a trimeric ELMO1 Dock180 Rac1 complex and ELMO augments the interaction between Dock180 and Rac. We propose that the Dock180 ELMO complex functions as an unconventional two-part exchange factor for Rac. 
 The N-terminal DH-PH domain of Trio induces cell spreading and   migration   by regulating lamellipodia dynamics in a Rac1-dependent fashion. The guanine-nucleotide exchange factor Trio encodes two DH-PH domains that catalyze nucleotide exchange on Rac1, RhoG and RhoA. The N-terminal DH-PH domain is known to activate Rac1 and RhoG, whereas the C-terminal DH-PH domain can activate RhoA. The current study shows that the N-terminal DH-PH domain, upon expression in HeLa cells, activates Rac1 and RhoG independently from each other. In addition, we show that the flanking SH3 domain binds to the proline-rich region of the C-terminus of Rac1, but not of RhoG. However, this SH3 domain is not required for Rac1 or RhoG GDP-GTP exchange. Rescue experiments in Trio-shRNA-expressing cells showed that the N-terminal DH-PH domain of Trio, but not the C-terminal DH-PH domain, restored fibronectin-mediated cell spreading and   migration   defects that are observed in Trio-silenced cells. Kymograph analysis revealed that the N-terminal DH-PH domain, independent of its SH3 domain, controls the dynamics of lamellipodia. Using siRNA against Rac1 or RhoG, we found that Trio-D1-induced lamellipodia formation required Rac1 but not RhoG expression. Together, we conclude that the GEF Trio is responsible for lamellipodia formation through its N-terminal DH-PH domain in a Rac1-dependent manner during fibronectin-mediated spreading and   migration  . 
 Rac1 links leading edge and uropod events through Rho and myosin activation during chemotaxis. Chemotactic responsiveness is crucial to neutrophil recruitment to sites of infection. During chemotaxis, highly divergent cytoskeletal programs are executed at the leading and trailing edge of motile neutrophils. The Rho family of small GTPases plays a critical role in cell   migration  , and recent work has focused on elucidating the specific roles played by Rac1, Rac2, Cdc42, and Rho during cellular chemotaxis. Rac GTPases regulate actin polymerization and extension of the leading edge, whereas Rho GTPases control myosin-based contraction of the trailing edge. Rac and Rho signaling are thought to crosstalk with one another, and previous research has focused on mutual inhibition of Rac and Rho signaling during chemotaxis. Indeed, polarization of neutrophils has been proposed to involve the activity of a negative feedback system where Rac activation at the front of the cell inhibits local Rho activation, and vice versa. Using primary human neutrophils and neutrophils derived from a Rac1/Rac2-null transgenic mouse model, we demonstrate here that Rac1 (and not Rac2) is essential for Rho and myosin activation at the trailing edge to regulate uropod function. We conclude that Rac plays both positive and negative roles in the organization of the Rhomyosin "backness" program, thereby promoting stable polarity in chemotaxing neutrophils. 
 Nucleophosmin1 is a negative regulator of the small GTPase Rac1. The Rac1 GTPase is a critical regulator of cytoskeletal dynamics and controls many biological processes, such as cell   migration  , cell-cell contacts, cellular growth and cell division. These complex processes are controlled by Rac1 signaling through effector proteins. We have previously identified several effector proteins of Rac1 that also act as Rac1 regulatory proteins, including caveolin-1 and PACSIN2. Here, we report that Rac1 interacts through its C-terminus with nucleophosmin1 (NPM1), a multifunctional nucleo-cytoplasmic shuttling protein with oncogenic properties. We show that Rac1 controls NPM1 subcellular localization. In cells expressing active Rac1, NPM1 translocates from the nucleus to the cytoplasm. In addition, Rac1 regulates the localization of the phosphorylated pool of NPM1 as this pool translocated from the nucleus to the cytosol in cells expressing activated Rac1. Conversely, we found that expression of NPM1 limits Rac1 GTP loading and cell spreading. In conclusion, this study identifies NPM1 as a novel, negative regulator of Rac1. 
 A neuronal transmembrane protein LRFN4 complexes with 14-3-3s and NCK1 to induce morphological change in monocytic cells via Rac1-mediated actin cytoskeleton reorganization. We previously reported that leucine-rich repeat and fibronectin type III domain-containing 4 (LRFN4) functioned in   migration   and morphological change (i.e. cell elongation) of monocytic cells. Here, we examined a molecular mechanism regulating LRFN4-mediated cell elongation. We found that 14-3-3 and NCK proteins complexed with LRFN4, and they were involved in LRFN4-mediated cell elongation. We also identified the regions of LRFN4 interacting with NCK1 and 14-3-3s. Finally, we demonstrated that a Rac1 small GTPase was involved in LRFN4-mediated cell elongation. These results indicated that LRFN4 complexed with 14-3-3s and NCK1 to mediate elongation in monocytic cells via Rac-1-mediated actin cytoskeleton reorganization. 
 Viability reduction and Rac1 gene downregulation of heterogeneous ex-vivo glioma acute slice infected by the oncolytic Newcastle disease virus strain V4UPM. Oncolytic viruses have been extensively evaluated for anticancer therapy because this virus preferentially infects cancer cells without interfering with normal cells. Newcastle Disease Virus (NDV) is an avian virus and one of the intensively studied oncolytic viruses affecting many types of cancer including glioma. Nevertheless, the capability of NDV infection on heterogeneous glioma tissue in a cerebrospinal fluid atmosphere has never been reported. Recently, Rac1 is reported to be required for efficient NDV replication in human cancer cells and established a link between tumourigenesis and sensitivity to NDV. Rac1 is a member of the Rho GTPases involved in the regulation of the cell   migration   and cell-cycle progression. Rac1 knockdown leads to significant inhibition of viral replication. In this work, we demonstrated that NDV treatment led to significant reduction of tumour tissue viability of freshly isolated heterogeneous human brain tumour slice, known as an ex vivo glioma acute slice (EGAS). Analysis of gene expression indicated that reduced tissue viability was associated with downregulation of Rac1. However, the viability reduction was not persistent. We conclude that NDV treatment induced EGAS viability suppression, but subsequent downregulation of Rac1 gene may reduce the NDV replication and lead to regrowth of EGAS tissue. 
 Interleukin-8 induces the endothelial cell   migration   through the activation of phosphoinositide 3-kinase-Rac1/RhoA pathway. Endothelial cell   migration   is essential for tumor angiogenesis, and interleukin-8 (IL-8) has been shown to play an important role in tumor growth, angiogenesis, and metastasis. This study aimed to investigate the molecular mechanism of IL-8 induced endothelial cell   migration  . Our results indicated that IL-8 induced a rapid rearrangement of the actin cytoskeleton in EA.Hy926 cells, generating extensions resembling membrane ruffling and stress fibers. These processes required parallel upregulation of the small GTPases Rac1 and RhoA. Moreover, we demonstrated that IL-8 activated PI3K following the same kinetics observed from IL-8 induction of cytoskeletal rearrangement, suggesting the participation of PI3K in these processes. Taken together, our study demonstrates that PI3K-Rac1/RhoA signaling pathway plays a vital role in IL-8 induced endothelial cell   migration  , and provides new insight into the molecular mechanisms by which IL-8 contributes to tumor angiogenesis and metastasis. 
 An anti-Wnt5a antibody suppresses metastasis of gastric cancer cells in vivo by inhibiting receptor-mediated endocytosis. Wnt5a is a representative ligand that activates the β-catenin-independent pathway in Wnt signaling. It was reported that the expression of Wnt5a in human gastric cancer is associated with aggressiveness and poor prognosis and that knockdown of Wnt5a reduces the ability of gastric cancer cells to metastasize in nude mice. Therefore, Wnt5a and its signaling pathway might be important targets for the therapy of gastric cancer. The aim of this study was to examine whether an anti-Wnt5a antibody affects metastasis of gastric cancer cells. One anti-Wnt5a polyclonal antibody (pAb5a-5) inhibited   migratio  n and invasion activities in vitro of gastric cancer cells with a high expression level of Wnt5a. Previously, it was shown that Wnt5a induces the internalization of receptors, which is required for Wnt5a-dependent activation of Rac1. pAb5a-5 inhibited Wnt5a-dependent internalization of receptors, thereby suppressed Wnt5a-dependent activation of Rac1. Laminin γ2 is one of target genes of Wnt5a signaling and Rac1 was involved in its expression. pAb5a-5 also inhibited Wnt5a-dependent expression of laminin γ2. In an experimental liver metastasis assay, gastric cancer cells were introduced into the spleens of nude mice. Laminin γ2 was required for liver metastatic ability of gastric cancer cells in vivo. Furthermore, intraperitoneal injection of pAb5a-5 inhibited the metastatic ability of gastric cancer cells. These results suggest that an anti-Wnt5a antibody was capable of suppressing Wnt5a-dependent internalization of receptors, resulting in the prevention of metastasis of gastric cancer cells by inhibiting the activation of Rac1 and the expression of laminin γ2. 
 MicroRNA-142-3p, a new regulator of RAC1, suppresses the   migration   and invasion of hepatocellular carcinoma cells. RAC1 regulates a diverse array of cellular events including   migration   and invasion. MicroRNAs (miRNAs) have a key role in the regulation of gene expression. In this study, we demonstrated that microRNA-142-3p (miR-142-3p) acted as a negative regulator of human RAC1. Overexpression of miR-142-3p decreased RAC1 mRNA and protein levels. Moreover, the overexpression of miR-142-3p suppressed, while blocking of miR-142-3p increased colony formation,   migration   and invasion in hepatocellular carcinoma (HCC) cell lines (QGY-7703 and SMMC-7721). RAC1 overexpression without the 3'untranslated region abolished the effect of miR-142-3p in the QGY-7703 and SMMC-7721 cells. These results demonstrated that miR-142-3p directly and negatively regulates RAC1 in HCC cells, which highlights the importance of miRNAs in tumorigenesis. 
 Prostaglandin E2-mediated   migration   of human trophoblast requires RAC1 and CDC42. The invasion of maternal decidua and uterine spiral arteries by a trophoblast subpopulation called extravillous trophoblast (EVT) is essential for the establishment of a normal placenta and an adequate blood flow toward the fetus. Derangements in these processes underlie pregnancy-related diseases like preeclampsia and intrauterine growth restriction. Many growth factors, growth factor binding proteins, and extracellular matrix components can positively or negatively regulate the proliferation,   migration  , and/or invasiveness of these EVT cells. RHO GTPases, including RHOA, RAC1, and CDC42, are ubiquitous proteins that control cytoskeletal changes by forming stress fibers and projecting lamellipodia and filopodia during cellular   migration  . We had previously shown that prostaglandin (PG) E(2) produced in abundance by the decidua promotes the   migration   of first-trimester human EVTs by increasing the intracellular concentration of calcium and activating calpain. Using our well-characterized immortalized EVT cell line, HTR-8/SVneo, as well as villus explants from first-trimester placentae, this study examined the role of RHO GTPases RAC1 and CDC42 in PGE(2)-mediated migratory responses of these cells. Though a RAC1 inhibitor, NSC23766 as well as RAC1 knockdown by siRNA decreased the   migration   of HTR-8/SVneo cells in a Transwell   migration   assay, this inhibition could not be restored by PGE(2) or 17-phenyl trinor PGE(2) (PGE receptor PTGER1 agonist) or PGE(1) Alcohol (PGE receptor PTGER4 agonist). Similar results were noted for EVT cell spreading in villus explants. Furthermore, CDC42 silencing using siRNA inhibited PGE(2)-induced   migration   of HTR-8/SVneo cells. Finally, the treatment of EVT cells with PGE(2), PTGER1 agonist, or PTGER4 agonist activated RAC1 and CDC42 at 10 min, suggesting that RAC1 and CDC42 play an essential role in PGE(2)-mediated   migration   of human EVTs. 
 Estrogen and resveratrol regulate Rac and Cdc42 signaling to the actin cytoskeleton of metastatic breast cancer cells. Estrogen and structurally related molecules play critical roles in breast cancer. We reported that resveratrol (50 microM), an estrogen-like phytosterol from grapes, acts in an antiestrogenic manner in breast cancer cells to reduce cell   migration   and to induce a global and sustained extension of actin structures called filopodia. Herein, we report that resveratrol-induced filopodia formation is time-dependent and concentration-dependent. In contrast to resveratrol at 50 microM, resveratrol at 5 microM acts in a manner similar to estrogen by increasing lamellipodia, as well as cell   migration   and invasion. Because Rho GTPases regulate the extension of actin structures, we investigated a role for Rac and Cdc42 in estrogen and resveratrol signaling. Our results demonstrate that 50 microM resveratrol decreases Rac and Cdc42 activity, whereas estrogen and 5 microM resveratrol increase Rac activity in breast cancer cells. MDA-MB-231 cells expressing dominant-negative Cdc42 or dominant-negative Rac retain filopodia response to 50 microM resveratrol. Lamellipodia response to 5 microM resveratrol, estrogen, or epidermal growth factor is inhibited in cells expressing dominant-negative Rac, indicating that Rac regulates estrogen and resveratrol (5 microM) signaling to the actin cytoskeleton. These results indicate that signaling to the actin cytoskeleton by low and high concentrations of resveratrol may be differentially regulated by Rac and Cdc42. 
 Rac1 targeting suppresses human non-small cell lung adenocarcinoma cancer stem cell activity. The cancer stem cell (CSC) theory predicts that a small fraction of cancer cells possess unique self-renewal activity and mediate tumor initiation and propagation. However, the molecular mechanisms involved in CSC regulation remains unclear, impinging on effective targeting of CSCs in cancer therapy. Here we have investigated the hypothesis that Rac1, a Rho GTPase implicated in cancer cell proliferation and invasion, is critical for tumor initiation and metastasis of human non-small cell lung adenocarcinoma (NSCLA). Rac1 knockdown by shRNA suppressed the tumorigenic activities of human NSCLA cell lines and primary patient NSCLA specimens, including effects on invasion, proliferation, anchorage-independent growth, sphere formation and lung colonization. Isolated side population (SP) cells representing putative CSCs from human NSCLA cells contained elevated levels of Rac1-GTP, enhanced in vitro   migration  , invasion, increased in vivo tumor initiating and lung colonizing activities in xenografted mice. However, CSC activity was also detected within the non-SP population, suggesting the importance of therapeutic targeting of all cells within a tumor. Further, pharmacological or shRNA targeting of Rac1 inhibited the tumorigenic activities of both SP and non-SP NSCLA cells. These studies indicate that Rac1 represents a useful target in NSCLA, and its blockade may have therapeutic value in suppressing CSC proliferation and metastasis. 
 Asef2 functions as a Cdc42 exchange factor and is stimulated by the release of an autoinhibitory module from a concealed C-terminal activation element. Asef (herein called Asef1) was identified as a Rac1-specific exchange factor stimulated by adenomatous polyposis coli (APC), contributing to colorectal cancer cell metastasis. We investigated Asef2, an Asef1 homologue having a similar N-terminal APC binding region (ABR) and Src-homology 3 (SH3) domain. Contrary to previous reports, we found that Asef1 and Asef2 exchange activity is Cdc42 specific. Moreover, the ABR of Asef2 did not function independently but acted in tandem with the SH3 domain to bind APC. The ABRSH3 also bound the C-terminal tail of Asef2, allowing it to function as an autoinhibitory module within the protein. Deletion of the C-terminal tail did not constitutively activate Asef2 as predicted; rather, a conserved C-terminal segment was required for augmented Cdc42 GDP/GTP exchange. Thus, Asef2 activation involves APC releasing the ABRSH3 from the C-terminal tail, resulting in Cdc42 exchange. These results highlight a novel exchange factor regulatory mechanism and establish Asef1 and Asef2 as Cdc42 exchange factors, providing a more appropriate context for understanding the contribution of APC in establishing cell polarity and   migration  . 
 TLR2 transmodulates monocyte adhesion and trans  migration   via Rac1- and PI3K-mediated inside-out signaling in response to Porphyromonas gingivalis fimbriae. We present evidence for a novel TLR2 function in transmodulating the adhesive activities of human monocytes in response to the fimbriae of Porphyromonas gingivalis, a pathogen implicated in chronic periodontitis and atherosclerosis. Monocyte recruitment into the subendothelium is a crucial step in atherosclerosis, and we investigated the role of P. gingivalis fimbriae in stimulating monocyte adhesion to endothelial cells and transendothelial   migration  . Fimbriae induced CD11b/CD18-dependent adhesion of human monocytes or mouse macrophages to endothelial receptor ICAM-1; these activities were inhibited by TLR2 blockade or deficiency or by pharmacological inhibitors of PI3K. Moreover, this inducible adhesive activity was sensitive to the action of Clostridium difficile toxin B, but was not affected by Clostridium botulinum C3 exoenzyme, pertussis toxin, or cholera toxin. Accordingly, we subsequently showed through the use of dominant negative signaling mutants of small GTPases, that Rac1 mediates the ability of fimbria-stimulated monocytes to bind ICAM-1. A dominant negative mutant of Rac1 also inhibited the lipid kinase activity of PI3K suggesting that Rac1 acts upstream of PI3K in this proadhesive pathway. Furthermore, fimbriae stimulated monocyte adhesion to HUVEC and trans  migration   across HUVEC monolayers; both activities required TLR2 and Rac1 signaling and were dependent upon ICAM-1 and the high-affinity state of CD11b/CD18. P. gingivalis-stimulated monocytes displayed enhanced transendothelial   migration   compared with monocytes stimulated with nonfimbriated isogenic mutants. Thus, P. gingivalis fimbriae activate a novel proadhesive pathway in human monocytes, involving TLR2, Rac1, PI3K, and CD11b/CD18, which may constitute a mechanistic basis linking P. gingivalis to inflammatory atherosclerotic processes. 
 srGAP1 regulates lamellipodial dynamics and cell migratory behavior by modulating Rac1 activity. The distinct levels of Rac activity differentially regulate the pattern of intrinsic cell   migration  . However, it remains unknown how Rac activity is modulated and how the level of Rac activity controls cell migratory behavior. Here we show that Slit-Robo GAP 1 (srGAP1) is a modulator of Rac activity in locomotive cells. srGAP1 possesses a GAP activity specific to Rac1 and is recruited to lamellipodia in a Rac1-dependent manner. srGAP1 limits Rac1 activity and allows concomitant activation of Rac1 and RhoA, which are mutually inhibitory. When both GTPases are activated, the protrusive structures caused by Rac1-dependent actin reorganization are spatially restricted and periodically destabilized, causing ruffling by RhoA-induced actomyosin contractility. Depletion of srGAP1 overactivates Rac1 and inactivates RhoA, resulting in continuous spatiotemporal spreading of lamellipodia and a modal shift of intrinsic cell motility from random to directionally persistent. Thus srGAP1 is a key determinant of lamellipodial dynamics and cell migratory behavior. 
 Balanced Tiam1-rac1 and RhoA drives proliferation and invasion of pancreatic cancer cells. Tiam1 is a rac1-specific guanine nucleotide exchange factor, and Tiam1-rac1 is involved in a number of cellular processes. Rac1 and RhoA act as molecular switches that cycle between GTP- and GDP-bound states to balance the activities of rac1 and RhoA. The downregulation of rac1 activity leads to upregulation of RhoA activity, which promotes invasion and   migration   of pancreatic cancers cells. At present, however, the role of Tiam1-rac1 and RhoA in pancreatic cancers is not fully understood. We found that Tiam1 was upregulated in pancreatic cancers and was significantly expressed in tumors without lymph node involvement or distant metastasis compared with cancers where there was involvement. Although Tiam1-rac1 signaling promoted pancreatic cancer cell proliferation and tumor growth via the Wnt signaling pathway in vitro and in vivo, inhibiting Tiam1-rac1 signaling did not prolong the overall survival time in vivo. This provided evidence that there was a balance between rac1 and RhoA activities in pancreatic cancers. Furthermore, only the combined inhibition of Tiam1-rac1 and RhoA had a beneficial effect on the growth of pancreatic cancers in vivo. Taken together, these results suggest that the progression of pancreatic tumors is partially controlled by the balance between Tiam1-rac1 and RhoA. 
 NEDD9 regulates 3D migratory activity independent of the Rac1 morphology switch in glioma and neuroblastoma. Metastasizing tumor cells must transmigrate the dense extracellular matrix that surrounds most organs. The use of three-dimensional (3D) collagen gels has revealed that many cancer cells can switch between different modes of invasion that are characterized by distinct morphologies (e.g., rounded vs. elongated). The adhesion protein NEDD9 has the potential to regulate the switch between elongated and rounded morphologies; therefore, its role was interrogated in the invasion switch of glioblastoma and neuroblastoma tumors that similarly derive from populations of neural crest cells. Interestingly, siRNA-mediated depletion of NEDD9 failed to induce cell rounding in glioma or neuroblastoma cells, contrasting the effects that have been described in other tumor model systems. Given that Rac1 GTPase has been suggested to mediate the switch between elongated and rounded invasion, the functionality of the Rac1 morphology switch was evaluated in the glioma and neuroblastoma cells. Using both dominant-negative Rac1 and Rac1-specific siRNA, the presence of this morphologic switch was confirmed in the neuroblastoma, but not in the glioma cells. However, in the absence of a morphologic change following NEDD9 depletion, a significant decrease in the cellular   migration   rate was observed. Thus, the data reveal that NEDD9 can regulate 3D   migration   speed independent of the Rac1 morphology switch.NEDD9 targeting is therapeutically viable as it does not stimulate adaptive changes in glioma and neuroblastoma invasion. 
 Rac1 and Rac3 have opposing functions in cell adhesion and differentiation of neuronal cells. Rac1 and Rac3 are highly homologous members of the Rho small GTPase family. Rac1 is ubiquitously expressed and regulates cell adhesion,   migration   and differentiation in various cell types. Rac3 is primarily expressed in brain and may therefore have a specific function in neuronal cells. We found that depletion of Rac1 by short interference RNA leads to decreased cell-matrix adhesions and cell rounding in neuronal N1E-115 cells. By contrast, depletion of Rac3 induces stronger cell adhesions and dramatically increases the outgrowth of neurite-like protrusions, suggesting opposite functions for Rac1 and Rac3 in neuronal cells. Consistent with this, overexpression of Rac1 induces cell spreading, whereas overexpression of Rac3 results in a contractile round morphology. Rac1 is mainly found at the plasma membrane, whereas Rac3 is predominantly localized in the perinuclear region. Residues 185-187, present in the variable polybasic rich region at the carboxyl terminus are responsible for the difference in phenotype induced by Rac1 and Rac3 as well as for their different intracellular localization. The Rac1-opposing function of Rac3 is not mediated by or dependent on components of the RhoA signaling pathway. It rather seems that Rac3 exerts its function through negatively affecting integrin-mediated cell-matrix adhesions. Together, our data reveal that Rac3 opposes Rac1 in the regulation of cell adhesion and differentiation of neuronal cells. 
 Decorin inhibits angiogenic potential of choroid-retinal endothelial cells by downregulating hypoxia-induced Met, Rac1, HIF-1α and VEGF expression in cocultured retinal pigment epithelial cells. Choroidal neovascularization (CNV) is one of the most common causes of severe vision loss. Decorin, a multiple receptor tyrosine kinase inhibitor, has been recently shown to play an important regulatory role in angiogenic response. This study aims to investigate whether the overexpression of decorin in retinal pigment epithelial (RPE) cells under hypoxia alters the in vitro angiogenic ability of cocultured choroid-retinal endothelial cells and to explore the possible mechanisms involved. Human RPE cells (ARPE-19) were subjected to hypoxia with or without decorin pretreatment, and RNA interference technique was used to knock down the Met gene in ARPE-19 cells. Cell viability was determined using the Cell Counting Kit-8 assay. Expression of Met, Rac1 and hypoxia-inducible factor-1 alpha (HIF-1α) was evaluated by western blot and quantitative real-time polymerase chain reaction (qRT-PCR). Vascular endothelial growth factor (VEGF) expression was evaluated by enzyme-linked immunosorbent assay (ELISA) and qRT-PCR. We then constructed a recombinant lentiviral vector carrying the decorin gene to transduce ARPE-19 cells. The overexpression of decorin in transduced RPE cells was confirmed by qRT-PCR and western blot. The transduced RPE cells were then cocultured with rhesus macaque choroid-retinal endothelial cells (RF/6A) in a transwell coculture system to observe the effects of decorin overexpression in ARPE-19 cells on the proliferatio  n, migrat  ion and tube formation of RF/6A cells. In response to hypoxia, the VEGF concentrations in the culture supernatants increased greatly at 24 and 48 h, and this effect was inhibited significantly and nearly equally in the presence of 50-200 nM decorin. Decorin pretreatment before hypoxia exposure effectively reduced the hypoxia-induced expression of Met, Rac1, HIF-1α and VEGF in ARPE-19 cells. Transfection of small interfering RNA against Met to ARPE-19 cells also resulted in significant downregulation of Rac1, HIF-1α and VEGF under hypoxia, and this effect was similar to that noted with decorin pretreatment alone or with their combination. Results from the coculture system showed that the overexpression of decorin in ARPE-19 cells significantly inhibited the prolifer  ation, mi  gration and tube formation of RF/6A cells. These results indicate that Met pathway activation plays an important role in the upregulation of VEGF in RPE cells under hypoxia. Decorin may interfere with angiogenesis by downregulating hypoxia-induced Met, Rac1, HIF-1α and VEGF expression in RPE cells, which suggests a potential strategy for the inhibition of CNV. 
 Type I PIPK-alpha regulates directed cell   migration   by modulating Rac1 plasma membrane targeting and activation. Phosphatidylinositol-4,5-bisphosphate (PI4,5P(2)) is a critical regulator of cell   migration  , but the roles of the type I phosphatidylinositol-4-phosphate 5-kinases (PIPKIs), which synthesize PI4,5P(2), have yet to be fully defined in this process. In this study, we report that one kinase, PIPKI-alpha, is a novel upstream regulator of Rac1 that links activated integrins to the regulation of cell   migration  . We show that PIPKI-alpha controls integrin-induced translocation of Rac1 to the plasma membrane and thereby regulates Rac1 activation. Strikingly, this function is not shared with other PIPKI isoforms, is independent of catalytic activity, and requires physical interaction of PIPKI-alpha with the Rac1 polybasic domain. Consistent with its role in Rac1 activation, depletion of PIPKI-alpha causes pronounced defects in membrane ruffling, actin organization, and focal adhesion formation, and ultimately affects the directional persistence of   migration  . Thus, our study defines the role of PIPKI-alpha in cell   migration   and describes a new mechanism for the spatial regulation of Rac1 activity that is critical for cell   migration  . 
 Activation of Rac1 by RhoG regulates cell   migration  . Cell   migration   is essential for normal development and many pathological processes. Rho-family small GTPases play important roles in this event. In particular, Rac regulates lamellipodia formation at the leading edge during   migration  . The small GTPase RhoG activates Rac through its effector ELMO and the ELMO-binding protein Dock180, which functions as a Rac-specific guanine nucleotide exchange factor. Here we investigated the role of RhoG in cell   migration  . RNA interference-mediated knockdown of RhoG in HeLa cells reduced cell   migration   in Transwell and scratch-wound   migration   assays. In RhoG-knockdown cells, activation of Rac1 and formation of lamellipodia at the leading edge in response to wounding were attenuated. By contrast, expression of active RhoG promoted cell   migration   through ELMO and Dock180. However, the interaction of Dock180 with Crk was dispensable for the activation of Rac1 and promotion of cell   migration   by RhoG. Taken together, these results suggest that RhoG contributes to the regulation of Rac activity in migrating cells. 
 Rac1 and Rho contribute to the migratory and invasive phenotype associated with somatic E-cadherin mutation. Recent evidence suggests a close association between extracellular E-cadherin mutation in diffuse-type gastric carcinoma and the acquisition of a migratory phenotype of tumour cells. To characterize the cellular machinery that mediates the gain of motility of tumour cells with mutant E-cadherin, we turned to the small Rho GTPases Rac1 and Rho because they have been implicated in pathological processes including tumour cell   migration   and invasion. In the present study, we analyse the activity of Rac1 and Rho in relation to E-cadherin harbouring an in-frame deletion of exon 8 and prove for the first time that the mutation reduces the ability of E-cadherin to activate Rac1 and to inhibit Rho. We provide evidence that the lack of Rac1 activation observed in response to mutant E-cadherin influences the downstream signalling of Rac1, as is shown by the decrease in the binding of the Rac1 effector protein IQGAP1 to Rac1-GTP. Moreover, reduced membranous localization of p120-catenin in mutant E-cadherin expressing cells provides an explanation for the lack of negative regulation of Rho by mutant E-cadherin. Further, we show by time-lapse laser scanning microscopy and invasion assay that the enhanced motility and invasion associated with mutant E-cadherin is sensitive to the inhibition of Rac1 and Rho. Together, these findings present evidence that the mutation of E-cadherin influences Rac1 and Rho activation in opposite directions and that Rac1 and Rho are involved in the establishment of the migratory and invasive phenotype of tumour cells that have an E-cadherin mutation. 
 Laminin-5-integrin interaction signals through PI 3-kinase and Rac1b to promote assembly of adherens junctions in HT-29 cells. Human intestinal cell differentiation is mediated by signaling pathways that remain largely undefined. We and others have shown that cell   migration   and differentiation along the crypt-villus axis is associated with temporal and spatial modulations of the repertoire, as well as with the function of integrins and E-cadherins and their substrates. Cross-talk between integrin and cadherin signaling was previously described and seems to coordinate this differentiation process. Here, we report that engagement of alpha6 and, to a lesser extent, alpha3 integrin subunits after HT-29 cell adhesion on laminin 5 increases the expression of E-cadherin, which then organizes into nascent adherens junctions. We further identify that phosphoinositide 3-kinase (PI 3-kinase) activation plays a key role in this cross-talk. Indeed, integrin-dependent adhesion on laminin 5 stimulates PI 3-kinase activity. Immunofluorescence and immunoprecipitation experiments revealed that activated PI 3-kinase is recruited at cell-cell contacts. Using LY294002, an inhibitor of PI 3-kinase activity, we found that this activation is essential for E-cadherin connection with the cytoskeleton and for biogenesis of adherens junctions. Finally, we demonstrated that PI 3-kinase could signal through Rac1b activation to control adherens junction assembly. Our results provide a mechanistic insight into integrin-cadherin cross-talk and identify a novel role for PI 3-kinase in the establishment of adherens junctions. 
 Phosphoinositide 3-Kinase C2beta regulates cytoskeletal organization and cell   migration   via Rac-dependent mechanisms. Receptor-linked class I phosphoinositide 3-kinases (PI3Ks) induce assembly of signal transduction complexes through protein-protein and protein-lipid interactions that mediate cell proliferation, survival, and   migration  . Although class II PI3Ks have the potential to make the same phosphoinositides as class I PI3Ks, their precise cellular role is currently unclear. In this report, we demonstrate that class II phosphoinositide 3-kinase C2beta (PI3KC2beta) associates with the Eps8/Abi1/Sos1 complex and is recruited to the EGF receptor as part of a multiprotein signaling complex also involving Shc and Grb2. Increased expression of PI3KC2beta stimulated Rac activity in A-431 epidermoid carcinoma cells, resulting in enhanced membrane ruffling and   migration   speed of the cells. Conversely, expression of dominant negative PI3KC2beta reduced Rac activity, membrane ruffling, and cell   migration  . Moreover, PI3KC2beta-overexpressing cells were protected from anoikis and displayed enhanced proliferation, independently of Rac function. Taken together, these findings suggest that PI3KC2beta regulates the   migration   and survival of human tumor cells by distinct molecular mechanisms. 
 Rac1-dependent lamellipodial motility in prostate cancer PC-3 cells revealed by optogenetic control of Rac1 activity. The lamellipodium, an essential structure for cell   migration  , plays an important role in the invasion and metastasis of cancer cells. Although Rac1 recognized as a key player in the formation of lamellipodia, the molecular mechanisms underlying lamellipodial motility are not fully understood. Optogenetic technology enabled us to spatiotemporally control the activity of photoactivatable Rac1 (PA-Rac1) in living cells. Using this system, we revealed the role of phosphatidylinositol 3-kinase (PI3K) in Rac1-dependent lamellipodial motility in PC-3 prostate cancer cells. Through local blue laser irradiation of PA-Rac1-expressing cells, lamellipodial motility was reversibly induced. First, outward extension of a lamellipodium parallel to the substratum was observed. The extended lamellipodium then showed ruffling activity at the periphery. Notably, PI(3,4,5)P3 and WAVE2 were localized in the extending lamellipodium in a PI3K-dependent manner. We confirmed that the inhibition of PI3K activity greatly suppressed lamellipodial extension, while the ruffling activity was less affected. These results suggest that Rac1-induced lamellipodial motility consists of two distinct activities, PI3K-dependent outward extension and PI3K-independent ruffling. 
 S-Adenosylmethionine suppresses the expression of Smad3/4 in activated human hepatic stellate cells via Rac1 promoter methylation. The aim of the present study was to investigate whether S-adenosylmethionine (SAM) was able to suppress activated human hepatic stellate cells (HSCs). Human LX-2 HSCs were cultured with SAM or NSC23766, and were transfected with plasmids encoding ras-related C3 botulinum toxin substrate 1 (Rac1) protein or an empty expression vector. Cell proliferation was detected by Cell Counting Kit-8. Cell   migratio  n and invasion were determined using the Transwell assay. The expression levels of Rac1 and Smad3/4 were detected by reverse transcription‑quantitative polymerase chain reaction (PCR) or western blotting. The methylation status of Rac1 promoters was measured by methylation‑specific PCR. The results demonstrated that SAM and NSC23766 suppressed the expression of Smad3/4 in LX‑2 cells. The overexpression of Rac1 enhanced the prolifer  ation, mi  gration and invasion of LX‑2 cells. In addition, compared with the control groups, a marked increase was observed in the protein expression levels of Smad3/4 in the LX‑2 cells transfected with Rac1 plasmids. The methylation-specific PCR findings showed that SAM increased the methylation of Rac1 promoters. The results of the present study suggested that Rac1 enhanced the expression of Smad3/4 in activated HSCs; however, this increase may be suppressed by SAM-induced methylation of Rac1 promoters. 
 Tuberous sclerosis complex 2 (TSC2) regulates cell   migration   and polarity through activation of CDC42 and RAC1. The phosphatidylinositol 3-kinase (PI3K)/AKT pathway plays important roles in regulating cell motility. TSC2, a downstream target of AKT, is a central player in negatively controlling cell proliferation and protein translation through suppressing the activity of mTOR (mammalian target of rapamycin). However, the function of TSC2 in regulating cell   migration   remains unclear. Here, we show that TSC2 plays a critical role in the control of cell spreading, polarity, and   migration  . TSC2-deficient fibroblast cells were impaired in their ability to spread and alter actin cytoskeleton upon stimulation with insulin-like growth factor-1. Using scratch-induced polarization assay, we demonstrate that TSC2((-/-)) fibroblast cells polarized poorly toward the wound compared with wild-type cells. Similarly, knockdown of TSC2 expression in colon cancer cells resulted in a marked decrease in cell motility. Functionally, the activation of CDC42- and RAC1-GTPase was largely reduced in TSC2 knock-out fibroblast and TSC2 knockdown cancer cells. Furthermore, overexpression of an activating p110alpha mutant or short term rapamycin treatment rescued the cell polarization defect in TSC2((-/-)) fibroblast cells. Concurrently, the activation of CDC42 and RAC1 increased. The defect in cell   migration   and CDC42 and RAC1 activation was reversed by reintroducing TSC2 back into TSC2((-/-)) fibroblast cells. Taken together, we identified a novel role of TSC2 in controlling cell polarity and   migration   by regulating CDC42 and RAC1 activation. 
 Novel Activities of Select NSAID R-Enantiomers against Rac1 and Cdc42 GTPases. Rho family GTPases (including Rac, Rho and Cdc42) collectively control cell proliferation, adhesion and   migration   and are of interest as functional therapeutic targets in numerous epithelial cancers. Based on high throughput screening of the Prestwick Chemical Library® and cheminformatics we identified the R-enantiomers of two approved drugs (naproxen and ketorolac) as inhibitors of Rac1 and Cdc42. The corresponding S-enantiomers are considered the active component in racemic drug formulations, acting as non-steroidal anti-inflammatory drugs (NSAIDs) with selective activity against cyclooxygenases. Here, we show that the S-enantiomers of naproxen and ketorolac are inactive against the GTPases. Additionally, more than twenty other NSAIDs lacked inhibitory action against the GTPases, establishing the selectivity of the two identified NSAIDs. R-naproxen was first identified as a lead compound and tested in parallel with its S-enantiomer and the non-chiral 6-methoxy-naphthalene acetic acid (active metabolite of nabumetone, another NSAID) as a structural series. Cheminformatics-based substructure analyses-using the rotationally constrained carboxylate in R-naproxen-led to identification of racemic [R/S] ketorolac as a suitable FDA-approved candidate. Cell based measurement of GTPase activity (in animal and human cell lines) demonstrated that the R-enantiomers specifically inhibit epidermal growth factor stimulated Rac1 and Cdc42 activation. The GTPase inhibitory effects of the R-enantiomers in cells largely mimic those of established Rac1 (NSC23766) and Cdc42 (CID2950007/ML141) specific inhibitors. Docking predicts that rotational constraints position the carboxylate moieties of the R-enantiomers to preferentially coordinate the magnesium ion, thereby destabilizing nucleotide binding to Rac1 and Cdc42. The S-enantiomers can be docked but are less favorably positioned in proximity to the magnesium. R-naproxen and R-ketorolac have potential for rapid translation and efficacy in the treatment of several epithelial cancer types on account of established human toxicity profiles and novel activities against Rho-family GTPases. 
 RhoA and Rac1 are both required for efficient wound closure of airway epithelial cells. Repair of the airway epithelium after injury is critical for restoring normal lung. The reepithelialization process involves spreading and   migration   followed later by cell proliferation. Rho-GTPases are key components of the wound healing process in many different types of tissues, but the specific roles for RhoA and Rac1 vary and have not been identified in lung epithelial cells. We investigated whether RhoA and Rac1 regulate wound closure of bronchial epithelial cells. RhoA and Rac1 proteins were efficiently expressed in a cell line of human bronchial epithelial cells (16HBE) by adenovirus-based gene transfer. We found that both constitutively active RhoA and dominant negative RhoA inhibited wound healing, suggesting that both activation and inhibition of RhoA interfere with normal wound healing. Overexpression of wild-type Rac1 induced upregulation of RhoA, disrupted intercellular junctions, and inhibited wound closure. Dominant negative Rac1 also inhibited wound closure. Inhibition of the downstream effector of RhoA, Rho-kinase, with Y-27632 suppressed actin stress fibers and focal adhesion formation, increased Rac1 activity, and stimulated wound closure. The activity of both RhoA and Rac1 are influenced by the polymerization state of microtubules, and cell   migration   involves coordinated action of actin and microtubules. Microtubule depolymerization upon nocodazole treatment led to an increase in focal adhesions and decreased wound closure. We conclude that coordination of both RhoA and Rac1 activity contributes to bronchial epithelial wound repair mechanisms in vitro, that inhibition of Rho-kinase accelerates wound closure, and that efficient repair involves intact microtubules. 
 Matricellular protein CCN3 (NOV) regulates actin cytoskeleton reorganization. CCN3 (NOV), a putative ligand for integrin receptors, is tightly associated with the extracellular matrix and mediates diverse cellular functions, including cell adhesion and proliferation. CCN3 has been shown to negatively regulate growth although it promotes   migration   in a cell type-specific manner. In this study, overexpression of CCN3 reduces growth and increases intercellular adhesion of breast cancer cells. Interestingly, CCN3 overexpression also led to the formation of multiple pseudopodia that are enriched in actin, CCN3, and vinculin. Breast cancer cells preincubated with exogenous CCN3 protein also induced the same phenotype, indicating that secreted CCN3 is sufficient to induce changes in cell morphology. Surprisingly, extracellular CCN3 is internalized to the early endosomes but not to the membrane protrusions, suggesting pseudopodia-enriched CCN3 may derive from a different source. The presence of an intracellular variant of CCN3 will be consistent with our finding that the cytoplasmic tail of the gap junction protein connexin43 (Cx43) associates with CCN3. Cx43 is a channel protein permitting intercellular communication to occur. However, neither the channel properties nor the protein levels of Cx43 are affected by the CCN3 protein. In contrast, CCN3 proteins are down-regulated in the absence of Cx43. Finally, we showed that overexpression of CCN3 increases the activity of the small GTPase Rac1, thereby revealing a pathway that links Cx43 directly to actin reorganization. 
 ARF6 promotes the formation of Rac1 and WAVE-dependent ventral F-actin rosettes in breast cancer cells in response to epidermal growth factor. Coordination between actin cytoskeleton assembly and localized polarization of intracellular trafficking routes is crucial for cancer cell   migration  . ARF6 has been implicated in the endocytic recycling of surface receptors and membrane components and in actin cytoskeleton remodeling. Here we show that overexpression of an ARF6 fast-cycling mutant in MDA-MB-231 breast cancer-derived cells to mimick ARF6 hyperactivation observed in invasive breast tumors induced a striking rearrangement of the actin cytoskeleton at the ventral cell surface. This phenotype consisted in the formation of dynamic actin-based podosome rosette-like structures expanding outward as wave positive for F-actin and actin cytoskeleton regulatory components including cortactin, Arp2/3 and SCAR/WAVE complexes and upstream Rac1 regulator. Ventral rosette-like structures were similarly induced in MDA-MB-231 cells in response to epidermal growth factor (EGF) stimulation and to Rac1 hyperactivation. In addition, interference with ARF6 expression attenuated activation and plasma membrane targeting of Rac1 in response to EGF treatment. Our data suggest a role for ARF6 in linking EGF-receptor signaling to Rac1 recruitment and activation at the plasma membrane to promote breast cancer cell directed   migration  . 
 Rac1-dependent cell cycle exit of MGE precursors and GABAergic interneuron   migration   to the cortex. Cortical γ-aminobutyric acid (GABA)ergic interneurons are characterized by extraordinary neurochemical and functional diversity. Although recent studies have uncovered some of the molecular components underlying interneuron development, including the cellular and molecular mechanisms guiding their   migratio  n to the cortex, the intracellular components involved are still unknown. Rac1, a member of the Rac subfamily of Rho-GTPases, has been implicated in various cellular processes such as cell cycle dynamics, axonogenesis, and   migratio  n. In this study, we have addressed the specific role of Rac1 in interneuron progenitors originating in the medial ganglionic eminence, via Cre/loxP technology. We show that ablation of Rac1 from Nkx2.1-positive progenitors, results in a migratory impairment. As a consequence, only half of GABAergic interneurons are found in the postnatal cortex. The rest remain aggregated in the ventral telencephalon and show morphological defects in their growing processes in vitro. Ablation of Rac1 from postmitotic progenitors does not result in similar defects, thus underlying a novel cell autonomous and stage-specific requirement for Rac1 activity, within proliferating progenitors of cortical interneurons. Rac1 is necessary for their transition from G1 to S phase, at least in part by regulating cyclin D levels and retinoblastoma protein phosphorylation. 
 A Steric-inhibition model for regulation of nucleotide exchange via the Dock180 family of GEFs. CDM (CED-5, Dock180, Myoblast city) family members have been recently identified as novel, evolutionarily conserved guanine nucleotide exchange factors (GEFs) for Rho-family GTPases . They regulate multiple processes, including embryonic development, cell   migration  , apoptotic-cell engulfment, tumor invasion, and HIV-1 infection, in diverse model systems . However, the mechanism(s) of regulation of CDM proteins has not been well understood. Here, our studies on the prototype member Dock180 reveal a steric-inhibition model for regulating the Dock180 family of GEFs. At basal state, the N-terminal SH3 domain of Dock180 binds to the distant catalytic Docker domain and negatively regulates the function of Dock180. Further studies revealed that the SH3:Docker interaction sterically blocks Rac access to the Docker domain. Interestingly, ELMO binding to the SH3 domain of Dock180 disrupted the SH3:Docker interaction, facilitated Rac access to the Docker domain, and contributed to the GEF activity of the Dock180/ELMO complex. Additional genetic rescue studies in C. elegans suggested that the regulation of the Docker-domain-mediated GEF activity by the SH3 domain and its adjoining region is evolutionarily conserved. This steric-inhibition model may be a general mechanism for regulating multiple SH3-domain-containing Dock180 family members and may have implications for a variety of biological processes. 
 Rac1-induced cell   migration   requires membrane recruitment of the nuclear oncogene SET. The Rho GTPase Rac1 controls cell adhesion and motility. The effector loop of Rac1 mediates interactions with downstream effectors, whereas its C-terminus binds the exchange factor beta-Pix, which mediates Rac1 targeting and activation. Here, we report that Rac1, through its C-terminus, also binds the nuclear oncogene SET/I2PP2A, an inhibitor of the serine/threonine phosphatase PP2A. We found that SET translocates to the plasma membrane in cells that express active Rac1 as well as in migrating cells. Membrane targeting of SET stimulates cell   migration   in a Rac1-dependent manner. Conversely, reduction of SET expression inhibits Rac1-induced   migration  , indicating that efficient Rac1 signalling requires membrane recruitment of SET. The recruitment of the SET oncogene to the plasma membrane represents a new feature of Rac1 signalling. Our results suggest a model in which Rac1-stimulated cell motility requires both effector loop-based downstream signalling and recruitment of a signalling amplifier, that is, SET, through the hypervariable C-terminus. 
 S100A10 is required for the organization of actin stress fibers and promotion of cell spreading. Dynamic remodeling of the actin cytoskeleton is crucial for biological processes such as cell   migration   and cell spreading. S100A10 is a member of the S100 protein family and is involved in intracellular trafficking and cell   migration  . In this study, we examined the role of S100A10 in actin cytoskeletal organization and cell spreading. Depletion of S100A10 induced disruption of stress fiber formation and delay in cell spreading. Rac1 activation during spreading was suppressed by S100A10 knockdown, and exogenous expression of active Rac1 restored the ability of cells to spread in the absence of S100A10. Our results demonstrate the crucial role of S100A10 in actin dynamics promoting cell spreading via Rac1 activation. 
 The tumor suppressor role of miR-124 in osteosarcoma. MicroRNAs have crucial roles in development and progression of human cancers, including osteosarcoma. Recent studies have shown that miR-124 was down-regulated in many cancers; however, the role of miR-124 in osteosarcoma development is unknown. In this study, we demonstrate that expression of miR-124 is significantly downregulated in osteosarcoma tissues and cell lines, compared to the adjacent tissues. The expression of miR-124 in the metastases osteosarcoma tissues was lower than that in non- metastases tissues. We identified and confirmed Rac1 as a novel, direct target of miR-124 using prediction algorithms and luciferase reporter gene assays. Overexpression of miR-124 suppressed Rac1 protein expression and attenuated cell proliferation,   migration  , and invasion and induced apoptosis in MG-63 and U2OS in vitro. Moreover, overexpression of Rac1 in miR-124-transfected osteosarcoma cells effectively rescued the inhibition of cell invasion caused by miR-124. Therefore, our results demonstrate that miR-124 is a tumor suppressor miRNA and suggest that this miRNA could be a potential target for the treatment of osteosarcoma in future. 
 Podocalyxin EBP50 ezrin molecular complex enhances the metastatic potential of renal cell carcinoma through recruiting Rac1 guanine nucleotide exchange factor ARHGEF7. Podocalyxin was initially identified in glomerular podocytes to critically maintain the structural and functional integrity of the glomerular ultrafiltrative apparatus. Lately, it has emerged as a malignant marker in tumors arising from a variety of tissue origins. By immunohistochemistry, we identified that 9.6% of renal cell carcinoma patients overexpress this protein. This subset of patients had significantly shorter disease-specific and overall survivals, and, importantly, we established podocalyxin overexpression as an independent prognostic factor for latent distant metastasis with multivariate analysis. Podocalyxin down-regulation by small interfering RNA led to defective   migration   in model renal tubular cells, which was corrected by re-expression of podocalyxin. The activity of the small GTPase Rac1, a well-characterized modulator of cell   migration  , was diminished by podocalyxin knock-down. Conversely, podocalyxin overexpression in human embryonic kidney cells up-regulated Rac1 activity, which depended on a complex formed by podocalyxin, ERM-binding phosphoprotein 50, ezrin, and ARHGEF7, a Rac1 activator. Therefore, podocalyxin can serve as a biomarker to identify renal cell carcinoma patients with higher metastatic potential for more aggressive intervention at earlier clinical stages. 
 A Trio-Rac1-Pak1 signalling axis drives invadopodia disassembly. Rho family GTPases control cell   migration   and participate in the regulation of cancer metastasis. Invadopodia, associated with invasive tumour cells, are crucial for cellular invasion and metastasis. To study Rac1 GTPase in invadopodia dynamics, we developed a genetically encoded, single-chain Rac1 fluorescence resonance energy (FRET) transfer biosensor. The biosensor shows Rac1 activity exclusion from the core of invadopodia, and higher activity when invadopodia disappear, suggesting that reduced Rac1 activity is necessary for their stability, and Rac1 activation is involved in disassembly. Photoactivating Rac1 at invadopodia confirmed this previously unknown Rac1 function. We describe here an invadopodia disassembly model, where a signalling axis involving TrioGEF, Rac1, Pak1, and phosphorylation of cortactin, causes invadopodia dissolution. This mechanism is critical for the proper turnover of invasive structures during tumour cell invasion, where a balance of proteolytic activity and locomotory protrusions must be carefully coordinated to achieve a maximally invasive phenotype. 
 Mutation of ARHGAP9 in patients with coronary spastic angina. Coronary artery spasm has an important function in the etiology of variant angina and other acute coronary syndromes. Abnormal activation of Rho-family GTPases has been observed in cardiovascular disorders, but the function of genetic variability in Rho-family GTPases remains to be evaluated in cardiovascular disorders. We examined the genetic variability of Rho-family GTPases and their regulators in coronary artery spasm. We performed a comprehensive candidate gene analysis of 67 single nucleotide polymorphisms with amino-acid substitution in Rho-family GTPases and their regulators in 103 unrelated Japanese patients with acetylcholine-induced coronary artery spasm and 102 control Japanese subjects without acetylcholine-induced coronary artery spasm. We noted an association of the single nucleotide polymorphism of ARHGAP9 (rs11544238, Ala370Ser) with coronary artery spasm (odds ratio =2.67). We found that ARHGAP9 inactivated Rac as RacGAP and that the mRNA level of ARHGAP9 was strongly detected in hematopoietic cells. ARHGAP9 negatively regulated cell   migration  . The Ala370Ser polymorphism counteracted ARHGAP9-reduced cell   migration  , spreading and adhesion. The Ala370Ser polymorphism in the ARHGAP9 gene is associated with coronary artery spasm. These data suggest that the polymorphism of ARHGAP9 has a critical function in the infiltration of hematopoietic cells into the endothelium and inflammation leading to endothelial dysfunction. 
 Thymosin beta 4 induces colon cancer cell   migration   and clinical metastasis via enhancing ILK/IQGAP1/Rac1 signal transduction pathway. Thymosin β(4) (Tβ(4)) overexpression increases cel  l migrati  on and tumor metastasis. Hence, understanding the mechanism of cancer cel  l migrati  on induced by Tβ(4) may provide means to inhibit their metastasis. We demonstrated higher Rac1 activities and expression levels of IQGAP1 and ILK in highly migrated Tβ(4)-overexpressing SW480 cells. In addition, IQGAP1 formed a complex with ILK and knockdown of Tβ(4) simultaneously reduced ILK and IQGAP1 protein levels as well as t  heir migr  ation ability. These findings suggest that Tβ(4) incr  eases mig  ration of colon cancer cells via activating Rac1 by elevating IQGAP1/ILK complexes and IHC results illustrated a similar mechanism occurring in vivo. 
 N-α-acetyltransferase 10 protein suppresses cancer cell metastasis by binding PIX proteins and inhibiting Cdc42/Rac1 activity. N-α-acetyltransferase 10 protein, Naa10p, is an N-acetyltransferase known to be involved in cell cycle control. We found that Naa10p was expressed lower in varieties of malignancies with lymph node metastasis compared with non-lymph node metastasis. Higher Naa10p expression correlates the survival of lung cancer patients. Naa10p significantly suppresse  d migrati  on, tumor growth, and metastasis independent of its enzymatic activity. Instead, Naa10p binds to the GIT-binding domain of PIX, thereby preventing the formation of the GIT-PIX-Paxillin complex, resulting in reduced intrinsic Cdc42/Rac1 activity and decreased cel  l migrati  on. Forced expression of PIX in Naa10-transfected tumor cells restored th  e migrati  on and metastasis ability. We suggest that Naa10p functions as a tumor metastasis suppressor by disrupting the migratory complex, PIX-GIT- Paxillin, in cancer cells. 
 Inhibition of tumor cell   migration   and invasion through knockdown of Rac1 expression in medulloblastoma cells. Medulloblastoma is one of the common malignant brain tumors in children or young adults and its overall disease-free 5-year survival rate is approximately 50% due to tumor progression, invasion, and metastasis. This study aimed to determine whether one of Rho GTPases, Rac1 can affect the morphology, motility, and invasion of medulloblastoma cells through knockdown of Rac1 expression. Medulloblastoma Daoy cells were used to manipulate Rac1 expression using Rac1 shRNA, Rac1N17, and Rac1L61 constructs. Reverse transcriptase-PCR and western blot were used to detect expression of Rac1 mRNA and protein, respectively. Invasion and   migration   assays were performed to assess invasion and   migration   capacity of Daoy cells, respectively. The data showed that Rac1 mRNA and protein were overexpressed in Daoy cells. Deletion of Rac1 decreased the cross-linked actin network and pseudopodia and also inhibited the number of   migration   cells migrated or invaded to the other side of the filter compared to control cells. These data indicated that the invasion and   migration   in Daoy cells were inhibited by deletion of Rac1, and suggest that targeting Rac1 by Rac1 shRNA may further be evaluated and used as a potential anticancer strategy to treat medulloblastoma. 
 Dissection of the interplay between class I PI3Ks and Rac signaling in phagocytic functions. Phagocytes, like neutrophils and macrophages, are specialized cells evolved to clear infectious pathogens. This function resides at the core of innate immunity and requires a series of concerted events that lead first to   migration   to the infected tissue and then to the killing of the invading pathogens. Molecular mechanisms underlying these processes are starting to emerge and point to the interplay between two families of crucial proteins: the PI3K lipid kinases and the Rac GTPases. This review focuses on how these two protein families contribute to   migration  , phagocytosis, and reactive oxygen species production, as well as their epistatic and feedback relations that finely tune these crucial aspects of the immune response. 
 Rac regulates the interaction of fascin with protein kinase C in cell   migration  . Fascin is an actin-bundling protein that is low or absent in normal epithelia; its upregulation correlates with poor prognosis in many human carcinomas. We have recently demonstrated in mouse xenograft models that fascin contributes to tumour development and metastasis through its dual actin-bundling and active PKC-binding activities. Rac was implicated as a regulator of fascin-dependent colon carcinoma cell   migration   in vitro. Here, we tested the hypothesis that Rac regulates the interaction of fascin with active PKC. The major conventional PKC in colon carcinoma cells is protein kinase Cgamma (PKCgamma). Endogenous PKCgamma, fascin and Rac1 colocalised at lamellipodial margins of migrating cells. Colocalisation of fascin and PKCgamma depended on Rac activity, and inhibition of Rac decreased PKCgamma activity in cell extracts but not in vitro. Fluorescence resonance energy transfer/fluorescence lifetime imaging microscopy uncovered that fascin and PKCgamma interact in protrusions and filopodia of migrating cells. Mechanistically, the interaction depended on phosphorylated fascin, active PKCgamma and active Rac, but not on active Cdc42. The activity of Rac on the fascin/PKC complex was mediated in part by Pak. Elucidation of this novel pathway for regulation of the fascin/PKCgamma complex in migrating carcinoma cells suggests novel targets for therapeutic intervention in metastasis. 
 Enhanced intrinsic   migration   of aggressive breast cancer cells by inhibition of Rac1 GTPase. Rac GTPases are known to play a crucial role in regulating cytoskeletal changes necessary for cell   migration  .   Migration   has been shown to be positively regulated by Rac in most cell types. However, there is also a large body of conflicting evidence in some other cell types with respect to the role of Rac in   migration  , suggesting that Rac GTPases regulate cell   migration   in a cell type-dependent manner. In the present study, we have characterized the effects of Rac1 GTPase inhibition on the migratory abilities of a number of breast cancer cell lines with differential degrees of tumorigenic and metastatic potentials. We show that Rac1 inhibition in non-metastatic (MCF-7, T-47D) or moderately metastatic (Hs578T) cell lines results in inhibition of   migration  , whereas in highly metastatic cell lines (MDA-MB-435, MDA-MB-231, and C3L5) Rac1 inhibition results in stimulation of   migration  . This stimulation of   migration   following Rac1 inhibition is also accompanied by the enhanced RhoA activity, suggesting a possible existence of a dominating role of RhoA over Rac1 in regulating intrinsic   migration   of the highly metastatic breast cancer cells. 
 Neuregulin mediates F-actin-driven cell   migration   through inhibition of protein kinase D1 via Rac1 protein. Neuregulin (NRG; heregulin) is overexpressed in ∼30% of breast cancers and mediates various processes involved in tumor progression, including tumor cel  l migrati  on and invasion. Here, we show that NRG mediates its effects on tumor cel  l migrati  on via PKD1. Downstream of RhoA, PKD1 can prevent directed cel  l migrati  on through phosphorylation of its substrate SSH1L. NRG exerts its inhibitory effects on PKD1 through Rac1/NADPH oxidase, leading to decreased PKD1 activation loop phosphorylation and decreased activity toward SSH1L. The consequence of PKD1 inhibition by NRG is decreased binding of 14-3-3 to SSH1L, localization of SSH1L to F-actin at the leading edge, and increased cofilin activity, resulting in increased reorganization of the actin cytoskeleton and cell motility. Our data provide a mechanism through which the Rho GTPase Rac1 cross-talks with PKD1 signaling pathways to facilitate directed cel  l migrati  on. 
 Rac1 GTPase-deficient HeLa cells present reduced DNA repair, proliferation, and survival under UV or gamma irradiation. Rac1 GTPase controls essential cellular functions related to the cytoskeleton, such as motility and adhesion. Rac1 is overexpressed in many tumor cells, including breast cancers, where it is also involved in the proliferation and checkpoint control necessary for the cell's recovery after exposure to ionizing radiation. However, its role in DNA damage and repair remains obscure in other tumor cells and under different genotoxic conditions. Here, we compare HeLa cells with mutants exogenously expressing a dominant-negative Rac1 (HeLa-Rac1-N17) by their responses to DNA damage induced by gamma or UV radiation. In HeLa cells, these treatments led to increased levels of active Rac1 (Rac1-GTP) and of stress fibers, with a diminished ability to migrate compared to untreated cells. However, the reduction of Rac1-GTP in Rac1-N17-deficient clones resulted in much higher levels of polymerized stress fibers accompanied by a strong impairment of cell   migration  , even after both radiation treatments. With regard to proliferation and genomic stability, dominant-negative Rac1 cells were more sensitive to gamma and UV radiation, exhibiting reduced proliferation and survival consistent with increased DNA damage and delayed or reduced DNA repair observed in this Rac1-deficient clone. The DNA damage response, as indicated by pH2AX and pChk1 levels, was increased in HeLa cells but was not effectively triggered in the Rac1-N17 clone after radiation treatment, which is likely the main cause of DNA damage accumulation. These data suggest that Rac1 GTPase plays an important role in signaling and contributes to the sensitivity of cervical cancer cells under UV or gamma radiation treatments. 
 Angiotensin-II and MARCKS: a hydrogen peroxide- and RAC1-dependent signaling pathway in vascular endothelium. MARCKS is an actin-binding protein that modulates vascular endothelial cell   migration   and cytoskeleton signaling (Kalwa, H., and Michel, T. (2011) J. Biol. Chem. 286, 2320-2330). Angiotensin-II is a vasoactive peptide implicated in vascular physiology as well as pathophysiology; the pathways connecting angiotensin-II and cytoskeletal remodeling are incompletely understood. Here we show that MARCKS is expressed in intact arterial preparations, with prominent staining of the endothelium. In endothelial cells, angiotensin-II-promoted MARCKS phosphorylation is abrogated by PEG-catalase, implicating endogenous H(2)O(2) in the angiotensin-II response. Studies using the H(2)O(2) biosensor HyPer2 reveal that angiotensin-II promotes increases in intracellular H(2)O(2). We used a Rac1 FRET biosensor to show that angiotensin-II promotes Rac1 activation that is attenuated by PEG-catalase. siRNA-mediated Rac1 knockdown blocks angiotensin-II-stimulated MARCKS phosphorylation. Cell imaging studies using a phosphoinositide 4,5-bisphosphate (PIP(2)) biosensor revealed that angiotensin-II PIP(2) regulation depends on MARCKS and H(2)O(2). siRNA-mediated knockdown of MARCKS or Rac1 attenuates receptor-mediated activation of the tyrosine kinase c-Abl and disrupts actin fiber formation. These studies establish a critical role for H(2)O(2) in angiotensin-II signaling to the endothelial cytoskeleton in a novel pathway that is critically dependent on MARCKS, Rac1, and c-Abl. 
 AIF-1 is an actin-polymerizing and Rac1-activating protein that promotes vascular smooth muscle cell   migration  . Development of vascular restenosis is a multifaceted process characterized by   migration   and proliferation of vascular smooth muscle cells (VSMCs), resulting in loss of lumen diameter. Characterization of proteins that mediate this process is essential in our understanding of the pathogenesis of arterial injury. Allograft inflammatory factor-1 (AIF-1) is a cytoplasmic, calcium-binding protein that is expressed in VSMCs by allograft and balloon angioplasty injury. AIF-1 is not present in cultured human VSMCs but is induced by cytokines, and overexpression of AIF-1 results in increased VSMC growth and cell-cycle gene expression. To characterize AIF-1 modulatory effects in primary human VSMCs, AIF-1-interacting proteins were identified by an AIF-1/glutathione S transferase fusion protein affinity assay. MALDI-TOF mass spectrophotometric amino analysis identified actin as an AIF-1 interacting protein. This interaction was verified by coimmunoprecipitation. This is a functional interaction, because AIF-1 binds to and polymerizes F-actin in vitro. In unstimulated VSMCs, AIF-1 colocalizes with F-actin but translocates to lamellipodia on stimulation with platelet-derived growth factor. VSMCs stably transduced with AIF-1 retrovirus migrate 2.6-fold more rapidly (85.1+/-2.9 versus 225.5+/-16.6; P<0.001) in response to platelet-derived growth factor versus control cells. AIF-1 colocalizes with Rac1, and AIF-1-transduced VSMCs show a constitutive and enhanced activation of Rac1, providing a mechanism for the increased   migration  . These data indicate that AIF-1 binds and polymerizes F actin and also regulates Rac1 activity and VSMC   migration  . Considering the AIF-1 expression pattern in injured arteries, this suggests that AIF-1 may be involved in the cytoskeletal signaling network leading to vascular remodeling. 
 Tiam1/Rac1 signals contribute to the proliferation and chemoresistance, but not motility, of chronic lymphocytic leukemia cells. Signals from the tumor microenvironment promote the   migration  , survival, and proliferation of chronic lymphocytic leukemia (CLL) cells. Rho GTPases control various signaling pathways downstream of microenvironmental cues. Here, we analyze the function of Rac1 in the motility and proliferation of CLL cells. We found decreased transcription of the Rac guanine nucleotide exchange factors Tiam1 and Vav1 in unstimulated peripheral blood CLL cells with almost complete loss of Tiam1 but increased transcription of the potential Rac antagonist RhoH. Consistently, stimulation of CLL cells with the chemokine CXCL12 induced RhoA but not Rac1 activation, whereas chemokine-induced CLL cell motility was Rac1-independent. Coculture of CLL cells with activated T cells induced their activation and subsequent proliferation. Here, Tiam1 expression was induced in the malignant cells in line with increased Ki-67 and c-Myc expression. Rac1 or Tiam1 knockdown using siRNA or treatment with the Tiam1/Rac inhibitor NSC-23766 attenuated c-Myc transcription. Furthermore, treatment of CLL cells with NSC-23766 reduced their proliferation. Rac inhibition also antagonized the chemoresistance of activated CLL cells toward fludarabine. Collectively, our data suggest a dynamic regulation of Rac1 function in the CLL microenvironment. Rac inhibition could be of clinical use by selectively interfering with CLL cell proliferation and chemoresistance. 
 14-3-3zeta Cooperates with ErbB2 to promote ductal carcinoma in situ progression to invasive breast cancer by inducing epithelial-mesenchymal transition. ErbB2, a metastasis-promoting oncoprotein, is overexpressed in approximately 25% of invasive/metastatic breast cancers, but in 50%-60% of noninvasive ductal carcinomas in situ (DCIS). It has been puzzling how a subset of ErbB2-overexpressing DCIS develops into invasive breast cancer (IBC). We found that co-overexpression of 14-3-3zeta in ErbB2-overexpressing DCIS conferred a higher risk of progression to IBC. ErbB2 and 14-3-3zeta overexpression, respectively, increased cell   migration   and decreased cell adhesion, two prerequisites of tumor cell invasion. 14-3-3zeta overexpression reduced cell adhesion by activating the TGF-beta/Smads pathway that led to ZFHX1B/SIP-1 upregulation, E-cadherin loss, and epithelial-mesenchymal transition. Importantly, patients whose breast tumors overexpressed both ErbB2 and 14-3-3zeta had higher rates of metastatic recurrence and death than those whose tumors overexpressed only one. 
 DOCK2 associates with CrkL and regulates Rac1 in human leukemia cell lines. The CDM (ced-5 of Caenorhabditis elegans, DOCK180 [downstream of Crk with molecular weight of 180 kDa] of humans, and myoblast city of Drosophila melanogaster) family of proteins has been shown to play a pivotal role in the integrin-mediated signaling pathway under the regulation of an adaptor molecule c-CT10-related kinase II (c-Crk-II) in adherent cells. Recently, hematopoietic cell-specific CDM protein DOCK2 has been shown to be indispensable for lymphocyte   migration  . However, the regulatory mechanism for DOCK2 is still unknown because DOCK2 lacks a c-Crk-II binding consensus motif. In this study, we demonstrated that DOCK2 bound to CrkL, which is present exclusively in hematopoietic cells both in vivo and in vitro, and we also found that 2 separate regions of DOCK2 contributed to its binding to Src homology 3 (SH3) domain of CrkL. Colocalization of DOCK2 with Crk-like (CrkL) and F-actin was shown by immunocytochemical analysis with the use of Jurkat cells. We also found that CrkL-induced activation of small guanine triphosphatase (GTPase) Rac1 was significantly inhibited by the DOCK2-dCS mutant in 293T cells. Furthermore, the association of DOCK2 and Vav, the guanine-nucleotide exchanging factor (GEF) for Rac1, was demonstrated in Jurkat cells. Finally, the stable expression of DOCK2-dCS mutant in Jurkat cells was shown to reduce cell attachment. These data suggest the presence of a novel protein complex of CrkL, DOCK2, and Vav to regulate Rac1 in leukemia cell lines. 
 Cell   migration   and signaling specificity is determined by the phosphatidylserine recognition motif of Rac1. The Rho guanosine triphosphatases (GTPases) control cell shape and motility and are frequently overexpressed during malignant growth. These proteins act as molecular switches cycling between active GTP- and inactive GDP-bound forms. Despite being membrane anchored via their isoprenylated C termini, Rho GTPases rapidly translocate between membrane and cytosolic compartments. Here, we show that the Rho GTPase Rac1 preferentially interacts with phosphatidylserine (PS)-containing bilayers through its polybasic motif (PBM). Rac1 isoprenylation contributes to membrane avidity but is not critical for PS recognition. The similar protein Cdc42 (cell division cycle 42), however, only associates with PS when prenylated. Conversely, other Rho GTPases such as Rac2, Rac3, and RhoA do not bind to PS even when they are prenylated. Cell stimulation with PS induces translocation of Rac1 toward the plasma membrane and stimulates GTP loading, membrane ruffling, and filopodia formation. This stimulation also promotes Cdc42 activation and phosphorylation of mitogen-activated protein kinase through Rac1/PS signaling. Consequently, the PBM specifically directs Rac1 to effect cytoskeletal rearrangement and cell   migration   by selective membrane phospholipid targeting. 
 Loss of WNK2 expression by promoter gene methylation occurs in adult gliomas and triggers Rac1-mediated tumour cell invasiveness. The gene encoding protein kinase WNK2 was recently identified to be silenced by promoter hypermethylation in gliomas and meningiomas, suggesting a tumour-suppressor role in these brain tumours. Following experimental depletion in cell lines, WNK2 was further found to control GTP-loading of Rac1, a signalling guanosine triphosphatase involved in cell   migration   and motility. Here we show that WNK2 promoter methylation also occurs in 17.5% (29 out of 166) of adult gliomas, whereas it is infrequent in its paediatric forms (1.6%; 1 out of 66). Re-expression of WNK2 in glioblastoma cells presenting WNK2 gene silencing reduced cell proliferation in vitro, tumour growth in vivo and also cell   migration   and invasion, an effect correlated with reduced activation of Rac1. In contrast, when endogenous WNK2 was depleted from glioblastoma cells with unmethylated WNK2 promoter, changes in cell morphology, an increase in invasion and activation of Rac1 were observed. Together, these results validate the WNK2 gene as a recurrent target for epigenetic silencing in glia-derived brain tumours and provide first mechanistic evidence for a tumour-suppressing role of WNK2 that is related to Rac1 signalling and tumour cell invasion and proliferation. 
 Adhesion molecule L1 stimulates neuronal   migration   through Vav2-Pak1 signaling. Organized neuronal   migration   and guided axon outgrowth are key determinants of the development of the functional nervous system. L1, a member of the Ig superfamily of cell surface receptors, stimulates cell   migration   and neurite outgrowth through the MAP kinases ERK1, 2. The signaling molecules participating in this signaling cascade have only partly been identified. Here it is shown that L1 clustering activates the guanine nucleotide exchange factor (GEF) Vav2 and the Rac1 effector p21 associated kinase 1 (Pak1). Also, we found that Pak1 kinase activity contributes to ERK activation by L1, and is necessary for L1-potentiated haptotactic cell   migration  . A signaling pathway is proposed from L1 through Vav2, Rac1, Pak1 and ERK that may be important for L1 mediated neuronal cell   migration  . 
 Immunohistochemical expression of Rho GTPases in ameloblastomas. Rho GTPases are proteins that regulate cell cycle, shape, polarization, invasion,   migration  , and apoptosis, which are important characteristics of normal and neoplastic cells. Rho GTPases expression has been reported in normal tooth germ and several pathologies; however, it has not been evaluated in ameloblastomas. The aim of this study was to analyze the expression and distribution of RhoA, RhoB, Rac1, and Cdc42 Rho GTPases in solid and unicystic ameloblastomas. Three-micrometer sections from paraffin-embedded specimens were evaluated by using an avidin-biotin immunohistochemical method with antibodies against the proteins mentioned above. RhoA and RhoB staining was observed in a high number of cells (P < 0.05) and greater intensity in non-polarized ones. Rac1 was not observed, and Cdc42 did not show any statistical differences between the number of non-polarized and basal positive cells (P > 0.05). Upon comparing the studied ameloblastomas, a higher number of positive cells in the unicystic variant was observed than that in the solid one (P < 0,05). The results obtained suggest that these GTPases could play a role in the ameloblastoma neoplastic epithelial cell phenotype determination (polarized or non-polarized), as well as in variant (solid or unicystic) and subtype (follicular or plexiform) determination. Furthermore, they could participate in solid ameloblastoma invasion mechanisms. 
 Astragaloside IV inhibits breast cancer cell invasion by suppressing Vav3 mediated Rac1/MAPK signaling. Astragaloside IV (AS-IV), the major active triterpenoid in Radix Astragali, has shown anti-tumorigenic properties in certain cancers; however, its role in breast cancer remains unclear. The present study investigated the effects of AS-IV on breast cancer in vitro and in vivo and examined the underlying mechanisms.The effects of AS-IV on MDA-MB-231 cell proliferation,   migration  , invasion and metastasis were investigated by MTT and Transwell assays, and western blotting. In addition, an orthotopic mouse tumor model was established for in vivo experiments.AS-IV inhibited the viability and invasive potential of MDA-MB-231 breast cancer cells, suppressed the activation of the mitogen activated protein kinase (MAPK) family members ERK1/2 and JNK, and downregulated matrix metalloproteases (MMP)-2 and -9. The effects of AS-IV were mediated by the downregulation of Vav3, a guanine nucleotide exchange factor, leading to decreased levels of activated Rac1, a Rho family GTPase. Vav3 overexpression promoted cell proliferation and invasion in vitro, whereas Vav3 silencing had the opposite effects. AS-IV suppressed orthotopic breast tumor growth and metastasis to the lungs, whereas ectopic expression of Vav3 reversed the inhibitory effect of AS-IV on cell viability, invasiveness, MAPK signaling and MMP expression.The present results provide a mechanistic explanation for the antitumor effects of AS-IV and suggest its potential in the treatment of metastatic breast cancer. 
 Guanine nucleotide exchange factor αPIX leads to activation of the Rac 1 GTPase/glycogen phosphorylase pathway in interleukin (IL)-2-stimulated T cells. Recently, we have reported that the active form of Rac 1 GTPase binds to the glycogen phosphorylase muscle isoform (PYGM) and modulates its enzymatic activity leading to T cell proliferation. In the lymphoid system, Rac 1 and in general other small GTPases of the Rho family participate in the signaling cascades that are activated after engagement of the T cell antigen receptor. However, little is known about the IL-2-dependent Rac 1 activator molecules. For the first time, a signaling pathway leading to the activation of Rac 1/PYGM in response to IL-2-stimulated T cell proliferation is described. More specifically, αPIX, a known guanine nucleotide exchange factor for the small GTPases of the Rho family, preferentially Rac 1, mediates PYGM activation in Kit 225 T cells stimulated with IL-2. Using directed mutagenesis, phosphorylation of αPIX Rho-GEF serines 225 and 488 is required for activation of the Rac 1/PYGM pathway. IL-2-stimulated serine phosphorylation was corroborated in Kit 225 T cells cultures. A parallel pharmacological and genetic approach identified PKCθ as the serine/threonine kinase responsible for αPIX serine phosphorylation. The phosphorylated state of αPIX was required to activate first Rac 1 and subsequently PYGM. These results demonstrate that the IL-2 receptor activation, among other early events, leads to activation of PKCθ. To activate Rac 1 and consequently PYGM, PKCθ phosphorylates αPIX in T cells. The biological significance of this PKCθ/αPIX/Rac 1 GTPase/PYGM signaling pathway seems to be the control of different cellular respons  es such a  s migration and proliferation. 
 VEGF-induced Rac1 activation in endothelial cells is regulated by the guanine nucleotide exchange factor Vav2. Vascular endothelial growth factor (VEGF) signaling is critical for both normal and disease-associated vascular development. Dysregulated VEGF signaling has been implicated in ischemic stroke, tumor angiogenesis, and many other vascular diseases. VEGF signals through several effectors, including the Rho family of small GTPases. As a member of this family, Rac1 promotes VEGF-induced endothelial cell   migration   by stimulating the formation of lamellipodia and membrane ruffles. To form these membrane protrusions, Rac1 is activated by guanine nucleotide exchange factors (GEFs) that catalyze the exchange of GDP for GTP. The goal of this study was to identify the GEF responsible for activating Rac1 in response to VEGF stimulation. We have found that VEGF stimulates biphasic activation of Rac1 and for these studies we focused on the peak of activation that occurs at 30 min. Inhibition of VEGFR-2 signaling blocks VEGF-induced Rac1 activation. Using a Rac1 nucleotide-free mutant (G15ARac1), which has a high affinity for binding activated GEFs, we show that the Rac GEF Vav2 associates with G15ARac1 after VEGF stimulation. Additionally, we show that depleting endothelial cells of endogenous Vav2 with siRNA prevents VEGF-induced Rac1 activation. Moreover, Vav2 is tyrosine phosphorylated upon VEGF treatment, which temporally correlates with Rac1 activation and requires VEGFR-2 signaling and Src kinase activity. Finally, we show that depressing Vav2 expression by siRNA impairs VEGF-induced endothelial cell   migration  . Taken together, our results provide evidence that Vav2 acts downstream of VEGF to activate Rac1. 
 FAT1 mutations cause a glomerulotubular nephropathy. Steroid-resistant nephrotic syndrome (SRNS) causes 15% of chronic kidney disease (CKD). Here we show that recessive mutations in FAT1 cause a distinct renal disease entity in four families with a combination of SRNS, tubular ectasia, haematuria and facultative neurological involvement. Loss of FAT1 results in decreased cell adhesion and   migration   in fibroblasts and podocytes and the decreased   migration   is partially reversed by a RAC1/CDC42 activator. Podocyte-specific deletion of Fat1 in mice induces abnormal glomerular filtration barrier development, leading to podocyte foot process effacement. Knockdown of Fat1 in renal tubular cells reduces   migration  , decreases active RAC1 and CDC42, and induces defects in lumen formation. Knockdown of fat1 in zebrafish causes pronephric cysts, which is partially rescued by RAC1/CDC42 activators, confirming a role of the two small GTPases in the pathogenesis. These findings provide new insights into the pathogenesis of SRNS and tubulopathy, linking FAT1 and RAC1/CDC42 to podocyte and tubular cell function. 
 Oligophrenin-1 encodes a rhoGAP protein involved in X-linked mental retardation. Primary or nonspecific X-linked mental retardation (MRX) is a heterogeneous condition in which affected patients do not have any distinctive clinical or biochemical features in common apart from cognitive impairment. Although it is present in approximately 0.15-0.3% of males, most of the genetic defects associated with MRX, which may involve more than ten different genes, remain unknown. Here we report the characterization of a new gene on the long arm of the X-chromosome (position Xq12) and the identification in unrelated individuals of different mutations that are predicted to cause a loss of function. This gene is highly expressed in fetal brain and encodes a protein of relative molecular mass 91K, named oligophrenin-1, which contains a domain typical of a Rho-GTPase-activating protein (rhoGAP). By enhancing their GTPase activity, GAP proteins inactivate small Rho and Ras proteins, so inactivation of rhoGAP proteins might cause constitutive activation of their GTPase targets. Such activation is known to affect cell   migration   and outgrowth of axons and dendrites in vivo. Our results demonstrate an association between cognitive impairment and a defect in a signalling pathway that depends on a Ras-like GTPase. 
 Association between Gαi2 and ELMO1/Dock180 connects chemokine signalling with Rac activation and metastasis. The chemokine CXCL12 and its G-protein-coupled receptor CXCR4 control the   migratio  n, invasiveness and metastasis of breast cancer cells. Binding of CXCL12 to CXCR4 triggers activation of heterotrimeric Gi proteins that regulate actin polymerization and   migratio  n. However, the pathways linking chemokine G-protein-coupled receptor/Gi signalling to actin polymerization and cancer cell   migratio  n are not known. Here we show that CXCL12 stimulation promotes interaction between Gαi2 and ELMO1. Gi signalling and ELMO1 are both required for CXCL12-mediated actin polymerization  , migrati  on and invasion of breast cancer cells. CXCL12 triggers a Gαi2-dependent membrane translocation of ELMO1, which associates with Dock180 to activate small G-proteins Rac1 and Rac2. In vivo, ELMO1 expression is associated with lymph node and distant metastasis, and knocking down ELMO1 impairs metastasis to the lung. Our findings indicate that a chemokine-controlled pathway, consisting of Gαi2, ELMO1/Dock180, Rac1 and Rac2, regulates the actin cytoskeleton during breast cancer metastasis. 
 PTP1B inhibitor promotes endothelial cell motility by activating the DOCK180/Rac1 pathway. Promoting endothelial cell (EC)   migration   is important not only for therapeutic angiogenesis, but also for accelerating re-endothelialization after vessel injury. Several recent studies have shown that inhibition of protein tyrosine phosphatase 1B (PTP1B) may promote EC   migration   and angiogenesis by enhancing the vascular endothelial growth factor receptor-2 (VEGFR2) signalling. In the present study, we demonstrated that PTP1B inhibitor could promote EC adhesion, spreading and   migration  , which were abolished by the inhibitor of Rac1 but not RhoA GTPase. PTP1B inhibitor significantly increased phosphorylation of p130Cas, and the interactions among p130Cas, Crk and DOCK180; whereas the phosphorylation levels of focal adhesion kinase, Src, paxillin, or Vav2 were unchanged. Gene silencing of DOCK180, but not Vav2, abrogated the effects of PTP1B inhibitor on EC motility. The effects of PTP1B inhibitor on EC motility and p130Cas/DOCK180 activation persisted in the presence of the VEGFR2 antagonist. In conclusion, we suggest that stimulation of the DOCK180 pathway represents an alternative mechanism of PTP1B inhibitor-stimulated EC motility, which does not require concomitant VEGFR2 activation as a prerequisite. Therefore, PTP1B inhibitor may be a useful therapeutic strategy for promoting EC   migration   in cardiovascular patients in which the VEGF/VEGFR functions are compromised. 
 The Rho-family GEF Asef2 activates Rac to modulate adhesion and actin dynamics and thereby regulate cell   migration  . Asef2 is a recently identified Rho-family guanine nucleotide exchange factor (GEF) that has been implicated in the modulation of actin, but its function in cell   migration   and adhesion dynamics is not well understood. In this study, we show that Asef2 is an important regulator of cell   migration   and adhesion assembly and disassembly (turnover). Asef2 localizes with actin at the leading edge of cells. Knockdown of endogenous Asef2 impairs   migration   and significantly slows the turnover of adhesions. Asef2 enhances both Rac1 and Cdc42 activity in HT1080 cells, but only Rac1 is crucial for the Asef2-promoted increase in   migration   and adhesion turnover. Phosphoinositide 3-kinase (PI3K) and the serine/threonine kinase Akt are also essential for the Asef2-mediated effects on   migration   and adhesion turnover. Consistent with this, Asef2 increases the amount of active Akt at the leading edge of cells. Asef2 signaling leads to an overall decrease in Rho activity, which is crucial for stimulating   migration   and adhesion dynamics. Thus, our results reveal an important new role for Asef2 in promoting cell   migration   and rapid adhesion turnover by coordinately regulating the activities of Rho-family GTPases. 
 RAC1 overexpression promotes the proliferation,   migration   and epithelial-mesenchymal transition of lens epithelial cells. Cataract is a main cause of blindness worldwide. RAC1 has been reported to have a close relationship with the proliferation and   migration   of cells. However, the relationship between RAC1 and cataract is not yet clear. The proliferation and   migration   of lens epithelial cells are key factors in the formation of cataract as well as in the complication of cataract surgery. In this study, the effect of RAC1 overexpression on the proliferation and   migration   of lens epithelial cells was explored. Results showed that RAC1 overexpression promoted the proliferation of lens epithelial cells and increased the protein level of proliferating cell nuclear antigen. RAC1 overexpression also promoted   migration   and invasion of lens epithelial cells and had an influence on the epithelial-mesenchymal transition process. These results indicate that RAC1 may become a therapeutic target of cataract and inhibition of RAC1 may become a promising way for the therapy of cataract. 
 Regulation of ephexin1, a guanine nucleotide exchange factor of Rho family GTPases, by fibroblast growth factor receptor-mediated tyrosine phosphorylation. Fibroblast growth factor (FGF) signal is implicated in not only cell proliferation, but cell   migration   and morphological changes. Several different Rho family GTPases downstream of the Ras/ERK pathway are postulated to mediate the latter functions. However, none have been recognized to be directly coupled to FGF receptors (FGFRs). We have previously reported that EphA4 and FGFRs hetero-oligomerize through their cytoplasmic domains, trans-activate each other, and transduce a signal for cell proliferation through a docking protein, FRS2alpha (Yokote, H., Fujita, K., Jing, X., Sawada, T., Liang, S., Yao, L., Yan, X., Zhang, Y., Schlessinger, J., and Sakaguchi, K. (2005) Proc. Natl. Acad. Sci. U. S. A. 102, 18866-18871). Here, we have found that ephexin1, a guanine nucleotide exchange factor for Rho family GTPases, constitutes another downstream component of the receptor complex. Ephexin1 directly binds to the kinase domain of FGFR mainly through its DH and PH domains. The binding appears to become weaker and limited to the DH domain when FGFRs become activated. FGFR-mediated phosphorylation of ephexin1 enhances the guanine nucleotide exchange activity toward RhoA without affecting the activity to Rac1 or Cdc42. The FGFR-mediated tyrosine phosphorylation includes, but is not limited to, the residue (Tyr-87) phosphorylated by Src family kinase, which is known to be activated following EphA4 activation. The Tyr-to-Asp mutations that mimic the tyrosine phosphorylation in some of the putative FGFR-mediated phosphorylation sites increase the nucleotide exchange activity for RhoA without changing the activity for Rac1 or Cdc42. From these results, we conclude that ephexin1 is located immediately downstream of the EphA4-FGFR complex and the function is altered by the FGFR-mediated tyrosine phosphorylation at multiple sites. 
 [Modulatory effect of Rac1 protein on epidermal stem cells   migration   during wound healing]. To investigate modulatory role of Rac1 protein in epidermal stem cell (ESC)   migration   during wound healing, in order to provide a reference for enriching basic theory of wound healing and guiding clinical application.Constitutively active mutant of Rac1 protein (Rac1Q61L) or dominant negative isoform of Rac1 protein (Rac1T17N) was transfected into ESC using a retroviral vector FUGW, and retroviral vector FUGW transfected into ESC in singles was used as blank control. The cells were divided into 3 parts according to the random number table and treated as follows. First, equal numbers of cells were inoculated into 24-well plates coated with collagen I (20 µg/mL), collagen IV (20 µg/mL) or fibronectin (10 µg/mL). Cells adhered to above matrices were quantitated using CytoTox 96 colorimetric kit. Second, 1000 cells adhered to collagen IV, after being stained with tetramethyl rhodamine isothiocyanate-phalloidin, were collected for observation of cell morphology and comparison of spreading area under confocal laser scanning microscope. Third, ESC with density of 2 × 10(5) cells per well were placed in upper compartment of Transwell chamber, DK-SFM culture medium alone or that containing stromal cell derived factor 1 (SDF-1) was added into lower compartment of Transwell chamb  er. Migra  tion of ESC was observed using inverted phase contrast microscope, and the result was denoted   as migra  tion rate. Lastly, ESC with density of 7.5 × 10(5) cells per well was inoculated into 6-well plates for 12 hours, and treated with 4 µg/mL mitomycin C for 2 hours. The remaining scratch width of monolayer was respectively measured 6 hours or 12 hours after scratching to calculate the percentage of remaining scratch width. Data were processed with t test.Compared with that of blank control, the number of Rac1Q61L-transfected cells adhered to collagen I was significantly increased (t = 5.302,P < 0.05), while the number of Rac1T17N-transfected cells adhered to collagen I, IV, and fibronectin were all obviously decreased (with t value respectively 13.741, 15.676, 8.256, P values all below 0.05). Confocal laser scanning microscope showed that spreading area of Rac1Q61L-transfected ESC (with laminate pseudopodia on edge) and Rac1T17N-transfected ESC was respectively larger and smaller as compared with that of blank control. With SDF-1 effect  , the mig  ration rate of Rac1T17N-transfected ESC was decreased by 78.0% and Rac1Q61L-transfected ESC was increased by 43.4% as compared with that of blank control. Without SDF-1 effect  , the mig  ration rate of Rac1T17N-transfected ESC was decreased by 55.2%, whil  e the mig  ration rate of Rac1Q61L-transfected ESC was close to that of blank control. Six or 12 hours after scratching, the percentage of remaining scratch width in Rac1Q61L-transfected ESC was lower as compared with that in blank control [(39 ± 9)% vs. (43 ± 5)%, (6 ± 5)% vs. (18 ± 7)%, with t value respectively 1.027, 4.389, with P value respectively above and below 0.05], while that in Rac1T17N-transfected ESC [(81 ± 9)%, (71 ± 11)%, respectively] was obviously higher as compared with that in blank control (with t value respectively 11.386, 11.726, P values all below 0.05).Rac1 protein may   control t  he migration of ESC by regulating its adhesion, spreading, and chemotaxis, and it plays an active role in wound healing accelerated by ESC. 
 Human neutrophils coordinate chemotaxis by differential activation of Rac1 and Rac2. Rac1 and Rac2, members of the small Rho GTPase family, play essential roles in coordinating directional   migration   and superoxide production during neutrophil responses to chemoattractants. Although earlier studies in Rac1 and Rac2 knockout mice have demonstrated unique roles for each Rac isoform in chemotaxis and NADPH oxidase activation, it is still unclear how human neutrophils use Rac1 and Rac2 to achieve their immunological responses to foreign agent stimulation. In the current study, we used TAT dominant-negative Rac1-T17N and Rac2-T17N fusion proteins to acutely alter the activity of Rac1 and Rac2 individually in human neutrophils. We demonstrate distinct activation kinetics and different roles for Rac1 and Rac2 in response to low vs high concentrations of fMLP. These observations were verified using neutrophils from mice in which Rac1 or Rac2 was genetically absent. Based on these results, we propose a model to explain how human neutrophils kill invading microbes while limiting oxidative damage to the adjacent surrounding healthy tissue through the differential activation of Rac1 and Rac2 in response to different concentrations of chemoattractant. 
 Rac1 and Aurora A regulate MCAK to polarize microtubule growth in migrating endothelial cells. Endothelial cells (ECs) migrate directionally during angiogenesis and wound healing by polarizing to extracellular cues to guide directional movement. EC polarization is controlled by microtubule (MT) growth dynamics, which are regulated by MT-associated proteins (MAPs) that alter MT stability. Mitotic centromere-associated kinesin (MCAK) is a MAP that promotes MT disassembly within the mitotic spindle, yet its function in regulating MT dynamics to promote EC polarity and   migration   has not been investigated. We used high-resolution fluorescence microscopy coupled with computational image analysis to elucidate the role of MCAK in regulating MT growth dynamics, morphology, and directional   migration   of ECs. Our results show that MCAK-mediated depolymerization of MTs is specifically targeted to the trailing edge of polarized wound-edge ECs. Regulation of MCAK function is dependent on Aurora A kinase, which is regionally enhanced by signaling from the small guanosine triphosphatase, Rac1. Thus, a Rac1-Aurora A-MCAK signaling pathway mediates EC polarization and directional   migration   by promoting regional differences in MT dynamics in the leading and trailing cell edges. 
 BPAG1e maintains keratinocyte polarity through beta4 integrin-mediated modulation of Rac1 and cofilin activities. alpha6beta4 integrin, a component of hemidesmosomes, also plays a role in keratinocyte   migration   via signaling through Rac1 to the actin-severing protein cofilin. Here, we tested the hypothesis that the beta4 integrin-associated plakin protein, bullous pemphigoid antigen 1e (BPAG1e) functions as a scaffold for Rac1/cofilin signal transduction. We generated keratinocyte lines exhibiting a stable knockdown in BPAG1e expression. Knockdown of BPAG1e does not affect expression levels of other hemidesmosomal proteins, nor the amount of beta4 integrin expressed at the cell surface. However, the amount of Rac1 associating with beta4 integrin and the activity of both Rac1 and cofilin are significantly lower in BPAG1e-deficient cells compared with wild-type keratinocytes. In addition, keratinocytes deficient in BPAG1e exhibit loss of front-to-rear polarity and display aberrant motility. These defects are rescued by inducing expression of constitutively active Rac1 or active cofilin. These data indicate that the BPAG1e is required for efficient regulation of keratinocyte polarity and   migration   by determining the activation of Rac1. 
 Elmo1 helps dock180 to regulate Rac1 activity and cell   migration   of ovarian cancer. Engulfment and cell motility 1 (Elmo1) has been reported to cooperate with dedicator of cytokinesis 1 (Dock180) and to be linked to the invasive phenotype of cancer cells through activating small G-protein Rac. We aimed to study the role of Elmo1 in the malignant   migration   of ovarian cancer.Engulfment and cell motility 1 expression was evaluated in specimens from 93 patients with serous ovarian cancer (SOC) by immunohistochemical staining. Next, Elmo1-RNAi cells were established by validated small interference RNAs. Cell proliferation and cell motility were observed and compared with Dock180-RNAi cells. To confirm their synergetic contribution to forming focal adhesion and activating Rac1, Rac1-GTP level was measured by GST pull-down assay and immunofluorescence was used to observe focal adhesion formation both in Elmo1-RNAi and Dock180-RNAi cells.Engulfment and cell motility 1 was mainly overexpressed in high-grade SOC tissues. Western blot analysis demonstrated that both Elmo1 and Dock180 expressions were hampered in Elmo1-RNAi cells. Compared with the negative control, decreased colony formation and cell invasion were observed in Elmo1-RNAi cells and Dock180-RNAi cells. Consistently, both exhibited reduced Rac1-GTP level and inhibited focal adhesion formation.Engulfment and cell motility 1 presents with synergetic action in helping Dock180 to activate Rac1 and promote cell motility, and thus promote untoward expansion and aggressiveness of SOC. 
 Semaphorin 5A and plexin-B3 inhibit human glioma cell motility through RhoGDIalpha-mediated inactivation of Rac1 GTPase. Semaphorins and plexins are implicated in the progression of various types of cancer, although the molecular basis has not been fully elucidated. Here, we report the expression of plexin-B3 in glioma cells, which upon stimulation by its ligand Sema5A results in significant inhibition of cell   migration   and invasion. A search for the underlying mechanism revealed direct interaction of plexin-B3 with RhoGDP dissociation inhibitor α (RhoGDIα), a negative regulator of RhoGTPases that blocks guanine nucleotide exchange and sequesters them away from the plasma membrane. Glioma cells challenged with Sema5A indeed showed a marked reduction in Rac1-GTP levels by 60%, with a concomitant disruption of lamellipodia. The inactivation of Rac1 was corroborated to contribute to the impediment of glioma cell invasion by Sema5A, as supported by the abolishment of effect upon forced expression of a constitutively active Rac1 mutant. Furthermore, silencing the endogenous expression of RhoGDIα in glioma cells was found to be sufficient in abrogating the down-regulation of Rac1-GTP and the ensuing suppression of glioma cell motility induced by Sema5A. Mechanistically, we provide evidence that Sema5A promotes Rac1 recruitment to RhoGDIα and reduces its membrane localization in a plexin-B3-dependent manner, thereby preventing Rac1 activation. This represents a novel signaling of semaphorin and plexin in the control of cell motility by indirect inactivation of Rac1 through RhoGDIα. 
 Protein kinase G signaling disrupts Rac1-dependent focal adhesion assembly in liver specific pericytes. Nitric oxide (NO) regulates the function of perivascular cells (pericytes), including hepatic stellate cells (HSC), mainly by activating cGMP and cGMP-dependent kinase (PKG) via NO/cGMP paracrine signaling. Although PKG is implicated in integrin-mediated cell adhesion to extracellular matrix, whether or how PKG signaling regulates the assembly of focal adhesion complexes (FA) and   migration   of HSC is not known. With the help of complementary molecular and cell biological approaches, we demonstrate here that activation of PKG signaling in HSC inhibits vascular tubulogenesis,   migration  /chemotaxis, and assembly of mature FA plaques, as assessed by vascular tubulogenesis assays and immunofluorescence localization of FA markers such as vinculin and vasodilator-stimulated phosphoprotein (VASP). To determine whether PKG inhibits FA assembly by phosphorylation of VASP at Ser-157, Ser-239, and Thr-278, we mutated these putative phosphorylation sites to alanine (VASP3A, phosphoresistant mutant) or aspartic acid (VASP3D, phosphomimetic), respectively. Data generated from these two mutants suggest that the effect of PKG on FA is independent of these three phosphorylation sites. In contrast, activation of PKG inhibits the activity of small GTPase Rac1 and its association with the effector protein IQGAP1. Moreover, PKG activation inhibits the formation of a trimeric protein complex containing Rac1, IQGAP1, and VASP. Finally, we found that expression of a constitutively active Rac1 mutant abolishes the inhibitory effects of PKG on FA formation. In summary, our data suggest that activation of PKG signaling in pericytes inhibits FA formation by inhibiting Rac1. 
 Neuronal chemorepellent Slit2 inhibits vascular smooth muscle cell   migration   by suppressing small GTPase Rac1 activation. The Slits are secreted proteins with roles in axonal guidance and leukocyte   migration  . On binding to Robo receptors, Slit2 repels developing axons and inhibits leukocyte chemotaxis. Slit2 is cleaved into Slit2-N, a protein tightly binding to cell membranes, and Slit2-C, a diffusible fragment. In the present study, we characterized the functional role of Slit2-N in vascular smooth muscle cells (VSMCs) and the cell association properties of 2 truncated versions of Slit2-N. Here, we document for the first time that Slit2-N is a chemorepellent of VSMCs. Intact blood vessels expressed Slit2 and Robo receptors as demonstrated by immunohistochemistry and quantitative real time PCR. Recombinant Slit2-N prevented the platelet-derived growth factor (PDGF)-stimulated   migration   of VSMCs. Slit2-N also abrogated PDGF-mediated activation of small guanosine triphosphatase (GTPase) Rac1, a member of the Rho GTPase superfamily of proteins involved in regulating the actin cytoskeleton. Furthermore, Slit2-N inhibited the PDGF-induced formation of lamellipodia, a crucial cytoskeletal reorganization event for cell motility. Slit2-N had no effect on the PDGF-mediated increase in DNA synthesis determined by [3H]thymidine uptake, suggesting that VSMC growth is unaffected by Slit2. Analysis of 2 engineered Slit2-N fragments (Slit2-N/1118 and Slit2-N/1121) indicated that 3 amino acids upstream of the putative cleavage site (Arg1121, Thr1122) are involved in the association of Slit2-N to the cell membrane. Our data assign a novel functional role to Slit2 in vascular function and show that cell guidance mechanisms that operate in the developing central nervous system are conserved in VSMCs. 
 Rac1 and Rac3 isoform activation is involved in the invasive and metastatic phenotype of human breast cancer cells. The metastatic progression of cancer is a direct result of the disregulation of numerous cellular signaling pathways, including those associated with adhesion,   migration  , and invasion. Members of the Rac family of small GTPases are known to act as regulators of actin cytoskeletal structures and strongly influence the cellular processes of integrin-mediated adhesion and   migration  . Even though hyperactivated Rac proteins have been shown to influence metastatic processes, these proteins have never been directly linked to metastatic progression.To investigate a role for Rac and Cdc42 in metastatic breast cancer cell invasion and   migration  , relative endogenous Rac or Cdc42 activity was determined in a panel of metastatic variants of the MDA-MB-435 metastatic human breast cancer cell line using a p21-binding domain-PAK pull down assay. To investigate the migratory and invasive potential of the Rac isoforms in human breast cancer, namely Rac1 and the subsequently cloned Rac3, we stably expressed either dominant active Rac1 or dominant active Rac3 into the least metastatic cell variant. Dominant negative Rac1 or dominant negative Rac3 were stably expressed in the most metastatic cell variant. Cell lines expressing mutant Rac1 or Rac3 were analyzed using in vitro adhesion,   migration   and invasion assays.We show that increased activation of Rac proteins directly correlates with increasing metastatic potential in a panel of cell variants derived from a single metastatic breast cancer cell line (MDA-MB-435). The same correlation could not be found with activated Cdc42. Expression of a dominant active Rac1 or a dominant active Rac3 resulted in a more invasive and motile phenotype. Moreover, expression of either dominant negative Rac1 or dominant negative Rac3 into the most metastatic cell variant resulted in decreased invasive and motile properties.This study correlates endogenous Rac activity with high metastatic potential and implicates Rac in the regulation of cell   migration   and invasion in metastatic breast cancer cells. Taken together, these results suggest a role for both the Rac1 and Rac3 GTPases in human breast cancer progression. 
 Invasion of breast cancer cells into collagen matrix requires TGF-α and Cdc42 signaling. Invasion of MDA-MB-231 breast cancer cells into three-dimensional (3-D) type I-collagen matrices depends on TGF-α. We characterized the steps of invasion mediated by TGF-α. Ce  ll migrat  ion, as observed by videomicroscopy, was effectively stimulated by collagen, suggesting that TGF-α may specifically participate in the invasion of a 3-D collagen matrix. We assessed the role of small GTPases of the Rho family in the invasion. Cdc42 was found to be necessary for invasion but dispensable for c  ell migra  tion. These results suggest that TGF-α mediates invasion into 3-D collagen matrices by initiating the formation of protrusions into collagen, likely through activation of Cdc42. 
 Specific roles of Rac1 and Rac2 in motile functions of HT1080 fibrosarcoma cells. Rho family proteins are constitutively activated in the highly invasive human fibrosarcoma HT1080 cells. We now investigated the specific roles of Rac1 and Rac2 in regulating morphology, F-actin organization, adhesion,   migration  , and chemotaxis of HT1080 cells. Downregulation of Rac1 using specific siRNA probes resulted in cell rounding, markedly decreased spreading, adhesion, and chemotaxis of HT1080 cells. 2D   migration   on laminin-coated surfaces in contrast was not markedly affected. Selective Rac2 depletion did not affect cell morphology, cell adhesion, and 2D   migration  , but significantly reduced chemotaxis. Downregulation of both Rac1 and Rac2 resulted in an even more marked reduction, but not complete abolishment, of chemotaxis indicating distinct as well as overlapping roles of both proteins in chemotaxis. Rac1 thus is selectively required for HT1080 cell spreading and adhesion whereas Rac1 and Rac2 are both required for efficient chemotaxis. 
 Implantation of the human embryo requires Rac1-dependent endometrial stromal cell   migration  . Failure of the human embryo to implant into the uterine wall during the early stages of pregnancy is a major cause of infertility. Implantation involves embryo apposition and adhesion to the endometrial epithelium followed by penetration through the epithelium and invasion of the embryonic trophoblast through the endometrial stroma. Although gene-knockdown studies have highlighted several molecules that are important for implantation in the mouse, the molecular mechanisms controlling implantation in the human are unknown. Here, we demonstrate in an in vitro model for human implantation that the Rho GTPases Rac1 and RhoA in human endometrial stromal cells modulate invasion of the human embryo through the endometrial stroma. We show that knockdown of Rac1 expression in human endometrial stromal cells inhibits human embryonic trophoblast invasion into stromal cell monolayers, whereas inhibition of RhoA activity promotes embryo invasion. Furthermore, we demonstrate that Rac1 is required for human endometrial stromal cell   migration   and that the motility of the stromal cells increases at implantation sites. This increased motility correlates with a localized increase in Rac1 activation and a reciprocal decrease in RacGAP1 levels. These results reveal embryo-induced and localized endometrial responses that may govern implantation of the human embryo. 
 Activation of Rac1 by Src-dependent phosphorylation of Dock180(Y1811) mediates PDGFRα-stimulated glioma tumorigenesis in mice and humans. Two hallmarks of glioblastoma multiforme, the most common malignant brain cancer in humans, are aggressive growth and the ability of single glioma cells to disperse throughout the brain. These characteristics render tumors resistant to current therapies and account for the poor prognosis of patients. Although it is known that oncogenic signaling caused by overexpression of genes such as PDGFRA is responsible for robust glioma growth and cell infiltration, the mechanisms underlying glioblastoma malignancy remain largely elusive. Here, we report that PDGFRα signaling in glioblastomas leads to Src-dependent phosphorylation of the guanine nucleotide exchange factor Dock180 at tyrosine 1811 (Dock180(Y1811)) that results in activation of the GTPase Rac1 and subsequent cell growth and invasion. In human glioma cells, knockdown of Dock180 and reversion with an RNAi-resistant Dock180(Y1811F) abrogated, whereas an RNAi-resistant Dock180(WT) rescued, PDGFRα-promoted glioma growth, survival, and invasion. Phosphorylation of Dock180(Y1811) enhanced its association with CrkII and p130(Cas), causing activation of Rac1 and consequent cell motility. Dock180 also associated with PDGFRα to promote c  ell migra  tion. Finally, phosphorylated Dock180(Y1811) was detected in clinical samples of gliomas and various types of human cancers, and coexpression of phosphorylated Dock180(Y1811), phosphorylated Src(Y418), and PDGFRα was predictive of extremely poor prognosis of patients with gliomas. Taken together, our findings provide insight into PDGFRα-stimulated gliomagenesis and suggest that phosphorylated Dock180(Y1811) contributes to activation of Rac1 in human cancers with PDGFRA amplification. 
 RhoG activates Rac1 by direct interaction with the Dock180-binding protein Elmo. The small GTPase Rac has a central role in regulating the actin cytoskeleton during cell   migration   and axon guidance. Elmo has been identified as an upstream regulator of Rac1 that binds to and functionally cooperates with Dock180 (refs 2-4). Dock180 does not contain a conventional catalytic domain for guanine nucleotide exchange on Rac, but possesses a domain that directly binds to and specifically activates Rac1 (refs 5, 6). The small GTPase RhoG mediates several cellular morphological processes, such as neurite outgrowth in neuronal cells, through a signalling cascade that activates Rac1 (refs 7-12); however, the downstream target of RhoG and the mechanism by which RhoG regulates Rac1 activity remain unclear. Here we show that RhoG interacts directly with Elmo in a GTP-dependent manner and forms a ternary complex with Dock180 to induce activation of Rac1. The RhoG-Elmo-Dock180 pathway is required for activation of Rac1 and cell spreading mediated by integrin, as well as for neurite outgrowth induced by nerve growth factor. We conclude that RhoG activates Rac1 through Elmo and Dock180 to control cell morphology. 
 Involvement of 14-3-3 proteins in the second epidermal growth factor-induced wave of Rac1 activation in the process of cell   migration  . Immense previous efforts have elucidated the core machinery in cell   migration  , actin remodeling regulated by Rho family small GTPases including RhoA, Cdc42, and Rac1; however, the spatiotemporal regulation of these molecules remains largely unknown. Here, we report that EGF induces biphasic Rac1 activation in the process of cell   migration  , and UTKO1, a cell   migration   inhibitor, inhibits the second EGF-induced wave of Rac1 activation but not the first wave. To address the regulation mechanism and role of the second wave of Rac1 activation, we identified 14-3-3ζ as a target protein of UTKO1 and also showed that UTKO1 abrogated the binding of 14-3-3ζ to Tiam1 that was responsible for the second wave of Rac1 activation, suggesting that the interaction of 14-3-3ζ with Tiam1 is involved in this event. To our knowledge, this is the first report to use a chemical genetic approach to demonstrate the mechanism of temporal activation of Rac1. 
 Rac1 controls the formation of midline commissures and the competency of tangential   migration   in ventral telencephalic neurons. Previous studies using dominant-mutant constructs have implicated Rac1 GTPase in neuritogenesis and neuronal   migration  . However, overexpression of dominant mutants generally blocks Rho-GTPase activity; thus, it may not reveal the specific or physiological functions of Rac1. To address this issue, we have applied a conditional gene-targeting strategy, using Foxg1-Cre mice to delete Rac1 in the ventricular zone (VZ) of telencephalon and Dlx5/6-Cre-IRES (internal ribosomal entry site)-EGFP (enhanced green fluorescent protein) (Dlx5/6-CIE) in the subventricular zone (SVZ) of ventral telencephalon, respectively. Surprisingly, the deletion of Rac1 in VZ progenitors did not prevent axonal outgrowth of telencephalic neurons. However, the anterior commissure was absent, and the corpus callosal as well as hippocampal commissural axons failed to cross the midline in Rac1/Foxg1-Cre knock-out embryos. The thalamocortical and corticothalamic axons also showed defasciculation or projection defects. These results suggest that Rac1 controls axon guidance rather than neuritogenesis. In addition, although Rac1/Foxg1-Cre knock-out embryos showed delayed radial   migration   of cortical projection neurons and severe impairment of tangential   migration   by the ventral telencephalon-derived interneurons, deletion of Rac1 in the SVZ by Dlx5/6-CIE mice produced no discernible defects in tangential   migration  . These contrasting effects of Rac1 deletion on tangential   migration   suggest that Rac1 is dispensable for cellular motility per se during neuronal   migration  . Together, these results underscore the challenge of deciphering the biological functions of Rac1, and Rho-GTPases in general, during mammalian brain development. Moreover, they indicate that Rac1 has a critical role in axon guidance and in acquisition of migratory competency during differentiation of the progenitors for the ventral telencephalon-derived interneurons. 
 A novel anti-CCN1 monoclonal antibody suppresses Rac-dependent cytoskeletal reorganization and migratory activities in breast cancer cells. CCN1, a secreted matrix-associated molecule, is involved in multiple cellular processes. Accumulating evidence supports that CCN1 plays an important role in tumorigenesis and progression of breast cancer. In this study, we have developed a novel CCN1 function-blocking monoclonal antibody (mAb), designated YM1B. YM1B binds to human CCN1 with high specificity, recognizing the native CCN1 structure with undisturbed disulfide linkages. Our analyses have mapped the YM1B recognition region to domain IV of CCN1, likely in proximity to the DM site. In breast cancer cells, CCN1 can induce actin reorganization, formation of lamellipodia, and cell   migration  /invasion through the αV integrins/Rac1/ERK signaling axis; these CCN1-dependent activities can be effectively suppressed by YM1B. Our results also suggest that YM1B may exert its CCN1-blocking effect by perturbing the interaction of CCN1 with vitronectin and fibronectin, which are ligands of αV integrins and instrumental for integrin activation. This CCN1-specific mAb may open a new potential avenue for therapeutic intervention of breast cancer progression. 
 Phospholipase D activity regulates integrin-mediated cell spreading and   migration   by inducing GTP-Rac translocation to the plasma membrane. Small GTPase Rac is a crucial regulator of actin cytoskeletal rearrangement, and it plays an important role in cell spreading,   migration  , mitogenesis, phagocytosis, superoxide generation, and axonal growth. It is generally accepted that Rac activity is regulated by the guanosine triphosphate (GTP)/guanosine diphosphate (GDP) cycle. But, it is suggested that in addition to Rac-GTP loading, membrane localization is required for the initiation of downstream effector signaling. However, the molecular mechanisms that control the targeting of GTP-Rac to the plasma membrane remain largely unknown. Here, we have uncovered a signaling pathway linking phospholipase D (PLD) to the localized functions of Rac1. We show that PLD product phosphatidic acid (PA) acts as a membrane anchor of Rac1. The C-terminal polybasic motif of Rac1 is responsible for direct interaction with PA, and Rac1 mutated in this region is incapable of translocating to the plasma membrane and of activating downstream target p21-activated kinase upon integrin activation. Finally, we show that PA induces dissociation of Rho-guanine nucleotide dissociation inhibitor from Rac1 and that PA-mediated Rac1 localization is important for integrin-mediated lamellipodia formation, cell spreading, and   migration  . These results provide a novel molecular mechanism for the GTP-Rac1 localization through the elevating PLD activity, and they suggest a general mechanism for diverse cellular functions that is required localized Rac activation. 
 Rhinovirus-induced barrier dysfunction in polarized airway epithelial cells is mediated by NADPH oxidase 1. Previously, we showed that rhinovirus (RV), which is responsible for the majority of common colds, disrupts airway epithelial barrier function, as evidenced by reduced transepithelial resistance (R(T)), dissociation of zona occludins 1 (ZO-1) from the tight junction complex, and bacterial trans  migration   across polarized cells. We also showed that RV replication is required for barrier function disruption. However, the underlying biochemical mechanisms are not known. In the present study, we found that a double-stranded RNA (dsRNA) mimetic, poly(I:C), induced tight junction breakdown and facilitated bacterial trans  migration   across polarized airway epithelial cells, similar to the case with RV. We also found that RV and poly(I:C) each stimulated Rac1 activation, reactive oxygen species (ROS) generation, and Rac1-dependent NADPH oxidase 1 (NOX1) activity. Inhibitors of Rac1 (NSC23766), NOX (diphenylene iodonium), and NOX1 (small interfering RNA [siRNA]) each blocked the disruptive effects of RV and poly(I:C) on R(T), as well as the dissociation of ZO-1 and occludin from the tight junction complex. Finally, we found that Toll-like receptor 3 (TLR3) is not required for either poly(I:C)- or RV-induced reductions in R(T). Based on these results, we concluded that Rac1-dependent NOX1 activity is required for RV- or poly(I:C)-induced ROS generation, which in turn disrupts the barrier function of polarized airway epithelia. Furthermore, these data suggest that dsRNA generated during RV replication is sufficient to disrupt barrier function. 
 Rac1-dependent phosphorylation and focal adhesion recruitment of myosin IIA regulates   migration   and mechanosensing. Cell   migration   requires coordinated formation of focal adhesions (FAs) and assembly and contraction of the actin cytoskeleton. Nonmuscle myosin II (MII) is a critical mediator of contractility and FA dynamics in cell   migration  . Signaling downstream of the small GTPase Rac1 also regulates FA and actin dynamics, but its role in regulation of MII during   migration   is less clear.We found that Rac1 promotes association of MIIA with FA. Live-cell imaging showed that, whereas most MIIA at the leading edge assembled into dorsal contractile arcs, a substantial subset assembled in or was captured within maturing FA, and this behavior was promoted by active Rac1. Protein kinase C (PKC) activation was necessary and sufficient for integrin- and Rac1-dependent phosphorylation of MIIA heavy chain (HC) on serine1916 (S1916) and recruitment to FA. S1916 phosphorylation of MIIA HC and localization in FA was enhanced during cell spreading and ECM stiffness mechanosensing, suggesting upregulation of this pathway during physiological Rac1 activation. Phosphomimic and nonphosphorylatable MIIA HC mutants demonstrated that S1916 phosphorylation was necessary and sufficient for the capture and assembly of MIIA minifilaments in FA. S1916 phosphorylation was also sufficient to promote the rapid assembly of FAs to enhance cell   migration   and for the modulation of traction force, spreading, and   migration   by ECM stiffness.Our study reveals for the first time that Rac1 and integrin activation regulates MIIA HC phosphorylation through a PKC-dependent mechanism that promotes MIIA association with FAs and acts as a critical modulator of cell   migration   and mechanosensing. 
 A novel high-content analysis tool reveals Rab8-driven cytoskeletal reorganization through Rho GTPases, calpain and MT1-MMP. Rab8 is a small Ras-related GTPase that regulates polarized membrane transport to the plasma membrane. Here, we developed a high-content analysis (HCA) tool to dissect Rab8-mediated actin and focal adhesion reorganization that revealed that Rab8 activation significantly induced Rac1 and Tiam1 to mediate cortical actin polymerization and RhoA-dependent stress fibre disassembly. Rab8 activation increased Rac1 activity, whereas its depletion activated RhoA, which led to reorganization of the actin cytoskeleton. Rab8 was also associated with focal adhesions, promoting their disassembly in a microtubule-dependent manner. This Rab8 effect involved calpain, MT1-MMP (also known as MMP14) and Rho GTPases. Moreover, we demonstrate the role of Rab8 in the cell   migration   process. Indeed, Rab8 is required for EGF-induced cell polarization and chemotaxis, as well as for the directional persistency of intrinsic cell motility. These data reveal that Rab8 drives cell motility by mechanisms both dependent and independent of Rho GTPases, thereby regulating the establishment of cell polarity, turnover of focal adhesions and actin cytoskeleton rearrangements, thus determining the directionality of cell   migration  . 
 srGAP2 arginine methylation regulates cell   migration   and cell spreading through promoting dimerization. The Slit-Robo GTPase-activating proteins (srGAPs) are critical for neuronal   migration   through inactivation of Rho GTPases Cdc42, Rac1, and RhoA. Here we report that srGAP2 physically interacts with protein arginine methyltransferase 5 (PRMT5). srGAP2 localizes to the cytoplasm and plasma membrane protrusion. srGAP2 knockdown reduces cell adhesion spreading and increases cell   migration  , but has no effect on cell proliferation. PRMT5 binds to the N terminus of srGAP2 (225-538 aa) and methylates its C-terminal arginine residue Arg-927. The methylation mutant srGAP2-R927A fails to rescue the cell spreading rate, is unable to localize to the plasma membrane leading edge, and perturbs srGAP2 homodimer formation mediated by the F-BAR domain. These results suggest that srGAP2 arginine methylation plays important roles in cell spreading and cell   migration   through influencing membrane protrusion. 
 The PI3K p110alpha isoform regulates endothelial adherens junctions via Pyk2 and Rac1. Endothelial cell-cell junctions control efflux of small molecules and leukocyte transendothelial   migration   (TEM) between blood and tissues. Inhibitors of phosphoinositide 3-kinases (PI3Ks) increase endothelial barrier function, but the roles of different PI3K isoforms have not been addressed. In this study, we determine the contribution of each of the four class I PI3K isoforms (p110alpha, -beta, -gamma, and -delta) to endothelial permeability and leukocyte TEM. We find that depletion of p110alpha but not other p110 isoforms decreases TNF-induced endothelial permeability, Tyr phosphorylation of the adherens junction protein vascular endothelial cadherin (VE-cadherin), and leukocyte TEM. p110alpha selectively mediates activation of the Tyr kinase Pyk2 and GTPase Rac1 to regulate barrier function. Additionally, p110alpha mediates the association of VE-cadherin with Pyk2, the Rac guanine nucleotide exchange factor Tiam-1 and the p85 regulatory subunit of PI3K. We propose that p110alpha regulates endothelial barrier function by inducing the formation of a VE-cadherin-associated protein complex that coordinates changes to adherens junctions with the actin cytoskeleton. 
 mTOR regulate EMT through RhoA and Rac1 pathway in prostate cancer. Recently, an increasing number of studies have suggested that mTOR plays a critical role in the regulation of tumor cell motility, invasion and cancer metastasis. However, little is known about the signaling mechanisms in regulating epithelial-mesenchymal transition (EMT) of prostate cancer. In this study, we found that the expression levels of Raptor and Rictor in prostate cancer tissues were elevated, which may suggest that Raptor and Rictor signaling pathways are associated with prostate cancer progression and metastasis. Inhibition of mTORC1 or mTORC2 by knock down of Raptor or Rictor, respectively,   migration   and invasion of prostate cancer were attenuated. Furthermore, EMT, a characterized by the changed expression levels of various markers like E-cadherin, β-catenin, N-cadherin, and vimentin emergend following inhibition of Raptor or Rictor. Finally, the small GTPases (RhoA and Rac1) which were crucial regulatory proteins in cell   migratio  n and invasion were inactivited after downregulating Raptor and Rictor. These results suggest that mTOR regulate EMT at least in part by down regulation of RhoA and Rac1 signaling pathways. Our findings provide novel very attractive target strategies that the inhibition of mTOR signaling pathways may retard prostate cancer   migratio  n and invasion at early stages. 
 Examining the role of Rac1 in tumor angiogenesis and growth: a clinically relevant RNAi-mediated approach. Angiogenesis, the sprouting of new blood vessels from the pre-existing vasculature, is a well established target in anti-cancer therapy. It is thought that the Rho GTPase Rac1 is required during vascular endothelial growth factor (VEGF)-mediated angiogenesis. In the present study, we have used a clinically relevant RNA interference approach to silence Rac1 expression. Human umbilical vein endothelial cells were transiently transfected with non-specific control siRNA (siNS) or Rac1 siRNA (siRac1) using electroporation or Lipofectamine 2000. Functional assays with transfected endothelial cells were performed to determine the effect of Rac1 knockdown on angiogenesis in vitro. Silencing of Rac1 inhibited VEGF-mediated tube formation, cell   migration  , invasion and proliferation. In addition, treatment with Rac1 siRNA inhibited angiogenesis in an in vivo Matrigel plug assay. Intratumoral injections of siRac1 almost completely inhibited the growth of grafted Neuro2a tumors and reduced tumor angiogenesis. Together, these data indicate that Rac1 is an important regulator of VEGF-mediated angiogenesis. Knockdown of Rac1 may represent an attractive approach to inhibit tumor angiogenesis and growth. 
 CCL2 induces prostate cancer transendothelial cell   migration   via activation of the small GTPase Rac. Nearly 85% of the men who will die of prostate cancer (PCa) have skeletal metastases present. The ability of PCa cells to interact with the microenvironment determines the success of the tumor cell to form metastatic lesions. The ability to bind to human bone marrow endothelial (HBME) cells and undergo transendothelial cell   migration   are key steps in allowing the PCa cell to extravasate from the bone microvasculature and invade the bone stroma. We have previously demonstrated that monoctyte chemoattractant protein 1 (MCP-1; CCL2) is expressed by HBME cells and promotes PCa proliferation and   migration  . In the current study, we demonstrate that the CCL2 stimulation of PCa cells activates the small GTPase, Rac through the actin-associated protein PCNT1. Activation of Rac GTPase is accompanied by morphologic changes and the ability of the cells to undergo diapedesis through HBME cells. These data suggest a role for HBME-secreted CCL2 in promoting PCa cell extravasation into the bone microenvironment. 
 Overexpression of Rac1 in leukemia patients and its role in leukemia cell   migration   and growth. Rac1 belongs to the Rho family that act as critical mediators of signaling pathways controlling cell   migration   and proliferation and contributes to the interactions of hematopoietic stem cells with their microenvironment. Alteration of Rac1 might result in unbalanced interactions and ultimately lead to leukemogenesis. In this study, we analyze the expression of Rac1 protein in leukemia patients and determine its role in the abnormal behaviours of leukemic cells. Rac1 protein is overexpressed in primary acute myeloid leukemia cells as compared to normal bone marrow mononuclear cells. siRNA-mediated silencing of Rac1 in leukemia cell lines induced inhibition of cell   migration  , proliferation, and colony formation. Additionally, blocking Rac1 activity by an inhibitor of Rac1-GTPase, NSC23766, suppressed cell   migration   and growth. We conclude that overexpression of Rac1 contributes to the accelerated   migration   and high proliferation potential of leukemia cells, which could be implicated in leukemia development and progression. 
 A MAPK-Driven Feedback Loop Suppresses Rac Activity to Promote RhoA-Driven Cancer Cell Invasion. Cell   migration   in 3D microenvironments is fundamental to development, homeostasis and the pathobiology of diseases such as cancer. Rab-coupling protein (RCP) dependent co-trafficking of α5β1 and EGFR1 promotes cancer cell invasion into fibronectin (FN) containing extracellular matrix (ECM), by potentiating EGFR1 signalling at the front of invasive cells. This promotes a switch in RhoGTPase signalling to inhibit Rac1 and activate a RhoA-ROCK-Formin homology domain-containing 3 (FHOD3) pathway and generate filopodial actin-spike protrusions which drive invasion. To further understand the signalling network that drives RCP-driven invasiv  e migrati  on, we generated a Boolean logical model based on existing network pathways/models, where each node can be interrogated by computational simulation. The model predicted an unanticipated feedback loop, whereby Raf/MEK/ERK signalling maintains suppression of Rac1 by inhibiting the Rac-activating Sos1-Eps8-Abi1 complex, allowing RhoA activity to predominate in invasive protrusions. MEK inhibition was sufficient to promote lamellipodia formation and oppose filopodial actin-spike formation, and led to activation of Rac and inactivation of RhoA at the leading edge of cells moving in 3D matrix. Furthermore, MEK inhibition abrogated RCP/α5β1/EGFR1-driven invas  ive migra  tion. However, upon knockdown of Eps8 (to suppress the Sos1-Abi1-Eps8 complex), MEK inhibition had no effect on RhoGTPase activity and did not oppose invas  ive migra  tion, suggesting that MEK-ERK signalling suppresses the Rac-activating Sos1-Abi1-Eps8 complex to maintain RhoA activity and promote filopodial actin-spike formation and invas  ive migra  tion. Our study highlights the predictive potential of mathematical modelling approaches, and demonstrates that a simple intervention (MEK-inhibition) could be of therapeutic benefit in preventing invas  ive migra  tion and metastasis. 
 A small GTPase‑like protein fragment of Mycoplasma promotes tumor cel  l migrati  on and proliferation in vitro via interaction with Rac1 and Stat3. The Mycoplasma genus comprises a group of microbes that cause persistent infection in humans and its role in promoting tumor development has long been a concern. Although mixtures of components isolated from Mycoplasma have been shown to activate host Rho family small GTPases and Stat3, no individual factor with this activity has been reported. In the current study, a conserved small GTPase-like protein fragment (SGLP) from Mycoplasma pulmonis chromosome partition protein, Smc, was identified as a virulence factor. SGLP was observed to interact with Rac1 and Stat3. The wild‑type (wt) SGLP, which contains a WxxxE motif, induced activation of Rac1 and phosphorylation of Stat3 at the tyrosine‑705 residue, while the SGLP mutant containing a mutation from WxxxE to AxxxA did not exert the same effects. Moreover, SGLP‑induced Stat3 phosphorylation was observed to be dependent upon Rac1 activity. Furthermore, wt SGLP was observed to prom  ote cell   migration and increase bromodeoxyuridine incorporation in HeLa cells and the SGLP mutant did not elicit these effects in HeLa cells. In conclusion, the current observations suggest that SGLP is an important virulence factor of Mycoplasma, which contributes to tu  mor cell   migration and proliferation in vitro via interaction with Rac1 and Stat3. 
 Inhibition of the Rac1-WAVE2-Arp2/3 signaling pathway promotes radiosensitivity via downregulation of cofilin-1 in U251 human glioma cells. The Ras-related C3 botulinum toxin substrate 1 (Rac1)-WASP-family verprolin-homologous protein-2 (WAVE2)-actin-related protein 2/3 (Arp2/3) signaling pathway has been identified to be involved in cell   migratio  n and invasion in various types of cancer cell. Cofilin‑1 (CFL‑1), which is regulated by the Rac1‑WAVE2‑Arp2/3 signaling pathway, may promote radioresistance in glioma. Therefore, the present study aimed to investigate the potential role of the Rac1‑WAVE2‑Arp2/3 signaling pathway in radioresistance in U251 human glioma cells and elucidate its affect on CFL‑1 expression. Western blot analysis was performed to evaluate the protein expression of CFL‑1. In the present study, Rac1 was inhibited by NSC 23766, WAVE2 was inhibited by transfection with short hairpin (sh)RNA‑WAVE2 using Lipofectamine™ 2000 and Arp2/3 was inhibited by CK‑666. Cell viability was measured using the 3‑(4,5‑dimethylthiazol‑2‑yl)-2,5‑diphenylt  etrazoliu  m bromide assay, the cell migration ability was examined by a wound‑healing assay, and the cell invasion ability was assessed using a Transwell culture chamber system. The results showed that inhibition of the Rac1‑WAVE2‑Arp2/3 signaling pathway using NSC 23766, shRNA‑  WAVE2 or   CK‑666 reduced the cell viability, migration and invasion abilities in U251 human glioma cells, concordant with a reduced expression of CFL‑1. Furthermore, the expression of CFL‑1 was significantly increased in radioresistant U251 glioma cells when compared with normal U251 human glioma cells. These findings indicate that inhibition of the Rac1‑WAVE2‑Arp2/3 signaling pathway may promote radiosensitivity, which may partially result from the downregulation of CFL‑1 in U251 human glioma cells. 
 16-kDa prolactin inhibits endothelial cell   migration   by down-regulating the Ras-Tiam1-Rac1-Pak1 signaling pathway. Angiogenesis plays a key role in promoting tumorigenesis and metastasis. The 16-kDa fragment of prolactin (16k PRL) is an NH(2)-terminal natural breakdown fragment of the intact 23-kDa prolactin and has been shown to have potent antiangiogenic and antitumor activities. The mechanism(s) involved in the action of 16k PRL in endothelial cells remains unclear. In this study, we showed that 16k PRL reduced rat aortic endothelial cell (RAEC)   migration   in a wound-healing assay and in a Matrigel tube formation assay, suggesting that 16k PRL inhibits endothelial cell   migration  , an important activity involved in angiogenesis and tumorigenesis. We further investigated how 16k PRL attenuates endothelial cell   migration  . We first showed that RAEC   migration   is mediated through the Rho GTPase Rac1, as Rac1 inhibition by the Rac1-specific inhibitor NSC27366 or Rac1 knockdown by small interfering RNA both blocked RAEC   migration  . We next showed that 16k PRL reduced the activation of Rac1 in a concentration-dependent manner. Furthermore, 16k PRL inhibition of Rac1 is mediated through the suppression of T lymphoma invasion and metastasis 1 (Tiam1) and its upstream activator Ras in a phosphoinositide-3-kinase-independent manner. 16k PRL also down-regulated the phosphorylation of the downstream effector of Rac1, p21-activating kinase 1 (Pak1), and inhibited its translocation to the leading edge of migrating cells. Thus, 16k PRL inhibits cell   migration   by blocking the Ras-Tiam1-Rac1-Pak1 signaling pathway in endothelial cells. 
 Small interfering RNA targeting Rac1 sensitizes colon cancer to dihydroartemisinin-induced cell cycle arrest and inhibited cell   migration   by suppressing NFκB activity. Dihydroartemisinin (DHA) has recently shown antitumor activity in various cancer cells. The small GTPase Rac1 regulates many cellular processes, including cytoskeletal reorganization, cell   migratio  n, proliferation, and survival. In addition, Rac1 plays a major role in activating NFκB-mediated transcription. Both Rac1 and NFκB regulate many properties of the malignant phenotype, including anchorage-independent proliferation and survival, metastasis, and angiogenesis. In this study, for the first time, we demonstrated that Rac1 knockdown can enhance DHA-induced growth inhibition, cell cycle arrest, apoptosis, a  nd migrat  ion in both HCT116 and RKO cell lines in vitro. The mechanism is due partially to DHA, and Rac1 siRNA deactivates NFκB activity, so as to decrease tremendously the expression of its target gene products, such as PCNA, cyclin D1, and CDK4; and increase the expression of p21, cleaved-caspase-3, and cleaved-PARP. In our in vivo studies, DHA also manifested remarkably enhanced antitumor effect when combined with Rac1 siRNA. We concluded that inhibition of NFκB activation is one of the mechanisms that Rac1 siRNA dramatically promotes DHAs antitumor effect on human colon cancer. 
 Phosphorylation and activation of the Rac1 and Cdc42 GEF Asef in A431 cells stimulated by EGF. Rac1 has a crucial role in epidermal growth factor (EGF)-induced membrane ruffling, lamellipodial protrusion, and cell   migration  . Several guanine nucleotide exchange factors (GEFs) including Sos1, Sos2, Tiam1 and Vav2 have been shown to transduce the growth signal from the EGF receptor to Rac1. To clarify the role of each GEF, we time-lapse imaged the EGF-induced activity change of Rac1 in A431 cells transfected with siRNA targeting each Rac1 GEF. Because knockdown of these GEFs suppressed EGF-induced Rac1 activation only partially, we looked for another Rac1 GEF downstream of the EGF receptor and found that Asef, a Rac1-Cdc42 GEF bound to the tumor suppressor APC, also contributed to EGF-induced Rac1 activation. Intriguingly, EGF stimulation induced phosphorylation of Tyr94 within the APC-binding region of Asef in a manner dependent on Src-family tyrosine kinases. The suppression of EGF-induced Rac1 activation in siRNA-treated cells was restored by wild-type Asef, but not by the Tyr94Phe mutant of Asef. This observation strongly argues for the positive role of Tyr94 phosphorylation in EGF-induced Asef activation following the activation of Rac1. 
 The role of PTEN in prostate cancer cell tropism to the bone micro-environment. Little is known about the role of the tumor suppressor gene phosphatase and tensin homolog deleted on chromosome 10 (PTEN) in prostate cancer bone metastasis. To explore this, we used a pTetOn PTEN cell line in which PTEN expression was reconstituted in a PTEN-null bone metastatic human prostate cancer cell line, LnCaP-C4-2. We found that C4-2 cells selectively migrated toward conditioned medium from primary mouse calvaria cells compared with that derived from lung fibroblasts. Further evaluation with conditioned medium from an established mouse calvaria osteoblast cell line and control non-osteoblast cell line indicates that osteoblastic characteristics convey this specific   migration   to C4-2 cells. We evaluated promiscuously metastatic PC-3 prostate as well as T24T and UMUC-3 bladder cells and found they did not have a specific migratory response to calvaria-conditioned medium as did C4-2. Induction of PTEN expression inhibited the motility of C4-2 cells toward calvaria-conditioned medium but had no effect on   migration   toward lung-conditioned medium and this inhibitory effect was dependent on the PTEN lipid phosphatase activity. Calvaria- but not lung-conditioned medium induced activation of the small GTPase Rac1. Constitutively active Rac1 but not focal adhesion kinase or Cdc42 could rescue cells from the inhibitory effect of PTEN on cell   migration   and PTEN induction was observed to inhibit Rac1 activation in response to calvaria-conditioned medium. Our results support the notion that loss of PTEN function in human prostate cancer may specifically facilitate bone rather than other organ metastasis and suggest that Rac1, as a PTEN effector, may contribute to this metastatic tropism. 
 CD81 is essential for the formation of membrane protrusions and regulates Rac1-activation in adhesion-dependent immune cell   migration  . CD81 (TAPA-1) is a member of the widely expressed and evolutionary conserved tetraspanin family that forms complexes with a variety of other cell surface receptors and facilitates hepatitis C virus entry. Here, we show that CD81 is specifically required for the formation of lamellipodia in migrating dendritic cells (DCs). Mouse CD81(-/-) DCs, or murine and human CD81 RNA interference knockdown DCs lacked the ability to form actin protrusions, thereby impairing their motility dramatically. Moreover, we observed a selective loss of Rac1 activity in the absence of CD81, the latter of which is exclusively required for integrin-dependent   migration   on 2-dimensional substrates. Neither integrin affinity for substrate nor the size of basal integrin clusters was affected by CD81 deficiency in adherent DCs. However, the use of total internal reflection fluorescence microscopy revealed an accumulation of integrin clusters above the basal layer in CD81 knockdown cells. Furthermore, β1- or β2-integrins, actin, and Rac are strongly colocalized at the leading edge of DCs, but the very fronts of these cells protrude CD81-containing membranes that project outward from the actin-integrin area. Taken together, these data suggest a thus far unappreciated role for CD81 in the mobilization of preformed integrin clusters into the leading edge of migratory DCs on 2-dimensional surfaces. 
 Clinical value of Tiam1-Rac1 signaling in primary gallbladder carcinoma. Tiam1-Rac1 signaling plays an important role in the   migration  , invasion and metastasis of tumor cells. The aim of the present study was to clarify the clinical value of Tiam1 and Rac1 expression in primary gallbladder carcinoma (PGC). Eighty-six PGC tissues were evaluated by immunohistochemistry for Tiam1 and Rac1 expression. The association of Tiam1 and Rac1 expression with clinicopathological characteristics and the univariate survival analysis for the influence of Tiam1 and Rac1 expression on the overall survival were analyzed. Tiam1 and Rac1 immunoreactivities were observed in 72 (83.7%) and 68 (79.1%) of the 86 PGC cases, but not in the non-neoplastic gallbladder tissues, respectively. The tumors with the positive expression of Tiam1 and Rac1 more frequently showed lymph node metastasis (P = 0.01 and 0.008), lymphovascular invasion (P = 0.009 and 0.01) and recurrence (both P = 0.01), respectively. In addition, the tumors with the positive expression of Tiam1 and Rac1 significantly tended to show deeper invasion depth (P = 0.007 and 0.005) and higher TNM stage (both P = 0.005). The Kaplan-Meier survival curves demonstrated that patients with the positive expression of Tiam1 and Rac1 had a significantly shorter survival time than those patients with their negative expression (both P = 0.01). Multivariate analysis revealed that the positive expression of Tiam1 (P = 0.02) and Rac1 (P = 0.02), as well as TNM stage (P = 0.04), to be independently associated with a poor prognosis of patients with PGC. The data from this study suggest for the first time that Tiam1 and Rac1 are frequently expressed in PGC and are associated with decreased survival of the patients. Therefore, Tiam1 and Rac1 expressions may be independent predictors for the poor prognosis in patients with PGC. 
 Direct interaction of Rnd1 with Plexin-B1 regulates PDZ-RhoGEF-mediated Rho activation by Plexin-B1 and induces cell contraction in COS-7 cells. Plexins are receptors for the axon guidance molecule semaphorins, and several lines of evidence suggest that Rho family small GTPases are implicated in the downstream signaling of Plexins. Recent studies have demonstrated that Plexin-B1 activates RhoA and induces growth cone collapse through Rho-specific guanine nucleotide exchange factor PDZ-RhoGEF. Here we show that Rnd1, a member of Rho family GTPases, directly interacted with the cytoplasmic domain of Plexin-B1. In COS-7 cells, coexpression of Rnd1 and Plexin-B1 induced cell contraction in response to semaphorin 4D (Sema4D), a ligand for Plexin-B1, whereas expression of Plexin-B1 alone or coexpression of Rnd1 and a Rnd1 interaction-defective mutant of Plexin-B1 did not. The Sema4D-induced contraction in Plexin-B1/Rnd1-expressing COS-7 cells was suppressed by dominant negative RhoA, a Rho-associated kinase inhibitor, a dominant negative form of PDZ-RhoGEF, or deletion of the carboxyl-terminal PDZ-RhoGEF-binding region of Plexin-B1, indicating that the PDZ-RhoGEF/RhoA/Rho-associated kinase pathway is involved in this morphological effect. We also found that Rnd1 promoted the interaction between Plexin-B1 and PDZ-RhoGEF and thereby dramatically potentiated the Plexin-B1-mediated RhoA activation. We propose that Rnd1 plays an important role in the regulation of Plexin-B1 signaling, leading to Rho activation during axon guidance and cell   migration  . 
 Mechanical stretch decreases   migration   of alveolar epithelial cells through mechanisms involving Rac1 and Tiam1. Mechanical ventilation can overdistend the lungs or generate shear forces in them during repetitive opening/closing, contributing to lung injury and inflammation in patients with acute respiratory distress syndrome (ARDS). Repair of the injured lung epithelium is important for restoring normal barrier and lung function. In the current study, we investigated the effects of cyclic mechanical strain (CS), constant distention strain (CD), and simulated positive end-expiratory pressure (PEEP) on activation of Rac1 and wound closure of rat primary alveolar type 2 (AT2) cells. Cyclic stretch inhibited the   migration   of wounded AT2 cells in a dose-dependent manner with no inhibition occurring with 5% CS, but significant inhibition with 10% and 15% CS. PEEP conditions were investigated by stretching AT2 cells to 15% maximum strain (at a frequency of 10 cycles/min) with relaxation to 10% strain. AT2 cells were also exposed to 20% CD. All three types of mechanical strain inhibited wound closure of AT2 cells compared with static controls. Since lamellipodial extensions in migrating cells at the wound edge were significantly smaller in stretched cells, we measured Rac1 activity and found it to be decreased in stretched cells. We also demonstrate that Tiam1, a Rac1-specific guanine nucleotide exchange factor, was expressed mainly in the cytosol of AT2 cells exposed to mechanical strain compared with membrane localization in static cells. Downregulation of Tiam1 with 100 microM NSC-23766 inhibited activation of Rac1 and   migration   of AT2 cells, suggesting its involvement in repair mechanisms of AT2 cells subjected to mechanical strain. 
 Exome sequencing identifies recurrent somatic RAC1 mutations in melanoma. We characterized the mutational landscape of melanoma, the form of skin cancer with the highest mortality rate, by sequencing the exomes of 147 melanomas. Sun-exposed melanomas had markedly more ultraviolet (UV)-like C>T somatic mutations compared to sun-shielded acral, mucosal and uveal melanomas. Among the newly identified cancer genes was PPP6C, encoding a serine/threonine phosphatase, which harbored mutations that clustered in the active site in 12% of sun-exposed melanomas, exclusively in tumors with mutations in BRAF or NRAS. Notably, we identified a recurrent UV-signature, an activating mutation in RAC1 in 9.2% of sun-exposed melanomas. This activating mutation, the third most frequent in our cohort of sun-exposed melanoma after those of BRAF and NRAS, changes Pro29 to serine (RAC1(P29S)) in the highly conserved switch I domain. Crystal structures, and biochemical and functional studies of RAC1(P29S) showed that the alteration releases the conformational restraint conferred by the conserved proline, causes an increased binding of the protein to downstream effectors, and promotes melanocyte proliferation and   migration  . These findings raise the possibility that pharmacological inhibition of downstream effectors of RAC1 signaling could be of therapeutic benefit. 
 Up-regulation of Tiam1 and Rac1 correlates with poor prognosis in hepatocellular carcinoma. T-cell lymphoma invasion and metastasis 1 (Tiam1) specifically activates Rho-like GTPases (e.g. Rac1) and Tiam1-Rac1 pathway affects the   migration   and invasion of many tumors, such as nasopharyngeal carcinoma, breast cancer and retinoblastoma. However, no studies have yet comprehensively examined the involvement of Tiam1-Rac1 pathway in hepatocellular carcinoma. In this study, we examined the relationship of the up-regulation of Tiam1 and Rac1 with clinicopathological features in patients with hepatocellular carcinoma.Expression of Tiam1 and Rac1 was assessed in 242 hepatocellular carcinoma tissues and their adjacent normal hepatic tissues by performing immunohistochemistry and was gauged regarding stage, grade and survival.Immunohistochemistry showed that patients with a high clinical stage hepatocellular carcinoma (III-IV) and α-fetoprotein levels had a higher tendency to express Tiam1 and Rac1 on tumor cells than the patients with low pathologic grade hepatocellular carcinoma (I-II) (P = 0.008 and 0.01, respectively) and low α-fetoprotein levels (P = 0.006 and 0.002, respectively). In addition, Tiam1 and Rac1 up-regulation was also significantly associated with vascular invasion status (both P = 0.02), intrahepatic metastasis status (P = 0.009 and 0.01, respectively) and histological differentiation (P = 0.008 and 0.009, respectively) of patients with hepatocellular carcinoma. Moreover, post-operative survival analysis indicated that hepatocellular carcinoma patients with strong Tiam1 (P = 0.01) and Rac1 (P = 0.02) expression had shorter disease-specific survival than those with weak expression. Multivariate analysis also showed that Tiam1 and Rac1 overexpression could be two predictors of poor prognosis (P = 0.02 and 0.03, respectively).The current study demonstrated for the first time that the Tiam1-Rac1 pathway may play a critical role in tumor progression of hepatocellular carcinoma. The expression of Tiam1 and Rac1 can be considered as the two useful indicators for predicting the prognosis of hepatocellular carcinoma. 
 ADP-ribosylation factor 6 regulates glioma cell invasion through the IQ-domain GTPase-activating protein 1-Rac1-mediated pathway. A common pathobiological feature of malignant gliomas is the insidious infiltration of single tumor cells into the brain parenchyma, rendering these deadly tumors virtually incurable with available therapies. In this study, we report that ADP-ribosylation factor 6 (ARF6), a Ras superfamily small GTPase, is abundantly expressed in invasive human glioma cells. Cellular depletion of ARF6 by small interfering RNA decreased Rac1 activation, impaired HGF-stimulated and serum-stimulated glioma cell   migration   in vitro, and markedly decreased the invasive capacity of invasive glioma in the brain. Furthermore, ectopic expression of ARF6 in glioma cells promoted cell   migration   via the activation of Rac1. Upon stimulation of glioma cells with HGF, we show that IQ-domain GTPase-activating protein 1 (IQGAP1) is recruited and overlaps with ARF6 at the leading edge of migrating cells. However, cellular depletion of ARF6 abrogated this recruitment of IQGAP1 and attenuated the formation of surface protrusions. ARF6 forms complexes with Rac1 and IQGAP1 in glioma cells upon HGF stimulation, and knockdown of IQGAP1 significantly inhibits ARF6-induced Rac1 activation and cell   migration  . Taken together, these data suggest that ARF6-mediated Rac1 activation is essential for glioma cell invasion via a signaling pathway that requires IQGAP1. 
 Cellular inhibitor of apoptosis protein 2 controls human colonic epithelial restitution,   migration  , and Rac1 activation. Identification of pathways involved in wound healing is important for understanding the pathogenesis of various intestinal diseases. Cellular inhibitor of apoptosis protein 2 (cIAP2) regulates proliferation and   migration   in nonepithelial cells and is expressed in human colonocytes. The aim of the study was to investigate the role of cIAP2 for wound healing in the normal human colon. Wound tissue was generated by taking rectosigmoidal biopsies across an experimental ulcer in healthy subjects after 5, 24, and 48 h. In experimental ulcers, the expression of cIAP2 in regenerating intestinal epithelial cells (IECs) was increased at the wound edge after 24 h (P < 0.05), returned to normal after reepithelialization, and correlated with the inflammatory reaction in the experimental wounds (P < 0.001). cIAP2 was induced in vitro in regenerating Caco2 IECs after wound infliction (P < 0.01). Knockdown of cIAP2 caused a substantial impairment of the IEC regeneration through inhibition of   migration   (P < 0.005). cIAP2 overexpression lead to formation of migrating IECs and upregulation of expression of RhoA and Rac1 as well as GTP-activation of Rac1. Transforming growth factor-β1 enhanced the expression of cIAP2 but was not upregulated in wounds in vivo and in vitro. NF-κB and MAPK pathways did not affect cIAP2 expression. cIAP2 is in conclusion a regulator of human intestinal wound healing through enhance  d migrati  on along with activation of Rac1, and the findings suggest that cIAP2 could be a future therapeutic target to improve intestinal wound healing. 
 PED interacts with Rac1 and regulates cell   migration  /invasion processes in human non-small cell lung cancer cells. PED (phosphoprotein enriched in diabetes) is a 15 kDa protein involved in many cellular pathways and human diseases including type II diabetes and cancer. We recently reported that PED is overexpressed in human cancers and mediates resistance to induced apoptosis. To better understand its role in cancer, we investigated on PED interactome in non-small cell lung cancer (NSCLC). By the Tandem Affinity Purification (TAP), we identified and characterized among others, Rac1, a member of mammalian Rho GTPase protein family, as PED-interacting protein. In this study we show that PED coadiuvates Rac1 activation by regulating AKT mediated Rac1-Ser(71) phosphorylation. Furthermore, we show that the expression of a constitutively active Rac, affected PED-Ser(104) phosphorylation, which is important for PED-regulated ERK 1/2 nuclear localization. Through specific Rac1-siRNA or its pharmacological inhibition, we demonstrate that PED augments   migration   and invasion in a Rac1-dependent manner in NSCLC. In conclusion, we show for the first time that PED and Rac1 interact and that this interaction modulates cell   migration  /invasion processes in cancer cells through ERK1/2 pathway. 
 Glycogen synthase kinase 3β sustains invasion of glioblastoma via the focal adhesion kinase, Rac1, and c-Jun N-terminal kinase-mediated pathway. The failure of current treatment options for glioblastoma stems from their inability to control tumor cell proliferation and invasion. Biologically targeted therapies offer great hope and one promising target is glycogen synthase kinase-3β (GSK3β), implicated in various diseases, including cancer. We previously reported that inhibition of GSK3β compromises the survival and proliferation of glioblastoma cells, induces their apoptosis, and sensitizes them to temozolomide and radiation. Here, we explore whether GSK3β also contributes to the highly invasive nature of glioblastoma. The effects of GSK3β inhibiti  on on mig  ration and invasion of glioblastoma cells were examined by wound-healing and Transwell assays, as well as in a mouse model of glioblastoma. We also investigated changes in cellular microarchitectures, cytoskeletal components, and proteins responsible for cell motility and invasion. Inhibition of GSK3β attenuat  ed the mi  gration and invasion of glioblastoma cells in vitro and that of tumor cells in a mouse model of glioblastoma. These effects were associated with suppression of the molecular axis involving focal adhesion kinase, guanine nucleotide exchange factors/Rac1 and c-Jun N-terminal kinase. Changes in cellular phenotypes responsible for cell motility and invasion were also observed, including decreased formation of lamellipodia and invadopodium-like microstructures and alterations in the subcellular localization, and activity of Rac1 and F-actin. These changes coincided with decreased expression of matrix metalloproteinases. Our results confirm the potential of GSK3β as an attractive therapeutic target against glioblastoma invasion, thus highlighting a second role in this tumor type in addition to its involvement in chemo- and radioresistance. 
 Urokinase-induced   migration   of human vascular smooth muscle cells requires coupling of the small GTPases RhoA and Rac1 to the Tyk2/PI3-K signalling pathway. Urokinase-type plasminogen activator (uPA) facilitates cell   migration   by localizing proteolisys on the cell surface and by inducing intracellular signalling pathways. In human vascular smooth muscle cell (VSMC) uPA stimulates   migration   via the uPA receptor (uPAR) signalling complex containing the Janus kinase Tyk2 and phosphatidylinositol 3-kinase (PI3-K). We report that active GTP-bound forms of small GTPases RhoA and Rac1, but not Cdc42, are directly associated with Tyk2 and PI3-K in an uPA/uPAR-dependent fashion. Endogenous RhoA, but not Rac1 or Cdc42, was significantly activated in response to uPA. RhoA activation was abolished by cell treatment with two unrelated, structurally distinct, specific inhibitors of PI3-K, wortmannin, and LY294002. Downstream of RhoA, phosphorylation of myosin light chain (MLC) was dramatically upregulated by uPA in a Rho kinase- and PI3-K-dependent manner. Thus, selective Rho kinase inhibitor Y27632 and PI3-K inhibitors wortmannin and LY294002 prevented the uPA-induced stimulation of MLC phosphorylation. Rho kinase inhibition also decreased uPA-stimulated VSMC   migration   as observed in a Boyden chamber. VSMC immunocytochemical staining demonstrated redistribution of RhoA and Rac1 active forms to the newly formed leading edge of migrating cell. VSMC microinjection with antibodies to either Rho or Rac1 decreased uPA-stimulated cell   migration  , indicating the involvement of both GTPases in the   migration   process. Our results provide evidence that the small GTPases RhoA and Rac1, together with Rho kinase, are necessary to mediate the uPA/uPAR-directed   migration   via the Tyk2/PI3-K signalling complex in human VSMC. 
 The slingshot family of phosphatases mediates Rac1 regulation of cofilin phosphorylation, laminin-332 organization, and motility behavior of keratinocytes. The motility of keratinocytes is an essential component of wound closure and the development of epidermal tumors. In vitro, the specific motile behavior of keratinocytes is dictated by the assembly of laminin-332 tracks, a process that is dependent upon alpha6beta4 integrin signaling to Rac1 and the actin-severing protein cofilin. Here we have analyzed how cofilin phosphorylation is regulated by phosphatases (slingshot (SSH) or chronophin (CIN)) downstream of signaling by alpha6beta4 integrin/Rac1 in human keratinocytes. Keratinocytes express all members of the SSH family (SSH1, SSH2, and SSH3) and CIN. However, expression of phosphatase-dead versions of all three SSH proteins, but not dominant inactive CIN, results in phosphorylation/inactivation of cofilin, changes in actin cytoskeleton organization, loss of cell polarity, and assembly of aberrant arrays of laminin-332 in human keratinocytes. SSH activity is regulated by 14-3-3 protein binding, and intriguingly, 14-3-3/alpha6beta4 integrin protein interaction is required for keratinocyte   migration  . We wondered whether 14-3-3 proteins function as regulators of Rac1-mediated keratinocyte   migration   patterns. In support of this hypothesis, inhibition of Rac1 results in an increase in 14-3-3 protein association with SSH. Thus, we propose a novel mechanism in which alpha6beta4 integrin signaling via Rac1, 14-3-3 proteins, and SSH family members regulates cofilin activation, cell polarity, and matrix assembly, leading to specific epidermal cell   migration   behavior. 
 Formyl peptide receptor-1 activation enhances intestinal epithelial cell restitution through phosphatidylinositol 3-kinase-dependent activation of Rac1 and Cdc42. Inflammatory disorders of the gastrointestinal tract result in the breakdown of the intestinal epithelial barrier in the form of erosion and ulceration. To reestablish the epithelial barrier, the epithelium must efficiently migrate to reseal wounds. Numerous signaling cascades are involved in the induction and regulation of this complex process. N-formyl peptide receptors comprise a group of Gi-coupled receptors that regulate innate immune responses. Previously, we identified the expression of functional N-formyl peptide receptors in model SK-CO15 intestinal epithelial cells and observed a role for activation of these receptors in regulating cellular invasive behavior. In these studies, we performed formyl peptide receptor-1 (FPR) localization and evaluated its role in regulating intestinal epithelial cell wound closure. Immunolocalization studies using a recently developed specific monoclonal anti-FPR Ab demonstrated its localization along the lateral membrane of crypt epithelial cells in normal human colonic epithelium. In vitro studies using the classical FPR agonist fMLF showed that FPR activation significantly enhances model intestinal epithelial cell restitution and that FPR localized along actin filaments in lamellipodial and filopodial extrusions. The increase in cell   migration   was associated with activation of PI3K, Rac1, and Cdc42. Pharmacologic inhibition of PI3K activity abrogated the fMLF-induced increase in wound closure and activation of both Rac1 and Cdc42. Inhibition of Rac1 and Cdc42 using pharmacologic inhibitors and dominant negative mutants also inhibited the fMLF-induced increase in cell   migration  . Taken together, theses results support a novel role for FPR stimulation in enhancing intestinal epithelial cell restitution through PI3K-dependent activation of Rac1 and Cdc42. 
 Reciprocal regulation of Rac1 and PAK-1 by HIF-1alpha: a positive-feedback loop promoting pulmonary vascular remodeling. Pulmonary vascular remodeling associated with pulmonary hypertension is characterized by media thickening, disordered proliferation, and in situ thrombosis. The p21-activated kinase-1 (PAK-1) can control growth,   migration  , and prothrombotic activity, and the hypoxia-inducible transcription factor HIF-1alpha was associated with pulmonary vascular remodeling. Here we studied whether PAK-1 and HIF-1alpha are linked in pulmonary vascular remodeling. PAK-1 was expressed in the media of remodeled pulmonary vessels from patients with pulmonary vasculopathy and was upregulated, together with its upstream regulator Rac1 and HIF-1alpha in lung tissue from lambs with pulmonary vascular remodeling. PAK-1 and Rac1 were activated by thrombin involving calcium, thus resulting in enhanced generation of reactive oxygen species (ROS) in human pulmonary artery smooth muscle cells (PASMCs). Activation of PAK-1 stimulated HIF activity and HIF-1alpha expression involving ROS and NF-kappaB, enhanced the expression of the HIF-1 target gene plasminogen activator inhibitor-1, and stimulated PASMC proliferation. Importantly, HIF-1 itself bound to the Rac1 promoter and enhanced Rac1 and PAK-1 transcription. Thus, PAK-1 and its activator Rac1 are novel HIF-1 targets that may constitute a positive-feedback loop for induction of HIF-1alpha by thrombin and ROS, thus explaining elevated levels of PAK-1, Rac1, and HIF-1alpha in remodeled pulmonary vessels. 
 Phosphorylation of Rac1 T108 by extracellular signal-regulated kinase in response to epidermal growth factor: a novel mechanism to regulate Rac1 function. Accumulating evidence has implicated Rho GTPases, including Rac1, in many aspects of cancer development. Recent findings suggest that phosphorylation might further contribute to the tight regulation of Rho GTPases. Interestingly, sequence analysis of Rac1 shows that Rac1 T108 within the (106)PNTP(109) motif is likely an extracellular signal-regulated kinase (ERK) phosphorylation site and that Rac1 also has an ERK docking site, (183)KKRKRKCLLL(192) (D site), at the C terminus. Indeed, we show here that both transfected and endogenous Rac1 interacts with ERK and that this interaction is mediated by its D site. Green fluorescent protein (GFP)-Rac1 is threonine (T) phosphorylated in response to epidermal growth factor (EGF), and EGF-induced Rac1 threonine phosphorylation is dependent on the activation of ERK. Moreover, mutant Rac1 with the mutation of T108 to alanine (A) is not threonine phosphorylated in response to EGF. In vitro ERK kinase assay further shows that pure active ERK phosphorylates purified Rac1 but not mutant Rac1 T108A. We also show that Rac1 T108 phosphorylation decreases Rac1 activity, partially due to inhibiting its interaction with phospholipase C-γ1 (PLC-γ1). T108 phosphorylation targets Rac1 to the nucleus, which isolates Rac1 from other guanine nucleotide exchange factors (GEFs) and hinders Rac1's role in cel  l migrati  on. We conclude that Rac1 T108 is phosphorylated by ERK in response to EGF, which plays an important role in regulating Rac1. 
 Sprouty regulates cell   migration   by inhibiting the activation of Rac1 GTPase. Sprouty (SPRY) protein negatively modulates fibroblast growth factor and epidermal growth factor actions. We showed that human SPRY2 inhibits cell growth and   migration   in response to serum and several growth factors. Using rat intestinal epithelial (IEC-6) cells, we investigated the involvement of the Rho family of GTPases, RhoA, Rac1, and cdc42 in SPRY2-mediated inhibition of cell   migration   and proliferation. The ability of TAT-tagged SPRY2 to inhibit proliferation and   migration   of IEC-6 cells transfected with constitutively active mutants of RhoA(G14V), Rac1(G12V), and cdc42 (F28L) was determined. Constitutively active RhoA(G14V), Rac1(G12V), or cdc42(F28L) did not protect cells from the anti-proliferative actions of TAT-SPRY2. The ability of TAT-hSPRY2 to inhibit   migration   was not altered by of RhoA(G14V) and cdc42(F28L). However, Rac1(G12V) obliterated the ability of SPRY2 to inhibit cell autonomous or serum-induced   migration  . Also, the activation of endogenous Rac1 was attenuated by TAT-SPRY2. Thus, SPRY2 mediates its anti-migratory actions by inhibiting Rac1 activation. 
 CD147 promotes Src-dependent activation of Rac1 signaling through STAT3/DOCK8 during the motility of hepatocellular carcinoma cells. Metastasis is considered a dynamic process in tumor development that is related to abnormal   migration   and invasion. Tumor cells can move as individual cells in two interconvertible modes: mesenchymal-type and amoeboid. Previously, we reported that the interaction between CD147 and Annexin II can inhibit the amoeboid movement in hepatocellular carcinoma (HCC) cells. However, the mechanism of CD147 involved in mesenchymal movement is still unclear. Notably, our results show overexpression of CD147 led to mesenchymal-type movement in HCC cells. Evidence indicated that the mesenchymal-type cell movement induced by CD147 was Src dependent, as observed by confocal microscopy and Rac1 activity assay. The phosphorylation of Src (pY416-Src) can be up-regulated by CD147, and this regulation is mediated by focal adhesion kinase (FAK). Next, we identified DOCK8 as a GEF for Rac1, a key molecule driving mesenchymal-type movement. We also found that Src promotes STAT3 phosphorylation and STAT3 facilitates DOCK8 transcription, thus enhancing DOCK8 expression and Rac1 activation. This study provides a novel mechanism of CD147 regulating mesenchymal-type movement in HCC cells. 
 Rac1 mutations produce aberrant epithelial differentiation in the developing and adult mouse small intestine. The mouse small intestinal epithelium undergoes continuous renewal throughout life. Previous studies suggest that differentiation of this epithelium is regulated by instructions that are received as cells migrate along crypt-villus units. The nature of the instructions and their intracellular processing remain largely undefined. In this report, we have used genetic mosaic analysis to examine the role of Rac1 GTPase-mediated signaling in controlling differentiation. A constitutively active mutation (Rac1Leu61) or a dominant negative mutation (Rac1Asn17) was expressed in the 129/Sv embryonic stem cell-derived component of the small intestine of C57Bl/6-ROSA26<->129/Sv mice. Rac1Leu61 induces precocious differentiation of members of the Paneth cell and enterocytic lineages in the proliferative compartment of the fetal gut, without suppressing cell division. Forced expression of the dominant negative mutation inhibits epithelial differentiation, without affecting cell division, and slows enterocytic   migration   along crypt-villus units. The effects produced by Rac1Leu61 or Rac1Asn17 in the 129/Sv epithelium do not spread to adjacent normal C57Bl/6 epithelial cells. These results provide in vivo evidence that Rac1 is involved in the import and intracellular processing of signals that control differentiation of a mammalian epithelium. 
 Role of SNTA1 in Rac1 activation, modulation of ROS generation, and migratory potential of human breast cancer cells. Alpha-1-syntrophin (SNTA1) has been implicated in the activation of Rac1. However, the underlying mechanism has not yet been explored. Here, we show that a novel complex, involving SNTA1, P66shc, and Grb2 proteins, is involved in Rac1 activation.Co-immunoprecipitation assays were used to show the complex formation, while siRNAs and shRNAs were used to downregulate expression of these proteins. Various Rac1 activation assays and functional assays, such as   migration   assays, in vitro wound healing assays, cell proliferation assays, and ROS generation assays, were also performed.The results showed a significant increase in activation of Rac1 when SNTA1 and P66shc were overexpressed, whereas depletion of SNTA1 and P66shc expression effectively reduced the levels of active Rac1. The results indicated a significant displacement of Sos1 protein from Grb2 when SNTA1 and P66shc are overexpressed in breast cancer cell lines, resulting in Sos1 predominantly forming a complex with Eps8 and E3b1. In addition, the SNTA1/P66shc-mediated Rac1 activation resulted in an increase in reactive oxygen species (ROS) production and migratory potential in human breast cancer cells.Together, our results present a possible mechanism of Rac1 activation involving SNTA1 and emphasise its role in ROS generation, cell   migration  , and acquisition of malignancy. 
 Essential role of class II phosphatidylinositol-3-kinase-C2α in sphingosine 1-phosphate receptor-1-mediated signaling and   migratio  n in endothelial cells. The phosphatidylinositol (PtdIns) 3-kinase (PI3K) family regulates diverse cellular processes, including cell proliferation,   migratio  n, and vesicular trafficking, through catalyzing 3'-phosphorylation of phosphoinositides. In contrast to class I PI3Ks, including p110α and p110β, functional roles of class II PI3Ks, comprising PI3K-C2α, PI3K-C2β, and PI3K-C2γ, are little understood. The lysophospholipid mediator sphingosine 1-phosphate (S1P) plays the important roles in regulating vascular functions, including vascular formation and barrier integrity, via the G-protein-coupled receptors S1P(1-3). We studied the roles of PI3K-C2α in S1P-induced endothelial cel  l (EC) mi  gration and tube formation. S1P stimulate  d cell mi  gration and activation of Akt, ERK, and Rac1, the latter of which acts as a signaling molecule essential fo  r cell mi  gration and tube formation, via S1P(1) in ECs. Knockdown of either PI3K-C2α or class I p110β markedly inhibited S1P  -induced   migration, lamellipodium formation, and tube formation, whereas that of p110α or Vps34 did not. Only p110β was necessary for S1P-iduced Akt activation, but both PI3K-C2α and p110β were required for Rac1 activation. FRET imaging showed that S1P induced Rac1 activation in both the plasma membrane and PtdIns 3-phosphate (PtdIns(3)P)-enriched endosomes. Knockdown of PI3K-C2α but not p110β markedly reduced PtdIns(3)P-enriched endosomes and suppressed endosomal Rac1 activation. Also, knockdown of PI3K-C2α but not p110β suppressed S1P-induced S1P(1) internalization into PtdIns(3)P-enriched endosomes. Finally, pharmacological inhibition of endocytosis suppressed S1P-induced S1P(1) internalization,   Rac1 acti  vation, migration, and tube formation. These observations indicate that PI3K-C2α plays the crucial role in S1P(1) internalization into the intracellular vesicular compartment, Rac1 activation on endos  omes, and   thereby migration through regulating vesicular trafficking in ECs. 
 RhoGDI2 is expressed in human trophoblasts and involved in their   migration   by inhibiting the activation of RAC1. The invasion of placental trophoblast cells into the maternal decidua is an essential aspect of placental embedment. The process of placentation bears several striking similarities to tumor cell metastasis. However, trophoblastic   migration   during implantation and placentation is stringently controlled both in space and time. RhoGDI2 belongs to a family of Rho guanosine diphosphate dissociation inhibitors (RhoGDIs), and RhoGDI2 is a metastasis suppressor gene and a marker of aggressive human cancer. We evaluated whether RhoGDI2 has a physiological role in embryo implantation, particularly trophoblast   migration  . The mRNA and protein expression levels of RhoGDI2 were higher in term placentas compared with first-trimester placentas as detected by real-time PCR and Western blot. Immunohistochemical studies indicated that RhoGDI2 localized to the cytotrophoblast layer and extravillous trophoblast in first-trimester placentas and was distributed in the syncytiotrophoblast layers of term placentas. Overexpression of RhoGDI2 in HTR-8/SVneo cells was associated with reduced RAC1-guanosine triphosphate (GTP) levels and inhibited cell   migration  . Conversely, small interfering RNA-mediated downregulation of RhoGDI2 resulted in significantly increased RAC1-GTP levels. Altered RhoGDI2 expression had no significant effects on cell proliferation. In conclusion, RhoGDI2 inhibits trophoblast cell   migration  , and this function may involve suppression of RAC1 activation. 
 The activation of the NF-κB-JNK pathway is independent of the PI3K-Rac1-JNK pathway involved in the bFGF-regulated human fibroblast cell   migratio  n. Skin wound healing is a complex process that repairs multiple organ-tissues. Fibroblasts are key players of skin cells, whose   migratio  n is important during wound healing process. bFGF has shown a great efficacy to promote cell   migratio  n, but the precise mechanism by which bFGF regulates cell   migratio  n remains elusive.The aim of this study was to find bFGF-regulated gene pools and further identify target molecules that participated in human fibroblast cell   migratio  n.Skin primary fibroblasts and rat skin wound model were used to demonstrate the novel mechanism of bFGF regulating cell   migratio  n to accelerate wound healing. Cell   migratio  n was determined using the wound healing scratch assay. The differentially expressed genes and numerous biochemical pathways after bFGF treatment were identified by RNA-Seq analysis, and differentially expressed genes were further verified by qRT-PCR. siRNA duplex target to interfering the expression of PI3-kinase (p110α) was transformed into NIH/3T3 cells. Western blotting analysis was used to determine marker protein expressions. The invasive activity of fibroblasts was measured using 3D spheroid cell invasion assay.RNA-Seq analysis identified numerous biochemical pathways including the NF-κB pathway under the control of FGF signaling. bFGF negatively regulates the phosphorylation of IκB-α, the most well studied NF-κB signaling regulator while bFGF induces JNK phosphorylation. Application of Bay11-7082, a representative NF-κB inhibitor promote  d cell mi  gration, invasion and enhanced the JNKs phosphorylation. However, inhibition of JNKs blocke  d cell mi  gration when NF-κB is inhibited. Moreover, application of the PI3K inhibitor LY294002 together with Bay11-7082 maintained norm  al cell m  igration and knocking-down PI3K (p110α) by a specific siRNA inhibited JNKs phosphorylation while maintaining normal IκBα phosphorylation, indicating that PI3K and NF-κB signaling independently regulate JNKs activation. In addition, administration of bFGF or Bay11-7082 promoted rat skin wound repair and accelerated the invasion of fibroblasts.This study sheds light on the mode of action of bFGF and identifies that the NF-κB-JNKs pathway is independent of the PI3K-JNKs pathway to accelerat  e fibrobl  ast migration. In addition, bFGF and the relief of inflammation could be a favorable therapeutic approach for skin wound healing. 
 TROY (TNFRSF19) is overexpressed in advanced glial tumors and promotes glioblastoma cell invasion via Pyk2-Rac1 signaling. A critical problem in the treatment of malignant gliomas is the extensive infiltration of individual tumor cells into adjacent brain tissues. This invasive phenotype severely limits all current therapies, and to date, no treatment is available to control the spread of this disease. Members of the tumor necrosis factor (TNF) ligand superfamily and their cognate receptors regulate various cellular responses including proliferation,   migration  , differentiation, and apoptosis. Specifically, the TNFRSF19/TROY gene encodes a type I cell surface receptor that is expressed on migrating or proliferating progenitor cells of the hippocampus, thalamus, and cerebral cortex. Here, we show that levels of TROY mRNA expression directly correlate with increasing glial tumor grade. Among malignant gliomas, TROY expression correlates inversely with overall patient survival. In addition, we show that TROY overexpression in glioma cells activates Rac1 signaling in a Pyk2-dependent manner to drive glioma cell invasion and   migration  . Pyk2 coimmunoprecipitates with the TROY receptor, and depletion of Pyk2 expression by short hairpin RNA interference oligonucleotides inhibits TROY-induced Rac1 activation and subsequent cellular   migration  . These findings position aberrant expression and/or signaling by TROY as a contributor, and possibly as a driver, of the malignant dispersion of glioma cells. 
 The microRNA -23b/-27b cluster suppresses the metastatic phenotype of castration-resistant prostate cancer cells. MicroRNAs (miRs) are small, endogenous, non-coding RNAs that regulate the stability and/or translation of complementary mRNA targets. MiRs have emerged not only as critical modulators of normal physiologic processes, but their deregulation may significantly impact prostate and other cancers. The expression of miR-23b and miR-27b, which are encoded by the same miR cluster (miR-23b/-27b), are downregulated in metastatic, castration-resistant tumors compared to primary prostate cancer and benign tissue; however, their possible role in prostate cancer progression is unknown. We found that ectopic expression of miR-23b/-27b in two independent castration-resistant prostate cancer cell lines resulted in suppression of invasion and   migration  , as well as reduced survival in soft agar (a measure of anoikis). However, there was no effect of miR-23b/-27b on cell proliferation suggesting that these miRs function as metastasis (but not growth) suppressors in prostate cancer. Conversely, inhibition of miR-23b/-27b in the less aggressive androgen-dependent LNCaP prostate cancer cell line resulted in enhanced invasion and   migration   also without affecting proliferation. Mechanistically, we found that introduction of miR-23b/-27b in metastatic, castration-resistant prostate cancer cell lines resulted in a significant attenuation of Rac1 activity without affecting total Rac1 levels and caused increased levels of the tumor suppressor E-cadherin. Inhibition of these miRs had the opposite effect in androgen-dependent LNCaP cells. These results suggest that miR-23b/-27b are metastasis suppressors that might serve as novel biomarkers and therapeutic agents for castration-resistant disease. 
 Endogenous ARF6 interacts with Rac1 upon angiotensin II stimulation to regulate membrane ruffling and cell   migration  . ARF6 and Rac1 are small GTPases known to regulate remodelling of the actin cytoskeleton. Here, we demonstrate that these monomeric G proteins are sequentially activated when HEK 293 cells expressing the angiotensin type 1 receptor (AT(1)R) are stimulated with angiotensin II (Ang II). After receptor activation, ARF6 and Rac1 transiently form a complex. Their association is, at least in part, direct and dependent on the nature of the nucleotide bound to both small G proteins. ARF6-GTP preferentially interacts with Rac1-GDP. AT(1)R expressing HEK293 cells ruffle, form membrane protrusions, and migrate in response to agonist treatment. ARF6, but not ARF1, depletion using small interfering RNAs recapitulates the ruffling and migratory phenotype observed after Ang II treatment. These results suggest that ARF6 depletion or Ang II treatment are functionally equivalent and point to a role for endogenous ARF6 as an inhibitor of Rac1 activity. Taken together, our findings reveal a novel function of endogenously expressed ARF6 and demonstrate that by interacting with Rac1, this small GTPase is a central regulator of the signaling pathways leading to actin remodeling. 
 The role of Crk/Dock180/Rac1 pathway in the malignant behavior of human ovarian cancer cell SKOV3. Small GTPases, particularly the Rho family, are key regulators of cell motility and   migration  . Dock180 was well known for the main target of signal adaptor protein Crk and acted as a guanine-nucleotide exchange factor for small GTPase Rac1. In the present study, Dock180 was found to combine primarily with CrkI other than CrkII, and its association with Elmo1 was also demonstrated in ovarian cancer cell SKOV3. To evaluate the role of Dock180 in human ovarian cancer cell, we performed RNAi-mediated knockdown of Dock180 in SKOV3 cells using small interfering RNA expression vector. In Dock180 knockdown cells, we found that Elmo1 expression and Rac1 activity were decreased simultaneously. By contrast, the expressions of both another Crk-combining molecule C3G and Rap1 activity were observed to increase obviously. Accordingly, all Dock180 knockdown cells present with evident change in cell morphology, reduced cell proliferation, and attenuated cell   migration  . Taken together, these results suggest that signal transfer of Crk/Dock180/Rac1 is implicated in actin cytoskeleton reorganization and thus in the cell proliferation, motility, invasion, and of human ovarian cancer cell line SKOV3. 
 A palmitoylation switch mechanism regulates Rac1 function and membrane organization. The small GTPase Rac1 plays important roles in many processes, including cytoskeletal reorganization, cell   migration  , cell-cycle progression and gene expression. The initiation of Rac1 signalling requires at least two mechanisms: GTP loading via the guanosine triphosphate (GTP)/guanosine diphosphate (GDP) cycle, and targeting to cholesterol-rich liquid-ordered plasma membrane microdomains. Little is known about the molecular mechanisms governing this specific compartmentalization. We show that Rac1 can incorporate palmitate at cysteine 178 and that this post-translational modification targets Rac1 for stabilization at actin cytoskeleton-linked ordered membrane regions. Palmitoylation of Rac1 requires its prior prenylation and the intact C-terminal polybasic region and is regulated by the triproline-rich motif. Non-palmitoylated Rac1 shows decreased GTP loading and lower association with detergent-resistant (liquid-ordered) membranes (DRMs). Cells expressing no Rac1 or a palmitoylation-deficient mutant have an increased content of disordered membrane domains, and markers of ordered membranes isolated from Rac1-deficient cells do not correctly partition in DRMs. Importantly, cells lacking Rac1 palmitoylation show spreading and   migration   defects. These data identify palmitoylation as a mechanism for Rac1 function in actin cytoskeleton remodelling by controlling its membrane partitioning, which in turn regulates membrane organization. 
 CD81 regulates cell   migration   through its association with Rac GTPase. CD81 is a member of the tetraspanin family that has been described to have a key role in cell   migration   of tumor and immune cells. To unravel the mechanisms of CD81-regulated cell   migration  , we performed proteomic analyses that revealed an interaction of the tetraspanin C-terminal domain with the small GTPase Rac. Direct interaction was confirmed biochemically. Moreover, microscopy cross-correlation analysis demonstrated the in situ integration of both molecules into the same molecular complex. Pull-down experiments revealed that CD81-Rac interaction was direct and independent of Rac activation status. Knockdown of CD81 resulted in enhanced protrusion rate, altered focal adhesion formation, and decreased cell   migration  , correlating with increased active Rac. Reexpression of wild-type CD81, but not its truncated form lacking the C-terminal cytoplasmic domain, rescued these effects. The phenotype of CD81 knockdown cells was mimicked by treatment with a soluble peptide with the C-terminal sequence of the tetraspanin. Our data show that the interaction of Rac with the C-terminal cytoplasmic domain of CD81 is a novel regulatory mechanism of the GTPase activity turnover. Furthermore, they provide a novel mechanism for tetraspanin-dependent regulation of cell motility and open new avenues for tetraspanin-targeted reagents by the use of cell-permeable peptides. 
 Denbinobin, a phenanthrene from Dendrobium nobile, impairs prostate cancer   migration   by inhibiting Rac1 activity. Prostate cancer is the most prevalent type of cancer in the United States. The most common site of prostate cancer metastasis is bone. CXCL12 is preferentially expressed in bone and is targeted by prostate cancer cells, which over-express the receptor for CXCL12, CXCR4. In response to CXCL12 stimulation, Rac1, a GTPase, along with its effectors, regulates actin polymerization to form lamellipodia, which is a critical event for cell   migration  . Cortactin, an actin-binding protein, is recruited to the lamellipodia and is phosphorylated at tyrosine residues. The phosphorylated cortactin is also involved in cell   migration  . The inhibition of Rac1 activity using a dominant negative Rac1 impairs lamellipodial protrusion as well as cortactin translocation and cortactin phosphorylation. Denbinobin, a substance extracted from Dendrobium nobile, has anticancer effects in many cancer cell lines. Whether denbinobin can inhibit prostate cancer cell   migration   is not clear. Here, we report that denbinobin inhibited Rac1 activity. The inhibition of Rac1 activity prevented lamellipodial formation. Cortactin phosphorylation and translocation to the lamellipodia were also impaired, and PC3 cells were unable to migrate. These results indicate that denbinobin prevents CXCL12-induced PC3 cell   migration   by inhibiting Rac1 activity. 
 DDX3 modulates cell adhesion and motility and cancer cell metastasis via Rac1-mediated signaling pathway. The DEAD-box RNA helicase DDX3 is a versatile protein involved in multiple steps of gene expression and various cellular signaling pathways. DDX3 mutations have been implicated in the wingless (Wnt) type of medulloblastoma. We show here that small interfering RNA-mediated DDX3 knockdown in various cell lines increased cell-cell adhesion but decreased cell-extracellular matrix adhesion. Moreover, DDX3 depletion suppressed cell motility and impaired directional   migration   in the wound-healing assay. Accordingly, DDX3-depleted cells exhibited reduced invasive capacities in vitro as well as reduced metastatic potential in mice. We also examined the mechanism underlying DDX3-regulated cell   migration  . DDX3 knockdown reduced the levels of both Rac1 and β-catenin proteins, and consequentially downregulated the expression of several β-catenin target genes. Moreover, we demonstrated that DDX3-regulated Rac1 mRNA translation, possibly through an interaction with its 5'-untranslated region, and affected β-catenin protein stability in an Rac1-dependent manner. Taken together, our results indicate the DDX3-Rac1-β-catenin regulatory axis in modulating the expression of Wnt/β-catenin target genes. Therefore, this report provides a mechanistic context for the role of DDX3 in Wnt-type tumors. 
 The human minor histocompatibility antigen 1 is a RhoGAP. The human minor Histocompatibility Antigen HMHA-1 is a major target of immune responses after allogeneic stem cell transplantation applied for the treatment of leukemia and solid tumors. The restriction of its expression to hematopoietic cells and many solid tumors raised questions regarding its cellular functions. Sequence analysis of the HMHA-1 encoding HMHA1 protein revealed the presence of a possible C-terminal RhoGTPase Activating Protein (GAP) domain and an N-terminal BAR domain. Rho-family GTPases, including Rac1, Cdc42, and RhoA are key regulators of the actin cytoskeleton and control cell spreading and   migration  . RhoGTPase activity is under tight control as aberrant signaling can lead to pathology, including inflammation and cancer. Whereas Guanine nucleotide Exchange Factors (GEFs) mediate the exchange of GDP for GTP resulting in RhoGTPase activation, GAPs catalyze the low intrinsic GTPase activity of active RhoGTPases, resulting in inactivation. Here we identify the HMHA1 protein as a novel RhoGAP. We show that HMHA1 constructs, lacking the N-terminal region, negatively regulate the actin cytoskeleton as well as cell spreading. Furthermore, we show that HMHA1 regulates RhoGTPase activity in vitro and in vivo. Finally, we demonstrate that the HMHA1 N-terminal BAR domain is auto-inhibitory as HMHA1 mutants lacking this region, but not full-length HMHA1, showed GAP activity towards RhoGTPases. In conclusion, this study shows that HMHA1 acts as a RhoGAP to regulate GTPase activity, cytoskeletal remodeling and cell spreading, which are crucial functions in normal hematopoietic and cancer cells. 
 p190 Rho-GTPase activating protein associates with plexins and it is required for semaphorin signalling. Plexins are transmembrane receptors for semaphorins, guiding cell   migration   and axon extension. Plexin activation leads to the disassembly of integrin-based focal adhesive structures and to actin cytoskeleton remodelling and inhibition of cell   migration  ; however, the underlying molecular mechanisms are unclear. We consistently observe a transient decrease of cellular RhoA-GTP levels upon plexin activation in adherent cells. One of the main effectors of RhoA downregulation is p190, a ubiquitously expressed GTPase activating protein (GAP). We show that, in p190-deficient fibroblasts, the typical functional activities mediated by plexins (such as cell collapse and inhibition of integrin-based adhesion) are blocked or greatly impaired. Notably, the functional response can be rescued in these cells by re-expressing exogenous p190, but not a mutant form specifically lacking RhoGAP activity. We furthermore demonstrate that semaphorin function is blocked in epithelial cells, primary endothelial cells and neuroblasts upon treatment with small interfering RNAs that knockdown p190 expression. Finally, we show that p190 transiently associates with plexins, and its RhoGAP activity is increased in response to semaphorin stimulation. We conclude that p190-RhoGAP is crucially involved in semaphorin signalling to the actin cytoskeleton, via interaction with plexins. 
 Human neutrophils utilize a Rac/Cdc42-dependent MAPK pathway to direct intracellular granule mobilization toward ingested microbial pathogens. Elevated levels of mitogen-activated protein kinase/extracellular regulatory kinase (MAPK/ERK) activity are frequently found in some cancer cells. In efforts to reduce tumor growth, attempts have been made to develop cancer therapeutic agents targeting the MAPK. Here, by use of biologic, biochemical, and gene manipulation methods in human polymorphonuclear neutrophils (PMNs), we have identified a key pathway important in normal cell function involving MAPK/ERK in PMNs for growth inhibition of Candida albicans. Contact with C albicans triggered MAPK/ERK activation in PMNs within 5 minutes, and blocking of MAPK/ERK activation, either by the pharmacologic reagent PD098059 or by dominant-negative MAPK kinase (MEK) expression via vaccinia viral delivery, suppressed antimicrobial activity. Rac and Cdc42, but not Ras or Rho, were responsible for this MAPK/ERK activation. Expression of dominant-negative Rac (N17Rac) or Cdc42 (N17Cdc42) eliminated not only C albicans- mediated ERK phosphorylation but also phagocytosis and granule   migration   toward the ingested microbes, whereas dominant-negative Ras (N17Ras) and Rho (N19Rho) did not. PAK1 (p21-activated kinase 1) activation is induced by C albicans, suggesting that PAK1 may also be involved in the Rac1 activation of MAPK/ERK. We conclude from these data that Rac/Cdc42-dependent activation of MAPK/ERK is a critical event in the immediate phagocytic response of PMNs to microbial challenge. Therefore, use of MAPK pharmacologic inhibitors for the treatment of cancer may result in the interruption of normal neutrophil function. A balance between therapeutic outcome and undesirable side effects must be attained to achieve successful and safe anticancer therapy. 
 Regulation of Rac1 and Cdc42 activation in thrombin- and collagen-stimulated CHRF-288-11 cells. Rac1 and Cdc42 are members of the Rho family of small GTPases and have been shown to promote the formation of lamellipodia and filopodia at the leading edge of motile cells and affect cell   migration  . In this study the authors have investigated the activation of Rac1 and Cdc42 by thrombin or collagen using the megakaryocytic cell line, CHRF-288-11. Maximal activation of Rac1 by thrombin or collagen was observed at 3 and 1 min, respectively. Similar results were obtained for the activation of Cdc42 by thrombin or collagen. The activation of Rac1 by thrombin and collagen was inhibited by the calmodulin-specific inhibitor, W7. However, W7 had no effect on the activation of Cdc42 by thrombin or collagen. The less potent calmodulin inhibitor, W5, did not have any effect on Rac1 or Cdc42 activation by thrombin or collagen. Transient over-expression of calmodulin in CHRF cells increased the basal and thrombin-mediated activation of Rac1 when compared to control but had no effect on the basal and thrombin-mediated activation of Cdc42 when compared to control. The results suggest that calmodulin regulates the activation of Rac1 in CHRF-288-11 cells in a manner similar to that in the human platelets. 
 Adiponectin promotes   migration   activities of endothelial progenitor cells via Cdc42/Rac1. Adiponectin has anti-atherosclerotic effects through its direct actions on vascular cells. The present study investigates the molecular mechanisms of adiponectin in the   migration   of endothelial progenitor cells (EPCs) which play an important role in neovascularization and re-endothelization. The phosphorylation of Akt and the activations of Cdc42 and Rac1 were significantly increased by adiponectin. Adiponectin increased the   migration   activity of EPCs, which was completely inhibited by a PI3-kinase inhibitor. siRNA of Cdc42 or Rac1 completely inhibited the adiponectin-induced   migration  , but siRNA of Akt had no effects, indicating that adiponectin promotes the   migration   activities of EPCs mainly through PI3-kinase/Cdc42/Rac1. 
 The small GTPase Rac1 is involved in the maintenance of stemness and malignancies in glioma stem-like cells. A subpopulation of cancer cells with stem cell properties is responsible for tumor formation, maintenance, and malignant progression; however, the molecular mechanisms underlying the maintenance of cancer stem-like cell properties have remained unclear. Here, we show that the Rho family GTPase Rac1 is involved in the glioma stem-like cell (GSLC) maintenance and tumorigenicity in human glioma. The Rac1-Pak signaling was markedly activated in GSLCs. Knockdown of Rac1 caused reduction of expression of GSLC markers, self-renewal-related proteins and neurosphere formation. Moreover, down-regulation of Rac1 suppressed the   migration  , invasion, and malignant transformation in GSLCs. Furthermore, inhibition of Rac1 enhanced radiation sensitivity of GSLCs. These results indicate that the small GTPase Rac1 is involved in the maintenance of stemness and malignancies in GSLCs. 
 Phosphoinositide-3 kinase-Akt pathway controls cellular entry of Ebola virus. The phosphoinositide-3 kinase (PI3K) pathway regulates diverse cellular activities related to cell growth,   migration  , survival, and vesicular trafficking. It is known that Ebola virus requires endocytosis to establish an infection. However, the cellular signals that mediate this uptake were unknown for Ebola virus as well as many other viruses. Here, the involvement of PI3K in Ebola virus entry was studied. A novel and critical role of the PI3K signaling pathway was demonstrated in cell entry of Zaire Ebola virus (ZEBOV). Inhibitors of PI3K and Akt significantly reduced infection by ZEBOV at an early step during the replication cycle. Furthermore, phosphorylation of Akt-1 was induced shortly after exposure of cells to radiation-inactivated ZEBOV, indicating that the virus actively induces the PI3K pathway and that replication was not required for this induction. Subsequent use of pseudotyped Ebola virus and/or Ebola virus-like particles, in a novel virus entry assay, provided evidence that activity of PI3K/Akt is required at the virus entry step. Class 1A PI3Ks appear to play a predominant role in regulating ZEBOV entry, and Rac1 is a key downstream effector in this regulatory cascade. Confocal imaging of fluorescently labeled ZEBOV indicated that inhibition of PI3K, Akt, or Rac1 disrupted normal uptake of virus particles into cells and resulted in aberrant accumulation of virus into a cytosolic compartment that was non-permissive for membrane fusion. We conclude that PI3K-mediated signaling plays an important role in regulating vesicular trafficking of ZEBOV necessary for cell entry. Disruption of this signaling leads to inappropriate trafficking within the cell and a block in steps leading to membrane fusion. These findings extend our current understanding of Ebola virus entry mechanism and may help in devising useful new strategies for treatment of Ebola virus infection. 
 Type XVII collagen regulates lamellipod stability, cell motility, and signaling to Rac1 by targeting bullous pemphigoid antigen 1e to alpha6beta4 integrin. Rac1 activity, polarity, lamellipodial dynamics, and directed motility are defective in keratinocytes exhibiting deficiency in β4 integrin or knockdown of the plakin protein Bullous Pemphigoid Antigen 1e (BPAG1e). The activity of Rac, formation of stable lamellipodia, and directed   migratio  n are restored in β4 integrin-deficient cells by inducing expression of a truncated form of β4 integrin, which lacks binding sites for BPAG1e and plectin. In these same cells, BPAG1e, the truncated β4 integrin, and type XVII collagen (Col XVII), a transmembrane BPAG1e-binding protein, but not plectin, colocalize along the substratum-attached surface. This finding suggested to us that Col XVII mediates the association of BPAG1e and α6β4 integrin containing the truncated β4 subunit and supports di  rected mi  gration. To test these possibilities, we knocked down Col XVII expression in keratinocytes expressing both full-length and truncated β4 integrin proteins. Col XVII-knockdown keratinocytes exhibit a loss in BPAG1e-α6β4 integrin interaction, a reduction in lamellipodial stability, an impairment in directional motility, and a decrease in Rac1 activity. These defects are rescued by a mutant Col XVII protein truncated at its carboxyl terminus. In summary, our results suggest that in motile cells Col XVII recruits BPAG1e to α6β4 integrin and is necessary for activation of signaling pathways, motile behavior, and lamellipodial stability. 
 Cancer cell-autonomous TRAIL-R signaling promotes KRAS-driven cancer progression, invasion, and metastasis. Many cancers harbor oncogenic mutations of KRAS. Effectors mediating cancer progression, invasion, and metastasis in KRAS-mutated cancers are only incompletely understood. Here we identify cancer cell-expressed murine TRAIL-R, whose main function ascribed so far has been the induction of apoptosis as a crucial mediator of KRAS-driven cancer progression, invasion, and metastasis and in vivo Rac-1 activation. Cancer cell-restricted genetic ablation of murine TRAIL-R in autochthonous KRAS-driven models of non-small-cell lung cancer (NSCLC) and pancreatic ductal adenocarcinoma (PDAC) reduces tumor growth, blunts metastasis, and prolongs survival by inhibiting cancer cell-autonomous   migratio  n, proliferation, and invasion. Consistent with this, high TRAIL-R2 expression correlates with invasion of human PDAC into lymph vessels and with shortened metastasis-free survival of KRAS-mutated colorectal cancer patients. 
 The IQGAP-related protein DGAP1 interacts with Rac and is involved in the modulation of the F-actin cytoskeleton and control of cell motility. DGAP1 of Dictyostelium discoideum is a cell cortex associated 95 kDa protein that shows homology to both RasGTPase-activating proteins (RasGAPs) and RasGAP-related proteins. When tested for RasGAP activity, recombinant DGAP1 protein did not promote the GTPase activity of human H-Ras or of Dictyostelium RasG in vitro. Instead, DGAP1 bound to Dictyostelium Rac1A and human Rac1, but not to human Cdc42. DGAP1 preferentially interacted with the activated GTP-bound forms of Rac1 and Rac1A, but did not affect the GTPase activities. Since Rho-type GTPases are implicated in the formation of specific F-actin structures and in the control of cell morphology, the microfilament system of mutants that either lack or overexpress DGAP1 has been analysed. DGAP1-null mutants showed elevated levels of F-actin that was organised in large leading edges, membrane ruffles or numerous large filopods. Expression of actin fused to green fluorescent protein (GFP) was used to monitor the actin dynamics in these cells, and revealed that the F-actin cytoskeleton of DGAP1-null cells was rapidly re-arranged to form ruffles and filopods. Conversely, in DGAP1-overexpressing cells, the formation of cellular projections containing F-actin was largely suppressed. Measurement of cell   migration   demonstrated that DGAP1 expression is inversely correlated with the speed of cell motility. 
 Differential roles of Smad2 and Smad3 in the regulation of TGF-β1-mediated growth inhibition and cell   migratio  n in pancreatic ductal adenocarcinoma cells: control by Rac1. Progression of pancreatic ductal adenocarcinoma (PDAC) is largely the result of genetic and/or epigenetic alterations in the transforming growth factor-beta (TGF-β)/Smad signalling pathway, eventually resulting in loss of TGF-β-mediated growth arrest and an increase in cellul  ar migrat  ion, invasion, and metastasis. These cellular responses to TGF-β are mediated solely or partially through the canonical Smad signalling pathway which commences with activation of receptor-regulated Smads (R-Smads) Smad2 and Smad3 by the TGF-β type I receptor. However, little is known on the relative contribution of each R-Smad, the possible existence of functional antagonism, or the crosstalk with other signalling pathways in the control of TGF-β1-induced growth inhibition and   cell mig  ration. Using genetic and pharmacologic approaches we have inhibited in PDAC cells endogenous Smad2 and Smad3, as well as a potential regulator, the small GTPase Rac1, and have analysed the consequences for TGF-β1-mediated growth inhibition an  d cell mi  gration (chemokinesis).SiRNA-mediated silencing of Smad3 in the TGF-β responsive PDAC cell line PANC-1 reduced TGF-β1-induced growth inhibition but increased the migratory response, while silencing of Smad2 enhanced growth inhibition but decreased chemokinesis. Interestingly, siRNA-mediated silencing of the small GTPase Rac1, or ectopic expression of a dominant-negative Rac1 mutant largely mimicked the effect of Smad2 silencing on both TGF-β1-induced growth inhibition, via upregulation of the cdk inhibitor p21WAF1,   and cell   migration. Inhibition of Rac1 activation reduced both TGF-β1-induction of a Smad2-specific transcriptional reporter and Smad2 C-terminal phosphorylation in PDAC cells while Smad3-specific transcriptional activity and Smad3 C-terminal phosphorylation appeared increased. Disruption of autocrine TGF-β signalling in PANC-1 cells rendered cells less susceptible to the growth-suppressive effect of Rac1 inhibition, suggesting that the decrease in "basal" proliferation upon Rac1 inhibition was caused by potentiation of autocrine TGF-β growth inhibition.In malignant cells with a functional TGF-β signalling pathway Rac1 antagonizes the TGF-β1 growth inhibitory response and   enhances   cell migration by antagonistically regulating Smad2 and Smad3 activation. This study reveals that Rac1 is prooncogenic in that it can alter TGF-β signalling at the R-Smad level from a tumour-suppressive towards a tumour-promoting outcome. Hence, Rac1 might represent a viable target for therapeutic intervention to inhibit PDAC progression. 
 Targeting and activation of Rac1 are mediated by the exchange factor beta-Pix. Rho guanosine triphosphatases (GTPases) are critical regulators of cytoskeletal dynamics and control complex functions such as cell adhesion, spreading,   migration  , and cell division. It is generally accepted that localized GTPase activation is required for the proper initiation of downstream signaling events, although the molecular mechanisms that control targeting of Rho GTPases are unknown. In this study, we show that the Rho GTPase Rac1, via a proline stretch in its COOH terminus, binds directly to the SH3 domain of the Cdc42/Rac activator beta-Pix (p21-activated kinase [Pak]-interacting exchange factor). The interaction with beta-Pix is nucleotide independent and is necessary and sufficient for Rac1 recruitment to membrane ruffles and to focal adhesions. In addition, the Rac1-beta-Pix interaction is required for Rac1 activation by beta-Pix as well as for Rac1-mediated spreading. Finally, using cells deficient for the beta-Pix-binding kinase Pak1, we show that Pak1 regulates the Rac1-beta-Pix interaction and controls cell spreading and adhesion-induced Rac1 activation. These data provide a model for the intracellular targeting and localized activation of Rac1 through its exchange factor beta-Pix. 
 Follicular fluid high-density lipoprotein-associated sphingosine 1-phosphate (S1P) promotes human granulosa lutein cell   migration   via S1P receptor type 3 and small G-protein RAC1. Coordinated   migration   and progesterone production by granulosa cells is critical to the development of the corpus luteum, but the underlying mechanisms remain obscure. Sphingosine 1-phosphate (S1P), which is associated with follicular fluid high-density lipoprotein (FF-HDL), was previously shown to regulate ovarian angiogenesis. We herein examined the effects of S1P and FF-HDL on the function of granulosa lutein cells. Both FF-HDL and S1P induced   migration   of primary human granulosa lutein cells (hGCs) and the granulosa lutein cell line HGL5. In addition, FF-HDL but not S1P promoted progesterone synthesis, and neither of the two compounds stimulated proliferation of granulosa lutein cells. Polymerase chain reaction and Western blot experiments demonstrated the expression of S1P receptor type 1 (S1PR1), S1PR2, S1PR3, and S1PR5 but not S1PR4 in hGCs and HGL5 cells. The FF-HDL- and S1P-induced granulosa lutein cell   migration   was emulated by FTY720, an agonist of S1PR1, S1PR3, S1PR4, and S1PR5, and by VPC24191, an agonist of S1PR1 and S1PR3, but not by SEW2871 and phytosphingosine 1-phosphate, agonists of S1PR1 and S1PR4, respectively. In addition, blockade of S1PR3 with CAY1044, suramine, or pertussis toxin inhibited hGC and HGL5 cell   migration   toward FF-HDL or S1P, while blockade of S1PR1 and S1PR2 with W146 and JTE013, respectively, had no effect. Both FF-HDL and S1P triggered activation of small G-protein RAC1 and actin polymerization in granulosa cells, and RAC1 inhibition with Clostridium difficile toxin B or NSC23766 abolished FF-HDL- and S1P-induced   migration  . The FF-HDL-associated S1P promotes granulosa lutein cell   migration   via S1PR3 and RAC1 activation. This may represent a novel mechanism contributing to the development of the corpus luteum. 
 Glycogen synthase kinase-3 regulates cytoskeleton and translocation of Rac1 in long cellular extensions of human keratinocytes. Wound keratinocytes form long cellular extensions that facilitate their   migration   from the wound edge into provisional matrix. We have previously shown that similar extensions can be induced by a long-term exposure to EGF or rapidly by staurosporine in cultured cells. This morphological change depends on the activity of glycogen synthase kinase-3 (GSK-3). Here, we have characterized the cytoskeletal changes involved in formation of these extended lamellipodia (E-lam) in human HaCaT keratinocytes. E-lams contained actin filaments, stable microtubules and keratin intermediate filaments. E-lam formation was prevented by cytochalasin D, colchicine and low concentrations of taxol and nocodazole, suggesting that actin and microtubule organization and dynamics are essential for E-lam formation. Staurosporine induced recruitment of filamentous actin (F-actin), cortactin, filamin, Arp2/3 complex, Rac1 GTPase and phospholipase C-gamma1 (PLC-gamma1) to lamellipodia. Treatment of cells with the GSK-3 inhibitors SB-415286 and LiCl(2) inhibited E-lam formation and prevented the accumulation of Rac1 and Arp2/3 complex at lamellipodia. The formation of E-lams was dependent on fibronectin-binding integrins and normally regulated Rac1, and expression of either dominant-negative or constitutively active forms of Rac1 prevented E-lam formation. Overexpression of either RhoA or Cdc42 GTPases suppressed E-lam formation. We conclude that extended lamellipodia formation in keratinocytes requires actin and tubulin assembly at the leading edge, and this process is regulated by Rac1 downstream of GSK-3. 
 The GTPase Rac regulates the proliferation and invasion of fibroblast-like synoviocytes from rheumatoid arthritis patients. Fibroblast-like synoviocytes (FLS) isolated from joints of rheumatoid arthritis (RA) patients display proliferative and invasive properties reminiscent of those of malignant tumor cells. Rac small GTPases play an important role in tumor cell proliferation and invasion. We therefore investigated the potential role of Rac proteins in the proliferative and invasive behavior of RA-FLS. We showed that inhibiting Rac activity with the Rac-specific small molecule inhibitor NSC23766 causes a strong inhibition of RA-FLS proliferation, without affecting cell survival. Rac inhibition also results in a strong reduction in RA-FLS invasion through reconstituted extracellular matrix and a less marked inhibition of two-dimensional   migration   as measured by monolayer wound healing. We also showed that small interfering RNA-mediated depletion of Rac1 inhibits RA-FLS proliferation and invasion to a similar extent as NSC23766. These results demonstrate for the first time that Rac proteins play an important role in the aggressive behavior of FLS isolated from RA patients. In addition, we observed that inhibiting Rac proteins prevents JNK activation and that the JNK inhibitor SP600125 strongly inhibits RA-FLS invasion, suggesting that Rac-mediated JNK activation contributes to the role of Rac proteins in the invasive behavior of RA-FLS. In conclusion, Rac-controlled signaling pathways may present a new source of drug targets for therapeutic intervention in RA. 
 PLC-gamma1 and Rac1 coregulate EGF-induced cytoskeleton remodeling and cell   migration  . It is well established that epidermal growth factor (EGF) induces the cytoskeleton reorganization and cell   migration   through two major signaling cascades: phospholipase C-gamma1 (PLC-gamma1) and Rho GTPases. However, little is known about the cross talk between PLC-gamma1 and Rho GTPases. Here we showed that PLC-gamma1 forms a complex with Rac1 in response to EGF. This interaction is direct and mediated by PLC-gamma1 Src homology 3 (SH3) domain and Rac1 (106)PNTP(109) motif. This interaction is critical for EGF-induced Rac1 activation in vivo, and PLC-gamma1 SH3 domain is actually a potent and specific Rac1 guanine nucleotide exchange factor in vitro. We have also demonstrated that the interaction between PLC-gamma1 SH3 domain and Rac1 play a significant role in EGF-induced F-actin formation and cell   migration  . We conclude that PLC-gamma1 and Rac1 coregulate EGF-induced cell cytoskeleton remodeling and cell   migration   by a direct functional interaction. 
 miR-320a suppresses colorectal cancer progression by targeting Rac1. MicroRNAs (miRNAs) have emerged as critical epigenetic regulators involved in cancer progression. miR-320a has been identified to be a novel tumour suppressive miRNA in colorectal cancer (CRC). However, the detailed molecular mechanisms are not fully understood. Here, we reported that miR-320a inversely associated with CRC aggressiveness in both cell lines and clinical specimens. Functional studies demonstrated that miR-320a significantly decreased the capability of cell   migration  /invasion and induced G0/G1 growth arrest in vitro and in vivo. Furthermore, Rac1 was identified as one of the direct downstream targets of miR-320a and miR-320a specifically binds to the conserved 8-mer at position 1140-1147 of Rac1 3'-untranslated region to regulate Rac1 protein expression. Over-expression of miR-320a in SW620 cells inhibited Rac1 expression, whereas reduction of miR-320a by anti-miR-320a in SW480 cells enhanced Rac1 expression. Re-expression of Rac1 in the SW620/miR-320a cells restored the cell   migration  /invasion inhibited by miR-320a, whereas knockdown of Rac1 in the SW480/anti-miR-320a cells repressed these cellular functions elevated by anti-miR-320a. Conclusively, our results demonstrate that miR-320a functions as a tumour-suppressive miRNA through targeting Rac1 in CRC. 
 P66Shc-rac1 pathway-mediated ROS production and cell   migration   is downregulated by ascorbic acid. The oxidative role(s) of p66Shc protein has been increasingly expanded over the last decade. However, its relation with the most potent antioxidant molecule, i.e. ascorbic acid has never been studied. We have previously shown that p66Shc mediates rac1 activation, reactive oxygen species (ROS) production and cell death. Here we studied the effect of ascorbic acid on the pathway involving p66Shc and rac1. Our results indicate a decrease in the expression of p66Shc in a dose- and time-dependent manner. We studied the effect of ascorbic acid on rac1 expression and its activity. Ascorbic acid has no effect on total rac1 expression; however, rac1 activation was inhibited in a dose-dependent manner. Results suggest that the decrease in rac1 activity is mediated through ascorbic acid-modulated p66Shc expression. The decrease in rac1 activity was evident in cells transfected with the p66shc mutant (proline motif mutant, at residues P47 to P50). Our studies indicate that p66Shc-mediated ROS upregulation is significantly decreased in the presence of ascorbic acid. Cell   migration   experiments point towards the inhibition of p66Shc-rac1-mediated   migration   in the presence of ascorbic acid. Finally, results are suggestive that ascorbic acid-mediated decrease in Shc expression occurs through an increased Shc ubiquitination. Overall, the study brings out the novel role of ascorbic acid in antioxidant signal transduction. 
 Interaction of the RAGE cytoplasmic domain with diaphanous-1 is required for ligand-stimulated cellular   migration   through activation of Rac1 and Cdc42. Cellular   migration   is a fundamental process linked to diverse pathological states such as diabetes and its complications, atherosclerosis, inflammation, and cancer. The receptor for advanced glycation end products (RAGE) is a multiligand cell surface macromolecule which binds distinct ligands that accumulate in these settings. RAGE-ligand interaction evokes central changes in key biological properties of cells, including proliferation, generation of inflammatory mediators, and   migration  . Although RAGE-dependent signal transduction is critically dependent on its short cytoplasmic domain, to date the proximate mechanism by which this RAGE domain engages and stimulates cytoplasmic signaling pathways has yet to be identified. Here we show that the RAGE cytoplasmic domain interacts with Diaphanous-1 (Dia-1) both in vitro and in vivo. We employed the human RAGE cytoplasmic domain as "bait" in the yeast two-hybrid assay and identified the formin homology (FH1) domain of Dia-1 as a potential binding partner of this RAGE domain. Immunoprecipitation studies revealed that the RAGE cytoplasmic domain interacts with the FH1 domain of Dia-1. Down-regulation of Dia-1 expression by RNA interference blocks RAGE-mediated activation of Rac-1 and Cdc42 and, in parallel, RAGE ligand-stimulated cellular   migration  . Taken together, these findings indicate that the interaction of the RAGE cytoplasmic domain with Dia-1 is required to transduce extracellular environmental cues evoked by binding of RAGE ligands to their cell surface receptor, a chief consequence of which is Rac-1 and Cdc42 activation and cellular   migration  . Because RAGE and Dia-1 are implicated in the regulation of inflammatory, vascular, and transformed cell   migration  , these findings highlight this interaction as a novel target for therapeutic intervention in inflammation, atherosclerosis, diabetes, and cancer. 
 HIV-1 induces cytoskeletal alterations and Rac1 activation during monocyte-blood-brain barrier interactions: modulatory role of CCR5. Most HIV strains that enter the brain are macrophage-tropic and use the CCR5 receptor to bind and infect target cells. Because the cytoskeleton is a network of protein filaments involved in cellular movement and   migration  , we investigated whether CCR5 and the cytoskeleton are involved in endothelial-mononuclear phagocytes interactions, adhesion, and HIV-1 infection.Using a cytoskeleton phospho-antibody microarray, we showed that after co-culture with human brain microvascular endothelial cells (HBMEC), HIV-1 infected monocytes increased expression and activation of cytoskeleton-associated proteins, including Rac1/cdc42 and cortactin, compared to non-infected monocytes co-cultured with HBMEC. Analysis of brain tissues from HIV-1-infected patients validated these findings, and showed transcriptional upregulation of Rac1 and cortactin, as well as increased activation of Rac1 in brain tissues of HIV-1-infected humans, compared to seronegative individuals and subjects with HIV-1-encephalitis. Confocal imaging showed that brain cells expressing phosphorylated Rac1 were mostly macrophages and blood vessels. CCR5 antagonists TAK-799 and maraviroc prevented HIV-induced upregulation and phosphorylation of cytoskeleton-associated proteins, prevented HIV-1 infection of macrophages, and diminished viral-induced adhesion of monocytes to HBMEC. Ingenuity pathway analysis suggests that during monocyte-endothelial interactions, HIV-1 alters protein expression and phosphorylation associated with integrin signaling, cellular morphology and cell movement, cellular assembly and organization, and post-translational modifications in monocytes. CCR5 antagonists prevented these HIV-1-induced alterations.HIV-1 activates cytoskeletal proteins during monocyte-endothelial interactions and increase transcription and activation of Rac1 in brain tissues. In addition to preventing macrophage infection, CCR5 antagonists could diminish viral-induced alteration and phosphorylation of cytoskeletal proteins, monocyte adhesion to the brain endothelium and viral entry into the central nervous system. 
 Small GTPase Rac1: structure, localization, and expression of the human gene. Rac1 is a member of the Rho family of small GTPases involved in signal transduction pathways that control proliferation, adhesion, and   migration   of cells during embryonic development and invasiveness of tumor cells. Here we present the complete structure of the human RAC1 gene and characterize its expression. The gene comprises 7 exons over a length of 29 kb and is localized to chromosome 7p22. The GC-rich gene promoter shows characteristics of a housekeeping gene and Northern blot studies revealed ubiquitous expression of two rac1 transcripts, 1.2 and 2.5 kb in size. The two transcripts are expressed in tissue-specific ratios, reflecting competition between two alternative polyadenylation sites. The RAC1 but not RAC2 gene contains an additional exon 3b that is included by alternative splicing into the variant Rac1b, a constitutively active mutant which induces the formation of lamellipodia in fibroblasts. These data indicate that the RAC1 gene encodes two signaling GTPases. The gene structure reported here will enable studies on the regulation of RAC1 expression during tumorigenesis and development. 
 RAC1 activation mediates Twist1-induced cancer cell   migration  . Epithelial-mesenchymal transition (EMT), which is characterized by the suppression of the adhesion protein E-cadherin, is a crucial process that promotes metastasis and stem-like properties of cancer cells. However, the dissociation of cellular aggregates is not sufficient to explain why cancer cells move, and the motile nature of cancer cells undergoing EMT remains elusive. Here, we identify a mechanism in which the EMT inducer Twist1 elicits cancer cell movement through activation of RAC1. Twist1 cooperates with BMI1 to suppress let-7i expression, which results in upregulation of NEDD9 and DOCK3, leading to RAC1 activation and enabling mesenchymal-mode movement in three-dimensional environments. Moreover, the suppression of let-7i contributes to Twist1-induced stem-like properties. Clinically, activation of the Twist1-let-7i-NEDD9 axis in head and neck cancer patients correlates with tumour invasiveness and worse outcome. Our results uncover an essential mechanism to explain how Twist1 induces the motile stem-like cancer cell phenotype beyond simply suppressing E-cadherin. 
 β-arrestin-dependent activation of the cofilin pathway is required for the scavenging activity of the atypical chemokine receptor D6. Chemokines promote the recruitment of leukocytes to sites of infection and inflammation by activating conventional heterotrimeric guanine nucleotide-binding protein (G protein)-coupled receptors (GPCRs). Chemokines are also recognized by a set of atypical chemokine receptors (ACRs), which cannot induce directional cell   migratio  n but are required for the generation of chemokine gradients in tissues. ACRs are presently considered "silent receptors" because no G protein-dependent signaling activity is observed after their engagement by cognate ligands. We report that engagement of the ACR D6 by its ligands activates a β-arrestin1-dependent, G protein-independent signaling pathway that results in the phosphorylation of the actin-binding protein cofilin through the Rac1-p21-activated kinase 1 (PAK1)-LIM kinase 1 (LIMK1) cascade. This signaling pathway is required for the increased abundance of D6 protein at the cell surface and for its chemokine-scavenging activity. We conclude that D6 is a signaling receptor that exerts its regulatory function on chemokine-mediated responses in inflammation and immunity through a distinct signaling pathway. 
 Rac1 and Rac2 regulate macrophage morphology but are not essential for   migration  . Rac GTPases are believed to contribute to   migration   in leukocytes by transducing signals from cell surface receptors to the actin and microtubule cytoskeletons. Mammals have three closely related Rac isoforms, Rac1, Rac2 and Rac3, and it is widely assumed that cell   migration   requires the activity of these Rac GTPases. We have previously shown that Rac1-null mouse macrophages have altered cell shape and reduced membrane ruffling but normal   migration   speed. Here we investigate the behaviour of macrophages lacking Rac2 (Rac2(-/-)) or Rac1 and Rac2 (Rac1/2(-/-)). Rac2(-/-) macrophages have reduced F-actin levels and lack podosomes, which are integrin-based adhesion sites, and their   migration   speed is similar to or slightly slower than wild-type macrophages, depending on the substrate. Unexpectedly, Rac1/2(-/-) macrophages, which do not express Rac1, Rac2 or Rac3, migrate at a similar speed to wild-type macrophages on a variety of substrates and perform chemotaxis normally, although their morphology and mode of   migration   is altered. However, Rac1(-/-) and Rac1/2(-/-) but not Rac2(-/-) macrophages are impaired in their ability to invade through Matrigel. Together, these data show that Rac1 and Rac2 have distinct roles in regulating cell morphology,   migration   and invasion, but are not essential for macrophage   migration   or chemotaxis. 
 Tyr724 phosphorylation of ELMO1 by Src is involved in cell spreading and   migration   via Rac1 activation. The complex of Dock180/ELMO1 that functions as a bipartite guanine nucleotide exchange factor for Rac is essential for diverse physiological and pathological processes of cells such as cell   migration  , phagocytosis, and invasion of cancer cells. Among the Src-family tyrosine kinases (SFKs), it has been reported that Hck directly phosphorylates ELMO1, regulating phagocytosis by promoting activation of Rac1; however, the involvement of other SFKs in ELMO1 phosphorylation has remained unknown. Here, we identified novel tyrosine (Y) residues of ELMO1 phosphorylated by SFKs, and examined the effects on Rac1 activity, cell adhesion, spreading, and cell motility on extracellular matrix (ECM).In this study, we unveiled that Src and Fyn can induce tyrosine phosphorylation of ELMO1 in in vivo and in vitro phosphorylation assays. Mutational analyses identified both Y720 and Y724 residues of ELMO1 as Src-mediated phosphorylation sites, preferentially on Y724. Single substitution of Y724 to Phe abrogated Rac1 activation triggered by Src. To elucidate the biological function of pY724, we established NIH3T3 cells stably expressing wild-type ELMO1 or its Y724F mutant together with Dock180. Among them, Y724-deficient cells exhibited a depletion of Rac1 activity with diminished phosphorylation of ELMO1 even upon the ECM-stimulation. It is noteworthy that NIH3T3 cells with ELMO1 Y724F were strikingly defective to promote cell spreading on fibronectin-coated dish, concomitantly exhibiting immature assemblies of actin stress fibers and focal adhesions. Eventually, ELMO1 Y724F significantly impaired cell   migration  .These results define that Src-mediated Y724 phosphorylation in ELMO1 plays a critical role for cell spreading via activation of Rac1, leading to promotion of cell   migration  . As the overexpression and/or hyperactivation of Src have been shown in a wide variety of human cancers, Src-mediated phosphorylation of Y724 in ELMO1 may regulate cancer cell adhesion to the ECM, invasion into surrounding tissues, and subsequent distant metastasis. 
 The requirement for polyamines for intestinal epithelial cell   migration   is mediated through Rac1. The rapid   migration   of intestinal epithelial cells is important to the healing of mucosal ulcers and wounds. This cell   migration   requires the presence of polyamines and the activation of RhoA. RhoA activity, however, is not sufficient for   migration   because polyamine depletion inhibited the   migration   of IEC-6 cells expressing constitutively active RhoA. The current study examines the role of Rac1 and Cdc42 in cell   migration   and whether their activities are polyamine-dependent. Polyamine depletion with alpha-difluoromethylornithine inhibited the activities of RhoA, Rac1, and Cdc42. This inhibition was prevented by supplying exogenous putrescine in the presence of alpha-difluoromethylornithine. IEC-6 cells transfected with constitutively active Rac1 and Cdc42 migrated more rapidly than vector-transfected cells, whereas cells expressing dominant negative Rac1 and Cdc42 migrated more slowly. Polyamine depletion had no effect on the   migration   of cells expressing Rac1 and only partially inhibited the   migration   of those expressing Cdc42. Although polyamine depletion caused the disappearance of actin stress fibers in cells transfected with empty vector, it had no effect on cells expressing Rac1. Constitutively active Rac1 increased RhoA and Cdc42 activity in both normal and polyamine-depleted cells. These results demonstrate that Rac1, RhoA, and Cdc42 are required for optimal epithelial cell   migration   and that Rac1 activity is sufficient for cell   migration   in the absence of polyamines due to its ability to activate RhoA and Cdc42 as well as its own effects on the process of cell   migration  . These data imply that the involvement of polyamines in cell   migration   occurs either at Rac1 itself or upstream from Rac1. 
 A Rac/Cdc42-specific exchange factor, GEFT, induces cell proliferation, transformation, and   migration  . The Rho family of small GTPases, including Rho, Rac, and Cdc42, play essential roles in diverse cellular functions. The ability of Rho family GTPases to participate in signaling events is determined by the ratio of inactive (GDP-bound) and active (GTP-bound) forms in the cell. The activation of Rho family proteins requires the exchange of bound GDP for GTP, a process catalyzed by the Dbl family of guanine nucleotide exchange factors (GEFs). The GEFs have high affinity for the guanine nucleotide-free state of the GTPases and are thought to promote GDP release by stabilizing an intermediate transition state. In this study, we have identified and characterized a new Rac/Cdc42-specific Dbl family guanine nucleotide exchange factor, named GEFT. GEFT is highly expressed in the excitable tissues, including brain, heart, and muscle. Low or very little expression was detected in other nonexcitable tissues. GEFT has specific exchange activity for Rac and Cdc42 in our in vitro GTPase exchange assays and glutathione S-transferase-PAK pull-down assays with GTP-bound Rac1 and Cdc42. Overexpression of GEFT leads to changes in cell morphology and actin cytoskeleton re-organization, including the formation of membrane microspikes, filopodia, and lamilliopodia. Furthermore, expression of GEFT in NIH3T3 cells promotes foci formation, cell proliferation, and cell   migration  , possibly through the activation of transcriptional factors involved in cell growth and proliferation. Together, our data suggest that GEFT is a Rac/Cdc42-specific GEF protein that regulates cell morphology, cell proliferation, and transformation. 
 Coronin1 proteins dictate rac1 intracellular dynamics and cytoskeletal output. Rac1 regulates lamellipodium formation, myosin II-dependent contractility, and focal adhesions during cell   migration  . While the spatiotemporal assembly of those processes is well characterized, the signaling mechanisms involved remain obscure. We report here that the cytoskeleton-related Coronin1A and -1B proteins control a myosin II inactivation-dependent step that dictates the intracellular dynamics and cytoskeletal output of active Rac1. This step is signaling-branch specific, since it affects the functional competence of active Rac1 only when forming complexes with downstream ArhGEF7 and Pak proteins in actomyosin-rich structures. The pathway is used by default unless Rac1 is actively rerouted away from the structures by upstream activators and signals from other Rho GTPases. These results indicate that Coronin1 proteins are at the center of a regulatory hub that coordinates Rac1 activation, effector exchange, and the F-actin organization state during cell signaling. Targeting this route could be useful to hamper   migration   of cancer cells harboring oncogenic RAC1 mutations. 
 Role of isoprenylcysteine carboxylmethyltransferase-catalyzed methylation in Rho function and   migration  . A number of proteins that play key roles in biological regulatory events undergo a process of post-translational modifications termed prenylation. The prenylation pathway consists of three enzymatic steps; the final processed protein is isoprenoid-modified and methylated on the C-terminal cysteine. This protein modification pathway plays a significant role in cancer biology because many oncogenic proteins undergo prenylation. Methylation of the C terminus by isoprenylcysteine carboxylmethyltransferase (Icmt) is the final step in the prenylation pathway. Cysmethynil, a specific Icmt inhibitor discovered in our laboratory, is able to inhibit Ras-mediated signaling, cell growth, and oncogenesis. We sought to examine the role of Icmt-mediated methylation on the behaviors of cancer cells associated with metastatic potential. Our results indicate that inhibition of methylation reduces   migration   of the highly metastatic MDA-MB-231 breast cancer cell line. In addition, cell adhesion and cell spreading are also significantly impacted by cysmethynil. To examine the mechanism of Icmt-dependent   migration   we focused on RhoA and Rac1, prenylated proteins that are important mediators of cell   migration   through their control of the actin cytoskeleton. Inhibition of Icmt significantly decreases the activation of both RhoA and Rac1; an increase in Rho GDP-dissociation inhibitor (RhoGDI) binding in the absence of methylation appears to contribute to this effect. Furthermore, in the absence of Icmt activity the addition of exogenous RhoA or Rac1 is able to partially rescue directed and random   migration  , respectively. These findings establish a role for Icmt-mediated methylation in cell   migration   and advance our understanding of the biological consequences of Rho methylation. 
 GIT2 represses Crk- and Rac1-regulated cell spreading and Cdc42-mediated focal adhesion turnover. G protein-coupled receptor kinase interactors (GITs) regulate focal adhesion (FA) turnover, cell spreading, and motility through direct interaction with paxillin and the Rac-exchange factor Pak-interacting exchange factor beta (betaPIX). However, it is not clear whether GITs function to activate or repress motility or if the predominant GIT forms, GIT1 and GIT2, serve distinct or redundant roles. Here we demonstrate an obligatory role for endogenous GIT2 in repression of lamellipodial extension and FA turnover by Rac1- and Cdc42-dependent signaling pathways, respectively. Moreover, we show that the SH2-SH3 adaptor protein Crk is an essential target of GIT2 inhibition. Unexpectedly, we find that betaPIX is dispensable for the effects elicited by knockdown of GIT2. Finally, we show that loss of GIT2 is sufficient to induce   migration   of the nontransformed epithelial cell line MCF10A. These results suggest that inactivation of GIT2 function is a required step for induction of cell motility and that GIT2 may be a target of oncogenic signaling pathways that regulate cell   migration  . 
 A putative novel protein, DEPDC1B, is overexpressed in oral cancer patients, and enhanced anchorage-independent growth in oral cancer cells that is mediated by Rac1 and ERK. The DEP domain is a globular domain containing approximately 90 amino acids, which was first discovered in 3 proteins: Drosophila disheveled, Caenorhabditis elegans EGL-10, and mammalian Pleckstrin; hence the term, DEP. DEPDC1B is categorized as a potential Rho GTPase-activating protein. The function of the DEP domain in signal transduction pathways is not fully understood. The DEPDC1B protein exhibits the characteristic features of a signaling protein, and contains 2 conserved domains (DEP and RhoGAP) that are involved in Rho GTPase signaling. Small GTPases, such as Rac, CDC42, and Rho, regulate a multitude of cell events, including cell motility, growth, differentiation, cytoskeletal reorganization and cell cycle progression.In this study, we found that it was a guanine nucleotide exchange factor and induced both cell   migration   in a cultured embryonic fibroblast cell line and cell invasion in cancer cell lines; moreover, it was observed to promote anchorage-independent growth in oral cancer cells. We also demonstrated that DEPDC1B plays a role in regulating Rac1 translocated onto cell membranes, suggesting that DEPDC1B exerts a biological function by regulating Rac1. We examined oral cancer tissue; 6 out of 7 oral cancer tissue test samples overexpressed DEPDC1B proteins, compared with normal adjacent tissue.DEPDC1B was a guanine nucleotide exchange factor and induced both cell   migration   in a cultured embryonic fibroblast cell line and cell invasion in cancer cell lines; moreover, it was observed to promote anchorage-independent growth in oral cancer cells. We also demonstrated that DEPDC1B exerts a biological function by regulating Rac1. We found that oral cancer samples overexpressed DEPDC1B proteins, compared with normal adjacent tissue. Suggest that DEPDC1B plays a role in the development of oral cancer. We revealed that proliferation was linked to a novel DEPDC1B-Rac1-ERK1/2 signaling axis in oral cancer cell lines. 
 Synthetic spatially graded Rac activation drives cell polarization and movement. Migrating cells possess intracellular gradients of active Rho GTPases, which serve as central hubs in transducing signals from extracellular receptors to cytoskeletal and adhesive machinery. However, it is unknown whether shallow exogenously induced intracellular gradients of Rho GTPases are sufficient to drive cell polarity and motility. Here, we use microfluidic control to generate gradients of a small molecule and thereby directly induce linear gradients of active, endogenous Rac without activation of chemotactic receptors. Gradients as low as 15% were sufficient not only to trigger cell   migration   up the chemical gradient but to induce both cell polarization and repolarization. Cellular response times were inversely proportional to the steepness of Rac inducer gradient in agreement with a mathematical model, suggesting a function for chemoattractant gradient amplification upstream of Rac. Increases in activated Rac levels beyond a well-defined threshold augmented polarization and decreased sensitivity to the imposed gradient. The threshold was governed by initial cell polarity and PI3K activity, supporting a role for both in defining responsiveness to Rac activation. Our results reveal that Rac can serve as a starting point in defining cell polarity. Furthermore, our methodology may serve as a template to investigate processes regulated by intracellular signaling gradients. 
 Kaempferol-3-O-rutinoside from Afgekia mahidoliae promotes keratinocyte   migration   through FAK and Rac1 activation. The restoration of the epidermal epithelium through re-epithelialization is a critical process in wound healing. Directed keratinocyte   migration   to the wound is required, and the retardation of this process may result in a chronic, non-healing wound. The present study contributes to research aiming to identify promising compounds that promote wound healing using a human keratinocyte model. The effects of three kaempferol glycosides from an Afgekia mahidoliae leaf extract, kaempferol-3-O-arabinoside, kaempferol-3-O-glucoside, and kaempferol-3-O-rutinoside, on keratinocyte   migration   were determined. Interestingly, kaempferol-3-O-rutinoside exhibited a pronounced effect on wound closure in comparison to the parental kaempferol and other glycosides. The mechanism by which kaempferol-3-O-rutinoside enhances cell   migration   involves the induction of filopodia and lamellipodia formation, increased cellular levels of phosphorylated FAK (Tyr 397) and phosphorylated Akt (Ser 473), and up-regulation of active Rac1-GTP. The data obtained in this study may support the development of this compound for use in wound healing therapies. 
 Dock4 is regulated by RhoG and promotes Rac-dependent cell   migration  . Cell   migration   is essential for normal development and many pathological processes including tumor metastasis. Rho family GTPases play important roles in this event. In particular, Rac is required for lamellipodia formation at the leading edge during   migration  . Dock4 is a member of the Dock180 family proteins, and Dock4 mutations are present in a subset of human cancer cell lines. However, the function and the regulatory mechanism of Dock4 remain unclear. Here we show that Dock4 is regulated by the small GTPase RhoG and its effector ELMO and promotes cell   migration   by activating Rac1. Dock4 formed a complex with ELMO, and expression of active RhoG induced translocation of the Dock4-ELMO complex from the cytoplasm to the plasma membrane and enhanced the Dock4- and ELMO-dependent Rac1 activation and cell   migration  . On the other hand, RNA interference-mediated knockdown of Dock4 in NIH3T3 cells reduced cell   migration  . Taken together, these results suggest that Dock4 plays an important role in the regulation of cell   migration   through activation of Rac1, and that RhoG is a key upstream regulator for Dock4. 
 IAPs regulate the plasticity of cell   migration   by directly targeting Rac1 for degradation. Inhibitors of apoptosis proteins (IAPs) are a highly conserved class of multifunctional proteins. Rac1 is a well-studied Rho GTPase that controls numerous basic cellular processes. While the regulation of nucleotide binding to Rac1 is well understood, the molecular mechanisms controlling Rac1 degradation are not known. Here, we demonstrate X-linked IAP (XIAP) and cellular IAP1 (c-IAP1) directly bind to Rac1 in a nucleotide-independent manner to promote its polyubiquitination at Lys147 and proteasomal degradation. These IAPs are also required for degradation of Rac1 upon CNF1 toxin treatment or RhoGDI depletion. Consistently, downregulation of XIAP or c-IAP1 by various strategies led to an increase in Rac1 protein levels in primary and tumour cells, leading to an elongated morphology and enhanced cell   migration  . Further, XIAP counteracts Rac1-dependent cellular polarization in the developing zebrafish hindbrain and promotes the delamination of neurons from the normal tissue architecture. These observations unveil an evolutionarily conserved role of IAPs in controlling Rac1 stability thereby regulating the plasticity of cell   migration   and morphogenesis. 
 Rac1 mediates phorbol 12-myristate 13-acetate-induced   migration   of glioblastoma cells via paxillin. Previously, we reported that phorbol 12-myristate 13-acetate (PMA)-activated protein kinase C (PKC) induced Rac1 activation in A172 glioblastoma cells. In this study, we investigated the mechanism of PMA-activated PKC-induced   migration   of glioblastoma cells by focusing on Rac1. PMA-induced formation of lamellipodia and focal complexes following   migration   were blocked by inhibiting Rac1 with small interfering RNA (siRNA), implicating Rac1 in PMA-induced glioblastoma cell   migration  . PMA-activated PKC induced phosphorylation of c-jun N-terminal kinase (JNK), one of the downstream effectors of Rac1. Immunohistochemical analysis showed that phosphorylated JNK was translocated to paxillin-containing focal complexes upon PMA stimulation and that Rac1 siRNA blocked these phenomena. These results suggest that phosphorylated JNK functions in cell   migration   and that JNK phosphorylation and translocation are mediated by Rac1. Furthermore, inhibition of Rac1 reduced phosphorylation of paxillin, a focal adhesion component and a downstream effector of JNK, at serine 178 (Ser178). Paxillin phosphorylation at this site has been shown to be involved in cell   migration  . Immunohistochemical analysis detected phosphorylation of paxillin (Ser178) in focal complexes upon PMA stimulation that was blocked by Rac1 siRNA. SP600125, a JNK inhibitor, also blocked PMA-induced phosphorylation of paxillin and aggregation of phosphorylated paxillin (Ser178) in focal complexes. In conclusion, paxillin is a critical downstream effector of Rac1 that may be involved in PMA-stimulated   migration   presumably by modulating the integrity of focal complex formation. 
 Sonic Hedgehog activates the GTPases Rac1 and RhoA in a Gli-independent manner through coupling of smoothened to Gi proteins. The vertebrate Hedgehog (Hh) pathway has essential functions during development and tissue homeostasis in normal physiology, and its dysregulation is a common theme in cancer. The Hh ligands (Sonic Hh, Indian Hh, and Desert Hh) bind to the receptors Patched1 and Patched2, resulting in inhibition of their repressive effect on Smoothened (Smo). Smo is a seven-transmembrane protein, which was only recently shown to function as a G protein-coupled receptor (GPCR) with specificity toward the heterotrimeric guanine nucleotide-binding protein G(i). In addition to activating G(i), Smo signals through its C-terminal tail to inhibit Suppressor of Fused, resulting in stabilization and activation of the Gli family of transcription factors, which execute a transcriptional response to so-called "canonical Hh signaling." In this Presentation, we illustrate two outcomes of Hh signaling that are independent of Gli transcriptional activity and, thus, are defined as "noncanonical." One outcome is dependent on Smo coupling to G(i) proteins and exerts changes to the actin cytoskeleton through stimulation of the small guanosine triphosphatases (GTPases) RhoA and Rac1. These cytoskeletal changes promote   migration   in fibroblasts and tubulogenesis in endothelial cells. Signaling through the other noncanonical Hh pathway is independent of Smo and inhibits Patched1-induced cell death. 
 ARAP2 signals through Arf6 and Rac1 to control focal adhesion morphology. Focal adhesions (FAs) are dynamic structures that connect the actin cytoskeleton with the extracellular matrix. At least six ADP-ribosylation factor (Arf) GTPase-activating proteins (GAPs), including ARAP2 (an Arf6 GAP), are implicated in regulation of FAs but the mechanisms for most are not well defined. Although Rac1 has been reported to function downstream of Arf6 to control membrane ruffling and cell   migration  , this pathway has not been directly examined as a regulator of FAs. Here we test the hypothesis that ARAP2 promotes the growth of FAs by converting Arf6·GTP to Arf6·GDP thereby preventing the activation of the Rho family GTP-binding protein Rac1. Reduced expression of ARAP2 decreased the number and size of FAs in cells and increased cellular Arf6·GTP and Rac1·GTP levels. Overexpression of ARAP2 had the opposite effects. The effects of ARAP2 on FAs and Rac1 were dependent on a functional ArfGAP domain. Constitutively active Arf6 affected FAs in the same way as did reduced ARAP2 expression and dominant negative mutants of Arf6 and Rac1 reversed the effect of reduced ARAP2 expression. However, neither dominant negative Arf6 nor Rac1 had the same effect as ARAP2 overexpression. We conclude that changes in Arf6 and Rac1 activities are necessary but not sufficient for ARAP2 to promote the growth of FAs and we speculate that ARAP2 has additional functions that are effector in nature to promote or stabilize FAs. 
 Endothelial-Rac1 is not required for tumor angiogenesis unless alphavbeta3-integrin is absent. Endothelial cell   migration   is an essential aspect of tumor angiogenesis. Rac1 activity is needed for cell   migration   in vitro implying a requirement for this molecule in angiogenesis in vivo. However, a precise role for Rac1 in tumor angiogenesis has never been addressed. Here we show that depletion of endothelial Rac1 expression in adult mice, unexpectedly, has no effect on tumor growth or tumor angiogenesis. In addition, repression of Rac1 expression does not inhibit VEGF-mediated angiogenesis in vivo or ex vivo, nor does it affect chemotactic migratory responses to VEGF in 3-dimensions. In contrast, the requirement for Rac1 in tumor growth and angiogenesis becomes important when endothelial beta3-integrin levels are reduced or absent: the enhanced tumor growth, tumor angiogenesis and VEGF-mediated responses in beta3-null mice are all Rac1-dependent. These data indicate that in the presence of alphavbeta3-integrin Rac1 is not required for tumor angiogenesis. 
 Rac1 mediates collapse of microvilli on chemokine-activated T lymphocytes. Lymphocytes circulate in the blood and upon chemokine activation rapidly bind, where needed, to microvasculature to mediate immune surveillance. Resorption of microvilli is an early morphological alteration induced by chemokines that facilitates lymphocyte e  migration  . However, the antecedent molecular mechanisms remain largely undefined. We demonstrate that Rac1 plays a fundamental role in chemokine-induced microvillar breakdown in human T lymphocytes. The supporting evidence includes: first, chemokine induces Rac1 activation within 5 s via a signaling pathway that involves Galphai. Second, constitutively active Rac1 mediates microvilli disintegration. Third, blocking Rac1 function by cell permeant C-terminal "Trojan" peptides corresponding to Rac1 (but not Rac2, Rho, or Cdc42) blocks microvillar loss induced by the chemokine stromal cell-derived factor 1alpha (SDF-1alpha). Furthermore, we demonstrate that the molecular mechanism of Rac1 action involves dephosphorylation-induced inactivation of the ezrin/radixin/moesin (ERM) family of actin regulators; such inactivation is known to detach the membrane from the underlying actin cytoskeleton, thereby facilitating disassembly of actin-based peripheral processes. Specifically, ERM dephosphorylation is induced by constitutively active Rac1 and stromal cell-derived factor 1alpha-induced ERM dephosphorylation is blocked by either the dominant negative Rac1 construct or by Rac1 C-terminal peptides. Importantly, the basic residues at the C terminus of Rac1 are critical to Rac1's participation in ERM dephosphorylation and in microvillar retraction. Together, these data elucidate new roles for Rac1 in early signal transduction and cytoskeletal rearrangement of T lymphocytes responding to chemokine. 
 Human tumor necrosis factor (TNF)-alpha-induced protein 8-like 2 suppresses hepatocellular carcinoma metastasis through inhibiting Rac1. Tumor invasion and metastasis are the major reasons for leading death of patients with hepatocellular carcinoma (HCC). Therefore, to identify molecules that can suppress invasion and metastasis of tumor will provide novel targets for HCC therapies. Tumor necrosis factor (TNF)-alpha-induced protein 8-like 2, TIPE2, is a novel immune negative molecule and an inhibitor of the oncogenic Ras in mice but its function in human is unclear. Our previous research has shown that TIPE2 is downregulated in human primary HCC compared with the paired adjacent non-tumor tissues.In present study, we provide evidence that TIPE2 inhibits effectively human hepatocellular carcinoma metastasis. The forced expression of TIPE2 in HCC-derived cell lines markedly inhibits tumor cell growth,   migration   and invasion in vitro and suppresses growth and metastasis of HCC in vivo. Clinical information from a cohort of 112 patients reveals that loss or reduced expression of TIPE2 in primary HCC tissues is significantly associated with tumor metastasis. Mechanically, TIPE2 inhibits the   migration   and invasion through targeting Rac1 and then reduces F-actin polymerization and expression of matrix metallopeptidase 9 (MMP9) and urokinase plasminogen activator (uPA).Our results indicate that human TIPE2 is endogenous inhibitor of Rac1 in HCC by which it attenuates invasion and metastasis of HCC. The data suggest that TIPE2 will be a new target for HCC therapy. 
 Ephexin4 and EphA2 mediate cell   migration   through a RhoG-dependent mechanism. EphA2, a member of the Eph receptor family, is frequently overexpressed in a variety of human cancers, including breast cancers, and promotes cancer cell motility and invasion independently of its ligand ephrin stimulation. In this study, we identify Ephexin4 as a guanine nucleotide exchange factor (GEF) for RhoG that interacts with EphA2 in breast cancer cells, and knockdown and rescue experiments show that Ephexin4 acts downstream of EphA2 to promote ligand-independent breast cancer cell   migration   and invasion toward epidermal growth factor through activation of RhoG. The activation of RhoG recruits its effector ELMO2 and a Rac GEF Dock4 to form a complex with EphA2 at the tips of cortactin-rich protrusions in migrating breast cancer cells. In addition, the Dock4-mediated Rac activation is required for breast cancer cell   migration  . Our findings reveal a novel link between EphA2 and Rac activation that contributes to the cell motility and invasiveness of breast cancer cells. 
 [The Rho protein family and its role in the cellular cytoskeleton]. Proteins of the Rho protein family (RhoA, Rac1, Cdc42) work as molecular switches in the regulation of many cellular processes. They are involved in cell   migration  , cell-cycle control, apoptosis, and the regulation of gene transcription. Rho proteins show their activity mainly in the cell's cytoskeleton by taking part in the reorganization of microfilaments and microtubules. In recent years, significant progress has been made in understanding the biochemical and genetic nature of many processes controlled by Rho proteins. Although there are still seveal unknowns and hypothesizes which require confirmation, newer and more precise experimental results allow us to better understand these processes. It has also been suggested to use Rho family proteins in the therapy of some diseases. The purpose of this study was to characterize the significance of Rho processes in the cellular cytoskeleton. 
 Automated screening of microtubule growth dynamics identifies MARK2 as a regulator of leading edge microtubules downstream of Rac1 in migrating cells. Polarized microtubule (MT) growth in the leading edge is critical to directed cell   migration  , and is mediated by Rac1 GTPase. To find downstream targets of Rac1 that affect MT assembly dynamics, we performed an RNAi screen of 23 MT binding and regulatory factors and identified RNAi treatments that suppressed changes in MT dynamics induced by constitutively activated Rac1. By analyzing fluorescent EB3 dynamics with automated tracking, we found that RNAi treatments targeting p150(glued), APC2, spastin, EB1, Op18, or MARK2 blocked Rac1-mediated MT growth in lamellipodia. MARK2 was the only protein whose RNAi targeting additionally suppressed Rac1 effects on MT orientation in lamellipodia, and thus became the focus of further study. We show that GFP-MARK2 rescued effects of MARK2 depletion on MT growth lifetime and orientation, and GFP-MARK2 localized in lamellipodia in a Rac1-activity-dependent manner. In a wound-edge motility assay, MARK2-depleted cells failed to polarize their centrosomes or exhibit oriented MT growth in the leading edge, and displayed defects in directional cell   migration  . Thus, automated image analysis of MT assembly dynamics identified MARK2 as a target regulated downstream of Rac1 that promotes oriented MT growth in the leading edge to mediate directed cell   migration  . 
 RAC1 inhibition as a therapeutic target for gefitinib-resistant non-small-cell lung cancer. Although epidermal growth factor receptor (EGFR)-tyrosine kinase inhibitors (EGFR-TKI), including gefitinib, provide a significant clinical benefit in non-small-cell lung cancer (NSCLC) patients, the acquisition of drug resistance has been known to limit the efficacy of EGFR-TKI therapy. In this study, we demonstrated the involvement of EGF-EGFR signaling in NSCLC cell   migration   and the requirement of RAC1 in EGFR-mediated progression of NSCLC. We showed the significant role of RAC1 pathway in the cell   migration   or lamellipodia formation by using gene silencing of RAC1 or induction of constitutive active RAC1 in EGFR-mutant NSCLC cells. Importantly, the RAC1 inhibition suppressed EGFR-mutant NSCLC cell   migration   and growth in vitro, and growth in vivo even in the gefitinib-resistant cells. In addition, these suppressions by RAC1 inhibition were mediated through MEK or PI3K independent mechanisms. Collectively, these results open up a new opportunity to control the cancer progression by targeting the RAC1 pathway to overcome the resistance to EGFR-TKI in NSCLC patients. 
 Cytoplasmic targeting of the proto-oncogene SET promotes cell spreading and   migration  . The RhoGTPase Rac1 is activated in a polarised fashion and controls cell motility. We previously showed that Rac1 binds the PP2A inhibitor SET and recruits nuclear SET to the cytosol. We show that a SET mutant, lacking a nuclear localization signal, SET(ΔNLS), promotes cell spreading and motility. This was accompanied by an increase in the number and frequency of membrane ruffles. Pharmacological inhibition of PP2A did not mimic the effects of SET(ΔNLS), however, we found that expression of SET and SET(ΔNLS) increases the levels of the MAP kinases ERK1 and ERK2. 
 Rac1 activation driven by 14-3-3ζ dimerization promotes prostate cancer cell-matrix interactions, motility and transendothelial   migratio  n. 14-3-3 proteins are ubiquitously expressed dimeric adaptor proteins that have emerged as key mediators of many cell signaling pathways in multiple cell types. Its effects are mainly mediated by binding to selective phosphoserine/threonine proteins. The importance of 14-3-3 proteins in cancer have only started to become apparent and its exact role in cancer progression as well as the mechanisms by which 14-3-3 proteins mediate cancer cell function remain unknown. While protein 14-3-3σ is widely accepted as a tumor suppressor, 14-3-3ζ, β and γ isoforms have been shown to have tumor promoting effects. Despite the importance of 14-3-3 family in mediating various cell processes, the exact role and mechanism of 14-3-3ζ remain unexplored. In the current study, we investigated the role of protein 14-3-3ζ in prostate cancer cell motility and transendot  helial mi  gration using biochemical, molecular biology and electric cell-substrate impedance sensing approaches as well as cell based functional assays. Our study indicated that expression with wild-type protein 14-3-3ζ significantly enhanced Rac activity in PC3 cells. In contrast, expression of dimer-resistant mutant of protein 14-3-3ζ (DM-14-3-3) inhibited Rac activity and associated phosphorylation of p21 activated kinase-1 and 2. Expression with wild-type 14-3-3ζ or constitutively active Rac1 enhanced extracellular matrix recognition, lamellipodia format  ion, cell   migration and trans-en  dothelial   migration by PC3 cells. In contrast, expression with DM 14-3-3ζ or DN-Rac1 in PC3 cells significantly inhibited these cell functions. Our results demonstrate for the first time that 14-3-3ζ enhances prostate cancer cell-matrix interactions, motility and trans  endotheli  al migration in vitro via activation of Rac1-GTPase and is an important target for therapeutic interventions for prostate cancer. 
 Forkhead box P3 regulates ARHGAP15 expression and affects   migration   of glioma cells through the Rac1 signaling pathway. Forkhead box P3 (FOXP3) plays a crucial role in the development and function of regulatory T cells and was recently identified as a tumor suppressor in different cancer types. Forkhead box P3 is expressed in normal brain tissues, but is strongly downregulated or absent in glioblastomas. In order to understand the FOXP3 adjustment mechanisms in glioma cells, we performed a DNA microarray in U87 cells overexpressing FOXP3 and validated the differences using quantitative real-time PCR, Western blot analysis, and immunohistochemistry in vitro and in vivo. We found that FOXP3 can regulate the expression of ARHGAP15. Expression of FOXP3 was also correlated with ARHGAP15 in glioma samples. Overexpression of FOXP3 inhibited glioma cell   migration   through ARHGAP15 upregulation and Rac1 inactivation. Silencing of FOXP3 promoted   migration   through ARHGAP15 downregulation and Rac1 activation. ARHGAP15, a GTPase-activating protein for Rac1, inhibits small GTPase signaling in a dual negative manner. We found that there is a correlation between expression of ARHGAP15 and glioma level. The small GTPase Rac1 plays an important role in cell   migration  . In addition, we found that FOXP3 regulates expression of epithelial-mesenchymal transition markers E-cadherin and N-cadherin, which is important given that epithelial-mesenchymal transition is critically involved in tumor spreading and dissemination. Thus, FOXP3 or ARHGAP15 may serve as a new molecular target for antimetastatic therapies in treating glioma. 
 MYADM regulates Rac1 targeting to ordered membranes required for cell spreading and   migration  . Membrane organization into condensed domains or rafts provides molecular platforms for selective recruitment of proteins. Cell   migration   is a general process that requires spatiotemporal targeting of Rac1 to membrane rafts. The protein machinery responsible for making rafts competent to recruit Rac1 remains elusive. Some members of the MAL family of proteins are involved in specialized processes dependent on this type of membrane. Because condensed membrane domains are a general feature of the plasma membrane of all mammalian cells, we hypothesized that MAL family members with ubiquitous expression and plasma membrane distribution could be involved in the organization of membranes for cell   migration  . We show that myeloid-associated differentiation marker (MYADM), a protein with unique features within the MAL family, colocalizes with Rac1 in membrane protrusions at the cell surface and distributes in condensed membranes. MYADM knockdown (KD) cells had altered membrane condensation and showed deficient incorporation of Rac1 to membrane raft fractions and, similar to Rac1 KD cells, exhibited reduced cell spreading and   migration  . Results of rescue-of-function experiments by expression of MYADM or active Rac1L61 in cells knocked down for Rac1 or MYADM, respectively, are consistent with the idea that MYADM and Rac1 act on parallel pathways that lead to similar functional outcomes. 
 The Phosphatidylinositol (3,4,5)-Trisphosphate-dependent Rac Exchanger 1·Ras-related C3 Botulinum Toxin Substrate 1 (P-Rex1·Rac1) Complex Reveals the Basis of Rac1 Activation in Breast Cancer Cells. The P-Rex (phosphatidylinositol (3,4,5)-trisphosphate (PIP3)-dependent Rac exchanger) family (P-Rex1 and P-Rex2) of the Rho guanine nucleotide exchange factors (Rho GEFs) activate Rac GTPases to regulate cel  l migrati  on, invasion, and metastasis in several human cancers. The family is unique among Rho GEFs, as their activity is regulated by the synergistic binding of PIP3 and Gβγ at the plasma membrane. However, the molecular mechanism of this family of multi-domain proteins remains unclear. We report the 1.95 Å crystal structure of the catalytic P-Rex1 DH-PH tandem domain in complex with its cognate GTPase, Rac1 (Ras-related C3 botulinum toxin substrate-1). Mutations in the P-Rex1·Rac1 interface revealed a critical role for this complex in signaling downstream of receptor tyrosine kinases and G protein-coupled receptors. The structural data indicated that the PIP3/Gβγ binding sites are on the opposite surface and markedly removed from the Rac1 interface, supporting a model whereby P-Rex1 binding to PIP3 and/or Gβγ releases inhibitory C-terminal domains to expose the Rac1 binding site. 
 Roles of the Rac1 and Rac3 GTPases in human tumor cell invasion. Members of the Rho family of small GTPases have been shown to be involved in tumorigenesis and metastasis. Currently, most of the available information on the function of Rho proteins in malignant transformation is based on the use of dominant-negative mutants of these GTPases. The specificity of these dominant-negative mutants is limited however. In this study, we used small interfering RNA directed against either Rac1 or Rac3 to reduce their expression specifically. In line with observations using dominant-negative Rac1 in other cell types, we show that RNA interference-mediated depletion of Rac1 strongly inhibits lamellipodia formation, cell   migration   and invasion in SNB19 glioblastoma cells. Surprisingly however, Rac1 depletion has a much smaller inhibitory effect on SNB19 cell proliferation and survival. Interestingly, whereas depletion of Rac3 strongly inhibits SNB19 cell invasion, it does not affect lamellipodia formation and has only minor effects on cell   migration   and proliferation. Similar results were obtained in BT549 breast carcinoma cells. Thus, functional analysis of Rac1 and Rac3 using RNA interference reveals a critical role for these GTPases in the invasive behavior of glioma and breast carcinoma cells. 
 Extension, retraction and contraction in the formation of a dendritic cell dendrite: distinct roles for Rho GTPases. The morphology of antigen-presenting dendritic cells (DC) is characterized by the possession of numerous long arborizing processes known as dendrites. The formation of these processes by DC, both in the periphery and in lymphoid organs, is believed to contribute to the remarkable efficiency with which they take up, process and present antigen to T cells. However, the process of dendrite formation and the signaling pathways that lead to the formation of these dendrites remain obscure. In this study we describe an in vitro model in which human immature DC form long processes similar to those formed in vivo. The formation of these processes involves initial attachment of a cell protrusion to the extracellular matrix substrate, and subsequent movement of the cell body away from the adhesion site, leaving behind a long slender dendrite. Dendrite formation, but not their maintenance, was found to be dependent on the activity of Rho GTPases. More specifically, Cdc42 and Rac1 were both required for the   migration   step of process formation, promoting cell spreading and extension. In contrast, Rho, and its downstream effector p160ROCK, regulated the release of adhesions to the substratum, and associated cellular contraction. Consequently, inhibition of Rho/p160ROCK leads to the formation of longer dendrites. DC therefore coordinate adhesion and protrusion to perform a specialized process of cellular morphogenesis, which differentiates these cells from all other cells of the immune system and may contribute to their distinctive function. 
 The role of SDF-1α/Rac pathway in the regulation of endothelial progenitor cell polarity; homing and expression of Rac1, Rac2 during endothelial repair. The Stromal cell-derived factor-1 (SDF-1)/CXC chemokine receptor 4 (CXCR4) system is considered to be of great importance in diseases involving cardiogenesis and angiogenesis. The SDF-1α-RAC signaling pathway plays a pivotal role in a cell's polarity and serves to activate cell morphology variation and to control the direction o  f migrati  on. We aimed to study whether the polarity of endothelial progenitor cells (EPCs) is changed by the induction of the SDF-1α-RAC signaling pathway, to investigate the mechanism of the effect of polarity on the homing action of EPCs, and to explore the gene and protein expression of Rac1/2 during endothelial repair. We measured the EPC characteristics of polarity induced by various final concentrations of SDF-1α; our observations included morphology variati  on, migra  tion direction, and excursion. Of the dynamic variation and cytoskeleton rearrangement of EPCs induced by different final concentrations of SDF-1α, the most obvious variation was exhibited at the SDF-1α concentration of 200 ng/ml. Obvious polarity variations were also found in the EPCs and signal receptors induced by the SDF-1α concentration of 200 ng/ml. The Western blot analysis of Rac1 and Rac2 showed that the addition of AMD 3100 significantly inhibited the expression of Rac. The SDF-1α pathway potentially regulates the expression of Rac1/2. The actual excursion vector and the direction   of the m  igration of EPCs induced by SDF-1α follows polarity, thus indicating the importance of further exploration regarding the homing induction of EPCs. 
 Engagement of CD44 promotes Rac activation and CD44 cleavage during tumor cell   migration  . CD44 is a major cell surface adhesion molecule for hyaluronan, a component of the extracellular matrix, and is implicated in tumor metastasis and invasion. We reported previously that hyaluronan oligosaccharides induce CD44 cleavage from tumor cells. Here we show that engagement of CD44 promotes CD44 cleavage and tumor cell   migration  , both of which were suppressed by a metalloproteinase inhibitor KB-R7785 and tissue inhibitor of metalloproteinases-1 (TIMP-1) but not by TIMP-2. We also present evidence that blockade of metalloproteinase-disintegrin ADAM10 (a disintegrin and metalloproteinase 10) by RNA interference suppresses CD44 cleavage induced by its ligation. Engagement of CD44 concurrently induced activation of the small GTPase Rac1 and led to drastic changes in cell morphology and actin cytoskeleton with redistribution of CD44 to newly generated membrane ruffling areas. A fluorescence resonance energy transfer approach to visualize GTP-bound Rac1 in living cells revealed the localization of the active Rac1 in the leading edge of the membrane ruffling areas upon ligation of CD44. Taken together, our results indicate that the cleavage of CD44 catalyzed by ADAM10 is augmented by the intracellular signaling elicited by engagement of CD44, through Rac-mediated cytoskeletal rearrangement, and suggest that CD44 cleavage contributes to the   migration   and invasion of tumor cells. 
 Assembly of non-contractile dorsal stress fibers requires α-actinin-1 and Rac1 in migrating and spreading cells. Cell   migratio  n and spreading is driven by actin polymerization and actin stress fibers. Actin stress fibers are considered to contain α-actinin crosslinkers and nonmuscle myosin II motors. Although several actin stress fiber subtypes have been identified in migrating and spreading cells, the degree of molecular diversity of their composition and the signaling pathways regulating fiber subtypes remain largely uncharacterized. In the present study we identify that dorsal stress fiber assembly requires α-actinin-1. Loss of dorsal stress fibers in α-actinin-1-depleted cells results in defective maturation of leading edge focal adhesions. This is accompanied by a delay in early cell spreading and slower c  ell migra  tion without noticeable alterations in myosin light chain phosphorylation. In agreement with the unaltered myosin II activity, dorsal stress fiber trunks lack myosin II and are resistant to myosin II ATPase inhibition. Furthermore, the non-contractility of dorsal stress fibers is supported by the finding that Rac1 induces dorsal stress fiber assembly whereas contractile ventral stress fibers are induced by RhoA. Loss of dorsal stress fibers either by depleting α-actinin-1 or Rac1 results in a β-actin accumulation at the leading edge in migrating and spreading cells. These findings molecularly specify dorsal stress fibers from other actin stress fiber subtypes. Furthermore, we propose that non-contractile dorsal stress fibers promote   cell mig  ration and early cell spreading through Rac1-induced actin polymerization. 
 The F-BAR domain protein PACSIN2 associates with Rac1 and regulates cell spreading and   migration  . The Rac1 GTPase controls cytoskeletal dynamics and is a key regulator of cell spreading and   migration   mediated by signaling through effector proteins, such as the PAK kinases and the Scar and WAVE proteins. We previously identified a series of regulatory proteins that associate with Rac1 through its hypervariable C-terminal domain, including the Rac1 activator β-Pix (also known as Rho guanine-nucleotide-exchange factor 7) and the membrane adapter caveolin-1. Here, we show that Rac1 associates, through its C-terminus, with the F-BAR domain protein PACSIN2, an inducer of membrane tubulation and a regulator of endocytosis. We show that Rac1 localizes with PACSIN2 at intracellular tubular structures and on early endosomes. Active Rac1 induces a loss of PACSIN2-positive tubular structures. By contrast, Rac1 inhibition results in an accumulation of PACSIN2-positive tubules. In addition, PACSIN2 appears to regulate Rac1 signaling; siRNA-mediated loss of PACSIN2 increases the levels of Rac1-GTP and promotes cell spreading and   migratio  n in a wound healing assay. Moreover, ectopic expression of PACSIN2 reduces Rac1-GTP levels in a fashion that is dependent on the PACSIN2-Rac1 interaction, on the membrane-tubulating capacity of PACSIN2 and on dynamin. These data identify the BAR-domain protein PACSIN2 as a Rac1 interactor that regulates Rac1-mediated cell spreading and   migratio  n. 
 Characterization and functional analysis of the calmodulin-binding domain of Rac1 GTPase. Rac1, a member of the Rho family of small GTPases, has been shown to promote formation of lamellipodia at the leading edge of motile cells and affect cell   migration  . We previously demonstrated that calmodulin can bind to a region in the C-terminal of Rac1 and that this interaction is important in the activation of platelet Rac1. Now, we have analyzed amino acid residue(s) in the Rac1-calmodulin binding domain that are essential for the interaction and assessed their functional contribution in Rac1 activation. The results demonstrated that region 151-164 in Rac1 is essential for calmodulin binding. Within the 151-164 region, positively-charged amino acids K153 and R163 were mutated to alanine to study impact on calmodulin binding. Mutant form of Rac1 (K153A) demonstrated significantly reduced binding to calmodulin while the double mutant K153A/R163A demonstrated complete lack of binding to calmodulin. Thrombin or EGF resulted in activation of Rac1 in CHRF-288-11 or HeLa cells respectively and W7 inhibited this activation. Immunoprecipitation studies demonstrated that higher amount of CaM was associated with Rac1 during EGF dependent activation. In cells expressing mutant forms of Rac1 (K153A or K153A/R163A), activation induced by EGF was significantly decreased in comparison to wild type or the R163A forms of Rac1. The lack of Rac1 activation in mutant forms was not due to an inability of GDP-GTP exchange or a change in subcelllular distribution. Moreover, Rac1 activation was decreased in cells where endogenous level of calmodulin was reduced using shRNA knockdown and increased in cells where calmodulin was overexpressed. Docking analysis and modeling demonstrated that K153 in Rac1 interacts with Q41 in calmodulin. These results suggest an important role for calmodulin in the activation of Rac1 and thus, in cytoskeleton reorganization and cell   migration  . 
 PIKfyve, MTMR3 and their product PtdIns5P regulate cancer cell   migration   and invasion through activation of Rac1. Previously, we have shown that the phosphoinositide metabolizing enzymes PIKfyve (phosphoinositide 5-kinase, FYVE finger containing) and MTMR3 (myotubularin-related protein 3), together with their lipid product PtdIns5P, are important for   migration   of normal human fibroblasts. As these proteins are a kinase and a phosphatase respectively, and thereby considered druggable, we wanted to test their involvement in cancer cell   migration   and invasion. First, we showed that PIKfyve and MTMR3 are expressed in most cancer cells. Next, we demonstrated that depletion of PIKfyve or MTMR3 resulted in decreased velocity in three different cancer cell lines by using new software for cell tracking. Inhibition of the enzymatic activity of PIKfyve by the inhibitor YM201636 also led to a strong reduction in cell velocity. Mechanistically, we show that PIKfyve and MTMR3 regulate the activation of the Rho family GTPase Rac1. Further experiments also implicated PtdIns5P in the activation of Rac1. The results suggest a model for the activation of Rac1 in cell   migration   where PIKfyve and MTMR3 produce PtdIns5P on cellular membranes which may then serve to recruit effectors to activate Rac1. Finally, in an invasion assay, we demonstrate that both PIKfyve and MTMR3 are implicated in invasive behaviour of cancer cells. Thus PIKfyve and MTMR3 could represent novel therapeutic targets in metastatic cancer. 
 [Role of Rac1 in the SDF-1-induced   migration   and invasion of human glioma cell line U251]. To explore the role of Rac1 in the SDF-1-induced   migration   and invasion of glioma cells with a specific Rac1 inhibitor.Human glioma cell lines U251 treated with SDF-1 or/and specific Rac1 inhibitor were used. The   migration   and invasion capacities of cells in 2D cell   migration  /3D invasion assay were assessed. Western blot was employed to detect the levels of Rac1 and GAPDH in cell lysates and the Rac1 activity measured by Rac1 activation assays. Immunofluorescence was used to identify the expression and intracellular location of Rac1 in U251 cells.SDF-1 significantly increased the   migration   and invasion capacities of U251 cells (P < 0.05). The stimulation of SDF-1 boosted the activity of Rac1 versus the unstimulated cells (P < 0.05). And Rac1 was recruited to protruding edge in SDF-1-stimulated cells. Inhibition of Rac1 with specific Rac1 inhibitor decreased the   migration   and invasion capacities of SDF-1-induced U251 cells (P < 0.05). In comparison with the SDF-1 treated group, the activity of Rac1 significantly decreased (P < 0.05) and the recruitment of Rac1 to protruding edge significantly decreased in the NSC23766 pre-treated group.This study provides novel evidence that Rac1 modulates the SDF-1-induced   migration   and invasion of glioma cells. It suggests that the inhibition of Rac1 activation may be a new therapeutic target for glioma. 
 RAC1 activity and intracellular ROS modulate the migratory potential of MCF-7 cells through a NADPH oxidase and NFkappaB-dependent mechanism. In the present study, we demonstrated that changes in Rac1 activity associated with the production of intracellular ROS modulate the migratory properties in MCF-7 and T47D human mammary cell lines. We also described that the NFkappaB pathway exerts a downstream control on the expression of the ROS-dependent cellular migratory potential. These results emphasize the importance of redox balance in the acquisition of malignancy and support previous data sustaining that an oxidative environment predisposes cells to activate signal-transduction pathways actively involved in cellular oncogenesis. Our data also provides evidence that NADPH oxidase could constitute the main source of intracellular ROS in response to changes in Rac1 activity. We suggest that Rac1 plays a role in cellular   migration   not only limited to its known function in reorganization of the actin cytoskeleton, but also as part of the intracellular machinery that controls the redox balance. 
 The Drosophila HEM-2/NAP1 homolog KETTE controls axonal pathfinding and cytoskeletal organization. In Drosophila, the correct formation of the segmental commissures depends on neuron-glial interactions at the midline. The VUM midline neurons extend axons along which glial cells migrate in between anterior and posterior commissures. Here, we show that the gene kette is required for the normal projection of the VUM axons and subsequently disrupts glial   migration  . Axonal projection defects are also found for many other moto- and interneurons. In addition, kette affects the cell morphology of mesodermal and epidermal derivatives, which show an abnormal actin cytoskeleton. The KETTE protein is homologous to the transmembrane protein HEM-2/NAP1 evolutionary conserved from worms to vertebrates. In vitro analysis has shown a specific interaction of the vertebrate HEM-2/NAP1 with the SH2-SH3 adapter protein NCK and the small GTPase RAC1, which both have been implicated in regulating cytoskeleton organization and axonal growth. Hypomorphic kette mutations lead to axonal defects similar to mutations in the Drosophila NCK homolog dreadlocks. Furthermore, we show that kette and dock mutants genetically interact. NCK is thought to interact with the small G proteins RAC1 and CDC42, which play a role in axonal growth. In line with these observations, a kette phenocopy can be obtained following directed expression of mutant DCDC42 or DRAC1 in the CNS midline. In addition, the kette mutant phenotype can be partially rescued by expression of an activated DRAC1 transgene. Our data suggest an important role of the HEM-2 protein in cytoskeletal organization during axonal pathfinding. 
 Clinical and pathological associations of the activating RAC1 P29S mutation in primary cutaneous melanoma. Activating mutations in the GTPase RAC1 are a recurrent event in cutaneous melanoma. We investigated the clinical and pathological associations of RAC1(P29S) in a cohort of 814 primary cutaneous melanomas with known BRAF and NRAS mutation status. The RAC1(P29S) mutation had a prevalence of 3.3% and was associated with increased thickness (OR=1.6 P = 0.001), increased mitotic rate (OR=1.3 P = 0.03), ulceration (OR=2.4 P = 0.04), nodular subtype (OR=3.4 P = 0.004), and nodal disease at diagnosis (OR=3.3 P = 0.006). BRAF mutant tumors were also associated with nodal metastases (OR=1.9 P = 0.004), despite being thinner at diagnosis than BRAF WT (median 1.2 mm versus 1.6 mm, P < 0.001). Immunohistochemical analysis of 51 melanomas revealed that 47% were immunoreactive for RAC1. Melanomas were more likely to show RAC1 immunoreactivity if they were BRAF mutant (OR=5.2 P = 0.01). RAC1 may therefore be important in reg  ulating t  he early migration of BRAF mutant tumors. RAC1 mutations are infrequent in primary melanomas but may accelerate disease progression. 
 Mammalian target of rapamycin and Rictor control neutrophil chemotaxis by regulating Rac/Cdc42 activity and the actin cytoskeleton. Chemotaxis allows neutrophils to seek out sites of infection and inflammation. The asymmetric accumulation of filamentous actin (F-actin) at the leading edge provides the driving force for protrusion and is essential for the development and maintenance of neutrophil polarity. The mechanism that governs actin cytoskeleton dynamics and assembly in neutrophils has been extensively explored and is still not fully understood. By using neutrophil-like HL-60 cells, we describe a pivotal role for Rictor, a component of mammalian target of rapamycin complex 2 (mTORC2), in regulating assembly of the actin cytoskeleton during neutrophil chemotaxis. Depletion of mTOR and Rictor, but not Raptor, impairs actin polymerization, leading-edge establishment, and directional   migration   in neutrophils stimulated with chemoattractants. Of interest, depletion of mSin1, an integral component of mTORC2, causes no detectable defects in neutrophil polarity and chemotaxis. In addition, experiments with chemical inhibition and kinase-dead mutants indicate that mTOR kinase activity and AKT phosphorylation are dispensable for chemotaxis. Instead, our results suggest that the small Rho GTPases Rac and Cdc42 serve as downstream effectors of Rictor to regulate actin assembly and organization in neutrophils. Together our findings reveal an mTORC2- and mTOR kinase-independent function and mechanism of Rictor in the regulation of neutrophil chemotaxis. 
 The C-terminal domain of Rac1 contains two motifs that control targeting and signaling specificity. Rho-like GTPases control a wide range of cellular functions such as integrin- and cadherin-mediated adhesion, cell motility, and gene expression. The hypervariable C-terminal domain of these GTPases has been implicated in membrane association and effector binding. We found that cell-permeable peptides, encoding the C termini of Rac1, Rac2, RhoA, and Cdc42, interfere with GTPase signaling in a specific fashion in a variety of cellular models. Pull-down assays showed that the C terminus of Rac1 does not associate to either RhoGDI or to Pak. In contrast, the C terminus of Rac1 (but not Rac2 or Cdc42) binds to phosphatidylinositol 4,5-phosphate kinase (PIP5K) via amino acids 185-187 (RKR). Moreover, Rac1 associates to the adapter protein Crk via the N-terminal Src homology 3 (SH3) domain of Crk and the proline-rich stretch in the Rac1 C terminus. These differential interactions mediate Rac1 localization, as well as Rac1 signaling, toward membrane ruffling, cell-cell adhesion, and   migration  . These data show that the C-terminal, hypervariable domain of Rac1 encodes two distinct binding motifs for signaling proteins and regulates intracellular targeting and differential signaling in a unique and non-redundant fashion. 
 Heregulin beta1 promotes breast cancer cell proliferation through Rac/ERK-dependent induction of cyclin D1 and p21Cip1. Accumulating evidence indicates that heregulins, EGF (epidermal growth factor)-like ligands, promote breast cancer cell proliferation and are involved in the progression of breast cancer towards an aggressive and invasive phenotype. However, there is limited information regarding the molecular mechanisms that mediate these effects. We have recently established that HRG (heregulin beta1) promotes breast cancer cell proliferation and   migration   via cross-talk with EGFR (EGF receptor) that involves the activation of the small GTPase Rac1. In the present paper we report that Rac1 is an essential player for mediating the induction of cyclin D1 and p21(Cip1) by HRG in breast cancer cells. Inhibition of Rac function by expressing either the Rac-GAP (GTPase-activating protein) beta2-chimaerin or the dominant-negative Rac mutant N17Rac1, or Rac1 depletion using RNAi (RNA interference), abolished the cyclin D1 and p21(Cip1) induction by HRG. Interestingly, the proliferative effect of HRG was impaired not only when the expression of Rac1 or cyclin D1 was inhibited, but also when cells were depleted of p21(Cip1) using RNAi. Inhibition of EGFR, PI3K (phosphoinositide 3-kinase; kinases required for Rac activation by HRG) or MEK [MAPK (mitogen-activated protein kinase)/ERK (extracellular-signal-regulated kinase) kinase] also blocked the up-regulation of cyclin D1 and p21(Cip1) by HRG. In addition, we found that HRG activates NF-kappaB (nuclear factor kappaB) in a Rac1- and MEK-dependent fashion, and inhibition of NF-kappaB abrogates cyclin D1/p21(Cip1) induction and proliferation by HRG. Taken together, these findings establish a central role for Rac1 in the control of HRG-induced breast cancer cell-cycle progression and proliferation through up-regulating the expression of cyclin D1 and p21(Cip1). 
 Smooth muscle cell expression of a constitutive active form of human Rac 1 accelerates cutaneous wound repair. Hyperoxia has been shown to improve wound healing; however, the mechanism for such therapeutic effects of oxygen remains hypothetical. Rac 1 regulates a wide variety of cellular activities, including cell proliferation and   migration  , and also is a key regulator for the activity of the nicotinamide dinucleotide phosphate oxidase the enzyme complex responsible for the production of a large fraction of cellular superoxide.We generated transgenic mice that express either the cDNA of a constitutively active mutant of human Rac 1 (V12 mutant or Rac CA) or the dominant negative isoform (V12 and N17 mutant or Rac DN) in the blood vessels using mouse vascular smooth muscle promoter for alpha-actin. We placed 2 wounds of 6 mm in diameter at the middorsal region of each mouse and allowed about 3 weeks for the wounds to heal.The size of the wounds in Rac CA transgenic mice was reduced relative to wild type mice; healing of Rac DN mice was slower than wild type and Rac CA ( P < .05). Blood vessel formation appeared faster in Rac CA mice, a finding associated with enhanced expression of some angiogenic growth factors.The current studies suggest that Rac 1 activation accelerates the wound healing process and is associated with more efficient angiogenesis at the wound site. 
 The sirtuins promote Dishevelled-1 scaffolding of TIAM1, Rac activation and cell   migration  . Rac1-GTPases serve as intermediary cellular switches, which conduct transient and constitutive signals from upstream cues, including those from Ras oncoproteins. Although the sirtuin1 (SIRT1) deacetylase is overexpressed in several human cancers and has recently been linked to cancer cell motility as a context-dependent regulator of multiple pathways, its role in Rac1 activation has not been reported. Similarly, SIRT2 has been demonstrated to be upregulated in some cancers; however, studies have also reported its role in tumor suppression. Here, we demonstrate that SIRT1 and SIRT2 positively regulate the levels of Rac1-GTP and the activity of T-cell lymphoma invasion and metastasis 1 (TIAM1), a Rac guanine nucleotide exchange factor (GEF). Transient inhibition of SIRT1 and SIRT2 resulted in increased acetylation of TIAM1, whereas chronic SIRT2 knockdown resulted in enhanced acetylation of TIAM1. SIRT1 regulates Dishevelled (DVL) protein levels in cancer cells, and DVL along with TIAM1 are known to augment Rac activation; however, SIRT1 or 2 has not been previously linked with TIAM1. We found that diminished sirtuin activity led to the disruption of the DVL1-TIAM1 interaction. We hence propose a model for Rac activation where SIRT1/2 positively modulates the DVL/TIAM1/Rac axis and promotes sustained pathway activation. 
 Rac1 protein regulates glycogen phosphorylase activation and controls interleukin (IL)-2-dependent T cell proliferation. Small GTPases of the Rho family have been implicated in important cellular processes such as cell   migration   and adhesion, protein secretion, and/or gene transcription. In the lymphoid system, these GTPases participate in the signaling cascades that are activated after engagement of antigen receptors. However, little is known about the role that Rho GTPases play in IL-2-mediated responses. Here, we show that IL-2 induces Rac1 activation in Kit 225 T cells. We identified by mass spectrometry the muscle isoform of glycogen phosphorylase (PYGM) as a novel Rac1 effector molecule in IL-2-stimulated cells. The interaction between the active form of Rac1 (Rac1-GTP) and PYGM was established directly through a domain comprising amino acids 191-270 of PYGM that exhibits significant homology with the Rac binding domain of PAK1. The integrity of this region was crucial for PYGM activation. Importantly, IL-2-dependent cellular proliferation was inhibited upon blocking both the activation of Rac1 and the activity of PYGM. These results reveal a new role for Rac1 in cell signaling, showing that this GTPase triggers T cell proliferation upon IL-2 stimulation by associating with PYGM and modulating its enzymatic activity. 
 Rho GTPases RhoA and Rac1 mediate effects of dietary folate on metastatic potential of A549 cancer cells through the control of cofilin phosphorylation. Folate, an important nutrient in the human diet, has been implicated in cancer, but its role in metastasis is not established. We have shown previously that the withdrawal of medium folate leads to the inhibition of   migration   and invasion of A549 lung carcinoma cells. Here we have demonstrated that medium folate regulates the function of Rho GTPases by enabling their carboxyl methylation and translocation to plasma membrane. Conversely, the lack of folate leads to the retention of these proteins in endoplasmic reticulum. Folate also promoted the switch from inactive (GDP-bound) to active (GTP-bound) GTPases, resulting in the activation of downstream kinases p21-activated kinase and LIM kinase and phosphorylation of the actin-depolymerizing factor cofilin. We have further demonstrated that in A549 cells two GTPases, RhoA and Rac1, but not Cdc42, are immediate sensors of folate status: the siRNA silencing of RhoA or Rac1 blocked effects of folate on cofilin phosphorylation and cellular   migration   and invasion. The finding that folate modulates metastatic potential of cancer cells was confirmed in an animal model of lung cancer using tail vein injection of A549 cells in SCID mice. A folate-rich diet enhanced lung colonization and distant metastasis to lymph nodes and decreased overall survival (35 versus 63 days for mice on a folate-restricted diet). High folate also promoted epithelial-mesenchymal transition in cancer cells and experimental mouse tumors. Our study provides experimental evidence for a mechanism of metastasis promotion by dietary folate and highlights the interaction between nutrients and metastasis-related signaling. 
 Rac1 and Cdc42 differentially modulate cigarette smoke-induced airway cell   migration   through p120-catenin-dependent and -independent pathways. The adherens junction protein p120-catenin (p120ctn) shuttles between E-cadherin-bound and cytoplasmic pools to regulate E-cadherin/catenin complex stability and cell   migration  , respectively. When released from the adherens junction, p120ctn promotes cell   migration   through modulation of the Rho GTPases Rac1, Cdc42, and RhoA. Accordingly, the down-regulation and cytoplasmic mislocalization of p120ctn has been reported in all subtypes of lung cancers and is associated with grave prognosis. Previously, we reported that cigarette smoke induced cytoplasmic translocation of p120ctn and cell   migration  , but the underlying mechanism was unclear. Using primary human bronchial epithelial cells exposed to smoke-concentrated medium (Smk), we observed the translocation of Rac1 and Cdc42, but not RhoA, to the leading edge of polarized and migrating human bronchial epithelial cells. Rac1 and Cdc42 were robustly activated by smoke, whereas RhoA was inhibited. Accordingly, siRNA knockdown of Rac1 or Cdc42 completely abolished Smk-induced cell   migration  , whereas knockdown of RhoA had no effect. p120ctn/Rac1 double knockdown completely abolished Smk-induced cell   migration  , whereas p120ctn/Cdc42 double knockdown did not. These data suggested that Rac1 and Cdc42 coactivation was essential to smoke-promoted cell   migration   in the presence of p120ctn, whereas   migration   proceeded via Rac1 alone in the absence of p120ctn. Thus, Rac1 may provide an omnipotent therapeutic target in reversing cell   migration   during the early (intact p120ctn) and late (loss of p120ctn) stages of lung carcinogenesis. 
 Integrative omics analysis of rheumatoid arthritis identifies non-obvious therapeutic targets. Identifying novel therapeutic targets for the treatment of disease is challenging. To this end, we developed a genome-wide approach of candidate gene prioritization. We independently collocated sets of genes that were implicated in rheumatoid arthritis (RA) pathogenicity through three genome-wide assays: (i) genome-wide association studies (GWAS), (ii) differentially expression in RA fibroblast-like synoviocytes (FLS), and (iii) differentially methylation in RA FLS. Integrated analysis of these complementary data sets identified a significant enrichment of multi-evidence genes (MEGs) within pathways relating to RA pathogenicity. One MEG is Engulfment and Cell Motility Protein-1 (ELMO1), a gene not previously considered as a therapeutic target in RA FLS. We demonstrated in RA FLS that ELMO1 is: (i) expressed, (ii) promotes cell   migration   and invasion, and (iii) regulates Rac1 activity. Thus, we created links between ELMO1 and RA pathogenicity, which in turn validates ELMO1 as a potential RA therapeutic target. This study illustrated the power of MEG-based approaches for therapeutic target identification. 
 Rac1 recruitment to the archipelago structure of the focal adhesion through the fluid membrane as revealed by single-molecule analysis. The focal adhesion (FA) is an integrin-based structure built in/on the plasma membrane (PM), linking the extracellular matrix to the actin stress-fibers, working as cell   migration   scaffolds. Previously, we proposed the archipelago architecture of the FA, in which FA largely consists of fluid membrane, dotted with small islands accumulating FA proteins: membrane molecules enter the inter-island channels in the FA zone rather freely, and the integrins in the FA-protein islands rapidly exchanges with those in the bulk membrane. Here, we examined how Rac1, a small G-protein regulating FA formation, and its activators αPIX and βPIX, are recruited to the FA zones. PIX molecules are recruited from the cytoplasm to the FA zones directly. In contrast, majorities of Rac1 molecules first arrive from the cytoplasm on the general inner PM surface, and then enter the FA zones via lateral diffusion on the PM, which is possible due to rapid Rac1 diffusion even within the FA zones, slowed only by a factor of two to four compared with that outside. The constitutively-active Rac1 mutant exhibited temporary and all-time immobilizations in the FA zone, suggesting that upon PIX-induced Rac1 activation at the FA-protein islands, Rac1 tends to be immobilized at the FA-protein islands. 
 Rac inhibition reverses the phenotype of fibrotic fibroblasts. Fibrosis, the excessive deposition of scar tissue by fibroblasts, is one of the largest groups of diseases for which there is no therapy. Fibroblasts from lesional areas of scleroderma patients possess elevated abilities to contract matrix and produce alpha-smooth muscle actin (alpha-SMA), type I collagen and CCN2 (connective tissue growth factor, CTGF). The basis for this phenomenon is poorly understood, and is a necessary prerequisite for developing novel, rational anti-fibrotic strategies.Compared to healthy skin fibroblasts, dermal fibroblasts cultured from lesional areas of scleroderma (SSc) patients possess elevated Rac activity. NSC23766, a Rac inhibitor, suppressed the persistent fibrotic phenotype of lesional SSc fibroblasts. NSC23766 caused a decrease in   migration   on and contraction of matrix, and alpha-SMA, type I collagen and CCN2 mRNA and protein expression. SSc fibroblasts possessed elevated Akt phosphorylation, which was also blocked by NSC23766. Overexpression of rac1 in normal fibroblasts induced matrix contraction and alpha-SMA, type I collagen and CCN2 mRNA and protein expression. Rac1 activity was blocked by PI3kinase/Akt inhibition. Basal fibroblast activity was not affected by NSC23766.Rac inhibition may be considered as a novel treatment for the fibrosis observed in SSc. 
 The tumour suppressor HACE1 controls cell   migration   by regulating Rac1 degradation. The small GTPase Rac1 is a key regulator of cell motility. Multiple mechanisms regulate Rac1 activity including its ubiquitylation and subsequent degradation. Here, we identify the tumour suppressor HACE1 (HECT domain and Ankyrin repeat Containing E3 ubiquitin-protein ligase 1) as an E3 ubiquitin ligase responsible for Rac1 degradation following activation by a   migration   stimulus. We show that HACE1 and Rac1 interaction is enhanced by hepatocyte growth factor (HGF) signalling, a Rac activator and potent stimulus of cell   migration  . Furthermore, HACE1 catalyses the poly-ubiquitylation of Rac1 at lysine 147 following its activation by HGF, resulting in its proteasomal degradation. This negative feedback mechanism likely restricts cell motility. Consistent with this, HACE1 depletion is accompanied by increased total Rac1 levels and accumulation of Rac1 in membrane ruffles. Moreover, HACE1-depletion enhances cell   migration   independently of growth factor stimulation, which may have significance for malignant conversion. A non-ubiquitylatable Rac1 rescues the   migration   defect of Rac1-null cells to a greater extent than wild-type Rac1. These findings identify HACE1 as an antagonist of cell   migration   through its ability to degrade active Rac1. 
 Overexpression of small GTPases directly correlates with expression of δ-catenin and their coexpression predicts a poor clinical outcome in nonsmall cell lung cancer. δ-catenin can affect cytoskeletal assembly, and promote cel  l migrati  on by regulating the activity of small GTPases. While many malignancies have been shown to be positive for δ-catenin, it is still unclear whether δ-catenin and small GTPases are coexpressed in tumor cells, and so is the relationship between their coexpression and prognosis in the tumor patients. In this study, immunohistochemistry was performed to examine expressive levels of δ-catenin, cdc42, and Rac1 in 135 cases of nonsmall cell lung cancer (NSCLC), including 60 cases with follow-up records. Thirty samples of paired lung cancer tissues and adjacent normal lung tissues were collected to analyze mRNA and protein expression of δ-catenin and small GTPases. The effects of δ-catenin on small GTPases expression and invasive ability of lung cancer cells were also evaluated. Compared with normal lung tissues, both mRNA and protein levels of δ-catenin and small GTPases were increased in lung cancer tissues (P < 0.05), and the expression of small GTPases directly correlated with that of δ-catenin (P < 0.001). In addition, δ-catenin and small GTPases tended to be coexpressed in lung adenocarcinoma, advanced stages, and primary tumors with lymph node metastasis (all P < 0.05). The patients with coexpression of δ-catenin and small GTPases had a shorter survival time than those without coexpression (P < 0.05). Furthermore, δ-catenin overexpression could enhance invasive ability of lung cancer cells by upregulating protein and transcriptional level of small GTPases. Therefore, δ-catenin likely upregulates the activity of small GTPases at transcriptional level, and their coexpression may predict a poor clinical outcome in NSCLC patients. 
 A Novel Pharmacologic Activity of Ketorolac for Therapeutic Benefit in Ovarian Cancer Patients. We previously identified the R-enantiomer of ketorolac as an inhibitor of the Rho-family GTPases Rac1 and Cdc42. Rac1 and Cdc42 regulate cancer-relevant functions, including cytoskeleton remodeling necessary for tumor cell adhesion and   migration  . This study investigated whether administration of racemic (R,S) ketorolac after ovarian cancer surgery leads to peritoneal distribution of R-ketorolac, target GTPase inhibition in cells retrieved from the peritoneal cavity, and measureable impact on patient outcomes.Eligible patients had suspected advanced-stage ovarian, fallopian tube or primary peritoneal cancer. Secondary eligibility was met when ovarian cancer was confirmed and optimally debulked, an intraperitoneal port was placed, and there were no contraindications for ketorolac administration. R- and S-ketorolac were measured in serum and peritoneal fluid, and GTPase activity was measured in peritoneal cells. A retrospective study correlated perioperative ketorolac and ovarian cancer-specific survival in ovarian cancer cases.Elevated expression and activity of Rac1 and Cdc42 was detected in ovarian cancer patient tissues, confirming target relevance. Ketorolac in peritoneal fluids was enriched in the R-enantiomer and peritoneal cell GTPase activity was inhibited after ketorolac administration when R-ketorolac was at peak levels. After adjusting for age, AJCC stage, completion of chemotherapy, and neoadjuvant therapy, women given perioperative ketorolac had a lower hazard of death (HR, 0.30; 95% confidence interval, 0.11-0.88).Ketorolac has a novel pharmacologic activity conferred by the R-enantiomer and R-ketorolac achieves sufficient levels in the peritoneal cavity to inhibit Rac1 and Cdc42, potentially contributing to the observed survival benefit in women who received ketorolac. 
  GeneRIFs  
 FOXP3 can regulate the expression of ARHGAP15 and affects   migration   of glioma cells through the Rac1 signaling pathway 
 Rab23 serves as an important oncoprotein in human astrocytoma by regulating cell invasion and   migration   through Rac1 activity 
 Activation of the NF-kappaB-JNK pathway is independent of the PIK3CA-Rac1-JNK pathway involved in the bFGF-regulated human fibroblast cell   migration   and wound healing. 
 we discovered distinct predictive performance of Rac1 and Cdc42 depending on the   migration   modes, indicating that Rac1 and Cdc42 contribute to persistent and random   migration  , respectively 
 RAC1 overexpression promoted the proliferation of lens epithelial cells. RAC1 overexpression also promoted   migration   and invasion of lens epithelial cells and had an influence on the epithelial-mesenchymal transition process. 
 Loss of FAT1 results in decreased cell adhesion and   migration   in fibroblasts and podocytes and the decreased   migration   is partially reversed by a RAC1/CDC42 activator. 
 Rap1- and Rac1-GTPase activation have roles in hypoxia/reoxygenation-experienced cancer cell   migration   and metastasis via the expression of thymosin beta-4 
 ELMO1 is expressed in rheumatoid arthritis synovium, promotes cell   migration   and invasion, and regulates Rac1 activity, thereby mediating rheumatoid arthritis pathogenicity. 
 Src-mediated Y724 phosphorylation in ELMO1 plays a critical role for cell spreading via activation of Rac1, leading to promotion of cell   migration  . 
 a role for ARF6 in linking EGF-receptor signaling to Rac1 recruitment and activation at the plasma membrane to promote breast cancer cell directed   migration   
 Inhibition of GSK3beta attenuated the   migration   and invasion of glioblastoma cells associated with suppression focal adhesion kinase, guanine nucleotide exchange factors/Rac1 and c-Jun N-terminal kinase. 
 It regulates actin polymerization to form lamellipodia, which is a critical event for cell   migration   of prostate cancer. 
 Results supported that miR-512-3p could inhibit tumor cell adhesion,   migration  , and invasion by regulating the RAC1 activity via DOCK3 in NSCLC A549 and H1299 cell lines. 
 The androgen-triggered AR/filamin A complex controls, through Rac 1, the decision of cells to halt cell cycle and   migration  . 
 biological significance of this PKCtheta;/alphaPIX/Rac 1 GTPase/PYGM signaling pathway seems to be the control of different cellular responses such as   migration   and proliferation 
 cIAP2 is in conclusion a regulator of human intestinal wound healing through enhanced   migration   along with activation of Rac1 
 RhoGDI2 inhibits trophoblast cell   migration  , and this function may involve suppression of RAC1 activation. 
 Data reveal that NEDD9 can regulate 3D   migration   speed independent of the Rac1 morphology switch. 
 RAC1 is an important downstream molecule of EGF-EGFR signaling involved in non-small-cell lung cancer cell   migration  . 
 MiR-200b suppresses arsenic-transformed cell   migration   by targeting PKCalpha and Wnt5b-PKCalpha positive feedback loop and inhibiting Rac1 activation. 
 A Rac1-Aurora A-MCAK signaling pathway mediates endothelial cell polarization and directional   migration   by promoting regional differences in microtubule dynamics. 
 Data suggest PIKFYVE (phosphoinositide kinase FYVE), MTMR3 (myotubularin related protein 3), and their product phosphatidylinositol 5-phosphate) are involved in activation of RAC1; this process regulates of   migration  /invasion of carcinoma/sarcoma. 
 These results indicated that Rac1 mediated shear stress-induced endothelial cells   migration   
 Coronin-1C overexpression is associated withand hepatocellular carcinoma growth through enhancement of tumor cell proliferation and   migration  , which are correlated with Rac-1 activation. 
 Rac1 differentially modulate cigarette smoke-induced airway cell   migration   through p120-catenin-dependent and -independent pathways 
 Cell   migration   experiments point towards the inhibition of p66Shc-rac1-mediated   migration   in the presence of ascorbic acid. Finally, results are suggestive that ascorbic acid-mediated decrease in Shc expression 
 Data indicate CCN1 can induce actin reorganization, formation of lamellipodia, and cell   migration  /invasion through the alphaV integrins/Rac1/ERK signaling axis; these CCN1-dependent activities can be effectively suppressed by monoclonal antibody YM1B. 
 study provides evidence the large GTPase Dyn2 regulates the small GTPase Rac1 to potentiate invasive   migration   of pancreatic tumor cells; Dyn2 plays an essential role in regulating Rac1-mediated pancreatic tumor cell   migration   through modulation of the Rac1 activator Vav1 via a direct interaction 
 HACE1 as an antagonist of cell   migration   through its ability to degrade active Rac1. 
 PI3K-C2alpha plays the crucial role in S1P(1) internalization into the intracellular vesicular compartment, Rac1 activation on endosomes, and thereby   migration   through regulating vesicular trafficking in ECs 
 Radixin plays an important role in promoting cell   migration   by regulating Rac1-mediated epithelial polarity and formation of adherens junctions through Vav GEFs. 
 Rac1 modulates the SDF-1-induced   migration   and invasion of glioma cells. 
 automated image analysis of MT assembly dynamics identified MARK2 as a target regulated downstream of Rac1 that promotes oriented MT growth in the leading edge to mediate directed cell   migration  . 
 Neuregulin mediates F-actin-driven cell   migration   through inhibition of protein kinase D1 via Rac1 protein 
 Glycogen synthase kinase 3beta inhibition prevents monocyte   migration   across brain endothelial cells via Rac1-GTPase suppression and down-regulation of active integrin conformation. 
 Tiam1, with the PAR complex, binds to integrins through talin and, together with the PAR complex, thereby regulates Rac1 activity and adhesion turnover for polarized   migration  . 
 14-3-3zeta enhances prostate cancer cell-matrix interactions, motility and transendothelial   migration   in vitro via activation of Rac1-GTPase and is an important target for therapeutic interventions for prostate cancer. 
 IL-8-Rac1/RhoA-p38MAPK signaling pathway plays a vital role in the IL-8-induced endothelial cell   migration  . 
 GEF Trio is responsible for lamellipodia formation through its N-terminal DH-PH domain in a Rac1-dependent manner during fibronectin-mediated spreading and   migration  . 
 Rac1 protein may control the   migration   of epidermal stem cells by regulating adhesion, spreading, and chemotaxis. It plays an active role in wound healing accelerated by ESC. 
 Involvement of 14-3-3 proteins in the second epidermal growth factor-induced wave of Rac1 activation in the process of cell   migration  . 
 Data identify the BAR-domain protein PACSIN2 as a Rac1 interactor that regulates Rac1-mediated cell spreading and   migration  . 
 The PI3K-Rac1/RhoA signaling pathway plays a vital role in IL-8 induced endothelial cell   migration   
 CD81 is required for the formation of actin membrane protrusions via RAC1 activation in adhesion-dependent immune cell   migration  . 
 Tbeta(4) increases   migration   of colon cancer cells via activating Rac1 by elevating IQGAP1/ILK complexes 
 Myeloid-associated differentiation marker (MYADM) regulates Rac1 targeting to ordered membranes required for cell spreading and   migration  . 
 These data indicated that the invasion and   migration   in Daoy cells were inhibited by deletion of Rac1. 
 The follicular fluid-HDL-associated S1P promotes granulosa lutein cell   migration   via S1PR3 and RAC1 activation. This may represent a novel mechanism contributing to the development of the corpus luteum. 
 Naa10p binds to the GIT-binding domain of PIX, thereby preventing the formation of the GIT-PIX-Paxillin complex, resulting in reduced intrinsic Cdc42/Rac1 activity and decreased cell   migration   
 TNFRSF19 overexpression in glioma cells activates Rac1 signaling in a Pyk2-dependent manner to drive glioma cell invasion and   migration   
 TSC2 has a role in controlling cell polarity and   migration   by regulating CDC42 and RAC1 activation 
 Data show that PED and Rac1 interact and that this interaction modulates cell   migration  /invasion processes in cancer cells through ERK1/2 pathway. 
 Results define the role of PIPKI-alpha in cell   migration   and describes a new mechanism for the spatial regulation of Rac1 activity that is critical for cell   migration  . 
 Asef2 activates Rac1 to modulate adhesion and actin dynamics and thereby regulate cell   migration   
 Dysregulation of Rac1 and Arf6 function by RCC2 knockdown also abolished persistent   migration   along fibronectin fibers, indicating a functional role for RCC2 in directional cell movement. 
 Inhibition of isoprenylcysteine carboxylmethyltransferase (Icmt) decreases activation of RhoA and Rac1, and in the absence of Icmt activity, addition of exogenous RhoA or Rac1 partially rescues directed and random   migration  , respectively. 
 BPAG1e is required for efficient regulation of keratinocyte polarity and   migration   by determining the activation of Rac1. 
 Results suggest that adiponectin promotes the   migration   activities of endothelial progenitor cells mainly through PI3-kinase/Cdc42/Rac1. 
 overexpression of Rac1 contributes to the accelerated   migration   and high proliferation potential of leukemia cells, which could be implicated in leukemia development and progression. 
 Phospholipase D activity regulates integrin-mediated cell spreading and   migration   by inducing GTP-Rac translocation to the plasma membrane 
 the interaction of the RAGE cytoplasmic domain with Dia-1 is required to transduce extracellular environmental cues evoked by binding of RAGE ligands to their cell surface receptor, resulting in Rac-1 and Cdc42 activation and cellular   migration   
 Rac1 is required for human endometrial stromal cell   migration   
 Positive regulation of   migration   and invasion by vasodilator-stimulated phosphoprotein via Rac1 pathway in human breast cancer cells. 
 paxillin is a critical downstream effector of Rac1 that may be involved in PMA-stimulated   migration   presumably by modulating the integrity of focal complex formation. 
 We suggest that Rac1 plays a role in cellular   migration   not only limited to its known function in reorganization of the actin cytoskeleton, but also as part of the intracellular machinery that controls the redox balance. 
 Protein kinase C-epsilon regulates sphingosine 1-phosphate-mediated   migration   of human lung endothelial cells through activation of phospholipase D2, protein kinase C-zeta, and Rac1 
 These results suggest that S1P(2) receptors/G(12/13)-proteins/Rho signaling pathways mediate S1P-induced inhibition of glioma cell   migration  . 
 A novel mechanism is proposed in which alpha6beta4 integrin signaling via Rac1, 14-3-3 proteins, and SSH family members regulates cofilin activation, cell polarity, and matrix assembly, leading to specific epidermal cell   migration   behavior. 
 16k PRL inhibits cell   migration   by blocking the Ras-Tiam1-Rac1-Pak1 signaling pathway in endothelial cells 
 Activation of Fn14 signaling by the ligand TNF-like weak inducer of apoptosis (TWEAK) stimulates   migration   and up-regulates expression of Fn14; this TWEAK effect requires Rac1 and nuclear factor-kappaB (NF-kappaB) activity. 
 evid,ence that the small GTPases RhoA and Rac1 but not Cdc42, are directly associated with Tyk2 and PI3-K in an uPA/uPAR-dependent fashion and are necessary to mediate the uPA/uPAR-directed   migration   via the Tyk2/PI3-K signalling complex in human VSMC 
 Akt1 and Akt2 have opposing roles in Rac/Pak signaling and cell   migration   
 Rac1 inhibition in non-metastatic or moderately metastatic cells results in inhibition of   migration  , whereas in highly metastatic cells Rac1 inhibition results in stimulation of   migration  . 
 The requirement for polyamines for intestinal epithelial cell   migration   is mediated through this protein. 
 Maspin controls mammary tumor cell   migration   through inhibiting Rac1 and Cdc42, but not the RhoA GTPase. 
 cleavage of CD44 catalyzed by ADAM10 is augmented by the intracellular signaling elicited by engagement of CD44, through Rac1-mediated cytoskeletal rearrangement, and suggest that CD44 cleavage contributes to the   migration   and invasion of tumor cells 
 Taken together, these results suggest that Dock4 plays an important role in the regulation of cell   migration   through activation of Rac1, and that RhoG is a key upstream regulator for Dock4. 
 The results suggest that oncogenic K-Ras enhances the malignant phenotype and identify the mitogen-activated protein kinase p38 as a target to inhibit oncogenic K-Ras-induced pancreatic tumor cell   migration  . 
 Both the beta4 integrin ligand-binding and cytoplasmic domains together with epidermal growth factor were required for the synergistic activation of a Rac-dependent signaling pathway that was essential for keratinocyte directional   migration  . 
 EGF induces ADAM10-mediated CD44 cleavage through Rac1 and mitogen-activated protein kinase activation, and thereby promotes tumour cell   migration   and invasion. 
 Rac1 and Rac2 have distinct roles in regulating cell morphology,   migration   and invasion, but are not essential for macrophage   migration   or chemotaxis. 
 the polybasic motif specifically directs Rac1 to effect cytoskeletal rearrangement and cell   migration   by selective membrane phospholipid targeting 
 Transgenic Rac1 has a critical role in axon guidance and cell   migration   during differentiation of the ventral telecephalon. 
 These findings suggest that PI3KC2beta regulates the   migration   and survival of human tumor cells by distinct molecular mechanisms. 
 AIF-1 binds and polymerizes F actin and also regulates Rac1 activity and vascular smooth muscle   migration   
 Slit2 inhibits vascular smooth muscle cell   migration   by suppressing small GTPase Rac1 activation. 
 Rac1 is involved in Porphyromonas gingivalis fimbria-stimulated monocytic cell adhesion to human vascular endothelium and transendothelial   migration   in vitro. 
  Alternative Name  
 cell   migration  -inducing gene 5 protein 
  ECM  PubMed  
 The roles of two distinct regions of PINCH-1 in the regulation of cell attachment and spreading. Cells attach to the extracellular matrix (  ECM  ) through integrins to form focal adhesion complexes, and this process is followed by the extension of lamellipodia to enable cell spreading. PINCH-1, an adaptor protein essential for the regulation of cell-  ECM   adhesion, consists of five tandem LIM domains and a small C-terminal region. PINCH-1 is known to interact with integrin-linked kinase (ILK) and Ras suppressor protein 1 (Rsu-1); however, the precise mechanism by which this complex regulates cell-  ECM   adhesion is not fully understood. We report here that the LIM1 domain of PINCH-1, which associates with ILK to stabilize the expression of this protein, is sufficient for cell attachment but not for cell spreading. In contrast, the C-terminal region of PINCH-1, which binds to Rsu-1, plays a pivotal role in cell spreading but not in cell attachment. We also show that PINCH-1 associates with Rsu-1 to activate Rac1 and that Rac1 activation is necessary for cell spreading. Thus, these data reveal how specific domains of PINCH-1 direct two independent pathways: one utilizing ILK to allow cell attachment, and the other recruiting Rsu-1 to activate Rac1 in order to promote cell spreading. 
 Prognostic role of integrin β1, E-cadherin, and rac1 expression in small cell lung cancer. Integrin β(1) mediates cellular adhesion to the extracellular matrix   (E  CM) and is correlated with highly invasive and metastatic behavior in small cell lung cancer (SCLC). E-cadherin (ECAD) is a calcium-dependent cell-cell adhesion receptor that restricts invasion of cells and reduces metastasis. Rac1 is involved in the regulation of the actin cytoskeleton, adhesion, migration, invasion, and tumor metastasis. The aim of this study was to examine integrin β(1) , ECAD and rac1 expression in SCLC and to analyze the prognostic value of these markers in patients with SCLC. We analyzed integrin β(1) , ECAD, and rac1 expression in 112 SCLC tissues by immunohistochemical staining. Correlative analyses between integrin β(1) , ECAD, and rac1 expression and cliniopathological factors were performed. A total of 65 patients had extensive disease (ED) (58%), and 47 had limited disease (LD) (42%). The median follow-up duration was 61 months (range: 14-117 months), and the median progression free survival (PFS) and overall survival (OS) were 6.1 months (range: 4.8-7.4 months) and 9.7 months (range: 8.1-11.3 months), respectively. The expression of integrin β(1) , ECAD, and rac1 protein was observed in 64, 73, and 99 of SCLC tissues, respectively. The correlative analyses between integrin β(1) , ECAD, or rac1 expression and various clinical parameters did not show any statistical significance. However, the ECAD expression was associated with OS in the entire cohort. In contrast, the expression of integrin β(1) and rac1 was not associated with PFS or OS. In a subgroup analysis, patients with less than two metastasis had significantly longer OS (p = 0.047) if their tumors expressed integrin β(1) compared to those without integrin β(1) expression. In addition, OS was longer for patients with ECAD positive tumors compared to those whose tumors did not express ECAD in males (p = 0.032) and patients who never smoked (p < 0.001). Multivariate analysis showed that LD (p = 0.004), overall response rate (p = 0.003), and expression of ECAD (p = 0.015) were the independent good prognostic factors for OS. LD (p = 0.024), overall response rate (p < 0.001), and less than two metastasis (p = 0.003) were prognostic factors for longer PFS. These results suggest that ECAD expression may be useful as a prognostic indicator in patients with SCLC. 
 Opposing roles for Akt1 and Akt2 in Rac/Pak signaling and cell migration. The Akt/PKB isoforms have different roles in animals, with Akt2 primarily regulating metabolic signaling and Akt1 regulating growth and survival. Here we show distinct roles for Akt1 and Akt2 in mouse embryo fibroblast cell migration and regulation of the cytoskeleton. Akt1-deficient cells responded poorly to platelet-derived growth factor while Akt2-deficient cells had a dramatically enhanced response, resulting in a substantial increase in dorsal ruffling. Swapping domains between Akt1 and Akt2 demonstrated that the N-terminal region containing the pleckstrin homology domain and a linker region distinguishes the two isoforms, while the catalytic domains are interchangeable. Akt2 knock-out cells also migrated faster than wild-type cells, especially through extracellular matrix (  ECM  ), while Akt1 knock-out cells migrated more slowly than wild-type cells. Consistently, Akt2 knock-out cells had elevated Pak1 and Rac activities, suggesting that Akt2 inhibits Rac and Pak1. Both Akt2 and Akt1 associated in complexes with Pak1, but only Akt2 inhibited Pak1 in kinase assays, suggesting an underlying molecular basis for the different cellular phenotypes. Together these data provide evidence for an unexpected functional link between Akt2 and Pak1 that opposes the actions of Akt1 on cell migration. 
 Prognostic role of integrin β1, E-cadherin, and rac1 expression in small cell lung cancer. Integrin β(1) mediates cellular adhesion to the extracellular matrix   (E  CM) and is correlated with highly invasive and metastatic behavior in small cell lung cancer (SCLC). E-cadherin (ECAD) is a calcium-dependent cell-cell adhesion receptor that restricts invasion of cells and reduces metastasis. Rac1 is involved in the regulation of the actin cytoskeleton, adhesion, migration, invasion, and tumor metastasis. The aim of this study was to examine integrin β(1) , ECAD and rac1 expression in SCLC and to analyze the prognostic value of these markers in patients with SCLC. We analyzed integrin β(1) , ECAD, and rac1 expression in 112 SCLC tissues by immunohistochemical staining. Correlative analyses between integrin β(1) , ECAD, and rac1 expression and cliniopathological factors were performed. A total of 65 patients had extensive disease (ED) (58%), and 47 had limited disease (LD) (42%). The median follow-up duration was 61 months (range: 14-117 months), and the median progression free survival (PFS) and overall survival (OS) were 6.1 months (range: 4.8-7.4 months) and 9.7 months (range: 8.1-11.3 months), respectively. The expression of integrin β(1) , ECAD, and rac1 protein was observed in 64, 73, and 99 of SCLC tissues, respectively. The correlative analyses between integrin β(1) , ECAD, or rac1 expression and various clinical parameters did not show any statistical significance. However, the ECAD expression was associated with OS in the entire cohort. In contrast, the expression of integrin β(1) and rac1 was not associated with PFS or OS. In a subgroup analysis, patients with less than two metastasis had significantly longer OS (p = 0.047) if their tumors expressed integrin β(1) compared to those without integrin β(1) expression. In addition, OS was longer for patients with ECAD positive tumors compared to those whose tumors did not express ECAD in males (p = 0.032) and patients who never smoked (p < 0.001). Multivariate analysis showed that LD (p = 0.004), overall response rate (p = 0.003), and expression of ECAD (p = 0.015) were the independent good prognostic factors for OS. LD (p = 0.024), overall response rate (p < 0.001), and less than two metastasis (p = 0.003) were prognostic factors for longer PFS. These results suggest that ECAD expression may be useful as a prognostic indicator in patients with SCLC. 
 Opposing roles for Akt1 and Akt2 in Rac/Pak signaling and cell migration. The Akt/PKB isoforms have different roles in animals, with Akt2 primarily regulating metabolic signaling and Akt1 regulating growth and survival. Here we show distinct roles for Akt1 and Akt2 in mouse embryo fibroblast cell migration and regulation of the cytoskeleton. Akt1-deficient cells responded poorly to platelet-derived growth factor while Akt2-deficient cells had a dramatically enhanced response, resulting in a substantial increase in dorsal ruffling. Swapping domains between Akt1 and Akt2 demonstrated that the N-terminal region containing the pleckstrin homology domain and a linker region distinguishes the two isoforms, while the catalytic domains are interchangeable. Akt2 knock-out cells also migrated faster than wild-type cells, especially through extracellular matrix (  ECM  ), while Akt1 knock-out cells migrated more slowly than wild-type cells. Consistently, Akt2 knock-out cells had elevated Pak1 and Rac activities, suggesting that Akt2 inhibits Rac and Pak1. Both Akt2 and Akt1 associated in complexes with Pak1, but only Akt2 inhibited Pak1 in kinase assays, suggesting an underlying molecular basis for the different cellular phenotypes. Together these data provide evidence for an unexpected functional link between Akt2 and Pak1 that opposes the actions of Akt1 on cell migration. 
 Vitreous-induced cytoskeletal rearrangements via the Rac1 GTPase-dependent signaling pathway in human retinal pigment epithelial cells. Proliferative vitreoretinopathy (PVR) is mainly caused by retinal pigment epithelial (RPE) cell migration, invasion, proliferation and transformation into fibroblast-like cells that produce the extracellular matrix (  ECM  ). The vitreous humor is known to play an important role in PVR. An epithelial-to-mesenchymal transdifferentiation (EMT) of human RPE cells induced by 25% vitreous treatment has been linked to stimulation of the mesenchymal phenotype, migration and invasion. Here, we characterized the effects of the vitreous on the cell morphology and cytoskeleton in human RPE cells. The signaling pathway that mediates these effects was investigated. Serum-starved RPE cells were incubated with 25% vitreous, and the morphological changes were examined by phase-contrast microscopy. Filamentous actin (F-actin) was examined by immunofluorescence and confocal microscopy. Protein phosphorylation of AKT, ERK1/2, Smad2/3, LIM kinase (LIMK) 1 and cofilin was analyzed by Western blot analysis. Vitreous treatment induced cytoskeletal rearrangements, activated Rac1 and enhanced the phosphorylation of AKT, ERK1/2 and Smad2/3. When the cells were treated with a Rac activation-specific inhibitor, the cytoskeletal rearrangements were prevented, and the phosphorylation of Smad2/3 was blocked. Vitreous treatment also enhanced the phosphorylation of LIMK1 and cofilin and the Rac inhibitor blocked this effect. We propose that vitreous-transformed human RPE cells undergo cytoskeletal rearrangements via Rac1 GTPase-dependent pathways that modulate LIMK1 and cofilin activity. The TGFβ-like activity of the vitreous may participate in this effect. Actin polymerization causes the cytoskeletal rearrangements that lead to the plasticity of vitreous-transformed RPE cells in PVR. 
 Extracellular matrix, Rac1 signaling, and estrogen-induced proliferation in MCF-7 breast cancer cells. Estrogen receptor positive (ER+), estrogen (E) responsive MCF-7 breast cancer cells cultured on the extracellular matrix (  ECM  ) protein laminin (LM), exhibit significantly reduced E-induced proliferation compared with cells cultured on collagen I (Col I) that is not due to a loss of ER. Based on reported differences in integrin-activated pathways on Col I vs. LM, we investigated the potential role of Rac1/c-jun-N-terminal kinase (JNK) activation and downstream regulation of cyclin D1 by E on Col I vs.LM. E-induced proliferation was increased on LM in MCF-7 cells expressing constitutively active Rac1 (CA Rac1) and decreased in dominant negative Rac1-(DN Rac1) expressing cells on Col I. siRNA knockdown established the specificity and requirement for Rac1 activation for E-induced regulation of cyclin D1. More robust c-Jun activation occurred on Col I than on LM and E-induced proliferation was abolished after treatment with a JNK inhibitor. These results provide evidence that Rac1/JNK/c-Jun activation promotes E-induced proliferation on Col I and reduced Rac1/JNK/c-Jun activation on LM contributes significantly to reduced E-induced proliferation in MCF-7 cells on LM. These results identify a novel role for extracellular matrix (  ECM  )-integrin regulation of Rac1-JNK pathway in E-regulated proliferation in ER+ breast cancer cells. These findings suggest that tumor stromal environment, i.e.,   ECM   composition, may contribute to loss of E regulation in ER+ breast cancers. Defining molecular markers for early identification of ER+ tumors that are ER+ but antiestrogen resistant would allow the design and use of alternative therapies to inhibit tumor growth and improve survival. 
 Maspin controls mammary tumor cell migration through inhibiting Rac1 and Cdc42, but not the RhoA GTPase. Rac1 and Cdc42 are members of the Rho family of small GTPases that play essential roles in diverse cellular functions, including cell migration. The activities of these Rho family proteins are controlled by growth factor receptor activation and cell-  ECM   interactions. Here, we show that maspin, a well-documented tumor suppressor gene, also controls cell motility through inhibiting Rac1/Cdc42 activity. Using the GST-PAK and GST-Rho binding protein pull-down assays for GTP-bound Rac1, Cdc42, and RhoA, we showed that treatment of MDA-MB-231 tumor cells with recombinant maspin for a short time period significantly inhibited the activity of Rac1 and Cdc42, but not RhoA. The reactive site loop (RSL) within maspin protein is the functional domain involved in the inhibition. Maspin mutants with the RSL deleted or a point mutation in the RSL region lost their inhibitory activity. We further examined the ability of maspin to inhibit Rac1- and Cdc42-mediated signaling pathways and transcription factors. Treatment of MDA-MB-231 cells with maspin led to the inhibition of JNK kinase activity as assayed by immuno-kinase assays. In addition, the AP-1 transcription activity downstream of JNK kinase pathway was also reduced. Together, we have identified Rac1 and Cdc42 as the downstream targets that mediate the inhibition of mammary tumor cell migration by maspin. 
 Tyrosine 397 phosphorylation is critical for FAK-promoted Rac1 activation and invasive properties in oral squamous cell carcinoma cells. Oral squamous cell carcinoma (OSCC) is a common cancer worldwide. Despite advances in diagnosis and therapy, treatment options for patients with metastatic OSCC are few, due in part to the limited understanding of the molecular events involved in the invasion and metastasis of OSCC. In this study, we investigated the expression of focal adhesion kinase (FAK) and its tyrosine 397 phosphorylation (pY397) in the tissue specimens of OSCC. The roles of pY397 in regulating the activities of Rac1 and cortactin and the invasive properties of OSCC cells were further determined. Results from immunohistochemical analyses in 9 benign, 19 premalignant, and 19 malignant oral tissues showed that the immunoreactivity of FAK was observed in 5 benign (56%), 19 premalignant (100%), and 18 malignant tissues (95%), whereas the immunoreactivity of pY397 was only found in 1 of 9 (11%) benign lesions but was observed in 9 premalignant (47%) and 12 malignant (63%) lesions. Compared with the low-invading SCC4 cells, the high-invading O  ECM  -1 cells exhibited higher levels of FAK expression and pY397, correlating with higher levels of GTP-bound Rac1 and cortactin phosphorylation. Manipulation of FAK expression or Y397 phosphorylation in SCC4, FaDu, O  ECM  -1, or HSC-3 cells regulated their Rac1 activities and invasive properties. Furthermore, treatment of NSC23766, a Rac1-specific inhibitor, in O  ECM  -1 and HSC-3 cells led to reduced invasive properties. Nevertheless, knockdown of FAK expression or suppression of pY397 had no effect on the cortactin activity in O  ECM  -1 cells. The data collectively suggest that pY397 plays critical roles in the FAK-promoted Rac1 activation and invasive properties in OSCC cells. Thus, the inhibition of FAK phosphorylation at Y397 or Rac1 activity can serve as a therapeutic strategy for treating patients with metastatic OSCC. 
 Rac1-dependent phosphorylation and focal adhesion recruitment of myosin IIA regulates migration and mechanosensing. Cell migration requires coordinated formation of focal adhesions (FAs) and assembly and contraction of the actin cytoskeleton. Nonmuscle myosin II (MII) is a critical mediator of contractility and FA dynamics in cell migration. Signaling downstream of the small GTPase Rac1 also regulates FA and actin dynamics, but its role in regulation of MII during migration is less clear.We found that Rac1 promotes association of MIIA with FA. Live-cell imaging showed that, whereas most MIIA at the leading edge assembled into dorsal contractile arcs, a substantial subset assembled in or was captured within maturing FA, and this behavior was promoted by active Rac1. Protein kinase C (PKC) activation was necessary and sufficient for integrin- and Rac1-dependent phosphorylation of MIIA heavy chain (HC) on serine1916 (S1916) and recruitment to FA. S1916 phosphorylation of MIIA HC and localization in FA was enhanced during cell spreading and   ECM   stiffness mechanosensing, suggesting upregulation of this pathway during physiological Rac1 activation. Phosphomimic and nonphosphorylatable MIIA HC mutants demonstrated that S1916 phosphorylation was necessary and sufficient for the capture and assembly of MIIA minifilaments in FA. S1916 phosphorylation was also sufficient to promote the rapid assembly of FAs to enhance cell migration and for the modulation of traction force, spreading, and migration by   ECM   stiffness.Our study reveals for the first time that Rac1 and integrin activation regulates MIIA HC phosphorylation through a PKC-dependent mechanism that promotes MIIA association with FAs and acts as a critical modulator of cell migration and mechanosensing. 
 A MAPK-Driven Feedback Loop Suppresses Rac Activity to Promote RhoA-Driven Cancer Cell Invasion. Cell migration in 3D microenvironments is fundamental to development, homeostasis and the pathobiology of diseases such as cancer. Rab-coupling protein (RCP) dependent co-trafficking of α5β1 and EGFR1 promotes cancer cell invasion into fibronectin (FN) containing extracellular matrix   (E  CM), by potentiating EGFR1 signalling at the front of invasive cells. This promotes a switch in RhoGTPase signalling to inhibit Rac1 and activate a RhoA-ROCK-Formin homology domain-containing 3 (FHOD3) pathway and generate filopodial actin-spike protrusions which drive invasion. To further understand the signalling network that drives RCP-driven invasive migration, we generated a Boolean logical model based on existing network pathways/models, where each node can be interrogated by computational simulation. The model predicted an unanticipated feedback loop, whereby Raf/MEK/ERK signalling maintains suppression of Rac1 by inhibiting the Rac-activating Sos1-Eps8-Abi1 complex, allowing RhoA activity to predominate in invasive protrusions. MEK inhibition was sufficient to promote lamellipodia formation and oppose filopodial actin-spike formation, and led to activation of Rac and inactivation of RhoA at the leading edge of cells moving in 3D matrix. Furthermore, MEK inhibition abrogated RCP/α5β1/EGFR1-driven invasive migration. However, upon knockdown of Eps8 (to suppress the Sos1-Abi1-Eps8 complex), MEK inhibition had no effect on RhoGTPase activity and did not oppose invasive migration, suggesting that MEK-ERK signalling suppresses the Rac-activating Sos1-Abi1-Eps8 complex to maintain RhoA activity and promote filopodial actin-spike formation and invasive migration. Our study highlights the predictive potential of mathematical modelling approaches, and demonstrates that a simple intervention (MEK-inhibition) could be of therapeutic benefit in preventing invasive migration and metastasis. 
 TGF-beta1-mediated activations of c-Src and Rac1 modulate levels of cyclins and p27(Kip1) CDK inhibitor in hepatoma cells replated on fibronectin. Integrin-mediated cell adhesion transduces signals to regulate actin cytoskeleton and cell proliferation. While understanding how integrin signals cross-talk with the TGF-beta1 pathways, we observed lamellipodia formation and cyclin regulation in Hep3B cells, following TGF-beta1 treatment. To answer if integrin signaling via actin organization might regulate cell cycle progression after TGF-beta1 treatment, we analyzed cross-talk between the two receptor-mediated pathways in hepatoma cells on specific   ECM  s. We found that basal and TGF-beta1-mediated activation of c-Src and Rac1, expression of cyclins E and A, and suppression of p27Kip1 were significant in cells replated on fibronectin, but not in cells on collagen I, indicating a different integrin-mediated cellular response to TGF-beta1 treatment. Levels of tyrosine phosphorylation and actin-enriched lamellipodia on fibronectin were also more prominent than in cells on collagen I. Studies using pharmacological inhibitors or transient transfections revealed that the preferential TGF-beta1 effects in cells on fibronectin required c-Src family kinase activity. These observations suggest that a specific cross-talk between TGF-beta1 and fibronectin-binding integrin signal pathways leads to the activation of c-Src/Rac1/actin-organization, leading to changes in cell cycle regulator levels in hepatoma cells. Therefore, this study represents another mechanism to regulate cell cycle regulators when integrin signaling is collaborative with TGF-beta1 pathways. 
 Tyr724 phosphorylation of ELMO1 by Src is involved in cell spreading and migration via Rac1 activation. The complex of Dock180/ELMO1 that functions as a bipartite guanine nucleotide exchange factor for Rac is essential for diverse physiological and pathological processes of cells such as cell migration, phagocytosis, and invasion of cancer cells. Among the Src-family tyrosine kinases (SFKs), it has been reported that Hck directly phosphorylates ELMO1, regulating phagocytosis by promoting activation of Rac1; however, the involvement of other SFKs in ELMO1 phosphorylation has remained unknown. Here, we identified novel tyrosine (Y) residues of ELMO1 phosphorylated by SFKs, and examined the effects on Rac1 activity, cell adhesion, spreading, and cell motility on extracellular matrix (  ECM  ).In this study, we unveiled that Src and Fyn can induce tyrosine phosphorylation of ELMO1 in in vivo and in vitro phosphorylation assays. Mutational analyses identified both Y720 and Y724 residues of ELMO1 as Src-mediated phosphorylation sites, preferentially on Y724. Single substitution of Y724 to Phe abrogated Rac1 activation triggered by Src. To elucidate the biological function of pY724, we established NIH3T3 cells stably expressing wild-type ELMO1 or its Y724F mutant together with Dock180. Among them, Y724-deficient cells exhibited a depletion of Rac1 activity with diminished phosphorylation of ELMO1 even upon the   ECM  -stimulation. It is noteworthy that NIH3T3 cells with ELMO1 Y724F were strikingly defective to promote cell spreading on fibronectin-coated dish, concomitantly exhibiting immature assemblies of actin stress fibers and focal adhesions. Eventually, ELMO1 Y724F significantly impaired cell migration.These results define that Src-mediated Y724 phosphorylation in ELMO1 plays a critical role for cell spreading via activation of Rac1, leading to promotion of cell migration. As the overexpression and/or hyperactivation of Src have been shown in a wide variety of human cancers, Src-mediated phosphorylation of Y724 in ELMO1 may regulate cancer cell adhesion to the   ECM  , invasion into surrounding tissues, and subsequent distant metastasis. 
  [Ee]xtracellular [Mm]atrix  PubMed  
 The roles of two distinct regions of PINCH-1 in the regulation of cell attachment and spreading. Cells attach to the   extracellular matrix   (ECM) through integrins to form focal adhesion complexes, and this process is followed by the extension of lamellipodia to enable cell spreading. PINCH-1, an adaptor protein essential for the regulation of cell-ECM adhesion, consists of five tandem LIM domains and a small C-terminal region. PINCH-1 is known to interact with integrin-linked kinase (ILK) and Ras suppressor protein 1 (Rsu-1); however, the precise mechanism by which this complex regulates cell-ECM adhesion is not fully understood. We report here that the LIM1 domain of PINCH-1, which associates with ILK to stabilize the expression of this protein, is sufficient for cell attachment but not for cell spreading. In contrast, the C-terminal region of PINCH-1, which binds to Rsu-1, plays a pivotal role in cell spreading but not in cell attachment. We also show that PINCH-1 associates with Rsu-1 to activate Rac1 and that Rac1 activation is necessary for cell spreading. Thus, these data reveal how specific domains of PINCH-1 direct two independent pathways: one utilizing ILK to allow cell attachment, and the other recruiting Rsu-1 to activate Rac1 in order to promote cell spreading. 
 Proteomic analysis of integrin-associated complexes identifies RCC2 as a dual regulator of Rac1 and Arf6. The binding of integrin adhesion receptors to their   extracellular matrix   ligands controls cell morphology, movement, survival, and differentiation in various developmental, homeostatic, and disease processes. Here, we report a methodology to isolate complexes associated with integrin adhesion receptors, which, like other receptor-associated signaling complexes, have been refractory to proteomic analysis. Quantitative, comparative analyses of the proteomes of two receptor-ligand pairs, alpha(4)beta(1)-vascular cell adhesion molecule-1 and alpha(5)beta(1)-fibronectin, defined both core and receptor-specific components. Regulator of chromosome condensation-2 (RCC2) was detected in the alpha(5)beta(1)-fibronectin signaling network at an intersection between the Rac1 and adenosine 5'-diphosphate ribosylation factor 6 (Arf6) subnetworks. RCC2 knockdown enhanced fibronectin-induced activation of both Rac1 and Arf6 and accelerated cell spreading, suggesting that RCC2 limits the signaling required for membrane protrusion and delivery. Dysregulation of Rac1 and Arf6 function by RCC2 knockdown also abolished persistent migration along fibronectin fibers, indicating a functional role for RCC2 in directional cell movement. This proteomics workflow now opens the way to further dissection and systems-level analyses of adhesion signaling. 
 The integrin cytoplasmic domain-associated protein ICAP-1 binds and regulates Rho family GTPases during cell spreading. Using two-hybrid screening, we isolated the integrin cytoplasmic domain-associated protein (ICAP-1), an interactor for the COOH terminal region of the beta1A integrin cytoplasmic domain. To investigate the role of ICAP-1 in integrin-mediated adhesive function, we expressed the full-length molecule in NIH3T3 cells. ICAP-1 expression strongly prevents NIH3T3 cell spreading on   extracellular matrix  . This inhibition is transient and can be counteracted by coexpression of a constitutively activated mutant of Cdc42, suggesting that ICAP-1 acts upstream of this GTPase. In addition, we found that ICAP-1 binds both to Cdc42 and Rac1 in vitro, and its expression markedly inhibits activation of these GTPases during integrin-mediated cell adhesion to fibronectin as detected by PAK binding assay. In the attempt to define the molecular mechanism of this inhibition, we show that ICAP-1 reduces both the intrinsic and the exchange factor-induced dissociation of GDP from Cdc42; moreover, purified ICAP-1 displaces this GTPase from cellular membranes. Together, these data show for the first time that ICAP-1 regulates Rho family GTPases during integrin-mediated cell matrix adhesion, acting as guanine dissociation inhibitor. 
 Regulation of endothelial cell cytoskeletal reorganization by a secreted frizzled-related protein-1 and frizzled 4- and frizzled 7-dependent pathway: role in neovessel formation. Consistent with findings of Wnt pathway members involved in vascular cells, a role for Wnt/Frizzled signaling has recently emerged in vascular cell development. Among the few Wnt family members implicated in vessel formation in adult, Wnt7b and Frizzled 4 have been shown as involved in vessel formation in the lung and in the retina, respectively. Our previous work has shown a role for secreted Frizzled-related protein-1 (sFRP-1), a proposed Wnt signaling inhibitor, in neovascularization after an ischemic event and demonstrated its role as a potent angiogenic factor. However the mechanisms involved have not been investigated. Here, we show that sFRP-1 treatment increases endothelial cell spreading on   extracellular matrix   as revealed by actin stress fiber reorganization in an integrin-dependent manner. We demonstrate that sFRP-1 can interact with Wnt receptors Frizzled 4 and 7 on endothelial cells to transduce downstream to cellular machineries requiring Rac-1 activity in cooperation with GSK-3beta. sFRP-1 overexpression in endothelium specifically reversed the inactivation of GSK-3 beta and increased neovascularization in ischemia-induced angiogenesis in mouse hindlimb. This study illustrates a regulated pathway by sFRP-1 involving GSK-3beta and Rac-1 in endothelial cell cytoskeletal reorganization and in neovessel formation. 
 Prognostic role of integrin β1, E-cadherin, and rac1 expression in small cell lung cancer. Integrin β(1) mediates cellular adhesion to th  e extracellular matr  ix (ECM) and is correlated with highly invasive and metastatic behavior in small cell lung cancer (SCLC). E-cadherin (ECAD) is a calcium-dependent cell-cell adhesion receptor that restricts invasion of cells and reduces metastasis. Rac1 is involved in the regulation of the actin cytoskeleton, adhesion, migration, invasion, and tumor metastasis. The aim of this study was to examine integrin β(1) , ECAD and rac1 expression in SCLC and to analyze the prognostic value of these markers in patients with SCLC. We analyzed integrin β(1) , ECAD, and rac1 expression in 112 SCLC tissues by immunohistochemical staining. Correlative analyses between integrin β(1) , ECAD, and rac1 expression and cliniopathological factors were performed. A total of 65 patients had extensive disease (ED) (58%), and 47 had limited disease (LD) (42%). The median follow-up duration was 61 months (range: 14-117 months), and the median progression free survival (PFS) and overall survival (OS) were 6.1 months (range: 4.8-7.4 months) and 9.7 months (range: 8.1-11.3 months), respectively. The expression of integrin β(1) , ECAD, and rac1 protein was observed in 64, 73, and 99 of SCLC tissues, respectively. The correlative analyses between integrin β(1) , ECAD, or rac1 expression and various clinical parameters did not show any statistical significance. However, the ECAD expression was associated with OS in the entire cohort. In contrast, the expression of integrin β(1) and rac1 was not associated with PFS or OS. In a subgroup analysis, patients with less than two metastasis had significantly longer OS (p = 0.047) if their tumors expressed integrin β(1) compared to those without integrin β(1) expression. In addition, OS was longer for patients with ECAD positive tumors compared to those whose tumors did not express ECAD in males (p = 0.032) and patients who never smoked (p < 0.001). Multivariate analysis showed that LD (p = 0.004), overall response rate (p = 0.003), and expression of ECAD (p = 0.015) were the independent good prognostic factors for OS. LD (p = 0.024), overall response rate (p < 0.001), and less than two metastasis (p = 0.003) were prognostic factors for longer PFS. These results suggest that ECAD expression may be useful as a prognostic indicator in patients with SCLC. 
 Opposing roles for Akt1 and Akt2 in Rac/Pak signaling and cell migration. The Akt/PKB isoforms have different roles in animals, with Akt2 primarily regulating metabolic signaling and Akt1 regulating growth and survival. Here we show distinct roles for Akt1 and Akt2 in mouse embryo fibroblast cell migration and regulation of the cytoskeleton. Akt1-deficient cells responded poorly to platelet-derived growth factor while Akt2-deficient cells had a dramatically enhanced response, resulting in a substantial increase in dorsal ruffling. Swapping domains between Akt1 and Akt2 demonstrated that the N-terminal region containing the pleckstrin homology domain and a linker region distinguishes the two isoforms, while the catalytic domains are interchangeable. Akt2 knock-out cells also migrated faster than wild-type cells, especially through   extracellular matrix   (ECM), while Akt1 knock-out cells migrated more slowly than wild-type cells. Consistently, Akt2 knock-out cells had elevated Pak1 and Rac activities, suggesting that Akt2 inhibits Rac and Pak1. Both Akt2 and Akt1 associated in complexes with Pak1, but only Akt2 inhibited Pak1 in kinase assays, suggesting an underlying molecular basis for the different cellular phenotypes. Together these data provide evidence for an unexpected functional link between Akt2 and Pak1 that opposes the actions of Akt1 on cell migration. 
 SILAC-based proteomics of human primary endothelial cell morphogenesis unveils tumor angiogenic markers. Proteomics has been successfully used for cell culture on dishes, but more complex cellular systems have proven to be challenging and so far poorly approached with proteomics. Because of the complexity of the angiogenic program, we still do not have a complete understanding of the molecular mechanisms involved in this process, and there have been no in depth quantitative proteomic studies. Plating endothelial cells on matrigel recapitulates aspects of vessel growth, and here we investigate this mechanism by using a spike-in SILAC quantitative proteomic approach. By comparing proteomic changes in primary human endothelial cells morphogenesis on matrigel to general adhesion mechanisms in cells spreading on culture dish, we pinpoint pathways and proteins modulated by endothelial cells. The cell-  extracellular matrix   adhesion proteome depends on the adhesion substrate, and a detailed proteomic profile of the   extracellular matrix   secreted by endothelial cells identified CLEC14A as a matrix component, which binds to MMRN2. We verify deregulated levels of these proteins during tumor angiogenesis in models of multistage carcinogenesis. This is the most in depth quantitative proteomic study of endothelial cell morphogenesis, which shows the potential of applying high accuracy quantitative proteomics to in vitro models of vessel growth to shed new light on mechanisms that accompany pathological angiogenesis. The mass spectrometry proteomics data have been deposited to the ProteomeXchange Consortium with the data set identifier PXD000359. 
 Prognostic role of integrin β1, E-cadherin, and rac1 expression in small cell lung cancer. Integrin β(1) mediates cellular adhesion to th  e extracellular matr  ix (ECM) and is correlated with highly invasive and metastatic behavior in small cell lung cancer (SCLC). E-cadherin (ECAD) is a calcium-dependent cell-cell adhesion receptor that restricts invasion of cells and reduces metastasis. Rac1 is involved in the regulation of the actin cytoskeleton, adhesion, migration, invasion, and tumor metastasis. The aim of this study was to examine integrin β(1) , ECAD and rac1 expression in SCLC and to analyze the prognostic value of these markers in patients with SCLC. We analyzed integrin β(1) , ECAD, and rac1 expression in 112 SCLC tissues by immunohistochemical staining. Correlative analyses between integrin β(1) , ECAD, and rac1 expression and cliniopathological factors were performed. A total of 65 patients had extensive disease (ED) (58%), and 47 had limited disease (LD) (42%). The median follow-up duration was 61 months (range: 14-117 months), and the median progression free survival (PFS) and overall survival (OS) were 6.1 months (range: 4.8-7.4 months) and 9.7 months (range: 8.1-11.3 months), respectively. The expression of integrin β(1) , ECAD, and rac1 protein was observed in 64, 73, and 99 of SCLC tissues, respectively. The correlative analyses between integrin β(1) , ECAD, or rac1 expression and various clinical parameters did not show any statistical significance. However, the ECAD expression was associated with OS in the entire cohort. In contrast, the expression of integrin β(1) and rac1 was not associated with PFS or OS. In a subgroup analysis, patients with less than two metastasis had significantly longer OS (p = 0.047) if their tumors expressed integrin β(1) compared to those without integrin β(1) expression. In addition, OS was longer for patients with ECAD positive tumors compared to those whose tumors did not express ECAD in males (p = 0.032) and patients who never smoked (p < 0.001). Multivariate analysis showed that LD (p = 0.004), overall response rate (p = 0.003), and expression of ECAD (p = 0.015) were the independent good prognostic factors for OS. LD (p = 0.024), overall response rate (p < 0.001), and less than two metastasis (p = 0.003) were prognostic factors for longer PFS. These results suggest that ECAD expression may be useful as a prognostic indicator in patients with SCLC. 
 Proteomic analysis of integrin-associated complexes identifies RCC2 as a dual regulator of Rac1 and Arf6. The binding of integrin adhesion receptors to their   extracellular matrix   ligands controls cell morphology, movement, survival, and differentiation in various developmental, homeostatic, and disease processes. Here, we report a methodology to isolate complexes associated with integrin adhesion receptors, which, like other receptor-associated signaling complexes, have been refractory to proteomic analysis. Quantitative, comparative analyses of the proteomes of two receptor-ligand pairs, alpha(4)beta(1)-vascular cell adhesion molecule-1 and alpha(5)beta(1)-fibronectin, defined both core and receptor-specific components. Regulator of chromosome condensation-2 (RCC2) was detected in the alpha(5)beta(1)-fibronectin signaling network at an intersection between the Rac1 and adenosine 5'-diphosphate ribosylation factor 6 (Arf6) subnetworks. RCC2 knockdown enhanced fibronectin-induced activation of both Rac1 and Arf6 and accelerated cell spreading, suggesting that RCC2 limits the signaling required for membrane protrusion and delivery. Dysregulation of Rac1 and Arf6 function by RCC2 knockdown also abolished persistent migration along fibronectin fibers, indicating a functional role for RCC2 in directional cell movement. This proteomics workflow now opens the way to further dissection and systems-level analyses of adhesion signaling. 
 Regulation of endothelial cell cytoskeletal reorganization by a secreted frizzled-related protein-1 and frizzled 4- and frizzled 7-dependent pathway: role in neovessel formation. Consistent with findings of Wnt pathway members involved in vascular cells, a role for Wnt/Frizzled signaling has recently emerged in vascular cell development. Among the few Wnt family members implicated in vessel formation in adult, Wnt7b and Frizzled 4 have been shown as involved in vessel formation in the lung and in the retina, respectively. Our previous work has shown a role for secreted Frizzled-related protein-1 (sFRP-1), a proposed Wnt signaling inhibitor, in neovascularization after an ischemic event and demonstrated its role as a potent angiogenic factor. However the mechanisms involved have not been investigated. Here, we show that sFRP-1 treatment increases endothelial cell spreading on   extracellular matrix   as revealed by actin stress fiber reorganization in an integrin-dependent manner. We demonstrate that sFRP-1 can interact with Wnt receptors Frizzled 4 and 7 on endothelial cells to transduce downstream to cellular machineries requiring Rac-1 activity in cooperation with GSK-3beta. sFRP-1 overexpression in endothelium specifically reversed the inactivation of GSK-3 beta and increased neovascularization in ischemia-induced angiogenesis in mouse hindlimb. This study illustrates a regulated pathway by sFRP-1 involving GSK-3beta and Rac-1 in endothelial cell cytoskeletal reorganization and in neovessel formation. 
 Opposing roles for Akt1 and Akt2 in Rac/Pak signaling and cell migration. The Akt/PKB isoforms have different roles in animals, with Akt2 primarily regulating metabolic signaling and Akt1 regulating growth and survival. Here we show distinct roles for Akt1 and Akt2 in mouse embryo fibroblast cell migration and regulation of the cytoskeleton. Akt1-deficient cells responded poorly to platelet-derived growth factor while Akt2-deficient cells had a dramatically enhanced response, resulting in a substantial increase in dorsal ruffling. Swapping domains between Akt1 and Akt2 demonstrated that the N-terminal region containing the pleckstrin homology domain and a linker region distinguishes the two isoforms, while the catalytic domains are interchangeable. Akt2 knock-out cells also migrated faster than wild-type cells, especially through   extracellular matrix   (ECM), while Akt1 knock-out cells migrated more slowly than wild-type cells. Consistently, Akt2 knock-out cells had elevated Pak1 and Rac activities, suggesting that Akt2 inhibits Rac and Pak1. Both Akt2 and Akt1 associated in complexes with Pak1, but only Akt2 inhibited Pak1 in kinase assays, suggesting an underlying molecular basis for the different cellular phenotypes. Together these data provide evidence for an unexpected functional link between Akt2 and Pak1 that opposes the actions of Akt1 on cell migration. 
 Vitreous-induced cytoskeletal rearrangements via the Rac1 GTPase-dependent signaling pathway in human retinal pigment epithelial cells. Proliferative vitreoretinopathy (PVR) is mainly caused by retinal pigment epithelial (RPE) cell migration, invasion, proliferation and transformation into fibroblast-like cells that produce the   extracellular matrix   (ECM). The vitreous humor is known to play an important role in PVR. An epithelial-to-mesenchymal transdifferentiation (EMT) of human RPE cells induced by 25% vitreous treatment has been linked to stimulation of the mesenchymal phenotype, migration and invasion. Here, we characterized the effects of the vitreous on the cell morphology and cytoskeleton in human RPE cells. The signaling pathway that mediates these effects was investigated. Serum-starved RPE cells were incubated with 25% vitreous, and the morphological changes were examined by phase-contrast microscopy. Filamentous actin (F-actin) was examined by immunofluorescence and confocal microscopy. Protein phosphorylation of AKT, ERK1/2, Smad2/3, LIM kinase (LIMK) 1 and cofilin was analyzed by Western blot analysis. Vitreous treatment induced cytoskeletal rearrangements, activated Rac1 and enhanced the phosphorylation of AKT, ERK1/2 and Smad2/3. When the cells were treated with a Rac activation-specific inhibitor, the cytoskeletal rearrangements were prevented, and the phosphorylation of Smad2/3 was blocked. Vitreous treatment also enhanced the phosphorylation of LIMK1 and cofilin and the Rac inhibitor blocked this effect. We propose that vitreous-transformed human RPE cells undergo cytoskeletal rearrangements via Rac1 GTPase-dependent pathways that modulate LIMK1 and cofilin activity. The TGFβ-like activity of the vitreous may participate in this effect. Actin polymerization causes the cytoskeletal rearrangements that lead to the plasticity of vitreous-transformed RPE cells in PVR. 
   Extracellular matrix  , Rac1 signaling, and estrogen-induced proliferation in MCF-7 breast cancer cells. Estrogen receptor positive (ER+), estrogen (E) responsive MCF-7 breast cancer cells cultured on the   extracellular matrix   (ECM) protein laminin (LM), exhibit significantly reduced E-induced proliferation compared with cells cultured on collagen I (Col I) that is not due to a loss of ER. Based on reported differences in integrin-activated pathways on Col I vs. LM, we investigated the potential role of Rac1/c-jun-N-terminal kinase (JNK) activation and downstream regulation of cyclin D1 by E on Col I vs.LM. E-induced proliferation was increased on LM in MCF-7 cells expressing constitutively active Rac1 (CA Rac1) and decreased in dominant negative Rac1-(DN Rac1) expressing cells on Col I. siRNA knockdown established the specificity and requirement for Rac1 activation for E-induced regulation of cyclin D1. More robust c-Jun activation occurred on Col I than on LM and E-induced proliferation was abolished after treatment with a JNK inhibitor. These results provide evidence that Rac1/JNK/c-Jun activation promotes E-induced proliferation on Col I and reduced Rac1/JNK/c-Jun activation on LM contributes significantly to reduced E-induced proliferation in MCF-7 cells on LM. These results identify a novel role for   extracellular matrix   (ECM)-integrin regulation of Rac1-JNK pathway in E-regulated proliferation in ER+ breast cancer cells. These findings suggest that tumor stromal environment, i.e., ECM composition, may contribute to loss of E regulation in ER+ breast cancers. Defining molecular markers for early identification of ER+ tumors that are ER+ but antiestrogen resistant would allow the design and use of alternative therapies to inhibit tumor growth and improve survival. 
 Role of Rac1 and oxygen radicals in collagenase-1 expression induced by cell shape change. Integrin-mediated reorganization of cell shape leads to an altered cellular phenotype. Disruption of the actin cytoskeleton, initiated by binding of soluble antibody to alpha5beta1 integrin, led to increased expression of the collagenase-1 gene in rabbit synovial fibroblasts. Activation of the guanosine triphosphate-binding protein Rac1, which was downstream of the integrin, was necessary for this process, and expression of activated Rac1 was sufficient to increase expression of collagenase-1. Rac1 activation generated reactive oxygen species that were essential for nuclear factor kappa B-dependent transcriptional regulation of interleukin-1alpha, which, in an autocrine manner, induced collagenase-1 gene expression. Remodeling of the   extracellular matrix   and consequent alterations of integrin-mediated adhesion and cytoarchitecture are central to development, wound healing, inflammation, and malignant disease. The resulting activation of Rac1 may lead to altered gene regulation and alterations in cellular morphogenesis, migration, and invasion. 
 Rac1 promotes TGF-beta-stimulated mesangial cell type I collagen expression through a PI3K/Akt-dependent mechanism. Transforming growth factor (TGF)-beta is a central mediator in the progression of glomerulosclerosis, leading to accumulation of aberrant   extracellular matrix   proteins and inappropriate expression of smooth muscle alpha-actin in the kidney. Previously, we reported that disrupting the cytoskeleton diminished TGF-beta-stimulated type I collagen accumulation in human mesangial cells. As cytoskeletal signaling molecules, including the Rho-family GTPases, have been implicated in fibrogenesis, we sought to determine the respective roles of RhoA and Rac1 in HMC collagen I expression. TGF-beta1 activated both RhoA and Rac1 within 5 min of treatment, and this activation was dependent on the kinase activity of the type I TGF-beta receptor. TGF-beta1-stimulated induction of type I collagen mRNA expression and promoter activity was diminished by inhibiting Rac1 activity and was increased by a constitutively active Rac1 mutant, whereas inhibiting RhoA activity had no such effect. Rac1 activation required phosphatidylinositol-3-kinase (PI3K) activity. Furthermore, the PI3K antagonist, LY294002, reduced TGF-beta1-stimulated COL1A2 promoter activity and Rac1 activation. It also partially blocked active Rac1-stimulated collagen promoter activity, suggesting that PI3K activity contributes to both TGF-beta activation of Rac1 and signal propagation downstream of Rac1. Thus, while both Rac1 and RhoA are rapidly activated in response to TGF-beta1 in human mesangial cells, only Rac1 activation enhances events that contribute to mesangial cell collagen expression, through a positive feedback loop involving PI3K. 
 Prostaglandin E2-mediated migration of human trophoblast requires RAC1 and CDC42. The invasion of maternal decidua and uterine spiral arteries by a trophoblast subpopulation called extravillous trophoblast (EVT) is essential for the establishment of a normal placenta and an adequate blood flow toward the fetus. Derangements in these processes underlie pregnancy-related diseases like preeclampsia and intrauterine growth restriction. Many growth factors, growth factor binding proteins, and   extracellular matrix   components can positively or negatively regulate the proliferation, migration, and/or invasiveness of these EVT cells. RHO GTPases, including RHOA, RAC1, and CDC42, are ubiquitous proteins that control cytoskeletal changes by forming stress fibers and projecting lamellipodia and filopodia during cellular migration. We had previously shown that prostaglandin (PG) E(2) produced in abundance by the decidua promotes the migration of first-trimester human EVTs by increasing the intracellular concentration of calcium and activating calpain. Using our well-characterized immortalized EVT cell line, HTR-8/SVneo, as well as villus explants from first-trimester placentae, this study examined the role of RHO GTPases RAC1 and CDC42 in PGE(2)-mediated migratory responses of these cells. Though a RAC1 inhibitor, NSC23766 as well as RAC1 knockdown by siRNA decreased the migration of HTR-8/SVneo cells in a Transwell migration assay, this inhibition could not be restored by PGE(2) or 17-phenyl trinor PGE(2) (PGE receptor PTGER1 agonist) or PGE(1) Alcohol (PGE receptor PTGER4 agonist). Similar results were noted for EVT cell spreading in villus explants. Furthermore, CDC42 silencing using siRNA inhibited PGE(2)-induced migration of HTR-8/SVneo cells. Finally, the treatment of EVT cells with PGE(2), PTGER1 agonist, or PTGER4 agonist activated RAC1 and CDC42 at 10 min, suggesting that RAC1 and CDC42 play an essential role in PGE(2)-mediated migration of human EVTs. 
 NEDD9 regulates 3D migratory activity independent of the Rac1 morphology switch in glioma and neuroblastoma. Metastasizing tumor cells must transmigrate the dense   extracellular matrix   that surrounds most organs. The use of three-dimensional (3D) collagen gels has revealed that many cancer cells can switch between different modes of invasion that are characterized by distinct morphologies (e.g., rounded vs. elongated). The adhesion protein NEDD9 has the potential to regulate the switch between elongated and rounded morphologies; therefore, its role was interrogated in the invasion switch of glioblastoma and neuroblastoma tumors that similarly derive from populations of neural crest cells. Interestingly, siRNA-mediated depletion of NEDD9 failed to induce cell rounding in glioma or neuroblastoma cells, contrasting the effects that have been described in other tumor model systems. Given that Rac1 GTPase has been suggested to mediate the switch between elongated and rounded invasion, the functionality of the Rac1 morphology switch was evaluated in the glioma and neuroblastoma cells. Using both dominant-negative Rac1 and Rac1-specific siRNA, the presence of this morphologic switch was confirmed in the neuroblastoma, but not in the glioma cells. However, in the absence of a morphologic change following NEDD9 depletion, a significant decrease in the cellular migration rate was observed. Thus, the data reveal that NEDD9 can regulate 3D migration speed independent of the Rac1 morphology switch.NEDD9 targeting is therapeutically viable as it does not stimulate adaptive changes in glioma and neuroblastoma invasion. 
 Non-small cell lung carcinoma cell motility, rac activation and metastatic dissemination are mediated by protein kinase C epsilon. Protein kinase C (PKC) ε, a key signaling transducer implicated in mitogenesis, survival, and cancer progression, is overexpressed in human primary non-small cell lung cancer (NSCLC). The role of PKCε in lung cancer metastasis has not yet been established.Here we show that RNAi-mediated knockdown of PKCε in H358, H1299, H322, and A549 NSCLC impairs activation of the small GTPase Rac1 in response to phorbol 12-myristate 13-acetate (PMA), serum, or epidermal growth factor (EGF). PKCε depletion markedly impaired the ability of NSCLC cells to form membrane ruffles and migrate. Similar results were observed by pharmacological inhibition of PKCε with εV1-2, a specific PKCε inhibitor. PKCε was also required for invasiveness of NSCLC cells and modulated the secre  tion of extracellula  r matrix proteases and protease inhibitors. Finally, we found that PKCε-depleted NSCLC cells fail to disseminate to lungs in a mouse model of metastasis.Our results implicate PKCε as a key mediator of Rac signaling and motility of lung cancer cells, highlighting its potential as a therapeutic target. 
 Matricellular protein CCN3 (NOV) regulates actin cytoskeleton reorganization. CCN3 (NOV), a putative ligand for integrin receptors, is tightly associated with the   extracellular matrix   and mediates diverse cellular functions, including cell adhesion and proliferation. CCN3 has been shown to negatively regulate growth although it promotes migration in a cell type-specific manner. In this study, overexpression of CCN3 reduces growth and increases intercellular adhesion of breast cancer cells. Interestingly, CCN3 overexpression also led to the formation of multiple pseudopodia that are enriched in actin, CCN3, and vinculin. Breast cancer cells preincubated with exogenous CCN3 protein also induced the same phenotype, indicating that secreted CCN3 is sufficient to induce changes in cell morphology. Surprisingly, extracellular CCN3 is internalized to the early endosomes but not to the membrane protrusions, suggesting pseudopodia-enriched CCN3 may derive from a different source. The presence of an intracellular variant of CCN3 will be consistent with our finding that the cytoplasmic tail of the gap junction protein connexin43 (Cx43) associates with CCN3. Cx43 is a channel protein permitting intercellular communication to occur. However, neither the channel properties nor the protein levels of Cx43 are affected by the CCN3 protein. In contrast, CCN3 proteins are down-regulated in the absence of Cx43. Finally, we showed that overexpression of CCN3 increases the activity of the small GTPase Rac1, thereby revealing a pathway that links Cx43 directly to actin reorganization. 
 Up-regulated type I collagen expression by the inhibition of Rac1 signaling pathway in human dermal fibroblasts. Tissue remodeling is known to play important roles in wound healing. Although Rac1 is reported to be one of the key signaling molecules in cutaneous wound healing process, the exact mechanisms of Rac1-mediated tissue remodeling is still unknown. This study investigated the role of Rac1 in the regulation of   extracellular matrix   in cultured human dermal fibroblasts obtained by skin biopsy from three healthy donors. Protein levels of type I collagen in cultured human fibroblasts were increased by the treatment with Rac1 inhibitor NSC23766 in a dose-dependent manner. However, the mRNA levels of alpha2(I) collagen was not altered by the inhibitor. On the other hand, by the addition of inhibitor, half-lives of type I collagen protein were increased and MMP1 levels were reduced. These data suggest that blockade of Rac1 signaling results in accumulation of type I collagen due to decreased collagenase activity. This study also suggests that controlling Rac1 signaling is a new therapeutic approach to chronic/untreatable ulcer. 
 Integrins regulate Rac targeting by internalization of membrane domains. Translocation of the small GTP-binding protein Rac1 to the cell plasma membrane is essential for activating downstream effectors and requires integrin-mediated adhesion of cells to   extracellular matrix  . We report that active Rac1 binds preferentially to low-density, cholesterol-rich membranes, and specificity is determined at least in part by membrane lipids. Cell detachment triggered internalization of plasma membrane cholesterol and lipid raft markers. Preventing internalization maintained Rac1 membrane targeting and effector activation in nonadherent cells. Regulation of lipid rafts by integrin signals may regulate the location of membrane domains such as lipid rafts and thereby control domain-specific signaling events in anchorage-dependent cells. 
 Rapid Remodeling of Invadosomes by Gi-coupled Receptors: DISSECTING THE ROLE OF Rho GTPases. Invadosomes are actin-rich membrane protrusions that degrade the   extracellular matrix   to drive tumor cell invasion. Key players in invadosome formation are c-Src and Rho family GTPases. Invadosomes can reassemble into circular rosette-like superstructures, but the underlying signaling mechanisms remain obscure. Here we show that Src-induced invadosomes in human melanoma cells (A375M and MDA-MB-435) undergo rapid remodeling into dynamic   extracellular matrix  -degrading rosettes by distinct G protein-coupled receptor agonists, notably lysophosphatidic acid (LPA; acting through the LPA1 receptor) and endothelin. Agonist-induced rosette formation is blocked by pertussis toxin, dependent on PI3K activity and accompanied by localized production of phosphatidylinositol 3,4,5-trisphosphate, whereas MAPK and Ca(2+) signaling are dispensable. Using FRET-based biosensors, we show that LPA and endothelin transiently activate Cdc42 through Gi, concurrent with a biphasic decrease in Rac activity and differential effects on RhoA. Cdc42 activity is essential for rosette formation, whereas G12/13-mediated RhoA-ROCK signaling suppresses the remodeling process. Our results reveal a Gi-mediated Cdc42 signaling axis by which G protein-coupled receptors trigger invadosome remodeling, the degree of which is dictated by the Cdc42-RhoA activity balance. 
 First step of the cell-penetrating peptide mechanism involves Rac1 GTPase-dependent actin-network remodelling. Application of CPPs (cell-penetrating peptides) constitutes a promising strategy for the intracellular delivery of therapeutic molecules. The non-covalent approach based on the amphipathic peptide MPG has been successfully used to improve the delivery of biologically active macromolecules, both in cellulo and in vivo, through a mechanism independent of the endosomal pathway and mediated by the membrane potential.In the present study, we have investigated the first step of the cellular uptake mechanism of MPG and shown that both MPG and MPG-cargo complexes interact with the   extracellular matrix   through the negatively charged heparan sulfate proteoglycans. We demonstrated that initiation of cellular uptake constitutes a highly dynamic mechanism where the binding of MPG or the MPG-cargo to the   extracellular matrix   is rapidly followed by a remodelling of the actin network associated with the activation of the GTPase Rac1. We suggest that MPG-induced clustering of the glycosaminoglycan platform constitutes the 'onset' of the cellular uptake mechanism, thereby increasing membrane dynamics and membrane fusion processes. This process favours cell entry of MPG or MPG-DNA complexes, which is further controlled by the ability of MPG to induce a local membrane destabilization.Although CPPs are taken up through different pathways and mechanisms, the initial step involves electrostatic interactions with the glycosaminoglycan platform, and the dynamics of associated membrane microdomains can be generalized to most non-viral delivery systems. 
 Protein kinase G signaling disrupts Rac1-dependent focal adhesion assembly in liver specific pericytes. Nitric oxide (NO) regulates the function of perivascular cells (pericytes), including hepatic stellate cells (HSC), mainly by activating cGMP and cGMP-dependent kinase (PKG) via NO/cGMP paracrine signaling. Although PKG is implicated in integrin-mediated cell adhesion to   extracellular matrix  , whether or how PKG signaling regulates the assembly of focal adhesion complexes (FA) and migration of HSC is not known. With the help of complementary molecular and cell biological approaches, we demonstrate here that activation of PKG signaling in HSC inhibits vascular tubulogenesis, migration/chemotaxis, and assembly of mature FA plaques, as assessed by vascular tubulogenesis assays and immunofluorescence localization of FA markers such as vinculin and vasodilator-stimulated phosphoprotein (VASP). To determine whether PKG inhibits FA assembly by phosphorylation of VASP at Ser-157, Ser-239, and Thr-278, we mutated these putative phosphorylation sites to alanine (VASP3A, phosphoresistant mutant) or aspartic acid (VASP3D, phosphomimetic), respectively. Data generated from these two mutants suggest that the effect of PKG on FA is independent of these three phosphorylation sites. In contrast, activation of PKG inhibits the activity of small GTPase Rac1 and its association with the effector protein IQGAP1. Moreover, PKG activation inhibits the formation of a trimeric protein complex containing Rac1, IQGAP1, and VASP. Finally, we found that expression of a constitutively active Rac1 mutant abolishes the inhibitory effects of PKG on FA formation. In summary, our data suggest that activation of PKG signaling in pericytes inhibits FA formation by inhibiting Rac1. 
 The Rho-family GTPase Rac1 regulates integrin localization in Drosophila immunosurveillance cells. When the parasitoid wasp Leptopilina boulardi lays an egg in a Drosophila larva, phagocytic cells called plasmatocytes and specialized cells known as lamellocytes encapsulate the egg. The Drosophila β-integrin Myospheroid (Mys) is necessary for lamellocytes to adhere to the cellular capsule surrounding L. boulardi eggs. Integrins are heterodimeric adhesion receptors consisting of α and β subunits, and similar to other plasma membrane receptors undergo ligand-dependent endocytosis. In mammalian cells it is known that integrin binding to t  he extracellular mat  rix induces the activation of Rac GTPases, and we have previously shown that Rac1 and Rac2 are necessary for a proper encapsulation response in Drosophila larvae. We wanted to test the possibility that Myospheroid and Rac GTPases interact during the Drosophila anti-parasitoid immune response.In the current study we demonstrate that Rac1 is required for the proper localization of Myospheroid to the cell periphery of haemocytes after parasitization. Interestingly, the mislocalization of Myospheroid in Rac1 mutants is rescued by hyperthermia, involving the heat shock protein Hsp83. From these results we conclude that Rac1 and Hsp83 are required for the proper localization of Mys after parasitization.We show for the first time that the small GTPase Rac1 is required for Mysopheroid localization. Interestingly, the necessity of Rac1 in Mys localization was negated by hyperthermia. This presents a problem, in Drosophila we quite often raise larvae at 29°C when using the GAL4/UAS misexpression system. If hyperthermia rescues receptor endosomal recycling defects, raising larvae in hyperthermic conditions may mask potentially interesting phenotypes. 
 A MAPK-Driven Feedback Loop Suppresses Rac Activity to Promote RhoA-Driven Cancer Cell Invasion. Cell migration in 3D microenvironments is fundamental to development, homeostasis and the pathobiology of diseases such as cancer. Rab-coupling protein (RCP) dependent co-trafficking of α5β1 and EGFR1 promotes cancer cell invasion into fibronectin (FN) containin  g extracellular matr  ix (ECM), by potentiating EGFR1 signalling at the front of invasive cells. This promotes a switch in RhoGTPase signalling to inhibit Rac1 and activate a RhoA-ROCK-Formin homology domain-containing 3 (FHOD3) pathway and generate filopodial actin-spike protrusions which drive invasion. To further understand the signalling network that drives RCP-driven invasive migration, we generated a Boolean logical model based on existing network pathways/models, where each node can be interrogated by computational simulation. The model predicted an unanticipated feedback loop, whereby Raf/MEK/ERK signalling maintains suppression of Rac1 by inhibiting the Rac-activating Sos1-Eps8-Abi1 complex, allowing RhoA activity to predominate in invasive protrusions. MEK inhibition was sufficient to promote lamellipodia formation and oppose filopodial actin-spike formation, and led to activation of Rac and inactivation of RhoA at the leading edge of cells moving in 3D matrix. Furthermore, MEK inhibition abrogated RCP/α5β1/EGFR1-driven invasive migration. However, upon knockdown of Eps8 (to suppress the Sos1-Abi1-Eps8 complex), MEK inhibition had no effect on RhoGTPase activity and did not oppose invasive migration, suggesting that MEK-ERK signalling suppresses the Rac-activating Sos1-Abi1-Eps8 complex to maintain RhoA activity and promote filopodial actin-spike formation and invasive migration. Our study highlights the predictive potential of mathematical modelling approaches, and demonstrates that a simple intervention (MEK-inhibition) could be of therapeutic benefit in preventing invasive migration and metastasis. 
 Temporal and spatial modulation of Rho GTPases during in vitro formation of capillary vascular network. Adherens junctions and myosin light chain as targets of Rac1 and RhoA. Endothelial cells (ECs) self-organize into capillary networks when plated on   extracellular matrix  . In this process, Rho GTPases-mediated cytoskeletal dynamics control cell movement and organization of cell-to-matrix and cell-to-cell contacts. Time course analysis of RhoA and Rac1 activation matches specific morphological aspects of nascent pattern. RhoA-GTP increases early during EC adhesion and accumulates at sites of membrane ruffling. Rac1 is activated later and localizes in lamellipodia and at cell-to-cell contacts of organized cell chains. When ECs stretch and remodel to form capillary structures, RhoA-GTP increases again and associates with stress fibers running along the major cell axis. N17Rac1 and N19RhoA mutants impair pattern formation. Cell-to-cell contacts and myosin light chains (MLC) are targets of Rac1 and RhoA, respectively. N17Rac1 reduces the shift of beta-catenin and vascular endothelial cadherin to Triton X-100-insoluble fraction and impairs beta-catenin distribution at adherens junctions, suggesting that Rac1 controls the dynamics of cadherin-catenin complex with F-actin. During the remodeling phase of network formation, ECs show an intense staining for phosphorylated MLC along the plasma membrane; in contrast, MLC is less phosphorylated and widely diffused in N19RhoA ECs. Both N17Rac1 and N19RhoA have been used to investigate the role of wild type molecules in the main steps characterizing in vitro angiogenesis: (i) cell adhesion to the substrate, (ii) cell movement, and (iii) mechanical remodeling of matrix. N17Rac1 has a striking inhibitory effect on haptotaxis, whereas N19RhoA slightly inhibits EC adhesion and motility but more markedly Matrigel contraction. We conclude that different Rho GTPases control distinct morphogenetic aspects of vascular morphogenesis. 
 DDX3 modulates cell adhesion and motility and cancer cell metastasis via Rac1-mediated signaling pathway. The DEAD-box RNA helicase DDX3 is a versatile protein involved in multiple steps of gene expression and various cellular signaling pathways. DDX3 mutations have been implicated in the wingless (Wnt) type of medulloblastoma. We show here that small interfering RNA-mediated DDX3 knockdown in various cell lines increased cell-cell adhesion but decreased cell-  extracellular matrix   adhesion. Moreover, DDX3 depletion suppressed cell motility and impaired directional migration in the wound-healing assay. Accordingly, DDX3-depleted cells exhibited reduced invasive capacities in vitro as well as reduced metastatic potential in mice. We also examined the mechanism underlying DDX3-regulated cell migration. DDX3 knockdown reduced the levels of both Rac1 and β-catenin proteins, and consequentially downregulated the expression of several β-catenin target genes. Moreover, we demonstrated that DDX3-regulated Rac1 mRNA translation, possibly through an interaction with its 5'-untranslated region, and affected β-catenin protein stability in an Rac1-dependent manner. Taken together, our results indicate the DDX3-Rac1-β-catenin regulatory axis in modulating the expression of Wnt/β-catenin target genes. Therefore, this report provides a mechanistic context for the role of DDX3 in Wnt-type tumors. 
 Altered adhesive structures and their relation to RhoGTPase activation in merlin-deficient Schwannoma. Schwannomas are Schwann cell tumors of the nervous system that occur spontaneously and in patients with neurofibromatosis 2 (NF2) and lack the tumor suppressor merlin. Merlin is known to bind paxillin, beta1 integrin and focal adhesion kinase, members of focal contacts, multi-protein complexes that mediate cell adhesion to the   extracellular matrix  . Moreover, merlin-deficient Schwannomas show pathological adhesion to the   extracellular matrix   making the characterization of focal contacts indispensable. Using our Schwannoma in vitro model of human primary Schwann and Schwannoma cells, we here show that Schwannoma cells display an increased number of mature and stable focal contacts. In addition to an involvement of RhoA signaling via the Rho kinase ROCK, Rac1 plays a significant role in the pathological adhesion of Schwannoma cells. The Rac1 guanine exchange factor- beta-Pix, localizes to focal contacts in human primary Schwannoma cells, and we show that part of the Rac1 activation, an effect of merlin-deficiency, occurs at the level of focal contacts in human primary Schwannoma cells. Our results help explaining the pathological adhesion of Schwannoma cells, further strengthen the importance of RhoGTPase signaling in Schwannoma development, and suggest that merlin's role in tumor suppression is linked to focal contacts. 
 The GTPase Rac regulates the proliferation and invasion of fibroblast-like synoviocytes from rheumatoid arthritis patients. Fibroblast-like synoviocytes (FLS) isolated from joints of rheumatoid arthritis (RA) patients display proliferative and invasive properties reminiscent of those of malignant tumor cells. Rac small GTPases play an important role in tumor cell proliferation and invasion. We therefore investigated the potential role of Rac proteins in the proliferative and invasive behavior of RA-FLS. We showed that inhibiting Rac activity with the Rac-specific small molecule inhibitor NSC23766 causes a strong inhibition of RA-FLS proliferation, without affecting cell survival. Rac inhibition also results in a strong reduction in RA-FLS invasion through reconstituted   extracellular matrix   and a less marked inhibition of two-dimensional migration as measured by monolayer wound healing. We also showed that small interfering RNA-mediated depletion of Rac1 inhibits RA-FLS proliferation and invasion to a similar extent as NSC23766. These results demonstrate for the first time that Rac proteins play an important role in the aggressive behavior of FLS isolated from RA patients. In addition, we observed that inhibiting Rac proteins prevents JNK activation and that the JNK inhibitor SP600125 strongly inhibits RA-FLS invasion, suggesting that Rac-mediated JNK activation contributes to the role of Rac proteins in the invasive behavior of RA-FLS. In conclusion, Rac-controlled signaling pathways may present a new source of drug targets for therapeutic intervention in RA. 
 Tyr724 phosphorylation of ELMO1 by Src is involved in cell spreading and migration via Rac1 activation. The complex of Dock180/ELMO1 that functions as a bipartite guanine nucleotide exchange factor for Rac is essential for diverse physiological and pathological processes of cells such as cell migration, phagocytosis, and invasion of cancer cells. Among the Src-family tyrosine kinases (SFKs), it has been reported that Hck directly phosphorylates ELMO1, regulating phagocytosis by promoting activation of Rac1; however, the involvement of other SFKs in ELMO1 phosphorylation has remained unknown. Here, we identified novel tyrosine (Y) residues of ELMO1 phosphorylated by SFKs, and examined the effects on Rac1 activity, cell adhesion, spreading, and cell motility on   extracellular matrix   (ECM).In this study, we unveiled that Src and Fyn can induce tyrosine phosphorylation of ELMO1 in in vivo and in vitro phosphorylation assays. Mutational analyses identified both Y720 and Y724 residues of ELMO1 as Src-mediated phosphorylation sites, preferentially on Y724. Single substitution of Y724 to Phe abrogated Rac1 activation triggered by Src. To elucidate the biological function of pY724, we established NIH3T3 cells stably expressing wild-type ELMO1 or its Y724F mutant together with Dock180. Among them, Y724-deficient cells exhibited a depletion of Rac1 activity with diminished phosphorylation of ELMO1 even upon the ECM-stimulation. It is noteworthy that NIH3T3 cells with ELMO1 Y724F were strikingly defective to promote cell spreading on fibronectin-coated dish, concomitantly exhibiting immature assemblies of actin stress fibers and focal adhesions. Eventually, ELMO1 Y724F significantly impaired cell migration.These results define that Src-mediated Y724 phosphorylation in ELMO1 plays a critical role for cell spreading via activation of Rac1, leading to promotion of cell migration. As the overexpression and/or hyperactivation of Src have been shown in a wide variety of human cancers, Src-mediated phosphorylation of Y724 in ELMO1 may regulate cancer cell adhesion to the ECM, invasion into surrounding tissues, and subsequent distant metastasis. 
 ARAP2 signals through Arf6 and Rac1 to control focal adhesion morphology. Focal adhesions (FAs) are dynamic structures that connect the actin cytoskeleton with the   extracellular matrix  . At least six ADP-ribosylation factor (Arf) GTPase-activating proteins (GAPs), including ARAP2 (an Arf6 GAP), are implicated in regulation of FAs but the mechanisms for most are not well defined. Although Rac1 has been reported to function downstream of Arf6 to control membrane ruffling and cell migration, this pathway has not been directly examined as a regulator of FAs. Here we test the hypothesis that ARAP2 promotes the growth of FAs by converting Arf6·GTP to Arf6·GDP thereby preventing the activation of the Rho family GTP-binding protein Rac1. Reduced expression of ARAP2 decreased the number and size of FAs in cells and increased cellular Arf6·GTP and Rac1·GTP levels. Overexpression of ARAP2 had the opposite effects. The effects of ARAP2 on FAs and Rac1 were dependent on a functional ArfGAP domain. Constitutively active Arf6 affected FAs in the same way as did reduced ARAP2 expression and dominant negative mutants of Arf6 and Rac1 reversed the effect of reduced ARAP2 expression. However, neither dominant negative Arf6 nor Rac1 had the same effect as ARAP2 overexpression. We conclude that changes in Arf6 and Rac1 activities are necessary but not sufficient for ARAP2 to promote the growth of FAs and we speculate that ARAP2 has additional functions that are effector in nature to promote or stabilize FAs. 
 Rac1 activation driven by 14-3-3ζ dimerization promotes prostate cancer cell-matrix interactions, motility and transendothelial migration. 14-3-3 proteins are ubiquitously expressed dimeric adaptor proteins that have emerged as key mediators of many cell signaling pathways in multiple cell types. Its effects are mainly mediated by binding to selective phosphoserine/threonine proteins. The importance of 14-3-3 proteins in cancer have only started to become apparent and its exact role in cancer progression as well as the mechanisms by which 14-3-3 proteins mediate cancer cell function remain unknown. While protein 14-3-3σ is widely accepted as a tumor suppressor, 14-3-3ζ, β and γ isoforms have been shown to have tumor promoting effects. Despite the importance of 14-3-3 family in mediating various cell processes, the exact role and mechanism of 14-3-3ζ remain unexplored. In the current study, we investigated the role of protein 14-3-3ζ in prostate cancer cell motility and transendothelial migration using biochemical, molecular biology and electric cell-substrate impedance sensing approaches as well as cell based functional assays. Our study indicated that expression with wild-type protein 14-3-3ζ significantly enhanced Rac activity in PC3 cells. In contrast, expression of dimer-resistant mutant of protein 14-3-3ζ (DM-14-3-3) inhibited Rac activity and associated phosphorylation of p21 activated kinase-1 and 2. Expression with wild-type 14-3-3ζ or constitutively active Rac1   enhanced extracellu  lar matrix recognition, lamellipodia formation, cell migration and trans-endothelial migration by PC3 cells. In contrast, expression with DM 14-3-3ζ or DN-Rac1 in PC3 cells significantly inhibited these cell functions. Our results demonstrate for the first time that 14-3-3ζ enhances prostate cancer cell-matrix interactions, motility and transendothelial migration in vitro via activation of Rac1-GTPase and is an important target for therapeutic interventions for prostate cancer. 
 Extension, retraction and contraction in the formation of a dendritic cell dendrite: distinct roles for Rho GTPases. The morphology of antigen-presenting dendritic cells (DC) is characterized by the possession of numerous long arborizing processes known as dendrites. The formation of these processes by DC, both in the periphery and in lymphoid organs, is believed to contribute to the remarkable efficiency with which they take up, process and present antigen to T cells. However, the process of dendrite formation and the signaling pathways that lead to the formation of these dendrites remain obscure. In this study we describe an in vitro model in which human immature DC form long processes similar to those formed in vivo. The formation of these processes involves initial attachment of a cell protrusion to the   extracellular matrix   substrate, and subsequent movement of the cell body away from the adhesion site, leaving behind a long slender dendrite. Dendrite formation, but not their maintenance, was found to be dependent on the activity of Rho GTPases. More specifically, Cdc42 and Rac1 were both required for the migration step of process formation, promoting cell spreading and extension. In contrast, Rho, and its downstream effector p160ROCK, regulated the release of adhesions to the substratum, and associated cellular contraction. Consequently, inhibition of Rho/p160ROCK leads to the formation of longer dendrites. DC therefore coordinate adhesion and protrusion to perform a specialized process of cellular morphogenesis, which differentiates these cells from all other cells of the immune system and may contribute to their distinctive function. 
 Engagement of CD44 promotes Rac activation and CD44 cleavage during tumor cell migration. CD44 is a major cell surface adhesion molecule for hyaluronan, a component of the   extracellular matrix  , and is implicated in tumor metastasis and invasion. We reported previously that hyaluronan oligosaccharides induce CD44 cleavage from tumor cells. Here we show that engagement of CD44 promotes CD44 cleavage and tumor cell migration, both of which were suppressed by a metalloproteinase inhibitor KB-R7785 and tissue inhibitor of metalloproteinases-1 (TIMP-1) but not by TIMP-2. We also present evidence that blockade of metalloproteinase-disintegrin ADAM10 (a disintegrin and metalloproteinase 10) by RNA interference suppresses CD44 cleavage induced by its ligation. Engagement of CD44 concurrently induced activation of the small GTPase Rac1 and led to drastic changes in cell morphology and actin cytoskeleton with redistribution of CD44 to newly generated membrane ruffling areas. A fluorescence resonance energy transfer approach to visualize GTP-bound Rac1 in living cells revealed the localization of the active Rac1 in the leading edge of the membrane ruffling areas upon ligation of CD44. Taken together, our results indicate that the cleavage of CD44 catalyzed by ADAM10 is augmented by the intracellular signaling elicited by engagement of CD44, through Rac-mediated cytoskeletal rearrangement, and suggest that CD44 cleavage contributes to the migration and invasion of tumor cells. 
 Rac1 recruitment to the archipelago structure of the focal adhesion through the fluid membrane as revealed by single-molecule analysis. The focal adhesion (FA) is an integrin-based structure built in/on the plasma membrane (PM), linking the   extracellular matrix   to the actin stress-fibers, working as cell migration scaffolds. Previously, we proposed the archipelago architecture of the FA, in which FA largely consists of fluid membrane, dotted with small islands accumulating FA proteins: membrane molecules enter the inter-island channels in the FA zone rather freely, and the integrins in the FA-protein islands rapidly exchanges with those in the bulk membrane. Here, we examined how Rac1, a small G-protein regulating FA formation, and its activators αPIX and βPIX, are recruited to the FA zones. PIX molecules are recruited from the cytoplasm to the FA zones directly. In contrast, majorities of Rac1 molecules first arrive from the cytoplasm on the general inner PM surface, and then enter the FA zones via lateral diffusion on the PM, which is possible due to rapid Rac1 diffusion even within the FA zones, slowed only by a factor of two to four compared with that outside. The constitutively-active Rac1 mutant exhibited temporary and all-time immobilizations in the FA zone, suggesting that upon PIX-induced Rac1 activation at the FA-protein islands, Rac1 tends to be immobilized at the FA-protein islands. 
  Component  
   extracellular matrix   
  [Ii]nvadosome  PubMed  
 Rapid Remodeling of   Invadosome  s by Gi-coupled Receptors: DISSECTING THE ROLE OF Rho GTPases.   Invadosome  s are actin-rich membrane protrusions that degrade the extracellular matrix to drive tumor cell invasion. Key players in   invadosome   formation are c-Src and Rho family GTPases.   Invadosome  s can reassemble into circular rosette-like superstructures, but the underlying signaling mechanisms remain obscure. Here we show that Src-induced   invadosome  s in human melanoma cells (A375M and MDA-MB-435) undergo rapid remodeling into dynamic extracellular matrix-degrading rosettes by distinct G protein-coupled receptor agonists, notably lysophosphatidic acid (LPA; acting through the LPA1 receptor) and endothelin. Agonist-induced rosette formation is blocked by pertussis toxin, dependent on PI3K activity and accompanied by localized production of phosphatidylinositol 3,4,5-trisphosphate, whereas MAPK and Ca(2+) signaling are dispensable. Using FRET-based biosensors, we show that LPA and endothelin transiently activate Cdc42 through Gi, concurrent with a biphasic decrease in Rac activity and differential effects on RhoA. Cdc42 activity is essential for rosette formation, whereas G12/13-mediated RhoA-ROCK signaling suppresses the remodeling process. Our results reveal a Gi-mediated Cdc42 signaling axis by which G protein-coupled receptors trigger   invadosome   remodeling, the degree of which is dictated by the Cdc42-RhoA activity balance. 
  GeneRIFs  
 Data suggest LPA (lysophosphatidic acid; acting via LPAR1) and endothelin activate Cdc42, concurrent with biphasic decrease in Rac1 activity, differential effects on RhoA; LPA/endothelin stim. leads to remodeling of   invadosome  s in melanoma cells. 
  [Ii]nvasive  PubMed  
 Genetic susceptibility to distinct bladder cancer subphenotypes. Clinical, pathologic, and molecular evidence indicate that bladder cancer is heterogeneous with pathologic/molecular features that define distinct subphenotypes with different prognoses. It is conceivable that specific patterns of genetic susceptibility are associated with particular subphenotypes.To examine evidence for the contribution of germline genetic variation to bladder cancer heterogeneity.The Spanish Bladder Cancer/EPICURO Study is a case-control study based in 18 hospitals located in five areas in Spain. Cases were patients with a newly diagnosed, histologically confirmed, urothelial cell carcinoma of the bladder from 1998 to 2001. Case diagnoses were reviewed and uniformly classified by pathologists following the World Health Organisation/International Society of Urological Pathology 1999 criteria. Controls were hospital-matched patients (n=1149).A total of 1526 candidate variants in 423 candidate genes were analysed. Three distinct subphenotypes were defined according to stage and grade: low-grade nonmuscle   invasive   (n=586), high-grade nonmuscle   invasive   (n=219), and muscle   invasive   (n=246). The association between each variant and subphenotype was assessed by polytomous risk models adjusting for potential confounders. Heterogeneity in genetic susceptibility among subphenotypes was also tested.Two established bladder cancer susceptibility genotypes, NAT2 slow-acetylation and GSTM1-null, exhibited similar associations among the subphenotypes, as did VEGF-rs25648, which was previously identified in our study. Other variants conferred risks for specific tumour subphenotypes such as PMS2-rs6463524 and CD4-rs3213427 (respective heterogeneity p values of 0.006 and 0.004), which were associated with muscle-  invasive   tumours (per-allele odds ratios [95% confidence interval] of 0.56 [0.41-0.77] and 0.71 [0.57-0.88], respectively) but not with non-muscle-  invasive   tumours. Heterogeneity p values were not robust in multiple testing according to their false-discovery rate.These exploratory analyses suggest that genetic susceptibility loci might be related to the molecular/pathologic diversity of bladder cancer. Validation through large-scale replication studies and the study of additional genes and single nucleotide polymorphisms are required. 
 Oncogenic K-Ras down-regulates Rac1 and RhoA activity and enhances migration and invasion of pancreatic carcinoma cells through activation of p38. Activating mutations in the K-ras gene are genetic alterations frequently found in human carcinomas, particularly in pancreatic adenocarcinomas. Mutation of the K-ras gene is thought to be an early and important event in pancreatic tumor initiation, but the precise role of the mutant K-Ras proteins in neoplastic progression is still unknown. In the present study, we have characterized the influence of oncogenic K-Ras on the phenotype and on the signal transduction of epitheloid PANC-1 pancreatic carcinoma cells by generating PANC-1 cell clones, which stably express EGFP(enhanced green fluorescent protein)-K-Ras (V12). EGFP-K-Ras (V12)-expressing cells exhibited a more fibroblastoid cellular phenotype with irregular cell shape and disorganized cytokeratin filaments. Moreover, these cells showed a marked enhancement of their migratory and   invasive   properties. Stable expression of EGFP-K-Ras (V12) down-regulated the activity of Rac1 and RhoA, resulting in reduced subcortical actin filaments and stress fibers, which might contribute to the epithelial dedifferentiation. Characterization of the activity of mitogen-activated protein kinases revealed that EGFP-K-Ras (V12) enhanced the activity of p38, but did not affect the activities of the Raf/MEK/ERK cascade and JNK. While inhibition of either MEK or JNK activity had no effect on EGFP-K-Ras (V12)-induced migration, inhibition of p38 activity markedly reduced EGFP-K-Ras (V12)-induced migration. Collectively, the results suggest that oncogenic K-Ras enhances the malignant phenotype and identify the mitogen-activated protein kinase p38 as a target to inhibit oncogenic K-Ras-induced pancreatic tumor cell migration. 
 Epidermal growth factor-regulated activation of Rac GTPase enhances CD44 cleavage by metalloproteinase disintegrin ADAM10.   Invasive   tumour cells, such as gliomas, frequently express EGF (epidermal growth factor) receptor at a high level and they exhibit enhanced cell migration in response to EGF. We reported previously that tumour cell migration is associated with ectodomain cleavage of CD44, the major adhesion molecule that is implicated in tumour invasion and metastasis, and that the cleavage is enhanced by ligation of CD44. In the present study, we show that EGF promotes CD44 cleavage and CD44-dependent cell migration. Introduction of a dominant-negative mutant of the small GTPase Rac1 or depletion of Rac1 by RNAi (RNA interference) abrogated CD44 cleavage induced by EGF. Treatment with PD98059, an inhibitor for MEK (mitogen-activated protein kinase/extracellular-signal-regulated kinase kinase), also suppressed the CD44 cleavage. Furthermore, RNAi studies showed that EGF induced ADAM10 (a disintegrin and metalloproteinase 10)-dependent CD44 cleavage and cell migration. These results indicate that EGF induces ADAM10-mediated CD44 cleavage through Rac1 and mitogen-activated protein kinase activation, and thereby promotes tumour cell migration and invasion. 
 Prognostic role of integrin β1, E-cadherin, and rac1 expression in small cell lung cancer. Integrin β(1) mediates cellular adhesion to the extracellular matrix (ECM) and is correlated with highl  y invasi  ve and metastatic behavior in small cell lung cancer (SCLC). E-cadherin (ECAD) is a calcium-dependent cell-cell adhesion receptor that restricts invasion of cells and reduces metastasis. Rac1 is involved in the regulation of the actin cytoskeleton, adhesion, migration, invasion, and tumor metastasis. The aim of this study was to examine integrin β(1) , ECAD and rac1 expression in SCLC and to analyze the prognostic value of these markers in patients with SCLC. We analyzed integrin β(1) , ECAD, and rac1 expression in 112 SCLC tissues by immunohistochemical staining. Correlative analyses between integrin β(1) , ECAD, and rac1 expression and cliniopathological factors were performed. A total of 65 patients had extensive disease (ED) (58%), and 47 had limited disease (LD) (42%). The median follow-up duration was 61 months (range: 14-117 months), and the median progression free survival (PFS) and overall survival (OS) were 6.1 months (range: 4.8-7.4 months) and 9.7 months (range: 8.1-11.3 months), respectively. The expression of integrin β(1) , ECAD, and rac1 protein was observed in 64, 73, and 99 of SCLC tissues, respectively. The correlative analyses between integrin β(1) , ECAD, or rac1 expression and various clinical parameters did not show any statistical significance. However, the ECAD expression was associated with OS in the entire cohort. In contrast, the expression of integrin β(1) and rac1 was not associated with PFS or OS. In a subgroup analysis, patients with less than two metastasis had significantly longer OS (p = 0.047) if their tumors expressed integrin β(1) compared to those without integrin β(1) expression. In addition, OS was longer for patients with ECAD positive tumors compared to those whose tumors did not express ECAD in males (p = 0.032) and patients who never smoked (p < 0.001). Multivariate analysis showed that LD (p = 0.004), overall response rate (p = 0.003), and expression of ECAD (p = 0.015) were the independent good prognostic factors for OS. LD (p = 0.024), overall response rate (p < 0.001), and less than two metastasis (p = 0.003) were prognostic factors for longer PFS. These results suggest that ECAD expression may be useful as a prognostic indicator in patients with SCLC. 
 p130Cas promotes   invasive  ness of three-dimensional ErbB2-transformed mammary acinar structures by enhanced activation of mTOR/p70S6K and Rac1. ErbB2 over-expression is detected in approximately 25% of   invasive   breast cancers and is strongly associated with poor patient survival. We have previously demonstrated that p130Cas adaptor is a crucial mediator of ErbB2 transformation. Here, we analysed the molecular mechanisms through which p130Cas controls ErbB2-dependent invasion in three-dimensional cultures of mammary epithelial cells. Concomitant p130Cas over-expression and ErbB2 activation enhance PI3K/Akt and Erk1/2 MAPK signalling pathways and promote invasion of mammary acini. By using pharmacological inhibitors, we demonstrate that both signalling cascades are required for the   invasive   behaviour of p130Cas over-expressing and ErbB2 activated acini. Erk1/2 MAPK and PI3K/Akt signalling triggers invasion through distinct downstream effectors involving mTOR/p70S6K and Rac1 activation, respectively. Moreover, in silico analyses indicate that p130Cas expression in ErbB2 positive human breast cancers significantly correlates with higher risk to develop distant metastasis, thus underlying the value of the p130Cas/ErbB2 synergism in regulating breast cancer invasion. In conclusion, high levels of p130Cas favour progression of ErbB2-transformed cells towards an   invasive   phenotype. 
 Prognostic role of integrin β1, E-cadherin, and rac1 expression in small cell lung cancer. Integrin β(1) mediates cellular adhesion to the extracellular matrix (ECM) and is correlated with highl  y invasi  ve and metastatic behavior in small cell lung cancer (SCLC). E-cadherin (ECAD) is a calcium-dependent cell-cell adhesion receptor that restricts invasion of cells and reduces metastasis. Rac1 is involved in the regulation of the actin cytoskeleton, adhesion, migration, invasion, and tumor metastasis. The aim of this study was to examine integrin β(1) , ECAD and rac1 expression in SCLC and to analyze the prognostic value of these markers in patients with SCLC. We analyzed integrin β(1) , ECAD, and rac1 expression in 112 SCLC tissues by immunohistochemical staining. Correlative analyses between integrin β(1) , ECAD, and rac1 expression and cliniopathological factors were performed. A total of 65 patients had extensive disease (ED) (58%), and 47 had limited disease (LD) (42%). The median follow-up duration was 61 months (range: 14-117 months), and the median progression free survival (PFS) and overall survival (OS) were 6.1 months (range: 4.8-7.4 months) and 9.7 months (range: 8.1-11.3 months), respectively. The expression of integrin β(1) , ECAD, and rac1 protein was observed in 64, 73, and 99 of SCLC tissues, respectively. The correlative analyses between integrin β(1) , ECAD, or rac1 expression and various clinical parameters did not show any statistical significance. However, the ECAD expression was associated with OS in the entire cohort. In contrast, the expression of integrin β(1) and rac1 was not associated with PFS or OS. In a subgroup analysis, patients with less than two metastasis had significantly longer OS (p = 0.047) if their tumors expressed integrin β(1) compared to those without integrin β(1) expression. In addition, OS was longer for patients with ECAD positive tumors compared to those whose tumors did not express ECAD in males (p = 0.032) and patients who never smoked (p < 0.001). Multivariate analysis showed that LD (p = 0.004), overall response rate (p = 0.003), and expression of ECAD (p = 0.015) were the independent good prognostic factors for OS. LD (p = 0.024), overall response rate (p < 0.001), and less than two metastasis (p = 0.003) were prognostic factors for longer PFS. These results suggest that ECAD expression may be useful as a prognostic indicator in patients with SCLC. 
 p130Cas promotes   invasive  ness of three-dimensional ErbB2-transformed mammary acinar structures by enhanced activation of mTOR/p70S6K and Rac1. ErbB2 over-expression is detected in approximately 25% of   invasive   breast cancers and is strongly associated with poor patient survival. We have previously demonstrated that p130Cas adaptor is a crucial mediator of ErbB2 transformation. Here, we analysed the molecular mechanisms through which p130Cas controls ErbB2-dependent invasion in three-dimensional cultures of mammary epithelial cells. Concomitant p130Cas over-expression and ErbB2 activation enhance PI3K/Akt and Erk1/2 MAPK signalling pathways and promote invasion of mammary acini. By using pharmacological inhibitors, we demonstrate that both signalling cascades are required for the   invasive   behaviour of p130Cas over-expressing and ErbB2 activated acini. Erk1/2 MAPK and PI3K/Akt signalling triggers invasion through distinct downstream effectors involving mTOR/p70S6K and Rac1 activation, respectively. Moreover, in silico analyses indicate that p130Cas expression in ErbB2 positive human breast cancers significantly correlates with higher risk to develop distant metastasis, thus underlying the value of the p130Cas/ErbB2 synergism in regulating breast cancer invasion. In conclusion, high levels of p130Cas favour progression of ErbB2-transformed cells towards an   invasive   phenotype. 
 Genetic susceptibility to distinct bladder cancer subphenotypes. Clinical, pathologic, and molecular evidence indicate that bladder cancer is heterogeneous with pathologic/molecular features that define distinct subphenotypes with different prognoses. It is conceivable that specific patterns of genetic susceptibility are associated with particular subphenotypes.To examine evidence for the contribution of germline genetic variation to bladder cancer heterogeneity.The Spanish Bladder Cancer/EPICURO Study is a case-control study based in 18 hospitals located in five areas in Spain. Cases were patients with a newly diagnosed, histologically confirmed, urothelial cell carcinoma of the bladder from 1998 to 2001. Case diagnoses were reviewed and uniformly classified by pathologists following the World Health Organisation/International Society of Urological Pathology 1999 criteria. Controls were hospital-matched patients (n=1149).A total of 1526 candidate variants in 423 candidate genes were analysed. Three distinct subphenotypes were defined according to stage and grade: low-grade nonmuscle   invasive   (n=586), high-grade nonmuscle   invasive   (n=219), and muscle   invasive   (n=246). The association between each variant and subphenotype was assessed by polytomous risk models adjusting for potential confounders. Heterogeneity in genetic susceptibility among subphenotypes was also tested.Two established bladder cancer susceptibility genotypes, NAT2 slow-acetylation and GSTM1-null, exhibited similar associations among the subphenotypes, as did VEGF-rs25648, which was previously identified in our study. Other variants conferred risks for specific tumour subphenotypes such as PMS2-rs6463524 and CD4-rs3213427 (respective heterogeneity p values of 0.006 and 0.004), which were associated with muscle-  invasive   tumours (per-allele odds ratios [95% confidence interval] of 0.56 [0.41-0.77] and 0.71 [0.57-0.88], respectively) but not with non-muscle-  invasive   tumours. Heterogeneity p values were not robust in multiple testing according to their false-discovery rate.These exploratory analyses suggest that genetic susceptibility loci might be related to the molecular/pathologic diversity of bladder cancer. Validation through large-scale replication studies and the study of additional genes and single nucleotide polymorphisms are required. 
 Oncogenic K-Ras down-regulates Rac1 and RhoA activity and enhances migration and invasion of pancreatic carcinoma cells through activation of p38. Activating mutations in the K-ras gene are genetic alterations frequently found in human carcinomas, particularly in pancreatic adenocarcinomas. Mutation of the K-ras gene is thought to be an early and important event in pancreatic tumor initiation, but the precise role of the mutant K-Ras proteins in neoplastic progression is still unknown. In the present study, we have characterized the influence of oncogenic K-Ras on the phenotype and on the signal transduction of epitheloid PANC-1 pancreatic carcinoma cells by generating PANC-1 cell clones, which stably express EGFP(enhanced green fluorescent protein)-K-Ras (V12). EGFP-K-Ras (V12)-expressing cells exhibited a more fibroblastoid cellular phenotype with irregular cell shape and disorganized cytokeratin filaments. Moreover, these cells showed a marked enhancement of their migratory and   invasive   properties. Stable expression of EGFP-K-Ras (V12) down-regulated the activity of Rac1 and RhoA, resulting in reduced subcortical actin filaments and stress fibers, which might contribute to the epithelial dedifferentiation. Characterization of the activity of mitogen-activated protein kinases revealed that EGFP-K-Ras (V12) enhanced the activity of p38, but did not affect the activities of the Raf/MEK/ERK cascade and JNK. While inhibition of either MEK or JNK activity had no effect on EGFP-K-Ras (V12)-induced migration, inhibition of p38 activity markedly reduced EGFP-K-Ras (V12)-induced migration. Collectively, the results suggest that oncogenic K-Ras enhances the malignant phenotype and identify the mitogen-activated protein kinase p38 as a target to inhibit oncogenic K-Ras-induced pancreatic tumor cell migration. 
 Epidermal growth factor-regulated activation of Rac GTPase enhances CD44 cleavage by metalloproteinase disintegrin ADAM10.   Invasive   tumour cells, such as gliomas, frequently express EGF (epidermal growth factor) receptor at a high level and they exhibit enhanced cell migration in response to EGF. We reported previously that tumour cell migration is associated with ectodomain cleavage of CD44, the major adhesion molecule that is implicated in tumour invasion and metastasis, and that the cleavage is enhanced by ligation of CD44. In the present study, we show that EGF promotes CD44 cleavage and CD44-dependent cell migration. Introduction of a dominant-negative mutant of the small GTPase Rac1 or depletion of Rac1 by RNAi (RNA interference) abrogated CD44 cleavage induced by EGF. Treatment with PD98059, an inhibitor for MEK (mitogen-activated protein kinase/extracellular-signal-regulated kinase kinase), also suppressed the CD44 cleavage. Furthermore, RNAi studies showed that EGF induced ADAM10 (a disintegrin and metalloproteinase 10)-dependent CD44 cleavage and cell migration. These results indicate that EGF induces ADAM10-mediated CD44 cleavage through Rac1 and mitogen-activated protein kinase activation, and thereby promotes tumour cell migration and invasion. 
 Invasion of endothelial cells by tissue-  invasive   M3 type group A streptococci requires Src kinase and activation of Rac1 by a phosphatidylinositol 3-kinase-independent mechanism. Streptococcus pyogenes can cause   invasive   diseases in humans, such as sepsis or necrotizing fasciitis. Among the various M serotypes of group A streptococci (GAS), M3 GAS lacks the major epithelial invasins SfbI/PrtF1 and M1 protein but has a high potential to cause   invasive   disease. We examined the uptake of M3 GAS into human endothelial cells and identified host signaling factors required to initiate streptococcal uptake. Bacterial uptake is accompanied by local F-actin accumulation and formation of membrane protrusions at the entry site. We found that Src kinases and Rac1 but not phosphatidylinositol 3-kinases (PI3Ks) are essential to mediate S. pyogenes internalization. Pharmacological inhibition of Src activity reduced bacterial uptake and abolished the formation of membrane protrusions and actin accumulation in the vicinity of adherent streptococci. We found that Src kinases are activated in a time-dependent manner in response to M3 GAS. We also demonstrated that PI3K is dispensable for internalization of M3 streptococci and the formation of F-actin accumulations at the entry site. Furthermore, Rac1 was activated in infected cells and accumulated with F-actin in a PI3K-independent manner at bacterial entry sites. Genetic interference with Rac1 function inhibited streptococcal internalization, demonstrating an essential role of Rac1 for the uptake process of streptococci into endothelial cells. In addition, we demonstrated for the first time accumulation of the actin nucleation complex Arp2/3 at the entry port of invading M3 streptococci. 
 Activation of Vav/Rho GTPase signaling by CXCL12 controls membrane-type matrix metalloproteinase-dependent melanoma cell invasion. Melanoma cells express the chemokine receptor CXCR4, which confers   invasive   signals on binding to its ligand CXCL12. We show here that knocking down membrane-type matrix metalloproteinase (MT1-MMP) expression translates into a blockade of invasion across reconstituted basement membranes and type I collagen gels in response to CXCL12, which is the result of lack of MMP-2 activation. Interference with MMP-2 expression further confirms its important role during this invasion. Vav proteins are guanine-nucleotide exchange factors for Rho GTPases that regulate actin dynamics and gene expression. We show that melanoma cells express Vav1 and Vav2, which are activated by CXCL12 involving Jak activity. Blocking Vav expression by RNA interference results in impaired activation of Rac and Rho by CXCL12 and in a remarkable inhibition of CXCL12-promoted invasion. Importantly, up-regulation of MT1-MMP expression by CXCL12, a mechanism contributing to melanoma cell invasion, is blocked by knocking down Vav expression or by inhibiting Jak. Together, these data indicate that activation of Jak/Vav/Rho GTPase pathway by CXCL12 is a key signaling event for MT1-MMP/MMP-2-dependent melanoma cell invasion. 
 MicroRNA-200b suppresses arsenic-transformed cell migration by targeting protein kinase Cα and Wnt5b-protein kinase Cα positive feedback loop and inhibiting Rac1 activation. MicroRNA-200b (miR-200b) is a member of miR-200 family that has been found to inhibit cell migration and cancer metastasis; however, the underlying mechanism is not well understood. We previously reported that miR-200 expression is depleted in arsenic-transformed human bronchial epithelial cells with highly migratory an  d invasi  ve characteristics, whereas stably re-expressing miR-200b strongly suppresses arsenic-transformed cell migration. This study was performed to investigate how miR-200b inhibits arsenic-transformed cell migration. We found that protein kinase Cα (PKCα) is significantly up-regulated in arsenic-transformed cells. Combining bioinformatics analysis with PKCα 3'-untranslated region vector luciferase reporter assays, we showed that PKCα is a direct target of miR-200b. Inhibiting PKCα activity or knocking down PKCα expression drastically reduced cell migration, phenocoping the inhibitory effect of overexpressing miR-200b. In contrast, forced expression of PKCα in miR-200b overexpressing cells impaired the inhibitory effect of miR-200b on cell migration. In addition, we also found a positive feedback loop between Wnt5b and PKCα in arsenic-transformed cells. Knocking down Wnt5b expression reduced phospho-PKC levels and cell migration; and knocking down PKCα expression decreased Wnt5b level and cell migration. Moreover, forced expression of PKCα increased Wnt5b and phospho-PKC levels and cell migration. Further mechanistic studies revealed that Rac1 is highly activated in arsenic-transformed cells and stably expressing miR-200b abolishes Rac1 activation changing actin cytoskeleton organization. Manipulating PKCα or Wnt5b expression levels significantly altered the level of active Rac1. Together, these findings indicate that miR-200b suppresses arsenic-transformed cell migration by targeting PKCα and Wnt5b-PKCα positive feedback loop and subsequently inhibiting Rac1 activation. 
 The FbaB-type fibronectin-binding protein of Streptococcus pyogenes promotes specific invasion into endothelial cells.   Invasive   serotype M3 Streptococcus pyogenes are among the most frequently isolated organisms from patients suffering from   invasive   streptococcal disease and have the potential to invade primary human endothelial cells (EC) via a rapid and efficient mechanism. FbaB protein, the fibronectin-binding protein expressed by M3 S. pyogenes, was herein identified as a potent invasin for EC. By combining heterologous gene expression with allelic replacement, we demonstrate that FbaB is essential and sufficient to trigger EC invasion via a Rac1-dependent phagocytosis-like uptake. FbaB-mediated uptake follows the classical endocytic pathway with lysosomal destination. FbaB is demonstrated to be a streptococcal invasin exhibiting EC tropism. FbaB thus initiates a process that may contribute to the deep tissue tropism and spread of   invasive   S. pyogenes isolates into the vascular EC lining. 
 Persistent activation of Rac1 in squamous carcinomas of the head and neck: evidence for an EGFR/Vav2 signaling axis involved in cell invasion. The poor prognosis associated with head and neck squamous cell carcinoma (HNSCC) is primarily due to both local invasion and the regional and/or distant metastatic spread. Recent findings have provided evidence that the acquisition of a motile and   invasive   phenotype by cancer cells involves the dysregulated function of key intracellular molecular mechanisms together with aberrant signaling events initiated by the surrounding microenvironment. These intrinsic and extrinsic biochemical pathways in turn often converge to stimulate the activity of members of the Rho family of Ras-related guanosine triphosphate (GTP)-binding proteins, including RhoA, Rac and Cdc42, which control the organization of the actin cytoskeleton thereby regulating cell adhesion, polarity and motility. In this study, we examined the status of activation of these GTPases in a representative collection of HNSCC cell lines. Surprisingly, we found that most HNSCC cells exhibit remarkably high levels of GTP-bound Rac1. Further analysis revealed that the activation of Rac1 in these HNSCC cells could be due to two independent signaling events, an epidermal growth factor receptor (EGFR)-based autocrine loop that leads to the activation of the Rac1 exchange factor Vav2 and an EGFR/Vav2-independent pathway that arises as a consequence of the oncogenic mutation of the H-ras proto-oncogene. Indeed, we provide evidence that the EGFR/Vav2/Rac1 axis is a crucial pathway for the acquisition of motile and   invasive   properties of most HNSCC cells. These findings shed light onto the molecular mechanisms involved in HNSCC cell invasion, and may reveal new therapeutic opportunities to halt the metastatic spread of these aggressive malignancies. 
 PI3K, Rac1 and pPAK1 are overexpressed in extramammary Paget's disease. Phosphatidylinositol 3-kinase (PI3K), Ras-related C3 botulinum toxin substrate 1 (Rac1) and P21-activated protein kinase 1 (PAK1) appear to play important roles in the pathogenesis of several tumors, but their expressions in extramammary Paget's disease (EMPD) have not been investigated yet.To investigate the potential contribution of the PI3K, Rac1 and PAK1 to the development of EMPD.Thirty-five paraffin-embedded EMPD specimens were subjected to immunohistochemical staining for PI3K (85α), Rac1 and pPAK1.All the 35 primary EMPD specimens, including 20 non  -invasiv  e EMPD, 13   invasiv  e EMPD and 2 metastatic lymph nodes, showed cytoplasm overexpression of PI3K (85α), Rac1 and pPAK1. The expression (% positive cells) of PI3K(85α), Rac1 and pPAK1 (90.1 ± 8.6, 91.4 ± 9.5 and 89.6 ± 10.8% ) in EMPD were significantly higher than in apocrine glands of normal skin ( 20.1 ± 11.9, 29.8 ± 8.9, 41.1 ± 13.4%), and the expre  ssion in   invasive EMPD with lymph node metastasis (98.2 ± 1.7, 98.8 ± 0.7 and 98.4 ± 0.9%) are significantly hig  her than   in invasive EMPD without lymph node metastasis (94.1 ± 2.6, 96.5 ± 1.7 and 95.3 ±   1.1%) a  nd non-invasive EMPD (85.2 ± 8.4, 87.1 ± 9.9 and 83.1 ± 10.6%). There were significant positive correlations of the expression levels between PI3K (85α) and Rac1, as well as between Rac1 and pPAK1 in EMPD.These results indicate that PI3K, Rac1 and PAK1 may play important roles in the pathogenesis of EMPD. 
 ARF1 controls Rac1 signaling to regulate migration of MDA-MB-231   invasive   breast cancer cells. ADP-ribosylation factors (ARFs) are monomeric G proteins that regulate many cellular processes such as reorganization of the actin cytoskeleton. We have previously shown that ARF1 is overexpressed in highly   invasive   breast cancer cells and contribute to their enhanced migration. In this study, we propose to define the molecular mechanism by which ARF1 regulates this complex cellular response by investigating the role of this ARF GTPase on the activation process of Rac1, a Rho GTPase, associated with lamellipodia formation during cell migration. Here, we first show that inhibition of ARF1 or Rac1 expression markedly impacts the ability of MDA-MB-231 cells to migrate upon EGF stimulation. However, the effect of ARF1 depletion can be reversed by overexpression of the Rac1 active mutant, Rac1 Q(61)L. Depletion of ARF1 also impairs the ability of EGF stimulation to promote GTP-loading of Rac1. To further investigate the possible cross-talk between ARF1 and Rac1, we next examined whether they could form a complex. We observed that the two GTPases could directly interact independently of the nature of the nucleotide bound to them. EGF treatment however resulted in the association of Rac1 with its effector IRSp53, which was completely abrogated in ARF1 depleted cells. We present evidences that this ARF isoform is responsible for the plasma membrane targeting of both Rac1 and IRSp53, a step essential for lamellipodia formation. In conclusion, this study provides a new mechanism by which ARF1 regulates cell migration and identifies this GTPase as a promising pharmacological target to reduce metastasis formation in breast cancer patients. 
 Dynamin 2 potentiates   invasive   migration of pancreatic tumor cells through stabilization of the Rac1 GEF Vav1. The large GTPase Dynamin 2 (Dyn2) is markedly upregulated in pancreatic cancer, is a potent activator of metastatic migration, and is required for Rac1-mediated formation of lamellipodia. Here we demonstrate an unexpected mechanism of Dyn2 action in these contexts via direct binding to the Rac1 guanine nucleotide exchange factor (GEF) Vav1. Surprisingly, disruption of the Dyn2-Vav1 interaction targets Vav1 to the lysosome for degradation via an interaction with the cytoplasmic chaperone Hsc70, resulting in a dramatic reduction of Vav1 protein stability. Importantly, a specific mutation in Vav1 near its Dyn2-binding C-terminal Src homology 3 (SH3) domain prevents Hsc70 binding, resulting in a stabilization of Vav1 levels. Dyn2 binding regulates the interaction of Vav1 with Hsc70 to control the stability and subsequent activity of this oncogenic GEF. These findings elucidate how Dyn2 activates Rac1, lamellipod protrusion, and   invasive   cellular migration and provide insight into how this specific Vav is ectopically expressed in pancreatic tumors. 
 Semaphorin 5A and plexin-B3 regulate human glioma cell motility and morphology through Rac1 and the actin cytoskeleton. Semaphorins are implicated in glioma progression, although little is known about the underlying mechanisms. We have reported plexin-B3 expression in human gliomas, which upon stimulation by Sema5A causes significant inhibition of cell migration and invasion. The concomitant inactivation of Rac1 is of mechanistic importance because forced expression of constitutively active Rac1 abolishes these inhibitory effects. Furthermore, Sema5A induces prominent cell collapse and ramification of processes reminiscent of astrocytic morphology, which temporally associate with extensive disassembly of actin stress fibers and disruption of focal adhesions, followed by accumulation of actin patches in protrusions. Mechanistically, Sema5A induces transient protein kinase C (PKC) phosphorylation of fascin-1, which can reduce its actin-binding/bundling activities and temporally parallels its translocation from cell body to extending processes. PKC inhibition or fascin-1 knockdown is sufficient to abrogate Sema5A-induced morphological differentiation, whereas the process is hastened by forced expression of fascin-1. Intriguingly, Sema5A induces re-expression of glial fibrillary acidic protein (GFAP), which when silenced restricts differentiation of glioma cells to bipolar instead of multipolar morphology. Therefore, we hypothesize complementary functions of fascin-1 and GFAP in the early and late phases of Sema5A-induced astrocytic differentiation of gliomas, respectively. In summary, Sema5A and plexin-B3 impede motility but promote differentiation of human gliomas. These effects are plausibly compromised in high-grade human astrocytomas in which Sema5A expression is markedly reduced, hence leading to infiltrative and anaplastic characteristics. This is evident by increased   invasive  ness of glioma cells when endogenous Sema5A is silenced. Therefore, Sema5A and plexin-B3 represent potential novel targets in counteracting glioma progression. 
 Positive regulation of migration and invasion by vasodilator-stimulated phosphoprotein via Rac1 pathway in human breast cancer cells. This study aimed to investigate the role of the cytoskeleton-associated protein vasodilator-stimulated phosphoprotein (VASP) on the migration and invasion of human breast cancer cells and its relationship to Rac1 which is a member of the Rho family and has been found to be implicated in tumorigenesis, invasion and metastasis. We detected the mRNA and protein expression levels of VASP and Rac1 of the non-  invasive   breast cancer cell line MCF-7 as well as the   invasive   cell line MDA-MB-231 by RT-PCR and Western blotting. GST pull-down assay was used to examine the activity of Rac1. Accordingly, the cell   invasive   migration ability was analyzed in a wound-healing assay (2D) and transwell assays (3D migration and invasion). We then used VASP-siRNA to inhibit the expression of VASP in breast cancer cells in order to study the relationship between the VASP expression level and the   invasive   migration ability of breast cancer cells. Rac1-siRNA was used to decrease the expression of Rac1, and observe its effect on the VASP expression level together with the migration and invasion ability of MCF-7 and MDA-MB-231 cells. Our results revealed that the   invasive   breast cancer cell line MDA-MB-231 showed a higher Rac1 activity and VASP expression level compared with the non-  invasive   MCF-7. Inhibition of Rac1 or VASP by siRNA, respectively, decreased the migration and invasion ability of breast cancer cells and the transfection of Rac1 siRNA-mediated reduction of VASP expression at mRNA and protein levels. Collectively, our data showed that the higher expression level of VASP contributed to a higher   invasive   migration capacity of human breast cancer cells which was controlled by the Rac1 pathway. 
 Rac1+ cells distributed in accordance with CD 133+ cells in glioblastomas and the elevated   invasive  ness of CD 133+ glioma cells with higher Rac1 activity. Recent studies have suggested that cancer stem cells are one of the major causes for tumor recurrence due to their resistance to radiotherapy and chemotherapy. Although the highly   invasive   nature of glioblastoma (GBM) cells is also implicated in the failure of current therapies, it is not clear how glioma stem cells (GSCs) are involved in   invasive  ness. Rac1 activity is necessary for inducing reorganization of actin cytoskeleton and cell movement. In this study, we aimed to investigate the distribution characteristics of CD133+ cells and Rac1+ cells in GBM as well as Rac1 activity in CD133+ GBM cells, and analyze the migration and invasion potential of these cells.A series of 21 patients with GBM were admitted consecutively and received tumor resection in Tianjin Medical University General Hospital during the first half of the year 2011. Tissue specimens were collected both from the peripheral and the central parts for each tumor under magnetic resonance imaging (MRI) navigation guidance. Immunohistochemical staining was used to detect the CD133+ cells and Rac1+ cells distribution in GBM specimens. Double-labeling immunofluorescence was further used to analyze CD133 and Rac1 co-expression and the relationship between CD133+ cells distribution and Rac1 expression. Serum-free medium culture and magnetic sorting were used to isolate CD133+ cells from U87 cell line. Rac1 activation assay was conducted to assess the activation of Rac1 in CD133+ and CD133 - U87 cells. The migration and   invasive   ability of CD133+ and CD133 - U87 cells were determined by cell migration and invasion assays in vitro. Student's t-test and one-way analysis of variance (ANOVA) test were used to determine statistical significance in this study.In the central parts of GBMs, CD133+ cells were found to cluster around necrosis and occasionally cluster around the vessels under the microscope by immunohistological staining. In the peripheral parts of the tumors, CD133+ cells were lined up along the basement membrane of the vessels and myelinated nerve fibers. Rac1 expression was high and diffused in the central parts of the GBMs, and the Rac1+ cells were distributed basically in accordance with CD133+ cells both in the central and peripheral parts of GBMs. In double-labeling immunofluorescence, Rac1 was expressed in (83.14 ± 4.23)% of CD133+ cells, and CD133 and Rac1 co-expressed cells were located around the vessels in GBMs. Significantly higher amounts of Rac1-GTP were expressed in the CD133+ cells (0.378 ± 0.007), compared to CD133- cells (0.195 ± 0.004) (t = 27.81; P < 0.05). CD133+ cells had stronger ability to migrate (74.34 ± 2.40 vs. 38.72 ± 2.60, t = 42.71, P < 0.005) and invade (52.00 ± 2.28 vs. 31.26 ± 1.82, t = 30.76, P < 0.005), compared to their counterpart CD133- cells in transwell cell migration/invasion assay.These data suggest that CD133+ GBM cells highly express Rac1 and have greater potential to migrate and invade through activated Rac1-GTP. The accordance of distribution between Rac1+ cells and CD133+ cells in GBMs implies that Rac1 might be an inhibited target to prevent invasion and migration and to avoid malignant glioma recurrence. 
 Receptor tyrosine kinase c-Met controls the cytoskeleton from different endosomes via different pathways. Receptor tyrosine kinases (RTKs) are increasingly recognized as having the capacity to signal post-internalization. Signalling outputs and/or duration, and subsequent cellular outcome, are thought to be distinct when emanating from endosomes compared with those from the plasma membrane. Here we show, in   invasive  , basal-like human breast cell models, that different mechanisms are engaged by the RTK c-Met in two different endosomes to control the actin cytoskeleton via the key migratory signal output Rac1. Despite an acute activation of Rac1 from peripheral endosomes (PEs), c-Met needs to traffic to a perinuclear endosome (PNE) to sustain Rac1 signalling, trigger optimal membrane ruffling, cell migration and invasion. Unexpectedly, in the PNE but not in the PE, PI3K and the Rac-GEF Vav2 are required. Thus we describe a novel endosomal signalling mechanism whereby one signal output, Rac1, is stimulated through distinct pathways by the same RTK depending on which endosome it is localized to in the cell. 
 Role of activated Rac1/Cdc42 in mediating endothelial cell proliferation and tumor angiogenesis in breast cancer. Angiogenesis is a well-established target in anti-cancer therapy. Although vascular endothelial growth factor (VEGF)-mediated angiogenesis apparently requires the Rho GTPases Rac1 and Cdc42, the relevant mechanisms are unclear. Here, we determined that activated Rac1/Cdc42 in MCF-7 breast cancer cells could decrease p53 protein levels and increase VEGF secretion to promote proliferation and tube formation of human umbilical vein endothelial cells (HUVECs). However, these effects are reversed after ubiquitin-proteasome breakage. In exploring potential mechanisms for this relationship, we confirmed that activated Rac1/Cdc42 could enhance p53 protein ubiquitination and weaken p53 protein stability to increase VEGF expression. Furthermore, in a xenograft model using nude mice that stably express active Rac1/Cdc42 protein, active Rac1/Cdc42 decreased p53 levels and increased VEGF expression. Additionally, tumor angiogenesis was inhibited, and p53 protein levels were augmented, by intratumoral injection of the ubiquitin-proteasome inhibitor MG132. Finally in 339 human breast cancer tissues, our analyses indicated that Rac1/Cdc42 expression was related to advanced TNM staging, high proliferation index, ER status, and positive   invasive   features. In particular, our data suggests that high Rac1/Cdc42 expression is correlated with low wt-p53 and high VEGF expression. We conclude that activated Rac1/Cdc42 is a vascular regulator of tumor angiogenesis and that it may reduce stability of the p53 protein to promote VEGF expression by enhancing p53 protein ubiquitin. 
 Vimentin regulates lung cancer cell adhesion through a VAV2-Rac1 pathway to control focal adhesion kinase activity. Vimentin is an intermediate filament protein whose expression correlates with increased metastatic disease, reduced patient survival and poor prognosis across multiple tumor types. Despite these well-characterized correlations, the molecular role of vimentin in cancer cell motility remains undefined. To approach this, we used an unbiased phosphoproteomics screen in lung cancer cell lines to discover cell motility proteins that show significant changes in phosphorylation upon vimentin depletion. We identified the guanine nucleotide exchange factor (GEF), VAV2, as having the greatest loss of phosphorylation owing to vimentin depletion. Since VAV2 serves as a GEF for the small Rho GTPase Rac1, a key player in cell motility and adhesion, we explored the vimentin-VAV2 pathway as a potential novel regulator of lung cancer cell motility. We show that VAV2 localizes to vimentin-positive focal adhesions (FAs) in lung cancer cells and complexes with vimentin and FA kinase (FAK). Vimentin loss impairs both pY142-VAV2 and downstream pY397-FAK activity showing that vimentin is critical for maintaining VAV2 and FAK activity. Importantly, vimentin depletion reduces the activity of the VAV2 target, Rac1, and a constitutively active Rac1 rescues defects in FAK and cell adhesion when vimentin or VAV2 is compromised. Based upon this data, we propose a model whereby vimentin promotes FAK stabilization through VAV2-mediated Rac1 activation. This model may explain why vimentin expressing metastatic lung cancer cells are more motile and   invasive  . 
 Prostaglandin E2-mediated migration of human trophoblast requires RAC1 and CDC42. The invasion of maternal decidua and uterine spiral arteries by a trophoblast subpopulation called extravillous trophoblast (EVT) is essential for the establishment of a normal placenta and an adequate blood flow toward the fetus. Derangements in these processes underlie pregnancy-related diseases like preeclampsia and intrauterine growth restriction. Many growth factors, growth factor binding proteins, and extracellular matrix components can positively or negatively regulate the proliferation, migration, and/or   invasive  ness of these EVT cells. RHO GTPases, including RHOA, RAC1, and CDC42, are ubiquitous proteins that control cytoskeletal changes by forming stress fibers and projecting lamellipodia and filopodia during cellular migration. We had previously shown that prostaglandin (PG) E(2) produced in abundance by the decidua promotes the migration of first-trimester human EVTs by increasing the intracellular concentration of calcium and activating calpain. Using our well-characterized immortalized EVT cell line, HTR-8/SVneo, as well as villus explants from first-trimester placentae, this study examined the role of RHO GTPases RAC1 and CDC42 in PGE(2)-mediated migratory responses of these cells. Though a RAC1 inhibitor, NSC23766 as well as RAC1 knockdown by siRNA decreased the migration of HTR-8/SVneo cells in a Transwell migration assay, this inhibition could not be restored by PGE(2) or 17-phenyl trinor PGE(2) (PGE receptor PTGER1 agonist) or PGE(1) Alcohol (PGE receptor PTGER4 agonist). Similar results were noted for EVT cell spreading in villus explants. Furthermore, CDC42 silencing using siRNA inhibited PGE(2)-induced migration of HTR-8/SVneo cells. Finally, the treatment of EVT cells with PGE(2), PTGER1 agonist, or PTGER4 agonist activated RAC1 and CDC42 at 10 min, suggesting that RAC1 and CDC42 play an essential role in PGE(2)-mediated migration of human EVTs. 
 Regulation of CXCR4/AKT-signaling-induced cell invasion and tumor metastasis by RhoA, Rac-1, and Cdc42 in human esophageal cancer. CXC chemokines and their cognate receptors have been implicated wildly in cancer pathogenesis. In the present study, we report a critical cause relationship between CXCR4 expression and tumorigenesis in the setting of human esophageal squamous cell carcinoma (ESCC). In ESCC cells, CXCR4 expression was significantly higher than in human esophageal epithelial cells (HEEC). Reduction of CXCR4 in ESCC cells reduced cell proliferation and invasion in vitro and tumor growth in vivo. Among the potential downstream targets of CXCR4-CXCL12 are RhoA, Rac-1, and Cdc42, which are likely to contribute to the   invasive  ness of ESCC cells. Finally, we found that CXCR4-CXCL12/AKT axis regulates RhoA, Rac-1, and Cdc42 to modulate cell invasion and tumor metastasis. Together, these results demonstrate a role for CXCR4 in ESCC metastasis and progression and suggest potential targets for therapeutic intervention. 
 Tyrosine 397 phosphorylation is critical for FAK-promoted Rac1 activation and   invasive   properties in oral squamous cell carcinoma cells. Oral squamous cell carcinoma (OSCC) is a common cancer worldwide. Despite advances in diagnosis and therapy, treatment options for patients with metastatic OSCC are few, due in part to the limited understanding of the molecular events involved in the invasion and metastasis of OSCC. In this study, we investigated the expression of focal adhesion kinase (FAK) and its tyrosine 397 phosphorylation (pY397) in the tissue specimens of OSCC. The roles of pY397 in regulating the activities of Rac1 and cortactin and the   invasive   properties of OSCC cells were further determined. Results from immunohistochemical analyses in 9 benign, 19 premalignant, and 19 malignant oral tissues showed that the immunoreactivity of FAK was observed in 5 benign (56%), 19 premalignant (100%), and 18 malignant tissues (95%), whereas the immunoreactivity of pY397 was only found in 1 of 9 (11%) benign lesions but was observed in 9 premalignant (47%) and 12 malignant (63%) lesions. Compared with the low-invading SCC4 cells, the high-invading OECM-1 cells exhibited higher levels of FAK expression and pY397, correlating with higher levels of GTP-bound Rac1 and cortactin phosphorylation. Manipulation of FAK expression or Y397 phosphorylation in SCC4, FaDu, OECM-1, or HSC-3 cells regulated their Rac1 activities and   invasive   properties. Furthermore, treatment of NSC23766, a Rac1-specific inhibitor, in OECM-1 and HSC-3 cells led to reduced   invasive   properties. Nevertheless, knockdown of FAK expression or suppression of pY397 had no effect on the cortactin activity in OECM-1 cells. The data collectively suggest that pY397 plays critical roles in the FAK-promoted Rac1 activation and   invasive   properties in OSCC cells. Thus, the inhibition of FAK phosphorylation at Y397 or Rac1 activity can serve as a therapeutic strategy for treating patients with metastatic OSCC. 
 Rac1 and Rho contribute to the migratory and   invasive   phenotype associated with somatic E-cadherin mutation. Recent evidence suggests a close association between extracellular E-cadherin mutation in diffuse-type gastric carcinoma and the acquisition of a migratory phenotype of tumour cells. To characterize the cellular machinery that mediates the gain of motility of tumour cells with mutant E-cadherin, we turned to the small Rho GTPases Rac1 and Rho because they have been implicated in pathological processes including tumour cell migration and invasion. In the present study, we analyse the activity of Rac1 and Rho in relation to E-cadherin harbouring an in-frame deletion of exon 8 and prove for the first time that the mutation reduces the ability of E-cadherin to activate Rac1 and to inhibit Rho. We provide evidence that the lack of Rac1 activation observed in response to mutant E-cadherin influences the downstream signalling of Rac1, as is shown by the decrease in the binding of the Rac1 effector protein IQGAP1 to Rac1-GTP. Moreover, reduced membranous localization of p120-catenin in mutant E-cadherin expressing cells provides an explanation for the lack of negative regulation of Rho by mutant E-cadherin. Further, we show by time-lapse laser scanning microscopy and invasion assay that the enhanced motility and invasion associated with mutant E-cadherin is sensitive to the inhibition of Rac1 and Rho. Together, these findings present evidence that the mutation of E-cadherin influences Rac1 and Rho activation in opposite directions and that Rac1 and Rho are involved in the establishment of the migratory and   invasive   phenotype of tumour cells that have an E-cadherin mutation. 
 Increased diacylglycerol kinase ζ expression in human metastatic colon cancer cells augments Rho GTPase activity and contributes to enhanced invasion. Unraveling the signaling pathways responsible for the establishment of a metastatic phenotype in carcinoma cells is critically important for understanding the pathology of cancer. The acquisition of cell motility is a key property of metastatic tumor cells and is a prerequisite for invasion. Rho GTPases regulate actin cytoskeleton reorganization and the cellular responses required for cell motility and invasion. Diacylglycerol kinase ζ (DGKζ), an enzyme that phosphorylates diacylglycerol to yield phosphatidic acid, regulates the activity of the Rho GTPases Rac1 and RhoA. DGKζ mRNA is highly expressed in several different colon cancer cell lines, as well as in colon cancer tissue relative to normal colonic epithelium, and thus may contribute to the metastatic process.To investigate potential roles of DGKζ in cancer metastasis, a cellular, isogenic model of human colorectal cancer metastatic transition was used. DGKζ protein levels, Rac1 and RhoA activity, and PAK phosphorylation were measured in the non-metastatic SW480 adenocarcinoma cell line and its highly metastatic variant, the SW620 line. The effect of DGKζ silencing on Rho GTPase activity and invasion through Matrigel-coated Transwell inserts was studied in SW620   cells. I  nvasiveness was also measured in PC-3 prostate cancer and MDA-MB-231 breast cancer cells depleted of DGKζ.DGKζ protein levels were elevated approximately 3-fold in SW620 cells compared to SW480 cells. There was a concomitant increase in active Rac1 in SW620 cells, as well as substantial increases in the expression and phosphorylation of the Rac1 effector PAK1. Similarly, RhoA activity and expression were increased in SW620 cells. Knockdown of DGKζ expression in SW620 cells by shRNA-mediated silencing significantly reduced Rac1 and RhoA activity and atten  uated th  e invasiveness of SW620 cells in vitro. DGKζ silencing in highly metastatic MDA-MB-231 breast cancer cells and PC-3 prostate cancer cells also significantly attenu  ated the  ir invasiveness.Elevated DGKζ expression contributes to increased Rho GTPase activation and the enhanced motility of metastatic cancer cells. These findings warrant further investigation of the clinical relevance of DGKζ upregulation in colon and other cancers. Interfering with DGKζ function could provide a means of inhibiting invasion and metastasis. 
 Non-small cell lung carcinoma cell motility, rac activation and metastatic dissemination are mediated by protein kinase C epsilon. Protein kinase C (PKC) ε, a key signaling transducer implicated in mitogenesis, survival, and cancer progression, is overexpressed in human primary non-small cell lung cancer (NSCLC). The role of PKCε in lung cancer metastasis has not yet been established.Here we show that RNAi-mediated knockdown of PKCε in H358, H1299, H322, and A549 NSCLC impairs activation of the small GTPase Rac1 in response to phorbol 12-myristate 13-acetate (PMA), serum, or epidermal growth factor (EGF). PKCε depletion markedly impaired the ability of NSCLC cells to form membrane ruffles and migrate. Similar results were observed by pharmacological inhibition of PKCε with εV1-2, a specific PKCε inhibitor. PKCε was also requi  red for   invasiveness of NSCLC cells and modulated the secretion of extracellular matrix proteases and protease inhibitors. Finally, we found that PKCε-depleted NSCLC cells fail to disseminate to lungs in a mouse model of metastasis.Our results implicate PKCε as a key mediator of Rac signaling and motility of lung cancer cells, highlighting its potential as a therapeutic target. 
 ARF6 promotes the formation of Rac1 and WAVE-dependent ventral F-actin rosettes in breast cancer cells in response to epidermal growth factor. Coordination between actin cytoskeleton assembly and localized polarization of intracellular trafficking routes is crucial for cancer cell migration. ARF6 has been implicated in the endocytic recycling of surface receptors and membrane components and in actin cytoskeleton remodeling. Here we show that overexpression of an ARF6 fast-cycling mutant in MDA-MB-231 breast cancer-derived cells to mimick ARF6 hyperactivation observed in   invasive   breast tumors induced a striking rearrangement of the actin cytoskeleton at the ventral cell surface. This phenotype consisted in the formation of dynamic actin-based podosome rosette-like structures expanding outward as wave positive for F-actin and actin cytoskeleton regulatory components including cortactin, Arp2/3 and SCAR/WAVE complexes and upstream Rac1 regulator. Ventral rosette-like structures were similarly induced in MDA-MB-231 cells in response to epidermal growth factor (EGF) stimulation and to Rac1 hyperactivation. In addition, interference with ARF6 expression attenuated activation and plasma membrane targeting of Rac1 in response to EGF treatment. Our data suggest a role for ARF6 in linking EGF-receptor signaling to Rac1 recruitment and activation at the plasma membrane to promote breast cancer cell directed migration. 
 A Trio-Rac1-Pak1 signalling axis drives invadopodia disassembly. Rho family GTPases control cell migration and participate in the regulation of cancer metastasis. Invadopodia, associated with   invasive   tumour cells, are crucial for cellular invasion and metastasis. To study Rac1 GTPase in invadopodia dynamics, we developed a genetically encoded, single-chain Rac1 fluorescence resonance energy (FRET) transfer biosensor. The biosensor shows Rac1 activity exclusion from the core of invadopodia, and higher activity when invadopodia disappear, suggesting that reduced Rac1 activity is necessary for their stability, and Rac1 activation is involved in disassembly. Photoactivating Rac1 at invadopodia confirmed this previously unknown Rac1 function. We describe here an invadopodia disassembly model, where a signalling axis involving TrioGEF, Rac1, Pak1, and phosphorylation of cortactin, causes invadopodia dissolution. This mechanism is critical for the proper turnover of   invasive   structures during tumour cell invasion, where a balance of proteolytic activity and locomotory protrusions must be carefully coordinated to achieve a maximally   invasive   phenotype. 
 Pro  invasive   activity of BMP-7 through SMAD4/src-independent and ERK/Rac/JNK-dependent signaling pathways in colon cancer cells. Recent data indicate that the Bone Morphogenetic Protein BMP-7 exhibits mucosal protection against experimental colitis in rats, suggesting that this cytokine exerts direct actions in intestinal epithelial cells during inflammatory bowel diseases and other precancerous lesions of the colon. In this study, we investigated the functional expression of BMP-7 and its receptors in normal human colon crypts, aberrant crypt foci (ACF) in sigmoiditis and colorectal tumors, and their derived cancer cell lines. Transcripts encoding BMP-7 receptors type II (BMPRII, ActRII, ActRIIB) and type I (ALK-2) were clearly detected by RT-PCR in several premalignant and carcinoma cell lines. The cytokine was identified by immunohistochemistry in surface epithelial cells and crypts in the normal colon mucosa, ACF in sigmoiditis, sporadic high grade dysplastic adenoma, and in 9 of 16 colon carcinomas (56.2%). In addition, the conditioned medium collected from the adenoma PC/AA/C1 and carcinoma HCT8/S11 and SW48 cell lines in culture contained significant levels of BMP-7 ranging from 0.17 to 0.38 ng/ml. We found that BMP-7 induced scattering and pro  invasive   responses (EC50=1 ng/ml) in kidney and colon cancer cell lines through SMAD4 and src -independent pathways and signaling cascades using FAK phosphorylation at Y925 and activation of ERK1/2, Rac1 and JNK. This phosphorylation of FAK on Y925 was also induced by the pro  invasive   agent EGF. Taken together, our findings suggest that BMP-7 exerts divergent effects in the colon mucosa, one counteracting transient inflammatory situations and the other linked to pejorative functions during chronic ulcerative diseases and the neoplastic progression. 
 HER2/Neu (ErbB2) signaling to Rac1-Pak1 is temporally and spatially modulated by transforming growth factor beta. In HER2 (ErbB2)-overexpressing cells, transforming growth factor beta (TGF-beta), via activation of phosphoinositide-3 kinase (PI3K), recruits actin and actinin to HER2, which then colocalizes with Vav2, activated Rac1, and Pak1 at cell protrusions. This results in prolonged Rac1 activation, enhanced motility and   invasive  ness, Bad phosphorylation, uncoupling of Bad/Bcl-2, and enhanced cell survival. The recruitment of the HER2/Vav2/Rac1/Pak1/actin/actinin complex to lamellipodia was abrogated by actinin siRNAs, dominant-negative (dn) p85, gefitinib, and dn-Rac1 or dn-Pak1, suggesting that the reciprocal interplay of PI3K, HER2 kinase, and Rac GTPases with the actin cytoskeleton is necessary for TGF-beta action in oncogene-overexpressing cells. Thus, by recruiting the actin skeleton, TGF-beta "cross-links" this signaling complex at cell lamellipodia; this prolongs Rac1 activation and increases metastatic properties and survival of HER2-overexpressing cells. 
 14-3-3zeta Cooperates with ErbB2 to promote ductal carcinoma in situ progression to   invasive   breast cancer by inducing epithelial-mesenchymal transition. ErbB2, a metastasis-promoting oncoprotein, is overexpressed in approximately 25% of   invasive  /metastatic breast cancers, but in 50%-60% of non  invasive   ductal carcinomas in situ (DCIS). It has been puzzling how a subset of ErbB2-overexpressing DCIS develops into   invasive   breast cancer (IBC). We found that co-overexpression of 14-3-3zeta in ErbB2-overexpressing DCIS conferred a higher risk of progression to IBC. ErbB2 and 14-3-3zeta overexpression, respectively, increased cell migration and decreased cell adhesion, two prerequisites of tumor cell invasion. 14-3-3zeta overexpression reduced cell adhesion by activating the TGF-beta/Smads pathway that led to ZFHX1B/SIP-1 upregulation, E-cadherin loss, and epithelial-mesenchymal transition. Importantly, patients whose breast tumors overexpressed both ErbB2 and 14-3-3zeta had higher rates of metastatic recurrence and death than those whose tumors overexpressed only one. 
 Basic fibroblast growth factor in the bone microenvironment enhances cell motility and invasion of Ewing's sarcoma family of tumours by activating the FGFR1-PI3K-Rac1 pathway. Ewing's sarcoma family of tumours (ESFT) is a malignant small round-cell tumour of the bone and soft tissues. It is characterised by a strong tendency to invade and form metastases. The microenvironment of the bone marrow is a large repository for many growth factors, including the basic fibroblast growth factor (bFGF). However, the role of bFGF in the   invasive   and metastatic phenotype of ESFT has not been investigated.The motility and invasion of ESFT cells were assessed by a wound-healing assay, chemotaxis assay, and invasion assay. The expression and activation of FGF receptors (FGFRs) in ESFT cell lines and clinical samples were detected by RT-PCR, western blotting, and immunohistochemistry. The morphology of ESFT cells was investigated by phase-contrast microscopy and fluorescence staining for actin. Activation of Rac1 was analysed by a pull-down assay.bFGF strongly induced the motility and invasion of ESFT cells. Furthermore, FGFR1 was found to be expressed and activated in clinical samples of ESFT. Basic FGF-induced cell motility was mediated through the FGFR1-phosphatidylinositol 3-kinase (PI3K)-Rac1 pathway. Conditioned medium from bone marrow stromal cells induced the motility of ESFT cells by activating bFGF/FGFR1 signalling.The bFGF-FGFR1-PI3K-Rac1 pathway in the bone microenvironment may have a significant role in the invasion and metastasis of ESFT. 
 Loss of WNK2 expression by promoter gene methylation occurs in adult gliomas and triggers Rac1-mediated tumour cell   invasive  ness. The gene encoding protein kinase WNK2 was recently identified to be silenced by promoter hypermethylation in gliomas and meningiomas, suggesting a tumour-suppressor role in these brain tumours. Following experimental depletion in cell lines, WNK2 was further found to control GTP-loading of Rac1, a signalling guanosine triphosphatase involved in cell migration and motility. Here we show that WNK2 promoter methylation also occurs in 17.5% (29 out of 166) of adult gliomas, whereas it is infrequent in its paediatric forms (1.6%; 1 out of 66). Re-expression of WNK2 in glioblastoma cells presenting WNK2 gene silencing reduced cell proliferation in vitro, tumour growth in vivo and also cell migration and invasion, an effect correlated with reduced activation of Rac1. In contrast, when endogenous WNK2 was depleted from glioblastoma cells with unmethylated WNK2 promoter, changes in cell morphology, an increase in invasion and activation of Rac1 were observed. Together, these results validate the WNK2 gene as a recurrent target for epigenetic silencing in glia-derived brain tumours and provide first mechanistic evidence for a tumour-suppressing role of WNK2 that is related to Rac1 signalling and tumour cell invasion and proliferation. 
 Podocalyxin-like 1 promotes invadopodia formation and metastasis through activation of Rac1/Cdc42/cortactin signaling in breast cancer cells. Metastatic disease is the leading cause of cancer mortality. Identifying biomarkers and regulatory mechanisms is important toward developing diagnostic and therapeutic tools against metastatic cancer. In this study, we demonstrated that podocalyxin-like 1 (PODXL) is overexpressed in breast tumor cells and increased in lymph node metastatic cancer. Mechanistically, we found that the expression of PODXL was associated with cell motility and   invasive  ness. Suppression of PODXL in MDA-MB-231 cells reduced lamellipodia formation and focal adhesion kinase (FAK) and paxillin phosphorylation. PODXL knockdown reduced the formation of invadopodia, such as inhibiting the colocalization of F-actin with cortactin and suppressing phosphorylation of cortactin and neural Wiskott-Aldrich syndrome protein. Conversely, overexpression of PODXL in MCF7 cells induced F-actin/cortactin colocalization and enhanced invadopodia formation and activation. Invadopodia activity and tumor invasion in PODXL-knockdown cells are similar to that in cortactin-knockdown cells. We further found that the DTHL motif in PODXL is crucial for regulating cortactin phosphorylation and Rac1/Cdc42 activation. Inhibition of Rac1/Cdc42 impeded PODXL-mediated cortactin activation and FAK and paxillin phosphorylation. Moreover, inhibition of PODXL in MDA-MB-231 cells significantly suppressed tumor colonization in the lungs and distant metastases, similar to those in cortactin-knockdown cells. These findings show that overexpression of PODXL enhanced invadopodia formation and tumor metastasis by inducing Rac1/Cdc42/cortactin signaling network. 
 Astragaloside IV inhibits breast cancer cell invasion by suppressing Vav3 mediated Rac1/MAPK signaling. Astragaloside IV (AS-IV), the major active triterpenoid in Radix Astragali, has shown anti-tumorigenic properties in certain cancers; however, its role in breast cancer remains unclear. The present study investigated the effects of AS-IV on breast cancer in vitro and in vivo and examined the underlying mechanisms.The effects of AS-IV on MDA-MB-231 cell proliferation, migration, invasion and metastasis were investigated by MTT and Transwell assays, and western blotting. In addition, an orthotopic mouse tumor model was established for in vivo experiments.AS-IV inhibited the viability and   invasive   potential of MDA-MB-231 breast cancer cells, suppressed the activation of the mitogen activated protein kinase (MAPK) family members ERK1/2 and JNK, and downregulated matrix metalloproteases (MMP)-2 and -9. The effects of AS-IV were mediated by the downregulation of Vav3, a guanine nucleotide exchange factor, leading to decreased levels of activated Rac1, a Rho family GTPase. Vav3 overexpression promoted cell proliferation and invasion in vitro, whereas Vav3 silencing had the opposite effects. AS-IV suppressed orthotopic breast tumor growth and metastasis to the lungs, whereas ectopic expression of Vav3 reversed the inhibitory effect of AS-IV on cell viability,   invasive  ness, MAPK signaling and MMP expression.The present results provide a mechanistic explanation for the antitumor effects of AS-IV and suggest its potential in the treatment of metastatic breast cancer. 
 Differential effects of invasion by and phagocytosis of Salmonella typhimurium on apoptosis in human macrophages: potential role of Rho-GTPases and Akt. In addition to direct activation of caspase-1 and induction of apoptosis by SipB,   invasive   Salmonella stimulates multiple signaling pathways that are key regulators of host cell survival. Nevertheless, little is known about the relative contributions of these pathways to Salmonella-mediated death of macrophages. We studied human monocytic U937 cells and found that apoptosis was induced by invading wild-type Salmonella typhimurium but not by phagocytosed, serum-opsonized, non  invasive   Salmonella mutants. Pretreating U937 cells with inhibitors of tyrosine kinases or phosphatidylinositol-3 kinase (PI-3K) completely blocked phagocytosis of opsonized Salmonella mutants but did not affect invasion by wild-type Salmonella or the apoptosis caused by invasion. However, pretreatment with GGTI-298, a geranylgeranyltransferase-1 inhibitor that prevents prenylation of Cdc42 and Rac1, suppressed Salmonella-induced apoptosis by approximately 70%. Transduction of Tat fusion constructs containing dominant-negative Cdc42 or Rac1 significantly inhibited Salmonella-induced cell death, indicating that the cytotoxicity of Salmonella requires activation of Cdc42 and Rac. In contrast to phagocytosis of opsonized bacteria, invasion by S. typhimurium stimulated Cdc42 and Rac1, regardless of the activities of tyrosine- or PI-3K. Moreover, Salmonella infection activated Akt protein in a tyrosine-kinase or PI-3K-dependent manner, and a reduced expression of Akt by antisense transfection rendered the cells more sensitive to apoptosis induced by opsonized Salmonella. These results indicate that direct activation of Cdc42 and Rac1 by   invasive   Salmonella is a prerequisite of Salmonella-mediated death of U937 cells, whereas the simultaneous activation of Akt by tyrosine kinase and PI-3K during receptor-mediated phagocytosis protects cells from apoptosis. 
 Association between Gαi2 and ELMO1/Dock180 connects chemokine signalling with Rac activation and metastasis. The chemokine CXCL12 and its G-protein-coupled receptor CXCR4 control the migration,   invasiv  eness and metastasis of breast cancer cells. Binding of CXCL12 to CXCR4 triggers activation of heterotrimeric Gi proteins that regulate actin polymerization and migration. However, the pathways linking chemokine G-protein-coupled receptor/Gi signalling to actin polymerization and cancer cell migration are not known. Here we show that CXCL12 stimulation promotes interaction between Gαi2 and ELMO1. Gi signalling and ELMO1 are both required for CXCL12-mediated actin polymerization, migration and invasion of breast cancer cells. CXCL12 triggers a Gαi2-dependent membrane translocation of ELMO1, which associates with Dock180 to activate small G-proteins Rac1 and Rac2. In vivo, ELMO1 expression is associated with lymph node and distant metastasis, and knocking down ELMO1 impairs metastasis to the lung. Our findings indicate that a chemokine-controlled pathway, consisting of Gαi2, ELMO1/Dock180, Rac1 and Rac2, regulates the actin cytoskeleton during breast cancer metastasis. 
 Elmo1 helps dock180 to regulate Rac1 activity and cell migration of ovarian cancer. Engulfment and cell motility 1 (Elmo1) has been reported to cooperate with dedicator of cytokinesis 1 (Dock180) and to be linked to the   invasive   phenotype of cancer cells through activating small G-protein Rac. We aimed to study the role of Elmo1 in the malignant migration of ovarian cancer.Engulfment and cell motility 1 expression was evaluated in specimens from 93 patients with serous ovarian cancer (SOC) by immunohistochemical staining. Next, Elmo1-RNAi cells were established by validated small interference RNAs. Cell proliferation and cell motility were observed and compared with Dock180-RNAi cells. To confirm their synergetic contribution to forming focal adhesion and activating Rac1, Rac1-GTP level was measured by GST pull-down assay and immunofluorescence was used to observe focal adhesion formation both in Elmo1-RNAi and Dock180-RNAi cells.Engulfment and cell motility 1 was mainly overexpressed in high-grade SOC tissues. Western blot analysis demonstrated that both Elmo1 and Dock180 expressions were hampered in Elmo1-RNAi cells. Compared with the negative control, decreased colony formation and cell invasion were observed in Elmo1-RNAi cells and Dock180-RNAi cells. Consistently, both exhibited reduced Rac1-GTP level and inhibited focal adhesion formation.Engulfment and cell motility 1 presents with synergetic action in helping Dock180 to activate Rac1 and promote cell motility, and thus promote untoward expansion and aggressiveness of SOC. 
 Rac1 and Rac3 isoform activation is involved in the   invasive   and metastatic phenotype of human breast cancer cells. The metastatic progression of cancer is a direct result of the disregulation of numerous cellular signaling pathways, including those associated with adhesion, migration, and invasion. Members of the Rac family of small GTPases are known to act as regulators of actin cytoskeletal structures and strongly influence the cellular processes of integrin-mediated adhesion and migration. Even though hyperactivated Rac proteins have been shown to influence metastatic processes, these proteins have never been directly linked to metastatic progression.To investigate a role for Rac and Cdc42 in metastatic breast cancer cell invasion and migration, relative endogenous Rac or Cdc42 activity was determined in a panel of metastatic variants of the MDA-MB-435 metastatic human breast cancer cell line using a p21-binding domain-PAK pull down assay. To investigate the migratory and   invasive   potential of the Rac isoforms in human breast cancer, namely Rac1 and the subsequently cloned Rac3, we stably expressed either dominant active Rac1 or dominant active Rac3 into the least metastatic cell variant. Dominant negative Rac1 or dominant negative Rac3 were stably expressed in the most metastatic cell variant. Cell lines expressing mutant Rac1 or Rac3 were analyzed using in vitro adhesion, migration and invasion assays.We show that increased activation of Rac proteins directly correlates with increasing metastatic potential in a panel of cell variants derived from a single metastatic breast cancer cell line (MDA-MB-435). The same correlation could not be found with activated Cdc42. Expression of a dominant active Rac1 or a dominant active Rac3 resulted in a more   invasive   and motile phenotype. Moreover, expression of either dominant negative Rac1 or dominant negative Rac3 into the most metastatic cell variant resulted in decreased   invasive   and motile properties.This study correlates endogenous Rac activity with high metastatic potential and implicates Rac in the regulation of cell migration and invasion in metastatic breast cancer cells. Taken together, these results suggest a role for both the Rac1 and Rac3 GTPases in human breast cancer progression. 
 Specific roles of Rac1 and Rac2 in motile functions of HT1080 fibrosarcoma cells. Rho family proteins are constitutively activated in the highly   invasive   human fibrosarcoma HT1080 cells. We now investigated the specific roles of Rac1 and Rac2 in regulating morphology, F-actin organization, adhesion, migration, and chemotaxis of HT1080 cells. Downregulation of Rac1 using specific siRNA probes resulted in cell rounding, markedly decreased spreading, adhesion, and chemotaxis of HT1080 cells. 2D migration on laminin-coated surfaces in contrast was not markedly affected. Selective Rac2 depletion did not affect cell morphology, cell adhesion, and 2D migration, but significantly reduced chemotaxis. Downregulation of both Rac1 and Rac2 resulted in an even more marked reduction, but not complete abolishment, of chemotaxis indicating distinct as well as overlapping roles of both proteins in chemotaxis. Rac1 thus is selectively required for HT1080 cell spreading and adhesion whereas Rac1 and Rac2 are both required for efficient chemotaxis. 
 Tumor cell-derived MMP3 orchestrates Rac1b and tissue alterations that promote pancreatic adenocarcinoma. Pancreatic ductal adenocarcinoma (PDA) arises at the convergence of genetic alterations in KRAS with a fostering microenvironment shaped by immune cell influx and fibrotic changes; identification of the earliest tumorigenic molecular mediators evokes the proverbial chicken and egg problem. Matrix metalloproteinases (MMP) are key drivers of tumor progression that originate primarily from stromal cells activated by the developing tumor. Here, MMP3, known to be expressed in PDA, was found to be associated with expression of Rac1b, a tumorigenic splice isoform of Rac1, in all stages of pancreatic cancer. Using a large cohort of human PDA tissue biopsies specimens, both MMP3 and Rac1b are expressed in PDA cells, that the expression levels of the two markers are highly correlated, and that the subcellular distribution of Rac1b in PDA is significantly associated with patient outcome. Using transgenic mouse models, coexpression of MMP3 with activated KRAS in pancreatic acinar cells stimulates metaplasia and immune cell infiltration, priming the stromal microenvironment for early tumor development. Finally, exposure of cultured pancreatic cancer cells to recombinant MMP3 stimulates expression of Rac1b, increases cellular   invasive  ness, and activation of tumorigenic transcriptional profiles.MMP3 acts as a coconspirator of oncogenic KRAS in pancreatic cancer tumorigenesis and progression, both through Rac1b-mediated phenotypic control of pancreatic cancer cells themselves, and by giving rise to the tumorigenic microenvironment; these findings also point to inhibition of this pathway as a potential therapeutic strategy for pancreatic cancer. 
 Loss of the E3 ubiquitin ligase HACE1 results in enhanced Rac1 signaling contributing to breast cancer progression. The transition from ductal carcinoma in situ (DCIS) to   invasive   breast cancer (IBC) is a crucial step in breast cancer progression. The specific alterations that govern this transition have not been elucidated. HER2/neu is frequently overexpressed in DCIS but is less common in IBC, thereby suggesting additional requirements for transformation. To identify genes capable of cooperating with HER2/neu to fully transform mammary epithelial cells, we used an insertional mutagenesis screen on cells isolated from wild-type neu expressing mice and identified the E3 ligase HACE1 as HER2 cooperative tumor suppressor gene. Loss of HACE1 expression is commonly seen in clinical breast cancer data sets. HACE1 downregulation in normal human mammary epithelial cells (HMECs) results in the accumulation of the activated GTP-bound Rac1 partially transforming these cells. Overexpression of HER2 activates Rac1, which further accumulates upon HACE1 loss resulting in Rac1 hyperactivation. Although the knockdown of HACE1 or overexpression of HER2 alone in HMECs is not sufficient for tumorigenesis, HER2 overexpression combined with HACE1 downregulation fully transforms HMECs resulting in robust tumor formation. The pharmaceutical interference of Rac function abrogates the effects of HACE1 loss both in vitro and in vivo, resulting in marked reduction in tumor burden. Our work supports a critical role for HACE1 in breast cancer progression and identifies patients that may benefit from Rac-targeted therapies. 
 Abnormal expression of p120-catenin, E-cadherin, and small GTPases is significantly associated with malignant phenotype of human lung cancer. Studies on a variety of cell lines have shown that p120-catenin can directly regulate the stability of E-cadherin complexes and control the activity of small GTPases to influence cell adhesion. Despite this data, clinical studies of human solid tumors have not been reported to investigate these protein interactions. To explore the correlation between p120-catenin, E-cadherin, and small GTPases in human lung cancer, we examined the expression patterns of p120-catenin, E-cadherin, RhoA, Cdc42, and Rac1, and their prognostic significance in 138 patients with non-small cell lung cancer (NSCLC). While normal bronchial epithelium showed strong membrane expression of p120-catenin and E-cadherin, lung cancer tissues had reduced membrane expression and ectopic cytoplasmic expression of p120-catenin and E-cadherin. Expression of RhoA, Cdc42, and Rac1 was also found to be higher in tumor tissue than in normal lung tissue. A correlation between abnormal p120-catenin, E-cadherin expression, and overexpression of specific small GTPases was also associated with poor differentiation, high TNM stage, and lymph node metastasis in NSCLC patients. We also used an in vitro model to evaluate their expression, and to determine whether protein expression correlated with the   invasive   capacity of lung cancer cell lines. Consistent with our in vivo data, abnormal expression of p120-catenin and E-cadherin with overexpression of specific small GTPases were significantly associated with the high metastatic capacity of BE1 cells. Based on our results, we conclude that abnormal p120-catenin expression correlates with abnormal E-cadherin expression and specific small GTPase overexpression, which contribute to the malignancy-related to NSCLC. 
 A MAPK-Driven Feedback Loop Suppresses Rac Activity to Promote RhoA-Driven Cancer Cell Invasion. Cell migration in 3D microenvironments is fundamental to development, homeostasis and the pathobiology of diseases such as cancer. Rab-coupling protein (RCP) dependent co-trafficking of α5β1 and EGFR1 promotes cancer cell invasion into fibronectin (FN) containing extracellular matrix (ECM), by potentiating EGFR1 signalling at the front o  f invasi  ve cells. This promotes a switch in RhoGTPase signalling to inhibit Rac1 and activate a RhoA-ROCK-Formin homology domain-containing 3 (FHOD3) pathway and generate filopodial actin-spike protrusions which drive invasion. To further understand the signalling network that drives RCP-drive  n invasi  ve migration, we generated a Boolean logical model based on existing network pathways/models, where each node can be interrogated by computational simulation. The model predicted an unanticipated feedback loop, whereby Raf/MEK/ERK signalling maintains suppression of Rac1 by inhibiting the Rac-activating Sos1-Eps8-Abi1 complex, allowing RhoA activity to predominate i  n invasi  ve protrusions. MEK inhibition was sufficient to promote lamellipodia formation and oppose filopodial actin-spike formation, and led to activation of Rac and inactivation of RhoA at the leading edge of cells moving in 3D matrix. Furthermore, MEK inhibition abrogated RCP/α5β1/EGFR1-dri  ven inva  sive migration. However, upon knockdown of Eps8 (to suppress the Sos1-Abi1-Eps8 complex), MEK inhibition had no effect on RhoGTPase activity and did not opp  ose inva  sive migration, suggesting that MEK-ERK signalling suppresses the Rac-activating Sos1-Abi1-Eps8 complex to maintain RhoA activity and promote filopodial actin-spike formation   and inva  sive migration. Our study highlights the predictive potential of mathematical modelling approaches, and demonstrates that a simple intervention (MEK-inhibition) could be of therapeutic benefit in prevent  ing inva  sive migration and metastasis. 
 ADP-ribosylation factor 6 regulates glioma cell invasion through the IQ-domain GTPase-activating protein 1-Rac1-mediated pathway. A common pathobiological feature of malignant gliomas is the insidious infiltration of single tumor cells into the brain parenchyma, rendering these deadly tumors virtually incurable with available therapies. In this study, we report that ADP-ribosylation factor 6 (ARF6), a Ras superfamily small GTPase, is abundantly expressed in   invasive   human glioma cells. Cellular depletion of ARF6 by small interfering RNA decreased Rac1 activation, impaired HGF-stimulated and serum-stimulated glioma cell migration in vitro, and markedly decreased the   invasive   capacity of   invasive   glioma in the brain. Furthermore, ectopic expression of ARF6 in glioma cells promoted cell migration via the activation of Rac1. Upon stimulation of glioma cells with HGF, we show that IQ-domain GTPase-activating protein 1 (IQGAP1) is recruited and overlaps with ARF6 at the leading edge of migrating cells. However, cellular depletion of ARF6 abrogated this recruitment of IQGAP1 and attenuated the formation of surface protrusions. ARF6 forms complexes with Rac1 and IQGAP1 in glioma cells upon HGF stimulation, and knockdown of IQGAP1 significantly inhibits ARF6-induced Rac1 activation and cell migration. Taken together, these data suggest that ARF6-mediated Rac1 activation is essential for glioma cell invasion via a signaling pathway that requires IQGAP1. 
 Glycogen synthase kinase 3β sustains invasion of glioblastoma via the focal adhesion kinase, Rac1, and c-Jun N-terminal kinase-mediated pathway. The failure of current treatment options for glioblastoma stems from their inability to control tumor cell proliferation and invasion. Biologically targeted therapies offer great hope and one promising target is glycogen synthase kinase-3β (GSK3β), implicated in various diseases, including cancer. We previously reported that inhibition of GSK3β compromises the survival and proliferation of glioblastoma cells, induces their apoptosis, and sensitizes them to temozolomide and radiation. Here, we explore whether GSK3β also contributes to the hi  ghly inv  asive nature of glioblastoma. The effects of GSK3β inhibition on migration and invasion of glioblastoma cells were examined by wound-healing and Transwell assays, as well as in a mouse model of glioblastoma. We also investigated changes in cellular microarchitectures, cytoskeletal components, and proteins responsible for cell motility and invasion. Inhibition of GSK3β attenuated the migration and invasion of glioblastoma cells in vitro and that of tumor cells in a mouse model of glioblastoma. These effects were associated with suppression of the molecular axis involving focal adhesion kinase, guanine nucleotide exchange factors/Rac1 and c-Jun N-terminal kinase. Changes in cellular phenotypes responsible for cell motility and invasion were also observed, including decreased formation of lamellipodia and invadopodium-like microstructures and alterations in the subcellular localization, and activity of Rac1 and F-actin. These changes coincided with decreased expression of matrix metalloproteinases. Our results confirm the potential of GSK3β as an attractive therapeutic target against glioblastoma invasion, thus highlighting a second role in this tumor type in addition to its involvement in chemo- and radioresistance. 
 Rac1-MKK3-p38-MAPKAPK2 pathway promotes urokinase plasminogen activator mRNA stability in   invasive   breast cancer cells. We reported previously that down-regulating or functionally blocking alphav integrins inhibits endogenous p38 mitogen-activated protein kinase (MAPK) activity and urokinase plasminogen activator (uPA) expression in   invasive   MDA-MB-231 breast cancer cells whereas engaging alphav integrins with vitronectin activates p38 MAPK and up-regulates uPA expression (Chen, J., Baskerville, C., Han, Q., Pan, Z., and Huang, S. (2001) J. Biol. Chem. 276, 47901-47905). Currently, it is not clear what upstream and downstream signaling molecules of p38 MAPK mediate alphav integrin-mediated uPA up-regulation. In the present study, we found that alphav integrin ligation activated small GTPase Rac1 preferentially, and dominant negative Rac1 inhibited alphav integrin-mediated p38 MAPK activation. Using constitutively active MAPK kinases, we found that both constitutively active MKK3 and MKK6 mutants were able to activate p38 MAPK and up-regulate uPA expression, but only dominant negative MKK3 blocked alphav integrin-mediated p38 MAPK activation and uPA up-regulation. These results suggest that MKK3, rather than MKK6, mediates alphav integrin-induced p38 MAPK activation. Among the potential downstream effectors of p38 MAPK, we found that only MAPK-activated protein kinase 2 affects alphav integrin-mediated uPA up-regulation significantly. Finally, using beta-globin reporter gene constructs containing uPA mRNA 3'-untranslated region (UTR) and adenosine/uridine-rich elements-deleted 3'-UTR, we demonstrated that p38 MAPK/MAPK-activated protein kinase 2 signaling pathway regulated uPA mRNA stability through a mechanism involving the adenosine/uridine-rich elements sequence in 3'-UTR of uPA mRNA. 
 Formyl peptide receptor-1 activation enhances intestinal epithelial cell restitution through phosphatidylinositol 3-kinase-dependent activation of Rac1 and Cdc42. Inflammatory disorders of the gastrointestinal tract result in the breakdown of the intestinal epithelial barrier in the form of erosion and ulceration. To reestablish the epithelial barrier, the epithelium must efficiently migrate to reseal wounds. Numerous signaling cascades are involved in the induction and regulation of this complex process. N-formyl peptide receptors comprise a group of Gi-coupled receptors that regulate innate immune responses. Previously, we identified the expression of functional N-formyl peptide receptors in model SK-CO15 intestinal epithelial cells and observed a role for activation of these receptors in regulating cellular   invasive   behavior. In these studies, we performed formyl peptide receptor-1 (FPR) localization and evaluated its role in regulating intestinal epithelial cell wound closure. Immunolocalization studies using a recently developed specific monoclonal anti-FPR Ab demonstrated its localization along the lateral membrane of crypt epithelial cells in normal human colonic epithelium. In vitro studies using the classical FPR agonist fMLF showed that FPR activation significantly enhances model intestinal epithelial cell restitution and that FPR localized along actin filaments in lamellipodial and filopodial extrusions. The increase in cell migration was associated with activation of PI3K, Rac1, and Cdc42. Pharmacologic inhibition of PI3K activity abrogated the fMLF-induced increase in wound closure and activation of both Rac1 and Cdc42. Inhibition of Rac1 and Cdc42 using pharmacologic inhibitors and dominant negative mutants also inhibited the fMLF-induced increase in cell migration. Taken together, theses results support a novel role for FPR stimulation in enhancing intestinal epithelial cell restitution through PI3K-dependent activation of Rac1 and Cdc42. 
 The activation of the NF-κB-JNK pathway is independent of the PI3K-Rac1-JNK pathway involved in the bFGF-regulated human fibroblast cell migration. Skin wound healing is a complex process that repairs multiple organ-tissues. Fibroblasts are key players of skin cells, whose migration is important during wound healing process. bFGF has shown a great efficacy to promote cell migration, but the precise mechanism by which bFGF regulates cell migration remains elusive.The aim of this study was to find bFGF-regulated gene pools and further identify target molecules that participated in human fibroblast cell migration.Skin primary fibroblasts and rat skin wound model were used to demonstrate the novel mechanism of bFGF regulating cell migration to accelerate wound healing. Cell migration was determined using the wound healing scratch assay. The differentially expressed genes and numerous biochemical pathways after bFGF treatment were identified by RNA-Seq analysis, and differentially expressed genes were further verified by qRT-PCR. siRNA duplex target to interfering the expression of PI3-kinase (p110α) was transformed into NIH/3T3 cells. Western blotting analysis was used to determine marker protein expressions. Th  e invasi  ve activity of fibroblasts was measured using 3D spheroid cell invasion assay.RNA-Seq analysis identified numerous biochemical pathways including the NF-κB pathway under the control of FGF signaling. bFGF negatively regulates the phosphorylation of IκB-α, the most well studied NF-κB signaling regulator while bFGF induces JNK phosphorylation. Application of Bay11-7082, a representative NF-κB inhibitor promoted cell migration, invasion and enhanced the JNKs phosphorylation. However, inhibition of JNKs blocked cell migration when NF-κB is inhibited. Moreover, application of the PI3K inhibitor LY294002 together with Bay11-7082 maintained normal cell migration and knocking-down PI3K (p110α) by a specific siRNA inhibited JNKs phosphorylation while maintaining normal IκBα phosphorylation, indicating that PI3K and NF-κB signaling independently regulate JNKs activation. In addition, administration of bFGF or Bay11-7082 promoted rat skin wound repair and accelerated the invasion of fibroblasts.This study sheds light on the mode of action of bFGF and identifies that the NF-κB-JNKs pathway is independent of the PI3K-JNKs pathway to accelerate fibroblast migration. In addition, bFGF and the relief of inflammation could be a favorable therapeutic approach for skin wound healing. 
 Activation of H-Ras and Rac1 correlates with epidermal growth factor-induced invasion in Hs578T and MDA-MB-231 breast carcinoma cells. There is considerable experimental evidence that hyperactive Ras proteins promote breast cancer growth and development including   invasive  ness, despite the low frequency of mutated forms of Ras in breast cancer. We have previously shown that H-Ras, but not N-Ras, induces an   invasive   phenotype mediated by small GTPase Rac1 in MCF10A human breast epithelial cells. Epidermal growth factor (EGF) plays an important role in aberrant growth and metastasis formation of many tumor types including breast cancer. The present study aims to investigate the correlation between EGF-induced   invasive  ness and Ras activation in four widely used breast cancer cell lines. Upon EGF stimulation,   invasive   abilities and H-Ras activation were significantly increased in Hs578T and MDA-MB-231 cell lines, but not in MDA-MB-453 and T47D cell lines. Using small interfering RNA (siRNA) to target H-Ras, we showed a crucial role of H-Ras in the   invasive   phenotype induced by EGF in Hs578T and MDA-MB-231 cells. Moreover, siRNA-knockdown of Rac1 significantly inhibited the EGF-induced   invasive  ness in these cells. Taken together, this study characterized human breast cancer cell lines with regard to the relationship between H-Ras activation and the   invasive   phenotype induced by EGF. Our data demonstrate that the activation of H-Ras and the downstream molecule Rac1 correlates with EGF-induced breast cancer cell invasion, providing important information on the regulation of malignant progression in mammary carcinoma cells. 
 TROY (TNFRSF19) is overexpressed in advanced glial tumors and promotes glioblastoma cell invasion via Pyk2-Rac1 signaling. A critical problem in the treatment of malignant gliomas is the extensive infiltration of individual tumor cells into adjacent brain tissues. This   invasive   phenotype severely limits all current therapies, and to date, no treatment is available to control the spread of this disease. Members of the tumor necrosis factor (TNF) ligand superfamily and their cognate receptors regulate various cellular responses including proliferation, migration, differentiation, and apoptosis. Specifically, the TNFRSF19/TROY gene encodes a type I cell surface receptor that is expressed on migrating or proliferating progenitor cells of the hippocampus, thalamus, and cerebral cortex. Here, we show that levels of TROY mRNA expression directly correlate with increasing glial tumor grade. Among malignant gliomas, TROY expression correlates inversely with overall patient survival. In addition, we show that TROY overexpression in glioma cells activates Rac1 signaling in a Pyk2-dependent manner to drive glioma cell invasion and migration. Pyk2 coimmunoprecipitates with the TROY receptor, and depletion of Pyk2 expression by short hairpin RNA interference oligonucleotides inhibits TROY-induced Rac1 activation and subsequent cellular migration. These findings position aberrant expression and/or signaling by TROY as a contributor, and possibly as a driver, of the malignant dispersion of glioma cells. 
 MT1-MMP modulates melanoma cell dissemination and metastasis through activation of MMP2 and RAC1. Metastatic melanoma remains the deadliest of all skin cancers with a survival rate at five years of less than 15%. MT1-MMP is a membrane-associated matrix metalloproteinase that controls pericellular proteolysis and is an important, invasion-promoting, pro-tumorigenic MMP in cancer. We show that deregulation of MT1-MMP expression happens as early as the transition from nevus to primary melanoma and continues to increase during melanoma progression. Furthermore, MT1-MMP expression is associated with poor melanoma patient outcome, underscoring a pivotal role of MT1-MMP in melanoma pathogenesis. We demonstrate that MT1-MMP is directly required for melanoma cells to metastasize, as cells deprived of MT1-MMP fail to form distant metastasis in an orthotopic mouse melanoma model. We show that MT1-MMP affects cell invasion by activating its target MMP2. Importantly, we demonstrate, for the first time, that activation of MMP2 by MT1-MMP is required to sustain RAC1 activity and promote MT1-MMP-dependent cell motility. These data highlight a novel MT1-MMP/MMP2/RAC1 signaling axis in melanoma that may represent an intriguing molecular target for the treatment of   invasive   melanoma. 
 DDX3 modulates cell adhesion and motility and cancer cell metastasis via Rac1-mediated signaling pathway. The DEAD-box RNA helicase DDX3 is a versatile protein involved in multiple steps of gene expression and various cellular signaling pathways. DDX3 mutations have been implicated in the wingless (Wnt) type of medulloblastoma. We show here that small interfering RNA-mediated DDX3 knockdown in various cell lines increased cell-cell adhesion but decreased cell-extracellular matrix adhesion. Moreover, DDX3 depletion suppressed cell motility and impaired directional migration in the wound-healing assay. Accordingly, DDX3-depleted cells exhibited reduced   invasive   capacities in vitro as well as reduced metastatic potential in mice. We also examined the mechanism underlying DDX3-regulated cell migration. DDX3 knockdown reduced the levels of both Rac1 and β-catenin proteins, and consequentially downregulated the expression of several β-catenin target genes. Moreover, we demonstrated that DDX3-regulated Rac1 mRNA translation, possibly through an interaction with its 5'-untranslated region, and affected β-catenin protein stability in an Rac1-dependent manner. Taken together, our results indicate the DDX3-Rac1-β-catenin regulatory axis in modulating the expression of Wnt/β-catenin target genes. Therefore, this report provides a mechanistic context for the role of DDX3 in Wnt-type tumors. 
 RASAL2 activates RAC1 to promote triple-negative breast cancer progression. Patients with triple-negative breast cancer (TNBC) have a high incidence of early relapse and metastasis; however, the molecular basis for recurrence in these individuals remains poorly understood. Here, we demonstrate that RASAL2, which encodes a RAS-GTPase-activating protein (RAS-GAP), is a functional target of anti-  invasive   microRNA-203 and is overexpressed in a subset of triple-negative or estrogen receptor-negative (ER-negative) breast tumors. As opposed to luminal B ER-positive breast cancers, in which RASAL2 has been shown to act as a RAS-GAP tumor suppressor, we found that RASAL2 is oncogenic in TNBC and drives mesenchymal invasion and metastasis. Moreover, high RASAL2 expression was predictive of poor disease outcomes in patients with TNBC. RASAL2 acted independently of its RAS-GAP catalytic activity in TNBC; however, RASAL2 promoted small GTPase RAC1 signaling, which promotes mesenchymal invasion, through binding and antagonizing the RAC1-GAP protein ARHGAP24. Together, these results indicate that activation of a RASAL2/ARHGAP24/RAC1 module contributes to TNBC tumorigenesis and identify a context-dependent role of RASAL2 in breast cancer. 
 The GTPase Rac regulates the proliferation and invasion of fibroblast-like synoviocytes from rheumatoid arthritis patients. Fibroblast-like synoviocytes (FLS) isolated from joints of rheumatoid arthritis (RA) patients display proliferative and   invasive   properties reminiscent of those of malignant tumor cells. Rac small GTPases play an important role in tumor cell proliferation and invasion. We therefore investigated the potential role of Rac proteins in the proliferative and   invasive   behavior of RA-FLS. We showed that inhibiting Rac activity with the Rac-specific small molecule inhibitor NSC23766 causes a strong inhibition of RA-FLS proliferation, without affecting cell survival. Rac inhibition also results in a strong reduction in RA-FLS invasion through reconstituted extracellular matrix and a less marked inhibition of two-dimensional migration as measured by monolayer wound healing. We also showed that small interfering RNA-mediated depletion of Rac1 inhibits RA-FLS proliferation and invasion to a similar extent as NSC23766. These results demonstrate for the first time that Rac proteins play an important role in the aggressive behavior of FLS isolated from RA patients. In addition, we observed that inhibiting Rac proteins prevents JNK activation and that the JNK inhibitor SP600125 strongly inhibits RA-FLS invasion, suggesting that Rac-mediated JNK activation contributes to the role of Rac proteins in the   invasive   behavior of RA-FLS. In conclusion, Rac-controlled signaling pathways may present a new source of drug targets for therapeutic intervention in RA. 
 Small GTPase Rac1: structure, localization, and expression of the human gene. Rac1 is a member of the Rho family of small GTPases involved in signal transduction pathways that control proliferation, adhesion, and migration of cells during embryonic development and   invasive  ness of tumor cells. Here we present the complete structure of the human RAC1 gene and characterize its expression. The gene comprises 7 exons over a length of 29 kb and is localized to chromosome 7p22. The GC-rich gene promoter shows characteristics of a housekeeping gene and Northern blot studies revealed ubiquitous expression of two rac1 transcripts, 1.2 and 2.5 kb in size. The two transcripts are expressed in tissue-specific ratios, reflecting competition between two alternative polyadenylation sites. The RAC1 but not RAC2 gene contains an additional exon 3b that is included by alternative splicing into the variant Rac1b, a constitutively active mutant which induces the formation of lamellipodia in fibroblasts. These data indicate that the RAC1 gene encodes two signaling GTPases. The gene structure reported here will enable studies on the regulation of RAC1 expression during tumorigenesis and development. 
 RAC1 activation mediates Twist1-induced cancer cell migration. Epithelial-mesenchymal transition (EMT), which is characterized by the suppression of the adhesion protein E-cadherin, is a crucial process that promotes metastasis and stem-like properties of cancer cells. However, the dissociation of cellular aggregates is not sufficient to explain why cancer cells move, and the motile nature of cancer cells undergoing EMT remains elusive. Here, we identify a mechanism in which the EMT inducer Twist1 elicits cancer cell movement through activation of RAC1. Twist1 cooperates with BMI1 to suppress let-7i expression, which results in upregulation of NEDD9 and DOCK3, leading to RAC1 activation and enabling mesenchymal-mode movement in three-dimensional environments. Moreover, the suppression of let-7i contributes to Twist1-induced stem-like properties. Clinically, activation of the Twist1-let-7i-NEDD9 axis in head and neck cancer patients correlates with tumour   invasive  ness and worse outcome. Our results uncover an essential mechanism to explain how Twist1 induces the motile stem-like cancer cell phenotype beyond simply suppressing E-cadherin. 
 Rac1 nucleocytoplasmic shuttling drives nuclear shape changes and tumor invasion. Nuclear membrane microdomains are proposed to act as platforms for regulation of nuclear function, but little is known about the mechanisms controlling their formation. Organization of the plasma membrane is regulated by actin polymerization, and the existence of an actin pool in the nucleus suggests that a similar mechanism might operate here. We show that nuclear membrane organization and morphology are regulated by the nuclear level of active Rac1 through actin polymerization-dependent mechanisms. Rac1 nuclear export is mediated by two internal nuclear export signals and through its interaction with nucleophosmin-1 (B23), which acts as a Rac1 chaperone inside the nucleus. Rac1 nuclear accumulation alters the balance between cytosolic Rac1 and Rho, increasing RhoA signaling in the cytoplasm and promoting a highly   invasive   phenotype. Nuclear Rac1 shuttling is a finely tuned mechanism for controlling nuclear shape and organization and cell   invasive  ness. 
 Rac1, RhoA, and Cdc42 participate in HeLa cell invasion by group B streptococcus. The group B streptococcus (GBS) is an important human pathogen with the ability to cause   invasive   disease. To do so, the bacteria must invade host cells. It has been well documented that GBS are able to invade a variety of nonphagocytic host cell types, and this process is thought to involve a number of pathogen-host cell interactions. While some of the molecular aspects of the GBS-host cell invasion process have been characterized, many events still remain unclear. The objective of this investigation was to evaluate the role of the Rho-family GTPases Rac, Rho, and Cdc42 in GBS invasion into epithelial cells. The epithelial cell invasion process was modeled using HeLa 229 cell culture. Treatment of HeLa cells with 10 microM compactin, a pan-GTPase inhibitor, abolished GBS internalization, suggesting that GTPases are involved in the GBS invasion process. The addition of Toxin B or exoenzyme C3 to HeLa cells before GBS infection reduced invasion by 50%, further suggesting that the Rho-family GTPases are involved in GBS entry. Examining invasion of GBS into HeLa cells with altered genetic backgrounds was used to confirm these findings; GBS invasion into HeLa cells transiently transfected with dominant negative Rac1, Cdc42, or RhoA reduced invasion by 75%, 51%, and 42%, respectively. Results of this study suggest that the Rho-family GTPases are required for efficient invasion of HeLa cells by GBS. 
 Ephexin4 and EphA2 mediate cell migration through a RhoG-dependent mechanism. EphA2, a member of the Eph receptor family, is frequently overexpressed in a variety of human cancers, including breast cancers, and promotes cancer cell motility and invasion independently of its ligand ephrin stimulation. In this study, we identify Ephexin4 as a guanine nucleotide exchange factor (GEF) for RhoG that interacts with EphA2 in breast cancer cells, and knockdown and rescue experiments show that Ephexin4 acts downstream of EphA2 to promote ligand-independent breast cancer cell migration and invasion toward epidermal growth factor through activation of RhoG. The activation of RhoG recruits its effector ELMO2 and a Rac GEF Dock4 to form a complex with EphA2 at the tips of cortactin-rich protrusions in migrating breast cancer cells. In addition, the Dock4-mediated Rac activation is required for breast cancer cell migration. Our findings reveal a novel link between EphA2 and Rac activation that contributes to the cell motility and   invasive  ness of breast cancer cells. 
 Roles of the Rac1 and Rac3 GTPases in human tumor cell invasion. Members of the Rho family of small GTPases have been shown to be involved in tumorigenesis and metastasis. Currently, most of the available information on the function of Rho proteins in malignant transformation is based on the use of dominant-negative mutants of these GTPases. The specificity of these dominant-negative mutants is limited however. In this study, we used small interfering RNA directed against either Rac1 or Rac3 to reduce their expression specifically. In line with observations using dominant-negative Rac1 in other cell types, we show that RNA interference-mediated depletion of Rac1 strongly inhibits lamellipodia formation, cell migration and invasion in SNB19 glioblastoma cells. Surprisingly however, Rac1 depletion has a much smaller inhibitory effect on SNB19 cell proliferation and survival. Interestingly, whereas depletion of Rac3 strongly inhibits SNB19 cell invasion, it does not affect lamellipodia formation and has only minor effects on cell migration and proliferation. Similar results were obtained in BT549 breast carcinoma cells. Thus, functional analysis of Rac1 and Rac3 using RNA interference reveals a critical role for these GTPases in the   invasive   behavior of glioma and breast carcinoma cells. 
 Rac1 activity regulates proliferation of aggressive metastatic melanoma. Molecular mechanisms underlying the different capacity of two in vivo selected human melanoma cell variants to form experimental metastases were studied. The doubling times of the FEMX-I and FEMX-V cell sublines in vitro were 15 and 25 h, respectively. The   invasive   capacity of FEMX-I cells was 8-fold higher than FEMX-V cells, and the time to form approximately 10 mm s.c. tumors in nude mice was 21 versus 35 days. FEMX-I displayed a spindle-like formation in vitro, whereas FEMX-V cells had a rounded shape. Hence, we examined known determinants of cell shape and proliferation, the small GTPases. The four studied showed equal expression in both cell types, but Rac1 activity was significantly decreased in FEMX-V cells. Rac1 stimulates NFkappaB, and we found that endogenous NFkappaB activity of FEMX-V cells was 2% of that of FEMX-I cells. Inhibition of Rac1 resulted in blocked NFkappaB activity. Specific inhibition of either Rac1 or NFkappaB significantly reduced proliferation and invasion of FEMX-I cells, the more pronounced effects observed with Rac1 inhibition. These data indicate that Rac1 activity in FEMX cells regulates cell proliferation and invasion, in part via its effect on NFkappaB, signifying Rac1 as a key molecule in melanoma progression and metastasis. 
 Phosphatidylinositol 5-phosphate regulates invasion through binding and activation of Tiam1. PtdIns5P is a lipid messenger acting as a stress-response mediator in the nucleus, and known to maintain cell activation through traffic alterations upon bacterial infection. Here, we show that PtdIns5P regulates actin dynamics and invasion via recruitment and activation of the exchange factor Tiam1 and Rac1. Restricted Rac1 activation results from the binding of Tiam1 DH-PH domains to PtdIns5P. Using an assay that mimics Rac1 membrane anchoring by using Rac1-His and liposomes containing Ni(2+)-NTA modified lipids, we demonstrate that intrinsic Tiam1 DH-PH activity increases when Rac1 is anchored in a PtdIns5P-enriched environment. This pathway appears to be general since it is valid in different pathophysiological models: receptor tyrosine kinase activation, bacterial phosphatase IpgD expression and the   invasive   NPM-ALK(+) lymphomas. The discovery that PtdIns5P could be a keystone of GTPases and cytoskeleton spatiotemporal regulation opens important research avenues towards unravelling new strategies counteracting cell invasion. 
 PIKfyve, MTMR3 and their product PtdIns5P regulate cancer cell migration and invasion through activation of Rac1. Previously, we have shown that the phosphoinositide metabolizing enzymes PIKfyve (phosphoinositide 5-kinase, FYVE finger containing) and MTMR3 (myotubularin-related protein 3), together with their lipid product PtdIns5P, are important for migration of normal human fibroblasts. As these proteins are a kinase and a phosphatase respectively, and thereby considered druggable, we wanted to test their involvement in cancer cell migration and invasion. First, we showed that PIKfyve and MTMR3 are expressed in most cancer cells. Next, we demonstrated that depletion of PIKfyve or MTMR3 resulted in decreased velocity in three different cancer cell lines by using new software for cell tracking. Inhibition of the enzymatic activity of PIKfyve by the inhibitor YM201636 also led to a strong reduction in cell velocity. Mechanistically, we show that PIKfyve and MTMR3 regulate the activation of the Rho family GTPase Rac1. Further experiments also implicated PtdIns5P in the activation of Rac1. The results suggest a model for the activation of Rac1 in cell migration where PIKfyve and MTMR3 produce PtdIns5P on cellular membranes which may then serve to recruit effectors to activate Rac1. Finally, in an invasion assay, we demonstrate that both PIKfyve and MTMR3 are implicated in   invasive   behaviour of cancer cells. Thus PIKfyve and MTMR3 could represent novel therapeutic targets in metastatic cancer. 
 Heregulin beta1 promotes breast cancer cell proliferation through Rac/ERK-dependent induction of cyclin D1 and p21Cip1. Accumulating evidence indicates that heregulins, EGF (epidermal growth factor)-like ligands, promote breast cancer cell proliferation and are involved in the progression of breast cancer towards an aggressive and   invasive   phenotype. However, there is limited information regarding the molecular mechanisms that mediate these effects. We have recently established that HRG (heregulin beta1) promotes breast cancer cell proliferation and migration via cross-talk with EGFR (EGF receptor) that involves the activation of the small GTPase Rac1. In the present paper we report that Rac1 is an essential player for mediating the induction of cyclin D1 and p21(Cip1) by HRG in breast cancer cells. Inhibition of Rac function by expressing either the Rac-GAP (GTPase-activating protein) beta2-chimaerin or the dominant-negative Rac mutant N17Rac1, or Rac1 depletion using RNAi (RNA interference), abolished the cyclin D1 and p21(Cip1) induction by HRG. Interestingly, the proliferative effect of HRG was impaired not only when the expression of Rac1 or cyclin D1 was inhibited, but also when cells were depleted of p21(Cip1) using RNAi. Inhibition of EGFR, PI3K (phosphoinositide 3-kinase; kinases required for Rac activation by HRG) or MEK [MAPK (mitogen-activated protein kinase)/ERK (extracellular-signal-regulated kinase) kinase] also blocked the up-regulation of cyclin D1 and p21(Cip1) by HRG. In addition, we found that HRG activates NF-kappaB (nuclear factor kappaB) in a Rac1- and MEK-dependent fashion, and inhibition of NF-kappaB abrogates cyclin D1/p21(Cip1) induction and proliferation by HRG. Taken together, these findings establish a central role for Rac1 in the control of HRG-induced breast cancer cell-cycle progression and proliferation through up-regulating the expression of cyclin D1 and p21(Cip1). 
 The Na+-H+ exchanger-1 induces cytoskeletal changes involving reciprocal RhoA and Rac1 signaling, resulting in motility and invasion in MDA-MB-435 cells. An increasing body of evidence shows that the tumour microenvironment is essential in driving neoplastic progression. The low serum component of this microenvironment stimulates motility/invasion in human breast cancer cells via activation of the Na+-H+ exchanger (NHE) isoform 1, but the signal transduction systems that underlie this process are still poorly understood. We undertook the present study to elucidate the role and pattern of regulation by the Rho GTPases of this serum deprivation-dependent activation of both NHE1 and subsequent   invasive   characteristics, such as pseudopodia and invadiopodia protrusion, directed cell motility and penetration of normal tissues.The present study was performed in a well characterized human mammary epithelial cell line representing late stage metastatic progression, MDA-MB-435. The activity of RhoA and Rac1 was modified using their dominant negative and constitutively active mutants and the activity of NHE1, cell motility/invasion, F-actin content and cell shape were measured.We show for the first time that serum deprivation induces NHE1-dependent morphological and cytoskeletal changes in metastatic cells via a reciprocal interaction of RhoA and Rac1, resulting in increased chemotaxis and invasion. Deprivation changed cell shape by reducing the amount of F-actin and inducing the formation of leading edge pseudopodia. Serum deprivation inhibited RhoA activity and stimulated Rac1 activity. Rac1 and RhoA were antagonistic regulators of both basal and stimulated tumour cell NHE1 activity. The regulation of NHE1 activity by RhoA and Rac1 in both conditions was mediated by an alteration in intracellular proton affinity of the exchanger. Interestingly, the role of each of these G-proteins was reversed during serum deprivation; basal NHE1 activity was regulated positively by RhoA and negatively by Rac1, whereas RhoA negatively and Rac1 positively directed the stimulation of NHE1 during serum deprivation. Importantly, the same pattern of RhoA and Rac1 regulation found for NHE1 activity was observed in both basal and serum deprivation dependent increases in motility, invasion and actin cytoskeletal organization.Our findings suggest that the reported antagonistic roles of RhoA and Rac1 in cell motility/invasion and cytoskeletal organization may be due, in part, to their concerted action on NHE1 activity as a convergence point. 
 Overexpression of small GTPases directly correlates with expression of δ-catenin and their coexpression predicts a poor clinical outcome in nonsmall cell lung cancer. δ-catenin can affect cytoskeletal assembly, and promote cell migration by regulating the activity of small GTPases. While many malignancies have been shown to be positive for δ-catenin, it is still unclear whether δ-catenin and small GTPases are coexpressed in tumor cells, and so is the relationship between their coexpression and prognosis in the tumor patients. In this study, immunohistochemistry was performed to examine expressive levels of δ-catenin, cdc42, and Rac1 in 135 cases of nonsmall cell lung cancer (NSCLC), including 60 cases with follow-up records. Thirty samples of paired lung cancer tissues and adjacent normal lung tissues were collected to analyze mRNA and protein expression of δ-catenin and small GTPases. The effects of δ-catenin on small GTPases expressi  on and i  nvasive ability of lung cancer cells were also evaluated. Compared with normal lung tissues, both mRNA and protein levels of δ-catenin and small GTPases were increased in lung cancer tissues (P < 0.05), and the expression of small GTPases directly correlated with that of δ-catenin (P < 0.001). In addition, δ-catenin and small GTPases tended to be coexpressed in lung adenocarcinoma, advanced stages, and primary tumors with lymph node metastasis (all P < 0.05). The patients with coexpression of δ-catenin and small GTPases had a shorter survival time than those without coexpression (P < 0.05). Furthermore, δ-catenin overexpression co  uld enha  nce invasive ability of lung cancer cells by upregulating protein and transcriptional level of small GTPases. Therefore, δ-catenin likely upregulates the activity of small GTPases at transcriptional level, and their coexpression may predict a poor clinical outcome in NSCLC patients. 
  GeneRIFs  
 A MAPK-Driven Feedback Loop Suppresses Rac Activity to Promote RhoA-Driven Cancer Cell Invasion. Our model predicted an unanticipated feedback loop, whereby Raf/MEK/ERK signalling maintains suppression of Rac1 by inhibiting the Rac-activating Sos1-Eps8-Abi1 complex, allowing RhoA activity to predominate in   invasive   protrusions. 
 Authors propose a model whereby vimentin promotes FAK stabilization through VAV2-mediated Rac1 activation. This model may explain why vimentin expressing metastatic lung cancer cells are more motile and   invasive  . 
 Nuclear Rac1 shuttling is a finely tuned mechanism for controlling nuclear shape and organization and cell   invasive  ness 
 Study demonstrates that increased DGKzeta protein levels in SW620 cells are associated with increased Rho GTPase activity and downstream signaling; silencing of DGKzeta expression in SW620 cells decreased Rac1 and RhoA activity and attenuated cell invasion; DGKzeta silencing also attenuated the   invasive  ness of PC-3 prostate cancer and MDA-MB-231 breast cancer cells. 
 study provides evidence the large GTPase Dyn2 regulates the small GTPase Rac1 to potentiate   invasive   migration of pancreatic tumor cells; Dyn2 plays an essential role in regulating Rac1-mediated pancreatic tumor cell migration through modulation of the Rac1 activator Vav1 via a direct interaction 
 The potential role of Rac proteins in the proliferative and   invasive   behavior of rheumatoid arthritis-fibroblast-like synoviocytes. 
 Pro  invasive   activity of BMP7 through SMAD4/src-independent and ERK/Rac/JNK-dependent signaling pathways in colon cancer cells is reported. 
 functional analysis of Rac1 and Rac3 using RNA interference reveals a critical role for these GTPases in the   invasive   behavior of glioma and breast carcinoma cells 
 The EGFR/Vav2/Rac1 axis is a crucial pathway for the acquisition of motile and   invasive   properties of most head and neck squamous cell carcinoma cells. 
 direct activation of Cdc42 and Rac1 by   invasive   Salmonella is a prerequisite of Salmonella-mediated death of U937 cells. 
 role in pathway that promotes urokinase plasminogen activator mRNA stability in   invasive   breast cancer cells 
  [Mm]etastasize  PubMed  
 An anti-Wnt5a antibody suppresses metastasis of gastric cancer cells in vivo by inhibiting receptor-mediated endocytosis. Wnt5a is a representative ligand that activates the β-catenin-independent pathway in Wnt signaling. It was reported that the expression of Wnt5a in human gastric cancer is associated with aggressiveness and poor prognosis and that knockdown of Wnt5a reduces the ability of gastric cancer cells to   metastasiz  e in nude mice. Therefore, Wnt5a and its signaling pathway might be important targets for the therapy of gastric cancer. The aim of this study was to examine whether an anti-Wnt5a antibody affects metastasis of gastric cancer cells. One anti-Wnt5a polyclonal antibody (pAb5a-5) inhibited migration and invasion activities in vitro of gastric cancer cells with a high expression level of Wnt5a. Previously, it was shown that Wnt5a induces the internalization of receptors, which is required for Wnt5a-dependent activation of Rac1. pAb5a-5 inhibited Wnt5a-dependent internalization of receptors, thereby suppressed Wnt5a-dependent activation of Rac1. Laminin γ2 is one of target genes of Wnt5a signaling and Rac1 was involved in its expression. pAb5a-5 also inhibited Wnt5a-dependent expression of laminin γ2. In an experimental liver metastasis assay, gastric cancer cells were introduced into the spleens of nude mice. Laminin γ2 was required for liver metastatic ability of gastric cancer cells in vivo. Furthermore, intraperitoneal injection of pAb5a-5 inhibited the metastatic ability of gastric cancer cells. These results suggest that an anti-Wnt5a antibody was capable of suppressing Wnt5a-dependent internalization of receptors, resulting in the prevention of metastasis of gastric cancer cells by inhibiting the activation of Rac1 and the expression of laminin γ2. 
 MT1-MMP modulates melanoma cell dissemination and metastasis through activation of MMP2 and RAC1. Metastatic melanoma remains the deadliest of all skin cancers with a survival rate at five years of less than 15%. MT1-MMP is a membrane-associated matrix metalloproteinase that controls pericellular proteolysis and is an important, invasion-promoting, pro-tumorigenic MMP in cancer. We show that deregulation of MT1-MMP expression happens as early as the transition from nevus to primary melanoma and continues to increase during melanoma progression. Furthermore, MT1-MMP expression is associated with poor melanoma patient outcome, underscoring a pivotal role of MT1-MMP in melanoma pathogenesis. We demonstrate that MT1-MMP is directly required for melanoma cells to   metastasize  , as cells deprived of MT1-MMP fail to form distant metastasis in an orthotopic mouse melanoma model. We show that MT1-MMP affects cell invasion by activating its target MMP2. Importantly, we demonstrate, for the first time, that activation of MMP2 by MT1-MMP is required to sustain RAC1 activity and promote MT1-MMP-dependent cell motility. These data highlight a novel MT1-MMP/MMP2/RAC1 signaling axis in melanoma that may represent an intriguing molecular target for the treatment of invasive melanoma. 
  [Mm]etastasis  PubMed  
 Rac activation and inactivation control plasticity of tumor cell movement. Tumor cells exhibit two different modes of individual cell movement. Mesenchymal-type movement is characterized by an elongated cellular morphology and requires extracellular proteolysis. In amoeboid movement, cells have a rounded morphology, are less dependent on proteases, and require high Rho-kinase signaling to drive elevated levels of actomyosin contractility. These two modes of cell movement are interconvertible. We show that mesenchymal-type movement in melanoma cells is driven by activation of the GTPase Rac through a complex containing NEDD9, a recently identified melanoma   metastasis   gene, and DOCK3, a Rac guanine nucleotide exchange factor. Rac signals through WAVE2 to direct mesenchymal movement and suppress amoeboid movement through decreasing actomyosin contractility. Conversely, in amoeboid movement, Rho-kinase signaling activates a Rac GAP, ARHGAP22, that suppresses mesenchymal movement by inactivating Rac. We demonstrate tight interplay between Rho and Rac in determining different modes of tumor cell movement, revealing how tumor cells switch between different modes of movement. 
 Interleukin-8 induces the endothelial cell migration through the Rac 1/RhoA-p38MAPK pathway. Endothelial cell migration is essential for tumor angiogenesis, and interleukin-8 (IL-8) has been shown to play an important role in tumor growth, angiogenesis, and   metastasis  . The objective of this study was to investigate the molecular mechanism of IL-8 induced endothelial cell migration in vitro.Fluorescence microscope was used to study the distribution of cytoskeleton. The expression of Rac1 and RhoA protein was detected by western blotting. After endothelial cells were transfected by lipofectamine 2000 reagent, the Transwell chamber motility assay was applied to observe the migration of endothelial cells induced by IL-8. The active p38MAPK (mitogen-activated protein kinase) was evaluated by the p38MAPK activation assay.We demonstrated that IL-8 activated cell migration can be impaired by p38MAPK inhibitor, suggesting the participation of p38MAPK in the cell migration. Our results indicated that p38MAPK signaling is required for membrane ruffles, lamellipodia extensions, and actin stress fibers formation induced by IL-8. Furthermore, p38MAPK inhibitor led to increased Rac1 and RhoA expression in IL-8 treated EA.hy926 cells. In addition, IL-8 induced p38MAPK activation was suppressed by dominant-negative mutant for Rac1 and RhoA.Our study demonstrates that IL-8-Rac1/RhoA-p38MAPK signaling pathway plays a vital role in the IL-8-induced endothelial cell migration, and it provides new insight into the molecular mechanisms by which IL-8 contributes to tumor angiogenesis and   metastasis  . 
 Upregulation of Eps8 in oral squamous cell carcinoma promotes cell migration and invasion through integrin-dependent Rac1 activation. Oral squamous cell carcinoma (OSCC) is a lethal disease and early death usually occurs as a result of local invasion and regional lymph node metastases. Current treatment regimens are, to a certain degree, inadequate, with a 5-year mortality rate of around 50% and novel therapeutic targets are urgently required. Using expression microarrays, we identified the eps8 gene as being overexpressed in OSCC cell lines relative to normal oral keratinocytes, and confirmed these findings using RT-PCR and western blotting. In human tissues, we found that Eps8 was upregulated in OSCC (32% of primary tumors) compared with normal oral mucosa, and that expression correlated significantly with lymph node   metastasis   (P=0.032), suggesting a disease-promoting effect. Using OSCC cell lines, we assessed the functional role of Eps8 in tumor cells. Although suppression of Eps8 produced no effect on cell proliferation, both cell spreading and migration were markedly inhibited. The latter cell functions may be modulated through the small GTP-ase, Rac1 and we used pull-down assays to investigate the role of Eps8 in Rac1 signaling. We found that alphavbeta6- and alpha5beta1-integrin-dependent activation of Rac1 was mediated through Eps8. Knockdown of either Eps8 or Rac1, inhibited integrin-dependent cell migration similarly and transient expression of constitutively active Rac1 restored migration of cells in which Eps8 expression had been suppressed. We also showed that knockdown of Eps8 inhibited tumor cell invasion in an organotypic model of OSCC. These data suggest that Eps8 and Rac1 are part of an integrated signaling pathway modulating integrin-dependent tumour cell motility and identify Eps8 as a possible therapeutic target. 
 Inhibition of RAC1-GEF DOCK3 by miR-512-3p contributes to suppression of   metastasis   in non-small cell lung cancer. MicroRNAs are a class of small non-coding RNAs regulating gene expression. In this study, we demonstrated that retinoic acid (RA) treatment increases the expression of miR-512-3p. Overexpression of miR-512-3p inhibited cell adhesion, migration, and invasion in non-small cell lung cancer (NSCLC) cell lines A549 and H1299. miR-512-3p inhibitor partially reversed these effects in H1299 cells stably expressing miR-512. We identified DOCK3, a RAC1-GEF (guanine nucleotide exchange factor), as a target gene of miR-512-3p. Overexpression of miR-512-3p led to the decrease of DOCK3 protein but not its mRNA. Knockdown of DOCK3 resulted in similar effects on adhesion, migration, and invasion as observed of miR-512-3p overexpression. Active RAC1 pull-down assay indicated that overexpression of miR-512-3p could decrease the activity of RAC1 with a higher efficiency than that of DOCK3 knockdown. Furthermore, expression of miR-512-3p was suppressed in most NSCLC patient tumor samples compared to its paired normal controls, suggesting that miR-512-3p might play a crucial role in lung cancer development. In conclusion, our results supported that miR-512-3p could inhibit tumor cell adhesion, migration, and invasion by regulating the RAC1 activity via DOCK3 in NSCLC A549 and H1299 cell lines. 
 Epidermal growth factor-regulated activation of Rac GTPase enhances CD44 cleavage by metalloproteinase disintegrin ADAM10. Invasive tumour cells, such as gliomas, frequently express EGF (epidermal growth factor) receptor at a high level and they exhibit enhanced cell migration in response to EGF. We reported previously that tumour cell migration is associated with ectodomain cleavage of CD44, the major adhesion molecule that is implicated in tumour invasion and   metastasis  , and that the cleavage is enhanced by ligation of CD44. In the present study, we show that EGF promotes CD44 cleavage and CD44-dependent cell migration. Introduction of a dominant-negative mutant of the small GTPase Rac1 or depletion of Rac1 by RNAi (RNA interference) abrogated CD44 cleavage induced by EGF. Treatment with PD98059, an inhibitor for MEK (mitogen-activated protein kinase/extracellular-signal-regulated kinase kinase), also suppressed the CD44 cleavage. Furthermore, RNAi studies showed that EGF induced ADAM10 (a disintegrin and metalloproteinase 10)-dependent CD44 cleavage and cell migration. These results indicate that EGF induces ADAM10-mediated CD44 cleavage through Rac1 and mitogen-activated protein kinase activation, and thereby promotes tumour cell migration and invasion. 
 Prognostic role of integrin β1, E-cadherin, and rac1 expression in small cell lung cancer. Integrin β(1) mediates cellular adhesion to the extracellular matrix (ECM) and is correlated with highly invasive and metastatic behavior in small cell lung cancer (SCLC). E-cadherin (ECAD) is a calcium-dependent cell-cell adhesion receptor that restricts invasion of cells and reduce  s metastas  is. Rac1 is involved in the regulation of the actin cytoskeleton, adhesion, migration, invasion, and tumo  r metastas  is. The aim of this study was to examine integrin β(1) , ECAD and rac1 expression in SCLC and to analyze the prognostic value of these markers in patients with SCLC. We analyzed integrin β(1) , ECAD, and rac1 expression in 112 SCLC tissues by immunohistochemical staining. Correlative analyses between integrin β(1) , ECAD, and rac1 expression and cliniopathological factors were performed. A total of 65 patients had extensive disease (ED) (58%), and 47 had limited disease (LD) (42%). The median follow-up duration was 61 months (range: 14-117 months), and the median progression free survival (PFS) and overall survival (OS) were 6.1 months (range: 4.8-7.4 months) and 9.7 months (range: 8.1-11.3 months), respectively. The expression of integrin β(1) , ECAD, and rac1 protein was observed in 64, 73, and 99 of SCLC tissues, respectively. The correlative analyses between integrin β(1) , ECAD, or rac1 expression and various clinical parameters did not show any statistical significance. However, the ECAD expression was associated with OS in the entire cohort. In contrast, the expression of integrin β(1) and rac1 was not associated with PFS or OS. In a subgroup analysis, patients with less t  han two me  tastasis had significantly longer OS (p = 0.047) if their tumors expressed integrin β(1) compared to those without integrin β(1) expression. In addition, OS was longer for patients with ECAD positive tumors compared to those whose tumors did not express ECAD in males (p = 0.032) and patients who never smoked (p < 0.001). Multivariate analysis showed that LD (p = 0.004), overall response rate (p = 0.003), and expression of ECAD (p = 0.015) were the independent good prognostic factors for OS. LD (p = 0.024), overall response rate (p < 0.001), and less   than two   metastasis (p = 0.003) were prognostic factors for longer PFS. These results suggest that ECAD expression may be useful as a prognostic indicator in patients with SCLC. 
 p130Cas promotes invasiveness of three-dimensional ErbB2-transformed mammary acinar structures by enhanced activation of mTOR/p70S6K and Rac1. ErbB2 over-expression is detected in approximately 25% of invasive breast cancers and is strongly associated with poor patient survival. We have previously demonstrated that p130Cas adaptor is a crucial mediator of ErbB2 transformation. Here, we analysed the molecular mechanisms through which p130Cas controls ErbB2-dependent invasion in three-dimensional cultures of mammary epithelial cells. Concomitant p130Cas over-expression and ErbB2 activation enhance PI3K/Akt and Erk1/2 MAPK signalling pathways and promote invasion of mammary acini. By using pharmacological inhibitors, we demonstrate that both signalling cascades are required for the invasive behaviour of p130Cas over-expressing and ErbB2 activated acini. Erk1/2 MAPK and PI3K/Akt signalling triggers invasion through distinct downstream effectors involving mTOR/p70S6K and Rac1 activation, respectively. Moreover, in silico analyses indicate that p130Cas expression in ErbB2 positive human breast cancers significantly correlates with higher risk to develop distant   metastasis  , thus underlying the value of the p130Cas/ErbB2 synergism in regulating breast cancer invasion. In conclusion, high levels of p130Cas favour progression of ErbB2-transformed cells towards an invasive phenotype. 
 Inhibition of RAC1-GEF DOCK3 by miR-512-3p contributes to suppression of   metastasis   in non-small cell lung cancer. MicroRNAs are a class of small non-coding RNAs regulating gene expression. In this study, we demonstrated that retinoic acid (RA) treatment increases the expression of miR-512-3p. Overexpression of miR-512-3p inhibited cell adhesion, migration, and invasion in non-small cell lung cancer (NSCLC) cell lines A549 and H1299. miR-512-3p inhibitor partially reversed these effects in H1299 cells stably expressing miR-512. We identified DOCK3, a RAC1-GEF (guanine nucleotide exchange factor), as a target gene of miR-512-3p. Overexpression of miR-512-3p led to the decrease of DOCK3 protein but not its mRNA. Knockdown of DOCK3 resulted in similar effects on adhesion, migration, and invasion as observed of miR-512-3p overexpression. Active RAC1 pull-down assay indicated that overexpression of miR-512-3p could decrease the activity of RAC1 with a higher efficiency than that of DOCK3 knockdown. Furthermore, expression of miR-512-3p was suppressed in most NSCLC patient tumor samples compared to its paired normal controls, suggesting that miR-512-3p might play a crucial role in lung cancer development. In conclusion, our results supported that miR-512-3p could inhibit tumor cell adhesion, migration, and invasion by regulating the RAC1 activity via DOCK3 in NSCLC A549 and H1299 cell lines. 
 Interleukin-8 induces the endothelial cell migration through the Rac 1/RhoA-p38MAPK pathway. Endothelial cell migration is essential for tumor angiogenesis, and interleukin-8 (IL-8) has been shown to play an important role in tumor growth, angiogenesis, and   metastasis  . The objective of this study was to investigate the molecular mechanism of IL-8 induced endothelial cell migration in vitro.Fluorescence microscope was used to study the distribution of cytoskeleton. The expression of Rac1 and RhoA protein was detected by western blotting. After endothelial cells were transfected by lipofectamine 2000 reagent, the Transwell chamber motility assay was applied to observe the migration of endothelial cells induced by IL-8. The active p38MAPK (mitogen-activated protein kinase) was evaluated by the p38MAPK activation assay.We demonstrated that IL-8 activated cell migration can be impaired by p38MAPK inhibitor, suggesting the participation of p38MAPK in the cell migration. Our results indicated that p38MAPK signaling is required for membrane ruffles, lamellipodia extensions, and actin stress fibers formation induced by IL-8. Furthermore, p38MAPK inhibitor led to increased Rac1 and RhoA expression in IL-8 treated EA.hy926 cells. In addition, IL-8 induced p38MAPK activation was suppressed by dominant-negative mutant for Rac1 and RhoA.Our study demonstrates that IL-8-Rac1/RhoA-p38MAPK signaling pathway plays a vital role in the IL-8-induced endothelial cell migration, and it provides new insight into the molecular mechanisms by which IL-8 contributes to tumor angiogenesis and   metastasis  . 
 Prognostic role of integrin β1, E-cadherin, and rac1 expression in small cell lung cancer. Integrin β(1) mediates cellular adhesion to the extracellular matrix (ECM) and is correlated with highly invasive and metastatic behavior in small cell lung cancer (SCLC). E-cadherin (ECAD) is a calcium-dependent cell-cell adhesion receptor that restricts invasion of cells and reduce  s metastas  is. Rac1 is involved in the regulation of the actin cytoskeleton, adhesion, migration, invasion, and tumo  r metastas  is. The aim of this study was to examine integrin β(1) , ECAD and rac1 expression in SCLC and to analyze the prognostic value of these markers in patients with SCLC. We analyzed integrin β(1) , ECAD, and rac1 expression in 112 SCLC tissues by immunohistochemical staining. Correlative analyses between integrin β(1) , ECAD, and rac1 expression and cliniopathological factors were performed. A total of 65 patients had extensive disease (ED) (58%), and 47 had limited disease (LD) (42%). The median follow-up duration was 61 months (range: 14-117 months), and the median progression free survival (PFS) and overall survival (OS) were 6.1 months (range: 4.8-7.4 months) and 9.7 months (range: 8.1-11.3 months), respectively. The expression of integrin β(1) , ECAD, and rac1 protein was observed in 64, 73, and 99 of SCLC tissues, respectively. The correlative analyses between integrin β(1) , ECAD, or rac1 expression and various clinical parameters did not show any statistical significance. However, the ECAD expression was associated with OS in the entire cohort. In contrast, the expression of integrin β(1) and rac1 was not associated with PFS or OS. In a subgroup analysis, patients with less t  han two me  tastasis had significantly longer OS (p = 0.047) if their tumors expressed integrin β(1) compared to those without integrin β(1) expression. In addition, OS was longer for patients with ECAD positive tumors compared to those whose tumors did not express ECAD in males (p = 0.032) and patients who never smoked (p < 0.001). Multivariate analysis showed that LD (p = 0.004), overall response rate (p = 0.003), and expression of ECAD (p = 0.015) were the independent good prognostic factors for OS. LD (p = 0.024), overall response rate (p < 0.001), and less   than two   metastasis (p = 0.003) were prognostic factors for longer PFS. These results suggest that ECAD expression may be useful as a prognostic indicator in patients with SCLC. 
 p130Cas promotes invasiveness of three-dimensional ErbB2-transformed mammary acinar structures by enhanced activation of mTOR/p70S6K and Rac1. ErbB2 over-expression is detected in approximately 25% of invasive breast cancers and is strongly associated with poor patient survival. We have previously demonstrated that p130Cas adaptor is a crucial mediator of ErbB2 transformation. Here, we analysed the molecular mechanisms through which p130Cas controls ErbB2-dependent invasion in three-dimensional cultures of mammary epithelial cells. Concomitant p130Cas over-expression and ErbB2 activation enhance PI3K/Akt and Erk1/2 MAPK signalling pathways and promote invasion of mammary acini. By using pharmacological inhibitors, we demonstrate that both signalling cascades are required for the invasive behaviour of p130Cas over-expressing and ErbB2 activated acini. Erk1/2 MAPK and PI3K/Akt signalling triggers invasion through distinct downstream effectors involving mTOR/p70S6K and Rac1 activation, respectively. Moreover, in silico analyses indicate that p130Cas expression in ErbB2 positive human breast cancers significantly correlates with higher risk to develop distant   metastasis  , thus underlying the value of the p130Cas/ErbB2 synergism in regulating breast cancer invasion. In conclusion, high levels of p130Cas favour progression of ErbB2-transformed cells towards an invasive phenotype. 
 Upregulation of Eps8 in oral squamous cell carcinoma promotes cell migration and invasion through integrin-dependent Rac1 activation. Oral squamous cell carcinoma (OSCC) is a lethal disease and early death usually occurs as a result of local invasion and regional lymph node metastases. Current treatment regimens are, to a certain degree, inadequate, with a 5-year mortality rate of around 50% and novel therapeutic targets are urgently required. Using expression microarrays, we identified the eps8 gene as being overexpressed in OSCC cell lines relative to normal oral keratinocytes, and confirmed these findings using RT-PCR and western blotting. In human tissues, we found that Eps8 was upregulated in OSCC (32% of primary tumors) compared with normal oral mucosa, and that expression correlated significantly with lymph node   metastasis   (P=0.032), suggesting a disease-promoting effect. Using OSCC cell lines, we assessed the functional role of Eps8 in tumor cells. Although suppression of Eps8 produced no effect on cell proliferation, both cell spreading and migration were markedly inhibited. The latter cell functions may be modulated through the small GTP-ase, Rac1 and we used pull-down assays to investigate the role of Eps8 in Rac1 signaling. We found that alphavbeta6- and alpha5beta1-integrin-dependent activation of Rac1 was mediated through Eps8. Knockdown of either Eps8 or Rac1, inhibited integrin-dependent cell migration similarly and transient expression of constitutively active Rac1 restored migration of cells in which Eps8 expression had been suppressed. We also showed that knockdown of Eps8 inhibited tumor cell invasion in an organotypic model of OSCC. These data suggest that Eps8 and Rac1 are part of an integrated signaling pathway modulating integrin-dependent tumour cell motility and identify Eps8 as a possible therapeutic target. 
 Rac activation and inactivation control plasticity of tumor cell movement. Tumor cells exhibit two different modes of individual cell movement. Mesenchymal-type movement is characterized by an elongated cellular morphology and requires extracellular proteolysis. In amoeboid movement, cells have a rounded morphology, are less dependent on proteases, and require high Rho-kinase signaling to drive elevated levels of actomyosin contractility. These two modes of cell movement are interconvertible. We show that mesenchymal-type movement in melanoma cells is driven by activation of the GTPase Rac through a complex containing NEDD9, a recently identified melanoma   metastasis   gene, and DOCK3, a Rac guanine nucleotide exchange factor. Rac signals through WAVE2 to direct mesenchymal movement and suppress amoeboid movement through decreasing actomyosin contractility. Conversely, in amoeboid movement, Rho-kinase signaling activates a Rac GAP, ARHGAP22, that suppresses mesenchymal movement by inactivating Rac. We demonstrate tight interplay between Rho and Rac in determining different modes of tumor cell movement, revealing how tumor cells switch between different modes of movement. 
 Epidermal growth factor-regulated activation of Rac GTPase enhances CD44 cleavage by metalloproteinase disintegrin ADAM10. Invasive tumour cells, such as gliomas, frequently express EGF (epidermal growth factor) receptor at a high level and they exhibit enhanced cell migration in response to EGF. We reported previously that tumour cell migration is associated with ectodomain cleavage of CD44, the major adhesion molecule that is implicated in tumour invasion and   metastasis  , and that the cleavage is enhanced by ligation of CD44. In the present study, we show that EGF promotes CD44 cleavage and CD44-dependent cell migration. Introduction of a dominant-negative mutant of the small GTPase Rac1 or depletion of Rac1 by RNAi (RNA interference) abrogated CD44 cleavage induced by EGF. Treatment with PD98059, an inhibitor for MEK (mitogen-activated protein kinase/extracellular-signal-regulated kinase kinase), also suppressed the CD44 cleavage. Furthermore, RNAi studies showed that EGF induced ADAM10 (a disintegrin and metalloproteinase 10)-dependent CD44 cleavage and cell migration. These results indicate that EGF induces ADAM10-mediated CD44 cleavage through Rac1 and mitogen-activated protein kinase activation, and thereby promotes tumour cell migration and invasion. 
 MicroRNA-200b suppresses arsenic-transformed cell migration by targeting protein kinase Cα and Wnt5b-protein kinase Cα positive feedback loop and inhibiting Rac1 activation. MicroRNA-200b (miR-200b) is a member of miR-200 family that has been found to inhibit cell migration and cance  r metastas  is; however, the underlying mechanism is not well understood. We previously reported that miR-200 expression is depleted in arsenic-transformed human bronchial epithelial cells with highly migratory and invasive characteristics, whereas stably re-expressing miR-200b strongly suppresses arsenic-transformed cell migration. This study was performed to investigate how miR-200b inhibits arsenic-transformed cell migration. We found that protein kinase Cα (PKCα) is significantly up-regulated in arsenic-transformed cells. Combining bioinformatics analysis with PKCα 3'-untranslated region vector luciferase reporter assays, we showed that PKCα is a direct target of miR-200b. Inhibiting PKCα activity or knocking down PKCα expression drastically reduced cell migration, phenocoping the inhibitory effect of overexpressing miR-200b. In contrast, forced expression of PKCα in miR-200b overexpressing cells impaired the inhibitory effect of miR-200b on cell migration. In addition, we also found a positive feedback loop between Wnt5b and PKCα in arsenic-transformed cells. Knocking down Wnt5b expression reduced phospho-PKC levels and cell migration; and knocking down PKCα expression decreased Wnt5b level and cell migration. Moreover, forced expression of PKCα increased Wnt5b and phospho-PKC levels and cell migration. Further mechanistic studies revealed that Rac1 is highly activated in arsenic-transformed cells and stably expressing miR-200b abolishes Rac1 activation changing actin cytoskeleton organization. Manipulating PKCα or Wnt5b expression levels significantly altered the level of active Rac1. Together, these findings indicate that miR-200b suppresses arsenic-transformed cell migration by targeting PKCα and Wnt5b-PKCα positive feedback loop and subsequently inhibiting Rac1 activation. 
 Rac1 expression in epithelial ovarian cancer: effect on cell EMT and clinical outcome. Ras-related C3 botulinum toxin substrate 1 (rac1) has been implicated in tumor epithelial-mesenchymal transition (EMT); however, limited information is available regarding the role of rac1 in epithelial ovarian cancer (EOC). This study aimed to evaluate the correlation of rac1 expression with EMT and EOC prognosis. Rac1 protein levels of 150 EOC specimens were evaluated by immunohistochemical staining. Survival analysis was performed to determine the correlation between rac1 expression and survival. Cellular and molecular changes were also examined after rac1 in ovarian cancer cells was silenced in vitro and in vivo. The mechanism of rac1 on EMT was investigated by Western blot analysis. Rac1 was highly expressed in EOC. Rac1 overexpression was closely associated with advanced stage based on International Federation of Gynecology and Obstetrics, poor grade, serum Ca-125, and residual tumor size. Survival analyses demonstrated that patients with high rac1 expression levels were more susceptible to early tumor recurrence with very poor prognosis. This study revealed that rac1 downregulation decreased cell EMT and proliferation capability in vitro and in vivo. Rac1 expression possibly altered cell EMT by interacting with p21-activated kinase 1 and p38 mitogen-activated protein kinase signaling pathways. The present study showed that rac1 overexpression is associated with cell EMT and poor EOC prognosis. Rac1 possibly plays an important role in predicting EOC   metastasis  . 
 Hypoxia/reoxygenation-experienced cancer cell migration and   metastasis   are regulated by Rap1- and Rac1-GTPase activation via the expression of thymosin beta-4. Signaling by small guanosine triphosphatases (GTPase), Rap1/Rac1, is one of the major pathways controlling cancer cell migration and tumor   metastasis  . Thymosin beta-4 (Tβ4), an actin-sequestering protein, has been shown to increase migration of cancer cells. Episodes of hypoxia and re-oxygenation (H/R) are an important phenomenon in tumor microenvironment (TME). We investigated whether Tβ4 could play as an intermediary to crosstalk between Rac1- and Rap1- GTPase activation under hypoxia/reoxygenation (H/R) conditions. Inhibition of Tβ4 expression using transcription activator-like effector nucleases (TALEN) significantly decreased lu  ng metasta  sis of B16F10 cells. Rac1 and Rap1 activity, as well as cancer cell migration, increased following induction of Tβ4 expression in normoxia- or H/R-experienced cells, but were barely detectable in Tβ4-depleted cells. Rap1-regulated Rac1 activity was decreased by a dominant negative Rap1 (Rap1N17), and increased by 8-(4-chloro-phenylthio)-2'-O-methyladenosine-3',5'-cyclic monophosphate (CPT), a Rap1 activator. In contrast, a Rac1-specific inhibitor, NSC23766, and dominant negative Rac1 (Rac1N17) enhanced Tβ4 expression and aberrant Rap1 activity. While NSC23766 and Rac1N17 incompletely inhibited   tumor meta  stasis in vivo, and H/R-experienced cancer cell migration in vitro, more efficient attenuation of cancer cell migration was accomplished by simultaneous inactivation of Rap1 and Rac1 with Rap1N17 and Rac1N17, respectively. These data suggest that a combination therapy targeting both Rap1 and Rac1 activity may be an effective method of inhibiting   tumor meta  stasis. 
 Rac1 is correlated with aggressiveness and a potential therapeutic target for gastric cancer. Rac1 is a member of the Rho GTPase family. Rac1 activity is critical in regulating cytoskeleton organization and thus, modulates a diverse spectrum of cellular functions in normal and malignant cells. The aims of the present study were to investigate the expression pattern and clinical significance of Rac1, as well as the role of Rac1 in gastric cancer tumorigenesis and   metastasis  . The expression of Rac1 in human gastric cancer was explored by immunohistochemistry. The correlation of Rac1 expression with the clinicopathological characteristics and the survival of patients were analyzed by Pearson's Chi-square and Kaplan-Meier analyses, respectively. Rac1 overexpression cell model was used to examine in vitro and in vivo effects of Rac1 in cell growth, migration and invasion. Rac1 was highly expressed in gastric cancer tissues and correlated with differentiation, local invasion, lymph node   metastasis   and Lauren's classification. Rac1 expression in gastric cancer predicted shorter survival. Overexpression of Rac1 in gastric cancer cells dramatically induced Rac1 activation and rendered a more aggressive phenotype such as increased cell growth and migration/invasion in vitro and in vivo. Inhibiting Rac1 activity by specific inhibitor abrogated the effects of Rac1 on the malignant phenotype. Our clinical findings demonstrated that Rac1 was well correlated with aggressiveness and a negative prognostic factor. In addition, our data on experimental cell models supported the fundamental role of Rac1 in gastric cancer. Given its pivotal role in gastric tumorigenesis and progression, Rac1 can serve as a promising therapeutic target for gastric cancer. 
 Methylation-mediated silencing of the miR-124 genes facilitates pancreatic cancer progression and   metastasis   by targeting Rac1. Previous studies have demonstrated that microRNA (miRNA) expression is altered in human cancer. However, the molecular mechanism underlying these changes in miRNA expression remains unclear. In this study, we investigated the epigenetic modification of miR-124 genes and the potential function of miR-124 in pancreatic cancer. Using pyrosequencing analysis, we found that miR-124 genes (including miR-124-1, miR-124-2 and miR-124-3) are highly methylated in pancreatic cancer tissues compared with in non-cancerous tissues. Hypermethylation mediated the silencing of miR-124, which was a frequent event in pancreatic duct adenocarcinoma (PDAC). Furthermore, miR-124 downregulation was significantly associated with worse survival of PDAC patients. Functional studies showed that miR-124 inhibited cell proliferation, invasion and   metastasis  . Furthermore, we characterized Rac1 as a direct target of miR-124, and miR-124 interacted with the 3'-untranslated region of Rac1, which we showed to be a putative tumor promoter in pancreatic cancer. Thus, the miR-124-mediated downregulation of Rac1 led to the inactivation of the MKK4-JNK-c-Jun pathway. Therefore, our study demonstrates that miR-124 is a tumor suppressor miRNA that is epigenetically silenced in pancreatic cancer. Our findings suggest a previously unidentified molecular mechanism involved in the progression and   metastasis   of pancreatic cancer. 
 PI3K, Rac1 and pPAK1 are overexpressed in extramammary Paget's disease. Phosphatidylinositol 3-kinase (PI3K), Ras-related C3 botulinum toxin substrate 1 (Rac1) and P21-activated protein kinase 1 (PAK1) appear to play important roles in the pathogenesis of several tumors, but their expressions in extramammary Paget's disease (EMPD) have not been investigated yet.To investigate the potential contribution of the PI3K, Rac1 and PAK1 to the development of EMPD.Thirty-five paraffin-embedded EMPD specimens were subjected to immunohistochemical staining for PI3K (85α), Rac1 and pPAK1.All the 35 primary EMPD specimens, including 20 non-invasive EMPD, 13 invasive EMPD and 2 metastatic lymph nodes, showed cytoplasm overexpression of PI3K (85α), Rac1 and pPAK1. The expression (% positive cells) of PI3K(85α), Rac1 and pPAK1 (90.1 ± 8.6, 91.4 ± 9.5 and 89.6 ± 10.8% ) in EMPD were significantly higher than in apocrine glands of normal skin ( 20.1 ± 11.9, 29.8 ± 8.9, 41.1 ± 13.4%), and the expression in invasive EMPD with ly  mph node m  etastasis (98.2 ± 1.7, 98.8 ± 0.7 and 98.4 ± 0.9%) are significantly higher than in invasive EMPD without   lymph nod  e metastasis (94.1 ± 2.6, 96.5 ± 1.7 and 95.3 ± 1.1%) and non-invasive EMPD (85.2 ± 8.4, 87.1 ± 9.9 and 83.1 ± 10.6%). There were significant positive correlations of the expression levels between PI3K (85α) and Rac1, as well as between Rac1 and pPAK1 in EMPD.These results indicate that PI3K, Rac1 and PAK1 may play important roles in the pathogenesis of EMPD. 
 ARF1 controls Rac1 signaling to regulate migration of MDA-MB-231 invasive breast cancer cells. ADP-ribosylation factors (ARFs) are monomeric G proteins that regulate many cellular processes such as reorganization of the actin cytoskeleton. We have previously shown that ARF1 is overexpressed in highly invasive breast cancer cells and contribute to their enhanced migration. In this study, we propose to define the molecular mechanism by which ARF1 regulates this complex cellular response by investigating the role of this ARF GTPase on the activation process of Rac1, a Rho GTPase, associated with lamellipodia formation during cell migration. Here, we first show that inhibition of ARF1 or Rac1 expression markedly impacts the ability of MDA-MB-231 cells to migrate upon EGF stimulation. However, the effect of ARF1 depletion can be reversed by overexpression of the Rac1 active mutant, Rac1 Q(61)L. Depletion of ARF1 also impairs the ability of EGF stimulation to promote GTP-loading of Rac1. To further investigate the possible cross-talk between ARF1 and Rac1, we next examined whether they could form a complex. We observed that the two GTPases could directly interact independently of the nature of the nucleotide bound to them. EGF treatment however resulted in the association of Rac1 with its effector IRSp53, which was completely abrogated in ARF1 depleted cells. We present evidences that this ARF isoform is responsible for the plasma membrane targeting of both Rac1 and IRSp53, a step essential for lamellipodia formation. In conclusion, this study provides a new mechanism by which ARF1 regulates cell migration and identifies this GTPase as a promising pharmacological target to reduce   metastasis   formation in breast cancer patients. 
 Positive regulation of migration and invasion by vasodilator-stimulated phosphoprotein via Rac1 pathway in human breast cancer cells. This study aimed to investigate the role of the cytoskeleton-associated protein vasodilator-stimulated phosphoprotein (VASP) on the migration and invasion of human breast cancer cells and its relationship to Rac1 which is a member of the Rho family and has been found to be implicated in tumorigenesis, invasion and   metastasis  . We detected the mRNA and protein expression levels of VASP and Rac1 of the non-invasive breast cancer cell line MCF-7 as well as the invasive cell line MDA-MB-231 by RT-PCR and Western blotting. GST pull-down assay was used to examine the activity of Rac1. Accordingly, the cell invasive migration ability was analyzed in a wound-healing assay (2D) and transwell assays (3D migration and invasion). We then used VASP-siRNA to inhibit the expression of VASP in breast cancer cells in order to study the relationship between the VASP expression level and the invasive migration ability of breast cancer cells. Rac1-siRNA was used to decrease the expression of Rac1, and observe its effect on the VASP expression level together with the migration and invasion ability of MCF-7 and MDA-MB-231 cells. Our results revealed that the invasive breast cancer cell line MDA-MB-231 showed a higher Rac1 activity and VASP expression level compared with the non-invasive MCF-7. Inhibition of Rac1 or VASP by siRNA, respectively, decreased the migration and invasion ability of breast cancer cells and the transfection of Rac1 siRNA-mediated reduction of VASP expression at mRNA and protein levels. Collectively, our data showed that the higher expression level of VASP contributed to a higher invasive migration capacity of human breast cancer cells which was controlled by the Rac1 pathway. 
 Constitutive RAC activation is not sufficient to initiate melanocyte neoplasia but accelerates malignant progression. Deregulated Ras signaling initiates and maintains melanocyte neoplasia. The Rho-like GTPase Rac has been implicated in Ras-induced neoplastic transformation. Moreover, a recurrent UV-induced mutation activating RAC1 has recently been detected in human melanoma. Here, a role for Rac in melanoma initiation and progression was investigated in human melanomas and zebrafish models of melanocyte neoplasia. Immunohistochemical analysis revealed RAC expression and activity restricted to melanocytes at the junction of the epidermis and dermis in benign neoplasms. Malignant melanocytes displayed elevated RAC activity that extended into the suprabasal epidermis, deeper into the dermis, and was maintained in metastases. Previously, we have used zebrafish transgenic models to demonstrate that deregulated Ras/Raf/mitogen-activated protein kinase signaling can initiate melanocyte neoplasia. Expression of a constitutively active RAC1 mutant (V12RAC1) was not sufficient to initiate melanocyte neoplasia in this organism. Furthermore, we did not detect an additive effect when combined with V600EBRAF, nor could V12RAC1 substitute for suppressed Pi3k signaling to restore melanoma progression. However, coexpression of V12RAC1 and oncogenic RAS accelerated tumor nodule formation. Immunohistochemical analysis revealed that the Rac activator Tiam1 (T-cell lymphoma invasion and   metastasis   1) is overexpressed in melanoma tumor nodules in both zebrafish and humans. Thus, our data suggest that Rac contributes to the progression of melanoma and that Tiam1 may activate Rac in nodular presentations. 
 Interleukin-8 induces the endothelial cell migration through the activation of phosphoinositide 3-kinase-Rac1/RhoA pathway. Endothelial cell migration is essential for tumor angiogenesis, and interleukin-8 (IL-8) has been shown to play an important role in tumor growth, angiogenesis, and   metastasis  . This study aimed to investigate the molecular mechanism of IL-8 induced endothelial cell migration. Our results indicated that IL-8 induced a rapid rearrangement of the actin cytoskeleton in EA.Hy926 cells, generating extensions resembling membrane ruffling and stress fibers. These processes required parallel upregulation of the small GTPases Rac1 and RhoA. Moreover, we demonstrated that IL-8 activated PI3K following the same kinetics observed from IL-8 induction of cytoskeletal rearrangement, suggesting the participation of PI3K in these processes. Taken together, our study demonstrates that PI3K-Rac1/RhoA signaling pathway plays a vital role in IL-8 induced endothelial cell migration, and provides new insight into the molecular mechanisms by which IL-8 contributes to tumor angiogenesis and   metastasis  . 
 An anti-Wnt5a antibody suppresses   metastasis   of gastric cancer cells in vivo by inhibiting receptor-mediated endocytosis. Wnt5a is a representative ligand that activates the β-catenin-independent pathway in Wnt signaling. It was reported that the expression of Wnt5a in human gastric cancer is associated with aggressiveness and poor prognosis and that knockdown of Wnt5a reduces the ability of gastric cancer cells to metastasize in nude mice. Therefore, Wnt5a and its signaling pathway might be important targets for the therapy of gastric cancer. The aim of this study was to examine whether an anti-Wnt5a antibody affects   metastasi  s of gastric cancer cells. One anti-Wnt5a polyclonal antibody (pAb5a-5) inhibited migration and invasion activities in vitro of gastric cancer cells with a high expression level of Wnt5a. Previously, it was shown that Wnt5a induces the internalization of receptors, which is required for Wnt5a-dependent activation of Rac1. pAb5a-5 inhibited Wnt5a-dependent internalization of receptors, thereby suppressed Wnt5a-dependent activation of Rac1. Laminin γ2 is one of target genes of Wnt5a signaling and Rac1 was involved in its expression. pAb5a-5 also inhibited Wnt5a-dependent expression of laminin γ2. In an experimental liv  er metasta  sis assay, gastric cancer cells were introduced into the spleens of nude mice. Laminin γ2 was required for liver metastatic ability of gastric cancer cells in vivo. Furthermore, intraperitoneal injection of pAb5a-5 inhibited the metastatic ability of gastric cancer cells. These results suggest that an anti-Wnt5a antibody was capable of suppressing Wnt5a-dependent internalization of receptors, resulting in the prevention   of metast  asis of gastric cancer cells by inhibiting the activation of Rac1 and the expression of laminin γ2. 
 Rac1 targeting suppresses human non-small cell lung adenocarcinoma cancer stem cell activity. The cancer stem cell (CSC) theory predicts that a small fraction of cancer cells possess unique self-renewal activity and mediate tumor initiation and propagation. However, the molecular mechanisms involved in CSC regulation remains unclear, impinging on effective targeting of CSCs in cancer therapy. Here we have investigated the hypothesis that Rac1, a Rho GTPase implicated in cancer cell proliferation and invasion, is critical for tumor initiation and   metastasis   of human non-small cell lung adenocarcinoma (NSCLA). Rac1 knockdown by shRNA suppressed the tumorigenic activities of human NSCLA cell lines and primary patient NSCLA specimens, including effects on invasion, proliferation, anchorage-independent growth, sphere formation and lung colonization. Isolated side population (SP) cells representing putative CSCs from human NSCLA cells contained elevated levels of Rac1-GTP, enhanced in vitro migration, invasion, increased in vivo tumor initiating and lung colonizing activities in xenografted mice. However, CSC activity was also detected within the non-SP population, suggesting the importance of therapeutic targeting of all cells within a tumor. Further, pharmacological or shRNA targeting of Rac1 inhibited the tumorigenic activities of both SP and non-SP NSCLA cells. These studies indicate that Rac1 represents a useful target in NSCLA, and its blockade may have therapeutic value in suppressing CSC proliferation and   metastasis  . 
 Asef2 functions as a Cdc42 exchange factor and is stimulated by the release of an autoinhibitory module from a concealed C-terminal activation element. Asef (herein called Asef1) was identified as a Rac1-specific exchange factor stimulated by adenomatous polyposis coli (APC), contributing to colorectal cancer cell   metastasis  . We investigated Asef2, an Asef1 homologue having a similar N-terminal APC binding region (ABR) and Src-homology 3 (SH3) domain. Contrary to previous reports, we found that Asef1 and Asef2 exchange activity is Cdc42 specific. Moreover, the ABR of Asef2 did not function independently but acted in tandem with the SH3 domain to bind APC. The ABRSH3 also bound the C-terminal tail of Asef2, allowing it to function as an autoinhibitory module within the protein. Deletion of the C-terminal tail did not constitutively activate Asef2 as predicted; rather, a conserved C-terminal segment was required for augmented Cdc42 GDP/GTP exchange. Thus, Asef2 activation involves APC releasing the ABRSH3 from the C-terminal tail, resulting in Cdc42 exchange. These results highlight a novel exchange factor regulatory mechanism and establish Asef1 and Asef2 as Cdc42 exchange factors, providing a more appropriate context for understanding the contribution of APC in establishing cell polarity and migration. 
 Regulation of CXCR4/AKT-signaling-induced cell invasion and tumor   metastasis   by RhoA, Rac-1, and Cdc42 in human esophageal cancer. CXC chemokines and their cognate receptors have been implicated wildly in cancer pathogenesis. In the present study, we report a critical cause relationship between CXCR4 expression and tumorigenesis in the setting of human esophageal squamous cell carcinoma (ESCC). In ESCC cells, CXCR4 expression was significantly higher than in human esophageal epithelial cells (HEEC). Reduction of CXCR4 in ESCC cells reduced cell proliferation and invasion in vitro and tumor growth in vivo. Among the potential downstream targets of CXCR4-CXCL12 are RhoA, Rac-1, and Cdc42, which are likely to contribute to the invasiveness of ESCC cells. Finally, we found that CXCR4-CXCL12/AKT axis regulates RhoA, Rac-1, and Cdc42 to modulate cell invasion and tumor   metastasis  . Together, these results demonstrate a role for CXCR4 in ESCC   metastasis   and progression and suggest potential targets for therapeutic intervention. 
 Tyrosine 397 phosphorylation is critical for FAK-promoted Rac1 activation and invasive properties in oral squamous cell carcinoma cells. Oral squamous cell carcinoma (OSCC) is a common cancer worldwide. Despite advances in diagnosis and therapy, treatment options for patients with metastatic OSCC are few, due in part to the limited understanding of the molecular events involved in the invasion and   metastasis   of OSCC. In this study, we investigated the expression of focal adhesion kinase (FAK) and its tyrosine 397 phosphorylation (pY397) in the tissue specimens of OSCC. The roles of pY397 in regulating the activities of Rac1 and cortactin and the invasive properties of OSCC cells were further determined. Results from immunohistochemical analyses in 9 benign, 19 premalignant, and 19 malignant oral tissues showed that the immunoreactivity of FAK was observed in 5 benign (56%), 19 premalignant (100%), and 18 malignant tissues (95%), whereas the immunoreactivity of pY397 was only found in 1 of 9 (11%) benign lesions but was observed in 9 premalignant (47%) and 12 malignant (63%) lesions. Compared with the low-invading SCC4 cells, the high-invading OECM-1 cells exhibited higher levels of FAK expression and pY397, correlating with higher levels of GTP-bound Rac1 and cortactin phosphorylation. Manipulation of FAK expression or Y397 phosphorylation in SCC4, FaDu, OECM-1, or HSC-3 cells regulated their Rac1 activities and invasive properties. Furthermore, treatment of NSC23766, a Rac1-specific inhibitor, in OECM-1 and HSC-3 cells led to reduced invasive properties. Nevertheless, knockdown of FAK expression or suppression of pY397 had no effect on the cortactin activity in OECM-1 cells. The data collectively suggest that pY397 plays critical roles in the FAK-promoted Rac1 activation and invasive properties in OSCC cells. Thus, the inhibition of FAK phosphorylation at Y397 or Rac1 activity can serve as a therapeutic strategy for treating patients with metastatic OSCC. 
 Balanced Tiam1-rac1 and RhoA drives proliferation and invasion of pancreatic cancer cells. Tiam1 is a rac1-specific guanine nucleotide exchange factor, and Tiam1-rac1 is involved in a number of cellular processes. Rac1 and RhoA act as molecular switches that cycle between GTP- and GDP-bound states to balance the activities of rac1 and RhoA. The downregulation of rac1 activity leads to upregulation of RhoA activity, which promotes invasion and migration of pancreatic cancers cells. At present, however, the role of Tiam1-rac1 and RhoA in pancreatic cancers is not fully understood. We found that Tiam1 was upregulated in pancreatic cancers and was significantly expressed in tumors without lymph node involvement or distant   metastasis   compared with cancers where there was involvement. Although Tiam1-rac1 signaling promoted pancreatic cancer cell proliferation and tumor growth via the Wnt signaling pathway in vitro and in vivo, inhibiting Tiam1-rac1 signaling did not prolong the overall survival time in vivo. This provided evidence that there was a balance between rac1 and RhoA activities in pancreatic cancers. Furthermore, only the combined inhibition of Tiam1-rac1 and RhoA had a beneficial effect on the growth of pancreatic cancers in vivo. Taken together, these results suggest that the progression of pancreatic tumors is partially controlled by the balance between Tiam1-rac1 and RhoA. 
 Rho regulates the hepatocyte growth factor/scatter factor-stimulated cell motility of human oral squamous cell carcinoma cells. To investigate the effects of hepatocyte growth factor/scatter factor (HGF/SF) on the invasion and   metastasis   of human oral squamous cell carcinoma (SCC) cells, we examined cell motility and intercellular signal transduction of a human oral SCC cell line (SAS) obtained from the primary lesion of a tongue carcinoma. HGF/SF stimulation significantly enhanced the motility of SAS cells in a dose-dependent manner. Clostridium botulinum C3 exoenzyme (C3), which is known to selectively impair the function of Ras-related small G-protein p21rho (Rho), significantly reduced the motility of SAS cells. HGF/SF stimulation also enhanced the tyrosine phosphorylation of HGF receptors (c-Met) and focal adhesion kinase (FAK) on SAS cells, but C3 completely inhibited the phosphorylation of FAK. Furthermore, it was observed that Rho A protein, normally located around the nuclear area, was translocated to the membrane and levels in the cytolysate increased following HGF/SF stimulation with no change in Rho A mRNA. These results suggest that the activation of FAK caused by phosphorylation of c-Met may mediate the HGF/SF-induced motility of human oral SCC cells, and that Rho protein regulates the tyrosine phosphorylation of FAK through translocation from the nucleus to the membrane. 
 Rac1-dependent lamellipodial motility in prostate cancer PC-3 cells revealed by optogenetic control of Rac1 activity. The lamellipodium, an essential structure for cell migration, plays an important role in the invasion and   metastasis   of cancer cells. Although Rac1 recognized as a key player in the formation of lamellipodia, the molecular mechanisms underlying lamellipodial motility are not fully understood. Optogenetic technology enabled us to spatiotemporally control the activity of photoactivatable Rac1 (PA-Rac1) in living cells. Using this system, we revealed the role of phosphatidylinositol 3-kinase (PI3K) in Rac1-dependent lamellipodial motility in PC-3 prostate cancer cells. Through local blue laser irradiation of PA-Rac1-expressing cells, lamellipodial motility was reversibly induced. First, outward extension of a lamellipodium parallel to the substratum was observed. The extended lamellipodium then showed ruffling activity at the periphery. Notably, PI(3,4,5)P3 and WAVE2 were localized in the extending lamellipodium in a PI3K-dependent manner. We confirmed that the inhibition of PI3K activity greatly suppressed lamellipodial extension, while the ruffling activity was less affected. These results suggest that Rac1-induced lamellipodial motility consists of two distinct activities, PI3K-dependent outward extension and PI3K-independent ruffling. 
 Increased diacylglycerol kinase ζ expression in human metastatic colon cancer cells augments Rho GTPase activity and contributes to enhanced invasion. Unraveling the signaling pathways responsible for the establishment of a metastatic phenotype in carcinoma cells is critically important for understanding the pathology of cancer. The acquisition of cell motility is a key property of metastatic tumor cells and is a prerequisite for invasion. Rho GTPases regulate actin cytoskeleton reorganization and the cellular responses required for cell motility and invasion. Diacylglycerol kinase ζ (DGKζ), an enzyme that phosphorylates diacylglycerol to yield phosphatidic acid, regulates the activity of the Rho GTPases Rac1 and RhoA. DGKζ mRNA is highly expressed in several different colon cancer cell lines, as well as in colon cancer tissue relative to normal colonic epithelium, and thus may contribute to the metastatic process.To investigate potential roles of DGKζ in ca  ncer metas  tasis, a cellular, isogenic model of human colorectal cancer metastatic transition was used. DGKζ protein levels, Rac1 and RhoA activity, and PAK phosphorylation were measured in the non-metastatic SW480 adenocarcinoma cell line and its highly metastatic variant, the SW620 line. The effect of DGKζ silencing on Rho GTPase activity and invasion through Matrigel-coated Transwell inserts was studied in SW620 cells. Invasiveness was also measured in PC-3 prostate cancer and MDA-MB-231 breast cancer cells depleted of DGKζ.DGKζ protein levels were elevated approximately 3-fold in SW620 cells compared to SW480 cells. There was a concomitant increase in active Rac1 in SW620 cells, as well as substantial increases in the expression and phosphorylation of the Rac1 effector PAK1. Similarly, RhoA activity and expression were increased in SW620 cells. Knockdown of DGKζ expression in SW620 cells by shRNA-mediated silencing significantly reduced Rac1 and RhoA activity and attenuated the invasiveness of SW620 cells in vitro. DGKζ silencing in highly metastatic MDA-MB-231 breast cancer cells and PC-3 prostate cancer cells also significantly attenuated their invasiveness.Elevated DGKζ expression contributes to increased Rho GTPase activation and the enhanced motility of metastatic cancer cells. These findings warrant further investigation of the clinical relevance of DGKζ upregulation in colon and other cancers. Interfering with DGKζ function could provide a means of inhibiting   invasion   and metastasis. 
 Non-small cell lung carcinoma cell motility, rac activation and metastatic dissemination are mediated by protein kinase C epsilon. Protein kinase C (PKC) ε, a key signaling transducer implicated in mitogenesis, survival, and cancer progression, is overexpressed in human primary non-small cell lung cancer (NSCLC). The role of PKCε in lung cance  r metastas  is has not yet been established.Here we show that RNAi-mediated knockdown of PKCε in H358, H1299, H322, and A549 NSCLC impairs activation of the small GTPase Rac1 in response to phorbol 12-myristate 13-acetate (PMA), serum, or epidermal growth factor (EGF). PKCε depletion markedly impaired the ability of NSCLC cells to form membrane ruffles and migrate. Similar results were observed by pharmacological inhibition of PKCε with εV1-2, a specific PKCε inhibitor. PKCε was also required for invasiveness of NSCLC cells and modulated the secretion of extracellular matrix proteases and protease inhibitors. Finally, we found that PKCε-depleted NSCLC cells fail to disseminate to lungs in a mouse   model of m  etastasis.Our results implicate PKCε as a key mediator of Rac signaling and motility of lung cancer cells, highlighting its potential as a therapeutic target. 
 Mechanisms of RhoGDI2 mediated lung cancer epithelial-mesenchymal transition suppression. The aim of this study was to evaluate the function of RhoGDI2 in lung cancer epithelial-mesenchymal transition (EMT) process and to illustrate the underlying mechanisms that will lead to improvement of lung cancer treatment.The RhoGDI2 knock-down and overexpressing A549 cell lines were first constructed. The influence of RhoGDI2 on cytoskeleton in A549 cells was studied using two approaches: G-LISA-based Rac1 activity measurement and immunostaining-based F-actin distribution. The expression levels of key EMT genes were analyzed using real time quantitative polymerase chain reaction (RT-qPCR), western blot and immunostaining in untreated and RhoGDI2 knock-down or overexpressing A549 cells in both in vivo and in vitro experimental settings.Our study showed that the activity of Rac1, a key gene that is crucial for the initiation and   metastasis   of human lung adenocarcinoma, causing the redistribution of F-actin with partial loss of cell-cell adhesions and stress fibers, was significantly suppressed by RhoGDI2. RhoGDI2 promoted the expression of EMT marker gene E-cadherin and repressed EMT promoting genes Slug, Snail, α-SMA in both A549 cells and lung and liver organs derived from the mouse models. Knocking-down RhoGDI2 induced abnormal morphology for lung organs.These findings indicate that RhoGDI2 repressed the activity of Rac1 and may be involved in the rearrangement of cytoskeleton in lung cancer cells. RhoGDI2 suppresses the   metastasi  s of lung cancer mediated through EMT by regulating the expression of key genes such as E-cadherin, Slug, Snail and α-SMA in both in vivo and in vitro models. 
 PKCα phosphorylation of RhoGDI2 at Ser31 disrupts interactions with Rac1 and decreases GDI activity. Rho family GTPases control a diverse range of cellular processes, and their deregulation has been implicated in human cancer. Guanine nucleotide dissociation inhibitors (GDIs) bind and sequester GTPases in the cytosol, restricting their actions. RhoGDI2 is a member of the GDI family that acts as a   metastasi  s suppressor in a variety of cancer types; however, very little is known about the regulation of this protein. Here, we present a mechanism for inactivation of RhoGDI2 via protein kinase C (PKC) phosphorylation of Ser31 in a region that contacts GTPases. In cells, RhoGDI2 becomes rapidly phosphorylated at Ser31 in response to phorbol 12-myristate 13-acetate stimulation. Based on the effects of pharmacological inhibitors and knockdown by siRNA, we determine that conventional type PKCα is responsible for this phosphorylation. Phospho-mimetic S31E-RhoGDI2 exhibits reduced binding to Rac1 relative to wild type, with a concomitant failure to reduce levels of activated endogenous Rac1 or remove Rac1 from membranes. These results reveal a mechanism of downregulation of RhoGDI2 activity through PKC-mediated phosphorylation of Ser31. We hypothesize that this mechanism may serve to neutralize RhoGDI2 function in tumors that express RhoGDI2 and active PKCα. 
 Podocalyxin EBP50 ezrin molecular complex enhances the metastatic potential of renal cell carcinoma through recruiting Rac1 guanine nucleotide exchange factor ARHGEF7. Podocalyxin was initially identified in glomerular podocytes to critically maintain the structural and functional integrity of the glomerular ultrafiltrative apparatus. Lately, it has emerged as a malignant marker in tumors arising from a variety of tissue origins. By immunohistochemistry, we identified that 9.6% of renal cell carcinoma patients overexpress this protein. This subset of patients had significantly shorter disease-specific and overall survivals, and, importantly, we established podocalyxin overexpression as an independent prognostic factor for latent distant   metastasis   with multivariate analysis. Podocalyxin down-regulation by small interfering RNA led to defective migration in model renal tubular cells, which was corrected by re-expression of podocalyxin. The activity of the small GTPase Rac1, a well-characterized modulator of cell migration, was diminished by podocalyxin knock-down. Conversely, podocalyxin overexpression in human embryonic kidney cells up-regulated Rac1 activity, which depended on a complex formed by podocalyxin, ERM-binding phosphoprotein 50, ezrin, and ARHGEF7, a Rac1 activator. Therefore, podocalyxin can serve as a biomarker to identify renal cell carcinoma patients with higher metastatic potential for more aggressive intervention at earlier clinical stages. 
 A Trio-Rac1-Pak1 signalling axis drives invadopodia disassembly. Rho family GTPases control cell migration and participate in the regulation of cancer   metastasis  . Invadopodia, associated with invasive tumour cells, are crucial for cellular invasion and   metastasis  . To study Rac1 GTPase in invadopodia dynamics, we developed a genetically encoded, single-chain Rac1 fluorescence resonance energy (FRET) transfer biosensor. The biosensor shows Rac1 activity exclusion from the core of invadopodia, and higher activity when invadopodia disappear, suggesting that reduced Rac1 activity is necessary for their stability, and Rac1 activation is involved in disassembly. Photoactivating Rac1 at invadopodia confirmed this previously unknown Rac1 function. We describe here an invadopodia disassembly model, where a signalling axis involving TrioGEF, Rac1, Pak1, and phosphorylation of cortactin, causes invadopodia dissolution. This mechanism is critical for the proper turnover of invasive structures during tumour cell invasion, where a balance of proteolytic activity and locomotory protrusions must be carefully coordinated to achieve a maximally invasive phenotype. 
 Thymosin beta 4 induces colon cancer cell migration and clinical   metastasis   via enhancing ILK/IQGAP1/Rac1 signal transduction pathway. Thymosin β(4) (Tβ(4)) overexpression increases cell migration and tumo  r metastas  is. Hence, understanding the mechanism of cancer cell migration induced by Tβ(4) may provide means to inhibit the  ir metasta  sis. We demonstrated higher Rac1 activities and expression levels of IQGAP1 and ILK in highly migrated Tβ(4)-overexpressing SW480 cells. In addition, IQGAP1 formed a complex with ILK and knockdown of Tβ(4) simultaneously reduced ILK and IQGAP1 protein levels as well as their migration ability. These findings suggest that Tβ(4) increases migration of colon cancer cells via activating Rac1 by elevating IQGAP1/ILK complexes and IHC results illustrated a similar mechanism occurring in vivo. 
 N-α-acetyltransferase 10 protein suppresses cancer cell   metastasi  s by binding PIX proteins and inhibiting Cdc42/Rac1 activity. N-α-acetyltransferase 10 protein, Naa10p, is an N-acetyltransferase known to be involved in cell cycle control. We found that Naa10p was expressed lower in varieties of malignancies with lymph nod  e metastas  is compared with non-lymph nod  e metastas  is. Higher Naa10p expression correlates the survival of lung cancer patients. Naa10p significantly suppressed migration, tumor growth, an  d metastas  is independent of its enzymatic activity. Instead, Naa10p binds to the GIT-binding domain of PIX, thereby preventing the formation of the GIT-PIX-Paxillin complex, resulting in reduced intrinsic Cdc42/Rac1 activity and decreased cell migration. Forced expression of PIX in Naa10-transfected tumor cells restored the migration an  d metastas  is ability. We suggest that Naa10p functions as a tumo  r metastas  is suppressor by disrupting the migratory complex, PIX-GIT- Paxillin, in cancer cells. 
 Inhibition of tumor cell migration and invasion through knockdown of Rac1 expression in medulloblastoma cells. Medulloblastoma is one of the common malignant brain tumors in children or young adults and its overall disease-free 5-year survival rate is approximately 50% due to tumor progression, invasion, and   metastasis  . This study aimed to determine whether one of Rho GTPases, Rac1 can affect the morphology, motility, and invasion of medulloblastoma cells through knockdown of Rac1 expression. Medulloblastoma Daoy cells were used to manipulate Rac1 expression using Rac1 shRNA, Rac1N17, and Rac1L61 constructs. Reverse transcriptase-PCR and western blot were used to detect expression of Rac1 mRNA and protein, respectively. Invasion and migration assays were performed to assess invasion and migration capacity of Daoy cells, respectively. The data showed that Rac1 mRNA and protein were overexpressed in Daoy cells. Deletion of Rac1 decreased the cross-linked actin network and pseudopodia and also inhibited the number of migration cells migrated or invaded to the other side of the filter compared to control cells. These data indicated that the invasion and migration in Daoy cells were inhibited by deletion of Rac1, and suggest that targeting Rac1 by Rac1 shRNA may further be evaluated and used as a potential anticancer strategy to treat medulloblastoma. 
 Rac regulates the interaction of fascin with protein kinase C in cell migration. Fascin is an actin-bundling protein that is low or absent in normal epithelia; its upregulation correlates with poor prognosis in many human carcinomas. We have recently demonstrated in mouse xenograft models that fascin contributes to tumour development and   metastasis   through its dual actin-bundling and active PKC-binding activities. Rac was implicated as a regulator of fascin-dependent colon carcinoma cell migration in vitro. Here, we tested the hypothesis that Rac regulates the interaction of fascin with active PKC. The major conventional PKC in colon carcinoma cells is protein kinase Cgamma (PKCgamma). Endogenous PKCgamma, fascin and Rac1 colocalised at lamellipodial margins of migrating cells. Colocalisation of fascin and PKCgamma depended on Rac activity, and inhibition of Rac decreased PKCgamma activity in cell extracts but not in vitro. Fluorescence resonance energy transfer/fluorescence lifetime imaging microscopy uncovered that fascin and PKCgamma interact in protrusions and filopodia of migrating cells. Mechanistically, the interaction depended on phosphorylated fascin, active PKCgamma and active Rac, but not on active Cdc42. The activity of Rac on the fascin/PKC complex was mediated in part by Pak. Elucidation of this novel pathway for regulation of the fascin/PKCgamma complex in migrating carcinoma cells suggests novel targets for therapeutic intervention in   metastasis  . 
 [Biological significance of relationship between nuclear localization of Rac1 and progression of gastric carcinoma]. To observe the subcellular localization of Rac1 and the expression of Tiam1 and Rac1 in gastric carcinoma, in order to reveal the relationship between the distribution of Rac1 and progression of gastric carcinoma.Both carcinoma and adjacent normal tissue of 48 patients with gastric carcinoma were studied in this study. Tissue distribution and expression of Rac1 and Tiam1 were analyzed by immunohistochemistry and real-time polymerase chain reaction (PCR).Compared with that of adjacent non-cancerous gastric mucosa, the expression of Rac1 in cancer tissues was significantly increased. The positive rate of Rac1 expression was 18.8% (9/48 cases) in adjacent non-neoplastic gastric and 79.2% (38/48 cases) in cancer tissues. The positive staining was mainly located in the cell nuclei (31 samples). The real-time PCR results demonstrated that the expression levels of Tiam1 and Rac1 mRNA in cancerous tissues with nuclear localization of Rac1 were evidently increased. Furthermore, nuclear localization of Rac1 was associated with tumor stage and   metastasis  .The majority of gastric cancer tissues show nuclear dislocalization of Rac1 expression, which may be a sign of abnormal activation of Tiam1-Rac1 pathway. It may suggest enhanced invasion ability of the gastric carcinoma. 
 14-3-3zeta Cooperates with ErbB2 to promote ductal carcinoma in situ progression to invasive breast cancer by inducing epithelial-mesenchymal transition. ErbB2, a   metastasis  -promoting oncoprotein, is overexpressed in approximately 25% of invasive/metastatic breast cancers, but in 50%-60% of noninvasive ductal carcinomas in situ (DCIS). It has been puzzling how a subset of ErbB2-overexpressing DCIS develops into invasive breast cancer (IBC). We found that co-overexpression of 14-3-3zeta in ErbB2-overexpressing DCIS conferred a higher risk of progression to IBC. ErbB2 and 14-3-3zeta overexpression, respectively, increased cell migration and decreased cell adhesion, two prerequisites of tumor cell invasion. 14-3-3zeta overexpression reduced cell adhesion by activating the TGF-beta/Smads pathway that led to ZFHX1B/SIP-1 upregulation, E-cadherin loss, and epithelial-mesenchymal transition. Importantly, patients whose breast tumors overexpressed both ErbB2 and 14-3-3zeta had higher rates of metastatic recurrence and death than those whose tumors overexpressed only one. 
 Basic fibroblast growth factor in the bone microenvironment enhances cell motility and invasion of Ewing's sarcoma family of tumours by activating the FGFR1-PI3K-Rac1 pathway. Ewing's sarcoma family of tumours (ESFT) is a malignant small round-cell tumour of the bone and soft tissues. It is characterised by a strong tendency to invade and form metastases. The microenvironment of the bone marrow is a large repository for many growth factors, including the basic fibroblast growth factor (bFGF). However, the role of bFGF in the invasive and metastatic phenotype of ESFT has not been investigated.The motility and invasion of ESFT cells were assessed by a wound-healing assay, chemotaxis assay, and invasion assay. The expression and activation of FGF receptors (FGFRs) in ESFT cell lines and clinical samples were detected by RT-PCR, western blotting, and immunohistochemistry. The morphology of ESFT cells was investigated by phase-contrast microscopy and fluorescence staining for actin. Activation of Rac1 was analysed by a pull-down assay.bFGF strongly induced the motility and invasion of ESFT cells. Furthermore, FGFR1 was found to be expressed and activated in clinical samples of ESFT. Basic FGF-induced cell motility was mediated through the FGFR1-phosphatidylinositol 3-kinase (PI3K)-Rac1 pathway. Conditioned medium from bone marrow stromal cells induced the motility of ESFT cells by activating bFGF/FGFR1 signalling.The bFGF-FGFR1-PI3K-Rac1 pathway in the bone microenvironment may have a significant role in the invasion and   metastasis   of ESFT. 
 KAI1/CD82 decreases Rac1 expression and cell proliferation through PI3K/Akt/mTOR pathway in H1299 lung carcinoma cells. Although the KAI1/CD82 protein has been reported to inhibit cell   metastasis   in many studies, its mechanism of action has not yet been fully elucidated. In the present study, we investigated the possible effects of KAI1/CD82 on the metastatic phenotype in H1299 lung carcinoma cells. These studies were based on the pivotal role that the acquisition of motile phenotype plays on the initial steps of   metastasis  . KAI1/CD82-mediated morphological changes were observed using phase contrast microscopy. We report here, that a KAI1/CD82-induced phenotypic change was involved in the decrease of Rac1 expression and GTPase activity. However, we found that KAI1/CD82 did not regulate Rac1 mRNA levels. This suggests the existence of another regulatory mechanism of Rac1 protein maturation or activation. To identify the signaling pathway of Rac1 regulation, we investigated the PI3K/Akt/mTOR pathway, since the PI3K/Akt pathway regulates Rac1 activation and mTOR is known to play a regulatory role in protein translation. H1299/CD82-transfectants showed lower mTOR expression and cell growth than the control group. The data obtained from this study suggested that KAI1/CD82 decreased the metastatic phenotype of H1299 lung carcinoma cells by down-regulating Rac1 expression through the PI3K/Akt/mTOR pathway. 
 Expression of Rac-1 related to tumor depth, lymph node   metastasis   and patient prognosis in esophageal squamous cell carcinoma. Rac-1, which is a member of the Rho guanosine triphosphatase (GTPase) family, has been demonstrated to play an important role in cancer invasion and   metastasis  . In this study, we investigated the clinical and prognostic significance of Rac-1 in esophageal squamous cell carcinoma (ESCC). The protein and messenger ribonucleic acid (mRNA) levels of Rac-1 in normal esophageal epithelia cells and paired ESCC tissues were examined by Western blot and reverse transcription polymerase chain reaction. The results showed that Rac-1 was upregulated at the protein and mRNA levels in ESCC cancerous specimens compared with normal esophageal tissues. We then examined the correlation between Rac-1 expression and clinicopathological features using immunochemical analysis of 233 surgically resected ESCC. Rac-1 protein was expressed in 228 (97.85%) cancer tissues with cytoplasm staining, and there were significant correlations between Rac-1 expression and tumor location (P = 0.045), tumor stage (P = 0.020), tumor depth (P = 0.023) and lymph node   metastasis   (P = 0.009). The overall survival and disease-free rates of ESCC patients with high Rac-1 expression were much lower than those with low Rac-1 expression (P < 0.001; P < 0.001, respectively). Multivariate analysis showed that high Rac-1 expression and lymph node   metastasis   were two independent factors for poor survival (P < 0.001; P < 0.001, respectively). The results in this study indicate, for the first time, that Rac-1 is involved in the invasion and metastatic progression of ESCC and may be a potential marker for evaluating the prognosis of ESCC patients and a therapy target for ESCC. 
 Podocalyxin-like 1 promotes invadopodia formation and   metastasis   through activation of Rac1/Cdc42/cortactin signaling in breast cancer cells. Metastatic disease is the leading cause of cancer mortality. Identifying biomarkers and regulatory mechanisms is important toward developing diagnostic and therapeutic tools against metastatic cancer. In this study, we demonstrated that podocalyxin-like 1 (PODXL) is overexpressed in breast tumor cells and increased in lymph node metastatic cancer. Mechanistically, we found that the expression of PODXL was associated with cell motility and invasiveness. Suppression of PODXL in MDA-MB-231 cells reduced lamellipodia formation and focal adhesion kinase (FAK) and paxillin phosphorylation. PODXL knockdown reduced the formation of invadopodia, such as inhibiting the colocalization of F-actin with cortactin and suppressing phosphorylation of cortactin and neural Wiskott-Aldrich syndrome protein. Conversely, overexpression of PODXL in MCF7 cells induced F-actin/cortactin colocalization and enhanced invadopodia formation and activation. Invadopodia activity and tumor invasion in PODXL-knockdown cells are similar to that in cortactin-knockdown cells. We further found that the DTHL motif in PODXL is crucial for regulating cortactin phosphorylation and Rac1/Cdc42 activation. Inhibition of Rac1/Cdc42 impeded PODXL-mediated cortactin activation and FAK and paxillin phosphorylation. Moreover, inhibition of PODXL in MDA-MB-231 cells significantly suppressed tumor colonization in the lungs and distant metastases, similar to those in cortactin-knockdown cells. These findings show that overexpression of PODXL enhanced invadopodia formation and tumor   metastasis   by inducing Rac1/Cdc42/cortactin signaling network. 
 Astragaloside IV inhibits breast cancer cell invasion by suppressing Vav3 mediated Rac1/MAPK signaling. Astragaloside IV (AS-IV), the major active triterpenoid in Radix Astragali, has shown anti-tumorigenic properties in certain cancers; however, its role in breast cancer remains unclear. The present study investigated the effects of AS-IV on breast cancer in vitro and in vivo and examined the underlying mechanisms.The effects of AS-IV on MDA-MB-231 cell proliferation, migration, invasion and   metastasis   were investigated by MTT and Transwell assays, and western blotting. In addition, an orthotopic mouse tumor model was established for in vivo experiments.AS-IV inhibited the viability and invasive potential of MDA-MB-231 breast cancer cells, suppressed the activation of the mitogen activated protein kinase (MAPK) family members ERK1/2 and JNK, and downregulated matrix metalloproteases (MMP)-2 and -9. The effects of AS-IV were mediated by the downregulation of Vav3, a guanine nucleotide exchange factor, leading to decreased levels of activated Rac1, a Rho family GTPase. Vav3 overexpression promoted cell proliferation and invasion in vitro, whereas Vav3 silencing had the opposite effects. AS-IV suppressed orthotopic breast tumor growth and   metastasis   to the lungs, whereas ectopic expression of Vav3 reversed the inhibitory effect of AS-IV on cell viability, invasiveness, MAPK signaling and MMP expression.The present results provide a mechanistic explanation for the antitumor effects of AS-IV and suggest its potential in the treatment of metastatic breast cancer. 
 Role of Rac1 and Cdc42 in hypoxia induced p53 and von Hippel-Lindau suppression and HIF1alpha activation. Low oxygen tension can influence tumor progression by enhancing angiogenesis, a process that may involve Rho GTPases whose activities have been implicated in tumorigenesis and   metastasis  . In the present study, we show that hypoxia can increase the mRNA levels and intracellular activities of Rac1 and Cdc42 in a time-dependent manner. The hypoxia-stimulated activities of Rac1 and Cdc42 could be blocked by the phosphatidylinositol 3'-kinase (PI3K) inhibitor LY294002 and the protein tyrosine kinase (PTK) inhibitor genistein but were not affected by the p38MAPK inhibitor SB203580 or the MEK-1 inhibitor PD98059, suggesting that the hypoxia-mediated signals were through PI3K and PTK. Correlating with the increased activities of Rac1 and Cdc42, the expression of the pro-angiogenesis factors HIF-1alpha and vascular endothelial growth factor (VEGF) was upregulated by hypoxia, whereas the expression of the tumor suppressors von Hippel-Lindau and p53 was down-regulated. Dominant negative N17Rac1 and N17Cdc42 could upregulate the expression of p53 and pVHL but downregulate that of HIF-1alpha and VEGF under hypoxia. Furthermore, the preconditioned medium from N17Rac1 or N17Cdc42-expressing gastric cancer cells was able to inhibit the proliferation of HUVECs. Our results indicate that PI3K and PTK-mediated activations of Rac1 and Cdc42 are involved in the hypoxia-induced production of angiogenesis-promoting factors and tumor suppressors, and suggest that the Rho family GTPases Rac1 and Cdc42 may contribute to the hypoxia-mediated angiogenesis. 
 Association between Gαi2 and ELMO1/Dock180 connects chemokine signalling with Rac activation and   metastasi  s. The chemokine CXCL12 and its G-protein-coupled receptor CXCR4 control the migration, invasiveness and   metastasi  s of breast cancer cells. Binding of CXCL12 to CXCR4 triggers activation of heterotrimeric Gi proteins that regulate actin polymerization and migration. However, the pathways linking chemokine G-protein-coupled receptor/Gi signalling to actin polymerization and cancer cell migration are not known. Here we show that CXCL12 stimulation promotes interaction between Gαi2 and ELMO1. Gi signalling and ELMO1 are both required for CXCL12-mediated actin polymerization, migration and invasion of breast cancer cells. CXCL12 triggers a Gαi2-dependent membrane translocation of ELMO1, which associates with Dock180 to activate small G-proteins Rac1 and Rac2. In vivo, ELMO1 expression is associated with lymph node and dista  nt metasta  sis, and knocking down ELMO1 impai  rs metasta  sis to the lung. Our findings indicate that a chemokine-controlled pathway, consisting of Gαi2, ELMO1/Dock180, Rac1 and Rac2, regulates the actin cytoskeleton during breast can  cer metast  asis. 
 Increased Rac1 activity and Pak1 overexpression are associated with lymphovascular invasion and lymph node   metastasis   of upper urinary tract cancer. Lymphovascular invasion (LVI) and lymph node   metastasis   are conventional pathological factors associated with an unfavorable prognosis of urothelial carcinoma of the upper urinary tract (UC-UUT), but little is known about the molecular mechanisms underlying LVI and nodal   metastasis   in this disease. Rac1 small GTPase (Rac1) is essential for tumor   metastasis  . Activated GTP-bound Rac1 (Rac1 activity) plays a key role in activating downstream effectors known as Pak (21-activated kinase), which are key regulators of cytoskeletal remolding, cell motility, and cell proliferation, and thus have a role in both carcinogenesis and tumor invasion.We analyzed Rac1 activity and Pak1 protein expression in matched sets of tumor tissue, non-tumor tissue, and metastatic lymph node tissue obtained from the surgical specimens of 108 Japanese patients with UC-UUT.Rac1 activity and Pak1 protein levels were higher in tumor tissue and metastatic lymph node tissue than in non-tumor tissue (both P < 0.0001). A high level of Rac1 activity and Pak1 protein expression in the primary tumor was related to poor differentiation (P < 0.05), muscle invasion (P < 0.01), LVI (P < 0.0001), and lymph node   metastasis   (P < 0.0001). Kaplan-Meier survival analysis showed that an increase of Rac1 activity and Pak1 protein was associated with a shorter disease-free survival time (P < 0.01) and shorter overall survival (P < 0.001). Cox proportional hazards analysis revealed that high Rac1 activity, Pak1 protein expression and LVI were independent prognostic factors for shorter overall and disease-free survival times (P < 0.01) on univariate analysis, although only Pak1 and LVI had an influence (P < 0.05) according to multivariate analysis.These findings suggest that Rac1 activity and Pak1 are involved in LVI and lymph node   metastasis   of UC-UUT, and may be prognostic markers for this disease. 
 Semaphorin-3F suppresses the stemness of colorectal cancer cells by inactivating Rac1. Tumor cell stemness has been recognized as a key contributor to tumor initiation, progression and recurrence. Our previous studies have found that semaphorin-3F (SEMA3F), an axon guidance molecule in the development of central nervous system, inhibited the growth and   metastasis   of colorectal cancer (CRC). However, a possible role for SEMA3F in regulating cancer cell stemness remains unknown. Here, we report a novel mechanism of the acquirement of stemness of CRC cells regulated by SEMA3F. Knockdown of SEMA3F significantly promoted the self-renewal and tumorigenicity of CRC cells, and increased the expression of stemness-associated genes, while overexpressing SEMA3F reduced the stemness of CRC cells. Mechanistically, GTP-Rac1 was involved in SEMA3F mediated regulation of CRC cell stemness by targeting the Wnt/β-catenin pathway. Clinically, GTP-Rac1 expression was inversely correlated with SEMA3F levels in CRC samples and patients with SEMA3F(low)/GTP-Rac1(high) CRC showed poorer prognosis. Our findings demonstrate the ability of SEMA3F to inhibit the stemness of human CRC cells by suppressing Rac1 activation, which suggests a novel therapeutic approach for CRC. 
 Crystal structure of Rac1 in complex with the guanine nucleotide exchange region of Tiam1. The principal guanine nucleotide exchange factors for Rho family G proteins contain tandem Dbl-homology (DH) and pleckstrin-homology (PH) domains that catalyse nucleotide exchange and the activation of G proteins. Here we have determined the crystal structure of the DH and PH domains of the T-lymphoma invasion and   metastasis   factor 1 (Tiam1) protein in complex with its cognate Rho family G protein, Rac1. The two switch regions of Rac1 are stabilized in conformations that disrupt both magnesium binding and guanine nucleotide interaction. The resulting cleft in Rac1 is devoid of nucleotide and highly exposed to solvent. The PH domain of Tiam1 does not contact Rac1, and the position and orientation of the PH domain is markedly altered relative to the structure of the uncomplexed, GTPase-free DH/PH element from Sos1. The Tiam1/Rac1 structure highlights the interactions that catalyse nucleotide exchange on Rho family G proteins, and illustrates structural determinants dictating specificity between individual Rho family members and their associated Dbl-related guanine nucleotide exchange factors. 
 Abnormal expression of p120-catenin, E-cadherin, and small GTPases is significantly associated with malignant phenotype of human lung cancer. Studies on a variety of cell lines have shown that p120-catenin can directly regulate the stability of E-cadherin complexes and control the activity of small GTPases to influence cell adhesion. Despite this data, clinical studies of human solid tumors have not been reported to investigate these protein interactions. To explore the correlation between p120-catenin, E-cadherin, and small GTPases in human lung cancer, we examined the expression patterns of p120-catenin, E-cadherin, RhoA, Cdc42, and Rac1, and their prognostic significance in 138 patients with non-small cell lung cancer (NSCLC). While normal bronchial epithelium showed strong membrane expression of p120-catenin and E-cadherin, lung cancer tissues had reduced membrane expression and ectopic cytoplasmic expression of p120-catenin and E-cadherin. Expression of RhoA, Cdc42, and Rac1 was also found to be higher in tumor tissue than in normal lung tissue. A correlation between abnormal p120-catenin, E-cadherin expression, and overexpression of specific small GTPases was also associated with poor differentiation, high TNM stage, and lymph node   metastasis   in NSCLC patients. We also used an in vitro model to evaluate their expression, and to determine whether protein expression correlated with the invasive capacity of lung cancer cell lines. Consistent with our in vivo data, abnormal expression of p120-catenin and E-cadherin with overexpression of specific small GTPases were significantly associated with the high metastatic capacity of BE1 cells. Based on our results, we conclude that abnormal p120-catenin expression correlates with abnormal E-cadherin expression and specific small GTPase overexpression, which contribute to the malignancy-related to NSCLC. 
 mTOR regulate EMT through RhoA and Rac1 pathway in prostate cancer. Recently, an increasing number of studies have suggested that mTOR plays a critical role in the regulation of tumor cell motility, invasion and cancer   metastasis  . However, little is known about the signaling mechanisms in regulating epithelial-mesenchymal transition (EMT) of prostate cancer. In this study, we found that the expression levels of Raptor and Rictor in prostate cancer tissues were elevated, which may suggest that Raptor and Rictor signaling pathways are associated with prostate cancer progression and   metastasis  . Inhibition of mTORC1 or mTORC2 by knock down of Raptor or Rictor, respectively, migration and invasion of prostate cancer were attenuated. Furthermore, EMT, a characterized by the changed expression levels of various markers like E-cadherin, β-catenin, N-cadherin, and vimentin emergend following inhibition of Raptor or Rictor. Finally, the small GTPases (RhoA and Rac1) which were crucial regulatory proteins in cell migration and invasion were inactivited after downregulating Raptor and Rictor. These results suggest that mTOR regulate EMT at least in part by down regulation of RhoA and Rac1 signaling pathways. Our findings provide novel very attractive target strategies that the inhibition of mTOR signaling pathways may retard prostate cancer migration and invasion at early stages. 
 A MAPK-Driven Feedback Loop Suppresses Rac Activity to Promote RhoA-Driven Cancer Cell Invasion. Cell migration in 3D microenvironments is fundamental to development, homeostasis and the pathobiology of diseases such as cancer. Rab-coupling protein (RCP) dependent co-trafficking of α5β1 and EGFR1 promotes cancer cell invasion into fibronectin (FN) containing extracellular matrix (ECM), by potentiating EGFR1 signalling at the front of invasive cells. This promotes a switch in RhoGTPase signalling to inhibit Rac1 and activate a RhoA-ROCK-Formin homology domain-containing 3 (FHOD3) pathway and generate filopodial actin-spike protrusions which drive invasion. To further understand the signalling network that drives RCP-driven invasive migration, we generated a Boolean logical model based on existing network pathways/models, where each node can be interrogated by computational simulation. The model predicted an unanticipated feedback loop, whereby Raf/MEK/ERK signalling maintains suppression of Rac1 by inhibiting the Rac-activating Sos1-Eps8-Abi1 complex, allowing RhoA activity to predominate in invasive protrusions. MEK inhibition was sufficient to promote lamellipodia formation and oppose filopodial actin-spike formation, and led to activation of Rac and inactivation of RhoA at the leading edge of cells moving in 3D matrix. Furthermore, MEK inhibition abrogated RCP/α5β1/EGFR1-driven invasive migration. However, upon knockdown of Eps8 (to suppress the Sos1-Abi1-Eps8 complex), MEK inhibition had no effect on RhoGTPase activity and did not oppose invasive migration, suggesting that MEK-ERK signalling suppresses the Rac-activating Sos1-Abi1-Eps8 complex to maintain RhoA activity and promote filopodial actin-spike formation and invasive migration. Our study highlights the predictive potential of mathematical modelling approaches, and demonstrates that a simple intervention (MEK-inhibition) could be of therapeutic benefit in preventing invasive migration   and metast  asis. 
 16-kDa prolactin inhibits endothelial cell migration by down-regulating the Ras-Tiam1-Rac1-Pak1 signaling pathway. Angiogenesis plays a key role in promoting tumorigenesis and   metastasis  . The 16-kDa fragment of prolactin (16k PRL) is an NH(2)-terminal natural breakdown fragment of the intact 23-kDa prolactin and has been shown to have potent antiangiogenic and antitumor activities. The mechanism(s) involved in the action of 16k PRL in endothelial cells remains unclear. In this study, we showed that 16k PRL reduced rat aortic endothelial cell (RAEC) migration in a wound-healing assay and in a Matrigel tube formation assay, suggesting that 16k PRL inhibits endothelial cell migration, an important activity involved in angiogenesis and tumorigenesis. We further investigated how 16k PRL attenuates endothelial cell migration. We first showed that RAEC migration is mediated through the Rho GTPase Rac1, as Rac1 inhibition by the Rac1-specific inhibitor NSC27366 or Rac1 knockdown by small interfering RNA both blocked RAEC migration. We next showed that 16k PRL reduced the activation of Rac1 in a concentration-dependent manner. Furthermore, 16k PRL inhibition of Rac1 is mediated through the suppression of T lymphoma invasion and   metastasis   1 (Tiam1) and its upstream activator Ras in a phosphoinositide-3-kinase-independent manner. 16k PRL also down-regulated the phosphorylation of the downstream effector of Rac1, p21-activating kinase 1 (Pak1), and inhibited its translocation to the leading edge of migrating cells. Thus, 16k PRL inhibits cell migration by blocking the Ras-Tiam1-Rac1-Pak1 signaling pathway in endothelial cells. 
 Small interfering RNA targeting Rac1 sensitizes colon cancer to dihydroartemisinin-induced cell cycle arrest and inhibited cell migration by suppressing NFκB activity. Dihydroartemisinin (DHA) has recently shown antitumor activity in various cancer cells. The small GTPase Rac1 regulates many cellular processes, including cytoskeletal reorganization, cell migration, proliferation, and survival. In addition, Rac1 plays a major role in activating NFκB-mediated transcription. Both Rac1 and NFκB regulate many properties of the malignant phenotype, including anchorage-independent proliferation and surviva  l, metasta  sis, and angiogenesis. In this study, for the first time, we demonstrated that Rac1 knockdown can enhance DHA-induced growth inhibition, cell cycle arrest, apoptosis, and migration in both HCT116 and RKO cell lines in vitro. The mechanism is due partially to DHA, and Rac1 siRNA deactivates NFκB activity, so as to decrease tremendously the expression of its target gene products, such as PCNA, cyclin D1, and CDK4; and increase the expression of p21, cleaved-caspase-3, and cleaved-PARP. In our in vivo studies, DHA also manifested remarkably enhanced antitumor effect when combined with Rac1 siRNA. We concluded that inhibition of NFκB activation is one of the mechanisms that Rac1 siRNA dramatically promotes DHAs antitumor effect on human colon cancer. 
 The role of PTEN in prostate cancer cell tropism to the bone micro-environment. Little is known about the role of the tumor suppressor gene phosphatase and tensin homolog deleted on chromosome 10 (PTEN) in prostate cancer bone   metastasis  . To explore this, we used a pTetOn PTEN cell line in which PTEN expression was reconstituted in a PTEN-null bone metastatic human prostate cancer cell line, LnCaP-C4-2. We found that C4-2 cells selectively migrated toward conditioned medium from primary mouse calvaria cells compared with that derived from lung fibroblasts. Further evaluation with conditioned medium from an established mouse calvaria osteoblast cell line and control non-osteoblast cell line indicates that osteoblastic characteristics convey this specific migration to C4-2 cells. We evaluated promiscuously metastatic PC-3 prostate as well as T24T and UMUC-3 bladder cells and found they did not have a specific migratory response to calvaria-conditioned medium as did C4-2. Induction of PTEN expression inhibited the motility of C4-2 cells toward calvaria-conditioned medium but had no effect on migration toward lung-conditioned medium and this inhibitory effect was dependent on the PTEN lipid phosphatase activity. Calvaria- but not lung-conditioned medium induced activation of the small GTPase Rac1. Constitutively active Rac1 but not focal adhesion kinase or Cdc42 could rescue cells from the inhibitory effect of PTEN on cell migration and PTEN induction was observed to inhibit Rac1 activation in response to calvaria-conditioned medium. Our results support the notion that loss of PTEN function in human prostate cancer may specifically facilitate bone rather than other organ   metastasis   and suggest that Rac1, as a PTEN effector, may contribute to this metastatic tropism. 
 Clinical value of Tiam1-Rac1 signaling in primary gallbladder carcinoma. Tiam1-Rac1 signaling plays an important role in the migration, invasion and   metastasis   of tumor cells. The aim of the present study was to clarify the clinical value of Tiam1 and Rac1 expression in primary gallbladder carcinoma (PGC). Eighty-six PGC tissues were evaluated by immunohistochemistry for Tiam1 and Rac1 expression. The association of Tiam1 and Rac1 expression with clinicopathological characteristics and the univariate survival analysis for the influence of Tiam1 and Rac1 expression on the overall survival were analyzed. Tiam1 and Rac1 immunoreactivities were observed in 72 (83.7%) and 68 (79.1%) of the 86 PGC cases, but not in the non-neoplastic gallbladder tissues, respectively. The tumors with the positive expression of Tiam1 and Rac1 more frequently showed lymph node   metastasis   (P = 0.01 and 0.008), lymphovascular invasion (P = 0.009 and 0.01) and recurrence (both P = 0.01), respectively. In addition, the tumors with the positive expression of Tiam1 and Rac1 significantly tended to show deeper invasion depth (P = 0.007 and 0.005) and higher TNM stage (both P = 0.005). The Kaplan-Meier survival curves demonstrated that patients with the positive expression of Tiam1 and Rac1 had a significantly shorter survival time than those patients with their negative expression (both P = 0.01). Multivariate analysis revealed that the positive expression of Tiam1 (P = 0.02) and Rac1 (P = 0.02), as well as TNM stage (P = 0.04), to be independently associated with a poor prognosis of patients with PGC. The data from this study suggest for the first time that Tiam1 and Rac1 are frequently expressed in PGC and are associated with decreased survival of the patients. Therefore, Tiam1 and Rac1 expressions may be independent predictors for the poor prognosis in patients with PGC. 
 Differential expression of Rac1 identifies its target genes and its contribution to progression of colorectal cancer. The small GTPase Rac1 is involved in the regulation of critical cellular functions, such as transcription control, cell cycle, and organization of actin cytoskeleton. Rac1 signalling modulates cancer progression since its overexpression leads to an increased tumour growth of xenografts of human colorectal tumour cells, while a drastic reduction of Rac1 expression by siRNA interferes with cancer progression (Espina et al., unpublished results). We aimed to study the molecular basis for the specific contribution of Rac1 in the progression of colorectal cancer. Comparative microarray analysis of a human colorectal carcinoma cell line genetically engineered to display different levels of Rac1 identified novel target genes for this GTPase. These results suggest that Rac1 plays a critical role in signalling transduction pathways relevant to human colorectal tumour progression, such as activation of Wnt signalling, inhibition of TGF-beta signalling, and enhancement of   metastasis  -inducing genes. 
 Up-regulation of Tiam1 and Rac1 correlates with poor prognosis in hepatocellular carcinoma. T-cell lymphoma invasion and   metastasis   1 (Tiam1) specifically activates Rho-like GTPases (e.g. Rac1) and Tiam1-Rac1 pathway affects the migration and invasion of many tumors, such as nasopharyngeal carcinoma, breast cancer and retinoblastoma. However, no studies have yet comprehensively examined the involvement of Tiam1-Rac1 pathway in hepatocellular carcinoma. In this study, we examined the relationship of the up-regulation of Tiam1 and Rac1 with clinicopathological features in patients with hepatocellular carcinoma.Expression of Tiam1 and Rac1 was assessed in 242 hepatocellular carcinoma tissues and their adjacent normal hepatic tissues by performing immunohistochemistry and was gauged regarding stage, grade and survival.Immunohistochemistry showed that patients with a high clinical stage hepatocellular carcinoma (III-IV) and α-fetoprotein levels had a higher tendency to express Tiam1 and Rac1 on tumor cells than the patients with low pathologic grade hepatocellular carcinoma (I-II) (P = 0.008 and 0.01, respectively) and low α-fetoprotein levels (P = 0.006 and 0.002, respectively). In addition, Tiam1 and Rac1 up-regulation was also significantly associated with vascular invasion status (both P = 0.02), intrahepati  c metastas  is status (P = 0.009 and 0.01, respectively) and histological differentiation (P = 0.008 and 0.009, respectively) of patients with hepatocellular carcinoma. Moreover, post-operative survival analysis indicated that hepatocellular carcinoma patients with strong Tiam1 (P = 0.01) and Rac1 (P = 0.02) expression had shorter disease-specific survival than those with weak expression. Multivariate analysis also showed that Tiam1 and Rac1 overexpression could be two predictors of poor prognosis (P = 0.02 and 0.03, respectively).The current study demonstrated for the first time that the Tiam1-Rac1 pathway may play a critical role in tumor progression of hepatocellular carcinoma. The expression of Tiam1 and Rac1 can be considered as the two useful indicators for predicting the prognosis of hepatocellular carcinoma. 
 Rac1 gene mutations in human brain tumours. Rac1 is a member of the Ras superfamily of small GTPase and plays a fundamental role in cytoskeleton reorganization, regulation of gene expression and cell proliferation, and cellular transformation. Though recent studies point to an involvement of rac1 in tumorigenesis, little is known about the alteration of rac1 gene in human brain tumours.Reverse transcription-polymerase chain reaction (RT-PCR), TA cloning, and DNA sequencing were performed to detect rac1 gene mutations in the surgical specimens of 45 human brain tumours.Twelve of 45 cases had base changes in the rac1 gene. The frequency of rac1 alterations was seven of 18 meningiomas, three of 14 astrocytomas, one of seven pituitary adenomas, and one of four metastatic brain tumours. No mutation was detected in acoustic neurilemomas. The subtypes of seven meningiomas include three meningotheliomatous, two atypical, one transitional and one angioblastic meningioma. Three astrocytomas had rac1 gene mutation, including one grade II, one grade III, and one grade IV astrocytoma. All of single base changes were transitions, five of them being T to C transitions. Sites of rac1 mutation were found in codons 34, 41 (two cases), 42 (two cases), 43, 44, 46 and 58. These mutations are mainly localized in the putative effector-domain of rac1 gene and may enhance the activity of rac1, which increases the survival of brain tumours.Our results suggest that rac1 gene may play a role in some brain tumours of divergent histogenesis and that the alterations of rac1 gene may contribute to tumorigenesis and/or   metastasis  . 
 TIAM1-RAC1 signalling axis-mediated activation of NADPH oxidase-2 initiates mitochondrial damage in the development of diabetic retinopathy. In diabetes, increased retinal oxidative stress is seen before the mitochondria are damaged. Phagocyte-like NADPH oxidase-2 (NOX2) is the predominant cytosolic source of reactive oxygen species (ROS). Activation of Ras-related C3 botulinum toxin substrate 1 (RAC1), a NOX2 holoenzyme member, is necessary for NOX2 activation and ROS generation. In this study we assessed the role of T cell lymphoma invasion and   metastasis   (TIAM1), a guanine nucleotide exchange factor for RAC1, in RAC1 and NOX2 activation and the onset of mitochondrial dysfunction in in vitro and in vivo models of glucotoxicity and diabetes.RAC1 and NOX2 activation, ROS generation, mitochondrial damage and cell apoptosis were quantified in bovine retinal endothelial cells exposed to high glucose concentrations, in the retina from normal and streptozotocin-induced diabetic rats and mice, and the retina from human donors with diabetic retinopathy.High glucose activated RAC1 and NOX2 (expression and activity) and increased ROS in endothelial cells before increasing mitochondrial ROS and mitochondrial DNA (mtDNA) damage. N6-[2-[[4-(diethylamino)-1-methylbutyl]amino]-6-methyl-4-pyrimidinyl]-2-methyl-4,6-quinolinediamine, trihydrochloride (NSC23766), a known inhibitor of TIAM1-RAC1, markedly attenuated RAC1 activation, total and mitochondrial ROS, mtDNA damage and cell apoptosis. An increase in NOX2 expression and membrane association of RAC1 and p47(phox) were also seen in diabetic rat retina. Administration of NSC23766 to diabetic mice attenuated retinal RAC1 activation and ROS generation. RAC1 activation and p47(phox) expression were also increased in the retinal microvasculature from human donors with diabetic retinopathy.The TIAM1-RAC1-NOX2 signalling axis is activated in the initial stages of diabetes to increase intracellular ROS leading to mitochondrial damage and accelerated capillary cell apoptosis. Strategies targeting TIAM1-RAC1 signalling could have the potential to halt the progression of diabetic retinopathy in the early stages of the disease. 
 Rac1 contributes to trastuzumab resistance of breast cancer cells: Rac1 as a potential therapeutic target for the treatment of trastuzumab-resistant breast cancer. Although treatment with trastuzumab improves outcomes for women with ErbB2-positive breast cancer, many patients who achieve an initial response to trastuzumab subsequently acquire resistance within 1 year. Rac1, a Ras-like small GTPase, has been implicated in the control of cell growth and morphology and is believed to be associated with breast cancer progression and   metastasis  . Here, we show that when parental SKBR3 cells become resistant to trastuzumab, Rac1 activity is increased, leading to altered cell morphology, which is accompanied by significant cytoskeleton disorganization. Furthermore, both trastuzumab-mediated down-regulation of ErbB2 and epidermal growth factor-induced down-regulation of epidermal growth factor receptor are impaired in the trastuzumab-resistant SKBR3 cells, indicating that the endocytic down-regulation of ErbB receptors is compromised in the resistant cells. This results in an aberrant accumulation of ErbB2 on the cell surface and enhanced ErbB2 and extracellular signal-regulated kinase activity in trastuzumab-resistant SKBR3 cells. Additionally, overexpression of constitutively active Rac1G12V in parental SKBR3 cells reduces sensitivity to trastuzumab. After reduction of Rac1 activity by NSC23766, a specific Rac1 inhibitor, trastuzumab-resistant SKBR3 cells display a cellular morphology similar to parental SKBR3 cells. Moreover, we show that NSC23766 restores trastuzumab-mediated endocytic down-regulation of ErbB2 and reduces extracellular signal-regulated kinase activity in resistant SKBR3 cells. Our findings highlight an important role for Rac1 in trastuzumab resistance of human breast cancer cells and identify the impaired trastuzumab-mediated endocytic down-regulation of ErbB2 as a novel mechanism of trastuzumab resistance. The significant effects of NSC23766 on trastuzumab-resistant SKBR3 cells warrant further study of NSC23766 as a potential treatment of trastuzumab-resistant breast cancers. 
 An EGFR/PI3K/AKT axis promotes accumulation of the Rac1-GEF Tiam1 that is critical in EGFR-driven tumorigenesis. Epidermal growth factor receptor (EGFR) signaling regulates cell growth and survival, and its overactivation drives cancer development. One important branch of EGFR signaling is through activation of GTPase Rac1, which further promotes cell proliferation, survival and cancer   metastasis  . Here, we show that EGFR activates Rac1 via inducing the accumulation of its specific guanine nucleotide exchange factor, T-cell lymphoma invasion and   metastasis   1 (Tiam1) in non-small-cell lung cancer and colon cancer cells. Conversely, elevated Tiam1 is required for EGFR-induced tumorigenesis. In human lung adenocarcinoma and colon cancer specimens, Tiam1 expression strongly correlates with EGFR expression. We further reveal that AKT, a key downstream protein kinase of EGFR, phosphorylates Tiam1 at several consensus sites, facilitates the interaction of Tiam1 with scaffold proteins 14-3-3 and leads to an increase of Tiam1 stability. Subsequently, Tiam1 is dephosporylated and destabilized by PP2A. Together, our study identifies a bidirectional (phosphorylation and dephosphorylation) regulatory mechanism controlling Tiam1 stability and provides new insights on how EGFR signaling triggers Rac1 activation and cancer development. 
 CD147 promotes Src-dependent activation of Rac1 signaling through STAT3/DOCK8 during the motility of hepatocellular carcinoma cells.   Metastasis   is considered a dynamic process in tumor development that is related to abnormal migration and invasion. Tumor cells can move as individual cells in two interconvertible modes: mesenchymal-type and amoeboid. Previously, we reported that the interaction between CD147 and Annexin II can inhibit the amoeboid movement in hepatocellular carcinoma (HCC) cells. However, the mechanism of CD147 involved in mesenchymal movement is still unclear. Notably, our results show overexpression of CD147 led to mesenchymal-type movement in HCC cells. Evidence indicated that the mesenchymal-type cell movement induced by CD147 was Src dependent, as observed by confocal microscopy and Rac1 activity assay. The phosphorylation of Src (pY416-Src) can be up-regulated by CD147, and this regulation is mediated by focal adhesion kinase (FAK). Next, we identified DOCK8 as a GEF for Rac1, a key molecule driving mesenchymal-type movement. We also found that Src promotes STAT3 phosphorylation and STAT3 facilitates DOCK8 transcription, thus enhancing DOCK8 expression and Rac1 activation. This study provides a novel mechanism of CD147 regulating mesenchymal-type movement in HCC cells. 
 RhoGDI2 is expressed in human trophoblasts and involved in their migration by inhibiting the activation of RAC1. The invasion of placental trophoblast cells into the maternal decidua is an essential aspect of placental embedment. The process of placentation bears several striking similarities to tumor cell   metastasis  . However, trophoblastic migration during implantation and placentation is stringently controlled both in space and time. RhoGDI2 belongs to a family of Rho guanosine diphosphate dissociation inhibitors (RhoGDIs), and RhoGDI2 is a   metastasis   suppressor gene and a marker of aggressive human cancer. We evaluated whether RhoGDI2 has a physiological role in embryo implantation, particularly trophoblast migration. The mRNA and protein expression levels of RhoGDI2 were higher in term placentas compared with first-trimester placentas as detected by real-time PCR and Western blot. Immunohistochemical studies indicated that RhoGDI2 localized to the cytotrophoblast layer and extravillous trophoblast in first-trimester placentas and was distributed in the syncytiotrophoblast layers of term placentas. Overexpression of RhoGDI2 in HTR-8/SVneo cells was associated with reduced RAC1-guanosine triphosphate (GTP) levels and inhibited cell migration. Conversely, small interfering RNA-mediated downregulation of RhoGDI2 resulted in significantly increased RAC1-GTP levels. Altered RhoGDI2 expression had no significant effects on cell proliferation. In conclusion, RhoGDI2 inhibits trophoblast cell migration, and this function may involve suppression of RAC1 activation. 
 Activation of H-Ras and Rac1 correlates with epidermal growth factor-induced invasion in Hs578T and MDA-MB-231 breast carcinoma cells. There is considerable experimental evidence that hyperactive Ras proteins promote breast cancer growth and development including invasiveness, despite the low frequency of mutated forms of Ras in breast cancer. We have previously shown that H-Ras, but not N-Ras, induces an invasive phenotype mediated by small GTPase Rac1 in MCF10A human breast epithelial cells. Epidermal growth factor (EGF) plays an important role in aberrant growth and   metastasis   formation of many tumor types including breast cancer. The present study aims to investigate the correlation between EGF-induced invasiveness and Ras activation in four widely used breast cancer cell lines. Upon EGF stimulation, invasive abilities and H-Ras activation were significantly increased in Hs578T and MDA-MB-231 cell lines, but not in MDA-MB-453 and T47D cell lines. Using small interfering RNA (siRNA) to target H-Ras, we showed a crucial role of H-Ras in the invasive phenotype induced by EGF in Hs578T and MDA-MB-231 cells. Moreover, siRNA-knockdown of Rac1 significantly inhibited the EGF-induced invasiveness in these cells. Taken together, this study characterized human breast cancer cell lines with regard to the relationship between H-Ras activation and the invasive phenotype induced by EGF. Our data demonstrate that the activation of H-Ras and the downstream molecule Rac1 correlates with EGF-induced breast cancer cell invasion, providing important information on the regulation of malignant progression in mammary carcinoma cells. 
 MT1-MMP modulates melanoma cell dissemination and   metastasis   through activation of MMP2 and RAC1. Metastatic melanoma remains the deadliest of all skin cancers with a survival rate at five years of less than 15%. MT1-MMP is a membrane-associated matrix metalloproteinase that controls pericellular proteolysis and is an important, invasion-promoting, pro-tumorigenic MMP in cancer. We show that deregulation of MT1-MMP expression happens as early as the transition from nevus to primary melanoma and continues to increase during melanoma progression. Furthermore, MT1-MMP expression is associated with poor melanoma patient outcome, underscoring a pivotal role of MT1-MMP in melanoma pathogenesis. We demonstrate that MT1-MMP is directly required for melanoma cells to metastasize, as cells deprived of MT1-MMP fail to form distant   metastasis   in an orthotopic mouse melanoma model. We show that MT1-MMP affects cell invasion by activating its target MMP2. Importantly, we demonstrate, for the first time, that activation of MMP2 by MT1-MMP is required to sustain RAC1 activity and promote MT1-MMP-dependent cell motility. These data highlight a novel MT1-MMP/MMP2/RAC1 signaling axis in melanoma that may represent an intriguing molecular target for the treatment of invasive melanoma. 
 The microRNA -23b/-27b cluster suppresses the metastatic phenotype of castration-resistant prostate cancer cells. MicroRNAs (miRs) are small, endogenous, non-coding RNAs that regulate the stability and/or translation of complementary mRNA targets. MiRs have emerged not only as critical modulators of normal physiologic processes, but their deregulation may significantly impact prostate and other cancers. The expression of miR-23b and miR-27b, which are encoded by the same miR cluster (miR-23b/-27b), are downregulated in metastatic, castration-resistant tumors compared to primary prostate cancer and benign tissue; however, their possible role in prostate cancer progression is unknown. We found that ectopic expression of miR-23b/-27b in two independent castration-resistant prostate cancer cell lines resulted in suppression of invasion and migration, as well as reduced survival in soft agar (a measure of anoikis). However, there was no effect of miR-23b/-27b on cell proliferation suggesting that these miRs function as   metastasis   (but not growth) suppressors in prostate cancer. Conversely, inhibition of miR-23b/-27b in the less aggressive androgen-dependent LNCaP prostate cancer cell line resulted in enhanced invasion and migration also without affecting proliferation. Mechanistically, we found that introduction of miR-23b/-27b in metastatic, castration-resistant prostate cancer cell lines resulted in a significant attenuation of Rac1 activity without affecting total Rac1 levels and caused increased levels of the tumor suppressor E-cadherin. Inhibition of these miRs had the opposite effect in androgen-dependent LNCaP cells. These results suggest that miR-23b/-27b are   metastasis   suppressors that might serve as novel biomarkers and therapeutic agents for castration-resistant disease. 
 RAC1: an emerging therapeutic option for targeting cancer angiogenesis and   metastasis  . Angiogenesis and   metastasis   are well recognized as processes fundamental to the development of malignancy. Both processes involve the coordination of multiple cellular and chemical activities through myriad signaling networks, providing a mass of potential targets for therapeutic intervention. This review will focus on one master regulator of cell motility, RAC1, and the existing data with regard to its role in cell motility, including particular roles for tumor angiogenesis and invasion/  metastasis  . We also emphasize the preclinical investigations carried out with RAC1 inhibitors to evaluate the therapeutic potential of this target. Herein, we explore potential future directions as well as the challenges of targeting RAC1 in the treatment of cancer. Recent insights at the molecular and cellular levels are paving the way for a more directed and detailed approach to target mechanisms of RAC1 regulating angiogenesis and   metastasis  . Understanding these mechanisms may provide insight into RAC1 signaling components as alternative therapeutic targets for tumor angiogenesis and   metastasis  . 
 Cucurbitacin I inhibits Rac1 activation in breast cancer cells by a reactive oxygen species-mediated mechanism and independently of Janus tyrosine kinase 2 and P-Rex1. The small GTPase Rac1 has been widely implicated in mammary tumorigenesis and   metastasis  . Previous studies established that stimulation of ErbB receptors in breast cancer cells activates Rac1 and enhances motility via the Rac-guanine nucleotide exchange factor P-Rex1. As the Janus tyrosine kinase 2 (Jak2)/signal transducer and activator of transcription 3 (Stat3) pathway has been shown to be functionally associated with ErbB receptors, we asked if this pathway could mediate P-Rex1/Rac1 activation in response to ErbB ligands. Here we found that the anticancer agent cucurbitacin I, a Jak2 inhibitor, reduced the activation of Rac1 and motility in response to the ErbB3 ligand heregulin in breast cancer cells. However, Rac1 activation was not affected by Jak2 or Stat3 RNA interference, suggesting that the effect of cucurbitacin I occurs through a Jak2-independent mechanism. Cucurbitacin I also failed to affect the activation of P-Rex1 by heregulin. Subsequent analysis revealed that cucurbitacin I strongly activates RhoA and the Rho effector Rho kinase (ROCK) in breast cancer cells and induces the formation of stress fibers. Interestingly, disruption of the RhoA-ROCK pathway prevented the inhibitory effect of cucurbitacin I on Rac1 activation by heregulin. Lastly, we found that RhoA activation by cucurbitacin I is mediated by reactive oxygen species (ROS). The ROS scavenger N-acetyl L-cysteine and the mitochondrial antioxidant Mito-TEMPO rescued the inhibitory effect of cucurbitacin I on Rac1 activation. In conclusion, these results indicate that ErbB-driven Rac1 activation in breast cancer cells proceeds independently of the Jak2 pathway. Moreover, they established that the inhibitory effect of cucurbitacin I on Rac1 activity involves the alteration of the balance between Rho and Rac. 
 Denbinobin, a phenanthrene from Dendrobium nobile, impairs prostate cancer migration by inhibiting Rac1 activity. Prostate cancer is the most prevalent type of cancer in the United States. The most common site of prostate cancer   metastasis   is bone. CXCL12 is preferentially expressed in bone and is targeted by prostate cancer cells, which over-express the receptor for CXCL12, CXCR4. In response to CXCL12 stimulation, Rac1, a GTPase, along with its effectors, regulates actin polymerization to form lamellipodia, which is a critical event for cell migration. Cortactin, an actin-binding protein, is recruited to the lamellipodia and is phosphorylated at tyrosine residues. The phosphorylated cortactin is also involved in cell migration. The inhibition of Rac1 activity using a dominant negative Rac1 impairs lamellipodial protrusion as well as cortactin translocation and cortactin phosphorylation. Denbinobin, a substance extracted from Dendrobium nobile, has anticancer effects in many cancer cell lines. Whether denbinobin can inhibit prostate cancer cell migration is not clear. Here, we report that denbinobin inhibited Rac1 activity. The inhibition of Rac1 activity prevented lamellipodial formation. Cortactin phosphorylation and translocation to the lamellipodia were also impaired, and PC3 cells were unable to migrate. These results indicate that denbinobin prevents CXCL12-induced PC3 cell migration by inhibiting Rac1 activity. 
 DDX3 modulates cell adhesion and motility and cancer cell   metastasis   via Rac1-mediated signaling pathway. The DEAD-box RNA helicase DDX3 is a versatile protein involved in multiple steps of gene expression and various cellular signaling pathways. DDX3 mutations have been implicated in the wingless (Wnt) type of medulloblastoma. We show here that small interfering RNA-mediated DDX3 knockdown in various cell lines increased cell-cell adhesion but decreased cell-extracellular matrix adhesion. Moreover, DDX3 depletion suppressed cell motility and impaired directional migration in the wound-healing assay. Accordingly, DDX3-depleted cells exhibited reduced invasive capacities in vitro as well as reduced metastatic potential in mice. We also examined the mechanism underlying DDX3-regulated cell migration. DDX3 knockdown reduced the levels of both Rac1 and β-catenin proteins, and consequentially downregulated the expression of several β-catenin target genes. Moreover, we demonstrated that DDX3-regulated Rac1 mRNA translation, possibly through an interaction with its 5'-untranslated region, and affected β-catenin protein stability in an Rac1-dependent manner. Taken together, our results indicate the DDX3-Rac1-β-catenin regulatory axis in modulating the expression of Wnt/β-catenin target genes. Therefore, this report provides a mechanistic context for the role of DDX3 in Wnt-type tumors. 
 RASAL2 activates RAC1 to promote triple-negative breast cancer progression. Patients with triple-negative breast cancer (TNBC) have a high incidence of early relapse and   metastasis  ; however, the molecular basis for recurrence in these individuals remains poorly understood. Here, we demonstrate that RASAL2, which encodes a RAS-GTPase-activating protein (RAS-GAP), is a functional target of anti-invasive microRNA-203 and is overexpressed in a subset of triple-negative or estrogen receptor-negative (ER-negative) breast tumors. As opposed to luminal B ER-positive breast cancers, in which RASAL2 has been shown to act as a RAS-GAP tumor suppressor, we found that RASAL2 is oncogenic in TNBC and drives mesenchymal invasion and   metastasis  . Moreover, high RASAL2 expression was predictive of poor disease outcomes in patients with TNBC. RASAL2 acted independently of its RAS-GAP catalytic activity in TNBC; however, RASAL2 promoted small GTPase RAC1 signaling, which promotes mesenchymal invasion, through binding and antagonizing the RAC1-GAP protein ARHGAP24. Together, these results indicate that activation of a RASAL2/ARHGAP24/RAC1 module contributes to TNBC tumorigenesis and identify a context-dependent role of RASAL2 in breast cancer. 
 A critical role for Rac1 in tumor progression of human colorectal adenocarcinoma cells. Colorectal adenocarcinoma is the second cause of cancer mortality in developed countries. Rac1 is a member of the family of Rho GTPases that regulates many intracellular signaling pathways, including those involved in tumorigenesis, invasion, and   metastasis  . We have investigated the role of Rac1 in colorectal tumor progression by genetic modification of the human colorectal adenocarcinoma cell line SW620 to either overexpress Rac1 or lack Rac1 expression. Tumor behavior was studied by orthotopic injection of stably modified cell lines into the cecal wall of athymic nude mice, a model that replicates the histopathological appearance and clinical behavior of human colorectal adenocarcinoma in humans. While overexpression of Rac1 resulted in an accelerated tumorigenic process, inducing a faster mortality rate, inhibition of Rac1 completely suppressed tumor formation. These results suggest that Rac1 plays a major role in colorectal adenocarcinoma progression. Finally, interference with Rac1 function may provide an important tool to block the malignant phenotype of colorectal adenocarcinoma cells. 
 Mtss1 promotes cell-cell junction assembly and stability through the small GTPase Rac1. Cell-cell junctions are an integral part of epithelia and are often disrupted in cancer cells during epithelial-to-mesenchymal transition (EMT), which is a main driver of metastatic spread. We show here that   Metastasis   suppressor-1 (Mtss1; Missing in   Metastasis  , MIM), a member of the IMD-family of proteins, inhibits cell-cell junction disassembly in wound healing or HGF-induced scatter assays by enhancing cell-cell junction strength. Mtss1 not only makes cells more resistant to cell-cell junction disassembly, but also accelerates the kinetics of adherens junction assembly. Mtss1 drives enhanced junction formation specifically by elevating Rac-GTP. Lastly, we show that Mtss1 depletion reduces recruitment of F-actin at cell-cell junctions. We thus propose that Mtss1 promotes Rac1 activation and actin recruitment driving junction maintenance. We suggest that the observed loss of Mtss1 in cancers may compromise junction stability and thus promote EMT and   metastasis  . 
 Cancer cell-autonomous TRAIL-R signaling promotes KRAS-driven cancer progression, invasion, and   metastasis  . Many cancers harbor oncogenic mutations of KRAS. Effectors mediating cancer progression, invasion, and   metastasis   in KRAS-mutated cancers are only incompletely understood. Here we identify cancer cell-expressed murine TRAIL-R, whose main function ascribed so far has been the induction of apoptosis as a crucial mediator of KRAS-driven cancer progression, invasion, and   metastasis   and in vivo Rac-1 activation. Cancer cell-restricted genetic ablation of murine TRAIL-R in autochthonous KRAS-driven models of non-small-cell lung cancer (NSCLC) and pancreatic ductal adenocarcinoma (PDAC) reduces tumor growth, blunts   metastasi  s, and prolongs survival by inhibiting cancer cell-autonomous migration, proliferation, and invasion. Consistent with this, high TRAIL-R2 expression correlates with invasion of human PDAC into lymph vessels and with shortened   metastasi  s-free survival of KRAS-mutated colorectal cancer patients. 
 Differential roles of Smad2 and Smad3 in the regulation of TGF-β1-mediated growth inhibition and cell migration in pancreatic ductal adenocarcinoma cells: control by Rac1. Progression of pancreatic ductal adenocarcinoma (PDAC) is largely the result of genetic and/or epigenetic alterations in the transforming growth factor-beta (TGF-β)/Smad signalling pathway, eventually resulting in loss of TGF-β-mediated growth arrest and an increase in cellular migration, invasion, a  nd metasta  sis. These cellular responses to TGF-β are mediated solely or partially through the canonical Smad signalling pathway which commences with activation of receptor-regulated Smads (R-Smads) Smad2 and Smad3 by the TGF-β type I receptor. However, little is known on the relative contribution of each R-Smad, the possible existence of functional antagonism, or the crosstalk with other signalling pathways in the control of TGF-β1-induced growth inhibition and cell migration. Using genetic and pharmacologic approaches we have inhibited in PDAC cells endogenous Smad2 and Smad3, as well as a potential regulator, the small GTPase Rac1, and have analysed the consequences for TGF-β1-mediated growth inhibition and cell migration (chemokinesis).SiRNA-mediated silencing of Smad3 in the TGF-β responsive PDAC cell line PANC-1 reduced TGF-β1-induced growth inhibition but increased the migratory response, while silencing of Smad2 enhanced growth inhibition but decreased chemokinesis. Interestingly, siRNA-mediated silencing of the small GTPase Rac1, or ectopic expression of a dominant-negative Rac1 mutant largely mimicked the effect of Smad2 silencing on both TGF-β1-induced growth inhibition, via upregulation of the cdk inhibitor p21WAF1, and cell migration. Inhibition of Rac1 activation reduced both TGF-β1-induction of a Smad2-specific transcriptional reporter and Smad2 C-terminal phosphorylation in PDAC cells while Smad3-specific transcriptional activity and Smad3 C-terminal phosphorylation appeared increased. Disruption of autocrine TGF-β signalling in PANC-1 cells rendered cells less susceptible to the growth-suppressive effect of Rac1 inhibition, suggesting that the decrease in "basal" proliferation upon Rac1 inhibition was caused by potentiation of autocrine TGF-β growth inhibition.In malignant cells with a functional TGF-β signalling pathway Rac1 antagonizes the TGF-β1 growth inhibitory response and enhances cell migration by antagonistically regulating Smad2 and Smad3 activation. This study reveals that Rac1 is prooncogenic in that it can alter TGF-β signalling at the R-Smad level from a tumour-suppressive towards a tumour-promoting outcome. Hence, Rac1 might represent a viable target for therapeutic intervention to inhibit PDAC progression. 
 RAC1 activation mediates Twist1-induced cancer cell migration. Epithelial-mesenchymal transition (EMT), which is characterized by the suppression of the adhesion protein E-cadherin, is a crucial process that promotes   metastasis   and stem-like properties of cancer cells. However, the dissociation of cellular aggregates is not sufficient to explain why cancer cells move, and the motile nature of cancer cells undergoing EMT remains elusive. Here, we identify a mechanism in which the EMT inducer Twist1 elicits cancer cell movement through activation of RAC1. Twist1 cooperates with BMI1 to suppress let-7i expression, which results in upregulation of NEDD9 and DOCK3, leading to RAC1 activation and enabling mesenchymal-mode movement in three-dimensional environments. Moreover, the suppression of let-7i contributes to Twist1-induced stem-like properties. Clinically, activation of the Twist1-let-7i-NEDD9 axis in head and neck cancer patients correlates with tumour invasiveness and worse outcome. Our results uncover an essential mechanism to explain how Twist1 induces the motile stem-like cancer cell phenotype beyond simply suppressing E-cadherin. 
 Rac activation is associated with hepatocellular carcinoma   metastasis   by up-regulation of vascular endothelial growth factor expression. Hepatocellular carcinoma (HCC) is associated with a propensity for vascular invasion and   metastasis  , which contribute to poor prognosis. Angiogenesis is a crucial process contributing to tumor growth and   metastasis  . Recently, Rac has been suggested to play a role in angiogenesis. However, the actual role of Rac in HCC angiogenesis remains unclear. Given that vascular endothelial growth factor (VEGF) is an important angiogenic factor in HCC, the purpose of this study was to evaluate the possible correlation between Rac activation and VEGF expression in HCC tumor samples, as well as the mechanism involved in Rac-induced HCC angiogenesis.We evaluated Rac and VEGF expression in the HCC tissue microarray of paired primary and metastatic HCC samples using immunohistochemical staining. The role of Rac-induced HCC angiogenesis was also evaluated in vitro in HCC cell lines.We first showed that activation of Rac was correlated with HCC   metastasis   (P<0.001), and its expression was significantly correlated with VEGF expression by tissue microarray. Ectopic Rac-dominant active transfection in Hep3B cells increased VEGF secretion, which induced the morphologic change and proliferation of human umbilical vein endothelial cells, resulting in the promotion of angiogenesis. Rac induced the transcriptional activation of VEGF by direct interaction with hypoxia-inducible factor-1alpha (HIF-1alpha) expression. In hypoxic conditions, Rac promoted angiogenesis through an increase in HIF-1alpha stabilization.This study shows that Rac is a novel angiogenic factor for HCC through the enhancement of HIF-1alpha protein stability, which provides a possible therapeutic target in the development of inhibitors of angiogenesis. 
 Tyr724 phosphorylation of ELMO1 by Src is involved in cell spreading and migration via Rac1 activation. The complex of Dock180/ELMO1 that functions as a bipartite guanine nucleotide exchange factor for Rac is essential for diverse physiological and pathological processes of cells such as cell migration, phagocytosis, and invasion of cancer cells. Among the Src-family tyrosine kinases (SFKs), it has been reported that Hck directly phosphorylates ELMO1, regulating phagocytosis by promoting activation of Rac1; however, the involvement of other SFKs in ELMO1 phosphorylation has remained unknown. Here, we identified novel tyrosine (Y) residues of ELMO1 phosphorylated by SFKs, and examined the effects on Rac1 activity, cell adhesion, spreading, and cell motility on extracellular matrix (ECM).In this study, we unveiled that Src and Fyn can induce tyrosine phosphorylation of ELMO1 in in vivo and in vitro phosphorylation assays. Mutational analyses identified both Y720 and Y724 residues of ELMO1 as Src-mediated phosphorylation sites, preferentially on Y724. Single substitution of Y724 to Phe abrogated Rac1 activation triggered by Src. To elucidate the biological function of pY724, we established NIH3T3 cells stably expressing wild-type ELMO1 or its Y724F mutant together with Dock180. Among them, Y724-deficient cells exhibited a depletion of Rac1 activity with diminished phosphorylation of ELMO1 even upon the ECM-stimulation. It is noteworthy that NIH3T3 cells with ELMO1 Y724F were strikingly defective to promote cell spreading on fibronectin-coated dish, concomitantly exhibiting immature assemblies of actin stress fibers and focal adhesions. Eventually, ELMO1 Y724F significantly impaired cell migration.These results define that Src-mediated Y724 phosphorylation in ELMO1 plays a critical role for cell spreading via activation of Rac1, leading to promotion of cell migration. As the overexpression and/or hyperactivation of Src have been shown in a wide variety of human cancers, Src-mediated phosphorylation of Y724 in ELMO1 may regulate cancer cell adhesion to the ECM, invasion into surrounding tissues, and subsequent distant   metastasis  . 
 Dock4 is regulated by RhoG and promotes Rac-dependent cell migration. Cell migration is essential for normal development and many pathological processes including tumor   metastasis  . Rho family GTPases play important roles in this event. In particular, Rac is required for lamellipodia formation at the leading edge during migration. Dock4 is a member of the Dock180 family proteins, and Dock4 mutations are present in a subset of human cancer cell lines. However, the function and the regulatory mechanism of Dock4 remain unclear. Here we show that Dock4 is regulated by the small GTPase RhoG and its effector ELMO and promotes cell migration by activating Rac1. Dock4 formed a complex with ELMO, and expression of active RhoG induced translocation of the Dock4-ELMO complex from the cytoplasm to the plasma membrane and enhanced the Dock4- and ELMO-dependent Rac1 activation and cell migration. On the other hand, RNA interference-mediated knockdown of Dock4 in NIH3T3 cells reduced cell migration. Taken together, these results suggest that Dock4 plays an important role in the regulation of cell migration through activation of Rac1, and that RhoG is a key upstream regulator for Dock4. 
 Human tumor necrosis factor (TNF)-alpha-induced protein 8-like 2 suppresses hepatocellular carcinoma   metastasis   through inhibiting Rac1. Tumor invasion and   metastasis   are the major reasons for leading death of patients with hepatocellular carcinoma (HCC). Therefore, to identify molecules that can suppress invasion and   metastasis   of tumor will provide novel targets for HCC therapies. Tumor necrosis factor (TNF)-alpha-induced protein 8-like 2, TIPE2, is a novel immune negative molecule and an inhibitor of the oncogenic Ras in mice but its function in human is unclear. Our previous research has shown that TIPE2 is downregulated in human primary HCC compared with the paired adjacent non-tumor tissues.In present study, we provide evidence that TIPE2 inhibits effectively human hepatocellular carcinoma   metastasis  . The forced expression of TIPE2 in HCC-derived cell lines markedly inhibits tumor cell growth, migration and invasion in vitro and suppresses growth and   metastasis   of HCC in vivo. Clinical information from a cohort of 112 patients reveals that loss or reduced expression of TIPE2 in primary HCC tissues is significantly associated with tumor   metastasis  . Mechanically, TIPE2 inhibits the migration and invasion through targeting Rac1 and then reduces F-actin polymerization and expression of matrix metallopeptidase 9 (MMP9) and urokinase plasminogen activator (uPA).Our results indicate that human TIPE2 is endogenous inhibitor of Rac1 in HCC by which it attenuates invasion and   metastasis   of HCC. The data suggest that TIPE2 will be a new target for HCC therapy. 
 The activity of RhoA is correlated with lymph node   metastasis   in human colorectal cancer. Rho family GTPases play a pivotal role in the regulation of numerous cellular functions associated with malignant transformation and   metastasis  . To evaluate the role of these GTPases in colorectal cancer, the protein expression levels and activities of these proteins in matched sets of tumor and non-tumor tissues of surgical specimens were analyzed. The relationship between the protein levels and activities in tumor tissues to the clinicopathological features was also assessed. The expression levels of RhoA, active RhoA, Rac1, and active Rac1 in tumor tissues were higher than in normal tissues. The amounts of active RhoA protein in primary tumors correlated with lymph nodes   metastasis  . No relationship was noted between the protein expression levels and other clinicopathological findings. These findings suggest that the Rho family small GTPases are related to malignant transformation and progression of colorectal cancer and the activation of RhoA is associated with the lymph node   metastasis  . 
 Expression of Rac1, HIF-1α, and VEGF in gastric carcinoma: correlation with angiogenesis and prognosis. The aim of this study was to investigate the relationship between the expression of hypoxia-inducible factor-1α (HIF-1α), Ras-related C3 botulinum toxin substrate 1 (Rac1), and vascular endothelial growth factor (VEGF), as well as their correlation with angiogenesis and prognosis in gastric carcinoma.The expression of Rac1, HIF-1α, VEGF, and CD34 (described in terms of microvessel density, MVD) was determined by immunohistochemical staining of tissues from 60 radically resected gastric cancer specimens.The proportion of specimens expressing Rac1, HIF-1α, and VEGF was 37/60 (61.7%), 35/60 (58.3%), and 40/60 (66.7%), respectively. The levels of Rac1, HIF-1α, and VEGF expression were significantly correlated with Lauren's classification, lymph   node meta  stasis, and pathologic staging (p < 0.05). There were positive correlations between MVD and the expression of Rac1, HIF-1α, and VEGF. The mean survival time and 5-year survival rate in cases with positive Rac1, HIF-1α, and VEGF expression and MVD ≥ 26.3 were significantly shorter than those with negative Rac1, HIF-1α, and VEGF expression and MVD < 26.3.Rac1, HIF-1α, and VEGF play an important role in tumor i  nvasion an  d metastasis, especially in tumor angiogenesis. Thus, testing the expression of Rac1, HIF-1α, and VEGF may be a useful index for treatment and prognosis. 
 Knockdown of BDNF suppressed invasion of HepG2 and HCCLM3 cells, a mechanism associated with inactivation of RhoA or Rac1 and actin skeleton disorganization. Brain-derived neurotrophic factor (BDNF) and its primary receptor tropomysin-related kinase B (TrkB) mediate critical signalings for supporting survival and growth of neurons. Even though we have previously confirmed that more expressions of BDNF and TrkB were closely correlated with multiple and advanced hepatocellular carcinoma (HCC), the exact mechanisms underlying have not been investigated. The expressions of BDNF and TrkB were examined by western blot and BDNF secretion was evaluated by ELISA in human HCC cell lines of HepG2 and HCCLM3 with high metastatic potential. BDNF knockdown was performed by specific BDNF-siRNA transfection in HCC cells, actin cytoskeleton was shown by FITC-phalloidin staining and the activations of RhoA, Rac1 or Cdc42 were determined using western blot. Cell apoptosis and invasion were examined by flow cytometry and transwell assay, respectively. More expressions of BDNF and TrkB were found in HCCLM3 than in HepG2 cells. Inhibited expression of BDNF by specific siRNA showed impaired actin polymerization and decreased activations of RhoA or Rac1 in both HepG2 and HCCLM3 cells. BDNF knockdown also induced apoptosis and suppressed invasion of both HepG2 and HCCLM3 cells. Our results suggested a role of BDNF/TrkB in confering HCCLM3 cells advantage of   metastasis  , and BDNF knockdown inhibited cell invasion probably through the blocked actin polymerization and the correlated inactivation of RhoA or Rac1. Aiming at BDNF/TrkB signaling interruption may be an effective strategy to prevent HCC progression. 
 The Phosphatidylinositol (3,4,5)-Trisphosphate-dependent Rac Exchanger 1·Ras-related C3 Botulinum Toxin Substrate 1 (P-Rex1·Rac1) Complex Reveals the Basis of Rac1 Activation in Breast Cancer Cells. The P-Rex (phosphatidylinositol (3,4,5)-trisphosphate (PIP3)-dependent Rac exchanger) family (P-Rex1 and P-Rex2) of the Rho guanine nucleotide exchange factors (Rho GEFs) activate Rac GTPases to regulate cell migration, invasion, an  d metastas  is in several human cancers. The family is unique among Rho GEFs, as their activity is regulated by the synergistic binding of PIP3 and Gβγ at the plasma membrane. However, the molecular mechanism of this family of multi-domain proteins remains unclear. We report the 1.95 Å crystal structure of the catalytic P-Rex1 DH-PH tandem domain in complex with its cognate GTPase, Rac1 (Ras-related C3 botulinum toxin substrate-1). Mutations in the P-Rex1·Rac1 interface revealed a critical role for this complex in signaling downstream of receptor tyrosine kinases and G protein-coupled receptors. The structural data indicated that the PIP3/Gβγ binding sites are on the opposite surface and markedly removed from the Rac1 interface, supporting a model whereby P-Rex1 binding to PIP3 and/or Gβγ releases inhibitory C-terminal domains to expose the Rac1 binding site. 
 Roles of the Rac1 and Rac3 GTPases in human tumor cell invasion. Members of the Rho family of small GTPases have been shown to be involved in tumorigenesis and   metastasis  . Currently, most of the available information on the function of Rho proteins in malignant transformation is based on the use of dominant-negative mutants of these GTPases. The specificity of these dominant-negative mutants is limited however. In this study, we used small interfering RNA directed against either Rac1 or Rac3 to reduce their expression specifically. In line with observations using dominant-negative Rac1 in other cell types, we show that RNA interference-mediated depletion of Rac1 strongly inhibits lamellipodia formation, cell migration and invasion in SNB19 glioblastoma cells. Surprisingly however, Rac1 depletion has a much smaller inhibitory effect on SNB19 cell proliferation and survival. Interestingly, whereas depletion of Rac3 strongly inhibits SNB19 cell invasion, it does not affect lamellipodia formation and has only minor effects on cell migration and proliferation. Similar results were obtained in BT549 breast carcinoma cells. Thus, functional analysis of Rac1 and Rac3 using RNA interference reveals a critical role for these GTPases in the invasive behavior of glioma and breast carcinoma cells. 
 Rac1 activity regulates proliferation of aggressive metastatic melanoma. Molecular mechanisms underlying the different capacity of two in vivo selected human melanoma cell variants to form experimental metastases were studied. The doubling times of the FEMX-I and FEMX-V cell sublines in vitro were 15 and 25 h, respectively. The invasive capacity of FEMX-I cells was 8-fold higher than FEMX-V cells, and the time to form approximately 10 mm s.c. tumors in nude mice was 21 versus 35 days. FEMX-I displayed a spindle-like formation in vitro, whereas FEMX-V cells had a rounded shape. Hence, we examined known determinants of cell shape and proliferation, the small GTPases. The four studied showed equal expression in both cell types, but Rac1 activity was significantly decreased in FEMX-V cells. Rac1 stimulates NFkappaB, and we found that endogenous NFkappaB activity of FEMX-V cells was 2% of that of FEMX-I cells. Inhibition of Rac1 resulted in blocked NFkappaB activity. Specific inhibition of either Rac1 or NFkappaB significantly reduced proliferation and invasion of FEMX-I cells, the more pronounced effects observed with Rac1 inhibition. These data indicate that Rac1 activity in FEMX cells regulates cell proliferation and invasion, in part via its effect on NFkappaB, signifying Rac1 as a key molecule in melanoma progression and   metastasis  . 
 Engagement of CD44 promotes Rac activation and CD44 cleavage during tumor cell migration. CD44 is a major cell surface adhesion molecule for hyaluronan, a component of the extracellular matrix, and is implicated in tumor   metastasis   and invasion. We reported previously that hyaluronan oligosaccharides induce CD44 cleavage from tumor cells. Here we show that engagement of CD44 promotes CD44 cleavage and tumor cell migration, both of which were suppressed by a metalloproteinase inhibitor KB-R7785 and tissue inhibitor of metalloproteinases-1 (TIMP-1) but not by TIMP-2. We also present evidence that blockade of metalloproteinase-disintegrin ADAM10 (a disintegrin and metalloproteinase 10) by RNA interference suppresses CD44 cleavage induced by its ligation. Engagement of CD44 concurrently induced activation of the small GTPase Rac1 and led to drastic changes in cell morphology and actin cytoskeleton with redistribution of CD44 to newly generated membrane ruffling areas. A fluorescence resonance energy transfer approach to visualize GTP-bound Rac1 in living cells revealed the localization of the active Rac1 in the leading edge of the membrane ruffling areas upon ligation of CD44. Taken together, our results indicate that the cleavage of CD44 catalyzed by ADAM10 is augmented by the intracellular signaling elicited by engagement of CD44, through Rac-mediated cytoskeletal rearrangement, and suggest that CD44 cleavage contributes to the migration and invasion of tumor cells. 
 Specific upregulation of RHOA and RAC1 in cancer-associated fibroblasts found at primary tumor and lymph node metastatic sites in breast cancer. The importance of tumor-stromal cell interactions in breast tumor progression and invasion is well established. Here, an evaluation of differential genomic profiles of carcinoma-associated fibroblasts (CAFs) compared to fibroblasts derived from tissues adjacent to fibroadenomas (NAFs) revealed altered focal adhesion pathways. These data were validated through confocal assays. To verify the possible role of fibroblasts in lymph node invasion, we constructed a tissue microarray consisting of primary breast cancer samples and corresponding lymph node   metastasis   and compared the expression of adhesion markers RhoA and Rac1 in fibroblasts located at these different locations. Two distinct tissue microarrays were constructed from the stromal component of 43 primary tumors and matched lymph node samples, respectively. Fibroblasts were characterized for their expression of α-smooth muscle actin (α-SMA) and vimentin. Moreover, we verified the level of these proteins in the stromal compartment from normal adjacent tissue and in non-compromised lymph nodes. Our immunohistochemistry revealed that 59 % of fibroblasts associated with primary tumors and 41 % of the respective metastatic lymph nodes (p = 0.271) displayed positive staining for RhoA. In line with this, 57.1 % of fibroblasts associated with primary tumors presented Rac1-positive staining, and the frequency of co-positivity within the lymph nodes was 42.9 % (p = 0.16). Expression of RhoA and Rac1 was absent in fibroblasts of adjacent normal tissue and in compromised lymph nodes. Based on our findings that no significant changes were observed between primary and metastatic lymph nodes, we suggest that fibroblasts are active participants in the invasion of cancer cells to lymph nodes and support the hypothesis that metastatic tumor cells continue to depend on their microenvironment. 
 Expression and significance of Rac1, Pak1 and Rock1 in gastric carcinoma. Rac1, Pak1 and Rock1 are indicators related to gastric cancer invasion and   metastasis  , but few reports discuss all three kinds of protein in research on gastric cancer invasion and   metastasis  . The aim of this study was to investigate the expression and clinical significance of Rac1, Pak1 and Rock1 in gastric carcinoma.Rac1, Pak1 and Rock1 expression in 158 cases of gastric carcinoma were investigated via immunohistochemical staining and clinical analysis.The positive expression rates of Rac1, Pak1 and Rock1 in normal tissue, intraepithelial neoplastic tissues and gastric carcinoma showed an increasing trend (P < 0.05). Their expression in lymph n  ode metast  asis was significantly higher than in patients with lymph-n  ode metast  asis than in those without lymph no  des metast  asis (P < 0.05). Their expression in tumor (TNM stages III and IV) were significantly higher than that in stages I and II (P < 0.05). Rac1, Pak1 and Rock1 expression did not differ significantly with patients' sex (P > 0.05).Positive rates of Rac1, Pak1 and Rock1 expression in normal tissue, dysplasia and gastric carcinoma show an increasing trend and are correlated with t  umor lymph   node metastasis and TNM stage. Rac1, Pak1 and Rock1 may be important biomarkers of gastric carcino  ma invasio  n and metastasis. 
 The sirtuins promote Dishevelled-1 scaffolding of TIAM1, Rac activation and cell migration. Rac1-GTPases serve as intermediary cellular switches, which conduct transient and constitutive signals from upstream cues, including those from Ras oncoproteins. Although the sirtuin1 (SIRT1) deacetylase is overexpressed in several human cancers and has recently been linked to cancer cell motility as a context-dependent regulator of multiple pathways, its role in Rac1 activation has not been reported. Similarly, SIRT2 has been demonstrated to be upregulated in some cancers; however, studies have also reported its role in tumor suppression. Here, we demonstrate that SIRT1 and SIRT2 positively regulate the levels of Rac1-GTP and the activity of T-cell lymphoma invasion and   metastasis   1 (TIAM1), a Rac guanine nucleotide exchange factor (GEF). Transient inhibition of SIRT1 and SIRT2 resulted in increased acetylation of TIAM1, whereas chronic SIRT2 knockdown resulted in enhanced acetylation of TIAM1. SIRT1 regulates Dishevelled (DVL) protein levels in cancer cells, and DVL along with TIAM1 are known to augment Rac activation; however, SIRT1 or 2 has not been previously linked with TIAM1. We found that diminished sirtuin activity led to the disruption of the DVL1-TIAM1 interaction. We hence propose a model for Rac activation where SIRT1/2 positively modulates the DVL/TIAM1/Rac axis and promotes sustained pathway activation. 
 A Rac1/PAK1 cascade controls β-catenin activation in colon cancer cells. P21-activated kinase 1 (PAK1) is associated with colon cancer progression and   metastasi  s, whereas the molecular mechanism remains elusive. Here, we show that downregulation of PAK1 in colon cancer cells reduces total β-catenin level, as well as cell proliferation. Mechanistically, PAK1 directly phosphorylates β-catenin proteins at Ser675 site and this leads to more stable and transcriptional active β-catenin. Corroborating these results, PAK1 is required for full Wnt signaling, and superactivation of β-catenin is achieved by simultaneous knockdown of adenomatous polyposis coli protein and activation of PAK1. Moreover, we show that Rac1 functions upstream of PAK1 in colon cancer cells and contributes to β-catenin phosphorylation and accumulation. We conclude that a Rac1/PAK1 cascade controls β-catenin S675 phosphorylation and full activation in colon cancer cells. Supporting this conclusion, overexpression of PAK1 is observed in 70% of colon cancer samples and is correlated with massive β-catenin accumulation. 
 Rho GTPases RhoA and Rac1 mediate effects of dietary folate on metastatic potential of A549 cancer cells through the control of cofilin phosphorylation. Folate, an important nutrient in the human diet, has been implicated in cancer, but its role in   metastasis   is not established. We have shown previously that the withdrawal of medium folate leads to the inhibition of migration and invasion of A549 lung carcinoma cells. Here we have demonstrated that medium folate regulates the function of Rho GTPases by enabling their carboxyl methylation and translocation to plasma membrane. Conversely, the lack of folate leads to the retention of these proteins in endoplasmic reticulum. Folate also promoted the switch from inactive (GDP-bound) to active (GTP-bound) GTPases, resulting in the activation of downstream kinases p21-activated kinase and LIM kinase and phosphorylation of the actin-depolymerizing factor cofilin. We have further demonstrated that in A549 cells two GTPases, RhoA and Rac1, but not Cdc42, are immediate sensors of folate status: the siRNA silencing of RhoA or Rac1 blocked effects of folate on cofilin phosphorylation and cellular migration and invasion. The finding that folate modulates metastatic potential of cancer cells was confirmed in an animal model of lung cancer using tail vein injection of A549 cells in SCID mice. A folate-rich diet enhanced lung colonization and distant   metastasis   to lymph nodes and decreased overall survival (35 versus 63 days for mice on a folate-restricted diet). High folate also promoted epithelial-mesenchymal transition in cancer cells and experimental mouse tumors. Our study provides experimental evidence for a mechanism of   metastasis   promotion by dietary folate and highlights the interaction between nutrients and   metastasis  -related signaling. 
 PI3K and ERK-induced Rac1 activation mediates hypoxia-induced HIF-1α expression in MCF-7 breast cancer cells. Hypoxia-inducible factor 1 (HIF-1α) expression induced by hypoxia plays a critical role in promoting tumor angiogenesis an  d metastas  is. However, the molecular mechanisms underlying the induction of HIF-1α in tumor cells remain unknown.In this study, we reported that hypoxia could induce HIF-1α and VEGF expression accompanied by Rac1 activation in MCF-7 breast cancer cells. Blockade of Rac1 activation with ectopic expression of an inactive mutant form of Rac1 (T17N) or Rac1 siRNA downregulated hypoxia-induced HIF-1α and VEGF expression. Furthermore, Hypoxia increased PI3K and ERK signaling activity. Both PI3K inhibitor LY294002 and ERK inhibitor U0126 suppressed hypoxia-induced Rac1 activation as well as HIF-1α expression. Moreover, hypoxia treatment resulted in a remarkable production of reactive oxygen species (ROS). N-acetyl-L-cysteine, a scavenger of ROS, inhibited hypoxia-induced ROS generation, PI3K, ERK and Rac1 activation as well as HIF-1α expression.Taken together, our study demonstrated that hypoxia-induced HIF-1α expression involves a cascade of signaling events including ROS generation, activation of PI3K and ERK signaling, and subsequent activation of Rac1. 
 Overexpression of small GTPases directly correlates with expression of δ-catenin and their coexpression predicts a poor clinical outcome in nonsmall cell lung cancer. δ-catenin can affect cytoskeletal assembly, and promote cell migration by regulating the activity of small GTPases. While many malignancies have been shown to be positive for δ-catenin, it is still unclear whether δ-catenin and small GTPases are coexpressed in tumor cells, and so is the relationship between their coexpression and prognosis in the tumor patients. In this study, immunohistochemistry was performed to examine expressive levels of δ-catenin, cdc42, and Rac1 in 135 cases of nonsmall cell lung cancer (NSCLC), including 60 cases with follow-up records. Thirty samples of paired lung cancer tissues and adjacent normal lung tissues were collected to analyze mRNA and protein expression of δ-catenin and small GTPases. The effects of δ-catenin on small GTPases expression and invasive ability of lung cancer cells were also evaluated. Compared with normal lung tissues, both mRNA and protein levels of δ-catenin and small GTPases were increased in lung cancer tissues (P < 0.05), and the expression of small GTPases directly correlated with that of δ-catenin (P < 0.001). In addition, δ-catenin and small GTPases tended to be coexpressed in lung adenocarcinoma, advanced stages, and primary tumors with l  ymph node   metastasis (all P < 0.05). The patients with coexpression of δ-catenin and small GTPases had a shorter survival time than those without coexpression (P < 0.05). Furthermore, δ-catenin overexpression could enhance invasive ability of lung cancer cells by upregulating protein and transcriptional level of small GTPases. Therefore, δ-catenin likely upregulates the activity of small GTPases at transcriptional level, and their coexpression may predict a poor clinical outcome in NSCLC patients. 
  GeneRIFs  
 we found that CXCR4-CXCL12/AKT axis regulates RhoA, Rac-1, and Cdc42 to modulate cell invasion and tumor   metastasis  . Together, these results demonstrate a role for CXCR4 in ESCC   metastasis   and progression and suggest potential targets for therapeutic intervention. 
 Rap1- and Rac1-GTPase activation have roles in hypoxia/reoxygenation-experienced cancer cell migration and   metastasis   via the expression of thymosin beta-4 
 Positive rates of Rac1, Pak1 and Rock1 expression in normal tissue, dysplasia and gastric carcinoma show an increasing trend and are correlated with tumor lymph node   metastasis   and TNM stage. 
 These findings show that overexpression of PODXL enhanced invadopodia formation and tumor   metastasis   by inducing Rac1/Cdc42/cortactin signaling network. 
 Dietary folate affects   metastasis   through the regulation of the carboxyl methylation of Rho family GTPases RhoA and Rac1. 
 High Rac-1 expression and lymph node   metastasis   were two independent factors for poor survival. 
 Human TIPE2 is an endogenous inhibitor of Rac1 in hepatocellular carcinoma (HCC) by which it attenuates invasion and   metastasis   of HCC. 
 Recent insights at the molecular and cellular levels are paving the way for a more directed and detailed approach to target mechanisms of RAC1 regulating angiogenesis and   metastasis   
 findings indicate that a chemokine-controlled pathway, consisting of Galphai2, ELMO1/Dock180, Rac1 and Rac2, regulates the actin cytoskeleton during breast cancer   metastasis   
 Rac1, HIF-1alpha, and VEGF play an important role in tumor invasion and   metastasis  , especially in tumor angiogenesis. 
 Data suggest that an anti-Wnt5a antibody was capable of suppressing Wnt5a-dependent internalization of Fz2 receptor, resulting in the prevention of   metastasis   of gastric cancer cells by inhibiting the activation of Rac1 and the expression of laminin gamma2. 
 Rac1 is critical for tumor initiation and   metastasis   of human non-small cell lung adenocarcinoma 
 Increased RAC1 is associated with vascular invasion and intrahepatic   metastasis   hepatocellular carcinoma. 
 The bFGF-FGFR1-PI3K-Rac1 pathway in the bone microenvironment may have a significant role in the invasion and   metastasis   of Ewing sarcoma. 
 Suggest that Rac1 activity and Pak1 are involved in lymphovascular invasion and lymph node   metastasis   of urothelial carcinoma of the upper urinary tract , and may be prognostic markers for this disease. 
 Results suggest that Rac1 plays a critical role in signalling transduction pathways relevant to colorectal tumour progression, such as activation of Wnt signalling, inhibition of TGF-beta signalling, and enhancement of   metastasis  -inducing genes. 
 These data indicate that Rac1 activity in FEMX cells regulates cell proliferation and invasion, in part via its effect on NFkappaB, signifying Rac1 as a key molecule in melanoma progression and   metastasis  . 
 loss of PTEN function in human prostate cancer may specifically facilitate bone rather than other organ   metastasis   and suggest that Rac1, as a PTEN effector, may contribute to this metastatic tropism. 
 alterations of rac1 gene may contribute to tumorigenesis and/or   metastasis   
  [Cc]ell [Ii]nvasion  PubMed  
 Upregulation of Eps8 in oral squamous cell carcinoma promotes cell migration and invasion through integrin-dependent Rac1 activation. Oral squamous cell carcinoma (OSCC) is a lethal disease and early death usually occurs as a result of local invasion and regional lymph node metastases. Current treatment regimens are, to a certain degree, inadequate, with a 5-year mortality rate of around 50% and novel therapeutic targets are urgently required. Using expression microarrays, we identified the eps8 gene as being overexpressed in OSCC cell lines relative to normal oral keratinocytes, and confirmed these findings using RT-PCR and western blotting. In human tissues, we found that Eps8 was upregulated in OSCC (32% of primary tumors) compared with normal oral mucosa, and that expression correlated significantly with lymph node metastasis (P=0.032), suggesting a disease-promoting effect. Using OSCC cell lines, we assessed the functional role of Eps8 in tumor cells. Although suppression of Eps8 produced no effect on cell proliferation, both cell spreading and migration were markedly inhibited. The latter cell functions may be modulated through the small GTP-ase, Rac1 and we used pull-down assays to investigate the role of Eps8 in Rac1 signaling. We found that alphavbeta6- and alpha5beta1-integrin-dependent activation of Rac1 was mediated through Eps8. Knockdown of either Eps8 or Rac1, inhibited integrin-dependent cell migration similarly and transient expression of constitutively active Rac1 restored migration of cells in which Eps8 expression had been suppressed. We also showed that knockdown of Eps8 inhibited tumor   cell invasion   in an organotypic model of OSCC. These data suggest that Eps8 and Rac1 are part of an integrated signaling pathway modulating integrin-dependent tumour cell motility and identify Eps8 as a possible therapeutic target. 
 Host cell kinases, α5 and β1 integrins, and Rac1 signalling on the microtubule cytoskeleton are important for non-typable Haemophilus influenzae invasion of respiratory epithelial cells. Non-typable Haemophilus influenzae (NTHi) is a common commensal of the human nasopharynx, but causes opportunistic infection when the respiratory tract is compromised by infection or disease. The ability of NTHi to invade epithelial cells has been described, but the underlying molecular mechanisms are poorly characterized. We previously determined that NTHi promotes phosphorylation of the serine-threonine kinase Akt in A549 human lung epithelial cells, and that Akt phosphorylation and NTH  i cell invasi  on are prevented by inhibition of phosphoinositide 3-kinase (PI3K). Because PI3K-Akt signalling is associated with several host cell networks, the purpose of the current study was to identify eukaryotic molecules important for NTHi epithelial invasion. We found that inhibition of Akt activity reduced NTHi internalization; differently, bacterial entry was increased by phospholipase Cγ1 inhibition but was not affected by protein kinase inhibition. We also found that α5 and β1 integrins, and the tyrosine kinases focal adhesion kinase and Src, are important for NTHi   A549 cell inv  asion. NTHi internalization was shown to be favoured by activation of Rac1 guanosine triphosphatase (GTPase), together with the guanine nucleotide exchange factor Vav2 and the effector Pak1. Also, Pak1 might be associated with inactivation of the microtubule destabilizing agent Op18/stathmin, to facilitate microtubule polymerization and NTHi entry. Conversely, inhibition of RhoA GTPase and its effector ROCK increased the number of internalized bacteria. Src and Rac1 were found to be important for NTHi-triggered Akt phosphorylation. An increase in host cyclic AMP reduced bacterial entry, which was linked to protein kinase A. These findings suggest that NTHi finely manipulates host signalling molecules to invade respiratory epithelial cells. 
 Host cell kinases, α5 and β1 integrins, and Rac1 signalling on the microtubule cytoskeleton are important for non-typable Haemophilus influenzae invasion of respiratory epithelial cells. Non-typable Haemophilus influenzae (NTHi) is a common commensal of the human nasopharynx, but causes opportunistic infection when the respiratory tract is compromised by infection or disease. The ability of NTHi to invade epithelial cells has been described, but the underlying molecular mechanisms are poorly characterized. We previously determined that NTHi promotes phosphorylation of the serine-threonine kinase Akt in A549 human lung epithelial cells, and that Akt phosphorylation and NTH  i cell invasi  on are prevented by inhibition of phosphoinositide 3-kinase (PI3K). Because PI3K-Akt signalling is associated with several host cell networks, the purpose of the current study was to identify eukaryotic molecules important for NTHi epithelial invasion. We found that inhibition of Akt activity reduced NTHi internalization; differently, bacterial entry was increased by phospholipase Cγ1 inhibition but was not affected by protein kinase inhibition. We also found that α5 and β1 integrins, and the tyrosine kinases focal adhesion kinase and Src, are important for NTHi   A549 cell inv  asion. NTHi internalization was shown to be favoured by activation of Rac1 guanosine triphosphatase (GTPase), together with the guanine nucleotide exchange factor Vav2 and the effector Pak1. Also, Pak1 might be associated with inactivation of the microtubule destabilizing agent Op18/stathmin, to facilitate microtubule polymerization and NTHi entry. Conversely, inhibition of RhoA GTPase and its effector ROCK increased the number of internalized bacteria. Src and Rac1 were found to be important for NTHi-triggered Akt phosphorylation. An increase in host cyclic AMP reduced bacterial entry, which was linked to protein kinase A. These findings suggest that NTHi finely manipulates host signalling molecules to invade respiratory epithelial cells. 
 Upregulation of Eps8 in oral squamous cell carcinoma promotes cell migration and invasion through integrin-dependent Rac1 activation. Oral squamous cell carcinoma (OSCC) is a lethal disease and early death usually occurs as a result of local invasion and regional lymph node metastases. Current treatment regimens are, to a certain degree, inadequate, with a 5-year mortality rate of around 50% and novel therapeutic targets are urgently required. Using expression microarrays, we identified the eps8 gene as being overexpressed in OSCC cell lines relative to normal oral keratinocytes, and confirmed these findings using RT-PCR and western blotting. In human tissues, we found that Eps8 was upregulated in OSCC (32% of primary tumors) compared with normal oral mucosa, and that expression correlated significantly with lymph node metastasis (P=0.032), suggesting a disease-promoting effect. Using OSCC cell lines, we assessed the functional role of Eps8 in tumor cells. Although suppression of Eps8 produced no effect on cell proliferation, both cell spreading and migration were markedly inhibited. The latter cell functions may be modulated through the small GTP-ase, Rac1 and we used pull-down assays to investigate the role of Eps8 in Rac1 signaling. We found that alphavbeta6- and alpha5beta1-integrin-dependent activation of Rac1 was mediated through Eps8. Knockdown of either Eps8 or Rac1, inhibited integrin-dependent cell migration similarly and transient expression of constitutively active Rac1 restored migration of cells in which Eps8 expression had been suppressed. We also showed that knockdown of Eps8 inhibited tumor   cell invasion   in an organotypic model of OSCC. These data suggest that Eps8 and Rac1 are part of an integrated signaling pathway modulating integrin-dependent tumour cell motility and identify Eps8 as a possible therapeutic target. 
 Activation of Vav/Rho GTPase signaling by CXCL12 controls membrane-type matrix metalloproteinase-dependent melanoma   cell invasion  . Melanoma cells express the chemokine receptor CXCR4, which confers invasive signals on binding to its ligand CXCL12. We show here that knocking down membrane-type matrix metalloproteinase (MT1-MMP) expression translates into a blockade of invasion across reconstituted basement membranes and type I collagen gels in response to CXCL12, which is the result of lack of MMP-2 activation. Interference with MMP-2 expression further confirms its important role during this invasion. Vav proteins are guanine-nucleotide exchange factors for Rho GTPases that regulate actin dynamics and gene expression. We show that melanoma cells express Vav1 and Vav2, which are activated by CXCL12 involving Jak activity. Blocking Vav expression by RNA interference results in impaired activation of Rac and Rho by CXCL12 and in a remarkable inhibition of CXCL12-promoted invasion. Importantly, up-regulation of MT1-MMP expression by CXCL12, a mechanism contributing to melanoma   cell invasion  , is blocked by knocking down Vav expression or by inhibiting Jak. Together, these data indicate that activation of Jak/Vav/Rho GTPase pathway by CXCL12 is a key signaling event for MT1-MMP/MMP-2-dependent melanoma   cell invasion  . 
 Involvement of Rac and Rho signaling in cancer cell motility in 3D substrates. The motility of cancer cells in 3D matrices is of two types: mesenchymal motility, in which the cells are elongated and amoeboid motility, in which the cells are round. Amoeboid motility is driven by an actomyosin-based contractile force, which is regulated by the Rho/ROCK pathway. However, the molecular mechanisms underlying the motility of elongated cells remain unknown. Here, we show that the motility of elongated cells is regulated by Rac signaling through the WAVE2/Arp2/3-dependent formation of elongated pseudopodia and cell-substrate adhesion in 3D substrates. The involvement of Rac signaling in cell motility was different in cell lines that displayed an elongated morphology in 3D substrates. In U87MG glioblastoma cells, most of which exhibit mesenchymal motility, inhibition of Rac signaling blocked the invasion of these cells in 3D substrates. In HT1080 fibrosarcoma cells, which display mixed cell motility involving both elongated and rounded cells, inhibition of Rac1 signaling not only blocked mesenchymal motility but also caused a mesenchymal-amoeboid transition. Additionally, Rac1 and RhoA signaling regulated the mesenchymal and amoeboid motility in these cells, respectively, and the inhibition of both pathways dramatically decreased   cell invasion  . Hence, we could conclude that Rac1 and RhoA signaling simultaneously regulate   cell invasion   in 3D matrices. 
 Increased fibroblast growth factor-inducible 14 expression levels promote glioma   cell invasion   via Rac1 and nuclear factor-kappaB and correlate with poor patient outcome. Glial tumors progress to malignant grades by heightened proliferation and relentless dispersion throughout the central nervous system. Understanding genetic and biochemical processes that foster these behaviors is likely to reveal specific and effective targets for therapeutic intervention. Our current report shows that the fibroblast growth factor-inducible 14 (Fn14), a member of the tumor necrosis factor (TNF) receptor superfamily, is expressed at high levels in migrating glioma cells in vitro and invading glioma cells in vivo. Forced Fn14 overexpression stimulates glioma cell migration and invasion, and depletion of Rac1 by small interfering RNA inhibits this cellular response. Activation of Fn14 signaling by the ligand TNF-like weak inducer of apoptosis (TWEAK) stimulates migration and up-regulates expression of Fn14; this TWEAK effect requires Rac1 and nuclear factor-kappaB (NF-kappaB) activity. The Fn14 promoter region contains NF-kappaB binding sites, which mediate positive feedback causing sustained overexpression of Fn14 and enduring glioma   cell invasion  . Furthermore, Fn14 gene expression levels increase with glioma grade and inversely correlate with patient survival. These results show that the Fn14 cascade operates as a positive feedback mechanism for elevated and sustained Fn14 expression. Such a feedback loop argues for aggressive targeting of the Fn14 axis as a unique and specific driver of glioma malignant behavior. 
 Persistent activation of Rac1 in squamous carcinomas of the head and neck: evidence for an EGFR/Vav2 signaling axis involved in   cell invasion  . The poor prognosis associated with head and neck squamous cell carcinoma (HNSCC) is primarily due to both local invasion and the regional and/or distant metastatic spread. Recent findings have provided evidence that the acquisition of a motile and invasive phenotype by cancer cells involves the dysregulated function of key intracellular molecular mechanisms together with aberrant signaling events initiated by the surrounding microenvironment. These intrinsic and extrinsic biochemical pathways in turn often converge to stimulate the activity of members of the Rho family of Ras-related guanosine triphosphate (GTP)-binding proteins, including RhoA, Rac and Cdc42, which control the organization of the actin cytoskeleton thereby regulating cell adhesion, polarity and motility. In this study, we examined the status of activation of these GTPases in a representative collection of HNSCC cell lines. Surprisingly, we found that most HNSCC cells exhibit remarkably high levels of GTP-bound Rac1. Further analysis revealed that the activation of Rac1 in these HNSCC cells could be due to two independent signaling events, an epidermal growth factor receptor (EGFR)-based autocrine loop that leads to the activation of the Rac1 exchange factor Vav2 and an EGFR/Vav2-independent pathway that arises as a consequence of the oncogenic mutation of the H-ras proto-oncogene. Indeed, we provide evidence that the EGFR/Vav2/Rac1 axis is a crucial pathway for the acquisition of motile and invasive properties of most HNSCC cells. These findings shed light onto the molecular mechanisms involved in HNSCC   cell invasion  , and may reveal new therapeutic opportunities to halt the metastatic spread of these aggressive malignancies. 
 Rab23 is overexpressed in human astrocytoma and promotes cell migration and invasion through regulation of Rac1. Rab23 overexpression has been implicated in several human cancers. However, its biological roles and molecular mechanism in astrocytoma have not been elucidated. The aim of this study is to explore clinical significance and biological roles of Rab23 in astrocytoma. We observed negative Rab23 staining in normal astrocytes and positive staining in 39 out of 86 (45 %) astrocytoma specimens using immunohistochemistry. The positive rate of Rab23 was higher in grades III and IV (56.5 %, 26/46) than grades I + II astrocytomas (32.5 %, 13/40, p < 0.05). Transfection of Rab23 plasmid was performed induced A172 cell proliferation, colony formation, invasion, and migration, while Rab23 depletion with siRNA reduced these abilities of U87 cells. In addition, we found that Rab23 transfection upregulated while its depletion reduced Rac1 activity. Treatment of transfected cells with a Rac1 inhibitor decreased Rac1 activity and invasion. In conclusion, Rab23 serves as an important oncoprotein in human astrocytoma by regu  lating cell i  nvasion and migration through Rac1 activity. 
 Regulation of CXCR4/AKT-signaling-induced   cell invasion   and tumor metastasis by RhoA, Rac-1, and Cdc42 in human esophageal cancer. CXC chemokines and their cognate receptors have been implicated wildly in cancer pathogenesis. In the present study, we report a critical cause relationship between CXCR4 expression and tumorigenesis in the setting of human esophageal squamous cell carcinoma (ESCC). In ESCC cells, CXCR4 expression was significantly higher than in human esophageal epithelial cells (HEEC). Reduction of CXCR4 in ESCC cells reduced cell proliferation and invasion in vitro and tumor growth in vivo. Among the potential downstream targets of CXCR4-CXCL12 are RhoA, Rac-1, and Cdc42, which are likely to contribute to the invasiveness of ESCC cells. Finally, we found that CXCR4-CXCL12/AKT axis regulates RhoA, Rac-1, and Cdc42 to modulate   cell invasion   and tumor metastasis. Together, these results demonstrate a role for CXCR4 in ESCC metastasis and progression and suggest potential targets for therapeutic intervention. 
 The Vibrio parahaemolyticus effector VopC mediates Cdc42-dependent invasion of cultured cells but is not required for pathogenicity in an animal model of infection. Vibrio parahaemolyticus is a Gram-negative marine bacterium that causes acute gastroenteritis in humans. The virulence of V. parahaemolyticus is dependent upon a type III secretion system (T3SS2). One effector for T3SS2, VopC, is a homologue of the catalytic domain of cytotoxic necrotizing factor (CNF), and was recently reported to be a Rho family GTPase activator and to be linked to internalization of V. parahaemolyticus by non-phagocytic cultured cells. Here, we provide direct evidence that VopC deamidates Rac1 and CDC42, but not RhoA, in vivo. Our results alsosuggest that VopC, through its activation of Rac1, contributes to formation of actin stress fibres in infected cells. Invasion of host cells, which occurs at a low frequency, does not seem linked to Rac1 activation, but instead appears to require CDC42. Finally, using an infant rabbit model of V. parahaemolyticus infection, we show that the virulence of V. parahaemolyticus is not dependent upon VopC-mediated invasion. Genetic inactivation of VopC did not impair intestinal colonization nor reduce signs of disease, including fluid accumulation, diarrhoea and tissue destruction. Thus, although VopC can promo  te host cell   invasion, such internalization is not a critical step of the disease process, consistent with the traditional view of V. parahaemolyticus as an extracellular pathogen. 
 The tumor suppressor role of miR-124 in osteosarcoma. MicroRNAs have crucial roles in development and progression of human cancers, including osteosarcoma. Recent studies have shown that miR-124 was down-regulated in many cancers; however, the role of miR-124 in osteosarcoma development is unknown. In this study, we demonstrate that expression of miR-124 is significantly downregulated in osteosarcoma tissues and cell lines, compared to the adjacent tissues. The expression of miR-124 in the metastases osteosarcoma tissues was lower than that in non- metastases tissues. We identified and confirmed Rac1 as a novel, direct target of miR-124 using prediction algorithms and luciferase reporter gene assays. Overexpression of miR-124 suppressed Rac1 protein expression and attenuated cell proliferation, migration, and invasion and induced apoptosis in MG-63 and U2OS in vitro. Moreover, overexpression of Rac1 in miR-124-transfected osteosarcoma cells effectively rescued the inhibition of   cell invasion   caused by miR-124. Therefore, our results demonstrate that miR-124 is a tumor suppressor miRNA and suggest that this miRNA could be a potential target for the treatment of osteosarcoma in future. 
 A Trio-Rac1-Pak1 signalling axis drives invadopodia disassembly. Rho family GTPases control cell migration and participate in the regulation of cancer metastasis. Invadopodia, associated with invasive tumour cells, are crucial for cellular invasion and metastasis. To study Rac1 GTPase in invadopodia dynamics, we developed a genetically encoded, single-chain Rac1 fluorescence resonance energy (FRET) transfer biosensor. The biosensor shows Rac1 activity exclusion from the core of invadopodia, and higher activity when invadopodia disappear, suggesting that reduced Rac1 activity is necessary for their stability, and Rac1 activation is involved in disassembly. Photoactivating Rac1 at invadopodia confirmed this previously unknown Rac1 function. We describe here an invadopodia disassembly model, where a signalling axis involving TrioGEF, Rac1, Pak1, and phosphorylation of cortactin, causes invadopodia dissolution. This mechanism is critical for the proper turnover of invasive structures during tumour   cell invasion  , where a balance of proteolytic activity and locomotory protrusions must be carefully coordinated to achieve a maximally invasive phenotype. 
 Rapid Remodeling of Invadosomes by Gi-coupled Receptors: DISSECTING THE ROLE OF Rho GTPases. Invadosomes are actin-rich membrane protrusions that degrade the extracellular matrix to drive tumor   cell invasion  . Key players in invadosome formation are c-Src and Rho family GTPases. Invadosomes can reassemble into circular rosette-like superstructures, but the underlying signaling mechanisms remain obscure. Here we show that Src-induced invadosomes in human melanoma cells (A375M and MDA-MB-435) undergo rapid remodeling into dynamic extracellular matrix-degrading rosettes by distinct G protein-coupled receptor agonists, notably lysophosphatidic acid (LPA; acting through the LPA1 receptor) and endothelin. Agonist-induced rosette formation is blocked by pertussis toxin, dependent on PI3K activity and accompanied by localized production of phosphatidylinositol 3,4,5-trisphosphate, whereas MAPK and Ca(2+) signaling are dispensable. Using FRET-based biosensors, we show that LPA and endothelin transiently activate Cdc42 through Gi, concurrent with a biphasic decrease in Rac activity and differential effects on RhoA. Cdc42 activity is essential for rosette formation, whereas G12/13-mediated RhoA-ROCK signaling suppresses the remodeling process. Our results reveal a Gi-mediated Cdc42 signaling axis by which G protein-coupled receptors trigger invadosome remodeling, the degree of which is dictated by the Cdc42-RhoA activity balance. 
 14-3-3zeta Cooperates with ErbB2 to promote ductal carcinoma in situ progression to invasive breast cancer by inducing epithelial-mesenchymal transition. ErbB2, a metastasis-promoting oncoprotein, is overexpressed in approximately 25% of invasive/metastatic breast cancers, but in 50%-60% of noninvasive ductal carcinomas in situ (DCIS). It has been puzzling how a subset of ErbB2-overexpressing DCIS develops into invasive breast cancer (IBC). We found that co-overexpression of 14-3-3zeta in ErbB2-overexpressing DCIS conferred a higher risk of progression to IBC. ErbB2 and 14-3-3zeta overexpression, respectively, increased cell migration and decreased cell adhesion, two prerequisites of tumor   cell invasion  . 14-3-3zeta overexpression reduced cell adhesion by activating the TGF-beta/Smads pathway that led to ZFHX1B/SIP-1 upregulation, E-cadherin loss, and epithelial-mesenchymal transition. Importantly, patients whose breast tumors overexpressed both ErbB2 and 14-3-3zeta had higher rates of metastatic recurrence and death than those whose tumors overexpressed only one. 
 Loss of WNK2 expression by promoter gene methylation occurs in adult gliomas and triggers Rac1-mediated tumour cell invasiveness. The gene encoding protein kinase WNK2 was recently identified to be silenced by promoter hypermethylation in gliomas and meningiomas, suggesting a tumour-suppressor role in these brain tumours. Following experimental depletion in cell lines, WNK2 was further found to control GTP-loading of Rac1, a signalling guanosine triphosphatase involved in cell migration and motility. Here we show that WNK2 promoter methylation also occurs in 17.5% (29 out of 166) of adult gliomas, whereas it is infrequent in its paediatric forms (1.6%; 1 out of 66). Re-expression of WNK2 in glioblastoma cells presenting WNK2 gene silencing reduced cell proliferation in vitro, tumour growth in vivo and also cell migration and invasion, an effect correlated with reduced activation of Rac1. In contrast, when endogenous WNK2 was depleted from glioblastoma cells with unmethylated WNK2 promoter, changes in cell morphology, an increase in invasion and activation of Rac1 were observed. Together, these results validate the WNK2 gene as a recurrent target for epigenetic silencing in glia-derived brain tumours and provide first mechanistic evidence for a tumour-suppressing role of WNK2 that is related to Rac1 signalling and tumour   cell invasion   and proliferation. 
 Astragaloside IV inhibits breast cancer   cell invasion   by suppressing Vav3 mediated Rac1/MAPK signaling. Astragaloside IV (AS-IV), the major active triterpenoid in Radix Astragali, has shown anti-tumorigenic properties in certain cancers; however, its role in breast cancer remains unclear. The present study investigated the effects of AS-IV on breast cancer in vitro and in vivo and examined the underlying mechanisms.The effects of AS-IV on MDA-MB-231 cell proliferation, migration, invasion and metastasis were investigated by MTT and Transwell assays, and western blotting. In addition, an orthotopic mouse tumor model was established for in vivo experiments.AS-IV inhibited the viability and invasive potential of MDA-MB-231 breast cancer cells, suppressed the activation of the mitogen activated protein kinase (MAPK) family members ERK1/2 and JNK, and downregulated matrix metalloproteases (MMP)-2 and -9. The effects of AS-IV were mediated by the downregulation of Vav3, a guanine nucleotide exchange factor, leading to decreased levels of activated Rac1, a Rho family GTPase. Vav3 overexpression promoted cell proliferation and invasion in vitro, whereas Vav3 silencing had the opposite effects. AS-IV suppressed orthotopic breast tumor growth and metastasis to the lungs, whereas ectopic expression of Vav3 reversed the inhibitory effect of AS-IV on cell viability, invasiveness, MAPK signaling and MMP expression.The present results provide a mechanistic explanation for the antitumor effects of AS-IV and suggest its potential in the treatment of metastatic breast cancer. 
 Role of the small Rho GTPases Rac1 and Cdc42 in host   cell invasion   of Campylobacter jejuni. Host   cell invasion   of the food-borne pathogen Campylobacter jejuni is one of the primary reasons of tissue damage in humans but molecular mechanisms are widely unclear. Here, we show that C. jejuni triggers membrane ruffling in the eukaryotic cell followed by invasion in a very specific manner first with its tip followed by the flagellar end. To pinpoint important signalling events involved in the C. jejuni invasion process, we examined the role of small Rho family GTPases. Using specific GTPase-modifying toxins, inhibitors and GTPase expression constructs we show that Rac1 and Cdc42, but not RhoA, are involved in C. jejuni invasion. In agreement with these observations, we found that internalization of C. jejuni is accompanied by a time-dependent activation of both Rac1 and Cdc42. Finally, we show that the activation of these GTPases involves different host cell kinases and the bacterial fibronectin-binding protein CadF. Thus, CadF is a bifunctional protein which triggers bacterial binding to host cells as well as signalling leading to GTPase activation. Collectively, our results suggest that C. jejuni invade host target cells by a unique mechanism and the activation of the Rho GTPase members Rac1 and Cdc42 plays a crucial role in this entry process. 
 Elmo1 helps dock180 to regulate Rac1 activity and cell migration of ovarian cancer. Engulfment and cell motility 1 (Elmo1) has been reported to cooperate with dedicator of cytokinesis 1 (Dock180) and to be linked to the invasive phenotype of cancer cells through activating small G-protein Rac. We aimed to study the role of Elmo1 in the malignant migration of ovarian cancer.Engulfment and cell motility 1 expression was evaluated in specimens from 93 patients with serous ovarian cancer (SOC) by immunohistochemical staining. Next, Elmo1-RNAi cells were established by validated small interference RNAs. Cell proliferation and cell motility were observed and compared with Dock180-RNAi cells. To confirm their synergetic contribution to forming focal adhesion and activating Rac1, Rac1-GTP level was measured by GST pull-down assay and immunofluorescence was used to observe focal adhesion formation both in Elmo1-RNAi and Dock180-RNAi cells.Engulfment and cell motility 1 was mainly overexpressed in high-grade SOC tissues. Western blot analysis demonstrated that both Elmo1 and Dock180 expressions were hampered in Elmo1-RNAi cells. Compared with the negative control, decreased colony formation and   cell invasion   were observed in Elmo1-RNAi cells and Dock180-RNAi cells. Consistently, both exhibited reduced Rac1-GTP level and inhibited focal adhesion formation.Engulfment and cell motility 1 presents with synergetic action in helping Dock180 to activate Rac1 and promote cell motility, and thus promote untoward expansion and aggressiveness of SOC. 
 Semaphorin 5A and plexin-B3 inhibit human glioma cell motility through RhoGDIalpha-mediated inactivation of Rac1 GTPase. Semaphorins and plexins are implicated in the progression of various types of cancer, although the molecular basis has not been fully elucidated. Here, we report the expression of plexin-B3 in glioma cells, which upon stimulation by its ligand Sema5A results in significant inhibition of cell migration and invasion. A search for the underlying mechanism revealed direct interaction of plexin-B3 with RhoGDP dissociation inhibitor α (RhoGDIα), a negative regulator of RhoGTPases that blocks guanine nucleotide exchange and sequesters them away from the plasma membrane. Glioma cells challenged with Sema5A indeed showed a marked reduction in Rac1-GTP levels by 60%, with a concomitant disruption of lamellipodia. The inactivation of Rac1 was corroborated to contribute to the impediment of gliom  a cell invasi  on by Sema5A, as supported by the abolishment of effect upon forced expression of a constitutively active Rac1 mutant. Furthermore, silencing the endogenous expression of RhoGDIα in glioma cells was found to be sufficient in abrogating the down-regulation of Rac1-GTP and the ensuing suppression of glioma cell motility induced by Sema5A. Mechanistically, we provide evidence that Sema5A promotes Rac1 recruitment to RhoGDIα and reduces its membrane localization in a plexin-B3-dependent manner, thereby preventing Rac1 activation. This represents a novel signaling of semaphorin and plexin in the control of cell motility by indirect inactivation of Rac1 through RhoGDIα. 
 Rac1 and Rac3 isoform activation is involved in the invasive and metastatic phenotype of human breast cancer cells. The metastatic progression of cancer is a direct result of the disregulation of numerous cellular signaling pathways, including those associated with adhesion, migration, and invasion. Members of the Rac family of small GTPases are known to act as regulators of actin cytoskeletal structures and strongly influence the cellular processes of integrin-mediated adhesion and migration. Even though hyperactivated Rac proteins have been shown to influence metastatic processes, these proteins have never been directly linked to metastatic progression.To investigate a role for Rac and Cdc42 in metastatic breast cancer   cell invasion   and migration, relative endogenous Rac or Cdc42 activity was determined in a panel of metastatic variants of the MDA-MB-435 metastatic human breast cancer cell line using a p21-binding domain-PAK pull down assay. To investigate the migratory and invasive potential of the Rac isoforms in human breast cancer, namely Rac1 and the subsequently cloned Rac3, we stably expressed either dominant active Rac1 or dominant active Rac3 into the least metastatic cell variant. Dominant negative Rac1 or dominant negative Rac3 were stably expressed in the most metastatic cell variant. Cell lines expressing mutant Rac1 or Rac3 were analyzed using in vitro adhesion, migration and invasion assays.We show that increased activation of Rac proteins directly correlates with increasing metastatic potential in a panel of cell variants derived from a single metastatic breast cancer cell line (MDA-MB-435). The same correlation could not be found with activated Cdc42. Expression of a dominant active Rac1 or a dominant active Rac3 resulted in a more invasive and motile phenotype. Moreover, expression of either dominant negative Rac1 or dominant negative Rac3 into the most metastatic cell variant resulted in decreased invasive and motile properties.This study correlates endogenous Rac activity with high metastatic potential and implicates Rac in the regulation of cell migration and invasion in metastatic breast cancer cells. Taken together, these results suggest a role for both the Rac1 and Rac3 GTPases in human breast cancer progression. 
 [Rab23 enhances invasion of Sa3 cutaneous squamous cell carcinoma cells via up-regulating the expression of Rac1]. To investigate the effect of Ras-related protein 23 (Rab23) on the invasion capacity of Sa3 cutaneous squamous cell carcinaoma (cSCC) cells and determine whether Rab23 can enhance the invasion of Sa3 cells through regulation of Ras-related C3 botulinum toxin substrate 1 (Rac1).Sa3 cells stably expressing exogenous Rab23 or Sa3 cells deprived of endogenous Rab23 were generated using lentivirus transfection. Transwell(TM) invasion assay was used to evaluate the invasion capacity in the above-mentioned cells. The effects of Rab23 overexpression or knockdown on Rac1 expression were assessed using Western blotting. In the last experimental setting, the engineered cells were pretreated with Rac1 inhibitor Z62954982 before being subjected to the assessment of invasion capacity by Transwell(TM) assay.Rab23 overexpression enhanced the invasion ability, whereas ablation of Rab23 attenuated the invasion in Sa3 cells. Rab23 could up-regulate the expression level of Rac1. Finally, using Rac1 inhibitor, we successfully suppressed the invasion capacity of Sa3 cells stably overexpressing Rab23.Rab23 may enhance cSCC   cell invasion   via up-regulating Rac1 signaling. 
 A MAPK-Driven Feedback Loop Suppresses Rac Activity to Promote RhoA-Driven Cancer   Cell Invasion  . Cell migration in 3D microenvironments is fundamental to development, homeostasis and the pathobiology of diseases such as cancer. Rab-coupling protein (RCP) dependent co-trafficking of α5β1 and EGFR1 promotes cance  r cell invasi  on into fibronectin (FN) containing extracellular matrix (ECM), by potentiating EGFR1 signalling at the front of invasive cells. This promotes a switch in RhoGTPase signalling to inhibit Rac1 and activate a RhoA-ROCK-Formin homology domain-containing 3 (FHOD3) pathway and generate filopodial actin-spike protrusions which drive invasion. To further understand the signalling network that drives RCP-driven invasive migration, we generated a Boolean logical model based on existing network pathways/models, where each node can be interrogated by computational simulation. The model predicted an unanticipated feedback loop, whereby Raf/MEK/ERK signalling maintains suppression of Rac1 by inhibiting the Rac-activating Sos1-Eps8-Abi1 complex, allowing RhoA activity to predominate in invasive protrusions. MEK inhibition was sufficient to promote lamellipodia formation and oppose filopodial actin-spike formation, and led to activation of Rac and inactivation of RhoA at the leading edge of cells moving in 3D matrix. Furthermore, MEK inhibition abrogated RCP/α5β1/EGFR1-driven invasive migration. However, upon knockdown of Eps8 (to suppress the Sos1-Abi1-Eps8 complex), MEK inhibition had no effect on RhoGTPase activity and did not oppose invasive migration, suggesting that MEK-ERK signalling suppresses the Rac-activating Sos1-Abi1-Eps8 complex to maintain RhoA activity and promote filopodial actin-spike formation and invasive migration. Our study highlights the predictive potential of mathematical modelling approaches, and demonstrates that a simple intervention (MEK-inhibition) could be of therapeutic benefit in preventing invasive migration and metastasis. 
 Inhibition of the Rac1-WAVE2-Arp2/3 signaling pathway promotes radiosensitivity via downregulation of cofilin-1 in U251 human glioma cells. The Ras-related C3 botulinum toxin substrate 1 (Rac1)-WASP-family verprolin-homologous protein-2 (WAVE2)-actin-related protein 2/3 (Arp2/3) signaling pathway has been identified to be involved in cell migration and invasion in various types of cancer cell. Cofilin‑1 (CFL‑1), which is regulated by the Rac1‑WAVE2‑Arp2/3 signaling pathway, may promote radioresistance in glioma. Therefore, the present study aimed to investigate the potential role of the Rac1‑WAVE2‑Arp2/3 signaling pathway in radioresistance in U251 human glioma cells and elucidate its affect on CFL‑1 expression. Western blot analysis was performed to evaluate the protein expression of CFL‑1. In the present study, Rac1 was inhibited by NSC 23766, WAVE2 was inhibited by transfection with short hairpin (sh)RNA‑WAVE2 using Lipofectamine™ 2000 and Arp2/3 was inhibited by CK‑666. Cell viability was measured using the 3‑(4,5‑dimethylthiazol‑2‑yl)-2,5‑diphenyltetrazolium bromide assay, the cell migration ability was examined   by a wound�  �healing assay, and the cell invasion ability was assessed using a Transwell culture chamber system. The results showed that inhibition of the Rac1‑WAVE2‑Arp2/3 signaling pathway using NSC 23766, shRNA‑WAVE2 or CK‑666 reduced the cell viability, migration and invasion abilities in U251 human glioma cells, concordant with a reduced expression of CFL‑1. Furthermore, the expression of CFL‑1 was significantly increased in radioresistant U251 glioma cells when compared with normal U251 human glioma cells. These findings indicate that inhibition of the Rac1‑WAVE2‑Arp2/3 signaling pathway may promote radiosensitivity, which may partially result from the downregulation of CFL‑1 in U251 human glioma cells. 
 ADP-ribosylation factor 6 regulates glioma   cell invasion   through the IQ-domain GTPase-activating protein 1-Rac1-mediated pathway. A common pathobiological feature of malignant gliomas is the insidious infiltration of single tumor cells into the brain parenchyma, rendering these deadly tumors virtually incurable with available therapies. In this study, we report that ADP-ribosylation factor 6 (ARF6), a Ras superfamily small GTPase, is abundantly expressed in invasive human glioma cells. Cellular depletion of ARF6 by small interfering RNA decreased Rac1 activation, impaired HGF-stimulated and serum-stimulated glioma cell migration in vitro, and markedly decreased the invasive capacity of invasive glioma in the brain. Furthermore, ectopic expression of ARF6 in glioma cells promoted cell migration via the activation of Rac1. Upon stimulation of glioma cells with HGF, we show that IQ-domain GTPase-activating protein 1 (IQGAP1) is recruited and overlaps with ARF6 at the leading edge of migrating cells. However, cellular depletion of ARF6 abrogated this recruitment of IQGAP1 and attenuated the formation of surface protrusions. ARF6 forms complexes with Rac1 and IQGAP1 in glioma cells upon HGF stimulation, and knockdown of IQGAP1 significantly inhibits ARF6-induced Rac1 activation and cell migration. Taken together, these data suggest that ARF6-mediated Rac1 activation is essential for glioma   cell invasion   via a signaling pathway that requires IQGAP1. 
 The activation of the NF-κB-JNK pathway is independent of the PI3K-Rac1-JNK pathway involved in the bFGF-regulated human fibroblast cell migration. Skin wound healing is a complex process that repairs multiple organ-tissues. Fibroblasts are key players of skin cells, whose migration is important during wound healing process. bFGF has shown a great efficacy to promote cell migration, but the precise mechanism by which bFGF regulates cell migration remains elusive.The aim of this study was to find bFGF-regulated gene pools and further identify target molecules that participated in human fibroblast cell migration.Skin primary fibroblasts and rat skin wound model were used to demonstrate the novel mechanism of bFGF regulating cell migration to accelerate wound healing. Cell migration was determined using the wound healing scratch assay. The differentially expressed genes and numerous biochemical pathways after bFGF treatment were identified by RNA-Seq analysis, and differentially expressed genes were further verified by qRT-PCR. siRNA duplex target to interfering the expression of PI3-kinase (p110α) was transformed into NIH/3T3 cells. Western blotting analysis was used to determine marker protein expressions. The invasive activity of fibroblasts was measured using 3D spheroi  d cell invasi  on assay.RNA-Seq analysis identified numerous biochemical pathways including the NF-κB pathway under the control of FGF signaling. bFGF negatively regulates the phosphorylation of IκB-α, the most well studied NF-κB signaling regulator while bFGF induces JNK phosphorylation. Application of Bay11-7082, a representative NF-κB inhibitor promoted cell migration, invasion and enhanced the JNKs phosphorylation. However, inhibition of JNKs blocked cell migration when NF-κB is inhibited. Moreover, application of the PI3K inhibitor LY294002 together with Bay11-7082 maintained normal cell migration and knocking-down PI3K (p110α) by a specific siRNA inhibited JNKs phosphorylation while maintaining normal IκBα phosphorylation, indicating that PI3K and NF-κB signaling independently regulate JNKs activation. In addition, administration of bFGF or Bay11-7082 promoted rat skin wound repair and accelerated the invasion of fibroblasts.This study sheds light on the mode of action of bFGF and identifies that the NF-κB-JNKs pathway is independent of the PI3K-JNKs pathway to accelerate fibroblast migration. In addition, bFGF and the relief of inflammation could be a favorable therapeutic approach for skin wound healing. 
 Activation of H-Ras and Rac1 correlates with epidermal growth factor-induced invasion in Hs578T and MDA-MB-231 breast carcinoma cells. There is considerable experimental evidence that hyperactive Ras proteins promote breast cancer growth and development including invasiveness, despite the low frequency of mutated forms of Ras in breast cancer. We have previously shown that H-Ras, but not N-Ras, induces an invasive phenotype mediated by small GTPase Rac1 in MCF10A human breast epithelial cells. Epidermal growth factor (EGF) plays an important role in aberrant growth and metastasis formation of many tumor types including breast cancer. The present study aims to investigate the correlation between EGF-induced invasiveness and Ras activation in four widely used breast cancer cell lines. Upon EGF stimulation, invasive abilities and H-Ras activation were significantly increased in Hs578T and MDA-MB-231 cell lines, but not in MDA-MB-453 and T47D cell lines. Using small interfering RNA (siRNA) to target H-Ras, we showed a crucial role of H-Ras in the invasive phenotype induced by EGF in Hs578T and MDA-MB-231 cells. Moreover, siRNA-knockdown of Rac1 significantly inhibited the EGF-induced invasiveness in these cells. Taken together, this study characterized human breast cancer cell lines with regard to the relationship between H-Ras activation and the invasive phenotype induced by EGF. Our data demonstrate that the activation of H-Ras and the downstream molecule Rac1 correlates with EGF-induced breast cancer   cell invasion  , providing important information on the regulation of malignant progression in mammary carcinoma cells. 
 TROY (TNFRSF19) is overexpressed in advanced glial tumors and promotes glioblastoma   cell invasion   via Pyk2-Rac1 signaling. A critical problem in the treatment of malignant gliomas is the extensive infiltration of individual tumor cells into adjacent brain tissues. This invasive phenotype severely limits all current therapies, and to date, no treatment is available to control the spread of this disease. Members of the tumor necrosis factor (TNF) ligand superfamily and their cognate receptors regulate various cellular responses including proliferation, migration, differentiation, and apoptosis. Specifically, the TNFRSF19/TROY gene encodes a type I cell surface receptor that is expressed on migrating or proliferating progenitor cells of the hippocampus, thalamus, and cerebral cortex. Here, we show that levels of TROY mRNA expression directly correlate with increasing glial tumor grade. Among malignant gliomas, TROY expression correlates inversely with overall patient survival. In addition, we show that TROY overexpression in glioma cells activates Rac1 signaling in a Pyk2-dependent manner to drive glioma   cell invasion   and migration. Pyk2 coimmunoprecipitates with the TROY receptor, and depletion of Pyk2 expression by short hairpin RNA interference oligonucleotides inhibits TROY-induced Rac1 activation and subsequent cellular migration. These findings position aberrant expression and/or signaling by TROY as a contributor, and possibly as a driver, of the malignant dispersion of glioma cells. 
 MT1-MMP modulates melanoma cell dissemination and metastasis through activation of MMP2 and RAC1. Metastatic melanoma remains the deadliest of all skin cancers with a survival rate at five years of less than 15%. MT1-MMP is a membrane-associated matrix metalloproteinase that controls pericellular proteolysis and is an important, invasion-promoting, pro-tumorigenic MMP in cancer. We show that deregulation of MT1-MMP expression happens as early as the transition from nevus to primary melanoma and continues to increase during melanoma progression. Furthermore, MT1-MMP expression is associated with poor melanoma patient outcome, underscoring a pivotal role of MT1-MMP in melanoma pathogenesis. We demonstrate that MT1-MMP is directly required for melanoma cells to metastasize, as cells deprived of MT1-MMP fail to form distant metastasis in an orthotopic mouse melanoma model. We show that MT1-MMP affects   cell invasion   by activating its target MMP2. Importantly, we demonstrate, for the first time, that activation of MMP2 by MT1-MMP is required to sustain RAC1 activity and promote MT1-MMP-dependent cell motility. These data highlight a novel MT1-MMP/MMP2/RAC1 signaling axis in melanoma that may represent an intriguing molecular target for the treatment of invasive melanoma. 
 Escherichia coli producing CNF1 toxin hijacks Tollip to trigger Rac1-dependent   cell invasion  . Rho GTPases, which are master regulators of both the actin cytoskeleton and membrane trafficking, are often hijacked by pathogens to enable their invasion of host cells. Here we report that the cytotoxic necrotizing factor-1 (CNF1) toxin of uropathogenic Escherichia coli (UPEC) promotes Rac1-dependent entry of bacteria into host cells. Our screen for proteins involved in Rac1-dependent UPEC entry identifies the Toll-interacting protein (Tollip) as a new interacting protein of Rac1 and its ubiquitinated forms. We show that knockdown of Tollip reduces CNF1-induced Rac1-dependent UPEC entry. Tollip depletion also reduces the Rac1-dependent entry of Listeria monocytogenes expressing InlB invasion protein. Moreover, knockdown of Tollip, Tom1 and clathrin, decreases CNF1 and Rac1-dependent internalization of UPEC. Finally, we show that Tollip, Tom1 and clathrin associate with Rac1 and localize at the site of bacterial entry. Collectively, these findings reveal a new link between Rac1 and Tollip, Tom1 and clathrin membrane trafficking components hijacked by pathogenic bacteria to allow their efficient invasion of host cells. 
 Effect of Rac1 downregulation mediated by shRNA on the biological behaviour of human cervical cancer cells. The function of Ras-related C3 botulinum toxin substrate1 (Rac1) in the progression of cervical cancer is unclear. This study used RNA interference technology to explore the involvement of Rac1 in the regulation of cervical cancer cells.A short hairpin (sh) RNA plasmid targeting Rac1 was constructed and transfected into HeLa cells. Rac1 mRNA and protein levels were investigated by reverse transcription-polymerase chain reaction and Western blot, respectively. Cell proliferation and cisplatin chemosensitivity were determined using the methyl thiazolyl tetrazolium assay. The Matrigel™ assay and flow cytometry were used to asses  s cell invasi  on and apoptosis, respectively. The concentration of matrix metalloproteinase (MMP)-2 in cell supernatants was detected by enzyme-linked immunosorbent assay.Rac1 expression was significantly downregulated at the mRNA and protein levels in HeLa cells transfected with Rac1 shRNA, and the cell proliferation and invasion capability of cells was decreased. Rac1 downregulation was associated with a decrease in MMP-2 secretion, and increased cell chemosensitivity to cisplatin and cisplatin-induced apoptosis.Rac1 may play an important role in cervical cancer progression and could be a potential target for anticancer therapy. 
 A putative novel protein, DEPDC1B, is overexpressed in oral cancer patients, and enhanced anchorage-independent growth in oral cancer cells that is mediated by Rac1 and ERK. The DEP domain is a globular domain containing approximately 90 amino acids, which was first discovered in 3 proteins: Drosophila disheveled, Caenorhabditis elegans EGL-10, and mammalian Pleckstrin; hence the term, DEP. DEPDC1B is categorized as a potential Rho GTPase-activating protein. The function of the DEP domain in signal transduction pathways is not fully understood. The DEPDC1B protein exhibits the characteristic features of a signaling protein, and contains 2 conserved domains (DEP and RhoGAP) that are involved in Rho GTPase signaling. Small GTPases, such as Rac, CDC42, and Rho, regulate a multitude of cell events, including cell motility, growth, differentiation, cytoskeletal reorganization and cell cycle progression.In this study, we found that it was a guanine nucleotide exchange factor and induced both cell migration in a cultured embryonic fibroblast cell line and   cell invasion   in cancer cell lines; moreover, it was observed to promote anchorage-independent growth in oral cancer cells. We also demonstrated that DEPDC1B plays a role in regulating Rac1 translocated onto cell membranes, suggesting that DEPDC1B exerts a biological function by regulating Rac1. We examined oral cancer tissue; 6 out of 7 oral cancer tissue test samples overexpressed DEPDC1B proteins, compared with normal adjacent tissue.DEPDC1B was a guanine nucleotide exchange factor and induced both cell migration in a cultured embryonic fibroblast cell line and   cell invasion   in cancer cell lines; moreover, it was observed to promote anchorage-independent growth in oral cancer cells. We also demonstrated that DEPDC1B exerts a biological function by regulating Rac1. We found that oral cancer samples overexpressed DEPDC1B proteins, compared with normal adjacent tissue. Suggest that DEPDC1B plays a role in the development of oral cancer. We revealed that proliferation was linked to a novel DEPDC1B-Rac1-ERK1/2 signaling axis in oral cancer cell lines. 
 Rac1, RhoA, and Cdc42 participate in HeLa   cell invasion   by group B streptococcus. The group B streptococcus (GBS) is an important human pathogen with the ability to cause invasive disease. To do so, the bacteria must invade host cells. It has been well documented that GBS are able to invade a variety of nonphagocytic host cell types, and this process is thought to involve a number of pathogen-host cell interactions. While some of the molecular aspects of the GBS-host   cell invasion   process have been characterized, many events still remain unclear. The objective of this investigation was to evaluate the role of the Rho-family GTPases Rac, Rho, and Cdc42 in GBS invasion into epithelial cells. The epithelial   cell invasion   process was modeled using HeLa 229 cell culture. Treatment of HeLa cells with 10 microM compactin, a pan-GTPase inhibitor, abolished GBS internalization, suggesting that GTPases are involved in the GBS invasion process. The addition of Toxin B or exoenzyme C3 to HeLa cells before GBS infection reduced invasion by 50%, further suggesting that the Rho-family GTPases are involved in GBS entry. Examining invasion of GBS into HeLa cells with altered genetic backgrounds was used to confirm these findings; GBS invasion into HeLa cells transiently transfected with dominant negative Rac1, Cdc42, or RhoA reduced invasion by 75%, 51%, and 42%, respectively. Results of this study suggest that the Rho-family GTPases are required for efficient invasion of HeLa cells by GBS. 
 LOX-1-MT1-MMP axis is crucial for RhoA and Rac1 activation induced by oxidized low-density lipoprotein in endothelial cells. RhoA and Rac1 activation plays a key role in endothelial dysfunction. Lectin-like oxidized low-density lipoprotein receptor-1 (LOX-1) is a major receptor for oxidized low-density lipoprotein (ox-LDL) in endothelial cells (ECs). Membrane type 1 matrix metalloproteinase (MT1-MMP) has been shown to be involved in atherogenesis. This study was conducted to investigate the role of the LOX-1-MT1-MMP axis in RhoA and Rac1 activation in response to ox-LDL in ECs.Ox-LDL induced rapid RhoA and Rac1 activation as well as MT1-MMP activity in cultured human aortic ECs. Inhibition of LOX-1 prevented ox-LDL-dependent RhoA and Rac1 activation. Knockdown of MT1-MMP by small interfering RNA prevented ox-LDL-induced RhoA and Rac1 activation, indicating that MT1-MMP is upstream of RhoA and Rac1. Fluorescent immunostaining revealed the colocalization of LOX-1 and MT1-MMP, and the formation of a complex of LOX-1 with MT1-MMP was detected by immunoprecipitation. Blockade of LOX-1 or MT1-MMP prevented RhoA-dependent endothelial NO synthase protein downregulation and   cell invasion  , Rac1-mediated NADPH oxidase activity, and reactive oxygen species generation.The present study provides evidence that the LOX-1-MT1-MMP axis plays a crucial role in RhoA and Rac1 activation signalling pathways in ox-LDL stimulation, suggesting that this axis may be a promising target for treating endothelial dysfunction. 
 Knockdown of BDNF suppressed invasion of HepG2 and HCCLM3 cells, a mechanism associated with inactivation of RhoA or Rac1 and actin skeleton disorganization. Brain-derived neurotrophic factor (BDNF) and its primary receptor tropomysin-related kinase B (TrkB) mediate critical signalings for supporting survival and growth of neurons. Even though we have previously confirmed that more expressions of BDNF and TrkB were closely correlated with multiple and advanced hepatocellular carcinoma (HCC), the exact mechanisms underlying have not been investigated. The expressions of BDNF and TrkB were examined by western blot and BDNF secretion was evaluated by ELISA in human HCC cell lines of HepG2 and HCCLM3 with high metastatic potential. BDNF knockdown was performed by specific BDNF-siRNA transfection in HCC cells, actin cytoskeleton was shown by FITC-phalloidin staining and the activations of RhoA, Rac1 or Cdc42 were determined using western blot. Cell apoptosis and invasion were examined by flow cytometry and transwell assay, respectively. More expressions of BDNF and TrkB were found in HCCLM3 than in HepG2 cells. Inhibited expression of BDNF by specific siRNA showed impaired actin polymerization and decreased activations of RhoA or Rac1 in both HepG2 and HCCLM3 cells. BDNF knockdown also induced apoptosis and suppressed invasion of both HepG2 and HCCLM3 cells. Our results suggested a role of BDNF/TrkB in confering HCCLM3 cells advantage of metastasis, and BDNF knockdown inhibited   cell invasion   probably through the blocked actin polymerization and the correlated inactivation of RhoA or Rac1. Aiming at BDNF/TrkB signaling interruption may be an effective strategy to prevent HCC progression. 
 Roles of the Rac1 and Rac3 GTPases in human tumor   cell invasion  . Members of the Rho family of small GTPases have been shown to be involved in tumorigenesis and metastasis. Currently, most of the available information on the function of Rho proteins in malignant transformation is based on the use of dominant-negative mutants of these GTPases. The specificity of these dominant-negative mutants is limited however. In this study, we used small interfering RNA directed against either Rac1 or Rac3 to reduce their expression specifically. In line with observations using dominant-negative Rac1 in other cell types, we show that RNA interference-mediated depletion of Rac1 strongly inhibits lamellipodia formation, cell migration and invasion in SNB19 glioblastoma cells. Surprisingly however, Rac1 depletion has a much smaller inhibitory effect on SNB19 cell proliferation and survival. Interestingly, whereas depletion of Rac3 strongly inhibits SNB19   cell invasion  , it does not affect lamellipodia formation and has only minor effects on cell migration and proliferation. Similar results were obtained in BT549 breast carcinoma cells. Thus, functional analysis of Rac1 and Rac3 using RNA interference reveals a critical role for these GTPases in the invasive behavior of glioma and breast carcinoma cells. 
 Phosphatidylinositol 5-phosphate regulates invasion through binding and activation of Tiam1. PtdIns5P is a lipid messenger acting as a stress-response mediator in the nucleus, and known to maintain cell activation through traffic alterations upon bacterial infection. Here, we show that PtdIns5P regulates actin dynamics and invasion via recruitment and activation of the exchange factor Tiam1 and Rac1. Restricted Rac1 activation results from the binding of Tiam1 DH-PH domains to PtdIns5P. Using an assay that mimics Rac1 membrane anchoring by using Rac1-His and liposomes containing Ni(2+)-NTA modified lipids, we demonstrate that intrinsic Tiam1 DH-PH activity increases when Rac1 is anchored in a PtdIns5P-enriched environment. This pathway appears to be general since it is valid in different pathophysiological models: receptor tyrosine kinase activation, bacterial phosphatase IpgD expression and the invasive NPM-ALK(+) lymphomas. The discovery that PtdIns5P could be a keystone of GTPases and cytoskeleton spatiotemporal regulation opens important research avenues towards unravelling new strategies counteracting   cell invasion  . 
  GeneRIFs  
 A MAPK-Driven Feedback Loop Suppresses Rac Activity to Promote RhoA-Driven Cancer   Cell Invasion  . Our model predicted an unanticipated feedback loop, whereby Raf/MEK/ERK signalling maintains suppression of Rac1 by inhibiting the Rac-activating Sos1-Eps8-Abi1 complex, allowing RhoA activity to predominate in invasive protrusions. 
 we found that CXCR4-CXCL12/AKT axis regulates RhoA, Rac-1, and Cdc42 to modulate   cell invasion   and tumor metastasis. Together, these results demonstrate a role for CXCR4 in ESCC metastasis and progression and suggest potential targets for therapeutic intervention. 
 Rab23 serves as an important oncoprotein in human astrocytoma by regulating   cell invasion   and migration through Rac1 activity 
 Rab23 enhance squamous cell carcinoma   cell invasion   via up-regulating Rac1. 
 TIAM1 is activated by phosphatidylinositol 5-phosphate which activates RAC1, regulating actin dynamics and   cell invasion  . 
 Study demonstrates that increased DGKzeta protein levels in SW620 cells are associated with increased Rho GTPase activity and downstream signaling; silencing of DGKzeta expression in SW620 cells decreased Rac1 and RhoA activity and attenuated   cell invasion  ; DGKzeta silencing also attenuated the invasiveness of PC-3 prostate cancer and MDA-MB-231 breast cancer cells. 
 results validate the WNK2 gene as a recurrent target for epigenetic silencing in glia-derived brain tumours and provide first mechanistic evidence for a tumour-suppressing role of WNK2 that is related to Rac1 signalling and tumour   cell invasion   and growth 
 BDNF knockdown inhibited   cell invasion   probably through the blocked actin polymerization and the correlated inactivation of RhoA or Rac1. 
 Data show that Semaphorin 5A (Sema5A) and plexin-B3 inhibit glioma   cell invasion   through Rac1 inactivation. 
 The authors demonstrate that the Streptococcus pyogenes FbaB is essential and sufficient to trigger endothelial   cell invasion   via a Rac1-dependent phagocytosis-like uptake. 
 these data demonstrate that the activation of H-Ras and the downstream molecule Rac1 correlates with EGF-induced breast cancer   cell invasion  . 
 TNFRSF19 overexpression in glioma cells activates Rac1 signaling in a Pyk2-dependent manner to drive glioma   cell invasion   and migration 
 Rac1 and RhoA signaling simultaneously regulate   cell invasion   in 3D matrices. 
 Data suggest that ARF6-mediated Rac1 activation is essential for glioma   cell invasion   via a signaling pathway that requires IQGAP1. 
   
  
 JUN 
 
  NCBI Gene  
  GeneRIF  
  Pubmed  
 
 
  Search Term  Rank  Fields  
  [Ff]ilopodia  1  PubMed(1)  
  [Mm]igration  6  Process(1); PubMed(50); GeneRIFs(8)  
  ECM  7  PubMed(2); GeneRIFs(1)  
  [Ee]xtracellular [Mm]atrix  8  PubMed(11)  
  [Ii]nvasive  10  PubMed(41); GeneRIFs(6)  
  [Mm]etastasis  12  PubMed(46); GeneRIFs(7)  
  [Cc]ell [Ii]nvasion  13  PubMed(16); GeneRIFs(5)  
 
 
 hide/show details for JUN 
  [Ff]ilopodia  PubMed  
 ARAP1: a point of convergence for Arf and Rho signaling. We have identified ARAP1 and ARAP2 and examined ARAP1 as a possible link between phosphoinositide-, Arf-, and Rho-mediated cell signaling. ARAP1 contains Arf GAP, Rho GAP, Ankyrin repeat, Ras-associating, and five PH domains. In vitro, ARAP1 had Rho GAP and phosphatidylinositol (3,4,5) trisphosphate (PIP3)-dependent Arf GAP activity. ARAP1 associated with the Golgi. The Rho GAP activity mediated cell rounding and loss of stress fibers when ARAP1 was overexpressed. The Arf GAP activity mediated changes in the Golgi apparatus and the formation of   filopodia  , the latter a consequence of increased cellular activity of Cdc42. The Arf GAP and Rho GAP activities both contributed to inhibiting cell spreading. Thus, ARAP1 is a PIP3-dependent Arf GAP that regulates Arf-, Rho-, and Cdc42-dependent cell activities. 
  [Mm]igration  Process  
 positive regulation of epithelial cell   migration   
  PubMed  
 Glucose-regulated protein 94 mediates metastasis by CCT8 and the JNK pathway in hepatocellular carcinoma. Hepatocellular carcinoma (HCC) is one of the leading causes of cancer death worldwide. Cancer metastasis is a major obstacle in clinical cancer therapy. The mechanisms underlying the metastasis of HCC remain unclear. Glucose-regulated protein 94 (GRP94) is a key protein involved in mediating cancer progression, and it is highly expressed in HCC specimens. However, the role of GRP94 in cancer metastasis is unclear. A specific short hairpin RNA (shRNA) was employed to knock down GRP94 gene expression in HCC cell lines. Wound-healing   migration  , transwell   migration  , and invasion assays were performed to determine the   migration   and invasive ability of HCC cells. We demonstrated that silencing GRP94 inhibited HCC cell wound healing,   migration  , and invasion. Furthermore, our findings indicated that GRP94 knockdown might attenuate HCC cell metastasis by inhibiting CCT8/c-Jun/EMT signaling. Our study indicated that silencing GRP94 significantly reduced the   migration   and invasion abilities of HCC cells. Moreover, depleting GRP94 inhibited cell   migration   and invasion by downregulating CCT8/c-Jun signaling. Thus, our data suggest that the GRP94/CCT8/c-Jun/EMT signaling cascade might be a new therapeutic target for HCC. 
 Glucose-regulated protein 94 mediates metastasis by CCT8 and the JNK pathway in hepatocellular carcinoma. Hepatocellular carcinoma (HCC) is one of the leading causes of cancer death worldwide. Cancer metastasis is a major obstacle in clinical cancer therapy. The mechanisms underlying the metastasis of HCC remain unclear. Glucose-regulated protein 94 (GRP94) is a key protein involved in mediating cancer progression, and it is highly expressed in HCC specimens. However, the role of GRP94 in cancer metastasis is unclear. A specific short hairpin RNA (shRNA) was employed to knock down GRP94 gene expression in HCC cell lines. Wound-healing   migration  , transwell   migration  , and invasion assays were performed to determine the   migration   and invasive ability of HCC cells. We demonstrated that silencing GRP94 inhibited HCC cell wound healing,   migration  , and invasion. Furthermore, our findings indicated that GRP94 knockdown might attenuate HCC cell metastasis by inhibiting CCT8/c-Jun/EMT signaling. Our study indicated that silencing GRP94 significantly reduced the   migration   and invasion abilities of HCC cells. Moreover, depleting GRP94 inhibited cell   migration   and invasion by downregulating CCT8/c-Jun signaling. Thus, our data suggest that the GRP94/CCT8/c-Jun/EMT signaling cascade might be a new therapeutic target for HCC. 
 Blimp1 activation by AP-1 in human lung cancer cells promotes a migratory phenotype and is inhibited by the lysyl oxidase propeptide. B lymphocyte-induced maturation protein 1 (Blimp1) is a master regulator of B cell differentiation, and controls   migration   of primordial germ cells. Recently we observed aberrant Blimp1 expression in breast cancer cells resulting from an NF-κB RelB to Ras signaling pathway. In order to address the question of whether the unexpected expression of Blimp1 is seen in other epithelial-derived tumors, we selected lung cancers as they are frequently driven by Ras signaling. Blimp1 was detected in all five lung cancer cell lines examined and shown to promote lung cancer cell   migratio  n and invasion. Interrogation of microarray datasets demonstrated elevated BLIMP1 RNA expression in lung adenocarcinoma, pancreatic ductal carcinomas, head and neck tumors as well as in glioblastomas. Involvement of Ras and its downstream kinase c-Raf was confirmed using mutant and siRNA strategies. We next addressed the issue of mechanism of Blimp1 activation in lung cancer. Using knockdown and ectopic expression, the role of the Activator Protein (AP)-1 family of transcription factors was demonstrated. Further, chromatin immunoprecipitation assays confirmed binding to identified AP-1 elements in the BLIMP1 promoter of ectopically expressed c-Jun and of endogenous AP-1 subunits following serum stimulation. The propeptide domain of lysyl oxidase (LOX-PP) was identified as a tumor suppressor, with ability to reduce Ras signaling in lung cancer cells. LOX-PP reduced expression of Blimp1 by binding to c-Raf and inhibiting activation of AP-1, thereby attenuating the migratory phenotype of lung cancer cells. Thus, Blimp1 is a mediator of Ras/Raf/AP-1 signaling that promotes cell   migratio  n, and is repressed by LOX-PP in lung cancer. 
 Mucin1 mediates autocrine transforming growth factor beta signaling through activating the c-Jun N-terminal kinase/activator protein 1 pathway in human hepatocellular carcinoma cells. In a previous study, we observed by global gene expression analysis that oncogene mucin1 (MUC1) silencing decreased transforming growth factor beta (TGF-β) signaling in the human hepatocellular carcinoma (HCC) cell line SMMC-7721. In this study, we report that MUC1 overexpression enhanced the levels of phosphorylated Smad3 linker region (p-Smad3L) (Ser-213) and its target gene MMP-9 in HCC cells, suggesting that MUC1 mediates TGF-β signaling. To investigate the effect of MUC1 on TGF-β signaling, we determined TGF-β secretion in MUC1 gene silencing and overexpressing cell lines. MUC1 expression enhanced not only TGF-β1 expression at the mRNA and protein levels but also luciferase activity driven by a TGF-β promoter, as well as elevated the activation of c-Jun N-terminal kinase (JNK) and c-Jun, a member of the activation protein 1 (AP-1) transcription factor family. Furthermore, pharmacological reduction of TGF-β receptor (TβR), JNK and c-Jun activity inhibited MUC1-induced autocrine TGF-β signaling. Moreover, a co-immunoprecipitation assay showed that MUC1 directly bound and activated JNK. In addition, both MUC1-induced TGF-β secretion and exogenous TGF-β1 significantly increased Smad signalin  g and cel  l migration, which were markedly inhibited by either TβR inhibitor or small interfering RNA silencing of TGF-β1 gene in HCC cells. The high correlation between MUC1 and TGF-β1 or p-Smad3L (Ser-213) expression was shown in tumor tissues from HCC patients by immunohistochemical staining analysis. Collectively, these results indicate that MUC1 mediates autocrine TGF-β signaling by activating the JNK/AP-1 pathway in HCC cells. Therefore, MUC1 plays a key role in HCC progression and could serve as an attractive target for HCC therapy. 
 AP-1 (Fra-1/c-Jun)-mediated induction of expression of matrix metalloproteinase-2 is required for 15S-hydroxyeicosatetraenoic acid-induced angiogenesis. To understand the involvement of matrix metalloproteinases (MMPs) in 15(S)-hydroxyeicosatetraenoic acid (15(S)-HETE)-induced angiogenesis, we have studied the role of MMP-2. 15(S)-HETE induced MMP-2 expression and activity in a time-dependent manner in human dermal microvascular endothelial cells (HDMVECs). Inhibition of MMP-2 activity or depletion of its levels attenuated 15(S)-HETE-induced HDMVEC   migration  , tube formation, and Matrigel plug angiogenesis. 15(S)-HETE also induced Fra-1 and c-Jun expression in a Rac1-MEK1-JNK1-dependent manner. In addition, 15(S)-HETE-induced MMP-2 expression and activity were mediated by Rac1-MEK1-JNK1-dependent activation of AP-1 (Fra-1/c-Jun). Cloning and site-directed mutagenesis of MMP-2 promoter revealed that AP-1 site proximal to the transcriptional start site is required for 15(S)-HETE-induced MMP-2 expression, and Fra-1 and c-Jun are the essential components of AP-1 that bind to MMP-2 promoter in response to 15(S)-HETE. Hind limb ischemia led to an increase in MEK1 and JNK1 activation and Fra-1, c-Jun, and MMP-2 expression resulting in enhanced neovascularization and recovery of blood perfusion in wild-type mice as compared with 12/15-Lox(-/-) mice. Together, these results provide the first direct evidence for a role of 12/15-Lox-12/15(S)-HETE axis in the regulation of ischemia-induced angiogenesis. 
 NAG7 promotes human nasopharyngeal carcinoma invasion through inhibition of estrogen receptor alpha and up-regulation of JNK2/AP-1/MMP1 pathways. The NAG7, an estrogen receptor repressor, is a negative regulator of nasopharyngeal carcinoma cell growth. Here, we report that NAG7 promotes human nasopharyngeal carcinoma invasion, and we identify the mechanisms underlying this function. As a consequence of elevated NAG7 expression, the adhesion,   migration  , and invasive capabilities of HNE1 cells in vitro and in vivo were enhanced. NAG7 was a significant negative regulator of protein expression of estrogen receptor alpha (ERalpha), and activated both the JNK2/AP-1/MMP1 and the upstream H-Ras/p-c-Raf pathways. None of these effects induced by NAG7 over-expression could be counteracted by estrogen. These observations indicate that NAG7 plays a potential role in promoting nasopharyngeal carcinoma invasion by regulation of ERalpha and the H-ras/p-c-Raf and JNK2/AP-1/MMP1 signaling pathways. 
 Disabled-2 (Dab2) mediates transforming growth factor beta (TGFbeta)-stimulated fibronectin synthesis through TGFbeta-activated kinase 1 and activation of the JNK pathway. The multifunctional cytokine transforming growth factor beta (TGFbeta) exerts many of its effects through its regulation of extracellular matrix components, including fibronectin (FN). Although expression of both TGFbeta and FN are essential for embryonic development and wound healing in the adult, overexpression leads to excessive deposition of extracellular matrix observed in many fibroproliferative disorders. We previously have demonstrated that TGFbeta-stimulated FN induction requires activation of the c-Jun N-terminal kinase (JNK) pathway; however, the signaling molecules that link the TGFbeta receptors to the JNK pathway remain unknown. We show here that the cytosolic adaptor protein disabled-2 (Dab2) directly stimulates JNK activity, whereas stable small interfering RNA-mediated ablation of Dab2 in NIH3T3 mouse fibroblasts and A10 rat aortic smooth muscle cells demonstrates that its expression is required for TGFbeta-mediated FN induction. We demonstrate that TGFbeta treatment stimulates the association of Dab2 with the mitogen-activated protein kinase kinase kinase, TAK1. Attenuation of cellular TAK1 levels by transient double-stranded RNA oligonucleotide transfection as well as overexpression of kinase-deficient TAK1 leads to abrogation of TGFbeta-stimulated FN induction. Furthermore, cell   migration  , another JNK-dependent response, is attenuated in NIH3T3-siDab2-expressing clones. We, therefore, delineate a signaling pathway proceeding from the TGFbeta receptors to Dab2 and TAK1, leading to TGFbeta-stimulated JNK activation, FN expression, and cell   migration  . 
 Epithelial-to-mesenchymal transition induced by TGF-β1 is mediated by AP1-dependent EpCAM expression in MCF-7 cells. The epithelial-to-mesenchymal transition (EMT), a process involving the breakdown of cell-cell junctions and loss of epithelial polarity, is closely related to cancer metastasis and invasion. The epithelial cell adhesion molecule (EpCAM) is a type I transmembrane protein expressed in the majority of normal epithelial tissues and overexpressed in the majority of human epithelial cancers including breast cancer. EpCAM plays an important role in cancer progression. We showed that EpCAM participated in TGF-β1-induced EMT. TGF-β1 treatment of MCF-7 breast cancer cells was shown to induce EpCAM expression, which promoted the EMT and ce  ll migrat  ion. EpCAM overexpression further enhanced TGF-β1-induced EMT and EpCAM knockdown inhibited TGF-β1-induced EMT. We further demonstrated that TGF-β1 treatment induced the phosphorylation of JNK that was in turn responsible for the increased expression of Jun and Fos. This result suggests an important role of the JNK to AP-1 signaling to EpCAM downstream of TGF-β1 for the induction of EMT in the breast cancer cells. Collectively, our study highlights a novel function for EpCAM in TGF-β1-induced EMT process and suggests that targeting of EpCAM may be an attractive strategy to treat breast cancer. This study implicates the potential value of EpCAM as a molecular marker for breast cancer. 
 Astrocyte elevated gene-1 (AEG-1) promotes osteosarcoma cell invasion through the JNK/c-Jun/MMP-2 pathway. Osteosarcoma is the most common primary malignant bone tumour in children and adolescents and is characterised by high malignant and metastatic potentials. However, the molecular mechanism underlying this invasiveness remains unclear. In this study, we determined that PD98059 and SP600125, the two mitogen-activated protein kinase (MAPK) family inhibitors, decreased the osteosarcoma cell U2OS-AEG-1   migration   and invasion that was enhanced by astrocyte elevated gene-1 (AEG-1) in an in vitro wound-healing and Matrigel invasion assay independently of cell viability. These findings indicate that AEG-1 promoted osteosarcoma cell invasion is relevant to the MAPK pathways. The up-regulation of AEG-1 increased the levels of phosphor-c-Jun N-terminal kinase (JNK) and phosphor-c-Jun; however, there were no marked changes in the levels of phosphor-extracellular regulated kinase (ERK) 1/2 or phosphor-c-Fos due to the activation of AEG-1 in U2OS. SP600125 (a JNK inhibitor) decreased phosphor-c-Jun and MMP-2 in U2OS-AEG-1, while PD98059 (a ERK1/2 inhibitor) had no influence on the levels of phosphor-c-Jun or MMP-2 in U2OS-AEG-1. Further study revealed that the down-regulation of phosphor-c-Jun not only obviously decreased the MMP-2 protein level and the MMP-2 transcriptional activity that were up-regulated by AEG-1 in Western-blot and luciferase reporter assays, but also inhibited the   migration   and invasion abilities of the U2OS-AEG-1 cells, which suggests that AEG-1 mediated U2OS invasion at least partially via the JNK/c-Jun/MMP-2 pathway. Consistent with these observations, immunohistochemical (IHC) staining revealed that AEG-1 expression was associated with the protein levels of phosphor-c-Jun and MMP-2 in needle biopsy paraffin-embedded archival human osteosarcoma tissues. Taken together, our findings suggest that AEG-1 plays a crucial role in the aggressiveness of osteosarcoma via the JNK/c-Jun/MMP-2 pathway. 
 Transcription factor Ap-1 mediates proangiogenic MIF expression in human endothelial cells exposed to Angiotensin II. Macrophage   migration   inhibitory factor (MIF) is an inflammatory cytokine associated with the atherosclerotic process and atherosclerotic plaque stability. MIF was shown to be highly expressed in advanced atherosclerotic lesions. Neutralizing MIF with a blocking antibody induced a regression of established atherosclerotic lesions. In this study, we investigated the mechanism underlying the proangiogenic effect of MIF in human umbilical vein endothelial cells (HUVECs). We showed that MIF induced the expression of angiogenesis-related genes in HUVECs. We also showed that MIF induced tube formation of HUVECs in vitro and in vivo. Angiotensin II (Ang II) could specifically up-regulate MIF expression in HUVECs. Using a luciferase reporter assay, we demonstrated that the AP-1 response element in the 5'-UTR of the MIF gene played a role in Ang II-induced MIF expression. Small hairpin RNA (shRNA) targeting c-Jun, a component of AP-1, and the AP-1 inhibitor CHX both efficiently inhibited MIF expression. The consistent result of electrophoretic mobility shift assay (EMSA) showed that Ang II specifically increased AP-1 activation in HUVECs. Our results suggest that AP-1 mediates Ang II-induced MIF expression which contributes to atherosclerotic plaque destabilization in human endothelial cells. 
 Podoplanin is regulated by AP-1 and promotes platelet aggregation and cell   migration   in osteosarcoma. Podoplanin is a type-I transmembrane sialomucin-like protein, which is expressed in a wide range of cell types and is involved in platelet aggregation and tumor metastasis. Here, we investigated the function, regulation, and expression of podoplanin in osteosarcoma. Podoplanin expression was observed in three osteosarcoma cell lines (MG-63, HOS, and U-2 OS) with platelet aggregation-inducing ability, which was blocked by podoplanin small-interfering RNA or a neutralizing antibody. Overexpression of podoplanin in nonmetastatic Dunn osteosarcoma cells promoted cell   migration   without attenuating cell proliferation. Both podoplanin and TGF-β1 were up-regulated by c-Fos induction in MC3T3-E1 osteoblastic cells, and were highly expressed in c-Fos transgenic mouse osteosarcomas and c-Fos-transformed osteosarcoma cell lines. Immunohistochemistry of human osteosarcoma tissue microarrays (n = 133) showed staining of tumor cells embedded in an excess of irregular neoplastic bone matrix in 100% of tumors undergoing so-called "normalization/maturation." Podoplanin was also expressed in osteosarcoma subtypes, with 65% of osteoblastic, 100% of chondroblastic, and 79% of fibroblastic tumors. CD44 and pERM immunohistochemistry showed coexpression with podoplanin in both mouse and human osteosarcoma. Podoplanin expression was significantly higher in metastatic osteosarcomas (n = 6) than in primary osteosarcomas (n = 10). Our data suggest that podoplanin, which is not expressed in normal osteoblasts but in osteocytes, is aberrantly expressed in transformed osteoblasts and in osteosarcoma, and is under AP-1 transcriptional control. Thus podoplanin is a candidate molecule for therapeutic targeting. 
 A feedback inhibition between miRNA-127 and TGFβ/c-Jun cascade in HCC cell   migratio  n via MMP13. Hepatocellular carcinoma (HCC) is the fifth most common cancer worldwide and is increasing in frequency in the U.S. The major reason for the low postoperative survival rate of HCC is widespread intrahepatic metastasis or invasion, and activation of TGFβ signaling is associated with the invasive phenotype. This study aims at determining the novel function of miR-127 in modulating HC  C migrati  on. Overexpression of miR-127 inhibits HCC cel  l migrati  on, invasion and tumor growth in nude mice. MiR-127 directly represses matrix metalloproteinase 13 (MMP13) 3'UTR activity and protein expression, and diminishes MMP13/TGFβ-induced H  CC migrat  ion. In turn, TGFβ decreases miR-127 expression by enhancing c-Jun-mediated inhibition of miR-127 promoter activity. In contrast, p53 transactivates miR-127 promoter and induces miR-127 expression, which is antagonized by c-Jun. The inhibition of miR-127 by c-Jun is through TGFβ-mediated ERK and JNK pathways. The lower miR-127 expression shows a negative correlation with the higher MMP13 expression in a subset of human HCC specimens. This is the first report elucidating a feedback regulation between miR-127 and the TGFβ/c-Jun cascade i  n HCC mig  ration via MMP13 that involves a crosstalk between the oncogene c-Jun and tumor suppressor p53. 
 Differential involvement of calmodulin-dependent protein kinase II-activated AP-1 and c-Jun N-terminal kinase-activated EGR-1 signaling pathways in tumor necrosis factor-alpha and lipopolysaccharide-induced CD44 expression in human monocytic cells. CD44 plays a crucial role in cell   migration  , inflammation, and immune responses. Alteration in the levels of CD44 expression on monocytic cells by endotoxins and immunoregulatory cytokines may modulate the   migration   of immune cells to inflammatory sites and the development of immune responses. Lipopolysaccharide (LPS) and the proinflammatory cytokine, tumor necrosis factor-alpha (TNF-alpha), act as important regulators of CD44 expression in human monocytic cells. We previously demonstrated that the c-Jun N-terminal kinase (JNK), a mitogen-activated protein kinase (MAPK), differentially regulated LPS- but not TNF-alpha-induced CD44 expression in monocytic cells. In this study, our results suggest that the calcium signaling pathway, in particular calmodulin (CaM) and CaM-dependent protein kinase II (CaMK-II), is involved in TNF-alpha- but not LPS-induced CD44 expression. CD44 promoter analysis suggested the participation of distinct transcription factors AP-1 and Egr-1 in TNF-alpha- and LPS-induced CD44 expression, respectively. Furthermore, TNF-alpha-induced CD44 expression was regulated by AP-1 through the activation of the CaMK-II pathway, whereas LPS-induced CD44 transcription was regulated specifically by Egr-1 through JNK activation. Overall, the results suggest the involvement of two distinct and independent signaling pathways involved in the regulation of CD44 transcription that may represent potential targets for anti-inflammatory agents capable of inhibiting CD44-mediated cell   migration  . 
 TGFβ-induced invasion of prostate cancer cells is promoted by c-Jun-dependent transcriptional activation of Snail1. High levels of transforming growth factor-β (TGFβ) correlate with poor prognosis for patients with prostate cancer and other cancers. TGFβ is a multifunctional cytokine and crucial regulator of cell fate, such as epithelial to mesenchymal transition (EMT), which is implicated in cancer invasion and progression. TGFβ conveys its signals upon binding to type I and type II serine/threonine kinase receptors (TβRI/II); phosphorylation of Smad2 and Smad3 promotes their association with Smad4, which regulates expression of targets genes, such as Smad7, p21, and c-Jun. TGFβ also activates the ubiquitin ligase tumor necrosis factor receptor-associated factor 6 (TRAF6), which associates with TβRI and activates the p38 mitogen-activated protein kinase (MAPK) pathway. Snail1 is a key transcription factor, induced by TGFβ that   promotes   migration and invasion of cancer cells. In this study, we have identified a novel binding site for c-Jun in the promoter of the Snail1 gene and report that the activation of the TGFβ-TRAF6-p38 MAPK pathway promotes both c-Jun expression and its activation via p38α-dependent phosphorylation of c-Jun at Ser63. The TRAF6-dependent activation of p38 also leads to increased stability of c-Jun, due to p38-dependent inactivation of glycogen synthase kinase (GSK) 3β by phosphorylation at Ser9. Thus, our findings elucidate a novel role for the p38 MAPK pathway in stimulated cells, leading to activation of c-Jun and its binding to the promoter of Snail1, thereby triggering motility and invasiveness of aggressive human prostate cancer cells. 
 Palbociclib inhibits epithelial-mesenchymal transition and metastasis in breast cancer via c-Jun/COX-2 signaling pathway. Palbociclib, a highly selective CDK4/6 inhibitor, has been shown to be a novel anti-tumor agent that suppresses breast cancer cell proliferation. However, its anti-metastasis activity remains controversial. In the present study, we evaluated whether palbociclib prevented breast cancer cell metastasis and revealed its regulatory mechanism. We found that palbociclib inhibited   migration   and invasion in the breast cancer cells MDA-MB-231 and T47D. The epithelial-mesenchymal transition (EMT) markers, vimentin and Snail, were down-regulated with palbociclib treatment. Moreover, we revealed that this inhibition was mediated by the c-Jun/COX-2 pathway. COX-2 was decreased after palbociclib treatment. The production of PGE2 was also reduced along with COX-2. Additionally, our data showed that c-Jun, a crucial transcriptional regulator of COX-2, was down-regulated by palbociclib. We found that palbociclib weakened the COX-2 promoter binding activity of c-Jun and prevented its translocation from the cytoplasm to cell nuclei. Bioluminescence imaging and tail intravenous injection were used to evaluate the anti-metastasis effect of palbociclib in vivo. The data demonstrated that palbociclib reduced breast cancer metastasis to the lung. These results therefore demonstrated that the anti-metastasis activity of palbociclib is mediated via the c-Jun/COX-2 signaling pathway by inhibiting EMT in breast cancer cells. 
 Human immunodeficiency virus tat modulates the Flk-1/KDR receptor, mitogen-activated protein kinases, and components of focal adhesion in Kaposi's sarcoma cells. Kaposi's sarcoma (KS) spindle cell growth and spread have been reported to be modulated by various cytokines as well as the human immunodeficiency virus (HIV) gene product Tat. Recently, HIV-1 Tat has been shown to act like a cytokine and bind to the Flk-1/KDR receptor for the vascular endothelial growth factor A (VEGF-A), which is expressed by KS cells. We have characterized signal transduction pathways stimulated by HIV-1 Tat upon its binding to surface receptors on KS cells. We observed that stimulation in KS 38 spindle cells resulted in tyrosine phosphorylation and activation of the Flk-1/KDR receptor. We also report that HIV-1 Tat treatment enhanced the phosphorylation and association of proteins found in focal adhesions, such as the related adhesion focal tyrosine kinase RAFTK, paxillin, and p130(cas). Further characterization revealed the activation of mitogen-activated protein kinase, c-Jun amino-terminal kinase (JNK), and Src kinase. HIV-1 Tat contains a basic domain which can interact with growth factor tyrosine kinase receptors and a classical RGD sequence which may bind to and activate the surface integrin receptors for fibronectin and vitronectin. We observed that stimulation of KS cells with basic as well as RGD sequence-containing Tat peptides resulted in enhanced phosphorylation of RAFTK and activation of MAP kinase. These studies reveal that Tat stimulation activates a number of signal transduction pathways that are associated with cell growth and   migration  . 
 Downregulation of non‑muscle myosin IIA expression inhibit  s migrati  on and invasion of gastric cancer cells via the c‑Jun N‑terminal kinase signaling pathway. Non‑muscle myosin IIA (NMIIA) has been found to be overexpressed in gastric cancer tissues. However, the roles of NMIIA   in the m  igration and invasion of gastric cancer cells have not yet been elucidated. The aim of the present study was to assess the effects of NMIIA knockdown on the migratory and invasive capacities of gastric cancer cells and to investigate the potential underlying mechanisms in vitro. First, the expression of NMIIA was assessed in gastric cancer tissues and non‑cancerous tissues using western blot analysis. The expression levels of NMIIA protein in 51 out of 63 gastric cancer tissue specimens were higher compared to those in their matched non‑tumoric gastric tissue specimens, and differences between the two groups were statistically significant (P<0.001). After downregulation of NMIIA using RNA interference, the migratory and invasive abilities of the SGC‑7901 and MGC‑803 gastric cancer cell lines were observed using a wound‑healing assay and a Transwell assay, respectively. Knockdown of NMIIA significantly decreased the amount of wound closure as well as the number of cells which transgressed through the Matrigel (P<0.01). Finally, the levels of c‑Jun N‑terminal kinase (JNK), phosphorylated (p)‑JNK, c‑Jun and p‑c‑Jun were detected using western blot analysis in order to explore the association between NMIIA and the JNK signaling pathway. Knockdown of NMIIA decreased the levels of p‑JNK and p‑c‑Jun in the two gastric cancer cell lines (P<0.05). In conclusion, the present study indicated th  at knockd  own of NMIIA inhibited the migration and invasion of gastric cancer cells, which may be, at least in part, mediated via the JNK signaling pathway. 
 MicroRNA miR-125b controls melanoma progression by direct regulation of c-Jun protein expression. A fundamental event in the development and progression of malignant melanoma is the deregulation of cancer-relevant transcription factors. We recently showed that c-Jun is a main regulator of tumor progression in melanoma and thus the most important member of the AP-1 transcription factor family for this disease. Interestingly, we revealed that c-Jun expression was regulated on the post-transcriptional level and therefore speculated that miRNAs could be involved in c-Jun regulation. We determined seed sequences for miR-125b and miR-527 in the coding region of c-Jun mRNA that hints at the direct involvement of miRNA-dependent regulation on the protein level. We found that the expression of miR-125b was significantly reduced in malignant melanoma cell lines and tissue samples compared with melanocytes, whereas miR-527 remained unchanged. In further functional experiments, treatment of melanoma cells with pre-miR-125b resulted in strong suppression of cellular proliferation and   migration  , supporting the role of miR-125b in melanoma. In addition, transfection of pre-miR-125b led to strong downregulation of c-Jun protein but not mRNA expression in melanoma cells. Luciferase assays using reporter plasmids containing the miR-125b seed sequence in the luciferase coding region confirmed the direct interaction with miR-125b. Furthermore, immunoprecipitation of Ago-2 revealed that c-Jun mRNA accumulated in the RNA-induced silencing complex after pre-miR-125b transfection in melanoma cells. In summary, we identified an important role for miR-125b in malignant melanoma. Moreover, we demonstrated post-transcriptional regulation of c-Jun by this miRNA and showed that c-Jun is a main mediator of the effects of miR-125b on melanoma cells. 
 p21-activated kinase 3 (PAK3) is an AP-1 regulated gene contributing to actin organisation and   migration   of transformed fibroblasts. Activating Protein 1 (AP-1) plays a vital role in cell proliferation, differentiation and apoptosis. While de-regulation of AP-1 has been linked to many cancers, little is known regarding its downstream transcriptional targets that associate with cellular transformation. Previous studies identified PAK3, a serine/threonine kinase, as a potential AP-1 target gene. PAK3 has been implicated in a variety of pathological disorders and over-expression of other PAK-family members has been linked to cancer. In this study, we investigate AP-1 regulation of PAK3 expression and the role of PAK3 in cJun/AP-1-associated cellular transformation. Our results showed elevated PAK3 expression at both the mRNA and protein level in cJun-over-expressing Rat1a fibroblasts, as well as in transformed human fibroblasts. Elevated PAK3 expression in cJun/AP-1 over-expressing cells associated with a significant increase in PAK3 promoter activation. This increased promoter activity was lost when a single putative Jun binding site, which can bind AP-1 directly both in vitro and in vivo, was mutated. Further, inhibition of PAK3 using siRNA showed a regression in the cell morphology, migratory potential and actin organisation associated with AP-1 transformed cells. Our study is a first to describe a role for AP-1 in regulating PAK3 expression and suggest that PAK3 is an AP-1 target required for actin organization and   migration   observed in transformed cells. 
 Twist1 and AP-1 cooperatively upregulate integrin α5 expression to induce invasion and the epithelial-mesenchymal transition. Epithelial-mesenchymal transition (EMT) is an important process implicated in tumor invasion and metastasis. Twist1 is a transcription factor that induces EMT, including E-cadherin suppression and cancer cell   migratio  n and invasion; hence it promotes cancer metastasis. Twist1 directly or indirectly regulates the expression of various genes and cellular functions involved in cancer progression. However, the underlying mechanisms remain largely unknown. In this study, we investigated the molecular basis for Twist1-mediated invasion and EMT. In human cancer cells, Twist1 was found to directly upregulate transcription of the mesenchymal gene integrin α5 in an E-box-independent, but activating protein-1 (AP-1) element-dependent, manner. Twist1 activated the integrin α5 promoter by interacting with and activating the transcription factor AP-1, composed of c-Jun and activating transcription factor-2 (ATF-2); it also enhanced the nuclear presence of ATF-2. AP-1 was critical for Twist1-induced cancer cell invasion, primarily through the induction of integrin α5, which activated c-Jun N-terminal kinase and focal adhesion kinase-signaling activities. Using data from The Cancer Genome Atlas, we found that Twist1 expression positively correlates with integrin α5 expression in human colorectal cancers. These findings suggest that cooperation between Twist1 and AP-1 represents a novel mechanism for EMT and tumor invasiveness. This study supports further investigation into the molecular basis underlying the diverse Twist1-mediated functions that occur during tumor progression. 
 Necrotic cells influence   migration   and invasion of glioblastoma via NF-κB/AP-1-mediated IL-8 regulation. Glioblastoma multiforme (GBM) is the most common primary intracranial tumor in adults and has poor prognosis. Diffuse infiltration into normal brain parenchyma, rapid growth, and the presence of necrosis are remarkable hallmarks of GBM. However, the effect of necrotic cells on GBM growth and metastasis is poorly understood at present. In this study, we examined the biological significance of necrotic tissues by exploring the molecular mechanisms underlying the signaling network between necrotic tissues and GBM cells. The   migratio  n and invasion of the GBM cell line CRT-MG was significantly enhanced by treatment with necrotic cells, as shown by assays for scratch wound healing and spheroid invasion. Incubation with necrotic cells induced IL-8 secretion in CRT-MG cells in a dose-dependent manner. In human GBM tissues, IL-8 positive cells were mainly distributed in the perinecrotic region, as seen in immunohistochemistry and immunofluorescence analysis. Necrotic cells induced NF-κB and AP-1 activation and their binding to the IL-8 promoter, leading to enhanced IL-8 production and secretion in GBM cells. Our data demonstrate that when GBM cells are exposed to and stimulated by necrotic cells, th  e migrati  on and invasion of GBM cells are enhanced and facilitated via NF-κB/AP-1 mediated IL-8 upregulation. 
 Expression of Jun and Fos proteins in ovarian tumors of different malignant potential and in ovarian cancer cell lines. Jun and Fos proteins form the transcription factor activating protein 1 (AP-1). They play a role in cell proliferation, malignant transformation and invasion in various tumors. The aim of the current study was to characterize the role of AP-1 in ovarian cancer. Fifty-six ovarian tumors of different invasive potential including 13 metastases as well as 5 ovarian cancer cell lines were analyzed by Western blot analysis regarding their expression of pc-Jun, Jun B, Jun D, c-Fos, Fos B, Fra-1 and Fra-2. In addition, invasion, cell proliferation and   migration   assays were performed. The expression of pc-Jun, Jun B, Jun D and Fra-2 was higher in invasive cancer compared to benign tumors. In metastases, c-Fos and Fos B expression was significantly lower than in the respective primary ovarian carcinomas. The invasive and proliferative potential of the cells was not associated with AP-1 protein expression. These results suggest that AP-1 proteins are differentially expressed in benign ovarian tumors, tumors with low malignant potential and epithelial ovarian carcinomas and metastases. No correlation with the proliferative and invasive potential of ovarian cancer cell lines could be found. 
 Blockade of the RhoA-JNK-c-Jun-MMP2 cascade by atorvastatin reduces osteosarcoma cell invasion. Osteosarcoma is characterized by a high malignant and metastatic potential, which points to the need for new therapeutic strategies to prevent cell metastasis. In this study, we show that statin-induced HMG-CoA reductase inhibition reduces cell   migration   and invasion in human and murine osteosarcoma cells, independently of the genotype. The statin-induced reduction of cell   migration   and invasion was independent of induction of apoptosis and was geranylgeranylpyrophosphate-dependent. The statin reduced matrix metalloproteinase (MMP) 2, 9, and 14 and TIMP2 expression or activity in invading cells. Forced expression of MMP2 and MMP14 overcame the inhibitory effect of the statin on cell invasion, suggesting a role for these MMPs in invasive potential. We also investigated the mechanisms involved in the reduced MMP2 activity and cell invasion. Inhibition of JNK, but not ERK1/2 signaling, reduced MMP2 activity. Pharmacological or constitutive activation of JNK overcame the reduced MMP2 activity and cell invasion induced by the statin. The statin decreased JNK phosphorylation and c-Jun nuclear translocation, suggesting that HMG-CoA reductase inhibition targets the JNK-c-Jun signaling pathway. We showed that mevalonate or geranylgeranylpyrophosphate treatment prevented the statin-induced reduction in JNK phosphorylation, MMP2 activity, and cell invasion. Forced expression of a constitutively active form of RhoA increased JNK phosphorylation and overcame the inhibitory effect of atorvastatin on MMP2 activity and cell invasion. The data establish a link between RhoA, JNK, c-Jun, and MMP2 activity that is functionally involved in the reduction in osteosarcoma cell invasion by the statin. This suggests a novel strategy targeting RhoA-JNK-c-Jun signaling to reduce osteosarcoma cell tumorigenesis. 
 c-Jun induces mammary epithelial cellular invasion and breast cancer stem cell expansion. The molecular mechanisms governing breast tumor cellular self-renewal contribute to breast cancer progression and therapeutic resistance. The ErbB2 oncogene is overexpressed in approximately 30% of human breast cancers. c-Jun, the first cellular proto-oncogene, is overexpressed in human breast cancer. However, the role of endogenous c-Jun in mammary tumor progression is unknown. Herein, transgenic mice expressing the mammary gland-targeted ErbB2 oncogene were crossed with c-jun(f/f) transgenic mice to determine the role of endogenous c-Jun in mammary tumor invasion and stem cell function. The excision of c-jun by Cre recombinase reduced cellular   migration  , invasion, and mammosphere formation of ErbB2-induced mammary tumors. Proteomic analysis identified a subset of secreted proteins (stem cell factor (SCF) and CCL5) induced by ErbB2 expression that were dependent upon endogenous c-Jun expression. SCF and CCL5 were identified as transcriptionally induced by c-Jun. CCL5 rescued the c-Jun-deficient breast tumor cellular invasion phenotype. SCF rescued the c-Jun-deficient mammosphere production. Endogenous c-Jun thus contributes to ErbB2-induced mammary tumor cell invasion and self-renewal. 
 c-Jun promotes cell   migration   and drives expression of the motility factor ENPP2 in soft tissue sarcomas. Genomic amplification of the c-Jun proto-oncogene has been identified in ∼30% of dedifferentiated liposarcomas (DDLPS), but the functional contribution of c-Jun to the progression of DDLPS remains poorly understood. In previous work we showed that knock-down of c-Jun by RNA interference impaired the in vitro proliferation and in vivo growth of a DDLPS cell line (LP6) with genomic amplification of the c-Jun locus. Here, we used gene expression analysis and functional studies in a broad panel of cell lines to further define the role of c-Jun in DDLPS and other soft tissue sarcomas. We show that c-Jun knock-down impairs transition through the G1 phase of the cell cycle in multiple DDLPS cell lines. We also found that high levels of c-Jun expression are both necessary and sufficient to promote DDLPS cel  l migrati  on and invasion in vitro. Our data suggest that high levels of c-Jun enhance motility in part by driving the expression of ENPP2/Autotaxin. c-Jun over-expression has minimal effects on in vitro proliferation but substantially enhances the in vivo growth of weakly tumourigenic DDLPS cell lines. Finally, we provide evidence that c-Jun genomic amplification and over-expression may have similar functional consequences in other types of soft tissue sarcoma. Our data suggest a model in which relatively low levels of c-Jun are sufficient for in vitro proliferation, but high levels of c-Jun enhance invasiveness and capacity for in vivo tumour growth. These observations provide an explanation for the selective advantage provided by c-Jun genomic amplification in vivo and suggest that sarcomas with elevated c-Jun levels are likely to have a particularly high malignant potential. Data from exon array and RNA-Seq experiments have been deposited in the GEO database (Accession No. GSE57531). 
 Involvement of aberrant miR-139/Jun feedback loop in human gastric cancer. Accumulating evidence indicates that some miRNAs could form feedback loops with their targets to fine-tune tissue homeostasis, while disruption of these loops constitutes an essential step towards human tumorigenesis. In this study, we report the identification of a novel negative feedback loop formed between miR-139 and its oncogenic target Jun. In this loop, miR-139 could inhibit Jun expression by targeting a conserved site on its 3'-UTR, whereas Jun could induce miR-139 expression in a dose dependent manner through a distant upstream regulatory element. Interestingly, aberration in this loop was found in human gastric cancer, where miR-139 was down-regulated and inversely correlated with Jun expression. Further functional analysis showed that restored expression of miR-139 in gastric cancer cells significantly induces apoptosis, and inhibits cell   migration   and proliferation as well as tumour growth through targeting Jun. Thus, our data strongly suggests a role of aberrant miR-139/Jun negative feedback loop in the development of human gastric cancer and miR-139 as a potential therapeutic target for gastric cancer. Given that miR-139 and Jun are deregulated in many cancers, our findings here might have broader implication in other types of human cancers. 
 Epstein-Barr virus latent membrane protein 2A regulates c-Jun protein through extracellular signal-regulated kinase. Epstein-Barr virus (EBV) latent membrane protein 2A (LMP2A) is widely expressed in both EBV-infected cells and EBV-associated malignancies. However, the function of LMP2A is still veiled. In this study, LMP2A was found to induce the kinase activities of extracellular signal-regulated kinase (ERK) and c-Jun N-terminal kinase/stress-activated protein kinase JNK/SAPK. Furthermore, the downstream effector c-Jun showed hyperphosphorylation under LMP2A expression. The phosphorylation could be inhibited by the ERK pathway inhibitor PD98059, indicating that ERK may contribute to the phosphorylation of c-Jun in LMP2A-expressing cells. The impact on c-Jun phosphorylation by mitogen-activated protein kinase (MAPK) is suggested to increase c-Jun protein stability, and this was also observed in LMP2A-expressing cells by a protein synthesis inhibition assay. Moreover, LMP2A-induced cell invasion was inhibited in the presence of the ERK pathway inhibitor. Taken together, we suggest that LMP2A may exploit MAPK kinases and affect both the phosphorylation and stability of c-Jun protein. Additionally, LMP2A may thereby promote the mobility of the cells. In doing so, it may enhance the mobility of EBV-infected cells and contribute to the metastatic process of malignant cells. Here we demonstrated the first evidence of LMP2A-induced   migration   and the underlying pathways accounting for it. 
 YB-1 represses AP1-dependent gene transactivation and interacts with an AP-1 DNA sequence. Involvement of the AP-1 (activator protein-1) transcription factor has been demonstrated previously in the regulation of cell proliferation and cell-cycle progression, in the control of cell   migration  , invasion and metastasis, and in signal transduction, stress responsiveness, DNA replication and DNA repair. YB-1 (Y-box-binding protein-1) has also been implicated in many of these processes. However, the mechanism by which YB-1 mediates these processes is poorly understood. In the present study, we report that overexpression of a transfected gene encoding YB-1 in human HeLa cervical carcinoma cells significantly represses the transactivation of a minimal AP-1 reporter construct in response to the tumour promoter PMA. YB-1 also represses mRNA expression and PMA-induced promoter transactivation of the endogenous AP-1 target gene encoding matrix metalloproteinase-12 (metalloelastase). YB-1 transrepression of both the minimal and matrix metalloproteinase-12 promoter reporter constructs is dependent on the AP-1 sequence. To identify new nuclear proteins that bind specifically to the AP-1 DNA-binding site, we devised a DNA-affinity-chromatography-based assay termed NAPSTER (nucleotide-affinity preincubation specificity test of recognition) and discovered a 49 kDa protein from human cancer cells that binds in a sequence-specific manner to the AP-1 DNA sequence. By tandem MS fragmentation sequencing analyses we determined that p49 is a YB-1. Immunoblotting of the NAPSTER-purified p49 protein using anti-YB-1 antibodies confirmed YB-1 binding to the AP-1 DNA sequence, as did gel mobility-supershift assays using YB-1 antibodies. This is the first report of YB-1 transrepression and interaction at the AP-1 DNA-binding site. 
 Hypoxia increases AP-1 binding activity by enhancing capacitative Ca2+ entry in human pulmonary artery endothelial cells. Activating protein (AP)-1 transcription factors modulate expression of genes involved in cell proliferation and   migration  . Chronic hypoxia increases pulmonary artery smooth muscle cell proliferation by upregulating AP-1-responsive genes encoding for endothelium-derived vasoactive and mitogenic factors implicated in pulmonary hypertension development. The expression of AP-1 transcription factors is sensitive to changes in cytosolic free [Ca2+] ([Ca2+]cyt). Capacitative Ca2+ entry (CCE) via store-operated Ca2+ channels (SOC) is an important mechanism for raising [Ca2+]cyt in pulmonary artery endothelial cells (PAEC). Using combined molecular biological, fluorescence microscopy, and biophysical approaches, we examined the effect of chronic hypoxia (3% O2, 72 h) on AP-1 DNA binding activity, CCE, and transient receptor potential (TRP) gene expression in human (h) PAEC. EMSA showed that AP-1 binding to hPAEC nuclear protein extracts was significantly enhanced by hypoxia, the increase being dependent on store-operated Ca2+ influx and sensitive to La3+, an SOC inhibitor. Hypoxia also increased basal [Ca2+]cyt, the amount of CCE produced by store depletion with cyclopiazonic acid, and the amplitude of SOC-mediated currents (ISOC). The increases of CCE amplitude and ISOC current density by hypoxia were paralleled by enhanced TRPC4 mRNA and protein expression. Hypoxia-enhanced CCE and TRPC4 expression were also attenuated by La3+. These data suggest that hypoxia increases AP-1 binding activity by enhancing Ca2+ influx via La3+-sensitive TRP-encoded SOC channels in hPAEC. The Ca2+-mediated increase in AP-1 binding may play an important role in upregulating AP-1-responsive gene expression, in stimulating pulmonary vascular cell proliferation and, ultimately, in pulmonary vascular remodeling in patients with hypoxia-mediated pulmonary hypertension. 
 c-Jun transcriptionally regulates alpha 1, 2-fucosyltransferase 1 (FUT1) in ovarian cancer. Alpha 1, 2-fucosyltransferase (FUT 1/2) is a rate-limiting enzyme that catalyzes the synthesis of Lewis y, a cell membrane-associated carbohydrate antigen. In human ovarian cancer, the upregulated expression of FUT1 and Lewis y is associated with advanced pathological stages and involved in cell proliferation,   migration   and invasion. However, the mechanism underlying the upregulation of FUT1 is largely unknown. Here, we identify an AP-1 binding site in FUT1 promoter in ovarian cancer cells. c-Jun promotes FUT1 expression, thereby enhancing Lewis y biosynthesis in various ovarian cancer cell lines. Moreover, EMSA, luciferase activity and ChIP assays demonstrate c-Jun directly interacts with FUT1 promoter. Furthermore, FUT1 mediates c-Jun-induced cell proliferation in ovarian cancer cells. In human ovarian cancer samples, c-Jun overexpression is linked to malignant degree and positively correlated to FUT1 and Lewis y expression. Taken together, c-Jun could transcriptionally modulate FUT1 expression in ovarian cancer, implicating the potential application of c-Jun inhibitors for human ovarian cancer therapy. 
 miR200c attenuates P-gp-mediated MDR and metastasis by targeting JNK2/c-Jun signaling pathway in colorectal cancer. MicroRNA-200c (miR200c) recently emerged as an important regulator of tumorigenicity and cancer metastasis; however, its role in regulating multidrug resistance (MDR) remains unknown. In the current study, we found that the expression levels of miR200c in recurrent and metastatic colorectal cancers were significantly lower, whereas the JNK2 expression was higher compared with primary tumors. We showed that in MDR colorectal cancer cells, miR200c targeted the 3' untranslated region of the JNK2 gene. Overexpression of miR200c attenuated the levels of p-JNK, p-c-Jun, P-gp, and MMP-2/-9, the downstream factors of the JNK signaling pathway, resulting in increased sensitivity to chemotherapeutic drugs, which was accompanied by heightened apoptosis and decreased cell invasion and   migration  . Moreover, in an orthotopic MDR colorectal cancer mouse model, we demonstrated that overexpression of miR200c effectively inhibited the tumor growth and metastasis. At last, in the tumor samples from patients with locally advanced colorectal cancer with routine postsurgical chemotherapy, we observed an inverse correlation between the levels of mRNA expression of miR200c and JNK2, ABCB1, and MMP-9, thus predicting patient therapeutic outcomes. In summary, we found that miR200c negatively regulated the expression of JNK2 gene and increased the sensitivity of MDR colorectal cancer cells to chemotherapeutic drugs, via inhibiting the JNK2/p-JNK/p-c-Jun/ABCB1 signaling. Restoration of miR200c expression in MDR colorectal cancer may serve as a promising therapeutic approach in MDR-induced metastasis. 
 Interleukin-5 enhances the   migration   and invasion of bladder cancer cells via ERK1/2-mediated MMP-9/NF-κB/AP-1 pathway: involvement of the p21WAF1 expression. Inflammatory cytokines may be a critical component of epithelial cancer progression. We examined the role of interleukin (IL)-5 in the   migratio  n of bladder cancer cells. The expression of IL-5 and its receptor IL-5Rα was enhanced in patients with muscle invasive bladder cancers (MIBC), and then it was detected in bladder cancer cell lines 5637 and T-24. IL-5 increase  d migrati  on and MMP-9 expression via activation of transcription factors NF-κB and AP-1, and induced activation of ERK1/2 and Jak-Stat signaling in both cells. Treatment with ERK1/2 inhibitor U0126 significantly inhibited induction   of migrat  ion, MMP-9 expression, and activation of NF-κB and AP-1 in IL-5-treated cells. However, none of the Jak inhibitors affected the IL-5-indu  ced migra  tion of bladder cancer cells. Moreover, gene knockdown for IL-5Rα, using siRNA transfection, suppre  ssed migr  ation, ERK1/2 activation, MMP-9 expression, as well as the binding activation of NF-κB and AP-1 in IL-5-treated bladder cancer cells. Similar results were observed in βc siRNA (si-βc) transfected cells. Unexpectedly, IL-5 treatment resulted in significant induction of p21WAF1 in both cell lines. The p21WAF1-specific small interfering RNA inhibited IL-5-induc  ed cell m  igration, ERK activity, MMP-9 expression, and activation of NF-κB and AP-1 in bladder cancer cells. The effects of IL-5-induced cell responses were confirmed by transfection of IL-5 gene, which demonstrated that p21WAF1 participates in the induction   of cell   migration, leading to an increase in ERK1/2-mediated MMP-9 expression through activation of NF-κB and AP-1 in IL-5-treated bladder cancer cells. These unexpected results provide a theoretical basis for the therapeutic targeting of IL-5 in bladder cancer. 
 Aberrant IKKα and IKKβ cooperatively activate NF-κB and induce EGFR/AP1 signaling to promote survival a  nd migrat  ion of head and neck cancer. The inhibitor-κB kinase-nuclear factor-κB (IKK-NF-κB) and epidermal growth factor receptor-activator protein-1 (EGFR-AP1) pathways are often co-activated and promote malignant behavior, but the underlying basis for this relationship is unclear. Resistance to inhibitors of IKKβ or EGFR is observed in head and neck squamous cell carcinomas (HNSCC). Here, we reveal that both IKKα and β contribute to nuclear activation of canonical and alternate NF-κB/REL family transcription factors, and overexpression of signal components that enhance co-activation of the EGFR-AP1 pathway. We observed that IKKα and IKKβ exhibit increased protein expression, nuclear localization, and phosphorylation in HNSCC tissues and cell lines. Individually, IKK activity varied among different cell lines, but overexpression of both IKKs induced the strongest NF-κB activation. Conversely, siRNA knock down of both IKKs significantly decreased nuclear localization and phosphorylation of canonical RELA and IκBα and alternative p52 and RELB subunits. Knock down of both IKKs more effectively inhibited NF-κB activation, broadly modulated gene expression and suppressed cell pr  oliferati  on and migration. Global expression profiling revealed that NF-κB, cytokine, inflammatory response and growth factor signaling are among the top pathways and networks regulated by IKKs. Importantly, IKKα and IKKβ together promoted the expression and activity of transforming growth factor α, EGFR and AP1 transcription factors cJun, JunB and Fra1. Knock down of AP1 subunits individually decreased 8/15 (53%) of IKK-targeted genes sampled and similarly inhibited cel  l prolife  ration and migration. Mutations of NF-κB and AP1-binding sites abolished or decreased IKK-induced interleukin-8 (IL-8) promoter activity. Compounds such as wedelactone with dual IKK inhibitory activity and geldanomycins that block IKKα/β and EGFR pathways were more active than IKKβ-specific inhibitors in suppressing NF-κB activation and proliferation and inducing cell death. We conclude that IKKα and IKKβ cooperatively activate NF-κB and EGFR/AP1 networks of signaling pathways and contribute to the malignant phenotype and the intrinsic or acquired therapeutic resistance of HNSCC. 
 The role of sLZIP in transcriptional regulation of c-Jun and involvement in   migration   and invasion of cervical cancer cells. Matrix metalloproteinase-9 (MMP-9) plays an important role in tumor invasion and metastasis through the breakdown of extracellular matrix. The c-Jun protein, a major component of the AP-1 transcription factor, is elevated in various cancers. Small leucine zipper protein (sLZIP) is a member of the leucine zipper transcription factor family. Although sLZIP is known to be involved in cancer cell   migration   and invasion, its biological roles in cancer development and the cellular target genes are not fully understood. In this study, we investigated the role of sLZIP in c-Jun expression, and their effects on expression of MMP-9 and   migration   of cervical cancer cells.sLZIP up-regulates transcription of c-Jun by binding directly to the CRE region in the c-Jun promoter. Elevated c-Jun due to sLZIP leads to activation of MMP-9 transcription by interaction with the AP-1 binding site in the MMP-9 promoter. c-Jun siRNA repressed   migration   and invasion of cervical cancer cells, whereas sLZIP recovered   migration   and invasion of cells transfected with c-Jun siRNA. Immunohistochemical analysis results revealed a significant correlation between the expressions of sLZIP and MMP-9 in clinical cervical specimens.These results indicate that sLZIP plays a role in expression of c-Jun, and   migration   and invasion of cervical cancer cells via regulation of MMP-9 transcription. 
 Expression of podoplanin in human astrocytic brain tumors is controlled by the PI3K-AKT-AP-1 signaling pathway and promoter methylation. Recently, we found strong overexpression of the mucin-type glycoprotein podoplanin (PDPN) in human astrocytic brain tumors, specifically in primary glioblastoma multiforme (GB). In the current study, we show an inverse correlation between PDPN expression and PTEN levels in primary human GB and glioma cell lines, and we report elevated PDPN protein levels in the subventricular zone of brain tissue sections of PTEN-deficient mice. In human glioma cells lacking functional PTEN, reintroduction of wild-type PTEN, inhibition of the PTEN downstream target protein kinase B/AKT, or interference with transcription factor AP-1 function resulted in efficient downregulation of PDPN expression. In addition, we observed hypoxia-dependent PDPN transcriptional control and demonstrated that PDPN expression is subject to negative transcriptional regulation by promoter methylation in human GB and in glioma cell lines. Treatment of PTEN-negative glioma cells with demethylating agents induced expression of PDPN. Together, our findings show that increased PDPN expression in human GB is caused by loss of PTEN function and activation of the PI3K-AKT-AP-1 signaling pathway, accompanied by epigenetic regulation of PDPN promoter activity. Silencing of PDPN expression leads to reduced proliferation and   migration   of glioma cells, suggesting a functional role of PDPN in glioma progression and malignancy. Thus, specific targeting of PDPN expression and/or function could be a promising strategy for the treatment of patients with primary GB. 
 Hydrogen peroxide stimulates proliferation and   migration   of human prostate cancer cells through activation of activator protein-1 and up-regulation of the heparin affin regulatory peptide gene. It is becoming increasingly recognized that hydrogen peroxide (HP) plays a role in cell proliferation and   migration  . In the present study we found that exogenous HP significantly induced human prostate cancer LNCaP cell proliferation and   migration  . Heparin affin regulatory peptide (HARP) seems to be involved in the stimulatory effect of HP, because the latter had no effect on stably transfected LNCaP cells that did not express HARP. Moreover, HP significantly increased HARP mRNA and protein amounts in a concentration- and time-dependent manner. Curcumin and activator protein-1 (AP-1) decoy oligonucleotides abrogated both HP-induced HARP expression and LNCaP cell proliferation and   migration  . HP increased luciferase activity of the 5'-flanking region of the HARP gene introduced in a reporter gene vector, an effect that was abolished when even one of the two putative AP-1 binding sites of the HARP promoter was mutated. The effect of HP seems to be due to the binding of Fra-1, JunD, and phospho-c-Jun to the HARP promoter. These results support the notion that HARP is important for human prostate cancer cell proliferation and   migration  , establish the role of AP-1 in the up-regulation of HARP expression by low concentrations of HP, and characterize the AP-1 dimers involved. 
 NRF-1, and AP-1 regulate the promoter of the human calpain small subunit 1 (CAPNS1) gene. Ubiquitously expressed micro- and m-calpain are cysteine proteases with broad functions in cell spreading,   migration  , proliferation, apoptosis, and in tumor invasion. They are heterodimers, with a distinct large 80-kDa catalytic, and a common small 28-kDa regulatory subunit (Capn4/CAPNS1). CAPNS1 is required to maintain stability and activity of both calpains. Despite its biological importance, the transcriptional regulation of this gene has not been studied, and the CAPNS1 promoter has not yet been characterized. In this study, we identified the main transcriptional start site, and cloned and characterized the ~2.0 kb upstream region of the CAPNS1 gene. Deletion analysis identified the core promoter located within region -187/+174. Site-directed mutagenesis, EMSA- and supershift analysis identified Sp1-, NRF-1-, and AP-1-binding elements within the CAPNS1 core promoter. Binding of NRF-1, Sp1 and AP-1 to the natural core promoter was confirmed by chromatin immunoprecipitation (ChIP). Site-directed mutagenesis at the NRF-1 site in HeLa and MCF7 cells substantially reduced core promoter activity by 70%, whereas mutation of the AP-1-binding and Sp1-binding site reduced promoter activity by 50% and 30%, respectively. Double mutation of the NRF-1 and the AP-1 site reduced promoter activity by 90%. In Drosophila SL2 cells, ectopic expression of NRF-1 led to a significant induction of CAPNS1 promoter activity. Furthermore, an siRNA against NRF-1 substantially reduced promoter activity in HeLa cells, which was paralleled by a significant downregulation of CAPNS1 mRNA. These results reveal that especially NRF-1, along with AP-1 and, to a minor extent, an Sp1 site, is essential for human CAPNS1 promoter activity and gene expression. 
 Critical role of c-Jun overexpression in liver metastasis of human breast cancer xenograft model. c-Jun/AP-1 has been linked to invasive properties of aggressive breast cancer. Recently, it has been reported that overexpression of c-Jun in breast cancer cell line MCF-7 resulted in increased AP-1 activity, motility and invasiveness of the cells in vitro and tumor formation in nude mice. However, the role of c-Jun in metastasis of human breast cancer in vivo is currently unknown.To further investigate the direct involvement of c-Jun in tumorigenesis and metastasis, in the present study, the effects of c-Jun overexpression were studied in both in vitro and in nude mice.Ectopic overexpression of c-Jun promoted the growth of MCF-7 cells and resulted in a significant increase in the percentage of cells in S phase and increased motility and invasiveness. Introduction of c-Jun gene alone into weakly invasive MCF-7 cells resulted in the transfected cells capable of metastasizing to the nude mouse liver following tail vein injection.The present study confirms that overexpression of c-Jun contributes to a more invasive phenotype in MCF-7 cells. It indicates an interesting relationship between c-Jun expression and increased property of adhesion,   migration   and in vivo liver metastasis of MCF-7/c-Jun cells. The results provide further evidence that c-Jun is involved in the metastasis of breast cancer. The finding also opens an opportunity for development of anti-c-Jun strategies in breast cancer therapy. 
 Interleukin-1β enhances cell   migratio  n through AP-1 and NF-κB pathway-dependent FGF2 expression in human corneal endothelial cells. Interleukin (IL)-1β is a major pro-inflammatory cytokine that plays a crucial role in the regulation of inflammation and wound healing in the cornea. Elucidation of IL-1β signalling may help identify therapeutic targets for corneal wound healing; however, mechanisms such as c  ell migra  tion, a component of IL-1β-induced wound healing response in human corneal endothelial cells (CEC), have not been well characterised.Stimulation of human CEC with IL-1β activated expression of fibroblast growth factor 2 (FGF2) and resulted in enhanced   cell mig  ration. This, in turn, was abolished by treatment with either IL-1 receptor antagonist or SU-5402, a pan-fibroblast growth factor signalling inhibitor. Phosphatidyl inositol (PI) 3-kinase or IL receptor-associated kinase 1/4 antagonists demonstrated that IL receptor-associated kinase 1/4 activates PI 3-kinase, which in turn phosphorylates p38 and inhibitor κB kinase α/β, leading to FGF2 expression through activation of activator protein 1 (AP-1) and nuclear factor kappa-light-chain enhancer of activated B cells (NF-κB) in human CEC. Treatment of IL-1β-stimulated human CEC with either AP-1 or NF-κB antagonists decreased FGF2 expression and resulted in reduced IL-1β-e  nhanced c  ell migration. Co-treatment of IL-1β-stimulated human CEC with both inhibitors completely blocked FGF2 expression and IL-1β  -enhanced   cell migration. Chromatin immunoprecipitation assays demonstrated that AP-1 and NF-κB directly bind to the FGF2 promoter following IL-1β stimulation.The results show that binding of IL-1β to its receptor in human CEC leads to parallel activation of AP-1 and NF-κB pathways, leading, in turn, to FGF2 expression   and enha  nced cell migration. 
 miR-221&222 regulate TRAIL resistance and enhance tumorigenicity through PTEN and TIMP3 downregulation. Lung and liver cancers are among the most deadly types of cancer. Despite improvements in treatment over the past few decades, patient survival remains poor, underlining the need for development of targeted therapies. MicroRNAs represent a class of small RNAs frequently deregulated in human malignancies. We now report that miR-221&222 are overexpressed in aggressive non-small cell lung cancer and hepatocarcinoma cells, as compared with less invasive and/or normal lung and liver cells. We show that miR-221&222, by targeting PTEN and TIMP3 tumor suppressors, induce TRAIL resistance and enhance cellular   migration   through the activation of the AKT pathway and metallopeptidases. Finally, we demonstrate that the MET oncogene is involved in miR-221&222 activation through the c-Jun transcription factor. 
 Tead and AP1 Coordinate Transcription and Motility. The Tead family transcription factors are the major intracellular mediators of the Hippo-Yap pathway. Despite the importance of Hippo signaling in tumorigenesis, Tead-dependent downstream oncogenic programs and target genes in cancer cells remain poorly understood. Here, we characterize Tead4-mediated transcriptional networks in a diverse range of cancer cells, including neuroblastoma, colorectal, lung, and endometrial carcinomas. By intersecting genome-wide chromatin occupancy analyses of Tead4, JunD, and Fra1/2, we find that Tead4 cooperates with AP1 transcription factors to coordinate target gene transcription. We find that Tead-AP1 interaction is JNK independent but engages the SRC1-3 co-activators to promote downstream transcription. Furthermore, we show that Tead-AP1 cooperation regulates the activity of the Dock-Rac/CDC42 module and drives the expression of a unique core set of target genes, thereby directing cell   migration   and invasion. Together, our data unveil a critical regulatory mechanism underlying Tead- and AP1-controlled transcriptional and functional outputs in cancer cells. 
 Cell specific CD44 expression in breast cancer requires the interaction of AP-1 and NFκB with a novel cis-element. Breast cancers contain a heterogeneous population of cells with a small percentage that possess properties similar to those found in stem cells. One of the widely accepted markers of breast cancer stem cells (BCSCs) is the cell surface marker CD44. As a glycoprotein, CD44 is involved in many cellular processes including cell adhesion,   migratio  n and proliferation, making it pro-oncogenic by nature. CD44 expression is highly up-regulated in BCSCs, and has been implicated in tumorigenesis and metastasis. However, the genetic mechanism that leads to a high level of CD44 expression in breast cancer cells and BCSCs is not well understood. Here, we identify a novel cis-element of the CD44 directs gene expression in breast cancer cells in a cell type specific manner. We have further identified key trans-acting factor binding sites and nuclear factors AP-1 and NFκB that are involved in the regulation of cell-specific CD44 expression. These findings provide new insight into the complex regulatory mechanism of CD44 expression, which may help identify more effective therapeutic targets against the breast cancer stem cells and metastatic tumors. 
 COP1 and GSK3β cooperate to promote c-Jun degradation and inhibit breast cancer cell tumorigenesis. High abundance of c-Jun is detected in invasive breast cancer cells and aggressive breast tumor malignancies. Here, we demonstrate that a major cause of high c-Jun abundance in invasive breast cancer cells is prolonged c-Jun protein stability owing to poor poly-ubiquitination of c-Jun. Among the known c-Jun-targeting E3 ligases, we identified constitutive photomorphogenesis protein 1 (COP1) as an E3 ligase responsible for c-Jun degradation in less invasive breast cancer cells because depletion of COP1 reduced c-Jun poly-ubiquitination leading to the stabilization of c-Jun protein. In a panel of breast cancer cell lines, we observed an inverse association between the levels of COP1 and c-Jun. However, overexpressing COP1 alone was unable to decrease c-Jun level in invasive breast cancer cells, indicating that efficient c-Jun protein degradation necessitates an additional event. Indeed, we found that glycogen synthase kinase 3 (GSK3) inhibitors elevated c-Jun abundance in less invasive breast cancer cells and that GSK3β nonphosphorylable c-Jun-T239A mutant displayed greater protein stability and poorer poly-ubiquitination compared to the wild-type c-Jun. The ability of simultaneously enforced expression of COP1 and constitutively active GSK3β to decrease c-Jun abundance in invasive breast cancer cells allowed us to conclude that c-Jun is negatively regulated through the coordinated action of COP1 and GSK3β. Importantly, co-expressing COP1 and active GSK3β blocked in vitro cell gr  owth/migr  ation and in vivo metastasis of invasive breast cancer cells. Gene expression profiling of breast tumor specimens further revealed that higher COP1 expression correlated with better recurrence-free survival. Our study supports the notion that COP1 is a suppressor of breast cancer progression. 
 Activation of TGF-β1 promoter by hepatitis C virus-induced AP-1 and Sp1: role of TGF-β1 in hepatic stellate cell activation and invasion. Our previous studies have shown the induction and maturation of transforming growth factor-beta 1 (TGF-β1) in HCV-infected human hepatoma cells. In this study, we have investigated the molecular mechanism of TGF-β1 gene expression in response to HCV infection. We demonstrate that HCV-induced transcription factors AP-1, Sp1, NF-κB and STAT-3 are involved in TGF-β1 gene expression. Using chromatin immunoprecipitation (ChIP) assay, we further show that AP-1 and Sp1 interact with TGF-b1 promoter in vivo in HCV-infected cells. In addition, we demonstrate that HCV-induced TGF-β1 gene expression is mediated by the activation of cellular kinases such as p38 MAPK, Src, JNK, and MEK1/2. Next, we determined the role of secreted bioactive TGF-β1 in human hepatic stellate cells (HSCs) activation and invasion. Using siRNA approach, we show that HCV-induced bioactive TGF-β1 is critical for the induction of alpha smooth muscle actin (α-SMA) and type 1 collagen, the markers of HSCs activation and proliferation. We further demonstrate the potential role of HCV-induced bioactive TGF-β1 in HSCs inv  asion/cel  l migration using a transwell Boyden chamber. Our results also suggest the role of HCV-induced TGF-β1 in HCV replication and release. Collectively, these observations provide insight into the mechanism of TGF-β1 promoter activation, as well as HSCs activation and invasion, which likely manifests in liver fibrosis associated with HCV infection. 
 Differential requirement of the epidermal growth factor receptor for G protein-mediated activation of transcription factors by lysophosphatidic acid. The role of the epidermal growth factor receptor (EGFR) and other receptor tyrosine kinases (RTKs) in provoking biological actions of G protein-coupled receptors (GPCRs) has been one of the most disputed subjects in the field of GPCR signal transduction. The purpose of the current study is to identify EGFR-mediated mechanisms involved in activation of G protein cascades and the downstream transcription factors by lysophosphatidic acid (LPA).In ovarian cancer cells highly responsive to LPA, activation of AP-1 by LPA was suppressed by inhibition of EGFR, an effect that could be reversed by co-stimulation of another receptor tyrosine kinase c-Met with hepatocyte growth factor, indicating that LPA-mediated activation of AP-1 requires activity of a RTK, not necessarily EGFR. Induction of AP-1 components by LPA lied downstream of Gi, G12/13, and Gq. Activation of the effectors of Gi, but not Gq or G12/13 was sensitive to inhibition of EGFR. In contrast, LPA stimulated another prominent transcription factor NF-kappaB via the Gq-PKC pathway in an EGFR-independent manner. Consistent with the importance of Gi-elicited signals in a plethora of biological processes, LPA-induced cytokine production, cell proliferation,   migration   and invasion require intact EGFR.An RTK activity is required for activation of the AP-1 transcription factor and other Gi-dependent cellular responses to LPA. In contrast, activation of G12/13, Gq and Gq-elicited NF-kappaB by LPA is independent of such an input. These results provide a novel insight into the role of RTK in GPCR signal transduction and biological functions. 
 TAF4b and Jun/activating protein-1 collaborate to regulate the expression of integrin alpha6 and cancer cell   migration   properties. The TAF4b subunit of the transcription factor IID, which has a central role in transcription by polymerase II, is involved in promoter recognition by selective recruitment of activators. The activating protein-1 (AP-1) family members participate in oncogenic transformation via gene regulation. Utilizing immunoprecipitation of endogenous protein complexes, we documented specific interactions between Jun family members and TATA box binding protein-associated factors (TAF) in colon HT29 adenocarcinoma cells. Particularly, TAF4b and c-Jun were found to colocalize and interact in the nucleus of advanced carcinoma cells and in cells with epithelial-to-mesenchymal transition (EMT) characteristics. TAF4b was found to specifically regulate the AP-1 target gene involved in EMT integrin alpha6, thus altering related cellular properties such as   migration   potential. Using a chromatin immunoprecipitation approach in colon adenocarcinoma cell lines, we further identified a synergistic role for TAF4b and c-Jun and other AP-1 family members on the promoter of integrin alpha6, underlining the existence of a specific mechanism related to gene expression control. We show evidence for the first time of an interdependence of TAF4b and AP-1 family members in cell type-specific promoter recognition and initiation of transcription in the context of cancer progression and EMT. 
 Mitochondrial dysfunction induces EMT through the TGF-β/Smad/Snail signaling pathway in Hep3B hepatocellular carcinoma cells. Mitochondrial dysfunction has been found to be associated with various pathological conditions, particularly cancer. However, the mechanisms underlying tumor malignancy induced by mitochondrial dysfunction are not fully understood. In the present study, the effects of mitochondrial dysfunction on epithelial-mesenchymal transition (EMT), were investigated using mitochondrial-depleted ρ(0) cells derived from the Hep3B hepatocarcinoma cell line. The Hep3B/ρ(0) cells displayed the EMT phenotype with more aggressi  ve migrat  ion and higher invasiveness compared to their parental cells. The Hep3B/ρ(0) cells also showed typical expression pattern of EMT markers such as vimentin and E-cadherin. These phenotypes in Hep3B/ρ(0) cells were mediated by increased transforming growth factor-β (TGF-β) through the canonical Smad-dependent signaling pathway. Additionally, TGF-β signaling was activated via induction of c-Jun/AP-1 expression and activity. Therefore, mitochondrial dysfunction induces EMT through TGF-β/Smad/Snail signaling via c-Jun/AP-1 activation. These results indicate that mitochondrial dysfunction plays an important role in the EMT process and could be a novel therapeutic target for malignant cancer therapy. 
 PMA-induced up-regulation of TBX3 is mediated by AP-1 and contributes to breast cancer cell   migration  . The T-box transcription factor TBX3 provides an important link between embryonic development and cancer. TBX3 mediates limb, mammary gland and heart development and, in humans, mutations resulting in haplo-insufficiency of TBX3 lead to ulnar-mammary syndrome. Importantly, the de-regulation of TBX3 gene expression has been linked to several cancers, where it acts to suppress senescence and promotes proliferation and tumour invasion. Despite the negative impact of de-regulated TBX3 expression as seen by developmental defects and cancer, surprisingly little is known about the regulation of the TBX3 gene. In the present paper, we show that the phorbol ester PMA increases TBX3 protein and mRNA levels in a protein kinase C-dependent manner via the AP-1 (activator protein 1) transcription factors c-Jun and JunB. Furthermore, these AP-1 factors are shown to mediate the activation of the TBX3 gene by binding a non-consensus PMA-response element in the TBX3 promoter in vitro and in vivo. We also demonstrate that TBX3 contributes to the PMA-induced   migration   previously observed for the MCF-7 breast epithelium cancer cell line. Our present results reveal a previously unidentified pathway that up-regulates TBX3 expression and provides additional evidence that increased levels of TBX3 contribute to metastasis. 
 The RNA-binding protein RBPMS1 represses AP-1 signaling and regulates breast cancer cell proliferation and   migration  . The activator protein-1 (AP-1) transcription factor complex plays a crucial role in tumor growth and progression. However, how AP-1 transcriptional activity is repressed is not fully understood. Here, we show that RNA-binding protein with multiple splicing 1 (RBPMS1) physically and functionally interacts with AP-1 in vitro and in vivo. The RNA-recognition motif (RRM) and C-terminus of the RBPMS1 isoforms RBPMS1A and RBPMS1C, but not RBPMS1B, interacted with cFos, a member of the AP-1 family that dimerizes with cJun to stimulate AP-1 transcriptional activity. RBPMS1 did not associate with Jun proteins. RBPMS1A and RBPMS1C bound to the basic leucine zipper (bZIP) domain of cFos that mediates dimerization of AP-1 proteins. In addition, RBPMS1A-C interacted with the transcription factor Smad3, which was shown to interact with cJun and increase AP-1 transcriptional activity. RBPMS1 inhibited c-Fos or Smad3-mediated AP-1 transactivation and the expression of AP-1 target genes known to be the key regulators of cancer growth and progression, including vascular endothelial growth factor (VEGF) and cyclin D1. Mechanistically, RBPMS1 blocks the formation of the cFos/cJun or Smad3/cJun complex as well as the recruitment of cFos or Smad3 to the promoters of AP-1 target genes. In cultured cells and a mouse xenograft model, RBPMS1 inhibited the growth and   migration   of breast cancer cells through c-Fos or Smad3. These data suggest that RBPMS1 is a critical repressor of AP-1 signaling and RBPMS1 activation may be a useful strategy for cancer treatment. 
 Inhibition of AP-1 by SARI negatively regulates transformation progression mediated by CCN1. Enhanced expression of the CCN family of secretory integrin-binding proteins correlates with many essential components of the cancerous state, including tumor cell adhesion, proliferation, invasion and   migration  . Consequently, CCN1 expression is elevated in various cancers, including breast cancer, and its expression directly correlates with poor patient prognosis. Using subtraction-hybridization, combined with induction of cancer cell terminal differentiation, we cloned SARI (suppressor of activator protein (AP)-1, regulated by interferon (IFN)), an IFN-beta-inducible, potent tumor suppressor gene that exerts cancer-selective growth inhibitory effects. Forced expression of SARI using an adenovirus (Ad.SARI) inhibits AP-1 function and downregulates CCN1 expression in multiple cancer lineages, resulting in a profound inhibition in anchorage-independent cell growth and tumor cell invasion. Overexpression of SARI reduces CCN1-promoter activity through inhibition of AP-1 binding. Accordingly, SARI selectively blocks expression of the transformed state in rat embryo fibroblast cells that stably overexpress c-Jun. These results illustrate that SARI inhibits AP-1 transactivating factor binding to the cis-element of the CCN1 promoter, possibly through its interaction with c-Jun. Overall, SARI can directly inhibit CCN1-induced transformation by inhibiting the transcription of CCN1, as well as indirectly by inhibiting the expression of c-Jun (and hence blocking AP-1 activity). In these contexts, transformed cells 'addicted' to AP-1 activity are rendered susceptible to SARI-mediated inhibition of expression of the transformed phenotype. 
  GeneRIFs  
 the present study indicated that knockdown of NMIIA inhibited the   migration   and invasion of gastric cancer cells, which may be, at least in part, mediated via the JNK signaling pathway. 
 These results indicate that sLZIP plays a role in expression of c-Jun, and   migration   and invasion of cervical cancer cells via regulation of MMP-9 transcription. 
 p21WAF1-specific small interfering RNA inhibited IL-5-induced cell   migration  , ERK activity, MMP-9 expression, and activation of NF-kappaB and AP-1 in bladder cancer cells. 
 This is the first report elucidating a feedback regulation between miR-127 and the TGFbeta/c-Jun cascade in hepatocellular carcinoma   migration   via MMP13. 
 Il-1beta enhances cell   migration   through AP-1 and NF-kappaB pathway-dependent FGF2 expression in human corneal endothelial cells. 
 Blimp1 is a mediator of Ras/Raf/AP-1 signaling that promotes cell   migration  , and is repressed by LOX-PP in lung cancer. 
 Podoplanin is regulated by AP-1 and promotes platelet aggregation and cell   migration   in osteosarcoma 
 results show that heparin affin regulatory peptide (HARP) is important for human prostate cancer cell proliferation and   migration   and establish role of activator protein-1 in up-regulation of HARP expression by low concentrations of hydrogen peroxide 
  ECM  PubMed  
 Pioglitazone inhibits high glucose-induced synthesis of extracellular matrix by NF-κB and AP-1 pathways in rat peritoneal mesothelial cells. High glucose (HG) in peritoneal dialysates has been demonstrated to induce extracellular matrix   (EC  M) synthesis by peritoneal mesothelial cells (PMCs) and to contribute to peritoneal fibrosis during continuous ambulatory peritoneal dialysis (CAPD). In the present study, we investigated the effects of pioglitazone, a peroxisome proliferator-activated receptor γ (PPARγ) agonist, on HG-induc  ed   ECM accumulation and the underlying mechanism in rat PMCs (RPMCs). In cultured RPMCs, HG treatment increased the expression of fibronectin (FN), collagen I and plasminogen activation inhibitor-1 (PAI-1) at the mRNA and protein levels, while it downregulated the expression of PPARγ in a time- and concentration-dependent manner. Pretreatment with pioglitazone not only decreased the expression of PAI-1 and matrix proteins (FN and collagen I), but prevented the downregulation of PPARγ in RPMCs under HG conditions. HG treatment activated the nuclear factor-κB (NF-κB) and activator protein-1 (AP-1) pathways. In addition, the NF-κB inhibitor, pyrrolidine dithiocarbamate (PDTC), and the AP-1 inhibitor, SP600125, decreased the protein levels of FN, collagen I and PAI-1, suggesting a role for the NF-κB and AP-1 pathways in the regul  ati  on of ECM accumulation induced by HG in RPMCs. Notably, we demonstrated that pretreatment with pioglitazone significantly inhibited HG-induced NF-κB and AP-1 activation. Collectively, these results suggest that pioglitazone inhibits H  G-i  nduced ECM accumulation in RPMCs by increasing PPARγ expression, and by inhibiting the NF-κB and AP-1 pathways. 
 AP-1 overexpression impairs corticosteroid inhibition of collagen production by fibroblasts isolated from asthmatic subjects. Asthma is characterized by airway remodeling associated with an increase in the deposition of   ECM   proteins such as type I collagen. These components are mainly produced by fibroblasts. Inhaled corticosteroids are considered the cornerstone of asthma therapy. Despite substantial evidence as to the anti-inflammatory action of corticosteroids, their effect on controlling   ECM   protein deposition in the airways is not completely understood. This study determined the effect of dexamethasone (Dex) on collagen production by bronchial fibroblasts derived from asthmatic and healthy subjects. Expression of procollagen mRNA in fibroblasts from asthmatics and normal controls was determined by quantitative PCR. Regulation of the procollagen-alpha(1)I promoter was evaluated by transient transfections. Transforming growth factor-beta (TGF-beta) protein expression was determined by ELISA. Protein expression of glucocorticoid receptor (GR) and interaction with activator protein-1 (AP-1), a collagen regulatory transcription factor, was assessed by Western blots, coimmunoprecipitations, and EMSA. AP-1 overexpression was performed by transient transfection using c-Fos/c-Jun expression plasmids. Dex significantly downregulated procollagen production and promoter activity in normal fibroblasts but had no effect on asthmatic fibroblasts. AP-1 and GR interaction increased after Dex stimulation in asthmatic fibroblasts. AP-1 overexpression in control fibroblasts abrogated collagen gene response to Dex. These results show that Dex failed to reduce collagen production in fibroblasts from asthmatic subjects. This impaired response may be related to AP-1 overexpression in these cells. 
  GeneRIFs  
 these results suggest that pioglitazone inhibits HG-induced   ECM   accumulation in RPMCs by increasing PPARgamma expression, and by inhibiting the NF-kappaB and AP-1 pathways. 
  [Ee]xtracellular [Mm]atrix  PubMed  
 Phosphorylation of c-Fos by members of the p38 MAPK family. Role in the AP-1 response to UV light. Exposure to sources of UV radiation, such as sunlight, induces a number of cellular alterations that are highly dependent on its ability to affect gene expression. Among them, the rapid activation of genes coding for two subfamilies of proto-oncoproteins, Fos and Jun, which constitute the AP-1 transcription factor, plays a key role in the subsequent regulation of expression of genes involved in DNA repair, cell proliferation, cell cycle arrest, death by apoptosis, and tissue and   extracellular matrix   remodeling proteases. Besides being regulated at the transcriptional level, Jun and Fos transcriptional activities are also regulated by phosphorylation as a result of the activation of intracellular signaling cascades. In this regard, the phosphorylation of c-Jun by UV-induced JNK has been readily documented, whereas a role for Fos proteins in UV-mediated responses and the identification of Fos-activating kinases has remained elusive. Here we identify p38 MAPKs as proteins that can associate with c-Fos and phosphorylate its transactivation domain both in vitro and in vivo. This phosphorylation is transduced into changes in its transcriptional ability as p38-activated c-Fos enhances AP1-driven gene expression. Our findings indicate that as a consequence of the activation of stress pathways induced by UV light, endogenous c-Fos becomes a substrate of p38 MAPKs and, for the first time, provide evidence that support a critical role for p38 MAPKs in mediating stress-induced c-Fos phosphorylation and gene transcription activation. Using a specific pharmacological inhibitor for p38alpha and -beta, we found that most likely these two isoforms mediate UV-induced c-Fos phosphorylation in vivo. Thus, these newly described pathways act concomitantly with the activation of c-Jun by JNK/MAPKs, thereby contributing to the complexity of AP1-driven gene transcription regulation. 
 Phosphorylation of c-Fos by members of the p38 MAPK family. Role in the AP-1 response to UV light. Exposure to sources of UV radiation, such as sunlight, induces a number of cellular alterations that are highly dependent on its ability to affect gene expression. Among them, the rapid activation of genes coding for two subfamilies of proto-oncoproteins, Fos and Jun, which constitute the AP-1 transcription factor, plays a key role in the subsequent regulation of expression of genes involved in DNA repair, cell proliferation, cell cycle arrest, death by apoptosis, and tissue and   extracellular matrix   remodeling proteases. Besides being regulated at the transcriptional level, Jun and Fos transcriptional activities are also regulated by phosphorylation as a result of the activation of intracellular signaling cascades. In this regard, the phosphorylation of c-Jun by UV-induced JNK has been readily documented, whereas a role for Fos proteins in UV-mediated responses and the identification of Fos-activating kinases has remained elusive. Here we identify p38 MAPKs as proteins that can associate with c-Fos and phosphorylate its transactivation domain both in vitro and in vivo. This phosphorylation is transduced into changes in its transcriptional ability as p38-activated c-Fos enhances AP1-driven gene expression. Our findings indicate that as a consequence of the activation of stress pathways induced by UV light, endogenous c-Fos becomes a substrate of p38 MAPKs and, for the first time, provide evidence that support a critical role for p38 MAPKs in mediating stress-induced c-Fos phosphorylation and gene transcription activation. Using a specific pharmacological inhibitor for p38alpha and -beta, we found that most likely these two isoforms mediate UV-induced c-Fos phosphorylation in vivo. Thus, these newly described pathways act concomitantly with the activation of c-Jun by JNK/MAPKs, thereby contributing to the complexity of AP1-driven gene transcription regulation. 
 Disabled-2 (Dab2) mediates transforming growth factor beta (TGFbeta)-stimulated fibronectin synthesis through TGFbeta-activated kinase 1 and activation of the JNK pathway. The multifunctional cytokine transforming growth factor beta (TGFbeta) exerts many of its effects through its regulation of   extracellular matrix   components, including fibronectin (FN). Although expression of both TGFbeta and FN are essential for embryonic development and wound healing in the adult, overexpression leads to excessive deposition of   extracellular matrix   observed in many fibroproliferative disorders. We previously have demonstrated that TGFbeta-stimulated FN induction requires activation of the c-Jun N-terminal kinase (JNK) pathway; however, the signaling molecules that link the TGFbeta receptors to the JNK pathway remain unknown. We show here that the cytosolic adaptor protein disabled-2 (Dab2) directly stimulates JNK activity, whereas stable small interfering RNA-mediated ablation of Dab2 in NIH3T3 mouse fibroblasts and A10 rat aortic smooth muscle cells demonstrates that its expression is required for TGFbeta-mediated FN induction. We demonstrate that TGFbeta treatment stimulates the association of Dab2 with the mitogen-activated protein kinase kinase kinase, TAK1. Attenuation of cellular TAK1 levels by transient double-stranded RNA oligonucleotide transfection as well as overexpression of kinase-deficient TAK1 leads to abrogation of TGFbeta-stimulated FN induction. Furthermore, cell migration, another JNK-dependent response, is attenuated in NIH3T3-siDab2-expressing clones. We, therefore, delineate a signaling pathway proceeding from the TGFbeta receptors to Dab2 and TAK1, leading to TGFbeta-stimulated JNK activation, FN expression, and cell migration. 
 Jun-regulated genes promote interaction of diffuse large B-cell lymphoma with the microenvironment. Diffuse large B-cell lymphoma (DLBCL) is an aggressive disease with a high proliferation rate. However, the molecular and genetic features that drive the aggressive clinical behavior of DLBCL are not fully defined. Here, we have demonstrated that activated Jun signaling is a frequent event in DLBCL that promotes dissemination of malignant cells. Downregulation of Jun dramatically reduces lymphoma cell adhesion to   extracellular matrix   proteins, subcutaneous tumor size in nude mice, and invasive behavior, including bone marrow infiltration and interaction with bone marrow stromal cells. Furthermore, using a combination of RNA interference and gene expression profiling, we identified Jun target genes that are associated with disseminated lymphoma. Among them, ITGAV, FoxC1, and CX3CR1 are significantly enriched in patients with 2 or more extranodal sites. Our results point to activated Jun signaling as a major driver of the aggressive phenotype of DLBCL. 
 Pioglitazone inhibits high glucose-induced synthesis of   extracellular matrix   by NF-κB and AP-1 pathways in rat peritoneal mesothelial cells. High glucose (HG) in peritoneal dialysates has been demonstrated to induce   extracellular matri  x (ECM) synthesis by peritoneal mesothelial cells (PMCs) and to contribute to peritoneal fibrosis during continuous ambulatory peritoneal dialysis (CAPD). In the present study, we investigated the effects of pioglitazone, a peroxisome proliferator-activated receptor γ (PPARγ) agonist, on HG-induced ECM accumulation and the underlying mechanism in rat PMCs (RPMCs). In cultured RPMCs, HG treatment increased the expression of fibronectin (FN), collagen I and plasminogen activation inhibitor-1 (PAI-1) at the mRNA and protein levels, while it downregulated the expression of PPARγ in a time- and concentration-dependent manner. Pretreatment with pioglitazone not only decreased the expression of PAI-1 and matrix proteins (FN and collagen I), but prevented the downregulation of PPARγ in RPMCs under HG conditions. HG treatment activated the nuclear factor-κB (NF-κB) and activator protein-1 (AP-1) pathways. In addition, the NF-κB inhibitor, pyrrolidine dithiocarbamate (PDTC), and the AP-1 inhibitor, SP600125, decreased the protein levels of FN, collagen I and PAI-1, suggesting a role for the NF-κB and AP-1 pathways in the regulation of ECM accumulation induced by HG in RPMCs. Notably, we demonstrated that pretreatment with pioglitazone significantly inhibited HG-induced NF-κB and AP-1 activation. Collectively, these results suggest that pioglitazone inhibits HG-induced ECM accumulation in RPMCs by increasing PPARγ expression, and by inhibiting the NF-κB and AP-1 pathways. 
 Induction of human LTBP-3 promoter activity by TGF-beta1 is mediated by Smad3/4 and AP-1 binding elements. Latent TGF-beta binding proteins (LTBPs) are   extracellular matrix   glycoproteins, which are essential for the targeting and activation of TGF-betas. LTBP-3 regulates the bioavailability of TGF-beta especially in the bone. To understand the regulation of LTBP-3 expression, we have isolated and characterized the promoter region of human LTBP-3 gene. The GC-rich TATA-less promoter contained several transcription initiation sites and putative binding sites for multiple sequence specific transcription factors including Sp1, AP-1, c-Ets, MZF-1, Runx1 and members of the GATA-family. Reporter gene analyses of the promoter indicated that it was more active in MG-63 than in Saos-2 osteosarcoma cells, suggesting that it is regulated as the endogenous gene. TGF-beta1 stimulated the transcriptional activity of LTBP-3 promoter in MG-63 cells, while certain other bone-derived growth factors and hormones were ineffective. TGF-beta1 increased LTBP-3 mRNA levels accordingly. Analyses of deletion constructs of the promoter and mutational deletion of specific transcription factor binding sites indicated that Smad3/4 and AP-1 binding sites mediated the TGF-beta1 response. The involvement of AP-1 activity was further indicated by decreased TGF-beta responsiveness of the LTBP-3 promoter in the presence of a MEK/Erk signaling pathway inhibitor. Our results suggest an important new role for TGF-beta1 in the regulation of its binding protein, LTBP-3. 
 AP-1 mediated transcriptional repression of matrix metalloproteinase-9 by recruitment of histone deacetylase 1 in response to interferon β. Matrix metalloproteinase-9 (MMP-9) is a 92 kDa zinc-dependant endopeptidase that degrades components of the   extracellular matri  x. Increased expression of MMP-9 is implicated in many pathological conditions including metastatic cancer, multiple sclerosis, and atherosclerosis. Although it has been widely noted that interferon-β (IFNβ) downregulates both the basal and phorbol 12-myristate 13-acetate (PMA)-induced MMP-9 expression at the transcriptional level, the molecular mechanism of this repression is poorly understood. In the present study we identify a novel mechanism for repression of MMP-9 transcription by IFNβ in HT1080 fibrosarcoma cells. Using reporter assays with promoter deletion constructs we show that IFNβ's inhibitory effects require a region of the promoter between -154 and -72, which contains an AP-1 binding site. Chromatin immunoprecipitation (ChIP) studies indicate that IFNβ increases histone deacetylase (HDAC)-1 recruitment to the MMP-9 promoter and reduces histone H3 acetylation, in addition to reduced NF-κB recruitment. ChIP analysis shows that IFNβ induced HDAC1 recruitment to the MMP-9 promoter and IFNβ mediated transcriptional repression is lost when the AP-1 binding site is inactivated by a point mutation. Altogether, our results establish that the repression of MMP-9 transcription in response to IFNβ occurs by the recruitment of HDAC1 via the proximal AP-1 binding site. 
 The role of sLZIP in transcriptional regulation of c-Jun and involvement in migration and invasion of cervical cancer cells. Matrix metalloproteinase-9 (MMP-9) plays an important role in tumor invasion and metastasis through the breakdown of   extracellular matrix  . The c-Jun protein, a major component of the AP-1 transcription factor, is elevated in various cancers. Small leucine zipper protein (sLZIP) is a member of the leucine zipper transcription factor family. Although sLZIP is known to be involved in cancer cell migration and invasion, its biological roles in cancer development and the cellular target genes are not fully understood. In this study, we investigated the role of sLZIP in c-Jun expression, and their effects on expression of MMP-9 and migration of cervical cancer cells.sLZIP up-regulates transcription of c-Jun by binding directly to the CRE region in the c-Jun promoter. Elevated c-Jun due to sLZIP leads to activation of MMP-9 transcription by interaction with the AP-1 binding site in the MMP-9 promoter. c-Jun siRNA repressed migration and invasion of cervical cancer cells, whereas sLZIP recovered migration and invasion of cells transfected with c-Jun siRNA. Immunohistochemical analysis results revealed a significant correlation between the expressions of sLZIP and MMP-9 in clinical cervical specimens.These results indicate that sLZIP plays a role in expression of c-Jun, and migration and invasion of cervical cancer cells via regulation of MMP-9 transcription. 
 Activator protein-1 in carotid plaques is related to cerebrovascular symptoms and cholesteryl ester content. Transcription factor activator protein-1 regulates genes involved in inflammation and repair. The aim of this study was to determine whether transcription factor activator protein-1 activity in carotid plaques is related to symptoms, lipid accumulation, or   extracellular matrix   composition.Twenty-eight atherosclerotic carotid plaques were removed by endarterectomy and divided into two groups based on the presence or absence of ipsilateral symptoms (<1 month ago). Activator protein-1 DNA binding activity was assessed, and subunit (c-Jun, JunD, JunB, c-Fos, FosB, Fra-1, Fra-2) protein levels analyzed by immunoblotting. Distribution of c-Jun in plaques was analyzed by immunohistochemistry.Plaques associated with symptoms had increased activator protein-1 activity and increased expression of c-Jun and JunD, as compared to asymptomatic plaques. Fra-1 and Fra-2 were present in equal amounts in both groups, whereas JunB, FosB, and c-Fos were undetectable. Activator protein-1 activity correlated with cholesteryl ester and elastin in plaques and decreased with age. Activator protein-1 activity did not correlate with collagen, calcified tissue, or proteoglycan content.Activator protein-1 is increased in plaques associated with symptoms. The correlation between activator protein-1 and cholesteryl esters suggests that high activator protein-1 is a marker of plaque vulnerability. Activator protein-1 expression can also reflect the activation of repair processes. 
 Mechanical force induces type I collagen expression in human periodontal ligament fibroblasts through activation of ERK/JNK and AP-1. Type I collagen (COL I) is the predominant collagen in the   extracellular matrix   of periodontal ligament (PDL), and its expression in PDL fibroblasts (PLF) is sensitive to mechanical force. However, the mechanism by which PLF induces COL I to respond to mechanical force is unclear. This study examined the nature of human PLF in mediating COL I expression in response to centrifugal force. Signal transduction pathways in the early stages of mechanotransduction involved in the force-driven regulation of COL I expression were also investigated. Centrifugal force up-regulated COL I without cytotoxicity and activated extracellular signal-regulated kinase (ERK), c-Jun N-terminal kinase (JNK), and p38 kinase. ERK and JNK inhibitor blocked the expression of COL I but p38 kinase inhibitor had no effect. Centrifugal force activated activator protein-1 (AP-1) through dimerization between c-Fos and c-Jun transcription factors. ERK and JNK inhibitors also inhibited AP-1-DNA binding, c-Fos nuclear translocation, and c-Jun phosphorylation that were increased in the force-exposed PLF. Further, transfecting the cells with c-Jun antisense oligonucleotides almost completely abolished the force-induced increase of c-Jun phosphorylation and COL I induction. Our findings suggest that mechanical signals are transmitted into the nucleus by ERK/JNK signaling pathways and then stimulate COL I expression through AP-1 activation in force-exposed human PLF. 
 Synergistic transcriptional activation of the tissue inhibitor of metalloproteinases-1 promoter via functional interaction of AP-1 and Ets-1 transcription factors. The tissue inhibitor of metalloproteinases-1 (TIMP-1) is an inhibitor of the   extracellular matrix  -degrading metalloproteinases. We characterized response elements that control TIMP-1 gene expression. One contains a binding site that selectively binds c-Fos and c-Jun in vitro and confers a response to multiple AP-1 family members in vivo. Adjacent to this is a binding site for Ets domain proteins. Although c-Ets-1 alone did not activate transcription from this element, it enhanced transcription synergistically with AP-1 either in the context of the natural promoter or when the sequence was linked upstream of a heterologous promoter. Furthermore, a complex of c-Jun and c-Fos interacted with c-Ets-1 in vitro. These results suggest that AP-1 tethers c-Ets-1 to the TIMP-1 promoter via protein-protein interaction to achieve Ets-dependent transcriptional regulation. Collectively, our results indicate that TIMP-1 expression is controlled by several DNA response elements that respond to variations in the level and activity of AP-1 and Ets transcriptional regulatory proteins. 
  [Ii]nvasive  PubMed  
 Glucose-regulated protein 94 mediates metastasis by CCT8 and the JNK pathway in hepatocellular carcinoma. Hepatocellular carcinoma (HCC) is one of the leading causes of cancer death worldwide. Cancer metastasis is a major obstacle in clinical cancer therapy. The mechanisms underlying the metastasis of HCC remain unclear. Glucose-regulated protein 94 (GRP94) is a key protein involved in mediating cancer progression, and it is highly expressed in HCC specimens. However, the role of GRP94 in cancer metastasis is unclear. A specific short hairpin RNA (shRNA) was employed to knock down GRP94 gene expression in HCC cell lines. Wound-healing migration, transwell migration, and invasion assays were performed to determine the migration and   invasive   ability of HCC cells. We demonstrated that silencing GRP94 inhibited HCC cell wound healing, migration, and invasion. Furthermore, our findings indicated that GRP94 knockdown might attenuate HCC cell metastasis by inhibiting CCT8/c-Jun/EMT signaling. Our study indicated that silencing GRP94 significantly reduced the migration and invasion abilities of HCC cells. Moreover, depleting GRP94 inhibited cell migration and invasion by downregulating CCT8/c-Jun signaling. Thus, our data suggest that the GRP94/CCT8/c-Jun/EMT signaling cascade might be a new therapeutic target for HCC. 
 Evaluation of candidate stromal epithelial cross-talk genes identifies association between risk of serous ovarian cancer and TERT, a cancer susceptibility "hot-spot". We hypothesized that variants in genes expressed as a consequence of interactions between ovarian cancer cells and the host micro-environment could contribute to cancer susceptibility. We therefore used a two-stage approach to evaluate common single nucleotide polymorphisms (SNPs) in 173 genes involved in stromal epithelial interactions in the Ovarian Cancer Association Consortium (OCAC). In the discovery stage, cases with epithelial ovarian cancer (n=675) and controls (n=1,162) were genotyped at 1,536 SNPs using an Illumina GoldenGate assay. Based on Positive Predictive Value estimates, three SNPs-PODXL rs1013368, ITGA6 rs13027811, and MMP3 rs522616-were selected for replication using TaqMan genotyping in up to 3,059 serous   invasive   cases and 8,905 controls from 16 OCAC case-control studies. An additional 18 SNPs with Pper-allele<0.05 in the discovery stage were selected for replication in a subset of five OCAC studies (n=1,233 serous   invasive   cases; n=3,364 controls). The discovery stage associations in PODXL, ITGA6, and MMP3 were attenuated in the larger replication set (adj. Pper-allele>or=0.5). However genotypes at TERT rs7726159 were associated with ovarian cancer risk in the smaller, five-study replication study (Pper-allele=0.03). Combined analysis of the discovery and replication sets for this TERT SNP showed an increased risk of serous ovarian cancer among non-Hispanic whites [adj. ORper-allele 1.14 (1.04-1.24) p=0.003]. Our study adds to the growing evidence that, like the 8q24 locus, the telomerase reverse transcriptase locus at 5p15.33, is a general cancer susceptibility locus. 
 Glucose-regulated protein 94 mediates metastasis by CCT8 and the JNK pathway in hepatocellular carcinoma. Hepatocellular carcinoma (HCC) is one of the leading causes of cancer death worldwide. Cancer metastasis is a major obstacle in clinical cancer therapy. The mechanisms underlying the metastasis of HCC remain unclear. Glucose-regulated protein 94 (GRP94) is a key protein involved in mediating cancer progression, and it is highly expressed in HCC specimens. However, the role of GRP94 in cancer metastasis is unclear. A specific short hairpin RNA (shRNA) was employed to knock down GRP94 gene expression in HCC cell lines. Wound-healing migration, transwell migration, and invasion assays were performed to determine the migration and   invasive   ability of HCC cells. We demonstrated that silencing GRP94 inhibited HCC cell wound healing, migration, and invasion. Furthermore, our findings indicated that GRP94 knockdown might attenuate HCC cell metastasis by inhibiting CCT8/c-Jun/EMT signaling. Our study indicated that silencing GRP94 significantly reduced the migration and invasion abilities of HCC cells. Moreover, depleting GRP94 inhibited cell migration and invasion by downregulating CCT8/c-Jun signaling. Thus, our data suggest that the GRP94/CCT8/c-Jun/EMT signaling cascade might be a new therapeutic target for HCC. 
 Evaluation of candidate stromal epithelial cross-talk genes identifies association between risk of serous ovarian cancer and TERT, a cancer susceptibility "hot-spot". We hypothesized that variants in genes expressed as a consequence of interactions between ovarian cancer cells and the host micro-environment could contribute to cancer susceptibility. We therefore used a two-stage approach to evaluate common single nucleotide polymorphisms (SNPs) in 173 genes involved in stromal epithelial interactions in the Ovarian Cancer Association Consortium (OCAC). In the discovery stage, cases with epithelial ovarian cancer (n=675) and controls (n=1,162) were genotyped at 1,536 SNPs using an Illumina GoldenGate assay. Based on Positive Predictive Value estimates, three SNPs-PODXL rs1013368, ITGA6 rs13027811, and MMP3 rs522616-were selected for replication using TaqMan genotyping in up to 3,059 serous   invasive   cases and 8,905 controls from 16 OCAC case-control studies. An additional 18 SNPs with Pper-allele<0.05 in the discovery stage were selected for replication in a subset of five OCAC studies (n=1,233 serous   invasive   cases; n=3,364 controls). The discovery stage associations in PODXL, ITGA6, and MMP3 were attenuated in the larger replication set (adj. Pper-allele>or=0.5). However genotypes at TERT rs7726159 were associated with ovarian cancer risk in the smaller, five-study replication study (Pper-allele=0.03). Combined analysis of the discovery and replication sets for this TERT SNP showed an increased risk of serous ovarian cancer among non-Hispanic whites [adj. ORper-allele 1.14 (1.04-1.24) p=0.003]. Our study adds to the growing evidence that, like the 8q24 locus, the telomerase reverse transcriptase locus at 5p15.33, is a general cancer susceptibility locus. 
 Cadmium induces matrix metalloproteinase-9 expression via ROS-dependent EGFR, NF-кB, and AP-1 pathways in human endothelial cells. Cadmium (Cd), a widespread cumulative pollutant, is a known human carcinogen, associated with inflammation and tumors. Matrix metalloproteinase-9 (MMP-9) plays a pivotal role in tumor metastasis; however, the mechanisms underlying the MMP-9 expression induced by Cd remain obscure in human endothelial cells. Here, Cd elevated MMP-9 expression in dose- and time-dependent manners in human endothelial cells. Cd increased ROS production and the ROS-producing NADPH oxidase. Cd translocates p47(phox), a key subunit of NADPH oxidase, to the cell membrane. Cd also activated the phosphorylation of EGFR, Akt, Erk1/2, and JNK1/2 in addition to promoting NF-кB and AP-1 binding activities. Specific inhibitor and mutagenesis studies showed that EGFR, Akt, Erk1/2, JNK1/2 and transcription factors NF-κB and AP-1 were related to Cd-induced MMP-9 expression in endothelial cells. Akt, Erk1/2, and JNK1/2 functioned as upstream signals in the activation of NF-κB and AP-1, respectively. In addition, N-acetyl-l-cystein (NAC), diphenyleneiodonium chloride (DPI) and apocynin (APO) inhibited the Cd-induced activation of EGFR, Akt, Erk1/2, JNK1/2, and p38 MAPK, indicating that ROS production by NADPH oxidase is the furthest upstream signal in MMP-9 expression. At present, it states that Cd displayed mar  ked inva  siveness in ECV304 cells, which was partially abrogated by MMP-9 neutralizing antibodies. These results demonstrated that Cd induces MMP-9 expression via ROS-dependent EGFR->Erk1/2, JNK1/2->AP-1 and EGFR->Akt->NF-κB signaling pathways and, in turn, stimul  ates inv  asiveness in human endothelial cells. 
 EGF stimulates uPAR expression and cell   invasive  ness through ERK, AP-1, and NF-kappaB signaling in human gastric carcinoma cells. Overexpression of epidermal growth factor (EGF) and urokinase plasminogen activator receptor (uPAR) have been observed in human gastric cancers. However, the interaction between EGF and uPAR in gastric cancer has not been well elucidated. In this study, we investigated the effect of EGF on uPAR expression and the underlying signal pathways in human gastric cancer AGS cells. EGF induced uPAR mRNA expression in a time- and concentration-dependent manner. EGF also induced uPAR promoter activity. In addition, EGF induced the activation of extracellular signal regulated kinase-1/2 (ERK-1/2) and P38 mitogen-activated protein kinase (MAPK) but not the activation of c-Jun amino terminal kinase. A specific inhibitor of MEK-1 (an upstream effector of ERK-1/2) and a dominant negative MEK-1 were able to suppress the EGF-induced uPAR promoter activity. Site-directed mutagenesis and electrophoretic mobility shift assays demonstrated that the binding sites of transcription factors, activator protein-1 (AP-1) and nuclear factor (NF)-kappaB, are involved in the EGF-induced uPAR transcription. Suppression of the EGF-induced uPAR promoter activity by the AP-1 decoy oligonuclotide, as well as expression vectors encoding mutated-type NF-kappaB-inducting kinase and I-kappaB, confirmed that the activation of AP-1 and NF-kappaB are essential for the EGF-induced uPAR upregulation. The AGS cells pretreated with EGF showed a remarkably enhanced   invasive  ness and this effect was partially abrogated by uPAR neutralizing antibodies and by the inhibitors of ERK-1/2, AP-1, and NF-kappaB. The above results suggest that EGF induces uPAR expression via ERK-1/2, AP-1, and NF-kappaB signaling pathways and, in turn, stimulates cell   invasive  ness in human gastric cancer AGS cells. 
 NAG7 promotes human nasopharyngeal carcinoma invasion through inhibition of estrogen receptor alpha and up-regulation of JNK2/AP-1/MMP1 pathways. The NAG7, an estrogen receptor repressor, is a negative regulator of nasopharyngeal carcinoma cell growth. Here, we report that NAG7 promotes human nasopharyngeal carcinoma invasion, and we identify the mechanisms underlying this function. As a consequence of elevated NAG7 expression, the adhesion, migration, and   invasive   capabilities of HNE1 cells in vitro and in vivo were enhanced. NAG7 was a significant negative regulator of protein expression of estrogen receptor alpha (ERalpha), and activated both the JNK2/AP-1/MMP1 and the upstream H-Ras/p-c-Raf pathways. None of these effects induced by NAG7 over-expression could be counteracted by estrogen. These observations indicate that NAG7 plays a potential role in promoting nasopharyngeal carcinoma invasion by regulation of ERalpha and the H-ras/p-c-Raf and JNK2/AP-1/MMP1 signaling pathways. 
 Jun-regulated genes promote interaction of diffuse large B-cell lymphoma with the microenvironment. Diffuse large B-cell lymphoma (DLBCL) is an aggressive disease with a high proliferation rate. However, the molecular and genetic features that drive the aggressive clinical behavior of DLBCL are not fully defined. Here, we have demonstrated that activated Jun signaling is a frequent event in DLBCL that promotes dissemination of malignant cells. Downregulation of Jun dramatically reduces lymphoma cell adhesion to extracellular matrix proteins, subcutaneous tumor size in nude mice, and   invasive   behavior, including bone marrow infiltration and interaction with bone marrow stromal cells. Furthermore, using a combination of RNA interference and gene expression profiling, we identified Jun target genes that are associated with disseminated lymphoma. Among them, ITGAV, FoxC1, and CX3CR1 are significantly enriched in patients with 2 or more extranodal sites. Our results point to activated Jun signaling as a major driver of the aggressive phenotype of DLBCL. 
 Astrocyte elevated gene-1 (AEG-1) promotes osteosarcoma cell invasion through the JNK/c-Jun/MMP-2 pathway. Osteosarcoma is the most common primary malignant bone tumour in children and adolescents and is characterised by high malignant and metastatic potentials. However, the molecular mechanism underlying this   invasive  ness remains unclear. In this study, we determined that PD98059 and SP600125, the two mitogen-activated protein kinase (MAPK) family inhibitors, decreased the osteosarcoma cell U2OS-AEG-1 migration and invasion that was enhanced by astrocyte elevated gene-1 (AEG-1) in an in vitro wound-healing and Matrigel invasion assay independently of cell viability. These findings indicate that AEG-1 promoted osteosarcoma cell invasion is relevant to the MAPK pathways. The up-regulation of AEG-1 increased the levels of phosphor-c-Jun N-terminal kinase (JNK) and phosphor-c-Jun; however, there were no marked changes in the levels of phosphor-extracellular regulated kinase (ERK) 1/2 or phosphor-c-Fos due to the activation of AEG-1 in U2OS. SP600125 (a JNK inhibitor) decreased phosphor-c-Jun and MMP-2 in U2OS-AEG-1, while PD98059 (a ERK1/2 inhibitor) had no influence on the levels of phosphor-c-Jun or MMP-2 in U2OS-AEG-1. Further study revealed that the down-regulation of phosphor-c-Jun not only obviously decreased the MMP-2 protein level and the MMP-2 transcriptional activity that were up-regulated by AEG-1 in Western-blot and luciferase reporter assays, but also inhibited the migration and invasion abilities of the U2OS-AEG-1 cells, which suggests that AEG-1 mediated U2OS invasion at least partially via the JNK/c-Jun/MMP-2 pathway. Consistent with these observations, immunohistochemical (IHC) staining revealed that AEG-1 expression was associated with the protein levels of phosphor-c-Jun and MMP-2 in needle biopsy paraffin-embedded archival human osteosarcoma tissues. Taken together, our findings suggest that AEG-1 plays a crucial role in the aggressiveness of osteosarcoma via the JNK/c-Jun/MMP-2 pathway. 
 MSP-induced RON activation upregulates uPAR expression and cell   invasive  ness via MAPK, AP-1 and NF-κB signals in gastric cancer cells. Overexpression of recepteur d'Origine nantais (RON) and urokinase plasminogen activator receptor (uPAR) have been observed in human gastric cancers. However, the interaction between RON and uPAR in gastric cancer is unclear. The present study investigated the effect of macrophage-stimulating protein (MSP, the RON ligand) on uPAR expression and the underlying signal pathways in human gastric cancer AGS cells. uPAR messenger RNA expression was induced by MSP in a time- and concentration-dependent manner. MSP also induced uPAR promoter activity. The introduction of RON-specific small interfering RNA (siRNA) significantly affected the MSP-induced uPAR transcription. Deleted and site-directed mutagenesis studies demonstrated the involvement of the binding sites of transcription factor nuclear factor-kappaB (NF-κB) and activator protein (AP)-1 in the MSP-induced uPAR expression. Studies with expression vectors encoding mutated-type NF-κB signaling molecules and AP-1 decoy confirmed that NF-κB and AP-1 were essential for the MSP-induced uPAR expression. In addition, MSP induced the activation of extracellular signal-regulated kinase-1/2 (Erk-1/2), c-Jun amino terminal kinase (JNK) and p38 mitogen-activated protein kinase (MAPK). Dominant-negative mutants (K97M and TAM67) and specific inhibitors of Erk-1/2 and JNK were able to suppress the MSP-induced uPAR expression. AGS cells pretreated with MSP showed a remarkably enhan  ced inva  siveness, which was partially abrogated by siRNA-targeted RON and uPAR-neutralizing antibodies. The above results suggest that MSP induces uPAR expression via MAPK, AP-1 and NF-κB signaling pathways and, in turn, stimulates   cell inv  asiveness in human gastric cancer AGS cells. 
 A feedback inhibition between miRNA-127 and TGFβ/c-Jun cascade in HCC cell migration via MMP13. Hepatocellular carcinoma (HCC) is the fifth most common cancer worldwide and is increasing in frequency in the U.S. The major reason for the low postoperative survival rate of HCC is widespread intrahepatic metastasis or invasion, and activation of TGFβ signaling is associated with th  e invasi  ve phenotype. This study aims at determining the novel function of miR-127 in modulating HCC migration. Overexpression of miR-127 inhibits HCC cell migration, invasion and tumor growth in nude mice. MiR-127 directly represses matrix metalloproteinase 13 (MMP13) 3'UTR activity and protein expression, and diminishes MMP13/TGFβ-induced HCC migration. In turn, TGFβ decreases miR-127 expression by enhancing c-Jun-mediated inhibition of miR-127 promoter activity. In contrast, p53 transactivates miR-127 promoter and induces miR-127 expression, which is antagonized by c-Jun. The inhibition of miR-127 by c-Jun is through TGFβ-mediated ERK and JNK pathways. The lower miR-127 expression shows a negative correlation with the higher MMP13 expression in a subset of human HCC specimens. This is the first report elucidating a feedback regulation between miR-127 and the TGFβ/c-Jun cascade in HCC migration via MMP13 that involves a crosstalk between the oncogene c-Jun and tumor suppressor p53. 
 TGFβ-induced invasion of prostate cancer cells is promoted by c-Jun-dependent transcriptional activation of Snail1. High levels of transforming growth factor-β (TGFβ) correlate with poor prognosis for patients with prostate cancer and other cancers. TGFβ is a multifunctional cytokine and crucial regulator of cell fate, such as epithelial to mesenchymal transition (EMT), which is implicated in cancer invasion and progression. TGFβ conveys its signals upon binding to type I and type II serine/threonine kinase receptors (TβRI/II); phosphorylation of Smad2 and Smad3 promotes their association with Smad4, which regulates expression of targets genes, such as Smad7, p21, and c-Jun. TGFβ also activates the ubiquitin ligase tumor necrosis factor receptor-associated factor 6 (TRAF6), which associates with TβRI and activates the p38 mitogen-activated protein kinase (MAPK) pathway. Snail1 is a key transcription factor, induced by TGFβ that promotes migration and invasion of cancer cells. In this study, we have identified a novel binding site for c-Jun in the promoter of the Snail1 gene and report that the activation of the TGFβ-TRAF6-p38 MAPK pathway promotes both c-Jun expression and its activation via p38α-dependent phosphorylation of c-Jun at Ser63. The TRAF6-dependent activation of p38 also leads to increased stability of c-Jun, due to p38-dependent inactivation of glycogen synthase kinase (GSK) 3β by phosphorylation at Ser9. Thus, our findings elucidate a novel role for the p38 MAPK pathway in stimulated cells, leading to activation of c-Jun and its binding to the promoter of Snail1, thereby triggering m  otility   and invasiveness of aggressive human prostate cancer cells. 
 Downregulation of non‑muscle myosin IIA expression inhibits migration and invasion of gastric cancer cells via the c‑Jun N‑terminal kinase signaling pathway. Non‑muscle myosin IIA (NMIIA) has been found to be overexpressed in gastric cancer tissues. However, the roles of NMIIA in the migration and invasion of gastric cancer cells have not yet been elucidated. The aim of the present study was to assess the effects of NMIIA knockdown on the migrat  ory and   invasive capacities of gastric cancer cells and to investigate the potential underlying mechanisms in vitro. First, the expression of NMIIA was assessed in gastric cancer tissues and non‑cancerous tissues using western blot analysis. The expression levels of NMIIA protein in 51 out of 63 gastric cancer tissue specimens were higher compared to those in their matched non‑tumoric gastric tissue specimens, and differences between the two groups were statistically significant (P<0.001). After downregulation of NMIIA using RNA interference, the mi  gratory   and invasive abilities of the SGC‑7901 and MGC‑803 gastric cancer cell lines were observed using a wound‑healing assay and a Transwell assay, respectively. Knockdown of NMIIA significantly decreased the amount of wound closure as well as the number of cells which transgressed through the Matrigel (P<0.01). Finally, the levels of c‑Jun N‑terminal kinase (JNK), phosphorylated (p)‑JNK, c‑Jun and p‑c‑Jun were detected using western blot analysis in order to explore the association between NMIIA and the JNK signaling pathway. Knockdown of NMIIA decreased the levels of p‑JNK and p‑c‑Jun in the two gastric cancer cell lines (P<0.05). In conclusion, the present study indicated that knockdown of NMIIA inhibited the migration and invasion of gastric cancer cells, which may be, at least in part, mediated via the JNK signaling pathway. 
 Decoding the regulatory landscape of melanoma reveals TEADS as regulators of the   invasive   cell state. Transcriptional reprogramming of proliferative melanoma cells into a phenotypically distinct   invasive   cell subpopulation is a critical event at the origin of metastatic spreading. Here we generate transcriptome, open chromatin and histone modification maps of melanoma cultures; and integrate this data with existing transcriptome and DNA methylation profiles from tumour biopsies to gain insight into the mechanisms underlying this key reprogramming event. This shows thousands of genomic regulatory regions underlying the proliferative and   invasive   states, identifying SOX10/MITF and AP-1/TEAD as regulators, respectively. Knockdown of TEADs shows a previously unrecognized role in the   invasive   gene network and establishes a causative link between these transcription factors, cell invasion and sensitivity to MAPK inhibitors. Using regulatory landscapes and in silico analysis, we show that transcriptional reprogramming underlies the distinct cellular states present in melanoma. Furthermore, it reveals an essential role for the TEADs, linking it to clinically relevant mechanisms such as invasion and resistance. 
 In situ proximity ligation detection of c-Jun/AP-1 dimers reveals increased levels of c-Jun/Fra1 complexes in aggressive breast cancer cell lines in vitro and in vivo. Genetic and biochemical studies have shown that selective interactions between the Jun, Fos, and activating transcription factor (ATF) components of transcription factor activating protein 1 (AP-1) exhibit specific and critical functions in the regulation of cell proliferation, differentiation, and survival. For instance, the ratio between c-Jun/c-Fos and c-Jun/ATF2 dimers in the cell can be a determining factor in the cellular response to oncogenic or apoptotic stimuli. Until recently, no methods were available to detect endogenous AP-1 complexes in cells and tissues in situ. Here, we validated the proximity ligation assay (PLA) for its ability to specifically visualize and quantify changes in endogenous c-Jun/c-Fos, c-Jun/ATF2, and c-Jun/Fra1 complexes by using, among others, partner-selective c-Jun mutants. Furthermore, we examined the levels of c-Jun/AP-1 dimers in cell lines representing different types of human breast cancer and found that aggressive basal-like breast cancer cells can be discriminated from much less   invasive   luminal-like cells by PLA detection of c-Jun/Fra1 rather than of c-Jun/ATF2 and c-Jun/c-Fos. Also in tumor tissue derived from highly metastatic basal-like MDA-MB231 cells, high levels of c-Jun/Fra1 complexes were detected. Together, these results demonstrate that in situ PLA is a powerful diagnostic tool to analyze and quantify the amounts of biologically critical AP-1 dimers in fixed cells and tissue material. 
 c-Jun, a crucial molecule in metastasis of breast cancer and potential target for biotherapy. Human breast cancer cell line SKBR3 expresses high level of the ErbB2 molecule, which has been associated with poor prognosis of breast cancer. Elevated ErbB2 functions as a transactivating co-receptor and promotes the formation of ErbB2 containing heterodimers, which are more mitogenic and transforming, and have a higher ligand affinity and signaling potency by virtue of the potent latent kinase activity of ErbB2. Interestingly, SKBR3 cells are non-tumorigenic in nude mice. By ectopic overexpression of c-Jun in SKBR3 cells, involvement of c-Jun in   invasive  ness and metastasis in vivo was investigated in this study. The critical role of c-Jun in the tumorigenesis and metastasis is demonstrated in nude mice. 
 Twist1 and AP-1 cooperatively upregulate integrin α5 expression to induce invasion and the epithelial-mesenchymal transition. Epithelial-mesenchymal transition (EMT) is an important process implicated in tumor invasion and metastasis. Twist1 is a transcription factor that induces EMT, including E-cadherin suppression and cancer cell migration and invasion; hence it promotes cancer metastasis. Twist1 directly or indirectly regulates the expression of various genes and cellular functions involved in cancer progression. However, the underlying mechanisms remain largely unknown. In this study, we investigated the molecular basis for Twist1-mediated invasion and EMT. In human cancer cells, Twist1 was found to directly upregulate transcription of the mesenchymal gene integrin α5 in an E-box-independent, but activating protein-1 (AP-1) element-dependent, manner. Twist1 activated the integrin α5 promoter by interacting with and activating the transcription factor AP-1, composed of c-Jun and activating transcription factor-2 (ATF-2); it also enhanced the nuclear presence of ATF-2. AP-1 was critical for Twist1-induced cancer cell invasion, primarily through the induction of integrin α5, which activated c-Jun N-terminal kinase and focal adhesion kinase-signaling activities. Using data from The Cancer Genome Atlas, we found that Twist1 expression positively correlates with integrin α5 expression in human colorectal cancers. These findings suggest that cooperation between Twist1 and AP-1 represents a novel mechanism for EMT and t  umor inv  asiveness. This study supports further investigation into the molecular basis underlying the diverse Twist1-mediated functions that occur during tumor progression. 
 Hypoxia protects HepG2 cells against etoposide-induced apoptosis via a HIF-1-independent pathway. Tumor hypoxia has been described to increase the resistance of cancer cells to radiation therapy and chemotherapy. It also supports the   invasive  ness and metastatic potential of the tumor. However, few data are available on the transduction pathway set up under hypoxia and leading to this resistance against anti-cancer therapies. HIF-1, the main transcription factor activated by hypoxia, has been recently shown to participate to this process although its role as an anti- or a pro-apoptotic protein is still controversy. In this study, we showed that hypoxia protected HepG2 cells against etoposide-induced apoptosis. The effect of hypoxia on cell death was assayed by measuring different parameters of the apoptotic pathway, like DNA fragmentation, caspase activity and PARP-1 cleavage. The possible implication of HIF-1 in the anti-apoptotic role of hypoxia was investigated using HIF-1alpha siRNA. Our results indicated that HIF-1 is not involved in the hypoxia-induced anti-apoptotic pathway. Another transcription factor, AP-1, was studied for its potential role in the hypoxia-induced protection against apoptosis. Specific inhibition of AP-1 decreased the protection effect of hypoxia against etoposide-induced apoptosis. Together, all these data underline that hypoxia could mediate its anti-apoptotic role via different transcription factors depending on the cellular context and pro-apoptotic stimuli. 
 Expression of Jun and Fos proteins in ovarian tumors of different malignant potential and in ovarian cancer cell lines. Jun and Fos proteins form the transcription factor activating protein 1 (AP-1). They play a role in cell proliferation, malignant transformation and invasion in various tumors. The aim of the current study was to characterize the role of AP-1 in ovarian cancer. Fifty-six ovarian tumors of different   invasive   potential including 13 metastases as well as 5 ovarian cancer cell lines were analyzed by Western blot analysis regarding their expression of pc-Jun, Jun B, Jun D, c-Fos, Fos B, Fra-1 and Fra-2. In addition, invasion, cell proliferation and migration assays were performed. The expression of pc-Jun, Jun B, Jun D and Fra-2 was higher in   invasive   cancer compared to benign tumors. In metastases, c-Fos and Fos B expression was significantly lower than in the respective primary ovarian carcinomas. The   invasive   and proliferative potential of the cells was not associated with AP-1 protein expression. These results suggest that AP-1 proteins are differentially expressed in benign ovarian tumors, tumors with low malignant potential and epithelial ovarian carcinomas and metastases. No correlation with the proliferative and   invasive   potential of ovarian cancer cell lines could be found. 
 Blockade of the RhoA-JNK-c-Jun-MMP2 cascade by atorvastatin reduces osteosarcoma cell invasion. Osteosarcoma is characterized by a high malignant and metastatic potential, which points to the need for new therapeutic strategies to prevent cell metastasis. In this study, we show that statin-induced HMG-CoA reductase inhibition reduces cell migration and invasion in human and murine osteosarcoma cells, independently of the genotype. The statin-induced reduction of cell migration and invasion was independent of induction of apoptosis and was geranylgeranylpyrophosphate-dependent. The statin reduced matrix metalloproteinase (MMP) 2, 9, and 14 and TIMP2 expression or activity in invading cells. Forced expression of MMP2 and MMP14 overcame the inhibitory effect of the statin on cell invasion, suggesting a role for these MMPs in   invasive   potential. We also investigated the mechanisms involved in the reduced MMP2 activity and cell invasion. Inhibition of JNK, but not ERK1/2 signaling, reduced MMP2 activity. Pharmacological or constitutive activation of JNK overcame the reduced MMP2 activity and cell invasion induced by the statin. The statin decreased JNK phosphorylation and c-Jun nuclear translocation, suggesting that HMG-CoA reductase inhibition targets the JNK-c-Jun signaling pathway. We showed that mevalonate or geranylgeranylpyrophosphate treatment prevented the statin-induced reduction in JNK phosphorylation, MMP2 activity, and cell invasion. Forced expression of a constitutively active form of RhoA increased JNK phosphorylation and overcame the inhibitory effect of atorvastatin on MMP2 activity and cell invasion. The data establish a link between RhoA, JNK, c-Jun, and MMP2 activity that is functionally involved in the reduction in osteosarcoma cell invasion by the statin. This suggests a novel strategy targeting RhoA-JNK-c-Jun signaling to reduce osteosarcoma cell tumorigenesis. 
 c-Jun promotes cell migration and drives expression of the motility factor ENPP2 in soft tissue sarcomas. Genomic amplification of the c-Jun proto-oncogene has been identified in ∼30% of dedifferentiated liposarcomas (DDLPS), but the functional contribution of c-Jun to the progression of DDLPS remains poorly understood. In previous work we showed that knock-down of c-Jun by RNA interference impaired the in vitro proliferation and in vivo growth of a DDLPS cell line (LP6) with genomic amplification of the c-Jun locus. Here, we used gene expression analysis and functional studies in a broad panel of cell lines to further define the role of c-Jun in DDLPS and other soft tissue sarcomas. We show that c-Jun knock-down impairs transition through the G1 phase of the cell cycle in multiple DDLPS cell lines. We also found that high levels of c-Jun expression are both necessary and sufficient to promote DDLPS cell migration and invasion in vitro. Our data suggest that high levels of c-Jun enhance motility in part by driving the expression of ENPP2/Autotaxin. c-Jun over-expression has minimal effects on in vitro proliferation but substantially enhances the in vivo growth of weakly tumourigenic DDLPS cell lines. Finally, we provide evidence that c-Jun genomic amplification and over-expression may have similar functional consequences in other types of soft tissue sarcoma. Our data suggest a model in which relatively low levels of c-Jun are sufficient for in vitro proliferation, but high levels of c-Jun enhanc  e invasi  veness and capacity for in vivo tumour growth. These observations provide an explanation for the selective advantage provided by c-Jun genomic amplification in vivo and suggest that sarcomas with elevated c-Jun levels are likely to have a particularly high malignant potential. Data from exon array and RNA-Seq experiments have been deposited in the GEO database (Accession No. GSE57531). 
 Phosphorylated c-Jun and Fra-1 induce matrix metalloproteinase-1 and thereby regulate invasion activity of 143B osteosarcoma cells. Osteosarcoma is the most common primary malignancy of bone and patients often develop pulmonary metastases. Despite the advances in surgical and medical management, the mechanisms underlying human osteosarcoma progression and metastasis remain to be elucidated. Gene expression profiles were compared by the cDNA microarray technique between two different human osteosarcoma sublines, MNNG/HOS and 143B, which differ greatly in spontaneous pulmonary metastatic potential. Here we report an enhanced expression of matrix metalloproteinase (MMP)-1 in the highly metastatic human osteosarcoma cell line 143B. Moreover, the in vitro invasion activity of 143B cells was MMP-1-dependent. The activator protein (AP)-1 binding site in the MMP-1 gene promoter was required for the constitutive expression of MMP-1 in 143B cells. Two AP-1 components, c-Jun and Fra-1, were phosphorylated, and bound to the AP-1 binding site of the MMP-1 promoter in 143B cells. Activated c-Jun and Fra-1 were essential for MMP-1 gene expression in 143B cells. Mitogen-activated protein kinase pathways including the c-Jun NH(2)-terminal kinase and the extracellular signal-regulated kinase activate c-Jun and Fra-1 and thereby regulate c-Jun/Fra-1 mediated events, establishing the mitogen-activated protein kinase/AP-1/MMP-1 axis as important in 143B cells. These data suggest that MMP-1 plays a central role in osteosarcoma invasion. Accordingly, MMP-1 might be a biomarker and therapeutic target for   invasive   osteosarcomas and pulmonary metastases. 
 Overexpression of MAPK15 in gastric cancer is associated with copy number gain and contributes to the stability of c-Jun. This study was aimed at understanding the functional and clinicopathological significance of MAPK15 alteration in gastric cancer. Genome-wide copy number alterations (CNAs) were first investigated in 40 gastric cancers using Agilent aCGH-244K or aCGH-400K, and copy number gains of MAPK15 found in aCGH were validated in another set of 48 gastric cancer tissues. The expression of MAPK15 was analyzed using immunohistochemistry in concurrent lesions of normal, adenoma, and carcinoma from additional 45 gastric cancer patients. The effects of MAPK15 on cell cycle, c-Jun phosphorylation, and mRNA stability were analyzed in gastric cancer cells. Copy number gains of MAPK15 were found in 15 (17%) of 88 tumor tissues. The mRNA levels of MAPK15 were relatively high in the gastric cancer tissues and gastric cancer cells with higher copy number gains than those without. Knockdown of MAPK15 using siRNA in gastric cancer cells significantly suppressed cell proliferation and resulted in cell cycle arrest at G1-S phase. Reduced c-Jun phosphorylation and c-Jun half-life were observed in MAPK15-knockdowned cells. In addition, transient transfection of MAPK15 into AGS gastric cancer cells with low copy number resulted in an increase of c-Jun phosphorylation and stability. The overexpression of MAPK15 occurred at a high frequency in carcinomas (37%) compared to concurrent normal tissues (2%) and adenomas (21%). In conclusion, the present study suggests that MAPK15 overexpression may contribute to the malignant transformation of gastric mucosa by prolonging the stability of c-Jun. And, patients with copy number gain of MAPK15 in normal or premalignant tissues of stomach may have a chance to progress to   invasive   cancer. 
 Interleukin-5 enhances the migration and invasion of bladder cancer cells via ERK1/2-mediated MMP-9/NF-κB/AP-1 pathway: involvement of the p21WAF1 expression. Inflammatory cytokines may be a critical component of epithelial cancer progression. We examined the role of interleukin (IL)-5 in the migration of bladder cancer cells. The expression of IL-5 and its receptor IL-5Rα was enhanced in patients with muscl  e invasi  ve bladder cancers (MIBC), and then it was detected in bladder cancer cell lines 5637 and T-24. IL-5 increased migration and MMP-9 expression via activation of transcription factors NF-κB and AP-1, and induced activation of ERK1/2 and Jak-Stat signaling in both cells. Treatment with ERK1/2 inhibitor U0126 significantly inhibited induction of migration, MMP-9 expression, and activation of NF-κB and AP-1 in IL-5-treated cells. However, none of the Jak inhibitors affected the IL-5-induced migration of bladder cancer cells. Moreover, gene knockdown for IL-5Rα, using siRNA transfection, suppressed migration, ERK1/2 activation, MMP-9 expression, as well as the binding activation of NF-κB and AP-1 in IL-5-treated bladder cancer cells. Similar results were observed in βc siRNA (si-βc) transfected cells. Unexpectedly, IL-5 treatment resulted in significant induction of p21WAF1 in both cell lines. The p21WAF1-specific small interfering RNA inhibited IL-5-induced cell migration, ERK activity, MMP-9 expression, and activation of NF-κB and AP-1 in bladder cancer cells. The effects of IL-5-induced cell responses were confirmed by transfection of IL-5 gene, which demonstrated that p21WAF1 participates in the induction of cell migration, leading to an increase in ERK1/2-mediated MMP-9 expression through activation of NF-κB and AP-1 in IL-5-treated bladder cancer cells. These unexpected results provide a theoretical basis for the therapeutic targeting of IL-5 in bladder cancer. 
 COP1D, an alternatively spliced constitutive photomorphogenic-1 (COP1) product, stabilizes UV stress-induced c-Jun through inhibition of full-length COP1. COP1 is an evolutionarily conserved RING-finger ubiquitin ligase acting within a Cullin-RING ligase (CRL) complex that promotes polyubiquitination of c-Jun and p53. Stability of the above substrates is affected by post-translational changes priming the proteins for polyubiquitination and proteasome-dependent degradation. However, degradation of both substrates is controlled indirectly by signaling pathways affecting the E3 ligases involved in their polyubiquitination. Here, we report the identification of COP1D, a ubiquitously expressed splice variant of COP1 lacking a portion of a coiled-coil region involved in intermolecular associations. While being unable to associate with other components of the CRL complex, COP1D exerts a dominant-negative function over the full-length protein, due to its ability to heterodimerize with COP1 and sequester it from the enzymatically active complex. Ectopic expression of COP1D antagonizes the function of COP1, while its selective downregulation by RNA interference promotes more efficient degradation of c-Jun and p53 by the full-length protein. The COP1/COP1D mRNA ratio is modulated by UV stress and a decreased COP1/COP1D ratio correlates with elevated c-Jun, but not p53 protein levels in   invasive   ductal breast cancer. Thus, dynamic changes of the COP1/COP1D ratio provide an additional level of regulation of the half-life of the substrates of this E3 ligase under homeostatic or pathological conditions. 
 Loss of E-cadherin-mediated cell-cell contacts activates a novel mechanism for up-regulation of the proto-oncogene c-Jun. Loss of E-cadherin-mediated cell-cell contacts can elicit a signaling pathway that leads to acquisition of an   invasive   phenotype. Here, we show that at the receiving end of this pathway is the proto-oncogene c-Jun, a member of the activator protein-1 family of transcription factors that play a key role in stimulation of cell proliferation and tumor promotion. Cell separation or abrogation of E-cadherin-mediated cell-cell contacts both cause a dramatic increase in accumulation of the c-Jun protein. Unlike growth factors that enhance the expression of c-Jun by activating the transcription of the c-jun gene, the cell contact-dependent increase in c-Jun accumulation is not accompanied by a corresponding increase in c-Jun mRNA or c-Jun protein stability but rather in the translatability of the c-Jun transcript. Consistently, the increase in c-Jun accumulation is not dependent on activation of the mitogen-activated protein kinase or beta-catenin pathways but is mediated by signals triggered by the restructured cytoskeleton. Depolymerization of the cytoskeleton can mimic the effect of cell separation and cause a dramatic increase in c-Jun accumulation, whereas Taxol inhibits the cell contact-dependent increase. This novel mechanism of c-Jun regulation seems to underlie the robust overexpression of c-Jun in tumor cells of patients with colon carcinoma. 
 Dimerumic acid inhibits SW620 cell invasion by attenuating H₂O₂-mediated MMP-7 expression via JNK/C-Jun and ERK/C-Fos activation in an AP-1-dependent manner. Reactive oxygen species (ROS) such as hydrogen peroxide (H₂O₂) in the tumor microenvironment play important roles in tumor invasion and metastasis. Recently, ROS have been reported to cause a significant increase in the production and expression of matrix metalloproteinase (MMP)-7, which is closely correlated with metastatic colorectal cancer. The present study was undertaken to evaluate the scavenging activity of dimerumic acid (DMA) for H₂O₂ isolated from Monascus-fermented rice to investigate the inhibitory effects of   DMA on   the invasive potential of SW620 human colon cancer cells, and to explore the mechanisms underlying both these phenomena. Our results showed that increased MMP-7 expression due to H₂O₂ exposure was mediated by activation of mitogen-activated protein kinases (MAPKs) such as Jun N-terminal kinase (JNK), extracellular-regulated kinase (ERK), and p38 kinase. DMA pretreatment suppressed activation of H₂O₂-mediated MAPK pathways and cell invasion. Moreover, H₂O₂-triggered MMP-7 production was demonstrated via JNK/c-Jun and ERK/c-Fos activation in an activating protein 1 (AP-1)-dependent manner. Taken together, these results suggest that DMA suppresses H₂O₂-induced cell invasion by inhibiting AP-1-mediated MMP-7 gene transcription via the JNK/c-Jun and ERK/c-Fos signaling pathways in SW620 human colon cancer cells. Our data suggest that DMA may be useful in minimizing the development of colorectal metastasis. In the future, DMA supplementation may be a beneficial antioxidant to enhance surgical outcomes. 
 Ascochlorin inhibits matrix metalloproteinase-9 expression by suppressing activator protein-1-mediated gene expression through the ERK1/2 signaling pathway: inhibitory effects of ascochlorin on the invasion of renal carcinoma cells. The expression of matrix metalloproteinases (MMPs) has been implicated in the invasion and metastasis of cancer cells. Here we examined the effect of ascochlorin, a prenyl-phenol anti-tumor compound from the fungus Ascochyta viciae, on the regulation of signaling pathways that control MMP-9 expression in human renal carcinoma (Caki-1) cells. Ascochlorin reduced the   invasive   activity of Caki-1 cells and inhibited phorbol 12-myristate 13-acetate-induced increases in MMP-9 expression and activity in a dose-dependent manner. Reporter gene, electrophoretic mobility shift, kinase inhibitor assays, and in vitro kinase assay showed that ascochlorin inhibits MMP-9 gene expression by suppressing activation of the nuclear transcription factor activator protein-1 (AP-1) via the extracellular signal-regulated kinase 1 and 2 pathway. The AP-1 family member most specifically affected by ascochlorin was Fra-1. Ascochlorin did not affect the activation of the c-Jun N-terminal or p38 kinase pathways. Moreover, transfection of Caki-1 cells with AP-1 decoy oligodeoxynucleotides resulted in the suppression of phorbol 12-myristate 13-acetate-induced MMP-9 expression and invasion. In conclusion, ascochlorin represents a unique natural anti-tumor compound that specifically inhibits MMP-9 activity through suppression of AP-1-dependent induction of MMP-9 gene expression. 
 c-Jun is involved in interstitial cystitis antiproliferative factor (APF)-induced growth inhibition of human bladder cancer T24 cells. To uncover the role of c-Jun, a proto-oncogene, in inhibitory effects of antiproliferative factor (APF) on tumor cell growth.Expression of c-Jun was analyzed by Western blotting in 45 clinical specimens (30 tumorous tissues and 15 paired non-tumorous tissues) and 3 bladder cancer cell lines. APF-responsive T24 transitional carcinoma bladder cells were treated with APF or mock control. Cell proliferation was measured by 3-(4,5-dimethylthiazolyl-2)-2,5-diphenyltetrazolium bromide (MTT) assay. Change of c-Jun expression was detected by RT-PCR and Western blotting. The influence of c-Jun on APF treatment was evaluated by transient transfection of c-Jun and MTT assay in T24 cells.c-Jun was significantly higher in   invasive   bladder cancer tissues and cell lines. T24 cells treated with APF had decreased c-Jun expression and suppressed cell growth. More importantly, ectopic c-Jun attenuated APF inhibitory effects on cell growth.These observations suggest that c-Jun is involved in APF-mediated inhibition for bladder tumor cell growth, as potential target of APF in patients with aggressive bladder carcinoma. 
 DYRK2 priming phosphorylation of c-Jun and c-Myc modulates cell cycle progression in human cancer cells. Dysregulation of the G(1)/S transition in the cell cycle contributes to tumor development. The oncogenic transcription factors c-Jun and c-Myc are indispensable regulators at this transition, and their aberrant expression is associated with many malignancies. Degradation of c-Jun/c-Myc is a critical process for the G(1)/S transition, which is initiated upon phosphorylation by glycogen synthase kinase 3 β (GSK3β). However, a specific kinase or kinases responsible for priming phosphorylation events that precede this GSK3β modification has not been definitively identified. Here, we found that the dual-specificity tyrosine phosphorylation-regulated kinase DYRK2 functions as a priming kinase of c-Jun and c-Myc. Knockdown of DYRK2 in human cancer cells shortened the G(1) phase and accelerated cell proliferation due to escape of c-Jun and c-Myc from ubiquitination-mediated degradation. In concert with these results, silencing DYRK2 increased cell proliferation in human cancer cells, and this promotion was completely impeded by codeprivation of c-Jun or c-Myc in vivo. We also found marked attenuation of DYRK2 expression in multiple human tumor samples. Downregulation of DYRK2 correlated with high levels of unphosphorylated c-Jun and c-Myc and, importantly, wi  th invas  iveness of human breast cancers. These results reveal that DYRK2 regulates tumor progression through modulation of c-Jun and c-Myc. 
 Genome-wide profiling of AP-1-regulated transcription provides insights into the   invasive  ness of triple-negative breast cancer. Triple-negative breast cancer (TNBC) is an aggressive clinical subtype accounting for up to 20% of all breast cancers, but its malignant determinants remain largely undefined. Here, we show that in TNBC the overexpression of Fra-1, a component of the transcription factor AP-1, offers prognostic potential. Fra-1 depletion or its heterodimeric partner c-Jun inhibits the proliferative and   invasive   phenotypes of TNBC cells in vitro. Similarly, RNAi-mediated attenuation of Fra-1 or c-Jun reduced cellular invasion in vivo in a zebrafish tumor xenograft model. Exploring the AP-1 cistrome and the AP-1-regulated transcriptome, we obtained insights into the transcriptional regulatory networks of AP-1 in TNBC cells. Among the direct targets identified for Fra-1/c-Jun involved in proliferation, adhesion, and cell-cell contact, we found that AP-1 repressed the expression of E-cadherin by transcriptional upregulation of ZEB2 to stimulate cell invasion. Overall, this work illuminates the pathways through which TNBC cells acquire   invasive   and proliferative properties. 
 Critical role of c-Jun overexpression in liver metastasis of human breast cancer xenograft model. c-Jun/AP-1 has been linked to   invasive   properties of aggressive breast cancer. Recently, it has been reported that overexpression of c-Jun in breast cancer cell line MCF-7 resulted in increased AP-1 activity, motility and   invasive  ness of the cells in vitro and tumor formation in nude mice. However, the role of c-Jun in metastasis of human breast cancer in vivo is currently unknown.To further investigate the direct involvement of c-Jun in tumorigenesis and metastasis, in the present study, the effects of c-Jun overexpression were studied in both in vitro and in nude mice.Ectopic overexpression of c-Jun promoted the growth of MCF-7 cells and resulted in a significant increase in the percentage of cells in S phase and increased motility and   invasive  ness. Introduction of c-Jun gene alone into weakly   invasive   MCF-7 cells resulted in the transfected cells capable of metastasizing to the nude mouse liver following tail vein injection.The present study confirms that overexpression of c-Jun contributes to a more   invasive   phenotype in MCF-7 cells. It indicates an interesting relationship between c-Jun expression and increased property of adhesion, migration and in vivo liver metastasis of MCF-7/c-Jun cells. The results provide further evidence that c-Jun is involved in the metastasis of breast cancer. The finding also opens an opportunity for development of anti-c-Jun strategies in breast cancer therapy. 
 miR-221&222 regulate TRAIL resistance and enhance tumorigenicity through PTEN and TIMP3 downregulation. Lung and liver cancers are among the most deadly types of cancer. Despite improvements in treatment over the past few decades, patient survival remains poor, underlining the need for development of targeted therapies. MicroRNAs represent a class of small RNAs frequently deregulated in human malignancies. We now report that miR-221&222 are overexpressed in aggressive non-small cell lung cancer and hepatocarcinoma cells, as compared with less   invasive   and/or normal lung and liver cells. We show that miR-221&222, by targeting PTEN and TIMP3 tumor suppressors, induce TRAIL resistance and enhance cellular migration through the activation of the AKT pathway and metallopeptidases. Finally, we demonstrate that the MET oncogene is involved in miR-221&222 activation through the c-Jun transcription factor. 
 (-)-Epigallocatechin-3-gallate blocks nicotine-induced matrix metalloproteinase-9 expression and   invasive  ness via suppression of NF-κB and AP-1 in endothelial cells. Cigarette smoke, specifically the nicotine contained within, has been shown to correlate closely with cell invasion and strategies to downregulate their expression may ultimately be of clinical utility. Matrix metalloproteinase-9 (MMP-9) is critically involved in the cell invasion and metastasis processes. Since nicotine plays a crucial role in the regulation of MMP-9 expression, the investigation of plant-derived compounds capable of modulating nicotine-induced signaling is an issue of concern. In this study, the effects of (-)-epigallocatechin-3-gallate (EGCG), a major green tea catechin, on nicotine-induced cell invasion and MMP-9 activity in ECV304 human endothelial cells were examined. EGCG treatment was found to reduce the MMP-9 expression and transcriptional activity in a dose-dependent manner. EGCG inhibited nicotine-activated production of reactive oxygen species (ROS), which are known as important signaling molecules to activate MMP-9. To further study the mechanisms for the EGCG-mediated regulation of MMP-9, the transcription factors NF-κB and AP-1 activities were examined. EGCG suppressed the nicotine-induced NF-κB and AP-1 activation. Studies with expression vectors encoding mutated NF-κB signaling molecules and AP-1 decoy confirmed that NF-κB and AP-1 were essential for the nicotine-stimulated MMP-9 expression. EGCG also abrogated the nicotine-induced activation of AP-1 subunits c-fos and c-jun. The above studies demonstrate that EGCG may exert at least part of its   anti-inv  asive effect in ECV304 human endothelial cells by controlling MMP-9 expression through the suppression of ROS, NF-κB and AP-1. 
 AP-1 transcription factors, mucin-type molecules and MMPs regulate the IL-11 mediated   invasive  ness of JEG-3 and HTR-8/SVneo trophoblastic cells. This study examines the IL-11 mediated activation of downstream signaling and expression of effector molecules to resolve the controversies associated with the IL-11 mediated regulation of the   invasive  ness of two commonly used trophoblastic cell models viz. JEG-3 and HTR-8/SVneo cells. It has been reported that IL-11 increases the   invasive  ness of JEG-3 cells while, reduces the   invasive  ness of HTR-8/SVneo cells. Invasion assay performed simultaneously for both the cell lines confirmed the above findings. In addition, HTR-8/SVneo cells showed a higher basal   invasive  ness than JEG-3 cells. Western blot showed the IL-11 mediated activation of STAT3(tyr705) and STAT1(tyr701) in both the cell lines. However, IL-11 activated the ERK1/2 phosphorylation in JEG-3 cells but, inhibited it in HTR-8/SVneo cells. Within 10 min of IL-11 treatment, p-STAT3(tyr705) was localized inside the nucleus of both the cell lines but, there was enhanced co-localization of protein inhibitor of activated STAT1/3 (PIAS1/3) and p-STAT3(tyr705) in HTR-8/SVneo cells and not in JEG-3 cells. This could be reason for the poor responsiveness of STAT3 responsive genes like mucin 1 (MUC1) in HTR-8/SVneo cells and not in JEG-3 cells. Further, microarray analysis of the IL-11 treated cells revealed differential responsiveness of JEG-3 as compared to HTR-8/SVneo cells. Several family of genes like activator protein-1 (AP-1) transcription factors (Jun and Fos), mucin-type molecules, MMP23B etc showed enhanced expression in IL-11 treated JEG-3 cells while, there was no response or decrease in their expression in IL-11 treated HTR-8/SVneo cells. Expression of these molecules was confirmed by quantitative RT-PCR. In addition, HTR-8/SVneo cells also showed a significant decrease in the expression of MMP2, MMP3 and MMP9 upon IL-11 treatment. Hence, IL-11 mediated differential activation of signaling and expression of effector molecules is responsible for the differential   invasive   response of JEG-3 and HTR-8/SVneo cells. 
 COP1 and GSK3β cooperate to promote c-Jun degradation and inhibit breast cancer cell tumorigenesis. High abundance of c-Jun is detected in   invasiv  e breast cancer cells and aggressive breast tumor malignancies. Here, we demonstrate that a major cause of high c-Jun abundance in   invasiv  e breast cancer cells is prolonged c-Jun protein stability owing to poor poly-ubiquitination of c-Jun. Among the known c-Jun-targeting E3 ligases, we identified constitutive photomorphogenesis protein 1 (COP1) as an E3 ligase responsible for c-Jun degradation in less   invasiv  e breast cancer cells because depletion of COP1 reduced c-Jun poly-ubiquitination leading to the stabilization of c-Jun protein. In a panel of breast cancer cell lines, we observed an inverse association between the levels of COP1 and c-Jun. However, overexpressing COP1 alone was unable to decrease c-Jun level in   invasiv  e breast cancer cells, indicating that efficient c-Jun protein degradation necessitates an additional event. Indeed, we found that glycogen synthase kinase 3 (GSK3) inhibitors elevated c-Jun abundance in less   invasiv  e breast cancer cells and that GSK3β nonphosphorylable c-Jun-T239A mutant displayed greater protein stability and poorer poly-ubiquitination compared to the wild-type c-Jun. The ability of simultaneously enforced expression of COP1 and constitutively active GSK3β to decrease c-Jun abundance   in invas  ive breast cancer cells allowed us to conclude that c-Jun is negatively regulated through the coordinated action of COP1 and GSK3β. Importantly, co-expressing COP1 and active GSK3β blocked in vitro cell growth/migration and in vivo metastasi  s of inv  asive breast cancer cells. Gene expression profiling of breast tumor specimens further revealed that higher COP1 expression correlated with better recurrence-free survival. Our study supports the notion that COP1 is a suppressor of breast cancer progression. 
 Lithocholic acid upregulates uPAR and cell   invasive  ness via MAPK and AP-1 signaling in colon cancer cells. The secondary bile acid lithocholic acid (LCA) induced expression of urokinase-type plasminogen activator receptor (uPAR) and enhanced cell   invasive  ness in colon cancer cells. A dominant negative mutant or a specific inhibitor of MEK-1 suppressed LCA-induced uPAR expression. Deletions and site-directed mutagenesis revealed that the AP-1 site was required for LCA-induced uPAR transcription. LCA-mediated enhanced cell   invasive  ness was partially abrogated by uPAR neutralizing antibody and inhibitors of both Erk-1/2 and AP-1. These results suggest that LCA induces uPAR expression via Erk-1/2 and AP-1 pathway and, in turn, stimulate   invasive  ness of human colon cancer cells. 
 The prognostic significance of Jun transcription factors in ovarian cancer. The Jun proteins (c-Jun, JunD and JunB) play an important role in the regulation of cell proliferation, apoptosis and angiogenesis. It is well established that these proteins participate in the carcinogenesis and progression in several tumour types. However, little is known about the prognostic significance of Jun proteins in patients with   invasive   epithelial ovarian carcinoma.We analysed fresh-frozen tissues of 161 ovarian cancer patients by using Western blot analysis to investigate protein levels of JunB, JunD, c-Jun and phosphorylated c-Jun (pc-Jun Ser63). The results were correlated with clinicopathologic prognostic parameters and survival data.A high pc-Jun expression was significantly associated with shorter progression-free survival (14 vs. 16 months, p = 0.017) and overall survival (25 vs. 41 months, p = 0.038). In case of JunD, moderate protein levels were associated with a better prognosis, leading to longer progression-free and overall survival compared to weak or strong JunD expression (PFS in cases with weak/moderate/strong JunD expression: 14 vs. 19.5 vs. 16 months, p = 0.011; OAS: 32 vs. 42 vs. 35.5 months, p = 0.009). Multivariate Cox regression analysis confirmed an independent and significant impact of pc-Jun and JunD on the patient's prognosis.Our results show that Jun proteins (pc-Jun and JunD) influence carcinogenesis and tumour progression, suggesting a significant role as prognostic predictors in human ovarian carcinoma. 
 Jun activation domain binding protein 1 expression is associated with low p27(Kip1)levels in node-negative breast cancer. The purpose is to evaluate expression levels of Jun activation domain-binding protein 1 (JAB1) in breast cancer tissue and adjacent normal tissue, to determine whether JAB1 expression is associated with p27(Kip1) expression in   invasive   breast carcinomas, and to evaluate the prognostic significance of JAB1 and p27(Kip1) in node-negative breast cancer.JAB1 levels were measured in 10 matched pairs of   invasive   breast tumor tissue and adjacent normal tissue using Western blot analysis. We also investigated the immunoreactivity of JAB1 and p27(Kip1) levels in paraffin-embedded tissue specimens from 220 patients with node-negative breast cancer who had not received adjuvant systemic therapy. The median follow-up was 15 years.JAB1 was expressed at higher levels in   invasive   tumors than in adjacent normal tissue (P = 0.01). JAB1 overexpression was observed in 57% of   invasive   breast cancers. Low levels of p27(Kip1) were noted in 70% of the tumor specimens. We found an inverse correlation between JAB1 and p27(Kip1) expression levels (P = 0.01). JAB1 overexpression was associated with patient age of at least 50 years (P = 0.03) and tumor size of </=2 cm (P = 0.01). Elevated levels of p27(Kip1) were associated with low nuclear grade (P = 0.01). At 5 years of follow-up, neither JAB1 nor p27(Kip1) expression was related to disease-free survival.These data indicate that JAB1 is commonly overexpressed in   invasive   breast carcinomas. JAB1 overexpression is associated with low levels of p27(Kip1) in node-negative breast cancer. In this study, JAB1 and p27(Kip1) were not independent prognostic factors. 
 Mitochondrial dysfunction induces EMT through the TGF-β/Smad/Snail signaling pathway in Hep3B hepatocellular carcinoma cells. Mitochondrial dysfunction has been found to be associated with various pathological conditions, particularly cancer. However, the mechanisms underlying tumor malignancy induced by mitochondrial dysfunction are not fully understood. In the present study, the effects of mitochondrial dysfunction on epithelial-mesenchymal transition (EMT), were investigated using mitochondrial-depleted ρ(0) cells derived from the Hep3B hepatocarcinoma cell line. The Hep3B/ρ(0) cells displayed the EMT phenotype with more aggressive migration and high  er invas  iveness compared to their parental cells. The Hep3B/ρ(0) cells also showed typical expression pattern of EMT markers such as vimentin and E-cadherin. These phenotypes in Hep3B/ρ(0) cells were mediated by increased transforming growth factor-β (TGF-β) through the canonical Smad-dependent signaling pathway. Additionally, TGF-β signaling was activated via induction of c-Jun/AP-1 expression and activity. Therefore, mitochondrial dysfunction induces EMT through TGF-β/Smad/Snail signaling via c-Jun/AP-1 activation. These results indicate that mitochondrial dysfunction plays an important role in the EMT process and could be a novel therapeutic target for malignant cancer therapy. 
 Divergent mechanisms underlie Smad4-mediated positive regulation of the three genes encoding the basement membrane component laminin-332 (laminin-5). Functional inactivation of the tumor suppressor Smad4 in colorectal and pancreatic carcinogenesis occurs coincident with the transition to   invasive   growth. Breaking the basement membrane (BM) barrier, a prerequisite for   invasive   growth, can be due to tumor induced proteolytic tissue remodeling or to reduced synthesis of BM molecules by incipient tumor cells. Laminin-332 (laminin-5), a heterotrimeric BM component composed of alpha 3-, beta 3- and gamma 2-chains, has recently been identified as a target structure of Smad4 and represents the first example for expression control of an essential BM component by a tumor and invasion suppressor. Biochemically Smad4 is a transmitter of signals of the TGFbeta superfamily of cytokines. We have reported previously, that Smad4 functions as a positive transcriptional regulator of constitutive and of TGFbeta-induced transcription of all three genes encoding Laminin-332, LAMA3, LAMB3 and LAMC2.Promoter-reporter constructs harboring 4 kb upstream regions, each of the three genes encoding Laminin-322 as well as deletion and mutations constructs were established. Promoter activities and TGFbeta induction were assayed through transient transfections in Smad4-negative human cancer cells and their stable Smad4-positive derivatives. Functionally relevant binding sites were subsequently confirmed through chromatin immunoprecipitation.Herein, we report that Smad4 mediates transcriptional regulation through three different mechanisms, namely through Smad4 binding to a functional SBE site exclusively in the LAMA3 promoter, Smad4 binding to AP1 (and Sp1) sites presumably via interaction with AP1 family components and lastly a Smad4 impact on transcription of AP1 factors. Whereas Smad4 is essential for positive regulation of all three genes, the molecular mechanisms are significantly divergent between the LAMA3 promoter as compared to the LAMB3 and LAMC2 promoters.We hypothesize that this divergence in modular regulation of the three promoters may lay the ground for uncoupled regulation of Laminin-332 in Smad4-deficient tumor cells in response to stromally expressed cytokines acting on budding tumor cells. 
  GeneRIFs  
 Melanoma reprogramming involves thousands of genomic regulatory regions underlying the proliferative and   invasive   states, identifying SOX10/MITF and AP-1/TEAD as regulators, respectively. 
 The above studies demonstrate that EGCG may exert at least part of its anti-  invasive   effect in ECV304 human endothelial cells by controlling MMP-9 expression through the suppression of ROS, NF-kappaB and AP-1 
 lithocholic acid induces uPAR expression via Erk-1/2 and AP-1 pathway and, in turn, stimulate   invasive  ness of human colon cancer cells. 
 AP-1 proteins (jun/fos) are differentially expressed in ovarian tumors of differing malignant potential. No correlation with the proliferative and   invasive   potential of ovarian cancer cell lines could be found. 
 EGF induces uPAR expression via ERK-1/2, AP-1, and NF-kappaB signaling pathways and, in turn, stimulates cell   invasive  ness in human gastric cancer AGS cells 
 overexpression of c-Jun is associated with   invasive  ness and metastasis in breast cancer 
  [Mm]etastasis  PubMed  
 Glucose-regulated protein 94 mediates   metastasis   by CCT8 and the JNK pathway in hepatocellular carcinoma. Hepatocellular carcinoma (HCC) is one of the leading causes of cancer death worldwide. Cancer   metastasis   is a major obstacle in clinical cancer therapy. The mechanisms underlying the   metastasis   of HCC remain unclear. Glucose-regulated protein 94 (GRP94) is a key protein involved in mediating cancer progression, and it is highly expressed in HCC specimens. However, the role of GRP94 in cancer   metastasis   is unclear. A specific short hairpin RNA (shRNA) was employed to knock down GRP94 gene expression in HCC cell lines. Wound-healing migration, transwell migration, and invasion assays were performed to determine the migration and invasive ability of HCC cells. We demonstrated that silencing GRP94 inhibited HCC cell wound healing, migration, and invasion. Furthermore, our findings indicated that GRP94 knockdown might attenuate HCC cell   metastasis   by inhibiting CCT8/c-Jun/EMT signaling. Our study indicated that silencing GRP94 significantly reduced the migration and invasion abilities of HCC cells. Moreover, depleting GRP94 inhibited cell migration and invasion by downregulating CCT8/c-Jun signaling. Thus, our data suggest that the GRP94/CCT8/c-Jun/EMT signaling cascade might be a new therapeutic target for HCC. 
 Glucose-regulated protein 94 mediates   metastasis   by CCT8 and the JNK pathway in hepatocellular carcinoma. Hepatocellular carcinoma (HCC) is one of the leading causes of cancer death worldwide. Cancer   metastasis   is a major obstacle in clinical cancer therapy. The mechanisms underlying the   metastasis   of HCC remain unclear. Glucose-regulated protein 94 (GRP94) is a key protein involved in mediating cancer progression, and it is highly expressed in HCC specimens. However, the role of GRP94 in cancer   metastasis   is unclear. A specific short hairpin RNA (shRNA) was employed to knock down GRP94 gene expression in HCC cell lines. Wound-healing migration, transwell migration, and invasion assays were performed to determine the migration and invasive ability of HCC cells. We demonstrated that silencing GRP94 inhibited HCC cell wound healing, migration, and invasion. Furthermore, our findings indicated that GRP94 knockdown might attenuate HCC cell   metastasis   by inhibiting CCT8/c-Jun/EMT signaling. Our study indicated that silencing GRP94 significantly reduced the migration and invasion abilities of HCC cells. Moreover, depleting GRP94 inhibited cell migration and invasion by downregulating CCT8/c-Jun signaling. Thus, our data suggest that the GRP94/CCT8/c-Jun/EMT signaling cascade might be a new therapeutic target for HCC. 
 Src induces urokinase receptor gene expression and invasion/intravasation via activator protein-1/p-c-Jun in colorectal cancer. The urokinase receptor [urokinase plasminogen activator receptor (u-PAR)] promotes invasion and   metastasis   and is associated with poor patient survival. Recently, it was shown that Src induces u-PAR gene expression via Sp1 bound to the u-PAR promoter region -152/-135. However, u-PAR is regulated by diverse promoter motifs, among them being an essential activator protein-1 (AP-1) motif at -190/-171. Moreover, an in vivo relevance of Src-induced transcriptional regulators of u-PAR-mediated invasion, in particular intravasation, and a relevance in resected patient tumors have not sufficiently been shown. The present study was conducted (a) to investigate if, in particular, AP-1-related transcriptional mediators are required for Src-induced u-PAR-gene expression, (b) to show in vivo relevance of AP-1-mediated Src-induced u-PAR gene expression for invasion/intravasation and for resected tissues from colorectal cancer patients. Src stimulation of the u-PAR promoter deleted for AP-1 region -190/-171 was reduced as compared with the wild-type promoter in cultured colon cancer cells. In gelshifts/chromatin immunoprecipitation, Src-transfected SW480 cells showed an increase of phospho-c-Jun, in addition to JunD and Fra-1, bound to region -190/-171. Src-transfected cells showed a significant increase in c-Jun phosphorylated at Ser(73) and also Ser(63), which was paralleled by increased phospho-c-jun-NH(2)-kinase. Significant decreases of invasion/in vivo intravasation (chorionallantoic membrane model) were observed in Src-overexpressing cells treated with Src inhibitors, u-PAR-small interfering RNA, and dominant negative c-Jun (TAM67). In resected tissues of 20 colorectal cancer patients, a significant correlation between Src activity, AP-1 complexes bound to u-PAR region -190/-171, and advanced pN stage were observed. These data suggest that Src-induced u-PAR gene expression and invasion/intravasation in vivo is also mediated via AP-1 region -190/-171, especially bound with c-Jun phosphorylated at Ser(73/63), and that this pathway is biologically relevant for colorectal cancer patients, suggesting therapeutic potential. 
 Microenvironmental regulation of BRCA1 gene expression by c-Jun and Fra2 in premalignant human ovarian surface epithelial cells. Reduced BRCA1 gene expression is common in the sporadic form of ovarian carcinoma. The spread of this highly lethal cancer often begins when tumor cell clusters are shed into the fluid of the abdominopelvic cavity such that they can float freely before seeding distant sites on the peritoneal walls and organs. Thus, the microenvironment that tumor cells find themselves in changes dramatically during these early shedding and floating stages of transperitoneal   metastasis  . To mimic this microenvironmental change in vitro, we released premalignant human ovarian surface epithelial cells from the substratum and forced them to cluster in suspension. Under these conditions, steady state levels of BRCA1 mRNA and protein fell significantly and the transcriptional activation state of the BRCA1 promoter was suppressed. Analysis of the promoter indicated that the previously identified "CRE" element located within the "positive regulatory region" (PRR) contributed to this suppression. More specifically, we show that the suppression was mediated, at least in part, by a suspension culture-driven decrease in the levels of two members of the AP1 transcription factor complex, c-Jun and Fra2, that bind to the CRE element. Therefore, a microenvironmental change that is manifested during the initial stages of ovarian carcinoma dissemination may, potentially, help suppress BRCA1 expression in sporadic tumors and thus promote their progression. 
 Cadmium induces matrix metalloproteinase-9 expression via ROS-dependent EGFR, NF-кB, and AP-1 pathways in human endothelial cells. Cadmium (Cd), a widespread cumulative pollutant, is a known human carcinogen, associated with inflammation and tumors. Matrix metalloproteinase-9 (MMP-9) plays a pivotal role in tumor   metastasi  s; however, the mechanisms underlying the MMP-9 expression induced by Cd remain obscure in human endothelial cells. Here, Cd elevated MMP-9 expression in dose- and time-dependent manners in human endothelial cells. Cd increased ROS production and the ROS-producing NADPH oxidase. Cd translocates p47(phox), a key subunit of NADPH oxidase, to the cell membrane. Cd also activated the phosphorylation of EGFR, Akt, Erk1/2, and JNK1/2 in addition to promoting NF-кB and AP-1 binding activities. Specific inhibitor and mutagenesis studies showed that EGFR, Akt, Erk1/2, JNK1/2 and transcription factors NF-κB and AP-1 were related to Cd-induced MMP-9 expression in endothelial cells. Akt, Erk1/2, and JNK1/2 functioned as upstream signals in the activation of NF-κB and AP-1, respectively. In addition, N-acetyl-l-cystein (NAC), diphenyleneiodonium chloride (DPI) and apocynin (APO) inhibited the Cd-induced activation of EGFR, Akt, Erk1/2, JNK1/2, and p38 MAPK, indicating that ROS production by NADPH oxidase is the furthest upstream signal in MMP-9 expression. At present, it states that Cd displayed marked invasiveness in ECV304 cells, which was partially abrogated by MMP-9 neutralizing antibodies. These results demonstrated that Cd induces MMP-9 expression via ROS-dependent EGFR->Erk1/2, JNK1/2->AP-1 and EGFR->Akt->NF-κB signaling pathways and, in turn, stimulates invasiveness in human endothelial cells. 
 TNF-alpha promotes lymphangiogenesis and lymphatic   metastasis   of gallbladder cancer through the ERK1/2/AP-1/VEGF-D pathway. Tumor necrosis factor-alpha (TNF-α), a key player in cancer-related inflammation, was recently demonstrated to be involved in the lymphatic   metastasi  s of gallbladder cancer (GBC). Vascular endothelial growth factor D (VEGF-D) is a key lymphangiogenic factor that is associated with lymphangiogenesis and lymph node   metastasi  s in GBC. However, whether VEGF-D is involved in TNF-α-induced lymphati  c metastas  is of GBC remains undetermined.The expression of VEGF-D in patient specimens was detected by immunohistochemistry and the relationship between VEGF-D in the tissue and TNF-α in the bile of the matching patients was analyzed. The VEGF-D mRNA and protein levels after treatment with exogenous TNF-α in NOZ, GBC-SD and SGC-996 cell lines were measured by real-time PCR and ELISA. The promoter activity and transcriptional regulation of VEGF-D were analyzed with the relative luciferase reporter assay, mutant constructs, electrophoretic mobility shift assay (EMSA), chromatin immunoprecipitation (ChIP) assay, RNA interference and Western blotting. Inhibitors of JNK, p38 MAPK and ERK1/2 were used to explore the upstream signaling effector of AP-1. We used lentiviral vector expressing a VEGF-D shRNA construct to knockdown VEGF-D gene in NOZ and GBC-SD cells. The role of the TNF-α-VEGF-D axis in the tube formation of human dermal lymphatic endothelial cells (HDLECs) was determined using a three-dimensional coculture system. The role of the TNF-α - VEGF-D axis in lymphangiogenesis and lymph   node meta  stasis was studied via animal experiment.TNF-α levels in the bile of GBC patients were positively correlated with VEGF-D expression in the clinical specimens. TNF-α can upregulate the protein expression and promoter activity of VEGF-D through the ERK1/2 - AP-1 pathway. Moreover, TNF-α can promote tube formation of HDLECs, lymphangiogenesis and ly  mph node m  etastasis of GBC by upregulation of VEGF-D in vitro and in vivo.Taken together, our data suggest that TNF-α can promote lymphangiogenesis and   lymphatic   metastasis of GBC through the ERK1/2/AP-1/VEGF-D pathway. 
 Epithelial-to-mesenchymal transition induced by TGF-β1 is mediated by AP1-dependent EpCAM expression in MCF-7 cells. The epithelial-to-mesenchymal transition (EMT), a process involving the breakdown of cell-cell junctions and loss of epithelial polarity, is closely related to cancer   metastasi  s and invasion. The epithelial cell adhesion molecule (EpCAM) is a type I transmembrane protein expressed in the majority of normal epithelial tissues and overexpressed in the majority of human epithelial cancers including breast cancer. EpCAM plays an important role in cancer progression. We showed that EpCAM participated in TGF-β1-induced EMT. TGF-β1 treatment of MCF-7 breast cancer cells was shown to induce EpCAM expression, which promoted the EMT and cell migration. EpCAM overexpression further enhanced TGF-β1-induced EMT and EpCAM knockdown inhibited TGF-β1-induced EMT. We further demonstrated that TGF-β1 treatment induced the phosphorylation of JNK that was in turn responsible for the increased expression of Jun and Fos. This result suggests an important role of the JNK to AP-1 signaling to EpCAM downstream of TGF-β1 for the induction of EMT in the breast cancer cells. Collectively, our study highlights a novel function for EpCAM in TGF-β1-induced EMT process and suggests that targeting of EpCAM may be an attractive strategy to treat breast cancer. This study implicates the potential value of EpCAM as a molecular marker for breast cancer. 
 NCOA1 promotes angiogenesis in breast tumors by simultaneously enhancing both HIF1α- and AP-1-mediated VEGFa transcription. Nuclear receptor coactivator 1 (NCOA1) is overexpressed in a subset of breast cancer and its increased expression positively correlates with disease recurrence and   metastasi  s. Although NCOA1 is known to promote breast cancer   metastasi  s through working with multiple transcription factors to upregulate the expression of Twist1, ITGA5, CSF-1, SDF1 and CXCR4, the role of NCOA1 in breast tumor angiogenesis has not been investigated. In this study, we found that the microvascular density (MVD) was significantly decreased and increased in Ncoa1-knockout and NCOA1-overexpressing mammary tumors, respectively, in several breast cancer mouse models. Knockout or knockdown of NCOA1 in breast cancer cell lines also markedly compromised their capability to induce angiogenesis in Matrigel plugs embedded subcutaneously in mice, while this compromised capability could be rescued by VEGFa treatment. At the molecular level, NCOA1 upregulates VEGFa expression in both mouse mammary tumors and cultured breast cancer cells, and it does so by associating with both c-Fos, which is recruited to the AP-1 site at bp -938 of the VEGFa promoter, and HIF1α, which is recruited to the HIF1α-binding element at bp -979 of the VEGFa promoter, to enhance VEGFa transcription. In 140 human breast tumors, high NCOA1 protein correlates with high MVD and patients with both high NCOA1 and high MVD showed significantly shorter survival time. In summary, this study revealed a novel mechanism that NCOA1 potentiates breast cancer angiogenesis through upregulating HIF1α and AP-1-mediated VEGFa expression, which reinforces the rational of targeting NCOA1 in controlling breast cancer progression   and metast  asis. 
 Podoplanin is regulated by AP-1 and promotes platelet aggregation and cell migration in osteosarcoma. Podoplanin is a type-I transmembrane sialomucin-like protein, which is expressed in a wide range of cell types and is involved in platelet aggregation and tumor   metastasis  . Here, we investigated the function, regulation, and expression of podoplanin in osteosarcoma. Podoplanin expression was observed in three osteosarcoma cell lines (MG-63, HOS, and U-2 OS) with platelet aggregation-inducing ability, which was blocked by podoplanin small-interfering RNA or a neutralizing antibody. Overexpression of podoplanin in nonmetastatic Dunn osteosarcoma cells promoted cell migration without attenuating cell proliferation. Both podoplanin and TGF-β1 were up-regulated by c-Fos induction in MC3T3-E1 osteoblastic cells, and were highly expressed in c-Fos transgenic mouse osteosarcomas and c-Fos-transformed osteosarcoma cell lines. Immunohistochemistry of human osteosarcoma tissue microarrays (n = 133) showed staining of tumor cells embedded in an excess of irregular neoplastic bone matrix in 100% of tumors undergoing so-called "normalization/maturation." Podoplanin was also expressed in osteosarcoma subtypes, with 65% of osteoblastic, 100% of chondroblastic, and 79% of fibroblastic tumors. CD44 and pERM immunohistochemistry showed coexpression with podoplanin in both mouse and human osteosarcoma. Podoplanin expression was significantly higher in metastatic osteosarcomas (n = 6) than in primary osteosarcomas (n = 10). Our data suggest that podoplanin, which is not expressed in normal osteoblasts but in osteocytes, is aberrantly expressed in transformed osteoblasts and in osteosarcoma, and is under AP-1 transcriptional control. Thus podoplanin is a candidate molecule for therapeutic targeting. 
 A feedback inhibition between miRNA-127 and TGFβ/c-Jun cascade in HCC cell migration via MMP13. Hepatocellular carcinoma (HCC) is the fifth most common cancer worldwide and is increasing in frequency in the U.S. The major reason for the low postoperative survival rate of HCC is widespread intrahepatic   metastasi  s or invasion, and activation of TGFβ signaling is associated with the invasive phenotype. This study aims at determining the novel function of miR-127 in modulating HCC migration. Overexpression of miR-127 inhibits HCC cell migration, invasion and tumor growth in nude mice. MiR-127 directly represses matrix metalloproteinase 13 (MMP13) 3'UTR activity and protein expression, and diminishes MMP13/TGFβ-induced HCC migration. In turn, TGFβ decreases miR-127 expression by enhancing c-Jun-mediated inhibition of miR-127 promoter activity. In contrast, p53 transactivates miR-127 promoter and induces miR-127 expression, which is antagonized by c-Jun. The inhibition of miR-127 by c-Jun is through TGFβ-mediated ERK and JNK pathways. The lower miR-127 expression shows a negative correlation with the higher MMP13 expression in a subset of human HCC specimens. This is the first report elucidating a feedback regulation between miR-127 and the TGFβ/c-Jun cascade in HCC migration via MMP13 that involves a crosstalk between the oncogene c-Jun and tumor suppressor p53. 
 Palbociclib inhibits epithelial-mesenchymal transition and   metastasis   in breast cancer via c-Jun/COX-2 signaling pathway. Palbociclib, a highly selective CDK4/6 inhibitor, has been shown to be a novel anti-tumor agent that suppresses breast cancer cell proliferation. However, its anti-  metastasis   activity remains controversial. In the present study, we evaluated whether palbociclib prevented breast cancer cell   metastasis   and revealed its regulatory mechanism. We found that palbociclib inhibited migration and invasion in the breast cancer cells MDA-MB-231 and T47D. The epithelial-mesenchymal transition (EMT) markers, vimentin and Snail, were down-regulated with palbociclib treatment. Moreover, we revealed that this inhibition was mediated by the c-Jun/COX-2 pathway. COX-2 was decreased after palbociclib treatment. The production of PGE2 was also reduced along with COX-2. Additionally, our data showed that c-Jun, a crucial transcriptional regulator of COX-2, was down-regulated by palbociclib. We found that palbociclib weakened the COX-2 promoter binding activity of c-Jun and prevented its translocation from the cytoplasm to cell nuclei. Bioluminescence imaging and tail intravenous injection were used to evaluate the anti-  metastasis   effect of palbociclib in vivo. The data demonstrated that palbociclib reduced breast cancer   metastasis   to the lung. These results therefore demonstrated that the anti-  metastasis   activity of palbociclib is mediated via the c-Jun/COX-2 signaling pathway by inhibiting EMT in breast cancer cells. 
 Downregulation of c-jun results in apoptosis-mediated anti-osteosarcoma activity in an orthotopic model. c-jun has been found to be upregulated in a variety of cancers including osteosarcoma. DNAzymes are oligonucleotides capable of specific downregulation of target genes. c-jun knockdown-mediated apoptosis in osteosarcoma cells involved caspases-1, -2 and -8, but not the Fas/FasL pathway. A c-jun DNAzyme, encapsulated within a novel cationic multilamellar vesicle liposome, inhibited the growth and   metastasis   of osteosarcoma in an orthotopic spontaneously   metastasis  ing model of the disease. The 60 nm DDAB:DOPE liposome was formulated using ethanol injection/extrusion. Clinically, downregulation of c-jun may proffer an improved treatment outcome for these tumours originating in bone. 
 NGX6 inhibits AP-1 and Ets-1 expression and down-regulates cyclin D1 in human colorectal cancer. Colorectal cancer (CRC) is a common malignant tumor that is associated with an increased incidence of morbidity and mortality. Nasopharyngeal carcinoma-associated gene 6 (NGX6) is a novel candidate suppressor gene of tumor   metastasis  , which is down-regulated in CRC. In the present study, we constructed a colorectal tissue microarray to examine the expression profiles of NGX6, phospho-c-Jun N-terminal kinase (p-JNK), and phospho-extracellular signal-regulated kinase (p-ERK ) in CRC tissues. We found that the NGX6 expression was lower in CRC tissues and metastatic lymph nodes, whereas the expressions of p-JNK and p-ERK were higher in CRC tissues, than in normal intestinal mucosa. The expressions of NGX6, p-JNK, and p-ERK were associated with the clinical pathological features of colorectal tissues. NGX6 overexpression inhibited the activation and nuclear translocation of JNK1, which led to an accumulation of p-JNK in the cytoplasm, but did not inhibit the activation and nuclear translocation of ERK1/2. NGX6 also inhibited the expression of the transcription factors AP-1 (c-jun and c-fos) and Ets-1. In addition, NGX6 overexpression decreased the expression of cyclin D1 and dramatically suppressed the transcriptional efficiency of the cyclin D1 promoter. We propose that NGX6 expression is lost in the multistep process of human colorectal carcinogenesis. Its overexpression can inhibit the expression of transcription factors AP-1 and Ets-1, and down-regulate the transcriptional activity of the cyclin D1 promoter in human CRC. 
 c-Jun, a crucial molecule in   metastasis   of breast cancer and potential target for biotherapy. Human breast cancer cell line SKBR3 expresses high level of the ErbB2 molecule, which has been associated with poor prognosis of breast cancer. Elevated ErbB2 functions as a transactivating co-receptor and promotes the formation of ErbB2 containing heterodimers, which are more mitogenic and transforming, and have a higher ligand affinity and signaling potency by virtue of the potent latent kinase activity of ErbB2. Interestingly, SKBR3 cells are non-tumorigenic in nude mice. By ectopic overexpression of c-Jun in SKBR3 cells, involvement of c-Jun in invasiveness and   metastasis   in vivo was investigated in this study. The critical role of c-Jun in the tumorigenesis and   metastasis   is demonstrated in nude mice. 
 Twist1 and AP-1 cooperatively upregulate integrin α5 expression to induce invasion and the epithelial-mesenchymal transition. Epithelial-mesenchymal transition (EMT) is an important process implicated in tumor invasion and   metastasi  s. Twist1 is a transcription factor that induces EMT, including E-cadherin suppression and cancer cell migration and invasion; hence it promotes cancer   metastasi  s. Twist1 directly or indirectly regulates the expression of various genes and cellular functions involved in cancer progression. However, the underlying mechanisms remain largely unknown. In this study, we investigated the molecular basis for Twist1-mediated invasion and EMT. In human cancer cells, Twist1 was found to directly upregulate transcription of the mesenchymal gene integrin α5 in an E-box-independent, but activating protein-1 (AP-1) element-dependent, manner. Twist1 activated the integrin α5 promoter by interacting with and activating the transcription factor AP-1, composed of c-Jun and activating transcription factor-2 (ATF-2); it also enhanced the nuclear presence of ATF-2. AP-1 was critical for Twist1-induced cancer cell invasion, primarily through the induction of integrin α5, which activated c-Jun N-terminal kinase and focal adhesion kinase-signaling activities. Using data from The Cancer Genome Atlas, we found that Twist1 expression positively correlates with integrin α5 expression in human colorectal cancers. These findings suggest that cooperation between Twist1 and AP-1 represents a novel mechanism for EMT and tumor invasiveness. This study supports further investigation into the molecular basis underlying the diverse Twist1-mediated functions that occur during tumor progression. 
 Specific interactions between Smad proteins and AP-1 components determine TGFβ-induced breast cancer cell invasion. Deregulation of the transforming growth factor β (TGFβ) signal transduction cascade is functionally linked to cancer. In early phases, TGFβ acts as a tumor suppressor by inhibiting tumor cell proliferation, whereas in late phases, it can act as a tumor promoter by stimulating tumor cell invasion   and metast  asis. Smad transcriptional effectors mediate TGFβ responses, but relatively little is known about the Smad-containing complexes that are important for epithelial-mesenchymal transition and invasion. In this study, we have tested the hypothesis that specific members of the AP-1 transcription factor family determine TGFβ signaling specificity in breast cancer cell invasion. Using a 3D model of collagen-embedded spheroids of MCF10A-MII premalignant human breast cancer cells, we identified the AP-1 transcription factor components c-Jun, JunB, c-Fos and Fra1 as essential factors for TGFβ-induced invasion and found that various mesenchymal and invasion-associated TGFβ-induced genes are co-regulated by these proteins. In situ proximity ligation assays showed that TGFβ signaling not only induces complexes between Smad3 and Smad4 in the nucleus but also complexes between Smad2/3 and Fra1, whereas complexes between Smad3, c-Jun and JunB could already be detected before TGFβ stimulation. Finally, chromatin immunoprecipitations showed that c-Jun, JunB and Fra1, but not c-Fos, are required for TGFβ-induced binding of Smad2/3 to the mmp-10 and pai-1 promoters. Together these results suggest that in particular formation of Smad2/3-Fra1 complexes may reflect activation of the Smad/AP-1-dependent TGFβ-induced invasion program. 
 The regulatory mechanism of the LY6K gene expression in human breast cancer cells. LY6K is a cancer biomarker and a therapeutic target that induces invasion and   metastasis  . However, the molecular mechanisms that determine human LY6K transcription are completely unknown. To elucidate the mechanisms involved in human LY6K gene regulation and expression, multiple cis-elements were predicted using TRANSFAC software, and the LY6K regulatory region was identified using the luciferase assay in the human LY6K gene promoter. We performed ChIP, EMSA, and supershift assays to investigate the transcription factor activity on the LY6K promoter, and the effect of a SNP and CpG site methylation on AP-1 transcription factor binding affinity. AP-1 and the CREB transcription factor bound to LY6K promoter within -550/-1, which was essential for LY6K expression, but only the AP-1 heterodimer, JunD, and Fra-1, modulates LY6K gene transcriptional level. A decrease in LY6K was associated with the SNP242 C allele, a polymorphic G/C-SNP at the 242 nucleotide in the LY6K promoter region (rs2585175), or methylation of the CpG site, which was closely located with the AP-1 site by interfering with binding of the AP-1 transcription factor to the LY6K promoter. Our findings reveal an important role for AP-1 activation in promoting LY6K gene expression that regulates cell mobility of breast cancer cells, whereas the SNP242 C allele or methylation of the CpG site may reduce the risk of invasion or   metastasis   by interfering AP-1 activation. 
 Necrotic cells influence migration and invasion of glioblastoma via NF-κB/AP-1-mediated IL-8 regulation. Glioblastoma multiforme (GBM) is the most common primary intracranial tumor in adults and has poor prognosis. Diffuse infiltration into normal brain parenchyma, rapid growth, and the presence of necrosis are remarkable hallmarks of GBM. However, the effect of necrotic cells on GBM growth and   metastasi  s is poorly understood at present. In this study, we examined the biological significance of necrotic tissues by exploring the molecular mechanisms underlying the signaling network between necrotic tissues and GBM cells. The migration and invasion of the GBM cell line CRT-MG was significantly enhanced by treatment with necrotic cells, as shown by assays for scratch wound healing and spheroid invasion. Incubation with necrotic cells induced IL-8 secretion in CRT-MG cells in a dose-dependent manner. In human GBM tissues, IL-8 positive cells were mainly distributed in the perinecrotic region, as seen in immunohistochemistry and immunofluorescence analysis. Necrotic cells induced NF-κB and AP-1 activation and their binding to the IL-8 promoter, leading to enhanced IL-8 production and secretion in GBM cells. Our data demonstrate that when GBM cells are exposed to and stimulated by necrotic cells, the migration and invasion of GBM cells are enhanced and facilitated via NF-κB/AP-1 mediated IL-8 upregulation. 
 Combined Expression of c-jun, c-fos, and p53 Improves Estimation of Prognosis in Oral Squamous Cell Carcinoma. To identify the prognostic value of c-jun, c-fos, and p53 in oral cancer, we examined the impact of immunohistochemical expression of these markers on tumor progression in 157 oral squamous cell carcinoma (OSCC). We found that c-jun or c-fos was significantly associated with lymph node   metastasis  , and coexpression of c-jun/c-fos, or c-jun/c-fos/p53 were significantly associated with lymph node   metastasis  , poor differentiation and clinical stage. The coexpression of c-jun/c-fos/p53 was identified as independent prognostic factors for overall survival. Simultaneous coexpression of these markers in OSCCs might prove to be a useful indicator for differentiation of low and high-risk patients. 
 Blockade of the RhoA-JNK-c-Jun-MMP2 cascade by atorvastatin reduces osteosarcoma cell invasion. Osteosarcoma is characterized by a high malignant and metastatic potential, which points to the need for new therapeutic strategies to prevent cell   metastasis  . In this study, we show that statin-induced HMG-CoA reductase inhibition reduces cell migration and invasion in human and murine osteosarcoma cells, independently of the genotype. The statin-induced reduction of cell migration and invasion was independent of induction of apoptosis and was geranylgeranylpyrophosphate-dependent. The statin reduced matrix metalloproteinase (MMP) 2, 9, and 14 and TIMP2 expression or activity in invading cells. Forced expression of MMP2 and MMP14 overcame the inhibitory effect of the statin on cell invasion, suggesting a role for these MMPs in invasive potential. We also investigated the mechanisms involved in the reduced MMP2 activity and cell invasion. Inhibition of JNK, but not ERK1/2 signaling, reduced MMP2 activity. Pharmacological or constitutive activation of JNK overcame the reduced MMP2 activity and cell invasion induced by the statin. The statin decreased JNK phosphorylation and c-Jun nuclear translocation, suggesting that HMG-CoA reductase inhibition targets the JNK-c-Jun signaling pathway. We showed that mevalonate or geranylgeranylpyrophosphate treatment prevented the statin-induced reduction in JNK phosphorylation, MMP2 activity, and cell invasion. Forced expression of a constitutively active form of RhoA increased JNK phosphorylation and overcame the inhibitory effect of atorvastatin on MMP2 activity and cell invasion. The data establish a link between RhoA, JNK, c-Jun, and MMP2 activity that is functionally involved in the reduction in osteosarcoma cell invasion by the statin. This suggests a novel strategy targeting RhoA-JNK-c-Jun signaling to reduce osteosarcoma cell tumorigenesis. 
 Inhibitor of Differentiation/DNA Binding 1 (ID1) Inhibits Etoposide-induced Apoptosis in a c-Jun/c-Fos-dependent Manner. ID1 (inhibitor of differentiation/DNA binding 1) acts an important role in   metastasis  , tumorigenesis, and maintenance of cell viability. It has been shown that the up-regulation of ID1 is correlated with poor prognosis and the resistance to chemotherapy of human cancers. However, the underlying molecular mechanism remains elusive. Here, we determined for the first time that up-regulating ID1 upon etoposide activation was mediated through AP-1 binding sites within theID1promoter and confirmed that ID1 enhanced cell resistance to DNA damage-induced apoptosis in esophageal squamous cell carcinoma cells. Ablation of c-Jun/c-Fos or ID1 expression enhanced etoposide-mediated apoptosis through increasing activity of caspase 3 and PARP cleavage. Moreover, c-Jun/c-Fos and ID1 were positively correlated in human cancers. More importantly, simultaneous high expression of ID1 and c-Jun or c-Fos was correlated with poor survival in cancer patients. Collectively, we demonstrate the importance of c-Jun/c-Fos-ID1 signaling pathway in chemoresistance of esophageal cancer cells and provide considerable insight into understanding the underlying molecular mechanisms in esophageal squamous cell carcinoma cell biology. 
 Peroxisome-proliferator-activated receptor alpha agonists inhibit cyclo-oxygenase 2 and vascular endothelial growth factor transcriptional activation in human colorectal carcinoma cells via inhibition of activator protein-1. Recent evidence indicates that PPAR (peroxisome-proliferator-activated receptor) alpha ligands possess anti-inflammatory and antitumoural properties owing to their inhibitory effects on the expression of genes that are involved in the inflammatory response. However, the precise molecular mechanisms underlying these effects are poorly understood. In the present study, we show that tumour promoter PMA-mediated induction of genes that are significantly associated with inflammation, tumour growth and   metastasis  , such as COX-2 (cyclo-oxygenase 2) and VEGF (vascular endothelial growth factor), is inhibited by PPARalpha ligands in the human colorectal carcinoma cell line SW620. PPARalpha activators LY-171883 and WY-14,643 were able to diminish transcriptional induction of COX-2 and VEGF by inhibiting AP-1 (activator protein-1)-mediated transcriptional activation induced by PMA or by c-Jun overexpression. The actions of these ligands on AP-1 activation and COX-2 and VEGF transcriptional induction were found to be dependent on PPARalpha expression. Our studies demonstrate the existence of a negative cross-talk between the PPARalpha- and AP-1-dependent signalling pathways in these cells. PPARalpha interfered with at least two steps within the pathway leading to AP-1 activation. First, PPARalpha activation impaired AP-1 binding to a consensus DNA sequence. Secondly, PPARalpha ligands inhibited c-Jun transactivating activity. Taken together, these findings provide new insight into the anti-inflammatory and anti-tumoural properties of PPARalpha activation, through the inhibition of the induction of AP-1-dependent genes that are involved in inflammation and tumour progression. 
 AP-1 Is a Key Regulator of Proinflammatory Cytokine TNFα-mediated Triple-negative Breast Cancer Progression. Triple-negative breast cancer (TNBC) represents a highly aggressive form of breast cancer with limited treatment options. Proinflammatory cytokines such as TNFα can facilitate tumor progression an  d metastas  is. However, the mechanistic aspects of inflammation mediated TNBC progression remain unclear. Using ChIP-seq, we demonstrate that the cistrome for the AP-1 transcription factor c-Jun is comprised of 13,800 binding regions in TNFα-stimulated TNBC cells. In addition, we show that c-Jun regulates nearly a third of the TNFα-regulated transcriptome. Interestingly, high expression level of the c-Jun-regulated pro-invasion gene program is associated with poor clinical outcome in TNBCs. We further demonstrate that c-Jun drives TNFα-mediated increase of malignant characteristics of TNBC cells by transcriptional regulation of Ninj1. As exemplified by the CXC chemokine genes clustered on chromosome 4, we demonstrate that NF-κB might be a pioneer factor required for the regulation of TNFα-inducible inflammatory genes, whereas c-Jun has little effect. Together, our results uncover AP-1 as an important determinant for inflammation-induced cancer progression, rather than inflammatory response. 
 YB-1 represses AP1-dependent gene transactivation and interacts with an AP-1 DNA sequence. Involvement of the AP-1 (activator protein-1) transcription factor has been demonstrated previously in the regulation of cell proliferation and cell-cycle progression, in the control of cell migration, invasion and   metastasis  , and in signal transduction, stress responsiveness, DNA replication and DNA repair. YB-1 (Y-box-binding protein-1) has also been implicated in many of these processes. However, the mechanism by which YB-1 mediates these processes is poorly understood. In the present study, we report that overexpression of a transfected gene encoding YB-1 in human HeLa cervical carcinoma cells significantly represses the transactivation of a minimal AP-1 reporter construct in response to the tumour promoter PMA. YB-1 also represses mRNA expression and PMA-induced promoter transactivation of the endogenous AP-1 target gene encoding matrix metalloproteinase-12 (metalloelastase). YB-1 transrepression of both the minimal and matrix metalloproteinase-12 promoter reporter constructs is dependent on the AP-1 sequence. To identify new nuclear proteins that bind specifically to the AP-1 DNA-binding site, we devised a DNA-affinity-chromatography-based assay termed NAPSTER (nucleotide-affinity preincubation specificity test of recognition) and discovered a 49 kDa protein from human cancer cells that binds in a sequence-specific manner to the AP-1 DNA sequence. By tandem MS fragmentation sequencing analyses we determined that p49 is a YB-1. Immunoblotting of the NAPSTER-purified p49 protein using anti-YB-1 antibodies confirmed YB-1 binding to the AP-1 DNA sequence, as did gel mobility-supershift assays using YB-1 antibodies. This is the first report of YB-1 transrepression and interaction at the AP-1 DNA-binding site. 
 Phosphorylated c-Jun and Fra-1 induce matrix metalloproteinase-1 and thereby regulate invasion activity of 143B osteosarcoma cells. Osteosarcoma is the most common primary malignancy of bone and patients often develop pulmonary metastases. Despite the advances in surgical and medical management, the mechanisms underlying human osteosarcoma progression and   metastasis   remain to be elucidated. Gene expression profiles were compared by the cDNA microarray technique between two different human osteosarcoma sublines, MNNG/HOS and 143B, which differ greatly in spontaneous pulmonary metastatic potential. Here we report an enhanced expression of matrix metalloproteinase (MMP)-1 in the highly metastatic human osteosarcoma cell line 143B. Moreover, the in vitro invasion activity of 143B cells was MMP-1-dependent. The activator protein (AP)-1 binding site in the MMP-1 gene promoter was required for the constitutive expression of MMP-1 in 143B cells. Two AP-1 components, c-Jun and Fra-1, were phosphorylated, and bound to the AP-1 binding site of the MMP-1 promoter in 143B cells. Activated c-Jun and Fra-1 were essential for MMP-1 gene expression in 143B cells. Mitogen-activated protein kinase pathways including the c-Jun NH(2)-terminal kinase and the extracellular signal-regulated kinase activate c-Jun and Fra-1 and thereby regulate c-Jun/Fra-1 mediated events, establishing the mitogen-activated protein kinase/AP-1/MMP-1 axis as important in 143B cells. These data suggest that MMP-1 plays a central role in osteosarcoma invasion. Accordingly, MMP-1 might be a biomarker and therapeutic target for invasive osteosarcomas and pulmonary metastases. 
 EGF-R is expressed and AP-1 and NF-kappaB are activated in stromal myofibroblasts surrounding colon adenocarcinomas paralleling expression of COX-2 and VEGF. COX-2 and VEGF are important triggers of colon cancer growth,   metastasis   and angiogenesis. Cox-2 promoter contains transcriptional regulatory elements for AP-1 and NF-kappaB transcription factors whilst vegf is a known AP-1 downstream target gene. We investigated whether stromal myofibroblasts surrounding colon adenocarcinomas express COX-2 and VEGF and whether activation of AP-1 and NF-kappaB, as well as expression of EGF-R parallel expression of COX-2 and VEGF in these cells.Immunohistochemical methodology was performed on archival sections from 40 patients with colon adenocarcinomas. We evaluated c-FOS, p-c-JUN (phosphorylated c-JUN), p-IkappaB-alpha (phosphorylated IkappaB-alpha), EGF-R, COX-2, NF-kappaB and VEGF expression in stromal myofibroblasts surrounding colon adenocarcinomas. Double immunostaining with a-smooth muscle actin and each antibody was done to verify the expression of these molecules in stromal myofibroblasts.VEGF, p-IkappaB-alpha, NF-kappaB, c-FOS, p-c-JUN, EGF-R and COX-2 were expressed in stromal myofibroblasts surrounding colon adenocarcinomas in the majority of cases. EGF-R, p-IkappaB-alpha, NF-kappaB, c-FOS and p-c-JUN correlated positively with COX-2 and VEGF expression.Stromal myofibroblasts surrounding colon adenocarcinomas are an important source of VEGF and COX-2 production, while AP-1 and NF-kappaB transcription factors are activated and EGF-R is expressed in these cells and associated with COX-2 and VEGF production. 
 miR200c attenuates P-gp-mediated MDR and   metastasis   by targeting JNK2/c-Jun signaling pathway in colorectal cancer. MicroRNA-200c (miR200c) recently emerged as an important regulator of tumorigenicity and cancer   metastasis  ; however, its role in regulating multidrug resistance (MDR) remains unknown. In the current study, we found that the expression levels of miR200c in recurrent and metastatic colorectal cancers were significantly lower, whereas the JNK2 expression was higher compared with primary tumors. We showed that in MDR colorectal cancer cells, miR200c targeted the 3' untranslated region of the JNK2 gene. Overexpression of miR200c attenuated the levels of p-JNK, p-c-Jun, P-gp, and MMP-2/-9, the downstream factors of the JNK signaling pathway, resulting in increased sensitivity to chemotherapeutic drugs, which was accompanied by heightened apoptosis and decreased cell invasion and migration. Moreover, in an orthotopic MDR colorectal cancer mouse model, we demonstrated that overexpression of miR200c effectively inhibited the tumor growth and   metastasis  . At last, in the tumor samples from patients with locally advanced colorectal cancer with routine postsurgical chemotherapy, we observed an inverse correlation between the levels of mRNA expression of miR200c and JNK2, ABCB1, and MMP-9, thus predicting patient therapeutic outcomes. In summary, we found that miR200c negatively regulated the expression of JNK2 gene and increased the sensitivity of MDR colorectal cancer cells to chemotherapeutic drugs, via inhibiting the JNK2/p-JNK/p-c-Jun/ABCB1 signaling. Restoration of miR200c expression in MDR colorectal cancer may serve as a promising therapeutic approach in MDR-induced   metastasis  . 
 Catecholamine up-regulates MMP-7 expression by activating AP-1 and STAT3 in gastric cancer. Stress, anxiety and depression can cause complex physiological and neuroendocrine changes, resulting in increased level of stress related hormone catecholamine, which may constitute a primary mechanism by which physiological factors impact gene expression in tumors. In the present study, we investigated the effects of catecholamine stimulation on MMP-7 expression in gastric cancer cells and elucidated the molecular mechanisms of the up-regulation of MMP-7 level by catecholamine through an adrenergic signaling pathway.Increased MMP-7 expression was identified at both mRNA and protein levels in the gastric cancer cells in response to isoproterenol stimulation. β2-AR antigonist effectively abrogated isoproterenol-induced MMP-7 expression. The activation of STAT3 and AP-1 was prominently induced by isoproterenol stimulation and AP-1 displayed a greater efficacy than STAT3 in isoproterenol-induced MMP-7 expression. Mutagenesis of three STAT3 binding sites in MMP-7 promoter failed to repress the transactivation of MMP-7 promoter and silencing STAT3 expression was not effective in preventing isoproterenol-induced MMP-7 expression. However, isoproterenol-induced MMP-7 promoter activities were completely disappeared when the AP-1 site was mutated. STAT3 and c-Jun could physically interact and bind to the AP-1 site, implicating that the interplay of both transcriptional factors on the AP-1 site is responsible for isoproterenol-stimulated MMP-7 expression in gastric cancer cells. The expression of MMP-7 in gastric cancer tissues was found to be at the site where β2-AR was overexpressed and the levels of MMP-7 and β2-AR were the highest in the metastatic locus of gastric cancer.Up-regulation of MMP-7 expression through β2-AR-mediated signaling pathway is involved in invasion   and metast  asis of gastric cancer. 
 Dimerumic acid inhibits SW620 cell invasion by attenuating H₂O₂-mediated MMP-7 expression via JNK/C-Jun and ERK/C-Fos activation in an AP-1-dependent manner. Reactive oxygen species (ROS) such as hydrogen peroxide (H₂O₂) in the tumor microenvironment play important roles in tumor invas  ion and me  tastasis. Recently, ROS have been reported to cause a significant increase in the production and expression of matrix metalloproteinase (MMP)-7, which is closely correlated with metastatic colorectal cancer. The present study was undertaken to evaluate the scavenging activity of dimerumic acid (DMA) for H₂O₂ isolated from Monascus-fermented rice to investigate the inhibitory effects of DMA on the invasive potential of SW620 human colon cancer cells, and to explore the mechanisms underlying both these phenomena. Our results showed that increased MMP-7 expression due to H₂O₂ exposure was mediated by activation of mitogen-activated protein kinases (MAPKs) such as Jun N-terminal kinase (JNK), extracellular-regulated kinase (ERK), and p38 kinase. DMA pretreatment suppressed activation of H₂O₂-mediated MAPK pathways and cell invasion. Moreover, H₂O₂-triggered MMP-7 production was demonstrated via JNK/c-Jun and ERK/c-Fos activation in an activating protein 1 (AP-1)-dependent manner. Taken together, these results suggest that DMA suppresses H₂O₂-induced cell invasion by inhibiting AP-1-mediated MMP-7 gene transcription via the JNK/c-Jun and ERK/c-Fos signaling pathways in SW620 human colon cancer cells. Our data suggest that DMA may be useful in minimizing th  e developm  ent of colorectal metastasis. In the future, DMA supplementation may be a beneficial antioxidant to enhance surgical outcomes. 
 Combination analysis of activator protein-1 family members, Sp1 and an activator protein-2alpha-related factor binding to different regions of the urokinase receptor gene in resected colorectal cancers. Studies on the transactivation of genes via promoter elements have mostly been done on cell lines rather than resected tissues. This, however, is essential to address an in vivo or clinical relevance. We have previously shown tumor-specific binding of Sp1 and an activator protein (AP)-2-related factor to promoter region -152/-135 of the   metastasis  -related u-PAR gene in 60% of in vivo-resected cancer tissues. Cell lines have implicated an additional role, and potential synergism, of an AP-1 region (-190/-171) in u-PAR regulation. This study was done to (a) analyze AP-1 binding to this region in resected tumor and normal tissues, and define subgroups in which it is tumor-specific, and (b) to analyze transcription factor-binding patterns to both promoter motifs in resected tissues, supporting synergism, and draw first prognostic conclusions.In 103 patients with colorectal cancer, electrophoretic mobility shift assay/supershift analysis for u-PAR promoter region -190/-171 was done in tumors and normal tissues. In 71 patients, region -152/-135 was also analyzed. U-PAR protein was measured by ELISA.Tumor-specific AP-1 binding to region -190/-171 of the u-PAR promoter was found in 40% of patients. Subgroup analysis showed tumor-specific binding for c-Fos in 58%, for c-Jun in 50%, for JunD in 39%, and for Fra-1 in 4% of cases. AP-1 binding correlated significantly with u-PAR protein amounts in both normal and tumor tissues (P<0.001), in contrast to a tumor-specific correlation with u-PAR of the AP-2/Sp1 region. In analyses for both promoter regions, 62% of cancers showed simultaneous binding for AP-1, AP-2, and Sp1, 11% for AP-1 and AP-2, 16% for AP-2 and Sp1, 4% for AP-2 only, 3% for AP-1 only, and 0% for Sp1 only. The binding of AP-1, AP-2, and Sp1 correlated significantly with each other (P<0.001), the combination of AP-1 and AP-2 showing the highest correlation with u-PAR (P=0.008). Preliminary survival analysis indicated a trend for poorer prognosis for binding of all three transcription factors.This is the first study differentiating transcription factor-binding to two important u-PAR promoter regions in a large series of resected tumors and normal tissues. The AP-1 site seems to be a less tumor-specific regulator than the Sp1/AP-2 motif. Nevertheless, data corroborate the hypothesis of synergism between both elements in resected tumors. 
 Galectin-3 modulates MUC2 mucin expression in human colon cancer cells at the level of transcription via AP-1 activation. Galectin-3 and MUC2 intestinal mucin each have been correlated with the malignant behavior of colon cancer cells. Galectin-3 modulates expression of MUC2 protein, but the specific regulatory mechanisms are unknown. This study sought to determine how galectin-3 increases MUC2 expression.Galectin-3 levels in human colon cancer cells of high and low metastatic ability were manipulated via expression of galectin-3 complementary DNA in sense or antisense orientation. Galectin-3 and MUC2 protein expression were determined by Western analysis and immunocytochemistry. Transient transfections of promoter reporter constructs were used to monitor MUC2 transcription and AP-1 activity. Electrophoretic mobility shift assays, site-directed mutagenesis, and chromatin immunoprecipitation were used to monitor the participation of AP-1 in MUC2 transcription.Alterations in galectin-3 levels correlated with both MUC2 protein expression and transcription. By using MUC2 promoter constructs of different lengths, galectin-3 responsiveness was found between 1500 and 2186 bp upstream of the translation start site, a region that contains 1 consensus AP-1 binding site. AP-1 activity paralleled MUC2 transcription in the different cell lines. Mutation in the AP-1 site markedly decreased MUC2 promoter activity, and MUC2 transcription was inhibited by cotransfection with a dominant-negative AP-1 vector. Electrophoretic mobility shift assays, co-immunoprecipitation, and chromatin immunoprecipitation analyses suggested an association between galectin-3, c-Jun, and Fra-1 in forming a complex at the AP-1 site on the MUC2 promoter.Galectin-3 up-regulation of MUC2 transcription occurs at the level of transcription through AP-1 activation. This may have important implications for understanding the role of galectin-3 and MUC2 in colon cancer   metastasis  . 
 SH3GL1 inhibition reverses multidrug resistance in colorectal cancer cells by downregulation of MDR1/P-glycoprotein via EGFR/ERK/AP-1 pathway. Multidrug resistance is one of the major reasons colorectal cancer (CRC) chemotherapy-based treatments fail, and novel biologically based therapies are urgently needed. Src homology 3 (SH3)-domain GRB2-like protein 1 (SH3GL1) is a membrane-bound protein which was found to be involved in tumor formation, progression, and   metastasis  . In this study, immunohistochemistry staining, quantitative real-time polymerase chain reaction (qRT-PCR), and western blot analysis revealed a high expression of SH3GL1 in human CRC tumor specimens and several CRC cells resistant to chemotherapeutics. Cell Counting Kit-8 (CCK-8) assay showed that transfection of pCDNA3.1(+)-SH3GL1 increased while transfection of SH3GL1 siRNA decreased cell viability in response to 5-fluorouracil (5-FU) treatment (P < 0.05). Further studies indicated that transfection of SH3GL1 siRNA significantly downregulated multidrug resistance protein 1 (MDR1)/P-glycoprotein expression (P < 0.05), decreased MDR1 promoter activity and activator protein-1 (AP-1) binding activity (P < 0.05), and inhibited the activation of epidermal growth factor receptor (EGFR) and extracellular signal-regulated kinases 1/2 (ERK1/2) signaling (P < 0.05) in CRC cells resistant to chemotherapeutics. Transfection of pCDNA3.1(+)-SH3GL1 caused the opposite effect. Additionally, pre-treatment with either EGFR kinase inhibitor PD153035 or ERK1/2 kinase inhibitor PD98059 in HCT116/5-FU cells partly inhibits P-glycoprotein expression and AP-1 binding activity (P < 0.05). In conclusion, we confirmed that inhibition of SH3GL1 reverses multidrug resistance through declining P-glycoprotein expression via the EGFR/ERK/AP-1 pathway. 
 Helicobacter pylori promotes invasion and   metastasis   of gastric cancer cells through activation of AP-1 and up-regulation of CACUL1. Infection with Helicobacter pylori is important in the development and progression of gastric cancer. However, the mechanisms that regulate this activation in gastric tumors remain elusive. CACUL1 has been cloned and identified as a novel gene that is expressed in many types of cancer and is involved in cell cycle regulation and tumor growth. The current study aimed to examine the expression of CACUL1 in gastric cancer samples and analyze its correlation with H. pylori infection. We found that CACUL1 was highly expressed in gastric cancer tissues and negatively correlated with gastric cancer differentiation and TNM stage. In addition, CACUL1 expression was high in H. pylori-infected tissues compared with H. pylori non-infected tissue. We found that H. pylori could up-regulate CACUL1 expression through activating protein 1. The up-regulation of CACUL1 expression could promote matrix metalloproteinase 9 and Slug expression to increase invasion and   metastasis   of tumor cells. These results suggested that H. pylori-triggered CACUL1 production occurred in an activating protein 1-dependent manner and regulated matrix metalloproteinase 9 and Slug expression to affect the invasion and   metastasis   of tumor cells. Therefore, CACUL1 is a potential therapeutic target for the treatment of aggressive gastric cancer. 
 Ascochlorin inhibits matrix metalloproteinase-9 expression by suppressing activator protein-1-mediated gene expression through the ERK1/2 signaling pathway: inhibitory effects of ascochlorin on the invasion of renal carcinoma cells. The expression of matrix metalloproteinases (MMPs) has been implicated in the invasion and   metastasis   of cancer cells. Here we examined the effect of ascochlorin, a prenyl-phenol anti-tumor compound from the fungus Ascochyta viciae, on the regulation of signaling pathways that control MMP-9 expression in human renal carcinoma (Caki-1) cells. Ascochlorin reduced the invasive activity of Caki-1 cells and inhibited phorbol 12-myristate 13-acetate-induced increases in MMP-9 expression and activity in a dose-dependent manner. Reporter gene, electrophoretic mobility shift, kinase inhibitor assays, and in vitro kinase assay showed that ascochlorin inhibits MMP-9 gene expression by suppressing activation of the nuclear transcription factor activator protein-1 (AP-1) via the extracellular signal-regulated kinase 1 and 2 pathway. The AP-1 family member most specifically affected by ascochlorin was Fra-1. Ascochlorin did not affect the activation of the c-Jun N-terminal or p38 kinase pathways. Moreover, transfection of Caki-1 cells with AP-1 decoy oligodeoxynucleotides resulted in the suppression of phorbol 12-myristate 13-acetate-induced MMP-9 expression and invasion. In conclusion, ascochlorin represents a unique natural anti-tumor compound that specifically inhibits MMP-9 activity through suppression of AP-1-dependent induction of MMP-9 gene expression. 
 miR-23a inhibits E-cadherin expression and is regulated by AP-1 and NFAT4 complex during Fas-induced EMT in gastrointestinal cancer. Fas signaling has been shown to induce the epithelial-mesenchymal transition (EMT) to promote gastrointestinal (GI) cancer   metastasis  , but the involvement of microRNA in this mechanism remains unknown. We found that Fas ligand (FasL) treatment inhibited E-cadherin expression and promoted cell invasion by upregulation of miR-23a, but overexpression of the miR-23a inhibitor could partially block this activity. FasL-induced extracellular signal-regulated kinase/mitogen-activated protein kinase signaling activated the activator protein 1 (AP-1) complex and repressed glycogen synthase kinase-3β activity, which contributed to nuclear translocation of AP-1 and nuclear factor of activated T cells (NFAT4). Nuclear accumulation and interaction of AP-1 and NFAT4 and subsequent binding to the miR-23a promoter led to increased miR-23a expression. Inhibition of Fas signaling by downregulation of the Fas receptor led to a decrease in miR-23a expression and cell invasion ability in vivo and in vitro, as well as an increase in E-cadherin. Evaluation of human GI precancerous and cancer specimens showed that the expression of FasL and miR-23a increased, whereas the expression of E-cadherin decreased during GI cancer progression. A significant correlation was noted between any two of these three molecules. An EMT phenotype was shown to correlate with an advanced cancer stage and worse prognosis. Taken together, our results show that miR-23a participates in the mechanism of the FasL-induced EMT process and may serve as a potential therapeutic target for cancer   metastasi  s. 
 Simultaneous blockade of AP-1 and phosphatidylinositol 3-kinase pathway in non-small cell lung cancer cells. c-Jun is a major constituent of AP-1 transcription factor that transduces multiple mitogen growth signals, and it is frequently overexpressed in non-small cell lung cancers (NSCLCs). Earlier, we showed that blocking AP-1 by the overexpression of a c-Jun dominant-negative mutant, TAM67, inhibited NSCLC cell growth. The phosphatidylinositol 3-kinase (PI3K)/Akt signal transduction pathway is important in transformation, proliferation, survival and   metastasis   of NSCLC cells. In this study, we used NCI-H1299 Tet-on clone cells that express TAM67 under the control of inducible promoter to determine the effects of inhibition of AP-1 and PI3K on cell growth. The PI3K inhibitor, LY294002, produced a dose-dependent inhibition of growth in H1299 cells and that inhibition was enhanced by TAM67. TAM67 increased dephosphorylation of Akt induced by LY294002 and reduced the TPA response element DNA-binding of phosphorylated c-Jun. TAM67 increased G1 cell cycle blockade induced by LY294002, which was partially associated with cyclin A decrease and p27(Kip1) accumulation. Furthermore, TAM67 and LY294002 act, at least additively, to inhibit anchorage-independent growth of the H1299 cells. These results suggest that AP-1 and PI3K/Akt pathways play an essential role in the growth of some NSCLC cells. 
 The role of sLZIP in transcriptional regulation of c-Jun and involvement in migration and invasion of cervical cancer cells. Matrix metalloproteinase-9 (MMP-9) plays an important role in tumor invasion and   metastasis   through the breakdown of extracellular matrix. The c-Jun protein, a major component of the AP-1 transcription factor, is elevated in various cancers. Small leucine zipper protein (sLZIP) is a member of the leucine zipper transcription factor family. Although sLZIP is known to be involved in cancer cell migration and invasion, its biological roles in cancer development and the cellular target genes are not fully understood. In this study, we investigated the role of sLZIP in c-Jun expression, and their effects on expression of MMP-9 and migration of cervical cancer cells.sLZIP up-regulates transcription of c-Jun by binding directly to the CRE region in the c-Jun promoter. Elevated c-Jun due to sLZIP leads to activation of MMP-9 transcription by interaction with the AP-1 binding site in the MMP-9 promoter. c-Jun siRNA repressed migration and invasion of cervical cancer cells, whereas sLZIP recovered migration and invasion of cells transfected with c-Jun siRNA. Immunohistochemical analysis results revealed a significant correlation between the expressions of sLZIP and MMP-9 in clinical cervical specimens.These results indicate that sLZIP plays a role in expression of c-Jun, and migration and invasion of cervical cancer cells via regulation of MMP-9 transcription. 
 ERK signaling regulates tumor promoter induced c-Jun recruitment at the Fra-1 promoter. Fra-1 as an integral part of AP-1 (Jun/Fos) drives transcriptional programs involved in several physiologic and pathologic processes. It is also critical for tumor cell motility and   metastasis  . We have previously shown that two critical elements of Fra-1 promoter, the upstream TPA response element (TRE) and the serum response element (SRE), are necessary for its induction in response to phorbol esters in human pulmonary epithelial cell lines. Here, we have investigated the roles of various MAP kinases in regulating Fra-1 expression in response to TPA. Using pharmacologic and genetic tools, we demonstrate a prominent role for ERK1/2, but not JNK1/2 and p38, signaling in the TPA-induced activation of specific transcription factors that bind to the AP1 site and the SRE. Inhibition of ERK1/2 pathway suppresses Elk1 activation, and c-Jun and Fra-2 recruitment to the promoter. 
 Critical role of c-Jun overexpression in liver   metastasis   of human breast cancer xenograft model. c-Jun/AP-1 has been linked to invasive properties of aggressive breast cancer. Recently, it has been reported that overexpression of c-Jun in breast cancer cell line MCF-7 resulted in increased AP-1 activity, motility and invasiveness of the cells in vitro and tumor formation in nude mice. However, the role of c-Jun in   metastasis   of human breast cancer in vivo is currently unknown.To further investigate the direct involvement of c-Jun in tumorigenesis and   metastasis  , in the present study, the effects of c-Jun overexpression were studied in both in vitro and in nude mice.Ectopic overexpression of c-Jun promoted the growth of MCF-7 cells and resulted in a significant increase in the percentage of cells in S phase and increased motility and invasiveness. Introduction of c-Jun gene alone into weakly invasive MCF-7 cells resulted in the transfected cells capable of metastasizing to the nude mouse liver following tail vein injection.The present study confirms that overexpression of c-Jun contributes to a more invasive phenotype in MCF-7 cells. It indicates an interesting relationship between c-Jun expression and increased property of adhesion, migration and in vivo liver   metastasis   of MCF-7/c-Jun cells. The results provide further evidence that c-Jun is involved in the   metastasis   of breast cancer. The finding also opens an opportunity for development of anti-c-Jun strategies in breast cancer therapy. 
 (-)-Epigallocatechin-3-gallate blocks nicotine-induced matrix metalloproteinase-9 expression and invasiveness via suppression of NF-κB and AP-1 in endothelial cells. Cigarette smoke, specifically the nicotine contained within, has been shown to correlate closely with cell invasion and strategies to downregulate their expression may ultimately be of clinical utility. Matrix metalloproteinase-9 (MMP-9) is critically involved in the cell invasion and   metastasi  s processes. Since nicotine plays a crucial role in the regulation of MMP-9 expression, the investigation of plant-derived compounds capable of modulating nicotine-induced signaling is an issue of concern. In this study, the effects of (-)-epigallocatechin-3-gallate (EGCG), a major green tea catechin, on nicotine-induced cell invasion and MMP-9 activity in ECV304 human endothelial cells were examined. EGCG treatment was found to reduce the MMP-9 expression and transcriptional activity in a dose-dependent manner. EGCG inhibited nicotine-activated production of reactive oxygen species (ROS), which are known as important signaling molecules to activate MMP-9. To further study the mechanisms for the EGCG-mediated regulation of MMP-9, the transcription factors NF-κB and AP-1 activities were examined. EGCG suppressed the nicotine-induced NF-κB and AP-1 activation. Studies with expression vectors encoding mutated NF-κB signaling molecules and AP-1 decoy confirmed that NF-κB and AP-1 were essential for the nicotine-stimulated MMP-9 expression. EGCG also abrogated the nicotine-induced activation of AP-1 subunits c-fos and c-jun. The above studies demonstrate that EGCG may exert at least part of its anti-invasive effect in ECV304 human endothelial cells by controlling MMP-9 expression through the suppression of ROS, NF-κB and AP-1. 
 Cell specific CD44 expression in breast cancer requires the interaction of AP-1 and NFκB with a novel cis-element. Breast cancers contain a heterogeneous population of cells with a small percentage that possess properties similar to those found in stem cells. One of the widely accepted markers of breast cancer stem cells (BCSCs) is the cell surface marker CD44. As a glycoprotein, CD44 is involved in many cellular processes including cell adhesion, migration and proliferation, making it pro-oncogenic by nature. CD44 expression is highly up-regulated in BCSCs, and has been implicated in tumorigenesis and   metastasi  s. However, the genetic mechanism that leads to a high level of CD44 expression in breast cancer cells and BCSCs is not well understood. Here, we identify a novel cis-element of the CD44 directs gene expression in breast cancer cells in a cell type specific manner. We have further identified key trans-acting factor binding sites and nuclear factors AP-1 and NFκB that are involved in the regulation of cell-specific CD44 expression. These findings provide new insight into the complex regulatory mechanism of CD44 expression, which may help identify more effective therapeutic targets against the breast cancer stem cells and metastatic tumors. 
 COP1 and GSK3β cooperate to promote c-Jun degradation and inhibit breast cancer cell tumorigenesis. High abundance of c-Jun is detected in invasive breast cancer cells and aggressive breast tumor malignancies. Here, we demonstrate that a major cause of high c-Jun abundance in invasive breast cancer cells is prolonged c-Jun protein stability owing to poor poly-ubiquitination of c-Jun. Among the known c-Jun-targeting E3 ligases, we identified constitutive photomorphogenesis protein 1 (COP1) as an E3 ligase responsible for c-Jun degradation in less invasive breast cancer cells because depletion of COP1 reduced c-Jun poly-ubiquitination leading to the stabilization of c-Jun protein. In a panel of breast cancer cell lines, we observed an inverse association between the levels of COP1 and c-Jun. However, overexpressing COP1 alone was unable to decrease c-Jun level in invasive breast cancer cells, indicating that efficient c-Jun protein degradation necessitates an additional event. Indeed, we found that glycogen synthase kinase 3 (GSK3) inhibitors elevated c-Jun abundance in less invasive breast cancer cells and that GSK3β nonphosphorylable c-Jun-T239A mutant displayed greater protein stability and poorer poly-ubiquitination compared to the wild-type c-Jun. The ability of simultaneously enforced expression of COP1 and constitutively active GSK3β to decrease c-Jun abundance in invasive breast cancer cells allowed us to conclude that c-Jun is negatively regulated through the coordinated action of COP1 and GSK3β. Importantly, co-expressing COP1 and active GSK3β blocked in vitro cell growth/migration and in   vivo metas  tasis of invasive breast cancer cells. Gene expression profiling of breast tumor specimens further revealed that higher COP1 expression correlated with better recurrence-free survival. Our study supports the notion that COP1 is a suppressor of breast cancer progression. 
 c-Jun Is critical for the progression of osteosarcoma: proof in an orthotopic spontaneously metastasizing model. The oncogene c-Jun has been found to be up-regulated in a variety of cancers including osteosarcoma. DNA enzymes (DNAzymes) are oligonucleotides capable of specific catalysis of target mRNA. A c-Jun DNAzyme inhibited the growth and   metastasis   of osteosarcoma in an orthotopic spontaneously metastasizing model of the disease. c-Jun down-regulation-mediated apoptosis in osteosarcoma cells involved caspase-1, caspase-2, and caspase-8, but not the Fas/FasL pathway. Clinically, knockdown of c-Jun with DNAzymes may proffer an improved treatment outcome for these tumors originating in bone. 
 PMA-induced up-regulation of TBX3 is mediated by AP-1 and contributes to breast cancer cell migration. The T-box transcription factor TBX3 provides an important link between embryonic development and cancer. TBX3 mediates limb, mammary gland and heart development and, in humans, mutations resulting in haplo-insufficiency of TBX3 lead to ulnar-mammary syndrome. Importantly, the de-regulation of TBX3 gene expression has been linked to several cancers, where it acts to suppress senescence and promotes proliferation and tumour invasion. Despite the negative impact of de-regulated TBX3 expression as seen by developmental defects and cancer, surprisingly little is known about the regulation of the TBX3 gene. In the present paper, we show that the phorbol ester PMA increases TBX3 protein and mRNA levels in a protein kinase C-dependent manner via the AP-1 (activator protein 1) transcription factors c-Jun and JunB. Furthermore, these AP-1 factors are shown to mediate the activation of the TBX3 gene by binding a non-consensus PMA-response element in the TBX3 promoter in vitro and in vivo. We also demonstrate that TBX3 contributes to the PMA-induced migration previously observed for the MCF-7 breast epithelium cancer cell line. Our present results reveal a previously unidentified pathway that up-regulates TBX3 expression and provides additional evidence that increased levels of TBX3 contribute to   metastasis  . 
 Prognostic value of HMGA2, CDK4, and JUN amplification in well-differentiated and dedifferentiated liposarcomas. HMGA2, CDK4, and JUN genes have been described as frequently coamplified with MDM2 in atypical lipomatous tumor, well-differentiated liposarcoma, and dedifferentiated liposarcoma. We studied the frequency of amplification of these genes in a series of 48 dedifferentiated liposarcomas and 68 atypical lipomatous tumors/well-differentiated liposarcomas. We correlated their amplification status with clinicopathological features and outcomes. Histologically, both CDK4 (P=0.007) and JUN (P=0.005) amplifications were associated with dedifferentiated liposarcoma, whereas amplification of the proximal parts of HMGA2 (5'-untranslated region (UTR) and exons 1-3) was associated with atypical lipomatous tumor/well-differentiated liposarcoma (P=0.01). CDK4 amplification was associated with axial tumors. Amplification of 5'-UTR and exons 1-3 of HMGA2 was associated with primary status and grade 1. Shorter overall survival was correlated with: age >64 years (P=0.03), chemotherapy used in first intent (P<0.001), no surgery (P=0.003), grade 3 (P<0.001), distant   metastasis   (P<0.001), node involvement (P=0.006), and CDK4 amplification (P=0.07). In multivariate analysis, distant   metastasis   (HR=8.8) and grade 3 (HR=18.2) were associated with shorter overall survival. A shorter recurrence-free survival was associated with dedifferentiated liposarcoma (P<0.001), grade 3 (P<0.001), node involvement (P<0.001), distant   metastasis   (P=0.02), recurrent status (P=0.009), axial location (P=0.001), and with molecular features such as CDK4 (P=0.05) and JUN amplification (P=0.07). Amplification of 5'-UTR and exons 1-3 (P=0.08) and 3'-UTR (P=0.01) of HMGA2 were associated with longer recurrence-free survival. Distant   metastasis   was associated with shorter recurrence-free survival (HR=5.8) in multivariate analysis. Dedifferentiated liposarcoma type was associated with axial location, grade 3 and recurrent status. In conclusion, we showed that the amplification of HMGA2 was associated with the atypical lipomatous tumor/well-differentiated liposarcoma histological type and a good prognosis, whereas CDK4 and JUN amplifications were associated with dedifferentiated liposarcoma histology and a bad prognosis. In addition, we also provided the first description of the molecular evolution of a well-differentiated liposarcoma into four successive dedifferentiated liposarcoma relapses, which was consistent with our general observations. 
 Cross-talk between different enhancer elements during mitogenic induction of the human stromelysin-1 gene. Platelet-derived growth factor (PDGF) induces the expression of human stromelysin-1, a matrix metalloproteinase involved in tumor invasion and   metastasis  . Here it is shown that stromelysin-1 gene induction by PDGF depends on Ras and involves three previously identified promoter elements (the stromelysin-1 PDGF-responsive element (SPRE) site, the two head-to-head polyomavirus enhancer A-binding protein-3 (PEA3) sites, and the activator protein-1 (AP-1) binding site). During mitogenic induction, these responsive elements appear to be organized in two independent transcriptional units, SPRE-AP-1 and PEA3-AP-1, which result from specific element cross-talking. Interestingly, expression of a dominant negative mutant of Raf-1 significantly interfered with the induction through PEA3-AP-1 but not with that operating through SPRE-AP-1. Conversely, only the induction operating through SPRE-AP-1 was affected significantly by the expression of a dominant negative mutant of the atypical lambda/iota protein kinase C (lambda/iotaPKC). These data strongly suggest that the signal triggered by PDGF flows through Ras and bifurcates toward two distinct pathways, one operating through Raf and involving PEA3-AP-1 and the other one Raf-independent, operating through lambda/iotaPKC and SPRE-AP-1. Furthermore, we present evidence suggesting that the novel SPRE-binding transcription factor SPBP cross-couples with c-Jun to transactivate the SPRE site. 
  GeneRIFs  
 We found that c-jun or c-fos was significantly associated with lymph node   metastasis  , and coexpression of c-jun/c-fos, or c-jun/c-fos/p53 were significantly associated with lymph node   metastasis  , poor differentiation and clinical stage. 
 Taken together, our data suggest that TNF-alpha can promote lymphangiogenesis and lymphatic   metastasis   of GBC through the ERK1/2/AP-1/VEGF-D pathway. 
 Palbociclib inhibits epithelial-mesenchymal transition and   metastasis   in breast cancer via c-Jun/COX-2 signaling pathway. 
 miR200c attenuates P-gp-mediated MDR and   metastasis   by targeting JNK2/c-Jun signaling pathway in colorectal cancer. 
 H. pylori-triggered CACUL1 production occurred in an activating protein 1-dependent manner and regulated matrix metalloproteinase 9 and Slug expression to affect the invasion and   metastasis   of tumor cells. 
 overexpression of c-Jun is associated with invasiveness and   metastasis   in breast cancer 
 The present study confirms that overexpression of c-Jun contributes to   metastasis   of breast cancer. 
  [Cc]ell [Ii]nvasion  PubMed  
 Activation of two distinct MAPK pathways governs constitutive expression of matrix metalloproteinase-1 in human pancreatic cancer cell lines. Elevated matrix metalloproteinase-1 (MMP-1) expression is known to correlate with poor prognosis of pancreatic cancer. We investigated the molecular mechanisms of constitutive expression of MMP-1 in pancreatic cancer cell lines. Expression of MMP-1 mRNA and protein as well as its enzymatic activity were observed in three pancreatic cancer cell lines. Transient transfection assays of two MMP-1 promoter/luciferase constructs (full-length 4.4-kb or proximal 0.6-kb region) showed high levels of transcription in pancreatic cancer cells compared with non-MMP-1 producing cells. The 0.6-kb promoter region of MMP-1 gene contained three activator protein-1 (AP-1) sites and the strong AP-1 activity was detected by electrophoretic mobility shift assays (EMSAs). In these cells, production and phosphorylation of c-Jun were commonly observed. Phosphorylated c-Jun NH2-terminal kinase (p-JNK) and activator transcription factor-2 (p-ATF-2) were also detected in two of the three cell lines. Phosphorylated extracellular signal-regulated kinase (p-ERK) was observed in one. The promoter activity, AP-1-binding activity and MMP-1 production were suppressed by a specific inhibitor of JNK or MEK. K-ras mutation, reported to be present in three cell lines used, is known to activate JNK and ERK pathways. Considering the facts together, our results revealed that activation of JNK/AP-1 or ERK/AP-1 pathway plays crucial roles in constitutive transactivation of MMP-1 in these cancer cells. This study contributes to provide new insights into strategies for inhibiting tumor   cell invasion   in pancreatic cancer. 
 Astrocyte elevated gene-1 (AEG-1) promotes osteosarcoma   cell invasion   through the JNK/c-Jun/MMP-2 pathway. Osteosarcoma is the most common primary malignant bone tumour in children and adolescents and is characterised by high malignant and metastatic potentials. However, the molecular mechanism underlying this invasiveness remains unclear. In this study, we determined that PD98059 and SP600125, the two mitogen-activated protein kinase (MAPK) family inhibitors, decreased the osteosarcoma cell U2OS-AEG-1 migration and invasion that was enhanced by astrocyte elevated gene-1 (AEG-1) in an in vitro wound-healing and Matrigel invasion assay independently of cell viability. These findings indicate that AEG-1 promoted osteosarcoma   cell invasion   is relevant to the MAPK pathways. The up-regulation of AEG-1 increased the levels of phosphor-c-Jun N-terminal kinase (JNK) and phosphor-c-Jun; however, there were no marked changes in the levels of phosphor-extracellular regulated kinase (ERK) 1/2 or phosphor-c-Fos due to the activation of AEG-1 in U2OS. SP600125 (a JNK inhibitor) decreased phosphor-c-Jun and MMP-2 in U2OS-AEG-1, while PD98059 (a ERK1/2 inhibitor) had no influence on the levels of phosphor-c-Jun or MMP-2 in U2OS-AEG-1. Further study revealed that the down-regulation of phosphor-c-Jun not only obviously decreased the MMP-2 protein level and the MMP-2 transcriptional activity that were up-regulated by AEG-1 in Western-blot and luciferase reporter assays, but also inhibited the migration and invasion abilities of the U2OS-AEG-1 cells, which suggests that AEG-1 mediated U2OS invasion at least partially via the JNK/c-Jun/MMP-2 pathway. Consistent with these observations, immunohistochemical (IHC) staining revealed that AEG-1 expression was associated with the protein levels of phosphor-c-Jun and MMP-2 in needle biopsy paraffin-embedded archival human osteosarcoma tissues. Taken together, our findings suggest that AEG-1 plays a crucial role in the aggressiveness of osteosarcoma via the JNK/c-Jun/MMP-2 pathway. 
 Decoding the regulatory landscape of melanoma reveals TEADS as regulators of the invasive cell state. Transcriptional reprogramming of proliferative melanoma cells into a phenotypically distinct invasive cell subpopulation is a critical event at the origin of metastatic spreading. Here we generate transcriptome, open chromatin and histone modification maps of melanoma cultures; and integrate this data with existing transcriptome and DNA methylation profiles from tumour biopsies to gain insight into the mechanisms underlying this key reprogramming event. This shows thousands of genomic regulatory regions underlying the proliferative and invasive states, identifying SOX10/MITF and AP-1/TEAD as regulators, respectively. Knockdown of TEADs shows a previously unrecognized role in the invasive gene network and establishes a causative link between these transcription factors,   cell invasion   and sensitivity to MAPK inhibitors. Using regulatory landscapes and in silico analysis, we show that transcriptional reprogramming underlies the distinct cellular states present in melanoma. Furthermore, it reveals an essential role for the TEADs, linking it to clinically relevant mechanisms such as invasion and resistance. 
 Twist1 and AP-1 cooperatively upregulate integrin α5 expression to induce invasion and the epithelial-mesenchymal transition. Epithelial-mesenchymal transition (EMT) is an important process implicated in tumor invasion and metastasis. Twist1 is a transcription factor that induces EMT, including E-cadherin suppression and cancer cell migration and invasion; hence it promotes cancer metastasis. Twist1 directly or indirectly regulates the expression of various genes and cellular functions involved in cancer progression. However, the underlying mechanisms remain largely unknown. In this study, we investigated the molecular basis for Twist1-mediated invasion and EMT. In human cancer cells, Twist1 was found to directly upregulate transcription of the mesenchymal gene integrin α5 in an E-box-independent, but activating protein-1 (AP-1) element-dependent, manner. Twist1 activated the integrin α5 promoter by interacting with and activating the transcription factor AP-1, composed of c-Jun and activating transcription factor-2 (ATF-2); it also enhanced the nuclear presence of ATF-2. AP-1 was critical for Twist1-induced canc  er cell invas  ion, primarily through the induction of integrin α5, which activated c-Jun N-terminal kinase and focal adhesion kinase-signaling activities. Using data from The Cancer Genome Atlas, we found that Twist1 expression positively correlates with integrin α5 expression in human colorectal cancers. These findings suggest that cooperation between Twist1 and AP-1 represents a novel mechanism for EMT and tumor invasiveness. This study supports further investigation into the molecular basis underlying the diverse Twist1-mediated functions that occur during tumor progression. 
 Specific interactions between Smad proteins and AP-1 components determine TGFβ-induced breast cancer   cell invasio  n. Deregulation of the transforming growth factor β (TGFβ) signal transduction cascade is functionally linked to cancer. In early phases, TGFβ acts as a tumor suppressor by inhibiting tumor cell proliferation, whereas in late phases, it can act as a tumor promoter by stimulating tu  mor cell inva  sion and metastasis. Smad transcriptional effectors mediate TGFβ responses, but relatively little is known about the Smad-containing complexes that are important for epithelial-mesenchymal transition and invasion. In this study, we have tested the hypothesis that specific members of the AP-1 transcription factor family determine TGFβ signaling specificity in breast c  ancer cell in  vasion. Using a 3D model of collagen-embedded spheroids of MCF10A-MII premalignant human breast cancer cells, we identified the AP-1 transcription factor components c-Jun, JunB, c-Fos and Fra1 as essential factors for TGFβ-induced invasion and found that various mesenchymal and invasion-associated TGFβ-induced genes are co-regulated by these proteins. In situ proximity ligation assays showed that TGFβ signaling not only induces complexes between Smad3 and Smad4 in the nucleus but also complexes between Smad2/3 and Fra1, whereas complexes between Smad3, c-Jun and JunB could already be detected before TGFβ stimulation. Finally, chromatin immunoprecipitations showed that c-Jun, JunB and Fra1, but not c-Fos, are required for TGFβ-induced binding of Smad2/3 to the mmp-10 and pai-1 promoters. Together these results suggest that in particular formation of Smad2/3-Fra1 complexes may reflect activation of the Smad/AP-1-dependent TGFβ-induced invasion program. 
 Pdcd4 knockdown up-regulates MAP4K1 expression and activation of AP-1 dependent transcription through c-Myc. Programmed cell death 4 (Pdcd4) is a novel tumor suppressor, whose expression is frequently down-regulated in several types of cancers. In the present study, we demonstrated that Pdcd4 knockdown up-regulates MAP kinase kinase kinase kinase 1 (MAP4K1) expression and increases phosphorylation of c-Jun. Over-expression of c-Myc in HEK293 cells increases the levels of MAP4K1, MAP4K1 promoter activity, and phospho-c-Jun. Mutation analysis showed that the c-Myc binding site at -536bp (relative to the initiation ATG) of map4k1 promoter responds to c-Myc regulation. In addition, chromatin immunoprecipitation demonstrated that c-Myc directly binds to map4k1 promoter at this site. Down-regulation of c-Myc reverses MAP4K1 expression and AP-1 activation in Pdcd4 knockdown cells. Moreover, over-expression of dominant negative Tcf4 decreases expression of c-Myc and MAP4K1, JNK activation, and AP-1 dependent transcription. Thus, activation of β-catenin/Tcf dependent transcription in Pdcd4 knockdown cells up-regulates MAP4K1 expression and AP-1 activity via c-Myc. The study presented here further reveals in detail the mechanism of how Pdcd4 inhibits tumor   cell invasio  n and provides a functional connection between β-catenin/Tcf and AP-1 dependent transcription. 
 Blockade of the RhoA-JNK-c-Jun-MMP2 cascade by atorvastatin reduces osteosarcoma   cell invasion  . Osteosarcoma is characterized by a high malignant and metastatic potential, which points to the need for new therapeutic strategies to prevent cell metastasis. In this study, we show that statin-induced HMG-CoA reductase inhibition reduces cell migration and invasion in human and murine osteosarcoma cells, independently of the genotype. The statin-induced reduction of cell migration and invasion was independent of induction of apoptosis and was geranylgeranylpyrophosphate-dependent. The statin reduced matrix metalloproteinase (MMP) 2, 9, and 14 and TIMP2 expression or activity in invading cells. Forced expression of MMP2 and MMP14 overcame the inhibitory effect of the statin on   cell invasion  , suggesting a role for these MMPs in invasive potential. We also investigated the mechanisms involved in the reduced MMP2 activity and   cell invasion  . Inhibition of JNK, but not ERK1/2 signaling, reduced MMP2 activity. Pharmacological or constitutive activation of JNK overcame the reduced MMP2 activity and   cell invasion   induced by the statin. The statin decreased JNK phosphorylation and c-Jun nuclear translocation, suggesting that HMG-CoA reductase inhibition targets the JNK-c-Jun signaling pathway. We showed that mevalonate or geranylgeranylpyrophosphate treatment prevented the statin-induced reduction in JNK phosphorylation, MMP2 activity, and   cell invasion  . Forced expression of a constitutively active form of RhoA increased JNK phosphorylation and overcame the inhibitory effect of atorvastatin on MMP2 activity and   cell invasion  . The data establish a link between RhoA, JNK, c-Jun, and MMP2 activity that is functionally involved in the reduction in osteosarcoma   cell invasion   by the statin. This suggests a novel strategy targeting RhoA-JNK-c-Jun signaling to reduce osteosarcoma cell tumorigenesis. 
 c-Jun induces mammary epithelial cellular invasion and breast cancer stem cell expansion. The molecular mechanisms governing breast tumor cellular self-renewal contribute to breast cancer progression and therapeutic resistance. The ErbB2 oncogene is overexpressed in approximately 30% of human breast cancers. c-Jun, the first cellular proto-oncogene, is overexpressed in human breast cancer. However, the role of endogenous c-Jun in mammary tumor progression is unknown. Herein, transgenic mice expressing the mammary gland-targeted ErbB2 oncogene were crossed with c-jun(f/f) transgenic mice to determine the role of endogenous c-Jun in mammary tumor invasion and stem cell function. The excision of c-jun by Cre recombinase reduced cellular migration, invasion, and mammosphere formation of ErbB2-induced mammary tumors. Proteomic analysis identified a subset of secreted proteins (stem cell factor (SCF) and CCL5) induced by ErbB2 expression that were dependent upon endogenous c-Jun expression. SCF and CCL5 were identified as transcriptionally induced by c-Jun. CCL5 rescued the c-Jun-deficient breast tumor cellular invasion phenotype. SCF rescued the c-Jun-deficient mammosphere production. Endogenous c-Jun thus contributes to ErbB2-induced mammary tumor   cell invasion   and self-renewal. 
 Epstein-Barr virus latent membrane protein 2A regulates c-Jun protein through extracellular signal-regulated kinase. Epstein-Barr virus (EBV) latent membrane protein 2A (LMP2A) is widely expressed in both EBV-infected cells and EBV-associated malignancies. However, the function of LMP2A is still veiled. In this study, LMP2A was found to induce the kinase activities of extracellular signal-regulated kinase (ERK) and c-Jun N-terminal kinase/stress-activated protein kinase JNK/SAPK. Furthermore, the downstream effector c-Jun showed hyperphosphorylation under LMP2A expression. The phosphorylation could be inhibited by the ERK pathway inhibitor PD98059, indicating that ERK may contribute to the phosphorylation of c-Jun in LMP2A-expressing cells. The impact on c-Jun phosphorylation by mitogen-activated protein kinase (MAPK) is suggested to increase c-Jun protein stability, and this was also observed in LMP2A-expressing cells by a protein synthesis inhibition assay. Moreover, LMP2A-induced   cell invasion   was inhibited in the presence of the ERK pathway inhibitor. Taken together, we suggest that LMP2A may exploit MAPK kinases and affect both the phosphorylation and stability of c-Jun protein. Additionally, LMP2A may thereby promote the mobility of the cells. In doing so, it may enhance the mobility of EBV-infected cells and contribute to the metastatic process of malignant cells. Here we demonstrated the first evidence of LMP2A-induced migration and the underlying pathways accounting for it. 
 miR200c attenuates P-gp-mediated MDR and metastasis by targeting JNK2/c-Jun signaling pathway in colorectal cancer. MicroRNA-200c (miR200c) recently emerged as an important regulator of tumorigenicity and cancer metastasis; however, its role in regulating multidrug resistance (MDR) remains unknown. In the current study, we found that the expression levels of miR200c in recurrent and metastatic colorectal cancers were significantly lower, whereas the JNK2 expression was higher compared with primary tumors. We showed that in MDR colorectal cancer cells, miR200c targeted the 3' untranslated region of the JNK2 gene. Overexpression of miR200c attenuated the levels of p-JNK, p-c-Jun, P-gp, and MMP-2/-9, the downstream factors of the JNK signaling pathway, resulting in increased sensitivity to chemotherapeutic drugs, which was accompanied by heightened apoptosis and decreased   cell invasion   and migration. Moreover, in an orthotopic MDR colorectal cancer mouse model, we demonstrated that overexpression of miR200c effectively inhibited the tumor growth and metastasis. At last, in the tumor samples from patients with locally advanced colorectal cancer with routine postsurgical chemotherapy, we observed an inverse correlation between the levels of mRNA expression of miR200c and JNK2, ABCB1, and MMP-9, thus predicting patient therapeutic outcomes. In summary, we found that miR200c negatively regulated the expression of JNK2 gene and increased the sensitivity of MDR colorectal cancer cells to chemotherapeutic drugs, via inhibiting the JNK2/p-JNK/p-c-Jun/ABCB1 signaling. Restoration of miR200c expression in MDR colorectal cancer may serve as a promising therapeutic approach in MDR-induced metastasis. 
 c-Jun has multiple enhancing activities in the novel cross talk between the androgen receptor and Ets variant gene 1 in prostate cancer. The multiple transcriptional roles of c-Jun are shown in a novel cross-talk between the androgen receptor (AR) and its new target gene, Ets variant gene 1 (ETV1). In this report, we show that c-Jun can mediate AR induction of ETV1 expression independent of c-Jun transactivation function. Interestingly, c-Jun can transactivate the cloned ETV1 promoter also in the absence of ligand-activated AR, suggesting two mechanisms by which c-Jun can induce ETV1 expression. In addition, both wild-type c-Jun and a transactivation-deficient mutant can enhance the transcriptional activity of ETV1, as measured by both reporter gene assay and endogenous expression of matrix metalloproteinase genes, well-known targets of Ets proteins. Overexpression of the c-Jun mutant protein also led to increased prostate cancer   cell invasion  . Immunoprecipitation and immunocytochemistry experiments showed copurification and colocalization of c-Jun with AR or ETV1, suggesting that c-Jun acts on AR or ETV1 via a physical association. Collectively, these results, together with a parallel overexpression of ETV1, c-Jun, and AR in prostate tumors, imply that c-Jun plays a pivotal role in the pathway that connects ligand-activated AR to elevated ETV1 expression, leading to enhanced expression of matrix metalloproteinases and prostate cancer   cell invasion  . 
 Dimerumic acid inhibits SW620   cell invasion   by attenuating H₂O₂-mediated MMP-7 expression via JNK/C-Jun and ERK/C-Fos activation in an AP-1-dependent manner. Reactive oxygen species (ROS) such as hydrogen peroxide (H₂O₂) in the tumor microenvironment play important roles in tumor invasion and metastasis. Recently, ROS have been reported to cause a significant increase in the production and expression of matrix metalloproteinase (MMP)-7, which is closely correlated with metastatic colorectal cancer. The present study was undertaken to evaluate the scavenging activity of dimerumic acid (DMA) for H₂O₂ isolated from Monascus-fermented rice to investigate the inhibitory effects of DMA on the invasive potential of SW620 human colon cancer cells, and to explore the mechanisms underlying both these phenomena. Our results showed that increased MMP-7 expression due to H₂O₂ exposure was mediated by activation of mitogen-activated protein kinases (MAPKs) such as Jun N-terminal kinase (JNK), extracellular-regulated kinase (ERK), and p38 kinase. DMA pretreatment suppressed activation of H₂O₂-mediate  d MAPK pathwa  ys and cell invasion. Moreover, H₂O₂-triggered MMP-7 production was demonstrated via JNK/c-Jun and ERK/c-Fos activation in an activating protein 1 (AP-1)-dependent manner. Taken together, these results suggest that DMA   suppresses H�  ��O₂-induced cell invasion by inhibiting AP-1-mediated MMP-7 gene transcription via the JNK/c-Jun and ERK/c-Fos signaling pathways in SW620 human colon cancer cells. Our data suggest that DMA may be useful in minimizing the development of colorectal metastasis. In the future, DMA supplementation may be a beneficial antioxidant to enhance surgical outcomes. 
 miR-23a inhibits E-cadherin expression and is regulated by AP-1 and NFAT4 complex during Fas-induced EMT in gastrointestinal cancer. Fas signaling has been shown to induce the epithelial-mesenchymal transition (EMT) to promote gastrointestinal (GI) cancer metastasis, but the involvement of microRNA in this mechanism remains unknown. We found that Fas ligand (FasL) treatment inhibited E-cadherin expression and promoted   cell invasion   by upregulation of miR-23a, but overexpression of the miR-23a inhibitor could partially block this activity. FasL-induced extracellular signal-regulated kinase/mitogen-activated protein kinase signaling activated the activator protein 1 (AP-1) complex and repressed glycogen synthase kinase-3β activity, which contributed to nuclear translocation of AP-1 and nuclear factor of activated T cells (NFAT4). Nuclear accumulation and interaction of AP-1 and NFAT4 and subsequent binding to the miR-23a promoter led to increased miR-23a expression. Inhibition of Fas signaling by downregulation of the Fas receptor led to a decrease in miR-23a expression and   cell invasio  n ability in vivo and in vitro, as well as an increase in E-cadherin. Evaluation of human GI precancerous and cancer specimens showed that the expression of FasL and miR-23a increased, whereas the expression of E-cadherin decreased during GI cancer progression. A significant correlation was noted between any two of these three molecules. An EMT phenotype was shown to correlate with an advanced cancer stage and worse prognosis. Taken together, our results show that miR-23a participates in the mechanism of the FasL-induced EMT process and may serve as a potential therapeutic target for cancer metastasis. 
 Genome-wide profiling of AP-1-regulated transcription provides insights into the invasiveness of triple-negative breast cancer. Triple-negative breast cancer (TNBC) is an aggressive clinical subtype accounting for up to 20% of all breast cancers, but its malignant determinants remain largely undefined. Here, we show that in TNBC the overexpression of Fra-1, a component of the transcription factor AP-1, offers prognostic potential. Fra-1 depletion or its heterodimeric partner c-Jun inhibits the proliferative and invasive phenotypes of TNBC cells in vitro. Similarly, RNAi-mediated attenuation of Fra-1 or c-Jun reduced cellular invasion in vivo in a zebrafish tumor xenograft model. Exploring the AP-1 cistrome and the AP-1-regulated transcriptome, we obtained insights into the transcriptional regulatory networks of AP-1 in TNBC cells. Among the direct targets identified for Fra-1/c-Jun involved in proliferation, adhesion, and cell-cell contact, we found that AP-1 repressed the expression of E-cadherin by transcriptional upregulation of ZEB2 to stimulate   cell invasion  . Overall, this work illuminates the pathways through which TNBC cells acquire invasive and proliferative properties. 
 (-)-Epigallocatechin-3-gallate blocks nicotine-induced matrix metalloproteinase-9 expression and invasiveness via suppression of NF-κB and AP-1 in endothelial cells. Cigarette smoke, specifically the nicotine contained within, has been shown to correlate closely with   cell invasio  n and strategies to downregulate their expression may ultimately be of clinical utility. Matrix metalloproteinase-9 (MMP-9) is critically involved in the   cell invasio  n and metastasis processes. Since nicotine plays a crucial role in the regulation of MMP-9 expression, the investigation of plant-derived compounds capable of modulating nicotine-induced signaling is an issue of concern. In this study, the effects of (-)-epigallocatechin-3-gallate (EGCG), a major green tea catechin, on nicotine-induced   cell invasio  n and MMP-9 activity in ECV304 human endothelial cells were examined. EGCG treatment was found to reduce the MMP-9 expression and transcriptional activity in a dose-dependent manner. EGCG inhibited nicotine-activated production of reactive oxygen species (ROS), which are known as important signaling molecules to activate MMP-9. To further study the mechanisms for the EGCG-mediated regulation of MMP-9, the transcription factors NF-κB and AP-1 activities were examined. EGCG suppressed the nicotine-induced NF-κB and AP-1 activation. Studies with expression vectors encoding mutated NF-κB signaling molecules and AP-1 decoy confirmed that NF-κB and AP-1 were essential for the nicotine-stimulated MMP-9 expression. EGCG also abrogated the nicotine-induced activation of AP-1 subunits c-fos and c-jun. The above studies demonstrate that EGCG may exert at least part of its anti-invasive effect in ECV304 human endothelial cells by controlling MMP-9 expression through the suppression of ROS, NF-κB and AP-1. 
 Inhibition of AP-1 by SARI negatively regulates transformation progression mediated by CCN1. Enhanced expression of the CCN family of secretory integrin-binding proteins correlates with many essential components of the cancerous state, including tumor cell adhesion, proliferation, invasion and migration. Consequently, CCN1 expression is elevated in various cancers, including breast cancer, and its expression directly correlates with poor patient prognosis. Using subtraction-hybridization, combined with induction of cancer cell terminal differentiation, we cloned SARI (suppressor of activator protein (AP)-1, regulated by interferon (IFN)), an IFN-beta-inducible, potent tumor suppressor gene that exerts cancer-selective growth inhibitory effects. Forced expression of SARI using an adenovirus (Ad.SARI) inhibits AP-1 function and downregulates CCN1 expression in multiple cancer lineages, resulting in a profound inhibition in anchorage-independent cell growth and tumor   cell invasion  . Overexpression of SARI reduces CCN1-promoter activity through inhibition of AP-1 binding. Accordingly, SARI selectively blocks expression of the transformed state in rat embryo fibroblast cells that stably overexpress c-Jun. These results illustrate that SARI inhibits AP-1 transactivating factor binding to the cis-element of the CCN1 promoter, possibly through its interaction with c-Jun. Overall, SARI can directly inhibit CCN1-induced transformation by inhibiting the transcription of CCN1, as well as indirectly by inhibiting the expression of c-Jun (and hence blocking AP-1 activity). In these contexts, transformed cells 'addicted' to AP-1 activity are rendered susceptible to SARI-mediated inhibition of expression of the transformed phenotype. 
  GeneRIFs  
 we found that AP-1 repressed the expression of E-cadherin by transcriptional upregulation of ZEB2 to stimulate   cell invasion  . 
 specific members of the AP-1 transcription factor family determine TGFbeta signaling specificity in breast cancer   cell invasion   
 Endogenous c-Jun contributes to ErbB2-induced mammary tumor   cell invasion   and self-renewal 
 a link between RhoA, JNK, c-Jun, and MMP2 activity that is functionally involved in the reduction in osteosarcoma   cell invasion   by the statin. This suggests a novel strategy targeting RhoA-JNK-c-Jun signaling to reduce osteosarcoma cell tumorigenesis. 
 These results imply that c-Jun plays a pivotal role in the pathway that connects ligand-activated AR to elevated ETV1 expression, leading to enhanced expression of matrix metalloproteinases and prostate cancer   cell invasion  . 
   
  
 JUND 
 
  NCBI Gene  
  GeneRIF  
  Pubmed  
 
 
  Search Term  Rank  Fields  
  [Mm]igration  6  PubMed(2)  
  ECM  7  PubMed(2)  
  [Ee]xtracellular [Mm]atrix  8  PubMed(3)  
  [Ii]nvasive  10  PubMed(2)  
  [Mm]etastasis  12  PubMed(1)  
 
 
 hide/show details for JUND 
  [Mm]igration  PubMed  
 BAG3 is upregulated by c-Jun and stabilizes JunD. BAG3 plays a regulatory role in a number of cellular processes, including cell proliferation, apoptosis, adhesion and   migration  , epithelial-mesenchymal transition (EMT), autophagy activation, and virus infection. The AP-1 transcription factors are implicated in a variety of important biological processes including cell differentiation, proliferation, apoptosis and oncogenesis. Recently, it has been reported that AP-1 protein c-Jun inhibits autophagy and enhances apoptotic cell death mediated by starvation. However, the molecular mechanisms remain unclear. For the first time, the current study demonstrated that serum starvation downregulated BAG3 at the transcriptional level via c-Jun. In addition, the current study reported that BAG3 stabilized JunD mRNA, which was, at least in part, responsible for the promotion of serum starvation mediated-growth inhibition by BAG3. 
 Human T-cell leukemia virus type 1 tax transactivates the matrix metalloproteinase 7 gene via JunD/AP-1 signaling. Adult T-cell leukemia (ATL) is a T-cell malignancy associated with human T-cell leukemia virus type 1 (HTLV-1) and characterized by visceral invasion. Degradation of the extracellular matrix by matrix metalloproteinases (MMPs) is a crucial process in invasion of tumors and metastasis. MMP-7 (or matrilysin), is a "minimal domain MMP" with proteolytic activity against components of the extracellular matrix. To determine the involvement of MMP-7 in visceral spread in ATL, this study investigated MMP-7 expression in ATL. MMP-7 expression was identified in HTLV-1-infected T-cell lines, peripheral blood ATL cells and ATL cells in lymph nodes, but not in uninfected T-cell lines or normal peripheral blood mononuclear cells. MMP-7 expression was induced following infection of a human T-cell line with HTLV-1, and specifically by the viral protein Tax. Functionally, MMP-7 promoted cell   migration   of HTLV-1-infected T cells. The MMP-7 promoter activity was increased by Tax and reduced by deletion of the activator protein-1 (AP-1) binding site. Electrophoretic mobility shift assay showed high levels of AP-1 binding proteins, including JunD, in HTLV-1-infected T-cell lines and ATL cells, and Tax elicited JunD binding to the MMP-7 AP-1 element. Tax-induced MMP-7 activation was inhibited by dominant negative JunD and augmented by JunD/JunD homodimers. Short interfering RNA against JunD inhibited MMP-7 mRNA expression in HTLV-1-infected T-cell lines. These results suggest that the induction of MMP-7 by Tax is regulated by JunD and that MMP-7 could facilitate visceral invasion in ATL. This article is part of a Special Issue entitled: 11th European Symposium on Calcium. 
  ECM  PubMed  
 SERPINE2 Inhibits IL-1α-Induced MMP-13 Expression in Human Chondrocytes: Involvement of ERK/NF-κB/AP-1 Pathways. Osteoarthritis (OA) is a chronic joint disease, characterized by a progressive loss of articular cartilage. During OA, proinflammatory cytokines, such as interleukin IL-1, induce the expression of matrix metalloproteinases (MMPs) in chondrocytes, contributing thus to the extracellular matrix   (E  CM) degradation. Members of Serpine family, including plasminogen activator inhibitors have been reported to participate i  n E  CM regulation. The aim of this study was to assess the expression of serpin peptidase inhibitor clade E member 2 (SERPINE2), under basal conditions and in response to increasing doses of IL-1α, in human cultured chondrocytes. We also examined the effects of SERPINE2 on IL-1α-induced MMP-13 expression. For completeness, the signaling pathway involved in this process was also explored.SERPINE2 mRNA and protein expression were evaluated by RT-qPCR and western blot analysis in human T/C-28a2 cell line and human primary chondrocytes. These cells were treated with human recombinant SERPINE2, alone or in combination with IL-1α. ERK 1/2, NFκB and AP-1 activation were assessed by western blot analysis.Human cultured chondrocytes express SERPINE2 in basal condition. This expression increased in response to IL-1α stimulation. In addition, recombinant SERPINE2 induced a clear inhibition of MMP-13 expression in IL-1α-stimulated chondrocytes. This inhibitory effect is likely regulated through a pathway involving ERK 1/2, NF-κB and AP-1.Taken together, these data demonstrate that SERPINE2 might prevent cartilage catabolism by inhibiting the expression of MMP-13, one of the most relevant collagenases, involved in cartilage breakdown in OA. 
 A time- and matrix-dependent TGFBR3-JUND-KRT5 regulatory circuit in single breast epithelial cells and basal-like premalignancies. Basal-like breast carcinoma is characterized by poor prognosis and high intratumour heterogeneity. In an immortalized basal-like breast epithelial cell line, we identified two anticorrelated gene-expression programs that arise among single extracellular matrix (  ECM  )-attached cells during organotypic three-dimensional culture. The first contains multiple TGF-β-related genes including TGFBR3, whereas the second contains JUND and the basal-like marker KRT5. TGFBR3 and JUND interconnect through four negative-feedback loops to form a circuit that exhibits spontaneous damped oscillations in three-dimensional culture. The TGFBR3-JUND circuit is conserved in some premalignant lesions that heterogeneously express KRT5. The circuit depends on   EC  M engagement, as detachment causes a rewiring that is triggered by RPS6 dephosphorylation and maintained by juxtacrine tenascin C, which is critical for intraductal colonization of basal-like breast cancer cells in vivo. Intratumour heterogeneity need not stem from partial differentiation and could instead reflect dynamic toggling of cells between expression states that are not cell autonomous. 
  [Ee]xtracellular [Mm]atrix  PubMed  
 Human T-cell leukemia virus type 1 tax transactivates the matrix metalloproteinase 7 gene via JunD/AP-1 signaling. Adult T-cell leukemia (ATL) is a T-cell malignancy associated with human T-cell leukemia virus type 1 (HTLV-1) and characterized by visceral invasion. Degradation of the   extracellular matrix   by matrix metalloproteinases (MMPs) is a crucial process in invasion of tumors and metastasis. MMP-7 (or matrilysin), is a "minimal domain MMP" with proteolytic activity against components of the   extracellular matrix  . To determine the involvement of MMP-7 in visceral spread in ATL, this study investigated MMP-7 expression in ATL. MMP-7 expression was identified in HTLV-1-infected T-cell lines, peripheral blood ATL cells and ATL cells in lymph nodes, but not in uninfected T-cell lines or normal peripheral blood mononuclear cells. MMP-7 expression was induced following infection of a human T-cell line with HTLV-1, and specifically by the viral protein Tax. Functionally, MMP-7 promoted cell migration of HTLV-1-infected T cells. The MMP-7 promoter activity was increased by Tax and reduced by deletion of the activator protein-1 (AP-1) binding site. Electrophoretic mobility shift assay showed high levels of AP-1 binding proteins, including JunD, in HTLV-1-infected T-cell lines and ATL cells, and Tax elicited JunD binding to the MMP-7 AP-1 element. Tax-induced MMP-7 activation was inhibited by dominant negative JunD and augmented by JunD/JunD homodimers. Short interfering RNA against JunD inhibited MMP-7 mRNA expression in HTLV-1-infected T-cell lines. These results suggest that the induction of MMP-7 by Tax is regulated by JunD and that MMP-7 could facilitate visceral invasion in ATL. This article is part of a Special Issue entitled: 11th European Symposium on Calcium. 
 SERPINE2 Inhibits IL-1α-Induced MMP-13 Expression in Human Chondrocytes: Involvement of ERK/NF-κB/AP-1 Pathways. Osteoarthritis (OA) is a chronic joint disease, characterized by a progressive loss of articular cartilage. During OA, proinflammatory cytokines, such as interleukin IL-1, induce the expression of matrix metalloproteinases (MMPs) in chondrocytes, contributing thus to th  e extracellular matr  ix (ECM) degradation. Members of Serpine family, including plasminogen activator inhibitors have been reported to participate in ECM regulation. The aim of this study was to assess the expression of serpin peptidase inhibitor clade E member 2 (SERPINE2), under basal conditions and in response to increasing doses of IL-1α, in human cultured chondrocytes. We also examined the effects of SERPINE2 on IL-1α-induced MMP-13 expression. For completeness, the signaling pathway involved in this process was also explored.SERPINE2 mRNA and protein expression were evaluated by RT-qPCR and western blot analysis in human T/C-28a2 cell line and human primary chondrocytes. These cells were treated with human recombinant SERPINE2, alone or in combination with IL-1α. ERK 1/2, NFκB and AP-1 activation were assessed by western blot analysis.Human cultured chondrocytes express SERPINE2 in basal condition. This expression increased in response to IL-1α stimulation. In addition, recombinant SERPINE2 induced a clear inhibition of MMP-13 expression in IL-1α-stimulated chondrocytes. This inhibitory effect is likely regulated through a pathway involving ERK 1/2, NF-κB and AP-1.Taken together, these data demonstrate that SERPINE2 might prevent cartilage catabolism by inhibiting the expression of MMP-13, one of the most relevant collagenases, involved in cartilage breakdown in OA. 
 A time- and matrix-dependent TGFBR3-JUND-KRT5 regulatory circuit in single breast epithelial cells and basal-like premalignancies. Basal-like breast carcinoma is characterized by poor prognosis and high intratumour heterogeneity. In an immortalized basal-like breast epithelial cell line, we identified two anticorrelated gene-expression programs that arise among single   extracellular matrix   (ECM)-attached cells during organotypic three-dimensional culture. The first contains multiple TGF-β-related genes including TGFBR3, whereas the second contains JUND and the basal-like marker KRT5. TGFBR3 and JUND interconnect through four negative-feedback loops to form a circuit that exhibits spontaneous damped oscillations in three-dimensional culture. The TGFBR3-JUND circuit is conserved in some premalignant lesions that heterogeneously express KRT5. The circuit depends on ECM engagement, as detachment causes a rewiring that is triggered by RPS6 dephosphorylation and maintained by juxtacrine tenascin C, which is critical for intraductal colonization of basal-like breast cancer cells in vivo. Intratumour heterogeneity need not stem from partial differentiation and could instead reflect dynamic toggling of cells between expression states that are not cell autonomous. 
  [Ii]nvasive  PubMed  
 The prognostic significance of Jun transcription factors in ovarian cancer. The Jun proteins (c-Jun, JunD and JunB) play an important role in the regulation of cell proliferation, apoptosis and angiogenesis. It is well established that these proteins participate in the carcinogenesis and progression in several tumour types. However, little is known about the prognostic significance of Jun proteins in patients with   invasive   epithelial ovarian carcinoma.We analysed fresh-frozen tissues of 161 ovarian cancer patients by using Western blot analysis to investigate protein levels of JunB, JunD, c-Jun and phosphorylated c-Jun (pc-Jun Ser63). The results were correlated with clinicopathologic prognostic parameters and survival data.A high pc-Jun expression was significantly associated with shorter progression-free survival (14 vs. 16 months, p = 0.017) and overall survival (25 vs. 41 months, p = 0.038). In case of JunD, moderate protein levels were associated with a better prognosis, leading to longer progression-free and overall survival compared to weak or strong JunD expression (PFS in cases with weak/moderate/strong JunD expression: 14 vs. 19.5 vs. 16 months, p = 0.011; OAS: 32 vs. 42 vs. 35.5 months, p = 0.009). Multivariate Cox regression analysis confirmed an independent and significant impact of pc-Jun and JunD on the patient's prognosis.Our results show that Jun proteins (pc-Jun and JunD) influence carcinogenesis and tumour progression, suggesting a significant role as prognostic predictors in human ovarian carcinoma. 
 The prognostic significance of Jun transcription factors in ovarian cancer. The Jun proteins (c-Jun, JunD and JunB) play an important role in the regulation of cell proliferation, apoptosis and angiogenesis. It is well established that these proteins participate in the carcinogenesis and progression in several tumour types. However, little is known about the prognostic significance of Jun proteins in patients with   invasive   epithelial ovarian carcinoma.We analysed fresh-frozen tissues of 161 ovarian cancer patients by using Western blot analysis to investigate protein levels of JunB, JunD, c-Jun and phosphorylated c-Jun (pc-Jun Ser63). The results were correlated with clinicopathologic prognostic parameters and survival data.A high pc-Jun expression was significantly associated with shorter progression-free survival (14 vs. 16 months, p = 0.017) and overall survival (25 vs. 41 months, p = 0.038). In case of JunD, moderate protein levels were associated with a better prognosis, leading to longer progression-free and overall survival compared to weak or strong JunD expression (PFS in cases with weak/moderate/strong JunD expression: 14 vs. 19.5 vs. 16 months, p = 0.011; OAS: 32 vs. 42 vs. 35.5 months, p = 0.009). Multivariate Cox regression analysis confirmed an independent and significant impact of pc-Jun and JunD on the patient's prognosis.Our results show that Jun proteins (pc-Jun and JunD) influence carcinogenesis and tumour progression, suggesting a significant role as prognostic predictors in human ovarian carcinoma. 
  [Mm]etastasis  PubMed  
 Human T-cell leukemia virus type 1 tax transactivates the matrix metalloproteinase 7 gene via JunD/AP-1 signaling. Adult T-cell leukemia (ATL) is a T-cell malignancy associated with human T-cell leukemia virus type 1 (HTLV-1) and characterized by visceral invasion. Degradation of the extracellular matrix by matrix metalloproteinases (MMPs) is a crucial process in invasion of tumors and   metastasis  . MMP-7 (or matrilysin), is a "minimal domain MMP" with proteolytic activity against components of the extracellular matrix. To determine the involvement of MMP-7 in visceral spread in ATL, this study investigated MMP-7 expression in ATL. MMP-7 expression was identified in HTLV-1-infected T-cell lines, peripheral blood ATL cells and ATL cells in lymph nodes, but not in uninfected T-cell lines or normal peripheral blood mononuclear cells. MMP-7 expression was induced following infection of a human T-cell line with HTLV-1, and specifically by the viral protein Tax. Functionally, MMP-7 promoted cell migration of HTLV-1-infected T cells. The MMP-7 promoter activity was increased by Tax and reduced by deletion of the activator protein-1 (AP-1) binding site. Electrophoretic mobility shift assay showed high levels of AP-1 binding proteins, including JunD, in HTLV-1-infected T-cell lines and ATL cells, and Tax elicited JunD binding to the MMP-7 AP-1 element. Tax-induced MMP-7 activation was inhibited by dominant negative JunD and augmented by JunD/JunD homodimers. Short interfering RNA against JunD inhibited MMP-7 mRNA expression in HTLV-1-infected T-cell lines. These results suggest that the induction of MMP-7 by Tax is regulated by JunD and that MMP-7 could facilitate visceral invasion in ATL. This article is part of a Special Issue entitled: 11th European Symposium on Calcium. 
   
  
 PPM1F 
 
  NCBI Gene  
  GeneRIF  
  Pubmed  
 
 
  Search Term  Rank  Fields  
  [Mm]igration  6  Process(1); PubMed(2)  
  [Ii]nvasive  10  PubMed(3); GeneRIFs(1)  
  [Mm]etastasis  12  PubMed(2); GeneRIFs(1)  
 
 
 hide/show details for PPM1F 
  [Mm]igration  Process  
 positive regulation of epithelial cell   migration   
  PubMed  
 MicroRNA-200c represses   migration   and invasion of breast cancer cells by targeting actin-regulatory proteins FHOD1 and PPM1F. MicroRNA-200c (miR-200c) has been shown to suppress epithelial-mesenchymal transition (EMT), which is attributed mainly to targeting of ZEB1/ZEB2, repressors of the cell-cell contact protein E-cadherin. Here we demonstrated that modulation of miR-200c in breast cancer cells regulates cell   migration  , cell elongation, and transforming growth factor β (TGF-β)-induced stress fiber formation by impacting the reorganization of cytoskeleton that is independent of the ZEB/E-cadherin axis. We identified FHOD1 and PPM1F, direct regulators of the actin cytoskeleton, as novel targets of miR-200c. Remarkably, expression levels of FHOD1 and PPM1F were inversely correlated with the level of miR-200c in breast cancer cell lines, breast cancer patient samples, and 58 cancer cell lines of various origins. Furthermore, individual knockdown/overexpression of these target genes phenocopied the effects of miR-200c overexpression/inhibition on cell elongation, stress fiber formation  , migrati  on, and invasion. Mechanistically, targeting of FHOD1 by miR-200c resulted in decreased expression and transcriptional activity of serum response factor (SRF), mediated by interference with the translocation of the SRF coactivator mycocardin-related transcription factor A (MRTF-A). This finally led to downregulation of the expression and phosphorylation of the SRF target myosin light chain 2 (MLC2) gene, required for stress fiber formation and contractility. Thus, miR-200c impacts on metastasis by regulating several EMT-related processes, including a novel mechanism involving the direct targeting of actin-regulatory proteins. 
 miR-149 represses metastasis of hepatocellular carcinoma by targeting actin-regulatory proteins PPM1F. microRNAs have been implicated in hepatocellular carcinoma (HCC) metastasis, which is predominant cause of high mortality in these patients. Although an increasing body of evidence indicates that miR-149 plays an important role in the growth and metastasis of multiple types of cancers, its role in the progression of HCC remains unknown. Here, we demonstrated that miR-149 was significantly down-regulated in HCC, which was correlated with distant metastasis and TNM stage with statistical significance. A survival analysis showed that decreased miR-149 expression was correlated with a poor prognosis of HCC as well. We found that over-expression of miR-149 suppressed   migration   and invasion of HCC cells in vitro. In addition, we identified PPM1F (protein phosphatase, Mg(2+)/Mn(2+)-dependent, 1F) as a direct target of miR-149 whose expression was negatively correlated with the expression of miR-149 in HCC tissues. The re-expression of PPM1F rescued the miR-149-mediated inhibition of cell   migration   and invasion. miR-149 regulated formation of stress fibers to inhibit   migration  , and re-expression of PPM1F reverted the miR-149-mediated loss of stress fibers. Moreover, we demonstrated that over-expression of miR-149 reduced pMLC2, a downstream effector of PPM1F, in MHCC-97H cells. In vivo studies confirm inhibition of HCC metastasis by miR-149. Taken together, our findings indicates that miR-149 is a potential prognostic biomarker of HCC and that the miR-149/PPM1F regulatory axis represents a novel therapeutic target for HCC treatment. 
  [Ii]nvasive  PubMed  
 Investigation of POPX2 phosphatase functions by comparative phosphoproteomic analysis. Identifying the substrates and biochemical pathway regulated by phosphatases has always been more challenging than finding those regulated by kinases. Here, we report the use of phosphoproteomic methods to analyse the pathways regulated by POPX2 (partner of PIX 2) phosphatase. POPX2 is a serine/threonine phosphatase, found in many cancer types. The levels of the POPX2 have been found to be up-regulated in the more   invasive   breast cancer cells compared with non-  invasive   ones. Our observations also suggest that POPX2 level is positively correlated with cell motility. Thus, finding substrates or pathways regulated by POPX2 will help to elucidate the regulatory mechanism of cancer cell motility and   invasive  ness. We have also developed and validated a protocol using electrostatic repulsion-hydrophilic interaction chromatography (ERLIC) to enrich the phosphopeptides followed by LC-MS/MS to allow comparison between the phosphoproteomes of control and POPX2 overexpressing cells. With this approach, we were able to identify a biochemical pathway through which POPX2 exerts its apparent cellular function: the regulation of activity of glycogen synthase kinase-3, which in turn modulates extracellular signal-regulated kinase and cell motility. 
 Integrative transcriptome and proteome study to identify the signaling network regulated by POPX2 phosphatase. POPX2 is a serine/threonine phosphatase belonging to the protein phosphatase 2C (PP2C) family that has been found to be elevated in   invasive   breast cancer cells. Silencing of POPX2 results in lower cell motility and   invasive  ness. The molecular mechanism of POPX2-regulated cell motility is not well understood. To identify the relevant signaling pathways, we investigated the transcriptome and proteome of POPX2-knockdown MDA-MB-231 breast cancer cells. Our data suggest that POPX2 might be involved in the regulation of focal adhesions and cytoskeleton dynamics through the regulation of MAP kinase (MAPK1/3) and glycogen synthase kinase 3 (GSK3α/β) activities. Silencing POPX2 alters phosphorylation levels of MAPK1/3 and GSK3α/β and results in reduced activity of these kinases. Both MAPK and GSK3 are known to regulate the activities of transcription factors. MAPK1/3 are also implicated in the phosphorylation of stathmin. The level of phospho-stathmin was found to be lower in POPX2 knockdown cells. As phosphorylation of stathmin inhibits its microtubule severing activity, we observed less stable microtubules in POPX2 knockdown cells. Taken together, our data suggest that POPX2 might regulate cell motility through its regulation of the MAPK1/3, leading to changes in the cytoskeleton and cell motility. 
 The POPX2 phosphatase regulates cancer cell motility and   invasive  ness. Rho GTP ases play major roles in the regulation of actin cytoskeleton, cell movement and cell cycle. PAK, one of the effector kinases of these small GTP ases, has long been associated with different types of cancer. Therefore, it is likely that deregulation of PAK activity or expression may contribute to the development of cancer. POP X2, a PP 2C serine/threonine phosphatase, is known to dephosphorylate PAK and downregulate its activity. We find that POPX2 is expressed in a wide variety of tumour cell lines, the levels being highest in the more   invasive   MDA-MB-231 and lowest in the non-  invasive   MCF7 breast cancer lines. We show that silencing of POPX2 reduces the amount of stress fibers and focal adhesions in both cells lines. Interestingly, POPX2 deficiency dramatically reduces cell motility and   invasive  ness in MDA-MB-231 cells, and cell motility in MCF7 cells. Conversely, overexpression of POP X2 in MDA-MB-231 and MCF7 cells increased their motility. The silencing of POP X2 also inhibits the expression of beta1 integrin implying that POP X2 may modulate cell attachment to the extra-cellular matrix, as reflected in diminished initial colonization of POPX2 knockdown cells in nude mice. Based on these results, we propose a mechanism by which POP X2 regulates the   invasive   behavior of the cells. 
  GeneRIFs  
 The POPX2 phosphatase regulates cancer cell motility and   invasive  ness. 
  [Mm]etastasis  PubMed  
 MicroRNA-200c represses migration and invasion of breast cancer cells by targeting actin-regulatory proteins FHOD1 and PPM1F. MicroRNA-200c (miR-200c) has been shown to suppress epithelial-mesenchymal transition (EMT), which is attributed mainly to targeting of ZEB1/ZEB2, repressors of the cell-cell contact protein E-cadherin. Here we demonstrated that modulation of miR-200c in breast cancer cells regulates cell migration, cell elongation, and transforming growth factor β (TGF-β)-induced stress fiber formation by impacting the reorganization of cytoskeleton that is independent of the ZEB/E-cadherin axis. We identified FHOD1 and PPM1F, direct regulators of the actin cytoskeleton, as novel targets of miR-200c. Remarkably, expression levels of FHOD1 and PPM1F were inversely correlated with the level of miR-200c in breast cancer cell lines, breast cancer patient samples, and 58 cancer cell lines of various origins. Furthermore, individual knockdown/overexpression of these target genes phenocopied the effects of miR-200c overexpression/inhibition on cell elongation, stress fiber formation, migration, and invasion. Mechanistically, targeting of FHOD1 by miR-200c resulted in decreased expression and transcriptional activity of serum response factor (SRF), mediated by interference with the translocation of the SRF coactivator mycocardin-related transcription factor A (MRTF-A). This finally led to downregulation of the expression and phosphorylation of the SRF target myosin light chain 2 (MLC2) gene, required for stress fiber formation and contractility. Thus, miR-200c impacts o  n metastas  is by regulating several EMT-related processes, including a novel mechanism involving the direct targeting of actin-regulatory proteins. 
 miR-149 represses   metastasis   of hepatocellular carcinoma by targeting actin-regulatory proteins PPM1F. microRNAs have been implicated in hepatocellular carcinoma (HCC)   metastasis  , which is predominant cause of high mortality in these patients. Although an increasing body of evidence indicates that miR-149 plays an important role in the growth and   metastasis   of multiple types of cancers, its role in the progression of HCC remains unknown. Here, we demonstrated that miR-149 was significantly down-regulated in HCC, which was correlated with distant   metastasis   and TNM stage with statistical significance. A survival analysis showed that decreased miR-149 expression was correlated with a poor prognosis of HCC as well. We found that over-expression of miR-149 suppressed migration and invasion of HCC cells in vitro. In addition, we identified PPM1F (protein phosphatase, Mg(2+)/Mn(2+)-dependent, 1F) as a direct target of miR-149 whose expression was negatively correlated with the expression of miR-149 in HCC tissues. The re-expression of PPM1F rescued the miR-149-mediated inhibition of cell migration and invasion. miR-149 regulated formation of stress fibers to inhibit migration, and re-expression of PPM1F reverted the miR-149-mediated loss of stress fibers. Moreover, we demonstrated that over-expression of miR-149 reduced pMLC2, a downstream effector of PPM1F, in MHCC-97H cells. In vivo studies confirm inhibition of HCC   metastasis   by miR-149. Taken together, our findings indicates that miR-149 is a potential prognostic biomarker of HCC and that the miR-149/PPM1F regulatory axis represents a novel therapeutic target for HCC treatment. 
  GeneRIFs  
 Overexpression of PPM1F is associated with   metastasis   of hepatocellular carcinoma. 
   
  
 PIP5K1A 
 
  NCBI Gene  
  GeneRIF  
  Pubmed  
 
 
  Search Term  Rank  Fields  
  [Ii]nvadopodia  3  PubMed(1); GeneRIFs(1)  
  [Mm]igration  6  Process(2); PubMed(4); GeneRIFs(2)  
  ECM  7  PubMed(1)  
  [Ee]xtracellular [Mm]atrix  8  PubMed(1)  
  [Ii]nvasive  10  PubMed(1)  
 
 
 hide/show details for PIP5K1A 
  [Ii]nvadopodia  PubMed  
 Phosphatidylinositol 4,5-bisphosphate and PIP5-kinase Ialpha are required for   invadopodia   formation in human breast cancer cells.   Invadopodia   are ventral cell protrusions formed in invasive cancer cells. Because   invadopodia   have extracellular matrix (ECM) degradation activity, they are thought to function in cancer invasion. In this study, we examined the roles of phosphatidylinositol 4,5-bisphosphate [PI(4,5)P(2)] and PI(4,5)P(2)-producing enzymes in   invadopodia   formation in MDA-MB-231 human breast cancer cells. Immunofluorescence analysis showed that PI(4,5)P(2) accumulates at   invadopodia   on the ventral cell surface. Injection of an anti-PI(4,5)P(2) antibody inhibited   invadopodia   formation along with gelatin degradation activity. Sequestering of PI(4,5)P(2) by overexpression of the phospholipase C (PLC) delta1-pleckstrin homology (PH) domain, a specific probe for PI(4,5)P(2), also blocked   invadopodia   formation, while a mutated PLCdelta1-PH domain that lacks PI(4,5)P(2)-binding activity had no effect. Cellular PI(4,5)P(2) production is mainly mediated by type-I phosphatidylinositol 4-phosphate 5-kinase (PIP5KI) family proteins, which include PIP5KIalpha, Ibeta, and Igamma. Real-time quantitative PCR analysis showed that PIP5KIalpha is a dominant isoform expressed in MDA-MB-231 cells. Knockdown of PIP5KIalpha by small-interfering RNA (siRNA) inhibited   invadopodia   formation and gelatin degradation. Immunofluorescence analysis revealed that endogenous PIP5KIalpha protein localizes at   invadopodia  , which is corroborated by the observation that exogenously expressed green fluorescent protein (GFP)-fused PIP5KIalpha protein also accumulates at gelatin degradation sites. These results indicate that localized production of PI(4,5)P(2) by PIP5KIalpha is required for   invadopodia   formation and ECM degradation by human breast cancer cells. 
  GeneRIFs  
 Localized production of Phosphatidylinositol 4,5-bisphosphate by PIP5K1A is required for   invadopodia   formation by breast cancer cells. 
  [Mm]igration  Process  
 cell   migration   
 fibroblast   migration   
  PubMed  
 Analysis of the myosin-II-responsive focal adhesion proteome reveals a role for β-Pix in negative regulation of focal adhesion maturation. Focal adhesions undergo myosin-II-mediated maturation wherein they grow and change composition to modulate integrin signalling for cell   migratio  n, growth and differentiation. To determine how focal adhesion composition is affected by myosin II activity, we performed proteomic analysis of isolated focal adhesions and compared protein abundance in focal adhesions from cells with and without myosin II inhibition. We identified 905 focal adhesion proteins, 459 of which changed in abundance with myosin II inhibition, defining the myosin-II-responsive focal adhesion proteome. The abundance of 73% of the proteins in the myosin-II-responsive focal adhesion proteome was enhanced by contractility, including proteins involved in Rho-mediated focal adhesion maturation and endocytosis- and calpain-dependent focal adhesion disassembly. During myosin II inhibition, 27% of proteins in the myosin-II-responsive focal adhesion proteome, including proteins involved in Rac-mediated lamellipodial protrusion, were enriched in focal adhesions, establishing that focal adhesion protein recruitment is also negatively regulated by contractility. We focused on the Rac guanine nucleotide exchange factor β-Pix, documenting its role in the negative regulation of focal adhesion maturation and the promotion of lamellipodial protrusion and focal adhesion turnover to drive cel  l migrati  on. 
 Type I PIPK-alpha regulates directed cell   migration   by modulating Rac1 plasma membrane targeting and activation. Phosphatidylinositol-4,5-bisphosphate (PI4,5P(2)) is a critical regulator of cell   migration  , but the roles of the type I phosphatidylinositol-4-phosphate 5-kinases (PIPKIs), which synthesize PI4,5P(2), have yet to be fully defined in this process. In this study, we report that one kinase, PIPKI-alpha, is a novel upstream regulator of Rac1 that links activated integrins to the regulation of cell   migration  . We show that PIPKI-alpha controls integrin-induced translocation of Rac1 to the plasma membrane and thereby regulates Rac1 activation. Strikingly, this function is not shared with other PIPKI isoforms, is independent of catalytic activity, and requires physical interaction of PIPKI-alpha with the Rac1 polybasic domain. Consistent with its role in Rac1 activation, depletion of PIPKI-alpha causes pronounced defects in membrane ruffling, actin organization, and focal adhesion formation, and ultimately affects the directional persistence of   migration  . Thus, our study defines the role of PIPKI-alpha in cell   migration   and describes a new mechanism for the spatial regulation of Rac1 activity that is critical for cell   migration  . 
 Type I PIPK-alpha regulates directed cell   migration   by modulating Rac1 plasma membrane targeting and activation. Phosphatidylinositol-4,5-bisphosphate (PI4,5P(2)) is a critical regulator of cell   migration  , but the roles of the type I phosphatidylinositol-4-phosphate 5-kinases (PIPKIs), which synthesize PI4,5P(2), have yet to be fully defined in this process. In this study, we report that one kinase, PIPKI-alpha, is a novel upstream regulator of Rac1 that links activated integrins to the regulation of cell   migration  . We show that PIPKI-alpha controls integrin-induced translocation of Rac1 to the plasma membrane and thereby regulates Rac1 activation. Strikingly, this function is not shared with other PIPKI isoforms, is independent of catalytic activity, and requires physical interaction of PIPKI-alpha with the Rac1 polybasic domain. Consistent with its role in Rac1 activation, depletion of PIPKI-alpha causes pronounced defects in membrane ruffling, actin organization, and focal adhesion formation, and ultimately affects the directional persistence of   migration  . Thus, our study defines the role of PIPKI-alpha in cell   migration   and describes a new mechanism for the spatial regulation of Rac1 activity that is critical for cell   migration  . 
 The LIM protein Ajuba regulates phosphatidylinositol 4,5-bisphosphate levels in migrating cells through an interaction with and activation of PIPKI alpha. The phosphoinositide phosphatidylinositol 4,5-bisphosphate [PI(4,5)P2] regulates the activity of many actin-binding proteins and as such is an important modulator of cytoskeleton organization during cell   migration  , for example. In migrating cells actin remodeling is tightly regulated and localized; therefore, how the PI(4,5)P2 level is spatially and temporally regulated is crucial to understanding how it controls cell   migration  . Here we show that the LIM protein Ajuba contributes to the cellular regulation of PI(4,5)P2 levels by interacting with and activating the enzymatic activity of the PI(4)P 5-kinase (PIPKIalpha), the predominant enzyme in the synthesis of PI(4,5)P2, in a   migration   stimulus-regulated manner. In migrating primary mouse embryonic fibroblasts (MEFs) from Ajuba(-/-) mice the level of PI(4,5)P2 was decreased with a corresponding increase in the level of the substrate PI(4)P. Reintroduction of Ajuba into these cells normalized PI(4,5)P2 levels. Localization of PI(4,5)P2 synthesis and PIPKIalpha in the leading lamellipodia and membrane ruffles, respectively, of migrating Ajuba(-/-) MEFs was impaired. In vitro, Ajuba dramatically activated the enzymatic activity of PIPKIalpha while inhibiting the activity of PIPKIIbeta. Thus, in addition to its effects upon Rac activity Ajuba can also influence cell   migration   through regulation of PI(4,5)P2 synthesis through direct activation of PIPKIalpha enzyme activity. 
  GeneRIFs  
 Results define the role of PIPKI-alpha in cell   migration   and describes a new mechanism for the spatial regulation of Rac1 activity that is critical for cell   migration  . 
 in addition to its effects upon Rac activity, Ajuba can also influence cell   migration   through regulation of PI(4,5)P2 synthesis through direct activation of PIPKIalpha enzyme activity 
  ECM  PubMed  
 Phosphatidylinositol 4,5-bisphosphate and PIP5-kinase Ialpha are required for invadopodia formation in human breast cancer cells. Invadopodia are ventral cell protrusions formed in invasive cancer cells. Because invadopodia have extracellular matrix (  ECM  ) degradation activity, they are thought to function in cancer invasion. In this study, we examined the roles of phosphatidylinositol 4,5-bisphosphate [PI(4,5)P(2)] and PI(4,5)P(2)-producing enzymes in invadopodia formation in MDA-MB-231 human breast cancer cells. Immunofluorescence analysis showed that PI(4,5)P(2) accumulates at invadopodia on the ventral cell surface. Injection of an anti-PI(4,5)P(2) antibody inhibited invadopodia formation along with gelatin degradation activity. Sequestering of PI(4,5)P(2) by overexpression of the phospholipase C (PLC) delta1-pleckstrin homology (PH) domain, a specific probe for PI(4,5)P(2), also blocked invadopodia formation, while a mutated PLCdelta1-PH domain that lacks PI(4,5)P(2)-binding activity had no effect. Cellular PI(4,5)P(2) production is mainly mediated by type-I phosphatidylinositol 4-phosphate 5-kinase (PIP5KI) family proteins, which include PIP5KIalpha, Ibeta, and Igamma. Real-time quantitative PCR analysis showed that PIP5KIalpha is a dominant isoform expressed in MDA-MB-231 cells. Knockdown of PIP5KIalpha by small-interfering RNA (siRNA) inhibited invadopodia formation and gelatin degradation. Immunofluorescence analysis revealed that endogenous PIP5KIalpha protein localizes at invadopodia, which is corroborated by the observation that exogenously expressed green fluorescent protein (GFP)-fused PIP5KIalpha protein also accumulates at gelatin degradation sites. These results indicate that localized production of PI(4,5)P(2) by PIP5KIalpha is required for invadopodia formation and   ECM   degradation by human breast cancer cells. 
  [Ee]xtracellular [Mm]atrix  PubMed  
 Phosphatidylinositol 4,5-bisphosphate and PIP5-kinase Ialpha are required for invadopodia formation in human breast cancer cells. Invadopodia are ventral cell protrusions formed in invasive cancer cells. Because invadopodia have   extracellular matrix   (ECM) degradation activity, they are thought to function in cancer invasion. In this study, we examined the roles of phosphatidylinositol 4,5-bisphosphate [PI(4,5)P(2)] and PI(4,5)P(2)-producing enzymes in invadopodia formation in MDA-MB-231 human breast cancer cells. Immunofluorescence analysis showed that PI(4,5)P(2) accumulates at invadopodia on the ventral cell surface. Injection of an anti-PI(4,5)P(2) antibody inhibited invadopodia formation along with gelatin degradation activity. Sequestering of PI(4,5)P(2) by overexpression of the phospholipase C (PLC) delta1-pleckstrin homology (PH) domain, a specific probe for PI(4,5)P(2), also blocked invadopodia formation, while a mutated PLCdelta1-PH domain that lacks PI(4,5)P(2)-binding activity had no effect. Cellular PI(4,5)P(2) production is mainly mediated by type-I phosphatidylinositol 4-phosphate 5-kinase (PIP5KI) family proteins, which include PIP5KIalpha, Ibeta, and Igamma. Real-time quantitative PCR analysis showed that PIP5KIalpha is a dominant isoform expressed in MDA-MB-231 cells. Knockdown of PIP5KIalpha by small-interfering RNA (siRNA) inhibited invadopodia formation and gelatin degradation. Immunofluorescence analysis revealed that endogenous PIP5KIalpha protein localizes at invadopodia, which is corroborated by the observation that exogenously expressed green fluorescent protein (GFP)-fused PIP5KIalpha protein also accumulates at gelatin degradation sites. These results indicate that localized production of PI(4,5)P(2) by PIP5KIalpha is required for invadopodia formation and ECM degradation by human breast cancer cells. 
  [Ii]nvasive  PubMed  
 Phosphatidylinositol 4,5-bisphosphate and PIP5-kinase Ialpha are required for invadopodia formation in human breast cancer cells. Invadopodia are ventral cell protrusions formed in   invasive   cancer cells. Because invadopodia have extracellular matrix (ECM) degradation activity, they are thought to function in cancer invasion. In this study, we examined the roles of phosphatidylinositol 4,5-bisphosphate [PI(4,5)P(2)] and PI(4,5)P(2)-producing enzymes in invadopodia formation in MDA-MB-231 human breast cancer cells. Immunofluorescence analysis showed that PI(4,5)P(2) accumulates at invadopodia on the ventral cell surface. Injection of an anti-PI(4,5)P(2) antibody inhibited invadopodia formation along with gelatin degradation activity. Sequestering of PI(4,5)P(2) by overexpression of the phospholipase C (PLC) delta1-pleckstrin homology (PH) domain, a specific probe for PI(4,5)P(2), also blocked invadopodia formation, while a mutated PLCdelta1-PH domain that lacks PI(4,5)P(2)-binding activity had no effect. Cellular PI(4,5)P(2) production is mainly mediated by type-I phosphatidylinositol 4-phosphate 5-kinase (PIP5KI) family proteins, which include PIP5KIalpha, Ibeta, and Igamma. Real-time quantitative PCR analysis showed that PIP5KIalpha is a dominant isoform expressed in MDA-MB-231 cells. Knockdown of PIP5KIalpha by small-interfering RNA (siRNA) inhibited invadopodia formation and gelatin degradation. Immunofluorescence analysis revealed that endogenous PIP5KIalpha protein localizes at invadopodia, which is corroborated by the observation that exogenously expressed green fluorescent protein (GFP)-fused PIP5KIalpha protein also accumulates at gelatin degradation sites. These results indicate that localized production of PI(4,5)P(2) by PIP5KIalpha is required for invadopodia formation and ECM degradation by human breast cancer cells. 
   
  
 CHAC1 
 
  NCBI Gene  
  GeneRIF  
  Pubmed  
 
 
  Search Term  Rank  Fields  
  [Mm]igration  6  PubMed(1)  
 
 
 hide/show details for CHAC1 
  [Mm]igration  PubMed  
 Elevated mRNA expression of CHAC1 splicing variants is associated with poor outcome for breast and ovarian cancer patients. The role of CHAC1 (cation transport regulator-like protein 1), a recently identified component of the unfolded protein response (UPR) pathway, in gynaecological cancers has not yet been characterised. Now, this work illustrates CHAC1 mRNA expression and associated clinical outcome in breast and ovarian cancer.The prognostic value of CHAC1 and its two transcript variants was investigated in 116 breast and 133 ovarian tissues using quantitative real-time reverse-transcriptase PCR. Subsequently, we conducted functional studies using short-interfering RNA-mediated knockdown and plasmid-mediated overexpression of CHAC1 in breast and ovarian cancer cells.Poorly differentiated tumours exhibited higher CHAC1 mRNA expression (breast cancer: P=0.004; ovarian cancer: P=0.024). Hormone receptor-negative breast tumours and advanced-staged ovarian cancers demonstrated elevated CHAC1 mRNA expression levels (P<0.001 and P=0.026, respectively). The multivariate survival analysis showed a prognostic value of both transcript variants in breast cancer (transcript variant 1: RR(death) 6.7 (2.4-18.9); P<0.001), RR(relapse) 6.7 (2.1-21.3); P=0.001); (transcript variant 2: RR(death) 4.9 (2.0-12.4); P<0.001), RR(relapse) 8.0 (2.4-26.8); P<0.001). Ovarian cancer patients aged younger than 62.6 years with high CHAC1 mRNA expression showed poorer relapse-free- and overall-survival (P=0.030 and P=0.012, respectively). In functional studies CHAC1 knockdown suppressed cell   migration  , whereas ectopic overexpression opposed these effects.High CHAC1 mRNA expression could be an independent indicator for elevated risk of cancer recurrence in breast and ovarian cancer. 
   
  
 PTPN21 
 
  NCBI Gene  
  GeneRIF  
  Pubmed  
 
 
  Search Term  Rank  Fields  
  [Mm]igration  6  PubMed(3)  
  [Ii]nvasive  10  PubMed(1)  
 
 
 hide/show details for PTPN21 
  [Mm]igration  PubMed  
 Expression of protein tyrosine phosphatase PTP-RL10 and its isoform in the mouse testis. The cytoplasmic-type protein tyrosine phosphatase PTP-RL10/PTPD1/PTP2E contains an ezrin-like domain and associates with the c-Src protein tyrosine kinase. Because tyrosine phosphorylation regulated by protein tyrosine kinases and phosphatases is involved in activation,   migration  , differentiation and proliferation of various cell types, the expression of PTP-RL10 and c-src in the mouse testis was investigated.Testes of wild-type mice and W/W(v) mutant mice that lack germ cells were analyzed by northern blotting, in situ hybridization histochemistry and reverse transcriptase-polymerase chain reaction for the expression of PTP-RL10, its isoform PTP-RL10b and c-src. Expression in the Sertoli cell lines, TM4 and TAMA26 was also analyzed.PTP-RL10 transcripts of 5.7kb and 2.9kb in size were detected in the testis. In situ hybridization histochemistry demonstrated that the 5.7kb transcripts were expressed in pachytene spermatocytes and somatic cells including Sertoli cells, in which c-src transcripts were detected. The 2.9kb transcript encoded a novel isoform, PTP-RL10b, that has the catalytic domain but not the domains that associate with c-Src. PTP-RL10b was expressed mainly in the testicular somatic cells. TM4 and TAMA26 cells were found to co-express PTP-RL10, PTP-RL10b and c-src transcripts.PTP-RL10 and its isoform are expressed in the Sertoli cells and are suggested to play roles in spermatogenesis by interacting with c-Src and/or other protein(s). 
 Protein-tyrosine phosphatase PTPD1 regulates focal adhesion kinase autophosphorylation and cell   migration  . PTPD1 is a cytosolic nonreceptor tyrosine phosphatase and a positive regulator of the Src-epidermal growth factor transduction pathway. We show that PTPD1 localizes along actin filaments and at adhesion plaques. PTPD1 forms a stable complex via distinct molecular modules with actin, Src tyrosine kinase, and focal adhesion kinase (FAK), a scaffold protein kinase enriched at adhesion plaques. Overexpression of PTPD1 promoted cell scattering and   migration  , short hairpin RNA-mediated silencing of endogenous PTPD1, or expression of PTPD1 mutants lacking either catalytic activity (PTPD1(C1108S)) or the FERM domain (PTPD1(Delta1-325)) significantly reduced cell motility. PTPD1 and Src catalytic activities were both required for epidermal growth factor-induced FAK autophosphorylation at its active site and for downstream propagation of ERK1/2 signaling. Our findings demonstrate that PTPD1 is a component of a multivalent scaffold complex nucleated by FAK at specific intracellular sites. By modulating Src-FAK signaling at adhesion sites, PTPD1 promotes the cytoskeleton events that induce cell adhesion and   migration  . 
 PTPD1 supports receptor stability and mitogenic signaling in bladder cancer cells. PTPD1, a cytosolic non-receptor protein-tyrosine phosphatase, stimulates the Src-EGF transduction pathway. Localization of PTPD1 at actin cytoskeleton and adhesion sites is required for cell scattering and   migration  . Here, we show that during EGF stimulation, PTPD1 is rapidly recruited to endocytic vesicles containing the EGF receptor. Endosomal localization of PTPD1 is mediated by interaction with KIF16B, an endosomal kinesin that modulates receptor recycling at the plasma membrane. Silencing of PTPD1 promotes degradation of EGF receptor and inhibits downstream ERK signaling. We also found that PTPD1 is markedly increased in bladder cancer tissue samples. PTPD1 levels positively correlated with the grading and invasiveness potential of these tumors. Transgenic expression of an inactive PTPD1 mutant or genetic knockdown of the endogenous PTPD1 severely inhibited both growth and motility of human bladder cancer cells. These findings identify PTPD1 as a novel component of the endocytic machinery that impacts on EGF receptor stability and on growth and motility of bladder cancer cells. 
  [Ii]nvasive  PubMed  
 PTPD1 supports receptor stability and mitogenic signaling in bladder cancer cells. PTPD1, a cytosolic non-receptor protein-tyrosine phosphatase, stimulates the Src-EGF transduction pathway. Localization of PTPD1 at actin cytoskeleton and adhesion sites is required for cell scattering and migration. Here, we show that during EGF stimulation, PTPD1 is rapidly recruited to endocytic vesicles containing the EGF receptor. Endosomal localization of PTPD1 is mediated by interaction with KIF16B, an endosomal kinesin that modulates receptor recycling at the plasma membrane. Silencing of PTPD1 promotes degradation of EGF receptor and inhibits downstream ERK signaling. We also found that PTPD1 is markedly increased in bladder cancer tissue samples. PTPD1 levels positively correlated with the grading and   invasive  ness potential of these tumors. Transgenic expression of an inactive PTPD1 mutant or genetic knockdown of the endogenous PTPD1 severely inhibited both growth and motility of human bladder cancer cells. These findings identify PTPD1 as a novel component of the endocytic machinery that impacts on EGF receptor stability and on growth and motility of bladder cancer cells. 
   
  
 ADAMTS9 
 
  NCBI Gene  
  GeneRIF  
  Pubmed  
 
 
  Search Term  Rank  Fields  
  [Ff]ilopodia  1  PubMed(1)  
  [Mm]igration  6  PubMed(2); GeneRIFs(1)  
  ECM  7  PubMed(5)  
  [Ee]xtracellular [Mm]atrix  8  PubMed(5); Component(2)  
  [Mm]etastasis  12  PubMed(1)  
 
 
 hide/show details for ADAMTS9 
  [Ff]ilopodia  PubMed  
 ADAMTS9 is a cell-autonomously acting, anti-angiogenic metalloprotease expressed by microvascular endothelial cells. The metalloprotease ADAMTS9 participates in melanoblast development and is a tumor suppressor in esophageal and nasopharyngeal cancer. ADAMTS9 null mice die before gastrulation, but, ADAMTS9+/- mice were initially thought to be normal. However, when congenic with the C57Bl/6 strain, 80% of ADAMTS9+/- mice developed spontaneous corneal neovascularization. beta-Galactosidase staining enabled by a lacZ cassette targeted to the ADAMTS9 locus showed that capillary endothelial cells (ECs) in embryonic and adult tissues and in capillaries growing into heterotopic tumors expressed ADAMTS9. Heterotopic B.16-F10 melanomas elicited greater vascular induction in ADAMTS9+/- mice than in wild-type littermates, suggesting a potential inhibitory role in tumor angiogenesis. Treatment of cultured human microvascular ECs with ADAMTS9 small-interfering RNA resulted in enhanced   filopodia  l extension, decreased cell adhesion, increased cell migration, and enhanced formation of tube-like structures on Matrigel. Conversely, overexpression of catalytically active, but not inactive, ADAMTS9 in ECs led to fewer tube-like structures, demonstrating that the proteolytic activity of ADAMTS9 was essential. However, unlike the related metalloprotease ADAMTS1, which exerts anti-angiogenic effects by cleavage of thrombospondins and sequestration of vascular endothelial growth factor165, ADAMTS9 neither cleaved thrombospondins 1 and 2, nor bound vascular endothelial growth factor165. Taken together, these data identify ADAMTS9 as a novel, constitutive, endogenous angiogenesis inhibitor that operates cell-autonomously in ECs via molecular mechanisms that are distinct from those used by ADAMTS1. 
  [Mm]igration  PubMed  
 ADAMTS9 is a cell-autonomously acting, anti-angiogenic metalloprotease expressed by microvascular endothelial cells. The metalloprotease ADAMTS9 participates in melanoblast development and is a tumor suppressor in esophageal and nasopharyngeal cancer. ADAMTS9 null mice die before gastrulation, but, ADAMTS9+/- mice were initially thought to be normal. However, when congenic with the C57Bl/6 strain, 80% of ADAMTS9+/- mice developed spontaneous corneal neovascularization. beta-Galactosidase staining enabled by a lacZ cassette targeted to the ADAMTS9 locus showed that capillary endothelial cells (ECs) in embryonic and adult tissues and in capillaries growing into heterotopic tumors expressed ADAMTS9. Heterotopic B.16-F10 melanomas elicited greater vascular induction in ADAMTS9+/- mice than in wild-type littermates, suggesting a potential inhibitory role in tumor angiogenesis. Treatment of cultured human microvascular ECs with ADAMTS9 small-interfering RNA resulted in enhanced filopodial extension, decreased cell adhesion, increased cell   migration  , and enhanced formation of tube-like structures on Matrigel. Conversely, overexpression of catalytically active, but not inactive, ADAMTS9 in ECs led to fewer tube-like structures, demonstrating that the proteolytic activity of ADAMTS9 was essential. However, unlike the related metalloprotease ADAMTS1, which exerts anti-angiogenic effects by cleavage of thrombospondins and sequestration of vascular endothelial growth factor165, ADAMTS9 neither cleaved thrombospondins 1 and 2, nor bound vascular endothelial growth factor165. Taken together, these data identify ADAMTS9 as a novel, constitutive, endogenous angiogenesis inhibitor that operates cell-autonomously in ECs via molecular mechanisms that are distinct from those used by ADAMTS1. 
 A new tumor suppressor LncRNA ADAMTS9-AS2 is regulated by DNMT1 and inhibits   migration   of glioma cells. Growing number of long noncoding RNAs (lncRNAs) are emerging as new modulators in cancer origination and progression. A lncRNA, ADAM metallopeptidase with thrombospondin type 1 motif, 9 (ADAMTS9) antisense RNA 2 (ADAMTS9-AS2), with unknown function, is the antisense transcript of tumor suppressor ADAMTS9. In the present study, we investigated the expression pattern and functional role of ADAMTS9-AS2 in glioma by using real-time PCR and gain-/loss-of-function studies. The results showed that the ADAMTS9-AS2 expression was significantly downregulated in tumor tissues compared with normal tissues and reversely associated with tumor grade and prognosis. Multivariate analysis of the prognosis factors showed that low ADAMTS9-AS2 expression was a significant independent predictor of poor survival in glioma. Overexpression of ADAMTS9-AS2 resulted in significant inhibition of cell   migration   in glioma, whereas knockdown of ADAMTS9-AS2 showed the opposite effect. We also found that ADAMTS9-AS2 expression was negatively correlated with DNA methyltransferase-1 (DNMT1). In addition, DNMT1 knockdown led to remarkable enhancement of ADAMTS9-AS2 expression. By 5-aza-dC treatment, the ADAMTS9-AS2 expression was also reactivated. The results suggested that ADAMTS9-AS2 is a novel tumor suppressor modulated by DNMT1 in glioma. LncRNA ADAMTS9-AS2 may serve as a potential biomarker and therapeutic target for glioma. 
  GeneRIFs  
 LncRNA ADAMTS9-AS2 is regulated by DNMT1 and inhibits   migration   of glioma cells. 
  ECM  PubMed  
 Proteomics analysis of cardiac extracellular matrix remodeling in a porcine model of ischemia/reperfusion injury. After myocardial ischemia, extracellular matrix (  ECM  ) deposition occurs at the site of the focal injury and at the border region.We have applied a novel proteomic method for the analysis of   ECM   in cardiovascular tissues to a porcine model of ischemia/reperfusion injury.   ECM   proteins were sequentially extracted and identified by liquid chromatography tandem mass spectrometry. For the first time,   ECM   proteins such as cartilage intermediate layer protein 1, matrilin-4, extracellular adipocyte enhancer binding protein 1, collagen α-1(XIV), and several members of the small leucine-rich proteoglycan family, including asporin and prolargin, were shown to contribute to cardiac remodeling. A comparison in 2 distinct cardiac regions (the focal injury in the left ventricle and the border region close to the occluded coronary artery) revealed a discordant regulation of protein and mRNA levels; although gene expression for selected   EC  M proteins was similar in both regions, the corresponding protein levels were much higher in the focal lesion. Further analysis based on >100   EC  M proteins delineated a signature of early- and late-stage cardiac remodeling with transforming growth factor-β1 signaling at the center of the interaction network. Finally, novel cardia  c E  CM proteins identified by proteomics were validated in human left ventricular tissue acquired from ischemic cardiomyopathy patients at cardiac transplantation.Our findings reveal a biosignature of early- and late-stag  e E  CM remodeling after myocardial ischemia/reperfusion injury, which may have clinical utility as a prognostic marker and modifiable target for drug discovery. 
 Identification of a novel ADAMTS9/GON-1 function for protein transport from the ER to the Golgi. A disintegrin-like and metalloprotease with thrombospondin type I motif (ADAMTS9) is a member of the secreted metalloprotease family that is believed to digest extracellular matrix (  ECM  ) proteins outside of cells. Its Caenorhabditis elegans orthologue, GON-1, is involved in   ECM   degradation and is required for gonad morphogenesis. ADAMTS9 and GON-1 have similar domain structures, and both have a unique C-terminal domain called the "GON domain," whose function remains unknown. Here we show that down-regulation of human ADAMTS9 and C. elegans GON-1 results in the inhibition of protein transport from the endoplasmic reticulum (ER) to the Golgi. This phenotype was rescued by the expression of the GON domain localizing in the ER in human cells and C. elegans. We propose a novel function of ADAMTS9 and GON-1 in the ER that promotes protein transport from the ER to the Golgi. This function is GON-domain dependent but protease activity independent. 
 ADAMTS: a novel family of extracellular matrix proteases. ADAMTS (a disintegrin and metalloprotease with thrombospondin motifs) is a novel family of extracellular proteases found in both mammals and invertebrates. Members of the family may be distinguished from the ADAM (a disintegrin and metalloprotease) family members based on the multiple copies of thrombospondin 1-like repeats they carry. With at least nine members in mammals alone, the ADAMTS family members are predicted by their structural domains to be extracellular matrix (  ECM  ) proteins with a wide range of activities and functions distinct from members of the ADAM family that are largely anchored on the cell surface. ADAMTS2 is a procollagen N-proteinase, and the mutations of its gene are responsible for Human Ehlers-Danlos syndrome type VII C and bovine dermatosparaxis. ADAMTS4 and ADAMTS5 are aggrecanases implicated in the degradation of cartilage aggrecan in arthritic diseases. Other members of the ADAMTS family have also been implicated in roles during embryonic development and angiogenesis. Current and future studies on this emerging group of   ECM   proteases may provide important insights into developmental or pathological processes involving   ECM   remodeling. 
 The expression and regulation of ADAMTS-1, -4, -5, -9, and -15, and TIMP-3 by TGFbeta1 in prostate cells: relevance to the accumulation of versican. Benign prostatic hyperplasia (BPH) is characterized by a proportional increase in the size of the stromal compartment of the gland, involving alterations to extracellular matrix (  ECM  ) components. Some of these changes have been associated with the activity and expression of transforming growth factor beta1 (TGFbeta1). Versican (chondroitin sulphate proteoglycan-2) is overexpressed in BPH and prostate cancer and potentially contributes to disease pathology. A sub-group of the ADAMTS lineage of metalloproteases possess versican-degrading properties and are potential regulators of proteoglycan accumulation associated with BPH. These enzymes have one major inhibitor in the   ECM  , tissue inhibitor of metalloproteinases (TIMP)-3.The effect of TGFbeta on mRNA expression in prostatic stromal cells was determined by real-time qRT-PCR using primers to ADAMTS-1, -4, -5, -9, -15, versican, and TIMP-3. MMP-inhibitory potential (TIMP activity) of conditioned medium was measured using a fluorometric peptide substrate.Prostatic stromal cell cultures consistently expressed ADAMTS-1, -4, -5, -9, -15 and TIMP-3, in contrast to PC3, DU145, and LNCaP cells which failed to express at least two ADAMTS transcripts. In stromal cells, TGFbeta1 decreased ADAMTS-1, -5, -9, and -15 transcripts and increased ADAMTS-4, versican, and TIMP-3. TGFbeta also increased TIMP activity in conditioned medium.The induction of versican expression by TGFbeta in BPH stromal cells is in agreement with histological studies. The negative effect of TGFbeta1 on ADAMTS-1, -5, -9, and -15 coupled with increases in their inhibitor, TIMP-3 may aid the accumulation of versican in the stromal compartment of the prostate in BPH and prostate cancer. 
 Characterization of ADAMTS-9 and ADAMTS-20 as a distinct ADAMTS subfamily related to Caenorhabditis elegans GON-1. We demonstrate that in humans, two metalloproteases, ADAMTS-9 (1935 amino acids) and ADAMTS-20 (1911 amino acids) are orthologs of GON-1, an ADAMTS protease required for gonadal morphogenesis in Caenorhabditis elegans. ADAMTS-9 and ADAMTS-20 have an identical modular structure, are distinct in possessing 15 TSRs and a unique C-terminal domain, and have a similar gene structure, suggesting that they comprise a new subfamily of human ADAMTS proteases. ADAMTS20 is very sparingly expressed, although it is detectable in epithelial cells of the breast and lung. However, ADAMTS9 is highly expressed in embryonic and adult tissues, and therefore we characterized the ADAMTS-9 protein further. Although the ADAMTS-9 zymogen has many proprotein convertase processing sites, pulse-chase analysis, site-directed mutagenesis, and amino acid sequencing demonstrated that maturation to the active form occurs by selective proprotein convertase (e.g. furin) cleavage of the Arg(287)-Phe(288) bond. Although lacking a transmembrane sequence, ADAMTS-9 is retained near the cell surface as well as in the   ECM   of transiently transfected COS-1 and 293 cells. COS-1 cells transfected with ADAMTS9 (but not vector-transfected cells) proteolytically cleaved bovine versican and aggrecan core proteins at the Glu(441)-Ala(442) bond of versican V1 and the Glu(1771)-Ala(1772) bond of aggrecan, respectively. In contrast, the ADAMTS-9 catalytic domain alone was neither localized to the cell surface nor able to confer these proteolytic activities on cells, demonstrating that the ancillary domains of ADAMTS-9, including the TSRs, are required both for specific extracellular localization and for its versicanase and aggrecanase activities. 
  [Ee]xtracellular [Mm]atrix  PubMed  
 Proteomics analysis of cardiac   extracellular matrix   remodeling in a porcine model of ischemia/reperfusion injury. After myocardial ischemia,   extracellular matrix   (ECM) deposition occurs at the site of the focal injury and at the border region.We have applied a novel proteomic method for the analysis of ECM in cardiovascular tissues to a porcine model of ischemia/reperfusion injury. ECM proteins were sequentially extracted and identified by liquid chromatography tandem mass spectrometry. For the first time, ECM proteins such as cartilage intermediate layer protein 1, matrilin-4, extracellular adipocyte enhancer binding protein 1, collagen α-1(XIV), and several members of the small leucine-rich proteoglycan family, including asporin and prolargin, were shown to contribute to cardiac remodeling. A comparison in 2 distinct cardiac regions (the focal injury in the left ventricle and the border region close to the occluded coronary artery) revealed a discordant regulation of protein and mRNA levels; although gene expression for selected ECM proteins was similar in both regions, the corresponding protein levels were much higher in the focal lesion. Further analysis based on >100 ECM proteins delineated a signature of early- and late-stage cardiac remodeling with transforming growth factor-β1 signaling at the center of the interaction network. Finally, novel cardiac ECM proteins identified by proteomics were validated in human left ventricular tissue acquired from ischemic cardiomyopathy patients at cardiac transplantation.Our findings reveal a biosignature of early- and late-stage ECM remodeling after myocardial ischemia/reperfusion injury, which may have clinical utility as a prognostic marker and modifiable target for drug discovery. 
 Identification of a novel ADAMTS9/GON-1 function for protein transport from the ER to the Golgi. A disintegrin-like and metalloprotease with thrombospondin type I motif (ADAMTS9) is a member of the secreted metalloprotease family that is believed to digest   extracellular matrix   (ECM) proteins outside of cells. Its Caenorhabditis elegans orthologue, GON-1, is involved in ECM degradation and is required for gonad morphogenesis. ADAMTS9 and GON-1 have similar domain structures, and both have a unique C-terminal domain called the "GON domain," whose function remains unknown. Here we show that down-regulation of human ADAMTS9 and C. elegans GON-1 results in the inhibition of protein transport from the endoplasmic reticulum (ER) to the Golgi. This phenotype was rescued by the expression of the GON domain localizing in the ER in human cells and C. elegans. We propose a novel function of ADAMTS9 and GON-1 in the ER that promotes protein transport from the ER to the Golgi. This function is GON-domain dependent but protease activity independent. 
 Seven new loci associated with age-related macular degeneration. Age-related macular degeneration (AMD) is a common cause of blindness in older individuals. To accelerate the understanding of AMD biology and help design new therapies, we executed a collaborative genome-wide association study, including >17,100 advanced AMD cases and >60,000 controls of European and Asian ancestry. We identified 19 loci associated at P < 5 × 10(-8). These loci show enrichment for genes involved in the regulation of complement activity, lipid metabolism,   extracellular matri  x remodeling and angiogenesis. Our results include seven loci with associations reaching P < 5 × 10(-8) for the first time, near the genes COL8A1-FILIP1L, IER3-DDR1, SLC16A8, TGFBR1, RAD51B, ADAMTS9 and B3GALTL. A genetic risk score combining SNP genotypes from all loci showed similar ability to distinguish cases and controls in all samples examined. Our findings provide new directions for biological, genetic and therapeutic studies of AMD. 
 ADAMTS: a novel family of   extracellular matrix   proteases. ADAMTS (a disintegrin and metalloprotease with thrombospondin motifs) is a novel family of extracellular proteases found in both mammals and invertebrates. Members of the family may be distinguished from the ADAM (a disintegrin and metalloprotease) family members based on the multiple copies of thrombospondin 1-like repeats they carry. With at least nine members in mammals alone, the ADAMTS family members are predicted by their structural domains to be   extracellular matrix   (ECM) proteins with a wide range of activities and functions distinct from members of the ADAM family that are largely anchored on the cell surface. ADAMTS2 is a procollagen N-proteinase, and the mutations of its gene are responsible for Human Ehlers-Danlos syndrome type VII C and bovine dermatosparaxis. ADAMTS4 and ADAMTS5 are aggrecanases implicated in the degradation of cartilage aggrecan in arthritic diseases. Other members of the ADAMTS family have also been implicated in roles during embryonic development and angiogenesis. Current and future studies on this emerging group of ECM proteases may provide important insights into developmental or pathological processes involving ECM remodeling. 
 The expression and regulation of ADAMTS-1, -4, -5, -9, and -15, and TIMP-3 by TGFbeta1 in prostate cells: relevance to the accumulation of versican. Benign prostatic hyperplasia (BPH) is characterized by a proportional increase in the size of the stromal compartment of the gland, involving alterations to   extracellular matrix   (ECM) components. Some of these changes have been associated with the activity and expression of transforming growth factor beta1 (TGFbeta1). Versican (chondroitin sulphate proteoglycan-2) is overexpressed in BPH and prostate cancer and potentially contributes to disease pathology. A sub-group of the ADAMTS lineage of metalloproteases possess versican-degrading properties and are potential regulators of proteoglycan accumulation associated with BPH. These enzymes have one major inhibitor in the ECM, tissue inhibitor of metalloproteinases (TIMP)-3.The effect of TGFbeta on mRNA expression in prostatic stromal cells was determined by real-time qRT-PCR using primers to ADAMTS-1, -4, -5, -9, -15, versican, and TIMP-3. MMP-inhibitory potential (TIMP activity) of conditioned medium was measured using a fluorometric peptide substrate.Prostatic stromal cell cultures consistently expressed ADAMTS-1, -4, -5, -9, -15 and TIMP-3, in contrast to PC3, DU145, and LNCaP cells which failed to express at least two ADAMTS transcripts. In stromal cells, TGFbeta1 decreased ADAMTS-1, -5, -9, and -15 transcripts and increased ADAMTS-4, versican, and TIMP-3. TGFbeta also increased TIMP activity in conditioned medium.The induction of versican expression by TGFbeta in BPH stromal cells is in agreement with histological studies. The negative effect of TGFbeta1 on ADAMTS-1, -5, -9, and -15 coupled with increases in their inhibitor, TIMP-3 may aid the accumulation of versican in the stromal compartment of the prostate in BPH and prostate cancer. 
  Component  
   extracellular matrix   
 proteinaceous   extracellular matrix   
  [Mm]etastasis  PubMed  
 High-resolution melting analysis of ADAMTS9 methylation levels in gastric, colorectal, and pancreatic cancers. ADAMTS (a disintegrin and metalloprotease with thrombospondin motifs) is a family of proteins characterized by the presence of a metalloproteinase domain linked to a variety of specialized ancillary domains. The ADAMTS9 gene (ADAM metallopeptidase with thrombospondin type 1 motif, 9); has been characterized as a novel tumor suppressor gene in and epigenetically silenced in association with lymph node metastases in nasopharyngeal carcinoma. High-resolution melting (HRM) analysis has been used as a tool for analysis of promoter methylation. Here, we report HRM analysis used to detect the methylation levels of ADAMTS9 gene in 100 gastric cancers, 100 colorectal cancers, 70 pancreatic cancers, and an equal number of adjacent normal tissues. The frequency of ADAMTS9 methylation in all three types of cancers was significantly higher than in normal tissues. Consistent with previous reports, expression levels of ADAMTS9 were inversely correlated with methylation levels. There was no significant association between ADAMTS9 methylation status and tumor-node-  metastasis   staging in all three types of cancers. In summary, application of HRM analysis to large numbers of clinical samples is a rapid and high-throughput way to investigate the epigenetic status of ADAMTS9. The present study is novel in evaluating the prevalence of ADAMTS9 methylation based on a large number of tumor samples and showing that epigenetic regulation of ADAMTS9 was associated with carcinogenesis. 
   
  
 ADAMTS20 
 
  NCBI Gene  
  GeneRIF  
  Pubmed  
 
 
  Search Term  Rank  Fields  
  ECM  7  PubMed(3)  
  [Ee]xtracellular [Mm]atrix  8  Process(1); PubMed(1); Component(2); Summary(1)  
 
 
 hide/show details for ADAMTS20 
  ECM  PubMed  
 Proteomics analysis of cardiac extracellular matrix remodeling in a porcine model of ischemia/reperfusion injury. After myocardial ischemia, extracellular matrix (  ECM  ) deposition occurs at the site of the focal injury and at the border region.We have applied a novel proteomic method for the analysis of   ECM   in cardiovascular tissues to a porcine model of ischemia/reperfusion injury.   ECM   proteins were sequentially extracted and identified by liquid chromatography tandem mass spectrometry. For the first time,   ECM   proteins such as cartilage intermediate layer protein 1, matrilin-4, extracellular adipocyte enhancer binding protein 1, collagen α-1(XIV), and several members of the small leucine-rich proteoglycan family, including asporin and prolargin, were shown to contribute to cardiac remodeling. A comparison in 2 distinct cardiac regions (the focal injury in the left ventricle and the border region close to the occluded coronary artery) revealed a discordant regulation of protein and mRNA levels; although gene expression for selected   EC  M proteins was similar in both regions, the corresponding protein levels were much higher in the focal lesion. Further analysis based on >100   EC  M proteins delineated a signature of early- and late-stage cardiac remodeling with transforming growth factor-β1 signaling at the center of the interaction network. Finally, novel cardia  c E  CM proteins identified by proteomics were validated in human left ventricular tissue acquired from ischemic cardiomyopathy patients at cardiac transplantation.Our findings reveal a biosignature of early- and late-stag  e E  CM remodeling after myocardial ischemia/reperfusion injury, which may have clinical utility as a prognostic marker and modifiable target for drug discovery. 
 Characterization of ADAMTS-9 and ADAMTS-20 as a distinct ADAMTS subfamily related to Caenorhabditis elegans GON-1. We demonstrate that in humans, two metalloproteases, ADAMTS-9 (1935 amino acids) and ADAMTS-20 (1911 amino acids) are orthologs of GON-1, an ADAMTS protease required for gonadal morphogenesis in Caenorhabditis elegans. ADAMTS-9 and ADAMTS-20 have an identical modular structure, are distinct in possessing 15 TSRs and a unique C-terminal domain, and have a similar gene structure, suggesting that they comprise a new subfamily of human ADAMTS proteases. ADAMTS20 is very sparingly expressed, although it is detectable in epithelial cells of the breast and lung. However, ADAMTS9 is highly expressed in embryonic and adult tissues, and therefore we characterized the ADAMTS-9 protein further. Although the ADAMTS-9 zymogen has many proprotein convertase processing sites, pulse-chase analysis, site-directed mutagenesis, and amino acid sequencing demonstrated that maturation to the active form occurs by selective proprotein convertase (e.g. furin) cleavage of the Arg(287)-Phe(288) bond. Although lacking a transmembrane sequence, ADAMTS-9 is retained near the cell surface as well as in the   ECM   of transiently transfected COS-1 and 293 cells. COS-1 cells transfected with ADAMTS9 (but not vector-transfected cells) proteolytically cleaved bovine versican and aggrecan core proteins at the Glu(441)-Ala(442) bond of versican V1 and the Glu(1771)-Ala(1772) bond of aggrecan, respectively. In contrast, the ADAMTS-9 catalytic domain alone was neither localized to the cell surface nor able to confer these proteolytic activities on cells, demonstrating that the ancillary domains of ADAMTS-9, including the TSRs, are required both for specific extracellular localization and for its versicanase and aggrecanase activities. 
 Characterization of ADAMTS-9 and ADAMTS-20 as a distinct ADAMTS subfamily related to Caenorhabditis elegans GON-1. We demonstrate that in humans, two metalloproteases, ADAMTS-9 (1935 amino acids) and ADAMTS-20 (1911 amino acids) are orthologs of GON-1, an ADAMTS protease required for gonadal morphogenesis in Caenorhabditis elegans. ADAMTS-9 and ADAMTS-20 have an identical modular structure, are distinct in possessing 15 TSRs and a unique C-terminal domain, and have a similar gene structure, suggesting that they comprise a new subfamily of human ADAMTS proteases. ADAMTS20 is very sparingly expressed, although it is detectable in epithelial cells of the breast and lung. However, ADAMTS9 is highly expressed in embryonic and adult tissues, and therefore we characterized the ADAMTS-9 protein further. Although the ADAMTS-9 zymogen has many proprotein convertase processing sites, pulse-chase analysis, site-directed mutagenesis, and amino acid sequencing demonstrated that maturation to the active form occurs by selective proprotein convertase (e.g. furin) cleavage of the Arg(287)-Phe(288) bond. Although lacking a transmembrane sequence, ADAMTS-9 is retained near the cell surface as well as in the   ECM   of transiently transfected COS-1 and 293 cells. COS-1 cells transfected with ADAMTS9 (but not vector-transfected cells) proteolytically cleaved bovine versican and aggrecan core proteins at the Glu(441)-Ala(442) bond of versican V1 and the Glu(1771)-Ala(1772) bond of aggrecan, respectively. In contrast, the ADAMTS-9 catalytic domain alone was neither localized to the cell surface nor able to confer these proteolytic activities on cells, demonstrating that the ancillary domains of ADAMTS-9, including the TSRs, are required both for specific extracellular localization and for its versicanase and aggrecanase activities. 
  [Ee]xtracellular [Mm]atrix  Process  
   extracellular matrix   organization 
  PubMed  
 Proteomics analysis of cardiac   extracellular matrix   remodeling in a porcine model of ischemia/reperfusion injury. After myocardial ischemia,   extracellular matrix   (ECM) deposition occurs at the site of the focal injury and at the border region.We have applied a novel proteomic method for the analysis of ECM in cardiovascular tissues to a porcine model of ischemia/reperfusion injury. ECM proteins were sequentially extracted and identified by liquid chromatography tandem mass spectrometry. For the first time, ECM proteins such as cartilage intermediate layer protein 1, matrilin-4, extracellular adipocyte enhancer binding protein 1, collagen α-1(XIV), and several members of the small leucine-rich proteoglycan family, including asporin and prolargin, were shown to contribute to cardiac remodeling. A comparison in 2 distinct cardiac regions (the focal injury in the left ventricle and the border region close to the occluded coronary artery) revealed a discordant regulation of protein and mRNA levels; although gene expression for selected ECM proteins was similar in both regions, the corresponding protein levels were much higher in the focal lesion. Further analysis based on >100 ECM proteins delineated a signature of early- and late-stage cardiac remodeling with transforming growth factor-β1 signaling at the center of the interaction network. Finally, novel cardiac ECM proteins identified by proteomics were validated in human left ventricular tissue acquired from ischemic cardiomyopathy patients at cardiac transplantation.Our findings reveal a biosignature of early- and late-stage ECM remodeling after myocardial ischemia/reperfusion injury, which may have clinical utility as a prognostic marker and modifiable target for drug discovery. 
  Component  
   extracellular matrix   
 proteinaceous   extracellular matrix   
  Summary  
 The protein encoded by this gene is a member of the ADAMTS family of zinc-dependent proteases. The encoded protein has a signal peptide that is cleaved to release the mature peptide, which is secreted and found in the   extracellular matrix  . This protein may be involved in tissue remodeling. [provided by RefSeq, Sep 2011] 
   
  
 ASAP2 
 
  NCBI Gene  
  GeneRIF  
  Pubmed  
 
 
  Search Term  Rank  Fields  
  [Ii]nvadopodia  3  PubMed(2)  
  [Mm]igration  6  PubMed(4)  
  [Ii]nvasive  10  PubMed(2)  
  [Mm]etastasis  12  PubMed(1)  
  [Cc]ell [Ii]nvasion  13  PubMed(1)  
 
 
 hide/show details for ASAP2 
  [Ii]nvadopodia  PubMed  
 CIN85, a Cbl-interacting protein, is a component of AMAP1-mediated breast cancer invasion machinery. Expression of AMAP1 correlates well with the invasive phenotypes and malignancy of human primary breast carcinomas. AMAP1 recruits its binding proteins, such as cortactin and paxillin, to sites of Arf6 activation to form   invadopodia  . A mouse ortholog of AMAP1, ASAP1, is known to bind to CIN85, a binding partner of an E3 ligase, Cbl. Here, we found that CIN85 colocalizes with AMAP1 at   invadopodia  , and binding of AMAP1 with CIN85 is important for the invasive activities of breast cancer cells, including MDA-MB-231. siRNA-mediated silencing of CIN85, as well as Cbl, also inhibited the invasion. We moreover found that AMAP1 is monoubiquitinated, rather than polyubiquitinated, by virtue of Cbl and provide evidence that the ability of AMAP1 to be monoubiquitinated is important for its involvement in invasion. Our results indicate that CIN85, as well as Cbl, which is a well-known suppressor of growth factor receptor signaling, can be positively involved in tumor invasion, and suggest that a complex epigenetic process is involved in AMAP1 function in breast cancer cell invasion. 
 Expression of AMAP1, an ArfGAP, provides novel targets to inhibit breast cancer invasive activities. Identification of the molecular machinery employed in cancer invasion, but not in normal adult cells, will greatly contribute to cancer therapeutics. Here we found that an ArfGAP, AMAP1/PAG2, is expressed at high levels in highly invasive breast cancer cells, but at very low levels in noninvasive breast cancer cells and normal mammary epithelial cells. siRNA-mediated silencing of AMAP1 effectively blocked the invasive activities. AMAP1 expression in human breast primary tumors also indicated its potential correlation with malignancy. Paxillin and cortactin have been shown to colocalize at   invadopodia   and play a pivotal role in breast cancer invasion. We found that AMAP1 is also localized at   invadopodia  , and acts to bridge paxillin and cortactin. This AMAP1-mediated trimeric protein complex was detected only in invasive cancer cells, and blocking this complex formation effectively inhibited their invasive activities in vitro and metastasis in mice. Our results indicate that AMAP1 is a component involved in invasive activities of different breast cancers, and provide new information regarding the possible therapeutic targets for prevention of breast cancer invasion and metastasis. 
  [Mm]igration  PubMed  
 The ASAP2 gene is a primary target of 1,25-dihydroxyvitamin D3 in human monocytes and macrophages. A genome-wide data set on vitamin D receptor (VDR) binding sites in human THP-1 cells (monocytes) led us to the genomic region around the ASAP2 (Arf-GAP with SH3 domain, ankyrin repeat and PH domain 2) gene, whose product is involved in the regulation of vesicular transport, cellular   migration   and autophagy. Using ENCODE data, we demonstrated that the ASAP2 gene is flanked by conserved binding sites of the insulating transcription factor CTCF. These sites define different chromosomal domains containing the ASAP2 gene, up to six additional genes and three VDR binding sites. In human monocytes (THP-1 cells) the ASAP2 gene is more weakly expressed but more and faster inducible by the biologically active form of vitamin D, 1α,25-dihydroxyvitamin D3 (1,25(OH)2D3), than in M2-type macrophages (phorbol ester-differentiated THP-1 cells). Within the investigated genomic region, the basal mRNA expressions of the neighboring genes are comparably high in both monocytes and macrophages, but the ASAP2 gene is the only primary 1,25(OH)2D3 target. The three VDR binding sites located 54, 436 and 574kb downstream of the ASAP2 transcription start site each carry a sequence formed by a direct repeat with three intervening nucleotides (DR3). Ligand-inducible VDR binding was confirmed to all three genomic sites in monocytes and macrophages. Taken together, the region around the ASAP2 gene is genome-wide highlighted as a special attraction point for the VDR, but the presently sole known functional consequence of the binding of VDR to three sites within this chromosomal region is that ASAP2 is a primary 1,25(OH)2D3 target gene in monocytes and macrophages. This article is part of a Special Issue entitled '16th Vitamin D Workshop'. 
 Interaction of POB1, a downstream molecule of small G protein Ral, with PAG2, a paxillin-binding protein, is involved in cell   migration  . POB1 was previously identified as a RalBP1-binding protein. POB1 and RalBP1 function downstream of small G protein Ral and regulate receptor-mediated endocytosis. To look for additional functions of POB1, we screened for POB1-binding proteins using a yeast two-hybrid method and found that POB1 interacts with mouse ASAP1, which is a human PAG2 homolog. PAG2 is a paxillin-associated protein with ADP-ribosylation factor GTPase-activating protein activity. POB1 formed a complex with PAG2 in intact cells. The carboxyl-terminal region containing the proline-rich motifs of POB1 directly bound to the carboxyl-terminal region including the SH3 domain of PAG2. Substitutions of Pro(423) and Pro(426) with Ala (POB1(PA)) impaired the binding of POB1 to PAG2. Expression of PAG2 inhibited fibronectin-dependent   migration   and paxillin recruitment to focal contacts of CHO-IR cells. Co-expression with POB1 but not with POB1(PA) suppressed the inhibitory action of PAG2 on cell   migration   and paxillin localization. These results suggest that POB1 interacts with PAG2 through its proline-rich motif, thereby regulating cell   migration  . 
 A new paxillin-binding protein, PAG3/Papalpha/KIAA0400, bearing an ADP-ribosylation factor GTPase-activating protein activity, is involved in paxillin recruitment to focal adhesions and cell   migration  . Paxillin acts as an adaptor molecule in integrin signaling. Paxillin is localized to focal contacts but seems to also exist in a relatively large cytoplasmic pool. Here, we report the identification of a new paxillin-binding protein, PAG3 (paxillin-associated protein with ADP-ribosylation factor [ARF] GTPase-activating protein [GAP] activity, number 3), which is involved in regulation of the subcellular localization of paxillin. PAG3 bound to all paxillin isoforms and was induced during monocyte maturation, at which time paxillin expression is also increased and integrins are activated. PAG3 was diffusely distributed in the cytoplasm in premature monocytes but became localized at cell periphery in mature monocytes, a fraction of which then colocalized with paxillin. PAG3, on the other hand, did not accumulate at focal adhesion plaques, suggesting that PAG3 is not an integrin assembly protein. PAG3 was identical to KIAA0400/Papalpha, which was previously identified as a Pyk2-binding protein bearing a GAP activity toward several ARFs in vitro. Mammalian ARFs fall into three classes, and we showed that all classes could affect subcellular localization of paxillin. We also examined possible interaction of PAG3 with ARFs and showed evidence that at least one of them, ARF6, seems to be an intracellular substrate for GAP activity of PAG3. Moreover, overexpression of PAG3, but not its GAP-inactive mutant, inhibited paxillin recruitment to focal contacts and hampered cell migratory activities, whereas cell adhesion activities were almost unaffected. Therefore, our results demonstrate that paxillin recruitment to focal adhesions is not mediated by simple cytoplasmic diffusion; rather, PAG3 appears to be involved in this process, possibly through its GAP activity toward ARF proteins. Our result thus delineates a new aspect of regulation of cell migratory activities. 
 Identification of a link between the SAMP repeats of adenomatous polyposis coli tumor suppressor and the Src homology 3 domain of DDEF. The adenomatous polyposis coli (APC) tumor suppressor protein is a multifunctional protein with a well characterized role in the Wnt signal transduction pathway and in cytoskeletal regulation. The SAMP repeats region of APC, an Axin-binding site, is known to be important for tumor suppression and for the developmental function of APC. We performed a yeast two-hybrid screening using the first SAMP motif-containing region of Xenopus APC as bait and obtained several SAMP binding candidates including DDEF2 (development and differentiation enhancing factor 2), which is an ADP-ribosylation factor (Arf) GTPase-activating protein (GAP (ArfGAP)) involved in the regulation of focal adhesions. In vitro and in cells the Src homology 3 (SH3) domain of DDEF2 and its close homolog, DDEF1, are associated with the SAMP motif of APC competitively with Axin1. Moreover, NMR chemical shift perturbation experiments revealed that the SAMP motif interacts at the same surface of the SH3 domain of DDEF as the known SH3 binding motif, PXXP. When fluorescent protein-tagged APC and DDEF are expressed in Xenopus A6 cells, co-localization at microtubule ends is observed. Overexpression and RNA interference experiments indicate that APC and DDEFs cooperatively regulate the distributions of microtubules and focal adhesions. Our findings reveal that the SAMP motif of APC specifically binds to the SH3 domains of DDEFs, providing new insights into the functions of APC in cell   migration  . 
  [Ii]nvasive  PubMed  
 CIN85, a Cbl-interacting protein, is a component of AMAP1-mediated breast cancer invasion machinery. Expression of AMAP1 correlates well with the   invasive   phenotypes and malignancy of human primary breast carcinomas. AMAP1 recruits its binding proteins, such as cortactin and paxillin, to sites of Arf6 activation to form invadopodia. A mouse ortholog of AMAP1, ASAP1, is known to bind to CIN85, a binding partner of an E3 ligase, Cbl. Here, we found that CIN85 colocalizes with AMAP1 at invadopodia, and binding of AMAP1 with CIN85 is important for the   invasive   activities of breast cancer cells, including MDA-MB-231. siRNA-mediated silencing of CIN85, as well as Cbl, also inhibited the invasion. We moreover found that AMAP1 is monoubiquitinated, rather than polyubiquitinated, by virtue of Cbl and provide evidence that the ability of AMAP1 to be monoubiquitinated is important for its involvement in invasion. Our results indicate that CIN85, as well as Cbl, which is a well-known suppressor of growth factor receptor signaling, can be positively involved in tumor invasion, and suggest that a complex epigenetic process is involved in AMAP1 function in breast cancer cell invasion. 
 Expression of AMAP1, an ArfGAP, provides novel targets to inhibit breast cancer   invasive   activities. Identification of the molecular machinery employed in cancer invasion, but not in normal adult cells, will greatly contribute to cancer therapeutics. Here we found that an ArfGAP, AMAP1/PAG2, is expressed at high levels in highly   invasive   breast cancer cells, but at very low levels in non  invasive   breast cancer cells and normal mammary epithelial cells. siRNA-mediated silencing of AMAP1 effectively blocked the   invasive   activities. AMAP1 expression in human breast primary tumors also indicated its potential correlation with malignancy. Paxillin and cortactin have been shown to colocalize at invadopodia and play a pivotal role in breast cancer invasion. We found that AMAP1 is also localized at invadopodia, and acts to bridge paxillin and cortactin. This AMAP1-mediated trimeric protein complex was detected only in   invasive   cancer cells, and blocking this complex formation effectively inhibited their   invasive   activities in vitro and metastasis in mice. Our results indicate that AMAP1 is a component involved in   invasive   activities of different breast cancers, and provide new information regarding the possible therapeutic targets for prevention of breast cancer invasion and metastasis. 
  [Mm]etastasis  PubMed  
 Expression of AMAP1, an ArfGAP, provides novel targets to inhibit breast cancer invasive activities. Identification of the molecular machinery employed in cancer invasion, but not in normal adult cells, will greatly contribute to cancer therapeutics. Here we found that an ArfGAP, AMAP1/PAG2, is expressed at high levels in highly invasive breast cancer cells, but at very low levels in noninvasive breast cancer cells and normal mammary epithelial cells. siRNA-mediated silencing of AMAP1 effectively blocked the invasive activities. AMAP1 expression in human breast primary tumors also indicated its potential correlation with malignancy. Paxillin and cortactin have been shown to colocalize at invadopodia and play a pivotal role in breast cancer invasion. We found that AMAP1 is also localized at invadopodia, and acts to bridge paxillin and cortactin. This AMAP1-mediated trimeric protein complex was detected only in invasive cancer cells, and blocking this complex formation effectively inhibited their invasive activities in vitro and   metastasis   in mice. Our results indicate that AMAP1 is a component involved in invasive activities of different breast cancers, and provide new information regarding the possible therapeutic targets for prevention of breast cancer invasion and   metastasis  . 
  [Cc]ell [Ii]nvasion  PubMed  
 CIN85, a Cbl-interacting protein, is a component of AMAP1-mediated breast cancer invasion machinery. Expression of AMAP1 correlates well with the invasive phenotypes and malignancy of human primary breast carcinomas. AMAP1 recruits its binding proteins, such as cortactin and paxillin, to sites of Arf6 activation to form invadopodia. A mouse ortholog of AMAP1, ASAP1, is known to bind to CIN85, a binding partner of an E3 ligase, Cbl. Here, we found that CIN85 colocalizes with AMAP1 at invadopodia, and binding of AMAP1 with CIN85 is important for the invasive activities of breast cancer cells, including MDA-MB-231. siRNA-mediated silencing of CIN85, as well as Cbl, also inhibited the invasion. We moreover found that AMAP1 is monoubiquitinated, rather than polyubiquitinated, by virtue of Cbl and provide evidence that the ability of AMAP1 to be monoubiquitinated is important for its involvement in invasion. Our results indicate that CIN85, as well as Cbl, which is a well-known suppressor of growth factor receptor signaling, can be positively involved in tumor invasion, and suggest that a complex epigenetic process is involved in AMAP1 function in breast cancer   cell invasion  . 
   
  
 ASAP1 
 
  NCBI Gene  
  GeneRIF  
  Pubmed  
 
 
  Search Term  Rank  Fields  
  [Ii]nvadopodia  3  PubMed(4)  
  [Pp]odosome(s)?  5  Process(1); PubMed(4); Component(1); GeneRIFs(1)  
  [Mm]igration  6  PubMed(11); GeneRIFs(1)  
  [Ii]nvasive  10  PubMed(9)  
  [Mm]etastasis  12  PubMed(11); GeneRIFs(3)  
  [Cc]ell [Ii]nvasion  13  PubMed(4); GeneRIFs(1)  
 
 
 hide/show details for ASAP1 
  [Ii]nvadopodia  PubMed  
 CIN85, a Cbl-interacting protein, is a component of AMAP1-mediated breast cancer invasion machinery. Expression of AMAP1 correlates well with the invasive phenotypes and malignancy of human primary breast carcinomas. AMAP1 recruits its binding proteins, such as cortactin and paxillin, to sites of Arf6 activation to form   invadopodia  . A mouse ortholog of AMAP1, ASAP1, is known to bind to CIN85, a binding partner of an E3 ligase, Cbl. Here, we found that CIN85 colocalizes with AMAP1 at   invadopodia  , and binding of AMAP1 with CIN85 is important for the invasive activities of breast cancer cells, including MDA-MB-231. siRNA-mediated silencing of CIN85, as well as Cbl, also inhibited the invasion. We moreover found that AMAP1 is monoubiquitinated, rather than polyubiquitinated, by virtue of Cbl and provide evidence that the ability of AMAP1 to be monoubiquitinated is important for its involvement in invasion. Our results indicate that CIN85, as well as Cbl, which is a well-known suppressor of growth factor receptor signaling, can be positively involved in tumor invasion, and suggest that a complex epigenetic process is involved in AMAP1 function in breast cancer cell invasion. 
 Autoinhibition of Arf GTPase-activating protein activity by the BAR domain in ASAP1. ASAP1 is an Arf GTPase-activating protein (GAP) that functions on membrane surfaces to catalyze the hydrolysis of GTP bound to Arf. ASAP1 contains a tandem of BAR, pleckstrin homology (PH), and Arf GAP domains and contributes to the formation of   invadopodia   and podosomes. The PH domain interacts with the catalytic domain influencing both the catalytic and Michaelis constants. Tandem BAR-PH domains have been found to fold into a functional unit. The results of sedimentation velocity studies were consistent with predictions from homology models in which the BAR and PH domains of ASAP1 fold together. We set out to test the hypothesis that the BAR domain of ASAP1 affects GAP activity by interacting with the PH and/or Arf GAP domains. Recombinant proteins composed of the BAR, PH, Arf GAP, and Ankyrin repeat domains (called BAR-PZA) and the PH, Arf GAP, and Ankyrin repeat domains (PZA) were compared. Catalytic power for the two proteins was determined using large unilamellar vesicles as a reaction surface. The catalytic power of PZA was greater than that of BAR-PZA. The effect of the BAR domain was dependent on the N-terminal loop of the BAR domain and was not the consequence of differential membrane association or changes in large unilamellar vesicle curvature. The Km for BAR-PZA was greater and the kcat was smaller than for PZA determined by saturation kinetics. Analysis of single turnover kinetics revealed a transition state intermediate that was affected by the BAR domain. We conclude that BAR domains can affect enzymatic activity through intraprotein interactions. 
 GEFH1 binds ASAP1 and regulates podosome formation.   Invadopodia   are cellular structures that are thought to mediate tumor invasion. ASAP1, an Arf GTPase-activating protein (GAP) containing a BAR domain, is a substrate of Src. ASAP1 is required for the assembly of   invadopodia   and podosomes, which are Src-induced structures related to   invadopodia   in NIH 3T3 fibroblasts. The BAR domain of ASAP1 is required for the assembly of podosomes. Using two-hybrid screening, we have identified GEFH1, a guanine nucleotide exchange factor for RhoA, as a binding partner of the BAR domain of ASAP1. We validated the interaction of endogenous GEFH1 with ASAP1 by immunoprecipitation, and found GEFH1 colocalized with ASAP1 in podosomes. The overexpression of GEFH1 inhibited podosome assembly and ASAP1 catalytic activity as a GAP. A mutant of GEFH1 lacking the domain that binds to the BAR domain of ASAP1 was less effective. Reduced expression of GEFH1, achieved with siRNA treatment, did not affect matrix degradation by podosomes but increased the rate of podosome assembly. Based on these results, we conclude that GEFH1 is a negative regulator of podosomes. 
 ASAP3 is a focal adhesion-associated Arf GAP that functions in cell migration and invasion. ASAP3, an Arf GTPase-activating protein previously called DDEFL1 and ACAP4, has been implicated in the pathogenesis of hepatocellular carcinoma. We have examined in vitro and in vivo functions of ASAP3 and compared it to the related Arf GAP ASAP1 that has also been implicated in oncogenesis. ASAP3 was biochemically similar to ASAP1: the pleckstrin homology domain affected function of the catalytic domain by more than 100-fold; catalysis was stimulated by phosphatidylinositol 4,5-bisphosphate; and Arf1, Arf5, and Arf6 were used as substrates in vitro. Like ASAP1, ASAP3 associated with focal adhesions and circular dorsal ruffles. Different than ASAP1, ASAP3 did not localize to   invadopodia   or podosomes. Cells, derived from a mammary carcinoma and from a glioblastoma, with reduced ASAP3 expression had fewer actin stress fiber, reduced levels of phosphomyosin, and migrated more slowly than control cells. Reducing ASAP3 expression also slowed invasion of mammary carcinoma cells. In contrast, reduction of ASAP1 expression had no effect on migration or invasion. We propose that ASAP3 functions nonredundantly with ASAP1 to control cell movement and may have a role in cancer cell invasion. In comparing ASAP1 and ASAP3, we also found that   invadopodia   are dispensable for the invasive behavior of cells derived from a mammary carcinoma. 
  [Pp]odosome(s)?  Process  
 positive regulation of   podosome   assembly 
  PubMed  
 Susceptibility to tuberculosis is associated with variants in the ASAP1 gene encoding a regulator of dendritic cell migration. Human genetic factors predispose to tuberculosis (TB). We studied 7.6 million genetic variants in 5,530 people with pulmonary TB and in 5,607 healthy controls. In the combined analysis of these subjects and the follow-up cohort (15,087 TB patients and controls altogether), we found an association between TB and variants located in introns of the ASAP1 gene on chromosome 8q24 (P = 2.6 × 10(-11) for rs4733781; P = 1.0 × 10(-10) for rs10956514). Dendritic cells (DCs) showed high ASAP1 expression that was reduced after Mycobacterium tuberculosis infection, and rs10956514 was associated with the level of reduction of ASAP1 expression. The ASAP1 protein is involved in actin and membrane remodeling and has been associated wit  h podosom  es. The ASAP1-depleted DCs showed impaired matrix degradation and migration. Therefore, genetically determined excessive reduction of ASAP1 expression in M. tuberculosis-infected DCs may lead to their impaired migration, suggesting a potential mechanism of predisposition to TB. 
 Autoinhibition of Arf GTPase-activating protein activity by the BAR domain in ASAP1. ASAP1 is an Arf GTPase-activating protein (GAP) that functions on membrane surfaces to catalyze the hydrolysis of GTP bound to Arf. ASAP1 contains a tandem of BAR, pleckstrin homology (PH), and Arf GAP domains and contributes to the formation of invadopodia and   podosomes  . The PH domain interacts with the catalytic domain influencing both the catalytic and Michaelis constants. Tandem BAR-PH domains have been found to fold into a functional unit. The results of sedimentation velocity studies were consistent with predictions from homology models in which the BAR and PH domains of ASAP1 fold together. We set out to test the hypothesis that the BAR domain of ASAP1 affects GAP activity by interacting with the PH and/or Arf GAP domains. Recombinant proteins composed of the BAR, PH, Arf GAP, and Ankyrin repeat domains (called BAR-PZA) and the PH, Arf GAP, and Ankyrin repeat domains (PZA) were compared. Catalytic power for the two proteins was determined using large unilamellar vesicles as a reaction surface. The catalytic power of PZA was greater than that of BAR-PZA. The effect of the BAR domain was dependent on the N-terminal loop of the BAR domain and was not the consequence of differential membrane association or changes in large unilamellar vesicle curvature. The Km for BAR-PZA was greater and the kcat was smaller than for PZA determined by saturation kinetics. Analysis of single turnover kinetics revealed a transition state intermediate that was affected by the BAR domain. We conclude that BAR domains can affect enzymatic activity through intraprotein interactions. 
 GEFH1 binds ASAP1 and regulates   podosome   formation. Invadopodia are cellular structures that are thought to mediate tumor invasion. ASAP1, an Arf GTPase-activating protein (GAP) containing a BAR domain, is a substrate of Src. ASAP1 is required for the assembly of invadopodia and   podosomes  , which are Src-induced structures related to invadopodia in NIH 3T3 fibroblasts. The BAR domain of ASAP1 is required for the assembly of   podosomes  . Using two-hybrid screening, we have identified GEFH1, a guanine nucleotide exchange factor for RhoA, as a binding partner of the BAR domain of ASAP1. We validated the interaction of endogenous GEFH1 with ASAP1 by immunoprecipitation, and found GEFH1 colocalized with ASAP1 in   podosomes  . The overexpression of GEFH1 inhibited   podosome   assembly and ASAP1 catalytic activity as a GAP. A mutant of GEFH1 lacking the domain that binds to the BAR domain of ASAP1 was less effective. Reduced expression of GEFH1, achieved with siRNA treatment, did not affect matrix degradation by   podosomes   but increased the rate of   podosome   assembly. Based on these results, we conclude that GEFH1 is a negative regulator of   podosomes  . 
 ASAP3 is a focal adhesion-associated Arf GAP that functions in cell migration and invasion. ASAP3, an Arf GTPase-activating protein previously called DDEFL1 and ACAP4, has been implicated in the pathogenesis of hepatocellular carcinoma. We have examined in vitro and in vivo functions of ASAP3 and compared it to the related Arf GAP ASAP1 that has also been implicated in oncogenesis. ASAP3 was biochemically similar to ASAP1: the pleckstrin homology domain affected function of the catalytic domain by more than 100-fold; catalysis was stimulated by phosphatidylinositol 4,5-bisphosphate; and Arf1, Arf5, and Arf6 were used as substrates in vitro. Like ASAP1, ASAP3 associated with focal adhesions and circular dorsal ruffles. Different than ASAP1, ASAP3 did not localize to invadopodia or   podosomes  . Cells, derived from a mammary carcinoma and from a glioblastoma, with reduced ASAP3 expression had fewer actin stress fiber, reduced levels of phosphomyosin, and migrated more slowly than control cells. Reducing ASAP3 expression also slowed invasion of mammary carcinoma cells. In contrast, reduction of ASAP1 expression had no effect on migration or invasion. We propose that ASAP3 functions nonredundantly with ASAP1 to control cell movement and may have a role in cancer cell invasion. In comparing ASAP1 and ASAP3, we also found that invadopodia are dispensable for the invasive behavior of cells derived from a mammary carcinoma. 
  Component  
   podosome   
  GeneRIFs  
 GEFH1 was identified as binding partner for the BAR domain of ASAP1.GEFH1 is a negative regulator of   podosomes  . 
  [Mm]igration  PubMed  
 GEP100-Arf6-AMAP1-cortactin pathway frequently used in cancer invasion is activated by VEGFR2 to promote angiogenesis. Angiogenesis and cancer invasiveness greatly contribute to cancer malignancy.Arf6 and its effector, AMAP1, are frequently overexpressed in breast cancer, and constitute a central pathway to induce the invasion and metastasis. In this pathway, Arf6 is activated by EGFR via GEP100. Arf6 is highly expressed also in human umbilical vein endothelial cells (HUVECs) and is implicated in angiogenesis. Here, we found that HUVECs also highly express AMAP1, and that vascular endothelial growth factor receptor-2 (VEGFR2) recruits GEP100 to activate Arf6. AMAP1 functions by binding to cortactin in cancer invasion and metastasis. We demonstrate that the same GEP100-Arf6-AMAP1-cortactin pathway is essential for angiogenesis activities, including cell   migration   and tubular formation, as well as for the enhancement of cell permeability and VE-cadherin endocytosis of VEGF-stimulated HUVECs. Components of this pathway are highly expressed in pathologic angiogenesis, and blocking of this pathway effectively inhibits VEGF- or tumor-induced angiogenesis and choroidal neovascularization. The GEP100-Arf6-AMAP1-cortactin pathway, activated by receptor tyrosine kinases, appears to be common in angiogenesis and cancer invasion and metastasis, and provides their new therapeutic targets. 
 Interaction of POB1, a downstream molecule of small G protein Ral, with PAG2, a paxillin-binding protein, is involved in cell   migration  . POB1 was previously identified as a RalBP1-binding protein. POB1 and RalBP1 function downstream of small G protein Ral and regulate receptor-mediated endocytosis. To look for additional functions of POB1, we screened for POB1-binding proteins using a yeast two-hybrid method and found that POB1 interacts with mouse ASAP1, which is a human PAG2 homolog. PAG2 is a paxillin-associated protein with ADP-ribosylation factor GTPase-activating protein activity. POB1 formed a complex with PAG2 in intact cells. The carboxyl-terminal region containing the proline-rich motifs of POB1 directly bound to the carboxyl-terminal region including the SH3 domain of PAG2. Substitutions of Pro(423) and Pro(426) with Ala (POB1(PA)) impaired the binding of POB1 to PAG2. Expression of PAG2 inhibited fibronectin-dependent   migration   and paxillin recruitment to focal contacts of CHO-IR cells. Co-expression with POB1 but not with POB1(PA) suppressed the inhibitory action of PAG2 on cell   migration   and paxillin localization. These results suggest that POB1 interacts with PAG2 through its proline-rich motif, thereby regulating cell   migration  . 
 Identification of a link between the SAMP repeats of adenomatous polyposis coli tumor suppressor and the Src homology 3 domain of DDEF. The adenomatous polyposis coli (APC) tumor suppressor protein is a multifunctional protein with a well characterized role in the Wnt signal transduction pathway and in cytoskeletal regulation. The SAMP repeats region of APC, an Axin-binding site, is known to be important for tumor suppression and for the developmental function of APC. We performed a yeast two-hybrid screening using the first SAMP motif-containing region of Xenopus APC as bait and obtained several SAMP binding candidates including DDEF2 (development and differentiation enhancing factor 2), which is an ADP-ribosylation factor (Arf) GTPase-activating protein (GAP (ArfGAP)) involved in the regulation of focal adhesions. In vitro and in cells the Src homology 3 (SH3) domain of DDEF2 and its close homolog, DDEF1, are associated with the SAMP motif of APC competitively with Axin1. Moreover, NMR chemical shift perturbation experiments revealed that the SAMP motif interacts at the same surface of the SH3 domain of DDEF as the known SH3 binding motif, PXXP. When fluorescent protein-tagged APC and DDEF are expressed in Xenopus A6 cells, co-localization at microtubule ends is observed. Overexpression and RNA interference experiments indicate that APC and DDEFs cooperatively regulate the distributions of microtubules and focal adhesions. Our findings reveal that the SAMP motif of APC specifically binds to the SH3 domains of DDEFs, providing new insights into the functions of APC in cell   migration  . 
 GEP100-Arf6-AMAP1-cortactin pathway frequently used in cancer invasion is activated by VEGFR2 to promote angiogenesis. Angiogenesis and cancer invasiveness greatly contribute to cancer malignancy.Arf6 and its effector, AMAP1, are frequently overexpressed in breast cancer, and constitute a central pathway to induce the invasion and metastasis. In this pathway, Arf6 is activated by EGFR via GEP100. Arf6 is highly expressed also in human umbilical vein endothelial cells (HUVECs) and is implicated in angiogenesis. Here, we found that HUVECs also highly express AMAP1, and that vascular endothelial growth factor receptor-2 (VEGFR2) recruits GEP100 to activate Arf6. AMAP1 functions by binding to cortactin in cancer invasion and metastasis. We demonstrate that the same GEP100-Arf6-AMAP1-cortactin pathway is essential for angiogenesis activities, including cell   migration   and tubular formation, as well as for the enhancement of cell permeability and VE-cadherin endocytosis of VEGF-stimulated HUVECs. Components of this pathway are highly expressed in pathologic angiogenesis, and blocking of this pathway effectively inhibits VEGF- or tumor-induced angiogenesis and choroidal neovascularization. The GEP100-Arf6-AMAP1-cortactin pathway, activated by receptor tyrosine kinases, appears to be common in angiogenesis and cancer invasion and metastasis, and provides their new therapeutic targets. 
 Interaction of POB1, a downstream molecule of small G protein Ral, with PAG2, a paxillin-binding protein, is involved in cell   migration  . POB1 was previously identified as a RalBP1-binding protein. POB1 and RalBP1 function downstream of small G protein Ral and regulate receptor-mediated endocytosis. To look for additional functions of POB1, we screened for POB1-binding proteins using a yeast two-hybrid method and found that POB1 interacts with mouse ASAP1, which is a human PAG2 homolog. PAG2 is a paxillin-associated protein with ADP-ribosylation factor GTPase-activating protein activity. POB1 formed a complex with PAG2 in intact cells. The carboxyl-terminal region containing the proline-rich motifs of POB1 directly bound to the carboxyl-terminal region including the SH3 domain of PAG2. Substitutions of Pro(423) and Pro(426) with Ala (POB1(PA)) impaired the binding of POB1 to PAG2. Expression of PAG2 inhibited fibronectin-dependent   migration   and paxillin recruitment to focal contacts of CHO-IR cells. Co-expression with POB1 but not with POB1(PA) suppressed the inhibitory action of PAG2 on cell   migration   and paxillin localization. These results suggest that POB1 interacts with PAG2 through its proline-rich motif, thereby regulating cell   migration  . 
 Identification of novel SH3 domain ligands for the Src family kinase Hck. Wiskott-Aldrich syndrome protein (WASP), WASP-interacting protein (WIP), and ELMO1. The importance of the SH3 domain of Hck in kinase regulation, substrate phosphorylation, and ligand binding has been established. However, few in vivo ligands are known for the SH3 domain of Hck. In this study, we used mass spectrometry to identify approximately 25 potential binding partners for the SH3 domain of Hck from the monocyte cell line U937. Two major interacting proteins were the actin binding proteins Wiskott-Aldrich syndrome protein (WASP) and WASP-interacting protein (WIP). We also focused on a novel interaction between Hck and ELMO1, an 84-kDa protein that was recently identified as the mammalian ortholog of the Caenorhabditis elegans gene, ced-12. In mammalian cells, ELMO1 interacts with Dock180 as a component of the CrkII/Dock180/Rac pathway responsible for phagocytosis and cell   migration  . Using purified proteins, we confirmed that WASP-interacting protein and ELMO1 interact directly with the SH3 domain of Hck. We also show that Hck and ELMO1 interact in intact cells and that ELMO1 is heavily tyrosine-phosphorylated in cells that co-express Hck, suggesting that it is a substrate of Hck. The binding of ELMO1 to Hck is specifically dependent on the interaction of a polyproline motif with the SH3 domain of Hck. Our results suggest that these proteins may be novel activators/effectors of Hck. 
 Susceptibility to tuberculosis is associated with variants in the ASAP1 gene encoding a regulator of dendritic cell   migration  . Human genetic factors predispose to tuberculosis (TB). We studied 7.6 million genetic variants in 5,530 people with pulmonary TB and in 5,607 healthy controls. In the combined analysis of these subjects and the follow-up cohort (15,087 TB patients and controls altogether), we found an association between TB and variants located in introns of the ASAP1 gene on chromosome 8q24 (P = 2.6 × 10(-11) for rs4733781; P = 1.0 × 10(-10) for rs10956514). Dendritic cells (DCs) showed high ASAP1 expression that was reduced after Mycobacterium tuberculosis infection, and rs10956514 was associated with the level of reduction of ASAP1 expression. The ASAP1 protein is involved in actin and membrane remodeling and has been associated with podosomes. The ASAP1-depleted DCs showed impaired matrix degradation an  d migrati  on. Therefore, genetically determined excessive reduction of ASAP1 expression in M. tuberculosis-infected DCs may lead to their impaire  d migrati  on, suggesting a potential mechanism of predisposition to TB. 
 DEF-1/ASAP1 is a GTPase-activating protein (GAP) for ARF1 that enhances cell motility through a GAP-dependent mechanism. DEF-1/ASAP1 is an ADP-ribosylation factor GTPase-activating protein (ARF GAP) that localizes to focal adhesions and is involved in cytoskeletal regulation. In this paper, we use a cell-based ARF GAP assay to demonstrate that DEF-1 functions as a GAP for ARF1 and not ARF6 in vivo. This degree of substrate preference was unique to DEF-1, as other ARF GAP proteins, ACAP1, ACAP2, and ARFGAP1, were able to function on both ARF1 and ARF6. Since transient overexpression of DEF-1 has been shown to interfere with focal adhesion formation and platelet-derived growth factor-induced membrane ruffling, we investigated whether NIH 3T3 cells stably expressing DEF-1 have altered cell motility. Here we report that ectopic DEF-1 enhances cell   migration   toward PDGF as well as IGF-1. This chemotactic effect appears to result from a general increase in cell motility, as DEF-1-expressing cells also exhibit enhanced levels of basal and chemokinetic motility. The increase in cell motility is dependent on DEF-1 GAP activity, since a DEF-1 mutant lacking the GAP domain failed to stimulate motility. This suggests that DEF-1 alters cell motility through the deactivation of ARF1. In contrast, the inhibition of cell spreading by DEF-1 was not dependent on GAP activity, indicating that spreading and motility are altered by DEF-1 through different pathways. 
 The Arf GTPase-activating Protein, ASAP1, Binds Nonmuscle Myosin 2A to Control Remodeling of the Actomyosin Network. ASAP1 regulates F-actin-based structures and functions, including focal adhesions (FAs) and circular dorsal ruffles (CDRs), cell spreading and   migration  . ASAP1 function requires its N-terminal BAR domain. We discovered that nonmuscle myosin 2A (NM2A) directly bound the BAR-PH tandem of ASAP1in vitro ASAP1 and NM2A co-immunoprecipitated and colocalized in cells. Knockdown of ASAP1 reduced colocalization of NM2A and F-actin in cells. Knockdown of ASAP1 or NM2A recapitulated each other's effects on FAs, cell   migration  , cell spreading, and CDRs. The NM2A-interacting BAR domain contributed to ASAP1 control of cell spreading and CDRs. Exogenous expression of NM2A rescued the effect of ASAP1 knockdown on CDRs but ASAP1 did not rescue NM2A knockdown defect in CDRs. Our results support the hypothesis that ASAP1 is a positive regulator of NM2A. Given other binding partners of ASAP1, ASAP1 may directly link signaling and the mechanical machinery of cell   migration  . 
 ASAP3 is a focal adhesion-associated Arf GAP that functions in cell   migration   and invasion. ASAP3, an Arf GTPase-activating protein previously called DDEFL1 and ACAP4, has been implicated in the pathogenesis of hepatocellular carcinoma. We have examined in vitro and in vivo functions of ASAP3 and compared it to the related Arf GAP ASAP1 that has also been implicated in oncogenesis. ASAP3 was biochemically similar to ASAP1: the pleckstrin homology domain affected function of the catalytic domain by more than 100-fold; catalysis was stimulated by phosphatidylinositol 4,5-bisphosphate; and Arf1, Arf5, and Arf6 were used as substrates in vitro. Like ASAP1, ASAP3 associated with focal adhesions and circular dorsal ruffles. Different than ASAP1, ASAP3 did not localize to invadopodia or podosomes. Cells, derived from a mammary carcinoma and from a glioblastoma, with reduced ASAP3 expression had fewer actin stress fiber, reduced levels of phosphomyosin, and migrated more slowly than control cells. Reducing ASAP3 expression also slowed invasion of mammary carcinoma cells. In contrast, reduction of ASAP1 expression had no effect on   migration   or invasion. We propose that ASAP3 functions nonredundantly with ASAP1 to control cell movement and may have a role in cancer cell invasion. In comparing ASAP1 and ASAP3, we also found that invadopodia are dispensable for the invasive behavior of cells derived from a mammary carcinoma. 
 ASAP1, a gene at 8q24, is associated with prostate cancer metastasis. Metastatic prostate cancer is a terminal disease, and the development of reliable prognostic tools and more effective therapy is critically important for improved disease survival and management. This study was aimed at identifying genes that are differentially expressed in metastatic and nonmetastatic prostate cancer cells and, as such, could be critical in the development of metastasis. Long-SAGE analysis was used to compare a transplantable human metastatic prostate cancer subline, PCa1-met, with a nonmetastatic counterpart, PCa2. Both sublines were developed from a patient's prostate cancer specimen via subrenal capsule grafting and subsequent orthotopic implantation into SCID mice. Among various differentially expressed genes identified, ASAP1, an 8q24 gene encoding an ADP-ribosylation factor GTPase-activating protein not previously associated with prostate cancer, was up-regulated in the metastatic subline as confirmed by quantitative real-time PCR. Immunohistochemistry of xenograft sections showed that cytoplasmic ASAP1 protein staining was absent or weak in benign tissue, significantly stronger in nonmetastatic PCa2 tissue, and strongest in PCa1-met tissue. In clinical specimens, ASAP1 protein staining was elevated in 80% of primary prostate cancers and substantially higher in metastatic lesions compared with benign prostate tissue. Moreover, additional ASAP1 gene copies were detected in 58% of the primary prostate cancer specimens. Small interfering RNA-induced reduction of ASAP1 protein expression markedly suppressed in vitro PC-3 cell   migration   (approximately 50%) and Matrigel invasion (approximately 67%). This study suggests that the ASAP1 gene plays a role in prostate cancer metastasis and may represent a therapeutic target and/or biomarker for metastatic disease. 
  GeneRIFs  
 These results suggest that POB1 interacts with PAG2 through its proline-rich motif, thereby regulating cell   migration   (PAG2). 
  [Ii]nvasive  PubMed  
 GEP100-Arf6-AMAP1-cortactin pathway frequently used in cancer invasion is activated by VEGFR2 to promote angiogenesis. Angiogenesis and cancer   invasive  ness greatly contribute to cancer malignancy.Arf6 and its effector, AMAP1, are frequently overexpressed in breast cancer, and constitute a central pathway to induce the invasion and metastasis. In this pathway, Arf6 is activated by EGFR via GEP100. Arf6 is highly expressed also in human umbilical vein endothelial cells (HUVECs) and is implicated in angiogenesis. Here, we found that HUVECs also highly express AMAP1, and that vascular endothelial growth factor receptor-2 (VEGFR2) recruits GEP100 to activate Arf6. AMAP1 functions by binding to cortactin in cancer invasion and metastasis. We demonstrate that the same GEP100-Arf6-AMAP1-cortactin pathway is essential for angiogenesis activities, including cell migration and tubular formation, as well as for the enhancement of cell permeability and VE-cadherin endocytosis of VEGF-stimulated HUVECs. Components of this pathway are highly expressed in pathologic angiogenesis, and blocking of this pathway effectively inhibits VEGF- or tumor-induced angiogenesis and choroidal neovascularization. The GEP100-Arf6-AMAP1-cortactin pathway, activated by receptor tyrosine kinases, appears to be common in angiogenesis and cancer invasion and metastasis, and provides their new therapeutic targets. 
 CIN85, a Cbl-interacting protein, is a component of AMAP1-mediated breast cancer invasion machinery. Expression of AMAP1 correlates well with the   invasive   phenotypes and malignancy of human primary breast carcinomas. AMAP1 recruits its binding proteins, such as cortactin and paxillin, to sites of Arf6 activation to form invadopodia. A mouse ortholog of AMAP1, ASAP1, is known to bind to CIN85, a binding partner of an E3 ligase, Cbl. Here, we found that CIN85 colocalizes with AMAP1 at invadopodia, and binding of AMAP1 with CIN85 is important for the   invasive   activities of breast cancer cells, including MDA-MB-231. siRNA-mediated silencing of CIN85, as well as Cbl, also inhibited the invasion. We moreover found that AMAP1 is monoubiquitinated, rather than polyubiquitinated, by virtue of Cbl and provide evidence that the ability of AMAP1 to be monoubiquitinated is important for its involvement in invasion. Our results indicate that CIN85, as well as Cbl, which is a well-known suppressor of growth factor receptor signaling, can be positively involved in tumor invasion, and suggest that a complex epigenetic process is involved in AMAP1 function in breast cancer cell invasion. 
 Rab5c promotes AMAP1-PRKD2 complex formation to enhance β1 integrin recycling in EGF-induced cancer invasion. Epidermal growth factor receptor (EGFR) signaling is one of the crucial factors in breast cancer malignancy. Breast cancer cells often overexpress Arf6 and its effector, AMAP1/ASAP1/DDEF1; in these cells, EGFR signaling may activate the Arf6 pathway to induce invasion and metastasis. Active recycling of some integrins is crucial for invasion and metastasis. Here, we show that the Arf6-AMAP1 pathway links to the machinery that recycles β1 integrins, such as α3β1, to promote cell invasion upon EGFR stimulation. We found that AMAP1 had the ability to bind directly to PRKD2 and hence to make a complex with the cytoplasmic tail of the β1 subunit. Moreover, GTP-Rab5c also bound to AMAP1, and activation of Rab5c by EGFR signaling was necessary to promote the intracellular association of AMAP1 and PRKD2. Our results suggest a novel mechanism by which EGFR signaling promotes   the inv  asiveness of some breast cancer cells via integrin recycling. 
 Rab5c promotes AMAP1-PRKD2 complex formation to enhance β1 integrin recycling in EGF-induced cancer invasion. Epidermal growth factor receptor (EGFR) signaling is one of the crucial factors in breast cancer malignancy. Breast cancer cells often overexpress Arf6 and its effector, AMAP1/ASAP1/DDEF1; in these cells, EGFR signaling may activate the Arf6 pathway to induce invasion and metastasis. Active recycling of some integrins is crucial for invasion and metastasis. Here, we show that the Arf6-AMAP1 pathway links to the machinery that recycles β1 integrins, such as α3β1, to promote cell invasion upon EGFR stimulation. We found that AMAP1 had the ability to bind directly to PRKD2 and hence to make a complex with the cytoplasmic tail of the β1 subunit. Moreover, GTP-Rab5c also bound to AMAP1, and activation of Rab5c by EGFR signaling was necessary to promote the intracellular association of AMAP1 and PRKD2. Our results suggest a novel mechanism by which EGFR signaling promotes   the inv  asiveness of some breast cancer cells via integrin recycling. 
 GEP100-Arf6-AMAP1-cortactin pathway frequently used in cancer invasion is activated by VEGFR2 to promote angiogenesis. Angiogenesis and cancer   invasive  ness greatly contribute to cancer malignancy.Arf6 and its effector, AMAP1, are frequently overexpressed in breast cancer, and constitute a central pathway to induce the invasion and metastasis. In this pathway, Arf6 is activated by EGFR via GEP100. Arf6 is highly expressed also in human umbilical vein endothelial cells (HUVECs) and is implicated in angiogenesis. Here, we found that HUVECs also highly express AMAP1, and that vascular endothelial growth factor receptor-2 (VEGFR2) recruits GEP100 to activate Arf6. AMAP1 functions by binding to cortactin in cancer invasion and metastasis. We demonstrate that the same GEP100-Arf6-AMAP1-cortactin pathway is essential for angiogenesis activities, including cell migration and tubular formation, as well as for the enhancement of cell permeability and VE-cadherin endocytosis of VEGF-stimulated HUVECs. Components of this pathway are highly expressed in pathologic angiogenesis, and blocking of this pathway effectively inhibits VEGF- or tumor-induced angiogenesis and choroidal neovascularization. The GEP100-Arf6-AMAP1-cortactin pathway, activated by receptor tyrosine kinases, appears to be common in angiogenesis and cancer invasion and metastasis, and provides their new therapeutic targets. 
 ASAP1 mediates the   invasive   phenotype of human laryngeal squamous cell carcinoma to affect survival prognosis. ASAP1 helps regulate cellular structures such as actin cytoskeletal remodeling and focal adhesions that have a pivotal function in tumor progression. Overexpression of ASAP1 has proven to be a malignant indicator for a variety of tumors. To further determine the potential involvement of ASAP1 in laryngeal squamous cell carcinoma (LSCC), we evaluated the expression levels of ASAP1 by quantitative real-time reverse-transcriptase polymerase chain reaction (qRT-PCR) and immunohistochemistry in tissue samples of 64 LSCC patients. We then analyzed and correlated the results with clinicopathological features. Furthermore, we used small interfering RNA (siRNA) to inhibit ASAP1 expression in vitro. The potential function of ASAP1 in   invasiv  eness was evaluated in the Hep-2 LSCC cell line. Kaplan-Meier method was utilized to determine the association of ASAP1 expression with survival of patients. We showed that ASAP1 was upregulated in primary LSCC tumors and was correlated with lymph node metastasis and clinical tumor stage. Similarly, higher levels of ASAP1 were detected in the Hep-2 cell line compared to the 16 human bronchial epithelial (16HBE) cell line. ASAP1 expression was downregulated by lentiviral vector transfection containing siRNA in vitro. Th  e invasi  ve potential of these cells was found to be significantly suppressed, while expression levels of Rac1 and Cdc42 positively correlated with the inhibition of ASAP1 expression. In Kaplan-Meier overall survival curves, higher ASAP1 mRNA levels were found to be associated with a shorter progression-free survival trend. Based on these results, ASAP1 appears to contribute to the malignant mechanism of LSCC and may represent a significant prognostic marker for LSCC patients. 
 Targeting AMAP1 and cortactin binding bearing an atypical src homology 3/proline interface for prevention of breast cancer invasion and metastasis.   Invasive   potentials of carcinomas greatly contribute to their metastasis, which is a major threat in most cancers. We have recently shown that Arf6 plays a pivotal role in breast cancer   invasive   activities and identified AMAP1 as an effector of GTP-Arf6 in invasion. Expression of AMAP1 correlates well with   invasive   phenotypes of primary tumors of the human breast. We also have shown that AMAP1 functions by forming a trimeric protein complex with cortactin and paxillin. In this complex, AMAP1 binds to the src homology 3 (SH3) domain of cortactin via its proline-rich peptide, SKKRPPPPPPGHKRT. SH3 domains are known to bind generally to the proline-rich ligands with a one-to-one stoichiometry. We found that AMAP1/cortactin binding is very atypical in its stoichiometry and interface structure, in which one AMAP1 proline-rich peptide binds to two cortactin SH3 domains simultaneously. We made a cell-permeable peptide derived from the AMAP1 peptide, and we show that this peptide specifically blocks AMAP1/cortactin binding, but not other canonical SH3/proline bindings, and effectively inhibits breast cancer invasion and metastasis. Moreover, this peptide was found to block invasion of other types of cancers, such as glioblastomas and lung carcinomas. We also found that a small-molecule compound, UCS15A, which was previously judged as a weak inhibitor against canonical SH3/proline bindings, effectively inhibits AMAP1/cortactin binding and breast cancer invasion and metastasis. Together with fine structural analysis, we propose that the AMAP1/cortactin complex, which is not detected in normal mammary epithelial cells, is an excellent drug target for cancer therapeutics. 
 ASAP1 promotes tumor cell motility and   invasive  ness, stimulates metastasis formation in vivo, and correlates with poor survival in colorectal cancer patients. We have previously performed an unbiased screen to identify genes whose expression is associated with the metastatic phenotype. Secondary screening of these genes using custom microarray chips identified ASAP1, a multi-domain adaptor protein with ADP-ribosylation factor-GAP activity, as being potentially involved in tumor progression. Here, we show that at least three different splice forms of ASAP1 are upregulated in rodent tumor models in a manner that correlates with metastatic potential. In human cancers, we found that ASAP1 expression is strongly upregulated in a variety of tumors in comparison with normal tissue and that this expression correlates with poor metastasis-free survival and prognosis in colorectal cancer patients. Using loss and gain of function approaches, we were able to show that ASAP1 promotes metastasis formation in vivo and stimulates tumor cell motility,   invasive  ness, and adhesiveness in vitro. Furthermore, we show that ASAP1 interacts with the metastasis-promoting protein h-prune and stimulates its phosphodiesterase activity. In addition, ASAP1 binds to the SH3 domains of several proteins, including SLK with which it co-immunoprecipitates. These data support the notion that ASAP1 can contribute to the dissemination of a variety of tumor types and represent a potential target for cancer therapy. 
 ASAP3 is a focal adhesion-associated Arf GAP that functions in cell migration and invasion. ASAP3, an Arf GTPase-activating protein previously called DDEFL1 and ACAP4, has been implicated in the pathogenesis of hepatocellular carcinoma. We have examined in vitro and in vivo functions of ASAP3 and compared it to the related Arf GAP ASAP1 that has also been implicated in oncogenesis. ASAP3 was biochemically similar to ASAP1: the pleckstrin homology domain affected function of the catalytic domain by more than 100-fold; catalysis was stimulated by phosphatidylinositol 4,5-bisphosphate; and Arf1, Arf5, and Arf6 were used as substrates in vitro. Like ASAP1, ASAP3 associated with focal adhesions and circular dorsal ruffles. Different than ASAP1, ASAP3 did not localize to invadopodia or podosomes. Cells, derived from a mammary carcinoma and from a glioblastoma, with reduced ASAP3 expression had fewer actin stress fiber, reduced levels of phosphomyosin, and migrated more slowly than control cells. Reducing ASAP3 expression also slowed invasion of mammary carcinoma cells. In contrast, reduction of ASAP1 expression had no effect on migration or invasion. We propose that ASAP3 functions nonredundantly with ASAP1 to control cell movement and may have a role in cancer cell invasion. In comparing ASAP1 and ASAP3, we also found that invadopodia are dispensable for the   invasive   behavior of cells derived from a mammary carcinoma. 
  [Mm]etastasis  PubMed  
 GEP100-Arf6-AMAP1-cortactin pathway frequently used in cancer invasion is activated by VEGFR2 to promote angiogenesis. Angiogenesis and cancer invasiveness greatly contribute to cancer malignancy.Arf6 and its effector, AMAP1, are frequently overexpressed in breast cancer, and constitute a central pathway to induce the invasion and   metastasis  . In this pathway, Arf6 is activated by EGFR via GEP100. Arf6 is highly expressed also in human umbilical vein endothelial cells (HUVECs) and is implicated in angiogenesis. Here, we found that HUVECs also highly express AMAP1, and that vascular endothelial growth factor receptor-2 (VEGFR2) recruits GEP100 to activate Arf6. AMAP1 functions by binding to cortactin in cancer invasion and   metastasis  . We demonstrate that the same GEP100-Arf6-AMAP1-cortactin pathway is essential for angiogenesis activities, including cell migration and tubular formation, as well as for the enhancement of cell permeability and VE-cadherin endocytosis of VEGF-stimulated HUVECs. Components of this pathway are highly expressed in pathologic angiogenesis, and blocking of this pathway effectively inhibits VEGF- or tumor-induced angiogenesis and choroidal neovascularization. The GEP100-Arf6-AMAP1-cortactin pathway, activated by receptor tyrosine kinases, appears to be common in angiogenesis and cancer invasion and   metastasis  , and provides their new therapeutic targets. 
 Rab5c promotes AMAP1-PRKD2 complex formation to enhance β1 integrin recycling in EGF-induced cancer invasion. Epidermal growth factor receptor (EGFR) signaling is one of the crucial factors in breast cancer malignancy. Breast cancer cells often overexpress Arf6 and its effector, AMAP1/ASAP1/DDEF1; in these cells, EGFR signaling may activate the Arf6 pathway to induce invasion and   metastasi  s. Active recycling of some integrins is crucial for invasion and   metastasi  s. Here, we show that the Arf6-AMAP1 pathway links to the machinery that recycles β1 integrins, such as α3β1, to promote cell invasion upon EGFR stimulation. We found that AMAP1 had the ability to bind directly to PRKD2 and hence to make a complex with the cytoplasmic tail of the β1 subunit. Moreover, GTP-Rab5c also bound to AMAP1, and activation of Rab5c by EGFR signaling was necessary to promote the intracellular association of AMAP1 and PRKD2. Our results suggest a novel mechanism by which EGFR signaling promotes the invasiveness of some breast cancer cells via integrin recycling. 
 Co-overexpression of GEP100 and AMAP1 proteins correlates with rapid local recurrence after breast conservative therapy. A major problem of current cancer research and therapy is prediction of tumor recurrence after initial treatment, rather than the simple biological characterization of the malignancy and proliferative properties of tumors. Breast conservation therapy (BCT) is a well-approved, standard treatment for patients with early stages of breast cancer, which consists of lumpectomy and whole-breast irradiation. In spite of extensive studies, only 'age' and 'Ki-67 positivity' have been identified to be well correlated with local recurrence after BCT. An Arf6 pathway, activated by GEP100 under receptor tyrosine kinases (RTKs) and employs AMAP1 as its effector, is crucial for invasion and   metastasis   of some breast cancer cells. This pathway activates β1 integrins and perturbs E-cadherin-based adhesions, hence appears to be integral for epithelial-mesenchymal transdifferentiation (EMT). We here show that expression of the Arf6 pathway components statistically correlates with rapid local recurrence after BCT. We retrospectively analyzed four hundred seventy-nine patients who received BCT in Hokkaido University Hospital, and found 20 patients had local recurrence. We then analyzed pathological samples of patients who experienced local recurrence by use of Kaplan-Meier analysis, Stepwise regression analysis and the t-test, coupled with immunostaining, and found that co-overexpression of GEP100 and AMAP1 correlates with rapidity of the local recurrence. Their margin-status, node-positivity, and estrogen receptor (ER)- or progesterone receptor (PgR)-positivity did not correlated with the rapidity. This study is the first to show that expression of a certain set of proteins correlates with the rapidity of local recurrence. Our results are useful not only for prediction, but highlight the possibility of developing novel strategies to block local recurrence. We also discuss why mRNAs encoding these proteins have not been identified to correlate with local recurrence by previous conventional gene expression profiling analyses. 
 Co-overexpression of GEP100 and AMAP1 proteins correlates with rapid local recurrence after breast conservative therapy. A major problem of current cancer research and therapy is prediction of tumor recurrence after initial treatment, rather than the simple biological characterization of the malignancy and proliferative properties of tumors. Breast conservation therapy (BCT) is a well-approved, standard treatment for patients with early stages of breast cancer, which consists of lumpectomy and whole-breast irradiation. In spite of extensive studies, only 'age' and 'Ki-67 positivity' have been identified to be well correlated with local recurrence after BCT. An Arf6 pathway, activated by GEP100 under receptor tyrosine kinases (RTKs) and employs AMAP1 as its effector, is crucial for invasion and   metastasis   of some breast cancer cells. This pathway activates β1 integrins and perturbs E-cadherin-based adhesions, hence appears to be integral for epithelial-mesenchymal transdifferentiation (EMT). We here show that expression of the Arf6 pathway components statistically correlates with rapid local recurrence after BCT. We retrospectively analyzed four hundred seventy-nine patients who received BCT in Hokkaido University Hospital, and found 20 patients had local recurrence. We then analyzed pathological samples of patients who experienced local recurrence by use of Kaplan-Meier analysis, Stepwise regression analysis and the t-test, coupled with immunostaining, and found that co-overexpression of GEP100 and AMAP1 correlates with rapidity of the local recurrence. Their margin-status, node-positivity, and estrogen receptor (ER)- or progesterone receptor (PgR)-positivity did not correlated with the rapidity. This study is the first to show that expression of a certain set of proteins correlates with the rapidity of local recurrence. Our results are useful not only for prediction, but highlight the possibility of developing novel strategies to block local recurrence. We also discuss why mRNAs encoding these proteins have not been identified to correlate with local recurrence by previous conventional gene expression profiling analyses. 
 Rab5c promotes AMAP1-PRKD2 complex formation to enhance β1 integrin recycling in EGF-induced cancer invasion. Epidermal growth factor receptor (EGFR) signaling is one of the crucial factors in breast cancer malignancy. Breast cancer cells often overexpress Arf6 and its effector, AMAP1/ASAP1/DDEF1; in these cells, EGFR signaling may activate the Arf6 pathway to induce invasion and   metastasi  s. Active recycling of some integrins is crucial for invasion and   metastasi  s. Here, we show that the Arf6-AMAP1 pathway links to the machinery that recycles β1 integrins, such as α3β1, to promote cell invasion upon EGFR stimulation. We found that AMAP1 had the ability to bind directly to PRKD2 and hence to make a complex with the cytoplasmic tail of the β1 subunit. Moreover, GTP-Rab5c also bound to AMAP1, and activation of Rab5c by EGFR signaling was necessary to promote the intracellular association of AMAP1 and PRKD2. Our results suggest a novel mechanism by which EGFR signaling promotes the invasiveness of some breast cancer cells via integrin recycling. 
 GEP100-Arf6-AMAP1-cortactin pathway frequently used in cancer invasion is activated by VEGFR2 to promote angiogenesis. Angiogenesis and cancer invasiveness greatly contribute to cancer malignancy.Arf6 and its effector, AMAP1, are frequently overexpressed in breast cancer, and constitute a central pathway to induce the invasion and   metastasis  . In this pathway, Arf6 is activated by EGFR via GEP100. Arf6 is highly expressed also in human umbilical vein endothelial cells (HUVECs) and is implicated in angiogenesis. Here, we found that HUVECs also highly express AMAP1, and that vascular endothelial growth factor receptor-2 (VEGFR2) recruits GEP100 to activate Arf6. AMAP1 functions by binding to cortactin in cancer invasion and   metastasis  . We demonstrate that the same GEP100-Arf6-AMAP1-cortactin pathway is essential for angiogenesis activities, including cell migration and tubular formation, as well as for the enhancement of cell permeability and VE-cadherin endocytosis of VEGF-stimulated HUVECs. Components of this pathway are highly expressed in pathologic angiogenesis, and blocking of this pathway effectively inhibits VEGF- or tumor-induced angiogenesis and choroidal neovascularization. The GEP100-Arf6-AMAP1-cortactin pathway, activated by receptor tyrosine kinases, appears to be common in angiogenesis and cancer invasion and   metastasis  , and provides their new therapeutic targets. 
 ASAP1 mediates the invasive phenotype of human laryngeal squamous cell carcinoma to affect survival prognosis. ASAP1 helps regulate cellular structures such as actin cytoskeletal remodeling and focal adhesions that have a pivotal function in tumor progression. Overexpression of ASAP1 has proven to be a malignant indicator for a variety of tumors. To further determine the potential involvement of ASAP1 in laryngeal squamous cell carcinoma (LSCC), we evaluated the expression levels of ASAP1 by quantitative real-time reverse-transcriptase polymerase chain reaction (qRT-PCR) and immunohistochemistry in tissue samples of 64 LSCC patients. We then analyzed and correlated the results with clinicopathological features. Furthermore, we used small interfering RNA (siRNA) to inhibit ASAP1 expression in vitro. The potential function of ASAP1 in invasiveness was evaluated in the Hep-2 LSCC cell line. Kaplan-Meier method was utilized to determine the association of ASAP1 expression with survival of patients. We showed that ASAP1 was upregulated in primary LSCC tumors and was correlated with lymph node   metastasi  s and clinical tumor stage. Similarly, higher levels of ASAP1 were detected in the Hep-2 cell line compared to the 16 human bronchial epithelial (16HBE) cell line. ASAP1 expression was downregulated by lentiviral vector transfection containing siRNA in vitro. The invasive potential of these cells was found to be significantly suppressed, while expression levels of Rac1 and Cdc42 positively correlated with the inhibition of ASAP1 expression. In Kaplan-Meier overall survival curves, higher ASAP1 mRNA levels were found to be associated with a shorter progression-free survival trend. Based on these results, ASAP1 appears to contribute to the malignant mechanism of LSCC and may represent a significant prognostic marker for LSCC patients. 
 Overexpression of ASAP1 is associated with poor prognosis in epithelial ovarian cancer. This study was conducted to analyze the clinical significance of ASAP1 in epithelial ovarian cancer (EOC).A total of 95 patients with EOC were included in the study. The expression profile of ASAP1 in 10 pairs of ovarian cancer and normal ovary tissues were detected by Real-time PCR. The expression level of ASAP1 in 95 paraffin-embedded EOC specimens was measured by immunohistochemistry staining. Statistical analysis was performed to evaluate the clinicopathologic significance of ASAP1.Levels of ASAP1 mRNA were higher in EOC than in normal ovary tissues. Patients with higher ASAP1 expression had shorter overall (P=0.019) and recurrence-free (P=0.030) survival time, whereas those with lower ASAP1 expression survived longer. In addition, high expression of ASAP1 was correlated with poor overall (P=0.044) and recurrence-free (P=0.006) survival in patients with advanced carcinomas. Moreover, statistical analysis displayed a significant correlation in ASAP1 expression with pelvic   metastasis   (P=0.015). Multivariate analysis revealed that an elevated ASAP1 expression was a significant independent prognostic factor for the overall (P=0.039) and recurrence-free (P=0.028) survival of EOC patients.These results indicated that elevated expression of ASAP1 plays an important role in the progression and   metastasis   of ovarian cancer, and that ASAP1 may be used as a biomarker in predicting patient outcome in EOC patients. 
 Targeting AMAP1 and cortactin binding bearing an atypical src homology 3/proline interface for prevention of breast cancer invasion and   metastasis  . Invasive potentials of carcinomas greatly contribute to their   metastasis  , which is a major threat in most cancers. We have recently shown that Arf6 plays a pivotal role in breast cancer invasive activities and identified AMAP1 as an effector of GTP-Arf6 in invasion. Expression of AMAP1 correlates well with invasive phenotypes of primary tumors of the human breast. We also have shown that AMAP1 functions by forming a trimeric protein complex with cortactin and paxillin. In this complex, AMAP1 binds to the src homology 3 (SH3) domain of cortactin via its proline-rich peptide, SKKRPPPPPPGHKRT. SH3 domains are known to bind generally to the proline-rich ligands with a one-to-one stoichiometry. We found that AMAP1/cortactin binding is very atypical in its stoichiometry and interface structure, in which one AMAP1 proline-rich peptide binds to two cortactin SH3 domains simultaneously. We made a cell-permeable peptide derived from the AMAP1 peptide, and we show that this peptide specifically blocks AMAP1/cortactin binding, but not other canonical SH3/proline bindings, and effectively inhibits breast cancer invasion and   metastasis  . Moreover, this peptide was found to block invasion of other types of cancers, such as glioblastomas and lung carcinomas. We also found that a small-molecule compound, UCS15A, which was previously judged as a weak inhibitor against canonical SH3/proline bindings, effectively inhibits AMAP1/cortactin binding and breast cancer invasion and   metastasis  . Together with fine structural analysis, we propose that the AMAP1/cortactin complex, which is not detected in normal mammary epithelial cells, is an excellent drug target for cancer therapeutics. 
 ASAP1 promotes tumor cell motility and invasiveness, stimulates   metastasis   formation in vivo, and correlates with poor survival in colorectal cancer patients. We have previously performed an unbiased screen to identify genes whose expression is associated with the metastatic phenotype. Secondary screening of these genes using custom microarray chips identified ASAP1, a multi-domain adaptor protein with ADP-ribosylation factor-GAP activity, as being potentially involved in tumor progression. Here, we show that at least three different splice forms of ASAP1 are upregulated in rodent tumor models in a manner that correlates with metastatic potential. In human cancers, we found that ASAP1 expression is strongly upregulated in a variety of tumors in comparison with normal tissue and that this expression correlates with poor   metastasis  -free survival and prognosis in colorectal cancer patients. Using loss and gain of function approaches, we were able to show that ASAP1 promotes   metastasis   formation in vivo and stimulates tumor cell motility, invasiveness, and adhesiveness in vitro. Furthermore, we show that ASAP1 interacts with the   metastasis  -promoting protein h-prune and stimulates its phosphodiesterase activity. In addition, ASAP1 binds to the SH3 domains of several proteins, including SLK with which it co-immunoprecipitates. These data support the notion that ASAP1 can contribute to the dissemination of a variety of tumor types and represent a potential target for cancer therapy. 
 ASAP1, a gene at 8q24, is associated with prostate cancer   metastasis  . Metastatic prostate cancer is a terminal disease, and the development of reliable prognostic tools and more effective therapy is critically important for improved disease survival and management. This study was aimed at identifying genes that are differentially expressed in metastatic and nonmetastatic prostate cancer cells and, as such, could be critical in the development of   metastasis  . Long-SAGE analysis was used to compare a transplantable human metastatic prostate cancer subline, PCa1-met, with a nonmetastatic counterpart, PCa2. Both sublines were developed from a patient's prostate cancer specimen via subrenal capsule grafting and subsequent orthotopic implantation into SCID mice. Among various differentially expressed genes identified, ASAP1, an 8q24 gene encoding an ADP-ribosylation factor GTPase-activating protein not previously associated with prostate cancer, was up-regulated in the metastatic subline as confirmed by quantitative real-time PCR. Immunohistochemistry of xenograft sections showed that cytoplasmic ASAP1 protein staining was absent or weak in benign tissue, significantly stronger in nonmetastatic PCa2 tissue, and strongest in PCa1-met tissue. In clinical specimens, ASAP1 protein staining was elevated in 80% of primary prostate cancers and substantially higher in metastatic lesions compared with benign prostate tissue. Moreover, additional ASAP1 gene copies were detected in 58% of the primary prostate cancer specimens. Small interfering RNA-induced reduction of ASAP1 protein expression markedly suppressed in vitro PC-3 cell migration (approximately 50%) and Matrigel invasion (approximately 67%). This study suggests that the ASAP1 gene plays a role in prostate cancer   metastasis   and may represent a therapeutic target and/or biomarker for metastatic disease. 
  GeneRIFs  
 results indicated that elevated expression of ASAP1 plays an important role in the progression and   metastasis   of ovarian cancer 
 Data suggest that GEP100-Arf6-AMAP1-cortactin pathway, activated by VEGFR2, appears to be common in angiogenesis and cancer invasion and   metastasis  , and provides their new therapeutic targets. 
 study suggests that the ASAP1 gene plays a role in prostate cancer   metastasis   and may represent a therapeutic target and/or biomarker for metastatic disease 
  [Cc]ell [Ii]nvasion  PubMed  
 CIN85, a Cbl-interacting protein, is a component of AMAP1-mediated breast cancer invasion machinery. Expression of AMAP1 correlates well with the invasive phenotypes and malignancy of human primary breast carcinomas. AMAP1 recruits its binding proteins, such as cortactin and paxillin, to sites of Arf6 activation to form invadopodia. A mouse ortholog of AMAP1, ASAP1, is known to bind to CIN85, a binding partner of an E3 ligase, Cbl. Here, we found that CIN85 colocalizes with AMAP1 at invadopodia, and binding of AMAP1 with CIN85 is important for the invasive activities of breast cancer cells, including MDA-MB-231. siRNA-mediated silencing of CIN85, as well as Cbl, also inhibited the invasion. We moreover found that AMAP1 is monoubiquitinated, rather than polyubiquitinated, by virtue of Cbl and provide evidence that the ability of AMAP1 to be monoubiquitinated is important for its involvement in invasion. Our results indicate that CIN85, as well as Cbl, which is a well-known suppressor of growth factor receptor signaling, can be positively involved in tumor invasion, and suggest that a complex epigenetic process is involved in AMAP1 function in breast cancer   cell invasion  . 
 Rab5c promotes AMAP1-PRKD2 complex formation to enhance β1 integrin recycling in EGF-induced cancer invasion. Epidermal growth factor receptor (EGFR) signaling is one of the crucial factors in breast cancer malignancy. Breast cancer cells often overexpress Arf6 and its effector, AMAP1/ASAP1/DDEF1; in these cells, EGFR signaling may activate the Arf6 pathway to induce invasion and metastasis. Active recycling of some integrins is crucial for invasion and metastasis. Here, we show that the Arf6-AMAP1 pathway links to the machinery that recycles β1 integrins, such as α3β1, to prom  ote cell inva  sion upon EGFR stimulation. We found that AMAP1 had the ability to bind directly to PRKD2 and hence to make a complex with the cytoplasmic tail of the β1 subunit. Moreover, GTP-Rab5c also bound to AMAP1, and activation of Rab5c by EGFR signaling was necessary to promote the intracellular association of AMAP1 and PRKD2. Our results suggest a novel mechanism by which EGFR signaling promotes the invasiveness of some breast cancer cells via integrin recycling. 
 Rab5c promotes AMAP1-PRKD2 complex formation to enhance β1 integrin recycling in EGF-induced cancer invasion. Epidermal growth factor receptor (EGFR) signaling is one of the crucial factors in breast cancer malignancy. Breast cancer cells often overexpress Arf6 and its effector, AMAP1/ASAP1/DDEF1; in these cells, EGFR signaling may activate the Arf6 pathway to induce invasion and metastasis. Active recycling of some integrins is crucial for invasion and metastasis. Here, we show that the Arf6-AMAP1 pathway links to the machinery that recycles β1 integrins, such as α3β1, to prom  ote cell inva  sion upon EGFR stimulation. We found that AMAP1 had the ability to bind directly to PRKD2 and hence to make a complex with the cytoplasmic tail of the β1 subunit. Moreover, GTP-Rab5c also bound to AMAP1, and activation of Rab5c by EGFR signaling was necessary to promote the intracellular association of AMAP1 and PRKD2. Our results suggest a novel mechanism by which EGFR signaling promotes the invasiveness of some breast cancer cells via integrin recycling. 
 ASAP3 is a focal adhesion-associated Arf GAP that functions in cell migration and invasion. ASAP3, an Arf GTPase-activating protein previously called DDEFL1 and ACAP4, has been implicated in the pathogenesis of hepatocellular carcinoma. We have examined in vitro and in vivo functions of ASAP3 and compared it to the related Arf GAP ASAP1 that has also been implicated in oncogenesis. ASAP3 was biochemically similar to ASAP1: the pleckstrin homology domain affected function of the catalytic domain by more than 100-fold; catalysis was stimulated by phosphatidylinositol 4,5-bisphosphate; and Arf1, Arf5, and Arf6 were used as substrates in vitro. Like ASAP1, ASAP3 associated with focal adhesions and circular dorsal ruffles. Different than ASAP1, ASAP3 did not localize to invadopodia or podosomes. Cells, derived from a mammary carcinoma and from a glioblastoma, with reduced ASAP3 expression had fewer actin stress fiber, reduced levels of phosphomyosin, and migrated more slowly than control cells. Reducing ASAP3 expression also slowed invasion of mammary carcinoma cells. In contrast, reduction of ASAP1 expression had no effect on migration or invasion. We propose that ASAP3 functions nonredundantly with ASAP1 to control cell movement and may have a role in cancer   cell invasion  . In comparing ASAP1 and ASAP3, we also found that invadopodia are dispensable for the invasive behavior of cells derived from a mammary carcinoma. 
  GeneRIFs  
 ASAP3 functions nonredundantly with ASAP1 to control cell movement and may have a role in cancer   cell invasion  . 
   
  
 DRAM1 
 
  NCBI Gene  
  GeneRIF  
  Pubmed  
 
 
  Search Term  Rank  Fields  
  [Mm]igration  6  PubMed(1); GeneRIFs(1)  
 
 
 hide/show details for DRAM1 
  [Mm]igration  PubMed  
 The autophagy-associated factors DRAM1 and p62 regulate cell   migration   and invasion in glioblastoma stem cells. The aggressiveness of glioblastoma multiforme (GBM) is defined by local invasion and resistance to therapy. Within established GBM, a subpopulation of tumor-initiating cells with stem-like properties (GBM stem cells, GSCs) is believed to underlie resistance to therapy. The metabolic pathway autophagy has been implicated in the regulation of survival in GBM. However, the status of autophagy in GBM and its role in the cancer stem cell fraction is currently unclear. We found that a number of autophagy regulators are highly expressed in GBM tumors carrying a mesenchymal signature, which defines aggressiveness and invasion, and are associated with components of the MAPK pathway. This autophagy signature included the autophagy-associated genes DRAM1 and SQSTM1, which encode a key regulator of selective autophagy, p62. High levels of DRAM1 were associated with shorter overall survival in GBM patients. In GSCs, DRAM1 and SQSTM1 expression correlated with activation of MAPK and expression of the mesenchymal marker c-MET. DRAM1 knockdown decreased p62 localization to autophagosomes and its autophagy-mediated degradation, thus suggesting a role for DRAM1 in p62-mediated autophagy. In contrast, autophagy induced by starvation or inhibition of mTOR/PI-3K was not affected by either DRAM1 or p62 downregulation. Functionally, DRAM1 and p62 regulate cell motility and invasion in GSCs. This was associated with alterations of energy metabolism, in particular reduced ATP and lactate levels. Taken together, these findings shed new light on the role of autophagy in GBM and reveal a novel function of the autophagy regulators DRAM1 and p62 in control of   migration  /invasion in cancer stem cells. 
  GeneRIFs  
 these findings shed new light on the role of autophagy in GBM and reveal a novel function of the autophagy regulators DRAM1 and p62 in control of   migration  /invasion in cancer stem cells. 
   
  
 TRIB2 
 
  NCBI Gene  
  GeneRIF  
  Pubmed  
 
 
  Search Term  Rank  Fields  
  [Mm]igration  6  PubMed(1)  
 
 
 hide/show details for TRIB2 
  [Mm]igration  PubMed  
 Survival factor withdrawal-induced apoptosis of TF-1 cells involves a TRB2-Mcl-1 axis-dependent pathway. Tribbles, an atypical protein kinase superfamily member, coordinates cell proliferation,   migration  , and morphogenesis during the development of Drosophila and Xenopus embryos. Although Tribbles are highly conserved throughout evolution, the physiological functions of mammalian Tribbles family remain largely unclear. Here we report that human TRB2 is a pro-apoptotic molecule that induces apoptosis of cells mainly of the hematopoietic origin. TRB2 mRNA is selectively induced by removal of granulocyte macrophage colony-stimulating factor (GM-CSF) or interleukin-2 from human erythroleukemia-derived TF-1 cell line or activated primary CD4(+) T cells, respectively. It is, however, not induced by many other treatments that trigger apoptosis of these two cell types. Overexpression of TRB2 activates many apoptotic events observed in GM-CSF-deprived TF-1 cells, including loss of mitochondrial membrane potential, Mcl-1 cleavage/degradation, and activation of Bax and a number of caspases. Specific knockdown of TRB2 significantly suppresses GM-CSF deprivation-induced apoptosis and all apoptotic events mentioned above. Finally, we demonstrate that TRB2-induced cleavage and degradation of Mcl-1 are mediated via a caspase-dependent but proteasome-independent mechanism, and overexpression of Mcl-1 or its upstream activator Akt can markedly overcome the apoptogenic effect of TRB2. Altogether, these results suggest that the TRB2-Mcl-1 axis plays an important role in survival factor withdrawal-induced apoptosis of TF-1 cells. 
   
  
 ARHGAP17 
 
  NCBI Gene  
  GeneRIF  
  Pubmed  
 
 
  Search Term  Rank  Fields  
  [Mm]igration  6  PubMed(1)  
 
 
 hide/show details for ARHGAP17 
  [Mm]igration  PubMed  
 Cyclic Nucleotide-dependent Protein Kinases Target ARHGAP17 and ARHGEF6 Complexes in Platelets. Endothelial cells release prostacyclin (PGI2) and nitric oxide (NO) to inhibit platelet functions. PGI2 and NO effects are mediated by cyclic nucleotides, cAMP- and cGMP-dependent protein kinases (PKA, PKG), and largely unknown PKA and PKG substrate proteins. The small G-protein Rac1 plays a key role in platelets and was suggested to be a target of cyclic nucleotide signaling. We confirm that PKA and PKG activation reduces Rac1-GTP levels. Screening for potential mediators of this effect resulted in the identification of the Rac1-specific GTPase-activating protein ARHGAP17 and the guanine nucleotide exchange factor ARHGEF6 as new PKA and PKG substrates in platelets. We mapped the PKA/PKG phosphorylation sites to serine 702 on ARHGAP17 using Phos-tag gels and to serine 684 on ARHGEF6. We show that ARHGAP17 binds to the actin-regulating CIP4 protein in platelets and that Ser-702 phosphorylation interferes with this interaction. Reduced CIP4 binding results in enhanced inhibition of cell   migration   by ARHGAP17. Furthermore, we show that ARHGEF6 is constitutively linked to GIT1, a GAP of Arf family small G proteins, and that ARHGEF6 phosphorylation enables binding of the 14-3-3 adaptor protein to the ARHGEF6/GIT1 complex. PKA and PKG induced rearrangement of ARHGAP17- and ARHGEF6-associated protein complexes might contribute to Rac1 regulation and platelet inhibition. 
   
  
 DDC 
 
  NCBI Gene  
  GeneRIF  
  Pubmed  
 
 
  Search Term  Rank  Fields  
  [Mm]etastasis  12  PubMed(1)  
 
 
 hide/show details for DDC 
  [Mm]etastasis  PubMed  
 L-dopa decarboxylase (DDC) gene expression is related to outcome in patients with prostate cancer. What's known on the subject? and What does the study add? L-dopa decarboxylase (DDC) has been documented as a novel co-activator of androgen receptor transcriptional activity. Recently, it was shown that DDC gene expression is significantly higher in patients with PCa than in those with BPH. In the present study, there was a significant association between the DDC gene expression levels and the pathological stage and Gleason score of patients with prostate cancer (PCa). Moreover, DDC expression was shown to be an unfavourable prognostic marker of biochemical recurrence and disease-free survival in patients with PCa treated by radical prostatectomy.To determine whether L-dopa decarboxylase gene (DDC) expression levels in patients with prostate cancer (PCa) correlate to biochemical recurrence and disease prognosis after radical prostatectomy (RP).The present study consisted of 56 samples with confirmed malignancy from patients with PCa who had undergone RP at a single tertiary academic centre. Total RNA was isolated from tissue specimens and a SYBR Green fluorescence-based quantitative real-time polymerase chain reaction methodology was developed for the determination of DDC mRNA expression levels of the tested tissues. Follow-up time ranged between 1.0 and 62.0 months (mean ± SE, 28.6 ± 2.1 month; median, 31.5 months). Time to biochemical recurrence was defined as the interval between the surgery and the measurement of two consecutive values of prostate-specific antigen (PSA) ≥0.2 ng/mL.DDC expression levels were found to be positively correlated with the tumour-n  ode-metast  asis stage (P = 0.021) and Gleason score (P = 0.036) of the patients with PCa. Patients with PCa with raised DDC expression levels run a significantly higher risk of biochemical recurrence after RP, as indicated by Cox proportional regression analysis (P = 0.021). Multivariate Cox proportional regression models revealed the preoperative PSA-, age- and digital rectal examination-independent prognostic value of DDC expression for the prediction of disease-free survival (DFS) among patients with PCa (P = 0.036). Kaplan-Meier survival analysis confirms the significantly shorter DFS after RP of PCa with higher DDC expression levels (P = 0.015).This is the first study indicating the potential of DDC expression as a novel prognostic biomarker in patients with PCa who have undergone RP. For further evaluation and clinical application of the findings of the present study, a direct analysis of mRNA and/or its protein expression level in preoperative biopsy, blood serum and urine should be conducted. 
   
  
 ERO1B 
 
  NCBI Gene  
  GeneRIF  
  Pubmed  
 
 
  Search Term  Rank  Fields  
  [Ee]xtracellular [Mm]atrix  8  Process(1)  
 
 
 hide/show details for ERO1B 
  [Ee]xtracellular [Mm]atrix  Process  
   extracellular matrix   organization 
   
  
 SPHK2 
 
  NCBI Gene  
  GeneRIF  
  Pubmed  
 
 
  Search Term  Rank  Fields  
  [Ff]ilopodia  1  PubMed(1)  
  [Mm]igration  6  PubMed(6); GeneRIFs(2); Summary(1)  
  [Ii]nvasive  10  PubMed(1)  
  [Mm]etastasis  12  PubMed(2)  
  [Cc]ell [Ii]nvasion  13  PubMed(2)  
 
 
 hide/show details for SPHK2 
  [Ff]ilopodia  PubMed  
 Sphingosine 1-phosphate induces   filopodia   formation through S1PR2 activation of ERM proteins. Previously we demonstrated that the sphingolipids ceramide and S1P (sphingosine 1-phosphate) regulate phosphorylation of the ERM (ezrin/radixin/moesin) family of cytoskeletal proteins [Canals, Jenkins, Roddy, Hernande-Corbacho, Obeid and Hannun (2010) J. Biol. Chem. 285, 32476-3285]. In the present article, we show that exogenously applied or endogenously generated S1P (in a sphingosine kinase-dependent manner) results in significant increases in phosphorylation of ERM proteins as well as   filopodia   formation. Using phosphomimetic and non-phosphorylatable ezrin mutants, we show that the S1P-induced cytoskeletal protrusions are dependent on ERM phosphorylation. Employing various pharmacological S1PR (S1P receptor) agonists and antagonists, along with siRNA (small interfering RNA) techniques and genetic knockout approaches, we identify the S1PR2 as the specific and necessary receptor to induce phosphorylation of ERM proteins and subsequent   filopodia   formation. Taken together, the results demonstrate a novel mechanism by which S1P regulates cellular architecture that requires S1PR2 and subsequent phosphorylation of ERM proteins. 
  [Mm]igration  PubMed  
 Distinct roles of sphingosine kinases 1 and 2 in human mast-cell functions. Sphingosine-1-phosphate (S1P) is now emerging as a potent lipid mediator produced by mast cells that contributes to inflammatory and allergic responses. In contrast to its weak effect on degranulation of murine mast cells, S1P potently induced degranulation of the human LAD2 mast-cell line and cord blood-derived human mast cells (hMCs). S1P also stimulated production and secretion of cytokines, TNF-alpha and IL-6, and markedly enhanced secretion of a chemokine, CCL2/MCP-1, important modulators of inflammation. S1P is produced in mast cells by the 2 sphingosine kinases, SphK1 and SphK2. SphK1 but not SphK2 plays a critical role in IgE/Ag-induced degranulation,   migration   toward antigen, and CCL2 secretion from hMCs, as determined by specifically down-regulating their expression. However, both isoenzymes were required for efficient TNF-alpha secretion. Taken together, our data suggest that differential formation of S1P by SphK1 and SphK2 has distinct and important actions in hMCs. 
 Down-regulation of Sphk2 suppresses bladder cancer progression. Bladder cancer is the second most common urological malignancy around the world and is by far the most frequent urological malignancy in China. The abnormal expression of sphingosine kinase 2 (SphK2) is associated with tumor progression and a poor patient survival rate, however, the effect of SphK2 on the bladder cancer cells remains unclear. The aim of the paper was to study the expression of SphK2 in bladder cancer and the role of SphK2 on the cell proliferation, metastasis, and apoptosis in bladder cancer in vitro. Our results showed that SphK2 is up-regulated in bladder cancer tissues compared with the corresponding adjacent non-neoplastic tissues, and the expression level of SphK2 was significantly higher in human bladder cancer cells in comparison with normal bladder epithelial cells. Silencing of SphK2 could inhibit the proliferation ability of T24 cells in vitro. In addition, SphK2 knockdown could induce a significant increase in the number of apoptotic cells. Furthermore, the transwell assay also showed significant cell   migration   inhibition in SphK2 siRNA transfectant compared with cell lines transfected with NC. Thus, this study suggested that SphK2 inhibition may provide a promising treatment for bladder cancer patients. 
 Increased mRNA Levels of Sphingosine Kinases and S1P Lyase and Reduced Levels of S1P Were Observed in Hepatocellular Carcinoma in Association with Poorer Differentiation and Earlier Recurrence. Although sphingosine 1-phosphate (S1P) has been reported to play an important role in cancer pathophysiology, little is known about S1P and hepatocellular carcinoma (HCC). To clarify the relationship between S1P and HCC, 77 patients with HCC who underwent surgical treatment were consecutively enrolled in this study. In addition, S1P and its metabolites were quantitated by LC-MS/MS. The mRNA levels of sphingosine kinases (SKs), which phosphorylate sphingosine to generate S1P, were increased in HCC tissues compared with adjacent non-HCC tissues. Higher mRNA levels of SKs in HCC were associated with poorer differentiation and microvascular invasion, whereas a higher level of SK2 mRNA was a risk factor for intra- and extra-hepatic recurrence. S1P levels, however, were unexpectedly reduced in HCC compared with non-HCC tissues, and increased mRNA levels of S1P lyase (SPL), which degrades S1P, were observed in HCC compared with non-HCC tissues. Higher SPL mRNA levels in HCC were associated with poorer differentiation. Finally, in HCC cell lines, inhibition of the expression of SKs or SPL by siRNA led to reduced proliferation, invasion and   migration  , whereas overexpression of SKs or SPL enhanced proliferation. In conclusion, increased SK and SPL mRNA expression along with reduced S1P levels were more commonly observed in HCC tissues compared with adjacent non-HCC tissues and were associated with poor differentiation and early recurrence. SPL as well as SKs may be therapeutic targets for HCC treatment. 
 Sphingosine kinase type 2 activation by ERK-mediated phosphorylation. Sphingosine 1-phosphate (S1P), a potent lipid mediator, is a ligand for a family of five G protein-coupled receptors (S1P(1-5)) that have been shown to regulate a variety of biological responses important for cancer progression. The cellular level of S1P is low and tightly regulated in a spatio-temporal manner through its synthesis catalyzed by two sphingosine kinases, denoted SphK1 and SphK2. Many stimuli activate and translocate SphK1 to the plasma membrane by mechanisms that are dependent on its phosphorylation. Much less is known about activation of SphK2. Here we demonstrate that epidermal growth factor (EGF) as well as the protein kinase C activator, phorbol ester, induce rapid phosphorylation of hSphK2 which was markedly reduced by inhibition of MEK1/ERK pathway. Down-regulation of ERK1 blocked EGF-induced phosphorylation of SphK2. Recombinant ERK1 phosphorylated hSphK2 in vitro and increased its enzymatic activity. ERK1 also was found to be in a complex with hSphK2 in vivo. Site-directed mutagenesis indicated that hSphK2 is phosphorylated on Ser-351 and Thr-578 by ERK1 and that phosphorylation of these residues is important for EGF-stimulated   migration   of MDA-MB-453 cells. These studies provide the first clues to the mechanism of agonist-mediated SphK2 activation and enhance understanding of the regulation of SphK2 activity by phosphorylation and its role in movement of human breast cancer cells toward EGF. 
 Sphingosine kinase 2 promotes colorectal cancer cell proliferation and invasion by enhancing MYC expression. Colorectal cancer (CRC) is one of the most commonly diagnosed cancers and causes of cancer death in developed countries. SphK2 is overexpressed in a number of aggressive human carcinomas; however, the expression profile and potential function of SphK2 in CRC are still unknown. In this study, we investigated the SphK2 expression in tumoral tissue and the matched normal mucosae using quantitative real-time PCR (qRT-PCR), Western blot, and immunohistochemistry. We also evaluated the impact of SphK2 knockdown on CRC cell proliferation and metastasis in vitro. SphK2 was significantly upregulated in CRC tissue as compared to the matched normal mucosae, and significant overexpression was found in the LoVo CRC cell line. SphK2 depletion by specific small interfering RNA (siRNA) in the CRC cell line was found to affect cell proliferation and cell   migration  . Our data suggest that the pathogenesis of CRC maybe mediated by SphK2, and SphK2 could represent a selective target for the molecularly targeted treatments of CRC. 
 Role of sphingosine kinase 2 in cell   migration   toward epidermal growth factor. Sphingosine 1-phosphate (S1P), produced by two sphingosine kinase isoenzymes, denoted SphK1 and SphK2, is the ligand for a family of five specific G protein-coupled receptors that regulate cytoskeletal rearrangements and cell motility. Whereas many growth factors stimulate SphK1, much less is known of the regulation of SphK2. Here we report that epidermal growth factor (EGF) stimulated SphK2 in HEK 293 cells. This is the first example of an agonist-dependent regulation of SphK2. Chemotaxis of HEK 293 cells toward EGF was inhibited by N,N-dimethylsphingosine, a competitive inhibitor of both SphKs, implicating S1P generation in this process. Down-regulating expression of SphK1 in HEK 293 cells with a specific siRNA abrogated   migration   toward EGF, whereas decreasing SphK2 expression had no effect. EGF contributes to the invasiveness of human breast cancer cells, and EGF receptor expression is associated with poor prognosis. EGF also stimulated SphK2 in MDA-MB-453 breast cancer cells. Surprisingly, however, down-regulation of SphK2 in these cells completely eliminated   migration   toward EGF without affecting fibronectin-induced haptotaxis. Our results suggest that SphK2 plays an important role in   migration   of MDA-MB-453 cells toward EGF. 
  GeneRIFs  
 Sphingosine kinase 2 plays an important role in   migration   of MDA-MB-453 cells toward EGF. 
 hSphK2 is phosphorylated on Ser-351 and Thr-578 by ERK1 and phosphorylation of these residues is important for EGF-stimulated   migration   of MDA-MB-453 cells 
  Summary  
 This gene encodes one of two sphingosine kinase isozymes that catalyze the phosphorylation of sphingosine into sphingosine 1-phosphate. Sphingosine 1-phosphate mediates many cellular processes including   migration  , proliferation and apoptosis, and also plays a role in several types of cancer by promoting angiogenesis and tumorigenesis. The encoded protein may play a role in breast cancer proliferation and chemoresistance. Alternatively spliced transcript variants encoding multiple isoforms have been observed for this gene. [provided by RefSeq, Aug 2011] 
  [Ii]nvasive  PubMed  
 Role of sphingosine kinase 2 in cell migration toward epidermal growth factor. Sphingosine 1-phosphate (S1P), produced by two sphingosine kinase isoenzymes, denoted SphK1 and SphK2, is the ligand for a family of five specific G protein-coupled receptors that regulate cytoskeletal rearrangements and cell motility. Whereas many growth factors stimulate SphK1, much less is known of the regulation of SphK2. Here we report that epidermal growth factor (EGF) stimulated SphK2 in HEK 293 cells. This is the first example of an agonist-dependent regulation of SphK2. Chemotaxis of HEK 293 cells toward EGF was inhibited by N,N-dimethylsphingosine, a competitive inhibitor of both SphKs, implicating S1P generation in this process. Down-regulating expression of SphK1 in HEK 293 cells with a specific siRNA abrogated migration toward EGF, whereas decreasing SphK2 expression had no effect. EGF contributes to the   invasive  ness of human breast cancer cells, and EGF receptor expression is associated with poor prognosis. EGF also stimulated SphK2 in MDA-MB-453 breast cancer cells. Surprisingly, however, down-regulation of SphK2 in these cells completely eliminated migration toward EGF without affecting fibronectin-induced haptotaxis. Our results suggest that SphK2 plays an important role in migration of MDA-MB-453 cells toward EGF. 
  [Mm]etastasis  PubMed  
 Down-regulation of Sphk2 suppresses bladder cancer progression. Bladder cancer is the second most common urological malignancy around the world and is by far the most frequent urological malignancy in China. The abnormal expression of sphingosine kinase 2 (SphK2) is associated with tumor progression and a poor patient survival rate, however, the effect of SphK2 on the bladder cancer cells remains unclear. The aim of the paper was to study the expression of SphK2 in bladder cancer and the role of SphK2 on the cell proliferation,   metastasis  , and apoptosis in bladder cancer in vitro. Our results showed that SphK2 is up-regulated in bladder cancer tissues compared with the corresponding adjacent non-neoplastic tissues, and the expression level of SphK2 was significantly higher in human bladder cancer cells in comparison with normal bladder epithelial cells. Silencing of SphK2 could inhibit the proliferation ability of T24 cells in vitro. In addition, SphK2 knockdown could induce a significant increase in the number of apoptotic cells. Furthermore, the transwell assay also showed significant cell migration inhibition in SphK2 siRNA transfectant compared with cell lines transfected with NC. Thus, this study suggested that SphK2 inhibition may provide a promising treatment for bladder cancer patients. 
 Sphingosine kinase 2 promotes colorectal cancer cell proliferation and invasion by enhancing MYC expression. Colorectal cancer (CRC) is one of the most commonly diagnosed cancers and causes of cancer death in developed countries. SphK2 is overexpressed in a number of aggressive human carcinomas; however, the expression profile and potential function of SphK2 in CRC are still unknown. In this study, we investigated the SphK2 expression in tumoral tissue and the matched normal mucosae using quantitative real-time PCR (qRT-PCR), Western blot, and immunohistochemistry. We also evaluated the impact of SphK2 knockdown on CRC cell proliferation and   metastasis   in vitro. SphK2 was significantly upregulated in CRC tissue as compared to the matched normal mucosae, and significant overexpression was found in the LoVo CRC cell line. SphK2 depletion by specific small interfering RNA (siRNA) in the CRC cell line was found to affect cell proliferation and cell migration. Our data suggest that the pathogenesis of CRC maybe mediated by SphK2, and SphK2 could represent a selective target for the molecularly targeted treatments of CRC. 
  [Cc]ell [Ii]nvasion  PubMed  
 Intracellular sphingosine kinase 2-derived sphingosine-1-phosphate mediates epidermal growth factor-induced ezrin-radixin-moesin phosphorylation and cancer   cell invasion  . The bioactive sphingolipid sphingosine-1-phosphate (S1P) mediates cellular proliferation, mitogenesis, inflammation, and angiogenesis. These biologies are mediated through S1P binding to specific GPCRs [sphingosine-1-phosphate receptor (S1PR)1-5] and some other less well-characterized intracellular targets. Ezrin-radixin-moesin (ERM) proteins, a family of adaptor molecules linking the cortical actin cytoskeleton to the plasma membrane, are emerging as critical regulators of cancer invasion via regulation of cell morphology and motility. Recently, we identified S1P as an acute ERM activator (via phosphorylation) through its action on S1PR2. In this work, we dissect the mechanism of S1P generation downstream of epidermal growth factor (EGF) leading to ERM phosphorylation and cancer invasion. Using pharmacologic inhibitors, small interfering RNA technologies, and genetic approaches, we demonstrate that sphingosine kinase (SK)2, and not SK1, is essential and sufficient in EGF-mediated ERM phosphorylation in HeLa cells. In fact, knocking down SK2 decreased ERM activation 2.5-fold. Furthermore, we provide evidence that SK2 is necessary to mediate EGF-induced invasion. In addition, overexpressing SK2 causes a 2-fold increase in HeLa   cell invasion  . Surprisingly, and for the first time, we find that this event, although dependent on S1PR2 activation, does not generate and does not require extracellular S1P secretion, therefore introducing a potential novel model of autocrine/intracrine action of S1P that still involves its GPCRs. These results define new mechanistic insights for EGF-mediated invasion and novel actions of SK2, therefore setting the stage for novel targets in the treatment of growth factor-driven malignancies. 
 Intracellular sphingosine kinase 2-derived sphingosine-1-phosphate mediates epidermal growth factor-induced ezrin-radixin-moesin phosphorylation and cancer   cell invasion  . The bioactive sphingolipid sphingosine-1-phosphate (S1P) mediates cellular proliferation, mitogenesis, inflammation, and angiogenesis. These biologies are mediated through S1P binding to specific GPCRs [sphingosine-1-phosphate receptor (S1PR)1-5] and some other less well-characterized intracellular targets. Ezrin-radixin-moesin (ERM) proteins, a family of adaptor molecules linking the cortical actin cytoskeleton to the plasma membrane, are emerging as critical regulators of cancer invasion via regulation of cell morphology and motility. Recently, we identified S1P as an acute ERM activator (via phosphorylation) through its action on S1PR2. In this work, we dissect the mechanism of S1P generation downstream of epidermal growth factor (EGF) leading to ERM phosphorylation and cancer invasion. Using pharmacologic inhibitors, small interfering RNA technologies, and genetic approaches, we demonstrate that sphingosine kinase (SK)2, and not SK1, is essential and sufficient in EGF-mediated ERM phosphorylation in HeLa cells. In fact, knocking down SK2 decreased ERM activation 2.5-fold. Furthermore, we provide evidence that SK2 is necessary to mediate EGF-induced invasion. In addition, overexpressing SK2 causes a 2-fold increase in HeLa   cell invasion  . Surprisingly, and for the first time, we find that this event, although dependent on S1PR2 activation, does not generate and does not require extracellular S1P secretion, therefore introducing a potential novel model of autocrine/intracrine action of S1P that still involves its GPCRs. These results define new mechanistic insights for EGF-mediated invasion and novel actions of SK2, therefore setting the stage for novel targets in the treatment of growth factor-driven malignancies. 
   
  
 SPHK1 
 
  NCBI Gene  
  GeneRIF  
  Pubmed  
 
 
  Search Term  Rank  Fields  
  [Ff]ilopodia  1  PubMed(1)  
  [Mm]igration  6  Process(1); PubMed(32); GeneRIFs(9)  
  ECM  7  PubMed(2)  
  [Ee]xtracellular [Mm]atrix  8  PubMed(4); GeneRIFs(1)  
  [Ii]nvasive  10  PubMed(7); GeneRIFs(2)  
  [Mm]etastasis  12  PubMed(13); GeneRIFs(4)  
  [Cc]ell [Ii]nvasion  13  PubMed(2)  
 
 
 hide/show details for SPHK1 
  [Ff]ilopodia  PubMed  
 Sphingosine 1-phosphate induces   filopodia   formation through S1PR2 activation of ERM proteins. Previously we demonstrated that the sphingolipids ceramide and S1P (sphingosine 1-phosphate) regulate phosphorylation of the ERM (ezrin/radixin/moesin) family of cytoskeletal proteins [Canals, Jenkins, Roddy, Hernande-Corbacho, Obeid and Hannun (2010) J. Biol. Chem. 285, 32476-3285]. In the present article, we show that exogenously applied or endogenously generated S1P (in a sphingosine kinase-dependent manner) results in significant increases in phosphorylation of ERM proteins as well as   filopodia   formation. Using phosphomimetic and non-phosphorylatable ezrin mutants, we show that the S1P-induced cytoskeletal protrusions are dependent on ERM phosphorylation. Employing various pharmacological S1PR (S1P receptor) agonists and antagonists, along with siRNA (small interfering RNA) techniques and genetic knockout approaches, we identify the S1PR2 as the specific and necessary receptor to induce phosphorylation of ERM proteins and subsequent   filopodia   formation. Taken together, the results demonstrate a novel mechanism by which S1P regulates cellular architecture that requires S1PR2 and subsequent phosphorylation of ERM proteins. 
  [Mm]igration  Process  
 positive regulation of cell   migration   
  PubMed  
 Increased mRNA Levels of Sphingosine Kinases and S1P Lyase and Reduced Levels of S1P Were Observed in Hepatocellular Carcinoma in Association with Poorer Differentiation and Earlier Recurrence. Although sphingosine 1-phosphate (S1P) has been reported to play an important role in cancer pathophysiology, little is known about S1P and hepatocellular carcinoma (HCC). To clarify the relationship between S1P and HCC, 77 patients with HCC who underwent surgical treatment were consecutively enrolled in this study. In addition, S1P and its metabolites were quantitated by LC-MS/MS. The mRNA levels of sphingosine kinases (SKs), which phosphorylate sphingosine to generate S1P, were increased in HCC tissues compared with adjacent non-HCC tissues. Higher mRNA levels of SKs in HCC were associated with poorer differentiation and microvascular invasion, whereas a higher level of SK2 mRNA was a risk factor for intra- and extra-hepatic recurrence. S1P levels, however, were unexpectedly reduced in HCC compared with non-HCC tissues, and increased mRNA levels of S1P lyase (SPL), which degrades S1P, were observed in HCC compared with non-HCC tissues. Higher SPL mRNA levels in HCC were associated with poorer differentiation. Finally, in HCC cell lines, inhibition of the expression of SKs or SPL by siRNA led to reduced proliferation, invasion and   migration  , whereas overexpression of SKs or SPL enhanced proliferation. In conclusion, increased SK and SPL mRNA expression along with reduced S1P levels were more commonly observed in HCC tissues compared with adjacent non-HCC tissues and were associated with poor differentiation and early recurrence. SPL as well as SKs may be therapeutic targets for HCC treatment. 
 Distinct roles of sphingosine kinases 1 and 2 in human mast-cell functions. Sphingosine-1-phosphate (S1P) is now emerging as a potent lipid mediator produced by mast cells that contributes to inflammatory and allergic responses. In contrast to its weak effect on degranulation of murine mast cells, S1P potently induced degranulation of the human LAD2 mast-cell line and cord blood-derived human mast cells (hMCs). S1P also stimulated production and secretion of cytokines, TNF-alpha and IL-6, and markedly enhanced secretion of a chemokine, CCL2/MCP-1, important modulators of inflammation. S1P is produced in mast cells by the 2 sphingosine kinases, SphK1 and SphK2. SphK1 but not SphK2 plays a critical role in IgE/Ag-induced degranulation,   migration   toward antigen, and CCL2 secretion from hMCs, as determined by specifically down-regulating their expression. However, both isoenzymes were required for efficient TNF-alpha secretion. Taken together, our data suggest that differential formation of S1P by SphK1 and SphK2 has distinct and important actions in hMCs. 
 Filamin A links sphingosine kinase 1 and sphingosine-1-phosphate receptor 1 at lamellipodia to orchestrate cell   migration  . Sphingosine kinase 1 (SphK1) catalyzes the phosphorylation of sphingosine to produce the potent lipid mediator sphingosine-1-phosphate (S1P), which plays a critical role in cell motility via its cell surface receptors. Here, we have identified filamin A (FLNa), an actin-cross-linking protein involved in cell movement, as a bona fide SphK1-interacting protein. Heregulin stimulated SphK1 activity only in FLNa-expressing A7 melanoma cells but not in FLNa-deficient cells and induced its translocation and colocalization with FLNa at lamellipodia. SphK1 was required for heregulin-induced   migration  , lamellipodia formation, activation of PAK1, and subsequent FLNa phosphorylation. S1P directly stimulated PAK1 kinase, suggesting that it may be a target of intracellularly generated S1P. Heregulin also induced colocalization of S1P(1) (promotility S1P receptor) but not S1P(2), with SphK1 and FLNa at membrane ruffles. Moreover, an S1P(1) antagonist inhibited the lamellipodia formation induced by heregulin. Hence, FLNa links SphK1 and S1P(1) to locally influence the dynamics of actin cytoskeletal structures by orchestrating the concerted actions of the triumvirate of SphK1, FLNa, and PAK1, each of which requires and/or regulates the actions of the others, at lamellipodia to promote cell movement. 
 Increased mRNA Levels of Sphingosine Kinases and S1P Lyase and Reduced Levels of S1P Were Observed in Hepatocellular Carcinoma in Association with Poorer Differentiation and Earlier Recurrence. Although sphingosine 1-phosphate (S1P) has been reported to play an important role in cancer pathophysiology, little is known about S1P and hepatocellular carcinoma (HCC). To clarify the relationship between S1P and HCC, 77 patients with HCC who underwent surgical treatment were consecutively enrolled in this study. In addition, S1P and its metabolites were quantitated by LC-MS/MS. The mRNA levels of sphingosine kinases (SKs), which phosphorylate sphingosine to generate S1P, were increased in HCC tissues compared with adjacent non-HCC tissues. Higher mRNA levels of SKs in HCC were associated with poorer differentiation and microvascular invasion, whereas a higher level of SK2 mRNA was a risk factor for intra- and extra-hepatic recurrence. S1P levels, however, were unexpectedly reduced in HCC compared with non-HCC tissues, and increased mRNA levels of S1P lyase (SPL), which degrades S1P, were observed in HCC compared with non-HCC tissues. Higher SPL mRNA levels in HCC were associated with poorer differentiation. Finally, in HCC cell lines, inhibition of the expression of SKs or SPL by siRNA led to reduced proliferation, invasion and   migration  , whereas overexpression of SKs or SPL enhanced proliferation. In conclusion, increased SK and SPL mRNA expression along with reduced S1P levels were more commonly observed in HCC tissues compared with adjacent non-HCC tissues and were associated with poor differentiation and early recurrence. SPL as well as SKs may be therapeutic targets for HCC treatment. 
 Filamin A links sphingosine kinase 1 and sphingosine-1-phosphate receptor 1 at lamellipodia to orchestrate cell   migration  . Sphingosine kinase 1 (SphK1) catalyzes the phosphorylation of sphingosine to produce the potent lipid mediator sphingosine-1-phosphate (S1P), which plays a critical role in cell motility via its cell surface receptors. Here, we have identified filamin A (FLNa), an actin-cross-linking protein involved in cell movement, as a bona fide SphK1-interacting protein. Heregulin stimulated SphK1 activity only in FLNa-expressing A7 melanoma cells but not in FLNa-deficient cells and induced its translocation and colocalization with FLNa at lamellipodia. SphK1 was required for heregulin-induced   migration  , lamellipodia formation, activation of PAK1, and subsequent FLNa phosphorylation. S1P directly stimulated PAK1 kinase, suggesting that it may be a target of intracellularly generated S1P. Heregulin also induced colocalization of S1P(1) (promotility S1P receptor) but not S1P(2), with SphK1 and FLNa at membrane ruffles. Moreover, an S1P(1) antagonist inhibited the lamellipodia formation induced by heregulin. Hence, FLNa links SphK1 and S1P(1) to locally influence the dynamics of actin cytoskeletal structures by orchestrating the concerted actions of the triumvirate of SphK1, FLNa, and PAK1, each of which requires and/or regulates the actions of the others, at lamellipodia to promote cell movement. 
 Distinct roles of sphingosine kinases 1 and 2 in human mast-cell functions. Sphingosine-1-phosphate (S1P) is now emerging as a potent lipid mediator produced by mast cells that contributes to inflammatory and allergic responses. In contrast to its weak effect on degranulation of murine mast cells, S1P potently induced degranulation of the human LAD2 mast-cell line and cord blood-derived human mast cells (hMCs). S1P also stimulated production and secretion of cytokines, TNF-alpha and IL-6, and markedly enhanced secretion of a chemokine, CCL2/MCP-1, important modulators of inflammation. S1P is produced in mast cells by the 2 sphingosine kinases, SphK1 and SphK2. SphK1 but not SphK2 plays a critical role in IgE/Ag-induced degranulation,   migration   toward antigen, and CCL2 secretion from hMCs, as determined by specifically down-regulating their expression. However, both isoenzymes were required for efficient TNF-alpha secretion. Taken together, our data suggest that differential formation of S1P by SphK1 and SphK2 has distinct and important actions in hMCs. 
 Sphingosine kinase-1 is a hypoxia-regulated gene that stimulates   migration   of human endothelial cells. Sphingosine kinases (SK) catalyze the production of sphingosine-1-phosphate which in turn regulates cell responses such as proliferation and   migration  . Here, we show that exposure of the human endothelial cell line EA.hy 926 to hypoxia stimulates a increased SK-1, but not SK-2, mRNA, protein expression, and activity. This effect was due to stimulated SK-1 promoter activity which contains two putative hypoxia-inducible factor-responsive-elements (HRE). By deletion of one of the two HREs, hypoxia-induced promoter activation was abrogated. Furthermore, hypoxia upregulated the expression of HIF-1alpha and HIF-2alpha, and both contributed to SK-1 gene transcription as shown by selective depletion of HIF-1alpha or HIF-2alpha by siRNA. The hypoxia-stimulated SK-1 upregulation was functionally coupled to increased   migration   since the selective depletion of SK-1, but not of SK-2, by siRNAs abolished the migratory response. In summary, these data show that hypoxia upregulates SK-1 activity and results in an accelerated migratory capacity of endothelial cells. SK-1 may thus serve as an attractive therapeutic target to treat diseases associated with increased endothelial   migration   and angiogenesis such as cancer growth and progression. 
 Factor-Xa-induced mitogenesis and   migration   require sphingosine kinase activity and S1P formation in human vascular smooth muscle cells. Sphingosine-1-phosphate (S1P) is a cellular signalling lipid generated by sphingosine kinase-1 (SPHK1). The aim of the study was to investigate whether the activated coagulation factor-X (FXa) regulates SPHK1 transcription and the formation of S1P and subsequent mitogenesis and   migration   of human vascular smooth muscle cells (SMC).FXa induced a time- (3-6 h) and concentration-dependent (3-30 nmol/L) increase of SPHK1 mRNA and protein expression in human aortic SMC, resulting in an increased synthesis of S1P. FXa-stimulated transcription of SPHK1 was mediated by the protease-activated receptor-1 (PAR-1) and PAR-2. In human carotid artery plaques, expression of SPHK1 was observed at SMC-rich sites and was co-localized with intraplaque FX/FXa content. FXa-induced SPHK1 transcription was attenuated by inhibitors of Rho kinase (Y27632) and by protein kinase C (PKC) isoforms (GF109203X). In addition, FXa rapidly induced the activation of the small GTPase Rho A. Inhibition of signalling pathways which regulate SPHK1 expression, inhibition of its activity or siRNA-mediated SPHK1 knockdown attenuated the mitogenic and chemotactic response of human SMC to FXa.These data suggest that FXa induces SPHK1 expression and increases S1P formation independent of thrombin and that this involves the activation of Rho A and PKC signalling. In addition to its key function in coagulation, this direct effect of FXa on human SMC may increase cell proliferation and   migration   at sites of vessel injury and thereby contribute to the progression of vascular lesions. 
 Comprehensive gene and microRNA expression profiling reveals a role for miRNAs in the oncogenic roles of SphK1 in papillary thyroid cancer. The oncogenic roles of sphingosine kinase 1 (SphK1) in various cancers, including thyroid cancer, have been well demonstrated. However, the microRNAs (miRNAs) associated with the oncogenic roles of SphK1 remain largely unknown.Global gene and miRNA expression in TPC1-Vector and TPC1-SphK1 cells was analyzed using digital gene expression (DGE) analysis and small RNA-seq, respectively. miRNA-mRNA interactions were explored by microT-CDS, and the predicted networks were visualized using CytoScape(®). Cell invasion and   migratio  n were assessed by performing Transwell invasion and wound-healing assays. Luciferase reporter and immunoblot assays were used to evaluate the targeting of fibronectin 1 (FN1) by miR-144-3p.In this study, we found that overexpression of SphK1 differentially regulates the expression of 46 miRNAs and 506 mRNAs in papillary thyroid cancer (PTC) TPC1 cells. Combining bioinformatics predictions of mRNA targets with DGE data on mRNA expression allowed us to identify the mRNA targets of deregulated miRNAs. The direct interaction between miR-144-3p and FN1, which mediates the pro-invasive role of SphK1 in PTC cells, was experimentally validated.Our results demonstrated that SphK1 overexpression drives a regulatory network governing miRNA and mRNA expression in PTC cells. We also demonstrated the roles played by miR-144-3p and FN1 in mediating the oncogenic function of SphK1, which enhanced the understanding of the etiology of PTC. 
 SphK1 promotes tumor cell   migration   and invasion in colorectal cancer. Colorectal cancer (CRC) is one of the most common cancers worldwide. Sphingosine kinase 1 (SphK1), which phosphorylates sphingosine to sphingosine-1-phosphate (S1P), is overexpressed in various types of cancers and may act as an oncogene in tumorigenesis. However, little is known about the role of SphK1 in CRC patients. We studied the expression of SphK1 in 85 cases of CRC tissues by immunohistochemistry, qRT-PCR, and western blot. We also evaluated the effect of SphK1 on cell proliferation and invasion by MTT and transwell invasion assay. SphK1 is overexpressed in CRC tissues and cell lines, and upregulation of SphK1 correlated significantly with the following parameters: lymph node metastasis, liver metastasis, and advanced TNM stage. SphK1 knockdown results in inhibition of cancer cell proliferation. Inhibition of CRC cell   migration   and invasion is also evident through reversal of EMT by increases in E-cadherin expression and decreases in vimentin expression. In conclusion, SphK1 is associated with the proliferation and invasiveness of CRC cells and the SphK1 gene may contribute to a novel therapeutic approach against CRC. 
 Dual role of sphingosine kinase-1 in promoting the differentiation of dermal fibroblasts and the dissemination of melanoma cells. Despite progress in the understanding of the biology and genetics of melanoma, no effective treatment against this cancer is available. The adjacent microenvironment has an important role in melanoma progression. Defining the molecular signals that control the bidirectional dialog between malignant cells and the surrounding stroma is crucial for efficient targeted therapy. Our study aimed at defining the role of sphingosine-1-phosphate (S1P) in melanoma-stroma interactions. Transcriptomic analysis of human melanoma cell lines showed increased expression of sphingosine kinase-1 (SPHK1), the enzyme that produces S1P, as compared with normal melanocytes. Such an increase was also observed by immunohistochemistry in melanoma specimens as compared with nevi, and occurred downstream of ERK activation because of BRAF or NRAS mutations. Importantly,   migration   of melanoma cells was not affected by changes in SPHK1 activity in tumor cells, but was stimulated by comparable modifications of S1P-metabolizing enzymes in cocultured dermal fibroblasts. Reciprocally, incubation of fibroblasts with the conditioned medium from SPHK1-expressing melanoma cells resulted in their differentiation to myofibroblasts, increased production of matrix metalloproteinases and enhanced SPHK1 expression and activity. In vivo tumorigenesis experiments showed that the lack of S1P in the microenvironment prevented the development of orthotopically injected melanoma cells. Finally, local tumor growth and dissemination were enhanced more efficiently by coinjection of wild-type skin fibroblasts than by fibroblasts from Sphk1(-/-) mice. This report is the first to document that SPHK1/S1P modulates the communication between melanoma cells and dermal fibroblasts. Altogether, our findings highlight SPHK1 as a potential therapeutic target in melanoma progression. 
 Sphingosine kinase as an oncogene: autocrine sphingosine 1-phosphate modulates ML-1 thyroid carcinoma cell   migration   by a mechanism dependent on protein kinase C-alpha and ERK1/2. Sphingosine 1-phosphate (S1P) induces   migration   of the human thyroid follicular carcinoma cell line ML-1 by activation of S1P(1) and S1P(3) receptors, G(i) proteins, and the phosphatidylinositol 3-kinase-Akt pathway. Because sphingosine kinase isoform 1 (SK) recently has been implicated as an oncogene in various cancer cell systems, we investigated the functions of SK in the   migration  , proliferation and adhesion of the ML-1 cell line. SK overexpressing ML-1 cells show an enhanced secretion of S1P, which can be attenuated, by inhibiting SK activity and a multidrug-resistant transport protein (ATP-binding cassette transporter). Furthermore, overexpression of SK enhances serum-induced   migration   of ML-1 cells, which can be attenuated by blocking ATP-binding cassette transporter and SK, suggesting that the   migration   is mediated by autocrine signaling through secretion of S1P. Inhibition of protein kinase C alpha, with both small interfering RNA (siRNA) and small molecular inhibitors attenuates   migration   in SK overexpressing cells. In addition, SK-overexpressing cells show an impaired adhesion, slower cell growth, and an up-regulation of ERK1/2 phosphorylation, as compared with cells expressing a dominant-negative SK. Taken together, we present evidence suggesting that SK enhances   migration   of ML-1 cells by an autocrine mechanism and that the S1P-evoked   migration   is dependent on protein kinase C alpha, ERK1/2, and SK. 
 SPHK1 inhibitor suppresses cell proliferation and invasion associated with the inhibition of NF-κB pathway in hepatocellular carcinoma. Sphingosine kinase 1 (SphK1) is an oncogenic enzyme promoting transformation, proliferation, and angiogenesis of a number of human tumors. However, its effect on hepatocellular carcinoma (HCC) behavior has not been fully clarified. The purpose of this study was to determine the correlation between HCC and SphK1, and to evaluate the effect of SphK1 inhibitor N,N-dimethylsphingosine (DMS) in HCC. The expression of SphK1 was measured in tissue samples from 76 HCC and paired adjacent noncancerous liver tissues (NT) by immunohistochemistry, quantitative real-time PCR, and Western blotting analysis. The effect of DMS was tested on HCC cells by evaluating cell viability in vitro. Transwell cell   migratio  n and invasion assay were carried out for functional analysis. Furthermore, Western blotting analysis was performed to examine the impact of DMS on the PI3K/Akt/NF-kB signaling. High expression of Sphk1 was observed in 84.21% (64/76) of the HCC versus 15.79% (12/76) of the adjacent non-tumorous liver tissues; the difference of Sphk1 expression between HCC and the adjacent non-tumorous liver tissues was statistically significant (P < 0.001). The results were confirmed by Western blot analyses and quantitative real-time PCR. DMS inhibited the proliferation of SK-Hep1 and MHCCLM3 cells which have a relatively high level of SphK1 in a time- and concentration-dependent manner, and the invasion   and migr  ation of SK-Hep1 cells were distinctly suppressed after undergoing treatment with DMS. Furthermore, DMS markedly suppressed the expression of phosphorylations of Akt and NF-κB in HCC cells. Our data suggest that the pathogenesis of human HCC maybe mediated by Sphk1, and the specific Sphk1 inhibitor DMS can play a therapeutic role in the treatment of HCC and thus, Sphk1 could represent selective targets for the molecularly targeted treatments of HCC. 
 [Effect of sphingosine kinase 1 on the apoptosis,   migration   and invasion of colon cancer HT-29 cells and its molecular mechanisms]. To investigate the effect of sphingosine kinase 1 (SphK1) on the proliferation, apoptosis,   migration   and invasion of colon cancer TH-29 cells and to explore its molecular mechanisms.Phorbol 12-myristate 13-acetate (PMA) was used to induce the activity of SphK1 and N, N-dimethylsphingosine (DMS) was used to suppress the activity of SphK1. Cell prolieration and apoptosis were detected by MTT assay and flow cytometry, respectively. The   migration   and invasion capabilities of the cells were assessed in Transwell chambers. The activity of SphK1 was assayed by autoradiography. Western blot was used to evaluate the protein expression of SphK1, p38, phosphorylated p38 (p-p38) and SAPK/JNK.PMA and DMS were able to induce and suppress the activity and protein expression of SphK1 in a time-dependent manner, respectively. PMA enhanced and DMS suppressed the cell viability in a time- and dose-dependent manner. Being treated with 100 nmol/L PMA or 50 µmol/L DMS for 0, 6, 12, 24 h, the cell apoptosis rates of PMA group were (9.35 ± 0.84)%, (7.61 ± 0.48)%, (5.53 ± 0.76)% and (0.56 ± 0.33)%, contrastly, that of DMS group were (9.18 ± 0.94)%, (12.06 ± 1.41)%, (19.80 ± 2.36)% and (31.85 ± 3.60)%, respectively. Compared with the control group,   the cell   migration and invasion capabilities of the PMA group were significantly enhanced, and that of the DMS group were significantly suppres  sed. The   migration cell number of control, PMA and DMS groups were 68.75 ± 6.15, 109.33 ± 11.63 and 10.83 ± 2.48, the invasion cell number of control, PMA and DMS groups were 55.42 ± 4.50, 90.58 ± 7.06 and 9.58 ± 2.39, respectively. With the elevating activity and expression of SphK1, the protein expressions of p38, p-p38 and SAPK/JNK were strikingly suppressed. On the contrary, after treating with DMS the protein expressions of p38, p-p38 and SAPK/JNK were enhanced.SphK1 potently enhances the   proliera  tion, migration and invasion of colon cancer HT-29 cells, meanwhile suppresses the cell apoptosis. The suppressing of the p38 and SAPK/JNK signalling pathways may be one of its molecular mechanisms. 
 Activation of sphingosine kinase-1 mediates induction of endothelial cell proliferation and angiogenesis by epoxyeicosatrienoic acids. Recent evidence suggests that the epoxyeicosatrienoic acids (EETs), which are products of cytochrome P450 (CYP) epoxygenases, possess mitogenic and angiogenic effects in vascular endothelial cells. However, the mechanisms underlying these effects are not fully elucidated. Because sphingosine kinase (SK) and its product S1P play essential roles in cell growth, survival and   migration  , we hypothesized that SK activation by EETs may mediate some of its angiogenic effects.We studied the effects of EETs on SK activity in human umbilical vein endothelial cells (HUVECs). Treatment with EETs, particularly 11,12-EET, markedly augmented SK activity in HUVECs. At the concentration of 1 micromol/L, 11,12-EET increased SK activity by 110% and the maximal effect on SK activation was observed at 20 min after 11,12-EET addition. Furthermore, inhibition of SK by a specific inhibitor, SKI-II, markedly attenuated 11,12-EET-induced EC proliferation. Importantly, 11,12-EET-induced activation of Akt kinase and transactivation of the epidermal growth factor (EGF) receptor was also inhibited by SKI-II. To investigate the isoform-specific role of SK in EET-induced angiogenesis, inhibition of SK1 by expression of dominant-negative SK1(G82D) substantially attenuated 11,12-EET-induced EC proliferation,   migration  , and tube formation in vitro and Matrigel plug angiogenesis in vivo. Furthermore, knockdown of SK1 expression by specific siRNA also inhibited 11,12-EET-induced EC proliferation and   migration  , whereas SK2 siRNA knockdown was without effect.These results suggest that SK1 is an important mediator of the 11,12-EET-induced angiogenic effects in human ECs. Thus, SK1 may represent a novel therapeutic modality for the treatment of angiogenesis-related diseases such as cancer and ischaemia. 
 Histamine increases sphingosine kinase-1 expression and activity in the human arterial endothelial cell line EA.hy 926 by a PKC-alpha-dependent mechanism. Sphingosine 1-phosphate (S1P) is a potent mitogenic signal generated from sphingosine by the action of sphingosine kinases (SKs). In this study, we show that in the human arterial endothelial cell line EA.hy 926 histamine induces a time-dependent upregulation of the SK-1 mRNA and protein expression which is followed by increased SK-1 activity. A similar upregulation of SK-1 is also observed with the direct protein kinase C activator 12-O-tetradecanoylphorbol-13-acetate (TPA). In contrast, SK-2 activity is not affected by neither histamine nor TPA. The increased SK-1 protein expression is due to stimulated de novo synthesis since cycloheximide inhibited the delayed SK-1 protein upregulation. Moreover, the increased SK-1 mRNA expression results from an increased promoter activation by histamine and TPA. In mechanistic terms, the transcriptional upregulation of SK-1 is dependent on PKC and the extracellular signal-regulated protein kinase (ERK) cascade since staurosporine and the MEK inhibitor U0126 abolish the TPA-induced SK-1 induction. Furthermore, the histamine effect is abolished by the H1-receptor antagonist diphenhydramine, but not by the H2-receptor antagonist cimetidine. Parallel to the induction of SK-1, histamine and TPA stimulate an increased   migration   of endothelial cells, which is prevented by depletion of the SK-1 by small interfering RNA (siRNA). To appoint this specific cell response to a specific PKC isoenzyme, siRNA of PKC-alpha, -delta, and -epsilon were used to selectively downregulate the respective isoforms. Interestingly, only depletion of PKC-alpha leads to a complete loss of TPA- and histamine-triggered SK-1 induction and cell   migration  . In summary, these data show that PKC-alpha activation in endothelial cells by histamine-activated H1-receptors, or by direct PKC activators leads to a sustained upregulation of the SK-1 protein expression and activity which, in turn, is critically involved in the mechanism of endothelial cell   migration  . 
 Intracellular S1P generation is essential for S1P-induced motility of human lung endothelial cells: role of sphingosine kinase 1 and S1P lyase. Earlier we have shown that extracellular sphingosine-1-phosphate (S1P) induces   migration   of human pulmonary artery endothelial cells (HPAECs) through the activation of S1P(1) receptor, PKCε, and PLD2-PKCζ-Rac1 signaling cascade. As endothelial cells generate intracellular S1P, here we have investigated the role of sphingosine kinases (SphKs) and S1P lyase (S1PL), that regulate intracellular S1P accumulation, in HPAEC motility.Inhibition of SphK activity with a SphK inhibitor 2-(p-Hydroxyanilino)-4-(p-Chlorophenyl) Thiazole or down-regulation of Sphk1, but not SphK2, with siRNA decreased S1P(int), and attenuated S1P(ext) or serum-induced motility of HPAECs. On the contrary, inhibition of S1PL with 4-deoxypyridoxine or knockdown of S1PL with siRNA increased S1P(int) and potentiated motility of HPAECs to S1P(ext) or serum. S1P(ext) mediates cell motility through activation of Rac1 and IQGAP1 signal transduction in HPAECs. Silencing of SphK1 by siRNA attenuated Rac1 and IQGAP1 translocation to the cell periphery; however, knockdown of S1PL with siRNA or 4-deoxypyridoxine augmented activated Rac1 and stimulated Rac1 and IQGAP1 translocation to cell periphery. The increased cell motility mediated by down-regulation was S1PL was pertussis toxin sensitive suggesting "inside-out" signaling of intracellularly generated S1P. Although S1P did not accumulate significantly in media under basal or S1PL knockdown conditions, addition of sodium vanadate increased S1P levels in the medium and inside the cells most likely by blocking phosphatases including lipid phosphate phosphatases (LPPs). Furthermore, addition of anti-S1P mAb to the incubation medium blocked S1P(ext) or 4-deoxypyridoxine-dependent endothelial cell motility.These results suggest S1P(ext) mediated endothelial cell motility is dependent on intracellular S1P production, which is regulated, in part, by SphK1 and S1PL. 
 Galpha(q)-mediated plasma membrane translocation of sphingosine kinase-1 and cross-activation of S1P receptors. Sphingosine-1-phosphate (S1P), formed by sphingosine kinases (SphKs), regulates cellular proliferation and   migration   by acting as an agonist at specific receptors or intracellularly. Since S1P's effects are probably dependent on subcellular localization of its formation and degradation, we have studied the influence of G protein-coupled receptors on the localization of SphK1. Activation of Gq-coupled receptors induced a profound, rapid (half-life 3-5 s) and long-lasting (> 2 h) translocation of SphK1 to the plasma membrane. This was mimicked by expression of constitutively active G protein alpha-subunits specifically of the Gq family. Classical Gq signalling pathways, or phosphorylation at Ser225, phospholipase D and Ca2+/calmodulin were not involved in M3 receptor-induced SphK1 translocation in HEK-293 cells. Translocation was associated with S1P receptor internalization, which was dependent on catalytic activity of SphK1 and S1P receptor binding and thus resulted from S1P receptor cross-activation. It is concluded that SphK1 is an important effector of Gq-coupled receptors, linking them via cross-activation of S1P receptors to G(i) and G12/13 signalling pathways. 
 Sphingosine kinases and sphingosine-1-phosphate are critical for transforming growth factor beta-induced extracellular signal-regulated kinase 1 and 2 activation and promotion of   migration   and invasion of esophageal cancer cells. Transforming growth factor beta (TGFbeta) plays a dual role in oncogenesis, acting as both a tumor suppressor and a tumor promoter. These disparate processes of suppression and promotion are mediated primarily by Smad and non-Smad signaling, respectively. A central issue in understanding the role of TGFbeta in the progression of epithelial cancers is the elucidation of the mechanisms underlying activation of non-Smad signaling cascades. Because the potent lipid mediator sphingosine-1-phosphate (S1P) has been shown to transactivate the TGFbeta receptor and activate Smad3, we examined its role in TGFbeta activation of extracellular signal-regulated kinases 1 and 2 (ERK1/2) and promotion of   migration   and invasion of esophageal cancer cells. Both S1P and TGFbeta activate ERK1/2, but only TGFbeta activates Smad3. Both ligands promoted ERK1/2-dependent   migration   and invasion. Furthermore, TGFbeta rapidly increased S1P, which was required for TGFbeta-induced ERK1/2 activation, as well as   migration   and invasion, since downregulation of sphingosine kinases, the enzymes that produce S1P, inhibited these responses. Finally, our data demonstrate that TGFbeta activation of ERK1/2, as well as induction of   migration   and invasion, is mediated at least in part by ligation of the S1P receptor, S1PR2. Thus, these studies provide the first evidence that TGFbeta activation of sphingosine kinases and formation of S1P contribute to non-Smad signaling and could be important for progression of esophageal cancer. 
 Sphingosine kinase 1 is required for TGF-β mediated fibroblastto- myofibroblast differentiation in ovarian cancer. Sphingosine kinase 1 (SPHK1), the enzyme that produces sphingosine 1 phosphate (S1P), is known to be highly expressed in many cancers. However, the role of SPHK1 in cells of the tumor stroma remains unclear. Here, we show that SPHK1 is highly expressed in the tumor stroma of high-grade serous ovarian cancer (HGSC), and is required for the differentiation and tumor promoting function of cancer-associated fibroblasts (CAFs). Knockout or pharmacological inhibition of SPHK1 in ovarian fibroblasts attenuated TGF-β-induced expression of CAF markers, and reduced their ability to promote ovarian cancer cel  l migrati  on and invasion in a coculture system. Mechanistically, we determined that SPHK1 mediates TGF-β signaling via the transactivation of S1P receptors (S1PR2 and S1PR3), leading to p38 MAPK phosphorylation. The importance of stromal SPHK1 in tumorigenesis was confirmed in vivo, by demonstrating a significant reduction of tumor growth and metastasis in SPHK1 knockout mice. Collectively, these findings demonstrate the potential of SPHK1 inhibition as a novel stroma-targeted therapy in HGSC. 
 Knockdown of sphingosine kinase 1 inhibits the   migration   and invasion of human rheumatoid arthritis fibroblast-like synoviocytes by down-regulating the PI3K/AKT activation and MMP-2/9 production in vitro. To investigate the potential regulation of sphingosine kinase 1 (SPHK1) on the   migration  , invasion, and matrix metalloproteinase (MMP) expression in human rheumatoid arthritis fibroblast-like synoviocytes (RA-FLS). RA-FLS were transfected control siRNA or SPHK1 siRNA. The   migration   and invasion of unmanipulated control, control siRNA or SPHK1 siRNA- transfected RA-FLS in vitro were measured by the transwell system. The relative levels of SPHK1, PI3K, and AKT as well as AKT phosphorylation in RA-FLS were determined by Western blot. The levels of MMP-2/9 secreted by RA-FLS were detected by ELISA. Knockdown of SPHK1 significantly inhibited the spontaneous   migration   and invasion of RA-FLS, accompanied by significantly reduced levels of PI3K expression and AKT phosphorylation. Similarly, treatment with LY294002, an inhibitor of the PI3K/AKT pathway, inhibited the   migration   and invasion of RA-FLS. Knockdown of SPHK1 and treatment with the inhibitor synergistically inhibited the   migration   and invasion of RA-FLS, by further reducing the levels of PI3K expression and AKT phosphorylation. In addition, knockdown of SPHK1 or treatment with LY294002 inhibited the secretion of MMP-2 and MMP-9, and both synergistically reduced the production of MMP-2 and MMP-9 in RA-FLS in vitro. Knockdown of SPHK1 expression inhibits the PI3K/AKT activation, MMP-2 and MMP-9 expression, and human RA-FLS   migration   and invasion in vitro. Potentially, SPHK1 may be a novel therapeutic target for RA. 
 Reduced sphingosine kinase-1 and enhanced sphingosine 1-phosphate lyase expression demonstrate deregulated sphingosine 1-phosphate signaling in Alzheimer's disease. The accumulation of beta amyloid (Aβ) peptides, a hallmark of Alzheimer's disease (AD) is related to mechanisms leading to neurodegeneration. Among its pleiotropic cellular effects, Aβ accumulation has been associated with a deregulation of sphingolipid metabolism. Sphingosine 1-phosphate (S1P) derived from sphingosine is emerging as a critical lipid mediator regulating various biological activities including cell proliferation, survival  , migrati  on, inflammation, or angiogenesis. S1P tissue level is low and kept under control through equilibrium between its synthesis mostly governed by sphingosine kinase-1 (SphK1) and its degradation by sphingosine 1-phosphate lyase (SPL). We have previously reported that Aβ peptides were able to decrease the activity of SphK1 in cell culture models, an effect that could be blocked by the prosurvival IGF-1/IGF-1R signaling.Herein, we report for the first time the expression of both SphK1 and SPL by immunohistochemistry in frontal and entorhinal cortices from 56 human AD brains. Immunohistochemical analysis revealed a decreased expression of SphK1 and an increased expression of SPL both correlated to amyloid deposits in the entorhinal cortex. Otherwise, analysis of brain tissue extracts showed a decrease of SphK1 expression in AD brains whereas SPL expression was increased. The content of IGF-1R, an activator of SphK1, was found decreased in AD brains as well as S1P1, the major receptor for S1P.Collectively, these results highlight the importance of S1P in AD suggesting the existence of a global deregulation of S1P signaling in this disease from its synthesis by SphK1 and degradation by SPL to its signaling by the S1P1 receptor. 
 High expression of sphingosine 1-phosphate receptors, S1P1 and S1P3, sphingosine kinase 1, and extracellular signal-regulated kinase-1/2 is associated with development of tamoxifen resistance in estrogen receptor-positive breast cancer patients. Various studies in cell lines have previously demonstrated that sphingosine kinase 1 (SK1) and extracellular signal-regulated kinase 1/2 (ERK-1/2) interact in an estrogen receptor (ER)-dependent manner to influence both breast cancer cell growth and   migration  . A cohort of 304 ER-positive breast cancer patients was used to investigate the prognostic significance of sphingosine 1-phosphate (S1P) receptors 1, 2, and 3 (ie, S1P1, S1P2, and S1P3), SK1, and ERK-1/2 expression levels. Expression levels of both SK1 and ERK-1/2 were already available for the cohort, and S1P1, S1P2, and S1P3 levels were established by immunohistochemical analysis. High membrane S1P1 expression was associated with shorter time to recurrence (P=0.008). High cytoplasmic S1P1 and S1P3 expression levels were also associated with shorter disease-specific survival times (P=0.036 and P=0.019, respectively). Those patients with tumors that expressed high levels of both cytoplasmic SK1 and ERK-1/2 had significantly shorter recurrence times than those that expressed low levels of cytoplasmic SK1 and cytoplasmic ERK-1/2 (P=0.00008), with a difference in recurrence time of 10.5 years. Similarly, high cytoplasmic S1P1 and cytoplasmic ERK-1/2 expression levels (P=0.004) and high cytoplasmic S1P3 expression and cytoplasmic ERK-1/2 expression levels (P=0.004) were associated with shorter recurrence times. These results support a model in which the interaction between SK1, S1P1, and/or S1P3 and ERK-1/2 might drive breast cancer progression, and these findings, therefore, warrant further investigation. 
 Sphingosine 1-phosphate as a regulator of osteoclast differentiation and osteoclast-osteoblast coupling. Sphingosine 1-phosphate (S1P), produced by sphingosine kinase (SPHK), acts both by intracellular and extracellular modes. We evaluated the role of SPHK1 and S1P in osteoclastogenesis using bone marrow-derived macrophage (BMM) single and BMM/osteoblast coculture systems. In BMM single cultures, the osteoclastogenic factor receptor activator of NF-kappaB ligand (RANKL) upregulated SPHK1 and increased S1P production and secretion. SPHK1 siRNA enhanced and SPHK1 overexpression attenuated osteoclastogenesis via modulation of p38 and ERK activities, and NFATc1 and c-Fos levels. Extracellular S1P had no effect in these cultures. These data suggest that intracellular S1P produced in response to RANKL forms a negative feedback loop in BMM single cultures. In contrast, S1P addition to BMM/osteoblast cocultures greatly increased osteoclastogenesis by increasing RANKL in osteoblasts via cyclooxygenase-2 and PGE(2) regulation. S1P also stimulated osteoblast   migration   and survival. The RANKL elevation and chemotactic effects were also observed with T cells. These results indicate that secreted S1P attracts and acts on osteoblasts and T cells to augment osteoclastogenesis. Taken together, S1P plays an important role in osteoclastogenesis regulation and in communication between osteoclasts and osteoblasts or T cells. 
 Sphingosine kinase activation regulates hepatocyte growth factor induced   migration   of endothelial cells. Hepatocyte growth factor (HGF)-induced   migration   of endothelial cells is critical for angiogenesis. Sphingosine kinase (SPK) is a key enzyme catalyzing the formation of sphingosine-1-phosphate (S1P), a lipid messenger that is implicated in the regulation of a wide variety of important cellular events through both intracellular and extracellular mechanisms. The aim of this study was to investigate whether activation of SPK is involved in the   migration   of endothelial cells induced by HGF. The biological functions of HGF are mediated through the activation of its high-affinity tyrosine kinase receptor, c-met protooncogene. In the present study, Treatment of ECV304 endothelial cells with HGF resulted in tyrosine phosphorylation of c-Met and activation of SPK in a concentration-dependent manner. Either Ly294002 or PD98059, specific inhibitor of the PI3K and ERK/MAPK pathways, respectively, blocked the HGF-induced activation of SPK. HGF stimulation significantly increased intracellular S1P level, but no detectable secretion of S1P into the cell culture medium was observed. Treatment of ECV304 cells with pertussis toxin (PTX) has no effect on the HGF-induced   migration  , indicating extracellular S1P is dispensable for this process. Overexpression of wild-type SPK gene in ECV 304 cells increased the intracellular S1P and enhanced the HGF-induced   migration  , whereas inhibition of cellular SPK activity by N,N-dimethylsphingosine (DMS), a potent inhibitor of SPK, or by expression of a dominant-negative SPK (DN-SK) blocked the HGF-induced   migration   of ECV 304 cells. It is suggested that PI3K and ERK/MAPK mediated the activation of SPK and would be involved in the HGF-induced   migration   of endothelial cells. These results elucidate a novel mechanism by which intracellularly generated S1P mediates signaling from HGF/c-Met to the endothelial cell   migration  . 
 Analysis of sphingosine kinase activity in single natural killer cells from peripheral blood. Sphingosine-1-phosphate (S1P), a lipid second messenger formed upon phosphorylation of sphingosine by sphingosine kinase (SK), plays a crucial role in natural killer (NK) cell proliferation,   migration  , and cytotoxicity. Dysregulation of the S1P pathway has been linked to a number of immune system disorders and therapeutic manipulation of the pathway has been proposed as a method of disease intervention. However, peripheral blood NK cells, as identified by surface markers (CD56(+)CD45(+)CD3(-)CD16) consist of a highly diverse population with distinct phenotypes and functions and it is unknown whether the S1P pathway is similarly diverse across peripheral blood NK cells. In this work, we measured the phosphorylation of sphingosine-fluorescein (SF) and subsequent metabolism of S1P fluorescein (S1PF) to form hexadecanoic acid fluorescein (HAF) in 111 single NK cells obtained from the peripheral blood of four healthy human subjects. The percentage of SF converted to S1PF or HAF was highly variable amongst the cells ranging from 0% to 100% (S1PF) and 0% to 97% (HAF). Subpopulations of cells with varying levels of S1PF formation and metabolism were readily identified. Across all subjects, the average percentage of SF converted to S1PF or HAF was 37 ± 36% and 12 ± 19%, respectively. NK cell metabolism of SF by the different subjects was also distinct with hierarchical clustering suggesting two possible phenotypes: low (<20%) or high (>50%) producers of S1PF. The heterogeneity of SK and downstream enzyme activity in NK cells may enable NK cells to respond effectively to a diverse array of pathogens as well as incipient tumor cells. NK cells from two subjects were also loaded with S1PF to assess the activity of S1P phosphatase (S1PP), which converts S1P to sphingosine. No NK cells (n = 41) formed sphingosine, suggesting that S1PP was minimally active in peripheral blood NK cells. In contrast to the SK activity, S1PP activity was homogeneous across the peripheral blood NK cells, suggesting a bias in the SK pathway towards proliferation an  d migrati  on, activities supported by S1P. 
 Hepatitis B virus X protein promotes human hepatoma cell growth via upregulation of transcription factor AP2α and sphingosine kinase 1. Sphingosine kinase 1 (SPHK1) is involved in various cellular functions, including cell growth,   migratio  n, apoptosis, cytoskeleton architecture and calcium homoeostasis, etc. As an oncogenic kinase, SPHK1 is associated with the development and progression of cancers. The aim of this study was to investigate whether SPHK1 was involved in hepatocarcinogenesis induced by the hepatitis B virus X protein (HBx).The expression of SPHK1 in hepatocellular carcinoma (HCC) tissue and hepatoma cells were measured using qRT-PCR and Western blot analysis. HBx expression levels in hepatoma cells were modulated by transiently transfected with HBx or psi-HBx plasmids. The SPHK1 promoter activity was measured using luciferase reporter gene assay, and the interaction of the transcription factor AP2α with the SPHK1 promoter was studied with chromatin immunoprecipitation assay. The growth of hepatoma cells was evaluated in vitro using MTT and colony formation assays, and in a tumor xenograft model.A positive correlation was found between the mRNA levels of SPHK1 and HBx in 38 clinical HCC samples (r=+0.727, P<0.01). Moreover, the expression of SPHK1 was markedly increased in the liver cancer tissue of HBx-transgenic mice. Overexpressing HBx in normal liver cells LO2 and hepatoma cells HepG2 dose-dependently increased the expression of SPHK1, whereas silencing HBx in HBx-expressing hepatoma cells HepG2-X and HepG2.2.15 suppressed SPHK1 expression. Furthermore, overexpressing HBx in HepG2 cells dose-dependently increased the SPHK1 promoter activity, whereas silencing HBx in HepG2-X cells suppressed this activity. In HepG2-X cells, AP2α was found to directly interact with the SPHK1 promoter, and silencing AP2α suppressed the SPHK1 promoter activity and SPHK1 expression. Silencing HBx in HepG2-X cells abolished the HBx-enhanced proliferation and colony formation in vitro, and tumor growth in vivo.HBx upregulates SPHK1 through the transcription factor AP2α, which promotes the growth of human hepatoma cells. 
 Overexpression of SphK1 enhances cell proliferation and invasion in triple-negative breast cancer via the PI3K/AKT signaling pathway. Sphingosine kinase 1 (SphK1) expression is elevated in various cancers and is associated with shorter survival times for patients. However, the molecular mechanism of SphK1 up-regulation in triple-negative breast cancer (TNBC) remains unclear. In this study, we assayed the expression level of SphK1 in TNBC tissues by qRT-PCR and immunohistochemistry. The level of S1P was quantified by ELISA in the serum of TNBC patients. Our results found that the levels of SphK1 and S1P were significantly increased in TNBC patients compared with normal control. Furthermore, knockdown of SphK1 with siRNA decreased TNBC cell proliferation and inhibited cell   migration  /invasion. These data suggest that SphK1 has an important role in TNBC and presents an attractive therapeutic target for the treatment for TNBC. 
 Sphingosine kinase 1 is required for   migration  , proliferation and survival of MCF-7 human breast cancer cells. Sphingosine-1-phosphate (S1P) is a potent lysolipid involved in a variety of biological responses important for cancer progression. Therefore, we investigated the role of sphingosine kinase type 1 (SphK1), the enzyme that makes S1P, in the motility, growth, and chemoresistance of MCF-7 breast cancer cells. Epidermal growth factor (EGF), an important growth factor for breast cancer progression, activated and translocated SphK1 to plasma membrane. SphK1 was required for EGF-directed motility. Downregulation of SphK1 in MCF-7 cells reduced EGF- and serum-stimulated growth and enhanced sensitivity to doxorubicin, a potent chemotherapeutic agent. These results suggest that SphK1 may be critical for growth, metastasis and chemoresistance of human breast cancers. 
 The CCT/TRiC chaperonin is required for maturation of sphingosine kinase 1. Sphingosine kinase 1 (SK1) catalyses the generation of sphingosine 1-phosphate (S1P), a bioactive phospholipid that influences a diverse range of cellular processes, including proliferation, survival, adhesion,   migration  , morphogenesis and differentiation. SK1 is controlled by various mechanisms, including transcriptional regulation, and post-translational activation by phosphorylation and protein-protein interactions which can regulate both the activity and localisation of this enzyme. To gain a better understanding of the regulatory mechanisms controlling SK1 activity and function we performed a yeast two-hybrid screen to identify SK1-interacting proteins. Using this approach we identified that SK1 interacts with subunit 7 (eta) of cytosolic chaperonin CCT (chaperonin containing t-complex polypeptide, also called TRiC for TCP-1 ring complex), a hexadecameric chaperonin that binds unfolded polypeptides and mediates their folding and release in an ATP-dependent manner. Further analysis of the SK1-CCTeta interaction demonstrated that other CCT/TRiC subunits also associated with SK1 in HEK293T cell lysates in an ATP-sensitive manner, suggesting that the intact, functional, multimeric CCT/TRiC complex associated with SK1. Furthermore, pulse-chase studies indicated that CCT/TRiC binds specifically to newly translated SK1. Finally, depletion of functional CCT/TRiC through the use of RNA interference in HeLa cells or temperature sensitive CCT yeast mutants reduced cellular SK1 activity. Thus, combined this data suggests that SK1 is a CCT/TRiC substrate, and that this chaperonin facilitates folding of newly translated SK1 into its mature active form. 
 Circulating sphingosine-1-phosphate and erythrocyte sphingosine kinase-1 activity as novel biomarkers for early prostate cancer detection. Current markers available for screening normal populations and for monitoring prostate cancer (PCa) treatment lack sensitivity and selectivity. Sphingosine-1-phosphate (S1P) is a circulating lipid second messenger involved in cell growth and   migration  , the immune response, angiogenesis, and malignant transformation.Eighty-eight patients with localised, locally advanced, or metastatic PCa were recruited into this prospective single-centre study. Plasma S1P levels were measured and compared with age-matched controls with benign prostate hyperplasia (BPH) (n=110) or with young healthy males with the very small chance of having PCa foci (n=20).Levels of circulating S1P were significantly higher in healthy subjects (10.36 ± 0.69 pmol per mg protein, P<0.0001) and patients with BPH (9.39 ± 0.75, P=0.0013) than in patients with PCa (6.89 ± 0.58, ANOVA, P=0.0019). Circulating S1P levels were an early marker of PCa progression to hormonal unresponsiveness and correlated with prostate-specific antigen (PSA) levels and lymph node metastasis. During the course of the study, nine patients have died of PCa. Importantly, their circulating S1P levels were significantly lower (5.11 ± 0.75) than in the surviving patients (7.02 ± 0.22, n=79, P=0.0439). Our data suggest that the decrease in circulating S1P during PCa progression may stem from a highly significant downregulation of erythrocyte sphingosine kinase-1 (SphK1) activity (2.14 ± 0.17 pmol per mg protein per minute in PCa patients vs 4.7 ± 0.42 in healthy individuals, P<0.0001), which may be a potential mechanism of cancer-induced anaemia.This current study has provided a potential mechanism for cancer-related anaemia and the first evidence that plasma S1P and erythrocyte SphK1 activity are the potential markers for the diagnosis, monitoring, and predicating for PCa mortality. 
 De novo biosynthesis of dihydrosphingosine-1-phosphate by sphingosine kinase 1 in mammalian cells. Sphingosine kinase 1 (SK1) is one of the two known kinases, which generates sphingosine-1-phosphate (S1P), a potent endogenous lipid mediator involved in cell survival, proliferation, and cell-cell interactions. Activation of SK1 and intracellular generation of S1P were suggested to be part of the growth and survival factor-induced signaling, and overexpression of SK1 provoked cell tumorigenic transformation. Using a highly selective and sensitive LC-MS/MS approach, here we show that SK1 overexpression, but not SK2, in different primary cells and cultured cell lines results in predominant upregulation of the synthesis of dihydrosphingosine-1-phosphate (DHS1P) compared to S1P. Stable isotope pulse-labeling experiments in conjunction with LC-MS/MS quantitation of different sphingolipids demonstrated strong interference of overexpressed SK1 with the de novo sphingolipid biosynthesis by deviating metabolic flow of newly formed sphingoid bases from ceramide formation toward the synthesis of DHS1P. On the contrary, S1P biosynthesis was not directly linked to the de novo sphingoid bases transformations and was dependent on catabolic generation of sphingosine from complex sphingolipids. As a result of SK1 overexpression,   migration   and Ca2+-response of human pulmonary artery endothelial cells (HPAEC) to stimulation with external S1P, but not thrombin, was strongly impaired. In contrast, selective increase in intracellular content of DHS1P or S1P through the uptake and phosphorylation of corresponding sphingoid bases had no effect on S1P-induced signaling or facilitation of wound healing. Furthermore, infection of human bronchial epithelial cells (HBEpC) with RSV A-2 virus increased SK1-mediated synthesis of DHS1P and S1P, whereas TNF-alpha enhanced only S1P production in HPAEC. These findings uncover a new functional role for SK1, which can control survival/death (DHS1P-S1P/ceramides) balance by targeting sphingolipid de novo biosynthesis and selectively generating DHS1P at a metabolic step preceding ceramide formation. 
  GeneRIFs  
 The levels of SphK1 were significantly increased in triple-negative breast cancer patients compared with normal controls. SphK1 knockdown with siRNA decreased TNBC cell proliferation and inhibited cell   migration  /invasion, suggesting that SphK1 has an important role in TNBC. 
 In contrast to the SK activity, S1PP activity was homogeneous across the peripheral blood NK cells, suggesting a bias in the SK pathway towards proliferation and   migration  , activities supported by S1P. 
 Knockdown of SPHK1 expression inhibits the PI3K/AKT activation, MMP-2 and MMP-9 expression, and human RA-FLS   migration   and invasion in vitro. Potentially, SPHK1 may be a novel therapeutic target for rheumatoid arthritis. 
 SphK1 potently enhances the prolieration,   migration   and invasion of colon cancer HT-29 cells, and suppresses cell apoptosis. 
 Evidence suggesting that SPHK enhances   migration   of ML-1 cells by an autocrine mechanism and that the S1P-evoked   migration   is dependent on protein kinase C alpha, ERK1/2, and SPHK. 
 Sphingosine kinases and sphingosine-1-phosphate are critical for transforming growth factor beta-induced ERK1 and ERK2 activation and promotion of   migration   and invasion of esophageal cancer cells 
 The hypoxia-stimulated SK-1 upregulation was functionally coupled to increased   migration   since the selective depletion of SK-1, but not of SK-2, by siRNAs abolished the migratory response. 
 PI3K and ERK/MAPK mediated the activation of sphingosine kinase and would be involved in the HGF-induced   migration   of endothelial cells. 
 Data show that protein kinase C-alpha activation in endothelial cells leads to upregulation of sphingosine kinase-1 expression and activity, which is critically involved in the mechanism of endothelial cell   migration  . 
  ECM  PubMed  
 Extracellular Matrix Rigidity-dependent Sphingosine-1-phosphate Secretion Regulates Metastatic Cancer Cell Invasion and Adhesion. Dynamic interaction between cancer cells and the surrounding microenvironment is critical for cancer progression via changes in cellular behavior including alteration of secreted molecules. However, the molecular mechanisms underlying the influence exerted by the cancer microenvironment on secretion of molecules during cancer progression remain largely unknown. In this study, we report that secretion of spingsine-1-phosphate (S1P) and its regulator, SphK1 expression is dependent of the substrate rigidity, which is critical for the balance between cancer cell invasion and adhesion. Conditioned media (CM) of MDA-MB-231, an aggressive breast cancer cell obtained from soft substrate (~0.5 kPa) induced chemo-attractive invasion, while CM obtained from stiff substrate (~2.5 kPa) increased cell adhesion instead. We found that the expression of SphK1 is upregulated in the stiff substrate, resulting in an increase in S1P levels in the CM. We also found that upregulation of SphK1 expression in the stiff substrate is dominant in metastatic cancer cells but not in primary cancer cells. These results suggest that alterations in the mechanical environment of   the   ECM surrounding the tumor cells actively regulate cellular properties such as secretion, which in turn, may contribute to cancer progression. 
 Transforming growth factor-β-sphingosine kinase 1/S1P signaling upregulates microRNA-21 to promote fibrosis in renal tubular epithelial cells. Renal fibrosis is a progressive pathological change characterized by tubular cell apoptosis, tubulointerstitial fibroblast proliferation, and excessive deposition of extracellular matrix   (EC  M). miR-21 has been implicated in transforming growth factor-β (TGF-β)-stimulated tissue fibrosis. Recent studies showed that sphingosine kinase/sphingosine-1-phosphate (SphK/S1P) are also critical for TGF-β-stimulated tissue fibrosis; however, it is not clear whether SphK/S1P interacts with miR-21 or not. In this study, we hypothesized that SphK/S1P signaling is linked to upregulation of miR-21 by TGF-β. To verify this hypothesis, we first determined that miR-21 was highly expressed in renal tubular epithelial cells (TECs) stimulated with TGF-β by using qRT-PCR and Northern blotting. Simultaneously, inhibition of miR-21, mediated by the corresponding antimir, markedly decreased the expression and deposition of type I collagen, fibronectin (Fn), cysteine-rich protein 61 (CCN1), α-smooth muscle actin, and fibroblast-specific protein1 in TGF-β-treated TECs. ELISA and qRT-PCR were used to measure the S1P and SphK1 levels in TECs. S1P production was induced by TGF-β through activation of SphK1. Furthermore, it was observed that TGF-β-stimulated upregulation of miR-21 was abolished by SphK1 siRNA and was restored by the addition of exogenous S1P. Blocking S1PR2 also inhibited upregulation of miR-21. Additionally, miR-21 overexpression attenuated the repression of TGF-β-  sti  mulated ECM deposition and epithelial-mesenchymal transition by SphK1 and S1PR2 siRNA. In summary, our study demonstrates a link between SphK1/S1P and TGF-β-induced miR-21 in renal TECs and may represent a novel therapeutic target in renal fibrosis. 
  [Ee]xtracellular [Mm]atrix  PubMed  
 Sphingosine kinase-1 associates with integrin {alpha}V{beta}3 to mediate endothelial cell survival. Sphingosine kinase (SK)-1 promotes endothelial cell (EC) survival through the cell junction molecule CD31 (platelet endothelial cell adhesion molecule-1). The integrin alpha(v)beta(3) is also essential for EC survival; inhibition of alpha(v)beta(3) ligation promotes apoptosis. Herein we demonstrate that under basal conditions, SK-1, alpha(v)beta(3), and CD31 exist as a heterotrimeric complex. Under conditions that affect EC survival such as loss of contact with the   extracellular matrix   or growth factor activation, more of this heterotrimeric complex forms. Overexpression studies demonstrate a requirement for SK-1 phosphorylation at serine 225 for increased heterotrimeric complex formation, activation of alpha(v)beta(3), and EC survival signals, including Bcl-X and nuclear factor-kappaB pathways. Moreover, beta(3) integrin depletion confirmed the requirement for this heterotrimeric complex in SK-1-mediated EC survival. Thus, with alpha(v)beta(3) integrin being identifiable primarily on angiogenic ECs and SK-1 being highly expressed in tumors, targeting SK-1 may affect multiple survival pathways, and its inhibition may be highly efficacious in controlling pathological EC survival. 
   Extracellular Matrix   Rigidity-dependent Sphingosine-1-phosphate Secretion Regulates Metastatic Cancer Cell Invasion and Adhesion. Dynamic interaction between cancer cells and the surrounding microenvironment is critical for cancer progression via changes in cellular behavior including alteration of secreted molecules. However, the molecular mechanisms underlying the influence exerted by the cancer microenvironment on secretion of molecules during cancer progression remain largely unknown. In this study, we report that secretion of spingsine-1-phosphate (S1P) and its regulator, SphK1 expression is dependent of the substrate rigidity, which is critical for the balance between cancer cell invasion and adhesion. Conditioned media (CM) of MDA-MB-231, an aggressive breast cancer cell obtained from soft substrate (~0.5 kPa) induced chemo-attractive invasion, while CM obtained from stiff substrate (~2.5 kPa) increased cell adhesion instead. We found that the expression of SphK1 is upregulated in the stiff substrate, resulting in an increase in S1P levels in the CM. We also found that upregulation of SphK1 expression in the stiff substrate is dominant in metastatic cancer cells but not in primary cancer cells. These results suggest that alterations in the mechanical environment of the ECM surrounding the tumor cells actively regulate cellular properties such as secretion, which in turn, may contribute to cancer progression. 
 Transforming growth factor-β-sphingosine kinase 1/S1P signaling upregulates microRNA-21 to promote fibrosis in renal tubular epithelial cells. Renal fibrosis is a progressive pathological change characterized by tubular cell apoptosis, tubulointerstitial fibroblast proliferation, and excessive deposition of   extracellular matri  x (ECM). miR-21 has been implicated in transforming growth factor-β (TGF-β)-stimulated tissue fibrosis. Recent studies showed that sphingosine kinase/sphingosine-1-phosphate (SphK/S1P) are also critical for TGF-β-stimulated tissue fibrosis; however, it is not clear whether SphK/S1P interacts with miR-21 or not. In this study, we hypothesized that SphK/S1P signaling is linked to upregulation of miR-21 by TGF-β. To verify this hypothesis, we first determined that miR-21 was highly expressed in renal tubular epithelial cells (TECs) stimulated with TGF-β by using qRT-PCR and Northern blotting. Simultaneously, inhibition of miR-21, mediated by the corresponding antimir, markedly decreased the expression and deposition of type I collagen, fibronectin (Fn), cysteine-rich protein 61 (CCN1), α-smooth muscle actin, and fibroblast-specific protein1 in TGF-β-treated TECs. ELISA and qRT-PCR were used to measure the S1P and SphK1 levels in TECs. S1P production was induced by TGF-β through activation of SphK1. Furthermore, it was observed that TGF-β-stimulated upregulation of miR-21 was abolished by SphK1 siRNA and was restored by the addition of exogenous S1P. Blocking S1PR2 also inhibited upregulation of miR-21. Additionally, miR-21 overexpression attenuated the repression of TGF-β-stimulated ECM deposition and epithelial-mesenchymal transition by SphK1 and S1PR2 siRNA. In summary, our study demonstrates a link between SphK1/S1P and TGF-β-induced miR-21 in renal TECs and may represent a novel therapeutic target in renal fibrosis. 
 Sphingosine kinase-1 enhances endothelial cell survival through a PECAM-1-dependent activation of PI-3K/Akt and regulation of Bcl-2 family members. Sphingosine-1-phosphate (S1P), the bioactive product of sphingosine kinase (SK) activation, is a survival factor for endothelial cells. The mechanism of SK-mediated survival was investigated in endothelial cells with moderately raised intracellular SK activity. Overexpression of SK mediated survival primarily through the activation of the phosphatidyl inositol 3-kinase (PI-3K)/protein kinase B (Akt/PKB) pathway and an associated up-regulation of the antiapoptotic protein B cell lymphoma gene 2 (Bcl-2) and down-regulation of the proapoptotic protein bisindolylmaleimide (Bcl-2 interacting mediator of cell death; Bim). In addition there was an up-regulation and dephosphorylation of the junctional molecule platelet endothelial cell adhesion molecule-1 (PECAM-1), which was obligatory for activation of the PI-3K/Akt pathway, for SK-induced cell survival, and for the changes in the apoptosis-related proteins. Thus, raised intracellular SK activity induced a molecule involved in cell-cell interactions to augment cell survival through a PI-3K/Akt-dependent pathway. This is distinct from the activation of both PI-3K/Akt and mitogen-activated protein kinase (MAPK) pathways seen with exogenously added S1P. Cells overexpressing SK showed enhanced survival under conditions of serum deprivation and absence of attachment to   extracellular matrix  , suggesting a role for SK in the regulation of vascular phenomena that occur under conditions of stress, such as angiogenesis and survival in unattached states, as would be required for a circulating endothelial cell. 
  GeneRIFs  
 Found that upregulation of SphK1 expression in the stiff substrate is dominant in metastatic cancer cells but not in primary cancer cells. These results suggest that alterations in the mechanical environment of the   extracellular matrix   surrounding the tumor cells actively regulate cellular properties such as secretion, which in turn, may contribute to cancer progression. 
  [Ii]nvasive  PubMed  
 Comprehensive gene and microRNA expression profiling reveals a role for miRNAs in the oncogenic roles of SphK1 in papillary thyroid cancer. The oncogenic roles of sphingosine kinase 1 (SphK1) in various cancers, including thyroid cancer, have been well demonstrated. However, the microRNAs (miRNAs) associated with the oncogenic roles of SphK1 remain largely unknown.Global gene and miRNA expression in TPC1-Vector and TPC1-SphK1 cells was analyzed using digital gene expression (DGE) analysis and small RNA-seq, respectively. miRNA-mRNA interactions were explored by microT-CDS, and the predicted networks were visualized using CytoScape(®). Cell invasion and migration were assessed by performing Transwell invasion and wound-healing assays. Luciferase reporter and immunoblot assays were used to evaluate the targeting of fibronectin 1 (FN1) by miR-144-3p.In this study, we found that overexpression of SphK1 differentially regulates the expression of 46 miRNAs and 506 mRNAs in papillary thyroid cancer (PTC) TPC1 cells. Combining bioinformatics predictions of mRNA targets with DGE data on mRNA expression allowed us to identify the mRNA targets of deregulated miRNAs. The direct interaction between miR-144-3p and FN1, which mediates the pro  -invasiv  e role of SphK1 in PTC cells, was experimentally validated.Our results demonstrated that SphK1 overexpression drives a regulatory network governing miRNA and mRNA expression in PTC cells. We also demonstrated the roles played by miR-144-3p and FN1 in mediating the oncogenic function of SphK1, which enhanced the understanding of the etiology of PTC. 
 SphK1 promotes tumor cell migration and invasion in colorectal cancer. Colorectal cancer (CRC) is one of the most common cancers worldwide. Sphingosine kinase 1 (SphK1), which phosphorylates sphingosine to sphingosine-1-phosphate (S1P), is overexpressed in various types of cancers and may act as an oncogene in tumorigenesis. However, little is known about the role of SphK1 in CRC patients. We studied the expression of SphK1 in 85 cases of CRC tissues by immunohistochemistry, qRT-PCR, and western blot. We also evaluated the effect of SphK1 on cell proliferation and invasion by MTT and transwell invasion assay. SphK1 is overexpressed in CRC tissues and cell lines, and upregulation of SphK1 correlated significantly with the following parameters: lymph node metastasis, liver metastasis, and advanced TNM stage. SphK1 knockdown results in inhibition of cancer cell proliferation. Inhibition of CRC cell migration and invasion is also evident through reversal of EMT by increases in E-cadherin expression and decreases in vimentin expression. In conclusion, SphK1 is associated with the proliferation and   invasive  ness of CRC cells and the SphK1 gene may contribute to a novel therapeutic approach against CRC. 
 An novel role of sphingosine kinase-1 (SPHK1) in the invasion and metastasis of esophageal carcinoma. Treatment failure for esophageal carcinoma is frequently due to lymph node metastasis and invasion to neighboring organs. The aim of the present study was to investigate invasion- and metastasis-related genes in esophageal carcinoma cells in vitro and in vivo.A metastasis model using a Matrigel invasion clonal selection approach was employed to establish a highly   invasive   subline EC9706-P4 from the esophageal carcinoma cell (ESCC) line EC9706. The differentially expressed genes of the subline and the parental cells determined by gene microarrays were further analyzed by RT-PCR and Western blotting.We identified sphingosine kinase 1 (SPHK1) as an invasion and metastasis-related gene of esophageal cancer. SPHK1 was overexpressed in the EC9706-P4 subline with high   invasive   capacity. Among six ESCC lines tested, KYSE2 and KYSE30 cells showed the highest SPHK1 mRNA and protein expressions as well as the most   invasive   phenotype. By Western blotting, in 7/12 cases (58%), SPHK1 expression was higher in esophageal carcinomas than in the companion normal tissue. In 23/30 cases (76%), SPHK1 protein expression was upregulated in the tumors compared to matched normal tissue by immunohistochemistry (IHC). Esophageal carcinoma tissue microarray analysis indicated that SPHK1 expression correlated with the depth of tumor invasion (P < 0.0001) and lymph node metastasis (P = 0.016). By Kaplan-Meier analysis, strong SPHK1 expression was significantly associated with clinical failure (P < 0.01), suggesting the involvement of SPHK1 in aggressiveness of human esophageal carcinoma. SPHK1 overexpression significantly increased the   invasive  ness of EC9706 cells in vitro and also increased EC9706 cell growth and spontaneous metastasis in vivo, promoting significant increases in tumor growth, tumor burden and spontaneous lung metastasis in nude mice. SPHK1 expression significantly correlated with the expression of many EGFR pathway genes associated with invasion of cancer cells. SPHK1 protein expression also significantly correlated with the phosphorylation of EGFR.In summary, our data implicate SPHK1 in the metastasis of esophageal cancer. Our study also identified downstream mediators of SPHK1 in esophageal cancer cells that may mediate enhanced malignant behavior, and several of these mediators may be useful as therapeutic targets. 
 Identification of novel functional and spatial associations between sphingosine kinase 1, sphingosine 1-phosphate receptors and other signaling proteins that affect prognostic outcome in estrogen receptor-positive breast cancer. Sphingosine kinase is an enzyme that catalyses the phosphorylation of sphingosine to form sphingosine 1-phosphate. Sphingosine 1-phosphate is a bioactive lipid, which has been shown to have an important role in promoting the survival, growth and   invasive  ness of cancer cells. Sphingosine 1-phosphate binds to five different plasma membrane sphingosine 1-phosphate receptors (S1P(1-5) ) and can regulate intracellular target proteins. We have used immunohistochemical analysis to determine the concurrent expression levels of sphingosine kinase 1 or S1P receptors and other signaling proteins in estrogen receptor-positive breast cancer tumors and have then assessed the impact of these combinations on clinical outcome. This approach has enabled identification of (i) novel biomarkers and (ii) several spatially controlled associations between either sphingosine kinase 1 or S1P(1-3) and other signaling proteins whose combination affect prognosis. For instance, the translocation of sphingosine kinase 1 to the plasma membrane has been shown to be a critical determinant in cancer progression. However, our findings identify an additional novel role for the nuclear localization of sphingosine kinase 1 combined with either ERK-1/2 or SFK or LYN or AKT or NF-κB, which significantly shortens disease-specific survival and/or recurrence. We also demonstrate that nuclear S1P(2) receptor and c-SRC are associated with improved prognosis and this is linked with a reduction in the nuclear localization of sphingosine kinase 1. These findings identify potential novel biomarker associations, which might serve as new targets for drug intervention designed to improve treatment of estrogen receptor-positive breast cancer. 
 Sphingosine kinase 1 promotes tumor progression and confers malignancy phenotypes of colon cancer by regulating the focal adhesion kinase pathway and adhesion molecules. Studies suggest a tumor-promoting function of sphingosine kinase 1 (SphK1) in some types of human tumors, however, its effect on colon cancer is still unclear. The aims of this study were to investigate the roles of SphK1 in the progression and tumor cell phenotypic changes in colon cancer. Moreover, the focal adhesion kinase (FAK) pathway and the expression of intercellular adhesion molecule‑1 (ICAM‑1) and vascular cell adhesion molecule‑1 (VCAM‑1) were detected to explore the mechanisms of SphK1 action. In this study, the expression of SphK1, FAK and phospho-FAK (p-FAK) was analyzed in 66 surgical specimens of primary colon cancer and matched adjacent normal tissues by immunohistochemistry and western blotting. In addition, N,N-dimethylsphingosine (DMS), SphK1 DNA and shRNA transfection were used to regulate the expression and activity of SphK1 in the LOVO colon cancer cell line. Tumor cell phenotypic changes were analyzed by cell viability, invasion and apoptosis assays. Results showed that the expression of SphK1, FAK and p-FAK in colon cancer tissues were significantly stronger compared to those in matched normal tissues. There was a close correlation between the expression of SphK1 and FAK or p-FAK and the co-expression of SphK1, FAK and p-FAK significantly associated with histological grade, Dukes' stage, lymph node metastasis and distant metastasis. Overexpression of SphK1 after DNA transfection enhanced tumor cell viabil  ity and   invasiveness, but suppressed cell apoptosis. In contrast, suppression of SphK1 by DMS and shRNA reduced tumor cell viabil  ity and   invasiveness, but promoted cell apoptosis. The expression of FAK, p-FAK, ICAM-1 and VCAM-1 in LOVO cells were increased with the overexpression of SphK1 but decreased with the suppression of SphK1. These findings indicate that SphK1 regulates tumor cell proliferation, apoptosis and invasion, which ultimately contributes to tumor progression and malignancy phenotype in colon cancer. FAK pathway, ICAM-1 and VCAM-1 may play critical roles in this SphK1‑mediated effect. 
 Interleukin-1 regulates the expression of sphingosine kinase 1 in glioblastoma cells. Chronic inflammation and inflammatory cytokines have recently been implicated in the development and progression of various types of cancer. In the brain, neuroinflammatory cytokines affect the growth and differentiation of both normal and malignant glial cells, with interleukin 1 (IL-1) shown to be secreted by the majority of glioblastoma cells. Recently, elevated levels of sphingosine kinase 1 (SphK1), but not SphK2, were correlated with a shorter survival prognosis for patients with glioblastoma multiforme. SphK1 is a lipid kinase that produces the pro-growth, anti-apoptotic sphingosine 1-phosphate, which can induce invasion of glioblastoma cells. Here, we show that the expression of IL-1 correlates with the expression of SphK1 in glioblastoma cells, and neutralizing anti-IL-1 antibodies inhibit both the growth and invasion of glioblastoma cells. Furthermore, IL-1 up-regulates SphK1 mRNA levels, protein expression, and activity in both primary human astrocytes and various glioblastoma cell lines; however, it does not affect SphK2 expression. The IL-1-induced SphK1 up-regulation can be blocked by the inhibition of JNK, the overexpression of the dominant-negative c-Jun(TAM67), and the down-regulation of c-Jun expression by small interference RNA. Activation of SphK1 expression by IL-1 occurs on the level of transcription and is mediated via a novel AP-1 element located within the first intron of the sphk1 gene. In summary, our results suggest that SphK1 expression is transcriptionally regulated by IL-1 in glioblastoma cells, and this pathway may be important in regulating survival and   invasive  ness of glioblastoma cells. 
 Sphingosine kinase 1 enhances colon cancer cell proliferation and invasion by upregulating the production of MMP-2/9 and uPA via MAPK pathways. Sphingosine kinase (SphK) 1 is an oncogenic enzyme promoting transformation, proliferation, and survival of a number of human tumor cells. However, its effect on colon cancer cell behavior has not been fully clarified.SphK1 plasmid or SphK1 shRNA transfection and N,N-dimethylsphingosine (DMS) was used to regulate the expression and activity of SphK1 in colon cancer line LOVO. Cell proliferation, apoptosis, invasion, and protein expression were detected by MTT, flow cytometry, transwell chambers model, and western blot. The levels of metalloproteinases-2/9 (MMP-2/9) and urokinase plasminogen activator (uPA) were detected by ELISA.Overexpression of SphK1 after plasmid transfection markedly enhanced LOVO cell viability and   invasive  ness and reduced cell apoptosis. In contrast, inhibition of SphK1 by DMS and shRNA significantly suppressed cell viability and   invasive  ness but promoted cell apoptosis. SphK1 increased the constitutive expression of extracellular signal-regulated kinase1/2 (ERK1/2) but reduced the constitutive expression of p38 mitogen-activated protein kinase (MAPK). Blocking ERK1/2 pathway inhibited the biological effects induced by overexpression of SphK1. Blocking p38 MAPK pathway reversed the effects of DMS and SphK1 shRNA. Moreover, SphK1 was required for the production of MMP-2/9 and uPA in tumor cells, which was suppressed by ERK1/2 inhibitor U0126, but enhanced by the p38 MAPK inhibitor SB203580.SphK1 enhances colon cancer cell proliferation and   invasive  ness, meanwhile suppressing cell apoptosis. SphK1 promoting the secretion of MMP-2/9 and uPA via activation of ERK1/2 and suppression of p38 MAPK pathways maybe the molecular mechanisms for its regulation of the malignant behavior of colon cancer cell. 
  GeneRIFs  
 SphK1 is associated with the proliferation and   invasive  ness of CRC cells and the SphK1 gene may contribute to a novel therapeutic approach against CRC. 
 SphK1 expression is transcriptionally regulated by IL-1 in glioblastoma cells, and this pathway may be important in regulating survival and   invasive  ness of glioblastoma cells. 
  [Mm]etastasis  PubMed  
 Role for sphingosine kinase 1 in colon carcinogenesis. Sphingosine kinase 1 (SphK1) phosphorylates sphingosine to form sphingosine-1-phosphate (S1P) and is a critical regulator of sphingolipid-mediated functions. Cell-based studies suggest a tumor-promoting function for the SphK1/S1P pathway. Also, our previous studies implicated the SphK1/S1P pathway in the induction of the arachidonic acid cascade, a major inflammatory pathway involved in colon carcinogenesis. Therefore, we investigated whether the SphK1/S1P pathway is necessary for mediating carcinogenesis in vivo. Here, we report that 89% (42/47) of human colon cancer samples stained positively for SphK1, whereas normal colon mucosa had negative or weak staining. Adenomas had higher expression of SphK1 vs. normal mucosa, and colon cancers with   metastasis   had higher expression of SphK1 than those without   metastasis  . In the azoxymethane (AOM) murine model of colon cancer, SphK1 and S1P were significantly elevated in colon cancer tissues compared to normal mucosa. Moreover, blood levels of S1P were higher in mice with colon cancers than in those without cancers. Notably, SphK1(-/-) mice subjected to AOM had significantly less aberrant crypt foci (ACF) formation and significantly reduced colon cancer development. These results are the first in vivo evidence that the SphK1/S1P pathway contributes to colon carcinogenesis and that inhibition of this pathway is a potential target for chemoprevention. 
 Microarray analysis of altered sphingolipid metabolism reveals prognostic significance of sphingosine kinase 1 in breast cancer. Beside their structural role for the cell membrane the family of sphingolipids act as effector molecules in signal transduction with links to various aspects of cancer initiation, progression and treatment response. The "sphingolipid rheostat" balances between apoptosis inducing ceramid and growth promoting sphingosine-1-phosphate. We analyzed gene expression of 43 proteins from this pathway in different subtypes of breast cancer using microarray data of 1,269 tumor samples (test set n=171; validation sets n=1098) and observed significant differences for several genes. Sphingosine kinase 1 (SPHK1), ceramide galactosyltransferase (UGT8), and Ganglioside GD3-Synthase (ST8SIA1) displayed higher expression among ER negative tumors. In contrast, glucosylceramidsynthase (GCS), dihydroceramidsynthases (LASS4, LASS 6) and acid ceramidase (ASAH1) were higher expressed in ER positive samples. Survival analysis revealed a worse outcome of patients with high SPHK1 expression. To avoid a confounding effect of the ER status we also restricted the analysis to 750 patients with ER positive tumors. Again a worse outcome was observed for tumors displaying high SPHK1 expression. While 75.8+/-1.9% of the patients with tumors low in SPHK1 expression were free of   metastasis   at 5 years, this was the case for only 64.9+/-3.6% of patients with tumors displaying high SPHK1 expression (P=0.008). Immunohistochemistry identified the carcinoma cells as the major source of SPHK1 expression in the tumor. The correlation of SPHK1 with a poor prognosis as well as its high expression among ER negative tumors are in line with the antiapoptotic and proliferative properties of its product sphingosine-1-phosphate. Targeting of the sphingolipid rheostat may thus open new treatment options. 
 SphK1 promotes tumor cell migration and invasion in colorectal cancer. Colorectal cancer (CRC) is one of the most common cancers worldwide. Sphingosine kinase 1 (SphK1), which phosphorylates sphingosine to sphingosine-1-phosphate (S1P), is overexpressed in various types of cancers and may act as an oncogene in tumorigenesis. However, little is known about the role of SphK1 in CRC patients. We studied the expression of SphK1 in 85 cases of CRC tissues by immunohistochemistry, qRT-PCR, and western blot. We also evaluated the effect of SphK1 on cell proliferation and invasion by MTT and transwell invasion assay. SphK1 is overexpressed in CRC tissues and cell lines, and upregulation of SphK1 correlated significantly with the following parameters: lymph node   metastasis  , liver   metastasis  , and advanced TNM stage. SphK1 knockdown results in inhibition of cancer cell proliferation. Inhibition of CRC cell migration and invasion is also evident through reversal of EMT by increases in E-cadherin expression and decreases in vimentin expression. In conclusion, SphK1 is associated with the proliferation and invasiveness of CRC cells and the SphK1 gene may contribute to a novel therapeutic approach against CRC. 
 An novel role of sphingosine kinase-1 (SPHK1) in the invasion and   metastasis   of esophageal carcinoma. Treatment failure for esophageal carcinoma is frequently due to lymph node   metastasis   and invasion to neighboring organs. The aim of the present study was to investigate invasion- and   metastasis  -related genes in esophageal carcinoma cells in vitro and in vivo.A   metastasis   model using a Matrigel invasion clonal selection approach was employed to establish a highly invasive subline EC9706-P4 from the esophageal carcinoma cell (ESCC) line EC9706. The differentially expressed genes of the subline and the parental cells determined by gene microarrays were further analyzed by RT-PCR and Western blotting.We identified sphingosine kinase 1 (SPHK1) as an invasion and   metastasis  -related gene of esophageal cancer. SPHK1 was overexpressed in the EC9706-P4 subline with high invasive capacity. Among six ESCC lines tested, KYSE2 and KYSE30 cells showed the highest SPHK1 mRNA and protein expressions as well as the most invasive phenotype. By Western blotting, in 7/12 cases (58%), SPHK1 expression was higher in esophageal carcinomas than in the companion normal tissue. In 23/30 cases (76%), SPHK1 protein expression was upregulated in the tumors compared to matched normal tissue by immunohistochemistry (IHC). Esophageal carcinoma tissue microarray analysis indicated that SPHK1 expression correlated with the depth of tumor invasion (P < 0.0001) and lymph node   metastasis   (P = 0.016). By Kaplan-Meier analysis, strong SPHK1 expression was significantly associated with clinical failure (P < 0.01), suggesting the involvement of SPHK1 in aggressiveness of human esophageal carcinoma. SPHK1 overexpression significantly increased the invasiveness of EC9706 cells in vitro and also increased EC9706 cell growth and spontaneous   metastasis   in vivo, promoting significant increases in tumor growth, tumor burden and spontaneous lung   metastasis   in nude mice. SPHK1 expression significantly correlated with the expression of many EGFR pathway genes associated with invasion of cancer cells. SPHK1 protein expression also significantly correlated with the phosphorylation of EGFR.In summary, our data implicate SPHK1 in the   metastasis   of esophageal cancer. Our study also identified downstream mediators of SPHK1 in esophageal cancer cells that may mediate enhanced malignant behavior, and several of these mediators may be useful as therapeutic targets. 
 Role of Sphk1 in the malignant transformation of breast epithelial cells and breast cancer progression. The ojective of the following study is to investigate the role of sphingosine kinase 1 (Sphk1) in the malignant transformation of breast epithelial cells and breast cancer progression and its mechanism.Immunohistochemistry was performed to detect Sphk1 and E-cadherin (E-cad) in resected breast samples. Sphk1 was transfected in normal human breast epithelial cell line (MCF-10A) by Lentivirus and silenced in breast cancer cell line (MCF-7) using small interfering ribonucleic acid. The effect of tumor necrosis factor alpha (TNF-α) and/or N, N-dimethylsphingosine (DMS) on the Sphk1 and E-cad expression, MCF-10A cell proliferation and invasion was investigated. Real time-polymerase chain reaction and western-blot was used to detect messenger ribonucleic acid and protein. Cell counting kit-8 and transwell were used to measure cell proliferation and invasion.Sphk1 was positive expression in 114 breast tumors (75.50%) but negative in fibroadenomas. The expression of E-cad and Sphk1 were negatively correlated and E-cad (-)/Sphk1 (+) carriers showed higher ratio of axillary lymph node   metastasi  s than E-cad (+)/Sphk1 (-) carriers. Overexpression of Sphk1 in MCF-10A reduced E-cad expression and improved cell proliferation and invasion, but knockdown of Sphk1 in MCF-7 decreased cell proliferation and invasion. TNF-α increased Sphk1 expression, enhanced the ability of Sphk1 in decreasing E-cad expression, which could be blocked by DMS. TNF-α promoted MCF-10A cell proliferation and invasion.Sphk1 plays an important role in the malignant transformation of breast epithelial cells and modulates breast canc  er metasta  sis through the regulation of E-cad expression. TNF-α can up-regulate Sphk1 expression and reduce E-cad expression through Sphk1, which can be blocked by DMS. TNF-α/Sphk1/E-cad pathway may be a newly discovered pathway and plays an important role in tumorigenesis   and metas  tasis. 
 Sphingosine kinase 1 promotes tumor progression and confers malignancy phenotypes of colon cancer by regulating the focal adhesion kinase pathway and adhesion molecules. Studies suggest a tumor-promoting function of sphingosine kinase 1 (SphK1) in some types of human tumors, however, its effect on colon cancer is still unclear. The aims of this study were to investigate the roles of SphK1 in the progression and tumor cell phenotypic changes in colon cancer. Moreover, the focal adhesion kinase (FAK) pathway and the expression of intercellular adhesion molecule‑1 (ICAM‑1) and vascular cell adhesion molecule‑1 (VCAM‑1) were detected to explore the mechanisms of SphK1 action. In this study, the expression of SphK1, FAK and phospho-FAK (p-FAK) was analyzed in 66 surgical specimens of primary colon cancer and matched adjacent normal tissues by immunohistochemistry and western blotting. In addition, N,N-dimethylsphingosine (DMS), SphK1 DNA and shRNA transfection were used to regulate the expression and activity of SphK1 in the LOVO colon cancer cell line. Tumor cell phenotypic changes were analyzed by cell viability, invasion and apoptosis assays. Results showed that the expression of SphK1, FAK and p-FAK in colon cancer tissues were significantly stronger compared to those in matched normal tissues. There was a close correlation between the expression of SphK1 and FAK or p-FAK and the co-expression of SphK1, FAK and p-FAK significantly associated with histological grade, Dukes' stage, lym  ph node me  tastasis and   distant me  tastasis. Overexpression of SphK1 after DNA transfection enhanced tumor cell viability and invasiveness, but suppressed cell apoptosis. In contrast, suppression of SphK1 by DMS and shRNA reduced tumor cell viability and invasiveness, but promoted cell apoptosis. The expression of FAK, p-FAK, ICAM-1 and VCAM-1 in LOVO cells were increased with the overexpression of SphK1 but decreased with the suppression of SphK1. These findings indicate that SphK1 regulates tumor cell proliferation, apoptosis and invasion, which ultimately contributes to tumor progression and malignancy phenotype in colon cancer. FAK pathway, ICAM-1 and VCAM-1 may play critical roles in this SphK1‑mediated effect. 
 [The expression and clinical significance of SphK1 and nuclear factor-κB p65 in human colon carcinoma]. To investigate the expression of sphingosine kinase 1 (SphK1) and NF-κB in colon carcinoma tissues and their correlation with clinicopathologic features.Sixty-six paraffin-embedded colon carcinoma samples and 66 fresh colon carcinoma samples were tested using immunohistochemistry, RT-PCR and Western blot, respectively.In 66 fresh colon carcinoma samples, the positive rate of SphK1 and NF-κB mRNA expression were 84.85%(56/66) and 74.24% (49/66), while the positive rate of SphK1 and NF-κB protein detected by Western blot were 78.79% (52/66) and 69.70% (46/66). The positive rates were higher than those in the adjacent tissues [mRNA: 63.64% (42/66), 48.49% (32/66); protein: 57.58% (38/66), 45.45% (30/66)] and the normal mucosa [mRNA: 42.42% (28/66), 25.76% (17/66); protein: 36.36% (24/66), 24.24% (16/66)], with statistical significances (all P values < 0.05). The mean expressive levels of SphK1 and NF-κB mRNA and protein in colon carcinoma were both significantly higher than those in the adjacent tissues and the normal mucosa (mRNA: 0.55 ± 0.06 vs 0.35 ± 0.05 vs 0.25 ± 0.05, 0.75 ± 0.06 vs 0.43 ± 0.05 vs 0.30 ± 0.04; protein: 0.77 ± 0.05 vs 0.38 ± 0.06 vs 0.12 ± 0.03, 0.45 ± 0.08 vs 0.23 ± 0.05 vs 0.13 ± 0.03; all P values < 0.05). There was a close correlation between SphK1 and NF-κB expression levels (r = 0.459, P = 0.036). The results of immunohistochemistry were similar to those of RT-PCR and Western blot. Overexpression of SphK1 and NF-κB in colon carcinoma was related with depth of invasion, dist  ant and ly  mph node metastasis and Dukes' stages (all P values < 0.05). The expression of SphK1 was also related with differentiation (P < 0.05).Overexpression of SphK1 and NF-κB may be involved in the pathogenesis and progression of colon carcinoma. Moreover, SphK1 and NF-κB may be correlated w  ith the in  vasion and metastasis of colon carcinoma. 
 High expression of sphingosine kinase 1 is associated with poor prognosis in nasopharyngeal carcinoma. It has been reported that sphingosine kinase 1 (SPHK1), an oncogenic enzyme, was involved in the development and progression of a number of human cancers. However, the role of SPHK1 in nasopharyngeal carcinoma (NPC) is largely unknown. The present study aimed to characterize the expression of SPHK1 in human NPC and evaluate its clinical significance. Real-time quantitative reverse transcriptase-polymerase chain reaction (qRT-PCR) and Western blot analyses showed that the expression of SPHK1 mRNA and protein in NPC specimens was significantly higher than that in non-tumorous nasopharyngeal mucosa biopsies. Immunohistochemistry (IHC) was conducted to characterize the expression pattern of SPHK1 in 142 archived paraffin-embedded NPC specimens. Statistical analyses revealed that high levels of SPHK1 expression were associated with the clinical stages, locoregional recurrence and distant   metastasis   of NPC. NPC patients with high levels of SPHK1 expression had shorter survival time, whereas those with lower levels of SPHK1 expression survived longer. Moreover, multivariate analysis suggested that SPHK1 up-regulation was an independent prognostic factor for NPC. Our results suggest for the first time that SPHK1 is involved in the development and progression of NPC, which can be used as a useful prognostic marker for NPC patients and may be an effective target for treating NPC. 
 Sphingosine kinase 1 is required for TGF-β mediated fibroblastto- myofibroblast differentiation in ovarian cancer. Sphingosine kinase 1 (SPHK1), the enzyme that produces sphingosine 1 phosphate (S1P), is known to be highly expressed in many cancers. However, the role of SPHK1 in cells of the tumor stroma remains unclear. Here, we show that SPHK1 is highly expressed in the tumor stroma of high-grade serous ovarian cancer (HGSC), and is required for the differentiation and tumor promoting function of cancer-associated fibroblasts (CAFs). Knockout or pharmacological inhibition of SPHK1 in ovarian fibroblasts attenuated TGF-β-induced expression of CAF markers, and reduced their ability to promote ovarian cancer cell migration and invasion in a coculture system. Mechanistically, we determined that SPHK1 mediates TGF-β signaling via the transactivation of S1P receptors (S1PR2 and S1PR3), leading to p38 MAPK phosphorylation. The importance of stromal SPHK1 in tumorigenesis was confirmed in vivo, by demonstrating a significant reduction of tumor growth a  nd metasta  sis in SPHK1 knockout mice. Collectively, these findings demonstrate the potential of SPHK1 inhibition as a novel stroma-targeted therapy in HGSC. 
 [Relationship between sphingosine kinase 1 expression and tumor invasion,   metastasis   and prognosis in gastric cancer]. To explore the clinical significance of sphingosine kinase 1 (SPHK1) in the invasion,   metastasis   and prognosis of gastric cancer.Immunohistochemistry was employed to analyze the expression of SPHK1 in 206 clinicopathologically characterized gastric cancer cases from January 2001 to December 2005 at Zhejiang Provincial People's Hospital.SPHK1 protein was detected in 3 (7.5%) of 40 human non-tumor mucosa. All samples expressed the protein at a low level. SPHK1 protein was detected in 181 (87.9%) of 206 human gastric cancer cases. An elevated expression of SPHK1 protein was detected in 126 (61.2%) tumors. And SPHK1 protein was up-regulated in gastric cancer lesions compared with adjacent noncancerous tissues (P = 0.001). The expression of SPHK1 was correlated with the depth of invasion, lymph node   metastasis  , distant   metastasis   and TNM stage (P = 0.039, 0.003, 0.020, 0.003). In stages I-II and III, the 5-year survival rate of the patients with a high expression of SPHK1 was significantly lower than those with a low expression (53.6% (15/28) vs 68.6% (24/35), 7.8% (6/77) vs 30.8% (12/39), P = 0.009, 0.006). In stage IV, the expression of SPHK1 was not correlated with the 5-year survival rate (P > 0.05). Further multivariate analysis suggested that lymph node   metastasis  , distant   metastasis  , TNM stage and the up-regulation of SPHK1 were independent prognostic indicators for gastric cancer.The expression of SPHK1 in gastric cancer is significantly associated with lymph node   metastasis  , distant   metastasis   and a poor prognosis. SPHK1 may become a useful marker of predicting tumor progression and prognosis. 
 Sphingosine kinase 1 is a reliable prognostic factor and a novel therapeutic target for uterine cervical cancer. Sphingosine kinase 1 (SPHK1), an oncogenic kinase, has previously been found to be upregulated in various types of human malignancy and to play a crucial role in tumor development and progression. Although SPHK1 has gained increasing prominence as an important enzyme in cancer biology, its potential as a predictive biomarker and a therapeutic target in cervical cancer remains unknown. SPHK1 expression was examined in 287 formalin-fixed, paraffin-embedded cervical cancer tissues using immunohistochemistry, and its clinical implications and prognostic significance were analyzed. Cervical cancer cell lines including HeLa and SiHa were treated with the SPHK inhibitors SKI-II or FTY720, and effects on cell survival, apoptosis, angiogenesis, and invasion were examined. Moreover, the effects of FTY720 on tumor growth were evaluated using a patient-derived xenograft (PDX) model of cervical cancer. Immunohistochemical analysis revealed that expression of SPHK1 was significantly increased in cervical cancer compared with normal tissues. SPHK1 expression was significantly associated with tumor size, invasion depth, FIGO stage, lymph node   metastasis  , and lymphovascular invasion. Patients with high SPHK1 expression had lower overall survival and recurrence-free survival rates than those with low expression. Treatment with SPHK inhibitors significantly reduced viability and increased apoptosis in cervical cancer cells. Furthermore, FTY720 significantly decreased in vivo tumor weight in the PDX model of cervical cancer. We provide the first convincing evidence that SPHK1 is involved in tumor development and progression of cervical cancer. Our data suggest that SPHK1 might be a potential prognostic marker and therapeutic target for the treatment of cervical cancer. 
 Sphingosine kinase 1 is required for migration, proliferation and survival of MCF-7 human breast cancer cells. Sphingosine-1-phosphate (S1P) is a potent lysolipid involved in a variety of biological responses important for cancer progression. Therefore, we investigated the role of sphingosine kinase type 1 (SphK1), the enzyme that makes S1P, in the motility, growth, and chemoresistance of MCF-7 breast cancer cells. Epidermal growth factor (EGF), an important growth factor for breast cancer progression, activated and translocated SphK1 to plasma membrane. SphK1 was required for EGF-directed motility. Downregulation of SphK1 in MCF-7 cells reduced EGF- and serum-stimulated growth and enhanced sensitivity to doxorubicin, a potent chemotherapeutic agent. These results suggest that SphK1 may be critical for growth,   metastasis   and chemoresistance of human breast cancers. 
 Circulating sphingosine-1-phosphate and erythrocyte sphingosine kinase-1 activity as novel biomarkers for early prostate cancer detection. Current markers available for screening normal populations and for monitoring prostate cancer (PCa) treatment lack sensitivity and selectivity. Sphingosine-1-phosphate (S1P) is a circulating lipid second messenger involved in cell growth and migration, the immune response, angiogenesis, and malignant transformation.Eighty-eight patients with localised, locally advanced, or metastatic PCa were recruited into this prospective single-centre study. Plasma S1P levels were measured and compared with age-matched controls with benign prostate hyperplasia (BPH) (n=110) or with young healthy males with the very small chance of having PCa foci (n=20).Levels of circulating S1P were significantly higher in healthy subjects (10.36 ± 0.69 pmol per mg protein, P<0.0001) and patients with BPH (9.39 ± 0.75, P=0.0013) than in patients with PCa (6.89 ± 0.58, ANOVA, P=0.0019). Circulating S1P levels were an early marker of PCa progression to hormonal unresponsiveness and correlated with prostate-specific antigen (PSA) levels and lymph no  de metasta  sis. During the course of the study, nine patients have died of PCa. Importantly, their circulating S1P levels were significantly lower (5.11 ± 0.75) than in the surviving patients (7.02 ± 0.22, n=79, P=0.0439). Our data suggest that the decrease in circulating S1P during PCa progression may stem from a highly significant downregulation of erythrocyte sphingosine kinase-1 (SphK1) activity (2.14 ± 0.17 pmol per mg protein per minute in PCa patients vs 4.7 ± 0.42 in healthy individuals, P<0.0001), which may be a potential mechanism of cancer-induced anaemia.This current study has provided a potential mechanism for cancer-related anaemia and the first evidence that plasma S1P and erythrocyte SphK1 activity are the potential markers for the diagnosis, monitoring, and predicating for PCa mortality. 
  GeneRIFs  
 Sphk1 plays an important role in the malignant transformation of breast epithelial cells and modulates breast cancer   metastasis   through the regulation of Ecadherin expression. 
 The expression of SPHK1 in gastric cancer is significantly associated with lymph node   metastasis  , distant   metastasis   and a poor prognosis. 
 SPHK1 is implicated in the   metastasis   of esophageal cancer. 
 results suggest that SphK1 may be critical for growth,   metastasis   and chemoresistance of human breast cancers 
  [Cc]ell [Ii]nvasion  PubMed  
 Comprehensive gene and microRNA expression profiling reveals a role for miRNAs in the oncogenic roles of SphK1 in papillary thyroid cancer. The oncogenic roles of sphingosine kinase 1 (SphK1) in various cancers, including thyroid cancer, have been well demonstrated. However, the microRNAs (miRNAs) associated with the oncogenic roles of SphK1 remain largely unknown.Global gene and miRNA expression in TPC1-Vector and TPC1-SphK1 cells was analyzed using digital gene expression (DGE) analysis and small RNA-seq, respectively. miRNA-mRNA interactions were explored by microT-CDS, and the predicted networks were visualized using CytoScape(®).   Cell invasio  n and migration were assessed by performing Transwell invasion and wound-healing assays. Luciferase reporter and immunoblot assays were used to evaluate the targeting of fibronectin 1 (FN1) by miR-144-3p.In this study, we found that overexpression of SphK1 differentially regulates the expression of 46 miRNAs and 506 mRNAs in papillary thyroid cancer (PTC) TPC1 cells. Combining bioinformatics predictions of mRNA targets with DGE data on mRNA expression allowed us to identify the mRNA targets of deregulated miRNAs. The direct interaction between miR-144-3p and FN1, which mediates the pro-invasive role of SphK1 in PTC cells, was experimentally validated.Our results demonstrated that SphK1 overexpression drives a regulatory network governing miRNA and mRNA expression in PTC cells. We also demonstrated the roles played by miR-144-3p and FN1 in mediating the oncogenic function of SphK1, which enhanced the understanding of the etiology of PTC. 
 Extracellular Matrix Rigidity-dependent Sphingosine-1-phosphate Secretion Regulates Metastatic Cancer   Cell Invasion   and Adhesion. Dynamic interaction between cancer cells and the surrounding microenvironment is critical for cancer progression via changes in cellular behavior including alteration of secreted molecules. However, the molecular mechanisms underlying the influence exerted by the cancer microenvironment on secretion of molecules during cancer progression remain largely unknown. In this study, we report that secretion of spingsine-1-phosphate (S1P) and its regulator, SphK1 expression is dependent of the substrate rigidity, which is critical for the balance between cancer   cell invasion   and adhesion. Conditioned media (CM) of MDA-MB-231, an aggressive breast cancer cell obtained from soft substrate (~0.5 kPa) induced chemo-attractive invasion, while CM obtained from stiff substrate (~2.5 kPa) increased cell adhesion instead. We found that the expression of SphK1 is upregulated in the stiff substrate, resulting in an increase in S1P levels in the CM. We also found that upregulation of SphK1 expression in the stiff substrate is dominant in metastatic cancer cells but not in primary cancer cells. These results suggest that alterations in the mechanical environment of the ECM surrounding the tumor cells actively regulate cellular properties such as secretion, which in turn, may contribute to cancer progression. 
   
  
 MAGI2 
 
  NCBI Gene  
  GeneRIF  
  Pubmed  
 
 
  Search Term  Rank  Fields  
  [Mm]igration  6  Process(1); PubMed(2); GeneRIFs(1)  
  [Mm]etastasis  12  PubMed(1)  
  [Cc]ell [Ii]nvasion  13  PubMed(1)  
 
 
 hide/show details for MAGI2 
  [Mm]igration  Process  
 negative regulation of cell   migration   
  PubMed  
 MAGI-2 Inhibits cell   migration   and proliferation via PTEN in human hepatocarcinoma cells. MAGI-2, a multidomain scaffolding protein, contains nine potential protein-protein interaction modules, including a GuK domain, two WW domains and six PDZ domains. In this study, we examined eight human hepatocarcinoma cell lines (HHCCs) and found that MAGI-2 was expressed only in 7721 cells. After 7721, 7404 and 97H cells were transfected with myc-MAGI-2 plasmid, their   migration   and proliferation was significantly inhibited, which was associated with downregulation of p-FAK and p-Akt. It is known that p-FAK is a substrate of PTEN and p-Akt can be regulated by PTEN via PIP(3). We demonstrated that PTEN was upregulated after myc-MAGI-2 transfection, which was due to the enhancement of PTEN protein stability rather than mRNA levels. Furthermore, MAGI-2-induced inhibition of cell   migration   and proliferation was attenuated in 7721 cells with PTEN silence or in PTEN-null cell line U87MG, and PTEN transfection could restore the effect of MAGI-2 in U87MG cells. Finally, the molecular association between PTEN and MAGI-2 was confirmed. Our results suggested that PTEN played a critical role in MAGI-2-induced inhibition of cell   migration   and proliferation in HHCCs. 
 MiR-134/487b/655 cluster regulates TGF-β-induced epithelial-mesenchymal transition and drug resistance to gefitinib by targeting MAGI2 in lung adenocarcinoma cells. Epithelial-mesenchymal transition (EMT) has recently been recognized as a key element of cell invasion,   migratio  n, metastasis, and drug resistance in several types of cancer, including non-small cell lung cancer (NSCLC). Our aim was to clarify microRNA (miRNA)-related mechanisms underlying EMT followed by acquired resistance to epidermal growth factor receptor tyrosine kinase inhibitor (EGFR-TKI) in NSCLC. miRNA expression profiles were examined before and after transforming growth factor β1 (TGF-β1) exposure in four human adenocarcinoma cell lines with or without EMT. Correlation between expressions of EMT-related miRNAs and resistance to EGFR-TKI gefitinib was evaluated. miRNA array and real-time quantitative reverse transcription PCR (qRT-PCR) revealed that TGF-β1 significantly induced overexpression of miR-134, miR-487b, and miR-655, which belong to the same cluster located on chromosome 14q32, in lung adenocarcinoma cells with EMT. MAGI2 (membrane-associated guanylate kinase, WW, and PDZ domain-containing protein 2), a predicted target of these miRNAs and a scaffold protein required for PTEN, was diminished in A549 cells with EMT after the TGF-β1 stimulation. Overexpression of miR-134 and miR-487b promoted the EMT phenomenon and affected the drug resistance to gefitinib, whereas knockdown of these miRNAs inhibited the EMT process and reversed TGF-β1-induced resistance to gefitinib. Our study demonstrated that the miR-134/487b/655 cluster contributed to the TGF-β1-induced EMT phenomenon and affected the resistance to gefitinib by directly targeting MAGI2, in which suppression subsequently caused loss of PTEN stability in lung cancer cells. The miR-134/miR-487b/miR-655 cluster may be a new therapeutic target in patients with advanced lung adenocarcinoma, depending on the EMT phenomenon. 
  GeneRIFs  
 PTEN plays a critical role in MAGI-2-induced inhibition of cell   migration   and proliferation in human hepatocarcinoma cells 
  [Mm]etastasis  PubMed  
 MiR-134/487b/655 cluster regulates TGF-β-induced epithelial-mesenchymal transition and drug resistance to gefitinib by targeting MAGI2 in lung adenocarcinoma cells. Epithelial-mesenchymal transition (EMT) has recently been recognized as a key element of cell invasion, migration,   metastasi  s, and drug resistance in several types of cancer, including non-small cell lung cancer (NSCLC). Our aim was to clarify microRNA (miRNA)-related mechanisms underlying EMT followed by acquired resistance to epidermal growth factor receptor tyrosine kinase inhibitor (EGFR-TKI) in NSCLC. miRNA expression profiles were examined before and after transforming growth factor β1 (TGF-β1) exposure in four human adenocarcinoma cell lines with or without EMT. Correlation between expressions of EMT-related miRNAs and resistance to EGFR-TKI gefitinib was evaluated. miRNA array and real-time quantitative reverse transcription PCR (qRT-PCR) revealed that TGF-β1 significantly induced overexpression of miR-134, miR-487b, and miR-655, which belong to the same cluster located on chromosome 14q32, in lung adenocarcinoma cells with EMT. MAGI2 (membrane-associated guanylate kinase, WW, and PDZ domain-containing protein 2), a predicted target of these miRNAs and a scaffold protein required for PTEN, was diminished in A549 cells with EMT after the TGF-β1 stimulation. Overexpression of miR-134 and miR-487b promoted the EMT phenomenon and affected the drug resistance to gefitinib, whereas knockdown of these miRNAs inhibited the EMT process and reversed TGF-β1-induced resistance to gefitinib. Our study demonstrated that the miR-134/487b/655 cluster contributed to the TGF-β1-induced EMT phenomenon and affected the resistance to gefitinib by directly targeting MAGI2, in which suppression subsequently caused loss of PTEN stability in lung cancer cells. The miR-134/miR-487b/miR-655 cluster may be a new therapeutic target in patients with advanced lung adenocarcinoma, depending on the EMT phenomenon. 
  [Cc]ell [Ii]nvasion  PubMed  
 MiR-134/487b/655 cluster regulates TGF-β-induced epithelial-mesenchymal transition and drug resistance to gefitinib by targeting MAGI2 in lung adenocarcinoma cells. Epithelial-mesenchymal transition (EMT) has recently been recognized as a key element of   cell invasio  n, migration, metastasis, and drug resistance in several types of cancer, including non-small cell lung cancer (NSCLC). Our aim was to clarify microRNA (miRNA)-related mechanisms underlying EMT followed by acquired resistance to epidermal growth factor receptor tyrosine kinase inhibitor (EGFR-TKI) in NSCLC. miRNA expression profiles were examined before and after transforming growth factor β1 (TGF-β1) exposure in four human adenocarcinoma cell lines with or without EMT. Correlation between expressions of EMT-related miRNAs and resistance to EGFR-TKI gefitinib was evaluated. miRNA array and real-time quantitative reverse transcription PCR (qRT-PCR) revealed that TGF-β1 significantly induced overexpression of miR-134, miR-487b, and miR-655, which belong to the same cluster located on chromosome 14q32, in lung adenocarcinoma cells with EMT. MAGI2 (membrane-associated guanylate kinase, WW, and PDZ domain-containing protein 2), a predicted target of these miRNAs and a scaffold protein required for PTEN, was diminished in A549 cells with EMT after the TGF-β1 stimulation. Overexpression of miR-134 and miR-487b promoted the EMT phenomenon and affected the drug resistance to gefitinib, whereas knockdown of these miRNAs inhibited the EMT process and reversed TGF-β1-induced resistance to gefitinib. Our study demonstrated that the miR-134/487b/655 cluster contributed to the TGF-β1-induced EMT phenomenon and affected the resistance to gefitinib by directly targeting MAGI2, in which suppression subsequently caused loss of PTEN stability in lung cancer cells. The miR-134/miR-487b/miR-655 cluster may be a new therapeutic target in patients with advanced lung adenocarcinoma, depending on the EMT phenomenon. 
   
  
 IFRD1 
 
  NCBI Gene  
  GeneRIF  
  Pubmed  
 
 
  Search Term  Rank  Fields  
  [Ee]xtracellular [Mm]atrix  8  PubMed(1)  
 
 
 hide/show details for IFRD1 
  [Ee]xtracellular [Mm]atrix  PubMed  
 Redifferentiation of dedifferentiated chondrocytes and chondrogenesis of human bone marrow stromal cells via chondrosphere formation with expression profiling by large-scale cDNA analysis. Characterization of dedifferentiated chondrocytes (DECs) and mesenchymal stem cells capable of differentiating into chondrocytes is of biological and clinical interest. We isolated DECs and bone marrow stromal cells (BMSCs), H4-1 and H3-4, and demonstrated that the cells started to produce extracellular matrices, such as type II collagen and aggrecan, at an early stage of chondrosphere formation. Furthermore, cDNA sequencing of cDNA libraries constricted by the oligocapping method was performed to analyze difference in mRNA expression profiling between DECs and marrow stromal cells. Upon redifferentiation of DECs, cartilage-related   extracellular matrix   genes, such as those encoding leucine-rich small proteoglycans, cartilage oligomeric matrix protein, and chitinase 3-like 1 (cartilage glycoprotein-39), were highly expressed. Growth factors such as FGF7 and CTGF were detected at a high frequency in the growth stage of monolayer stromal cultures. By combining the expression profile and flow cytometry, we demonstrated that isolated stromal cells, defined by CD34(-), c-kit(-), and CD140alpha(- or low), have chondrogenic potential. The newly established human mesenchymal cells with expression profiling provide a powerful model for a study of chondrogenic differentiation and further understanding of cartilage regeneration in the means of redifferentiated DECs and BMSCs. 
   
  
 LARS 
 
  NCBI Gene  
  GeneRIF  
  Pubmed  
 
 
  Search Term  Rank  Fields  
  [Mm]igration  6  PubMed(1); GeneRIFs(1)  
 
 
 hide/show details for LARS 
  [Mm]igration  PubMed  
 Implication of leucyl-tRNA synthetase 1 (LARS1) over-expression in growth and   migration   of lung cancer cells detected by siRNA targeted knock-down analysis. Molecular mechanism of lung carcinogenesis and its aggressive nature is still largely elusive. To uncover the biomarkers related with tumorigenesis and behavior of lung cancer, we screened novel differentially expressed genes (DEG) in A549 lung cancer cell line by comparison with CCD-25Lu, normal pulmonary epithelial cell line, using annealing control primer(ACP)-based GeneFishing system. Of the DEGs, over-expression of leucyl-tRNA synthetase 1 (LARS1) was prominent and this up-regulation was confirmed by immunoblotting and real-time quantitative RT-PCR analysis. In addition to A549 cell line, primary lung cancer tissues also expressed higher level of LARS1 mRNA than their normal counter tissues. To explore the oncogenic potential of LARS1 over-expression in lung cancer, we knocked-down LARS1 by treating siRNA and observed the tumor behavior. LARS1 knock-down cells showed reduced ability to migrate through transwell membrane and to form colonies in both soft agar and culture plate. Taken together, these findings suggest that LARS1 may play roles in   migration   and growth of lung cancer cells, which suggest its potential implication in lung tumorigenesis. 
  GeneRIFs  
 findings suggest that LARS1 may play roles in   migration   and growth of lung cancer cells, which suggest its potential implication in lung tumorigenesis 
   
  
 PTBP1 
 
  NCBI Gene  
  GeneRIF  
  Pubmed  
 
 
  Search Term  Rank  Fields  
  [Ff]ilopodia  1  PubMed(1)  
  [Mm]igration  6  PubMed(3)  
  [Ii]nvasive  10  PubMed(2)  
  [Mm]etastasis  12  PubMed(1)  
  [Cc]ell [Ii]nvasion  13  PubMed(1)  
 
 
 hide/show details for PTBP1 
  [Ff]ilopodia  PubMed  
 Regulation and functional significance of CDC42 alternative splicing in ovarian cancer. Our previous study found that splicing factor polypyrimidine tract-binding protein 1 (PTBP1) had a role in tumorigenesis but the underlying mechanism remained unclear. In this study, we observed that knockdown of PTBP1 inhibited   filopodia   formation. Subsequently, we found that PTBP1 regulated the alternative splicing of CDC42, a major regulator of   filopodia   formation. Two CDC42 variants, CDC42-v1 and CDC42-v2, can be generated through alternative splicing. Knockdown of PTBP1 increased the expression of CDC42-v2. Ectopic expression of individual variants showed that CDC42-v2 suppressed   filopodia   formation, opposite to the effect of CDC42-v1. Quantitative RT-PCR revealed that CDC42-v2 was expressed at lower levels in ovarian cancer cell lines and ovarian tumor tissues than in normal control cells and tissues. Further, CDC42-v2 was observed to have inhibitory effects on ovarian tumor cell growth, colony formation in soft agar and invasiveness. In contrast, these inhibitory effects were not found with CDC42-v1. Taken together, above results suggest that the role of PTBP1 in tumorigenesis may be partly mediated by its regulation of CDC42 alternative splicing and CDC42-v2 might function as a tumor suppressor. 
  [Mm]igration  PubMed  
 PTB deficiency causes the loss of adherens junctions in the dorsal telencephalon and leads to lethal hydrocephalus. Polypyrimidine tract-binding protein (PTB) is a well-characterized RNA-binding protein and known to be preferentially expressed in neural stem cells (NSCs) in the central nervous system; however, its role in NSCs in the developing brain remains unclear. To explore the role of PTB in embryonic NSCs in vivo, Nestin-Cre-mediated conditional Ptb knockout mice were generated for this study. In the mutant forebrain, despite the depletion of PTB protein, neither abnormal neurogenesis nor flagrant morphological abnormalities were observed at embryonic day 14.5 (E14.5). Nevertheless, by 10 weeks, nearly all mutant mice succumbed to hydrocephalus (HC), which was caused by a lack of the ependymal cell layer in the dorsal cortex. Upon further analysis, a gradual loss of adherens junctions (AJs) was observed in the ventricular zone (VZ) of the dorsal telencephalon in the mutant brains, beginning at E14.5. In the AJs-deficient VZ, impaired interkinetic nuclear   migration   and precocious differentiation of NSCs were observed after E14.5. These findings demonstrated that PTB depletion in the dorsal telencephalon is causally involved in the development of HC and that PTB is important for the maintenance of AJs in the NSCs of the dorsal telencephalon. 
 Nuclear relocalization of the pre-mRNA splicing factor PSF during apoptosis involves hyperphosphorylation, masking of antigenic epitopes, and changes in protein interactions. The spatial nuclear organization of regulatory proteins often reflects their functional state. PSF, a factor essential for pre-mRNA splicing, is visualized by the B92 mAb as discrete nuclear foci, which disappeared during apoptosis. Because this mode of cell death entails protein degradation, it was considered that PSF, which like other splicing factors is sensitive to proteolysis, might be degraded. Nonetheless, during the apoptotic process, PSF remained intact and was N-terminally hyperphosphorylated on serine and threonine residues. Retarded gel   migration   profiles suggested differential phosphorylation of the molecule in mitosis vs. apoptosis and under-phosphorylation during blockage of cells at G1/S. Experiments with the use of recombinant GFP-tagged PSF provided evidence that in the course of apoptosis the antigenic epitopes of PSF are masked and that PSF reorganizes into globular nuclear structures. In apoptotic cells, PSF dissociated from PTB and bound new partners, including the U1--70K and SR proteins and therefore may acquire new functions. 
 PTBP1-dependent regulation of USP5 alternative RNA splicing plays a role in glioblastoma tumorigenesis. Aberrant RNA splicing is thought to play a key role in tumorigenesis. The assessment of its specific contributions is limited by the complexity of information derived from genome-wide array-based approaches. We describe how performing splicing factor-specific comparisons using both tumor and cell line data sets may more readily identify physiologically relevant tumor-specific splicing events. Affymetrix exon array data derived from glioblastoma (GBM) tumor samples with defined polypyrimidine tract-binding protein 1 (PTBP1) levels were compared with data from U251 GBM cells with and without PTBP1 knockdown. This comparison yielded overlapping gene sets that comprised only a minor fraction of each data set. The identification of a novel GBM-specific splicing event involving the USP5 gene led us to further examine its role in tumorigenesis. In GBM, USP5 generates a shorter isoform 2 through recognition of a 5' splice site within exon 15. Production of the USP5 isoform 2 was strongly correlated with PTBP1 expression in GBM tumor samples and cell lines. Splicing regulation was consistent with the presence of an intronic PTBP1 binding site and could be modulated through antisense targeting of the isoform 2 splice site to force expression of isoform 1 in GBM cells. The forced expression of USP5 isoform 1 in two GBM cell lines inhibited cell growth and   migration  , implying an important role for USP5 splicing in gliomagenesis. These results support a role for aberrant RNA splicing in tumorigenesis and suggest that changes in relatively few genes may be sufficient to drive the process. 
  [Ii]nvasive  PubMed  
 Polypyrimidine tract-binding protein (PTB) differentially affects malignancy in a cell line-dependent manner. RNA processing is altered during malignant transformation, and expression of the polypyrimidine tract-binding protein (PTB) is often increased in cancer cells. Although some data support that PTB promotes cancer, the functional contribution of PTB to the malignant phenotype remains to be clarified. Here we report that although PTB levels are generally increased in cancer cell lines from multiple origins and in endometrial adenocarcinoma tumors, there appears to be no correlation between PTB levels and disease severity or metastatic capacity. The three isoforms of PTB increase heterogeneously among different tumor cells. PTB knockdown in transformed cells by small interfering RNA decreases cellular growth in monolayer culture and to a greater extent in semi-solid media without inducing apoptosis. Down-regulation of PTB expression in a normal cell line reduces proliferation even more significantly. Reduction of PTB inhibits the   invasive   behavior of two cancer cell lines in Matrigel invasion assays but enhances the   invasive   behavior of another. At the molecular level, PTB in various cell lines differentially affects the alternative splicing pattern of the same substrates, such as caspase 2. Furthermore, overexpression of PTB does not enhance proliferation, anchorage-independent growth, or invasion in immortalized or normal cells. These data demonstrate that PTB is not oncogenic and can either promote or antagonize a malignant trait dependent upon the specific intra-cellular environment. 
 Regulation and functional significance of CDC42 alternative splicing in ovarian cancer. Our previous study found that splicing factor polypyrimidine tract-binding protein 1 (PTBP1) had a role in tumorigenesis but the underlying mechanism remained unclear. In this study, we observed that knockdown of PTBP1 inhibited filopodia formation. Subsequently, we found that PTBP1 regulated the alternative splicing of CDC42, a major regulator of filopodia formation. Two CDC42 variants, CDC42-v1 and CDC42-v2, can be generated through alternative splicing. Knockdown of PTBP1 increased the expression of CDC42-v2. Ectopic expression of individual variants showed that CDC42-v2 suppressed filopodia formation, opposite to the effect of CDC42-v1. Quantitative RT-PCR revealed that CDC42-v2 was expressed at lower levels in ovarian cancer cell lines and ovarian tumor tissues than in normal control cells and tissues. Further, CDC42-v2 was observed to have inhibitory effects on ovarian tumor cell growth, colony formation in soft agar and   invasive  ness. In contrast, these inhibitory effects were not found with CDC42-v1. Taken together, above results suggest that the role of PTBP1 in tumorigenesis may be partly mediated by its regulation of CDC42 alternative splicing and CDC42-v2 might function as a tumor suppressor. 
  [Mm]etastasis  PubMed  
 Significance of Polypyrimidine Tract-Binding Protein 1 Expression in Colorectal Cancer. Polypyrimidine tract-binding protein (PTBP1) is an RNA-binding protein with various molecular functions related to RNA metabolism and a major repressive regulator of alternative splicing, causing exon skipping in numerous alternatively spliced pre-mRNAs. Here, we have investigated the role of PTBP1 in colorectal cancer. PTBP1 expression levels were significantly overexpressed in cancerous tissues compared with corresponding normal mucosal tissues. We also observed that PTBP1 expression levels, c-MYC expression levels, and PKM2:PKM1 ratio were positively correlated in colorectal cancer specimens. Moreover, PTBP1 expression levels were positively correlated to poor prognosis and lymph node   metastasis  . In analyses of colorectal cancer cells using siRNA for PTBP1, we observed that PTBP1 affects cell invasion, which was partially correlated to CD44 splicing, and this correlation was also confirmed in clinical samples. PTBP1 expression also affected anchorage-independent growth in colorectal cancer cell lines. PTBP1 expression also affected cell proliferation. Using time-lapse imaging analysis, PTBP1 was implicated in prolonged G2-M phase in HCT116 cells. As for the mechanism of prolonged G2-M phase in HCT116 siPTBP1 cells, Western blotting revealed that PTBP1 expression level was correlated to CDK11(p58) expression level, which was reported to play an important role on progression to complete mitosis. These findings indicated that PTBP1 is a potential therapeutic target for colorectal cancer. 
  [Cc]ell [Ii]nvasion  PubMed  
 Significance of Polypyrimidine Tract-Binding Protein 1 Expression in Colorectal Cancer. Polypyrimidine tract-binding protein (PTBP1) is an RNA-binding protein with various molecular functions related to RNA metabolism and a major repressive regulator of alternative splicing, causing exon skipping in numerous alternatively spliced pre-mRNAs. Here, we have investigated the role of PTBP1 in colorectal cancer. PTBP1 expression levels were significantly overexpressed in cancerous tissues compared with corresponding normal mucosal tissues. We also observed that PTBP1 expression levels, c-MYC expression levels, and PKM2:PKM1 ratio were positively correlated in colorectal cancer specimens. Moreover, PTBP1 expression levels were positively correlated to poor prognosis and lymph node metastasis. In analyses of colorectal cancer cells using siRNA for PTBP1, we observed that PTBP1 affects   cell invasion  , which was partially correlated to CD44 splicing, and this correlation was also confirmed in clinical samples. PTBP1 expression also affected anchorage-independent growth in colorectal cancer cell lines. PTBP1 expression also affected cell proliferation. Using time-lapse imaging analysis, PTBP1 was implicated in prolonged G2-M phase in HCT116 cells. As for the mechanism of prolonged G2-M phase in HCT116 siPTBP1 cells, Western blotting revealed that PTBP1 expression level was correlated to CDK11(p58) expression level, which was reported to play an important role on progression to complete mitosis. These findings indicated that PTBP1 is a potential therapeutic target for colorectal cancer. 
   
  
 POU3F2 
 
  NCBI Gene  
  GeneRIF  
  Pubmed  
 
 
  Search Term  Rank  Fields  
  [Mm]igration  6  Process(1); PubMed(4)  
  [Ii]nvasive  10  PubMed(5); GeneRIFs(2)  
  [Mm]etastasis  12  PubMed(2)  
 
 
 hide/show details for POU3F2 
  [Mm]igration  Process  
 cerebral cortex radially oriented cell   migration   
  PubMed  
 Capsaicin Inhibited Aggressive Phenotypes through Downregulation of Tumor-Associated NADH Oxidase (tNOX) by POU Domain Transcription Factor POU3F2. Capsaicin has been reported to preferentially inhibit the activity of tumor-associated NADH oxidase (tNOX), which belongs to a family of growth-related plasma membrane hydroquinone oxidases in cancer/transformed cells. The inhibitory effect of capsaicin on tNOX is associated with cell growth attenuation and apoptosis. However, no previous study has examined the transcriptional regulation of tNOX protein expression. Bioinformatic analysis has indicated that the tNOX promoter sequence harbors a binding motif for POU3F2, which is thought to play important roles in neuronal differentiation, melanocytes growth/differentiation and tumorigenesis. In this study, we found that capsaicin-mediated tNOX downregulation and cell   migration   inhibition were through POU3F2. The protein expression levels of POU3F2 and tNOX are positively correlated, and that overexpression of POU3F2 (and the corresponding upregulation of tNOX) enhanced the proliferation,   migration   and invasion in AGS (human gastric carcinoma) cells. In contrast, knockdown of POU3F2 downregulates tNOX, and the cancer phenotypes are affected. These findings not only shed light on the molecular mechanism of the anticancer properties of capsaicin, but also the transcription regulation of tNOX expression that may potentially explain how POU3F2 is associated with tumorigenesis. 
 NFIB Mediates BRN2 Driven Melanoma Cell   Migration   and Invasion Through Regulation of EZH2 and MITF. While invasion and metastasis of tumour cells are the principle factor responsible for cancer related deaths, the mechanisms governing the process remain poorly defined. Moreover, phenotypic divergence of sub-populations of tumour cells is known to underpin alternative behaviors linked to tumour progression such as proliferation, survival and invasion. In the context of melanoma, heterogeneity between two transcription factors, BRN2 and MITF, has been associated with phenotypic switching between predominantly invasive and proliferative behaviors respectively. Epigenetic changes, in response to external cues, have been proposed to underpin this process, however the mechanism by which the phenotypic switch occurs is unclear. Here we report the identification of the NFIB transcription factor as a novel downstream effector of BRN2 function in melanoma cells linked to the migratory and invasive characteristics of these cells. Furthermore, the function of NFIB appears to drive an invasive phenotype through an epigenetic mechanism achieved via the upregulation of the polycomb group protein EZH2. A notable target of NFIB mediated up-regulation of EZH2 is decreased MITF expression, which further promotes a less proliferative, more invasive phenotype. Together our data reveal that NFIB has the ability to promote dynamic changes in the chromatin state of melanoma cells to facilitate   migration  , invasion and metastasis. 
 Brn-2 represses microphthalmia-associated transcription factor expression and marks a distinct subpopulation of microphthalmia-associated transcription factor-negative melanoma cells. The origin of tumor heterogeneity is poorly understood, yet it represents a major barrier to effective therapy. In melanoma and in melanocyte development, the microphthalmia-associated transcription factor (Mitf) controls survival, differentiation, proliferation, and   migration  /metastasis. The Brn-2 (N-Oct-3, POU3F2) transcription factor also regulates melanoma proliferation and is up-regulated by BRAF and beta-catenin, two key melanoma-associated signaling molecules. Here, we show that Brn-2 also regulates invasiveness and directly represses Mitf expression. Remarkably, in melanoma biopsies, Mitf and Brn-2 each mark a distinct subpopulation of melanoma cells, providing a striking illustration of melanoma tumor heterogeneity with implications for melanoma therapy. 
 BRN2 is a transcriptional repressor of CDH13 (T-cadherin) in melanoma cells. T-cadherin (cadherin 13, H-cadherin, gene name CDH13) has been proposed to act as a tumor-suppressor gene as its expression is significantly diminished in several types of carcinomas, including melanomas. Allelic loss and promoter hypermethylation have been proposed as mechanisms for silencing of CDH13. However, they do not account for loss of T-cadherin expression in all carcinomas, and other genetic or epigenetic alterations can be presumed. The present study investigated transcriptional regulation of CDH13 in melanoma. Bioinformatical analysis pointed to the presence of known BRN2 (also known as POU3F2 and N-Oct-3)-binding motifs in the CDH13 promoter sequence. We found an inverse correlation between BRN2 and T-cadherin protein and transcript expression. Reporter gene analysis and electrophoretic mobility shift assays in melanoma cells demonstrated that CDH13 is a direct target of BRN2 and that BRN2 is a functional transcriptional repressor of CDH13 promoter activity. The regulatory binding element of BRN2 was located -219 bp of the CDH13 promoter proximal to the start codon and was identified as 5'-CATGCAAAA-3'. Ectopic expression of BRN2 in BRN2-negative/T-cadherin-positive melanoma cells resulted in suppression of CDH13 promoter activity, whereas BRN2 knockdown in BRN2-positive/T-cadherin-negative melanoma cells resulted in re-expression of T-cadherin transcripts and protein. Transcriptional repression of CDH13 by BRN2 may participate in malignant transformation of melanoma by increasing invasion an  d migrati  on potentials of melanoma cells. The study has identified CDH13 as a novel direct BRN2 transcriptional target gene and has advanced knowledge of mechanisms underlying loss of T-cadherin expression in melanoma. 
  [Ii]nvasive  PubMed  
 NFIB Mediates BRN2 Driven Melanoma Cell Migration and Invasion Through Regulation of EZH2 and MITF. While invasion and metastasis of tumour cells are the principle factor responsible for cancer related deaths, the mechanisms governing the process remain poorly defined. Moreover, phenotypic divergence of sub-populations of tumour cells is known to underpin alternative behaviors linked to tumour progression such as proliferation, survival and invasion. In the context of melanoma, heterogeneity between two transcription factors, BRN2 and MITF, has been associated with phenotypic switching between predominantly   invasive   and proliferative behaviors respectively. Epigenetic changes, in response to external cues, have been proposed to underpin this process, however the mechanism by which the phenotypic switch occurs is unclear. Here we report the identification of the NFIB transcription factor as a novel downstream effector of BRN2 function in melanoma cells linked to the migratory and   invasive   characteristics of these cells. Furthermore, the function of NFIB appears to drive an   invasive   phenotype through an epigenetic mechanism achieved via the upregulation of the polycomb group protein EZH2. A notable target of NFIB mediated up-regulation of EZH2 is decreased MITF expression, which further promotes a less proliferative, more   invasive   phenotype. Together our data reveal that NFIB has the ability to promote dynamic changes in the chromatin state of melanoma cells to facilitate migration, invasion and metastasis. 
 A phosphatidylinositol 3-kinase-Pax3 axis regulates Brn-2 expression in melanoma. Deregulation of transcription arising from mutations in key signaling pathways is a hallmark of cancer. In melanoma, the most aggressive and lethal form of skin cancer, the Brn-2 transcription factor (POU3F2) regulates proliferation and   invasive  ness and lies downstream from mitogen-activated protein kinase (MAPK) and Wnt/β-catenin, two melanoma-associated signaling pathways. In vivo Brn-2 represses expression of the microphthalmia-associated transcription factor, MITF, to drive cells to a more stem cell-like and   invasiv  e phenotype. Given the key role of Brn-2 in regulating melanoma biology, understanding the signaling pathways that drive Brn-2 expression is an important issue. Here, we show that inhibition of phosphatidylinositol 3-kinase (PI3K) signaling reduces   invasiv  eness of melanoma cells in culture and strongly inhibits Brn-2 expression. Pax3, a transcription factor regulating melanocyte lineage-specific genes, directly binds and regulates the Brn-2 promoter, and Pax3 expression is also decreased upon PI3K inhibition. Collectively, our results highlight a crucial role for PI3K in regulating Brn-2 and Pax3 expression, reveal a mechanism by which PI3K can regulate   invasiv  eness, and imply that PI3K signaling is a key determinant of melanoma subpopulation diversity. Together with our previous work, the results presented here now place Brn-2 downstream of three melanoma-associated signaling pathways. 
 Melanoma cell   invasive  ness is regulated by miR-211 suppression of the BRN2 transcription factor. To identify microRNAs potentially involved in melanomagenesis, we compared microRNA expression profiles between melanoma cell lines and cultured melanocytes. The most differentially expressed microRNA between the normal and tumor cell lines was miR-211. We focused on this pigment-cell-enriched miRNA as it is derived from the microphthalmia-associated transcription factor (MITF)-regulated gene, TRPM1 (melastatin). We find that miR-211 expression is greatly decreased in melanoma cells and melanoblasts compared to melanocytes. Bioinformatic analysis identified a large number of potential targets of miR-211, including POU3F2 (BRN2). Inhibition of miR-211 in normal melanocytes resulted in increased BRN2 protein, indicating that endogenous miR-211 represses BRN2 in differentiated cells. Over-expression of miR-211 in melanoma cell lines changed the   invasive   potential of the cells in vitro through directly targeting BRN2 translation. We propose a model for the apparent non-overlapping expression levels of BRN2 and MITF in melanoma, mediated by miR-211 expression. 
 Brn-2 represses microphthalmia-associated transcription factor expression and marks a distinct subpopulation of microphthalmia-associated transcription factor-negative melanoma cells. The origin of tumor heterogeneity is poorly understood, yet it represents a major barrier to effective therapy. In melanoma and in melanocyte development, the microphthalmia-associated transcription factor (Mitf) controls survival, differentiation, proliferation, and migration/metastasis. The Brn-2 (N-Oct-3, POU3F2) transcription factor also regulates melanoma proliferation and is up-regulated by BRAF and beta-catenin, two key melanoma-associated signaling molecules. Here, we show that Brn-2 also regulates   invasive  ness and directly represses Mitf expression. Remarkably, in melanoma biopsies, Mitf and Brn-2 each mark a distinct subpopulation of melanoma cells, providing a striking illustration of melanoma tumor heterogeneity with implications for melanoma therapy. 
 Genome-wide analysis of POU3F2/BRN2 promoter occupancy in human melanoma cells reveals Kitl as a novel regulated target gene. POU3F2 is a POU-Homeodomain transcription factor expressed in neurons and melanoma cells. In melanoma lesions, cells expressing high levels of POU3F2 show enhanced   invasive   and metastatic capacity that can in part be explained by repression of Micropthalmia-associated Transcription Factor (MITF) expression via POU3F2 binding to its promoter. To identify other POU3F2 target genes that may be involved in modulating the properties of melanoma cells, we performed ChIP-chip experiments in 501Mel melanoma cells. 2108 binding loci located in the regulatory regions of 1700 potential target genes were identified. Bioinformatic and experimental assays showed the presence of known POU3F2-binding motifs, but also many AT-rich sequences with only partial similarity to the known motifs at the occupied loci. Functional analysis indicates that POU3F2 regulates the stem cell factor (Kit ligand, Kitl) promoter via a cluster of four closely spaced binding sites located in the proximal promoter. Our results suggest that POU3F2 may regulate the properties of melanoma cells via autocrine KIT ligand signalling. 
  GeneRIFs  
 PI3K has a crucial role in regulating Brn-2 and Pax3 expression and neoplasm   invasive  ness, and it is a key determinant of melanoma subpopulation diversity. 
 Brn-2 regulates   invasive  ness and directly represses Mitf expression. 
  [Mm]etastasis  PubMed  
 NFIB Mediates BRN2 Driven Melanoma Cell Migration and Invasion Through Regulation of EZH2 and MITF. While invasion and   metastasis   of tumour cells are the principle factor responsible for cancer related deaths, the mechanisms governing the process remain poorly defined. Moreover, phenotypic divergence of sub-populations of tumour cells is known to underpin alternative behaviors linked to tumour progression such as proliferation, survival and invasion. In the context of melanoma, heterogeneity between two transcription factors, BRN2 and MITF, has been associated with phenotypic switching between predominantly invasive and proliferative behaviors respectively. Epigenetic changes, in response to external cues, have been proposed to underpin this process, however the mechanism by which the phenotypic switch occurs is unclear. Here we report the identification of the NFIB transcription factor as a novel downstream effector of BRN2 function in melanoma cells linked to the migratory and invasive characteristics of these cells. Furthermore, the function of NFIB appears to drive an invasive phenotype through an epigenetic mechanism achieved via the upregulation of the polycomb group protein EZH2. A notable target of NFIB mediated up-regulation of EZH2 is decreased MITF expression, which further promotes a less proliferative, more invasive phenotype. Together our data reveal that NFIB has the ability to promote dynamic changes in the chromatin state of melanoma cells to facilitate migration, invasion and   metastasis  . 
 Brn-2 represses microphthalmia-associated transcription factor expression and marks a distinct subpopulation of microphthalmia-associated transcription factor-negative melanoma cells. The origin of tumor heterogeneity is poorly understood, yet it represents a major barrier to effective therapy. In melanoma and in melanocyte development, the microphthalmia-associated transcription factor (Mitf) controls survival, differentiation, proliferation, and migration/  metastasis  . The Brn-2 (N-Oct-3, POU3F2) transcription factor also regulates melanoma proliferation and is up-regulated by BRAF and beta-catenin, two key melanoma-associated signaling molecules. Here, we show that Brn-2 also regulates invasiveness and directly represses Mitf expression. Remarkably, in melanoma biopsies, Mitf and Brn-2 each mark a distinct subpopulation of melanoma cells, providing a striking illustration of melanoma tumor heterogeneity with implications for melanoma therapy. 
   
  
 POU3F3 
 
  NCBI Gene  
  GeneRIF  
  Pubmed  
 
 
  Search Term  Rank  Fields  
  [Mm]igration  6  Process(1); PubMed(2)  
  [Mm]etastasis  12  PubMed(1)  
 
 
 hide/show details for POU3F3 
  [Mm]igration  Process  
 cerebral cortex radially oriented cell   migration   
  PubMed  
 Knockdown of linc-POU3F3 suppresses the proliferation, apoptosis, and   migration   resistance of colorectal cancer. Long intergenic noncoding RNAs (lincRNAs) play important roles in regulating the biological functions and underlying molecular mechanisms of colorectal cancer (CRC). Here, we investigated the association of linc-POU3F3 and prognosis in CRC. We demonstrated that linc-POU3F3 was overexpressed in CRC tissues and positively correlated with tumor grade and N stage. Inhibition of linc-POU3F3 resulted in inhibition of cell proliferation and G1 cell cycle arrest, which was mediated by cyclin D1, CDK4, p18, Rb, and phosphorylated Rb. Inhibition of linc-POU3F3 induced apoptosis, and suppressed   migration   and invasion in LOVO and SW480 cell lines. This inhibition also increased the expressions of epithelial markers and decreased the expressions of mesenchymal markers, thus inhibiting the cancer epithelial-mesenchymal transition. The decreased   migration   and invasion following linc-POU3F3 knockdown were mediated by an increased BMP signal. Furthermore, autophagy was enhanced by linc-POU3F3 knockdown, suggesting the involvement of autophagy in the induced apoptosis. Collectively, linc-POU3F3 might be crucial in pro-proliferation, anti-apoptosis, and metastasis in LOVO and SW480 cells by regulating the cell cycle, intrinsic apoptosis, BMP signaling and autophagy. Thus, linc-POU3F3 is a potential therapeutic target and novel molecular biomarker for CRC. 
 POU-III transcription factors (Brn1, Brn2, and Oct6) influence neurogenesis, molecular identity, and migratory destination of upper-layer cells of the cerebral cortex. The upper layers (II-IV) are the most prominent distinguishing feature of mammalian neocortex compared with avian or reptilian dorsal cortex, and are vastly expanded in primates. Although the time-dependent embryonic generation of upper-layer cells is genetically instructed within their parental progenitors, mechanisms governing cell-intrinsic fate transitions remain obscure. POU-homeodomain transcription factors Pou3f3 and Pou3f2 (Brn1 and Brn2) are known to label postmitotic upper-layer cells, and are redundantly required for their production. We find that the onset of Pou3f3/2 expression actually occurs in ventricular zone (VZ) progenitors, and that Pou3f3/2 subsequently label neural progeny switching from deep-layer Ctip2(+) identity to Satb2(+) upper-layer fate as they migrate to proper superficial positions. By using an Engrailed dominant-negative repressor, we show that sustained neurogenesis after the deep- to upper-layer transition requires the proneual action of Pou3fs in VZ progenitors. Conversely, single-gene overexpression of any Pou3f in early neural progenitors is sufficient to specify the precocious birth of Satb2(+) daughter neurons that extend axons to the contralateral hemisphere, as well as exhibit robust pia-directed   migration   that is characteristic of upper-layer cells. Finally, we demonstrate that Pou3fs influence multiple stages of neurogenesis by suppressing Notch effector Hes5, and promoting the expression of proneural transcription factors Tbr2 and Tbr1. 
  [Mm]etastasis  PubMed  
 Knockdown of linc-POU3F3 suppresses the proliferation, apoptosis, and migration resistance of colorectal cancer. Long intergenic noncoding RNAs (lincRNAs) play important roles in regulating the biological functions and underlying molecular mechanisms of colorectal cancer (CRC). Here, we investigated the association of linc-POU3F3 and prognosis in CRC. We demonstrated that linc-POU3F3 was overexpressed in CRC tissues and positively correlated with tumor grade and N stage. Inhibition of linc-POU3F3 resulted in inhibition of cell proliferation and G1 cell cycle arrest, which was mediated by cyclin D1, CDK4, p18, Rb, and phosphorylated Rb. Inhibition of linc-POU3F3 induced apoptosis, and suppressed migration and invasion in LOVO and SW480 cell lines. This inhibition also increased the expressions of epithelial markers and decreased the expressions of mesenchymal markers, thus inhibiting the cancer epithelial-mesenchymal transition. The decreased migration and invasion following linc-POU3F3 knockdown were mediated by an increased BMP signal. Furthermore, autophagy was enhanced by linc-POU3F3 knockdown, suggesting the involvement of autophagy in the induced apoptosis. Collectively, linc-POU3F3 might be crucial in pro-proliferation, anti-apoptosis, and   metastasis   in LOVO and SW480 cells by regulating the cell cycle, intrinsic apoptosis, BMP signaling and autophagy. Thus, linc-POU3F3 is a potential therapeutic target and novel molecular biomarker for CRC. 
   
  
 ARAP3 
 
  NCBI Gene  
  GeneRIF  
  Pubmed  
 
 
  Search Term  Rank  Fields  
  [Mm]igration  6  Process(1); PubMed(1)  
  ECM  7  PubMed(1)  
  [Ee]xtracellular [Mm]atrix  8  PubMed(2)  
  [Ii]nvasive  10  PubMed(1)  
 
 
 hide/show details for ARAP3 
  [Mm]igration  Process  
 negative regulation of cell   migration   
  PubMed  
 Identification and structural basis for a novel interaction between Vav2 and Arap3. Vav2 is a ubiquitous guanine nucleotide exchange factor (GEF) for the small GTPase Rac1. It regulates processes including cell   migration  , neuronal development and phagocytosis through interactions with different proteins. In this study, Arap3, a dual GTPase-activating protein (GAP) for RhoA and Arf6, was first identified to be a novel interaction partner for Vav2 both in vitro and in vivo. ITC and NMR chemical shift perturbation experiments demonstrated that Vav2 SH2 domain was able to interact directly with phosphorylated Y1403 and Y1408 within the C-terminal region of Arap3 with high affinities, with the dissociation constants (Kd) of ≈ 0.27 and ≈ 1.40 μM, respectively. In addition, using different phosphotyrosine peptides, the pY +3 specificity of Vav2 SH2 domain was discovered. The solution structures of Vav2 SH2 domain in free and in complex with the phosphotyrosine peptide pY1408 were therefore determined to understand the structural basis of this recognition specificity. Structural analysis revealed that the presence of a Phe residue in the BG loop (BG6) leads to the formation of a shallow hydrophobic pY +3 pocket on the surface of Vav2 SH2 domain, which determines the pY +3 specificity of Vav2 SH2 domain. 
  ECM  PubMed  
 ARAP3 is transiently tyrosine phosphorylated in cells attaching to fibronectin and inhibits cell spreading in a RhoGAP-dependent manner. ARAP3 is a GTPase activating protein (GAP) for Rho and Arf GTPases that is implicated in phosphoinositide 3-kinase (PI 3-kinase) signalling pathways controlling lamellipodia formation and actin stress fibre assembly. We have identified ARAP3 as a phosphorylated target of protein tyrosine kinases. In cells, ARAP3 was tyrosine phosphorylated when co-expressed with Src-family kinases (SFKs), upon stimulation with growth factors and during adhesion to the extracellular matrix (  ECM  ) substrate fibronectin. Adhesion-induced phosphorylation of ARAP3 was suppressed by selective inhibitors of Src-family kinases and PI 3-kinase and by a Src dominant interfering mutant. Inducible expression of ARAP3 in HEK293 epithelial cells resulted in increased cell rounding, membrane process formation and cell clustering on   ECM   substrates. In contrast, ARAP3 dramatically slowed the kinetics of cell spreading on fibronectin but had no effect on cell adhesion. These effects of ARAP3 required a functional Rho GAP domain and were associated with reduced cellular levels of active RhoA and Rac1 but did not require the sterile alpha motif (SAM) or Arf GAP domains. Mutation of two phosphorylation sites, Y1399 and Y1404, enhanced some ARAP3 activities, suggesting that ARAP3 may be negatively regulated by phosphorylation on these tyrosine residues. These results implicate ARAP3 in integrin-mediated tyrosine kinase signalling pathways controlling Rho GTPases and cell spreading. 
  [Ee]xtracellular [Mm]atrix  PubMed  
 ARAP3 inhibits peritoneal dissemination of scirrhous gastric carcinoma cells by regulating cell adhesion and invasion. During the analysis of phosphotyrosine-containing proteins in scirrhous gastric carcinoma cell lines, we observed an unusual expression of Arf-GAP with Rho-GAP domain, ankyrin repeat and PH domain 3 (ARAP3), a multimodular signaling protein that is a substrate of Src family kinases. Unlike other phosphotyrosine proteins, such as CUB domain-containing protein 1 (CDCP1) and Homo sapiens chromosome 9 open reading frame 10/oxidative stress-associated Src activator (C9orf10/Ossa), which are overexpressed and hyperphosphorylated in scirrhous gastric carcinoma cell lines, ARAP3 was underexpressed in cancerous human gastric tissues. In this study, we found that overexpression of ARAP3 in the scirrhous gastric carcinoma cell lines significantly reduced peritoneal dissemination. In vitro studies also showed that ARAP3 regulated cell attachment to the   extracellular matrix  , as well as invasive activities. These effects were suppressed by mutations in the Rho-GTPase-activating protein (GAP) domain or in the C-terminal two tyrosine residues that are phosphorylated by Src. Thus, the expression and phosphorylation state of ARAP3 may affect the invasiveness of cancer by modulating cell adhesion and motility. Our results suggest that ARAP3 is a unique Src substrate that suppresses peritoneal dissemination of scirrhous gastric carcinoma cells. 
 ARAP3 is transiently tyrosine phosphorylated in cells attaching to fibronectin and inhibits cell spreading in a RhoGAP-dependent manner. ARAP3 is a GTPase activating protein (GAP) for Rho and Arf GTPases that is implicated in phosphoinositide 3-kinase (PI 3-kinase) signalling pathways controlling lamellipodia formation and actin stress fibre assembly. We have identified ARAP3 as a phosphorylated target of protein tyrosine kinases. In cells, ARAP3 was tyrosine phosphorylated when co-expressed with Src-family kinases (SFKs), upon stimulation with growth factors and during adhesion to the   extracellular matrix   (ECM) substrate fibronectin. Adhesion-induced phosphorylation of ARAP3 was suppressed by selective inhibitors of Src-family kinases and PI 3-kinase and by a Src dominant interfering mutant. Inducible expression of ARAP3 in HEK293 epithelial cells resulted in increased cell rounding, membrane process formation and cell clustering on ECM substrates. In contrast, ARAP3 dramatically slowed the kinetics of cell spreading on fibronectin but had no effect on cell adhesion. These effects of ARAP3 required a functional Rho GAP domain and were associated with reduced cellular levels of active RhoA and Rac1 but did not require the sterile alpha motif (SAM) or Arf GAP domains. Mutation of two phosphorylation sites, Y1399 and Y1404, enhanced some ARAP3 activities, suggesting that ARAP3 may be negatively regulated by phosphorylation on these tyrosine residues. These results implicate ARAP3 in integrin-mediated tyrosine kinase signalling pathways controlling Rho GTPases and cell spreading. 
  [Ii]nvasive  PubMed  
 ARAP3 inhibits peritoneal dissemination of scirrhous gastric carcinoma cells by regulating cell adhesion and invasion. During the analysis of phosphotyrosine-containing proteins in scirrhous gastric carcinoma cell lines, we observed an unusual expression of Arf-GAP with Rho-GAP domain, ankyrin repeat and PH domain 3 (ARAP3), a multimodular signaling protein that is a substrate of Src family kinases. Unlike other phosphotyrosine proteins, such as CUB domain-containing protein 1 (CDCP1) and Homo sapiens chromosome 9 open reading frame 10/oxidative stress-associated Src activator (C9orf10/Ossa), which are overexpressed and hyperphosphorylated in scirrhous gastric carcinoma cell lines, ARAP3 was underexpressed in cancerous human gastric tissues. In this study, we found that overexpression of ARAP3 in the scirrhous gastric carcinoma cell lines significantly reduced peritoneal dissemination. In vitro studies also showed that ARAP3 regulated cell attachment to the extracellular matrix, as well as   invasive   activities. These effects were suppressed by mutations in the Rho-GTPase-activating protein (GAP) domain or in the C-terminal two tyrosine residues that are phosphorylated by Src. Thus, the expression and phosphorylation state of ARAP3 may affect the   invasive  ness of cancer by modulating cell adhesion and motility. Our results suggest that ARAP3 is a unique Src substrate that suppresses peritoneal dissemination of scirrhous gastric carcinoma cells. 
   
  
 KCNJ2 
 
  NCBI Gene  
  GeneRIF  
  Pubmed  
 
 
  Search Term  Rank  Fields  
  [Mm]igration  6  PubMed(1)  
  [Ee]xtracellular [Mm]atrix  8  PubMed(2)  
 
 
 hide/show details for KCNJ2 
  [Mm]igration  PubMed  
 Effects of BKCa and Kir2.1 Channels on Cell Cycling Progression and   Migration   in Human Cardiac c-kit+ Progenitor Cells. Our previous study demonstrated that a large-conductance Ca2+-activated K+ current (BKCa), a voltage-gated TTX-sensitive sodium current (INa.TTX), and an inward rectifier K+ current (IKir) were heterogeneously present in most of human cardiac c-kit+ progenitor cells. The present study was designed to investigate the effects of these ion channels on cell cycling progression and   migration   of human cardiac c-kit+ progenitor cells with approaches of cell proliferation and mobility assays, siRNA, RT-PCR, Western blots, flow cytometry analysis, etc. It was found that inhibition of BKCa with paxilline, but not INa.TTX with tetrodotoxin, decreased both cell proliferation and   migration  . Inhibition of IKir with Ba2+ had no effect on cell proliferation, while enhanced cell mobility. Silencing KCa.1.1 reduced cell proliferation by accumulating the cells at G0/G1 phase and decreased cell mobility. Interestingly, silencing Kir2.1 increased the cell   migration   without affecting cell cycling progression. These results demonstrate the novel information that blockade or silence of BKCa channels, but not INa.TTX channels, decreases cell cycling progression and mobility, whereas inhibition of Kir2.1 channels increases cell mobility without affecting cell cycling progression in human cardiac c-kit+ progenitor cells. 
  [Ee]xtracellular [Mm]atrix  PubMed  
 Genome-wide meta-analyses of multiancestry cohorts identify multiple new susceptibility loci for refractive error and myopia. Refractive error is the most common eye disorder worldwide and is a prominent cause of blindness. Myopia affects over 30% of Western populations and up to 80% of Asians. The CREAM consortium conducted genome-wide meta-analyses, including 37,382 individuals from 27 studies of European ancestry and 8,376 from 5 Asian cohorts. We identified 16 new loci for refractive error in individuals of European ancestry, of which 8 were shared with Asians. Combined analysis identified 8 additional associated loci. The new loci include candidate genes with functions in neurotransmission (GRIA4), ion transport (KCNQ5), retinoic acid metabolism (RDH5),   extracellular matrix   remodeling (LAMA2 and BMP2) and eye development (SIX6 and PRSS56). We also confirmed previously reported associations with GJD2 and RASGRF1. Risk score analysis using associated SNPs showed a tenfold increased risk of myopia for individuals carrying the highest genetic load. Our results, based on a large meta-analysis across independent multiancestry studies, considerably advance understanding of the mechanisms involved in refractive error and myopia. 
 The inward rectifier potassium channel Kir2.1 is required for osteoblastogenesis. Andersen's syndrome (AS) is a rare and dominantly inherited pathology, linked to the inwardly rectifying potassium channel Kir2.1. AS patients exhibit a triad of symptoms that include periodic paralysis, cardiac dysrhythmia and bone malformations. Some progress has been made in understanding the contribution of the Kir2.1 channel to skeletal and cardiac muscle dysfunctions, but its role in bone morphogenesis remains unclear. We isolated myoblast precursors from muscle biopsies of healthy individuals and typical AS patients with dysmorphic features. Myoblast cultures underwent osteogenic differentiation that led to   extracellular matrix   mineralization. Osteoblastogenesis was monitored through the activity of alkaline phosphatase, and through the hydroxyapatite formation using Alizarin Red and Von Kossa staining techniques. Patch-clamp recordings revealed the presence of an inwardly rectifying current in healthy cells that was absent in AS osteoblasts, showing the dominant-negative effect of the Kir2.1 mutant allele in osteoblasts. We also found that while control cells actively synthesize hydroxyapatite, AS osteoblasts are unable to efficiently form any   extracellular matrix  . To further demonstrate the role of the Kir2.1 channels during the osteogenesis, we inhibited Kir2.1 channel activity in healthy patient cells by applying extracellular Ba(2+) or using adenoviruses carrying mutant Kir2.1 channels. In both cases, cells were no longer able to produce   extracellular matrix  es. Moreover, osteogenic activity of AS osteoblasts was restored by rescue experiments, via wild-type Kir2.1 channel overexpression. These observations provide a proof that normal Kir2.1 channel function is essential during osteoblastogenesis. 
   
  
 ANXA11 
 
  NCBI Gene  
  GeneRIF  
  Pubmed  
 
 
  Search Term  Rank  Fields  
  [Mm]igration  6  PubMed(1)  
  [Ii]nvasive  10  PubMed(2)  
  [Mm]etastasis  12  PubMed(1)  
 
 
 hide/show details for ANXA11 
  [Mm]igration  PubMed  
 Suppression of annexin A11 in ovarian cancer: implications in chemoresistance. Ovarian cancer patients treated with cisplatin-based chemotherapy often develop acquired cisplatin resistance and, consequently, cancer recurrence. We have previously reported that annexin A11 is associated with cisplatin resistance and related to tumor recurrence in ovarian cancer patients. In this study, we used small interfering RNA to suppress annexin A11 expression in ovarian cancer cells followed by various in vitro assays. We showed that knockdown of annexin A11 expression reduced cell proliferation and colony formation ability of ovarian cancer cells. Epigenetic silencing of annexin A11 conferred cisplatin resistance to ovarian cancer cells. Through a comprehensive time course study of cisplatin response in ovarian cancer cells with/without suppression of annexin A11 expression using whole-genome oligonucleotide microarrays, we identified a set of differentially expressed genes associated with annexin A11 expression and some patterns of gene expressions in response to cisplatin exposure. These identified genes/patterns were further validated by real-time polymerase chain reaction and immunoblot analysis. Many of them such as HMOX1, TGFBI, LY6D, S100P, EIF4EBP2, DHRS2, and PCSK9 have been involved in apoptosis, cell cycling/proliferation, cell adhesion/  migration  , transcription regulation, and signal transduction. In addition, immunohistochemistry analyses indicated that annexin A11 immunointensity inversely correlated with HMOX1 immunoreactivity in 142 ovarian cancer patients. In contrast to annexin A11, HMOX1 immunoreactivity positively correlated with in vitro cisplatin resistance in ovarian cancers. Collectively, annexin A11 is directly involved in cell proliferation and cisplatin resistance of ovarian cancer. Manipulation of annexin A11 and its associated genes may represent a novel therapeutic strategy in human ovarian cancers. 
  [Ii]nvasive  PubMed  
 Prostate cancer-associated autoantibodies in serum against tumor-associated antigens as potential new biomarkers. The limitations of the current prostate cancer (PCa) screening tests demands new biomarkers for early diagnosis of PCa. In this study, we aim to investigate serum autoantibody signatures as PCa specific biomarkers. PCa proteins were resolved by 2-DE and then transferred onto polyvinylidene difluoride membrane, which were subsequently incubated with either pooled serum from PCa patients or from normal controls. Mass spectrometry results have identified 18 antigens from 21 different 2-DE spots associated with PCa. Autoantibody response to antigens PRDX2, PRDX6 and ANXA11 in PCa patient's sera was confirmed using recombinant antigens. Further validation with an independent set of PCa patient's sera have shown relatively increased abundance of PRDX6 and ANXA11 antibodies in PCa patients. Formal concept analysis method was applied to assess whether the abundance of these autoantibodies could influence the classification of patients. However, sensitivity of the single antibody to discriminate prostate tumor and healthy controls varies from 70% to 80%, whereas combination of both PRDX6 and ANXA11 antibodies increased sensitivity to 90% for tumors and 100% for healthy controls. Therefore, we hereby report that the detection of these antibodies in PCa patient's serum in combination with the existing non-  invasive   diagnostic procedures may have significance in PCa diagnosis.The present study aimed to investigate serum autoantibody signatures as new biomarkers for early diagnosis of prostate cancer (PCa). To investigate serum autoantibodies in patients with PCa, we used proteomics approach based on two-dimensional gel electrophoresis (2-DE) and mass spectrometry. Total tissue proteins extracted from prostate were separated by 2-DE and then transferred onto polyvinylidene difluoride (PVDF) membrane, which were subsequently incubated with either pooled serum from PCa patients or from normal controls with no history for PCa. Proteomic analysis results have identified 18 antigens that showed antibody response specifically to cancer patient's serum. For validation experiments using recombinant antigens, confirmed autoantibody response to three antigens PRDX2, PRDX6 and ANXA11. Further validation using a second independent set of PCa patient's sera has shown relatively increased abundance of PRDX6 and ANXA11 antibodies specifically in PCa patients. Partition analysis of patients based on abundance of autoantibodies highlighted a combination of both PRDX6 and ANXA11 antibodies in serum with 90% sensitivity in case of tumors and 100% in case of healthy controls. Therefore, we hereby report that the detection of these antibodies in PCa patient's serum in combination with known markers may have significance in diagnosis of PCa with further validation in larger cohort of samples. 
 S100A6 binds to annexin 2 in pancreatic cancer cells and promotes pancreatic cancer cell motility. High levels of S100A6 have been associated with poor outcome in pancreatic cancer patients. The functional role of S100A6 is, however, poorly understood.Immunoprecipitation followed by two-dimensional gel electrophoresis and mass spectrometry were undertaken to identify S100A6 interacting proteins in pancreatic cancer cells. Immunohistochemistry and coimmunofluorescence were performed to examine expression or colocalisation of proteins. siRNA was used to deplete specific proteins and effects on motility were measured using Boyden Chamber and wound healing assays.Our proteomic screen to identify S100A6 interacting proteins revealed annexin 11, annexin 2, tropomyosin beta and a candidate novel interactor lamin B1. Of these, annexin 2 was considered particularly interesting, as, like S100A6, it is expressed early in the development of pancreatic cancer and overexpression occurs with high frequency in   invasive   cancer. Reciprocal immunoprecipitation confirmed the interaction between annexin 2 and S100A6 and the proteins colocalised, particularly in the plasma membrane of cultured pancreatic cancer cells and primary pancreatic tumour tissue. Analysis of primary pancreatic cancer specimens (n=55) revealed a strong association between high levels of cytoplasmic S100A6 and the presence of annexin 2 in the plasma membrane of cancer cells (P=0.009). Depletion of S100A6 was accompanied by diminished levels of membrane annexin 2 and caused a pronounced reduction in the motility of pancreatic cancer cells.These findings point towards a functional role for S100A6 that may help explain the link between S100A6 expression in pancreatic cancer and aggressive disease. 
  [Mm]etastasis  PubMed  
 Annexin A11 in disease. Ubiquitously expressed in many cell types, annexin A11 (Anxa11) is a member of the multigene family of Ca(2+)-regulated phospholipid-dependent and membrane-binding annexin proteins. Studies have shown that Anxa11 plays an important role in cell division, Ca(2+) signaling, vesicle trafficking and apoptosis. The deregulation and mutation of Anxa11 are involved in systemic autoimmune diseases, sarcoidosis and the development, chemoresistance and recurrence of cancers. Malfunction of Anxa11 may lead to or enhance the   metastasis  , invasion and drug resistance of cancers through the platelet-derived growth factor receptor (PDGFR) pathway and/or the mitogen-activated protein kinase (MAPK)/p53 pathway. In a variety of diseases, Anxa11 is most commonly reported to function through interactions with apoptosis-linked gene-2 protein (ALG-2) and/or calcyclin (S100A6). Although it has been little studied, Anxa11 is a promising biomarker for the diagnosis, treatment and prognosis of certain diseases. In this review, the associations of Anxa11 with Ca(2+)-regulated exocytosis, cytokinesis, sex differentiation, autoimmune diseases, thrombolysis and cancers are summarized and interpreted. 
   
  
 QPCT 
 
  NCBI Gene  
  GeneRIF  
  Pubmed  
 
 
  Search Term  Rank  Fields  
  [Mm]igration  6  PubMed(1)  
  [Mm]etastasis  12  PubMed(1)  
 
 
 hide/show details for QPCT 
  [Mm]igration  PubMed  
 Role of glutaminyl cyclases in thyroid carcinomas. CCL2 is a chemokine known to recruit monocytes/macrophages to sites of inflammation. CCL2 is also associated with tumor progression in several cancer types. Recently, we showed that the N-terminus of CCL2 is modified to a pyroglutamate (pE)-residue by both glutaminyl cyclases (QC (QPCT)) and its isoenzyme (isoQC (QPCTL)). The pE-residue increases stability against N-terminal degradation by aminopeptidases. Here, we report an upregulation of QPCT expression in tissues of patients with thyroid carcinomas compared with goiter tissues, whereas QPCTL was not regulated. In thyroid carcinoma cell lines, QPCT gene expression correlates with the mRNA levels of its substrate CCL2. Both QPCT and CCL2 are regulated in a NF-κB-dependent pathway shown by stimulation with TNFa and IL1b as well as by inhibition with the IKK2 inhibitor and RNAi of p50. In the culture supernatant of thyroid carcinoma cells, equal amounts of pECCL2 and total CCL2 were detected by two ELISAs discriminating between total CCL2 and pECCL2, concluding that all CCL2 is secreted as pECCL2. Activation of the CCL2/CCR2 pathway by recombinant CCL2 increased tumor cell   migratio  n of FTC238 cells in scratch assays as well as thyroid carcinoma cell-derived CCL2-induced   migratio  n of monocytic THP1 cells. Suppression of CCL2 signaling by CCR2 antagonist, IKK2 inhibitor, and QPCT RNAi reduced FTC238 cell growth measured by WST8 proliferation assays. Our results reveal new evidence for a novel role of QC in thyroid carcinomas and provide an intriguing rationale for the use of QC inhibitors as a means of blocking pECCL2 formation and preventing thyroid cancer metastasis. 
  [Mm]etastasis  PubMed  
 Role of glutaminyl cyclases in thyroid carcinomas. CCL2 is a chemokine known to recruit monocytes/macrophages to sites of inflammation. CCL2 is also associated with tumor progression in several cancer types. Recently, we showed that the N-terminus of CCL2 is modified to a pyroglutamate (pE)-residue by both glutaminyl cyclases (QC (QPCT)) and its isoenzyme (isoQC (QPCTL)). The pE-residue increases stability against N-terminal degradation by aminopeptidases. Here, we report an upregulation of QPCT expression in tissues of patients with thyroid carcinomas compared with goiter tissues, whereas QPCTL was not regulated. In thyroid carcinoma cell lines, QPCT gene expression correlates with the mRNA levels of its substrate CCL2. Both QPCT and CCL2 are regulated in a NF-κB-dependent pathway shown by stimulation with TNFa and IL1b as well as by inhibition with the IKK2 inhibitor and RNAi of p50. In the culture supernatant of thyroid carcinoma cells, equal amounts of pECCL2 and total CCL2 were detected by two ELISAs discriminating between total CCL2 and pECCL2, concluding that all CCL2 is secreted as pECCL2. Activation of the CCL2/CCR2 pathway by recombinant CCL2 increased tumor cell migration of FTC238 cells in scratch assays as well as thyroid carcinoma cell-derived CCL2-induced migration of monocytic THP1 cells. Suppression of CCL2 signaling by CCR2 antagonist, IKK2 inhibitor, and QPCT RNAi reduced FTC238 cell growth measured by WST8 proliferation assays. Our results reveal new evidence for a novel role of QC in thyroid carcinomas and provide an intriguing rationale for the use of QC inhibitors as a means of blocking pECCL2 formation and preventing thyroid cancer   metastasi  s. 
   
  
 ANO1 
 
  NCBI Gene  
  GeneRIF  
  Pubmed  
 
 
  Search Term  Rank  Fields  
  [Mm]igration  6  PubMed(10); GeneRIFs(5)  
  [Ee]xtracellular [Mm]atrix  8  PubMed(1)  
  [Ii]nvasive  10  PubMed(2)  
  [Mm]etastasis  12  PubMed(8); GeneRIFs(3)  
 
 
 hide/show details for ANO1 
  [Mm]igration  PubMed  
 Functional role of the Ca²⁺-activated Cl⁻ channel DOG1/TMEM16A in gastrointestinal stromal tumor cells. DOG1, a Ca(2+)-activated Cl(-) channel (CaCC), was identified in 2004 to be robustly expressed in gastrointestinal stromal tumors (GIST). It was rapidly included as a tumor marker in routine diagnostics, but the functional role remained unknown. CaCCs are important regulators of normal physiological functions, but also implicated in tumorigenesis, cancer progression, metastasis,   cell migr  ation, apoptosis, proliferation and viability in several malignancies. We therefore investigated whether DOG1 plays a role in the three latter in GIST by utilizing in vitro cell model systems. Confocal microscopy identified different subcellular localizations of DOG1 in imatinib-sensitive and imatinib-resistant cells. Electrophysiological studies confirmed that DOG1-specific pharmacological agents possess potent activating and inhibiting properties. Proliferation assays showed small effects up to 72 h, and flow cytometric analysis of adherent cells with 7-AAD/Annexin V detected no pharmacological effects on viable GIST cells. However, inhibition of DOG1 conveyed pro-apoptotic effects among early apoptotic imatinib-resistant cells. In conclusion, DOG1 generates Cl(-) currents in GIST that can be regulated pharmacologically, with small effects on cell viability and proliferation in vitro. Inhibition of DOG1 might act pro-apoptotic on some early apoptotic GIST cell populations. Further studies are warranted to fully illuminate the function of DOG1 and its potential as therapeutic target. 
 Transmembrane protein with unknown function 16A overexpression promotes glioma formation through the nuclear factor-κB signaling pathway. Ion channels have been suggested to be important in the development and progression of tumors, however, chloride channels have rarely been analyzed in tumorigenesis. More recently, transmembrane protein with unknown function 16A (TMEM16A), hypothesized to be a candidate calcium‑activated Cl‑ channel, has been found to be overexpressed in a number of tumor types. Although several studies have implicated the overexpression of TMEM16A in certain tumor types, the exact role of TMEM16A in gliomas and the underlying mechanisms in tumorigenesis, remain poorly understood. In the present study, the role of TMEM16A in gliomas and the potential underlying mechanisms were analyzed. TMEM16A was highly abundant in various grades of gliomas and cultured glioma cells. Knockdown of TMEM16A suppressed cell proliferat  ion, migr  ation and invasion. Furthermore, nuclear factor‑κB (NF‑κB) was activated by overexpression of TMEM16A. In addition, TMEM16A regulated the expression of NF‑κB‑mediated genes, including cyclin D1, cyclin E and c‑myc, involved in cell proliferation, and matrix metalloproteinases (MMPs)‑2 and MMP‑9, which ar  e associa  ted with the migration and invasion of glioma cells. Collectively, results of the present study provide evidence for the involvement of TMEM16A in gliomas and the potential mechanism through which TMEM16A promotes glioma formation. 
 Enhanced expression of ANO1 in head and neck squamous cell carcinoma causes cell   migration   and correlates with poor prognosis. Head and neck squamous cell carcinoma (HNSCC) has the potential for early metastasis and is associated with poor survival. Ano1 (Dog1) is an established and sensitive marker for the diagnosis of gastrointestinal stromal tumors (GIST) and has recently been identified as a Ca(2+) activated Cl(-) channel. Although the ANO1 gene is located on the 11q13 locus, a region which is known to be amplified in different types of human carcinomas, a detailed analysis of Ano1 amplification and expression in HNSCC has not been performed. It is thus still unclear how Ano1 contributes to malignancy in HNSCC. We analyzed genomic amplification of the 11q13 locus and Ano1 together with Ano1-protein expression in a large collection of HNSCC samples. We detected a highly significant correlation between amplification and expression of Ano1 and showed that HNSCC patients with Ano1 protein expression have a poor overall survival. We further analyzed the expression of the Ano1 protein in more than 4'000 human samples from 80 different tumor types and 76 normal tissue types and detected that besides HNSCC and GISTs, Ano1 was rarely expressed in other tumor samples or healthy human tissues. In HNSCC cell lines, expression of Ano1 caused Ca(2+) activated Cl(-) currents, which induced cell motility and cell   migration   in wound healing and in real time   migration   assays, respectively. In contrast, knockdown of Ano1 did not affect intracellular Ca(2+) signaling and surprisingly did not reduce cell proliferation in BHY cells. Further, expression and activity of Ano1 strongly correlated with the ability of HNSCC cells to regulate their volume. Thus, poor survival in HNSCC patients is correlated with the presence of Ano1. Our results further suggest that Ano1 facilitates regulation of the cell volume and causes cell   migration  , which both can contribute to metastatic progression in HNSCC. 
 TMEM16A overexpression contributes to tumor invasion and poor prognosis of human gastric cancer through TGF-β signaling. TMEM16A is a newly identified calcium activated chloride channel, and has been reported to be overexpressed by various solid malignant cancers to promote proliferation and invasion, yet little is known about its role in gastric cancer(GC). Therefore, we investigated the role of TMEM16A in GC and its clinical significance by a retrospective analysis of 367 GC patients, and in vitro study was performed for validation and underlying molecular mechanism.TMEM16A was significantly upregulated and amplified in GC tissues, and its overexpression was positively correlated with disease stage, negatively with patient survival and identified as an independent prognostic factor for patient outcome. A negative correlation between TMEM16A and E-cadherin was found in 367 GC specimens. TMEM16A silencing significantly decreased calcium activated chloride currents, impaired TGF-β secretion, reduced E-cadherin expression, and inhibited th  e migrati  on and invasion without affecting proliferation of GC cells (AGS and BGC-823). Supplement of TGF-β reverted the effects of TMEM16A silencing on E-cadherin expression, ce  ll migrat  ion and invasion.In conclusion, TMEM16A promotes invasion and metastasis in GC, and might be a novel prognostic biomarker and potential therapeutic target in the treatment of GC. 
 Anoctamin 1 dysregulation alters bronchial epithelial repair in cystic fibrosis. Cystic fibrosis (CF) airway epithelium is constantly subjected to injury events due to chronic infection and inflammation. Moreover, abnormalities in CF airway epithelium repair have been described and contribute to the lung function decline seen in CF patients. In the last past years, it has been proposed that anoctamin 1 (ANO1), a Ca(2+)-activated Cl(-) channel, might offset the CFTR deficiency but this protein has not been characterized in CF airways. Interestingly, recent evidence indicates a role for ANO1 in cell proliferation and tumor growth. Our aims were to study non-CF and CF bronchial epithelial repair and to determine whether ANO1 is involved in airway epithelial repair. Here, we showed, with human bronchial epithelial cell lines and primary cells, that both cell proliferation and   migration   during epithelial repair are delayed in CF compared to non-CF cells. We then demonstrated that ANO1 Cl(-) channel activity was significantly decreased in CF versus non-CF cells. To explain this decreased Cl(-) channel activity in CF context, we compared ANO1 expression in non-CF vs. CF bronchial epithelial cell lines and primary cells, in lung explants from wild-type vs. F508del mice and non-CF vs. CF patients. In all these models, ANO1 expression was markedly lower in CF compared to non-CF. Finally, we established that ANO1 inhibition or overexpression was associated respectively with decreases and increases in cell proliferation and   migration  . In summary, our study demonstrates involvement of ANO1 decreased activity and expression in abnormal CF airway epithelial repair and suggests that ANO1 correction may improve this process. 
 ANO1 (TMEM16A) in pancreatic ductal adenocarcinoma (PDAC). Pancreatic ductal adenocarcinoma (PDAC) has one of the worst survival rates of all cancers. ANO1 (TMEM16A) is a recently identified Ca(2+)-activated Cl(-) channel (CaCC) that is upregulated in several tumors. Although ANO1 was subject to extensive studies in the recent years, its pathophysiological function has only been poorly understood. The aim of the present study is to establish the significance of ANO1 in PDAC behavior and demarcate its roles in PDAC from those of the volume-regulated anion channel (VRAC). We performed qPCR and Western blot measurements on different PDAC cell lines (Panc-1, Mia PaCa 2, Capan-1, AsPC-1, BxPC-3) and compared the results to those obtained in a human pancreatic ductal epithelium (HPDE) cell line. All cancer cell lines showed an upregulation of ANO1 on mRNA and protein levels. Whole-cell patch-clamp recordings identified large Ca(2+) and voltage-dependent Cl(-) currents in PDAC cells. Using siRNA knockdown of ANO1 and three ANO1 inhibitors (T16Ainh-A01, CaCCinh-A01, and NS3728), we found that ANO1 is the main constituent of CaCC current in PDAC cells. We further characterized these three inhibitors and found that they had unspecific effects on the free intracellular calcium concentration. Functional studies on PDAC behavior showed that surprisingly inhibition of ANO1 did not influence cellular proliferation. On the other hand, we found ANO1 channel to be pivotal in PDAC cell   migration   as assessed in wound healing experiments. 
 Inhibition of Calcium-Activated Chloride Channel ANO1/TMEM16A Suppresses Tumor Growth and Invasion in Human Lung Cancer. Lung cancer or pulmonary carcinoma is primarily derived from epithelial cells that are thin and line on the alveolar surfaces of the lung for gas exchange. ANO1/TMEM16A, initially identified from airway epithelial cells, is a member of Ca2+-activated Cl- channels (CaCCs) that function to regulate epithelial secretion and cell volume for maintenance of ion and tissue homeostasis. ANO1/TMEM16A has recently been shown to be highly expressed in several epithelium originated carcinomas. However, the role of ANO1 in lung cancer remains unknown. In this study, we show that inhibition of calcium-activated chloride channel ANO1/TMEM16A suppresses tumor growth and invasion in human lung cancer. ANO1 is upregulated in different human lung cancer cell lines. Knocking-down ANO1 by small hairpin RNAs inhibited proliferation,   migration   and invasion of GLC82 and NCI-H520 cancel cells evaluated by CCK-8, would-healing, transwell and 3D soft agar assays. ANO1 protein is overexpressed in 77.3% cases of human lung adenocarcinoma tissues detected by immunohistochemistry. Furthermore, the tumor growth in nude mice implanted with GLC82 cells was significantly suppressed by ANO1 silencing. Taken together, our findings provide evidence that ANO1 overexpression contributes to tumor growth and invasion of lung cancer; and suppressing ANO1 overexpression may have therapeutic potential in lung cancer therapy. 
 TMEM16A alternative splicing coordination in breast cancer. TMEM16A, also known as Anoctamin-1, is a calcium-activated chloride channel gene overexpressed in many tumors. The role of TMEM16A in cancer is not completely understood and no data are available regarding the potential tumorigenic properties of the multiple isoforms generated by alternative splicing (AS).We evaluated TMEM16A AS pattern, isoforms distribution and Splicing Coordination (SC), in normal tissues and breast cancers, through a semi-quantitative PCR-assay that amplifies transcripts across three AS exons, 6b, 13 and 15.In breast cancer, we did not observe an association either to AS of individual exons or to specific TMEM16A isoforms, and induced expression of the most common isoforms present in tumors in the HEK293 Flp-In Tet-ON system had no effect on cellular proliferation and   migration  . The analysis of splicing coordination, a mechanism that regulates AS of distant exons, showed a preferential association of exon 6b and 15 in several normal tissues and tumors: isoforms that predominantly include exon 6b tend to exclude exon 15 and vice versa. Interestingly, we found an increase in SC in breast tumors compared to matched normal tissues.As the different TMEM16A isoforms do not affect proliferation or   migration   and do not associate with tumors, our results suggest that the resulting channel activities are not directly involved in cell growth and motility. Conversely, the observed increase in SC in breast tumors suggests that the maintenance of the regulatory mechanism that coordinates distant alternative spliced exons in multiple genes other than TMEM16A is necessary for cancer cell viability. 
 Inhibition of TMEM16A expression suppresses growth and invasion in human colorectal cancer cells. Metastasis leads to poor prognosis in colorectal cancer patients, and there is a growing need for new therapeutic targets. TMEM16A (ANO1, DOG1 or TAOS2) has recently been identified as a calcium-activated chloride channel (CaCC) and is reported to be overexpressed in several malignancies; however, its expression and function in colorectal cancer (CRC) remains unclear. In this study, we found expression of TMEM16A mRNA and protein in high-metastatic-potential SW620, HCT116 and LS174T cells, but not in primary HCT8 and SW480 cells, using RT-PCR, western blotting and immunofluorescence labeling. Patch-clamp recordings detected CaCC currents regulated by intracellular Ca(2+) and voltage in SW620 cells. Knockdown of TMEM16A by short hairpin RNAs (shRNA) resulted in the suppression of growth,   migration   and invasion of SW620 cells as detected by MTT, wound-healing and transwell assays. Mechanistically, TMEM16A depletion was accompanied by the dysregulation of phospho-MEK, phospho-ERK1/2 and cyclin D1 expression. Flow cytometry analysis showed that SW620 cells were inhibited from the G1 to S phase of the cell cycle in the TMEM16A shRNA group compared with the control group. In conclusion, our results indicate that TMEM16A CaCC is involved in growth,   migration   and invasion of metastatic CRC cells and provide evidence for TMEM16A as a potential drug target for treating metastatic colorectal carcinoma. 
 ANO1 as a marker of oral squamous cell carcinoma and silencing ANO1 suppresses   migration   of human SCC-25 cells. The purpose of this study is to confirm that ANO1 correlates with occurrence and metastasis of OSCC.Immunohistochemistry was used to detect the expression of ANO1 in 160 specimens of OSCC and normal tissues. Lentiviral silencing ANO1 was used in SCC-25 cell line to study the cell   migration   and cell detachment.Immunohistochemical staining revealed that ANO1 was expressed in a large majority (132 out of 160, 82.5%) of OSCC specimens and that the rate of ANO1 expression in OSCC was significantly higher than that of normal tissue (P<0.05); The rate of ANO1 expression was higher in metastatic tumors than in non-metastatic tumors, and the difference was significant (P<0.05). The results of cell   migration   assay showed that the percentage of cells through the membrane was 26.61 ±0.81 in assay group, and 54.26 ±3.74 in control group, respectively (t=-16.22,P<0.0001). The results of cell detachment assay showed that the percentage of cells detachment was 37.42 ±0.90 in assay group, and 87.38 ±1.59 in control group, respectively (t=-62.34, P<0.0001). The results of wound healing assay showed the assay group had a redu  ced migra  tion rate compared with the control group in 32 h (F=1038.78, P<0.0001). Wound closure was no significantly different between the assay and control cells when DIDS was used in wound healing assay (F=4.61,P>0.05).Our study shows that abnormal expression of ANO1 correlated with the occurrence and metastasis of OSCC in clinical specimens and that silencing ANO1 greatly redu  ced migra  tion ability of scc-25 cells. Calcium activated chloride channel activity of ANO1 promoted the c  ell migra  tion. Thus, ANO1 could represent a new diagnostic biomarker and a potentially important therapeutic target of OSCC. 
  GeneRIFs  
 Results indicate that TMEM16A is involved in growth,   migration   and invasion of metastatic colorectal cancer cells. 
 investigated whether DOG1 plays a role in cell   migration  , apoptosis, proliferation and viability in gastrointestinal stromal tumors 
 TMEM16A regulated the expression of NFkappaBmediated genes, including cyclin D1, cyclin E and cmyc, involved in cell proliferation, and matrix metalloproteinases (MMPs)2 and MMP9, which are associated with the   migration   and invasion of glioma cells. 
 As the different TMEM16A isoforms do not affect proliferation or   migration   and do not associate with tumors, our results suggest that the resulting channel activities are not directly involved in cell growth and motility. 
 Enhanced expression of ANO1 in head and neck squamous cell carcinoma causes cell   migration   and correlates with poor prognosis. 
  [Ee]xtracellular [Mm]atrix  PubMed  
 Proteomic analysis of human parotid gland exosomes by multidimensional protein identification technology (MudPIT). Human ductal saliva contributes over a thousand unique proteins to whole oral fluids. The mechanism by which most of these proteins are secreted by salivary glands remains to be determined. The present study used a mass spectrometry-based, shotgun proteomics approach to explore the possibility that a subset of the proteins found in saliva are derived from exosomes, membrane-bound vesicles of endosomal origin within multivesicular endosomes. Using MudPIT (multidimensional protein identification technology) mass spectrometry, we catalogued 491 proteins in the exosome fraction of human parotid saliva. Many of these proteins were previously observed in ductal saliva from parotid glands (265 proteins). Furthermore, 72 of the proteins in parotid exosomes overlap with those previously identified as urinary exosome proteins, proteins which are also frequently associated with exosomes from other tissues and cell types. Gene Ontology (GO) and KEGG pathway analyses found that cytosolic proteins comprise the largest category of proteins in parotid exosomes (43%), involved in such processes as phosphatidylinositol signaling system, calcium signaling pathway, inositol metabolism, protein export, and signal transduction, among others; whereas the integral plasma membrane proteins and associated/peripheral plasma membrane proteins (26%) were associated with   extracellular matrix  -receptor interaction, epithelial cell signaling, T-cell and B-cell receptor signaling, cytokine receptor interaction, and antigen processing and presentation, among other biological functions. In addition, these putative saliva exosomal proteins were linked to specific diseases (e.g., neurodegenerative disorders, prion disease, cancers, type I and II diabetes). Consequently, parotid glands secrete exosomes that reflect the metabolic and functional status of the gland and may also carry informative protein markers useful in the diagnosis and treatment of systemic diseases. 
  [Ii]nvasive  PubMed  
 Novel Use for DOG1 in Discriminating Breast   Invasive   Carcinoma from Non  invasive   Breast Lesions. DOG1 has proven to be a useful marker of gastrointestinal stromal tumors (GISTs). Recently, DOG1 expression has also been reported in some non-GIST malignant tumors, but the details related to DOG1 expression in breast tissue remain unclear. The aim of this study was to detect the expression of DOG1 in the human breast and to evaluate the feasibility of using DOG1 to discriminate between   invasive   breast carcinoma and non  invasive   breast lesions.A total of 210 cases, including both   invasive   and non  invasive   breast lesions, were collected to assess DOG1 expression immunohistochemically. DOG1 expression was consistently positive in breast myoepithelial cells (MECs), which was similar to the results obtained for three other MEC markers: calponin, smooth muscle myosin heavy chain (SMMHC), and P63 (P > 0.05 in all). Importantly, DOG1 was useful in discriminating   invasive   breast carcinoma from non  invasive   breast lesions (P < 0.05).DOG1 is a useful marker of breast MECs, and adding DOG1 to the MEC identification panel will provide more sophisticated information when diagnosing uncertain cases in the breast. 
 Correlation of angiogenesis with other immunohistochemical markers in cutaneous basal and squamous cell carcinomas. The aim of this study was to establish an immunoprofile of squamous cell carcinoma (SCC) and basal cell carcinoma (BCC), and to explore as first time in literature the possible correlation between maspin, DOG-1, p16 protein and angiogenesis in these tumors. For SCCs, the histological grade of differentiation was also taken into account. The angiogenesis was quantified in 38 randomly selected cases of SCCs and 17 BCCc, respectively, using the antibodies vascular endothelial growth factor (VEGF-A) and COX-2, while the microvessel density (MVD) was evaluated with the CD31.In SCCs, maspin cytoplasm to nuclear shift was an indicator of a deeper tissue invasion and dedifferentiation in the invasion front. The poorly differentiated cases, compared to G1÷G2-SCCs, expressed more frequent the markers p16 (30.77% vs. 8%) and VEGF-A (53.85% vs. 32%), regardless the MVD. However, the p16 positivity was more frequent in BCCs than SCCs (52.94% vs. 15.79%). All of the p16-positive carcinomas were located in the head and neck area. DOG-1 marked 21.05% of SCCs and 5.88% of BCCs, being directly correlated with COX-2 positivity. Eccrine glands and hair follicles also expressed DOG-1.In cutaneous SCCs located in the head and neck area, sun-dependent p16÷VEGF interaction seems to be responsible by tumor dedifferentiation, whereas maspin cytoplasm to nuclear shift might indicate a high degree o  f invasi  veness. This is the first report about DOG-1 positivity in BCCs and eccrine glands, the significance of this pattern being unknown. 
  [Mm]etastasis  PubMed  
 Functional role of the Ca²⁺-activated Cl⁻ channel DOG1/TMEM16A in gastrointestinal stromal tumor cells. DOG1, a Ca(2+)-activated Cl(-) channel (CaCC), was identified in 2004 to be robustly expressed in gastrointestinal stromal tumors (GIST). It was rapidly included as a tumor marker in routine diagnostics, but the functional role remained unknown. CaCCs are important regulators of normal physiological functions, but also implicated in tumorigenesis, cancer progress  ion, metas  tasis, cell migration, apoptosis, proliferation and viability in several malignancies. We therefore investigated whether DOG1 plays a role in the three latter in GIST by utilizing in vitro cell model systems. Confocal microscopy identified different subcellular localizations of DOG1 in imatinib-sensitive and imatinib-resistant cells. Electrophysiological studies confirmed that DOG1-specific pharmacological agents possess potent activating and inhibiting properties. Proliferation assays showed small effects up to 72 h, and flow cytometric analysis of adherent cells with 7-AAD/Annexin V detected no pharmacological effects on viable GIST cells. However, inhibition of DOG1 conveyed pro-apoptotic effects among early apoptotic imatinib-resistant cells. In conclusion, DOG1 generates Cl(-) currents in GIST that can be regulated pharmacologically, with small effects on cell viability and proliferation in vitro. Inhibition of DOG1 might act pro-apoptotic on some early apoptotic GIST cell populations. Further studies are warranted to fully illuminate the function of DOG1 and its potential as therapeutic target. 
 Enhanced expression of ANO1 in head and neck squamous cell carcinoma causes cell migration and correlates with poor prognosis. Head and neck squamous cell carcinoma (HNSCC) has the potential for early   metastasis   and is associated with poor survival. Ano1 (Dog1) is an established and sensitive marker for the diagnosis of gastrointestinal stromal tumors (GIST) and has recently been identified as a Ca(2+) activated Cl(-) channel. Although the ANO1 gene is located on the 11q13 locus, a region which is known to be amplified in different types of human carcinomas, a detailed analysis of Ano1 amplification and expression in HNSCC has not been performed. It is thus still unclear how Ano1 contributes to malignancy in HNSCC. We analyzed genomic amplification of the 11q13 locus and Ano1 together with Ano1-protein expression in a large collection of HNSCC samples. We detected a highly significant correlation between amplification and expression of Ano1 and showed that HNSCC patients with Ano1 protein expression have a poor overall survival. We further analyzed the expression of the Ano1 protein in more than 4'000 human samples from 80 different tumor types and 76 normal tissue types and detected that besides HNSCC and GISTs, Ano1 was rarely expressed in other tumor samples or healthy human tissues. In HNSCC cell lines, expression of Ano1 caused Ca(2+) activated Cl(-) currents, which induced cell motility and cell migration in wound healing and in real time migration assays, respectively. In contrast, knockdown of Ano1 did not affect intracellular Ca(2+) signaling and surprisingly did not reduce cell proliferation in BHY cells. Further, expression and activity of Ano1 strongly correlated with the ability of HNSCC cells to regulate their volume. Thus, poor survival in HNSCC patients is correlated with the presence of Ano1. Our results further suggest that Ano1 facilitates regulation of the cell volume and causes cell migration, which both can contribute to metastatic progression in HNSCC. 
 TMEM16A overexpression contributes to tumor invasion and poor prognosis of human gastric cancer through TGF-β signaling. TMEM16A is a newly identified calcium activated chloride channel, and has been reported to be overexpressed by various solid malignant cancers to promote proliferation and invasion, yet little is known about its role in gastric cancer(GC). Therefore, we investigated the role of TMEM16A in GC and its clinical significance by a retrospective analysis of 367 GC patients, and in vitro study was performed for validation and underlying molecular mechanism.TMEM16A was significantly upregulated and amplified in GC tissues, and its overexpression was positively correlated with disease stage, negatively with patient survival and identified as an independent prognostic factor for patient outcome. A negative correlation between TMEM16A and E-cadherin was found in 367 GC specimens. TMEM16A silencing significantly decreased calcium activated chloride currents, impaired TGF-β secretion, reduced E-cadherin expression, and inhibited the migration and invasion without affecting proliferation of GC cells (AGS and BGC-823). Supplement of TGF-β reverted the effects of TMEM16A silencing on E-cadherin expression, cell migration and invasion.In conclusion, TMEM16A promotes invasion a  nd metasta  sis in GC, and might be a novel prognostic biomarker and potential therapeutic target in the treatment of GC. 
 Downregulation of Ca2+-activated Cl- channel TMEM16A by the inhibition of histone deacetylase in TMEM16A-expressing cancer cells. The Ca(2+)-activated Cl(-) channel transmembrane proteins with unknown function 16 A (TMEM16A; also known as anoctamin 1 or discovered on gastrointestinal stromal tumor 1) plays an important role in facilitating the cell growth and   metastasis   of TMEM16A-expressing cancer cells. Histone deacetylase (HDAC) inhibitors (HDACi) are useful agents for cancer therapy, but it remains unclear whether ion channels are epigenetically regulated by them. Using real-time polymerase chain reaction, Western blot analysis, and whole-cell patch-clamp assays, we found a significant decrease in TMEM16A expression and its functional activity was induced by the vorinostat, a pan-HDACi in TMEM16A-expressing human cancer cell lines, the prostatic cancer cell line PC-3, and the breast cancer cell line YMB-1. TMEM16A downregulation was not induced by the chemotherapy drug paclitaxel in either cell type. Pharmacologic blockade of HDAC3 by 1 μM T247 [N-(2-aminophenyl)-4-[1-(2-thiophen-3-ylethyl)-1H-[1],[2],[3]triazol-4-yl]benzamide], a HDAC3-selective HDACi, elicited a large decrease in TMEM16A expression and functional activity in both cell types, and pharmacologic blockade of HDAC2 by AATB [4-(acetylamino)-N-[2-amino-5-(2-thienyl)phenyl]-benzamide; 300 nM] elicited partial inhibition of TMEM16A expression (∼40%) in both. Pharmacologic blockade of HDAC1 or HDAC6 did not elicit any significant change in TMEM16A expression, respectively. In addition, inhibition of HDAC3 induced by small interfering RNA elicited a large decrease in TMEM16A transcripts in both cell types. Taken together, in malignancies with a frequent gene amplification of TMEM16A, HDAC3 inhibition may exert suppressive effects on cancer cell viability via downregulation of TMEM16A. 
 To "grow" or "go": TMEM16A expression as a switch between tumor growth and   metastasis   in SCCHN. Tumor   metastasis   is the leading cause of death in patients with cancer. However, the mechanisms that underlie metastatic progression remain unclear. We examined TMEM16A (ANO1) expression as a key factor shifting tumors between growth and   metastasis  .We evaluated 26 pairs of primary and metastatic lymph node (LN) tissue from patients with squamous cell carcinoma of the head and neck (SCCHN) for differential expression of TMEM16A. In addition, we identified mechanisms by which TMEM16A expression influences tumor cell motility via proteomic screens of cell lines and in vivo mouse studies of   metastasis  .Compared with primary tumors, TMEM16A expression decreases in metastatic LNs of patients with SCCHN. Stable reduction of TMEM16A expression enhances cell motility and increases metastases while decreasing tumor proliferation in an orthotopic mouse model. Evaluation of human tumor tissues suggests an epigenetic mechanism for decreasing TMEM16A expression through promoter methylation that correlated with a transition between an epithelial and a mesenchymal phenotype. These effects of TMEM16A expression on tumor cell size and epithelial-to-mesenchymal transition (EMT) required the amino acid residue serine 970 (S970); however, mutation of S970 to alanine does not disrupt the proliferative advantages of TMEM16A overexpression. Furthermore, S970 mediates the association of TMEM16A with Radixin, an actin-scaffolding protein implicated in EMT.Together, our results identify TMEM16A, an eight transmembrane domain Ca2+-activated Cl- channel, as a primary driver of the "Grow" or "Go" model for cancer progression, in which TMEM16A expression acts to balance tumor proliferation and   metastasis   via its promoter methylation. 
 Utility of DOG1 Immunomarker in Fine Needle Aspirates of Gastrointestinal Stromal Tumor. Gastrointestinal stromal tumor (GIST) is the most common mesenchymal tumor of the gastrointestinal tract. DOG1 is a sensitive and specific immunohistochemical marker for the diagnosis of GIST. To date, no study has reported the utility of DOG1 immunocytochemistry on aspirate smears.Aspirates with a cytological diagnosis of GIST were retrieved. DOG1 immunocytochemistry was performed on aspirates with adequate material.23 cases were included (11 primary, 2 recurrent, 10 metastatic). Primary tumors were most frequently located in the stomach; most metastatic tumors were in the liver. Tumor cells were arranged in cohesive clusters with high cellularity. Cells were spindled, had a low N:C ratio, and a moderate amount of cytoplasm, which was elongated and tapering. Characteristic nuclear features included elongated nuclei with blunt or tapering ends, fine chromatin, mild anisonucleosis, and longitudinal grooves. The mitotic count was low, including in metastatic tumors. DOG1 immunopositivity was noted in 57% of the cases examined. Histopathology was available in 5 cases, all diagnosed as GIST.Cytology is a sensitive investigative modality for the preoperative diagnosis and confirmation of   metastasis   of GISTs. In ambiguous cases, DOG1 immunocytochemistry can serve as a valuable adjunct. Cytologic assessment, however, cannot predict malignant potential of GISTs as even metastatic tumors display bland nuclear features. 
 Inhibition of TMEM16A expression suppresses growth and invasion in human colorectal cancer cells.   Metastasis   leads to poor prognosis in colorectal cancer patients, and there is a growing need for new therapeutic targets. TMEM16A (ANO1, DOG1 or TAOS2) has recently been identified as a calcium-activated chloride channel (CaCC) and is reported to be overexpressed in several malignancies; however, its expression and function in colorectal cancer (CRC) remains unclear. In this study, we found expression of TMEM16A mRNA and protein in high-metastatic-potential SW620, HCT116 and LS174T cells, but not in primary HCT8 and SW480 cells, using RT-PCR, western blotting and immunofluorescence labeling. Patch-clamp recordings detected CaCC currents regulated by intracellular Ca(2+) and voltage in SW620 cells. Knockdown of TMEM16A by short hairpin RNAs (shRNA) resulted in the suppression of growth, migration and invasion of SW620 cells as detected by MTT, wound-healing and transwell assays. Mechanistically, TMEM16A depletion was accompanied by the dysregulation of phospho-MEK, phospho-ERK1/2 and cyclin D1 expression. Flow cytometry analysis showed that SW620 cells were inhibited from the G1 to S phase of the cell cycle in the TMEM16A shRNA group compared with the control group. In conclusion, our results indicate that TMEM16A CaCC is involved in growth, migration and invasion of metastatic CRC cells and provide evidence for TMEM16A as a potential drug target for treating metastatic colorectal carcinoma. 
 ANO1 as a marker of oral squamous cell carcinoma and silencing ANO1 suppresses migration of human SCC-25 cells. The purpose of this study is to confirm that ANO1 correlates with occurrence and   metastasis   of OSCC.Immunohistochemistry was used to detect the expression of ANO1 in 160 specimens of OSCC and normal tissues. Lentiviral silencing ANO1 was used in SCC-25 cell line to study the cell migration and cell detachment.Immunohistochemical staining revealed that ANO1 was expressed in a large majority (132 out of 160, 82.5%) of OSCC specimens and that the rate of ANO1 expression in OSCC was significantly higher than that of normal tissue (P<0.05); The rate of ANO1 expression was higher in metastatic tumors than in non-metastatic tumors, and the difference was significant (P<0.05). The results of cell migration assay showed that the percentage of cells through the membrane was 26.61 ±0.81 in assay group, and 54.26 ±3.74 in control group, respectively (t=-16.22,P<0.0001). The results of cell detachment assay showed that the percentage of cells detachment was 37.42 ±0.90 in assay group, and 87.38 ±1.59 in control group, respectively (t=-62.34, P<0.0001). The results of wound healing assay showed the assay group had a reduced migration rate compared with the control group in 32 h (F=1038.78, P<0.0001). Wound closure was no significantly different between the assay and control cells when DIDS was used in wound healing assay (F=4.61,P>0.05).Our study shows that abnormal expression of ANO1 correlated with the occurrence   and metast  asis of OSCC in clinical specimens and that silencing ANO1 greatly reduced migration ability of scc-25 cells. Calcium activated chloride channel activity of ANO1 promoted the cell migration. Thus, ANO1 could represent a new diagnostic biomarker and a potentially important therapeutic target of OSCC. 
  GeneRIFs  
 TMEM16A overexpression promotes invasion and   metastasis   in gastric cancer. 
 DOG1 immunocytochemistry can serve as a valuable adjunct for the preoperative diagnosis and confirmation of   metastasis   of Gastrointestinal Stromal Tumors. 
 Together, our results identify TMEM16A as a primary driver of the "Grow" or "Go" model for cancer progression, in which TMEM16A expression acts to balance tumor proliferation and   metastasis   via its promoter methylation. 
   
  
 SLC16A10 
 
  NCBI Gene  
  GeneRIF  
  Pubmed  
 
 
  Search Term  Rank  Fields  
  [Mm]etastasis  12  PubMed(1); GeneRIFs(1)  
 
 
 hide/show details for SLC16A10 
  [Mm]etastasis  PubMed  
 Monitoring the treatment outcome in endometrial cancer patients by CEA and TATI. An attempt was made to compare the usefulness of determining markers carcinoembryonic antigen (CEA) and tumor-associated trypsin inhibitor (TATI) in endometrial cancer patients in whom recurrence or distant   metastasis   was diagnosed in observation after treatment. The study included 316 patients aged 32-81, average age of 61 years, SD = 8.72, with diagnosed endometrial cancer, treated between 1994 and 1995 at the Oncology Center in Warsaw and then under observation from 4 months to 17 years after completion of treatment. The levels of the markers TATI and CEA were assessed from the first five serum samples taken during postoperative radiotherapy and in the initial period of observation after completed treatment. Receiver operating characteristic (ROC) curves were generated, determining the sensitivity and specificity of both CEA and TATI in patients who experienced treatment failure, i.e., recurrence and d  istant met  astasis. Assessing the sensitivity of the marker CEA, it was found that if in the third sample, i.e., during radiation therapy, the marker level increased by more than 20 % compared with the first sample, then recurrence of cancer occurred during the observation period in 75.9 % of patients and metastatic occurred in 69.7 % of patients. In the evaluation of the marker TATI, it was found that if the level of TATI between the first and the third sample increases by 10.6 % from the initial level, then in 84.4 % (sensitivity) of cases, this means the occurrence of cancer recurrence and in 75.7 % (sensitivity) of cases, the o  ccurrence   of metastasis. The specificity of both markers is low and not useful diagnostically. 
  GeneRIFs  
 High TAT1 expression is associated with   metastasis   and recurrence in endometrial cancer. 
   
  
 FAP 
 
  NCBI Gene  
  GeneRIF  
  Pubmed  
 
 
  Search Term  Rank  Fields  
  [Ii]nvadopodia  3  PubMed(6); GeneRIFs(1)  
  [Ii]nvadopodium  4  Component(1)  
  [Mm]igration  6  Process(1); PubMed(17); GeneRIFs(9)  
  ECM  7  PubMed(9); GeneRIFs(3)  
  [Ee]xtracellular [Mm]atrix  8  Process(2); PubMed(13)  
  [Ii]nvasive  10  PubMed(16); GeneRIFs(4)  
  [Mm]etastasis  12  PubMed(18); GeneRIFs(5)  
  [Cc]ell [Ii]nvasion  13  PubMed(8)  
 
 
 hide/show details for FAP 
  [Ii]nvadopodia  PubMed  
 Molecular proximity of seprase and the urokinase-type plasminogen activator receptor on malignant melanoma cell membranes: dependence on beta1 integrins and the cytoskeleton. Previous studies have shown that several proteolytic enzymes are associated with membrane protrusions at the leading edge of migrating tumor cells. In this study we demonstrate that seprase and the urokinase plasminogen activator receptor (uPAR), co-localize in the plasma membrane of LOX malignant melanoma cells. Cells were labeled with fluorochrome-conjugated monoclonal antibodies (mAb) directed against seprase and uPAR. Proximity between these two molecules was detected with resonance energy transfer (RET) imaging, single-cell emission spectrophotometry, and single-cell excitation spectrophotometry. Significant RET signals were detected on LOX cells when adherent to uncoated and extracellular matrix (ECM)-coated surfaces. This indicates that seprase and uPAR are within approximately 7 nm in the plasma membrane of LOX cells. When LOX cells adhered to a 3D extracellular-like matrix, seprase-uPAR complexes were found to be associated with   invadopodia  . Further microscopy experiments demonstrated gelatinolytic activity, a functional attribute of seprase, in association with seprase-uPAR membrane domains. Formation of seprase-uPAR membrane complexes is dependent upon both the cytoskeleton and integrins. Specifically, the involvement of beta(1)-integrins was demonstrated by the inhibition of RET by an inhibitory anti-beta(1)-integrin mAb. Based on these findings, we speculate that formation of heterogeneous lytic domains in the invading membranes of LOX cells increases the efficiency of directed pericellular proteolysis. 
 A novel protease-docking function of integrin at   invadopodia  .   Invadopodia   are membrane extensions of aggressive tumor cells that function in the activation of membrane-bound proteases occurring during tumor cell invasion. We explore a novel and provocative activity of integrins in docking proteases to sites of invasion, termed   invadopodia  . In the absence of collagen, alpha(3)beta(1) integrin and the gelatinolytic enzyme, seprase, exist as nonassociating membrane proteins. Type I collagen substratum induces the association of alpha(3)beta(1) integrin with seprase as a complex on   invadopodia  . The results show that alpha(3)beta(1) integrin is a docking protein for seprase to form functional   invadopodia  . In addition, alpha(5)beta(1) integrin may participate in the adhesion process necessary for   invadopodia  l formation. Thus, alpha(3)beta(1) and alpha(5)beta(1) integrins play major organizational roles in the adhesion and formation of   invadopodia  , promoting invasive cell behavior. 
 Molecular proximity of seprase and the urokinase-type plasminogen activator receptor on malignant melanoma cell membranes: dependence on beta1 integrins and the cytoskeleton. Previous studies have shown that several proteolytic enzymes are associated with membrane protrusions at the leading edge of migrating tumor cells. In this study we demonstrate that seprase and the urokinase plasminogen activator receptor (uPAR), co-localize in the plasma membrane of LOX malignant melanoma cells. Cells were labeled with fluorochrome-conjugated monoclonal antibodies (mAb) directed against seprase and uPAR. Proximity between these two molecules was detected with resonance energy transfer (RET) imaging, single-cell emission spectrophotometry, and single-cell excitation spectrophotometry. Significant RET signals were detected on LOX cells when adherent to uncoated and extracellular matrix (ECM)-coated surfaces. This indicates that seprase and uPAR are within approximately 7 nm in the plasma membrane of LOX cells. When LOX cells adhered to a 3D extracellular-like matrix, seprase-uPAR complexes were found to be associated with   invadopodia  . Further microscopy experiments demonstrated gelatinolytic activity, a functional attribute of seprase, in association with seprase-uPAR membrane domains. Formation of seprase-uPAR membrane complexes is dependent upon both the cytoskeleton and integrins. Specifically, the involvement of beta(1)-integrins was demonstrated by the inhibition of RET by an inhibitory anti-beta(1)-integrin mAb. Based on these findings, we speculate that formation of heterogeneous lytic domains in the invading membranes of LOX cells increases the efficiency of directed pericellular proteolysis. 
 The protease complex consisting of dipeptidyl peptidase IV and seprase plays a role in the migration and invasion of human endothelial cells in collagenous matrices. Dipeptidyl peptidase IV (DPP4/CD26) and seprase/fibroblast activation protein alpha are homologous type II transmembrane, homodimeric glycoproteins that exhibit unique prolyl peptidase activities. Human DPP4 is ubiquitously expressed in epithelial and endothelial cells and serves multiple functions in cleaving the penultimate positioned prolyl bonds at the NH(2) terminus of a variety of physiologically important peptides in the circulation. Recent studies showed a linkage between DPP4 and down-regulation of certain chemokines and mitogenic growth factors, and degradation of denatured collagens (gelatin), suggesting a role of DPP4 in the cell invasive phenotype. Here, we found the existence of a novel protease complex consisting of DPP4 and seprase in human endothelial cells that were activated to migrate and invade in the extracellular matrix in vitro. DPP4 and seprase were coexpressed with the three major protease systems (matrix metalloproteinase, plasminogen activator, and type II transmembrane serine protease) at the cell surface and organize as a complex at   invadopodia  -like protrusions. Both proteases were colocalized at the endothelial cells of capillaries, but not large blood vessels, in invasive breast ductal carcinoma in vivo. Importantly, monoclonal antibodies against the gelatin-binding domain of DPP4 blocked the local gelatin degradation by endothelial cells in the presence of the major metallo- and serine protease systems that modified pericellular collagenous matrices and subsequent cell migration and invasion. Thus, we have identified a novel mechanism involving the DPP4 gelatin-binding domain of the DPP4-seprase complex that facilitates the local degradation of the extracellular matrix and the invasion of the endothelial cells into collagenous matrices. 
 Regulation of fibroblast migration on collagenous matrix by a cell surface peptidase complex. The invasion of migratory cells through connective tissues involves metallo- and serine types of cell surface proteases. We show that formation of a novel protease complex, consisting of the membrane-bound prolyl peptidases seprase and dipeptidyl peptidase IV (DPPIV), at   invadopodia   of migratory fibroblasts is a prerequisite for cell invasion and migration on a collagenous matrix. Seprase and DPPIV form a complex on the cell surface that elicits both gelatin binding and gelatinase activities localized at   invadopodia   of cells migrating on collagenous fibers. The protease complex participates in the binding to gelatin and localized gelatin degradation, cellular migration, and monolayer wound closure. Serine protease inhibitors can block the gelatinase activity and the localized gelatin degradation by cells. Antibodies to the gelatin-binding domain of DPPIV reduce the cellular abilities of the proteases to degrade gelatin but do not affect cellular adhesion or spreading on type I collagen. Furthermore, expression of the seprase-DPPIV complex is restricted to migratory cells involved in wound closure in vitro and in connective tissue cells during closure of gingival wounds but not in differentiated tissue cells. Thus, we have identified cell surface proteolytic activities, which are non-metalloproteases, seprase and DPPIV, that are responsible for the tissue-invasive phenotype. 
 The stromal cell-surface protease fibroblast activation protein-α localizes to lipid rafts and is recruited to   invadopodi  a. Fibroblast activation protein alpha (FAPα) is a cell surface protease expressed by cancer-associated fibroblasts in the microenvironment of most solid tumors. As there is increasing evidence for proteases having non-catalytic functions, we determined the FAPα interactome in cancer-associated fibroblasts using the quantitative immunoprecipitation combined with knockdown (QUICK) method. Complex formation with adenosin deaminase, erlin-2, stomatin, prohibitin, Thy-1 membrane glycoprotein, and caveolin-1 was further validated by immunoblotting. Co-immunoprecipitation (co-IP) of the known stoichiometric FAPα binding partner dipeptidyl-peptidase IV (DPPIV) corroborated the proteomic strategy. Reverse co-IPs validated the FAPα interaction with caveolin-1, erlin-2, and stomatin while co-IP upon RNA-interference mediated knock-down of DPPIV excluded adenosin deaminase as a direct FAPα interaction partner. Many newly identified FAPα interaction partners localize to lipid rafts, including caveolin-1, a widely-used marker for lipid raft localization. We hypothesized that this indicates a recruitment of FAPα to lipid raft structures. In density gradient centrifugation, FAPα co-fractionates with caveolin-1. Immunofluorescence optical sectioning microscopy of FAPα and lipid raft markers further corroborates recruitment of FAPα to lipid   rafts and   invadopodia. FAPα is therefore an integral component of stromal lipid rafts in solid tumors. In essence, we provide one of the first interactome analyses of a cell surface protease and translate these results into novel biological aspects of a marker protein for cancer-associated fibroblasts. 
  GeneRIFs  
 Immunofluorescence optical sectioning microscopy of FAPalpha and lipid raft markers further corroborates recruitment of FAPalpha to lipid rafts and   invadopodia  . 
  [Ii]nvadopodium  Component  
   invadopodium   membrane 
  [Mm]igration  Process  
 endothelial cell   migration   
  PubMed  
 Analysis of the myosin-II-responsive focal adhesion proteome reveals a role for β-Pix in negative regulation of focal adhesion maturation. Focal adhesions undergo myosin-II-mediated maturation wherein they grow and change composition to modulate integrin signalling for cell   migratio  n, growth and differentiation. To determine how focal adhesion composition is affected by myosin II activity, we performed proteomic analysis of isolated focal adhesions and compared protein abundance in focal adhesions from cells with and without myosin II inhibition. We identified 905 focal adhesion proteins, 459 of which changed in abundance with myosin II inhibition, defining the myosin-II-responsive focal adhesion proteome. The abundance of 73% of the proteins in the myosin-II-responsive focal adhesion proteome was enhanced by contractility, including proteins involved in Rho-mediated focal adhesion maturation and endocytosis- and calpain-dependent focal adhesion disassembly. During myosin II inhibition, 27% of proteins in the myosin-II-responsive focal adhesion proteome, including proteins involved in Rac-mediated lamellipodial protrusion, were enriched in focal adhesions, establishing that focal adhesion protein recruitment is also negatively regulated by contractility. We focused on the Rac guanine nucleotide exchange factor β-Pix, documenting its role in the negative regulation of focal adhesion maturation and the promotion of lamellipodial protrusion and focal adhesion turnover to drive cel  l migrati  on. 
 Fibroblast activation protein alpha expression identifies activated fibroblasts after myocardial infarction. Fibroblast activation protein α (FAP) is a membrane-bound serine protease expressed by activated fibroblasts during wound healing in the skin. Expression of FAP after myocardial infarction (MI) and potential effects on cardiac wound healing are largely unknown.MI was induced in rats and FAP expression was analyzed at 3, 7 and 28 days post-MI by microarray, Western blot and immunohistochemistry. In human hearts after MI, a FAP(+) fibroblast population was identified, and characterized by immunohistochemistry for prolyl-4-hydroxylase β, α-smooth muscle actin, Thy-1 and vimentin. Signaling pathways leading to FAP expression were studied in human cardiac fibroblasts by Western blot and ELISA using TGFβ1, TGF-beta type I-receptor (TGFbR1)-inhibitor SB431542 or the MAPK-inhibitor U0126 as well as siRNA targeting SMAD2 and SMAD3. Finally, fibroblasts were assayed for FAP-depend  ent migra  tion (modified Boyden-chamber), proliferation (BrdU-assay) and gelatinolytic activity by gelatin zymography.In rats, FAP expression was increased after MI especially in the peri-infarct area peaking at 7 days post-MI. Co-localization analysis identified the majority of FAP(+) cells as activated proto-myofibroblasts and myofibroblasts. Concordantly, FAP(+) fibroblasts were abundant in ischemic tissue of human hearts after MI, but not in healthy control hearts. In vitro, FAP was induced by TGFβ1 via the canonical SMAD2/SMAD3 pathway. Depletion of FAP in fibroblasts reduced migratory capacity, while proliferation was not affected. Gelatin zymography revealed gelatinase activity by fibroblast-derived FAP.In this study, we show for the first time the expression of FAP in activated fibroblasts after MI and its activation by TGFβ1. Effects of FAP on fibro  blast mig  ration and gelatinolytic activity indicate a potential role in cardiac wound healing and remodeling. 
 The protease complex consisting of dipeptidyl peptidase IV and seprase plays a role in the   migration   and invasion of human endothelial cells in collagenous matrices. Dipeptidyl peptidase IV (DPP4/CD26) and seprase/fibroblast activation protein alpha are homologous type II transmembrane, homodimeric glycoproteins that exhibit unique prolyl peptidase activities. Human DPP4 is ubiquitously expressed in epithelial and endothelial cells and serves multiple functions in cleaving the penultimate positioned prolyl bonds at the NH(2) terminus of a variety of physiologically important peptides in the circulation. Recent studies showed a linkage between DPP4 and down-regulation of certain chemokines and mitogenic growth factors, and degradation of denatured collagens (gelatin), suggesting a role of DPP4 in the cell invasive phenotype. Here, we found the existence of a novel protease complex consisting of DPP4 and seprase in human endothelial cells that were activated to migrate and invade in the extracellular matrix in vitro. DPP4 and seprase were coexpressed with the three major protease systems (matrix metalloproteinase, plasminogen activator, and type II transmembrane serine protease) at the cell surface and organize as a complex at invadopodia-like protrusions. Both proteases were colocalized at the endothelial cells of capillaries, but not large blood vessels, in invasive breast ductal carcinoma in vivo. Importantly, monoclonal antibodies against the gelatin-binding domain of DPP4 blocked the local gelatin degradation by endothelial cells in the presence of the major metallo- and serine protease systems that modified pericellular collagenous matrices and subsequent cell   migration   and invasion. Thus, we have identified a novel mechanism involving the DPP4 gelatin-binding domain of the DPP4-seprase complex that facilitates the local degradation of the extracellular matrix and the invasion of the endothelial cells into collagenous matrices. 
 Fibroblast activation protein increases apoptosis, cell adhesion, and   migration   by the LX-2 human stellate cell line. Injury and repair in chronic liver disease involve cell adhesion,   migration  , apoptosis, proliferation, and a wound healing response. In liver, fibroblast activation protein (FAP) has both collagenase and dipeptidyl peptidase IV (DPIV) activities and is expressed only by activated hepatic stellate cells (HSC) and myofibroblasts, which produce and degrade extracellular matrix (ECM). FAP was colocalized with collagen fibers, fibronectin, and collagen type I in human liver. FAP function was examined in vitro by expressing green fluorescent protein FAP fusion protein in cell lines cultured on collagen-I, fibronectin, and Matrigel. Glutamates at 203 and 204 as well as serine624 of FAP were essential for peptidase activity. Human embryonic kidney 293T cells overexpressing FAP showed reduced adhesion and   migration  . FAP overexpression in the human HSC line LX-2 caused increased cell adhesion and   migration   on ECM proteins as well as invasion across transwells in the absence or presence of transforming growth factor beta-1. FAP overexpression enhanced staurosporine streptomyces-stimulated apoptosis in both cell lines. Interestingly, the enzyme activity of FAP was not required for these functions. Overexpressing FAP increased the expression of matrix metalloproteinase-2 and CD44 and reduced integrin-beta1 expression in 293T cells, suggesting potential pathways of FAP-mediated impairment of cell adhesion and   migration   in this epithelial cell line. In conclusion, these findings further support a pro-fibrogenic role for FAP by indicating that, in addition to its enzymatic functions, FAP has important nonenzymatic functions that in chronic liver injury may facilitate tissue remodeling through FAP-mediated enhancement of HSC cell adhesion,   migration  , and apoptosis. 
 Downregulation of FAP suppresses cell proliferation and metastasis through PTEN/PI3K/AKT and Ras-ERK signaling in oral squamous cell carcinoma. It is largely recognized that fibroblast activation protein (FAP) is expressed in cancer-associated fibroblasts (CAFs) of many human carcinomas. Furthermore, FAP was recently also reported to be expressed in carcinoma cells of the breast, stomach, pancreatic ductal adenocarcinoma, colorectum, and uterine cervix. The carcinoma cell expression pattern of FAP has been described in several types of cancers, but the role of FAP in oral squamous cell carcinoma (OSCC) is unknown. The role of endogenous FAP in epithelium-derived tumors and molecular mechanisms has also not been reported. In this study, FAP was found to be expressed in carcinoma cells of OSCC and was upregulated in OSCC tissue samples compared with benign tissue samples using immunohistochemistry. In addition, its expression level was closely correlated with overall survival of patients with OSCC. Silencing FAP inhibited the growth and metastasis of OSCC cells in vitro and in vivo. Mechanistically, knockdown of FAP inactivated PTEN/PI3K/AKT and Ras-ERK and its downstream signaling regulating proliferation,   migration  , and invasion in OSCC cells, as the inhibitory effects of FAP on the proliferation and metastasis could be rescued by PTEN silencing. Our study suggests that FAP acts as an oncogene and may be a potential therapeutic target for patients with OSCC. 
 Regulation of fibroblast   migration   on collagenous matrix by a cell surface peptidase complex. The invasion of migratory cells through connective tissues involves metallo- and serine types of cell surface proteases. We show that formation of a novel protease complex, consisting of the membrane-bound prolyl peptidases seprase and dipeptidyl peptidase IV (DPPIV), at invadopodia of migratory fibroblasts is a prerequisite for cell invasion and   migration   on a collagenous matrix. Seprase and DPPIV form a complex on the cell surface that elicits both gelatin binding and gelatinase activities localized at invadopodia of cells migrating on collagenous fibers. The protease complex participates in the binding to gelatin and localized gelatin degradation, cellular   migration  , and monolayer wound closure. Serine protease inhibitors can block the gelatinase activity and the localized gelatin degradation by cells. Antibodies to the gelatin-binding domain of DPPIV reduce the cellular abilities of the proteases to degrade gelatin but do not affect cellular adhesion or spreading on type I collagen. Furthermore, expression of the seprase-DPPIV complex is restricted to migratory cells involved in wound closure in vitro and in connective tissue cells during closure of gingival wounds but not in differentiated tissue cells. Thus, we have identified cell surface proteolytic activities, which are non-metalloproteases, seprase and DPPIV, that are responsible for the tissue-invasive phenotype. 
 Fibroblast activation protein-α promotes ovarian cancer cell proliferation and invasion via extracellular and intracellular signaling mechanisms. Fibroblast activation protein-α (FAPα) is secreted by activated stromal fibroblasts and can promote ovarian cancer cell proliferatio  n, migrat  ion and invasion. However, the molecular mechanism by which FAPα promotes tumor cell proliferation and invasion is unknown. The role of the non-enzymatic activities of FAPα in t  umor migr  ation and invasion and the intracellular and extracellular signaling mechanisms of FAPα were investigated. In this study, we confirm that FAPα promote ovarian cancer cell prolifer  ation, mi  gration and invasion by extracellular and intracellular signaling mechanisms. These results provide evidence that FAPα, together with integrin α3β1 and the uPAR signaling complex, mediate ca  ncer cell   migration in the HO-8910PM cell line via the small GTPase Rac1. FAPα-mediated upregulation of p-ERK occurred in a time-dependent manner. 
 Ultraviolet exposure of melanoma cells induces fibroblast activation protein-α in fibroblasts: Implications for melanoma invasion. Fibroblast activation protein-α (FAP-α) promotes tumor growth and cell invasiveness through extracellular matrix degradation. How ultraviolet radiation (UVR), the major risk factor for malignant melanoma, influences the expression of FAP-α is unknown. We examined the effect of UVR on FAP-α expression in melanocytes, keratinocytes and fibroblasts from the skin and in melanoma cells. UVR induces upregulation of FAP-α in fibroblasts, melanocytes and primary melanoma cells (PM) whereas keratinocytes and metastatic melanoma cells remained FAP-α negative. UVA and UVB stimulated FAP-α  -driven m  igration and invasion in fibroblasts, melanocytes and PM. In co-culture systems UVR of melanocytes, PM and cells from regional metastases upregulated FAP-α in fibroblasts but only supernatants from non-irradiated PM were able to induce FAP-α in fibroblasts. Further, UV-radiated melanocytes and PM significantly increased FAP-α expression in fibroblasts through secretory crosstalk via Wnt5a, PDGF-BB and TGF-β1. Moreover, UV radiated melanocytes and PM increased collagen I i  nvasion a  nd migration of fibroblasts. The FAP-α/DPPIV inhibitor Gly-ProP(OPh)2 significantly decreased this response implicating FAP-α/DPPIV as an important protein co  mplex in   cell migration and invasion. These experiments suggest a functional association between UVR and FAP-α expression in fibroblasts, melanocytes and melanoma cells implicating that UVR of malignant melanoma converts fibroblasts into FAP-α expressing and ECM degrading fibroblasts thus facilitati  ng invasi  on and migration. The secretory crosstalk between melanoma and tumor surrounding fibroblasts is mediated via PDGF-BB, TGF-β1 and Wnt5a and these factors should be evaluated as targets to reduce FAP-α activity and prevent early melanoma dissemination. 
 Over-expression of fibroblast activation protein alpha increases tumor growth in xenografts of ovarian cancer cells. Fibroblast activation protein alpha (FAPα) is a 95-kDa serine protease of post-prolyl peptidase family on cell surface. FAPα is widely expressed in tumor microenvironment. The wide spread association of FAPα expression with cancer suggests that it has important functions in the disease. However, the nature of FAPα's roles in cancer cell activity is not well-determined. It has been showed that FAPα silencing in SKOV3 cells induces ovarian tumors but significantly reduces tumor growth in a xenograft mouse model. To further determine the role of FAPα in epithelial ovarian cancer cells, SKOV3-FAPα and HO8910-FAPα cell lines, which over-expressed FAPα stably, were constructed and then their biological behaviors were investigated. It was found that FAPα promoted ovarian cancer cell proliferation, drug resistance, invasive  ness, and   migration in vitro. Immunochemistry assay showed that FAPα significantly facilitated tumor growth in xenograft tumor tissues. These results suggested that FAPα might directly promote tumor growth and invasiveness in ovarian cancer cells. 
 TGF-beta induces fibroblast activation protein expression; fibroblast activation protein expression increases the proliferation, adhesion, and   migration   of HO-8910PM [corrected]. Several studies recognize cancer-stromal fibroblasts' role in cancer-cell invasion and metastasis. Through paracrine signaling molecules, TGF-beta and IL-1beta, cancer cells activate stromal fibroblasts and induce the expression of fibroblast activation protein (FAP). FAP, in turn, affects the proliferation, invasion and   migration   of the cancer cells. We report that TGF-beta and IL-1beta are important factors in inducing differentiation of myofibroblasts and expression of functional markers, notably alpha-SMA. We discover that TGF-beta is the dominant factor in promoting FAPalpha protein expression. This study also examines FAP's function in vitro by assaying the proliferation,   migration   and invasion of ovarian cancer cell line HO-8910PM. 
 Fibroblast activation protein (FAP) is essential for the   migration   of bone marrow mesenchymal stem cells through RhoA activation. The ability of human bone marrow mesenchymal stem cells (BM-MSCs) to migrate and localize specifically to injured tissues is central in developing therapeutic strategies for tissue repair and regeneration. Fibroblast activation protein (FAP) is a cell surface serine protease expressed at sites of tissue remodeling during embryonic development. It is also expressed in BM-MSCs, but not in normal tissues or cells. The function of FAP in BM-MSCs is not known.We found that depletion of FAP proteins significantly inhibited the   migration   of BM-MSCs in a transwell chemotaxis assay. Such impaired   migration   ability of BM-MSCs could be rescued by re-expressing FAP in these cells. We then demonstrated that depletion of FAP activated intracellular RhoA GTPase. Consistently, inhibition of RhoA activity using a RhoA inhibitor rescued its   migration   ability. Inhibition of FAP activity with an FAP-specific inhibitor did not affect the activation of RhoA or the   migration   of BM-MSCs. Furthermore, the inflammatory cytokines interleukin-1beta (IL-1β) and transforming growth factor-beta (TGF-β) upregulated FAP expression, which coincided with better BM-MS  C migrati  on.Our results indicate FAP plays an important role in th  e migrati  on of BM-MSCs through modulation of RhoA GTPase activity. The peptidase activity of FAP is not essential for suc  h migrati  on. Cytokines IL-1β and TGF-β upregulate the expression level of FAP and thus enhance BM-  MSC migra  tion. 
 Deficiency of fibroblast activation protein alpha ameliorates cartilage destruction in inflammatory destructive arthritis. Inflammatory destructive arthritis, like rheumatoid arthritis (RA), is characterized by invasion of synovial fibroblasts (SF) into the articular cartilage and erosion of the underlying bone, leading to progressive joint destruction. Because fibroblast activation protein alpha (FAP) has been associated with cell   migration   and cell invasiveness, we studied the function of FAP in joint destruction in RA.Expression of FAP in synovial tissues and fibroblasts from patients with osteoarthritis (OA) and RA as well as from wild-type and arthritic mice was evaluated by immunohistochemistry, fluorescence microscopy and polymerase chain reaction (PCR). Fibroblast adhesion and   migration   capacity was assessed using cartilage attachment assays and wound-healing assays, respectively. For in vivo studies, FAP-deficient mice were crossed into the human tumor necrosis factor transgenic mice (hTNFtg), which develop a chronic inflammatory arthritis. Beside clinical assessment, inflammation, cartilage damage, and bone erosion were evaluated by histomorphometric analyses.RA synovial tissues demonstrated high expression of FAP whereas in OA samples only marginal expression was detectable. Consistently, a higher expression was detected in arthritis SF compared to non-arthritis OA SF in vitro. FAP-deficiency in hTNFtg mice led to less cartilage degradation despite unaltered inflammation and bone erosion. Accordingly, FAP(-/-) hTNFtg SF demonstrated a lower cartilage adhesion capacity compared to hTNFtg SF in vitro.These data point to a so far unknown role of FAP in the attachment of SF to cartilage, promoting proteoglycan loss and subsequently cartilage degradation in chronic inflammatory arthritis. 
 Effects of the fibroblast activation protein on the invasion and   migration   of gastric cancer. Cancer-associated fibroblasts (CAFs) are one of the most important components of tumor microenvironment. CAFs are believed to play an important role in tumor invasion and metastasis. Recently, fibroblast activation protein (FAP), a type II integral membrane glycoprotein belonging to the serine protease family, has emerged as a specific marker of CAFs. FAP was overexpressed in stromal fibroblasts of solid malignancies, however, the role of FAP on the process of invasion and metastasis of gastric carcinomas is still unknown.Expression of FAP level was detected by immunohistochemistry in 60 gastric cancer surgical specimens (28 with omentum metastasis and 32 without), 20 normal human gastric tissues and omentum of 10 nonneoplastic gastric diseases. Fibroblasts were isolated from patient's tissues in the distal normal zones and tumor zones respectively, which were correspondingly designated as normal zone fibroblasts (NFs) and cancer-associated fibroblasts (CAFs). To explore the effects of FAP on NFs or CAFs, fibroblasts were co-cultured with human gastric cancer cell line MGC-803 cells. The ability of invasion and   migration   of MGC-803 cells was evaluated after transfecting FAP siRNA into CAFs of gastric carcinomas.We investigated the level of expression of FAP in surgical specimens, and found overexpressed in CAFs and non-expressed in NFs. Expression of FAP level in CAFs is significantly associated with Lauren classification,the degree of differentiation, depth of tumor invasion and TNM stage, but it is not correlated to age and gender in gastric carcinoma patients. There was positive correlation between the FAP level with metastasis to the omentum(p < 0.05, R(2) = 0.2736, p < 0.05, R(2) = 0.1479). In addition, the invasion and   migration   abilities of MGC-803 cells were significantly increased when cells were co-cultured with CAFs. On the other hand, invasion and   migration   abilities were significantly decreased by 46.9 and 50.3%, respectively, after knocking down FAP in CAFs.Further, NFs did not have appreciable effect on the invasion and   migration   of MGC-803 cells.Our findings showed that FAP was overexpressed in CAFs of gastric carcinomas, and siRNA-mediated knock down of FAP significantly suppressed invasion and   migration   of MGC-803 cells. FAP may be an important regulator in the invasion and   migration   of gastric cancer and may provide a novel therapeutic target in gastric carcinomas. 
 Elevation of seprase expression and promotion of an invasive phenotype by collagenous matrices in ovarian tumor cells. Tumor cells do not constitutively exhibit invasive activity, but rather, can be transiently induced to adhere and form lesions. We report here that the expression of seprase, a dominant EDTA-resistant gelatinase in malignant tumors, is dependent on tumor cell exposure to type I collagen gel (TICg). The induced seprase expression of ovarian tumor cells influences their collagen contraction and invasion capability. Importantly, tumor cells with reduced seprase expression, due to manipulation by RNA interference, showed a reduction of TICg contraction in the gel contractility assay, inhibition of tumor cell invasion through TICg as shown by a transwell   migration   assay and inhibition of peritoneal membrane tumor lesion in a mouse model. In addition, mAb C27, an antibody against beta1 integrin, which blocks cellular avidity to TICg, can induce seprase RNA expression and promote the invasive phenotype and metastatic potential of ovarian tumor cells. Thus, collagenous matrices in the tumor cell niche induce the expression of seprase and initiate tumor invasion and metastatic cascades. 
 Fibroblast activation protein increases metastatic potential of fibrosarcoma line HT1080 through upregulation of integrin-mediated signaling pathways. The serine protease fibroblast activation protein (FAP) is selectively expressed on tumour-associated fibroblasts in most human epithelial tumours, as well as on some mesenchymal tumours such as sarcoma. High FAP expression is most often associated with poor outcome and increased metastasis. Here, we compare the in vitro metastatic potential of HT1080 fibrosarcoma cells with and without FAP expression in order to elucidate the mechanism by which FAP may influence metastasis. In the presence of FAP, cells were more adhesive to extracellular matrix proteins and migrated and invaded through Matrigel to a greater degree. The anti-FAP antibody ESC11, which caused internalization of FAP, decreased adhesion and   migration  , but only when cells expressed FAP. It was also found that blocking activity of integrins β1 and αvβ3 reduced both cell adhesion a  nd migrat  ion and this effect was much more marked in FAP-expressing HT1080 cells than mock-transfected HT1080 cells. The expression or activation of intracellular proteins that form part of the downstream signaling of integrins, including integrin-linked kinase, Rac1 and focal adhesion kinase, was also upregulated when FAP was expressed, suggesting that FAP not only upregulates metastatic-like cell behaviours through interaction with integrins, but also influences the intracellular signaling of integrins. This was confirmed using both PI3 kinase and Src kinase inhibitors, which decreased adhesion a  nd migrat  ion in FAP-expressing cells, but did not affect mock-transfected HT1080 cells. FAP is therefore a useful target for anti-cancer therapy, as not only is its expression tumour-selective, but its downregulation has the potential to reduce incidence of metastasis. 
 FAP-α (Fibroblast activation protein-α) is involved in the control of human breast cancer cell line growth and motility via the FAK pathway. Fibroblast Activation Protein alpha (FAP-α) or seprase is an integral membrane serine peptidase. Previous work has not satisfactorily explained both the suppression and promotion effects that have been observed in cancer. The purpose of this work was to investigate the role of FAP-α in human breast cancer. Expression of FAP-α was characterized in primary tumour samples and in cell lines, along with the effects of FAP-α expression on in vitro growth, invasion, attachmen  t and mig  ration. Furthermore the potential interaction of FAP-α with other signalling pathways was investigated.FAP-α was significantly increased in patients with poor outcome and survival. In vitro results showed that breast cancer cells over expressing FAP-α had increased growth ability and impaired migratory ability. The growth of MDA-MB-231 cells and the adhesion and invasion ability of both MCF-7 cells and MDA-MB-231 cells were not dramatically influenced by FAP-α expression. Over-expression of FAP-α resulted in a reduction of phosphorylated focal adhesion kinase (FAK) level in both cells cultured in normal media and serum-free media. An inhibitor to FAK restored the reduced motility ability of both MCF-7exp cells and MDA-MB-231exp cells and prevented the change in phosphorylated FAK levels. However, inhibitors to PI3K, ERK, PLCΥ, NWASP, ARP2/3, and ROCK had no influence this.FAP-α in significantly associated with poor outcome in patients with breast cancer. In vitro, FAP-α promotes proliferation   and inhi  bits migration of breast cancer cells, potentially by regulating the FAK pathway. These results suggest FAP-α could be a target for future therapies. 
 Fibroblast activation protein, a dual specificity serine protease expressed in reactive human tumor stromal fibroblasts. Proteolytic degradation of extracellular matrix (ECM) components during tissue remodeling plays a pivotal role in normal and pathological processes including wound healing, inflammation, tumor invasion, and metastasis. Proteolytic enzymes in tumors may activate or release growth factors from the ECM or act directly on the ECM itself, thereby facilitating angiogenesis or tumor cell   migration  . Fibroblast activation protein (FAP) is a cell surface antigen of reactive tumor stromal fibroblasts found in epithelial cancers and in granulation tissue during wound healing. It is absent from most normal adult human tissues. FAP is conserved throughout chordate evolution, with homologues in mouse and Xenopus laevis, whose expression correlates with tissue remodeling events. Using recombinant and purified natural FAP, we show that FAP has both dipeptidyl peptidase activity and a collagenolytic activity capable of degrading gelatin and type I collagen; by sequence, FAP belongs to the serine protease family rather than the matrix metalloprotease family. Mutation of the putative catalytic serine residue of FAP to alanine abolishes both enzymatic activities. Consistent with its in vivo expression pattern determined by immunohistochemistry, FAP enzyme activity was detected by an immunocapture assay in human cancerous tissues but not in matched normal tissues. This study demonstrates that FAP is present as an active cell surface-bound collagenase in epithelial tumor stroma and opens up investigation into physiological substrates of its novel, tumor-associated dipeptidyl peptidase activity. 
  GeneRIFs  
 In this study, we show for the first time the expression of FAP in activated fibroblasts after MI and its activation by TGFbeta1. Effects of FAP on fibroblast   migration   and gelatinolytic activity indicate a potential role in cardiac wound healing 
 knockdown of FAP inactivated PTEN/PI3K/AKT and Ras-ERK and its downstream signaling regulating proliferation,   migration  , and invasion in oral squamous cell carcinoma cells. 
 In vitro, FAP-alpha promotes proliferation and inhibits   migration   of breast cancer cells, potentially by regulating the FAK pathway. 
 FAP is essential for the   migration   of bone marrow mesenchymal stem cells through RhoA activation. 
 High fibroblast activation protein is associated with invasion and   migration   of gastric cancer. 
 These experiments suggest a functional association between UVR and FAP-alpha expression that UVR of malignant melanoma converts fibroblasts into FAP-alpha expressing and ECM degrading fibroblasts thus facilitating invasion and   migration  . 
 This study examines FAP's function in vitro by assaying the proliferation,   migration   and invasion of ovarian cancer cell line HO-8910PM.. 
 overexpression in HEK293 cells reduced cell adhesion,   migration   and invasion on ECM components, increased proliferation and promoted apoptosis, independently of enzyme activity 
 FAP has important nonenzymatic functions that in chronic liver injury may facilitate tissue remodeling through FAP-mediated enhancement of HSC cell adhesion,   migration  , and apoptosis 
  ECM  PubMed  
 Molecular proximity of seprase and the urokinase-type plasminogen activator receptor on malignant melanoma cell membranes: dependence on beta1 integrins and the cytoskeleton. Previous studies have shown that several proteolytic enzymes are associated with membrane protrusions at the leading edge of migrating tumor cells. In this study we demonstrate that seprase and the urokinase plasminogen activator receptor (uPAR), co-localize in the plasma membrane of LOX malignant melanoma cells. Cells were labeled with fluorochrome-conjugated monoclonal antibodies (mAb) directed against seprase and uPAR. Proximity between these two molecules was detected with resonance energy transfer (RET) imaging, single-cell emission spectrophotometry, and single-cell excitation spectrophotometry. Significant RET signals were detected on LOX cells when adherent to uncoated and extracellular matrix (  ECM  )-coated surfaces. This indicates that seprase and uPAR are within approximately 7 nm in the plasma membrane of LOX cells. When LOX cells adhered to a 3D extracellular-like matrix, seprase-uPAR complexes were found to be associated with invadopodia. Further microscopy experiments demonstrated gelatinolytic activity, a functional attribute of seprase, in association with seprase-uPAR membrane domains. Formation of seprase-uPAR membrane complexes is dependent upon both the cytoskeleton and integrins. Specifically, the involvement of beta(1)-integrins was demonstrated by the inhibition of RET by an inhibitory anti-beta(1)-integrin mAb. Based on these findings, we speculate that formation of heterogeneous lytic domains in the invading membranes of LOX cells increases the efficiency of directed pericellular proteolysis. 
 Molecular proximity of seprase and the urokinase-type plasminogen activator receptor on malignant melanoma cell membranes: dependence on beta1 integrins and the cytoskeleton. Previous studies have shown that several proteolytic enzymes are associated with membrane protrusions at the leading edge of migrating tumor cells. In this study we demonstrate that seprase and the urokinase plasminogen activator receptor (uPAR), co-localize in the plasma membrane of LOX malignant melanoma cells. Cells were labeled with fluorochrome-conjugated monoclonal antibodies (mAb) directed against seprase and uPAR. Proximity between these two molecules was detected with resonance energy transfer (RET) imaging, single-cell emission spectrophotometry, and single-cell excitation spectrophotometry. Significant RET signals were detected on LOX cells when adherent to uncoated and extracellular matrix (  ECM  )-coated surfaces. This indicates that seprase and uPAR are within approximately 7 nm in the plasma membrane of LOX cells. When LOX cells adhered to a 3D extracellular-like matrix, seprase-uPAR complexes were found to be associated with invadopodia. Further microscopy experiments demonstrated gelatinolytic activity, a functional attribute of seprase, in association with seprase-uPAR membrane domains. Formation of seprase-uPAR membrane complexes is dependent upon both the cytoskeleton and integrins. Specifically, the involvement of beta(1)-integrins was demonstrated by the inhibition of RET by an inhibitory anti-beta(1)-integrin mAb. Based on these findings, we speculate that formation of heterogeneous lytic domains in the invading membranes of LOX cells increases the efficiency of directed pericellular proteolysis. 
 Cleavage of Type I Collagen by Fibroblast Activation Protein-α Enhances Class A Scavenger Receptor Mediated Macrophage Adhesion. Pathophysiological conditions such as fibrosis, inflammation, and tumor progression are associated with modification of the extracellular matrix   (EC  M). These modifications create ligands that differentially interact with cells to promote responses that drive pathological processes. Within the tumor stroma, fibroblasts are activated and increase the expression of type I collagen. In addition, activated fibroblasts specifically express fibroblast activation protein-α (FAP), a post-prolyl peptidase. Although FAP reportedly cleaves type I collagen and contributes to tumor progression, the specific pathophysiologic role of FAP is not clear. In this study, the possibility that FAP-mediated cleavage of type I collagen modulates macrophage interaction with collagen was examined using macrophage adhesion assays. Our results demonstrate that FAP selectively cleaves type I collagen resulting in increased macrophage adhesion. Increased macrophage adhesion to FAP-cleaved collagen was not affected by inhibiting integrin-mediated interactions, but was abolished in macrophages lacking the class A scavenger receptor (SR-A/CD204). Further, SR-A expressing macrophages localize with activated fibroblasts in breast tumors of MMTV-PyMT mice. Together, these results demonstrate that FAP-cleaved collagen is a substrate for SR-A-dependent macrophage adhesion, and suggest that by modifying th  e E  CM, FAP plays a novel role in mediating communication between activated fibroblasts and macrophages. 
 Fibroblast activation protein increases apoptosis, cell adhesion, and migration by the LX-2 human stellate cell line. Injury and repair in chronic liver disease involve cell adhesion, migration, apoptosis, proliferation, and a wound healing response. In liver, fibroblast activation protein (FAP) has both collagenase and dipeptidyl peptidase IV (DPIV) activities and is expressed only by activated hepatic stellate cells (HSC) and myofibroblasts, which produce and degrade extracellular matrix (  ECM  ). FAP was colocalized with collagen fibers, fibronectin, and collagen type I in human liver. FAP function was examined in vitro by expressing green fluorescent protein FAP fusion protein in cell lines cultured on collagen-I, fibronectin, and Matrigel. Glutamates at 203 and 204 as well as serine624 of FAP were essential for peptidase activity. Human embryonic kidney 293T cells overexpressing FAP showed reduced adhesion and migration. FAP overexpression in the human HSC line LX-2 caused increased cell adhesion and migration on   ECM   proteins as well as invasion across transwells in the absence or presence of transforming growth factor beta-1. FAP overexpression enhanced staurosporine streptomyces-stimulated apoptosis in both cell lines. Interestingly, the enzyme activity of FAP was not required for these functions. Overexpressing FAP increased the expression of matrix metalloproteinase-2 and CD44 and reduced integrin-beta1 expression in 293T cells, suggesting potential pathways of FAP-mediated impairment of cell adhesion and migration in this epithelial cell line. In conclusion, these findings further support a pro-fibrogenic role for FAP by indicating that, in addition to its enzymatic functions, FAP has important nonenzymatic functions that in chronic liver injury may facilitate tissue remodeling through FAP-mediated enhancement of HSC cell adhesion, migration, and apoptosis. 
 Ultraviolet exposure of melanoma cells induces fibroblast activation protein-α in fibroblasts: Implications for melanoma invasion. Fibroblast activation protein-α (FAP-α) promotes tumor growth and cell invasiveness through extracellular matrix degradation. How ultraviolet radiation (UVR), the major risk factor for malignant melanoma, influences the expression of FAP-α is unknown. We examined the effect of UVR on FAP-α expression in melanocytes, keratinocytes and fibroblasts from the skin and in melanoma cells. UVR induces upregulation of FAP-α in fibroblasts, melanocytes and primary melanoma cells (PM) whereas keratinocytes and metastatic melanoma cells remained FAP-α negative. UVA and UVB stimulated FAP-α-driven migration and invasion in fibroblasts, melanocytes and PM. In co-culture systems UVR of melanocytes, PM and cells from regional metastases upregulated FAP-α in fibroblasts but only supernatants from non-irradiated PM were able to induce FAP-α in fibroblasts. Further, UV-radiated melanocytes and PM significantly increased FAP-α expression in fibroblasts through secretory crosstalk via Wnt5a, PDGF-BB and TGF-β1. Moreover, UV radiated melanocytes and PM increased collagen I invasion and migration of fibroblasts. The FAP-α/DPPIV inhibitor Gly-ProP(OPh)2 significantly decreased this response implicating FAP-α/DPPIV as an important protein complex in cell migration and invasion. These experiments suggest a functional association between UVR and FAP-α expression in fibroblasts, melanocytes and melanoma cells implicating that UVR of malignant melanoma converts fibroblasts into FAP-α   ex  pressing and ECM degrading fibroblasts thus facilitating invasion and migration. The secretory crosstalk between melanoma and tumor surrounding fibroblasts is mediated via PDGF-BB, TGF-β1 and Wnt5a and these factors should be evaluated as targets to reduce FAP-α activity and prevent early melanoma dissemination. 
 Increased expression of fibroblast activation protein-alpha in keloid fibroblasts: implications for development of a novel treatment option. Keloid scars are common benign fibroproliferative reticular dermal lesions with unknown etiology and ill-defined management with high rate of recurrence post surgery. The progression of keloids is characterized by increased deposition of extracellular matrix proteins, invasion into the surrounding healthy skin and inflammation. Fibroblasts are considered to be the key cellular mediators of fibrogenesis in keloid scars. Fibroblast activation protein alpha (FAP-α) and dipeptidyl peptidase IV (DPPIV) are proteases located at the plasma membrane promoting cell invasiveness and tumor growth and have been previously associated with keloid scars. Therefore, in this study we analyzed in further detail the expression of FAP-α in keloid fibroblasts compared to control skin fibroblasts. Dermal fibroblasts were obtained from punch-biopsies from the active margin of four keloids and four control skin samples. Flow cytometry was used to analyze FAP-α expression and the CytoSelect 24-Well Collagen I Cell Invasion Assay was applied to study fibroblast invasion. Secretion of extracellular matri  x (  ECM) proteins was investigated by multiplexed particle-based flow cytometric assay and enzyme-linked immunosorbent assay. We found an increased expression of FAP-α in keloid fibroblasts compared to control skin fibroblasts (p < 0.001). Inhibition of FAP-α/DPPIV activity using the irreversible inhibitor H(2)N-Gly-Pro diphenylphosphonate reduced the increased invasiveness of keloid fibroblasts (p < 0.001) indicating that keloid invasion may be partly FAP-α/DPPIV mediated. FAP-α/DPPIV inhibition had no effect, (a) on the synthesis   of   the ECM proteins procollagen type I C-terminal peptide and fibronectin, (b) on the production of fibroblast growth factor or vascular endothelial growth factor, (c) on the expression of the proinflammatory cytokines interleukin-6 (IL-6), interleukin 8 (IL-8) or monocyte chemotactic protein-1. These results suggest a potential role for FAP-α and DPPIV in the invasive behavior of keloids. FAP-α and DPPIV may increase the invasive capacity of keloid fibroblasts rather than by modulating inflamm  ati  on or ECM production. Since FAP-α expression is restricted to reactive fibroblasts in wound healing and normal adult tissues are generally FAP-α negative, inhibiting FAP-α/DPPIV activity may be a novel treatment option to prevent keloid progression. 
 Upregulated expression and activation of membrane‑associated proteases in esophageal squamous cell carcinoma. To better understand the role of membrane-associated proteolytic systems in the development of esophageal cancer, we studied the expression of two serine proteases, fibroblast activation protein-α (FAP-α) and dipeptidyl peptidase IV (DPPIV) and three metalloproteinases, matrix metalloproteinase (MMP)-2, MMP-9 and MT1-MMP in 24 primary esophageal squamous cell carcinoma (ESCC) tissues and paired non-cancer tissues. Using reverse-transcription PCR, western blotting and zymography, we showed that both serine proteases and all three metalloproteinases were highly altered in ESCC. A positive correlation between the expression of FAP-α and DPPIV and the activity of both gelatinases was found. This may indicate that these proteolytic systems are tightly linked to each other and collectively are involved in the proc  ess   of ECM degradation that facilitates cancer cell invasion and metastasis. 
 Fibroblast activation protein, a dual specificity serine protease expressed in reactive human tumor stromal fibroblasts. Proteolytic degradation of extracellular matrix (  ECM  ) components during tissue remodeling plays a pivotal role in normal and pathological processes including wound healing, inflammation, tumor invasion, and metastasis. Proteolytic enzymes in tumors may activate or release growth factors from the   ECM   or act directly on the   ECM   itself, thereby facilitating angiogenesis or tumor cell migration. Fibroblast activation protein (FAP) is a cell surface antigen of reactive tumor stromal fibroblasts found in epithelial cancers and in granulation tissue during wound healing. It is absent from most normal adult human tissues. FAP is conserved throughout chordate evolution, with homologues in mouse and Xenopus laevis, whose expression correlates with tissue remodeling events. Using recombinant and purified natural FAP, we show that FAP has both dipeptidyl peptidase activity and a collagenolytic activity capable of degrading gelatin and type I collagen; by sequence, FAP belongs to the serine protease family rather than the matrix metalloprotease family. Mutation of the putative catalytic serine residue of FAP to alanine abolishes both enzymatic activities. Consistent with its in vivo expression pattern determined by immunohistochemistry, FAP enzyme activity was detected by an immunocapture assay in human cancerous tissues but not in matched normal tissues. This study demonstrates that FAP is present as an active cell surface-bound collagenase in epithelial tumor stroma and opens up investigation into physiological substrates of its novel, tumor-associated dipeptidyl peptidase activity. 
 FAP-overexpressing fibroblasts produce an extracellular matrix that enhances invasive velocity and directionality of pancreatic cancer cells. Alterations towards a permissive stromal microenvironment provide important cues for tumor growth, invasion, and metastasis. In this study, Fibroblast activation protein (FAP), a serine protease selectively produced by tumor-associated fibroblasts in over 90% of epithelial tumors, was used as a platform for studying tumor-stromal interactions. We tested the hypothesis that FAP enzymatic activity locally modifies stromal   ECM   (extracellular matrix) components thus facilitating the formation of a permissive microenvironment promoting tumor invasion in human pancreatic cancer.We generated a tetracycline-inducible FAP overexpressing fibroblastic cell line to synthesize an in vivo-like 3-dimensional (3D) matrix system which was utilized as a stromal landscape for studying matrix-induced cancer cell behaviors. A FAP-dependent topographical and compositional alteration of the   ECM   was characterized by measuring the relative orientation angles of fibronectin fibers and by Western blot analyses. The role of FAP in the matrix-induced permissive tumor behavior was assessed in Panc-1 cells in assorted matrices by time-lapse acquisition assays. Also, FAP+ matrix-induced regulatory molecules in cancer cells were determined by Western blot analyses.We observed that FAP remodels the   ECM   through modulating protein levels, as well as through increasing levels of fibronectin and collagen fiber organization. FAP-dependent architectural/compositional alterations of the   ECM   promote tumor invasion along characteristic parallel fiber orientations, as demonstrated by enhanced directionality and velocity of pancreatic cancer cells on FAP+ matrices. This phenotype can be reversed by inhibition of FAP enzymatic activity during matrix production resulting in the disorganization of the   ECM   and impeded tumor invasion. We also report that the FAP+ matrix-induced tumor invasion phenotype is β1-integrin/FAK mediated.Cancer cell invasiveness can be affected by alterations in the tumor microenvironment. Disruption of FAP activity and β1-integrins may abrogate the invasive capabilities of pancreatic and other tumors by disrupting the FAP-directed organization of stroma  l E  CM and blocking β1-integrin dependent cell-matrix interactions. This provides a novel preclinical rationale for therapeutics aimed at interfering with the architectural organization of tumor-associat  ed   ECM. Better understanding of the stromal influences that fuel progressive tumorigenic behaviors may allow the effective future use of targeted therapeutics aimed at disrupting specific tumor-stromal interactions. 
  GeneRIFs  
 FAP remodels the   ECM   by modulating protein levels & increasing fibronectin levels & collagen fiber organization. FAP-dependent architectural/compositional   ECM   alterations promote tumor invasion along characteristic parallel fiber orientations. 
 These experiments suggest a functional association between UVR and FAP-alpha expression that UVR of malignant melanoma converts fibroblasts into FAP-alpha expressing and   ECM   degrading fibroblasts thus facilitating invasion and migration. 
 overexpression in HEK293 cells reduced cell adhesion, migration and invasion on   ECM   components, increased proliferation and promoted apoptosis, independently of enzyme activity 
  [Ee]xtracellular [Mm]atrix  Process  
 negative regulation of   extracellular matrix   disassembly 
 negative regulation of   extracellular matrix   organization 
  PubMed  
 Molecular proximity of seprase and the urokinase-type plasminogen activator receptor on malignant melanoma cell membranes: dependence on beta1 integrins and the cytoskeleton. Previous studies have shown that several proteolytic enzymes are associated with membrane protrusions at the leading edge of migrating tumor cells. In this study we demonstrate that seprase and the urokinase plasminogen activator receptor (uPAR), co-localize in the plasma membrane of LOX malignant melanoma cells. Cells were labeled with fluorochrome-conjugated monoclonal antibodies (mAb) directed against seprase and uPAR. Proximity between these two molecules was detected with resonance energy transfer (RET) imaging, single-cell emission spectrophotometry, and single-cell excitation spectrophotometry. Significant RET signals were detected on LOX cells when adherent to uncoated and   extracellular matrix   (ECM)-coated surfaces. This indicates that seprase and uPAR are within approximately 7 nm in the plasma membrane of LOX cells. When LOX cells adhered to a 3D extracellular-like matrix, seprase-uPAR complexes were found to be associated with invadopodia. Further microscopy experiments demonstrated gelatinolytic activity, a functional attribute of seprase, in association with seprase-uPAR membrane domains. Formation of seprase-uPAR membrane complexes is dependent upon both the cytoskeleton and integrins. Specifically, the involvement of beta(1)-integrins was demonstrated by the inhibition of RET by an inhibitory anti-beta(1)-integrin mAb. Based on these findings, we speculate that formation of heterogeneous lytic domains in the invading membranes of LOX cells increases the efficiency of directed pericellular proteolysis. 
 Molecular proximity of seprase and the urokinase-type plasminogen activator receptor on malignant melanoma cell membranes: dependence on beta1 integrins and the cytoskeleton. Previous studies have shown that several proteolytic enzymes are associated with membrane protrusions at the leading edge of migrating tumor cells. In this study we demonstrate that seprase and the urokinase plasminogen activator receptor (uPAR), co-localize in the plasma membrane of LOX malignant melanoma cells. Cells were labeled with fluorochrome-conjugated monoclonal antibodies (mAb) directed against seprase and uPAR. Proximity between these two molecules was detected with resonance energy transfer (RET) imaging, single-cell emission spectrophotometry, and single-cell excitation spectrophotometry. Significant RET signals were detected on LOX cells when adherent to uncoated and   extracellular matrix   (ECM)-coated surfaces. This indicates that seprase and uPAR are within approximately 7 nm in the plasma membrane of LOX cells. When LOX cells adhered to a 3D extracellular-like matrix, seprase-uPAR complexes were found to be associated with invadopodia. Further microscopy experiments demonstrated gelatinolytic activity, a functional attribute of seprase, in association with seprase-uPAR membrane domains. Formation of seprase-uPAR membrane complexes is dependent upon both the cytoskeleton and integrins. Specifically, the involvement of beta(1)-integrins was demonstrated by the inhibition of RET by an inhibitory anti-beta(1)-integrin mAb. Based on these findings, we speculate that formation of heterogeneous lytic domains in the invading membranes of LOX cells increases the efficiency of directed pericellular proteolysis. 
 Structural and kinetic analysis of the substrate specificity of human fibroblast activation protein alpha. Fibroblast activation protein alpha (FAPalpha) is highly expressed in epithelial cancers and has been implicated in   extracellular matrix   remodeling, tumor growth, and metastasis. We present the first high resolution structure for the apoenzyme as well as kinetic data toward small dipeptide substrates. FAPalpha exhibits a dipeptidyl peptidase IV (DPPIV)-like fold, featuring an alpha/beta-hydrolase domain and an eight-bladed beta-propeller domain. Known DPPIV dipeptides are cleaved by FAPalpha with an approximately 100-fold decrease in catalytic efficiency compared with DPPIV. Moreover, FAPalpha, but not DPPIV, possesses endopeptidase activity toward N-terminal benzyloxycarbonyl (Z)-blocked peptides. Comparison of the crystal structures of FAPalpha and DPPIV revealed one major difference in the vicinity of the Glu motif (Glu(203)-Glu(204) for FAPalpha; Glu(205)-Glu(206) for DPPIV) within the active site of the enzyme. Ala(657) in FAPalpha, instead of Asp(663) as in DP-PIV, reduces the acidity in this pocket, and this change could explain the lower affinity for N-terminal amines by FAPalpha. This hypothesis was tested by kinetic analysis of the mutant FAPalpha/A657D, which shows on average an approximately 60-fold increase in the catalytic efficiency, as measured by k(cat)/K(m), for the cleavage of dipeptide substrates. Furthermore, the catalytic efficiency of the mutant is reduced by approximately 350-fold for cleavage of Z-Gly-Pro-7-amino-4-methylcoumarin. Our data provide a clear understanding of the molecular determinants responsible for the substrate specificity and endopeptidase activity of FAPalpha. 
 Cleavage of Type I Collagen by Fibroblast Activation Protein-α Enhances Class A Scavenger Receptor Mediated Macrophage Adhesion. Pathophysiological conditions such as fibrosis, inflammation, and tumor progression are associated with modification of the   extracellular matri  x (ECM). These modifications create ligands that differentially interact with cells to promote responses that drive pathological processes. Within the tumor stroma, fibroblasts are activated and increase the expression of type I collagen. In addition, activated fibroblasts specifically express fibroblast activation protein-α (FAP), a post-prolyl peptidase. Although FAP reportedly cleaves type I collagen and contributes to tumor progression, the specific pathophysiologic role of FAP is not clear. In this study, the possibility that FAP-mediated cleavage of type I collagen modulates macrophage interaction with collagen was examined using macrophage adhesion assays. Our results demonstrate that FAP selectively cleaves type I collagen resulting in increased macrophage adhesion. Increased macrophage adhesion to FAP-cleaved collagen was not affected by inhibiting integrin-mediated interactions, but was abolished in macrophages lacking the class A scavenger receptor (SR-A/CD204). Further, SR-A expressing macrophages localize with activated fibroblasts in breast tumors of MMTV-PyMT mice. Together, these results demonstrate that FAP-cleaved collagen is a substrate for SR-A-dependent macrophage adhesion, and suggest that by modifying the ECM, FAP plays a novel role in mediating communication between activated fibroblasts and macrophages. 
 The protease complex consisting of dipeptidyl peptidase IV and seprase plays a role in the migration and invasion of human endothelial cells in collagenous matrices. Dipeptidyl peptidase IV (DPP4/CD26) and seprase/fibroblast activation protein alpha are homologous type II transmembrane, homodimeric glycoproteins that exhibit unique prolyl peptidase activities. Human DPP4 is ubiquitously expressed in epithelial and endothelial cells and serves multiple functions in cleaving the penultimate positioned prolyl bonds at the NH(2) terminus of a variety of physiologically important peptides in the circulation. Recent studies showed a linkage between DPP4 and down-regulation of certain chemokines and mitogenic growth factors, and degradation of denatured collagens (gelatin), suggesting a role of DPP4 in the cell invasive phenotype. Here, we found the existence of a novel protease complex consisting of DPP4 and seprase in human endothelial cells that were activated to migrate and invade in the   extracellular matrix   in vitro. DPP4 and seprase were coexpressed with the three major protease systems (matrix metalloproteinase, plasminogen activator, and type II transmembrane serine protease) at the cell surface and organize as a complex at invadopodia-like protrusions. Both proteases were colocalized at the endothelial cells of capillaries, but not large blood vessels, in invasive breast ductal carcinoma in vivo. Importantly, monoclonal antibodies against the gelatin-binding domain of DPP4 blocked the local gelatin degradation by endothelial cells in the presence of the major metallo- and serine protease systems that modified pericellular collagenous matrices and subsequent cell migration and invasion. Thus, we have identified a novel mechanism involving the DPP4 gelatin-binding domain of the DPP4-seprase complex that facilitates the local degradation of the   extracellular matrix   and the invasion of the endothelial cells into collagenous matrices. 
 Fibroblast activation protein increases apoptosis, cell adhesion, and migration by the LX-2 human stellate cell line. Injury and repair in chronic liver disease involve cell adhesion, migration, apoptosis, proliferation, and a wound healing response. In liver, fibroblast activation protein (FAP) has both collagenase and dipeptidyl peptidase IV (DPIV) activities and is expressed only by activated hepatic stellate cells (HSC) and myofibroblasts, which produce and degrade   extracellular matrix   (ECM). FAP was colocalized with collagen fibers, fibronectin, and collagen type I in human liver. FAP function was examined in vitro by expressing green fluorescent protein FAP fusion protein in cell lines cultured on collagen-I, fibronectin, and Matrigel. Glutamates at 203 and 204 as well as serine624 of FAP were essential for peptidase activity. Human embryonic kidney 293T cells overexpressing FAP showed reduced adhesion and migration. FAP overexpression in the human HSC line LX-2 caused increased cell adhesion and migration on ECM proteins as well as invasion across transwells in the absence or presence of transforming growth factor beta-1. FAP overexpression enhanced staurosporine streptomyces-stimulated apoptosis in both cell lines. Interestingly, the enzyme activity of FAP was not required for these functions. Overexpressing FAP increased the expression of matrix metalloproteinase-2 and CD44 and reduced integrin-beta1 expression in 293T cells, suggesting potential pathways of FAP-mediated impairment of cell adhesion and migration in this epithelial cell line. In conclusion, these findings further support a pro-fibrogenic role for FAP by indicating that, in addition to its enzymatic functions, FAP has important nonenzymatic functions that in chronic liver injury may facilitate tissue remodeling through FAP-mediated enhancement of HSC cell adhesion, migration, and apoptosis. 
 Ultraviolet exposure of melanoma cells induces fibroblast activation protein-α in fibroblasts: Implications for melanoma invasion. Fibroblast activation protein-α (FAP-α) promotes tumor growth and cell invasiveness throu  gh extracellular mat  rix degradation. How ultraviolet radiation (UVR), the major risk factor for malignant melanoma, influences the expression of FAP-α is unknown. We examined the effect of UVR on FAP-α expression in melanocytes, keratinocytes and fibroblasts from the skin and in melanoma cells. UVR induces upregulation of FAP-α in fibroblasts, melanocytes and primary melanoma cells (PM) whereas keratinocytes and metastatic melanoma cells remained FAP-α negative. UVA and UVB stimulated FAP-α-driven migration and invasion in fibroblasts, melanocytes and PM. In co-culture systems UVR of melanocytes, PM and cells from regional metastases upregulated FAP-α in fibroblasts but only supernatants from non-irradiated PM were able to induce FAP-α in fibroblasts. Further, UV-radiated melanocytes and PM significantly increased FAP-α expression in fibroblasts through secretory crosstalk via Wnt5a, PDGF-BB and TGF-β1. Moreover, UV radiated melanocytes and PM increased collagen I invasion and migration of fibroblasts. The FAP-α/DPPIV inhibitor Gly-ProP(OPh)2 significantly decreased this response implicating FAP-α/DPPIV as an important protein complex in cell migration and invasion. These experiments suggest a functional association between UVR and FAP-α expression in fibroblasts, melanocytes and melanoma cells implicating that UVR of malignant melanoma converts fibroblasts into FAP-α expressing and ECM degrading fibroblasts thus facilitating invasion and migration. The secretory crosstalk between melanoma and tumor surrounding fibroblasts is mediated via PDGF-BB, TGF-β1 and Wnt5a and these factors should be evaluated as targets to reduce FAP-α activity and prevent early melanoma dissemination. 
 Increased expression of fibroblast activation protein-alpha in keloid fibroblasts: implications for development of a novel treatment option. Keloid scars are common benign fibroproliferative reticular dermal lesions with unknown etiology and ill-defined management with high rate of recurrence post surgery. The progression of keloids is characterized by increased deposition of   extracellular matrix   proteins, invasion into the surrounding healthy skin and inflammation. Fibroblasts are considered to be the key cellular mediators of fibrogenesis in keloid scars. Fibroblast activation protein alpha (FAP-α) and dipeptidyl peptidase IV (DPPIV) are proteases located at the plasma membrane promoting cell invasiveness and tumor growth and have been previously associated with keloid scars. Therefore, in this study we analyzed in further detail the expression of FAP-α in keloid fibroblasts compared to control skin fibroblasts. Dermal fibroblasts were obtained from punch-biopsies from the active margin of four keloids and four control skin samples. Flow cytometry was used to analyze FAP-α expression and the CytoSelect 24-Well Collagen I Cell Invasion Assay was applied to study fibroblast invasion. Secretion   of extracellular mat  rix (ECM) proteins was investigated by multiplexed particle-based flow cytometric assay and enzyme-linked immunosorbent assay. We found an increased expression of FAP-α in keloid fibroblasts compared to control skin fibroblasts (p < 0.001). Inhibition of FAP-α/DPPIV activity using the irreversible inhibitor H(2)N-Gly-Pro diphenylphosphonate reduced the increased invasiveness of keloid fibroblasts (p < 0.001) indicating that keloid invasion may be partly FAP-α/DPPIV mediated. FAP-α/DPPIV inhibition had no effect, (a) on the synthesis of the ECM proteins procollagen type I C-terminal peptide and fibronectin, (b) on the production of fibroblast growth factor or vascular endothelial growth factor, (c) on the expression of the proinflammatory cytokines interleukin-6 (IL-6), interleukin 8 (IL-8) or monocyte chemotactic protein-1. These results suggest a potential role for FAP-α and DPPIV in the invasive behavior of keloids. FAP-α and DPPIV may increase the invasive capacity of keloid fibroblasts rather than by modulating inflammation or ECM production. Since FAP-α expression is restricted to reactive fibroblasts in wound healing and normal adult tissues are generally FAP-α negative, inhibiting FAP-α/DPPIV activity may be a novel treatment option to prevent keloid progression. 
 Fibroblast activation protein increases metastatic potential of fibrosarcoma line HT1080 through upregulation of integrin-mediated signaling pathways. The serine protease fibroblast activation protein (FAP) is selectively expressed on tumour-associated fibroblasts in most human epithelial tumours, as well as on some mesenchymal tumours such as sarcoma. High FAP expression is most often associated with poor outcome and increased metastasis. Here, we compare the in vitro metastatic potential of HT1080 fibrosarcoma cells with and without FAP expression in order to elucidate the mechanism by which FAP may influence metastasis. In the presence of FAP, cells were more adhesive to   extracellular matrix   proteins and migrated and invaded through Matrigel to a greater degree. The anti-FAP antibody ESC11, which caused internalization of FAP, decreased adhesion and migration, but only when cells expressed FAP. It was also found that blocking activity of integrins β1 and αvβ3 reduced both cell adhesion and migration and this effect was much more marked in FAP-expressing HT1080 cells than mock-transfected HT1080 cells. The expression or activation of intracellular proteins that form part of the downstream signaling of integrins, including integrin-linked kinase, Rac1 and focal adhesion kinase, was also upregulated when FAP was expressed, suggesting that FAP not only upregulates metastatic-like cell behaviours through interaction with integrins, but also influences the intracellular signaling of integrins. This was confirmed using both PI3 kinase and Src kinase inhibitors, which decreased adhesion and migration in FAP-expressing cells, but did not affect mock-transfected HT1080 cells. FAP is therefore a useful target for anti-cancer therapy, as not only is its expression tumour-selective, but its downregulation has the potential to reduce incidence of metastasis. 
 Fibroblast activation protein alpha is expressed by chondrocytes following a pro-inflammatory stimulus and is elevated in osteoarthritis. Arthritis is characterised by the proteolytic degradation of articular cartilage leading to a loss of joint function. Articular cartilage is composed of an   extracellular matrix   of proteoglycans and collagens. We have previously shown that serine proteinases are involved in the activation cascades leading to cartilage collagen degradation. The aim of this study was to use an active-site probe, biotinylated fluorophosphonate, to identify active serine proteinases present on the chondrocyte membrane after stimulation with the pro-inflammatory cytokines IL-1 and oncostatin M (OSM), agents that promote cartilage resorption. Fibroblast activation protein alpha (FAPalpha), a type II integral membrane serine proteinase, was identified on chondrocyte membranes stimulated with IL-1 and OSM. Real-time PCR analysis shows that FAPalpha gene expression is up-regulated by this cytokine combination in both isolated chondrocytes and cartilage explant cultures and is significantly higher in cartilage from OA patients compared to phenotypically normal articular cartilage. Immunohistochemistry analysis shows FAPalpha expression on chondrocytes in the superficial zone of OA cartilage tissues. This is the first report demonstrating the expression of active FAPalpha on the chondrocyte membrane and elevated levels in cartilage from OA patients. Its cell surface location and expression profile suggest that it may have an important pathological role in the cartilage turnover prevalent in arthritic diseases. 
 Expression of dipeptidyl peptidase-IV activity and/or structure homologs in human meningiomas. Meningiomas are tumors derived from arachnoid cap cells that represent approximately 30% of all intracranial tumors. In this study, we investigated 22 human meningiomas for the expression of dipeptidyl peptidase (DPP)-IV activity and/or structure homologs (DASH), including canonical DPP-IV/CD26, fibroblast activation protein-alpha (FAPalpha), DPP8 and DPP9. DPP-IV-like enzymatic activity, including all enzymatically-active DASH molecules, was found in all 18 benign meningiomas WHO grade I and IV atypical meningiomas WHO grade II by continuous rate fluorimetric assay in tissue homogenates and catalytic enzyme histochemistry in situ. In atypical meningiomas, this activity was significantly higher and was associated with higher cell proliferation as detected by Ki67 antigen immunohistochemistry. The expression of DPP-IV/CD26 and FAPalpha demonstrated by real-time RT-PCR and immunohistochemistry was low. As shown histochemically, it occurred most often on the surface of fibrous bundles and whorls rich in   extracellular matrix  . Compared to DPP-IV/CD26 and FAPalpha, the expression of DPP8 and DPP9 was higher and, in addition, it was present also in the cells inside these structures. Expression of CXCR4, the receptor of pro-proliferative chemokine stromal cell-derived factor-1alpha (SDF-1alpha), DPP-IV substrate, was found in all tumors, suggesting higher values in atypical grade II samples. This is the first report on the expression status of dipeptidyl peptidase-IV and related molecules in meningiomas. It shows that DPP8 and DPP9 prevail over canonical DPP-IV/CD26 and FAPalpha in all examined patients. In addition, the study suggests an increase of DPP-IV-like enzymatic activity in these tumors of WHO grade II. 
 Fibroblast activation protein, a dual specificity serine protease expressed in reactive human tumor stromal fibroblasts. Proteolytic degradation of   extracellular matrix   (ECM) components during tissue remodeling plays a pivotal role in normal and pathological processes including wound healing, inflammation, tumor invasion, and metastasis. Proteolytic enzymes in tumors may activate or release growth factors from the ECM or act directly on the ECM itself, thereby facilitating angiogenesis or tumor cell migration. Fibroblast activation protein (FAP) is a cell surface antigen of reactive tumor stromal fibroblasts found in epithelial cancers and in granulation tissue during wound healing. It is absent from most normal adult human tissues. FAP is conserved throughout chordate evolution, with homologues in mouse and Xenopus laevis, whose expression correlates with tissue remodeling events. Using recombinant and purified natural FAP, we show that FAP has both dipeptidyl peptidase activity and a collagenolytic activity capable of degrading gelatin and type I collagen; by sequence, FAP belongs to the serine protease family rather than the matrix metalloprotease family. Mutation of the putative catalytic serine residue of FAP to alanine abolishes both enzymatic activities. Consistent with its in vivo expression pattern determined by immunohistochemistry, FAP enzyme activity was detected by an immunocapture assay in human cancerous tissues but not in matched normal tissues. This study demonstrates that FAP is present as an active cell surface-bound collagenase in epithelial tumor stroma and opens up investigation into physiological substrates of its novel, tumor-associated dipeptidyl peptidase activity. 
 FAP-overexpressing fibroblasts produce an   extracellular matrix   that enhances invasive velocity and directionality of pancreatic cancer cells. Alterations towards a permissive stromal microenvironment provide important cues for tumor growth, invasion, and metastasis. In this study, Fibroblast activation protein (FAP), a serine protease selectively produced by tumor-associated fibroblasts in over 90% of epithelial tumors, was used as a platform for studying tumor-stromal interactions. We tested the hypothesis that FAP enzymatic activity locally modifies stromal ECM (  extracellular matrix  ) components thus facilitating the formation of a permissive microenvironment promoting tumor invasion in human pancreatic cancer.We generated a tetracycline-inducible FAP overexpressing fibroblastic cell line to synthesize an in vivo-like 3-dimensional (3D) matrix system which was utilized as a stromal landscape for studying matrix-induced cancer cell behaviors. A FAP-dependent topographical and compositional alteration of the ECM was characterized by measuring the relative orientation angles of fibronectin fibers and by Western blot analyses. The role of FAP in the matrix-induced permissive tumor behavior was assessed in Panc-1 cells in assorted matrices by time-lapse acquisition assays. Also, FAP+ matrix-induced regulatory molecules in cancer cells were determined by Western blot analyses.We observed that FAP remodels the ECM through modulating protein levels, as well as through increasing levels of fibronectin and collagen fiber organization. FAP-dependent architectural/compositional alterations of the ECM promote tumor invasion along characteristic parallel fiber orientations, as demonstrated by enhanced directionality and velocity of pancreatic cancer cells on FAP+ matrices. This phenotype can be reversed by inhibition of FAP enzymatic activity during matrix production resulting in the disorganization of the ECM and impeded tumor invasion. We also report that the FAP+ matrix-induced tumor invasion phenotype is β1-integrin/FAK mediated.Cancer cell invasiveness can be affected by alterations in the tumor microenvironment. Disruption of FAP activity and β1-integrins may abrogate the invasive capabilities of pancreatic and other tumors by disrupting the FAP-directed organization of stromal ECM and blocking β1-integrin dependent cell-matrix interactions. This provides a novel preclinical rationale for therapeutics aimed at interfering with the architectural organization of tumor-associated ECM. Better understanding of the stromal influences that fuel progressive tumorigenic behaviors may allow the effective future use of targeted therapeutics aimed at disrupting specific tumor-stromal interactions. 
  [Ii]nvasive  PubMed  
 A novel protease-docking function of integrin at invadopodia. Invadopodia are membrane extensions of aggressive tumor cells that function in the activation of membrane-bound proteases occurring during tumor cell invasion. We explore a novel and provocative activity of integrins in docking proteases to sites of invasion, termed invadopodia. In the absence of collagen, alpha(3)beta(1) integrin and the gelatinolytic enzyme, seprase, exist as nonassociating membrane proteins. Type I collagen substratum induces the association of alpha(3)beta(1) integrin with seprase as a complex on invadopodia. The results show that alpha(3)beta(1) integrin is a docking protein for seprase to form functional invadopodia. In addition, alpha(5)beta(1) integrin may participate in the adhesion process necessary for invadopodial formation. Thus, alpha(3)beta(1) and alpha(5)beta(1) integrins play major organizational roles in the adhesion and formation of invadopodia, promoting   invasive   cell behavior. 
 Antiplasmin-cleaving enzyme is a soluble form of fibroblast activation protein. Circulating antiplasmin-cleaving enzyme (APCE) has a role in fibrinolysis and appears structurally similar to fibroblast activation protein (FAP), a cell-surface proteinase that promotes   invasive  ness of certain epithelial cancers. To explore this potential relationship, we performed comparative structure/function analyses of the 2 enzymes. APCE from human plasma and recombinant FAP (rFAP) exhibited identical pH optima of 7.5, extinction coefficients (in(280 nm)(1%)) of 20.2 and 20.5, common sequences of tryptic peptides, and cross-reactivity with FAP antibody. APCE and rFAP are homodimers with monomeric subunits of 97 and 93 kDa. Only homodimers appear to have enzymatic activity, with essentially identical kinetics toward Met-alpha2-antiplasmin (Met-alpha2AP) and peptide substrates. APCE and rFAP cleave both Pro3-Leu4 and Pro12-Asn13 bonds of Met-alpha2AP, but relative kcat/Km values for Pro12-Asn13 are about 16-fold higher than for Pro3-Leu4. APCE and rFAP demonstrate higher kcat/Km values toward a peptide modeled on P4-P4' sequence surrounding the Pro12-Asn13 primary cleavage site than for Z-Gly-Pro-AMC and Ala-Pro-AFC substrates. These data support APCE as a soluble derivative of FAP and Met-alpha2AP as its physiologic substrate. Conversion of Met-alpha2AP by membrane or soluble FAP to the more easily fibrin-incorporable form, Asn-alpha2AP, may increase plasmin inhibition within fibrin surrounding certain neoplasms and have an impact on growth and therapeutic susceptibility. 
 Expression and role of fibroblast activation protein-alpha in micro  invasive   breast carcinoma. Diagnosis of ductal carcinoma in situ (DCIS) in breast cancer cases is challenging for pathologist due to a variety of in situ patterns and artefacts, which could be misinterpreted as stromal invasion. Microinvasion is detected by the presence of cytologically malignant cells outside the confines of the basement membrane and myoepithelium. When malignant cells invade the stroma, there is tissue remodeling induced by perturbed stromal-epithelial interactions. Carcinoma-associated fibroblasts (CAFs) are main cells in the microenvironment of the remodeled tumor-host interface. They are characterized by the expression of the specific fibroblast activation protein-alpha (FAP-α), and differ from that of normal fibroblasts exhibiting an immunophenotype of CD34. We hypothesized that staining for FAP-α may be helpful in determining whether DCIS has microinvasion.349 excised breast specimens were immunostained for smooth muscle actin SMA, CD34, FAP-α, and Calponin. Study material was divided into 5 groups: group 1: normal mammary tissues of healthy women after plastic surgery; group 2: usual ductal hyperplasia (UDH); group 3: DCIS without microinvasion on H & E stain; group 4: DCIS with microinvasion on H & E stain (DCIS-MI), and group   5: invas  ive ductal carcinoma (IDC). A comparative evaluation of the four immunostains was conducted.Our results demonstrated that using FAP-α and Calponin adjunctively improved the sensitivity of pathological diagnosis of DCIS-MI by 11.29%, whereas the adjunctive use of FAP-α and Calponin improved the sensitivity of pathological diagnosis of DCIS by 13.6%.This study provides the first evidence that immunostaining with FAP-α and Calponin can serve as a novel marker for pathologically diagnosing whether DCIS has microinvasion. 
 The protease complex consisting of dipeptidyl peptidase IV and seprase plays a role in the migration and invasion of human endothelial cells in collagenous matrices. Dipeptidyl peptidase IV (DPP4/CD26) and seprase/fibroblast activation protein alpha are homologous type II transmembrane, homodimeric glycoproteins that exhibit unique prolyl peptidase activities. Human DPP4 is ubiquitously expressed in epithelial and endothelial cells and serves multiple functions in cleaving the penultimate positioned prolyl bonds at the NH(2) terminus of a variety of physiologically important peptides in the circulation. Recent studies showed a linkage between DPP4 and down-regulation of certain chemokines and mitogenic growth factors, and degradation of denatured collagens (gelatin), suggesting a role of DPP4 in the cell   invasive   phenotype. Here, we found the existence of a novel protease complex consisting of DPP4 and seprase in human endothelial cells that were activated to migrate and invade in the extracellular matrix in vitro. DPP4 and seprase were coexpressed with the three major protease systems (matrix metalloproteinase, plasminogen activator, and type II transmembrane serine protease) at the cell surface and organize as a complex at invadopodia-like protrusions. Both proteases were colocalized at the endothelial cells of capillaries, but not large blood vessels, in   invasive   breast ductal carcinoma in vivo. Importantly, monoclonal antibodies against the gelatin-binding domain of DPP4 blocked the local gelatin degradation by endothelial cells in the presence of the major metallo- and serine protease systems that modified pericellular collagenous matrices and subsequent cell migration and invasion. Thus, we have identified a novel mechanism involving the DPP4 gelatin-binding domain of the DPP4-seprase complex that facilitates the local degradation of the extracellular matrix and the invasion of the endothelial cells into collagenous matrices. 
 Molecular cloning of seprase: a serine integral membrane protease from human melanoma. Seprase is a homodimeric 170 kDa integral membrane gelatinase whose expression correlates with the   invasive  ness of the human melanoma cell line LOX. Here, we report the molecular cloning of a cDNA that encodes the 97 kDa subunit of seprase. Its deduced amino acid sequence predicts a type II integral membrane protein with a cytoplasmic tail of 6 amino acids, followed by a transmembrane domain of 20 amino acids and an extracellular domain of 734 amino acids. The carboxyl terminus contains a putative catalytic region (approximately 200 amino acids) which is homologous (68% identity) to that of the nonclassical serine protease dipeptidyl peptidase IV (DPPIV). The conserved serine protease motif G-X-S-X-G is present as G-W-S-Y-G. However, sequence analysis of seprase cDNA from LOX and other cell lines strongly suggests that seprase and human fibroblast activation protein alpha (FAP alpha) are products of the same gene. We propose that seprase/FAP alpha and DPPIV represent a new subfamily of serine integral membrane proteases (SIMP). 
 Regulation of fibroblast migration on collagenous matrix by a cell surface peptidase complex. The invasion of migratory cells through connective tissues involves metallo- and serine types of cell surface proteases. We show that formation of a novel protease complex, consisting of the membrane-bound prolyl peptidases seprase and dipeptidyl peptidase IV (DPPIV), at invadopodia of migratory fibroblasts is a prerequisite for cell invasion and migration on a collagenous matrix. Seprase and DPPIV form a complex on the cell surface that elicits both gelatin binding and gelatinase activities localized at invadopodia of cells migrating on collagenous fibers. The protease complex participates in the binding to gelatin and localized gelatin degradation, cellular migration, and monolayer wound closure. Serine protease inhibitors can block the gelatinase activity and the localized gelatin degradation by cells. Antibodies to the gelatin-binding domain of DPPIV reduce the cellular abilities of the proteases to degrade gelatin but do not affect cellular adhesion or spreading on type I collagen. Furthermore, expression of the seprase-DPPIV complex is restricted to migratory cells involved in wound closure in vitro and in connective tissue cells during closure of gingival wounds but not in differentiated tissue cells. Thus, we have identified cell surface proteolytic activities, which are non-metalloproteases, seprase and DPPIV, that are responsible for the tissue-  invasive   phenotype. 
 Expression patterns of seprase, a membrane serine protease, in cervical carcinoma and cervical intraepithelial neoplasm. Seprase is a membrane-bound serine proteinase with gelatinase activity, which may be involved in cancer invasion and metastasis.We examined the seprase expression patterns in   invasive   or pre  invasive   squamous epithelial lesions.No seprase immunoreactivity was found in the atypical epithelial cells and subepithelial stromal cells of CIN1 and 2 lesions. No seprase expression could be detected even in CIN3 lesions. However, in contrast to CIN1 and 2, seprase immunoreactivity was occasionally observed in the subepithelial stromal cells that were associated with moderate or severe inflammatory infiltrates. Some micro  invasive   carcinomas and all   invasive   carcinomas showed seprase immunoreactivity in the cancer cells with various degrees of seprase-positive stromal cells. Seprase may be an early marker of tumor invasion in squamous lesions of the uterine cervix. 
 Ultraviolet exposure of melanoma cells induces fibroblast activation protein-α in fibroblasts: Implications for melanoma invasion. Fibroblast activation protein-α (FAP-α) promotes tumor growth and ce  ll invas  iveness through extracellular matrix degradation. How ultraviolet radiation (UVR), the major risk factor for malignant melanoma, influences the expression of FAP-α is unknown. We examined the effect of UVR on FAP-α expression in melanocytes, keratinocytes and fibroblasts from the skin and in melanoma cells. UVR induces upregulation of FAP-α in fibroblasts, melanocytes and primary melanoma cells (PM) whereas keratinocytes and metastatic melanoma cells remained FAP-α negative. UVA and UVB stimulated FAP-α-driven migration and invasion in fibroblasts, melanocytes and PM. In co-culture systems UVR of melanocytes, PM and cells from regional metastases upregulated FAP-α in fibroblasts but only supernatants from non-irradiated PM were able to induce FAP-α in fibroblasts. Further, UV-radiated melanocytes and PM significantly increased FAP-α expression in fibroblasts through secretory crosstalk via Wnt5a, PDGF-BB and TGF-β1. Moreover, UV radiated melanocytes and PM increased collagen I invasion and migration of fibroblasts. The FAP-α/DPPIV inhibitor Gly-ProP(OPh)2 significantly decreased this response implicating FAP-α/DPPIV as an important protein complex in cell migration and invasion. These experiments suggest a functional association between UVR and FAP-α expression in fibroblasts, melanocytes and melanoma cells implicating that UVR of malignant melanoma converts fibroblasts into FAP-α expressing and ECM degrading fibroblasts thus facilitating invasion and migration. The secretory crosstalk between melanoma and tumor surrounding fibroblasts is mediated via PDGF-BB, TGF-β1 and Wnt5a and these factors should be evaluated as targets to reduce FAP-α activity and prevent early melanoma dissemination. 
 Over-expression of fibroblast activation protein alpha increases tumor growth in xenografts of ovarian cancer cells. Fibroblast activation protein alpha (FAPα) is a 95-kDa serine protease of post-prolyl peptidase family on cell surface. FAPα is widely expressed in tumor microenvironment. The wide spread association of FAPα expression with cancer suggests that it has important functions in the disease. However, the nature of FAPα's roles in cancer cell activity is not well-determined. It has been showed that FAPα silencing in SKOV3 cells induces ovarian tumors but significantly reduces tumor growth in a xenograft mouse model. To further determine the role of FAPα in epithelial ovarian cancer cells, SKOV3-FAPα and HO8910-FAPα cell lines, which over-expressed FAPα stably, were constructed and then their biological behaviors were investigated. It was found that FAPα promoted ovarian cancer cell proliferation, drug re  sistance  , invasiveness, and migration in vitro. Immunochemistry assay showed that FAPα significantly facilitated tumor growth in xenograft tumor tissues. These results suggested that FAPα might directly promote tumor   growth   and invasiveness in ovarian cancer cells. 
 Identification of the 170-kDa melanoma membrane-bound gelatinase (seprase) as a serine integral membrane protease. The 170-kDa membrane-bound gelatinase, seprase, is a cell surface protease, the expression of which correlates with the   invasive   phenotype of human melanoma and carcinoma cells. We have isolated seprase from cell membranes and shed vesicles of LOX human melanoma cells. The active enzyme is a dimer of N-glycosylated 97-kDa subunits. Sequence analysis of three internal proteolytic fragments of the 97-kDa polypeptide revealed up to 87.5% identity to the 95-kDa fibroblast activation protein alpha (FAPalpha), the function of which is unknown. Thus, we used reverse transcription-polymerase chain reaction to generate a 2.4-kilobase cDNA from LOX mRNA with FAPalpha primers. COS-7 cells transfected with this cDNA expressed a 170-kDa gelatinase that is recognized by monoclonal antibodies directed against seprase. Sequence analysis also showed similarities to the 110-kDa subunit of dipeptidyl peptidase IV (DPPIV). Like DPPIV, the gelatinase activity of seprase was completely blocked by serine-protease inhibitors, including diisopropyl fluorophosphate. Seprase could be affinity-labeled by [3H]diisopropyl fluorophosphate, but the proteolytically inactive 97-kDa subunit could not, confirming the existence of a serine protease active site on the dimeric form. Proteolytic activity is lost upon dissociation into its 97-kDa subunit following treatment with acid, heat, or cysteine and histidine-modifying agents. We conclude that seprase, FAPalpha, and DPPIV are related serine integral membrane proteases and that seprase is similar to DPPIV, the proteolytic activities of which are dependent upon subunit association. 
 Deficiency of fibroblast activation protein alpha ameliorates cartilage destruction in inflammatory destructive arthritis. Inflammatory destructive arthritis, like rheumatoid arthritis (RA), is characterized by invasion of synovial fibroblasts (SF) into the articular cartilage and erosion of the underlying bone, leading to progressive joint destruction. Because fibroblast activation protein alpha (FAP) has been associated with cell migration and cell   invasive  ness, we studied the function of FAP in joint destruction in RA.Expression of FAP in synovial tissues and fibroblasts from patients with osteoarthritis (OA) and RA as well as from wild-type and arthritic mice was evaluated by immunohistochemistry, fluorescence microscopy and polymerase chain reaction (PCR). Fibroblast adhesion and migration capacity was assessed using cartilage attachment assays and wound-healing assays, respectively. For in vivo studies, FAP-deficient mice were crossed into the human tumor necrosis factor transgenic mice (hTNFtg), which develop a chronic inflammatory arthritis. Beside clinical assessment, inflammation, cartilage damage, and bone erosion were evaluated by histomorphometric analyses.RA synovial tissues demonstrated high expression of FAP whereas in OA samples only marginal expression was detectable. Consistently, a higher expression was detected in arthritis SF compared to non-arthritis OA SF in vitro. FAP-deficiency in hTNFtg mice led to less cartilage degradation despite unaltered inflammation and bone erosion. Accordingly, FAP(-/-) hTNFtg SF demonstrated a lower cartilage adhesion capacity compared to hTNFtg SF in vitro.These data point to a so far unknown role of FAP in the attachment of SF to cartilage, promoting proteoglycan loss and subsequently cartilage degradation in chronic inflammatory arthritis. 
 Inhibition of fibroblast activation protein and dipeptidylpeptidase 4 increases cartilage invasion by rheumatoid arthritis synovial fibroblasts. Since fibroblasts in the synovium of patients with rheumatoid arthritis (RA) express the serine proteases fibroblast activation protein (FAP) and dipeptidylpeptidase 4 (DPP-4)/CD26, we undertook the current study to determine the functional role of both enzymes in the invasion of RA synovial fibroblasts (RASFs) into articular cartilage.Expression of FAP and DPP-4/CD26 by RASFs was analyzed using fluorescence-activated cell sorting and immunocytochemistry. Serine protease activity was measured by cleavage of fluorogenic substrates and inhibited upon treatment with L-glutamyl L-boroproline. The induction and expression of matrix metalloproteinases (MMPs) and tissue inhibitors of metalloproteinases (TIMPs) in RASFs were detected using real-time polymerase chain reaction. Densitometric measurements of MMPs using immunoblotting confirmed our findings on the messenger RNA level. Stromal cell-derived factor 1 (SDF-1 [CXCL12]), MMP-1, and MMP-3 protein levels were measured using enzyme-linked immunosorbent assay. The impact of FAP and DPP-4/CD26 inhibition on the   invasive  ness of RASFs was analyzed in the SCID mouse coimplantation model of RA using immunohistochemistry.Inhibition of serine protease activity of FAP and DPP-4/CD26 in vitro led to increased levels of SDF-1 in concert with MMP-1 and MMP-3, which are downstream effectors of SDF-1 signaling. Using the SCID mouse coimplantation model, inhibition of enzymatic activity in vivo significantly promoted invasion of xenotransplanted RASFs into cotransplanted human cartilage. Zones of cartilage resorption were infiltrated by FAP-expressing RASFs and marked by a significantly higher accumulation of MMP-1 and MMP-3, when compared with controls.Our results indicate a central role for the serine protease activity of FAP and DPP-4/CD26 in protecting articular cartilage against invasion by synovial fibroblasts in RA. 
 Transcriptional regulation of seprase in   invasive   melanoma cells by transforming growth factor-β signaling. The tumor   invasiv  e phenotype driven by seprase expression/activity has been widely examined in an array of malignant tumor cell types; however, very little is known about the transcriptional regulation of this critical protease. Seprase (also named fibroblast activation protein-α, antiplasmin-cleaving enzyme, and dipeptidyl prolyl peptidase 5) is expressed at high levels by stromal fibroblast, endothelial, and tumor cells in a variety o  f invasi  ve tumors but is undetectable in the majority of normal adult tissues. To examine the transcriptional regulation of the gene, we cloned the human seprase promoter and demonstrated that endogenous seprase expression and exogenous seprase promoter activity are high i  n invasi  ve melanoma cells but not in no  n-invasi  ve melanoma cells/primary melanocytes. In addition, we identified a crucial TGF-β-responsive cis-regulatory element in the proximal seprase promoter region that enabled robust transcriptional activation of the gene. Treatment of metastatic but not normal/n  on-invas  ive cells with TGF-β1 caused a rapid and profound up-regulation of endogenous seprase mRNA, which coincided with an abolishment of the negative regulator c-Ski, and an increase in binding of Smad3/4 to the seprase promoter in vivo. Blocking TGF-β signalin  g in inv  asive melanoma cells through overexpression of c-Ski, chemically using SB-431542, or with a neutralizing antibody against TGF-β significantly reduced seprase mRNA levels. Strikingly, RNAi of sepra  se in in  vasive cells greatly diminished   their in  vasive potential in vitro as did blocking TGF-β signaling using SB-431542. Altogether, we found that seprase is transcriptionally up-regula  ted in i  nvasive melanoma cells via the canonical TGF-β signaling pathway, supporting the roles of both TGF-β and seprase in tumor invasion and metastasis. 
 Elevation of seprase expression and promotion of an   invasive   phenotype by collagenous matrices in ovarian tumor cells. Tumor cells do not constitutively exhibit   invasive   activity, but rather, can be transiently induced to adhere and form lesions. We report here that the expression of seprase, a dominant EDTA-resistant gelatinase in malignant tumors, is dependent on tumor cell exposure to type I collagen gel (TICg). The induced seprase expression of ovarian tumor cells influences their collagen contraction and invasion capability. Importantly, tumor cells with reduced seprase expression, due to manipulation by RNA interference, showed a reduction of TICg contraction in the gel contractility assay, inhibition of tumor cell invasion through TICg as shown by a transwell migration assay and inhibition of peritoneal membrane tumor lesion in a mouse model. In addition, mAb C27, an antibody against beta1 integrin, which blocks cellular avidity to TICg, can induce seprase RNA expression and promote the   invasive   phenotype and metastatic potential of ovarian tumor cells. Thus, collagenous matrices in the tumor cell niche induce the expression of seprase and initiate tumor invasion and metastatic cascades. 
 Increased expression of fibroblast activation protein-alpha in keloid fibroblasts: implications for development of a novel treatment option. Keloid scars are common benign fibroproliferative reticular dermal lesions with unknown etiology and ill-defined management with high rate of recurrence post surgery. The progression of keloids is characterized by increased deposition of extracellular matrix proteins, invasion into the surrounding healthy skin and inflammation. Fibroblasts are considered to be the key cellular mediators of fibrogenesis in keloid scars. Fibroblast activation protein alpha (FAP-α) and dipeptidyl peptidase IV (DPPIV) are proteases located at the plasma membrane promoting cell   invasiv  eness and tumor growth and have been previously associated with keloid scars. Therefore, in this study we analyzed in further detail the expression of FAP-α in keloid fibroblasts compared to control skin fibroblasts. Dermal fibroblasts were obtained from punch-biopsies from the active margin of four keloids and four control skin samples. Flow cytometry was used to analyze FAP-α expression and the CytoSelect 24-Well Collagen I Cell Invasion Assay was applied to study fibroblast invasion. Secretion of extracellular matrix (ECM) proteins was investigated by multiplexed particle-based flow cytometric assay and enzyme-linked immunosorbent assay. We found an increased expression of FAP-α in keloid fibroblasts compared to control skin fibroblasts (p < 0.001). Inhibition of FAP-α/DPPIV activity using the irreversible inhibitor H(2)N-Gly-Pro diphenylphosphonate reduced the incre  ased inv  asiveness of keloid fibroblasts (p < 0.001) indicating that keloid invasion may be partly FAP-α/DPPIV mediated. FAP-α/DPPIV inhibition had no effect, (a) on the synthesis of the ECM proteins procollagen type I C-terminal peptide and fibronectin, (b) on the production of fibroblast growth factor or vascular endothelial growth factor, (c) on the expression of the proinflammatory cytokines interleukin-6 (IL-6), interleukin 8 (IL-8) or monocyte chemotactic protein-1. These results suggest a potential role for FAP-α and DPPIV   in the   invasive behavior of keloids. FAP-α and DPPIV may incr  ease the   invasive capacity of keloid fibroblasts rather than by modulating inflammation or ECM production. Since FAP-α expression is restricted to reactive fibroblasts in wound healing and normal adult tissues are generally FAP-α negative, inhibiting FAP-α/DPPIV activity may be a novel treatment option to prevent keloid progression. 
 FAP-overexpressing fibroblasts produce an extracellular matrix that enhances   invasive   velocity and directionality of pancreatic cancer cells. Alterations towards a permissive stromal microenvironment provide important cues for tumor growth, invasion, and metastasis. In this study, Fibroblast activation protein (FAP), a serine protease selectively produced by tumor-associated fibroblasts in over 90% of epithelial tumors, was used as a platform for studying tumor-stromal interactions. We tested the hypothesis that FAP enzymatic activity locally modifies stromal ECM (extracellular matrix) components thus facilitating the formation of a permissive microenvironment promoting tumor invasion in human pancreatic cancer.We generated a tetracycline-inducible FAP overexpressing fibroblastic cell line to synthesize an in vivo-like 3-dimensional (3D) matrix system which was utilized as a stromal landscape for studying matrix-induced cancer cell behaviors. A FAP-dependent topographical and compositional alteration of the ECM was characterized by measuring the relative orientation angles of fibronectin fibers and by Western blot analyses. The role of FAP in the matrix-induced permissive tumor behavior was assessed in Panc-1 cells in assorted matrices by time-lapse acquisition assays. Also, FAP+ matrix-induced regulatory molecules in cancer cells were determined by Western blot analyses.We observed that FAP remodels the ECM through modulating protein levels, as well as through increasing levels of fibronectin and collagen fiber organization. FAP-dependent architectural/compositional alterations of the ECM promote tumor invasion along characteristic parallel fiber orientations, as demonstrated by enhanced directionality and velocity of pancreatic cancer cells on FAP+ matrices. This phenotype can be reversed by inhibition of FAP enzymatic activity during matrix production resulting in the disorganization of the ECM and impeded tumor invasion. We also report that the FAP+ matrix-induced tumor invasion phenotype is β1-integrin/FAK mediated.Cancer cell   invasiv  eness can be affected by alterations in the tumor microenvironment. Disruption of FAP activity and β1-integrins may abrogate th  e invasi  ve capabilities of pancreatic and other tumors by disrupting the FAP-directed organization of stromal ECM and blocking β1-integrin dependent cell-matrix interactions. This provides a novel preclinical rationale for therapeutics aimed at interfering with the architectural organization of tumor-associated ECM. Better understanding of the stromal influences that fuel progressive tumorigenic behaviors may allow the effective future use of targeted therapeutics aimed at disrupting specific tumor-stromal interactions. 
  GeneRIFs  
 We found that seprase is transcriptionally up-regulated in   invasive   melanoma cells via the canonical TGF-beta signaling pathway, supporting the roles of both TGF-beta and seprase in tumor invasion and metastasis. 
 results suggested that FAPalpha might directly promote tumor growth and   invasive  ness in ovarian cancer cells 
 role in the   invasive   behavior of keloids 
 seprase may have a role in promotion of an   invasive   phenotype by collagenous matrices in ovarian tumor cells 
  [Mm]etastasis  PubMed  
 FAP-alpha and uPA show different expression patterns in premalignant and malignant esophageal lesions. Fibroblast activation protein-alpha (FAP-alpha) and urokinase-type plasminogen activator (uPA) are serine proteases involved in cancer invasion and   metastasis  . The authors examined FAP-alpha and uPA expression in premalignant and malignant stages of esophageal adenocarcinoma by immunohistochemistry. Additionally, Western blotting was performed on fresh-frozen tissue samples. FAP-alpha and uPA were detected in metaplastic, dysplastic, and carcinoma cells, as well as in adjacent stroma. Stromal FAP-alpha expression was associated with depth of tumor invasion, while stromal uPA expression correlated with lymph node metastases in adenocarcinomas. Stromal uPA expression in cells with premalignant changes correlated with histological grading. Immunoblotting showed higher protease expression in carcinoma tissues than in normal esophageal epithelium. These results suggest that FAP-alpha and uPA expression in metaplastic, dysplastic, and esophageal cancer tissue is associated with neoplastic progression of esophageal lesions. 
 Structural and kinetic analysis of the substrate specificity of human fibroblast activation protein alpha. Fibroblast activation protein alpha (FAPalpha) is highly expressed in epithelial cancers and has been implicated in extracellular matrix remodeling, tumor growth, and   metastasis  . We present the first high resolution structure for the apoenzyme as well as kinetic data toward small dipeptide substrates. FAPalpha exhibits a dipeptidyl peptidase IV (DPPIV)-like fold, featuring an alpha/beta-hydrolase domain and an eight-bladed beta-propeller domain. Known DPPIV dipeptides are cleaved by FAPalpha with an approximately 100-fold decrease in catalytic efficiency compared with DPPIV. Moreover, FAPalpha, but not DPPIV, possesses endopeptidase activity toward N-terminal benzyloxycarbonyl (Z)-blocked peptides. Comparison of the crystal structures of FAPalpha and DPPIV revealed one major difference in the vicinity of the Glu motif (Glu(203)-Glu(204) for FAPalpha; Glu(205)-Glu(206) for DPPIV) within the active site of the enzyme. Ala(657) in FAPalpha, instead of Asp(663) as in DP-PIV, reduces the acidity in this pocket, and this change could explain the lower affinity for N-terminal amines by FAPalpha. This hypothesis was tested by kinetic analysis of the mutant FAPalpha/A657D, which shows on average an approximately 60-fold increase in the catalytic efficiency, as measured by k(cat)/K(m), for the cleavage of dipeptide substrates. Furthermore, the catalytic efficiency of the mutant is reduced by approximately 350-fold for cleavage of Z-Gly-Pro-7-amino-4-methylcoumarin. Our data provide a clear understanding of the molecular determinants responsible for the substrate specificity and endopeptidase activity of FAPalpha. 
 Seprase, a membrane-type serine protease, has different expression patterns in intestinal- and diffuse-type gastric cancer. Seprase is an integral membrane serine proteinase with gelatinase activity that may be involved in cancer invasion and   metastasis  . However, the pathophysiologic significance of its expression in gastric cancer tissue has not been fully elucidated.Seprase expression and distribution in gastric cancer specimens obtained from 133 patients were examined by immunohistochemistry and immunoblotting.Immunohistochemistry showed that in intestinal-type cancer, which includes well and moderately differentiated adenocarcinoma, seprase immunoreactivity was mainly recognized in the moderately differentiated cells and not in the well differentiated cells. In the diffuse type, which includes poorly differentiated adenocarcinoma and signet ring cell carcinoma, seprase immunoreactivity was seen mainly in cells with poor cell-to-cell junctions. The reactive pattern in the cells was different between moderately differentiated adenocarcinoma and diffuse-type carcinoma. Besides the cytoplasm, the cell membrane also apparently reacted in the former, while only the cytoplasm reacted diffusely in the latter. Seprase immunoreactivity was also recognized in endothelial cells and stromal cells especially adjacent to tumor nests. The immunoreactivity of the stromal cells was more abundant in the intestinal type than in the diffuse type, and these stromal expressions of seprase in the intestinal type correlated with the liver (13/13 = 100% of cases with metastases) or lymph node metastases (33/34 = 97% of cases with metastases). Immunoblotting showed that the levels of seprase protein were higher in intestinal-type cancer than in diffuse-type cancer.These results suggested that there is a difference in seprase expression between intestinal- and diffuse-type gastric cancer; this difference may reflect distinct biological features of these types of cancer. 
 Expression of CAF-Related Proteins Is Associated with Histologic Grade of Breast Phyllodes Tumor. Purpose. The purpose of this study was to investigate the expression of cancer-associated fibroblast- (CAF-) related proteins and the implications in breast phyllodes tumor (PT). Methods. Tissue microarrays of 194 PT cases (151 benign PT, 27 borderline PT, and 16 malignant PT) were constructed. We performed immunohistochemical staining for CAF-related proteins (podoplanin, prolyl 4-hydroxylase, FAPα, S100A4, PDGFR α/β, and NG2) and analyzed the results according to clinicopathologic parameters. Results. Expression of PDGFRα and PDGFRβ in the stromal component increased with increasing histologic grade of PT (p = 0.003 and p = 0.034, resp.). Among clinicopathologic parameters, only expression of FAPα in stroma was associated with di  stant meta  stasis (p = 0.002). In univariate analysis, stromal expression of PDGFRα was associated with shorter overall survival (p = 0.002). In Cox multivariate analysis, stromal overgrowth and PDGFRα stromal positivity were associated with shorter overall survival (p = 0.006 and p = 0.050, resp.). Furthermore, expression of PDGFRβ in stroma was associated with shorter overall survival in patients with malignant PT (p = 0.041). Conclusion. Stromal expression of PDGFRα and PDGFRβ increased with increasing histologic grade of PT. In addition, PDGFR stromal positivity was associated with shorter overall survival. These results suggest that CAFs are associated with breast PT progression. 
 High intratumoral expression of fibroblast activation protein (FAP) in colon cancer is associated with poorer patient prognosis. An active stroma is important for cancer cell invasion and   metastasis  . We investigated the expression of fibroblast activation protein (FAP) in relation to patient prognosis in colorectal cancer. Colorectal cancer specimens from 449 patients were immunohistochemically stained with a FAP antibody and evaluated in the tumor center and tumor front using a semiquantitative four-level scale. FAP was expressed by fibroblasts in 85-90 % of the tumors examined. High versus no/low expression in the tumor center was associated with poor prognosis (multivariate hazard ratio, HR = 1.72; 95 % CI 1.07-2.77, p = 0.025). FAP expression in the tumor front, though more frequent than in the tumor center, was not associated with prognosis. FAP expression in the tumor center was more common in specimens with positive microsatellite instability (MSI) screening status and in patients with high CpG island methylator phenotype (CIMP) status. However, inclusion of MSI screening status and CIMP status in the multivariate analysis strengthened the risk estimates for high FAP expression in the tumor center (HR = 1.89; 95 % CI 1.13-3.14; p = 0.014), emphasizing the role of FAP as an independent prognostic factor. Stromal FAP expression is common in colorectal cancer, and we conclude that high FAP expression in the tumor center, but not the tumor front, is an independent negative prognostic factor. 
 Downregulation of FAP suppresses cell proliferation and   metastasis   through PTEN/PI3K/AKT and Ras-ERK signaling in oral squamous cell carcinoma. It is largely recognized that fibroblast activation protein (FAP) is expressed in cancer-associated fibroblasts (CAFs) of many human carcinomas. Furthermore, FAP was recently also reported to be expressed in carcinoma cells of the breast, stomach, pancreatic ductal adenocarcinoma, colorectum, and uterine cervix. The carcinoma cell expression pattern of FAP has been described in several types of cancers, but the role of FAP in oral squamous cell carcinoma (OSCC) is unknown. The role of endogenous FAP in epithelium-derived tumors and molecular mechanisms has also not been reported. In this study, FAP was found to be expressed in carcinoma cells of OSCC and was upregulated in OSCC tissue samples compared with benign tissue samples using immunohistochemistry. In addition, its expression level was closely correlated with overall survival of patients with OSCC. Silencing FAP inhibited the growth and   metastasis   of OSCC cells in vitro and in vivo. Mechanistically, knockdown of FAP inactivated PTEN/PI3K/AKT and Ras-ERK and its downstream signaling regulating proliferation, migration, and invasion in OSCC cells, as the inhibitory effects of FAP on the proliferation and   metastasis   could be rescued by PTEN silencing. Our study suggests that FAP acts as an oncogene and may be a potential therapeutic target for patients with OSCC. 
 Expression patterns of seprase, a membrane serine protease, in cervical carcinoma and cervical intraepithelial neoplasm. Seprase is a membrane-bound serine proteinase with gelatinase activity, which may be involved in cancer invasion and   metastasis  .We examined the seprase expression patterns in invasive or preinvasive squamous epithelial lesions.No seprase immunoreactivity was found in the atypical epithelial cells and subepithelial stromal cells of CIN1 and 2 lesions. No seprase expression could be detected even in CIN3 lesions. However, in contrast to CIN1 and 2, seprase immunoreactivity was occasionally observed in the subepithelial stromal cells that were associated with moderate or severe inflammatory infiltrates. Some microinvasive carcinomas and all invasive carcinomas showed seprase immunoreactivity in the cancer cells with various degrees of seprase-positive stromal cells. Seprase may be an early marker of tumor invasion in squamous lesions of the uterine cervix. 
 'Increased expression of seprase, a membrane-type serine protease, is associated with lymph node   metastasis   in human colorectal cancer'. Seprase is a membrane-bound serine proteinase with gelatinase activity, which may be involved in cancer invasion and   metastasis  . We examined seprase expression in colorectal cancer specimens obtained from 109 patients. Seprase immunoreactivity was found in cancer cells and adjacent stromal cells. Immunoblotting showed higher levels of seprase protein in colorectal cancer tissue than in normal colorectal tissue. A semiquantitative assessment of the immunohistochemistry results revealed a significant correlation between seprase expression and lymph node   metastasis  . These results suggested that an abundant expression of seprase in colorectal cancer tissue is associated with lymph node   metastasis  . 
 TGF-beta induces fibroblast activation protein expression; fibroblast activation protein expression increases the proliferation, adhesion, and migration of HO-8910PM [corrected]. Several studies recognize cancer-stromal fibroblasts' role in cancer-cell invasion and   metastasis  . Through paracrine signaling molecules, TGF-beta and IL-1beta, cancer cells activate stromal fibroblasts and induce the expression of fibroblast activation protein (FAP). FAP, in turn, affects the proliferation, invasion and migration of the cancer cells. We report that TGF-beta and IL-1beta are important factors in inducing differentiation of myofibroblasts and expression of functional markers, notably alpha-SMA. We discover that TGF-beta is the dominant factor in promoting FAPalpha protein expression. This study also examines FAP's function in vitro by assaying the proliferation, migration and invasion of ovarian cancer cell line HO-8910PM. 
 Transcriptional regulation of seprase in invasive melanoma cells by transforming growth factor-β signaling. The tumor invasive phenotype driven by seprase expression/activity has been widely examined in an array of malignant tumor cell types; however, very little is known about the transcriptional regulation of this critical protease. Seprase (also named fibroblast activation protein-α, antiplasmin-cleaving enzyme, and dipeptidyl prolyl peptidase 5) is expressed at high levels by stromal fibroblast, endothelial, and tumor cells in a variety of invasive tumors but is undetectable in the majority of normal adult tissues. To examine the transcriptional regulation of the gene, we cloned the human seprase promoter and demonstrated that endogenous seprase expression and exogenous seprase promoter activity are high in invasive melanoma cells but not in non-invasive melanoma cells/primary melanocytes. In addition, we identified a crucial TGF-β-responsive cis-regulatory element in the proximal seprase promoter region that enabled robust transcriptional activation of the gene. Treatment of metastatic but not normal/non-invasive cells with TGF-β1 caused a rapid and profound up-regulation of endogenous seprase mRNA, which coincided with an abolishment of the negative regulator c-Ski, and an increase in binding of Smad3/4 to the seprase promoter in vivo. Blocking TGF-β signaling in invasive melanoma cells through overexpression of c-Ski, chemically using SB-431542, or with a neutralizing antibody against TGF-β significantly reduced seprase mRNA levels. Strikingly, RNAi of seprase in invasive cells greatly diminished their invasive potential in vitro as did blocking TGF-β signaling using SB-431542. Altogether, we found that seprase is transcriptionally up-regulated in invasive melanoma cells via the canonical TGF-β signaling pathway, supporting the roles of both TGF-β and seprase in tumor inva  sion and m  etastasis. 
 Effects of the fibroblast activation protein on the invasion and migration of gastric cancer. Cancer-associated fibroblasts (CAFs) are one of the most important components of tumor microenvironment. CAFs are believed to play an important role in tumor invasion and   metastasis  . Recently, fibroblast activation protein (FAP), a type II integral membrane glycoprotein belonging to the serine protease family, has emerged as a specific marker of CAFs. FAP was overexpressed in stromal fibroblasts of solid malignancies, however, the role of FAP on the process of invasion and   metastasis   of gastric carcinomas is still unknown.Expression of FAP level was detected by immunohistochemistry in 60 gastric cancer surgical specimens (28 with omentum   metastasis   and 32 without), 20 normal human gastric tissues and omentum of 10 nonneoplastic gastric diseases. Fibroblasts were isolated from patient's tissues in the distal normal zones and tumor zones respectively, which were correspondingly designated as normal zone fibroblasts (NFs) and cancer-associated fibroblasts (CAFs). To explore the effects of FAP on NFs or CAFs, fibroblasts were co-cultured with human gastric cancer cell line MGC-803 cells. The ability of invasion and migration of MGC-803 cells was evaluated after transfecting FAP siRNA into CAFs of gastric carcinomas.We investigated the level of expression of FAP in surgical specimens, and found overexpressed in CAFs and non-expressed in NFs. Expression of FAP level in CAFs is significantly associated with Lauren classification,the degree of differentiation, depth of tumor invasion and TNM stage, but it is not correlated to age and gender in gastric carcinoma patients. There was positive correlation between the FAP level with   metastasis   to the omentum(p < 0.05, R(2) = 0.2736, p < 0.05, R(2) = 0.1479). In addition, the invasion and migration abilities of MGC-803 cells were significantly increased when cells were co-cultured with CAFs. On the other hand, invasion and migration abilities were significantly decreased by 46.9 and 50.3%, respectively, after knocking down FAP in CAFs.Further, NFs did not have appreciable effect on the invasion and migration of MGC-803 cells.Our findings showed that FAP was overexpressed in CAFs of gastric carcinomas, and siRNA-mediated knock down of FAP significantly suppressed invasion and migration of MGC-803 cells. FAP may be an important regulator in the invasion and migration of gastric cancer and may provide a novel therapeutic target in gastric carcinomas. 
 Increased expression of seprase, a membrane-type serine protease, is associated with lymph node   metastasis   in human colorectal cancer. Seprase is a membrane-bound serine proteinase with gelatinase activity, which may be involved in cancer invasion and   metastasis  . We examined seprase expression in colorectal cancer specimens obtained from 109 patients. Seprase immunoreactivity was found in cancer cells and adjacent stromal cells. Immunoblotting showed higher levels of seprase protein in colorectal cancer tissue than in normal colorectal tissue. A semiquantitative assessment of the immunohistochemistry results revealed a significant correlation between seprase expression and lymph node   metastasis  . These results suggested that an abundant expression of seprase in colorectal cancer tissue is associated with lymph node   metastasis  . 
 Fibroblast activation protein overexpression and clinical implications in solid tumors: a meta-analysis. Fibroblast activation protein (FAP) plays a vital role in tumor invasion and   metastasis  . Previous studies have reported its prognostic value in different tumors. However, the results of these reports remain controversial. In this study, a meta-analysis was performed to clarify this issue.A search of the PubMed, Embase and CNKI databases was conducted to analyze relevant articles. The outcomes included the relations between FAP expression and histological differentiation, tumor invasion, lymph node   metastasis  , distant   metastasis   and overall survival (OS). Sensitivity analysis by FAP expression in different cells and tumor types were further subjected to sensitivity analyses as subgroups. Pooled odds ratios (ORs) and hazard ratios (HRs) were evaluated using the random-effects model.The global analysis included 15 studies concerning various solid tumors. For global analysis, FAP overexpression in tumor tissue displayed significant associations with poor OS and tumor progression (OS: HR = 2.18, P = 0.004; tumor invasion: OR = 4.48, P = 0.007; and lymph node   metastasis  : OR = 3.80, P = 0.004). The subgroup analyses yielded two notable results. First, the relation between FAP overexpression and poor OS and tumor lymph node   metastasis   was closer in the patients with FAP expression in tumor cells. Second, the pooled analyses of colorectal cancers or pancreatic cancers all indicated that FAP overexpression was associated with a detrimental OS (HR: 1.72, P = 0.009; HR: 3.18, P = 0.005, respectively). The magnitude of this effect was not statistically significant compared with that in patients with non-colorectal cancers or non-pancreatic cancers. These analyses did not display a statistically significant correlation between FAP expression and histological differentiation and distant   metastasis   in all of the groups.FAP expression is associated with worse prognosis in solid tumors, and this association is particularly pronounced if FAP overexpression is found in the tumor cells rather than the stroma. 
 Fibroblast activation protein increases metastatic potential of fibrosarcoma line HT1080 through upregulation of integrin-mediated signaling pathways. The serine protease fibroblast activation protein (FAP) is selectively expressed on tumour-associated fibroblasts in most human epithelial tumours, as well as on some mesenchymal tumours such as sarcoma. High FAP expression is most often associated with poor outcome and increased   metastasis  . Here, we compare the in vitro metastatic potential of HT1080 fibrosarcoma cells with and without FAP expression in order to elucidate the mechanism by which FAP may influence   metastasis  . In the presence of FAP, cells were more adhesive to extracellular matrix proteins and migrated and invaded through Matrigel to a greater degree. The anti-FAP antibody ESC11, which caused internalization of FAP, decreased adhesion and migration, but only when cells expressed FAP. It was also found that blocking activity of integrins β1 and αvβ3 reduced both cell adhesion and migration and this effect was much more marked in FAP-expressing HT1080 cells than mock-transfected HT1080 cells. The expression or activation of intracellular proteins that form part of the downstream signaling of integrins, including integrin-linked kinase, Rac1 and focal adhesion kinase, was also upregulated when FAP was expressed, suggesting that FAP not only upregulates metastatic-like cell behaviours through interaction with integrins, but also influences the intracellular signaling of integrins. This was confirmed using both PI3 kinase and Src kinase inhibitors, which decreased adhesion and migration in FAP-expressing cells, but did not affect mock-transfected HT1080 cells. FAP is therefore a useful target for anti-cancer therapy, as not only is its expression tumour-selective, but its downregulation has the potential to reduce incidence   of metasta  sis. 
 Upregulated expression and activation of membrane‑associated proteases in esophageal squamous cell carcinoma. To better understand the role of membrane-associated proteolytic systems in the development of esophageal cancer, we studied the expression of two serine proteases, fibroblast activation protein-α (FAP-α) and dipeptidyl peptidase IV (DPPIV) and three metalloproteinases, matrix metalloproteinase (MMP)-2, MMP-9 and MT1-MMP in 24 primary esophageal squamous cell carcinoma (ESCC) tissues and paired non-cancer tissues. Using reverse-transcription PCR, western blotting and zymography, we showed that both serine proteases and all three metalloproteinases were highly altered in ESCC. A positive correlation between the expression of FAP-α and DPPIV and the activity of both gelatinases was found. This may indicate that these proteolytic systems are tightly linked to each other and collectively are involved in the process of ECM degradation that facilitates cancer cell invasi  on and met  astasis. 
 Fibroblast activation protein, a dual specificity serine protease expressed in reactive human tumor stromal fibroblasts. Proteolytic degradation of extracellular matrix (ECM) components during tissue remodeling plays a pivotal role in normal and pathological processes including wound healing, inflammation, tumor invasion, and   metastasis  . Proteolytic enzymes in tumors may activate or release growth factors from the ECM or act directly on the ECM itself, thereby facilitating angiogenesis or tumor cell migration. Fibroblast activation protein (FAP) is a cell surface antigen of reactive tumor stromal fibroblasts found in epithelial cancers and in granulation tissue during wound healing. It is absent from most normal adult human tissues. FAP is conserved throughout chordate evolution, with homologues in mouse and Xenopus laevis, whose expression correlates with tissue remodeling events. Using recombinant and purified natural FAP, we show that FAP has both dipeptidyl peptidase activity and a collagenolytic activity capable of degrading gelatin and type I collagen; by sequence, FAP belongs to the serine protease family rather than the matrix metalloprotease family. Mutation of the putative catalytic serine residue of FAP to alanine abolishes both enzymatic activities. Consistent with its in vivo expression pattern determined by immunohistochemistry, FAP enzyme activity was detected by an immunocapture assay in human cancerous tissues but not in matched normal tissues. This study demonstrates that FAP is present as an active cell surface-bound collagenase in epithelial tumor stroma and opens up investigation into physiological substrates of its novel, tumor-associated dipeptidyl peptidase activity. 
 FAP-overexpressing fibroblasts produce an extracellular matrix that enhances invasive velocity and directionality of pancreatic cancer cells. Alterations towards a permissive stromal microenvironment provide important cues for tumor growth, invasion, and   metastasis  . In this study, Fibroblast activation protein (FAP), a serine protease selectively produced by tumor-associated fibroblasts in over 90% of epithelial tumors, was used as a platform for studying tumor-stromal interactions. We tested the hypothesis that FAP enzymatic activity locally modifies stromal ECM (extracellular matrix) components thus facilitating the formation of a permissive microenvironment promoting tumor invasion in human pancreatic cancer.We generated a tetracycline-inducible FAP overexpressing fibroblastic cell line to synthesize an in vivo-like 3-dimensional (3D) matrix system which was utilized as a stromal landscape for studying matrix-induced cancer cell behaviors. A FAP-dependent topographical and compositional alteration of the ECM was characterized by measuring the relative orientation angles of fibronectin fibers and by Western blot analyses. The role of FAP in the matrix-induced permissive tumor behavior was assessed in Panc-1 cells in assorted matrices by time-lapse acquisition assays. Also, FAP+ matrix-induced regulatory molecules in cancer cells were determined by Western blot analyses.We observed that FAP remodels the ECM through modulating protein levels, as well as through increasing levels of fibronectin and collagen fiber organization. FAP-dependent architectural/compositional alterations of the ECM promote tumor invasion along characteristic parallel fiber orientations, as demonstrated by enhanced directionality and velocity of pancreatic cancer cells on FAP+ matrices. This phenotype can be reversed by inhibition of FAP enzymatic activity during matrix production resulting in the disorganization of the ECM and impeded tumor invasion. We also report that the FAP+ matrix-induced tumor invasion phenotype is β1-integrin/FAK mediated.Cancer cell invasiveness can be affected by alterations in the tumor microenvironment. Disruption of FAP activity and β1-integrins may abrogate the invasive capabilities of pancreatic and other tumors by disrupting the FAP-directed organization of stromal ECM and blocking β1-integrin dependent cell-matrix interactions. This provides a novel preclinical rationale for therapeutics aimed at interfering with the architectural organization of tumor-associated ECM. Better understanding of the stromal influences that fuel progressive tumorigenic behaviors may allow the effective future use of targeted therapeutics aimed at disrupting specific tumor-stromal interactions. 
 Overexpression of fibroblast activation protein and its clinical implications in patients with osteosarcoma. Fibroblast activation protein (FAP) expression has been detected in fibroblastic component of osteosarcomas. The aim of this study was to analyze the correlation of FAP expression with the clinicopathological features of osteosarcoma.FAP mRNA and protein expression levels in human osteosarcoma tissues were, respectively detected by RT-PCR, Western blot, and immunohistochemistry assays.FAP mRNA and protein expression were both higher in osteosarcoma than in corresponding noncancerous bone tissues (both P < 0.001). In addition, the immunohistochemistry assay found that all patients showed positive FAP expression. Higher FAP expression was significantly correlated with advanced clinical stage (P = 0.006), high histological grade (P = 0.02), positive metastatic status (P = 0.01), shorter overall (P < 0.001), and disease-free (P < 0.001) survival in osteosarcoma patients. Furthermore, Cox multivariate analysis showed that FAP overexpression was an independent prognostic factor for predicting both overall and disease-free survival of osteosarcoma patients.Expression of FAP in osteosarcoma could be adopted as a candidate biomarker for the diagnosis of clinical stage,   histologi  cal grade and metastasis, and for assessing prognosis, indicating for the first time that FAP may play an important role in tumor development and progression in osteosarcoma. FAP might be considered as a novel therapeutic target against this cancer. 
  GeneRIFs  
 Expression of FAPalpha in stroma was associated with distant   metastasis   of breast phyllodes tumor. 
 High FAP expression is associated with increased   metastasis  . 
 We found that seprase is transcriptionally up-regulated in invasive melanoma cells via the canonical TGF-beta signaling pathway, supporting the roles of both TGF-beta and seprase in tumor invasion and   metastasis  . 
 An abundant expression of seprase in colorectal cancer tissue is associated with lymph node   metastasis  . 
 Increased expression of seprase is associated with lymph node   metastasis   in colorectal cancer 
  [Cc]ell [Ii]nvasion  PubMed  
 A novel protease-docking function of integrin at invadopodia. Invadopodia are membrane extensions of aggressive tumor cells that function in the activation of membrane-bound proteases occurring during tumor   cell invasion  . We explore a novel and provocative activity of integrins in docking proteases to sites of invasion, termed invadopodia. In the absence of collagen, alpha(3)beta(1) integrin and the gelatinolytic enzyme, seprase, exist as nonassociating membrane proteins. Type I collagen substratum induces the association of alpha(3)beta(1) integrin with seprase as a complex on invadopodia. The results show that alpha(3)beta(1) integrin is a docking protein for seprase to form functional invadopodia. In addition, alpha(5)beta(1) integrin may participate in the adhesion process necessary for invadopodial formation. Thus, alpha(3)beta(1) and alpha(5)beta(1) integrins play major organizational roles in the adhesion and formation of invadopodia, promoting invasive cell behavior. 
 Fibroblast activation protein-α: a key modulator of the microenvironment in multiple pathologies. Fibroblast activation protein-α (FAP) is a serine protease that can provide target specificity to therapeutic agents because in adults its expression is restricted to pathologic sites, including cancer, fibrosis, arthritis, wounding, or inflammation. It is not expressed in most normal tissues. The majority of FAP is expressed by activated fibroblasts responding to the pathologic situations. FAP is typically found as a type II transmembrane protein physically attached to cells and with the bulk of the protein, including the catalytic domain, exposed to the extracellular space and accessible to small molecules. In this chapter, we review the structure, substrate specificities, signaling functions, and current design of FAP inhibitors. Evidence indicating the presence of FAP in multiple cancers, arthritis, fibrosis, keloids, and other pathologies is described and indicates possible roles for FAP in facilitatin  g cell invasi  on and growth. Separate sections are devoted to the role of FAP in coordinating the stromal response to cancer, including a role in angiogenesis and a potential role in modulation of the antitumor immune response. Finally studies attempting to demonstrate the clinical potential of FAP are discussed, as well as some novel applications employing FAP in therapy or diagnosis. Throughout this review, effort is made to highlight areas where information is lacking and to highlight important questions that require further investigation. 
 High intratumoral expression of fibroblast activation protein (FAP) in colon cancer is associated with poorer patient prognosis. An active stroma is important for cancer   cell invasion   and metastasis. We investigated the expression of fibroblast activation protein (FAP) in relation to patient prognosis in colorectal cancer. Colorectal cancer specimens from 449 patients were immunohistochemically stained with a FAP antibody and evaluated in the tumor center and tumor front using a semiquantitative four-level scale. FAP was expressed by fibroblasts in 85-90 % of the tumors examined. High versus no/low expression in the tumor center was associated with poor prognosis (multivariate hazard ratio, HR = 1.72; 95 % CI 1.07-2.77, p = 0.025). FAP expression in the tumor front, though more frequent than in the tumor center, was not associated with prognosis. FAP expression in the tumor center was more common in specimens with positive microsatellite instability (MSI) screening status and in patients with high CpG island methylator phenotype (CIMP) status. However, inclusion of MSI screening status and CIMP status in the multivariate analysis strengthened the risk estimates for high FAP expression in the tumor center (HR = 1.89; 95 % CI 1.13-3.14; p = 0.014), emphasizing the role of FAP as an independent prognostic factor. Stromal FAP expression is common in colorectal cancer, and we conclude that high FAP expression in the tumor center, but not the tumor front, is an independent negative prognostic factor. 
 Regulation of fibroblast migration on collagenous matrix by a cell surface peptidase complex. The invasion of migratory cells through connective tissues involves metallo- and serine types of cell surface proteases. We show that formation of a novel protease complex, consisting of the membrane-bound prolyl peptidases seprase and dipeptidyl peptidase IV (DPPIV), at invadopodia of migratory fibroblasts is a prerequisite for   cell invasion   and migration on a collagenous matrix. Seprase and DPPIV form a complex on the cell surface that elicits both gelatin binding and gelatinase activities localized at invadopodia of cells migrating on collagenous fibers. The protease complex participates in the binding to gelatin and localized gelatin degradation, cellular migration, and monolayer wound closure. Serine protease inhibitors can block the gelatinase activity and the localized gelatin degradation by cells. Antibodies to the gelatin-binding domain of DPPIV reduce the cellular abilities of the proteases to degrade gelatin but do not affect cellular adhesion or spreading on type I collagen. Furthermore, expression of the seprase-DPPIV complex is restricted to migratory cells involved in wound closure in vitro and in connective tissue cells during closure of gingival wounds but not in differentiated tissue cells. Thus, we have identified cell surface proteolytic activities, which are non-metalloproteases, seprase and DPPIV, that are responsible for the tissue-invasive phenotype. 
 TGF-beta induces fibroblast activation protein expression; fibroblast activation protein expression increases the proliferation, adhesion, and migration of HO-8910PM [corrected]. Several studies recognize cancer-stromal fibroblasts' role in cancer-  cell invasion   and metastasis. Through paracrine signaling molecules, TGF-beta and IL-1beta, cancer cells activate stromal fibroblasts and induce the expression of fibroblast activation protein (FAP). FAP, in turn, affects the proliferation, invasion and migration of the cancer cells. We report that TGF-beta and IL-1beta are important factors in inducing differentiation of myofibroblasts and expression of functional markers, notably alpha-SMA. We discover that TGF-beta is the dominant factor in promoting FAPalpha protein expression. This study also examines FAP's function in vitro by assaying the proliferation, migration and invasion of ovarian cancer cell line HO-8910PM. 
 Elevation of seprase expression and promotion of an invasive phenotype by collagenous matrices in ovarian tumor cells. Tumor cells do not constitutively exhibit invasive activity, but rather, can be transiently induced to adhere and form lesions. We report here that the expression of seprase, a dominant EDTA-resistant gelatinase in malignant tumors, is dependent on tumor cell exposure to type I collagen gel (TICg). The induced seprase expression of ovarian tumor cells influences their collagen contraction and invasion capability. Importantly, tumor cells with reduced seprase expression, due to manipulation by RNA interference, showed a reduction of TICg contraction in the gel contractility assay, inhibition of tumor   cell invasion   through TICg as shown by a transwell migration assay and inhibition of peritoneal membrane tumor lesion in a mouse model. In addition, mAb C27, an antibody against beta1 integrin, which blocks cellular avidity to TICg, can induce seprase RNA expression and promote the invasive phenotype and metastatic potential of ovarian tumor cells. Thus, collagenous matrices in the tumor cell niche induce the expression of seprase and initiate tumor invasion and metastatic cascades. 
 Increased expression of fibroblast activation protein-alpha in keloid fibroblasts: implications for development of a novel treatment option. Keloid scars are common benign fibroproliferative reticular dermal lesions with unknown etiology and ill-defined management with high rate of recurrence post surgery. The progression of keloids is characterized by increased deposition of extracellular matrix proteins, invasion into the surrounding healthy skin and inflammation. Fibroblasts are considered to be the key cellular mediators of fibrogenesis in keloid scars. Fibroblast activation protein alpha (FAP-α) and dipeptidyl peptidase IV (DPPIV) are proteases located at the plasma membrane promoting cell invasiveness and tumor growth and have been previously associated with keloid scars. Therefore, in this study we analyzed in further detail the expression of FAP-α in keloid fibroblasts compared to control skin fibroblasts. Dermal fibroblasts were obtained from punch-biopsies from the active margin of four keloids and four control skin samples. Flow cytometry was used to analyze FAP-α expression and the CytoSelect 24-Well Collagen   I Cell Invas  ion Assay was applied to study fibroblast invasion. Secretion of extracellular matrix (ECM) proteins was investigated by multiplexed particle-based flow cytometric assay and enzyme-linked immunosorbent assay. We found an increased expression of FAP-α in keloid fibroblasts compared to control skin fibroblasts (p < 0.001). Inhibition of FAP-α/DPPIV activity using the irreversible inhibitor H(2)N-Gly-Pro diphenylphosphonate reduced the increased invasiveness of keloid fibroblasts (p < 0.001) indicating that keloid invasion may be partly FAP-α/DPPIV mediated. FAP-α/DPPIV inhibition had no effect, (a) on the synthesis of the ECM proteins procollagen type I C-terminal peptide and fibronectin, (b) on the production of fibroblast growth factor or vascular endothelial growth factor, (c) on the expression of the proinflammatory cytokines interleukin-6 (IL-6), interleukin 8 (IL-8) or monocyte chemotactic protein-1. These results suggest a potential role for FAP-α and DPPIV in the invasive behavior of keloids. FAP-α and DPPIV may increase the invasive capacity of keloid fibroblasts rather than by modulating inflammation or ECM production. Since FAP-α expression is restricted to reactive fibroblasts in wound healing and normal adult tissues are generally FAP-α negative, inhibiting FAP-α/DPPIV activity may be a novel treatment option to prevent keloid progression. 
 Upregulated expression and activation of membrane‑associated proteases in esophageal squamous cell carcinoma. To better understand the role of membrane-associated proteolytic systems in the development of esophageal cancer, we studied the expression of two serine proteases, fibroblast activation protein-α (FAP-α) and dipeptidyl peptidase IV (DPPIV) and three metalloproteinases, matrix metalloproteinase (MMP)-2, MMP-9 and MT1-MMP in 24 primary esophageal squamous cell carcinoma (ESCC) tissues and paired non-cancer tissues. Using reverse-transcription PCR, western blotting and zymography, we showed that both serine proteases and all three metalloproteinases were highly altered in ESCC. A positive correlation between the expression of FAP-α and DPPIV and the activity of both gelatinases was found. This may indicate that these proteolytic systems are tightly linked to each other and collectively are involved in the process of ECM degradation that facilitates   cancer cell i  nvasion and metastasis. 
   
  
 FLT1 
 
  NCBI Gene  
  GeneRIF  
  Pubmed  
 
 
  Search Term  Rank  Fields  
  [Mm]igration  6  Process(2); PubMed(59); GeneRIFs(9)  
  [Ee]xtracellular [Mm]atrix  8  PubMed(8)  
  [Ii]nvasive  10  PubMed(16); GeneRIFs(2)  
  [Mm]etastasize  11  PubMed(1)  
  [Mm]etastasis  12  PubMed(42); GeneRIFs(7)  
  [Cc]ell [Ii]nvasion  13  PubMed(4)  
 
 
 hide/show details for FLT1 
  [Mm]igration  Process  
 cell   migration   
 positive regulation of cell   migration   
  PubMed  
 Vascular endothelial growth factor receptor-1 is deposited in the extracellular matrix by endothelial cells and is a ligand for the alpha 5 beta 1 integrin. Vascular endothelial growth factor receptor-1 (VEGFR-1) is a tyrosine kinase receptor for several growth factors of the VEGF family. Endothelial cells express a membrane-spanning form of VEGFR-1 and secrete a soluble variant of the receptor comprising only the extracellular region. The role of this variant has not yet been completely defined. In this study, we report that the secreted VEGFR-1 is present within the extracellular matrix deposited by endothelial cells in culture, suggesting a possible involvement in endothelial cell adhesion and   migration  . In adhesion assays, VEGFR-1 extracellular region specifically promoted endothelial cell attachment. VEGFR-1-mediated cell adhesion was divalent cation-dependent, and inhibited by antibodies directed against the alpha 5 beta 1 integrin. Moreover, VEGFR-1 promoted endothelial cell   migration  , and this effect was inhibited by anti-alpha 5 beta 1 antibodies. Direct binding of VEGFR-1 to the alpha 5 beta 1 integrin was also detected. Finally, binding to VEGFR-1 initiated endothelial cell spreading. Altogether these results indicate that the soluble VEGFR-1 secreted by endothelial cells becomes a matrix-associated protein that is able to interact with the alpha 5 beta 1 integrin, suggesting a new role of VEGFR-1 in angiogenesis, in addition to growth factor binding. 
 Analysis of the myosin-II-responsive focal adhesion proteome reveals a role for β-Pix in negative regulation of focal adhesion maturation. Focal adhesions undergo myosin-II-mediated maturation wherein they grow and change composition to modulate integrin signalling for cell   migratio  n, growth and differentiation. To determine how focal adhesion composition is affected by myosin II activity, we performed proteomic analysis of isolated focal adhesions and compared protein abundance in focal adhesions from cells with and without myosin II inhibition. We identified 905 focal adhesion proteins, 459 of which changed in abundance with myosin II inhibition, defining the myosin-II-responsive focal adhesion proteome. The abundance of 73% of the proteins in the myosin-II-responsive focal adhesion proteome was enhanced by contractility, including proteins involved in Rho-mediated focal adhesion maturation and endocytosis- and calpain-dependent focal adhesion disassembly. During myosin II inhibition, 27% of proteins in the myosin-II-responsive focal adhesion proteome, including proteins involved in Rac-mediated lamellipodial protrusion, were enriched in focal adhesions, establishing that focal adhesion protein recruitment is also negatively regulated by contractility. We focused on the Rac guanine nucleotide exchange factor β-Pix, documenting its role in the negative regulation of focal adhesion maturation and the promotion of lamellipodial protrusion and focal adhesion turnover to drive cel  l migrati  on. 
 Vascular endothelial growth factor-induced   migration   of multiple myeloma cells is associated with beta 1 integrin- and phosphatidylinositol 3-kinase-dependent PKC alpha activation. In multiple myeloma (MM),   migration   is necessary for the homing of tumor cells to bone marrow (BM), for expansion within the BM microenvironment, and for egress into the peripheral blood. In the present study we characterize the role of vascular endothelial growth factor (VEGF) and beta(1) integrin (CD29) in MM cell   migration  . We show that protein kinase C (PKC) alpha is translocated to the plasma membrane and activated by adhesion of MM cells to fibronectin and VEGF. We identify beta(1) integrin modulating VEGF-triggered MM cell   migration   on fibronectin. We show that transient enhancement of MM cell adhesion to fibronectin triggered by VEGF is dependent on the activity of both PKC and beta(1) integrin. Moreover, we demonstrate that PKC alpha is constitutively associated with beta(1) integrin. These data are consistent with PKC alpha-dependent exocytosis of activated beta(1) integrin to the plasma membrane, where its increased surface expression mediates binding to fibronectin; conversely, catalytically active PKC alpha-driven internalization of beta(1) integrin results in MM cell de-adhesion. We show that the regulatory subunit of phosphatidylinositol (PI) 3-kinase (p85) is constitutively associated with FMS-like tyrosine kinase-1 (Flt-1). VEGF stimulates activation of PI 3-kinase, and both MM cell adhesion and   migration   are PI 3-kinase-dependent. Moreover, both VEGF-induced PI 3-kinase activation and beta(1) integrin-mediated binding to fibronectin are required for the recruitment and activation of PKC alpha. Time-lapse phase contrast video microscopy (TLVM) studies confirm the importance of these signaling components in VEGF-triggered MM cell   migration   on fibronectin. 
 Flt-1 in colorectal cancer cells is required for the tumor invasive effect of placental growth factor through a p38-MMP9 pathway. Placenta growth factor (PlGF), a dimeric glycoprotein with 53% homology to VEGF, binds to VEGF receptor-1 (Flt-1), but not to VEGF receptor-2 (Flk-1), and may function by modulating VEGF activity. We previously have showed that PlGF displays prognostic value in colorectal cancer (CRC) but the mechanism remains elucidated.Overexpression of PlGF increased the invasive/  migration   ability and decreased apoptosis in CRC cells showing Flt-1 expression. Increased   migration   was associated with increasing MMP9 via p38 MAPK activation. Tumors grew faster, larger; with higher vascularity from PlGF over-expression cells in xenograft assay. In two independent human CRC tissue cohorts, PlGF, MMP9, and Flt-1 expressions were higher in the advanced than the localized disease group. PlGF expression correlated with MMP9, and Flt-1 expression. CRC patients with high PlGF and high Flt-1 expression in tissue had poor prognosis.PlGF/Flt-1 signaling plays an important role in CRC progression, blocking PlGF/Flt-1 signaling maybe an alternative therapy for CRC. 
 Analysis of protein-protein interactions in cross-talk pathways reveals CRKL protein as a novel prognostic marker in hepatocellular carcinoma. Deciphering the network of signaling pathways in cancer via protein-protein interactions (PPIs) at the cellular level is a promising approach but remains incomplete. We used an in situ proximity ligation assay to identify and quantify 67 endogenous PPIs among 21 interlinked pathways in two hepatocellular carcinoma (HCC) cells, Huh7 (minimally migratory cells) and Mahlavu (highly migratory cells). We then applied a differential network biology analysis and determined that the novel interaction, CRKL-FLT1, has a high centrality ranking, and the expression of this interaction is strongly correlated with the migratory ability of HCC and other cancer cell lines. Knockdown of CRKL and FLT1 in HCC cells leads to a decrease in cell   migration   via ERK signaling and the epithelial-mesenchymal transition process. Our immunohistochemical analysis shows high expression levels of the CRKL and CRKL-FLT1 pair that strongly correlate with reduced disease-free and overall survival in HCC patient samples, and a multivariate analysis further established CRKL and the CRKL-FLT1 as novel prognosis markers. This study demonstrated that functional exploration of a disease network with interlinked pathways via PPIs can be used to discover novel biomarkers. 
 Flt-1 in colorectal cancer cells is required for the tumor invasive effect of placental growth factor through a p38-MMP9 pathway. Placenta growth factor (PlGF), a dimeric glycoprotein with 53% homology to VEGF, binds to VEGF receptor-1 (Flt-1), but not to VEGF receptor-2 (Flk-1), and may function by modulating VEGF activity. We previously have showed that PlGF displays prognostic value in colorectal cancer (CRC) but the mechanism remains elucidated.Overexpression of PlGF increased the invasive/  migration   ability and decreased apoptosis in CRC cells showing Flt-1 expression. Increased   migration   was associated with increasing MMP9 via p38 MAPK activation. Tumors grew faster, larger; with higher vascularity from PlGF over-expression cells in xenograft assay. In two independent human CRC tissue cohorts, PlGF, MMP9, and Flt-1 expressions were higher in the advanced than the localized disease group. PlGF expression correlated with MMP9, and Flt-1 expression. CRC patients with high PlGF and high Flt-1 expression in tissue had poor prognosis.PlGF/Flt-1 signaling plays an important role in CRC progression, blocking PlGF/Flt-1 signaling maybe an alternative therapy for CRC. 
 Vascular endothelial growth factor receptor-1 is deposited in the extracellular matrix by endothelial cells and is a ligand for the alpha 5 beta 1 integrin. Vascular endothelial growth factor receptor-1 (VEGFR-1) is a tyrosine kinase receptor for several growth factors of the VEGF family. Endothelial cells express a membrane-spanning form of VEGFR-1 and secrete a soluble variant of the receptor comprising only the extracellular region. The role of this variant has not yet been completely defined. In this study, we report that the secreted VEGFR-1 is present within the extracellular matrix deposited by endothelial cells in culture, suggesting a possible involvement in endothelial cell adhesion and   migration  . In adhesion assays, VEGFR-1 extracellular region specifically promoted endothelial cell attachment. VEGFR-1-mediated cell adhesion was divalent cation-dependent, and inhibited by antibodies directed against the alpha 5 beta 1 integrin. Moreover, VEGFR-1 promoted endothelial cell   migration  , and this effect was inhibited by anti-alpha 5 beta 1 antibodies. Direct binding of VEGFR-1 to the alpha 5 beta 1 integrin was also detected. Finally, binding to VEGFR-1 initiated endothelial cell spreading. Altogether these results indicate that the soluble VEGFR-1 secreted by endothelial cells becomes a matrix-associated protein that is able to interact with the alpha 5 beta 1 integrin, suggesting a new role of VEGFR-1 in angiogenesis, in addition to growth factor binding. 
 The phosphorylated 1169-tyrosine containing region of flt-1 kinase (VEGFR-1) is a major binding site for PLCgamma. Flt-1, a tyrosine kinase receptor for vascular endothelial growth factor (VEGF), plays important roles in the angiogenesis required for embryogenesis and in monocyte/macrophage   migration  . However, the signal transduction of Flt-1 is poorly understood due to its very weak tyrosine kinase activity. Therefore, we overexpressed Flt-1 in insect cells using the Baculovirus system in order to examine for autophosphorylation sites and association with adapter molecules such as phospholipase Cgamma-1 (PLCgamma). Tyr-1169 and Tyr-1213 on Flt-1 were found to be auto-phosphorylated, but only a phenylalanine mutant of Tyr-1169 strongly suppressed its association with PLCgamma. In Flt-1 overexpressing NIH3T3 cells, VEGF induced autophosphorylation of Flt-1, tyrosine-phosphorylation of PLCgamma and protein kinase C-dependent activation of MAP kinase. These results strongly suggest that Tyr-1169 on Flt-1 is a major binding site for PLCgamma and important for Flt-1 signal transduction within the cell. 
 VEGFR-1 activation-induced MMP-9-dependent invasion in hepatocellular carcinoma. VEGFR-1 can promote invasion through epithelial-mesenchymal transition induction in hepatocellular carcinoma (HCC). This study aims to elucidate VEGFR-1 impact on proteolytic enzymes profile involved with invasion.The effect on cell invasion was evaluated by invasive and   migration   assays with and without VEGFR-1 activation. The mechanism was investigated by real-time PCR, western blot and gelatin zymography using inhibitors for MMP-9. In total, 95 HCC patients were enrolled for its clinical value evaluation.VEGFR-1 activation induced invasion in HCC cells with an increase in the expression and activity of MMP-9 and Snail. MMP-9 blockage effectively inhibited VEGFR-1-induced invasion. High coexpression of both in HCC predicted a worse clinical outcome.Data show a novel VEGFR-1 activation-to-MMP-9 mechanism promoting HCC invasion. 
 Galectin-1 induces vascular permeability through the neuropilin-1/vascular endothelial growth factor receptor-1 complex. Galectin-1 (Gal-1) is a β-galactoside-binding lectin that regulates endothelial cell   migratio  n, proliferation, and adhesion. However, the effect of Gal-1 on vascular permeability and the underlying mechanisms are unclear. We found that high Gal-1 expression was associated with elevated tumor vascular permeability in specimens of oral squamous cell carcinoma. Using transendothelial passage of FITC-dextran and a Miles assay, we demonstrated that Gal-1 increased vascular permeability extracellularly through its carbohydrate recognition domain. Mechanism dissection revealed that the neuropilin (NRP)-1/vascular endothelial growth factor receptor- (VEGFR)-1 complex was required for Gal-1-regulated vascular permeability. Activation of VEGFR-1 triggered activation of Akt which led to a reduction in vascular endothelial-cadherin at cell-cell junctions and resulted in cytoskeletal rearrangement. Both inhibition of Gal-1 secreted from cancer cells and administration of an anti-Gal-1 antibody in the tumor microenvironment suppressed tumor growth and vascular permeability in xenograft models. In conclusion, our results demonstrate a novel function of Gal-1 of increasing vascular permeability through the NRP-1/VEGFR1 and Akt signaling pathway and indicate that targeting Gal-1 by an anti-Gal-1 antibody is a feasible therapy for vascular hyperpermeability and cancer. 
 Glioma cells enhance endothelial progenitor cell angiogenesis via VEGFR-2, not VEGFR-1. Although potential contribution of endothelial progenitor cells (EPCs) to angiogenesis in glioma has been proposed, the molecular mechanisms of EPCs recruitment to vasculature have not been fully elucidated. Here, we show that the supernatant from glioma cells promotes EPCs angiogenesis via VEGFR-2, not VEGFR-1. Moreover, VEGFR-2 siRNA inhibits VEGFR-2 expression in EPCs, tube formation on matrigel and cell   migration  . MMP-9 activity and expression and the Akt and ERK phosphorylations are decreased by VEGFR-2 siRNA. Thus, these results indicate that glioma cells enhance EPC angiogenesis via VEGFR-2, not VEGFR-1, mediated by the MMP-9, Akt and ERK signal pathways. 
 Vascular endothelial growth factor receptor-1 mediates   migration   of human colorectal carcinoma cells by activation of Src family kinases. Vascular endothelial growth factor (VEGF) is the predominant pro-angiogenic cytokine in human malignancy, and its expression correlates with disease recurrence and poor outcomes in patients with colorectal cancer. Recently, expression of vascular endothelial growth factor receptors (VEGFRs) has been observed on tumours of epithelial origin, including those arising in the colon, but the molecular mechanisms governing potential VEGF-driven biologic functioning in these tumours are not well characterised. In this report, we investigated the role of Src family kinases (SFKs) in VEGF-mediated signalling in human colorectal carcinoma (CRC) cell lines. Vascular endothelial growth factor specifically activated SFKs in HT29 and KM12L4 CRC cell lines. Further, VEGF stimulation resulted in enhanced cellular   migration  , which was effectively blocked by pharmacologic inhibition of VEGFR-1 or Src kinase. Correspondingly,   migration   studies using siRNA clones with reduced Src expression confirmed the requirement for Src in VEGF-induced   migration   in these cells. Furthermore, VEGF treatment enhanced VEGFR-1/SFK complex formation and increased tyrosine phosphorylation of focal adhesion kinase, p130 cas and paxillin. Finally, we demonstrate that VEGF-induced   migration   is not due, at least in part, to VEGF acting as a mitogen. These results suggest that VEGFR-1 promotes   migration   of tumour cells through a Src-dependent pathway linked to activation of focal adhesion components that regulate this process. 
 Immunolocalization and expression of vascular endothelial growth factor receptors (VEGFRs) and neuropilins (NRPs) on keratinocytes in human epidermis. Vascular endothelial growth factor (VEGF) plays an important role in normal and pathological angiogenesis. VEGF receptors (VEGFRs, including VEGFR-1, VEGFR-2, and VEGFR-3) and neuropilins (NRPs, including NRP-1 and NRP-2) are high-affinity receptors for VEGF and are typically considered to be specific for endothelial cells. Here we showed expression of VEGFRs and NRPs on cultured epidermal keratinocytes at both mRNA and protein levels. We further localized these receptors by immunofluorescence (IF) staining in the epidermis of surgical skin specimens. We found positive staining for VEGFRs and NRPs in all layers of the epidermis except for the stratum corneum. VEGFR-1 and VEGFR-2 are primarily expressed on the cytoplasmic membrane of basal cells and the adjacent spinosum keratinocytes. All layers of the epidermis except for the horny cell layer demonstrated a uniform pattern of VEGFR-3, NRP-1, and NRP-2. Sections staining for NRP-1 and NRP-2 also showed diffuse intense fluorescence and were localized to the cell membrane and cytoplasm of keratinocytes. In another panel of experiments, keratinocytes were treated with different concentrations of VEGF, with or without VEGFR-2 neutralizing antibody in culture. VEGF enhanced the proliferation and   migration   of keratinocytes, and these effects were partially inhibited by pretreatment with VEGFR-2 neutralizing antibody. Adhesion of keratinocytes to type IV collagen-coated culture plates was decreased by VEGF treatment, but this reduction could be completely reversed by pretreatment with VEGFR-2 neutralizing antibody. Taken together, our results suggest that the expression of VEGFRs and NRPs on keratinocytes may constitute important regulators for its activity and may possibly be responsible for the autocrine signaling in the epidermis. 
 VEGF mediated signaling in oral cancer. Increasingly it is being realized that oral cancer arises from genetic/epigenetic mutations, dysregulations of spatio-temporally controlled signal transduction cascades and loss of apoptosis. Epidemiological studies have provided a stronger association between tobacco use (chewed and smoked) and oral cancer. Nevertheless, alcohol has also gained attention as a significant risk factor, having a multiplicative synergistic cancer promoting effect with tobacco. Vascular Endothelial Growth Factor (VEGF) mediated signaling has gained limelight because of its instrumental role in endothelial cell proliferation, survival, invasion,   migration  , chemotaxis of bone marrow (BM)-derived progenitor cells, vasodilation and vascular permeability. In this review we provide most recent updates on involvement of VEGF/VEGFR signaling axis in oral cancer. We partition this multi-component review into different sections and summarize latest advancements related to therapies against VEGF/VEGFR signaling axis and how microRNAs tactfully modulate VEGF and VEGFR in oral cancers. Data obtained through preclinical and clinical studies has revealed that therapeutic benefits associated with VEGF-targeted therapy are complicated in different cancers and involve myriad of mechanisms. A better understanding of VEGF/VEGFR mediated signaling in oral cancers and testing of novel therapeutic agents in preclinical models will prove to be helpful in effective translation of safest drugs from benchtop to the bedside. 
 VEGF-A165 induces human aortic smooth muscle cell   migration   by activating neuropilin-1-VEGFR1-PI3K axis. Vascular smooth muscle cells (SMCs), one of the major cell types of the vascular wall, play a critical role in the process of angiogenesis under both physiological and pathophysiological conditions, including the cancer microenvironment. Previous studies have shown that VEGF-A 165 augments vascular SMC   migration   via VEGFR2 (KDR/Flk1) pathways. In this study, we found that VEGF-A 165 (recombinant protein or breast tumor cell-secreted) is also capable of inducing   migration   of VEGFR2-negative human aortic smooth muscle cells (hAOSMCs), and this induction is mediated through a molecular cross-talk of neuropilin-1 (NRP-1), VEGFR1 (Flt-1), and phosphoinositide 3-kinase (PI3K)/Akt signaling kinase. We found that VEGF-A 165 induces hAOSMC   migration   parallel with the induction of NRP-1 and VEGFR1 expressions and their associations along with the activation of PI3K/Akt. Neutralization of VEGF action by its antibody or inhibition of VEGF-induced PI3K/Akt kinase activation by wortmannin, a PI3K/Akt specific inhibitor, results in inhibition of VEGF-induced hAOSMC   migration  . Moreover, RNAi-mediated elimination of the NRP-1 expression or blocking of the activity of VEGFR1 by its antibody in hAOSMCs impairs the VEGF-A 165-induced   migration   of these cells as well as activation of PI3K/Akt kinase. Collectively, these results establish, for the first time, a mechanistic link among VEGF-A 165, NRP-1, VEGFR1, and PI3K/Akt in the regulation of   migration   of human vascular smooth muscle cells that eventually could be involved in the angiogenic switch. 
 Elevated placental soluble vascular endothelial growth factor receptor-1 inhibits angiogenesis in preeclampsia. Preeclampsia is an inflammatory disorder in which serum levels of vascular endothelial growth factor (VEGF) and its soluble receptor-1 (sVEGFR-1, also known as sFlt-1) are elevated. We hypothesize that VEGF and placenta growth factor (PlGF) are dysregulated in preeclampsia due to high levels of sVEGFR-1, which leads to impaired placental angiogenesis. Analysis of supernatants taken from preeclamptic placental villous explants showed a four-fold increase in sVEGFR-1 than normal pregnancies, suggesting that villous explants in vitro retain a hypoxia memory reflecting long-term fetal programming. The relative ratios of VEGF to sVEGFR-1 and PlGF to sVEGFR-1 released from explants decreased by 53% and 70%, respectively, in preeclampsia compared with normal pregnancies. Exposure of normal villous explants to hypoxia increased sVEGFR-1 release compared with tissue normoxia (P<0.001), as did stimulation with tumor necrosis factor-alpha (P<0.01). Conditioned medium (CM) from normal villous explants induced endothelial cell   migration   and in vitro tube formation, which were both attenuated by pre-incubation with exogenous sVEGFR-1 (P<0.001). In contrast, endothelial cells treated with preeclamptic CM showed substantially reduced angiogenesis compared with normal CM (P<0.001), which was not further decreased by the addition of exogenous sVEGFR-1, indicating a saturation of the soluble receptor. Removal of sVEGFR-1 by immunoprecipitation from preeclamptic CM significantly restored   migration   (P<0.001) and tube formation (P<0.001) to levels comparable to that induced by normal CM, demonstrating that elevated levels of sVEGFR-1 in preeclampsia are responsible for inhibiting angiogenesis. Our finding demonstrates the dysregulation of the VEGF/PlGF axis in preeclampsia and offers an entirely new therapeutic approach to its treatment. 
 Vascular endothelial growth factor receptor-1 promotes   migration   and invasion in pancreatic carcinoma cell lines. Vascular endothelial growth factor receptor-1 (VEGFR-1) is one of three receptor tyrosine kinases for VEGF, a key regulator of angiogenesis in cancer. Although VEGFRs initially were believed to be expressed exclusively on endothelial cells (ECs), recent studies have demonstrated the presence of VEGFR-1 on non-EC types. The authors hypothesized that VEGFR-1 is present and functional in pancreatic carcinoma cells, contributing to the malignant phenotype.The authors assessed the expression of VEGFR-1 and its ligands in 11 pancreatic carcinoma cell lines by reverse-transcriptase-polymerase chain reaction, enzyme-linked immunosorbent assay, and/or Western blot analysis. The function of VEGFR-1 was evaluated by treating two representative cell lines with VEGF-B, a selective ligand for VEGFR-1, and/or a specific anti-VEGFR-1 antibody and assessing the effects on signaling,   migration  , invasion, and proliferation.All 11 pancreatic carcinoma cell lines expressed VEGFR-1 mRNA and protein, as well as the VEGFR-1 ligands VEGF-A and VEGF-B. Two representative cell lines (L3.6 and Panc-1) exhibited VEGF-B-induced mitogen-activated protein kinase signaling. A VEGFR-1 neutralizing antibody abrogated signaling, confirming that the ligand effect was mediated through VEGFR-1. VEGFR-1 stimulation by VEGF-A or VEGF-B was found to promote   migration   in both cell lines. Panc-1 cells also demonstrated enhanced Matrigel invasion after VEGFR-1 stimulation. VEGFR-1-dependent   migration   and invasion were blocked by the VEGFR-1 neutralizing antibody. VEGFR-1 activation did not appear to enhance cell proliferation.VEGFR-1 appears to be expressed ubiquitously in pancreatic carcinoma cell lines, in which it induces signaling and promotes   migration   and invasion. Overexpression of VEGF in tumors may activate tumor cells bearing VEGFR-1 via an autocrine pathway. Agents targeting VEGF or its receptors may have a dual inhibitory effect on tumor growth by suppressing both angiogenesis and tumor cell function. 
 Urokinase-type Plasminogen Activator (uPA) Promotes Angiogenesis by Attenuating Proline-rich Homeodomain Protein (PRH) Transcription Factor Activity and De-repressing Vascular Endothelial Growth Factor (VEGF) Receptor Expression. Urokinase-type plasminogen activator (uPA) regulates angiogenesis and vascular permeability through proteolytic degradation of extracellular matrix and intracellular signaling initiated upon its binding to uPAR/CD87 and other cell surface receptors. Here, we describe an additional mechanism by which uPA regulates angiogenesis. Ex vivo VEGF-induced vascular sprouting from Matrigel-embedded aortic rings isolated from uPA knock-out (uPA(-/-)) mice was impaired compared with vessels emanating from wild-type mice. Endothelial cells isolated from uPA(-/-) mice show less proliferation and   migration   in response to VEGF than their wild type counterparts or uPA(-/-) endothelial cells in which expression of wild type uPA had been restored. We reported previously that uPA is transported from cell surface receptors to nuclei through a mechanism that requires its kringle domain. Intranuclear uPA modulates gene transcription by binding to a subset of transcription factors. Here we report that wild type single-chain uPA, but not uPA variants incapable of nuclear transport, increases the expression of cell surface VEGF receptor 1 (VEGFR1) and VEGF receptor 2 (VEGFR2) by translocating to the nuclei of ECs. Intranuclear single-chain uPA binds directly to and interferes with the function of the transcription factor hematopoietically expressed homeodomain protein or proline-rich homeodomain protein (HHEX/PRH), which thereby lose their physiologic capacity to repress the activity of vehgr1 and vegfr2 gene promoters. These studies identify uPA-dependent de-repression of vegfr1 and vegfr2 gene transcription through binding to HHEX/PRH as a novel mechanism by which uPA mediates the pro-angiogenic effects of VEGF and identifies a potential new target for control of pathologic angiogenesis. 
 VEGFR-1 (FLT-1) activation modulates acute lymphoblastic leukemia localization and survival within the bone marrow, determining the onset of extramedullary disease. The presence of persistent circulating leukemia cells, or engrafted into extramedullary tissues, is a bad prognostic factor for patients with acute leukemia. However, little is known about the mechanisms that regulate the exit of leukemia cells from the bone marrow (BM) microenvironment. We reveal that vascular endothelial growth factor receptor 1 (FLT-1) modulates acute leukemia distribution within the BM, along VEGF and PlGF gradients, regulating leukemia survival and exit into the peripheral circulation. FLT-1 activation on acute lymphoblastic leukemia (ALL) cells results in cell   migration   and proliferation in vitro, whereas in vivo FLT-1-overexpressing cells accumulate in the BM epiphysis of nonobese diabetic-severe combined immunodeficient (NOD-SCID) recipients and are detected in circulation 2 weeks after inoculation. In turn, FLT-1 neutralization affects leukemia localization (now in the BM diaphysis), increases leukemia apoptosis, and impedes the exit of ALL cells, prolonging the survival of inoculated mice. We demonstrate further that FLT-1-induced cell   migration   involves actin polymerization and lipid raft formation. Taken together, we show that FLT-1 regulates the BM localization of ALL cells, determining their survival and exit into the circulation and ultimately the survival of inoculated recipients. FLT-1 targeting on subsets of acute leukemias may delay the onset of extramedullary disease, which may be advantageous in combinatorial therapeutic settings. 
 Hypoxia differentially regulates VEGFR1 and VEGFR2 levels and alters intracellular signaling and cell   migration   in endothelial cells. The role of hypoxia on endothelial cell function and response to growth factors is unknown. Here, we tested the hypothesis that hypoxia re-programs endothelial function by modulating vascular endothelial growth factor receptor levels which in turn alter intracellular signaling and cell function. Hypoxia stimulated VEGF-A and VEGFR1 expression but decreased VEGFR2 levels in endothelial cells. During hypoxia, plasma membrane VEGFR1 levels were elevated whereas VEGFR2 levels were depleted. One functional consequence of hypoxia is a reduction in VEGF-A-stimulated and VEGFR2-regulated intracellular signaling including lowered endothelial nitric oxide synthase activation. Venous, arterial and capillary endothelial cells subjected to hypoxia all exhibited reduced cell   migration   in response to VEGF-A. A mechanistic explanation is that VEGFR1:VEGFR2 ratio is substantially increased during hypoxia to block VEGF-A-stimulated and VEGFR2-regulated endothelial responses to maximize cell viability and recovery. 
 A SNP in the flt-1 promoter integrates the VEGF system into the p53 transcriptional network. The VEGF system is essential for angiogenesis. VEGF overexpression frequently correlates with increased microvascularity and metastasis and decreased spontaneous apoptosis. Although a precise mechanism has not been established, studies suggest that VEGF expression is negatively regulated by p53, a master regulator and tumor suppressor. There are no reports of additional components of the VEGF signal transduction pathway being part of the p53 transcriptional network. A target of VEGF, the VEGF receptor 1/flt-1, can regulate growth and   migration   of endothelial cells and modulate angiogenesis. VEGF appears to be up-regulated in various cancers in which flt-1 may have a role in tumor progression and metastasis. We identified a C-to-T SNP upstream of the transcriptional start site in approximately 6% of the people examined. The SNP is located within a putative p53 response element. Only the promoter with the T SNP (FLT1-T) was responsive to p53 when examined with reporter assays or by endogenous gene expression analysis in cell lines with different SNP status. In response to doxorubicin-induced DNA damage, there was clear allele discrimination based on p53 binding at the FLT1-T but not FLT1-C promoters as well as p53-dependent induction of flt-1 mRNA, which required the presence of FLT1-T. Our results establish that p53 can differentially stimulate transcription at a polymorphic variant of the flt-1 promoter and directly places the VEGF system in the p53 stress-response network via flt-1 in a significant fraction of the human population. We suggest that the p53-VEGF-flt-1 interaction is relevant to risks in angiogenesis-associated diseases, including cancer. 
 Cholesterol regulates VEGFR-1 (FLT-1) expression and signaling in acute leukemia cells. VEGF receptors 1 (FLT-1) and 2 (KDR) are expressed on subsets of acute myeloid leukemia (AML) and acute lymphoid leukemia cells, in which they induce cell survival, proliferation, and   migration  . However, little is known about possible cofactors that regulate VEGF receptor expression and activation on leukemia cells. Here we show that cholesterol accumulates in leukemia-rich sites within bone marrow of xenotransplanted severe combined immunodeficient (SCID) mice. Therefore, we hypothesized that cholesterol-rich domains might regulate FLT-1 signaling and chemotaxis of acute leukemias. We then showed that FLT-1 accumulates in discrete cholesterol-rich membrane domains where it associates with caveolin-1 and that placenta growth factor (PlGF)/VEGF stimulation promotes FLT-1 localization in such cholesterol-rich domains. Accordingly, FLT-1 localization and its phosphorylation are abrogated by methyl-β-cyclodextrin (MβCD), which removes cellular cholesterol, and by nystatin, an inhibitor of lipid-raft endocytosis. Mechanistically, cholesterol increases FLT-1 expression and promotes PlGF/VEGF-induced leukemia cells viability and also induces VEGF production by the leukemia cells in vitro. Taken together, we conclude that cholesterol regulates VEGF:VEGFR-1 signaling on subsets of acute leukemias, modulating cel  l migrati  on, and viability, which may be crucial for disease progression. Finally, we provide evidence obtained from human AML samples that primary leukemia cells accumulate significantly more cholesterol than do normal cells and that cholesterol accumulation correlates with disease aggressiveness. 
 RACK1 regulates VEGF/Flt1-mediated cell   migration   via activation of a PI3K/Akt pathway. Vascular endothelial growth factor (VEGF) is vital to physiological as well as pathological angiogenesis, and regulates a variety of cellular functions, largely by activating its 2 receptors, fms-like tyrosine kinase (Flt1) and kinase domain receptor (KDR). KDR plays a critical role in the proliferation of endothelial cells by controlling VEGF-induced phospholipase Cγ-protein kinase C (PLCγ-PKC) signaling. The function of Flt1, however, remains to be clarified. Recent evidence has indicated that Flt1 regulates the VEGF-triggere  d migrati  on of endothelial cells and macrophages. Here, we show that RACK1, a ubiquitously expressed scaffolding protein, functions as an important regulator of this process. We found that RACK1 (receptor for activated protein kinase C 1) binds to Flt1 in vitro. When the endogenous expression of RACK1 was attenuated by RNA interference, the VEGF-drive  n migrati  on was remarkably suppressed whereas the proliferation was unaffected in a stable Flt1-expressing cell line, AG1-G1-Flt1. Further, we demonstrated that the VEGF/Flt-mediate  d migrati  on of AG1-G1-Flt1 cells occurred mainly via the activation of the PI3 kinase (PI3K)/Akt and Rac1 pathways, and that RACK1 plays a crucial regulatory role in promoting PI3K/Akt-Rac1 activation. 
 Early endocardial morphogenesis requires Scl/Tal1. The primitive heart tube is composed of an outer myocardial and an inner endocardial layer that will give rise to the cardiac valves and septa. Specification and differentiation of these two cell layers are among the earliest events in heart development, but the embryonic origins and genetic regulation of early endocardial development remain largely undefined. We have analyzed early endocardial development in the zebrafish using time-lapse confocal microscopy and show that the endocardium seems to originate from a region in the lateral plate mesoderm that will give rise to hematopoietic cells of the primitive myeloid lineage. Endocardial precursors appear to rapidly migrate to the site of heart tube formation, where they arrive prior to the bilateral myocardial primordia. Analysis of a newly discovered zebrafish Scl/Tal1 mutant showed an additional and previously undescribed role of this transcription factor during the development of the endocardium. In Scl/Tal1 mutant embryos, endocardial precursors are specified, but   migration   is severely defective and endocardial cells aggregate at the ventricular pole of the heart. We further show that the initial fusion of the bilateral myocardial precursor populations occurs independently of the endocardium and tal1 function. Our results suggest early separation of the two components of the primitive heart tube and imply Scl/Tal1 as an indispensable component of the molecular hierarchy that controls endocardium morphogenesis. 
 Vascular endothelial growth factor receptor-1 contributes to resistance to anti-epidermal growth factor receptor drugs in human cancer cells. The resistance to selective EGFR inhibitors involves the activation of alternative signaling pathways, and Akt activation and VEGF induction have been described in EGFR inhibitor-resistant tumors. Combined inhibition of EGFR and other signaling proteins has become a successful therapeutic approach, stimulating the search for further determinants of resistance as basis for novel therapeutic strategies.We established human cancer cell lines with various degrees of EGFR expression and sensitivity to EGFR inhibitors and analyzed signal transducers under the control of EGFR-dependent and EGFR-independent pathways.Multitargeted inhibitor vandetanib (ZD6474) inhibited the growth and the phosphorylation of Akt and its effector p70S6 kinase in both wild-type and EGFR inhibitor-resistant human colon, prostate, and breast cancer cells. We found that the resistant cell lines exhibit, as common feature, VEGFR-1/Flt-1 overexpression, increased secretion of VEGF and placental growth factor, and augmented   migration   capabilities and that vandetanib is able to antagonize them. Accordingly, a new kinase assay revealed that in addition to VEGF receptor (VEGFR)-2, RET, and EGFR, vandetanib efficiently inhibits also VEGFR-1. The contribution of VEGFR-1 to the resistant phenotype was further supported by the demonstration that VEGFR-1 silencing in resistant cells restored sensitivity to anti-EGFR drugs and impaired   migration   capabilities, whereas exogenous VEGFR-1 overexpression in wild-type cells conferred resistance to these agents.This study shows that VEGFR-1 contributes to anti-EGFR drug resistance in different human cancer cells. Moreover, vandetanib inhibits VEGFR-1 activation, cell proliferation, and   migration  , suggesting its potential utility in patients resistant to EGFR inhibitors. 
 Role of serum concentration of VEGFR1 and TIMP2 on clinical outcome in primary cervical cancer: results of a companion protocol of the randomized, NOGGO-AGO phase III adjuvant trial of simultaneous cisplatin-based radiochemotherapy vs. carboplatin and paclitaxel containing sequential radiotherapy. Aim of the present study was to analyze the expression-profile of IGF1, IGFBP3, sICAM1, sVCAM1, MMP2, MMP9, TIMP2, VEGFA, VEGFD, VEGFC and VEGFR1 in patients with high-risk FIGO-stage Ib-IIb cervical cancer.Serum from 68 cervical cancer patients treated within a phase-III-trial with either simultaneous cisplatin radiochemotherapy or sequential systemic carboplatin and paclitaxel followed by percutaneous irradiation was analyzed by ELISA. Both target expression and correlation with important clinicopathological factors were analyzed following standard statistic procedures.All 68 patients underwent a primary radical hysterectomy with pelvic and/or paraaortic lymphadenectomy. 85.3% of the extirpated tumors had clear surgical margins (R0). Increased levels of VEGFR1, TIMP2 and MMP2 were significantly associated with positive surgical margins (p=0.004, p=0.018 and p=0.004, respectively). High concentration of MMP2 and TIMP2 correlated additionally with an advanced age at time of diagnosis (p=0.001 and p=0.007, respectively). For the cut-off value of 100 pg/ml, an increased VEGFR1 was significantly associated with poor overall (OS) and progression-free (PFS) survival (p=0.017 and p=0.015, respectively). A TIMP2 concentration of lower than 90 ng/ml was significantly associated with poorer OS and PFS (p=0.009 and p=0.043, respectively). In the multivariate analysis, TIMP2 expression in serum was the only independent prognostic factor for OS (p=0.032, HR=6.51, 95% CI=1.17-36.01).Expression-profile of specific biomarkers associated with tumor invasion, cell   migration   and angiogenesis seems to be of prognostic value for both OS and PFS in patients undergoing surgery due to primary cervical cancer. Further analyses are warranted to allow an implementation of such markers into clinical practice. 
 VEGF-A and PlGF-1 stimulate chemotactic   migration   of human mesenchymal progenitor cells. Vascular endothelial growth factor (VEGF) has been indicated to play a role during endochondral ossification by stimulation of blood vessel invasion into hypertrophic cartilage resulting in its replacement by trabecular bone. We could demonstrate a dose-dependent chemoattractive effect of VEGF-A and PlGF-1, but not VEGF-E or VEGF-C, on human mesenchymal progenitor cells. Quantitative realtime PCR revealed the expression of VEGFR-1 (Flt-1), VEGFR-2 (KDR/Flk-1), and VEGFR-3 (Flt-4), which markedly declined during osteogenic differentiation. In addition, expression of neuropilin-1 and -2 was detected by RT-PCR. In an in vitro kinase assay, we could demonstrate activation of VEGFR-1 and VEGFR-2 upon stimulation with specific ligands. These findings are consistent with the idea that the chemotactic effect of VEGF-A on MPC is mediated via VEGFR-1, and that VEGF-A and PlGF-1, have a functional role for recruitment of osteoprogenitor cells in the course of endochondral bone formation or remodeling. 
 Glutaredoxin-1 up-regulation induces soluble vascular endothelial growth factor receptor 1, attenuating post-ischemia limb revascularization. Glutaredoxin-1 (Glrx) is a cytosolic enzyme that regulates diverse cellular function by removal of GSH adducts from S-glutathionylated proteins including signaling molecules and transcription factors. Glrx is up-regulated during inflammation and diabetes, and Glrx overexpression inhibits VEGF-induced EC   migration  . The aim was to investigate the role of up-regulated Glrx in EC angiogenic capacities and in vivo revascularization in the setting of hind limb ischemia. Glrx-overexpressing EC from Glrx transgenic (TG) mice showed impaired   migration   and network formation and secreted higher levels of soluble VEGF receptor 1 (sFlt), an antagonizing factor to VEGF. After hind limb ischemia surgery Glrx TG mice demonstrated impaired blood flow recovery, associated with lower capillary density and poorer limb motor function compared with wild type littermates. There were also higher levels of anti-angiogenic sFlt expression in the muscle and plasma of Glrx TG mice after surgery. Noncanonical Wnt5a is known to induce sFlt. Wnt5a was highly expressed in ischemic muscles and EC from Glrx TG mice, and exogenous Wnt5a induced sFlt expression and inhibited network formation in human microvascular EC. Adenoviral Glrx-induced sFlt in EC was inhibited by a competitive Wnt5a inhibitor. Furthermore, Glrx overexpression removed GSH adducts on p65 in ischemic muscle and EC and enhanced NF-κB activity, which was responsible for Wnt5a-sFlt induction. Taken together, up-regulated Glrx induces sFlt in EC via NF-κB-dependent Wnt5a, resulting in attenuated revascularization in hind limb ischemia. The Glrx-induced sFlt explains part of the mechanism of redox-regulated VEGF signaling. 
 Degradation of soluble VEGF receptor-1 by MMP-7 allows VEGF access to endothelial cells. Vascular endothelial growth factor (VEGF) signaling in endothelial cells serves a critical role in physiologic and pathologic angiogenesis. Endothelial cells secrete soluble VEGF receptor-1 (sVEGFR-1/sFlt-1), an endogenous VEGF inhibitor that sequesters VEGF and blocks its access to VEGF receptors. This raises the question of how VEGF passes through this endogenous VEGF trap to reach its membrane receptors on endothelial cells, a step required for VEGF-driven angiogenesis. Here, we show that matrix metalloproteinase-7 (MMP-7) degrades human sVEGFR-1, which increases VEGF bioavailability around the endothelial cells. Using a tube formation assay,   migration   assay, and coimmunoprecipitation assay with human umbilical vein endothelial cells (HUVECs), we show that the degradation of sVEGFR-1 by MMP-7 liberates the VEGF(165) isoform from sVEGFR-1. The presence of MMP-7 abrogates the inhibitory effect of sVEGFR-1 on VEGF-induced phosphorylation of VEGF receptor-2 on HUVECs. These data suggest that VEGF escapes the sequestration by endothelial sVEGFR-1 and promotes angiogenesis in the presence of MMP-7. 
 Flt-1, vascular endothelial growth factor receptor 1, is a novel cell surface marker for the lineage of monocyte-macrophages in humans. Flt-1, also known as vascular endothelial growth factor receptor 1 (VEGFR-1), is a high-affinity tyrosine kinase receptor for VEGF and is expressed almost exclusively on vascular endothelial cells. As an exception, Flt-1 transcript was recently found to be expressed in human peripheral blood monocytes. However, the protein of the Flt-1 receptor on the cell surface of monocytes is yet to be identified, and whether the Flt-1 protein is expressed during the differentiation of monocyte-macrophage lineage cells has not been examined. Using monoclonal antibodies against 2 different antigenic epitopes on the Flt-1 extracellular domain, this study found that the major population of the monocyte-marker CD97+ cells in human peripheral blood express Flt-1 as a cell surface molecule. VEGFR-2 (KDR/Flk-1) was not expressed at detectable levels in these cells. An Flt-1 neutralizing monoclonal antibody significantly suppressed VEGF-induced   migration   of the monocytes, suggesting an important role for Flt-1 in the biologic function of monocytes. Furthermore, CD34+ cells in human cord blood, originally negative for the Flt-1 expression, differentiated into Flt-1+ cells in association with the appearance of monocyte-macrophage markers after a 2-week culture in the presence of hematopoietic cytokines. In addition, the Flt-1+ CD11b+ cell fraction from CD34+ cells was found to efficiently differentiate into multinuclear osteoclasts in the presence of macrophage colony-stimulating factor and osteoclast differentiation factor. These results strongly suggest that Flt-1 is a novel cell surface marker as well as a biologically functional molecule for monocyte-macrophage lineages in humans. 
 Lysophosphatidic acid upregulates vascular endothelial growth factor-C and tube formation in human endothelial cells through LPA(1/3), COX-2, and NF-kappaB activation- and EGFR transactivation-dependent mechanisms. Lysophosphatidic acid (LPA) is a lipid bioactive mediator which binds to G-protein-coupled receptors and activates a variety of cellular functions. LPA modulates multiple behaviors in endothelial cells, including cell proliferation and   migration  , capillary-like tube formation in vitro, activation of proteases, interactions with leukocytes, and expressions of inflammation-related genes, thereby regulating vessel formation. LPA has been reported to modulate the angiogenesis process. However, the role of LPA in the lymphangiogenesis process has not been studied. In this study, we showed that LPA upregulated vascular endothelial growth factor-C (VEGF-C) mRNA expression in human umbilical vein endothelial cells (HUVECs) and subsequent endothelial cell tube formation in vitro and in vivo. These enhancement effects were LPA(1)- and LPA(3)-dependent and required cyclooxygenase-2 (COX-2), endothelial growth factor receptor (EGFR) transactivation and activation of nuclear factor kappaB (NF-kappaB). Moreover, LPA induced the protein expressions of the lymphatic markers, Prox-1, LYVE-1, and podoplanin, in HUVECs, and these enhancement effects were dependent on LPA(1) and LPA(3) activation and EGFR transactivation. Our results demonstrated that LPA might regulate VEGF-C and lymphatic marker expression in endothelial cells, which contributes to endothelial cell tube formation in vitro and in vivo, thus facilitating endothelial cell participation in the lymphangiogenesis process. This study clarifies the signaling mechanism of LPA-regulated VEGF-C expression and lymphatic marker expressions in endothelial cells, which suggest that LPA may be a suitable target for generating therapeutics against lymphangiogenesis and tumor metastasis. 
 Genetic inhibition of vascular endothelial growth factor receptor-1 significantly inhibits the   migration   and proliferation of leukemia cells and increases their sensitivity to chemotherapy. Little is known about the role of vascular endothelial growth factor (VEGF) receptor-1 (VEGFR-1) in acute leukemia. In this study, using real-time PCR and ELISA, we found that VEGF and VEGFR-1 are highly expressed in U937 leukemia cells and primary leukemia cells (M4/M5 subtypes), which are associated with an increased   migration   rate and extramedullary disease. In order to elucidate the role of VEGFR-1 in acute leukemia, we used a lentivirus-mediated shRNA expression system to specifically inhibit VEGFR-1 expression in the U937 cell line. In addition, a series of in vitro experiments were conducted, including cell proliferation and   migration   assays and drug treatments. Our results showed that shRNA reduced the proliferation and   migration   of U937 cells. RNA interference targeting VEGFR-1 in combination with bevacizumab did not exert synergistic antitumor effects. However, shRNA enhanced the sensitivity of the U937 cells to cytarabine by decreasing the IC50 of cytarabine, reducing the number of cells in the S phase and suppressing the expression of the survivin gene. Taken together, these results suggest that VEGFR-1 interference may serve as a novel antitumor therapeutic strategy for the treatment of leukemia. 
 Trafficking of multipotent mesenchymal stromal cells from maternal circulation through the placenta involves vascular endothelial growth factor receptor-1 and integrins. Maternal cells can become engrafted in various fetal organs during pregnancy. The nature of the cells and the mechanisms of maternofetal cell trafficking are not clear. We demonstrate that human lineage-negative, CD34-negative (Lin(-)CD34(-)) multipotent mesenchymal stromal cells express alpha(2), alpha(4), alpha(5), and beta(1) integrins, which mediate their adhesion to endothelium, and vascular endothelial growth factor receptor-1 (VEGFR-1), which mediates their response to vascular endothelial growth factor A (VEGF-A). A maternal-fetal VEGF-A concentration gradient exists across the placental barrier, and cord blood plasma induces transendothelial and trans-Matrigel   migration   of stem cells in vitro.   Migration   is inhibited by a VEGF-A-neutralizing antibody or antibodies against VEGFR-1 or integrin alpha(2), alpha(4), alpha(5), or beta(1). When Lin(-)CD34(-) multipotent mesenchymal stromal cells are transferred to rat maternal venous blood, they traffic through the placenta, engraft in various fetal organs, and persist in offspring for at least 12 weeks. Cell proliferation ability is retained in the xenogeneic placenta. Maternofetal trafficking is significantly reduced by blocking antibodies against integrins alpha(2), alpha(4), alpha(5), and beta(1) or VEGFR-1. These results suggest that maternal microchimerism arises by the trafficking of multipotent mesenchymal stromal cells via VEGF-A- and integrin-dependent pathways across the hemochorial placenta to fetal tissues. 
 Placental-Specific sFLT-1 e15a Protein Is Increased in Preeclampsia, Antagonizes Vascular Endothelial Growth Factor Signaling, and Has Antiangiogenic Activity. In preeclampsia, the antiangiogenic factor soluble fms-like tyrosine kinase-1 (sFLT-1) is released from placenta into the maternal circulation, causing endothelial dysfunction and organ injury. A recently described splice variant, sFLT-1 e15a, is primate specific and the most abundant placentally derived sFLT-1. Therefore, it may be the major sFLT-1 isoform contributing to the pathophysiology of preeclampsia. sFLT-1 e15a protein remains poorly characterized: its bioactivity has not been comprehensively examined, and serum levels in normal and preeclamptic pregnancy have not been reported. We generated and validated an sFLT-1 e15a-specific ELISA to further characterize serum levels during pregnancy, and in the presence of preeclampsia. Furthermore, we performed assays to examine the bioactivity and antiangiogenic properties of sFLT-1 e15a protein. sFLT-1 e15a was expressed in the syncytiotrophoblast, and serum levels rose across pregnancy. Strikingly, serum levels were increased 10-fold in preterm preeclampsia compared with normotensive controls. We confirmed sFLT-1 e15a is bioactive and is able to inhibit vascular endothelial growth factor signaling of vascular endothelial growth factor receptor 2 and block downstream Akt phosphorylation. Furthermore, sFLT-1 e15a has antiangiogenic properties. sFLT-1 e15a decreased endothelial cell   migration  , invasion, and inhibited endothelial cell tube formation. Administering sFLT-1 e15a blocked vascular endothelial growth factor induced sprouts from mouse aortic rings ex vivo. We have demonstrated that sFLT-1 e15a is increased in preeclampsia, antagonizes vascular endothelial growth factor signaling, and has antiangiogenic activity. Future development of diagnostics and therapeutics for preeclampsia should consider targeting placentally derived sFLT-1 e15a. 
 Herbal compound farnesiferol C exerts antiangiogenic and antitumor activity and targets multiple aspects of VEGFR1 (Flt1) or VEGFR2 (Flk1) signaling cascades. Farnesiferol C (FC) is one of the major compounds isolated from Ferula assafoetida, an Asian herbal spice used for cancer treatment as a folk remedy. Here, we examined the hypothesis that novel antiangiogenic activities of FC contribute to anticancer efficacy. In human umbilical vein endothelial cells (HUVEC), exposure to the 10 to 40 mumol/L concentration range of FC inhibited vascular endothelial growth factor (VEGF)-induced cell proliferation,   migration  , invasion, tube formation, and the expression of matrix metalloproteinase-2. In addition, FC inhibited the angiogenic sprouting of VEGF-treated rat aorta in an ex vivo model. Furthermore, FC inhibited the in vivo growth of mouse Lewis lung cancer allograft model by 60% (P < 0.001) at a daily i.p. dosage of 1 mg/kg body weight without any negative effect on the weight of the host mice. Immunohistochemistry staining showed decreased microvessel density (CD34) and proliferative index (Ki-67) without affecting the apoptotic (terminal deoxynucleotidyl transferase-mediated dUTP nick end labeling) index. Mechanistically, FC decreased the binding of VEGF to VEGFR1/Flt-1, but not to VEGFR2/KDR/Flk-1. In terms of early signaling, FC exerted a rapid inhibitory action (examined within 10 minutes) on VEGF-induced autophosphorylation of VEGFR1 without affecting that of VEGFR2. Nevertheless, FC decreased the phosphorylation of most of the kinases downstream of VEGFR2: focal adhesion kinase, Src, extracellular signal-regulated kinase 1/2, p38 mitogen-activated protein kinase, and c-jun-NH(2)-kinase without affecting AKT. Computer simulation suggests that FC may inhibit Src or focal adhesion kinase protein activities directly through its docking to their ATP-binding sites. Taken together, the multitargeting actions of FC, particularly VEGFR1 inhibition, may make it a novel drug candidate to complement current VEGF/VEGFR2-targeting antiangiogenic modalities for cancer. 
 Expression and function of the vascular endothelial growth factor receptor FLT-1 in human eosinophils. Vascular endothelial growth factor (VEGF) is highly expressed in the airway of patients with asthma. Whether VEGF affects eosinophil function in vitro and if VEGF receptors are involved was tested. Eosinophils were from venous blood of healthy donors. Cell   migration   was studied by micropore filter assays. Signaling mechanisms required for VEGF-dependent   migration   were tested using signaling enzyme blockers. Expression of flt-1 and KDR/flk-1 mRNA in eosinophils was demonstrated in reverse transcriptase-polymerase chain reaction, and receptor expression was investigated by fluorescence-activated cell sorting analysis. Eosinophil cationic protein release was measured in eosinophil supernatants by enzyme-linked immunosorbent assay. VEGF significantly stimulated eosinophil chemotaxis via activation of protein kinase C and phosphatidylinositol 3'-kinase. The effect on   migration   was reversed by an antibody against VEGF receptor flt-1, but not by an antibody against KDR/flk-1. Expression of VEGF receptor flt-1 mRNA was shown and synthesis of VEGF receptor in eosinophils is suggested by detection of VEGF receptor immunoreactivity on the cell surface. Data suggest that VEGF receptor flt-1 is expressed by eosinophils whose activation with VEGF stimulates directed   migration   and release of eosinophil cationic protein. Thus, VEGF may play an important role in the modulation of eosinophilic inflammation. 
 Angiogenesis in systemic sclerosis: impaired expression of vascular endothelial growth factor receptor 1 in endothelial progenitor-derived cells under hypoxic conditions. To assess angiogenesis and explore the expression and regulation of vascular endothelial growth factor (VEGF), VEGF receptor 1 (VEGFR-1), and VEGFR-2, the leading mediators of angiogenesis, in SSc patients and controls.Late-outgrowth endothelial progenitor cells (EPCs), isolated from the peripheral blood of systemic sclerosis (SSc) patients and controls, and human umbilical vein endothelial cells (HUVECs) were assessed under normal and hypoxic conditions. Genomic background was evaluated in a large case-control study (including 659 patients with SSc and 511 controls) using tag single-nucleotide polymorphisms on VEGFR1 and VEGFR2 genes.EPCs from SSc patients had the phenotype of genuine endothelial cells and displayed in vitro angiogenic properties similar to those of HUVECs and control EPCs under basal conditions, as determined by flow cytometry, tube formation, and   migration   assay. However, after 6 hours of hypoxic exposure, EPCs from SSc patients exhibited lower induced expression of VEGFR-1 at the messenger RNA and protein levels, but similar VEGF and VEGFR-2 expression, compared with HUVECs or EPCs from healthy controls. There was no evidence of defective expression of hypoxia-inducible factor 1alpha. These results were supported by the lower serum levels of soluble VEGFR-1 found in SSc patients (n = 187) compared with healthy controls (n = 48) (mean +/- SD 163.7 +/- 98.5 versus 210.4 +/- 109.5 pg/ml; P = 0.0042). These abnormalities did not seem to be related to genomic background.Our findings shed new light on the possible role of VEGFR-1 in the main vascular disturbances that occur in SSc and lead to more severe disease. 
 Tat-human immunodeficiency virus-1 induces human monocyte chemotaxis by activation of vascular endothelial growth factor receptor-1. Human immunodeficiency virus-1 (HIV-1) Tat protein can be released by infected cells and activates mesenchymal cells. Among these, monocytes respond to Tat by migrating into tissues and releasing inflammatory mediators. In the present study, we have examined the molecular mechanism of monocyte activation by Tat, showing that this viral protein signals inside the cells through the tyrosine kinase receptor for vascular endothelial growth factor encoded by fms-like tyrosine kinase gene (VEGFR-1/Flt-1). Subnanomolar concentrations of Tat induced monocyte chemotaxis, which was inhibited by cell preincubation with vascular-endothelial growth factor-A (VEGF-A). This desensitisation was specific for VEGF-A, because it not was observed with FMLP. In addition, the soluble form of VEGFR-1 specifically inhibited polarization and   migration   induced by Tat and VEGF-A, thus confirming the common use of this receptor. Binding studies performed at equilibrium by using radiolabeled Tat showed that monocytes expressed a unique class of binding site, with a kd of approximately 0.2 nmol/L. The binding of radiolabeled Tat to monocyte surface and the cross-linking to a protein of 150 kD was inhibited specifically by an excess of cold Tat or VEGF-A. Western blot analysis with an antibody anti-VEGFR-1/Flt-1 performed on monocyte phosphoproteins immunoprecipitated by an monoclonal antibody anti-phosphotyrosine showed that Tat induced a rapid phosphorylation in tyrosine residue of the 150-kD VEGFR-1/Flt-1. Taken together, these results suggest that biologic activities of HIV-1 Tat in human monocytes may, at least in part, be elicited by activation of VEGFR-1/Flt-1. 
 Soluble forms of VEGF receptor-1 and -2 promote vascular maturation via mural cell recruitment. Two soluble forms of vascular endothelial growth factor (VEGF) receptors, sVEGFR-1 and sVEGFR-2, are physiologically released and overproduced in some pathologies. They are known to act as anti-VEGF agents. Here we report that these soluble receptors contribute to vessel maturation by mediating a dialogue between endothelial cells (ECs) and mural cells that leads to blood vessel stabilization. Through a multidisciplinary approach, we provide evidence that these soluble VEGF receptors promote mural cell   migration   through a paracrine mechanism involving interplay in ECs between VEGF/VEGFR-2 and sphingosine-1-phosphate type-1 (S1P)/S1P1 pathways that leads to endothelial nitric oxyde synthase (eNOS) activation. This new paradigm is supported by the finding that sVEGFR-1 and -2 perform the following actions: 1) induce an eNOS-dependent outgrowth of a mural cell network in an ex vivo model of angiogenesis, 2) increase the mural cell coverage of neovessels in vitro and in vivo, 3) promote mural cell   migration   toward ECs, and 4) stimulate endothelial S1P1 overproduction and eNOS activation that promote the   migration   and the recruitment of neighboring mural cells. These findings provide new insights into mechanisms regulating physiological and pathological angiogenesis and vessel stabilization. 
 Altered angiogenesis in low birth weight individuals: a role for anti-angiogenic circulating factors. Low birth weight (LBW) is a risk factor for hypertension at adulthood. Endothelial progenitor cells (EPCs) dysfunction has been characterized in LBW neonates. We hypothesized that changes in soluble, plasma pro- or anti-angiogenic factors are associated with EPCs dysfunction and impaired angiogenesis in LBW neonates.Venous umbilical cord blood was collected from 42 normal, term neonates and 75 LBW neonates. Cord blood endothelial colony forming cells (ECFC) from control patients were cultured in the presence of 10% of serum obtained from both groups.The proliferation and the   migration   of ECFC were significantly reduced when cultured with 10% of serum of LBW neonates compared to serum of control neonates. Matrigel invasion assay was not significantly altered. Umbilical vein plasma VEGF concentration was significantly reduced in LBW neonates while that of sVEGFR and PF4 were significantly higher. Addition of VEGF corrected the inhibitory effect of LBW serum on normal ECFC proliferation.Serum obtained from LBW babies contains factors that exhibit an antiangiogenic effect on ECFC proliferation and   migration  . VEGF/sVEGF/PF4 pathway seems to be involved in the EPCs dysfunction in LBW neonates. 
   Migration   of human monocytes in response to vascular endothelial growth factor (VEGF) is mediated via the VEGF receptor flt-1. Treatment of human monocytes with vascular endothelial growth factor (VEGF) isolated from tumor cell supernatants was reported to induce monocyte activation and   migration  . In this study we show that recombinant human VEGF165, and VEGF121 had a maximal effect on human monocyte   migration   at 65 to 250 pmol/L. Chemotactic activity of VEGF165 was inhibited by a specific antiserum against VEGF, by heat treatment of VEGF165, and by protein kinase inhibitors. In addition, we could show that VEGF-stimulated monocyte   migration   is mediated by a pertussis toxin-sensitive GTP-binding protein. Placenta growth factor (PlGF152), a heparin-binding growth factor related to VEGF, was also chemotactic for monocytes at concentrations between 2.5 and 25 pmol/L. In accordance with these findings, human monocytes showed specific and saturable binding for 125I-VEGF165 (half-maximal binding at 1 to 1.5 nmol/L). Using Northern blot analysis, we further could show that human monocytes express only the gene for the VEGF receptor type, flt-1, but not for the second known VEGF receptor, KDR. Resting monocytes expressed low levels of flt-1 gene only. Brief exposure (2 to 4 hours) of human monocytes to lipopolysaccharide, a prototypic monocyte activator, led to a significant upregulation of the flt-1 mRNA level. The results presented here suggest that monocyte chemotaxis in response to VEGF and most likely to PlGF152 is mediated by flt-1 and thus show a possible function for the VEGF-receptor flt-1. 
 Integrin activation is required for VEGF and FGF receptor protein presence on human microvascular endothelial cells. Endothelial cell proliferation and   migration   is initiated by growth factors including FGF and VEGF that bind to specific transmembrane receptor tyrosine kinases. Mechanisms that regulate in vivo expression of fibroblast growth factor receptors (FGFR) and vascular endothelial growth factor receptors (VEGFR) are not well understood. Since it is well known that different matrices influence the proliferation and   migration   of endothelial cells in culture, we hypothesized that changes in the extracellular matrix environment can regulate growth factor receptors on endothelial cells. We cultured human microvascular endothelial cells on different matrices (vitronectin, laminin, fibronectin, fibrin, and collagen IV) and examined for the presence of growth factor receptors (FGFR-1, FGFR-2, VEGFR-1, and VEGFR-2). We show that vitronectin increased the presence of all four growth factor receptors and most notably, VEGFR-1. In contrast, fibrin decreased all four receptors, especially FGFR-1 and FGFR-2. Inhibiting phosphotyrosine signaling abolished immunostaining for all four receptors, regardless of the matrix, but was not dependent on activating the Fyn-Shc pathway. Cells plated on vitronectin in the presence of blocking antibodies to integrins alphavbeta3 and alphavbeta5 similarly decreased presence of these growth factor receptors. Our data suggests a possible mechanism of how matrix-integrin interactions regulate endothelial cell responsiveness to growth factors and anchorage-dependent cell growth. 
 Soluble vascular endothelial growth factor (VEGF) receptor-1 inhibits   migration   of human monocytic THP-1 cells in response to VEGF. We aimed to investigate the regulation and contribution of vascular endothelial growth factor (VEGF) and sFlt-1(1-3) to human monocytic THP-1   migration  .Ad-sFlt-1/FLAG, a recombinant adenovirus carrying the human sFlt-1(1-3) (the first three extracellular domains of FLT-1, the hVEGF receptor-1) gene, was constructed. L929 cells were infected with Ad-sFlt-1/FLAG and the expression of sFlt-1 was detected by immunofluorescent assay and ELISA. Corning(®) Transwell(®) Filter Inserts containing polyethylene terephthalate (PET) membranes with pore sizes of 3 μm were used as an experimental model to simulate TH  P-1 migra  tion. Five VEGF concentrations (0, 0.1, 1, 10 and 100 ng/ml), four concentrations of sFlt-1(1-3)/FLAG expression supernatants (0.1, 1, 10 and 100 ng/ml), and monocyte chemoattractant protein-1 (MCP-1, 10 ng/ml) were used to test the ability of THP-1 cells to migrate through PET membranes.The sFlt-1(1-3) gene was successfully recombined into Ad-sFlt-1/FLAG. sFlt-1(1-3) was expressed in L929 cells transfected with Ad-sFlt-1/FLAG. THP-  1 cell mi  gration increased with increasing concentrations of VEGF, whil  e cell mi  gration decreased with increasing concentrations of sFlt1(1-3)/FLAG. sFlt1(1-3)/FLAG had no effect on MCP-1-induce  d cell mi  gration.This study demonstrated that VEGF is able to elicit a migratory response in THP-1 cells, and that sFlt-1(1-3) is an effective inhibitor of   THP-1 mi  gration towards VEGF. 
 Malignant hematopoietic cells induce an increased expression of VEGFR-1 and VEGFR-3 on bone marrow endothelial cells via AKT and mTOR signalling pathways. Angiogenesis plays a significant role in a variety of malignant hematologic diseases, and it is recognized that it has prognostic value. However, the cellular mechanisms by which malignant hematologic cells induce angiogenesis are not well understood. In order to investigate the role of cells from B-cell chronic lymphocytic leukemia (B-CLL) and multiple myeloma (MM) in angiogenesis on human bone marrow endothelial cells (HBMEC), we analyzed the impact of factors secreted by B-CLL cells and by MM cells on HBMEC capillary tube formation on matrigel. It was found that, in addition to the secretion of angiogenic factors VEGF and b-FGF by B-CLL and MM cells, MM cells (but not B-CLL cells) induced a dramatic increase in expression of VEGFR-1 and VEGFR-3 on human bone marrow endothelial cells (HBMEC). It would seem that this increase in VEGFR-3 occurred via the ERK and mTOR pathways, since their respective inhibitors U0126, LY294002 or rapamycin were responsible for a decrease of VEGFR-3. In response to MM cells-increased VEGF receptors on HBMEC, endothelial cell   migration   was enhanced in a wound artificially produced in a semi-confluent HBMEC culture, a phenomenon which was also down-regulated by the same inhibitors that reversed the increase in VEGF receptors. The present study suggests that, in addition to the classic angiogenic pathway, another mechanism related to an increased expression of VEGFRs on HBMEC might exist in malignant hematopoietic angiogenesis. 
 GM-CSF induces expression of soluble VEGF receptor-1 from human monocytes and inhibits angiogenesis in mice. GM-CSF promotes homeostasis of myeloid cells. We report that GM-CSF upregulates mRNA and protein production of the soluble form of membrane bound VEGF receptor-1 (sVEGFR-1) in human monocytes. This sVEGFR-1 was biologically active, as cell-free supernatants from GM-CSF-stimulated monocytes blocked detection of endogenously expressed VEGF and inhibited endothelial cell   migration   and tube formation, even in the presence of exogenous rhVEGF. VEGF activity was recovered by neutralizing sVEGFR-1. To determine whether these events were important in vivo, Matrigel plugs were incubated with rhVEGF, rhGM-CSF, or rhGM-CSF/rhVEGF and injected into mice. Plugs containing GM-CSF or GM-CSF/VEGF had less endothelial cell invasion than plugs containing rhVEGF and were similar to plugs incubated with PBS alone. Neutralizing antibodies specific for sVEGFR-1 injected in these plugs reversed the effects of GM-CSF or GM-CSF/VEGF, while an isogenic antibody did not. Thus, GM-CSF and monocytes play a vital role in angiogenesis through the regulation of VEGF and sVEGFR-1. 
 Vascular endothelial growth factor and its receptor system: physiological functions in angiogenesis and pathological roles in various diseases. Vascular endothelial growth factors (VEGFs) belong to the platelet-derived growth factor supergene family, and they play central roles in the regulation of angiogenesis and lymphangiogenesis. VEGF-A, the major factor for angiogenesis, binds to two tyrosine kinase (TK) receptors, VEGFR-1 (Flt-1) and VEGFR-2 (KDR/Flk-1), and regulates endothelial cell proliferation,   migration  , vascular permeability, secretion and other endothelial functions. VEGFR-2 exhibits a strong TK activity towards pro-angiogenic signals, whereas the soluble VEGFR-1 (sFlt-1) functions as an endogenous VEGF inhibitor. sFlt-1 is abnormally overexpressed in the placenta of preeclampsia patients, resulting in the major symptoms of the disease due to abnormal trapping of VEGFs. The VEGF-VEGFR system is crucial for tumour angiogenesis, and anti-VEGF-VEGFR molecules are now widely used in the clinical field to treat cancer patients. The efficacy of these molecules in prolonging the overall survival of patients has been established; however, some cancers do not respond well and reduced tumour sensitivity to anti-VEGF signals may occur after long-term treatment. The molecular basis of tumour refractoriness should be determined to improve anti-angiogenic therapy. 
 The inhibition of tyrosine kinase receptor signalling in leiomyosarcoma cells using the small molecule kinase inhibitor PTK787/ZK222584 (Vatalanib®). Leiomyosarcomas remain challenging tumors to manage and novel therapy strategies besides radiation and conventional chemotherapy are needed. Targeting angiogenesis by inhibition of vascular endothelial growth factor (VEGF) receptor tyrosine kinases (RTKs) of the tumor vasculature with small molecules is a promising new therapy. It has been shown recently that these receptors are not only expressed on tumor endothelium but also on tumor cells themselves. Thus, we investigated the expression of members of the VEGF receptor (VEGFR) family and corresponding growth factors in leiomyosarcoma tissue specimens and in the leiomyosarcoma cell lines SK-LMS-1 and SK-UT-1. We evaluated the influence of the VEGFR inhibitor PTK787/ZK222584 (PTK787) on cell growth  , migrati  on, apoptosis and phosphorylation of intracellular signalling molecules. In human leiomyosarcoma tissue specimens VEGFR‑1/-2 and platelet-derived growth factor receptor (PDGFR-β) were strongly expressed. Both leiomyosarcoma cell lines expressed VEGFR‑1/-3 and PDGFR-β but VEGFR-2 protein expression was positive only in SK-UT-1. SK-LMS-1 and SK-UT-1 cells secreted high and low amounts of VEGF-A, respectively, whereas PDGF-BB secretion was similar in both cell lines. Application of PTK787 led to partial inhibition of PDGF-BB-activated AKT/p90RSK and ERK1/2 signalling pathways. In contrast, protein phosphorylation was not affected by PTK787 in VEGF-A-treated cells. PTK787 turned out to inhib  it cell m  igration even though no effects were observed upon stimulation with VEGF-A or PDGF-BB. In line, cell growth in leiomyosarcoma cell lines remained unchanged upon PTK787 treatment alone and with subsequent VEGF-A- or PDGF-BB-stimulation. However, VEGF-A, but not PDGF-BB-treated cells showed increased cell death upon PTK787 treatment. VEGFR family members are expressed in leiomyosarcomas in vivo and in vitro. Upon receptor stimulation, PTK787 is able to inhibit subsequent phosphorylation events and influences cell survival but not metabolic activ  ity and m  igration. Thus, the inhibitor is possibly an additional option in the treatment of leiomyosarcomas. 
 Angiotensin II-induced process of angiogenesis is mediated by spleen tyrosine kinase via VEGF receptor-1 phosphorylation. Spleen tyrosine kinase (Syk), expressed in endothelial cells, has been implicated in   migration   and proliferation and in vasculogenesis. This study was conducted to determine the contribution of Syk and the underlying mechanism to the angiogenic effect of ANG II and VEGF. Angiogenesis was determined by tube formation from the endothelial cell line EA.hy926 (EA) and human umbilical vein endothelial cells (HUVECs) and microvessel sprouting in rat aortic rings. ANG II (10 nM), EGF (30 ng/ml), and VEGF (50 ng/ml) stimulated EA cells and HUVECs to form tubular networks and increased aortic sprouting; these effects were blocked by VEGF receptor-1 and Flt-1 antibody (Flt-1/Fc) but not by the VEGF receptor-2 (Flk-1) antagonist SU-1498. ANG II increased the phosphorylation of Flt-1 but not Flk-1, whereas VEGF increased the phosphorylation of both receptors in EA cells and HUVECs. VEGF expression elicited by ANG II was not altered by Flt-1/Fc or SU-1498. EGF stimulated tube formation from EA cells and HUVECs and Flt-1 phosphorylation and aortic sprouting, which were blocked by the EGF receptor antagonist AG-1478 and Flt-1/Fc but not by SU-1498. ANG II-, EGF-, and VEGF-induced tube formation and aortic sprouting were attenuated by the Syk inhibitor piceatannol and by Syk short hairpin interfering (sh)RNA and small interfering RNA, respectively. ANG II, EGF, and VEGF increased Syk phosphorylation, which was inhibited by piceatannol and Syk shRNA in EA cells and HUVECs. Neither piceatannol nor Syk shRNA altered ANG II-, EGF-, or VEGF-induced phosphorylation of Flt-1. These data suggest that ANG II stimulates angiogenesis via transactivation of the EGF receptor, which promotes the phosphorylation of Flt-1 and activation of Syk independent of VEGF expression. 
 Soluble VEGFR-1 secreted by endothelial cells and monocytes is present in human serum and plasma from healthy donors. It was shown before that the soluble form of VEGFR-1 (sVEGFR-1) is present in serum of pregnant women. The aim of the present study was to investigate the presence of this endogenous vascular endothelial growth factor-A (VEGF-A) antagonist in human serum in more detail. sVEGFR-1 was detected in human serum and plasma from normal healthy male and female donors by ELISA. sVEGFR-1 levels ranged from non-detectable up to 440 pg/ml, with no significant difference between male and female donors. In addition, vein endothelial cells (ECs) from an intact vascular bed, the umbilical cord, were shown to secrete sVEGFR-1. Furthermore, human peripheral blood monocytes, a non-EC type expressing VEGFR-1, were shown to contribute to the sVEGFR-1 detectable in human serum and plasma for the first time. EC- and monocyte-derived sVEGFR-1 proved capable of inhibiting the VEGF-induced proliferation and   migration   of ECs in vitro. Finally, secretion of sVEGFR-1 was increased by the angiogenic factor basic fibroblast growth factor (bFGF) in human ECs and was also enhanced in lipopolysaccharide-activated human monocytes. In human umbilical vein endothelial cells, both the membrane-bound and the sVEGFR-1 seem to be equally regulated on the mRNA as well as the protein level. The presence of an sVEGFR-1 in human serum and plasma of normal male and female donors strongly suggests that it plays an important role as a naturally occurring VEGF antagonist in the regulation and availability of VEGF-mediated biological activities in vivo. 
 Vascular endothelial growth factor B (VEGF-B) binds to VEGF receptor-1 and regulates plasminogen activator activity in endothelial cells. The vascular endothelial growth factor (VEGF) family has recently expanded by the identification and cloning of three additional members, namely VEGF-B, VEGF-C, and VEGF-D. In this study we demonstrate that VEGF-B binds selectively to VEGF receptor-1/Flt-1. This binding can be blocked by excess VEGF, indicating that the interaction sites on the receptor are at least partially overlapping. Mutating the putative VEGF receptor-1/Flt-1 binding determinants Asp63, Asp64, and Glu67 to alanine residues in VEGF-B reduced the affinity to VEGF receptor-1 but did not abolish binding. Mutational analysis of conserved cysteines contributing to VEGF-B dimer formation suggest a structural conservation with VEGF and platelet-derived growth factor. Proteolytic processing of the 60-kDa VEGF-B186 dimer results in a 34-kDa dimer containing the receptor-binding epitopes. The binding of VEGF-B to its receptor on endothelial cells leads to increased expression and activity of urokinase type plasminogen activator and plasminogen activator inhibitor 1, suggesting a role for VEGF-B in the regulation of extracellular matrix degradation, cell adhesion, and   migration  . 
 Recombinant pigment epithelium-derived factor PEDF binds vascular endothelial growth factor receptors 1 and 2. Angiogenesis, or the formation of new blood vessels, is stimulated by angiogenic factors such as vascular endothelial growth factor (VEGF). Pigment epithelium-derived factor (PEDF) is a potent inhibitor of angiogenesis. To explore the mechanism by which PEDF acts, recombinant PEDF was expressed with a 6x-His tag (for purification) and a green fluorescent protein (GFP) tag. The PEDF fusion protein was confirmed to be active in inhibition of endothelial cell proliferation and   migration  . Direct binding of PEDF to both vascular endothelial growth factor receptor-1 (VEGFR-1) and VEGFR-2 was demonstrated in an in vitro assay similar to an enzyme-linked immunosorbent assay (ELISA). PEDF was shown by immune-confocal microscopy to be localized within treated endothelial cells. When VEGF-stimulated endothelial cells were incubated with PEDF the VEGF receptors showed intracellular localization. These data suggest that the interaction between PEDF and VEGFR-1 or VEGFR-2 may be a possible mechanism for inhibiting angiogenesis. PEDF may be binding to the VEGF receptors to promote their internalization and/or degradation to limit VEGF responses in treated cells. 
 Increased expression of vascular endothelial growth factor and its receptors, VEGFR-1 and VEGFR-2, in medullary thyroid carcinoma. Vascular endothelial growth factor (VEGF-A) expression is upregulated in the majority of human tumors, where it stimulates proliferation,   migration  , and survival of endothelial cells. Studies have suggested that VEGF inhibitors can be used as an alternative therapy in medullary thyroid carcinoma (MTC), but data about expression of VEGF-A and its receptor in this tumor are scarce. The aims of this study were to evaluate VEGF-A, VEGF receptor (VEGFR)-1, VEGFR-2, and microvessel density (MVD) expression in MTC samples and correlate it with clinical parameters.Paraffin-embedded samples from 38 MTC patients were evaluated for VEGF-A, VEGFR-1, VEGFR-2, and MVD expression by immunohistochemistry. Clinical data were retrospectively reviewed in medical records.Thirty-eight patients aged 31.8 +/- 17.1 years were enrolled. Twenty-seven patients had hereditary disease (71.1%). Twenty-five of them were found to have multiple endocrine neoplasia (MEN) 2A and two were found to have MEN 2B. VEGF-A immunohistochemical staining was detected in 95% (36/38), VEGFR-1 in 96% (36/37), and VEGFR-2 in 91% (31/34) of MTC samples. Age at surgery was positively correlated with VEGFR-2 (p = 0.003). There was no correlation between VEGF-A, VEGFR-2, and tumor stage (tumor node metastasis). Nevertheless, VEGFR-1 was found to be inversely correlated with tumor node metastasis (p = 0.034). We also observed a trend toward an association between VEGFR-1 signal intensity and cure of disease, although this did not reach statistical significance (p = 0.054). Neither VEGF-A nor VEGFR-2 was associated with disease outcome after a median follow-up period of 5 years (p = 0.882 and p = 0.236, respectively). As expected, MVD was correlated with age at surgery (p = 0.005) and tumor size (p = 0.03). Patients with the hereditary form of the disease had a stronger intensity for VEGFR-1 (p = 0.039), whereas patients with sporadic disease displayed higher MVD counts (44 [27-63] vs. 21 [9-49], p = 0.018).The VEGF-A, VEGFR-1, and VEGFR-2 immunoreactive proteins are overexpressed in MTC lesions and might be implicated in tumor progression. It is not clear, however, if expression of these molecules provides prognostic information regarding the spread or outcome of MTC. 
 The role of heterodimerization between VEGFR-1 and VEGFR-2 in the regulation of endothelial cell homeostasis. VEGF-A activity is tightly regulated by ligand and receptor availability. Here we investigate the physiological function of heterodimers between VEGF receptor-1 (VEGFR-1; Flt-1) and VEGFR-2 (KDR; Flk-1) (VEGFR(1-2)) in endothelial cells with a synthetic ligand that binds specifically to VEGFR(1-2). The dimeric ligand comprises one VEGFR-2-specific monomer (VEGF-E) and a VEGFR-1-specific monomer (PlGF-1). Here we show that VEGFR(1-2) activation mediates VEGFR phosphorylation, endothelial cell   migration  , sustained in vitro tube formation and vasorelaxation via the nitric oxide pathway. VEGFR(1-2) activation does not mediate proliferation or elicit endothelial tissue factor production, confirming that these functions are controlled by VEGFR-2 homodimers. We further demonstrate that activation of VEGFR(1-2) inhibits VEGF-A-induced prostacyclin release, phosphorylation of ERK1/2 MAP kinase and mobilization of intracellular calcium from primary endothelial cells. These findings indicate that VEGFR-1 subunits modulate VEGF activity predominantly by forming heterodimer receptors with VEGFR-2 subunits and such heterodimers regulate endothelial cell homeostasis. 
 Properties of two VEGF receptors, Flt-1 and KDR, in signal transduction. The properties of two VEGF receptors, Flt-1 and KDR, in the signal transduction of VEGF in human umbilical vein endothelial cells (HUVECs) were investigated by using two newly developed blocking monoclonal antibodies (mAbs) against Flt-1 and KDR. VEGF stimulated the expression of transcription factor Ets-1 as well as matrix metalloproteinase-1 (MMP-1) and Flt-1 in HUVECs. The KDR/Flt-1 heterodimer and the KDR homodimer mediate the expression of Ets-1, MMP-1, and Flt-1. VEGF also stimulated DNA synthesis and   migration   of HUVECs. DNA synthesis is mediated by the same signaling system as the expression of Ets-1. In contrast, cell   migration   is regulated by two distinct signaling systems. The Flt-1 homodimer is required for actin reorganization. The KDR/Flt-1 heterodimer and the KDR homodimer are required for the assembly of vinculin in focal adhesion plaque by regulating the phosphorylation of focal adhesion kinase (FAK) and paxillin. 
 Thrombospondin-1 is part of a Slug-independent motility and metastatic program in cutaneous melanoma, in association with VEGFR-1 and FGF-2. Differently from most transformed cells, cutaneous melanoma expresses the pleiotropic factor thrombospondin-1 (TSP-1). Herein, we show that TSP-1 (RNA and protein), undetectable in four cultures of melanocytes and a RGP melanoma, was variously present in 13 cell lines from advanced melanomas or metastases. Moreover, microarray analysis of 55 human lesions showed higher TSP-1 expression in primary melanomas and metastases than in common and dysplastic nevi. In a functional enrichment analysis, the expression of TSP-1 correlated with motility-related genes. Accordingly, TSP-1 production was associated with melanoma cell motility in vitro and lung colonization potential in vivo. VEGF/VEGFR-1 and FGF-2, involved in melanoma progression, regulated TSP-1 production. These factors were coexpressed with TSP-1 and correlated negatively with Slug (SNAI2), a cell   migration   master gene implicated in melanoma metastasis. We conclude that TSP-1 cooperates with FGF-2 and VEGF/VEGFR-1 in determining melanoma invasion and metastasis, as part of a Slug-independent motility program. 
 Prognostic significance of VEGFR1/Flt-1 immunoexpression in colorectal carcinoma. Colorectal carcinoma (CRC) is a major cause of morbidity and mortality. Vascular endothelial growth factor 1/Fms-like tyrosine kinase 1 (VEGFR1/Flt-1) regulates monocyte   migration  , recruits endothelial cell progenitors, increases the adhesive properties of natural killer cells and induces of growth factors. Flt-1 is expressed on tumour cells and has been implicated in tumour growth and progression. The objective of this study is to address the relation of Flt-1 expression to tumour prognostication. Paraffin blocks from 143 primary CRC and 48 regional nodal metastases were retrieved from the archives of the Department of Pathology at King Abdulaziz University. Tissue microarrays were designed and constructed. Immunohistochemistry for Flt-1 was performed. Staining intensity and extent of staining were assessed and combined. Results were dichotomised as low expression and high expression. Flt-1 was overexpressed in primary tumours and nodal metastasis (p < 0.001 and 0.001) with no difference between primary and nodal metastasis (p = 0.690). Flt-1 immunoexpression was not associated with the clinicopathological parameters. Flt-1 overexpression was an independent predictor of positive margin status, positive lymphovascular invasion and local disease recurrence (p < 0.001, p < 0.001 and p = 0.003, respectively). Flt-1 was not associated with survival (log-rank = 0.003, p = 0.959). Flt-1 was overexpressed in primary CRC and their nodal metastases. Flt-1 expression was an independent predictor of margin status, lymphovascular invasion and local disease recurrence. Therefore, expression profiling of Flt-1 seems to have a prognostic potential in CRC. However, to elucidate the association of overexpression of Flt-1 with tumour characteristics and prognostication, more in vivo and in vitro molecular investigations are recommended. 
 [Inhibition effect of short hairpin RNA on VEGF receptor flt-1 gene expression in leukemia cell line K562]. This study was purposed to investigate the inhibitory effect of short hairpin RNA (shRNA) on expression of vascular endothelial growth factor (VEGF) receptor flt-1 gene in leukemia cells line K562, and to explore the influence of shRNA invasive ability on leukemia cells and its mechanism. The recombinant eukaryotic expression plasmid containing flt-1 shRNA gene was transfected into K562 cells by lipofectamine mediation and positive clones were screened by G418. shRNA gene in K562 cells was confirmed by PCR. RT-PCR and Western blot were employed to detect the expression of flt-1 mRNA and protein in leukemia cells. The invasive ability of K562 cells was studied by Boyden chamber invasion assay before and after flt-1 shRNA transfection. MMP-2 and MMP-9 mRNA expressions were detected by RT-PCR after transfection of the recombinant plasmid C1/U6/FltS2 into K562 cells through liposome. The results showed that the recombinant eukaryotic expression plasmid had been transfected into the human leukemia cell line K562 and positive clones had been screened by G418 for 2 weeks. PCR detection revealed the stable expression of the shRNA gene in K562 cells. Flt-1 gene and protein expressions were inhibited by plasmid-expressed shRNA after transfection of recombinant vetors C1/U6/FltS into K562 cells. The inhibitory efficiency of two different shRNA sequences targeting Flt-1 gene were 46.1% and 65.4% respectively. The expression of MMP-2 and MMP-9 mRNA decreased, and the mean invasion rate in C1/U6/fltS2-transfected K562 cells was lower than that in nontransfected cells. It is concluded that shRNA eukaryotic expression vector specific to VEGF receptor flt-1 gene can high efficiently be transfected into leukemia cell line K562, effectively inhibits the expression of flt-1 gene, weakens the in vitro invasive ability of leukemia cells and the expression levels of MMP-2 and MMP-9 mRNA, which suggests that the VEGF involves in the   migration   of leukemia cells by regulating the MMP-2 and MMP-9 through joints with the receptor. 
 Thymosin beta10 inhibits cell   migration   and capillary-like tube formation of human coronary artery endothelial cells. Thymosin beta10 is a cytoplasm G-actin sequestering protein whose functions are largely unknown. To determine the direct effects of exogenous thymosin beta10 on angiogenic potentials as endothelial cell   migration   and capillary-like tube formation, human coronary artery endothelial cells (HCAECs) were incubated with increasing doses of thymosin beta10 (25-100 ng/ml). By using a modified Boyden chamber assay, thymosin beta10 inhibited cell   migration   in a dose- and time-dependent manner with the maximal effect being a 36% reduction at 100 ng/ml as compared to controls (P < 0.01). In addition, thymosin beta10 (100 ng/ml) significantly inhibited the capillary-like tube-formation of HCAECs on Matrigel, showing a 21% reduction of the total tube length as compared to negative controls (P < 0.01). Furthermore, by using real time PCR analysis, thymosin beta10 significantly decreased mRNA levels of vascular endothelial growth factor (VEGF), VEGF receptor-1 (VEGFR-1) and integrin alphaV after 24 h treatment in HCAECs. By contrast, thymosin beta4 significantly increased HCAEC   migration  . These results indicate that thymosin beta10, but not thymosin beta4, have direct inhibitive effects on endothelial   migration   and tube formation that might be mediated via downregulation of VEGF, VEGFR-1 and integrin alphaV in HCAECs. This study suggests a potential therapeutic application of thymosin beta10 to the diseases with excessive angiogenesis such as cancer. 
 Combined vascular endothelial growth factor and platelet-derived growth factor inhibition in rat cardiac allografts: beneficial effects on inflammation and smooth muscle cell proliferation. Perivascular inflammation and subsequent smooth muscle cell (SMC) proliferation are central in the development of cardiac allograft arteriosclerosis. We examined the effect of combined inhibition of proinflammatory vascular endothelial growth factor (VEGF) and SMC mitogen platelet-derived growth factor (PDGF) in rat cardiac allografts.Heterotopic cardiac transplantations were performed between fully major histocompatibility mismatched rat strains receiving cyclosporine A immunosuppression. In situ hybridization and immunohistochemistry were performed to examine VEGF and PDGF ligand and receptor (R) expression. Protein tyrosine kinase inhibitors PTK787 and imatinib were used to inhibit VEGFR and PDGFR activity, respectively. Rat coronary artery SMC   migration   and proliferation assays were used to examine the effect of VEGF and PDGF and tyrosine kinase inhibitors in vitro.Both ligand and receptor expression of VEGF and PDGF were detected in chronically rejecting allografts. In vitro, PDGF-BB mediated rat coronary artery SMC   migration   and proliferation was completely inhibited with imatinib and partially with PTK787. In vivo, combined treatment with PTK787 and imatinib significantly reduced the formation of neointimal lesions in arteries of cardiac allografts at 8 weeks, producing a greater effect than either drug alone. PTK787, in contrast with imatinib, reduced the number of ED1 macrophages and PDGF-B immunoreactivity in the allografts at 4 weeks.Blocking VEGF and PDGF receptor signaling in cardiac allografts has distinctive effects on inflammation and SMC proliferation, suggesting that targeting both inflammation and pathologic vascular remodeling may be needed to inhibit cardiac allograft arteriosclerosis. 
  GeneRIFs  
 VEGFR family members are expressed in leiomyosarcomas in vivo and in vitro. Upon receptor stimulation, PTK787 is able to inhibit subsequent phosphorylation events and influences cell survival but not metabolic activity and   migration   
 Data demonstrate that VEGF is able to elicit a migratory response in THP-1 cells, and that sFlt-1(1-3)/VEGFR1 is an effective inhibitor of THP-1   migration   towards VEGF. 
 Cholesterol regulates VEGF:VEGFR-1 signaling on subsets of acute leukemias, modulating cell   migration  , and viability, which may be crucial for disease progression. 
 RACK1 regulates VEGF/Flt1-mediated cell   migration   via activation of a PI3K/Akt pathway. 
 provide evidence that soluble VEGF receptors promote mural cell   migration   through a paracrine mechanism involving interplay in ECs between VEGF/VEGFR-2 and S1P/S1P1 pathways that leads to endothelial nitric oxide synthase activation 
 thymosin beta10, but not thymosin beta4, have direct inhibitive effects on endothelial   migration   and tube formation that might be mediated via downregulation of VEGF, VEGFR-1 and integrin alphaV in HCAECs. 
 VEGF-A induction of VEGFR2-negative human aortic smooth muscle cell   migration   is mediated through a molecular cross-talk of VEGFR1 (Flt-1), neuropilin-1 (NRP-1), and phosphoinositide 3-kinase (PI3K)/Akt signaling kinase. 
 VEGFR-1 promotes   migration   of tumour cells through a Src-dependent pathway linked to activation of focal adhesion components that regulate this process in colonic adenocarcinoma. 
 Data suggest that vascular endothelial growth factor (VEGF) receptor flt-1 is expressed by eosinophils whose activation with VEGF stimulates directed   migration   and release of eosinophil cationic protein. 
  [Ee]xtracellular [Mm]atrix  PubMed  
 Vascular endothelial growth factor receptor-1 is deposited in the   extracellular matrix   by endothelial cells and is a ligand for the alpha 5 beta 1 integrin. Vascular endothelial growth factor receptor-1 (VEGFR-1) is a tyrosine kinase receptor for several growth factors of the VEGF family. Endothelial cells express a membrane-spanning form of VEGFR-1 and secrete a soluble variant of the receptor comprising only the extracellular region. The role of this variant has not yet been completely defined. In this study, we report that the secreted VEGFR-1 is present within the   extracellular matrix   deposited by endothelial cells in culture, suggesting a possible involvement in endothelial cell adhesion and migration. In adhesion assays, VEGFR-1 extracellular region specifically promoted endothelial cell attachment. VEGFR-1-mediated cell adhesion was divalent cation-dependent, and inhibited by antibodies directed against the alpha 5 beta 1 integrin. Moreover, VEGFR-1 promoted endothelial cell migration, and this effect was inhibited by anti-alpha 5 beta 1 antibodies. Direct binding of VEGFR-1 to the alpha 5 beta 1 integrin was also detected. Finally, binding to VEGFR-1 initiated endothelial cell spreading. Altogether these results indicate that the soluble VEGFR-1 secreted by endothelial cells becomes a matrix-associated protein that is able to interact with the alpha 5 beta 1 integrin, suggesting a new role of VEGFR-1 in angiogenesis, in addition to growth factor binding. 
 Vascular endothelial growth factor receptor-1 is deposited in the   extracellular matrix   by endothelial cells and is a ligand for the alpha 5 beta 1 integrin. Vascular endothelial growth factor receptor-1 (VEGFR-1) is a tyrosine kinase receptor for several growth factors of the VEGF family. Endothelial cells express a membrane-spanning form of VEGFR-1 and secrete a soluble variant of the receptor comprising only the extracellular region. The role of this variant has not yet been completely defined. In this study, we report that the secreted VEGFR-1 is present within the   extracellular matrix   deposited by endothelial cells in culture, suggesting a possible involvement in endothelial cell adhesion and migration. In adhesion assays, VEGFR-1 extracellular region specifically promoted endothelial cell attachment. VEGFR-1-mediated cell adhesion was divalent cation-dependent, and inhibited by antibodies directed against the alpha 5 beta 1 integrin. Moreover, VEGFR-1 promoted endothelial cell migration, and this effect was inhibited by anti-alpha 5 beta 1 antibodies. Direct binding of VEGFR-1 to the alpha 5 beta 1 integrin was also detected. Finally, binding to VEGFR-1 initiated endothelial cell spreading. Altogether these results indicate that the soluble VEGFR-1 secreted by endothelial cells becomes a matrix-associated protein that is able to interact with the alpha 5 beta 1 integrin, suggesting a new role of VEGFR-1 in angiogenesis, in addition to growth factor binding. 
 Urokinase-type Plasminogen Activator (uPA) Promotes Angiogenesis by Attenuating Proline-rich Homeodomain Protein (PRH) Transcription Factor Activity and De-repressing Vascular Endothelial Growth Factor (VEGF) Receptor Expression. Urokinase-type plasminogen activator (uPA) regulates angiogenesis and vascular permeability through proteolytic degradation of   extracellular matrix   and intracellular signaling initiated upon its binding to uPAR/CD87 and other cell surface receptors. Here, we describe an additional mechanism by which uPA regulates angiogenesis. Ex vivo VEGF-induced vascular sprouting from Matrigel-embedded aortic rings isolated from uPA knock-out (uPA(-/-)) mice was impaired compared with vessels emanating from wild-type mice. Endothelial cells isolated from uPA(-/-) mice show less proliferation and migration in response to VEGF than their wild type counterparts or uPA(-/-) endothelial cells in which expression of wild type uPA had been restored. We reported previously that uPA is transported from cell surface receptors to nuclei through a mechanism that requires its kringle domain. Intranuclear uPA modulates gene transcription by binding to a subset of transcription factors. Here we report that wild type single-chain uPA, but not uPA variants incapable of nuclear transport, increases the expression of cell surface VEGF receptor 1 (VEGFR1) and VEGF receptor 2 (VEGFR2) by translocating to the nuclei of ECs. Intranuclear single-chain uPA binds directly to and interferes with the function of the transcription factor hematopoietically expressed homeodomain protein or proline-rich homeodomain protein (HHEX/PRH), which thereby lose their physiologic capacity to repress the activity of vehgr1 and vegfr2 gene promoters. These studies identify uPA-dependent de-repression of vegfr1 and vegfr2 gene transcription through binding to HHEX/PRH as a novel mechanism by which uPA mediates the pro-angiogenic effects of VEGF and identifies a potential new target for control of pathologic angiogenesis. 
 Systemic production of vascular endothelial growth factor and fms-like tyrosine kinase-1 receptor in acute Kawasaki disease. Increased vascular permeability is an important event during the initial process of Kawasaki disease (KD). One potential responsible candidate for the induction of vascular hyperpermeability is vascular endothelial growth factor (VEGF).We investigated the expression of VEGF and its receptors (flt-1, KDR) in acute KD tissues at 7 days to 5 weeks of illness. Neuropilin-1, which enhances the binding of VEGF(165) to KDR, was also studied. Abundant expression of VEGF and flt-1 was documented immunohistochemically in many organs from acute KD, including heart and lung. VEGF and flt-1 were colocalized in all vessels that showed edema. These molecules resided in endothelium and vascular media and also in migrating smooth muscle cells in neointima and infiltrating macrophages. Compared with controls, coronary vessels of acute KD had upregulation of VEGF and flt-1 but not KDR or neuropilin-1. KDR was expressed by vessels at 7 days of illness but not later in the illness. Plasma proteins were more extensively bound to the   extracellular matrix   in coronary vessels in acute KD than controls. Furthermore, elevation of serum VEGF levels was correlated with low serum albumin in acute KD (n=220, r=-0.53, P<0.001).These findings suggest that VEGF and flt-1 are upregulated in blood vessels in many organs of acute KD. Expression of KDR was limited to the early stage of acute KD. The roles of VEGF in acute KD may involve promotion of vascular permeability and macrophage activation. Low serum albumin may indicate overproduction of VEGF in acute KD. 
 Integrin activation is required for VEGF and FGF receptor protein presence on human microvascular endothelial cells. Endothelial cell proliferation and migration is initiated by growth factors including FGF and VEGF that bind to specific transmembrane receptor tyrosine kinases. Mechanisms that regulate in vivo expression of fibroblast growth factor receptors (FGFR) and vascular endothelial growth factor receptors (VEGFR) are not well understood. Since it is well known that different matrices influence the proliferation and migration of endothelial cells in culture, we hypothesized that changes in the   extracellular matrix   environment can regulate growth factor receptors on endothelial cells. We cultured human microvascular endothelial cells on different matrices (vitronectin, laminin, fibronectin, fibrin, and collagen IV) and examined for the presence of growth factor receptors (FGFR-1, FGFR-2, VEGFR-1, and VEGFR-2). We show that vitronectin increased the presence of all four growth factor receptors and most notably, VEGFR-1. In contrast, fibrin decreased all four receptors, especially FGFR-1 and FGFR-2. Inhibiting phosphotyrosine signaling abolished immunostaining for all four receptors, regardless of the matrix, but was not dependent on activating the Fyn-Shc pathway. Cells plated on vitronectin in the presence of blocking antibodies to integrins alphavbeta3 and alphavbeta5 similarly decreased presence of these growth factor receptors. Our data suggests a possible mechanism of how matrix-integrin interactions regulate endothelial cell responsiveness to growth factors and anchorage-dependent cell growth. 
 A genetic association study of maternal and fetal candidate genes that predispose to preterm prelabor rupture of membranes (PROM). We sought to determine whether maternal/fetal single-nucleotide polymorphisms (SNPs) in candidate genes are associated with preterm prelabor rupture of membranes (pPROM).A case-control study was conducted in patients with pPROM (225 mothers and 155 fetuses) and 599 mothers and 628 fetuses with a normal pregnancy; 190 candidate genes and 775 SNPs were studied. Single locus/haplotype association analyses were performed; false discovery rate was used to correct for multiple testing (q* = 0.15).First, a SNP in tissue inhibitor of metalloproteinase 2 in mothers was significantly associated with pPROM (odds ratio, 2.12; 95% confidence interval, 1.47-3.07; P = .000068), and this association remained significant after correction for multiple comparisons. Second, haplotypes for Alpha 3 type IV collagen isoform precursor in the mother were associated with pPROM (global P = .003). Third, multilocus analysis identified a 3-locus model, which included maternal SNPs in collagen type I alpha 2, defensin alpha 5 gene, and endothelin 1.DNA variants in a maternal gene involved in   extracellular matrix   metabolism doubled the risk of pPROM. 
 Vascular endothelial growth factor B (VEGF-B) binds to VEGF receptor-1 and regulates plasminogen activator activity in endothelial cells. The vascular endothelial growth factor (VEGF) family has recently expanded by the identification and cloning of three additional members, namely VEGF-B, VEGF-C, and VEGF-D. In this study we demonstrate that VEGF-B binds selectively to VEGF receptor-1/Flt-1. This binding can be blocked by excess VEGF, indicating that the interaction sites on the receptor are at least partially overlapping. Mutating the putative VEGF receptor-1/Flt-1 binding determinants Asp63, Asp64, and Glu67 to alanine residues in VEGF-B reduced the affinity to VEGF receptor-1 but did not abolish binding. Mutational analysis of conserved cysteines contributing to VEGF-B dimer formation suggest a structural conservation with VEGF and platelet-derived growth factor. Proteolytic processing of the 60-kDa VEGF-B186 dimer results in a 34-kDa dimer containing the receptor-binding epitopes. The binding of VEGF-B to its receptor on endothelial cells leads to increased expression and activity of urokinase type plasminogen activator and plasminogen activator inhibitor 1, suggesting a role for VEGF-B in the regulation of   extracellular matrix   degradation, cell adhesion, and migration. 
 Identification of fetal and maternal single nucleotide polymorphisms in candidate genes that predispose to spontaneous preterm labor with intact membranes. The purpose of this study was to determine whether maternal/fetal single nucleotide polymorphisms (SNPs) in candidate genes are associated with spontaneous preterm labor/delivery.A genetic association study was conducted in 223 mothers and 179 fetuses (preterm labor with intact membranes who delivered <37 weeks of gestation [preterm birth (PTB)]), and 599 mothers and 628 fetuses (normal pregnancy); 190 candidate genes and 775 SNPs were studied. Single locus/haplotype association analyses were performed; the false discovery rate was used to correct for multiple testing.The strongest single locus associations with PTB were interleukin-6 receptor 1 (fetus; P=.000148) and tissue inhibitor of metalloproteinase 2 (mother; P=.000197), which remained significant after correction for multiple comparisons. Global haplotype analysis indicated an association between a fetal DNA variant in insulin-like growth factor F2 and maternal alpha 3 type IV collagen isoform 1 (global, P=.004 and .007, respectively).An SNP involved in controlling fetal inflammation (interleukin-6 receptor 1) and DNA variants in maternal genes encoding for proteins involved in   extracellular matrix   metabolism approximately doubled the risk of PTB. 
  [Ii]nvasive  PubMed  
 Flt-1 in colorectal cancer cells is required for the tumor   invasive   effect of placental growth factor through a p38-MMP9 pathway. Placenta growth factor (PlGF), a dimeric glycoprotein with 53% homology to VEGF, binds to VEGF receptor-1 (Flt-1), but not to VEGF receptor-2 (Flk-1), and may function by modulating VEGF activity. We previously have showed that PlGF displays prognostic value in colorectal cancer (CRC) but the mechanism remains elucidated.Overexpression of PlGF increased the   invasive  /migration ability and decreased apoptosis in CRC cells showing Flt-1 expression. Increased migration was associated with increasing MMP9 via p38 MAPK activation. Tumors grew faster, larger; with higher vascularity from PlGF over-expression cells in xenograft assay. In two independent human CRC tissue cohorts, PlGF, MMP9, and Flt-1 expressions were higher in the advanced than the localized disease group. PlGF expression correlated with MMP9, and Flt-1 expression. CRC patients with high PlGF and high Flt-1 expression in tissue had poor prognosis.PlGF/Flt-1 signaling plays an important role in CRC progression, blocking PlGF/Flt-1 signaling maybe an alternative therapy for CRC. 
 Flt-1 in colorectal cancer cells is required for the tumor   invasive   effect of placental growth factor through a p38-MMP9 pathway. Placenta growth factor (PlGF), a dimeric glycoprotein with 53% homology to VEGF, binds to VEGF receptor-1 (Flt-1), but not to VEGF receptor-2 (Flk-1), and may function by modulating VEGF activity. We previously have showed that PlGF displays prognostic value in colorectal cancer (CRC) but the mechanism remains elucidated.Overexpression of PlGF increased the   invasive  /migration ability and decreased apoptosis in CRC cells showing Flt-1 expression. Increased migration was associated with increasing MMP9 via p38 MAPK activation. Tumors grew faster, larger; with higher vascularity from PlGF over-expression cells in xenograft assay. In two independent human CRC tissue cohorts, PlGF, MMP9, and Flt-1 expressions were higher in the advanced than the localized disease group. PlGF expression correlated with MMP9, and Flt-1 expression. CRC patients with high PlGF and high Flt-1 expression in tissue had poor prognosis.PlGF/Flt-1 signaling plays an important role in CRC progression, blocking PlGF/Flt-1 signaling maybe an alternative therapy for CRC. 
 [The expression and significance of vascular endothelial growth factor and its receptors in hepatocellular carcinoma cell lines with various metastatic potentialities]. To examine whether or not vascular endothelial growth factor (VEGF) and its receptors were expressed in hepatocellular carcinoma cell lines with various metastatic potentialities.Reverse transcription-polymerase chain reaction (RT-PCR), enzyme linked immunosorbent assay (ELISA) and Western blot were employed to study the expressions of VEGFR-1, VEGFR-2, VEGF-A and VEGF-B in four hepatocellular carcinoma cell lines MHCC97-H, MHCC97-L, SMMC7721 and HepG-2 and one normal liver cell line L-02.Three hepatocellular carcinoma cell lines (MHCC97-H, MHCC97-L, SMMC7721) expressed VEGFR-1 mRNA and their proteins. The expression level of VEGFR-1 in MHCC97-H was higher than that in MHCC97-L (P less than 0.05) and the expression level of VEGFR-1 in MHCC97-L was higher than that in SMMC7721 (P less than 0.05). No expression of VEGFR-1 was found in HepG-2 or L-02. All four hepatocellular carcinoma cell lines and the L-02 cell line expressed VEGFR-2 mRNA and the protein, as well as the VEGFR-1 ligands VEGF-A and VEGF-B. The expression level of VEGFR-2 in all tested hepatocellular carcinoma cell lines and normal liver cell line L-02 showed no significant differences (P more than 0.05).VEGFR-1 was expressed in 4 hepatocellular carcinoma cell lines with various metastatic potentialities. The expression levels appeared to be positively correlated with the potentialities of metastasis of the hepatocellular carcinoma cell lines. VEGFR-1 may relate to the   invasive  ness and metastatic potential of the hepatocellular carcinoma. 
 VEGFR-1 activation-induced MMP-9-dependent invasion in hepatocellular carcinoma. VEGFR-1 can promote invasion through epithelial-mesenchymal transition induction in hepatocellular carcinoma (HCC). This study aims to elucidate VEGFR-1 impact on proteolytic enzymes profile involved with invasion.The effect on cell invasion was evaluated by   invasive   and migration assays with and without VEGFR-1 activation. The mechanism was investigated by real-time PCR, western blot and gelatin zymography using inhibitors for MMP-9. In total, 95 HCC patients were enrolled for its clinical value evaluation.VEGFR-1 activation induced invasion in HCC cells with an increase in the expression and activity of MMP-9 and Snail. MMP-9 blockage effectively inhibited VEGFR-1-induced invasion. High coexpression of both in HCC predicted a worse clinical outcome.Data show a novel VEGFR-1 activation-to-MMP-9 mechanism promoting HCC invasion. 
 Expression and prognostic significance of vascular endothelial growth factor receptor 1 in hepatocellular carcinoma. Activation of vascular endothelial growth factor receptor 1 (VEGFR-1) promotes   invasive  ness in some cancer cells. However, VEGFR-1 expression and its relationship with clinical features and prognosis in hepatocellular carcinoma (HCC) remain unclear. Therefore, this study investigated the expression pattern of VEGFR-1 in HCC cell lines and tissue specimens in order to evaluate the role of VEGFR-1 in prognosis of HCC.Expression and localisation of VEGFR-1 in cell lines were determined by western blot and immunofluorescence, respectively. Expression of VEGFR-1 in tissue specimens from 135 HCC patients with curative resections was determined by immunohistochemistry. Overall survival (OS) and recurrence-free survival (RFS) were determined by Kaplan-Meier analysis and a Cox regression model. The relationships between VEGFR-1 expression and clinicopathological features were also analysed.VEGFR-1 expression in more   invasive   HCC cell lines is higher than that in less   invasive   cell lines. VEGFR-1 expression in HCC tissues was significantly higher than that in peritumoral tissues (p<0.001). Patients with high expression of VEGFR-1 had significantly worse RFS and OS after curative resections (p<0.001). Strong expression of VEGFR-1 in HCC tissues was correlated with the most prominent clinicopathological features associated with progression, and poor differentiation was an independent prognosticator for RFS and OS (RFS HR 2.397, 95% CI 1.686 to 3.409; OS HR 2.44, 95% CI 1.518 to 3.922; p<0.001 for both).High expression and distinctive cytomembrane localisation of VEGFR-1 in HCC cells is associated with HCC progression and worse outcome; it may serve as a novel prognostic marker for patients with HCC. 
 Expression and function of vascular endothelial growth factor receptor-1 on human colorectal cancer cells. Vascular endothelial growth factor (VEGF) is associated with tumor angiogenesis and poor prognosis in human colorectal cancer (CRC). VEGF receptor-1 (VEGFR-1 or Flt-1) is a high-affinity receptor for VEGF and is typically considered specific to endothelial cells. Here we report the expression and function of VEGFR-1 in CRC cell lines. VEGFR-1 was expressed in all CRC cell lines studied as determined by RT-PCR, Western blot analysis, FACS, and ELISA. Treatment of the human CRC cell lines HT-29 and SW480 with VEGF-A (a ligand for both VEGFR-1 and -2) or VEGF-B (a ligand specific for VEGFR-1) led to activation of Erk-1/2, SAPK/JNK, and translocation of the p65 subunit of nuclear factor-kappaB into the nucleus. Both VEGF-A and -B led to significant induction of cell motility and   invasive  ness of CRC cells. Stimulation of cells with VEGF-A or -B also led to larger and more numerous colonies in soft agar. However, activation of VEGFR-1 did not increase CRC cell proliferation. In contrast to the previous paradigm that VEGFRs are not present on tumor cells of epithelial origin, we found that VEGFR-1 is present and functional on CRC cells, and activation by VEGF family ligands can activate processes involved in tumor progression and metastasis. 
 Role of the VEGF ligand to receptor ratio in the progression of mismatch repair-proficient colorectal cancer. The VEGF family of ligands and receptors are intimately involved in tumor angiogenesis, lymphangiogenesis and metastasis. The evaluation of VEGF ligand/receptor ratios may provide a more profound understanding of the involvement of these proteins in colorectal tumour progression. The aim of this study was to elucidate the role of the VEGF ligand/receptor ratios on tumour progression and metastasis in patients with mismatch repair-proficient colorectal cancer.Immunohistochemistry for VEGF-A, VEGF-B, VEGF-C, VEGF-D, VEGFR1, VEGFR2 and VEGF3 was carried out on 387 mismatch repair-proficient colorectal cancers using a tissue microarray. Evaluation of immunoreactivity was performed semi-quantitatively and the ligand/receptor expression ratio was obtained.An increased VEGF-A/VEGFR1 ratio, VEGF-A and VEGFR1 was linked to the presence of peritumoral lymphocytic inflammation at the   invasive   front (p = 0.032; p = 0.005; p = 0.032, respectively). VEGFR1 expression was related to poorer outcome in multivariable analysis with pT stage, pN stage, vascular invasion, and post-operative therapy. A higher ratio of VEGF-A/VEGFR2 was linked to advanced TNM stage (p = 0.005) while VEGF-A and VEGFR2 were elevated in tumours with an infiltrating tumour growth pattern (p = 0.006; p = 0.014; p = 0.006). No effect of VEGF-A/VEGFR2, VEGF-A or VEGFR2 on survival time was noted.Our findings highlight an involvement of VEGF-A, VEGR1 and VEGFR2 in events occurring at the   invasive   tumour front and a potential prognostic role of VEGFR1 expression in mismatch repair-proficient colorectal cancers. The VEGF-A ligand to VEGFR1 or VEGFR2 ratio may represent an alternative evaluation system for identifying patients with poorer clinical outcome. 
 Growth factor receptor expression in anal squamous lesions: modifications associated with oncogenic human papillomavirus and human immunodeficiency virus. High prevalence of squamous anal lesions is linked to oncogenic human papillomavirus (HPV). Human immunodeficiency virus (HIV) promotes anal carcinogenesis. Epidermal growth factor receptor (EGFR), HER2/neu, c-Met, and vascular endothelial growth factor receptor-1 (VEGFR1) (tyrosine kinase growth factor receptors) are implicated in tumor progression, but little is known about their role in anal lesions. We investigated their expression and distribution in normal, dysplastic, and carcinomatous anal epithelium and then tried to analyze the effects on these variables of HPV and the HIV-positive status. Seventy-one HIV-positive and 47 HIV-negative patients were selected. We studied growth factor receptors, p16 and Ki67 expression, by in situ hybridization, fluorescent in situ hybridization (FISH) and chromogen in situ hybridization (CISH), immunocytochemistry, and morphological quantification in 226 lesions, either infected by HPV6 and 11 (31 condylomas acuminata) or infected with oncogenic HPVs (48   invasive   cancers, 147 anal intraepithelial neoplasias). No HER2/neu was detected. Strong EGFR immunolabeling was not accompanied by gene amplification. The number and intensity of EGFR- and c-Met-immunoreactive cells increased significantly during lesion progression, highlighting the effects of oncogenic HPVs. EGFR, c-Met, VEGFR1, and p16 were coexpressed in 96% of   invasive   cancers. HIV-modified c-Met expression in condyloma acuminata (P < .008) and   invasive   cancers (P < .02). Strong HIV-related immunodeficiency and an absence of antiretroviral therapy increased c-Met and/or EGFR expression. HIV-positive anal cancers showed correlated c-Met and VEGFR1 (P < .003), strong p16 labeling, and an increased Ki67 proliferation. The finding that EGFR, c-Met, and VEGFR1 involved in carcinogenesis are well-represented and coexpressed in anal cancers, especially in HIV-positive population, suggests possible novel targeted treatments for anal diseases. 
 High co-expression of vascular endothelial growth factor receptor-1 and Snail is associated with poor prognosis after curative resection of hepatocellular carcinoma. VEGFR-1-mediated signaling promotes   invasive  ness by direct tumor activation in some cancers. However, VEGFR-1 expression and its relationship with clinical features and prognosis in HCC remained unclear. Overexpression of Snail is common in HCC and associated with poorer prognosis. Therefore, expression of VEGFR-1 and Snail was investigated in HCC cell lines and tissue specimens in our study, and special attention was paid to evaluating the role of VEGFR-1 expression in prognosis of HCC. Western blot and immunofluorescence were used to detect expression of VEGFR-1, Snail and MMP-9 in 4 HCC cell lines, respectively. Moreover, expression of these proteins was confirmed on the samples from 95 HCC patients who underwent curative resection using immunohistochemistry. ROC and survival analysis determined the predictive values of parameters and the association with survival. HCC cell lines with a higher VEGFR-1 expression were more   invasive  . Both VEGFR-1 and Snail expressions were significantly higher in HCC tissues than in non-cancerous tissues. High-expression VEGFR-1 and Snail, associated with adverse clinical features, were independent prognostic factors for RFS and OS (P=0.023 and P=0.044, respectively). Positive correlation was found between VEGFR-1 and Snail or MMP-9 (r=0.418 and r=0.232, respectively, P<0.05 for both) in cancerous tissues. The combination of VEGFR-1 and Snail gave a better power to predict patients' recurrence and death (P<0.001 for both). High VEGFR-1 expression, distinctively expressed in the cytomembrane of HCC cells, was associated with HCC progression and worse outcome. High co-expression of VEGFR-1 and Snail may be a novel prognostic marker for HCC, especially in recurrence. 
 Association of polymorphisms of angiogenesis genes with breast cancer. Few studies have systematically explored a pathway approach: to test the association of multiple polymorphisms from multiple genes important to angiogenesis simultaneously with risk of breast cancer. We report our preliminary data evaluating the association of polymorphisms from seven genes known to influence angiogenesis with the likelihood of having breast cancer.We recruited 715 controls and 520 subjects with breast cancer. Subjects provided a blood specimen and completed a questionnaire that included common breast cancer risk factors and breast cancer status. We evaluated candidate polymorphisms in the following genes: Hypoxia Inducible Factor-1 alpha (HIF1alpha), Vascular Endothelial Growth Factor (VEGF), VEGF Receptor 1 (VEGFR-1), VEGFR-2, endothelial Nitric Oxide Synthase (eNOS), Neuropilin-1 (NRP-1) and Neuropilin-2 (NRP-2). Testing for associations between each polymorphism and the presence or absence of breast cancer was performed.VEGF-2578 AA and -1498 CC genotypes were more common in cancer cases than controls (P = 0.06 and P = 0.04, respectively). These two genotypes remained significant predictors of breast cancer status after adjusting for non-genetic risk factors estimated by the Gail model (P = 0.03 and P = 0.03, respectively). When comparing women with   invasive   versus pre-  invasive   breast cancer, the eNOS-786 TT and eNOS 894 GG genotypes were associated with a greater likelihood of   invasive   disease and the eNOS 894 GG genotype was associated with a greater likelihood of having metastatic disease.There is an association of the VEGF-2578A and -1498C alleles with increased breast cancer risk. This association remains significant when adjusted for Gail score-related risk factors. 
 Cell type- and tumor zone-specific expression of pVEGFR-1 and its ligands influence colon cancer metastasis. Detailed knowledge of the essential pro-angiogenic biomolecules, the vascular endothelial growth factor (VEGF) family and its receptors, in the characteristically heterogeneous tumor tissue is a pre-requisite for an effective personalized target therapy. The effects of VEGF receptors after ligand binding are mediated through receptor tyrosine autophosphorylation. We determined the relevance of the VEGFR-1 activating pathway for colon cancer (CC) metastasis.The expression profiles of VEGFR-1, phosphorylated (activated) VEGFR-1 (pVEGFR-1(Tyr1048), pVEGFR-1(Tyr1213) and pVEGFR-1(Tyr1333)) and the VEGFR-1 ligands (VEGF, PlGF and VEGF-B) were investigated using immunohistochemistry in different tumor compartments (intratumoral -   invasive   front - extratumoral), cell types (tumor cells - macro- (large and small vessels) and the microvasculature (capillaries) - inflammatory cells) in human sporadic non-metastatic, lymphogenous metastatic and haematogenous metastatic CC.VEGF and PlGF produced by tumor cells have an autocrine affinity for their receptor VEGFR-1. Subsequent PlGF-mediated receptor activation by autophosphorylation at Tyr1048 and Tyr1213 is a potential signaling pathway, which in turn seems to protect against distant metastasis and, in regions of tumor budding, additionally against lymph node metastasis. This autocrine link could be supported by possible formation of PlGF-VEGF heterodimers and PlGF-PlGF homodimers, which are known to have anti-metastatic properties. In contrast, in order to enhance their potential for distant metastasis tumor cells produce paracrine-acting VEGF-B. VEGFR-1 activation in tumor-associated macrovasculature but not capillaries appears to affect metastatic ability. Paracrine-mediated receptor autophosphorylation at Tyr1048 and Tyr1213 in small vessels located intratumorally and along the   invasive   front appears to be inversely correlated with metastasis, especially distant metastasis. Additionally, macrovessels are able to produce VEGFR-1 ligands, which influence the metastatic potential. Paracrine-acting VEGF-B production by intratumorally located small vessels and autocrine-acting PlGF production by extratumorally located small vessels seem to be associated with the non-metastatic phenotype. In contrast, VEGF-B-expressing extratumoral large and small vessels correlate with distant metastasis. Lymphocyte-associated VEGFR-1 expression in the   invasive   front without accompanying autophosphorylation could prevent against distant metastasis possibly by acting as a decoy and scavenger receptor.VEGFR-1 and its ligands participate in vascular, tumor cell-mediated and immuno-inflammatory processes in a complex biomolecule-dependent and tumor zone-specific manner and hence could influence metastatic behavior in CC. 
 Expression of VEGF and its receptors VEGFR1/VEGFR2 is associated with   invasive  ness of bladder cancer. Vascular endothelial growth factor (VEGF) signaling is frequently altered in   invasive   tumor cells and is associated with patient outcome. In the present study, we examined VEGF, VEGFR1, and VEGFR2 expression in non-muscle   invasive   bladder cancer (NMIBC) and muscle   invasive   bladder cancer (MIBC), and evaluated the association between VEGF and its receptors with disease characteristics and bladder cancer recurrence.Tissue microarrays containing bladder cancer specimens (n=212) and adjacent normal bladder mucosa (n=131) were immunostained using antibodies against VEGF, VEGFR1, and VEGFR2. The association between the expression of these proteins and clinical parameters including stage, lymph node metastasis, and recurrence-free survival were statistically evaluated. VEGF mRNA expression data were extracted from the public Oncomine database.VEGF and VEGFR1 mRNA levels were significantly higher in bladder cancer specimens than that of normal mucosa (for VEGF, p<0.001; for VEGFR1, p=0.02). Analysis of their expression at protein levels showed that levels of VEGF and VEGFR1 were significantly higher in NMIBC than in MIBC (p<0.001), while that of VEGFR2 was significantly higher in all cancer specimens compared to benign urothelial mucosa (p=0.001). Further-more, the expression of VEGFR2 was significantly higher in MIBC, as compared to NMIBC (p<0.001). Patients with higher levels of VEGF, VEGFR1, and VEGFR2 tended to have poorer recurrence-free survival than those with lower levels, but this was not statistically significant.Our results suggest that alterations in the expression of VEGF and VEGF receptors are associated with disease stage and recurrence. 
 Expression of vascular endothelial growth factor and vascular endothelial growth factor receptors 1 and 2 in   invasive   breast carcinoma: prognostic significance and relationship with markers for aggressiveness. Vascular endothelial growth factor (VEGF), VEGF receptor 1 (VEGFR-1) and VEGF receptor 2 (VEGFR-2) play a role in breast cancer growth and angiogenesis. We examined the expression and relationship with clinical outcome and other prognostic factors.Tumour sections from 468 breast cancer patients were immunostained for VEGF, VEGFR-1, and VEGFR-2, and their relationships with tumour vascularity, disseminated tumour cells (DTCs) in bone marrow and other clinicopathological parameters were evaluated. VEGF, VEGFR-1 and VEGFR-2 immunoreactivities were observed in   invasive   breast carcinoma cells. VEGF expression was significantly associated with VEGFR-1 and VEGFR-2 expression (P < 0.001). High-level cytoplasmic expression of VEGFR-1 was associated with significantly reduced distant disease-free survival (DDFS) (P = 0.017, log-rank) and breast cancer-specific survival (BCSS) (P = 0.005, log-rank) for all patients, and for node-negative patients without systemic treatment (DDFS, P = 0.03, log-rank; BCSS, P = 0.009, log-rank). VEGFR-1 expression was significantly associated with histopathological markers of aggressiveness (P < 0.05). Significantly reduced survival was observed in DTC-positive patients as compared with DTC-negative patients in the combined moderate/high VEGFR-1 group (P < 0.001 for DDFS and BCSS), and the same was true for DDFS in the moderate VEGFR-2 group (P = 0.006).High-level expression of VEGFR-1 indicates reduced survival. Higher-level expression of VEGFR-1 or VEGFR-2 in primary breast carcinomas combined with the presence of DTC selects a prognostically unfavourable patient group. 
 A novel intracellular isoform of VEGFR-1 activates Src and promotes cell invasion in MDA-MB-231 breast cancer cells. Two types of VEGFR-1 receptors have been characterized: a full-length transmembrane receptor and a truncated extracellular soluble isoform (sVEGFR-1). We report here the characterization, in normal and cancer cells, of a new family of intracellular isoforms of VEGFR-1 resulting from alternative initiation of transcription in intronic sequences of the gene. While the classical isoforms of VEGFR-1 were barely detectable in MDA-MB-231 breast cancer cells, one of the intracellular isoforms transcribed from intron 21 (i(21)VEGFR-1) was the main isoform expressed in these cells. The new transcript encodes for a protein that contains only the phosphotransferase domain and the carboxyterminal tail of VEGFR-1. Treatment of MDA-MB-231 cells with siRNA specific for the tyrosine domain of VEGFR-1 suppressed the expression of i(21)VEGFR-1, downregulated phosphorylation of Src at tyrosine 418, and reduced markedly the invasion capacity of these cells in vitro. Accordingly, overexpression of transfected i(21)VEGFR-1 in MDA-MB-231 cells upregulated the active form of Src and increased   invasive  ness of MDA-MB-231 cells. The expression of i(21)VEGFR-1 in MDA-MB-231 cells was inhibited by retinoic acid. Both, activation of Src and downregulation by retinoic acid, have been reported in other intracellular members of the Fms/Kit/PDGFR family of tyrosine kinases, particularly in the intracellular isoform of c-kit, analogous structurally to i(21)VEGFR-1 and frequently expressed in cancer cells. 
 Expression of vascular endothelial growth factor and its receptors flt and KDR in ovarian carcinoma. Two thirds of patients with ovarian carcinoma have advanced disease at diagnosis and have poor prognoses because of the presence of highly   invasive   carcinoma cells and rapidly accumulating ascitic fluid. Vascular endothelial growth factor (VEGF), a potent mitogen of endothelial cells, is produced in elevated amounts by many tumors, including ovarian carcinomas. The known human receptors for VEGF, flt and KDR, are both cell surface tyrosine kinases and are expressed predominantly on endothelial cells. Acting through these receptors, VEGF may stimulate angiogenesis and promote tumor progression.We aimed to clarify the function of VEGF in tumor development by identifying the cells in ovarian carcinoma tissue that express VEGF and its receptors.VEGF, flt, and KDR expression was localized by in situ hybridization and immunohistochemistry in frozen sections of primary tumors from five patients with ovarian carcinoma and from metastases of ovarian carcinoma from three different patients. Reverse transcription followed by polymerase chain reaction (RT-PCR) and an enzyme-linked immunosorbent assay were used to analyze VEGF, flt, and KDR expression in six epithelial cell lines derived from ovarian carcinoma ascites from five additional patients.Messenger RNAs (mRNAs) encoding VEGF, flt, and KDR were detected in primary ascitic cells and in three of four ovarian carcinoma cell lines examined by RT-PCR. Two novel complementary DNAs that may encode truncated, soluble forms of flt were cloned from one primary source. VEGF levels of 20-120 pM were found in culture media conditioned by the cell lines. Elevated expression of VEGF mRNA was found in all primary tumors and metastases, especially at the margins of tumor acini. VEGF immunoreactivity was concentrated in clusters of tumor cells and patches of stromal matrix. flt immunoreactivity was confined to tumor blood vessels, but flt mRNA was not detected by in situ hybridization. In contrast, KDR mRNA was detected not only in vascular endothelial cells but also in tumor cells at primary malignant sites.VEGF is expressed by tumor cells in primary and metastatic ovarian carcinoma and accumulates in the stromal matrix. Its receptors, flt and KDR, are expressed by some tumor cells that coexpress VEGF. This is the first localization of KDR expression in nonendothelial cells.Coexpression of VEGF and KDR by tumor cells in ovarian carcinoma raises the possibility of autocrine stimulation and of therapeutic strategies targeting this receptor-ligand interaction. 
 [Inhibition effect of short hairpin RNA on VEGF receptor flt-1 gene expression in leukemia cell line K562]. This study was purposed to investigate the inhibitory effect of short hairpin RNA (shRNA) on expression of vascular endothelial growth factor (VEGF) receptor flt-1 gene in leukemia cells line K562, and to explore the influence of shRNA   invasive   ability on leukemia cells and its mechanism. The recombinant eukaryotic expression plasmid containing flt-1 shRNA gene was transfected into K562 cells by lipofectamine mediation and positive clones were screened by G418. shRNA gene in K562 cells was confirmed by PCR. RT-PCR and Western blot were employed to detect the expression of flt-1 mRNA and protein in leukemia cells. The   invasive   ability of K562 cells was studied by Boyden chamber invasion assay before and after flt-1 shRNA transfection. MMP-2 and MMP-9 mRNA expressions were detected by RT-PCR after transfection of the recombinant plasmid C1/U6/FltS2 into K562 cells through liposome. The results showed that the recombinant eukaryotic expression plasmid had been transfected into the human leukemia cell line K562 and positive clones had been screened by G418 for 2 weeks. PCR detection revealed the stable expression of the shRNA gene in K562 cells. Flt-1 gene and protein expressions were inhibited by plasmid-expressed shRNA after transfection of recombinant vetors C1/U6/FltS into K562 cells. The inhibitory efficiency of two different shRNA sequences targeting Flt-1 gene were 46.1% and 65.4% respectively. The expression of MMP-2 and MMP-9 mRNA decreased, and the mean invasion rate in C1/U6/fltS2-transfected K562 cells was lower than that in nontransfected cells. It is concluded that shRNA eukaryotic expression vector specific to VEGF receptor flt-1 gene can high efficiently be transfected into leukemia cell line K562, effectively inhibits the expression of flt-1 gene, weakens the in vitro   invasive   ability of leukemia cells and the expression levels of MMP-2 and MMP-9 mRNA, which suggests that the VEGF involves in the migration of leukemia cells by regulating the MMP-2 and MMP-9 through joints with the receptor. 
  GeneRIFs  
 Silencing flt-1 weakens the in vitro   invasive   ability of leukemia cells and reduces the expression levels of MMP-2 and MMP-9 mRNA. 
 VEGF-A, VEGFR1 and VEGFR2 have roles in events occurring at the   invasive   tumour front 
  [Mm]etastasize  PubMed  
 Hematogenous metastasis in gastric cancer requires isolated tumor cells and expression of vascular endothelial growth factor receptor-1. Recent studies of cancer metastasis have focused on the role of premetastatic gene expression and circulating tumor cells. We did a blind prospective study in gastric cancer to assess the significance of isolated tumor cells (ITC) and to test the hypothesis that vascular endothelial growth factor receptor-1 (VEGFR-1) is expressed within the bone marrow at tumor-specific, premetastatic sites.Both bone marrow and peripheral blood samples from 810 gastric cancer patients were collected at the Central Hospital, National Cancer Center (Tokyo, Japan). The samples were transferred to Kyushu University Hospital (Beppu, Japan) where they were analyzed by quantitative real-time reverse transcription-PCR for three epithelial cell markers, carcinoembryonic antigen, cytokeratin-19, and cytokeratin-7, as well as VEGFR-1.ITCs were observed in peripheral blood and bone marrow even in early stages of gastric cancer. The frequency of ITC in bone marrow was significantly associated with the stage of disease by ANOVA (P < 0.01). Gastric cancer   metastasize  d when ITCs were observed in the presence of VEGFR-1. In the 380 patients who were ITC negative and showed low VEGFR-1 expression, synchronous (at the time of surgery) and heterochronous (recurrent) metastases were not observed.ITCs circulate even in early stages of disease. Furthermore, elevated expression of VEGFR-1 facilitates the establishment of hematogenous metastases in gastric cancer. This study indicates that the simultaneous presence of ITC and VEGFR-1 expression at premetastatic sites is clinically significant for disease progression. 
  [Mm]etastasis  PubMed  
 [The expression and significance of vascular endothelial growth factor and its receptors in hepatocellular carcinoma cell lines with various metastatic potentialities]. To examine whether or not vascular endothelial growth factor (VEGF) and its receptors were expressed in hepatocellular carcinoma cell lines with various metastatic potentialities.Reverse transcription-polymerase chain reaction (RT-PCR), enzyme linked immunosorbent assay (ELISA) and Western blot were employed to study the expressions of VEGFR-1, VEGFR-2, VEGF-A and VEGF-B in four hepatocellular carcinoma cell lines MHCC97-H, MHCC97-L, SMMC7721 and HepG-2 and one normal liver cell line L-02.Three hepatocellular carcinoma cell lines (MHCC97-H, MHCC97-L, SMMC7721) expressed VEGFR-1 mRNA and their proteins. The expression level of VEGFR-1 in MHCC97-H was higher than that in MHCC97-L (P less than 0.05) and the expression level of VEGFR-1 in MHCC97-L was higher than that in SMMC7721 (P less than 0.05). No expression of VEGFR-1 was found in HepG-2 or L-02. All four hepatocellular carcinoma cell lines and the L-02 cell line expressed VEGFR-2 mRNA and the protein, as well as the VEGFR-1 ligands VEGF-A and VEGF-B. The expression level of VEGFR-2 in all tested hepatocellular carcinoma cell lines and normal liver cell line L-02 showed no significant differences (P more than 0.05).VEGFR-1 was expressed in 4 hepatocellular carcinoma cell lines with various metastatic potentialities. The expression levels appeared to be positively correlated with the potentialities of   metastasis   of the hepatocellular carcinoma cell lines. VEGFR-1 may relate to the invasiveness and metastatic potential of the hepatocellular carcinoma. 
 [Expression of vascular endothelial growth factor and its receptor (Flt-1) in breast carcinoma]. To investigate the expression of vascular endothelial cell growth factor (VEGF) and its receptor (Flt-1) in breast carcinoma and the relationship of such expression with angiogenesis, tumor invasion and   metastasis  .In situ hybridization and immunohistochemistry technique were used to test the expression of mRNA and protein expression of VEGF and flt-1 in 48 specimens of breast carcinoma. 48 specimens of tissues near cancer, 3 specimens of fibroadenoma of breast, and 3 specimens of normal breast were used as controls.VEGF mRNA and its protein were expressed highly in breast carcinoma cells with the positive rate of 75% and 70.8% respectively. There was almost no expression of VEGF in vascular endothelial cells. Flt-1 mRNA and its protein were expressed only in a small quantity in a few tumor cells. The amount of blood vessel positive in Fit-1 mRNA and its protein was (26 +/- 12) piece/0.72 mm(2) and (24 +/- 12) piece/0.72 mm(2) respectively. The microvascular density (MVD) was significantly greater in VEGF and Flt-1 higher expression groups than in lower groups (P < 0.01). Both VEGF expression and Flt-1 expression were well correlated with the histological grade and lymph node   metastasis  .VEGF promotes angiogenesis by paracrining in breast carcinoma, and takes part in tumor invasion and lymph node   metastasis  . Test of the expression of VEGF and Flt-1 may act as a reliable index to determine angiogenesis, malignancy, invasion and   metastasis  . Blocking their secretion and effect may act as a new treatment for breast carcinoma. 
 Angiogenesis-related protein expression in bevacizumab-treated metastatic colorectal cancer: NOTCH1 detrimental to overall survival. The development of targeted therapies has undoubtedly broadened therapeutic options for patients with colorectal cancer (CRC). The use of bevacizumab to reduce angiogenesis has been associated with improved clinical outcomes. However, an urgent need for prognostic/predictive biomarkers for anti-angiogenic therapies still exists.Clinical data of 105 CRC patients treated with bevacizumab in conjunction with chemotherapy were analyzed. The expression of vascular endothelial growth factor (VEGF) receptors, NOTCH1 receptor and its ligand DLL4 were determined by immunohistochemistry. Tumor samples were arranged on a tissue microarray. The association between protein expression and clinicopathological characteristics and outcomes was determined.Bevacizumab was administered as a first-line of treatment in 70.5 % of our cases. The median progression-free survival (PFS) was 10.2 months. The median overall survival (OS) of the total cohort was 24.4 months. Bevacizumab, as the first-line of treatment, and the presence of liver   metastasis   were independently associated with objective response rate. Membrane VEGFR1 and VEGFR3 expressions were associated with the presence of lung   metastasis  ; interestingly, VEGFR3 was associated with less liver   metastasis  . NOTCH1 expression was associated with lymph node   metastasis  . There was a trend toward association between improved PFS and lower NOTCH1 expression (p = 0.06). Improved OS was significantly associated with lower NOTCH1 expression (p = 0.01). In a multivariate analysis, ECOG (Eastern Cooperative Oncology Group) performance status  , liver me  tastasis, histological grade, and NOTCH1 expression were independently associated with OS.Our findings illustrated the expression profile of angiogenesis-related proteins and their association with clinicopathological characteristics and outcomes. NOTCH1 expression is a detrimental prognostic factor in metastatic CRC patients treated with chemotherapy plus bevacizumab. 
 VEGF and sVEGFR-1 in malignant pleural effusions: association with survival and pleurodesis outcomes. VEGF is a key mediator of tumour growth and   metastasis   and is considered central to the formation of exudative pleural effusions. This study examined the relationship between levels of VEGF and its soluble receptor, sVEGFR-1 in the pleural fluid and plasma of patients with malignant pleural effusions and their association with pleurodesis outcomes and survival. 103 patients with malignant pleural effusions were recruited at their first presentation. Follow-up was to 6 months or death. Survival and pleurodesis outcomes were robustly ascertained. VEGF and sVEGFR-1 were measured in pleural fluid and plasma by ELISA. VEGF and sVEGFR-1 were present in significantly higher concentrations in pleural fluid than plasma. There was no significant correlation between mediators within or between sample types. There was no association between baseline pleural fluid VEGF or sVEGFR-1 levels and pleurodesis failure. In both sample types, survival was inversely associated with sVEGFR-1 and within the non-small cell lung cancer sub-group (n=26), a highly significant association between increased pleural fluid VEGF and sVEGFR-1 and reduced survival was demonstrated (p=0.02 and 0.004 respectively). In conclusion, we have shown for the first time that sVEGFR-1 can be reproducibly measured in pleural fluid from malignant effusions. High levels at presentation in those with non-small cell carcinoma are strongly associated with poor outcomes. 
 Vascular endothelial growth factor receptor-1 (VEGFR-1/Flt-1): a dual regulator for angiogenesis. Vascular endothelial growth factor receptor-1 (VEGFR-1) is a member of the VEGFR family, and binds VEGF-A, PlGF, and VEGF-B. An important feature of VEGFR-1 is that, unlike other VEGFR genes, it expresses two types of mRNA, one for a full-length receptor and another for a soluble short protein known as soluble VEGFR-1 (sFlt-1). The binding-affinity of VEGFR-1 for VEGF-A is one order of magnitude higher than that of VEGFR-2, whereas the kinase activity of VEGFR-1 is about 10-fold weaker than that of VEGFR-2. Through its ligand-binding region and by trapping ligands, VEGFR-1 plays a negative role in angiogenesis at embryogenesis. In adulthood, however, VEGFR-1 is expressed not only on endothelial cells but also on macrophages, and promotes the function of macrophages, inflammatory diseases, cancer   metastasis  , and atherosclerosis via its kinase activity. Soluble VEGFR-1 is abnormally overexpressed in the placenta of preeclamptic patients, and suggested to cause the major pathological symptoms on the maternal side such as hypertension and renal dysfunction, most likely by blocking the physiological VEGF-A. VEGFR-1 including its soluble form is involved in a variety of human illnesses, making it an important target in the development of new strategies to suppress disease. 
 Vascular endothelial growth factor receptor-1 activation mediates epithelial to mesenchymal transition in hepatocellular carcinoma cells. To explore the molecular mechanism of Vascular endothelial growth factor receptor-1 (VEGFR-1) in invasion and   metastasis   of hepatocellular carcinoma.Reverse transcription polymerase chain reaction was performed to test expression of VEGFR-1 and its ligand VEGF-B19 in four hepatoma carcinoma cell. Fluorescent immunohistochemistry and western blotting were used to test the change of expression of E-cadherin or α-catenin.VEGF-B-treated cells exhibited a change in E-cadherin from an organized, membrane-bound structure to a disorganized state that was dispersed throughout the cytoplasm. The maximal changes in E-cadherin were observed 24 hr after treatment of cells with VEGF-B. α-catenin was observed to translocate to the nucleus from its usual membrane-bound location 24 hr after treatment with either VEGF-B. Expression of the epithelial adhesion molecules E-cadherin was observed to decrease 48 hours after VEGF-B treatment. The nuclear expression of α-catenin was observed to increase 24 hr after treatment with VEGF-B.VEGFR-1 on tumor cells may contribute to the aggressive behavior of hepatocellular carcinoma cells by inducing epithelial to mesenchymal transition (EMT). Targeting VEGFR-1 and downstream mediators of EMT may provide the foundation for the development of novel therapeutic approaches for this morbid and lethal disease. 
 BCL-6 oncoprotein in breast cancer: loss of expression in disease progression. To investigate the biological role of BCL-6 oncoprotein in breast cancer disease progression (recurrence and   metastasis  ).The series consisted of 93 consecutive female patients with primary breast cancer and median follow-up of 10 years. BCL-6 expression was assessed in vivo by immunohistochemistry and real-time PCR. Breast cancer cell lines and some   metastasis  -related genes (CXCR4, Itgbeta-3 and FLT-1) were also analysed by molecular techniques. Prognostic evaluation was performed by fitting a multivariate Cox regression model.BCL-6 immunoexpression was positive in 22 (23.7%) tumours and negative in 71 (76.3%). All axillary lymph node metastases of 47 node-positive patients were negative, including 12 cases showing BCL-6-positive primary tumours. Likewise, in 9 recurrence cases, BCL-6 expression was similar or decreased compared with primary tumours. No correlation between immunoexpression and gene expression of BCL-6 was observed. BCL-6 was significantly reduced both in derived metastases of a breast cancer cell line (M435) and when the latter was treated with a demethylation agent (5-azacytidine). However, BCL-6-transfected breast cancer cell lines expressed significantly higher levels of CXCR4, Itgbeta-3 and FLT-1. Co-expression of the 4 genes was found in 4 of 17 tumours evaluated, but lacking prognostic significance. BCL-6 oncoprotein revealed no significant influence on outcome.The results strongly suggest the loss of BCL-6 expression in breast cancer progression, which might be related with methylation status alterations of still unknown partner gene(s). 
 Expression and function of vascular endothelial growth factor receptor-1 on human colorectal cancer cells. Vascular endothelial growth factor (VEGF) is associated with tumor angiogenesis and poor prognosis in human colorectal cancer (CRC). VEGF receptor-1 (VEGFR-1 or Flt-1) is a high-affinity receptor for VEGF and is typically considered specific to endothelial cells. Here we report the expression and function of VEGFR-1 in CRC cell lines. VEGFR-1 was expressed in all CRC cell lines studied as determined by RT-PCR, Western blot analysis, FACS, and ELISA. Treatment of the human CRC cell lines HT-29 and SW480 with VEGF-A (a ligand for both VEGFR-1 and -2) or VEGF-B (a ligand specific for VEGFR-1) led to activation of Erk-1/2, SAPK/JNK, and translocation of the p65 subunit of nuclear factor-kappaB into the nucleus. Both VEGF-A and -B led to significant induction of cell motility and invasiveness of CRC cells. Stimulation of cells with VEGF-A or -B also led to larger and more numerous colonies in soft agar. However, activation of VEGFR-1 did not increase CRC cell proliferation. In contrast to the previous paradigm that VEGFRs are not present on tumor cells of epithelial origin, we found that VEGFR-1 is present and functional on CRC cells, and activation by VEGF family ligands can activate processes involved in tumor progression and   metastasis  . 
 A SNP in the flt-1 promoter integrates the VEGF system into the p53 transcriptional network. The VEGF system is essential for angiogenesis. VEGF overexpression frequently correlates with increased microvascularity and   metastasis   and decreased spontaneous apoptosis. Although a precise mechanism has not been established, studies suggest that VEGF expression is negatively regulated by p53, a master regulator and tumor suppressor. There are no reports of additional components of the VEGF signal transduction pathway being part of the p53 transcriptional network. A target of VEGF, the VEGF receptor 1/flt-1, can regulate growth and migration of endothelial cells and modulate angiogenesis. VEGF appears to be up-regulated in various cancers in which flt-1 may have a role in tumor progression and   metastasis  . We identified a C-to-T SNP upstream of the transcriptional start site in approximately 6% of the people examined. The SNP is located within a putative p53 response element. Only the promoter with the T SNP (FLT1-T) was responsive to p53 when examined with reporter assays or by endogenous gene expression analysis in cell lines with different SNP status. In response to doxorubicin-induced DNA damage, there was clear allele discrimination based on p53 binding at the FLT1-T but not FLT1-C promoters as well as p53-dependent induction of flt-1 mRNA, which required the presence of FLT1-T. Our results establish that p53 can differentially stimulate transcription at a polymorphic variant of the flt-1 promoter and directly places the VEGF system in the p53 stress-response network via flt-1 in a significant fraction of the human population. We suggest that the p53-VEGF-flt-1 interaction is relevant to risks in angiogenesis-associated diseases, including cancer. 
 Role of the VEGF ligand to receptor ratio in the progression of mismatch repair-proficient colorectal cancer. The VEGF family of ligands and receptors are intimately involved in tumor angiogenesis, lymphangiogenesis and   metastasis  . The evaluation of VEGF ligand/receptor ratios may provide a more profound understanding of the involvement of these proteins in colorectal tumour progression. The aim of this study was to elucidate the role of the VEGF ligand/receptor ratios on tumour progression and   metastasis   in patients with mismatch repair-proficient colorectal cancer.Immunohistochemistry for VEGF-A, VEGF-B, VEGF-C, VEGF-D, VEGFR1, VEGFR2 and VEGF3 was carried out on 387 mismatch repair-proficient colorectal cancers using a tissue microarray. Evaluation of immunoreactivity was performed semi-quantitatively and the ligand/receptor expression ratio was obtained.An increased VEGF-A/VEGFR1 ratio, VEGF-A and VEGFR1 was linked to the presence of peritumoral lymphocytic inflammation at the invasive front (p = 0.032; p = 0.005; p = 0.032, respectively). VEGFR1 expression was related to poorer outcome in multivariable analysis with pT stage, pN stage, vascular invasion, and post-operative therapy. A higher ratio of VEGF-A/VEGFR2 was linked to advanced TNM stage (p = 0.005) while VEGF-A and VEGFR2 were elevated in tumours with an infiltrating tumour growth pattern (p = 0.006; p = 0.014; p = 0.006). No effect of VEGF-A/VEGFR2, VEGF-A or VEGFR2 on survival time was noted.Our findings highlight an involvement of VEGF-A, VEGR1 and VEGFR2 in events occurring at the invasive tumour front and a potential prognostic role of VEGFR1 expression in mismatch repair-proficient colorectal cancers. The VEGF-A ligand to VEGFR1 or VEGFR2 ratio may represent an alternative evaluation system for identifying patients with poorer clinical outcome. 
 Identification of the high-risk group for   metastasis   of gastric cancer cases by vascular endothelial growth factor receptor-1 overexpression in peripheral blood. Identification of an isolated tumour cell with metastatic ability is important for predicting the recurrence and prognosis of gastric cancer. A biological marker for evaluating the metastatic ability of gastric cancer cells has not yet been identified. We assessed vascular endothelial growth factor receptor-1 mRNA expression by quantitative real-time reverse transcriptase-polymerase chain reaction. Vascular endothelial growth factor receptor-1 mRNA in peripheral blood was more highly expressed in perioperative   metastasis  -positive and postoperative recurrence cases than in normal control cases, early cancer cases and nonmetastatic advanced cancer cases. The peripheral blood vascular endothelial growth factor receptor-1 mRNA-positive group was associated with advanced clinical stage, deep invasion beyond the muscularis propria, lymphatic involvement, vascular involvement, lymph node   metastasis  , positive peritoneal lavage cytology, preoperative   metastasis   and postoperative recurrence. Flow cytometry analysis disclosed that vascular endothelial growth factor receptor-1 expressing cells in the peripheral blood were more abundant in cancer cases with metastases than in cases without metastases. Our data suggest that the amount of positive cells may provide information on the clinical features of gastric cancer, especially in regard to gastric cancer   metastasis  . 
 The soluble VEGF receptor 1 and 2 expression in cerebral spinal fluid as an indicator for leukemia central nervous system   metastasis  . Over-expression of vascular endothelial growth factor A (VEGF-A) is correlated with leukemia   metastasis  . VEGF-A acts by binding to its membrane receptors R1 and R2 present in soluble forms (sVEGFR1, sVEGFR2) with different functions. sVEGFR could inhibit VEGF-A bioactivities, associated with favorable prognosis in solid tumors. However, its role is obscure in central nervous system leukemia (CNSL). The aim of this study was to investigate sVEGFR1, R2 as biomarkers in CNSL. Paired cerebrospinal fluid (CSF) and serum samples were collected from 35 leukemia cases with or without CNS   metastasis  . Levels of sVEGFR1 and sVEGFR2 in both CSF (sVEGFR1CSF, sVEGFR2CSF) and serum (sVEGFR1Serum, sVEGFR2Serum) were detected by ELISA. Other risk factors related to CNSL prognosis were also analyzed. sVEGFRSerum levels were 2.54-fold (sVEGFR1) and 25.6-fold (sVEGFR2) higher than sVEGFRCSF in both leukemic groups. sVEGFR1CSF in CNSL were 33 % higher than in the non-CNSL, and the levels of sVEGFR2CSF and sVEGFR2Serum had the same trend. Elevated sVEGFR1CSF and sVEGFR2CSF is closely correlated with blood-brain barrier (BBB) values and WBCCSF that is an indicator of CNSL disease burden. Cox regression analysis showed that the sVEGFR2CSF had a positive effect on event-free survival. Our data suggest that sVEGFR2CSF may be more potent than sVEGFR1CSF in predicting the outcome of leukemia patients, the balance between sVEGFR2CSF and VEGF-ACSF levels might be crucial for the progression of CNSL. 
 Immunolocalizations of VEGF, its receptors flt-1, KDR and TGF-beta's in epithelial ovarian tumors. Angiogenesis is an essential factor for growth, differentiation, invasion and   metastasis   of tumors. In this study, we aimed to evaluate the immunolocalizations of vascular endothelial growth factor (VEGF), its receptors flt-1, KDR/flk-1, and transforming growth factor-beta's (TGF-beta) in epithelial ovarian tumors, utilizing indirect immunohistochemistry to understand the role of the angiogenic events in ovarian neoplasia.Tissue blocks from 40 patients who had ovarian pathology (borderline serous-mucinous tumor and malignant serous-mucinous adenocarcinoma of the ovary) were included in this study. All formalin-fixed, paraffin-embedded tissue sections were stained with hematoxylin-eosin or primary antibodies against VEGF, flt-1, KDR/flk-1, TGF-beta1, TGF-beta2 and TGF-beta3 using the avidin-biotin-peroxidase method. H-SCORE, a semi-quantitative grading system, was used to compare immunohistochemical staining intensities.Positive VEGF immunoreactivity was concentrated in the epithelial and stromal parts of all the ovarian samples and the endothelial cells in the stroma were also stained. Increased immunoreactivity of VEGF was observed in malignant ovarian adenocarcinomas compared to the borderline tumors of the ovary. VEGF receptors, flt-1 and KDR/flk-1 immunoreactivities were detected not only in vascular endothelial cells, but also in tumor cells at malignant sites. Immunoreactivities of VEGF and its receptors were coexpressed in tumor cells of the ovarian carcinoma. While immunoreactivities of TGF-beta1 and TGF-beta2 were both overexpressed in malignant ovarian carcinomas, immunoreactivity of TGF-beta3 was still mild.Our results suggest that overexpression of VEGF, its receptors flt-1, KDR/flk-1 and TGF-beta interaction may play an important role in the ovarian cancer biology, with potential effects on tumor growth and angiogenesis. New therapeutic strategies using VEGF and TGF-beta antagonists could obtain an additional approach to the treatment ovarian carcinoma by inhibiting angiogenesis. 
 Prognostic roles of cross-talk between peritumoral hepatocytes and stromal cells in hepatocellular carcinoma involving peritumoral VEGF-C, VEGFR-1 and VEGFR-3. Peritumoral liver tissue could play a potential role in hepatocellular carcinoma (HCC) progression and patient survival via angiogenesis- and lymphangiogensis-related factors. The prognostic role of these factors in hepatocytes and stromal cells in HCC patients after curative resection remains to be explored.Tumor tissue and surrounding peritumoral tissue were obtained from 145 resected HCC patients without lymph node   metastasis   (LNM) and 37 resected HCC patients with LNM. Tissue microarrays were constructed from duplicate cores of tumor tissue and surrounding peritumoral tissue from each resected specimen. Immunohistochemistry and real-time polymerase chain reaction were used to evaluate the expression of vascular endothelial growth factor-A (VEGF-A), VEGF-C, VEGF receptor-1(VEGFR-1), VEGFR-2, and VEGFR-3. Macrophage infiltration was determined by CD68 staining. Correlations between the expression of these factors and overall survival (OS) and time to recurrence (TTR) were studied.The peritumoral expression of VEGF-A, VEGF-C, VEGFR-1, VEGFR-2, and VEGFR-3 were significantly higher than expression of these factors in tumors. VEGFR-1 was mostly located in peritumoral macrophages, while VEGF-C and VEGFR-3 were mostly located in peritumoral hepatocytes. HCC with high peritumoral co-expression of VEGF-C, VEGFR-1, and VEGFR-3 was associated with higher peritumoral distribution of macrophages (0.87%±0.26% versus 0.45%±0.20%), LNM (32.4% versus 12.0%), shorter TTR (10.2 months versus 34.5 months), and poor prognosis (19.4 months versus 49.3 months).Expression of VEGF-C, VEGFR-1, and VEGFR-3 in peritumoral liver tissue is associated with a unique type of HCC that has a poorer outcome after hepatectomy. 
 FLT1 signaling in   metastasis  -associated macrophages activates an inflammatory signature that promotes breast cancer   metastasis  . Although the link between inflammation and cancer initiation is well established, its role in metastatic diseases, the primary cause of cancer deaths, has been poorly explored. Our previous studies identified a population of   metastasis  -associated macrophages (MAMs) recruited to the lung that promote tumor cell seeding and growth. Here we show that FMS-like tyrosine kinase 1 (Flt1, also known as VEGFR1) labels a subset of macrophages in human breast cancers that are significantly enriched in metastatic sites. In mouse models of breast cancer pulmonary   metastasis  , MAMs uniquely express FLT1. Using several genetic models, we show that macrophage FLT1 signaling is critical for   metastasis  . FLT1 inhibition does not affect MAM recruitment to metastatic lesions but regulates a set of inflammatory response genes, including colony-stimulating factor 1 (CSF1), a central regulator of macrophage biology. Using a gain-of-function approach, we show that CSF1-mediated autocrine signaling in MAMs is downstream of FLT1 and can restore the tumor-promoting activity of FLT1-inhibited MAMs. Thus, CSF1 is epistatic to FLT1, establishing a link between FLT1 and inflammatory responses within breast tumor metastases. Importantly, FLT1 inhibition reduces tumor metastatic efficiency even after initial seeding, suggesting that these pathways represent therapeutic targets in metastatic disease. 
 Lysophosphatidic acid upregulates vascular endothelial growth factor-C and tube formation in human endothelial cells through LPA(1/3), COX-2, and NF-kappaB activation- and EGFR transactivation-dependent mechanisms. Lysophosphatidic acid (LPA) is a lipid bioactive mediator which binds to G-protein-coupled receptors and activates a variety of cellular functions. LPA modulates multiple behaviors in endothelial cells, including cell proliferation and migration, capillary-like tube formation in vitro, activation of proteases, interactions with leukocytes, and expressions of inflammation-related genes, thereby regulating vessel formation. LPA has been reported to modulate the angiogenesis process. However, the role of LPA in the lymphangiogenesis process has not been studied. In this study, we showed that LPA upregulated vascular endothelial growth factor-C (VEGF-C) mRNA expression in human umbilical vein endothelial cells (HUVECs) and subsequent endothelial cell tube formation in vitro and in vivo. These enhancement effects were LPA(1)- and LPA(3)-dependent and required cyclooxygenase-2 (COX-2), endothelial growth factor receptor (EGFR) transactivation and activation of nuclear factor kappaB (NF-kappaB). Moreover, LPA induced the protein expressions of the lymphatic markers, Prox-1, LYVE-1, and podoplanin, in HUVECs, and these enhancement effects were dependent on LPA(1) and LPA(3) activation and EGFR transactivation. Our results demonstrated that LPA might regulate VEGF-C and lymphatic marker expression in endothelial cells, which contributes to endothelial cell tube formation in vitro and in vivo, thus facilitating endothelial cell participation in the lymphangiogenesis process. This study clarifies the signaling mechanism of LPA-regulated VEGF-C expression and lymphatic marker expressions in endothelial cells, which suggest that LPA may be a suitable target for generating therapeutics against lymphangiogenesis and tumor   metastasis  . 
 Expression of vascular endothelial growth factor and its receptors KDR and Flt-1 in gastric cancer cells. The expression of vascular endothelial growth factor (VEGF) and its receptors KDR and Flt-1 by gastric carcinoma tissues and different gastric carcinoma cell lines was detected to elucidate the molecular mechanism of this growth factor in promoting tumor growth.The expression of VEGF, Flt-1 and KDR was determined by reverse transcription-polymerase chain reaction (RT-PCR) in gastric cancer cell lines RF-1, RF-48, AGS-1, NCI-N87, NCI-SNU-1, NCI-SNU-5, NCI-SNU-16 and KATO-III. The expression of Flt-1 and KDR in paraffin-embedded specimens of gastric cancer was determined by immunohistochemistry. The 3-(4,5-dimethylthiazol-2-yl)-2, 5-diphenyltetrazolium bromide (MTT) assay was used to assess the role of VEGF in tumor cell proliferation.All 8 gastric cancer cell lines analyzed expressed VEGF(121) and VEGF(165) and six of them expressed both Flt-1 and KDR, while cell line NCI-SNU-5 expressed Flt-1 only and cell line KATOIII expressed neither Flt-1 nor KDR. The gastric carcinoma tissues expressed Flt-1 and KDR widely, with the positive rate of expression of Flt-1 and KDR being 84.6 % and 70 % respectively. The exogenous VEGF stimulated the growth of KDR-positive cell lines NCI-N87 and AGS-1 in a dose-dependent manner but exhibited no effect on the growth of KDR-negative cell line NCI-N87.VEGF and its receptors KDR and Flt-1 were expressed widely in gastric carcinoma cells and the VEGF stimulated KDR-positive tumor cell growth directly. These results suggest that VEGF may play a role in promoting tumor growth and   metastasis   by participating in both paracrine and autocrine pathways. 
 Vascular endothelial growth factor receptors 1,3 and caveolin-1 are implicated in colorectal cancer aggressiveness and prognosis--correlations with epidermal growth factor receptor, CD44v6, focal adhesion kinase, and c-Met. Vascular endothelial growth factor receptor-1 (VEGFR-1) and caveolin-1 have been shown to act both as tumor-promoting and tumor-suppressing proteins in various malignancies as well as in colorectal cancer (CRC), while VEGFR-3's lymphagiogenic involvement and connection to tumor parameters has yielded heterogenic results. This study was designed to investigate the expression of these molecules in 183 human CRC tissue specimens and explore their effect in both clinicopathological parameters and disease prognosis. We also utilize our previous results regarding epidermal growth factor receptor (EGFR), c-Met, CD44v6, and focal adhesion kinase, in an attempt to further clarify their distinct role in tumor prognosis and their crosstalk. Caveolin-1 was more freely distributed in the neoplasms of the right colon and restricted towards the left and the rectal cancer samples (p = 0.022); VEGFR-3 was associated with higher nodal   metastasis  ' status (p = 0.001) and staging (p = 0.006), and loss of VEGFR-1 predicted distant   metastasis   (p = 0.026) and advanced stage (p = 0.049). Prompted by previous reports, we performed all analyses also in the patient group of early (I and II) tumor stage where it was evident that VEGFR-1 was more frequently expressed in patients under 60 years old (p = 0.014) and VEGFR-3 was significantly elevated in left colon cancers (p = 0.039) and female patients (p = 0.038). Within the advanced stage (III and IV), the absence of VEGFR-1 exhibited a tendency for higher M status (p = 0.067) and lack of caveolin-1 signified worse AJCC classification (p = 0.053). Additionally, patient survival was influenced from VEGFR-3 (p = 0.019) for the whole sample, whereas subgroup analyses provided a correlation between caveolin-1 expression and improved survival in the early detection group of patients (p = 0.022). Using Cox regression for all available markers, FAK, CD44v6, and Caveolin-1 [corrected] emerged in this study as potential surrogate markers, the latter having positive prognostic significance. We further explored the multiple receptor correlations that were identified. 
 VEGFR1-positive haematopoietic bone marrow progenitors initiate the pre-metastatic niche. The cellular and molecular mechanisms by which a tumour cell undergoes   metastasis   to a predetermined location are largely unknown. Here we demonstrate that bone marrow-derived haematopoietic progenitor cells that express vascular endothelial growth factor receptor 1 (VEGFR1; also known as Flt1) home to tumour-specific pre-metastatic sites and form cellular clusters before the arrival of tumour cells. Preventing VEGFR1 function using antibodies or by the removal of VEGFR1(+) cells from the bone marrow of wild-type mice abrogates the formation of these pre-metastatic clusters and prevents tumour   metastasis  , whereas reconstitution with selected Id3 (inhibitor of differentiation 3)-competent VEGFR1+ cells establishes cluster formation and tumour   metastasis   in Id3 knockout mice. We also show that VEGFR1+ cells express VLA-4 (also known as integrin alpha4beta1), and that tumour-specific growth factors upregulate fibronectin--a VLA-4 ligand--in resident fibroblasts, providing a permissive niche for incoming tumour cells. Conditioned media obtained from distinct tumour types with unique patterns of metastatic spread redirected fibronectin expression and cluster formation, thereby transforming the metastatic profile. These findings demonstrate a requirement for VEGFR1+ haematopoietic progenitors in the regulation of   metastasis  , and suggest that expression patterns of fibronectin and VEGFR1+VLA-4+ clusters dictate organ-specific tumour spread. 
 Soluble vascular endothelial growth factor receptor-1 (sVEGFR-1) is decreased in lung cancer patients showing progression: a pilot study. Tumor growth and   metastasis   depend on angiogenesis, and the vascular endothelial growth factor (VEGF) is known to be one of the most important angiogenic factors although the knowledge about its receptors is limited. We, therefore, investigated the treatment-related changes both in the level of the soluble vascular endothelial growth factor receptor-1 (sVEGFR-1) in the serum by ELISA and the expression of VEGFR-1 in cancer tissues by immunohistochemistry. The serum levels were studied in 38 lung cancer patients, and 55 control subjects (21 benign disease and 34 healthy subjects) before the chemotherapy. The treatment-related changes in serum sVEGFR-1 were evaluated in 15 patients 24 and 48 hours after treatment. In addition to serum analysis, the tissue expressions were evaluated in 32 patients before treatment. The treatment-related changes in tissue VEGFR-1 expressions were evaluated in only 12 patients 24 hours after treatment. We observed no significant difference in terms of serum sVEGFR-1 levels between malignant and nonmalignant groups (p > 0.05). There were no significant differences in the levels of sVEGFR-1 before and after treatment (p > 0.05). However, there was a significant difference between sVEGFR-1 levels in the groups (regressive, stable, progressive) classified according to the response to therapy (p = 0.043). A significant difference also was present between the expression levels of tissue VEGFR-1 in the same groups (p = 0.037). As a conclusion, we suggest that prechemotherapy sVEGFR-1 can be helpful for prediction of long-term response to therapy, but it should be studied in larger groups to elucidate its benefit in clinics. 
 [Expression of vascular endothelial growth factor and its receptors in laryngeal carcinoma cell and its significance]. To study the expression of vascular endothelial growth factor (VEGF) and both the Flt-1 and KDR high affinity VEGF receptors in human laryngeal carcinoma cells and its significance.In this study, we investigated the expression of VEGF mRNA and both the Fit-1 mRNA and KDR mRNA high affinity VEGF receptors in human laryngeal carcinoma cells using techniques of semi-quantify RT-PCR.It was showed that the expression level of VEGF mRNA was significantly increased in human laryngeal carcinoma cells as compared with the normal cells, Hacat cells (P <0.05), and there was the high expression level of Flt-1 mRNA in the Hep-2 cells, but not KDR mRNA.These data raise the possibility that VEGF and its receptors many play key roles in the growth, invasion and   metastasis   of human laryngeal carcinoma cells through either a paracrine or an autocrine mechanism. 
 Vascular endothelial growth factor receptor-1 mRNA overexpression in peripheral blood as a useful prognostic marker in breast cancer. Identification of useful markers associated with poor prognosis in breast cancer patients is critically needed. We previously showed that expression of vascular endothelial growth factor receptor-1 mRNA in peripheral blood may be useful to predict distant   metastasis   in gastric cancer patients. However, expression of vascular endothelial growth factor receptor-1 mRNA in peripheral blood of breast cancer patients has not yet been studied.Real-time reverse transcriptase-PCR was used to analyze vascular endothelial growth factor receptor-1 mRNA expression status with respect to various clinical parameters in 515 patients with breast cancer and 25 controls.Expression of vascular endothelial growth factor receptor-1 mRNA in peripheral blood was higher in breast cancer patients than in controls. Increased vascular endothelial growth factor receptor-1 mRNA expression was associated with large tumor size, lymph node   metastasis   and clinical stage. Patients with high vascular endothelial growth factor receptor-1 mRNA expression also experienced a poorer survival rate than those with low expression levels, including those patients with triple-negative type and luminal-HER2(-) type disease.Expression of vascular endothelial growth factor receptor-1 mRNA in peripheral blood may be useful for prediction of poor prognosis in breast cancer, especially in patients with triple-negative type and luminal-HER2(-) type disease. 
 [Relationship between the expression of vascular endothelial growth factor, fms-like tyrosine kinase-1 and biological behavior in gastric carcinoma]. To investigate the relationship between the expression of vascular endothelial growth factor (VEGF) and fms-like tyrosine kinase (Flt-1) mRNA and tumor progression, microvessel density and survival time in gastric carcinoma.In situ hybridization and immunohistochemical techniques were used to detect the gene expression of VEGF, Flt-1 and CD34 in 118 gastric carcinoma specimens.In situ hybridization revealed that positive expression rates of VEGF and Flt-1 mRNA in gastric carcinoma were 54.24% and 55.9% respectively. There was a significant correlation between the expression of VEGF and Flt-1 mRNA and growth pattern, the depth of tumor invasion, vessel invasion, lymph node and distant   metastasis   (P < 0.01). The mean tumor microvessel densities (MVD) in patients of stage T3-T4 or those with vessel invasion, lymph node and distant metastases were significantly higher than those of stage T1-T2 and without metastases (P < 0.01). MVD value was correlated with the expression levels of VEGF and Flt-1 mRNA (P < 0.01). The mean survival time and survival rate of patients with positive mRNA expression and mean MVD value >or=54.9/mm2 were significantly lower than those of patients with negative mRNA expression and mean MVD value < 54.9/mm2.The expression of VEGF and Flt-1 can promote tumor angiogenesis and contribute to tumor invasion and   metastasis   in gastric carcinoma. VEGF and Flt-1 may serve as valuable indicators of biological behaviour, prognosis and target of gene therapy in gastric carcinoma. 
 Inhibition of prostate cancer osteoblastic progression with VEGF121/rGel, a single agent targeting osteoblasts, osteoclasts, and tumor neovasculature. A hallmark of prostate cancer (PCa) progression is the development of osteoblastic bone metastases, which respond poorly to available therapies. We previously reported that VEGF(121)/rGel targets osteoclast precursors and tumor neovasculature. Here we tested the hypothesis that targeting nontumor cells expressing these receptors can inhibit tumor progression in a clinically relevant model of osteoblastic PCa.Cells from MDA PCa 118b, a PCa xenograft obtained from a bone   metastasis   in a patient with castrate-resistant PCa, were injected into the femurs of mice. Osteoblastic progression was monitored following systemic administration of VEGF(121)/rGel.VEGF(121)/rGel was cytotoxic in vitro to osteoblast precursor cells. This cytotoxicity was specific as VEGF(121)/rGel internalization into osteoblasts was VEGF(121) receptor driven. Furthermore, VEGF(121)/rGel significantly inhibited PCa-induced bone formation in a mouse calvaria culture assay. In vivo, VEGF(121)/rGel significantly inhibited the osteoblastic progression of PCa cells in the femurs of nude mice. Microcomputed tomographic analysis revealed that VEGF(121)/rGel restored the bone volume fraction of tumor-bearing femurs to values similar to those of the contralateral (non-tumor-bearing) femurs. VEGF(121)/rGel significantly reduced the number of tumor-associated osteoclasts but did not change the numbers of peritumoral osteoblasts. Importantly, VEGF(121)/rGel-treated mice had significantly less tumor burden than control mice. Our results thus indicate that VEGF(121)/rGel inhibits osteoblastic tumor progression by targeting angiogenesis, osteoclastogenesis, and bone formation.Targeting VEGF receptor (VEGFR)-1- or VEGFR-2-expressing cells is effective in controlling the osteoblastic progression of PCa in bone. These findings provide the basis for an effective multitargeted approach for metastatic PCa. 
 Cell type- and tumor zone-specific expression of pVEGFR-1 and its ligands influence colon cancer   metastasis  . Detailed knowledge of the essential pro-angiogenic biomolecules, the vascular endothelial growth factor (VEGF) family and its receptors, in the characteristically heterogeneous tumor tissue is a pre-requisite for an effective personalized target therapy. The effects of VEGF receptors after ligand binding are mediated through receptor tyrosine autophosphorylation. We determined the relevance of the VEGFR-1 activating pathway for colon cancer (CC)   metastasis  .The expression profiles of VEGFR-1, phosphorylated (activated) VEGFR-1 (pVEGFR-1(Tyr1048), pVEGFR-1(Tyr1213) and pVEGFR-1(Tyr1333)) and the VEGFR-1 ligands (VEGF, PlGF and VEGF-B) were investigated using immunohistochemistry in different tumor compartments (intratumoral - invasive front - extratumoral), cell types (tumor cells - macro- (large and small vessels) and the microvasculature (capillaries) - inflammatory cells) in human sporadic non-metastatic, lymphogenous metastatic and haematogenous metastatic CC.VEGF and PlGF produced by tumor cells have an autocrine affinity for their receptor VEGFR-1. Subsequent PlGF-mediated receptor activation by autophosphorylation at Tyr1048 and Tyr1213 is a potential signaling pathway, which in turn seems to protect against distant   metastasis   and, in regions of tumor budding, additionally against lymph node   metastasis  . This autocrine link could be supported by possible formation of PlGF-VEGF heterodimers and PlGF-PlGF homodimers, which are known to have anti-metastatic properties. In contrast, in order to enhance their potential for distant   metastasis   tumor cells produce paracrine-acting VEGF-B. VEGFR-1 activation in tumor-associated macrovasculature but not capillaries appears to affect metastatic ability. Paracrine-mediated receptor autophosphorylation at Tyr1048 and Tyr1213 in small vessels located intratumorally and along the invasive front appears to be inversely correlated with   metastasis  , especially distant   metastasis  . Additionally, macrovessels are able to produce VEGFR-1 ligands, which influence the metastatic potential. Paracrine-acting VEGF-B production by intratumorally located small vessels and autocrine-acting PlGF production by extratumorally located small vessels seem to be associated with the non-metastatic phenotype. In contrast, VEGF-B-expressing extratumoral large and small vessels correlate with distant   metastasis  . Lymphocyte-associated VEGFR-1 expression in the invasive front without accompanying autophosphorylation could prevent against distant   metastasis   possibly by acting as a decoy and scavenger receptor.VEGFR-1 and its ligands participate in vascular, tumor cell-mediated and immuno-inflammatory processes in a complex biomolecule-dependent and tumor zone-specific manner and hence could influence metastatic behavior in CC. 
 Circulating serum levels of angiogenic factors and vascular endothelial growth factor receptors 1 and 2 in melanoma patients. Angiogenesis is essential for tumor progression and   metastasis  ; however, the angiogenesis regulators that are biologically relevant for melanoma are still unknown. In this study, we analyzed the circulating serum levels of potent angiogenic factors, including vascular endothelial growth factor (VEGF), angiogenin, transforming growth factor-beta1 and VEGF receptors, VEGFR1 and VEGFR2, in human melanoma patients. One hundred and fourteen patients with histopathologically verified cutaneous melanoma at different stages and 30 healthy controls were investigated. Serum levels of angiogenic factors and VEGF receptors were quantitatively analyzed by solid-phase enzyme-linked immunosorbent assay. The age of the patients (61 men and 53 women) ranged from 18 to 80 years; median age was 51 years. Serum transforming growth factor-beta1 (P < 0.001), VEGF (P = 0.006) and VEGFR1 (P = 0.007) levels were significantly higher in patients with melanoma than in the control group. No significant differences, however, exist in the serum angiogenin and VEGFR2 levels between melanoma patients and the controls. The positive correlations of elevated serum levels of transforming growth factor-beta1, VEGF and VEGFR1 with advanced stages of disease were found. Significant relationship was found only between serum levels of VEGF and VEGFR2. Elevated serum transforming growth factor-beta1 (P < 0.001) and VEGF levels (P = 0.0012) were found to be poor prognostic factors. Serum level of angiogenin and VEGF receptors, however, had no effect on survival. Our data suggest that the angiogenic serum factors, including VEGF, transforming growth factor-beta1 and VEGFR1, but not angiogenin and VEGFR2 were increased in melanoma patients, especially associated with advanced disease stages. The mechanism of VEGF regulation of angiogenesis may in part be due to enhanced proliferation of VEGFRs, especially VEGFR1. 
 Functional expression and localization of vascular endothelial growth factor and its receptors in the human epididymis. Blood supply is essential for the maintenance of epididymal function. Since there is no considerable neovascularization in the epididymis, this tissue could represent a suitable model to study the vascular endothelial growth factor (VEGF) effect for vascular permeability. We studied the expression and function of VEGF and its receptors fms-like tyrosine kinase (Flt-1) and fetal liver kinase (designated as kinase insert domain-containing receptor, KDR in the human) in the human epididymis. VEGF and VEGF receptors mRNA were detected in the human epididymal tissue. VEGF protein was localized in peritubular and in ciliated cells of efferent ducts as well as in peritubular and basal cells of the epididymal duct. Vascular endothelial cells did not express VEGF. Flt-1 protein was localized in ciliated cells of efferent ducts and in lymphatic vessels. Vascular endothelial cells were negative for Flt-1 but positive for KDR. In vitro VEGF165 treatment of epididymal tissue induced endothelial fenestrations and opening of interendothelial junctions. Additionally, we observed for the first time that VEGF could induce transendothelial gaps. We conclude that these gaps might be of importance not only for molecular transport but also for cell passage across the vessel wall, which may be significant for tumor   metastasis  . VEGF may act as a paracrine effector to influence the permeability of lymphatic vessels via Flt-1, and of blood vessels via KDR. 
 Measurement of circulating levels of VEGF-A, -C, and -D and their receptors, VEGFR-1 and -2 in gastric adenocarcinoma. To analyze the serum levels and prognostic significance of vascular endothelial growth factor (VEGF) -A, -C, and -D, and their receptors, VEGFR-1 and -2 in gastric adenocarcinomas.The serum levels of VEGF family members were measured in 76 control subjects and 76 patients with gastric adenocarcinoma using an enzyme-linked immunosorbent assay (ELISA). These measurements were correlated with clinco-pathological features and survival rates.The serum levels of VEGF-A and its receptor, VEGFR-1, were significantly higher in patients with gastric cancer than in healthy donors (t = 2.3, P = 0.02 and t = 4.2, P < 0.0001, respectively). In contrast, the serum levels of VEGF-D were significantly higher in control subjects than in patients (t = 2.9, P = 0.004). There was no significant difference in serum levels of VEGF-C and VEGFR-2 between patients and controls. VEGF-C was associated with advanced tumor stage and presence of   metastasis  . VEGFR-1 was associated with   metastasis  , advanced overall stage, tumor differentiation and survival. VEGFR-2 levels were associated with poor tumor differentiation. There was no significant prognostic value for any of the VEGF family members or their receptors except for VEGFR-1 where high levels were associated with a poor overall survival.Serum VEGF levels vary significantly in the same cohort of patients with variable clinico-pathological features and prognostic values. The simultaneous measurement of VEGF receptors levels in sera may overcome the limitations of a single biomarker assay. 
 Vascular endothelial growth factor receptor 1 expression in pelvic lymph nodes predicts the risk of cancer progression after radical prostatectomy. Recent studies suggest that vascular endothelial growth factor receptor (VEGFR) 1-positive hematopoietic progenitor cells precede the arrival of tumor cells and form clusters that may portend sites of future metastatic disease. The aim of the present study was to clarify whether VEGFR1 expression in pelvic lymph nodes predicts the risk of prostate cancer progression after radical prostatectomy. VEGFR1 expression in pelvic lymph nodes was examined by immunohistochemistry in 95 patients who underwent radical prostatectomy for prostate cancer. A cluster of VEGFR1-positive cells was considered positive. Expression of VEGFR1 in pelvic lymph nodes and biochemical recurrence after radical prostatectomy were examined by univariate survival analysis and multivariate Cox proportional hazards regression analysis. Thirty-seven of 79 lymph node-negative patients (46.8%) were found to have VEGFR1-positive cells in their pelvic lymph nodes, whereas 16 of 16 lymph node   metastasis  -positive patients (100%) had VEGFR1 clusters. There was a significant correlation between pathological stage and VEGFR1 staining (P = 0.002). Univariate analysis showed that pathological stage > or = pT3 and VEGFR1 expression in pelvic lymph nodes were each significantly associated with biochemical recurrence after radical prostatectomy. Multivariate analysis showed VEGFR1 expression to be an independent predictor of biochemical recurrence after radical prostatectomy (risk ratio = 5.715, P = 0.010), as was preoperative prostate-specific antigen (PSA) level > or = 10 ng/mL. Although larger validation studies are required, our results suggest that VEGFR1 expression in pelvic lymph nodes predicts the risk of biochemical PSA recurrence after radical prostatectomy. 
 Overproduction of VEGF concomitantly expressed with its receptors promotes growth and survival of melanoma cells through MAPK and PI3K signaling. Vascular endothelial growth factor (VEGF) is an important mediator of tumor-associated angiogenesis, and consequently it has been associated with   metastasis  . We report here that the overexpression of VEGF(165) in melanoma xenografts promotes an acceleration of tumor growth and an increase in angiogenesis as well as the spontaneous   metastasis   formation. In addition, VEGF receptors (VEGFR)1, VEGFR2 and neurophilin-1 are expressed in A375 melanoma cells. Forced overexpression of VEGF in these cells induces cell growth and triggers survival activity in serum-starved cultures, by a mechanism dependent on the mitogen-activating protein kinase signaling pathway. Furthermore, these effects are dependent MEK 1/2 activity. Kinase domain region-specific tyrosine kinase inhibitors dramatically reduced DNA synthesis to 20% with respect to the controls, although they did not completely suppress either the p44 or p42-phosphorylated forms of extracellular signal-regulated protein kinase. These inhibitors also provoked a decrease in Akt phosphorylation. We observed a dramatic reduction in survival after treatment with phosphatidylinositol 3'-kinase (PI3K)-specific inhibitor in the presence of specific tyrosinase inhibitors. We suggest that the overproduction of VEGF(165) concomitantly expressed with its receptors favors cell growth and survival of melanoma cells through MAPK and PI3K signaling pathways. These data support the involvement in melanoma growth and survival of a VEGF-dependent internal autocrine loop mechanism, at least in vitro. 
 Signals from Eph and ephrin proteins: a developmental tool kit. Interactions between Eph receptors and their ligands the ephrin proteins are critically important in many key developmental processes. Emerging evidence also supports a role for these molecules in postembryonic tissues, particularly in pathological processes, including tissue injury and tumor   metastasis  . We review the signaling mechanisms that allow the 14 Eph and nine ephrin proteins to deliver intracellular signals that regulate cell shape and movement. What emerges is that the initiation of these signals is critically dependent on which Eph and ephrin proteins are expressed, the level of their expression, and, in some cases, which splice variants are expressed. Diversity at the level of initial interaction and in the downstream signaling processes regulated by Eph-ephrin signaling provides a subtle, versatile system of regulation of intercellular adhesion, cell shape, and cell motility. 
 Expression of VEGF and its receptors VEGFR1/VEGFR2 is associated with invasiveness of bladder cancer. Vascular endothelial growth factor (VEGF) signaling is frequently altered in invasive tumor cells and is associated with patient outcome. In the present study, we examined VEGF, VEGFR1, and VEGFR2 expression in non-muscle invasive bladder cancer (NMIBC) and muscle invasive bladder cancer (MIBC), and evaluated the association between VEGF and its receptors with disease characteristics and bladder cancer recurrence.Tissue microarrays containing bladder cancer specimens (n=212) and adjacent normal bladder mucosa (n=131) were immunostained using antibodies against VEGF, VEGFR1, and VEGFR2. The association between the expression of these proteins and clinical parameters including stage, lymph node   metastasis  , and recurrence-free survival were statistically evaluated. VEGF mRNA expression data were extracted from the public Oncomine database.VEGF and VEGFR1 mRNA levels were significantly higher in bladder cancer specimens than that of normal mucosa (for VEGF, p<0.001; for VEGFR1, p=0.02). Analysis of their expression at protein levels showed that levels of VEGF and VEGFR1 were significantly higher in NMIBC than in MIBC (p<0.001), while that of VEGFR2 was significantly higher in all cancer specimens compared to benign urothelial mucosa (p=0.001). Further-more, the expression of VEGFR2 was significantly higher in MIBC, as compared to NMIBC (p<0.001). Patients with higher levels of VEGF, VEGFR1, and VEGFR2 tended to have poorer recurrence-free survival than those with lower levels, but this was not statistically significant.Our results suggest that alterations in the expression of VEGF and VEGF receptors are associated with disease stage and recurrence. 
 VEGFR1 expression is related to lymph node   metastasis   and serum VEGF may be a marker of progression in the follow-up of patients with differentiated thyroid carcinoma. Vascular endothelial growth factor (VEGF) and VEGF receptor 1 (VEGFR1) are known to be related to thyroid tumorigenesis. The aim of the study was to examine the expressions and serum levels of VEGF, VEGFR1, IGF1, and IGF1 receptor (IGF1R) in patients with differentiated thyroid carcinoma (DTC) compared with patients with nodular goiter (NG).We examined 39 patients with DTC who had a clinical history of at least 2 years and compared them with 25 patients who had a pathological diagnosis of NG after thyroidectomy. Serum VEGF, VEGFR1, and IGF1 levels were measured in both patient groups. The expressions of VEGF, VEGFR1, IGF1, and IGF1R were analyzed by the immunohistochemical method in the paraffin blocks of patients' thyroidectomy samples of the patients.The immunostainings scores for VEGF, VEGFR1, IGF1, and IGF1R were found to be higher in patients with DTC than in those with NG. Only VEGFR1 expression was related to lymph node   metastasis   at the time of surgery. None of the expressions were related to the long-term prognosis of the patients. Serum VEGF was found to be higher in patients with progressive DTC than in patients in clinical remission.The expressions of VEGF and VEGFR1 were shown to be correlated with the expression of IGF1 and IGF1R. VEGFR1 expression may be an important index for the presence of lymph node   metastasis   at the time of thyroidectomy. Increased serum levels of VEGF may reflect disease recurrence in DTC. 
 [Bone marrow-derived cells contribute to niche formation in cancer progression]. Bone marrow-derived cells, which are recruited to peripheral tissues, have been shown to play a major role as angiogenic factor supplier. Moreover, bone marrow-derived "hemangiocytes" through graded chemokines production, can function as the molecular hub for hematopoietic cytokine-induced neovascularization. Current focus on cancer   metastasis   has centered on the intrinsic factors regulating the cell autonomous homing of the tumor cells to the metastatic site : Bone marrow-derived cells expressing vascular endothelial growth factor receptor 1 can be mobilized into the circulation due to growth factors released by the primary tumor. These cells cluster at distant sites, "the premetastatic niche" , where they set up a microenvironment ideal for tumor cells to seed, thereby completing the metastatic spread. Focus on the early cellular and molecular events in cancer infiltration,   metastasis   and proliferation will likely lead to new approaches to detect and prevent   metastasis   at its earliest inception. 
 Increased expression of vascular endothelial growth factor and its receptors, VEGFR-1 and VEGFR-2, in medullary thyroid carcinoma. Vascular endothelial growth factor (VEGF-A) expression is upregulated in the majority of human tumors, where it stimulates proliferation, migration, and survival of endothelial cells. Studies have suggested that VEGF inhibitors can be used as an alternative therapy in medullary thyroid carcinoma (MTC), but data about expression of VEGF-A and its receptor in this tumor are scarce. The aims of this study were to evaluate VEGF-A, VEGF receptor (VEGFR)-1, VEGFR-2, and microvessel density (MVD) expression in MTC samples and correlate it with clinical parameters.Paraffin-embedded samples from 38 MTC patients were evaluated for VEGF-A, VEGFR-1, VEGFR-2, and MVD expression by immunohistochemistry. Clinical data were retrospectively reviewed in medical records.Thirty-eight patients aged 31.8 +/- 17.1 years were enrolled. Twenty-seven patients had hereditary disease (71.1%). Twenty-five of them were found to have multiple endocrine neoplasia (MEN) 2A and two were found to have MEN 2B. VEGF-A immunohistochemical staining was detected in 95% (36/38), VEGFR-1 in 96% (36/37), and VEGFR-2 in 91% (31/34) of MTC samples. Age at surgery was positively correlated with VEGFR-2 (p = 0.003). There was no correlation between VEGF-A, VEGFR-2, and tumor stage (tumor node   metastasis  ). Nevertheless, VEGFR-1 was found to be inversely correlated with tumor node   metastasis   (p = 0.034). We also observed a trend toward an association between VEGFR-1 signal intensity and cure of disease, although this did not reach statistical significance (p = 0.054). Neither VEGF-A nor VEGFR-2 was associated with disease outcome after a median follow-up period of 5 years (p = 0.882 and p = 0.236, respectively). As expected, MVD was correlated with age at surgery (p = 0.005) and tumor size (p = 0.03). Patients with the hereditary form of the disease had a stronger intensity for VEGFR-1 (p = 0.039), whereas patients with sporadic disease displayed higher MVD counts (44 [27-63] vs. 21 [9-49], p = 0.018).The VEGF-A, VEGFR-1, and VEGFR-2 immunoreactive proteins are overexpressed in MTC lesions and might be implicated in tumor progression. It is not clear, however, if expression of these molecules provides prognostic information regarding the spread or outcome of MTC. 
 Functional significance of vascular endothelial growth factor and its receptor (receptor-1) in various lung cancer types. VEGF is one of the key factors in tumor angiogenesis that may be involved in tumor growth and   metastasis  . VEGF receptor, a naturally occurring soluble form of the VEGFR-1 (sVEGFR-1. flt-1), is produced by endothelial cells by differential splicing of the flt-1 gene, and is a negative counterpart of the VEGF signaling pathway.We investigated the levels of VEGF and sVEGFR-1, a known intrinsic inhibitor of VEGF, in 42 patients with various types of lung cancers before beginning treatment and 18 healthy subjects.Serum sVEGFR-1 levels (mean +/- SD; pg/ml) were 465.17 +/- 158.34 in patients and were significantly higher than those of the healthy subjects (156.39 +/- 89.17) (P < 0.0001). Serum VEGF levels of patients (449.48 +/- 175.54 pg/ml) were significantly higher in patients than in healthy subjects (77.06 +/- 47.26 pg/ml) (P < 0.0001).We conclude that increased sVEGFR-1 and VEGF levels are important parameters in lung cancers. 
 Differential expression of VEGF ligands and receptors in prostate cancer. Prostate cancer disseminates to regional lymph nodes, however the molecular mechanisms responsible for lymph node   metastasis   are poorly understood. The vascular endothelial growth factor (VEGF) ligand and receptor family have been implicated in the growth and spread of prostate cancer via activation of the blood vasculature and lymphatic systems. The purpose of this study was to comprehensively examine the expression pattern of VEGF ligands and receptors in the glandular epithelium, stroma, lymphatic vasculature and blood vessels in prostate cancer.The localization of VEGF-A, VEGF-C, VEGF-D, VEGF receptor (VEGFR)-1, VEGFR-2, and VEGFR-3 was examined in cancerous and adjacent benign prostate tissue from 52 subjects representing various grades of prostate cancer.Except for VEGFR-2, extensive staining was observed for all ligands and receptors in the prostate specimens. In epithelial cells, VEGF-A and VEGFR-1 expression was higher in tumor tissue compared to benign tissue. VEGF-D and VEGFR-3 expression was significantly higher in benign tissue compared to tumor in the stroma and the endothelium of lymphatic and blood vessels. In addition, the frequency of lymphatic vessels, but not blood vessels, was lower in tumor tissue compared with benign tissue.These results suggest that activation of VEGFR-1 by VEGF-A within the carcinoma, and activation of lymphatic endothelial cell VEGFR-3 by VEGF-D within the adjacent benign stroma may be important signaling mechanisms involved in the progression and subsequent metastatic spread of prostate cancer. Thus inhibition of these pathways may contribute to therapeutic strategies for the management of prostate cancer. 
 miR-200 Inhibits lung adenocarcinoma cell invasion and   metastasis   by targeting Flt1/VEGFR1. The microRNA-200 (miR-200) family is part of a gene expression signature that predicts poor prognosis in lung cancer patients. In a mouse model of K-ras/p53-mutant lung adenocarcinoma, miR-200 levels are suppressed in   metastasis  -prone tumor cells, and forced miR-200 expression inhibits tumor growth and   metastasis  , but the miR-200 target genes that drive lung tumorigenesis have not been fully elucidated. Here, we scanned the genome for putative miR-200 binding sites and found them in the 3'-untranslated region (3'-UTR) of 35 genes that are amplified in human cancer. Mining of a database of resected human lung adenocarcinomas revealed that the levels of one of these genes, Flt1/VEGFR1, correlate inversely with duration of survival. Forced miR-200 expression suppressed Flt1 levels in   metastasis  -prone lung adenocarcinoma cells derived from K-ras/p53-mutant mice, and negatively regulated the Flt1 3'-UTR in reporter assays. Cancer-associated fibroblasts (CAFs) isolated from murine lung adenocarcinomas secreted abundant VEGF and enhanced tumor cell invasion in coculture studies. CAF-induced tumor cell invasion was abrogated by VEGF neutralization or Flt1 knockdown in tumor cells. Flt1 knockdown decreased the growth and   metastasis   of tumor cells in syngeneic mice. We conclude that miR-200 suppresses lung tumorigenesis by targeting Flt1. 
 Hematogenous   metastasis   in gastric cancer requires isolated tumor cells and expression of vascular endothelial growth factor receptor-1. Recent studies of cancer   metastasis   have focused on the role of premetastatic gene expression and circulating tumor cells. We did a blind prospective study in gastric cancer to assess the significance of isolated tumor cells (ITC) and to test the hypothesis that vascular endothelial growth factor receptor-1 (VEGFR-1) is expressed within the bone marrow at tumor-specific, premetastatic sites.Both bone marrow and peripheral blood samples from 810 gastric cancer patients were collected at the Central Hospital, National Cancer Center (Tokyo, Japan). The samples were transferred to Kyushu University Hospital (Beppu, Japan) where they were analyzed by quantitative real-time reverse transcription-PCR for three epithelial cell markers, carcinoembryonic antigen, cytokeratin-19, and cytokeratin-7, as well as VEGFR-1.ITCs were observed in peripheral blood and bone marrow even in early stages of gastric cancer. The frequency of ITC in bone marrow was significantly associated with the stage of disease by ANOVA (P < 0.01). Gastric cancer metastasized when ITCs were observed in the presence of VEGFR-1. In the 380 patients who were ITC negative and showed low VEGFR-1 expression, synchronous (at the time of surgery) and heterochronous (recurrent) metastases were not observed.ITCs circulate even in early stages of disease. Furthermore, elevated expression of VEGFR-1 facilitates the establishment of hematogenous metastases in gastric cancer. This study indicates that the simultaneous presence of ITC and VEGFR-1 expression at premetastatic sites is clinically significant for disease progression. 
 Vegf, Vegf-B, Vegf-C and their receptors KDR, FLT-1 and FLT-4 during the neoplastic progression of human colonic mucosa. Because the crucial role of angiogenesis has been demonstrated in tumor growth and   metastasis  , the present study was undertaken to characterize the relative expression of vascular endothelial growth factors VEGF (vascular endothelial growth factor), VEGF-B, VEGF-C, and their receptors KDR (kinase insert domain-containing receptor), FLT-1 (fms-like tyrosine kinase), and FLT-4 in human colonic cancers, in relation to the Astler-Coller pathological classification, and to prognosis. VEGF and VEGF-B gene expression was quantified by Northern blot in 72 tumor samples matched with control tissues. VEGF gene expression was 1.4 times higher in adenocarcinomas than in control tissues (p = 0.02), but did not increase further between Astler-Coller tumor stages A and D, and did not correlate with disease recurrence for patients at stages B2 or C. In adenomas, VEGF mRNA levels were not significantly different from those in the paired control colonic mucosa. The expression pattern of VEGF isoforms, mainly identified by RT-PCR (reverse-transcriptase-coupled polymerase chain reaction) as VEGF121 and VEGF165 and to a lesser extent VEGF189, was comparable in tumor and control tissues. VEGF-B mRNA levels were unchanged during the neoplastic progression of colonic mucosa. In contrast to KDR and FLT-4, the expression of VEGF-C and FLT-1 genes increased in some pathological tissues. These results provide evidence that the early and sustained increase in VEGF transcripts and the expression of multiple angiogenic factors and receptors contribute to the development of colon cancer, and thus constitute a putative target for anti-angiogenic drug therapy. 
 Thrombospondin-1 is part of a Slug-independent motility and metastatic program in cutaneous melanoma, in association with VEGFR-1 and FGF-2. Differently from most transformed cells, cutaneous melanoma expresses the pleiotropic factor thrombospondin-1 (TSP-1). Herein, we show that TSP-1 (RNA and protein), undetectable in four cultures of melanocytes and a RGP melanoma, was variously present in 13 cell lines from advanced melanomas or metastases. Moreover, microarray analysis of 55 human lesions showed higher TSP-1 expression in primary melanomas and metastases than in common and dysplastic nevi. In a functional enrichment analysis, the expression of TSP-1 correlated with motility-related genes. Accordingly, TSP-1 production was associated with melanoma cell motility in vitro and lung colonization potential in vivo. VEGF/VEGFR-1 and FGF-2, involved in melanoma progression, regulated TSP-1 production. These factors were coexpressed with TSP-1 and correlated negatively with Slug (SNAI2), a cell migration master gene implicated in melanoma   metastasis  . We conclude that TSP-1 cooperates with FGF-2 and VEGF/VEGFR-1 in determining melanoma invasion and   metastasis  , as part of a Slug-independent motility program. 
 Prognostic significance of VEGFR1/Flt-1 immunoexpression in colorectal carcinoma. Colorectal carcinoma (CRC) is a major cause of morbidity and mortality. Vascular endothelial growth factor 1/Fms-like tyrosine kinase 1 (VEGFR1/Flt-1) regulates monocyte migration, recruits endothelial cell progenitors, increases the adhesive properties of natural killer cells and induces of growth factors. Flt-1 is expressed on tumour cells and has been implicated in tumour growth and progression. The objective of this study is to address the relation of Flt-1 expression to tumour prognostication. Paraffin blocks from 143 primary CRC and 48 regional nodal metastases were retrieved from the archives of the Department of Pathology at King Abdulaziz University. Tissue microarrays were designed and constructed. Immunohistochemistry for Flt-1 was performed. Staining intensity and extent of staining were assessed and combined. Results were dichotomised as low expression and high expression. Flt-1 was overexpressed in primary tumours and nodal   metastasis   (p < 0.001 and 0.001) with no difference between primary and no  dal metast  asis (p = 0.690). Flt-1 immunoexpression was not associated with the clinicopathological parameters. Flt-1 overexpression was an independent predictor of positive margin status, positive lymphovascular invasion and local disease recurrence (p < 0.001, p < 0.001 and p = 0.003, respectively). Flt-1 was not associated with survival (log-rank = 0.003, p = 0.959). Flt-1 was overexpressed in primary CRC and their nodal metastases. Flt-1 expression was an independent predictor of margin status, lymphovascular invasion and local disease recurrence. Therefore, expression profiling of Flt-1 seems to have a prognostic potential in CRC. However, to elucidate the association of overexpression of Flt-1 with tumour characteristics and prognostication, more in vivo and in vitro molecular investigations are recommended. 
  GeneRIFs  
 High expression levels of VEGFR1 and VEGFR3 were associated with a higher rate of lung   metastasis  . 
 TSP-1 cooperates with FGF-2 and VEGF/VEGFR-1 in determining melanoma invasion and   metastasis  , as part of a Slug-independent motility program. 
 VEGF and its receptors many play key roles in the growth, invasion and   metastasis   of human laryngeal carcinoma cells. 
 In differentiated thyroid carcinoma, VEGFR1 expression may be an important index for the presence of lymph node   metastasis   at the time of thyroidectomy. 
 VEGFR1 expression levels appeared to be positively correlated with the potentialities of   metastasis   of the hepatocellular carcinoma cell lines. 
 The expression of VEGF and Flt-1 can promote tumor angiogenesis and contribute to tumor invasion and   metastasis   in gastric carcinoma. 
 findings demonstrate a requirement for VEGFR1+ haematopoietic progenitors in the regulation of   metastasis  , and suggest that expression patterns of fibronectin and VEGFR1+VLA-4+ clusters dictate organ-specific tumour spread 
  [Cc]ell [Ii]nvasion  PubMed  
 VEGFR-1 activation-induced MMP-9-dependent invasion in hepatocellular carcinoma. VEGFR-1 can promote invasion through epithelial-mesenchymal transition induction in hepatocellular carcinoma (HCC). This study aims to elucidate VEGFR-1 impact on proteolytic enzymes profile involved with invasion.The effect on   cell invasion   was evaluated by invasive and migration assays with and without VEGFR-1 activation. The mechanism was investigated by real-time PCR, western blot and gelatin zymography using inhibitors for MMP-9. In total, 95 HCC patients were enrolled for its clinical value evaluation.VEGFR-1 activation induced invasion in HCC cells with an increase in the expression and activity of MMP-9 and Snail. MMP-9 blockage effectively inhibited VEGFR-1-induced invasion. High coexpression of both in HCC predicted a worse clinical outcome.Data show a novel VEGFR-1 activation-to-MMP-9 mechanism promoting HCC invasion. 
 GM-CSF induces expression of soluble VEGF receptor-1 from human monocytes and inhibits angiogenesis in mice. GM-CSF promotes homeostasis of myeloid cells. We report that GM-CSF upregulates mRNA and protein production of the soluble form of membrane bound VEGF receptor-1 (sVEGFR-1) in human monocytes. This sVEGFR-1 was biologically active, as cell-free supernatants from GM-CSF-stimulated monocytes blocked detection of endogenously expressed VEGF and inhibited endothelial cell migration and tube formation, even in the presence of exogenous rhVEGF. VEGF activity was recovered by neutralizing sVEGFR-1. To determine whether these events were important in vivo, Matrigel plugs were incubated with rhVEGF, rhGM-CSF, or rhGM-CSF/rhVEGF and injected into mice. Plugs containing GM-CSF or GM-CSF/VEGF had less endothelial   cell invasion   than plugs containing rhVEGF and were similar to plugs incubated with PBS alone. Neutralizing antibodies specific for sVEGFR-1 injected in these plugs reversed the effects of GM-CSF or GM-CSF/VEGF, while an isogenic antibody did not. Thus, GM-CSF and monocytes play a vital role in angiogenesis through the regulation of VEGF and sVEGFR-1. 
 A novel intracellular isoform of VEGFR-1 activates Src and promotes   cell invasion   in MDA-MB-231 breast cancer cells. Two types of VEGFR-1 receptors have been characterized: a full-length transmembrane receptor and a truncated extracellular soluble isoform (sVEGFR-1). We report here the characterization, in normal and cancer cells, of a new family of intracellular isoforms of VEGFR-1 resulting from alternative initiation of transcription in intronic sequences of the gene. While the classical isoforms of VEGFR-1 were barely detectable in MDA-MB-231 breast cancer cells, one of the intracellular isoforms transcribed from intron 21 (i(21)VEGFR-1) was the main isoform expressed in these cells. The new transcript encodes for a protein that contains only the phosphotransferase domain and the carboxyterminal tail of VEGFR-1. Treatment of MDA-MB-231 cells with siRNA specific for the tyrosine domain of VEGFR-1 suppressed the expression of i(21)VEGFR-1, downregulated phosphorylation of Src at tyrosine 418, and reduced markedly the invasion capacity of these cells in vitro. Accordingly, overexpression of transfected i(21)VEGFR-1 in MDA-MB-231 cells upregulated the active form of Src and increased invasiveness of MDA-MB-231 cells. The expression of i(21)VEGFR-1 in MDA-MB-231 cells was inhibited by retinoic acid. Both, activation of Src and downregulation by retinoic acid, have been reported in other intracellular members of the Fms/Kit/PDGFR family of tyrosine kinases, particularly in the intracellular isoform of c-kit, analogous structurally to i(21)VEGFR-1 and frequently expressed in cancer cells. 
 miR-200 Inhibits lung adenocarcinoma   cell invasion   and metastasis by targeting Flt1/VEGFR1. The microRNA-200 (miR-200) family is part of a gene expression signature that predicts poor prognosis in lung cancer patients. In a mouse model of K-ras/p53-mutant lung adenocarcinoma, miR-200 levels are suppressed in metastasis-prone tumor cells, and forced miR-200 expression inhibits tumor growth and metastasis, but the miR-200 target genes that drive lung tumorigenesis have not been fully elucidated. Here, we scanned the genome for putative miR-200 binding sites and found them in the 3'-untranslated region (3'-UTR) of 35 genes that are amplified in human cancer. Mining of a database of resected human lung adenocarcinomas revealed that the levels of one of these genes, Flt1/VEGFR1, correlate inversely with duration of survival. Forced miR-200 expression suppressed Flt1 levels in metastasis-prone lung adenocarcinoma cells derived from K-ras/p53-mutant mice, and negatively regulated the Flt1 3'-UTR in reporter assays. Cancer-associated fibroblasts (CAFs) isolated from murine lung adenocarcinomas secreted abundant VEGF and enhanced tumor   cell invasion   in coculture studies. CAF-induced tumor   cell invasion   was abrogated by VEGF neutralization or Flt1 knockdown in tumor cells. Flt1 knockdown decreased the growth and metastasis of tumor cells in syngeneic mice. We conclude that miR-200 suppresses lung tumorigenesis by targeting Flt1. 
   
  
 VAMP2 
 
  NCBI Gene  
  GeneRIF  
  Pubmed  
 
 
  Search Term  Rank  Fields  
  [Mm]igration  6  PubMed(2); GeneRIFs(1)  
  ECM  7  PubMed(2)  
  [Ee]xtracellular [Mm]atrix  8  PubMed(2)  
 
 
 hide/show details for VAMP2 
  [Mm]igration  PubMed  
 Vesicle-associated membrane protein 2 mediates trafficking of alpha5beta1 integrin to the plasma membrane. Integrins are major receptors for cell adhesion to the extracellular matrix (ECM). As transmembrane proteins, the levels of integrins at the plasma membrane or the cell surface are ultimately determined by the balance between two vesicle trafficking events: endocytosis of integrins at the plasma membrane and exocytosis of the vesicles that transport integrins. Here, we report that vesicle-associated membrane protein 2 (VAMP2), a SNARE protein that mediates vesicle fusion with the plasma membrane, is involved in the trafficking of alpha5beta1 integrin. VAMP2 was present on vesicles containing endocytosed beta1 integrin. Small interfering RNA (siRNA) silencing of VAMP2 markedly reduced cell surface alpha5beta1 and inhibited cell adhesion and chemotactic   migration   to fibronectin, the ECM ligand of alpha5beta1, without altering cell surface expression of alpha2beta1 integrin or alpha3beta1 integrin. By contrast, silencing of VAMP8, another SNARE protein, had no effect on cell surface expression of the integrins or cell adhesion to fibronectin. In addition, VAMP2-mediated trafficking is involved in cell adhesion to collagen but not to laminin. Consistent with disruption of integrin functions in cell proliferation and survival, VAMP2 silencing diminished proliferation and triggered apoptosis. Collectively, these data indicate that VAMP2 mediates the trafficking of alpha5beta1 integrin to the plasma membrane and VAMP2-dependent integrin trafficking is critical in cell adhesion,   migration   and survival. 
 Vesicle-associated membrane protein 2 mediates trafficking of alpha5beta1 integrin to the plasma membrane. Integrins are major receptors for cell adhesion to the extracellular matrix (ECM). As transmembrane proteins, the levels of integrins at the plasma membrane or the cell surface are ultimately determined by the balance between two vesicle trafficking events: endocytosis of integrins at the plasma membrane and exocytosis of the vesicles that transport integrins. Here, we report that vesicle-associated membrane protein 2 (VAMP2), a SNARE protein that mediates vesicle fusion with the plasma membrane, is involved in the trafficking of alpha5beta1 integrin. VAMP2 was present on vesicles containing endocytosed beta1 integrin. Small interfering RNA (siRNA) silencing of VAMP2 markedly reduced cell surface alpha5beta1 and inhibited cell adhesion and chemotactic   migration   to fibronectin, the ECM ligand of alpha5beta1, without altering cell surface expression of alpha2beta1 integrin or alpha3beta1 integrin. By contrast, silencing of VAMP8, another SNARE protein, had no effect on cell surface expression of the integrins or cell adhesion to fibronectin. In addition, VAMP2-mediated trafficking is involved in cell adhesion to collagen but not to laminin. Consistent with disruption of integrin functions in cell proliferation and survival, VAMP2 silencing diminished proliferation and triggered apoptosis. Collectively, these data indicate that VAMP2 mediates the trafficking of alpha5beta1 integrin to the plasma membrane and VAMP2-dependent integrin trafficking is critical in cell adhesion,   migration   and survival. 
  GeneRIFs  
 VAMP2 mediates the trafficking of alpha5beta1 integrin to the plasma membrane and VAMP2-dependent integrin trafficking is critical in cell adhesion,   migration   and survival. 
  ECM  PubMed  
 Vesicle-associated membrane protein 2 mediates trafficking of alpha5beta1 integrin to the plasma membrane. Integrins are major receptors for cell adhesion to the extracellular matrix (  ECM  ). As transmembrane proteins, the levels of integrins at the plasma membrane or the cell surface are ultimately determined by the balance between two vesicle trafficking events: endocytosis of integrins at the plasma membrane and exocytosis of the vesicles that transport integrins. Here, we report that vesicle-associated membrane protein 2 (VAMP2), a SNARE protein that mediates vesicle fusion with the plasma membrane, is involved in the trafficking of alpha5beta1 integrin. VAMP2 was present on vesicles containing endocytosed beta1 integrin. Small interfering RNA (siRNA) silencing of VAMP2 markedly reduced cell surface alpha5beta1 and inhibited cell adhesion and chemotactic migration to fibronectin, the   ECM   ligand of alpha5beta1, without altering cell surface expression of alpha2beta1 integrin or alpha3beta1 integrin. By contrast, silencing of VAMP8, another SNARE protein, had no effect on cell surface expression of the integrins or cell adhesion to fibronectin. In addition, VAMP2-mediated trafficking is involved in cell adhesion to collagen but not to laminin. Consistent with disruption of integrin functions in cell proliferation and survival, VAMP2 silencing diminished proliferation and triggered apoptosis. Collectively, these data indicate that VAMP2 mediates the trafficking of alpha5beta1 integrin to the plasma membrane and VAMP2-dependent integrin trafficking is critical in cell adhesion, migration and survival. 
 Vesicle-associated membrane protein 2 mediates trafficking of alpha5beta1 integrin to the plasma membrane. Integrins are major receptors for cell adhesion to the extracellular matrix (  ECM  ). As transmembrane proteins, the levels of integrins at the plasma membrane or the cell surface are ultimately determined by the balance between two vesicle trafficking events: endocytosis of integrins at the plasma membrane and exocytosis of the vesicles that transport integrins. Here, we report that vesicle-associated membrane protein 2 (VAMP2), a SNARE protein that mediates vesicle fusion with the plasma membrane, is involved in the trafficking of alpha5beta1 integrin. VAMP2 was present on vesicles containing endocytosed beta1 integrin. Small interfering RNA (siRNA) silencing of VAMP2 markedly reduced cell surface alpha5beta1 and inhibited cell adhesion and chemotactic migration to fibronectin, the   ECM   ligand of alpha5beta1, without altering cell surface expression of alpha2beta1 integrin or alpha3beta1 integrin. By contrast, silencing of VAMP8, another SNARE protein, had no effect on cell surface expression of the integrins or cell adhesion to fibronectin. In addition, VAMP2-mediated trafficking is involved in cell adhesion to collagen but not to laminin. Consistent with disruption of integrin functions in cell proliferation and survival, VAMP2 silencing diminished proliferation and triggered apoptosis. Collectively, these data indicate that VAMP2 mediates the trafficking of alpha5beta1 integrin to the plasma membrane and VAMP2-dependent integrin trafficking is critical in cell adhesion, migration and survival. 
  [Ee]xtracellular [Mm]atrix  PubMed  
 Vesicle-associated membrane protein 2 mediates trafficking of alpha5beta1 integrin to the plasma membrane. Integrins are major receptors for cell adhesion to the   extracellular matrix   (ECM). As transmembrane proteins, the levels of integrins at the plasma membrane or the cell surface are ultimately determined by the balance between two vesicle trafficking events: endocytosis of integrins at the plasma membrane and exocytosis of the vesicles that transport integrins. Here, we report that vesicle-associated membrane protein 2 (VAMP2), a SNARE protein that mediates vesicle fusion with the plasma membrane, is involved in the trafficking of alpha5beta1 integrin. VAMP2 was present on vesicles containing endocytosed beta1 integrin. Small interfering RNA (siRNA) silencing of VAMP2 markedly reduced cell surface alpha5beta1 and inhibited cell adhesion and chemotactic migration to fibronectin, the ECM ligand of alpha5beta1, without altering cell surface expression of alpha2beta1 integrin or alpha3beta1 integrin. By contrast, silencing of VAMP8, another SNARE protein, had no effect on cell surface expression of the integrins or cell adhesion to fibronectin. In addition, VAMP2-mediated trafficking is involved in cell adhesion to collagen but not to laminin. Consistent with disruption of integrin functions in cell proliferation and survival, VAMP2 silencing diminished proliferation and triggered apoptosis. Collectively, these data indicate that VAMP2 mediates the trafficking of alpha5beta1 integrin to the plasma membrane and VAMP2-dependent integrin trafficking is critical in cell adhesion, migration and survival. 
 Vesicle-associated membrane protein 2 mediates trafficking of alpha5beta1 integrin to the plasma membrane. Integrins are major receptors for cell adhesion to the   extracellular matrix   (ECM). As transmembrane proteins, the levels of integrins at the plasma membrane or the cell surface are ultimately determined by the balance between two vesicle trafficking events: endocytosis of integrins at the plasma membrane and exocytosis of the vesicles that transport integrins. Here, we report that vesicle-associated membrane protein 2 (VAMP2), a SNARE protein that mediates vesicle fusion with the plasma membrane, is involved in the trafficking of alpha5beta1 integrin. VAMP2 was present on vesicles containing endocytosed beta1 integrin. Small interfering RNA (siRNA) silencing of VAMP2 markedly reduced cell surface alpha5beta1 and inhibited cell adhesion and chemotactic migration to fibronectin, the ECM ligand of alpha5beta1, without altering cell surface expression of alpha2beta1 integrin or alpha3beta1 integrin. By contrast, silencing of VAMP8, another SNARE protein, had no effect on cell surface expression of the integrins or cell adhesion to fibronectin. In addition, VAMP2-mediated trafficking is involved in cell adhesion to collagen but not to laminin. Consistent with disruption of integrin functions in cell proliferation and survival, VAMP2 silencing diminished proliferation and triggered apoptosis. Collectively, these data indicate that VAMP2 mediates the trafficking of alpha5beta1 integrin to the plasma membrane and VAMP2-dependent integrin trafficking is critical in cell adhesion, migration and survival. 
   
  
 VAMP1 
 
  NCBI Gene  
  GeneRIF  
  Pubmed  
 
 
  Search Term  Rank  Fields  
  [Ee]xtracellular [Mm]atrix  8  PubMed(1)  
 
 
 hide/show details for VAMP1 
  [Ee]xtracellular [Mm]atrix  PubMed  
 Redifferentiation of dedifferentiated chondrocytes and chondrogenesis of human bone marrow stromal cells via chondrosphere formation with expression profiling by large-scale cDNA analysis. Characterization of dedifferentiated chondrocytes (DECs) and mesenchymal stem cells capable of differentiating into chondrocytes is of biological and clinical interest. We isolated DECs and bone marrow stromal cells (BMSCs), H4-1 and H3-4, and demonstrated that the cells started to produce extracellular matrices, such as type II collagen and aggrecan, at an early stage of chondrosphere formation. Furthermore, cDNA sequencing of cDNA libraries constricted by the oligocapping method was performed to analyze difference in mRNA expression profiling between DECs and marrow stromal cells. Upon redifferentiation of DECs, cartilage-related   extracellular matrix   genes, such as those encoding leucine-rich small proteoglycans, cartilage oligomeric matrix protein, and chitinase 3-like 1 (cartilage glycoprotein-39), were highly expressed. Growth factors such as FGF7 and CTGF were detected at a high frequency in the growth stage of monolayer stromal cultures. By combining the expression profile and flow cytometry, we demonstrated that isolated stromal cells, defined by CD34(-), c-kit(-), and CD140alpha(- or low), have chondrogenic potential. The newly established human mesenchymal cells with expression profiling provide a powerful model for a study of chondrogenic differentiation and further understanding of cartilage regeneration in the means of redifferentiated DECs and BMSCs. 
   
  
 ACTB 
 
  NCBI Gene  
  GeneRIF  
  Pubmed  
 
 
  Search Term  Rank  Fields  
  [Ff]ilopodia  1  PubMed(3)  
  [Pp]odosome(s)?  5  PubMed(1)  
  [Mm]igration  6  PubMed(13); GeneRIFs(1)  
  ECM  7  PubMed(2)  
  [Ee]xtracellular [Mm]atrix  8  PubMed(9)  
  [Ii]nvasive  10  PubMed(6)  
  [Mm]etastasis  12  PubMed(4); GeneRIFs(1)  
  [Cc]ell [Ii]nvasion  13  PubMed(3)  
 
 
 hide/show details for ACTB 
  [Ff]ilopodia  PubMed  
 Microtubule-binding proteins CLASP1 and CLASP2 interact with actin filaments. Cell morphogenesis requires dynamic communication between actin filaments and microtubules which is mediated, at least in part, by direct structural links between the two cytoskeletal systems. Here, we examined interaction between the CLIP-associated proteins (CLASP) CLASP1 and CLASP2, and actin filaments. We demonstrate that, in addition to a well-established association with the distal ends of microtubules, CLASP2alpha co-localizes with stress fibers, and that both CLASP1alpha and CLASP2alpha co-immunoprecipitate with actin. GFP-CLASP2alpha exhibits retrograde flow in the lamellipodia of Xenopus primary fibroblasts and in the   filopodia   of Xenopus spinal cord neurons. A deletion mapping analysis reveals that both the microtubule-binding domain of CLASP2 (which is homologous between all CLASPs) and the N-terminal dis1/TOG domain of CLASP2alpha (which is homologous between alpha isoforms) possess actin-binding activity. Fluorescence resonance energy transfer experiments demonstrate significant energy transfer between YFP-CLASP2alpha and CFP-actin. Our results indicate that CLASPs function as actin/microtubule crosslinkers in interphase cells. We propose that CLASPs facilitate recognition of actin filaments by the plus ends of growing microtubules at the initial stages of actin-microtubule interaction. Cell Motil. 
 PtdIns(4,5)P-restricted plasma membrane localization of FAN is involved in TNF-induced actin reorganization. The WD-repeat protein factor associated with nSMase activity (FAN) is a member of the family of TNF receptor adaptor proteins that are coupled to specific signaling cascades. However, the precise functional involvement of FAN in specific cellular TNF responses remain unclear. Here, we report the involvement of FAN in TNF-induced actin reorganization and   filopodia   formation mediated by activation of Cdc42. The pleckstrin-homology (PH) domain of FAN specifically binds to phosphatidylinositol-4,5-bisphosphate (PtdIns(4,5)P), which targets FAN to the plasma membrane. Site-specific mutagenesis revealed that the ability of FAN to mediate   filopodia   formation was blunted either by the destruction of the PtdIns(4,5)P binding motif, or by the disruption of intramolecular interactions between the PH domain and the adjacent beige and Chediak-Higashi (BEACH) domain. Furthermore, FAN was shown to interact with the actin cytoskeleton in TNF-stimulated cells via direct filamentous actin (F-actin) binding. The results of this study suggest that PH-mediated plasma membrane targeting of FAN is critically involved in TNF-induced Cdc42 activation and cytoskeleton reorganization. 
 Contribution of rearranged actin structures to the spread of Ectromelia virus infection in vitro. We describe here a contribution of virus-induced actin tails and   filopodia   in transmission of Ectromelia virus (ECTV) infection in permissive cells detected by the immunofluorescence and confocal microscopy. Immunoblot analysis revealed profoundly decreased beta-actin levels during ECTV replicative cycle in the infected cells 24 hrs post infection (p.i.). These results provided a basis for the further analysis of ECTV motion in the infected cells as well as for impact of ECTV infection on the cytoskeletal proteins. 
  [Pp]odosome(s)?  PubMed  
 HGAL, a lymphoma prognostic biomarker, interacts with the cytoskeleton and mediates the effects of IL-6 on cell migration. HGAL is a newly identified germinal center (GC)-specific gene whose expression by the tumor cells correlates with a favorable prognosis in patients with diffuse large B-cell and classical Hodgkin lymphomas. The function of HGAL is unknown. Previous studies demonstrated that HGAL is dispensable for GC formation, immunoglobulin gene class-switch recombination, and somatic hypermutation. Herein, we identify a role for HGAL in the regulation of cell motility. We demonstrate that IL-6 induces the phosphorylation of the C-terminal tyrosine residue of the HGAL protein via the Lyn kinase, and promotes its relocalization from the cytoplasm to   podosome  -like structures. Further, IL-6-induced HGAL phosphorylation increases its interaction with myosin II and is associated with inhibition of cell migration. Knockdown of endogenous HGAL ameliorates IL-6-induced inhibition of cell migration, whereas overexpression of HGAL imparts inhibitory effects of IL-6 on cell migration. Taken together, our results suggest that HGAL is involved in negative regulation of lymphocyte migration, thus constraining lymphocytes to the GC. Inhibition of lymphocyte migration might contribute to the less aggressive clinical behavior of HGAL-expressing lymphomas. 
  [Mm]igration  PubMed  
 Analysis of the myosin-II-responsive focal adhesion proteome reveals a role for β-Pix in negative regulation of focal adhesion maturation. Focal adhesions undergo myosin-II-mediated maturation wherein they grow and change composition to modulate integrin signalling for cell   migratio  n, growth and differentiation. To determine how focal adhesion composition is affected by myosin II activity, we performed proteomic analysis of isolated focal adhesions and compared protein abundance in focal adhesions from cells with and without myosin II inhibition. We identified 905 focal adhesion proteins, 459 of which changed in abundance with myosin II inhibition, defining the myosin-II-responsive focal adhesion proteome. The abundance of 73% of the proteins in the myosin-II-responsive focal adhesion proteome was enhanced by contractility, including proteins involved in Rho-mediated focal adhesion maturation and endocytosis- and calpain-dependent focal adhesion disassembly. During myosin II inhibition, 27% of proteins in the myosin-II-responsive focal adhesion proteome, including proteins involved in Rac-mediated lamellipodial protrusion, were enriched in focal adhesions, establishing that focal adhesion protein recruitment is also negatively regulated by contractility. We focused on the Rac guanine nucleotide exchange factor β-Pix, documenting its role in the negative regulation of focal adhesion maturation and the promotion of lamellipodial protrusion and focal adhesion turnover to drive cel  l migrati  on. 
 Identification of novel SH3 domain ligands for the Src family kinase Hck. Wiskott-Aldrich syndrome protein (WASP), WASP-interacting protein (WIP), and ELMO1. The importance of the SH3 domain of Hck in kinase regulation, substrate phosphorylation, and ligand binding has been established. However, few in vivo ligands are known for the SH3 domain of Hck. In this study, we used mass spectrometry to identify approximately 25 potential binding partners for the SH3 domain of Hck from the monocyte cell line U937. Two major interacting proteins were the actin binding proteins Wiskott-Aldrich syndrome protein (WASP) and WASP-interacting protein (WIP). We also focused on a novel interaction between Hck and ELMO1, an 84-kDa protein that was recently identified as the mammalian ortholog of the Caenorhabditis elegans gene, ced-12. In mammalian cells, ELMO1 interacts with Dock180 as a component of the CrkII/Dock180/Rac pathway responsible for phagocytosis and cell   migration  . Using purified proteins, we confirmed that WASP-interacting protein and ELMO1 interact directly with the SH3 domain of Hck. We also show that Hck and ELMO1 interact in intact cells and that ELMO1 is heavily tyrosine-phosphorylated in cells that co-express Hck, suggesting that it is a substrate of Hck. The binding of ELMO1 to Hck is specifically dependent on the interaction of a polyproline motif with the SH3 domain of Hck. Our results suggest that these proteins may be novel activators/effectors of Hck. 
 The mitotic kinase Aurora-A induces mammary cell   migration   and breast cancer metastasis by activating the Cofilin-F-actin pathway. The mitotic kinase Aurora-A (Aur-A) is required to form the bipolar spindle and ensure accurate chromosome segregation before cell division. Aur-A dysregulation represents an oncogenic event that promotes tumor formation. Here, we report that Aur-A promotes breast cancer metastasis. Aur-A overexpression enhanced mammary cell   migration   by dephosphorylation and activation of cofilin, which facilitates actin reorganization and polymerization. Cofilin knockdown impaired Aur-A-driven cell   migration   and protrusion of the cell membrane. Conversely, overexpression of activated cofilin abrogated the effects of Aur-A knockdown on cell   migration  . Moreover, Aur-A overexpession increased the expression of the cofilin phosphatase Slingshot-1 (SSH1), contributing to cofilin activation and cell   migration  . We found that phosphatidylinositol 3-kinase (PI3K) inhibition blocked Aur-A-induced cofilin dephosphorylation, actin reorganization, and cell   migration  , suggesting crosstalk with PI3K signaling and a potential benefit of PI3K inhibition in tumors with deregulated Aur-A. Additionally, we found an association between Aur-A overexpression and cofilin activity in breast cancer tissues. Our findings indicate that activation of the cofilin-F-actin pathway contributes to tumor cell   migration   and metastasis enhanced by Aur-A, revealing a novel function for mitotic Aur-A kinase in tumor progression. 
 Tyrosine phosphorylation of cofilin at Y68 by v-Src leads to its degradation through ubiquitin-proteasome pathway. Cofilin is a major regulator of actin dynamics involved in the regulation of cell spreading and   migration   through its actin depolymerizing and severing activities. v-Src is an activated Src tyrosine kinase and a potent oncogene known to phosphorylate a variety of cellular proteins in cell transformation process including altered cell adhesion, spreading and   migration  . Recently, it has been suggested that cofilin is a potential substrate of v-Src (Rush et al., 2005). Here, we show direct tyrosine phosphorylation of cofilin by v-Src and identify Y68 as the major phosphorylation site. Cofilin phosphorylation at Y68 did not change its activity per se, but induced increased ubiquitination of cofilin and its degradation through the proteosome pathway. Furthermore, the negative effect of cofilin on cellular F-actin contents was inhibited by coexpression of v-Src, whereas that of cofilin mutant Y68F (Y68 mutated to F) was not affected, suggesting that v-Src-mediated cofilin phosphorylation at Y68 is required for the degradation of cofilin in vivo. Lastly, inhibition of cell spreading by v-Src was rescued partially by coexpression of cofilin, and to a greater extent by the Y68F mutant, which is not subjected to v-Src-induced degradation through phosphorylation, suggesting that v-Src-mediated changes in cell spreading is, at least in part, through inhibiting the function of cofilin through phosphorylating it at Y68. Together, these results suggest a novel mechanism by which cofilin is regulated by v-Src through tyrosine phosphorylation at Y68 that triggers the degradation of cofilin through ubiquitination-proteosome pathway and consequently inhibits cofilin activity in reducing cellular F-actin contents and cell spreading. 
 Silencing of the Tropomyosin-1 gene by DNA methylation alters tumor suppressor function of TGF-beta. Loss of actin stress fibers has been associated with cell transformation and metastasis. TGF-beta induction of stress fibers in epithelial cells requires high molecular weight tropomyosins encoded by TPM1 and TPM2 genes. Here, we investigated the mechanism underlying the failure of TGF-beta to induce stress fibers and inhibit cell   migration   in metastatic cells. RT-PCR analysis in carcinoma cell lines revealed a significant reduction in TPM1 transcripts in metastatic MDA-MB-231, MDA-MB-435 and SW620 cell lines. Treatment of these cells with demethylating agent 5-aza-2'-deoxycytidine (5-aza-dC) increased mRNA levels of TPM1 with no effect on TPM2. Importantly, 5-aza-dC treatment of MDA-MB-231 cells restored TGF-beta induction of TPM1 and formation of stress fibers. Forced expression of TPM1 by using Tet-Off system increased stress fibers in MDA-MB-231 cells and reduced cell   migration  . A potential CpG island spanning the TPM1 proximal promoter, exon 1, and the beginning of intron 1 was identified. Bisulfite sequencing showed significant cytosine methylation in metastatic cell lines that correlated with a reduced expression of TPM1. Together these results suggest that epigenetic suppression of TPM1 may alter TGF-beta tumor suppressor function and contribute to metastatic properties of tumor cells. 
 Expression of CD 73 (ecto-5'-nucleotidase) in 165 glioblastomas by immunohistochemistry and electronmicroscopic histochemistry. CD 73 (5'-nucleotidase) is an ectoenzyme, which is expressed on normal and neoplastic glial plasma membranes. The enzyme binds to intracellular filamentous actin and the extracellular matrix proteins laminin and fibronectin. CD 73 is a signalling pathway metabolite in the immune response of lymphocytes. The ectoenzyme catalyzes the conversion of purine and pyrimidine ribo- and deoxyribo-nucleoside monophosphates (AMP, GMP, IMP) and leads to elevation of the corresponding nucleosides (adenosine) in the extracellular space and might therefore modulate neuronal signalling and vascular perfusion. CD 73 has also been called a cellular motility factor. There is an increasing amount of evidence for the modulatory role of PKC-mediated CD 73 activity in ischemia, regeneration and repair, glioma cell proliferation and a possible invasion promoting feature of the ectoenzyme. The aim of the present study was to investigate the expression patterns of CD 73 together with the labelling of PKC and EGFR. The latter is known as a marker for primary glioblastomas.We investigated the expression of CD 73 in 165 glioblastoma specimens together with the expression patterns of PKC and EGFR by immunocytochemistry on cryosections with a 4-step grading evaluation by two independent observers. CD 73 was further investigated morphologically by electron-microscopic histochemistry in cell cultures of glioblastoma specimens.With these methods it was possible to demonstrate a dense labelling pattern of glioblastoma specimens with anti-CD 73. 95.7% of the glioblastomas were identified with staining products, 63% with labelling grades 2 and 3. The dense staining of the endoplasmatic reticulum, vesicles, caveolar structures and glial membranes was demonstrated by electron-microscopic histochemistry. Some free enzymatic activity was located bound to the ECM components. We observed a significant coexpressions of CD 73 with PKC (p = 0.001) and CD 73 with EGFR (p = 0.022), which is a prospective marker for a high rate of early recurrency.The CD 73 activity was densely distributed on the membranes of glioblastoma cells in vivo and in cell cultures. The electron-microscopic histochemical studies could demonstrate enzymatic activity at the cell membranes and in vesicular structures and caveolae. Free staining deposits located on ECM components may result in a   migration  - and infiltration-promoting activity. The CD 73 expression could be correlated with the expression grades of PKC and EGFR. The latter has been identified as a prognostic factor which is expressed mainly on primary glioblastomas. PKC is a known tumour metabolite in several proliferation promoting pathways of EGF receptor signalling. 
 De novo mutations in the actin genes ACTB and ACTG1 cause Baraitser-Winter syndrome. Brain malformations are individually rare but collectively common causes of developmental disabilities. Many forms of malformation occur sporadically and are associated with reduced reproductive fitness, pointing to a causative role for de novo mutations. Here, we report a study of Baraitser-Winter syndrome, a well-defined disorder characterized by distinct craniofacial features, ocular colobomata and neuronal   migration   defect. Using whole-exome sequencing of three proband-parent trios, we identified de novo missense changes in the cytoplasmic actin-encoding genes ACTB and ACTG1 in one and two probands, respectively. Sequencing of both genes in 15 additional affected individuals identified disease-causing mutations in all probands, including two recurrent de novo alterations (ACTB, encoding p.Arg196His, and ACTG1, encoding p.Ser155Phe). Our results confirm that trio-based exome sequencing is a powerful approach to discover genes causing sporadic developmental disorders, emphasize the overlapping roles of cytoplasmic actin proteins in development and suggest that Baraitser-Winter syndrome is the predominant phenotype associated with mutation of these two genes. 
 The RNA-binding protein HuR promotes cell   migration   and cell invasion by stabilizing the beta-actin mRNA in a U-rich-element-dependent manner. A high expression level of the beta-actin protein is required for important biological mechanisms, such as maintaining cell shape, growth, and motility. Although the elevated cellular level of the beta-actin protein is directly linked to the long half-life of its mRNA, the molecular mechanisms responsible for this effect are unknown. Here we show that the RNA-binding protein HuR stabilizes the beta-actin mRNA by associating with a uridine-rich element within its 3' untranslated region. Using RNA interference to knock down the expression of HuR in HeLa cells, we demonstrate that HuR plays an important role in the stabilization but not in the nuclear/cytoplasmic distribution of the beta-actin mRNA. HuR depletion in HeLa cells alters key beta-actin-based cytoskeleton functions, such as cell adhesion,   migration  , and invasion, and these defects correlate with a loss of the actin stress fiber network. Together our data establish that the posttranscriptional event involving HuR-mediated beta-actin mRNA stabilization could be a part of the regulatory mechanisms responsible for maintaining cell integrity, which is a prerequisite for avoiding transformation and tumor formation. 
 Severe forms of Baraitser-Winter syndrome are caused by ACTB mutations rather than ACTG1 mutations. ACTB and ACTG1 mutations have recently been reported to cause Baraitser-Winter syndrome (BRWS) - a rare condition characterized by ptosis, colobomata, neuronal   migration   disorder, distinct facial anomalies and intellectual disability. One of the patients carrying an ACTB mutation was previously diagnosed with Fryns-Aftimos syndrome (FAS), which is a rare and severe, multiple congenital anomaly (MCA) syndrome whose symptoms partially overlap with that of BRWS. However, several patients with Fryns-Aftimos were considered not to fit into the ACTB and ACTG1 spectrum because of their severe impairment and additional malformations. We report on three patients who had been diagnosed with FAS. All three patients carry a mutation in the ACTB gene. On the basis of the ACTB mutations and analysis of the clinical findings, we reclassify the diagnosis of these patients as severe BRWS. We suggest that mutations in ACTB cause a distinctly more severe phenotype than ACTG1 mutations, despite the structural similarity of beta- and gamma-actins and their overlapping expression pattern. We expand the spectrum of BRWS and confirm that FAS is not a separate entity but an early and severe manifestation of BRWS. 
 Downregulation of β-actin and its regulatory gene HuR affect cell   migratio  n of human corneal fibroblasts. In an earlier study, we showed that human antigen R (HuR) and β-actin expression levels were downregulated in fibroblasts isolated from human keratoconus stroma compared to normal corneal stroma. To further extend the finding, we determined whether HuR expression affects β-actin gene expression and in turn affects corneal fibrobla  st migrat  ion and wound healing.Stromal keratocytes from normal human corneas were cultured in the presence of serum. Cells were transfected with siRNA specific for β-actin or HuR. SiRNAs specific for GAPDH or a scrambled sequence were used as positive and negative controls (siCTR) for transfection, respectively. The effects of gene silencing were analyzed at the transcriptional and translational levels. Specific proteins were immunohistochemically localized using confocal imaging. The effects of gene silencing on c  ell migra  tion and cell proliferation were analyzed using a modified Boyden chamber and with a wound healing assay, respectively.Reverse-transcription PCR (RT-PCR) and western blot analyses showed that when the HuR gene was silenced, β-actin expression was significantly downregulated. This was further confirmed at the translational level with immunohistochemical-confocal analysis. However, when the β-actin gene was silenced, its expression was significantly decreased but showed no effect on HuR gene expression. When the β-actin or HuR gene was individually silenced, the motility and proliferation of corneal fibroblasts were significantly reduced.The results show that downregulation of the HuR gene results in decreased β-actin gene expression, which in turn results in decreased motility and proliferation of corneal fibroblasts. We conclude that decreased β-actin expression in normal corneal stroma clearly disrupts the cytoskeletal structure and functions, including keratocyte motility and wound healing. 
 HGAL, a lymphoma prognostic biomarker, interacts with the cytoskeleton and mediates the effects of IL-6 on cell   migration  . HGAL is a newly identified germinal center (GC)-specific gene whose expression by the tumor cells correlates with a favorable prognosis in patients with diffuse large B-cell and classical Hodgkin lymphomas. The function of HGAL is unknown. Previous studies demonstrated that HGAL is dispensable for GC formation, immunoglobulin gene class-switch recombination, and somatic hypermutation. Herein, we identify a role for HGAL in the regulation of cell motility. We demonstrate that IL-6 induces the phosphorylation of the C-terminal tyrosine residue of the HGAL protein via the Lyn kinase, and promotes its relocalization from the cytoplasm to podosome-like structures. Further, IL-6-induced HGAL phosphorylation increases its interaction with myosin II and is associated with inhibition of cell   migration  . Knockdown of endogenous HGAL ameliorates IL-6-induced inhibition of cell   migration  , whereas overexpression of HGAL imparts inhibitory effects of IL-6 on cell   migration  . Taken together, our results suggest that HGAL is involved in negative regulation of lymphocyte   migration  , thus constraining lymphocytes to the GC. Inhibition of lymphocyte   migration   might contribute to the less aggressive clinical behavior of HGAL-expressing lymphomas. 
 Molecular mechanisms of disease-related human β-actin mutations p.R183W and p.E364K. Cytoplasmic β-actin supports fundamental cellular processes in healthy and diseased cells including cell adhesion  , migrati  on, cytokinesis and maintenance of cell polarity. Mutations in ACTB, the gene encoding cytoplasmic β-actin, lead to severe disorders with a broad range of symptoms. The two dominant heterozygous gain-of-function β-actin mutations p.R183W and p.E364K were identified in patients with developmental malformations, deafness and juvenile-onset dystonia (p.R183W) and neutrophil dysfunction (p.E364K). Here, we report the recombinant production and functional characterization of the two mutant proteins. Arg183 is located near the nucleotide-binding pocket of actin. Our results from biochemical studies and molecular dynamics simulations show that replacement by a tryptophan residue at position 183 establishes an unusual stacking interaction with Tyr69 that perturbs nucleotide release from actin monomers and polymerization behavior by inducing a closed state conformation. The replacement of Glu364 by a lysine residue appears to act as an allosteric trigger event leading to the preferred formation of the closed state. Thus, our approach indicates that both mutations affect interdomain mobility and nucleotide interactions as a basis for the formation of disease phenotypes in patients. 
 p39 activates cdk5 in neurons, and is associated with the actin cytoskeleton. Cyclin-dependent kinase 5 (cdk5) is a small serine/threonine kinase that displays close sequence homology to the mitotically active cyclin-dependent kinases. Cdk5 has been shown to play an essential role in the development of the nervous system, including neuronal   migration   and neurite outgrowth. Cdk5 activation requires the presence of a regulatory activator such as p35. cdk5 -/- mice have much more extensive defects in the development of the nervous system than p35 -/- mice, leading to the speculation that other regulatory activators of cdk5 exist. Indeed, p39 is a p35 related protein isolated by sequence homology to p35. We show here that p39 associates with cdk5 in brain lysates, and that this complex is active in phosphorylation of histone H1. By extensive characterization of p39 subcellular localization in different cell types, we demonstrate the presence of p39 in lamellipodial and fillopodial structures of cells and in growth cones of neurons. We show that p39 colocalizes with actin, and cofractionates with the detergent insoluble cytoskeleton from brain. Further, p39 coimmunoprecipitates with actin in brain lysates. Finally, disruption of the actin cytoskeleton alters p39 subcellular localization as well as kinase activity of the p39/cdk5 complex. Therefore, our results reveal the existence of the p39/cdk5 complex in vivo and suggest that it might play a role in regulating actin cytoskeletal dynamics in cells. 
  GeneRIFs  
 Findings indicate that activation of the cofilin-F-actin pathway contributes to tumor cell   migration   and metastasis enhanced by Aur-A, revealing a novel function for mitotic Aur-A kinase in tumor progression. 
  ECM  PubMed  
 Depletion of nuclear actin is a key mediator of quiescence in epithelial cells. Functional differentiation is orchestrated by precise growth-regulatory controls conveyed by the tissue microenvironment. Cues from laminin 111 (LN1) lower transcription and suppress mammary epithelial cell growth in culture, but how LN1 induces quiescence is unknown. Recent literature points to involvement of nuclear β-actin in transcriptional regulation. Here, we show that quiescence induced by growth factor withdrawal, or LN1 addition, rapidly decreases nuclear β-actin. LN1, but not other extracellular matrix   (E  CM) molecules, decreases the levels of nuclear β-actin and destabilizes RNA polymerase (RNA Pol) II and III binding to transcription sites, leading to a dramatic drop in transcription and DNA synthesis. Constitutive overexpression of globular β-actin in the nucleus reverses the effect of LN1 on transcription and RNA Pol II association and prevents the cells from becoming quiescent in the presence of LN1. The physiological relevance of our findings was verified by identifying a clear spatial separation of LN1 and β-actin in developing mammary end buds. These data indicate a novel role for nuclear β-actin in growth arrest of epithelial cells and underscore the importance of the integrity of the basement membrane in homeostasis. 
 Expression of CD 73 (ecto-5'-nucleotidase) in 165 glioblastomas by immunohistochemistry and electronmicroscopic histochemistry. CD 73 (5'-nucleotidase) is an ectoenzyme, which is expressed on normal and neoplastic glial plasma membranes. The enzyme binds to intracellular filamentous actin and the extracellular matrix proteins laminin and fibronectin. CD 73 is a signalling pathway metabolite in the immune response of lymphocytes. The ectoenzyme catalyzes the conversion of purine and pyrimidine ribo- and deoxyribo-nucleoside monophosphates (AMP, GMP, IMP) and leads to elevation of the corresponding nucleosides (adenosine) in the extracellular space and might therefore modulate neuronal signalling and vascular perfusion. CD 73 has also been called a cellular motility factor. There is an increasing amount of evidence for the modulatory role of PKC-mediated CD 73 activity in ischemia, regeneration and repair, glioma cell proliferation and a possible invasion promoting feature of the ectoenzyme. The aim of the present study was to investigate the expression patterns of CD 73 together with the labelling of PKC and EGFR. The latter is known as a marker for primary glioblastomas.We investigated the expression of CD 73 in 165 glioblastoma specimens together with the expression patterns of PKC and EGFR by immunocytochemistry on cryosections with a 4-step grading evaluation by two independent observers. CD 73 was further investigated morphologically by electron-microscopic histochemistry in cell cultures of glioblastoma specimens.With these methods it was possible to demonstrate a dense labelling pattern of glioblastoma specimens with anti-CD 73. 95.7% of the glioblastomas were identified with staining products, 63% with labelling grades 2 and 3. The dense staining of the endoplasmatic reticulum, vesicles, caveolar structures and glial membranes was demonstrated by electron-microscopic histochemistry. Some free enzymatic activity was located bound to the   ECM   components. We observed a significant coexpressions of CD 73 with PKC (p = 0.001) and CD 73 with EGFR (p = 0.022), which is a prospective marker for a high rate of early recurrency.The CD 73 activity was densely distributed on the membranes of glioblastoma cells in vivo and in cell cultures. The electron-microscopic histochemical studies could demonstrate enzymatic activity at the cell membranes and in vesicular structures and caveolae. Free staining deposits located on   ECM   components may result in a migration- and infiltration-promoting activity. The CD 73 expression could be correlated with the expression grades of PKC and EGFR. The latter has been identified as a prognostic factor which is expressed mainly on primary glioblastomas. PKC is a known tumour metabolite in several proliferation promoting pathways of EGF receptor signalling. 
  [Ee]xtracellular [Mm]atrix  PubMed  
 Migfilin and Mig-2 link focal adhesions to filamin and the actin cytoskeleton and function in cell shape modulation. Cell-  extracellular matrix   adhesion is an important determinant of cell morphology. We show here that migfilin, a LIM-containing protein, localizes to cell-matrix adhesions, associates with actin filaments, and is essential for cell shape modulation. Migfilin interacts with the cell-matrix adhesion protein Mig-2 (mitogen inducible gene-2), a mammalian homolog of UNC-112, and the actin binding protein filamin through its C- and N-terminal domains, respectively. Loss of Mig-2 or migfilin impairs cell shape modulation. Mig-2 recruits migfilin to cell-matrix adhesions, while the interaction with filamin mediates the association of migfilin with actin filaments. Migfilin therefore functions as an important scaffold at cell-matrix adhesions. Together, Mig-2, migfilin and filamin define a connection between cell matrix adhesions and the actin cytoskeleton and participate in the orchestration of actin assembly and cell shape modulation. 
 Proteomic analysis of human parotid gland exosomes by multidimensional protein identification technology (MudPIT). Human ductal saliva contributes over a thousand unique proteins to whole oral fluids. The mechanism by which most of these proteins are secreted by salivary glands remains to be determined. The present study used a mass spectrometry-based, shotgun proteomics approach to explore the possibility that a subset of the proteins found in saliva are derived from exosomes, membrane-bound vesicles of endosomal origin within multivesicular endosomes. Using MudPIT (multidimensional protein identification technology) mass spectrometry, we catalogued 491 proteins in the exosome fraction of human parotid saliva. Many of these proteins were previously observed in ductal saliva from parotid glands (265 proteins). Furthermore, 72 of the proteins in parotid exosomes overlap with those previously identified as urinary exosome proteins, proteins which are also frequently associated with exosomes from other tissues and cell types. Gene Ontology (GO) and KEGG pathway analyses found that cytosolic proteins comprise the largest category of proteins in parotid exosomes (43%), involved in such processes as phosphatidylinositol signaling system, calcium signaling pathway, inositol metabolism, protein export, and signal transduction, among others; whereas the integral plasma membrane proteins and associated/peripheral plasma membrane proteins (26%) were associated with   extracellular matrix  -receptor interaction, epithelial cell signaling, T-cell and B-cell receptor signaling, cytokine receptor interaction, and antigen processing and presentation, among other biological functions. In addition, these putative saliva exosomal proteins were linked to specific diseases (e.g., neurodegenerative disorders, prion disease, cancers, type I and II diabetes). Consequently, parotid glands secrete exosomes that reflect the metabolic and functional status of the gland and may also carry informative protein markers useful in the diagnosis and treatment of systemic diseases. 
 The identification of myocilin-associated proteins in the human trabecular meshwork. Myocilin forms high molecular weight complexes in vivo presumably due to interaction with itself and other myocilin binding proteins. To identify myocilin interacting proteins, yeast 2-hybrid analysis was performed on >1x10(6) human trabecular meshwork cDNA clones. Coimmunoprecipitation and Far Western analysis were also performed on cell lysates obtained from fresh human trabecular meshworks or cultured human monolayer trabecular cell lines. Among the different methods, 46 candidate myocilin-associated proteins were identified, including molecules associated with the   extracellular matrix  , cytoskeleton, signaling, and metabolism. The most consistent interaction was myocilin-myocilin binding. Yeast-2 hybrid and Far Western analysis also found an association between myocilin and glyceraldehyde-3-phosphate dehydrogenase (GAPDH). None of the other candidate myocilin interacting proteins were identified in more than one method. Characterization of these potential interacting proteins may help to better understand the function of myocilin in the trabecular meshwork and aqueous outflow pathway. 
 Depletion of nuclear actin is a key mediator of quiescence in epithelial cells. Functional differentiation is orchestrated by precise growth-regulatory controls conveyed by the tissue microenvironment. Cues from laminin 111 (LN1) lower transcription and suppress mammary epithelial cell growth in culture, but how LN1 induces quiescence is unknown. Recent literature points to involvement of nuclear β-actin in transcriptional regulation. Here, we show that quiescence induced by growth factor withdrawal, or LN1 addition, rapidly decreases nuclear β-actin. LN1, but not othe  r extracellular matr  ix (ECM) molecules, decreases the levels of nuclear β-actin and destabilizes RNA polymerase (RNA Pol) II and III binding to transcription sites, leading to a dramatic drop in transcription and DNA synthesis. Constitutive overexpression of globular β-actin in the nucleus reverses the effect of LN1 on transcription and RNA Pol II association and prevents the cells from becoming quiescent in the presence of LN1. The physiological relevance of our findings was verified by identifying a clear spatial separation of LN1 and β-actin in developing mammary end buds. These data indicate a novel role for nuclear β-actin in growth arrest of epithelial cells and underscore the importance of the integrity of the basement membrane in homeostasis. 
 The effect of epidermal growth factor on matrix metalloproteinases and tissue inhibitors of metalloproteinase gene expression in cultured human gingival fibroblasts. Matrix metalloproteinases (MMPs) and tissue inhibitors of metalloproteinases (TIMPs) play a role in the breakdown of the   extracellular matrix   during normal physiological processes, and in pathological processes, including periodontitis. The aim of this study was to evaluate the effect of epidermal growth factor (EGF) on the expression of MMPs and TIMPs in cultured human gingival fibroblasts.Fibroblasts were stimulated with 10(-3), 10(-6) or 10(-12)M EGF for 24h; untreated fibroblasts served as controls. Alterations in the expression of MMP-1, 2, 3, 7, 11, TIMP-1 and 2 were evaluated using real-time PCR and Western blotting. beta-Actin expression was used as a reference to normalize gene expression.Increased MMP-1, 3, 7 and 11 expressions were observed at all EGF concentrations (p<0.05). At the lowest EGF concentration, MMP-1, 3 and 7 presented the lowest expression and MMP-11 presented the greatest expression; at higher EGF concentrations, MMP-1, 3 and 7 presented greater up-regulation, and MMP-11 lower up-regulation (p<0.05). Protein expression was similarly regulated by EGF: increased up-regulation of MMP-1, 3 and 7 was observed with increasing EGF concentrations, except for MMP-11 that exhibited greater up-regulation at the lower EGF concentration. The gene expression of MMP-2, TIMP-1 and 2 was not affected by EGF (p<0.05).We conclude that EGF regulates expression for MMP-1, 3, 7 and 11 in a dose-dependent manner, suggesting that EGF may play a role in periodontal destruction and wound repair. 
 Complete cDNA sequence and tissue localization of N-RAP, a novel nebulin-related protein of striated muscle. We have cloned and sequenced the full-length cDNA of N-RAP, a novel nebulin-related protein, from mouse skeletal muscle. The N-RAP message is specifically expressed in skeletal and cardiac muscle, but is not detected by Northern blot in non-muscle tissues. The full-length N-RAP cDNA contains an open reading frame of 3,525 base pairs which is predicted to encode a protein of 133 kDa. A 587 amino acid region near the C-terminus is 45% identical to the actin binding region of human nebulin, containing more than 2 complete 245 residue nebulin super repeats. The N-terminus contains the consensus sequence of a cysteine-rich LIM domain, which may function in mediating protein-protein interactions. These data suggest that the encoded protein may link actin filaments to some other proteins or structure. We expressed full-length N-RAP in Escherichia coli, as well as the nebulin-like super repeat region of N-RAP (N-RAP-SR) and the region between the LIM domain and N-RAP-SR (N-RAP-IB). An anti-N-RAP antibody raised against a 30 amino acid peptide corresponding to sequence from N-RAP-IB detected recombinant N-RAP and N-RAP-IB, but failed to detect N-RAP-SR. This antibody specifically identified a 185 kDa band as N-RAP on immunoblots of mouse skeletal and cardiac muscle proteins. In an assay of actin binding to electrophoresed and blotted proteins, we detected significant actin binding to expressed nebulin super repeats and N-RAP-SR, but only a trace amount of binding to N-RAP-IB. In immunofluorescence experiments, N-RAP was found to be localized at the myotendinous junction in mouse skeletal muscle and at the intercalated disc in cardiac muscle. Based on its domain organization, actin binding properties, and tissue localization, we propose that N-RAP plays a role in anchoring the terminal actin filaments in the myofibril to the membrane and may be important in transmitting tension from the myofibrils to the   extracellular matrix  . 
 Expression of CD 73 (ecto-5'-nucleotidase) in 165 glioblastomas by immunohistochemistry and electronmicroscopic histochemistry. CD 73 (5'-nucleotidase) is an ectoenzyme, which is expressed on normal and neoplastic glial plasma membranes. The enzyme binds to intracellular filamentous actin and the   extracellular matrix   proteins laminin and fibronectin. CD 73 is a signalling pathway metabolite in the immune response of lymphocytes. The ectoenzyme catalyzes the conversion of purine and pyrimidine ribo- and deoxyribo-nucleoside monophosphates (AMP, GMP, IMP) and leads to elevation of the corresponding nucleosides (adenosine) in the extracellular space and might therefore modulate neuronal signalling and vascular perfusion. CD 73 has also been called a cellular motility factor. There is an increasing amount of evidence for the modulatory role of PKC-mediated CD 73 activity in ischemia, regeneration and repair, glioma cell proliferation and a possible invasion promoting feature of the ectoenzyme. The aim of the present study was to investigate the expression patterns of CD 73 together with the labelling of PKC and EGFR. The latter is known as a marker for primary glioblastomas.We investigated the expression of CD 73 in 165 glioblastoma specimens together with the expression patterns of PKC and EGFR by immunocytochemistry on cryosections with a 4-step grading evaluation by two independent observers. CD 73 was further investigated morphologically by electron-microscopic histochemistry in cell cultures of glioblastoma specimens.With these methods it was possible to demonstrate a dense labelling pattern of glioblastoma specimens with anti-CD 73. 95.7% of the glioblastomas were identified with staining products, 63% with labelling grades 2 and 3. The dense staining of the endoplasmatic reticulum, vesicles, caveolar structures and glial membranes was demonstrated by electron-microscopic histochemistry. Some free enzymatic activity was located bound to the ECM components. We observed a significant coexpressions of CD 73 with PKC (p = 0.001) and CD 73 with EGFR (p = 0.022), which is a prospective marker for a high rate of early recurrency.The CD 73 activity was densely distributed on the membranes of glioblastoma cells in vivo and in cell cultures. The electron-microscopic histochemical studies could demonstrate enzymatic activity at the cell membranes and in vesicular structures and caveolae. Free staining deposits located on ECM components may result in a migration- and infiltration-promoting activity. The CD 73 expression could be correlated with the expression grades of PKC and EGFR. The latter has been identified as a prognostic factor which is expressed mainly on primary glioblastomas. PKC is a known tumour metabolite in several proliferation promoting pathways of EGF receptor signalling. 
 Protein disulfide isomerase directly interacts with β-actin Cys374 and regulates cytoskeleton reorganization. Recent studies support the role of cysteine oxidation in actin cytoskeleton reorganization during cell adhesion. The aim of this study was to explain whether protein disulfide isomerase (PDI) is responsible for the thiol-disulfide rearrangement in the β-actin molecule of adhering cells. First, we showed that PDI forms a disulfide-bonded complex with β-actin with a molecular mass of 110 kDa. Specific interaction of both proteins was demonstrated by a solid phase binding assay, surface plasmon resonance analysis, and immunoprecipitation experiments. Second, using confocal microscopy, we found that both proteins colocalized when spreading MEG-01 cells on fibronectin. Colocalization of PDI and β-actin could be abolished by the membrane-permeable sulfhydryl blocker, N-ethylmaleimide, by the RGD peptide, and by anti-αIIbβ3 antibodies. Consequently, down-regulation of PDI expression by antisense oligonucleotides impaired the spreading of cells and initiated reorganization of the cytoskeleton. Third, because of transfection experiments followed by immunoprecipitation and confocal analysis, we provided evidence that PDI binds to the β-actin Cys(374) thiol. Formation of the β-actin-PDI complex was mediated by integrin-dependent signaling in response to the adhesion of cells   to the extracellula  r matrix. Our data suggest that PDI is released from subcellular compartments to the cytosol and translocated toward the periphery of the cell, where it forms a disulfide bond with β-actin when MEG-01 cells adhere via the αIIbβ3 integrin to fibronectin. Thus, PDI appears to regulate cytoskeletal reorganization by the thiol-disulfide exchange in β-actin via a redox-dependent mechanism. 
 Regulation of mRNA localization by transmembrane signalling: local interaction of HB-GAM (heparin-binding growth-associated molecule) with the cell surface localizes beta-actin mRNA. Localization of mRNAs is currently thought to be partially responsible for molecular sorting to specific compartments within the cell. In mammalian cells the best-studied example is the beta-actin mRNA that is localized to the cell processes, and its localization is necessary in migratory responses of cells. It is reasonable to assume that mRNA localization within cells is coupled to transmembrane signalling due to extracellular factors, but little is known about such putative mechanisms. We show here that HB-GAM, an   extracellular matrix  -associated factor that enhances migratory responses in cells, is able to localize beta-actin mRNA when locally applied to cells via microbeads. The HB-GAM-induced mRNA localization is specifically inhibited by low concentrations of heparin and by heparitinase treatment of cells, showing that cell-surface heparin-type glycans are required for the effect. The finding that soluble N-syndecan is also inhibitory suggests that the transmembrane proteoglycan N-syndecan, previously identified as an HB-GAM receptor, is involved in the mRNA-localizing effect of HB-GAM. Inhibition of the mRNA localization by the src-kinase inhibitor PP1 is compatible with an N-syndecan-mediated effect since the receptor function of N-syndecan has been recently found to depend on the src-kinase signalling pathway. The mRNA-localizing activity of N-syndecan is also suggested by the finding that affinity-purified anti-N-syndecan antibodies coated on microbeads are able to localize beta-actin mRNA. 
  [Ii]nvasive  PubMed  
 Comparison of tear protein levels in breast cancer patients and healthy controls using a de novo proteomic approach. Non  invasive   biomarkers are urgently needed for early detection of breast cancer since the risk of recurrence, morbidity and mortality are closely related to disease stage at the time of primary surgery. In the past decade, many proteomics-based approaches were developed that utilize the protein profiling of human body fluids or identification of putative biomarkers to obtain more knowledge on the effects of cancer emergence and progression. Herein, we report on an analysis of proteins in the tear fluid from breast carcinoma patients and healthy women using a de novo proteomic approach and 25 mixed samples from each group. This study included 25 patients with primary   invasiv  e breast carcinoma and 25 age-matched healthy controls. We performed a MALDI-TOF-TOF-driven semi-quantitative comparison of tear protein levels in cancer (CA) and control (CTRL) using a de novo approach in pooled samples. Over 150 proteins in the tear fluid of CTRL and CA were identified. Using an in-house-developed algorithm we found more than 20 proteins distinctly upregulated or downregulated in the CTRL and CA groups. We identified several proteins that had modified expression in breast cancer patients. These proteins are involved in host immune system pathways (e.g., C1Q1 or S100A8) and different metabolic cascades (ALDH3A or TPI). Further validation of the results in an independent population combined with individual protein profiling of participants is needed to confirm the specificity of our findings and may lead to a better understanding of the pathological mechanism of breast cancer. 
 Sublocalization of an invasion-inducing locus and other genes on human chromosome 7. By somatic cell fusion studies between non  invasive   mouse T-lymphoma cells and   invasive   human activated normal T-cells we have previously shown that the genetic information responsible for the induction of   invasive   and metastatic potential in interspecies T-cell hybrids is located on human chromosome 7. Apparently, genes derived from normal activated T-cells are dominantly expressed in the hybrids and control the   invasive   and, as a consequence, metastatic potential of these T-lymphoma cells. To sublocalize the invasion-inducing locus on chromosome 7 we have generated hybrids that harbor only specific regions of human chromosome 7 with or without a small fragment of human chromosome 21. Analysis of these hybrids revealed that the invasion-inducing locus maps to 7p12----cen. The human DNA complement of the hybrids was confirmed by Southern blot analysis using a large panel of chromosome 7-specific DNA probes. Several of these genes could be further sublocalized. These included: ARAF2 to 7p12----cen, D7S21 to 7pter----p12, ACTB to 7p15----p12, EGFR to 7p12, MDH2 to 7cen----q22, and PDGFA to 7pter----p15. 
 Specific changes to the mechanism of cell locomotion induced by overexpression of beta-actin. Overexpression of beta-actin is known to alter cell morphology, though its effect on cell motility has not been documented previously. Here we show that overexpressing beta-actin in myoblasts has striking effects on motility, increasing cell speed to almost double that of control cells. This occurs by increasing the areas of protrusion and retraction and is accompanied by raised levels of beta-actin in the newly protruded regions. These regions of the cell margin, however, show decreased levels of polymerised actin, indicating that protrusion can outpace the rate of actin polymerisation in these cells. Moreover, the expression of beta*-actin (a G244D mutant, which shows defective polymerisation in vitro) is equally effective at increasing speed and protrusion. Concomitant changes in actin binding proteins show no evidence of a consistent mechanism for increasing the rate of actin polymerisation in these actin overexpressing cells. The increase in motility is confined to poorly spread cells in both cases and the excess motility can be abolished by blocking myosin function with butanedione monoxime (BDM). Our observations on normal myoblasts are consistent with the view that they protrude by the assembly and cross linking of actin filaments. In contrast, the additional motility shown by cells overexpressing beta-actin appears not to result from an increase in the rate of actin polymerisation but to depend on myosin function. This suggests that the additional protrusion arises from a different mechanism. We discuss the possibility that it is related to retraction-induced protrusion in fibroblasts. In this phenomenon, a wave of increased protrusion follows a sudden collapse in cell spreading. This view could explain why it is only the additional motility that depends on spreading, and has implications for understanding the differences in locomotion that distinguish tissue cells from highly   invasive   cell types such as leucocytes and malignant cells. 
 Structural basis for parasite-specific functions of the divergent profilin of Plasmodium falciparum. Profilins are key regulators of actin dynamics. They sequester actin monomers, forming a pool for rapid polymer formation stimulated by proteins such as formins. Apicomplexan parasites utilize a highly specialized microfilament system for motility and host cell invasion. Their genomes encode only a small number of divergent actin regulators. We present the first crystal structure of an apicomplexan profilin, that of the malaria parasite Plasmodium falciparum, alone and in complex with a polyproline ligand peptide. The most striking feature of Plasmodium profilin is a unique minidomain consisting of a large beta-hairpin extension common to all apicomplexan parasites, and an acidic loop specific for Plasmodium species. Reverse genetics in the rodent malaria model, Plasmodium berghei, suggests that profilin is essential for the   invasive   blood stages of the parasite. Together, our data establish the structural basis for understanding the functions of profilin in the malaria parasite. 
 ACTB in cancer. Beta-actin (ACTB) has traditionally been regarded as an endogenous housekeeping gene and has been widely used as a reference gene/protein in quantifying expression levels in tumors. However, ACTB is closely associated with a variety of cancers and accumulating evidence indicates that ACTB is de-regulated in liver, melanoma, renal, colorectal, gastric, pancreatic, esophageal, lung, breast, prostate, ovarian cancers, leukemia and lymphoma. ACTB is generally found to be up-regulated in the majority of tumor cells and tissues. The abnormal expression and polymerization of ACTB and the resulting changes to the cytoskeleton are revealed to be associated with the   invasive  ness and metastasis of cancers. The current review explores relevant mechanisms, integrates current understandings, and provides suggestions for future studies of the roles of ACTB in tumors. 
 Myopodin, a synaptopodin homologue, is frequently deleted in   invasive   prostate cancers. Prostate cancer is one of the leading causes of cancer-related deaths for men in the United States. Like other malignancies, prostate cancer is underscored by a variety of aberrant genetic alterations during its development. Although loss of heterozygosity or allelic loss is frequently identified among prostate cancers, few genes have been identified thus far as critical to the development of   invasive   prostate cancers. In this report, we used the recently developed technology, the "differential subtraction chain," to perform a genome-wide search for sequences that are deleted in an aggressive prostate cancer. Among the deleted sequences, we found that one sequence was deleted in >50% of prostate cancers we tested. We mapped this sequence to chromosome 4q25 by screening the Genebridge 4 hamster radiation panel with primers specific to this probe, and subsequently identify a 54-kb minimal common deletion region that contains the sequence encoding myopodin. Sequence analysis indicates that myopodin shares significant homology with synaptopodin, a protein closely associated with podocyte and neuron differentiation. Further study shows that frequent complete or partial deletions of the myopodin gene occurred among   invasive   prostate cancer cases (25 of 31 cases, or 80%). Statistical analysis indicates that deletion of myopodin is highly correlated with the   invasive  ness of prostate cancers, and thus may hold promise as an important prognostic marker for prostate cancers. 
  [Mm]etastasis  PubMed  
 The mitotic kinase Aurora-A induces mammary cell migration and breast cancer   metastasis   by activating the Cofilin-F-actin pathway. The mitotic kinase Aurora-A (Aur-A) is required to form the bipolar spindle and ensure accurate chromosome segregation before cell division. Aur-A dysregulation represents an oncogenic event that promotes tumor formation. Here, we report that Aur-A promotes breast cancer   metastasis  . Aur-A overexpression enhanced mammary cell migration by dephosphorylation and activation of cofilin, which facilitates actin reorganization and polymerization. Cofilin knockdown impaired Aur-A-driven cell migration and protrusion of the cell membrane. Conversely, overexpression of activated cofilin abrogated the effects of Aur-A knockdown on cell migration. Moreover, Aur-A overexpession increased the expression of the cofilin phosphatase Slingshot-1 (SSH1), contributing to cofilin activation and cell migration. We found that phosphatidylinositol 3-kinase (PI3K) inhibition blocked Aur-A-induced cofilin dephosphorylation, actin reorganization, and cell migration, suggesting crosstalk with PI3K signaling and a potential benefit of PI3K inhibition in tumors with deregulated Aur-A. Additionally, we found an association between Aur-A overexpression and cofilin activity in breast cancer tissues. Our findings indicate that activation of the cofilin-F-actin pathway contributes to tumor cell migration and   metastasis   enhanced by Aur-A, revealing a novel function for mitotic Aur-A kinase in tumor progression. 
 Silencing of the Tropomyosin-1 gene by DNA methylation alters tumor suppressor function of TGF-beta. Loss of actin stress fibers has been associated with cell transformation and   metastasis  . TGF-beta induction of stress fibers in epithelial cells requires high molecular weight tropomyosins encoded by TPM1 and TPM2 genes. Here, we investigated the mechanism underlying the failure of TGF-beta to induce stress fibers and inhibit cell migration in metastatic cells. RT-PCR analysis in carcinoma cell lines revealed a significant reduction in TPM1 transcripts in metastatic MDA-MB-231, MDA-MB-435 and SW620 cell lines. Treatment of these cells with demethylating agent 5-aza-2'-deoxycytidine (5-aza-dC) increased mRNA levels of TPM1 with no effect on TPM2. Importantly, 5-aza-dC treatment of MDA-MB-231 cells restored TGF-beta induction of TPM1 and formation of stress fibers. Forced expression of TPM1 by using Tet-Off system increased stress fibers in MDA-MB-231 cells and reduced cell migration. A potential CpG island spanning the TPM1 proximal promoter, exon 1, and the beginning of intron 1 was identified. Bisulfite sequencing showed significant cytosine methylation in metastatic cell lines that correlated with a reduced expression of TPM1. Together these results suggest that epigenetic suppression of TPM1 may alter TGF-beta tumor suppressor function and contribute to metastatic properties of tumor cells. 
 ACTB in cancer. Beta-actin (ACTB) has traditionally been regarded as an endogenous housekeeping gene and has been widely used as a reference gene/protein in quantifying expression levels in tumors. However, ACTB is closely associated with a variety of cancers and accumulating evidence indicates that ACTB is de-regulated in liver, melanoma, renal, colorectal, gastric, pancreatic, esophageal, lung, breast, prostate, ovarian cancers, leukemia and lymphoma. ACTB is generally found to be up-regulated in the majority of tumor cells and tissues. The abnormal expression and polymerization of ACTB and the resulting changes to the cytoskeleton are revealed to be associated with the invasiveness and   metastasis   of cancers. The current review explores relevant mechanisms, integrates current understandings, and provides suggestions for future studies of the roles of ACTB in tumors. 
 A novel interplay between oncogenic PFTK1 protein kinase and tumor suppressor TAGLN2 in the control of liver cancer cell motility. The PFTK1 gene encodes a cdc2-related serine/threonine protein kinase that has been shown to confer cell migratory properties in hepatocellular carcinoma (HCC). However, the prognostic value and biological mechanism by which PFTK1 promotes HCC motility remain largely unknown. Here, we showed from tissue microarray that common upregulations of PFTK1 in primary HCC tumors (n=133/180) correlated significantly with early age onset (40 years), advance tumor grading and presence of microvascular invasion (P0.05). To understand downstream phosphorylated substrate(s) of PFTK1, phospho-proteins in PFTK1 expressing and knockdown Hep3B cells were profiled by two-dimensional-polyacrylamide gel electrophoresis mass spectrometric analysis. Protein identification of differential spots revealed β-actin (ACTB) and transgelin2 (TAGLN2) as the two most profound phosphorylated changes affected by PFTK1. We verified the presence of TAGLN2 serine phosphorylation and ACTB tyrosine phosphorylation. Moreover, reduced TAGLN2 and ACTB phosphorylations in PFTK1-suppressed Hep3B corresponded to distinct actin depolymerizations and marked inhibition on cell invasion and motility. Given that TAGLN2 is a tumor suppressor whose function has been ascribed in ca  ncer metas  tasis, we examined if TAGLN2 is an intermediate substrate in the biological path of PFTK1. We showed in PFTK1-suppressed cells that knockdown of TAGLN2 over-rode the inhibitory effect on cell invasion and motility, and a recovery on actin polymerization was evident. Interestingly, we also found that unphosphorylated TAGLN2 in PFTK1-suppressed cells elicited strong actin-binding ability, a mechanism that possibly halts the actin cytoskeleton dynamics. Site-directed mutagenesis of TAGLN2 suggested that PFTK1 regulates the actin-binding affinity of TAGLN2 through the S83 and S163 residues, which if mutated can significantly affect HCC cell motility. Taken together, our data propose a novel, oncogene-tumor suppressor interplay, where oncogenic PFTK1 confers HCC cell motility through inactivating the actin-binding motile suppressing function of TAGLN2 via phosphorylation. 
  GeneRIFs  
 Findings indicate that activation of the cofilin-F-actin pathway contributes to tumor cell migration and   metastasis   enhanced by Aur-A, revealing a novel function for mitotic Aur-A kinase in tumor progression. 
  [Cc]ell [Ii]nvasion  PubMed  
 The RNA-binding protein HuR promotes cell migration and   cell invasion   by stabilizing the beta-actin mRNA in a U-rich-element-dependent manner. A high expression level of the beta-actin protein is required for important biological mechanisms, such as maintaining cell shape, growth, and motility. Although the elevated cellular level of the beta-actin protein is directly linked to the long half-life of its mRNA, the molecular mechanisms responsible for this effect are unknown. Here we show that the RNA-binding protein HuR stabilizes the beta-actin mRNA by associating with a uridine-rich element within its 3' untranslated region. Using RNA interference to knock down the expression of HuR in HeLa cells, we demonstrate that HuR plays an important role in the stabilization but not in the nuclear/cytoplasmic distribution of the beta-actin mRNA. HuR depletion in HeLa cells alters key beta-actin-based cytoskeleton functions, such as cell adhesion, migration, and invasion, and these defects correlate with a loss of the actin stress fiber network. Together our data establish that the posttranscriptional event involving HuR-mediated beta-actin mRNA stabilization could be a part of the regulatory mechanisms responsible for maintaining cell integrity, which is a prerequisite for avoiding transformation and tumor formation. 
 Structural basis for parasite-specific functions of the divergent profilin of Plasmodium falciparum. Profilins are key regulators of actin dynamics. They sequester actin monomers, forming a pool for rapid polymer formation stimulated by proteins such as formins. Apicomplexan parasites utilize a highly specialized microfilament system for motility and host   cell invasion  . Their genomes encode only a small number of divergent actin regulators. We present the first crystal structure of an apicomplexan profilin, that of the malaria parasite Plasmodium falciparum, alone and in complex with a polyproline ligand peptide. The most striking feature of Plasmodium profilin is a unique minidomain consisting of a large beta-hairpin extension common to all apicomplexan parasites, and an acidic loop specific for Plasmodium species. Reverse genetics in the rodent malaria model, Plasmodium berghei, suggests that profilin is essential for the invasive blood stages of the parasite. Together, our data establish the structural basis for understanding the functions of profilin in the malaria parasite. 
 A novel interplay between oncogenic PFTK1 protein kinase and tumor suppressor TAGLN2 in the control of liver cancer cell motility. The PFTK1 gene encodes a cdc2-related serine/threonine protein kinase that has been shown to confer cell migratory properties in hepatocellular carcinoma (HCC). However, the prognostic value and biological mechanism by which PFTK1 promotes HCC motility remain largely unknown. Here, we showed from tissue microarray that common upregulations of PFTK1 in primary HCC tumors (n=133/180) correlated significantly with early age onset (40 years), advance tumor grading and presence of microvascular invasion (P0.05). To understand downstream phosphorylated substrate(s) of PFTK1, phospho-proteins in PFTK1 expressing and knockdown Hep3B cells were profiled by two-dimensional-polyacrylamide gel electrophoresis mass spectrometric analysis. Protein identification of differential spots revealed β-actin (ACTB) and transgelin2 (TAGLN2) as the two most profound phosphorylated changes affected by PFTK1. We verified the presence of TAGLN2 serine phosphorylation and ACTB tyrosine phosphorylation. Moreover, reduced TAGLN2 and ACTB phosphorylations in PFTK1-suppressed Hep3B corresponded to distinct actin depolymerizations and marked inhibitio  n on cell inv  asion and motility. Given that TAGLN2 is a tumor suppressor whose function has been ascribed in cancer metastasis, we examined if TAGLN2 is an intermediate substrate in the biological path of PFTK1. We showed in PFTK1-suppressed cells that knockdown of TAGLN2 over-rode the inhibitory effec  t on cell inv  asion and motility, and a recovery on actin polymerization was evident. Interestingly, we also found that unphosphorylated TAGLN2 in PFTK1-suppressed cells elicited strong actin-binding ability, a mechanism that possibly halts the actin cytoskeleton dynamics. Site-directed mutagenesis of TAGLN2 suggested that PFTK1 regulates the actin-binding affinity of TAGLN2 through the S83 and S163 residues, which if mutated can significantly affect HCC cell motility. Taken together, our data propose a novel, oncogene-tumor suppressor interplay, where oncogenic PFTK1 confers HCC cell motility through inactivating the actin-binding motile suppressing function of TAGLN2 via phosphorylation. 
   
  
 ACTG1 
 
  NCBI Gene  
  GeneRIF  
  Pubmed  
 
 
  Search Term  Rank  Fields  
  [Ff]ilopodia  1  PubMed(1)  
  [Ff]ilopodium  2  PubMed(1)  
  [Mm]igration  6  PubMed(9)  
  [Ee]xtracellular [Mm]atrix  8  PubMed(3); Component(1)  
  [Ii]nvasive  10  PubMed(2)  
  [Mm]etastasis  12  PubMed(1)  
 
 
 hide/show details for ACTG1 
  [Ff]ilopodia  PubMed  
 Targeting protein phosphatase 1 (PP1) to the actin cytoskeleton: the neurabin I/PP1 complex regulates cell morphology. Neurabin I, a neuronal actin-binding protein, binds protein phosphatase 1 (PP1) and p70 ribosomal S6 protein kinase (p70S6K), both proteins implicated in cytoskeletal dynamics. We expressed wild-type and mutant neurabins fused to green fluorescent protein in Cos7, HEK293, and hippocampal neurons. Biochemical and cellular studies showed that an N-terminal F-actin-binding domain dictated neurabin I localization at actin cytoskeleton and promoted disassembly of stress fibers. Deletion of the C-terminal coiled-coil and sterile alpha motif domains abolished neurabin I dimerization and induced filopodium extension. Immune complex assays showed that neurabin I recruited an active PP1 via a PP1-docking sequence,(457)KIKF(460). Mutation of the PP1-binding motif or PP1 inhibition by okadaic acid and calyculin A abolished   filopodia   and restored stress fibers in cells expressing neurabin I. In vitro and in vivo studies suggested that the actin-binding domain attenuated protein kinase A (PKA) phosphorylation of neurabin I. Modification of a major PKA site, serine-461, impaired PP1 binding. Finally, p70S6K was excluded from neurabin I/PP1 complexes and required the displacement of PP1 for recruitment to neurabin I. These studies provided new insights into the assembly and regulation of a neurabin I/PP1 complex that controls actin rearrangement to promote spine development in mammalian neurons. 
  [Ff]ilopodium  PubMed  
 Targeting protein phosphatase 1 (PP1) to the actin cytoskeleton: the neurabin I/PP1 complex regulates cell morphology. Neurabin I, a neuronal actin-binding protein, binds protein phosphatase 1 (PP1) and p70 ribosomal S6 protein kinase (p70S6K), both proteins implicated in cytoskeletal dynamics. We expressed wild-type and mutant neurabins fused to green fluorescent protein in Cos7, HEK293, and hippocampal neurons. Biochemical and cellular studies showed that an N-terminal F-actin-binding domain dictated neurabin I localization at actin cytoskeleton and promoted disassembly of stress fibers. Deletion of the C-terminal coiled-coil and sterile alpha motif domains abolished neurabin I dimerization and induced   filopodium   extension. Immune complex assays showed that neurabin I recruited an active PP1 via a PP1-docking sequence,(457)KIKF(460). Mutation of the PP1-binding motif or PP1 inhibition by okadaic acid and calyculin A abolished filopodia and restored stress fibers in cells expressing neurabin I. In vitro and in vivo studies suggested that the actin-binding domain attenuated protein kinase A (PKA) phosphorylation of neurabin I. Modification of a major PKA site, serine-461, impaired PP1 binding. Finally, p70S6K was excluded from neurabin I/PP1 complexes and required the displacement of PP1 for recruitment to neurabin I. These studies provided new insights into the assembly and regulation of a neurabin I/PP1 complex that controls actin rearrangement to promote spine development in mammalian neurons. 
  [Mm]igration  PubMed  
 De novo mutations in the actin genes ACTB and ACTG1 cause Baraitser-Winter syndrome. Brain malformations are individually rare but collectively common causes of developmental disabilities. Many forms of malformation occur sporadically and are associated with reduced reproductive fitness, pointing to a causative role for de novo mutations. Here, we report a study of Baraitser-Winter syndrome, a well-defined disorder characterized by distinct craniofacial features, ocular colobomata and neuronal   migration   defect. Using whole-exome sequencing of three proband-parent trios, we identified de novo missense changes in the cytoplasmic actin-encoding genes ACTB and ACTG1 in one and two probands, respectively. Sequencing of both genes in 15 additional affected individuals identified disease-causing mutations in all probands, including two recurrent de novo alterations (ACTB, encoding p.Arg196His, and ACTG1, encoding p.Ser155Phe). Our results confirm that trio-based exome sequencing is a powerful approach to discover genes causing sporadic developmental disorders, emphasize the overlapping roles of cytoplasmic actin proteins in development and suggest that Baraitser-Winter syndrome is the predominant phenotype associated with mutation of these two genes. 
 Severe forms of Baraitser-Winter syndrome are caused by ACTB mutations rather than ACTG1 mutations. ACTB and ACTG1 mutations have recently been reported to cause Baraitser-Winter syndrome (BRWS) - a rare condition characterized by ptosis, colobomata, neuronal   migration   disorder, distinct facial anomalies and intellectual disability. One of the patients carrying an ACTB mutation was previously diagnosed with Fryns-Aftimos syndrome (FAS), which is a rare and severe, multiple congenital anomaly (MCA) syndrome whose symptoms partially overlap with that of BRWS. However, several patients with Fryns-Aftimos were considered not to fit into the ACTB and ACTG1 spectrum because of their severe impairment and additional malformations. We report on three patients who had been diagnosed with FAS. All three patients carry a mutation in the ACTB gene. On the basis of the ACTB mutations and analysis of the clinical findings, we reclassify the diagnosis of these patients as severe BRWS. We suggest that mutations in ACTB cause a distinctly more severe phenotype than ACTG1 mutations, despite the structural similarity of beta- and gamma-actins and their overlapping expression pattern. We expand the spectrum of BRWS and confirm that FAS is not a separate entity but an early and severe manifestation of BRWS. 
 Severe forms of Baraitser-Winter syndrome are caused by ACTB mutations rather than ACTG1 mutations. ACTB and ACTG1 mutations have recently been reported to cause Baraitser-Winter syndrome (BRWS) - a rare condition characterized by ptosis, colobomata, neuronal   migration   disorder, distinct facial anomalies and intellectual disability. One of the patients carrying an ACTB mutation was previously diagnosed with Fryns-Aftimos syndrome (FAS), which is a rare and severe, multiple congenital anomaly (MCA) syndrome whose symptoms partially overlap with that of BRWS. However, several patients with Fryns-Aftimos were considered not to fit into the ACTB and ACTG1 spectrum because of their severe impairment and additional malformations. We report on three patients who had been diagnosed with FAS. All three patients carry a mutation in the ACTB gene. On the basis of the ACTB mutations and analysis of the clinical findings, we reclassify the diagnosis of these patients as severe BRWS. We suggest that mutations in ACTB cause a distinctly more severe phenotype than ACTG1 mutations, despite the structural similarity of beta- and gamma-actins and their overlapping expression pattern. We expand the spectrum of BRWS and confirm that FAS is not a separate entity but an early and severe manifestation of BRWS. 
 De novo mutations in the actin genes ACTB and ACTG1 cause Baraitser-Winter syndrome. Brain malformations are individually rare but collectively common causes of developmental disabilities. Many forms of malformation occur sporadically and are associated with reduced reproductive fitness, pointing to a causative role for de novo mutations. Here, we report a study of Baraitser-Winter syndrome, a well-defined disorder characterized by distinct craniofacial features, ocular colobomata and neuronal   migration   defect. Using whole-exome sequencing of three proband-parent trios, we identified de novo missense changes in the cytoplasmic actin-encoding genes ACTB and ACTG1 in one and two probands, respectively. Sequencing of both genes in 15 additional affected individuals identified disease-causing mutations in all probands, including two recurrent de novo alterations (ACTB, encoding p.Arg196His, and ACTG1, encoding p.Ser155Phe). Our results confirm that trio-based exome sequencing is a powerful approach to discover genes causing sporadic developmental disorders, emphasize the overlapping roles of cytoplasmic actin proteins in development and suggest that Baraitser-Winter syndrome is the predominant phenotype associated with mutation of these two genes. 
 IQGAP1 regulates reactive oxygen species-dependent endothelial cell   migration   through interacting with Nox2. Endothelial cell (EC)   migration   is a key event for repair process after vascular injury and angiogenesis. EC   migration   is regulated by reorganization of the actin cytoskeleton at the leading edge and localized production of reactive oxygen species (ROS) at the site of injury. However, underlying mechanisms are unclear. We reported that IQGAP1, an actin binding scaffold protein, mediates VEGF-induced activation of gp91phox (Nox2)-dependent NAD(P)H oxidase and EC   migration  . We thus hypothesized that Nox2 and IQGAP1 may play important roles in ROS-dependent EC   migration   in response to injury.Using a monolayer scratch assay with confluent ECs, we show that ROS production is increased at the margin of scratch area and Nox2 translocates to the leading edge, where it colocalizes and associates with both actin and IQGAP1 in migrating ECs. Knockdown of IQGAP1 using siRNA and inhibition of the actin cytoskeleton blocked scratch injury-induced H2O2 production, Nox2 translocation and its interaction with actin, and EC   migration   toward the injured site.These suggest that IQGAP1 may function to link Nox2 to actin at the leading edge, thereby facilitating ROS production at the site of injury, which may contribute to EC   migration  . 
 The formin-homology-domain-containing protein FHOD1 enhances cell   migration  . Formin-homology-domain-containing proteins interact with Rho-family GTPases and regulate actin cytoskeleton organization and gene transcription. FHOD1 is a member of this family, interacts with Rac1 and induces transcription from the serum response element. In this study, we examined the effects of FHOD1 expression on cytoskeletal organization and function in mammalian cells. FHOD1 proteins were stably expressed in WM35 melanoma cells and NIH-3T3 fibroblasts. Cells expressing full-length FHOD1 demonstrated an elongated phenotype compared with vector-transfected cells and cells expressing a truncated FHOD1 (1-421) that lacks the conserved FH1 and FH2 domains. Full-length FHOD1 co-localized with filamentous actin at cell peripheries. Cells transiently expressing a C-terminal FHOD1 truncation mutant (DeltaC, residues 1-1010), which lacks an autoinhibitory protein-protein interaction domain, displayed prominent stress fibers. FHOD1 (1-421) did not induce stress fibers but localized to membrane ruffles in a manner similar to the full-length protein, indicating that the FH1 and FH2 domains are required for stress fiber appearance. FHOD1 DeltaC (1-1010)-dependent stress fibers were sensitive to dominant-negative RacN17 and the RhoA and ROCK inhibitors, C3 transferase and Y-27632. Stable overexpression of full-length FHOD1 enhanced the   migration   of WM35 and NIH-3T3 cells to type-I collagen and fibronectin, respectively. Cells expressing FHOD1 (1-421) migrated similar to control cells. Integrin expression and activation were not affected by FHOD1 expression. Moreover, FHOD1 overexpression did not alter integrin usage during adhesion or   migration  . These data demonstrate that FHOD1 interacts with and regulates the structure of the cytoskeleton and stimulates cell   migration   in an integrin-independent manner. 
 Measurements of spatiotemporal changes in G-actin concentration reveal its effect on stimulus-induced actin assembly and lamellipodium extension. To understand the intracellular role of G-actin concentration in stimulus-induced actin assembly and lamellipodium extension during cell   migration  , we developed a novel technique for quantifying spatiotemporal changes in G-actin concentration in live cells, consisting of sequential measurements of fluorescent decay after photoactivation (FDAP) of Dronpa-labeled actin. Cytoplasmic G-actin concentrations decreased by ∼40% immediately after cell stimulation and thereafter the cell area extended. The extent of stimulus-induced G-actin loss and cell extension correlated linearly with G-actin concentration in unstimulated cells, even at concentrations much higher than the critical concentration of actin filaments, indicating that cytoplasmic G-actin concentration is a critical parameter for determining the extent of stimulus-induced G-actin assembly and cell extension. Multipoint FDAP analysis revealed that G-actin concentration in lamellipodia was comparable to that in the cell body. We also assessed the cellular concentrations of free G-actin, profilin- and thymosin-β4-bound G-actin, and free barbed and pointed ends of actin filaments by model fitting of jasplakinolide-induced temporal changes in G-actin concentration. 
 Loss of ASAP3 destabilizes cytoskeletal protein ACTG1 to suppress cancer cell   migration  . ArfGAP with SH3 domain, ankyrin repeat and PH domain 3 (ASAP3), previously known as ACAP4, DDEFL1 and UPLC1, is considered to be an important regulator in cancer cell   migration  /invasion and actin-based cytoskeletal remodeling. However, the underlying mechanisms through which ASAP3 mediates these processes are not well-elucidated. This study reported that in certain types of cancer cells, loss of ASAP3 suppressed cell   migration  /invasion, in part by destabilizing γ-actin-1 (ACTG1), a cytoskeletal protein considered to be an integral component of the cell migratory machinery, essential for the rearrangement of the dynamic cytoskeletal networks and important in diseases, such as brain malformation, hearing loss and cancer development. The data, for the first time, link ASAP3 with ACTG1 in the regulation of cytoskeletal maintenance and cell motility. 
 γ-Actin regulates cell   migratio  n and modulates the ROCK signaling pathway. Cell   migratio  n plays a crucial role in numerous cellular functions, and alterations in the regulation of cell   migratio  n are required for invasive transformation of a tumor cell. While the mechanistic process of actin-based   migratio  n has been well documented, little is known as to the specific function of the nonmuscle actin isoforms in mammalian cells. Here, we present a comprehensive examination of γ-actin's role in cel  l migrati  on using an RNAi approach. The partial suppression of γ-actin expression in SH-EP neuroblastoma cells resulted in a significant decrease in wound healing and transwe  ll migrat  ion. Similarly, the knockdown of γ-actin significantly reduced speed of motility and severely affected the cell's ability to explore, which was, in part, due to a loss of cell polarity. Moreover, there was a significant increase in the size and number of paxillin-containing focal adhesions, coupled with a significant decrease in phosphorylated paxillin in γ-actin-knockdown cells. In addition, there was a significant increase in the phosphorylation of cofilin and myosin regulatory light chain, suggesting an overactivated Rho-associated kinase (ROCK) signaling pathway in γ-actin-knockdown cells. The alterations in the phosphorylation of paxillin and myosin regulatory light chain were unique to γ-actin and not β-actin knockdown. Inhibition of the ROCK pathway with the inhibitor Y-27632 restored the ability of γ-actin-knockdown cells to migrate. This study demonstrates γ-actin as a potential upstream regulator of ROCK medi  ated cell   migration. 
  [Ee]xtracellular [Mm]atrix  PubMed  
 Migfilin and Mig-2 link focal adhesions to filamin and the actin cytoskeleton and function in cell shape modulation. Cell-  extracellular matrix   adhesion is an important determinant of cell morphology. We show here that migfilin, a LIM-containing protein, localizes to cell-matrix adhesions, associates with actin filaments, and is essential for cell shape modulation. Migfilin interacts with the cell-matrix adhesion protein Mig-2 (mitogen inducible gene-2), a mammalian homolog of UNC-112, and the actin binding protein filamin through its C- and N-terminal domains, respectively. Loss of Mig-2 or migfilin impairs cell shape modulation. Mig-2 recruits migfilin to cell-matrix adhesions, while the interaction with filamin mediates the association of migfilin with actin filaments. Migfilin therefore functions as an important scaffold at cell-matrix adhesions. Together, Mig-2, migfilin and filamin define a connection between cell matrix adhesions and the actin cytoskeleton and participate in the orchestration of actin assembly and cell shape modulation. 
 SILAC-based proteomics of human primary endothelial cell morphogenesis unveils tumor angiogenic markers. Proteomics has been successfully used for cell culture on dishes, but more complex cellular systems have proven to be challenging and so far poorly approached with proteomics. Because of the complexity of the angiogenic program, we still do not have a complete understanding of the molecular mechanisms involved in this process, and there have been no in depth quantitative proteomic studies. Plating endothelial cells on matrigel recapitulates aspects of vessel growth, and here we investigate this mechanism by using a spike-in SILAC quantitative proteomic approach. By comparing proteomic changes in primary human endothelial cells morphogenesis on matrigel to general adhesion mechanisms in cells spreading on culture dish, we pinpoint pathways and proteins modulated by endothelial cells. The cell-  extracellular matrix   adhesion proteome depends on the adhesion substrate, and a detailed proteomic profile of the   extracellular matrix   secreted by endothelial cells identified CLEC14A as a matrix component, which binds to MMRN2. We verify deregulated levels of these proteins during tumor angiogenesis in models of multistage carcinogenesis. This is the most in depth quantitative proteomic study of endothelial cell morphogenesis, which shows the potential of applying high accuracy quantitative proteomics to in vitro models of vessel growth to shed new light on mechanisms that accompany pathological angiogenesis. The mass spectrometry proteomics data have been deposited to the ProteomeXchange Consortium with the data set identifier PXD000359. 
 The actin/MKL1 signalling pathway influences cell growth and gene expression through large-scale chromatin reorganization and histone post-translational modifications. In addition to soluble factors, mechanical constraints and   extracellular matrix   stiffness are important regulators of cell fate that are mediated by cytoskeletal modifications. The EMT (epithelial-mesenchymal transition) that occurs during normal development and malignant progression is a typical example of the phenotypic switch associated with profound actin remodelling and changes in gene expression. For instance, actin dynamics control motile cell functions in EMT, in part, through regulating the subcellular localization of the myocardin-related transcription factor MKL1 (megakaryoblastic leukaemia translocation 1), a co-activator of SRF (serum-responsive factor). In the present paper, we show that MKL1 participates also to the control of the cellular switch between growth and quiescence. Experimental disconnection between MKL1 and G-actin (globular actin), by using an MKL1 mutant or enhancing the F (filamentous)-/G-actin ratio, generates a widely open chromatin state and a global increase in biosynthetic activity, classically associated with cell growth. Conversely, G-actin accumulation favours nuclear condensation and cell quiescence. These large-scale chromatin changes rely upon extensive histone modifications, exemplified by that of H3K9 (H3 Lys9) shifting from trimethylation, a heterochromatin mark, to acetylation, a mark of euchromatin. The present study provides the first evidence for a global reversible hetero/euchromatinization phenomenon triggered by the actin/MKL1 signalling pathway. 
  Component  
   extracellular matrix   
  [Ii]nvasive  PubMed  
 Proteomic identification of exosomal LRG1: a potential urinary biomarker for detecting NSCLC. In the present research, we aimed to screen for non-small cell lung cancer (NSCLC)-related proteins in urinary exosomes by comparing urinary exosomes proteome of normal controls and NSCLC patients. Urinary exosomes were isolated by ultracentrifugation and identified by electron microscopy. Exosomal proteins were separated by 1-D SDS-PAGE and the differentially expressed bands between healthy controls and NSCLC patients ranging in size from 35 to 45 kD were cut from the gel. After tryptic digestion, 18 proteins were identified by nano-HPLC-chip-MS/MS. The differential expression of leucine-rich α-2-glycoprotein (LRG1) was further validated in urinary exosomes by Western blot and in lung tissue by immunohistochemistry. The LRG1 was found to be expressed at higher levels in urinary exosomes and lung tissue of NSCLC patients. These results suggested that LRG1 may be a candidate biomarker for n  on-invas  ive diagnosis of NSCLC in urine. 
 γ-Actin regulates cell migration and modulates the ROCK signaling pathway. Cell migration plays a crucial role in numerous cellular functions, and alterations in the regulation of cell migration are required for   invasiv  e transformation of a tumor cell. While the mechanistic process of actin-based migration has been well documented, little is known as to the specific function of the nonmuscle actin isoforms in mammalian cells. Here, we present a comprehensive examination of γ-actin's role in cell migration using an RNAi approach. The partial suppression of γ-actin expression in SH-EP neuroblastoma cells resulted in a significant decrease in wound healing and transwell migration. Similarly, the knockdown of γ-actin significantly reduced speed of motility and severely affected the cell's ability to explore, which was, in part, due to a loss of cell polarity. Moreover, there was a significant increase in the size and number of paxillin-containing focal adhesions, coupled with a significant decrease in phosphorylated paxillin in γ-actin-knockdown cells. In addition, there was a significant increase in the phosphorylation of cofilin and myosin regulatory light chain, suggesting an overactivated Rho-associated kinase (ROCK) signaling pathway in γ-actin-knockdown cells. The alterations in the phosphorylation of paxillin and myosin regulatory light chain were unique to γ-actin and not β-actin knockdown. Inhibition of the ROCK pathway with the inhibitor Y-27632 restored the ability of γ-actin-knockdown cells to migrate. This study demonstrates γ-actin as a potential upstream regulator of ROCK mediated cell migration. 
  [Mm]etastasis  PubMed  
 Proteomics analysis of the interactome of N-myc downstream regulated gene 1 and its interactions with the androgen response program in prostate cancer cells. NDRG1 is known to play important roles in both androgen-induced cell differentiation and inhibition of prostate cancer   metastasis  . However, the proteins associated with NDRG1 function are not fully enumerated. Using coimmunoprecipitation and mass spectrometry analysis, we identified 58 proteins that interact with NDRG1 in prostate cancer cells. These proteins include nuclear proteins, adhesion molecules, endoplasmic reticulum (ER) chaperons, proteasome subunits, and signaling proteins. Integration of our data with protein-protein interaction data from the Human Proteome Reference Database allowed us to build a comprehensive interactome map of NDRG1. This interactome map consists of several modules such as a nuclear module and a cell membrane module; these modules explain the reported versatile functions of NDRG1. We also determined that serine 330 and threonine 366 of NDRG1 were phosphorylated and demonstrated that the phosphorylation of NDRG1 was prominently mediated by protein kinase A (PKA). Further, we showed that NDRG1 directly binds to beta-catenin and E-cadherin. However, the phosphorylation of NDRG1 did not interrupt the binding of NDRG1 to E-cadherin and beta-catenin. Finally, we showed that the inhibition of NDRG1 expression by RNA interference decreased the ER inducible chaperon GRP94 expression, directly proving that NDRG1 is involved in the ER stress response. Intriguingly, we observed that many members of the NDRG1 interactome are androgen-regulated and that the NDRG1 interactome links to the androgen response network through common interactions with beta-catenin and heat shock protein 90. Therefore we overlaid the transcriptomic expression changes in the NDRG1 interactome in response to androgen treatment and built a dual dynamic picture of the NDRG1 interactome in response to androgen. This interactome map provides the first road map for understanding the functions of NDRG1 in cells and its roles in human diseases, such as prostate cancer, which can progress from androgen-dependent curable stages to androgen-independent incurable stages. 
   
  
 IGF1R 
 
  NCBI Gene  
  GeneRIF  
  Pubmed  
 
 
  Search Term  Rank  Fields  
  [Mm]igration  6  Process(1); PubMed(81); GeneRIFs(10)  
  ECM  7  PubMed(1)  
  [Ee]xtracellular [Mm]atrix  8  PubMed(13)  
  [Ii]nvasive  10  PubMed(40); GeneRIFs(4)  
  [Mm]etastasize  11  PubMed(2)  
  [Mm]etastasis  12  PubMed(79); GeneRIFs(23)  
  [Cc]ell [Ii]nvasion  13  PubMed(6); GeneRIFs(1)  
 
 
 hide/show details for IGF1R 
  [Mm]igration  Process  
 positive regulation of cell   migration   
  PubMed  
 Decorin regulates endothelial cell motility on collagen I through activation of insulin-like growth factor I receptor and modulation of alpha2beta1 integrin activity. The proteoglycan decorin is expressed by sprouting but not quiescent endothelial cells, and angiogenesis is dysregulated in its absence. Previously, we have shown that decorin core protein can bind to and activate insulin-like growth factor-I receptor (IGF-IR) in endothelial cells. In this study, we show that decorin promotes alpha2beta1 integrin-dependent endothelial cell adhesion and   migration   on fibrillar collagen type I. We provide evidence that decorin modulates cell-matrix interaction in this context by stimulating cytoskeletal and focal adhesion reorganization through activation of the IGF-IR and the small GTPase Rac. Further, the glycosaminoglycan moiety of decorin interacts with alpha2beta1, but not alpha1beta1 integrin, at a site distinct from the collagen I-binding A-domain, to allosterically modulate collagen I-binding activity of the integrin. We propose that induction of decorin expression in angiogenic, as opposed to quiescent, endothelial cells promotes a motile phenotype in an interstitial collagen I-rich environment by both signaling through IGF-IR and influencing alpha2beta1 integrin activity. 
 Integrin expression regulates neuroblastoma attachment and   migration  . Neuroblastoma (NBL) is the most common malignant disease of infancy, and children with bone metastasis have a mortality rate greater than 90%. Two major classes of proteins, integrins and growth factors, regulate the metastatic process. We have previously shown that tumorigenic NBL cells express higher levels of the type I insulin-like growth factor receptor (IGF-IR) and that beta1 integrin expression is inversely proportional to tumorigenic potential in NBL. In the current study, we analyze the effect of beta1 integrin and IGF-IR on NBL cell attachment and   migration  . Nontumorigenic S-cells express high levels of beta1 integrin, whereas tumorigenic N-cells express little beta1 integrin. Alterations in beta1 integrin are due to regulation at the protein level, as translation is decreased in N-type cells. Moreover, inhibition of protein synthesis shows that beta1 integrin is degraded more slowly in S-type cells (SHEP) than in N-type cells (SH-SY5Y and IMR32). Inhibition of alpha5beta1 integrin prevents SHEP (but not SH-SY5Y or IMR32) cell attachment to fibronectin and increases SHEP cell   migration  . Increases in IGF-IR decrease beta1 integrin expression, and enhance SHEP cell   migration  , potentially through increased expression of alphavbeta3. These data suggest that specific classes of integrins in concert with IGF-IR regulate NBL attachment and   migration  . 
 Insulin-like growth factor-1 induces adhesion and   migration   in human multiple myeloma cells via activation of beta1-integrin and phosphatidylinositol 3'-kinase/AKT signaling. Insulin-like growth factor-1 (IGF-I) is a growth and survival factor in human multiple myeloma (MM) cells. Here we examine the effect of IGF-I on MM cell adhesion and   migration  , and define the role of beta1 integrin in these processes. IGF-I increases adhesion of MM.1S and OPM6 MM cells to fibronectin (FN) in a time- and dose-dependent manner, as a consequence of IGF-IR activation. Conversely, blocking anti-beta1 integrin monoclonal antibody, RGD peptide, and cytochalasin D inhibit IGF-I-induced cell adhesion to FN. IGF-I rapidly and transiently induces association of IGF-IR and beta1 integrin, with phosphorylation of IGF-IR, IRS-1, and p85(PI3-K). IGF-I also triggers phosphorylation of AKT and ERK significantly. Both IGF-IR and beta1 integrin colocalize to lipid rafts on the plasma membrane after IGF-I stimulation. In addition, IGF-I triggers polymerization of F-actin, induces phosphorylation of p125(FAK) and paxillin, and enhances beta1 integrin interaction with these focal adhesion proteins. Importantly, using pharmacological inhibitors of phosphatidylinositol 3'-kinase (PI3-K) (LY294002 and wortmannin) and extracellular signal-regulated kinase (PD98059), we demonstrate that IGF-I-induced MM cell adhesion to FN is achieved only when PI3-K/AKT is activated. IGF-I induces a 1.7-2.2 (MM.1S) and 2-2.5-fold (OPM6) increase in   migration  , whereas blocking anti-IGF-I and anti-beta1 integrin monoclonal antibodies, PI3-K inhibitors, as well as cytochalasin D abrogate IGF-I-induced MM cell trans  migration  . Finally, IGF-I induces adhesion of CD138+ patient MM cells. Therefore, these studies suggest a role for IGF-I in trafficking and localization of MM cells in the bone marrow microenvironment. Moreover, they define the functional association of IGF-IR and beta1 integrin in mediating MM cell homing, providing the preclinical rationale for novel treatment strategies targeting IGF-I/IGF-IR in MM. 
 Decorin regulates endothelial cell motility on collagen I through activation of insulin-like growth factor I receptor and modulation of alpha2beta1 integrin activity. The proteoglycan decorin is expressed by sprouting but not quiescent endothelial cells, and angiogenesis is dysregulated in its absence. Previously, we have shown that decorin core protein can bind to and activate insulin-like growth factor-I receptor (IGF-IR) in endothelial cells. In this study, we show that decorin promotes alpha2beta1 integrin-dependent endothelial cell adhesion and   migration   on fibrillar collagen type I. We provide evidence that decorin modulates cell-matrix interaction in this context by stimulating cytoskeletal and focal adhesion reorganization through activation of the IGF-IR and the small GTPase Rac. Further, the glycosaminoglycan moiety of decorin interacts with alpha2beta1, but not alpha1beta1 integrin, at a site distinct from the collagen I-binding A-domain, to allosterically modulate collagen I-binding activity of the integrin. We propose that induction of decorin expression in angiogenic, as opposed to quiescent, endothelial cells promotes a motile phenotype in an interstitial collagen I-rich environment by both signaling through IGF-IR and influencing alpha2beta1 integrin activity. 
 Integrin expression regulates neuroblastoma attachment and   migration  . Neuroblastoma (NBL) is the most common malignant disease of infancy, and children with bone metastasis have a mortality rate greater than 90%. Two major classes of proteins, integrins and growth factors, regulate the metastatic process. We have previously shown that tumorigenic NBL cells express higher levels of the type I insulin-like growth factor receptor (IGF-IR) and that beta1 integrin expression is inversely proportional to tumorigenic potential in NBL. In the current study, we analyze the effect of beta1 integrin and IGF-IR on NBL cell attachment and   migration  . Nontumorigenic S-cells express high levels of beta1 integrin, whereas tumorigenic N-cells express little beta1 integrin. Alterations in beta1 integrin are due to regulation at the protein level, as translation is decreased in N-type cells. Moreover, inhibition of protein synthesis shows that beta1 integrin is degraded more slowly in S-type cells (SHEP) than in N-type cells (SH-SY5Y and IMR32). Inhibition of alpha5beta1 integrin prevents SHEP (but not SH-SY5Y or IMR32) cell attachment to fibronectin and increases SHEP cell   migration  . Increases in IGF-IR decrease beta1 integrin expression, and enhance SHEP cell   migration  , potentially through increased expression of alphavbeta3. These data suggest that specific classes of integrins in concert with IGF-IR regulate NBL attachment and   migration  . 
 Vascular endothelial-cadherin stimulates syndecan-1-coupled insulin-like growth factor-1 receptor and cross-talk between αVβ3 integrin and vascular endothelial growth factor receptor 2 at the onset of endothelial cell dissemination during angiogenesis. Vascular endothelial growth factor (VEGF)-stimulated angiogenesis depends on a cross-talk mechanism involving VEGF receptor 2 (VEGFR2), vascular endothelial (VE)-cadherin and the αVβ3 integrin. Because we have shown that αVβ3 integrin activation is dependent on its incorporation, along with the insulin-like growth factor-1 receptor (IGF1R) kinase, into a ternary receptor complex organized by the matrix receptor syndecan-1 (Sdc1), we questioned the role of this core complex in VEGF-stimulated angiogenesis. We find that the Sdc1-coupled ternary receptor complex is required for VEGF signalling and for stimulation of vascular endothelial   cell mig  ration by vascular endothelial cadherin (VE-cadherin) engagement. VE-cadherin binding to Fc/VE-cadherin extracellular domain chimera activates Sdc1-coupled IGF1R and αvβ3 integrin; this depends on VEGFR2 and c-Src activated by the cadherin. Blocking homotypic VE-cadherin engagement disrupts VEGF-stimulat  ed cell m  igration, which is restored by clustering the cadherin in the absence of cell-cell adhesion. This cadherin-dependent stimulation requires VEGFR2 and IGF1R and is blocked by synstatin (SSTN)(92-119), a peptide that competitively disrupts the Sdc1-coupled ternary complex and prevents the αVβ3 integrin activation required for VEGFR2 activation. VEGFR2-stimulated angiogenesis in the mouse aortic ring explant assay is disrupted by SSTN, although only early in the process, suggesting that IGF1R coupling to Sdc1 and αVβ3 integrin comprises a core activation mechanism activated by VE-cadherin that is necessary for VEGFR2 and integrin activation in the initial stages of endothelial cell dissemination during angiogenesis. 
 Expression of the Insulin-like Growth Factor-1 Receptor in Odontogenic Myxoma. Odontogenic myxoma (OM) is a rare mesenchymal tumour arising in the jaws. The origin and pathogenesis of OM is poorly understood. The aim of this study was to characterize OM by immunolocalization of certain antigens in the tumour that are relevant for cellular differentiation,   migration   and maintenance.Five OMs were immunohistochemically investigated for expression of nestin, CD133, podoplanin, and insulin-like growth factor 1 receptor (IGF-1R).OM failed to react with antibodies applied in this study, with the exception of IGF-1R in tumour cells.OM is a poorly characterized benign, invasive tumour of the jaws. The absence of stem cell marker in OM does not exclude possible temporary expression of these antigens during certain phases of tumour development. The identification of IGF-1R in OM is shared with numerous tumours and indicates the ability of these tumour cells to respond to growth factors. 
 Decorin antagonizes IGF receptor I (IGF-IR) function by interfering with IGF-IR activity and attenuating downstream signaling. We have recently discovered that the insulin-like growth factor receptor I (IGF-IR) is up-regulated in human invasive bladder cancer and promotes   migration   and invasion of transformed urothelial cells. The proteoglycan decorin, a key component of the tumor stroma, can positively regulate the IGF-IR system in normal cells. However, there are no available data on the role of decorin in modulating IGF-IR activity in transformed cells or in tumor models. Here we show that the expression of decorin inversely correlated with IGF-IR expression in low and high grade bladder cancers (n = 20 each). Decorin bound with high affinity IGF-IR and IGF-I at distinct sites and negatively regulated IGF-IR activity in urothelial cancer cells. Nanomolar concentrations of decorin promoted down-regulation of IRS-1, one of the critical proteins of the IGF-IR pathway, and attenuated IGF-I-dependent activation of Akt and MAPK. This led to decorin-evoked inhibition of   migration   and invasion upon IGF-I stimulation. Notably, decorin did not cause down-regulation of the IGF-IR in bladder, breast, and squamous carcinoma cells. This indicates that decorin action on the IGF-IR differs from its known activity on other receptor tyrosine kinases such as the EGF receptor and Met. Our results provide a novel mechanism for decorin in negatively modulating both IGF-I and its receptor. Thus, decorin loss may contribute to increased IGF-IR activity in the progression of bladder cancer and perhaps other forms of cancer where IGF-IR plays a role. 
 Focal adhesion kinase (FAK) activates and stabilizes IGF-1 receptor. Recent studies have shown a direct association between IGF-1R and FAK, two important mediators of cell growth, survival and   migration  . However, the mechanism by which FAK affects IGF-1R function remains unknown. This study investigates the potential role of FAK in mediating activation and stability of IGF-1R. Autophosphorylation and phosphorylation capacities of wild type and mutant IGF-1R were studied. Surprisingly, we found that the mutant IGF-1R lacking the three core tyrosine residues in the activation-loop can be phosphorylated although it is unable to undergo autophosphorylation, suggesting that another kinase possesses the ability to phosphorylate IGF-1R. By using wild type MEFs and FAK-/- MEFs we could demonstrate that FAK mediates activation-loop independent phosphorylation, as well as Akt and ERK activation. Furthermore, the stability of IGF-1R was decreased upon FAK siRNA or inactivation. Taken together, our data suggest a role for FAK in phosphorylation, signaling and stability of the IGF-1R. 
 Targeting insulin-like growth factor 1 receptor inhibits pancreatic cancer growth and metastasis. Pancreatic cancer is one of the most lethal cancers. Increasing incidence and mortality indicates that there is still much lacking in detection and management of the disease. This is partly due to a lack of specific symptoms during early stages of the disease. Several growth factor receptors have been associated with pancreatic cancer. Here, we have investigated if an RNA interference approach targeted to IGF-IR could be effective and efficient against pancreatic cancer growth and metastasis. For that, we evaluated the effects of IGF-1R inhibition using small interfering RNA (siRNAs) on tumor growth and metastasis in HPAC and PANC-1 pancreatic cancer cell lines. We found that silencing IGF-1R inhibits pancreatic cancer growth and metastasis by blocking key signaling pathways such AKT/PI3K, MAPK, JAK/STAT and EMT. Silencing IGF-1R resulted in an anti-proliferative effect in PANC-1 and HPAC pancreatic cancer cell lines. Matrigel invasion, transwell   migration   and wound healing assays also revealed a role for IGF-1R in metastatic properties of pancreatic cancer. These results were further confirmed using Western blotting analysis of key intermediates involved in proliferation, epithelial mesenchymal transition,   migration  , and invasion. In addition, soft agar assays showed that silencing IGF-1R also blocks the colony forming capabilities of pancreatic cancer cells in vitro. Western blots, as well as, flow cytometric analysis revealed the induction of apoptosis in IGF-1R silenced cells. Interestingly, silencing IGF-1R also suppressed the expression of insulin receptor β. All these effects together significantly control pancreatic cancer cell growth and metastasis. To conclude, our results demonstrate the significance of IGF-1R in pancreatic cancer. 
 MiR-323-5p acts as a tumor suppressor by targeting the insulin-like growth factor 1 receptor in human glioma cells. MicroRNAs, small noncoding RNA molecules, can regulate mammalian cell growth, apoptosis and differentiation by controlling the expression of target genes. The aim of this study was to investigate the function of miR-323-5p in the glioma cell line, U251.After over-expression of miR-323- 5p using miR-323-5p mimics, cell growth, apoptosis and   migration   were tested by MTT, flow cytometry and cell wound healing assay, respectively. We also assessed the influence of miR-323-5p on the mRNA expression of IGF- 1R by quantitative real-time reverse transcriptase PCR (qRT-PCR), and on the protein levels by Western blot analysi. In addition, dual-luciferase reporter assays were performed to determine the target site of miR-323-5p to IGF-1R 3'UTR.Our findings showed that over-expression of miR-323-5p could promote apoptosis of U251 and inhibit the proliferation and   migration   of the glioma cells.This study demonstrated that increased expression of miR-323-5p might be related to glioma progression, which indicates a potential role of miR-323-5p for clinical therapy. 
 The effect of IGF-I receptor blockade for human esophageal squamous cell carcinoma and adenocarcinoma. Insulin-like growth factor-I receptor (IGF-IR) signaling is required for carcinogenicity and tumor development, and this pathway has not been well studied in human esophageal carcinomas. Esophageal cancer is one of the human cancers with the worst prognosis and has two main histologies: squamous cell carcinomas (ESCC) and adenocarcinoma (EAC). Previously, we have reported that detection of the IGF axis may be useful for the prediction of recurrence and poor prognosis of ESCC. We have also shown the successful therapy for several gastrointestinal cancers using recombinant adenoviruses expressing dominant negative IGF-IR (ad-IGF-IR/dn). The aim of this study is to develop potential targeted therapeutics to IGF-IR and to assess the effect of IGF-IR blockade in both of these types of esophageal cancer. We determined immunohistochemical expression of IGF-IR in a tissue microarray. We then assessed the effect of IGF-IR blockade on signal transduction, proliferation, apoptosis, and motility. Ad-IGF-IR/dn, a tyrosine kinase inhibitor, BMS-536924, and adenovirus expressing shRNA for IGF-IR were used. IGF-IR expression was common in both tumor types but not in normal tissues. IGF-IR was detected in metastatic sites at similar levels compared to the primary site. IGF-IR inhibition suppressed proliferation and colony formation in both cancers. IGF-IR blockades up-regulated both stress- and chemotherapy-induced apoptosis and reduced   migration  . Although IGF-IR/dn blocked ligand-induced activation of Akt-1 mainly, BMS-536924 effectively blocked both activation of Akt and MAPK. The IGF axis might play a key role in tumor progression of esophageal carcinomas. The IGF-IR targeting strategies might thus be useful anticancer therapeutics for human esophageal malignancies. 
 DNp73 exerts function in metastasis initiation by disconnecting the inhibitory role of EPLIN on IGF1R-AKT/STAT3 signaling. Dissemination of cancer cells from primary tumors is the key event in metastasis, but specific determinants are widely unknown. Here, we show that DNp73, an inhibitor of the p53 tumor suppressor family, drives   migration   and invasion of nonmetastatic melanoma cells. Knockdown of endogenous DNp73 reduces this behavior in highly metastatic cell lines. Tumor xenografts expressing DNp73 show a higher ability to invade and metastasize, while growth remains unaffected. DNp73 facilitates an EMT-like phenotype with loss of E-cadherin and Slug upregulation. We provide mechanistic insight toward regulation of LIMA1/EPLIN by p73/DNp73 and demonstrate a direct link between the DNp73-EPLIN axis and IGF1R-AKT/STAT3 activation. These findings establish initiation of the invasion-metastasis cascade via EPLIN-dependent IGF1R regulation as major activity of DNp73. 
 Tumor suppressor role of miR-133a in gastric cancer by repressing IGF1R. To investigate the function and mechanism of miR-133a in gastric cancer (GC) and its relationship with clinicopathological characteristics of GC.A total of 105 GC patients who underwent surgical resection as primary treatment were selected for this study. Real-time quantitative reverse transcriptase polymerase chain (qRT-PCR) was used to examine the expression levels of miR-133a in human GC and adjacent non-tumor tissues, as well as in GC cell lines (SGC-7901, BGC-823, MGC-803, and AGS) and a human gastric mucosal epithelial cell line (GES-1). The biological role of miRNA (miR)-133a was assessed in the GC cell lines using MTT, apoptosis,   migration   and invasion, and colony formation assays, and xenograft tumorigenesis. qRT-PCR and western blot analyses were used to evaluate the potential target gene expression of miR-133a. Pearson's correlation was calculated to evaluate the correlation between miR-133a and insulin-like growth factor 1 receptor (IGF1R) expression. The regulation of IGF1R by miR-133a was verified using the luciferase reporter assay.In 80% of the 105 GC patients, the mean expression of miR-133a was significantly downregulated in tumor tissues compared with adjacent normal tissues (1.215 ± 0.1477 vs 3.093 ± 0.4104, P < 0.0001). Downregulation of miR-133a was significantly correlated with the degree of differentiation (P = 0.01), local invasion (P = 0.001) and TNM stage (P = 0.02) in GC patients. Compared with a control construct, forced expression of miR-133a in GC cell lines inhibited proliferation (0.4787 ± 0.0219 vs 0.7050 ± 0.0147, P = 0.0013 in SGC-7901 cells; and 0.5448 ± 0.0085 vs 0.7270 ± 0.0084, P = 0.001 in MGC-803 ce  lls); mig  ration (0.6333 ± 0.0233 vs 1.037 ± 0.0584, P = 0.003 in SGC-7901 cells; 0.6126 ± 0.0311 vs 1.024 ± 0.0456, P = 0.0017 in MGC-803 cells); and invasion (0.613 ± 0.0399 vs 1.033 ± 0.0278, P = 0.0013 in SGC-7901 cells; 0.7433 ± 0.0221 vs 1.017 ± 0.0311, P = 0.002 in MGC-803 cells). It also induced apoptosis (18.19% ± 0.2483% vs 5.887% ± 0.3837%, P < 0.0001 in SGC-7901 cells; 22.69% ± 0.7846% vs 9.347% ± 0.3012%, P < 0.0001 in MGC-803 cells). Furthermore, miR-133a inhibited tumor growth and xenograft tumorigenesis of SGC -7901 cells in vivo. In addition, we identified IGF1R as a regulatory target of miR-133a in GC.This study suggests that miR-133a is downregulated in GC and functions as a tumor suppressor in vitro and in vivo partly by repressing IGF1R. 
 MicroRNA-497 is a potential prognostic marker in human cervical cancer and functions as a tumor suppressor by targeting the insulin-like growth factor 1 receptor. Increasing evidence has shown that microRNAs function as oncogenes or tumor suppressors in human malignancies, but the roles of microRNA (miR)-497 in human cervical cancer still remain unclear. Our aim was to analyze the clinicopathologic and prognostic significance of miR-497 in human cervical cancer and to investigate the effects of miR-497 on the malignant phenotype of cervical cancer cells.First, we detected miR-497 expression in the HPV-16-immortalized cervical epithelial cell lines and 4 other cervical cancer cell lines (HeLa, Caski, SiHa, and HeLa-S3). Then the expression of miR-497 was analyzed in cervical cancer tissues and paired nontumor tissues, and its correlation with clinicopathologic features and survival was analyzed. Finally, the roles of miR-497 in regulation of tumor proliferation, apoptosis,   migration  , invasion, and target gene expression were further investigated.MiR-497 was downregulated in cervical cancer cells or tissues compared with HPV-16-immortalized cervical epithelial cell lines or the paired nontumor tissues. Also, the decrease in miR-497 correlated closely with the criteria of the International Federation of Gynaecology and Obstetrics stage and lymph node metastases in patients with cervical cancer. Multivariate Cox analysis showed that low miR-497 expression appeared to be an unfavorable prognostic factor. Transient forced expression of miR-497 decreased the growth and colony-formation capacity of HeLa and SiHa cells by inducing Caspase-3-dependent apoptosis. Forced expression of miR-497 suppressed the   migration   and invasiveness of cervical cancer cells. By computational miRNA target prediction and functional analysis, miR-497 was demonstrated to bind to the 3' untranslated regions of IGF-1R mRNA, and upregulation of miR-497 downregulated IGF-1R protein expression. Further investigation showed that small interfering RNA-mediated IGF-1R knockdown could mimic the effect of enforced miR-497 expression on the malignant phenotypes of cervical cancer cells.MiR-497 may be a potential prognostic marker and functions as a tumor suppressor in human cervical cancer by post-transcriptionally targeting IGF-1R. 
 HRD1 suppresses the growth and metastasis of breast cancer cells by promoting IGF-1R degradation. HRD1 (3-hydroxy-3-methylglutaryl reductase degradation) is an E3 ubiquitin ligase. We found that HRD1 was significantly downregulated in 170 breast cancer tissues. Low tumoral HRD1 expression was correlated with clinicopathological characteristics and a shorter survival in breast cancer patients. P65 specifically bound to the HRD1 promoter and inhibited HRD1 expression. Suppression of NF-κB activity reversed IL-6-induced downregulation of HRD1 expression. HRD1 interacted with IGF-1R and promoted its ubiquitination and degradation by the proteasome. Overexpression of HRD1 resulted in the inhibition of growth,   migratio  n and invasion of breast cancer cells in vitro and in vivo. Furthermore, HRD1 attenuated IL-6-induced epithelial-mesenchymal transition in MCF10A cells. These findings uncover a novel role for HRD1 in breast cancer. 
 Tumor necrosis factor-alpha (TNF-alpha) inhibits insulin-like growth factor-I (IGF-I) activities in human trophoblast cell cultures through IGF-I/insulin hybrid receptors. Tumor necrosis factor-alpha (TNF-alpha) in placenta is believed to be involved in pathogenesis of intrauterine growth restriction. In contrast, insulin-like growth factors (IGFs) are believed to be important for stimulation of fetal and placental growth. IGF-I stimulates metabolic and growth-promoting actions directly through its receptors: IGF-I receptor (IGF-IR), insulin receptor (IR) and IGF-I/insulin hybrid receptor (HR). However, it has not been elucidated which receptor mediates the growth promoting effects in fetal and placental growth. The current studies were undertaken to test whether TNF-alpha affects IGF-I action on placenta using human trophoblast cell cultures, and to test which receptor mediates growth promoting effects of IGF-I in placenta. Primary culture of trophoblast cells, which express IGF-IR, IR, and HR, were exposed to TNF-alpha, and the effects of IGF-I in stimulating trophoblast cell proliferation and   migration   were determined. Exposure to TNF-alpha attenuated the effects of IGF-I on cell proliferation and   migration  . To determine which receptors are involved in this inhibitory effect, the ability of IGF-I to stimulate phosphorylation of its receptors was analyzed in the presence of TNF-alpha. TNF-alpha exposure neither attenuated the phosphorylation of IGF-IR homodimer by IGF-I nor changed receptor abundance. In contrast, TNF-alpha reduced the ability of IGF-I to stimulate phosphorylation of HR with reducing amounts of HR. Exposure to TNF-alpha also attenuated phosphorylation of insulin receptor substrate-1 (IRS-1) and the association of IRS-1 with phosphatydilinositol-3 kinase (PI-3 kinase). Taken together, these findings indicate that TNF-alpha induces a loss of sensitivity to stimulation by IGF-I, through reducing amounts of HR and the stimulation of HR tyrosine kinase activity by IGF-I. 
 Metformin inhibits the proliferation of human prostate cancer PC-3 cells via the downregulation of insulin-like growth factor 1 receptor. Metformin is a biguanide drug that is widely used for the treatment of type 2 diabetes. Recent studies have shown that metformin inhibits cancer cell proliferation and tumor growth both in vitro and in vivo. The anti-tumor mechanisms of metformin include activation of the AMP-activated protein kinase/mTOR pathway and direct inhibition of insulin/insulin-like growth factor (IGF)-mediated cellular proliferation. However, the anti-tumor mechanism in prostate cancer remains unclear. Because activation of the IGF-1 receptor (IGF-1R) is required for prostate cell proliferation, IGF-1R inhibitors may be of therapeutic value. Accordingly, we examined the effects of metformin on IGF-1R signaling in prostate cancer cells. Metformin significantly inhibited PC-3 cell proliferation  , migrati  on, and invasion. IGF-1R mRNA expression decreased significantly after 48 h of treatment, and IGF-1R protein expression decreased in a similar manner. IGF-1R knockdown by siRNA transfection led to inhibited proliferatio  n, migrat  ion and invasion of PC-3 cells. IGF-1 activated both ERK1/2 and Akt, but these effects were attenuated by metformin treatment. In addition, intraperitoneal treatment with metformin significantly reduced tumor growth and IGF-1R mRNA expression in PC-3 xenografts. Our results suggest that metformin is a potent inhibitor of the IGF-1/IGF-1R system and may be beneficial in prostate cancer treatment. 
 MiRNA-181b suppresses IGF-1R and functions as a tumor suppressor gene in gliomas. MicroRNAs (miRNAs) are single-stranded, 18- to 23-nt RNA molecules that function as regulators of gene expression. Previous studies have shown that microRNAs play important roles in human cancers, including gliomas. Here, we found that expression levels of miR-181b were decreased in gliomas, and we identified IGF-1R as a novel direct target of miR-181b. MiR-181b overexpression inhibited cell proliferation,   migration  , invasion, and tumorigenesis by targeting IGF-1R and its downstream signaling pathways, PI3K/AKT and MAPK/ERK1/2. Overexpression of IGF-1R rescued the inhibitory effects of miR-181b. In clinical specimens, IGF-1R was overexpressed, and its protein levels were inversely correlated with miR-181b expression. Taken together, our results indicate that miR-181b functions in gliomas to suppress growth by targeting the IGF-1R oncogene and that miR-181b may serve as a novel therapeutic target for gliomas. 
 MicroRNA-503 acts as a tumor suppressor in glioblastoma for multiple antitumor effects by targeting IGF-1R. microRNA (miRNA) dysregulation is associated with various types of human cancer by regulating cancer cell survival, proliferation and invasion. Aberrant expression of microRNA-503 (miR-503) has been reported in several cancer profiles. However, potential linkage of miR-503 levels and the underlying regulatory mechanisms in human glioblastoma multiforme (GBM) remain unclear. In the present study, we showed for the first time that the expression of miR-503 was significantly reduced in GBM tissues and cell lines (U251 and U87MG) relative to normal brain tissues. Furthermore, our results demonstrated that overexpression of miR-503 in GBM cell lines not only suppressed cell proliferation through inducing G0/G1 cell cycle arrest and apoptosis, but also inhibited cancer cell   migration   and tumor invasion. In addition, we identified insulin-like growth factor-1 (IGF‑1R) receptor mRNA is a bona fide target of miR-503 by computational analysis followed by luciferase reporter assays. Of note, upregulation of miR-503 in GBM cells suppressed endogenous IGF-1R protein expression. Further mechanistic analysis revealed that forced expression of miR-503 inhibited AKT activation, suggesting the tumor suppressive effect of miR-503 in GBM cells is partially mediated by phosphatidylinositol 3-kinase/AKT signaling. Taken together, the results of the present study demonstrated that miR-503 is a tumor suppressor for GBM and a favorable factor against glioma progression through targeting IGF-1R, thus providing a new evidence-supported prognostic marker for GBM diagnosis. 
 Functional insulin-like growth factor-1/insulin-like growth factor-1 receptor-mediated circuit in human and murine thymic epithelial cells. Interactions between thymocytes and thymic epithelial cell (TEC) can be modulated by growth hormone via insulin-like growth factor-1 (IGF-1). In this study, we showed IGF-1 and IGF-1 receptor mRNA expression by human and murine TEC and thymocytes. Functionally, IGF-1 stimulates extracellular matrix production by human TEC. Moreover, pretreatment of murine TEC with IGF-1 increases their adhesion to thymocytes. Interestingly, we observed an increase in the frequency of CD4-CD8-CD90+ T cells which adhered to pretreated TEC, supporting the concept that IGF-1 may also act indirectly on intrathymic T cell differentiation and   migration   through the thymic epithelium. 
 miR-422a Inhibits Glioma Proliferation and Invasion by Targeting IGF1 and IGF1R. Glioma is a common type of malignant brain tumor characterized by aggressive metastasis capability. Recent evidence has suggested that noncoding RNAs, including microRNAs, have important functions in the pathophysiology of glioma development. In this study, we investigated the biological function of miR-422a in human glioma. We found that miR-422a was downregulated in glioma tissues. We also demonstrated that expression of miR-422a in glioma cells markedly suppressed cell proliferation,   migration  , and invasion. In addition, we identified insulin-like growth factor 1 (IGF1) and IGF1 receptor (IGF1R) as inhibitory targets of miR-422a in glioma cells. We established that the expression levels of miR-422a were negatively correlated with the expression levels of IGF1/IGF1R and the clinical parameters in glioma patients. An IGFR inhibitor, AG1024, completely blocked the activity of miR-442a on glioma cell proliferation and invasion, which further confirmed that miR-422a functions through IGF1 and IGF1R. 
 Role of estrogen receptor alpha in modulating IGF-I receptor signaling and function in breast cancer. The insulin-like growth factor I (IGF-I) receptor (IGF-IR) is a multifunctional transmembrane tyrosine kinase that has been implicated in neoplastic transformation. The tumorigenic potential of IGF-IR relies on its strong anti-apoptotic and mitogenic activity. The growth and survival signals of IGF-IR are mediated through multiple intracellular pathways, many of which emanate from insulin receptor substrate 1 (IRS-1). In hormone-dependent breast cancer cells, IGF-IR and IRS-1 are often co-expressed with the estrogen receptor alpha (ERalpha), and IGF-I and ER systems are engaged in a powerful functional cross-talk. Most notably, activation of ERalpha upregulates the expression of IRS-1, IGF-IR, and IGF-1, which results in amplification of IGF-I responses. Reciprocally, stimulation of IGF-IR increases the phosphorylation and activity of ERalpha. In contrast, in ERalpha-negative breast cancer cells and tumors, the levels of IGF-IR and IRS-1 are often decreased and IGF-I is non-mitogenic. Our data suggest that defective IGF-IR signaling in ERalpha-negative cells is related, at least in part, to improper activation of the IRS-1/PI-3K/Akt/GSK-3 pathway and lack of Rb1 phosphorylation. These defects are partially reversed by re-expression of ERalpha. Interestingly, some non-mitogenic IGF-I responses, such as   migration   and invasion are retained in the absence of ERalpha, suggesting that IGF-IR function in breast cancer cells might depend on the ERalpha status. The understanding of how ERalpha may dictate IGF-I responses will help in devising rational anti-IGF-IR strategies for breast cancer treatment. 
 Molecular and functional characterizations of the association and interactions between nucleophosmin-anaplastic lymphoma kinase and type I insulin-like growth factor receptor. Nucleophosmin-anaplastic lymphoma kinase (NPM-ALK) is aberrantly expressed in a subset of T cell lymphoma that commonly affects children and young adults. NPM-ALK possesses significant oncogenic potential that was previously documented using in vitro and in vivo experimental models. The exact mechanisms by which NPM-ALK induces its effects are poorly understood. We have recently demonstrated that NPM-ALK is physically associated with type I insulin-like growth factor receptor (IGF-IR). A positive feedback loop appears to exist between NPM-ALK and IGF-IR through which these two kinases interact to potentiate their effects. We have also found that a single mutation of the Tyr(644) or Tyr(664) residue of the C terminus of NPM-ALK to phenylalanine decreases significantly, but does not completely abolish, the association between NPM-ALK and IGF-IR. The purpose of this study was to determine whether the dual mutation of Tyr(644) and Tyr(664) abrogates the association and interactions between NPM-ALK and IGF-IR. We also examined the impact of this dual mutation on the oncogenic potential of NPM-ALK. Our results show that NPM-ALK(Y644,664F) completely lacks association with IGF-IR. Importantly, we found that the dual mutation of Tyr(644) and Tyr(664) diminishes the oncogenic effects of NPM-ALK, including its ability to induce anchorage-independent colony formation and to sustain cellular transformation, proliferation, and   migration  . Furthermore, the association between NPM-ALK and IGF-IR through Tyr(644) and Tyr(664) appears to contribute to maintaining the stability of NPM-ALK protein. Our results provide novel insights into the mechanisms by which NPM-ALK induces its oncogenic effects through interactions with IGF-IR in this aggressive lymphoma. 
 IGF-IR promotes prostate cancer growth by stabilizing α5β1 integrin protein levels. Dynamic crosstalk between growth factor receptors, cell adhesion molecules and extracellular matrix is essential for cancer cel  l migrati  on and invasion. Integrins are transmembrane receptors that bind extracellular matrix proteins and enable cell adhesion and cytoskeletal organization. They also mediate signal transduction to regulate cell proliferation and survival. The type 1 insulin-like growth factor receptor (IGF-IR) mediates tumor cell growth, adhesion and inhibition of apoptosis in several types of cancer. We have previously demonstrated that β1 integrins regulate anchorage-independent growth of prostate cancer (PrCa) cells by regulating IGF-IR expression and androgen receptor-mediated transcriptional functions. Furthermore, we have recently reported that IGF-IR regulates the expression of β1 integrins in PrCa cells. We have dissected the mechanism through which IGF-IR regulates β1 integrin expression in PrCa. Here we report that IGF-IR is crucial for PrCa cell growth and that β1 integrins contribute to the regulation of proliferation by IGF-IR. We demonstrate that β1 integrin regulation by IGF-IR does not occur at the mRNA level. Exogenous expression of a CD4 - β1 integrin cytoplasmic domain chimera does not interfere with such regulation and fails to stabilize β1 integrin expression in the absence of IGF-IR. This appears to be due to the lack of interaction between the β1 cytoplasmic domain and IGF-IR. We demonstrate that IGF-IR stabilizes the β1 subunit by protecting it from proteasomal degradation. The α5 subunit, one of the binding partners of β1, is also downregulated along with β1 upon IGF-IR knockdown while no change is observed in the expression of the α2, α3, α4, α6 and α7 subunits. Our results reveal a crucial mechanistic role for the α5β1 integrin, downstream of IGF-IR, in regulating cancer growth. 
 Inhibition of type I insulin-like growth factor receptor signaling attenuates the development of breast cancer brain metastasis. Brain metastasis is a common cause of mortality in cancer patients, yet potential therapeutic targets remain largely unknown. The type I insulin-like growth factor receptor (IGF-IR) is known to play a role in the progression of breast cancer and is currently being investigated in the clinical setting for various types of cancer. The present study demonstrates that IGF-IR is constitutively autophosphorylated in brain-seeking breast cancer sublines. Knockdown of IGF-IR results in a decrease of phospho-AKT and phospho-p70s6k, as well as decreased   migration   and invasion of MDA-MB-231Br brain-seeking cells. In addition, transient ablation of IGFBP3, which is overexpressed in brain-seeking cells, blocks IGF-IR activation. Using an in vivo experimental brain metastasis model, we show that IGF-IR knockdown brain-seeking cells have reduced potential to establish brain metastases. Finally, we demonstrate that the malignancy of brain-seeking cells is attenuated by pharmacological inhibition with picropodophyllin, an IGF-IR-specific tyrosine kinase inhibitor. Together, our data suggest that the IGF-IR is an important mediator of brain metastasis and its ablation delays the onset of brain metastases in our model system. 
 MicroRNA-7 functions as an anti-metastatic microRNA in gastric cancer by targeting insulin-like growth factor-1 receptor. Metastasis is a major clinical obstacle in the treatment of gastric cancer (GC) and it accounts for the majority of cancer-related mortality. MicroRNAs have recently emerged as regulators of metastasis by acting on multiple signaling pathways. In this study, we found that miR-7 is significantly downregulated in highly metastatic GC cell lines and metastatic tissues. Both gain-of-function and loss-of-function experiments showed that increased miR-7 expression significantly reduced GC cell   migration   and invasion, whereas decreased miR-7 expression dramatically enhanced cell   migration   and invasion. In vivo metastasis assays also demonstrated that overexpression of miR-7 markedly inhibited GC metastasis. Moreover, the insulin-like growth factor-1 receptor (IGF1R) oncogene, which is often mutated or amplified in human cancers and functions as an important regulator of cell growth and tumor invasion, was identified as a direct target of miR-7. Silencing of IGF1R using small interefering RNA (siRNA) recapitulated the anti-metastatic function of miR-7, whereas restoring the IGF1R expression attenuated the function of miR-7 in GC cells. Furthermore, we found that suppression of Snail by miR-7, through targeting IGF1R, increased E-cadherin expression and partially reversed the epithelial-mesenchymal transition (EMT). Finally, analyses of miR-7 and IGF1R levels in human primary GC with matched lymph node metastasis tissue arrays revealed that miR-7 is inversely correlated with IGF1R expression. The present study provides insight into the specific biological behavior of miR-7 in EMT and tumor metastasis. Targeting this novel miR-7/IGF1R/Snail axis would be helpful as a therapeutic approach to block GC metastasis. 
 Ligand-independent activation of MET through IGF-1/IGF-1R signaling. The receptor tyrosine kinase, MET, has been implicated in tumorigenesis and metastasis of many solid tumors, by multiple mechanisms, including cross talk with epidermal growth factor receptor. In this study, we examined the role of insulin-like growth factor receptor-1 (IGF-1R) signaling in MET activation, focusing on prostate cancer cells. Stimulation of the prostate cancer cell line PC3 with IGF-1 induces a delayed phosphorylation of MET at multiple sites (indicative of full activation), reaching a maximum 18 hr after IGF-1 addition. MET activation does not require the sole MET ligand hepatocyte growth factor (HGF), but does require transcription to occur. Furthermore, direct injection of IGF-1 is sufficient to induce MET activation in vivo, in a PC3 xenograft model. Pharmacologic or genetic inhibition of the tyrosine kinase, Src, abolishes MET phosphorylation, and expression of activated Src is sufficient to induce Met phosphorylation in the absence of IGF-1 stimulation. Activated MET is essential for IGF-1-mediated increased   migration   of PC3 cells, demonstrating an important biologic effect of IGF-1-mediated MET activation. Finally, we demonstrate that IGF-1-induced delayed MET activation occurs in multiple cell lines which express both the receptors, suggesting that IGF-1R-mediated MET activation may contribute to tumorigenic properties of multiple cancer types when both growth factor receptors are expressed. The results further suggest that MET may be activated by multiple receptor tyrosine kinase receptors, and dual targeting of these receptors may be important therapeutically. 
 Oncogenic functions of IGF1R and INSR in prostate cancer include enhanced tumor growth, cell   migration   and angiogenesis. We scrutinized the effect of insulin receptor (INSR) in addition to IGF1R in PCa using in vitro and in vivo models. In-vitro overexpression of IGF1R and INSRA, but not INSRB increased cell proliferation, colony formation,   migration  , invasion and resistance to apoptosis in prostate cancer cells (DU145, LNCaP, PC3). Opposite effects were induced by downregulation of IGF1R and total INSR, but not INSRB. In contrast to tumor cells, non-cancerous epithelial cells of the prostate (EP156T, RWPE-1) were inhibited on overexpression and stimulated by knockdown of receptors. In-vivo analyses using the chicken allantoic membrane assay confirmed the tumorigenic effects of IGF1R and INSR. Apart of promoting tumor growth, IGF1R and INSR overexpression also enhanced angiogenesis indicated by higher vessel density and increased number of desmin-immunoreactive pericytes. Our study underscores the oncogenic impact of IGF1R including significant effects on tumor growth, cell   migration  , sensitivity to apoptotic/chemotherapeutic agents and angiogenesis, and characterizes the INSR, in particular the isoform INSRA, as additional cancer-promoting receptor in prostate cancer. Both, the insulin-like growth factor receptor 1 and the insulin receptor exert oncogenic functions, thus proposing that both receptors need to be considered in therapeutic settings. 
 MicroRNA-139-5p inhibits cell proliferation and invasion by targeting insulin-like growth factor 1 receptor in human non-small cell lung cancer. Increasing evidence suggested that microRNAs (miRNAs) play a critical role in tumorigenesis. Decreased expression of miRNA-139-5p has been observed in various types of cancers. However, the biological function of miRNA-139-5p in non-small cell lung cancer (NSCLC) is still largely unknown.Quantitative real-time PCR (qRT-PCR) was used to explore the expression level of miRNA-139-5p in NSCLC tissues and cell lines. Then, we investigated the role of miRNA-139-5p to determine its potential roles on lung cancer cell proliferation,   migration   and invasion in vitro. A luciferase reporter assay was performed to confirm the target gene of miRNA-139-5p and the results were validated in renal cancer cells.miRNA-139-5p was significantly decreased in NSCLC tissues and cell lines. Over-expression of miRNA-139-5p could inhibit lung cancer cell proliferation,   migration  , and invasion in vitro. Furthermore, we identified insulin-like growth factor 1 receptor (IGF1R) as a target of miR-139-5p and miR-139-5p function as a tumor suppressor via targeting IGF1R in NSCLC.Our results indicated that miR-139-5p acts as a tumor suppressor in NSCLC partially via down-regulating IGF1R expression. 
 Reciprocal regulation of microRNA-99a and insulin-like growth factor I receptor signaling in oral squamous cell carcinoma cells. MicroRNAs (miRNAs), small noncoding RNA molecules can function as oncogenes or tumor suppressors in tumorigenesis. Oral squamous cell carcinoma (OSCC) is one of the most prevalent cancers worldwide with a 5-year survival rate of approximately 50%.The expression of microRNA-99a (miR-99a) in OSCC tissues and cell lines was investigated using quantitative reverse transcription-polymerase chain reaction (qRT-PCR) analysis. The functions of miR-99a in   migration  /invasion and lung colonization were determined by transwell and tail vein injection assays, respectively. Specific targets of miR-99a were determined by software prediction, correlation with target protein expression, and luciferase reporter assay. The signaling pathways involved in regulation of miR-99a were investigated using the kinase inhibitors.We observed reduced levels of miR-99a, identified as one of the most downregulated miRNA in OSCC and all tested OSCC cell lines compared to normal oral keratinocytes. Ectopic miR-99a expression in OSCC cells markedly reduced   migration   and invasion in vitro as well as lung colonization in vivo. When evaluating the specific targets of miR-99a, we found that ectopic miR-99a expression downregulates insulin-like growth factor 1 receptor (IGF1R) protein and that the expression of miR-99a correlates negatively with IGF1R protein in OSCC cells. Insertion of the 3'UTR of IGF1R mRNA into the 3'UTR of a reporter gene markedly reduced luciferase activity in OSCC cells expressing miR-99a, suggesting that miR-99a reduces luciferase activity by targeting the 3'UTR of IGF1R mRNA. When evaluating the mechanisms of miR-99a downregulation, we observed the upregulation of miR-99a expression in serum-starved conditions and its suppression in response to insulin-like growth factor (IGF1) stimulation. Inhibitors of phosphatidylinositol 3-kinase (PI3K) and mitogen-activated protein kinase (MAPK) kinase inhibited IGF1-induced suppression of miR-99a, suggesting the negative regulation of miR-99a expression by IGF1R signaling.Overall, results indicate that miR-99a functions as a tumor metastasis suppressor in OSCC cells and mutually regulates IGF1R expression in a reciprocal regulation. 
 miR-98 suppresses tumor cell growth and metastasis by targeting IGF1R in oral squamous cell carcinoma. Increasing evidences indicate that dysregulation of miRNAs contributes to the pathogenesis of oral squamous cell carcinoma (OSCC). However, little is known about the potential role of miR-98 in OSCC. Here, we found that miR-98 was downregulated in OSCC tissues and cell lines. Overexpression of miR-98 inhibited proliferation, colony formation,   migration  , and invasion of OSCC cells. IGF1R was identified as the potential target of miR-98 using dual luciferase assay, qRT-PCR and western blot. Furthermore, restoration of IGF1R remarkably reversed the tumor-suppressive effects of miR-98 on OSCC cells. Moreover, miR-98 expression was inversely correlated with IGF1R expression in 19 cases of OSCC. These findings suggest that miR-98 inhibits cancer cell growth and metastasis by direct targeting IGF1R, implicating miR-98 as a novel potential therapeutic target for OSCC. 
 Identification of novel predictive markers for the prognosis of pancreatic ductal adenocarcinoma. This study demonstrated that klotho gene expression was significantly lower whereas miR-504 and phospho-IGF-1R levels were significantly higher in PDAC than in normal pancreatic tissues. miR-504 level significantly correlated with klotho mRNA and promoter methylation level in PDACs. Loss of Klotho protein expression, klotho promoter hypermethylation, high miR-504 levels, and high phospho-IGF-1R levels significantly correlated with poor survival, high clinical and pathological stages in PDAC patients. The demethylation reagent and miR-504 inhibitor increased klotho gene expression, invasion, and   migration   in BxPC-3 and Panc-1 cells. Klotho is a tumor suppressor whose expression in PDAC correlated with progression and prognosis of PDAC. 
 Insulin/insulin-like growth factor I hybrid receptors have different biological characteristics depending on the insulin receptor isoform involved. The insulin receptor (IR) and the insulin-like growth factor I receptor (IGF-IR) have a highly homologous structure, but different biological effects. Insulin and IGF-I half-receptors can heterodimerize, leading to the formation of insulin/IGF-I hybrid receptors (Hybrid-Rs) that bind IGF-I with high affinity. As the IR exists in two isoforms (IR-A and IR-B), we evaluated whether the assembly of the IGF-IR with either IR-A or IR-B moieties may differently affect Hybrid-R signaling and biological role. Three different models were studied: (a) 3T3-like mouse fibroblasts with a disrupted IGF-IR gene (R(-) cells) cotransfected with the human IGF-IR and with either the IR-A or IR-B cDNA; (b) a panel of human cell lines variably expressing the two IR isoforms; and (c) HepG2 human hepatoblastoma cells predominantly expressing either IR-A or IR-B, depending on their differentiation state. We found that Hybrid-Rs containing IR-A (Hybrid-Rs(A)) bound to and were activated by IGF-I, IGF-II, and insulin. By binding to Hybrid-Rs(A), insulin activated the IGF-I half-receptor beta-subunit and the IGF-IR-specific substrate CrkII. In contrast, Hybrid-Rs(B) bound to and were activated with high affinity by IGF-I, with low affinity by IGF-II, and insignificantly by insulin. As a consequence, cell proliferation and   migration   in response to both insulin and IGFs were more effectively stimulated in Hybrid-R(A)-containing cells than in Hybrid-R(B)-containing cells. The relative abundance of IR isoforms therefore affects IGF system activation through Hybrid-Rs, with important consequences for tissue-specific responses to both insulin and IGFs. 
 Computational model of EGFR and IGF1R pathways in lung cancer: a Systems Biology approach for Translational Oncology. In this paper we propose a Systems Biology approach to understand the molecular biology of the Epidermal Growth Factor Receptor (EGFR, also known as ErbB1/HER1) and type 1 Insulin-like Growth Factor (IGF1R) pathways in non-small cell lung cancer (NSCLC). This approach, combined with Translational Oncology methodologies, is used to address the experimental evidence of a close relationship among EGFR and IGF1R protein expression, by immunohistochemistry (IHC) and gene amplification, by in situ hybridization (FISH) and the corresponding ability to develop a more aggressive behavior. We develop a detailed in silico model, based on ordinary differential equations, of the pathways and study the dynamic implications of receptor alterations on the time behavior of the MAPK cascade down to ERK, which in turn governs proliferation and cell   migration  . In addition, an extensive sensitivity analysis of the proposed model is carried out and a simplified model is proposed which allows us to infer a similar relationship among EGFR and IGF1R activities and disease outcome. 
 Substrate-bound insulin-like growth factor (IGF)-I-IGF binding protein-vitronectin-stimulated breast cell   migration   is enhanced by coactivation of the phosphatidylinositide 3-Kinase/AKT pathway by alphav-integrins and the IGF-I receptor. IGF-I can bind to the extracellular matrix protein vitronectin (VN) through the involvement of IGF-binding proteins-2, -3, -4, and -5. Because IGF-I and VN have established roles in tumor cell dissemination, we were keen to investigate the functional consequences of the interaction of IGF-I, IGF binding proteins (IGFBPs), and VN in tumor cell biology. Hence, functional responses of MCF-7 breast carcinoma cells and normal nontumorgenic MCF-10A mammary epithelial cells were investigated to allow side-by-side comparisons of these complexes in both cancerous and normal breast cells. We demonstrate that substrate-bound IGF-I-IGFBP-VN complexes stimulate synergistic increases in cellular   migration   in both cell types. Studies using IGF-I analogs determined this stimulation to be dependent on both heterotrimeric IGF-I-IGFBP-VN complex formation and the involvement of the IGF-I receptor (IGF-IR). Furthermore, the enhanced cellular   migration   was abolished on incubation of MCF-7 and MCF-10A cells with function blocking antibodies directed at VN-binding integrins and the IGF-IR. Analysis of the signal transduction pathways underlying the enhanced cell   migration   revealed that the complexes stimulate a transient activation of the ERK/MAPK signaling pathway while simultaneously producing a sustained activation of the phosphatidylinositide 3-kinase/AKT pathway. Experiments using pharmacological inhibitors of these pathways determined a requirement for phosphatidylinositide 3-kinase/AKT activation in the observed response. Overexpression of wild type and activated AKT further increases substrate-bound IGF-I-IGFBP-VN-stimulated   migration  . This study provides the first mechanistic insights into the action of IGF-I-IGFBP-VN complexes and adds further evidence to support the involvement of VN-binding integrins and their cooperativity with the IGF-IR in the promotion of tumor cell   migration  . 
 Genomic and molecular characterization of malignant peripheral nerve sheath tumor identifies the IGF1R pathway as a primary target for treatment. Malignant peripheral nerve sheath tumor (MPNST) is a rare sarcoma that lacks effective therapeutic strategies. We gain insight into the most recurrent genetically altered pathways with the purpose of scanning possible therapeutic targets.We conducted a microarray-based comparative genomic hybridization profiling of two cohorts of primary MPNST tissue samples including 25 patients treated at The University of Texas MD Anderson Cancer Center and 26 patients from Tianjin Cancer Hospital. Immunohistochemistry (IHC) and cell biology detection and validation were carried out on human MPNST tissues and cell lines.Genomic characterization of 51 MPNST tissue samples identified several frequently amplified regions harboring 2,599 genes and regions of deletion including 4,901 genes. At the pathway level, we identified a significant enrichment of copy number-altering events in the insulin-like growth factor 1 receptor (IGF1R) pathway, including frequent amplifications of the IGF1R gene itself. To validate the IGF1R pathway as a potential target in MPNSTs, we first confirmed that high IGF1R protein correlated with worse tumor-free survival in an independent set of samples using IHC. Two MPNST cell lines (ST88-14 and STS26T) were used to determine the effect of attenuating IGF1R. Inhibition of IGF1R in ST88-14 cells using siRNAs or an IGF1R inhibitor, MK-0646, led to significant decreases in cell proliferation, invasion, and   migration   accompanied by attenuation of the PI3K/AKT and mitogen-activated protein kinase pathways.These integrated genomic and molecular studies provide evidence that the IGF1R pathway is a potential therapeutic target for patients with MPNST. 
 Growth factor signaling pathways as targets for prevention of epithelial carcinogenesis. Growth factor receptor (GFR) signaling controls epithelial cell growth by responding to various endogenous or exogenous stimuli and subsequently activating downstream signaling pathways including Stat3, PI3K/Akt/mTOR, MAPK, and c-Src. Environmental chemical toxicants and UVB irradiation cause enhanced and prolonged activation of GFR signaling and downstream pathways that contributes to epithelial cancer development including skin cancer. Recent studies, especially those with tissue-specific transgenic mouse models, have demonstrated that GFRs and their downstream signaling pathways contribute to all three stages of epithelial carcinogenesis by regulating a wide variety of biological functions including proliferation, apoptosis, angiogenesis, cell adhesion, and   migration  . Inhibiting these signaling pathways early in the carcinogenic process results in reduced cell proliferation and survival, leading to decreased tumor formation. Collectively, these studies suggest that GFR signaling and subsequent downstream signaling pathways are potential targets for the prevention of epithelial cancers including skin cancer. 
 Regulatory role of c-Met in insulin-like growth factor-I receptor-mediated   migration   and invasion of human pancreatic carcinoma cells. Pancreatic carcinoma cells overexpress the insulin-like growth factor-I (IGF-I) receptor (IGF-IR) and the hepatocyte growth factor (HGF) receptor, c-Met, which are both known to mediate tumor cell   migration   and invasion. We hypothesized that IGF-IR and c-Met cooperate to induce   migration   and invasion of human pancreatic carcinoma cells and that IGF-I-mediated   migration   and invasion depend on c-Met.   Migration   and invasion assays were done with the human pancreatic cancer cell line L3.6pl treated with PBS, IGF-I, HGF, or IGF-I plus HGF. To determine if c-Met is necessary for IGF-IR-mediated   migration   and invasion, c-Met was down-regulated in L3.6pl cells via adenoviral infection with a c-Met ribozyme before IGF-I treatment. IGF-I and HGF increased cell   migration   and invasion. Furthermore, IGF-I plus HGF had a greater than additive effect on cell   migration   and invasion compared with either growth factor alone. Down-regulation of c-Met nearly completely inhibited IGF-I-mediated   migration   and invasion. Our findings suggest that IGF-IR and c-Met cooperate to induce   migration   and invasion of human pancreatic carcinoma cells. Furthermore, c-Met is required for both HGF- and IGF-I-mediated   migration   and invasion. Elucidation of the signaling pathways that contribute to tumor progression and metastasis should provide a foundation for the development of targeted therapies for pancreatic carcinoma. 
 MicroRNA-133a Inhibits Osteosarcoma Cells Proliferation and Invasion via Targeting IGF-1R. MicroRNAs (miRNAs) are a class of small noncoding RNAs that regulate gene expression by repressing translation or cleaving RNA transcripts in a sequence-specific manner. Downregulated microRNAs and their roles in cancer development have attracted much attention. A growing body of evidence showed that microRNA-133a (miR-133a) has inhibitory effects on cell proliferation,   migration  , invasion, and metastasis of osteosarcoma.MiR-133a expression in human osteosarcoma cell lines and human normal osteoblastic cell line hFOB was investigated by real-time PCR (RT-PCR). The role of miR-133a in human osteosarcoma growth and invasion was assessed in cell lines in vitro and in vivo. Then, luciferase reporter assay validated IGF-1R as a downstream and functional target of miR-133a, and functional studies revealed that the anti-tumor effect of miR-133a was probably due to targeting and repressing of IGF-1R expression.MiR-133a was lower expressed in human osteosarcoma cell lines than human normal osteoblastic cell line hFOB and its effect on inhibiting proliferation, invasion and metastasis is mediated by its direct interaction with the IGF-1R. Furthermore, the tumour-suppressive function of miR-133a probably contributed to inhibiting the activation AKT and ERK signaling pathway.MiR-133a suppresses osteosarcoma progression and metastasis by targeting IGF-1R in human osteosarcoma cells, providing a novel candidate prognostic factor and a potential anti-metastasis therapeutic target in osteosarcoma. 
 MicroRNA-133a functions as a tumor suppressor by targeting IGF-1R in hepatocellular carcinoma. MicroRNAs (miRNAs) are a class of small non-coding RNAs and have critical roles in tumorigenesis and metastasis. A growing body of evidence showed that microRNA-133a (miR-133a) was downregulated and played tumor suppressor roles in gastric, colorectal, bladder, and lung cancer. However, the role and underlying molecular mechanism of miR-133a in hepatocellular carcinoma (HCC) remain unclear. In this study, we analyzed the expression of miR-133a in HCC tissues and HCC cell lines. We find that miR-133a was downregulated in HCC tissues and cell lines and that miR-133a expression negatively correlated with tumor differentiation (P < 0.01), TNM stage (P < 0.01), and lymph node metastasis (P < 0.01). Then, functional studies demonstrate that restoration of miR-133a in HepG2 cells significantly suppressed proliferation, colony   formatio  n, migration, and invasion, induced cell cycle arrest at G0/G1 stage and cell apoptosis in vitro, and decreased tumor size and weight in a nude mouse HepG2 xenograft model. Using bioinformatics method and dual luciferase assays identified insulin-like growth factor 1 receptor (IGF-1R) as a direct target of miR-133a in HCC cells. Furthermore, overexpression of miR-133a inhibited activation AKT and ERK signal pathway, which contributed to suppression of HCC cell growth. These findings suggest that miR-133a may act as a tumor suppressor and inhibited survival of HCC cells by targeting IGF-1R. 
 microRNA-194 suppresses osteosarcoma cell proliferation and metastasis in vitro and in vivo by targeting CDH2 and IGF1R. Studies have shown that miR-194 functions as a tumor suppressor and is associated with tumor growth and metastasis. We studied the effects of miR-194 in osteosarcoma and the possible mechanism by which miR-194 affected the survival, apoptosis and metastasis of osteosarcoma. Both human osteosarcoma cell lines SOSP-9607 and U2-OS were transfected with recombinant lentiviruses to regulate miR-194 expression. Overexpression of miR-194 partially inhibited the proliferation,   migration  , and invasion of osteosarcoma cells in vitro, as well as tumor growth and pulmonary metastasis of osteosarcoma cells in vivo. Potential miR-194 target genes were predicted using bioinformatics. Luciferase reporter assay, real-time quantitative PCR and western blotting confirmed that CDH2 (N-cadherin) and IGF1R were targets of miR-194. Using real-time quantitative PCR, we evaluated the expression of miR-194 and two miR-194 target genes, CDH2 and IGF1R in osteosarcoma samples from 107 patients and 99 formalin- or paraformalin-fixed paraffin-embedded tissues. The expressions of the target genes were also examined in osteosarcoma samples using immunohistochemistry. Overexpression of miR-194 inhibited tumor growth and metastasis of osteosarcoma probably by downregulating CDH2 and IGF1R. miR-194 may prove to be a promising therapeutic agent for osteosarcoma. 
 Protein-tyrosine phosphatase 1B antagonized signaling by insulin-like growth factor-1 receptor and kinase BRK/PTK6 in ovarian cancer cells. Ovarian cancer, which is the leading cause of death from gynecological malignancies, is a heterogeneous disease known to be associated with disruption of multiple signaling pathways. Nevertheless, little is known regarding the role of protein phosphatases in the signaling events that underlie the disease; such knowledge will be essential to gain a complete understanding of the etiology of the disease and how to treat it. We have demonstrated that protein-tyrosine phosphatase 1B (PTP1B) was underexpressed in a panel of ovarian carcinoma-derived cell lines, compared with immortalized human ovarian surface epithelial cell lines. Stable restoration of PTP1B in those cancer cell lines substantially decreased cell   migration   and invasion, as well as proliferation and anchorage-independent survival. Mechanistically, the pro-survival IGF-1R signaling pathway was attenuated upon ectopic expression of PTP1B. This was due to dephosphorylation by PTP1B of IGF-1R β-subunit and BRK/PTK6, an SRC-like protein-tyrosine kinase that physically and functionally interacts with the IGF-1R β-subunit. Restoration of PTP1B expression led to enhanced activation of BAD, one of the major pro-death members of the BCL-2 family, which triggered cell death through apoptosis. Conversely, inhibition of PTP1B with a small molecular inhibitor, MSI-1436, increased proliferation an  d migrati  on of immortalized HOSE cell lines. These data reveal an important role for PTP1B as a negative regulator of BRK and IGF-1Rβ signaling in ovarian cancer cells. 
 Sphingosine 1-phosphate induces membrane ruffling and increases motility of human umbilical vein endothelial cells via vascular endothelial growth factor receptor and CrkII. Sphingosine 1-phosphate (S1P), a ligand for endothelial differentiation gene family proteins, is one of the most potent signal mediators released from activated platelets. Here, we report that S1P induces membrane ruffling of human umbilical vein endothelial cells (HUVECs) via the vascular endothelial growth factor receptor (VEGFR), Src family tyrosine kinase(s), and the CrkII adaptor protein. S1P induced prominent phosphorylation of CrkII in HUVECs, indicating that CrkII was involved in the S1P-induced signaling pathway. S1P-induced CrkII phosphorylation was blocked by pertussis toxin and overexpression of the carboxyl terminus of beta-adrenergic receptor kinase, indicating that the betagamma subunit of G(i) was required for the phosphorylation. Notably, the S1P-induced CrkII phosphorylation was also abolished by inhibitors of VEGFR or Src family tyrosine kinases. By using Picchu, a real time monitoring protein for CrkII phosphorylation, we found that S1P induced rapid CrkII phosphorylation at membrane ruffles. Finally, we observed that expression of a dominant negative mutant of CrkII inhibited the S1P-induced membrane ruffling and cell   migration  . These results delineated a novel S1P signaling pathway that involves sequential activation of G(i)-coupled receptor(s), VEGFR, Src family tyrosine kinase(s), and the CrkII adaptor protein, and which is responsible for both the induction of membrane ruffling and the increase in cell motility. 
 [Insulin-like growth factor receptor I signaling in a breast cancer cell line]. In vitro studies strongly suggest that proliferation,   migration   and cell survival of breast cancer cell lines are significantly affected by activation of the IGF type 1 receptor (IGF-IR).The phosphorylation by IGF-I of IGF-IR and the intracellular signaling molecules Akt (PI-3K pathway) and Erk1/2 (MAPK pathway) was characterized in a human breast cancer cell lines.The study compared a standard breast adenocarcinoma line (MCF-7) cell line with a line (CSC 1595) derived from an infiltrating ductal breast cancer in a Colombian patient. The CSC 1595 and MCF-7 cell lines were cultured in DMEM supplemented with 10% fetal bovine serum, 2 mM glutamine, 100 U/ml penicillin, and 100 µg/ml streptomycin and grown at 37 °C in 5% CO₂ atmosphere and 95% humidity. Cell extracts were prepared, followed by immunoprecipitation and immunoblotting with specific anti-pIGF-IR, anti-pERK1/2 and anti-pAkt antibodies.After 5 minute stimulation with IGF-I, 70% of the IGF-IR was phosphorylated in the cell line CSC 1595 and 25% in MCF-7. In addition, Akt (oncogene protein v-akt) and ERK1/2 (extracellular signal-regulated MAP kinases) were phosphorylated. Basal and stimulated levels of phosphorylated ERK1/2 were substantially higher compared to those in the MCF-7 cell line.The IGF-IR and MAPK kinase pathway involving proteins ERK1/2 showed more significant phosphorylation in the 1,595 cells compared to the observed in the MCF-7 cell line. Since the IGF-IR is the major activator of this pathway it may play an important role in ductal infiltrating breast cancer tumor growth and metastases. 
 Transactivation of CXCR4 by the insulin-like growth factor-1 receptor (IGF-1R) in human MDA-MB-231 breast cancer epithelial cells. In the multimolecular environment in tissues and organs, cross-talk between growth factor and G protein-coupled receptors is likely to play an important role in both normal and pathological responses. In this report, we demonstrate transactivation of the chemokine receptor CXCR4 by the growth factor insulin-like growth factor (IGF)-1 is required for IGF-1-induced cell   migration   in metastatic MDA-MB-231 cells. The induction of chemotaxis in MDA-MB-231 cells by IGF-1 was inhibited by pretreatment of the cells with pertussis toxin (PTX) and by RNAi-mediated knockdown of CXCR4. Transactivation of the CXCR4 pathway by IGF-1 occurred independently of CXCL12, the chemokine ligand of CXCR4. Neither CXCR4 knockdown nor PTX had any effect on the ability of IGF-1 to activate IGF-1R, suggesting that CXCR4 and G proteins are activated subsequent to, or independently of, phosphorylation of IGF-1R by IGF-1. Coprecipitation studies revealed the presence of a constitutive complex containing IGF-1R, CXCR4, and the G protein subunits, G(i)alpha2 and Gbeta, and stimulation of MDA-MB-231 cells with IGF-1 led to the release of G(i)alpha2 and Gbeta from CXCR4. Based on our findings, we propose that CXCR4 constitutively forms a complex with IGF-1R in MDA-MB-231 cells, and that this interaction allows IGF-1 to activate   migration  al signaling pathways through CXCR4, G(i)alpha2 and Gbeta. 
 Evaluation of the coordinated actions of estrogen receptors with epidermal growth factor receptor and insulin-like growth factor receptor in the expression of cell surface heparan sulfate proteoglycans and cell motility in breast cancer cells. Estradiol (E2)-estrogen receptor (ER) actions are implicated in initiation, growth and progression of hormone-dependent breast cancer. Crosstalk between ERs, epidermal growth factor receptor (EGFR) and/or insulin-like growth factor receptor (IGFR) is critical for the observed resistance to endocrine therapies. Cell surface heparan sulfate proteoglycans (HSPGs) are principal mediators of cancer cell properties and the E2-ER pathway as well as those activated by EGFR and IGFR have significant roles in regulating the expression of certain cell surface HSPGs, such as syndecan-2 (SDC-2), syndecan-4 (SDC-4) and glypican-1. In this study, we therefore evaluated the role of EGFR-IGFR signaling on the constitutive expression and E2-mediated expression of ERs and HSPGs as well as the effect of E2-ERs and IGFR/EGFR-mediated cell   migration   in ERα+ (MCF-7) and ERβ+ (MDA-MB-231) breast cancer cells using specific intracellular inhibitors of EGFR and IGFR. We report that the expression of ERα is mainly enhanced by IGFR, whereas ERβ expression is mainly coordinated by EGFR. Moreover, constitutive SDC-2 expression in ERα+ and ERβ+ cells is mainly mediated through the IGFR, whereas in ERα+ E2-treated cells EGFR is the active one. In contrast, SDC-4 expression is regulated by IGFR in the presence and absence of E2. E2 also seems to diminish the inhibitory effect of EGFR and IGFR inhibitors in breast cance  r cell mi  gration. These data suggest that the coordinated action of ERs with EGFR and/or IGFR is of crucial importance, providing potential targets for designing and developing novel multi-potent agents for endocrine therapies. 
 EGFR and IGF-1R in regulation of prostate cancer cell phenotype and polarity: opposing functions and modulation by T-cadherin. T-cadherin is an atypical glycosylphosphatidylinsoitol-anchored member of the cadherin superfamily of adhesion molecules. We found that T-cadherin overexpression in malignant (DU145) and benign (BPH-1) prostatic epithelial cell lines or silencing in the BPH-1 cell line, respectively, promoted or inhibited   migration   and spheroid invasion in collagen I gel and Matrigel. T-cadherin-dependent effects were associated with changes in cell phenotype: overexpression caused cell dissemination and loss of polarity evaluated by relative positioning of the Golgi/nuclei in cell groups, whereas silencing caused formation of compact polarized epithelial-like clusters. Epidermal growth factor receptor (EGFR) and IGF factor-1 receptor (IGF-1R) were identified as mediators of T-cadherin effects. These receptors per se had opposing influences on cell phenotype. EGFR activation with EGF or IGF-1R inhibition with NVP-AEW541 promoted dissemination, invasion, and polarity loss. Conversely, inhibition of EGFR with gefitinib or activation of IGF-1R with IGF-1 rescued epithelial morphology and decreased invasion. T-cadherin silencing enhanced both EGFR and IGF-1R phosphorylation, yet converted cells to the morphology typical for activated IGF-1R. T-cadherin effects were sensitive to modulation of EGFR or IGF-1R activity, suggesting direct involvement of both receptors. We conclude that T-cadherin regulates prostate cancer cell behavior by tuning the balance in EGFR/IGF-1R activity and enhancing the impact of IGF-1R. 
 Fibulin-3-mediated inhibition of epithelial-to-mesenchymal transition and self-renewal of ALDH+ lung cancer stem cells through IGF1R signaling. Fibulins (FBLNs), a family of extracellular matrix proteins, have recently been shown to act as tumor suppressors or activators in different cancers, and the underlying molecular mechanisms of their action in cancer remain unclear. We have previously shown that the expression of FBLN3 is suppressed by promoter hypermethylation and is associated with invasiveness in aggressive non-small cell lung cancer. In this study, we evaluated the roles and signaling mechanism of FBLN3 in lung cancer stem cells (CSCs). Forced expression of FBLN3 suppressed invasion and   migration   of lung adenocarcinoma cells and decreased the expression of epithelial-to-mesenchymal transition (EMT) activators, including N-cadherin and Snail. Stemness activities of lung adenocarcinoma cells were also suppressed by FBLN3 as indicated by a decrease in spheroid formation and the levels of stemness markers such as Sox2 and β-catenin. These effects of FBLN3 were mediated by the glycogen synthase kinase-3β, GSK3β/β-catenin pathway, and the upstream regulators of GSK3β, including phosphoinositide 3-kinase (PI3K)/AKT and insulin-like growth factor-1 receptor (IGF1R), were inactivated by FBLN3. Moreover, IGF1R was shown to be a direct target of FBLN3, which competitively inhibited insulin-like growth factor (IGF) action. To confirm the effect of FBLN3 on lung CSCs, aldehyde dehydrogenase-positive (ALDH+) A549 lung CSCs were sorted and treated with recombinant FBLN3 protein. FBLN3 clearly suppressed EMT, stemness activity and the over-activated IGF1R/PI3K/AKT/GSK3β pathway of the ALDH+ CSC subpopulation. In addition, injection of recombinant FBLN3 protein around subcutaneous xenografts established with ALDH+ CSCs in athymic nude mice significantly suppressed tumor growth and progression. Overall, our results show that FBLN3 suppresses both EMT and self-renewal of the lung CSCs by modulating the IGF1R/PI3K/AKT/GSK3β pathway and that FBLN3 would be useful as an alternative CSC therapy. 
 Autocrine insulin-like growth factor-II stimulation of tumor cell   migration   is a progression step in human hepatocarcinogenesis. The protumorigenic insulin-like growth factor (IGF)-II is highly expressed in a significant fraction of human hepatocellular carcinomas (HCC). However, a functional dissection that clarifies the contribution of IGF-II-binding receptors in tumor progression and a respective molecular characterization of IGF-II signaling has not been performed. Therefore, expression of IGF-II and its receptors IGF-receptor type I (IGF-IR) and insulin receptor (IR) was efficiently blocked using small interfering RNA (siRNA) in HCC cells. Despite functional IR-signaling, oncogenic IGF-II effects such as tumor cell viability, proliferation, and anti-apoptosis were solely transmitted by IGF-IR. Although IGF-II signaling was previously not described in the context of HCC cell   migration  , the IGF-II-dependent expression profile displayed a high percentage of genes involved in cell motility and adhesion. Indeed, IGF-II overexpression promoted HCC cell   migration  , especially in synergy with hepatocyte growth factor (HGF). The therapeutic relevance of IGF-II/IGF-IR signaling was tested in vitro and in a murine xenograft transplantation model using the IGF-IR inhibitor picropodophyllin (PPP). IGF-IR inhibition by small molecule treatment efficiently reduced IGF-II-dependent signaling and all protumorigenic properties of the IGF-II/IGF-IR pathway.In human HCC cells, IGF-IR but not IR is involved in oncogenic IGF-II signaling. Autocrine stimulation of IGF-II induces HCC motility by integration of paracrine signals for full malignant competence. Thus, activation of IGF-II/IGF-IR signaling is likely a progression switch selected by function that promotes tumor cell dissemination and aggressive tumor behavior. 
 A PDZ domain protein interacts with the C-terminal tail of the insulin-like growth factor-1 receptor but not with the insulin receptor. In this study, we report on the isolation of a PDZ domain protein, here designated as IIP-1, insulin-like growth factor-1 (IGF-1) receptor-interacting protein-1, which binds to the IGF-1 receptor, but not to the related insulin receptor, and which is involved in the regulation of cell motility. The interaction between the IGF-1 receptor and IIP-1 as well as a splice variant IIP-1/p26 was demonstrated in the yeast two-hybrid system. Using co-precipitation experiments, we confirmed the interaction in transfected cells as well as in vitro. Analysis of deletion mutants indicates that the PDZ domain of IIP-1 mediates interaction with the C-terminal tail of the IGF-1 receptor (serine-threonine-cysteine). This finding demonstrates that the C terminus of the IGF-1 receptor acts as novel PDZ domain binding site. Immunofluorescence analysis revealed an overlapping localization of IIP-1 and the IGF-1 receptor in the breast cancer cell line MCF-7. A functional connection between IIP-1 and the IGF-1 receptor is further supported by the finding that the level of expression of IIP-1 and the IGF-1 receptor strongly correlates in different normal and cancer cells. Furthermore, overexpression of IIP-1 resulted in an attenuation of   migration   of MCF-7 cells, which is one of the biological activities mediated by the IGF-1 signaling system. 
 LMP1 promotes expression of insulin-like growth factor 1 (IGF1) to selectively activate IGF1 receptor and drive cell proliferation. Epstein-Barr Virus (EBV) is a gammaherpesvirus that infects the majority of the human population and is linked to the development of multiple cancers, including nasopharyngeal carcinoma. Latent membrane protein 1 (LMP1) is considered the primary oncoprotein of EBV, and in epithelial cells it induces the expression and activation, or phosphorylation, of the epidermal growth factor receptor kinase. To identify effects on additional kinases, an unbiased screen of receptor tyrosine kinases potentially activated by LMP1 was performed. Using a protein array, it was determined that LMP1 selectively activates insulin-like growth factor 1 receptor (IGF1R). This activation takes place in fibroblast, epithelial, and nasopharyngeal cell lines that express LMP1 stably and transiently. Of note, LMP1 altered the phosphorylation, but not the expression, of IGF1R. The use of LMP1 mutants with defective signaling domains revealed that the C-terminal activating region 2 domain of LMP1 increased the mRNA expression and the secretion of the ligand IGF1, which promoted phosphorylation of IGF1R. IGF1R phosphorylation was dependent upon activation of canonical NF-κB signaling and was suppressed by IκBα and a dominant negative form of TRAF6. Inhibition of IGF1R activation with two small-molecule inhibitors, AG1024 and picropodophyllin (PPP), or with short hairpin RNA (shRNA) directed against IGF1R selectively reduced proliferation, focus formation, and Akt activation in LMP1-positive cells but did not impair LMP1-induced ce  ll migrat  ion. Expression of constitutively active Akt rescued cell proliferation in the presence of IGF1R inhibitors. These findings suggest that LMP1-mediated activation of IGF1R contributes to the ability of LMP1 to transform epithelial cells.EBV is linked to the development of multiple cancers in both lymphoid and epithelial cells, including nasopharyngeal carcinoma. Nasopharyngeal carcinoma is a major cancer that develops in specific populations, with nearly 80,000 new cases reported annually. LMP1 is consistently expressed in early lesions and continues to be detected within 50 to 80% of these cancers at later stages. It is therefore of paramount importance to understand the mechanisms through which LMP1 alters cell growth and contributes to tumorigenesis. This study is the first to determine that LMP1 activates the IGF1R tyrosine kinase by regulating expression of the ligand IGF1. Additionally, the data in this paper reveal that specific targeting of IGF1R selectively impacts LMP1-positive cells. These findings suggest that therapies directed against IGF1R may specifically impair the growth of EBV-infected cells. 
 MicroRNA-145 directly targets the insulin-like growth factor receptor I in human bladder cancer cells. The insulin-like growth factor receptor I (IGF-IR) is a proto-oncogene with potent mitogenic and antiapoptotic activities. It has been reported that expression of IGF-IR is up-regulated in bladder cancer. Here, we assessed whether microRNA-145 (miR-145) regulates IGF-IR expression in bladder cancer. In our study, miR-145 was shown to directly target IGF-IR 3'-untranslated region (UTR) in human bladder cancer cells. Small interfering RNA (siRNA)- and miR-145-mediated IGF-IR knockdown experiments revealed that miR-145 promotes cell apoptosis, and suppresses cell proliferation and   migration   through suppression of IGF-IR expression. Taken together, our data suggest that miR-145 may inhibit bladder cancer initiation by affecting IGF-IR signaling. 
 Germline polymorphisms in genes involved in the IGF1 pathway predict efficacy of cetuximab in wild-type KRAS mCRC patients. The insulin-like growth factor 1 (IGF1) signaling pathway is an important growth-regulatory pathway, which plays a crucial role in colorectal cancer (CRC) proliferation, differentiation,   migration  , angiogenesis, and apoptosis. Previous studies showed that hyperactivation of the IGF1 receptor (IGF1R) may result in resistance to anti-epidermal growth factor receptor-targeted treatment. We tested whether germline variations within the IGF1 pathway are associated with clinical outcome in wild-type (wt) KRAS drug-refractory metastatic CRC (mCRC) patients who were treated with cetuximab monotherapy (IMC-0144).Formalin-fixed, paraffin-embedded (FFPE) tissue samples of 130 drug-refractory mCRC patients enrolled in IMC-0144, a phase II clinical trial of cetuximab monotherapy, were analyzed. gDNA was extracted from dissected FFPE tumor tissue, and KRAS mutation status and six potentially functional IGF1 and IGF1R polymorphisms were analyzed using direct DNA sequencing or PCR-RFLP. Tumor response analysis was based on recursive partitioning, and survival analyses were based on univariate and multivariate hazard regression models.In univariate and multivariate analyses, five IGF pathway single-nucleotide polymorphisms were significantly associated with progression-free survival (PFS) and/or overall survival (OS). In multivariate combined risk allele analysis, the additive model for PFS and OS was significantly associated with the number of risk alleles in wt KRAS patients (P = 0.001 and P = 0.02, respectively). In addition, wt KRAS patients harboring IGF1 rs2946834 A/A genotype had a 50% objective response rate compared with 0% for A/G genotype.These results indicate that IGF1 pathway polymorphisms are potential predictive/prognostic molecular markers for cetuximab efficacy in wt KRAS mCRC patients. Prospective biomarker-embedded clinical trials are warranted to validate our findings. Clin Cancer Res; 16(22); 5591-602. ©2010 AACR. 
 Insulin-like growth factor receptor as a therapeutic target in head and neck cancer. Insulin-like growth factor type I receptor (IGF-IR) plays critical roles in epithelial cancer cell development, proliferation, motility, and survival, and new therapeutic agents targeting IGF-IR are in development. Another receptor tyrosine kinase, the epidermal growth factor receptor (EGFR), is an established therapeutic target in head and neck cancer and IGF-IR/EGFR heterodimerization has been reported in other epithelial cancers. The present study was undertaken to determine the effects of anti-IGF-IR therapeutic targeting on cell signaling and cancer cell phenotypes in squamous cell carcinomas of the head and neck (SCCHN).The therapeutic efficacy of the human anti-IGF-IR antibody IMC-A12 alone and in combination with the EGFR blocking antibody cetuximab (C225) was tested in SCCHN cell lines and in tumor xenografts.IGF-IR was overexpressed in human head and neck cancer cell lines and tumors. Pretreatment of serum-starved 183A or TU159 SCCHN cell lines with A12 (10 microg/mL) blocked IGF-stimulated activation of IGF-IR, insulin receptor substrate (IRS)-1 and IRS-2, mitogen-activated protein kinase, and phosphatidylinositol 3-kinase. A12 induced G(0)-G(1) cell cycle arrest and blocked cell growth, motility, and anchorage-independent growth. Stimulation of head and neck cancer cells with either IGF or EGF resulted in IGF-IR and EGFR heterodimerization, but only IGF caused activating phosphorylation of both receptors. Combined treatment with A12 and the EGFR blocking antibody C225 was more effective at reducing cell proliferation and   migration   than either agent alone. Finally, TU159 tongue cancer cell xenografts grown in athymic nude mice were treated thrice weekly for 4 weeks with vehicle, A12 (40 mg/kg i.p.), C225 (40 mg/kg i.p.), or both agents (n=8 mice per group; 2 tumors per mouse). Linear regression slope analysis showed significant differences in median tumor volume over time between all three treatment groups and the control group. Complete regression was seen in 31% (A12), 31% (C225), and 44% (A12 + C225) of tumors.Here we found the overexpression of IGF-IR, the functional heterodimerization of IGF-IR and EGFR, and effective therapeutic targeting of these receptors in human head and neck cancer xenografts. 
 The reciprocal regulation of gamma-synuclein and IGF-I receptor expression creates a circuit that modulates IGF-I signaling. Insulin-like growth factor (IGF) system plays important roles in carcinogenesis and maintenance of the malignant phenotype. Signaling through the IGF-I receptor (IGF-IR) has been shown to stimulate the growth and motility of a wide range of cancer cells. γ-synuclein (SNCG) is primarily expressed in peripheral neurons but also overexpressed in various cancer cells. Overexpression of SNCG correlates with tumor progression. In the present study we demonstrated a reciprocal regulation of IGF-I signaling and SNCG expression. IGF-I induced SNCG expression in various cancer cells. IGF-IR knockdown or IGF-IR inhibitor repressed SNCG expression. Both phosphatidylinositol 3-kinase and mitogen-activated protein kinase were involved in IGF-I induction of SNCG expression. Interestingly, SNCG knockdown led to proteasomal degradation of IGF-IR, thereby decreasing the steady-state levels of IGF-IR. Silencing of SNCG resulted in a decrease in ligand-induced phosphorylation of IGF-IR and its downstream signaling components, including insulin receptor substrate (IRS), Akt, and ERK1/2. Strikingly, SNCG physically interacted with IGF-IR and IRS-2. Silencing of IRS-2 impaired the interaction between SNCG and IGF-IR. Finally, SNCG knockdown suppressed IGF-I-induced cell proliferation and   migratio  n. These data reveal that SNCG and IGF-IR are mutually regulated by each other. SNCG blockade may suppress IGF-I-induced cell proliferation and   migratio  n. Conversely, IGF-IR inhibitors may be of utility in suppressing the aberrant expression of SNCG in cancer cells and thereby block its pro-tumor effects. 
 Selective tyrosine kinase inhibition of insulin-like growth factor-1 receptor inhibits human and mouse breast cancer-induced bone cell activity, bone remodeling, and osteolysis. Insulin-like growth factor 1 (IGF-1) plays an important role in both bone metabolism and breast cancer. In this study, we investigated the effects of the novel IGF-1 receptor tyrosine kinase inhibitor cis-3-[3-(4-methyl-piperazin-l-yl)-cyclobutyl]-1-(2-phenyl-quinolin-7-yl)-imidazo[1,5-a]pyrazin-8-ylamine (PQIP) on osteolytic bone disease associated with breast cancer. Human MDA-MB-231 and mouse 4T1 breast cancer cells enhanced osteoclast formation in receptor activator of NF-κB ligand (RANKL) and macrophage colony-stimulating factor (M-CSF) stimulated bone marrow cultures, and these effects were significantly inhibited by PQIP. Functional studies in osteoclasts showed that PQIP inhibited both IGF-1 and conditioned medium-induced osteoclast formation by preventing phosphatidylinositol 3-kinase (PI3K)/protein kinase B (Akt) activation without interfering with RANKL or M-CSF signaling. Treatment of osteoblasts with PQIP significantly inhibited the increase in RANKL/osteoprotegerin (OPG) ratio by IGF-1 and conditioned medium and totally prevented conditioned medium-induced osteoclast formation in osteoblast-bone marrow (BM) cell cocultures, thereby suggesting an inhibitory effect on osteoblast-osteoclast coupling. PQIP also inhibited IGF-1-induced osteoblast differentiation, spreading,   migratio  n, and bone nodule formation. Treatment with PQIP significantly reduced MDA-MB-231 conditioned medium-induced osteolytic bone loss in a mouse calvarial organ culture system ex vivo and in adult mice in vivo. Moreover, once daily oral administration of PQIP significantly decreased trabecular bone loss and reduced the size of osteolytic bone lesions following 4T1 intratibial injection in mice. Quantitative histomorphometry showed a significant reduction in bone resorption and formation indices, indicative of a reduced rate of cancer-associated bone turnover. We conclude that inhibition of IGF-1 receptor tyrosine kinase activity by PQIP suppresses breast cancer-induced bone turnover and osteolysis. Therefore, PQIP, and its novel derivatives that are currently in advanced clinical development for the treatment of a number of solid tumors, may be of value in the treatment of osteolytic bone disease associated with breast cancer. 
 The role of EphB4 and IGF-IR expression in breast cancer cells. to investigate the role of EphB4 and IGF-1R in the proliferation and   migration   of breast cancer.The relative mRNA levels of EphB4 were measured by RT-PCR. The proliferation of the cells was determined by MTT assay, and cells   migration   and invasive ability was analyzed using the scratch   migration   assay.The expression of EphB4 in control group was significantly decreased when compared with IGF-I group (P<0.001). The expression of EphB4 in IGF-I+LY and LY group were lower than that of the control group (P<0.001).The cell proliferation and   migration   ability of the cells in IGF-I group increased significantly compared to the cells in the control group (P<0.001), while the cells in IGF-1+LY group and LY group showed a decreased proliferation and   migration   ability compared to the control group (P<0.001).IGF-IR might be a upstream gene of EphB4. Besides, higher expression of EphB4 shows increased tumor proliferation and   migration   in breast cells. The study of EphB4 upstream gene and signaling pathway can provide more targeted anti-tumor point selection for targeted therapy. 
 P-cadherin cooperates with insulin-like growth factor-1 receptor to promote metastatic signaling of gonadotropin-releasing hormone in ovarian cancer via p120 catenin. Gonadotropin-releasing hormone (GnRH) is a potent prometastatic factor in ovarian cancer, but the intracellular signaling events are not well understood. The classical Gα(q)-phospholipase C signal transduction pathway known to operate in the pituitary is not involved in GnRH actions at non-pituitary targets. Here we showed that GnRH treatment of ovarian cancer cells led to a rapid and remarkable tyrosine phosphorylation of p120 catenin (p120(ctn)), which was mediated by P-cadherin. The use of P-cadherin small interfering RNA or neutralizing antibodies to inhibit P-cadherin expression and function resulted in diminished p120(ctn) activation, confirming that the effect was P-cadherin specific. On exploring how P-cadherin, which lacks intrinsic kinase activity, might regulate the activation of p120(ctn), we found that P-cadherin could induce the ligand-independent activation of insulin-like growth factor-1 receptor (IGF-1R). Inhibition of IGF-1R expression or its activity significantly inhibited GnRH-induced p120(ctn) activation, and the subsequent cell   migratio  n and invasion. In addition, we showed that IGF-1R regulation by P-cadherin was associated with complex formation between IGF-1R and P-cadherin, and this regulation was also observed to be in vivo correlated with metastasis. Furthermore, using a mouse model of ovarian cancer metastasis, GnRH receptor knockdown was shown to diminish peritoneal dissemination of tumors and ascites formation. These findings suggest for the first time that GnRH can initiate an outside-in p120(ctn) signal transduction through the cross-talk between P-cadherin and IGF-1R, thus providing a novel molecular mechanism by which GnRH may control the high level of aggressiveness and invasion and metastasis potential that are characteristic of ovarian cancer. 
 Rho guanosine 5'-triphosphatases differentially regulate insulin-like growth factor I (IGF-I) receptor-dependent and -independent actions of IGF-II on human trophoblast   migration  . Both IGF-I and IGF-II stimulate   migration   of human extravillous trophoblast (EVT) cells. Although IGF-I is known to signal through IGF type 1 receptor (IGF1R), IGF-II signals through IGF1R as well as in an IGF1R-independent manner. The purpose of this study was to investigate the roles of Rho GTPases in IGF1R-independent and -dependent actions of IGF-II on EVT cell   migration  . To distinguish IGF1R-dependent and -independent actions, we used picropodophyllin, a selective inhibitor of IGF1R tyrosine kinase, and IGF analogs with differential affinities for IGF1R, IGF-II/cation-independent mannose 6-phosphate receptor, and IGF-binding proteins. IGF1R-dependent actions of IGF-II were confirmed by showing the effects of IGF1R-selective agonist Des1-3 IGF-I. We used pharmacological inhibitors or selective small interfering RNAs to investigate the roles of RhoA, RhoC, Rac1, Cdc42, and Rho effector kinases called ROCK-I and -II in IGF-induced EVT cell   migration  . Although basal   migration   of EVT cells required each member of the Rho GTPase family studied, IGF1R-dependent and -independent EVT cell   migration   exhibited differential requirements for these enzymes. IGF1R-mediated EVT cell   migration   was found to depend on RhoA and RhoC but not on Rac1 or Cdc42. However, IGF1R-independent effect of IGF-II on EVT cell   migration   required ROCKs but not RhoA, RhoC, Rac1, or Cdc42. Most importantly, IGF1R-independent action of IGF-II was found to be exaggerated when RhoA or RhoC was down-regulated. Thus, different members of the Rho GTPase family regulate IGF-II-mediated EVT cell   migration   differentially, depending upon whether it signals through IGF1R or in an IGF1R-independent manner. 
 c-Met, epidermal growth factor receptor, and insulin-like growth factor-1 receptor are important for growth in uveal melanoma and independently contribute to   migration   and metastatic potential. Uveal melanoma (UM) has a high propensity to develop hepatic metastases. We sought to define the mechanisms required for preferential liver homing and to understand further the biologic behavior of this disease. The Met tyrosine kinase receptor and its ligand hepatocyte growth factor are expressed in hepatocytes. We therefore considered Met/hepatocyte growth factor signaling as a candidate   migration  /growth factor for UM cells. We further explored the relationship between c-Met and other growth factor receptors prevalent in the liver and their roles in UM metastatic potential. UM cell lines were evaluated for c-Met, epidermal growth factor receptor (EGFR), and insulin-like growth factor-1R (IGF-1R) expression by immunoblotting, and gene amplification by comparative genomic hybridization and fluorescence in-situ hybridization. High c-Met, phosphorylated c-Met, and EGFR expression were noted in two of nine cell lines, independent of IGF-1R levels. Knockdown of c-Met decreased proliferation of high c-Met-expressing UM cells but did not induce apoptosis. Selective inhibitors of EGFR and IGF-1R decreased proliferation and induced apoptosis in UM cells regardless of the expression levels of c-Met, EGFR, and IGF-1R. Although c-Met, EGFR, and IGF-1R play proliferative roles, EGFR and IGF-1R are also critical for UM cell survival. High c-Met/EGFR-expressing cell lines possessed the greatest   migration   potential. c-Met knockdown and selective inhibitors of c-Met, EGFR, and IGF-1R revealed independent contribution of these receptors to   migration  . UM can be categorized by levels of c-Met and EGFR expression which are associated with migratory/invasiveness responses to soluble factors present at high levels in the liver. This provides biologic relevance for UM clinical behavior with potential therapeutic implications. 
 Constitutively active type I insulin-like growth factor receptor causes transformation and xenograft growth of immortalized mammary epithelial cells and is accompanied by an epithelial-to-mesenchymal transition mediated by NF-kappaB and snail. Type I insulin-like growth factor receptor (IGF-IR) can transform mouse fibroblasts; however, little is known about the transforming potential of IGF-IR in human fibroblasts or epithelial cells. We found that overexpression of a constitutively activated IGF-IR (CD8-IGF-IR) was sufficient to cause transformation of immortalized human mammary epithelial cells and growth in immunocompromised mice. Furthermore, CD8-IGF-IR caused cells to undergo an epithelial-to-mesenchymal transition (EMT) which was associated with dramatically increased   migration   and invasion. The EMT was mediated by the induction of the transcriptional repressor Snail and downregulation of E-cadherin. NF-kappaB was highly active in CD8-IGF-IR-MCF10A cells, and both increased levels of Snail and the EMT were partially reversed by blocking NF-kappaB or IGF-IR activity. This study places IGF-IR among a small group of oncogenes that, when overexpressed alone, can confer in vivo tumorigenic growth of MCF10A cells and indicates the hierarchy in the mechanism of IGF-IR-induced EMT. 
 Suppression of type 1 Insulin-like growth factor receptor expression by small interfering RNA inhibits A549 human lung cancer cell invasion in vitro and metastasis in xenograft nude mice. Cancer invasion and metastasis, involving a variety of pathological processes and cytophysiological changes, contribute to the high mortality of lung cancer. The type 1 insulin-like growth factor receptor (IGF-1R), associated with cancer progression and invasion, is a potential anti-invasion and anti-metastasis target in lung cancer. To inhibit the invasive properties of lung cancer cells, we successfully down-regulated IGF-1R gene expression in A549 human lung cancer cells by small interfering RNA (siRNA) technology, and evaluated its effects on invasion-related gene expression, tumor cell in vitro invasion, and metastasis in xenograft nude mice. A549 cells transfected with a plasmid expressing hairpin siRNA for IGF-1R showed a significantly decreased IGF-1R expression at the mRNA level as well as the protein level. In biological assays, transfected A549 cells showed a significant reduction of cell-matrix adhesion,   migration   and invasion. Consistent with these results, we found that down-regulation of IGR-1R concomitantly accompanied by a large reduction in invasion-related gene expressions, including MMP-2, MMP-9, u-PA, and IGF-1R specific downstream p-Akt. Direct tail vein injections of plasmid expressing hairpin siRNA for IGF-1R significantly inhibited the formation of lung metastases in nude mice. Our results showed the therapeutic potential of siRNA as a method for gene therapy in inhibiting lung cancer invasion and metastasis. 
 Identification of the cathelicidin peptide LL-37 as agonist for the type I insulin-like growth factor receptor. The human cathelicidin antimicrobial protein-18 and its C terminal peptide, LL-37, displays broad antimicrobial activity that is mediated through direct contact with the microbial cell membrane. In addition, recent studies reveal that LL-37 is involved in diverse biological processes such as immunomodulation, apoptosis, angiogenesis and wound healing. An intriguing role for LL-37 in carcinogenesis is also beginning to emerge and the aim of this paper was to explore if and how LL-37 contributes to the signaling involved in tumor development. To this end, we investigated the putative interaction between LL-37 and growth factor receptors known to be involved in tumor growth and progression. Among several receptors tested, LL-37 bound with the highest affinity to insulin-like growth factor 1 receptor (IGF-1R), a receptor that is strongly linked to malignant cellular transformation. Furthermore, this interaction resulted in a dose-dependent phosphorylation and ubiquitination of IGF-1R, with downstream signaling confined to the mitogen-activated protein kinase/extracellular signal-regulated kinase (ERK)-pathway but not affecting phosphatidylinositol 3 kinase/Akt signaling. We found that signaling induced by LL-37 was dependent on the recruitment of β-arrestin to the fully functional IGF-1R and by using mutant receptors we demonstrated that LL-37 signaling is dependent on β-arrestin-1 binding to the C-terminus of IGF-1R. When analyzing the biological consequences of increased ERK activation induced by LL-37, we found that it resulted in enhance  d migrati  on and invasion of malignant cells in an IGF-1R/β-arrestin manner, but did not affect cell proliferation. These results indicate that LL-37 may act as a partial agonist for IGF-1R, with subsequent intra-cellular signaling activation driven by the binding of β-arrestin-1 to the IGF-1R. Functional experiments show that LL-37-dependent activation of the IGF-1R signaling resulted in increased migratory and invasive potential of malignant cells. 
 Superior antitumor activity of a novel bispecific antibody cotargeting human epidermal growth factor receptor 2 and type I insulin-like growth factor receptor. The humanized anti-HER2 monoclonal antibody (mAb) trastuzumab (Herceptin; Genentech) effectively inhibits human epidermal growth factor receptor 2 (HER2)-positive breast tumors. However, many patients responding to treatment often develop resistance. Cross-talk between type I insulin-like growth factor receptor (IGF-IR) and HER2 and elevated IGF-IR signaling have been implicated in tumor cell resistance to trastuzumab therapy. Previously, we reported that the anti-IGF-IR mAb m590 inhibits proliferation and   migration   of breast cancer MCF-7 cells in vitro. Here, we generated a "knobs-into-holes" bispecific antibody (Bi-Ab) against HER2 and IGF-IR by engineering trastuzumab and m590. We compared the effects of Bi-Ab treatment in vitro and in SKOV-3 HER2- and IGF-IR-overexpressing cancer xenograft mouse model with those of m590 and trastuzumab treatment alone or in combination. Bi-Ab effectively inhibited proliferation of HER2- and IGF-IR-overexpressing ovarian cancer SKOV-3 cells in vitro by ablating receptor phosphorylation and downstream PI3K/Akt and mitogen-activated protein kinase signaling. Bi-Ab more effectively inhibited cancer growth in SKOV-3 HER2- and IGF-IR-overexpressing cancer xenograft mouse model than m590 and trastuzumab alone or in combination. Mice bearing SKOV-3 HER2- and IGF-IR-overexpressing xenografts showed extensive and sustainable tumor regression when treated with Bi-Ab. Our results suggest that Bi-Ab has superior antitumor activity compared with monospecific antibodies, and cotargeting HER2 and IGF-IR may be clinically beneficial in minimizing the acquired resistance to trastuzumab therapy. 
 Tumor suppressor role of miR-217 in human epithelial ovarian cancer by targeting IGF1R. Accumulating evidence shows that microRNA-217 (miR-217) is frequently dysregulated in various cancers, and plays crucial roles in tumorigenesis and metastasis; however, the role and underlying molecular mechanism of miR-217 in human epithelial ovarian cancer (EOC) remains unclear. Here, we report that miR-217 expression was downregulated in EOC tissue and inversely correlated with advanced FIGO stage, high histological grading and lymph node metastasis (P<0.01). Function analysis revealed that the ectopic expression of miR-217 in EOC cells inhibited cell proliferation,   migration   and invasion in vitro, as well as suppressed tumor growth in vivo. Bioinformatics analysis and dual luciferase assays identified insulin-like growth factor 1 receptor (IGF1R) as a direct target of miR-217 in EOC cells. Western blot assay showed that overexpression of miR-217 in EOC cells inhibited IGF1R expression. In addition, downregulation of IGF1R mimicked the tumor-suppressive effects of miR-217 in EOC cells, whereas the reintroduction of IGF1R partially abrogated the suppression effect induced by miR-217 on EOC cells. Collectively, these results demonstrated that miR-217 plays a tumor suppressor role in human epithelial ovarian cancer by directly targeting IGF1R gene, suggesting a new potential therapeutic target in EOC. 
 Precise mapping of an IGF-I-binding site on the IGF-1R. The IGF-1R [type 1 IGF (insulin-like growth factor) receptor] is activated upon binding to IGF-I and IGF-II leading to cell growth, survival and   migration   of both normal and cancerous cells. We have characterized the binding interaction between the IGF-1R and its ligands using two high-affinity mouse anti-IGF-1R mAbs (monoclonal antibodies), 7C2 and 9E11. These mAbs both block IGF-I binding to the IGF-1R but have no effect on IGF-II binding. Epitope mapping using chimaeras of the IGF-1R and insulin receptor revealed that the mAbs bind to the CR (cysteine-rich) domain of IGF-1R. The epitope was finely mapped using single point mutations in the IGF-1R. Mutation of Phe241, Phe251 or Phe266 completely abolished 7C2 and 9E11 binding. The three-dimensional structure showed that these residues cluster on the surface of the CR-domain. BIAcore analyses revealed that IGF-I and a chimaeric IGF-II with the IGF-I C-domain competed for the binding of both mAbs with the IGF-1R, whereas neither IGF-II nor a chimaeric IGF-I with the IGF-II C-domain affected antibody binding. We therefore conclude the IGF-I C-domain interacts with the CR (cysteine-rich) domain of the receptor at the cluster of residues Phe241, Phe251 and Phe266. These results allow precise orientation of IGF-I within the IGF-I-IGF-1R complex involving the IGF-I C-domain binding to the IGF-1R CR domain. In addition, mAbs 7C2 and 9E11 inhibited both IGF-I- and IGF-II-induced cancer cell proliferation,   migration   and IGF-1R down-regulation, demonstrating that targeting the IGF-1R is an effective strategy for inhibition of cancer cell growth. 
 MicroRNA-320a suppresses in GBM patients and modulates glioma cell functions by targeting IGF-1R. Glioblastoma (GBM) is the most aggressive and malignant glioma. Currently, a few modern surgical and medical therapeutic strategies are applied for GBM, but the prognosis of GBM patients remains poor, and the average median survival time is only 14.6 months. In this study, we for the first time found that the levels of miR-320a were decreased in both GBM patients and glioma cells. In GBM patients, elevated miR-320a expression was associated with better prognosis. In addition, insulin-like growth factor-1 receptor (IGF-1R) was identified as a key direct target of miR-320a. Overexpression of miR-320a led to the inhibition of cell proliferation,   migratio  n, invasion, as well as tumorigenesis by targeting IGF-1R, and thus regulated the signaling pathways downstream, including PI3K/AKT and MAPK/ERK. In tumor orthotopic xenograft experiment, the tumor growth was depressed and survival time of mice model was prolonged when miR-320a was overexpressed. Therefore, our results suggested that miR-320a could suppress tumor development and growth by targeting IGF-1R, and miR-320a might serve as a new effective target for anti-cancer therapy strategies. 
 Expression of insulin-like growth factor receptor type 1 correlate with lymphatic metastases in human gastric cancer. Most patients with gastric cancer are diagnosed at advanced clinical stages with a high frequency of lymph node metastasis. It is very important to find novel factors for the early diagnostic and prognostic evaluation of gastric cancer. It has been shown that IGF-1R activates mitotic division and inhibits apoptosis of cancer cells through the activation of signaling MAP/ERK and PI3K/Akt-1 pathways. IGF-1R plays a role in cell transformation and maintenance of the phenotype in modified cells. Moreover, an IGF-1 receptor effect influences the processes of adhesion,   migration  , invasion and metastasis of tumor cells. The aim of the study was to assess the expression of IGF-1R in gastric carcinoma in correlation with selected anatomo-clinical parameters. The study enrolled a group of 49 patients treated surgically for gastric cancer. 28 patients had no lymph node metastases. The expression of the studied proteins was assessed using the immunohistochemical method. We found that the expression of IGF-1R in gastric cancer is associated with lymph node metastasis (p < 0.001), is correlated with worse prognosis and high histological malignancy grade, and is an independent predictor of survival in patients with gastric cancer (p < 0.001). IGF-1R may play an important role in tumor growth and metastasis via the lymphatic pathway. 
 Tyrosine 221 in Crk regulates adhesion-dependent membrane localization of Crk and Rac and activation of Rac signaling. The adaptor protein CrkII plays a central role in signal transduction cascades downstream of a number of different stimuli. We and others have previously shown that CrkII mediates attachment-induced JNK activation, membrane ruffling and cell motility in a Rac-dependent manner. We report here that cell attachment leads to tyrosine phosphorylation of CrkII on Y221, and that CrkII-Y221F mutant demonstrates enhanced association with the Crk-binding partners C3G and paxillin. Despite this enhanced signaling complex formation, CrkII-Y221F fails to induce JNK and PAK activation, membrane ruffling and cell   migration  , suggesting that it is defective in activating Rac signaling. Wild-type CrkII has no effect on adhesion-induced GTP loading of Rac, but its expression results in enhanced membrane localization of Rac, which is known to be required for Rac signaling. In contrast, CrkII-Y221F is deficient in enhancing membrane localization of Rac. Mutations in Rac and CrkII-Y221F that force membrane targeting of these molecules restore Rac signaling in adherent cells. Together, these results indicate that the Y221 site in CrkII regulates Rac membrane translocation upon cell adhesion, which is necessary for activation of downstream Rac signaling pathways. 
 The insulin-like growth factor receptor I promotes motility and invasion of bladder cancer cells through Akt- and mitogen-activated protein kinase-dependent activation of paxillin. The insulin-like growth factor receptor I (IGF-IR) plays an essential role in transformation by promoting cell growth and protecting cancer cells from apoptosis. Aberrant IGF-IR signaling is implicated in several types of tumors, including carcinomas of the lung, breast, prostate, pancreas, liver, and colon. However, the contribution of the IGF-IR to the development of the transformed phenotype in urothelial cells has not been clearly established. In this study we demonstrated that the IGF-IR is overexpressed in invasive bladder cancer tissues compared with nonmalignant controls. We have investigated the role of the IGF-IR in bladder cancer by using urothelial carcinoma-derived 5637 and T24 cells. Although activation of the IGF-IR did not appreciably affect their growth, it did promote   migration   and stimulate in vitro wound closure and invasion. These effects required the activation of the Akt and Mitogen-activated protein kinase (MAPK) pathways as well as IGF-I-induced Akt- and MAPK-dependent phosphorylation of paxillin, which relocated at dynamic focal adhesions and was necessary for promoting motility in bladder cancer cells. Our results provide the first evidence for a role of the IGF-IR in motility and invasion of bladder cancer cells and support the hypothesis that the IGF-IR may play a critical role in the establishment of the invasive phenotype in urothelial neoplasia. Thus, the IGF-IR may also serve as a novel biomarker for bladder cancer. 
 Additive impact of HER2-/PTK6-RNAi on interactions with HER3 or IGF-1R leads to reduced breast cancer progression in vivo. The human epidermal growth factor receptor 2 (HER2) and the protein tyrosine kinase 6 (PTK6) are often co- and over-expressed in invasive breast cancers. At early diagnosis, only distinct groups, such as HER2-or hormone receptor-positive benefit from a targeted therapy. However, a part of these tumours develops resistance within a year of administration of the drug but the majority of the patients depends on general therapies with severe side effects. A PTK6-directed approach does not yet exist. In our present study, we successfully demonstrate, in vitro and in vivo, a significantly additive reduction of tumourigenesis of breast cancer cells simultaneously depleted of both HER2 and PTK6. In comparison with single RNAi approaches, the combined RNAi (co-RNAi) led to a stronger reduced phosphorylation of tumour-promoting proteins. Moreover, the co-RNAi additively decreased cel  l migrati  on as well as two and three dimensional cell proliferation in vitro. The in vivo experiments showed an additive reduction (p < 0.00001) in the growth of xenografts due to the co-RNAi compared with HER2 or PTK6 RNAi alone. Interestingly, the complexes of HER2 or PTK6 with tumour-relevant interaction partners, such as HER3 or the insulin-like growth factor receptor 1 (IGF-1R), respectively, were also reduced in xenografts although their protein expression levels were not affected following the co-RNAi of HER2 and PTK6. Our present study reveals the potential of using combined HER2- and PTK6- knockdown as a powerful strategy for the treatment of breast cancers. Therefore, the combined inhibition of these proteins may represent an attractive tool for efficient therapy of breast cancers. 
 Evaluation of the expression and role of IGF pathway biomarkers in human sarcomas. Recent studies have shown that insulin-like growth factor (IGF) signaling components have been involved in the pathogenesis and progression of different types of sarcomas. There has been some evidence to indicate the differential expression of IGF2 and insulin-like growth factor 1 receptor (IGF1R) in human sarcomas. The present study utilized immunohistochemistry (IHC) and in situ hybridization (ISH) to determine the expression of IGF2 and IGF1R in eighty-two cases of human sarcoma specimens and eight cases of non-tumor tissue (NTT). IGF2/IGF1R signaling was blocked by recombinant adenovirus-mediated IGF1R small hairpin RNA (shIGF1R), which was used to transfect into human osteosarcoma (OS) MG-63 cells. The expression of IGF2, IGF1R, matrix metallopeptidase-2 (MMP-2) and MMP-9 was detected by Real-time PCR. Cell   migration   was evaluated by wound healing assay. As a consequence, the expression of IGF1R and IGF2 was found in human OS with higher strong reactivity rate compared with the NTT (85.0 percent vs 50.0 percent, P=0.022; 95.0 percent vs 100.0 percent, P=0.042), elevating with the ascending order of tumor malignancy. Also, IGF1R had differential expression in different types of sarcomas (P=0.002), while IGF2 had no significant difference (P=0.105). Targeted blockade of IGF2/IGF1R signaling decreased the expression of IGF2, IGF1R, and MMP-2/-9, and diminished the   migration   capabilities of MG-63 cells. In conclusion, IGF1R is differentially-expressed in different types of human sarcomas, and targeted blockade of IGF1R pathway may inhibit human OS   migration   through down-regulation of MMP-2/-9 expression. IGF1R pathway may represent a significant therapeutic modality for the treatment of sarcomas. 
 Increased expression of insulin-like growth factor-1 receptor is correlated with tumor metastasis and prognosis in patients with osteosarcoma. The aim of this study was to investigate the association of insulin-like growth factor-1 receptor (IGF-1R) with metastasis and prognosis of osteosarcoma patients.RT-PCR and Western blot assays were performed to detect IGF-1R mRNA and protein expression in 26 osteosarcoma and noncancerous bone tissues. Immunohistochemistry was performed to analyze the correlation of IGF-1R expression in 84 osteosarcoma tissues with clinicopathological factors or survival of patients. Lentivirus-mediated RNA interference system was employed to downregulate IGF-1R expression and analyze the effects of IGF-1R downregulation on invasion and metastasis of osteosarcoma cells.The relative levels of IGF-1R mRNA and protein expression were significantly higher in osteosarcoma tissues than in corresponding noncancerous bone tissues. The expression of IGF-1R protein was closely associated with surgical stage and distant metastasis of osteosarcoma patients. Osteosarcoma patients with high IGF-1R expression had poorer survival, and multivariate Cox analyses showed that high IGF-1R expression was an independent prognostic maker. Lentivirus-mediated targeting IGF-1R could significantly inhibit adhesion,   migration  , invasion, and metastasis of osteosarcoma cells, which might be correlated with of inactivation of Akt signaling pathway.IGF-1R is an independent prognostic marker for osteosarcoma patients and increased expression of this molecular is correlated with metastasis of osteosarcoma. 
 miR-494 suppresses tumor growth of epithelial ovarian carcinoma by targeting IGF1R. A growing body of evidence suggests that microRNA-494 (miR-494) could act as tumor-suppressive or oncogenic microRNAs (miRNAs) in different types of tumors. However, the biological roles and underlying mechanisms of miR-494 remain unknown in human epithelial ovarian carcinoma (EOC). Therefore, the aims of this study were to investigate the miR-494 expression and the significance of its clinical diagnosis in patients suffering EOC and to analyze its role and underlying molecular mechanism on the carcinogenesis of EOC. Here, we found that miR-494 was significantly decreased in EOC cell lines and tissues and its expression was negatively correlated with advanced International Federation of Gynecology and Obstetrics (FIGO) stage, high pathological grade, and lymph node metastasis (all P < 0.01). Functional studies showed that overexpression of miR-494 in EOC cells could remarkably inhibit proliferation, colony formati  on, migra  tion, and invasion and induce cell apoptosis, G0/G1 phase arrest. An in vivo analysis revealed that the overexpression of miR-494 suppressed tumor growth in a nude mouse xenograft model system. Bioinformatic assay and dual-luciferase assay confirmed that insulin-like growth factor 1 receptor (IGF1R) was as a direct target of miR-494 in EOC cells. Western blot assay showed that overexpression of miR-494 inhibited IGF1R expression and its downstream signal protein expression. In addition, downregulation of IGF1R has similar effects with miR-494 overexpression on EOC cells and overexpression of IGF1R effectively rescued the inhibition of overexpressed miR-494 in EOC cells. These data suggested that miR-494 functions as a tumor suppressor in EOC by targeting IGF1R. 
 TM4SF4 overexpression in radiation-resistant lung carcinoma cells activates IGF1R via elevation of IGF1. Transmembrane 4 L six family member 4 (TM4SF4) is a member of the tetraspanin L6 domain family. Other members of this family, TM4SF1 (also known as L6-Ag) and TM4SF5, have been shown to be upregulated in multiple tumors and involved in epithelial-to-mesenchymal transition and cell   migration  . However, unlike its homologs, little is known about TM4SF4. Here, we show that TM4SF4 was highly expressed in radiation-resistant lung adenocarcinoma cells, such as A549 and Calu-3 cells, and its expression activated cell growth,   migration  , and invasion. Overexpression of TM4SF4 in A549 cells increased the activation of PI3K, AKT, and NF-kappaB and the expression of PTEN. IGF1R was clearly activated by overexpression of TM4SF4, although EGFR was also slightly activated. TM4SF4 expression was correlated with the increased expression of IGF1, consequently resulting in IGF1R activation. Tumorigenic activity of TM4SF4 in lung adenocarcinoma cells was also demonstrated by xenograft assay; however, this activity was almost completely suppressed by treatment with anti-TM4SF4 antibody. Our results suggest that TM4SF4 overexpression in lung carcinoma cells results in resistance to radiotherapy via IGF1-induced IGF1R activation and blocking the activity of TM4SF4 using specific antibody can be a promising therapeutics against TM4SF4-overexpressing lung adenocarcinoma. 
 The transcription factors Ik-1 and MZF1 downregulate IGF-IR expression in NPM-ALK⁺ T-cell lymphoma. The type I insulin-like growth factor receptor (IGF-IR) tyrosine kinase promotes the survival of an aggressive subtype of T-cell lymphoma by interacting with nucleophosmin-anaplastic lymphoma kinase (NPM-ALK) oncogenic protein. NPM-ALK(+) T-cell lymphoma exhibits much higher levels of IGF-IR than normal human T lymphocytes. The mechanisms underlying increased expression of IGF-IR in this lymphoma are not known. We hypothesized that upregulation of IGF-IR could be attributed to previously unrecognized defects that inherently exist in the transcriptional machinery in NPM-ALK(+) T-cell lymphoma.Screening studies showed substantially lower levels of the transcription factors Ikaros isoform 1 (Ik-1) and myeloid zinc finger 1 (MZF1) in NPM-ALK(+) T-cell lymphoma cell lines and primary tumor tissues from patients than in human T lymphocytes. A luciferase assay supported that Ik-1 and MZF1 suppress IGF-IR gene promoter. Furthermore, ChIP assay showed that these transcription factors bind specific sites located within the IGF-IR gene promoter. Forced expression of Ik-1 or MZF1 in the lymphoma cells decreased IGF-IR mRNA and protein. This decrease was associated with downregulation of pIGF-IR, and the phosphorylation of its interacting proteins IRS-1, AKT, and NPM-ALK. In addition, overexpression of Ik-1 and MZF1 decreased the viability, proliferation  , migrati  on, and anchorage-independent colony formation of the lymphoma cells.Our results provide novel evidence that the aberrant decreases in Ik-1 and MZF1 contribute significantly to the pathogenesis of NPM-ALK(+) T-cell lymphoma through the upregulation of IGF-IR expression. These findings could be exploited to devise new strategies to eradicate this lymphoma. 
 IGF-I stimulates cooperative interaction between the IGF-I receptor and CSK homologous kinase that regulates SHPS-1 phosphorylation in vascular smooth muscle cells. IGF-I plays an important role in smooth muscle cell proliferation and   migration  . In vascular smooth muscle cells cultured in 25 mm glucose, IGF-I stimulated a significant increase in Src homology 2 domain containing protein tyrosine phosphatase substrate-1 (SHPS-1) phosphorylation compared with 5 mm glucose and this increase was required for smooth muscle cell proliferation. A proteome-wide screen revealed that carboxyl-terminal SRC kinase homologous kinase (CTK) bound directly to phosphotyrosines in the SHPS-1 cytoplasmic domain. Because the kinase(s) that phosphorylates these tyrosines in response to IGF-I is unknown, we determined the roles of IGF-I receptor (IGF-IR) and CTK in mediating SHPS-1 phosphorylation. After IGF-I stimulation, CTK was recruited to IGF-IR and subsequently to phospho-SHPS-1. Expression of an IGF-IR mutant that eliminated CTK binding reduced CTK transfer to SHPS-1, SHPS-1 phosphorylation, and cell proliferation. IGF-IR phosphorylated SHPS-1, which provided a binding site for CTK. CTK recruitment to SHPS-1 resulted in a further enhancement of SHPS-1 phosphorylation. CTK knockdown also impaired IGF-I-stimulated SHPS-1 phosphorylation and downstream signaling. Analysis of specific tyrosines showed that mutation of tyrosines 428/452 in SHPS-1 to phenylalanine reduced SHPS-1 phosphorylation but allowed CTK binding. In contrast, the mutation of tyrosines 469/495 inhibited IGF-IR-mediated the phosphorylation of SHPS-1 and CTK binding, suggesting that IGF-IR phosphorylated Y469/495, allowing CTK binding, and that CTK subsequently phosphorylated Y428/452. Based on the above findings, we conclude that after IGF-I stimulation, CTK is recruited to IGF-IR and its recruitment facilitates CTK's subsequent association with phospho-SHPS-1. This results in the enhanced CTK transfer to SHPS-1, and the two kinases then fully phosphorylate SHPS-1, which is necessary for IGF-I stimulated cellular proliferation. 
 Differential expression of IGF-I and insulin receptor isoforms in HPV positive and negative human cervical cancer cell lines. Human papillomavirus (HPV) is the main risk factor for cervical cancer; however, some carcinomas occur in the absence of the virus. IGF-IR and an isoform of the insulin receptor, IR-A, play important roles in cancer. In this study we assessed the role of the IGF/insulin receptors in cervical cancer cell lines with different HPV status, SiHa (HPV positive), and C33a (HPV negative). Different patterns of receptor expression were found; while SiHa expressed IGF-IR, IR-A and IR-B, and IR/IGF-IR hybrid receptors, C33a cells expressed the IR-A only. Tyrosine phosphorylation of these receptors in response to their corresponding ligands correlated with the expression level of these receptors in the cell lines. Activation of PI3-K and MAPK pathways was revealed in both cell lines, however, no effects on proliferation,   migration  , or invasion were observed. Here we show that cervical cancer cell lines--positive and negative for HPV--differ in the type of insulin and IGF-1 receptors expressed. Additional studies are needed for characterization of the role of IR-A in cervical carcinogenesis. 
 miR-140 suppresses tumor growth and metastasis of non-small cell lung cancer by targeting insulin-like growth factor 1 receptor. MicroRNAs (miRNAs) are a class of small noncoding RNA molecules that play important roles in carcinogenesis and tumor progression. In this study, we investigated the roles and mechanisms of miR-140 in human non-small cell lung cancer (NSCLC). We found that miR-140 is significantly downregulated in NSCLC tissues and cell lines. Both gain-of-function and loss-of-function studies demonstrated that miR-140 suppresses NSCLC cell proliferation,   migration  , and invasion in vitro. Importantly, overexpression of miR-140 effectively repressed tumor growth and metastasis in nude mouse models. Integrated analysis identified IGF1R as a direct and functional target of miR-140. Knockdown of IGF1R inhibited cell proliferation and invasion resembling that of miR-140 overexpression, while overexpression of IGF1R attenuated the function of miR-140 in NSCLC cells. Together, our results highlight the significance of miR-140 and IGF1R in the development and progression of NSCLC. 
 Anti-tumor effect in human breast cancer by TAE226, a dual inhibitor for FAK and IGF-IR in vitro and in vivo. Focal adhesion kinase (FAK) is a 125-kDa non-receptor type tyrosine kinase that localizes to focal adhesions. FAK overexpression is frequently found in invasive and metastatic cancers of the breast, colon, thyroid, and prostate, but its role in osteolytic metastasis is not well understood. In this study, we have analyzed anti-tumor effects of the novel FAK Tyr(397) inhibitor TAE226 against bone metastasis in breast cancer by using TAE226. Oral administration of TAE226 in mice significantly decreased bone metastasis and osteoclasts involved which were induced by MDA-MB-231 breast cancer cells and increased the survival rate of the mouse models of bone metastasis. TAE226 also suppressed the growth of subcutaneous tumors in vivo and the proliferation and   migration   of MDA-MB-231 cells in vitro. Significantly, TAE226 inhibited the osteoclast formation in murine pre-osteoclastic RAW264.7 cells, and actin ring and pit formation in mature osteoclasts. Moreover, TAE226 inhibited the receptor activator for nuclear factor κ B Ligand (RANKL) gene expression induced by parathyroid hormone-related protein (PTHrP) in bone stromal ST2 cells and blood free calcium concentration induced by PTHrP administration in vivo. These findings suggest that FAK was critically involved in osteolytic metastasis and activated in tumors, pre-osteoclasts, mature osteoclasts, and bone stromal cells and TAE226 can be effectively used for the treatment of cancer induced bone metastasis and other bone diseases. 
  GeneRIFs  
 Suggest role for EphB4/IGF1R signaling in regulation proliferation/  migration   of breast cancer cells. 
 IGF1R is a direct target of mir-323-5p and its targeting results in increased apoptosis and decreased cell proliferation and   migration   in a glioma cell line. 
 Overexpression of IGF1R increased cell proliferation, colony formation,   migration  , invasion and resistance to apoptosis in prostate cancer. 
 IGF1R is differentially-expressed in different types of human sarcomas, and targeted blockade of IGF1R pathway may inhibit human OS   migration   through down-regulation of MMP-2/-9 expression 
 MiR-181b overexpression inhibited cell proliferation,   migration  , invasion, and tumorigenesis by targeting IGF-1R and its downstream signaling pathways. 
 c-Met, epidermal growth factor receptor, and insulin-like growth factor-1 receptor are important for growth in uveal melanoma and independently contribute to   migration   and metastatic potential 
 Signal transduction pathways underlying the enhanced cell   migration   reveal that the IGF-I-IGFBP-VN complex stimulates a transient activation of the ERK/MAPK signaling pathway and a sustained activation of the phosphatidylinositide 3-kinase/AKT pathway. 
 Different members of Rho GTPase family regulate IGF-II-mediated EVT cell   migration   differentially, depending upon whether it signals through IGF1R or in an IGF1R-independent manner. 
 Increases in IGF-IR decrease beta1 integrin expression, and enhance cell   migration   in neuroblastoma cells. 
 IGF-IR and c-Met cooperate to induce   migration   and invasion of human pancreatic carcinoma cells. 
  ECM  PubMed  
 Insulin-like growth factor-I receptor mediates the prosurvival effect of fibronectin. We recently showed that extracellular matrix (  ECM  ) proteins, which are abundant in desmoplastic pancreatic tumor, are as potent as growth factors in inhibiting apoptosis in pancreatic cancer (PaCa) cells. Here we show that fibronectin, a major   ECM   component, engages insulin-like growth factor-I receptor (IGF-IR) to inhibit PaCa cell death. We found that fibronectin-induced protection from apoptosis is fully mediated by IGF-IR and is independent of IGF-I. Pharmacologic and molecular inhibitions of IGF-IR stimulated apoptosis and prevented the prosurvival effect of fibronectin in PaCa cells. Our data indicate that fibronectin protects from apoptosis through trans-activation of IGF-IR. We showed that fibronectin stimulated complex formation between its receptor beta3 integrin and protein-tyrosine phosphatase SHP-2. This process of complex formation, in turn, prevents SHP-2 from dephosphorylating IGF-IR resulting in sustained phosphorylation of IGF-IR and leading to the downstream activation of Akt kinase, up-regulation of antiapoptotic Bcl(xL), and inhibition of apoptosis. Among   ECM   proteins tested only fibronectin and laminin but not vitronectin and collagen I stimulated trans-activation of IGF-IR. Interaction of fibronectin with beta3 but not beta1 integrin receptors mediates the survival pathway. In contrast, fibronectin-induced adhesion is mediated through beta1 integrin receptor and is IGF-IR-independent. Thus, our results indicate that the prosurvival effect of fibronectin in PaCa cells is mediated by trans-activation of IGF-IR induced by the beta3 integrin receptor. The data suggest IGF-IR as a key target for prevention of the prosurvival effects of   ECM   proteins and growth factors in pancreatic cancer. 
  [Ee]xtracellular [Mm]atrix  PubMed  
 RACK1, an insulin-like growth factor I (IGF-I) receptor-interacting protein, modulates IGF-I-dependent integrin signaling and promotes cell spreading and contact with   extracellular matrix  . The insulin-like growth factor I (IGF-I) receptor (IGF-IR) is known to regulate a variety of cellular processes including cell proliferation, cell survival, cell differentiation, and cell transformation. IRS-1 and Shc, substrates of the IGF-IR, are known to mediate IGF-IR signaling pathways such as those of mitogen-activated protein kinase (MAPK) and phosphatidylinositol 3-kinase (PI3K), which are believed to play important roles in some of the IGF-IR-dependent biological functions. We used the cytoplasmic domain of IGF-IR in a yeast two-hybrid interaction trap to identify IGF-IR-interacting molecules that may potentially mediate IGF-IR-regulated functions. We identified RACK1, a WD repeat family member and a Gbeta homologue, and demonstrated that RACK1 interacts with the IGF-IR but not with the closely related insulin receptor (IR). In several types of mammalian cells, RACK1 interacted with IGF-IR, protein kinase C, and beta1 integrin in response to IGF-I and phorbol 12-myristate 13-acetate stimulation. Whereas most of RACK1 resides in the cytoskeletal compartment of the cytoplasm, transformation of fibroblasts and epithelial cells by v-Src, oncogenic IR or oncogenic IGF-IR, but not by Ros or Ras, resulted in a significantly increased association of RACK1 with the membrane. We examined the role of RACK1 in IGF-IR-mediated functions by stably overexpressing RACK1 in NIH 3T3 cells that expressed an elevated level of IGF-IR. RACK1 overexpression resulted in reduced IGF-I-induced cell growth in both anchorage-dependent and anchorage-independent conditions. Overexpression of RACK1 also led to enhanced cell spreading, increased stress fibers, and increased focal adhesions, which were accompanied by increased tyrosine phosphorylation of focal adhesion kinase and paxillin. While IGF-I-induced activation of IRS-1, Shc, PI3K, and MAPK pathways was unaffected, IGF-I-inducible beta1 integrin-associated kinase activity and association of Crk with p130(CAS) were significantly inhibited by RACK1 overexpression. In RACK1-overexpressing cells, delayed cell cycle progression in G(1) or G(1)/S was correlated with retinoblastoma protein hypophophorylation, increased levels of p21(Cip1/WAF1) and p27(Kip1), and reduced IGF-I-inducible Cdk2 activity. Reduction of RACK1 protein expression by antisense oligonucleotides prevented cell spreading and suppressed IGF-I-dependent monolayer growth. Our data suggest that RACK1 is a novel IGF-IR signaling molecule that functions as a positive mediator of cell spreading and contact with   extracellular matrix  , possibly through a novel IGF-IR signaling pathway involving integrin and focal adhesion signaling molecules. 
 A genetic association study of maternal and fetal candidate genes that predispose to preterm prelabor rupture of membranes (PROM). We sought to determine whether maternal/fetal single-nucleotide polymorphisms (SNPs) in candidate genes are associated with preterm prelabor rupture of membranes (pPROM).A case-control study was conducted in patients with pPROM (225 mothers and 155 fetuses) and 599 mothers and 628 fetuses with a normal pregnancy; 190 candidate genes and 775 SNPs were studied. Single locus/haplotype association analyses were performed; false discovery rate was used to correct for multiple testing (q* = 0.15).First, a SNP in tissue inhibitor of metalloproteinase 2 in mothers was significantly associated with pPROM (odds ratio, 2.12; 95% confidence interval, 1.47-3.07; P = .000068), and this association remained significant after correction for multiple comparisons. Second, haplotypes for Alpha 3 type IV collagen isoform precursor in the mother were associated with pPROM (global P = .003). Third, multilocus analysis identified a 3-locus model, which included maternal SNPs in collagen type I alpha 2, defensin alpha 5 gene, and endothelin 1.DNA variants in a maternal gene involved in   extracellular matrix   metabolism doubled the risk of pPROM. 
 Identification of fetal and maternal single nucleotide polymorphisms in candidate genes that predispose to spontaneous preterm labor with intact membranes. The purpose of this study was to determine whether maternal/fetal single nucleotide polymorphisms (SNPs) in candidate genes are associated with spontaneous preterm labor/delivery.A genetic association study was conducted in 223 mothers and 179 fetuses (preterm labor with intact membranes who delivered <37 weeks of gestation [preterm birth (PTB)]), and 599 mothers and 628 fetuses (normal pregnancy); 190 candidate genes and 775 SNPs were studied. Single locus/haplotype association analyses were performed; the false discovery rate was used to correct for multiple testing.The strongest single locus associations with PTB were interleukin-6 receptor 1 (fetus; P=.000148) and tissue inhibitor of metalloproteinase 2 (mother; P=.000197), which remained significant after correction for multiple comparisons. Global haplotype analysis indicated an association between a fetal DNA variant in insulin-like growth factor F2 and maternal alpha 3 type IV collagen isoform 1 (global, P=.004 and .007, respectively).An SNP involved in controlling fetal inflammation (interleukin-6 receptor 1) and DNA variants in maternal genes encoding for proteins involved in   extracellular matrix   metabolism approximately doubled the risk of PTB. 
 Identification of fetal and maternal single nucleotide polymorphisms in candidate genes that predispose to spontaneous preterm labor with intact membranes. The purpose of this study was to determine whether maternal/fetal single nucleotide polymorphisms (SNPs) in candidate genes are associated with spontaneous preterm labor/delivery.A genetic association study was conducted in 223 mothers and 179 fetuses (preterm labor with intact membranes who delivered <37 weeks of gestation [preterm birth (PTB)]), and 599 mothers and 628 fetuses (normal pregnancy); 190 candidate genes and 775 SNPs were studied. Single locus/haplotype association analyses were performed; the false discovery rate was used to correct for multiple testing.The strongest single locus associations with PTB were interleukin-6 receptor 1 (fetus; P=.000148) and tissue inhibitor of metalloproteinase 2 (mother; P=.000197), which remained significant after correction for multiple comparisons. Global haplotype analysis indicated an association between a fetal DNA variant in insulin-like growth factor F2 and maternal alpha 3 type IV collagen isoform 1 (global, P=.004 and .007, respectively).An SNP involved in controlling fetal inflammation (interleukin-6 receptor 1) and DNA variants in maternal genes encoding for proteins involved in   extracellular matrix   metabolism approximately doubled the risk of PTB. 
 A genetic association study of maternal and fetal candidate genes that predispose to preterm prelabor rupture of membranes (PROM). We sought to determine whether maternal/fetal single-nucleotide polymorphisms (SNPs) in candidate genes are associated with preterm prelabor rupture of membranes (pPROM).A case-control study was conducted in patients with pPROM (225 mothers and 155 fetuses) and 599 mothers and 628 fetuses with a normal pregnancy; 190 candidate genes and 775 SNPs were studied. Single locus/haplotype association analyses were performed; false discovery rate was used to correct for multiple testing (q* = 0.15).First, a SNP in tissue inhibitor of metalloproteinase 2 in mothers was significantly associated with pPROM (odds ratio, 2.12; 95% confidence interval, 1.47-3.07; P = .000068), and this association remained significant after correction for multiple comparisons. Second, haplotypes for Alpha 3 type IV collagen isoform precursor in the mother were associated with pPROM (global P = .003). Third, multilocus analysis identified a 3-locus model, which included maternal SNPs in collagen type I alpha 2, defensin alpha 5 gene, and endothelin 1.DNA variants in a maternal gene involved in   extracellular matrix   metabolism doubled the risk of pPROM. 
 Functional insulin-like growth factor-1/insulin-like growth factor-1 receptor-mediated circuit in human and murine thymic epithelial cells. Interactions between thymocytes and thymic epithelial cell (TEC) can be modulated by growth hormone via insulin-like growth factor-1 (IGF-1). In this study, we showed IGF-1 and IGF-1 receptor mRNA expression by human and murine TEC and thymocytes. Functionally, IGF-1 stimulates   extracellular matrix   production by human TEC. Moreover, pretreatment of murine TEC with IGF-1 increases their adhesion to thymocytes. Interestingly, we observed an increase in the frequency of CD4-CD8-CD90+ T cells which adhered to pretreated TEC, supporting the concept that IGF-1 may also act indirectly on intrathymic T cell differentiation and migration through the thymic epithelium. 
 IGF-IR promotes prostate cancer growth by stabilizing α5β1 integrin protein levels. Dynamic crosstalk between growth factor receptors, cell adhesion molecules an  d extracellular matr  ix is essential for cancer cell migration and invasion. Integrins are transmembrane receptors that bin  d extracellular matr  ix proteins and enable cell adhesion and cytoskeletal organization. They also mediate signal transduction to regulate cell proliferation and survival. The type 1 insulin-like growth factor receptor (IGF-IR) mediates tumor cell growth, adhesion and inhibition of apoptosis in several types of cancer. We have previously demonstrated that β1 integrins regulate anchorage-independent growth of prostate cancer (PrCa) cells by regulating IGF-IR expression and androgen receptor-mediated transcriptional functions. Furthermore, we have recently reported that IGF-IR regulates the expression of β1 integrins in PrCa cells. We have dissected the mechanism through which IGF-IR regulates β1 integrin expression in PrCa. Here we report that IGF-IR is crucial for PrCa cell growth and that β1 integrins contribute to the regulation of proliferation by IGF-IR. We demonstrate that β1 integrin regulation by IGF-IR does not occur at the mRNA level. Exogenous expression of a CD4 - β1 integrin cytoplasmic domain chimera does not interfere with such regulation and fails to stabilize β1 integrin expression in the absence of IGF-IR. This appears to be due to the lack of interaction between the β1 cytoplasmic domain and IGF-IR. We demonstrate that IGF-IR stabilizes the β1 subunit by protecting it from proteasomal degradation. The α5 subunit, one of the binding partners of β1, is also downregulated along with β1 upon IGF-IR knockdown while no change is observed in the expression of the α2, α3, α4, α6 and α7 subunits. Our results reveal a crucial mechanistic role for the α5β1 integrin, downstream of IGF-IR, in regulating cancer growth. 
 Decreased proliferative capacity of aged dermal fibroblasts in a three dimensional matrix is associated with reduced IGF1R expression and activation. Skin aging results in increased susceptibility to injury and impaired wound healing. Proliferation of fibroblasts is reduced in aged dermis, which contributes to delays in wound closure. Age-associated differences are regulated, in part, by local or systemic factors such as the IGF-1/IGF1R system. The aim of this study was to determine if expression and activation of IGF1R in aged human dermal fibroblasts, when compared to young fibroblasts, is associated with altered proliferative capacity in a 3D collagen matrix that better simulates the dermal   extracellular matrix   in vivo. The proliferation of young and aged human dermal fibroblasts in 3D collagen and its association with baseline levels of IGF1R expression were measured. The effect of stimulation and inhibition of Erk phosphorylation on the proliferative capacity of fibroblasts in a 3D collagen matrix was defined. Our results show that proliferation and Erk phosphorylation is reduced in aged dermal fibroblasts relative to young fibroblasts. Activation of Erk phosphorylation in aged fibroblasts is associated with a significant increase in fibroblast proliferation in 3D collagen. 
 Substrate-bound insulin-like growth factor (IGF)-I-IGF binding protein-vitronectin-stimulated breast cell migration is enhanced by coactivation of the phosphatidylinositide 3-Kinase/AKT pathway by alphav-integrins and the IGF-I receptor. IGF-I can bind to the   extracellular matrix   protein vitronectin (VN) through the involvement of IGF-binding proteins-2, -3, -4, and -5. Because IGF-I and VN have established roles in tumor cell dissemination, we were keen to investigate the functional consequences of the interaction of IGF-I, IGF binding proteins (IGFBPs), and VN in tumor cell biology. Hence, functional responses of MCF-7 breast carcinoma cells and normal nontumorgenic MCF-10A mammary epithelial cells were investigated to allow side-by-side comparisons of these complexes in both cancerous and normal breast cells. We demonstrate that substrate-bound IGF-I-IGFBP-VN complexes stimulate synergistic increases in cellular migration in both cell types. Studies using IGF-I analogs determined this stimulation to be dependent on both heterotrimeric IGF-I-IGFBP-VN complex formation and the involvement of the IGF-I receptor (IGF-IR). Furthermore, the enhanced cellular migration was abolished on incubation of MCF-7 and MCF-10A cells with function blocking antibodies directed at VN-binding integrins and the IGF-IR. Analysis of the signal transduction pathways underlying the enhanced cell migration revealed that the complexes stimulate a transient activation of the ERK/MAPK signaling pathway while simultaneously producing a sustained activation of the phosphatidylinositide 3-kinase/AKT pathway. Experiments using pharmacological inhibitors of these pathways determined a requirement for phosphatidylinositide 3-kinase/AKT activation in the observed response. Overexpression of wild type and activated AKT further increases substrate-bound IGF-I-IGFBP-VN-stimulated migration. This study provides the first mechanistic insights into the action of IGF-I-IGFBP-VN complexes and adds further evidence to support the involvement of VN-binding integrins and their cooperativity with the IGF-IR in the promotion of tumor cell migration. 
 Fibulin-3-mediated inhibition of epithelial-to-mesenchymal transition and self-renewal of ALDH+ lung cancer stem cells through IGF1R signaling. Fibulins (FBLNs), a family of   extracellular matrix   proteins, have recently been shown to act as tumor suppressors or activators in different cancers, and the underlying molecular mechanisms of their action in cancer remain unclear. We have previously shown that the expression of FBLN3 is suppressed by promoter hypermethylation and is associated with invasiveness in aggressive non-small cell lung cancer. In this study, we evaluated the roles and signaling mechanism of FBLN3 in lung cancer stem cells (CSCs). Forced expression of FBLN3 suppressed invasion and migration of lung adenocarcinoma cells and decreased the expression of epithelial-to-mesenchymal transition (EMT) activators, including N-cadherin and Snail. Stemness activities of lung adenocarcinoma cells were also suppressed by FBLN3 as indicated by a decrease in spheroid formation and the levels of stemness markers such as Sox2 and β-catenin. These effects of FBLN3 were mediated by the glycogen synthase kinase-3β, GSK3β/β-catenin pathway, and the upstream regulators of GSK3β, including phosphoinositide 3-kinase (PI3K)/AKT and insulin-like growth factor-1 receptor (IGF1R), were inactivated by FBLN3. Moreover, IGF1R was shown to be a direct target of FBLN3, which competitively inhibited insulin-like growth factor (IGF) action. To confirm the effect of FBLN3 on lung CSCs, aldehyde dehydrogenase-positive (ALDH+) A549 lung CSCs were sorted and treated with recombinant FBLN3 protein. FBLN3 clearly suppressed EMT, stemness activity and the over-activated IGF1R/PI3K/AKT/GSK3β pathway of the ALDH+ CSC subpopulation. In addition, injection of recombinant FBLN3 protein around subcutaneous xenografts established with ALDH+ CSCs in athymic nude mice significantly suppressed tumor growth and progression. Overall, our results show that FBLN3 suppresses both EMT and self-renewal of the lung CSCs by modulating the IGF1R/PI3K/AKT/GSK3β pathway and that FBLN3 would be useful as an alternative CSC therapy. 
 Insulin-like growth factor-I receptor mediates the prosurvival effect of fibronectin. We recently showed that   extracellular matrix   (ECM) proteins, which are abundant in desmoplastic pancreatic tumor, are as potent as growth factors in inhibiting apoptosis in pancreatic cancer (PaCa) cells. Here we show that fibronectin, a major ECM component, engages insulin-like growth factor-I receptor (IGF-IR) to inhibit PaCa cell death. We found that fibronectin-induced protection from apoptosis is fully mediated by IGF-IR and is independent of IGF-I. Pharmacologic and molecular inhibitions of IGF-IR stimulated apoptosis and prevented the prosurvival effect of fibronectin in PaCa cells. Our data indicate that fibronectin protects from apoptosis through trans-activation of IGF-IR. We showed that fibronectin stimulated complex formation between its receptor beta3 integrin and protein-tyrosine phosphatase SHP-2. This process of complex formation, in turn, prevents SHP-2 from dephosphorylating IGF-IR resulting in sustained phosphorylation of IGF-IR and leading to the downstream activation of Akt kinase, up-regulation of antiapoptotic Bcl(xL), and inhibition of apoptosis. Among ECM proteins tested only fibronectin and laminin but not vitronectin and collagen I stimulated trans-activation of IGF-IR. Interaction of fibronectin with beta3 but not beta1 integrin receptors mediates the survival pathway. In contrast, fibronectin-induced adhesion is mediated through beta1 integrin receptor and is IGF-IR-independent. Thus, our results indicate that the prosurvival effect of fibronectin in PaCa cells is mediated by trans-activation of IGF-IR induced by the beta3 integrin receptor. The data suggest IGF-IR as a key target for prevention of the prosurvival effects of ECM proteins and growth factors in pancreatic cancer. 
 The direct binding of insulin-like growth factor-1 (IGF-1) to integrin alphavbeta3 is involved in IGF-1 signaling. It has been proposed that ligand occupancy of integrin alphavbeta3 with   extracellular matrix   ligands (e.g. vitronectin) plays a critical role in insulin-like growth factor-1 (IGF-1) signaling. We found that expression of alphavbeta3 enhanced IGF-1-induced proliferation of Chinese hamster ovary cells in serum-free conditions (in the absence of vitronectin). We hypothesized that the direct integrin binding to IGF-1 may play a role in IGF-1 signaling. We demonstrated that alphavbeta3 specifically and directly bound to IGF-1 in cell adhesion, enzyme-linked immunosorbent assay-type binding, and surface plasmon resonance studies. We localized the amino acid residues of IGF-1 that are critical for integrin binding by docking simulation and mutagenesis. We found that mutating two Arg residues at positions 36 and 37 in the C-domain of IGF-1 to Glu (the R36E/R37E mutation) effectively reduced integrin binding. Interestingly, although the mutant still bound to IGF1R, it was defective in inducing IGF1R phosphorylation, AKT and ERK1/2 activation, and cell proliferation. Furthermore wild type IGF-1 mediated co-precipitation of alphavbeta3 and IGF1R, whereas the R36E/R37E mutant did not, suggesting that IGF-1 mediates the interaction between alphavbeta3 and IGF1R. These results suggest that the direct binding to IGF-1 to integrin alphavbeta3 plays a role in IGF-1 signaling through ternary complex formation (alphavbeta3-IGF-IGF1R), and integrin-IGF-1 interaction is a novel target for drug discovery. 
 Insulin-like growth factors are essential to prevent anoikis in oestrogen-responsive breast cancer cells: importance of the type I IGF receptor and PI3-kinase/Akt pathway. Detachment of epithelial cells from the   extracellular matrix   initiates programmed cell death by a process termed anoikis. Malignant cells must acquire anoikis resistance to leave the primary tumour and metastasise. Multiple signal transduction pathways can activate anoikis and confer anoikis resistance, but these are not understood in breast cancer.Models for anoikis of oestrogen-responsive breast cancer cells were established and the protective effects of IGF-1 tested. Cleaved PARP was measured by western transfer and cleaved caspase 3 by flow cytometry. Pathways involved in anoikis and in anoikis resistance were investigated with PI3-kinase, Akt, and MEK1 and MEK2 inhibitors. The importance of the type I IGF receptor was investigated by IGF-concentration dependence, siRNA knockdown and pharmacological inhibition. Association between IGF-1R expression and relapse with distant metastasis was analysed in 1609 patients by log rank test.Unattached breast cancer cells required culture in serum-free medium to induce anoikis. Rapid loss of FAK, Akt and Bad phosphorylation was concurrent with anoiks induction, but ERK1 and ERK2 phosphorylation increased which suggested that anoikis resistance is mediated by the PI3-kinase/Akt rather than the Grb2/Ras/MAP-kinase pathway. IGF-1 conferred anoikis resistance in serum-free medium. IGF-1 activated the PI3-kinase/Akt and Grb2/Ras/MAP-kinase pathways but experiments with PI3-kinase, Akt and MEK1 and MEK2 inhibitors showed that IGF protection is via the PI3-kinase/Akt pathway. The concentration dependence of IGF protection, knockdown experiments with siRNA and pharmacological inhibition with figitumumab, showed that IGF-1 signals through the type I IGF receptor. The crucial role of the type I IGF receptor was demonstrated by induction of anoikis in full serum by figitumumab. High IGF-1R expression was associated with reduced time to relapse with distant metastases in oestrogen receptor-positive patients, especially those with aggressive disease which confirms its relevance in vivo.Anoikis resistance of oestrogen-responsive breast cancer cells depends upon IGF activation of the type I IGF receptor and PI3-kinase/Akt pathway. Because IGF-dependent evasion of anoikis will facilitate metastasis by malignant breast cancer cells, effective inhibition of IGF signal transduction should be included in combinations of targeted drugs designed to treat metastatic oestrogen receptor-positive breast cancers. 
  [Ii]nvasive  PubMed  
 Genetic susceptibility to distinct bladder cancer subphenotypes. Clinical, pathologic, and molecular evidence indicate that bladder cancer is heterogeneous with pathologic/molecular features that define distinct subphenotypes with different prognoses. It is conceivable that specific patterns of genetic susceptibility are associated with particular subphenotypes.To examine evidence for the contribution of germline genetic variation to bladder cancer heterogeneity.The Spanish Bladder Cancer/EPICURO Study is a case-control study based in 18 hospitals located in five areas in Spain. Cases were patients with a newly diagnosed, histologically confirmed, urothelial cell carcinoma of the bladder from 1998 to 2001. Case diagnoses were reviewed and uniformly classified by pathologists following the World Health Organisation/International Society of Urological Pathology 1999 criteria. Controls were hospital-matched patients (n=1149).A total of 1526 candidate variants in 423 candidate genes were analysed. Three distinct subphenotypes were defined according to stage and grade: low-grade nonmuscle   invasive   (n=586), high-grade nonmuscle   invasive   (n=219), and muscle   invasive   (n=246). The association between each variant and subphenotype was assessed by polytomous risk models adjusting for potential confounders. Heterogeneity in genetic susceptibility among subphenotypes was also tested.Two established bladder cancer susceptibility genotypes, NAT2 slow-acetylation and GSTM1-null, exhibited similar associations among the subphenotypes, as did VEGF-rs25648, which was previously identified in our study. Other variants conferred risks for specific tumour subphenotypes such as PMS2-rs6463524 and CD4-rs3213427 (respective heterogeneity p values of 0.006 and 0.004), which were associated with muscle-  invasive   tumours (per-allele odds ratios [95% confidence interval] of 0.56 [0.41-0.77] and 0.71 [0.57-0.88], respectively) but not with non-muscle-  invasive   tumours. Heterogeneity p values were not robust in multiple testing according to their false-discovery rate.These exploratory analyses suggest that genetic susceptibility loci might be related to the molecular/pathologic diversity of bladder cancer. Validation through large-scale replication studies and the study of additional genes and single nucleotide polymorphisms are required. 
 Genetic susceptibility to distinct bladder cancer subphenotypes. Clinical, pathologic, and molecular evidence indicate that bladder cancer is heterogeneous with pathologic/molecular features that define distinct subphenotypes with different prognoses. It is conceivable that specific patterns of genetic susceptibility are associated with particular subphenotypes.To examine evidence for the contribution of germline genetic variation to bladder cancer heterogeneity.The Spanish Bladder Cancer/EPICURO Study is a case-control study based in 18 hospitals located in five areas in Spain. Cases were patients with a newly diagnosed, histologically confirmed, urothelial cell carcinoma of the bladder from 1998 to 2001. Case diagnoses were reviewed and uniformly classified by pathologists following the World Health Organisation/International Society of Urological Pathology 1999 criteria. Controls were hospital-matched patients (n=1149).A total of 1526 candidate variants in 423 candidate genes were analysed. Three distinct subphenotypes were defined according to stage and grade: low-grade nonmuscle   invasive   (n=586), high-grade nonmuscle   invasive   (n=219), and muscle   invasive   (n=246). The association between each variant and subphenotype was assessed by polytomous risk models adjusting for potential confounders. Heterogeneity in genetic susceptibility among subphenotypes was also tested.Two established bladder cancer susceptibility genotypes, NAT2 slow-acetylation and GSTM1-null, exhibited similar associations among the subphenotypes, as did VEGF-rs25648, which was previously identified in our study. Other variants conferred risks for specific tumour subphenotypes such as PMS2-rs6463524 and CD4-rs3213427 (respective heterogeneity p values of 0.006 and 0.004), which were associated with muscle-  invasive   tumours (per-allele odds ratios [95% confidence interval] of 0.56 [0.41-0.77] and 0.71 [0.57-0.88], respectively) but not with non-muscle-  invasive   tumours. Heterogeneity p values were not robust in multiple testing according to their false-discovery rate.These exploratory analyses suggest that genetic susceptibility loci might be related to the molecular/pathologic diversity of bladder cancer. Validation through large-scale replication studies and the study of additional genes and single nucleotide polymorphisms are required. 
 Expression of the Insulin-like Growth Factor-1 Receptor in Odontogenic Myxoma. Odontogenic myxoma (OM) is a rare mesenchymal tumour arising in the jaws. The origin and pathogenesis of OM is poorly understood. The aim of this study was to characterize OM by immunolocalization of certain antigens in the tumour that are relevant for cellular differentiation, migration and maintenance.Five OMs were immunohistochemically investigated for expression of nestin, CD133, podoplanin, and insulin-like growth factor 1 receptor (IGF-1R).OM failed to react with antibodies applied in this study, with the exception of IGF-1R in tumour cells.OM is a poorly characterized benign,   invasive   tumour of the jaws. The absence of stem cell marker in OM does not exclude possible temporary expression of these antigens during certain phases of tumour development. The identification of IGF-1R in OM is shared with numerous tumours and indicates the ability of these tumour cells to respond to growth factors. 
 Decorin antagonizes IGF receptor I (IGF-IR) function by interfering with IGF-IR activity and attenuating downstream signaling. We have recently discovered that the insulin-like growth factor receptor I (IGF-IR) is up-regulated in human   invasive   bladder cancer and promotes migration and invasion of transformed urothelial cells. The proteoglycan decorin, a key component of the tumor stroma, can positively regulate the IGF-IR system in normal cells. However, there are no available data on the role of decorin in modulating IGF-IR activity in transformed cells or in tumor models. Here we show that the expression of decorin inversely correlated with IGF-IR expression in low and high grade bladder cancers (n = 20 each). Decorin bound with high affinity IGF-IR and IGF-I at distinct sites and negatively regulated IGF-IR activity in urothelial cancer cells. Nanomolar concentrations of decorin promoted down-regulation of IRS-1, one of the critical proteins of the IGF-IR pathway, and attenuated IGF-I-dependent activation of Akt and MAPK. This led to decorin-evoked inhibition of migration and invasion upon IGF-I stimulation. Notably, decorin did not cause down-regulation of the IGF-IR in bladder, breast, and squamous carcinoma cells. This indicates that decorin action on the IGF-IR differs from its known activity on other receptor tyrosine kinases such as the EGF receptor and Met. Our results provide a novel mechanism for decorin in negatively modulating both IGF-I and its receptor. Thus, decorin loss may contribute to increased IGF-IR activity in the progression of bladder cancer and perhaps other forms of cancer where IGF-IR plays a role. 
 ImmunoSPECT and immunoPET of IGF-1R expression with the radiolabeled antibody R1507 in a triple-negative breast cancer model. The insulinlike growth factor 1 receptor (IGF-1R) is a new target for the treatment of breast cancer. Patients with breast cancer lesions that express IGF-1R may benefit from treatment with anti-IGF-1R antibodies. Therefore, the aim of the present study was to develop a non  invasive  , in vivo imaging method, using radiolabeled antibodies, to visualize IGF-1R expression.R1507 is a monoclonal antibody directed against the IGF-1R. In vitro, the affinity and internalization kinetics of (111)In-R1507 were determined using the IGF-1R-expressing triple-negative breast cancer cell line SUM149. In vivo, the pharmacodynamics of (111)In-R1507 and (125)I-R1507 were determined in mice with subcutaneous SUM149 tumors. (111)In-R1507 SPECT and (89)Zr-R1507 PET images of mice with subcutaneous SUM149 tumors were acquired at 1, 3, and 7 d after injection.(111)In-R1507 (concentration required to inhibit binding by 50%, 0.1 nM) was slowly internalized by SUM149 cells. (111)In-R1507 specifically and efficiently accumulated in the SUM149 xenografts: the tumor uptake was 20 percentage injected dose per gram (%ID/g), 33 %ID/g, and 31 %ID/g at 1, 3, and 7 d after injection, respectively. (125)I-R1507 accumulated in the tumor less efficiently. Small-animal SPECT and small-animal PET of mice clearly visualized the subcutaneous SUM149 xenograft, with increasing contrast at later time points.(111)In-R1507 and (89)Zr-R1507 are new tracers to non  invasive  ly determine IGF-1R expression in vivo in breast cancer xenografts using SPECT and PET. In the future, these techniques may enable patient selection for IGF-1R-targeted therapy. 
 Insulin-like growth factor receptor I mediates resistance to anti-epidermal growth factor receptor therapy in primary human glioblastoma cells through continued activation of phosphoinositide 3-kinase signaling. Overexpression of the epidermal growth factor receptor (EGFR) has been shown previously to correlate with enhanced malignant potential of many human tumor types, including glioblastoma multiforme (GBM). Anti-EGFR targeting has been demonstrated to enhance apoptosis and reduce both cellular invasion and angiogenic potential. It remains unclear whether absolute EGFR expression levels are sufficient to predict which tumors will respond best to anti-EGFR therapy. We have identified two primary GBM cell lines with equivalent EGFR expression levels with very different sensitivities to the EGFR receptor tyrosine kinase inhibitor, AG1478. This was apparent despite similar reductions in EGFR signaling in both cell lines, as measured by phospho-EGFR levels. AG1478 enhanced both spontaneous and radiation-induced apoptosis and reduced   invasive   potential in the GBM(S), but not in the GBM(R), cell line. The resistant GBM(R) cell line demonstrated an up-regulation of insulin-like growth factor receptor I (IGFR-I) levels on AG1478 administration. This resulted in sustained signaling through the phosphoinositide 3-kinase pathway, resulting in potent antiapoptotic and proinvasion effects. Cotargeting IGFR-I with EGFR greatly enhanced both spontaneous and radiation-induced apoptosis of the GBM(R) cells and reduced their   invasive   potential. Akt1 and p70(s6k) appeared to be important downstream targets of IGFR-I-mediated resistance to anti-EGFR targeting. These findings suggest that IGFR-I signaling through phosphoinositide 3-kinase may represent a novel and potentially important mechanism of resistance to anti-EGFR therapy. 
 MicroRNA-497 is a potential prognostic marker in human cervical cancer and functions as a tumor suppressor by targeting the insulin-like growth factor 1 receptor. Increasing evidence has shown that microRNAs function as oncogenes or tumor suppressors in human malignancies, but the roles of microRNA (miR)-497 in human cervical cancer still remain unclear. Our aim was to analyze the clinicopathologic and prognostic significance of miR-497 in human cervical cancer and to investigate the effects of miR-497 on the malignant phenotype of cervical cancer cells.First, we detected miR-497 expression in the HPV-16-immortalized cervical epithelial cell lines and 4 other cervical cancer cell lines (HeLa, Caski, SiHa, and HeLa-S3). Then the expression of miR-497 was analyzed in cervical cancer tissues and paired nontumor tissues, and its correlation with clinicopathologic features and survival was analyzed. Finally, the roles of miR-497 in regulation of tumor proliferation, apoptosis, migration, invasion, and target gene expression were further investigated.MiR-497 was downregulated in cervical cancer cells or tissues compared with HPV-16-immortalized cervical epithelial cell lines or the paired nontumor tissues. Also, the decrease in miR-497 correlated closely with the criteria of the International Federation of Gynaecology and Obstetrics stage and lymph node metastases in patients with cervical cancer. Multivariate Cox analysis showed that low miR-497 expression appeared to be an unfavorable prognostic factor. Transient forced expression of miR-497 decreased the growth and colony-formation capacity of HeLa and SiHa cells by inducing Caspase-3-dependent apoptosis. Forced expression of miR-497 suppressed the migration and   invasive  ness of cervical cancer cells. By computational miRNA target prediction and functional analysis, miR-497 was demonstrated to bind to the 3' untranslated regions of IGF-1R mRNA, and upregulation of miR-497 downregulated IGF-1R protein expression. Further investigation showed that small interfering RNA-mediated IGF-1R knockdown could mimic the effect of enforced miR-497 expression on the malignant phenotypes of cervical cancer cells.MiR-497 may be a potential prognostic marker and functions as a tumor suppressor in human cervical cancer by post-transcriptionally targeting IGF-1R. 
 P-cadherin potentiates ligand-dependent EGFR and IGF-1R signaling in dysplastic and malignant oral keratinocytes. Oral and oropharyngeal cancer together constitute the sixth most common cancer worldwide, with over 400,000 new cases diagnosed each year. Early detection is paramount, as the 5-year survival rate for these cancers decreases markedly once tumors have become regionally   invasive  . In many tissues, including oral epithelia, neoplastic progression is accompanied by alterations in expression of the epithelial cell adhesion molecules E-cadherin and P-cadherin. Oral epithelia is one of only a few tissues in which P-cadherin levels have been noted to increase in dysplasia and well-differentiated carcinomas and decrease in advanced malignancies. In the present study, P-cadherin was overexpressed in both dysplastic and malignant oral keratinocytes to characterize the mechanisms by which aberrantly expressed P-cadherin may modulate tumor progression. We found that P-cadherin was able to potentiate ligand-dependent signaling of insulin-like growth factor 1 receptor (IGF-1R) in malignant keratinocytes and epidermal growth factor receptor (EGFR) in dysplastic cells. P-cadherin prolonged activation of the mitogen-activated protein kinase (MAPK) in both cell lines and also increased the magnitude of AKT phosphorylation in dysplastic cells. P-cadherin overexpression alone was sufficient to increase steady-state levels of the mesenchymal transcription factor Snail, increase cell motility and also induce morphological changes in dysplastic keratinocytes. Taken together, these data suggest that the aberrantly elevated levels of P-cadherin which occur in early oral tumor development may play a critical role in the augmentation of neoplastic signaling networks and in the further acquisition of aggressive phenotypes. 
 Absence or low IGF-1R-expression in esophageal adenocarcinoma is associated with tumor   invasive  ness and radicality of surgical resection. Esophageal adenocarcinoma (EAC) incidence increases, maybe due to increasing prevalences of obesity and diabetes. Concurrent hyperinsulinemia might promote carcinogenesis via the insulin-like growth factor-I receptor (IGF-1R). Expression of the IGF-1R was studied in correlation with diabetes and prognostic parameters.Patients with EAC undergoing esophagectomy were prospectively selected. From resected tumors a tissue microarray was constructed. Immunohistochemistry evaluated IGF-1R-expression. Logistic-, cox regression models and survival analyses assessed if diabetes and IGF-1R-expression were associated with prognostic parameters. IGF-1R-expression in normal and Barrett tissues was studied.Absence or low IGF-1R-expression was associated with T3-, grade 3 tumors and R1 resections (P = 0.001, P = 0.025, P < 0.001, respectively). Logistic regression showed that this was associated with R1 resections (HR 0.24, 95%CI 0.11-0.52). Diabetes was not associated with IGF-1R-expression (P = 0.612). Absence or low IGF-1R-expression decreased 5-year overall survival (P = 0.023) univariably, but not multivariably. IGF-1R-expression was present in Barrett tissues, but diminished in high-grade dysplasia.Absence or low expression of IGF-1R was associated with high grade- and advanced tumors and less radical resections. IGF-1R might be a tumor marker in Barrett's esophagus since a change in expression patterns was found in the course from normal esophageal tissue to adenocarcinoma. 
 Regulation of receptor for activated C kinase 1 protein by the von Hippel-Lindau tumor suppressor in IGF-I-induced renal carcinoma cell   invasive  ness. von Hippel-Lindau (VHL) tumor suppressor loss is associated with renal cell carcinoma (RCC) pathogenesis. Meanwhile, aberrant activation of the insulin-like growth factor-I (IGF-I) signaling has been implicated in the development of highly   invasive   metastatic RCC. However, the link between VHL inactivation and RCC   invasive  ness is still unexplored. Here, we show that the receptor for activated C kinase 1 (RACK1) is a novel pVHL-interacting protein. pVHL competes with IGF-I receptor (IGF-IR) for binding to RACK1 thus potentially modulating the downstream IGF-I signal pathway. Upon IGF-I stimulation, pVHL-deficient RCC cells exhibit increased RACK1/IGF-IR binding and increased IGF-IR tyrosine kinase activity. pVHL-deficient RCC cells also demonstrate elevated PI3K/Akt signaling and matrix metalloproteinase-2 activity that culminates in enhanced cellular   invasive  ness, which can be partially suppressed by RACK1 small interfering RNA. Domain mapping analysis showed that the pVHL α-domain and the RACK1 WD 6-7 domains are critical for the interaction. Additionally, the RACK1 expression level is not regulated by pVHL expression status, suggesting that pVHL modifies RACK1 functions independent of the VHL/elongin E3 ubiquitin ligase complex. Our data indicate that RACK1 serves as a direct mediator between loss of pVHL function and enhanced IGF-IR signaling pathway in RCC. 
 Insulin-like growth factor-1 receptor expression in thymic malignancies. Thymic epithelial tumors are rare mediastinal malignancies that can be   invasive   and difficult to treat. Insulin-like growth factor-1 receptor (IGF-1R) is a transmembrane receptor implicated in the regulation of cell metabolism, growth, and survival. As higher levels of IGF-1R protein expression may be associated with relative sensitivity to anti-IGF-1R antibody treatment, we investigated IGF-1R expression in thymic malignancies.Sixty-three thymic tumors (56 thymomas and seven thymic carcinomas) were analyzed for total IGF-1R expression using immunohistochemistry with a specific antibody (clone G11, Roche-Ventana, Tucson, AZ). Expression levels were correlated with relevant clinical and pathologic variables, including epidermal growth factor receptor and KIT expression, and patient outcome.IGF-1R staining was negative in 13 (21%) cases, low (1+) in 20 (32%) cases, moderate (2+) in 20 (32%) cases, and high (3+) in 10 (16%) cases. Moderate to high IGF-1R staining was observed in 6 of 7 (86%) thymic carcinomas and in 24 of 56 (43%) thymomas (p = 0.039). Moderate to high IGF-1R staining was associated with high epidermal growth factor receptor staining (p = 0.015). By multivariate analysis, only tumor stage and histologic type were significant prognostic factors on time to progression (hazard ratio [HR] = 4.12, 95% confidence interval [CI]: 1.98-14.23; p = 0.010 and HR = 2.79, 95% CI: 1.62-12.50; p = 0.018, respectively). There was no association between IGF-1R expression and time to progression (HR = 3.07, 95% CI: 0.38-24.59; p = 0.291).A majority of thymic malignancies display moderate to high expression of IGF-1R. The lack of preclinical models prevented us to further study the functional consequences of anti-IGF-1R therapy in this setting. However, given correlations in other cancers, these data support the evaluation of anti-IGF-1R inhibitors in thymic tumors. 
 Requirement of different signaling pathways mediated by insulin-like growth factor-I receptor for proliferation, invasion, and VPF/VEGF expression in a pancreatic carcinoma cell line. Several oncogenes and growth factors are found to be mutated and overexpressed in adenocarcinoma of the pancreas, and may correlate with its highly aggressive nature. Insulin-like growth factor (IGF-I) and its receptor (IGF-IR) are highly expressed in this tumor type. We examined the IGF-IR-mediated signaling pathways in relation to cell proliferation,   invasive  ness, and expression pattern of vascular permeability factor/vascular endothelial growth factor (VPF/VEGF) in the pancreatic cancer line ASPC-1. Our findings show that IGF-IR is an important growth factor receptor for cell proliferation and invasion, and VPF/VEGF expression in ASPC-1. Further experiments indicate that IGF-IR mediates different signaling pathways to execute its functions. Activation of Ras by IGF-IR was found to be required for the cell invasion. On the other hand Src activation through IGF-IR is required for the cell proliferation, invasion, and also VPF/VEGF expression. Taken together, our data indicate the importance of IGF-IR in growth and   invasive  ness of the pancreatic cancer cell lines and also point out the multiple signaling pathways channeled through this receptor. 
 IGF-1 and its receptor in esophageal cancer: association with adenocarcinoma and visceral obesity. The insulin-like growth factor (IGF) pathway and visceral obesity have been independently linked with esophageal cancer. This study aimed to delineate the differential and interlinked role of visceral obesity and the IGF-1 system in esophageal adenocarcinoma and esophageal squamous-cell carcinoma (SCC).IGF-1 receptor (IGF-1R) mRNA and protein were examined in esophageal SCC (KYSE 410, OE21) and esophageal adenocarcinoma (OE19, OE33) cell lines by western blotting. Tumor cell proliferation in response to IGF-1 was assessed by bromodeoxyuridine incorporation assay. In esophageal tumor sections, expression of IGF-1R and CD68(+) cell numbers were assessed by immunohistochemistry. IGF-1 was measured in serum from esophageal cancer patients, Barrett's esophagus patients, and healthy controls by enzyme-linked immunosorbent assay.Higher IGF-1R protein expressions were observed in SCC cells compared with esophageal adenocarcinoma cells however only adenocarcinoma cell lines significantly increased proliferation in response to IGF-1 (P<0.01). Serum IGF-1 levels were highest in esophageal adenocarcinoma patients (P<0.01) and higher in viscerally obese vs. nonobese (P<0.05) patients. In resected esophageal cancer, increased expression of IGF-1R was observed in the tumor and   invasive   edge compared with tumor-associated stroma (P<0.05), which coincided with increased CD68(+) cells in stromal tissue surrounding   invasive   tumor edge (P<0.01).This novel study examined the differential role of the IGF system in esophageal adenocarcinoma and SCC, and its association with visceral obesity. These results indicate that the IGF-1 axis has a key role in malignant progression of esophageal cancer, and represents a plausible mechanism through which visceral obesity impacts on esophageal adenocarcinoma risk and tumor biology. 
 Differentiated thyroid carcinomas from children and adolescents express IGF-I and the IGF-I receptor (IGF-I-R). Cancers with the most intense IGF-I-R expression may be more aggressive. Adult thyroid cancers express IGF and IGF-I receptor (IGF-I-R), but the clinical impact is not clear. No previous study examined any childhood thyroid cancers that are well-differentiated and have a favorable prognosis. We used immunohistochemistry to determine IGF-I and IGF-I-R in 23 papillary thyroid cancers (PTC) and 6 follicular thyroid cancers (FTC) from children and adolescents. IGF-I was detected in 45% and IGF-I-R in 43% of cancers. IGF-I and IGF-I-R were found more often in PTC (IGF-I = 9/23, IGF-I-R = 8/19) than normal surrounding thyroid (IGF-I = 0/10, p = 0.032 and IGF-I-R = 0/10, p = 0.030). There were too few FTC to support independent statistical analysis, but IGF-I was found in 4 of 6 FTC (0/10 normal), and IGF-I-R was found in 2 of 4 FTC (0/10 normal). IGF-I-R staining was more intense in aggressive (  invasive  , metastatic, recurrent, or persistent) than indolent tumors (confined to the gland, p = 0.029). Over time, six tumors recurred, five of which expressed IGF-I-R. Overall recurrence risk was significantly greater for tumors that expressed IGF-I-R (p = 0.05) but only approached statistical significance (p = 0.08) when disease-free survival was determined. We conclude that differentiated thyroid cancers of children and adolescents express IGF-I and IGF-I-R. Tumors that express IGF-I-R are more likely to show aggressive clinical features (invasion beyond the capsule, metastasis, or recurrence) and persistence despite treatment. 
 Analysis of expression of membrane-bound tumor markers in ductal carcinoma in situ of the breast: paving the way for molecular imaging. Achieving radicality during breast conserving surgery for pure ductal carcinoma in situ (DCIS) of the breast and   invasive   cancer surrounded by DCIS is challenging. Molecular imaging holds promise here, when applied as a tool for image-guided surgery of DCIS.Tissue microarrays containing 24 pure DCIS and 63 DCIS with adjacent   invasive   breast cancer cases were stained by immunohistochemistry for a panel of membrane-bound targets.GLUT1 expression was present in 60.9%, IGF1-R in 55.2% HER2 in 28.7%, MET in 18.4%, EGFR in 16.1%, CD44v6 in 69%, carbonic anhydrase XII (CAXII) in 24.1% and Mammaglobin in 14.9% of DCIS cases. No expression differences between pure DCIS and DCIS with adjacent cancer were observed. Further, HER2 and EGFR expression were correlated with high grade DCIS (p=0.001) and CAXII with low grade DCIS (p=0.027). A putative panel containing HER2, EGFR, GLUT1 and IGF1-R had a detection rate of 90.2% for DCIS and 78.3% for adjacent breast cancer.We found that membrane-bound targets are more frequently expressed in DCIS than in   invasive   breast cancer, but that single membrane proteins are too infrequently expressed to serve as single imaging targets for the detection of DCIS. However, a panel of markers consisting of IGF1-R, CD44v6, GLUT1, EGFR, and HER2 was found to be positive in 96.3% of DICS based on marker expression in the adjacent   invasive   breast cancer as described earlier. This implies that detection of DCIS based on marker expression in the adjacent   invasive   breast cancer during breast conserving surgery should be possible with a panel of molecular imaging tracers targeting CD44v6, GLUT1, HER2, IGF1-R, and EGFR. 
 Low activation of Insulin-like Growth Factor 1-Receptor (IGF1R) is associated with local recurrence in early breast carcinoma. Background The predictive value of IGF1R on local recurrence in   invasive   breast carcinoma (BC) is not well known. Methods In a series of 197 lymph-node negative BC patients treated with breast-conserving surgery and radiation therapy, we performed immunohistochemistry for alpha-IGF1R, beta-IGF1R (phosphorylated/active form) and Estrogen/Progesterone receptors. We further evaluated the IGF1R mRNA expression by quantitative RT-PCR and IGF1R mutations by direct DNA sequencing (exons 19 and 21) in 85 primary BC (42 control cases, 31 with local recurrence and 12 with distant metastasis) and in 31 local recurrences. Unconditional logistic regression analyses were performed to identify risk factors for recurrence. Results Local recurrences were associated with high-grade tumors, PR-negative and low active-IGF1R, which emerged as independent breast relapse predictors by multivariate analysis. Conclusion Patients with early BC treated with lumpectomy and radiation who have low-grade tumors and favorable markers (increased content of active IGF1R and PR-positive) have a low risk of local recurrence. Therefore, do not benefit from a boost dose on the surgical scar. 
 Insulin-like growth factor-1 receptor gene expression is associated with survival in breast cancer: a comprehensive analysis of gene copy number, mRNA and protein expression. Insulin-like growth factor-1 receptor (IGF1R) plays a key role in the initiation and progression of breast cancer. However, its prognostic relevance to breast cancer patients has long been a matter of debate. In a series of 325 primary   invasive   breast cancer patients, we performed a comprehensive analysis of IGF1R at the levels of gene copy number, mRNA expression and protein expression. The relationship between the IGF1R status and the clinicopathological characteristics and prognosis was evaluated. IGF1R mRNA levels not only correlated with protein expression, but also were significantly associated with several clinicopathological parameters and prognosis. Patients with low nuclear grade, negative axillary lymph nodes, positive hormone receptor, negative Her2, negative Ki67, and luminal subtype tumors showed higher expression levels of IGF1R mRNA, which was shown to be a significant univariate parameter for both relapse-free survival and breast cancer-specific survival (BCSS) as well as a significant multivariate parameter for BCSS. IGF1R protein expression showed an association with a prolonged BCSS in univariate analysis. In contrast, IGF1R gene copy number was not correlated with mRNA and protein expression, and harbored no prognostic value. When studied in the luminal tumor subtype groups, IGF1R mRNA level was still significantly associated with a better BCSS. Overall, our data indicated a correlation between IGF1R mRNA expression and protein expression in primary breast cancer. In particular, IGF1R mRNA expression appeared to be a good prognostic marker both in the entire cohort and in the luminal subtype group. These data may serve as background information for IGF1R-targeted therapy. 
 Fibulin-3-mediated inhibition of epithelial-to-mesenchymal transition and self-renewal of ALDH+ lung cancer stem cells through IGF1R signaling. Fibulins (FBLNs), a family of extracellular matrix proteins, have recently been shown to act as tumor suppressors or activators in different cancers, and the underlying molecular mechanisms of their action in cancer remain unclear. We have previously shown that the expression of FBLN3 is suppressed by promoter hypermethylation and is associated with   invasive  ness in aggressive non-small cell lung cancer. In this study, we evaluated the roles and signaling mechanism of FBLN3 in lung cancer stem cells (CSCs). Forced expression of FBLN3 suppressed invasion and migration of lung adenocarcinoma cells and decreased the expression of epithelial-to-mesenchymal transition (EMT) activators, including N-cadherin and Snail. Stemness activities of lung adenocarcinoma cells were also suppressed by FBLN3 as indicated by a decrease in spheroid formation and the levels of stemness markers such as Sox2 and β-catenin. These effects of FBLN3 were mediated by the glycogen synthase kinase-3β, GSK3β/β-catenin pathway, and the upstream regulators of GSK3β, including phosphoinositide 3-kinase (PI3K)/AKT and insulin-like growth factor-1 receptor (IGF1R), were inactivated by FBLN3. Moreover, IGF1R was shown to be a direct target of FBLN3, which competitively inhibited insulin-like growth factor (IGF) action. To confirm the effect of FBLN3 on lung CSCs, aldehyde dehydrogenase-positive (ALDH+) A549 lung CSCs were sorted and treated with recombinant FBLN3 protein. FBLN3 clearly suppressed EMT, stemness activity and the over-activated IGF1R/PI3K/AKT/GSK3β pathway of the ALDH+ CSC subpopulation. In addition, injection of recombinant FBLN3 protein around subcutaneous xenografts established with ALDH+ CSCs in athymic nude mice significantly suppressed tumor growth and progression. Overall, our results show that FBLN3 suppresses both EMT and self-renewal of the lung CSCs by modulating the IGF1R/PI3K/AKT/GSK3β pathway and that FBLN3 would be useful as an alternative CSC therapy. 
 Downregulation of miR-383 promotes glioma cell invasion by targeting insulin-like growth factor 1 receptor.   Invasive  ness is a major clinical feature of glioma, an aggressive brain tumor with poor prognosis. Although there is emerging evidence that some microRNAs are involved in the glioma cell invasion process, it remains necessary to find functional microRNAs and elucidate the underlying molecular mechanisms. Here, we reported that a microRNA, miR-383, was downregulated in gliomas and inversely correlated with glioma pathological grades. Downregulation of miR-383 enhanced, whereas upregulation of miR-383 inhibited, the glioma cell   invasive   ability. Furthermore, we found that downregulation of miR-383 activated the AKT signaling following upregulation of MMP2 expression by directly targeting insulin-like growth factor 1 receptor (IGF1R). Importantly, we demonstrated that IGF1R expression is critical for miR-383 downregulation-induced cell invasion. Taken together, these findings uncover a novel regulatory mechanism for constitutive IGF1R signaling activation in glioma cancer and may provide miR-383 as a useful diagnostic marker or therapeutic target. 
 Ras-related protein 1 and the insulin-like growth factor type I receptor are associated with risk of progression in patients diagnosed with carcinoma in situ. Currently, there are no applied molecular markers to aid in predicting risk of carcinoma in situ (CIS) progression to   invasive   cancer, and therefore, all women diagnosed with CIS undergo surgery. Standard assessment of protein expression in fixed tissue by immunohistochemistry (IHC) is not quantitative and hence is not well suited for measuring biomarkers. In this study, we developed an original analytical method for IHC quantification. Using our novel image-based uniplex (IBU) method, quantitative protein profiling was performed on 90 samples of the breast (17 histologically normal tissues, 16 benign lesions, 15 CIS, and 42   invasive   carcinomas). Differences between groups were assessed using analysis of variance (ANOVA) and mixed effects models. Measuring protein expression on a continuous scale revealed a significant increase in Ras-related protein 1 (Rap1) and the insulin-like growth factor type I receptor (IGF-IR) in conjunction with the presence of cancer invasion. Women with   invasive   cancers were four times more likely to have increased levels of Rap1 [odds ratio (OR) = 3.91; P = 0.0002] and IGF-IR (OR=4.33; P<0.0001) than women with non-  invasive   lesions. Furthermore, expression of both proteins was also increased significantly in CIS adjacent to   invasive   tumors compared with non-cancerous tissue. These novel findings of a significant up-regulation of Rap1 and IGF-IR in CIS progressing to   invasive   cancers warrant further investigation of Rap1 and IGF-IR together as a dual biomarker to aid in predicting risk of progression and ultimately providing non-surgical treatment options to those at lower risk. 
 Prognostic significance of insulin growth factor-I receptor and insulin growth factor binding protein-3 expression in primary breast cancer. We analyzed insulin-like growth factor receptor I (IGF-IR) and insulin growth factor binding protein-3 (IGFBP-3) expression with respect to overall survival and relapse-free survival (RFS) in 460 patients with primary   invasive   breast cancer using immunohistochemistry. IGF-IR expression had a significant positive correlation with positive estrogen receptor (ER), positive progesterone receptor (PR) and Bcl-2 expression. Univariate analysis showed significantly better overall survival (p=0.000) and RFS (p=0.004), in the IGF-IR-positive group. Multivariate analysis showed a significant reduction in relative risk for overall survival (p=0.019, HR=0.221, 95% CI=0.062-0.780) and RFS (p=0.026, HR=0.462, 95% CI=0.234-0.913) in the IGF-IR-positive group. IGF-IR expression in primary breast cancer is an independent favorable prognostic factor. IGFBP-3 expression in breast cancer is associated with poor outcome. 
 Insulin-like growth factor receptor-1 (IGF-1R) expression in normal breast, proliferative breast lesions, and breast carcinoma. Insulin-like growth factor receptor 1 (IGF-1R) is a receptor protein tyrosine kinase that is activated by ligand (IGF-1) binding and promotes mitogenic, metastatic, and antiapoptotic phenotypes of breast cancer. There is a dearth of studies analyzing IGF-1R expression by immunohistochemistry in breast carcinoma. This biomarker analysis will be important for pharmacologic interventions that target the IGF system. IGF-1R expression pattern was first analyzed in normal breast tissue and a variety of breast lesions (71 diagnoses from 35 patients), followed by analysis in 191 consecutive   invasive   breast carcinomas. Furthermore, 86 carcinomas treated with neoadjuvant chemotherapy were also analyzed. The carcinomas were classified using immunohistochemical surrogate (to molecular classes) markers-estrogen receptors (ER), progesterone receptors, and human epidermal growth factor receptor 2. IGF-1R is expressed at moderate level in normal breast tissue which was considered as normal expression. Overexpression and lower expression were defined as higher than normal or lower than normal expression, respectively. Among the benign and non  invasive   breast lesions, IGF-1R expression was slightly increased in lesions that are hormonally driven (such as atypical ductal hyperplasia and columnar cells changes) whereas it was significantly reduced in ER-negative lesions (such as apocrine metaplasia). Similarly, in 191 consecutive breast carcinomas, IGF-1R overexpression was predominantly seen in ER-positive+ tumors. The tumor group that consistently showed reduced expression was the ERBB2 group (ER negative/progesterone receptors negative/human epidermal growth factor receptor 2 positive). The expression was somewhat heterogeneous in the triple-negative group. IGF-1R expression was not predictive of pathologic complete response or tumor volume reduction in ER-negative tumors, but reduced IGF-1R was associated with pathologic complete response and significant tumor volume reduction in ER+ tumors. Therapies targeting IGF-1R will be useful in majority of ER+ and a subset of triple-negative tumors that express IGF-1R. The consistent presence of IGF-1R in ER+ tumors may explain the lower response rate to neoadjuvant chemotherapy in luminal tumors. 
 Insulin receptor substrate-2 mediated insulin-like growth factor-I receptor overexpression in pancreatic adenocarcinoma through protein kinase Cdelta. Pancreatic adenocarcinoma (PCA) is an almost invariably fatal disease. Recently, it has been shown by several groups as well as ours that insulin-like growth factor-I receptor (IGF-IR) overexpression is related to higher proliferation, survival, angiogenesis, and highly   invasive   pancreatic tumors. Several studies have been carried out to understand the pathways that lead to growth factor-mediated signaling, but the molecular mechanism of receptor overexpression remains mostly unknown. Treatment with neutralizing antibodies or a specific kinase inhibitor against IGF-IR could block the receptor expression in PCA cells. Furthermore, we also showed that insulin receptor substrate (IRS)-2, but not IRS-1, is involved in regulation of IGF-IR expression, which is most likely not transcriptional control. By blocking mammalian target of rapamycin (mTOR) pathway with rapamycin as well as other biochemical analysis, we defined a unique regulation of IGF-IR expression mediated by protein kinase Cdelta (PKCdelta) and mTOR pathway. Moreover, we showed that the down-regulation of IGF-IR expression due to IRS-2 small interfering RNA can be compensated by overexpression of dominant-active mutant of PKCdelta, suggesting that PKCdelta is downstream of IGF-IR/IRS-2 axis. Overall, these findings suggest a novel regulatory role of IRS-2 on the expression of IGF-IR through PKCdelta and mTOR in pancreatic cancer cells. 
 IL-6 promotes prostate tumorigenesis and progression through autocrine cross-activation of IGF-IR. As an established mediator of inflammation, interleukin-6 (IL-6) is implicated to facilitate prostate cancer progression to androgen independence through transactivation of the androgen receptor. However, whether IL-6 has a causative role in de novo prostate tumorigenesis was never investigated. We now provide the first evidence that IL-6 can induce tumorigenic conversion and further progression to an   invasive   phenotype of non-tumorigenic benign prostate epithelial cells. Moreover, we find that paracrine IL-6 stimulates the autocrine IL-6 loop and autocrine activation of insulin-like type I growth factor receptor (IGF-IR) to confer the tumorigenic property and also that activation of signal transducer and activator of transcription 3 (STAT3) is critical in these processes. Inhibition of STAT3 activation or IGF-IR signaling suppresses IL-6-mediated malignant conversion and the associated   invasive   phenotype. Inhibition of STAT3 activation suppresses IL-6-induced upregulation of IGF-IR and its ligands, namely IGF-I and IGF-II. These findings indicate that IL-6 signaling cooperates with IGF-IR signaling in the prostate microenvironment to promote prostate tumorigenesis and progression to aggressiveness. Our findings suggest that STAT3 and IGF-IR may represent potential effective targets for prevention or treatment of prostate cancer. 
 Immunohistochemical evaluation of insulin-like growth factor I receptor status in cervical cancer specimens. The insulin-like growth factor I receptor (IGF-IR) is exceptionally overexpressed in many cervical-cancer-derived cell lines. It is postulated that a decrease of p53 protein levels due to human papillomavirus (HPV) infection may contribute to the up-regulation of IGF-IR expression in cervical cancer cells because transcription of IGF-IR is strictly down-regulated by p53. To evaluate this fact in clinical cervical cancer specimens, we checked the expression levels and activated status of IGF-IR by immunohistochemistry. Formalin-fixed and paraffin-embedded specimens obtained by conization or hysterectomy were stained with anti-IGF-IR and with an antibody recognizing phosphorylated tyrosine at its c-terminus. The expression levels of IGF-IR were significantly high in cervical intraepithelial neoplasia (CIN) III and   invasive   cancer specimens. Phosphorylation of IGF-IR was promoted in all CIN and   invasive   cancer specimens, and its intensity was related to the promotion of lesions. Interestingly, IGF-IR overexpression was missing in the basal layer of CIN I and II lesions, whereas it was evenly distributed in CIN III and   invasive   cancer lesions. This IGF-IR overexpression pattern may be utilized in the diagnosis of HPV infection status in CIN lesions. 
 Insulin-like growth factor 1 receptor enhances invasion and induces resistance to apoptosis of colon cancer cells through the Akt/Bcl-x(L) pathway. Colon cancer overexpresses insulin-like growth factor 1 (IGF1) and insulin-like growth factor 1 receptor (IGF1-R), as compared with normal or adenomatous mucosa, and it has been postulated that colorectal cancer cells depend on the IGF1/IGF1-R pathway for their growth and progression. In this study, using the human colon cancer cell line HCT116, we find that established HCT116/IGF1-R transfectants exhibit a more aggressive transformed phenotype than the parental cell line, as demonstrated by their higher proliferation rate in response to IGF1, higher degree of anchorage-independent growth, resistance to serum deprivation-induced apoptosis, and higher migratory capability in a monolayer "wounding assay." When injected into nude mice, HCT116/IGF1-R transfectants were highly   invasive   and produced distant metastases, whereas the parental cell did not. Moreover, the overexpression of IGF1-R in these cells was associated with IGF1-R-induced activation of Akt and up-regulation of the antiapoptotic protein Bcl-x(L). We also show that Akt pathway mediates IGF1-R-induced Bcl-x(L) expression at transcriptional level. Our data demonstrate, for the first time, that IGF1-R/Akt/Bcl-x(L) pathway may contribute to a more aggressive malignant phenotype, in a subset of colorectal cancers. 
 The role of EphB4 and IGF-IR expression in breast cancer cells. to investigate the role of EphB4 and IGF-1R in the proliferation and migration of breast cancer.The relative mRNA levels of EphB4 were measured by RT-PCR. The proliferation of the cells was determined by MTT assay, and cells migration and   invasive   ability was analyzed using the scratch migration assay.The expression of EphB4 in control group was significantly decreased when compared with IGF-I group (P<0.001). The expression of EphB4 in IGF-I+LY and LY group were lower than that of the control group (P<0.001).The cell proliferation and migration ability of the cells in IGF-I group increased significantly compared to the cells in the control group (P<0.001), while the cells in IGF-1+LY group and LY group showed a decreased proliferation and migration ability compared to the control group (P<0.001).IGF-IR might be a upstream gene of EphB4. Besides, higher expression of EphB4 shows increased tumor proliferation and migration in breast cells. The study of EphB4 upstream gene and signaling pathway can provide more targeted anti-tumor point selection for targeted therapy. 
 c-Met, epidermal growth factor receptor, and insulin-like growth factor-1 receptor are important for growth in uveal melanoma and independently contribute to migration and metastatic potential. Uveal melanoma (UM) has a high propensity to develop hepatic metastases. We sought to define the mechanisms required for preferential liver homing and to understand further the biologic behavior of this disease. The Met tyrosine kinase receptor and its ligand hepatocyte growth factor are expressed in hepatocytes. We therefore considered Met/hepatocyte growth factor signaling as a candidate migration/growth factor for UM cells. We further explored the relationship between c-Met and other growth factor receptors prevalent in the liver and their roles in UM metastatic potential. UM cell lines were evaluated for c-Met, epidermal growth factor receptor (EGFR), and insulin-like growth factor-1R (IGF-1R) expression by immunoblotting, and gene amplification by comparative genomic hybridization and fluorescence in-situ hybridization. High c-Met, phosphorylated c-Met, and EGFR expression were noted in two of nine cell lines, independent of IGF-1R levels. Knockdown of c-Met decreased proliferation of high c-Met-expressing UM cells but did not induce apoptosis. Selective inhibitors of EGFR and IGF-1R decreased proliferation and induced apoptosis in UM cells regardless of the expression levels of c-Met, EGFR, and IGF-1R. Although c-Met, EGFR, and IGF-1R play proliferative roles, EGFR and IGF-1R are also critical for UM cell survival. High c-Met/EGFR-expressing cell lines possessed the greatest migration potential. c-Met knockdown and selective inhibitors of c-Met, EGFR, and IGF-1R revealed independent contribution of these receptors to migration. UM can be categorized by levels of c-Met and EGFR expression which are associated with migratory/  invasive  ness responses to soluble factors present at high levels in the liver. This provides biologic relevance for UM clinical behavior with potential therapeutic implications. 
 Suppression of type 1 Insulin-like growth factor receptor expression by small interfering RNA inhibits A549 human lung cancer cell invasion in vitro and metastasis in xenograft nude mice. Cancer invasion and metastasis, involving a variety of pathological processes and cytophysiological changes, contribute to the high mortality of lung cancer. The type 1 insulin-like growth factor receptor (IGF-1R), associated with cancer progression and invasion, is a potential anti-invasion and anti-metastasis target in lung cancer. To inhibit the   invasive   properties of lung cancer cells, we successfully down-regulated IGF-1R gene expression in A549 human lung cancer cells by small interfering RNA (siRNA) technology, and evaluated its effects on invasion-related gene expression, tumor cell in vitro invasion, and metastasis in xenograft nude mice. A549 cells transfected with a plasmid expressing hairpin siRNA for IGF-1R showed a significantly decreased IGF-1R expression at the mRNA level as well as the protein level. In biological assays, transfected A549 cells showed a significant reduction of cell-matrix adhesion, migration and invasion. Consistent with these results, we found that down-regulation of IGR-1R concomitantly accompanied by a large reduction in invasion-related gene expressions, including MMP-2, MMP-9, u-PA, and IGF-1R specific downstream p-Akt. Direct tail vein injections of plasmid expressing hairpin siRNA for IGF-1R significantly inhibited the formation of lung metastases in nude mice. Our results showed the therapeutic potential of siRNA as a method for gene therapy in inhibiting lung cancer invasion and metastasis. 
 Identification of the cathelicidin peptide LL-37 as agonist for the type I insulin-like growth factor receptor. The human cathelicidin antimicrobial protein-18 and its C terminal peptide, LL-37, displays broad antimicrobial activity that is mediated through direct contact with the microbial cell membrane. In addition, recent studies reveal that LL-37 is involved in diverse biological processes such as immunomodulation, apoptosis, angiogenesis and wound healing. An intriguing role for LL-37 in carcinogenesis is also beginning to emerge and the aim of this paper was to explore if and how LL-37 contributes to the signaling involved in tumor development. To this end, we investigated the putative interaction between LL-37 and growth factor receptors known to be involved in tumor growth and progression. Among several receptors tested, LL-37 bound with the highest affinity to insulin-like growth factor 1 receptor (IGF-1R), a receptor that is strongly linked to malignant cellular transformation. Furthermore, this interaction resulted in a dose-dependent phosphorylation and ubiquitination of IGF-1R, with downstream signaling confined to the mitogen-activated protein kinase/extracellular signal-regulated kinase (ERK)-pathway but not affecting phosphatidylinositol 3 kinase/Akt signaling. We found that signaling induced by LL-37 was dependent on the recruitment of β-arrestin to the fully functional IGF-1R and by using mutant receptors we demonstrated that LL-37 signaling is dependent on β-arrestin-1 binding to the C-terminus of IGF-1R. When analyzing the biological consequences of increased ERK activation induced by LL-37, we found that it resulted in enhanced migration and invasion of malignant cells in an IGF-1R/β-arrestin manner, but did not affect cell proliferation. These results indicate that LL-37 may act as a partial agonist for IGF-1R, with subsequent intra-cellular signaling activation driven by the binding of β-arrestin-1 to the IGF-1R. Functional experiments show that LL-37-dependent activation of the IGF-1R signaling resulted in increased migratory   and inva  sive potential of malignant cells. 
 A polymorphic variant of the insulin-like growth factor type I receptor gene modifies risk of obesity for esophageal adenocarcinoma. To investigate potential biologic mechanisms underlying the association between obesity and risk for esophageal adenocarcinoma (EADC), we studied the frequency of a common polymorphism of the insulin-like growth factor I receptor (IGF-IR) gene in patients with either gastroesophageal reflux disease (GERD), premalignant Barrett esophagus (BE) and or   invasive   EADC.Using a well characterized series of 431 individuals enrolled in a case-control study, we studied the frequency of the IGF-IR gene polymorphism, G1013A.On multivariate analysis controlling for age and gender, in comparison to asymptomatic controls, obese individuals with the polymorphic A-variant (G/A, A/A) were found to have significantly increased risk for EADC (OR 4.81; 95%CI 1.09-21.15), whereas obese individuals with the G/G variant were not at statistically significant increased risk (OR 2.69; 95%CI 0.41-17.62). Similarly, compared to asymptomatic controls, only obese individuals with the A-variant (G/A, A/A) were at increased risk for BE (OR 3.11; 95%CI 1.12-8.63), while obese individuals with the G/G variant were not at increased risk for BE (OR 2.91; 95%CI 0.69-12.15).We conclude that the common IGF-IR gene polymorphism G1013A modulates the risk of obesity for EADC, an effect most likely mediated by altered the receptor function by influencing gene transcription or mRNA stability. These findings further implicate the insulin-like growth factor axis in the molecular pathogenesis of EADC, and represent a plausible mechanistic link underlying the association between obesity and malignancy. 
 Insulin-like growth factor-1 receptor signaling increases the   invasive   potential of human epidermal growth factor receptor 2-overexpressing breast cancer cells via Src-focal adhesion kinase and forkhead box protein M1. Resistance to the human epidermal growth factor receptor (HER2)-targeted antibody trastuzumab is a major clinical concern in the treatment of HER2-positive metastatic breast cancer. Increased expression or signaling from the insulin-like growth factor-1 receptor (IGF-1R) has been reported to be associated with trastuzumab resistance. However, the specific molecular and biologic mechanisms through which IGF-1R promotes resistance or disease progression remain poorly defined. In this study, we found that the major biologic effect promoted by IGF-1R was invasion, which was mediated by both Src-focal adhesion kinase (FAK) signaling and Forkhead box protein M1 (FoxM1). Cotargeting IGF-1R and HER2 using either IGF-1R antibodies or IGF-1R short hairpin RNA in combination with trastuzumab resulted in significant but modest growth inhibition. Reduced invasion was the most significant biologic effect achieved by cotargeting IGF-1R and HER2 in trastuzumab-resistant cells. Constitutively active Src blocked the anti-  invasive   effect of IGF-1R/HER2 cotargeted therapy. Furthermore, knockdown of FoxM1 blocked IGF-1-mediated invasion, and dual targeting of IGF-1R and HER2 reduced expression of FoxM1. Re-expression of FoxM1 restored the   invasive   potential of IGF-1R knockdown cells treated with trastuzumab. Overall, our results strongly indicate that therapeutic combinations that cotarget IGF-1R and HER2 may reduce the   invasive   potential of cancer cells that are resistant to trastuzumab through mechanisms that depend in part on Src and FoxM1. 
 Insulin-like growth factor-1 receptor overexpression is associated with outcome in   invasive   urothelial carcinoma of urinary bladder: a retrospective study of patients treated using radical cystectomy. To assess the insulin-like growth factor-1 receptor (IGF1R) expression in urothelial carcinoma (UC) and its prognostic role in relation to clinicopathologic parameters.A total of 100 cases of   invasive   UC were evaluated using tissue microarrays. Membranous IGF1R staining was evaluated using immunohistochemistry. A scoring method analogous to that of HER2 expression in breast carcinoma was used, and the highest score was assigned in each tumor. IGF1R was considered overexpressed in cases with score≥1.We found IGF1R overexpression in 62% o  f invasi  ve UC. IGF1R overexpression was associated with race (P=.04) and pT category (P=.03). Median follow-up was 29 months (range, 0.5-212). Progression rate was 60%, and overall mortality and cancer-specific mortality rates were 69% and 51%, respectively. I  n invasi  ve UC, IGF1R overexpression was significantly associated with overall mortality and cancer-specific mortality (Mantel Cox P=.0002 and P=.006, respectively). IGF1R overexpression was associated with increased hazard ratios (HRs) for overall mortality (HR=2.63, P=.001) and cancer-specific mortality (HR=2.45, P=.01), independently and after adjusting for clinicopathologic features and treatment modalities.We found IGF1R overexpression in 62% of bladder UC. More importantly, IGF1R overexpression was a significant predictor of overall mortality and cancer-specific mortality, suggesting its potential role as a prognosticator in UC of bladder. 
 The insulin-like growth factor receptor I promotes motility and invasion of bladder cancer cells through Akt- and mitogen-activated protein kinase-dependent activation of paxillin. The insulin-like growth factor receptor I (IGF-IR) plays an essential role in transformation by promoting cell growth and protecting cancer cells from apoptosis. Aberrant IGF-IR signaling is implicated in several types of tumors, including carcinomas of the lung, breast, prostate, pancreas, liver, and colon. However, the contribution of the IGF-IR to the development of the transformed phenotype in urothelial cells has not been clearly established. In this study we demonstrated that the IGF-IR is overexpressed in   invasive   bladder cancer tissues compared with nonmalignant controls. We have investigated the role of the IGF-IR in bladder cancer by using urothelial carcinoma-derived 5637 and T24 cells. Although activation of the IGF-IR did not appreciably affect their growth, it did promote migration and stimulate in vitro wound closure and invasion. These effects required the activation of the Akt and Mitogen-activated protein kinase (MAPK) pathways as well as IGF-I-induced Akt- and MAPK-dependent phosphorylation of paxillin, which relocated at dynamic focal adhesions and was necessary for promoting motility in bladder cancer cells. Our results provide the first evidence for a role of the IGF-IR in motility and invasion of bladder cancer cells and support the hypothesis that the IGF-IR may play a critical role in the establishment of the   invasive   phenotype in urothelial neoplasia. Thus, the IGF-IR may also serve as a novel biomarker for bladder cancer. 
 p73 and IGF1R Regulate Emergence of Aggressive Cancer Stem-like Features via miR-885-5p Control. Cancer stem-like cells (CSC) have been proposed to promote cancer progression by initiating tumor growth at distant sites, suggesting that stem-like cell features can support metastatic efficiency. Here, we demonstrate that oncogenic DNp73, a dominant-negative variant of the tumor-suppressor p73, confers cancer cells with enhanced stem-like properties. DNp73 overexpression in non  invasive   melanoma and lung cancer cells increased anchorage-independent growth and elevated the expression of the pluripotency factors CD133, Nanog, and Oct4. Conversely, DNp73 depletion in metastatic cells downregulated stemness genes, attenuated sphere formation and reduced the tumor-initiating capability of spheroids in tumor xenograft models. Mechanistic investigations indicated that DNp73 acted by attenuating expression of miR-885-5p, a direct regulator of the IGF1 receptor (IGF1R) responsible for stemness marker expression. Modulating this pathway was sufficient to enhance chemosensitivity, overcoming DNp73-mediated drug resistance. Clinically, we established a correlation between low p73 function and high IGF1R/CD133/Nanog/Oct4 levels in melanoma specimens that associated with reduced patient survival. Our work shows how DNp73 promotes cancer stem-like features and provides a mechanistic rationale to target the DNp73-IGF1R cascade as a therapeutic strategy to eradicate CSC. 
 Additive impact of HER2-/PTK6-RNAi on interactions with HER3 or IGF-1R leads to reduced breast cancer progression in vivo. The human epidermal growth factor receptor 2 (HER2) and the protein tyrosine kinase 6 (PTK6) are often co- and over-expressed in   invasive   breast cancers. At early diagnosis, only distinct groups, such as HER2-or hormone receptor-positive benefit from a targeted therapy. However, a part of these tumours develops resistance within a year of administration of the drug but the majority of the patients depends on general therapies with severe side effects. A PTK6-directed approach does not yet exist. In our present study, we successfully demonstrate, in vitro and in vivo, a significantly additive reduction of tumourigenesis of breast cancer cells simultaneously depleted of both HER2 and PTK6. In comparison with single RNAi approaches, the combined RNAi (co-RNAi) led to a stronger reduced phosphorylation of tumour-promoting proteins. Moreover, the co-RNAi additively decreased cell migration as well as two and three dimensional cell proliferation in vitro. The in vivo experiments showed an additive reduction (p < 0.00001) in the growth of xenografts due to the co-RNAi compared with HER2 or PTK6 RNAi alone. Interestingly, the complexes of HER2 or PTK6 with tumour-relevant interaction partners, such as HER3 or the insulin-like growth factor receptor 1 (IGF-1R), respectively, were also reduced in xenografts although their protein expression levels were not affected following the co-RNAi of HER2 and PTK6. Our present study reveals the potential of using combined HER2- and PTK6- knockdown as a powerful strategy for the treatment of breast cancers. Therefore, the combined inhibition of these proteins may represent an attractive tool for efficient therapy of breast cancers. 
 Biological effects of insulin and its analogs on cancer cells with different insulin family receptor expression. Hyperinsulinemia is a likely cause of the increased cancer incidence and mortality in diabetic patients, but its role is difficult to define in vivo. Previous in vitro studies testing the mitogenic potential of insulin and its analogs provided incomplete and sometimes contradictory results. To better evaluate cancer cell responsiveness to insulin, to its analogs and to IGF-I, we measured under identical experimental conditions cell proliferation,   invasive  ness, and foci formation in six cancer cell lines with different insulin receptor family expression levels. The cancer cells studied have a different expression of insulin receptor (IR), its isoforms (IR-A and IR-B), and of the IGF-I receptor. The data indicate that insulin stimulates proliferation in all cancer cell lines,   invasive  ness in some, and foci formation in none. Cancer cell responses to insulin (and IGF-I) are not related to receptor expression levels; moreover, hormone-stimulated proliferation and   invasive  ness are not correlated. IGF-I is a more potent stimulator than insulin in most but not all cancer cell lines. Insulin analogs including M1 and M2 Glargine metabolites stimulate cancer cells similar to insulin. However, exceptions occur for specific analogs in particular cancer cells. In conclusion, in vitro insulin is an effective growth factor for all cancer cells but the biological response to insulin cannot be predicted on the basis of receptor expression levels. In the clinical setting, these observations should be taken in account when deciding treatment for diabetic patients who are at risk of undiscovered cancer or survivors of oncological diseases. 
 Influence of insulin resistance on adiponectin receptor expression in breast cancer. Adipositas and insulin resistance are modifiable risk factors for breast cancer. Adiponectin seems to be an important linkage of these associations. In this study, we investigated the relationship between intratumoral adiponectin receptor expression and insulin resistance as well as intratumoral insulin/IGF receptor expression in breast cancer specimen.Breast cancer tissue and fasting serum were collected from 26 female patients. After microdissection of frozen samples, RNA was isolated and expression of insulin receptor, IGFR1, IGFR2, AdipoR1 and AdipoR2 was measured on mRNA level by means of real time RT-PCR. Fasting insulin, glucose and c-peptide serum levels were analysed by ELISA. Insulin resistance was calculated using the HOMA model.We were able to confirm AdipoR1 and AdipoR2 expression, respectively, in breast cancer specimen. Actually, neither insulin serum level nor whole-body insulin resistance showed any effect on insulin/IGF or adiponectin receptor expression in breast cancer. A strong positive correlation between insulin as well as IGF1 receptor and AdipoR1, but not AdipoR2, expression could be observed. Interestingly, AdipoR2 expression significantly correlated with vascular and lymphovascular invasion of breast cancer.We propose a close relationship between the intratumoral insulin signalling system and AdipoR1 but not AdipoR2 expression. As AdipoR2 but not AdipoR1 expression seems to correlate with   invasive  ness, we assume different functions of the two adiponectin receptors in breast cancer. 
 Heat shock protein 90-sheltered overexpression of insulin-like growth factor 1 receptor contributes to malignancy of thymic epithelial tumors. The underlying molecular mechanisms of thymic epithelial malignancies (TEMs) are poorly understood. Consequently, there is a lack of efficacious targeted therapies and patient prognosis remains dismal, particularly for advanced TEMs. We sought to investigate protumorigenic mechanism relevant to this understudied cancer.Recently established cell lines derived from thymic epithelial tumors were used as a model system. The antitumor activity of specific heat shock protein 90 (Hsp90) inhibitors was investigated by an analysis of cell viability, cell cycle, and apoptosis using MTT-assays and flow cytometry. Western blotting was used to investigate the altered expression of Hsp90 clients. Pharmacological inhibitors against select Hsp90 clients, as well as RNAi, were employed to test the relevance of each client independently. Tissue microarray analysis was performed to match the in vitro findings with observations obtained from patient-derived samples.Hsp90 inhibition significantly reduces cell viability of thymic carcinoma cells, induces cell cycle arrest and apoptosis, and blocks   invasive  ness. Hsp90 inhibition triggers the degradation of multiple oncogenic clients, for example insulin-like growth factor 1 receptor (IGF-1R), CDK4, and the inactivation of PI3K/Akt and RAF/Erk signaling. Mechanistically, the IGF/IGF-1R-signaling axis contributes to the establishment of the antiapoptotic phenotype of thymic cancer cells. Finally, IGF-1R is overexpressed in advanced TEMs.We have unraveled a novel protumorigenic mechanism in TEMs, namely Hsp90-capacitated overexpression of IGF-1R, which confers apoptosis evasion in malignant thymic epithelial cells. Our data indicate that Hsp90 inhibition, which simultaneously blocks multiple cancer hallmarks, represents a therapeutic strategy in TEMs that may merit evaluation in clinical trials. 
 Anti-tumor effect in human breast cancer by TAE226, a dual inhibitor for FAK and IGF-IR in vitro and in vivo. Focal adhesion kinase (FAK) is a 125-kDa non-receptor type tyrosine kinase that localizes to focal adhesions. FAK overexpression is frequently found in   invasive   and metastatic cancers of the breast, colon, thyroid, and prostate, but its role in osteolytic metastasis is not well understood. In this study, we have analyzed anti-tumor effects of the novel FAK Tyr(397) inhibitor TAE226 against bone metastasis in breast cancer by using TAE226. Oral administration of TAE226 in mice significantly decreased bone metastasis and osteoclasts involved which were induced by MDA-MB-231 breast cancer cells and increased the survival rate of the mouse models of bone metastasis. TAE226 also suppressed the growth of subcutaneous tumors in vivo and the proliferation and migration of MDA-MB-231 cells in vitro. Significantly, TAE226 inhibited the osteoclast formation in murine pre-osteoclastic RAW264.7 cells, and actin ring and pit formation in mature osteoclasts. Moreover, TAE226 inhibited the receptor activator for nuclear factor κ B Ligand (RANKL) gene expression induced by parathyroid hormone-related protein (PTHrP) in bone stromal ST2 cells and blood free calcium concentration induced by PTHrP administration in vivo. These findings suggest that FAK was critically involved in osteolytic metastasis and activated in tumors, pre-osteoclasts, mature osteoclasts, and bone stromal cells and TAE226 can be effectively used for the treatment of cancer induced bone metastasis and other bone diseases. 
  GeneRIFs  
 Report significant up-regulation of Rap1 and IGF-IR in carcinoma in situ progressing to   invasive   breast cancers. 
 Functional experiments showed that LL-37-dependent activation of the IGF-1 receptor signaling resulted in increased migratory and   invasive   potential of malignant cells. 
 (111)In-R1507 and (89)Zr-R1507 are new tracers to non  invasive  ly determine IGF-1R expression in vivo in breast cancer xenografts using SPECT and PET. 
 Local recurrences of   invasive   breast carcinoma were associated with high-grade tumors, PR-negative and low active-IGF1R 
  [Mm]etastasize  PubMed  
 DNp73 exerts function in metastasis initiation by disconnecting the inhibitory role of EPLIN on IGF1R-AKT/STAT3 signaling. Dissemination of cancer cells from primary tumors is the key event in metastasis, but specific determinants are widely unknown. Here, we show that DNp73, an inhibitor of the p53 tumor suppressor family, drives migration and invasion of nonmetastatic melanoma cells. Knockdown of endogenous DNp73 reduces this behavior in highly metastatic cell lines. Tumor xenografts expressing DNp73 show a higher ability to invade and   metastasize  , while growth remains unaffected. DNp73 facilitates an EMT-like phenotype with loss of E-cadherin and Slug upregulation. We provide mechanistic insight toward regulation of LIMA1/EPLIN by p73/DNp73 and demonstrate a direct link between the DNp73-EPLIN axis and IGF1R-AKT/STAT3 activation. These findings establish initiation of the invasion-metastasis cascade via EPLIN-dependent IGF1R regulation as major activity of DNp73. 
 MicroRNA-100 regulates IGF1-receptor expression in metastatic pancreatic cancer cells. Patients with pancreatic adenocarcinoma have the lowest 5 year survival rate and yearly rates of incidence are nearly equal to the mortality rates. Long term cure rates by standard therapies are disappointing owing to disseminated disease at diagnosis and chemotherapeutic resistance. New therapeutic targets are necessary to decrease the progression of pancreatic cancer and the ability to identify targets specific to metastasis would improve patient care. We evaluated the levels of microRNA of metastatic and non-metastatic cell lines. The expression levels of microRNAs and mRNAs were determined using microarray analysis to examine and compare five pancreatic cancer cell lines, two that can   metastasize   in vivo (S2VP10 and S2CP9) and three that do not   metastasize   (MiaPaCa2, Panc-1 and ASPC-1). MicroRNA analysis indicated an increase in miR-100 and a decrease in miR-138 expression in metastatic cancer cells. Microarray analysis of different expressions of mRNAs in metastatic and non-metastatic pancreatic cell lines also indicated significantly increased insulin growth factor-1 receptor (IGF1-R) expression in metastatic pancreatic cancer cell lines compared to non-metastatic pancreatic cancer cell lines. To confirm microarray analysis results, western blot and immunocytochemistry were performed. Western blot revealed that IGF1-R expression exhibited in metastatic cancer cell lines a seven-fold increase compared to non-metastatic cell lines. In addition, downstream expressions of the proteins, GRB2 and phosphorylated PI3K, also were increased in aggressive cancer cell lines. Immunocytochemistry confirmed the linkage of IGF1-R to miR-100, because cells transfected with miR-100 inhibitor showed a decrease in IGF1-R. Cells transfected with a miR-138 mimic, however, did not affect IGF1-R expression. 
  [Mm]etastasis  PubMed  
 Integrin expression regulates neuroblastoma attachment and migration. Neuroblastoma (NBL) is the most common malignant disease of infancy, and children with bone   metastasis   have a mortality rate greater than 90%. Two major classes of proteins, integrins and growth factors, regulate the metastatic process. We have previously shown that tumorigenic NBL cells express higher levels of the type I insulin-like growth factor receptor (IGF-IR) and that beta1 integrin expression is inversely proportional to tumorigenic potential in NBL. In the current study, we analyze the effect of beta1 integrin and IGF-IR on NBL cell attachment and migration. Nontumorigenic S-cells express high levels of beta1 integrin, whereas tumorigenic N-cells express little beta1 integrin. Alterations in beta1 integrin are due to regulation at the protein level, as translation is decreased in N-type cells. Moreover, inhibition of protein synthesis shows that beta1 integrin is degraded more slowly in S-type cells (SHEP) than in N-type cells (SH-SY5Y and IMR32). Inhibition of alpha5beta1 integrin prevents SHEP (but not SH-SY5Y or IMR32) cell attachment to fibronectin and increases SHEP cell migration. Increases in IGF-IR decrease beta1 integrin expression, and enhance SHEP cell migration, potentially through increased expression of alphavbeta3. These data suggest that specific classes of integrins in concert with IGF-IR regulate NBL attachment and migration. 
 Integrin expression regulates neuroblastoma attachment and migration. Neuroblastoma (NBL) is the most common malignant disease of infancy, and children with bone   metastasis   have a mortality rate greater than 90%. Two major classes of proteins, integrins and growth factors, regulate the metastatic process. We have previously shown that tumorigenic NBL cells express higher levels of the type I insulin-like growth factor receptor (IGF-IR) and that beta1 integrin expression is inversely proportional to tumorigenic potential in NBL. In the current study, we analyze the effect of beta1 integrin and IGF-IR on NBL cell attachment and migration. Nontumorigenic S-cells express high levels of beta1 integrin, whereas tumorigenic N-cells express little beta1 integrin. Alterations in beta1 integrin are due to regulation at the protein level, as translation is decreased in N-type cells. Moreover, inhibition of protein synthesis shows that beta1 integrin is degraded more slowly in S-type cells (SHEP) than in N-type cells (SH-SY5Y and IMR32). Inhibition of alpha5beta1 integrin prevents SHEP (but not SH-SY5Y or IMR32) cell attachment to fibronectin and increases SHEP cell migration. Increases in IGF-IR decrease beta1 integrin expression, and enhance SHEP cell migration, potentially through increased expression of alphavbeta3. These data suggest that specific classes of integrins in concert with IGF-IR regulate NBL attachment and migration. 
 Detection and downregulation of type I IGF receptor expression by antibody-conjugated quantum dots in breast cancer cells. The type I insulin-like growth factor (IGF) receptor (IGF1R) is a transmembrane tyrosine kinase involved in breast cancer proliferation, survival, and   metastasis  . Several monoclonal antibodies directed against the receptor are in clinical trials. In order to develop a methodology to detect and measure IGF1R levels in breast cancer cells, we covalently conjugated an IGF1R antibody, AVE-1642, with quantum dots (Qdots), which are nanocrystals that emit fluorescence upon excitation. AVE-1642 Qdots only bound to cells that express IGF1R, and measured IGF1R levels by fluorescence emission at 655 nm. After binding to the cell surface, AVE-1642 Qdots underwent receptor mediated endocytosis, localized to endosome, and later translocated into the nucleus. Treating MCF-7 cells with AVE-1642 Qdots, but not unconjugated Qdots alone, downregulated IGF1R levels and rendered cells refractory to IGF-I stimulation. Furthermore, cell proliferation was slightly inhibited by AVE-1642 Qdots, but not the unconjugated Qdots. Our data suggest that AVE-1642 Qdots can be used to detect IGF1R expression and measure changes in cell surface receptor levels. In addition, the inhibitory effect of AVE-1642 Qdots to cell proliferation implies that it may serve as a traceable therapeutic agent. 
 [Expression and significance of IGF-IR and PKC in laryngeal squamous cell carcinoma]. To investigate the expression of IGF-IR and PKC and their clinical significance in laryngeal squamous cell carcinoma(LSCC).The expression of IGF-IR and PKC protein were detected by immunohistochemistry and Western blot in 60 cases of LSCC, 25 cases of adjacent non-tumorous laryngeal epithelium and 10 cases of normal laryngeal epithelium.The positive rate of IGF-IR and PKC expression in LSCC were significantly higher than that in normal laryngeal epithelium and adjacent non-tumorous laryngeal epithelium (P < 0.05). IGF-IR positive expression were increased more in cases with stage III-IV, cervical lymph node   metastasis   and well differentiation than that in cases with stage I-II, non-lymph node   metastasis   and poor/mediate differentiation, respectively (P < 0.05). The positive rate of PKC in LSCC were correlated with clinical stage and cervical lymph node   metastasis  . There was a correlation between the expression of IGF-IR and PKC in LSCC.The overexpression of IGF-IR and PKC in the laryngeal carcinoma may play an important role in the carcinogenesis and development of LSCC. It is suggested that detecting the expression of IGF-IR and PKC can be used for early diagnosis and treatment of LSCC. 
 Targeting insulin-like growth factor 1 receptor inhibits pancreatic cancer growth and   metastasis  . Pancreatic cancer is one of the most lethal cancers. Increasing incidence and mortality indicates that there is still much lacking in detection and management of the disease. This is partly due to a lack of specific symptoms during early stages of the disease. Several growth factor receptors have been associated with pancreatic cancer. Here, we have investigated if an RNA interference approach targeted to IGF-IR could be effective and efficient against pancreatic cancer growth and   metastasis  . For that, we evaluated the effects of IGF-1R inhibition using small interfering RNA (siRNAs) on tumor growth and   metastasis   in HPAC and PANC-1 pancreatic cancer cell lines. We found that silencing IGF-1R inhibits pancreatic cancer growth and   metastasis   by blocking key signaling pathways such AKT/PI3K, MAPK, JAK/STAT and EMT. Silencing IGF-1R resulted in an anti-proliferative effect in PANC-1 and HPAC pancreatic cancer cell lines. Matrigel invasion, transwell migration and wound healing assays also revealed a role for IGF-1R in metastatic properties of pancreatic cancer. These results were further confirmed using Western blotting analysis of key intermediates involved in proliferation, epithelial mesenchymal transition, migration, and invasion. In addition, soft agar assays showed that silencing IGF-1R also blocks the colony forming capabilities of pancreatic cancer cells in vitro. Western blots, as well as, flow cytometric analysis revealed the induction of apoptosis in IGF-1R silenced cells. Interestingly, silencing IGF-1R also suppressed the expression of insulin receptor β. All these effects together significantly control pancreatic cancer cell growth and   metastasi  s. To conclude, our results demonstrate the significance of IGF-1R in pancreatic cancer. 
 Id1-induced IGF-II and its autocrine/endocrine promotion of esophageal cancer progression and chemoresistance--implications for IGF-II and IGF-IR-targeted therapy. To investigate the autocrine/endocrine role of Id1-induced insulin-like growth factor-II (IGF-II) in esophageal cancer, and evaluate the potential of IGF-II- and IGF-type I receptor (IGF-IR)-targeted therapies.Antibody array-based screening was used to identify differentially secreted growth factors from Id1-overexpressing esophageal cancer cells. In vitro and in vivo assays were performed to confirm the induction of IGF-II by Id1, and to study the autocrine and endocrine effects of IGF-II in promoting esophageal cancer progression. Human esophageal cancer tissue microarray was analyzed for overexpression of IGF-II and its correlation with that of Id1 and phosphorylated AKT (p-AKT). The efficacy of intratumorally injected IGF-II antibody and intraperitoneally injected cixutumumab (fully human monoclonal IGF-IR antibody) was evaluated using in vivo tumor xenograft and experimental   metastasis   models.Id1 overexpression induced IGF-II secretion, which promoted cancer cell proliferation, survival, and invasion by activating AKT in an autocrine manner. Overexpression of IGF-II was found in 21 of 35 (60%) esophageal cancer tissues and was associated with upregulation of Id1 and p-AKT. IGF-II secreted by Id1-overexpressing esophageal cancer xenograft could instigate the growth of distant esophageal tumors, as well as promote   metastasis   of circulating cancer cells. Targeting IGF-II and IGF-IR had significant suppressive effects on tumor growth and   metastasis   in mice. Cixutumumab treatment enhanced the chemosensitivity of tumor xenografts to fluorouracil and cisplatin.The Id1-IGF-II-IGF-IR-AKT signaling cascade plays an important role in esophageal cancer progression. Blockade of IGF-II/IGF-IR signaling has therapeutic potential in the management of esophageal cancer. 
 Expression of the insulin-like growth factor-I receptor in primary breast cancer and lymph node metastases: correlations with estrogen receptors alpha and beta. Numerous laboratory studies and some epidemiological data have suggested the involvement of the insulin-like growth factor-I receptor (IGF-IR) in breast cancer development and progression. However, data on IGF-IR expression in human tissues, including breast cancer sections, are limited and often inconsistent. We therefore examined by immunohistochemistry the expression of IGF-IR in primary tumors and breast cancer metastases to lymph nodes, and correlated IGF-IR positivity with estrogen receptor (ER) status and selected clinicopathological features. We found that 1) IGF-IR was expressed in primary tumors as well as in lymph node metastases, but the expression in primary tumors was more frequent (56 % vs. 44.4 %); 2) IGF-IR expression in primary tumors was associated with negative node status (p < 0.033); 3) in node-negative primary tumors, IGF-IR positively correlated with ERbeta (p < 0.008; r = 0.538), but not with ERalpha, tumor size or grade; 4) both IGF-IR-positive and IGF-IR-negative primary tumors were found to produce IGF-IR-positive as well as IGF-IR-negative metastases; 5) in metastases, IGF-IR expression did not associate with ERalpha, ERbeta or any of the studied pathobiological markers. The results suggest that IGF-IR could become a viable pharmaceutical target in breast cancer therapy, but such therapy should be based on IGF-IR assessment in primary tumor and   metastasis   in each potential patient. 
 DNp73 exerts function in   metastasis   initiation by disconnecting the inhibitory role of EPLIN on IGF1R-AKT/STAT3 signaling. Dissemination of cancer cells from primary tumors is the key event in   metastasis  , but specific determinants are widely unknown. Here, we show that DNp73, an inhibitor of the p53 tumor suppressor family, drives migration and invasion of nonmetastatic melanoma cells. Knockdown of endogenous DNp73 reduces this behavior in highly metastatic cell lines. Tumor xenografts expressing DNp73 show a higher ability to invade and metastasize, while growth remains unaffected. DNp73 facilitates an EMT-like phenotype with loss of E-cadherin and Slug upregulation. We provide mechanistic insight toward regulation of LIMA1/EPLIN by p73/DNp73 and demonstrate a direct link between the DNp73-EPLIN axis and IGF1R-AKT/STAT3 activation. These findings establish initiation of the invasion-  metastasis   cascade via EPLIN-dependent IGF1R regulation as major activity of DNp73. 
 Molecular biomarkers for prediction of response to treatment and survival in triple negative breast cancer patients from Egypt. Triple negative breast cancer (TNBC) is an aggressive phenotype of breast cancer with reduced survival and poor prognosis. Increased VEGF-A, IGF-I, IGF-IR and TGF-β1 expressions were detected in breast cancer. However, little is known about their prognostic and predictive roles in TNBC.We assessed the possible prognostic and predictive values of VEGF-A, IGF-I/IGF-IR and TGF-β1 in TNBC cases by measuring their protein and mRNA expression in TNBC and non-TNBC cases.VEGF-A, IGF-I, IGF-IR and TGF-β1 RNA and their corresponding proteins were assessed in 43 TNBCs, 53 non-TNBCs and 30 normal breast tissues (NBT) by real time PCR (qPCR) and immunohistochemistry (IHC); respectively. Results were related to clinico-pathological factors, response to treatment and survival rates.Increased mRNA expression of VEGF-A, IGF-I, and IGF-IR was significantly higher in TNBC (65.1%, 65.1%, and 72.1%) than non-TNBC (28.1%, 33.96% and 28.3%) and NBT (0.00%) (P<0.001). Similarly, TNBC patients were significantly associated with high expression of VEGF-A, IGF-I, and IGF-IR proteins (67.44%, 62.79% and 83.72%) than non-TNBC (20.75%, 35.86% and 20.75%) and NBT (0.00%) (P<0.001). Protein and RNA expression levels of all studied markers showed high concordance in all investigated patients (correlation coefficient exceeding 0.5 and 0.4, respectively). In the TNBC grou  p, metasta  sis and poor response to treatment were significantly associated with VEGF-A (P<0.001, P=0.007, respectively), IGF-I (P<0.001, P<0.001, respectively), IGF-IR (P=0.001, P=0.015, respectively) and TGF-β1 (P<0.001, P=0.007, respectively) protein levels. Multivariate logistic regression showed that IGF-I was the only independent prognostic factor for reduced OS (P=0.034) and DFS (P=0.026) in the TNBC patients.VEGF-A, IGF-I and IGF-IR play an important role in the development and progression of TNBC compared to non-TNBC. Therefore, they could be used as prognostic and predictive biomarkers as well as candidates for targeted therapy. However, only IGF-I can predict survival in those patients. 
 HRD1 suppresses the growth and   metastasis   of breast cancer cells by promoting IGF-1R degradation. HRD1 (3-hydroxy-3-methylglutaryl reductase degradation) is an E3 ubiquitin ligase. We found that HRD1 was significantly downregulated in 170 breast cancer tissues. Low tumoral HRD1 expression was correlated with clinicopathological characteristics and a shorter survival in breast cancer patients. P65 specifically bound to the HRD1 promoter and inhibited HRD1 expression. Suppression of NF-κB activity reversed IL-6-induced downregulation of HRD1 expression. HRD1 interacted with IGF-1R and promoted its ubiquitination and degradation by the proteasome. Overexpression of HRD1 resulted in the inhibition of growth, migration and invasion of breast cancer cells in vitro and in vivo. Furthermore, HRD1 attenuated IL-6-induced epithelial-mesenchymal transition in MCF10A cells. These findings uncover a novel role for HRD1 in breast cancer. 
 The IGF-I receptor can alter the matrix metalloproteinase repertoire of tumor cells through transcriptional regulation of PKC-{alpha}. The IGF-I receptor (IGF-IR) was identified as a tumor progression factor, but its role in invasion and   metastasis   has been the subject of some controversy. Previously we reported that in murine lung carcinoma M-27 cells, overexpression of IGF-IR increased the synthesis and activation of matrix metalloproteinase (MMP)-2 via Akt/phosphatidylinositol 3-kinase signaling. In contrast, we show here that in these and other cells, IGF-IR overexpression reduced the constitutive and phorbol 12-myristate 13-acetate (PMA)-inducible expression of three protein kinase C (PKC)-regulated metalloproteinases, MMP-3, MMP-9, and MMP-13, in cultured cells as well as in vivo in sc tumors. To elucidate the underlying mechanism, we analyzed the effect of IGF-IR on PKC expression and activity using wild-type and IGF-IR-overexpressing (M-27(IGFIR)) tumor cells. Our results show that overexpression and activation of IGF-IR reduced PKC-alpha expression, PKC activity, and downstream ERK1/2 signaling, and these effects were reversed in cells expressing kinase (Y(1131,1135,1136)F) or C-terminal (Y(1250/51)F) domain mutants of IGF-IR. This reduction was due to transcriptional down-regulation of PKC-alpha as evidenced by reduced PKC-alpha mRNA expression in a phosphatidylinositol 3-kinase-dependent manner and a blockade of PKC-alpha promoter activation as revealed by a reporter gene assay. Finally, reconstitution of PKC-alpha levels could restore MMP-9 expression levels in these cells. Collectively, these results show that IGF-IR can inhibit PKC-alpha gene transcription and thereby block the synthesis of PMA-regulated MMPs, suggesting that within the same cells, IGF-IR can act as both a positive and negative regulator of MMP expression and function. 
 The expression and significance of insulin-like growth factor-1 receptor and its pathway on breast cancer stem/progenitors. Dysregulation of the insulin-like growth factor-1 receptor (IGF-1R)/phosphatidylinositol-3-kinase (PI3K)/Akt pathway was shown to correlate with breast cancer disease progression. Cancer stem cells are a subpopulation within cancer cells that participate in tumor initiation, radio/chemoresistance and   metastasis  . In breast cancer, breast cancer stem cells (BCSCs) were identified as CD24-CD44+ cells or cells with high intracellular aldehyde dehydrogenase activity (ALDH+). Elucidation of the role of IGF-1R in BCSCs is crucial to the design of breast cancer therapies targeting BCSCs.IGF-1R expression in BCSCs and noncancer stem cells sorted from xenografts of human primary breast cancers was examined by fluorescence-activated cell sorting (FACS), western blot analysis and immunoprecipitation. The role of IGF-1R in BCSCs was assessed by IGF-1R blockade with chemical inhibitor and gene silencing. Involvement of PI3K/Akt/mammalian target of rapamycin (mTOR) as the downstream pathway was studied by their phosphorylation status upon IGF-1R inhibition and the effects of chemical inhibitors of these signaling molecules on BCSCs. We also studied 16 clinical specimens of breast cancer for the expression of phosphor-Akt in the BCSCs by FACS.Expression of phosphorylated IGF-1R was greater in BCSCs than in non-BCSCs from xenografts of human breast cancer, which were supported by western blot and immunoprecipitation experiments. The sorted IGF-1R-expressing cells displayed features of cancer stem/progenitors such as mammosphere formation in vitro and tumorigenicity in vivo, both of which were suppressed by knockdown of IGF-1R. A specific inhibitor of the IGF-1R, picropodophyllin suppressed phospho-AktSer473 and preferentially decreased ALDH+ BCSC populations of human breast cancer cells. Furthermore, picropodophyllin inhibited the capacity of CD24-CD44+ BCSCs to undergo the epithelial-mesenchymal transition process with downregulation of mesenchymal markers. Inhibitors of signal molecules downstream of IGF-1R including PI3K/Akt/mTOR also reduced the ALDH+ population of breast cancer cells. Furthermore, the mTOR inhibitor, rapamycin, suppressed BCSCs in vitro and in vivo.Our data support the notion that IGF-1R is a marker of stemness, and IGF-1R and its downstream PI3K/Akt/mTOR pathway are attractive targets for therapy directed against breast cancer stem/progenitors. 
 Association of IGFBP-6 Expression with Metabolic Syndrome and Adiponectin and IGF-IR Receptor Levels in Colorectal Cancer. To assess IGFBP-6 expression in relation with the presence of the metabolic syndrome, adiponectin receptors (AdipoR1 and AdipoR2) and IGF-IR levels in colorectal adenocarcinoma cases.IGFBP-6 mRNA and protein levels were analyzed using real-time quantitative PCR and Western blotting in 46 patients. ELISA and ow cytometry were used for evaluation of AdipoR1, AdipoR2 and IGF-IR.The results showed that IGFBP-6 mRNA expression and the IGFBP-6 content were higher in tumor tissue samples of colorectal cancer patients with and without the metabolic syndrome. In addition, IGFBP-6 mRNA expression was associated with tumor invasion (tumor size) and the IGFBP-6 protein level was associated with nodal status. Positive correlations and positive nonlinear relations were found between the IGFBP-6 level and the AdipoR1 and AdipoR2 contents in colorectal cancer patients.The IGFBP-6 mRNA level and protein level were found to be associated with presence of the metabolic syndrome. Positive correlations indicated probable cross-talk between the IGF-IR-mediated and adiponectin-mediated signaling pathways in colorectal carcinomas. IGFBP-6 may be considered as a potential biomarker associated with lymphogenous   metastasis   and the metabolic syndrome in colorectal cancer. 
 miR-422a Inhibits Glioma Proliferation and Invasion by Targeting IGF1 and IGF1R. Glioma is a common type of malignant brain tumor characterized by aggressive   metastasis   capability. Recent evidence has suggested that noncoding RNAs, including microRNAs, have important functions in the pathophysiology of glioma development. In this study, we investigated the biological function of miR-422a in human glioma. We found that miR-422a was downregulated in glioma tissues. We also demonstrated that expression of miR-422a in glioma cells markedly suppressed cell proliferation, migration, and invasion. In addition, we identified insulin-like growth factor 1 (IGF1) and IGF1 receptor (IGF1R) as inhibitory targets of miR-422a in glioma cells. We established that the expression levels of miR-422a were negatively correlated with the expression levels of IGF1/IGF1R and the clinical parameters in glioma patients. An IGFR inhibitor, AG1024, completely blocked the activity of miR-442a on glioma cell proliferation and invasion, which further confirmed that miR-422a functions through IGF1 and IGF1R. 
 MiR-195 inhibits the growth and   metastasis   of NSCLC cells by targeting IGF1R. MicroRNAs (miRNAs) are small, non-coding RNAs which act as oncogenes or tumor suppressors in multiple human cancers. Accumulating evidence reveals that aberrant expression of miRNAs contributes to the development and progression of non-small cell lung cancer (NSCLC). Here, we identified miR-195 as a tumor suppressor in NSCLC cells, whose expression level was dramatically decreased in both NSCLC tissues and cell lines. Ectopic expression of miR-195 suppressed NSCLC cell proliferation and   metastasis  -related traits in vitro. Insulin-like growth factor 1 receptor (IGF1R) was identified as a direct target of miR-195 in NSCLC cells. Furthermore, restoration of IGF1R remarkably attenuated the tumor suppressive effects of miR-195 on NSCLC cells. Our data suggest that miR-195 may be involved in the carcinogenesis of NSCLC partially by targeting IGF1R. 
 Caveolin-1 Confers Resistance of Hepatoma Cells to Anoikis by Activating IGF-1 Pathway. Anoikis resistance is a prerequisite for hepatocellular carcinoma (HCC)   metastasis  . The role of Caveolin-1 (CAV1) in anoikis resistance of HCC remains unclear.The oncogenic effect of CAV1 on anchor-independent growth and anoikis resistance was investigated by overexpression and knockdown of CAV1 in hepatoma cells. IGF-1 pathway and its downstream signals were detected by immunoblot analysis. Caveolae invagination and IGF-1R internalization was studied by electron microscopy and (125)I-IGF1 internalization assay, respectively. The role of IGF-1R and tyrosine-14 residue (Y-14) of CAV1 was explored by deletion experiment and mutation experiment, respectively. The correlation of CAV1 and IGF-1R was further examined by immunochemical analysis in 120 HCC specimens.CAV1 could promote anchor-independent growth and anoikis resistance in hepatoma cells. CAV1-overexpression increased the expression of IGF-1R and subsequently activated PI3K/Akt and RAF/MEK/ERK pathway, while CAV1 knockdown showed the opposite effect. The mechanism study revealed that CAV1 facilitated caveolae invagination and (125)I-IGF1 internalization. IGF-1R deletion or Y-14 mutation reversed CAV1 mediated anchor-independent growth and anoikis resistance. In addition, CAV1 expression was positively related to IGF-1R expression in human HCC tissues.CAV1 confers resistance of hepatoma cells to anoikis by activating IGF-1 pathway, providing a potential therapeutic target for HCC   metastasis  . 
 A high expression level of insulin-like growth factor I receptor is associated with increased expression of transcription factor Sp1 and regional lymph node   metastasis   of human gastric cancer. Insulin-like growth factor I receptor (IGF-IR) is critical to cell survival and growth and altered IGF-IR expression is found in many human cancers. However, its expression and potential role in gastric cancer development and progression has not been explored. The IGF-IR expression level was determined via immunohistochemistry in primary tumor and lymph node   metastasis   of 86 cases of resected gastric cancer. Relationships of IGF-IR expression with transcription factor Spl expression and clinicopathological features were analyzed. The impact of altered Sp1 expression on IGF-IR expression and gastric cancer biology was further determined using small inhibitory RNA for Sp1 mRNA. We found that IGF-IR was overexpressed in 62% of the tumor samples when compared with adjacent tumor-free gastric mucosa. Patients with lymph node metastases had strong expression of IGF-IR in both primary and metastatic tumor cells. IGF-IR overexpression in the primary tumor correlated with increased lymph node   metastasis  . Furthermore, the level of IGF-IR expression directly correlated with that of Spl, an important transcription factor for IGF-IR regulation. Knocking-down of Spl expression by small inhibitory RNA led to decreased IGF-IR expression and attenuated growth and   metastasis   of gastric cancer cells. Therefore, dysregulated expression of IGF-IR and/or Sp1 may contribute to the growth and   metastasis   of gastric cancer and potentially can be a target of therapeutic intervention. 
 Inhibition of type I insulin-like growth factor receptor signaling attenuates the development of breast cancer brain   metastasis  . Brain   metastasis   is a common cause of mortality in cancer patients, yet potential therapeutic targets remain largely unknown. The type I insulin-like growth factor receptor (IGF-IR) is known to play a role in the progression of breast cancer and is currently being investigated in the clinical setting for various types of cancer. The present study demonstrates that IGF-IR is constitutively autophosphorylated in brain-seeking breast cancer sublines. Knockdown of IGF-IR results in a decrease of phospho-AKT and phospho-p70s6k, as well as decreased migration and invasion of MDA-MB-231Br brain-seeking cells. In addition, transient ablation of IGFBP3, which is overexpressed in brain-seeking cells, blocks IGF-IR activation. Using an in vivo experimental brain   metastasis   model, we show that IGF-IR knockdown brain-seeking cells have reduced potential to establish brain metastases. Finally, we demonstrate that the malignancy of brain-seeking cells is attenuated by pharmacological inhibition with picropodophyllin, an IGF-IR-specific tyrosine kinase inhibitor. Together, our data suggest that the IGF-IR is an important mediator of brain   metastasis   and its ablation delays the onset of brain metastases in our model system. 
 The Clinical Significance of the Insulin-Like Growth Factor-1 Receptor Polymorphism in Non-Small-Cell Lung Cancer with Epidermal Growth Factor Receptor Mutation. The insulin-like growth factor 1 (IGF1) signaling pathway mediates multiple cancer cell biological processes. IGF1 receptor (IGF1R) expression has been used as a reporter of the clinical significance of non-small-cell lung carcinoma (NSCLC). However, the association between IGF1R genetic variants and the clinical utility of NSCLC positive for epidermal growth factor receptor (EGFR) mutation is not clear. The current study investigated the association between the IGF1R genetic variants, the occurrence of EGFR mutations, and clinicopathological characteristics in NSCLC patients. A total of 452 participants, including 362 adenocarcinoma lung cancer and 90 squamous cell carcinoma lung cancer patients, were selected for analysis of IGF1R genetic variants (rs7166348, rs2229765, and rs8038415) using real-time polymerase chain reaction (PCR)genotyping. The results indicated that GA + AA genotypes of IGF1R rs2229765 were significantly associated with EGFR mutation in female lung adenocarcinoma patients (odds ratio (OR) = 0.39, 95% confidence interval (CI) = 0.17-0.87). Moreover, The GA + AA genotype IGF1R rs2229765 was significantly associated with EGFR L858R mutation (p = 0.02) but not with the exon 19 in-frame deletion. Furthermore, among patients without EGFR mutation, those who have at least one polymorphic A allele of IGF1R rs7166348 have an increased incidence of lymph node   metastasis   when compared with those patients homozygous for GG (OR, 2.75; 95% CI, 1.20-2.31). Our results showed that IGF1R genetic variants are related to EGFR mutation in female lung adenocarcinoma patients and may be a predictive factor for tumor lymph node   metastasis   in Taiwanese patients with NSCLC. 
 MicroRNA-7 functions as an anti-metastatic microRNA in gastric cancer by targeting insulin-like growth factor-1 receptor.   Metastasis   is a major clinical obstacle in the treatment of gastric cancer (GC) and it accounts for the majority of cancer-related mortality. MicroRNAs have recently emerged as regulators of   metastasis   by acting on multiple signaling pathways. In this study, we found that miR-7 is significantly downregulated in highly metastatic GC cell lines and metastatic tissues. Both gain-of-function and loss-of-function experiments showed that increased miR-7 expression significantly reduced GC cell migration and invasion, whereas decreased miR-7 expression dramatically enhanced cell migration and invasion. In vivo   metastasis   assays also demonstrated that overexpression of miR-7 markedly inhibited GC   metastasis  . Moreover, the insulin-like growth factor-1 receptor (IGF1R) oncogene, which is often mutated or amplified in human cancers and functions as an important regulator of cell growth and tumor invasion, was identified as a direct target of miR-7. Silencing of IGF1R using small interefering RNA (siRNA) recapitulated the anti-metastatic function of miR-7, whereas restoring the IGF1R expression attenuated the function of miR-7 in GC cells. Furthermore, we found that suppression of Snail by miR-7, through targeting IGF1R, increased E-cadherin expression and partially reversed the epithelial-mesenchymal transition (EMT). Finally, analyses of miR-7 and IGF1R levels in human primary GC with matched lymph node   metastasis   tissue arrays revealed that miR-7 is inversely correlated with IGF1R expression. The present study provides insight into the specific biological behavior of miR-7 in EMT and tumor   metastasis  . Targeting this novel miR-7/IGF1R/Snail axis would be helpful as a therapeutic approach to block GC   metastasis  . 
 [Clinicopathological significance of expression of IGF-1R in uveal melanoma and its association with expression of p-AKT Thr308]. To investigate the clinicopathological significance of expression of insulin-like growth factor-1 receptor (IGF-1R) and its association with the expression of p-AKT Thr308 in uveal melanoma.Experimental study. Twenty-four patients with uveal melanoma were included from January 2000 to December 2008. The levels of IGF-1R and p-AKT Thr308 were detected by immunohistochemical methods, and their association with clinicopathological parameters including localization of tumor, tumor size, largest tumor diameters, cell type, necrosis, degree of pigmentation, lymphocyte infiltration, mitosis rate, scleral invasion and liver   metastasis   were statistically analyzed. The relationship of expression of IGF-1R with clinicopathological parameters or with the expression of p-AKT Thr308 was analyzed by chi-square test.The positive rate of expression of IGF-1R in 24 cases of uveal melanoma was 75%. The expression of IGF-1R were associated with the largest tumor diameters, degree of pigmentation, liver   metastasis   and lymphocyte infiltration (χ(2) = 15.569, P = 0.016; χ(2) = 11.348, P = 0.010; χ(2) = 8.738, P = 0.033; χ(2) = 8.362, P = 0.039). The positive rate of expression of p-AKT Thr308 was 58%. The expression of IGF-1R and p-AKT Thr308 was positively correlated (χ(2) = 17.108, P = 0.009).IGF-1R plays a role in the development of uveal melanoma which may be induced by activation in PI3K/AKT pathway. 
 Ligand-independent activation of MET through IGF-1/IGF-1R signaling. The receptor tyrosine kinase, MET, has been implicated in tumorigenesis and   metastasis   of many solid tumors, by multiple mechanisms, including cross talk with epidermal growth factor receptor. In this study, we examined the role of insulin-like growth factor receptor-1 (IGF-1R) signaling in MET activation, focusing on prostate cancer cells. Stimulation of the prostate cancer cell line PC3 with IGF-1 induces a delayed phosphorylation of MET at multiple sites (indicative of full activation), reaching a maximum 18 hr after IGF-1 addition. MET activation does not require the sole MET ligand hepatocyte growth factor (HGF), but does require transcription to occur. Furthermore, direct injection of IGF-1 is sufficient to induce MET activation in vivo, in a PC3 xenograft model. Pharmacologic or genetic inhibition of the tyrosine kinase, Src, abolishes MET phosphorylation, and expression of activated Src is sufficient to induce Met phosphorylation in the absence of IGF-1 stimulation. Activated MET is essential for IGF-1-mediated increased migration of PC3 cells, demonstrating an important biologic effect of IGF-1-mediated MET activation. Finally, we demonstrate that IGF-1-induced delayed MET activation occurs in multiple cell lines which express both the receptors, suggesting that IGF-1R-mediated MET activation may contribute to tumorigenic properties of multiple cancer types when both growth factor receptors are expressed. The results further suggest that MET may be activated by multiple receptor tyrosine kinase receptors, and dual targeting of these receptors may be important therapeutically. 
 Impact of IGF-1, IGF-1R, and IGFBP-3 promoter methylation on the risk and prognosis of esophageal carcinoma. The aim of this study is to investigate IGF-1, IGF-1R, and IGFBP-3 methylations in esophageal carcinoma (EC) patients and their relationship with the development and prognosis of EC. This study population consisted of 264 patients (case group) whom EC radical resection was performed and 283 healthy individuals (control group). Methylation-specific PCR (MSP) detected the methylation status of IGF-1, IGF-1R, and IGFBP-3 in the peripheral blood in both groups. The expressions of IGF-1, IGF-1R, and IGFBP-3 in EC and adjacent normal tissues were detected by immunohistochemistry (IHC). The methylation rates of IGF-1, IGF-1R, IGFBP3, and IGF-1 + IGF1R + IGFBP3 in the case group were higher than those in the control group (all P < 0.05). Additionally, there were statistical significances for the methylation rates of IGF-1, IGF-1R, IGFBP3, and IGF-1 + IGF1R + IGFBP3 IGF-1 among patients of different clinicopathological features (all P < 0.05). The positive expression rates of IGF-1 and IGF-1R in EC were significantly higher than those in adjacent normal tissues (both P < 0.001), and the rate of IGFBP-3 in EC was significantly lower than that in adjacent normal tissues (P < 0.05). Correlation analysis showed that IGF-1 and IGF1R gene promoter methylation was positively correlated with the positive expressions of IGF-1 (r = 0.139, P = 0.024) and IGF-1R (r = 0.135, P = 0.028), while the IGFBP3 methylation was negatively correlated with the positive expression of IGFBP3 (r = -0.133, P = 0.031). The positive expressions of IGF-1, IGF-1R, and IGFBP-3 were related to different clinicopathological features (all P < 0.05). Cox multivariate analysis results showed that methylation status of IGF-1, IGF-1R, and IGF-1 + IGF1R + IGFBP3 ; expressi  ons of IGF  -1 and IGF-1R protein; infiltration depth; and lymph node metastasis (LNM) were independent factors of EC prognosis. Our study demonstrated that methylation of IGF-1, IGF1R, IGFBP3, and IGF-1 + IGF1R + IGFBP3 was closely linked with the occurrence of EC and patients' clinicopathological features. Besides, the methylation status of the target genes and the expressions of IGF-1 and IGF-1R protein were independent factors of EC prognosis, which could provide a direction for the prognosis and treatment of EC. 
 Reciprocal regulation of microRNA-99a and insulin-like growth factor I receptor signaling in oral squamous cell carcinoma cells. MicroRNAs (miRNAs), small noncoding RNA molecules can function as oncogenes or tumor suppressors in tumorigenesis. Oral squamous cell carcinoma (OSCC) is one of the most prevalent cancers worldwide with a 5-year survival rate of approximately 50%.The expression of microRNA-99a (miR-99a) in OSCC tissues and cell lines was investigated using quantitative reverse transcription-polymerase chain reaction (qRT-PCR) analysis. The functions of miR-99a in migration/invasion and lung colonization were determined by transwell and tail vein injection assays, respectively. Specific targets of miR-99a were determined by software prediction, correlation with target protein expression, and luciferase reporter assay. The signaling pathways involved in regulation of miR-99a were investigated using the kinase inhibitors.We observed reduced levels of miR-99a, identified as one of the most downregulated miRNA in OSCC and all tested OSCC cell lines compared to normal oral keratinocytes. Ectopic miR-99a expression in OSCC cells markedly reduced migration and invasion in vitro as well as lung colonization in vivo. When evaluating the specific targets of miR-99a, we found that ectopic miR-99a expression downregulates insulin-like growth factor 1 receptor (IGF1R) protein and that the expression of miR-99a correlates negatively with IGF1R protein in OSCC cells. Insertion of the 3'UTR of IGF1R mRNA into the 3'UTR of a reporter gene markedly reduced luciferase activity in OSCC cells expressing miR-99a, suggesting that miR-99a reduces luciferase activity by targeting the 3'UTR of IGF1R mRNA. When evaluating the mechanisms of miR-99a downregulation, we observed the upregulation of miR-99a expression in serum-starved conditions and its suppression in response to insulin-like growth factor (IGF1) stimulation. Inhibitors of phosphatidylinositol 3-kinase (PI3K) and mitogen-activated protein kinase (MAPK) kinase inhibited IGF1-induced suppression of miR-99a, suggesting the negative regulation of miR-99a expression by IGF1R signaling.Overall, results indicate that miR-99a functions as a tumor   metastasis   suppressor in OSCC cells and mutually regulates IGF1R expression in a reciprocal regulation. 
 miR-98 suppresses tumor cell growth and   metastasis   by targeting IGF1R in oral squamous cell carcinoma. Increasing evidences indicate that dysregulation of miRNAs contributes to the pathogenesis of oral squamous cell carcinoma (OSCC). However, little is known about the potential role of miR-98 in OSCC. Here, we found that miR-98 was downregulated in OSCC tissues and cell lines. Overexpression of miR-98 inhibited proliferation, colony formation, migration, and invasion of OSCC cells. IGF1R was identified as the potential target of miR-98 using dual luciferase assay, qRT-PCR and western blot. Furthermore, restoration of IGF1R remarkably reversed the tumor-suppressive effects of miR-98 on OSCC cells. Moreover, miR-98 expression was inversely correlated with IGF1R expression in 19 cases of OSCC. These findings suggest that miR-98 inhibits cancer cell growth and   metastasis   by direct targeting IGF1R, implicating miR-98 as a novel potential therapeutic target for OSCC. 
 Phosphorylated insulin-like growth factor-1 receptor expression predicts poor prognosis of Chinese patients with gastric cancer. Previous studies have established the role of phosphorylated form of insulin-like growth factor type 1 receptor (p-IGF1R) as a good candidate for tumor biomarker. The aims of this study were to investigate p-IGF1R expression status in gastric cancer (GC) specimens and to clarify its clinical significance. A total of 78 GC patients treated with radical resection were enrolled in this study. Immunohistochemistry was used to detect p-IGF1R and phosphatase and tensin homolog deleted on chromosome ten (PTEN) protein expression in paired tumor and adjacent normal tissues. Results showed a higher level of p-IGF1R protein expression in tumor tissues than that in normal tissues, and the rate of p-IGF1R protein high/moderate expression in GC and normal tissues was 52.6% (41/78) and 6.4% (5/78), respectively (p < 0.001). In contrast, PTEN protein expression was downregulated in GC, as compared with normal tissues (negative/low expression 49/78 vs. 8/78, p < 0.001). Moreover, PTEN protein downregulation was consistent with p-IGF1R upregulation. Overexpression of p-IGF1R protein was associated with lymph   metastasis  , clinical stage, and adverse 3-year progression-free survival (PFS). Survival analysis and Cox proportional hazards model revealed that p-IGF1R overexpression was an independent factor in predicting PFS for GC patients, apart from lymph   metastasis  . In conclusion, p-IGF1R is highly expressed in GC, which may be a novel biomarker to predict the clinical outcome of GC patients. 
 Regulatory role of c-Met in insulin-like growth factor-I receptor-mediated migration and invasion of human pancreatic carcinoma cells. Pancreatic carcinoma cells overexpress the insulin-like growth factor-I (IGF-I) receptor (IGF-IR) and the hepatocyte growth factor (HGF) receptor, c-Met, which are both known to mediate tumor cell migration and invasion. We hypothesized that IGF-IR and c-Met cooperate to induce migration and invasion of human pancreatic carcinoma cells and that IGF-I-mediated migration and invasion depend on c-Met. Migration and invasion assays were done with the human pancreatic cancer cell line L3.6pl treated with PBS, IGF-I, HGF, or IGF-I plus HGF. To determine if c-Met is necessary for IGF-IR-mediated migration and invasion, c-Met was down-regulated in L3.6pl cells via adenoviral infection with a c-Met ribozyme before IGF-I treatment. IGF-I and HGF increased cell migration and invasion. Furthermore, IGF-I plus HGF had a greater than additive effect on cell migration and invasion compared with either growth factor alone. Down-regulation of c-Met nearly completely inhibited IGF-I-mediated migration and invasion. Our findings suggest that IGF-IR and c-Met cooperate to induce migration and invasion of human pancreatic carcinoma cells. Furthermore, c-Met is required for both HGF- and IGF-I-mediated migration and invasion. Elucidation of the signaling pathways that contribute to tumor progression and   metastasis   should provide a foundation for the development of targeted therapies for pancreatic carcinoma. 
 MicroRNA-133a Inhibits Osteosarcoma Cells Proliferation and Invasion via Targeting IGF-1R. MicroRNAs (miRNAs) are a class of small noncoding RNAs that regulate gene expression by repressing translation or cleaving RNA transcripts in a sequence-specific manner. Downregulated microRNAs and their roles in cancer development have attracted much attention. A growing body of evidence showed that microRNA-133a (miR-133a) has inhibitory effects on cell proliferation, migration, invasion, and   metastasis   of osteosarcoma.MiR-133a expression in human osteosarcoma cell lines and human normal osteoblastic cell line hFOB was investigated by real-time PCR (RT-PCR). The role of miR-133a in human osteosarcoma growth and invasion was assessed in cell lines in vitro and in vivo. Then, luciferase reporter assay validated IGF-1R as a downstream and functional target of miR-133a, and functional studies revealed that the anti-tumor effect of miR-133a was probably due to targeting and repressing of IGF-1R expression.MiR-133a was lower expressed in human osteosarcoma cell lines than human normal osteoblastic cell line hFOB and its effect on inhibiting proliferation, invasion and   metastasis   is mediated by its direct interaction with the IGF-1R. Furthermore, the tumour-suppressive function of miR-133a probably contributed to inhibiting the activation AKT and ERK signaling pathway.MiR-133a suppresses osteosarcoma progression and   metastasis   by targeting IGF-1R in human osteosarcoma cells, providing a novel candidate prognostic factor and a potential anti-  metastasis   therapeutic target in osteosarcoma. 
 Differentiated thyroid carcinomas from children and adolescents express IGF-I and the IGF-I receptor (IGF-I-R). Cancers with the most intense IGF-I-R expression may be more aggressive. Adult thyroid cancers express IGF and IGF-I receptor (IGF-I-R), but the clinical impact is not clear. No previous study examined any childhood thyroid cancers that are well-differentiated and have a favorable prognosis. We used immunohistochemistry to determine IGF-I and IGF-I-R in 23 papillary thyroid cancers (PTC) and 6 follicular thyroid cancers (FTC) from children and adolescents. IGF-I was detected in 45% and IGF-I-R in 43% of cancers. IGF-I and IGF-I-R were found more often in PTC (IGF-I = 9/23, IGF-I-R = 8/19) than normal surrounding thyroid (IGF-I = 0/10, p = 0.032 and IGF-I-R = 0/10, p = 0.030). There were too few FTC to support independent statistical analysis, but IGF-I was found in 4 of 6 FTC (0/10 normal), and IGF-I-R was found in 2 of 4 FTC (0/10 normal). IGF-I-R staining was more intense in aggressive (invasive, metastatic, recurrent, or persistent) than indolent tumors (confined to the gland, p = 0.029). Over time, six tumors recurred, five of which expressed IGF-I-R. Overall recurrence risk was significantly greater for tumors that expressed IGF-I-R (p = 0.05) but only approached statistical significance (p = 0.08) when disease-free survival was determined. We conclude that differentiated thyroid cancers of children and adolescents express IGF-I and IGF-I-R. Tumors that express IGF-I-R are more likely to show aggressive clinical features (invasion beyond the capsule,   metastasis  , or recurrence) and persistence despite treatment. 
 MicroRNA-133a functions as a tumor suppressor by targeting IGF-1R in hepatocellular carcinoma. MicroRNAs (miRNAs) are a class of small non-coding RNAs and have critical roles in tumorigenesis and   metastasis  . A growing body of evidence showed that microRNA-133a (miR-133a) was downregulated and played tumor suppressor roles in gastric, colorectal, bladder, and lung cancer. However, the role and underlying molecular mechanism of miR-133a in hepatocellular carcinoma (HCC) remain unclear. In this study, we analyzed the expression of miR-133a in HCC tissues and HCC cell lines. We find that miR-133a was downregulated in HCC tissues and cell lines and that miR-133a expression negatively correlated with tumor differentiation (P < 0.01), TNM stage (P < 0.01), and lym  ph node me  tastasis (P < 0.01). Then, functional studies demonstrate that restoration of miR-133a in HepG2 cells significantly suppressed proliferation, colony formation, migration, and invasion, induced cell cycle arrest at G0/G1 stage and cell apoptosis in vitro, and decreased tumor size and weight in a nude mouse HepG2 xenograft model. Using bioinformatics method and dual luciferase assays identified insulin-like growth factor 1 receptor (IGF-1R) as a direct target of miR-133a in HCC cells. Furthermore, overexpression of miR-133a inhibited activation AKT and ERK signal pathway, which contributed to suppression of HCC cell growth. These findings suggest that miR-133a may act as a tumor suppressor and inhibited survival of HCC cells by targeting IGF-1R. 
 Low levels of insulin-like growth factor type 1 receptor expression at cancer cell membrane predict liver   metastasis   in Dukes' C human colorectal cancers. The aim of this study was to evaluate the prognostic significance of insulin-like growth factor type 1 receptor (IGF-1R) expression in Dukes' C human colorectal cancers (CRCs).Immunohistochemical staining for IGF-1R was done on formalin-fixed, paraffin-embedded specimens from 161 patients with curatively resected Dukes' C CRC and at least 5-year follow-up periods. We investigated the association between the levels of IGF-1R expression and the clinicopathologic parameters. To evaluate the accurate prognostic value of IGF-1R expression, we investigated two patterns of recurrence-free survival (RFS) according to the mode of recurrence, the hepatic-RFS (H-RFS), and the nonhepatic-RFS (nH-RFS). The influence of the pattern of IGF-1R immunostaining (membranous or cytoplasmic) on RFS was also estimated.High (diffuse staining) and low (focal staining) levels of IGF-1R expression were found in 45 (28%) and 116 (72%) specimens, respectively. The recurrence rate was significantly higher in the latter group (49 of 116) than the former group (9 of 45; P = 0.01). H-RFS was significantly longer for the former group than the latter group (P = 0.021), whereas no difference was found in nH-RFS between the two groups (P = 0.121). In multivariate analysis, the level of IGF-1R expression was an independent factor for H-RFS (P = 0.015) as were the depth of invasion and lymph vessel invasion (P = 0.006 and 0.022, respectively). Using a combination of the level of IGF-1R expression and these two factors, the prognostic value was further increased. When IGF-1R staining patterns (membranous or cytoplasmic) were compared, membrane staining of IGF-1R possessed prognostic significance.In Dukes' C CRC, focal membrane expression of IGF-1R in the primary tumor can predict a high risk of recurrence, especially liver   metastasis  . Understanding the mechanisms involved could lead to new therapeutic approaches for advanced CRC. 
 microRNA-194 suppresses osteosarcoma cell proliferation and   metastasis   in vitro and in vivo by targeting CDH2 and IGF1R. Studies have shown that miR-194 functions as a tumor suppressor and is associated with tumor growth and   metastasis  . We studied the effects of miR-194 in osteosarcoma and the possible mechanism by which miR-194 affected the survival, apoptosis and   metastasis   of osteosarcoma. Both human osteosarcoma cell lines SOSP-9607 and U2-OS were transfected with recombinant lentiviruses to regulate miR-194 expression. Overexpression of miR-194 partially inhibited the proliferation, migration, and invasion of osteosarcoma cells in vitro, as well as tumor growth and pulmonary   metastasi  s of osteosarcoma cells in vivo. Potential miR-194 target genes were predicted using bioinformatics. Luciferase reporter assay, real-time quantitative PCR and western blotting confirmed that CDH2 (N-cadherin) and IGF1R were targets of miR-194. Using real-time quantitative PCR, we evaluated the expression of miR-194 and two miR-194 target genes, CDH2 and IGF1R in osteosarcoma samples from 107 patients and 99 formalin- or paraformalin-fixed paraffin-embedded tissues. The expressions of the target genes were also examined in osteosarcoma samples using immunohistochemistry. Overexpression of miR-194 inhibited tumor growth an  d metastas  is of osteosarcoma probably by downregulating CDH2 and IGF1R. miR-194 may prove to be a promising therapeutic agent for osteosarcoma. 
 Low activation of Insulin-like Growth Factor 1-Receptor (IGF1R) is associated with local recurrence in early breast carcinoma. Background The predictive value of IGF1R on local recurrence in invasive breast carcinoma (BC) is not well known. Methods In a series of 197 lymph-node negative BC patients treated with breast-conserving surgery and radiation therapy, we performed immunohistochemistry for alpha-IGF1R, beta-IGF1R (phosphorylated/active form) and Estrogen/Progesterone receptors. We further evaluated the IGF1R mRNA expression by quantitative RT-PCR and IGF1R mutations by direct DNA sequencing (exons 19 and 21) in 85 primary BC (42 control cases, 31 with local recurrence and 12 with distant   metastasis  ) and in 31 local recurrences. Unconditional logistic regression analyses were performed to identify risk factors for recurrence. Results Local recurrences were associated with high-grade tumors, PR-negative and low active-IGF1R, which emerged as independent breast relapse predictors by multivariate analysis. Conclusion Patients with early BC treated with lumpectomy and radiation who have low-grade tumors and favorable markers (increased content of active IGF1R and PR-positive) have a low risk of local recurrence. Therefore, do not benefit from a boost dose on the surgical scar. 
 Insulin-like growth factor receptor I (IGF-IR) and vascular endothelial growth factor receptor 2 (VEGFR-2) are expressed on the circulating epithelial tumor cells of breast cancer patients. Circulating epithelial tumor cell (CETC) analysis is a promising diagnostic field for estimating the risk for metastatic relapse and progression in patients with malignant disease. CETCs characterization can be used as a liquid biopsy for prognostic and predictive purposes in breast and other cancers. IGF-IR and VEGFR-2 play an important role in tumor growth and the progression of cancer disease. The purpose of the current study was therefore to investigate their expression on CETCs.CETCs were determined from the blood of 50 patients suffering from breast cancer. The number of vital CETCs and the expression of IGF-IR and VEGFR-2 were investigated using the maintrac® method.IGF-IR and VEGFR-2 expression on the surface of CETCs were detected in 84% of patients. A statistically high correlation was found between IGF-IR and VEGFR-2 (r = 0.745 and p<0.001) on the CETCs. The co-expression of both receptors was confirmed in some experiments and ranged between 70% and 100%. Statistically significant correlations were observed between the number of CETCs and IGF-IR (r = 0.315 and p<0.05) and VEGFR-2 (r = 0.310 and p<0.05) expression. The presence of CETCs and the level of IGF-IR and VEGFR-2 expression were not associated with tumor stage, hormone receptor status or n  odal/dista  nt metastasis.In this study, a parallel and co-expression of IGF-IR and VEGFR-2 was examined on the surface of CETCs in breast cancer patients for the first time. Characterization of CETCs may be a promising approach for the rational design of targeted anticancer therapies. 
 Differential localization of MT1-MMP in human prostate cancer tissue: role of IGF-1R in MT1-MMP expression. MT1-MMP is a metalloproteinase involved in prostate cancer   metastasis  . The IGF-1R is a tyrosine kinase receptor involved with tumor progression and   metastasis  . The purpose of this investigation was to examine MT1-MMP and IGF-1R expression and localization in prostate cancer tissues and explore the role of IGF-1R in regulating MT1-MMP in prostate cancer cell lines.Immunohistochemistry was utilized to study MT1-MMP and IGF-1R expression in human prostate tissues. IGF-1R regulation of MT1-MMP expression was determined by gene promoter analysis, quantitative RT-PCR and Western blot analysis following pharmacological inhibition of the receptor in PC-3N cells and treatment of LNCaP cells with androgen and IGF-1.MT1-MMP expression was high in the apical regions of the luminal cells in PIN and prostate cancer and less intense in the basalateral regions of benign tissues. IGF-1R was expressed primarily in the basal cells of normal glands and highly expressed in prostate cancer. Inhibition of IGF-1R in PC-3N cells decreased MT1-MMP expression and treatment of LNCaP cells with a synthetic androgen and IGF-1 increased MT1-MMP expression.These data demonstrate that MT1-MMP is highly expressed in the apical cytoplasmic regions of the luminal cells in PIN and prostate cancer when compared to basalateral cytoplasmic membrane staining in benign glands. Additionally, we demonstrate that IGF-1R is highly expressed in human prostate carcinoma. These findings suggest that MT1-MMP localization and IGF-1R expression in prostate carcinoma could be predictive biomarkers for aggressive disease and support IGF-1R as a promising therapeutic target to decrease processes of prostate cancer   metastasis  . 
 Dependence receptors: the trophic theory revisited. Most transmembrane receptors are believed to be inactive in the absence of their cognate ligand and initiate downstream signal transduction only upon ligand binding. However, research has shown that some receptors are also active in the absence of their ligand and induce a "negative" signal to trigger apoptosis. Thus, these receptors, dubbed dependence receptors, create a cellular state of dependence on ligand availability. The dependence receptor theory postulates key roles for these receptors during embryonic development, neurodegenerative diseases, or cancer progression and   metastasis   and is bolstered by new evidence. 
 Expression of insulin-like growth factor 1 and insulin-like growth factor 1 receptor is associated with the favorable clinicopathologic parameters in small intestinal carcinomas. The insulin-like growth factor (IGF) system has been known to play a critical role in tumor development and progression in many human cancers. However, the role of the IGF system in small intestinal carcinoma (SIC) has not been studied yet.We evaluated the expression of IGF1 and IGF1 receptor (IFG1R) in a total of 194 cases of SIC.IGF1 expression was associated with well/moderate differentiation, better survival, lower pT, lower stage and no lymph node   metastasis  . IGF1R was more diffusely and strongly expressed in tumors with lower pT and lower stage.IGF1 and IGF1R expression is associated with favorable clinicopathologic parameters and may involve early carcinogenesis of SICs. Target therapy for the IGF1R signaling pathway may not have a major therapeutic role in treating SIC. 
 Expression and significance of IGF-1 and IGF-1R in thyroid nodules. There are limitations in the diagnosis of thyroid nodules, especially follicular lesions, and their pathogenesis remains unclear. Insulin-like growth factor-1 (IGF-1) has been implicated in tumor cell apoptosis, transformation, invasion, and   metastasis  ; however, its role in thyroid nodules is undetermined. The aim of this study is to investigate the relationship between expression of IGF-1 and thyroid nodule, and evaluate the role of IGF-1 in differential diagnosis and pathogenetic function of benign and malignant thyroid nodules. Sixty-two paraffin-embedded thyroid tissues from patients with thyroid nodules were selected, including 18 follicular adenomas (FA), 17 nodular goiters, 13 papillary thyroid carcinomas (PTC), 2 follicular thyroid carcinomas, and 12 normal controls. IGF-1 and IGF-1R protein and mRNA expression was detected by immunohistochemistry (IHC) and real-time quantitative PCR (qRT-PCR), respectively. Grouping comparisons were employed among the above groups based on the clinical and pathological subtypes. IGF-1 and IGF-1R expression was significantly higher in FA, nodular goiters, and PTC than that in the controls (all P < 0.01). Similarly, IGF-1 mRNA expression was also significantly higher in FA (P < 0.05), nodular goiters (P < 0.01), and PTC (P < 0.01) compared with the controls. IGF-1R mRNA expression was also significantly higher in FA, nodular goiters, and PTC (all P < 0.01) compared with the controls. Compared with FA and nodular goiters, PTC showed significantly higher IGF-1 and IGF-1R protein (P < 0.01, P < 0.05, respectively) and mRNA expression (P < 0.01, P < 0.05, respectively). IGF-1 probably plays an important role in the genesis and development of certain solid cold thyroid nodules, including PTC, nodular goiters, and FA. Detection of IGF-1 and IGF-1R expression in thyroid tissues by IHC or qRT-PCR is hard to distinguish malignant from benign lesions. 
 siRNA-mediated type 1 insulin-like growth factor receptor silencing induces chemosensitization of a human liver cancer cell line with mutant P53. Overexpression of type 1 insulin-like growth factor receptor (IGF1R) contributes to the progression and   metastasis   of liver cancer, implying that IGF1R gene is a suitable target of RNA interference (RNAi) for liver cancer therapy. To investigate the possible regulation of IGF1R by P53, we examined the level of IGF1R expression in liver cancer cell lines in response to adriamycin. Levels of IGF1R mRNA and protein in cell lines with wild-type P53 decreased dramatically after P53 induction, but no such reduction of IGF1R was observed in cell lines with mutated P53. Inhibition of wild-type P53 in HEPG2 cells by small interfering RNA (siRNA) significantly upregulated the expression of IGF1R. IGF1R inhibition by siRNA in Huh7 cells with mutated P53 significantly depressed cell proliferation. To investigate the sensitivity of cancer cells to adriamycin after inhibition of IGF1R, we depressed IGF1R expression using siRNA, and then added adriamycin at an IC50 dose. After a further 48 h incubation with adriamycin, proliferation was significantly depressed in the cells treated with siRNA targeting IGF1R, in comparison with siRNA targeting scramble. Furthermore, both TUNEL and pro-caspase-3 expression assay showed a significant increase in apoptosis after combined treatment with adriamycin and siRNA targeting IGF1R. Our results demonstrate that IGF1R is downregulated by P53, and that siRNA targeting of IGF1R increases liver cancer cells sensitivity to adriamycin and promotes apoptosis. siRNA targeting of IGF1R could be potentially useful for increasing sensitivity to anti-cancer drugs, especially in drug-resistant cells with mutated P53. 
 [Expression and significance of IGF-1R and VEGF in gastric carcinoma]. To analyze the expression of IGF-1R and VEGF in human gastric carcinoma, and evaluate the relationship between their expression and tumor's invasion and   metastasis  .56 cases with gastric carcinoma, 20 cases of atypical hyperplasia tissues of adjacent carcinoma and 28 normal gastric mucosa membrane tissues were examined for the expression of IGF-1R and VEGF by SABC immunohistochemistry.The positive rates of IGF-1R protein in gastric carcinoma tissues, atypical hyperplasia tissues of adjacent carcinoma and normal gastric mucosa membrane tissues are 87.50%, 55.00% and 17.86%, respectively, and the difference between any two groups was significant (P<0.01); the positive rates of IGF-1R protein in the tissues of   metastasis   and non-  metastasis   are 97.06% and 72.73%, respectively(P<0.05); the positive rates of IGF-1R protein in the gastric carcinoma tissues of invaded submucosa, muscular and outer layers are 66.67%, 92.59% and 100%, respectively (P<0.05). The positive rates of VEGF protein in gastric carcinoma tissues, atypical hyperplasia tissues of adjacent carcinoma and normal gastric mucosa are 69.64%, 45.00% and 25.00%, respectively, and the difference between any two groups is significant (P<0.05); the positive rates of VEGF protein in the tissues of   metastasis   and non-  metastasis   are 85.29% and 45.45%, respectively (P<0.01); the positive rates of VEGF protein in the gastric carcinoma tissues of invaded submucosa, muscular and outer layers are 40.00%, 74.07% and 85.71%, respectively (P<0.05).The results suggest that the expression of IGF-1R or VEGF may be related to the development, invasion and   metastasis   of gastric carcinoma. 
 Association of insulin-like growth factor-I receptor polymorphism with colorectal cancer development. The insulin-like growth factor (IGF) system plays a prominent role in the cancer development. The IGF-1 receptor (IGF-1R) and its associated signalling pathway is an important growth regulatory pathway that has been implicated in colorectal carcinogenesis. This study was designed to compare +3179G/A IGF1R (rs 2229765) genotype distribution in 110 colorectal cancer (CRC) patients to a group of 143 healthy controls (HCs). We also investigated serum IGF-1 levels in CRC patients and HCs in an association to genotype. IGF-1 serum levels were measured by enzyme-linked immunosorbent assay and genotyping for the +3179G/A polymorphism was performed by restriction fragment length polymorphisms-polymerase chain reaction assay. Although the genotype frequencies were comparable in both groups, higher frequency of dominant genotypes [AA/AG; 71 vs. 62 %; odds ratio (OR) = 1.52] and lower frequency of GG genotype (29 vs. 38 %) was seen in cases versus controls. When CRC patient's group was divided into stages of disease by tumor-node-  metastasis   classification we observed the significantly highest frequency of AA genotype in III stage compared to controls: 22.5 versus 15 %; OR = 3.37, p = 0.026. There was a significant association between IGF-1R rs2229765 polymorphism and advanced CRC (AA/AG vs. GG: OR = 3.06, p = 0.004). The frequency of A-allele in advanced CRC was significantly higher then early CRC (52 vs. 37.7, OR = 1.78). According to genotype serum IGF-1 levels was significantly decreased in patients with GG genotype then patients with dominant genotypes. Our results showed a relationship between the +3179G>A polymorphism of the IGF-1R and serum IGF-1 with the progression of colorectal carcinoma. A dominant genetic model was established for IGF-1R rs2229765 polymorphism and CRC progression. 
 A dominant negative type I insulin-like growth factor receptor inhibits   metastasis   of human cancer cells. We have previously shown that LCC6 wild-type (WT) cells, a metastatic variant of MDA-MB-435 cancer cells originally derived from a breast cancer patient, exhibit enhanced motility in response to IGF-I compared with the parent MDA-MB-435 cells. To further understand the role of the type I insulin-like growth factor (IGF) receptor (IGF1R) in cancer   metastasis   we inhibited signaling via IGF1R using a C-terminal-truncated IGF1R. The truncated receptor retains the ligand binding domain but lacks the autophosphorylated tyrosine residues in the carboxyl terminus. Cells stably transfected with this truncated receptor (LCC6-DN cells) overexpressed the truncated IGF1R messenger RNA nearly 50-fold over endogenous receptor. The truncated receptor in the LCC6-DN cells behaved in a dominant negative manner to inhibit endogenous IGF1R activation by IGF-I. Compared with the LCC6-WT cells, LCC6-DN cells failed to phosphorylate the adaptor proteins insulin receptor substrate-1 and -2 in response to IGF-I and did not activate Akt after exposure to IGF-I. Unlike LCC6-WT cells, LCC6-DN cells did not show enhanced motility in response to IGF-I. To assay for   metastasis  , LCC6-WT and LCC6-DN cells were injected into the mammary fat pads of mice, and the primary xenograft tumors were removed after 21 days. Mice sacrificed 5 weeks later showed multiple lung metastases derived from LCC-WT xenografts, whereas mice harboring LCC6-DN xenografts showed no lung metastases. Our data show that IGF1R can regulate several aspects of the malignant phenotype. In these cells,   metastasis   but not proliferation requires IGF1R function. 
 Pancreatic cancer cells activate CCL5 expression in mesenchymal stromal cells through the insulin-like growth factor-I pathway. Mesenchymal stromal cells (MSCs) have a critical role in cancer progression and   metastasis  . Despite extensive studies of the physiological responses in cancer cells, the molecular mechanisms regulating gene expression in MSCs by cancer cells remain undefined. Here we demonstrate that CC chemokine ligand 5 (CCL5) expression was increased in MSCs co-cultured with pancreatic cancer cells (PCCs), and this activation was dependent on extracellular insulin-like growth factor (IGF-I). Moreover, CCL5 induction in MSCs was required for the activation of IGF-I pathway in PCCs. These results reveal a link between the IGF-I pathway in PCCs and CCL5 pathway in MSCs through the interaction of those cells. 
 Northwestern profiling of potential translation-regulatory proteins in human breast epithelial cells and malignant breast tissues: evidence for pathological activation of the IGF1R IRES. Genes involved in the control of cell proliferation and survival (those genes most important to cancer pathogenesis) are often specifically regulated at the translational level, through RNA-protein interactions involving the 5'-untranslated region of the mRNA. IGF1R is a proto-oncogene strongly implicated in human breast cancer, promoting survival and proliferation of tumor cells, as well as   metastasis   and chemoresistance. Our lab has focused on the molecular mechanisms regulating IGF1R expression at the translational level. We previously discovered an internal ribosome entry site (IRES) within the 5'-untranslated region of the human IGF1R mRNA, and identified and functionally characterized two individual RNA-binding proteins, HuR and hnRNP C, which bind the IGF1R 5'-UTR and differentially regulate IRES activity. Here we have developed and implemented a high-resolution northwestern profiling strategy to characterize, as a group, the full spectrum of sequence-specific RNA-binding proteins potentially regulating IGF1R translational efficiency through interaction with the 5'-untranslated sequence. The putative IGF1R IRES trans-activating factors (ITAFs) are a heterogeneous group of RNA-binding proteins including hnRNPs originating in the nucleus as well as factors tightly associated with ribosomes in the cytoplasm. The IGF1R ITAFs can be categorized into three distinct groups: (a) high molecular weight external ITAFs, which likely modulate the overall conformation of the 5'-untranslated region of the IGF1R mRNA and thereby the accessibility of the core functional IRES; (b) low molecular weight external ITAFs, which may function as general chaperones to unwind the RNA, and (c) internal ITAFs which may directly facilitate or inhibit the fundamental process of ribosome recruitment to the IRES. We observe dramatic changes in the northwestern profile of non-malignant breast cells downregulating IGF1R expression in association with acinar differentiation in 3-D culture. Most importantly, we are able to assess the RNA-binding activities of these putative translation-regulatory proteins in primary human breast surgical specimens, and begin to discern positive correlations between individual ITAFs and the malignant phenotype. Together with our previous findings, these new data provide further evidence that pathological dysregulation of IGF1R translational control may contribute to development and progression of human breast cancer, and breast   metastasis   in particular. 
 Treatment with insulin-like growth factor 1 receptor inhibitor reverses hypoxia-induced epithelial-mesenchymal transition in non-small cell lung cancer. Insulin-like growth factor 1 receptor (IGF1R) is expressed in many types of solid tumors including non-small cell lung cancer (NSCLC), and enhanced activation of IGF1R is thought to reflect cancer progression. Epithelial-mesenchymal transition (EMT) has been established as one of the mechanisms responsible for cancer progression and   metastasis  , and microenvironment conditions, such as hypoxia, have been shown to induce EMT. The purposes of this study were to address the role of IGF1R activation in hypoxia-induced EMT in NSCLC and to determine whether inhibition of IGF1R might reverse hypoxia-induced EMT. Human NSCLC cell lines A549 and HCC2935 were exposed to hypoxia to investigate the expression of EMT-related genes and phenotypes. Gene expression analysis was performed by quantitative real-time PCR and cell phenotypes were studied by morphology assessment, scratch wound assay, and immunofluorescence. Hypoxia-exposed cells exhibited a spindle-shaped morphology with increased cell motility reminiscent of EMT, and demonstrated the loss of E-cadherin and increased expression of fibronectin and vimentin. Hypoxia also led to increased expression of IGF1, IGF binding protein-3 (IGFBP3), and IGF1R, but not transforming growth factor β1 (TGFβ1). Inhibition of hypoxia-inducible factor 1α (HIF1α) with YC-1 abrogated activation of IGF1R, and reduced IGF1 and IGFBP3 expression in hypoxic cells. Furthermore, inhibition of IGF1R using AEW541 in hypoxic condition restored E-cadherin expression, and reduced expression of fibronectin and vimentin. Finally, IGF1 stimulation of normoxic cells induced EMT. Our findings indicated that hypoxia induced EMT in NSCLC cells through activation of IGF1R, and that IGF1R inhibition reversed these phenomena. These results suggest a potential role for targeting IGF1R in the prevention of hypoxia-induced cancer progression   and metast  asis mediated by EMT. 
 Expression of IGF-1R, VEGF-C and D2-40 and their correlation with lymph node   metastasis   in endometrial adenocarcinoma. Insulin-like growth factor-1 receptor (IGF-1R) regulates carcinogenesis and tumor development and is expressed in normal endometrium. Vascular endothelial growth factor C (VEGF-C) promotes lymph node   metastasis  . We investigated IGF-1R, VEGF-C and D2-40 in endometrial adenocarcinoma and the association between IGF-1R and lymphatic   metastasis  , using an immunohistochemical S-P method with 40 cases of endometrial adenocarcinoma and 14 of normal endometrium. IGF-1R expression differed significantly between normal endometrium and adenocarcinoma; it was associated with histological grade but not surgical stage. IGF-1R overexpression was associated with   metastasis  , but expression was not. VEGF-C expression was greater in normal endometrium than in adenocarcinoma and was associated with   metastasis   but not with surgical stage or histological grade. IGF-IR and VEGF-C expression were correlated in endometrial adenocarcinoma, and lymphatic vessel density was closely related to both. Abnormal IGF-IR and VEGF-C expression may be important in lymph node   metastasis   of endometrial adenocarcinoma and might be used to evaluate the prognosis. 
 Lack of any prognostic role of insulin-like growth factor-1 receptor in non-small cell lung cancer. The purpose of this study is to determine whether the IGF1R expression has a prognostic role in non-small cell lung cancer.Forty-seven patients histopathologically diagnosed with small cell lung cancer upon bronchoscopic biopsy or resection materials were included in the study. IGF1R expression was examined via immunohistochemical methods. In samples, >10% staining were assessed as positive and ≤10% as negative. Information about demographic datas and treatments was obtained by retrospective searches of patient files.IGF1R expression was determined as positive in 38 (80.9%) and as negative in 9 (19.1%) patients. There was no significant relation between IGF1R expression and histological sub-type, local invasion, lymph node an  d metastas  is status (p=0.842, p=0.437, 0.064, 0.447, respectively). There was also no correlation with IGF1R expression and survival (p=0.141).There are conflicting results between IGF1R and its prognostic effects in the various studies. It has been claimed in some studies it is not related to prognosis as in our study, and in some studies it has been claimed that it is a good prognostic factor whereas in some studies it has been claimed as being a factor for worse prognosis. We think that IGF1R expression in non-small cell lung carcinoma patients deserves further analysis, because of its potential prognostic and predictive roles. 
 Tyrosine kinase signalling in breast cancer: insulin-like growth factors and their receptors in breast cancer. The insulin-like growth factor (IGF) system exerts pleiotropic effects on mammalian cells. This review focuses on type I IGF receptor (IGF1R)-mediated signal transduction and its relevance in breast cancer. Upon activation by the IGFs, IGF1R, a transmembrane tyrosine kinase receptor, undergoes autophosphorylation, and then binds and phosphorylates additional signaling molecules. These intermediates initiate a series of downstream signaling events that are involved in multiple physiologic processes for cells. Recent data demonstrate that the IGF receptor system actively interacts with the estrogen receptor and integrin receptor systems. Cross-talk among these pathways regulates breast cancer proliferation, protection from cell death, and   metastasis  . Better understanding of IGF biochemical signaling pathways is of utmost importance for developing therapies for breast cancer. 
 Metastatic cells can escape the proapoptotic effects of TNF-α through increased autocrine IL-6/STAT3 signaling. The liver is a common site for cancer metastases in which the entrance of tumor cells has been shown to trigger a rapid inflammatory response. In considering how an inflammatory response may affect metastatic colonization in this setting, we hypothesized that tumor cells may acquire resistance to the proapoptotic and tumoricidal effects of TNF-α, a cytokine that is elevated in a proinflammatory tissue microenvironment. In this study, we investigated molecular mechanisms by which such resistance may emerge using tumor cells in which the overexpression of the type I insulin-like growth factor receptor (IGF-IR) enhanced the inflammatory and metastatic capacities of poorly metastatic cells in the liver. Mechanistic investigations in vitro revealed that IGF-IR overexpression increased cell survival in the presence of high levels of TNF-α, in a manner associated with increased autocrine production of interleukin-6 (IL)-6. In turn, tumor cell-derived IL-6 induced gp130 and IL-6R-dependent activation of STAT3, leading to reduced caspase-3 activation and apoptosis. We found that IL-6 production and cell death resistance were dose dependent with increasing TNF-α levels. In addition, RNA interference-mediated knockdown of either IL-6 or gp130 that established a blockade to autocrine STAT3 induction was sufficient to abolish the prosurvival effect of TNF-α and to inhibit l  iver metas  tasis. Taken together, our findings define an IGF-IR-mediated mechanism of cancer cell survival that is critical for metastatic colonization of the liver. 
 The type I insulin-like growth factor receptor regulates cancer   metastasis   independently of primary tumor growth by promoting invasion and survival. The type I insulin-like growth factor receptor (IGF1R) regulates multiple aspects of malignancy and is the target of several drugs currently in clinical trials. Although the function of IGF1R in proliferation and survival is well studied, the regulation of   metastasis   by IGF1R is not as clearly delineated. Previous work showed that disruption of IGF1R signaling by overexpression of a dominant-negative IGF1R inhibited   metastasis  . To establish a clinically applicable approach to inhibition of   metastasis   by targeting IGF1R, we examined the effect of an inhibitory antibody against IGF1R, EM164 and its humanized version, AVE1642, on   metastasis   of cancer cells. EM164 and AVE1642 did not affect primary tumor growth of MDA-435A/LCC6 cells but inhibited   metastasis   of these cells. Consistent with this inhibition in the formation of metastatic nodules, disruption of IGF1R also resulted in a decreased number of circulating tumor cells in blood of tumor-bearing mice. Disruption of IGF1R with a dominant-negative construct or antibody inhibited invasion across Matrigel in vitro. When tumor cells were directly injected into the circulation through the lateral tail vein of mice, IGF1R disruption also resulted in significant reduction of pulmonary nodules, suggesting that regulation of invasion is not the only function of IGF1R signaling. Further, disruption of IGF1R rendered cells more susceptible to anoikis. Thus, IGF1R regulated   metastasis   independently of tumor growth. The multiple phenotypes regulated by IGF1R must be considered during development of this therapeutic strategy as inhibition of   metastasis   independent of inhibition of tumor growth is not easily assessed in phase II clinical trials. 
 Overexpression of the insulin-like growth factor 1 receptor (IGF-1R) is associated with malignancy in familial pheochromocytomas and paragangliomas. Pheochromocytomas and paragangliomas (pheo/pgl) are neuroendocrine tumours derived from chromaffin cells. Although mostly benign, up to 26% of pheo/pgl will undergo malignant transformation. Reliable histological signs to differentiate benign pheo/pgl from malignant tumours are currently lacking. Increased IGF-1R expression has been shown during progression to metastatic phenotypes of several types of cancer.To analyse the distribution and expression of the IGF-1R in pheo/pgl of different genetic origin and degree of malignancy.We studied the expression of the IGF-1R protein by immunohistochemistry, in 40 primary tumours from patients with pheo/pgl from different genetic aetiology (11 of 29 metastatic/nonmetastatic diseases).We found a strong association between increased expression of IGF-1R and malignant behaviour regardless of the age at diagnosis and the genetic aetiology. IGF-1R labelling was mostly weak in primary tumours from patients with nonmetastatic pheo/pgl. Conversely, intense IGF-1R labelling was predominant in cases of pheo/pgl with confirmed metastatic disease. The risk of metastases was 11·7 times higher if tumour IGF-1R labelling was intense independently of age at diagnosis. The probability of remaining free of metastases was higher in patients with pheo/pgl scored weak for IGF-1R at 60 months and more than twofold higher at 120 months of follow-up than in patients with intense IGF-1R labelling in their primary tumours.Our results strongly suggest that IGF-1R is associated with malignancy in familial pheo/pgl and that IGF-1R expression in the primary tumour might be a useful tool to detect those patients harbouring pheo/pgl who have an increased risk   of metasta  sis. 
 P-cadherin cooperates with insulin-like growth factor-1 receptor to promote metastatic signaling of gonadotropin-releasing hormone in ovarian cancer via p120 catenin. Gonadotropin-releasing hormone (GnRH) is a potent prometastatic factor in ovarian cancer, but the intracellular signaling events are not well understood. The classical Gα(q)-phospholipase C signal transduction pathway known to operate in the pituitary is not involved in GnRH actions at non-pituitary targets. Here we showed that GnRH treatment of ovarian cancer cells led to a rapid and remarkable tyrosine phosphorylation of p120 catenin (p120(ctn)), which was mediated by P-cadherin. The use of P-cadherin small interfering RNA or neutralizing antibodies to inhibit P-cadherin expression and function resulted in diminished p120(ctn) activation, confirming that the effect was P-cadherin specific. On exploring how P-cadherin, which lacks intrinsic kinase activity, might regulate the activation of p120(ctn), we found that P-cadherin could induce the ligand-independent activation of insulin-like growth factor-1 receptor (IGF-1R). Inhibition of IGF-1R expression or its activity significantly inhibited GnRH-induced p120(ctn) activation, and the subsequent cell migration and invasion. In addition, we showed that IGF-1R regulation by P-cadherin was associated with complex formation between IGF-1R and P-cadherin, and this regulation was also observed to be in vivo correlated with   metastasi  s. Furthermore, using a mouse model of ovarian cancer   metastasi  s, GnRH receptor knockdown was shown to diminish peritoneal dissemination of tumors and ascites formation. These findings suggest for the first time that GnRH can initiate an outside-in p120(ctn) signal transduction through the cross-talk between P-cadherin and IGF-1R, thus providing a novel molecular mechanism by which GnRH may control the high level of aggressiveness and invasion and   metastasi  s potential that are characteristic of ovarian cancer. 
 [Correlations between IGF-IR expression and clinicopathological factors and prognosis in patients with lung adenocarcinoma]. the incidence of lung adenocarcinoma increases rapidly, and IGF-IR is the key mediator of several growth factors signal transduction, therefore it plays an important role in the proliferation and differentiation of cancer cell. The aim of this study is to detect the expression of IGF-IR in lung adenocarcinoma and to evaluate its implication for the clinicopathological factors and prognosis of patients with this disease.the IGF-IR expression was detected by immunohistochemical staining. Correlations between IGF-IR expression with clinicopathological factors were analyzed using the Chi-squared test. The Kaplan-Meier method was used to calculate the overall patient survival rate, and the difference in survival curves was evaluated using a Log-rank test. Univariate and multivariate analysis was carried out using the Cox proportional-hazard model.in 126 cases of tumor sections tested, IGF-IR were detected in 89 cases. Statistical analysis revealed that the IGF-IR expression was related to tumor size and T stage, while there were no relations between IGFIR expression and age, gender, smoking, pathological stages, and differentiation. Cox analysis indicated that   metastasis   and chemotherapy efficacy were the prognostic factors in these patients, while IGF-IR expression was not the independent prognostic factor.the IGF-IR expression is related to tumor size and T stage, while there is no relation between IGF-IR expression and prognosis. 
 Expression of insulin-like growth factor-1 receptor in metastatic uveal melanoma and implications for potential autocrine and paracrine tumor cell growth. We investigated the importance of the insulin-like growth factor-1 receptor (IGF-1R) in hepatic metastases of uveal melanoma. The expression pattern of IGF-1R in archival tissue samples of hepatic   metastasis   from 24 patients was analyzed by immunohistochemistry. All the samples of hepatic metastases stained positive for IGF-1R. To investigate the biological role of IGF-1R on the growth of metastatic uveal melanoma, a long-term cell line obtained from a hepatic   metastasis   (TJU-UM001) was evaluated. TJU-UM001 expressed cell surface IGF-1R (>90%) and proliferated in response to exogenous and endogenous insulin-like growth factor-1 (IGF-1). Correlatively, anti-IGF-1R antibody completely blocked IGF-1-induced growth of TJU-UM001 cells. IGF-1 preferentially induced phosphorylation of Akt (S473) in quiescent TJU-UM001 cells, and this was blocked by anti-IGF-1R antibody. This study suggests that autocrine and paracrine mechanisms underlie IGF-1-induced growth of metastatic uveal melanoma and underscore the potential benefit of IGF-1 or IGF-1R antagonism in treatment for metastatic uveal melanoma. 
 Suppression of type 1 Insulin-like growth factor receptor expression by small interfering RNA inhibits A549 human lung cancer cell invasion in vitro and   metastasis   in xenograft nude mice. Cancer invasion and   metastasis  , involving a variety of pathological processes and cytophysiological changes, contribute to the high mortality of lung cancer. The type 1 insulin-like growth factor receptor (IGF-1R), associated with cancer progression and invasion, is a potential anti-invasion and anti-  metastasis   target in lung cancer. To inhibit the invasive properties of lung cancer cells, we successfully down-regulated IGF-1R gene expression in A549 human lung cancer cells by small interfering RNA (siRNA) technology, and evaluated its effects on invasion-related gene expression, tumor cell in vitro invasion, and   metastasis   in xenograft nude mice. A549 cells transfected with a plasmid expressing hairpin siRNA for IGF-1R showed a significantly decreased IGF-1R expression at the mRNA level as well as the protein level. In biological assays, transfected A549 cells showed a significant reduction of cell-matrix adhesion, migration and invasion. Consistent with these results, we found that down-regulation of IGR-1R concomitantly accompanied by a large reduction in invasion-related gene expressions, including MMP-2, MMP-9, u-PA, and IGF-1R specific downstream p-Akt. Direct tail vein injections of plasmid expressing hairpin siRNA for IGF-1R significantly inhibited the formation of lung metastases in nude mice. Our results showed the therapeutic potential of siRNA as a method for gene therapy in inhibiting lung cancer invasion and   metastasis  . 
 Phosphorylated insulin-like growth factor-1 receptor (pIGF1R) is a poor prognostic factor in brain metastases from lung adenocarcinomas. A greater understanding of brain metastases is imperative for developing novel therapeutic strategies. Our previous study showed that insulin-like growth factor (IGF) signaling pathway was activated in brain-tropic cancer cells. In this study, we investigated the clinical relevance of activated (phosphorylated) IGF-1 receptor (pIGF1R) expression in brain metastases originating from lung adenocarcinomas. All pathologically confirmed brain metastases from lung adenocarcinomas, with available archived specimens from January 1998 to December 2009 at National Taiwan University Hospital, were assessed immunohistochemically for pIGF1R expression using H-score criteria. A median H-score was used as a cutoff point to define high or low pIGF1R expression. The mutation status in the tyrosine kinase domain of the epidermal growth factor receptor (EGFR) was examined using direct sequencing. The prognostic significance of pIGF1R expression, its correlations with clinicopathological characteristics, and EGFR status were evaluated. In the 86 cases, high membranous/cytoplasmic pIGF1R expression in brain metastases correlated with a shorter median survival (10.8 vs 27.8 mo, P = 0.003). This correlation was more significant in patients with EGFR mutations [hazard ratio (HR) 2.38, 95 % confidence interval (CI) 1.19-4.77 for EGFR mutations; HR 1.99, 95 % CI 0.95-4.15 for EGFR wild type] and remained statistically significant in multivariate analysis after adjusting for the effects of other potential prognostic factors, including the graded prognostic assessment score, solitary b  rain metas  tasis, extracranial metastatic status, EGFR mutations, and treatment using EGFR tyrosine kinase inhibitors. Although we also identified nuclear pIGF1R expression, this result was prognostically non-significant. Our study results showed that high membranous/cytoplasmic pIGF1R expression in brain metastases was a poor prognostic factor, more significantly in patients with EGFR mutations than in those with wild-type EGFRs. 
 Tumor suppressor role of miR-217 in human epithelial ovarian cancer by targeting IGF1R. Accumulating evidence shows that microRNA-217 (miR-217) is frequently dysregulated in various cancers, and plays crucial roles in tumorigenesis and   metastasis  ; however, the role and underlying molecular mechanism of miR-217 in human epithelial ovarian cancer (EOC) remains unclear. Here, we report that miR-217 expression was downregulated in EOC tissue and inversely correlated with advanced FIGO stage, high histological grading and lymph node   metastasis   (P<0.01). Function analysis revealed that the ectopic expression of miR-217 in EOC cells inhibited cell proliferation, migration and invasion in vitro, as well as suppressed tumor growth in vivo. Bioinformatics analysis and dual luciferase assays identified insulin-like growth factor 1 receptor (IGF1R) as a direct target of miR-217 in EOC cells. Western blot assay showed that overexpression of miR-217 in EOC cells inhibited IGF1R expression. In addition, downregulation of IGF1R mimicked the tumor-suppressive effects of miR-217 in EOC cells, whereas the reintroduction of IGF1R partially abrogated the suppression effect induced by miR-217 on EOC cells. Collectively, these results demonstrated that miR-217 plays a tumor suppressor role in human epithelial ovarian cancer by directly targeting IGF1R gene, suggesting a new potential therapeutic target in EOC. 
 Insulin-like growth factor type 1 receptor expression correlates to good prognosis in highly malignant soft tissue sarcoma. To evaluate known and suggested prognostic markers, especially insulin-like growth factor type 1 receptor (IGF-1R), in highly malignant soft tissue sarcomas (STS).A cohort of 101 patients with primary STS of high malignancy grade was studied with respect to development of   metastasis  , local recurrence, and survival during a minimum of 5 years follow-up. All tumors were analyzed by immunohistochemistry for expression of Ki-67, p53, p27, Bcl-2, IGF-1R, and microvessel density. The traditional clinical variables size, malignancy grade (3 or 4), necrosis, mitotic frequency, infiltrative tumor growth, vascular invasion, depth, and surgical margins were also evaluated.A significant association was shown between high expression of IGF-1R and favorable outcome. Among STS with positive IGF-1R immunoreactivity, cases with high expression (76-100% positive cells) had the best outcome, whereas cases with the lowest expression (1-25% positive cells) had the worst. As expected, large tumor size (>11 cm), presence of necrosis, high mitotic count, intralesional surgery, and deep location were all significantly associated with poor outcome, both in univariate and multivariate analyses. No difference in outcome was observed between cases of malignancy grade 3 versus 4, whereas the included and more objective variables necrosis and mitotic count were found to be reliable prognostic markers.IGF-1R expression is a common feature of highly malignant STS. Further elucidation of the role of IGF-1R and the IGF system in STS may both provide a basis for development of new prognostic tools in STS, as well as shed light on the basic mechanisms of the STS development. 
 Expression of insulin-like growth factor receptor type 1 correlate with lymphatic metastases in human gastric cancer. Most patients with gastric cancer are diagnosed at advanced clinical stages with a high frequency of lymph node   metastasis  . It is very important to find novel factors for the early diagnostic and prognostic evaluation of gastric cancer. It has been shown that IGF-1R activates mitotic division and inhibits apoptosis of cancer cells through the activation of signaling MAP/ERK and PI3K/Akt-1 pathways. IGF-1R plays a role in cell transformation and maintenance of the phenotype in modified cells. Moreover, an IGF-1 receptor effect influences the processes of adhesion, migration, invasion and   metastasis   of tumor cells. The aim of the study was to assess the expression of IGF-1R in gastric carcinoma in correlation with selected anatomo-clinical parameters. The study enrolled a group of 49 patients treated surgically for gastric cancer. 28 patients had no lymph node metastases. The expression of the studied proteins was assessed using the immunohistochemical method. We found that the expression of IGF-1R in gastric cancer is associated with lymph node   metastasis   (p < 0.001), is correlated with worse prognosis and high histological malignancy grade, and is an independent predictor of survival in patients with gastric cancer (p < 0.001). IGF-1R may play an important role in tumor growth and   metastasis   via the lymphatic pathway. 
 Transforming growth factor-β, insulin-like growth factor I/insulin-like growth factor I receptor and vascular endothelial growth factor-A: prognostic and predictive markers in triple-negative and non-triple-negative breast cancer. In the current study, the prognostic and predictive values of serum transforming growth factor-β1 (TGF-β1), insulin-like growth factor I (IGF-I)/IGF-I receptor (IGF-IR) and vascular endothelial growth factor-A (VEGF-A) were evaluated in triple-negative and non-triple-negative breast cancer (TNBC and non-TNBC). The aim was to identify a group of serological biomarkers and to identify possible candidates for targeted therapy in patients with TNBC and non-TNBC. Protein levels of TGF-β1, IGF-I/IGF-IR and VEGF-A in the serum were measured in 43 TNBC, 53 non‑TNBC and 20 normal control participants using quantitative ELISA assays. Results were correlated against standard prognostic factors, response to treatment and survival. TNBC was identified to be associated with poor prognosis and serum levels of VEGF-A and IGF/IGF-IR were significantly higher in the TNBC group compared with the non-TNBC group. IGF-IR and VEGF-A overexpression was observed to be correlated with TGF-β1 expression and all of the markers investigated were associate  d with met  astasis and disease progression. In the multivariate analysis, VEGF-A, IGF-I and IGF-IR were observed to be independent predictors for overall survival, whereas TGF-β1 and lymph node status were identified as independent predictors for disease-free survival. The overall response rate was significantly lower in patients with TNBC and those with high levels of TGF-β1, IGF-I/IGF-IR and VEGF-A. In view of the present results, it was concluded that TGF-β1, IGF-I/IGF-IR and VEGF-A overexpression is associated with the presence of aggressive tumors, which exhibit an increased proba  bility of   metastasis, a poor response to treatment and reduced survival rate. This indicates that VEGF-A, IGF-IR and IGF-I have the potential to be used as surrogate biomarkers and are promising candidates for targeted therapy, particularly in patients with TNBC. 
 MicroRNA-100 regulates IGF1-receptor expression in metastatic pancreatic cancer cells. Patients with pancreatic adenocarcinoma have the lowest 5 year survival rate and yearly rates of incidence are nearly equal to the mortality rates. Long term cure rates by standard therapies are disappointing owing to disseminated disease at diagnosis and chemotherapeutic resistance. New therapeutic targets are necessary to decrease the progression of pancreatic cancer and the ability to identify targets specific to   metastasis   would improve patient care. We evaluated the levels of microRNA of metastatic and non-metastatic cell lines. The expression levels of microRNAs and mRNAs were determined using microarray analysis to examine and compare five pancreatic cancer cell lines, two that can metastasize in vivo (S2VP10 and S2CP9) and three that do not metastasize (MiaPaCa2, Panc-1 and ASPC-1). MicroRNA analysis indicated an increase in miR-100 and a decrease in miR-138 expression in metastatic cancer cells. Microarray analysis of different expressions of mRNAs in metastatic and non-metastatic pancreatic cell lines also indicated significantly increased insulin growth factor-1 receptor (IGF1-R) expression in metastatic pancreatic cancer cell lines compared to non-metastatic pancreatic cancer cell lines. To confirm microarray analysis results, western blot and immunocytochemistry were performed. Western blot revealed that IGF1-R expression exhibited in metastatic cancer cell lines a seven-fold increase compared to non-metastatic cell lines. In addition, downstream expressions of the proteins, GRB2 and phosphorylated PI3K, also were increased in aggressive cancer cell lines. Immunocytochemistry confirmed the linkage of IGF1-R to miR-100, because cells transfected with miR-100 inhibitor showed a decrease in IGF1-R. Cells transfected with a miR-138 mimic, however, did not affect IGF1-R expression. 
 High frequency of loss of allelic integrity at Wilms' tumor suppressor gene-1 locus in advanced breast tumors associated with aggressiveness of the tumor. The product of Wilms' tumor suppressor gene (WT1), a nuclear transcription factor, regulates the expression of the insulin-like growth factor (IGF) and transforming growth factor (TGF) systems, both of which are implicated in breast tumorigenesis and are known to facilitate angiogenesis. In the present study, WT1 allelic integrity was examined by Loss of Heterozygosity (LOH) studies in infiltrating breast carcinoma (n=60), ductal carcinoma in situ (DCIS) (n=10) and benign breast disease (n=5) patients, to determine its possible association with tumor progression.LOH at the WT1 locus (11p13) as determined by PCR-RFLP for Hinf1 restriction site and was subsequently examined for its association with intratumoral expression of various growth factors i.e. TGF-beta1, IGF-II, IGF-1R and angiogenesis (VEGF and Intratumoral micro-vessel density) in breast carcinoma.Six of 22 (27.2%) genetically heterozygous of infiltrating breast carcinoma and 1 of 4 DCIS cases showed loss of one allele at WT1 locus. Histologically, the tumors with LOH at WT1 were Intraductal carcinoma (IDC) and were of grade II and III. There was no correlation in the appearance of LOH at WT1 locus with age, tumor stage, menopausal status, chemotherapy status and lymph node   metastasis  . The expression of factor IGF-II and its receptor, IGF-1R was significantly higher in carcinoma having LOH at WT1 locus. A positive correlation was observed between the TGF-beta1, VEGF expression and IMD scores in infiltrating carcinoma.The current study indicates that the high frequency of loss of allelic integrity at Wilms' tumor suppressor gene-1 locus in high-graded breast tumors is associated with aggressiveness of the tumor. 
 Activated cMET and IGF1R-driven PI3K signaling predicts poor survival in colorectal cancers independent of KRAS mutational status. Oncogenic mutational analysis provides predictive guidance for therapeutics such as anti-EGFR antibodies, but it is successful only for a subset of colorectal cancer (CRC) patients.A comprehensive molecular profiling of 120 CRC patients, including 116 primary, 15 liver   metastasis  , and 1 peritoneal seeding tissue samples was performed to identify the relationship between v-Ki-ras2 Kirsten rat sarcoma viral oncogene homolog (KRAS) WT and mutant CRC tumors and clinical outcomes. This included determination of the protein activation patterns of human epidermal receptor 1 (HER1), HER2, HER3, c-MET, insulin-like growth factor 1 receptor (IGF1R), phosphatidylinositide 3-kinase (PI3K), Src homology 2 domain containing (Shc), protein kinase B (AKT), and extracellular signal-regulated kinase (ERK) kinases using multiplexed collaborative enzyme enhanced reactive (CEER) immunoassay.KRAS WT and mutated CRCs were not different with respect to the expression of the various signaling molecules. Poor prognosis in terms of early relapse (<2 years) and shorter disease-free survival (DFS) correlated with enhanced activation of PI3K signaling relative to the HER kinase pathway signaling, but not with the KRAS mutational status. KRAS WT CRCs were identified as a mixed prognosis population depending on their level of PI3K signaling. KRAS WT CRCs with high HER1/c-MET index ratio demonstrated a better DFS post-surgery. c-MET and IGF1R activities relative to HER axis activity were considerably higher in early relapse CRCs, suggesting a role for these alternative receptor tyrosine kinases (RTKs) in driving high PI3K signaling.The presented data subclassified CRCs based on their activated signaling pathways and identify a role for c-MET and IGF1R-driven PI3K signaling in CRCs, which is superior to KRAS mutational tests alone. The results from this study can be utilized to identify aggressive CRCs, explain failure of currently approved therapeutics in specific CRC subsets, and, most importantly, generate hypotheses for pathway-guided therapeutic strategies that can be tested clinically. 
 [Expression of IGF-1R in esophageal squamous cell carcinoma and the effect of its silencing by siRNA on the proliferation of esophageal cancer EC9706 cells in vitro]. To explore the correlation of IGF-1R expression with clinical features of esophageal squamous cell carcinoma (ESCC) and to investigate the effect of silencing IGF-1R by siRNA on the proliferation of esophageal cancer cell line EC9706 cells.Immunohistochemistry was used to detect the expresion of IGF-1R in 80 specimens of ESCC and 18 specimens of normal esophageal mucosa. IGF-1R siRNA was transfected into esophageal squamous cell carcinoma EC9706 cells, and the effect of RNAi was assessed by Western blot. The proliferation of EC9706 cells was determined by drawing growth curve, MTT assay and plate colony-forming assay.The total and strong positive rates of IGF-1R expression were 86.3% and 51.3% in ESCC, and 61.1% and 11.1% in normal esophageal epithelium, respectively. The total and strong positive rates of IGF-1R expression in patients with lymph node   metastasis   were 94.4% and 74.1%, significantly higher than 69.2% and 3.9%, respectively, in those without lymph node   metastasis   (P<0.01). A significantly higher IGF-1R expression was associated with lower histological grade (P<0.05). The total and strong rates of IGF-1R expression in 39 patients of stages III and IV were 97.4% and 71.8% , significantly higher than the 75.6% and 31.7%, respectively, in 41 cases of stages I and II (P<0.01). IGF-1R RNAi significantly inhibited IGF-1R expression and the growth of EC9706 cells. The clone formation rate of RNAi-IGF-1R transfected cells was 19.1%, significantly lower than that of 52.3% in non-transfected cells and 49.0% in empty vector-transfected EC9706 cells (P<0.05).The overexpression of IGF-1R is colerated with lymph node   metastasis  , differentiation and clinical stage. Down-regulation of IGF-1R can inhibit the proliferation of esophageal cancer EC9706 cells in vitro. 
 Insulin-like growth factors are essential to prevent anoikis in oestrogen-responsive breast cancer cells: importance of the type I IGF receptor and PI3-kinase/Akt pathway. Detachment of epithelial cells from the extracellular matrix initiates programmed cell death by a process termed anoikis. Malignant cells must acquire anoikis resistance to leave the primary tumour and   metastasis  e. Multiple signal transduction pathways can activate anoikis and confer anoikis resistance, but these are not understood in breast cancer.Models for anoikis of oestrogen-responsive breast cancer cells were established and the protective effects of IGF-1 tested. Cleaved PARP was measured by western transfer and cleaved caspase 3 by flow cytometry. Pathways involved in anoikis and in anoikis resistance were investigated with PI3-kinase, Akt, and MEK1 and MEK2 inhibitors. The importance of the type I IGF receptor was investigated by IGF-concentration dependence, siRNA knockdown and pharmacological inhibition. Association between IGF-1R expression and relapse with distant   metastasis   was analysed in 1609 patients by log rank test.Unattached breast cancer cells required culture in serum-free medium to induce anoikis. Rapid loss of FAK, Akt and Bad phosphorylation was concurrent with anoiks induction, but ERK1 and ERK2 phosphorylation increased which suggested that anoikis resistance is mediated by the PI3-kinase/Akt rather than the Grb2/Ras/MAP-kinase pathway. IGF-1 conferred anoikis resistance in serum-free medium. IGF-1 activated the PI3-kinase/Akt and Grb2/Ras/MAP-kinase pathways but experiments with PI3-kinase, Akt and MEK1 and MEK2 inhibitors showed that IGF protection is via the PI3-kinase/Akt pathway. The concentration dependence of IGF protection, knockdown experiments with siRNA and pharmacological inhibition with figitumumab, showed that IGF-1 signals through the type I IGF receptor. The crucial role of the type I IGF receptor was demonstrated by induction of anoikis in full serum by figitumumab. High IGF-1R expression was associated with reduced time to relapse with distant metastases in oestrogen receptor-positive patients, especially those with aggressive disease which confirms its relevance in vivo.Anoikis resistance of oestrogen-responsive breast cancer cells depends upon IGF activation of the type I IGF receptor and PI3-kinase/Akt pathway. Because IGF-dependent evasion of anoikis will facilitate   metastasis   by malignant breast cancer cells, effective inhibition of IGF signal transduction should be included in combinations of targeted drugs designed to treat metastatic oestrogen receptor-positive breast cancers. 
 Relevance of insulin-like growth factor 1 receptor gene expression as a prognostic factor in non-small-cell lung cancer. Signalling through the insulin-like growth factor 1 receptor (IGF-1R) is implicated in carcinogenesis,   metastasis  , and resistance to cytotoxic cancer therapies. The purpose of this study was to investigate the prognostic role of IGF-1R expression in surgically resected non-small-cell lung cancer (NSCLC), and responses to IGF-1R tyrosine kinase inhibitor NVP-ADW742 in a panel of lung cancer cell lines.Insulin-like growth factor 1 receptor (IGF-1R) expression was evaluated by quantitative RT-PCR in 115 NSCLC samples and in a panel of 6 NSCLC cell lines. Cytotoxicity experiments with IGF-1R inhibitor and conventional systemic drugs such as paclitaxel in cell lines were realised.Insulin-like growth factor 1 receptor (IGF-1R) was differentially expressed across histologic subtypes, with the lowest levels observed in squamous cell tumours. Median survival was longer in patients with squamous tumour histology expressing low IGF-1R levels. In multivariable analysis, ageing and high tumour stage were significant predictors of worse overall survival. The hazard of death was lower in patients with squamous histology and low IGF-1R gene expression. There was no correlation between IGF-1R expression and response to tyrosine kinase inhibitor in cell lines tested. However, combination drug treatment resulted in synergistically enhanced antiproliferative effects on several cell lines.These findings suggest that IGF-1R is a potential target for therapy in NSCLC patients. Combination therapies will have an important role in treatment. 
 [Expression and clinical significance of insulin like growth factor-1 protein in nasopharyngeal carcinoma]. To evaluate the expression of IGF-1R protein and its clinical signification in nasopharyngeal carcinoma.SP immunohistochemical staining was performed to detect the expression of IGF-1R in 63 cases of NPC and 21 inflammatory nasopharyngeal mucosa; and the expression of IGF-1R in three NPC cell lines and chronic nasopharyngitis tissues was examined by western blots.The positive rates of IGF-1R were 82.5% and 14.3% respectively in 63 cases of NPC and 21 inflammatory nasopharyngeal mucosa; it also showed stronger positive expression in Stage III-IV of NPC than that in stage I-II (P < 0 01); IGF-1R positive expression in cases of NPC with cervical lymph node   metastasis   was significantly higher than that of NPC without lymph node   metastasis   (P < 0.05); it more often appears in recurrent cases than in primary cases (P < 0.05); IGF-1R positive expression had a significantly stronger in patients whose survival time less than 5 years than that in patients whose survival time over 5 years (P < 0.01).Over-expression of IGF-1R is related to poor differentiation of NPC, also may be linked with oncogenesis and development of NPC. The over-expression of IGF-1R may be one of a prognostic factors in NPC. The IGF-1R might be a potential therapeutic target in nasopharyngeal cancer. 
 IGF/STAT3/NANOG/Slug Signaling Axis Simultaneously Controls Epithelial-Mesenchymal Transition and Stemness Maintenance in Colorectal Cancer. Discovery of epithelial-mesenchymal transition (EMT) and cancer stem cells (CSCs) are two milestones in people exploring the nature of malignant tumor in recent decades. Although some studies have presented the potential connections between them, the link details, underneath their superficial correlation, are largely unknown. In this study, we identified a small subpopulation of NANOG-positive colorectal cancer (CRC) cells, and demonstrated that they exhibited characteristics of CSCs and EMT traits simultaneously. Furthermore, we found that NANOG was a core factor in regulating both of EMT and stemness in CRC cells, NANOG modulate EMT and   metastasis   by binding to Slug promoter and transcriptionally regulate Slug expression. For the first time, we demonstrated that NANOG was regulated by extracellular IGF signaling pathway via STAT3 phosphorylation in CRC. This coincides with that IGF receptor IGF-1R is often increasing expressed in malignant   metastasis   colon cancer. Taken together, our data define the crucial functions of IGF/STAT3/NANOG/Slug signaling axis in the progression of CRC by operating EMT and CSCs properties, which make them served as potential therapeutic targets for treatment of CRC. 
 Matrix metalloproteinase-7 facilitates insulin-like growth factor bioavailability through its proteinase activity on insulin-like growth factor binding protein 3. Matrix metalloproteinase-7 (MMP-7) secreted by cancer cells has been implicated classically in the basement membrane destruction associated with tumor cell invasion and   metastasis  . Recent epidemiologic studies have established a correlation between high levels of circulating insulin-like growth factor (IGF) and low levels of IGF binding protein 3 (IGFBP-3), and relative risk of developing colon, breast, prostate, and lung cancer, which are known to produce MMP-7. In this study, IGFBP-3 was assessed as a candidate for the physiologic substrate of MMP-7. MMP-7 proteolysis generated four major fragments (26 kDa, 17 kDa, 15.5 kDa, and 15.5 kDa), and two cleavage sites were identified: one at the site of hydrolysis of the K(144)-I(145) peptide bond and one at the R(95)-L(96) peptide bond. The former site is different from the previously reported site of cleavage of IGFBP-3 by other proteases. Addition of IGFBP-3 inhibited IGF-I-mediated IGF type 1 receptor (IGF-IR) phosphorylation and activation of the downstream molecule Akt in BALB/c 3T3 fibroblasts overexpressing human IGF-IR (3T3-IGF-IR) and in two human colon cancer cell lines (COLO201 and HT29). Coincubation of the IGF-I/IGFBP-3 complex with MMP-7 restored IGF-I-mediated IGF-IR phosphorylation and activation of Akt in these cell lines. The IGF-I signal recovered by MMP-7 protected against apoptosis induced by anoikis in 3T3-IGF-IR cells. These results indicate that MMP-7 proteolysis of IGFBP-3 plays a crucial role in regulating IGF-I bioavailability, thereby promoting cell survival. This mechanism may contribute to the tumorigenesis of MMP-7-producing IGF-IR-expressing tumors in the primary site and to organ-specific   metastasis   in a paracrine manner. 
 Prognostic value of insulin- like growth factor-I receptor expression in renal cell carcinoma. The insulin-like growth factor-I receptor (IGF-IR), a tyrosine kinase receptor over expressed in many tumor cell lines and in some human tumors, plays a critical role in transformation, tumorigenicity and   metastasis  . The aim of the present study is to investigate the role of IGF-IR expression as a prognostic factor in RCC. This study was conducted in a historical cohort of 82 patients who had RCC treated with radical nephrectomy from 1994 to 2005. Specimens were reevaluated with regard to histological subtype, nuclear grade, stage and IGF-IR expression. The IGF-IR stain was semi-quantitatively evaluated using the Allred score system. Kaplan-Meier analysis demonstrated a significant positive correlation between Fuhrman nuclear grade and IGF-IR Allred score (P< 0.0001). Survival in patients with score IGF-I < or = 4 was 90.21 month and in patients with score IGF-1R> 4 was 33.39 month (P Value < 0.0001). Cox regression analysis indicated that expression of IGF-IR is a prognostic factor in patients with RCC (P Value < 0.0001, odds Ratio = 2.38). In conclusion, a statistically significant correlation was demonstrated between IGF-IR expression and Fuhrman nuclear grading and survival in patients with RCC. In stage-by-stage and grade-by-grade analysis; however, it seems that we cannot consider IGF-IR as an independent prognostic factor. 
 Clinicopathological significance of the gene expression of matrix metalloproteinase-7, insulin-like growth factor-1, insulin-like growth factor-2 and insulin-like growth factor-1 receptor in patients with colorectal cancer: insulin-like growth factor-1 receptor gene expression is a useful predictor of liver   metastasis   from colorectal cancer. Matrix metalloproteinase-7 (MMP-7), secreted by cancer cells, has been implicated classically in the basement membrane destruction associated with tumor cell invasion and   metastasis  . Epidemiological studies have established a correlation between high levels of circulating insulin-like growth factor-1 (IGF-1) and the relative risk of colorectal cancer, which is known to produce MMP-7. We examined the clinicopathological significance of the relative expression of MMP-7, IGF-1, IGF-2 and IGF-1 receptor genes in patients with colorectal cancer, especially with regard to   metastasis  . We studied surgical specimens of cancer tissue and adjacent normal mucosa obtained from 205 patients with untreated colorectal carcinoma. MMP-7, IGF-1, IGF-2, IGF-1R and beta-actin mRNA in cancer tissue and adjacent normal mucosa were measured by quantitative real-time reverse-transcriptase polymerase chain reaction. MMP-7 and IGF-1R gene expression levels were higher in cancer tissue than in adjacent normal mucosa. In contrast, IGF-1 gene expression was lower in cancer tissue than in adjacent normal mucosa. As for the relationship of gene expression to clinicopathological factors, IGF-1R expression correlated with venous invasion and liver   metastasis  . IGF-1R gene expression is thus considered a useful predictor of liver   metastasis   from colorectal cancer. 
 Expression and clinical significance of epidermal growth factor receptor and insulin-like growth factor receptor 1 in patients with ampullary adenocarcinoma. Epidermal growth factor receptor (EGFR) and insulin-like growth factor receptor 1 (IGF-1R) play important roles in cell proliferation, antiapoptosis, angiogenesis, and   metastasis   and have been used for targeted therapies for patients with advanced colorectal and lung cancers. However, the expression and function of EGFR and IGF-1R in ampullary adenocarcinoma (AA) have not been examined in detail. We examined the expression of EGFR and IGF-1R in 106 AA patients at our institution using tissue microarrays and immunohistochemistry. The results were correlated with the clinicopathological parameters and survival. Overexpression of EGFR and IGF-1R was detected in 18 (17%) and 26 (25%) of AAs, respectively. Patients with EGFR-high tumors had shorter overall survival (mean, 109.8 ± 22.3 months) than those patients whose tumors were EGFR-low in overall study population (mean, 164.2 ± 10.6 months; P = .04). Overexpression of EGFR correlated with poor overall survival in patients with intestinal-type AA (P = .01) but not in those with pancreaticobiliary-type AAs (P = .47). In multivariate analysis, EGFR overexpression was an independent prognostic factor for overall survival (P = .02). In addition, we found that overexpression of IGF-1R correlated with AAs of pancreaticobiliary histology. No additional correlation between the expression of EGFR or IGF-1R and other clinicopathological factors was observed in our patient population. Our study demonstrates that EGFR and IGF-1R appear to be overexpressed in a subset of AAs and that strong membranous expression of EGFR is an independent predictor for overall survival in patients with AA. EGFR and IGF-1R represent potential therapeutic targets for treatment of patient with AAs. 
 Increased expression of insulin-like growth factor-1 receptor is correlated with tumor   metastasis   and prognosis in patients with osteosarcoma. The aim of this study was to investigate the association of insulin-like growth factor-1 receptor (IGF-1R) with   metastasis   and prognosis of osteosarcoma patients.RT-PCR and Western blot assays were performed to detect IGF-1R mRNA and protein expression in 26 osteosarcoma and noncancerous bone tissues. Immunohistochemistry was performed to analyze the correlation of IGF-1R expression in 84 osteosarcoma tissues with clinicopathological factors or survival of patients. Lentivirus-mediated RNA interference system was employed to downregulate IGF-1R expression and analyze the effects of IGF-1R downregulation on invasion and   metastasis   of osteosarcoma cells.The relative levels of IGF-1R mRNA and protein expression were significantly higher in osteosarcoma tissues than in corresponding noncancerous bone tissues. The expression of IGF-1R protein was closely associated with surgical stage and distant   metastasis   of osteosarcoma patients. Osteosarcoma patients with high IGF-1R expression had poorer survival, and multivariate Cox analyses showed that high IGF-1R expression was an independent prognostic maker. Lentivirus-mediated targeting IGF-1R could significantly inhibit adhesion, migration, invasion, and   metastasis   of osteosarcoma cells, which might be correlated with of inactivation of Akt signaling pathway.IGF-1R is an independent prognostic marker for osteosarcoma patients and increased expression of this molecular is correlated with   metastasis   of osteosarcoma. 
 Impact of insulin-like growth factor receptor-I function on angiogenesis, growth, and   metastasis   of colon cancer. Insulin-like growth factors and their principal receptor, IGF-I receptor (IGF-IR), are frequently expressed in human colon cancers and play a role in preventing apoptosis, enhancing cell proliferation, and inducing expression of vascular endothelial growth factor (VEGF). The role of IGF-IR in regulating angiogenesis and metastases of human colon cancer has not been elucidated. To determine the in vitro and in vivo effects of IGF-IR in human colon cancer growth and angiogenesis, human KM12L4 colon cancer cells were transfected with a truncated dominant-negative form of IGF-IR (IGF-IR dom-neg). IGF-IR dom-neg-transfected cells demonstrated markedly decreased constitutive expression of VEGF mRNA and protein. Subcutaneous injections of IGF-IR dom-neg-transfected cells in nude mice led to significantly decreased tumor growth (p < 0.05) that was associated with decreased tumor cell proliferation, VEGF expression, and vessel count and with increased tumor cell apoptosis (p < 0.05 for all parameters compared with controls). In addition, pericyte coverage of endothelial cells was significantly decreased in tumors from IGF-IR dom-neg-transfected cells. Following this observation, we demonstrated in vitro that vascular smooth muscle cells migrated significantly less in conditioned medium derived from IGF-IR dom-neg-transfected cells compared with medium from control cells. After splenic injections, IGF-IR dom-neg transfectants failed to produce liver metastases, in contrast to parental cells and mock transfectants (p < 0.05). In addition, IGF-IR dom-neg-transfected cells failed to form liver tumors after direct injection into the liver. These studies demonstrate that the IGF-IR plays an important role in multiple mechanisms that mediate the growth, angiogenesis, and   metastasis   of human colon cancer. IGF-IR is a valid target for the therapy of human colon cancer. 
 miR-494 suppresses tumor growth of epithelial ovarian carcinoma by targeting IGF1R. A growing body of evidence suggests that microRNA-494 (miR-494) could act as tumor-suppressive or oncogenic microRNAs (miRNAs) in different types of tumors. However, the biological roles and underlying mechanisms of miR-494 remain unknown in human epithelial ovarian carcinoma (EOC). Therefore, the aims of this study were to investigate the miR-494 expression and the significance of its clinical diagnosis in patients suffering EOC and to analyze its role and underlying molecular mechanism on the carcinogenesis of EOC. Here, we found that miR-494 was significantly decreased in EOC cell lines and tissues and its expression was negatively correlated with advanced International Federation of Gynecology and Obstetrics (FIGO) stage, high pathological grade, and lymph node   metastasis   (all P < 0.01). Functional studies showed that overexpression of miR-494 in EOC cells could remarkably inhibit proliferation, colony formation, migration, and invasion and induce cell apoptosis, G0/G1 phase arrest. An in vivo analysis revealed that the overexpression of miR-494 suppressed tumor growth in a nude mouse xenograft model system. Bioinformatic assay and dual-luciferase assay confirmed that insulin-like growth factor 1 receptor (IGF1R) was as a direct target of miR-494 in EOC cells. Western blot assay showed that overexpression of miR-494 inhibited IGF1R expression and its downstream signal protein expression. In addition, downregulation of IGF1R has similar effects with miR-494 overexpression on EOC cells and overexpression of IGF1R effectively rescued the inhibition of overexpressed miR-494 in EOC cells. These data suggested that miR-494 functions as a tumor suppressor in EOC by targeting IGF1R. 
 Selective inhibition of proprotein convertases represses the metastatic potential of human colorectal tumor cells. The proprotein convertases (PCs) are implicated in the activation of various precursor proteins that play an important role in tumor cell   metastasis  . Here, we report their involvement in the regulation of the metastatic potential of colorectal tumor cells. PC function in the human and murine colon carcinoma cell lines HT-29 and CT-26, respectively, was inhibited using siRNA targeting the PCs furin, PACE4, PC5, and PC7 or by overexpression of the general PC inhibitor alpha1-antitrypsin Portland (alpha1-PDX). We found that overexpression of alpha1-PDX and knockdown of furin expression inhibited processing of IGF-1 receptor and its subsequent activation by IGF-1 to induce IRS-1 and Akt phosphorylation, all important in colon carcinoma   metastasis  . These data suggest that the PC furin is a major IGF-1 receptor convertase. Expression of alpha1-PDX reduced the production of TNF-alpha and IL-1alpha by human colon carcinoma cells, and incubation of murine liver endothelial cells with conditioned media derived from these cells failed to induce tumor cell adhesion to activated murine endothelial cells, a critical step in metastatic invasion. Furthermore, colon carcinoma cells in which PC activity was inhibited by overexpression of alpha1-PDX when injected into the portal vein of mice showed a significantly reduced ability to form liver metastases. This suggests that inhibition of PCs is a potentially promising strategy for the prevention of colorectal liver   metastasis  . 
 Increased insulin-like growth factor 1 receptor (IGF1R) expression in small cell lung cancer and the effect of inhibition of IGF1R expression by RNAi on growth of human small cell lung cancer NCI-H446 cell. Insulin-like growth factor 1 receptor (IGF1R) is a tyrosine kinase receptor implicated in tumourigenesis that may be an attractive target for anti-cancer treatment. In this study, the expression and clinical significance of IGF1R were investigated in serum and lung cancer tissues from small cell lung cancinoma (SCLC). We also compared the effect of IGF1R up-regulation and IGF1R inhibition on viability and apoptosis of NCI-H446 cells. We found the concentration of IGF1R in blood serum was significantly increased and positive IGF1R protein in cancer tissue was more prevalent in SCLC. A statistically significant correlation among IGF1R-positve tumors, lymph node   metastasis   and local invasion was discussed. Furthermore, IGF1R overexpression lead to an increase of cell survival and suppressed cell apoptosis, IGF1R silencing mediated by RNAi abrogate this response of NCI-H446 cells. Our results further demonstrated that the effects of these treatments may be assigned to the effective inhibition of lung cancer cells from Akt/P27(Kip1) pathway in IGF-1R signaling. These features may have important implications for future anti-IGF1R therapeutic approaches. 
 miR-140 suppresses tumor growth and   metastasis   of non-small cell lung cancer by targeting insulin-like growth factor 1 receptor. MicroRNAs (miRNAs) are a class of small noncoding RNA molecules that play important roles in carcinogenesis and tumor progression. In this study, we investigated the roles and mechanisms of miR-140 in human non-small cell lung cancer (NSCLC). We found that miR-140 is significantly downregulated in NSCLC tissues and cell lines. Both gain-of-function and loss-of-function studies demonstrated that miR-140 suppresses NSCLC cell proliferation, migration, and invasion in vitro. Importantly, overexpression of miR-140 effectively repressed tumor growth and   metastasis   in nude mouse models. Integrated analysis identified IGF1R as a direct and functional target of miR-140. Knockdown of IGF1R inhibited cell proliferation and invasion resembling that of miR-140 overexpression, while overexpression of IGF1R attenuated the function of miR-140 in NSCLC cells. Together, our results highlight the significance of miR-140 and IGF1R in the development and progression of NSCLC. 
 Anti-tumor effect in human breast cancer by TAE226, a dual inhibitor for FAK and IGF-IR in vitro and in vivo. Focal adhesion kinase (FAK) is a 125-kDa non-receptor type tyrosine kinase that localizes to focal adhesions. FAK overexpression is frequently found in invasive and metastatic cancers of the breast, colon, thyroid, and prostate, but its role in osteolytic   metastasis   is not well understood. In this study, we have analyzed anti-tumor effects of the novel FAK Tyr(397) inhibitor TAE226 against bone   metastasis   in breast cancer by using TAE226. Oral administration of TAE226 in mice significantly decreased bone   metastasis   and osteoclasts involved which were induced by MDA-MB-231 breast cancer cells and increased the survival rate of the mouse models of bone   metastasis  . TAE226 also suppressed the growth of subcutaneous tumors in vivo and the proliferation and migration of MDA-MB-231 cells in vitro. Significantly, TAE226 inhibited the osteoclast formation in murine pre-osteoclastic RAW264.7 cells, and actin ring and pit formation in mature osteoclasts. Moreover, TAE226 inhibited the receptor activator for nuclear factor κ B Ligand (RANKL) gene expression induced by parathyroid hormone-related protein (PTHrP) in bone stromal ST2 cells and blood free calcium concentration induced by PTHrP administration in vivo. These findings suggest that FAK was critically involved in osteolytic   metastasi  s and activated in tumors, pre-osteoclasts, mature osteoclasts, and bone stromal cells and TAE226 can be effectively used for the treatment of cancer induced bone   metastasi  s and other bone diseases. 
  GeneRIFs  
 Results showed that IGF1R genetic variants are related to EGFR mutation in female lung adenocarcinoma patients and may be a predictive factor for tumor lymph node   metastasis   in Taiwanese patients with non-small-cell lung carcinoma. 
 NANOG was regulated by extracellular IGF signaling pathway via STAT3 phosphorylation in colorectal cancer (CRC). This coincides with that IGF receptor IGF-1R is often increasing expressed in malignant   metastasis   colon cancer. Taken together, our data define the crucial functions of IGF/STAT3/NANOG/Slug signaling axis in the progression of CRC 
 The findings suggest that miR-98 inhibits cancer cell growth and   metastasis   by direct targeting IGF1R, implicating miR-98 as a novel potential therapeutic target for oral squamous cell carcinoma (OSCC). 
 MiR-133a suppresses osteosarcoma progression and   metastasis   by targeting IGF-1R in human osteosarcoma cell. 
 it was concluded that TGF-beta1, IGF-I/IGF-IR and VEGF-A overexpression is associated with the presence of aggressive tumors, which exhibit an increased probability of   metastasis  , a poor response to treatment and reduced survival rate. 
 Results demonstrate that miR-194 affected the growth and   metastasis   of osteosarcoma cells both in vitro and in vivo suggesting that miR-194 functions as tumor suppressor gene probably by downregulating CDH2 and IGF1R. 
 IGF1R may have a role in hypoxia-induced cancer progression and   metastasis   mediated by epithelial-mesenchymal transition 
 The expression pattern of IGF-1R in archival tissue samples of hepatic   metastasis   from 24 patients was analyzed by immunohistochemistry. 
 Expression of IGF-1R in gastric cancer is associated with lymph node   metastasis  , is correlated with worse prognosis and high histological malignancy grade, and is an independent predictor of survival in patients with gastric cancer. 
 miR-99a functions as a tumor   metastasis   suppressor in Oral squamous cell carcinoma cells and mutually regulates IGF1R 
 Results strongly suggest that IGF-1R is associated with malignancy in familial pheo/pgl and that IGF-1R expression in the primary tumour might be a useful tool to detect those patients harbouring pheo/pgl who have an increased risk of   metastasis   
 a major activity of DNp73 is to establish initiation of the invasion-  metastasis   cascade via EPLIN-dependent IGF1R regulation 
 The overexpression of IGF-1R is colerated with lymph node   metastasis  , differentiation and clinical stage of esophageal squamous cell carcinoma. Down-regulation of IGF-1R can inhibit the proliferation of esophageal cancer EC9706 cells in vitro. 
 IGF-1R is an independent prognostic marker for osteosarcoma patients and increased expression of this molecular is correlated with   metastasis   of osteosarcoma. 
 Abnormal IGF-IR and VEGF-C expression may be important in lymph node   metastasis   of endometrial adenocarcinoma and might be used to evaluate the prognosis. 
 Data suggest that FAK is critically involved in osteolytic   metastasis   and activated in tumors, pre-osteoclasts, mature osteoclasts, and bone stromal cells, and the FAK/IGF-1R inhibitor TAE226 may be used for cancer induced bone   metastasis  . 
 pathological dysregulation of IGF1R translational control may contribute to development and progression of breast cancer, and breast   metastasis   in particular 
 Data suggest that the expression of IGF-1R or VEGF may be related to the development, invasion and   metastasis   of gastric carcinoma. 
 Higher IGF-I Receptor is associated with venous invasion and liver   metastasis   in colorectal cancer. 
 affects angiogenesis, growth, and   metastasis   of colon cancer 
 Low levels of insulin-like growth factor type 1 receptor expression at cancer cell membrane is associated with liver   metastasis   in colorectal cancers 
 high expression level of insulin-like growth factor I receptor is associated with increased expression of transcription factor Sp1 and regional lymph node   metastasis   of human gastric cancer 
 To further understand the role of the type I insulin-like growth factor (IGF) receptor (IGF1R) in cancer   metastasis   we inhibited signaling via IGF1R using a C-terminal-truncated IGF1R. 
  [Cc]ell [Ii]nvasion  PubMed  
 Requirement of different signaling pathways mediated by insulin-like growth factor-I receptor for proliferation, invasion, and VPF/VEGF expression in a pancreatic carcinoma cell line. Several oncogenes and growth factors are found to be mutated and overexpressed in adenocarcinoma of the pancreas, and may correlate with its highly aggressive nature. Insulin-like growth factor (IGF-I) and its receptor (IGF-IR) are highly expressed in this tumor type. We examined the IGF-IR-mediated signaling pathways in relation to cell proliferation, invasiveness, and expression pattern of vascular permeability factor/vascular endothelial growth factor (VPF/VEGF) in the pancreatic cancer line ASPC-1. Our findings show that IGF-IR is an important growth factor receptor for cell proliferation and invasion, and VPF/VEGF expression in ASPC-1. Further experiments indicate that IGF-IR mediates different signaling pathways to execute its functions. Activation of Ras by IGF-IR was found to be required for the   cell invasion  . On the other hand Src activation through IGF-IR is required for the cell proliferation, invasion, and also VPF/VEGF expression. Taken together, our data indicate the importance of IGF-IR in growth and invasiveness of the pancreatic cancer cell lines and also point out the multiple signaling pathways channeled through this receptor. 
 Downregulation of miR-383 promotes glioma   cell invasion   by targeting insulin-like growth factor 1 receptor. Invasiveness is a major clinical feature of glioma, an aggressive brain tumor with poor prognosis. Although there is emerging evidence that some microRNAs are involved in the glioma   cell invasion   process, it remains necessary to find functional microRNAs and elucidate the underlying molecular mechanisms. Here, we reported that a microRNA, miR-383, was downregulated in gliomas and inversely correlated with glioma pathological grades. Downregulation of miR-383 enhanced, whereas upregulation of miR-383 inhibited, the glioma cell invasive ability. Furthermore, we found that downregulation of miR-383 activated the AKT signaling following upregulation of MMP2 expression by directly targeting insulin-like growth factor 1 receptor (IGF1R). Importantly, we demonstrated that IGF1R expression is critical for miR-383 downregulation-induced   cell invasion  . Taken together, these findings uncover a novel regulatory mechanism for constitutive IGF1R signaling activation in glioma cancer and may provide miR-383 as a useful diagnostic marker or therapeutic target. 
 Suppression of type 1 Insulin-like growth factor receptor expression by small interfering RNA inhibits A549 human lung cancer   cell invasion   in vitro and metastasis in xenograft nude mice. Cancer invasion and metastasis, involving a variety of pathological processes and cytophysiological changes, contribute to the high mortality of lung cancer. The type 1 insulin-like growth factor receptor (IGF-1R), associated with cancer progression and invasion, is a potential anti-invasion and anti-metastasis target in lung cancer. To inhibit the invasive properties of lung cancer cells, we successfully down-regulated IGF-1R gene expression in A549 human lung cancer cells by small interfering RNA (siRNA) technology, and evaluated its effects on invasion-related gene expression, tumor cell in vitro invasion, and metastasis in xenograft nude mice. A549 cells transfected with a plasmid expressing hairpin siRNA for IGF-1R showed a significantly decreased IGF-1R expression at the mRNA level as well as the protein level. In biological assays, transfected A549 cells showed a significant reduction of cell-matrix adhesion, migration and invasion. Consistent with these results, we found that down-regulation of IGR-1R concomitantly accompanied by a large reduction in invasion-related gene expressions, including MMP-2, MMP-9, u-PA, and IGF-1R specific downstream p-Akt. Direct tail vein injections of plasmid expressing hairpin siRNA for IGF-1R significantly inhibited the formation of lung metastases in nude mice. Our results showed the therapeutic potential of siRNA as a method for gene therapy in inhibiting lung cancer invasion and metastasis. 
 Ovarian cancer: Stat3, RhoA and IGF-IR as therapeutic targets. Seeking to improve ovarian cancer therapy, we compared biological characteristics of the moderately-aggressive OVCAR-3 cell line with two highly aggressive ovarian cancer cell populations: the SK-OV-3 cell line, and HASCJ primary cells isolated from the ascitic fluid of a patient with FIGO stage IV ovarian cancer. Secretion of angiogenic factors was not discriminative, whereas   cell invasion   through Matrigel and vasculogenic mimicry were much greater in the more aggressive cells. Among 10 agents tested for their ability to decrease cancer cell aggressivity using these two models, inhibitors of Stat3, IGF-IR and Rho GTPase were found to be the most promising. 
 Matrix metalloproteinase-7 facilitates insulin-like growth factor bioavailability through its proteinase activity on insulin-like growth factor binding protein 3. Matrix metalloproteinase-7 (MMP-7) secreted by cancer cells has been implicated classically in the basement membrane destruction associated with tumor   cell invasion   and metastasis. Recent epidemiologic studies have established a correlation between high levels of circulating insulin-like growth factor (IGF) and low levels of IGF binding protein 3 (IGFBP-3), and relative risk of developing colon, breast, prostate, and lung cancer, which are known to produce MMP-7. In this study, IGFBP-3 was assessed as a candidate for the physiologic substrate of MMP-7. MMP-7 proteolysis generated four major fragments (26 kDa, 17 kDa, 15.5 kDa, and 15.5 kDa), and two cleavage sites were identified: one at the site of hydrolysis of the K(144)-I(145) peptide bond and one at the R(95)-L(96) peptide bond. The former site is different from the previously reported site of cleavage of IGFBP-3 by other proteases. Addition of IGFBP-3 inhibited IGF-I-mediated IGF type 1 receptor (IGF-IR) phosphorylation and activation of the downstream molecule Akt in BALB/c 3T3 fibroblasts overexpressing human IGF-IR (3T3-IGF-IR) and in two human colon cancer cell lines (COLO201 and HT29). Coincubation of the IGF-I/IGFBP-3 complex with MMP-7 restored IGF-I-mediated IGF-IR phosphorylation and activation of Akt in these cell lines. The IGF-I signal recovered by MMP-7 protected against apoptosis induced by anoikis in 3T3-IGF-IR cells. These results indicate that MMP-7 proteolysis of IGFBP-3 plays a crucial role in regulating IGF-I bioavailability, thereby promoting cell survival. This mechanism may contribute to the tumorigenesis of MMP-7-producing IGF-IR-expressing tumors in the primary site and to organ-specific metastasis in a paracrine manner. 
 Clinicopathological significance of the gene expression of matrix metalloproteinase-7, insulin-like growth factor-1, insulin-like growth factor-2 and insulin-like growth factor-1 receptor in patients with colorectal cancer: insulin-like growth factor-1 receptor gene expression is a useful predictor of liver metastasis from colorectal cancer. Matrix metalloproteinase-7 (MMP-7), secreted by cancer cells, has been implicated classically in the basement membrane destruction associated with tumor   cell invasion   and metastasis. Epidemiological studies have established a correlation between high levels of circulating insulin-like growth factor-1 (IGF-1) and the relative risk of colorectal cancer, which is known to produce MMP-7. We examined the clinicopathological significance of the relative expression of MMP-7, IGF-1, IGF-2 and IGF-1 receptor genes in patients with colorectal cancer, especially with regard to metastasis. We studied surgical specimens of cancer tissue and adjacent normal mucosa obtained from 205 patients with untreated colorectal carcinoma. MMP-7, IGF-1, IGF-2, IGF-1R and beta-actin mRNA in cancer tissue and adjacent normal mucosa were measured by quantitative real-time reverse-transcriptase polymerase chain reaction. MMP-7 and IGF-1R gene expression levels were higher in cancer tissue than in adjacent normal mucosa. In contrast, IGF-1 gene expression was lower in cancer tissue than in adjacent normal mucosa. As for the relationship of gene expression to clinicopathological factors, IGF-1R expression correlated with venous invasion and liver metastasis. IGF-1R gene expression is thus considered a useful predictor of liver metastasis from colorectal cancer. 
  GeneRIFs  
 We report that miR-383 was downregulated in gliomas and inversely correlated with glioma pathological grades; we demonstrated that IGF1R expression is critical for miR-383 downregulation-induced   cell invasion  . 
   
  
 TIMP2 
 
  NCBI Gene  
  GeneRIF  
  Pubmed  
 
 
  Search Term  Rank  Fields  
  [Mm]igration  6  PubMed(27); GeneRIFs(6)  
  ECM  7  PubMed(31); GeneRIFs(1)  
  [Ee]xtracellular [Mm]atrix  8  Conserved Domains(1); Process(1); Component(2); Summary(1); PubMed(87); GeneRIFs(4)  
  [Ii]nvasive  10  PubMed(31); GeneRIFs(12)  
  [Mm]etastasize  11  PubMed(1)  
  [Mm]etastasis  12  PubMed(55); GeneRIFs(9)  
  [Cc]ell [Ii]nvasion  13  PubMed(8); GeneRIFs(1)  
 
 
 hide/show details for TIMP2 
  [Mm]igration  PubMed  
 Role of serum concentration of VEGFR1 and TIMP2 on clinical outcome in primary cervical cancer: results of a companion protocol of the randomized, NOGGO-AGO phase III adjuvant trial of simultaneous cisplatin-based radiochemotherapy vs. carboplatin and paclitaxel containing sequential radiotherapy. Aim of the present study was to analyze the expression-profile of IGF1, IGFBP3, sICAM1, sVCAM1, MMP2, MMP9, TIMP2, VEGFA, VEGFD, VEGFC and VEGFR1 in patients with high-risk FIGO-stage Ib-IIb cervical cancer.Serum from 68 cervical cancer patients treated within a phase-III-trial with either simultaneous cisplatin radiochemotherapy or sequential systemic carboplatin and paclitaxel followed by percutaneous irradiation was analyzed by ELISA. Both target expression and correlation with important clinicopathological factors were analyzed following standard statistic procedures.All 68 patients underwent a primary radical hysterectomy with pelvic and/or paraaortic lymphadenectomy. 85.3% of the extirpated tumors had clear surgical margins (R0). Increased levels of VEGFR1, TIMP2 and MMP2 were significantly associated with positive surgical margins (p=0.004, p=0.018 and p=0.004, respectively). High concentration of MMP2 and TIMP2 correlated additionally with an advanced age at time of diagnosis (p=0.001 and p=0.007, respectively). For the cut-off value of 100 pg/ml, an increased VEGFR1 was significantly associated with poor overall (OS) and progression-free (PFS) survival (p=0.017 and p=0.015, respectively). A TIMP2 concentration of lower than 90 ng/ml was significantly associated with poorer OS and PFS (p=0.009 and p=0.043, respectively). In the multivariate analysis, TIMP2 expression in serum was the only independent prognostic factor for OS (p=0.032, HR=6.51, 95% CI=1.17-36.01).Expression-profile of specific biomarkers associated with tumor invasion, cell   migration   and angiogenesis seems to be of prognostic value for both OS and PFS in patients undergoing surgery due to primary cervical cancer. Further analyses are warranted to allow an implementation of such markers into clinical practice. 
 HIV-1-Tat protein promotes chemotaxis and invasive behavior by monocytes. Monocytes are susceptible to HIV infection and to activation by a regulatory gene product of the HIV genome, HIV-Tat. Recently, we have demonstrated that treatment with HIV-Tat up-regulates monocyte adhesion to the endothelium and increases metalloproteinase production. in the present study, we have examined the ability of the HIV-Tat protein to alter the migratory and invasive behavior of monocytes. Monocytes pretreated for 24 h with 10 ng/ml HIV-Tat exhibited enhanced migratory behavior compared with untreated monocytes in chemotaxis assays, both in the absence of a chemoattractant as well as in response to FMLP. in addition, HIV-Tat itself induced the   migration   of both untreated and HIV-Tat pretreated monocytes. Checkerboard analysis showed that monocytes migrated in response to an HIV-Tat concentration gradient, thus confirming the chemotactic characteristics of the HIV-Tat protein. Pretreatment of monocytes with 10 ng/ml HIV-Tat for 24 h also increased their ability to invade reconstituted extracellular membrane (Matrigel)-coated filters by 5-fold in the absence of chemoattractant. The presence of FMLP or HIV-Tat further enhanced invasion by both untreated and HIV-Tat-pretreated monocytes by more than 10-fold. Monocyte invasion was partially inhibited by the inclusion of anti-beta integrin Ab or tissue inhibitor of metalloproteinase (TIMP). Thus, for the first time, we present evidence that HIV-Tat can enhance the chemotactic and invasive behaviors of monocytes and propose an active role for HIV-Tat in the recruitment of monocytes into extravascular tissues, a process which may contribute to the destruction of tissues and cellular architecture often seen in patients with acquired immunodeficiency syndrome. 
 Role of serum concentration of VEGFR1 and TIMP2 on clinical outcome in primary cervical cancer: results of a companion protocol of the randomized, NOGGO-AGO phase III adjuvant trial of simultaneous cisplatin-based radiochemotherapy vs. carboplatin and paclitaxel containing sequential radiotherapy. Aim of the present study was to analyze the expression-profile of IGF1, IGFBP3, sICAM1, sVCAM1, MMP2, MMP9, TIMP2, VEGFA, VEGFD, VEGFC and VEGFR1 in patients with high-risk FIGO-stage Ib-IIb cervical cancer.Serum from 68 cervical cancer patients treated within a phase-III-trial with either simultaneous cisplatin radiochemotherapy or sequential systemic carboplatin and paclitaxel followed by percutaneous irradiation was analyzed by ELISA. Both target expression and correlation with important clinicopathological factors were analyzed following standard statistic procedures.All 68 patients underwent a primary radical hysterectomy with pelvic and/or paraaortic lymphadenectomy. 85.3% of the extirpated tumors had clear surgical margins (R0). Increased levels of VEGFR1, TIMP2 and MMP2 were significantly associated with positive surgical margins (p=0.004, p=0.018 and p=0.004, respectively). High concentration of MMP2 and TIMP2 correlated additionally with an advanced age at time of diagnosis (p=0.001 and p=0.007, respectively). For the cut-off value of 100 pg/ml, an increased VEGFR1 was significantly associated with poor overall (OS) and progression-free (PFS) survival (p=0.017 and p=0.015, respectively). A TIMP2 concentration of lower than 90 ng/ml was significantly associated with poorer OS and PFS (p=0.009 and p=0.043, respectively). In the multivariate analysis, TIMP2 expression in serum was the only independent prognostic factor for OS (p=0.032, HR=6.51, 95% CI=1.17-36.01).Expression-profile of specific biomarkers associated with tumor invasion, cell   migration   and angiogenesis seems to be of prognostic value for both OS and PFS in patients undergoing surgery due to primary cervical cancer. Further analyses are warranted to allow an implementation of such markers into clinical practice. 
 Matrix metalloprotease activity is an essential link between mechanical stimulus and mesenchymal stem cell behavior. Progenitor cells are involved in the regeneration of the musculoskeletal system, which is known to be influenced by mechanical boundary conditions. Furthermore, matrix metalloproteases (MMPs) and tissue-specific inhibitors of metalloproteases (TIMPs) are crucial for matrix remodelling processes that occur during regeneration of bone and other tissues. This study has therefore investigated whether MMP activity affects mesenchymal stem cell (MSC) behavior and how MMP activity is influenced by the mechanical stimulation of these cells. Broad spectrum inhibition of MMPs altered the   migration  , proliferation, and osteogenic differentiation of MSCs. Expression analysis detected MMP-2, -3, -10, -11, -13, and -14, as well as TIMP-2, in MSCs at the mRNA and protein levels. Mechanical stimulation of MSCs led to an upregulation of their extracellular gelatinolytic activity, which was consistent with the increased protein levels seen for MMP-2, -3, -13, and TIMP-2. However, mRNA expression levels of MMPs/TIMPs showed no changes in response to mechanical stimulation, indicating an involvement of post-transcriptional regulatory processes such as alterations in MMP secretion or activation. One potential regulatory molecule might be the furin protease. Specific inhibition of MMP-2, -3, and -13 showed MMP-13 to be involved in osteogenic differentiation. The results of this study suggest that MSC function is controlled by MMP activity, which in turn is regulated by mechanical stimulation of cells. Thus, MMP/TIMP balance seems to play an essential role in transferring mechanical signals into MSC function. Disclosure of potential conflicts of interest is found at the end of this article. 
 NUR77 inhibits the expression of TIMP2 and increases the   migration   and invasion of HTR-8/SVneo cells induced by CYR61. To investigate the function and mechanism of CYR61 on the   migration   and invasion of the trophoblast cell line, HTR-8/SVneo cells.The mRNA and protein levels of NUR77 in the placentas of normal and preeclampsia (PE) women were evaluated using real-time PCR and Western blot, respectively. Paraffin-embedded tissues were processed for localization of NUR77 protein in placental villus by immunohistochemistry. HTR-8/SVneo cells were cultured in the presence of CYR61, Ad-NUR77 or a small interfering RNA for NUR77 (Ad-sinur77). The expression of NUR77 in the HTR-8/SVneo cells was detected and the effects of CYR61 on the   migration   and invasion of HTR-8/SVneo cells were assessed in wound-healing and transwell experiments, respectively. Gelatin zymography was used to measure the MMP2 release in HTR-8/SVneo cells.NUR77 is significantly decreased in the placenta of women with PE compared with the levels during a normal pregnancy. CYR61 can significantly increase the expression of NUR77 in HTR-8/SVneo cells. CYR61, as well as NUR77, can promote HTR-8/SVneo cells   migration   and invasion, which can be blocked by Ad-sinur77. Both CYR61 and Ad-nur77 reduced the mRNA expression of TIMP2 in HTR-8/SVneo cells.CYR61 may promote HTR-8/SVneo cells   migration   and invasion through the upregulation of NUR77, leading to the increase of MMP2 release and the downregulation of TIMP2 expression. 
 MicroRNA-106a targets TIMP2 to regulate invasion and metastasis of gastric cancer. Emerging evidence has shown that microRNA plays an important role in tumor development and progression. Here, we report that miR-106a is frequently up-regulated in gastric cancer tissues and positively correlates with metastasis. Restrained expression of miR-106a in gastric cancer cells significantly reduces their capacity of proliferation,   migration   and invasion. In tissue sections, the positive signal of miR-106a localized in metastasis-associated regions confirmed this result. Moreover, we show that TIMP2 is a direct downstream target for miR-106a and knockdown of TIMP2 strengthens the beneficial effects of miR-106a. Our study adds miR-106a to the complex mechanisms of tumor metastasis. 
 miRNA-221 promotes proliferation,   migration   and invasion by targeting TIMP2 in renal cell carcinoma. MicroRNAs (miRNAs) play important roles in tumorigenesis. In this study, we investigated the role of miR-221 in the development and progression of clear cell renal cell carcinoma (ccRCC).Quantitative real-time PCR (qRT-PCR) was used to measure the expression level of miR-221 in ccRCC tissues and cell lines. Then, we investigated the role of miR-221 to determine its potential roles on renal cancer cell proliferation,   migration   and invasion in vitro. A luciferase reporter assay was conducted to confirm the target gene of miR-221 and the results were validated in renal cancer cells.In the present study, we found that miR-221 was significantly increased in ccRCC tissues and cell lines. Knocked-down expression of miR-221 remarkably inhibited cell proliferation,   migration   and invasion of renal cancer cells. Moreover, at the molecular level, our results suggested that TIMP2 as a direct target of miR-221 through which miR-221 promoted tumor cell proliferation,   migration   and invasion.These findings suggested that miR-221 play an oncogenic role in the renal cancer cell proliferation,   migration   and invasion by directly inhibiting the tumor suppressor TIMP2, indicating miR-221 act as a potential new therapeutic target for the treatment of ccRCC. 
 Transient RNA silencing of tissue factor pathway inhibitor-2 modulates lung cancer cell invasion. Tissue Factor Pathway Inhibitor-2 (TFPI-2) is a potent inhibitor of plasmin which activates metalloproteinases (MMPs) involved in extracellular matrix (ECM) degradation. Its secretion in ECM makes TFPI-2 a potential inhibitor to regulate tumour invasion and metastasis. Moreover, TFPI-2 is frequently downregulated, particularly in aggressive cancers. In this study, we silenced TFPI-2 in the NCI-H460 non-small cell lung cancer cell line and evaluated the role of TFPI-2 in cell invasion and its impact on MMPs expression. As the effects of siRNA are transient, the consequences of both gene silencing and restoration to normal expression could be studied kinetically in the same cells. We showed that TFPI-2 expression by NCI-H460 cells was effectively downregulated using specific small interfering RNA and this silencing was associated with an increase in the invasive potential of tumour cells while   migration   was not affected. We also showed that mRNA levels and protein expression of MMP-2, -3, -9, -14 were not influenced by TFPI-2 silencing. Moreover, the gelatinase activity of MMP-2 and MMP-9 was unmodified. In contrast, MMP-1 mRNA levels and protein were significantly and similarly increased in cells transfected with TFPI-2 siRNA. In conclusion, this study confirms that TFPI-2 downregulation can contribute to tumour invasion of lung cancer cells. 
 Clinical significance of serum levels of matrix metalloproteinase 2 (MMP-2) and its tissue inhibitor (TIMP-2) in gastric cancer. Matrix metalloproteinase 2 (MMP-2) is able to degrade type IV collagen, and thus plays a key role in the   migration   of tumor cells. MMP-2 activity is inhibited by its tissue inhibitor (TIMP-2). The imbalance between MMPs and TIMPs may facilitate progression of cancer cells. The aim of this study was to compare the clinical importance of MMP-2 and TIMP-2 to that of classical tumor markers, namely carcinoembryonic antigen (CEA) and carbohydrate antigen (CA 19-9) in the diagnosis of gastric cancer (GC) by calculating the diagnostic criteria and estimating the levels of MMP-2, TIMP-2, CEA and CA 19-9 in GC patients in relation to clinicopathological features of cancer. We found that serum levels of MMP-2 and TIMP-2 were significantly lower, whereas serum tumor markers were higher, in GC patients than in healthy subjects. Moreover, concentrations of TIMP-2 and CEA correlated with gastric wall infiltration, while CA 19-9 levels correlated with gastric wall infiltration and the presence of nodal metastasis. None of the proteins tested was found to be an independent prognostic factor for GC patients' survival. The percentage of true positive results of TIMP-2 (61%) was higher than those of MMP-2 (54%) and the classical tumor markers CEA (21%) and CA 19-9 (31%). The highest diagnostic sensitivity was observed for the combined use of TIMP-2 with MMP-2 (77%). The results suggest the greater importance of serum MMP-2 and TIMP-2 than of the classical tumor markers CEA and CA 19-9 in the diagnosis of GC. But this issue requires further investigation. 
 mRNA expression of matrix metalloproteinases (MMPs) 2 and 9 and tissue inhibitor of matrix metalloproteinases (TIMPs) 1 and 2 in childhood acute lymphoblastic leukemia: potential role of TIMP1 as an adverse prognostic factor. This study evaluates the mRNA expression profile of genes TIMP1, TIMP2, MMP2 and MMP9 in diagnostic bone marrow samples from 134 consecutive ALL children by real-time quantitative PCR. A significant association was observed between higher expression levels of MMP9 and low risk group and absence of extramedullary infiltration and higher expression levels of TIMP2 and MMP2 with T-ALL. TIMP1 gene expression values higher than the median were associated with a significantly lower 5-year event free-survival in univariable (P=0.04) and multivariable analysis (P=0.01). Our data address new information in the complex interaction of the   migration  /adhesion genes and childhood ALL. 
 Timp-2 binding with cellular MT1-MMP stimulates invasion-promoting MEK/ERK signaling in cancer cells. Both invasion-promoting MT1-MMP and its physiological inhibitor TIMP-2 play a significant role in tumorigenesis and are identified in the most aggressive cancers. Despite its antiproteolytic effects in vitro, clinical data suggest that TIMP-2 expression is positively associated with tumor recurrence, thus emphasizing the wide-ranging role of TIMP-2 in malignancies. To shed light on this role of TIMP-2, we report that low concentrations of TIMP-2, by interacting with MT1-MMP (a specific membrane receptor of TIMP-2), induce the MEK/ERK signaling cascade in fibrosarcoma HT1080 cells which express MT1-MMP naturally. TIMP-2 binding with cell surface-associated MT1-MMP stimulates phosphorylation of MEK1/2, which is upstream of ERK1/2, and the ERK1/2 substrate p90RSK. Consistent with volumes of literature, we confirmed that the activation of ERK stimulated cell   migration  . Both the transcriptional silencing of MT1-MMP and the inhibition of MEK1/2 reversed the signaling effects of TIMP-2/MT1-MMP while the active site-targeting MMP inhibitor GM6001 did not. Our data suggest that both the interactions of TIMP-2 with MT1-MMP, which activate the pro-migratory ERK signaling cascade,and the conventional inhibition of MT1-MMP's catalytic activity by TIMP-2, play a role in the invasion-promoting function of MT1-MMP. The TIMP-2-induced stimulation of ERK signaling in cancer cells explains the direct, as opposed to the inverse, association of TIMP-2 expression with poor prognosis in cancer. 
 Differential growth regulation in human melanoma cell lines by TIMP-1 and TIMP-2. We investigated the role of tissue inhibitor of metalloproteinase (TIMP)-1 and TIMP-2 in the growth regulation. Primary (PM-WK and KHm-4), recurrent primary (RPM-EP and RPM-MC), lymph node metastatic (MM-AN, MM-BP and MM-RU), and a visceral metastatic (MM-LH) melanoma cell lines were used. Reverse transcriptase-coupled polymerase chain reaction and Western blotting revealed that all expressed and produced TIMP-1 and TIMP-2 except for PM-WK, which neither expressed nor produced TIMP-1. TIMP-1 and TIMP-2 secretion levels were measured by enzyme-linked immunosorbent assay in supernatants of cells. We found that the TIMP-1 production level was correlated with the cell   migration   rate. Moreover, TIMP-1 enhanced the cell   migration   of PM-WK. The growth of the primary melanoma cell lines was stimulated by TIMP-1 and inhibited by TIMP-2. In contrast, the growth of the visceral metastatic melanoma cell line was stimulated by TIMP-2. 
 Clinicopathological significance of MMP-2 and its specific inhibitor TIMP-2 in gastric cancer. Matrix metalloproteinases (MMPs) can degrade type IV collagen of extracellular matrices and basal membranes and thus play a key role in the   migration   of malignant cells. In vivo, MMPs are inhibited by tissue inhibitors of metalloproteinases (TIMPs). Since in a previous study we showed that the expression of MMP-2 correlates with clinicopathological parameters in gastric cancer, we have now investigated a possible correlation of MMP-2 and TIMP-2 expression with survival in gastric cancer, as well as the possible association of TIMP-2 with clinicopathological parameters. Tissue samples were obtained from 116 gastric cancer patients who underwent gastrectomy with extended lymphadenectomy. MMP-2 and TIMP-2 expression was analysed using immunohistochemical staining and was graded semiquantitatively (score 0 - 3). High epithelial MMP-2 immunoreactivity was significantly associated with tumor stage and poor survival using the Kaplan-Meier log-rank statistical method (log-rank statistics). However, using Cox regression analysis, high epithelial MMP-2 immunoreactivity was not an independent prognostic factor. TIMP-2 showed no association with survival in gastric cancer, but the intensity of TIMP-2 staining in tumor cells correlated significantly with tumor differentiation based on the WHO and Lauren and Ming classifications, as well as with presence of distant metastasis. Our results show that high epithelial MMP-2 expression in gastric cancer is associated with poor survival, although it is not an independent prognostic factor, and that aggressive forms of gastric cancer are associated with low TIMP-2 expression. 
 In vitro study of matrix metalloproteinase/tissue inhibitor of metalloproteinase production by mesenchymal stromal cells in response to inflammatory cytokines: the role of their   migration   in injured tissues. The transmigratory capacity of bone marrow (BM) mesenchymal stromal cells (MSC) through the endothelial cell barrier into various tissues and their differentiation potential makes them ideal candidates for cell therapy. Nevertheless, the mechanisms and agents promoting their   migration   are not fully understood. We evaluated the effects of several inflammatory cytokines on the   migration   of BM MSC and matrix metalloproteinase (MMP)/tissue inhibitor of metalloproteinase (TIMP) production.The migratory potential of BM MSC was evaluated using a Boyden chamber coated with Matrigel in the presence and absence of stromal cell-derived (SDF)-1alpha, platelet-derived growth factor (PDGF)bb, insulin-like growth factor (IGF)-I and interleukin (IL)-6. The ability of inflammatory cytokines to induce MSC   migration   was tested in presence of their respective Ab or blocking peptide. We used immunofluorescence to check the expression of cytokine receptors, and MMP/TIMP production was analyzed at the protein (human cytokine array, enzyme-linked immunosorbent assay (ELISA), gelatine zymography and Western blot) and mRNA quantitative real-time polymerase chain reaction (qRT-PCR) levels.We have demonstrated that inflammatory cytokines promote the migratory capacity of BM MSC according to the expression of their respective receptors. Higher   migration   through Matrigel was observed in response to IL-6 and PDGFbb. qRT-PCR and cytokine array revealed that   migration   was the result of the variable level of MMP/TIMP in response to inflammatory stimuli.Our observations suggest that chemokines and cytokines involved in the regulation of the immunity or inflammatory process promote the   migration   of MSC into BM or damaged tissues. One of the mechanisms used by MSC to promote their   migration   though the extracellular matrix is modulation of the production of MMP-1, MMP-2, MMP-13, TIMP-1 and TIMP-2. 
 Gravitational unloading induces an anti-angiogenic phenotype in human microvascular endothelial cells. Creating conditions similar to those occurring during exposure of cells to microgravity modulates endothelial functions. We have previously demonstrated that human macrovascular endothelial cells in simulated hypogravity proliferate faster than controls, partly because they downregulate interleukin 1alpha. On the contrary, murine microvascular endothelial cells are growth inhibited in simulated hypogravity, and this is due, at least in part, to the decrease of interleukin 6. Since endothelial cells are very heterogeneous and differences between various species have been reported, we exposed human microvascular cells to gravitational unloading and found that it retards cell growth without affecting cell   migration  . Interestingly, we detected the induction of Tissue Inhibitor of Metalloprotease-2, which inhibits endothelial growth in vitro and angiogenesis in vivo. Together with the finding that hypogravity stimulates the synthesis of nitric oxide, involved also in neovascularization, our results underscore a modulation of the angiogenic properties of microvascular human endothelial cells. We also show that hypogravity inhibits proteasome activity, thus suggesting that post-translational mechanisms are involved in the adaptations of these cells to hypogravity. These results underscore that hypogravity differently impacts on micro- and macro-vascular human endothelial cells. In particular, these results may shed some light on the molecular mechanisms contributing to the impairment of angiogenesis observed in different models in space. Our data might also explain why bioengineered tissues to be used for regenerative medicine fail to neovascularize when assembled in simulated hypogravity. 
 Reduced TIMP-2 in hypoxia enhances angiogenesis. Hypoxia, which characterizes ischemia, trauma, inflammation, and solid tumors, recruits monocytes, immobilizes them, and alters their function, leading to an anti-inflammatory and proangiogenic phenotype. Monocyte extravasation from the circulation and their   migration   in tissues are partially mediated by the balance between matrix metalloproteinases (MMPs) and their tissue inhibitors (TIMPs). The mechanisms evoked by hypoxia that regulate monocyte   migration   and activation are not entirely clear. Specifically, the effect of hypoxia on TIMPs in these cells has hardly been investigated. We show that hypoxia reduces TIMP-2 secretion from human primary monocytes and from the monocyte-like cell lines U937 and THP-1 by three- to fourfold (P < 0.01), by inhibiting TIMP-2 transcription through mechanisms that involve the transcription factor SP-1. Hypoxia also lowers TIMP-2 protein secretion from human endothelial cells (by 2-fold, P < 0.05). TIMP-2 levels do not influence the reduced   migration   of THP-1 cells in hypoxia; however, low TIMP-2 levels enhance endothelial cell   migration  /proliferation, their ability to form tubelike structures in vitro, and the appearance of mature blood vessels in a Matrigel plug assay in vivo. Thus we conclude that reduced TIMP-2 levels secreted from both hypoxic monocytes and endothelial cells are proangiogenic. 
 Lysophosphatidic acid downregulates tissue inhibitor of metalloproteinases, which are negatively involved in lysophosphatidic acid-induced cell invasion. Ovarian cancer is a highly metastatic disease. Lysophosphatidic acid (LPA) levels are elevated in ascites from ovarian cancer patients, but its potential role in ovarian cancer metastasis has just begun to be revealed. In this work, we show that LPA stimulates invasion of primary ovarian cancer cells, but not ovarian epithelial or borderline ovarian tumor cells, although these benign cells indeed respond to LPA in cell   migration  . We have found that LPA downregulates tissue inhibitor of metalloproteinases (TIMPs). TIMP2 and TIMP3 play functional role in LPA-induced invasion as negative regulators. G(i) protein, phosphatidylinositol-3 kinase (PI3K), p38 mitogen-activated protein kinase (MAPK), cytosolic phospholipase A(2) and urokinase type plasminogen activator (uPA) are required for LPA-induced cells invasion. TIMP3 may affect two independent downstream targets, vascular endothelial growth factor receptor and p38 MAPK. In vivo, LPA stimulates tumor metastasis in an orthotopic ovarian tumor model, which can be inhibited by a PI3K inhibitor, LY294002. In summary, LPA is likely a key component for promoting ovarian metastasis in vivo. LPA downregulates TIMP3, which may have targets other than metalloproteinases. Our in vivo metastasis mouse model is useful for studying the efficacy of therapeutic regimes of ovarian cancer. 
 TIMP-2 promotes cell spreading and adhesion via upregulation of Rap1 signaling. We previously demonstrated that TIMP-2 treatment of human microvascular endothelial cells (hMVECs) activates Rap1 via the pathway of paxillin-Crk-C3G. Here, we show that TIMP-2 overexpression in hMVECs by adenoviral infection enhances Rap1 expression, leading to further increase in Rap1-GTP. TIMP-2 expression, previously reported to inhibit cell   migration  , also leads to cell spreading accompanied with increased cell adhesion. HMVECs stably expressing Rap1 display a similar phenotype as hMVECs-TIMP-2, whereas the expression of inactive Rap1 mutant, Rap1(38N), leads to elongated appearance with greatly reduced cell adhesion. Furthermore, the phenotype of hMVECs-Rap1(38N) was not reversed by TIMP-2 overexpression. TIMP-2 greatly promotes the association of Rap1 with actin. Therefore, these findings suggest that TIMP-2 mediated alteration in cell morphology requires Rap1, TIMP-2 may recruit Rap1 to sites of actin cytoskeleton remodeling necessary for cell spreading, and enhanced cell adhesion by TIMP-2 expression may hinder cell   migration  . 
 Comparative study of the immunohistochemical expression of tissue inhibitors of metalloproteinases 1 and 2 between clearly invasive carcinomas and "in situ" trophoblast invasion. Tissue inhibitors of metalloproteinases (TIMPs) play an important role in extracellular matrix homeostasis by regulating MMP activity. Although they were initially considered inhibitors of tumor growth and metastasis, recently their role in cancer progression has been controversial. The aim of our study was to compare the immunohistochemical expression of TIMP1 and TIMP2 between an uncontrollably invasive phenomenon (cancer) and an "in situ" process (trophoblast invasion) in an effort to assess any differential role of these molecules between these two distinct phenomena and therefore to understand better their contribution in cancer invasion and   migration  . We performed an immunohistochemical analysis of 50 carcinomas (colorectal, gastric, breast, pulmonary, and renal) and 40 first trimester gestations. The marker expression was evaluated semiquantitatively, separately in cancer parenchymal and trophoblastic cells as well as in malignant stromal and decidual cells, according to a percentage scale (0, <10, 10-50, and >50%) and according to staining intensity (0, +, ++, and +++). Our results showed that there was no statistically significant difference in TIMP1 expression between cancer parenchymal cells and trophoblastic cells. On the other hand, TIMP1 was expressed more often in decidual cells than in cancer stromal cells. Immunostaining for TIMP2 was more extensive and intense both in trophoblastic and decidual cells than in cancer parenchymal and stromal cells, respectively. The reduced expression of TIMP2 in metastatic carcinomas by comparison with non-metastatic gestation specimens underlines its importance in cancer invasion and   migration  . On the other hand, TIMP1 was more expressed in decidua than cancer stroma, but at the same time showed no statistically significant difference between cancer parenchyma and trophoblasts, highlighting its multifunctional activity in cancer progression. 
 Tissue inhibitor of metalloproteinases-2 binding to membrane-type 1 matrix metalloproteinase induces MAPK activation and cell growth by a non-proteolytic mechanism. Membrane-type 1 matrix metalloproteinase (MT1-MMP), a transmembrane proteinase with a short cytoplasmic domain and an extracellular catalytic domain, controls a variety of physiological and pathological processes through the proteolytic degradation of extracellular or transmembrane proteins. MT1-MMP forms a complex on the cell membrane with its physiological protein inhibitor, tissue inhibitor of metalloproteinases-2 (TIMP-2). Here we show that, in addition to extracellular proteolysis, MT1-MMP and TIMP-2 control cell proliferation and   migration   through a non-proteolytic mechanism. TIMP-2 binding to MT1-MMP induces activation of ERK1/2 by a mechanism that does not require the proteolytic activity and is mediated by the cytoplasmic tail of MT1-MMP. MT1-MMP-mediated activation of ERK1/2 up-regulates cell   migration   and proliferation in vitro independently of extracellular matrix proteolysis. Proteolytically inactive MT1-MMP promotes tumor growth in vivo, whereas proteolytically active MT1-MMP devoid of cytoplasmic tail does not have this effect. These findings illustrate a novel role for MT1-MMP-TIMP-2 interaction, which controls cell functions by a mechanism independent of extracellular matrix degradation. 
 Shp-1 mediates the antiproliferative activity of tissue inhibitor of metalloproteinase-2 in human microvascular endothelial cells. The tissue inhibitors of metalloproteinases (TIMPs) regulate matrix metalloproteinase activity required for cell   migration  /invasion associated with cancer progression and angiogenesis. TIMPs also modulate cell proliferation in vitro and angiogenesis in vivo independent of their matrix metalloproteinase inhibitory activity. Here, we show that TIMP-2 mediates G1 growth arrest in human endothelial cells through de novo synthesis of the cyclin-dependent kinase inhibitor p27Kip1. TIMP-2-mediated inhibition of Cdk4 and Cdk2 activity is associated with increased binding of p27Kip1 to these complexes in vivo. Protein-tyrosine phosphatase inhibitors or expression of a dominant negative Shp-1 mutant ablates TIMP-2 induction of p27Kip1. Finally, angiogenic responses to fibroblast growth factor-2 and vascular endothelial growth factor-A in "motheaten viable" Shp-1-deficient mice are resistant to TIMP-2 inhibition, demonstrating that Shp-1 is an important negative regulator of angiogenesis in vivo. 
 CAPN 7 promotes the   migration   and invasion of human endometrial stromal cell by regulating matrix metalloproteinase 2 activity. Matrix metalloproteinase 2 (MMP-2) has been reported to be an important regulator of cell   migration   and invasion through degradation of the extracellular matrix (ECM) in many diseases, such as cancer and endometriosis. Here, we found calcium-activated neutral protease 7 (CAPN 7) expression was markedly upregulated in the eutopic endometrium and endometrial stromal cells of women diagnosed with endometriosis. Our studies were carried out to detect the effects of CAPN 7 on human endometrial stromal cell (hESC)   migration   and invasion.Western blotting and quantitative real-time PCR were used to detect the expression of CAPN 7 in endometriosis patients and normal fertile women. Scratch-wound-healing and invasion chamber assay were used to investigate the role of CAPN 7 in hESC   migration   and invasion. Western blotting, quantitative real-time PCR and zymography were carried out to detect the effect of CAPN 7 on the expressions and activity of MMP-2.CAPN 7 was markedly up-regulated in endometriosis, thereby promoting the   migration   and invasion of hESC. CAPN 7 overexpression led to increased expression of MMP-2 and tissue inhibitor of metalloproteinases 2 (TIMP-2); CAPN 7 knockdown reversed these changes. CAPN 7 increased MMP-2 activity by increasing the ratio of MMP-2 to TIMP-2. We also found that OA-Hy (an MMP-2 inhibitor) decreased the effects of CAPN 7 overexpression on hESC   migration   and invasion by approximately 50% and 55%, respectively. Additionally, a coimmunoprecipitation assay demonstrated that CAPN 7 interacted with activator protein 2α (AP-2α): an important transcription factor of MMP-2.CAPN 7 promotes hES  C migrati  on and invasion by increasing the activity of MMP-2 via an increased ratio of MMP-2 to TIMP-2. 
 Differential regulation of membrane type 1-matrix metalloproteinase activity by ERK 1/2- and p38 MAPK-modulated tissue inhibitor of metalloproteinases 2 expression controls transforming growth factor-beta1-induced pericellular collagenolysis. Acquisition of matrix metalloproteinase-2 (MMP-2) activity is temporally associated with increased   migration   and invasiveness of cancer cells. ProMMP-2 activation requires multimolecular complex assembly involving proMMP-2, membrane type 1-MMP (MT1-MMP, MMP-14), and tissue inhibitor of metalloproteinases-2 (TIMP-2). Because transforming growth factor-beta1 (TGF-beta1) promotes tumor invasion in advanced squamous cell carcinomas, the role of TGF-beta1 in the regulation of MMP activity in a cellular model of invasive oral squamous cell carcinoma was examined. Treatment of oral squamous cell carcinoma cells with TGF-beta1 promoted MMP-dependent cell scattering and collagen invasion, increased expression of MMP-2 and MT1-MMP, and enhanced MMP-2 activation. TGF-beta1 induced concomitant activation of ERK1/2 and p38 MAPK, and kinase inhibition studies revealed a negative regulatory role for ERK1/2 in modulating acquisition of MMP-2 activity. Thus, a reciprocal effect on proMMP-2 activation was observed whereupon blocking ERK1/2 phosphorylation promoted proMMP-2 activation and MT1-MMP activity, whereas inhibiting p38 MAPK activity decreased proteolytic potential. The cellular mechanism for the control of MT1-MMP catalytic activity involved concurrent reciprocal modulation of TIMP-2 expression by ERK1/2 and p38 MAPKs, such that inhibition of ERK1/2 phosphorylation decreased TIMP-2 production, and down-regulation of p38 MAPK activity enhanced TIMP-2 synthesis. Further, p38 MAPK inhibition promoted ERK1/2 phosphorylation, providing additional evidence for cross-talk between MAPK pathways. These observations demonstrate the complex reciprocal effects of ERK1/2 and p38 MAPK in the regulation of MMP activity, which could complicate the use of MAPK-specific inhibitors as therapeutic agents to down-regulate the biologic effects of TGF-beta1 on pericellular collagen degradation and tumor invasion. 
 Mesenchymal stem cells inhibit breast cancer cell   migration   and invasion through secretion of tissue inhibitor of metalloproteinase-1 and -2. Mesenchymal stem cells (MSCs) form part of tumor stroma, and are typically considered to be pro-tumorigenic, promoting continuing tumor growth and metastasis. Here, we describe a mechanism by which MSCs may be anti-tumorigenic, through inhibition of breast cancer cell   migration   and invasion, an important part of metastasis. MDA-MB-231 and T47D cells were co-cultured in a Transwell insert above MSCs or MSC conditioned media and their   migration   or invasion through Matrigel measured. The conditioned media was found to inhibit breast cancer cell movement. TIMP-1 and TIMP-2, inhibitors of matrix metalloproteinases (MMPs), were identified as candidates for this inhibition and enhanced secretion of MMPs was not sufficient to counter the anti-migratory effects of TIMP expression. Inhibition of TIMP activity showed that TIMP-1 and TIMP-2 together were largely responsible for the reduction of   migration   and invasion by MSCs. Therefore, MSCs may play anti-tumorigenic, anti-metastatic roles in tumor development, with the overall outcome dependent upon the balance of pro-and anti-tumorigenic molecules secreted. 
 mRNA expressions of TIMP-1, -2, and -3 and 92-KD type IV collagenase in early human placenta and decidual membrane as studied by in situ hybridization. Cytotrophoblasts of early placenta invade the decidual membrane, gestational endometrium, and spiral arteries during early pregnancy. Unlike tumor invasion, this physiological invasion is well controlled, although its molecular mechanisms are largely unknown. We have previously shown that cytotrophoblasts synthesize significant mRNAs for 72-KD Type IV collagenase, laminin, and Type IV collagen, proteins implicated in extracellular matrix turnover and   migration  . In this study we used in situ hybridization and immunohistochemistry to investigate the mRNA expression pattern of 92-KD Type IV collagenase and the matix metalloproteinase inhibitors TIMP-1, TIMP-2, and TIMP-3 in early human placenta and decidual membrane. mRNAs for 92-KD Type IV collagenase, TIMP-1, TIMP-2, and TIMP-3 were found in the cells of cytotrophoblastic columns, the endothelial and fibroblastic stromal cells of villi, and the large decidualized cells of decidual membrane. TIMP-1 expression was notably accentuated in the fibroblasts of fibrotic villi. In the decidual membrane, the signals for 92-KD Type IV collagenase and TIMP-1 mRNA were particularly strong around the glandular structures. The trophoblastic epithelium of villi and the epithelial cells of decidual glands showed a signal for 92-KD Type IV collagenase and TIMP-2, but not for TIMP-1 or TIMP-3. The coincidental expression of the proteolytic 92-KD Type IV collagenase and inhibitors TIMP-1, TIMP-2, and TIMP-3 generally in the same cells suggests that the activity of 92-KD Type IV collagenase, which is regulated by TIMPs, plays an important role in placental tissue organization and in the invasion of trophoblastic cells into the uterine wall. 
 TIMP-2 Interaction with MT1-MMP Activates the AKT Pathway and Protects Tumor Cells from Apoptosis. Membrane-type 1 matrix metalloproteinase (MT1-MMP), a transmembrane proteinase with an extracellular catalytic domain and a short cytoplasmic tail, degrades a variety of extracellular matrix (ECM) components. In addition, MT1-MMP activates intracellular signaling through proteolysis-dependent and independent mechanisms. We have previously shown that binding of tissue inhibitor of metalloproteinases-2 (TIMP-2) to MT1-MMP controls cell proliferation and   migration  , as well as tumor growth in vivo by activating the Ras-extracellular signal regulated kinase-1 and -2 (ERK1/2) pathway through a mechanism that requires the cytoplasmic but not the proteolytic domain of MT1-MMP. Here we show that in MT1-MMP expressing cells TIMP-2 also induces rapid and sustained activation of AKT in a dose- and time-dependent manner and by a mechanism independent of the proteolytic activity of MT1-MMP. Fibroblast growth factor receptor-1 mediates TIMP-2 induction of ERK1/2 but not of AKT activation; however, Ras activation is necessary to transduce the TIMP-2-activated signal to both the ERK1/2 and AKT pathways. ERK1/2 and AKT activation by TIMP-2 binding to MT1-MMP protects tumor cells from apoptosis induced by serum starvation. Conversely, TIMP-2 upregulates apoptosis induced by three-dimensional type I collagen in epithelial cancer cells. Thus, TIMP-2 interaction with MT1-MMP provides tumor cells with either pro- or anti-apoptotic signaling depending on the extracellular environment and apoptotic stimulus. 
 Norcantharidin enhances TIMP‑2 anti‑vasculogenic mimicry activity for human gallbladder cancers through downregulating MMP‑2 and MT1‑MMP. Vasculogenic mimicry (VM) is a tumor microcirculation pattern in highly aggressive gallbladder cancers (GBCs). We recently reported the anti‑VM activity of norcantharidin (NCTD) in highly aggressive GBC‑SD cells and xenografts. In this study, we further investigated that NCTD enhanced tissue inhibitor of matrix metalloproteinase‑2 (TIMP‑2) anti‑VM activity for GBCs and the underlying mechanisms. In vivo and in vitro experiments were performed to determine the effects of NCTD in combination with TIMP‑2 on tumor growth, host survival, VM formation, hemodynamic of GBC‑SD xenografts, and VM‑like networks and malignant phenotypes of GBC‑SD cells. Expression of matrix metalloproteinase (MMP)‑2 and membrane type 1‑MMP (MT1‑MMP) among human GBCs, GBC‑SD cells and xenografts were determined, respectively. The results showed that expression of MMP‑2 and MT1‑MMP in human GBCs, GBC‑SD cells and xenografts was significantly related to VM in GBCs; a shorter survival time of VM‑positive patients with high expression of MMP‑2 or MT1‑MMP compared to that of the patients with low expression. After treatment with NCTD+TIMP‑2, tumor growth, VM formation, VM hemodynamic of the xenografts in vivo were significantly inhibited as compared to control, NCTD or TIMP‑2 group, with a prolonged survival time of the xenograft mice (log‑rank test, P=0.0115); and these observations were confirmed by VM‑like networks by 3‑D matr  ices and   showed that proliferation, apoptosis, invasion, migration of GBC‑SD cells in vitro were markedly affected. Furthermore, expression of MMP‑2 and MT1‑MMP in VM formation of the xenografts in vivo and GBC‑SD cells in vitro was downregulated as compared to control, NCTD or TIMP‑2 group. Thus, we concluded that NCTD enhances TIMP‑2 antitumor and anti‑VM activities in GBCs through downregulating MMP‑2 and MT1‑MMP. 
  GeneRIFs  
 Suggest that miR-221 plays an oncogenic role in renal cancer cell proliferation,   migration   and invasion by directly inhibiting the tumor suppressor TIMP2. 
 Inhibition of TIMP activity showed that TIMP-1 and TIMP-2 together were largely responsible for the reduction of   migration   and invasion by MSCs. 
 Data indicate that CAPN 7 promotes endometrial stromal cell (hESC)   migration   and invasion by increasing the activity of MMP-2 via an increased ratio of MMP-2 to TIMP-2. 
 cysteine-rich angiogenic inducer 61 may promote trophoblast cell   migration   and invasion through upregulation of NUR77 protein leading to the increase of matrix metalloproteinase 2(MMP2) release and the downregulation of tissue inhibitor of MMP2 expression 
 The   migration   of bone marrow (BM) mesenchymal stromal cells was the result of the variable level of MMP/TIMP in response to inflammatory stimuli. 
 TIMP-2 mediated alteration in cell morphology requires Rap1, TIMP-2 may recruit Rap1 to sites of actin cytoskeleton remodeling necessary for cell spreading, and enhanced cell adhesion by TIMP-2 expression may hinder cell   migration   
  ECM  PubMed  
 Knockdown of glucose-regulated protein 78 decreases the invasion, metalloproteinase expression and   ECM   degradation in hepatocellular carcinoma cells. We have reported previously that overexpression of glucose-regulated protein 78 (GRP78) promotes the invasion of hepatocellular carcinoma. However, whether GRP78 knockdown affects the extracellular matrix degradation has not been elucidated. Here we are going to determine whether GRP78 knockdown affect the   ECM   degradation and the role of MMP-2 and MMP-9 in these process in hepatocellular carcinoma cells.Human hepatocellular carcinoma cell line SMMC7721 and HepG2 were cultured in DMEM supplemented with 10% FBS, RT-PCR and western blot were used to detect the endogenous expression of GRP78, MMP-2, MMP-9 and TIMP-2 in SMMC7721 and HepG2. GRP78 shRNAs were transfected using lipofection2000. Transwell assay and wound healing assay were used to analyze the invasion of each transfectant. Gelatin zymography and FITC-gelatin degradation assay were employed to investigate the capabilities of   ECM   degradation of each transfectant. MTT assay was used to determine the proliferation status. Western blot was employed to detect the expression of matrix metalloproteinase 2(MMP-2), MMP-9, MMP-14, and tissue inhibitor of metalloproteinases 2(TIMP-2), focal adhesion kinase (FAK), ERK1/2, JNK and Src.According to the expression levels of GRP78, MMP-2, MMP-9, MMP-14 and TIMP-1 in hepatocellular carcinoma cell lines SMMC7721 and hepG2, we used SMMC7721 as the in vitro invasion model for further functional analysis. Using this model, we found that GRP78 knockdown decreased the invasion of tumor cells, and this inhibitory effect was independent of cell proliferation. In hepatocellular carcinoma cells, Grp78 knockdown inhibited   ECM   degradation and the decreased activity and expression of MMP-2, but not MMP-9 contributed largely to this impact. Further analysis revealed that the decreased activity and expression of MMP-2 is mediated by JNK.Knockdown of GRP78 decreases   ECM   degradation, and downregulates the expression and activity of MMP-2 and TIMP-2. These results further demonstrate that GRP78 is a potential target for inhibiting the invasion of hepatocellular carcinoma cells. 
 Knockdown of glucose-regulated protein 78 decreases the invasion, metalloproteinase expression and   ECM   degradation in hepatocellular carcinoma cells. We have reported previously that overexpression of glucose-regulated protein 78 (GRP78) promotes the invasion of hepatocellular carcinoma. However, whether GRP78 knockdown affects the extracellular matrix degradation has not been elucidated. Here we are going to determine whether GRP78 knockdown affect the   ECM   degradation and the role of MMP-2 and MMP-9 in these process in hepatocellular carcinoma cells.Human hepatocellular carcinoma cell line SMMC7721 and HepG2 were cultured in DMEM supplemented with 10% FBS, RT-PCR and western blot were used to detect the endogenous expression of GRP78, MMP-2, MMP-9 and TIMP-2 in SMMC7721 and HepG2. GRP78 shRNAs were transfected using lipofection2000. Transwell assay and wound healing assay were used to analyze the invasion of each transfectant. Gelatin zymography and FITC-gelatin degradation assay were employed to investigate the capabilities of   ECM   degradation of each transfectant. MTT assay was used to determine the proliferation status. Western blot was employed to detect the expression of matrix metalloproteinase 2(MMP-2), MMP-9, MMP-14, and tissue inhibitor of metalloproteinases 2(TIMP-2), focal adhesion kinase (FAK), ERK1/2, JNK and Src.According to the expression levels of GRP78, MMP-2, MMP-9, MMP-14 and TIMP-1 in hepatocellular carcinoma cell lines SMMC7721 and hepG2, we used SMMC7721 as the in vitro invasion model for further functional analysis. Using this model, we found that GRP78 knockdown decreased the invasion of tumor cells, and this inhibitory effect was independent of cell proliferation. In hepatocellular carcinoma cells, Grp78 knockdown inhibited   ECM   degradation and the decreased activity and expression of MMP-2, but not MMP-9 contributed largely to this impact. Further analysis revealed that the decreased activity and expression of MMP-2 is mediated by JNK.Knockdown of GRP78 decreases   ECM   degradation, and downregulates the expression and activity of MMP-2 and TIMP-2. These results further demonstrate that GRP78 is a potential target for inhibiting the invasion of hepatocellular carcinoma cells. 
 TIMP1, TIMP2, and TIMP4 are increased in aqueous humor from primary open angle glaucoma patients. Elevated intraocular pressure (IOP) is the only known modifiable risk factor for primary open angle glaucoma (POAG), and it can be caused by reduced aqueous humor outflow from the anterior chamber. Outflow is predominantly regulated by the trabecular meshwork, consisting of specialized cells within a complex extracellular matrix (  ECM  ). An imbalance between   ECM  -degrading matrix metalloproteinases (MMPs) and the tissue inhibitors of MMPs (TIMPs) within the trabecular meshwork is thought to contribute to POAG. This study aimed to quantify levels of TIMPs and MMPs in aqueous humor samples from glaucomatous and non-glaucomatous eyes, analyze MMP/TIMP ratios, and correlate results with age, IOP, and Humphrey's visual field pattern standard deviation (PSD).Aqueous humor samples were collected from 26 non-glaucomatous control subjects before cataract surgery and 23 POAG patients undergoing trabeculectomy or cataract surgery. Analyte concentrations were measured using multiplexed immunoassays. Statistical significance was assessed with Mann-Whitney U tests, and Spearman's method was used to assess correlations with age, IOP, and PSD.Concentrations of TIMP1 (p = 0.0008), TIMP2 (p = 0.002), TIMP4 (p = 0.002), and MMP2 (p = 0.020) were significantly increased in aqueous humor samples from POAG versus cataract samples. For the majority of MMP/TIMP molar ratios calculated for the cataract group, TIMPs outweighed MMPs. In POAG, molar ratios of MMP2/TIMP1 (p = 0.007) and MMP9/TIMP1 (p = 0.005) showed a significant decrease, corresponding to an elevated excess of TIMPs over MMPs in POAG compared to cataract samples. Conversely, MMP2/TIMP3 (p = 0.045) and MMP3/TIMP3 (p = 0.032) molar ratios increased. Several MMP/TIMP molar ratios correlated with IOP (r = 0.476-0.609, p = 0.007-0.034) and PSD (r = -0.482 to -0.655, p = 0.005-0.046) in POAG samples and with age in cataract control samples.An imbalance among MMPs and TIMPs was found in glaucomatous aqueous humor samples, with a shift toward raised TIMP levels. This may result in the inhibition of MMP activity, leading to an altered   ECM   composition in the TM and thereby contributing to increased outflow resistance. 
 May High MMP-2 and TIMP-2 Expressions Increase or Decrease the Aggressivity of Oral Cancer? Matrix metalloproteinases-2 (MMP-2) and the tissue inhibitor of metalloproteinase-2 (TIMP-2), may presumably have an important role on the invasion and metastatic spread of malignancies attributed to an uncontrolled degradation of the extracellular matrix (  ECM  ). A retrospective chart analysis was carried out to study the expression of MMP-2 and TIMP-2 on the archival samples of oral squamous cell carcinoma (OSCC) (n = 30) and normal mucosa (n = 10) by immunohistochemistry and compared with the clinicopathologic parameters of cases. Both MMP-2 and TIMP-2 expressions showed a positive correlation with the grades, stages and metastatic capacities of tumors (Spearman's correlation, p < 0.05). Concomitant increase in the expression of TIMP-2 and MMP-2 suggested that the rate of MMP-2/TIMP-2 expression is a better marker for characterization of MMP-2 concentration. High expression and/or activity of MMP-2 were linked with poorer survival in OSCC cases, while TIMPs have been shown to apparently act as either growth-stimulating or suppressor factors for tumors. It was also revealed that MMP-2 and TIMP-2 were secreted by both tumor cells and stromal cells. A new concept, supposing the dynamic, anticancer partnership between the residual genome stabilizer machinery of tumor cells and defensive cells adjacent to tumors, may illuminate the controversial results. In conclusion, the stronger the infiltrative and metastatic capacity of cancers, the higher is the rate of MMP-2/TIMP-2 expression helping the arrival of humoral and cellular anticancer forces. 
 [Expression of matrix metalloproteinase-2, 9 and their inhibitor-TIMP 1,2 in human squamous cell carcinoma of uterine cervix]. Studies of tumor invasion and metastasis have focused on the degradation of extracellular matrix (  ECM  ). Matrix metalloproteinases (MMPs), which could degrade   ECM  , were implicated in cancer invasion and metastasis. Activated MMPs were controlled by specific tissue inhibitors of metalloproteinases (TIMPs). This study was designed to investigate the expression of MMP-2, MMP-9 and TIMP-1, TIMP-2 in human squamous cell carcinomas of uterine cervix, and the association with invasion and metastasis of human squamous cell carcinoma of uterine cervix.Tissue samples from 40 cases of squamous cell carcinoma of the uterine cervix, 29 cases of cervical intraepithelial neoplasia (CIN), and 16 cases of normal cervices were stained immunohistochemically.Immunohistochemical staining of tumor cells for MMP-2, MMP-9, TIMP-1, and TIMP-2 were noted 82.5%, 70.0%, 62.5%, and 97.5% in carcinomas, 44.8%, 62.1%, 86.21%, and 96.55% in CIN lesions respectively, for MMP-2, MMP-9, TIMP-1 in 25.0% normal cervices, but for TIMP-2 in 56.25% normal cervices. There was significant difference for MMP-2 among carcinomas, normal cervices, and CIN respectively (P < 0.01). There was significant difference for MMP-9 among normal cervices, carcinomas, and CIN, respectively (P < 0.05). The positive rates of TIMP-1 and TIMP-2 in the cervical carcinoma group and CIN group were obviously higher than that in normal cervical group(P < 0.01). The positive rates of MMP-2 and MMP-9 in lymph node metastasis were obviously higher than that in without lymph node metastasis(P < 0.01), but for TIMP-2 was contrary and there was no significant difference for TIMP-1. No significant correlation could be established among the expression of these markers and the tumor diameter, the clinical stage, and pathologic grade.The over-expression of MMP-2, MMP-9, TIMP-1, and TIMP-2 may play a key role in invasion and lymph-node metastasis of in squamous carcinoma of the cervix. 
 HPV16 oncoproteins induce MMPs/RECK-TIMP-2 imbalance in primary keratinocytes: possible implications in cervical carcinogenesis. Cervical cancer is the third most common cancer in women worldwide. Persistent infection with high-risk HPV types, principally HPV16 and 18 is the main risk factor for the development of this malignancy. However, the onset of invasive tumor occurs many years after initial exposure in a minority of infected women. This suggests that other factors beyond viral infection are necessary for tumor establishment and progression. Tumor progression is characterized by an increase in secretion and activation of matrix metalloproteinases (MMPs) produced by either the tumor cells themselves or tumor-associated fibroblasts or macrophages. Increased MMPs expression, including MMP-2, MMP-9 and MT1-MMP, has been observed during cervical carcinoma progression. These proteins have been associated with degradation of   ECM   components, tumor invasion, metastasis and recurrence. However, few studies have evaluated the interplay between HPV infection and the expression and activity of MMPs and their regulators in cervical cancer. We analyzed the effect of HPV16 oncoproteins on the expression and activity of MMP-2, MMP-9, MT1-MMP, and their inhibitors TIMP-2 and RECK in cultures of human keratinocytes. We observed that E7 expression is associated with increased pro-MMP-9 activity in the epithelial component of organotypic cultures, while E6 and E7 oncoproteins co-expression down-regulates RECK and TIMP-2 levels in organotypic and monolayers cultures. Finally, a study conducted in human cervical tissues showed a decrease in RECK expression levels in precancer and cancer lesions. Our results indicate that HPV oncoproteins promote MMPs/RECK-TIMP-2 imbalance which may be involved in HPV-associated lesions outcome. 
 Transient RNA silencing of tissue factor pathway inhibitor-2 modulates lung cancer cell invasion. Tissue Factor Pathway Inhibitor-2 (TFPI-2) is a potent inhibitor of plasmin which activates metalloproteinases (MMPs) involved in extracellular matrix (  ECM  ) degradation. Its secretion in   ECM   makes TFPI-2 a potential inhibitor to regulate tumour invasion and metastasis. Moreover, TFPI-2 is frequently downregulated, particularly in aggressive cancers. In this study, we silenced TFPI-2 in the NCI-H460 non-small cell lung cancer cell line and evaluated the role of TFPI-2 in cell invasion and its impact on MMPs expression. As the effects of siRNA are transient, the consequences of both gene silencing and restoration to normal expression could be studied kinetically in the same cells. We showed that TFPI-2 expression by NCI-H460 cells was effectively downregulated using specific small interfering RNA and this silencing was associated with an increase in the invasive potential of tumour cells while migration was not affected. We also showed that mRNA levels and protein expression of MMP-2, -3, -9, -14 were not influenced by TFPI-2 silencing. Moreover, the gelatinase activity of MMP-2 and MMP-9 was unmodified. In contrast, MMP-1 mRNA levels and protein were significantly and similarly increased in cells transfected with TFPI-2 siRNA. In conclusion, this study confirms that TFPI-2 downregulation can contribute to tumour invasion of lung cancer cells. 
 The role of EZH2 in the regulation of the activity of matrix metalloproteinases in prostate cancer cells. Degradation of the extracellular matrix (  ECM  ), a critical step in cancer metastasis, is determined by the balance between MMPs (matrix metalloproteinases) and their inhibitors TIMPs (tissue inhibitors of metalloproteinases). In cancer cells, this balance is shifted towards MMPs, promoting   ECM   degradation. Here, we show that EZH2 plays an active role in this process by repressing the expression of TIMP2 and TIMP3 in prostate cancer cells. The TIMP genes are derepressed by knockdown of EZH2 expression in human prostate cancer cells but repressed by overexpression of EZH2 in benign human prostate epithelial cells. EZH2 catalyzes H3K27 trimethylation and subsequent DNA methylation of the TIMP gene promoters. Overexpression of EZH2 confers an invasive phenotype on benign prostate epithelial cells; however, this phenotype is suppressed by cooverexpression of TIMP3. EZH2 knockdown markedly reduces the proteolytic activity of MMP-9, thereby decreasing the invasive activity of prostate cancer cells. These results suggest that the transcriptional repression of the TIMP genes by EZH2 may be a major mechanism to shift the MMPs/TIMPs balance in favor of MMP activity and thus to promote   ECM   degradation and subsequent invasion of prostate cancer cells. 
 siRNA knockdown of tissue inhibitor of metalloproteinase-1 in keloid fibroblasts leads to degradation of collagen type I. Keloids are defined as overgrowths of scar tissue resulting from abnormal wound healing. They are characterized by excessive dermal deposition of thick, hyalinized collagen bundles resulting from an imbalance between the production and degradation of extracellular matrix (  ECM  ) components. Matrix metalloproteinases (MMPs) and tissue inhibitors of metalloproteinases (TIMPs) are two important regulators of   ECM   degradation and remodeling. To evaluate the role played by knockdown of TIMPs in keloid formation, we transduced human keloid-derived fibroblasts (KFs) with small interfering RNAs targeting TIMP-1 or -2 (siTIMP-1 or siTIMP-2) using a lentiviral vector and assessed the biological effects. We found that MMP-1/TIMP-1 and MMP-1/TIMP-2 complexes were suppressed and that MMP-2 activity was upregulated in KFs expressing siTIMP-1 or siTIMP-2. In addition, increased degradation of collagen type I was observed in the supernatant of KFs expressing siTIMP-1, but not siTIMP-2, with the suppression of cell viability and induction of apoptosis. These results suggest that targeting TIMP-1 using small interfering RNA has significant therapeutic potential as an approach to treating keloids through degradation of their thick collagen bundles. 
 Relationship between matrix metalloproteinases/tissue inhibitors of matrix metalloproteinases systems and autoantibody patterns in systemic sclerosis. Systemic sclerosis (SSc) is a connective tissue disease characterized by tissue fibrosis that reflects an imbalance between collagen production and degradation. Matrix metalloproteinases (MMPs) are a family of endopeptidases involved in the remodelling of extracellular matrix (  ECM  ). This activity is controlled by tissue inhibitors of MMP (TIMPs). Aim of this study was the evaluation of MMP-9/TIMP-1 and MMP-2/TIMP-2 systems in patients with SSc.SearchLight Human MMP Array 1 was used to measure MMPs and TIMPs in 32 SSc patients and 32 matched healthy controls.SSc patients showed higher values of both MMP-9 and TIMP-1 in comparison with controls. The patients with anticentromere antibodies (ACA) positivity showed higher values of MMPs and TIMPs in comparison with either controls or the patients with anti-Scl70-positive antibodies.Results of this investigation suggest that SSc patients with ACA positivity, after a primary fibrogenetic noxa, react with a more abundant release of MMP/TIMP, whereas patients with anti-Scl70 antibody show a normal response. 
 Expression of MMP-2, TIMP-2, TGF-β1, and decorin in Dupuytren's contracture. To investigate the mechanisms underlying matrix deposition in Dupuytren's disease, the expression of gelatinase A (MMP-2), the tissue inhibitor of metalloproteinase-2 (TIMP-2), transforming growth factor beta 1 (TGF-β1), decorin (DCN), and periostin was studied. The level of relative MMP-2 activation was investigated using zymography. The mRNA expression of MMP-2, TIMP-2, TGF-β1, and DCN was detected using reverse transcription polymerase chain reaction (RT-PCR), while the presence of protein was detected using immunohistochemical (IHC) and Western blot techniques. The level of MMP-2 activation was significantly elevated in tissues with Dupuytren's contracture. RT-PCR demonstrated significantly higher expression of MMP-2, TIMP-2, TGF-β1, and DCN mRNA in the pathological tissues; and the IHC and immunoblotting studies revealed elevated expression of TGF-β1, DCN, and periostin. The balance between MMP-2 and TIMP-2 was disrupted in patients with Dupuytren's disease. TGF-β1, DCN, and periostin are involved in extracellular ma  tri  x (ECM) homeostasis in Dupuytren's contracture. 
 Polymorphisms in MMP-9 and TIMP-2 in Chinese patients with varicose veins. Varicose veins (VVs), a common vascular disease, are functionally characterized by dilation and tortuosity and are widely prevalent in the adult population. The pathophysiology and molecular mechanism of VVs are still unclear. A genetic risk for VVs has been demonstrated, although no genetic variant pertaining to VVs has been identified. Matrix metalloproteinases (MMPs) and their endogenous tissue inhibitors (TIMPs), which can prevent excessive extracellular matrix (  ECM  ) degradation, greatly impact vascular remodeling and may play a vital role in patients with VVs. We evaluated a potential association between polymorphisms in the promoters of MMP-9 and TIMP-2 and the risk for VVs in the Chinese population.Genotyping of the promoter region polymorphisms -1562C/T in MMP-9 and -418G/C in TIMP-2 was performed with PCR and restriction fragment length polymorphism (PCR-RFLP) assays with a group of 60 patients with VVs and 60 healthy controls. Purified PCR products were sequenced.A significant correlation was found between patients with VVs and controls at -1562C/T in MMP-9. The TIMP-2 gene polymorphism -418G/C was also associated with VVs.Our results suggest that polymorphisms in the promoter region of MMP-9 and TIMP-2 are associated with VVs in the Chinese population. 
 Aberrant tubulointerstitial immunoexpression of matrix metalloproteinases MMP-2, MMP-9 and tissue inhibitor of matrix proteinase-2 (TIMP-2) in acute cellular rejection of human renal allograft. Acute cellular rejection (ACR) may initiate chronic allograft dysfunction with alterations in the extracellular matrix compartment (  ECM  ). Turnover of   ECM   proteins is regulated by matrix metalloproteinases (MMPs). The aim of the present study was to determine the immunoexpression of MMP-2, MMP-9 and TIMP-2 in ACR, and to examine the relationship between expression of MMPs and monocytes/macrophages, transforming growth factor beta-1 (TGFbeta-1), and alpha-smooth muscle actin (alpha-SMA). Immunoperoxidase study with antibodies against MMP-2, MMP-9, TIMP-2, CD68, TGFbeta-1 and alpha-SMA was carried out on 24 renal allograft biopsy specimens from patients with ACR and 11 allograft biopsy specimens from patients with no signs of rejection. Our study revealed increased immunoexpression of MMP-2, MMP-9 and TIM-2 in ACR as compared with controls, and significant positive correlations between immunostaining of MMP-2 and TGF-beta-1, as well as between MMP-2 and alpha-SMA. Increased immunoexpression of MMP-9 was positively correlated with alpha-SMA, and the number of interstitial CD68+cells. In conclusion our study supports a role of gelatinases in tissue damage in human renal acute cellular allograft rejection and provides some interesting insights into early renal remodeling which may lead to chronic allograft dysfunction. 
 [Analysis of tissue inhibitor of metalloproteinase-2 gene polymorphisms in a caucasian population with abdominal aortic aneurysms]. The formation of sporadic abdominal aortic aneurysm (AAA) is explained by a remodelling of the extracellular matrix (  ECM  ) and breakdown of structural components of the vascular wall. Matrix metalloproteinases are the principle matrix-degrading proteases and are known to play a major role in the remodelling of the extracellular matrix in arterial vessels. Their activity is controlled by tissue inhibitors of metalloproteinases (TIMPs). Decreased TIMP-1 and TIMP-2 expression in the extracellular matrix of the walls of AAAs has been demonstrated in several studies. This case-control study was designed to investigate the possible impact of genetic variants of the TIMP-2 gene in the aetiology of AAA and to reproduce a recently described significant difference in allele frequency of the SNP 303G>A in a German population.TIMP-2 single nucleotide polymorphisms (SNPs) were analysed in a study sample of 50 patients with AAA and 41 controls. Differences in genotype and allele frequencies of the identified polymorphisms were determined after sequencing the entire coding region and selected parts of the promoter using the automated laser fluorescence technique.Six polymorphisms were identified, one of which is described for the first time, located in the intron, (231+23C>T). An association of the SNP 303G>A with the phenotype was not confirmed in our study (p=0.648). However, the CT genotype of the SNP -479C>T was more frequent in patients with AAA than in the control group (p=0.054).In our analysis of the TIMP-2 gene, we identified one new SNP. A previously published association of the SNP 303G>A with the phenotype could not be validated in our population. However, we detected an association for the CT genotype of one polymorphism in the promoter region (g-479C>T) and AAA. This result has to be proved in a second study sample. 
 NDRG2 ameliorates hepatic fibrosis by inhibiting the TGF-β1/Smad pathway and altering the MMP2/TIMP2 ratio in rats. Liver fibrosis is a worldwide clinical issue. It has been well established that activated hepatic stellate cells (HSCs) are responsible for excessive extracellular matrix   (EC  M) deposition in chronically damaged livers. The identification of key elements that control HSCs activation will help to further our understanding of liver fibrosis and improve the outcome of clinical treatment. This study demonstrates that N-Myc downstream-regulated gene 2 (NDRG2) is a potential regulator of liver fibrosis as NDRG2 mRNA and protein levels were reduced during HSCs activation. In addition, enhanced NDRG2 expression reduced Smad3 transcription and phosphorylation, which inhibited HSCs activation by blocking the TGF-β1 signal. Moreover, NDRG2 contributed to an increase in the ratio of matrix metalloproteinase 2 (MMP2) to tissue inhibitor of matrix metalloproteinase 2 (TIMP2), which may facilitate the degradation of th  e E  CM. In dimethylnitrosamine (DMN)-induced fibrotic rat livers, adenovirus-mediated NDRG2 overexpression resulted in decrease  d E  CM deposition and improved liver function compared with controls. In conclusion, the present findings indicate that the modulation of NDRG2 is a promising strategy for the treatment of liver fibrosis. 
 Polymorphisms of MMP9 and TIMP2 in Patients with Varicose Veins. Genetic predisposition is a suggested risk factor in the etiology of varicose veins. The matrix metalloproteinase (MMP) family degrades extracellular matrix (  ECM  ) and may lead to disturbances in vein wall structure. The activity of MMPs in the   ECM   are controlled by specific tissue inhibitors of MMPs (TIMP). The present study aimed to investigate the relationship between MMP9 and TIMP2 gene polymorphisms and varicose vein risk.Genotyping of the polymorphisms of MMP9 (1562 C/T) and TIMP2 (418G/C) was performed using polymerase chain reaction and restriction-fragment length polymorphism assays in a group of patients with varicose veins (n=63) and healthy controls (n=70).The frequencies of MMP9 alleles and genotypes did not differ significantly between patient and control groups. However, TIMP2 -418 C allele was associated with increased risk for varicose vein formation (p=0.007). It was also shown that the frequency of the GG genotype was significantly higher in the control group than in the patient group (odds ratio=0.333, 95% confidence interval=0.14-0.78, p=0.012).TIMP2 -418 C allele is associated with susceptibility for varicose vein formation and individuals with GG genotype may have a lower risk for varicose vein formation. 
 Circulating matrix metalloproteinase-9 and tissue inhibitors of metalloproteinases-1 and -2 levels in gestational hypertension. Gestational hypertension (GH) is dangerous to both mother and child. Arterial invasiveness and growth are dependent on successful extracellular matrix (  ECM  ) breakdown, which may be abnormal in GH. We hypothesized abnormalities in circulating matrix metalloproteinase-9 (MMP-9) and tissue inhibitors of metalloproteinases-1 and -2 (TIMP-1 and TIMP-2, respectively) in patients with GH, when compared with normotensive women with normal pregnancies and healthy nonpregnant control subjects.Plasma MMP-9, TIMP-1, and TIMP-2 were measured by ELISA in 23 women with GH, 30 normotensive pregnant women, and 28 nonpregnant women who were matched for age, gestational age, and parity.Levels of circulating MMP-9, TIMP-1 and TIMP-2, and the MMP-9/TIMP-1 and MMP-9/TIMP-2 ratios were significantly different among the three groups (P = .026, P = .006, P = .007, P = .001 and P = .008 respectively). Within the GH group, MMP-9 and the MMP-9/TIMP-1 ratio correlated negatively with age (r = -0.581, P = .004 and r = -0.563, P = .005, respectively) and levels of diastolic blood pressure (r = -0.432, P = .040 and r = -0.461, P = .027, respectively). With multiple regression analysis, only age independently correlated with circulating levels of MMP-9 (P = .010); neither age nor levels of diastolic blood pressure had any effect on the MMP-9/TIMP-1 ratio.We have demonstrated altered MMP/TIMP ratios in maternal blood during GH. These observations suggest pregnancy-related changes in   ECM   breakdown and turnover. Given the importance of changes in   ECM   composition to vascular and cardiac structure in hypertension, we suggest that these observations may be related to the pathophysiology of human GH. 
 Human cardiac fibroblast extracellular matrix remodeling: dual effects of tissue inhibitor of metalloproteinase-2. Tissue inhibitor of metalloproteinase-2 (TIMP-2) is an endogenous inhibitor of matrix metalloproteinases (MMPs) that attenuates maladaptive cardiac remodeling in ischemic heart failure. We examined the effects of TIMP-2 on human cardiac fibroblast activation and extracellular matrix (  ECM  ) remodeling.Human cardiac fibroblasts within a three-dimensional collagen matrix were assessed for phenotype conversion,   ECM   architecture and key molecular regulators of   ECM   remodeling after differential exposure to TIMP-2 and Ala+TIMP-2 (a modified TIMP-2 analogue devoid of MMP inhibitory activity).TIMP-2 induced opposite effects on human cardiac fibroblast activation and   ECM   remodeling depending on concentration. TIMP-2 activated fibroblasts into contractile myofibroblasts that remodeled   ECM  . At higher concentrations (>10 nM), TIMP-2 inhibited fibroblast activation and prevented   ECM   remodeling. As compared to profibrotic cytokine transforming growth factor (TGF)-beta1, TIMP-2 activated fibroblasts and remodeled   ECM   without a net accumulation of matrix elements. TIMP-2 increased total protease activity as compared to TGF-beta1. Ala+TIMP-2 exposure revealed that the actions of TIMP-2 on cardiac fibroblast activation are independent of its effects on MMP inhibition. In the presence of GM6001, a broad-spectrum MMP inhibitor, TIMP-2-mediated   ECM   contraction was completely abolished, indicating that TIMP-2-mediated fibroblast activation is MMP dependent.TIMP-2 functions in a contextual fashion such that the effect on cardiac fibroblasts depends on the tissue microenvironment. These observations highlight potential clinical challenges in using TIMP-2 as a therapeutic strategy to attenuate postinjury cardiac remodeling. 
 Expression of MMP-7, MMP-9, TIMP-1 and TIMP-2 mRNA in lung tissue of patients with non-small cell lung cancer (NSCLC) and benign pulmonary disease. The expression of matrix metallo-proteinases (MMP-7 and MMP-9) and tissue inhibitors of metalloproteinases (TIMP-1 and TIMP-2), which are involved in the degradation of the extracellular matrix (  ECM  ) and tumor growth, was investigated in normal lung tissue, tissue of benign pulmonary diseases and non-small cell lung cancer (NSCLC) tissue.Tumor tissue and surrounding carcinoma-free lung tissue samples were obtained from 91 patients with NSCLC who had undergone surgery in the years 2005-2007 as well as lung tissue from 12 patients operated on for 'benign' bullous emphysema or interstitial lung disease. The mRNA was isolated from the tissues and the expression of mRNA was assessed using a real-time RT PCR method.Significantly higher expression of MMP-7, MMP-9 and TIMP-1 mRNA was demonstrated in the NSCLC tissue in comparison with the normal lung tissue from the same patients (p=0.0003, p<0.0001 and p=0.0018, respectively). Similar results for MMP-7, MMP-9 and TIMP-1 were found in the histological subgroups: squamous cell lung cancer vs. normal tissue (p=0.0198, p=0.0015 and p=0.0366, respectively), and adenocarcinoma vs. normal tissue (p=0.0045, p<0.0001 and p=0.0140, respectively). The expression of MMP-7 was found to be significantly higher in tumor tissue vs. lung tissue of the benign diseases (p=0.0086) and similar results were also recorded in the histological subgroups: squamous cell lung cancer vs. benign tissue (p=0.0171) and adenocarcinoma vs. benign tissue (p=0.0135). The expression of MMP-9 was significantly higher only in the adenocarcinoma subgroup vs. the benign tissue (p=0.0412). No differences in the expression of mRNA between stage IA and stages IB-IIIB of NSCLC were recorded.Significantly higher expression of MMP-7 and MMP-9 in tumor tissue than in the surrounding tissue or in benign lung disease tissue supports the notion of an important role of these metalloproteinases in the growth of lung carcinoma. TIMP-1 expression is increased only in carcinoma, but not in benign lung disease. 
 Higher risk of matrix metalloproteinase (MMP-2, 7, 9) and tissue inhibitor of metalloproteinase (TIMP-2) genetic variants to gallbladder cancer. Matrix metalloproteinase belong to family of pericellular collagenases which degrade extracellular matrix (  ECM  ), and is involved in the modulation and susceptibility of various cancers.The present study included 410 gallbladder (GBC) cases and 230 healthy controls from North India. Study examined the associations of polymorphisms of MMP-2c.735C>T (rs2285053), MMP-2c.1306 C>T (rs243865), MMP7c.181A>G (rs11568818), MMP-9p.R279Q (rs17556) MMP-9p.P574R (rs2250889), MMP-9 p.R668Q (rs17577) and TIMP2c.418 G>C (rs8179090) to GBC susceptibility. Genotyping was carried out by PCR-RFLP. Statistical analysis was performed by using SPSS ver16.The MMP-2 c.735 [CT+TT], MMP-2c.1306 [CT+TT], MMP7 c.181 [AG+GG] and MMP-9 p.668 [RQ+QQ],TIMP2c.418 [GG+GC] genotypes were significantly associated with increased risk of GBC (P = 0.01; [OR]1.87, P = 0.02; [OR] 1.68, P = 0.02; [OR]=1.61, P = 0.002; [OR]=1.91,P = 0.01; [OR]=1.78 and (P = 0.03; [OR]=1.68; P = 0.01; [OR]=1.78 respectively). Haplotypes [C(-735) -T(-1306) ] and [T(-1306) -C(-735) ] of MMP-2 (P = <0.005; [OR] =1.78 P = <0.0001; [OR] =2.09) and haplotype [Q(279) -P(574) -Q(668) ]of the MMP-9 (P = 0.04; [OR] =2.75) were significantly associated with GBC risk. On stratification of GBC patients with/without gallstones, MMP-2 haplotypes were associated with higher GBC risk in patients accompanying gallstones whereas MMP-9 haplotypes showed risk in patients without stones. Combined effect of > 3 MMP/TIMP variant containing genotypes imparted increased risk of GBC (P < 0.0001; [OR] =3.36). Multivariate logistic regression results also supported association of MMP-2 (c.735C>T, c.1306 C>T), MMP-9 p.R668Q and TIMP2c.418G>C variants with GBC susceptibility.This study suggests that genetic variants in MMP-2,7,9 and TIMP-2genes are associated with higher susceptibility of gallbladder cancer. 
 Urinary Metalloproteinases-9 and -2 and Their Inhibitors TIMP-1 and TIMP-2 are Markers of Early and Long-Term Graft Function After Renal Transplantation. Renal ischemia-reperfusion (I-R) injury (IRI) is an inseparable feature of organ transplantation and may have a negative impact on the graft, its function and survival. Acute tubular necrosis, which is reversible thanks to the regenerative capacity of renal tubular epithelial cells, is the main cause of acute renal failure secondary to IRI. MMP-2 and MMP-9 are proteolytic enzymes involved in digesting proteins that are components of the extracellular matrix (  ECM  ) and the basement membrane of the nephrons. This way post-reperfusion MMP activation allows the inflammatory process to spread.In our studies, we focused on identifying whether the concentrations of MMP-2 and MMP-9 and their natural inhibitors TIMP-1 and TIMP-2 in urine sample at day 1 and day 30 as well as after 12 months following renal transplantation are markers of early and long-term renal function during meanly five-years observation. Moreover, in urine sampled at months 6 and 12 after renal transplantation, we determined the content of TGF-β as a graft fibrosis indicator.MMP-9 concentration in the early post-transplant period is a major marker of early and long-term function of the transplanted kidney. Its increased concentration was correlated with lesions related to tubular atrophy and fibrosis in renal biopsies performed at months 3 and 12 after transplantation. Its concentration is correlated with TGF-β content in a later period.TIMP-1 and-2 are primarily markers of an early function of the transplanted kidney. Early post-transplant concentration of MMP-2 is a marker of proteinuria in early and long-term post-transplant periods. 
 Polymorphisms in MMP-2 and TIMP-2 in Turkish patients with prostate cancer. Prostate cancer is the most commonly diagnosed malignancy and the second most common cause of cancer deaths in the Western male population. Matrix metalloproteinases (MMPs) and tissue inhibitors of MMPs (TIMPs) modulate the remodeling of the extracellular matrix (  ECM  ). The imbalance between MMPs and TIMPs may lead to an emergence of pathological processes such as cancer. In this study, the association between TIMP-2 (-418 G/C) and MMP-2 (-1306 C/T) polymorphisms and prostate cancer in the Turkish population was investigated.Sixty-one prostate cancer patients and 46 healthy subjects were included in the study. DNA was isolated from 2 mL of peripheral blood taken from subjects, and genotypes were analyzed by the polymerase chain reaction-restriction fragment length polymorphism method.The TIMP-2 -418 (GC) genotype was found in 15 cases (32.6%) in the control group and in 9 cases (14.8%) in the patients group, and statistical significance was determined (P = 0.037, OR = 0.346). The MMP-2 -1306 (CT) genotype was found 2.17 times more in the patient group than in the control group (P = 0.149, OR = 2.17).Our results show that the TIMP-2 -418 (GC) genotype had a putative protective effect against prostate cancer. 
 Promoted activation of matrix metalloproteinase (MMP)-2 in keloid fibroblasts and increased expression of MMP-2 in collagen bundle regions: implications for mechanisms of keloid progression. Keloid is characterized by excessive deposition of collagen, resulting from aberrant extracellular matrix (  ECM  ) production and degradation. The aim was to investigate the role of matrix metalloproteinases (MMPs) and tissue inhibitor of metalloproteinases (TIMPs) in pathological wound healing in keloids.Semiquantitative analysis of 60 keloid tissue samples and 25 mature scar tissue samples demonstrated significantly increased expression of MMP-2, TIMP-2 and TIMP-3 in keloids compared with mature scars. Within keloid regions, MMP-2 expression was significantly higher in collagen bundle regions than in non-collagen bundle regions. Double immunofluorescence revealed that keloid fibroblasts between collagen bundles exhibited MMP-2, TIMP-2 and membrane-type 1 MMP (MT1-MMP) co-expression, whereas only MMP-2 expression was evident on the edge of collagen bundles. Western blot analysis and gelatin zymography of 13 keloid-derived fibroblasts (KFbs) and six normal skin dermal-derived fibroblasts (NFbs) demonstrated that unstimulated KFbs exhibited significantly increased MMP-2 activity and expression compared with NFbs under the same conditions.These results together indicate that MMP-2 activity can be promoted in keloid fibroblasts between collagen bundles in cooperation with TIMP-2 and MT1-MMP. This could contribute to remodelling of collagen bundle regions and invasion of fibroblasts into peripheral normal regions through promoted degradation of   ECM  . 
 CAPN 7 promotes the migration and invasion of human endometrial stromal cell by regulating matrix metalloproteinase 2 activity. Matrix metalloproteinase 2 (MMP-2) has been reported to be an important regulator of cell migration and invasion through degradation of the extracellular matrix (  ECM  ) in many diseases, such as cancer and endometriosis. Here, we found calcium-activated neutral protease 7 (CAPN 7) expression was markedly upregulated in the eutopic endometrium and endometrial stromal cells of women diagnosed with endometriosis. Our studies were carried out to detect the effects of CAPN 7 on human endometrial stromal cell (hESC) migration and invasion.Western blotting and quantitative real-time PCR were used to detect the expression of CAPN 7 in endometriosis patients and normal fertile women. Scratch-wound-healing and invasion chamber assay were used to investigate the role of CAPN 7 in hESC migration and invasion. Western blotting, quantitative real-time PCR and zymography were carried out to detect the effect of CAPN 7 on the expressions and activity of MMP-2.CAPN 7 was markedly up-regulated in endometriosis, thereby promoting the migration and invasion of hESC. CAPN 7 overexpression led to increased expression of MMP-2 and tissue inhibitor of metalloproteinases 2 (TIMP-2); CAPN 7 knockdown reversed these changes. CAPN 7 increased MMP-2 activity by increasing the ratio of MMP-2 to TIMP-2. We also found that OA-Hy (an MMP-2 inhibitor) decreased the effects of CAPN 7 overexpression on hESC migration and invasion by approximately 50% and 55%, respectively. Additionally, a coimmunoprecipitation assay demonstrated that CAPN 7 interacted with activator protein 2α (AP-2α): an important transcription factor of MMP-2.CAPN 7 promotes hESC migration and invasion by increasing the activity of MMP-2 via an increased ratio of MMP-2 to TIMP-2. 
 MMP-2 protein in invasive breast cancer and the impact of MMP-2/TIMP-2 phenotype on overall survival. Crucial event in the metastasis of cancer cells is the secretion of matrix metalloproteinases (MMPs), which are responsible for the degradation of extracellular matrix (  ECM  ). Among them, matrix metalloproteinase-2 (MMP-2) is a gelatinase, which degrades basement membrane type-IV collagen. Immunohistochemistry was performed to detect MMP-2 protein in 135 infiltrative breast carcinomas. MMP-2 was studied along with clinicopathological parameters (tumor size, histological type, nuclear and histological grade, stage, lymph node status, ER, and PR), patients' survival and tissue inhibitor metalloproteinase-2 (TIMP-2), Ki-67, and p53 proteins. MMP-2 immunoreactivity was detected in the cytoplasm in cancer cells in 102 (75.6%) and in both tumor and tumor stromal cells in 37 (27.4%) of 135 cases respectively. MMP-2 reactivity in cancer cells displayed a statistically significant association with tumor size > 2 cm (p = 0.022). In tumor stromal cells a strong parallel association was observed between the expression of MMP-2 and TIMP-2 (p = 0.015), while an inverse correlation was found between MMP-2 and both Ki-67 and p53 (p = 0.033 and p = 0.034 respectively). In the subgroup with negative lymph nodes MMP-2 was also inversely associated with p53 in cancer cells (p = 0.045). Finally a statistically significant association was revealed using Kaplan-Meier and Cox's proportional hazard regression model between the MMP-2/TIMP-2 phenotype and patients' better survival (p = 0.021). Our results point out the strong relation between MMP-2 and TIMP-2 and the effect of the MMP-2/TIMP-2 phenotype in the patients' overall survival. The inverse correlation between MMP-2 and both Ki-67 and p53 can be explained by the potential inhibition of MMP-2 by TIMP-2. These results suggest the necessity of further investigation. 
 The distribution of matrix metalloproteinases and tissue inhibitors of metalloproteinases in the lungs of congenital diaphragmatic hernia patients and age-matched controls. In congenital diaphragmatic hernia (CDH), the pathogenesis of abnormal pulmonary morphology is still incompletely understood. Matrix metalloproteinases (MMPs) and tissue inhibitors of metalloproteinases (TIMPs) are known to play an important role in the turnover of the extracellular matrix (  ECM  ) during development and in remodelling of tissue. The aim of this study was to investigate differences in the expression of MMPs and TIMPs between CDH lungs and controls, against the background of the abnormal pulmonary vasculature in CDH.We studied 12 lungs of term CDH patients who died < 24 h after birth and 11 normal age-matched control lungs, by immunohistochemistry with antibodies against human MMP-1, -2, -9, TIMP-1 and -2.There was a clear increase in the number of MMP-1-reactive capillaries and fibroblasts in CDH lungs compared with controls. In contrast, TIMP-2 reactivity in these structures was decreased in CDH lungs. The arterial endothelium and medial smooth muscle expressed MMP-2, -9 and TIMP-2 in both CDH and control lungs. In small arteries (< 100 microm in diameter), the positive surface area of MMP-2, -9 and TIMP-2 was significantly larger in CDH lungs than in controls. There was no difference in the distribution and expression of TIMP-1 between CDH lungs and normal controls.The differences in staining pattern of MMPs and TIMPs between normal and CDH lungs suggest that these enzymes might play a role in the abnormal remodelling of the interstitium and the pulmonary arteries in CDH lungs. This could contribute to our understanding of the abnormal lung morphology and the occurrence of pulmonary hypertension, which forms one of the major obstacles to the successful treatment of these patients. 
 Gelatinases and their tissue inhibitors during human ovulation: increased expression of tissue inhibitor of matrix metalloproteinase-1. Remodelling of the extracellular matrix (  ECM  ) of the follicular wall by matrix metalloproteinases (MMPs) and tissue inhibitors of matrix metalloproteinases (TIMPs) has been suggested to be crucial in ovulation. To investigate the expression of the gelatinases, MMP-2 and MMP-9, together with their inhibitors, TIMP-2 and TIMP-1, in the perifollicular ovarian stroma from women just before and during ovulation, we obtained biopsies of the stroma adjacent to the leading follicle. Laparoscopic surgery was performed either before the LH peak or at any of three intervals after ovulation triggering by hCG. Immunoblotting, immunohistochemistry and quantitative RT-PCR were performed. All four proteins were expressed by immunoblots, with no detectable changes in the expression of MMP-2, MMP-9 and TIMP-2. Scattered immunostaining for MMP-9 and TIMP-2 was seen, and MMP-2 was demonstrated in a concentric layer. A significant increase in TIMP-1 protein and mRNA was seen during the three ovulatory phases, and a strong and patchy immunostaining for TIMP-1 was shown. This is the first study that has demonstrated an ovulation-associated expression of these   ECM  -remodelling enzymes around the human follicle at ovulation. The increased expression of TIMP-1 may reflect a specific temporal inhibition of collagenolysis and thereby a time-dependent regulation of   ECM   breakdown in areas surrounding the apex of the follicle. 
 Polymorphic variation within the ADAMTS2, ADAMTS14, ADAMTS5, ADAM12 and TIMP2 genes and the risk of Achilles tendon pathology: a genetic association study. Achilles tendon pathology (ATP) is a multifactorial condition for which genetic risk factors have been identified. The ADAMTS, ADAM12 and TIMP2 genes encode enzymes that are important regulators of tendon homeostasis. ADAMTS2 and ADAMTS14 proteins are procollagen N-propeptidases for pro-collagen type I, type II, and type III. ADAMTS2, like COL5A1, has been linked to Ehlers-Danlos syndrome. Variants within ADAMTS5 and ADAM12 have been associated with osteoarthritis. TIMP2, a metalloprotease inhibitor, maintains homeostasis in the   ECM   by inhibiting ADAM, ADAMTS and MMP functions. We sought to determine whether single nucleotide polymorphisms (SNPs) within the ADAMTS2, ADAMTS5, ADAMTS14, ADAM12 and TIMP2 genes were associated with the risk of ATP in two independent populations.213 (115 ATP cases and 98 asymptomatic controls) South African Caucasian participants and 209 (60 ATP cases and 149 asymptomatic controls) Australian Caucasian participants were recruited for this case-control genetic association study.All participants were genotyped using TaqMan technology for the ADAMTS2 rs1054480, ADAMTS5 rs226794, ADAMTS14 rs4747096, ADAM12 rs3740199, and TIMP2 rs4789932 SNPs.We report for the first time a significant (p=0.016) genotypic association between the TIMP2 rs4789932 variant and ATP in a combined Caucasian cohort. We also identify an interaction between the ADAMTS14 rs4747096 variant and age of onset of ATP (p=0.024).Our data show that DNA sequence variation within the TIMP2 gene is a risk factor for ATP in Caucasians. Furthermore, carriage of the ADAMTS14 rs4747096 GG variant appears to delay onset of the injury in the ATP group. 
 Matrix metalloproteinases and tissue inhibitors of metalloproteinases in human fetal testis and ovary. Matrix metalloproteinases (MMP) and tissue inhibitors of metalloproteinases (TIMP) are major regulators of tissue remodelling of the extracellular matrix (  ECM  ) and may also be involved in the control of growth factor availability. We have investigated their production and localization in the developing human gonad during mid-gestation using zymographic techniques and immunohistochemistry. The secretion of MMP-2, MMP-9 and all four TIMP was demonstrated from both testis and ovary, with the predominant gelatinase produced by both being MMP-2. In the testis, MMP-1, MMP-2, MMP-9 and all TIMP family members were localized to the interstitium and to varying degrees within the tubules. MMP-9 and TIMP-4 were abundant in both Sertoli cells and gonocytes and MMP-1 and TIMP-1 were localized in particular to Sertoli cells. In the ovary, all TIMP and MMP-1, MMP-2 and MMP-9 were localized to the oogonium/oocyte cytoplasm with varying intensities and MMP-1, TIMP-2 and TIMP-3 were also detected in the ovarian stroma. This study demonstrates that MMP-1, MMP-2, MMP-9 and all TIMP family members are secreted by the developing ovary and testis and are localized to specific cell and tissue sites. MMP and TIMP are likely to play a role in   ECM   remodelling during gonadal development and also in the cell and matrix interactions that control a range of cellular functions. 
 Growth-stimulatory activity of TIMP-2 is mediated through c-Src activation followed by activation of FAK, PI3-kinase/AKT, and ERK1/2 independent of MMP inhibition in lung adenocarcinoma cells. Tissue inhibitors of metalloproteinases (TIMPs) control extracellular matrix (  ECM  ) homeostasis by inhibiting the activity of matrix metalloproteinases (MMPs), which are associated with   ECM   turnover. Recent studies have revealed that TIMPs are implicated in tumorigenesis in both MMP-dependent and MMP-independent manners. We examined a mechanism by which TIMP-2 stimulated lung adenocarcinoma cell proliferation, independent of MMP inhibition. The stimulation of growth by TIMP-2 in A549 cells required c-Src kinase activation. c-Src kinase activity, induced by TIMP-2, concomitantly increased FAK, phosphoinositide 3-kinase (PI3-kinase)/AKT, and ERK1/2 activation. Selective knockdown of integrin α3β1, known as a TIMP-2 receptor, did not significantly change TIMP-2 growth promoting activity. Furthermore, we showed that high TIMP-2 expression in lung adenocarcinomas is associated with a worse prognosis from multiple cohorts, especially for stage I lung adenocarcinoma. Through integrated analysis of The Cancer Genome Atlas data, TIMP-2 expression was significantly associated with the alteration of driving genes, c-Src activation, and PI3-kinase/AKT pathway activation. Taken together, our results demonstrate that TIMP-2 stimulates lung adenocarcinoma cell proliferation through c-Src, FAK, PI3-kinase/AKT, and ERK1/2 pathway activation in an MMP-independent manner. 
 TIMP-2 Interaction with MT1-MMP Activates the AKT Pathway and Protects Tumor Cells from Apoptosis. Membrane-type 1 matrix metalloproteinase (MT1-MMP), a transmembrane proteinase with an extracellular catalytic domain and a short cytoplasmic tail, degrades a variety of extracellular matrix (  ECM  ) components. In addition, MT1-MMP activates intracellular signaling through proteolysis-dependent and independent mechanisms. We have previously shown that binding of tissue inhibitor of metalloproteinases-2 (TIMP-2) to MT1-MMP controls cell proliferation and migration, as well as tumor growth in vivo by activating the Ras-extracellular signal regulated kinase-1 and -2 (ERK1/2) pathway through a mechanism that requires the cytoplasmic but not the proteolytic domain of MT1-MMP. Here we show that in MT1-MMP expressing cells TIMP-2 also induces rapid and sustained activation of AKT in a dose- and time-dependent manner and by a mechanism independent of the proteolytic activity of MT1-MMP. Fibroblast growth factor receptor-1 mediates TIMP-2 induction of ERK1/2 but not of AKT activation; however, Ras activation is necessary to transduce the TIMP-2-activated signal to both the ERK1/2 and AKT pathways. ERK1/2 and AKT activation by TIMP-2 binding to MT1-MMP protects tumor cells from apoptosis induced by serum starvation. Conversely, TIMP-2 upregulates apoptosis induced by three-dimensional type I collagen in epithelial cancer cells. Thus, TIMP-2 interaction with MT1-MMP provides tumor cells with either pro- or anti-apoptotic signaling depending on the extracellular environment and apoptotic stimulus. 
  GeneRIFs  
 Knockdown of GRP78 decreases   ECM   degradation, and downregulates the expression and activity of TIMP-2 
  [Ee]xtracellular [Mm]atrix  Conserved Domains  
 cd03585: NTR_TIMP; NTR domain, TIMP subfamily; TIMPs, or tissue inibitors of metalloproteases, are essential regulators of   extracellular matrix   turnover and remodeling. They form complexes with matrix metalloproteases (MMPs) and inactivate them irreversibly by ... 
  Process  
   extracellular matrix   disassembly 
  Component  
   extracellular matrix   
 proteinaceous   extracellular matrix   
  Summary  
 This gene is a member of the TIMP gene family. The proteins encoded by this gene family are natural inhibitors of the matrix metalloproteinases, a group of peptidases involved in degradation of the   extracellular matrix  . In addition to an inhibitory role against metalloproteinases, the encoded protein has a unique role among TIMP family members in its ability to directly suppress the proliferation of endothelial cells. As a result, the encoded protein may be critical to the maintenance of tissue homeostasis by suppressing the proliferation of quiescent tissues in response to angiogenic factors, and by inhibiting protease activity in tissues undergoing remodelling of the   extracellular matrix  . [provided by RefSeq, Jul 2008] 
  PubMed  
 A genetic association study of maternal and fetal candidate genes that predispose to preterm prelabor rupture of membranes (PROM). We sought to determine whether maternal/fetal single-nucleotide polymorphisms (SNPs) in candidate genes are associated with preterm prelabor rupture of membranes (pPROM).A case-control study was conducted in patients with pPROM (225 mothers and 155 fetuses) and 599 mothers and 628 fetuses with a normal pregnancy; 190 candidate genes and 775 SNPs were studied. Single locus/haplotype association analyses were performed; false discovery rate was used to correct for multiple testing (q* = 0.15).First, a SNP in tissue inhibitor of metalloproteinase 2 in mothers was significantly associated with pPROM (odds ratio, 2.12; 95% confidence interval, 1.47-3.07; P = .000068), and this association remained significant after correction for multiple comparisons. Second, haplotypes for Alpha 3 type IV collagen isoform precursor in the mother were associated with pPROM (global P = .003). Third, multilocus analysis identified a 3-locus model, which included maternal SNPs in collagen type I alpha 2, defensin alpha 5 gene, and endothelin 1.DNA variants in a maternal gene involved in   extracellular matrix   metabolism doubled the risk of pPROM. 
 Identification of fetal and maternal single nucleotide polymorphisms in candidate genes that predispose to spontaneous preterm labor with intact membranes. The purpose of this study was to determine whether maternal/fetal single nucleotide polymorphisms (SNPs) in candidate genes are associated with spontaneous preterm labor/delivery.A genetic association study was conducted in 223 mothers and 179 fetuses (preterm labor with intact membranes who delivered <37 weeks of gestation [preterm birth (PTB)]), and 599 mothers and 628 fetuses (normal pregnancy); 190 candidate genes and 775 SNPs were studied. Single locus/haplotype association analyses were performed; the false discovery rate was used to correct for multiple testing.The strongest single locus associations with PTB were interleukin-6 receptor 1 (fetus; P=.000148) and tissue inhibitor of metalloproteinase 2 (mother; P=.000197), which remained significant after correction for multiple comparisons. Global haplotype analysis indicated an association between a fetal DNA variant in insulin-like growth factor F2 and maternal alpha 3 type IV collagen isoform 1 (global, P=.004 and .007, respectively).An SNP involved in controlling fetal inflammation (interleukin-6 receptor 1) and DNA variants in maternal genes encoding for proteins involved in   extracellular matrix   metabolism approximately doubled the risk of PTB. 
 SILAC-based proteomics of human primary endothelial cell morphogenesis unveils tumor angiogenic markers. Proteomics has been successfully used for cell culture on dishes, but more complex cellular systems have proven to be challenging and so far poorly approached with proteomics. Because of the complexity of the angiogenic program, we still do not have a complete understanding of the molecular mechanisms involved in this process, and there have been no in depth quantitative proteomic studies. Plating endothelial cells on matrigel recapitulates aspects of vessel growth, and here we investigate this mechanism by using a spike-in SILAC quantitative proteomic approach. By comparing proteomic changes in primary human endothelial cells morphogenesis on matrigel to general adhesion mechanisms in cells spreading on culture dish, we pinpoint pathways and proteins modulated by endothelial cells. The cell-  extracellular matrix   adhesion proteome depends on the adhesion substrate, and a detailed proteomic profile of the   extracellular matrix   secreted by endothelial cells identified CLEC14A as a matrix component, which binds to MMRN2. We verify deregulated levels of these proteins during tumor angiogenesis in models of multistage carcinogenesis. This is the most in depth quantitative proteomic study of endothelial cell morphogenesis, which shows the potential of applying high accuracy quantitative proteomics to in vitro models of vessel growth to shed new light on mechanisms that accompany pathological angiogenesis. The mass spectrometry proteomics data have been deposited to the ProteomeXchange Consortium with the data set identifier PXD000359. 
 Knockdown of glucose-regulated protein 78 decreases the invasion, metalloproteinase expression and ECM degradation in hepatocellular carcinoma cells. We have reported previously that overexpression of glucose-regulated protein 78 (GRP78) promotes the invasion of hepatocellular carcinoma. However, whether GRP78 knockdown affects the   extracellular matrix   degradation has not been elucidated. Here we are going to determine whether GRP78 knockdown affect the ECM degradation and the role of MMP-2 and MMP-9 in these process in hepatocellular carcinoma cells.Human hepatocellular carcinoma cell line SMMC7721 and HepG2 were cultured in DMEM supplemented with 10% FBS, RT-PCR and western blot were used to detect the endogenous expression of GRP78, MMP-2, MMP-9 and TIMP-2 in SMMC7721 and HepG2. GRP78 shRNAs were transfected using lipofection2000. Transwell assay and wound healing assay were used to analyze the invasion of each transfectant. Gelatin zymography and FITC-gelatin degradation assay were employed to investigate the capabilities of ECM degradation of each transfectant. MTT assay was used to determine the proliferation status. Western blot was employed to detect the expression of matrix metalloproteinase 2(MMP-2), MMP-9, MMP-14, and tissue inhibitor of metalloproteinases 2(TIMP-2), focal adhesion kinase (FAK), ERK1/2, JNK and Src.According to the expression levels of GRP78, MMP-2, MMP-9, MMP-14 and TIMP-1 in hepatocellular carcinoma cell lines SMMC7721 and hepG2, we used SMMC7721 as the in vitro invasion model for further functional analysis. Using this model, we found that GRP78 knockdown decreased the invasion of tumor cells, and this inhibitory effect was independent of cell proliferation. In hepatocellular carcinoma cells, Grp78 knockdown inhibited ECM degradation and the decreased activity and expression of MMP-2, but not MMP-9 contributed largely to this impact. Further analysis revealed that the decreased activity and expression of MMP-2 is mediated by JNK.Knockdown of GRP78 decreases ECM degradation, and downregulates the expression and activity of MMP-2 and TIMP-2. These results further demonstrate that GRP78 is a potential target for inhibiting the invasion of hepatocellular carcinoma cells. 
 Knockdown of glucose-regulated protein 78 decreases the invasion, metalloproteinase expression and ECM degradation in hepatocellular carcinoma cells. We have reported previously that overexpression of glucose-regulated protein 78 (GRP78) promotes the invasion of hepatocellular carcinoma. However, whether GRP78 knockdown affects the   extracellular matrix   degradation has not been elucidated. Here we are going to determine whether GRP78 knockdown affect the ECM degradation and the role of MMP-2 and MMP-9 in these process in hepatocellular carcinoma cells.Human hepatocellular carcinoma cell line SMMC7721 and HepG2 were cultured in DMEM supplemented with 10% FBS, RT-PCR and western blot were used to detect the endogenous expression of GRP78, MMP-2, MMP-9 and TIMP-2 in SMMC7721 and HepG2. GRP78 shRNAs were transfected using lipofection2000. Transwell assay and wound healing assay were used to analyze the invasion of each transfectant. Gelatin zymography and FITC-gelatin degradation assay were employed to investigate the capabilities of ECM degradation of each transfectant. MTT assay was used to determine the proliferation status. Western blot was employed to detect the expression of matrix metalloproteinase 2(MMP-2), MMP-9, MMP-14, and tissue inhibitor of metalloproteinases 2(TIMP-2), focal adhesion kinase (FAK), ERK1/2, JNK and Src.According to the expression levels of GRP78, MMP-2, MMP-9, MMP-14 and TIMP-1 in hepatocellular carcinoma cell lines SMMC7721 and hepG2, we used SMMC7721 as the in vitro invasion model for further functional analysis. Using this model, we found that GRP78 knockdown decreased the invasion of tumor cells, and this inhibitory effect was independent of cell proliferation. In hepatocellular carcinoma cells, Grp78 knockdown inhibited ECM degradation and the decreased activity and expression of MMP-2, but not MMP-9 contributed largely to this impact. Further analysis revealed that the decreased activity and expression of MMP-2 is mediated by JNK.Knockdown of GRP78 decreases ECM degradation, and downregulates the expression and activity of MMP-2 and TIMP-2. These results further demonstrate that GRP78 is a potential target for inhibiting the invasion of hepatocellular carcinoma cells. 
 A genetic association study of maternal and fetal candidate genes that predispose to preterm prelabor rupture of membranes (PROM). We sought to determine whether maternal/fetal single-nucleotide polymorphisms (SNPs) in candidate genes are associated with preterm prelabor rupture of membranes (pPROM).A case-control study was conducted in patients with pPROM (225 mothers and 155 fetuses) and 599 mothers and 628 fetuses with a normal pregnancy; 190 candidate genes and 775 SNPs were studied. Single locus/haplotype association analyses were performed; false discovery rate was used to correct for multiple testing (q* = 0.15).First, a SNP in tissue inhibitor of metalloproteinase 2 in mothers was significantly associated with pPROM (odds ratio, 2.12; 95% confidence interval, 1.47-3.07; P = .000068), and this association remained significant after correction for multiple comparisons. Second, haplotypes for Alpha 3 type IV collagen isoform precursor in the mother were associated with pPROM (global P = .003). Third, multilocus analysis identified a 3-locus model, which included maternal SNPs in collagen type I alpha 2, defensin alpha 5 gene, and endothelin 1.DNA variants in a maternal gene involved in   extracellular matrix   metabolism doubled the risk of pPROM. 
 Identification of fetal and maternal single nucleotide polymorphisms in candidate genes that predispose to spontaneous preterm labor with intact membranes. The purpose of this study was to determine whether maternal/fetal single nucleotide polymorphisms (SNPs) in candidate genes are associated with spontaneous preterm labor/delivery.A genetic association study was conducted in 223 mothers and 179 fetuses (preterm labor with intact membranes who delivered <37 weeks of gestation [preterm birth (PTB)]), and 599 mothers and 628 fetuses (normal pregnancy); 190 candidate genes and 775 SNPs were studied. Single locus/haplotype association analyses were performed; the false discovery rate was used to correct for multiple testing.The strongest single locus associations with PTB were interleukin-6 receptor 1 (fetus; P=.000148) and tissue inhibitor of metalloproteinase 2 (mother; P=.000197), which remained significant after correction for multiple comparisons. Global haplotype analysis indicated an association between a fetal DNA variant in insulin-like growth factor F2 and maternal alpha 3 type IV collagen isoform 1 (global, P=.004 and .007, respectively).An SNP involved in controlling fetal inflammation (interleukin-6 receptor 1) and DNA variants in maternal genes encoding for proteins involved in   extracellular matrix   metabolism approximately doubled the risk of PTB. 
 [Expression of matrix metalloproteinases 2 and 9 and their tissue inhibitors 1 and 2 in papillary thyroid cancer: an association with the clinical, morphological and ultrastructural characteristics of a tumor]. Papillary thyroid cancer (PTC) was ascertained to be characterized by a significant expression of matrix metalloproteinases (MMP-2 and 9) and a TIMP-2 inhibitor and decreased TIMP-1 accumulation in the cytoplasm of tumor cells; at the same time a combination of high cytoplasmic MMP-9 expression with no TIMP-1, as well as triple positive co-expression of MMP-2, MMP-9, and TIMP-2 are poor immunohistochemical indicators. The results of the performed study suggest that the detection of the types of expression of MMP-9+/TIMP-1 and MMP-2+/MMP-9+/TIMP-2+ is associated with the possible progression of a tumor process and concurrent with poor clinical and morphological signs, such as the large size of the tumor, the presence of intravascular invasion and regional metastases, organ capsular invasion, and low ultrastructural tumor cell differentiation. Thus, changes in the tumor expression of the   extracellular matrix   enzymes MMR-2 and MMP-9 and their inhibitors TIMP-1 and TIMP-2 along with the major clinical, morphological, and ultrastructural signs of the disease are important additional indicators for analyzing the malignant potential of PTC. 
 Tissue inhibitor of metalloproteinase 1 is an independent predictor of prognosis in patients with nonsmall cell lung carcinoma who undergo resection with curative intent. Matrix metalloproteinases (MMPs) and tissue inhibitors of metalloproteinases (TIMPs) play a role in the processes of   extracellular matrix   degradation. Changes in their expression levels have been observed in various tumor types, including lung carcinoma. However, their clinical significance and their prognostic importance in the progression of nonsmall cell lung carcinoma (NSCLC) remain to be specified. In this study, mRNA expression levels of MMP-1, MMP-9, TIMP-1, and TIMP-2 were evaluated in patients with resected NSCLC, and their associations with disease progression and prognosis were determined.Between June 1996 and December 1999, 116 patients underwent resection for NSCLC. Expression levels of MMPs and TIMPs were evaluated using Northern blot analysis in these NSCLC tissue samples and in 39 matched samples of normal lung tissue.MMP-1, MMP-9, and TIMP-1 expression levels were increased in tumor samples compared with matched, corresponding normal tissues. In contrast, TIMP-2 expression was decreased in tumor samples. MMP-1 tumor expression was correlated significantly with the evolution of lymph node status and tumor-lymph node-metastasis (TNM) stage. In contrast, MMP-9 tumor expression was correlated significantly with increased T stage. TIMP-1 overexpression was an independent predictor of worse survival in patients with NSCLC that was not associated with other prognosis factors, such as TNM stage.The overexpression of TIMP-1 was an independent prognostic marker in patients with NSCLC, and evaluating TIMP-1 may be important for identifying patients who are at greater risk of disease recurrence. 
 Hematopoiesis in mice is extremely resilient to wide variation in TIMP/MMP balance. Tissue inhibitors of matrix metalloproteinases (TIMPs) are natural inhibitors of matrix metalloproteinases (MMPs) and are associated with normal and pathologic   extracellular matrix   turnover. Because the microenvironment is critical for normal hematopoietic stem/progenitor cell function, we aimed to determine whether alterations in the TIMP/MMP balance impact upon normal hematopoiesis in mice. We have used both overexpression and knockout mouse models to determine whether early hematopoiesis is susceptible to potentially pathologic changes in TIMP/MMP level. These studies used TIMP-1(-/-) mice and retroviral vectors co-expressing human TIMP-1 or TIMP-2 linked with the green fluorescent protein (GFP) transduced into bone marrow (BM) cells and transplanted into lethally-irradiated recipient mice. Loss of TIMP-1 in knockout mice or retroviral overexpression of TIMP-1 or TIMP-2 did not alter hematopoietic stem/progenitor function during steady-state hematopoiesis. Surprisingly, even when applying hematopoietic stress through mobilization, chemotaxis, or myelosuppression, murine hematopoiesis was not adversely affected by TIMP-1 or TIMP-2 level. We conclude that TIMP/MMP balance alone does not exert significant influence on blood cell development and homeostasis. An important corollary of these studies is that specific modulation using MMP inhibitors for cancer or immunologic therapy is unlikely to have adverse hematopoietic side effects. 
 Alterations in biochemical components of   extracellular matrix   in intervertebral disc herniation: role of MMP-2 and TIMP-2 in type II collagen loss. Alterations in the composition of intervertebral disc   extracellular matrix  , mainly collagen and proteoglycans, may cause changes in mechanical properties of the disc, leading to dysfunction, nerve root compression, and herniation with severe clinical manifestations. Matrix metalloproteinases may be involved in degradation by hydrolysing   extracellular matrix   components. Inhibitors of matrix metalloproteinases, in contrast, function in the maintenance of degradation control. In this study, we investigated: (i) whether the level of matrix degradation correlated with the duration of the symptomatic disease, (ii) roles of matrix metalloproteinase-2 (MMP-2) and tissue inhibitor of matrix metalloproteinases-2 (TIMP-2) in intervertebral disc degeneration. Nucleus pulposus of intervertebral discs were obtained from 22 patients and analysed for collagen and proteoglycan contents, and pro-MMP-2, TIMP-2 levels. Collagen content was determined as hydroxyproline and proteoglycan content was measured as glycosaminoglycans. The loss in matrix components did not correlate with the duration of the degenerative disc disease. Pro-MMP-2 levels were higher at early stages of the degenerative disc disease (r = -0.495, P < 0.05). TIMP-2 levels were similar in all samples. Pro-MMP-2 and TIMP-2 levels negatively correlated in herniated discs samples (r = -0.855, P < 0.01). Pro- MMP-2 levels negatively correlated with the collagen content in herniated disc material. Our findings may suggest a silent period of active disease prior to symptomatic outcome during which irreversible matrix loss occurs. Involvement of other proteolytic enzymes at different stages of the disease should also be investigated to help to control the degradation cascade at relatively early stages of disc degeneration before the clinical onset of disease. 
 TIMP1, TIMP2, and TIMP4 are increased in aqueous humor from primary open angle glaucoma patients. Elevated intraocular pressure (IOP) is the only known modifiable risk factor for primary open angle glaucoma (POAG), and it can be caused by reduced aqueous humor outflow from the anterior chamber. Outflow is predominantly regulated by the trabecular meshwork, consisting of specialized cells within a complex   extracellular matrix   (ECM). An imbalance between ECM-degrading matrix metalloproteinases (MMPs) and the tissue inhibitors of MMPs (TIMPs) within the trabecular meshwork is thought to contribute to POAG. This study aimed to quantify levels of TIMPs and MMPs in aqueous humor samples from glaucomatous and non-glaucomatous eyes, analyze MMP/TIMP ratios, and correlate results with age, IOP, and Humphrey's visual field pattern standard deviation (PSD).Aqueous humor samples were collected from 26 non-glaucomatous control subjects before cataract surgery and 23 POAG patients undergoing trabeculectomy or cataract surgery. Analyte concentrations were measured using multiplexed immunoassays. Statistical significance was assessed with Mann-Whitney U tests, and Spearman's method was used to assess correlations with age, IOP, and PSD.Concentrations of TIMP1 (p = 0.0008), TIMP2 (p = 0.002), TIMP4 (p = 0.002), and MMP2 (p = 0.020) were significantly increased in aqueous humor samples from POAG versus cataract samples. For the majority of MMP/TIMP molar ratios calculated for the cataract group, TIMPs outweighed MMPs. In POAG, molar ratios of MMP2/TIMP1 (p = 0.007) and MMP9/TIMP1 (p = 0.005) showed a significant decrease, corresponding to an elevated excess of TIMPs over MMPs in POAG compared to cataract samples. Conversely, MMP2/TIMP3 (p = 0.045) and MMP3/TIMP3 (p = 0.032) molar ratios increased. Several MMP/TIMP molar ratios correlated with IOP (r = 0.476-0.609, p = 0.007-0.034) and PSD (r = -0.482 to -0.655, p = 0.005-0.046) in POAG samples and with age in cataract control samples.An imbalance among MMPs and TIMPs was found in glaucomatous aqueous humor samples, with a shift toward raised TIMP levels. This may result in the inhibition of MMP activity, leading to an altered ECM composition in the TM and thereby contributing to increased outflow resistance. 
 Distribution of matrix metalloproteinases MMP-1, MMP-2, MMP-8 and tissue inhibitor of matrix metalloproteinases-2 in nasal polyposis and chronic rhinosinusitis. Nasal polyposis (NP), a chronic inflammatory disease of the upper airway, is a subgroup of chronic rhinosinusitis (CRS). Matrix metallo-proteinases (MMPs) and their tissue inhibitors (TIMPs) are considered to play important roles in the pathogenesis of nasal polyposis. The aim of the current study was to evaluate and compare the levels of MMP-1, MMP-2, MMP-8 and TIMP-2 in NP and CRS with normal nasal mucosa by using immunohistochemistry. Twenty-five patients with NP and fifteen patients with CRS underwent endoscopic sinus surgery. Diseased mucosal samples were obtained from ethmoidal sinuses. Control nasal mucosa (n=10) was obtained from inferior nasal turbinate. Immunohistochemistry for MMP-1, MMP-2, MMP-8 and TIMP-2 was performed. The expression of MMP-1, MMP-2 and MMP-8 significantly increased in NP and CRS compared with control (p<0.05). The distribution of TIMP-2 was higher in CRS than control and NP respectively (p<0.05). MMP-1 immunoreactivity was distributed in the   extracellular matrix   whereas MMP-2, MMP-8 and TIMP-2 immunostaining was present in the epithelium, submucosal glands, vascular endothelium and inflammatory cells in CRS and NP. We suggest that differences in histological features between CRS and NP might be related to the expression of MMP-1, MMP-2, MMP-8 and their tissue inhibitor-2. 
 Overexpression of matrix metalloproteinases in lung tissue of patients with primary spontaneous pneumothorax. Although blebs and bullae are frequently found in the apexes of lungs of patients with primary spontaneous pneumothorax (PSP), its pathogens remain unclear.To examine the role of proteases [matrix metalloproteinase (MMP)-2, MMP-7 and MMP-9] and antiproteases [tissue inhibitors of metalloproteinase (TIMP)-1, TIMP-2, TIMP-3 and TIMP-4] in the pathogenesis of PSP.Fifty consecutive PSP patients who received standard surgical care were enrolled in the study. Lung tissues from 20 patients with stage I non-small cell lung cancer were used as a control. Immunohistochemistry (IHC), reverse transcription-polymerase chain reaction (RT-PCR) and gelatin zymography were used to evaluate the expression of MMP and TIMP in the lung tissue of patients with PSP.Overexpression of MMP-2, MMP-7 and MMP-9 was found in the afflicted lung by IHC, zymography and RT-PCR. By IHC, higher expression of MMP-2 and MMP-9 in PSP patients was identified in alveolar macrophages and type II pneumocytes (88 and 92% of patients in macrophages, and 72 and 70% of patients in type II pneumocytes, respectively). MMP-2, MMP-7 and MMP-9 expression in patients was higher in mesothelial cells (66, 76 and 76%). Overexpression of TIMP-2 was detected in the   extracellular matrix   around bullae and blebs. Expression levels of TIMP-1, TIMP-3 and TIMP-4 were negligible (<10% of cells) in both PSP patients and controls.MMP-2, MMP-9, MMP-7 and TIMP-2 were upregulated in PSP lesions. These results suggest that an imbalance between the expression of proteases and antiproteases may be involved in the pathogeneses of PSP. 
 Strong association of the tissue inhibitor of metalloproteinase-2 polymorphism with an increased risk of oral squamous cell carcinoma in Europeans. The present study was performed in order to investigate the possible association of the -418 G/C polymorphism in the tissue inhibitor of metalloproteinase-2 (TIMP-2) gene, which affects its expression, with the risk of developing oral cancer. PCR-based restriction analysis was performed in DNA samples from 158 patients with oral squamous cell carcinoma (OSCC) and 168 healthy controls of equivalent sex, age and ethnicity (Greeks and Germans). Statistical analyses were performed with Fisher's exact test and the calculation of odds ratios with a 95% confidence interval (CI). The frequency of the low C allele expression was ten times greater in the patients than the controls (31% vs 2.7%, respectively; P<0.001). The C/C and G/C genotypes were associated with an increased risk of developing OSCC (P<0.001, OR=40.88, 95% CI=2.24-744.40, and P<0.001, OR=21.31, 95%=9.82-46.21, respectively). The same pattern of significant differences with the controls was also observed in the subgroups of patients in regard to the initial or advanced stages of oral cancer, family history of any type of cancer or thrombosis, and smoking habits or alcohol abuse. These findings are consistent with the reduced levels of TIMP-2 in the presence of the low expression C allele, which are insufficient to inhibit the matrix metalloproteinase-driven degradation of the   extracellular matrix   (leading to cancer invasion) and mitogen-driven neoangiogenesis (leading to tumor growth and metastasis). In conclusion, the studied TIMP-2 polymorphism is strongly associated with an increased risk of OSCC in Europeans carrying the low C allele expression. These results indicate that this polymorphism could serve as a genetic marker for the susceptibility of cancer in the oral cavity. 
 Polymorphisms in the Promoters of the MMP-2 and TIMP-2 Genes Are Associated with Spontaneous Deep Intracerebral Hemorrhage in the Taiwan Population. Spontaneous intracerebral hemorrhage (ICH) is a devastating stroke subtype. Matrix metalloproteinases (MMPs) function in the degradation of   extracellular matrix   and the activities of MMPs are modulated by their endogenous inhibitors, tissue inhibitors of metalloproteinases (TIMPs). This study aimed to discuss relationship of MMP-2 and TIMP-2 to spontaneous deep ICH (SDICH) susceptibility and hematoma size.Associations were tested by logistic regression and general linear models (GLM) where appropriate, adjusting with covariables of age, sex, hypertension, diabetes mellitus, smoking, and alcohol consumption. Association analyses were performed first by stratification of genders and then by the age of 65 years old (y/o). Elder population was defined as subjects who were older than 65 y/o.There were 396 SDICH patients and 376 control subjects in this study. In the elder group, rs7503607 C>A variant in TIMP-2 was associated with SDICH in male and overall patients (OR = 3.49, 95% CI 1.45 to 8.40, P = 0.005 and OR = 2.45, 95% CI 1.37 to 4.38, P = 0.003, respectively) in additive genetic model. In recessive genetic model, rs2285053 TT genotype in MMP-2 was correlated to SDICH in male patients and overall elder group (OR = 7.30, 95% CI 1.3 to 40, P = 0.02 and OR = 2.91, 95% CI 1.02 to 8.31, P = 0.046, respectively), and rs7503726 AA genotype in TIMP-2 was associated with SDICH in female patients (OR = 0.29, 95% CI 0.1 to 0.84, P = 0.02). In younger male and overall younger patients, SDICH patients who had supratentorial hemorrhage had significantly lower frequency of AA genotypes in rs7503726 than those with infratentorial hemorrhage (OR = 0.36, 95% CI 0.17 to 0.75, P = 0.006 and OR = 0.43, 95% CI 0.22 to 0.84, P = 0.014, respectively). Hemorrhage size increased by 9.7 (95% CI 2.1 to 43, P = 0.004) cm3 per minor allele (A) of the rs7503607 variant in the elder female patients and increased by 4.3 (95% CI 1.4 to 12.9, P = 0.009) cm3 per minor allele (A) in all elder patients. In younger patients, the hemorrhage size decreased by 3.3 (95% CI 1.2 to 9.5, P = 0.03) cm3 per minor allele of the s7503726 variant in the female patients.This study showed a significant association between the variants of MMP-2 and TIMP-2 promoters and SDICH susceptibility with significant age and gender differences. Hemorrhage location and size might be affected by TIMP-2 promoter variants in the SDICH patients. 
 EMMPRIN-induced MMP-2 activation cascade in human cervical squamous cell carcinoma. Tumor progression and recurrence of cervical cancer is associated with upregulation of matrix metalloproteinase 2 (MMP-2). We evaluated the location, origin and activity of MMP-2 in cervical squamous cell carcinomas in comparison with MT1-MMP (MMP-14), TIMP-2 and   extracellular matrix   metalloproteinase inducer (EMMPRIN). Positive immunostaining for MMP-2 in malignant cells was detected in 83% of the patients. Two patterns of tumor cell MMP-2 staining were observed: either homogenous in all tumor cells or confined to the cells neighboring the stroma (tumor-border staining pattern, TBS). Fluorescence in situ zymography showed active MMP-2 mainly around tumor nodules displaying TBS. The MMP-2 staining of TBS tumors correlated significantly with the presence of TIMP-2 and MT1-MMP, proteins involved in docking MMP-2 to the cell surface and essential for MMP-2 activation. In situ mRNA hybridization in TBS tumors demonstrated more abundant presence of MMP-2 mRNA in neighboring myofibroblasts than in the adjacent tumor cells. Moreover, the TBS MMP-2 pattern correlated with the presence of EMMPRIN (p = 0.023), suggesting that tumor cells induce MMP-2 production in nearby stromal cells. This pro-MMP-2 could subsequently be activated on tumor cells via the presence of MT1-MMP and TIMP-2. The biological relevance of this locally activated MMP-2 was underscored by the observation that only the TBS pattern of MMP-2 significantly correlated with decreased survival. In conclusion, the colocalization of EMMPRIN, MT1-MMP and TIMP-2 in human cervical carcinomas seems to be involved in a specific distribution pattern of tumor cell bound MMP-2, which is related with local proteolytic activity and therefore might be associated with worse prognosis of the patients. 
 May High MMP-2 and TIMP-2 Expressions Increase or Decrease the Aggressivity of Oral Cancer? Matrix metalloproteinases-2 (MMP-2) and the tissue inhibitor of metalloproteinase-2 (TIMP-2), may presumably have an important role on the invasion and metastatic spread of malignancies attributed to an uncontrolled degradation of the   extracellular matrix   (ECM). A retrospective chart analysis was carried out to study the expression of MMP-2 and TIMP-2 on the archival samples of oral squamous cell carcinoma (OSCC) (n = 30) and normal mucosa (n = 10) by immunohistochemistry and compared with the clinicopathologic parameters of cases. Both MMP-2 and TIMP-2 expressions showed a positive correlation with the grades, stages and metastatic capacities of tumors (Spearman's correlation, p < 0.05). Concomitant increase in the expression of TIMP-2 and MMP-2 suggested that the rate of MMP-2/TIMP-2 expression is a better marker for characterization of MMP-2 concentration. High expression and/or activity of MMP-2 were linked with poorer survival in OSCC cases, while TIMPs have been shown to apparently act as either growth-stimulating or suppressor factors for tumors. It was also revealed that MMP-2 and TIMP-2 were secreted by both tumor cells and stromal cells. A new concept, supposing the dynamic, anticancer partnership between the residual genome stabilizer machinery of tumor cells and defensive cells adjacent to tumors, may illuminate the controversial results. In conclusion, the stronger the infiltrative and metastatic capacity of cancers, the higher is the rate of MMP-2/TIMP-2 expression helping the arrival of humoral and cellular anticancer forces. 
 [Expression of matrix metalloproteinase-2, 9 and their inhibitor-TIMP 1,2 in human squamous cell carcinoma of uterine cervix]. Studies of tumor invasion and metastasis have focused on the degradation of   extracellular matrix   (ECM). Matrix metalloproteinases (MMPs), which could degrade ECM, were implicated in cancer invasion and metastasis. Activated MMPs were controlled by specific tissue inhibitors of metalloproteinases (TIMPs). This study was designed to investigate the expression of MMP-2, MMP-9 and TIMP-1, TIMP-2 in human squamous cell carcinomas of uterine cervix, and the association with invasion and metastasis of human squamous cell carcinoma of uterine cervix.Tissue samples from 40 cases of squamous cell carcinoma of the uterine cervix, 29 cases of cervical intraepithelial neoplasia (CIN), and 16 cases of normal cervices were stained immunohistochemically.Immunohistochemical staining of tumor cells for MMP-2, MMP-9, TIMP-1, and TIMP-2 were noted 82.5%, 70.0%, 62.5%, and 97.5% in carcinomas, 44.8%, 62.1%, 86.21%, and 96.55% in CIN lesions respectively, for MMP-2, MMP-9, TIMP-1 in 25.0% normal cervices, but for TIMP-2 in 56.25% normal cervices. There was significant difference for MMP-2 among carcinomas, normal cervices, and CIN respectively (P < 0.01). There was significant difference for MMP-9 among normal cervices, carcinomas, and CIN, respectively (P < 0.05). The positive rates of TIMP-1 and TIMP-2 in the cervical carcinoma group and CIN group were obviously higher than that in normal cervical group(P < 0.01). The positive rates of MMP-2 and MMP-9 in lymph node metastasis were obviously higher than that in without lymph node metastasis(P < 0.01), but for TIMP-2 was contrary and there was no significant difference for TIMP-1. No significant correlation could be established among the expression of these markers and the tumor diameter, the clinical stage, and pathologic grade.The over-expression of MMP-2, MMP-9, TIMP-1, and TIMP-2 may play a key role in invasion and lymph-node metastasis of in squamous carcinoma of the cervix. 
 Differential regulation of MMP-2, TIMP-2 and IL-6 in valve replacement versus CABG patients.   Extracellular matrix   degradation may play an important role in left ventricular (LV) remodeling. It has been reported that matrix metalloproteinase-2 (MMP-2) is activated under mechanical stress conditions. Therefore, we examined the release of MMP-2, its inhibitor and interleukin-6 (IL-6), which affects MMPs, in patients undergoing cardiac surgery with cardiopulmonary bypass (CPB).Arterial blood samples were obtained from 20 patients undergoing cardiac surgery and six patients with descending aortic replacement (as noncardiac control) with CPB. Samples were assayed for plasma MMP-2, tissue inhibitors of matrix metalloproteinase-2 (TIMP-2) and IL-6 concentration.Plasma MMP-2 concentrations in the valvular disease patients were greater than in other patients (p < 0.05) and correlated with the LV mass (r=0.810, p < 0.0001) prior to the operation. Plasma MMP-2 concentrations decreased during CPB and gradually recovered to the baseline levels after CPB. Plasma TIMP-2 concentrations increased significantly during and after CPB in a biphasic manner. Plasma IL-6 concentrations also increased significantly during CPB (p < 0.05 versus baseline levels).Plasma MMP-2 concentrations reflect the state of the left ventricle, and changes in plasma MMP-2 and TIMP-2 concentrations during CPB may play an important role in LV remodeling after cardiac surgery. 
 The regulating role of mutant IkappaBalpha in expression of TIMP-2 and MMP-9 in human glioblastoma multiform. Our previous studies demonstrated that mutant IkappaBalpha (IkappaBalphaM) inhibited the occurrence, growth and angiogenesis of human glioblastoma multiform (GBM). However, the specific mechanism by which IkappaBalphaM regulates protein-degrading enzymes secreted from GBM to inhibit invasion and metastasis has remained unclear. The aim of the present study was to investigate the regulatory role and significance of IkappaBalphaM genes in the expression of tissue inhibitor of metalloproteinase (TIMP)-2 and matrix metalloproteinase (MMP)-9 in human GBM.We established the following four GBM cell lines stably expressing IkappaBalphaM by plasmid construction, gene transfection and screening for IkappaBalphaM protein expression: mutant IkappaBalpha-transfected cells (G36Delta-M), wild-type IkappaBalpha-transfected cells (G36Delta-W), empty plasmid transfected cells (G36Delta-P) and untransfected cells (G36Delta). The TIMP-2 and MMP-9 expression was detected by RT-PCR and Western blotting. Tumor cells were then implanted subcutaneously into nude mice to establish an animal model of ectopic tumor growth, and TIMP-2 and MMP-9 expression was determined by immunohistochemical methods.The results showed that there was a significant increase in TIMP-2 expression and a significant decrease in MMP-9 expression in the G36Delta-M group at both the RNA and protein levels compared with the G36Delta-W group, G36Delta-P group and G36Delta group. Similar results were observed in the immunohistochemical staining analysis of tumor tissues. In the G36Delta-M group, TIMP-2 expression was significantly higher while MMP-9 expression was significantly lower than in the other three groups.Our findings indicate that IkappaBalphaM inhibits the activation of NF-kappaB. It significantly up-regulates TIMP-2 expression in human malignant glioma cells and down-regulates the expression of MMP-9. Thus, IkappaBalphaM maintains the integrity of the   extracellular matrix   and further inhibits the growth and metastasis of tumor tissues. 
 Matrix metalloproteinases-2, -3 and tissue inhibitors of metalloproteinases-1, -2 in placentas from preterm pregnancies and their association with one-carbon metabolites. Maternal nutrition is an important determinant of one-carbon metabolism and defects in the one-carbon metabolism may lead to poor obstetric outcomes. This study was designed to test the hypothesis that altered intake/metabolism of micronutrients (folic acid and vitamin B12) and docosahexaenoic acid (DHA) contributes to increased homocysteine and oxidative stress leading to altered levels of matrix metalloproteinases (MMPs) and tissue inhibitors of metalloproteinases (TIMPs) in women delivering preterm. We have earlier reported increased vitamin B12, homocysteine, and oxidative stress along with reduced placental DHA in women delivering preterm. In this study, we further examine the placental levels of MMP2, MMP3, TIMP1, and TIMP2 in 75 women delivering at term and 73 women delivering preterm. Placental levels of MMPs and TIMPs were determined by ELISA. Placental MMP2 and MMP3 levels were higher (P<0.01) in women delivering preterm as compared with term. There was no difference in the placental TIMP1 and TIMP2 levels in women delivering preterm and at term. Further placental MMP2 and MMP3 levels were higher (P<0.01) in women with preterm labor as compared with those in labor at term, suggesting that MMPs may favor degradation of   extracellular matrix   in the placenta during preterm labor. Our study for the first time suggests a crucial role of micronutrients and MMPs in preterm birth. Future studies need to examine if epigenetic modifications through the one-carbon cycle contribute to increased levels of MMPs leading to preterm deliveries. 
 Matrix metalloproteinases MMP-2, -9 and tissue inhibitors TIMP-1, -2 expression and secretion by primary human osteoblast cells in response to titanium, zirconia, and alumina ceramics. Osteogenic properties of bone cells are a key parameter governing osseointegration of implant devices. In this context, osteoblasts have a central role via   extracellular matrix   synthesis and remodeling that they regulate through different protease activity. In this study, we have analyzed the expression of two matrix metalloproteinases (MMPs): MMP-2 (72 kDa) and MMP-9 (92 kDa) and their specific tissue inhibitors TIMP-1 and TIMP-2 in primary human osteoblastic cells. The effect of titanium, zirconia, and alumina ceramics on the synthesis of these proteases was assessed using reverse transcriptase-polymerase chain reaction, enzyme-linked immunosorbent assay, and zymographic analysis. Our results showed that osteoblasts express MMP-2 and -9 mRNA. Furthermore, MMP-2 mRNA expression was decreased by titanium and increased by alumina whereas zirconia did not have any significant effect. Conversely, MMP-9 mRNA expression was stimulated by titanium but decreased with zirconia, whereas alumina induced no significant changes. Zymographic analysis has evidenced pro-MMP-2 gelatinolytic activity in all cell populations with time-dependent increase profile; pro-MMP-9, however, was not detected. Enzyme-linked immunosorbent assay data confirmed the production of MMP-2 and very low levels of MMP-9. In addition, TIMP-1 was secreted in 24-h-cultured cells and increased to maximal level at 48-72 h whereas TIMP-2 levels were very low. The interactions between human osteoblasts and the studied biomaterials altered both MMP-2, -9 and TIMP-1expression indicating that biomaterials may influence osseointegration and bone remodeling. 
 TIMP-2: an endogenous inhibitor of angiogenesis. Remodeling of the   extracellular matrix  --regulated by the matrix metalloproteinases (MMPs) and their endogenous inhibitors--is an important component of disease progression in many chronic disease states. Unchecked MMP activity can result in significant tissue damage, facilitate disease progression and is associated with host responses to pathologic injury, such as angiogenesis. The tissue inhibitors of metalloproteinases (TIMPs) have been shown to regulate MMP activity. However, recent findings demonstrate that an MMP-independent effect of TIMP-2 inhibits the mitogenic response of human microvascular endothelial cells to growth factors. This is the first demonstration of a cell-surface signaling receptor for a member of the TIMP family and suggests that TIMP-2 functions to regulate cellular responses to growth factors. These new findings are integrated in a comprehensive model of TIMP-2 function in tissue homeostasis. 
 Tissue inhibitor of metalloproteinase-2 as a multifunctional molecule of which the expression is associated with adverse prognosis of patients with urothelial bladder carcinomas. Tissue inhibitors of metalloproteinases (TIMPs) regulate matrix metalloproteinase (MMP) activity controlling the breakdown of   extracellular matrix   components and, thus, play an important role in the process of invasion and metastasis. Moreover, there are several new functions, growth control, apoptosis, and angiogenesis-, in which TIMPs seem to be involved. The aim of this study was to elucidate the role of TIMP-2 in human urothelial cancer assessing TIMP-2 protein expression in 106 urothelial bladder carcinomas and evaluating its importance relative to clinicopathologic parameters (age, gender, histological grade, and stage) and patient survival, as well as to markers associated with cell growth and apoptosis (Ki-67, p53, and bcl-2).Immunohistochemistry (avidin-biotin complex method-horseradish peroxidase) was performed to detect TIMP-2, Ki-67, p53, and bcl-2 proteins using monoclonal and polyclonal antibodies. Statistical analysis was univariate and multivariate.TIMP-2 immunohistochemical expression was observed in stromal fibroblasts and in cancerous cells in 26.4% and 69.8% of cases, respectively. TIMP-2 stromal but not cancerous cell expression associated significantly with the high histological grade of carcinomas (P < 0.0001) and the advanced stage of the disease (P = 0.001). TIMP-2 either stromal or cancerous cell expression correlated significantly with the expression of Ki-67 proliferation indice (P = 0.02 and P = 0.044, respectively) and the mutant p53 protein (P = 0.043 and P = 0.045, respectively). In univariate survival analysis patients with positive TIMP-2 stromal cell immunohistochemical expression had a significantly worse overall survival in comparison with TIMP-2 stromal cell-negative patients (log rank test: P = 0.0002). However, in multivariate survival analysis the only independent survival factors were the stage of the disease and patient age.TIMP-2 protein expression in either the stromal or cancerous cells is associated with the proliferation index Ki-67 and the apoptosis-related protein p53. These findings are in keeping with in vitro studies reporting a growth-promoting ability of TIMP-2 and its involvement in apoptosis regulation. On the other hand, TIMP-2 stromal cell expression only was associated with adverse prognosis of urothelial bladder cancer patients. 
 Matrix metalloproteinases and their natural inhibitors in fibrovascular membranes of proliferative diabetic retinopathy. To examine epiretinal membranes of proliferative diabetic retinopathy (PDR) for the presence of selective matrix metalloproteinases (MMPs) and their natural inhibitors (TIMPs), in order to determine whether neovascularisation and fibrosis, characteristic of this complication of diabetes mellitus, are associated with specific anomalies of MMP or TIMP expression.The presence of selected MMPs and TIMPs was investigated in 24 fibrovascular epiretinal membranes of PDR, and the findings compared with that observed in 21 avascular epiretinal membranes of proliferative vitreoretinopathy (PVR) and five normal retinas. Specimens were examined for deposition of interstitial collagenase (MMP-1), stromelysin-1 (MMP-3), gelatinase A (MMP-2), gelatinase B (MMP-9), and three tissue inhibitors of metalloproteinases (TIMP-1, TIMP-2, and TIMP-3).The results showed that unlike normal retina, which constitutively expresses MMP-1 and TIMP-2, a large proportion of PDR membranes (> 62%) stained for MMP-1, MMP-2, MMP-3, MMP-9, TIMP-1, TIMP-2, and TIMP-3. There were no differences in the expression of these molecules when compared with PVR membranes. A characteristic staining for MMP-9 was observed within the perivascular matrix of PDR membranes, and there was a significant increase in TIMP-2 expression by PDR membranes (p= 0.036) when compared with PVR membranes.The findings that MMPs involved in degradation of fibrovascular tissue matrix, as well as TIMP-1 and TIMP-2, are found in a large proportion of PDR membranes, and that their expression does not differ from that of PVR membranes, suggest the existence of common pathways of   extracellular matrix   degradation in pathological processes leading to retinal neovascularisation and fibrosis. 
 Transient RNA silencing of tissue factor pathway inhibitor-2 modulates lung cancer cell invasion. Tissue Factor Pathway Inhibitor-2 (TFPI-2) is a potent inhibitor of plasmin which activates metalloproteinases (MMPs) involved in   extracellular matrix   (ECM) degradation. Its secretion in ECM makes TFPI-2 a potential inhibitor to regulate tumour invasion and metastasis. Moreover, TFPI-2 is frequently downregulated, particularly in aggressive cancers. In this study, we silenced TFPI-2 in the NCI-H460 non-small cell lung cancer cell line and evaluated the role of TFPI-2 in cell invasion and its impact on MMPs expression. As the effects of siRNA are transient, the consequences of both gene silencing and restoration to normal expression could be studied kinetically in the same cells. We showed that TFPI-2 expression by NCI-H460 cells was effectively downregulated using specific small interfering RNA and this silencing was associated with an increase in the invasive potential of tumour cells while migration was not affected. We also showed that mRNA levels and protein expression of MMP-2, -3, -9, -14 were not influenced by TFPI-2 silencing. Moreover, the gelatinase activity of MMP-2 and MMP-9 was unmodified. In contrast, MMP-1 mRNA levels and protein were significantly and similarly increased in cells transfected with TFPI-2 siRNA. In conclusion, this study confirms that TFPI-2 downregulation can contribute to tumour invasion of lung cancer cells. 
 Genetic analysis of polymorphisms in biologically relevant candidate genes in patients with abdominal aortic aneurysms. Abdominal aortic aneurysms (AAAs) are characterized by histologic signs of chronic inflammation, destructive remodeling of   extracellular matrix  , and depletion of vascular smooth muscle cells. We investigated the process of   extracellular matrix   remodeling by performing a genetic association study with polymorphisms in the genes for matrix metalloproteinases (MMPs), tissue inhibitors of metalloproteinases (TIMPs), and structural   extracellular matrix   molecules in AAA. Our hypothesis was that genetic variations in one or more of these genes contribute to greater or lesser activity of these gene products, and thereby contribute to susceptibility for developing AAAs.DNA samples from 812 unrelated white subject (AAA, n = 387; controls, n = 425) were genotyped for 14 polymorphisms in 13 different candidate genes: MMP1(nt-1607), MMP2(nt-955), MMP3(nt-1612), MMP9(nt-1562), MMP10(nt+180), MMP12(nt-82), MMP13(nt-77), TIMP1(nt+434), TIMP1(rs2070584), TIMP2(rs2009196), TIMP3(nt-1296), TGFB1(nt-509), ELN(nt+422), and COL3A1(nt+581). Odds ratios and P values adjusted for gender and country of origin using logistic regression and stratified by family history of AAA were calculated to test for association between genotype and disease status. Haplotype analysis was carried out for the two TIMP1 polymorphisms in male subjects.Analyses with one polymorphism per test without interactions showed an association with the two TIMP1 gene polymorphisms (nt+434, P = .0047; rs2070584, P = .015) in male subjects without a family history of AAA. The association remained significant when analyzing TIMP1 haplotypes (chi 2 P = .014 and empirical P = .009). In addition, we found a significant interaction between the polymorphism and gender for MMP10 ( P = .037) in cases without a family history of AAA, as well as between the polymorphism and country of origin for ELN ( P = .0169) and TIMP3 ( P = .0023) in cases with a family history of AAA.These findings suggest that genetic variations in TIMP1, TIMP3, MMP10, and ELN genes may contribute to the pathogenesis of AAAs. Further work is needed to confirm the findings in an independent set of samples and to study the functional role of these variants in AAA. It is noteworthy that contrary to a previous study, we did not find an association between the MMP9 (nt-1562) polymorphism and AAA, suggesting genetic heterogeneity of the disease.Abdominal aortic aneurysms (AAAs) are an important cardiovascular disease, but the genetic and environmental risk factors, which contribute to individual's risk to develop an aneurysm, are poorly understood. Histologically, AAAs are characterized by signs of chronic inflammation, destructive remodeling of the   extracellular matrix  , and depletion of vascular smooth muscle cells. We hypothesized that genes involved in these events could harbor changes that make individuals more susceptible to developing aneurysms. This study identified significant genetic associations between DNA sequence changes in tissue inhibitor of metalloproteinase 1 (TIMP1), TIMP3, matrix metalloproteinase 10 (MMP10) and elastin (ELN) genes, and AAA. The results will require confirmation using an independent set of samples. After replication it is possible that these sequence changes in combination with other risk factors could be used in the future to identify individuals who are at increased risk for developing an AAA. 
 Temporal investigation of matrix metalloproteinases and their inhibitors in human cervicovaginal fluid in late pregnancy and labor. Temporal expression of matrix metalloproteinase (MMP)-1, -2, -3, -7, -8, -9, -12, and -13, and tissue inhibitors of metalloproteinases (TIMPs)-1 and -2 in human cervicovaginal fluid (CVF) in term pregnancy and labor was investigated. Term parous women provided CVF samples that were grouped into labor, 1 to 3, 6 to 8, and 12 to 16 days before labor onset. Both MMPs and TIMPs (n = 60) were quantified using multiplex solution array and enzyme-linked immunosorbent assays, respectively. Further analysis of TIMP-1 (n = 180) was undertaken. All MMPs and TIMPs except MMP-12 and -13 were detected in the CVF. Matrix metalloproteinase 7, TIMP-1, and TIMP-2 were significantly increased in labor. Tissue inhibitors of metalloproteinase 1 was significantly increased up to 7 days before spontaneous labor onset. The data suggest a role of MMP-7 in the remodeling and rupture of fetal membranes and may reflect the homeostatic regulation of   extracellular matrix   remodeling of MMP-7 by TIMP-1 and TIMP-2. 
 The role of EZH2 in the regulation of the activity of matrix metalloproteinases in prostate cancer cells. Degradation of the   extracellular matrix   (ECM), a critical step in cancer metastasis, is determined by the balance between MMPs (matrix metalloproteinases) and their inhibitors TIMPs (tissue inhibitors of metalloproteinases). In cancer cells, this balance is shifted towards MMPs, promoting ECM degradation. Here, we show that EZH2 plays an active role in this process by repressing the expression of TIMP2 and TIMP3 in prostate cancer cells. The TIMP genes are derepressed by knockdown of EZH2 expression in human prostate cancer cells but repressed by overexpression of EZH2 in benign human prostate epithelial cells. EZH2 catalyzes H3K27 trimethylation and subsequent DNA methylation of the TIMP gene promoters. Overexpression of EZH2 confers an invasive phenotype on benign prostate epithelial cells; however, this phenotype is suppressed by cooverexpression of TIMP3. EZH2 knockdown markedly reduces the proteolytic activity of MMP-9, thereby decreasing the invasive activity of prostate cancer cells. These results suggest that the transcriptional repression of the TIMP genes by EZH2 may be a major mechanism to shift the MMPs/TIMPs balance in favor of MMP activity and thus to promote ECM degradation and subsequent invasion of prostate cancer cells. 
 The gene for tissue inhibitor of metalloproteinases-2 is localized on human chromosome arm 17q25. Tissue inhibitor of metalloproteinases-2 (TIMP2) is a natural inhibitor of several proteinases that are involved in the degradation of the   extracellular matrix  . By means of somatic cell hybrids segregating human chromosomes, the gene encoding this inhibitor was assigned to human chromosome 17. Fluorescence in situ hybridization confirmed this assignment and allowed mapping of the gene to the terminal region (17q25) of the chromosome. 
 siRNA knockdown of tissue inhibitor of metalloproteinase-1 in keloid fibroblasts leads to degradation of collagen type I. Keloids are defined as overgrowths of scar tissue resulting from abnormal wound healing. They are characterized by excessive dermal deposition of thick, hyalinized collagen bundles resulting from an imbalance between the production and degradation of   extracellular matrix   (ECM) components. Matrix metalloproteinases (MMPs) and tissue inhibitors of metalloproteinases (TIMPs) are two important regulators of ECM degradation and remodeling. To evaluate the role played by knockdown of TIMPs in keloid formation, we transduced human keloid-derived fibroblasts (KFs) with small interfering RNAs targeting TIMP-1 or -2 (siTIMP-1 or siTIMP-2) using a lentiviral vector and assessed the biological effects. We found that MMP-1/TIMP-1 and MMP-1/TIMP-2 complexes were suppressed and that MMP-2 activity was upregulated in KFs expressing siTIMP-1 or siTIMP-2. In addition, increased degradation of collagen type I was observed in the supernatant of KFs expressing siTIMP-1, but not siTIMP-2, with the suppression of cell viability and induction of apoptosis. These results suggest that targeting TIMP-1 using small interfering RNA has significant therapeutic potential as an approach to treating keloids through degradation of their thick collagen bundles. 
 Relationship between matrix metalloproteinases/tissue inhibitors of matrix metalloproteinases systems and autoantibody patterns in systemic sclerosis. Systemic sclerosis (SSc) is a connective tissue disease characterized by tissue fibrosis that reflects an imbalance between collagen production and degradation. Matrix metalloproteinases (MMPs) are a family of endopeptidases involved in the remodelling of   extracellular matrix   (ECM). This activity is controlled by tissue inhibitors of MMP (TIMPs). Aim of this study was the evaluation of MMP-9/TIMP-1 and MMP-2/TIMP-2 systems in patients with SSc.SearchLight Human MMP Array 1 was used to measure MMPs and TIMPs in 32 SSc patients and 32 matched healthy controls.SSc patients showed higher values of both MMP-9 and TIMP-1 in comparison with controls. The patients with anticentromere antibodies (ACA) positivity showed higher values of MMPs and TIMPs in comparison with either controls or the patients with anti-Scl70-positive antibodies.Results of this investigation suggest that SSc patients with ACA positivity, after a primary fibrogenetic noxa, react with a more abundant release of MMP/TIMP, whereas patients with anti-Scl70 antibody show a normal response. 
 Expression of MMP-2, TIMP-2, TGF-β1, and decorin in Dupuytren's contracture. To investigate the mechanisms underlying matrix deposition in Dupuytren's disease, the expression of gelatinase A (MMP-2), the tissue inhibitor of metalloproteinase-2 (TIMP-2), transforming growth factor beta 1 (TGF-β1), decorin (DCN), and periostin was studied. The level of relative MMP-2 activation was investigated using zymography. The mRNA expression of MMP-2, TIMP-2, TGF-β1, and DCN was detected using reverse transcription polymerase chain reaction (RT-PCR), while the presence of protein was detected using immunohistochemical (IHC) and Western blot techniques. The level of MMP-2 activation was significantly elevated in tissues with Dupuytren's contracture. RT-PCR demonstrated significantly higher expression of MMP-2, TIMP-2, TGF-β1, and DCN mRNA in the pathological tissues; and the IHC and immunoblotting studies revealed elevated expression of TGF-β1, DCN, and periostin. The balance between MMP-2 and TIMP-2 was disrupted in patients with Dupuytren's disease. TGF-β1, DCN, and periostin are involv  ed in extracellular   matrix (ECM) homeostasis in Dupuytren's contracture. 
 Matrix metalloproteinase expression in the ascending aorta and aortic valve.   Extracellular matrix   degradation and increased proteolytic enzyme (matrix metalloproteinase (MMP)) activity characterise abdominal aortic aneurysm formation. Post-stenotic dilatation of ascending aorta is associated with aortic stenosis and regurgitation, haemodynamically normal bicuspid aortic valve (BAV) and following AV replacement. We aimed to determine an association between ascending aortic pathology and abnormal AV, with particular reference to MMPs, and ascertain differences between BAV and tricuspid (TAV) AV. Subset of the study population (n=19) with a preoperative ascending aorta of >4 cm was analysed. Samples of ascending aorta and AV were obtained from 82 patients (TAV, n=54, BAV, n=28) undergoing surgery. Gene expression of MMP-1, -2, -9 and tissue inhibitor of metalloproteinase (TIMP)-1 and -2 was quantified by real-time RT-PCR. No significant difference was seen in gene expression level of MMPs, TIMPs and ratio of MMPs/TIMPs in ascending aorta and AV between patients with BAV and TAV. MMP-2/TIMP-1 in ascending aorta was greater in BAV, in the subset of patients with preoperative aortic dilatation (P<0.05). No difference exists in gene expression of MMPs in ascending aorta and AV between patients with BAV and TAV. However, patients with larger aortic diameters have increased MMP-2/TIMP-1. Modifying MMP expression may have a role in development of aneurysms. 
 Enhanced immunoreactivity of TIMP-2 in the stromal compartment of tumor as a marker of favorable prognosis in ovarian cancer patients. Degradation of the   extracellular matrix   and basement membrane is a critical step in tumor progression. Matrix metalloproteinase 2 (MMP-2) and tissue inhibitor of metalloproteinase 2 (TIMP 2) act in a coordinated manner to form an integrated system involved in ovarian cancer (OC) progression. In this study, the authors describe the expression of TIMP-2 detected by immunohistochemistry in 6 OC cell lines and in 43 malignant epithelial ovarian tumors (in tumor and stromal compartments) in sections originating from primary laparotomies. No significant correlations between overall and progression-free survival and TIMP-2 expression in tumor compartment were observed. The analysis demonstrated a significant association between enhanced stromal expression of TIMP-2 and better clinical response to cisplatin- and paclitaxel-based chemotherapy. Increased expression of TIMP-2 in the stromal compartment and simultaneous overexpression in both stromal and tumor compartments strongly correlated with increased survival. No significant correlations were found in vitro between resistance to cisplatin, paclitaxel, or topotecan and the expression of TIMP-2 in the OC cell lines, suggesting stromal influences on tumor chemoresistance in the physiological environment. This study supports the concept of TIMP-2 expression in the stromal compartment of OC as a promising marker of prognosis and response to cisplatin- and paclitaxel-based chemotherapy in OC patients. 
 Polymorphisms in MMP-9 and TIMP-2 in Chinese patients with varicose veins. Varicose veins (VVs), a common vascular disease, are functionally characterized by dilation and tortuosity and are widely prevalent in the adult population. The pathophysiology and molecular mechanism of VVs are still unclear. A genetic risk for VVs has been demonstrated, although no genetic variant pertaining to VVs has been identified. Matrix metalloproteinases (MMPs) and their endogenous tissue inhibitors (TIMPs), which can prevent excessive   extracellular matrix   (ECM) degradation, greatly impact vascular remodeling and may play a vital role in patients with VVs. We evaluated a potential association between polymorphisms in the promoters of MMP-9 and TIMP-2 and the risk for VVs in the Chinese population.Genotyping of the promoter region polymorphisms -1562C/T in MMP-9 and -418G/C in TIMP-2 was performed with PCR and restriction fragment length polymorphism (PCR-RFLP) assays with a group of 60 patients with VVs and 60 healthy controls. Purified PCR products were sequenced.A significant correlation was found between patients with VVs and controls at -1562C/T in MMP-9. The TIMP-2 gene polymorphism -418G/C was also associated with VVs.Our results suggest that polymorphisms in the promoter region of MMP-9 and TIMP-2 are associated with VVs in the Chinese population. 
 Tissue inhibitor of metalloproteinases-2 improves antitumor efficacy of a replicating adenovirus in vivo. Clinical studies of replicating adenoviruses for the treatment of cancer have demonstrated their safety but have yielded disappointing results, indicating the need for new strategies to improve their efficacy. We hypothesized that the efficacy of a replicating adenovirus could be improved by expression of tissue inhibitor of metalloproteinases-2 (TIMP-2), a 21-kDa unglycosylated secretory protein. TIMP-2 specifically inhibits the active forms of a number of matrix metalloproteinases (MMPs) that play a role in the degradation of basement membranes and the   extracellular matrix   and are therefore involved in the control of the growth, invasion and metastasis of tumor cells, as well as angiogenesis. In addition, TIMP-2 can abrogate tumor growth and angiogenesis by a variety of mechanisms independent of MMP inhibition. In this study, we demonstrate that expression of TIMP-2 enhanced the antitumor efficacy of a replicating adenovirus in vivo, by reducing both tumor growth and angiogenesis. 
 Aberrant tubulointerstitial immunoexpression of matrix metalloproteinases MMP-2, MMP-9 and tissue inhibitor of matrix proteinase-2 (TIMP-2) in acute cellular rejection of human renal allograft. Acute cellular rejection (ACR) may initiate chronic allograft dysfunction with alterations in the   extracellular matrix   compartment (ECM). Turnover of ECM proteins is regulated by matrix metalloproteinases (MMPs). The aim of the present study was to determine the immunoexpression of MMP-2, MMP-9 and TIMP-2 in ACR, and to examine the relationship between expression of MMPs and monocytes/macrophages, transforming growth factor beta-1 (TGFbeta-1), and alpha-smooth muscle actin (alpha-SMA). Immunoperoxidase study with antibodies against MMP-2, MMP-9, TIMP-2, CD68, TGFbeta-1 and alpha-SMA was carried out on 24 renal allograft biopsy specimens from patients with ACR and 11 allograft biopsy specimens from patients with no signs of rejection. Our study revealed increased immunoexpression of MMP-2, MMP-9 and TIM-2 in ACR as compared with controls, and significant positive correlations between immunostaining of MMP-2 and TGF-beta-1, as well as between MMP-2 and alpha-SMA. Increased immunoexpression of MMP-9 was positively correlated with alpha-SMA, and the number of interstitial CD68+cells. In conclusion our study supports a role of gelatinases in tissue damage in human renal acute cellular allograft rejection and provides some interesting insights into early renal remodeling which may lead to chronic allograft dysfunction. 
 Age- and gender-dependent changes in connective tissue remodeling: physiological differences in circulating MMP-3, MMP-10, TIMP-1 and TIMP-2 level. the mechanisms which cause age-dependent remodeling of connective tissue are still not fully understood. Matrix metalloproteinases (MMPs) and tissue inhibitors of metalloproteinases (TIMPs) constitute an important proteolytic pathway affecting physiological matrix remodeling.the way in which changes in the   extracellular matrix   metabolism during the ageing process influence the level of circulating MMP-3 and MMP-10, as well as their tissue inhibitors TIMP-1 and TIMP-2, in a healthy population was investigated in this study.blood samples were taken from 81 healthy individuals aged 6-62 years and measured for MMP-3, MMP-10, TIMP-1 and TIMP-2 levels using enzyme-linked immunosorbent assays. Polyacrylamide gel electrophoresis followed by Western immunoblotting allowed for the detection of pro- and active forms of both MMPs.Serum MMP-3 and TIMP-1 values were positively correlated with age (r = 0.44, p = 0.00001 and r = 0.28, p = 0.012, respectively). A contrary tendency was found for MMP-10 and TIMP-2 serum levels. A strong age-related decrease in MMP-10 (-0.53; p = 0.000) and TIMP-2 (-0.52; p = 0.000) was noticed in our study. Gender was a significant factor modifying MMP/TIMP potential, except for the MMP-10 level.the data presented indicate that changes in MMP/TIMP balance occur in physiological ageing. Moreover, these findings highlight the necessity of utilizing age- and sex-matched values for analysis of MMPs and TIMPs in the pathological conditions. 
 In vitro study of matrix metalloproteinase/tissue inhibitor of metalloproteinase production by mesenchymal stromal cells in response to inflammatory cytokines: the role of their migration in injured tissues. The transmigratory capacity of bone marrow (BM) mesenchymal stromal cells (MSC) through the endothelial cell barrier into various tissues and their differentiation potential makes them ideal candidates for cell therapy. Nevertheless, the mechanisms and agents promoting their migration are not fully understood. We evaluated the effects of several inflammatory cytokines on the migration of BM MSC and matrix metalloproteinase (MMP)/tissue inhibitor of metalloproteinase (TIMP) production.The migratory potential of BM MSC was evaluated using a Boyden chamber coated with Matrigel in the presence and absence of stromal cell-derived (SDF)-1alpha, platelet-derived growth factor (PDGF)bb, insulin-like growth factor (IGF)-I and interleukin (IL)-6. The ability of inflammatory cytokines to induce MSC migration was tested in presence of their respective Ab or blocking peptide. We used immunofluorescence to check the expression of cytokine receptors, and MMP/TIMP production was analyzed at the protein (human cytokine array, enzyme-linked immunosorbent assay (ELISA), gelatine zymography and Western blot) and mRNA quantitative real-time polymerase chain reaction (qRT-PCR) levels.We have demonstrated that inflammatory cytokines promote the migratory capacity of BM MSC according to the expression of their respective receptors. Higher migration through Matrigel was observed in response to IL-6 and PDGFbb. qRT-PCR and cytokine array revealed that migration was the result of the variable level of MMP/TIMP in response to inflammatory stimuli.Our observations suggest that chemokines and cytokines involved in the regulation of the immunity or inflammatory process promote the migration of MSC into BM or damaged tissues. One of the mechanisms used by MSC to promote their migration though the   extracellular matrix   is modulation of the production of MMP-1, MMP-2, MMP-13, TIMP-1 and TIMP-2. 
 The influence of MMP-14, TIMP-2 and MMP-2 expression on breast cancer prognosis. Matrix metalloproteinase (MMP)-2 is very active at degrading   extracellular matrix  . It is under the influence of an activator, membrane type 1 MMP (MMP-14), and the tissue inhibitor of metalloproteases (TIMP)-2. We hypothesized that the individual expression of these three markers or their balance may help to predict breast cancer prognosis.MMP-2, MMP-14 and TIMP-2 expression has been evaluated by 35S mRNA in situ hybridization on paraffin material of 539 breast cancers without distant metastasis at diagnosis and with a median follow-up of 9.2 years.MMP-2 and MMP-14 mRNA was detected primarily in reactive stromal cells whereas TIMP-2 mRNA was expressed by both stromal and cancer cells. Of the three molecules, an adjusted Cox model revealed that high MMP-14 mRNA (> or = 10% cells) alone predicted a significantly shorter overall survival (p = 0.031) when adjusted for clinical factors (tumor size and number of involved lymph nodes). Prognostic significance was lost when further adjusted for Her-2/neu and urokinase-type plasminogen activator (p = 0.284). Furthermore, when all three components were analyzed together, the survival was worst for patients with high MMP-2/high MMP-14/low TIMP-2 (5 year survival = 60%) and best with low MMP-2/low MMP-14/high TIMP-2 (5 year survival = 74%), but the difference did not reach statistical significance (p = 0.3285).Of the MMP-14/TIMP-2/MMP-2 complex, MMP-14 was the factor most significantly associated with the outcome of breast cancer and was an independent factor of poor overall survival when adjusted for clinical prognostic factors, but not for certain ancillary markers. 
 Increased expression of metalloproteinase-2 and -9 (MMP-2, MMP-9), tissue inhibitor of metalloproteinase-1 and -2 (TIMP-1, TIMP-2), and EMMPRIN (CD147) in multiple myeloma. Activity of metalloproteinases (MMP) is controlled both by specific tissue inhibitors (TIMP) and activators (  extracellular matrix   metalloproteinase inducer, EMMPRIN). There are few data available concerning concentration the bone marrow of MMP-2, MMP-9, TIMP-1, and TIMP-2, or EMMPRIM expression by bone marrow mesenchymal stromal cells (BMSCs) in patients with multiple myeloma (MM).We studied 40 newly diagnosed, untreated patients: 18 males and 22 females with de novo MM and 11 healthy controls. Bone marrow was collected prior to therapy. BMSCs were derived by culturing bone marrow cells on MesenCult. Protein concentrations were determined in bone marrow plasma and culture supernatants by ELISA. EMMPRIN expression by BMSCs was assessed by flow cytometry.The median concentrations of MMP-9, TIMP-1, and TIMP-2 in both marrow plasma and culture supernatants were significantly higher in MM patients than controls.EMMPRIN expression and ratios MMP-9/TIMP-1 and MMP-2/TIMP-2 were higher in MM patients, our results demonstrate that in MM patients MMP-2 and MMP-9 are secreted in higher amounts and are not balanced by inhibitors. 
 [Analysis of tissue inhibitor of metalloproteinase-2 gene polymorphisms in a caucasian population with abdominal aortic aneurysms]. The formation of sporadic abdominal aortic aneurysm (AAA) is explained by a remodelling of the   extracellular matrix   (ECM) and breakdown of structural components of the vascular wall. Matrix metalloproteinases are the principle matrix-degrading proteases and are known to play a major role in the remodelling of the   extracellular matrix   in arterial vessels. Their activity is controlled by tissue inhibitors of metalloproteinases (TIMPs). Decreased TIMP-1 and TIMP-2 expression in the   extracellular matrix   of the walls of AAAs has been demonstrated in several studies. This case-control study was designed to investigate the possible impact of genetic variants of the TIMP-2 gene in the aetiology of AAA and to reproduce a recently described significant difference in allele frequency of the SNP 303G>A in a German population.TIMP-2 single nucleotide polymorphisms (SNPs) were analysed in a study sample of 50 patients with AAA and 41 controls. Differences in genotype and allele frequencies of the identified polymorphisms were determined after sequencing the entire coding region and selected parts of the promoter using the automated laser fluorescence technique.Six polymorphisms were identified, one of which is described for the first time, located in the intron, (231+23C>T). An association of the SNP 303G>A with the phenotype was not confirmed in our study (p=0.648). However, the CT genotype of the SNP -479C>T was more frequent in patients with AAA than in the control group (p=0.054).In our analysis of the TIMP-2 gene, we identified one new SNP. A previously published association of the SNP 303G>A with the phenotype could not be validated in our population. However, we detected an association for the CT genotype of one polymorphism in the promoter region (g-479C>T) and AAA. This result has to be proved in a second study sample. 
 Analysis of MMP-9 and TIMP-2 gene promoter polymorphisms in individuals with hypodontia. Hypodontia, the congenital absence of one or a few teeth, is one of the most common developmental alterations of human dentition. It may cause masticatory and speech dysfunctions and create esthetic problems with orthodontic and prosthetic implications. MMP-9 is an important member of the matrix metalloproteinase (MMP) family that participates in remodeling of   extracellular matrix   during tooth development. A C-to-T base exchange at position -1562 creates two different alleles, and the CT and TT genotypes promote high activity of the MMP-9 gene promoter. Tissue inhibitor of metalloproteinase-2 (TIMP-2) regulates the activity of MMPs in the   extracellular matrix   and is co-expressed with gelatinases A (MMP-2) and B (MMP-9) during mouse tooth morphogenesis. A polymorphism in the TIMP-2 gene promoter at position -418 has been found in a Sp-1 binding site. In this study, the association between these DNA polymorphisms and hypodontia has been investigated. The significance of differences in frequencies of polymorphisms in control and test groups was assessed by Chi-square test (p<0.05). Data suggested that MMP-9 gene promoter polymorphism was not associated with hypodontia. The high frequency of GG genotype in the TIMP-2 gene promoter showed that this site was unsuitable for studies of DNA polymorphism-disease associations in the studied population. 
 NDRG2 ameliorates hepatic fibrosis by inhibiting the TGF-β1/Smad pathway and altering the MMP2/TIMP2 ratio in rats. Liver fibrosis is a worldwide clinical issue. It has been well established that activated hepatic stellate cells (HSCs) are responsible for excessive   extracellular matri  x (ECM) deposition in chronically damaged livers. The identification of key elements that control HSCs activation will help to further our understanding of liver fibrosis and improve the outcome of clinical treatment. This study demonstrates that N-Myc downstream-regulated gene 2 (NDRG2) is a potential regulator of liver fibrosis as NDRG2 mRNA and protein levels were reduced during HSCs activation. In addition, enhanced NDRG2 expression reduced Smad3 transcription and phosphorylation, which inhibited HSCs activation by blocking the TGF-β1 signal. Moreover, NDRG2 contributed to an increase in the ratio of matrix metalloproteinase 2 (MMP2) to tissue inhibitor of matrix metalloproteinase 2 (TIMP2), which may facilitate the degradation of the ECM. In dimethylnitrosamine (DMN)-induced fibrotic rat livers, adenovirus-mediated NDRG2 overexpression resulted in decreased ECM deposition and improved liver function compared with controls. In conclusion, the present findings indicate that the modulation of NDRG2 is a promising strategy for the treatment of liver fibrosis. 
 Polymorphisms of MMP9 and TIMP2 in Patients with Varicose Veins. Genetic predisposition is a suggested risk factor in the etiology of varicose veins. The matrix metalloproteinase (MMP) family degrades   extracellular matrix   (ECM) and may lead to disturbances in vein wall structure. The activity of MMPs in the ECM are controlled by specific tissue inhibitors of MMPs (TIMP). The present study aimed to investigate the relationship between MMP9 and TIMP2 gene polymorphisms and varicose vein risk.Genotyping of the polymorphisms of MMP9 (1562 C/T) and TIMP2 (418G/C) was performed using polymerase chain reaction and restriction-fragment length polymorphism assays in a group of patients with varicose veins (n=63) and healthy controls (n=70).The frequencies of MMP9 alleles and genotypes did not differ significantly between patient and control groups. However, TIMP2 -418 C allele was associated with increased risk for varicose vein formation (p=0.007). It was also shown that the frequency of the GG genotype was significantly higher in the control group than in the patient group (odds ratio=0.333, 95% confidence interval=0.14-0.78, p=0.012).TIMP2 -418 C allele is associated with susceptibility for varicose vein formation and individuals with GG genotype may have a lower risk for varicose vein formation. 
 Tissue inhibitor of metalloproteinases-1, -2, and -3 polymorphisms in a white population with intracranial aneurysms. Remodeling of the   extracellular matrix   seems to be a crucial event in the pathogenesis of cerebral aneurysms. Matrix metalloproteinases are the most important degrading enzymes in the   extracellular matrix  . Their activity is controlled predominantly by tissue inhibitors of metalloproteinases (TIMPs). To investigate the possible impact of genetic variants within the genes encoding TIMP-1, -2, and -3, we conducted this case-control study.A study sample was analyzed that comprised 44 patients with intracranial aneurysms and 44, 41, and 40 controls for the analysis of TIMP-1, -2, and -3, respectively. Differences in genotype and allele frequencies of identified polymorphisms were determined. The entire coding regions and parts of the promoter sequences of the TIMP-1, -2, and -3 genes were with using the automated laser fluorescence technique.Nine polymorphisms were identified, 3 located in TIMP-1 (-19C>T, 261C>T, 372T>C), 4 in TIMP-2 (-621C>T, -596A>C, -261G>A, 303G>A), and 2 in TIMP-3 (249T>C, 261C>T), whereas -621C>T, -596A>C, and -261G>A of the TIMP-2 gene are newly identified polymorphisms. We detected no deviation from Hardy-Weinberg equilibrium in any of the groups. The C allele of the 372T>C polymorphism was more frequently found in female than in male controls (exact nominal P=0.0012). However, this finding could not be validated by analysis of a second sample of 113 controls (exact nominal P=1.0000). There were no differences in genotype and allele frequencies between any of the other groups.Our analysis of the entire coding region of 3 TIMPs, which are the main inhibitors of metalloproteinase activity in the   extracellular matrix  , failed to show an association between genetic polymorphisms and an intracranial aneurysm. These data do not support the hypothesis that genetic variants within these genes have an impact on aneurysm development in the white population. 
 NF-Y and Sp1 cooperate for the transcriptional activation and cAMP response of human tissue inhibitor of metalloproteinases-2. The balance between matrix metalloproteinases (MMPs) and tissue inhibitors of MMPs (TIMPs) is a key determinant in the homeostasis of the   extracellular matrix  . We have identified two cis-acting elements involved in the transcriptional regulation of TIMP-2. The first is an inverted CCAAT box located at position -73 to -69 in the TIMP-2 promoter that binds the transcription factor NF-Y. The second is a GAGGAGGGGG motif located at position -107 to -98, that binds the transcription factors Sp1 and Sp3. NF-Y and Sp1 cooperate for the basal transcription activity of the promoter. We then determined that TIMP-2 is transcriptionally up-regulated by cAMP analogs. Up-regulation of TIMP-2 by dibutyryl cAMP is a delayed response that requires de novo protein synthesis and does not affect RNA stability. The NF-Y and the Sp1 binding site are both involved in cAMP-dependent up-regulation of TIMP-2. Whereas NF-Y is essential for cAMP mediated regulation, Sp1 alone is not sufficient but enhances the activity of NF-Y. Dibutyryl cAMP has no effect on the expression of MMP-2 and MMP-9 and switches the MMP-TIMP balance in favor of the inhibitor. 
 Circulating matrix metalloproteinase-9 and tissue inhibitors of metalloproteinases-1 and -2 levels in gestational hypertension. Gestational hypertension (GH) is dangerous to both mother and child. Arterial invasiveness and growth are dependent on successful   extracellular matrix   (ECM) breakdown, which may be abnormal in GH. We hypothesized abnormalities in circulating matrix metalloproteinase-9 (MMP-9) and tissue inhibitors of metalloproteinases-1 and -2 (TIMP-1 and TIMP-2, respectively) in patients with GH, when compared with normotensive women with normal pregnancies and healthy nonpregnant control subjects.Plasma MMP-9, TIMP-1, and TIMP-2 were measured by ELISA in 23 women with GH, 30 normotensive pregnant women, and 28 nonpregnant women who were matched for age, gestational age, and parity.Levels of circulating MMP-9, TIMP-1 and TIMP-2, and the MMP-9/TIMP-1 and MMP-9/TIMP-2 ratios were significantly different among the three groups (P = .026, P = .006, P = .007, P = .001 and P = .008 respectively). Within the GH group, MMP-9 and the MMP-9/TIMP-1 ratio correlated negatively with age (r = -0.581, P = .004 and r = -0.563, P = .005, respectively) and levels of diastolic blood pressure (r = -0.432, P = .040 and r = -0.461, P = .027, respectively). With multiple regression analysis, only age independently correlated with circulating levels of MMP-9 (P = .010); neither age nor levels of diastolic blood pressure had any effect on the MMP-9/TIMP-1 ratio.We have demonstrated altered MMP/TIMP ratios in maternal blood during GH. These observations suggest pregnancy-related changes in ECM breakdown and turnover. Given the importance of changes in ECM composition to vascular and cardiac structure in hypertension, we suggest that these observations may be related to the pathophysiology of human GH. 
 Prognostic impact of polymorphism of matrix metalloproteinase-2 and metalloproteinase tissue inhibitor-2 promoters in breast cancer in Tunisia: case-control study. Matrix metalloproteinases (MMPs) are proteolytic enzymes that play important roles in tumor invasion and metastasis by degrading   extracellular matrix   components. Genetic variations in promoter regions of MMP genes, affecting their expression, have been associated with susceptibility to cancers. The aim of this study was to investigate the susceptibility and prognostic implications of the matrix metalloproteinase-2 (MMP-2) and tissue inhibitor of metalloproteinase-2 (TIMP-2) polymorphism in Tunisian breast cancer patients. MMP-2 genotypes were determined by real-time polymerase chain reaction (RT-PCR), and TIMP-2 genotypes were identified using a PCR-restriction fragment length polymorphism (RFLP) method in 210 breast cancer patients and 250 frequency-matched control women. Association of the clinicopathological parameters and the genetic markers with risk of breast cancer was assessed using univariate analyses. We found that the variant MMP-2 genotype (-1306CT or TT) was associated with substantially reduced risk of breast cancer [odds ratio (OR), 0.49; 95 % confidence interval (95 % CI), 0.033-0.73], compared with the CC genotype. For TIMP-2, a moderately reduced risk of the cancer (OR, 0.57; 95 % CI, 0.37-0.87) was also associated with the variant allele (-418GC or CC), compared with the GG common allele. Furthermore, polymorphisms in both genes seem to have additive effects and the highest risk for breast cancer has been observed in those with MMP-2 CC genotype and TIMP-2 GC or CC genotype (p = 0.006). A significant association was also found between the CC genotype and the aggressive forms of breast cancer as defined by advanced stages at the time of diagnosis and metastasis. This is the first report on the association of MMP-2 and TIMP-2 gene polymorphisms in breast cancer in Tunisian population. Our results suggest that the presence of the variant allele in the promoter of MMP-2 or TIMP-2 may be a protective factor for the development of breast cancer. 
 Human cardiac fibroblast   extracellular matrix   remodeling: dual effects of tissue inhibitor of metalloproteinase-2. Tissue inhibitor of metalloproteinase-2 (TIMP-2) is an endogenous inhibitor of matrix metalloproteinases (MMPs) that attenuates maladaptive cardiac remodeling in ischemic heart failure. We examined the effects of TIMP-2 on human cardiac fibroblast activation and   extracellular matrix   (ECM) remodeling.Human cardiac fibroblasts within a three-dimensional collagen matrix were assessed for phenotype conversion, ECM architecture and key molecular regulators of ECM remodeling after differential exposure to TIMP-2 and Ala+TIMP-2 (a modified TIMP-2 analogue devoid of MMP inhibitory activity).TIMP-2 induced opposite effects on human cardiac fibroblast activation and ECM remodeling depending on concentration. TIMP-2 activated fibroblasts into contractile myofibroblasts that remodeled ECM. At higher concentrations (>10 nM), TIMP-2 inhibited fibroblast activation and prevented ECM remodeling. As compared to profibrotic cytokine transforming growth factor (TGF)-beta1, TIMP-2 activated fibroblasts and remodeled ECM without a net accumulation of matrix elements. TIMP-2 increased total protease activity as compared to TGF-beta1. Ala+TIMP-2 exposure revealed that the actions of TIMP-2 on cardiac fibroblast activation are independent of its effects on MMP inhibition. In the presence of GM6001, a broad-spectrum MMP inhibitor, TIMP-2-mediated ECM contraction was completely abolished, indicating that TIMP-2-mediated fibroblast activation is MMP dependent.TIMP-2 functions in a contextual fashion such that the effect on cardiac fibroblasts depends on the tissue microenvironment. These observations highlight potential clinical challenges in using TIMP-2 as a therapeutic strategy to attenuate postinjury cardiac remodeling. 
 Expression of MMP-9/TIMP-2 in nasal polyps and its functional implications. Nasal polyps (NP) involve tissue repair and structural remodel, both of which require the   extracellular matrix  . Matrix metalloproteinase (MMP) and tissue inhibitor of metalloproteinase (TIMP) are known regulators for tissue reconstruction. This study therefore aimed to analyze the expressional profile of MMP-9 and TIMP-2 in NP patients, with further investigation of their roles in pathogenesis. A total of 60 NP tissue samples (including 15 type I, 21 type II and 24 type III) were collected from surgeries in our hospital, in addition to 6 normal ethmoid sinus mucosa samples. The mRNA and protein expression levels of MMP-9/TIMP-2 were quantified by real-time PCR and Western blotting, respectively. Serum levels were also checked by enzyme-linked immunosorbent assay (ELISA). Both mRNA and protein levels of MMP-9 in NP tissues or serum were significantly elevated compared to those in control ones (P<0.05) while the TIMP-2 expression was suppressed (P<0.05). In patients with more advanced stage, MMP-9 expression was further elevated, with lowered TIMP-2 levels (P<0.05 in both cases). Pathogenesis and progression of NP is closely related with elevated MMP-9 and suppressed TIMP-2 expression, suggesting the role of those factors as indexes for evaluating NP stage. Our results also provide evidences for further studies of pathogenesis and drug targets of NP. 
 TIMP-2 mediated inhibition of angiogenesis: an MMP-independent mechanism. Tissue inhibitors of metalloproteinases (TIMPs) suppress matrix metalloproteinase (MMP) activity critical for   extracellular matrix   turnover associated with both physiologic and pathologic tissue remodeling. We demonstrate here that TIMP-2 abrogates angiogenic factor-induced endothelial cell proliferation in vitro and angiogenesis in vivo independent of MMP inhibition. These effects require alpha 3 beta 1 integrin-mediated binding of TIMP-2 to endothelial cells. Further, TIMP-2 induces a decrease in total protein tyrosine phosphatase (PTP) activity associated with beta1 integrin subunits as well as dissociation of the phosphatase SHP-1 from beta1. TIMP-2 treatment also results in a concomitant increase in PTP activity associated with tyrosine kinase receptors FGFR-1 and KDR. Our findings establish an unexpected, MMP-independent mechanism for TIMP-2 inhibition of endothelial cell proliferation in vitro and reveal an important component of the antiangiogenic effect of TIMP2 in vivo. 
 Expression of MMP-7, MMP-9, TIMP-1 and TIMP-2 mRNA in lung tissue of patients with non-small cell lung cancer (NSCLC) and benign pulmonary disease. The expression of matrix metallo-proteinases (MMP-7 and MMP-9) and tissue inhibitors of metalloproteinases (TIMP-1 and TIMP-2), which are involved in the degradation of the   extracellular matrix   (ECM) and tumor growth, was investigated in normal lung tissue, tissue of benign pulmonary diseases and non-small cell lung cancer (NSCLC) tissue.Tumor tissue and surrounding carcinoma-free lung tissue samples were obtained from 91 patients with NSCLC who had undergone surgery in the years 2005-2007 as well as lung tissue from 12 patients operated on for 'benign' bullous emphysema or interstitial lung disease. The mRNA was isolated from the tissues and the expression of mRNA was assessed using a real-time RT PCR method.Significantly higher expression of MMP-7, MMP-9 and TIMP-1 mRNA was demonstrated in the NSCLC tissue in comparison with the normal lung tissue from the same patients (p=0.0003, p<0.0001 and p=0.0018, respectively). Similar results for MMP-7, MMP-9 and TIMP-1 were found in the histological subgroups: squamous cell lung cancer vs. normal tissue (p=0.0198, p=0.0015 and p=0.0366, respectively), and adenocarcinoma vs. normal tissue (p=0.0045, p<0.0001 and p=0.0140, respectively). The expression of MMP-7 was found to be significantly higher in tumor tissue vs. lung tissue of the benign diseases (p=0.0086) and similar results were also recorded in the histological subgroups: squamous cell lung cancer vs. benign tissue (p=0.0171) and adenocarcinoma vs. benign tissue (p=0.0135). The expression of MMP-9 was significantly higher only in the adenocarcinoma subgroup vs. the benign tissue (p=0.0412). No differences in the expression of mRNA between stage IA and stages IB-IIIB of NSCLC were recorded.Significantly higher expression of MMP-7 and MMP-9 in tumor tissue than in the surrounding tissue or in benign lung disease tissue supports the notion of an important role of these metalloproteinases in the growth of lung carcinoma. TIMP-1 expression is increased only in carcinoma, but not in benign lung disease. 
 Expression of matrix metalloproiteinases (MMPs) and tissue inhibitors of metalloproteinases (TIMPs) in hepatocellular carcinoma tissue, compared with the surrounding non-tumor tissue. Matrix metalloproteinases (MMPs) have been implicated in the tumor invasion and growth through the degradation of   extracellular matrix  . In this study, we selected 46 hepatocellular carcinoma (HCC) cases, at random, and we immunohistologically examined the expression of MMP-1, MMP-2, MMP-3, MMP-7, MMP-9, tissue inhibitor of metalloproteinases (TIMP)-1, TIMP-2, in cancerous and non-cancerous areas using avidin-biotin-peroxidase complex method. In all cases, cancer cells, hepatocytes, sinusoidal lining cells, leukocytes, and bile ducts were positive for all the primary antibodies. The expressions of MMPs and TIMPs in most of the HCC tissues were equal or low compared with those in the surrounding non-tumor tissues, although mixed expression pattern were recognized in some HCC tissues. The difference of MMP and TIMP expression was not related with the histological differentiation of HCC and the condition of non-cancerous area. These findings suggested little association of the clinicopathological findings of HCC with the histological expression of MMPs and TIMPs. 
 The role of matrix metalloproteinases (MMPs) and their inhibitors (TIMPs) in the development of esophageal cancer. Esophageal cancer (EC) is one of the most aggressive malignant tumors of the gastrointestinal tract. There are two distinct histological types of EC: esophageal squamous cell carcinoma and adenocarcinoma of the esophagus. Etiologic factors and the patterns of incidence of both subtypes are different. Matrix metalloproteinases (MMPs) and their tissue inhibitors (TIMPs) play an important role in esophageal carcinogenesis. Gellatinases MMP-2 and MMP-9 are able to degrade collagen IV from basement membranes and   extracellular matrix   which is related to tumor progression, including invasion, metastasis, growth and angiogenesis. It has been shown that increased expression of MMPs plays a crucial role in the development of several human malignancies, including esophageal cancer. The activity of MMPs is regulated by their endogenous natural inhibitors (TIMPs). Among these, the roles of TIMP-1 and TIMP-2 in EC development, tumor progression and formation of metastases have been most extensively characterized and best recognized. 
 TIMP-1 expression in human colorectal cancer is associated with TGF-B1, LOXL2, INHBA1, TNF-AIP6 and TIMP-2 transcript profiles. The balance of activity between the endogenous enzyme inhibitors known as tissue inhibitors of metalloproteinases and their targets, the matrix metalloproteinases, in the   extracellular matrix   is thought to play an important role in tumour cell invasion. Supporting this notion, we have shown that colorectal cancer patients have increased plasma levels of the tissue inhibitor of metalloproteinases-1 (TIMP-1), and that high plasma TIMP-1 levels are associated with short colorectal cancer patient survival. However, although TIMP-1 has been extensively studied in cancer, very little is known about how it is regulated. To further elucidate potential mechanisms of regulation of this protein, we did a number of experiments to look at associations between the transcript profile of TIMP-1 with known matrix metalloproteinases (MMPs) as well as with expression profiles of other genes differentially regulated in human colorectal cancer (CRC) and the other TIMPs 2-4, which have also been associated with the progression of colorectal cancer. Genome-wide expression profiling of 172 CRC and normal mucosa samples was used to identify transcript changes for the genes under investigation. We found that TIMP-1 was up-regulated in CRC samples compared with normal tissue, while TIMP-2 was down-regulated. Eight MMPs were up-regulated in CRC compared with normal tissue. Correlating up-regulated genes with the TIMP-1 transcript, we identified 13 that were also up-regulated in cancerous tissue. Among these were genes associated with the synthesis of extracellullar matrix, genes involved in the TGF-beta signalling pathway, and genes that are likely transcribed by the tumour cells. These insights add to the complex picture emerging about the regulation of TIMPs in colorectal cancer. 
 Tissue inhibitor of metalloproteinases-2 growth-stimulatory activity is mediated by nuclear factor-kappa B in A549 lung epithelial cells. Tissue inhibitors of metalloproteinases (TIMPs) are pleiotropic factors that function as key regulators of   extracellular matrix   remodeling. They exhibit multifunctional roles including cell growth-stimulating activities and protection from apoptosis. In the present study, we showed that human recombinant TIMP-2 (hrTIMP-2) promotes growth of A549 lung cells. This effect was accompanied by increase in nuclear factor-kappa B (NF-kappaB) activity 24h after exposure as determined by electrophoretic mobility shift assay (EMSA) analysis. This effect was correlated with downregulation of IkappaBalpha and beta proteins and later increases in Bcl-3, IkappaB, and cyclin D1 proteins. Blocking induction of NF-kappaB activity using a dominant-negative mutated version of IkappaBalpha abrogated NF-kappaB activation and cell proliferation. 
 Comparative study of the immunohistochemical expression of tissue inhibitors of metalloproteinases 1 and 2 between clearly invasive carcinomas and "in situ" trophoblast invasion. Tissue inhibitors of metalloproteinases (TIMPs) play an important role in   extracellular matrix   homeostasis by regulating MMP activity. Although they were initially considered inhibitors of tumor growth and metastasis, recently their role in cancer progression has been controversial. The aim of our study was to compare the immunohistochemical expression of TIMP1 and TIMP2 between an uncontrollably invasive phenomenon (cancer) and an "in situ" process (trophoblast invasion) in an effort to assess any differential role of these molecules between these two distinct phenomena and therefore to understand better their contribution in cancer invasion and migration. We performed an immunohistochemical analysis of 50 carcinomas (colorectal, gastric, breast, pulmonary, and renal) and 40 first trimester gestations. The marker expression was evaluated semiquantitatively, separately in cancer parenchymal and trophoblastic cells as well as in malignant stromal and decidual cells, according to a percentage scale (0, <10, 10-50, and >50%) and according to staining intensity (0, +, ++, and +++). Our results showed that there was no statistically significant difference in TIMP1 expression between cancer parenchymal cells and trophoblastic cells. On the other hand, TIMP1 was expressed more often in decidual cells than in cancer stromal cells. Immunostaining for TIMP2 was more extensive and intense both in trophoblastic and decidual cells than in cancer parenchymal and stromal cells, respectively. The reduced expression of TIMP2 in metastatic carcinomas by comparison with non-metastatic gestation specimens underlines its importance in cancer invasion and migration. On the other hand, TIMP1 was more expressed in decidua than cancer stroma, but at the same time showed no statistically significant difference between cancer parenchyma and trophoblasts, highlighting its multifunctional activity in cancer progression. 
 Higher risk of matrix metalloproteinase (MMP-2, 7, 9) and tissue inhibitor of metalloproteinase (TIMP-2) genetic variants to gallbladder cancer. Matrix metalloproteinase belong to family of pericellular collagenases which degrade   extracellular matrix   (ECM), and is involved in the modulation and susceptibility of various cancers.The present study included 410 gallbladder (GBC) cases and 230 healthy controls from North India. Study examined the associations of polymorphisms of MMP-2c.735C>T (rs2285053), MMP-2c.1306 C>T (rs243865), MMP7c.181A>G (rs11568818), MMP-9p.R279Q (rs17556) MMP-9p.P574R (rs2250889), MMP-9 p.R668Q (rs17577) and TIMP2c.418 G>C (rs8179090) to GBC susceptibility. Genotyping was carried out by PCR-RFLP. Statistical analysis was performed by using SPSS ver16.The MMP-2 c.735 [CT+TT], MMP-2c.1306 [CT+TT], MMP7 c.181 [AG+GG] and MMP-9 p.668 [RQ+QQ],TIMP2c.418 [GG+GC] genotypes were significantly associated with increased risk of GBC (P = 0.01; [OR]1.87, P = 0.02; [OR] 1.68, P = 0.02; [OR]=1.61, P = 0.002; [OR]=1.91,P = 0.01; [OR]=1.78 and (P = 0.03; [OR]=1.68; P = 0.01; [OR]=1.78 respectively). Haplotypes [C(-735) -T(-1306) ] and [T(-1306) -C(-735) ] of MMP-2 (P = <0.005; [OR] =1.78 P = <0.0001; [OR] =2.09) and haplotype [Q(279) -P(574) -Q(668) ]of the MMP-9 (P = 0.04; [OR] =2.75) were significantly associated with GBC risk. On stratification of GBC patients with/without gallstones, MMP-2 haplotypes were associated with higher GBC risk in patients accompanying gallstones whereas MMP-9 haplotypes showed risk in patients without stones. Combined effect of > 3 MMP/TIMP variant containing genotypes imparted increased risk of GBC (P < 0.0001; [OR] =3.36). Multivariate logistic regression results also supported association of MMP-2 (c.735C>T, c.1306 C>T), MMP-9 p.R668Q and TIMP2c.418G>C variants with GBC susceptibility.This study suggests that genetic variants in MMP-2,7,9 and TIMP-2genes are associated with higher susceptibility of gallbladder cancer. 
 Tissue inhibitor of metalloproteinases-2 binding to membrane-type 1 matrix metalloproteinase induces MAPK activation and cell growth by a non-proteolytic mechanism. Membrane-type 1 matrix metalloproteinase (MT1-MMP), a transmembrane proteinase with a short cytoplasmic domain and an extracellular catalytic domain, controls a variety of physiological and pathological processes through the proteolytic degradation of extracellular or transmembrane proteins. MT1-MMP forms a complex on the cell membrane with its physiological protein inhibitor, tissue inhibitor of metalloproteinases-2 (TIMP-2). Here we show that, in addition to extracellular proteolysis, MT1-MMP and TIMP-2 control cell proliferation and migration through a non-proteolytic mechanism. TIMP-2 binding to MT1-MMP induces activation of ERK1/2 by a mechanism that does not require the proteolytic activity and is mediated by the cytoplasmic tail of MT1-MMP. MT1-MMP-mediated activation of ERK1/2 up-regulates cell migration and proliferation in vitro independently of   extracellular matrix   proteolysis. Proteolytically inactive MT1-MMP promotes tumor growth in vivo, whereas proteolytically active MT1-MMP devoid of cytoplasmic tail does not have this effect. These findings illustrate a novel role for MT1-MMP-TIMP-2 interaction, which controls cell functions by a mechanism independent of   extracellular matrix   degradation. 
 Urinary Metalloproteinases-9 and -2 and Their Inhibitors TIMP-1 and TIMP-2 are Markers of Early and Long-Term Graft Function After Renal Transplantation. Renal ischemia-reperfusion (I-R) injury (IRI) is an inseparable feature of organ transplantation and may have a negative impact on the graft, its function and survival. Acute tubular necrosis, which is reversible thanks to the regenerative capacity of renal tubular epithelial cells, is the main cause of acute renal failure secondary to IRI. MMP-2 and MMP-9 are proteolytic enzymes involved in digesting proteins that are components of the   extracellular matrix   (ECM) and the basement membrane of the nephrons. This way post-reperfusion MMP activation allows the inflammatory process to spread.In our studies, we focused on identifying whether the concentrations of MMP-2 and MMP-9 and their natural inhibitors TIMP-1 and TIMP-2 in urine sample at day 1 and day 30 as well as after 12 months following renal transplantation are markers of early and long-term renal function during meanly five-years observation. Moreover, in urine sampled at months 6 and 12 after renal transplantation, we determined the content of TGF-β as a graft fibrosis indicator.MMP-9 concentration in the early post-transplant period is a major marker of early and long-term function of the transplanted kidney. Its increased concentration was correlated with lesions related to tubular atrophy and fibrosis in renal biopsies performed at months 3 and 12 after transplantation. Its concentration is correlated with TGF-β content in a later period.TIMP-1 and-2 are primarily markers of an early function of the transplanted kidney. Early post-transplant concentration of MMP-2 is a marker of proteinuria in early and long-term post-transplant periods. 
 Low density lipoprotein receptor-related protein mediates endocytic clearance of pro-MMP-2.TIMP-2 complex through a thrombospondin-independent mechanism. The low density lipoprotein receptor-related protein (LRP) mediates the endocytic clearance of various proteinases and proteinase.inhibitor complexes, including thrombospondin (TSP)-dependent endocytosis of matrix metalloproteinase (MMP)-2 (or gelatinase A), a key effector of   extracellular matrix   remodeling and cancer progression. However, the zymogen of MMP-2 (pro-MMP-2) mostly occurs in tissues as a complex with the tissue inhibitor of MMPs (TIMP-2). Here we show that clearance of the pro-MMP-2.TIMP-2 complex is also mediated by LRP, because addition of receptor-associated protein (RAP), a natural LRP ligand antagonist, inhibited endocytosis and lysosomal degradation of (125)I-pro-MMP-2.TIMP-2. Both TIMP-2 and the pro-MMP-2 collagen-binding domain independently competed for endocytosis of (125)I-pro-MMP-2.TIMP-2 complex. Surface plasmon resonance studies indicated that pro-MMP-2, TIMP-2, and pro-MMP-2.TIMP-2 directly interact with LRP in the absence of TSP. LRP-mediated endocytic clearance of (125)I-pro-MMP-2 was inhibited by anti-TSP antibodies and accelerated upon complexing with TSP-1, but these treatments had no effect on (125)I-pro-MMP-2.TIMP-2 uptake. This implies that mechanisms of clearance by LRP of pro-MMP-2 and pro-MMP-2.TIMP-2 complex are different. Interestingly, RAP did not inhibit binding of (125)I-pro-MMP-2.TIMP-2 to the cell surface. We conclude that clearance of pro-MMP-2.TIMP-2 complex is a TSP-independent two-step process, involving (i) initial binding to the cell membrane in a RAP-insensitive manner and (ii) subsequent LRP-dependent (RAP-sensitive) internalization and degradation. 
 Polymorphisms in MMP-2 and TIMP-2 in Turkish patients with prostate cancer. Prostate cancer is the most commonly diagnosed malignancy and the second most common cause of cancer deaths in the Western male population. Matrix metalloproteinases (MMPs) and tissue inhibitors of MMPs (TIMPs) modulate the remodeling of the   extracellular matrix   (ECM). The imbalance between MMPs and TIMPs may lead to an emergence of pathological processes such as cancer. In this study, the association between TIMP-2 (-418 G/C) and MMP-2 (-1306 C/T) polymorphisms and prostate cancer in the Turkish population was investigated.Sixty-one prostate cancer patients and 46 healthy subjects were included in the study. DNA was isolated from 2 mL of peripheral blood taken from subjects, and genotypes were analyzed by the polymerase chain reaction-restriction fragment length polymorphism method.The TIMP-2 -418 (GC) genotype was found in 15 cases (32.6%) in the control group and in 9 cases (14.8%) in the patients group, and statistical significance was determined (P = 0.037, OR = 0.346). The MMP-2 -1306 (CT) genotype was found 2.17 times more in the patient group than in the control group (P = 0.149, OR = 2.17).Our results show that the TIMP-2 -418 (GC) genotype had a putative protective effect against prostate cancer. 
 Promoted activation of matrix metalloproteinase (MMP)-2 in keloid fibroblasts and increased expression of MMP-2 in collagen bundle regions: implications for mechanisms of keloid progression. Keloid is characterized by excessive deposition of collagen, resulting from aberrant   extracellular matrix   (ECM) production and degradation. The aim was to investigate the role of matrix metalloproteinases (MMPs) and tissue inhibitor of metalloproteinases (TIMPs) in pathological wound healing in keloids.Semiquantitative analysis of 60 keloid tissue samples and 25 mature scar tissue samples demonstrated significantly increased expression of MMP-2, TIMP-2 and TIMP-3 in keloids compared with mature scars. Within keloid regions, MMP-2 expression was significantly higher in collagen bundle regions than in non-collagen bundle regions. Double immunofluorescence revealed that keloid fibroblasts between collagen bundles exhibited MMP-2, TIMP-2 and membrane-type 1 MMP (MT1-MMP) co-expression, whereas only MMP-2 expression was evident on the edge of collagen bundles. Western blot analysis and gelatin zymography of 13 keloid-derived fibroblasts (KFbs) and six normal skin dermal-derived fibroblasts (NFbs) demonstrated that unstimulated KFbs exhibited significantly increased MMP-2 activity and expression compared with NFbs under the same conditions.These results together indicate that MMP-2 activity can be promoted in keloid fibroblasts between collagen bundles in cooperation with TIMP-2 and MT1-MMP. This could contribute to remodelling of collagen bundle regions and invasion of fibroblasts into peripheral normal regions through promoted degradation of ECM. 
 CAPN 7 promotes the migration and invasion of human endometrial stromal cell by regulating matrix metalloproteinase 2 activity. Matrix metalloproteinase 2 (MMP-2) has been reported to be an important regulator of cell migration and invasion through degradation of the   extracellular matrix   (ECM) in many diseases, such as cancer and endometriosis. Here, we found calcium-activated neutral protease 7 (CAPN 7) expression was markedly upregulated in the eutopic endometrium and endometrial stromal cells of women diagnosed with endometriosis. Our studies were carried out to detect the effects of CAPN 7 on human endometrial stromal cell (hESC) migration and invasion.Western blotting and quantitative real-time PCR were used to detect the expression of CAPN 7 in endometriosis patients and normal fertile women. Scratch-wound-healing and invasion chamber assay were used to investigate the role of CAPN 7 in hESC migration and invasion. Western blotting, quantitative real-time PCR and zymography were carried out to detect the effect of CAPN 7 on the expressions and activity of MMP-2.CAPN 7 was markedly up-regulated in endometriosis, thereby promoting the migration and invasion of hESC. CAPN 7 overexpression led to increased expression of MMP-2 and tissue inhibitor of metalloproteinases 2 (TIMP-2); CAPN 7 knockdown reversed these changes. CAPN 7 increased MMP-2 activity by increasing the ratio of MMP-2 to TIMP-2. We also found that OA-Hy (an MMP-2 inhibitor) decreased the effects of CAPN 7 overexpression on hESC migration and invasion by approximately 50% and 55%, respectively. Additionally, a coimmunoprecipitation assay demonstrated that CAPN 7 interacted with activator protein 2α (AP-2α): an important transcription factor of MMP-2.CAPN 7 promotes hESC migration and invasion by increasing the activity of MMP-2 via an increased ratio of MMP-2 to TIMP-2. 
 Gene polymorphism -418 G/C of tissue inhibitor of metalloproteinases 2 is associated with abdominal aortic aneurysm. The pathogenesis of abdominal aortic aneurysm (AAA) is connected with abnormal   extracellular matrix   remodeling, with the assistance of   extracellular matrix   metalloproteinases and tissue inhibitors of metalloproteinases (TIMPs). A decrease in tissue inhibitor of metalloproteinases 2 (TIMP2) gene expression was detected in AAA patients. Recently, a -418 G/C (rs8179090) polymorphism of the TIMP2 gene promoter, influencing the transcription rate of the gene, has been described. This study investigated whether the -418 G/C gene polymorphism is associated with AAA in the Polish population.The TIMP2 gene promoter polymorphism was evaluated by polymerase chain reaction, followed by restriction enzyme analysis and pyrosequencing in 128 patients affected by AAA and in 180 individuals included as references. The control group was directly matched to patients according to known risk factors for vascular diseases.The frequency of the C allele was significantly higher in AAA patients than in the control group (odds ratio [OR], 2.516; P = .0005). The distribution of genotypes also differed significantly between the groups (CC + CG vs GG: OR, 2.906; P = .0037) or was close to being significantly different (CC vs GG + GC: OR, 2.144; P = .0501). A similar trend was observed in men but not in women. The multivariate logistic regression analysis indicated the TIMP2 gene promoter polymorphism is risk factor of AAA in the Polish population, with an adjusted OR of 4.99, when applied to a dominant inheritance model.Our study supports the hypothesis that TIMP2 and the -418G/C polymorphism located in the promoter of the TIMP2 gene are important in AAA pathophysiology. 
 Clinical and prognostic role of circulating MMP-2 and its inhibitor TIMP-2 in HCC patients prior to and after trans-hepatic arterial chemo-embolization. Trans-hepatic arterial chemo-embolization is the most commonly used treatment for unresectable hepatocellular carcinoma. The prognostic impact of tumor biomarkers has not therefore been evaluated in this treatment. Imbalance between matrix metalloproteinase-2 and tissue inhibitor metalloproteinase-2 is considered to play an important role in   extracellular matrix   remodeling and degradation. Higher serum levels of MMP-2 have been shown to predict a poor prognosis and shorter overall survival in HCC after TACE. The objective of this study was to evaluate the serum levels of MMP-2 and TIMP-2 in HCC patients before and after TACE to evaluate their clinical significance and usefulness as prognostic biomarkers.MMP-2 and TIMP-2 levels were measured by ELISA in 75 HCC patients and 30 healthy controls. Sera MMP-2 and TIMP-2 were correlated with clinico-pathological features.The mean serum MMP-2 and TIMP-2 levels of HCC patients before TACE were 1700±71ng/mL and 89±45ng/mL respectively, significantly higher than that of the control group: 771±60ng/mL (p<0.0001, t-test) and 25.7±20ng/mL respectively (p<0.0001, t-test). A significant decrease of MMP-2 levels after 1 and 3months compared to baseline time was observed (p<0.0001), while with TIMP-2 a gradual increase in serum before and after TACE (p<0.01) was detected. No significant correlation between serum MMP-2 levels and other clinico-pathological features was observed. Patients with serum MMP-2 >1500ng/mL (median value) had worse overall and recurrence-free survival compared with those with serum MMP-2 levels <1500ng/mL before treatment.Higher serum MMP-2 levels and MMP-2/TIMP-2 ratio could predict poor prognosis after TACE, suggesting prognostic role of these biomarkers in HCC. 
 MMP-2 protein in invasive breast cancer and the impact of MMP-2/TIMP-2 phenotype on overall survival. Crucial event in the metastasis of cancer cells is the secretion of matrix metalloproteinases (MMPs), which are responsible for the degradation of   extracellular matrix   (ECM). Among them, matrix metalloproteinase-2 (MMP-2) is a gelatinase, which degrades basement membrane type-IV collagen. Immunohistochemistry was performed to detect MMP-2 protein in 135 infiltrative breast carcinomas. MMP-2 was studied along with clinicopathological parameters (tumor size, histological type, nuclear and histological grade, stage, lymph node status, ER, and PR), patients' survival and tissue inhibitor metalloproteinase-2 (TIMP-2), Ki-67, and p53 proteins. MMP-2 immunoreactivity was detected in the cytoplasm in cancer cells in 102 (75.6%) and in both tumor and tumor stromal cells in 37 (27.4%) of 135 cases respectively. MMP-2 reactivity in cancer cells displayed a statistically significant association with tumor size > 2 cm (p = 0.022). In tumor stromal cells a strong parallel association was observed between the expression of MMP-2 and TIMP-2 (p = 0.015), while an inverse correlation was found between MMP-2 and both Ki-67 and p53 (p = 0.033 and p = 0.034 respectively). In the subgroup with negative lymph nodes MMP-2 was also inversely associated with p53 in cancer cells (p = 0.045). Finally a statistically significant association was revealed using Kaplan-Meier and Cox's proportional hazard regression model between the MMP-2/TIMP-2 phenotype and patients' better survival (p = 0.021). Our results point out the strong relation between MMP-2 and TIMP-2 and the effect of the MMP-2/TIMP-2 phenotype in the patients' overall survival. The inverse correlation between MMP-2 and both Ki-67 and p53 can be explained by the potential inhibition of MMP-2 by TIMP-2. These results suggest the necessity of further investigation. 
 Circulating matrix metalloproteinase MMP-9 and MMP-2/TIMP-2 complex are associated with spontaneous early pregnancy failure. Trophoblast cell (CTB) invasion into the maternal endometrium plays a crucial role during human embryo implantation and placentation. This invasion is facilitated by the activity of matrix metalloproteinases, which are regulated by tissue inhibitors of MMPs (TIMPs).This study compares the serum levels of MMP-9, MMP-2/TIMP-2 complex, TIMP-1 and TIMP-2 in 129 patients with ongoing pregnancy (n = 40) or spontaneous early pregnancy failure (n = 89).MMP-9 was markedly (p < 0.0001) elevated in missed abortions, as was MMP-2/TIMP-2 complex (p < 0.0005). However, the serum levels of TIMP-1 and TIMP-2 were markedly elevated (p < 0.0001) in ongoing pregnancies.Human placentation is mediated by fetal trophoblastic cells that invade the maternal uterine endometrium. Trophoblast invasion requires a precisely regulated secretion of specific proteolytic enzymes able to degrade the endometrial basement membrane and   extracellular matrix  . The elevated levels of MMP-9 and MMP-2/TIMP-2 complex may play a role in spontaneous termination of pregnancy. 
 Phage display of tissue inhibitor of metalloproteinases-2 (TIMP-2): identification of selective inhibitors of collagenase-1 (metalloproteinase 1 (MMP-1)). Tissue inhibitor of metalloproteinases-2 (TIMP-2) is a broad spectrum inhibitor of the matrix metalloproteinases (MMPs), which function in   extracellular matrix   catabolism. Here, phage display was used to identify variants of human TIMP-2 that are selective inhibitors of human MMP-1, a collagenase whose unregulated action is linked to cancer, arthritis, and fibrosis. Using hard randomization of residues 2, 4, 5, and 6 (L1) and soft randomization of residues 34-40 (L2) and 67-70 (L3), a library was generated containing 2 × 10(10) variants of TIMP-2. Five clones were isolated after five rounds of selection with MMP-1, using MMP-3 as a competitor. The enriched phages selectively bound MMP-1 relative to MMP-3 and contained mutations only in L1. The most selective variant (TM8) was used to generate a second library in which residues Cys(1)-Gln(9) were soft-randomized. Four additional clones, selected from this library, showed a similar affinity for MMP-1 as wild-type TIMP-2 but reduced affinity for MMP-3. Variants of the N-terminal domain of TIMP-2 (N-TIMP-2) with the sequences of the most selective clones were expressed and characterized for inhibitory activity against eight MMPs. All were effective inhibitors of MMP-1 with nanomolar K(i) values, but TM8, containing Ser(2) to Asp and Ser(4) to Ala substitutions, was the most selective having a nanomolar K(i) value for MMP-1 but no detectable inhibitory activity toward MMP-3 and MMP-14 up to 10 μM. This study suggests that phage display and selection with other MMPs may be an effective method for discovering tissue inhibitor of metalloproteinase variants that discriminate between specified MMPs as targets. 
 The N-terminus of collagenase MMP-8 determines superactivity and inhibition: a relation of structure and function analyzed by biomolecular interaction analysis. Tissue inhibitors of metalloproteinases (TIMPs) are the physiological, specific inhibitors of matrix metalloproteinases (MMPs) forming tight, noncovalent complexes. Therefore they control the proteolytic activity of MMPs toward the   extracellular matrix  . To analyze the inhibition of the "activated" and "superactivated" variants of human neutrophil collagenase (MMP-8) by TIMP-2, we determined complex dissociation constants using biomolecular interaction analysis (BIA). As it is known that the association rate constants can exceed the limits of the BIA instruments, the biomolecular interaction analysis was used to examine the equlibrium situation. The dissociation constants were determined by fitting the parameters of the mathematical term for the binding of collagenase onto the TIMP-coupled sensor chip surface to the saturation curve derived from individual sensorgrams. The resulting values are in the nanomolar range and correlate with the results of fluorescence kinetics. These data reveal that TIMP-2 (the recombinant inhibitory domain of human TIMP-2 and bovine TIMP-2 isolated from seminal plama) is a better inhibitor of the activated neutrophil collagenase than of the superactivated variant (the recombinant catalytic domain of human MMP-8). It has been demonstrated by X-ray analysis that the N-terminal heptapeptide only of superactivated MMP-8 is attached by a salt bridge and hydrophobic interaction to the C-terminal helix. Because these interactions have to be disrupted in the complex formation with TIMP we assume that the activated variant enables higher flexibility and a tighter induced fit in the complex formation. Therefore superactivation of MMP-8 correlates with weaker inhibition by TIMP-2. 
 Analysis of the MMP-9 (C-1562 T) and TIMP-2 (G-418C) gene promoter polymorphisms in patients with chronic periodontitis. Matrix metalloproteinases (MMP)-9 is an important member of the matrix metalloproteinase family. A functional polymorphism has been described in the promoter region of the human MMP-9 gene. A C-to-T base exchange at -1562 creates two different alleles, and the C/T and T/T genotypes promote high activity of the MMP-9 gene promoter, increasing the risk for inflammatory diseases. The metalloproteinase-2 tissue inhibitor (TIMP-2) regulates the activity of MMPs in the   extracellular matrix  , and a polymorphism at the -418 position of the TIMP-2 gene promoter has been found in a Sp-1 binding site. In this study we have investigated the association between the above-mentioned polymorphisms and chronic periodontitis severity.Genomic DNA from oral mucosa of 100 subjects was amplified by polymerase chain reaction and analysed by restriction endonuclease digestion. The significance of the differences in observed frequencies of polymorphisms in moderate and severe disease and healthy groups was assessed by chi(2) test (p<0.05).No association was observed between the polymorphism in the promoter region of MMP-9 (p=0.6693) and chronic periodontitis. The analysis of TIMP-2 showed that the G/G genotype was found at a frequency of 99%.The results show that the polymorphism in the promoter region of MMP-9 gene is not associated with chronic periodontitis. The high frequency of GG genotype in the TIMP-2 gene promoter in the population studied did not allow any conclusion regarding its effect on chronic periodontitis. 
 Expression of matrix metalloproteinase and its tissue inhibitor in haemangioma. The action mechanism of matrix metalloproteinases-2 (MMP-2) and tissue inhibitor of metalloproteinases-2 (TIMP-2) in the genesis, development and degeneration of haemangioma was investigated by detecting their expression in the tissue of haemangioma in different phases by using the immunohistochemistry. Fifty paraffin-embedded specimens of skin capillary haemangioma were collected, which were documented in the Department of Pathology, Renmin Hospital of Wuhan University from 2000 to 2006. All samples were stained by regular HE method, and proliferative cell nuclear antigen (PCNA) was tested by immunohistochemical S-P method. The samples were classified according to the Mulliken criteria and the expression pattern of PCNA. Immunohistochemical S-P method was applied to detect the expression of MMP-2 and TIMP-2 in proliferative and degenerative phases of cutaneous capillary haemangioma, and in normal skin tissues. In combination with the detection of the expression of factor VIII-related antigen, it was verified that in haemangioma tissues, the cells expressing MMP-2 and TIMP-2 were vascular endothelial cells. The MMP-2 and TIMP-2 expression was quantitatively analyzed by image analysis system (HPIAS-1000), and one-way ANOVA(107) and SNK(q) test were done to analyze average absorbance (A) and positive area rate of immunohistochemically positive particles by using SPSS11.5. The results showed: (1) Among 50 samples of haemangioma, there were 26 proliferative haemangiomas, and 24 degenerative haemangiomas, respectively; (2) The expression of MMP-2 was weak in normal vascular endothelial cells, cytoplasm of connective tissues and   extracellular matrix   around blood vessels. The expression of MMP-2 in proliferative group was significantly higher than in degenerative group and control group (normal skin) (P<0.05), but there was no statistically significant difference between the latter two groups; (3) TIMP-2 was highly expressed in normal tissues, degenerative vascular endothelial cells, cytoplasm of connective tissues and   extracellular matrix   around blood vessels. The expression level of TIMP-2 in proliferative phase was significantly lower than in degenerative phase (P<0.05), and the expression of TIMP-2 in proliferative phase was significantly different from that in degenerative phase and normal tissues (P<0.05). It was concluded that in proliferative phase of haemangioma, MMP-2 may promote over-proliferation of endothelial cells of haemangioma, and in degenerative phase, TIMP-2 can inhibit the proliferation of endothelial cells of haemangioma. The two substances play important roles in the genesis, development and degeneration of haemangiomas. 
 The distribution of matrix metalloproteinases and tissue inhibitors of metalloproteinases in the lungs of congenital diaphragmatic hernia patients and age-matched controls. In congenital diaphragmatic hernia (CDH), the pathogenesis of abnormal pulmonary morphology is still incompletely understood. Matrix metalloproteinases (MMPs) and tissue inhibitors of metalloproteinases (TIMPs) are known to play an important role in the turnover of the   extracellular matrix   (ECM) during development and in remodelling of tissue. The aim of this study was to investigate differences in the expression of MMPs and TIMPs between CDH lungs and controls, against the background of the abnormal pulmonary vasculature in CDH.We studied 12 lungs of term CDH patients who died < 24 h after birth and 11 normal age-matched control lungs, by immunohistochemistry with antibodies against human MMP-1, -2, -9, TIMP-1 and -2.There was a clear increase in the number of MMP-1-reactive capillaries and fibroblasts in CDH lungs compared with controls. In contrast, TIMP-2 reactivity in these structures was decreased in CDH lungs. The arterial endothelium and medial smooth muscle expressed MMP-2, -9 and TIMP-2 in both CDH and control lungs. In small arteries (< 100 microm in diameter), the positive surface area of MMP-2, -9 and TIMP-2 was significantly larger in CDH lungs than in controls. There was no difference in the distribution and expression of TIMP-1 between CDH lungs and normal controls.The differences in staining pattern of MMPs and TIMPs between normal and CDH lungs suggest that these enzymes might play a role in the abnormal remodelling of the interstitium and the pulmonary arteries in CDH lungs. This could contribute to our understanding of the abnormal lung morphology and the occurrence of pulmonary hypertension, which forms one of the major obstacles to the successful treatment of these patients. 
 Functional polymorphisms in matrix metalloproteinases-1, -3, -9 are associated with arteriovenous fistula patency in hemodialysis patients. Matrix metalloproteinases (MMPs) are risk factors for cardiovascular diseases. This study evaluated the association of genotype polymorphisms of MMPs and tissue inhibitors of metalloproteinases (TIMPs) in hemodialysis (HD) patients with arteriovenous fistula (AVF) failure.Genotype polymorphism of MMP-1, MMP-2, MMP-3, and MMP-9 and TIMP-1 and TIMP-2 and clinical and laboratory parameters were compared between Chinese HD patients with (n = 170) and without (n = 426) AVF failure.Significant associations were found between AVF failure and the following factors (hazard ratio): longer HD duration (1.007 per month), lower pump flow (0.991 per ml/min), higher dynamic venous pressure (1.016 per mmHg), location of AVF on right side (1.630 versus left side) and upper arm (2.385 versus forearm), history of cardiovascular disease (1.656 versus absence of history), 1G/1G genotype of MMP-1 -1607 1G >2G SNP (2.315 versus 1G/2G + 2G/2G genotypes), 6A/6A genotype of MMP-3 -1612 5A >6A SNP (1.712 versus 5A/6A + 5A/5A), and C/C genotype of MMP-9 -1562 C>T SNP (1.650 versus C/T+T/T). The positive predictive rates for AVF failure were 63.0% and 6.7% for patients with the highest-risk (1G1G/6A6A/CC) and lowest-risk (2G2G or 2G1G/5A5A or 6A6A/TT or TC) composite MMP-1/MMP-3/MMP-9 genotype, respectively. The unassisted patency of AVF at 5 years decreased significantly from 93.3% to 38.4% for the composite MMP-1/MMP-3/MMP-9 genotypes (lowest versus highest risk, P < 0.001).Specific genotypes of MMP-1, MMP-3 and MMP-9 with lower transcriptional activity are associated with higher frequencies of AVF failure, which may result from more accumulation of   extracellular matrix  , leading to AVF stenosis. 
 [Assessment of tissue inhibitor of metalloproteinases-2 (TIMP-2) in laryngeal cancer]. Metalloproteinases are the proteolytic enzymes that digest components of the   extracellular matrix   in many physiological and pathological conditions. Their activity is regulated by their natural inhibitors: non-specific alpha2-macroglobulin and specific tissue inhibitors of metalloproteinases (TIMP). There are four TIMPs. TIMP-2 is the only enzyme that is expressed in constitutive manner and has the ability to inhibit activity of all metalloproteinases. TIMP-2 has been identified in many carcinomas including cancers of lung, oral cavity, breast and colon. There was correlation between TIMP-2 expression and clinical course of the disease observed in most of the neoplasm.The aim of the research was to evaluate the expression of TIMP-2 in laryngeal cancer and to assess the prognostic significance of this factor.104 patients with laryngeal cancer, that underwent surgical treatment were included in the study. Only cases with at least a 5-year follow-up were included. Immunohistochemical studies were performed on formalin fixed, paraffin embedded sections by using monoclonal antibodies against TIMP-2 antigen and ABC detection system.TIMP-2 expression was cytoplasmatic, mainly in cancer cells, but also in some stromal cells. There was correlation between TIMP-2 expression and tumor size and grading observed. We didn't find any correlation between TIMP-2 and nodal metastases, recurrence and survival.Our results don't suggest that TIMP-2 expression may be used as a prognostic factor in patients with laryngeal cancer. Nevertheless there are more researches needed to explain the role of TIMPs in growth and progression of neoplastic tumors. 
 Gelatinases and their tissue inhibitors during human ovulation: increased expression of tissue inhibitor of matrix metalloproteinase-1. Remodelling of the   extracellular matrix   (ECM) of the follicular wall by matrix metalloproteinases (MMPs) and tissue inhibitors of matrix metalloproteinases (TIMPs) has been suggested to be crucial in ovulation. To investigate the expression of the gelatinases, MMP-2 and MMP-9, together with their inhibitors, TIMP-2 and TIMP-1, in the perifollicular ovarian stroma from women just before and during ovulation, we obtained biopsies of the stroma adjacent to the leading follicle. Laparoscopic surgery was performed either before the LH peak or at any of three intervals after ovulation triggering by hCG. Immunoblotting, immunohistochemistry and quantitative RT-PCR were performed. All four proteins were expressed by immunoblots, with no detectable changes in the expression of MMP-2, MMP-9 and TIMP-2. Scattered immunostaining for MMP-9 and TIMP-2 was seen, and MMP-2 was demonstrated in a concentric layer. A significant increase in TIMP-1 protein and mRNA was seen during the three ovulatory phases, and a strong and patchy immunostaining for TIMP-1 was shown. This is the first study that has demonstrated an ovulation-associated expression of these ECM-remodelling enzymes around the human follicle at ovulation. The increased expression of TIMP-1 may reflect a specific temporal inhibition of collagenolysis and thereby a time-dependent regulation of ECM breakdown in areas surrounding the apex of the follicle. 
 [Expression of metalloproteinase MMP-9 and tissue inhibitor of metalloproteinase TIMP-2 in placenta of pregnant women with intrauterine growth restriction]. The development of pregnancy depends on many factors, among other the implantation and the development of the placenta and placental vascularity The correct course of these processes depends on the proper activity of the enzymes degradating elements of   extracellular matrix   and basal membrane - metalloproteinases.Assessment of the expression of MMP-9 and TIMP-2 in the placenta of women with intrauterine fetal growth restriction.Two study groups were distinguished - with untreated IUGR and with IUGR treated with the acetylsalicylic acid and L-arginine and the control group with correct fetal growth and course of pregnancy Marked microscope preparations from placental samples were made with specific antibodies for MMP-9 and TIMP-2.The greatest intensity of the reaction and the villous TIMP-2 expression was characteristic of the group with untreated IUGR. Strongest expression MMP-9 was observed in the control group. The smallest surface of the trophoblast with positive MMP-9 reaction appeared in the group with untreated IUGR.High MMP-9 activity in the placenta correlates with its correct structure and function. Application of IUGR treatment reduces the expression of TIMP-2 in the placental villi. 
 The ERK/MAPK pathway regulates the activity of the human tissue factor pathway inhibitor-2 promoter. Human tissue factor pathway inhibitor-2 (hTFPI-2) is a 32-kDa serine protease inhibitor that is associated with the   extracellular matrix  . hTFPI-2 inhibits several   extracellular matrix  -degrading serine proteases and may play a role in tumor invasion and metastasis. To study the signal transduction pathway that leads to the activation of the hTFPI-2, we cloned the potential promoter region of this gene adjacent to a heterologous luciferase reporter gene. Phorbol 12-myristate 13-acetate (PMA) induced the luciferase reporter gene in HEK293 cells and other epithelial cell lines, such as the human lung carcinoma A549 cells, the breast carcinoma MCF7 cells, and the cervical HeLa cells. This PMA induction was blocked with the MEK inhibitor UO126, suggesting that the PMA-induced activation of the hTFPI-2 promoter is mediated through MEK. Furthermore, epidermal growth factor induced the luciferase reporter gene in HeLa cells. Cotransfection of the luciferase construct with constitutively active components of the Ras/Raf/MEK/ERK pathway in EcR-293 cells lead to a 7- to 92-fold induction of the luciferase reporter gene, indicating that regulation of hTFPI-2 is mediated through this pathway. A series of luciferase reporter gene constructs with progressive deletions of the 5'-flanking region suggested that the minimal basal promoter activity is located between nucleotide positions -89 and -384, whereas the minimal inducible promoter activity is between -89 and -222. We have used the computer program TFSEARCH and mutagenesis to analyze potential transcription factor binding sites. We identified an AP-1 binding site at nucleotide position -156 (inducible activity) and a Sp1 site at position -134 (basal activity) as potential cis-acting elements in the promoter region of the hTFPI-2. 
 Expression of metalloproteinases and their tissue inhibitors in squamous cell laryngeal carcinoma. The hallmark of cancer invasion is the degradation of   extracellular matrix   components. Matrix metalloproteinases are the major enzymes participating in this event and their activity is regulated extracellularly by their presence as proenzymes and the concomitant presence of the specific tissue inhibitors. The present study describes the immunohistochemical localization of gelatinases, matrix metalloproteinase (MMP)-2 and -9 and tissue inhibitor of metalloproteinase (TIMP)-1 and -2 in human laryngeal carcinoma and their expression with respect to tumor classification and compared with the respective healthy subjects. MMP-2 was immunolocalized in the cytoplasm of the epithelial cells and in the loose connective tissue, whereas MMP-9 was also observed in basement membrane and chondrocytes. Both were also found in tumor cells, but staining was decreased with increasing stage of cancer. TIMP-1 was present exclusively in stroma and totally absent from tumor cells and it was overexpressed in normal cells surrounding the tumor. TIMP-2 was identified in the cytoplasm of epithelial cells, in stroma and sometimes in chondrocytes. In addition, it was present in tumor cells of only stage IV samples. The expression level of both gelatinases and TIMPs increased as the stage of cancer increased, suggesting the possible post-transcriptional removal of their mRNA. These observations, performed in a given head and neck site, suggest that the behavior of head and neck tumors seems to depend on the site and additional studies should be performed to obtain a general understanding of the disease and ascertain the role of the constituents examined. 
 Matrix metalloproteinases and tissue inhibitors of metalloproteinases in human fetal testis and ovary. Matrix metalloproteinases (MMP) and tissue inhibitors of metalloproteinases (TIMP) are major regulators of tissue remodelling of the   extracellular matrix   (ECM) and may also be involved in the control of growth factor availability. We have investigated their production and localization in the developing human gonad during mid-gestation using zymographic techniques and immunohistochemistry. The secretion of MMP-2, MMP-9 and all four TIMP was demonstrated from both testis and ovary, with the predominant gelatinase produced by both being MMP-2. In the testis, MMP-1, MMP-2, MMP-9 and all TIMP family members were localized to the interstitium and to varying degrees within the tubules. MMP-9 and TIMP-4 were abundant in both Sertoli cells and gonocytes and MMP-1 and TIMP-1 were localized in particular to Sertoli cells. In the ovary, all TIMP and MMP-1, MMP-2 and MMP-9 were localized to the oogonium/oocyte cytoplasm with varying intensities and MMP-1, TIMP-2 and TIMP-3 were also detected in the ovarian stroma. This study demonstrates that MMP-1, MMP-2, MMP-9 and all TIMP family members are secreted by the developing ovary and testis and are localized to specific cell and tissue sites. MMP and TIMP are likely to play a role in ECM remodelling during gonadal development and also in the cell and matrix interactions that control a range of cellular functions. 
 mRNA expressions of TIMP-1, -2, and -3 and 92-KD type IV collagenase in early human placenta and decidual membrane as studied by in situ hybridization. Cytotrophoblasts of early placenta invade the decidual membrane, gestational endometrium, and spiral arteries during early pregnancy. Unlike tumor invasion, this physiological invasion is well controlled, although its molecular mechanisms are largely unknown. We have previously shown that cytotrophoblasts synthesize significant mRNAs for 72-KD Type IV collagenase, laminin, and Type IV collagen, proteins implicated in   extracellular matrix   turnover and migration. In this study we used in situ hybridization and immunohistochemistry to investigate the mRNA expression pattern of 92-KD Type IV collagenase and the matix metalloproteinase inhibitors TIMP-1, TIMP-2, and TIMP-3 in early human placenta and decidual membrane. mRNAs for 92-KD Type IV collagenase, TIMP-1, TIMP-2, and TIMP-3 were found in the cells of cytotrophoblastic columns, the endothelial and fibroblastic stromal cells of villi, and the large decidualized cells of decidual membrane. TIMP-1 expression was notably accentuated in the fibroblasts of fibrotic villi. In the decidual membrane, the signals for 92-KD Type IV collagenase and TIMP-1 mRNA were particularly strong around the glandular structures. The trophoblastic epithelium of villi and the epithelial cells of decidual glands showed a signal for 92-KD Type IV collagenase and TIMP-2, but not for TIMP-1 or TIMP-3. The coincidental expression of the proteolytic 92-KD Type IV collagenase and inhibitors TIMP-1, TIMP-2, and TIMP-3 generally in the same cells suggests that the activity of 92-KD Type IV collagenase, which is regulated by TIMPs, plays an important role in placental tissue organization and in the invasion of trophoblastic cells into the uterine wall. 
 Association of matrix metalloproteinase gene polymorphisms with refractive error in Amish and Ashkenazi families. Matrix metalloproteinases (MMPs) and tissue inhibitors of metalloproteinases (TIMPs) are involved in scleral   extracellular matrix   remodeling and have shown differential expression in experimental myopia. The genetic association of refractive error and polymorphisms in MMP and TIMP genes in Old Order Amish (AMISH) and Ashkenazi Jewish (ASHK) families was investigated.Individuals from 55 AMISH and 63 ASHK families participated in the study. Ascertainment was designed to enrich the families for myopia; the mean spherical equivalent (MSE) refractive error (SD) was -1.61 (2.72) D in the AMISH, and -3.56 (3.32) D in the ASHK. One hundred forty-six common haplotype tagging SNPs covering 14 MMP and 4 TIMP genes were genotyped in 358 AMISH and 535 ASHK participants. Association analyses of MSE and the spherical component of refraction (SPH) were performed separately for the AMISH and the ASHK. Bonferroni-corrected significance thresholds and local false discovery rates were used to account for multiple testing.After they were filtered for quality-control, 127 SNPs were included in the analyses. No polymorphisms showed statistically significant association to refraction in the ASHK (minimum P = 0.0132). In AMISH, two SNPs showed evidence of association with refractive phenotypes: rs1939008 (P = 0.00016 for SPH); and rs9928731 (P = 0.00026 for SPH). These markers were each estimated to explain <5% of the variance of SPH in the AMISH sample.Statistically significant genetic associations of ocular refraction to polymorphisms near MMP1 and within MMP2 were identified in the AMISH but not among the ASHK families. The results suggest that the MMP1 and MMP2 genes are involved in refractive variation in the AMISH. Genetic and/or environmental heterogeneity most likely contribute to differences in association results between ethnic groups. 
 Growth-stimulatory activity of TIMP-2 is mediated through c-Src activation followed by activation of FAK, PI3-kinase/AKT, and ERK1/2 independent of MMP inhibition in lung adenocarcinoma cells. Tissue inhibitors of metalloproteinases (TIMPs) control   extracellular matrix   (ECM) homeostasis by inhibiting the activity of matrix metalloproteinases (MMPs), which are associated with ECM turnover. Recent studies have revealed that TIMPs are implicated in tumorigenesis in both MMP-dependent and MMP-independent manners. We examined a mechanism by which TIMP-2 stimulated lung adenocarcinoma cell proliferation, independent of MMP inhibition. The stimulation of growth by TIMP-2 in A549 cells required c-Src kinase activation. c-Src kinase activity, induced by TIMP-2, concomitantly increased FAK, phosphoinositide 3-kinase (PI3-kinase)/AKT, and ERK1/2 activation. Selective knockdown of integrin α3β1, known as a TIMP-2 receptor, did not significantly change TIMP-2 growth promoting activity. Furthermore, we showed that high TIMP-2 expression in lung adenocarcinomas is associated with a worse prognosis from multiple cohorts, especially for stage I lung adenocarcinoma. Through integrated analysis of The Cancer Genome Atlas data, TIMP-2 expression was significantly associated with the alteration of driving genes, c-Src activation, and PI3-kinase/AKT pathway activation. Taken together, our results demonstrate that TIMP-2 stimulates lung adenocarcinoma cell proliferation through c-Src, FAK, PI3-kinase/AKT, and ERK1/2 pathway activation in an MMP-independent manner. 
 TIMP-2 Interaction with MT1-MMP Activates the AKT Pathway and Protects Tumor Cells from Apoptosis. Membrane-type 1 matrix metalloproteinase (MT1-MMP), a transmembrane proteinase with an extracellular catalytic domain and a short cytoplasmic tail, degrades a variety of   extracellular matrix   (ECM) components. In addition, MT1-MMP activates intracellular signaling through proteolysis-dependent and independent mechanisms. We have previously shown that binding of tissue inhibitor of metalloproteinases-2 (TIMP-2) to MT1-MMP controls cell proliferation and migration, as well as tumor growth in vivo by activating the Ras-extracellular signal regulated kinase-1 and -2 (ERK1/2) pathway through a mechanism that requires the cytoplasmic but not the proteolytic domain of MT1-MMP. Here we show that in MT1-MMP expressing cells TIMP-2 also induces rapid and sustained activation of AKT in a dose- and time-dependent manner and by a mechanism independent of the proteolytic activity of MT1-MMP. Fibroblast growth factor receptor-1 mediates TIMP-2 induction of ERK1/2 but not of AKT activation; however, Ras activation is necessary to transduce the TIMP-2-activated signal to both the ERK1/2 and AKT pathways. ERK1/2 and AKT activation by TIMP-2 binding to MT1-MMP protects tumor cells from apoptosis induced by serum starvation. Conversely, TIMP-2 upregulates apoptosis induced by three-dimensional type I collagen in epithelial cancer cells. Thus, TIMP-2 interaction with MT1-MMP provides tumor cells with either pro- or anti-apoptotic signaling depending on the extracellular environment and apoptotic stimulus. 
  GeneRIFs  
 changes in the   extracellular matrix   metabolism during the ageing process influence the level of circulating MMP-3 and MMP-10, as well as their tissue inhibitors TIMP-1 and TIMP-2, in a healthy population 
 TIMP-2 regulates the activity of MMPs in the   extracellular matrix  . The high frequency of GG genotype in the TIMP-2 gene promoter showed this site was unsuitable for studies of DNA polymorphism-disease associations in the studied population. 
 Findings illustrate a novel role for MT1-MMP-TIMP-2 interaction, which controls cell functions by a mechanism independent of   extracellular matrix   degradation. 
 Our analysis of the entire coding region of TIMP-1, -2, and -3, which are the main inhibitors of metalloproteinase activity in the   extracellular matrix  , failed to show an association between genetic polymorphisms and an intracranial aneurysm 
  [Ii]nvasive  PubMed  
 HIV-1-Tat protein promotes chemotaxis and   invasive   behavior by monocytes. Monocytes are susceptible to HIV infection and to activation by a regulatory gene product of the HIV genome, HIV-Tat. Recently, we have demonstrated that treatment with HIV-Tat up-regulates monocyte adhesion to the endothelium and increases metalloproteinase production. in the present study, we have examined the ability of the HIV-Tat protein to alter the migratory and   invasive   behavior of monocytes. Monocytes pretreated for 24 h with 10 ng/ml HIV-Tat exhibited enhanced migratory behavior compared with untreated monocytes in chemotaxis assays, both in the absence of a chemoattractant as well as in response to FMLP. in addition, HIV-Tat itself induced the migration of both untreated and HIV-Tat pretreated monocytes. Checkerboard analysis showed that monocytes migrated in response to an HIV-Tat concentration gradient, thus confirming the chemotactic characteristics of the HIV-Tat protein. Pretreatment of monocytes with 10 ng/ml HIV-Tat for 24 h also increased their ability to invade reconstituted extracellular membrane (Matrigel)-coated filters by 5-fold in the absence of chemoattractant. The presence of FMLP or HIV-Tat further enhanced invasion by both untreated and HIV-Tat-pretreated monocytes by more than 10-fold. Monocyte invasion was partially inhibited by the inclusion of anti-beta integrin Ab or tissue inhibitor of metalloproteinase (TIMP). Thus, for the first time, we present evidence that HIV-Tat can enhance the chemotactic and   invasive   behaviors of monocytes and propose an active role for HIV-Tat in the recruitment of monocytes into extravascular tissues, a process which may contribute to the destruction of tissues and cellular architecture often seen in patients with acquired immunodeficiency syndrome. 
 Transcriptional control of cell density dependent regulation of matrix metalloproteinase and TIMP expression in breast cancer cell lines. Our recent studies on breast carcinoma cell lines with differing tumorigenicity/  invasive  ness (MCF-7<MDA-MB-468<MDAMB-231<MDA-MB-435) had shown significantly decreasing expression levels of MMPs-1,-2,-3,-8,-9,-10,-11 and -13 with increasing cell density while the levels of TIMP-1 and -2 increased. This correlated well with a lower   invasive  ness of confluent cells. In the present study, we extend our in vitro studies on three-dimensional cultures of breast cancer cell lines MCF-7 and MDAMB-435 and the transcriptional control of MMP and TIMP-expression in two-dimensional cultures of MDA-MB-231 and -435 cells. The tumor spheroid model showed that MMP expression and proteolytic activity were considerably higher in loosely structured tumor groups as compared to densely growing "compact" cell complexes. These data suggested that cell density regulates MMP and TIMP transcription and therefore, we tested whether AP-1, NF kappa B and CRE are involved in this process. Gene silencing of c-jun in sparse cultures had an inhibitory effect on MMP-3, -9 and -13 expression, on proteolytic activity as well as on the   invasive   potential of the cells, thus confirming a role for AP-1.TIMP-1, and -2 expression was up-regulated as compared to control cells. Consistent with this, overexpression of c-jun and c-fos in confluent breast cancer cell lines leads to up-regulation of MMP expression, proteolytic activity and invasion as well as down-regulation of TIMP-1. In summary, we provide evidence that cell density influences the   invasive   potential of tumor cells via regulation of MMPs and TIMPs by AP-1, NF kappa B and CRE transcription factors. Overexpression of MMPs in sparse cultures could help explain early dissemination of potentially metastatic cells. 
 Expression and role of metalloproteinase-2 and endogenous tissue regulator in ameloblastoma. Ameloblastoma is a frequently encountered odontogenic benign tumor characterized by local   invasive  ness and high risk of recurrence. Matrix metalloproteinase (MMP)-2 can degrade type IV collagen, one of the major components in the basement membrane, resulting in the promotion of tumor invasion, whereas it is under the influence of an activator, membrane type 1-MMP-14 and the tissue inhibitor of metalloproteases (TIMP)-2. The aim of this study was to investigate combinatorial role played by MMP-2, TIMP-2 and MMP-14 in ameloblastoma.Transcriptional expression of MMP-2, TIMP-2 and MMP-14 in tissue extracts of 42 ameloblastomas and 10 dental follicles was detected using reverse transcription-polymerase chain reaction, and compared difference in clinical type and local recurrence of ameloblastoma.MMP-2, TIMP-2 and MMP-14 mRNA were detected in all investigated ameloblastoma tissues. While MMP-2 mRNA was only expressed in eight of 10 odontotheca tissues and TIMP- 2 and MMP-14 were not found in all odontotheca tissues. The mRNA expression of MMP-2, TIMP-2 and MMP-14 were significantly higher in ameloblastoma tissues than in odontotheca tissues. The mRNA levels of TIMP-2 and MMP-14 were significantly higher in recurrent and solid/multicystic ameloblastoma tissues than in primary and unicystic ameloblastoma tissues respectively.Of the MMP-2/TIMP-2/MMP-14 complex, a high expression of MMP-2, TIMP-2 and MMP-14 mRNA levels may contribute to the local   invasive   characteristics of ameloblastoma, whereas the local   invasive   capacity of ameloblastoma is more likely related to a high transcriptional levels of TIMP-2 and MMP-14. 
 Clinical implications of HER-2/neu overexpression and proteolytic activity imbalance in breast cancer. The aggressive biological behavior of   invasive   and metastatic cancer is considered to be the most insidious and life-threatening aspect for breast cancer patients. It is mostly the result of changes in many molecular characteristics of tumor cells, including alterations in gene expression and the balance of proteolytic activity. The objective of this study was to determine the level of MMP-2, its natural inhibitor TIMP-2, their ratio and HER-2/neu as diagnostic and prognostic factors. Markers were analyzed in 240 tissue samples categorized into 96 benign breast disease and 144 breast cancer patients. Enzyme linked immunosorbent assay procedure was used to evaluate the level of MMP-2 and TIMP-2 in the cell lysate, HER-2/neu in the membrane fraction, and steroid hormone receptors (ER and PgR) in the cytosol fraction. Breast cancer patients were followed-up for three years. Receiver operating characteristic curves were used to determine the cutoff points for the investigated factors. Positive values for all investigated factors were significantly increased in breast cancer patients compared to benign ones. Mean levels for all investigated factors were significantly correlated with lymph node and hormone receptor status, while MMP-2 and TIMP-2 were correlated with tumor grade (P < 0.05). In Univariate analysis, positive MMP-2, MMP-2/TIMP-2, HER-2/neu overexpression, higher tumor grade, late clinical stages and positive lymph nodes status were significantly associated with relapse. By multivariate analysis, all aforementioned factors apart from tumor grade were independent variables. Thus, the investigated markers are constructive for biologic aggressiveness of breast cancer and MMP-2/TIMP-2 ratio might be a new significant marker in early diagnosis and estimate prognosis in breast cancer. 
 HPV16 oncoproteins induce MMPs/RECK-TIMP-2 imbalance in primary keratinocytes: possible implications in cervical carcinogenesis. Cervical cancer is the third most common cancer in women worldwide. Persistent infection with high-risk HPV types, principally HPV16 and 18 is the main risk factor for the development of this malignancy. However, the onset of   invasive   tumor occurs many years after initial exposure in a minority of infected women. This suggests that other factors beyond viral infection are necessary for tumor establishment and progression. Tumor progression is characterized by an increase in secretion and activation of matrix metalloproteinases (MMPs) produced by either the tumor cells themselves or tumor-associated fibroblasts or macrophages. Increased MMPs expression, including MMP-2, MMP-9 and MT1-MMP, has been observed during cervical carcinoma progression. These proteins have been associated with degradation of ECM components, tumor invasion, metastasis and recurrence. However, few studies have evaluated the interplay between HPV infection and the expression and activity of MMPs and their regulators in cervical cancer. We analyzed the effect of HPV16 oncoproteins on the expression and activity of MMP-2, MMP-9, MT1-MMP, and their inhibitors TIMP-2 and RECK in cultures of human keratinocytes. We observed that E7 expression is associated with increased pro-MMP-9 activity in the epithelial component of organotypic cultures, while E6 and E7 oncoproteins co-expression down-regulates RECK and TIMP-2 levels in organotypic and monolayers cultures. Finally, a study conducted in human cervical tissues showed a decrease in RECK expression levels in precancer and cancer lesions. Our results indicate that HPV oncoproteins promote MMPs/RECK-TIMP-2 imbalance which may be involved in HPV-associated lesions outcome. 
 Inhibition of cyclooxygenase-2 suppresses the   invasive  ness of oral squamous cell carcinoma cell lines via down-regulation of matrix metalloproteinase-2 production and activation. Increased cyclooxygenase (COX-2) expression in tumors is known to be correlated with tumor invasion, angiogenesis, resistance to apoptosis, and suppression of host immunity. We previously reported that the   invasive  ness of human oral squamous cell carcinoma (OSCC) cell lines NA and HSC-4 was suppressed by treatment with either NS-398, a selective COX-2 inhibitor, or COX-2 antisense oligonucleotide (AS). In the present study, to explore the effects of COX-2 inhibition on the interaction between cancer cells and fibroblasts, we examined the effects of these anti-COX-2 reagents on the expression of matrix metalloproteinases (MMPs) in fibroblast cell lines WI-38 and MRC-5. Western blotting and enzyme-linked immunosorbent assay revealed that NS-398 and COX-2 AS down-regulated the expression and secretion of MMP-2 and the tissue inhibitor of matrix metalloproteinase-2 (TIMP-2) in human fibroblast cell lines. Furthermore, invasion activity of OSCC cells was down-regulated by the addition of culture supernatant from fibroblasts treated with anti-COX-2 reagents in a Matrigel invasion assay. These results suggest that selective COX-2 inhibition suppresses the invasion activity of OSCC cells via down-regulation of an MMP-2-activating mechanism involving TIMP-2 and production of the MMP-2 protein by an interaction between cancer cells and stromal fibroblasts. Genetic or pharmacological inhibition of COX-2 may therefore be a beneficial strategy in the treatment of OSCC. 
 [Matrix metalloproteinases 2 and 9, their endogenous regulators, and angiotensin-converting enzyme in cervical squamous cell carcinoma]. to investigate the specific features of the expression of matrix metalloproteinases 2 and 9 (MMP-2, MMP-9), tissue inhibitor of metalloptoteinase 2 (TIMP-2), urokinase-type plasminogen activator (uPA), and angiotensin-converting enzyme (ACE) in cervical squamous cell carcinoma (CSCC).The samples of tumor tissue and morphologically normal tissue adjacent to the tumor were investigated. Enzymatic assays applying specific substrates, as well as zymographic and immunohistochemical studies were used.The   invasive   potential of CSCC has been established to be substantially influenced by the increased expression of MMP-9 and uPA and by the decreased expression of TIMP-2, as well as to a lesser extent by a change in MMP-2 expression. MMP-9 may serve as a marker for   invasive   growth. Enhanced ACE activity in cancer confirms the involvement of this enzyme in tumor progression. The morphologically normal tissue adjacent to the tumor shows the substantial expression of MMP-2 and MMP-9 and in some cases the enhanced activity of uPA and ACE, which makes an additional contribution to the increased   invasive   potential of tumor.The findings are important in understanding the mechanisms of cancer progression and may affect therapeutic strategies for the patient. 
 Tissue Inhibitors of Metalloproteinase-1 and 2 and Obesity Related Non-Alcoholic Fatty Liver Disease: Is There a Relationship. Non-alcoholic fatty liver disease is a spectrum of clinical conditions, including simple steatosis and non-alcoholic steatohepatitis (NASH). The aim of the study is to evaluate the tissue inhibitors of metalloproteinase-1 and 2 (TIMPs) as non  invasive   predictors of NASH.Three groups were included in the study. Obese patients (n = 30) with normal liver enzymes were included in group I and obese patients (n = 30) with elevated liver enzymes with liver biopsy-based diagnosis of NASH were included in group II. Age-matched subjects (n = 30) formed the control as group III. The lipid profile, liver enzyme levels and levels of TIMPs were compared among all the patients and subjects.Comparison of groups I and II showed significantly elevated levels of TIMP-1 and TIMP-2 in group II as compared to group I (p < 0.05). Similarly, comparison between groups II and III showed significantly increased levels of TIMP-1 and TIMP-2 in group II as compared to group III (p < 0.05). TIMP-1 (sensitivity 96.7%, specificity 100%) and TIMP-2 (sensitivity 93.3%, specificity 100%) showed high accuracy in NASH diagnosis.TIMP-1 and TIMP-2 may be considered non  invasive   markers for the diagnosis of NASH. 
 Transient RNA silencing of tissue factor pathway inhibitor-2 modulates lung cancer cell invasion. Tissue Factor Pathway Inhibitor-2 (TFPI-2) is a potent inhibitor of plasmin which activates metalloproteinases (MMPs) involved in extracellular matrix (ECM) degradation. Its secretion in ECM makes TFPI-2 a potential inhibitor to regulate tumour invasion and metastasis. Moreover, TFPI-2 is frequently downregulated, particularly in aggressive cancers. In this study, we silenced TFPI-2 in the NCI-H460 non-small cell lung cancer cell line and evaluated the role of TFPI-2 in cell invasion and its impact on MMPs expression. As the effects of siRNA are transient, the consequences of both gene silencing and restoration to normal expression could be studied kinetically in the same cells. We showed that TFPI-2 expression by NCI-H460 cells was effectively downregulated using specific small interfering RNA and this silencing was associated with an increase in the   invasive   potential of tumour cells while migration was not affected. We also showed that mRNA levels and protein expression of MMP-2, -3, -9, -14 were not influenced by TFPI-2 silencing. Moreover, the gelatinase activity of MMP-2 and MMP-9 was unmodified. In contrast, MMP-1 mRNA levels and protein were significantly and similarly increased in cells transfected with TFPI-2 siRNA. In conclusion, this study confirms that TFPI-2 downregulation can contribute to tumour invasion of lung cancer cells. 
 The role of EZH2 in the regulation of the activity of matrix metalloproteinases in prostate cancer cells. Degradation of the extracellular matrix (ECM), a critical step in cancer metastasis, is determined by the balance between MMPs (matrix metalloproteinases) and their inhibitors TIMPs (tissue inhibitors of metalloproteinases). In cancer cells, this balance is shifted towards MMPs, promoting ECM degradation. Here, we show that EZH2 plays an active role in this process by repressing the expression of TIMP2 and TIMP3 in prostate cancer cells. The TIMP genes are derepressed by knockdown of EZH2 expression in human prostate cancer cells but repressed by overexpression of EZH2 in benign human prostate epithelial cells. EZH2 catalyzes H3K27 trimethylation and subsequent DNA methylation of the TIMP gene promoters. Overexpression of EZH2 confers an   invasive   phenotype on benign prostate epithelial cells; however, this phenotype is suppressed by cooverexpression of TIMP3. EZH2 knockdown markedly reduces the proteolytic activity of MMP-9, thereby decreasing the   invasive   activity of prostate cancer cells. These results suggest that the transcriptional repression of the TIMP genes by EZH2 may be a major mechanism to shift the MMPs/TIMPs balance in favor of MMP activity and thus to promote ECM degradation and subsequent invasion of prostate cancer cells. 
 Advanced colorectal carcinomas measuring 20 mm or less exhibit markedly higher   invasive  ness despite their size. To analyze the biology of small (20 mm or less) advanced colorectal carcinomas (SAC), 24 cases, 22 small early colorectal carcinomas (SEC) of similar size, and 52 advanced colorectal carcinomas (AC) were studied. The proliferative (Ki-67) labeling index for SAC was 65.9+/-17.1%, significantly higher than those for SEC (30.9+/-13.7%) or AC (43.0+/-17.1%) (P < 0.01). Matrix metalloproteinase (MMP)-9 and tissue inhibitors of metalloproteinase (TIMP)-2 expressions for SAC were 62.5 and 79.2%, respectively, significantly higher than those for SEC (4.5, 13.6%) or AC (21, 33%) (P < 0.01). Small advanced carcinomas have higher   invasive  ness than SEC or AC and may represent a different type of cancer. 
 [Membrane type 1 matrix metalloproteinase (MT1-MMP) and the regulators of its activity as   invasive   factors in squamous cell cervical carcinomas]. Membrane type 1 matrix metalloproteinase (MT1MMP) is one of matrix metalloproteinases (MMP), which play а key role in tumor invasion and metastasis. The aim of this study was to elucidate the peculiarities of expression of MT1MMP and endogenous regulators of its activity: the activator - furin and the inhibitor - TIMP-2, as   invasiv  e factors of squamous cell cervical carcinomas (SCC). The study was carried out using 11 specimens of SCC and 11 specimens of morphologically normal tissue adjacent to the tumor. It was shown that the increase of MT1-MMP and furin expression and low of TIMP-2 expression makes the main contribution to the destructive   (invasiv  e) potential of SCC. Moreover, substantial expression of MT1-MMP was registered in the specimens of morphologically normal adjoining to tumor tissue. This expression was found to make an additional contribution to the destructive potential of the cervical tumor. 
 Immunoreactivity for TIMP-2 is associated with a favorable prognosis in endometrial carcinoma. Tissue inhibitors of metalloproteinases are important regulators of metalloproteinase activity, and the balance of active enzyme and inhibitor is a critical determinant of tumor cell   invasive  ness. This study aimed to evaluate the prognostic and clinical implications of the two main inhibitors of matrix metalloproteinases, TIMP-1 and TIMP-2, in endometrial carcinoma. The material consisted of 241 patients with primary endometrial carcinoma. The median follow-up time was 77 months. Expressions of TIMP-1 and TIMP-2 proteins were examined in paraffin-embedded tumor sections by immunohistochemical methods. Positive staining for TIMP-1 and -2 was observed in 88% and 86% of the primary tumors, respectively. The Kaplan-Meier analysis showed that the 5-year cancer-specific survival rate of the patients with TIMP-2 positive immunostaining was 89% and that of the TIMP-2 negative patients 78%. Positive immunoreaction for TIMP-2 correlated with favorable cancer-specific and overall survival. When including only endometrioid adenocarcinomas, a similar trend towards favorable survival was seen. Excluding stage IA carcinomas, the difference became again statistically significant. For TIMP-1, there was no statistically significant association with overall or cancer-specific survival. The Cox regression analysis showed stage, grade and TIMP-2 to be significant predictors of survival. We suggest that TIMP-2 may have a more important role in endometrial carcinoma progression than TIMP-1 and might serve as a potential marker for favorable prognosis in this type of cancer. 
 NEDD9 depletion leads to MMP14 inactivation by TIMP2 and prevents invasion and metastasis. The scaffolding protein NEDD9 is an established prometastatic marker in several cancers. Nevertheless, the molecular mechanisms of NEDD9-driven metastasis in cancers remain ill-defined. Here, using a comprehensive breast cancer tissue microarray, it was shown that increased levels of NEDD9 protein significantly correlated with the transition from carcinoma in situ to   invasive   carcinoma. Similarly, it was shown that NEDD9 overexpression is a hallmark of highly   invasive   breast cancer cells. Moreover, NEDD9 expression is crucial for the protease-dependent mesenchymal invasion of cancer cells at the primary site but not at the metastatic site. Depletion of NEDD9 is sufficient to suppress invasion of tumor cells in vitro and in vivo, leading to decreased circulating tumor cells and lung metastases in xenograft models. Mechanistically, NEDD9 localized to   invasive   pseudopods and was required for local matrix degradation. Depletion of NEDD9 impaired invasion of cancer cells through inactivation of membrane-bound matrix metalloproteinase MMP14 by excess TIMP2 on the cell surface. Inactivation of MMP14 is accompanied by reduced collagenolytic activity of soluble metalloproteinases MMP2 and MMP9. Reexpression of NEDD9 is sufficient to restore the activity of MMP14 and the   invasive   properties of breast cancer cells in vitro and in vivo. Collectively, these findings uncover critical steps in NEDD9-dependent invasion of breast cancer cells.This study provides a mechanistic basis for potential therapeutic interventions to prevent metastasis. 
 Matrix metalloproteinase-9 and tissue inhibitor of matrix metalloproteinase-2: Prognostic biological markers in   invasive   prolactinomas. In this study, the predictive roles of matrix metalloproteinase (MMP)-9 and tissue inhibitor of matrix metalloproteinase (TIMP)-1 and 2 in   invasive   and non  invasive   prolactinomas were examined. Prognostic biomarkers to distinguish between   invasive   and non  invasive   pituitary adenomas are required for the effective treatment of pituitary adenoma patients. We analyzed 57 prolactinoma patients classified as having   invasive   and non  invasive   adenomas for MMP-9, TIMP-1 and TIMP-2 expression using immunohistochemical methods. Significantly higher MMP-9 expression was detected in   invasive   prolactinomas (p=0.004). There was also a significant relationship between TIMP-2 expression and   invasive   behavior (p=0.004) and TIMP-2 expression and recurrence (p=0.005). Because MMP-9 expression is significantly increased in   invasive   prolactinomas, MMP-9 has potential as a marker for invasion. TIMP-2 may be a marker for both invasion and recurrence. These findings require further examination in large scale prospective studies. 
 Circulating matrix metalloproteinase-9 and tissue inhibitors of metalloproteinases-1 and -2 levels in gestational hypertension. Gestational hypertension (GH) is dangerous to both mother and child. Arterial   invasive  ness and growth are dependent on successful extracellular matrix (ECM) breakdown, which may be abnormal in GH. We hypothesized abnormalities in circulating matrix metalloproteinase-9 (MMP-9) and tissue inhibitors of metalloproteinases-1 and -2 (TIMP-1 and TIMP-2, respectively) in patients with GH, when compared with normotensive women with normal pregnancies and healthy nonpregnant control subjects.Plasma MMP-9, TIMP-1, and TIMP-2 were measured by ELISA in 23 women with GH, 30 normotensive pregnant women, and 28 nonpregnant women who were matched for age, gestational age, and parity.Levels of circulating MMP-9, TIMP-1 and TIMP-2, and the MMP-9/TIMP-1 and MMP-9/TIMP-2 ratios were significantly different among the three groups (P = .026, P = .006, P = .007, P = .001 and P = .008 respectively). Within the GH group, MMP-9 and the MMP-9/TIMP-1 ratio correlated negatively with age (r = -0.581, P = .004 and r = -0.563, P = .005, respectively) and levels of diastolic blood pressure (r = -0.432, P = .040 and r = -0.461, P = .027, respectively). With multiple regression analysis, only age independently correlated with circulating levels of MMP-9 (P = .010); neither age nor levels of diastolic blood pressure had any effect on the MMP-9/TIMP-1 ratio.We have demonstrated altered MMP/TIMP ratios in maternal blood during GH. These observations suggest pregnancy-related changes in ECM breakdown and turnover. Given the importance of changes in ECM composition to vascular and cardiac structure in hypertension, we suggest that these observations may be related to the pathophysiology of human GH. 
 Promotion of astrocytoma cell invasion by micro RNA-22 targeting of tissue inhibitor of matrix metalloproteinase-2. OBJECTIVE Diffuse astrocytomas (DAs) have a high recurrence rate due to diffuse infiltration into the brain and spinal cord. Micro RNAs (miRNAs) are small noncoding RNAs that regulate gene expression by binding to complementary sequences of target messenger RNA (mRNA). It has been reported that miRNA-22 (miR-22) is involved in the invasion of some cancer cell lines. The aim of this study was to identify the biological effects of miR-22 in regard to the invasion of human DAs. METHODS The authors evaluated whether the level of miR-22 is elevated in human spinal DAs by using miRNA chips. Next, the role of miR-22 in 1321N1 human astrocytoma cells was investigated. Finally, to elucidate whether miR-22 promotes invasion by astrocytoma cells in vivo, the authors transplanted miR-22 overexpressed astrocytoma cells into mouse thoracic spinal cord. RESULTS The miR-22 significantly upregulated the invasion capacity of 1321N1 cells. Computational in silico analysis predicted that tissue inhibitor of matrix metalloproteinase-2 (TIMP2) is a target gene of miR-22. This was confirmed by quantitative reverse transcription polymerase chain reaction and Western blotting, which showed that miR-22 inhibited TIMP2 mRNA and protein expression, respectively. Luciferase reporter assays demonstrated that miR-22 directly bound the 3'-untranslated regions of TIMP2. The authors further showed that miR-22 promoted   invasive  ness in 1321N1 astrocytoma cells when transplanted into mouse spinal cord. CONCLUSIONS These data suggest that miR-22 acts to regulate invasion of 1321N1 astrocytoma cells by targeting TIMP2 expression. Additional studies with more cases and cell lines are required to elucidate the findings of this study for a novel treatment target for spinal DAs. 
 Downregulation of reversion-inducing cysteine-rich protein with Kazal motifs in malignant melanoma: inverse correlation with membrane-type 1-matrix metalloproteinase and tissue inhibitor of metalloproteinase 2. The   invasive   phenotype of many tumors is associated with an imbalance between the matrix metalloproteinases (MMPs) and their inhibitors, tissue inhibitors of metalloproteinases (TIMPs), and the membrane-anchored reversion-inducing cysteine-rich protein with Kazal motifs (RECK). RECK inhibits MMP-2, MMP-9, and MT1-MMP, and has been linked to patient survival and better prognosis in several types of tumors. However, despite the wide implication of these MMPs in melanoma establishment and progression, the role of RECK in this type of tumor is still unknown. Here, we analyzed the expression of RECK, TIMP1, TIMP2, TIMP3, MT1MMP, MMP2, and MMP9 in two publicly available melanoma microarray datasets and in a panel of human melanoma cell lines. We found that RECK is downregulated in malignant melanoma, accompanied by upregulation of MT1MMP and TIMP2. In both datasets, we observed that the group of samples displaying higher RECK levels show lower median expression levels of MT1MMP and TIMP2 and higher levels of TIMP3. When tested in a sample-wise manner, these correlations were statistically significant. Inverse correlations between RECK, MT1MMP, and TIMP2 were verified in a panel of human melanoma cell lines and in a further reduced model that includes a pair of matched primary tumor-derived and metastasis-derived cell lines. Taken together, our data indicate a consistent correlation between RECK, MT1MMP, and TIMP2 across different models of clinical samples and cell lines and suggest evidence of the potential use of this subset of genes as a gene signature for diagnosing melanoma. 
 High variability in MMP2/TIMP2 and MMP9/TIMP1 expression in secondary liver tumors. The assessment of MMPs/TIMPs expression in various primary tumors has potential prognostic values. Considering the paucity of studies in secondary liver tumors, our aim was to study the expression of MMP2, MMP9, TIMP1, and TIMP2 in secondary hepatic cancer, focusing on their variability in the malignant cells. The study group included 25 cases of liver metastases of colorectal cancer, diagnosed and surgically treated at "Sf. Spiridon" University Hospital of Iassy, Romania. Immunohistochemistry for MMP2, MMP9, TIMP1, and TIMP2 has been performed, followed by the semi-quantitative assessment of the markers using a scoring system based on the positive tumoral cells percentage and the staining intensity. The expression of investigated markers revealed an increased staining variability. The scores showed that MMP2/TIMP2 and MMP9/TIMP1 immunoreactivity was extremely heterogeneous within the analyzed group, with a dominant weak expression for MMP2 and MMP9, in contrast to strong TIMP2 and TIMP1 expression. Ten different patterns of expression of the investigated markers have been identified. No major differences between the expression of MMP2 (14 positive cases) and MMP9 (16 positive cases) could be detected. Our results sustain the inverse correlation between MMP and the correspondent TIMP expression, supporting the hypothesis that MMPs/TIMPs balance has mainly an inhibitory effect on   invasive  ness. Our study demonstrated that tumoral cells are adapted to MMPs:TIMPs production in the liver microenvironment. The lack of significant differences between MMP2 and MMP9 expression shows that the activity of both MMPs is independent, without reciprocal influences. 
 Epigenetic inactivation of the tissue inhibitor of metalloproteinase-2 (TIMP-2) gene in human prostate tumors. Gene silencing via CpG island methylation in the promoter region is one of the mechanisms by which tumor suppressor genes are inactivated in human cancers. Previous studies have shown that the tissue inhibitors of metalloproteinases (TIMP)-2 gene, which is an endogenous inhibitor of matrix metalloproteinases involved in cell invasion and tumorigenesis, is downregulated or silenced in a variety of human cancer cell lines. Here, we investigated the mechanism underlying TIMP-2 expression in prostate cancer cell lines and primary prostate tumor samples. We observed a strong correlation between promoter hypermethylation and lost expression of TIMP-2 gene, which was supported by other results demonstrating that promoter demethylation by 5-aza-2'-deoxycytidine and trichostatin A reactivated TIMP-2 and restored its expression in TIMP-2-silenced metastatic prostate cell lines. These results were further substantiated by a chromatin immunoprecipitation assay, showing the preferential binding of MeCP2 to methylated CpG island in TIMP-2-silenced metastatic prostate cell lines. In vitro Matrigel invasion assays showed that re-expression of TIMP-2 after a combined treatment with 5-aza and trichostatin-A in metastatic prostate cells resulted in a significant reduction of tumor cell invasion. Furthermore, CpG methylation of TIMP-2 promoter was also shown in primary prostate tumors that expressed decreased TIMP-2 protein levels. These results suggest that the downregulation of the TIMP-2 gene is associated with promoter methylation and that this may play an important role in prostate cancer progression during the   invasive   and metastatic stages of the disease. 
 [Interstitial collagenase, gelatinases A and B and their endogenous inhibitors in squamous cell cervical carcinomas]. Interstitial collagenase and gelatinases are matrix metalloproteinases (MMP), which play the key role in tumor invasion and metastasis. The aim of this study was to elucidate the peculiarities of expression of interstitial collagenase (MMP-1), gelatinases A and B (MMP-2 and MMP-9) and their endogenous tissue inhibitors TIMP-1 and TIMP-2 as   invasive   factors of squamous cell carcinomas (SCC) of human cervical cancer. The study was carried out using 24 specimens of SCC and 11 specimens of adjacent to tumor morphologically normal tissue. All carcinoma specimens expressed E7 HPV-16 gene. It was shown that the increase of MMP-1 and MMP-9 expression and low of TIMP-1 and TIMP-2 expression makes the main contribution to the destructive (  invasive  ) potential of SCC. The change of MMP-2 expression is not so significant and it is less influenced to the destructive potential. Moreover, substantial expression of MMP-1, MMP-2 and MMP-9 was registered in the specimens of morphologically normal adjoining to tumor tissue. This expression was found to make an additional contribution to the destructive potential of cervical tumor. 
 Comparative study of the immunohistochemical expression of tissue inhibitors of metalloproteinases 1 and 2 between clearly   invasive   carcinomas and "in situ" trophoblast invasion. Tissue inhibitors of metalloproteinases (TIMPs) play an important role in extracellular matrix homeostasis by regulating MMP activity. Although they were initially considered inhibitors of tumor growth and metastasis, recently their role in cancer progression has been controversial. The aim of our study was to compare the immunohistochemical expression of TIMP1 and TIMP2 between an uncontrollably   invasive   phenomenon (cancer) and an "in situ" process (trophoblast invasion) in an effort to assess any differential role of these molecules between these two distinct phenomena and therefore to understand better their contribution in cancer invasion and migration. We performed an immunohistochemical analysis of 50 carcinomas (colorectal, gastric, breast, pulmonary, and renal) and 40 first trimester gestations. The marker expression was evaluated semiquantitatively, separately in cancer parenchymal and trophoblastic cells as well as in malignant stromal and decidual cells, according to a percentage scale (0, <10, 10-50, and >50%) and according to staining intensity (0, +, ++, and +++). Our results showed that there was no statistically significant difference in TIMP1 expression between cancer parenchymal cells and trophoblastic cells. On the other hand, TIMP1 was expressed more often in decidual cells than in cancer stromal cells. Immunostaining for TIMP2 was more extensive and intense both in trophoblastic and decidual cells than in cancer parenchymal and stromal cells, respectively. The reduced expression of TIMP2 in metastatic carcinomas by comparison with non-metastatic gestation specimens underlines its importance in cancer invasion and migration. On the other hand, TIMP1 was more expressed in decidua than cancer stroma, but at the same time showed no statistically significant difference between cancer parenchyma and trophoblasts, highlighting its multifunctional activity in cancer progression. 
 CD133 affects the   invasive   ability of HCT116 cells by regulating TIMP-2. CD133 is widely expressed in colorectal cancer (CRC) tissues and cell lines. This protein has been used as a marker of CRC cancer stem cells, although the function and mechanism of CD133 in CRC invasion and metastasis remain unclear. In our study, we examined the role of CD133 in CRC invasion in vitro and investigated the mechanism involved in CD133-related invasion. CD133(high) and CD133(low) HCT116 cells were isolated, and the proliferation and   invasiv  e ability of these two subpopulations were tested. CD133(high) HCT116 cells exhibited greater proliferation and invasion compared with CD133(low) HCT116 cells. CD133 knockdown (using CD133 small-interfering [si]RNA) inhibited the   invasiv  e activity of CD133si-HCT116 cells. For the first time, we found that the expression of tissue inhibitor of matrix metalloproteinases-2 (TIMP-2) was down-regulated in CD133si-HCT116 cells. In addition, for the TIMP-2si-HCT116 cells (transfected with TIMP-2 siRNA), in vitro invasion was significantly decreased, whereas the expression of CD133 remained unchanged. Finally, the metalloproteinase 2 and MEK/ERK signaling pathways were examined, and no significant change was observed after the knockdown of CD133 or TIMP-2 in HCT116 cells. In conclusion, we demonstrated that CD133 plays an important role in HCT116 cell invasion, and for the first time, we found that CD133 knockdown significantly down-regulated TIMP-2 expression, which suggests that CD133 affects th  e invasi  ve ability of HCT116 cells by regulating TIMP-2. 
 Concentrations and activities of metalloproteinases 2 and 9 and their inhibitors (TIMPS) in chronic pancreatitis and pancreatic adenocarcinoma. Pancreatic cancer (PC) and chronic pancreatitis (CP) are still significant problems. The aim of this study was a comparative analysis of the activity and concentrations of matrix metalloproteinases 2 and 9 and the concentrations of their tissue inhibitors (TIMP 1 and 2) in the PC compared to CP tissue homogenates. The study was performed in a group of 63 patients with pancreatic cancer or chronic pancreatitis selected for resection procedures. Group 1 consisted of 31 patients with CP, group 2 consisted of 32 patients with PC. There was no coincidence of pancreatic cancer in CP group. The pancreatic tumor samples have been properly prepared in order to perform electrophoresis and immunoassay testing. The activity of MMPs and the concentrations of MMPs and TIMPs were evaluated.the revealed activities of gelatinases and concentrations levels of the gelatinases and their inhibitors were significantly higher in the PC tissue samples compared to CP. In both groups, higher concentrations of MMP9 compared to MMP2 and TIMP2 compared to TIMP1 were shown. High potential for tumor   invasive  ness demonstrated by the formation of lymph node metastases was characterized by the higher concentrations of MMP9 and TIMP2. However, in the case of infiltration of the nerve fibers, a decrease in the concentration of MMP2 was found.gelatinases and their inhibitors play important role in the pathogenesis of the CP as well as PC. The activity and concentration of gelatinases and the concentration of their inhibitors were all significantly higher in the PC group. 
 Activation ratio of MMP-2 and expression of MT1-MMP are correlated in thymic epithelial tumours. To study the relationships between the activation of pro-MMP-2 and the mRNA levels of MT1-MMP and TIMP-2 in thymic tissues and thymic epithelial tumours.We examined the mRNA expressions of MMP-2, MT1-MMP and TIMP-2 with real-time reverse transcription-polymerase chain reaction (RT-PCR). The gelatinolytic activity and MMP-2 activation ratio of thymic tissues, thymomas, and thymic carcinomas were determined with gelatin zymography. The cellular localisation of MMP-2 was detected with film in situ gelatin zymography. We then examined the mRNA expression of MMP-2 in the epithelial tumour cells or lymphocytes and stromal cells distant from the tumour (obtained from two   invasive   thymomas) using laser-capture microdissection (LCM).The mRNA levels of MMP-2, MT1-MMP and TIMP-2 were significantly increased from thymic tissue, Stage I-II, III-IV thymomas to thymic carcinomas (p<0.005). They were also significantly increased from AB-B1 (lymphocyte rich and mixed types), B2-B3 (cortical and predominantly polygonal cells types) thymomas to thymic carcinomas (p<0.05). The MMP-2 activation ratio also had the same tendency among the above groups (p<0.05) and was directly correlated to MT1-MMP and TIMP-2 mRNA expression (Spearman rank correlation: r = 0.8627, r = 0.8314; p<0.005). Film in situ zymography demonstrated that positive expression is mainly localised in tumour cell nests and adjacent stroma cells. LCM and real-time RT-PCR results confirmed that the expression of MMP-2 was higher in epithelial tumour cells than in lymphocytes and stromal cells.MMP-2, MT1-MMP and TIMP-2 mRNA expression levels were correlated with clinical stages and histological subtypes of thymic epithelial tumours. The activation of pro-MMP-2 might be mediated by MT1-MMP and TIMP-2 up-regulation. 
 MMP-2 protein in   invasive   breast cancer and the impact of MMP-2/TIMP-2 phenotype on overall survival. Crucial event in the metastasis of cancer cells is the secretion of matrix metalloproteinases (MMPs), which are responsible for the degradation of extracellular matrix (ECM). Among them, matrix metalloproteinase-2 (MMP-2) is a gelatinase, which degrades basement membrane type-IV collagen. Immunohistochemistry was performed to detect MMP-2 protein in 135 infiltrative breast carcinomas. MMP-2 was studied along with clinicopathological parameters (tumor size, histological type, nuclear and histological grade, stage, lymph node status, ER, and PR), patients' survival and tissue inhibitor metalloproteinase-2 (TIMP-2), Ki-67, and p53 proteins. MMP-2 immunoreactivity was detected in the cytoplasm in cancer cells in 102 (75.6%) and in both tumor and tumor stromal cells in 37 (27.4%) of 135 cases respectively. MMP-2 reactivity in cancer cells displayed a statistically significant association with tumor size > 2 cm (p = 0.022). In tumor stromal cells a strong parallel association was observed between the expression of MMP-2 and TIMP-2 (p = 0.015), while an inverse correlation was found between MMP-2 and both Ki-67 and p53 (p = 0.033 and p = 0.034 respectively). In the subgroup with negative lymph nodes MMP-2 was also inversely associated with p53 in cancer cells (p = 0.045). Finally a statistically significant association was revealed using Kaplan-Meier and Cox's proportional hazard regression model between the MMP-2/TIMP-2 phenotype and patients' better survival (p = 0.021). Our results point out the strong relation between MMP-2 and TIMP-2 and the effect of the MMP-2/TIMP-2 phenotype in the patients' overall survival. The inverse correlation between MMP-2 and both Ki-67 and p53 can be explained by the potential inhibition of MMP-2 by TIMP-2. These results suggest the necessity of further investigation. 
 Differential regulation of membrane type 1-matrix metalloproteinase activity by ERK 1/2- and p38 MAPK-modulated tissue inhibitor of metalloproteinases 2 expression controls transforming growth factor-beta1-induced pericellular collagenolysis. Acquisition of matrix metalloproteinase-2 (MMP-2) activity is temporally associated with increased migration and   invasive  ness of cancer cells. ProMMP-2 activation requires multimolecular complex assembly involving proMMP-2, membrane type 1-MMP (MT1-MMP, MMP-14), and tissue inhibitor of metalloproteinases-2 (TIMP-2). Because transforming growth factor-beta1 (TGF-beta1) promotes tumor invasion in advanced squamous cell carcinomas, the role of TGF-beta1 in the regulation of MMP activity in a cellular model of   invasive   oral squamous cell carcinoma was examined. Treatment of oral squamous cell carcinoma cells with TGF-beta1 promoted MMP-dependent cell scattering and collagen invasion, increased expression of MMP-2 and MT1-MMP, and enhanced MMP-2 activation. TGF-beta1 induced concomitant activation of ERK1/2 and p38 MAPK, and kinase inhibition studies revealed a negative regulatory role for ERK1/2 in modulating acquisition of MMP-2 activity. Thus, a reciprocal effect on proMMP-2 activation was observed whereupon blocking ERK1/2 phosphorylation promoted proMMP-2 activation and MT1-MMP activity, whereas inhibiting p38 MAPK activity decreased proteolytic potential. The cellular mechanism for the control of MT1-MMP catalytic activity involved concurrent reciprocal modulation of TIMP-2 expression by ERK1/2 and p38 MAPKs, such that inhibition of ERK1/2 phosphorylation decreased TIMP-2 production, and down-regulation of p38 MAPK activity enhanced TIMP-2 synthesis. Further, p38 MAPK inhibition promoted ERK1/2 phosphorylation, providing additional evidence for cross-talk between MAPK pathways. These observations demonstrate the complex reciprocal effects of ERK1/2 and p38 MAPK in the regulation of MMP activity, which could complicate the use of MAPK-specific inhibitors as therapeutic agents to down-regulate the biologic effects of TGF-beta1 on pericellular collagen degradation and tumor invasion. 
 Matrix metalloproteinase-2, membranous type 1 matrix metalloproteinase, and tissue inhibitor of metalloproteinase-2 expression in ectopic and eutopic endometrium. To investigate expression of matrix metalloproteinase-2 (MMP-2), membranous type 1 matrix metalloproteinase (MT1-MMP), and tissue inhibitor of metalloproteinase-2 (TIMP-2) in ectopic and eutopic endometrium from women with and without endometriosis throughout the menstrual cycle.Molecular studies in human tissue.Reproductive immunology laboratory of a university medical center.Fifty-three premenopausal woman (23 with endometriosis and 30 without endometriosis) undergoing laparoscopic surgery. Endometrium and ectopic endometriosis tissue were obtained at the time of surgery.Messenger RNA and protein expression from eutopic and ectopic endometrium was analyzed by using quantitative competitive polymerase chain reaction, zymography, and Western blot assay.Uterine endometrium from women with endometriosis expressed higher levels of MMP-2 and MT1-MMP and lower levels of TIMP-2 than did endometrium from normal women.Eutopic endometrium from patients with endometriosis may be more   invasive   and prone to peritoneal implantation because of greater expression of MMP-2 and MT1-MMP and lower expression of TIMP-2 messenger RNA, compared with endometrium from women without endometriosis. Thus, increased proteolytic activity may help to explain the   invasive   factors that result in endometriosis. 
 TIMP-2 mediates the anti-  invasive   effects of the nitric oxide-releasing prodrug JS-K in breast cancer cells. Tumor invasion and metastasis remain a major cause of mortality in breast cancer patients. High concentrations of nitric oxide (NO) suppress tumor invasion and metastasis in vivo. NO prodrugs generate large amounts of NO upon metabolism by appropriate intracellular enzymes, and therefore could have potential in the prevention and therapy of metastatic breast cancer.The present study was designed to determine the effects of the NO-releasing prodrug O2-(2,4-dinitrophenyl) 1- [(4-ethoxycarbonyl)piperazin-1-yl]diazen-1-ium-1,2-diolate (JS-K) on breast cancer invasion and the mechanisms involved. MDA-MB-231, MDA-MB-231/F10, and MCF-7/COX-2 were the three breast cancer cell lines tested. NO levels were determined spectrophotometrically using a NO assay kit. Invasion and the expression of matrix metalloproteinases (MMPs) and tissue inhibitor of MMPs were determined using Matrigel invasion assays, an MMP array kit and ELISAs. The activity and expression of extracellular signal-regulated kinase 1/2, p38, and c-Jun N-terminal kinase mitogen-activated protein kinases were determined using western blot analyses.Under conditions by which JS-K was not cytotoxic, JS-K significantly decreased (P < 0.05) the   invasive  ness of breast cancer cells across the Matrigel basement membrane, which was directly correlated with NO production. JS-43-126, a non-NO-releasing analog of JS-K, had no effect on NO levels or invasion. JS-K increased (P < 0.05) TIMP-2 production, and blocking TIMP-2 activity with a neutralizing antibody significantly increased (P < 0.05) the   invasive   activity of JS-K-treated cells across Matrigel. JS-K decreased p38 activity, whereas the activity and the expression of extracellular signal-regulated kinase 1/2 and c-Jun N-terminal kinase were unaffected.We report the novel findings that JS-K inhibits breast cancer invasion across the Matrigel basement membrane, and NO production is vital for this activity. Upregulation of TIMP-2 production is one mechanism by which JS-K mediates its anti-  invasive   effects. JS-K and other NO prodrugs may represent an innovative biological approach in the prevention and treatment of metastatic breast cancer. 
 TIMP-2 gene methylation in cervical precursor and   invasive   lesions. To analyze the presence of HPV-DNA and TIMP-2 gene methylation in cervical precursor and   invasive   lesions, as well as to study the associations among the latter, the presence of HPV-DNA, and the clinical evolution of such lesions.Cross-sectional study that includes 49 biopsy or brush smear samples from women with a normal cervix, LSIL, HSIL, micro  invasive   carcinoma and   invasive   carcinoma. The presence of HPV-DNA and specific methylation was analyzed using PCR. Thirty-eight biopsy samples for HSIL, micro  invasive   carcinoma and frank   invasive   carcinoma as well as 11 brush smear samples for LSIL and normal cervices were analyzed.TIMP-2 gene methylation was detected in 86.8% (33/38) of the samples from the group with lesions and 50% (4/8) of the normal samples (p=0.03). HPV-DNA was detected in 81.6% (31/38) of the samples from the group with lesions and 25% (2/8) of the normal samples (p=0.003). HPV-DNA was more frequent in the methylated samples (50%), and the group with methylation had a higher risk of unfavorable evolution than the group without methylation; however, such observations were not statistically significant (p=0.19).TIMP-2 gene methylation and the presence of HPV-DNA were characteristic of the group with cervical lesions. Methylation was not associated with the presence of HPV-DNA or an unfavorable clinical evolution. 
 Frequent hypermethylation of 5' flanking region of TIMP-2 gene in cervical cancer. Tissue inhibitor of metalloproteinase-2 (TIMP-2) is an endogenous inhibitor of matrix metalloproteinases (MMPs). This multifunctional protein regulates activities of MMPs and possesses growth promoting effect in cell culture, anti-tumoral, anti-apoptotic and anti-angiogenic effects in animal model systems in vivo. It has been shown that this gene is downregulated in cervical carcinomas. The mechanism of inhibition of TIMP-2 expression remains obscure. We have examined whether aberrant DNA methylation of the 5'CpG island of the TIMP-2 gene is involved in its inhibition during cervical carcinogenesis. Bisulfite-modified DNA sequencing and MSP assay showed aberrant methylation of TIMP-2 5'-CpG island in 17 of 36 (47%)   invasive   cervical carcinomas and in 2 of 3 cervical cancer cell lines. TIMP-2 gene was mostly unmethylated in the morphologically normal tissues adjacent to the tumors, whereas methylated alleles of this gene were found in 4 samples. Each tumor and each cell line DNA was characterized by unique methylation pattern, however a discrete region of TIMP-2 CpG island upstream to the transcription start site was densely methylated in all hypermethylated DNA samples examined. The expression of TIMP-2 mRNA can be restored in the cell lines, in which this discrete region of TIMP-2 CpG island is methylated, by treatment with demethylating agents, 5-azacytidine and 5-aza-2'-deoxycytidine. Our data suggest that the aberrant methylation of TIMP-2 favors the development of primary cervical tumors. We describe for the first time the aberrant hypermethylation of TIMP-2 gene in human cancer. 
  GeneRIFs  
 The   invasive   potential of CSCC has been established to be substantially influenced by the increased expression of MMP-9 and uPA and by the decreased expression of TIMP-2, as well as to a lesser extent by a change in MMP-2 expression 
 TIMP-2 may be a marker for both invasion and recurrence of   invasive   prolactinomas 
 TIMP-2 gene methylation is associated with cervical precursor and   invasive   lesions. 
 Up-regulation of MT1-MMP and furin expression and low TIMP-2 expression make the main contribution to the   invasive  ness potential of squamous cell cervical carcinomas. 
 CD133 affects the   invasive   ability of HCT116 cells by regulating TIMP-2. 
 The aim of our study was to compare the immunohistochemical expression of TIMP1 and TIMP2 between an uncontrollably   invasive   phenomenon (cancer) and an "in situ" process (trophoblast invasion) 
 the increase of MMP-1 and MMP-9 expression and low of TIMP-1 and TIMP-2 expression makes the main contribution to the destructive (  invasive  ) potential of SCC. 
 Of MMP-2/TIMP-2/MMP-14 complex, high expression of MMP-2, TIMP-2 & MMP-14 mRNA may contribute to local   invasive  ness of ameloblastoma; local   invasive   capacity of ameloblastoma more likely related to high transcription levels of TIMP-2 and MMP-14. 
 Upregulation of TIMP-2 production is one mechanism by which prodrug JS-K mediates its anti-  invasive   effects 
 downregulation of the TIMP-2 gene is associated with promoter methylation and that this may play an important role in prostate cancer progression during the   invasive   and metastatic stages of the disease 
 Expression in colorectal carcinomas relating to size and   invasive  ness of the tumor. 
 evidence that cell density influences the   invasive   potential of tumor cells via regulation of MMPs and TIMP-1 and TIMP-2 by AP-1, NF kappa B and CRE transcription factors 
  [Mm]etastasize  PubMed  
 Alterations in enhancer of zeste homolog 2, matrix metalloproteinase-2 and tissue inhibitor of metalloproteinase-2 expression are associated with ex vivo and in vitro bone metastasis in renal cell carcinoma. Renal cell carcinoma (RCC) has a high potential for bone metastasis; however, the molecular mechanisms underlying this metastasis have remained to be elucidated. The present study aimed to explore the expression levels of enhancer of zeste homolog 2 (EZH2), matrix metalloproteinase-2 (MMP2) and tissue inhibitor of metalloproteinase-2 (TIMP2) as determinants of RCC-associated bone metastasis. Their expression was evaluated in a newly generated RCC cell subline that has a high potential for bone metastasis, in tissue specimens from   metastasize  d bone tissues from patients with RCC and in RCC tissues without metastasis. A total of 25 RCC tissue specimens without metastasis and 13 RCC tissue specimens with bone metastasis were acquired for immunohistochemical analysis of EZH2, MMP2 and TIMP2 protein expression. The expression levels of EZH2, MMP2 and TIMP2 mRNA and protein were analyzed in the ACHN and ACHN-BO5 cell lines using western blot and reverse transcription polymerase chain reaction (PCR) analyses. Methylation-specific PCR was also used to analyze TIMP2 promoter methylation. EZH2 and MMP2 proteins were found to be expressed at higher levels in tissues from patients where RCC had   metastasize  d to the bone as compared with those in RCC patients without metastasis, whereas there was no significant difference in the expression of TIMP2 protein between the two tissues. Furthermore, the expression of EZH2 protein was correlated with MMP2 expression, but there was no significant correlation between the expression of EZH2 and TIMP2 proteins. The in vitro results using cell lines confirmed the ex vivo findings, indicating that the expression levels of EZH2 and MMP2 protein and mRNA were higher in ACHN-BO5 cells than those in ACHN cells. By contrast, TIMP2 protein and mRNA expression levels were lower in ACHN-BO5 cells than those in the parental ACHN cells. The TIMP2 promoter was highly methylated in ACHN-BO5 cells compared with that in ACHN cells. Upregulation of EZH2, MMP2 and TIMP2 expression was correlated with metastasis of RCC to bone tissues ex vivo and in vitro. Further studies are required in order to elucidate the mechanism underlying the altered expression of these genes. 
  [Mm]etastasis  PubMed  
 Tissue inhibitor of metalloproteinase 1 is an independent predictor of prognosis in patients with nonsmall cell lung carcinoma who undergo resection with curative intent. Matrix metalloproteinases (MMPs) and tissue inhibitors of metalloproteinases (TIMPs) play a role in the processes of extracellular matrix degradation. Changes in their expression levels have been observed in various tumor types, including lung carcinoma. However, their clinical significance and their prognostic importance in the progression of nonsmall cell lung carcinoma (NSCLC) remain to be specified. In this study, mRNA expression levels of MMP-1, MMP-9, TIMP-1, and TIMP-2 were evaluated in patients with resected NSCLC, and their associations with disease progression and prognosis were determined.Between June 1996 and December 1999, 116 patients underwent resection for NSCLC. Expression levels of MMPs and TIMPs were evaluated using Northern blot analysis in these NSCLC tissue samples and in 39 matched samples of normal lung tissue.MMP-1, MMP-9, and TIMP-1 expression levels were increased in tumor samples compared with matched, corresponding normal tissues. In contrast, TIMP-2 expression was decreased in tumor samples. MMP-1 tumor expression was correlated significantly with the evolution of lymph node status and tumor-lymph node-  metastasis   (TNM) stage. In contrast, MMP-9 tumor expression was correlated significantly with increased T stage. TIMP-1 overexpression was an independent predictor of worse survival in patients with NSCLC that was not associated with other prognosis factors, such as TNM stage.The overexpression of TIMP-1 was an independent prognostic marker in patients with NSCLC, and evaluating TIMP-1 may be important for identifying patients who are at greater risk of disease recurrence. 
 Clinical impact of MMP and TIMP gene polymorphisms in gastric cancer. Recent studies suggest that single-nucleotide polymorphisms (SNPs) within matrix metalloproteinase (MMP) genes and genes of tissue inhibitors of metalloproteinases (TIMPs) have an impact on the expression of these genes and on the prognosis for gastric cancer.Genomic DNA was extracted from paraffin-embedded tissues of 135 patients who were treated surgically for primary gastric carcinoma. Genotyping was performed for MMP-2(-1306C>T), TIMP-2(303C>T), and MMP-7(-181A>G). MMP-2 and TIMP-2 antigen expression in resected tumor tissues was detected immunohistochemically. Genotyping was correlated with antigen expression, histopathologic parameters, and prognosis.The SNPs did not correlate with tumor differentiation, pT, R category, or the classifications according to the International Union Against Cancer (UICC), the World Health Organization (WHO), and Laurén and Ming. A significant correlation was observed for TIMP-2(303C>T) with higher pN stages (p = 0.01) and more dista  nt metasta  sis (p = 0.02) for patients with the CC genotypes. In univariate analysis, patients with the TIMP-2(303C>T) CC genotype had an inferior survival, that was not significant (p = 0.2). However, among the gastric cancer patients in the present study, MMP-2(-1306C>T) significantly correlated with gender, with men having more CC genotypes than women (p = 0.025). There were no significant correlations between genotype and protein levels of MMP-2 (p = 0.766) and TIMP-2 (p = 0.684).The TIMP-2(303C>T) CC genotype is associated with higher pN and pM categories and, in contrast to previous studies, with worse survival in gastric cancer. 
 Alterations in enhancer of zeste homolog 2, matrix metalloproteinase-2 and tissue inhibitor of metalloproteinase-2 expression are associated with ex vivo and in vitro bone   metastasis   in renal cell carcinoma. Renal cell carcinoma (RCC) has a high potential for bone   metastasis  ; however, the molecular mechanisms underlying this   metastasis   have remained to be elucidated. The present study aimed to explore the expression levels of enhancer of zeste homolog 2 (EZH2), matrix metalloproteinase-2 (MMP2) and tissue inhibitor of metalloproteinase-2 (TIMP2) as determinants of RCC-associated bone   metastasis  . Their expression was evaluated in a newly generated RCC cell subline that has a high potential for bone   metastasis  , in tissue specimens from metastasized bone tissues from patients with RCC and in RCC tissues without   metastasis  . A total of 25 RCC tissue specimens without   metastasis   and 13 RCC tissue specimens with bone   metastasis   were acquired for immunohistochemical analysis of EZH2, MMP2 and TIMP2 protein expression. The expression levels of EZH2, MMP2 and TIMP2 mRNA and protein were analyzed in the ACHN and ACHN-BO5 cell lines using western blot and reverse transcription polymerase chain reaction (PCR) analyses. Methylation-specific PCR was also used to analyze TIMP2 promoter methylation. EZH2 and MMP2 proteins were found to be expressed at higher levels in tissues from patients where RCC had metastasized to the bone as compared with those in RCC patients without   metastasis  , whereas there was no significant difference in the expression of TIMP2 protein between the two tissues. Furthermore, the expression of EZH2 protein was correlated with MMP2 expression, but there was no significant correlation between the expression of EZH2 and TIMP2 proteins. The in vitro results using cell lines confirmed the ex vivo findings, indicating that the expression levels of EZH2 and MMP2 protein and mRNA were higher in ACHN-BO5 cells than those in ACHN cells. By contrast, TIMP2 protein and mRNA expression levels were lower in ACHN-BO5 cells than those in the parental ACHN cells. The TIMP2 promoter was highly methylated in ACHN-BO5 cells compared with that in ACHN cells. Upregulation of EZH2, MMP2 and TIMP2 expression was correlated with   metastasis   of RCC to bone tissues ex vivo and in vitro. Further studies are required in order to elucidate the mechanism underlying the altered expression of these genes. 
 Strong association of the tissue inhibitor of metalloproteinase-2 polymorphism with an increased risk of oral squamous cell carcinoma in Europeans. The present study was performed in order to investigate the possible association of the -418 G/C polymorphism in the tissue inhibitor of metalloproteinase-2 (TIMP-2) gene, which affects its expression, with the risk of developing oral cancer. PCR-based restriction analysis was performed in DNA samples from 158 patients with oral squamous cell carcinoma (OSCC) and 168 healthy controls of equivalent sex, age and ethnicity (Greeks and Germans). Statistical analyses were performed with Fisher's exact test and the calculation of odds ratios with a 95% confidence interval (CI). The frequency of the low C allele expression was ten times greater in the patients than the controls (31% vs 2.7%, respectively; P<0.001). The C/C and G/C genotypes were associated with an increased risk of developing OSCC (P<0.001, OR=40.88, 95% CI=2.24-744.40, and P<0.001, OR=21.31, 95%=9.82-46.21, respectively). The same pattern of significant differences with the controls was also observed in the subgroups of patients in regard to the initial or advanced stages of oral cancer, family history of any type of cancer or thrombosis, and smoking habits or alcohol abuse. These findings are consistent with the reduced levels of TIMP-2 in the presence of the low expression C allele, which are insufficient to inhibit the matrix metalloproteinase-driven degradation of the extracellular matrix (leading to cancer invasion) and mitogen-driven neoangiogenesis (leading to tumor growth and   metastasis  ). In conclusion, the studied TIMP-2 polymorphism is strongly associated with an increased risk of OSCC in Europeans carrying the low C allele expression. These results indicate that this polymorphism could serve as a genetic marker for the susceptibility of cancer in the oral cavity. 
 MicroRNA-106a targets TIMP2 to regulate invasion and   metastasis   of gastric cancer. Emerging evidence has shown that microRNA plays an important role in tumor development and progression. Here, we report that miR-106a is frequently up-regulated in gastric cancer tissues and positively correlates with   metastasis  . Restrained expression of miR-106a in gastric cancer cells significantly reduces their capacity of proliferation, migration and invasion. In tissue sections, the positive signal of miR-106a localized in   metastasis  -associated regions confirmed this result. Moreover, we show that TIMP2 is a direct downstream target for miR-106a and knockdown of TIMP2 strengthens the beneficial effects of miR-106a. Our study adds miR-106a to the complex mechanisms of tumor   metastasis  . 
 Significant relationship of matrix metalloproteinase 9 with nuclear grade and prognostic impact of tissue inhibitor of metalloproteinase 2 for incidental clear cell renal cell carcinoma. To examine the immunoreactivity of tumors for matrix metalloproteinases 2 and 9 (MMP-2, MMP-9), tissue inhibitors of metalloproteinases 1 and 2 (TIMP-1, TIMP-2), and membrane type MMP 1 (MT-MMP-1), to evaluate the optimum assessment of incidental renal cell carcinoma.Tumor samples from 120 incidental clear cell renal cell carcinoma (ICRCC) patients without distant   metastasis   or invasion beyond Gerota's fascia were obtained by surgery. They were immunohistochemically stained for MMP-2, MMP-9, TIMP-1, TIMP-2, and MT-MMP-1. Immunoreactivity for these factors was analyzed by semiquantitative multivariate analysis for cancer-specific survival.The cancer-specific 5 and 10-year survival rates were 91.4% and 91.4%, respectively. Univariate analysis revealed that nuclear grade (P = 0.0064) and TIMP-2 (P = 0.034) were significant prognostic factors. Matrix metalloproteinase 9 has a significant relationship with high nuclear grade RCC (P = 0.017) and was found to be an independent prognosticator by Cox multiple regression analysis (P = 0.0028).Nuclear grade and TIMP-2 were significant prognostic factors of ICRCC. Multivariate analysis showed that a nuclear grade greater than 3 was associated with a 566% significant increase in the odds of cancer death. Strong expression of MMP-9 was associated with a 774% increase in the odds of high nuclear grade, with statistical significance. Although ICRCC is well known for having a favorable prognosis, patients with tumors having a high nuclear grade and strongly expressed MMP-9 and TIMP-2 should undergo strict postoperative follow-up. 
 [Expression of matrix metalloproteinase-2, 9 and their inhibitor-TIMP 1,2 in human squamous cell carcinoma of uterine cervix]. Studies of tumor invasion and   metastasis   have focused on the degradation of extracellular matrix (ECM). Matrix metalloproteinases (MMPs), which could degrade ECM, were implicated in cancer invasion and   metastasis  . Activated MMPs were controlled by specific tissue inhibitors of metalloproteinases (TIMPs). This study was designed to investigate the expression of MMP-2, MMP-9 and TIMP-1, TIMP-2 in human squamous cell carcinomas of uterine cervix, and the association with invasion and   metastasis   of human squamous cell carcinoma of uterine cervix.Tissue samples from 40 cases of squamous cell carcinoma of the uterine cervix, 29 cases of cervical intraepithelial neoplasia (CIN), and 16 cases of normal cervices were stained immunohistochemically.Immunohistochemical staining of tumor cells for MMP-2, MMP-9, TIMP-1, and TIMP-2 were noted 82.5%, 70.0%, 62.5%, and 97.5% in carcinomas, 44.8%, 62.1%, 86.21%, and 96.55% in CIN lesions respectively, for MMP-2, MMP-9, TIMP-1 in 25.0% normal cervices, but for TIMP-2 in 56.25% normal cervices. There was significant difference for MMP-2 among carcinomas, normal cervices, and CIN respectively (P < 0.01). There was significant difference for MMP-9 among normal cervices, carcinomas, and CIN, respectively (P < 0.05). The positive rates of TIMP-1 and TIMP-2 in the cervical carcinoma group and CIN group were obviously higher than that in normal cervical group(P < 0.01). The positive rates of MMP-2 and MMP-9 in lymph node   metastasis   were obviously higher than that in without lymph node   metastasis  (P < 0.01), but for TIMP-2 was contrary and there was no significant difference for TIMP-1. No significant correlation could be established among the expression of these markers and the tumor diameter, the clinical stage, and pathologic grade.The over-expression of MMP-2, MMP-9, TIMP-1, and TIMP-2 may play a key role in invasion and lymph-node   metastasis   of in squamous carcinoma of the cervix. 
 Clinicopathological significance of MMP-2 and TIMP-2 genotypes in gastric cancer. Single nucleotide polymorphisms in matrix metalloproteinase-2 (MMP-2) -1306 C/T and tissue inhibitor of metalloproteinase-2 (TIMP-2) -418 G/C abolish the Sp-1 binding site and down-regulate expression of these genes. We aim to elucidate the role of MMP-2 and TIMP-2 in clinicopathological manifestations of gastric cancer.We enrolled 240 gastric cancer patients and 283 controls. DNA was extracted from peripheral blood leucocytes. MMP-2 and TIMP-2 genotypes were analysed by PCR-direct sequencing and PCR-RFLP method, respectively.MMP-2 and TIMP-2 genotypes were not associated with gastric cancer development. However, patients with MMP-2 -1306 C/C genotype showed higher risk of lymphatic invasion (odds ratio (OR)=2.77, p=0.01) and venous invasion (OR=2.93, p=0.012). TIMP-2 G/G genotype was associated with serosal invasion (OR=1.89, p=0.009), lymph node   metastasis   (OR=2.19, p=0.021), lymphatic invasion (OR=2.87, p=0.016) and venous invasion (OR=2.65, p=0.033).Our results suggest MMP-2 and TIMP-2 genotypes play a crucial role in gastric cancer invasion, but not with development of gastric cancer. 
 HPV16 oncoproteins induce MMPs/RECK-TIMP-2 imbalance in primary keratinocytes: possible implications in cervical carcinogenesis. Cervical cancer is the third most common cancer in women worldwide. Persistent infection with high-risk HPV types, principally HPV16 and 18 is the main risk factor for the development of this malignancy. However, the onset of invasive tumor occurs many years after initial exposure in a minority of infected women. This suggests that other factors beyond viral infection are necessary for tumor establishment and progression. Tumor progression is characterized by an increase in secretion and activation of matrix metalloproteinases (MMPs) produced by either the tumor cells themselves or tumor-associated fibroblasts or macrophages. Increased MMPs expression, including MMP-2, MMP-9 and MT1-MMP, has been observed during cervical carcinoma progression. These proteins have been associated with degradation of ECM components, tumor invasion,   metastasis   and recurrence. However, few studies have evaluated the interplay between HPV infection and the expression and activity of MMPs and their regulators in cervical cancer. We analyzed the effect of HPV16 oncoproteins on the expression and activity of MMP-2, MMP-9, MT1-MMP, and their inhibitors TIMP-2 and RECK in cultures of human keratinocytes. We observed that E7 expression is associated with increased pro-MMP-9 activity in the epithelial component of organotypic cultures, while E6 and E7 oncoproteins co-expression down-regulates RECK and TIMP-2 levels in organotypic and monolayers cultures. Finally, a study conducted in human cervical tissues showed a decrease in RECK expression levels in precancer and cancer lesions. Our results indicate that HPV oncoproteins promote MMPs/RECK-TIMP-2 imbalance which may be involved in HPV-associated lesions outcome. 
 The regulating role of mutant IkappaBalpha in expression of TIMP-2 and MMP-9 in human glioblastoma multiform. Our previous studies demonstrated that mutant IkappaBalpha (IkappaBalphaM) inhibited the occurrence, growth and angiogenesis of human glioblastoma multiform (GBM). However, the specific mechanism by which IkappaBalphaM regulates protein-degrading enzymes secreted from GBM to inhibit invasion and   metastasis   has remained unclear. The aim of the present study was to investigate the regulatory role and significance of IkappaBalphaM genes in the expression of tissue inhibitor of metalloproteinase (TIMP)-2 and matrix metalloproteinase (MMP)-9 in human GBM.We established the following four GBM cell lines stably expressing IkappaBalphaM by plasmid construction, gene transfection and screening for IkappaBalphaM protein expression: mutant IkappaBalpha-transfected cells (G36Delta-M), wild-type IkappaBalpha-transfected cells (G36Delta-W), empty plasmid transfected cells (G36Delta-P) and untransfected cells (G36Delta). The TIMP-2 and MMP-9 expression was detected by RT-PCR and Western blotting. Tumor cells were then implanted subcutaneously into nude mice to establish an animal model of ectopic tumor growth, and TIMP-2 and MMP-9 expression was determined by immunohistochemical methods.The results showed that there was a significant increase in TIMP-2 expression and a significant decrease in MMP-9 expression in the G36Delta-M group at both the RNA and protein levels compared with the G36Delta-W group, G36Delta-P group and G36Delta group. Similar results were observed in the immunohistochemical staining analysis of tumor tissues. In the G36Delta-M group, TIMP-2 expression was significantly higher while MMP-9 expression was significantly lower than in the other three groups.Our findings indicate that IkappaBalphaM inhibits the activation of NF-kappaB. It significantly up-regulates TIMP-2 expression in human malignant glioma cells and down-regulates the expression of MMP-9. Thus, IkappaBalphaM maintains the integrity of the extracellular matrix and further inhibits the growth and   metastasis   of tumor tissues. 
 Expression of matrix metalloproteinase-2 and its tissue inhibitor-2 in fetal and neoplastic thyroid tissue and their significance as diagnostic and prognostic markers in papillary carcinoma. Matrix metalloproteinases (MMPs) and their tissue inhibitors (TIMPs) have roles in physiological and pathological processes. We evaluated immunohistochemical expression of MMP-2 and TIMP-2 in paraffin sections of 12 human fetal thyroids at mid-term gestation and 79 thyroid tumors of follicular origin. Besides evaluating expression of these proteins during fetal development and neoplastic transformation, we determined whether expression of MMP-2 and TIMP-2 may help to differentiate papillary thyroid carcinoma (PTC) from follicular thyroid adenoma (FTA) and/or peritumoral tissue (PT). We also investigated their relationship with prognostically important clinicopathological parameters of PTC. Immunoreactive MMP-2 and TIMP-2 were found in all fetal thyroid tissues examined. Tumor tissues contained variable amounts of MMP-2 and TIMP-2, with overexpression of these proteins in PTC compared to FTA and PT tissue. According to the statistical analysis, MMP-2 distinguished follicular variant of PTC from FTA and overall PTC from total nonmalignant lesions. In PTC, high MMP-2 expression correlated with lymph node   metastasis   (P=0.022), while high TIMP-2 expression was positively correlated with tumor size (P=0.049) and extrathyroid invasion (P=0.017). Overall, these results indicate a role for MMP-2 and TIMP-2 in both thyroid development and malignant transformation and suggest that positive immunohistochemistry for MMP-2 and TIMP-2 might support diagnosis of PTC and predict unfavorable biological behavior. 
 [Association of polymorphism in matrix metalloproteinase-2 and tissue inhibitor of metalloproteinase-2 with genetic susceptibility of gastric cancer]. This study was to explore the correlations between genetic variants in MMP2-1306C/T, TIMP2 2379C/T, TIMP2 303G/A and the genetic susceptibility to gastric carcinoma (GC) in Fujian province, China.A case-control study was conducted. Polymorphisms of MMP2-1306C/T, TIMP2 2379C/T, TIMP2 303G/A in 479 gastric carcinoma patients and 469 cancer-free controls, frequency-matched by age and sex, were determined by Matrix-Assisted Laser Desorption/Ionization Time of Flight Mass Spectrometry (MALDI-TOF-MS). Multivariate logistic regression analysis was used to evaluate the correlations between the polymorphisms with the susceptibility to gastric cancer. Tests for an interaction between the MMP2-1306C/T and TIMP2 2379C/T or TIMP2 303G/A were performed using the likelihood ratio test.The frequencies of GG, AG, AA of TIMP2 303G/A in gastric cancer group were 55.9% (267/478), 38.7% (185/478) and 5.4% (26/478); and those in control group were 53.1% (243/458), 36.9% (169/458) and 10.0% (46/458), which showed a significant difference between the patient and control groups (χ(2) = 7.0, P = 0.03). As compared with AA genotype, patients with GG + AG had a significantly higher risk of the cancer with OR of 1.94 (95%CI: 1.2 - 3.2). The frequencies of GG + AG and AA of TIMP2 303G/A in the muscle group were 87.5% (70/80), 12.5% (10/80), however, those in the serosa and beyond group were 96.0% (382/398), 4.0% (16/398), respectively, which showed a significant difference between the patient in muscle group and the serosa and beyond group (χ(2) = 9.32, P = 0.002). As compared with AA genotype, patients with GG + AG had a significantly higher risk in tumor invasion with OR of 3.4 (95%CI: 1.5 - 7.8). The frequencies of CC + CT, TT of TIMP2 2379C/T in the lymphoma node no  n-metastas  is group were 93.4% (113/121), and 6.6% (8/121), but those in the lymphoma nod  e metastas  is group were 98.6% (349/354) and 1.4% (5/354), respectively, which showed a significant difference between the patient in the lymphoma node no  n-metastas  is group and in the lymphoma nod  e metastas  is group in genotype distributions of TIMP2 2379C/T) χ(2) = 9.16, P = 0.002). As compared with TT genotype, patients with CC + CT had a significantly higher risk in lymph no  de metasta  sis with OR of 4.9 (95%CI: 1.6 - 15.3). MMP2-1306C/T genotype was not associated with tumor size, tissue differentiation, invasion, TNM nor lymph no  de metasta  sis of gastric carcinoma (χ(2)(tumor size) = 0.05, P = 0.98; χ(2)(depth of invasion) = 1.87, P = 0.39; χ(2)(histological type) = 0.55, P = 0.76; χ  (2)(LN met  astasis) = 0.44, P = 0.80; χ(2)(TNM) = 2.6, P = 0.28). There was no significant interaction between the polymorphisms of the two genes observed (χ(2) values were 0.98 and 0.80, P values were 0.81 and 0.85).Polymorphism of TIMP2 is significantly related with occurrence and development of GC and maybe acts as a new biomarker of GC in prognosis. 
 MiR-761 Promotes Progression and   Metastasis   of Non-Small Cell Lung Cancer by Targeting ING4 and TIMP2. The aim of this study was to investigate the role of microRNA miR-761 in the progression and   metastasis   of non-small cell lung cancer (NSCLC), and the mechanisms by which miR-761 regulates cell proliferation and metastatic activity of NSCLC cell lines.Quantitative real-time PCR (qRT-PCR) was used to assess miR-761 expression in NSCLC serum and tissue. MTT, wound healing, and transwell assays were performed to examine the role of miR-761 in regulation of cell proliferation and metastatic activity in NSCLC cell lines. In addition, the correlations of miR-761 expression with clinical-pathologic factors were statistically analyzed. Finally, we investigated whether miR-761 promotes proliferation and   metastasis   in NSCLC cell lines by targeting ING4 (inhibitor of growth family, member 4) and TIMP2 (tissue inhibitor of metalloproteinase 2).MiR-761 was significantly upregulated in both NSCLC serum and tissues as compared to normal participants and paired noncancerous tissues respectively. Ectopic expression of miR-761 promoted cell proliferation and   metastasis   in H460 cells, while miR-761 inhibitor reduced proliferation rates and   metastasis   in H23 cells. Furthermore, luciferase reporter assay and functional analyses indicated that miR-761 directly targeted ING4 and TIMP2.miR-761 promotes progression and   metastasis   of NSCLC by targeting ING4 and TIMP2. 
 Tissue inhibitor of metalloproteinase-2 as a multifunctional molecule of which the expression is associated with adverse prognosis of patients with urothelial bladder carcinomas. Tissue inhibitors of metalloproteinases (TIMPs) regulate matrix metalloproteinase (MMP) activity controlling the breakdown of extracellular matrix components and, thus, play an important role in the process of invasion and   metastasis  . Moreover, there are several new functions, growth control, apoptosis, and angiogenesis-, in which TIMPs seem to be involved. The aim of this study was to elucidate the role of TIMP-2 in human urothelial cancer assessing TIMP-2 protein expression in 106 urothelial bladder carcinomas and evaluating its importance relative to clinicopathologic parameters (age, gender, histological grade, and stage) and patient survival, as well as to markers associated with cell growth and apoptosis (Ki-67, p53, and bcl-2).Immunohistochemistry (avidin-biotin complex method-horseradish peroxidase) was performed to detect TIMP-2, Ki-67, p53, and bcl-2 proteins using monoclonal and polyclonal antibodies. Statistical analysis was univariate and multivariate.TIMP-2 immunohistochemical expression was observed in stromal fibroblasts and in cancerous cells in 26.4% and 69.8% of cases, respectively. TIMP-2 stromal but not cancerous cell expression associated significantly with the high histological grade of carcinomas (P < 0.0001) and the advanced stage of the disease (P = 0.001). TIMP-2 either stromal or cancerous cell expression correlated significantly with the expression of Ki-67 proliferation indice (P = 0.02 and P = 0.044, respectively) and the mutant p53 protein (P = 0.043 and P = 0.045, respectively). In univariate survival analysis patients with positive TIMP-2 stromal cell immunohistochemical expression had a significantly worse overall survival in comparison with TIMP-2 stromal cell-negative patients (log rank test: P = 0.0002). However, in multivariate survival analysis the only independent survival factors were the stage of the disease and patient age.TIMP-2 protein expression in either the stromal or cancerous cells is associated with the proliferation index Ki-67 and the apoptosis-related protein p53. These findings are in keeping with in vitro studies reporting a growth-promoting ability of TIMP-2 and its involvement in apoptosis regulation. On the other hand, TIMP-2 stromal cell expression only was associated with adverse prognosis of urothelial bladder cancer patients. 
 Immunoexpression of metalloproteinases 2 and 14 and TIMP-2 inhibitor in main types of primary gastric carcinomas and lymph node   metastasis  . Metalloproteinase-2 (MMP-2) and -14 (MMP-14) and the tissue inhibitor of metalloproteinases type 2 (TIMP-2) participate in epithelial-mesenchymal transition and tumor progression in many cancers. However, the correlation between these enzymes in gastric cancer and the metastatic potential to their respective lymph node needs to be determined. Here, we evaluated the expression of these enzymes in gastric carcinoma and lymph node metastases and their possible involvement in tumor progression. Histological samples from 83 patients with gastric cancer and their respective lymph nodes were used. MMP-2, MMP-14 and TIMP-2 immunoexpression was scored. TIMP-2 expression in tumor-associated macrophages occurred more frequently than in normal mucosa (P = 0.0128). Female tumor samples presented higher MMP-2 expression (P = 0.0248), while TIMP-2 occurred mainly in patients over 50 years old (P = 0.0034). MMP-2 was higher expressed in primary tumor macrophages than in neoplastic cells (P = 0.0118), and was also seen in macrophages from metastatic-affected lymph nodes of intestinal and diffuse histotypes (P = 0.0006). MMP-2, MMP-14 and TIMP-2 expression in mononuclear cells might be correlated with progression of gastric cancer. MMP-14 production by macrophages appears to be more involved in diffuse gastric cancer progression. 
 Transient RNA silencing of tissue factor pathway inhibitor-2 modulates lung cancer cell invasion. Tissue Factor Pathway Inhibitor-2 (TFPI-2) is a potent inhibitor of plasmin which activates metalloproteinases (MMPs) involved in extracellular matrix (ECM) degradation. Its secretion in ECM makes TFPI-2 a potential inhibitor to regulate tumour invasion and   metastasis  . Moreover, TFPI-2 is frequently downregulated, particularly in aggressive cancers. In this study, we silenced TFPI-2 in the NCI-H460 non-small cell lung cancer cell line and evaluated the role of TFPI-2 in cell invasion and its impact on MMPs expression. As the effects of siRNA are transient, the consequences of both gene silencing and restoration to normal expression could be studied kinetically in the same cells. We showed that TFPI-2 expression by NCI-H460 cells was effectively downregulated using specific small interfering RNA and this silencing was associated with an increase in the invasive potential of tumour cells while migration was not affected. We also showed that mRNA levels and protein expression of MMP-2, -3, -9, -14 were not influenced by TFPI-2 silencing. Moreover, the gelatinase activity of MMP-2 and MMP-9 was unmodified. In contrast, MMP-1 mRNA levels and protein were significantly and similarly increased in cells transfected with TFPI-2 siRNA. In conclusion, this study confirms that TFPI-2 downregulation can contribute to tumour invasion of lung cancer cells. 
 Expression of MMP-2,TIMP-2 protein and the ratio of MMP-2/TIMP-2 in gallbladder carcinoma and their significance. To study the correlation between expression of MMP-2, TIMP-2 protein and the ratio of MMP-2/TIMP-2 and clinical-pathological parameters of patients with gallbladder carcinoma.Carcinomas (n=45) and polypoid lesions (n=15) of the gallbladder were studied for the expression of MMP-2 and TIMP-2 protein by immunohistochemical avidin-biotin-complex method and image analysis. Clinical-pathological data of patients with gallbladder carcinoma such as histological type, grade of differentiation, level of infiltration, liver invasion and lymph node involvement, etc, were recorded.There was significant difference between the average level (1.123+/-0.108 vs 1.030+/-0.054, P=0.002) of MMP-2, the ratio (1.050+/-0.013 vs 0.937+/-0.078, P=0.003) of MMP-2/TIMP-2 in gallbladder carcinomas and in polypoid lesions of the gallbladder. Significant difference was found between the expression of MMP-2 in early stage and advanced tumors, but there was no correlation between MMP-2 protein expression and histological type, differentiation degree, infiltration level, lymph node involvement or liver invasion. Although no difference was observed between TIMP-2 expression and histological type or differentiation degree, significant difference was found between TIMP-2 expression and different Nevin stage, infiltration level, local lymph node involvement or liver invasion (1.168+/-0.067 vs 1.048+/-0.075, 1.170+/-0.062 vs 1.039+/-0.069, 1.039+/-0.076 vs 1.147+/-0.083, 1.048+/-0.074 vs 1.103+/-0.095, P<0.05). MMP-2/TIMP-2 ratio did not correlate with histological type, grade of differentiation and liver invasion, but significant differences were found between MMP-2/TIMP-2 ratio and different Nevin stage, infiltration level and lymph node involvement in patients with carcinoma of gallbladder.TIMP-2 and MMP-2/TIMP-2 ratio could reflect more accurately biological characteristic of gallbladder carcinoma and MMP-2/TIMP-2 ratio might be a new significant marker in early diagnosis, in the judgment of invasion or   metastasis   and the estimate of prognosis in patients with gallbladder carcinomas. 
 The role of EZH2 in the regulation of the activity of matrix metalloproteinases in prostate cancer cells. Degradation of the extracellular matrix (ECM), a critical step in cancer   metastasis  , is determined by the balance between MMPs (matrix metalloproteinases) and their inhibitors TIMPs (tissue inhibitors of metalloproteinases). In cancer cells, this balance is shifted towards MMPs, promoting ECM degradation. Here, we show that EZH2 plays an active role in this process by repressing the expression of TIMP2 and TIMP3 in prostate cancer cells. The TIMP genes are derepressed by knockdown of EZH2 expression in human prostate cancer cells but repressed by overexpression of EZH2 in benign human prostate epithelial cells. EZH2 catalyzes H3K27 trimethylation and subsequent DNA methylation of the TIMP gene promoters. Overexpression of EZH2 confers an invasive phenotype on benign prostate epithelial cells; however, this phenotype is suppressed by cooverexpression of TIMP3. EZH2 knockdown markedly reduces the proteolytic activity of MMP-9, thereby decreasing the invasive activity of prostate cancer cells. These results suggest that the transcriptional repression of the TIMP genes by EZH2 may be a major mechanism to shift the MMPs/TIMPs balance in favor of MMP activity and thus to promote ECM degradation and subsequent invasion of prostate cancer cells. 
 Clinical significance of serum levels of matrix metalloproteinase 2 (MMP-2) and its tissue inhibitor (TIMP-2) in gastric cancer. Matrix metalloproteinase 2 (MMP-2) is able to degrade type IV collagen, and thus plays a key role in the migration of tumor cells. MMP-2 activity is inhibited by its tissue inhibitor (TIMP-2). The imbalance between MMPs and TIMPs may facilitate progression of cancer cells. The aim of this study was to compare the clinical importance of MMP-2 and TIMP-2 to that of classical tumor markers, namely carcinoembryonic antigen (CEA) and carbohydrate antigen (CA 19-9) in the diagnosis of gastric cancer (GC) by calculating the diagnostic criteria and estimating the levels of MMP-2, TIMP-2, CEA and CA 19-9 in GC patients in relation to clinicopathological features of cancer. We found that serum levels of MMP-2 and TIMP-2 were significantly lower, whereas serum tumor markers were higher, in GC patients than in healthy subjects. Moreover, concentrations of TIMP-2 and CEA correlated with gastric wall infiltration, while CA 19-9 levels correlated with gastric wall infiltration and the presence of nodal   metastasis  . None of the proteins tested was found to be an independent prognostic factor for GC patients' survival. The percentage of true positive results of TIMP-2 (61%) was higher than those of MMP-2 (54%) and the classical tumor markers CEA (21%) and CA 19-9 (31%). The highest diagnostic sensitivity was observed for the combined use of TIMP-2 with MMP-2 (77%). The results suggest the greater importance of serum MMP-2 and TIMP-2 than of the classical tumor markers CEA and CA 19-9 in the diagnosis of GC. But this issue requires further investigation. 
 Expression of metalloproteases and their inhibitors in primary tumors and in local recurrences after mastectomy for breast cancer. To investigate the expression of matrix metalloproteases (MMPs) and their inhibitors (TIMPs) in patients who develop local recurrence (LR) after mastectomy.We analyzed the expressions of MMP-1, -2, -7, -9, -11, -13, -14, TIMP-1, -2, and -3, using immunohistochemical techniques, in primary tumors from patients without tumoral recurrence (n = 50), patients who developed distant   metastasis   (n = 50), and from patients who develop LRs (n = 25). LRs of the latter group were also analyzed for MMPs expression. All the patients underwent mastectomy.Score values for all MMPs and TIMPs were significantly higher in primary tumors of patients with distant   metastasis  . Primary tumors from patients with LR have lower expressions of MMPs and TIMPs compared with those from patients who developed distant   metastasis  , and with patients without recurrence for some MMPs. Remarkably, however, primary tumors from patients with LR showed significantly higher percentage of TIMP-1 and 2 expression in stromal cells compared to primary tumors from patients with distant   metastasis   or primary tumors from patients without tumoral progression. Furthermore, LRs had significantly higher MMP-9 expression than their corresponding primary tumors.Our data indicate differences in MMPs/TIMPs expression between primary tumors of patients with LRs and of those with distant   metastasis  , both after mastectomy for breast cancer. 
 Clinicopathological significance of MMP-2 and its specific inhibitor TIMP-2 in gastric cancer. Matrix metalloproteinases (MMPs) can degrade type IV collagen of extracellular matrices and basal membranes and thus play a key role in the migration of malignant cells. In vivo, MMPs are inhibited by tissue inhibitors of metalloproteinases (TIMPs). Since in a previous study we showed that the expression of MMP-2 correlates with clinicopathological parameters in gastric cancer, we have now investigated a possible correlation of MMP-2 and TIMP-2 expression with survival in gastric cancer, as well as the possible association of TIMP-2 with clinicopathological parameters. Tissue samples were obtained from 116 gastric cancer patients who underwent gastrectomy with extended lymphadenectomy. MMP-2 and TIMP-2 expression was analysed using immunohistochemical staining and was graded semiquantitatively (score 0 - 3). High epithelial MMP-2 immunoreactivity was significantly associated with tumor stage and poor survival using the Kaplan-Meier log-rank statistical method (log-rank statistics). However, using Cox regression analysis, high epithelial MMP-2 immunoreactivity was not an independent prognostic factor. TIMP-2 showed no association with survival in gastric cancer, but the intensity of TIMP-2 staining in tumor cells correlated significantly with tumor differentiation based on the WHO and Lauren and Ming classifications, as well as with presence of distant   metastasis  . Our results show that high epithelial MMP-2 expression in gastric cancer is associated with poor survival, although it is not an independent prognostic factor, and that aggressive forms of gastric cancer are associated with low TIMP-2 expression. 
 MiR-19a is correlated with prognosis and apoptosis of laryngeal squamous cell carcinoma by regulating TIMP-2 expression. MiRNAs are small, noncoding RNA molecules that act as posttranscriptional regulators of gene expression and function as important regulators in cancer-related processes. The miR-19a is overexpressed in various cancers and has been causally related to cellular proliferation and growth. To determine whether miR-19a plays a role in laryngeal squamous cell carcinoma (LSCC), we used quantitative real time PCR to detect miR-19a expression in LSCC tissues. We found that miR-19a is overexpressed in LSCC and correlated with neck nodal   metastasis  , poor differentiation and advanced stage. Statistical analysis suggests that higher level of miR-19a was associated with reduced overall survival. In vitro functional study showed that inhibition of miR-19a by antisense oligonucleotides (ASO) led to apoptosis and reduction of cell proliferation in LSCC cells. Furthermore, growth of LSCC xenograft tumors was significantly suppressed by repeated injection of ASO-miR-19a lentivirus. The TUNEL stain and transmission electron microscopy also detected increased apoptotic cells in ASO-miR-19a treated LSCC xenografts. In addition, both realtime PCR and western blot showed ASO-miR-19a can upregulate TIMP-2 expression and this suggests miR-19a is related with TIMP-2 pathway in LSCC cells. Taken together, these data suggest that miR-19a plays an oncogenic role in the progression of LSCC, and may serve as a biomarker or therapeutic target for patients with LSCC. 
 The metalloproteinase inhibitor TIMP-2 is down-regulated by androgens in LNCaP prostate carcinoma cells. Tissue inhibitors of metalloproteinases (TIMPs) have been shown to perform several biological functions in tumor promotion, principally by their action of inhibiting matrix metalloproteinases (MMPs) at different steps of the metastatic process. In particular, TIMP-2 is involved in a functional complex with the membrane-type 1 (MT1) MMP to convert the secreted MMP-2 progelatinase into the fully active proteolytic enzyme. We used the human, androgen-sensitive prostate carcinoma cell line LNCaP in coculture with the human osteosarcoma cell line OHS to experimentally address the possibility of androgen-dependent regulatory effects on the functional MT1-MMP/TIMP-2/MMP-2 complex upon interaction between prostate carcinoma and osteoblastic cells in   metastasis   of prostate cancer to bone. In the LNCaP cells a gradual, time-dependent decline in TIMP-2 mRNA expression was observed in the presence of the synthetic androgen analogue R1881 (100 nM), reaching approximately 25% of the control level after 48 h of incubation. Consistent with this, the accumulation of secreted TIMP-2 in media from R1881-treated cells was significantly inhibited already after 3 h. Neither MMP-2 gelatinolytic activity nor expression of MT1-MMP was detected in LNCaP cells. In contrast, the OHS cells showed membrane-associated MT1-MMP expression as well as MMP-2 secretion. However, R1881 treatment of the LNCaP/OHS coculture model did not seem to change the overall proteolytic activity of the MT1 -MMP/TIMP-2/MMP-2 complex. Hormonal control of TIMP-2 expression in prostate carcinoma cells has not been previously reported, but whether such regulation has any functional role in the development of osteoblastic metastases in prostate cancer is still unclear. 
 MiR-221/222 promote human glioma cell invasion and angiogenesis by targeting TIMP2. miR-221/222 are two highly homologous microRNAs that are frequently upregulated in solid tumors. However, the effects of miR-221/222 in malignant gliomas have not been investigated thoroughly. In this study, we found that miR-221/222 were significantly upregulated in human glioma samples and glioma cell lines. Both gain- and loss-of-function studies showed that miR-221/222 regulate cell proliferation, the cell cycle and apoptosis, in addition to, invasion,   metastasis  , and angiogenesis in glioma cell lines. Subsequent investigations revealed that TIMP2 is a direct target of miR-221/222, and overexpression of TIMP2 reduced the miR-221/222-mediated invasion,   metastasis  , and angiogenesis of glioma cells. Taken together, our results suggest that the suppression of miR-221/222 may be a feasible approach for inhibiting the malignant behaviors of glioma. 
 Tissue inhibitor of metalloproteinases-2 improves antitumor efficacy of a replicating adenovirus in vivo. Clinical studies of replicating adenoviruses for the treatment of cancer have demonstrated their safety but have yielded disappointing results, indicating the need for new strategies to improve their efficacy. We hypothesized that the efficacy of a replicating adenovirus could be improved by expression of tissue inhibitor of metalloproteinases-2 (TIMP-2), a 21-kDa unglycosylated secretory protein. TIMP-2 specifically inhibits the active forms of a number of matrix metalloproteinases (MMPs) that play a role in the degradation of basement membranes and the extracellular matrix and are therefore involved in the control of the growth, invasion and   metastasis   of tumor cells, as well as angiogenesis. In addition, TIMP-2 can abrogate tumor growth and angiogenesis by a variety of mechanisms independent of MMP inhibition. In this study, we demonstrate that expression of TIMP-2 enhanced the antitumor efficacy of a replicating adenovirus in vivo, by reducing both tumor growth and angiogenesis. 
 The influence of MMP-14, TIMP-2 and MMP-2 expression on breast cancer prognosis. Matrix metalloproteinase (MMP)-2 is very active at degrading extracellular matrix. It is under the influence of an activator, membrane type 1 MMP (MMP-14), and the tissue inhibitor of metalloproteases (TIMP)-2. We hypothesized that the individual expression of these three markers or their balance may help to predict breast cancer prognosis.MMP-2, MMP-14 and TIMP-2 expression has been evaluated by 35S mRNA in situ hybridization on paraffin material of 539 breast cancers without distant   metastasis   at diagnosis and with a median follow-up of 9.2 years.MMP-2 and MMP-14 mRNA was detected primarily in reactive stromal cells whereas TIMP-2 mRNA was expressed by both stromal and cancer cells. Of the three molecules, an adjusted Cox model revealed that high MMP-14 mRNA (> or = 10% cells) alone predicted a significantly shorter overall survival (p = 0.031) when adjusted for clinical factors (tumor size and number of involved lymph nodes). Prognostic significance was lost when further adjusted for Her-2/neu and urokinase-type plasminogen activator (p = 0.284). Furthermore, when all three components were analyzed together, the survival was worst for patients with high MMP-2/high MMP-14/low TIMP-2 (5 year survival = 60%) and best with low MMP-2/low MMP-14/high TIMP-2 (5 year survival = 74%), but the difference did not reach statistical significance (p = 0.3285).Of the MMP-14/TIMP-2/MMP-2 complex, MMP-14 was the factor most significantly associated with the outcome of breast cancer and was an independent factor of poor overall survival when adjusted for clinical prognostic factors, but not for certain ancillary markers. 
 [Membrane type 1 matrix metalloproteinase (MT1-MMP) and the regulators of its activity as invasive factors in squamous cell cervical carcinomas]. Membrane type 1 matrix metalloproteinase (MT1MMP) is one of matrix metalloproteinases (MMP), which play а key role in tumor invasion and   metastasi  s. The aim of this study was to elucidate the peculiarities of expression of MT1MMP and endogenous regulators of its activity: the activator - furin and the inhibitor - TIMP-2, as invasive factors of squamous cell cervical carcinomas (SCC). The study was carried out using 11 specimens of SCC and 11 specimens of morphologically normal tissue adjacent to the tumor. It was shown that the increase of MT1-MMP and furin expression and low of TIMP-2 expression makes the main contribution to the destructive (invasive) potential of SCC. Moreover, substantial expression of MT1-MMP was registered in the specimens of morphologically normal adjoining to tumor tissue. This expression was found to make an additional contribution to the destructive potential of the cervical tumor. 
 The ratio of MMP-2 to TIMP-2 in hilar cholangiocarcinoma: a semi-quantitative study. Hilar cholangiocarcinoma is associated with low resectability and poor survival. The aim of this study was to evaluate the roles of matrix metalloproteinases-2 (MMP-2) and its tissue inhibitor of metalloproteinase-2(TIMP-2) in tumor invasion or as a prognostic factor in patients with human hilar cholangiocarcinoma.The expressions of MMP-2 and TIMP-2 were investigated in patients. Paraffinized tissue sections obtained from 50 patients with human hilar cholangiocarcinoma were analysed. The expressions of MMP-2 and TIMP-2 were examined immunohistochemically. Image analysis with image-pro plus analysis software was used to semiquantitatively determine the ratio of MMP-2 to TIMP-2.The expression levels of MMP-2 and TIMP-2 were strongly associated with tumor hepatic invasion in patients with hilar cholangiocarcinoma. Significant differences in the ratio of MMP-2 to TIMP-2 between some pathologic factors were observed in patients with hilar cholangiocarcinoma.MMP-2 plays an essential role in tumor invasion and   metastasis  , while TIMP-2 is shown to strongly inhibit cancer invasion and   metastasis  . The ratio of MMP-2 to TIMP-2 may be a prognostic indicator for patients with hilar cholangiocarcinoma. 
 Inactivation of the tissue inhibitor of metalloproteinases-2 gene by promoter hypermethylation in lymphoid malignancies. The tissue inhibitor of metalloproteinases-2 (TIMP-2) is known to antagonize matrix metalloproteinase activity and to suppress tumor growth, angiogenesis, invasion and   metastasis  . We analysed the methylation status of the CpG island in the TIMP-2 promoter region by methylation-specific polymerase chain reaction (MSP) in hematopoietic cell lines. TIMP-2 promoter hypermethylation in the lymphoma cell line Raji and the leukemia cell line KG1a was associated with transcriptional repression. Treatment with the demethylating agent 5-aza-2'-deoxycytidine resulted in TIMP-2 upregulation in both cell lines. TIMP-2 was expressed in the cell lines HL60, U266 and XG1, which carry an unmethylated promoter region. MSP analysis of primary patient samples revealed aberrant methylation of TIMP-2 in 33/90 (36.7%) cases of non-Hodgkin's lymphoma (NHL), but not in normal peripheral blood lymphocytes as well as in nonmalignant bone marrow and lymph nodes. The frequency of TIMP-2 methylation was slightly higher in aggressive NHL subtypes compared to those with an indolent subtype (38.6 versus 33.3%). In contrast, TIMP-2 was not hypermethylated in any of the 40 cases of acute myelogenous leukemia examined. We conclude that promoter hypermethylation of TIMP-2 is a novel epigenetic event in the pathogenesis of lymphoid malignancies and may contribute to a more aggressive NHL phenotype. 
 NEDD9 depletion leads to MMP14 inactivation by TIMP2 and prevents invasion and   metastasis  . The scaffolding protein NEDD9 is an established prometastatic marker in several cancers. Nevertheless, the molecular mechanisms of NEDD9-driven   metastasis   in cancers remain ill-defined. Here, using a comprehensive breast cancer tissue microarray, it was shown that increased levels of NEDD9 protein significantly correlated with the transition from carcinoma in situ to invasive carcinoma. Similarly, it was shown that NEDD9 overexpression is a hallmark of highly invasive breast cancer cells. Moreover, NEDD9 expression is crucial for the protease-dependent mesenchymal invasion of cancer cells at the primary site but not at the metastatic site. Depletion of NEDD9 is sufficient to suppress invasion of tumor cells in vitro and in vivo, leading to decreased circulating tumor cells and lung metastases in xenograft models. Mechanistically, NEDD9 localized to invasive pseudopods and was required for local matrix degradation. Depletion of NEDD9 impaired invasion of cancer cells through inactivation of membrane-bound matrix metalloproteinase MMP14 by excess TIMP2 on the cell surface. Inactivation of MMP14 is accompanied by reduced collagenolytic activity of soluble metalloproteinases MMP2 and MMP9. Reexpression of NEDD9 is sufficient to restore the activity of MMP14 and the invasive properties of breast cancer cells in vitro and in vivo. Collectively, these findings uncover critical steps in NEDD9-dependent invasion of breast cancer cells.This study provides a mechanistic basis for potential therapeutic interventions to prevent   metastasis  . 
 Prognostic impact of polymorphism of matrix metalloproteinase-2 and metalloproteinase tissue inhibitor-2 promoters in breast cancer in Tunisia: case-control study. Matrix metalloproteinases (MMPs) are proteolytic enzymes that play important roles in tumor invasion and   metastasis   by degrading extracellular matrix components. Genetic variations in promoter regions of MMP genes, affecting their expression, have been associated with susceptibility to cancers. The aim of this study was to investigate the susceptibility and prognostic implications of the matrix metalloproteinase-2 (MMP-2) and tissue inhibitor of metalloproteinase-2 (TIMP-2) polymorphism in Tunisian breast cancer patients. MMP-2 genotypes were determined by real-time polymerase chain reaction (RT-PCR), and TIMP-2 genotypes were identified using a PCR-restriction fragment length polymorphism (RFLP) method in 210 breast cancer patients and 250 frequency-matched control women. Association of the clinicopathological parameters and the genetic markers with risk of breast cancer was assessed using univariate analyses. We found that the variant MMP-2 genotype (-1306CT or TT) was associated with substantially reduced risk of breast cancer [odds ratio (OR), 0.49; 95 % confidence interval (95 % CI), 0.033-0.73], compared with the CC genotype. For TIMP-2, a moderately reduced risk of the cancer (OR, 0.57; 95 % CI, 0.37-0.87) was also associated with the variant allele (-418GC or CC), compared with the GG common allele. Furthermore, polymorphisms in both genes seem to have additive effects and the highest risk for breast cancer has been observed in those with MMP-2 CC genotype and TIMP-2 GC or CC genotype (p = 0.006). A significant association was also found between the CC genotype and the aggressive forms of breast cancer as defined by advanced stages at the time of diagnosis and   metastasis  . This is the first report on the association of MMP-2 and TIMP-2 gene polymorphisms in breast cancer in Tunisian population. Our results suggest that the presence of the variant allele in the promoter of MMP-2 or TIMP-2 may be a protective factor for the development of breast cancer. 
 Role of matrix metalloproteinase (MMP) 2 and MMP-9 in soft tissue sarcoma. We investigated the expression of matrix metalloproteinases (MMPs) and tissue inhibitors of metalloproteinases (TIMPs) in malignant fibrous histiocytoma (MFH), and determined whether these could be useful as prognostic factors.Among patients treated from 1993 to 2007, 30 cases of MFH were evaluated. Immunohistochemical staining was performed for MMP-2, MMP-9, TIMP-1, and TIMP-2 using paraffin wax-embedded blocks of MFH tissues. Reverse transcriptase polymerase chain reaction (RT-PCR) and Western blot and zymography were performed using fresh tissues obtained from 17 of the 30 cases. The levels of MMP and TIMP expression were compared between the MFH and normal control groups, and between non-metastatic and metastatic MFH groups.Expression levels of MMP-2, MMP-9, TIMP-1, and TIMP-2 were higher in the MFH group than the control group by RT-PCR, Western blotting, and zymography. Immunohistochemical staining revealed that MMP-2 and MMP-9 protein expression was higher in the metastatic than in the non-metastatic group. The expression levels of MMP-2 and TIMP-1 were significantly higher in the metastatic than in the non-metastatic group (p < 0.05) by RT-PCR. By Western blot analysis, the expression levels of MMP-2, TIMP-1, and TIMP-2 were higher in the metastatic group (p < 0.05), but MMP-9 showed only a slight increase in the metastatic group compared with the non-metastatic group (p > 0.05). Finally, gelatin zymography analysis showed that the expression levels of the pro- and active forms of MMP-2 were significantly higher in the metastatic group (p < 0.05), but the expression of the pro- and active forms of MMP-9 showed a slight decrease in the metastatic group (p > 0.05).These results suggest that MMP-2, MMP-9, TIMP-1, and TIMP-2 may have important roles in the development and progression of MFH, and that the degree of expression of these metalloproteinases and their inhibitors, especially MMP-2, could be useful as prognostic factors related to   metastasis   in MFH. 
 Downregulation of reversion-inducing cysteine-rich protein with Kazal motifs in malignant melanoma: inverse correlation with membrane-type 1-matrix metalloproteinase and tissue inhibitor of metalloproteinase 2. The invasive phenotype of many tumors is associated with an imbalance between the matrix metalloproteinases (MMPs) and their inhibitors, tissue inhibitors of metalloproteinases (TIMPs), and the membrane-anchored reversion-inducing cysteine-rich protein with Kazal motifs (RECK). RECK inhibits MMP-2, MMP-9, and MT1-MMP, and has been linked to patient survival and better prognosis in several types of tumors. However, despite the wide implication of these MMPs in melanoma establishment and progression, the role of RECK in this type of tumor is still unknown. Here, we analyzed the expression of RECK, TIMP1, TIMP2, TIMP3, MT1MMP, MMP2, and MMP9 in two publicly available melanoma microarray datasets and in a panel of human melanoma cell lines. We found that RECK is downregulated in malignant melanoma, accompanied by upregulation of MT1MMP and TIMP2. In both datasets, we observed that the group of samples displaying higher RECK levels show lower median expression levels of MT1MMP and TIMP2 and higher levels of TIMP3. When tested in a sample-wise manner, these correlations were statistically significant. Inverse correlations between RECK, MT1MMP, and TIMP2 were verified in a panel of human melanoma cell lines and in a further reduced model that includes a pair of matched primary tumor-derived and   metastasis  -derived cell lines. Taken together, our data indicate a consistent correlation between RECK, MT1MMP, and TIMP2 across different models of clinical samples and cell lines and suggest evidence of the potential use of this subset of genes as a gene signature for diagnosing melanoma. 
 Lysophosphatidic acid downregulates tissue inhibitor of metalloproteinases, which are negatively involved in lysophosphatidic acid-induced cell invasion. Ovarian cancer is a highly metastatic disease. Lysophosphatidic acid (LPA) levels are elevated in ascites from ovarian cancer patients, but its potential role in ovarian cancer   metastasis   has just begun to be revealed. In this work, we show that LPA stimulates invasion of primary ovarian cancer cells, but not ovarian epithelial or borderline ovarian tumor cells, although these benign cells indeed respond to LPA in cell migration. We have found that LPA downregulates tissue inhibitor of metalloproteinases (TIMPs). TIMP2 and TIMP3 play functional role in LPA-induced invasion as negative regulators. G(i) protein, phosphatidylinositol-3 kinase (PI3K), p38 mitogen-activated protein kinase (MAPK), cytosolic phospholipase A(2) and urokinase type plasminogen activator (uPA) are required for LPA-induced cells invasion. TIMP3 may affect two independent downstream targets, vascular endothelial growth factor receptor and p38 MAPK. In vivo, LPA stimulates tumor   metastasis   in an orthotopic ovarian tumor model, which can be inhibited by a PI3K inhibitor, LY294002. In summary, LPA is likely a key component for promoting ovarian   metastasis   in vivo. LPA downregulates TIMP3, which may have targets other than metalloproteinases. Our in vivo   metastasis   mouse model is useful for studying the efficacy of therapeutic regimes of ovarian cancer. 
 The role of matrix metalloproteinases (MMPs) and their inhibitors (TIMPs) in the development of esophageal cancer. Esophageal cancer (EC) is one of the most aggressive malignant tumors of the gastrointestinal tract. There are two distinct histological types of EC: esophageal squamous cell carcinoma and adenocarcinoma of the esophagus. Etiologic factors and the patterns of incidence of both subtypes are different. Matrix metalloproteinases (MMPs) and their tissue inhibitors (TIMPs) play an important role in esophageal carcinogenesis. Gellatinases MMP-2 and MMP-9 are able to degrade collagen IV from basement membranes and extracellular matrix which is related to tumor progression, including invasion,   metastasis  , growth and angiogenesis. It has been shown that increased expression of MMPs plays a crucial role in the development of several human malignancies, including esophageal cancer. The activity of MMPs is regulated by their endogenous natural inhibitors (TIMPs). Among these, the roles of TIMP-1 and TIMP-2 in EC development, tumor progression and formation of metastases have been most extensively characterized and best recognized. 
 Expression of matrix metalloproteinases MMP-2, MMP-9 and their tissue inhibitors TIMP-1 and TIMP-2 in the epithelium and stroma of salivary gland pleomorphic adenomas. The balance between matrix metalloproteinases (MMPs) and their tissue inhibitors (TIMPs) is involved in the morphogenesis of normal salivary gland as well as in the mechanisms of tumour invasion and   metastasis  . The role of MMPs and TIMPs in pleomorphic adenoma has not been elucidated sufficiently. Our aim was to analyse the mRNA and protein expression of MMP-2, MMP-9, TIMP-1 and TIMP-2 in the epithelium and stroma of pleomorphic adenoma and to evaluate their roles.In each sample from six patients, cells from the epithelium and stroma were obtained by laser microdissection. The mRNA expression of MMPs and TIMPs was determined by real-time quantitative reverse transcriptase-polymerase chain reaction and protein expression was confirmed by immunohistochemistry. Results showed that mRNA expression of MMPs and TIMPs was significantly higher in stroma than in epithelium in most patients. MMPs and TIMPs were immunoreactive mainly in epithelium rather than in stroma.Our results provide preliminary evidence that stromal myoepithelium may be the primary source of MMPs and that the stroma has the potential to play a more important role than ductal epithelium in biological behaviour of pleomorphic adenomas. These findings seem worthy of further investigation. 
 Matrix metalloproteinase 2 (MMP-2) and their tissue inhibitor 2 (TIMP-2) in gastric cancer patients. Matrix metalloproteinase 2 (MMP-2) is able to degrade type IV collagen and its activity is mostly regulated by tissue inhibitor of matrix metalloproteinase 2 (TIMP-2). These proteins might play a role in tumor progression, including gastric cancer (GC).The study included 108 individuals, GC patients and healthy subjects. Serum levels of all analyzed markers were evaluated by the immunological methods, while immunohistochemistry was used to assess the expression of these proteins in GC, interstitial inflammatory cells and normal tissues.The percentage of positive reactions of MMP-2 and TIMP-2 was higher in GC and inflammatory cells compared to normal tissue, while serum levels of these proteins were statistically lower in GC patients in comparison to healthy subjects. There was a significant positive correlation between TIMP-2 immunoreactivity in inflammatory cells and the presence of lymph node   metastasis  . Area under ROC curve (AUC) for TIMP-2 was higher than MMP-2, while serum MMP-2 was an independent prognostic factor of GC patients' survival.Our findings suggest that TIMP-2 seems to be a predictor of tumor progression, especially for nodal involvement, whereas serum MMP-2 might be useful as an independent prognostic factor of patients' survival. 
 Substantial reduction in risk of breast cancer associated with genetic polymorphisms in the promoters of the matrix metalloproteinase-2 and tissue inhibitor of metalloproteinase-2 genes. The matrix metalloproteinases (MMPs) and tissue inhibitor of metalloproteinases (TIMPs) have been shown to play important roles in multiple ways in all stages of cancer initiation and development. Single nucleotide polymorphisms identified in the promoters of MMP2 (-1306C-->T) and TIMP2 (-418G-->C) abolish the Sp1-binding site and thus may down-regulate expression of the genes. This case-control study examined the contribution of these functional polymorphisms to susceptibility to development and   metastasis   of breast cancer. MMP2 genotypes were determined by PCR-based denaturing high performance liquid chromatography analysis and TIMP2 genotypes identified by a PCR-RFLP method in 462 breast cancer patients and 509 frequency matched control women. We found that the variant MMP2 genotype (-1306CT or TT) was associated with substantially reduced risk of breast cancer [odds ratio (OR), 0.46; 95% confidence interval (95% CI), 0.34-0.63], compared with the CC genotype. For TIMP2, a moderately reduced risk of the cancer (OR, 0.76; 95% CI, 0.58-0.99) was also associated with the variant allele (-418GC or CC), compared with the GG common allele. Furthermore, it seemed that the polymorphisms in the two genes had some additive effect and the reduced risk related to the polymorphisms appeared to be more pronounced in younger subjects. However, no significant associations were observed between the polymorphisms in the two genes and tumor stage and   metastasis   of the cancer at the time of diagnosis. These findings suggest that the presence of the variant allele in the promoter of MMP2 or TIMP2 may be a protective factor for the development of breast cancer. 
 DNA ploidy analysis and expression of MMP-9, TIMP-2, and E-cadherin in gastric carcinoma. To investigate DNA ploidy and expression of MMP-9, TIMP-2, and E-cadherin in gastric carcinoma and to explore the mechanism of invasion and   metastasis   of gastric carcinoma.Immunohistochemical methods were used to detect the expressions of MMP-9, TIMP-2, and E-cadherin in 156 cases, including 99 cases of gastric carcinoma, 16 cases of adjacent noncancerous mucosa, 16 cases of distant metastases and 25 cases of metastatic lymph node (LN) from gastric carcinoma. Flow cytometry DNA ploidy and S-phase fraction (SPF) analysis were performed on 57 cases, including 47 cases of gastric cancer, 6 cases of adjacent noncancerous mucosa, and 4 cases of distant metastatic cancer.The expression of MMP-9 was significantly correlated with Lauren's classification, Borrmann's classification, LN   metastasis  , tumor   metastasis  , and TNM stage, as well as depth of invasion (all P<0.05). The positive rate was lower in noncarcinoma than in carcinoma (31.3% vs 66.7%, P<0.01). The expression of TIMP-2 was significantly correlated with Borrmann's classification, LN   metastasis  , and the depth of invasion (all P<0.05). The expression of E-cadherin was significantly correlated with differentiation, Lauren's classification, Borrmann's classification, and LN   metastasis  , as well as the depth of invasion (P<0.01 or P<0.05). E-cadherin was less expressed in carcinoma than in noncarcinoma (42.4% vs 87.5%, P<0.01). There was a positive correlation between MMP-9 and TIMP-2 and a negative correlation between MMP-9 and E-cadherin, but no correlation between TIMP-2 and E-cadherin. Also there was a positive correlation between DNA aneuploid rate and differentiation and LN   metastasis  . SPF that was higher than 15% was positively correlated with tumor size, differentiation and LN   metastasis  . And there was a significant difference between carcinoma and noncarcinoma in DNA aneuploid rate and SPF.With tumor progression and development of heterogeneity, the abnormal expressions of MMP-9, TIMP-2, and E-cadherin or DNA aneuploid rate or high SPF gradually increases, suggesting that they play a crucial role in gastric carcinoma progression. 
 Clinical characteristics of TIMP2, MMP2, and MMP9 gene polymorphisms in colorectal cancer. Genetic variations and the expression profile of matrix metalloproteinases (MMP) and tissue inhibitors of metalloproteinases (TIMP) are involved in the invasion and   metastasis   of colorectal cancer.The gene profiles of TIMP2 and MMP were assayed from 333 colorectal cancer using polymerase chain reaction-restriction fragment length polymorphism.TIMP2-418*G/*G, TIMP2 303*G/*G and MMP9-1562*C/*C were more frequent in patients than in controls (P = 0.020, P < 0.0001 and P < 0.044, respectively). Frequency of TIMP2-418*G/*G was higher in patients with   metastasis   than in those without   metastasis  , and that of TIMP2 303*G/*G was higher in patients with rectal cancer than in those with colon cancer (P = 0.008 and P =0.022, respectively). TIMP2-303*A/*A and MMP2-1575*G/*G were less frequent in patients than in controls (P = 0.001 and P = 0.005, respectively). The TIMP2-418*G303*G haplotype was more frequent (P < 0.0001) and MMP2-1575*G-735*C haplotype was less frequent in patients than in controls (P= 0.005).Specific single-nucleotide polymorphism in TIMP2 and MMP appeared to be associated with tumorigenesis and biological behavior in colorectal cancer, which is expected be further verified in a larger cohort in the future. 
 [Interstitial collagenase, gelatinases A and B and their endogenous inhibitors in squamous cell cervical carcinomas]. Interstitial collagenase and gelatinases are matrix metalloproteinases (MMP), which play the key role in tumor invasion and   metastasis  . The aim of this study was to elucidate the peculiarities of expression of interstitial collagenase (MMP-1), gelatinases A and B (MMP-2 and MMP-9) and their endogenous tissue inhibitors TIMP-1 and TIMP-2 as invasive factors of squamous cell carcinomas (SCC) of human cervical cancer. The study was carried out using 24 specimens of SCC and 11 specimens of adjacent to tumor morphologically normal tissue. All carcinoma specimens expressed E7 HPV-16 gene. It was shown that the increase of MMP-1 and MMP-9 expression and low of TIMP-1 and TIMP-2 expression makes the main contribution to the destructive (invasive) potential of SCC. The change of MMP-2 expression is not so significant and it is less influenced to the destructive potential. Moreover, substantial expression of MMP-1, MMP-2 and MMP-9 was registered in the specimens of morphologically normal adjoining to tumor tissue. This expression was found to make an additional contribution to the destructive potential of cervical tumor. 
 Comparative study of the immunohistochemical expression of tissue inhibitors of metalloproteinases 1 and 2 between clearly invasive carcinomas and "in situ" trophoblast invasion. Tissue inhibitors of metalloproteinases (TIMPs) play an important role in extracellular matrix homeostasis by regulating MMP activity. Although they were initially considered inhibitors of tumor growth and   metastasis  , recently their role in cancer progression has been controversial. The aim of our study was to compare the immunohistochemical expression of TIMP1 and TIMP2 between an uncontrollably invasive phenomenon (cancer) and an "in situ" process (trophoblast invasion) in an effort to assess any differential role of these molecules between these two distinct phenomena and therefore to understand better their contribution in cancer invasion and migration. We performed an immunohistochemical analysis of 50 carcinomas (colorectal, gastric, breast, pulmonary, and renal) and 40 first trimester gestations. The marker expression was evaluated semiquantitatively, separately in cancer parenchymal and trophoblastic cells as well as in malignant stromal and decidual cells, according to a percentage scale (0, <10, 10-50, and >50%) and according to staining intensity (0, +, ++, and +++). Our results showed that there was no statistically significant difference in TIMP1 expression between cancer parenchymal cells and trophoblastic cells. On the other hand, TIMP1 was expressed more often in decidual cells than in cancer stromal cells. Immunostaining for TIMP2 was more extensive and intense both in trophoblastic and decidual cells than in cancer parenchymal and stromal cells, respectively. The reduced expression of TIMP2 in metastatic carcinomas by comparison with non-metastatic gestation specimens underlines its importance in cancer invasion and migration. On the other hand, TIMP1 was more expressed in decidua than cancer stroma, but at the same time showed no statistically significant difference between cancer parenchyma and trophoblasts, highlighting its multifunctional activity in cancer progression. 
 CD133 affects the invasive ability of HCT116 cells by regulating TIMP-2. CD133 is widely expressed in colorectal cancer (CRC) tissues and cell lines. This protein has been used as a marker of CRC cancer stem cells, although the function and mechanism of CD133 in CRC invasion and   metastasis   remain unclear. In our study, we examined the role of CD133 in CRC invasion in vitro and investigated the mechanism involved in CD133-related invasion. CD133(high) and CD133(low) HCT116 cells were isolated, and the proliferation and invasive ability of these two subpopulations were tested. CD133(high) HCT116 cells exhibited greater proliferation and invasion compared with CD133(low) HCT116 cells. CD133 knockdown (using CD133 small-interfering [si]RNA) inhibited the invasive activity of CD133si-HCT116 cells. For the first time, we found that the expression of tissue inhibitor of matrix metalloproteinases-2 (TIMP-2) was down-regulated in CD133si-HCT116 cells. In addition, for the TIMP-2si-HCT116 cells (transfected with TIMP-2 siRNA), in vitro invasion was significantly decreased, whereas the expression of CD133 remained unchanged. Finally, the metalloproteinase 2 and MEK/ERK signaling pathways were examined, and no significant change was observed after the knockdown of CD133 or TIMP-2 in HCT116 cells. In conclusion, we demonstrated that CD133 plays an important role in HCT116 cell invasion, and for the first time, we found that CD133 knockdown significantly down-regulated TIMP-2 expression, which suggests that CD133 affects the invasive ability of HCT116 cells by regulating TIMP-2. 
 [Expression of tissue inhibitor of metalloproteinase-2 in nasopharyngeal carcinoma]. To study the expression of tissue inhibitor of matrix metalloproteinase 2 (TIMP2)in the nasopharyngeal carcinoma and to evaluate the relationships between the expression of TIMP2 and biological behavior of NPC.The expression of TIMP2 was observed by immunohistochemical technique in 50 cases with nasopharyngeal carcinoma and 15 cases of Inflammation nasopharyngeal tissues.The positive rates of TIMP2 are significantly higher in NPC than in Inflammation tissues (P < 0.05). The expression of TIMP2 increase significantly with lymph node   metastasis   (P < 0.01), but had no relevant to the rise clinical invasion stage (P > 0.05).The results indicate that TIMP2 may play a critical role in assisting carcinoma cell to degrade basement membrane to invade surrounding tissues and to form metastatic colonies in lymph node. Especially the unbalance in MMP2/TIMP2 has probably a prognostic value in nasopharyngeal carcinoma. 
 MMP-2 protein in invasive breast cancer and the impact of MMP-2/TIMP-2 phenotype on overall survival. Crucial event in the   metastasis   of cancer cells is the secretion of matrix metalloproteinases (MMPs), which are responsible for the degradation of extracellular matrix (ECM). Among them, matrix metalloproteinase-2 (MMP-2) is a gelatinase, which degrades basement membrane type-IV collagen. Immunohistochemistry was performed to detect MMP-2 protein in 135 infiltrative breast carcinomas. MMP-2 was studied along with clinicopathological parameters (tumor size, histological type, nuclear and histological grade, stage, lymph node status, ER, and PR), patients' survival and tissue inhibitor metalloproteinase-2 (TIMP-2), Ki-67, and p53 proteins. MMP-2 immunoreactivity was detected in the cytoplasm in cancer cells in 102 (75.6%) and in both tumor and tumor stromal cells in 37 (27.4%) of 135 cases respectively. MMP-2 reactivity in cancer cells displayed a statistically significant association with tumor size > 2 cm (p = 0.022). In tumor stromal cells a strong parallel association was observed between the expression of MMP-2 and TIMP-2 (p = 0.015), while an inverse correlation was found between MMP-2 and both Ki-67 and p53 (p = 0.033 and p = 0.034 respectively). In the subgroup with negative lymph nodes MMP-2 was also inversely associated with p53 in cancer cells (p = 0.045). Finally a statistically significant association was revealed using Kaplan-Meier and Cox's proportional hazard regression model between the MMP-2/TIMP-2 phenotype and patients' better survival (p = 0.021). Our results point out the strong relation between MMP-2 and TIMP-2 and the effect of the MMP-2/TIMP-2 phenotype in the patients' overall survival. The inverse correlation between MMP-2 and both Ki-67 and p53 can be explained by the potential inhibition of MMP-2 by TIMP-2. These results suggest the necessity of further investigation. 
 TIMP-2 mediates the anti-invasive effects of the nitric oxide-releasing prodrug JS-K in breast cancer cells. Tumor invasion and   metastasis   remain a major cause of mortality in breast cancer patients. High concentrations of nitric oxide (NO) suppress tumor invasion and   metastasis   in vivo. NO prodrugs generate large amounts of NO upon metabolism by appropriate intracellular enzymes, and therefore could have potential in the prevention and therapy of metastatic breast cancer.The present study was designed to determine the effects of the NO-releasing prodrug O2-(2,4-dinitrophenyl) 1- [(4-ethoxycarbonyl)piperazin-1-yl]diazen-1-ium-1,2-diolate (JS-K) on breast cancer invasion and the mechanisms involved. MDA-MB-231, MDA-MB-231/F10, and MCF-7/COX-2 were the three breast cancer cell lines tested. NO levels were determined spectrophotometrically using a NO assay kit. Invasion and the expression of matrix metalloproteinases (MMPs) and tissue inhibitor of MMPs were determined using Matrigel invasion assays, an MMP array kit and ELISAs. The activity and expression of extracellular signal-regulated kinase 1/2, p38, and c-Jun N-terminal kinase mitogen-activated protein kinases were determined using western blot analyses.Under conditions by which JS-K was not cytotoxic, JS-K significantly decreased (P < 0.05) the invasiveness of breast cancer cells across the Matrigel basement membrane, which was directly correlated with NO production. JS-43-126, a non-NO-releasing analog of JS-K, had no effect on NO levels or invasion. JS-K increased (P < 0.05) TIMP-2 production, and blocking TIMP-2 activity with a neutralizing antibody significantly increased (P < 0.05) the invasive activity of JS-K-treated cells across Matrigel. JS-K decreased p38 activity, whereas the activity and the expression of extracellular signal-regulated kinase 1/2 and c-Jun N-terminal kinase were unaffected.We report the novel findings that JS-K inhibits breast cancer invasion across the Matrigel basement membrane, and NO production is vital for this activity. Upregulation of TIMP-2 production is one mechanism by which JS-K mediates its anti-invasive effects. JS-K and other NO prodrugs may represent an innovative biological approach in the prevention and treatment of metastatic breast cancer. 
 [Expression and significance of matrix metalloproteinase and tissue inhibitor of matrix metalloproteinase in non-melanoma skin cancer]. To investigate the expression and significance of matrix metalloproteinases (MMP-2, MMP-9) and tissue inhibitor of matrix metalloproteinase (TIMP-2, TIMP-1) in non-melanoma skin cancer (NMSC).Thirty six patients with squamous cell carcinoma (SCC) and 32 patients with basal cell carcinoma (BCC), confirmed by pathology, were selected, and 30 cases of normal skin were selected as control. The expression of MMP-2, MMP-9, TIMP-1 and TIMP-2 in all samples were examined by immunohistochemistry and reverse transcription polymerase chain reaction (RT-PCR). The expression rate, expression intensity and expression level of each factor were recorded. The results were compared between the groups.The expression rates of MMP-2 and MMP-9 in the control group were 30.0% and 36.7%, the expression levels of MMP-2 and MMP-9 in the control group were 57.216 ± 12.785 and 59.318 ± 13.262, all significantly lower than those in the tumor edge and center of the SCC and BCC groups (P < 0.01). The expression rates of TIMP-1 and TIMP-2 in the control group were 96.7% and 100%, their expression levels were 121.738 ± 25.516 and 122.612 ± 25.964, all significantly higher than those in the SCC and BCC groups (P < 0.01). The expression levels of MMP-2 and MMP-9 in the tumor center and edge of SCC group were significantly higher than those in the corresponding parts of the BCC group, while the expression levels of TIMP-1 and TIMP-2 were significantly lower than those in the BCC group (P < 0.01). The expression levels of MMP-2 and MMP-9 in the tumor edge of the SCC and BCC groups were significantly higher than those in the tumor centers (P < 0.01), while the expression levels of TIMP-1and TIMP-2 were significantly lower than those in the tumor centers (P < 0.01).MMP-2, MMP-9 and TIMP-2, TIMP-1 may play an important role in the development, progression, invasion   and metast  asis of non-melanoma skin cancer. 
 Mesenchymal stem cells inhibit breast cancer cell migration and invasion through secretion of tissue inhibitor of metalloproteinase-1 and -2. Mesenchymal stem cells (MSCs) form part of tumor stroma, and are typically considered to be pro-tumorigenic, promoting continuing tumor growth and   metastasis  . Here, we describe a mechanism by which MSCs may be anti-tumorigenic, through inhibition of breast cancer cell migration and invasion, an important part of   metastasis  . MDA-MB-231 and T47D cells were co-cultured in a Transwell insert above MSCs or MSC conditioned media and their migration or invasion through Matrigel measured. The conditioned media was found to inhibit breast cancer cell movement. TIMP-1 and TIMP-2, inhibitors of matrix metalloproteinases (MMPs), were identified as candidates for this inhibition and enhanced secretion of MMPs was not sufficient to counter the anti-migratory effects of TIMP expression. Inhibition of TIMP activity showed that TIMP-1 and TIMP-2 together were largely responsible for the reduction of migration and invasion by MSCs. Therefore, MSCs may play anti-tumorigenic, anti-metastatic roles in tumor development, with the overall outcome dependent upon the balance of pro-and anti-tumorigenic molecules secreted. 
 Expression of matrix metalloproteinase 2 and its tissue inhibitor in oral squamous cell carcinoma. The aim of this study is to investigate the expression of matrix metalloproteinase 2 (MMP-2) and the tissue inhibitor of metalloproteinase 2 (TIMP-2) in oral squamous cell carcinoma (OSCC) and adjacent normal tissues, and explore the role of MMP-2 and TIMP-2 in carcinoma   metastasis   and invasion. Expression of MMP-2 and TIMP-2 was evaluated in 40 cases with semi-quantitative reverse transcriptase polymerase chain reaction (RT-PCR) and immunohistochemical techniques. The values of MMP-2/beta-actin and TIMP-2/beta-actin in OSCC were significantly higher than those in adjacent normal tissues 2 cm and > or = 5 cm from carcinoma tissues (p<0.05). The results of immunohistochemical analysis show that the positive expression of MMP-2 and TIMP-2 protein in tumor tissues (60.0 and 52.5%) was higher than that in adjacent normal tissues 2 cm (30.0 and 22.5%) and 5 cm (22.5 and 25.0%) from cancer tissues, and the difference was significant p<0.05). The expression of MMP-2 and TIMP-2 was related to the differentiation degree of tumor cells, metastatic lymph node status and stage of carcinoma, but not related to the age and gender of patients, or location of the carcinoma. Therefore, MMP-2 and TIMP-2 may play important roles in the invasion and   metastasis   of OSCC. 
 The ERK/MAPK pathway regulates the activity of the human tissue factor pathway inhibitor-2 promoter. Human tissue factor pathway inhibitor-2 (hTFPI-2) is a 32-kDa serine protease inhibitor that is associated with the extracellular matrix. hTFPI-2 inhibits several extracellular matrix-degrading serine proteases and may play a role in tumor invasion and   metastasis  . To study the signal transduction pathway that leads to the activation of the hTFPI-2, we cloned the potential promoter region of this gene adjacent to a heterologous luciferase reporter gene. Phorbol 12-myristate 13-acetate (PMA) induced the luciferase reporter gene in HEK293 cells and other epithelial cell lines, such as the human lung carcinoma A549 cells, the breast carcinoma MCF7 cells, and the cervical HeLa cells. This PMA induction was blocked with the MEK inhibitor UO126, suggesting that the PMA-induced activation of the hTFPI-2 promoter is mediated through MEK. Furthermore, epidermal growth factor induced the luciferase reporter gene in HeLa cells. Cotransfection of the luciferase construct with constitutively active components of the Ras/Raf/MEK/ERK pathway in EcR-293 cells lead to a 7- to 92-fold induction of the luciferase reporter gene, indicating that regulation of hTFPI-2 is mediated through this pathway. A series of luciferase reporter gene constructs with progressive deletions of the 5'-flanking region suggested that the minimal basal promoter activity is located between nucleotide positions -89 and -384, whereas the minimal inducible promoter activity is between -89 and -222. We have used the computer program TFSEARCH and mutagenesis to analyze potential transcription factor binding sites. We identified an AP-1 binding site at nucleotide position -156 (inducible activity) and a Sp1 site at position -134 (basal activity) as potential cis-acting elements in the promoter region of the hTFPI-2. 
 A role for matrix metalloproteinases and tumor host interaction in hepatocellular carcinomas. Hepatocellular carcinoma (HCC) occur in livers with injury-remodeling, accomplished by enzymes called matrix metalloproteinases (MMP).   Metastasis   involves basement membrane invasion also caused by MMP activity. Alterations in MMP expression and their endogenous inhibitor (TIMP) may factor in HCC   metastasis  .HCC specimens and lymph nodes (n = 7), and normal lymph tissue from organ donors (n = 8), were snap-frozen in liquid nitrogen and the mRNA precipitated. A series of reverse transcription-polymerase chain reactions (RT-PCR) were performed using MMP (MMP2, MMP7, MMP9) primers and TIMP (TIMP1, TIMP2) primers. These were semiquantitatively analyzed by comparing concentration with constitutive GADPH expression.There is an increase in MMP2:TIMP2 mRNA expression ratio in the normal and tumor margin tissue compared to the tumor. There are increases in all MMP and TIMP mRNA expression (except TIMP1) and alterations in all of the MMP:TIMP expression ratios in the draining lymph node.Alterations exist in MMP2:TIMP2: expression at the margin, and all of the MMPs in the draining lymph nodes. This likely reflects a host-tumor interaction that regulates tumor   metastasis  . 
 MicroRNA-200b is overexpressed in endometrial adenocarcinomas and enhances MMP2 activity by downregulating TIMP2 in human endometrial cancer cell line HEC-1A cells. MicroRNAs (miRNAs) play important roles in tumorigenesis and   metastasis  . In this study, we investigated miR-200b expression in endometrial adenocarcinomas and normal adjacent tissues and found that miR-200b is more highly expressed in cancer tissues than in normal adjacent tissues. A novel target of miR-200b, tissue inhibitor of metalloproteinase 2 (TIMP2), was predicted using a bioinformatics approach and was confirmed in human endometrial cancer cell line HEC-1A cells by luciferase assay, quantitative real-time polymerase chain reaction, western blotting, and enzyme-linked immunosorbent assay. We found that miR-200b repressed TIMP2 expression at both the messenger RNA and protein levels, although a family member, miR-200a, had no such effect. Using reverse gelatin zymography, we showed that miR-200b enhances matrix metallopeptidase 2 (MMP2) activity by downregulating TIMP2 expression in HEC-1A cells. These data suggest that miR-200b may play an important role in the   metastasis   of endometrial adenocarcinomas. 
 Serum levels and tissue expression of matrix metalloproteinase 2 (MMP-2) and tissue inhibitor of metalloproteinases 2 (TIMP-2) in colorectal cancer patients. The objective of the study was the assessment of serum levels and tissue expression of matrix metalloproteinase 2 (MMP-2) and tissue inhibitor of matrix metalloproteinases 2 (TIMP-2) in patients with colorectal cancer (CRC). The study included 72 CRC patients and 68 healthy subjects. The serum levels of MMP-2 and TIMP-2 were measured using enzyme-linked immunosorbent assay (ELISA) method, whereas tissue expression of MMP-2 and TIMP-2 in cancer cells, interstitial inflammatory cells, and adjacent normal colorectal mucosa were examined by immunohistochemical staining of tumor samples. The serum levels of MMP-2 and TIMP-2 in cancer patients were significantly lower than those in control group, but the percentage of positive immunoreactivity of these proteins were higher in malignant and inflammatory cells as compared to normal tissue. There was a significant correlation between MMP-2 immunoreactivity in inflammatory cells and the presence of distant metastases and between TIMP-2 expression in inflammatory cells and tumor size, nodal involvement, and distant metastases. Area under receiver operating characteristic (ROC) curve (AUC) for serum MMP-2 was higher than for serum TIMP-2. Moreover, positive tissue expression of MMP-2 was a significant prognostic factor for CRC patients' survival. Our findings suggest that MMP-2 and TIMP-2 might play a role in the process of colorectal cancer invasion and   metastasis  , but the significance of their interactions with tumor stroma and interstitial inflammatory infiltration in colorectal neoplasia require further elucidation. 
 TIMP-2 modulates cancer cell transcriptional profile and enhances E-cadherin/beta-catenin complex expression in A549 lung cancer cells. Tissue Inhibitor of Metalloproteinase 2 (TIMP-2) plays an essential role in regulating matrix remodeling, cell growth, differentiation, angiogenesis and apoptosis in vitro and in vivo. We have recently shown that TIMP-2-mediated inhibition of tumor growth is independent of matrix metalloproteinase-mediated mechanisms, and is a consequence of modulating both the tumor cells and the tumor microenvironment. In the current study we aim to identify the molecular pathways associated with these effects. We analyzed the transcriptional profile of the human lung cancer cell line A549 upon overexpression of TIMP-2 and Ala+TIMP-2 (mutant that does not inhibit MMP activity), and we found changes in gene expression predominantly related to decreased tumor development and   metastasis  . Increased E-cadherin expression in response to both TIMP-2 and Ala+TIMP-2 expression was confirmed by real time quantitative RT-PCR and immunoblotting. A549 cells treated with epidermal growth factor (EGF) displayed loss of cobblestone morphology and cell-cell contact, while cells overexpressing TIMP-2 or Ala+TIMP-2 were resistant to EGF-induced morphological changes. Moreover, exogenous treatment with recombinant Ala+TIMP-2 blocked EGF induced down-regulation of E-cadherin. In vivo, immunohistochemistry of A549 xenografts expressing either TIMP-2 or Ala+TIMP-2 demonstrated increased E-cadherin protein levels. More importantly, transcriptional profile analysis of tumor tissue revealed critical pathways associated with effects on tumor-host interaction and inhibition of tumor growth. In conclusion, we show that TIMP-2 promotes an anti-tumoral transcriptional profile in vitro and in vivo, including upregulation of E-cadherin, in A549 lung cancer cells. 
 MMP-2, TIMP-2 and CD44v6 expression in non-small-cell lung carcinomas. Factors that emerge as crucial participants in tumour invasion and metastases are matrix metalloproteinases (MMPs), tissue inhibitor of metalloproteinase (TIMP) inhibitors and cellular adhesion molecules (CD44 and similar molecules). They play important roles in tumour invasion and   metastasis   in non-small-cell lung carcinomas (NSCLCs).The study was performed using the data of 33 patients. MMP-2 from the metalloproteinase family, TIMP-2 from the metalloproteinase inhibitor family and the adhesion molecule CD44v6 expression were investigated immunohistochemically to search their role in the   metastasis   and the clinical outcome of the patients with NSCLCs.Twenty-three tumours (70%) were squamous cell carcinoma (SCC), 9 (27%) were adenocarcinoma (AC), and 1 (3%) was large cell carcinoma (LCC). MMP-2 and TIMP-2 were expressed in high rates in NSCLC but CD44v6 expression was about 50%. Lymphatic invasion was less frequent in TIMP-2- positive patients and this difference was statistically significant (P = 0.005). There was a statistically significant difference between SCCs and ACs with respect to CD44v6 tumoral expression (P = 0.004). Also, there was a negative correlation between lymphatic invasion and the extent of CD44v6; lymphatic invasion was significantly less in CD44v6-positive cases (P = 0.013).We found that TIMP-2 and CD44v6 can decrease the lymphatic invasion in NSCLCs. Also there was observed histiotype-related pattern of CD44v6 variant expression in SCCs. 
  GeneRIFs  
 Upregulation of EZH2, MMP2 and TIMP2 expression was correlated with   metastasis   of RCC to bone tissues ex vivo and in vitro. 
 Subsequent investigations revealed that TIMP2 is a direct target of miR-221/222, and overexpression of TIMP2 reduced the miR-221/222-mediated invasion,   metastasis  , and angiogenesis of glioma cells. 
 MMP-2 and TIMP-2 might play a role in the process of colorectal cancer invasion and   metastasis   
 MMP-2, MMP-9 and TIMP-2, TIMP-1 may play an important role in the development, progression, invasion and   metastasis   of non-melanoma skin cancer. 
 The primary tumors from patients with LR showed significantly higher percentage of TIMP-2 expression in stromal cells compared to primary tumors from patients with distant   metastasis   or primary tumors from patients without tumoral progression. 
 Crucial participants in tumour invasion and metastases are matrix metalloproteinases, tissue inhibitor of metalloproteinase inhibitors and cellular adhesion molecules. They play roles in tumour invasion and   metastasis   in non-small-cell lung carcinomas. 
 The over-expression of MMP-2, MMP-9, TIMP-1, and TIMP-2 may play a key role in invasion and lymph-node   metastasis   of in squamous carcinoma of the cervix. 
 MMP-2 plays an essential role in tumor invasion and   metastasis  , while TIMP-2 is shown to strongly inhibit cancer invasion and   metastasis  . 
 TIMP-2 may play an important role in the invasion and   metastasis   of oral squamous cell carcinoma. 
  [Cc]ell [Ii]nvasion  PubMed  
 Transient RNA silencing of tissue factor pathway inhibitor-2 modulates lung cancer   cell invasion  . Tissue Factor Pathway Inhibitor-2 (TFPI-2) is a potent inhibitor of plasmin which activates metalloproteinases (MMPs) involved in extracellular matrix (ECM) degradation. Its secretion in ECM makes TFPI-2 a potential inhibitor to regulate tumour invasion and metastasis. Moreover, TFPI-2 is frequently downregulated, particularly in aggressive cancers. In this study, we silenced TFPI-2 in the NCI-H460 non-small cell lung cancer cell line and evaluated the role of TFPI-2 in   cell invasion   and its impact on MMPs expression. As the effects of siRNA are transient, the consequences of both gene silencing and restoration to normal expression could be studied kinetically in the same cells. We showed that TFPI-2 expression by NCI-H460 cells was effectively downregulated using specific small interfering RNA and this silencing was associated with an increase in the invasive potential of tumour cells while migration was not affected. We also showed that mRNA levels and protein expression of MMP-2, -3, -9, -14 were not influenced by TFPI-2 silencing. Moreover, the gelatinase activity of MMP-2 and MMP-9 was unmodified. In contrast, MMP-1 mRNA levels and protein were significantly and similarly increased in cells transfected with TFPI-2 siRNA. In conclusion, this study confirms that TFPI-2 downregulation can contribute to tumour invasion of lung cancer cells. 
 MiR-221/222 promote human glioma   cell invasion   and angiogenesis by targeting TIMP2. miR-221/222 are two highly homologous microRNAs that are frequently upregulated in solid tumors. However, the effects of miR-221/222 in malignant gliomas have not been investigated thoroughly. In this study, we found that miR-221/222 were significantly upregulated in human glioma samples and glioma cell lines. Both gain- and loss-of-function studies showed that miR-221/222 regulate cell proliferation, the cell cycle and apoptosis, in addition to, invasion, metastasis, and angiogenesis in glioma cell lines. Subsequent investigations revealed that TIMP2 is a direct target of miR-221/222, and overexpression of TIMP2 reduced the miR-221/222-mediated invasion, metastasis, and angiogenesis of glioma cells. Taken together, our results suggest that the suppression of miR-221/222 may be a feasible approach for inhibiting the malignant behaviors of glioma. 
 Promotion of astrocytoma   cell invasion   by micro RNA-22 targeting of tissue inhibitor of matrix metalloproteinase-2. OBJECTIVE Diffuse astrocytomas (DAs) have a high recurrence rate due to diffuse infiltration into the brain and spinal cord. Micro RNAs (miRNAs) are small noncoding RNAs that regulate gene expression by binding to complementary sequences of target messenger RNA (mRNA). It has been reported that miRNA-22 (miR-22) is involved in the invasion of some cancer cell lines. The aim of this study was to identify the biological effects of miR-22 in regard to the invasion of human DAs. METHODS The authors evaluated whether the level of miR-22 is elevated in human spinal DAs by using miRNA chips. Next, the role of miR-22 in 1321N1 human astrocytoma cells was investigated. Finally, to elucidate whether miR-22 promotes invasion by astrocytoma cells in vivo, the authors transplanted miR-22 overexpressed astrocytoma cells into mouse thoracic spinal cord. RESULTS The miR-22 significantly upregulated the invasion capacity of 1321N1 cells. Computational in silico analysis predicted that tissue inhibitor of matrix metalloproteinase-2 (TIMP2) is a target gene of miR-22. This was confirmed by quantitative reverse transcription polymerase chain reaction and Western blotting, which showed that miR-22 inhibited TIMP2 mRNA and protein expression, respectively. Luciferase reporter assays demonstrated that miR-22 directly bound the 3'-untranslated regions of TIMP2. The authors further showed that miR-22 promoted invasiveness in 1321N1 astrocytoma cells when transplanted into mouse spinal cord. CONCLUSIONS These data suggest that miR-22 acts to regulate invasion of 1321N1 astrocytoma cells by targeting TIMP2 expression. Additional studies with more cases and cell lines are required to elucidate the findings of this study for a novel treatment target for spinal DAs. 
 Lysophosphatidic acid downregulates tissue inhibitor of metalloproteinases, which are negatively involved in lysophosphatidic acid-induced   cell invasion  . Ovarian cancer is a highly metastatic disease. Lysophosphatidic acid (LPA) levels are elevated in ascites from ovarian cancer patients, but its potential role in ovarian cancer metastasis has just begun to be revealed. In this work, we show that LPA stimulates invasion of primary ovarian cancer cells, but not ovarian epithelial or borderline ovarian tumor cells, although these benign cells indeed respond to LPA in cell migration. We have found that LPA downregulates tissue inhibitor of metalloproteinases (TIMPs). TIMP2 and TIMP3 play functional role in LPA-induced invasion as negative regulators. G(i) protein, phosphatidylinositol-3 kinase (PI3K), p38 mitogen-activated protein kinase (MAPK), cytosolic phospholipase A(2) and urokinase type plasminogen activator (uPA) are required for LPA-induced cells invasion. TIMP3 may affect two independent downstream targets, vascular endothelial growth factor receptor and p38 MAPK. In vivo, LPA stimulates tumor metastasis in an orthotopic ovarian tumor model, which can be inhibited by a PI3K inhibitor, LY294002. In summary, LPA is likely a key component for promoting ovarian metastasis in vivo. LPA downregulates TIMP3, which may have targets other than metalloproteinases. Our in vivo metastasis mouse model is useful for studying the efficacy of therapeutic regimes of ovarian cancer. 
 TIMP-1 expression in human colorectal cancer is associated with TGF-B1, LOXL2, INHBA1, TNF-AIP6 and TIMP-2 transcript profiles. The balance of activity between the endogenous enzyme inhibitors known as tissue inhibitors of metalloproteinases and their targets, the matrix metalloproteinases, in the extracellular matrix is thought to play an important role in tumour   cell invasion  . Supporting this notion, we have shown that colorectal cancer patients have increased plasma levels of the tissue inhibitor of metalloproteinases-1 (TIMP-1), and that high plasma TIMP-1 levels are associated with short colorectal cancer patient survival. However, although TIMP-1 has been extensively studied in cancer, very little is known about how it is regulated. To further elucidate potential mechanisms of regulation of this protein, we did a number of experiments to look at associations between the transcript profile of TIMP-1 with known matrix metalloproteinases (MMPs) as well as with expression profiles of other genes differentially regulated in human colorectal cancer (CRC) and the other TIMPs 2-4, which have also been associated with the progression of colorectal cancer. Genome-wide expression profiling of 172 CRC and normal mucosa samples was used to identify transcript changes for the genes under investigation. We found that TIMP-1 was up-regulated in CRC samples compared with normal tissue, while TIMP-2 was down-regulated. Eight MMPs were up-regulated in CRC compared with normal tissue. Correlating up-regulated genes with the TIMP-1 transcript, we identified 13 that were also up-regulated in cancerous tissue. Among these were genes associated with the synthesis of extracellullar matrix, genes involved in the TGF-beta signalling pathway, and genes that are likely transcribed by the tumour cells. These insights add to the complex picture emerging about the regulation of TIMPs in colorectal cancer. 
 Epigenetic inactivation of the tissue inhibitor of metalloproteinase-2 (TIMP-2) gene in human prostate tumors. Gene silencing via CpG island methylation in the promoter region is one of the mechanisms by which tumor suppressor genes are inactivated in human cancers. Previous studies have shown that the tissue inhibitors of metalloproteinases (TIMP)-2 gene, which is an endogenous inhibitor of matrix metalloproteinases involved in   cell invasion   and tumorigenesis, is downregulated or silenced in a variety of human cancer cell lines. Here, we investigated the mechanism underlying TIMP-2 expression in prostate cancer cell lines and primary prostate tumor samples. We observed a strong correlation between promoter hypermethylation and lost expression of TIMP-2 gene, which was supported by other results demonstrating that promoter demethylation by 5-aza-2'-deoxycytidine and trichostatin A reactivated TIMP-2 and restored its expression in TIMP-2-silenced metastatic prostate cell lines. These results were further substantiated by a chromatin immunoprecipitation assay, showing the preferential binding of MeCP2 to methylated CpG island in TIMP-2-silenced metastatic prostate cell lines. In vitro Matrigel invasion assays showed that re-expression of TIMP-2 after a combined treatment with 5-aza and trichostatin-A in metastatic prostate cells resulted in a significant reduction of tumor   cell invasion  . Furthermore, CpG methylation of TIMP-2 promoter was also shown in primary prostate tumors that expressed decreased TIMP-2 protein levels. These results suggest that the downregulation of the TIMP-2 gene is associated with promoter methylation and that this may play an important role in prostate cancer progression during the invasive and metastatic stages of the disease. 
 CD133 affects the invasive ability of HCT116 cells by regulating TIMP-2. CD133 is widely expressed in colorectal cancer (CRC) tissues and cell lines. This protein has been used as a marker of CRC cancer stem cells, although the function and mechanism of CD133 in CRC invasion and metastasis remain unclear. In our study, we examined the role of CD133 in CRC invasion in vitro and investigated the mechanism involved in CD133-related invasion. CD133(high) and CD133(low) HCT116 cells were isolated, and the proliferation and invasive ability of these two subpopulations were tested. CD133(high) HCT116 cells exhibited greater proliferation and invasion compared with CD133(low) HCT116 cells. CD133 knockdown (using CD133 small-interfering [si]RNA) inhibited the invasive activity of CD133si-HCT116 cells. For the first time, we found that the expression of tissue inhibitor of matrix metalloproteinases-2 (TIMP-2) was down-regulated in CD133si-HCT116 cells. In addition, for the TIMP-2si-HCT116 cells (transfected with TIMP-2 siRNA), in vitro invasion was significantly decreased, whereas the expression of CD133 remained unchanged. Finally, the metalloproteinase 2 and MEK/ERK signaling pathways were examined, and no significant change was observed after the knockdown of CD133 or TIMP-2 in HCT116 cells. In conclusion, we demonstrated that CD133 plays an important role in HCT11  6 cell invasi  on, and for the first time, we found that CD133 knockdown significantly down-regulated TIMP-2 expression, which suggests that CD133 affects the invasive ability of HCT116 cells by regulating TIMP-2. 
 Up-regulation of tissue inhibitor of metalloproteinase-2 promotes SHI-1   cell invasion   in nude mice. The role of tissue inhibitor of metalloproteinase-2 (TIMP-2) in extramedullary infiltration of acute leukemia is unclear. We demonstrated in our previous study that the up-regulation of TIMP-2 promoted SHI-1   cell invasion   in vitro. We investigated in the present study whether TIMP-2 would have the same effect in vivo. A retroviral vector carrying human TIMP-2 cDNA was constructed and transfected into SHI-1 cells. Three subclone cells (S1, S2 and S3) that highly expressed TIMP-2 were selected to establish nude mouse models of acute leukemia. Times of leukemic onset in mice of S1, S2 and S3 groups were all earlier than that of the SHI-1 group, whereas the survival times of S1, S2 and S3 groups were all shorter than that of the SHI-1 group (p < 0.05). Histopathological results demonstrated severe leukemic infiltration in numerous organs in each group. Reverse transcription polymerase chain reaction (RT-PCR) assay showed that several organs expressed the MLL/F6 fusion gene. Moreover, the numbers of organs infiltrated by leukemic cells in S1, S2 and S3 groups were more than those in the SHI-1 group (p < 0.05). Up-regulating TIMP-2 expression enhanced SHI-1   cell invasion   in nude mice and resulted in more severe leukemia infiltration. This phenomenon suggests that targeted therapy with TIMP-2 for acute leukemia should be performed with prudence. 
  GeneRIFs  
 Up-regulation of tissue inhibitor of metalloproteinase-2 promotes SHI-1   cell invasion   in nude mice. 
   
  
 TIMP1 
 
  NCBI Gene  
  GeneRIF  
  Pubmed  
 
 
  Search Term  Rank  Fields  
  [Mm]igration  6  Process(1); PubMed(34); GeneRIFs(10)  
  ECM  7  PubMed(45); GeneRIFs(3)  
  [Ee]xtracellular [Mm]atrix  8  Process(1); PubMed(147); Component(1); GeneRIFs(10); Summary(1)  
  [Ii]nvasive  10  PubMed(57); GeneRIFs(15)  
  [Mm]etastasize  11  PubMed(2)  
  [Mm]etastasis  12  PubMed(68); GeneRIFs(12)  
  [Cc]ell [Ii]nvasion  13  PubMed(21); GeneRIFs(5)  
 
 
 hide/show details for TIMP1 
  [Mm]igration  Process  
 negative regulation of trophoblast cell   migration   
  PubMed  
 mRNA expression of matrix metalloproteinases (MMPs) 2 and 9 and tissue inhibitor of matrix metalloproteinases (TIMPs) 1 and 2 in childhood acute lymphoblastic leukemia: potential role of TIMP1 as an adverse prognostic factor. This study evaluates the mRNA expression profile of genes TIMP1, TIMP2, MMP2 and MMP9 in diagnostic bone marrow samples from 134 consecutive ALL children by real-time quantitative PCR. A significant association was observed between higher expression levels of MMP9 and low risk group and absence of extramedullary infiltration and higher expression levels of TIMP2 and MMP2 with T-ALL. TIMP1 gene expression values higher than the median were associated with a significantly lower 5-year event free-survival in univariable (P=0.04) and multivariable analysis (P=0.01). Our data address new information in the complex interaction of the   migration  /adhesion genes and childhood ALL. 
 Differential growth regulation in human melanoma cell lines by TIMP-1 and TIMP-2. We investigated the role of tissue inhibitor of metalloproteinase (TIMP)-1 and TIMP-2 in the growth regulation. Primary (PM-WK and KHm-4), recurrent primary (RPM-EP and RPM-MC), lymph node metastatic (MM-AN, MM-BP and MM-RU), and a visceral metastatic (MM-LH) melanoma cell lines were used. Reverse transcriptase-coupled polymerase chain reaction and Western blotting revealed that all expressed and produced TIMP-1 and TIMP-2 except for PM-WK, which neither expressed nor produced TIMP-1. TIMP-1 and TIMP-2 secretion levels were measured by enzyme-linked immunosorbent assay in supernatants of cells. We found that the TIMP-1 production level was correlated with the cell   migration   rate. Moreover, TIMP-1 enhanced the cell   migration   of PM-WK. The growth of the primary melanoma cell lines was stimulated by TIMP-1 and inhibited by TIMP-2. In contrast, the growth of the visceral metastatic melanoma cell line was stimulated by TIMP-2. 
 TIMP-1 modulates chemotaxis of human neural stem cells through CD63 and integrin signalling. We recently reported that hNSCs (human neural stem cells) have the interesting characteristic of   migration   towards an intracranial glioma. However, the molecules and mechanisms responsible for tumour tropism are unclear. In the present study, we used microarray and proteomics analyses to identify a novel chemoattractant molecule, TIMP-1 (tissue inhibitor of metalloproteinase-1), secreted from human brain tumour tissues. We demonstrate that TIMP-1 significantly enhances hNSC adhesion and   migration   in a cell culture system. These effects were critically dependent on CD63, as shRNA-mediated ablation of CD63 expression attenuated the response. TIMP-1 significantly increased the number of FAs (focal adhesions) and cytoskeletal reorganization for cell   migration   in hNSCs, whereas knockdown of CD63 resulted in decreased hNSC spreading, FAs and   migration  , even after TIMP-1 treatment. In addition, TIMP-1 binding to CD63 activated β1 integrin-mediated signalling through Akt and FAK phosphorylation, leading to pattern changes in distribution of vinculin and F-actin (filamentous actin). Furthermore, inactivation of β1 integrin by use of a blocking antibody or inhibition of PI3K (phosphoinositide 3-kinase) signalling impaired th  e migrati  on of hNSCs towards TIMP-1. Collectively, our results underline TIMP-1 as a novel and effective key regulator of CD63 and β1 integrin-mediated signalling, which regulates hNSC adhesion a  nd migrat  ion. 
 In vitro study of matrix metalloproteinase/tissue inhibitor of metalloproteinase production by mesenchymal stromal cells in response to inflammatory cytokines: the role of their   migration   in injured tissues. The transmigratory capacity of bone marrow (BM) mesenchymal stromal cells (MSC) through the endothelial cell barrier into various tissues and their differentiation potential makes them ideal candidates for cell therapy. Nevertheless, the mechanisms and agents promoting their   migration   are not fully understood. We evaluated the effects of several inflammatory cytokines on the   migration   of BM MSC and matrix metalloproteinase (MMP)/tissue inhibitor of metalloproteinase (TIMP) production.The migratory potential of BM MSC was evaluated using a Boyden chamber coated with Matrigel in the presence and absence of stromal cell-derived (SDF)-1alpha, platelet-derived growth factor (PDGF)bb, insulin-like growth factor (IGF)-I and interleukin (IL)-6. The ability of inflammatory cytokines to induce MSC   migration   was tested in presence of their respective Ab or blocking peptide. We used immunofluorescence to check the expression of cytokine receptors, and MMP/TIMP production was analyzed at the protein (human cytokine array, enzyme-linked immunosorbent assay (ELISA), gelatine zymography and Western blot) and mRNA quantitative real-time polymerase chain reaction (qRT-PCR) levels.We have demonstrated that inflammatory cytokines promote the migratory capacity of BM MSC according to the expression of their respective receptors. Higher   migration   through Matrigel was observed in response to IL-6 and PDGFbb. qRT-PCR and cytokine array revealed that   migration   was the result of the variable level of MMP/TIMP in response to inflammatory stimuli.Our observations suggest that chemokines and cytokines involved in the regulation of the immunity or inflammatory process promote the   migration   of MSC into BM or damaged tissues. One of the mechanisms used by MSC to promote their   migration   though the extracellular matrix is modulation of the production of MMP-1, MMP-2, MMP-13, TIMP-1 and TIMP-2. 
 HIV-1-Tat protein promotes chemotaxis and invasive behavior by monocytes. Monocytes are susceptible to HIV infection and to activation by a regulatory gene product of the HIV genome, HIV-Tat. Recently, we have demonstrated that treatment with HIV-Tat up-regulates monocyte adhesion to the endothelium and increases metalloproteinase production. in the present study, we have examined the ability of the HIV-Tat protein to alter the migratory and invasive behavior of monocytes. Monocytes pretreated for 24 h with 10 ng/ml HIV-Tat exhibited enhanced migratory behavior compared with untreated monocytes in chemotaxis assays, both in the absence of a chemoattractant as well as in response to FMLP. in addition, HIV-Tat itself induced the   migration   of both untreated and HIV-Tat pretreated monocytes. Checkerboard analysis showed that monocytes migrated in response to an HIV-Tat concentration gradient, thus confirming the chemotactic characteristics of the HIV-Tat protein. Pretreatment of monocytes with 10 ng/ml HIV-Tat for 24 h also increased their ability to invade reconstituted extracellular membrane (Matrigel)-coated filters by 5-fold in the absence of chemoattractant. The presence of FMLP or HIV-Tat further enhanced invasion by both untreated and HIV-Tat-pretreated monocytes by more than 10-fold. Monocyte invasion was partially inhibited by the inclusion of anti-beta integrin Ab or tissue inhibitor of metalloproteinase (TIMP). Thus, for the first time, we present evidence that HIV-Tat can enhance the chemotactic and invasive behaviors of monocytes and propose an active role for HIV-Tat in the recruitment of monocytes into extravascular tissues, a process which may contribute to the destruction of tissues and cellular architecture often seen in patients with acquired immunodeficiency syndrome. 
 Comparative study of the immunohistochemical expression of tissue inhibitors of metalloproteinases 1 and 2 between clearly invasive carcinomas and "in situ" trophoblast invasion. Tissue inhibitors of metalloproteinases (TIMPs) play an important role in extracellular matrix homeostasis by regulating MMP activity. Although they were initially considered inhibitors of tumor growth and metastasis, recently their role in cancer progression has been controversial. The aim of our study was to compare the immunohistochemical expression of TIMP1 and TIMP2 between an uncontrollably invasive phenomenon (cancer) and an "in situ" process (trophoblast invasion) in an effort to assess any differential role of these molecules between these two distinct phenomena and therefore to understand better their contribution in cancer invasion and   migration  . We performed an immunohistochemical analysis of 50 carcinomas (colorectal, gastric, breast, pulmonary, and renal) and 40 first trimester gestations. The marker expression was evaluated semiquantitatively, separately in cancer parenchymal and trophoblastic cells as well as in malignant stromal and decidual cells, according to a percentage scale (0, <10, 10-50, and >50%) and according to staining intensity (0, +, ++, and +++). Our results showed that there was no statistically significant difference in TIMP1 expression between cancer parenchymal cells and trophoblastic cells. On the other hand, TIMP1 was expressed more often in decidual cells than in cancer stromal cells. Immunostaining for TIMP2 was more extensive and intense both in trophoblastic and decidual cells than in cancer parenchymal and stromal cells, respectively. The reduced expression of TIMP2 in metastatic carcinomas by comparison with non-metastatic gestation specimens underlines its importance in cancer invasion and   migration  . On the other hand, TIMP1 was more expressed in decidua than cancer stroma, but at the same time showed no statistically significant difference between cancer parenchyma and trophoblasts, highlighting its multifunctional activity in cancer progression. 
 Mesenchymal stem cells inhibit breast cancer cell   migration   and invasion through secretion of tissue inhibitor of metalloproteinase-1 and -2. Mesenchymal stem cells (MSCs) form part of tumor stroma, and are typically considered to be pro-tumorigenic, promoting continuing tumor growth and metastasis. Here, we describe a mechanism by which MSCs may be anti-tumorigenic, through inhibition of breast cancer cell   migration   and invasion, an important part of metastasis. MDA-MB-231 and T47D cells were co-cultured in a Transwell insert above MSCs or MSC conditioned media and their   migration   or invasion through Matrigel measured. The conditioned media was found to inhibit breast cancer cell movement. TIMP-1 and TIMP-2, inhibitors of matrix metalloproteinases (MMPs), were identified as candidates for this inhibition and enhanced secretion of MMPs was not sufficient to counter the anti-migratory effects of TIMP expression. Inhibition of TIMP activity showed that TIMP-1 and TIMP-2 together were largely responsible for the reduction of   migration   and invasion by MSCs. Therefore, MSCs may play anti-tumorigenic, anti-metastatic roles in tumor development, with the overall outcome dependent upon the balance of pro-and anti-tumorigenic molecules secreted. 
 Mesenchymal stem cells inhibit breast cancer cell   migration   and invasion through secretion of tissue inhibitor of metalloproteinase-1 and -2. Mesenchymal stem cells (MSCs) form part of tumor stroma, and are typically considered to be pro-tumorigenic, promoting continuing tumor growth and metastasis. Here, we describe a mechanism by which MSCs may be anti-tumorigenic, through inhibition of breast cancer cell   migration   and invasion, an important part of metastasis. MDA-MB-231 and T47D cells were co-cultured in a Transwell insert above MSCs or MSC conditioned media and their   migration   or invasion through Matrigel measured. The conditioned media was found to inhibit breast cancer cell movement. TIMP-1 and TIMP-2, inhibitors of matrix metalloproteinases (MMPs), were identified as candidates for this inhibition and enhanced secretion of MMPs was not sufficient to counter the anti-migratory effects of TIMP expression. Inhibition of TIMP activity showed that TIMP-1 and TIMP-2 together were largely responsible for the reduction of   migration   and invasion by MSCs. Therefore, MSCs may play anti-tumorigenic, anti-metastatic roles in tumor development, with the overall outcome dependent upon the balance of pro-and anti-tumorigenic molecules secreted. 
 TIMP-1 modulates chemotaxis of human neural stem cells through CD63 and integrin signalling. We recently reported that hNSCs (human neural stem cells) have the interesting characteristic of   migration   towards an intracranial glioma. However, the molecules and mechanisms responsible for tumour tropism are unclear. In the present study, we used microarray and proteomics analyses to identify a novel chemoattractant molecule, TIMP-1 (tissue inhibitor of metalloproteinase-1), secreted from human brain tumour tissues. We demonstrate that TIMP-1 significantly enhances hNSC adhesion and   migration   in a cell culture system. These effects were critically dependent on CD63, as shRNA-mediated ablation of CD63 expression attenuated the response. TIMP-1 significantly increased the number of FAs (focal adhesions) and cytoskeletal reorganization for cell   migration   in hNSCs, whereas knockdown of CD63 resulted in decreased hNSC spreading, FAs and   migration  , even after TIMP-1 treatment. In addition, TIMP-1 binding to CD63 activated β1 integrin-mediated signalling through Akt and FAK phosphorylation, leading to pattern changes in distribution of vinculin and F-actin (filamentous actin). Furthermore, inactivation of β1 integrin by use of a blocking antibody or inhibition of PI3K (phosphoinositide 3-kinase) signalling impaired th  e migrati  on of hNSCs towards TIMP-1. Collectively, our results underline TIMP-1 as a novel and effective key regulator of CD63 and β1 integrin-mediated signalling, which regulates hNSC adhesion a  nd migrat  ion. 
 Comparative study of the immunohistochemical expression of tissue inhibitors of metalloproteinases 1 and 2 between clearly invasive carcinomas and "in situ" trophoblast invasion. Tissue inhibitors of metalloproteinases (TIMPs) play an important role in extracellular matrix homeostasis by regulating MMP activity. Although they were initially considered inhibitors of tumor growth and metastasis, recently their role in cancer progression has been controversial. The aim of our study was to compare the immunohistochemical expression of TIMP1 and TIMP2 between an uncontrollably invasive phenomenon (cancer) and an "in situ" process (trophoblast invasion) in an effort to assess any differential role of these molecules between these two distinct phenomena and therefore to understand better their contribution in cancer invasion and   migration  . We performed an immunohistochemical analysis of 50 carcinomas (colorectal, gastric, breast, pulmonary, and renal) and 40 first trimester gestations. The marker expression was evaluated semiquantitatively, separately in cancer parenchymal and trophoblastic cells as well as in malignant stromal and decidual cells, according to a percentage scale (0, <10, 10-50, and >50%) and according to staining intensity (0, +, ++, and +++). Our results showed that there was no statistically significant difference in TIMP1 expression between cancer parenchymal cells and trophoblastic cells. On the other hand, TIMP1 was expressed more often in decidual cells than in cancer stromal cells. Immunostaining for TIMP2 was more extensive and intense both in trophoblastic and decidual cells than in cancer parenchymal and stromal cells, respectively. The reduced expression of TIMP2 in metastatic carcinomas by comparison with non-metastatic gestation specimens underlines its importance in cancer invasion and   migration  . On the other hand, TIMP1 was more expressed in decidua than cancer stroma, but at the same time showed no statistically significant difference between cancer parenchyma and trophoblasts, highlighting its multifunctional activity in cancer progression. 
 mRNA expression of matrix metalloproteinases (MMPs) 2 and 9 and tissue inhibitor of matrix metalloproteinases (TIMPs) 1 and 2 in childhood acute lymphoblastic leukemia: potential role of TIMP1 as an adverse prognostic factor. This study evaluates the mRNA expression profile of genes TIMP1, TIMP2, MMP2 and MMP9 in diagnostic bone marrow samples from 134 consecutive ALL children by real-time quantitative PCR. A significant association was observed between higher expression levels of MMP9 and low risk group and absence of extramedullary infiltration and higher expression levels of TIMP2 and MMP2 with T-ALL. TIMP1 gene expression values higher than the median were associated with a significantly lower 5-year event free-survival in univariable (P=0.04) and multivariable analysis (P=0.01). Our data address new information in the complex interaction of the   migration  /adhesion genes and childhood ALL. 
 In vitro study of matrix metalloproteinase/tissue inhibitor of metalloproteinase production by mesenchymal stromal cells in response to inflammatory cytokines: the role of their   migration   in injured tissues. The transmigratory capacity of bone marrow (BM) mesenchymal stromal cells (MSC) through the endothelial cell barrier into various tissues and their differentiation potential makes them ideal candidates for cell therapy. Nevertheless, the mechanisms and agents promoting their   migration   are not fully understood. We evaluated the effects of several inflammatory cytokines on the   migration   of BM MSC and matrix metalloproteinase (MMP)/tissue inhibitor of metalloproteinase (TIMP) production.The migratory potential of BM MSC was evaluated using a Boyden chamber coated with Matrigel in the presence and absence of stromal cell-derived (SDF)-1alpha, platelet-derived growth factor (PDGF)bb, insulin-like growth factor (IGF)-I and interleukin (IL)-6. The ability of inflammatory cytokines to induce MSC   migration   was tested in presence of their respective Ab or blocking peptide. We used immunofluorescence to check the expression of cytokine receptors, and MMP/TIMP production was analyzed at the protein (human cytokine array, enzyme-linked immunosorbent assay (ELISA), gelatine zymography and Western blot) and mRNA quantitative real-time polymerase chain reaction (qRT-PCR) levels.We have demonstrated that inflammatory cytokines promote the migratory capacity of BM MSC according to the expression of their respective receptors. Higher   migration   through Matrigel was observed in response to IL-6 and PDGFbb. qRT-PCR and cytokine array revealed that   migration   was the result of the variable level of MMP/TIMP in response to inflammatory stimuli.Our observations suggest that chemokines and cytokines involved in the regulation of the immunity or inflammatory process promote the   migration   of MSC into BM or damaged tissues. One of the mechanisms used by MSC to promote their   migration   though the extracellular matrix is modulation of the production of MMP-1, MMP-2, MMP-13, TIMP-1 and TIMP-2. 
 Differential growth regulation in human melanoma cell lines by TIMP-1 and TIMP-2. We investigated the role of tissue inhibitor of metalloproteinase (TIMP)-1 and TIMP-2 in the growth regulation. Primary (PM-WK and KHm-4), recurrent primary (RPM-EP and RPM-MC), lymph node metastatic (MM-AN, MM-BP and MM-RU), and a visceral metastatic (MM-LH) melanoma cell lines were used. Reverse transcriptase-coupled polymerase chain reaction and Western blotting revealed that all expressed and produced TIMP-1 and TIMP-2 except for PM-WK, which neither expressed nor produced TIMP-1. TIMP-1 and TIMP-2 secretion levels were measured by enzyme-linked immunosorbent assay in supernatants of cells. We found that the TIMP-1 production level was correlated with the cell   migration   rate. Moreover, TIMP-1 enhanced the cell   migration   of PM-WK. The growth of the primary melanoma cell lines was stimulated by TIMP-1 and inhibited by TIMP-2. In contrast, the growth of the visceral metastatic melanoma cell line was stimulated by TIMP-2. 
 Cannabinoids inhibit angiogenic capacities of endothelial cells via release of tissue inhibitor of matrix metalloproteinases-1 from lung cancer cells. Cannabinoids inhibit tumor neovascularization as part of their tumorregressive action. However, the underlying mechanism is still under debate. In the present study the impact of cannabinoids on potential tumor-to-endothelial cell communication conferring anti-angiogenesis was studied. Cellular behavior of human umbilical vein endothelial cells (HUVEC) associated with angiogenesis was evaluated by Boyden chamber, two-dimensional tube formation and fibrin bead assay, with the latter assessing three-dimensional sprout formation. Viability was quantified by the WST-1 test. Conditioned media (CM) from A549 lung cancer cells treated with cannabidiol, Δ(9)-tetrahydrocannabinol, R(+)-methanandamide or the CB2 agonist JWH-133 elicited decreased   migratio  n as well as tube and sprout formation of HUVEC as compared to CM of vehicle-treated cancer cells. Inhibition of sprout formation was further confirmed for cannabinoid-treated A549 cells co-cultured with HUVEC. Using antagonists to cannabinoid-activated receptors the antimigratory action was shown to be mediated via cannabinoid receptors or transient receptor potential vanilloid 1. SiRNA approaches revealed a cannabinoid-induced expression of tissue inhibitor of matrix metalloproteinases-1 (TIMP-1) as well as its upstream trigger, the intercellular adhesion molecule-1, to be causally linked to the observed decrease of HUVEC   migratio  n. Comparable anti-angiogenic effects were not detected following direct exposure of HUVEC to cannabinoids, but occurred after addition of recombinant TIMP-1 to HUVEC. Finally, antimigratory effects were confirmed for CM of two other cannabinoid-treated lung cancer cell lines (H460 and H358). Collectively, our data suggest a pivotal role of the anti-angiogenic factor TIMP-1 in intercellular tumor-endothelial cell communication resulting in anti-angiogenic features of endothelial cells. 
 Increased expression ratio of matrix metalloproteinase-9 (MMP9) and tissue inhibitor of matrix metalloproteinase (TIMP-1) RNA levels in Iranian multiple sclerosis patients. Multiple sclerosis (MS) is an autoimmune disease involving the central nervous system (CNS) with unknown immunopathogenic mechanisms. Matrix metalloproteinase-9 (MMP-9) facilitates T-cell   migration   into the CNS while the tissue inhibitor matrix metalloproteinase-1 (TIMP-1) inhibits MMP-9 actions. The aim of this study was to evaluate the expression of TIMP-1 RNA and MMP-9/TIMP-1 RNA ratio in blood cells of Iranian patients with relapsing-remitting multiple sclerosis (RRMS) treated with IFNb.The study compared the expression level of TIMP-1 gene in RRMS samples with normal individuals in Iran and the results were compared using a ratio of MMP-9 to TIMP-1. All patients were HLA-DRB1*15 negative and were responders to interferon-beta with a normal vitamin D level.The RRMS patients manifested a lower expression level of TIMP-1 RNA than their normal counterparts although the result was not significant (P= 0.06). Also, the ratio of MMP-9 to TIMP-1 RNA increased significantly (P= 0.009). There was no linear correlation between TIMP-1 expression level and risk of Expanded Disability Status Scale of Kurtzke (EDSS); nor was there any significant correlation between expression status of TIMP-1 and duration of the disease. Although there was no significant decrease in TIMP-1 expression level, the MMP-9/TIMP-1 RNA ratio in RRMS was significantly higher than normal subjects.Further studies are recommended to compare MMP-9/TIMP-1 RNA ratio in patients before and after taking IFN-beta in order to find out if MMP-9/TIMP-1 RNA ratio can function as a proper marker of the bio efficacy of IFN-beta treatment of MS. 
 OncomiR-196 promotes an invasive phenotype in oral cancer through the NME4-JNK-TIMP1-MMP signaling pathway. MicroRNA-196 (miR-196), which is highly up-regulated in oral cancer cells, has been reported to be aberrantly expressed in several cancers; however, the significance of miR-196 in oral cancer has not yet been addressed.Cellular functions in response to miR-196 modulation were examined, including cell growth,   migration  , invasion and radio/chemosensitivity. Algorithm-based studies were used to identify the regulatory target of miR-196. The miR-196 target gene and downstream molecular mechanisms were confirmed by RT-qPCR, western blot, luciferase reporter and confocal microscopy analyses. miR-196 expression was determined in paired cancer and adjacent normal tissues from oral cancer patients.Both miR-196a and miR-196b were highly over-expressed in the cancer tissue and correlated with lymph node metastasis (P = 0.001 and P = 0.006, respectively). Functionally, miR-196 actively promot  ed cell m  igration and invasion without affecting cell growth. Mechanistically, miR-196 performed it's their function by inhibiting NME4 expression and further activating p-JNK, suppressing TIMP1, and augmenting MMP1/9.miR-196 contributes to oral cancer by promoti  ng cell m  igration and invasion. Clinically, miR-196a/b was significantly over-expressed in the cancer tissues and correlated with lymph node metastasis. Thus, our findings provide new knowledge of the underlying mechanism of cancer metastasis. miR-196 may serve as a promising marker for better oral cancer management. 
 Diagnostic value of matrix metalloproteinase 9 and tissue inhibitor of matrix metalloproteinases 1 in cholesteatoma. Matrix metalloproteinase 9 (MMP-9), able to degrade type IV collagen, plays a key role in inflammatory cell   migration   as well as in the destructive behaviour of cholesteatoma. The aim of our study was to compare the expression of MMP-9 and TIMP-1 in cholesteatoma tissue and in the concentrations in serum and plasma concentrations.Twenty five adult patients suffering from cholesteatoma (a study group) were included in the study. A comparison group consisted of 25 adult patients admitted to hospital due to nasal septum deviation. MM-9 and TIMP-1 serum and plasma concentrations as well as proteins' expressions in cholesteatoma tissues (study group) and normal retroauricular skin specimens (control group) were evaluated. MMP-9 and TIMP-1 concentrations were measured using enzyme-linked immunosorbent assay (ELISA). Cholesteatoma tissues and normal retroauricular skin specimens were evaluated immunohistochemically.In the study and a comparison groups, MMP-9 and TIMP-1 concentrations were similar with no significant difference within the groups. In cholesteatoma tissues, the expression of the investigated enzyme and its inhibitor was higher than in normal skin specimens, limited mostly to cholesteatoma perimatrix.Cholesteatoma may be limited to the middle ear or parts of the temporal bones. Our findings suggest better clinical usefulness of MMP-9 and TIMP-1 expression in cholesteatoma tissues than either serum or plasma levels of these proteins. It might suggest that the higher the expression of MMP-9 the stronger the inflammation -accompanied cholesteatoma. 
 Down-regulation of TIMP-1 inhibits cell   migration  , invasion, and metastatic colonization in lung adenocarcinoma. Tissue inhibitor metalloproteinase-1 (TIMP-1) is clinically associated with a poor prognosis for various cancers, but the roles of TIMP-1 in lung cancer metastasis are controversial. Our previous secretomic study revealed that TIMP-1 is highly abundant in high invasiveness cells of lung adenocarcinoma. In the current study, TIMP-1 abundances in primary lung adenocarcinoma tissues, as revealed by immunohistochemistry, are significantly higher in patients with lymph invasion and distant metastasis than in those without. Receiver operating characteristic curve analyses suggest 73.7 and 86.2 % accuracy to separate patients with lymph node and distant metastasis and those without, respectively. Moreover, we demonstrate that the expression level of TIMP-1 positively associates with cell mobility, invasiveness, and metastatic colonization. Most notably, the novel mechanism in which TIMP-1 facilitates metastatic colonization through the mediation of pericellular polyFN1 assembly was revealed. In summary, this study presents novel functions of TIMP-1 in promoting cancer metastasis and suggests TIMP-1 is a potential tissue biomarker for lymph invasion and distant metastasis of lung adenocarcinoma. 
 The cytokine-protease connection: identification of a 96-kD THP-1 gelatinase and regulation by interleukin-1 and cytokine inducers. The induction of proteolytic enzymes is an important mechanism in the   migration   of monocytes into tissues and body fluids. The monocytic cell line THP-1 was used as a model system to study the production of a particular gelatinase. Upon stimulation with phorbol myristate acetate (PMA) the cells differentiated to the adherent phenotype and produced significant amounts of a 96-kD gelatinase in a dose-dependent way. The secretion rate was maximal between 12 and 24 h after induction. Study of gelatinase mRNA steady state levels showed that the synthesis of THP-1 gelatinase is regulated by PMA at transcriptional or posttranscriptional levels. Stimulation of signal transduction pathways with other substances, including calcium ionophore A 23187, dibutyryl cyclic AMP, and dexamethasone, were ineffective in inducing gelatinase mRNA or enzyme activity. However, THP-1 cells were responsive to the cytokine interleukin (IL)-1 beta, to bacterial lipopolysaccharide (LPS), and the lectin concanavalin A (Con A), the kinetics of gelatinase induction being similar to those of induction by PMA. The THP-1 cells did not synthesize and/or secrete detectable levels of IL-6 after stimulation with PMA, Con A, LPS, or IL-1 beta. The 96-kD monocytic THP-1 gelatinase was shown to be a neutral metalloproteinase that cross-reacted with hepatoma-derived and neutrophil gelatinases in immunoprecipitation experiments. The active enzyme produced by THP-1 cells consistently showed, however, a molecular mass different from that of normal granulocyte-, monocyte-, and tumor cell-derived gelatinases.(ABSTRACT TRUNCATED AT 250 WORDS) 
 TIMP-1 expression induced by IL-32 is mediated through activation of AP-1 signal pathway. Hepatic fibrosis is a necessarily stage from the progression of chronic liver diseases to cirrhosis, even hepatocellular carcinoma (HCC). Hepatic fibrosis is characterized by the progressive accumulation of extracellular matrix (ECM). The balance between ECM production and degradation is mediated by matrix metalloproteinases (MMPs) and tissue inhibitors of metalloproteinases (TIMPs). TIMP-1 is an important regulator in the synthesis and degradation of ECM. IL-32, a multi-function cytokine, could induce IL-1β, IL-6, IL-8 and other cytokine expressions by activating AP-1, NF-κβ, p38MAPK signal pathways. IL-32 expression is increased in liver tissues of hepatic fibrosis. However, the role of IL-32 in the pathogenesis of liver fibrosis is not thoroughly clear. Recently, it is demonstrated that TIMP-1 expression is induced by the activation of AP-1 signal pathway. So we assayed the effect of IL-32 on TIMP-1 expression by LX-2 cells (one of HSCs cell lines) in the present study. We found that IL-32 could induce TIMP-1 expression by LX-2 cells at a dose-dependent manner. IL-32 could increase TIMP-1 promoter activity and induce TIMP-1 expression by activating AP-1 signal pathway. Moreover, the increase of TIMP-1 expression could promote t  he migrat  ion of LX-2 cells. In conclusion, we believe that IL-32 might be involved in the pathogenesis of hepatic fibrosis by inducing TIMP-1 expression. 
 Tissue inhibitor of metalloproteinase 1 activates normal human granulocytes, protects them from apoptosis, and blocks their trans  migration   during inflammation. Urinary levels of tissue inhibitor of metalloproteinase 1 (TIMP-1) higher than those of matrix metalloproteinase 9 (MMP-9) during acute pyelonephritis have previously been associated with a higher degree of acute inflammation and of postinfective renal scarring. The aim of the present study was to evaluate possible mechanisms by which TIMP-1 could affect the scarring process already during the acute phase of inflammation. The growth of Escherichia coli, bactericidal activity of fresh human blood, and respiratory burst, spontaneous apoptosis, and trans-basement membrane   migration   of normal human granulocytes were studied in vitro in the presence of different concentrations of recombinant human TIMP-1. To imitate the "normal" environment during inflammation in the kidney, granulocytes were also incubated with a conditioned medium from E. coli-stimulated renal epithelial cells. In order to compare our data with the in vivo situation, blood and urinary leukocyte levels were analyzed for 40 children with acute pyelonephritis, together with urinary MMP-9 and TIMP-1 levels. TIMP-1 at a concentration of 500 ng/ml increased the bactericidal activity of blood, increased the respiratory burst of granulocytes, decreased phosphatidylserine exposure and caspase 3 activity, which are features of spontaneous apoptosis, and inhibited granulocyte trans  migration  . Moreover, in the patients with pyelonephritis, MMP-9/TIMP-1 ratios in urine correlated with the degree of leukocyte trans  migration  . Thus, our data suggest that TIMP-1 specifically blocks the trans  migration   of granulocytes into urine. Entrapped and activated granulocytes, protected from apoptosis, might excessively destroy renal parenchyma and thus contribute to the pathogenesis of renal scarring following acute pyelonephritis. 
 TIMP-1 promotes accumulation of cancer associated fibroblasts and cancer progression. Treatment options for late stage prostate and colon cancer are limited and there is an urgent need to develop more effective and targeted novel therapies, which starts with identification and validation of novel therapeutic targets. Recent clinical studies have demonstrated that tissue inhibitor matrix metalloproteinase-1 (TIMP-1) levels are elevated in cancer patient plasma and elevated TIMP-1 levels are associated with worse clinical outcomes. However, it is unknown whether TIMP-1 serves merely as a biomarker of cancer progression or has a functional role in promoting cancer progression and can serve as a cancer therapeutic target, which is the main objective of this study. Here, we show that stroma of human prostate and colon cancer express higher levels of TIMP-1 compared to their normal counterparts and increased expression of TIMP-1 promotes in vivo growth of both cancer types. We demonstrate for the first time that increased TIMP-1 expression stimulates accumulation of cancer associated fibroblasts (CAFs) within prostate and colon cancer tissues and that TIMP-1 enhances prostate CAF proliferation and   migration   in vitro and promotes ERK1/2 kinase activation in these CAF cells. Our results establish the novel promotive effects of TIMP-1 on cancer progression and on accumulation of CAFs that in turn provides a pro-tumor microenvironment. Together, these results establish the potential of TIMP-1 as a novel target for cancer therapy and the mechanism underlying the pro-tumor activity of TIMP-1. 
 Nodal signals through activin receptor-like kinase 7 to inhibit trophoblast   migration   and invasion: implication in the pathogenesis of preeclampsia. Trophoblast cell invasion into the uterus is an essential process for successful pregnancy, and shallow invasion of trophoblasts into the maternal decidua is linked to preeclampsia. We have reported that Nodal, a member of the transforming growth factor-β superfamily, acts through activin receptor-like kinase 7 (ALK7) to inhibit trophoblast proliferation and to induce apoptosis. In this study, we examined the spatial and temporal expression patterns of Nodal and ALK7 in human placenta from normal and preeclamptic pregnancies and investigated whether Nodal regulated trophoblast   migratio  n and invasion. Nodal and ALK7 were detected in villous and extravillous trophoblast cell populations in early gestation, and their levels were strongly up-regulated in preeclamptic placenta. Overexpression of Nodal or constitutively active ALK7 decreased cell   migratio  n and invasion, whereas knockdown of Nodal and ALK7 had the opposite effects. In placental explant culture, treatment with Nodal inhibited trophoblast outgrowth, whereas Nodal small-interfering RNA strongly induced the expansion of explants and the   migratio  n of extravillous trophoblast cells. Nodal stimulated the secretion of tissue inhibitor of metalloproteinase-1 and inhibited matrix metalloproteinase (MMP)-2 and MMP-9 activity. These findings suggest that the Nodal/ALK7 pathway plays important roles in human placentation and that its abnormal signaling may contribute to the development of preeclampsia. 
 TIMP-1 inhibits microvascular endothelial cell   migration   by MMP-dependent and MMP-independent mechanisms. It was reported over a decade ago that tissue inhibitor of metalloproteinases-1 (TIMP-1) suppresses angiogenesis in experimental models but the mechanism is still incompletely understood. This in vitro study focused on the molecular basis of TIMP-1-mediated inhibition of endothelial cell (EC)   migration  , a key step in the angiogenic process. Both recombinant human TIMP-1 and the synthetic MMP inhibitors, GM6001 and MMP-2-MMP-9 Inhibitor III, suppressed   migration   of human dermal microvascular endothelial cells (HDMVEC) in a dose-dependent fashion. The MMP-dependent inhibition of   migration   was associated with increased expression of the junctional adhesion proteins, VE-cadherin and PECAM-1, and VE-cadherin accumulation at cell-cell junctions. TIMP-1 also caused MMP-independent dephosphorylation of focal adhesion kinase (FAK) (pY397) and paxillin, which was associated with reduced number of F-actin stress fibers and focal adhesions. Moreover, TIMP-1 stimulated expression of PTEN that has been shown to reduce phosphorylation of FAK and inhibit cell   migration  . Our data suggest that TIMP-1 inhibits HDMVEC   migration   through MMP-dependent stimulation of VE-cadherin and MMP-independent stimulation of PTEN with subsequent dephosphorylation of FAK and cytoskeletal remodeling. 
 Identification of positive and negative regulator elements for the tissue inhibitor of metalloproteinase 1 gene. We have identified an IL-10 inducible enhancer (HTE) (5'-CACGATGACTCATCACTGTTGAAAGACA-3') (-864 to -836 bp) and associated silencer element (HTS) (5'-CCACTGGCCCATCGTATAT-3') (-1284 to -1266 bp) in the 5' promoter region of the human tissue inhibitor of metalloproteinase-1 (TIMP-1) gene. Chloramphenicol acetyl transferase (CAT), electrophoretic   migration   shift assays (EMSAs), and DNase footprinting revealed that IL-10 (15 ng/ml for 1-2 h) induced the HTE enhancer. In comparison, phorbol ester stimulated the HTS silencer and blocked IL-10's effects in a dose-dependent, orientation- and position-independent fashion, suggesting that HTS is a true silencer element. EMSAs combined with deletion and mutation analysis of the HTE and HTS elements confirmed these observations. Finally, Northern blot, Western blot, immunoprecipitation, and ELISA analysis showed that IL-10 (15 ng/ml) induced TIMP-1 expression (approximately 10-fold by 18 h), whereas PMA (100 ng/ml) inhibited the stimulatory effects of IL-10 on TIMP-1 expression. The data indicate that HTE and HTS function as positive and negative regulatory elements that control human TIMP-1 expression. 
 Differential effects of sulfated triterpene glycosides, holothurin A1, and 24-dehydroechinoside A, on antimetastasic activity via regulation of the MMP-9 signal pathway. Two sulfated triterpene glycosides, holothurin A(1) (HA(1)) and 24-dehydroechinoside A (DHEA), isolated from the sea cucumber Pearsonothuria graeffei, are of the holostane type with 18(20)-lactone and identical carbohydrate chains. DHEA has a side chain 23 (24)-double bond, while HA(1) has a hydroxyl group at C-21. In this study, we compared the effects of DHEA and HA(1) on metastasis in vitro and in vivo. The results show that HA(1) and DHEA treatment significantly suppressed adhesion of human hepatocellular liver carcinoma cells (HepG2) to both matrigel and human endothelial cells (ECV-304) and inhibited HepG2 cell   migration   and invasion in a dose-dependant manner. HA(1) and DHEA reduced tube formation of ECV-304 cells on the matrigel in vitro and attenuated neovascularization in the chick embryo using the chorioallantoic membrane (CAM) assay in vivo. Immunocytochemistry analyses revealed that both HA(1) and DHEA significantly decreased the expression of the matrix metallo-proteinase-9 (MMP-9) and increased the expression level of tissue inhibitor of metalloproteinase-1 (TIMP-1), an important regulator of MMP-9 activation. Western blot analyses demonstrated that HA(1) and DHEA remarkably abolished the expression of vascular endothelial growth factor (VEGF). The expression of nuclear factor-kappa B (NF-κB) was significantly decreased by HA(1), while DHEA treatment had no effect on the down regulation of NF-κB expression. These data suggest that both DHEA and HA(1) exert significant antimetastatic activities by inhibiting MMP-9 and VEGF expression. DHEA-induced antimetastasis was more potent than HA(1). In addition, only HA(1) treatment downregulated the expression level of NF-κB, suggesting that the antimetastatic activity of triterpene glycosides derived from P. graeffei can be either NF-κB-dependent or -independent, depending on their structure. 
 The tissue inhibitor of metalloproteinases-1 improves   migration   and adhesion of hematopoietic stem and progenitor cells. Homing and engraftment of hematopoietic stem and progenitor cells (HSPCs) during bone marrow transplantation are critically dependent on integrins such as β1-integrin. In the present study, we show that β1-integrin and the tetraspanin CD63 form a cell surface receptor complex for the soluble serum protein tissue inhibitor of metalloproteinases-1 (TIMP-1) on human CD34⁺ HSPCs. Through binding to this receptor complex, TIMP-1 activates β1-integrin, increases adhesion   and migr  ation of human CD34⁺ cells, and protects these cells from induced apoptosis. TIMP-1 stimulation in murine bone marrow mononuclear cells also pr  omotes mi  gration and adhesion; this is associated with augmented homing of murine mononuclear cells and of murine LSK⁺ cells during bone marrow transplantation. These results not only indicate that TIMP-1 is conducive to HSPC homing; they also identify CD63 and β1-integrin as a TIMP-1 receptor complex on HSPCs. 
 Differential expression of stromal MMP-1, MMP-9 and TIMP-1 in basal cell carcinomas of immunosuppressed patients and controls. Matrix metalloproteinases (MMPs) have an important role in the initiation, growth, and invasion of malignant tumors. Basal cell cancer (BCC) is the most common human malignancy. The risk of BCC is 10-16 times higher among organ transplant recipients compared with the nontransplanted population. The aim of this study was to compare the expression of several MMPs and their tissue inhibitors (TIMPs) in BCCs from kidney transplant recipients and controls. Expression of MMPs-1, -7, -8, -9, -10, -13, -26, and TIMPs-1 and -3 was evaluated by immunohistochemistry in 25 samples of BCC of kidney transplant recipients and 25 matched controls representing superficial and nodular subtypes. No significant differences were detected in MMP expression of BCC tumor cells between immunocompetent and immunodeficient patients. However, MMPs-1 and -9 and TIMP-1 were expressed more frequently in stromal macrophages in the BCCs of immunocompetent patients. When tumor subtypes were compared irrespective of the patient group, more MMP-1-positive fibroblasts and MMP-9-positive neutrophils were detected in the superficial subtype, while stromal MMP-10 expression was more abundant in nodular tumors. Our results suggest that abundant peritumoral expression of TIMP-1 in non-immunocompromised patients limits ECM degradation permissive for cancer cell   migration  . 
 Prostaglandin E2-dependent enhancement of tissue inhibitors of metalloproteinases-1 production limits dendritic cell   migration   through extracellular matrix. Dendritic cell (DC)   migration   is crucial for the initiation of immune responses. The balance between metalloproteinases (MMP) and tissue inhibitors of metalloproteinases (TIMP) has been shown to modulate DC   migration  . PGE2, which is overproduced in a wide variety of human malignancies, has been implicated in MMP and TIMP regulation in various cells, including monocytes. In the present study, we hypothesized that tumor-derived PGE2 would affect DC migratory capacity through the extracellular matrix (ECM) by altering MMP and TIMP balance. Treatment of monocyte-derived immature DC with exogenous PGE2 induced TIMP-1 secretion but not MMP-9 production and was correlated with reduced DC   migration   through ECM. Because recombinant TIMP-1 replicated PGE2 inhibition of DC   migration   while anti-TIMP-1 neutralizing Ab reversed it, we conclude that PGE2-mediated induction of TIMP-1 was responsible for the reduced   migration   of PGE2-treated DC. Similarly, DC cultured for 48 h in supernatants from cyclooxygenase-2 overexpressing lung cancer cells that secrete high levels of PGE2, exhibited decreased   migration   through ECM. Finally, analysis of E prostanoid receptor expression and their selective inhibition revealed that the enhanced TIMP-1 secretion in PGE2-treated DC was mediated predominantly by the E prostanoid receptor 2. These findings indicate that PGE2-dependent enhancement of TIMP-1 production causes reduced   migration   of DC through ECM. 
 Enhanced   migration   of tissue inhibitor of metalloproteinase overexpressing hepatoma cells is attributed to gelatinases: relevance to intracellular signaling pathways. To study the effect of gelatinases (especially MMP-9) on   migration   of tissue inhibitor of metalloproteinase (TIMP-1) overexpressing hepatoma cells.Wild type HepG2 cells, cells stably transfected with TIMP-1 and TIMP-1 antagonist (MMP-9-H401A, a catalytically inactive matrix metalloproteinase (MMP) which still binds and neutralizes TIMP-1) were incubated in Boyden chambers either with or without Galardin (a synthetic inhibitor of MMP-1, -2, -3, -8, -9) or a specific inhibitor of gelatinases.Compared to wild type HepG2 cells, the cells overexpressing TIMP-1 showed 115%   migration   (P<0.05) and the cells overexpressing MMP-9-H401A showed 62%   migration   (P<0.01). Galardin reduced cell   migration   dose dependently in all cases. The gelatinase inhibitor reduced   migration   in TIMP-1 overexpressing cells predominantly. Furthermore, we examined intracellular signal transduction pathways of TIMP-1-dependent HepG2 cells. TIMP-1 deactivates cell signaling pathways of MMP-2 and MMP-9 involving p38 mitogen-activated protein kinase. Specific blockade of the ERK pathway suppresses gelatinase expression either in the presence or absence of TIMP-1.Overexpressing functional TIMP-1- enhanced   migration   of HepG2-TIMP-1 cells depends on enhanced MMP-activity, especially MMP-9. 
 Tissue inhibitor of metalloproteinase-1 moderates airway re-epithelialization by regulating matrilysin activity. Obliterative bronchiolitis (OB) is the histopathological finding in chronic lung allograft rejection. Mounting evidence suggests that epithelial damage drives the development of airway fibrosis in OB. Tissue inhibitor of metalloproteinase (TIMP)-1 expression increases in lung allografts and is associated with the onset of allograft rejection. Furthermore, in a mouse model of OB, airway obliteration is reduced in TIMP-1-deficient mice. Matrilysin (matrix metallproteinase-7) is essential for airway epithelial repair and is required for the re-epithelialization of airway wounds by facilitating cell   migration  ; therefore, the goal of this study was to determine whether TIMP-1 inhibits re-epithelialization through matrilysin. We found that TIMP-1 and matrilysin co-localized in the epithelium of human lungs with OB and both co-localized and co-immunoprecipitated in wounded primary airway epithelial cultures. TIMP-1-deficient cultures migrated faster, and epithelial cells spread to a greater extent compared with wild-type cultures. TIMP-1 also inhibited matrilysin-mediated cell   migration   and spreading in vitro. In vivo, TIMP-1 deficiency enhanced airway re-epithelialization after naphthalene injury. Furthermore, TIMP-1 and matrilysin co-localized in airway epithelial cells adjacent to the wound edge. Our data demonstrate that TIMP-1 interacts with matrix metalloproteinases and regulates matrilysin activity during airway epithelial repair. Furthermore, we speculate that TIMP-1 overexpression restricts airway re-epithelialization by inhibiting matrilysin activity, contributing to a stereotypic injury response that promotes airway fibrosis via bronchiole airway epithelial damage and obliteration. 
 Inhibition of cancer cell invasion by cannabinoids via increased expression of tissue inhibitor of matrix metalloproteinases-1. Cannabinoids, in addition to having palliative benefits in cancer therapy, have been associated with anticarcinogenic effects. Although the antiproliferative activities of cannabinoids have been intensively investigated, little is known about their effects on tumor invasion.Matrigel-coated and uncoated Boyden chambers were used to quantify invasiveness and   migration  , respectively, of human cervical cancer (HeLa) cells that had been treated with cannabinoids (the stable anandamide analog R(+)-methanandamide [MA] and the phytocannabinoid delta9-tetrahydrocannabinol [THC]) in the presence or absence of antagonists of the CB1 or CB2 cannabinoid receptors or of transient receptor potential vanilloid 1 (TRPV1) or inhibitors of p38 or p42/44 mitogen-activated protein kinase (MAPK) pathways. Reverse transcriptase-polymerase chain reaction (RT-PCR) and immunoblotting were used to assess the influence of cannabinoids on the expression of matrix metalloproteinases (MMPs) and endogenous tissue inhibitors of MMPs (TIMPs). The role of TIMP-1 in the anti-invasive action of cannabinoids was analyzed by transfecting HeLa, human cervical carcinoma (C33A), or human lung carcinoma cells (A549) cells with siRNA targeting TIMP-1. All statistical tests were two-sided.Without modifying   migration  , MA and THC caused a time- and concentration-dependent suppression of HeLa cell invasion through Matrigel that was accompanied by increased expression of TIMP-1. At the lowest concentrations tested, MA (0.1 microM) and THC (0.01 microM) led to a decrease in invasion (normalized to that observed with vehicle-treated cells) of 61.5% (95% CI = 38.7% to 84.3%, P < .001) and 68.1% (95% CI = 31.5% to 104.8%, P = .0039), respectively. The stimulation of TIMP-1 expression and suppression of cell invasion were reversed by pretreatment of cells with antagonists to CB1 or CB2 receptors, with inhibitors of MAPKs, or, in the case of MA, with an antagonist to TRPV1. Knockdown of cannabinoid-induced TIMP-1 expression by siRNA led to a reversal of the cannabinoid-elicited decrease in tumor cell invasiveness in HeLa, A549, and C33A cells.Increased expression of TIMP-1 mediates an anti-invasive effect of cannabinoids. Cannabinoids may therefore offer a therapeutic option in the treatment of highly invasive cancers. 
 Recombinant GPI-anchored TIMP-1 stimulates growth and   migration   of peritoneal mesothelial cells. Mesothelial cells are critical in the pathogenesis of post-surgical intraabdominal adhesions as well as in the deterioration of the peritoneal membrane associated with long-term peritoneal dialysis. Mesothelial denudation is a pathophysiolocigally important finding in these processes. Matrix metalloproteinase (MMP) biology underlies aspects of mesothelial homeostasis as well as wound repair. The endogenous tissue inhibitors of metalloproteinases (TIMPs) moderate MMP activity.By modifying human TIMP-1 through the addition of a glycosylphosphatidylinositol (GPI) anchor, a recombinant protein was generated that efficiently focuses TIMP-1 on the cell surface. Treatment of primary mesothelial cells with TIMP-1-GPI facilitates their mobilization and   migration   leading to a dramatic increase in the rate of wound experimental closure. Mesothelial cells treated with TIMP-1-GPI showed a dose dependent increase in cell proliferation, reduced secretion of MMP-2, MMP-9, TNF-α and urokinase-type plasminogen activator (uPA), but increased tissue plasminogen activator (t-PA). Treatment resulted in reduced expression and processing of latent TGF-β1.TIMP-1-GPI stimulated rapid and efficient in vitro wound closure. The agent enhanced mesothelial cell proliferation an  d migrati  on and was bioactive in the nanogram range. The application of TIMP-1-GPI may represent a new approach for limiting or repairing damaged mesothelium. 
 Small GTPase Rab37 targets tissue inhibitor of metalloproteinase 1 for exocytosis and thus suppresses tumour metastasis. Rab small GTPases are master regulators of membrane trafficking and guide vesicle targeting. Recent publications show that Rab-controlled trafficking pathways are altered during tumorigenesis. However, whether any of the Rabs plays a metastasis suppressor role is least explored. Here we address the metastasis suppressive function of human Rab37 (hRAB37) using secretomics, cell, animal and clinical analyses. We show that tissue inhibitor of metalloproteinase 1 (TIMP1), a secreted glycoprotein that inhibits extracellular matrix turnover, is a novel cargo of hRAB37. hRAB37 regulates the exocytosis of TIMP1 in a nucleotide-dependent manner to inactivate matrix metalloproteinase 9 (MMP9)   migration   axis in vitro and in vivo. Dysfunction of hRAB37 or TIMP1 abrogates metastasis suppression. Lung cancer patients with metastasis and poor survival show low hRAB37 protein expression coinciding with low TIMP1 in tumours. Our findings identify hRAB37 as a novel metastasis suppressor Rab that functions through the TIMP1-MMP9 pathway and has significant prognostic power. 
  GeneRIFs  
 this study shows that increased TIMP-1 expression could promote the   migration   of hepatocellular carcinoma cells 
 Inhibition of TIMP activity showed that TIMP-1 and TIMP-2 together were largely responsible for the reduction of   migration   and invasion by MSCs. 
 RAB37 regulates the exocytosis of TIMP1 in a nucleotide-dependent manner to inactivate MMP9   migration   axis in vitro and in vivo and to suppress tumor metastasis. 
 Data suggest that TIMP1 functions as chemoattractant for neural stem cells (NSC); TIMP1 enhances NSC adhesion,   migration  , and cytoskeletal reorganization; these effects are dependent on CD63 antigen (granulophysin) and CD29 (integrin beta1). 
 The   migration   of bone marrow (BM) mesenchymal stromal cells was the result of the variable level of MMP/TIMP in response to inflammatory stimuli. 
 Abundant peritumoral expression of TIMP-1 in non-immunocompromised patients limits extracellular matrix degradation permissive for cancer cell   migration  . 
 data suggest that TIMP-1 inhibits HDMVEC   migration   through MMP-dependent stimulation of VE-cadherin and MMP-independent stimulation of PTEN with subsequent dephosphorylation of FAK and cytoskeletal remodeling. 
 Enhancement of TIMP-1 production by tumor-derived prostaglandin E2 correlates with reduced dendritic cell   migration   through the tumor extracellular matrix. 
 Overexpressing functional TIMP-1- enhanced   migration   of HepG2-TIMP-1 cells depends on enhanced MMP-activity, especially MMP-9. 
 blocks trans  migration   of granulocytes into urine; entrapped and activated granulocytes, protected from apoptosis, might destroy renal parenchyma and contribute to the pathogenesis of renal scarring following acute pyelonephritis 
  ECM  PubMed  
 TIMP1, TIMP2, and TIMP4 are increased in aqueous humor from primary open angle glaucoma patients. Elevated intraocular pressure (IOP) is the only known modifiable risk factor for primary open angle glaucoma (POAG), and it can be caused by reduced aqueous humor outflow from the anterior chamber. Outflow is predominantly regulated by the trabecular meshwork, consisting of specialized cells within a complex extracellular matrix (  ECM  ). An imbalance between   ECM  -degrading matrix metalloproteinases (MMPs) and the tissue inhibitors of MMPs (TIMPs) within the trabecular meshwork is thought to contribute to POAG. This study aimed to quantify levels of TIMPs and MMPs in aqueous humor samples from glaucomatous and non-glaucomatous eyes, analyze MMP/TIMP ratios, and correlate results with age, IOP, and Humphrey's visual field pattern standard deviation (PSD).Aqueous humor samples were collected from 26 non-glaucomatous control subjects before cataract surgery and 23 POAG patients undergoing trabeculectomy or cataract surgery. Analyte concentrations were measured using multiplexed immunoassays. Statistical significance was assessed with Mann-Whitney U tests, and Spearman's method was used to assess correlations with age, IOP, and PSD.Concentrations of TIMP1 (p = 0.0008), TIMP2 (p = 0.002), TIMP4 (p = 0.002), and MMP2 (p = 0.020) were significantly increased in aqueous humor samples from POAG versus cataract samples. For the majority of MMP/TIMP molar ratios calculated for the cataract group, TIMPs outweighed MMPs. In POAG, molar ratios of MMP2/TIMP1 (p = 0.007) and MMP9/TIMP1 (p = 0.005) showed a significant decrease, corresponding to an elevated excess of TIMPs over MMPs in POAG compared to cataract samples. Conversely, MMP2/TIMP3 (p = 0.045) and MMP3/TIMP3 (p = 0.032) molar ratios increased. Several MMP/TIMP molar ratios correlated with IOP (r = 0.476-0.609, p = 0.007-0.034) and PSD (r = -0.482 to -0.655, p = 0.005-0.046) in POAG samples and with age in cataract control samples.An imbalance among MMPs and TIMPs was found in glaucomatous aqueous humor samples, with a shift toward raised TIMP levels. This may result in the inhibition of MMP activity, leading to an altered   ECM   composition in the TM and thereby contributing to increased outflow resistance. 
 Circulating matrix metalloproteinase-9 and tissue inhibitors of metalloproteinases-1 and -2 levels in gestational hypertension. Gestational hypertension (GH) is dangerous to both mother and child. Arterial invasiveness and growth are dependent on successful extracellular matrix (  ECM  ) breakdown, which may be abnormal in GH. We hypothesized abnormalities in circulating matrix metalloproteinase-9 (MMP-9) and tissue inhibitors of metalloproteinases-1 and -2 (TIMP-1 and TIMP-2, respectively) in patients with GH, when compared with normotensive women with normal pregnancies and healthy nonpregnant control subjects.Plasma MMP-9, TIMP-1, and TIMP-2 were measured by ELISA in 23 women with GH, 30 normotensive pregnant women, and 28 nonpregnant women who were matched for age, gestational age, and parity.Levels of circulating MMP-9, TIMP-1 and TIMP-2, and the MMP-9/TIMP-1 and MMP-9/TIMP-2 ratios were significantly different among the three groups (P = .026, P = .006, P = .007, P = .001 and P = .008 respectively). Within the GH group, MMP-9 and the MMP-9/TIMP-1 ratio correlated negatively with age (r = -0.581, P = .004 and r = -0.563, P = .005, respectively) and levels of diastolic blood pressure (r = -0.432, P = .040 and r = -0.461, P = .027, respectively). With multiple regression analysis, only age independently correlated with circulating levels of MMP-9 (P = .010); neither age nor levels of diastolic blood pressure had any effect on the MMP-9/TIMP-1 ratio.We have demonstrated altered MMP/TIMP ratios in maternal blood during GH. These observations suggest pregnancy-related changes in   ECM   breakdown and turnover. Given the importance of changes in   ECM   composition to vascular and cardiac structure in hypertension, we suggest that these observations may be related to the pathophysiology of human GH. 
 siRNA knockdown of tissue inhibitor of metalloproteinase-1 in keloid fibroblasts leads to degradation of collagen type I. Keloids are defined as overgrowths of scar tissue resulting from abnormal wound healing. They are characterized by excessive dermal deposition of thick, hyalinized collagen bundles resulting from an imbalance between the production and degradation of extracellular matrix (  ECM  ) components. Matrix metalloproteinases (MMPs) and tissue inhibitors of metalloproteinases (TIMPs) are two important regulators of   ECM   degradation and remodeling. To evaluate the role played by knockdown of TIMPs in keloid formation, we transduced human keloid-derived fibroblasts (KFs) with small interfering RNAs targeting TIMP-1 or -2 (siTIMP-1 or siTIMP-2) using a lentiviral vector and assessed the biological effects. We found that MMP-1/TIMP-1 and MMP-1/TIMP-2 complexes were suppressed and that MMP-2 activity was upregulated in KFs expressing siTIMP-1 or siTIMP-2. In addition, increased degradation of collagen type I was observed in the supernatant of KFs expressing siTIMP-1, but not siTIMP-2, with the suppression of cell viability and induction of apoptosis. These results suggest that targeting TIMP-1 using small interfering RNA has significant therapeutic potential as an approach to treating keloids through degradation of their thick collagen bundles. 
 Relationship between matrix metalloproteinases/tissue inhibitors of matrix metalloproteinases systems and autoantibody patterns in systemic sclerosis. Systemic sclerosis (SSc) is a connective tissue disease characterized by tissue fibrosis that reflects an imbalance between collagen production and degradation. Matrix metalloproteinases (MMPs) are a family of endopeptidases involved in the remodelling of extracellular matrix (  ECM  ). This activity is controlled by tissue inhibitors of MMP (TIMPs). Aim of this study was the evaluation of MMP-9/TIMP-1 and MMP-2/TIMP-2 systems in patients with SSc.SearchLight Human MMP Array 1 was used to measure MMPs and TIMPs in 32 SSc patients and 32 matched healthy controls.SSc patients showed higher values of both MMP-9 and TIMP-1 in comparison with controls. The patients with anticentromere antibodies (ACA) positivity showed higher values of MMPs and TIMPs in comparison with either controls or the patients with anti-Scl70-positive antibodies.Results of this investigation suggest that SSc patients with ACA positivity, after a primary fibrogenetic noxa, react with a more abundant release of MMP/TIMP, whereas patients with anti-Scl70 antibody show a normal response. 
 Expression of MMP-7, MMP-9, TIMP-1 and TIMP-2 mRNA in lung tissue of patients with non-small cell lung cancer (NSCLC) and benign pulmonary disease. The expression of matrix metallo-proteinases (MMP-7 and MMP-9) and tissue inhibitors of metalloproteinases (TIMP-1 and TIMP-2), which are involved in the degradation of the extracellular matrix (  ECM  ) and tumor growth, was investigated in normal lung tissue, tissue of benign pulmonary diseases and non-small cell lung cancer (NSCLC) tissue.Tumor tissue and surrounding carcinoma-free lung tissue samples were obtained from 91 patients with NSCLC who had undergone surgery in the years 2005-2007 as well as lung tissue from 12 patients operated on for 'benign' bullous emphysema or interstitial lung disease. The mRNA was isolated from the tissues and the expression of mRNA was assessed using a real-time RT PCR method.Significantly higher expression of MMP-7, MMP-9 and TIMP-1 mRNA was demonstrated in the NSCLC tissue in comparison with the normal lung tissue from the same patients (p=0.0003, p<0.0001 and p=0.0018, respectively). Similar results for MMP-7, MMP-9 and TIMP-1 were found in the histological subgroups: squamous cell lung cancer vs. normal tissue (p=0.0198, p=0.0015 and p=0.0366, respectively), and adenocarcinoma vs. normal tissue (p=0.0045, p<0.0001 and p=0.0140, respectively). The expression of MMP-7 was found to be significantly higher in tumor tissue vs. lung tissue of the benign diseases (p=0.0086) and similar results were also recorded in the histological subgroups: squamous cell lung cancer vs. benign tissue (p=0.0171) and adenocarcinoma vs. benign tissue (p=0.0135). The expression of MMP-9 was significantly higher only in the adenocarcinoma subgroup vs. the benign tissue (p=0.0412). No differences in the expression of mRNA between stage IA and stages IB-IIIB of NSCLC were recorded.Significantly higher expression of MMP-7 and MMP-9 in tumor tissue than in the surrounding tissue or in benign lung disease tissue supports the notion of an important role of these metalloproteinases in the growth of lung carcinoma. TIMP-1 expression is increased only in carcinoma, but not in benign lung disease. 
 [Expression of matrix metalloproteinase-2, 9 and their inhibitor-TIMP 1,2 in human squamous cell carcinoma of uterine cervix]. Studies of tumor invasion and metastasis have focused on the degradation of extracellular matrix (  ECM  ). Matrix metalloproteinases (MMPs), which could degrade   ECM  , were implicated in cancer invasion and metastasis. Activated MMPs were controlled by specific tissue inhibitors of metalloproteinases (TIMPs). This study was designed to investigate the expression of MMP-2, MMP-9 and TIMP-1, TIMP-2 in human squamous cell carcinomas of uterine cervix, and the association with invasion and metastasis of human squamous cell carcinoma of uterine cervix.Tissue samples from 40 cases of squamous cell carcinoma of the uterine cervix, 29 cases of cervical intraepithelial neoplasia (CIN), and 16 cases of normal cervices were stained immunohistochemically.Immunohistochemical staining of tumor cells for MMP-2, MMP-9, TIMP-1, and TIMP-2 were noted 82.5%, 70.0%, 62.5%, and 97.5% in carcinomas, 44.8%, 62.1%, 86.21%, and 96.55% in CIN lesions respectively, for MMP-2, MMP-9, TIMP-1 in 25.0% normal cervices, but for TIMP-2 in 56.25% normal cervices. There was significant difference for MMP-2 among carcinomas, normal cervices, and CIN respectively (P < 0.01). There was significant difference for MMP-9 among normal cervices, carcinomas, and CIN, respectively (P < 0.05). The positive rates of TIMP-1 and TIMP-2 in the cervical carcinoma group and CIN group were obviously higher than that in normal cervical group(P < 0.01). The positive rates of MMP-2 and MMP-9 in lymph node metastasis were obviously higher than that in without lymph node metastasis(P < 0.01), but for TIMP-2 was contrary and there was no significant difference for TIMP-1. No significant correlation could be established among the expression of these markers and the tumor diameter, the clinical stage, and pathologic grade.The over-expression of MMP-2, MMP-9, TIMP-1, and TIMP-2 may play a key role in invasion and lymph-node metastasis of in squamous carcinoma of the cervix. 
 Urinary Metalloproteinases-9 and -2 and Their Inhibitors TIMP-1 and TIMP-2 are Markers of Early and Long-Term Graft Function After Renal Transplantation. Renal ischemia-reperfusion (I-R) injury (IRI) is an inseparable feature of organ transplantation and may have a negative impact on the graft, its function and survival. Acute tubular necrosis, which is reversible thanks to the regenerative capacity of renal tubular epithelial cells, is the main cause of acute renal failure secondary to IRI. MMP-2 and MMP-9 are proteolytic enzymes involved in digesting proteins that are components of the extracellular matrix (  ECM  ) and the basement membrane of the nephrons. This way post-reperfusion MMP activation allows the inflammatory process to spread.In our studies, we focused on identifying whether the concentrations of MMP-2 and MMP-9 and their natural inhibitors TIMP-1 and TIMP-2 in urine sample at day 1 and day 30 as well as after 12 months following renal transplantation are markers of early and long-term renal function during meanly five-years observation. Moreover, in urine sampled at months 6 and 12 after renal transplantation, we determined the content of TGF-β as a graft fibrosis indicator.MMP-9 concentration in the early post-transplant period is a major marker of early and long-term function of the transplanted kidney. Its increased concentration was correlated with lesions related to tubular atrophy and fibrosis in renal biopsies performed at months 3 and 12 after transplantation. Its concentration is correlated with TGF-β content in a later period.TIMP-1 and-2 are primarily markers of an early function of the transplanted kidney. Early post-transplant concentration of MMP-2 is a marker of proteinuria in early and long-term post-transplant periods. 
 Gelatinases and their tissue inhibitors during human ovulation: increased expression of tissue inhibitor of matrix metalloproteinase-1. Remodelling of the extracellular matrix (  ECM  ) of the follicular wall by matrix metalloproteinases (MMPs) and tissue inhibitors of matrix metalloproteinases (TIMPs) has been suggested to be crucial in ovulation. To investigate the expression of the gelatinases, MMP-2 and MMP-9, together with their inhibitors, TIMP-2 and TIMP-1, in the perifollicular ovarian stroma from women just before and during ovulation, we obtained biopsies of the stroma adjacent to the leading follicle. Laparoscopic surgery was performed either before the LH peak or at any of three intervals after ovulation triggering by hCG. Immunoblotting, immunohistochemistry and quantitative RT-PCR were performed. All four proteins were expressed by immunoblots, with no detectable changes in the expression of MMP-2, MMP-9 and TIMP-2. Scattered immunostaining for MMP-9 and TIMP-2 was seen, and MMP-2 was demonstrated in a concentric layer. A significant increase in TIMP-1 protein and mRNA was seen during the three ovulatory phases, and a strong and patchy immunostaining for TIMP-1 was shown. This is the first study that has demonstrated an ovulation-associated expression of these   ECM  -remodelling enzymes around the human follicle at ovulation. The increased expression of TIMP-1 may reflect a specific temporal inhibition of collagenolysis and thereby a time-dependent regulation of   ECM   breakdown in areas surrounding the apex of the follicle. 
 Urinary Metalloproteinases-9 and -2 and Their Inhibitors TIMP-1 and TIMP-2 are Markers of Early and Long-Term Graft Function After Renal Transplantation. Renal ischemia-reperfusion (I-R) injury (IRI) is an inseparable feature of organ transplantation and may have a negative impact on the graft, its function and survival. Acute tubular necrosis, which is reversible thanks to the regenerative capacity of renal tubular epithelial cells, is the main cause of acute renal failure secondary to IRI. MMP-2 and MMP-9 are proteolytic enzymes involved in digesting proteins that are components of the extracellular matrix (  ECM  ) and the basement membrane of the nephrons. This way post-reperfusion MMP activation allows the inflammatory process to spread.In our studies, we focused on identifying whether the concentrations of MMP-2 and MMP-9 and their natural inhibitors TIMP-1 and TIMP-2 in urine sample at day 1 and day 30 as well as after 12 months following renal transplantation are markers of early and long-term renal function during meanly five-years observation. Moreover, in urine sampled at months 6 and 12 after renal transplantation, we determined the content of TGF-β as a graft fibrosis indicator.MMP-9 concentration in the early post-transplant period is a major marker of early and long-term function of the transplanted kidney. Its increased concentration was correlated with lesions related to tubular atrophy and fibrosis in renal biopsies performed at months 3 and 12 after transplantation. Its concentration is correlated with TGF-β content in a later period.TIMP-1 and-2 are primarily markers of an early function of the transplanted kidney. Early post-transplant concentration of MMP-2 is a marker of proteinuria in early and long-term post-transplant periods. 
 TIMP1, TIMP2, and TIMP4 are increased in aqueous humor from primary open angle glaucoma patients. Elevated intraocular pressure (IOP) is the only known modifiable risk factor for primary open angle glaucoma (POAG), and it can be caused by reduced aqueous humor outflow from the anterior chamber. Outflow is predominantly regulated by the trabecular meshwork, consisting of specialized cells within a complex extracellular matrix (  ECM  ). An imbalance between   ECM  -degrading matrix metalloproteinases (MMPs) and the tissue inhibitors of MMPs (TIMPs) within the trabecular meshwork is thought to contribute to POAG. This study aimed to quantify levels of TIMPs and MMPs in aqueous humor samples from glaucomatous and non-glaucomatous eyes, analyze MMP/TIMP ratios, and correlate results with age, IOP, and Humphrey's visual field pattern standard deviation (PSD).Aqueous humor samples were collected from 26 non-glaucomatous control subjects before cataract surgery and 23 POAG patients undergoing trabeculectomy or cataract surgery. Analyte concentrations were measured using multiplexed immunoassays. Statistical significance was assessed with Mann-Whitney U tests, and Spearman's method was used to assess correlations with age, IOP, and PSD.Concentrations of TIMP1 (p = 0.0008), TIMP2 (p = 0.002), TIMP4 (p = 0.002), and MMP2 (p = 0.020) were significantly increased in aqueous humor samples from POAG versus cataract samples. For the majority of MMP/TIMP molar ratios calculated for the cataract group, TIMPs outweighed MMPs. In POAG, molar ratios of MMP2/TIMP1 (p = 0.007) and MMP9/TIMP1 (p = 0.005) showed a significant decrease, corresponding to an elevated excess of TIMPs over MMPs in POAG compared to cataract samples. Conversely, MMP2/TIMP3 (p = 0.045) and MMP3/TIMP3 (p = 0.032) molar ratios increased. Several MMP/TIMP molar ratios correlated with IOP (r = 0.476-0.609, p = 0.007-0.034) and PSD (r = -0.482 to -0.655, p = 0.005-0.046) in POAG samples and with age in cataract control samples.An imbalance among MMPs and TIMPs was found in glaucomatous aqueous humor samples, with a shift toward raised TIMP levels. This may result in the inhibition of MMP activity, leading to an altered   ECM   composition in the TM and thereby contributing to increased outflow resistance. 
 siRNA knockdown of tissue inhibitor of metalloproteinase-1 in keloid fibroblasts leads to degradation of collagen type I. Keloids are defined as overgrowths of scar tissue resulting from abnormal wound healing. They are characterized by excessive dermal deposition of thick, hyalinized collagen bundles resulting from an imbalance between the production and degradation of extracellular matrix (  ECM  ) components. Matrix metalloproteinases (MMPs) and tissue inhibitors of metalloproteinases (TIMPs) are two important regulators of   ECM   degradation and remodeling. To evaluate the role played by knockdown of TIMPs in keloid formation, we transduced human keloid-derived fibroblasts (KFs) with small interfering RNAs targeting TIMP-1 or -2 (siTIMP-1 or siTIMP-2) using a lentiviral vector and assessed the biological effects. We found that MMP-1/TIMP-1 and MMP-1/TIMP-2 complexes were suppressed and that MMP-2 activity was upregulated in KFs expressing siTIMP-1 or siTIMP-2. In addition, increased degradation of collagen type I was observed in the supernatant of KFs expressing siTIMP-1, but not siTIMP-2, with the suppression of cell viability and induction of apoptosis. These results suggest that targeting TIMP-1 using small interfering RNA has significant therapeutic potential as an approach to treating keloids through degradation of their thick collagen bundles. 
 Expression of MMP-7, MMP-9, TIMP-1 and TIMP-2 mRNA in lung tissue of patients with non-small cell lung cancer (NSCLC) and benign pulmonary disease. The expression of matrix metallo-proteinases (MMP-7 and MMP-9) and tissue inhibitors of metalloproteinases (TIMP-1 and TIMP-2), which are involved in the degradation of the extracellular matrix (  ECM  ) and tumor growth, was investigated in normal lung tissue, tissue of benign pulmonary diseases and non-small cell lung cancer (NSCLC) tissue.Tumor tissue and surrounding carcinoma-free lung tissue samples were obtained from 91 patients with NSCLC who had undergone surgery in the years 2005-2007 as well as lung tissue from 12 patients operated on for 'benign' bullous emphysema or interstitial lung disease. The mRNA was isolated from the tissues and the expression of mRNA was assessed using a real-time RT PCR method.Significantly higher expression of MMP-7, MMP-9 and TIMP-1 mRNA was demonstrated in the NSCLC tissue in comparison with the normal lung tissue from the same patients (p=0.0003, p<0.0001 and p=0.0018, respectively). Similar results for MMP-7, MMP-9 and TIMP-1 were found in the histological subgroups: squamous cell lung cancer vs. normal tissue (p=0.0198, p=0.0015 and p=0.0366, respectively), and adenocarcinoma vs. normal tissue (p=0.0045, p<0.0001 and p=0.0140, respectively). The expression of MMP-7 was found to be significantly higher in tumor tissue vs. lung tissue of the benign diseases (p=0.0086) and similar results were also recorded in the histological subgroups: squamous cell lung cancer vs. benign tissue (p=0.0171) and adenocarcinoma vs. benign tissue (p=0.0135). The expression of MMP-9 was significantly higher only in the adenocarcinoma subgroup vs. the benign tissue (p=0.0412). No differences in the expression of mRNA between stage IA and stages IB-IIIB of NSCLC were recorded.Significantly higher expression of MMP-7 and MMP-9 in tumor tissue than in the surrounding tissue or in benign lung disease tissue supports the notion of an important role of these metalloproteinases in the growth of lung carcinoma. TIMP-1 expression is increased only in carcinoma, but not in benign lung disease. 
 Relationship between matrix metalloproteinases/tissue inhibitors of matrix metalloproteinases systems and autoantibody patterns in systemic sclerosis. Systemic sclerosis (SSc) is a connective tissue disease characterized by tissue fibrosis that reflects an imbalance between collagen production and degradation. Matrix metalloproteinases (MMPs) are a family of endopeptidases involved in the remodelling of extracellular matrix (  ECM  ). This activity is controlled by tissue inhibitors of MMP (TIMPs). Aim of this study was the evaluation of MMP-9/TIMP-1 and MMP-2/TIMP-2 systems in patients with SSc.SearchLight Human MMP Array 1 was used to measure MMPs and TIMPs in 32 SSc patients and 32 matched healthy controls.SSc patients showed higher values of both MMP-9 and TIMP-1 in comparison with controls. The patients with anticentromere antibodies (ACA) positivity showed higher values of MMPs and TIMPs in comparison with either controls or the patients with anti-Scl70-positive antibodies.Results of this investigation suggest that SSc patients with ACA positivity, after a primary fibrogenetic noxa, react with a more abundant release of MMP/TIMP, whereas patients with anti-Scl70 antibody show a normal response. 
 Gelatinases and their tissue inhibitors during human ovulation: increased expression of tissue inhibitor of matrix metalloproteinase-1. Remodelling of the extracellular matrix (  ECM  ) of the follicular wall by matrix metalloproteinases (MMPs) and tissue inhibitors of matrix metalloproteinases (TIMPs) has been suggested to be crucial in ovulation. To investigate the expression of the gelatinases, MMP-2 and MMP-9, together with their inhibitors, TIMP-2 and TIMP-1, in the perifollicular ovarian stroma from women just before and during ovulation, we obtained biopsies of the stroma adjacent to the leading follicle. Laparoscopic surgery was performed either before the LH peak or at any of three intervals after ovulation triggering by hCG. Immunoblotting, immunohistochemistry and quantitative RT-PCR were performed. All four proteins were expressed by immunoblots, with no detectable changes in the expression of MMP-2, MMP-9 and TIMP-2. Scattered immunostaining for MMP-9 and TIMP-2 was seen, and MMP-2 was demonstrated in a concentric layer. A significant increase in TIMP-1 protein and mRNA was seen during the three ovulatory phases, and a strong and patchy immunostaining for TIMP-1 was shown. This is the first study that has demonstrated an ovulation-associated expression of these   ECM  -remodelling enzymes around the human follicle at ovulation. The increased expression of TIMP-1 may reflect a specific temporal inhibition of collagenolysis and thereby a time-dependent regulation of   ECM   breakdown in areas surrounding the apex of the follicle. 
 Circulating matrix metalloproteinase-9 and tissue inhibitors of metalloproteinases-1 and -2 levels in gestational hypertension. Gestational hypertension (GH) is dangerous to both mother and child. Arterial invasiveness and growth are dependent on successful extracellular matrix (  ECM  ) breakdown, which may be abnormal in GH. We hypothesized abnormalities in circulating matrix metalloproteinase-9 (MMP-9) and tissue inhibitors of metalloproteinases-1 and -2 (TIMP-1 and TIMP-2, respectively) in patients with GH, when compared with normotensive women with normal pregnancies and healthy nonpregnant control subjects.Plasma MMP-9, TIMP-1, and TIMP-2 were measured by ELISA in 23 women with GH, 30 normotensive pregnant women, and 28 nonpregnant women who were matched for age, gestational age, and parity.Levels of circulating MMP-9, TIMP-1 and TIMP-2, and the MMP-9/TIMP-1 and MMP-9/TIMP-2 ratios were significantly different among the three groups (P = .026, P = .006, P = .007, P = .001 and P = .008 respectively). Within the GH group, MMP-9 and the MMP-9/TIMP-1 ratio correlated negatively with age (r = -0.581, P = .004 and r = -0.563, P = .005, respectively) and levels of diastolic blood pressure (r = -0.432, P = .040 and r = -0.461, P = .027, respectively). With multiple regression analysis, only age independently correlated with circulating levels of MMP-9 (P = .010); neither age nor levels of diastolic blood pressure had any effect on the MMP-9/TIMP-1 ratio.We have demonstrated altered MMP/TIMP ratios in maternal blood during GH. These observations suggest pregnancy-related changes in   ECM   breakdown and turnover. Given the importance of changes in   ECM   composition to vascular and cardiac structure in hypertension, we suggest that these observations may be related to the pathophysiology of human GH. 
 [Expression of matrix metalloproteinase-2, 9 and their inhibitor-TIMP 1,2 in human squamous cell carcinoma of uterine cervix]. Studies of tumor invasion and metastasis have focused on the degradation of extracellular matrix (  ECM  ). Matrix metalloproteinases (MMPs), which could degrade   ECM  , were implicated in cancer invasion and metastasis. Activated MMPs were controlled by specific tissue inhibitors of metalloproteinases (TIMPs). This study was designed to investigate the expression of MMP-2, MMP-9 and TIMP-1, TIMP-2 in human squamous cell carcinomas of uterine cervix, and the association with invasion and metastasis of human squamous cell carcinoma of uterine cervix.Tissue samples from 40 cases of squamous cell carcinoma of the uterine cervix, 29 cases of cervical intraepithelial neoplasia (CIN), and 16 cases of normal cervices were stained immunohistochemically.Immunohistochemical staining of tumor cells for MMP-2, MMP-9, TIMP-1, and TIMP-2 were noted 82.5%, 70.0%, 62.5%, and 97.5% in carcinomas, 44.8%, 62.1%, 86.21%, and 96.55% in CIN lesions respectively, for MMP-2, MMP-9, TIMP-1 in 25.0% normal cervices, but for TIMP-2 in 56.25% normal cervices. There was significant difference for MMP-2 among carcinomas, normal cervices, and CIN respectively (P < 0.01). There was significant difference for MMP-9 among normal cervices, carcinomas, and CIN, respectively (P < 0.05). The positive rates of TIMP-1 and TIMP-2 in the cervical carcinoma group and CIN group were obviously higher than that in normal cervical group(P < 0.01). The positive rates of MMP-2 and MMP-9 in lymph node metastasis were obviously higher than that in without lymph node metastasis(P < 0.01), but for TIMP-2 was contrary and there was no significant difference for TIMP-1. No significant correlation could be established among the expression of these markers and the tumor diameter, the clinical stage, and pathologic grade.The over-expression of MMP-2, MMP-9, TIMP-1, and TIMP-2 may play a key role in invasion and lymph-node metastasis of in squamous carcinoma of the cervix. 
 Identification of the extracellular matrix (  ECM  ) binding motifs of tissue inhibitor of metalloproteinases (TIMP)-3 and effective transfer to TIMP-1. Tissue inhibitor of metalloproteinases (TIMPs) are the endogenous inhibitors of the zinc-dependent endopeptidases of the matrix metalloproteinase families. There are four mammalian TIMPs (TIMP-1 to -4) but only TIMP-3 is sequestered to the extracellular matrix (  ECM  ). The molecular basis for the TIMP-3:  ECM   association has never been fully investigated until now. In this report, we identify the unique amino acid configuration that constitutes the basis of the   ECM   binding motif in TIMP-3. By systematically exchanging the subdomains of the TIMPs and exhaustive mutation of TIMP-3, we have identified the surface residues directly responsible for   ECM   association. Contrary to the accepted view, we have found that TIMP-3 interacts with the   ECM   via both its N- and C-terminal domains. The amino acids involved in   ECM   binding are all basic in nature: Lys-26, Lys-27, Lys-30, Lys-76 of the N-terminal domain and Arg-163, Lys-165 of the C-terminal domain. Replacement of these residues with glutamate (E) and glutamine (Q) (K26/27/30/76E + R163/K165Q) resulted in a soluble TIMP-3 devoid of   ECM  -adhering ability. Using the   ECM   binding motif derived from TIMP-3, we have also created a TIMP-1 mutant (K26/27/30 + K76 transplant) capable of   ECM   association. This is the first instance of TIMPs being intentionally rendered soluble or   ECM  -bound. The ability to prepare TIMPs in soluble or   ECM  -bound forms also opens new avenues for future TIMP research. 
 Expression of vascular endothelial factor-A, gelatinases (MMP-2, MMP-9) and TIMP-1 in uterine leiomyomas. Leiomyomas growth involves cellular hypertrophy, modulation of mitotic activity and upregulation of extracellular matrix (  ECM  ). Vascular factors and matrix metalloproteinases (MMPs) play a coordinated role during neoplasia and tissue remodeling. The present study investigates the role of angiogenic factor vascular endothelial growth factor (VEGF)-A with the activity of main gelatinases, MMP-2/MMP-9 and their tissue inhibitor TIMP-1 in patients with leiomyomas.Peripheral blood of 46 women with uterine leiomyomas was obtained prior hysterectomy to assess VEGF-A, MMP-2, -9, TIMP-1 levels by enzyme-linked immunosorbent assay compared to 39 healthy controls. Protein expression levels of VEGF-A, MMP-2 and MMP-9 were evaluated by western immunoblotting and immunohistochemistry in leiomyomas tissue specimens after hysterectomy. Furthermore, the activity of gelatinases in leiomyoma tissue extracts and control myometrium was evaluated by semi-quantitative zymography.Circulating levels of VEGF-A, MMP-2 and TIMP-1 were significantly elevated in leiomyoma patients compared to controls (p<0.001, p=0.004, p=0.003, respectively). A positive correlation was found between VEGF-A and MMP-2 (p=0.021) as well as MMP-9 (p=0.001) peripheral levels in the patient's group. Furthermore, increased VEGF-A protein levels were detected in leiomyoma tissue compared to control myometrium, followed by increased localization of both VEGF-A and MMP-2 in the   ECM   embedding bundles of smooth muscle cells of leiomyomas. The activity of MMP-2 was significantly higher in leiomyomas than normal myometrium in all investigated tissues.This study demonstrates a possible coordinated role of VEGF-A and MMP-2 during uterine leiomyomas growth and angiogenesis with potential prognostic significance. 
 Evaluation of metalloproteinases 2 and 9 and their inhibitors in physiologic and pre-eclamptic pregnancy. Matrix metalloproteinases (MMPs) are a family of zinc and calcium-dependent endopeptidases involved in remodeling and physiological homeostasis of extracellular matrix (  ECM  ). The metalloproteinases activity is predominantly modulated by specific tissue inhibitors of matrix metalloproteinases (TIMPs). The balance between MMPs and TIMPs is likely to play an important role in remodeling uterine arteries in pregnancy, and it may represent means by which vasodilatation is maintained in later pregnancy. Moreover, increased levels of MMPs and in particular MMP-2 play a role in the vascular alterations induced by hypertension. The aim of this study was the evaluation of MMP-2 and -9, along with their inhibitors TIMP-1 and -2, in pre-eclamptic women compared with normotensive pregnancy and non-pregnant women. Fourteen pre-eclamptic women were compared with 37 normotensive women in different gestational age and 21 non-pregnant women. Multiplexed sandwich enzyme-linked immunosorbent assay was used to measure MMPs and TIMPs simultaneously. MMP-2 levels were significantly higher in pre-eclamptic women vs. both non-pregnant and physiologic pregnant women. MMP-9 concentrations were significantly higher in physiologic pregnant vs. non-pregnant women. The serum levels of TIMP-1 were significantly higher in pre-eclamptic vs. both non-pregnant and physiologic pregnant women. TIMP-2 values were higher in physiologic pregnant women and pre-eclamptic women vs. non-pregnant women. A positive correlation between MMP-9 values and gestational age was observed in normal pregnant women. Results of the present investigation confirm that MMP-2 and TIMP-1 values are significantly higher in preeclampsia. We confirm that the modification of the fine balance between MMPs and their inhibitors plays a greater role in the structural and functional vascular changes of women with complicated pregnancies. 
 TIMP-1 regulates cell proliferation by interacting with the ninth zinc finger domain of PLZF. The tissue inhibitors of metalloproteinases (TIMPs) are multifunctional proteins that specifically inhibit matrix metalloproteinases (MMPs) and regulate extracellular matrix (  ECM  ) turnover and tissue remodeling. This is directed by forming tightly bound inhibitory complexes with MMPs. Recent years have revealed important differences of various biological activities between TIMP families but molecular mechanisms are not clear. To define the molecular mechanisms of TIMP-1-dependent biological processes, we used TIMP-1 as bait in a yeast two-hybrid screen, along with a human ovary cDNA library. Further characterization revealed the ninth zinc finger domain as an interacting domain of the promyelocytic leukemia zinc finger protein (PLZF). Interaction of PLZF with TIMP-1 in mammalian cells was also confirmed by co-immunoprecipitation and with in vitro binding assays. We investigated whether TIMP-1-mediated anti-apoptotic activity could promote the growth of ovarian cancer in an experimental model system. TIMP-1 treatment was found to be more effective at increasing ovarian cancer growth when compared with PLZF in parallel experiments. Subsequently, the efficacy of a combined treatment with TIMP-1 and PLZF was investigated. In the presence of both of these proteins, TIMP-1 significantly reduced apoptosis induced by PLZF in cervical carcinoma cells. These combined results indicate that TIMP-1 functions as an anti-activator of the transcriptional repressive activity of PLZF. 
 Levels of matrix metalloproteinase-9 and its inhibitor in bronchoalveolar lavage cells of asthmatic children. Our objective was to investigate the role of matrix metalloproteinase-9 (MMP-9) and tissue inhibitor of matrix metalloproteinase-1 (TIMP-1) and their cellular sources in childhood asthma. We used 12 controls and 16 asthmatic children. The levels of MMP-9 and TIMP-1 in bronchoalveolar lavage (BAL) cells of asthmatic children were measured immunocytochemically. The positive level index, defined as the percentage of positive-stained cells x average optical density, was used to assess the expressing levels of MMP-9 and TIMP-1. The percentages of eosinophils and mast cells in bronchoalveolar lavage fluid (BALF) of asthmatic children were increased. Levels of MMP-9 and TIMP-1 in BAL cell of asthmatic children were increased significantly at about 30- and 35-fold relative to the controls, respectively. These results suggest that both MMP-9 and TIMP-1 contribute to tissue remodeling. MMP-9, which mediates the degradation of extracellular matrix (  ECM  ), is increased significantly in the early or acute stage and may play a role in   ECM   degeneration. Excessive TIMP-1 may be synthesized following MMP-9 production when the body tries to repair the damage, which results in excessive deposition of   ECM   component. 
 ERK2-regulated TIMP1 induces hyperproliferation of K-Ras(G12D)-transformed pancreatic ductal cells. Pancreatic ductal adenocarcinoma (PDAC) commonly contains a mutation in K-Ras(G12D) and is characterized by a desmoplastic reaction composed of deregulated, proliferating cells embedded in an abnormal extracellular matrix (  ECM  ). Our previous observations imply that inhibiting the mitogen-activated protein kinase (MAPK)-extracellular signal-regulated kinase (ERK2) kinase signal pathway reverses a matrix metalloproteinase 1-specific invasive phenotype. Here, we investigated the specific genes downstream of MAPK-ERK2 responsible for the hyperproliferative abilities of human and murine primary ductal epithelial cells (PDCs) within an   ECM  . Compared with control, DNA synthesis and total cell proliferation was significantly increased in human PDCs harboring the PDAC common p53, Rb/p16(INK4a), and K-Ras (G12D) mutations. Both of these effects were readily reversed following small-molecule inhibition or lentiviral silencing of ERK2. Microarray analysis of PDCs in three-dimensional (3D) culture revealed a unique, MAPK-influenced gene signature downstream of K-Ras (G12D). Unbiased hierarchical analysis permitted filtration of tissue inhibitor of matrix metalloproteinase 1 (TIMP1). Pancreatic cells isolated from Pdx1-Cre; LSL-K-ras(G12D/+)-mutated mice exhibit increased TIMP1 RNA transcription compared to wild-type littermate controls. Analyses of both 3D, in vitro human K-Ras (G12D) PDCs and data mining of publicly annotated human pancreatic data sets correlatively indicate increased levels of TIMP1 RNA. While silencing TIMP1 did not significantly effect PDC proliferation, exogenous addition of human recombinant TIMP1 significantly increased proliferation but only in transformed K-Ras (G12D) PDCs in 3D. Overall, TIMP1 is an upregulated gene product and a proliferative inducer of K-Ras(G12D)-mutated PDCs through the ERK2 signaling pathway. 
 Proteasome blockade exerts an antifibrotic activity by coordinately down-regulating type I collagen and tissue inhibitor of metalloproteinase-1 and up-regulating metalloproteinase-1 production in human dermal fibroblasts. Tissue fibrosis results when dysregulation of extracellular matrix (  ECM  ) turnover favors deposition of collagen and other   ECM   proteins over degradation. Fibrosis may then lead to organ dysfunction and pathology as observed in systemic sclerosis (SSc). In the present study, we investigated the antifibrotic properties of proteasome blockade. A dose- and time-dependent reduction in type-I collagen and tissue inhibitor of metalloproteinase-1 (TIMP-1) production was observed in normal fibroblasts exposed to proteasome inhibitors (PI). In the same culture conditions, metalloproteinase-1 (MMP-1) protein and the collagenolytic activity on type I collagen was increased. The steady-state mRNA levels of COL1A1, TIMP-1, and MMP-1 paralleled protein levels. These effects were dominant over the profibrotic properties of TGF-beta and were observed with fibroblasts generated from normal and SSc skin. PI decreased type I collagen mRNA levels with kinetics similar to those observed with DRB, a specific RNA polymerase II inhibitor, thus indicating transcriptional inhibition. Of interest, PI induced c-Jun phosphorylation and c-Jun nuclear accumulation. The specific N-terminal Jun-kinase inhibitor SP-600125 selectively abrogated c-Jun phosphorylation and, in a dose-dependent fashion, the up-regulated synthesis of MMP-1 induced by PI. Finally, PI did not affect fibroblast viability. Thus, the coordinated down-regulation of collagen and TIMP-1 and up-regulation of MMP-1 renders proteasome blockade an attractive strategy for treating conditions as SSc, characterized by excessive fibrosis. 
 MiR-29a reduces TIMP-1 production by dermal fibroblasts via targeting TGF-β activated kinase 1 binding protein 1, implications for systemic sclerosis. Systemic sclerosis (SSc) is an autoimmune connective tissue disease characterised by skin and internal organs fibrosis due to accumulation of extra cellular matrix   (EC  M) proteins. Tissue inhibitor of metalloproteinases 1 (TIMP-1) plays a key role in   EC  M deposition.To investigate the role of miR-29a in regulation of TAB1-mediated TIMP-1 production in dermal fibroblasts in systemic sclerosis.Healthy control (HC) and SSc fibroblasts were cultured from skin biopsies. The expression of TIMP-1, MMP-1 and TGF-β activated kinase 1 binding protein 1 (TAB1) was measured following miR-29a transfection using ELISA, qRT-PCR, and Western Blotting. The functional effect of miR-29a on dermal fibroblasts was assessed in collagen gel assay. In addition, HeLa cells were transfected with 3'UTR of TAB1 plasmid cloned downstream of firefly luciferase gene to assess TAB1 activity. HC fibroblasts and HeLa cells were also transfected with Target protectors in order to block the endogenous miR-29a activity.We found that TAB1 is a novel target gene of miR-29a, also regulating downstream TIMP-1 production. TAB1 is involved in TGF-β signal transduction, a key cytokine triggering TIMP-1 production. To confirm that TAB1 is a bona fide target gene of miR-29a, we used a TAB1 3'UTR luciferase assay and Target protector system. We showed that miR-29a not only reduced TIMP-1 secretion via TAB1 repression, but also increased functional MMP-1 production resulting in collagen degradation. Blocking TAB1 activity by pharmacological inhibition or TAB1 knockdown resulted in TIMP-1 reduction, confirming TAB1-dependent TIMP-1 regulation. Enhanced expression of miR-29a was able to reverse the profibrotic phenotype of SSc fibroblasts via downregulation of collagen and TIMP-1.miR-29a repressed TAB1-mediated TIMP-1 production in dermal fibroblasts, demonstrating that miR-29a may be a therapeutic target in SSc. 
 Analysis of TIMP-1 gene polymorphisms in Italian sclerodermic patients. Systemic sclerosis (SSc) is an autoimmune disease characterized by skin and internal organs fibrosis due to an extracellular matrix (  ECM  ) accumulation of type I collagen. The turnover of the   ECM   is dependent on the balance between matrix metalloproteinases (MMPs) and tissue inhibitors of matrix metalloproteinases (TIMPs). The disruption of this balance is involved in SSc because higher serum TIMP-1 levels have been demonstrated in SSc patients than in controls. On this basis, we analyzed three polymorphisms: -19A>G, +261C>T, and +372T>C of the TIMP-1 gene in SSc patients (67 females, eight males) and controls (29 females, nine males). The C allele of the +372T>C single nucleotide polymorphism (SNP) was observed at a higher frequency in male patients than in healthy individuals (P=0.02), while no differences were observed in the female subjects. Our findings suggest that the +372T>C polymorphism of the TIMP-1 gene is associated with SSc in male individuals. No association with the clinical characteristics of SSc Italian patients and TIMP-1 gene polymorphisms was observed. Thus, the role of TIMP-1 gene in predisposition to SSc remains controversial. 
 The endogenous protease inhibitor TIMP-1 mediates protection and recovery from cutaneous photodamage. UVB exposure is well known to induce skin photodamage and photoaging that correlates with qualitative and quantitative deterioration of the dermal extracellular matrix (  ECM  ) because of the upregulation of matrix metalloproteinases (MMPs). Although inhibitory effects of tissue inhibitor of metalloproteinases (TIMPs) on most MMPs have been reported, the protective role of TIMP-1 against photodamage is poorly understood. To address this, TIMP-1 function was augmented or abolished in a human skin xenograft photodamage model after the confirmation of significantly diminished TIMP-1 expression both in photoaged and intrinsically aged skins. During a chronic UVB exposure regimen, pre-treatment with a lentiviral vector overexpressing TIMP-1 or concomitant administration of an anti-TIMP-1-neutralizing antibody (NAB) led to photoprotection or more severe photodamage, respectively. Overexpression of TIMP-1 resulted in significant inhibition of UVB-induced   ECM   degradation, as well as suppression of decreased skin elasticity and roughness, whereas the NAB-mediated inhibition of TIMP-1 had opposite effects. Furthermore, UVB-induced production of the pro-inflammatory cytokine, tumor necrosis factor α, was inhibited by TIMP-1 treatment of human keratinocytes. Taken together, these data shed light on the important role of TIMP-1 in protection and recovery from cutaneous photodamage because of its suppression of   EC  M degradation and inflammation. 
 TIMP-1 expression induced by IL-32 is mediated through activation of AP-1 signal pathway. Hepatic fibrosis is a necessarily stage from the progression of chronic liver diseases to cirrhosis, even hepatocellular carcinoma (HCC). Hepatic fibrosis is characterized by the progressive accumulation of extracellular matrix (  ECM  ). The balance between   ECM   production and degradation is mediated by matrix metalloproteinases (MMPs) and tissue inhibitors of metalloproteinases (TIMPs). TIMP-1 is an important regulator in the synthesis and degradation of   ECM  . IL-32, a multi-function cytokine, could induce IL-1β, IL-6, IL-8 and other cytokine expressions by activating AP-1, NF-κβ, p38MAPK signal pathways. IL-32 expression is increased in liver tissues of hepatic fibrosis. However, the role of IL-32 in the pathogenesis of liver fibrosis is not thoroughly clear. Recently, it is demonstrated that TIMP-1 expression is induced by the activation of AP-1 signal pathway. So we assayed the effect of IL-32 on TIMP-1 expression by LX-2 cells (one of HSCs cell lines) in the present study. We found that IL-32 could induce TIMP-1 expression by LX-2 cells at a dose-dependent manner. IL-32 could increase TIMP-1 promoter activity and induce TIMP-1 expression by activating AP-1 signal pathway. Moreover, the increase of TIMP-1 expression could promote the migration of LX-2 cells. In conclusion, we believe that IL-32 might be involved in the pathogenesis of hepatic fibrosis by inducing TIMP-1 expression. 
 Increased expression of matrix metalloproteinase-9 in the eutopic endometrial tissue of women with endometriosis. Endometriosis is a disease where endometrial tissue implants in ectopic locations. Remodelling of the extracellular matrix (  ECM  ) is a prerequisite for the implantation of this tissue to be possible.In this study, we detected immunoreactive matrix metalloproteinase-9 (MMP-9) throughout endometrial tissue and identified von Willebrand factor (vWF)-positive endothelial cells, CD45-positive leukocytes, CD3-positive T lymphocytes and CD68-positive macrophages as cells expressing MMP-9 in the stroma.We found an increased expression of MMP-9 in the uterine endometrial tissue of women with endometriosis, as assessed by zymography and enzyme-linked immunosorbent assay (ELISA) (P < 0.05). However, RT-PCR did not show a statistically significant increase in MMP-9 mRNA expression in these tissues (P = 0.14). There was no significant difference between women with and without endometriosis in the expression of tissue inhibitor of MMPs (TIMP)-1, a known natural inhibitor of the pro- and active forms of MMP-9, whether tested by ELISA or by RT-PCR (P = 0.46 and 0.37, respectively). Interestingly, the ratio of MMP-9/TIMP-1 expression was significantly higher in women with endometriosis than in normal women both at the protein and the mRNA levels (P < 0.05).These findings make plausible the involvement of MMP-9/TIMP-1 imbalance in the invasiveness of the endometrial tissue of patients with endometriosis and the ectopic development of the disease. 
 Association of a high activity of matrix metalloproteinase-9 to low levels of tissue inhibitors of metalloproteinase-1 and -3 in human hepatitis B-viral hepatoma cells. Matrix metalloproteinases (MMPs) play a major role in the turnover of extracellular matrix (  ECM  ) during cancer invasion and metastasis, and tissue inhibitors of metalloproteinases (TIMPs) control MMPs, thus maintaining a balanced   ECM   catabolism under physiological conditions. The aim of this study was to assess the behavior of some MMPs (FASEB J., 7, 1993, 1434; Cancer Metastasis Rev., 9(4) 1990, 289) and TIMPs (Biochem. Biophys. Res. Commun., 301, 2003, 1069; FASEB J., 7, 1993, 1434; Nature, 370, 1994, 61). Competitive RT-PCR, gelatin-substrate zymography, and ELISA techniques were used for quantification. The hepatitis B virus (HBV)-DNA-containing hepatocellular carcinoma cell lines, Hep3B, HepG2-HBV and HFF-T2 contain highly activated matrix metallproteinase-9 (MMP-9), which is rarely found in normal liver cell lines such as the Chang lines. MMP-9 activities of HFH-T2, HepG2-HBV and Hep3B were significantly higher than that of non-HBV-hepatocellular carcinoma SK-Hep1 and HepG2 (HCC origin, HBV not detected), as assayed by gelatin zymography. Low levels of TIMP-1 and TIMP-3 were observed in HFH-T2, HepG2-HBV and Hep3B, while the TIMP-2 level was high, as evidenced by reverse zymography. In contrast, 3 TIMP-1, -2 and -3 were largely detected in Chang, HepG2 and SK-Hep1 cells. To investigate the nature of the quantitative regulation of MMPs and TIMPs for these cell lines at the transcriptional levels, a reverse transcriptase-polymerase chain reaction (RT-PCR) was carried out. Not only MMP-9 mRNAs of HFH-T2, HepG2-HBV and Hep3B but also MMP-9 mRNA of SK-Hep1 and HepG2 were highly expressed with no major differences among these four cell lines. However, TIMP-1 and TIMP-3 mRNAs of HFH-T2, HepG2-HBV and Hep3B were markedly reduced, while those of SK-Hep1, HepG2 and Chang cells were maintained at high levels. Finally, an invasion assay using matrigel indicated in an increase in invasiveness in HFH-T2, HepG2-HBV and Hep3B cells, but a decrease in invasiveness of Chang, HepG2 and SK-Hep1 cells. These results indicate that the overexpression of MMP-9 mRNAs and the suppression of TIMP-1 and TIMP-3 in HFH-T2, HepG2-HBV and Hep3B were the result of HBV transfection. Based on these results, it is concluded that HBV affects the malignance of hepatocellular cancer by elevating MMP-9 activity, and suppressing TIMP-1 and TIMP-3. 
 Modulation of u-PA, MMPs and their inhibitors by a novel nutrient mixture in pediatric human sarcoma cell lines. Pediatric sarcomas are highly aggressive tumors that are characterized by high levels of matrix metalloproteinase (MMP)-2 and -9 secretions that degrade the   ECM   and basement membrane, allowing cancer cells to spread to distal organs. Proteases play a key role in tumor cell invasion and metastasis by digesting the basement membrane and   ECM   components. Strong clinical and experimental evidence demonstrates association of elevated levels of u-PA and MMPs with cancer progression, metastasis and shortened patient survival. MMP activities are regulated by specific tissue inhibitors of metalloproteinases (TIMPs). Our main objective was to study the effect of a nutrient mixture (NM) on activity of u-PA, MMPs and TIMPs in various human pediatric sarcomas. Human osteosarcoma MNNG-HOS, osteosarcoma U-2OS and rhabdomyosarcoma RD cell lines (ATCC) were cultured in their respective media and treated at confluence with NM at 0, 50, 100, 250, 500 and 1,000 µg/ml. Analysis of u-PA activity was carried out by fibrin zymography, MMPs by gelatinase zymography and TIMPs by reverse zymography. All sarcoma cell lines studied expressed u-PA, which was inhibited by NM in a dose-dependent manner. On gelatinase zymography, osteosarcoma MNNG-HOS showed a band corresponding to MMP-2 and induction of MMP-9 with PMA (100 ng/ml) treatment. U-2OS osteosarcoma cells showed strong bands corresponding to inactive MMP-2 and MMP-9 and faint bands corresponding to active MMP-2 and MMP-9 dimer; PMA treatment enhanced MMP-9 and MMP-9 dimer activity. Rhabdomyosarcoma showed MMP-2 and faint MMP-9 bands; PMA treatment enhanced MMP-9 expression. NM inhibited their expression in a dose-dependent manner. Activity of TIMPs was upregulated by NM in all cancer cell lines in a dose-dependent manner. Analysis revealed a positive correlation between u-PA and MMPs and a negative correlation between u-PA/MMPs and TIMPs. These findings suggest the therapeutic potential of NM in treatment of pediatric sarcomas. 
 Expression of tissue inhibitors of metalloproteinase 1 (TIMP-1) in gastric cancer tissue. Degradation of extracellular matrix (  ECM  ) is an essential step of invasion and metastasis of gastric cancer. The proteolysis of basement membranes depends on the balance between activities of matrix metalloproteinases (MMPs) and their tissue inhibitors (TIMPs). The aim of the study was to assess the expression of TIMP-1 in gastric cancer (GC) and interstitial inflammatory infiltrate cells within GC tissue in relation to clinico-pathological features of tumor and to estimate the prognostic significance of TIMP-1 expression for patients' survival. The presence of TIMP-1 in 54 cases of gastric cancer samples was investigated by immunohistochemistry. The expression of TIMP-1 in cancer and interstitial inflammatory infiltrate cells was evaluated in semi-quantitative scale. The immunoreactivity of TIMP-1 in cancer and inflammatory cells was positive in 100% of cases and varied from weak to intense reaction. The intensity of TIMP-1 expression increased with more advanced tumor stages and in patients who died of cancer during 2-year observation. TIMP-1 expression in interstitial inflammatory infiltrate cells was the independent prognostic factor for patients' survival. The results suggest the role of TIMP-1 in gastric tumorigenesis, although this issue requires further investigtions. 
 A novel mechanism of tissue inhibitor of metalloproteinases-1 activation by interleukin-1 in primary human astrocytes. Reactive astrogliosis is the gliotic response to brain injury with activated astrocytes and microglia being the major effector cells. These cells secrete inflammatory cytokines, proteinases, and proteinase inhibitors that influence extracellular matrix (  ECM  ) remodeling. In astrocytes, the expression of tissue inhibitor of metalloproteinases-1 (TIMP-1) is up-regulated by interleukin-1 (IL-1), which is a major neuroinflammatory cytokine. We report that IL-1 activates TIMP-1 expression via both the IKK/NF-kappaB and MEK3/6/p38/ATF-2 pathways in astrocytes. The activation of the TIMP-1 gene can be blocked by using pharmacological inhibitors, including BAY11-7082 and SB202190, overexpression of the dominant-negative inhibitor of NF-kappaB (IkappaBalphaSR), or by the knock-down of p65 subunit of NF-kappaB. Binding of activated NF-kappaB (p50/p65 heterodimer) and ATF-2 (homodimer) to two novel regulatory elements located -2.7 and -2.2 kb upstream of the TIMP-1 transcription start site, respectively, is required for full IL-1-responsiveness. Mutational analysis of these regulatory elements and their weak activity when linked to the minimal tk promoter suggest that cooperative binding is required to activate transcription. In contrast to astrocytes, we observed that TIMP-1 is expressed at lower levels in gliomas and is not regulated by IL-1. We provide evidence that the lack of TIMP-1 activation in gliomas results from either dysfunctional IKK/NF-kappaB or MEK3/6/p38/ATF-2 activation by IL-1. In summary, we propose a novel mechanism of TIMP-1 regulation, which ensures an increased supply of the inhibitor after brain injury, and limits   ECM   degradation. This mechanism does not function in gliomas, and may in part explain the increased invasiveness of glioma cells. 
 Genetic polymorphisms of the matrix metalloproteinase-3 (MMP-3) and tissue inhibitors of matrix metalloproteinases-1 (TIMP-1) modulate the development of ankylosing spondylitis. The aetiology of ankylosing spondylitis (AS) remains unclear. Inflammation progresses to fibrosis and calcification of the spine and sacroiliac joints in AS development. Fibrosis results from excessive accumulations of the extracellular matrix (  ECM  ).   ECM   turnover depends on the balance between matrix metalloproteinases (MMPs) and their tissue inhibitors (TIMPs).To evaluate the effects of the MMP-3 -1171 and TIMP-1 372 T>C polymorphisms on the modified risk of AS.Genotypes of 241 patients with AS and 241 controls were identified by PCR. Disease activity and functional status were assessed by the Bath Ankylosing Spondylitis Activity Index (BASDAI), the Bath Ankylosing Spondylitis Functional Index (BASFI) and the Bath Ankylosing Spondylitis Global (BAS-G) Score.MMP-3 6A/6A carriers had a 2.41-fold (95% confidence interval (CI) 1.55 to 3.74) increased risk of AS compared with 6A/5A and 5A/5A carriers. TIMP-1 C alleles had a greater risk of AS, but this was not significant (odds ratio (OR) = 1.28, 95% CI 0.92 to 1.77). Pairwise analysis of the MMP-3/TIMP-1 alleles showed that 6A/C (OR = 3.23, 95% CI 1.50 to 6.95) and 6A/T (OR = 2.55, 95% CI 1.17 to 5.54) had a significantly greater risk of AS than the 5A/T alleles. After adjustment for the effects of age, gender and disease duration, the MMP-3/TIMP-1 5A/T alleles had the lowest BASDAI (p = 0.02), BASFI (p = 0.05) and BAS-G (p = 0.02) among all MMP-3/TIMP-1 alleles.The findings highlight the importance of the MMP-3 and TIMP-1 genes as crucial elements in AS development. 
 Expression profiles of MMP-1 and TIMP-1 in lumbar intervertebral disc degeneration. Lumbar intervertebral disc degeneration (IDD) is a common clinical pathology and has become a focus for research in recent years. Matrix metalloproteinases (MMPs) are enzymes responsible for the degradation of almost all extracellular matrix proteins (  ECM  ). The over-expression of MMPs or tissue inhibitors of metalloproteinases (TIMPs) may disrupt the dynamic balance of the   ECM  . Therefore, in the current study, the expression levels of MMP-1 and TIMP-1 in lumbar IDD patients were evaluated in an attempt to elucidate their role in IDD pathogenesis and progression. In total, 60 IDD patients were recruited as the experimental group, along with 20 cases of lumbar vertebral injury without disc degeneration as the control group. Preoperative venous blood samples were collected, and intervertebral disc tissues were collected from the lesion during surgery. Serum and tissue levels of MMP-1 and TIMP-1 were quantified by enzyme-linked immunosorbent assay and immunohistochemical staining, respectively. Serum and tissue MMP-1 levels in IDD patients were significantly higher than those in the control group (P < 0.05). Additionally, sub-group analysis revealed that severe IDD patients had higher MMP-1 levels compared with mild or moderate IDD patients (P < 0.05). However, there were no significant differences in TIMP- 1 levels in either the serum or tissues of IDD patients compared to patients in the control group (P > 0.05). These results demonstrate that MMP-1 expression is increased in IDD, with higher expression observed in more severe cases, whereas TIMP-1 expression was similarly expressed in both normal and degenerated discs. 
 Differential expression of stromal MMP-1, MMP-9 and TIMP-1 in basal cell carcinomas of immunosuppressed patients and controls. Matrix metalloproteinases (MMPs) have an important role in the initiation, growth, and invasion of malignant tumors. Basal cell cancer (BCC) is the most common human malignancy. The risk of BCC is 10-16 times higher among organ transplant recipients compared with the nontransplanted population. The aim of this study was to compare the expression of several MMPs and their tissue inhibitors (TIMPs) in BCCs from kidney transplant recipients and controls. Expression of MMPs-1, -7, -8, -9, -10, -13, -26, and TIMPs-1 and -3 was evaluated by immunohistochemistry in 25 samples of BCC of kidney transplant recipients and 25 matched controls representing superficial and nodular subtypes. No significant differences were detected in MMP expression of BCC tumor cells between immunocompetent and immunodeficient patients. However, MMPs-1 and -9 and TIMP-1 were expressed more frequently in stromal macrophages in the BCCs of immunocompetent patients. When tumor subtypes were compared irrespective of the patient group, more MMP-1-positive fibroblasts and MMP-9-positive neutrophils were detected in the superficial subtype, while stromal MMP-10 expression was more abundant in nodular tumors. Our results suggest that abundant peritumoral expression of TIMP-1 in non-immunocompromised patients limits   ECM   degradation permissive for cancer cell migration. 
 Prostaglandin E2-dependent enhancement of tissue inhibitors of metalloproteinases-1 production limits dendritic cell migration through extracellular matrix. Dendritic cell (DC) migration is crucial for the initiation of immune responses. The balance between metalloproteinases (MMP) and tissue inhibitors of metalloproteinases (TIMP) has been shown to modulate DC migration. PGE2, which is overproduced in a wide variety of human malignancies, has been implicated in MMP and TIMP regulation in various cells, including monocytes. In the present study, we hypothesized that tumor-derived PGE2 would affect DC migratory capacity through the extracellular matrix (  ECM  ) by altering MMP and TIMP balance. Treatment of monocyte-derived immature DC with exogenous PGE2 induced TIMP-1 secretion but not MMP-9 production and was correlated with reduced DC migration through   ECM  . Because recombinant TIMP-1 replicated PGE2 inhibition of DC migration while anti-TIMP-1 neutralizing Ab reversed it, we conclude that PGE2-mediated induction of TIMP-1 was responsible for the reduced migration of PGE2-treated DC. Similarly, DC cultured for 48 h in supernatants from cyclooxygenase-2 overexpressing lung cancer cells that secrete high levels of PGE2, exhibited decreased migration through   ECM  . Finally, analysis of E prostanoid receptor expression and their selective inhibition revealed that the enhanced TIMP-1 secretion in PGE2-treated DC was mediated predominantly by the E prostanoid receptor 2. These findings indicate that PGE2-dependent enhancement of TIMP-1 production causes reduced migration of DC through   ECM  . 
 Forkhead factor, FOXO3a, induces apoptosis of endothelial cells through activation of matrix metalloproteinases. The forkhead factor, FOXO3a, is known to induce apoptosis in endothelial cells (ECs). However, its effects on extracellular matrices (  ECM  ), which are important in EC survival, remained unknown. Here, we evaluated the role of FOXO3a on EC-  ECM   interaction.Constitutively active FOXO3a was transduced to human umbilical vein endothelial cells by adenoviral vector (Ad-TM-FOXO3a). Ad-TM-FOXO3a transfection led to dehiscence of ECs from fibronectin-coated plates, resulting in anoikis, which was significantly reversed by matrix metalloproteinase (MMP) inhibitor, GM6001. FOXO3a increased the expression of MMP-3 (stromelysin-1) but decreased the expression of tissue inhibitors of metalloproteinases-1 (TIMP-1), which was associated with increased MMP enzymatic activity in zymography. Pathophysiologic conditions such as serum starvation or heat shock also induced activation of endogenous FOXO3a, leading to activation of MMP-3 and apoptosis, which was reversed by GM6001. Delivery of Ad-TM-FOXO3a to the intraluminal surface in vivo led to EC denudation, disrupted vascular integrity, and impaired endothelium-dependent vasorelaxation.Activation of MMPs and possible   ECM   disruption represent novel mechanisms of FOXO3a-mediated apoptosis in ECs. 
 Matrix metalloproteinase-2, -9, and tissue inhibitor of metalloproteinase-1 in patients with hypertension. There are conflicting data in the literature regarding the expression pattern of the vascular matrix metalloproteinase (MMP) system and their inhibitors (TIMPs) in human hypertension. The authors hypothesized that MMP-2, MMP-9, and TIMP-1 would be abnormal in hypertension, reflecting alterations in extracellular matrix (  ECM  ) turnover. The authors measured plasma levels and activities of MMP-2, MMP-9, and TIMP-1 in 44 hypertensive patients and 44 controls. MMP-2 levels and activity were significantly higher in hypertensive group (p < .0001). Significant increase was also observed for MMP-9 level and activity (p < .0001) and for TIMP-1 (p < .0001) in hypertensive patients. Plasma levels and activities of MMP-2, MMP-9, and TIMP-1 are increased in hypertensive patients, which may reflect abnormal   ECM   metabolism. 
 TIMP-1 polymorphisms in a Chinese Han population with intracerebral hemorrhage. Remodeling of extracellular matrix (  ECM  ) and breakdown of blood-brain barrier (BBB) are crucial events in the pathogenesis of intracerebral hemorrhage (ICH). Matrix metalloproteinases (MMPs), particularly MMP-9 and MMP-2, are the most important degrading enzymes in the   ECM   and BBB. These proteolytic effects are controlled predominantly by tissue inhibitors of metalloproteinases (TIMPs). TIMP-1 is the main endogenous inhibitor of MMP-9. Two polymorphisms in the TIMP-1 gene (rs4898 and rs2070584) were selected through a literature review and successfully genotyped in a study sample of 410 ICH patients and 305 controls. Differences in genotype and allele frequencies of identified polymorphisms were determined. Furthermore, the serum levels of TIMP-1 were measured in a subgroup of 96 ICH patients on days 1 after ICH onset and 76 controls. Analyses showed that C allele of rs2070584 was significantly associated with the development of ICH in male subjects (p = 0.037, OR = 1.535, 95%CI 1.025-2.300). Multiple logistic regression analysis under three genetic models demonstrated both rs4898 and rs2070584 were not risk factors for ICH in female subjects. Furthermore, serum levels of TIMP-1 were significantly higher in ICH patients than those in normal controls. However, the serum levels of TIMP-1 showed a nonsignificant decrease, depending on the alleles and genotypes of rs2070584 both in male and female cases. In conclusion, this is the first association study of the TIMP-1 gene variants with ICH. Our data suggest that C allele of rs2070584 is a risk factor for ICH development in the Chinese male population. However, the precise function of this variant needs further investigation. 
 Novel association patterns of cardiac remodeling markers in patients with essential hypertension and atrial fibrillation. Matrix metalloproteinases (MMPs) and their tissue inhibitors (TIMPs) are essential for the cardiac extracellular matrix (  ECM  ) remodeling. We investigated differences in serum levels of these markers between patients with atrial fibrillation (AF) and sinus rhythm (SR).Serum levels of MMP-2, MMP-3, MMP-9 and TIMP-1 were measured in 86 patients: 27 on SR without any AF history, 33 with paroxysmal and 26 with permanent AF. All subjects had essential hypertension, normal systolic function and no coronary artery disease.Patients with AF had higher MMP-2, MMP-3 and MMP-9 and lower TIMP-1 compared to SR subjects (all p < 0.001). Paroxysmal AF was associated with higher MMP-2 levels compared to permanent AF (p < 0.001). Matrix metalloproteinase-9 but not MMP-3 was higher in permanent compared to paroxysmal AF group (p < 0.001). Patients with AF had lower levels of TIMP-1 compared to those with SR while permanent AF subjects had lower TIMP-1 levels than those with paroxysmal AF (p < 0.001 for both comparisons). Lower TIMP-1 was the only independent factor associated with AF (OR: 0.259, 95%CI: 0.104-0.645, p = 0.004).In hypertensives, paroxysmal AF and permanent AF differ with respect to serum MMPs. Increased MMP-2 is associated with paroxysmal, whereas increased MMP-9 with permanent AF. Additionally, lower levels of TIMP-1 had a strong association with AF incidence. 
 Inhibition of histone deacetylase activity in human endometrial stromal cells promotes extracellular matrix remodelling and limits embryo invasion. Invasion of the trophoblast into the maternal decidua is regulated by both the trophoectoderm and the endometrial stroma, and entails the action of tissue remodeling enzymes. Trophoblast invasion requires the action of metalloproteinases (MMPs) to degrade extracellular matrix (  ECM  ) proteins and in turn, decidual cells express tissue inhibitors of MMPs (TIMPs). The balance between these promoting and restraining factors is a key event for the successful outcome of pregnancy. Gene expression is post-transcriptionally regulated by histone deacetylases (HDACs) that unpacks condensed chromatin activating gene expression. In this study we analyze the effect of histone acetylation on the expression of tissue remodeling enzymes and activity of human endometrial stromal cells (hESCs) related to trophoblast invasion control. Treatment of hESCs with the HDAC inhibitor trichostatin A (TSA) increased the expression of TIMP-1 and TIMP-3 while decreased MMP-2, MMP-9 and uPA and have an inhibitory effect on trophoblast invasion. Moreover, histone acetylation is detected at the promoters of TIMP-1 and TIMP-3 genes in TSA-treated. In addition, in an in vitro decidualized hESCs model, the increase of TIMP-1 and TIMP-3 expression is associated with histone acetylation at the promoters of these genes. Our results demonstrate that histone acetylation disrupt the balance of   ECM   modulators provoking a restrain of trophoblast invasion. These findings are important as an epigenetic mechanism that can be used to control trophoblast invasion. 
 Tissue inhibitor of metalloproteinase-1 (TIMP-1) polymorphisms in a Caucasian population with abdominal aortic aneurysm. The formation of a sporadic abdominal aortic aneurysm (AAA) is explained by the remodeling of the extracellular matrix (  ECM  ) and breakdown of structural components of the vascular wall. Matrix metalloproteinases are the principal matrix-degrading proteases and are known to play a major role in the remodeling of the extracellular matrix in arterial vessels. Their activity is controlled by tissue inhibitors of metalloproteinases (TIMPs). Decreased TIMP-1 and TIMP-2 expression in the extracellular matrix of the walls of AAAs has been shown in several studies. This case control study was designed to investigate the possible impact of genetic variants of the TIMP-1 gene in the etiology of AAA.TIMP-1 single nucleotide polymorphisms (SNPs) were analyzed in a primary study sample of 50 patients with AAA and 44 controls. Differences in genotype and allele frequencies of identified polymorphisms were determined after sequencing the entire coding region and selected parts of the promoter using the automated laser fluorescence technique. A second sample (96 patients vs. 89 controls) was investigated by single-base sequencing to confirm significant results.Three polymorphisms were identified, one of which, described for the first time in this article, is located in intron 4 (TIMP-1: 328 + 16C > T). A statistically significant difference in allele frequencies for SNP TIMP-1 372T>C was detected in the primary study group. The C allele was more frequent in male patients with AAA than in the control group [23 vs. 4, p = 0.029, OR (95% CI) 4.38 (1.13-20.47)]. However, this result could not be confirmed in a second sample of males [52 vs. 45, p = 0.624, OR (95% CI) 1.16 (0.65-2.06)]. There were no statistically significant differences in genotype or allele frequencies of the other detected SNPs between the two groups.Our analysis of the entire coding region and selected parts of the promoter of the TIMP-1 gene failed to show an association between genetic polymorphisms and AAA, suggesting that variations in the TIMP-1 gene do not contribute to the development of AAA. 
 Matrix metalloproteinase-9 and tissue inhibitor of metalloproteinase-1 in hypertension and their relationship to cardiovascular risk and treatment: a substudy of the Anglo-Scandinavian Cardiac Outcomes Trial (ASCOT). Hypertension results in structural changes to the cardiac and vascular extracellular matrix (  ECM  ). Matrix metalloproteinases (MMP) and their inhibitors (TIMP) may play a central role in the modulation of this matrix. We hypothesized that both MMP-9 and TIMP-1 would be abnormal in hypertension, reflecting alterations in   ECM   turnover, and that their circulating levels should be linked to cardiovascular (CHD) and stroke (CVA) risk scores using the Framingham equation. Second, we hypothesized that treatment would result in changes in   ECM   indices.Plasma MMP-9 and TIMP-1 were measured before and after treatment (median 3 years) from 96 patients with uncontrolled hypertension participating in the Anglo-Scandinavian Cardiac Outcomes Trial (ASCOT). Pretreatment values were compared to circulating MMP-9 and TIMP-1 levels in 45 age- and sex-matched healthy controls.Circulating pretreatment MMP-9 and TIMP-1 levels were significantly higher in patients with hypertension than in the normotensive controls (P =.0041 and P =.0166, respectively). Plasma MMP-9 levels decreased, and TIMP-1 levels increased after treatment (P =.035 and P =.005, respectively). Levels of MMP-9 correlated with CHD risk (r = 0.317, P =.007) and HDL cholesterol (r = -0.237, P =.022), but not CVA risk. There were no significant correlations between TIMP-1 and CVA or CHD scores.Increased circulating MMP-9 and TIMP-1 at baseline in patients with hypertension could reflect an increased deposition and retention of type I collagen at the expense of other components of   ECM   within the cardiac and vascular   ECM  . After cardiovascular risk management, MMP-9 levels decreased and TIMP-1 levels increased. Elevated levels of MMP-9 also appeared to be associated with higher Framingham cardiovascular risk scores. Our observations suggest a possible role for these surrogate markers of tissue   ECM   composition and the prognosis of cardiovascular events in hypertension. 
 Prognostic value of serum PIIINP, MMP1 and TIMP1 levels in hypertensive patients: a community-based prospective cohort study. The purpose of this study was to examine the prognostic value of serum   ECM   biomarkers in hypertensive patients with no history of cardiovascular events. In a community-based cohort study of 125 hypertensive patients free of cardiovascular events, we collected clinical data and blood samples to assess serum levels of amino-terminal propeptide of type III procollagen (PIIINP), matrix metalloproteinase type 1(MMP1) and tissue inhibitor of MMPs type 1(TIMP1). Left ventricular hypertrophy (LVH) was assessed using the ECG Cornell product. Patients were followed up for death or cardiovascular hospitalisation. We used Cox regression models to assess the prognostic value of   ECM   biomarkers. The sample included 60.8% women; the mean (±SD) age was 62.9 (±11.4) years. Patients were followed up for a median of 5.5 years, during which 23 events (five deaths) occurred. PIIINP (3.2 ± 1.0 vs. 2.6 ± 0.8 μg/L, P = 0.001) and TIMP1 (886 ± 168 vs. 751 ± 202 μg/L, P < 0.001) levels were higher in the presence of LVH than with no LVH. Basal MMP1 serum levels were significantly associated with CV events (MMP1: HR, 1.06; 95%CI [1.02-1.09]). Adjusting for confounders did not modify this result. Cardiac fibrosis, as assessed wit  h s  erum ECM biomarkers, might develop early in hypertensive patients and is predictive of cardiovascular events or death. 
 Expression of matrix metalloproteinase-1 and tissue inhibitor of metalloproteinase-1 in ulcerative colitis. To examine the expression of metalloproteinase-1 (MMP-1) and tissue inhibitor of metalloproteinase-1 (TIMP-1) in the colonic mucosa of patients with ulcerative colitis (UC).Reverse transcription-polymerase chain reaction (RT-PCR) and immunohistochemistry were used to study the expression of MMP-1 and TIMP-1 at both mRNA and protein levels in patients with UC and controls. The relationship between MMP-1 mRNA, TIMP-1 mRNA, MMP-1 mRNA/TIMP-1 mRNA ratio and the severity of clinical symptoms of the patients with UC were also analyzed.The expression of MMP-1 mRNA and TIMP-1 mRNA in the ulcerated and inflamed colonic mucosa was significantly higher than that in the non-inflamed colonic mucosa (P < 0.001), but there was no statistically significant difference in the non-inflamed colonic mucosa of UC patients and normal controls (P > 0.05). The mRNA expression of MMP-1 and TIMP-1 in ulcerated colonic mucosa of UC patients was increased by 80-fold and 2.2-fold, respectively when compared with the normal controls. In the inflamed colonic mucosa, the increase was 30-fold and 1.6-fold, respectively. Immunohistochemical analysis showed that among the ulcerated, inflamed, and non-inflamed colonic mucosae of UC patients and the normal controls, the positive rate of MMP-1 expression was 87%, 87%, 40% and 35% respectively, and the positive rate of TIMP-1 expression was 89%, 89%, 80% and 75%, respectively. Furthermore, the expression of MMP-1 mRNA, TIMP-1 mRNA and the MMP-1 mRNA/ TIMP-1 mRNA ratio were correlated with the severity of clinical symptoms (P <0.05).Excessive expression of MMP-1 in the diseased colonic mucosa causes excessive hydrolysis of the extracellular matrix (  ECM  ) and ulceration in UC patients. MMP-1 mRNA, TIMP-1 mRNA and MMP-1 mRNA/TIMP-1 mRNA ratio can be used as biomarkers to judge the severity of clinical symptoms in patients with UC. Exogenous TIMP-1 or MMP-1 inhibitor therapy is a novel treatment for patients with UC. 
  GeneRIFs  
 a TIMP-1 mutant (K26/27/30 + K76 transplant) capable of   ECM   association. 
 Excessive expression of MMP-1 and TIMP-1 in the diseased colonic mucosa causes excessive hydrolysis of the extracellular matrix (  ECM  ) and ulceration in ulcerative colitis (UC) patients. 
 Increased expression of TIMP-1 may reflect a specific temporal inhibition of collagenolysis and thereby a time-dependent regulation of   ECM   breakdown in areas surrounding the apex of the follicle. 
  [Ee]xtracellular [Mm]atrix  Process  
   extracellular matrix   disassembly 
  PubMed  
 [Expression of matrix metalloproteinases 2 and 9 and their tissue inhibitors 1 and 2 in papillary thyroid cancer: an association with the clinical, morphological and ultrastructural characteristics of a tumor]. Papillary thyroid cancer (PTC) was ascertained to be characterized by a significant expression of matrix metalloproteinases (MMP-2 and 9) and a TIMP-2 inhibitor and decreased TIMP-1 accumulation in the cytoplasm of tumor cells; at the same time a combination of high cytoplasmic MMP-9 expression with no TIMP-1, as well as triple positive co-expression of MMP-2, MMP-9, and TIMP-2 are poor immunohistochemical indicators. The results of the performed study suggest that the detection of the types of expression of MMP-9+/TIMP-1 and MMP-2+/MMP-9+/TIMP-2+ is associated with the possible progression of a tumor process and concurrent with poor clinical and morphological signs, such as the large size of the tumor, the presence of intravascular invasion and regional metastases, organ capsular invasion, and low ultrastructural tumor cell differentiation. Thus, changes in the tumor expression of the   extracellular matrix   enzymes MMR-2 and MMP-9 and their inhibitors TIMP-1 and TIMP-2 along with the major clinical, morphological, and ultrastructural signs of the disease are important additional indicators for analyzing the malignant potential of PTC. 
 Tissue inhibitor of metalloproteinase 1 is an independent predictor of prognosis in patients with nonsmall cell lung carcinoma who undergo resection with curative intent. Matrix metalloproteinases (MMPs) and tissue inhibitors of metalloproteinases (TIMPs) play a role in the processes of   extracellular matrix   degradation. Changes in their expression levels have been observed in various tumor types, including lung carcinoma. However, their clinical significance and their prognostic importance in the progression of nonsmall cell lung carcinoma (NSCLC) remain to be specified. In this study, mRNA expression levels of MMP-1, MMP-9, TIMP-1, and TIMP-2 were evaluated in patients with resected NSCLC, and their associations with disease progression and prognosis were determined.Between June 1996 and December 1999, 116 patients underwent resection for NSCLC. Expression levels of MMPs and TIMPs were evaluated using Northern blot analysis in these NSCLC tissue samples and in 39 matched samples of normal lung tissue.MMP-1, MMP-9, and TIMP-1 expression levels were increased in tumor samples compared with matched, corresponding normal tissues. In contrast, TIMP-2 expression was decreased in tumor samples. MMP-1 tumor expression was correlated significantly with the evolution of lymph node status and tumor-lymph node-metastasis (TNM) stage. In contrast, MMP-9 tumor expression was correlated significantly with increased T stage. TIMP-1 overexpression was an independent predictor of worse survival in patients with NSCLC that was not associated with other prognosis factors, such as TNM stage.The overexpression of TIMP-1 was an independent prognostic marker in patients with NSCLC, and evaluating TIMP-1 may be important for identifying patients who are at greater risk of disease recurrence. 
 Hematopoiesis in mice is extremely resilient to wide variation in TIMP/MMP balance. Tissue inhibitors of matrix metalloproteinases (TIMPs) are natural inhibitors of matrix metalloproteinases (MMPs) and are associated with normal and pathologic   extracellular matrix   turnover. Because the microenvironment is critical for normal hematopoietic stem/progenitor cell function, we aimed to determine whether alterations in the TIMP/MMP balance impact upon normal hematopoiesis in mice. We have used both overexpression and knockout mouse models to determine whether early hematopoiesis is susceptible to potentially pathologic changes in TIMP/MMP level. These studies used TIMP-1(-/-) mice and retroviral vectors co-expressing human TIMP-1 or TIMP-2 linked with the green fluorescent protein (GFP) transduced into bone marrow (BM) cells and transplanted into lethally-irradiated recipient mice. Loss of TIMP-1 in knockout mice or retroviral overexpression of TIMP-1 or TIMP-2 did not alter hematopoietic stem/progenitor function during steady-state hematopoiesis. Surprisingly, even when applying hematopoietic stress through mobilization, chemotaxis, or myelosuppression, murine hematopoiesis was not adversely affected by TIMP-1 or TIMP-2 level. We conclude that TIMP/MMP balance alone does not exert significant influence on blood cell development and homeostasis. An important corollary of these studies is that specific modulation using MMP inhibitors for cancer or immunologic therapy is unlikely to have adverse hematopoietic side effects. 
 TIMP1, TIMP2, and TIMP4 are increased in aqueous humor from primary open angle glaucoma patients. Elevated intraocular pressure (IOP) is the only known modifiable risk factor for primary open angle glaucoma (POAG), and it can be caused by reduced aqueous humor outflow from the anterior chamber. Outflow is predominantly regulated by the trabecular meshwork, consisting of specialized cells within a complex   extracellular matrix   (ECM). An imbalance between ECM-degrading matrix metalloproteinases (MMPs) and the tissue inhibitors of MMPs (TIMPs) within the trabecular meshwork is thought to contribute to POAG. This study aimed to quantify levels of TIMPs and MMPs in aqueous humor samples from glaucomatous and non-glaucomatous eyes, analyze MMP/TIMP ratios, and correlate results with age, IOP, and Humphrey's visual field pattern standard deviation (PSD).Aqueous humor samples were collected from 26 non-glaucomatous control subjects before cataract surgery and 23 POAG patients undergoing trabeculectomy or cataract surgery. Analyte concentrations were measured using multiplexed immunoassays. Statistical significance was assessed with Mann-Whitney U tests, and Spearman's method was used to assess correlations with age, IOP, and PSD.Concentrations of TIMP1 (p = 0.0008), TIMP2 (p = 0.002), TIMP4 (p = 0.002), and MMP2 (p = 0.020) were significantly increased in aqueous humor samples from POAG versus cataract samples. For the majority of MMP/TIMP molar ratios calculated for the cataract group, TIMPs outweighed MMPs. In POAG, molar ratios of MMP2/TIMP1 (p = 0.007) and MMP9/TIMP1 (p = 0.005) showed a significant decrease, corresponding to an elevated excess of TIMPs over MMPs in POAG compared to cataract samples. Conversely, MMP2/TIMP3 (p = 0.045) and MMP3/TIMP3 (p = 0.032) molar ratios increased. Several MMP/TIMP molar ratios correlated with IOP (r = 0.476-0.609, p = 0.007-0.034) and PSD (r = -0.482 to -0.655, p = 0.005-0.046) in POAG samples and with age in cataract control samples.An imbalance among MMPs and TIMPs was found in glaucomatous aqueous humor samples, with a shift toward raised TIMP levels. This may result in the inhibition of MMP activity, leading to an altered ECM composition in the TM and thereby contributing to increased outflow resistance. 
 Overexpression of matrix metalloproteinases in lung tissue of patients with primary spontaneous pneumothorax. Although blebs and bullae are frequently found in the apexes of lungs of patients with primary spontaneous pneumothorax (PSP), its pathogens remain unclear.To examine the role of proteases [matrix metalloproteinase (MMP)-2, MMP-7 and MMP-9] and antiproteases [tissue inhibitors of metalloproteinase (TIMP)-1, TIMP-2, TIMP-3 and TIMP-4] in the pathogenesis of PSP.Fifty consecutive PSP patients who received standard surgical care were enrolled in the study. Lung tissues from 20 patients with stage I non-small cell lung cancer were used as a control. Immunohistochemistry (IHC), reverse transcription-polymerase chain reaction (RT-PCR) and gelatin zymography were used to evaluate the expression of MMP and TIMP in the lung tissue of patients with PSP.Overexpression of MMP-2, MMP-7 and MMP-9 was found in the afflicted lung by IHC, zymography and RT-PCR. By IHC, higher expression of MMP-2 and MMP-9 in PSP patients was identified in alveolar macrophages and type II pneumocytes (88 and 92% of patients in macrophages, and 72 and 70% of patients in type II pneumocytes, respectively). MMP-2, MMP-7 and MMP-9 expression in patients was higher in mesothelial cells (66, 76 and 76%). Overexpression of TIMP-2 was detected in the   extracellular matrix   around bullae and blebs. Expression levels of TIMP-1, TIMP-3 and TIMP-4 were negligible (<10% of cells) in both PSP patients and controls.MMP-2, MMP-9, MMP-7 and TIMP-2 were upregulated in PSP lesions. These results suggest that an imbalance between the expression of proteases and antiproteases may be involved in the pathogeneses of PSP. 
 Matrix metalloproteinases-2, -3 and tissue inhibitors of metalloproteinases-1, -2 in placentas from preterm pregnancies and their association with one-carbon metabolites. Maternal nutrition is an important determinant of one-carbon metabolism and defects in the one-carbon metabolism may lead to poor obstetric outcomes. This study was designed to test the hypothesis that altered intake/metabolism of micronutrients (folic acid and vitamin B12) and docosahexaenoic acid (DHA) contributes to increased homocysteine and oxidative stress leading to altered levels of matrix metalloproteinases (MMPs) and tissue inhibitors of metalloproteinases (TIMPs) in women delivering preterm. We have earlier reported increased vitamin B12, homocysteine, and oxidative stress along with reduced placental DHA in women delivering preterm. In this study, we further examine the placental levels of MMP2, MMP3, TIMP1, and TIMP2 in 75 women delivering at term and 73 women delivering preterm. Placental levels of MMPs and TIMPs were determined by ELISA. Placental MMP2 and MMP3 levels were higher (P<0.01) in women delivering preterm as compared with term. There was no difference in the placental TIMP1 and TIMP2 levels in women delivering preterm and at term. Further placental MMP2 and MMP3 levels were higher (P<0.01) in women with preterm labor as compared with those in labor at term, suggesting that MMPs may favor degradation of   extracellular matrix   in the placenta during preterm labor. Our study for the first time suggests a crucial role of micronutrients and MMPs in preterm birth. Future studies need to examine if epigenetic modifications through the one-carbon cycle contribute to increased levels of MMPs leading to preterm deliveries. 
 Matrix metalloproteinases MMP-2, -9 and tissue inhibitors TIMP-1, -2 expression and secretion by primary human osteoblast cells in response to titanium, zirconia, and alumina ceramics. Osteogenic properties of bone cells are a key parameter governing osseointegration of implant devices. In this context, osteoblasts have a central role via   extracellular matrix   synthesis and remodeling that they regulate through different protease activity. In this study, we have analyzed the expression of two matrix metalloproteinases (MMPs): MMP-2 (72 kDa) and MMP-9 (92 kDa) and their specific tissue inhibitors TIMP-1 and TIMP-2 in primary human osteoblastic cells. The effect of titanium, zirconia, and alumina ceramics on the synthesis of these proteases was assessed using reverse transcriptase-polymerase chain reaction, enzyme-linked immunosorbent assay, and zymographic analysis. Our results showed that osteoblasts express MMP-2 and -9 mRNA. Furthermore, MMP-2 mRNA expression was decreased by titanium and increased by alumina whereas zirconia did not have any significant effect. Conversely, MMP-9 mRNA expression was stimulated by titanium but decreased with zirconia, whereas alumina induced no significant changes. Zymographic analysis has evidenced pro-MMP-2 gelatinolytic activity in all cell populations with time-dependent increase profile; pro-MMP-9, however, was not detected. Enzyme-linked immunosorbent assay data confirmed the production of MMP-2 and very low levels of MMP-9. In addition, TIMP-1 was secreted in 24-h-cultured cells and increased to maximal level at 48-72 h whereas TIMP-2 levels were very low. The interactions between human osteoblasts and the studied biomaterials altered both MMP-2, -9 and TIMP-1expression indicating that biomaterials may influence osseointegration and bone remodeling. 
 Circulating matrix metalloproteinase-9 and tissue inhibitors of metalloproteinases-1 and -2 levels in gestational hypertension. Gestational hypertension (GH) is dangerous to both mother and child. Arterial invasiveness and growth are dependent on successful   extracellular matrix   (ECM) breakdown, which may be abnormal in GH. We hypothesized abnormalities in circulating matrix metalloproteinase-9 (MMP-9) and tissue inhibitors of metalloproteinases-1 and -2 (TIMP-1 and TIMP-2, respectively) in patients with GH, when compared with normotensive women with normal pregnancies and healthy nonpregnant control subjects.Plasma MMP-9, TIMP-1, and TIMP-2 were measured by ELISA in 23 women with GH, 30 normotensive pregnant women, and 28 nonpregnant women who were matched for age, gestational age, and parity.Levels of circulating MMP-9, TIMP-1 and TIMP-2, and the MMP-9/TIMP-1 and MMP-9/TIMP-2 ratios were significantly different among the three groups (P = .026, P = .006, P = .007, P = .001 and P = .008 respectively). Within the GH group, MMP-9 and the MMP-9/TIMP-1 ratio correlated negatively with age (r = -0.581, P = .004 and r = -0.563, P = .005, respectively) and levels of diastolic blood pressure (r = -0.432, P = .040 and r = -0.461, P = .027, respectively). With multiple regression analysis, only age independently correlated with circulating levels of MMP-9 (P = .010); neither age nor levels of diastolic blood pressure had any effect on the MMP-9/TIMP-1 ratio.We have demonstrated altered MMP/TIMP ratios in maternal blood during GH. These observations suggest pregnancy-related changes in ECM breakdown and turnover. Given the importance of changes in ECM composition to vascular and cardiac structure in hypertension, we suggest that these observations may be related to the pathophysiology of human GH. 
 Genetic analysis of polymorphisms in biologically relevant candidate genes in patients with abdominal aortic aneurysms. Abdominal aortic aneurysms (AAAs) are characterized by histologic signs of chronic inflammation, destructive remodeling of   extracellular matrix  , and depletion of vascular smooth muscle cells. We investigated the process of   extracellular matrix   remodeling by performing a genetic association study with polymorphisms in the genes for matrix metalloproteinases (MMPs), tissue inhibitors of metalloproteinases (TIMPs), and structural   extracellular matrix   molecules in AAA. Our hypothesis was that genetic variations in one or more of these genes contribute to greater or lesser activity of these gene products, and thereby contribute to susceptibility for developing AAAs.DNA samples from 812 unrelated white subject (AAA, n = 387; controls, n = 425) were genotyped for 14 polymorphisms in 13 different candidate genes: MMP1(nt-1607), MMP2(nt-955), MMP3(nt-1612), MMP9(nt-1562), MMP10(nt+180), MMP12(nt-82), MMP13(nt-77), TIMP1(nt+434), TIMP1(rs2070584), TIMP2(rs2009196), TIMP3(nt-1296), TGFB1(nt-509), ELN(nt+422), and COL3A1(nt+581). Odds ratios and P values adjusted for gender and country of origin using logistic regression and stratified by family history of AAA were calculated to test for association between genotype and disease status. Haplotype analysis was carried out for the two TIMP1 polymorphisms in male subjects.Analyses with one polymorphism per test without interactions showed an association with the two TIMP1 gene polymorphisms (nt+434, P = .0047; rs2070584, P = .015) in male subjects without a family history of AAA. The association remained significant when analyzing TIMP1 haplotypes (chi 2 P = .014 and empirical P = .009). In addition, we found a significant interaction between the polymorphism and gender for MMP10 ( P = .037) in cases without a family history of AAA, as well as between the polymorphism and country of origin for ELN ( P = .0169) and TIMP3 ( P = .0023) in cases with a family history of AAA.These findings suggest that genetic variations in TIMP1, TIMP3, MMP10, and ELN genes may contribute to the pathogenesis of AAAs. Further work is needed to confirm the findings in an independent set of samples and to study the functional role of these variants in AAA. It is noteworthy that contrary to a previous study, we did not find an association between the MMP9 (nt-1562) polymorphism and AAA, suggesting genetic heterogeneity of the disease.Abdominal aortic aneurysms (AAAs) are an important cardiovascular disease, but the genetic and environmental risk factors, which contribute to individual's risk to develop an aneurysm, are poorly understood. Histologically, AAAs are characterized by signs of chronic inflammation, destructive remodeling of the   extracellular matrix  , and depletion of vascular smooth muscle cells. We hypothesized that genes involved in these events could harbor changes that make individuals more susceptible to developing aneurysms. This study identified significant genetic associations between DNA sequence changes in tissue inhibitor of metalloproteinase 1 (TIMP1), TIMP3, matrix metalloproteinase 10 (MMP10) and elastin (ELN) genes, and AAA. The results will require confirmation using an independent set of samples. After replication it is possible that these sequence changes in combination with other risk factors could be used in the future to identify individuals who are at increased risk for developing an AAA. 
 Temporal investigation of matrix metalloproteinases and their inhibitors in human cervicovaginal fluid in late pregnancy and labor. Temporal expression of matrix metalloproteinase (MMP)-1, -2, -3, -7, -8, -9, -12, and -13, and tissue inhibitors of metalloproteinases (TIMPs)-1 and -2 in human cervicovaginal fluid (CVF) in term pregnancy and labor was investigated. Term parous women provided CVF samples that were grouped into labor, 1 to 3, 6 to 8, and 12 to 16 days before labor onset. Both MMPs and TIMPs (n = 60) were quantified using multiplex solution array and enzyme-linked immunosorbent assays, respectively. Further analysis of TIMP-1 (n = 180) was undertaken. All MMPs and TIMPs except MMP-12 and -13 were detected in the CVF. Matrix metalloproteinase 7, TIMP-1, and TIMP-2 were significantly increased in labor. Tissue inhibitors of metalloproteinase 1 was significantly increased up to 7 days before spontaneous labor onset. The data suggest a role of MMP-7 in the remodeling and rupture of fetal membranes and may reflect the homeostatic regulation of   extracellular matrix   remodeling of MMP-7 by TIMP-1 and TIMP-2. 
 siRNA knockdown of tissue inhibitor of metalloproteinase-1 in keloid fibroblasts leads to degradation of collagen type I. Keloids are defined as overgrowths of scar tissue resulting from abnormal wound healing. They are characterized by excessive dermal deposition of thick, hyalinized collagen bundles resulting from an imbalance between the production and degradation of   extracellular matrix   (ECM) components. Matrix metalloproteinases (MMPs) and tissue inhibitors of metalloproteinases (TIMPs) are two important regulators of ECM degradation and remodeling. To evaluate the role played by knockdown of TIMPs in keloid formation, we transduced human keloid-derived fibroblasts (KFs) with small interfering RNAs targeting TIMP-1 or -2 (siTIMP-1 or siTIMP-2) using a lentiviral vector and assessed the biological effects. We found that MMP-1/TIMP-1 and MMP-1/TIMP-2 complexes were suppressed and that MMP-2 activity was upregulated in KFs expressing siTIMP-1 or siTIMP-2. In addition, increased degradation of collagen type I was observed in the supernatant of KFs expressing siTIMP-1, but not siTIMP-2, with the suppression of cell viability and induction of apoptosis. These results suggest that targeting TIMP-1 using small interfering RNA has significant therapeutic potential as an approach to treating keloids through degradation of their thick collagen bundles. 
 Relationship between matrix metalloproteinases/tissue inhibitors of matrix metalloproteinases systems and autoantibody patterns in systemic sclerosis. Systemic sclerosis (SSc) is a connective tissue disease characterized by tissue fibrosis that reflects an imbalance between collagen production and degradation. Matrix metalloproteinases (MMPs) are a family of endopeptidases involved in the remodelling of   extracellular matrix   (ECM). This activity is controlled by tissue inhibitors of MMP (TIMPs). Aim of this study was the evaluation of MMP-9/TIMP-1 and MMP-2/TIMP-2 systems in patients with SSc.SearchLight Human MMP Array 1 was used to measure MMPs and TIMPs in 32 SSc patients and 32 matched healthy controls.SSc patients showed higher values of both MMP-9 and TIMP-1 in comparison with controls. The patients with anticentromere antibodies (ACA) positivity showed higher values of MMPs and TIMPs in comparison with either controls or the patients with anti-Scl70-positive antibodies.Results of this investigation suggest that SSc patients with ACA positivity, after a primary fibrogenetic noxa, react with a more abundant release of MMP/TIMP, whereas patients with anti-Scl70 antibody show a normal response. 
 Matrix metalloproteinase expression in the ascending aorta and aortic valve.   Extracellular matrix   degradation and increased proteolytic enzyme (matrix metalloproteinase (MMP)) activity characterise abdominal aortic aneurysm formation. Post-stenotic dilatation of ascending aorta is associated with aortic stenosis and regurgitation, haemodynamically normal bicuspid aortic valve (BAV) and following AV replacement. We aimed to determine an association between ascending aortic pathology and abnormal AV, with particular reference to MMPs, and ascertain differences between BAV and tricuspid (TAV) AV. Subset of the study population (n=19) with a preoperative ascending aorta of >4 cm was analysed. Samples of ascending aorta and AV were obtained from 82 patients (TAV, n=54, BAV, n=28) undergoing surgery. Gene expression of MMP-1, -2, -9 and tissue inhibitor of metalloproteinase (TIMP)-1 and -2 was quantified by real-time RT-PCR. No significant difference was seen in gene expression level of MMPs, TIMPs and ratio of MMPs/TIMPs in ascending aorta and AV between patients with BAV and TAV. MMP-2/TIMP-1 in ascending aorta was greater in BAV, in the subset of patients with preoperative aortic dilatation (P<0.05). No difference exists in gene expression of MMPs in ascending aorta and AV between patients with BAV and TAV. However, patients with larger aortic diameters have increased MMP-2/TIMP-1. Modifying MMP expression may have a role in development of aneurysms. 
 Age- and gender-dependent changes in connective tissue remodeling: physiological differences in circulating MMP-3, MMP-10, TIMP-1 and TIMP-2 level. the mechanisms which cause age-dependent remodeling of connective tissue are still not fully understood. Matrix metalloproteinases (MMPs) and tissue inhibitors of metalloproteinases (TIMPs) constitute an important proteolytic pathway affecting physiological matrix remodeling.the way in which changes in the   extracellular matrix   metabolism during the ageing process influence the level of circulating MMP-3 and MMP-10, as well as their tissue inhibitors TIMP-1 and TIMP-2, in a healthy population was investigated in this study.blood samples were taken from 81 healthy individuals aged 6-62 years and measured for MMP-3, MMP-10, TIMP-1 and TIMP-2 levels using enzyme-linked immunosorbent assays. Polyacrylamide gel electrophoresis followed by Western immunoblotting allowed for the detection of pro- and active forms of both MMPs.Serum MMP-3 and TIMP-1 values were positively correlated with age (r = 0.44, p = 0.00001 and r = 0.28, p = 0.012, respectively). A contrary tendency was found for MMP-10 and TIMP-2 serum levels. A strong age-related decrease in MMP-10 (-0.53; p = 0.000) and TIMP-2 (-0.52; p = 0.000) was noticed in our study. Gender was a significant factor modifying MMP/TIMP potential, except for the MMP-10 level.the data presented indicate that changes in MMP/TIMP balance occur in physiological ageing. Moreover, these findings highlight the necessity of utilizing age- and sex-matched values for analysis of MMPs and TIMPs in the pathological conditions. 
 In vitro study of matrix metalloproteinase/tissue inhibitor of metalloproteinase production by mesenchymal stromal cells in response to inflammatory cytokines: the role of their migration in injured tissues. The transmigratory capacity of bone marrow (BM) mesenchymal stromal cells (MSC) through the endothelial cell barrier into various tissues and their differentiation potential makes them ideal candidates for cell therapy. Nevertheless, the mechanisms and agents promoting their migration are not fully understood. We evaluated the effects of several inflammatory cytokines on the migration of BM MSC and matrix metalloproteinase (MMP)/tissue inhibitor of metalloproteinase (TIMP) production.The migratory potential of BM MSC was evaluated using a Boyden chamber coated with Matrigel in the presence and absence of stromal cell-derived (SDF)-1alpha, platelet-derived growth factor (PDGF)bb, insulin-like growth factor (IGF)-I and interleukin (IL)-6. The ability of inflammatory cytokines to induce MSC migration was tested in presence of their respective Ab or blocking peptide. We used immunofluorescence to check the expression of cytokine receptors, and MMP/TIMP production was analyzed at the protein (human cytokine array, enzyme-linked immunosorbent assay (ELISA), gelatine zymography and Western blot) and mRNA quantitative real-time polymerase chain reaction (qRT-PCR) levels.We have demonstrated that inflammatory cytokines promote the migratory capacity of BM MSC according to the expression of their respective receptors. Higher migration through Matrigel was observed in response to IL-6 and PDGFbb. qRT-PCR and cytokine array revealed that migration was the result of the variable level of MMP/TIMP in response to inflammatory stimuli.Our observations suggest that chemokines and cytokines involved in the regulation of the immunity or inflammatory process promote the migration of MSC into BM or damaged tissues. One of the mechanisms used by MSC to promote their migration though the   extracellular matrix   is modulation of the production of MMP-1, MMP-2, MMP-13, TIMP-1 and TIMP-2. 
 Increased expression of metalloproteinase-2 and -9 (MMP-2, MMP-9), tissue inhibitor of metalloproteinase-1 and -2 (TIMP-1, TIMP-2), and EMMPRIN (CD147) in multiple myeloma. Activity of metalloproteinases (MMP) is controlled both by specific tissue inhibitors (TIMP) and activators (  extracellular matrix   metalloproteinase inducer, EMMPRIN). There are few data available concerning concentration the bone marrow of MMP-2, MMP-9, TIMP-1, and TIMP-2, or EMMPRIM expression by bone marrow mesenchymal stromal cells (BMSCs) in patients with multiple myeloma (MM).We studied 40 newly diagnosed, untreated patients: 18 males and 22 females with de novo MM and 11 healthy controls. Bone marrow was collected prior to therapy. BMSCs were derived by culturing bone marrow cells on MesenCult. Protein concentrations were determined in bone marrow plasma and culture supernatants by ELISA. EMMPRIN expression by BMSCs was assessed by flow cytometry.The median concentrations of MMP-9, TIMP-1, and TIMP-2 in both marrow plasma and culture supernatants were significantly higher in MM patients than controls.EMMPRIN expression and ratios MMP-9/TIMP-1 and MMP-2/TIMP-2 were higher in MM patients, our results demonstrate that in MM patients MMP-2 and MMP-9 are secreted in higher amounts and are not balanced by inhibitors. 
 Expression of MMP-7, MMP-9, TIMP-1 and TIMP-2 mRNA in lung tissue of patients with non-small cell lung cancer (NSCLC) and benign pulmonary disease. The expression of matrix metallo-proteinases (MMP-7 and MMP-9) and tissue inhibitors of metalloproteinases (TIMP-1 and TIMP-2), which are involved in the degradation of the   extracellular matrix   (ECM) and tumor growth, was investigated in normal lung tissue, tissue of benign pulmonary diseases and non-small cell lung cancer (NSCLC) tissue.Tumor tissue and surrounding carcinoma-free lung tissue samples were obtained from 91 patients with NSCLC who had undergone surgery in the years 2005-2007 as well as lung tissue from 12 patients operated on for 'benign' bullous emphysema or interstitial lung disease. The mRNA was isolated from the tissues and the expression of mRNA was assessed using a real-time RT PCR method.Significantly higher expression of MMP-7, MMP-9 and TIMP-1 mRNA was demonstrated in the NSCLC tissue in comparison with the normal lung tissue from the same patients (p=0.0003, p<0.0001 and p=0.0018, respectively). Similar results for MMP-7, MMP-9 and TIMP-1 were found in the histological subgroups: squamous cell lung cancer vs. normal tissue (p=0.0198, p=0.0015 and p=0.0366, respectively), and adenocarcinoma vs. normal tissue (p=0.0045, p<0.0001 and p=0.0140, respectively). The expression of MMP-7 was found to be significantly higher in tumor tissue vs. lung tissue of the benign diseases (p=0.0086) and similar results were also recorded in the histological subgroups: squamous cell lung cancer vs. benign tissue (p=0.0171) and adenocarcinoma vs. benign tissue (p=0.0135). The expression of MMP-9 was significantly higher only in the adenocarcinoma subgroup vs. the benign tissue (p=0.0412). No differences in the expression of mRNA between stage IA and stages IB-IIIB of NSCLC were recorded.Significantly higher expression of MMP-7 and MMP-9 in tumor tissue than in the surrounding tissue or in benign lung disease tissue supports the notion of an important role of these metalloproteinases in the growth of lung carcinoma. TIMP-1 expression is increased only in carcinoma, but not in benign lung disease. 
 The identification of myocilin-associated proteins in the human trabecular meshwork. Myocilin forms high molecular weight complexes in vivo presumably due to interaction with itself and other myocilin binding proteins. To identify myocilin interacting proteins, yeast 2-hybrid analysis was performed on >1x10(6) human trabecular meshwork cDNA clones. Coimmunoprecipitation and Far Western analysis were also performed on cell lysates obtained from fresh human trabecular meshworks or cultured human monolayer trabecular cell lines. Among the different methods, 46 candidate myocilin-associated proteins were identified, including molecules associated with the   extracellular matrix  , cytoskeleton, signaling, and metabolism. The most consistent interaction was myocilin-myocilin binding. Yeast-2 hybrid and Far Western analysis also found an association between myocilin and glyceraldehyde-3-phosphate dehydrogenase (GAPDH). None of the other candidate myocilin interacting proteins were identified in more than one method. Characterization of these potential interacting proteins may help to better understand the function of myocilin in the trabecular meshwork and aqueous outflow pathway. 
 Tissue inhibitor of metalloproteinases-1, -2, and -3 polymorphisms in a white population with intracranial aneurysms. Remodeling of the   extracellular matrix   seems to be a crucial event in the pathogenesis of cerebral aneurysms. Matrix metalloproteinases are the most important degrading enzymes in the   extracellular matrix  . Their activity is controlled predominantly by tissue inhibitors of metalloproteinases (TIMPs). To investigate the possible impact of genetic variants within the genes encoding TIMP-1, -2, and -3, we conducted this case-control study.A study sample was analyzed that comprised 44 patients with intracranial aneurysms and 44, 41, and 40 controls for the analysis of TIMP-1, -2, and -3, respectively. Differences in genotype and allele frequencies of identified polymorphisms were determined. The entire coding regions and parts of the promoter sequences of the TIMP-1, -2, and -3 genes were with using the automated laser fluorescence technique.Nine polymorphisms were identified, 3 located in TIMP-1 (-19C>T, 261C>T, 372T>C), 4 in TIMP-2 (-621C>T, -596A>C, -261G>A, 303G>A), and 2 in TIMP-3 (249T>C, 261C>T), whereas -621C>T, -596A>C, and -261G>A of the TIMP-2 gene are newly identified polymorphisms. We detected no deviation from Hardy-Weinberg equilibrium in any of the groups. The C allele of the 372T>C polymorphism was more frequently found in female than in male controls (exact nominal P=0.0012). However, this finding could not be validated by analysis of a second sample of 113 controls (exact nominal P=1.0000). There were no differences in genotype and allele frequencies between any of the other groups.Our analysis of the entire coding region of 3 TIMPs, which are the main inhibitors of metalloproteinase activity in the   extracellular matrix  , failed to show an association between genetic polymorphisms and an intracranial aneurysm. These data do not support the hypothesis that genetic variants within these genes have an impact on aneurysm development in the white population. 
 Expression of matrix metalloproiteinases (MMPs) and tissue inhibitors of metalloproteinases (TIMPs) in hepatocellular carcinoma tissue, compared with the surrounding non-tumor tissue. Matrix metalloproteinases (MMPs) have been implicated in the tumor invasion and growth through the degradation of   extracellular matrix  . In this study, we selected 46 hepatocellular carcinoma (HCC) cases, at random, and we immunohistologically examined the expression of MMP-1, MMP-2, MMP-3, MMP-7, MMP-9, tissue inhibitor of metalloproteinases (TIMP)-1, TIMP-2, in cancerous and non-cancerous areas using avidin-biotin-peroxidase complex method. In all cases, cancer cells, hepatocytes, sinusoidal lining cells, leukocytes, and bile ducts were positive for all the primary antibodies. The expressions of MMPs and TIMPs in most of the HCC tissues were equal or low compared with those in the surrounding non-tumor tissues, although mixed expression pattern were recognized in some HCC tissues. The difference of MMP and TIMP expression was not related with the histological differentiation of HCC and the condition of non-cancerous area. These findings suggested little association of the clinicopathological findings of HCC with the histological expression of MMPs and TIMPs. 
 The role of matrix metalloproteinases (MMPs) and their inhibitors (TIMPs) in the development of esophageal cancer. Esophageal cancer (EC) is one of the most aggressive malignant tumors of the gastrointestinal tract. There are two distinct histological types of EC: esophageal squamous cell carcinoma and adenocarcinoma of the esophagus. Etiologic factors and the patterns of incidence of both subtypes are different. Matrix metalloproteinases (MMPs) and their tissue inhibitors (TIMPs) play an important role in esophageal carcinogenesis. Gellatinases MMP-2 and MMP-9 are able to degrade collagen IV from basement membranes and   extracellular matrix   which is related to tumor progression, including invasion, metastasis, growth and angiogenesis. It has been shown that increased expression of MMPs plays a crucial role in the development of several human malignancies, including esophageal cancer. The activity of MMPs is regulated by their endogenous natural inhibitors (TIMPs). Among these, the roles of TIMP-1 and TIMP-2 in EC development, tumor progression and formation of metastases have been most extensively characterized and best recognized. 
 [Expression of matrix metalloproteinase-2, 9 and their inhibitor-TIMP 1,2 in human squamous cell carcinoma of uterine cervix]. Studies of tumor invasion and metastasis have focused on the degradation of   extracellular matrix   (ECM). Matrix metalloproteinases (MMPs), which could degrade ECM, were implicated in cancer invasion and metastasis. Activated MMPs were controlled by specific tissue inhibitors of metalloproteinases (TIMPs). This study was designed to investigate the expression of MMP-2, MMP-9 and TIMP-1, TIMP-2 in human squamous cell carcinomas of uterine cervix, and the association with invasion and metastasis of human squamous cell carcinoma of uterine cervix.Tissue samples from 40 cases of squamous cell carcinoma of the uterine cervix, 29 cases of cervical intraepithelial neoplasia (CIN), and 16 cases of normal cervices were stained immunohistochemically.Immunohistochemical staining of tumor cells for MMP-2, MMP-9, TIMP-1, and TIMP-2 were noted 82.5%, 70.0%, 62.5%, and 97.5% in carcinomas, 44.8%, 62.1%, 86.21%, and 96.55% in CIN lesions respectively, for MMP-2, MMP-9, TIMP-1 in 25.0% normal cervices, but for TIMP-2 in 56.25% normal cervices. There was significant difference for MMP-2 among carcinomas, normal cervices, and CIN respectively (P < 0.01). There was significant difference for MMP-9 among normal cervices, carcinomas, and CIN, respectively (P < 0.05). The positive rates of TIMP-1 and TIMP-2 in the cervical carcinoma group and CIN group were obviously higher than that in normal cervical group(P < 0.01). The positive rates of MMP-2 and MMP-9 in lymph node metastasis were obviously higher than that in without lymph node metastasis(P < 0.01), but for TIMP-2 was contrary and there was no significant difference for TIMP-1. No significant correlation could be established among the expression of these markers and the tumor diameter, the clinical stage, and pathologic grade.The over-expression of MMP-2, MMP-9, TIMP-1, and TIMP-2 may play a key role in invasion and lymph-node metastasis of in squamous carcinoma of the cervix. 
 TIMP-1 expression in human colorectal cancer is associated with TGF-B1, LOXL2, INHBA1, TNF-AIP6 and TIMP-2 transcript profiles. The balance of activity between the endogenous enzyme inhibitors known as tissue inhibitors of metalloproteinases and their targets, the matrix metalloproteinases, in the   extracellular matrix   is thought to play an important role in tumour cell invasion. Supporting this notion, we have shown that colorectal cancer patients have increased plasma levels of the tissue inhibitor of metalloproteinases-1 (TIMP-1), and that high plasma TIMP-1 levels are associated with short colorectal cancer patient survival. However, although TIMP-1 has been extensively studied in cancer, very little is known about how it is regulated. To further elucidate potential mechanisms of regulation of this protein, we did a number of experiments to look at associations between the transcript profile of TIMP-1 with known matrix metalloproteinases (MMPs) as well as with expression profiles of other genes differentially regulated in human colorectal cancer (CRC) and the other TIMPs 2-4, which have also been associated with the progression of colorectal cancer. Genome-wide expression profiling of 172 CRC and normal mucosa samples was used to identify transcript changes for the genes under investigation. We found that TIMP-1 was up-regulated in CRC samples compared with normal tissue, while TIMP-2 was down-regulated. Eight MMPs were up-regulated in CRC compared with normal tissue. Correlating up-regulated genes with the TIMP-1 transcript, we identified 13 that were also up-regulated in cancerous tissue. Among these were genes associated with the synthesis of extracellullar matrix, genes involved in the TGF-beta signalling pathway, and genes that are likely transcribed by the tumour cells. These insights add to the complex picture emerging about the regulation of TIMPs in colorectal cancer. 
 Comparative study of the immunohistochemical expression of tissue inhibitors of metalloproteinases 1 and 2 between clearly invasive carcinomas and "in situ" trophoblast invasion. Tissue inhibitors of metalloproteinases (TIMPs) play an important role in   extracellular matrix   homeostasis by regulating MMP activity. Although they were initially considered inhibitors of tumor growth and metastasis, recently their role in cancer progression has been controversial. The aim of our study was to compare the immunohistochemical expression of TIMP1 and TIMP2 between an uncontrollably invasive phenomenon (cancer) and an "in situ" process (trophoblast invasion) in an effort to assess any differential role of these molecules between these two distinct phenomena and therefore to understand better their contribution in cancer invasion and migration. We performed an immunohistochemical analysis of 50 carcinomas (colorectal, gastric, breast, pulmonary, and renal) and 40 first trimester gestations. The marker expression was evaluated semiquantitatively, separately in cancer parenchymal and trophoblastic cells as well as in malignant stromal and decidual cells, according to a percentage scale (0, <10, 10-50, and >50%) and according to staining intensity (0, +, ++, and +++). Our results showed that there was no statistically significant difference in TIMP1 expression between cancer parenchymal cells and trophoblastic cells. On the other hand, TIMP1 was expressed more often in decidual cells than in cancer stromal cells. Immunostaining for TIMP2 was more extensive and intense both in trophoblastic and decidual cells than in cancer parenchymal and stromal cells, respectively. The reduced expression of TIMP2 in metastatic carcinomas by comparison with non-metastatic gestation specimens underlines its importance in cancer invasion and migration. On the other hand, TIMP1 was more expressed in decidua than cancer stroma, but at the same time showed no statistically significant difference between cancer parenchyma and trophoblasts, highlighting its multifunctional activity in cancer progression. 
 A genetic association study of maternal and fetal candidate genes that predispose to preterm prelabor rupture of membranes (PROM). We sought to determine whether maternal/fetal single-nucleotide polymorphisms (SNPs) in candidate genes are associated with preterm prelabor rupture of membranes (pPROM).A case-control study was conducted in patients with pPROM (225 mothers and 155 fetuses) and 599 mothers and 628 fetuses with a normal pregnancy; 190 candidate genes and 775 SNPs were studied. Single locus/haplotype association analyses were performed; false discovery rate was used to correct for multiple testing (q* = 0.15).First, a SNP in tissue inhibitor of metalloproteinase 2 in mothers was significantly associated with pPROM (odds ratio, 2.12; 95% confidence interval, 1.47-3.07; P = .000068), and this association remained significant after correction for multiple comparisons. Second, haplotypes for Alpha 3 type IV collagen isoform precursor in the mother were associated with pPROM (global P = .003). Third, multilocus analysis identified a 3-locus model, which included maternal SNPs in collagen type I alpha 2, defensin alpha 5 gene, and endothelin 1.DNA variants in a maternal gene involved in   extracellular matrix   metabolism doubled the risk of pPROM. 
 Urinary Metalloproteinases-9 and -2 and Their Inhibitors TIMP-1 and TIMP-2 are Markers of Early and Long-Term Graft Function After Renal Transplantation. Renal ischemia-reperfusion (I-R) injury (IRI) is an inseparable feature of organ transplantation and may have a negative impact on the graft, its function and survival. Acute tubular necrosis, which is reversible thanks to the regenerative capacity of renal tubular epithelial cells, is the main cause of acute renal failure secondary to IRI. MMP-2 and MMP-9 are proteolytic enzymes involved in digesting proteins that are components of the   extracellular matrix   (ECM) and the basement membrane of the nephrons. This way post-reperfusion MMP activation allows the inflammatory process to spread.In our studies, we focused on identifying whether the concentrations of MMP-2 and MMP-9 and their natural inhibitors TIMP-1 and TIMP-2 in urine sample at day 1 and day 30 as well as after 12 months following renal transplantation are markers of early and long-term renal function during meanly five-years observation. Moreover, in urine sampled at months 6 and 12 after renal transplantation, we determined the content of TGF-β as a graft fibrosis indicator.MMP-9 concentration in the early post-transplant period is a major marker of early and long-term function of the transplanted kidney. Its increased concentration was correlated with lesions related to tubular atrophy and fibrosis in renal biopsies performed at months 3 and 12 after transplantation. Its concentration is correlated with TGF-β content in a later period.TIMP-1 and-2 are primarily markers of an early function of the transplanted kidney. Early post-transplant concentration of MMP-2 is a marker of proteinuria in early and long-term post-transplant periods. 
 Functional polymorphisms in matrix metalloproteinases-1, -3, -9 are associated with arteriovenous fistula patency in hemodialysis patients. Matrix metalloproteinases (MMPs) are risk factors for cardiovascular diseases. This study evaluated the association of genotype polymorphisms of MMPs and tissue inhibitors of metalloproteinases (TIMPs) in hemodialysis (HD) patients with arteriovenous fistula (AVF) failure.Genotype polymorphism of MMP-1, MMP-2, MMP-3, and MMP-9 and TIMP-1 and TIMP-2 and clinical and laboratory parameters were compared between Chinese HD patients with (n = 170) and without (n = 426) AVF failure.Significant associations were found between AVF failure and the following factors (hazard ratio): longer HD duration (1.007 per month), lower pump flow (0.991 per ml/min), higher dynamic venous pressure (1.016 per mmHg), location of AVF on right side (1.630 versus left side) and upper arm (2.385 versus forearm), history of cardiovascular disease (1.656 versus absence of history), 1G/1G genotype of MMP-1 -1607 1G >2G SNP (2.315 versus 1G/2G + 2G/2G genotypes), 6A/6A genotype of MMP-3 -1612 5A >6A SNP (1.712 versus 5A/6A + 5A/5A), and C/C genotype of MMP-9 -1562 C>T SNP (1.650 versus C/T+T/T). The positive predictive rates for AVF failure were 63.0% and 6.7% for patients with the highest-risk (1G1G/6A6A/CC) and lowest-risk (2G2G or 2G1G/5A5A or 6A6A/TT or TC) composite MMP-1/MMP-3/MMP-9 genotype, respectively. The unassisted patency of AVF at 5 years decreased significantly from 93.3% to 38.4% for the composite MMP-1/MMP-3/MMP-9 genotypes (lowest versus highest risk, P < 0.001).Specific genotypes of MMP-1, MMP-3 and MMP-9 with lower transcriptional activity are associated with higher frequencies of AVF failure, which may result from more accumulation of   extracellular matrix  , leading to AVF stenosis. 
 Identification of fetal and maternal single nucleotide polymorphisms in candidate genes that predispose to spontaneous preterm labor with intact membranes. The purpose of this study was to determine whether maternal/fetal single nucleotide polymorphisms (SNPs) in candidate genes are associated with spontaneous preterm labor/delivery.A genetic association study was conducted in 223 mothers and 179 fetuses (preterm labor with intact membranes who delivered <37 weeks of gestation [preterm birth (PTB)]), and 599 mothers and 628 fetuses (normal pregnancy); 190 candidate genes and 775 SNPs were studied. Single locus/haplotype association analyses were performed; the false discovery rate was used to correct for multiple testing.The strongest single locus associations with PTB were interleukin-6 receptor 1 (fetus; P=.000148) and tissue inhibitor of metalloproteinase 2 (mother; P=.000197), which remained significant after correction for multiple comparisons. Global haplotype analysis indicated an association between a fetal DNA variant in insulin-like growth factor F2 and maternal alpha 3 type IV collagen isoform 1 (global, P=.004 and .007, respectively).An SNP involved in controlling fetal inflammation (interleukin-6 receptor 1) and DNA variants in maternal genes encoding for proteins involved in   extracellular matrix   metabolism approximately doubled the risk of PTB. 
 Gelatinases and their tissue inhibitors during human ovulation: increased expression of tissue inhibitor of matrix metalloproteinase-1. Remodelling of the   extracellular matrix   (ECM) of the follicular wall by matrix metalloproteinases (MMPs) and tissue inhibitors of matrix metalloproteinases (TIMPs) has been suggested to be crucial in ovulation. To investigate the expression of the gelatinases, MMP-2 and MMP-9, together with their inhibitors, TIMP-2 and TIMP-1, in the perifollicular ovarian stroma from women just before and during ovulation, we obtained biopsies of the stroma adjacent to the leading follicle. Laparoscopic surgery was performed either before the LH peak or at any of three intervals after ovulation triggering by hCG. Immunoblotting, immunohistochemistry and quantitative RT-PCR were performed. All four proteins were expressed by immunoblots, with no detectable changes in the expression of MMP-2, MMP-9 and TIMP-2. Scattered immunostaining for MMP-9 and TIMP-2 was seen, and MMP-2 was demonstrated in a concentric layer. A significant increase in TIMP-1 protein and mRNA was seen during the three ovulatory phases, and a strong and patchy immunostaining for TIMP-1 was shown. This is the first study that has demonstrated an ovulation-associated expression of these ECM-remodelling enzymes around the human follicle at ovulation. The increased expression of TIMP-1 may reflect a specific temporal inhibition of collagenolysis and thereby a time-dependent regulation of ECM breakdown in areas surrounding the apex of the follicle. 
 Association of matrix metalloproteinase gene polymorphisms with refractive error in Amish and Ashkenazi families. Matrix metalloproteinases (MMPs) and tissue inhibitors of metalloproteinases (TIMPs) are involved in scleral   extracellular matrix   remodeling and have shown differential expression in experimental myopia. The genetic association of refractive error and polymorphisms in MMP and TIMP genes in Old Order Amish (AMISH) and Ashkenazi Jewish (ASHK) families was investigated.Individuals from 55 AMISH and 63 ASHK families participated in the study. Ascertainment was designed to enrich the families for myopia; the mean spherical equivalent (MSE) refractive error (SD) was -1.61 (2.72) D in the AMISH, and -3.56 (3.32) D in the ASHK. One hundred forty-six common haplotype tagging SNPs covering 14 MMP and 4 TIMP genes were genotyped in 358 AMISH and 535 ASHK participants. Association analyses of MSE and the spherical component of refraction (SPH) were performed separately for the AMISH and the ASHK. Bonferroni-corrected significance thresholds and local false discovery rates were used to account for multiple testing.After they were filtered for quality-control, 127 SNPs were included in the analyses. No polymorphisms showed statistically significant association to refraction in the ASHK (minimum P = 0.0132). In AMISH, two SNPs showed evidence of association with refractive phenotypes: rs1939008 (P = 0.00016 for SPH); and rs9928731 (P = 0.00026 for SPH). These markers were each estimated to explain <5% of the variance of SPH in the AMISH sample.Statistically significant genetic associations of ocular refraction to polymorphisms near MMP1 and within MMP2 were identified in the AMISH but not among the ASHK families. The results suggest that the MMP1 and MMP2 genes are involved in refractive variation in the AMISH. Genetic and/or environmental heterogeneity most likely contribute to differences in association results between ethnic groups. 
 Expression of metalloproteinases and their tissue inhibitors in squamous cell laryngeal carcinoma. The hallmark of cancer invasion is the degradation of   extracellular matrix   components. Matrix metalloproteinases are the major enzymes participating in this event and their activity is regulated extracellularly by their presence as proenzymes and the concomitant presence of the specific tissue inhibitors. The present study describes the immunohistochemical localization of gelatinases, matrix metalloproteinase (MMP)-2 and -9 and tissue inhibitor of metalloproteinase (TIMP)-1 and -2 in human laryngeal carcinoma and their expression with respect to tumor classification and compared with the respective healthy subjects. MMP-2 was immunolocalized in the cytoplasm of the epithelial cells and in the loose connective tissue, whereas MMP-9 was also observed in basement membrane and chondrocytes. Both were also found in tumor cells, but staining was decreased with increasing stage of cancer. TIMP-1 was present exclusively in stroma and totally absent from tumor cells and it was overexpressed in normal cells surrounding the tumor. TIMP-2 was identified in the cytoplasm of epithelial cells, in stroma and sometimes in chondrocytes. In addition, it was present in tumor cells of only stage IV samples. The expression level of both gelatinases and TIMPs increased as the stage of cancer increased, suggesting the possible post-transcriptional removal of their mRNA. These observations, performed in a given head and neck site, suggest that the behavior of head and neck tumors seems to depend on the site and additional studies should be performed to obtain a general understanding of the disease and ascertain the role of the constituents examined. 
 Urinary Metalloproteinases-9 and -2 and Their Inhibitors TIMP-1 and TIMP-2 are Markers of Early and Long-Term Graft Function After Renal Transplantation. Renal ischemia-reperfusion (I-R) injury (IRI) is an inseparable feature of organ transplantation and may have a negative impact on the graft, its function and survival. Acute tubular necrosis, which is reversible thanks to the regenerative capacity of renal tubular epithelial cells, is the main cause of acute renal failure secondary to IRI. MMP-2 and MMP-9 are proteolytic enzymes involved in digesting proteins that are components of the   extracellular matrix   (ECM) and the basement membrane of the nephrons. This way post-reperfusion MMP activation allows the inflammatory process to spread.In our studies, we focused on identifying whether the concentrations of MMP-2 and MMP-9 and their natural inhibitors TIMP-1 and TIMP-2 in urine sample at day 1 and day 30 as well as after 12 months following renal transplantation are markers of early and long-term renal function during meanly five-years observation. Moreover, in urine sampled at months 6 and 12 after renal transplantation, we determined the content of TGF-β as a graft fibrosis indicator.MMP-9 concentration in the early post-transplant period is a major marker of early and long-term function of the transplanted kidney. Its increased concentration was correlated with lesions related to tubular atrophy and fibrosis in renal biopsies performed at months 3 and 12 after transplantation. Its concentration is correlated with TGF-β content in a later period.TIMP-1 and-2 are primarily markers of an early function of the transplanted kidney. Early post-transplant concentration of MMP-2 is a marker of proteinuria in early and long-term post-transplant periods. 
 Increased expression of metalloproteinase-2 and -9 (MMP-2, MMP-9), tissue inhibitor of metalloproteinase-1 and -2 (TIMP-1, TIMP-2), and EMMPRIN (CD147) in multiple myeloma. Activity of metalloproteinases (MMP) is controlled both by specific tissue inhibitors (TIMP) and activators (  extracellular matrix   metalloproteinase inducer, EMMPRIN). There are few data available concerning concentration the bone marrow of MMP-2, MMP-9, TIMP-1, and TIMP-2, or EMMPRIM expression by bone marrow mesenchymal stromal cells (BMSCs) in patients with multiple myeloma (MM).We studied 40 newly diagnosed, untreated patients: 18 males and 22 females with de novo MM and 11 healthy controls. Bone marrow was collected prior to therapy. BMSCs were derived by culturing bone marrow cells on MesenCult. Protein concentrations were determined in bone marrow plasma and culture supernatants by ELISA. EMMPRIN expression by BMSCs was assessed by flow cytometry.The median concentrations of MMP-9, TIMP-1, and TIMP-2 in both marrow plasma and culture supernatants were significantly higher in MM patients than controls.EMMPRIN expression and ratios MMP-9/TIMP-1 and MMP-2/TIMP-2 were higher in MM patients, our results demonstrate that in MM patients MMP-2 and MMP-9 are secreted in higher amounts and are not balanced by inhibitors. 
 TIMP1, TIMP2, and TIMP4 are increased in aqueous humor from primary open angle glaucoma patients. Elevated intraocular pressure (IOP) is the only known modifiable risk factor for primary open angle glaucoma (POAG), and it can be caused by reduced aqueous humor outflow from the anterior chamber. Outflow is predominantly regulated by the trabecular meshwork, consisting of specialized cells within a complex   extracellular matrix   (ECM). An imbalance between ECM-degrading matrix metalloproteinases (MMPs) and the tissue inhibitors of MMPs (TIMPs) within the trabecular meshwork is thought to contribute to POAG. This study aimed to quantify levels of TIMPs and MMPs in aqueous humor samples from glaucomatous and non-glaucomatous eyes, analyze MMP/TIMP ratios, and correlate results with age, IOP, and Humphrey's visual field pattern standard deviation (PSD).Aqueous humor samples were collected from 26 non-glaucomatous control subjects before cataract surgery and 23 POAG patients undergoing trabeculectomy or cataract surgery. Analyte concentrations were measured using multiplexed immunoassays. Statistical significance was assessed with Mann-Whitney U tests, and Spearman's method was used to assess correlations with age, IOP, and PSD.Concentrations of TIMP1 (p = 0.0008), TIMP2 (p = 0.002), TIMP4 (p = 0.002), and MMP2 (p = 0.020) were significantly increased in aqueous humor samples from POAG versus cataract samples. For the majority of MMP/TIMP molar ratios calculated for the cataract group, TIMPs outweighed MMPs. In POAG, molar ratios of MMP2/TIMP1 (p = 0.007) and MMP9/TIMP1 (p = 0.005) showed a significant decrease, corresponding to an elevated excess of TIMPs over MMPs in POAG compared to cataract samples. Conversely, MMP2/TIMP3 (p = 0.045) and MMP3/TIMP3 (p = 0.032) molar ratios increased. Several MMP/TIMP molar ratios correlated with IOP (r = 0.476-0.609, p = 0.007-0.034) and PSD (r = -0.482 to -0.655, p = 0.005-0.046) in POAG samples and with age in cataract control samples.An imbalance among MMPs and TIMPs was found in glaucomatous aqueous humor samples, with a shift toward raised TIMP levels. This may result in the inhibition of MMP activity, leading to an altered ECM composition in the TM and thereby contributing to increased outflow resistance. 
 Overexpression of matrix metalloproteinases in lung tissue of patients with primary spontaneous pneumothorax. Although blebs and bullae are frequently found in the apexes of lungs of patients with primary spontaneous pneumothorax (PSP), its pathogens remain unclear.To examine the role of proteases [matrix metalloproteinase (MMP)-2, MMP-7 and MMP-9] and antiproteases [tissue inhibitors of metalloproteinase (TIMP)-1, TIMP-2, TIMP-3 and TIMP-4] in the pathogenesis of PSP.Fifty consecutive PSP patients who received standard surgical care were enrolled in the study. Lung tissues from 20 patients with stage I non-small cell lung cancer were used as a control. Immunohistochemistry (IHC), reverse transcription-polymerase chain reaction (RT-PCR) and gelatin zymography were used to evaluate the expression of MMP and TIMP in the lung tissue of patients with PSP.Overexpression of MMP-2, MMP-7 and MMP-9 was found in the afflicted lung by IHC, zymography and RT-PCR. By IHC, higher expression of MMP-2 and MMP-9 in PSP patients was identified in alveolar macrophages and type II pneumocytes (88 and 92% of patients in macrophages, and 72 and 70% of patients in type II pneumocytes, respectively). MMP-2, MMP-7 and MMP-9 expression in patients was higher in mesothelial cells (66, 76 and 76%). Overexpression of TIMP-2 was detected in the   extracellular matrix   around bullae and blebs. Expression levels of TIMP-1, TIMP-3 and TIMP-4 were negligible (<10% of cells) in both PSP patients and controls.MMP-2, MMP-9, MMP-7 and TIMP-2 were upregulated in PSP lesions. These results suggest that an imbalance between the expression of proteases and antiproteases may be involved in the pathogeneses of PSP. 
 siRNA knockdown of tissue inhibitor of metalloproteinase-1 in keloid fibroblasts leads to degradation of collagen type I. Keloids are defined as overgrowths of scar tissue resulting from abnormal wound healing. They are characterized by excessive dermal deposition of thick, hyalinized collagen bundles resulting from an imbalance between the production and degradation of   extracellular matrix   (ECM) components. Matrix metalloproteinases (MMPs) and tissue inhibitors of metalloproteinases (TIMPs) are two important regulators of ECM degradation and remodeling. To evaluate the role played by knockdown of TIMPs in keloid formation, we transduced human keloid-derived fibroblasts (KFs) with small interfering RNAs targeting TIMP-1 or -2 (siTIMP-1 or siTIMP-2) using a lentiviral vector and assessed the biological effects. We found that MMP-1/TIMP-1 and MMP-1/TIMP-2 complexes were suppressed and that MMP-2 activity was upregulated in KFs expressing siTIMP-1 or siTIMP-2. In addition, increased degradation of collagen type I was observed in the supernatant of KFs expressing siTIMP-1, but not siTIMP-2, with the suppression of cell viability and induction of apoptosis. These results suggest that targeting TIMP-1 using small interfering RNA has significant therapeutic potential as an approach to treating keloids through degradation of their thick collagen bundles. 
 Matrix metalloproteinases-2, -3 and tissue inhibitors of metalloproteinases-1, -2 in placentas from preterm pregnancies and their association with one-carbon metabolites. Maternal nutrition is an important determinant of one-carbon metabolism and defects in the one-carbon metabolism may lead to poor obstetric outcomes. This study was designed to test the hypothesis that altered intake/metabolism of micronutrients (folic acid and vitamin B12) and docosahexaenoic acid (DHA) contributes to increased homocysteine and oxidative stress leading to altered levels of matrix metalloproteinases (MMPs) and tissue inhibitors of metalloproteinases (TIMPs) in women delivering preterm. We have earlier reported increased vitamin B12, homocysteine, and oxidative stress along with reduced placental DHA in women delivering preterm. In this study, we further examine the placental levels of MMP2, MMP3, TIMP1, and TIMP2 in 75 women delivering at term and 73 women delivering preterm. Placental levels of MMPs and TIMPs were determined by ELISA. Placental MMP2 and MMP3 levels were higher (P<0.01) in women delivering preterm as compared with term. There was no difference in the placental TIMP1 and TIMP2 levels in women delivering preterm and at term. Further placental MMP2 and MMP3 levels were higher (P<0.01) in women with preterm labor as compared with those in labor at term, suggesting that MMPs may favor degradation of   extracellular matrix   in the placenta during preterm labor. Our study for the first time suggests a crucial role of micronutrients and MMPs in preterm birth. Future studies need to examine if epigenetic modifications through the one-carbon cycle contribute to increased levels of MMPs leading to preterm deliveries. 
 Comparative study of the immunohistochemical expression of tissue inhibitors of metalloproteinases 1 and 2 between clearly invasive carcinomas and "in situ" trophoblast invasion. Tissue inhibitors of metalloproteinases (TIMPs) play an important role in   extracellular matrix   homeostasis by regulating MMP activity. Although they were initially considered inhibitors of tumor growth and metastasis, recently their role in cancer progression has been controversial. The aim of our study was to compare the immunohistochemical expression of TIMP1 and TIMP2 between an uncontrollably invasive phenomenon (cancer) and an "in situ" process (trophoblast invasion) in an effort to assess any differential role of these molecules between these two distinct phenomena and therefore to understand better their contribution in cancer invasion and migration. We performed an immunohistochemical analysis of 50 carcinomas (colorectal, gastric, breast, pulmonary, and renal) and 40 first trimester gestations. The marker expression was evaluated semiquantitatively, separately in cancer parenchymal and trophoblastic cells as well as in malignant stromal and decidual cells, according to a percentage scale (0, <10, 10-50, and >50%) and according to staining intensity (0, +, ++, and +++). Our results showed that there was no statistically significant difference in TIMP1 expression between cancer parenchymal cells and trophoblastic cells. On the other hand, TIMP1 was expressed more often in decidual cells than in cancer stromal cells. Immunostaining for TIMP2 was more extensive and intense both in trophoblastic and decidual cells than in cancer parenchymal and stromal cells, respectively. The reduced expression of TIMP2 in metastatic carcinomas by comparison with non-metastatic gestation specimens underlines its importance in cancer invasion and migration. On the other hand, TIMP1 was more expressed in decidua than cancer stroma, but at the same time showed no statistically significant difference between cancer parenchyma and trophoblasts, highlighting its multifunctional activity in cancer progression. 
 The role of matrix metalloproteinases (MMPs) and their inhibitors (TIMPs) in the development of esophageal cancer. Esophageal cancer (EC) is one of the most aggressive malignant tumors of the gastrointestinal tract. There are two distinct histological types of EC: esophageal squamous cell carcinoma and adenocarcinoma of the esophagus. Etiologic factors and the patterns of incidence of both subtypes are different. Matrix metalloproteinases (MMPs) and their tissue inhibitors (TIMPs) play an important role in esophageal carcinogenesis. Gellatinases MMP-2 and MMP-9 are able to degrade collagen IV from basement membranes and   extracellular matrix   which is related to tumor progression, including invasion, metastasis, growth and angiogenesis. It has been shown that increased expression of MMPs plays a crucial role in the development of several human malignancies, including esophageal cancer. The activity of MMPs is regulated by their endogenous natural inhibitors (TIMPs). Among these, the roles of TIMP-1 and TIMP-2 in EC development, tumor progression and formation of metastases have been most extensively characterized and best recognized. 
 Temporal investigation of matrix metalloproteinases and their inhibitors in human cervicovaginal fluid in late pregnancy and labor. Temporal expression of matrix metalloproteinase (MMP)-1, -2, -3, -7, -8, -9, -12, and -13, and tissue inhibitors of metalloproteinases (TIMPs)-1 and -2 in human cervicovaginal fluid (CVF) in term pregnancy and labor was investigated. Term parous women provided CVF samples that were grouped into labor, 1 to 3, 6 to 8, and 12 to 16 days before labor onset. Both MMPs and TIMPs (n = 60) were quantified using multiplex solution array and enzyme-linked immunosorbent assays, respectively. Further analysis of TIMP-1 (n = 180) was undertaken. All MMPs and TIMPs except MMP-12 and -13 were detected in the CVF. Matrix metalloproteinase 7, TIMP-1, and TIMP-2 were significantly increased in labor. Tissue inhibitors of metalloproteinase 1 was significantly increased up to 7 days before spontaneous labor onset. The data suggest a role of MMP-7 in the remodeling and rupture of fetal membranes and may reflect the homeostatic regulation of   extracellular matrix   remodeling of MMP-7 by TIMP-1 and TIMP-2. 
 Age- and gender-dependent changes in connective tissue remodeling: physiological differences in circulating MMP-3, MMP-10, TIMP-1 and TIMP-2 level. the mechanisms which cause age-dependent remodeling of connective tissue are still not fully understood. Matrix metalloproteinases (MMPs) and tissue inhibitors of metalloproteinases (TIMPs) constitute an important proteolytic pathway affecting physiological matrix remodeling.the way in which changes in the   extracellular matrix   metabolism during the ageing process influence the level of circulating MMP-3 and MMP-10, as well as their tissue inhibitors TIMP-1 and TIMP-2, in a healthy population was investigated in this study.blood samples were taken from 81 healthy individuals aged 6-62 years and measured for MMP-3, MMP-10, TIMP-1 and TIMP-2 levels using enzyme-linked immunosorbent assays. Polyacrylamide gel electrophoresis followed by Western immunoblotting allowed for the detection of pro- and active forms of both MMPs.Serum MMP-3 and TIMP-1 values were positively correlated with age (r = 0.44, p = 0.00001 and r = 0.28, p = 0.012, respectively). A contrary tendency was found for MMP-10 and TIMP-2 serum levels. A strong age-related decrease in MMP-10 (-0.53; p = 0.000) and TIMP-2 (-0.52; p = 0.000) was noticed in our study. Gender was a significant factor modifying MMP/TIMP potential, except for the MMP-10 level.the data presented indicate that changes in MMP/TIMP balance occur in physiological ageing. Moreover, these findings highlight the necessity of utilizing age- and sex-matched values for analysis of MMPs and TIMPs in the pathological conditions. 
 [Expression of matrix metalloproteinases 2 and 9 and their tissue inhibitors 1 and 2 in papillary thyroid cancer: an association with the clinical, morphological and ultrastructural characteristics of a tumor]. Papillary thyroid cancer (PTC) was ascertained to be characterized by a significant expression of matrix metalloproteinases (MMP-2 and 9) and a TIMP-2 inhibitor and decreased TIMP-1 accumulation in the cytoplasm of tumor cells; at the same time a combination of high cytoplasmic MMP-9 expression with no TIMP-1, as well as triple positive co-expression of MMP-2, MMP-9, and TIMP-2 are poor immunohistochemical indicators. The results of the performed study suggest that the detection of the types of expression of MMP-9+/TIMP-1 and MMP-2+/MMP-9+/TIMP-2+ is associated with the possible progression of a tumor process and concurrent with poor clinical and morphological signs, such as the large size of the tumor, the presence of intravascular invasion and regional metastases, organ capsular invasion, and low ultrastructural tumor cell differentiation. Thus, changes in the tumor expression of the   extracellular matrix   enzymes MMR-2 and MMP-9 and their inhibitors TIMP-1 and TIMP-2 along with the major clinical, morphological, and ultrastructural signs of the disease are important additional indicators for analyzing the malignant potential of PTC. 
 In vitro study of matrix metalloproteinase/tissue inhibitor of metalloproteinase production by mesenchymal stromal cells in response to inflammatory cytokines: the role of their migration in injured tissues. The transmigratory capacity of bone marrow (BM) mesenchymal stromal cells (MSC) through the endothelial cell barrier into various tissues and their differentiation potential makes them ideal candidates for cell therapy. Nevertheless, the mechanisms and agents promoting their migration are not fully understood. We evaluated the effects of several inflammatory cytokines on the migration of BM MSC and matrix metalloproteinase (MMP)/tissue inhibitor of metalloproteinase (TIMP) production.The migratory potential of BM MSC was evaluated using a Boyden chamber coated with Matrigel in the presence and absence of stromal cell-derived (SDF)-1alpha, platelet-derived growth factor (PDGF)bb, insulin-like growth factor (IGF)-I and interleukin (IL)-6. The ability of inflammatory cytokines to induce MSC migration was tested in presence of their respective Ab or blocking peptide. We used immunofluorescence to check the expression of cytokine receptors, and MMP/TIMP production was analyzed at the protein (human cytokine array, enzyme-linked immunosorbent assay (ELISA), gelatine zymography and Western blot) and mRNA quantitative real-time polymerase chain reaction (qRT-PCR) levels.We have demonstrated that inflammatory cytokines promote the migratory capacity of BM MSC according to the expression of their respective receptors. Higher migration through Matrigel was observed in response to IL-6 and PDGFbb. qRT-PCR and cytokine array revealed that migration was the result of the variable level of MMP/TIMP in response to inflammatory stimuli.Our observations suggest that chemokines and cytokines involved in the regulation of the immunity or inflammatory process promote the migration of MSC into BM or damaged tissues. One of the mechanisms used by MSC to promote their migration though the   extracellular matrix   is modulation of the production of MMP-1, MMP-2, MMP-13, TIMP-1 and TIMP-2. 
 Expression of MMP-7, MMP-9, TIMP-1 and TIMP-2 mRNA in lung tissue of patients with non-small cell lung cancer (NSCLC) and benign pulmonary disease. The expression of matrix metallo-proteinases (MMP-7 and MMP-9) and tissue inhibitors of metalloproteinases (TIMP-1 and TIMP-2), which are involved in the degradation of the   extracellular matrix   (ECM) and tumor growth, was investigated in normal lung tissue, tissue of benign pulmonary diseases and non-small cell lung cancer (NSCLC) tissue.Tumor tissue and surrounding carcinoma-free lung tissue samples were obtained from 91 patients with NSCLC who had undergone surgery in the years 2005-2007 as well as lung tissue from 12 patients operated on for 'benign' bullous emphysema or interstitial lung disease. The mRNA was isolated from the tissues and the expression of mRNA was assessed using a real-time RT PCR method.Significantly higher expression of MMP-7, MMP-9 and TIMP-1 mRNA was demonstrated in the NSCLC tissue in comparison with the normal lung tissue from the same patients (p=0.0003, p<0.0001 and p=0.0018, respectively). Similar results for MMP-7, MMP-9 and TIMP-1 were found in the histological subgroups: squamous cell lung cancer vs. normal tissue (p=0.0198, p=0.0015 and p=0.0366, respectively), and adenocarcinoma vs. normal tissue (p=0.0045, p<0.0001 and p=0.0140, respectively). The expression of MMP-7 was found to be significantly higher in tumor tissue vs. lung tissue of the benign diseases (p=0.0086) and similar results were also recorded in the histological subgroups: squamous cell lung cancer vs. benign tissue (p=0.0171) and adenocarcinoma vs. benign tissue (p=0.0135). The expression of MMP-9 was significantly higher only in the adenocarcinoma subgroup vs. the benign tissue (p=0.0412). No differences in the expression of mRNA between stage IA and stages IB-IIIB of NSCLC were recorded.Significantly higher expression of MMP-7 and MMP-9 in tumor tissue than in the surrounding tissue or in benign lung disease tissue supports the notion of an important role of these metalloproteinases in the growth of lung carcinoma. TIMP-1 expression is increased only in carcinoma, but not in benign lung disease. 
 TIMP-1 expression in human colorectal cancer is associated with TGF-B1, LOXL2, INHBA1, TNF-AIP6 and TIMP-2 transcript profiles. The balance of activity between the endogenous enzyme inhibitors known as tissue inhibitors of metalloproteinases and their targets, the matrix metalloproteinases, in the   extracellular matrix   is thought to play an important role in tumour cell invasion. Supporting this notion, we have shown that colorectal cancer patients have increased plasma levels of the tissue inhibitor of metalloproteinases-1 (TIMP-1), and that high plasma TIMP-1 levels are associated with short colorectal cancer patient survival. However, although TIMP-1 has been extensively studied in cancer, very little is known about how it is regulated. To further elucidate potential mechanisms of regulation of this protein, we did a number of experiments to look at associations between the transcript profile of TIMP-1 with known matrix metalloproteinases (MMPs) as well as with expression profiles of other genes differentially regulated in human colorectal cancer (CRC) and the other TIMPs 2-4, which have also been associated with the progression of colorectal cancer. Genome-wide expression profiling of 172 CRC and normal mucosa samples was used to identify transcript changes for the genes under investigation. We found that TIMP-1 was up-regulated in CRC samples compared with normal tissue, while TIMP-2 was down-regulated. Eight MMPs were up-regulated in CRC compared with normal tissue. Correlating up-regulated genes with the TIMP-1 transcript, we identified 13 that were also up-regulated in cancerous tissue. Among these were genes associated with the synthesis of extracellullar matrix, genes involved in the TGF-beta signalling pathway, and genes that are likely transcribed by the tumour cells. These insights add to the complex picture emerging about the regulation of TIMPs in colorectal cancer. 
 Hematopoiesis in mice is extremely resilient to wide variation in TIMP/MMP balance. Tissue inhibitors of matrix metalloproteinases (TIMPs) are natural inhibitors of matrix metalloproteinases (MMPs) and are associated with normal and pathologic   extracellular matrix   turnover. Because the microenvironment is critical for normal hematopoietic stem/progenitor cell function, we aimed to determine whether alterations in the TIMP/MMP balance impact upon normal hematopoiesis in mice. We have used both overexpression and knockout mouse models to determine whether early hematopoiesis is susceptible to potentially pathologic changes in TIMP/MMP level. These studies used TIMP-1(-/-) mice and retroviral vectors co-expressing human TIMP-1 or TIMP-2 linked with the green fluorescent protein (GFP) transduced into bone marrow (BM) cells and transplanted into lethally-irradiated recipient mice. Loss of TIMP-1 in knockout mice or retroviral overexpression of TIMP-1 or TIMP-2 did not alter hematopoietic stem/progenitor function during steady-state hematopoiesis. Surprisingly, even when applying hematopoietic stress through mobilization, chemotaxis, or myelosuppression, murine hematopoiesis was not adversely affected by TIMP-1 or TIMP-2 level. We conclude that TIMP/MMP balance alone does not exert significant influence on blood cell development and homeostasis. An important corollary of these studies is that specific modulation using MMP inhibitors for cancer or immunologic therapy is unlikely to have adverse hematopoietic side effects. 
 Genetic analysis of polymorphisms in biologically relevant candidate genes in patients with abdominal aortic aneurysms. Abdominal aortic aneurysms (AAAs) are characterized by histologic signs of chronic inflammation, destructive remodeling of   extracellular matrix  , and depletion of vascular smooth muscle cells. We investigated the process of   extracellular matrix   remodeling by performing a genetic association study with polymorphisms in the genes for matrix metalloproteinases (MMPs), tissue inhibitors of metalloproteinases (TIMPs), and structural   extracellular matrix   molecules in AAA. Our hypothesis was that genetic variations in one or more of these genes contribute to greater or lesser activity of these gene products, and thereby contribute to susceptibility for developing AAAs.DNA samples from 812 unrelated white subject (AAA, n = 387; controls, n = 425) were genotyped for 14 polymorphisms in 13 different candidate genes: MMP1(nt-1607), MMP2(nt-955), MMP3(nt-1612), MMP9(nt-1562), MMP10(nt+180), MMP12(nt-82), MMP13(nt-77), TIMP1(nt+434), TIMP1(rs2070584), TIMP2(rs2009196), TIMP3(nt-1296), TGFB1(nt-509), ELN(nt+422), and COL3A1(nt+581). Odds ratios and P values adjusted for gender and country of origin using logistic regression and stratified by family history of AAA were calculated to test for association between genotype and disease status. Haplotype analysis was carried out for the two TIMP1 polymorphisms in male subjects.Analyses with one polymorphism per test without interactions showed an association with the two TIMP1 gene polymorphisms (nt+434, P = .0047; rs2070584, P = .015) in male subjects without a family history of AAA. The association remained significant when analyzing TIMP1 haplotypes (chi 2 P = .014 and empirical P = .009). In addition, we found a significant interaction between the polymorphism and gender for MMP10 ( P = .037) in cases without a family history of AAA, as well as between the polymorphism and country of origin for ELN ( P = .0169) and TIMP3 ( P = .0023) in cases with a family history of AAA.These findings suggest that genetic variations in TIMP1, TIMP3, MMP10, and ELN genes may contribute to the pathogenesis of AAAs. Further work is needed to confirm the findings in an independent set of samples and to study the functional role of these variants in AAA. It is noteworthy that contrary to a previous study, we did not find an association between the MMP9 (nt-1562) polymorphism and AAA, suggesting genetic heterogeneity of the disease.Abdominal aortic aneurysms (AAAs) are an important cardiovascular disease, but the genetic and environmental risk factors, which contribute to individual's risk to develop an aneurysm, are poorly understood. Histologically, AAAs are characterized by signs of chronic inflammation, destructive remodeling of the   extracellular matrix  , and depletion of vascular smooth muscle cells. We hypothesized that genes involved in these events could harbor changes that make individuals more susceptible to developing aneurysms. This study identified significant genetic associations between DNA sequence changes in tissue inhibitor of metalloproteinase 1 (TIMP1), TIMP3, matrix metalloproteinase 10 (MMP10) and elastin (ELN) genes, and AAA. The results will require confirmation using an independent set of samples. After replication it is possible that these sequence changes in combination with other risk factors could be used in the future to identify individuals who are at increased risk for developing an AAA. 
 Tissue inhibitor of metalloproteinases-1, -2, and -3 polymorphisms in a white population with intracranial aneurysms. Remodeling of the   extracellular matrix   seems to be a crucial event in the pathogenesis of cerebral aneurysms. Matrix metalloproteinases are the most important degrading enzymes in the   extracellular matrix  . Their activity is controlled predominantly by tissue inhibitors of metalloproteinases (TIMPs). To investigate the possible impact of genetic variants within the genes encoding TIMP-1, -2, and -3, we conducted this case-control study.A study sample was analyzed that comprised 44 patients with intracranial aneurysms and 44, 41, and 40 controls for the analysis of TIMP-1, -2, and -3, respectively. Differences in genotype and allele frequencies of identified polymorphisms were determined. The entire coding regions and parts of the promoter sequences of the TIMP-1, -2, and -3 genes were with using the automated laser fluorescence technique.Nine polymorphisms were identified, 3 located in TIMP-1 (-19C>T, 261C>T, 372T>C), 4 in TIMP-2 (-621C>T, -596A>C, -261G>A, 303G>A), and 2 in TIMP-3 (249T>C, 261C>T), whereas -621C>T, -596A>C, and -261G>A of the TIMP-2 gene are newly identified polymorphisms. We detected no deviation from Hardy-Weinberg equilibrium in any of the groups. The C allele of the 372T>C polymorphism was more frequently found in female than in male controls (exact nominal P=0.0012). However, this finding could not be validated by analysis of a second sample of 113 controls (exact nominal P=1.0000). There were no differences in genotype and allele frequencies between any of the other groups.Our analysis of the entire coding region of 3 TIMPs, which are the main inhibitors of metalloproteinase activity in the   extracellular matrix  , failed to show an association between genetic polymorphisms and an intracranial aneurysm. These data do not support the hypothesis that genetic variants within these genes have an impact on aneurysm development in the white population. 
 Identification of fetal and maternal single nucleotide polymorphisms in candidate genes that predispose to spontaneous preterm labor with intact membranes. The purpose of this study was to determine whether maternal/fetal single nucleotide polymorphisms (SNPs) in candidate genes are associated with spontaneous preterm labor/delivery.A genetic association study was conducted in 223 mothers and 179 fetuses (preterm labor with intact membranes who delivered <37 weeks of gestation [preterm birth (PTB)]), and 599 mothers and 628 fetuses (normal pregnancy); 190 candidate genes and 775 SNPs were studied. Single locus/haplotype association analyses were performed; the false discovery rate was used to correct for multiple testing.The strongest single locus associations with PTB were interleukin-6 receptor 1 (fetus; P=.000148) and tissue inhibitor of metalloproteinase 2 (mother; P=.000197), which remained significant after correction for multiple comparisons. Global haplotype analysis indicated an association between a fetal DNA variant in insulin-like growth factor F2 and maternal alpha 3 type IV collagen isoform 1 (global, P=.004 and .007, respectively).An SNP involved in controlling fetal inflammation (interleukin-6 receptor 1) and DNA variants in maternal genes encoding for proteins involved in   extracellular matrix   metabolism approximately doubled the risk of PTB. 
 A genetic association study of maternal and fetal candidate genes that predispose to preterm prelabor rupture of membranes (PROM). We sought to determine whether maternal/fetal single-nucleotide polymorphisms (SNPs) in candidate genes are associated with preterm prelabor rupture of membranes (pPROM).A case-control study was conducted in patients with pPROM (225 mothers and 155 fetuses) and 599 mothers and 628 fetuses with a normal pregnancy; 190 candidate genes and 775 SNPs were studied. Single locus/haplotype association analyses were performed; false discovery rate was used to correct for multiple testing (q* = 0.15).First, a SNP in tissue inhibitor of metalloproteinase 2 in mothers was significantly associated with pPROM (odds ratio, 2.12; 95% confidence interval, 1.47-3.07; P = .000068), and this association remained significant after correction for multiple comparisons. Second, haplotypes for Alpha 3 type IV collagen isoform precursor in the mother were associated with pPROM (global P = .003). Third, multilocus analysis identified a 3-locus model, which included maternal SNPs in collagen type I alpha 2, defensin alpha 5 gene, and endothelin 1.DNA variants in a maternal gene involved in   extracellular matrix   metabolism doubled the risk of pPROM. 
 Association of matrix metalloproteinase gene polymorphisms with refractive error in Amish and Ashkenazi families. Matrix metalloproteinases (MMPs) and tissue inhibitors of metalloproteinases (TIMPs) are involved in scleral   extracellular matrix   remodeling and have shown differential expression in experimental myopia. The genetic association of refractive error and polymorphisms in MMP and TIMP genes in Old Order Amish (AMISH) and Ashkenazi Jewish (ASHK) families was investigated.Individuals from 55 AMISH and 63 ASHK families participated in the study. Ascertainment was designed to enrich the families for myopia; the mean spherical equivalent (MSE) refractive error (SD) was -1.61 (2.72) D in the AMISH, and -3.56 (3.32) D in the ASHK. One hundred forty-six common haplotype tagging SNPs covering 14 MMP and 4 TIMP genes were genotyped in 358 AMISH and 535 ASHK participants. Association analyses of MSE and the spherical component of refraction (SPH) were performed separately for the AMISH and the ASHK. Bonferroni-corrected significance thresholds and local false discovery rates were used to account for multiple testing.After they were filtered for quality-control, 127 SNPs were included in the analyses. No polymorphisms showed statistically significant association to refraction in the ASHK (minimum P = 0.0132). In AMISH, two SNPs showed evidence of association with refractive phenotypes: rs1939008 (P = 0.00016 for SPH); and rs9928731 (P = 0.00026 for SPH). These markers were each estimated to explain <5% of the variance of SPH in the AMISH sample.Statistically significant genetic associations of ocular refraction to polymorphisms near MMP1 and within MMP2 were identified in the AMISH but not among the ASHK families. The results suggest that the MMP1 and MMP2 genes are involved in refractive variation in the AMISH. Genetic and/or environmental heterogeneity most likely contribute to differences in association results between ethnic groups. 
 Functional polymorphisms in matrix metalloproteinases-1, -3, -9 are associated with arteriovenous fistula patency in hemodialysis patients. Matrix metalloproteinases (MMPs) are risk factors for cardiovascular diseases. This study evaluated the association of genotype polymorphisms of MMPs and tissue inhibitors of metalloproteinases (TIMPs) in hemodialysis (HD) patients with arteriovenous fistula (AVF) failure.Genotype polymorphism of MMP-1, MMP-2, MMP-3, and MMP-9 and TIMP-1 and TIMP-2 and clinical and laboratory parameters were compared between Chinese HD patients with (n = 170) and without (n = 426) AVF failure.Significant associations were found between AVF failure and the following factors (hazard ratio): longer HD duration (1.007 per month), lower pump flow (0.991 per ml/min), higher dynamic venous pressure (1.016 per mmHg), location of AVF on right side (1.630 versus left side) and upper arm (2.385 versus forearm), history of cardiovascular disease (1.656 versus absence of history), 1G/1G genotype of MMP-1 -1607 1G >2G SNP (2.315 versus 1G/2G + 2G/2G genotypes), 6A/6A genotype of MMP-3 -1612 5A >6A SNP (1.712 versus 5A/6A + 5A/5A), and C/C genotype of MMP-9 -1562 C>T SNP (1.650 versus C/T+T/T). The positive predictive rates for AVF failure were 63.0% and 6.7% for patients with the highest-risk (1G1G/6A6A/CC) and lowest-risk (2G2G or 2G1G/5A5A or 6A6A/TT or TC) composite MMP-1/MMP-3/MMP-9 genotype, respectively. The unassisted patency of AVF at 5 years decreased significantly from 93.3% to 38.4% for the composite MMP-1/MMP-3/MMP-9 genotypes (lowest versus highest risk, P < 0.001).Specific genotypes of MMP-1, MMP-3 and MMP-9 with lower transcriptional activity are associated with higher frequencies of AVF failure, which may result from more accumulation of   extracellular matrix  , leading to AVF stenosis. 
 Matrix metalloproteinase expression in the ascending aorta and aortic valve.   Extracellular matrix   degradation and increased proteolytic enzyme (matrix metalloproteinase (MMP)) activity characterise abdominal aortic aneurysm formation. Post-stenotic dilatation of ascending aorta is associated with aortic stenosis and regurgitation, haemodynamically normal bicuspid aortic valve (BAV) and following AV replacement. We aimed to determine an association between ascending aortic pathology and abnormal AV, with particular reference to MMPs, and ascertain differences between BAV and tricuspid (TAV) AV. Subset of the study population (n=19) with a preoperative ascending aorta of >4 cm was analysed. Samples of ascending aorta and AV were obtained from 82 patients (TAV, n=54, BAV, n=28) undergoing surgery. Gene expression of MMP-1, -2, -9 and tissue inhibitor of metalloproteinase (TIMP)-1 and -2 was quantified by real-time RT-PCR. No significant difference was seen in gene expression level of MMPs, TIMPs and ratio of MMPs/TIMPs in ascending aorta and AV between patients with BAV and TAV. MMP-2/TIMP-1 in ascending aorta was greater in BAV, in the subset of patients with preoperative aortic dilatation (P<0.05). No difference exists in gene expression of MMPs in ascending aorta and AV between patients with BAV and TAV. However, patients with larger aortic diameters have increased MMP-2/TIMP-1. Modifying MMP expression may have a role in development of aneurysms. 
 Expression of metalloproteinases and their tissue inhibitors in squamous cell laryngeal carcinoma. The hallmark of cancer invasion is the degradation of   extracellular matrix   components. Matrix metalloproteinases are the major enzymes participating in this event and their activity is regulated extracellularly by their presence as proenzymes and the concomitant presence of the specific tissue inhibitors. The present study describes the immunohistochemical localization of gelatinases, matrix metalloproteinase (MMP)-2 and -9 and tissue inhibitor of metalloproteinase (TIMP)-1 and -2 in human laryngeal carcinoma and their expression with respect to tumor classification and compared with the respective healthy subjects. MMP-2 was immunolocalized in the cytoplasm of the epithelial cells and in the loose connective tissue, whereas MMP-9 was also observed in basement membrane and chondrocytes. Both were also found in tumor cells, but staining was decreased with increasing stage of cancer. TIMP-1 was present exclusively in stroma and totally absent from tumor cells and it was overexpressed in normal cells surrounding the tumor. TIMP-2 was identified in the cytoplasm of epithelial cells, in stroma and sometimes in chondrocytes. In addition, it was present in tumor cells of only stage IV samples. The expression level of both gelatinases and TIMPs increased as the stage of cancer increased, suggesting the possible post-transcriptional removal of their mRNA. These observations, performed in a given head and neck site, suggest that the behavior of head and neck tumors seems to depend on the site and additional studies should be performed to obtain a general understanding of the disease and ascertain the role of the constituents examined. 
 Relationship between matrix metalloproteinases/tissue inhibitors of matrix metalloproteinases systems and autoantibody patterns in systemic sclerosis. Systemic sclerosis (SSc) is a connective tissue disease characterized by tissue fibrosis that reflects an imbalance between collagen production and degradation. Matrix metalloproteinases (MMPs) are a family of endopeptidases involved in the remodelling of   extracellular matrix   (ECM). This activity is controlled by tissue inhibitors of MMP (TIMPs). Aim of this study was the evaluation of MMP-9/TIMP-1 and MMP-2/TIMP-2 systems in patients with SSc.SearchLight Human MMP Array 1 was used to measure MMPs and TIMPs in 32 SSc patients and 32 matched healthy controls.SSc patients showed higher values of both MMP-9 and TIMP-1 in comparison with controls. The patients with anticentromere antibodies (ACA) positivity showed higher values of MMPs and TIMPs in comparison with either controls or the patients with anti-Scl70-positive antibodies.Results of this investigation suggest that SSc patients with ACA positivity, after a primary fibrogenetic noxa, react with a more abundant release of MMP/TIMP, whereas patients with anti-Scl70 antibody show a normal response. 
 Matrix metalloproteinases MMP-2, -9 and tissue inhibitors TIMP-1, -2 expression and secretion by primary human osteoblast cells in response to titanium, zirconia, and alumina ceramics. Osteogenic properties of bone cells are a key parameter governing osseointegration of implant devices. In this context, osteoblasts have a central role via   extracellular matrix   synthesis and remodeling that they regulate through different protease activity. In this study, we have analyzed the expression of two matrix metalloproteinases (MMPs): MMP-2 (72 kDa) and MMP-9 (92 kDa) and their specific tissue inhibitors TIMP-1 and TIMP-2 in primary human osteoblastic cells. The effect of titanium, zirconia, and alumina ceramics on the synthesis of these proteases was assessed using reverse transcriptase-polymerase chain reaction, enzyme-linked immunosorbent assay, and zymographic analysis. Our results showed that osteoblasts express MMP-2 and -9 mRNA. Furthermore, MMP-2 mRNA expression was decreased by titanium and increased by alumina whereas zirconia did not have any significant effect. Conversely, MMP-9 mRNA expression was stimulated by titanium but decreased with zirconia, whereas alumina induced no significant changes. Zymographic analysis has evidenced pro-MMP-2 gelatinolytic activity in all cell populations with time-dependent increase profile; pro-MMP-9, however, was not detected. Enzyme-linked immunosorbent assay data confirmed the production of MMP-2 and very low levels of MMP-9. In addition, TIMP-1 was secreted in 24-h-cultured cells and increased to maximal level at 48-72 h whereas TIMP-2 levels were very low. The interactions between human osteoblasts and the studied biomaterials altered both MMP-2, -9 and TIMP-1expression indicating that biomaterials may influence osseointegration and bone remodeling. 
 Gelatinases and their tissue inhibitors during human ovulation: increased expression of tissue inhibitor of matrix metalloproteinase-1. Remodelling of the   extracellular matrix   (ECM) of the follicular wall by matrix metalloproteinases (MMPs) and tissue inhibitors of matrix metalloproteinases (TIMPs) has been suggested to be crucial in ovulation. To investigate the expression of the gelatinases, MMP-2 and MMP-9, together with their inhibitors, TIMP-2 and TIMP-1, in the perifollicular ovarian stroma from women just before and during ovulation, we obtained biopsies of the stroma adjacent to the leading follicle. Laparoscopic surgery was performed either before the LH peak or at any of three intervals after ovulation triggering by hCG. Immunoblotting, immunohistochemistry and quantitative RT-PCR were performed. All four proteins were expressed by immunoblots, with no detectable changes in the expression of MMP-2, MMP-9 and TIMP-2. Scattered immunostaining for MMP-9 and TIMP-2 was seen, and MMP-2 was demonstrated in a concentric layer. A significant increase in TIMP-1 protein and mRNA was seen during the three ovulatory phases, and a strong and patchy immunostaining for TIMP-1 was shown. This is the first study that has demonstrated an ovulation-associated expression of these ECM-remodelling enzymes around the human follicle at ovulation. The increased expression of TIMP-1 may reflect a specific temporal inhibition of collagenolysis and thereby a time-dependent regulation of ECM breakdown in areas surrounding the apex of the follicle. 
 Tissue inhibitor of metalloproteinases-1, -2, and -3 polymorphisms in a white population with intracranial aneurysms. Remodeling of the   extracellular matrix   seems to be a crucial event in the pathogenesis of cerebral aneurysms. Matrix metalloproteinases are the most important degrading enzymes in the   extracellular matrix  . Their activity is controlled predominantly by tissue inhibitors of metalloproteinases (TIMPs). To investigate the possible impact of genetic variants within the genes encoding TIMP-1, -2, and -3, we conducted this case-control study.A study sample was analyzed that comprised 44 patients with intracranial aneurysms and 44, 41, and 40 controls for the analysis of TIMP-1, -2, and -3, respectively. Differences in genotype and allele frequencies of identified polymorphisms were determined. The entire coding regions and parts of the promoter sequences of the TIMP-1, -2, and -3 genes were with using the automated laser fluorescence technique.Nine polymorphisms were identified, 3 located in TIMP-1 (-19C>T, 261C>T, 372T>C), 4 in TIMP-2 (-621C>T, -596A>C, -261G>A, 303G>A), and 2 in TIMP-3 (249T>C, 261C>T), whereas -621C>T, -596A>C, and -261G>A of the TIMP-2 gene are newly identified polymorphisms. We detected no deviation from Hardy-Weinberg equilibrium in any of the groups. The C allele of the 372T>C polymorphism was more frequently found in female than in male controls (exact nominal P=0.0012). However, this finding could not be validated by analysis of a second sample of 113 controls (exact nominal P=1.0000). There were no differences in genotype and allele frequencies between any of the other groups.Our analysis of the entire coding region of 3 TIMPs, which are the main inhibitors of metalloproteinase activity in the   extracellular matrix  , failed to show an association between genetic polymorphisms and an intracranial aneurysm. These data do not support the hypothesis that genetic variants within these genes have an impact on aneurysm development in the white population. 
 Tissue inhibitor of metalloproteinase 1 is an independent predictor of prognosis in patients with nonsmall cell lung carcinoma who undergo resection with curative intent. Matrix metalloproteinases (MMPs) and tissue inhibitors of metalloproteinases (TIMPs) play a role in the processes of   extracellular matrix   degradation. Changes in their expression levels have been observed in various tumor types, including lung carcinoma. However, their clinical significance and their prognostic importance in the progression of nonsmall cell lung carcinoma (NSCLC) remain to be specified. In this study, mRNA expression levels of MMP-1, MMP-9, TIMP-1, and TIMP-2 were evaluated in patients with resected NSCLC, and their associations with disease progression and prognosis were determined.Between June 1996 and December 1999, 116 patients underwent resection for NSCLC. Expression levels of MMPs and TIMPs were evaluated using Northern blot analysis in these NSCLC tissue samples and in 39 matched samples of normal lung tissue.MMP-1, MMP-9, and TIMP-1 expression levels were increased in tumor samples compared with matched, corresponding normal tissues. In contrast, TIMP-2 expression was decreased in tumor samples. MMP-1 tumor expression was correlated significantly with the evolution of lymph node status and tumor-lymph node-metastasis (TNM) stage. In contrast, MMP-9 tumor expression was correlated significantly with increased T stage. TIMP-1 overexpression was an independent predictor of worse survival in patients with NSCLC that was not associated with other prognosis factors, such as TNM stage.The overexpression of TIMP-1 was an independent prognostic marker in patients with NSCLC, and evaluating TIMP-1 may be important for identifying patients who are at greater risk of disease recurrence. 
 Genetic analysis of polymorphisms in biologically relevant candidate genes in patients with abdominal aortic aneurysms. Abdominal aortic aneurysms (AAAs) are characterized by histologic signs of chronic inflammation, destructive remodeling of   extracellular matrix  , and depletion of vascular smooth muscle cells. We investigated the process of   extracellular matrix   remodeling by performing a genetic association study with polymorphisms in the genes for matrix metalloproteinases (MMPs), tissue inhibitors of metalloproteinases (TIMPs), and structural   extracellular matrix   molecules in AAA. Our hypothesis was that genetic variations in one or more of these genes contribute to greater or lesser activity of these gene products, and thereby contribute to susceptibility for developing AAAs.DNA samples from 812 unrelated white subject (AAA, n = 387; controls, n = 425) were genotyped for 14 polymorphisms in 13 different candidate genes: MMP1(nt-1607), MMP2(nt-955), MMP3(nt-1612), MMP9(nt-1562), MMP10(nt+180), MMP12(nt-82), MMP13(nt-77), TIMP1(nt+434), TIMP1(rs2070584), TIMP2(rs2009196), TIMP3(nt-1296), TGFB1(nt-509), ELN(nt+422), and COL3A1(nt+581). Odds ratios and P values adjusted for gender and country of origin using logistic regression and stratified by family history of AAA were calculated to test for association between genotype and disease status. Haplotype analysis was carried out for the two TIMP1 polymorphisms in male subjects.Analyses with one polymorphism per test without interactions showed an association with the two TIMP1 gene polymorphisms (nt+434, P = .0047; rs2070584, P = .015) in male subjects without a family history of AAA. The association remained significant when analyzing TIMP1 haplotypes (chi 2 P = .014 and empirical P = .009). In addition, we found a significant interaction between the polymorphism and gender for MMP10 ( P = .037) in cases without a family history of AAA, as well as between the polymorphism and country of origin for ELN ( P = .0169) and TIMP3 ( P = .0023) in cases with a family history of AAA.These findings suggest that genetic variations in TIMP1, TIMP3, MMP10, and ELN genes may contribute to the pathogenesis of AAAs. Further work is needed to confirm the findings in an independent set of samples and to study the functional role of these variants in AAA. It is noteworthy that contrary to a previous study, we did not find an association between the MMP9 (nt-1562) polymorphism and AAA, suggesting genetic heterogeneity of the disease.Abdominal aortic aneurysms (AAAs) are an important cardiovascular disease, but the genetic and environmental risk factors, which contribute to individual's risk to develop an aneurysm, are poorly understood. Histologically, AAAs are characterized by signs of chronic inflammation, destructive remodeling of the   extracellular matrix  , and depletion of vascular smooth muscle cells. We hypothesized that genes involved in these events could harbor changes that make individuals more susceptible to developing aneurysms. This study identified significant genetic associations between DNA sequence changes in tissue inhibitor of metalloproteinase 1 (TIMP1), TIMP3, matrix metalloproteinase 10 (MMP10) and elastin (ELN) genes, and AAA. The results will require confirmation using an independent set of samples. After replication it is possible that these sequence changes in combination with other risk factors could be used in the future to identify individuals who are at increased risk for developing an AAA. 
 Expression of matrix metalloproiteinases (MMPs) and tissue inhibitors of metalloproteinases (TIMPs) in hepatocellular carcinoma tissue, compared with the surrounding non-tumor tissue. Matrix metalloproteinases (MMPs) have been implicated in the tumor invasion and growth through the degradation of   extracellular matrix  . In this study, we selected 46 hepatocellular carcinoma (HCC) cases, at random, and we immunohistologically examined the expression of MMP-1, MMP-2, MMP-3, MMP-7, MMP-9, tissue inhibitor of metalloproteinases (TIMP)-1, TIMP-2, in cancerous and non-cancerous areas using avidin-biotin-peroxidase complex method. In all cases, cancer cells, hepatocytes, sinusoidal lining cells, leukocytes, and bile ducts were positive for all the primary antibodies. The expressions of MMPs and TIMPs in most of the HCC tissues were equal or low compared with those in the surrounding non-tumor tissues, although mixed expression pattern were recognized in some HCC tissues. The difference of MMP and TIMP expression was not related with the histological differentiation of HCC and the condition of non-cancerous area. These findings suggested little association of the clinicopathological findings of HCC with the histological expression of MMPs and TIMPs. 
 Circulating matrix metalloproteinase-9 and tissue inhibitors of metalloproteinases-1 and -2 levels in gestational hypertension. Gestational hypertension (GH) is dangerous to both mother and child. Arterial invasiveness and growth are dependent on successful   extracellular matrix   (ECM) breakdown, which may be abnormal in GH. We hypothesized abnormalities in circulating matrix metalloproteinase-9 (MMP-9) and tissue inhibitors of metalloproteinases-1 and -2 (TIMP-1 and TIMP-2, respectively) in patients with GH, when compared with normotensive women with normal pregnancies and healthy nonpregnant control subjects.Plasma MMP-9, TIMP-1, and TIMP-2 were measured by ELISA in 23 women with GH, 30 normotensive pregnant women, and 28 nonpregnant women who were matched for age, gestational age, and parity.Levels of circulating MMP-9, TIMP-1 and TIMP-2, and the MMP-9/TIMP-1 and MMP-9/TIMP-2 ratios were significantly different among the three groups (P = .026, P = .006, P = .007, P = .001 and P = .008 respectively). Within the GH group, MMP-9 and the MMP-9/TIMP-1 ratio correlated negatively with age (r = -0.581, P = .004 and r = -0.563, P = .005, respectively) and levels of diastolic blood pressure (r = -0.432, P = .040 and r = -0.461, P = .027, respectively). With multiple regression analysis, only age independently correlated with circulating levels of MMP-9 (P = .010); neither age nor levels of diastolic blood pressure had any effect on the MMP-9/TIMP-1 ratio.We have demonstrated altered MMP/TIMP ratios in maternal blood during GH. These observations suggest pregnancy-related changes in ECM breakdown and turnover. Given the importance of changes in ECM composition to vascular and cardiac structure in hypertension, we suggest that these observations may be related to the pathophysiology of human GH. 
 [Expression of matrix metalloproteinase-2, 9 and their inhibitor-TIMP 1,2 in human squamous cell carcinoma of uterine cervix]. Studies of tumor invasion and metastasis have focused on the degradation of   extracellular matrix   (ECM). Matrix metalloproteinases (MMPs), which could degrade ECM, were implicated in cancer invasion and metastasis. Activated MMPs were controlled by specific tissue inhibitors of metalloproteinases (TIMPs). This study was designed to investigate the expression of MMP-2, MMP-9 and TIMP-1, TIMP-2 in human squamous cell carcinomas of uterine cervix, and the association with invasion and metastasis of human squamous cell carcinoma of uterine cervix.Tissue samples from 40 cases of squamous cell carcinoma of the uterine cervix, 29 cases of cervical intraepithelial neoplasia (CIN), and 16 cases of normal cervices were stained immunohistochemically.Immunohistochemical staining of tumor cells for MMP-2, MMP-9, TIMP-1, and TIMP-2 were noted 82.5%, 70.0%, 62.5%, and 97.5% in carcinomas, 44.8%, 62.1%, 86.21%, and 96.55% in CIN lesions respectively, for MMP-2, MMP-9, TIMP-1 in 25.0% normal cervices, but for TIMP-2 in 56.25% normal cervices. There was significant difference for MMP-2 among carcinomas, normal cervices, and CIN respectively (P < 0.01). There was significant difference for MMP-9 among normal cervices, carcinomas, and CIN, respectively (P < 0.05). The positive rates of TIMP-1 and TIMP-2 in the cervical carcinoma group and CIN group were obviously higher than that in normal cervical group(P < 0.01). The positive rates of MMP-2 and MMP-9 in lymph node metastasis were obviously higher than that in without lymph node metastasis(P < 0.01), but for TIMP-2 was contrary and there was no significant difference for TIMP-1. No significant correlation could be established among the expression of these markers and the tumor diameter, the clinical stage, and pathologic grade.The over-expression of MMP-2, MMP-9, TIMP-1, and TIMP-2 may play a key role in invasion and lymph-node metastasis of in squamous carcinoma of the cervix. 
 NMR structure of tissue inhibitor of metalloproteinases-1 implicates localized induced fit in recognition of matrix metalloproteinases. A high quality solution structure of the matrix metalloproteinase inhibitory N-terminal domain of recombinant human tissue inhibitor of metalloproteinases-1 (N-TIMP-1) has been determined. For the rigidly packed residues, the average RMSD to the mean structure is 0. 57 A for the backbone atoms and 1.00 A for all heavy atoms. Comparison of the solution structure of free N-TIMP-1 with the crystal structure of TIMP-1 bound to the catalytic domain of MMP-3 ( Gomis-R]uth et al., 1997 ) shows that the structural core of the beta barrel flanked by helices is nearly unchanged by the association with MMP-3, evident from a backbone RMSD of 1.15 A. However, clear differences in the conformation of the MMP-binding ridge of free and MMP-bound TIMP-1 suggest induced fit throughout the ridge. The MMP-dependent conformational changes in the ridge include a dramatic bending of AB loop residues Glu28 through Leu34, moderate hinge bending of the CD-loop about residues Ala65 and Cys70, and modest bending of the Cys1 through Pro6 segment. A large number of interresidue Nuclear Overhauser enhancements (NOEs) augmented by stereospecific assignments, torsion restraints, and dipolar couplings (an average of 18 non-trivial restraints per residue) engender confidence in these structural inferences. A tight cluster of three lysine residues and one arginine residue atop beta-strands A and B, and identical among TIMP sequences, form the heart of a highly conserved electropositive patch that may interact with anionic components of the   extracellular matrix  . 
 Inhibition of apoptosis in rat mesangial cells by tissue inhibitor of metalloproteinase-1. Tissue inhibitor of metalloproteinase-1 (TIMP-1) is an important inhibitor of   extracellular matrix   degradation. Recently, it was reported that TIMP-1 also could inhibit apoptosis in B type lymphocyte. This study was designed to examine the effects of TIMP-1 on mesangial cell apoptosis.The full-length cDNA of TIMP-1 was cloned and used to construct two recombinant vectors, TIMP-1S and TIMP-1AS, encoding sense TIMP-1 and antisense TIMP-1, respectively. The vectors were transfected into rat mesangial cells (RMC) and their expressions detected by Northern and Western blotting. Apoptosis was induced by serum deprivation, and was monitored for DNA fragmentation by TUNEL assay and DNA laddering. In addition, the expression of endogenous TIMP-1, matrix metalloprotein-2 (MMP-2), and MMP-9, as well as apoptosis-related genes Bcl-2 and Bax were investigated.TIMP-1AS transfection induced a suppression of TIMP-1 expression accompanied by an earlier onset of apoptosis, and TIMP-1S transfection induced TIMP-1 over-expression accompanied by a much later onset of apoptosis. A neutralizing antibody of TIMP-1 restored the sensitivity of TIMP-1S-transfected RMC to serum deprivation, but a synthetic matrix metalloproteinase inhibitor BB-94 did not influence the sensitivity of TIMP-1S-transfected RMC to serum deprivation. Finally, TIMP-1 over-expression inhibited the expression of Bax but with no effect on the expression of Bcl-2.TIMP-1 inhibits the serum deprivation-induced apoptosis in RMC, in which Bax might be involved. 
 Identification of the   extracellular matrix   (ECM) binding motifs of tissue inhibitor of metalloproteinases (TIMP)-3 and effective transfer to TIMP-1. Tissue inhibitor of metalloproteinases (TIMPs) are the endogenous inhibitors of the zinc-dependent endopeptidases of the matrix metalloproteinase families. There are four mammalian TIMPs (TIMP-1 to -4) but only TIMP-3 is sequestered to the   extracellular matrix   (ECM). The molecular basis for the TIMP-3:ECM association has never been fully investigated until now. In this report, we identify the unique amino acid configuration that constitutes the basis of the ECM binding motif in TIMP-3. By systematically exchanging the subdomains of the TIMPs and exhaustive mutation of TIMP-3, we have identified the surface residues directly responsible for ECM association. Contrary to the accepted view, we have found that TIMP-3 interacts with the ECM via both its N- and C-terminal domains. The amino acids involved in ECM binding are all basic in nature: Lys-26, Lys-27, Lys-30, Lys-76 of the N-terminal domain and Arg-163, Lys-165 of the C-terminal domain. Replacement of these residues with glutamate (E) and glutamine (Q) (K26/27/30/76E + R163/K165Q) resulted in a soluble TIMP-3 devoid of ECM-adhering ability. Using the ECM binding motif derived from TIMP-3, we have also created a TIMP-1 mutant (K26/27/30 + K76 transplant) capable of ECM association. This is the first instance of TIMPs being intentionally rendered soluble or ECM-bound. The ability to prepare TIMPs in soluble or ECM-bound forms also opens new avenues for future TIMP research. 
 Expression of vascular endothelial factor-A, gelatinases (MMP-2, MMP-9) and TIMP-1 in uterine leiomyomas. Leiomyomas growth involves cellular hypertrophy, modulation of mitotic activity and upregulation of   extracellular matrix   (ECM). Vascular factors and matrix metalloproteinases (MMPs) play a coordinated role during neoplasia and tissue remodeling. The present study investigates the role of angiogenic factor vascular endothelial growth factor (VEGF)-A with the activity of main gelatinases, MMP-2/MMP-9 and their tissue inhibitor TIMP-1 in patients with leiomyomas.Peripheral blood of 46 women with uterine leiomyomas was obtained prior hysterectomy to assess VEGF-A, MMP-2, -9, TIMP-1 levels by enzyme-linked immunosorbent assay compared to 39 healthy controls. Protein expression levels of VEGF-A, MMP-2 and MMP-9 were evaluated by western immunoblotting and immunohistochemistry in leiomyomas tissue specimens after hysterectomy. Furthermore, the activity of gelatinases in leiomyoma tissue extracts and control myometrium was evaluated by semi-quantitative zymography.Circulating levels of VEGF-A, MMP-2 and TIMP-1 were significantly elevated in leiomyoma patients compared to controls (p<0.001, p=0.004, p=0.003, respectively). A positive correlation was found between VEGF-A and MMP-2 (p=0.021) as well as MMP-9 (p=0.001) peripheral levels in the patient's group. Furthermore, increased VEGF-A protein levels were detected in leiomyoma tissue compared to control myometrium, followed by increased localization of both VEGF-A and MMP-2 in the ECM embedding bundles of smooth muscle cells of leiomyomas. The activity of MMP-2 was significantly higher in leiomyomas than normal myometrium in all investigated tissues.This study demonstrates a possible coordinated role of VEGF-A and MMP-2 during uterine leiomyomas growth and angiogenesis with potential prognostic significance. 
 The balance of serum matrix metalloproteinase-8 and its tissue inhibitor in acute coronary syndrome and its recurrence. Matrix metalloproteinase-8 (MMP-8) is involved in the breakdown of the   extracellular matrix   increasing the vulnerability of atherosclerotic lesions. We analysed the diagnostic value of serum MMP-8 and tissue inhibitor of metalloproteinase-1 (TIMP-1) concentrations in acute coronary syndrome (ACS) and their prognostic value in ACS recurrence.The population comprised 343 patients with ACS [including 108 unstable angina pectoris and 235 acute myocardial infarctions (AMI)] and 326 healthy controls. Additionally, 157 (45.8%) patients were resampled during the recovery. The ACS patients were followed up for 6 years.MMP-8, TIMP-1, and their molar ratio distinguished the cases from the controls; C-statistic of the multivariate model (95% CI, p-value) including the MMP-8/TIMP-1 ratio regarding its discriminating ability for AMI was 0.922 (0.893-0.950, p<0.001). After the acute phase of ACS, median MMP-8 and TIMP-1 concentrations decreased (p<0.001) by 34.5 and 28.7%, respectively, but ended up on a different level than those found in the controls. In the follow-up, acute phase and recovery period TIMP-1 concentrations associated with cardiovascular death with hazard ratios 4.31 (2.00-9.26, p<0.001) and 4.69 (1.10-20.01, p=0.037), respectively.The increase of serum MMP-8 and TIMP-1 concentrations may reflect plaque instability and tissue damage. TIMP-1 concentrations are associated with poor outcome in patients with ACS. The findings may have practical implications in both diagnostics and therapeutics. 
 Evaluation of metalloproteinases 2 and 9 and their inhibitors in physiologic and pre-eclamptic pregnancy. Matrix metalloproteinases (MMPs) are a family of zinc and calcium-dependent endopeptidases involved in remodeling and physiological homeostasis of   extracellular matrix   (ECM). The metalloproteinases activity is predominantly modulated by specific tissue inhibitors of matrix metalloproteinases (TIMPs). The balance between MMPs and TIMPs is likely to play an important role in remodeling uterine arteries in pregnancy, and it may represent means by which vasodilatation is maintained in later pregnancy. Moreover, increased levels of MMPs and in particular MMP-2 play a role in the vascular alterations induced by hypertension. The aim of this study was the evaluation of MMP-2 and -9, along with their inhibitors TIMP-1 and -2, in pre-eclamptic women compared with normotensive pregnancy and non-pregnant women. Fourteen pre-eclamptic women were compared with 37 normotensive women in different gestational age and 21 non-pregnant women. Multiplexed sandwich enzyme-linked immunosorbent assay was used to measure MMPs and TIMPs simultaneously. MMP-2 levels were significantly higher in pre-eclamptic women vs. both non-pregnant and physiologic pregnant women. MMP-9 concentrations were significantly higher in physiologic pregnant vs. non-pregnant women. The serum levels of TIMP-1 were significantly higher in pre-eclamptic vs. both non-pregnant and physiologic pregnant women. TIMP-2 values were higher in physiologic pregnant women and pre-eclamptic women vs. non-pregnant women. A positive correlation between MMP-9 values and gestational age was observed in normal pregnant women. Results of the present investigation confirm that MMP-2 and TIMP-1 values are significantly higher in preeclampsia. We confirm that the modification of the fine balance between MMPs and their inhibitors plays a greater role in the structural and functional vascular changes of women with complicated pregnancies. 
 TIMP-1 regulates cell proliferation by interacting with the ninth zinc finger domain of PLZF. The tissue inhibitors of metalloproteinases (TIMPs) are multifunctional proteins that specifically inhibit matrix metalloproteinases (MMPs) and regulate   extracellular matrix   (ECM) turnover and tissue remodeling. This is directed by forming tightly bound inhibitory complexes with MMPs. Recent years have revealed important differences of various biological activities between TIMP families but molecular mechanisms are not clear. To define the molecular mechanisms of TIMP-1-dependent biological processes, we used TIMP-1 as bait in a yeast two-hybrid screen, along with a human ovary cDNA library. Further characterization revealed the ninth zinc finger domain as an interacting domain of the promyelocytic leukemia zinc finger protein (PLZF). Interaction of PLZF with TIMP-1 in mammalian cells was also confirmed by co-immunoprecipitation and with in vitro binding assays. We investigated whether TIMP-1-mediated anti-apoptotic activity could promote the growth of ovarian cancer in an experimental model system. TIMP-1 treatment was found to be more effective at increasing ovarian cancer growth when compared with PLZF in parallel experiments. Subsequently, the efficacy of a combined treatment with TIMP-1 and PLZF was investigated. In the presence of both of these proteins, TIMP-1 significantly reduced apoptosis induced by PLZF in cervical carcinoma cells. These combined results indicate that TIMP-1 functions as an anti-activator of the transcriptional repressive activity of PLZF. 
 Levels of matrix metalloproteinase-9 and its inhibitor in bronchoalveolar lavage cells of asthmatic children. Our objective was to investigate the role of matrix metalloproteinase-9 (MMP-9) and tissue inhibitor of matrix metalloproteinase-1 (TIMP-1) and their cellular sources in childhood asthma. We used 12 controls and 16 asthmatic children. The levels of MMP-9 and TIMP-1 in bronchoalveolar lavage (BAL) cells of asthmatic children were measured immunocytochemically. The positive level index, defined as the percentage of positive-stained cells x average optical density, was used to assess the expressing levels of MMP-9 and TIMP-1. The percentages of eosinophils and mast cells in bronchoalveolar lavage fluid (BALF) of asthmatic children were increased. Levels of MMP-9 and TIMP-1 in BAL cell of asthmatic children were increased significantly at about 30- and 35-fold relative to the controls, respectively. These results suggest that both MMP-9 and TIMP-1 contribute to tissue remodeling. MMP-9, which mediates the degradation of   extracellular matrix   (ECM), is increased significantly in the early or acute stage and may play a role in ECM degeneration. Excessive TIMP-1 may be synthesized following MMP-9 production when the body tries to repair the damage, which results in excessive deposition of ECM component. 
 ERK2-regulated TIMP1 induces hyperproliferation of K-Ras(G12D)-transformed pancreatic ductal cells. Pancreatic ductal adenocarcinoma (PDAC) commonly contains a mutation in K-Ras(G12D) and is characterized by a desmoplastic reaction composed of deregulated, proliferating cells embedded in an abnormal   extracellular matrix   (ECM). Our previous observations imply that inhibiting the mitogen-activated protein kinase (MAPK)-extracellular signal-regulated kinase (ERK2) kinase signal pathway reverses a matrix metalloproteinase 1-specific invasive phenotype. Here, we investigated the specific genes downstream of MAPK-ERK2 responsible for the hyperproliferative abilities of human and murine primary ductal epithelial cells (PDCs) within an ECM. Compared with control, DNA synthesis and total cell proliferation was significantly increased in human PDCs harboring the PDAC common p53, Rb/p16(INK4a), and K-Ras (G12D) mutations. Both of these effects were readily reversed following small-molecule inhibition or lentiviral silencing of ERK2. Microarray analysis of PDCs in three-dimensional (3D) culture revealed a unique, MAPK-influenced gene signature downstream of K-Ras (G12D). Unbiased hierarchical analysis permitted filtration of tissue inhibitor of matrix metalloproteinase 1 (TIMP1). Pancreatic cells isolated from Pdx1-Cre; LSL-K-ras(G12D/+)-mutated mice exhibit increased TIMP1 RNA transcription compared to wild-type littermate controls. Analyses of both 3D, in vitro human K-Ras (G12D) PDCs and data mining of publicly annotated human pancreatic data sets correlatively indicate increased levels of TIMP1 RNA. While silencing TIMP1 did not significantly effect PDC proliferation, exogenous addition of human recombinant TIMP1 significantly increased proliferation but only in transformed K-Ras (G12D) PDCs in 3D. Overall, TIMP1 is an upregulated gene product and a proliferative inducer of K-Ras(G12D)-mutated PDCs through the ERK2 signaling pathway. 
 Alterations in peripheral blood levels of TIMP-1, MMP-2, and MMP-9 in patients with type-2 diabetes. In vivo and in vitro experimental findings indicate that the hyperglycemic diabetic milieu can induce altered expression of the matrix metalloproteinase (MMP) genes and contribute to imbalances in vascular matrix homeostasis. We examined the plasma levels of enzymes and inhibitors involved in   extracellular matrix   turnover.We measured the plasma concentrations of MMP-2, MMP-9, and tissue inhibitor of metalloproteinase 1 (TIMP-1) in 80 type-2 diabetic subjects without uremia and in 80 age-matched controls. In addition, we determined the plasma levels of intercellular adhesion molecule 1 (ICAM-1), vascular cell adhesion molecule 1 (VCAM-1) and high sensitive(hs) C-reactive protein (CRP) in both groups.Plasma MMP-2, TIMP-1, and hs-CRP concentrations were significantly elevated in diabetic patients as compared to the control subjects (p<0.05). Plasma levels of MMP-2, MMP-9, TIMP-1, VCAM-1, ICAM-1, and hs-CRP were found not to be significantly associated with age, duration of diabetes, blood pressure, or serum lipid concentrations.Plasma MMP-2, TIMP-1 and hs-CRP concentrations were significantly increased in type-2 diabetic patients. 
 Prognostic significance of adipocytokines and   extracellular matrix   activity in heart failure patients with high B-type natriuretic peptide. To perform risk stratification by serum adipocytokines and serum markers of   extracellular matrix   in heart failure patients with high b-type natriuretic peptide (BNP).Patients with heart failure were enrolled in this study. Serum adipocytokines and serum markers of   extracellular matrix   were analyzed.In total, 131 patients were enrolled and followed-up for 240+/-174 days. Mortality was significantly associated with adiponectin, resistin, type III amioterminal propeptide of procollagen (PIIINP), matrix metalloproteinase-2 (MMP-2), tissue inhibitor of metalloproteinase 1 (TIMP-1), and serum creatinine. Heart failure-related admission was significantly associated with apelin, and PIIINP. Cox regression analysis identified that mortality and heart failure-related admissions were significantly associated with MMP-2 (P=0.008) and PIIINP (P=0.011), respectively.Serum markers of   extracellular matrix   rather than adipocytokines may warrant further risk stratification for impacting the prognosis of patients with heart failure with high BNP. 
 Imbalance between expression of matrix metalloproteinase-9 and tissue inhibitor of metalloproteinase-1 in invasiveness and metastasis of human gastric carcinoma. The expressive balance between matrix metalloproteinase-9 (MMP-9) and its tissue inhibitor of metalloproteinase-1 (TIMP-1) plays a critical role in maintaining the degradation and synthesis of   extracellular matrix  . Loss of such balance is associated with invasion and metastasis of tumors. This study aimed to determine the expression of MMP-9 and TIMP-1 in gastric carcinoma, and the association of the expressive imbalance between MMP-9 and TIMP-1 with the invasion and metastasis and prognosis of gastric carcinoma.We used immunohistochemistry to determine the expressions of MMP-9, TIMP-1 and proliferating cell nuclear antigen Ki-67 in the gastric specimens taken from 256 patients with primary gastric carcinoma. The patients were followed-up for up to 96 months.No association between the expression of MMP-9 and TIMP-1 and patients' sex and age, tumor size and location of gastric carcinoma was observed. The incidence of the positive expression of MMP-9 in cases with tumors invasion to muscularis propria and visceral peritoneum (70.13 % and 69.09 %, respectively) was significantly higher than that in cases with tumor invasion only to lamina propria or submucosa (42.50 %, P=0.0162). The positive correlation between MMP-9 expression and the depth of tumor invasion was observed (Pearson correlation coefficient=0.2129, P=0.016). Along with the increase of the metastatic station of lymph nodes, the incidence of the MMP-9 expression was increased by degrees; a positive correlation between them was observed (Pearson correlation coefficient=0.2910, P=0.0001). There was also a significant correlation between MMP-9 expression and the TNM stage in gastric carcinoma (Pearson correlation coefficient=0.3027, P<0.0001). The incidence of MMP-9 expression in stage II and III/IV (75.00 % and 76.15 %, respectively) was significantly higher than those in stage I (46.15 %, P<0.0001). A negative correlation between TIMP-1 immunoreactivity and the depth of invasion, status of lymph node metastasis and TNM stage was observed (Pearson correlation coefficient =-0.1688, -0.3556 and -0.3004, P=0.023, <0.0001 and <0.0001, respectively). Four types of co-expression of MMP-9 and TIMP-1 were observed; i.e. MMP-9 positive but TIMP-1 negative (n=115), both positive (n=52), both negative (n=62) and MMP-9 negative but TIMP-1 positive (n=27). The frequency of serosal invasiveness was significant higher in patients with MMP-9 but without TIMP-1 expression than those with other types of the co-expression (P=0.0303). The incidence of lymph node metastasis was highest in patients with MMP-9 but without TIMP-1 expression, and lowest in those with TIMP-1 but without MMP-9 expression (P<0.0001). The survival rate in patients with MMP-9 but without TIMP-1 expression was lower than that in those with TIMP-1 but without MMP-9 expression (P=0.0014).Our results in gastric carcinoma demonstrated a significant positive association of MMP-9 over-expression with proliferation of tumor cells, the depth of invasiveness, lymph node metastasis and TNM stage, suggesting MMP-9 can serve as a molecular marker of tumor invasion and metastasis. We also demonstrate a significant negative relationship of TIMP-1 expression with the depth of invasiveness and lymph node metastasis, which provide a new idea in the tumor biological and genetic treatment. The interaction between MMP-9 and TIMP-1 in the processes of tumor invasion and metastasis is that MMP-9 mainly promotes tumor invasion and metastasis and TIMP-1 inhibits functions of MMP-9. The imbalance between MMP-9 and TIMP-1 expression may suggest the occurrence of tumor invasion and metastasis, predict poor prognosis. For patients with imbalanced MMP-9 and TIMP-1 expression, the optimal treatment scheme needs to be selected. 
 Proteasome blockade exerts an antifibrotic activity by coordinately down-regulating type I collagen and tissue inhibitor of metalloproteinase-1 and up-regulating metalloproteinase-1 production in human dermal fibroblasts. Tissue fibrosis results when dysregulation of   extracellular matrix   (ECM) turnover favors deposition of collagen and other ECM proteins over degradation. Fibrosis may then lead to organ dysfunction and pathology as observed in systemic sclerosis (SSc). In the present study, we investigated the antifibrotic properties of proteasome blockade. A dose- and time-dependent reduction in type-I collagen and tissue inhibitor of metalloproteinase-1 (TIMP-1) production was observed in normal fibroblasts exposed to proteasome inhibitors (PI). In the same culture conditions, metalloproteinase-1 (MMP-1) protein and the collagenolytic activity on type I collagen was increased. The steady-state mRNA levels of COL1A1, TIMP-1, and MMP-1 paralleled protein levels. These effects were dominant over the profibrotic properties of TGF-beta and were observed with fibroblasts generated from normal and SSc skin. PI decreased type I collagen mRNA levels with kinetics similar to those observed with DRB, a specific RNA polymerase II inhibitor, thus indicating transcriptional inhibition. Of interest, PI induced c-Jun phosphorylation and c-Jun nuclear accumulation. The specific N-terminal Jun-kinase inhibitor SP-600125 selectively abrogated c-Jun phosphorylation and, in a dose-dependent fashion, the up-regulated synthesis of MMP-1 induced by PI. Finally, PI did not affect fibroblast viability. Thus, the coordinated down-regulation of collagen and TIMP-1 and up-regulation of MMP-1 renders proteasome blockade an attractive strategy for treating conditions as SSc, characterized by excessive fibrosis. 
 Tissue inhibitor of matrix metalloproteinase-1 expression in colorectal cancer liver metastases is associated with vascular structures. Metastatic growth by colorectal cancer cells in the liver requires the ability of the cancer cells to interact with the new microenvironment. This interaction results in three histological growth patterns of liver metastases: desmoplastic, pushing, and replacement. In primary colorectal cancer several proteases, involved in the degradation of   extracellular matrix   components, are up-regulated. In liver metastases, their expression is growth pattern dependent. Tissue inhibitor of matrix metalloproteinase-1 (TIMP-1) is a strong prognostic marker in plasma from colorectal cancer patients, with significant higher levels in patients with metastatic disease. We therefore wanted to determine the expression pattern of TIMP-1 in primary colorectal cancers and their matching liver metastases. TIMP-1 mRNA was primarily seen in α-smooth-muscle actin (α-SMA)-positive cells. In all primary tumors and liver metastases with desmoplastic growth pattern, TIMP-1 mRNA was primarily found in α-SMA-positive myofibroblasts located at the invasive front. Some α-SMA-positive cells with TIMP-1 mRNA were located adjacent to CD34-positive endothelial cells, identifying them as pericytes. This indicates that TIMP-1 in primary tumors and liver metastases with desmoplastic growth pattern has dual functions; being an MMP-inhibitor at the cancer periphery and involved in tumor-induced angiogenesis in the pericytes. In the liver metastases with pushing or replacement growth patterns, TIMP-1 was primarily expressed by activated hepatic stellate cells at the metastasis/liver parenchyma interface. These cells were located adjacent to CD34-positive endothelial cells, suggesting a function in tumor-induced angiogenesis. We therefore conclude that TIMP-1 expression is growth pattern dependent in colorectal cancer liver metastases. 
 Relations of serum MMP-9 and TIMP-1 levels to left ventricular measures and cardiovascular risk factors: a population-based study.   Extracellular matrix   remodeling is a hallmark of pathological left ventricular (LV) hypertrophy and heart failure. This process is tightly controlled by the degrading matrix metalloproteinases (MMPs) and their tissue inhibitors (TIMPs). We hypothesized that circulating MMP-9 and TIMP-1 levels are altered already in persons with the signs of LV remodeling that forego clinical heart failure.Cross-sectional study in the Prospective Investigation of the Vasculature in Uppsala Seniors, a community-based cohort of 891 70-year-old men and women free from valvular disease, heart failure, and myocardial infarction.We examined relations of serum MMP-9 and TIMP-1 to echocardiographic LV geometry and function. All models were adjusted for sex, height, intra-arterial systolic and diastolic blood pressures, antihypertensive medication use, and serum freezer time.Serum TIMP-1 was positively related to LV mass and wall thickness (r=0.15, P<0.0001 and r=0.16, P<0.0001, respectively), with a 32 g higher LV mass and 2.2 mm thicker walls in the fourth compared with the first quartile of serum TIMP-1. Serum TIMP-1 was also inversely related to LV ejection fraction (r=-0.10, P=0.009), but not to LV dimension or diastolic function indices. Serum MMP-9 was only weakly related to LV wall thickness and isovolumic relaxation time (r=0.08, P=0.04 and r=-0.08, P=0.04).In this large population-based sample, serum TIMP-1 levels were related to LV mass, wall thickness, and inversely to systolic function. This may imply that   extracellular matrix   remodeling is involved already in the earliest stages of the process leading to heart failure. 
 Prognostic significance of TIMP-1 in non-small cell lung cancer. Tissue inhibitor of metalloproteinases 1 (TIMP1) regulates not only   extracellular matrix   catabolism but the major effect in tumor tissue is promotion of cell growth and anti-apoptotic activity. The aim of our study was to evaluate plasma TIMP1 levels and tissue TIMP1 mRNA expression as prognostic markers in NSCLC patients.We studied a group of 108 patients with NSCLC who had undergone lung surgery. Estimation of TIMP1 mRNA was performed by quantitative polymerase chain reaction (qPCR) and estimation of plasma TIMP1 protein using enzyme-linked immunosorbent assay (ELISA).There was shorter disease-free interval (DFI) for NSCLC patients at stage II, with a higher expression of TIMP1 mRNA in tumor tissue (p=0.0246). We recorded a relationship between tumor tissue TIMP1 mRNA expression and DFI in squamous cell carcinoma (SCC) (p=0.0117). Shorter overall survival was found in patients at stages IIIa+IIIb+IV, with a higher expression of TIMP1 mRNA (p=0.0389). We found differences in plasma TIMP1 levels between patients with SCC and those with adenocarcinoma (p=0.0491).A higher tissue level of TIMP1 mRNA is related to an adverse prognosis of patients. However, our results did not show any relation of TIMP1 protein in blood plasma to prognosis. 
 Biomarkers of   extracellular matrix   metabolism (MMP-9 and TIMP-1) and risk of stroke, myocardial infarction, and cause-specific mortality: cohort study. Turnover of the   extracellular matrix   in all solid organs is governed mainly by a balance between the degrading matrix metalloproteinases (MMPs) and their tissue inhibitors (TIMPs). An altered   extracellular matrix   metabolism has been implicated in a variety of diseases. We investigated relations of serum levels of MMP-9 and TIMP-1 to mortality risk from an etiological perspective.The prospective Uppsala Longitudinal Study of Adult Men (ULSAM) cohort, followed from 1991-1995 for up to 18.1 years. A random population-based sample of 1,082 71-year-old men, no loss to follow-up. Endpoints were all-cause (n = 628), cardiovascular (n = 230), non-cardiovascular (n = 398) and cancer mortality (n = 178), and fatal or non-fatal myocardial infarction (n = 138) or stroke (n = 163).Serum MMP-9 and TIMP-1 levels were associated with risk of all-cause mortality (Cox proportional hazard ratio [HR] per standard deviation 1.10, 95% confidence interval [CI] 1.03-1.19; and 1.11, 1.02-1.20; respectively). TIMP-1 levels were mainly related to risks of cardiovascular mortality and stroke (HR per standard deviation 1.22, 95% CI 1.09-1.37; and 1.18, 1.04-1.35; respectively). All relations except those of TIMP-1 to stroke risk were attenuated by adjustment for cardiovascular disease risk factors. Relations in a subsample without cardiovascular disease or cancer were similar to those in the total sample.In this community-based cohort of elderly men, serum MMP-9 and TIMP-1 levels were related to mortality risk. An altered   extracellular matrix   metabolism may be involved in several detrimental pathways, and circulating MMP-9 or TIMP-1 levels may be relevant markers thereof. 
 Matrix metalloproteinase-9, tissue inhibitor of metalloproteinase-1, B⁺ tenascin-C and ED-A⁺ fibronectin in dilated cardiomyopathy: potential impact on disease progression and patients' prognosis. Dilated cardiomyopathy (DCM) is associated with heart failure and increased mortality and there is no reliable biomarker to estimate patients' prognosis. During cardiac remodeling, an extensive reorganization of   the extracellular ma  trix occurs. The study was aimed to investigate matrix metalloproteinase 9 (MMP-9), tissue inhibitor of metalloproteinase 1 (TIMP-1) and fetal tenascin-C (B(+) Tn-C) and fibronectin (ED-A(+) Fn) variants known to be involved in that process.In 187 patients with DCM, levels of MMP-9, TIMP-1 and B(+) Tn-C in serum as well as B(+) Tn-C and ED-A(+) Fn in tissue were quantified and subjected to univariate analysis. For all serum markers, concentrations above a calculated threshold were associated with decreased survival (MMP-9: p = 0.008, TIMP-1: p = 0.001, B(+) Tn-C: p < 0.001) and a significantly higher risk to die or undergo transplantation. In tissue, a reexpression of B(+) Tn-C and ED-A(+) Fn could be shown. Protein deposition levels of ≥4.5% for B(+) Tn-C and ≥2.1% for ED-A(+) Fn were associated with a significantly decreased survival (p = 0.001 for B(+) Tn-C, p = 0.031 for ED-A(+) Fn) and an increased risk to die or undergo transplantation. In a multivariate analysis, TIMP-1 is the superior parameter to predict transplantation free survival (p = 0.027).Serum levels of MMP-9, TIMP-1 and B(+) Tn-C and tissue levels of B(+) Tn-C and ED-A(+) Fn are promising markers for risk assessment. The reoccurrence of ED-A(+) Fn and the availability of a human antibody usable as a vehicle for targeted drug delivery might be the basis for novel therapeutic strategies. 
 Broncho-alveolar lavage matrix metalloproteases as a sensitive measure of bronchiolitis obliterans. Bronchiolitis obliterans (BO) is a survival-limiting factor in lung transplantation. There are no common BO markers in use. Since BO is associated with   extracellular matrix   remodeling, we asked whether matrix metalloproteases (MMPs) and their tissue inhibitors (TIMPs) could serve as BO markers. In 72 lung transplant patients (34 BO syndrome (BOS) 0, 15 BOS 0-p, and 23 BOS 1) serum and broncho-alveolar lavage (BAL) MMP and TIMP levels were examined by ELISA. The BAL cell counts were additionally analyzed. The serum MMP-2, MMP-8, MMP-9 and TIMP-2 levels were not different in all groups. In contrast, the BAL MMP-8, -9 and TIMP-1 levels were significantly elevated in BOS 0-p (p = 0.003; p = 0.007; p = 0.0003, respectively) and BOS 1 (p = 0.003; p = 0.001; p = 0.0004, respectively) as compared to BOS 0 patients. The BAL MMP-8, -9 and TIMP-1 levels were significant predictors of BOS 0-p (p = 0.01; p = 0.01; p = 0.01, respectively) and BOS-1 (p = 0.007; p = 0.01; p = 0.006, respectively) in receiver operating characteristic analysis. Except for BAL macrophages that were significantly decreased in BOS 0-p versus BOS 0 patients; other cell counts were not different between the groups. BAL MMP-8, -9 and TIMP-1 might be useful markers to detect BO in lung transplant patients. 
 Tissue inhibitor of metalloproteinase (TIMP)-1: the TIMPed balance of matrix metalloproteinases in the central nervous system. Astrocytes are intimately involved in the mechanisms of neural injury and repair. They participate in a variety of homeostatic functions and elicit repair responses as balance mechanisms. Currently, there is a growing appreciation of a more active role of astrocytes in neuronal signaling and function. One key homeostatic mechanism of astrocytes in tissue repair is maintained through their production of tissue inhibitors of metalloproteinases (TIMPs). The family of TIMPs (1-4) plays a central regulatory role as inhibitors of matrix metalloproteinases (MMPs), enzymes involved in   extracellular matrix   maintenance and remodeling. Recently, TIMP-1, the inducible form, has been identified as a multifunctional molecule with divergent functions. It participates in wound healing and regeneration, cell morphology and survival, tumor metastasis, angiogenesis, and inflammatory responses. An imbalance of MMP/TIMP regulation has been implicated in several inflammatory diseases of the central nervous system (CNS). Here we review the conundrums of TIMP-1 regulation in CNS pathophysiology. We propose that astrocyte-TIMP-1 may play an important role in CNS homeostasis and disease. Astrocyte TIMP-1 expression is differentially regulated in inflammatory neurodegenerative diseases and may have significant therapeutic relevance. 
 Glomerular protein levels of matrix metalloproteinase-1 and tissue inhibitor of metalloproteinase-1 are lower in diabetic subjects. Matrix metalloproteinases (MMPs) and tissue inhibitors of metalloproteinases (TIMPs) regulate   extracellular matrix   turnover throughout the body, including in renal glomeruli. We investigated protein levels of multiple MMPs (MMP-1, MMP-2, MMP-3, and MMP-9) and TIMP-1 in glomeruli and investigated whether disease phenotypes were associated with levels of these proteins. Renal cortex was collected from 100 adult autopsy subjects arrayed across 17 tissue microarrays. Immunohistochemical staining intensity for each MMP and TIMP-1 was determined using quantitative color deconvolution techniques. We observed significantly decreased glomerular MMP-1 and TIMP-1 staining in subjects with diabetes, hypertension, and an estimated glomerular filtration rate <30 ml/min/1.73 m(2) in univariate analyses. MMP-1 staining, but not TIMP-1 staining, was inversely correlated with increased glomerular fibrosis (r = -0.40). In multivariable analysis, diabetes was robustly associated with decreased staining intensity. This study indicates that in human subjects, the long-term sequelae of diseases such as diabetes that cause chronic renal failure result in decreased TIMP-1 and MMP-1 proteins in renal glomeruli. This manuscript contains online supplemental material at http://www.jhc.org. Please visit this article online to view these materials. 
 Thrombospondin-1 (TSP-1) up-regulates tissue inhibitor of metalloproteinase-1 (TIMP-1) production in human tumor cells: exploring the functional significance in tumor cell invasion. Thrombospondin-1 (TSP-1), a matrix-bound adhesive glycoprotein, has been shown to modulate tumor progression. We previously demonstrated that TSP-1 up-regulates matrix metalloproteinases MMP-2 and MMP-9. Our studies suggested that the balance between MMPs and tissue inhibitors of metalloproteinases (TIMPs) is a key determinant in tumor cell invasion. We now report that TSP-1 up-regulates TIMP-1 expression in both human breast and prostate cancer cell lines. The effect of TSP-1 on TIMP-1 expression was examined in human breast adenocarcinoma cell lines (MDA-MB-231) and human prostate cancer cell lines (PC3-NI and PC3-ML) treated with exogenous TSP-1. TIMP-1 expression was also examined in TSP-1 stably transfected breast cancer cell line (MDA-MB-435). Northern and western blot analysis revealed TIMP-1 mRNA and TIMP-1 protein expression increased with increasing concentrations of TSP-1. This effect was inhibited by antibodies against the type I repeat domain of TSP-1 further suggesting that TSP-1 mediates TIMP-1 secretion. Inhibition of TSP-1 induced TIMP-1 levels increased tumor cell invasion. We conclude that TSP-1 is involved in influencing the critical balance between MMPs and their inhibitors, maintaining the controlled degradation of the   extracellular matrix   needed to support metastasis and our results may provide an explanation for the divergent activities reported for TSP-1 in tumor progression. 
 Analysis of TIMP-1 gene polymorphisms in Italian sclerodermic patients. Systemic sclerosis (SSc) is an autoimmune disease characterized by skin and internal organs fibrosis due to an   extracellular matrix   (ECM) accumulation of type I collagen. The turnover of the ECM is dependent on the balance between matrix metalloproteinases (MMPs) and tissue inhibitors of matrix metalloproteinases (TIMPs). The disruption of this balance is involved in SSc because higher serum TIMP-1 levels have been demonstrated in SSc patients than in controls. On this basis, we analyzed three polymorphisms: -19A>G, +261C>T, and +372T>C of the TIMP-1 gene in SSc patients (67 females, eight males) and controls (29 females, nine males). The C allele of the +372T>C single nucleotide polymorphism (SNP) was observed at a higher frequency in male patients than in healthy individuals (P=0.02), while no differences were observed in the female subjects. Our findings suggest that the +372T>C polymorphism of the TIMP-1 gene is associated with SSc in male individuals. No association with the clinical characteristics of SSc Italian patients and TIMP-1 gene polymorphisms was observed. Thus, the role of TIMP-1 gene in predisposition to SSc remains controversial. 
 The endogenous protease inhibitor TIMP-1 mediates protection and recovery from cutaneous photodamage. UVB exposure is well known to induce skin photodamage and photoaging that correlates with qualitative and quantitative deterioration of the dermal   extracellular matrix   (ECM) because of the upregulation of matrix metalloproteinases (MMPs). Although inhibitory effects of tissue inhibitor of metalloproteinases (TIMPs) on most MMPs have been reported, the protective role of TIMP-1 against photodamage is poorly understood. To address this, TIMP-1 function was augmented or abolished in a human skin xenograft photodamage model after the confirmation of significantly diminished TIMP-1 expression both in photoaged and intrinsically aged skins. During a chronic UVB exposure regimen, pre-treatment with a lentiviral vector overexpressing TIMP-1 or concomitant administration of an anti-TIMP-1-neutralizing antibody (NAB) led to photoprotection or more severe photodamage, respectively. Overexpression of TIMP-1 resulted in significant inhibition of UVB-induced ECM degradation, as well as suppression of decreased skin elasticity and roughness, whereas the NAB-mediated inhibition of TIMP-1 had opposite effects. Furthermore, UVB-induced production of the pro-inflammatory cytokine, tumor necrosis factor α, was inhibited by TIMP-1 treatment of human keratinocytes. Taken together, these data shed light on the important role of TIMP-1 in protection and recovery from cutaneous photodamage because of its suppression of ECM degradation and inflammation. 
 [Clinical value of serum matrix metalloproteinase-9 and tissue inhibitor of metalloproteinase-1 for the prediction and early diagnosis of coronary artery lesion in patients with Kawasaki disease]. Kawasaki disease (KD) complicated with coronary artery lesion (CAL) seriously threatens survival quality and life of patients, suggesting that it is very important to early predict the risk of CAL and to early diagnose the disease. Nevertheless, up to now there has been no specific clinical biochemical marker for it because of the poor understanding of the pathological process of KD with CAL. Matrix metalloproteinases (MMPs) and their specific tissue inhibitor of metalloproteinases (TIMPs) play an important role in arterial wall   extracellular matrix   breakdown and remodeling and are involved in CAL in other diseases. In the present study the practical value of serum MMP-9 and TIMP-1 levels in the prediction and early diagnosis of CAL in KD patients was investigated.All subjects were from Chinese population. Serum levels of MMP-9 and TIMP-1 were measured by rapid one-step sandwich enzyme immunoassay in 59 KD patients including 15 with CAL and 44 without CAL by 2 D-echocardiography and coronary angiography, and 20 normal healthy children as controls. Blood samples of patients were obtained before and after intravenous immunoglobulin (IVIG) treatment in acute stage, subacute stage and convalescent stage as well. Serum MMP-9 and TIMP-1 levels and the ratio of MMP-9/TIMP-1 were compared by statistical method in KD patients and controls, as well as in KD with CAL and without CAL.In acute stage serum MMP-9 and TIMP-1 levels and the ratio of MMP-9/TIMP-1 were higher (P < 0.01) in patients with KD than those in healthy children. After IVIG treatment in KD patients serum MMP-9 level and the ratio of MMP-9/TIMP-1 decreased (P < 0.01). Before IVIG treatment serum MMP-9 level and the ratio of MMP-9/TIMP-1 were higher (P < 0.01) in patients with CAL than those in patients without CAL, and in acute stage after IVIG treatment serum MMP-9 level of KD patients with CAL was still higher than that of KD patients without CAL.In acute stage of KD serum MMP-9 level and the ratio of MMP-9/TIMP-1 were higher in patients with CAL than those in patients without CAL, suggesting that during acute phase of KD the great increase in serum MMP-9 and the imbalance of the MMP-9/TIMP-1 ratio might be high risk factors of KD coronary artery lesion. Therefore, the measurement of serum MMP-9 and TIMP-1 might be of important clinical value in the prediction and the early diagnosis of KD with coronary artery lesion. 
 TIMP-1 expression induced by IL-32 is mediated through activation of AP-1 signal pathway. Hepatic fibrosis is a necessarily stage from the progression of chronic liver diseases to cirrhosis, even hepatocellular carcinoma (HCC). Hepatic fibrosis is characterized by the progressive accumulation of   extracellular matrix   (ECM). The balance between ECM production and degradation is mediated by matrix metalloproteinases (MMPs) and tissue inhibitors of metalloproteinases (TIMPs). TIMP-1 is an important regulator in the synthesis and degradation of ECM. IL-32, a multi-function cytokine, could induce IL-1β, IL-6, IL-8 and other cytokine expressions by activating AP-1, NF-κβ, p38MAPK signal pathways. IL-32 expression is increased in liver tissues of hepatic fibrosis. However, the role of IL-32 in the pathogenesis of liver fibrosis is not thoroughly clear. Recently, it is demonstrated that TIMP-1 expression is induced by the activation of AP-1 signal pathway. So we assayed the effect of IL-32 on TIMP-1 expression by LX-2 cells (one of HSCs cell lines) in the present study. We found that IL-32 could induce TIMP-1 expression by LX-2 cells at a dose-dependent manner. IL-32 could increase TIMP-1 promoter activity and induce TIMP-1 expression by activating AP-1 signal pathway. Moreover, the increase of TIMP-1 expression could promote the migration of LX-2 cells. In conclusion, we believe that IL-32 might be involved in the pathogenesis of hepatic fibrosis by inducing TIMP-1 expression. 
 Plasma TIMP1 level is a prognostic factor in patients with liver metastases. TIMP1 (tissue inhibitor of metalloproteinases 1) regulates   extracellular matrix   turnover and also promotes cell growth and has anti-apoptotic activity, which promotes malignant processes in tumor tissue. The aim of our study was to evaluate the relation of plasma TIMP1 protein levels with prognosis in patients with liver metastases, with particular regard to possible early-prediction of recurrence of the disease.We studied a group of 87 patients with metastatic liver disease (mostly from colorectal cancer) who underwent surgery for liver metastases, and assessed their preoperative plasma TIMP1 levels. These levels were evaluated according to prognosis. Furthermore, we measured plasma TIMP1 in the post-operative period and tried to relate the changes with the diagnosis of relapse.We found preoperative plasma TIMP1 levels to be related to overall survival in the group of all patients with metastatic liver disease (p=0.0047), with a higher level being associated with an adverse outcome; the cut-off value was set at 165 ng/ml. This applied to all patients, regardless of the type of surgery. Assessment of the post-operative dynamics of TIMP1 was not found to be statistically significant to indicate disease recurrence.We found there to be a relationship between higher plasma levels of TIMP1 and an adverse prognosis in patients with liver metastases. The assessment of plasma TIMP1 levels could help the detection of patients with worse outcome. 
 Tissue inhibitor of matrix metalloproteinase-1 overexpression in M1 myeloblasts impairs IL-6-induced differentiation. The balance between matrix metalloproteinase (MMP) and tissue inhibitor of matrix metalloproteinase (TIMP) is important for   extracellular matrix   interactions of hematopoietic cells. MMP-independent growth modulating activity for TIMP-1 on B lymphocytes and erythroid progenitors has also been described, but a role for TIMP-1 in myelomonocytic differentiation has not been previously reported. In this study, we demonstrate that TIMP-1 overexpression impairs differentiation of the myeloblastic M1 cell line following interleukin (IL)-6 stimulation. We generated retroviral vectors coexpressing human TIMP-1 and the green fluorescent protein (GFP) and stably transduced murine M1 myeloid cells. TIMP-1 expressing cells showed a large reduction in IL-6-induced macrophage differentiation in vitro that was reversible with a specific monoclonal antibody. The differentiation delay in M1/TIMP-1 cells was also specifically reversible by pharmacologic phosphatidylinositol-3 kinase (PI3-K) inhibition. Additionally, overexpression of a TIMP-1/GFP fusion protein also impaired M1 differentiation and this protein was localized to the cell surface, consistent with an autocrine receptor-mediated mechanism. Surprisingly, TIMP-1 transduced cells had a selective advantage for growth in IL-6, indicating that functional effects on growth and differentiation of M1 cells were primarily through an autocrine mechanism. Intrinsic TIMP-1 expression in myeloid leukemia cells might thus impact upon survival or differentiation. 
 The gp130 ligand oncostatin M regulates tissue inhibitor of metalloproteinases-1 through ERK1/2 and p38 in human adult cardiac myocytes and in human adult cardiac fibroblasts: a possible role for the gp130/gp130 ligand system in the modulation of   extracellular matrix   degradation in the human heart. There is ample evidence supporting the view that alterations in the balance between matrix deposition and matrix degradation brought about by changes in the respective activities of matrix metalloproteinases (MMPs) and tissue inhibitors of matrix metalloproteinases (TIMPs) contribute significantly to cardiac dysfunction and disease. Here we show that TIMP-1 was upregulated up to threefold after treatment with the inflammatory mediator and gp130 ligand oncostatin M (OSM) in human adult cardiac myocytes and fibroblasts. The Erk1/2 inhibitor PD98059 and the p38 inhibitor SD202190 abolished the effect of OSM on TIMP-1 production in both cell types. Human cardiac myocytes and human cardiac fibroblasts also express MMP-1, 2, 3 and 9, and TIMP-2 constitutively. OSM, however, did not affect the expression of these proteins. In addition also the other gp130 ligands tested, cardiotrophin-1 (CT-1), interleukin-6 (IL-6) and leukemia inhibitory factor (LIF) had no effect on the expression of TIMPs and MMPs studied. We speculate that OSM by inducing TIMP-1 expression counteracts excessive proteolysis and unrestricted matrix degradation during inflammatory processes in the heart. The notion that OSM favors matrix stabilization in the human heart is further supported by our earlier observation that OSM also upregulates PAI-1, the physiological inhibitor of the protease urokinase-type PA (u-PA), which in turn is essential for extracellular proteolysis. Therefore we propose a role for the gp130 ligand OSM in the modulation of cardiac remodeling and repair processes. 
 Gene expression changes during the development of acute lung injury: role of transforming growth factor beta. Acute lung injury can occur from multiple causes, resulting in high mortality. The pathophysiology of nickel-induced acute lung injury in mice is remarkably complex, and the molecular mechanisms are uncertain.To integrate molecular pathways and investigate the role of transforming growth factor beta (TGF-beta) in acute lung injury in mice.cDNA microarray analyses were used to identify lung gene expression changes after nickel exposure. MAPPFinder analysis of the microarray data was used to determine significantly altered molecular pathways. TGF-beta1 protein in bronchoalveolar lavage fluid, as well as the effect of inhibition of TGF-beta, was assessed in nickel-exposed mice. The effect of TGF-beta on surfactant-associated protein B (Sftpb) promoter activity was measured in mouse lung epithelial cells.Genes that decreased the most after nickel exposure play important roles in lung fluid absorption or surfactant and phospholipid synthesis, and genes that increased the most were involved in TGF-beta signaling. MAPPFinder analysis further established TGF-beta signaling to be significantly altered. TGF-beta-inducible genes involved in the regulation of   extracellular matrix   function and fibrinolysis were significantly increased after nickel exposure, and TGF-beta1 protein was also increased in the lavage fluid. Pharmacologic inhibition of TGF-beta attenuated nickel-induced protein in bronchoalveolar lavage. In addition, treatment with TGF-beta1 dose-dependently repressed Sftpb promoter activity in vitro, and a novel TGF-beta-responsive region in the Sftpb promoter was identified.These data suggest that TGF-beta acts as a central mediator of acute lung injury through the alteration of several different molecular pathways. 
 Increased expression of matrix metalloproteinase-9 in the eutopic endometrial tissue of women with endometriosis. Endometriosis is a disease where endometrial tissue implants in ectopic locations. Remodelling of the   extracellular matrix   (ECM) is a prerequisite for the implantation of this tissue to be possible.In this study, we detected immunoreactive matrix metalloproteinase-9 (MMP-9) throughout endometrial tissue and identified von Willebrand factor (vWF)-positive endothelial cells, CD45-positive leukocytes, CD3-positive T lymphocytes and CD68-positive macrophages as cells expressing MMP-9 in the stroma.We found an increased expression of MMP-9 in the uterine endometrial tissue of women with endometriosis, as assessed by zymography and enzyme-linked immunosorbent assay (ELISA) (P < 0.05). However, RT-PCR did not show a statistically significant increase in MMP-9 mRNA expression in these tissues (P = 0.14). There was no significant difference between women with and without endometriosis in the expression of tissue inhibitor of MMPs (TIMP)-1, a known natural inhibitor of the pro- and active forms of MMP-9, whether tested by ELISA or by RT-PCR (P = 0.46 and 0.37, respectively). Interestingly, the ratio of MMP-9/TIMP-1 expression was significantly higher in women with endometriosis than in normal women both at the protein and the mRNA levels (P < 0.05).These findings make plausible the involvement of MMP-9/TIMP-1 imbalance in the invasiveness of the endometrial tissue of patients with endometriosis and the ectopic development of the disease. 
 Association of a high activity of matrix metalloproteinase-9 to low levels of tissue inhibitors of metalloproteinase-1 and -3 in human hepatitis B-viral hepatoma cells. Matrix metalloproteinases (MMPs) play a major role in the turnover of   extracellular matrix   (ECM) during cancer invasion and metastasis, and tissue inhibitors of metalloproteinases (TIMPs) control MMPs, thus maintaining a balanced ECM catabolism under physiological conditions. The aim of this study was to assess the behavior of some MMPs (FASEB J., 7, 1993, 1434; Cancer Metastasis Rev., 9(4) 1990, 289) and TIMPs (Biochem. Biophys. Res. Commun., 301, 2003, 1069; FASEB J., 7, 1993, 1434; Nature, 370, 1994, 61). Competitive RT-PCR, gelatin-substrate zymography, and ELISA techniques were used for quantification. The hepatitis B virus (HBV)-DNA-containing hepatocellular carcinoma cell lines, Hep3B, HepG2-HBV and HFF-T2 contain highly activated matrix metallproteinase-9 (MMP-9), which is rarely found in normal liver cell lines such as the Chang lines. MMP-9 activities of HFH-T2, HepG2-HBV and Hep3B were significantly higher than that of non-HBV-hepatocellular carcinoma SK-Hep1 and HepG2 (HCC origin, HBV not detected), as assayed by gelatin zymography. Low levels of TIMP-1 and TIMP-3 were observed in HFH-T2, HepG2-HBV and Hep3B, while the TIMP-2 level was high, as evidenced by reverse zymography. In contrast, 3 TIMP-1, -2 and -3 were largely detected in Chang, HepG2 and SK-Hep1 cells. To investigate the nature of the quantitative regulation of MMPs and TIMPs for these cell lines at the transcriptional levels, a reverse transcriptase-polymerase chain reaction (RT-PCR) was carried out. Not only MMP-9 mRNAs of HFH-T2, HepG2-HBV and Hep3B but also MMP-9 mRNA of SK-Hep1 and HepG2 were highly expressed with no major differences among these four cell lines. However, TIMP-1 and TIMP-3 mRNAs of HFH-T2, HepG2-HBV and Hep3B were markedly reduced, while those of SK-Hep1, HepG2 and Chang cells were maintained at high levels. Finally, an invasion assay using matrigel indicated in an increase in invasiveness in HFH-T2, HepG2-HBV and Hep3B cells, but a decrease in invasiveness of Chang, HepG2 and SK-Hep1 cells. These results indicate that the overexpression of MMP-9 mRNAs and the suppression of TIMP-1 and TIMP-3 in HFH-T2, HepG2-HBV and Hep3B were the result of HBV transfection. Based on these results, it is concluded that HBV affects the malignance of hepatocellular cancer by elevating MMP-9 activity, and suppressing TIMP-1 and TIMP-3. 
 Potential mechanisms for astrocyte-TIMP-1 downregulation in chronic inflammatory diseases. The pathogenesis of many neurodegenerative disorders, including human immunodeficiency virus (HIV)-1 associated dementia, is exacerbated by an imbalance between matrix metalloproteinases (MMPs) and their inhibitors, tissue inhibitors of metalloproteinases (TIMPs). In the context of disease, TIMP-1 has emerged as an important multifunctional protein capable of regulating inflammation. We previously reported differential TIMP-1 expression in acute versus chronic activation of astrocytes. This study investigates possible mechanisms underlying TIMP-1 downregulation in chronic neuroinflammation. We used interleukin (IL)-1beta as a model pro-inflammatory stimulus and measured TIMP-1 binding to   extracellular matrix  , cell death, receptor downregulation, TIMP-1 mRNA stability and transcriptional regulation in activated astrocytes. TIMP-1 remained localized to the cell body or was secreted into the cell supernatant. DNA fragmentation ELISA and MTT assay showed that prolonged IL-1beta activation of astrocytes induced significant astrocyte death. In acute and chronic IL-1beta-activated astrocytes, IL-1 receptor levels were not significantly different. TIMP-1 mRNA stability was measured in astrocytes and U87 astroglioma cells by real-time PCR, and TIMP-1 promoter activation was studied using TIMP-1-luciferase reporter constructs in transfected astrocytes. Our results indicated that TIMP-1 expression is regulated through multiple mechanisms. Transcriptional control and loss of mRNA stabilization are, however, the most likely primary contributors to chronic downregulation of TIMP-1. These data are important for unraveling the mechanisms underlying astrocyte responses during chronic neuroinflammation and have broader implications in other inflammatory diseases that involve MMP/TIMP imbalance. 
 Sphingosine kinase 1 (SPHK1) is induced by transforming growth factor-beta and mediates TIMP-1 up-regulation. Transforming growth factor-beta (TGF-beta) signaling plays a pivotal role in   extracellular matrix   deposition by stimulating collagen production and other   extracellular matrix   proteins and by inhibiting matrix degradation. The present study was undertaken to define the role of sphingosine kinase (SphK) in TGF-beta signaling. TGF-beta markedly up-regulated SphK1 mRNA and protein amounts and caused a prolonged increase in SphK activity in dermal fibroblasts. Concomitantly, TGF-beta reduced sphingosine-1-phosphate phosphatase activity. Consistent with the changes in enzyme activity, corresponding changes in sphingolipid levels were observed such that sphingosine 1-phosphate (S1P) was increased (approximately 2-fold), whereas sphingosine and ceramide were reduced after 24 h of TGF-beta treatment. Given the relatively early induction of SphK gene expression in response to TGF-beta, we examined whether SphK1 may be involved in the regulation of TGF-beta-inducible genes that exhibit compatible kinetics, e.g. tissue inhibitor of metalloproteinase-1 (TIMP-1). We demonstrate that decreasing SphK1 expression by small interfering RNA (siRNA) blocked TGF-beta-mediated up-regulation of TIMP-1 protein suggesting that up-regulation of SphK1 contributes to the induction of TIMP-1 in response to TGF-beta. The role of SphK1 as a positive regulator of TIMP-1 gene expression was further corroborated by using ectopically expressed SphK1 in the absence of TGF-beta. Adenovirally expressed SphK1 led to a 2-fold increase of endogenous S1P and to increased TIMP-1 mRNA and protein production. In addition, ectopic SphK1 and TGF-beta cooperated in TIMP-1 up-regulation. Mechanistically, experiments utilizing TIMP-1 promoter constructs demonstrated that the action of SphK1 on the TIMP-1 promoter is through the AP1-response element, consistent with the SphK1-mediated up-regulation of phospho-c-Jun levels, a key component of AP1. Together, these experiments demonstrate that SphK/S1P are important components of the TGF-beta signaling pathway involved in up-regulation of the TIMP-1 gene. 
 Expression of tissue inhibitors of metalloproteinase 1 (TIMP-1) in gastric cancer tissue. Degradation of   extracellular matrix   (ECM) is an essential step of invasion and metastasis of gastric cancer. The proteolysis of basement membranes depends on the balance between activities of matrix metalloproteinases (MMPs) and their tissue inhibitors (TIMPs). The aim of the study was to assess the expression of TIMP-1 in gastric cancer (GC) and interstitial inflammatory infiltrate cells within GC tissue in relation to clinico-pathological features of tumor and to estimate the prognostic significance of TIMP-1 expression for patients' survival. The presence of TIMP-1 in 54 cases of gastric cancer samples was investigated by immunohistochemistry. The expression of TIMP-1 in cancer and interstitial inflammatory infiltrate cells was evaluated in semi-quantitative scale. The immunoreactivity of TIMP-1 in cancer and inflammatory cells was positive in 100% of cases and varied from weak to intense reaction. The intensity of TIMP-1 expression increased with more advanced tumor stages and in patients who died of cancer during 2-year observation. TIMP-1 expression in interstitial inflammatory infiltrate cells was the independent prognostic factor for patients' survival. The results suggest the role of TIMP-1 in gastric tumorigenesis, although this issue requires further investigtions. 
 Unveiling the surface epitopes that render tissue inhibitor of metalloproteinase-1 inactive against membrane type 1-matrix metalloproteinase. Membrane type 1-matrix metalloproteinase (MT1-MMP) is a zinc-dependent, membrane-associated endoproteinase of the metzincin family. The enzyme regulates   extracellular matrix   remodeling and is capable of cleaving a wide variety of transmembrane proteins. The enzymatic activity of MT1-MMP is regulated by endogenous inhibitors, the tissue inhibitor of metalloproteinases (TIMP). To date, four variants of mammalian TIMP have been identified. Whereas TIMP-2-4 are potent inhibitors against MT1-MMP, TIMP-1 displays negligible inhibitory activity against the enzyme. The rationale for such selectivity is hitherto unknown. Here we identify the surface epitopes that render TIMP-1 inactive against MT1-MMP. We show that TIMP-1 can be transformed into an active inhibitor against MT1-MMP by the mutation of a single residue, namely threonine 98 to leucine (T98L). The resultant mutant displayed inhibitory characteristics of a typical slow, tight binding inhibitor. The potency of the mutant could be further enhanced by the introduction of valine 4 to alanine (V4A) and proline 6 to valine (P6V) mutations. Indeed, the inhibitory profile of the triple mutant (V4A/P6V/T98L) is indistinguishable from those of other TIMPs. Our findings suggest that threonine 98 is critical in initiating MMP binding and complex stabilization. Our findings also provide a potential mechanistic explanation for MMP-TIMP selectivity. 
 Expression of matrix metalloproteinase-1, -9, -13, and tissue inhibitor of metalloproteinases-1 in basal cell carcinomas of the eyelid. Matrix metalloproteinases (MMPs) and tissue inhibitors of metalloproteinases (TIMPs) function in the remodelling of the   extracellular matrix   in morphogenesis, angiogenesis, tissue repair, and tumor invasion. Elevated levels of distinct MMPs in tumor tissue are related to worse prognosis. However, no overall consistent pattern of expression in human cancer has been identified. The aim of the present study was to evaluate the expression of MMP-1, -9, -13 and TIMP-1 in tumor epithelial cells and surrounding connective tissue in primary basal cell carcinomas (BCC) of the eyelid, and to assess their role as prognostic markers for tumor recurrence.Surgical specimens of 49 histologically proven primary BBCs of the eyelid of different histological subtypes were included. Immunohistological studies were performed using antibodies against MMP-1, MMP-9, MMP-13 and TIMP-1, and staining intensity was analyzed semi-quantitatively.MMP-1, -9, -13, and TIMP-1 were expressed at various intensities in epithelial tumor cells and surrounding stromal cells including fibroblasts, inflammatory cells, and vascular endothelial cells in all tumor subtypes. Staining was especially prominent at the invading edge of the BCC. A statistically significant correlation was seen between increased TIMP-1 expression in tumor and/or stromal cells with the presence of MMP-13 (p = 0.007 and p < 0.0001 respectively). Moreover, TIMP-1 expression in tumor and/or stroma was significantly associated with relapse (p = 0.012 and p = 0.042 respectively).MMP-9, MMP-13 and TIMP-1 expression may serve as a prognostic marker for early tumor invasiveness. Moreover, up-regulation of TIMP-1 in tumor and/or surrounding stromal cells may indicate an increased risk for BCC recurrence. 
 [To study of the nasal mucosa remodeling of allergic rhinitis patients]. To explore whether there was tissue remodeling in the nasal mucosa of allergic rhinitis (AR) patients and detect the protein expressions of transforming growth factor beta1 (TGF-beta1), matrix metalloproteinases 9 (MMP-9) and tissue inhibitors of matrix metalloproteinases 1 (TIMP-1) in the nasal mucosa of these patients.Pathologic staining was used to explore the mucosa of the middle turbinate tissues from 16 patients with mild AR, from 12 patients with severe AR, and from 15 non-AR, respect. The infiltrating of eosinophils and damage of epithelium were examined by the hematoxylin-eosin staining; goblet cells were counted by the alcian blue-periodic acid-schiff staining; the percentage area of   extracellular matrix   was determined by the MT; the protein expressions were measured by ELISA of TGF-beta1, MMP-9 and TIMP-1 in the middle turbinate tissues.Compared with the control group, significant eosinophil infiltration and goblet cells were observed in both AR groups (P < 0.05). Evident epithelial damage and   extracellular matrix   deposition were observed in severe-AR group (P < 0.05). The expressions of TGF-beta1, MMP-9 and TIMP-1 in AR tissues were significant increased (P < 0.05).The nasal mucosa remodeling was observed in AR groups. The characteristics were as follows: eosinophils infiltration, epithelial damage, goblet cells hyperplasia and   extracellular matrix   deposition. TGF-beta1, MMP-9, TIMP-1 may play a role in the tissue remodeling processes. 
 Serum levels of MMP-9 and TIMP-1 in primary hypertension and effect of antihypertensive treatment. Matrix metalloproteinases, a family of proteolytic enzymes are thought to be involved in   extracellular matrix   accumulation during development of hypertensive target organ disease. The present study was designed to compare hypertensive patients with normotensive individuals with respect to serum levels of matrix metalloproteinase (MMP)-9 and tissue inhibitor of metalloproteinase (TIMP)-1 and to search for the effect of antihypertensive treatment on the serum enzyme levels.Thirty-three patients with stage 1 primary hypertension and sixteen age- and sexmatched control subjects were enrolled into the study. Serum MMP-9 and TIMP-1 levels were assessed in the hypertensive group before and after a 3-month-antihypertensive treatment (candesartan 8 mg/day to 17 patients and lisinopril 10 mg/day to 16 patients).Pre-treatment serum MMP-9 levels were higher in the hypertensive group (p=0.309) while serum TIMP-1 levels were lower (p=0.296). Serum MMP-9 levels were decreased (p<0.001) and TIMP-1 levels were increased (p=0.022) after the antihypertensive treatment.In hypertensive patients, increased MMP-9 activity could result in increased degradation of elastin relative to collagen and non-elasticity, while decreased TIMP-1 activity could lead to accumulation of poorly cross-linked, immature and unstable fibril degradation products, which result in misdirected deposition of collagen. Our study is important for revealing the role of the MMP enzyme system in the pathogenesis of hypertensive target organ disease. 
 Metalloproteinase 9 and TIMP-1 expression in retina and optic nerve in absolute angle closure glaucoma. Glaucoma is one of the most important reason causes of the blindness, associated with retinal ganglion cells (RGC) death. This process is not fully understood, however apoptosis due to hypoxia is one of the most important processes leading to RGC death. Glaucomatous optic neuropathy is characterized by remodeling of the   extracellular matrix   due to metalloproteinase activation, which leads to loss of RGC and axons at the optic nerve head. The aim of the study was to evaluate metalloproteinase 9 (MMP-9) and tissue metalloproteinase inhibitor-1 (TIMP-1) expression in the retinal ganglion cells and optic nerve axons in 33 eyes with absolute primary glaucoma.To evaluate MMP-9 and TIMP-1 expression primary polyclonal goat antibodies against MMP-9 and TIMP-1 were used. The control group was composed of 8 cases of eyes enucleated and fixed in the first day after trauma.MMP-9 expression was observed in retinal ganglion cells and in the inner nuclear layer of the retina in all the examined cases. In 28 out of 33 glaucomatous eyes, MMP-9 expression was observed in the proliferating glial cells surrounding the optic nerve axons. TIMP-1 expression was observed in 10 out of 33 glaucomatous eyes, only in retinal ganglion cells. None of the examined injured eyes showed MMP-9 and TIMP-1 expression.MMP-9 activation rather than TIMP-1 may by associated with the pathomechanism of retinal ganglion cell and optic nerve damage in absolute glaucoma. 
 A novel mechanism of tissue inhibitor of metalloproteinases-1 activation by interleukin-1 in primary human astrocytes. Reactive astrogliosis is the gliotic response to brain injury with activated astrocytes and microglia being the major effector cells. These cells secrete inflammatory cytokines, proteinases, and proteinase inhibitors that influence   extracellular matrix   (ECM) remodeling. In astrocytes, the expression of tissue inhibitor of metalloproteinases-1 (TIMP-1) is up-regulated by interleukin-1 (IL-1), which is a major neuroinflammatory cytokine. We report that IL-1 activates TIMP-1 expression via both the IKK/NF-kappaB and MEK3/6/p38/ATF-2 pathways in astrocytes. The activation of the TIMP-1 gene can be blocked by using pharmacological inhibitors, including BAY11-7082 and SB202190, overexpression of the dominant-negative inhibitor of NF-kappaB (IkappaBalphaSR), or by the knock-down of p65 subunit of NF-kappaB. Binding of activated NF-kappaB (p50/p65 heterodimer) and ATF-2 (homodimer) to two novel regulatory elements located -2.7 and -2.2 kb upstream of the TIMP-1 transcription start site, respectively, is required for full IL-1-responsiveness. Mutational analysis of these regulatory elements and their weak activity when linked to the minimal tk promoter suggest that cooperative binding is required to activate transcription. In contrast to astrocytes, we observed that TIMP-1 is expressed at lower levels in gliomas and is not regulated by IL-1. We provide evidence that the lack of TIMP-1 activation in gliomas results from either dysfunctional IKK/NF-kappaB or MEK3/6/p38/ATF-2 activation by IL-1. In summary, we propose a novel mechanism of TIMP-1 regulation, which ensures an increased supply of the inhibitor after brain injury, and limits ECM degradation. This mechanism does not function in gliomas, and may in part explain the increased invasiveness of glioma cells. 
 Regulation of TIMP-1 phenotypic expression in Epstein--Barr virus-immortalized B lymphocytes. Normal B lymphocytes as well as malignant B cells extravasate from blood circulation during physiological and pathological processes and require matrix metalloproteinases (MMPs) to facilitate trafficking through the subendothelial basal lamina and the   extracellular matrix  . We have previously shown that Epstein-Barr virus (EBV)-immortalized B lymphocytes constitutively synthesized low levels of MMP-9 and huge amounts of its preferential inhibitor, tissue inhibitor of matrix metalloproteinase-1 (TIMP-1). In the present study, TIMP-1 phenotypic expression was extensively investigated in response to various mediators including interleukins, chemokines, growth factors and tumor promotor, and was compared to MMP-9 synthesis. Results showed a roughly constitutive TIMP-1 expression opposed to an inducible MMP-9 synthesis. Nevertheless, further analysis of TIMP-1 synthesis showed the existence of regulation mechanisms: modulation of intracellular Ca(2+) concentration as well as cation ionophore monensin were demonstrated to influence TIMP-1 production and secretion. The precise pathways implicated in these regulation mechanisms are currently under survey. 
 Genetic polymorphisms of the matrix metalloproteinase-3 (MMP-3) and tissue inhibitors of matrix metalloproteinases-1 (TIMP-1) modulate the development of ankylosing spondylitis. The aetiology of ankylosing spondylitis (AS) remains unclear. Inflammation progresses to fibrosis and calcification of the spine and sacroiliac joints in AS development. Fibrosis results from excessive accumulations of the   extracellular matrix   (ECM). ECM turnover depends on the balance between matrix metalloproteinases (MMPs) and their tissue inhibitors (TIMPs).To evaluate the effects of the MMP-3 -1171 and TIMP-1 372 T>C polymorphisms on the modified risk of AS.Genotypes of 241 patients with AS and 241 controls were identified by PCR. Disease activity and functional status were assessed by the Bath Ankylosing Spondylitis Activity Index (BASDAI), the Bath Ankylosing Spondylitis Functional Index (BASFI) and the Bath Ankylosing Spondylitis Global (BAS-G) Score.MMP-3 6A/6A carriers had a 2.41-fold (95% confidence interval (CI) 1.55 to 3.74) increased risk of AS compared with 6A/5A and 5A/5A carriers. TIMP-1 C alleles had a greater risk of AS, but this was not significant (odds ratio (OR) = 1.28, 95% CI 0.92 to 1.77). Pairwise analysis of the MMP-3/TIMP-1 alleles showed that 6A/C (OR = 3.23, 95% CI 1.50 to 6.95) and 6A/T (OR = 2.55, 95% CI 1.17 to 5.54) had a significantly greater risk of AS than the 5A/T alleles. After adjustment for the effects of age, gender and disease duration, the MMP-3/TIMP-1 5A/T alleles had the lowest BASDAI (p = 0.02), BASFI (p = 0.05) and BAS-G (p = 0.02) among all MMP-3/TIMP-1 alleles.The findings highlight the importance of the MMP-3 and TIMP-1 genes as crucial elements in AS development. 
 Estradiol-estrogen receptor: a key interplay of the expression of syndecan-2 and metalloproteinase-9 in breast cancer cells. Estrogens are related with the growth and development of target tissues and play a critical role in breast cancer progression. The effects of estrogens are mediated by the estrogen receptors ERalpha and ERbeta, which are members of the nuclear steroid receptor superfamily. To date, it is not known how these hormones elicit many of their effects on   extracellular matrix   molecules and how these effects can be connected with ER expression. For this purpose, the effect of estradiol on ER expression as well as on proteoglycan and metalloproteinase expression was studied. The effect of E2 on   extracellular matrix   molecule expression has been studied using ERalpha suppression in breast cancer cells. Our studies using ERalpha-positive MCF-7 cells show that estradiol affects the expression of syndecan-2, but not of syndecan-4, through ERalpha. Furthermore, the ability of estradiol to affect MMP-9 and TIMP-1 expression is connected with ERalpha status. Together, these data demonstrate the significant role of ERalpha on mediating the effect of estradiol on   extracellular matrix   molecules. 
 Association of carotid artery atherosclerosis with circulating biomarkers of   extracellular matrix   remodeling: the Framingham Offspring Study. We sought to relate circulating biomarkers of   extracellular matrix   turnover to site-specific measures of carotid artery atherosclerosis on duplex ultrasound.Matrix metalloproteinases (MMPs) and tissue inhibitors of MMPs (TIMPs) regulate   extracellular matrix   remodeling, a key feature of atherosclerosis, and their circulating concentrations can be assayed. MMP-9, TIMP-1, and protocollagen-III n-terminal propeptide (PIIINP) may relate differentially to the severity of atherosclerosis at different carotid artery sites. However, data examining this premise are sparse.We related circulating MMP-9, TIMP-1, and/or PIIINP concentrations to carotid atherosclerosis on duplex ultrasound in 1006 Framingham offspring (mean age 58 years, 56% women) who attended a routine examination from 1995 to 1998. We used multivariable regression to relate MMP-9 (detectable v undetectable), TIMP-1, and PIIINP (age- and sex-specific quartiles) to internal carotid artery (IC) stenosis (>25%) and log-transformed common carotid artery and IC intima-media thickness (IMT).Detectable MMP-9 was associated with carotid stenosis (odds ratio [OR] 1.71, P = .032) but not with IMT. Higher TIMP-1 was associated with carotid stenosis (OR for Quartiles (Q)4 v Q1-3, 1.63, P = .022) and a higher IC IMT (beta 0.057 +/- 0.025, Q4 v Q1-3, P = .023). Higher PIIINP (Q4 v Q1-3) showed a borderline association with carotid stenosis (OR 1.45 for Q4 v Q1-3, P = .095) but not with IMT. TIMP-1 was not associated with common carotid artery IMT.In our community-based sample of middle-aged to older adults, higher circulating biomarkers of matrix remodeling were associated with a greater prevalence of carotid stenosis and subclinical atherosclerosis in the IC. Our findings are consistent with regional differences in matrix remodeling in the carotid artery. 
 Impact of the metalloproteinase-9/tissue inhibitor of metalloproteinase-1 system on large arterial stiffness in patients with essential hypertension. The   extracellular matrix   is vital for maintaining tissue integrity, and the matrix metalloproteinases/tissue inhibitors of metalloproteinases (MMPs/TIMPs) system is involved in the regulation of   extracellular matrix   metabolism.   Extracellular matrix   turnover plays an important role in the change of large arterial mechanical properties in hypertension. However, the association of the metalloproteinase-9/tissue inhibitor of metalloproteinase-1 (MMP-9/TIMP-1) system and arterial stiffness is not straightforward and existing data are rather limited. Our objective is to explore the impact of the MMP-9/TIMP-1 system on large arterial stiffness in patients with essential hypertension. An automatic pulse wave velocity (PWV) measuring system was used to examine carotid-femoral PWV (CFPWV) and carotid-radial PWV (CRPWV) as the parameters reflecting central elastic large arterial and peripheral muscular medium-sized arterial elasticity, respectively; and serum MMP-9 and TIMP-1 levels, along with a number of other established biomarkers, were measured by enzyme-linked immunosorbent assay (ELISA) in 202 essential hypertensive patients and 54 age and gender-matched control subjects. Compared with the control subjects, hypertensive patients exhibited higher levels of MMP-9 (p=0.001) and TIMP-1 (p=0.002). Spearman's correlation analysis showed that serum levels of MMP-9 (p=0.014) and TIMP-1 (p=0.005) were significantly and positively correlated with CFPWV in hypertensive patients. A stepwise multiple regressive analysis demonstrated that age, systolic blood pressure, heart rate and TIMP-1 were independent predictors of CFPWV in patients with essential hypertension (adjusted r2=0.458). In conclusion, our results imply that the MMP-9/TIMP-1 system may play an important role in the determination of arterial function, and these findings may have implications for the involvement of MMP-9/TIMP-1 system in the pathophysiology of cardiovascular disease. 
 Expression profiles of MMP-1 and TIMP-1 in lumbar intervertebral disc degeneration. Lumbar intervertebral disc degeneration (IDD) is a common clinical pathology and has become a focus for research in recent years. Matrix metalloproteinases (MMPs) are enzymes responsible for the degradation of almost all   extracellular matrix   proteins (ECM). The over-expression of MMPs or tissue inhibitors of metalloproteinases (TIMPs) may disrupt the dynamic balance of the ECM. Therefore, in the current study, the expression levels of MMP-1 and TIMP-1 in lumbar IDD patients were evaluated in an attempt to elucidate their role in IDD pathogenesis and progression. In total, 60 IDD patients were recruited as the experimental group, along with 20 cases of lumbar vertebral injury without disc degeneration as the control group. Preoperative venous blood samples were collected, and intervertebral disc tissues were collected from the lesion during surgery. Serum and tissue levels of MMP-1 and TIMP-1 were quantified by enzyme-linked immunosorbent assay and immunohistochemical staining, respectively. Serum and tissue MMP-1 levels in IDD patients were significantly higher than those in the control group (P < 0.05). Additionally, sub-group analysis revealed that severe IDD patients had higher MMP-1 levels compared with mild or moderate IDD patients (P < 0.05). However, there were no significant differences in TIMP- 1 levels in either the serum or tissues of IDD patients compared to patients in the control group (P > 0.05). These results demonstrate that MMP-1 expression is increased in IDD, with higher expression observed in more severe cases, whereas TIMP-1 expression was similarly expressed in both normal and degenerated discs. 
 MMP-9 overexpression due to TIMP-1 and RECK underexpression is associated with prognosis in prostate cancer.   Extracellular matrix   homeostasis is strictly maintained by a coordinated balance between the expression of metalloproteinases (MMPs) and their inhibitors. The purpose of this study was to investigate whether the expression of MMP-9 and its specific inhibitors, TIMP-1 and RECK, are expressed in a reproducible, specific pattern and if the profiles are related to prognosis and clinical outcome in prostate cancer (PC).MMP-9, TIMP-1, and RECK expression levels were analyzed by quantitative real-time polymerase chain reaction (qRT-PCR) in fresh-frozen malignant tissue specimens collected from 79 patients with clinically localized PC submitted to radical prostatectomy (RP). Frozen benign prostatic tissue from another 10 men with prostate cancer, also submitted to RP, was analyzed to determine if the profile of gene expression was maintained. The control group consisted of 11 patients with benign prostate hyperplasia (BPH).In the tumor samples, MMP-9 was overexpressed by 9.2 times, and TIMP-1 and RECK were underexpressed (0.75 and 0.80 times, respectively). Overexpression of MMP-9 was significantly related to PSA levels above 10 ng/mL (p=0.033). In addition, MMP-9 overexpression was related to biochemical recurrence, with a marginal statistical significance (p=0.089). MMP-9 was also overexpressed in benign tissues of patients with PC, as were TIMP-1 and RECK, in contrast to their underexpression in tumor samples.Our results show that MMP-9 is overexpressed and its negative regulators are underexpressed in PC tissue, emphasizing a possible role of MMP-9 in the carcinogenesis process. Additionally, we noticed a relationship between MMP-9 overexpression and increased levels of PSA, an important prognostic factor. In benign tissue adjacent to tumors, the MMP-9 equilibrium is likely maintained because the expression of its negative regulators is preserved. 
 Change in proteoglycan metabolism is a characteristic of human patellar tendinopathy. To determine differences in the metabolism of proteoglycans and the gene expression of proteinases and their inhibitors between patellar tendons exhibiting chronic overuse tendinopathy and normal patellar tendons in humans.Rates of loss and synthesis of proteoglycans were determined. Radiolabeled and total proteoglycans retained in and lost from the tissue were analyzed by fluorography and Western blotting. Levels of messenger RNA for matrix metalloproteinase 1 (MMP-1), MMP-2, MMP-3, MMP-9, MMP-13, ADAMTS-1, ADAMTS-4, ADAMTS-5, tissue inhibitor of metalloproteinases 1 (TIMP-1), TIMP-2, TIMP-3, and TIMP-4 were determined in fresh tissue.The rate of loss of (35)S-labeled proteoglycans was greater in abnormal tendons, as was the rate of synthesis of proteoglycans. Fluorography and Western blotting revealed the presence of greater amounts of large proteoglycans (aggrecan and versican) in abnormal tendons, and these proteoglycans were rapidly lost from the matrix of abnormal tendons. There was no significant difference in the expression of ADAMTS-1, ADAMTS-4, ADAMTS-5, MMP-1, MMP-2, MMP-3, MMP-13, TIMP-2, TIMP-3, or TIMP-4. There was a significant increase in the expression of MMP-9 and TIMP-1 in abnormal tendons.Our findings suggest that a change in the proteoglycan content of the   extracellular matrix   in abnormal tendons results from the altered metabolism of the cells, reflected in the enhanced synthesis of the large proteoglycans aggrecan and versican, and does not appear to result from changes at the level of gene expression. 
 Prognostic values of matrix metalloproteinase family expression in human colorectal carcinoma. We examined expression patterns of matrix metalloproteinase (MMP), tissue inhibitor of metalloproteinase (TIMP), and reversion-inducing cysteine-rich protein with Kazal motifs (RECK) in colorectal cancer tissues to assess their prognostic significance.mRNA expressions of 17 MMPs, 4 TIMPs, and RECK were measured in 112 colorectal cancerous tissues, 20 normal mucosa tissues, and 11 metastatic liver lesions by real-time reverse-transcriptional-polymerase chain reaction. The protein level expressions were confirmed with immunohistochemistry.Cancers and normal mucosa displayed highly significant differences (P < 0.01) in expression of nine genes (MMP-1, -3, -7, -9, -10, -11, -12, -14, and RECK). Primary cancers and metastatic lesions showed highly significant differences (P < 0.01) in MMP-1, -10, -11, and TIMP-1. MMP-12 expression was higher in the primary tumors that were associated without hepatic metastasis than those with metastasis (P < 0.01). High expression of MMP-15 was related to longer disease-free survival (generalized Wilcoxon test, P < 0.0062; Cox hazard model, P < 0.028, hazard ratio, 0.099).MMP, TIMP, RECK expression patterns may provide an insight into   extracellular matrix   degrading (which is characteristic of colorectal cancers) and its role in metastasis. 
 Tissue inhibitor of metalloproteinases (TIMP-1), genetic markers of insulin resistance and cardiomyopathy in patients with kidney failure. Left ventricular hypertrophy (LVH) is a major cardiovascular (CV) complication in patients with kidney failure, and an association between polymorphisms in the ectonucleotide pyrophosphatase/phosphodiesterase 1 (ENPP1) gene, a genetic marker of insulin resistance, and LVH and Left ventricular (LV) concentric remodelling has been recently documented in these patients. Aims. Since myocardial fibrosis is a prominent feature in LVH induced by insulin resistance, we tested the hypothesis that the interaction between ENPP1 rs1974201 and rs9402349 polymorphisms and the tissue inhibitor of metalloproteinases (TIMP-1)--a pro-fibrotic protein which inhibits   extracellular matrix   degradation--is implicated in concentric LVH and diastolic dysfunction in a cohort of 223 dialysis patients.Both ENPP1 polymorphisms rs1974201 and rs9402349 were in Hardy-Weinberg equilibrium in dialysis patients. In an analysis stratified by ENPP1, rs1974201 polymorphism, circulating levels of TIMP-1 in GG patients were coherently associated with two markers of concentric remodelling [relative wall thickness (RWT) and LV mass-to-volume ratio] as well as with a marker of diastolic dysfunction (E/A ratio) (P ranging from 0.005 to 0.02), whereas no such associations existed in CC or CG patients. These observations suggest that the rs1974201 modifies the relationship between TIMP-1 and LV geometry and diastolic dysfunction. Accordingly, in a multiple regression model, an identical increase of TIMP-1 (100 ng/mL) was associated with an increase of 22% in RWT, 14% in LV mass-to-volume ratio and 29% in E/A ratio in GG patients but with almost no change (from -0.22 to -3.78%) in these echocardiographic indices in the remaining patients (P for the effect modification ≤ 0.024). The rs9402349 did not modify the relationship between TIMP-1 and LV geometry and function.In dialysis patients, the ENPP1 rs1974201 polymorphism modifies the association between TIMP-1 and LV geometry and diastolic function. These results are consistent with the hypothesis that insulin resistance is involved not only in LVH but also in myocardial fibrosis, an alteration of primary importance in the high risk of this population. 
 Crystal structure of the catalytic domain of matrix metalloproteinase-1 in complex with the inhibitory domain of tissue inhibitor of metalloproteinase-1. The mammalian collagenases are a subgroup of the matrix metalloproteinases (MMPs) that are uniquely able to cleave triple helical fibrillar collagens. Collagen breakdown is an essential part of   extracellular matrix   turnover in key physiological processes including morphogenesis and wound healing; however, unregulated collagenolysis is linked to important diseases such as arthritis and cancer. The tissue inhibitors of metalloproteinases (TIMPs) function in controlling the activity of MMPs, including collagenases. We report here the structure of a complex of the catalytic domain of fibroblast collagenase (MMP-1) with the N-terminal inhibitory domain of human TIMP-1 (N-TIMP-1) at 2.54 A resolution. Comparison with the previously reported structure of the TIMP-1/stromelysin-1 (MMP-3) complex shows that the mechanisms of inhibition of both MMPs are generally similar, yet there are significant differences in the protein-protein interfaces in the two complexes. Specifically, the loop between beta-strands A and B of TIMP-1 makes contact with MMP-3 but not with MMP-1, and there are marked differences in the roles of individual residues in the C-D connector of TIMP-1 in binding to the two MMPs. Structural rearrangements in the bound MMPs are also strikingly different. This is the first crystallographic structure that contains the truncated N-terminal domain of a TIMP, which shows only minor differences from the corresponding region of the full-length protein. Differences in the interactions in the two TIMP-1 complexes provide a structural explanation for the results of previous mutational studies and a basis for designing new N-TIMP-1 variants with restricted specificity. 
 Characterization of a human corneal metalloproteinase inhibitor (TIMP-1). The gradual corneal thinning seen in keratoconus may be due to altered degradation of the corneal   extracellular matrix  . Studies have shown that human keratocytes produce matrix metalloproteinase-2 (MMP-2) and two proteins (28 kDa and 21 kDa) that are capable of inhibiting the activity of MMP-2. In the present study, the 28 kDa inhibitor from keratoconus keratocyte cultures has been characterized as it may be important to the elevated MMP-2 activity seen in these cultures. Biochemical analyses indicated that this keratoconus corneal inhibitor was similar to TIMP-1 from other sources. Oligonucleotides to the reported sequence of human tumor cell TIMP-1 were used for reverse-transcriptase PCR to generate a 700 bp clone of the 28 kDa inhibitor from keratoconus keratocyte cytoplasmic RNA. Sequence analysis verified that the clone was nearly identical to the reported human TIMP-1 with a single base substitution that did not affect the predicted amino acid sequence. In addition, protein translated from the clone corresponded to the expected size. This data suggests that the elevated levels of gelatinolytic activity in these keratoconus keratocyte cultures is not due to a primary alteration of the TIMP-1 molecule. Protein expression studies of the TIMP-1 clone are currently underway. 
 Differential expression of matrix metalloproteinases and tissue-derived inhibitors of metalloproteinase in fetal and adult skins. Matrix metalloproteinases and their tissue-derived inhibitors determine the architecture of the   extracellular matrix  . In early gestation, the amount and organization of   extracellular matrix   may be associated with scarless repair of fetal skin wounds. To elucidate the part of the mechanism(s) underlying the phenotypic transition from scarless to scar-forming healing observed during fetal gestation, the ontogeny of matrix metalloproteinase-2, -9, -14 and their tissue inhibitors was characterized in non-wounded fetal human skin with different gestational ages from 13 to 33 weeks and adult skin using reverse transcriptase-polymerase chain reaction, immunohistochemical staining and western blot protocols. We showed that the levels of gene expressions for matrix metalloproteinase-2, -9, -14 and their endogenous inhibitors were significantly more in late gestational and adult skins than that in early gestational skin. Similar results were noted in terms of protein contents of these enzymes and inhibitors in fetal and adult skins. We concluded that the endogenous matrix metalloproteinase-2, -9, -14 and their endogenous inhibitors might be involved in skin development and in maintenance of cutaneous structure and function. Lower protein contents of tissue-derived inhibitor-1, -2 in early gestational skin might provide a predominantly antiscarring signal while higher protein expression of these two inhibitors might be associated with scar-forming healing in late gestational and adult skins. 
 Prostaglandin E2-dependent enhancement of tissue inhibitors of metalloproteinases-1 production limits dendritic cell migration through   extracellular matrix  . Dendritic cell (DC) migration is crucial for the initiation of immune responses. The balance between metalloproteinases (MMP) and tissue inhibitors of metalloproteinases (TIMP) has been shown to modulate DC migration. PGE2, which is overproduced in a wide variety of human malignancies, has been implicated in MMP and TIMP regulation in various cells, including monocytes. In the present study, we hypothesized that tumor-derived PGE2 would affect DC migratory capacity through the   extracellular matrix   (ECM) by altering MMP and TIMP balance. Treatment of monocyte-derived immature DC with exogenous PGE2 induced TIMP-1 secretion but not MMP-9 production and was correlated with reduced DC migration through ECM. Because recombinant TIMP-1 replicated PGE2 inhibition of DC migration while anti-TIMP-1 neutralizing Ab reversed it, we conclude that PGE2-mediated induction of TIMP-1 was responsible for the reduced migration of PGE2-treated DC. Similarly, DC cultured for 48 h in supernatants from cyclooxygenase-2 overexpressing lung cancer cells that secrete high levels of PGE2, exhibited decreased migration through ECM. Finally, analysis of E prostanoid receptor expression and their selective inhibition revealed that the enhanced TIMP-1 secretion in PGE2-treated DC was mediated predominantly by the E prostanoid receptor 2. These findings indicate that PGE2-dependent enhancement of TIMP-1 production causes reduced migration of DC through ECM. 
 Elevated plasma levels of tissue inhibitors of metalloproteinase-1 and their overexpression in muscle in human and mouse muscular dystrophy. To investigate the role of tissue inhibitors of metalloproteinases (TIMPs) in muscular dystrophy, we examined the expression of TIMP-1 using plasma and biopsied muscle from patients with various muscular dystrophies by ELISA, immunohistochemistry, and Western blot analysis. TIMP-1 immunolocalization was also studied in mouse models of muscular dystrophy. Plasma TIMP-1 was elevated and correlated with TGF-β1 in Duchenne muscular dystrophy (DMD) and congenital muscular dystrophy (CMD), but not in Becker muscular dystrophy. In dystrophic human muscles, TIMP-1 was immunopositive in the regenerating and non-regenerating muscle fibers, and interstitial cells that consist of activated fibroblasts and macrophages. TIMP-1 immunoreactivity was also closely associated with TGF-β1. Western blot analysis showed elevated TIMP-1 protein in muscles in DMD. The semiquantitative analysis of TIMP-1 staining intensity and tissue fibrosis showed that TIMP-1 immunoreactivity is closely associated with the extent of tissue fibrosis in human and mouse dystrophic muscles. In conclusion, the present study implied that the TGF-β1-TIMP-1 pathway is activated in dystrophic muscles and the overexpression of TIMP-1 may result in increased deposition   of extracellular mat  rix leading to tissue fibrosis. 
 Tissue inhibitor of metalloproteinase-1 is responsible for residual pleural thickening in pleural tuberculosis. Residual pleural thickening (RPT) is the most frequent complication associated with pleural tuberculosis, and may occur even after successful anti-tuberculosis medications. Matrix metalloproteinases (MMPs) are zinc-dependent proteinases capable of degrading all components of the   extracellular matrix  . The proteolytic action of MMPs may be involved in the pathogenesis of tuberculosis. MMP-9, secreted by monocytes and lymphocyte, may lead to long-term fibrosis. The aim of the present study was to determine whether MMP-2 and/or MMP-9 and their specific inhibitors, tissue inhibitors of metalloproteinase 1 (TIMP-1) and TIMP-2, could be used to predict RPT. This retrospective study enrolled 52 patients diagnosed with pleural tuberculosis. Levels of MMP-2, MMP-9, TIMP-1, and TIM-2 were determined in the pleural fluid by ELISA. The RPT was measured on chest X-ray at the completion of treatment and the final follow-up. The average periods of anti-tuberculosis medication and the follow-up after completion of treatment were 6.7 and 7.6 months, respectively. MMP-2 or MMP-9 levels had no significant correlation to RPT. The patients with RPT > 2 mm at the completion of anti-tuberculosis medication and the final follow-up had higher TIMP-1 levels (p = 0.00 and p = 0.001, respectively). However, patients with RPT > 2 mm at the completion of anti-tuberculosis medication had lower TIMP-2 levels (p = 0.005). In a logistic regression model, elevated TIMP-1 levels at the completion of anti-tuberculosis medications were associated with RPT. In conclusion, higher TIMP-1 levels are responsible for the development of RPT and may be helpful for predicting RPT in pleural tuberculosis. 
 Lack of relationship between TIMP-1 tumour cell immunoreactivity, treatment efficacy and prognosis in patients with advanced epithelial ovarian cancer. Tissue inhibitor of metalloproteinase 1 (TIMP-1) is a natural inhibitor of the matrix metalloproteinases (MMPs) which are proteolytic enzymes involved in degradation of   extracellular matrix   thereby favoring tumour cell invasion and metastasis. TIMP-1 activity in tumour tissue may therefore play an essential role in the progression of a malignant tumour.The primary aim of the present study was to evaluate TIMP-1 protein immunoreactivity in tissue from primary ovarian cancer patients and associate these findings with the course of the disease including response to treatment in the individual patient.TIMP-1 was assessed by immunohistochemistry (in tissue micro arrays) in a total of 163 ovarian cancer specimens obtained from primary debulking surgery during 1991-1994 as part of a randomized clinical protocol.Positive TIMP-1 immunoreactivity was found in 12.3% of the tumours. The median survival time for the 143 patients with TIMP-1 negative tumours was 23.7 months [19.0-29.4] 95% CI, while the median survival time for the 20 patients with TIMP-1 positive tumours was 15.9 months [12.3-27.4] 95% CI. Although a difference of 7.8 months in median overall survival in favor of the TIMP-1 tumour negative patients was found, this difference did not reach statistical significance (p = 0.28, Kaplan-Meier, log-rank test). Moreover, TIMP-1 immunoreactivity was not associated with CA125 response (p = 0.53) or response at second look surgery (p = 0.72).TIMP-1 immunoreactivity in tumour tissue from patients with primary epithelial ovarian cancer did not correlate with patient survival or response to combination platinum/cyclophosphamide therapy. 
 Matrix metalloproteinase-2, -9, and tissue inhibitor of metalloproteinase-1 in patients with hypertension. There are conflicting data in the literature regarding the expression pattern of the vascular matrix metalloproteinase (MMP) system and their inhibitors (TIMPs) in human hypertension. The authors hypothesized that MMP-2, MMP-9, and TIMP-1 would be abnormal in hypertension, reflecting alterations in   extracellular matrix   (ECM) turnover. The authors measured plasma levels and activities of MMP-2, MMP-9, and TIMP-1 in 44 hypertensive patients and 44 controls. MMP-2 levels and activity were significantly higher in hypertensive group (p < .0001). Significant increase was also observed for MMP-9 level and activity (p < .0001) and for TIMP-1 (p < .0001) in hypertensive patients. Plasma levels and activities of MMP-2, MMP-9, and TIMP-1 are increased in hypertensive patients, which may reflect abnormal ECM metabolism. 
 TIMP-1 interaction with αvβ3 integrin confers resistance to human osteosarcoma cell line MG-63 against TNF-α-induced apoptosis. Tumor necrosis factor-α (TNF-α) is a pleiotropic cytokine affecting diverse cellular responses. TNF-α is cytotoxic in many systems, but it can also act as an anti-apoptotic signal to promote cell survival pathways activated through integrin  s and extracellular   matrix components. This is particularly evident in cancer cells. To unravel the basis of resistance to TNF-α-induced apoptosis, human osteosarcoma MG-63 cell line was used. Our data showed that resistance to apoptosis was accompanied by high levels of TIMP-1 expression in part mediated by NF-κB activation, whereas under apoptotic conditions, in the presence of cycloheximide (CHX), TIMP-1 and αvβ3 integrin protein levels were significantly reduced. Silencing TIMP-1 using siRNA led to increased apoptosis following treatment with TNF-α, whereas exogenously-added recombinant TIMP-1 reduced the extent of apoptosis. Immunoprecipitation and confocal microscopy experiments demonstrated that TIMP-1 interacted with αvβ3 integrins. The biological role of this interaction was revealed by the use of echistatin, an antagonist of αvβ3 integrin. In the presence of echistatin, decreased protection against apoptosis by recombinant TIMP-1 was observed. 
 Tissue inhibitor of matrix metalloproteinase 1 (TIMP1) controls adipogenesis in obesity in mice and in humans.   Extracellular matrix   reorganisation is a crucial step of adipocyte differentiation and is controlled by the matrix metalloproteinase-tissue inhibitor of matrix metalloproteinase (TIMP) enzyme system. We therefore sought to define the role of TIMP1 in adipogenesis and to elucidate whether upregulation of TIMP1 in obesity has direct effects on adipocyte formation.TIMP1 protein levels and mRNA were measured in lean and obese mice with a focus on levels in adipose tissue. We also analysed the effect of recombinant murine TIMP1 on adipogenesis, adipocyte size and metabolic control in vitro and in vivo.TIMP1 levels were increased in the serum and adipose tissue of obese mouse models. Recombinant murine TIMP1 inhibited adipocyte differentiation in 3T3-L1 as well as in subcutaneous primary pre-adipocytes. Conversely, neutralising TIMP1 with a specific antibody enhanced adipocyte differentiation. In vivo, injection of recombinant TIMP1 in mice challenged with a high-fat diet led to enlarged adipocytes. TIMP1-treated mice developed an impaired metabolic profile with increased circulating NEFA levels, hepatic triacylglycerol accumulation and accelerated insulin resistance. Altered glucose clearance in TIMP1-injected mice was due to changes in adipose tissue glucose uptake, whereas muscle glucose clearance remained unaffected.TIMP1 is a negative regulator of adipogenesis. In vivo, TIMP1 leads to enlarged adipocytes in the state of overnutrition. This might contribute to the detrimental metabolic consequences seen in TIMP1-injected mice, such as systemic fatty acid overload, hepatic lipid accumulation and insulin resistance. 
 TIMP-1 polymorphisms in a Chinese Han population with intracerebral hemorrhage. Remodeling of   extracellular matrix   (ECM) and breakdown of blood-brain barrier (BBB) are crucial events in the pathogenesis of intracerebral hemorrhage (ICH). Matrix metalloproteinases (MMPs), particularly MMP-9 and MMP-2, are the most important degrading enzymes in the ECM and BBB. These proteolytic effects are controlled predominantly by tissue inhibitors of metalloproteinases (TIMPs). TIMP-1 is the main endogenous inhibitor of MMP-9. Two polymorphisms in the TIMP-1 gene (rs4898 and rs2070584) were selected through a literature review and successfully genotyped in a study sample of 410 ICH patients and 305 controls. Differences in genotype and allele frequencies of identified polymorphisms were determined. Furthermore, the serum levels of TIMP-1 were measured in a subgroup of 96 ICH patients on days 1 after ICH onset and 76 controls. Analyses showed that C allele of rs2070584 was significantly associated with the development of ICH in male subjects (p = 0.037, OR = 1.535, 95%CI 1.025-2.300). Multiple logistic regression analysis under three genetic models demonstrated both rs4898 and rs2070584 were not risk factors for ICH in female subjects. Furthermore, serum levels of TIMP-1 were significantly higher in ICH patients than those in normal controls. However, the serum levels of TIMP-1 showed a nonsignificant decrease, depending on the alleles and genotypes of rs2070584 both in male and female cases. In conclusion, this is the first association study of the TIMP-1 gene variants with ICH. Our data suggest that C allele of rs2070584 is a risk factor for ICH development in the Chinese male population. However, the precise function of this variant needs further investigation. 
 Novel association patterns of cardiac remodeling markers in patients with essential hypertension and atrial fibrillation. Matrix metalloproteinases (MMPs) and their tissue inhibitors (TIMPs) are essential for the cardiac   extracellular matrix   (ECM) remodeling. We investigated differences in serum levels of these markers between patients with atrial fibrillation (AF) and sinus rhythm (SR).Serum levels of MMP-2, MMP-3, MMP-9 and TIMP-1 were measured in 86 patients: 27 on SR without any AF history, 33 with paroxysmal and 26 with permanent AF. All subjects had essential hypertension, normal systolic function and no coronary artery disease.Patients with AF had higher MMP-2, MMP-3 and MMP-9 and lower TIMP-1 compared to SR subjects (all p < 0.001). Paroxysmal AF was associated with higher MMP-2 levels compared to permanent AF (p < 0.001). Matrix metalloproteinase-9 but not MMP-3 was higher in permanent compared to paroxysmal AF group (p < 0.001). Patients with AF had lower levels of TIMP-1 compared to those with SR while permanent AF subjects had lower TIMP-1 levels than those with paroxysmal AF (p < 0.001 for both comparisons). Lower TIMP-1 was the only independent factor associated with AF (OR: 0.259, 95%CI: 0.104-0.645, p = 0.004).In hypertensives, paroxysmal AF and permanent AF differ with respect to serum MMPs. Increased MMP-2 is associated with paroxysmal, whereas increased MMP-9 with permanent AF. Additionally, lower levels of TIMP-1 had a strong association with AF incidence. 
 Expression and regulation of tissue inhibitor of metalloproteinase-1 and matrix metalloproteinases by intestinal myofibroblasts in inflammatory bowel disease. Intestinal fibrosis and strictures frequently occur in Crohn's disease but not ulcerative colitis. We have recently shown that, compared to myofibroblasts obtained from normal and ulcerative colitis tissue, myofibroblasts isolated from fibrotic Crohn's disease mucosal samples express significantly lower amounts of transforming growth factor (TGF)-beta 3, but the expression of TGF-beta 2 was significantly greater. We now report that in myofibroblast cultures established from fibrotic Crohn's disease mucosal samples there is significantly higher constitutive expression of tissue inhibitor of metalloproteinase (TIMP)-1 compared to similar cells isolated from normal or ulcerative colitis tissue. Myofibroblasts derived from normal mucosa and from mucosa affected by ulcerative colitis or Crohn's disease also expressed matrix metalloproteinase (MMP)-1, MMP-2, and MMP-3 but did not express MMP-9. Recombinant (r) TGF-beta 1 and rTGF-beta 2, but not rTGF-beta 3, induced expression of TIMP-1 in normal intestinal myofibroblasts. These studies illustrate a potential mechanism by which differential expression of isoforms of TGF-beta may lead to excessive deposition of   extracellular matrix   and stricture formation via TIMP-1-mediated inhibition of MMP activity. 
 Novel role of TGF-beta in differential astrocyte-TIMP-1 regulation: implications for HIV-1-dementia and neuroinflammation. Astrocyte production of tissue inhibitor of metalloproteinase (TIMP)-1 is important in central nervous system (CNS) homeostasis and inflammatory diseases such as HIV-1-associated dementia (HAD). TIMPs and matrix metalloproteinases (MMPs) regulate the remodeling of the   extracellular matrix  . An imbalance between TIMPs and MMPs is associated with many pathologic conditions. Our recently published studies uniquely demonstrate that HAD patients have reduced levels of TIMP-1 in the brain. Astrocyte-TIMP-1 expression is differentially regulated in acute and chronic inflammatory conditions. In this and the adjoining report (Gardner et al., 2006), we investigate the mechanisms that may be involved in differential TIMP-1 regulation. One mechanism for TIMP-1 downregulation is the production of anti-inflammatory molecules, which can activate signaling pathways during chronic inflammation. We investigated the contribution of transforming growth factor (TGF)-signaling in astrocyte-MMP/TIMP-1-astrocyte regulation. TGF-beta1 and beta2 levels were upregulated in HAD brain tissues. Co-stimulation of astrocytes with IL-1beta and TGF-beta mimicked the TIMP-1 downregulation observed with IL-1beta chronic activation. Measurement of astrocyte-MMP protein levels showed that TGF-beta combined with IL-1beta increased MMP-2 and decreased proMMP-1 expression compared to IL-1beta alone. We propose that one of the mechanisms involved in TIMP-1 downregulation may be through TGF-signaling in chronic immune activation. These studies show a novel extracellular regulatory loop in astrocyte-TIMP-1 regulation. 
 Inhibition of histone deacetylase activity in human endometrial stromal cells promotes   extracellular matrix   remodelling and limits embryo invasion. Invasion of the trophoblast into the maternal decidua is regulated by both the trophoectoderm and the endometrial stroma, and entails the action of tissue remodeling enzymes. Trophoblast invasion requires the action of metalloproteinases (MMPs) to degrade   extracellular matrix   (ECM) proteins and in turn, decidual cells express tissue inhibitors of MMPs (TIMPs). The balance between these promoting and restraining factors is a key event for the successful outcome of pregnancy. Gene expression is post-transcriptionally regulated by histone deacetylases (HDACs) that unpacks condensed chromatin activating gene expression. In this study we analyze the effect of histone acetylation on the expression of tissue remodeling enzymes and activity of human endometrial stromal cells (hESCs) related to trophoblast invasion control. Treatment of hESCs with the HDAC inhibitor trichostatin A (TSA) increased the expression of TIMP-1 and TIMP-3 while decreased MMP-2, MMP-9 and uPA and have an inhibitory effect on trophoblast invasion. Moreover, histone acetylation is detected at the promoters of TIMP-1 and TIMP-3 genes in TSA-treated. In addition, in an in vitro decidualized hESCs model, the increase of TIMP-1 and TIMP-3 expression is associated with histone acetylation at the promoters of these genes. Our results demonstrate that histone acetylation disrupt the balance of ECM modulators provoking a restrain of trophoblast invasion. These findings are important as an epigenetic mechanism that can be used to control trophoblast invasion. 
 Gingival crevicular fluid matrix metalloproteinase (MMP)-7, extracellular MMP inducer, and tissue inhibitor of MMP-1 levels in periodontal disease. During periodontal inflammation, matrix metalloproteinases (MMPs) are under the control of several regulatory mechanisms including the upregulation of expression by inducers and downregulation by inhibitors. Our study aimed to examine the levels and molecular forms of MMP-7, tissue inhibitor of MMP (TIMP)-1, and   extracellular matrix   metalloproteinase inducer (EMMPRIN) in gingival crevicular fluid (GCF) from patients with different periodontal diseases.A total of 80 subjects (20 patients with generalized aggressive periodontitis [GAgP], 20 with chronic periodontitis [CP], 20 with gingivitis, and 20 periodontally healthy subjects) were included in this study. Periodontal status was evaluated by measuring probing depth, clinical attachment loss, presence of bleeding on probing, and plaque. GCF MMP-7, TIMP-1, and EMMPRIN levels and molecular forms were analyzed by enzyme-linked immunosorbent assay (ELISA) and Western immunoblot techniques using specific antibodies.Total amounts of GCF MMP-7 were found to be similar between the study groups. GAgP, CP, and gingivitis groups had significantly higher total amounts of GCF EMMPRIN compared to healthy subjects (P <0.008). Among the patient groups, the GAgP group had the highest total amount of GCF EMMPRIN relative to the gingivitis group (P = 0.0004). Soluble EMMPRIN existed in GCF in multiple molecular-weight species especially in periodontitis-affected GCF under non-reducing conditions, i.e., 30-, 55-, 100-, 180-, and 200-kDa species. All patient groups had significantly elevated total amounts of GCF TIMP-1 relative to the healthy group (P <0.0001). GAgP and CP groups also had a higher total amount of GCF TIMP-1 compared to the gingivitis group (P <0.0001 and P <0.0001, respectively). The GAgP group had higher GCF TIMP-1 and EMMPRIN levels compared to the CP group, but this elevation did not reach statistical significance.Our data indicate that MMP-7 is associated with the innate host defense in periodontal tissues. Increased EMMPRIN and TIMP-1 levels in GCF are associated with the enhanced severity of periodontal inflammation, indicating that these molecules can participate in the regulation of progression of periodontal diseases. To our knowledge, the present study demonstrated the presence of soluble forms of EMMPRIN in GCF of patients with different periodontal diseases for the first time. 
 The low density lipoprotein receptor-related protein modulates levels of matrix metalloproteinase 9 (MMP-9) by mediating its cellular catabolism. Members of the matrix metalloproteinase (MMP) family of enzymes participate in matrix remodeling and share a number of structural and functional features. The activity of this family of proteinases is carefully regulated at the level of zymogen activation and by a family of specific inhibitors termed tissue inhibitors of metalloproteinases (TIMP). It is now becoming clear that levels of certain MMPs are modulated by their association with cellular receptors that mediate their rapid internalization and degradation. In the current investigation we report that the amount of MMP-9 in conditioned cell culture medium is significantly increased when mouse embryonic fibroblasts are grown in the presence of the 39-kDa receptor-associated protein (RAP), an antagonist of ligand binding to low density lipoprotein receptor-related protein (LRP). In vitro assays reveal that the MMP-9.TIMP-1 complex binds to LRP with high affinity and that the binding determinant for LRP appears to reside on MMP-9. Cell lines expressing LRP mediate the internalization of 125I-labeled MMP-9.TIMP-1 complexes, whereas cell lines genetically deficient in LRP show a diminished capacity to mediate the cellular catabolism of MMP-9.TIMP-1 complexes. The results demonstrate that LRP is a functional receptor for MMP-9 and suggest a major role for LRP in modulating remodeling of the   extracellular matrix   by regulating extracellular proteinase activity. 
 Tissue inhibitor of metalloproteinase-1 (TIMP-1) polymorphisms in a Caucasian population with abdominal aortic aneurysm. The formation of a sporadic abdominal aortic aneurysm (AAA) is explained by the remodeling of the   extracellular matrix   (ECM) and breakdown of structural components of the vascular wall. Matrix metalloproteinases are the principal matrix-degrading proteases and are known to play a major role in the remodeling of the   extracellular matrix   in arterial vessels. Their activity is controlled by tissue inhibitors of metalloproteinases (TIMPs). Decreased TIMP-1 and TIMP-2 expression in the   extracellular matrix   of the walls of AAAs has been shown in several studies. This case control study was designed to investigate the possible impact of genetic variants of the TIMP-1 gene in the etiology of AAA.TIMP-1 single nucleotide polymorphisms (SNPs) were analyzed in a primary study sample of 50 patients with AAA and 44 controls. Differences in genotype and allele frequencies of identified polymorphisms were determined after sequencing the entire coding region and selected parts of the promoter using the automated laser fluorescence technique. A second sample (96 patients vs. 89 controls) was investigated by single-base sequencing to confirm significant results.Three polymorphisms were identified, one of which, described for the first time in this article, is located in intron 4 (TIMP-1: 328 + 16C > T). A statistically significant difference in allele frequencies for SNP TIMP-1 372T>C was detected in the primary study group. The C allele was more frequent in male patients with AAA than in the control group [23 vs. 4, p = 0.029, OR (95% CI) 4.38 (1.13-20.47)]. However, this result could not be confirmed in a second sample of males [52 vs. 45, p = 0.624, OR (95% CI) 1.16 (0.65-2.06)]. There were no statistically significant differences in genotype or allele frequencies of the other detected SNPs between the two groups.Our analysis of the entire coding region and selected parts of the promoter of the TIMP-1 gene failed to show an association between genetic polymorphisms and AAA, suggesting that variations in the TIMP-1 gene do not contribute to the development of AAA. 
 Associations of Matrix Metalloproteinase-9 and Tissue Inhibitory Factor-1 Polymorphisms With Parkinson Disease in Taiwan. Matrix metalloproteinases (MMPs) function in the degradation of   extracellular matrix   and are considered to play a role in the pathogenesis of neurodegenerative diseases including Parkinson disease (PD). MMPs activities are modulated by tissue inhibitors of metalloproteinases (TIMPs). This study examined whether the genetic polymorphisms of MMP-3, gelatinase (MMP-2 and MMP-9), TIMP-2, and TIMP-1 were associated with PD in Taiwan.A total of 359 PD patients and 332 controls were enrolled. The candidate genetic variants included MMP-2 rs2285053 (-735 C > T), MMP-3 rs3025058 (-1171 5A > 6A), MMP-9 rs3918241 (-1831 T > A), rs17576 (G > A, R279Q), and rs3787268 (G > A, intron), TIMP-1 rs4898 (T > C, F124F), and TIMP-2 rs7503607 (-269 G > T). Associations were tested by logistic regression, adjusted with gender and age at onset.Minor allele frequency of TIMP-1 rs4898 (36.0%) was significantly lower in the male PD patients than in the male controls (51.2%) (χ test, P = 0.004). When adjusted with gender and age at onset, MMP-9 rs17576 AA genotype was associated with PD susceptibility in a recessive fashion (odds ratios [OR] = 2.28, 95% confidence intervals [95% CI] = 1.12-4.62, P = 0.02). In males, TIMP-1 rs4898 C allele was associated with a protective effect on PD (OR = 0.75, 95% CI = 0.60-0.94, P = 0.014). We did not find association between the examined genetic variants of MMP-2, MMP-3, and TIMP-2 and PD susceptibility.This is the first study that demonstrated a protective effect of TIMP-1 rs4898 C allele on male PD and a modest association of MMP-9 rs17576 AA genotype with PD susceptibility in the Taiwan population. Further replication is needed for confirmation. 
 Circulating beta-chemokines and matrix metalloproteinase-9/tissue inhibitor of metalloproteinase-1 system in hemodialyzed patients--role of oxidative stress. Matrix metalloproteinases (MMPs) and their tissue inhibitors (TIMPs), beta-chemokines, increased oxidative stress (SOX) and inflammation have been implicated as important factors in atherosclerosis and vascular remodeling. We hypothesized the possible roles of beta-chemokines [monocyte chemoattractant protein-1 (MCP-1), macrophage inflammatory proteins (MIP-1alpha, MIP-1beta) and regulated upon activation, normal T-cell expressed and secreted (RANTES)] as regulators of the metabolism of the vascular   extracellular matrix   in conditions of increased SOX in hemodialysis (HD) patients. We compared pre-dialysis levels of MMP-9/TIMP-1 system, beta-chemokines, Cu/Zn superoxide dismutase (Cu/Zn SOD) as a marker of SOX and C-reactive protein (CRP) as a marker of inflammation in HD patients with and without cardiovascular disease (CVD) to those of controls. HD patients, particularly those with CVD, showed a significant increase in values of Cu/Zn SOD, CRP, TIMP-1, TIMP-1/MMP-9 ratio, MCP-1 and MIP-1beta, whereas RANTES levels were lower than in the controls. The levels of MIP-1alpha as well as MMP-9 in all HD groups were similar to the controls. The positive correlations were observed between the MMP-9/TIMP-1 system and beta-chemokines, SOX and inflammation in whole HD group and in the subgroup with CVD. Multivariate analysis showed that the duration of dialysis followed by Cu/Zn SOD, MIP-1alpha and beta levels were the significant positive predictors of TIMP-1. In conclusion, our data show that MMP-9/TIMP-1 system and beta-chemokines could cooperate in conditions of elevated SOX, which ultimately predisposes hemodialysis patients to accelerated atherosclerosis. 
 Increased expression of   extracellular matrix   regulators TIMP1 and MMP1 in deteriorating heart failure. The authors previously identified and compared alterations in gene expression in the myocardia of patients with deteriorating heart failure who underwent left ventricular assist device (LVAD) implantation with those of patients with stable end-stage failure (ESF). We hypothesized that matrix metalloproteinases (MMPs) and their endogenous inhibitors, the tissue inhibitors of MMPs (TIMPs), would be implicated in the mechanisms that underlie deteriorating heart failure.Gridded macro-array filters were used to provide a broad overview of MMP and TIMP mRNA expression in heart failure. Precise mRNA levels of TIMP1, MMP1, and beta-spectrin were determined using quantitative real-time reverse-transcription polymerase chain reaction (RT-PCR) of myocardial samples from 27 patients with deteriorating heart failure who underwent LVAD implantation, from 17 patients with stable ESF who underwent elective heart transplantation, and from 28 donor organs with good hemodynamic function.Gridded macro-arrays analysis of pooled failing heart samples determined that TIMP1 mRNA was the most readily detectable TIMP in failing myocardium. Quantitative RT-PCR showed that expression levels in individual patients were similar in patients with stable ESF (1.00 +/- 0.24, n = 17) and in donor organ samples (1.49 +/- 0.22, n = 28) but were significantly increased in the deteriorating heart failure group (5.38 +/- 0.32, n = 26, p < 0.0001 compared with patients with ESF). Similarly, MMP1 levels did not differ between donor and ESF groups but increased in the deteriorating failure group (6.04 +/- 0.50, n = 27, p < 0.001 compared with the ESF group). Levels of beta-II spectrin were the same in all 3 groups. Both TIMP1 and MMP1 showed positive correlation with each other and with previously determined levels of mRNA for both interleukin-1beta (IL-1beta) and IL-6 in this patient series when considering all patients individually, but neither correlated with tumor necrosis factor alpha.Patients with deteriorating heart failure have increased expression of TIMP1 and MMP1 mRNA. Correlation with pro-inflammatory cytokines suggests common pathways of regulation and potential activation by IL-6 and IL1-beta. 
 Matrix metalloproteinase-9 and tissue inhibitor of metalloproteinase-1 in hypertension and their relationship to cardiovascular risk and treatment: a substudy of the Anglo-Scandinavian Cardiac Outcomes Trial (ASCOT). Hypertension results in structural changes to the cardiac and vascular   extracellular matrix   (ECM). Matrix metalloproteinases (MMP) and their inhibitors (TIMP) may play a central role in the modulation of this matrix. We hypothesized that both MMP-9 and TIMP-1 would be abnormal in hypertension, reflecting alterations in ECM turnover, and that their circulating levels should be linked to cardiovascular (CHD) and stroke (CVA) risk scores using the Framingham equation. Second, we hypothesized that treatment would result in changes in ECM indices.Plasma MMP-9 and TIMP-1 were measured before and after treatment (median 3 years) from 96 patients with uncontrolled hypertension participating in the Anglo-Scandinavian Cardiac Outcomes Trial (ASCOT). Pretreatment values were compared to circulating MMP-9 and TIMP-1 levels in 45 age- and sex-matched healthy controls.Circulating pretreatment MMP-9 and TIMP-1 levels were significantly higher in patients with hypertension than in the normotensive controls (P =.0041 and P =.0166, respectively). Plasma MMP-9 levels decreased, and TIMP-1 levels increased after treatment (P =.035 and P =.005, respectively). Levels of MMP-9 correlated with CHD risk (r = 0.317, P =.007) and HDL cholesterol (r = -0.237, P =.022), but not CVA risk. There were no significant correlations between TIMP-1 and CVA or CHD scores.Increased circulating MMP-9 and TIMP-1 at baseline in patients with hypertension could reflect an increased deposition and retention of type I collagen at the expense of other components of ECM within the cardiac and vascular ECM. After cardiovascular risk management, MMP-9 levels decreased and TIMP-1 levels increased. Elevated levels of MMP-9 also appeared to be associated with higher Framingham cardiovascular risk scores. Our observations suggest a possible role for these surrogate markers of tissue ECM composition and the prognosis of cardiovascular events in hypertension. 
 Regulation of tissue inhibitor of metalloproteinase-1 by astrocytes: links to HIV-1 dementia. The neuropathogenesis of HIV-1-associated dementia (HAD) revolves around the secretion of toxic molecules from infected and immune-competent mononuclear phagocytes. Astrocyte activation occurs in parallel but limited insights are available for its role in neurotoxicity and cognitive dysfunction. One means in which astrocytes may affect disease is through their production of tissue inhibitors of metalloproteinases (TIMPs). TIMPs are regulators of matrix metalloproteinases, enzymes that affect blood-brain barrier integrity through altering the   extracellular matrix  . We hypothesized that in response to injury and inflammation in HAD, astrocytes regulate the production of TIMP-1, the inducible type of TIMP that is important in inflammation. To address astrocyte-mediated TIMP-1 regulation in HAD, we evaluated the responses of primary human to IL-1beta and HIV-1. TIMP-1 levels in plasma, CSF, and brain tissue of control, HIV-1 infected patients without cognitive impairment, and HAD patients were also studied. Our data show that an upregulation of TIMP-1 results from astrocytes acutely activated with IL-1beta. In contrast, CSF and brain tissue samples from HAD patients showed reduced TIMP-1 levels compared to seronegative controls. MMP-2 levels in brains showed the opposite. Consistent with this, prolonged activation of astrocytes led to a reduction in TIMP-1 and MMP-2, but a sustained elevation in MMP-1. Our data suggest that in diseased brain tissue, the ability of astrocytes to counteract the destructive effects of MMP through expression of TIMP-1 is diminished by chronic activation. Our studies reveal new opportunities for repair-based therapeutic strategies in HAD. 
 Tissue inhibitor of metalloproteinase-1 and matrix metalloproteinase-9 levels in patients with hypertension Relationship to tissue Doppler indices of diastolic relaxation. Hypertension, hypertensive heart disease, and left ventricular (LV) hypertrophy are integral to symptomatic diastolic heart failure. Tissue inhibitor of metalloproteinase-1 (TIMP-1) is linked to   extracellular matrix   fibrosis and is elevated in hypertension. We hypothesized a link between circulating TIMP-1, matrix metalloproteinase-9 (MMP-9), and resting echocardiographic LV filling parameters using tissue Doppler parameters of diastolic (dys)function.Circulating MMP-9 and TIMP-1 levels were measured in citrated plasma by ELISA in 74 patients with hypertension (58 men, mean age 58 +/- 11 years) and 34 controls (23 men, mean age 53 +/- 13 years). All had confirmed normal short axis systolic contractility, with no significant wall motion abnormalities; the LV mass and standard resting tissue Doppler echocardiographic indices of diastolic function were also recorded.Both MMP-9 and TIMP-1 levels were higher in the hypertensive group (P =.0039 and P =.0054, respectively). When compared to controls, hypertensive patients had a greater LV mass (P =.0054), and differences in many of the parameters reflecting diastolic dysfunction (controls versus hypertensives: E: 0.71 +/- 0.15 v 0.81 +/- 0.15 m/sec, P =.004; A: 0.66 +/- 0.12 v 0.81 +/- 0.16 m/sec, P <.0001; e': 0.12 (0.09-0.14) v 0.09 (0.07-0.10) m/sec, P =.0017; e'/a': 1.20 (1.00-1.80) v 0.88 (0.71-1.05), P <.0001; E/e': 6.54 (4.75-7.14) v 8.89 (7.55-10.75), P <.0001, respectively). Within the hypertensive cohort, only TIMP-1 levels correlated with LV mass (r = 0.271, P =.024), LV mass index (r = 0.323, P =.007), and tissue Doppler parameters of diastolic dysfunction, including e' (r = -0.338, P =.005), a' (r = -0.350, P =.005), and E/e' (r = 0.334, P =.005).TIMP-1 is thought to increase tissue concentrations of collagen type I by preventing its breakdown by MMPs. Our findings therefore add weight to a hypothesis suggesting that TIMP-1 may be a key mediator of LV diastolic dysfunction through definition of ventricular matrix composition. 
 Mechanism of inhibition of the human matrix metalloproteinase stromelysin-1 by TIMP-1. Matrix metalloproteinases (MMPs) are zinc endopeptidases that are required for the degradation of   extracellular matrix   components during normal embryo development, morphogenesis and tissue remodelling. Their proteolytic activities are precisely regulated by endogenous tissue inhibitors of metalloproteinases (TIMPs). Disruption of this balance results in diseases such as arthritis, atherosclerosis, tumour growth and metastasis. Here we report the crystal structure of an MMP-TIMP complex formed between the catalytic domain of human stromelysin-1 (MMP-3) and human TIMP-1. TIMP-1, a 184-residue protein, has the shape of an elongated, contiguous wedge. With its long edge, consisting of five different chain regions, it occupies the entire length of the active-site cleft of MMP-3. The central disulphide-linked segments Cys 1-Thr 2-Cys 3-Val 4 and Ser 68-Val 69 bind to either side of the catalytic zinc. Cys 1 bidentally coordinates this zinc, and the Thr-2 side chain extends into the large specificity pocket of MMP-3. This unusual architecture of the interface between MMP-3 and TIMP-1 suggests new possibilities for designing TIMP variants and synthetic MMP inhibitors with potential therapeutic applications. 
 Urinary Emmprin, matrix metalloproteinase 9 and tissue inhibitor of metalloproteinase 1 as potential biomarkers in children with ureteropelvic junction narrowing on conservative treatment. Rapid diagnosis and initiation of the treatment on congenital obstructive nephropathy are important for young children to slow down renal injury. The aim of our study was to investigate the role of urinary   extracellular matrix   metalloproteinase inducer (Emmprin), matrix metalloproteinase 9 (MMP-9) and tissue inhibitor of metalloproteinase 1 (TIMP-1) in the long-term follow-up of children with ureteropelvic junction (UPJ) narrowing on conservative treatment.The study included 40 children with non-obstructed hydronephrosis due to unilateral UPJ narrowing who were treated conservatively and followed up for 24 months. Voided urine samples were collected at diagnosis and at 3, 9, 15 and 24 months of follow-up, respectively. Three enzymes concentrations were measured in urine.During the follow-up, 25 children showed renal function stabilization (non-obstructed group) and 15 children renal function deterioration (obstructed group). In the non-obstructed group, a comparison between urine three enzymes levels at the last follow-up and at baseline showed no significant differences (all P > 0.05). Glomerular filtration rate (GFR) and split renal function (SRF) showed similar trends. In the obstructed group, a comparison between the three enzymes levels at diagnosis and at basal condition showed a significant increase (all P < 0.01). But GFR and SRF showed a marked reduction at diagnosis (all P < 0.001). Receiver operator characteristic (ROC) analyses revealed a better diagnostic profile for uEmmprin, uMMP-9 and uTIMP-1 in identifying children with abnormal SRF (<40%) at 24 months of follow-up [area under the curve (AUC) 0.877, 0.727 and 0.823, respectively].Urinary Emmprin, MMP-9 and TIMP-1 may be noninvasive potential biomarkers that could be used for long-term follow-up of children with UPJ narrowing on conservative treatment to determine those who might develop obstruction. 
 [Changes of matrix metalloproteinase-9 and tissue inhibitor of metalloproteinase-1 in the bronchoalveolar lavage fluid and the serum of patients with idiopathic pulmonary fibrosis]. To detect the levels of matrix metalloproteinase-9 (MMP-9) and tissue inhibitor of metalloproteinase-1 (TIMP-1) in the bronchoalveolar lavage fluid (BALF) and the serum of patients with idiopathic pulmonary fibrosis (IPF), and to evaluate the significance of the changes in the pathogenesis of IPF.Enzyme-linked immunoadsorbent assay (ELISA) was used to detect MMP-9 and TIMP-1 in the BALF and serum of 30 patients with IPF.The levels of MMP-9 in the BALF and serum of the patients showed no significant difference as compared with those of the control group. The levels of TIMP-1 in the BALF [(522 +/- 81) ng/L] and serum [(166 +/- 29) ng/L] of the patients were higher than those [(201 +/- 31), (87 +/- 16) ng/L] of the control group (P < 0.01). The ratios of MMP-9/TIMP-1 in the BALF (0.53 +/- 0.18) and serum (1.5 +/- 0.3) of patients with IPF were lower than those (1.06 +/- 0.38, 2.6 +/- 0.5) of the control group (P < 0.01, < 0.05). TIMP-1 in the BALF of the patients showed a strong positive correlation with chest CT fibrosis and pulmonary function test scores (P < 0.01), while MMP-9/TIMP-1 in the BALF had negative correlation with them (P < 0.01).Pulmonary fibrosis may be associated with increased TIMP-1 and decreased MMP-9/TIMP-1, which is able to inhibit the degradation of   extracellular matrix  . 
 Elevated serum levels of proinflammatory cytokines and biomarkers of matrix remodeling in never-treated patients with familial hypercholesterolemia. Familial hypercholesterolemia (FH) is a common inherited disorder of lipoprotein metabolism, whose origin involves mutations in the gene coding for the low-density lipoprotein receptor protein. Although FH is monogenic, wide variation occurs in the onset and severity of atherosclerosis in these patients.Since data on levels of inflammatory proteins and/or active factors in FH patients who have never received lipid-lowering treatment are lacking, serum levels of MMP-3, active MMP-9 and TIMP-1 as well as pro-inflammatory cytokines (TNF-alpha, IL-18) were determined in never-treated homozygous FH Moroccan patients (n=4) and compared to those of heterozygous FH subjects (n=7) and of healthy control subjects (n=5).When compared to controls, homozygous FH patients exhibited levels of active MMP-9 and TIMP-1 (p<0.05), and of both high sensitive-CRP and IL-18 which were significantly elevated (p<0.05 and p<0.01, respectively). In heterozygous FH patients, intermediate values between FH homozygotes and healthy controls were observed for these markers, with the exception of MMP-9 activity whose levels were significantly elevated (p<0.05). Multivariate analysis revealed a positive correlation between apolipoprotein B, TIMP-1 and IL-18 levels, and between hs-CRP and IL-18 (p<0.01).Although the sample size of this FH group was limited, our data suggest that nontreated homozygous FH patients, and to a lesser degree heterozygous FH patients, exhibit not only a markedly proinflammatory vascular state but also pronounced   extracellular matrix   remodeling, as reflected by elevated circulating levels of inflammatory cytokines and MMPs. 
 Novel expression and regulation of TIMP-1 in Epstein Barr virus-infected cells and its impact on cell survival. Epstein Barr virus (EBV) uses various strategies to manipulate host cytokine production in favor of the survival of infected B-cells. Microarray and cytokine protein array assays revealed that tissue inhibitor of metalloproteinase-1 (TIMP-1) was significantly up-regulated in EBV-infected primary B cells and maintained in abundance in EBV-immortalized lymphoblastoid cell lines (LCLs). TIMP-1 plays critical roles in   extracellular matrix   homeostasis and regulates signaling pathways. In this study, we demonstrated that the EBV-encoded immediate early lytic protein, Zta, upregulates mainly TIMP-1 expression by binding to the AP-1 site within the TIMP-1 promoter. Moreover, knockdown of TIMP-1 expression promoted cisplastin and cold shock-induced death of LCLs. This study provides a mechanistic link between EBV-induced TIMP-1 expression and its impact on LCL survival. 
 Expression of matrix metalloproteinase-1 and tissue inhibitor of metalloproteinase-1 in ulcerative colitis. To examine the expression of metalloproteinase-1 (MMP-1) and tissue inhibitor of metalloproteinase-1 (TIMP-1) in the colonic mucosa of patients with ulcerative colitis (UC).Reverse transcription-polymerase chain reaction (RT-PCR) and immunohistochemistry were used to study the expression of MMP-1 and TIMP-1 at both mRNA and protein levels in patients with UC and controls. The relationship between MMP-1 mRNA, TIMP-1 mRNA, MMP-1 mRNA/TIMP-1 mRNA ratio and the severity of clinical symptoms of the patients with UC were also analyzed.The expression of MMP-1 mRNA and TIMP-1 mRNA in the ulcerated and inflamed colonic mucosa was significantly higher than that in the non-inflamed colonic mucosa (P < 0.001), but there was no statistically significant difference in the non-inflamed colonic mucosa of UC patients and normal controls (P > 0.05). The mRNA expression of MMP-1 and TIMP-1 in ulcerated colonic mucosa of UC patients was increased by 80-fold and 2.2-fold, respectively when compared with the normal controls. In the inflamed colonic mucosa, the increase was 30-fold and 1.6-fold, respectively. Immunohistochemical analysis showed that among the ulcerated, inflamed, and non-inflamed colonic mucosae of UC patients and the normal controls, the positive rate of MMP-1 expression was 87%, 87%, 40% and 35% respectively, and the positive rate of TIMP-1 expression was 89%, 89%, 80% and 75%, respectively. Furthermore, the expression of MMP-1 mRNA, TIMP-1 mRNA and the MMP-1 mRNA/ TIMP-1 mRNA ratio were correlated with the severity of clinical symptoms (P <0.05).Excessive expression of MMP-1 in the diseased colonic mucosa causes excessive hydrolysis of the   extracellular matrix   (ECM) and ulceration in UC patients. MMP-1 mRNA, TIMP-1 mRNA and MMP-1 mRNA/TIMP-1 mRNA ratio can be used as biomarkers to judge the severity of clinical symptoms in patients with UC. Exogenous TIMP-1 or MMP-1 inhibitor therapy is a novel treatment for patients with UC. 
 Expression of matrix metalloproteinase-9 (MMP-9) and tissue inhibitor of matrix metalloproteinase (TIMP-1) in tissues with a diagnosis of childhood lymphoma. Matrix metalloproteinases (MMP) are enzymes involved in the reconfiguration of the microenvironment by means of degrading the   extracellular matrix   and have more than 20 subgroups containing zinc. Proteins that serve as the inhibitors of these enzymes are called tissue inhibitors of matrix metalloproteinase (TIMP). These enzymes have been shown to be active in a wide range of processes, from wound recovery to fetus development, heart diseases, and spread of malignant diseases. The aim of this study was to investigate whether there is a relationship between the type, stage, and prognosis of childhood lymphoma subjects and matrix metalloproteinase type-9 (MMP-9) and its inhibitor, tissue inhibitor of matrix metalloproteinase type-1 (TIMP-1). Paraffin blocks of childhood patients diagnosed with non-Hodgkin lymphoma (n = 23), Hodgkin lymphoma (n = 14), or reactive lymphadenopathy (n = 12) were retrospectively immunohistochemically stained with MMP-9 and TIMP-1 stains and whether there was a relationship between the degree of staining and the type, tumor stage, and prognosis of the disease was investigated. Moderate and high degrees of MMP-9 staining were detected in 94.6% of the lymphoma patient tissues and a slight TIMP-1 staining was detected in 21.6% of the lymphoma patient tissues. No relationship was observed between the degree of these staining patterns and the type, tumor stage, and prognosis of the disease. This study indicates that the equilibrium between MMP-9 and TIMP-1 is important in lymphomas in addition to all the physiological and pathologic events although MMP-9 and the TIMP-1 staining patterns are not related to the tumor stage, prognosis, and type of the disease. Larger series of patients are needed to determine the prognostic value of MMP-9 and TIMP-1 in childhood lymphoma. 
 [Metalloproteinase-9/tissue inhibitor of metalloproteinase-1 in induced sputum in patients with asthma and chronic obstructive pulmonary disease and their relationship to airway inflammation and airflow limitation]. To investigate the levels of metalloproteinase-9 (MMP-9) and tissue inhibitor of metalloproteinase-1 (TIMP-1) in induced sputum in patients with asthma and chronic obstructive pulmonary disease (COPD), and its relationship to the number of inflammation cells and lung function.Fourteen patients with asthma in remission stages, 12 patients with stable COPD and 10 normal control subjects were included in this study. Lung function was measured. Induced sputum was obtained and processed for cell differential and the supernatant was assayed for the concentrations of interleukin-4 (IL-4), MMP-9 and TIMP-1 by enzyme-linked immunosorbent assay (ELISA).The percentage of eosinophils in induced sputum in asthmatics (0.181 +/- 0.067) was significantly higher than that in normal control subjects (0.007 +/- 0.005) and in COPD (0.042 +/- 0.017, F = 4.32, P < 0.05). The percentage of neutrophils in induced sputum in patients with COPD (0.500 +/- 0.101) was significantly higher than that in asthmatics (0.30 +/- 0.07) and in normal control subjects (0.26 +/- 0.06, F = 4.13, P < 0.05). The concentrations of IL-4 in asthmatics, COPD and normal control subjects [respectively, (19 +/- 7) x 10(-3) g/L, (14 +/- 6) x 10(-3) g/L, (11 +/- 4) x 10(-3) g/L] did not show significant difference (F = 1.56, all P > 0.05) and did not correlate with the number of eosinophils (r = 0.33, 0.11, 0.19, all P > 0.05) and neutrophil (r = 0.25, 0.39, 0.40, all P > 0.05) and FEV(1) values (predicted r = 0.21, 0.35, 0.17, all P > 0.05). The concentrations of MMP-9 and TIMP-1 in induced sputum in asthmatics [(15.9 +/- 6.0) g/L, (19.8 +/- 8.5) g/L, respectively] and COPD [(13.4 +/- 5.1) g/L, (16.7 +/- 7.6) g/L, respectively] were significantly higher than those in normal control subjects [(1.8 +/- 1.1) g/L, (1.3 +/- 0.9) g/L, respectively] (F = 2.99, 4.22, respectively, all P < 0.05). Increased concentration of MMP-9 correlated positively with the percentage of eosinophils in asthmatics (r = 0.71, P < 0.05) and with the percentage of neutrophils in COPD (r = 0.59, P < 0.05), but did not correlate with FEV(1) values (predicted r = 0.22, 0.16, all P > 0.05) and FEV(1)/FVC (r = 0.25, 0.30, all P > 0.05). Increased concentration of TIMP-1 did not correlate with the number of eosinophils (r = 0.27, 0.31, all P > 0.05) and neutrophil (r = 0.20, 0.35, all P > 0.05) in asthmatics and COPD, but correlated inversely with FEV(1) values (predicted, respectively, r = -0.58, -0.62, all P < 0.05). The ratio of MMP-9/TIMP-1 was significantly lower in asthmatics 0.8 +/- 0.7 and COPD 0.8 +/- 0.6 than that in normal control subjects (1.5 +/- 0.6, F = 3.70, P < 0.05). The ratio was not statistically different between asthmatics and COPD (F = 1.78, P > 0.05). In asthmatics and COPD patients, the ratio of MMP-9/TIMP-1 in induced sputum correlated positively with FEV(1%) (respectively, r = 0.56, 0.61, all P < 0.05).An imbalance between MMP-9 and TIMP-1 in induced sputum in asthmatics and COPD is associated with airway inflammation and airflow limitation, which may play a role in the pathogenesis of   extracellular matrix   remodeling and airflow limitation. 
 Small GTPase Rab37 targets tissue inhibitor of metalloproteinase 1 for exocytosis and thus suppresses tumour metastasis. Rab small GTPases are master regulators of membrane trafficking and guide vesicle targeting. Recent publications show that Rab-controlled trafficking pathways are altered during tumorigenesis. However, whether any of the Rabs plays a metastasis suppressor role is least explored. Here we address the metastasis suppressive function of human Rab37 (hRAB37) using secretomics, cell, animal and clinical analyses. We show that tissue inhibitor of metalloproteinase 1 (TIMP1), a secreted glycoprotein that inhibits   extracellular matrix   turnover, is a novel cargo of hRAB37. hRAB37 regulates the exocytosis of TIMP1 in a nucleotide-dependent manner to inactivate matrix metalloproteinase 9 (MMP9) migration axis in vitro and in vivo. Dysfunction of hRAB37 or TIMP1 abrogates metastasis suppression. Lung cancer patients with metastasis and poor survival show low hRAB37 protein expression coinciding with low TIMP1 in tumours. Our findings identify hRAB37 as a novel metastasis suppressor Rab that functions through the TIMP1-MMP9 pathway and has significant prognostic power. 
  Component  
 proteinaceous   extracellular matrix   
  GeneRIFs  
 Data suggest that cardiac fibrosis, as assessed by serum   extracellular matrix   protein biomarkers (including TIMP1), develops early in hypertensive patients and is predictive of cardiovascular events or death. 
 These data shed light on the important role of TIMP-1 in protection and recovery from cutaneous photodamage because of its suppression of   extracellular matrix   degradation and inflammation. 
 changes in the   extracellular matrix   metabolism during the ageing process influence the level of circulating MMP-3 and MMP-10, as well as their tissue inhibitors TIMP-1 and TIMP-2, in a healthy population 
 Abundant peritumoral expression of TIMP-1 in non-immunocompromised patients limits   extracellular matrix   degradation permissive for cancer cell migration. 
 Coordinated down-regulation of collagen & TIMP-1 and up-regulation of MMP-1 occurs in fibroblasts exposed to proteasome inhibitors, indicating the proteasome's role in fibrosis and   extracellular matrix   remodeling. 
 Enhancement of TIMP-1 production by tumor-derived prostaglandin E2 correlates with reduced dendritic cell migration through the tumor   extracellular matrix  . 
 Excessive expression of MMP-1 and TIMP-1 in the diseased colonic mucosa causes excessive hydrolysis of the   extracellular matrix   (ECM) and ulceration in ulcerative colitis (UC) patients. 
 plasma levels and activities of MMP-2, MMP-9, and TIMP-1 are increased in hypertensive patients, which may reflect abnormal   extracellular matrix   metabolism 
 Our analysis of the entire coding region of TIMP-1, -2, and -3, which are the main inhibitors of metalloproteinase activity in the   extracellular matrix  , failed to show an association between genetic polymorphisms and an intracranial aneurysm 
 This study proposes a novel mechanism of TIMP-1 regulation, which ensures an increased supply of the inhibitor after brain injury, and limits   extracellular matrix   degradation. 
  Summary  
 This gene belongs to the TIMP gene family. The proteins encoded by this gene family are natural inhibitors of the matrix metalloproteinases (MMPs), a group of peptidases involved in degradation of the   extracellular matrix  . In addition to its inhibitory role against most of the known MMPs, the encoded protein is able to promote cell proliferation in a wide range of cell types, and may also have an anti-apoptotic function. Transcription of this gene is highly inducible in response to many cytokines and hormones. In addition, the expression from some but not all inactive X chromosomes suggests that this gene inactivation is polymorphic in human females. This gene is located within intron 6 of the synapsin I gene and is transcribed in the opposite direction. [provided by RefSeq, Jul 2008] 
  [Ii]nvasive  PubMed  
 Circulating matrix metalloproteinase-9 and tissue inhibitors of metalloproteinases-1 and -2 levels in gestational hypertension. Gestational hypertension (GH) is dangerous to both mother and child. Arterial   invasive  ness and growth are dependent on successful extracellular matrix (ECM) breakdown, which may be abnormal in GH. We hypothesized abnormalities in circulating matrix metalloproteinase-9 (MMP-9) and tissue inhibitors of metalloproteinases-1 and -2 (TIMP-1 and TIMP-2, respectively) in patients with GH, when compared with normotensive women with normal pregnancies and healthy nonpregnant control subjects.Plasma MMP-9, TIMP-1, and TIMP-2 were measured by ELISA in 23 women with GH, 30 normotensive pregnant women, and 28 nonpregnant women who were matched for age, gestational age, and parity.Levels of circulating MMP-9, TIMP-1 and TIMP-2, and the MMP-9/TIMP-1 and MMP-9/TIMP-2 ratios were significantly different among the three groups (P = .026, P = .006, P = .007, P = .001 and P = .008 respectively). Within the GH group, MMP-9 and the MMP-9/TIMP-1 ratio correlated negatively with age (r = -0.581, P = .004 and r = -0.563, P = .005, respectively) and levels of diastolic blood pressure (r = -0.432, P = .040 and r = -0.461, P = .027, respectively). With multiple regression analysis, only age independently correlated with circulating levels of MMP-9 (P = .010); neither age nor levels of diastolic blood pressure had any effect on the MMP-9/TIMP-1 ratio.We have demonstrated altered MMP/TIMP ratios in maternal blood during GH. These observations suggest pregnancy-related changes in ECM breakdown and turnover. Given the importance of changes in ECM composition to vascular and cardiac structure in hypertension, we suggest that these observations may be related to the pathophysiology of human GH. 
 Transcriptional control of cell density dependent regulation of matrix metalloproteinase and TIMP expression in breast cancer cell lines. Our recent studies on breast carcinoma cell lines with differing tumorigenicity/  invasive  ness (MCF-7<MDA-MB-468<MDAMB-231<MDA-MB-435) had shown significantly decreasing expression levels of MMPs-1,-2,-3,-8,-9,-10,-11 and -13 with increasing cell density while the levels of TIMP-1 and -2 increased. This correlated well with a lower   invasive  ness of confluent cells. In the present study, we extend our in vitro studies on three-dimensional cultures of breast cancer cell lines MCF-7 and MDAMB-435 and the transcriptional control of MMP and TIMP-expression in two-dimensional cultures of MDA-MB-231 and -435 cells. The tumor spheroid model showed that MMP expression and proteolytic activity were considerably higher in loosely structured tumor groups as compared to densely growing "compact" cell complexes. These data suggested that cell density regulates MMP and TIMP transcription and therefore, we tested whether AP-1, NF kappa B and CRE are involved in this process. Gene silencing of c-jun in sparse cultures had an inhibitory effect on MMP-3, -9 and -13 expression, on proteolytic activity as well as on the   invasive   potential of the cells, thus confirming a role for AP-1.TIMP-1, and -2 expression was up-regulated as compared to control cells. Consistent with this, overexpression of c-jun and c-fos in confluent breast cancer cell lines leads to up-regulation of MMP expression, proteolytic activity and invasion as well as down-regulation of TIMP-1. In summary, we provide evidence that cell density influences the   invasive   potential of tumor cells via regulation of MMPs and TIMPs by AP-1, NF kappa B and CRE transcription factors. Overexpression of MMPs in sparse cultures could help explain early dissemination of potentially metastatic cells. 
 HIV-1-Tat protein promotes chemotaxis and   invasive   behavior by monocytes. Monocytes are susceptible to HIV infection and to activation by a regulatory gene product of the HIV genome, HIV-Tat. Recently, we have demonstrated that treatment with HIV-Tat up-regulates monocyte adhesion to the endothelium and increases metalloproteinase production. in the present study, we have examined the ability of the HIV-Tat protein to alter the migratory and   invasive   behavior of monocytes. Monocytes pretreated for 24 h with 10 ng/ml HIV-Tat exhibited enhanced migratory behavior compared with untreated monocytes in chemotaxis assays, both in the absence of a chemoattractant as well as in response to FMLP. in addition, HIV-Tat itself induced the migration of both untreated and HIV-Tat pretreated monocytes. Checkerboard analysis showed that monocytes migrated in response to an HIV-Tat concentration gradient, thus confirming the chemotactic characteristics of the HIV-Tat protein. Pretreatment of monocytes with 10 ng/ml HIV-Tat for 24 h also increased their ability to invade reconstituted extracellular membrane (Matrigel)-coated filters by 5-fold in the absence of chemoattractant. The presence of FMLP or HIV-Tat further enhanced invasion by both untreated and HIV-Tat-pretreated monocytes by more than 10-fold. Monocyte invasion was partially inhibited by the inclusion of anti-beta integrin Ab or tissue inhibitor of metalloproteinase (TIMP). Thus, for the first time, we present evidence that HIV-Tat can enhance the chemotactic and   invasive   behaviors of monocytes and propose an active role for HIV-Tat in the recruitment of monocytes into extravascular tissues, a process which may contribute to the destruction of tissues and cellular architecture often seen in patients with acquired immunodeficiency syndrome. 
 High variability in MMP2/TIMP2 and MMP9/TIMP1 expression in secondary liver tumors. The assessment of MMPs/TIMPs expression in various primary tumors has potential prognostic values. Considering the paucity of studies in secondary liver tumors, our aim was to study the expression of MMP2, MMP9, TIMP1, and TIMP2 in secondary hepatic cancer, focusing on their variability in the malignant cells. The study group included 25 cases of liver metastases of colorectal cancer, diagnosed and surgically treated at "Sf. Spiridon" University Hospital of Iassy, Romania. Immunohistochemistry for MMP2, MMP9, TIMP1, and TIMP2 has been performed, followed by the semi-quantitative assessment of the markers using a scoring system based on the positive tumoral cells percentage and the staining intensity. The expression of investigated markers revealed an increased staining variability. The scores showed that MMP2/TIMP2 and MMP9/TIMP1 immunoreactivity was extremely heterogeneous within the analyzed group, with a dominant weak expression for MMP2 and MMP9, in contrast to strong TIMP2 and TIMP1 expression. Ten different patterns of expression of the investigated markers have been identified. No major differences between the expression of MMP2 (14 positive cases) and MMP9 (16 positive cases) could be detected. Our results sustain the inverse correlation between MMP and the correspondent TIMP expression, supporting the hypothesis that MMPs/TIMPs balance has mainly an inhibitory effect on   invasive  ness. Our study demonstrated that tumoral cells are adapted to MMPs:TIMPs production in the liver microenvironment. The lack of significant differences between MMP2 and MMP9 expression shows that the activity of both MMPs is independent, without reciprocal influences. 
 [Interstitial collagenase, gelatinases A and B and their endogenous inhibitors in squamous cell cervical carcinomas]. Interstitial collagenase and gelatinases are matrix metalloproteinases (MMP), which play the key role in tumor invasion and metastasis. The aim of this study was to elucidate the peculiarities of expression of interstitial collagenase (MMP-1), gelatinases A and B (MMP-2 and MMP-9) and their endogenous tissue inhibitors TIMP-1 and TIMP-2 as   invasive   factors of squamous cell carcinomas (SCC) of human cervical cancer. The study was carried out using 24 specimens of SCC and 11 specimens of adjacent to tumor morphologically normal tissue. All carcinoma specimens expressed E7 HPV-16 gene. It was shown that the increase of MMP-1 and MMP-9 expression and low of TIMP-1 and TIMP-2 expression makes the main contribution to the destructive (  invasive  ) potential of SCC. The change of MMP-2 expression is not so significant and it is less influenced to the destructive potential. Moreover, substantial expression of MMP-1, MMP-2 and MMP-9 was registered in the specimens of morphologically normal adjoining to tumor tissue. This expression was found to make an additional contribution to the destructive potential of cervical tumor. 
 Comparative study of the immunohistochemical expression of tissue inhibitors of metalloproteinases 1 and 2 between clearly   invasive   carcinomas and "in situ" trophoblast invasion. Tissue inhibitors of metalloproteinases (TIMPs) play an important role in extracellular matrix homeostasis by regulating MMP activity. Although they were initially considered inhibitors of tumor growth and metastasis, recently their role in cancer progression has been controversial. The aim of our study was to compare the immunohistochemical expression of TIMP1 and TIMP2 between an uncontrollably   invasive   phenomenon (cancer) and an "in situ" process (trophoblast invasion) in an effort to assess any differential role of these molecules between these two distinct phenomena and therefore to understand better their contribution in cancer invasion and migration. We performed an immunohistochemical analysis of 50 carcinomas (colorectal, gastric, breast, pulmonary, and renal) and 40 first trimester gestations. The marker expression was evaluated semiquantitatively, separately in cancer parenchymal and trophoblastic cells as well as in malignant stromal and decidual cells, according to a percentage scale (0, <10, 10-50, and >50%) and according to staining intensity (0, +, ++, and +++). Our results showed that there was no statistically significant difference in TIMP1 expression between cancer parenchymal cells and trophoblastic cells. On the other hand, TIMP1 was expressed more often in decidual cells than in cancer stromal cells. Immunostaining for TIMP2 was more extensive and intense both in trophoblastic and decidual cells than in cancer parenchymal and stromal cells, respectively. The reduced expression of TIMP2 in metastatic carcinomas by comparison with non-metastatic gestation specimens underlines its importance in cancer invasion and migration. On the other hand, TIMP1 was more expressed in decidua than cancer stroma, but at the same time showed no statistically significant difference between cancer parenchyma and trophoblasts, highlighting its multifunctional activity in cancer progression. 
 Concentrations and activities of metalloproteinases 2 and 9 and their inhibitors (TIMPS) in chronic pancreatitis and pancreatic adenocarcinoma. Pancreatic cancer (PC) and chronic pancreatitis (CP) are still significant problems. The aim of this study was a comparative analysis of the activity and concentrations of matrix metalloproteinases 2 and 9 and the concentrations of their tissue inhibitors (TIMP 1 and 2) in the PC compared to CP tissue homogenates. The study was performed in a group of 63 patients with pancreatic cancer or chronic pancreatitis selected for resection procedures. Group 1 consisted of 31 patients with CP, group 2 consisted of 32 patients with PC. There was no coincidence of pancreatic cancer in CP group. The pancreatic tumor samples have been properly prepared in order to perform electrophoresis and immunoassay testing. The activity of MMPs and the concentrations of MMPs and TIMPs were evaluated.the revealed activities of gelatinases and concentrations levels of the gelatinases and their inhibitors were significantly higher in the PC tissue samples compared to CP. In both groups, higher concentrations of MMP9 compared to MMP2 and TIMP2 compared to TIMP1 were shown. High potential for tumor   invasive  ness demonstrated by the formation of lymph node metastases was characterized by the higher concentrations of MMP9 and TIMP2. However, in the case of infiltration of the nerve fibers, a decrease in the concentration of MMP2 was found.gelatinases and their inhibitors play important role in the pathogenesis of the CP as well as PC. The activity and concentration of gelatinases and the concentration of their inhibitors were all significantly higher in the PC group. 
 Tissue Inhibitors of Metalloproteinase-1 and 2 and Obesity Related Non-Alcoholic Fatty Liver Disease: Is There a Relationship. Non-alcoholic fatty liver disease is a spectrum of clinical conditions, including simple steatosis and non-alcoholic steatohepatitis (NASH). The aim of the study is to evaluate the tissue inhibitors of metalloproteinase-1 and 2 (TIMPs) as non  invasive   predictors of NASH.Three groups were included in the study. Obese patients (n = 30) with normal liver enzymes were included in group I and obese patients (n = 30) with elevated liver enzymes with liver biopsy-based diagnosis of NASH were included in group II. Age-matched subjects (n = 30) formed the control as group III. The lipid profile, liver enzyme levels and levels of TIMPs were compared among all the patients and subjects.Comparison of groups I and II showed significantly elevated levels of TIMP-1 and TIMP-2 in group II as compared to group I (p < 0.05). Similarly, comparison between groups II and III showed significantly increased levels of TIMP-1 and TIMP-2 in group II as compared to group III (p < 0.05). TIMP-1 (sensitivity 96.7%, specificity 100%) and TIMP-2 (sensitivity 93.3%, specificity 100%) showed high accuracy in NASH diagnosis.TIMP-1 and TIMP-2 may be considered non  invasive   markers for the diagnosis of NASH. 
 Evaluation of candidate stromal epithelial cross-talk genes identifies association between risk of serous ovarian cancer and TERT, a cancer susceptibility "hot-spot". We hypothesized that variants in genes expressed as a consequence of interactions between ovarian cancer cells and the host micro-environment could contribute to cancer susceptibility. We therefore used a two-stage approach to evaluate common single nucleotide polymorphisms (SNPs) in 173 genes involved in stromal epithelial interactions in the Ovarian Cancer Association Consortium (OCAC). In the discovery stage, cases with epithelial ovarian cancer (n=675) and controls (n=1,162) were genotyped at 1,536 SNPs using an Illumina GoldenGate assay. Based on Positive Predictive Value estimates, three SNPs-PODXL rs1013368, ITGA6 rs13027811, and MMP3 rs522616-were selected for replication using TaqMan genotyping in up to 3,059 serous   invasive   cases and 8,905 controls from 16 OCAC case-control studies. An additional 18 SNPs with Pper-allele<0.05 in the discovery stage were selected for replication in a subset of five OCAC studies (n=1,233 serous   invasive   cases; n=3,364 controls). The discovery stage associations in PODXL, ITGA6, and MMP3 were attenuated in the larger replication set (adj. Pper-allele>or=0.5). However genotypes at TERT rs7726159 were associated with ovarian cancer risk in the smaller, five-study replication study (Pper-allele=0.03). Combined analysis of the discovery and replication sets for this TERT SNP showed an increased risk of serous ovarian cancer among non-Hispanic whites [adj. ORper-allele 1.14 (1.04-1.24) p=0.003]. Our study adds to the growing evidence that, like the 8q24 locus, the telomerase reverse transcriptase locus at 5p15.33, is a general cancer susceptibility locus. 
 Tissue Inhibitors of Metalloproteinase-1 and 2 and Obesity Related Non-Alcoholic Fatty Liver Disease: Is There a Relationship. Non-alcoholic fatty liver disease is a spectrum of clinical conditions, including simple steatosis and non-alcoholic steatohepatitis (NASH). The aim of the study is to evaluate the tissue inhibitors of metalloproteinase-1 and 2 (TIMPs) as non  invasive   predictors of NASH.Three groups were included in the study. Obese patients (n = 30) with normal liver enzymes were included in group I and obese patients (n = 30) with elevated liver enzymes with liver biopsy-based diagnosis of NASH were included in group II. Age-matched subjects (n = 30) formed the control as group III. The lipid profile, liver enzyme levels and levels of TIMPs were compared among all the patients and subjects.Comparison of groups I and II showed significantly elevated levels of TIMP-1 and TIMP-2 in group II as compared to group I (p < 0.05). Similarly, comparison between groups II and III showed significantly increased levels of TIMP-1 and TIMP-2 in group II as compared to group III (p < 0.05). TIMP-1 (sensitivity 96.7%, specificity 100%) and TIMP-2 (sensitivity 93.3%, specificity 100%) showed high accuracy in NASH diagnosis.TIMP-1 and TIMP-2 may be considered non  invasive   markers for the diagnosis of NASH. 
 High variability in MMP2/TIMP2 and MMP9/TIMP1 expression in secondary liver tumors. The assessment of MMPs/TIMPs expression in various primary tumors has potential prognostic values. Considering the paucity of studies in secondary liver tumors, our aim was to study the expression of MMP2, MMP9, TIMP1, and TIMP2 in secondary hepatic cancer, focusing on their variability in the malignant cells. The study group included 25 cases of liver metastases of colorectal cancer, diagnosed and surgically treated at "Sf. Spiridon" University Hospital of Iassy, Romania. Immunohistochemistry for MMP2, MMP9, TIMP1, and TIMP2 has been performed, followed by the semi-quantitative assessment of the markers using a scoring system based on the positive tumoral cells percentage and the staining intensity. The expression of investigated markers revealed an increased staining variability. The scores showed that MMP2/TIMP2 and MMP9/TIMP1 immunoreactivity was extremely heterogeneous within the analyzed group, with a dominant weak expression for MMP2 and MMP9, in contrast to strong TIMP2 and TIMP1 expression. Ten different patterns of expression of the investigated markers have been identified. No major differences between the expression of MMP2 (14 positive cases) and MMP9 (16 positive cases) could be detected. Our results sustain the inverse correlation between MMP and the correspondent TIMP expression, supporting the hypothesis that MMPs/TIMPs balance has mainly an inhibitory effect on   invasive  ness. Our study demonstrated that tumoral cells are adapted to MMPs:TIMPs production in the liver microenvironment. The lack of significant differences between MMP2 and MMP9 expression shows that the activity of both MMPs is independent, without reciprocal influences. 
 Concentrations and activities of metalloproteinases 2 and 9 and their inhibitors (TIMPS) in chronic pancreatitis and pancreatic adenocarcinoma. Pancreatic cancer (PC) and chronic pancreatitis (CP) are still significant problems. The aim of this study was a comparative analysis of the activity and concentrations of matrix metalloproteinases 2 and 9 and the concentrations of their tissue inhibitors (TIMP 1 and 2) in the PC compared to CP tissue homogenates. The study was performed in a group of 63 patients with pancreatic cancer or chronic pancreatitis selected for resection procedures. Group 1 consisted of 31 patients with CP, group 2 consisted of 32 patients with PC. There was no coincidence of pancreatic cancer in CP group. The pancreatic tumor samples have been properly prepared in order to perform electrophoresis and immunoassay testing. The activity of MMPs and the concentrations of MMPs and TIMPs were evaluated.the revealed activities of gelatinases and concentrations levels of the gelatinases and their inhibitors were significantly higher in the PC tissue samples compared to CP. In both groups, higher concentrations of MMP9 compared to MMP2 and TIMP2 compared to TIMP1 were shown. High potential for tumor   invasive  ness demonstrated by the formation of lymph node metastases was characterized by the higher concentrations of MMP9 and TIMP2. However, in the case of infiltration of the nerve fibers, a decrease in the concentration of MMP2 was found.gelatinases and their inhibitors play important role in the pathogenesis of the CP as well as PC. The activity and concentration of gelatinases and the concentration of their inhibitors were all significantly higher in the PC group. 
 Comparative study of the immunohistochemical expression of tissue inhibitors of metalloproteinases 1 and 2 between clearly   invasive   carcinomas and "in situ" trophoblast invasion. Tissue inhibitors of metalloproteinases (TIMPs) play an important role in extracellular matrix homeostasis by regulating MMP activity. Although they were initially considered inhibitors of tumor growth and metastasis, recently their role in cancer progression has been controversial. The aim of our study was to compare the immunohistochemical expression of TIMP1 and TIMP2 between an uncontrollably   invasive   phenomenon (cancer) and an "in situ" process (trophoblast invasion) in an effort to assess any differential role of these molecules between these two distinct phenomena and therefore to understand better their contribution in cancer invasion and migration. We performed an immunohistochemical analysis of 50 carcinomas (colorectal, gastric, breast, pulmonary, and renal) and 40 first trimester gestations. The marker expression was evaluated semiquantitatively, separately in cancer parenchymal and trophoblastic cells as well as in malignant stromal and decidual cells, according to a percentage scale (0, <10, 10-50, and >50%) and according to staining intensity (0, +, ++, and +++). Our results showed that there was no statistically significant difference in TIMP1 expression between cancer parenchymal cells and trophoblastic cells. On the other hand, TIMP1 was expressed more often in decidual cells than in cancer stromal cells. Immunostaining for TIMP2 was more extensive and intense both in trophoblastic and decidual cells than in cancer parenchymal and stromal cells, respectively. The reduced expression of TIMP2 in metastatic carcinomas by comparison with non-metastatic gestation specimens underlines its importance in cancer invasion and migration. On the other hand, TIMP1 was more expressed in decidua than cancer stroma, but at the same time showed no statistically significant difference between cancer parenchyma and trophoblasts, highlighting its multifunctional activity in cancer progression. 
 [Interstitial collagenase, gelatinases A and B and their endogenous inhibitors in squamous cell cervical carcinomas]. Interstitial collagenase and gelatinases are matrix metalloproteinases (MMP), which play the key role in tumor invasion and metastasis. The aim of this study was to elucidate the peculiarities of expression of interstitial collagenase (MMP-1), gelatinases A and B (MMP-2 and MMP-9) and their endogenous tissue inhibitors TIMP-1 and TIMP-2 as   invasive   factors of squamous cell carcinomas (SCC) of human cervical cancer. The study was carried out using 24 specimens of SCC and 11 specimens of adjacent to tumor morphologically normal tissue. All carcinoma specimens expressed E7 HPV-16 gene. It was shown that the increase of MMP-1 and MMP-9 expression and low of TIMP-1 and TIMP-2 expression makes the main contribution to the destructive (  invasive  ) potential of SCC. The change of MMP-2 expression is not so significant and it is less influenced to the destructive potential. Moreover, substantial expression of MMP-1, MMP-2 and MMP-9 was registered in the specimens of morphologically normal adjoining to tumor tissue. This expression was found to make an additional contribution to the destructive potential of cervical tumor. 
 Evaluation of candidate stromal epithelial cross-talk genes identifies association between risk of serous ovarian cancer and TERT, a cancer susceptibility "hot-spot". We hypothesized that variants in genes expressed as a consequence of interactions between ovarian cancer cells and the host micro-environment could contribute to cancer susceptibility. We therefore used a two-stage approach to evaluate common single nucleotide polymorphisms (SNPs) in 173 genes involved in stromal epithelial interactions in the Ovarian Cancer Association Consortium (OCAC). In the discovery stage, cases with epithelial ovarian cancer (n=675) and controls (n=1,162) were genotyped at 1,536 SNPs using an Illumina GoldenGate assay. Based on Positive Predictive Value estimates, three SNPs-PODXL rs1013368, ITGA6 rs13027811, and MMP3 rs522616-were selected for replication using TaqMan genotyping in up to 3,059 serous   invasive   cases and 8,905 controls from 16 OCAC case-control studies. An additional 18 SNPs with Pper-allele<0.05 in the discovery stage were selected for replication in a subset of five OCAC studies (n=1,233 serous   invasive   cases; n=3,364 controls). The discovery stage associations in PODXL, ITGA6, and MMP3 were attenuated in the larger replication set (adj. Pper-allele>or=0.5). However genotypes at TERT rs7726159 were associated with ovarian cancer risk in the smaller, five-study replication study (Pper-allele=0.03). Combined analysis of the discovery and replication sets for this TERT SNP showed an increased risk of serous ovarian cancer among non-Hispanic whites [adj. ORper-allele 1.14 (1.04-1.24) p=0.003]. Our study adds to the growing evidence that, like the 8q24 locus, the telomerase reverse transcriptase locus at 5p15.33, is a general cancer susceptibility locus. 
 Transcriptional control of cell density dependent regulation of matrix metalloproteinase and TIMP expression in breast cancer cell lines. Our recent studies on breast carcinoma cell lines with differing tumorigenicity/  invasive  ness (MCF-7<MDA-MB-468<MDAMB-231<MDA-MB-435) had shown significantly decreasing expression levels of MMPs-1,-2,-3,-8,-9,-10,-11 and -13 with increasing cell density while the levels of TIMP-1 and -2 increased. This correlated well with a lower   invasive  ness of confluent cells. In the present study, we extend our in vitro studies on three-dimensional cultures of breast cancer cell lines MCF-7 and MDAMB-435 and the transcriptional control of MMP and TIMP-expression in two-dimensional cultures of MDA-MB-231 and -435 cells. The tumor spheroid model showed that MMP expression and proteolytic activity were considerably higher in loosely structured tumor groups as compared to densely growing "compact" cell complexes. These data suggested that cell density regulates MMP and TIMP transcription and therefore, we tested whether AP-1, NF kappa B and CRE are involved in this process. Gene silencing of c-jun in sparse cultures had an inhibitory effect on MMP-3, -9 and -13 expression, on proteolytic activity as well as on the   invasive   potential of the cells, thus confirming a role for AP-1.TIMP-1, and -2 expression was up-regulated as compared to control cells. Consistent with this, overexpression of c-jun and c-fos in confluent breast cancer cell lines leads to up-regulation of MMP expression, proteolytic activity and invasion as well as down-regulation of TIMP-1. In summary, we provide evidence that cell density influences the   invasive   potential of tumor cells via regulation of MMPs and TIMPs by AP-1, NF kappa B and CRE transcription factors. Overexpression of MMPs in sparse cultures could help explain early dissemination of potentially metastatic cells. 
 Circulating matrix metalloproteinase-9 and tissue inhibitors of metalloproteinases-1 and -2 levels in gestational hypertension. Gestational hypertension (GH) is dangerous to both mother and child. Arterial   invasive  ness and growth are dependent on successful extracellular matrix (ECM) breakdown, which may be abnormal in GH. We hypothesized abnormalities in circulating matrix metalloproteinase-9 (MMP-9) and tissue inhibitors of metalloproteinases-1 and -2 (TIMP-1 and TIMP-2, respectively) in patients with GH, when compared with normotensive women with normal pregnancies and healthy nonpregnant control subjects.Plasma MMP-9, TIMP-1, and TIMP-2 were measured by ELISA in 23 women with GH, 30 normotensive pregnant women, and 28 nonpregnant women who were matched for age, gestational age, and parity.Levels of circulating MMP-9, TIMP-1 and TIMP-2, and the MMP-9/TIMP-1 and MMP-9/TIMP-2 ratios were significantly different among the three groups (P = .026, P = .006, P = .007, P = .001 and P = .008 respectively). Within the GH group, MMP-9 and the MMP-9/TIMP-1 ratio correlated negatively with age (r = -0.581, P = .004 and r = -0.563, P = .005, respectively) and levels of diastolic blood pressure (r = -0.432, P = .040 and r = -0.461, P = .027, respectively). With multiple regression analysis, only age independently correlated with circulating levels of MMP-9 (P = .010); neither age nor levels of diastolic blood pressure had any effect on the MMP-9/TIMP-1 ratio.We have demonstrated altered MMP/TIMP ratios in maternal blood during GH. These observations suggest pregnancy-related changes in ECM breakdown and turnover. Given the importance of changes in ECM composition to vascular and cardiac structure in hypertension, we suggest that these observations may be related to the pathophysiology of human GH. 
 Serum levels of hyaluronic acid and tissue metalloproteinase inhibitor-1 combined with age predict the presence of nonalcoholic steatohepatitis in a pilot cohort of subjects with nonalcoholic fatty liver disease. Hyaluronic acid (HA) and tissue inhibitor of metalloproteinase 1 (TIMP-1) are reliable markers of liver fibrosis and are closely linked to the proinflammatory status. In this pilot cohort study, we attempted to identify a clinical score that would predict the severity of nonalcoholic fatty liver disease (NAFLD) based on clinical variables and serum markers of fibrosis and inflammation. The cohort included 46 patients with histologically confirmed NAFLD (76.1% male; mean age, 43+/-13 years; mean body mass index [BMI], 27.8+/-3.5). Serum transforming growth factor beta (TGF-beta), HA, TIMP, and matrix metalloproteinase (MMP) levels were measured with commercial enzyme-linked immunoassay (ELISA) kits. Demographic features and clinical and laboratory findings were subjected to univariate and multivariate binary logistic regression analysis to construct the mathematical model. Receiver operating characteristic curve (ROC) analysis was used to identify a threshold value for diagnosis of NASH and to assess its sensitivity and specificity. Serum levels of HA and TIMP-1 were statistically different in patients with nonalcoholic steatohepatitis (NASH) (P<0.05). Logistic regression analysis of several clinical variables indicated patient age as the only independent predictor of NASH (odds ratio [OR], 1.129, 95% confidence interval [CI], 1.019-1.251, P=0.020). The mathematical model constructed on the basis of these results included age, TIMP-1, and HA levels. A value of 148.27 or more identified patients with NASH with 85.7% sensitivity, 87.1% specificity, and negative and positive predictive values of 96.4% and 60%, respectively. This model seems to represent a reliable non  invasive   tool for excluding the presence of NASH. If validated in larger prospective cohort studies, it might be useful for determining when a liver biopsy is actually warranted in patients with NAFLD. 
 BRAFV600E mutation, TIMP-1 upregulation, and NF-κB activation: closing the loop on the papillary thyroid cancer trilogy. BRAF(V600E) is the most common mutation found in papillary thyroid carcinoma (PTC). Tissue inhibitor of metalloproteinases (TIMP-1) and nuclear factor (NF)-κB have been shown to play an important role in thyroid cancer. In particular, TIMP-1 binds its receptor CD63 on cell surface membrane and activates Akt signaling pathway, which is eventually responsible for its anti-apoptotic activity. The aim of our study was to evaluate whether interplay among these three factors exists and exerts a functional role in PTCs. To this purpose, 56 PTC specimens were analyzed for BRAF(V600E) mutation, TIMP-1 expression, and NF-κB activation. We found that BRAF(V600E) mutation occurs selectively in PTC nodules and is associated with hyperactivation of NF-κB and upregulation of both TIMP-1 and its receptor CD63. To assess the functional relationship among these factors, we first silenced BRAF gene in BCPAP cells, harboring BRAF(V600E) mutation. We found that silencing causes a marked decrease in TIMP-1 expression and NF-κB binding activity, as well as decre  ased inv  asiveness. After treatment with specific inhibitors of MAPK pathway, we found that only sorafenib was able to increase IκB-α and reduce both TIMP-1 expression and Akt phosphorylation in BCPAP cells, indicating that BRAF(V600E) activates NF-κB and this pathway is MEK-independent. Taken together, our findings demonstrate that BRAF(V600E) causes upregulation of TIMP-1 via NF-κB. TIMP-1 binds then its surface receptor CD63, leading eventually to Akt activation, which in turn confers antiapoptotic behavior and promotion of cell invasion. The recognition of this functional trilogy provides insight on how BRAF(V600E) determines cancer initiation, progress  ion, and   invasiveness in PTC, also identifying new therapeutic targets for the treatment of highly aggressive forms. 
 Functional proteomics study reveals that N-Acetylglucosaminyltransferase V reinforces the   invasive  /metastatic potential of colon cancer through aberrant glycosylation on tissue inhibitor of metalloproteinase-1. N-Acetylglucosaminyltransferase-V (GnT-V) has been reported to be up-regulated in   invasive  /metastatic cancer cells, but a comprehensive understanding of how the transferase correlates with the   invasive  /metastatic potential is not currently available. Through a glycomics approach, we identified 30 proteins, including tissue inhibitor of metalloproteinase-1 (TIMP-1), as a target protein for GnT-V in human colon cancer cell WiDr. TIMP-1 was aberrantly glycosylated as characterized by the addition of beta1,6-N-acetylglucosamine, polylactosaminylation, and sialylation in GnT-V-overexpressing WiDr cells. Compared with normal TIMP-1, the aberrantly glycosylated TIMP-1 showed the weaker inhibition on both matrix metalloproteinase (MMP)-2 and MMP-9, and this aberrancy was closely associated with cancer cell invasion and metastasis in vivo as well as in vitro. Integrated data, both of TIMP-1 expression level and aberrant glycosylation, could provide important information to aid to improve the clinical outcome of colon cancer patients. 
 OncomiR-196 promotes an   invasive   phenotype in oral cancer through the NME4-JNK-TIMP1-MMP signaling pathway. MicroRNA-196 (miR-196), which is highly up-regulated in oral cancer cells, has been reported to be aberrantly expressed in several cancers; however, the significance of miR-196 in oral cancer has not yet been addressed.Cellular functions in response to miR-196 modulation were examined, including cell growth, migration, invasion and radio/chemosensitivity. Algorithm-based studies were used to identify the regulatory target of miR-196. The miR-196 target gene and downstream molecular mechanisms were confirmed by RT-qPCR, western blot, luciferase reporter and confocal microscopy analyses. miR-196 expression was determined in paired cancer and adjacent normal tissues from oral cancer patients.Both miR-196a and miR-196b were highly over-expressed in the cancer tissue and correlated with lymph node metastasis (P = 0.001 and P = 0.006, respectively). Functionally, miR-196 actively promoted cell migration and invasion without affecting cell growth. Mechanistically, miR-196 performed it's their function by inhibiting NME4 expression and further activating p-JNK, suppressing TIMP1, and augmenting MMP1/9.miR-196 contributes to oral cancer by promoting cell migration and invasion. Clinically, miR-196a/b was significantly over-expressed in the cancer tissues and correlated with lymph node metastasis. Thus, our findings provide new knowledge of the underlying mechanism of cancer metastasis. miR-196 may serve as a promising marker for better oral cancer management. 
 The role of urinary TIMP1 and MMP9 levels in predicting vesicoureteral reflux in neonates with antenatal hydronephrosis. Antenatal hydronephrosis (AH) is commonly found on evaluation of pregnant women and 20-30 % of neonates have vesicoureteral reflux (VUR). In order to diagnose VUR, we required   invasive   testing and exposure of the neonate to radiation. The concentrations of a matrix metalloproteinase, MMP9, and its inhibitor TIMP1, were analyzed in hydronephrotic newborns with VUR and were compared to those without reflux.The neonates with a history of prenatal hydronephrosis were enrolled in two groups based on imaging study results, the neonates with VUR and without VUR. Neonates with a normal prenatal history and postnatal ultrasound were placed in a third group. We measured the random urinary levels of MMP9, TIMP1, and creatinine, their cut-off values and the MMP9/Cr and MMP9/TIMP1/Cr ratio was calculated, and an ROC curve was drawn.Sixty-nine neonates were enrolled in three groups; 27 patients (20 male, seven female) with AH and VUR were in group 1, 23 neonates (19 male, four female) without VUR were placed in group 2, and 19 (15 male, four female) acted as controls in group 3. The differences between the three groups and the normal and total hydronephrotic groups were statistically significant for MMP9, the MMP9/Cr, MMP9/TIMP1, and MMP9/TIMP1/Cr ratios. The urinary TIMP1 and TIMP1/Cr ratios were not significantly different between the groups. A cut-off value of MMP9 was measured as 358.5 ng/ml (sensitivity [sens] 74 %, specificity [spec] 78 %) and was used to compare groups 1 and 2. For groups 2 and 3, this cut-off was 181.00 pg/ml (sens 91 %, spec 89 %). The cut-off values measured for the MMP9/TIMP1 ratio were 30.32 (sens 70 %, spec 61 %) and 9.85 (sens 96 %, spec 89 %) to compare groups 1 and 2, and 2 and 3, respectively. We found no valuable cut-offs for the TIMP1 and TIMP1/Cr values. There was no difference between neonates with mild, moderate, and severe VUR according to urinary biomarker concentrations.Evaluation of urinary levels of MMP9, or the MMP9/Cr, MMP9/TIMP1, or MMP9/TIMP1/Cr ratios may help us to differentiate the newborns with hydronephrosis and VUR from those without reflux. 
 Prognostic significance of matrix metalloproteinase-1 and tissue inhibitor of metalloproteinase-1 in voided urine samples from patients with transitional cell carcinoma of the bladder. To study the role of urinary matrix metalloproteinase-1 (MMP-1) and tissue inhibitor of metalloproteinase-1 (TIMP-1) in bladder cancer and their relationship to tumor progression.MMP-1 and TIMP-1 were measured by ELISA in urine samples from 131 patients with bladder tumors (7 cis, 74 Ta, 29 T1, and 21 T2-T4; 46 G(1), 41 G(2), and 37 G(3)), 5 patients with prostate cancer, 33 patients with benign lower urinary tract disorders, and 36 healthy volunteers. Complete clinical data were available for 100 patients with bladder cancer with a median follow-up time of 24 months (range: 4-39 months).MMP-1 was detected in urine samples from 21 of 131 (16%) patients with bladder cancer but was undetectable in samples from all other groups (P < 0.0001). Urinary MMP-1 was detected in a higher percentage of patients with T2-T4 tumors and G(3) tumors than patients with cis/Ta/T1 or G(1)-G(2) tumors (P = 0.04 and P = 0.0074, respectively). Patients with detectable concentrations of urinary MMP-1 had higher rates of disease progression (P = 0.04) and death from bladder cancer (P = 0.02) than patients with undetectable urinary MMP-1. All patient groups had higher urinary TIMP-1 concentrations than healthy volunteers (P = 0.02). Patients with muscle-  invasive   tumors had higher concentrations of urinary TIMP-1 than patients with cis/Ta/T1 tumors (P = 0.037), but there was no association between TIMP-1 and tumor grade. Urinary TIMP-1 levels strongly correlated with tumor size (P = 0.0002). Progression-free survival rates were lower for patients with urinary TIMP-1 concentrations above the median (1.8 ng/ml, P = 0.04), but urinary TIMP-1 levels were not related to disease-specific survival. Patients with T2-T4 tumors and G(3) tumors had significantly lower urinary MMP-1:TIMP-1 ratios than patients with Ta/T1 bladder tumors (P = 0.039) or G(1)-G(2) tumors (P = 0.0415).Where urinary MMP-1 is detectable, the patient is more likely to have a bladder tumor of advanced stage or grade and may be at increased risk of disease progression and death of bladder cancer. The relationship between urinary TIMP-1, muscle-invasion, and disease progression in bladder cancer is at variance with its role as an inhibitor of MMPs and warrants additional evaluation. 
 ERK2-regulated TIMP1 induces hyperproliferation of K-Ras(G12D)-transformed pancreatic ductal cells. Pancreatic ductal adenocarcinoma (PDAC) commonly contains a mutation in K-Ras(G12D) and is characterized by a desmoplastic reaction composed of deregulated, proliferating cells embedded in an abnormal extracellular matrix (ECM). Our previous observations imply that inhibiting the mitogen-activated protein kinase (MAPK)-extracellular signal-regulated kinase (ERK2) kinase signal pathway reverses a matrix metalloproteinase 1-specific   invasive   phenotype. Here, we investigated the specific genes downstream of MAPK-ERK2 responsible for the hyperproliferative abilities of human and murine primary ductal epithelial cells (PDCs) within an ECM. Compared with control, DNA synthesis and total cell proliferation was significantly increased in human PDCs harboring the PDAC common p53, Rb/p16(INK4a), and K-Ras (G12D) mutations. Both of these effects were readily reversed following small-molecule inhibition or lentiviral silencing of ERK2. Microarray analysis of PDCs in three-dimensional (3D) culture revealed a unique, MAPK-influenced gene signature downstream of K-Ras (G12D). Unbiased hierarchical analysis permitted filtration of tissue inhibitor of matrix metalloproteinase 1 (TIMP1). Pancreatic cells isolated from Pdx1-Cre; LSL-K-ras(G12D/+)-mutated mice exhibit increased TIMP1 RNA transcription compared to wild-type littermate controls. Analyses of both 3D, in vitro human K-Ras (G12D) PDCs and data mining of publicly annotated human pancreatic data sets correlatively indicate increased levels of TIMP1 RNA. While silencing TIMP1 did not significantly effect PDC proliferation, exogenous addition of human recombinant TIMP1 significantly increased proliferation but only in transformed K-Ras (G12D) PDCs in 3D. Overall, TIMP1 is an upregulated gene product and a proliferative inducer of K-Ras(G12D)-mutated PDCs through the ERK2 signaling pathway. 
 Imbalance between expression of matrix metalloproteinase-9 and tissue inhibitor of metalloproteinase-1 in   invasive  ness and metastasis of human gastric carcinoma. The expressive balance between matrix metalloproteinase-9 (MMP-9) and its tissue inhibitor of metalloproteinase-1 (TIMP-1) plays a critical role in maintaining the degradation and synthesis of extracellular matrix. Loss of such balance is associated with invasion and metastasis of tumors. This study aimed to determine the expression of MMP-9 and TIMP-1 in gastric carcinoma, and the association of the expressive imbalance between MMP-9 and TIMP-1 with the invasion and metastasis and prognosis of gastric carcinoma.We used immunohistochemistry to determine the expressions of MMP-9, TIMP-1 and proliferating cell nuclear antigen Ki-67 in the gastric specimens taken from 256 patients with primary gastric carcinoma. The patients were followed-up for up to 96 months.No association between the expression of MMP-9 and TIMP-1 and patients' sex and age, tumor size and location of gastric carcinoma was observed. The incidence of the positive expression of MMP-9 in cases with tumors invasion to muscularis propria and visceral peritoneum (70.13 % and 69.09 %, respectively) was significantly higher than that in cases with tumor invasion only to lamina propria or submucosa (42.50 %, P=0.0162). The positive correlation between MMP-9 expression and the depth of tumor invasion was observed (Pearson correlation coefficient=0.2129, P=0.016). Along with the increase of the metastatic station of lymph nodes, the incidence of the MMP-9 expression was increased by degrees; a positive correlation between them was observed (Pearson correlation coefficient=0.2910, P=0.0001). There was also a significant correlation between MMP-9 expression and the TNM stage in gastric carcinoma (Pearson correlation coefficient=0.3027, P<0.0001). The incidence of MMP-9 expression in stage II and III/IV (75.00 % and 76.15 %, respectively) was significantly higher than those in stage I (46.15 %, P<0.0001). A negative correlation between TIMP-1 immunoreactivity and the depth of invasion, status of lymph node metastasis and TNM stage was observed (Pearson correlation coefficient =-0.1688, -0.3556 and -0.3004, P=0.023, <0.0001 and <0.0001, respectively). Four types of co-expression of MMP-9 and TIMP-1 were observed; i.e. MMP-9 positive but TIMP-1 negative (n=115), both positive (n=52), both negative (n=62) and MMP-9 negative but TIMP-1 positive (n=27). The frequency of serosal   invasive  ness was significant higher in patients with MMP-9 but without TIMP-1 expression than those with other types of the co-expression (P=0.0303). The incidence of lymph node metastasis was highest in patients with MMP-9 but without TIMP-1 expression, and lowest in those with TIMP-1 but without MMP-9 expression (P<0.0001). The survival rate in patients with MMP-9 but without TIMP-1 expression was lower than that in those with TIMP-1 but without MMP-9 expression (P=0.0014).Our results in gastric carcinoma demonstrated a significant positive association of MMP-9 over-expression with proliferation of tumor cells, the depth of   invasive  ness, lymph node metastasis and TNM stage, suggesting MMP-9 can serve as a molecular marker of tumor invasion and metastasis. We also demonstrate a significant negative relationship of TIMP-1 expression with the depth of   invasive  ness and lymph node metastasis, which provide a new idea in the tumor biological and genetic treatment. The interaction between MMP-9 and TIMP-1 in the processes of tumor invasion and metastasis is that MMP-9 mainly promotes tumor invasion and metastasis and TIMP-1 inhibits functions of MMP-9. The imbalance between MMP-9 and TIMP-1 expression may suggest the occurrence of tumor invasion and metastasis, predict poor prognosis. For patients with imbalanced MMP-9 and TIMP-1 expression, the optimal treatment scheme needs to be selected. 
 Tissue inhibitor of matrix metalloproteinase-1 expression in colorectal cancer liver metastases is associated with vascular structures. Metastatic growth by colorectal cancer cells in the liver requires the ability of the cancer cells to interact with the new microenvironment. This interaction results in three histological growth patterns of liver metastases: desmoplastic, pushing, and replacement. In primary colorectal cancer several proteases, involved in the degradation of extracellular matrix components, are up-regulated. In liver metastases, their expression is growth pattern dependent. Tissue inhibitor of matrix metalloproteinase-1 (TIMP-1) is a strong prognostic marker in plasma from colorectal cancer patients, with significant higher levels in patients with metastatic disease. We therefore wanted to determine the expression pattern of TIMP-1 in primary colorectal cancers and their matching liver metastases. TIMP-1 mRNA was primarily seen in α-smooth-muscle actin (α-SMA)-positive cells. In all primary tumors and liver metastases with desmoplastic growth pattern, TIMP-1 mRNA was primarily found in α-SMA-positive myofibroblasts located at t  he invas  ive front. Some α-SMA-positive cells with TIMP-1 mRNA were located adjacent to CD34-positive endothelial cells, identifying them as pericytes. This indicates that TIMP-1 in primary tumors and liver metastases with desmoplastic growth pattern has dual functions; being an MMP-inhibitor at the cancer periphery and involved in tumor-induced angiogenesis in the pericytes. In the liver metastases with pushing or replacement growth patterns, TIMP-1 was primarily expressed by activated hepatic stellate cells at the metastasis/liver parenchyma interface. These cells were located adjacent to CD34-positive endothelial cells, suggesting a function in tumor-induced angiogenesis. We therefore conclude that TIMP-1 expression is growth pattern dependent in colorectal cancer liver metastases. 
 Nitric oxide-mediated invasion in Barrett's high-grade dysplasia and adenocarcinoma. Nitric oxide (NO) has been shown to induce double strand DNA breaks in Barrett's oesophagus (BO) and in other cancers has a role in invasion. The specific aims of this study were to investigate whether NO can induce invasion in cells representative of different stages of Barrett's progression and to determine possible underlying mechanisms. Physiological concentrations of NO that mimic luminal production of NO from dietary sources enhanced invasion in cell lines from high-grade dysplasia (GihTERT) and oesophageal adenocarcinoma (FLO) but not a non-dysplastic Barrett's cell line (QhTERT). Real-time reverse transcription-polymerase chain reaction revealed that NO induced expression of matrix metalloproteinase (MMP)-1, -3, -7, -9 and -10 and tissue inhibitor of metalloproteinase (TIMP)-1, -2 and -3 in these cell lines. Furthermore, ex vivo treatment of Barrett's biopsy samples with NO induced increases in MMP-1 and TIMP-1 expression, suggesting that NO enhances invasion through deregulating MMP and TIMP expression in epithelial cells. In keeping with these findings, microarray analysis and immunohistochemistry performed on biopsy samples showed enhanced expression of MMP-1, -3, -7 and -10 and TIMP-1 in the progression from non-dysplastic BO to adenocarcinoma, although this could not be directly attributed to the effect of NO. Thus, NO may play a role in Barrett's carcinogenesis through deregulating MMP and TIMP expression to enhance   invasive   potential. 
 Hyaluronan synthase 2 (HAS2) promotes breast cancer cell invasion by suppression of tissue metalloproteinase inhibitor 1 (TIMP-1). Invasion and metastasis are the primary causes of breast cancer mortality, and increased knowledge about the molecular mechanisms involved in these processes is highly desirable. High levels of hyaluronan in breast tumors have been correlated with poor patient survival. The involvement of hyaluronan in the early   invasive   phase of a clone of breast cancer cell line MDA-MB-231 that forms bone metastases was studied using an in vivo-like basement membrane model. The metastatic to bone tumor cells exhibited a 7-fold higher hyaluronan-synthesizing capacity compared with MDA-MB-231 cells predominately due to an increased expression of hyaluronan synthase 2 (HAS2). We found that knockdown of HAS2 completely suppressed the   invasive   capability of these cells by the induction of tissue metalloproteinase inhibitor 1 (TIMP-1) and dephosphorylation of focal adhesion kinase. HAS2 knockdown-mediated inhibition of basement membrane remodeling was rescued by HAS2 overexpression, transfection with TIMP-1 siRNA, or addition of TIMP-1-blocking antibodies. Moreover, knockdown of HAS2 suppressed the EGF-mediated induction of the focal adhesion kinase/PI3K/Akt signaling pathway. Thus, this study provides new insights into a possible mechanism whereby HAS2 enhances breast cancer invasion. 
 Immunohistochemical expression of matrix metalloproteinase-9 and inhibitor of matrix metalloproteinase-1 in prostate adenocarcinoma. An immunohistochemical study on the cells proliferative activity by Ki-67 protein and localization of the matrix metalloproteinase-9 and the inhibitor of matrix metalloproteinase-1 was carried out at the benign prostatic hyperplasia (BPH) and the adenocarcinoma (AC) of different Gleason's grades. A significant decrease of the MMP-9 and TIMP-1 level in the AC of different gradations was observed. A moderate positive correlation between the Gleason score and cell proliferation Ki-67 index (rs = 0.674) and a moderate negative correlation with the level of such a score and expression of MMP-9 (rs = -0.660) was detected. A weak negative correlation exists also between the level of proliferative activity of secretory cells and the expression of MMP-9 by tumor cells (rs = -0.369). The   invasive   properties of AC cells that promote a degradation of the basal membrane and connective tissue in prostate may be explained by the imbalance between the MMP-9 and TIMP-1, which expression is significantly reduced in AC, in comparison with BPH. 
 Staurosporine enhances the expression of tissue inhibitor of metalloproteinase-1 in human prostate cancer cells. We reported previously that human prostate cancer cell line TSU-Pr1 can differentiate into neuronal cells by staurosporine treatment. In this process, reduction of   invasive   abilities was observed in staurosporine treated TSU-Pr1 cells. In the present study, we investigated the effect of staurosporine on tissue inhibitor of metalloproteinases (TIMPs) in prostate cancer cells. We show that treatment of TSU-Pr1 cells with staurosporine results in induction of TIMP-1 mRNA and protein secretion. The induction of TIMP-1 mRNA expression by staurosporine is likely to be caused by increased transcriptional activity and this mechanism is indirect. Furthermore, recombinant human TIMP-1 reduces the   invasive   activity of TSU-Pr1 cells. We are the first to report that mRNA expression and protein secretion of TIMP-1 are enhanced by staurosporine treatment in prostate cancer cells. These findings suggest that enhancement of TIMP-1 is associated with suppression of   invasive   activity caused by staurosporine treatment. 
 Down-regulation of TIMP-1 inhibits cell migration, invasion, and metastatic colonization in lung adenocarcinoma. Tissue inhibitor metalloproteinase-1 (TIMP-1) is clinically associated with a poor prognosis for various cancers, but the roles of TIMP-1 in lung cancer metastasis are controversial. Our previous secretomic study revealed that TIMP-1 is highly abundant in high   invasive  ness cells of lung adenocarcinoma. In the current study, TIMP-1 abundances in primary lung adenocarcinoma tissues, as revealed by immunohistochemistry, are significantly higher in patients with lymph invasion and distant metastasis than in those without. Receiver operating characteristic curve analyses suggest 73.7 and 86.2 % accuracy to separate patients with lymph node and distant metastasis and those without, respectively. Moreover, we demonstrate that the expression level of TIMP-1 positively associates with cell mobility,   invasive  ness, and metastatic colonization. Most notably, the novel mechanism in which TIMP-1 facilitates metastatic colonization through the mediation of pericellular polyFN1 assembly was revealed. In summary, this study presents novel functions of TIMP-1 in promoting cancer metastasis and suggests TIMP-1 is a potential tissue biomarker for lymph invasion and distant metastasis of lung adenocarcinoma. 
 Gambogic acid inhibits invasion of osteosarcoma via upregulation of TIMP-1. Gambogic acid (GA), the natural product, has been demonstrated to be a promising chemotherapeutic drug for osteosarcoma (OS) due to its ability to induce apoptosis and cell cycle arrest. To date, no studies have examined the role of GA in metastatic bone disease. Matrix metalloproteinases (MMPs) play critical roles in invasion and metastasis, and the tissue inhibitors of metalloproteinase (TIMP) family regulates the activity of multifunctional metalloproteinases. In this study, we investigated the gene expression of matrix metalloproteinase-9 (MMP-9) and tissue inhibitor of matrix metalloproteinase-1 (TIMP-1) in OS cell lines treated by the GA. The expression of MMP-9 and TIMP-1 were studied by reverse transcription-polymerase chain reaction (RT-PCR) and western blotting. In vitro invasion of OS cell lines (Saos-2, MG-63) were investigated by the Matrigel invasion assay. Mean MMP-9 protein and mRNA expression was significantly suppressed; in addition, mean TIMP-1 protein mRNA expression were upregulated by increasing GA concentrations. GA reduced the   invasiv  eness of OS cell lines dose-dependently. Furthermore, specific inhibition of TIMP-1 secretion with siRNA against TIMP-1 significantly reduced the effect of GA on OS cell lines. Overall, our findings suggest that GA reduces the   invasiv  e potential of OS cells via attenuation of MMP-9 and upregulation of TIMP-1. Moreover, TIMP-1 played an important role in the reduction of   invasiv  e potential of the OS cells which were treated by GA. 
 Matrix metalloproteinase-9 and tissue inhibitor of metalloproteinase-1 in diagnosis of pleural effusion of malignant origin. The aim of the present study was to evaluate the diagnostic accuracy of matrix metalloproteinase-9 and tissue inhibitor of metalloproteinase-1 in differentiating benign from malignant exudative pleural effusions.This is a unicentre observational study including 97 consecutive patients with exudative pleural effusions. Metalloproteinase-9, tissue inhibitor of metalloproteinase-1, lactate dehydrogenase, ferritin, carcinoembryonic antigen and carbohydrate antigen 15-3 were measured in pleural effusion and serum by enzyme-linked immunosorbent assay. The activity of metalloproteinase-9 was also evaluated by substrate zymography. The data were correlated with final diagnosis of pleural effusions to evaluate the diagnostic accuracy.Of the 97 eligible patients, 6 were excluded. Of the 91 patients included in the study, 70 had malignant pleural effusions and 21 had benign pleural effusions. Both in sera and pleural effusions, matrix metalloproteinase-9 (P < 0.0001), tissue inhibitor of metalloproteinase-1 (P < 0.0001) and carcinoembryonic antigen (P < 0.0001) levels were higher in neoplastic patients than in benign group. Zymography analysis showed a most prominent band at a molecular weight of 92 kDa (metalloproteinase-9) whereas a less intense band was observed at 72 kDa (metalloproteinase-2). A significant correlation was found between metalloproteinase-9 and tissue inhibitor of metalloproteinase-1 levels in pleural effusion (P < 0.0001; r = 0.8) and serum (P < 0.03; r = 0.2). Pleural effusion metalloproteinase-9 and tissue inhibitor of metalloproteinase-1 levels showed higher value of sensitivity (97 and 91%, respectively) and specificity (90 and 95%, respectively) compared with other standard markers. Serum metalloproteinase-9 and tissue inhibitor of metalloproteinase-1 levels showed similar results. Among 70 neoplastic patients, 29 had negative pleural cytology. Of these, 25 presented elevated levels of metalloproteinase-9 and tissue inhibitor of metalloproteinase-1, whereas 4 patients had elevated levels of one of the two markers.Our results showed that metalloproteinase-9 and tissue inhibitor of metalloproteinase-1 might be valuable markers in differentiating benign from malignant pleural effusions. Their levels are neither influenced by the histology and tumour origin nor by the presence of tumour cells in pleural effusions. Thus, their use in clinical practice could help in the selection of patients needing more   invasive   procedures, such as thoracoscopic biopsy. 
 Signal transducers and activators of transcription-3 up-regulates tissue inhibitor of metalloproteinase-1 expression and decreases   invasive  ness of breast cancer. Signal transducers and activators of transcription (STAT)-3 is an oncogenic protein that is constitutively activated in many human cancers, including 30 to 60% of primary breast cancer. The biological significance of STAT3 activation in breast cancer is not fully understood. We have previously shown that STAT3 up-regulates tissue inhibitors of metalloproteinase (TIMP)-1, a cytokine known to block metalloproteinases and decrease   invasive  ness in certain cancer cell types. We hypothesize that STAT3 activation may modulate tumor   invasive  ness of breast cancer by regulating TIMP1 expression. Using MCF-7 cells transfected with tetracycline-off STAT3C (constitutively active STAT3), we generated an in vitro system in which STAT3C levels can be tightly controlled in breast cancer cells. Increasing tetracycline levels gradually decreased STAT3C and TIMP1 in a dose-dependent manner, and down-regulation of these proteins led to a reciprocal decrease in   invasive  ness of these cells in Matrigel. Addition of a neutralizing anti-TIMP1 antibody increased   invasive  ness in the same experimental system. Using immunohistochemistry and 142 primary breast tumors, we found a significant association between the expression of the phosphorylated/active form of STAT3 (pSTAT3) and that of TIMP1. Importantly, STAT3 activation correlated significantly with a lower frequency of vascular and lymphatic invasion (P = 0.015 and P = 0.0002, respectively). Our data support the concept that STAT3 activation significantly modulates the biological and clinical behavior of breast cancer. 
 Expression of matrix metalloproteinases (MMP-2 and -9) and tissue inhibitors of metalloproteinases (TIMP-1 and -2) in acute myelogenous leukaemia blasts: comparison with normal bone marrow cells. We compared the expression of matrix metalloproteinases (MMP-2 and MMP-9) and tissue inhibitors of metalloproteinases (TIMP-1 and TIMP-2) in bone marrow acute myelogenous leukaemia (AML) blasts and leukaemic cell lines (HEL, HL-60, K-562 and KG-1) with their expression in normal bone marrow cells. All AML samples and leukaemic cell lines tested expressed MMP-9 and/or MMP-2 mRNA and, accordingly, these gelatinases were secreted into media. Moreover, TIMP-1 and TIMP-2 mRNA and secreted proteins were demonstrated in all the AML samples. Although all the leukaemic cell lines expressed TIMP-1, the HL-60 cells also expressed TIMP-2. In contrast, normal steady-state bone marrow immature progenitor cells (CD34+ cells) did not express or secrete either MMP-2 or MMP-9, but more mature mononuclear cells from normal bone marrow expressed and secreted MMP-9. Also, normal bone marrow CD34+ cells and mononuclear cells expressed TIMP-1 and TIMP-2 mRNA, but these proteins were not detectable by reverse zymography. Furthermore, whereas bone marrow fibroblasts and endothelial cells secreted only latent MMP-2, the activated form of this enzyme was found in media conditioned by cells obtained from long-term cultures of normal and AML bone marrow adherent layers. Our finding of up-regulated production of gelatinases, TIMP-1 and TIMP-2 by leukaemic cells suggests that these proteins may be implicated in the   invasive   phenotype of AML. 
 The TIMP-1 gene transferred through adenovirus mediation shows a suppressive effect on peritoneal metastases from gastric cancer. It has become clear in recent years that peritoneal metastasis takes place as the result of a multistep process involving attachment, invasion, proliferation, and angiogenesis. The aim of the present study was to evaluate the suppressive effect of tissue inhibitor of metalloproteinase-1 (TIMP-1) gene transfer on peritoneal dissemination.We established a high-potential peritoneal metastasis cell line (MKN-45P), using the gastric cancer cell line MKN-45, and developed a peritoneal metastasis model in nude mice. The TIMP-1 gene was transferred to MKN-45 or MKN-45P by adenoviral transfection, and we performed an in vitro invasion assay and an in vivo study, using the peritoneal metastasis model. The TIMP-1 transfected group was compared with a non-virus group and a Lac-Z transfected group.The in vitro invasion assay showed that the number of   invasive   cells was significantly reduced in the TIMP-1 transfected group compared with that in the non-virus group and the Lac-Z transfected group, Moreover, the in vivo studies showed that the number and the weight of the peritoneal nodes in the TIMP-1 transfected group were significantly less than those in the Lac-Z transfected group, and less than those in the non-viral group. No bloody ascites was recognized in the TIMP-1 transfected group. The mean number of tumor vessels in the non-virus group and the Lac-Z group was significantly higher than that in the TIMP-1 group.TIMP-1 demonstrated an inhibitory effect on angiogenesis, and may be worthwhile investigating for use as a future therapy for peritoneal dissemination. 
 Expression of metalloproteinase-2, metalloproteinase-9, and tissue inhibitor of metalloproteinase-1 in transitional cell carcinoma of upper urinary tract: correlation with tumor stage and survival. To investigate the relationship between the expression of matrix metalloproteinase (MMP)-2, MMP-9, tissue inhibitor of metalloproteinase (TIMP)-1, and TIMP-2 and pT stage or survival in patients with transitional cell carcinoma of the upper urinary tract. MMP-2 and MMP-9 are associated with tumor invasion in several malignancies. TIMPs exert an anti-  invasive   effect by blocking MMP activity. Recent studies have shown, however, that TIMPs can also stimulate cell proliferation and angiogenesis.Tumor sections surgically removed from 91 patients were examined for expression of MMP-2, MMP-9, TIMP-1, and TIMP-2 by immunohistochemistry. We also determined the proliferation index and microvessel density in each tumor and investigated the independent roles of these factors in tumor stage and survival using multivariate analysis.Of 91 tissue samples, 50, 51, 45, and 39 were positive for MMP-2, MMP-9, TIMP-1, and TIMP-2 expression, respectively. Tumors positive for MMP-2, MMP-9, and TIMP-1 exhibited a greater proliferation index than tumors with negative expression (P <0.001, P = 0.013, and P <0.001, respectively). The microvessel density of tumors positive for MMP-2 and TIMP-1 was greater than that of negative tumors (P <0.001). The expression of MMP-2, MMP-9, and TIMP-1 was an independent predictor of high pT stage. Cox proportional hazard analysis identified TIMP-1 expression as an independent factor for cause-specific survival (odds ratio 5.2, P = 0.011), similar to microvessel density, pT4, and lymph node metastasis.TIMP-1 expression correlated with pT stage and was an independent predictor of cause-specific survival. Our results suggest that TIMP-1 expression is a potentially useful tool for the selection of postoperative observation strategies in patients with transitional cell carcinoma of the upper urinary tract. 
 [Pathologic study of expression and significance of matrix metalloproteinases-9, tissue inhibitor of metalloproteinase-1, vascular endothelial growth factor and transforming growth factor beta-1 in papillary carcinoma and follicular carcinoma of thyroid]. To study the roles of matrix metalloproteinases-9 (MMP-9), tissue inhibitor of metalloproteinase-1 (TIMP-1), vascular endothelial growth factor (VEGF) and transforming growth factor β-1 (TGFβ-1) in differentiation  , invasi  veness and metastatic potential of papillary carcinoma and follicular carcinoma of thyroid.Eighty-five cases of papillary thyroid carcinoma and 59 cases of follicular thyroid carcinoma were enrolled into the study. Immunohistochemistry using EnVision method was carried out for assessment of the expression of MMP-9, TIMP-1, VEGF and TGFβ-1 in the tumor tissue.MMP-9, TIMP-1, VEGF and TGFβ-1 were expressed in the cytoplasm of tumor cells. The positivity rates of MMP-9, TIMP-1, VEGF and TGFβ-1 in papillary thyroid carcinoma (83.5%, 81.2%, 90.6% and 75.3%, respectively) were similar to or lower than those in follicular thyroid carcinoma (93.2%, 86.4%, 89.9% and 78.0%, respectively). The expression rates in papillary thyroid carcinoma with lymph node metastasis were also higher than those in tumors without lymph node metastasis. The expression rates of MMP-9, VEGF and TGFβ-1 in poorly-differentiated follicular thyroid carcinoma were higher than those in well-differentiated follicular thyroid carcinoma. The expression of TIMP-1 however showed a negative correlation with the tumor cell differentiation. In general, the expression of VEGF and MMP-9 was higher than that of TIMP-1 and TGFβ-1 in papillary thyroid carcinoma and follicular thyroid carcinoma.Immunohistochemical detection of MMP-9, TIMP-1, VEGF and TGFβ-1 expression may carry clinical significance in evaluating the degree of different  iation,   invasiveness, metastatic potential and prognosis of papillary thyroid carcinoma and follicular thyroid carcinoma. 
 Increased expression of matrix metalloproteinase-9 in the eutopic endometrial tissue of women with endometriosis. Endometriosis is a disease where endometrial tissue implants in ectopic locations. Remodelling of the extracellular matrix (ECM) is a prerequisite for the implantation of this tissue to be possible.In this study, we detected immunoreactive matrix metalloproteinase-9 (MMP-9) throughout endometrial tissue and identified von Willebrand factor (vWF)-positive endothelial cells, CD45-positive leukocytes, CD3-positive T lymphocytes and CD68-positive macrophages as cells expressing MMP-9 in the stroma.We found an increased expression of MMP-9 in the uterine endometrial tissue of women with endometriosis, as assessed by zymography and enzyme-linked immunosorbent assay (ELISA) (P < 0.05). However, RT-PCR did not show a statistically significant increase in MMP-9 mRNA expression in these tissues (P = 0.14). There was no significant difference between women with and without endometriosis in the expression of tissue inhibitor of MMPs (TIMP)-1, a known natural inhibitor of the pro- and active forms of MMP-9, whether tested by ELISA or by RT-PCR (P = 0.46 and 0.37, respectively). Interestingly, the ratio of MMP-9/TIMP-1 expression was significantly higher in women with endometriosis than in normal women both at the protein and the mRNA levels (P < 0.05).These findings make plausible the involvement of MMP-9/TIMP-1 imbalance in the   invasive  ness of the endometrial tissue of patients with endometriosis and the ectopic development of the disease. 
 Thioredoxin stimulates MMP-9 expression, de-regulates the MMP-9/TIMP-1 equilibrium and promotes MMP-9 dependent invasion in human MDA-MB-231 breast cancer cells. Increased expression of thioredoxin (Trx)-1 and matrix metalloproteinase (MMP)-9 associates with malignant breast cancer progression. Here, we describe a functional relationship between Trx-1 and MMP-9 in promoting MDA-MB-231 breast cancer cell   invasive   behaviour. Trx-1 overexpression stimulated MMP-9 expression, de-regulated the MMP-9/TIMP-1 equilibrium and augmented MMP-9 involvement in a more   invasive   phenotype. Trx-1 augmented MMP-9 transcription through NF-κB, AP-1 and SP1 elements; stimulated p50/p65 NF-κB activity and recruitment to the MMP-9 promoter; and facilitated MMP-9 promoter-accessibility to NF-κB by preventing HDAC recruitment and maintaining MMP-9 promoter histone acetylation. Our data provide a functional basis for Trx-1 and MMP-9 association in malignant breast cancer and identify Trx-1 and NF-κB as potentially druggable targets for reducing MMP-9 involvement in malignant behaviour. 
 Hypoxia induces FGF2 production by vascular endothelial cells and alters MMP9 and TIMP1 expression in extravillous trophoblasts and their   invasive  ness in a cocultured model. The role of fibroblast growth factor 2 (FGF2) secretion by vascular endothelial cells during trophoblast invasion was assessed. The human extravillous trophoblast cell line, TEV-1, and umbilical vein endothelial cell line, HUVE-12, were cocultured under normal and hypoxic conditions. FGF2 expression in HUVE-12 cells and matrix metalloproteinase 9 (MMP9) and tissue inhibitor of metalloproteinase 1 (TIMP1) expression in TEV-1 cells were analyzed using quantitative RT-PCR and Western blot analyses. TEV-1 cell invasion was also examined. FGF2 expression in the HUVE-12 cells cocultured with TEV-1 cells was significantly increased under hypoxic conditions. In the TEV-1 cells cocultured with HUVE-12, hypoxia reduced MMP9 expression and increased TIMP1 expression; it also reduced cell invasion by 43%. However, the expression of MMP9 and TIMP1 and ratio of MMP9/TIMP1 were increased when the TEV-1 cells were cultured alone under hypoxic conditions. These findings suggest that FGF2 release by stressed endothelial cells of uterine spiral arteries play roles in decreasing MMP9 and increasing TIMP1 production in extravillous trophoblasts (EVT) in response to stress, resulting in reduced EVT invasion and possibly shallow implantation of the placenta. 
 Association of a high activity of matrix metalloproteinase-9 to low levels of tissue inhibitors of metalloproteinase-1 and -3 in human hepatitis B-viral hepatoma cells. Matrix metalloproteinases (MMPs) play a major role in the turnover of extracellular matrix (ECM) during cancer invasion and metastasis, and tissue inhibitors of metalloproteinases (TIMPs) control MMPs, thus maintaining a balanced ECM catabolism under physiological conditions. The aim of this study was to assess the behavior of some MMPs (FASEB J., 7, 1993, 1434; Cancer Metastasis Rev., 9(4) 1990, 289) and TIMPs (Biochem. Biophys. Res. Commun., 301, 2003, 1069; FASEB J., 7, 1993, 1434; Nature, 370, 1994, 61). Competitive RT-PCR, gelatin-substrate zymography, and ELISA techniques were used for quantification. The hepatitis B virus (HBV)-DNA-containing hepatocellular carcinoma cell lines, Hep3B, HepG2-HBV and HFF-T2 contain highly activated matrix metallproteinase-9 (MMP-9), which is rarely found in normal liver cell lines such as the Chang lines. MMP-9 activities of HFH-T2, HepG2-HBV and Hep3B were significantly higher than that of non-HBV-hepatocellular carcinoma SK-Hep1 and HepG2 (HCC origin, HBV not detected), as assayed by gelatin zymography. Low levels of TIMP-1 and TIMP-3 were observed in HFH-T2, HepG2-HBV and Hep3B, while the TIMP-2 level was high, as evidenced by reverse zymography. In contrast, 3 TIMP-1, -2 and -3 were largely detected in Chang, HepG2 and SK-Hep1 cells. To investigate the nature of the quantitative regulation of MMPs and TIMPs for these cell lines at the transcriptional levels, a reverse transcriptase-polymerase chain reaction (RT-PCR) was carried out. Not only MMP-9 mRNAs of HFH-T2, HepG2-HBV and Hep3B but also MMP-9 mRNA of SK-Hep1 and HepG2 were highly expressed with no major differences among these four cell lines. However, TIMP-1 and TIMP-3 mRNAs of HFH-T2, HepG2-HBV and Hep3B were markedly reduced, while those of SK-Hep1, HepG2 and Chang cells were maintained at high levels. Finally, an invasion assay using matrigel indicated in an increase in   invasive  ness in HFH-T2, HepG2-HBV and Hep3B cells, but a decrease in   invasive  ness of Chang, HepG2 and SK-Hep1 cells. These results indicate that the overexpression of MMP-9 mRNAs and the suppression of TIMP-1 and TIMP-3 in HFH-T2, HepG2-HBV and Hep3B were the result of HBV transfection. Based on these results, it is concluded that HBV affects the malignance of hepatocellular cancer by elevating MMP-9 activity, and suppressing TIMP-1 and TIMP-3. 
 Immunohistochemical study of matrix metalloproteinase 9 and tissue inhibitor of matrix metalloproteinase 1 in benign and malignant breast tissue--strong expression in intraductal carcinomas of the breast. Matrix metalloproteinases (MMPs) are involved in carcinogenesis due to their tissue remodeling capability, and there is convincing evidence linking gelatinase B (MMP-9) with malignant cell invasion. Tissue inhibitor 1 of MMP (TIMP-1) is a strong inhibitor of MMP-9 but has also tumor-enhancing effects. Only few data exist on MMP-9 or TIMP-1 expression in tissue samples of different breast histology.MMP-9 and TIMP-1 immunoreactivity was examined in a wide range of breast tissue samples differing in histology from usual ductal hyperplasia (UDH) to fully developed ductal breast carcinoma. Immunohistochemical expression of MMP-9 was studied in 178 samples: 31 UDH samples, 29 atypical ductal hyperplasia (ADH) samples, 28 ductal carcinoma in situ (DCIS) samples and 90 ductal   invasive   carcinoma samples (30 samples of malignancy grades I, II and III, respectively). TIMP-1 expression was also analyzed in 178 breast tissue samples: 41 UDH, 21 ADH and 34 DCIS lesions, and 82   invasive   ductal breast carcinomas (25 in grade I, 30 in grade II and 27 in grade III).A significantly distinctive pattern of MMP-9 protein expression was shown in DCIS samples, where 85.7% of the cases showed moderate or strong positivity and negative staining was rare (p = 0.021). Negative or weakly positive MMP-9 staining was the most prominent finding in UDH (71%), ADH (69%) as well as in   invasive   carcinoma samples (64.4%). Various degrees of TIMP-1 expression were seen in 86.5% of all cases. DCIS and   invasive   carcinoma samples revealed similar immunostaining: at least some positivity was seen in 91.1% of the DCIS samples and 91.5% of infiltrative carcinomas. Thus, TIMP-1 negativity (22.2%) was significantly associated with hyperplastic lesions (p = 0.026).These results suggest that MMP-9 and TIMP-1 overexpression are early markers of breast carcinogenesis preceding tumor invasion. Apparently, DCIS carries the risk to evolve into a malignant phenotype according to these markers. The clinical importance of these findings is discussed. 
 Expression of matrix metalloproteinase-1, -9, -13, and tissue inhibitor of metalloproteinases-1 in basal cell carcinomas of the eyelid. Matrix metalloproteinases (MMPs) and tissue inhibitors of metalloproteinases (TIMPs) function in the remodelling of the extracellular matrix in morphogenesis, angiogenesis, tissue repair, and tumor invasion. Elevated levels of distinct MMPs in tumor tissue are related to worse prognosis. However, no overall consistent pattern of expression in human cancer has been identified. The aim of the present study was to evaluate the expression of MMP-1, -9, -13 and TIMP-1 in tumor epithelial cells and surrounding connective tissue in primary basal cell carcinomas (BCC) of the eyelid, and to assess their role as prognostic markers for tumor recurrence.Surgical specimens of 49 histologically proven primary BBCs of the eyelid of different histological subtypes were included. Immunohistological studies were performed using antibodies against MMP-1, MMP-9, MMP-13 and TIMP-1, and staining intensity was analyzed semi-quantitatively.MMP-1, -9, -13, and TIMP-1 were expressed at various intensities in epithelial tumor cells and surrounding stromal cells including fibroblasts, inflammatory cells, and vascular endothelial cells in all tumor subtypes. Staining was especially prominent at the invading edge of the BCC. A statistically significant correlation was seen between increased TIMP-1 expression in tumor and/or stromal cells with the presence of MMP-13 (p = 0.007 and p < 0.0001 respectively). Moreover, TIMP-1 expression in tumor and/or stroma was significantly associated with relapse (p = 0.012 and p = 0.042 respectively).MMP-9, MMP-13 and TIMP-1 expression may serve as a prognostic marker   for earl  y tumor invasiveness. Moreover, up-regulation of TIMP-1 in tumor and/or surrounding stromal cells may indicate an increased risk for BCC recurrence. 
 A novel mechanism of tissue inhibitor of metalloproteinases-1 activation by interleukin-1 in primary human astrocytes. Reactive astrogliosis is the gliotic response to brain injury with activated astrocytes and microglia being the major effector cells. These cells secrete inflammatory cytokines, proteinases, and proteinase inhibitors that influence extracellular matrix (ECM) remodeling. In astrocytes, the expression of tissue inhibitor of metalloproteinases-1 (TIMP-1) is up-regulated by interleukin-1 (IL-1), which is a major neuroinflammatory cytokine. We report that IL-1 activates TIMP-1 expression via both the IKK/NF-kappaB and MEK3/6/p38/ATF-2 pathways in astrocytes. The activation of the TIMP-1 gene can be blocked by using pharmacological inhibitors, including BAY11-7082 and SB202190, overexpression of the dominant-negative inhibitor of NF-kappaB (IkappaBalphaSR), or by the knock-down of p65 subunit of NF-kappaB. Binding of activated NF-kappaB (p50/p65 heterodimer) and ATF-2 (homodimer) to two novel regulatory elements located -2.7 and -2.2 kb upstream of the TIMP-1 transcription start site, respectively, is required for full IL-1-responsiveness. Mutational analysis of these regulatory elements and their weak activity when linked to the minimal tk promoter suggest that cooperative binding is required to activate transcription. In contrast to astrocytes, we observed that TIMP-1 is expressed at lower levels in gliomas and is not regulated by IL-1. We provide evidence that the lack of TIMP-1 activation in gliomas results from either dysfunctional IKK/NF-kappaB or MEK3/6/p38/ATF-2 activation by IL-1. In summary, we propose a novel mechanism of TIMP-1 regulation, which ensures an increased supply of the inhibitor after brain injury, and limits ECM degradation. This mechanism does not function in gliomas, and may in part explain the increased   invasive  ness of glioma cells. 
 TIMP-1 overexpression in lung carcinoma enhances tumor kinetics and angiogenesis in brain metastasis. Tissue inhibitors of matrix metalloproteinase (TIMP) orchestrate many biologic activities, including inhibition of matrix metalloproteinase activity, activation of pro-matrix metalloproteinases, and regulation of cell proliferation, angiogenesis, and apoptosis induction. Tissue inhibitors of matrix metalloproteinase can play a protective role during tumor invasion and metastasis, but elevated TIMP messenger RNA levels have also been associated with aggressive cancers and poor clinical outcome. We examined the potential roles of TIMP-1 in H2009 lung adenocarcinoma cells and in cells transfected with a human TIMP-1-overexpressing vector (HB-6 and HB-1). Tumors resulting from the implantation of parental cell lines and transfected HB-1 cells into the brains of nude mice had a typical carcinoma profile, but human TIMP-1-overexpressing tumors showed enhanced tumor kinetics and focally more infiltrative features; vessel density assessed with anti-CD31 immunohistochemistry was also greater within HB-1 tumor implants. Similar effects on HB-6 and HB-1 cells versus parental cell lines and empty vector clones were observed in endothelial cell assays. Anchorage-independent growth and invasion through Matrigel were also increased in TIMP-1-overexpressing cells. Together, these results indicate tumor-promoting functions of TIMP-1 through alterations in angiogenesis, increased tumorigenicity, and   invasive   behavior. Although matrix metalloproteinase inhibition has been the traditionally identified function of TIMP-1, matrix metalloproteinase-independent interactions may contribute to the growth of metastatic carcinomas in the brain. 
 Regulating effect of MMP-9 and TIMP-1 in pituitary adenoma invasion. Pituitary adenomas can cause endocrine disorder and organ damage, with some aggressive ones leading to a high postoperative recurrence rate. The occurrence and development of these type of tumors is closely related with matrix metalloproteinases (MMPs) and endogenous specific tissue inhibitor of MMPs (TIMPs). In this study, the relationship between pituitary adenoma invasion and the changes in MMP-8 and TIMP-1 expressions is analyzed. Specimens from sixty patients with pituitary adenoma were collected in our hospital after surgery, including thirty cases of   invasive   pituitary adenomas and thirty cases of non  invasive   pituitary adenomas. Western blotting and real-time PCR were used to detect MMP-8/TIMP-1 protein and mRNA levels, respectively, in the two types of pituitary adenomas, while ELISA was used to detect both compounds' levels in the patient's serum. Compared with non  invasive   pituitary adenomas, MMP-8 was significantly overexpressed in   invasive   pituitary adenomas, while TIMP-1 was obviously lower (P < 0.05 for both). Moreover, MMP-8 mRNA expression in   invasive   pituitary adenomas was significantly higher than in non  invasive   pituitary adenomas, while TIMP-1 mRNA expression was markedly lower (P < 0.05 for both). Finally, MMP-8 expression in the serum is upregulated in patients with   invasive   pituitary adenomas relative to the non  invasive   ones, and the expression of TIMP-1 significantly reduced (P < 0.05 for both). These results show that increased MMP-8 and decreased TIMP-1 expressions are closely related to the   invasive   pituitary adenoma, and can be helpful for the evaluation. 
 Invasion front-specific overexpression of tissue inhibitor of metalloproteinase-1 in liver metastases from colorectal cancer. Matrix metalloproteinases (MMPs) and their inhibitors, the tissue inhibitors of metalloproteinases (TIMPs), have been implicated in invasion and metastasis. The distribution of MMPs and TIMPs in the invasion front of liver metastases from colorectal cancer were investigated in order to understand their potential role in   invasive  ness.Freshly frozen material of colorectal metastases of the liver was microdissected into four separate compartments, namely pure liver, liver invasion, tumour invasion and pure tumour. RNA was isolated and analyzed on Affymetrix microarrays. Expression of TIMP-1 was confirmed by quantitative polymerase chain reaction in 10 colorectal liver metastases. Cellular localisation of TIMP-1 was examined by immunohistochemistry.Affymetrix microarray data revealed that several MMP and TIMP genes including MMP-2, -3, -7, -9, -11, -12, -14, -15, -16, -19 and -24, and TIMP-1, -2 and -3 were generally up-regulated in both invasion front compartments. Among these genes, TIMP-1 showed the highest expression. The qPCR results indicated an average 15-fold upregulation of TIMP-1 in the liver   invasive   front and an average 13-fold up-regulation in the tumor   invasive   front, each compared to normal liver tissue. Immunohistochemistry revealed expression of TIMP-1 in tumour epithelia as well as in host tissue cells, including fibroblastic cells.Our data indicate that tumour invasion in colorectal liver metastasis is associated with increased TIMP-1 RNA and protein levels in both tumour and host cells. 
 Leukemia inhibitory factor increases the   invasive  ness of trophoblastic cells through integrated increase in the expression of adhesion molecules and pappalysin 1 with a concomitant decrease in the expression of tissue inhibitor of matrix metalloproteinases. To identify the invasion-associated molecules during leukemia inhibitory factor-(LIF-)mediated increase in the invasion of trophoblast cells.Experimental study.Research institution.None.Cultured trophoblastic HTR-8/SVneo cells were treated with LIF.Matrigel matrix-based invasion assay of HTR-8/SVneo cells. Signaling molecules associated with LIF-mediated increase in invasion were investigated by Western blot, cDNA microarray, quantitative reverse transcriptase polymerase chain reaction, immunofluorescence, immunohistochemistry, and gene silencing by siRNA.Treatment of HTR-8/SVneo cells with LIF (50 ng/mL) led to a significant increase in invasion. Treatment with LIF also led to an increase in nuclear localization of activated STAT1 and STAT3. Among 237 differentially expressed genes after LIF treatment, expression of pappalysin 1, SERPINB3, podoplanin, integrin β3, ID1, ICAM1, and so on went up, while tissue inhibitor of matrix metalloproteinase 1 (TIMP1), TIMP2, and TIMP3 went down significantly. The presence of several of these proteins has also been demonstrated in human trophoblast cells. Silencing of pappalysin 1 led to a significant reduction in basal as well as LIF-mediated   invasiv  eness of HTR-8/SVneo cells.Identification of novel molecules associated with a LIF-mediated increase in trophoblastic cell invasion may facilitate our understanding of implantation biology. 
 The role of serum biomarkers in predicting fibrosis progression in pediatric and adult hepatitis C virus chronic infection. Liver biopsy represents the gold standard for damage evaluation, but non  invasive   serum markers that mirror liver fibrosis progression are actual goals both in adults and especially in children. The aim was to determine specific serum markers that correlate with liver fibrosis progression during chronic HCV infection.Liver biopsies and concomitant serum samples from 22 pediatric and 22 adult HCV patients were analyzed. Histological parameters were evaluated. On serum TGF-ß1, tissue inhibitor of matrix metalloprotein inhibitor-1 (TIMP-1), hyaluronic acid (HA) and aminoterminal peptide of procollagen type III (PIIINP) were tested.Significant fibrosis (F≥2) and advanced fibrosis (F≥3) represented 64% and 20%, respectively in children; while 54% F≥2 and 23% F≥3 in adults. Hyaluronic acid (p = 0.011) and PIIINP (p = 0.016) were related to worse fibrosis stages only in adults, along with TIMP-1 (p = 0.039) just in children; but TGF-ß1 was associated with mild fibrosis (p = 0.022) in adults. The AUROC of TIMP-1 in children to discriminate advanced fibrosis was 0.800 (95%IC 0.598-0.932). In adults, the best AUROCs were that of HA, PIIINP and TGF-ß1 [0.929 (IC95% 0.736-0.994), 0.894 (IC95% 0.689-0.984) and 0.835 (IC95% 0.617-0.957)], respectively. In children, according to the cut off (165.7 ng/mL) value for TIMP-1, biopsies could have been avoided in 72% (18/25). Considering the cut off for HA (109.7 ng/mL), PIIINP (9.1 µg/L), and TGF-ß1 (10,848.3 pg/mL), biopsies could have been avoided in 87% (19/22) of adult patients by using HA and 73% (16/22) using PIIINP or TGF-ß1.In adults given the diagnostic accuracy of HA, PIIINP, TGF-ß1, their combination may provide a potential useful tool to assess liver fibrosis. This first pediatric study suggests that TIMP-1 is clinically useful for predicting liver fibrosis in HCV patients. 
 Effects of recombinant H2 relaxin on the expression of matrix metalloproteinases and tissue inhibitor metalloproteinase in cultured early placental extravillous trophoblasts. Relaxin promotes softening of the uterine cervix and inhibits uterine contractility in rats, mice and pigs. Little information, however, is available about the role of relaxin in humans. In 2002, LGR7 and LGR8 were discovered to be receptors for relaxin and those receptors were identified in the human placenta. Thus, in this study, effects of recombinant H2 (rH2) relaxin on human early placental extravillous trophoblasts (EVTs) were examined. Isolation of EVTs from early placental trophoblasts was performed using the procedures established in our laboratory. After 48-h subculture, the presence of relaxin receptors in cultured EVTs was characterized by RT-PCR and immunoblotting. The cultured EVTs were treated with different doses (0.3-3 ng/ml) of rH2 relaxin for 24 h. The effects of rH2 relaxin on MMP-2, -3, -9 and TIMP-1 mRNAs levels were examined by real-time RT-PCR. RT-PCR and immunoblotting revealed that relaxin receptors are present in early placental EVTs. Treatment with rH2 relaxin increased MMP-2 and -9 mRNAs levels and decreased TIMP-1 mRNA levels in cultured EVTs, whereas rH2 relaxin did not affect MMP-3 mRNA levels. These results suggest that relaxin may promote the   invasive   potential of early placental EVTs through up-regulating MMP-2, -9 mRNAs and down-regulating TIMP-1 mRNA in EVTs. 
 Imbalance between MMP-2, 9 and TIMP-1 promote the invasion and metastasis of renal cell carcinoma via SKP2 signaling pathways. S-phase kinase-associated protein-2 (Skp2) is overexpressed in human cancers and acted as an oncogenic protein associated with poor prognosis by enhancing tumor metastasis. The present study has demonstrated that Skp2 overexpresses stable transfectants from 786-0 human renal cancer cells. We found that these stable transfectants exhibited increased migratory and   invasive   abilities. In addition, expression of matrix metalloproteinase-2 (MMP-2) and MMP-9 was upregulated and tissue inhibitor of matrix metalloproteinase-1 (TIMP-1) was downregulated. In contrast, RNA interference-mediated knockdown Skp2 expression suppressed the ability of ACHN cells to migratory and   invasive  . Skp2 depletion increased P27 and decreased cyclin E activity, and then induced cell cycle arrest in the G0/G1 phase. Skp2 depletion also downregulated MMP-2 and MMP-9, while upregulated the TIMP-1 activity and expression. The results suggest that Skp2 signaling pathways promoted the ability to metastasize, by stimulating cell proliferation and increasing the ratio of MMP-2 and MMP-9/TIMP-1. So, in conclusion, we provide the first evidence that the imbalance of MMP/TIMP, including upregulation of MMP-2 and MMP-9 and downregulation of TIMP-1, is one of the mechanisms by which Skp2 promotes cell invasion. 
 Increased matrix metalloproteinase-2 and tissue inhibitor of metalloproteinase-1 secretion but unaffected   invasive  ness of endometrial stromal cells in adenomyosis. To investigate cell   invasive  ness and the production of matrix metalloproteinase-2 (MMP-2), MMP-9, tissue inhibitor of metalloproteinase-1 (TIMP-1) and TIMP-2 in endometrial stromal cells (ESCs) of adenomyosis.Prospective study.Tertiary university hospital.Twenty-one women with adenomyosis (study group) and 25 women without adenomyosis (control group).Endometrial stromal cells were purified from eutopic endometrium and were cultured in vitro.Matrigel invasion assay and measurement of MMP-2, MMP-9, TIMP-1, and TIMP-2 concentrations.Endometrial stromal cell   invasive  ness of adenomyosis was not different from that of the control group (0.95 vs. 1, using the latter as the bench mark) in ESCs cultured alone. After the ESCs were cultured with IL-6, anti-IL-6, or GM6001 for 24 hours, the indices of cell invasion were not different between the two groups. MMP-2 (median 7.3 vs. 3.5 ng/mL) and TIMP-1 (median 12.1 vs. 3.9 ng/mL), but not MMP-9 and TIMP-2, secreted by ESCs in women with adenomyosis were much higher than those in women without adenomyosis.The formation of adenomyosis does not result from altered   invasive  ness of ESCs. The concomitant elevation of MMP-2 and TIMP-1 may partially justify why   invasive  ness was not increased, but other enzymes should also be considered. 
 Inhibition of cancer cell invasion by cannabinoids via increased expression of tissue inhibitor of matrix metalloproteinases-1. Cannabinoids, in addition to having palliative benefits in cancer therapy, have been associated with anticarcinogenic effects. Although the antiproliferative activities of cannabinoids have been intensively investigated, little is known about their effects on tumor invasion.Matrigel-coated and uncoated Boyden chambers were used to quantify   invasive  ness and migration, respectively, of human cervical cancer (HeLa) cells that had been treated with cannabinoids (the stable anandamide analog R(+)-methanandamide [MA] and the phytocannabinoid delta9-tetrahydrocannabinol [THC]) in the presence or absence of antagonists of the CB1 or CB2 cannabinoid receptors or of transient receptor potential vanilloid 1 (TRPV1) or inhibitors of p38 or p42/44 mitogen-activated protein kinase (MAPK) pathways. Reverse transcriptase-polymerase chain reaction (RT-PCR) and immunoblotting were used to assess the influence of cannabinoids on the expression of matrix metalloproteinases (MMPs) and endogenous tissue inhibitors of MMPs (TIMPs). The role of TIMP-1 in the anti-  invasive   action of cannabinoids was analyzed by transfecting HeLa, human cervical carcinoma (C33A), or human lung carcinoma cells (A549) cells with siRNA targeting TIMP-1. All statistical tests were two-sided.Without modifying migration, MA and THC caused a time- and concentration-dependent suppression of HeLa cell invasion through Matrigel that was accompanied by increased expression of TIMP-1. At the lowest concentrations tested, MA (0.1 microM) and THC (0.01 microM) led to a decrease in invasion (normalized to that observed with vehicle-treated cells) of 61.5% (95% CI = 38.7% to 84.3%, P < .001) and 68.1% (95% CI = 31.5% to 104.8%, P = .0039), respectively. The stimulation of TIMP-1 expression and suppression of cell invasion were reversed by pretreatment of cells with antagonists to CB1 or CB2 receptors, with inhibitors of MAPKs, or, in the case of MA, with an antagonist to TRPV1. Knockdown of cannabinoid-induced TIMP-1 expression by siRNA led to a reversal of the cannabinoid-elicited decrease in tumor cell   invasive  ness in HeLa, A549, and C33A cells.Increased expression of TIMP-1 mediates an anti-  invasive   effect of cannabinoids. Cannabinoids may therefore offer a therapeutic option in the treatment of highly   invasive   cancers. 
 [Expression of matrix metalloproteinase-1 and tissue inhibitor of metalloproteinase-1 in human and nude mouse ectopic endometrium and the effect of estrogen and progestin on their expression]. To explore the roles of matrix metalloproteinase-1(MMP-1) and tissue inhibitor of metalloproteinase-1(TIMP-1) in the pathogenesis of endometriosis and the effects of estrogen and progestin on their expression.Immunohistochemistry and RT-PCR were employed to detect the expression of MMP-1 and TIMP-1 in the ectopic tissues of 35 patients with endometriosis, 22 eutopic endometrium tissues from women with endometriosis and 28 normal controls. Fifty-nine nude mice were injected with human late secretory endometrial chippings and randomized into estrogen group, progestin group, estrogen-progestin group and control group with corresponding treatments. The implantation rates and graft morphology were observed and MMP-1 and TIMP-1 expressions in the grafts detected by immunohistochemistry.Typical endometrial glands and stroma were observed in all the groups with comparable implantation rates. The administration of progestin was associated with multiple peritoneal implantation sites and significantly larger implants. The transplanted endometria showed proliferative or secretory changes with estrogen or progestin administration. MMP-1 expression significantly increased and TIMP-1 expression decreased with increased MMP-1/TIMP-1 ratio in human and nude mouse ectopic endometria in comparison with those in normal endometria (P<0.05, P<0.01). MMP-1 expression was higher in estrogen and estrogen-progestin groups than in the control group, and was lower in the 3 sexual hormone-treated groups than in the control group. MMP-1 mRNA expression in the eutopic endometrium was significantly higher than that in the normal endometria.Progestrin can not inhibit MMP-1 expression or the effect of estrogen on ectopic endometrium known as progestin resistance. The high expression of MMP-1 and low expression of TIMP-1 in endometriotic tissues confer strong   invasive  ness of ectopic endometrial tissue, especially in eutopic endometrial tissue, and may play an important role in the pathogenesis of endometriosis. 
 [Relationship of expression unbalance of matrix metalloproteinase and tissue inhibitor of metalloproteinase to   invasive  ness and metastasis in gastric carcinomas]. Matrix metalloproteinase-9 (MMPs) and its tissue inhibitor of metalloproteinase-1 (TIMPs) have the important regulatory roles in the processes during tumor invasion and metastasis. Observing the coexpression of matrix metalloproteinase 9(MMP-9) and tissue inhibitor of metalloproteinase 1(TIMP-1) in gastric carcinomas, this study was designed to investigate the relationship of the expression unbalance of MMP-9 and TIMP-1 to the   invasive  ness, metastasis, and prognosis in gastric carcinomas.By using immunohistochemistry, the expression of MMP-9, TIMP-1, and proliferating cell nuclear antigen Ki-67 was detected in 256 gastric cancers, and the patients were followed-up.The expressive degree of MMP-9 in the tumor invasion beyond musculariis propria(70.13%) was higher than that within lamina propria or submucosa(42.50%, P < 0.05) and its expression in tumor was positively associated with tumor cell proliferation index and lymph node metastasis, and its expression in high TNM stage(75.00%) was higher than those in low TNM stage(46.15%, P < 0.05). The relation of the expression of TIMP-1 to the depth of invasion, lymph node metastasis, and TNM stage was negative(Pearson contingency coefficient were 0.1688, 0.3556, and 0.3004, respectively P < 0.05). There were four groups of the unbalance expression of MMP-9 and TIMP-1 in gastric carcinomas. While the frequency of the serosa invasion and lymph node metastasis in the group of the expression of MMP-9 over TIMP-1 was significantly higher than those in the groups of the expression of TIMP-1 over MMP-9 or the balance expression of MMP-9 and TIMP-1. However, prognosis of the patients with the expression of MMP-9 over TIMP-1 was significantly worse than that with expression of TIMP-1 over MMP-9(P < 0.05).The expression of MMP-9 was positively related to the tumor   invasive  ness and metastasis in gastric cancers, however the association between the positive expression of TIMP-1 and the   invasive  ness and metastasis was negative. The relationship of MMP-9 and TIMP-1 to the   invasive  ness and metastasis in gastric carcinomas was that MMP-9 promotes and TIMP-1 inhibits tumor metastasis. The patients with MMP-9 positive expression and TIMP-1 negative expression are poorer prognosis. 
 Urinary Emmprin, matrix metalloproteinase 9 and tissue inhibitor of metalloproteinase 1 as potential biomarkers in children with ureteropelvic junction narrowing on conservative treatment. Rapid diagnosis and initiation of the treatment on congenital obstructive nephropathy are important for young children to slow down renal injury. The aim of our study was to investigate the role of urinary extracellular matrix metalloproteinase inducer (Emmprin), matrix metalloproteinase 9 (MMP-9) and tissue inhibitor of metalloproteinase 1 (TIMP-1) in the long-term follow-up of children with ureteropelvic junction (UPJ) narrowing on conservative treatment.The study included 40 children with non-obstructed hydronephrosis due to unilateral UPJ narrowing who were treated conservatively and followed up for 24 months. Voided urine samples were collected at diagnosis and at 3, 9, 15 and 24 months of follow-up, respectively. Three enzymes concentrations were measured in urine.During the follow-up, 25 children showed renal function stabilization (non-obstructed group) and 15 children renal function deterioration (obstructed group). In the non-obstructed group, a comparison between urine three enzymes levels at the last follow-up and at baseline showed no significant differences (all P > 0.05). Glomerular filtration rate (GFR) and split renal function (SRF) showed similar trends. In the obstructed group, a comparison between the three enzymes levels at diagnosis and at basal condition showed a significant increase (all P < 0.01). But GFR and SRF showed a marked reduction at diagnosis (all P < 0.001). Receiver operator characteristic (ROC) analyses revealed a better diagnostic profile for uEmmprin, uMMP-9 and uTIMP-1 in identifying children with abnormal SRF (<40%) at 24 months of follow-up [area under the curve (AUC) 0.877, 0.727 and 0.823, respectively].Urinary Emmprin, MMP-9 and TIMP-  1 may be   noninvasive potential biomarkers that could be used for long-term follow-up of children with UPJ narrowing on conservative treatment to determine those who might develop obstruction. 
  GeneRIFs  
 These results show that increased MMP-8 and decreased TIMP-1 expressions are closely related to the   invasive   pituitary adenoma, and can be helpful for the evaluation. 
 Urinary Emmprin, MMP-9 and TIMP-1 may be non  invasive   potential biomarkers that could be used for long-term follow-up of children with UPJ narrowing on conservative treatment to determine those who might develop obstruction. 
 Report imbalance between MMP-9 and TIMP-1 levels in prostatic adenocarcinoma which may contribute to neoplasm   invasive  ness. 
 The aim of our study was to compare the immunohistochemical expression of TIMP1 and TIMP2 between an uncontrollably   invasive   phenomenon (cancer) and an "in situ" process (trophoblast invasion) 
 the increase of MMP-1 and MMP-9 expression and low of TIMP-1 and TIMP-2 expression makes the main contribution to the destructive (  invasive  ) potential of SCC. 
 Gambogic acid reduces the   invasive   potential of osteosarcoma cells via attenuation of MMP-9 and upregulation of TIMP-1. 
 MMP-9, MMP-13 and TIMP-1 expression may serve as a prognostic marker for early tumor   invasive  ness. Moreover, up-regulation of TIMP-1 in tumor and/or surrounding stromal cells may indicate an increased risk for BCC recurrence. 
 The high expression of MMP-1 and low expression of TIMP-1 in endometriotic tissues confer strong   invasive  ness of ectopic endometrial tissue, and may play an important role in the pathogenesis of endometriosis. 
 Formation of adenomyosis does not result from altered   invasive  ness of endometrial stromal cells, but may result in elevation of MMP-2 and TIMP-1. 
 results suggest that relaxin may promote the   invasive   potential of early placental EVTs through up-regulating MMP-2, -9 mRNAs and down-regulating TIMP-1 mRNA in EVTs. 
 Increased expression of TIMP-1 mediates an anti-  invasive   effect of cannabinoids. Cannabinoids may therefore offer a therapeutic option in the treatment of highly   invasive   cancers. 
 enhancement of TIMP-1 is associated with suppression of prostate neoplasm   invasive  ness caused by staurosporine treatment 
 evidence that cell density influences the   invasive   potential of tumor cells via regulation of MMPs and TIMP-1 and TIMP-2 by AP-1, NF kappa B and CRE transcription factors 
 These findings make plausible the involvement of MMP-9/TIMP-1 imbalance in the   invasive  ness of the endometrial tissue of patients with endometriosis and the ectopic development of the disease. 
 the association between the positive expression of TIMP-1 and the   invasive  ness and metastasis was negative. 
  [Mm]etastasize  PubMed  
 Diagnostic significance of TIMP-1 level in serum and its immunohistochemical expression in colorectal cancer patients. Tissue inhibitor of metalloproteinase-1 (TIMP-1) inhibits the ability of cancer cells to   metastasize  , but it can also stimulate cancer development. The aim of this study was to assess the level of TIMP-1 in serum and its expression in patients with colorectal cancer (CRC). The study group consisted of 43 patients diagnosed with colorectal cancer and 24 healthy volunteers. The level of TIMP-1 was assessed by the ELISA method while the expression of this protein was performed immunohistochemically. The concentration of TIMP-1 in the sera of colorectal cancer patients was significantly higher than in the healthy control group (p = 0.004). Higher level of TIMP-1 in the sera correlated with female gender (p = 0.045), tumor location in colon (p = 0.016), poorly differentiated tumor (p = 0.034) and higher platelet count in whole blood (p < 0.004). A positive reaction of the protein in cancer cells was observed in 31 cases and was found to correlate negatively with its reaction in peritumoral stroma (p < 0.001). According to this study, TIMP-1 protein may play an important role in cancer development. The assessment of this molecule in serum and tissue can be useful at the time of diagnosis and can help us to understand the nature of colorectal pathogenesis. 
 Imbalance between MMP-2, 9 and TIMP-1 promote the invasion and metastasis of renal cell carcinoma via SKP2 signaling pathways. S-phase kinase-associated protein-2 (Skp2) is overexpressed in human cancers and acted as an oncogenic protein associated with poor prognosis by enhancing tumor metastasis. The present study has demonstrated that Skp2 overexpresses stable transfectants from 786-0 human renal cancer cells. We found that these stable transfectants exhibited increased migratory and invasive abilities. In addition, expression of matrix metalloproteinase-2 (MMP-2) and MMP-9 was upregulated and tissue inhibitor of matrix metalloproteinase-1 (TIMP-1) was downregulated. In contrast, RNA interference-mediated knockdown Skp2 expression suppressed the ability of ACHN cells to migratory and invasive. Skp2 depletion increased P27 and decreased cyclin E activity, and then induced cell cycle arrest in the G0/G1 phase. Skp2 depletion also downregulated MMP-2 and MMP-9, while upregulated the TIMP-1 activity and expression. The results suggest that Skp2 signaling pathways promoted the ability to   metastasize  , by stimulating cell proliferation and increasing the ratio of MMP-2 and MMP-9/TIMP-1. So, in conclusion, we provide the first evidence that the imbalance of MMP/TIMP, including upregulation of MMP-2 and MMP-9 and downregulation of TIMP-1, is one of the mechanisms by which Skp2 promotes cell invasion. 
  [Mm]etastasis  PubMed  
 Tissue inhibitor of metalloproteinase 1 is an independent predictor of prognosis in patients with nonsmall cell lung carcinoma who undergo resection with curative intent. Matrix metalloproteinases (MMPs) and tissue inhibitors of metalloproteinases (TIMPs) play a role in the processes of extracellular matrix degradation. Changes in their expression levels have been observed in various tumor types, including lung carcinoma. However, their clinical significance and their prognostic importance in the progression of nonsmall cell lung carcinoma (NSCLC) remain to be specified. In this study, mRNA expression levels of MMP-1, MMP-9, TIMP-1, and TIMP-2 were evaluated in patients with resected NSCLC, and their associations with disease progression and prognosis were determined.Between June 1996 and December 1999, 116 patients underwent resection for NSCLC. Expression levels of MMPs and TIMPs were evaluated using Northern blot analysis in these NSCLC tissue samples and in 39 matched samples of normal lung tissue.MMP-1, MMP-9, and TIMP-1 expression levels were increased in tumor samples compared with matched, corresponding normal tissues. In contrast, TIMP-2 expression was decreased in tumor samples. MMP-1 tumor expression was correlated significantly with the evolution of lymph node status and tumor-lymph node-  metastasis   (TNM) stage. In contrast, MMP-9 tumor expression was correlated significantly with increased T stage. TIMP-1 overexpression was an independent predictor of worse survival in patients with NSCLC that was not associated with other prognosis factors, such as TNM stage.The overexpression of TIMP-1 was an independent prognostic marker in patients with NSCLC, and evaluating TIMP-1 may be important for identifying patients who are at greater risk of disease recurrence. 
 Expression of metalloproteases and their inhibitors in primary tumors and in local recurrences after mastectomy for breast cancer. To investigate the expression of matrix metalloproteases (MMPs) and their inhibitors (TIMPs) in patients who develop local recurrence (LR) after mastectomy.We analyzed the expressions of MMP-1, -2, -7, -9, -11, -13, -14, TIMP-1, -2, and -3, using immunohistochemical techniques, in primary tumors from patients without tumoral recurrence (n = 50), patients who developed distant   metastasis   (n = 50), and from patients who develop LRs (n = 25). LRs of the latter group were also analyzed for MMPs expression. All the patients underwent mastectomy.Score values for all MMPs and TIMPs were significantly higher in primary tumors of patients with distant   metastasis  . Primary tumors from patients with LR have lower expressions of MMPs and TIMPs compared with those from patients who developed distant   metastasis  , and with patients without recurrence for some MMPs. Remarkably, however, primary tumors from patients with LR showed significantly higher percentage of TIMP-1 and 2 expression in stromal cells compared to primary tumors from patients with distant   metastasis   or primary tumors from patients without tumoral progression. Furthermore, LRs had significantly higher MMP-9 expression than their corresponding primary tumors.Our data indicate differences in MMPs/TIMPs expression between primary tumors of patients with LRs and of those with distant   metastasis  , both after mastectomy for breast cancer. 
 Timp1 interacts with beta-1 integrin and CD63 along melanoma genesis and confers anoikis resistance by activating PI3-K signaling pathway independently of Akt phosphorylation. Anoikis resistance is one of the abilities acquired along tumor progression. This characteristic is associated with   metastasis   development, since tumorigenic cells must survive independently of cell-matrix interactions in this process. In our laboratory, it was developed a murine melanocyte malignant transformation model associated with a sustained stressful condition. After subjecting melan-a melanocytes to 1, 2, 3 and 4 cycles of anchorage impediment, anoikis resistant cells were established and named 1C, 2C, 3C and 4C, respectively. These cells showed altered morphology and PMA independent cell growth, but were not tumorigenic, corresponding to pre-malignant cells. After limiting dilution of 4C pre-malignant cells, melanoma cell lines with different characteristics were obtained. Previous data from our group showed that increased Timp1 expression correlated with anoikis-resistant phenotype. Timp1 was shown to confer anchorage-independent growth capability to melan-a melanocytes and render melanoma cells more aggressive when injected into mice. However, the mechanisms involved in anoikis regulation by Timp1 in tumorigenic cells are not clear yet.The β1-integrin and Timp1 expression were evaluated by Western blotting and CD63 protein expression by flow cytometry using specific antibodies. To analyze the interaction among Timp1, CD63 and β1-integrin, immunoprecipitation assays were performed, anoikis resistance capability was evaluated in the presence or not of the PI3-K inhibitors, Wortmannin and LY294002. Relative expression of TIMP1 and CD63 in human metastatic melanoma cells was analyzed by real time PCR.Differential association among Timp1, CD63 and β1-integrins was observed in melan-a melanocytes, 4C pre-malignant melanocytes and 4C11- and 4C11+ melanoma cells. Timp1 present in conditioned medium of melanoma cells rendered melan-a melanocytes anoikis-resistant through PI3-K signaling pathway independently of Akt activation. In human melanoma cell lines, in which TIMP1 and beta-1 integrin were also found to be interacting, TIMP1 and CD63 levels together was shown to correlate significantly with colony formation capacity.Our results show that Timp1 is assembled in a supramolecular complex containing CD63 and β1-integrins along melanoma genesis and confers anoikis resistance by activating PI3-K signaling pathway, independently of Akt phosphorylation. In addition, our data point TIMP1, mainly together with CD63, as a potential biomarker of melanoma. 
 Role of matrix metalloproteinase (MMP) 2 and MMP-9 in soft tissue sarcoma. We investigated the expression of matrix metalloproteinases (MMPs) and tissue inhibitors of metalloproteinases (TIMPs) in malignant fibrous histiocytoma (MFH), and determined whether these could be useful as prognostic factors.Among patients treated from 1993 to 2007, 30 cases of MFH were evaluated. Immunohistochemical staining was performed for MMP-2, MMP-9, TIMP-1, and TIMP-2 using paraffin wax-embedded blocks of MFH tissues. Reverse transcriptase polymerase chain reaction (RT-PCR) and Western blot and zymography were performed using fresh tissues obtained from 17 of the 30 cases. The levels of MMP and TIMP expression were compared between the MFH and normal control groups, and between non-metastatic and metastatic MFH groups.Expression levels of MMP-2, MMP-9, TIMP-1, and TIMP-2 were higher in the MFH group than the control group by RT-PCR, Western blotting, and zymography. Immunohistochemical staining revealed that MMP-2 and MMP-9 protein expression was higher in the metastatic than in the non-metastatic group. The expression levels of MMP-2 and TIMP-1 were significantly higher in the metastatic than in the non-metastatic group (p < 0.05) by RT-PCR. By Western blot analysis, the expression levels of MMP-2, TIMP-1, and TIMP-2 were higher in the metastatic group (p < 0.05), but MMP-9 showed only a slight increase in the metastatic group compared with the non-metastatic group (p > 0.05). Finally, gelatin zymography analysis showed that the expression levels of the pro- and active forms of MMP-2 were significantly higher in the metastatic group (p < 0.05), but the expression of the pro- and active forms of MMP-9 showed a slight decrease in the metastatic group (p > 0.05).These results suggest that MMP-2, MMP-9, TIMP-1, and TIMP-2 may have important roles in the development and progression of MFH, and that the degree of expression of these metalloproteinases and their inhibitors, especially MMP-2, could be useful as prognostic factors related to   metastasis   in MFH. 
 The role of matrix metalloproteinases (MMPs) and their inhibitors (TIMPs) in the development of esophageal cancer. Esophageal cancer (EC) is one of the most aggressive malignant tumors of the gastrointestinal tract. There are two distinct histological types of EC: esophageal squamous cell carcinoma and adenocarcinoma of the esophagus. Etiologic factors and the patterns of incidence of both subtypes are different. Matrix metalloproteinases (MMPs) and their tissue inhibitors (TIMPs) play an important role in esophageal carcinogenesis. Gellatinases MMP-2 and MMP-9 are able to degrade collagen IV from basement membranes and extracellular matrix which is related to tumor progression, including invasion,   metastasis  , growth and angiogenesis. It has been shown that increased expression of MMPs plays a crucial role in the development of several human malignancies, including esophageal cancer. The activity of MMPs is regulated by their endogenous natural inhibitors (TIMPs). Among these, the roles of TIMP-1 and TIMP-2 in EC development, tumor progression and formation of metastases have been most extensively characterized and best recognized. 
 Expression of matrix metalloproteinases MMP-2, MMP-9 and their tissue inhibitors TIMP-1 and TIMP-2 in the epithelium and stroma of salivary gland pleomorphic adenomas. The balance between matrix metalloproteinases (MMPs) and their tissue inhibitors (TIMPs) is involved in the morphogenesis of normal salivary gland as well as in the mechanisms of tumour invasion and   metastasis  . The role of MMPs and TIMPs in pleomorphic adenoma has not been elucidated sufficiently. Our aim was to analyse the mRNA and protein expression of MMP-2, MMP-9, TIMP-1 and TIMP-2 in the epithelium and stroma of pleomorphic adenoma and to evaluate their roles.In each sample from six patients, cells from the epithelium and stroma were obtained by laser microdissection. The mRNA expression of MMPs and TIMPs was determined by real-time quantitative reverse transcriptase-polymerase chain reaction and protein expression was confirmed by immunohistochemistry. Results showed that mRNA expression of MMPs and TIMPs was significantly higher in stroma than in epithelium in most patients. MMPs and TIMPs were immunoreactive mainly in epithelium rather than in stroma.Our results provide preliminary evidence that stromal myoepithelium may be the primary source of MMPs and that the stroma has the potential to play a more important role than ductal epithelium in biological behaviour of pleomorphic adenomas. These findings seem worthy of further investigation. 
 [Expression of matrix metalloproteinase-2, 9 and their inhibitor-TIMP 1,2 in human squamous cell carcinoma of uterine cervix]. Studies of tumor invasion and   metastasis   have focused on the degradation of extracellular matrix (ECM). Matrix metalloproteinases (MMPs), which could degrade ECM, were implicated in cancer invasion and   metastasis  . Activated MMPs were controlled by specific tissue inhibitors of metalloproteinases (TIMPs). This study was designed to investigate the expression of MMP-2, MMP-9 and TIMP-1, TIMP-2 in human squamous cell carcinomas of uterine cervix, and the association with invasion and   metastasis   of human squamous cell carcinoma of uterine cervix.Tissue samples from 40 cases of squamous cell carcinoma of the uterine cervix, 29 cases of cervical intraepithelial neoplasia (CIN), and 16 cases of normal cervices were stained immunohistochemically.Immunohistochemical staining of tumor cells for MMP-2, MMP-9, TIMP-1, and TIMP-2 were noted 82.5%, 70.0%, 62.5%, and 97.5% in carcinomas, 44.8%, 62.1%, 86.21%, and 96.55% in CIN lesions respectively, for MMP-2, MMP-9, TIMP-1 in 25.0% normal cervices, but for TIMP-2 in 56.25% normal cervices. There was significant difference for MMP-2 among carcinomas, normal cervices, and CIN respectively (P < 0.01). There was significant difference for MMP-9 among normal cervices, carcinomas, and CIN, respectively (P < 0.05). The positive rates of TIMP-1 and TIMP-2 in the cervical carcinoma group and CIN group were obviously higher than that in normal cervical group(P < 0.01). The positive rates of MMP-2 and MMP-9 in lymph node   metastasis   were obviously higher than that in without lymph node   metastasis  (P < 0.01), but for TIMP-2 was contrary and there was no significant difference for TIMP-1. No significant correlation could be established among the expression of these markers and the tumor diameter, the clinical stage, and pathologic grade.The over-expression of MMP-2, MMP-9, TIMP-1, and TIMP-2 may play a key role in invasion and lymph-node   metastasis   of in squamous carcinoma of the cervix. 
 [Interstitial collagenase, gelatinases A and B and their endogenous inhibitors in squamous cell cervical carcinomas]. Interstitial collagenase and gelatinases are matrix metalloproteinases (MMP), which play the key role in tumor invasion and   metastasis  . The aim of this study was to elucidate the peculiarities of expression of interstitial collagenase (MMP-1), gelatinases A and B (MMP-2 and MMP-9) and their endogenous tissue inhibitors TIMP-1 and TIMP-2 as invasive factors of squamous cell carcinomas (SCC) of human cervical cancer. The study was carried out using 24 specimens of SCC and 11 specimens of adjacent to tumor morphologically normal tissue. All carcinoma specimens expressed E7 HPV-16 gene. It was shown that the increase of MMP-1 and MMP-9 expression and low of TIMP-1 and TIMP-2 expression makes the main contribution to the destructive (invasive) potential of SCC. The change of MMP-2 expression is not so significant and it is less influenced to the destructive potential. Moreover, substantial expression of MMP-1, MMP-2 and MMP-9 was registered in the specimens of morphologically normal adjoining to tumor tissue. This expression was found to make an additional contribution to the destructive potential of cervical tumor. 
 Comparative study of the immunohistochemical expression of tissue inhibitors of metalloproteinases 1 and 2 between clearly invasive carcinomas and "in situ" trophoblast invasion. Tissue inhibitors of metalloproteinases (TIMPs) play an important role in extracellular matrix homeostasis by regulating MMP activity. Although they were initially considered inhibitors of tumor growth and   metastasis  , recently their role in cancer progression has been controversial. The aim of our study was to compare the immunohistochemical expression of TIMP1 and TIMP2 between an uncontrollably invasive phenomenon (cancer) and an "in situ" process (trophoblast invasion) in an effort to assess any differential role of these molecules between these two distinct phenomena and therefore to understand better their contribution in cancer invasion and migration. We performed an immunohistochemical analysis of 50 carcinomas (colorectal, gastric, breast, pulmonary, and renal) and 40 first trimester gestations. The marker expression was evaluated semiquantitatively, separately in cancer parenchymal and trophoblastic cells as well as in malignant stromal and decidual cells, according to a percentage scale (0, <10, 10-50, and >50%) and according to staining intensity (0, +, ++, and +++). Our results showed that there was no statistically significant difference in TIMP1 expression between cancer parenchymal cells and trophoblastic cells. On the other hand, TIMP1 was expressed more often in decidual cells than in cancer stromal cells. Immunostaining for TIMP2 was more extensive and intense both in trophoblastic and decidual cells than in cancer parenchymal and stromal cells, respectively. The reduced expression of TIMP2 in metastatic carcinomas by comparison with non-metastatic gestation specimens underlines its importance in cancer invasion and migration. On the other hand, TIMP1 was more expressed in decidua than cancer stroma, but at the same time showed no statistically significant difference between cancer parenchyma and trophoblasts, highlighting its multifunctional activity in cancer progression. 
 [Expression and significance of matrix metalloproteinase and tissue inhibitor of matrix metalloproteinase in non-melanoma skin cancer]. To investigate the expression and significance of matrix metalloproteinases (MMP-2, MMP-9) and tissue inhibitor of matrix metalloproteinase (TIMP-2, TIMP-1) in non-melanoma skin cancer (NMSC).Thirty six patients with squamous cell carcinoma (SCC) and 32 patients with basal cell carcinoma (BCC), confirmed by pathology, were selected, and 30 cases of normal skin were selected as control. The expression of MMP-2, MMP-9, TIMP-1 and TIMP-2 in all samples were examined by immunohistochemistry and reverse transcription polymerase chain reaction (RT-PCR). The expression rate, expression intensity and expression level of each factor were recorded. The results were compared between the groups.The expression rates of MMP-2 and MMP-9 in the control group were 30.0% and 36.7%, the expression levels of MMP-2 and MMP-9 in the control group were 57.216 ± 12.785 and 59.318 ± 13.262, all significantly lower than those in the tumor edge and center of the SCC and BCC groups (P < 0.01). The expression rates of TIMP-1 and TIMP-2 in the control group were 96.7% and 100%, their expression levels were 121.738 ± 25.516 and 122.612 ± 25.964, all significantly higher than those in the SCC and BCC groups (P < 0.01). The expression levels of MMP-2 and MMP-9 in the tumor center and edge of SCC group were significantly higher than those in the corresponding parts of the BCC group, while the expression levels of TIMP-1 and TIMP-2 were significantly lower than those in the BCC group (P < 0.01). The expression levels of MMP-2 and MMP-9 in the tumor edge of the SCC and BCC groups were significantly higher than those in the tumor centers (P < 0.01), while the expression levels of TIMP-1and TIMP-2 were significantly lower than those in the tumor centers (P < 0.01).MMP-2, MMP-9 and TIMP-2, TIMP-1 may play an important role in the development, progression, invasion   and metast  asis of non-melanoma skin cancer. 
 Mesenchymal stem cells inhibit breast cancer cell migration and invasion through secretion of tissue inhibitor of metalloproteinase-1 and -2. Mesenchymal stem cells (MSCs) form part of tumor stroma, and are typically considered to be pro-tumorigenic, promoting continuing tumor growth and   metastasis  . Here, we describe a mechanism by which MSCs may be anti-tumorigenic, through inhibition of breast cancer cell migration and invasion, an important part of   metastasis  . MDA-MB-231 and T47D cells were co-cultured in a Transwell insert above MSCs or MSC conditioned media and their migration or invasion through Matrigel measured. The conditioned media was found to inhibit breast cancer cell movement. TIMP-1 and TIMP-2, inhibitors of matrix metalloproteinases (MMPs), were identified as candidates for this inhibition and enhanced secretion of MMPs was not sufficient to counter the anti-migratory effects of TIMP expression. Inhibition of TIMP activity showed that TIMP-1 and TIMP-2 together were largely responsible for the reduction of migration and invasion by MSCs. Therefore, MSCs may play anti-tumorigenic, anti-metastatic roles in tumor development, with the overall outcome dependent upon the balance of pro-and anti-tumorigenic molecules secreted. 
 Mesenchymal stem cells inhibit breast cancer cell migration and invasion through secretion of tissue inhibitor of metalloproteinase-1 and -2. Mesenchymal stem cells (MSCs) form part of tumor stroma, and are typically considered to be pro-tumorigenic, promoting continuing tumor growth and   metastasis  . Here, we describe a mechanism by which MSCs may be anti-tumorigenic, through inhibition of breast cancer cell migration and invasion, an important part of   metastasis  . MDA-MB-231 and T47D cells were co-cultured in a Transwell insert above MSCs or MSC conditioned media and their migration or invasion through Matrigel measured. The conditioned media was found to inhibit breast cancer cell movement. TIMP-1 and TIMP-2, inhibitors of matrix metalloproteinases (MMPs), were identified as candidates for this inhibition and enhanced secretion of MMPs was not sufficient to counter the anti-migratory effects of TIMP expression. Inhibition of TIMP activity showed that TIMP-1 and TIMP-2 together were largely responsible for the reduction of migration and invasion by MSCs. Therefore, MSCs may play anti-tumorigenic, anti-metastatic roles in tumor development, with the overall outcome dependent upon the balance of pro-and anti-tumorigenic molecules secreted. 
 Role of matrix metalloproteinase (MMP) 2 and MMP-9 in soft tissue sarcoma. We investigated the expression of matrix metalloproteinases (MMPs) and tissue inhibitors of metalloproteinases (TIMPs) in malignant fibrous histiocytoma (MFH), and determined whether these could be useful as prognostic factors.Among patients treated from 1993 to 2007, 30 cases of MFH were evaluated. Immunohistochemical staining was performed for MMP-2, MMP-9, TIMP-1, and TIMP-2 using paraffin wax-embedded blocks of MFH tissues. Reverse transcriptase polymerase chain reaction (RT-PCR) and Western blot and zymography were performed using fresh tissues obtained from 17 of the 30 cases. The levels of MMP and TIMP expression were compared between the MFH and normal control groups, and between non-metastatic and metastatic MFH groups.Expression levels of MMP-2, MMP-9, TIMP-1, and TIMP-2 were higher in the MFH group than the control group by RT-PCR, Western blotting, and zymography. Immunohistochemical staining revealed that MMP-2 and MMP-9 protein expression was higher in the metastatic than in the non-metastatic group. The expression levels of MMP-2 and TIMP-1 were significantly higher in the metastatic than in the non-metastatic group (p < 0.05) by RT-PCR. By Western blot analysis, the expression levels of MMP-2, TIMP-1, and TIMP-2 were higher in the metastatic group (p < 0.05), but MMP-9 showed only a slight increase in the metastatic group compared with the non-metastatic group (p > 0.05). Finally, gelatin zymography analysis showed that the expression levels of the pro- and active forms of MMP-2 were significantly higher in the metastatic group (p < 0.05), but the expression of the pro- and active forms of MMP-9 showed a slight decrease in the metastatic group (p > 0.05).These results suggest that MMP-2, MMP-9, TIMP-1, and TIMP-2 may have important roles in the development and progression of MFH, and that the degree of expression of these metalloproteinases and their inhibitors, especially MMP-2, could be useful as prognostic factors related to   metastasis   in MFH. 
 Timp1 interacts with beta-1 integrin and CD63 along melanoma genesis and confers anoikis resistance by activating PI3-K signaling pathway independently of Akt phosphorylation. Anoikis resistance is one of the abilities acquired along tumor progression. This characteristic is associated with   metastasis   development, since tumorigenic cells must survive independently of cell-matrix interactions in this process. In our laboratory, it was developed a murine melanocyte malignant transformation model associated with a sustained stressful condition. After subjecting melan-a melanocytes to 1, 2, 3 and 4 cycles of anchorage impediment, anoikis resistant cells were established and named 1C, 2C, 3C and 4C, respectively. These cells showed altered morphology and PMA independent cell growth, but were not tumorigenic, corresponding to pre-malignant cells. After limiting dilution of 4C pre-malignant cells, melanoma cell lines with different characteristics were obtained. Previous data from our group showed that increased Timp1 expression correlated with anoikis-resistant phenotype. Timp1 was shown to confer anchorage-independent growth capability to melan-a melanocytes and render melanoma cells more aggressive when injected into mice. However, the mechanisms involved in anoikis regulation by Timp1 in tumorigenic cells are not clear yet.The β1-integrin and Timp1 expression were evaluated by Western blotting and CD63 protein expression by flow cytometry using specific antibodies. To analyze the interaction among Timp1, CD63 and β1-integrin, immunoprecipitation assays were performed, anoikis resistance capability was evaluated in the presence or not of the PI3-K inhibitors, Wortmannin and LY294002. Relative expression of TIMP1 and CD63 in human metastatic melanoma cells was analyzed by real time PCR.Differential association among Timp1, CD63 and β1-integrins was observed in melan-a melanocytes, 4C pre-malignant melanocytes and 4C11- and 4C11+ melanoma cells. Timp1 present in conditioned medium of melanoma cells rendered melan-a melanocytes anoikis-resistant through PI3-K signaling pathway independently of Akt activation. In human melanoma cell lines, in which TIMP1 and beta-1 integrin were also found to be interacting, TIMP1 and CD63 levels together was shown to correlate significantly with colony formation capacity.Our results show that Timp1 is assembled in a supramolecular complex containing CD63 and β1-integrins along melanoma genesis and confers anoikis resistance by activating PI3-K signaling pathway, independently of Akt phosphorylation. In addition, our data point TIMP1, mainly together with CD63, as a potential biomarker of melanoma. 
 Comparative study of the immunohistochemical expression of tissue inhibitors of metalloproteinases 1 and 2 between clearly invasive carcinomas and "in situ" trophoblast invasion. Tissue inhibitors of metalloproteinases (TIMPs) play an important role in extracellular matrix homeostasis by regulating MMP activity. Although they were initially considered inhibitors of tumor growth and   metastasis  , recently their role in cancer progression has been controversial. The aim of our study was to compare the immunohistochemical expression of TIMP1 and TIMP2 between an uncontrollably invasive phenomenon (cancer) and an "in situ" process (trophoblast invasion) in an effort to assess any differential role of these molecules between these two distinct phenomena and therefore to understand better their contribution in cancer invasion and migration. We performed an immunohistochemical analysis of 50 carcinomas (colorectal, gastric, breast, pulmonary, and renal) and 40 first trimester gestations. The marker expression was evaluated semiquantitatively, separately in cancer parenchymal and trophoblastic cells as well as in malignant stromal and decidual cells, according to a percentage scale (0, <10, 10-50, and >50%) and according to staining intensity (0, +, ++, and +++). Our results showed that there was no statistically significant difference in TIMP1 expression between cancer parenchymal cells and trophoblastic cells. On the other hand, TIMP1 was expressed more often in decidual cells than in cancer stromal cells. Immunostaining for TIMP2 was more extensive and intense both in trophoblastic and decidual cells than in cancer parenchymal and stromal cells, respectively. The reduced expression of TIMP2 in metastatic carcinomas by comparison with non-metastatic gestation specimens underlines its importance in cancer invasion and migration. On the other hand, TIMP1 was more expressed in decidua than cancer stroma, but at the same time showed no statistically significant difference between cancer parenchyma and trophoblasts, highlighting its multifunctional activity in cancer progression. 
 [Interstitial collagenase, gelatinases A and B and their endogenous inhibitors in squamous cell cervical carcinomas]. Interstitial collagenase and gelatinases are matrix metalloproteinases (MMP), which play the key role in tumor invasion and   metastasis  . The aim of this study was to elucidate the peculiarities of expression of interstitial collagenase (MMP-1), gelatinases A and B (MMP-2 and MMP-9) and their endogenous tissue inhibitors TIMP-1 and TIMP-2 as invasive factors of squamous cell carcinomas (SCC) of human cervical cancer. The study was carried out using 24 specimens of SCC and 11 specimens of adjacent to tumor morphologically normal tissue. All carcinoma specimens expressed E7 HPV-16 gene. It was shown that the increase of MMP-1 and MMP-9 expression and low of TIMP-1 and TIMP-2 expression makes the main contribution to the destructive (invasive) potential of SCC. The change of MMP-2 expression is not so significant and it is less influenced to the destructive potential. Moreover, substantial expression of MMP-1, MMP-2 and MMP-9 was registered in the specimens of morphologically normal adjoining to tumor tissue. This expression was found to make an additional contribution to the destructive potential of cervical tumor. 
 [Expression and significance of matrix metalloproteinase and tissue inhibitor of matrix metalloproteinase in non-melanoma skin cancer]. To investigate the expression and significance of matrix metalloproteinases (MMP-2, MMP-9) and tissue inhibitor of matrix metalloproteinase (TIMP-2, TIMP-1) in non-melanoma skin cancer (NMSC).Thirty six patients with squamous cell carcinoma (SCC) and 32 patients with basal cell carcinoma (BCC), confirmed by pathology, were selected, and 30 cases of normal skin were selected as control. The expression of MMP-2, MMP-9, TIMP-1 and TIMP-2 in all samples were examined by immunohistochemistry and reverse transcription polymerase chain reaction (RT-PCR). The expression rate, expression intensity and expression level of each factor were recorded. The results were compared between the groups.The expression rates of MMP-2 and MMP-9 in the control group were 30.0% and 36.7%, the expression levels of MMP-2 and MMP-9 in the control group were 57.216 ± 12.785 and 59.318 ± 13.262, all significantly lower than those in the tumor edge and center of the SCC and BCC groups (P < 0.01). The expression rates of TIMP-1 and TIMP-2 in the control group were 96.7% and 100%, their expression levels were 121.738 ± 25.516 and 122.612 ± 25.964, all significantly higher than those in the SCC and BCC groups (P < 0.01). The expression levels of MMP-2 and MMP-9 in the tumor center and edge of SCC group were significantly higher than those in the corresponding parts of the BCC group, while the expression levels of TIMP-1 and TIMP-2 were significantly lower than those in the BCC group (P < 0.01). The expression levels of MMP-2 and MMP-9 in the tumor edge of the SCC and BCC groups were significantly higher than those in the tumor centers (P < 0.01), while the expression levels of TIMP-1and TIMP-2 were significantly lower than those in the tumor centers (P < 0.01).MMP-2, MMP-9 and TIMP-2, TIMP-1 may play an important role in the development, progression, invasion   and metast  asis of non-melanoma skin cancer. 
 The role of matrix metalloproteinases (MMPs) and their inhibitors (TIMPs) in the development of esophageal cancer. Esophageal cancer (EC) is one of the most aggressive malignant tumors of the gastrointestinal tract. There are two distinct histological types of EC: esophageal squamous cell carcinoma and adenocarcinoma of the esophagus. Etiologic factors and the patterns of incidence of both subtypes are different. Matrix metalloproteinases (MMPs) and their tissue inhibitors (TIMPs) play an important role in esophageal carcinogenesis. Gellatinases MMP-2 and MMP-9 are able to degrade collagen IV from basement membranes and extracellular matrix which is related to tumor progression, including invasion,   metastasis  , growth and angiogenesis. It has been shown that increased expression of MMPs plays a crucial role in the development of several human malignancies, including esophageal cancer. The activity of MMPs is regulated by their endogenous natural inhibitors (TIMPs). Among these, the roles of TIMP-1 and TIMP-2 in EC development, tumor progression and formation of metastases have been most extensively characterized and best recognized. 
 Expression of metalloproteases and their inhibitors in primary tumors and in local recurrences after mastectomy for breast cancer. To investigate the expression of matrix metalloproteases (MMPs) and their inhibitors (TIMPs) in patients who develop local recurrence (LR) after mastectomy.We analyzed the expressions of MMP-1, -2, -7, -9, -11, -13, -14, TIMP-1, -2, and -3, using immunohistochemical techniques, in primary tumors from patients without tumoral recurrence (n = 50), patients who developed distant   metastasis   (n = 50), and from patients who develop LRs (n = 25). LRs of the latter group were also analyzed for MMPs expression. All the patients underwent mastectomy.Score values for all MMPs and TIMPs were significantly higher in primary tumors of patients with distant   metastasis  . Primary tumors from patients with LR have lower expressions of MMPs and TIMPs compared with those from patients who developed distant   metastasis  , and with patients without recurrence for some MMPs. Remarkably, however, primary tumors from patients with LR showed significantly higher percentage of TIMP-1 and 2 expression in stromal cells compared to primary tumors from patients with distant   metastasis   or primary tumors from patients without tumoral progression. Furthermore, LRs had significantly higher MMP-9 expression than their corresponding primary tumors.Our data indicate differences in MMPs/TIMPs expression between primary tumors of patients with LRs and of those with distant   metastasis  , both after mastectomy for breast cancer. 
 Expression of matrix metalloproteinases MMP-2, MMP-9 and their tissue inhibitors TIMP-1 and TIMP-2 in the epithelium and stroma of salivary gland pleomorphic adenomas. The balance between matrix metalloproteinases (MMPs) and their tissue inhibitors (TIMPs) is involved in the morphogenesis of normal salivary gland as well as in the mechanisms of tumour invasion and   metastasis  . The role of MMPs and TIMPs in pleomorphic adenoma has not been elucidated sufficiently. Our aim was to analyse the mRNA and protein expression of MMP-2, MMP-9, TIMP-1 and TIMP-2 in the epithelium and stroma of pleomorphic adenoma and to evaluate their roles.In each sample from six patients, cells from the epithelium and stroma were obtained by laser microdissection. The mRNA expression of MMPs and TIMPs was determined by real-time quantitative reverse transcriptase-polymerase chain reaction and protein expression was confirmed by immunohistochemistry. Results showed that mRNA expression of MMPs and TIMPs was significantly higher in stroma than in epithelium in most patients. MMPs and TIMPs were immunoreactive mainly in epithelium rather than in stroma.Our results provide preliminary evidence that stromal myoepithelium may be the primary source of MMPs and that the stroma has the potential to play a more important role than ductal epithelium in biological behaviour of pleomorphic adenomas. These findings seem worthy of further investigation. 
 Tissue inhibitor of metalloproteinase 1 is an independent predictor of prognosis in patients with nonsmall cell lung carcinoma who undergo resection with curative intent. Matrix metalloproteinases (MMPs) and tissue inhibitors of metalloproteinases (TIMPs) play a role in the processes of extracellular matrix degradation. Changes in their expression levels have been observed in various tumor types, including lung carcinoma. However, their clinical significance and their prognostic importance in the progression of nonsmall cell lung carcinoma (NSCLC) remain to be specified. In this study, mRNA expression levels of MMP-1, MMP-9, TIMP-1, and TIMP-2 were evaluated in patients with resected NSCLC, and their associations with disease progression and prognosis were determined.Between June 1996 and December 1999, 116 patients underwent resection for NSCLC. Expression levels of MMPs and TIMPs were evaluated using Northern blot analysis in these NSCLC tissue samples and in 39 matched samples of normal lung tissue.MMP-1, MMP-9, and TIMP-1 expression levels were increased in tumor samples compared with matched, corresponding normal tissues. In contrast, TIMP-2 expression was decreased in tumor samples. MMP-1 tumor expression was correlated significantly with the evolution of lymph node status and tumor-lymph node-  metastasis   (TNM) stage. In contrast, MMP-9 tumor expression was correlated significantly with increased T stage. TIMP-1 overexpression was an independent predictor of worse survival in patients with NSCLC that was not associated with other prognosis factors, such as TNM stage.The overexpression of TIMP-1 was an independent prognostic marker in patients with NSCLC, and evaluating TIMP-1 may be important for identifying patients who are at greater risk of disease recurrence. 
 [Expression of matrix metalloproteinase-2, 9 and their inhibitor-TIMP 1,2 in human squamous cell carcinoma of uterine cervix]. Studies of tumor invasion and   metastasis   have focused on the degradation of extracellular matrix (ECM). Matrix metalloproteinases (MMPs), which could degrade ECM, were implicated in cancer invasion and   metastasis  . Activated MMPs were controlled by specific tissue inhibitors of metalloproteinases (TIMPs). This study was designed to investigate the expression of MMP-2, MMP-9 and TIMP-1, TIMP-2 in human squamous cell carcinomas of uterine cervix, and the association with invasion and   metastasis   of human squamous cell carcinoma of uterine cervix.Tissue samples from 40 cases of squamous cell carcinoma of the uterine cervix, 29 cases of cervical intraepithelial neoplasia (CIN), and 16 cases of normal cervices were stained immunohistochemically.Immunohistochemical staining of tumor cells for MMP-2, MMP-9, TIMP-1, and TIMP-2 were noted 82.5%, 70.0%, 62.5%, and 97.5% in carcinomas, 44.8%, 62.1%, 86.21%, and 96.55% in CIN lesions respectively, for MMP-2, MMP-9, TIMP-1 in 25.0% normal cervices, but for TIMP-2 in 56.25% normal cervices. There was significant difference for MMP-2 among carcinomas, normal cervices, and CIN respectively (P < 0.01). There was significant difference for MMP-9 among normal cervices, carcinomas, and CIN, respectively (P < 0.05). The positive rates of TIMP-1 and TIMP-2 in the cervical carcinoma group and CIN group were obviously higher than that in normal cervical group(P < 0.01). The positive rates of MMP-2 and MMP-9 in lymph node   metastasis   were obviously higher than that in without lymph node   metastasis  (P < 0.01), but for TIMP-2 was contrary and there was no significant difference for TIMP-1. No significant correlation could be established among the expression of these markers and the tumor diameter, the clinical stage, and pathologic grade.The over-expression of MMP-2, MMP-9, TIMP-1, and TIMP-2 may play a key role in invasion and lymph-node   metastasis   of in squamous carcinoma of the cervix. 
 Functional proteomics study reveals that N-Acetylglucosaminyltransferase V reinforces the invasive/metastatic potential of colon cancer through aberrant glycosylation on tissue inhibitor of metalloproteinase-1. N-Acetylglucosaminyltransferase-V (GnT-V) has been reported to be up-regulated in invasive/metastatic cancer cells, but a comprehensive understanding of how the transferase correlates with the invasive/metastatic potential is not currently available. Through a glycomics approach, we identified 30 proteins, including tissue inhibitor of metalloproteinase-1 (TIMP-1), as a target protein for GnT-V in human colon cancer cell WiDr. TIMP-1 was aberrantly glycosylated as characterized by the addition of beta1,6-N-acetylglucosamine, polylactosaminylation, and sialylation in GnT-V-overexpressing WiDr cells. Compared with normal TIMP-1, the aberrantly glycosylated TIMP-1 showed the weaker inhibition on both matrix metalloproteinase (MMP)-2 and MMP-9, and this aberrancy was closely associated with cancer cell invasion and   metastasis   in vivo as well as in vitro. Integrated data, both of TIMP-1 expression level and aberrant glycosylation, could provide important information to aid to improve the clinical outcome of colon cancer patients. 
 OncomiR-196 promotes an invasive phenotype in oral cancer through the NME4-JNK-TIMP1-MMP signaling pathway. MicroRNA-196 (miR-196), which is highly up-regulated in oral cancer cells, has been reported to be aberrantly expressed in several cancers; however, the significance of miR-196 in oral cancer has not yet been addressed.Cellular functions in response to miR-196 modulation were examined, including cell growth, migration, invasion and radio/chemosensitivity. Algorithm-based studies were used to identify the regulatory target of miR-196. The miR-196 target gene and downstream molecular mechanisms were confirmed by RT-qPCR, western blot, luciferase reporter and confocal microscopy analyses. miR-196 expression was determined in paired cancer and adjacent normal tissues from oral cancer patients.Both miR-196a and miR-196b were highly over-expressed in the cancer tissue and correlated with lymph node   metastasis   (P = 0.001 and P = 0.006, respectively). Functionally, miR-196 actively promoted cell migration and invasion without affecting cell growth. Mechanistically, miR-196 performed it's their function by inhibiting NME4 expression and further activating p-JNK, suppressing TIMP1, and augmenting MMP1/9.miR-196 contributes to oral cancer by promoting cell migration and invasion. Clinically, miR-196a/b was significantly over-expressed in the cancer tissues and correlated with lym  ph node me  tastasis. Thus, our findings provide new knowledge of the underlying mechanism of   cancer me  tastasis. miR-196 may serve as a promising marker for better oral cancer management. 
 Concentrations of TIMP1 mRNA splice variants and TIMP-1 protein are differentially associated with prognosis in primary breast cancer. TIMP-1 protein is a prognostic factor for recurrence-free and overall survival (OS) time in breast cancer. We evaluated the prognostic value of TIMP1 mRNA and a novel TIMP1 mRNA splice variant in 1301 primary breast cancer patients.We measured mRNA transcripts of full-length TIMP1 (TIMP1-v1) and the novel splice variant lacking exon 2 (TIMP1-v2) by use of real-time RT-PCR in frozen primary tumor samples. Transcript concentrations are correlated with histomorphological and biological factors, TIMP-1 protein, and distant   metastasis  -free survival (MFS) and OS time.TIMP1-v1 and TIMP1-v2 alone were not informative with respect to predicting prognosis. However, the PCR assay designed to measure the combination of v1 + v2 showed that high concentrations of this combination were associated with good prognosis. In Cox multivariate regression analysis, which also included the traditional prognostic factors, increasing concentrations were independently associated with prolonged MFS (P = 0.004) and OS (P = 0.048). Including TIMP-1 protein and TIMP1-v1+v2 mRNA together in the multivariate model revealed that protein and mRNA were both independently associated with prognosis, with hazard ratios pointing in opposite directions.High concentrations of TIMP1-v1+2 mRNA are associated with good prognosis in patients with primary breast cancer. Since high concentrations of TIMP-1 protein are associated with poor prognosis, the presence of possible posttranscriptional mechanisms requires further investigation. 
 [Significance of TIMP-1 and nm-23 expressions in HER-2-positive breast cancer]. Trastuzmab combined therapy has been recommended as standard therapy for HER-2-positive breast cancer. However, more than 70% HER-2-positive cases do not recur and might thus be with overtreatment risk. The aim of this study was to explore the significance of TIMP-1 and nm-23 expression in identifying the good prognosis subtype in HER-2-positive breast cancer.One hundred and sixty-five cases of HER-2-positive breast cancer treated in Tianjin Medical University Cancer Hospital from Jan. 1987 to Dec. 1988 were studied. Expressions of TIMP-1 and nm-23 were detected by immunohistochemistry and the correlation with clinicopathologic characteristics and long-term outcome was analyzed.53.3% (88/165) of TIMP-1 and 72.7% (120/165) of nm-23 expression were detected in the patients. The TIMP-1-negative patients classified as good prognosis group had a 10-year   metastasis   free survival (MFS) of 58.4% and 10-year overall survival (OS) of 68.8%, compared with the 10-year MFS of 43.2% (P = 0.041) and 10-year OS of 52.0% in the positive group (P = 0.020). The nm-23-positive patients classified as good prognosis group had a 10-year MFS of 54.2% and 10-year OS of 65.7%, compared with the 10-year MFS of 40.0% (P = 0.049) and 44.1% (P = 0.015) in the negative group. The multivariate analysis showed that expressions of TIMP-1 and nm-23, tumor size and lymph node involvement were independent prognostic factors.In HER-2-positive breast cancer, TIMP-1 and nm-23 can be used to further distinguish between low-risk and high-risk subgroups, and they are independent factors in prognosis. 
 Tissue inhibitor of metalloproteinases-1-induced scattered liver   metastasis   is mediated by host-derived urokinase-type plasminogen activator. Paradoxically, not only proteinases but also their inhibitors can correlate with bad prognosis of cancer patients, underlining the evolving concept of the protease web as the complex interplay between proteinases, their inhibitors and effector molecules. Elevated levels of tissue inhibitor of metalloproteinases-1 (TIMP-1) render the liver more susceptible to   metastasis   by triggering urokinase plasminogen activator (uPA) expression as well as hepatocyte growth factor (HGF) signalling, thereby leading to the fatal scattered infiltration of metastasizing tumour cells throughout the parenchyma of the target organ. Here, we investigated whether host uPA is a crucial protagonist for the TIMP-1-induced modulation of a pro-metastatic microenvironment in the liver. Indeed, in livers of uPA-ablated mice elevated TIMP-1 levels did not trigger HGF signalling and did not promote   metastasis   of a murine T-lymphoma cell line. In contrast, lack of tumour cell-derived uPA induced by gene silencing did not interfere with this pro-metastatic pathway. Furthermore, host uPA was necessary for the recruitment of neutrophilic granulocytes and the associated increase of HGF in livers with elevated TIMP-1 levels. This newly identified co-operation between TIMP-1 and host uPA suggests that therapies, simultaneously interfering with pro- and anti-proteolytic pathways may be beneficial for patients with metastatic disease. 
 Imbalance between expression of matrix metalloproteinase-9 and tissue inhibitor of metalloproteinase-1 in invasiveness and   metastasis   of human gastric carcinoma. The expressive balance between matrix metalloproteinase-9 (MMP-9) and its tissue inhibitor of metalloproteinase-1 (TIMP-1) plays a critical role in maintaining the degradation and synthesis of extracellular matrix. Loss of such balance is associated with invasion and   metastasis   of tumors. This study aimed to determine the expression of MMP-9 and TIMP-1 in gastric carcinoma, and the association of the expressive imbalance between MMP-9 and TIMP-1 with the invasion and   metastasis   and prognosis of gastric carcinoma.We used immunohistochemistry to determine the expressions of MMP-9, TIMP-1 and proliferating cell nuclear antigen Ki-67 in the gastric specimens taken from 256 patients with primary gastric carcinoma. The patients were followed-up for up to 96 months.No association between the expression of MMP-9 and TIMP-1 and patients' sex and age, tumor size and location of gastric carcinoma was observed. The incidence of the positive expression of MMP-9 in cases with tumors invasion to muscularis propria and visceral peritoneum (70.13 % and 69.09 %, respectively) was significantly higher than that in cases with tumor invasion only to lamina propria or submucosa (42.50 %, P=0.0162). The positive correlation between MMP-9 expression and the depth of tumor invasion was observed (Pearson correlation coefficient=0.2129, P=0.016). Along with the increase of the metastatic station of lymph nodes, the incidence of the MMP-9 expression was increased by degrees; a positive correlation between them was observed (Pearson correlation coefficient=0.2910, P=0.0001). There was also a significant correlation between MMP-9 expression and the TNM stage in gastric carcinoma (Pearson correlation coefficient=0.3027, P<0.0001). The incidence of MMP-9 expression in stage II and III/IV (75.00 % and 76.15 %, respectively) was significantly higher than those in stage I (46.15 %, P<0.0001). A negative correlation between TIMP-1 immunoreactivity and the depth of invasion, status of lymph node   metastasis   and TNM stage was observed (Pearson correlation coefficient =-0.1688, -0.3556 and -0.3004, P=0.023, <0.0001 and <0.0001, respectively). Four types of co-expression of MMP-9 and TIMP-1 were observed; i.e. MMP-9 positive but TIMP-1 negative (n=115), both positive (n=52), both negative (n=62) and MMP-9 negative but TIMP-1 positive (n=27). The frequency of serosal invasiveness was significant higher in patients with MMP-9 but without TIMP-1 expression than those with other types of the co-expression (P=0.0303). The incidence of lymph node   metastasis   was highest in patients with MMP-9 but without TIMP-1 expression, and lowest in those with TIMP-1 but without MMP-9 expression (P<0.0001). The survival rate in patients with MMP-9 but without TIMP-1 expression was lower than that in those with TIMP-1 but without MMP-9 expression (P=0.0014).Our results in gastric carcinoma demonstrated a significant positive association of MMP-9 over-expression with proliferation of tumor cells, the depth of invasiveness, lymph node   metastasis   and TNM stage, suggesting MMP-9 can serve as a molecular marker of tumor invasion and   metastasis  . We also demonstrate a significant negative relationship of TIMP-1 expression with the depth of invasiveness and lymph node   metastasis  , which provide a new idea in the tumor biological and genetic treatment. The interaction between MMP-9 and TIMP-1 in the processes of tumor invasion and   metastasis   is that MMP-9 mainly promotes tumor invasion and   metastasis   and TIMP-1 inhibits functions of MMP-9. The imbalance between MMP-9 and TIMP-1 expression may suggest the occurrence of tumor invasion and   metastasis  , predict poor prognosis. For patients with imbalanced MMP-9 and TIMP-1 expression, the optimal treatment scheme needs to be selected. 
 Tissue inhibitor of matrix metalloproteinase-1 expression in colorectal cancer liver metastases is associated with vascular structures. Metastatic growth by colorectal cancer cells in the liver requires the ability of the cancer cells to interact with the new microenvironment. This interaction results in three histological growth patterns of liver metastases: desmoplastic, pushing, and replacement. In primary colorectal cancer several proteases, involved in the degradation of extracellular matrix components, are up-regulated. In liver metastases, their expression is growth pattern dependent. Tissue inhibitor of matrix metalloproteinase-1 (TIMP-1) is a strong prognostic marker in plasma from colorectal cancer patients, with significant higher levels in patients with metastatic disease. We therefore wanted to determine the expression pattern of TIMP-1 in primary colorectal cancers and their matching liver metastases. TIMP-1 mRNA was primarily seen in α-smooth-muscle actin (α-SMA)-positive cells. In all primary tumors and liver metastases with desmoplastic growth pattern, TIMP-1 mRNA was primarily found in α-SMA-positive myofibroblasts located at the invasive front. Some α-SMA-positive cells with TIMP-1 mRNA were located adjacent to CD34-positive endothelial cells, identifying them as pericytes. This indicates that TIMP-1 in primary tumors and liver metastases with desmoplastic growth pattern has dual functions; being an MMP-inhibitor at the cancer periphery and involved in tumor-induced angiogenesis in the pericytes. In the liver metastases with pushing or replacement growth patterns, TIMP-1 was primarily expressed by activated hepatic stellate cells at   the metast  asis/liver parenchyma interface. These cells were located adjacent to CD34-positive endothelial cells, suggesting a function in tumor-induced angiogenesis. We therefore conclude that TIMP-1 expression is growth pattern dependent in colorectal cancer liver metastases. 
 Hyaluronan synthase 2 (HAS2) promotes breast cancer cell invasion by suppression of tissue metalloproteinase inhibitor 1 (TIMP-1). Invasion and   metastasis   are the primary causes of breast cancer mortality, and increased knowledge about the molecular mechanisms involved in these processes is highly desirable. High levels of hyaluronan in breast tumors have been correlated with poor patient survival. The involvement of hyaluronan in the early invasive phase of a clone of breast cancer cell line MDA-MB-231 that forms bone metastases was studied using an in vivo-like basement membrane model. The metastatic to bone tumor cells exhibited a 7-fold higher hyaluronan-synthesizing capacity compared with MDA-MB-231 cells predominately due to an increased expression of hyaluronan synthase 2 (HAS2). We found that knockdown of HAS2 completely suppressed the invasive capability of these cells by the induction of tissue metalloproteinase inhibitor 1 (TIMP-1) and dephosphorylation of focal adhesion kinase. HAS2 knockdown-mediated inhibition of basement membrane remodeling was rescued by HAS2 overexpression, transfection with TIMP-1 siRNA, or addition of TIMP-1-blocking antibodies. Moreover, knockdown of HAS2 suppressed the EGF-mediated induction of the focal adhesion kinase/PI3K/Akt signaling pathway. Thus, this study provides new insights into a possible mechanism whereby HAS2 enhances breast cancer invasion. 
 Serum Biomarkers Associated with Clinical Outcomes Fail to Predict Brain Metastases in Patients with Stage IV Non-Small Cell Lung Cancers. Lung cancers account for the majority of brain metastases which pose major therapeutic challenges. Biomarkers prognosticating for the development of brain metastases in patients with non-small cell lung cancers (NSCLC) may improve personalized care. Six serum proteomic biomarkers were previously investigated at Memorial Sloan Kettering but their associations with brain metastases were unknown.Serum NSE, CYFRA 21-1, ProGRP, SCC-Ag, TIMP1, and HE4 by ELISA-based proteomic assays were prospectively collected from consecutive patients with stage IV NSCLC. Pre-treatment serum biomarker levels as well as age, histology, and epidermal growth factor receptor (EGFR) mutation status were evaluated for association with the baseline presence of brain metastases using logistic regression and multivariable analysis. For patients without brain metastases at baseline, the cumulative incidence of subsequent brain metastases were compared according to baseline biomarkers and clinical factors using Gray's test.A total of 118 patients were enrolled, 31 (26%; 95% CI 0.19-0.35) had brain metastases at baseline and a further 26 (22%; 95% CI 0.15-0.30) developed brain metastases subsequently. Pre-treatment serum biomarker levels were available in 104 patients. There was no significant association between the six serum biomarkers and the baseline presence or subsequent development of brain metastases. Age younger than 65 years was the only clinical factor significantly associated with brain   metastasis   at baseline (OR 3.00; 95% CI 1.22-7.34, P = 0.02) by multivariable analysis. A trend toward increased cumulative incidence of subsequent brain metastases was observed in patients with EGFR mutation (p = 0.2), but this was not statistically significant possibly due to small sample size.Serum NSE, CYFRA 21-1, Pro-GRP, SCC-Ag, TIMP1, and HE4 are not significantly associated with brain metastases. Our methods taking into account follow-up time may be applied to independent datasets to identify a patient cohort with a higher biologic propensity for developing brain metastases. Such information may be useful for the study of agents targeting the development of brain metastases. 
 Association of E-cadherin, matrix metalloproteinases, and tissue inhibitors of metalloproteinases with the progression and   metastasis   of hepatocellular carcinoma. Molecular markers can provide additional information to traditional histomorphological evaluation for the assessment of tumor progression and predicting the likelihood of invasion and   metastasis   in various types of malignancies. We studied the association of E-cadherin, matrix metalloproteinases (MMPs), and tissue inhibitors of metalloproteinase with the progression and   metastasis   of hepatocellular carcinoma. Tissue microarray including six normal livers, 14 cirrhotic livers, 39 macroregenerative nodules, 16 dysplastic nodules, 22 grade I hepatocellular carcinomas, 43 grade II hepatocellular carcinomas, seven grade III hepatocellular carcinomas, and 10 metastatic hepatocellular carcinomas were stained immunohistochemically with antibodies against MMPs -1, -2, -3, -7, -9, tissue inhibitors of metalloproteinase-1, -2, -3, and E-cadherin. The intensities of staining were scored manually by two pathologists and verified by the Chromavision Automated Cellular Imaging System. Compared with normal liver, cirrhotic liver had significantly lower E-cadherin and tissue inhibitors of metalloproteinase-1 but higher MMP-1 and -7, which suggest a more favorable environment for tumor invasion and   metastasis  . Grade I and grade II hepatocellular carcinomas demonstrated high E-cadherin and decreased MMP-3 and -9, which may explain the rarity of extrahepatic   metastasis   in low-grade hepatocellular carcinomas despite the high circulatory volume of the liver. The histological progression from dysplastic nodule to well-differentiated hepatocellular carcinoma and to less differentiated tumors was associated with a gradual decrease in tissue expression of E-cadherin, tissue inhibitors of metalloproteinase-2 and -3. Metastatic hepatocellular carcinomas showed significantly lower level of tissue inhibitors of metalloproteinase-1, -2, -3 but higher level of MMP-7. These data suggest that tissue expression of E-cadherin, certain MMPs, and tissue inhibitors of metalloproteinases could be useful markers to predict the progression and   metastasis   of hepatocellular carcinoma. 
 Clinical implications of proteolytic activity imbalance in breast cancer diagnosis. Matrix metalloproteinase-9 and its tissue inhibitor TIMP-1 have been documented as putative tumor markers because of their involvement in cancer invasion and   metastasis  .The aim of our study was to elucidate the diagnostic efficacy of proteolytic activity markers among traditional tumor markers (CEA and CA15.3) and clinicopathological variables.Serum samples were withdrawn from 160 individuals (80 patients with primary breast cancer, 40 patients with benign breast lesions and 40 individuals serve as healthy controls). MMP-9 and TIMP-1 were measured by ELISA and gelatin zymography.The best cutoff points for MMP-9 and TIMP-1 were depicted by receiver operating characteristic (ROC) curve. The positivity rates and the median levels for MMP-9 and TIMP-1 showed significant difference among the three investigated groups (P< 0.0001). MMP-9 and MMP-9/TIMP-1 were inversely related to clinical stage and lymph node involvement (P< 0.0001). TIMP-1 was significantly correlated with hormonal receptor status (ER, and PgR). MMP zymography results were comparable to those from ELISA. The sensitivity and the specificity of MMP-9, TIMP-1 and MMP-9/TIMP-1 were superior to traditional tumor markers (CEA and CA15.3) especially for early stages (T1) and low grade breast cancer patients.These findings indicate that investigated biomarkers are constructive for early diagnosis of breast cancer and MMP-9/TIMP-1 ratio might be a new significant marker in predicting breast cancer development. 
 Negative regulation of TIMP1 is mediated by transcription factor TWIST1. TWIST1 is involved in tumor invasion and   metastasis   by promoting epithelial-to-mesenchymal transition. However, the molecular target of TWIST1 involving this mechanism is poorly understood. To identify the TWIST1-regulated genes, we established the Saos-2 cells stably expressing TWIST1 gene by transfecting TWIST1 cDNA into the cells and performed a differential display approach by using annealing control primers. Here, we report that one of the genes that were down-regulated in TWIST1 expressing cells is predicted to be TIMP1. TIMP1 has been reported as the naturally occurring specific inhibitor of matrix metalloproteinases (MMPs). Overexpression of TWIST1 gene suppressed the expression of TIMP1 mRNA but had no effect on TIMP2 and MMP-2 expression, as determined by semi-quantitative RT-PCR. We showed that TWIST1 was up-regulated in SCCBHY cells, which have a strong capacity of invasion into mandibular bone compared with SCCHN cells. Our present results suggest that TWIST1 is involved in tumor invasion by regulating the expression of TIMP1. 
 [Expression of MMP-9 and TIMP-1 in nasopharyngeal carcinoma and its signification at Xi'an and Shenzhen]. To compare the expression of matrix metalloproteinase-9 and tissue inhibitor of matrix metalloproteinase-1 of nasopharyngeal carcinoma at Xi'an and Shenzhen area.The protein expression of MMP 9 and TIMP 1 were studied by immunohistochemistry staining in 21 cases nasopharyngeal carcinoma of Xi'an and 22 cases of Shenzhen.The expression positive rates of MMP-9 in nasopharyngeal carcinoma tissues at Xi'an and Shenzhen were 61.90% and 68.18%. The positive rates of TIMP-1 in nasopharyngeal carcinoma tissues at two regions were 28.57% and 31.82%. The expression between MMP-9 and TIMP-1 were negative relationship either Xi'an or Shenzhen. The expression of MMP-9 was positively correlated to lymph node   metastasis  , and TIMP-1 was no relationship with lymph node   metastasis  . The expression of MMP-9 and TIMP-1 was no difference between Xi'an and Shenzhen.The expression of MMP-9 and TIMP-1 in nasopharyngeal carcinoma 2 tissues was no difference between Xi'an and Shenzhen. Increase of MMP-9 play an important role in progression and   metastasis   of nasopharyngeal carcinoma. 
 Runx3 suppresses gastric cancer   metastasis   through inactivation of MMP9 by upregulation of TIMP-1. Recent studies have suggested that loss of RUNX3 expression is involved with gastric tumor   metastasis  . However, the precise mechanism of RUNX3-mediated suppression of tumor   metastasis   remains elusive. We aimed to clarify the effect of RUNX3 on tumor   metastasis   in gastric cancer cell lines and tumors. Immunohistochemistry revealed that RUNX3 was significantly decreased in metastatic gastric cancer. Gelatin zymography and Western blot showed that instead of regulating matrix metalloproteinase 9 (MMP9) expression, RUNX3 expression inhibited MMP9 enzyme activity, and this was consistent with the upregulation of tissue inhibitor of metalloproteinases 1 (TIMP1) by RUNX3. TIMP1 siRNA treatment impaired RUNX3-mediated suppression of gastric cancer cell invasion. Reporter assays demonstrated regulation of TIMP-1 by RUNX3. Two RUNX3 binding sites were identified in the TIMP-1 promoter and direct interaction of RUNX3 with the TIMP-1 promoter was confirmed in vitro and in vivo. These findings provide evidence for RUNX3-mediated suppression of gastric cancer invasion and   metastasis   and define a novel molecular mechanism that for the   metastasis  -inhibiting activity of RUNX3. These data may be applied in the development of RUNX3 for gastric cancer   metastasis   diagnostics and therapeutics. 
 Tissue inhibitor of metalloproteinase (TIMP)-1: the TIMPed balance of matrix metalloproteinases in the central nervous system. Astrocytes are intimately involved in the mechanisms of neural injury and repair. They participate in a variety of homeostatic functions and elicit repair responses as balance mechanisms. Currently, there is a growing appreciation of a more active role of astrocytes in neuronal signaling and function. One key homeostatic mechanism of astrocytes in tissue repair is maintained through their production of tissue inhibitors of metalloproteinases (TIMPs). The family of TIMPs (1-4) plays a central regulatory role as inhibitors of matrix metalloproteinases (MMPs), enzymes involved in extracellular matrix maintenance and remodeling. Recently, TIMP-1, the inducible form, has been identified as a multifunctional molecule with divergent functions. It participates in wound healing and regeneration, cell morphology and survival, tumor   metastasis  , angiogenesis, and inflammatory responses. An imbalance of MMP/TIMP regulation has been implicated in several inflammatory diseases of the central nervous system (CNS). Here we review the conundrums of TIMP-1 regulation in CNS pathophysiology. We propose that astrocyte-TIMP-1 may play an important role in CNS homeostasis and disease. Astrocyte TIMP-1 expression is differentially regulated in inflammatory neurodegenerative diseases and may have significant therapeutic relevance. 
 Down-regulation of TIMP-1 inhibits cell migration, invasion, and metastatic colonization in lung adenocarcinoma. Tissue inhibitor metalloproteinase-1 (TIMP-1) is clinically associated with a poor prognosis for various cancers, but the roles of TIMP-1 in lung cancer   metastasis   are controversial. Our previous secretomic study revealed that TIMP-1 is highly abundant in high invasiveness cells of lung adenocarcinoma. In the current study, TIMP-1 abundances in primary lung adenocarcinoma tissues, as revealed by immunohistochemistry, are significantly higher in patients with lymph invasion and distant   metastasis   than in those without. Receiver operating characteristic curve analyses suggest 73.7 and 86.2 % accuracy to separate patients with lymph node and distant   metastasis   and those without, respectively. Moreover, we demonstrate that the expression level of TIMP-1 positively associates with cell mobility, invasiveness, and metastatic colonization. Most notably, the novel mechanism in which TIMP-1 facilitates metastatic colonization through the mediation of pericellular polyFN1 assembly was revealed. In summary, this study presents novel functions of TIMP-1 in promoting cancer   metastasis   and suggests TIMP-1 is a potential tissue biomarker for lymph invasion and distant   metastasis   of lung adenocarcinoma. 
 Integrin alphavbeta3-mediated transcriptional regulation of TIMP-1 in a human ovarian cancer cell line. We have previously reported that a disintegrin inhibits solid tumor growth and   metastasis   in mouse model [I.C. Kang, Y.D. Lee, D.S. Kim, A novel disintegrin salmosin inhibits tumor angiogenesis, Cancer Res. 59 (1999) 3754-3760; S.I. Kim, K.S. Kim, H.S. Kim, D.S. Kim, Y. Jang, K.H. Chung, Y.S. Park, Inhibitory effect of the salmosin gene transferred by cationic liposomes on the progression of B16BL6 tumors, Cancer Res. 63 (2003) 6458-462]. In this study, we have investigated the modulatory effect of a disintegrin, saxatilin, on the balance between MMP-9 and tissue inhibitor of metalloproteinase-1 (TIMP-1) in human ovarian cancer cell line MDAH 2774. Functional mechanism of the disintegrin-mediated transcriptional regulation of MMP-9 and TIMP-1 was examined in the ovarian cancer cell line. Saxatilin strongly induced TIMP-1 expression in dose- and time-dependent manners, while the disintegrin suppressed MMP-9 expression. Further analyses clearly indicated that interaction of the disintegrin and integrin alphavbeta3 results in the TIMP-1 promoter activation via c-fos to suppress TNF-alpha-induced cancer cell invasion. These results demonstrate that integrin alphavbeta3-mediated transcriptional regulation of MMP-9 and TIMP-1 is critical for suppressing the ovarian cancer cell invasion. 
 Thrombospondin-1 (TSP-1) up-regulates tissue inhibitor of metalloproteinase-1 (TIMP-1) production in human tumor cells: exploring the functional significance in tumor cell invasion. Thrombospondin-1 (TSP-1), a matrix-bound adhesive glycoprotein, has been shown to modulate tumor progression. We previously demonstrated that TSP-1 up-regulates matrix metalloproteinases MMP-2 and MMP-9. Our studies suggested that the balance between MMPs and tissue inhibitors of metalloproteinases (TIMPs) is a key determinant in tumor cell invasion. We now report that TSP-1 up-regulates TIMP-1 expression in both human breast and prostate cancer cell lines. The effect of TSP-1 on TIMP-1 expression was examined in human breast adenocarcinoma cell lines (MDA-MB-231) and human prostate cancer cell lines (PC3-NI and PC3-ML) treated with exogenous TSP-1. TIMP-1 expression was also examined in TSP-1 stably transfected breast cancer cell line (MDA-MB-435). Northern and western blot analysis revealed TIMP-1 mRNA and TIMP-1 protein expression increased with increasing concentrations of TSP-1. This effect was inhibited by antibodies against the type I repeat domain of TSP-1 further suggesting that TSP-1 mediates TIMP-1 secretion. Inhibition of TSP-1 induced TIMP-1 levels increased tumor cell invasion. We conclude that TSP-1 is involved in influencing the critical balance between MMPs and their inhibitors, maintaining the controlled degradation of the extracellular matrix needed to support   metastasis   and our results may provide an explanation for the divergent activities reported for TSP-1 in tumor progression. 
 Gambogic acid inhibits invasion of osteosarcoma via upregulation of TIMP-1. Gambogic acid (GA), the natural product, has been demonstrated to be a promising chemotherapeutic drug for osteosarcoma (OS) due to its ability to induce apoptosis and cell cycle arrest. To date, no studies have examined the role of GA in metastatic bone disease. Matrix metalloproteinases (MMPs) play critical roles in invasion and   metastasis  , and the tissue inhibitors of metalloproteinase (TIMP) family regulates the activity of multifunctional metalloproteinases. In this study, we investigated the gene expression of matrix metalloproteinase-9 (MMP-9) and tissue inhibitor of matrix metalloproteinase-1 (TIMP-1) in OS cell lines treated by the GA. The expression of MMP-9 and TIMP-1 were studied by reverse transcription-polymerase chain reaction (RT-PCR) and western blotting. In vitro invasion of OS cell lines (Saos-2, MG-63) were investigated by the Matrigel invasion assay. Mean MMP-9 protein and mRNA expression was significantly suppressed; in addition, mean TIMP-1 protein mRNA expression were upregulated by increasing GA concentrations. GA reduced the invasiveness of OS cell lines dose-dependently. Furthermore, specific inhibition of TIMP-1 secretion with siRNA against TIMP-1 significantly reduced the effect of GA on OS cell lines. Overall, our findings suggest that GA reduces the invasive potential of OS cells via attenuation of MMP-9 and upregulation of TIMP-1. Moreover, TIMP-1 played an important role in the reduction of invasive potential of the OS cells which were treated by GA. 
 Targeting TR4 nuclear receptor suppresses prostate cancer invasion via reduction of infiltrating macrophages with alteration of the TIMP-1/MMP2/MMP9 signals. TR4 nuclear receptor 4 (TR4) plays an important role in macrophages-associated foam cell formation of cardiovascular diseases and infiltrating macrophages are critical for prostate cancer (PCa) progression. However, the linkage of macrophages and TR4 and their impacts on PCa   metastasis   remains unclear.Knocking-down TR4 in human PCa cells (C4-2, CWR22Rv1), but not in human macrophages cells (THP-1), led to suppress the macrophages infiltration to PCa cells. The consequences of such suppression of the recruitment of macrophages toward PCa then resulted in suppressing the PCa cell invasion. Mechanism dissection found that knocking-down TR4 in PCa cells suppressed   metastasis  -related genes including MMP2, with induction of TIMP-1. Interruption assays using TIMP-1 neutralizing antibody could then reverse TR4-macrophage-mediated PCa invasion. IHC staining showed higher TR4 level, more macrophage infiltration, lower TIMP-1 and stronger MMP2/MMP9 in tumor tissues of the Gleason score 5 + 4 patients compared with the Gleason score 3 + 3 patients.Targeting TR4 in prostate tumor microenvironment might represent a potential new therapeutic approach to better bat  tle PCa me  tastasis. 
 The TIMP-1 gene transferred through adenovirus mediation shows a suppressive effect on peritoneal metastases from gastric cancer. It has become clear in recent years that peritoneal   metastasis   takes place as the result of a multistep process involving attachment, invasion, proliferation, and angiogenesis. The aim of the present study was to evaluate the suppressive effect of tissue inhibitor of metalloproteinase-1 (TIMP-1) gene transfer on peritoneal dissemination.We established a high-potential peritoneal   metastasis   cell line (MKN-45P), using the gastric cancer cell line MKN-45, and developed a peritoneal   metastasis   model in nude mice. The TIMP-1 gene was transferred to MKN-45 or MKN-45P by adenoviral transfection, and we performed an in vitro invasion assay and an in vivo study, using the peritoneal   metastasis   model. The TIMP-1 transfected group was compared with a non-virus group and a Lac-Z transfected group.The in vitro invasion assay showed that the number of invasive cells was significantly reduced in the TIMP-1 transfected group compared with that in the non-virus group and the Lac-Z transfected group, Moreover, the in vivo studies showed that the number and the weight of the peritoneal nodes in the TIMP-1 transfected group were significantly less than those in the Lac-Z transfected group, and less than those in the non-viral group. No bloody ascites was recognized in the TIMP-1 transfected group. The mean number of tumor vessels in the non-virus group and the Lac-Z group was significantly higher than that in the TIMP-1 group.TIMP-1 demonstrated an inhibitory effect on angiogenesis, and may be worthwhile investigating for use as a future therapy for peritoneal dissemination. 
 Expression of metalloproteinase-2, metalloproteinase-9, and tissue inhibitor of metalloproteinase-1 in transitional cell carcinoma of upper urinary tract: correlation with tumor stage and survival. To investigate the relationship between the expression of matrix metalloproteinase (MMP)-2, MMP-9, tissue inhibitor of metalloproteinase (TIMP)-1, and TIMP-2 and pT stage or survival in patients with transitional cell carcinoma of the upper urinary tract. MMP-2 and MMP-9 are associated with tumor invasion in several malignancies. TIMPs exert an anti-invasive effect by blocking MMP activity. Recent studies have shown, however, that TIMPs can also stimulate cell proliferation and angiogenesis.Tumor sections surgically removed from 91 patients were examined for expression of MMP-2, MMP-9, TIMP-1, and TIMP-2 by immunohistochemistry. We also determined the proliferation index and microvessel density in each tumor and investigated the independent roles of these factors in tumor stage and survival using multivariate analysis.Of 91 tissue samples, 50, 51, 45, and 39 were positive for MMP-2, MMP-9, TIMP-1, and TIMP-2 expression, respectively. Tumors positive for MMP-2, MMP-9, and TIMP-1 exhibited a greater proliferation index than tumors with negative expression (P <0.001, P = 0.013, and P <0.001, respectively). The microvessel density of tumors positive for MMP-2 and TIMP-1 was greater than that of negative tumors (P <0.001). The expression of MMP-2, MMP-9, and TIMP-1 was an independent predictor of high pT stage. Cox proportional hazard analysis identified TIMP-1 expression as an independent factor for cause-specific survival (odds ratio 5.2, P = 0.011), similar to microvessel density, pT4, and lymph node   metastasis  .TIMP-1 expression correlated with pT stage and was an independent predictor of cause-specific survival. Our results suggest that TIMP-1 expression is a potentially useful tool for the selection of postoperative observation strategies in patients with transitional cell carcinoma of the upper urinary tract. 
 [Pathologic study of expression and significance of matrix metalloproteinases-9, tissue inhibitor of metalloproteinase-1, vascular endothelial growth factor and transforming growth factor beta-1 in papillary carcinoma and follicular carcinoma of thyroid]. To study the roles of matrix metalloproteinases-9 (MMP-9), tissue inhibitor of metalloproteinase-1 (TIMP-1), vascular endothelial growth factor (VEGF) and transforming growth factor β-1 (TGFβ-1) in differentiation, invasiveness and metastatic potential of papillary carcinoma and follicular carcinoma of thyroid.Eighty-five cases of papillary thyroid carcinoma and 59 cases of follicular thyroid carcinoma were enrolled into the study. Immunohistochemistry using EnVision method was carried out for assessment of the expression of MMP-9, TIMP-1, VEGF and TGFβ-1 in the tumor tissue.MMP-9, TIMP-1, VEGF and TGFβ-1 were expressed in the cytoplasm of tumor cells. The positivity rates of MMP-9, TIMP-1, VEGF and TGFβ-1 in papillary thyroid carcinoma (83.5%, 81.2%, 90.6% and 75.3%, respectively) were similar to or lower than those in follicular thyroid carcinoma (93.2%, 86.4%, 89.9% and 78.0%, respectively). The expression rates in papillary thyroid carcinoma with lymph   node metas  tasis were also higher than those in tumors without lymph   node metas  tasis. The expression rates of MMP-9, VEGF and TGFβ-1 in poorly-differentiated follicular thyroid carcinoma were higher than those in well-differentiated follicular thyroid carcinoma. The expression of TIMP-1 however showed a negative correlation with the tumor cell differentiation. In general, the expression of VEGF and MMP-9 was higher than that of TIMP-1 and TGFβ-1 in papillary thyroid carcinoma and follicular thyroid carcinoma.Immunohistochemical detection of MMP-9, TIMP-1, VEGF and TGFβ-1 expression may carry clinical significance in evaluating the degree of differentiation, invasiveness, metastatic potential and prognosis of papillary thyroid carcinoma and follicular thyroid carcinoma. 
 Association of a high activity of matrix metalloproteinase-9 to low levels of tissue inhibitors of metalloproteinase-1 and -3 in human hepatitis B-viral hepatoma cells. Matrix metalloproteinases (MMPs) play a major role in the turnover of extracellular matrix (ECM) during cancer invasion and   metastasis  , and tissue inhibitors of metalloproteinases (TIMPs) control MMPs, thus maintaining a balanced ECM catabolism under physiological conditions. The aim of this study was to assess the behavior of some MMPs (FASEB J., 7, 1993, 1434; Cancer   Metastasis   Rev., 9(4) 1990, 289) and TIMPs (Biochem. Biophys. Res. Commun., 301, 2003, 1069; FASEB J., 7, 1993, 1434; Nature, 370, 1994, 61). Competitive RT-PCR, gelatin-substrate zymography, and ELISA techniques were used for quantification. The hepatitis B virus (HBV)-DNA-containing hepatocellular carcinoma cell lines, Hep3B, HepG2-HBV and HFF-T2 contain highly activated matrix metallproteinase-9 (MMP-9), which is rarely found in normal liver cell lines such as the Chang lines. MMP-9 activities of HFH-T2, HepG2-HBV and Hep3B were significantly higher than that of non-HBV-hepatocellular carcinoma SK-Hep1 and HepG2 (HCC origin, HBV not detected), as assayed by gelatin zymography. Low levels of TIMP-1 and TIMP-3 were observed in HFH-T2, HepG2-HBV and Hep3B, while the TIMP-2 level was high, as evidenced by reverse zymography. In contrast, 3 TIMP-1, -2 and -3 were largely detected in Chang, HepG2 and SK-Hep1 cells. To investigate the nature of the quantitative regulation of MMPs and TIMPs for these cell lines at the transcriptional levels, a reverse transcriptase-polymerase chain reaction (RT-PCR) was carried out. Not only MMP-9 mRNAs of HFH-T2, HepG2-HBV and Hep3B but also MMP-9 mRNA of SK-Hep1 and HepG2 were highly expressed with no major differences among these four cell lines. However, TIMP-1 and TIMP-3 mRNAs of HFH-T2, HepG2-HBV and Hep3B were markedly reduced, while those of SK-Hep1, HepG2 and Chang cells were maintained at high levels. Finally, an invasion assay using matrigel indicated in an increase in invasiveness in HFH-T2, HepG2-HBV and Hep3B cells, but a decrease in invasiveness of Chang, HepG2 and SK-Hep1 cells. These results indicate that the overexpression of MMP-9 mRNAs and the suppression of TIMP-1 and TIMP-3 in HFH-T2, HepG2-HBV and Hep3B were the result of HBV transfection. Based on these results, it is concluded that HBV affects the malignance of hepatocellular cancer by elevating MMP-9 activity, and suppressing TIMP-1 and TIMP-3. 
 RUNX3 mediates suppression of tumor growth and   metastasis   of human CCRCC by regulating cyclin related proteins and TIMP-1. Here we presented that the expression of RUNX3 was significantly decreased in 75 cases of clear cell renal cell carcinoma (CCRCC) tissues (p<0.05). Enforced RUNX3 expression mediated 786-O cells to exhibit inhibition of growth, G1 cell-cycle arrest and   metastasis   in vitro, and to lost tumorigenicity in nude mouse model in vivo. RUNX3-induced growth suppression was found partially to regulate various proteins, including inhibition of cyclinD1, cyclinE, cdk2, cdk4 and p-Rb, but increase of p27(Kip1), Rb and TIMP-1. Therefore, RUNX3 had the function of inhibiting the proliferative and metastatic abilities of CCRCC cells by regulating cyclins and TIMP1. 
 Differential effects of sulfated triterpene glycosides, holothurin A1, and 24-dehydroechinoside A, on antimetastasic activity via regulation of the MMP-9 signal pathway. Two sulfated triterpene glycosides, holothurin A(1) (HA(1)) and 24-dehydroechinoside A (DHEA), isolated from the sea cucumber Pearsonothuria graeffei, are of the holostane type with 18(20)-lactone and identical carbohydrate chains. DHEA has a side chain 23 (24)-double bond, while HA(1) has a hydroxyl group at C-21. In this study, we compared the effects of DHEA and HA(1) on   metastasis   in vitro and in vivo. The results show that HA(1) and DHEA treatment significantly suppressed adhesion of human hepatocellular liver carcinoma cells (HepG2) to both matrigel and human endothelial cells (ECV-304) and inhibited HepG2 cell migration and invasion in a dose-dependant manner. HA(1) and DHEA reduced tube formation of ECV-304 cells on the matrigel in vitro and attenuated neovascularization in the chick embryo using the chorioallantoic membrane (CAM) assay in vivo. Immunocytochemistry analyses revealed that both HA(1) and DHEA significantly decreased the expression of the matrix metallo-proteinase-9 (MMP-9) and increased the expression level of tissue inhibitor of metalloproteinase-1 (TIMP-1), an important regulator of MMP-9 activation. Western blot analyses demonstrated that HA(1) and DHEA remarkably abolished the expression of vascular endothelial growth factor (VEGF). The expression of nuclear factor-kappa B (NF-κB) was significantly decreased by HA(1), while DHEA treatment had no effect on the down regulation of NF-κB expression. These data suggest that both DHEA and HA(1) exert significant antimetastatic activities by inhibiting MMP-9 and VEGF expression. DHEA-induced an  timetastas  is was more potent than HA(1). In addition, only HA(1) treatment downregulated the expression level of NF-κB, suggesting that the antimetastatic activity of triterpene glycosides derived from P. graeffei can be either NF-κB-dependent or -independent, depending on their structure. 
 Modulation of u-PA, MMPs and their inhibitors by a novel nutrient mixture in pediatric human sarcoma cell lines. Pediatric sarcomas are highly aggressive tumors that are characterized by high levels of matrix metalloproteinase (MMP)-2 and -9 secretions that degrade the ECM and basement membrane, allowing cancer cells to spread to distal organs. Proteases play a key role in tumor cell invasion and   metastasis   by digesting the basement membrane and ECM components. Strong clinical and experimental evidence demonstrates association of elevated levels of u-PA and MMPs with cancer progression,   metastasis   and shortened patient survival. MMP activities are regulated by specific tissue inhibitors of metalloproteinases (TIMPs). Our main objective was to study the effect of a nutrient mixture (NM) on activity of u-PA, MMPs and TIMPs in various human pediatric sarcomas. Human osteosarcoma MNNG-HOS, osteosarcoma U-2OS and rhabdomyosarcoma RD cell lines (ATCC) were cultured in their respective media and treated at confluence with NM at 0, 50, 100, 250, 500 and 1,000 µg/ml. Analysis of u-PA activity was carried out by fibrin zymography, MMPs by gelatinase zymography and TIMPs by reverse zymography. All sarcoma cell lines studied expressed u-PA, which was inhibited by NM in a dose-dependent manner. On gelatinase zymography, osteosarcoma MNNG-HOS showed a band corresponding to MMP-2 and induction of MMP-9 with PMA (100 ng/ml) treatment. U-2OS osteosarcoma cells showed strong bands corresponding to inactive MMP-2 and MMP-9 and faint bands corresponding to active MMP-2 and MMP-9 dimer; PMA treatment enhanced MMP-9 and MMP-9 dimer activity. Rhabdomyosarcoma showed MMP-2 and faint MMP-9 bands; PMA treatment enhanced MMP-9 expression. NM inhibited their expression in a dose-dependent manner. Activity of TIMPs was upregulated by NM in all cancer cell lines in a dose-dependent manner. Analysis revealed a positive correlation between u-PA and MMPs and a negative correlation between u-PA/MMPs and TIMPs. These findings suggest the therapeutic potential of NM in treatment of pediatric sarcomas. 
 The diagnostic value of matrix metalloproteinase 9 (MMP-9) and tissue inhibitor of matrix metalloproteinases 1 (TIMP-1) determination in the sera of colorectal adenoma and cancer patients. Tumor cells, including colorectal cancer (CRC), are able to produce and release matrix metalloproteinase 9 (MMP-9) which is involved in tumor invasion and   metastasis  . Natural tissue inhibitors of matrix metalloproteinases (TIMPs) regulate activity of MMPs and stimulate tumor growth and malignant transformation. The aim of the present study was to compare the clinical significance of serum MMP-9 with TIMP-1 in the diagnosis of CRC patients and in the differentiation between colorectal adenoma (CA) and cancer.Serum MMP-9 and TIMP-1 were measured in 75 CRC patients, 35 CA, and 70 healthy subjects using enzyme-linked immunosorbent assay. Concentrations of carcinoembryonic antigen (CEA) and carbohydrate antigen 19-9 (CA 19-9) were determined by microparticle enzyme immunoassay.Serum levels of all proteins tested were significantly higher in CRC patients than in healthy subjects. Additionally, serum TIMP-1 was significantly higher in patients with CRC than in CA patients. Concentrations of TIMP-1 correlated with tumor stage, nodal involvement, presence of distant metastases, patients' survival, and tumor resectability. Diagnostic sensitivity of TIMP-1 was higher (61%) than those of other biomarkers (MMP-9, 55%; CEA, 39%; CA 19-9, 11%), and increased in combined use with MMP-9 (75%) or CEA (73%). The areas under receiver operating characteristic curves of TIMP-1 were larger than those of MMP-9.Our findings suggest better usefulness of serum TIMP-1 than MMP-9 in the diagnosis of CRC, especially in the assessment of Duke's classification of tumor stage, survival of cancer patients, resectability of tumor, and in the differentiation between CA and cancer. 
 MicroRNA-377 predicts poor clinical outcome of gastric cancer and induces tumorigenesis by targeting multiple tumor-suppressor genes. Gastric cancer (GC) is a major cause of cancer mortality worldwide. MicroRNAs are evolutionally conserved small non-coding RNAs that are critical for the regulation of gene expression. The aberrant expression of microRNA (miRNA) is involved in tumorigenesis and prognosis. In the present study, the clinical significance of miR-377 was assessed using RT-qPCR and MTT assay. The results showed that the expression of miR-377 was upregulated in GC compared with normal gastric tissues, and its expression level was increased in GC cell lines compared with normal gastric cells. In addition, there was a significant association between miR-377 expression and clinicopathological characteristics, in particular distant   metastasis  , TNM stage and early recurrence. GC patients with a higher miR-377 expression showed significantly poorer overall survival (OR) and shorter time to recurrence than those with a lower miR-377 expression. The Cox regression analysis identified miR-377 overexpression as an independent prognostic factor for GC. Overexpression of miR-377 in MKN-45 GC cells significantly promoted cell proliferation, whereas the suppression of miR-377 inhibited these effects. Furthermore, miR-377 downregulated p53, PTEN and TIMP1 expression by directly targeting the 3'-untranslated region of these target genes. Collectively, miR-377 potentially served as a new molecular predictive biomarker of GC tumorigenesis and prognosis, which may be useful in targeted therapy and the prognosis of GC patients. 
 Expression of tissue inhibitors of metalloproteinase 1 (TIMP-1) in gastric cancer tissue. Degradation of extracellular matrix (ECM) is an essential step of invasion and   metastasis   of gastric cancer. The proteolysis of basement membranes depends on the balance between activities of matrix metalloproteinases (MMPs) and their tissue inhibitors (TIMPs). The aim of the study was to assess the expression of TIMP-1 in gastric cancer (GC) and interstitial inflammatory infiltrate cells within GC tissue in relation to clinico-pathological features of tumor and to estimate the prognostic significance of TIMP-1 expression for patients' survival. The presence of TIMP-1 in 54 cases of gastric cancer samples was investigated by immunohistochemistry. The expression of TIMP-1 in cancer and interstitial inflammatory infiltrate cells was evaluated in semi-quantitative scale. The immunoreactivity of TIMP-1 in cancer and inflammatory cells was positive in 100% of cases and varied from weak to intense reaction. The intensity of TIMP-1 expression increased with more advanced tumor stages and in patients who died of cancer during 2-year observation. TIMP-1 expression in interstitial inflammatory infiltrate cells was the independent prognostic factor for patients' survival. The results suggest the role of TIMP-1 in gastric tumorigenesis, although this issue requires further investigtions. 
 High expression of TIMP-1 in human breast cancer tissues is a predictive of resistance to paclitaxel-based chemotherapy. For breast cancer patients with lymph node   metastasis  , paclitaxel is the first-line chemotherapy drug. Clinical studies showed that some patients with breast cancer were insensitive to paclitaxel, which led to chemotherapy failure. Today, no validated markers exist for the prediction of chemotherapy sensitivity in this patient group. Tissue inhibitor of metalloproteinases-1 (TIMP-1) has been shown to protect against apoptosis. Epidemiological studies have also associated elevated tumor tissue TIMP-1 levels with a poor response to cyclophosphamide/methotrexate/5-fluorouracil and anthracycline-based chemotherapy. Additionally, our previous study proved that TIMP-1 significantly decreased the sensitivity of breast cancer cells to paclitaxel-induced apoptosis by enhancing degradation of cyclin B1. These data imply that TIMP-1 may be a useful predictive biomarker for chemotherapy resistance. In this retrospective study, we investigated the association between expression levels of TIMP-1 protein in the primary tumor and objective response to paclitaxel-based chemotherapy in 99 patients with breast cancer. With Kaplan-Meier survival analysis, the patients with high TIMP-1 levels were found to have significantly worse 5-year DFS (71.1 %) than the patients with low levels (88.5 %; P = 0.020). Similarly, the patients with high TIMP-1 levels had significantly worse 5-year OS (78.9 %) than patients with low levels (96.7 %; P = 0.004). In Cox's univariate and multivariate analyses, TIMP-1 was prognostic for both DFS and OS. Our data showed that elevated tumor tissue TIMP-1 levels were significantly associated with a poor response to paclitaxel-based chemotherapy, and TIMP-1 might be a potential biomarker for predicting response of breast cancer patients to paclitaxel-based chemotherapy. 
 Prognostic values of matrix metalloproteinase family expression in human colorectal carcinoma. We examined expression patterns of matrix metalloproteinase (MMP), tissue inhibitor of metalloproteinase (TIMP), and reversion-inducing cysteine-rich protein with Kazal motifs (RECK) in colorectal cancer tissues to assess their prognostic significance.mRNA expressions of 17 MMPs, 4 TIMPs, and RECK were measured in 112 colorectal cancerous tissues, 20 normal mucosa tissues, and 11 metastatic liver lesions by real-time reverse-transcriptional-polymerase chain reaction. The protein level expressions were confirmed with immunohistochemistry.Cancers and normal mucosa displayed highly significant differences (P < 0.01) in expression of nine genes (MMP-1, -3, -7, -9, -10, -11, -12, -14, and RECK). Primary cancers and metastatic lesions showed highly significant differences (P < 0.01) in MMP-1, -10, -11, and TIMP-1. MMP-12 expression was higher in the primary tumors that were associated without hepatic   metastasis   than those with   metastasis   (P < 0.01). High expression of MMP-15 was related to longer disease-free survival (generalized Wilcoxon test, P < 0.0062; Cox hazard model, P < 0.028, hazard ratio, 0.099).MMP, TIMP, RECK expression patterns may provide an insight into extracellular matrix degrading (which is characteristic of colorectal cancers) and its role in   metastasis  . 
 TIMP-1 overexpression in lung carcinoma enhances tumor kinetics and angiogenesis in brain   metastasis  . Tissue inhibitors of matrix metalloproteinase (TIMP) orchestrate many biologic activities, including inhibition of matrix metalloproteinase activity, activation of pro-matrix metalloproteinases, and regulation of cell proliferation, angiogenesis, and apoptosis induction. Tissue inhibitors of matrix metalloproteinase can play a protective role during tumor invasion and   metastasis  , but elevated TIMP messenger RNA levels have also been associated with aggressive cancers and poor clinical outcome. We examined the potential roles of TIMP-1 in H2009 lung adenocarcinoma cells and in cells transfected with a human TIMP-1-overexpressing vector (HB-6 and HB-1). Tumors resulting from the implantation of parental cell lines and transfected HB-1 cells into the brains of nude mice had a typical carcinoma profile, but human TIMP-1-overexpressing tumors showed enhanced tumor kinetics and focally more infiltrative features; vessel density assessed with anti-CD31 immunohistochemistry was also greater within HB-1 tumor implants. Similar effects on HB-6 and HB-1 cells versus parental cell lines and empty vector clones were observed in endothelial cell assays. Anchorage-independent growth and invasion through Matrigel were also increased in TIMP-1-overexpressing cells. Together, these results indicate tumor-promoting functions of TIMP-1 through alterations in angiogenesis, increased tumorigenicity, and invasive behavior. Although matrix metalloproteinase inhibition has been the traditionally identified function of TIMP-1, matrix metalloproteinase-independent interactions may contribute to the growth of metastatic carcinomas in the brain. 
 Upregulated TIMP-1 correlates with poor prognosis of laryngeal squamous cell carcinoma. The tissue inhibitor of metalloproteinase-1 (TIMP-1) is an endogenous inhibitor of matrix metalloproteinases and potential biomarker of various types of human cancers. However, the association between TIMP-1 expression and the clinical features of laryngeal squamous cell carcinoma (LSCC) is barely investigated. In this study, one-step quantitative reverse transcription-polymerase chain reaction and immunohistochemical staining with tissue microarrays were employed to evaluate the relationship between TIMP-1 expression and the clinicopathological characteristics of LSCC. Results showed that the TIMP-1 mRNA and protein expression levels were significantly higher in LSCC than in the corresponding non-cancerous tissues (p<0.05). TIMP-1 protein expression in LSCC was associated with tumor differentiation (p=0.012) and overall survival (p=0.043). Kaplan-Meier method and Cox multi-factor analysis suggested that high TIMP-1 expression (p=0.008) and positive lymph node   metastasis   (p=0.029) were significantly associated with the poor survival of patients with LSCC. These data indicated that TIMP-1 may be identified as a prognostic marker of LSCC. 
 Invasion front-specific overexpression of tissue inhibitor of metalloproteinase-1 in liver metastases from colorectal cancer. Matrix metalloproteinases (MMPs) and their inhibitors, the tissue inhibitors of metalloproteinases (TIMPs), have been implicated in invasion and   metastasis  . The distribution of MMPs and TIMPs in the invasion front of liver metastases from colorectal cancer were investigated in order to understand their potential role in invasiveness.Freshly frozen material of colorectal metastases of the liver was microdissected into four separate compartments, namely pure liver, liver invasion, tumour invasion and pure tumour. RNA was isolated and analyzed on Affymetrix microarrays. Expression of TIMP-1 was confirmed by quantitative polymerase chain reaction in 10 colorectal liver metastases. Cellular localisation of TIMP-1 was examined by immunohistochemistry.Affymetrix microarray data revealed that several MMP and TIMP genes including MMP-2, -3, -7, -9, -11, -12, -14, -15, -16, -19 and -24, and TIMP-1, -2 and -3 were generally up-regulated in both invasion front compartments. Among these genes, TIMP-1 showed the highest expression. The qPCR results indicated an average 15-fold upregulation of TIMP-1 in the liver invasive front and an average 13-fold up-regulation in the tumor invasive front, each compared to normal liver tissue. Immunohistochemistry revealed expression of TIMP-1 in tumour epithelia as well as in host tissue cells, including fibroblastic cells.Our data indicate that tumour invasion in colorectal liver   metastasis   is associated with increased TIMP-1 RNA and protein levels in both tumour and host cells. 
 Tissue inhibitor of matrix metalloproteinase-1 is prognostic in head and neck squamous cell carcinoma: comparison of the circulating and tissue immunoreactive protein. Tissue inhibitors of metalloproteinases (TIMP) are capable of inhibiting the matrix metalloproteinases, but they also possess other biological functions. Little is known about the role of TIMP-1 in the progression and spreading of cancer cells among patients with head and neck squamous cell carcinoma (HNSCC). In this study, the pretreatment serum levels of TIMP-1 or the overexpression of TIMP-1 immunoreactive protein in the primary tumor was correlated to the clinical course in patients with HNSCC.The TIMP-1 immunoreactive protein was studied in 74 cases representing HNSCC. The tissue immunoreactive protein was evaluated from paraffin-embedded tumor sections in 68 cases using immunohistologic staining with a specific antibody, and in 68 cases the pretreatment serum levels of TIMP-1 were quantitatively measured by ELISA assay. The results were compared with the clinicopathologic factors of the disease and the patients' outcome.A positive correlation was found between the size of the primary tumor (T) and the circulating TIMP-1 level (P = 0.021) or the positive immunoreaction of TIMP-1 in tumor (P = 0.039). The 5-year cause-specific survival was significantly lower in patients presenting with a high serum TIMP-1 level than in those with a low level of TIMP-1 (38% versus 64%, P = 0.034). They also had an unfavorable 5-year relapse-free survival rate (37% versus 56%, respectively). Similarly, the expression of TIMP-1 in tumor was prognostic for shortened survival, the 5-year cumulative relapse-free survival being 42% in patients with a TIMP-1-positive tumor versus 75% in cases with a negative tumor (P = 0.035). Tissue TIMP-1 positivity also seemed associated to the cause-specific survival (P = 0.075) and to be connected with later lymph node or hematogenic relapses.This study shows for the first time that both circulating and tissue TIMP-1 immunoreactive protein predicts the clinical course and dissemination in HNSCC, suggesting that TIMP-1 might be related to both tumor growth and   metastasis   in HNSCC. 
 Lack of relationship between TIMP-1 tumour cell immunoreactivity, treatment efficacy and prognosis in patients with advanced epithelial ovarian cancer. Tissue inhibitor of metalloproteinase 1 (TIMP-1) is a natural inhibitor of the matrix metalloproteinases (MMPs) which are proteolytic enzymes involved in degradation of extracellular matrix thereby favoring tumour cell invasion and   metastasis  . TIMP-1 activity in tumour tissue may therefore play an essential role in the progression of a malignant tumour.The primary aim of the present study was to evaluate TIMP-1 protein immunoreactivity in tissue from primary ovarian cancer patients and associate these findings with the course of the disease including response to treatment in the individual patient.TIMP-1 was assessed by immunohistochemistry (in tissue micro arrays) in a total of 163 ovarian cancer specimens obtained from primary debulking surgery during 1991-1994 as part of a randomized clinical protocol.Positive TIMP-1 immunoreactivity was found in 12.3% of the tumours. The median survival time for the 143 patients with TIMP-1 negative tumours was 23.7 months [19.0-29.4] 95% CI, while the median survival time for the 20 patients with TIMP-1 positive tumours was 15.9 months [12.3-27.4] 95% CI. Although a difference of 7.8 months in median overall survival in favor of the TIMP-1 tumour negative patients was found, this difference did not reach statistical significance (p = 0.28, Kaplan-Meier, log-rank test). Moreover, TIMP-1 immunoreactivity was not associated with CA125 response (p = 0.53) or response at second look surgery (p = 0.72).TIMP-1 immunoreactivity in tumour tissue from patients with primary epithelial ovarian cancer did not correlate with patient survival or response to combination platinum/cyclophosphamide therapy. 
 Expression of matrix metalloproteinases in ovarian endometriomas: immunohistochemical study and enzyme immunoassay. Like carcinoma, endometriosis has the unique characteristics, of invasion and   metastasis  , though pathologically, it is a benign tumor. However, the mechanism of destruction of the surrounding tissue in endometriosis is still unclear. In this study, the expression and localization of matrix metalloproteinases (MMP)-1, -2, -3, -7, -9 and tissue inhibitors of metalloproteinases-1 (TIMP-1) were evaluated by immunohistochemistry for 20 cases and the amounts of MMP-1, TIMP-1 and MMP-1/TIMP-1 complex in the fluid of endometrioma, were analyzed by ELISA and western blotting for 20 cases, which were analyzed by immunohistochemical study. MMP-1, -2 and -9 were detected strongly in both stromal and epithelial cells and MMP-7 in the epithelial cells in the menstrual period. MMP-3 was mainly expressed in macrophage containing hemosiderin but the change of expression was not clear. TIMP-1 was intensively detected in both stromal and epithelial cells in the menstrual period but the expression decreased in other stages of the menstrual cycle. ELISA for MMP-1 also showed results similar to immunohistochemistry, suggesting that it was released to the cyst in the menstrual period when it was released to the extracellular space from the cytoplasm. The expression of TIMP-1 was not clearly changed during the menstrual cycle. From these results, it was suggested that the destruction of the surrounding matrix by endometriosis might be caused by various MMPs, which are mainly produced in stromal cells. 
 Levels of matrix metalloproteinase-1 and tissue inhibitors of metalloproteinase-1 in gastric cancer. To evaluate the levels of preoperative serum matrix metalloproteinase-1 (MMP-1) and tissue inhibitor of metalloproteinase-1 (TIMP-1) in gastric cancer.One hundred gastric cancer patients who underwent gastrectomy were enrolled in this study. The serum concentrations of MMP-1 and TIMP-1 in these patients and in fifty healthy controls were determined using an enzyme-linked immunosorbent assay.Higher serum MMP-1 and TIMP-1 levels were observed in patients than in controls (P < 0.001). Serum MMP-1 and TIMP-1 levels were positively associated with morphological appearance, tumor size, depth of wall invasion, lymph node   metastasis  , liver   metastasis  , perineural invasion, and pathological stage. They were not significantly associated with age, gender, tumor location, or histological type.Increased MMP-1 and TIMP-1 were associated with gastric cancer. Although these markers are not good markers for diagnosis, these markers show in advanced gastric cancer. 
 Imbalance between MMP-2, 9 and TIMP-1 promote the invasion and   metastasis   of renal cell carcinoma via SKP2 signaling pathways. S-phase kinase-associated protein-2 (Skp2) is overexpressed in human cancers and acted as an oncogenic protein associated with poor prognosis by enhancing tumor   metastasis  . The present study has demonstrated that Skp2 overexpresses stable transfectants from 786-0 human renal cancer cells. We found that these stable transfectants exhibited increased migratory and invasive abilities. In addition, expression of matrix metalloproteinase-2 (MMP-2) and MMP-9 was upregulated and tissue inhibitor of matrix metalloproteinase-1 (TIMP-1) was downregulated. In contrast, RNA interference-mediated knockdown Skp2 expression suppressed the ability of ACHN cells to migratory and invasive. Skp2 depletion increased P27 and decreased cyclin E activity, and then induced cell cycle arrest in the G0/G1 phase. Skp2 depletion also downregulated MMP-2 and MMP-9, while upregulated the TIMP-1 activity and expression. The results suggest that Skp2 signaling pathways promoted the ability to metastasize, by stimulating cell proliferation and increasing the ratio of MMP-2 and MMP-9/TIMP-1. So, in conclusion, we provide the first evidence that the imbalance of MMP/TIMP, including upregulation of MMP-2 and MMP-9 and downregulation of TIMP-1, is one of the mechanisms by which Skp2 promotes cell invasion. 
 Protein levels of tissue inhibitor of metalloproteinase-1 in tumor extracts as a marker for prognosis and recurrence in patients with gastric cancer. Tissue inhibitor of metalloproteinase-1 (TIMP-1) correlates with tumor progression in patients with gastric cancer; however, the clinical significance of TIMP-1 as a marker for prognosis and recurrence has not been fully clarified.TIMP-1 protein was measured by an enzyme-linked immunosorbent assay in tumor samples from 86 patients who had undergone surgical resection. An intratumoral TIMP-1 value of 10.0 ng/mg protein or more was defined as positive. Patients were followed up for more than 5 years prospectively.Thirty-one of the 86 patients (36.0%) were positive for TIMP-1. Kaplan-Meier curves for overall survival were significantly different between patients who were positive and those who were negative for TIMP-1. Univariate analysis of factors affecting overall survival showed that depth of tumor invasion; lymph node   metastasis  ; peritoneal dissemination; lymphatic invasion; venous invasion; Lauren classification of histology; curability; and TIMP-1 were statistically significant. Stepwise multivariate analysis for overall survival demonstrated that depth of tumor invasion, nodal   metastasis  , peritoneal dissemination, and TIMP-1 remained independent prognostic factors. Kaplan-Meier curves for disease-free survival were significantly different between patients who were positive and those who were negative for TIMP-1. The incidence of recurrence was significantly higher in patients positive for TIMP-1 than in those who were negative for TIMP-1. The frequency at each site of recurrence was higher in patients positive for TIMP-1.These results suggested that the protein concentration of TIMP-1 in the tumor extracts was a useful marker for overall survival, disease-free survival, and disease recurrence in patients with gastric cancer. Thus, tumor TIMP-1 may serve to identify a high-risk group, for whom optimal surgical and medical treatment can be given. 
 Increased levels of tissue inhibitor of metalloproteinase-1 in human hepatocellular carcinoma. Invasion and   metastasis   of hepatocellular carcinoma (HCC) are regulated by matrix metalloproteinases (MMPs) and tissue inhibitors of metalloproteinases (TIMPs). However, the role of TIMPs in these processes is not clear.To examine the potential involvement of TIMP-1 in HCC and the association between TIMP-1 and clinical outcome of patients with HCC.The study included 91 patients who underwent surgical removal of HCC. TIMP-1 concentrations in the supernatant of tissue homogenates of HCC and non-neoplastic liver were measured by enzyme immunoassay. The relationships between TIMP-1 concentration and various clinicopathological features and recurrence of HCC after surgical operation were examined.The mean level of TIMP-1 in HCC (486 +/- 610 ng/mg protein, +/- SD) was significantly higher than in the non-neoplastic liver (75 +/- 69, P < 0.0001). The median level of TIMP-1 in poorly differentiated HCCs (701 ng/mg protein) was significantly higher than in well- (80) and moderately (172) differentiated HCCs (P = 0.0047 and P = 0.0082, respectively). TIMP-1 level in liver cirrhosis was higher than in chronic hepatitis (P = 0.0015). TIMP-1 levels in HCC did not influence the recurrence rate of HCC.TIMP-1 concentration in HCC was higher than in non-neoplastic liver and correlated with the differentiation grade of HCCs. However, tissue TIMP-1 concentration does not seem to be an important determinant of HCC recurrence. 
 [Relationship of expression unbalance of matrix metalloproteinase and tissue inhibitor of metalloproteinase to invasiveness and   metastasis   in gastric carcinomas]. Matrix metalloproteinase-9 (MMPs) and its tissue inhibitor of metalloproteinase-1 (TIMPs) have the important regulatory roles in the processes during tumor invasion and   metastasis  . Observing the coexpression of matrix metalloproteinase 9(MMP-9) and tissue inhibitor of metalloproteinase 1(TIMP-1) in gastric carcinomas, this study was designed to investigate the relationship of the expression unbalance of MMP-9 and TIMP-1 to the invasiveness,   metastasis  , and prognosis in gastric carcinomas.By using immunohistochemistry, the expression of MMP-9, TIMP-1, and proliferating cell nuclear antigen Ki-67 was detected in 256 gastric cancers, and the patients were followed-up.The expressive degree of MMP-9 in the tumor invasion beyond musculariis propria(70.13%) was higher than that within lamina propria or submucosa(42.50%, P < 0.05) and its expression in tumor was positively associated with tumor cell proliferation index and lymph node   metastasis  , and its expression in high TNM stage(75.00%) was higher than those in low TNM stage(46.15%, P < 0.05). The relation of the expression of TIMP-1 to the depth of invasion, lymph node   metastasis  , and TNM stage was negative(Pearson contingency coefficient were 0.1688, 0.3556, and 0.3004, respectively P < 0.05). There were four groups of the unbalance expression of MMP-9 and TIMP-1 in gastric carcinomas. While the frequency of the serosa invasion and lymph node   metastasis   in the group of the expression of MMP-9 over TIMP-1 was significantly higher than those in the groups of the expression of TIMP-1 over MMP-9 or the balance expression of MMP-9 and TIMP-1. However, prognosis of the patients with the expression of MMP-9 over TIMP-1 was significantly worse than that with expression of TIMP-1 over MMP-9(P < 0.05).The expression of MMP-9 was positively related to the tumor invasiveness and   metastasis   in gastric cancers, however the association between the positive expression of TIMP-1 and the invasiveness and   metastasis   was negative. The relationship of MMP-9 and TIMP-1 to the invasiveness and   metastasis   in gastric carcinomas was that MMP-9 promotes and TIMP-1 inhibits tumor   metastasis  . The patients with MMP-9 positive expression and TIMP-1 negative expression are poorer prognosis. 
 Mechanism of inhibition of the human matrix metalloproteinase stromelysin-1 by TIMP-1. Matrix metalloproteinases (MMPs) are zinc endopeptidases that are required for the degradation of extracellular matrix components during normal embryo development, morphogenesis and tissue remodelling. Their proteolytic activities are precisely regulated by endogenous tissue inhibitors of metalloproteinases (TIMPs). Disruption of this balance results in diseases such as arthritis, atherosclerosis, tumour growth and   metastasis  . Here we report the crystal structure of an MMP-TIMP complex formed between the catalytic domain of human stromelysin-1 (MMP-3) and human TIMP-1. TIMP-1, a 184-residue protein, has the shape of an elongated, contiguous wedge. With its long edge, consisting of five different chain regions, it occupies the entire length of the active-site cleft of MMP-3. The central disulphide-linked segments Cys 1-Thr 2-Cys 3-Val 4 and Ser 68-Val 69 bind to either side of the catalytic zinc. Cys 1 bidentally coordinates this zinc, and the Thr-2 side chain extends into the large specificity pocket of MMP-3. This unusual architecture of the interface between MMP-3 and TIMP-1 suggests new possibilities for designing TIMP variants and synthetic MMP inhibitors with potential therapeutic applications. 
 Tamoxifen lowers the MMP-9/TIMP-1 ratio and inhibits the invasion capacity of ER-positive non-small cell lung cancer cells. Tamoxifen (TAM) serves for decades as a therapy drug for the prevention and treatment of breast cancers, especially effective for the estrogen receptor (ER)-positive ones. An increasing number of studies are trying to explore its potential application in treating other types of tumor including lung cancer. However, the effects of TAM on lung cancer cells, especially ER-positive ones, remain unclear. Thus, the present study was undertaken to assess the impact of TAM on the invasion capacity of an ER-positive human lung cancer cell model. In this study, the immunohistochemical staining was applied to verify the expression of estrogen receptors in SPC-A-1, a human lung adenocarcinoma cell line. The real-time PCR analysis was performed to test the expressions of MMP-9 and TIMP-1 in SPC-A-1 cells treated with different doses of TAM, while the invasion capacity was determined using transwell assays. In TAM-treated SPC-A-1 cells, which are ER-positive, an impaired ratio of MMP-9/TIMP-1 was observed as a net result of an increased transcriptional level of TIMP-1 as well as a reduced one of MMP-9. Furthermore, TAM suppressed the invasion of SPC-A-1 cells in vitro. Thus, we propose that TAM could work as an anti-  metastasis   drug inhibiting the invasion of human lung cancer cells. 
 Small GTPase Rab37 targets tissue inhibitor of metalloproteinase 1 for exocytosis and thus suppresses tumour   metastasis  . Rab small GTPases are master regulators of membrane trafficking and guide vesicle targeting. Recent publications show that Rab-controlled trafficking pathways are altered during tumorigenesis. However, whether any of the Rabs plays a   metastasis   suppressor role is least explored. Here we address the   metastasis   suppressive function of human Rab37 (hRAB37) using secretomics, cell, animal and clinical analyses. We show that tissue inhibitor of metalloproteinase 1 (TIMP1), a secreted glycoprotein that inhibits extracellular matrix turnover, is a novel cargo of hRAB37. hRAB37 regulates the exocytosis of TIMP1 in a nucleotide-dependent manner to inactivate matrix metalloproteinase 9 (MMP9) migration axis in vitro and in vivo. Dysfunction of hRAB37 or TIMP1 abrogates   metastasis   suppression. Lung cancer patients with   metastasis   and poor survival show low hRAB37 protein expression coinciding with low TIMP1 in tumours. Our findings identify hRAB37 as a novel   metastasis   suppressor Rab that functions through the TIMP1-MMP9 pathway and has significant prognostic power. 
  GeneRIFs  
 Results show that TIMP-1 expression is growth pattern dependent in colorectal cancer liver metastases. At the   metastasis   periphery, TIMP-1 could have MMP inhibitory activity, and in the pericytes it could be involved in tumor angiogenesis. 
 RAB37 regulates the exocytosis of TIMP1 in a nucleotide-dependent manner to inactivate MMP9 migration axis in vitro and in vivo and to suppress tumor   metastasis  . 
 this study presents novel functions of TIMP-1 in promoting cancer   metastasis   and suggests TIMP-1 is a potential tissue biomarker for lymph invasion and distant   metastasis   of lung adenocarcinoma. 
 MMP-2, MMP-9 and TIMP-2, TIMP-1 may play an important role in the development, progression, invasion and   metastasis   of non-melanoma skin cancer. 
 TIMP-1 expression had no relationship with lymph node   metastasis   in nasopharyngeal carcinoma. 
 The primary tumors from patients with LR showed significantly higher percentage of TIMP-1 expression in stromal cells compared to primary tumors from patients with distant   metastasis   or primary tumors from patients without tumoral progression. 
 Tumour invasion in colorectal liver   metastasis   is associated with increased TIMP-1 RNA and protein levels in both tumour and host cells. 
 Study suggest that TIMP-1 might be related to both tumor growth and   metastasis   in head and neck squamous cell carcinoma. 
 Data suggest that tissue expression of TIMP1 could be a useful marker to predict the progression and   metastasis   of hepatocellular carcinoma. 
 The interaction between MMP-9 and TIMP-1 in the processes of gastric tumor invasion and   metastasis   is that MMP-9 mainly promotes tumor invasion and   metastasis   and TIMP-1 inhibits functions of MMP-9. 
 the association between the positive expression of TIMP-1 and the invasiveness and   metastasis   was negative. 
 The over-expression of MMP-2, MMP-9, TIMP-1, and TIMP-2 may play a key role in invasion and lymph-node   metastasis   in squamous carcinoma of the cervix. 
  [Cc]ell [Ii]nvasion  PubMed  
 TIMP-1 expression in human colorectal cancer is associated with TGF-B1, LOXL2, INHBA1, TNF-AIP6 and TIMP-2 transcript profiles. The balance of activity between the endogenous enzyme inhibitors known as tissue inhibitors of metalloproteinases and their targets, the matrix metalloproteinases, in the extracellular matrix is thought to play an important role in tumour   cell invasion  . Supporting this notion, we have shown that colorectal cancer patients have increased plasma levels of the tissue inhibitor of metalloproteinases-1 (TIMP-1), and that high plasma TIMP-1 levels are associated with short colorectal cancer patient survival. However, although TIMP-1 has been extensively studied in cancer, very little is known about how it is regulated. To further elucidate potential mechanisms of regulation of this protein, we did a number of experiments to look at associations between the transcript profile of TIMP-1 with known matrix metalloproteinases (MMPs) as well as with expression profiles of other genes differentially regulated in human colorectal cancer (CRC) and the other TIMPs 2-4, which have also been associated with the progression of colorectal cancer. Genome-wide expression profiling of 172 CRC and normal mucosa samples was used to identify transcript changes for the genes under investigation. We found that TIMP-1 was up-regulated in CRC samples compared with normal tissue, while TIMP-2 was down-regulated. Eight MMPs were up-regulated in CRC compared with normal tissue. Correlating up-regulated genes with the TIMP-1 transcript, we identified 13 that were also up-regulated in cancerous tissue. Among these were genes associated with the synthesis of extracellullar matrix, genes involved in the TGF-beta signalling pathway, and genes that are likely transcribed by the tumour cells. These insights add to the complex picture emerging about the regulation of TIMPs in colorectal cancer. 
 TIMP-1 expression in human colorectal cancer is associated with TGF-B1, LOXL2, INHBA1, TNF-AIP6 and TIMP-2 transcript profiles. The balance of activity between the endogenous enzyme inhibitors known as tissue inhibitors of metalloproteinases and their targets, the matrix metalloproteinases, in the extracellular matrix is thought to play an important role in tumour   cell invasion  . Supporting this notion, we have shown that colorectal cancer patients have increased plasma levels of the tissue inhibitor of metalloproteinases-1 (TIMP-1), and that high plasma TIMP-1 levels are associated with short colorectal cancer patient survival. However, although TIMP-1 has been extensively studied in cancer, very little is known about how it is regulated. To further elucidate potential mechanisms of regulation of this protein, we did a number of experiments to look at associations between the transcript profile of TIMP-1 with known matrix metalloproteinases (MMPs) as well as with expression profiles of other genes differentially regulated in human colorectal cancer (CRC) and the other TIMPs 2-4, which have also been associated with the progression of colorectal cancer. Genome-wide expression profiling of 172 CRC and normal mucosa samples was used to identify transcript changes for the genes under investigation. We found that TIMP-1 was up-regulated in CRC samples compared with normal tissue, while TIMP-2 was down-regulated. Eight MMPs were up-regulated in CRC compared with normal tissue. Correlating up-regulated genes with the TIMP-1 transcript, we identified 13 that were also up-regulated in cancerous tissue. Among these were genes associated with the synthesis of extracellullar matrix, genes involved in the TGF-beta signalling pathway, and genes that are likely transcribed by the tumour cells. These insights add to the complex picture emerging about the regulation of TIMPs in colorectal cancer. 
 BRAFV600E mutation, TIMP-1 upregulation, and NF-κB activation: closing the loop on the papillary thyroid cancer trilogy. BRAF(V600E) is the most common mutation found in papillary thyroid carcinoma (PTC). Tissue inhibitor of metalloproteinases (TIMP-1) and nuclear factor (NF)-κB have been shown to play an important role in thyroid cancer. In particular, TIMP-1 binds its receptor CD63 on cell surface membrane and activates Akt signaling pathway, which is eventually responsible for its anti-apoptotic activity. The aim of our study was to evaluate whether interplay among these three factors exists and exerts a functional role in PTCs. To this purpose, 56 PTC specimens were analyzed for BRAF(V600E) mutation, TIMP-1 expression, and NF-κB activation. We found that BRAF(V600E) mutation occurs selectively in PTC nodules and is associated with hyperactivation of NF-κB and upregulation of both TIMP-1 and its receptor CD63. To assess the functional relationship among these factors, we first silenced BRAF gene in BCPAP cells, harboring BRAF(V600E) mutation. We found that silencing causes a marked decrease in TIMP-1 expression and NF-κB binding activity, as well as decreased invasiveness. After treatment with specific inhibitors of MAPK pathway, we found that only sorafenib was able to increase IκB-α and reduce both TIMP-1 expression and Akt phosphorylation in BCPAP cells, indicating that BRAF(V600E) activates NF-κB and this pathway is MEK-independent. Taken together, our findings demonstrate that BRAF(V600E) causes upregulation of TIMP-1 via NF-κB. TIMP-1 binds then its surface receptor CD63, leading eventually to Akt activation, which in turn confers antiapoptotic behavior and prom  otion of cell   invasion. The recognition of this functional trilogy provides insight on how BRAF(V600E) determines cancer initiation, progression, and invasiveness in PTC, also identifying new therapeutic targets for the treatment of highly aggressive forms. 
 Functional proteomics study reveals that N-Acetylglucosaminyltransferase V reinforces the invasive/metastatic potential of colon cancer through aberrant glycosylation on tissue inhibitor of metalloproteinase-1. N-Acetylglucosaminyltransferase-V (GnT-V) has been reported to be up-regulated in invasive/metastatic cancer cells, but a comprehensive understanding of how the transferase correlates with the invasive/metastatic potential is not currently available. Through a glycomics approach, we identified 30 proteins, including tissue inhibitor of metalloproteinase-1 (TIMP-1), as a target protein for GnT-V in human colon cancer cell WiDr. TIMP-1 was aberrantly glycosylated as characterized by the addition of beta1,6-N-acetylglucosamine, polylactosaminylation, and sialylation in GnT-V-overexpressing WiDr cells. Compared with normal TIMP-1, the aberrantly glycosylated TIMP-1 showed the weaker inhibition on both matrix metalloproteinase (MMP)-2 and MMP-9, and this aberrancy was closely associated with cancer   cell invasion   and metastasis in vivo as well as in vitro. Integrated data, both of TIMP-1 expression level and aberrant glycosylation, could provide important information to aid to improve the clinical outcome of colon cancer patients. 
 Hyaluronan synthase 2 (HAS2) promotes breast cancer   cell invasion   by suppression of tissue metalloproteinase inhibitor 1 (TIMP-1). Invasion and metastasis are the primary causes of breast cancer mortality, and increased knowledge about the molecular mechanisms involved in these processes is highly desirable. High levels of hyaluronan in breast tumors have been correlated with poor patient survival. The involvement of hyaluronan in the early invasive phase of a clone of breast cancer cell line MDA-MB-231 that forms bone metastases was studied using an in vivo-like basement membrane model. The metastatic to bone tumor cells exhibited a 7-fold higher hyaluronan-synthesizing capacity compared with MDA-MB-231 cells predominately due to an increased expression of hyaluronan synthase 2 (HAS2). We found that knockdown of HAS2 completely suppressed the invasive capability of these cells by the induction of tissue metalloproteinase inhibitor 1 (TIMP-1) and dephosphorylation of focal adhesion kinase. HAS2 knockdown-mediated inhibition of basement membrane remodeling was rescued by HAS2 overexpression, transfection with TIMP-1 siRNA, or addition of TIMP-1-blocking antibodies. Moreover, knockdown of HAS2 suppressed the EGF-mediated induction of the focal adhesion kinase/PI3K/Akt signaling pathway. Thus, this study provides new insights into a possible mechanism whereby HAS2 enhances breast cancer invasion. 
 TIMP-1 overexpression promotes tumorigenesis of MDA-MB-231 breast cancer cells and alters expression of a subset of cancer promoting genes in vivo distinct from those observed in vitro. TIMP-1 (Tissue inhibitor of matrix metalloproteinase-1) is typically associated with inhibition of matrix metalloproteinases (MMP) induced invasion. However, TIMP-1 is overexpressed in many malignancies and is associated with poor prognosis in breast cancer. The mechanisms by which TIMP-1 promotes tumorigenesis are unclear. Reduced levels of TIMP-1 mediated by shRNA in MDA-MB-231 breast cancer cells had no effect on cellular physiology in vitro or tumor growth in SCID mice compared to vector control MDA-MB-231 cells. However, overexpression of TIMP-1 in MDA-MB-231 cells resulted in inhibition of   cell invasion   and enhanced phosphorylation of p38 MAPK and AKT in vitro. Additionally, treatment of parental MDA-MB-231 cells with purified TIMP-1 protein led to activation of p38 MAPK and MKK 3/6. cDNA array analysis demonstrated that high expression of TIMP-1 in MDA-MB-231 cells resulted in alterations in expression of approximately 200 genes, 1.5 fold or greater compared to vector control cells (P < 0.1). Real-time RT-PCR confirmed changes in expression of several genes associated with cancer progression including DAPK1, FGFR4 and MAPK13. In vivo, high TIMP-1 expression induced tumor growth in SCID mice compared to vector control cells and increased tumor vessel density. Affymetrix array analysis of vector control and TIMP-1 MDA-MB-231 xenograft tumors revealed that TIMP-1 altered expression of approximately 600 genes in vivo, including MMP1, MMP13, S100A14, S100P, Rab25 and ID4. These combined observations suggest that the effects of TIMP-1 differ significantly in a 2-D environment compared to the 3-D environment and that TIMP-1 stimulates tumor growth. 
 Runx3 suppresses gastric cancer metastasis through inactivation of MMP9 by upregulation of TIMP-1. Recent studies have suggested that loss of RUNX3 expression is involved with gastric tumor metastasis. However, the precise mechanism of RUNX3-mediated suppression of tumor metastasis remains elusive. We aimed to clarify the effect of RUNX3 on tumor metastasis in gastric cancer cell lines and tumors. Immunohistochemistry revealed that RUNX3 was significantly decreased in metastatic gastric cancer. Gelatin zymography and Western blot showed that instead of regulating matrix metalloproteinase 9 (MMP9) expression, RUNX3 expression inhibited MMP9 enzyme activity, and this was consistent with the upregulation of tissue inhibitor of metalloproteinases 1 (TIMP1) by RUNX3. TIMP1 siRNA treatment impaired RUNX3-mediated suppression of gastric cancer   cell invasion  . Reporter assays demonstrated regulation of TIMP-1 by RUNX3. Two RUNX3 binding sites were identified in the TIMP-1 promoter and direct interaction of RUNX3 with the TIMP-1 promoter was confirmed in vitro and in vivo. These findings provide evidence for RUNX3-mediated suppression of gastric cancer invasion and metastasis and define a novel molecular mechanism that for the metastasis-inhibiting activity of RUNX3. These data may be applied in the development of RUNX3 for gastric cancer metastasis diagnostics and therapeutics. 
 TIMP-1 Induces α-Smooth Muscle Actin in Fibroblasts to Promote Urethral Scar Formation. Tissue inhibitor of metalloproteinases-1 (TIMP-1) has been reported to upregulate in urethral scar. However, the underlying molecular mechanisms remain undefined.Here, we studied levels of TIMP-1 and α-smooth muscle actin (α-SMA) in the fibroblasts isolated from urethral scar tissues, compared to the fibroblasts isolated from normal urethra. Then we either overexpressed TIMP-1, or inhibited TIMP-1 by lentiviruses carrying a transgene or a short hairpin small interfering RNA for TIMP-1 in human fibroblasts. We examined the effects of modulation of TIMP-1 on α-SMA, and on epithelial-mesenchymal transition (EMT)-related genes. We also studied the underlying mechanisms.We detected significantly higher levels of TIMP-1 and α-smooth muscle actin (α-SMA) in the fibroblasts isolated from urethral scar tissues, compared to the fibroblasts isolated from normal urethra. Moreover, the levels of TIMP-1 and α-SMA strongly correlated. Moreover, we found that TIMP-1 significantly increased levels of α-SMA, transforming growth factor β 1 (TGFβ1), Collagen I and some other key factors related to an enhanced EMT, suggesting that TIMP-1 may induce transformation of fibroblasts into myofibroblasts to promote tissue EMT to enhance the formation of urethral scar. Moreover, increases in TIMP-1 also induced an increase in fibroblast cell g  rowth and cel  l invasion, in an ERK/MAPK-signaling-dependent manner.Our study thus highlights a pivotal role of TIMP-1 in urethral scar formation. 
 Integrin alphavbeta3-mediated transcriptional regulation of TIMP-1 in a human ovarian cancer cell line. We have previously reported that a disintegrin inhibits solid tumor growth and metastasis in mouse model [I.C. Kang, Y.D. Lee, D.S. Kim, A novel disintegrin salmosin inhibits tumor angiogenesis, Cancer Res. 59 (1999) 3754-3760; S.I. Kim, K.S. Kim, H.S. Kim, D.S. Kim, Y. Jang, K.H. Chung, Y.S. Park, Inhibitory effect of the salmosin gene transferred by cationic liposomes on the progression of B16BL6 tumors, Cancer Res. 63 (2003) 6458-462]. In this study, we have investigated the modulatory effect of a disintegrin, saxatilin, on the balance between MMP-9 and tissue inhibitor of metalloproteinase-1 (TIMP-1) in human ovarian cancer cell line MDAH 2774. Functional mechanism of the disintegrin-mediated transcriptional regulation of MMP-9 and TIMP-1 was examined in the ovarian cancer cell line. Saxatilin strongly induced TIMP-1 expression in dose- and time-dependent manners, while the disintegrin suppressed MMP-9 expression. Further analyses clearly indicated that interaction of the disintegrin and integrin alphavbeta3 results in the TIMP-1 promoter activation via c-fos to suppress TNF-alpha-induced cancer   cell invasion  . These results demonstrate that integrin alphavbeta3-mediated transcriptional regulation of MMP-9 and TIMP-1 is critical for suppressing the ovarian cancer   cell invasion  . 
 Thrombospondin-1 (TSP-1) up-regulates tissue inhibitor of metalloproteinase-1 (TIMP-1) production in human tumor cells: exploring the functional significance in tumor   cell invasion  . Thrombospondin-1 (TSP-1), a matrix-bound adhesive glycoprotein, has been shown to modulate tumor progression. We previously demonstrated that TSP-1 up-regulates matrix metalloproteinases MMP-2 and MMP-9. Our studies suggested that the balance between MMPs and tissue inhibitors of metalloproteinases (TIMPs) is a key determinant in tumor   cell invasion  . We now report that TSP-1 up-regulates TIMP-1 expression in both human breast and prostate cancer cell lines. The effect of TSP-1 on TIMP-1 expression was examined in human breast adenocarcinoma cell lines (MDA-MB-231) and human prostate cancer cell lines (PC3-NI and PC3-ML) treated with exogenous TSP-1. TIMP-1 expression was also examined in TSP-1 stably transfected breast cancer cell line (MDA-MB-435). Northern and western blot analysis revealed TIMP-1 mRNA and TIMP-1 protein expression increased with increasing concentrations of TSP-1. This effect was inhibited by antibodies against the type I repeat domain of TSP-1 further suggesting that TSP-1 mediates TIMP-1 secretion. Inhibition of TSP-1 induced TIMP-1 levels increased tumor   cell invasion  . We conclude that TSP-1 is involved in influencing the critical balance between MMPs and their inhibitors, maintaining the controlled degradation of the extracellular matrix needed to support metastasis and our results may provide an explanation for the divergent activities reported for TSP-1 in tumor progression. 
 Targeting TR4 nuclear receptor suppresses prostate cancer invasion via reduction of infiltrating macrophages with alteration of the TIMP-1/MMP2/MMP9 signals. TR4 nuclear receptor 4 (TR4) plays an important role in macrophages-associated foam cell formation of cardiovascular diseases and infiltrating macrophages are critical for prostate cancer (PCa) progression. However, the linkage of macrophages and TR4 and their impacts on PCa metastasis remains unclear.Knocking-down TR4 in human PCa cells (C4-2, CWR22Rv1), but not in human macrophages cells (THP-1), led to suppress the macrophages infiltration to PCa cells. The consequences of such suppression of the recruitment of macrophages toward PCa then resulted in suppressing the PCa   cell invasion  . Mechanism dissection found that knocking-down TR4 in PCa cells suppressed metastasis-related genes including MMP2, with induction of TIMP-1. Interruption assays using TIMP-1 neutralizing antibody could then reverse TR4-macrophage-mediated PCa invasion. IHC staining showed higher TR4 level, more macrophage infiltration, lower TIMP-1 and stronger MMP2/MMP9 in tumor tissues of the Gleason score 5 + 4 patients compared with the Gleason score 3 + 3 patients.Targeting TR4 in prostate tumor microenvironment might represent a potential new therapeutic approach to better battle PCa metastasis. 
 Nodal signals through activin receptor-like kinase 7 to inhibit trophoblast migration and invasion: implication in the pathogenesis of preeclampsia. Trophoblast   cell invasion   into the uterus is an essential process for successful pregnancy, and shallow invasion of trophoblasts into the maternal decidua is linked to preeclampsia. We have reported that Nodal, a member of the transforming growth factor-β superfamily, acts through activin receptor-like kinase 7 (ALK7) to inhibit trophoblast proliferation and to induce apoptosis. In this study, we examined the spatial and temporal expression patterns of Nodal and ALK7 in human placenta from normal and preeclamptic pregnancies and investigated whether Nodal regulated trophoblast migration and invasion. Nodal and ALK7 were detected in villous and extravillous trophoblast cell populations in early gestation, and their levels were strongly up-regulated in preeclamptic placenta. Overexpression of Nodal or constitutively active ALK7 decreased cell migration and invasion, whereas knockdown of Nodal and ALK7 had the opposite effects. In placental explant culture, treatment with Nodal inhibited trophoblast outgrowth, whereas Nodal small-interfering RNA strongly induced the expansion of explants and the migration of extravillous trophoblast cells. Nodal stimulated the secretion of tissue inhibitor of metalloproteinase-1 and inhibited matrix metalloproteinase (MMP)-2 and MMP-9 activity. These findings suggest that the Nodal/ALK7 pathway plays important roles in human placentation and that its abnormal signaling may contribute to the development of preeclampsia. 
 Hypoxia induces FGF2 production by vascular endothelial cells and alters MMP9 and TIMP1 expression in extravillous trophoblasts and their invasiveness in a cocultured model. The role of fibroblast growth factor 2 (FGF2) secretion by vascular endothelial cells during trophoblast invasion was assessed. The human extravillous trophoblast cell line, TEV-1, and umbilical vein endothelial cell line, HUVE-12, were cocultured under normal and hypoxic conditions. FGF2 expression in HUVE-12 cells and matrix metalloproteinase 9 (MMP9) and tissue inhibitor of metalloproteinase 1 (TIMP1) expression in TEV-1 cells were analyzed using quantitative RT-PCR and Western blot analyses. TEV-1   cell invasion   was also examined. FGF2 expression in the HUVE-12 cells cocultured with TEV-1 cells was significantly increased under hypoxic conditions. In the TEV-1 cells cocultured with HUVE-12, hypoxia reduced MMP9 expression and increased TIMP1 expression; it also reduced   cell invasion   by 43%. However, the expression of MMP9 and TIMP1 and ratio of MMP9/TIMP1 were increased when the TEV-1 cells were cultured alone under hypoxic conditions. These findings suggest that FGF2 release by stressed endothelial cells of uterine spiral arteries play roles in decreasing MMP9 and increasing TIMP1 production in extravillous trophoblasts (EVT) in response to stress, resulting in reduced EVT invasion and possibly shallow implantation of the placenta. 
 Modulation of u-PA, MMPs and their inhibitors by a novel nutrient mixture in pediatric human sarcoma cell lines. Pediatric sarcomas are highly aggressive tumors that are characterized by high levels of matrix metalloproteinase (MMP)-2 and -9 secretions that degrade the ECM and basement membrane, allowing cancer cells to spread to distal organs. Proteases play a key role in tumor   cell invasion   and metastasis by digesting the basement membrane and ECM components. Strong clinical and experimental evidence demonstrates association of elevated levels of u-PA and MMPs with cancer progression, metastasis and shortened patient survival. MMP activities are regulated by specific tissue inhibitors of metalloproteinases (TIMPs). Our main objective was to study the effect of a nutrient mixture (NM) on activity of u-PA, MMPs and TIMPs in various human pediatric sarcomas. Human osteosarcoma MNNG-HOS, osteosarcoma U-2OS and rhabdomyosarcoma RD cell lines (ATCC) were cultured in their respective media and treated at confluence with NM at 0, 50, 100, 250, 500 and 1,000 µg/ml. Analysis of u-PA activity was carried out by fibrin zymography, MMPs by gelatinase zymography and TIMPs by reverse zymography. All sarcoma cell lines studied expressed u-PA, which was inhibited by NM in a dose-dependent manner. On gelatinase zymography, osteosarcoma MNNG-HOS showed a band corresponding to MMP-2 and induction of MMP-9 with PMA (100 ng/ml) treatment. U-2OS osteosarcoma cells showed strong bands corresponding to inactive MMP-2 and MMP-9 and faint bands corresponding to active MMP-2 and MMP-9 dimer; PMA treatment enhanced MMP-9 and MMP-9 dimer activity. Rhabdomyosarcoma showed MMP-2 and faint MMP-9 bands; PMA treatment enhanced MMP-9 expression. NM inhibited their expression in a dose-dependent manner. Activity of TIMPs was upregulated by NM in all cancer cell lines in a dose-dependent manner. Analysis revealed a positive correlation between u-PA and MMPs and a negative correlation between u-PA/MMPs and TIMPs. These findings suggest the therapeutic potential of NM in treatment of pediatric sarcomas. 
 In papillary thyroid carcinoma, TIMP-1 expression correlates with BRAF (V600E) mutation status and together with hypoxia-related proteins predicts aggressive behavior. BRAF (V600E) causes upregulation of tissue inhibitor of metalloproteinase-1 (TIMP-1), which promotes   cell invasion   in papillary thyroid carcinoma (PTC). Hypoxia-inducible factor-1α (HIF- α) is regulated by hypoxia and also by the BRAF-mediated signaling pathway in PTC. We assessed the association of expression of TIMP-1, HIF-1α, and hypoxia-inducible carbonic anhydrase IX (CAIX) and XII (CAXII) with clinical parameters in PTC. TPC-1/BRAF (WT) wild-type and BcPAP/BRAF (V600E) -mutated PTC cell lines were selected to study the effects of the BRAF (V600E) mutation and hypoxia on expression in vitro of TIMP-1, CAIX, and CAXII proteins by immunoblotting. Higher expression of all proteins was detected in BcPAP cells exposed to hypoxia. Tissue microarray immunohistochemistry analysis was performed to study protein expression in 114 BRAF-genotyped PTC samples. Expression data on tumor tissue were compared with clinicopathological variables. TIMP-1 expression had a sensitivity of 87 % and a specificity of 83 % in identifying a BRAF mutation (P < 0.001) and was associated with pT stage (P = 0.001), pN stage (P = 0.02), and multifocality (P = 0.03). HIF-1α expression correlated with pT stage (P = 0.05). CAIX expression was associated with pN stage (P = 0.02), and both CAIX (P = 0.004) and CAXII (P = 0.05) were strongly associated with vascular invasion. We conclude that TIMP-1 protein expression is a reliable surrogate marker for BRAF-mutated status in PTC. TIMP-1 and hypoxia-regulated proteins are promising as predictors of aggressiveness in PTC and warrant further investigation as new therapeutic targets for the treatment of highly aggressive forms of PTC. 
 Immunohistochemical study of matrix metalloproteinase 9 and tissue inhibitor of matrix metalloproteinase 1 in benign and malignant breast tissue--strong expression in intraductal carcinomas of the breast. Matrix metalloproteinases (MMPs) are involved in carcinogenesis due to their tissue remodeling capability, and there is convincing evidence linking gelatinase B (MMP-9) with malignant   cell invasion  . Tissue inhibitor 1 of MMP (TIMP-1) is a strong inhibitor of MMP-9 but has also tumor-enhancing effects. Only few data exist on MMP-9 or TIMP-1 expression in tissue samples of different breast histology.MMP-9 and TIMP-1 immunoreactivity was examined in a wide range of breast tissue samples differing in histology from usual ductal hyperplasia (UDH) to fully developed ductal breast carcinoma. Immunohistochemical expression of MMP-9 was studied in 178 samples: 31 UDH samples, 29 atypical ductal hyperplasia (ADH) samples, 28 ductal carcinoma in situ (DCIS) samples and 90 ductal invasive carcinoma samples (30 samples of malignancy grades I, II and III, respectively). TIMP-1 expression was also analyzed in 178 breast tissue samples: 41 UDH, 21 ADH and 34 DCIS lesions, and 82 invasive ductal breast carcinomas (25 in grade I, 30 in grade II and 27 in grade III).A significantly distinctive pattern of MMP-9 protein expression was shown in DCIS samples, where 85.7% of the cases showed moderate or strong positivity and negative staining was rare (p = 0.021). Negative or weakly positive MMP-9 staining was the most prominent finding in UDH (71%), ADH (69%) as well as in invasive carcinoma samples (64.4%). Various degrees of TIMP-1 expression were seen in 86.5% of all cases. DCIS and invasive carcinoma samples revealed similar immunostaining: at least some positivity was seen in 91.1% of the DCIS samples and 91.5% of infiltrative carcinomas. Thus, TIMP-1 negativity (22.2%) was significantly associated with hyperplastic lesions (p = 0.026).These results suggest that MMP-9 and TIMP-1 overexpression are early markers of breast carcinogenesis preceding tumor invasion. Apparently, DCIS carries the risk to evolve into a malignant phenotype according to these markers. The clinical importance of these findings is discussed. 
 Leukemia inhibitory factor increases the invasiveness of trophoblastic cells through integrated increase in the expression of adhesion molecules and pappalysin 1 with a concomitant decrease in the expression of tissue inhibitor of matrix metalloproteinases. To identify the invasion-associated molecules during leukemia inhibitory factor-(LIF-)mediated increase in the invasion of trophoblast cells.Experimental study.Research institution.None.Cultured trophoblastic HTR-8/SVneo cells were treated with LIF.Matrigel matrix-based invasion assay of HTR-8/SVneo cells. Signaling molecules associated with LIF-mediated increase in invasion were investigated by Western blot, cDNA microarray, quantitative reverse transcriptase polymerase chain reaction, immunofluorescence, immunohistochemistry, and gene silencing by siRNA.Treatment of HTR-8/SVneo cells with LIF (50 ng/mL) led to a significant increase in invasion. Treatment with LIF also led to an increase in nuclear localization of activated STAT1 and STAT3. Among 237 differentially expressed genes after LIF treatment, expression of pappalysin 1, SERPINB3, podoplanin, integrin β3, ID1, ICAM1, and so on went up, while tissue inhibitor of matrix metalloproteinase 1 (TIMP1), TIMP2, and TIMP3 went down significantly. The presence of several of these proteins has also been demonstrated in human trophoblast cells. Silencing of pappalysin 1 led to a significant reduction in basal as well as LIF-mediated invasiveness of HTR-8/SVneo cells.Identification of novel molecules associated with a LIF-mediated increase in trophoblastic   cell invasio  n may facilitate our understanding of implantation biology. 
 Lack of relationship between TIMP-1 tumour cell immunoreactivity, treatment efficacy and prognosis in patients with advanced epithelial ovarian cancer. Tissue inhibitor of metalloproteinase 1 (TIMP-1) is a natural inhibitor of the matrix metalloproteinases (MMPs) which are proteolytic enzymes involved in degradation of extracellular matrix thereby favoring tumour   cell invasion   and metastasis. TIMP-1 activity in tumour tissue may therefore play an essential role in the progression of a malignant tumour.The primary aim of the present study was to evaluate TIMP-1 protein immunoreactivity in tissue from primary ovarian cancer patients and associate these findings with the course of the disease including response to treatment in the individual patient.TIMP-1 was assessed by immunohistochemistry (in tissue micro arrays) in a total of 163 ovarian cancer specimens obtained from primary debulking surgery during 1991-1994 as part of a randomized clinical protocol.Positive TIMP-1 immunoreactivity was found in 12.3% of the tumours. The median survival time for the 143 patients with TIMP-1 negative tumours was 23.7 months [19.0-29.4] 95% CI, while the median survival time for the 20 patients with TIMP-1 positive tumours was 15.9 months [12.3-27.4] 95% CI. Although a difference of 7.8 months in median overall survival in favor of the TIMP-1 tumour negative patients was found, this difference did not reach statistical significance (p = 0.28, Kaplan-Meier, log-rank test). Moreover, TIMP-1 immunoreactivity was not associated with CA125 response (p = 0.53) or response at second look surgery (p = 0.72).TIMP-1 immunoreactivity in tumour tissue from patients with primary epithelial ovarian cancer did not correlate with patient survival or response to combination platinum/cyclophosphamide therapy. 
 Imbalance between MMP-2, 9 and TIMP-1 promote the invasion and metastasis of renal cell carcinoma via SKP2 signaling pathways. S-phase kinase-associated protein-2 (Skp2) is overexpressed in human cancers and acted as an oncogenic protein associated with poor prognosis by enhancing tumor metastasis. The present study has demonstrated that Skp2 overexpresses stable transfectants from 786-0 human renal cancer cells. We found that these stable transfectants exhibited increased migratory and invasive abilities. In addition, expression of matrix metalloproteinase-2 (MMP-2) and MMP-9 was upregulated and tissue inhibitor of matrix metalloproteinase-1 (TIMP-1) was downregulated. In contrast, RNA interference-mediated knockdown Skp2 expression suppressed the ability of ACHN cells to migratory and invasive. Skp2 depletion increased P27 and decreased cyclin E activity, and then induced cell cycle arrest in the G0/G1 phase. Skp2 depletion also downregulated MMP-2 and MMP-9, while upregulated the TIMP-1 activity and expression. The results suggest that Skp2 signaling pathways promoted the ability to metastasize, by stimulating cell proliferation and increasing the ratio of MMP-2 and MMP-9/TIMP-1. So, in conclusion, we provide the first evidence that the imbalance of MMP/TIMP, including upregulation of MMP-2 and MMP-9 and downregulation of TIMP-1, is one of the mechanisms by which Skp2 promotes   cell invasion  . 
 Increased matrix metalloproteinase-2 and tissue inhibitor of metalloproteinase-1 secretion but unaffected invasiveness of endometrial stromal cells in adenomyosis. To investigate cell invasiveness and the production of matrix metalloproteinase-2 (MMP-2), MMP-9, tissue inhibitor of metalloproteinase-1 (TIMP-1) and TIMP-2 in endometrial stromal cells (ESCs) of adenomyosis.Prospective study.Tertiary university hospital.Twenty-one women with adenomyosis (study group) and 25 women without adenomyosis (control group).Endometrial stromal cells were purified from eutopic endometrium and were cultured in vitro.Matrigel invasion assay and measurement of MMP-2, MMP-9, TIMP-1, and TIMP-2 concentrations.Endometrial stromal cell invasiveness of adenomyosis was not different from that of the control group (0.95 vs. 1, using the latter as the bench mark) in ESCs cultured alone. After the ESCs were cultured with IL-6, anti-IL-6, or GM6001 for 24 hours, the indices of   cell invasion   were not different between the two groups. MMP-2 (median 7.3 vs. 3.5 ng/mL) and TIMP-1 (median 12.1 vs. 3.9 ng/mL), but not MMP-9 and TIMP-2, secreted by ESCs in women with adenomyosis were much higher than those in women without adenomyosis.The formation of adenomyosis does not result from altered invasiveness of ESCs. The concomitant elevation of MMP-2 and TIMP-1 may partially justify why invasiveness was not increased, but other enzymes should also be considered. 
 Inhibition of cancer   cell invasion   by cannabinoids via increased expression of tissue inhibitor of matrix metalloproteinases-1. Cannabinoids, in addition to having palliative benefits in cancer therapy, have been associated with anticarcinogenic effects. Although the antiproliferative activities of cannabinoids have been intensively investigated, little is known about their effects on tumor invasion.Matrigel-coated and uncoated Boyden chambers were used to quantify invasiveness and migration, respectively, of human cervical cancer (HeLa) cells that had been treated with cannabinoids (the stable anandamide analog R(+)-methanandamide [MA] and the phytocannabinoid delta9-tetrahydrocannabinol [THC]) in the presence or absence of antagonists of the CB1 or CB2 cannabinoid receptors or of transient receptor potential vanilloid 1 (TRPV1) or inhibitors of p38 or p42/44 mitogen-activated protein kinase (MAPK) pathways. Reverse transcriptase-polymerase chain reaction (RT-PCR) and immunoblotting were used to assess the influence of cannabinoids on the expression of matrix metalloproteinases (MMPs) and endogenous tissue inhibitors of MMPs (TIMPs). The role of TIMP-1 in the anti-invasive action of cannabinoids was analyzed by transfecting HeLa, human cervical carcinoma (C33A), or human lung carcinoma cells (A549) cells with siRNA targeting TIMP-1. All statistical tests were two-sided.Without modifying migration, MA and THC caused a time- and concentration-dependent suppression of HeLa   cell invasion   through Matrigel that was accompanied by increased expression of TIMP-1. At the lowest concentrations tested, MA (0.1 microM) and THC (0.01 microM) led to a decrease in invasion (normalized to that observed with vehicle-treated cells) of 61.5% (95% CI = 38.7% to 84.3%, P < .001) and 68.1% (95% CI = 31.5% to 104.8%, P = .0039), respectively. The stimulation of TIMP-1 expression and suppression of   cell invasion   were reversed by pretreatment of cells with antagonists to CB1 or CB2 receptors, with inhibitors of MAPKs, or, in the case of MA, with an antagonist to TRPV1. Knockdown of cannabinoid-induced TIMP-1 expression by siRNA led to a reversal of the cannabinoid-elicited decrease in tumor cell invasiveness in HeLa, A549, and C33A cells.Increased expression of TIMP-1 mediates an anti-invasive effect of cannabinoids. Cannabinoids may therefore offer a therapeutic option in the treatment of highly invasive cancers. 
  GeneRIFs  
 upregulation of MMP-2 and MMP-9 and downregulation of TIMP-1, is one of the mechanisms by which Skp2 promotes   cell invasion   of human renal cancer cells 
 Identification of novel molecules associated with a LIF-mediated increase in trophoblastic   cell invasion   may facilitate our understanding of implantation biology. 
 BRAF(V600E) causes upregulation of TIMP-1 via NF-kappaB. TIMP-1 binds then its surface receptor CD63, leading eventually to Akt activation, which in turn confers antiapoptotic behavior and promotion of   cell invasion  . 
 Hyaluronan synthase 2 (HAS2) promotes breast cancer   cell invasion   by suppression of tissue metalloproteinase inhibitor 1 (TIMP-1) 
 These results demonstrate that integrin alphavbeta3-mediated transcriptional regulation of MMP-9 and TIMP-1 is critical for suppressing the ovarian cancer   cell invasion  . 
   
  
 TIMP3 
 
  NCBI Gene  
  GeneRIF  
  Pubmed  
 
 
  Search Term  Rank  Fields  
  [Mm]igration  6  PubMed(19); GeneRIFs(5)  
  ECM  7  PubMed(27); GeneRIFs(1); Summary(1)  
  [Ee]xtracellular [Mm]atrix  8  Component(2); PubMed(76); Conserved Domains(1); GeneRIFs(6); Summary(1)  
  [Ii]nvasive  10  PubMed(24); GeneRIFs(4)  
  [Mm]etastasis  12  PubMed(32); GeneRIFs(6)  
  [Cc]ell [Ii]nvasion  13  PubMed(9); GeneRIFs(1)  
 
 
 hide/show details for TIMP3 
  [Mm]igration  PubMed  
 Lysophosphatidic acid downregulates tissue inhibitor of metalloproteinases, which are negatively involved in lysophosphatidic acid-induced cell invasion. Ovarian cancer is a highly metastatic disease. Lysophosphatidic acid (LPA) levels are elevated in ascites from ovarian cancer patients, but its potential role in ovarian cancer metastasis has just begun to be revealed. In this work, we show that LPA stimulates invasion of primary ovarian cancer cells, but not ovarian epithelial or borderline ovarian tumor cells, although these benign cells indeed respond to LPA in cell   migration  . We have found that LPA downregulates tissue inhibitor of metalloproteinases (TIMPs). TIMP2 and TIMP3 play functional role in LPA-induced invasion as negative regulators. G(i) protein, phosphatidylinositol-3 kinase (PI3K), p38 mitogen-activated protein kinase (MAPK), cytosolic phospholipase A(2) and urokinase type plasminogen activator (uPA) are required for LPA-induced cells invasion. TIMP3 may affect two independent downstream targets, vascular endothelial growth factor receptor and p38 MAPK. In vivo, LPA stimulates tumor metastasis in an orthotopic ovarian tumor model, which can be inhibited by a PI3K inhibitor, LY294002. In summary, LPA is likely a key component for promoting ovarian metastasis in vivo. LPA downregulates TIMP3, which may have targets other than metalloproteinases. Our in vivo metastasis mouse model is useful for studying the efficacy of therapeutic regimes of ovarian cancer. 
 miR-221&222 regulate TRAIL resistance and enhance tumorigenicity through PTEN and TIMP3 downregulation. Lung and liver cancers are among the most deadly types of cancer. Despite improvements in treatment over the past few decades, patient survival remains poor, underlining the need for development of targeted therapies. MicroRNAs represent a class of small RNAs frequently deregulated in human malignancies. We now report that miR-221&222 are overexpressed in aggressive non-small cell lung cancer and hepatocarcinoma cells, as compared with less invasive and/or normal lung and liver cells. We show that miR-221&222, by targeting PTEN and TIMP3 tumor suppressors, induce TRAIL resistance and enhance cellular   migration   through the activation of the AKT pathway and metallopeptidases. Finally, we demonstrate that the MET oncogene is involved in miR-221&222 activation through the c-Jun transcription factor. 
 miR-221&222 regulate TRAIL resistance and enhance tumorigenicity through PTEN and TIMP3 downregulation. Lung and liver cancers are among the most deadly types of cancer. Despite improvements in treatment over the past few decades, patient survival remains poor, underlining the need for development of targeted therapies. MicroRNAs represent a class of small RNAs frequently deregulated in human malignancies. We now report that miR-221&222 are overexpressed in aggressive non-small cell lung cancer and hepatocarcinoma cells, as compared with less invasive and/or normal lung and liver cells. We show that miR-221&222, by targeting PTEN and TIMP3 tumor suppressors, induce TRAIL resistance and enhance cellular   migration   through the activation of the AKT pathway and metallopeptidases. Finally, we demonstrate that the MET oncogene is involved in miR-221&222 activation through the c-Jun transcription factor. 
 Lysophosphatidic acid downregulates tissue inhibitor of metalloproteinases, which are negatively involved in lysophosphatidic acid-induced cell invasion. Ovarian cancer is a highly metastatic disease. Lysophosphatidic acid (LPA) levels are elevated in ascites from ovarian cancer patients, but its potential role in ovarian cancer metastasis has just begun to be revealed. In this work, we show that LPA stimulates invasion of primary ovarian cancer cells, but not ovarian epithelial or borderline ovarian tumor cells, although these benign cells indeed respond to LPA in cell   migration  . We have found that LPA downregulates tissue inhibitor of metalloproteinases (TIMPs). TIMP2 and TIMP3 play functional role in LPA-induced invasion as negative regulators. G(i) protein, phosphatidylinositol-3 kinase (PI3K), p38 mitogen-activated protein kinase (MAPK), cytosolic phospholipase A(2) and urokinase type plasminogen activator (uPA) are required for LPA-induced cells invasion. TIMP3 may affect two independent downstream targets, vascular endothelial growth factor receptor and p38 MAPK. In vivo, LPA stimulates tumor metastasis in an orthotopic ovarian tumor model, which can be inhibited by a PI3K inhibitor, LY294002. In summary, LPA is likely a key component for promoting ovarian metastasis in vivo. LPA downregulates TIMP3, which may have targets other than metalloproteinases. Our in vivo metastasis mouse model is useful for studying the efficacy of therapeutic regimes of ovarian cancer. 
 TIMP3 regulates osteosarcoma cell   migration  , invasion, and chemotherapeutic resistances. Tissue inhibitors of metalloproteinases (TIMPs) inhibit matrix metalloproteinases (MMPs) to limit degradation of the extracellular matrix. Low levels of TIMP3 have been demonstrated in cancer tissues at advanced clinical stages, with positive distant metastasis and chemotherapeutic resistance. We examined the role of TIMP3 in osteosarcoma (OS) cell invasiveness and chemoresistance. TIMP3 was overexpressed or knocked down in the human OS cell lines Saos2 and MG63. Cell   migration   and invasion capacities were then evaluated using Transwell assays, and resistance to cisplatin was assessed by CCK-8 assay and flow cytometry. Real-time PCR and western blotting were used to investigate activation of signaling pathways downstream of TIMP3. Overexpression of TIMP3 inhibited the   migration   and invasion of Saos2 and MG63 cells, while knockdown of TIMP3 had the opposite effect. Cell survival after exposure to cisplatin was inhibited by TIMP3 overexpression in both Saos2 and MG63 cells. Consistently, downregulation of TIMP3 gene expression significantly decreased the sensitivity of OS cells to cisplatin treatment. MMP1, MMP2, Bcl-2, and Akt1 were all downregulated following TIMP3 overexpression, while Bax and cleaved caspase-3 were upregulated. TIMP3 knockdown had opposite effects on the regulation of these genes. Taken together, our findings suggest TIMP3 as a new target for inhibition of OS progression and chemotherapeutic resistance. 
 Identification and characterization of human tissue inhibitor of metalloproteinase-3 and detection of three additional metalloproteinase inhibitor activities in extracellular matrix. We have identified and characterized a novel human tissue inhibitor of metalloproteinase (TIMP). It is found exclusively in the extracellular matrix of a large number of cultured human cells, including: primary embryonal kidney (293), neuroblastoma (SK-N-SH), normal whole embryo (FHs 173We), cervical carcinoma (HeLa S3), colon adenocarcinoma (Caco-2), ileocecal adenocarcinoma (HCT-8), fibrosarcomas (SW 684 and Hs 913T) and normal gingival fibroblasts (GF11 and 1292). It was not detected in the conditioned media from any of these cell lines. Its apparent molecular mass of 24-25 kDa, as determined by its   migration   on protease-substrate gels, is intermediate between TIMP-1 (28.5 kDa) and TIMP-2 (21 kDa). Like the latter two proteins, human TIMP-3 contains intrachain disulfide bonds and displays altered electrophoretic mobility in the presence of beta-mercaptoethanol. The N-terminal, amino acid sequence of the protein is identical to that of chicken TIMP-3 (ChIMP-3), and its amino acid composition is similar. The protein is not N-glycosylated, as determined by treatment with N-glycosidase-F. Finally, it is recognized by antisera raised against pure ChIMP-3 but not by anti-human TIMP-1 or anti-human TIMP-2 antibodies. Based on these properties, we propose that this protein is TIMP-3 and is the human counterpart of ChIMP-3 (Pavloff et al., J. Biol. Chem. 267: 17321-17326, 1992). Two additional inhibitors detected in the matrix of human cell lines, designated inhibitor of metalloproteinase (IMP)-a and IMP-b, migrate with apparent masses of 29 kDa and 30 kDa. Both are N-glycosylated. A fourth inhibitor activity, which is smaller in mass than TIMP-3 and is also pecifically located in the matrix, is detectable in some cell lines. 
 P14ARF inhibits human glioblastoma-induced angiogenesis by upregulating the expression of TIMP3. Malignant gliomas are the most common and the most lethal primary brain tumors in adults. Among malignant gliomas, 60%-80% show loss of P14ARF tumor suppressor activity due to somatic alterations of the INK4A/ARF genetic locus. The tumor suppressor activity of P14ARF is in part a result of its ability to prevent the degradation of P53 by binding to and sequestering HDM2. However, the subsequent finding of P14ARF loss in conjunction with TP53 gene loss in some tumors suggests the protein may have other P53-independent tumor suppressor functions. Here, we report what we believe to be a novel tumor suppressor function for P14ARF as an inhibitor of tumor-induced angiogenesis. We found that P14ARF mediates antiangiogenic effects by upregulating expression of tissue inhibitor of metalloproteinase-3 (TIMP3) in a P53-independent fashion. Mechanistically, this regulation occurred at the gene transcription level and was controlled by HDM2-SP1 interplay, where P14ARF relieved a dominant negative interaction of HDM2 with SP1. P14ARF-induced expression of TIMP3 inhibited endothelial cell   migration   and vessel formation in response to angiogenic stimuli produced by cancer cells. The discovery of this angiogenesis regulatory pathway may provide new insights into P53-independent P14ARF tumor-suppressive mechanisms that have implications for the development of novel therapies directed at tumors and other diseases characterized by vascular pathology. 
 Differential expression and regulation of ADAM17 and TIMP3 in acute inflamed intestinal epithelia. The acute phase of Crohn's disease (CD) is characterized by a large afflux of polymorphonuclear leukocytes (PMNL) into the mucosa and by the release of TNF-alpha. Conversion of inactive TNF-alpha into an active form requires the cleavage of a transmembrane TNF-alpha precursor by the TNF-alpha-converting enzyme (ADAM17), a protease mainly regulated by the tissue inhibitor of metalloproteinase 3 (TIMP3). The aim of the present study was to investigate in an in vitro model of PMNL transepithelial   migration   and in the intestinal mucosa of patients with CD the expression and regulation of ADAM17 and TIMP3 in intestinal epithelial cells (IEC). ADAM17 and TIMP3 expression was analyzed by Western blotting, RT-PCR, confocal microscopy, and immunohistochemistry by using the T84 model and digestive biopsies. ADAM17 expression in IEC was increased at a posttranscriptional level during the early phase (from 2 to 4 h) of PMNL transepithelial   migration   whereas TIMP3 was only increased 24 h later. TNF-alpha induced an early upregulation of ADAM17 in T84 cells, whereas PMNL adhesion, H(2)O(2), or epithelial tight junction opening alone did not affect the amount of ADAM17. Immunohistochemistry of intestinal biopsies revealed that strong expression of ADAM17 was associated with a high activity of CD. In contrast, TIMP3 was very poorly expressed in these biopsies. ADAM17 and TIMP3 profiling did not correlated with the NOD2/CARD15 status. The ADAM17 activity was higher both in the early phase of PMNL transepithelial   migration   and in active CD. These results showed early posttranscriptional upregulation of ADAM17 in IEC linked to PMNL transepithelial   migration   and a high activity of CD. 
 TIMP3 regulates   migration  , invasion and in vivo tumorigenicity of thyroid tumor cells. Papillary thyroid carcinoma (PTC) arises from the thyroid follicular epithelium and represents the most frequent thyroid malignancy. PTC is associated with gene rearrangements generating RET/PTC and TRK oncogenes, and to the BRAFV600E activating point mutation. A role of tumor-suppressor genes in the pathogenesis of PTC has not been assessed yet. The tissue inhibitor of metalloproteinase-3 (TIMP3) gene, encoding a metalloproteinases inhibitor and capable of inhibiting growth, angiogenesis, invasion and metastasis of several cancers, was found to be silenced by promoter methylation in a consistent fraction of PTCs, in association with tumor aggressiveness and BRAFV600E mutation, thus suggesting an oncosuppressor role. To explore this possibility, in this study we performed gene expression and functional studies. Analysis of gene expression data produced in our laboratory as well as meta-analysis of publicly available data sets confirmed the downregulation of TIMP3 gene expression in PTC with respect to normal thyroid. The functional consequences of TIMP3 downregulation were investigated in the PTC-derived NIM1 cell line, in which the expression of TIMP3 is silenced. Restoration of TIMP3 expression by exposure to soluble TIMP3 protein or by complementary DNA transfection had no effect on the growth rate of NIM1 cells. Instead, it affected the adhesive, migratory and invasive capabilities of NIM1 cells by modulating several proteins involved in these processes. A striking effect was observed in vivo, as TIMP3 reduced the tumorigenicity of NIM1 cells by repressing angiogenesis and macrophage infiltration. Our data indicate that the loss of TIMP3 expression exerts a functional role in the pathogenesis of PTC. 
 Polymorphisms and Plasma Levels of Tissue Inhibitor of Metalloproteinase-3: Impact on Genetic Susceptibility and Clinical Outcome of Oral Cancer. Oral cancer, the fourth most common cancer among men in Taiwan, is associated with environmental carcinogens. Tissue inhibitor of metalloproteinase-3 (TIMP3), a member of the TIMP family, is the only protein that binds to the extracellular matrix for suppressing cancer cell growth, angiogenesis,   migration  , and invasion. The association of TIMP3 polymorphism with oral cancer susceptibility, however, has not yet been reported. In this study, 1947 participants-1200 healthy male controls and 747 male patients with oral cancer-were recruited. Allelic discrimination of TIMP3 -1296 T > C (rs9619311), TIMP3 C > T (rs9862), and TIMP3 C > T (rs11547635) polymorphisms were assessed through real-time polymerase chain reaction. The authors discovered that individuals carrying the polymorphic rs9862 allele are more susceptible to oral cancer [odds ratio (OR), 1.5; 95% confidence interval (CI), 1.2-1.9; adjusted OR (AOR), 1.6; 95% CI, 1.2-2.1] after adjustment for betel quid chewing, alcohol, and tobacco consumption. Among 601 betel quid chewers, the TIMP3 polymorphism rs9862 T/T carriers had a 32.2-fold (95% CI, 20.2-51.3) increased oral cancer risk compared with those carrying C/C and not chewing betel quid. In addition, the authors observed a significant association between rs9862 variants and large tumors (OR, 1.5; 95% CI, 1.0-2.3) development. Moreover, TIMP3 plasma levels significantly increased in oral cancer patients who have large tumor or carry T allele rs9862 polymorphism. In conclusion, these results suggest that gene-environment interactions between the TIMP3 rs9862 polymorphisms and betel quid may alter oral cancer susceptibility and tumor growth in Taiwanese men. 
 Smad2/3/4 Pathway Contributes to TGF-β-Induced MiRNA-181b Expression to Promote Gastric Cancer Metastasis by Targeting Timp3. Transforming growth factor beta (TGF-β) plays a major role in tumorigenesis. MicroRNA-181b (miRNA-181b) is a multifaceted miRNA that has been implicated in many cellular processes such as cell fate determination and cellular invasion. This study aimed to confirm the relationship of miRNA-181b and the TGF-β-Smad2/3/4 pathway with the induction of the epithelial-to-mesenchymal transition (EMT) in gastric cancer.This study investigated the ability of TGF-β to ind  uce migra  tion by wound healing and transwell invasion assays in human gastric cancer cell lines. miRNA expression was altered using miRNA-181b mimic and inhibitor in the same system. Expression of miRNA-181b, the hypothetical target gene Timp3 and EMT-related markers were analyzed by real-time real-time quantitative RT-PCR. Immunoblotting was used to investigate the levels of phospho-Smad2 and Smad4. Dual-luciferase reporter assays were performed to confirm the direct binding of miRNA-181b to Timp3.miRNA-181b was significantly upregulated in response to TGF-β treatment in gastric cancer cell lines. Overexpression of miR-181b mimic induced an in vitro EMT-like change to a phenotype similar to that following TGF-β treatment alone and was reversed by miRNA-181b inhibitor. Inhibition of TGF-β-Smad2/3 signaling with SD-208 significantly attenuated the upregulation of miRNA-181b. Knockdown of Smad4 in gastric cancer cells strongly attenuated the upregulation of miRNA-181b. Moreover, miR-181b was found to directly target the 3' untranslated region (3'UTR) of Timp3 mRNA affecting TGF-β-induced EMT.Our results elucidate a novel mechanism through which the TGF-β pathway regulates the EMT of gastric cancer cells by increasing the levels of miRNA-181b to target Timp3 via the Smad2/3/4-dependent pathway. These findings provide insights into the cellular and environmental factors regulating EMT, which may guide future studies on therapeutic strategies targeting these cells. 
 Tissue inhibitor of metalloproteinase-3 (TIMP3) promotes endothelial apoptosis via a caspase-independent mechanism. Tissue inhibitor of metalloproteinases-3 (TIMP3) is a tumor suppressor and a potent inhibitor of angiogenesis. TIMP3 exerts its anti-angiogenic effect via a direct interaction with vascular endothelial growth factor (VEGF) receptor-2 (KDR) and inhibition of proliferation,   migration   and tube formation of endothelial cells (ECs). TIMP3 has also been shown to induce apoptosis in some cancer cells and vascular smooth muscle cells via MMP inhibition and caspase-dependent mechanisms. In this study, we examined the molecular mechanisms of TIMP3-mediated apoptosis in endothelial cells. We have previously demonstrated that mice developed smaller tumors with decreased vascularity when injected with breast carcinoma cells overexpressing TIMP3, than with control breast carcinoma cells. TIMP3 overexpression resulted in increased apoptosis in human breast carcinoma (MDA-MB435) in vivo but not in vitro. However, TIMP3 could induce apoptosis in ECs in vitro. The apoptotic activity of TIMP3 in ECs appears to be independent of MMP inhibitory activity. Furthermore, the equivalent expression of functional TIMP3 promoted apoptosis and caspase activation in ECs expressing KDR (PAE/KDR), but not in ECs expressing PDGF beta-receptor (PAE/β-R). Surprisingly, the apoptotic activity of TIMP3 appears to be independent of caspases. TIMP3 inhibited matrix-induced focal adhesion kinase (FAK) tyrosine phosphorylation and association with paxillin and disrupted the incorporation of β3 integrin, FAK and paxillin into focal adhesion contacts on the matrix, which were not affected by caspase inhibitors. Thus, TIMP3 may induce apoptosis in ECs by triggering a caspase-independent cell death pathway and targeting a FAK-dependent survival pathway. 
 Expression and inhibitory role of TIMP-3 in hepatocellular carcinoma. As a member of the tissue inhibitor of metallo-proteinases (TIMP) family, it has been reported that TIMP-3 is involved in human cancer development. However, the function of TIMP-3 in hepatocellular carcinoma (HCC) development is unclear. We aimed to determine the biological role of TIMP-3 in HCC by evaluating the effects of its methylation status and expression on HCC cell function. TIMP-3 expression in HCC tissues was visibly analyzed by immunohistochemistry. Methylation of the TIMP-3 promoter was evaluated by methylation-specific PCR. Effects of TIMP-3 on HCC cell growth, apoptosis,   migration  , and invasion were examined by transfecting the TIMP-3-expressing plasmid, pCMV6. TIMP-3 was expressed in non-tumorous live tissue, but silenced or downregulated in 60% of HCC cases (P<0.05). Reduced protein expression of TIMP-3 was associated with reduced tumor differentiation (P=0.003) and increased metastatic activity (P=0.005) in HCC cell lines. Promoter methylation contributed to the TIMP-3 inactivation. Overexpression of TIMP-3 in HCC cell lines suppressed cell proliferation, induced apoptosis, and inhibited   migration   and invasion in vitro. TIMP-3 expression is suppressed by promoter methylation in HCC. This inhibitory protein acts as a functional tumor suppressor by inhibiting HCC cell proliferation, invasion, and   migratio  n and by inducing apoptosis and cell cycle arrest at the G2/M phase. 
 MiR-21 enhances melanoma invasiveness via inhibition of tissue inhibitor of metalloproteinases 3 expression: in vivo effects of MiR-21 inhibitor. Metastatic melanoma is the most aggressive form of this cancer. It is important to understand factors that increase or decrease metastatic activity in order to more effectively research and implement treatments for melanoma. Increased cell invasion through the extracellular matrix is required for metastasis and is enhanced by matrix metalloproteinases (MMPs). Tissue inhibitor of metalloproteinases 3 (TIMP3) inhibits MMP activity. It was previously shown by our group that miR-21, a potential regulator of TIMP3, is over-expressed in cutaneous melanoma. It was therefore hypothesized that increased levels of miR-21 expression would lead to decreased expression of TIMP3 and thereby enhance the invasiveness of melanoma cells. miR-21 over-expression in the melanoma cell lines WM1552c, WM793b, A375 and MEL 39 was accomplished via transfection with pre-miR-21. Immunoblot analysis of miR-21-overexpressing cell lines revealed reduced expression of TIMP3 as compared to controls. This in turn led to a significant increase in the invasiveness of the radial growth phase cell line WM1552c and the vertical growth phase cell line WM793b (p < 0.05), but not in the metastatic cell lines A375 or MEL 39. The proliferation and   migration   of miR-21 over-expressing cell lines was not affected. Reduced expression of TIMP3 was achieved by siRNA knockdown and significantly enhanced invasion of melanoma cell lines, mimicking the effects of miR-21 over-expression. Treatment of tumor cells with a linked nucleic acid antagomir to miR-21 inhibited tumor growth and increased tumor expression of TIMP3 in vivo in 01B74 Athymic NCr-nu/nu mice. Intra-tumoral injections of anti-miR-21 produced similar effects. This data shows that increased expression of miR-21 enhanced the invasive potential of melanoma cell lines through TIMP3 inhibition. Therefore, inhibition of miR-21 in melanoma may reduce melanoma invasiveness. 
 The Angiogenic Effect of microRNA-21 Targeting TIMP3 through the Regulation of MMP2 and MMP9. microRNAs are a novel set of small, non-protein-coding nucleotide RNAs that negatively regulate the expression of target mRNAs. miRNA-21 is a microRNA that is highly enriched in endothelial cells. miRNA-21 has been shown to be a potential pro-angiogenic factor in some biological systems. Our previous study showed that the expression of miRNA-21 was up-regulated after spinal cord injury. However, the effect of miRNA-21 on angiogenesis in the spinal cord was unclear. In this study, to understand the role of miRNA-21 on injured endothelial cells exclusively, an oxygen and glucose deprivation model of endothelial cells was constructed, and the up-regulation of miRNA-21 was discovered in this model. An increased level of miRNA-21 by mimics promoted the survival,   migration   and tube formation of endothelial cells, which simultaneously inhibited tissue inhibitor of metalloproteinase-3 (TIMP3) expression and promoted matrix metalloproteinase-2 (MMP2) and matrix metalloproteinase-9 (MMP9) expression and secretion. A decreased level of miRNA-21 by antagomir exerted an opposite effect. As is well known, survival,   migration   and tube formation of endothelial cells are necessary prerequisites for angiogenesis after injury. TIMP3 was validated as a direct target of miRNA-21 by dual-luciferase reporter assay. Silencing with small interfering RNA against TIMP3 promoted tube formation and increased MMP2 and MMP9 expression at the protein level. In vivo, we found that decreased levels of miRNA-21 inhibited angiogenesis after spinal cord injury in rats using synchrotron radiation micro-computed tomography. In summary, these findings suggest that miRNA-21 has a protective effect on angiogenesis by reducing cell death and promoting cell survival,   migration   and tube formation via partially targeting the TIMP3 by potentially regulating MMP2 and MMP9. TIMP3 is a functional target gene. Identifying the role of miRNA-21 in the protection of angiogenesis might offer a novel therapeutic target for secondary spinal cord injury, in which angiogenesis is indispensable. 
 Differential TIMP3 expression affects tumor progression and angiogenesis in melanomas through regulation of directionally persistent endothelial cell   migration  . The angiogenic potential of solid tumors, or the ability to initiate neovasculature development from pre-existing host vessels, is facilitated by soluble factors secreted by tumor cells and involves breaching of extracellular matrix barriers, endothelial cell (EC) proliferation,   migration   and reassembly. We evaluated the angiogenic potential of human melanoma cell lines differing in their degree of aggressiveness, based on their ability to regulate directionally persistent EC   migration  . We observed that conditioned-medium (CM) of the aggressive melanoma cell line BLM induced a high effective migratory response in ECs, while CMs of Mel57 and 1F6 had an inhibitory effect. Further, the melanoma cell lines exhibited a varied expression profile of tissue inhibitor of metalloproteinase-3 (TIMP3), detectable in the CM. TIMP3 expression inversely correlated with aggressiveness of the melanoma cell line, and ability of the respective CMs to induce directed EC   migration  . Interestingly, TIMP3 expression was found to be silenced in the BLM cell line, concurrent with its role as a tumor suppressor. Treatment with recombinant human TIMP3 and CM of modified, TIMP3 expressing, BLM cells mitigated directional EC   migration  , while CM of TIMP3 silenced 1F6 cells induced directed EC   migration  . The functional implication of TIMP3 expression on tumor growth and angiogenic potential in melanoma was evaluated in vivo. We observed that TIMP3 expression reduced tumor growth, angiogenesis and macrophage infiltration of BLM tumors while silencing TIMP3 increased tumor growth and angiogenesis of 1F6 tumors. Taken together, our results demonstrate that TIMP3 expression correlates with inhibition of directionally persistent EC   migration   and adversely affects the angiogenic potential and growth of melanomas. 
 EZH2 regulates cancer cell   migration   through repressing TIMP-3 in non-small cell lung cancer. Histone methylations play important roles in human cancer metastasis. Enhancer of zeste homolog 2 (EZH2) is a key component of the polycomb repressor complex 2, which is responsible for histone H3K27 methylation. EZH2 is overexpressed in lung cancer and epigenetically silences tumor suppressor genes. Here, we showed that EZH2 was up-regulated in lung cancer and had a positive correlation with pathologic stage, nodal involvement in lung cancer patients. Moreover, overexpression of EZH2 was correlated with reduced tissue inhibitor of metalloproteinase-3 (TIMP-3) expression, which was shown to be negatively associated with tumor metastasis. Of note, overall survival time of patients with high EZH2/low TIMP-3 expression was significantly shorter than that of patients with low EZH2/high TIMP-3 (P = 0.031). RNA interfering and pharmacologic inhibition of EZH2 reduced histone H3 lysine 27 tri-methylation level and increased TIMP-3 expression level. Knockdown of EZH2 by siRNA significantly reduced A549 cancer cell   migration  . In contrast, reduction of TIMP-3 in A549 cells partially rescued EZH2 deficiency-induced loss of cell   migration   capacity. Taken together, our findings indicate that EZH2 accelerates cancer cell   migration  , in part, via the repression of TIMP-3 expression, suggesting a potential mechanism by which EZH2 promotes lung cancer progression and metastasis. 
   Migration   of culture-expanded human mesenchymal stem cells through bone marrow endothelium is regulated by matrix metalloproteinase-2 and tissue inhibitor of metalloproteinase-3. Mesenchymal stem cells (MSC) are adult stem cells that can be expanded many fold in vitro and have the therapeutic potential to restore the bone marrow microenvironment and support hematopoietic recovery after myeloablative conditioning for hematopoietic stem cell transplantation. Successful homing to the target tissue, such as bone marrow, implies that MSC are able to extravasate after systemic administration. However, the extravasation capacity of MSC and the underlying mechanisms are poorly understood to date. We studied in vitro the capacity of MSC to migrate through bone marrow endothelium.In vitro invasion and transendothelial   migration   assays were performed. The expression of matrix metalloproteinase (MMP) was analyzed by reverse transcriptase polymerase chain reaction (RT-PCR) and zymography.   Migration   of cells cultured at high or low confluence was compared and differential gene expression in these conditions was analyzed with microarray and real-time RT-PCR. The functional involvement in MSC   migration   was assessed using neutralizing anti-MMP-2 antibody, MMP-2 short interfering RNA or recombinant tissue inhibitor of metalloproteinase (TIMP-3).We demonstrated that MSC can invade reconstituted basement membrane and that bone marrow endothelial cells stimulate this process. We also showed that the transendothelial   migration   of MSC is at least partially regulated by MMP-2. High culture confluence was found to increase production of the natural MMP-inhibitor TIMP-3 and decrease transendothelial   migration   of MSC.We show that MSC have the potential to migrate through bone marrow endothelium and that this process involves MMP-2. Moreover, the   migration   of MSC is significantly influenced by the level of culture confluence. Increased culture confluence impairs   migration   and is related to an upregulation of TIMP-3. The therapeutic use of MSC would benefit from a selection of culture conditions that allow optimal extravasation of these cells. 
 S156C mutation in tissue inhibitor of metalloproteinases-3 induces increased angiogenesis. Tissue Inhibitor of metalloproteinases-3 (TIMP-3) is a potent matrix-bound angiogenesis inhibitor. Mutations in TIMP-3 cause Sorsby Fundus Dystrophy, a dominant inherited, early onset macular degenerative disease, with choroidal neovascularization causing a loss of vision in the majority of patients. Here we report that expression of S156C TIMP-3 mutation in endothelial cells results in an abnormal localization of the protein, increased glycosylation, decreased matrix metalloproteinase inhibitory activity, and increased vascular endothelial growth factor (VEGF) binding with a consequent increase in VEGF-dependent   migration   and tube formation. These enhanced signaling events appear to be mediated as a consequence of a post-transcriptionally regulated increase in the expression of membrane-associated VEGFR-2 in endothelial cells of Timp-3(156/156) mutant mice as well as in human Sorsby fundus dystrophy eyes. Understanding the mechanism(s) by which mutant TIMP-3 can induce abnormal neovascularization provides important insight into the pathophysiology of a number of diseases with increased angiogenesis. 
  GeneRIFs  
 EZH2 accelerates cancer cell   migration  , in part, via the repression of TIMP-3 expression. 
 results demonstrate that TIMP3 expression correlates with inhibition of directionally persistent EC   migration   and adversely affects the angiogenic potential and growth of melanomas. 
 show that miR-221&222, by targeting PTEN and TIMP3 tumor suppressors, induce TRAIL resistance and enhance cellular   migration   through the activation of the AKT pathway and metallopeptidases 
 Results show, in contrast to TIMP3, early posttranscriptional upregulation of ADAM17 in intestinal epithelial cells linked to PMNL transepithelial   migration   and a high activity of Crohn's disease. 
 Increased mesenchymal stem cell culture confluence impairs   migration   and is related to an upregulation of TIMP-3. 
  ECM  PubMed  
 Association of a high activity of matrix metalloproteinase-9 to low levels of tissue inhibitors of metalloproteinase-1 and -3 in human hepatitis B-viral hepatoma cells. Matrix metalloproteinases (MMPs) play a major role in the turnover of extracellular matrix (  ECM  ) during cancer invasion and metastasis, and tissue inhibitors of metalloproteinases (TIMPs) control MMPs, thus maintaining a balanced   ECM   catabolism under physiological conditions. The aim of this study was to assess the behavior of some MMPs (FASEB J., 7, 1993, 1434; Cancer Metastasis Rev., 9(4) 1990, 289) and TIMPs (Biochem. Biophys. Res. Commun., 301, 2003, 1069; FASEB J., 7, 1993, 1434; Nature, 370, 1994, 61). Competitive RT-PCR, gelatin-substrate zymography, and ELISA techniques were used for quantification. The hepatitis B virus (HBV)-DNA-containing hepatocellular carcinoma cell lines, Hep3B, HepG2-HBV and HFF-T2 contain highly activated matrix metallproteinase-9 (MMP-9), which is rarely found in normal liver cell lines such as the Chang lines. MMP-9 activities of HFH-T2, HepG2-HBV and Hep3B were significantly higher than that of non-HBV-hepatocellular carcinoma SK-Hep1 and HepG2 (HCC origin, HBV not detected), as assayed by gelatin zymography. Low levels of TIMP-1 and TIMP-3 were observed in HFH-T2, HepG2-HBV and Hep3B, while the TIMP-2 level was high, as evidenced by reverse zymography. In contrast, 3 TIMP-1, -2 and -3 were largely detected in Chang, HepG2 and SK-Hep1 cells. To investigate the nature of the quantitative regulation of MMPs and TIMPs for these cell lines at the transcriptional levels, a reverse transcriptase-polymerase chain reaction (RT-PCR) was carried out. Not only MMP-9 mRNAs of HFH-T2, HepG2-HBV and Hep3B but also MMP-9 mRNA of SK-Hep1 and HepG2 were highly expressed with no major differences among these four cell lines. However, TIMP-1 and TIMP-3 mRNAs of HFH-T2, HepG2-HBV and Hep3B were markedly reduced, while those of SK-Hep1, HepG2 and Chang cells were maintained at high levels. Finally, an invasion assay using matrigel indicated in an increase in invasiveness in HFH-T2, HepG2-HBV and Hep3B cells, but a decrease in invasiveness of Chang, HepG2 and SK-Hep1 cells. These results indicate that the overexpression of MMP-9 mRNAs and the suppression of TIMP-1 and TIMP-3 in HFH-T2, HepG2-HBV and Hep3B were the result of HBV transfection. Based on these results, it is concluded that HBV affects the malignance of hepatocellular cancer by elevating MMP-9 activity, and suppressing TIMP-1 and TIMP-3. 
 Inhibition of histone deacetylase activity in human endometrial stromal cells promotes extracellular matrix remodelling and limits embryo invasion. Invasion of the trophoblast into the maternal decidua is regulated by both the trophoectoderm and the endometrial stroma, and entails the action of tissue remodeling enzymes. Trophoblast invasion requires the action of metalloproteinases (MMPs) to degrade extracellular matrix (  ECM  ) proteins and in turn, decidual cells express tissue inhibitors of MMPs (TIMPs). The balance between these promoting and restraining factors is a key event for the successful outcome of pregnancy. Gene expression is post-transcriptionally regulated by histone deacetylases (HDACs) that unpacks condensed chromatin activating gene expression. In this study we analyze the effect of histone acetylation on the expression of tissue remodeling enzymes and activity of human endometrial stromal cells (hESCs) related to trophoblast invasion control. Treatment of hESCs with the HDAC inhibitor trichostatin A (TSA) increased the expression of TIMP-1 and TIMP-3 while decreased MMP-2, MMP-9 and uPA and have an inhibitory effect on trophoblast invasion. Moreover, histone acetylation is detected at the promoters of TIMP-1 and TIMP-3 genes in TSA-treated. In addition, in an in vitro decidualized hESCs model, the increase of TIMP-1 and TIMP-3 expression is associated with histone acetylation at the promoters of these genes. Our results demonstrate that histone acetylation disrupt the balance of   ECM   modulators provoking a restrain of trophoblast invasion. These findings are important as an epigenetic mechanism that can be used to control trophoblast invasion. 
 The expression and regulation of ADAMTS-1, -4, -5, -9, and -15, and TIMP-3 by TGFbeta1 in prostate cells: relevance to the accumulation of versican. Benign prostatic hyperplasia (BPH) is characterized by a proportional increase in the size of the stromal compartment of the gland, involving alterations to extracellular matrix (  ECM  ) components. Some of these changes have been associated with the activity and expression of transforming growth factor beta1 (TGFbeta1). Versican (chondroitin sulphate proteoglycan-2) is overexpressed in BPH and prostate cancer and potentially contributes to disease pathology. A sub-group of the ADAMTS lineage of metalloproteases possess versican-degrading properties and are potential regulators of proteoglycan accumulation associated with BPH. These enzymes have one major inhibitor in the   ECM  , tissue inhibitor of metalloproteinases (TIMP)-3.The effect of TGFbeta on mRNA expression in prostatic stromal cells was determined by real-time qRT-PCR using primers to ADAMTS-1, -4, -5, -9, -15, versican, and TIMP-3. MMP-inhibitory potential (TIMP activity) of conditioned medium was measured using a fluorometric peptide substrate.Prostatic stromal cell cultures consistently expressed ADAMTS-1, -4, -5, -9, -15 and TIMP-3, in contrast to PC3, DU145, and LNCaP cells which failed to express at least two ADAMTS transcripts. In stromal cells, TGFbeta1 decreased ADAMTS-1, -5, -9, and -15 transcripts and increased ADAMTS-4, versican, and TIMP-3. TGFbeta also increased TIMP activity in conditioned medium.The induction of versican expression by TGFbeta in BPH stromal cells is in agreement with histological studies. The negative effect of TGFbeta1 on ADAMTS-1, -5, -9, and -15 coupled with increases in their inhibitor, TIMP-3 may aid the accumulation of versican in the stromal compartment of the prostate in BPH and prostate cancer. 
 Identification of the extracellular matrix (  ECM  ) binding motifs of tissue inhibitor of metalloproteinases (TIMP)-3 and effective transfer to TIMP-1. Tissue inhibitor of metalloproteinases (TIMPs) are the endogenous inhibitors of the zinc-dependent endopeptidases of the matrix metalloproteinase families. There are four mammalian TIMPs (TIMP-1 to -4) but only TIMP-3 is sequestered to the extracellular matrix (  ECM  ). The molecular basis for the TIMP-3:  ECM   association has never been fully investigated until now. In this report, we identify the unique amino acid configuration that constitutes the basis of the   ECM   binding motif in TIMP-3. By systematically exchanging the subdomains of the TIMPs and exhaustive mutation of TIMP-3, we have identified the surface residues directly responsible for   ECM   association. Contrary to the accepted view, we have found that TIMP-3 interacts with the   ECM   via both its N- and C-terminal domains. The amino acids involved in   ECM   binding are all basic in nature: Lys-26, Lys-27, Lys-30, Lys-76 of the N-terminal domain and Arg-163, Lys-165 of the C-terminal domain. Replacement of these residues with glutamate (E) and glutamine (Q) (K26/27/30/76E + R163/K165Q) resulted in a soluble TIMP-3 devoid of   ECM  -adhering ability. Using the   ECM   binding motif derived from TIMP-3, we have also created a TIMP-1 mutant (K26/27/30 + K76 transplant) capable of   ECM   association. This is the first instance of TIMPs being intentionally rendered soluble or   ECM  -bound. The ability to prepare TIMPs in soluble or   ECM  -bound forms also opens new avenues for future TIMP research. 
 Proteomics characterization of extracellular space components in the human aorta. The vascular extracellular matrix (  ECM  ) is essential for the structural integrity of the vessel wall and also serves as a substrate for the binding and retention of secreted products of vascular cells as well as molecules coming from the circulation. Although proteomics has been previously applied to vascular tissues, few studies have specifically targeted the vascular   ECM   and its associated proteins. Thus, its detailed composition remains to be characterized. In this study, we describe a methodology for the extraction of extracellular proteins from human aortas and their identification by proteomics. The approach is based on (a) effective decellularization to enrich for scarce extracellular proteins, (b) successful solubilization and deglycosylation of   ECM   proteins, and (c) relative estimation of protein abundance using spectral counting. Our three-step extraction approach resulted in the identification of 103 extracellular proteins of which one-third have never been reported in the proteomics literature of vascular tissues. In particular, three glycoproteins (podocan, sclerostin, and agrin) were identified for the first time in human aortas at the protein level. We also identified extracellular adipocyte enhancer-binding protein 1, the cartilage glycoprotein asporin, and a previously hypothetical protein, retinal pigment epithelium (RPE) spondin. Moreover, our methodology allowed us to screen for proteolysis in the aortic samples based on the identification of proteolytic enzymes and their corresponding degradation products. For instance, we were able to detect matrix metalloproteinase-9 by mass spectrometry and relate its presence to degradation of fibronectin in a clinical specimen. We expect this proteomics methodology to further our understanding of the composition of the vascular extracellular environment, shed light on   ECM   remodeling and degradation, and provide insights into important pathological processes, such as plaque rupture, aneurysm formation, and restenosis. 
 The role of EZH2 in the regulation of the activity of matrix metalloproteinases in prostate cancer cells. Degradation of the extracellular matrix (  ECM  ), a critical step in cancer metastasis, is determined by the balance between MMPs (matrix metalloproteinases) and their inhibitors TIMPs (tissue inhibitors of metalloproteinases). In cancer cells, this balance is shifted towards MMPs, promoting   ECM   degradation. Here, we show that EZH2 plays an active role in this process by repressing the expression of TIMP2 and TIMP3 in prostate cancer cells. The TIMP genes are derepressed by knockdown of EZH2 expression in human prostate cancer cells but repressed by overexpression of EZH2 in benign human prostate epithelial cells. EZH2 catalyzes H3K27 trimethylation and subsequent DNA methylation of the TIMP gene promoters. Overexpression of EZH2 confers an invasive phenotype on benign prostate epithelial cells; however, this phenotype is suppressed by cooverexpression of TIMP3. EZH2 knockdown markedly reduces the proteolytic activity of MMP-9, thereby decreasing the invasive activity of prostate cancer cells. These results suggest that the transcriptional repression of the TIMP genes by EZH2 may be a major mechanism to shift the MMPs/TIMPs balance in favor of MMP activity and thus to promote   ECM   degradation and subsequent invasion of prostate cancer cells. 
 Promoted activation of matrix metalloproteinase (MMP)-2 in keloid fibroblasts and increased expression of MMP-2 in collagen bundle regions: implications for mechanisms of keloid progression. Keloid is characterized by excessive deposition of collagen, resulting from aberrant extracellular matrix (  ECM  ) production and degradation. The aim was to investigate the role of matrix metalloproteinases (MMPs) and tissue inhibitor of metalloproteinases (TIMPs) in pathological wound healing in keloids.Semiquantitative analysis of 60 keloid tissue samples and 25 mature scar tissue samples demonstrated significantly increased expression of MMP-2, TIMP-2 and TIMP-3 in keloids compared with mature scars. Within keloid regions, MMP-2 expression was significantly higher in collagen bundle regions than in non-collagen bundle regions. Double immunofluorescence revealed that keloid fibroblasts between collagen bundles exhibited MMP-2, TIMP-2 and membrane-type 1 MMP (MT1-MMP) co-expression, whereas only MMP-2 expression was evident on the edge of collagen bundles. Western blot analysis and gelatin zymography of 13 keloid-derived fibroblasts (KFbs) and six normal skin dermal-derived fibroblasts (NFbs) demonstrated that unstimulated KFbs exhibited significantly increased MMP-2 activity and expression compared with NFbs under the same conditions.These results together indicate that MMP-2 activity can be promoted in keloid fibroblasts between collagen bundles in cooperation with TIMP-2 and MT1-MMP. This could contribute to remodelling of collagen bundle regions and invasion of fibroblasts into peripheral normal regions through promoted degradation of   ECM  . 
 The role of EZH2 in the regulation of the activity of matrix metalloproteinases in prostate cancer cells. Degradation of the extracellular matrix (  ECM  ), a critical step in cancer metastasis, is determined by the balance between MMPs (matrix metalloproteinases) and their inhibitors TIMPs (tissue inhibitors of metalloproteinases). In cancer cells, this balance is shifted towards MMPs, promoting   ECM   degradation. Here, we show that EZH2 plays an active role in this process by repressing the expression of TIMP2 and TIMP3 in prostate cancer cells. The TIMP genes are derepressed by knockdown of EZH2 expression in human prostate cancer cells but repressed by overexpression of EZH2 in benign human prostate epithelial cells. EZH2 catalyzes H3K27 trimethylation and subsequent DNA methylation of the TIMP gene promoters. Overexpression of EZH2 confers an invasive phenotype on benign prostate epithelial cells; however, this phenotype is suppressed by cooverexpression of TIMP3. EZH2 knockdown markedly reduces the proteolytic activity of MMP-9, thereby decreasing the invasive activity of prostate cancer cells. These results suggest that the transcriptional repression of the TIMP genes by EZH2 may be a major mechanism to shift the MMPs/TIMPs balance in favor of MMP activity and thus to promote   ECM   degradation and subsequent invasion of prostate cancer cells. 
 Inhibition of histone deacetylase activity in human endometrial stromal cells promotes extracellular matrix remodelling and limits embryo invasion. Invasion of the trophoblast into the maternal decidua is regulated by both the trophoectoderm and the endometrial stroma, and entails the action of tissue remodeling enzymes. Trophoblast invasion requires the action of metalloproteinases (MMPs) to degrade extracellular matrix (  ECM  ) proteins and in turn, decidual cells express tissue inhibitors of MMPs (TIMPs). The balance between these promoting and restraining factors is a key event for the successful outcome of pregnancy. Gene expression is post-transcriptionally regulated by histone deacetylases (HDACs) that unpacks condensed chromatin activating gene expression. In this study we analyze the effect of histone acetylation on the expression of tissue remodeling enzymes and activity of human endometrial stromal cells (hESCs) related to trophoblast invasion control. Treatment of hESCs with the HDAC inhibitor trichostatin A (TSA) increased the expression of TIMP-1 and TIMP-3 while decreased MMP-2, MMP-9 and uPA and have an inhibitory effect on trophoblast invasion. Moreover, histone acetylation is detected at the promoters of TIMP-1 and TIMP-3 genes in TSA-treated. In addition, in an in vitro decidualized hESCs model, the increase of TIMP-1 and TIMP-3 expression is associated with histone acetylation at the promoters of these genes. Our results demonstrate that histone acetylation disrupt the balance of   ECM   modulators provoking a restrain of trophoblast invasion. These findings are important as an epigenetic mechanism that can be used to control trophoblast invasion. 
 Promoted activation of matrix metalloproteinase (MMP)-2 in keloid fibroblasts and increased expression of MMP-2 in collagen bundle regions: implications for mechanisms of keloid progression. Keloid is characterized by excessive deposition of collagen, resulting from aberrant extracellular matrix (  ECM  ) production and degradation. The aim was to investigate the role of matrix metalloproteinases (MMPs) and tissue inhibitor of metalloproteinases (TIMPs) in pathological wound healing in keloids.Semiquantitative analysis of 60 keloid tissue samples and 25 mature scar tissue samples demonstrated significantly increased expression of MMP-2, TIMP-2 and TIMP-3 in keloids compared with mature scars. Within keloid regions, MMP-2 expression was significantly higher in collagen bundle regions than in non-collagen bundle regions. Double immunofluorescence revealed that keloid fibroblasts between collagen bundles exhibited MMP-2, TIMP-2 and membrane-type 1 MMP (MT1-MMP) co-expression, whereas only MMP-2 expression was evident on the edge of collagen bundles. Western blot analysis and gelatin zymography of 13 keloid-derived fibroblasts (KFbs) and six normal skin dermal-derived fibroblasts (NFbs) demonstrated that unstimulated KFbs exhibited significantly increased MMP-2 activity and expression compared with NFbs under the same conditions.These results together indicate that MMP-2 activity can be promoted in keloid fibroblasts between collagen bundles in cooperation with TIMP-2 and MT1-MMP. This could contribute to remodelling of collagen bundle regions and invasion of fibroblasts into peripheral normal regions through promoted degradation of   ECM  . 
 Identification of the extracellular matrix (  ECM  ) binding motifs of tissue inhibitor of metalloproteinases (TIMP)-3 and effective transfer to TIMP-1. Tissue inhibitor of metalloproteinases (TIMPs) are the endogenous inhibitors of the zinc-dependent endopeptidases of the matrix metalloproteinase families. There are four mammalian TIMPs (TIMP-1 to -4) but only TIMP-3 is sequestered to the extracellular matrix (  ECM  ). The molecular basis for the TIMP-3:  ECM   association has never been fully investigated until now. In this report, we identify the unique amino acid configuration that constitutes the basis of the   ECM   binding motif in TIMP-3. By systematically exchanging the subdomains of the TIMPs and exhaustive mutation of TIMP-3, we have identified the surface residues directly responsible for   ECM   association. Contrary to the accepted view, we have found that TIMP-3 interacts with the   ECM   via both its N- and C-terminal domains. The amino acids involved in   ECM   binding are all basic in nature: Lys-26, Lys-27, Lys-30, Lys-76 of the N-terminal domain and Arg-163, Lys-165 of the C-terminal domain. Replacement of these residues with glutamate (E) and glutamine (Q) (K26/27/30/76E + R163/K165Q) resulted in a soluble TIMP-3 devoid of   ECM  -adhering ability. Using the   ECM   binding motif derived from TIMP-3, we have also created a TIMP-1 mutant (K26/27/30 + K76 transplant) capable of   ECM   association. This is the first instance of TIMPs being intentionally rendered soluble or   ECM  -bound. The ability to prepare TIMPs in soluble or   ECM  -bound forms also opens new avenues for future TIMP research. 
 The expression and regulation of ADAMTS-1, -4, -5, -9, and -15, and TIMP-3 by TGFbeta1 in prostate cells: relevance to the accumulation of versican. Benign prostatic hyperplasia (BPH) is characterized by a proportional increase in the size of the stromal compartment of the gland, involving alterations to extracellular matrix (  ECM  ) components. Some of these changes have been associated with the activity and expression of transforming growth factor beta1 (TGFbeta1). Versican (chondroitin sulphate proteoglycan-2) is overexpressed in BPH and prostate cancer and potentially contributes to disease pathology. A sub-group of the ADAMTS lineage of metalloproteases possess versican-degrading properties and are potential regulators of proteoglycan accumulation associated with BPH. These enzymes have one major inhibitor in the   ECM  , tissue inhibitor of metalloproteinases (TIMP)-3.The effect of TGFbeta on mRNA expression in prostatic stromal cells was determined by real-time qRT-PCR using primers to ADAMTS-1, -4, -5, -9, -15, versican, and TIMP-3. MMP-inhibitory potential (TIMP activity) of conditioned medium was measured using a fluorometric peptide substrate.Prostatic stromal cell cultures consistently expressed ADAMTS-1, -4, -5, -9, -15 and TIMP-3, in contrast to PC3, DU145, and LNCaP cells which failed to express at least two ADAMTS transcripts. In stromal cells, TGFbeta1 decreased ADAMTS-1, -5, -9, and -15 transcripts and increased ADAMTS-4, versican, and TIMP-3. TGFbeta also increased TIMP activity in conditioned medium.The induction of versican expression by TGFbeta in BPH stromal cells is in agreement with histological studies. The negative effect of TGFbeta1 on ADAMTS-1, -5, -9, and -15 coupled with increases in their inhibitor, TIMP-3 may aid the accumulation of versican in the stromal compartment of the prostate in BPH and prostate cancer. 
 Association of a high activity of matrix metalloproteinase-9 to low levels of tissue inhibitors of metalloproteinase-1 and -3 in human hepatitis B-viral hepatoma cells. Matrix metalloproteinases (MMPs) play a major role in the turnover of extracellular matrix (  ECM  ) during cancer invasion and metastasis, and tissue inhibitors of metalloproteinases (TIMPs) control MMPs, thus maintaining a balanced   ECM   catabolism under physiological conditions. The aim of this study was to assess the behavior of some MMPs (FASEB J., 7, 1993, 1434; Cancer Metastasis Rev., 9(4) 1990, 289) and TIMPs (Biochem. Biophys. Res. Commun., 301, 2003, 1069; FASEB J., 7, 1993, 1434; Nature, 370, 1994, 61). Competitive RT-PCR, gelatin-substrate zymography, and ELISA techniques were used for quantification. The hepatitis B virus (HBV)-DNA-containing hepatocellular carcinoma cell lines, Hep3B, HepG2-HBV and HFF-T2 contain highly activated matrix metallproteinase-9 (MMP-9), which is rarely found in normal liver cell lines such as the Chang lines. MMP-9 activities of HFH-T2, HepG2-HBV and Hep3B were significantly higher than that of non-HBV-hepatocellular carcinoma SK-Hep1 and HepG2 (HCC origin, HBV not detected), as assayed by gelatin zymography. Low levels of TIMP-1 and TIMP-3 were observed in HFH-T2, HepG2-HBV and Hep3B, while the TIMP-2 level was high, as evidenced by reverse zymography. In contrast, 3 TIMP-1, -2 and -3 were largely detected in Chang, HepG2 and SK-Hep1 cells. To investigate the nature of the quantitative regulation of MMPs and TIMPs for these cell lines at the transcriptional levels, a reverse transcriptase-polymerase chain reaction (RT-PCR) was carried out. Not only MMP-9 mRNAs of HFH-T2, HepG2-HBV and Hep3B but also MMP-9 mRNA of SK-Hep1 and HepG2 were highly expressed with no major differences among these four cell lines. However, TIMP-1 and TIMP-3 mRNAs of HFH-T2, HepG2-HBV and Hep3B were markedly reduced, while those of SK-Hep1, HepG2 and Chang cells were maintained at high levels. Finally, an invasion assay using matrigel indicated in an increase in invasiveness in HFH-T2, HepG2-HBV and Hep3B cells, but a decrease in invasiveness of Chang, HepG2 and SK-Hep1 cells. These results indicate that the overexpression of MMP-9 mRNAs and the suppression of TIMP-1 and TIMP-3 in HFH-T2, HepG2-HBV and Hep3B were the result of HBV transfection. Based on these results, it is concluded that HBV affects the malignance of hepatocellular cancer by elevating MMP-9 activity, and suppressing TIMP-1 and TIMP-3. 
 TIMP-3 inhibits the procollagen N-proteinase ADAMTS-2. ADAMTS-2 is an extracellular metalloproteinase responsible for cleaving the N-propeptides of procollagens I-III; an activity necessary for the formation of collagenous   ECM   (extracellular matrix). The four TIMPs (tissue inhibitors of metalloproteinases) regulate the activities of matrix metalloproteinases, which are involved in degrading   ECM   components. Here we delineate the abilities of the TIMPs to affect biosynthetic processing of procollagens. TIMP-1, -2 and -4 show no inhibitory activity towards ADAMTS-2, in addition none of the TIMPs showed inhibitory activity towards bone morphogenetic protein 1, which is responsible for cleaving procollagen C-propeptides. In contrast, TIMP-3 is demonstrated to inhibit ADAMTS-2 in vitro with apparent Ki values of 160 and 602 nM, in the presence of heparin or without respectively; and TIMP-3 is shown to inhibit procollagen processing by cells. 
 TGF-beta-induced expression of tissue inhibitor of metalloproteinases-3 gene in chondrocytes is mediated by extracellular signal-regulated kinase pathway and Sp1 transcription factor. Transforming growth factor (TGF-beta1) is a potent inducer of chondrogenesis and stimulant of cartilage extracellular matrix (  ECM  ) synthesis. Tissue inhibitor of metalloproteinases-3 (TIMP-3) is located in   ECM   and is the major inhibitor of matrix metalloproteinases (MMPs) and aggrecanase, the principal enzymes implicated in collagen and aggrecan degradation in arthritis. We investigated the role of extracellular-signal-regulated kinase (ERK)-mitogen-activated protein kinases (MAPK) and Sp1 transcription factor in TGF-beta-induced TIMP-3 gene in chondrocytes and chondrosarcoma cells. TGF-beta time-dependently induced a sustained phosphorylation of ERK-MAPKs in primary human or bovine chondrocytes. Inhibitors of this pathway, PD98059 and U0126, downregulated TGF-beta-induced expression of TIMP-3 RNA and protein. Since the ERKs can phosphorylate Sp1, and the promoter of human TIMP-3 gene contains four Sp1-binding sites, we investigated whether Sp1 is a downstream target of this pathway. Mithramycin and WP631, the agents that prevent binding of Sp1 to its consensus site, downregulated TGF-beta-inducible TIMP-3 expression. Indeed, mithramycin blocked TGF-beta-stimulated Sp1 binding activity. Transfection of cytomegalovirus (CMV) promoter-Sp1 plasmid increased TIMP-3 promoter (-940 to +376)-driven luciferase activity. Depletion of Sp1 by transfection of an antisense phosphorothioate oligonucleotide suppressed TGF-beta-induced TIMP-3 protein expression, while its sense homolog had no effect. These results suggest that activation of ERK-MAPK pathway and Sp1 transcription factor play a pivotal role in the induction of TIMP-3 by TGF-beta in chondrocytes. 
 Sorsby's fundus dystrophy tissue inhibitor of metalloproteinases-3 (TIMP-3) mutants have unimpaired matrix metalloproteinase inhibitory activities, but affect cell adhesion to the extracellular matrix. The TIMP family of matrix metalloproteinase inhibitors consists of four members, of which TIMP-1, -2 and -4 are secreted, freely diffusible proteins, whereas TIMP-3 is   ECM  -associated. Mutations in the TIMP3 gene have been linked to Sorsby's fundus dystrophy (SFD), an autosomal dominant inherited retinal degenerative disease that leads to blindness. The SFD mutations characterized result in introduction of an unpaired cysteine residue in the C-terminal domain of TIMP-3. We have expressed four SFD mutant TIMP-3 proteins in baby hamster kidney (BHK) cells and evaluated their characteristics alongside wild-type TIMP-3. Analysis of the mutant proteins (Ser156Cys, Gly167Cys, Tyr168Cys and Ser181Cys) by SDS-PAGE and reverse zymography revealed that each of the mutants retained gelatinase A and gelatinase B inhibitory activity, and were localized to the   ECM  . Association rate constants for Ser156Cys TIMP-3 with gelatinase-A, gelatinase-B, stromelysin-1 and collagenase-3 were only moderately reduced compared to wild-type TIMP-3. However, all of the mutants displayed aberrant protein-protein interactions, resulting in the presence of additional proteins or complexes in   ECM   preparations. Two of the mutants (Ser156Cys and Ser181Cys) showed a marked propensity to form multiple higher molecular-weight complexes that retained TIMP activity on reverse zymography. Expression of the SFD mutant TIMP-3 (and to a lesser extent, wild-type TIMP-3) proteins in BHK cells conferred increased cell adhesiveness to the   ECM  . Our findings indicate that the pathogenesis of Sorsby's fundus dystrophy cannot be attributed to a failure to localize SFD TIMP-3 proteins to the   ECM   or defects in MMP inhibition, but may involve the formation of aberrant TIMP-3-containing protein complexes and altered cell adhesion. 
 Gene expression analysis of drug-resistant MCF-7 cells: implications for relation to extracellular matrix proteins. Since multidrug resistance is a multifactorial phenomenon, a large-scale expression analysis of drug-resistant cells by using high-density oligonucleotide microarrays may provide information about new candidate genes contributing to resistance. Extracellular matrix (  ECM  ) is responsible for many aspects of proliferation and invasive/metastatic behavior of tumor cells. This study demonstrates alterations in gene expression levels of several   ECM   components, matrix metalloproteinases (MMPs), adamalysins (ADAMs and ADAMTSs) and tissue inhibitors of metalloproteinases (TIMPs) in paclitaxel, docetaxel, vincristine and doxorubicin-resistant MCF-7 cells.Resistant MCF-7 cells were developed by stepwise selection of cells in increasing concentrations of drugs. Affymetrix GeneChip Human Genome U133 Plus 2.0 Array was used for hybridizations. Statistical significance was determined by independent sample t test. The genes having altered expression levels in drug-resistant sublines were selected and filtered by volcano plots.Genes up/downregulated more than twofolds were selected and listed. Expression of 25 genes encoding   ECM   proteins (including collagen, finronectin and syndecan) and integrin receptor subunits were found to be upregulated in drug-resistant cells. In addition, expression levels of, 13 genes encoding MMPs, ADAMs, ADAMTSs and TIMPs (including MMP1, MMP9, ADAM9 and TIMP3) were found to be altered in drug-resistant sublines when compared with sensitive MCF-7.Based on the expression analysis profiles, this report provides a preliminary insight into the relationship between drug resistance and   ECM   components, which are related to invasion and metastasis. Correlation of each specific   ECM   component with drug resistance requires further analysis. 
 Tissue inhibitor of matrix metalloproteinase-3 levels in the extracellular matrix of lung, kidney, and eye increase with age. Tissue inhibitor of matrix metalloproteinase-3 (TIMP-3) is an important regulator of matrix metalloproteinase activity in many types of disease, including atherosclerosis, neoplasia, and inflammatory conditions. Among TIMPs, TIMP-3 uniquely binds the extracellular matrix (  ECM  ). We performed IHC staining on 17 tissue microarrays containing >1500 samples to determine the location of   ECM   TIMP-3 staining in a variety of predominantly vascular tissues. We found a unique pattern of TIMP-3 staining in the   ECM   of renal arterioles, small pulmonary vessels and parenchyma, and Bruch's membrane in the retina. There was no staining in larger caliber arteries including coronary and internal mammary arteries. TIMP-3 protein accumulation was found to be an age-dependent phenomenon, with staining appearing in all three tissues in early adulthood and becoming more robust among the elderly. These findings may help to explain the late onset of the TIMP-3-associated ocular diseases Sorsby fundus dystrophy and age-related macular degeneration and suggest a similar phenomenon could be at work in other age-related conditions. 
 Genomic organization of the human tissue inhibitor of metalloproteinases-3 (TIMP3) The tissue inhibitors of metalloproteinases (TIMPs) play a crucial role in the physiological turnover of the extracellular matrix (  ECM  ) by tightly regulating matrix metalloproteinase (MMP) activities. Disturbances in the TIMP/MMP system have been implicated in many disease processes where loss of   ECM   integrity is a principal feature. More recently, we have shown that mutations in TIMP3 cause the autosomal dominant disorder Sorsby's fundus dystrophy (SFD). This is a macular degeneration disorder with characteristic   ECM   irregularities in Bruch's membrane. To further facilitate mutational analysis and to provide a basis for functional studies, we now report the genomic organization of the human TIMP3 gene. 
 Early activation of matrix metalloproteinases underlies the exacerbated systolic and diastolic dysfunction in mice lacking TIMP3 following myocardial infarction. Extracellular matrix (  ECM  ) remodeling is a critical aspect of cardiac remodeling following myocardial infarction. Tissue inhibitors of metalloproteinases (TIMPs) are physiological inhibitors of matrix metalloproteinases (MMPs) that degrade the   ECM   proteins. TIMP3 is highly expressed in the heart, and is markedly downregulated in patients with ischemic cardiomyopathy. We therefore examined the time- and region-dependent role of TIMP3 in the cardiac response to myocardial infarction (MI). TIMP3(-/-) and wild-type (WT) mice were subjected to MI by ligation of the left anterior descending artery. TIMP3(-/-)-MI mice exhibited a significantly compromised rate of survival compared with WT-MI mice, primarily due to increased left ventricular (LV) rupture, greater infarct expansion, exacerbated LV dilation, and greater systolic and diastolic dysfunction. Second harmonic generation imaging of unfixed and unstained hearts revealed greater collagen disarray and reduced density in the TIMP3(-/-) infarct myocardium compared with the WT group. Gelatinolytic and collagenolytic activities increased in TIMP3(-/-) compared with WT hearts at 1 day post-MI but not at 3 days or 1 wk post-MI. Neutrophil infiltration and inflammatory MMPs were significantly increased in the infarct and peri-infarct regions of TIMP3(-/-)-MI hearts. Treatment of TIMP3(-/-) mice with a broad-spectrum MMP inhibitor (PD-166793) for 2 days before and 2 days after MI markedly improved post-MI infarct expansion, LV rupture incident, LV dilation, and systolic dysfunction in these mice up to 1 wk post-MI. Our data demonstrate that the initial rise in proteolytic activities early post-MI is a triggering factor for subsequent LV adverse remodeling, LV rupture, and dilated cardiomyopathy. Hence, timing of treatments to improve cardiac response to MI may be critical in producing favorable outcome. 
 TGF-β-elicited induction of tissue inhibitor of metalloproteinases (TIMP)-3 expression in fibroblasts involves complex interplay between Smad3, p38α, and ERK1/2. Transforming growth factor-β (TGF-β) promotes extracellular matrix deposition by down-regulating the expression of matrix degrading proteinases and upregulating their inhibitors. Tissue inhibitor of metalloproteinases (TIMP)-3 is   an   ECM-associated specific inhibitor of matrix degrading metalloproteinases. Here, we have characterized the signaling pathways mediating TGF-β-induced expression of TIMP-3. Basal and TGF-β-induced TIMP-3 mRNA expression was abolished in Smad4-deficient mouse embryonic fibroblasts and restoring Smad4 expression rescued the response. Inhibition of Smad signaling by expression of Smad7 and dominant negative Smad3 completely abolished TGF-β-elicited expression of TIMP-3 in human fibroblasts, whereas overexpression of Smad3 enhanced it. Inhibition of extracellular signal-regulated kinase 1/2 (ERK1/2) activation with PD98059 and p38 mitogen-activated protein kinase activity by SB203580 resulted in suppression of TGF-β-induced TIMP-3 expression, indicating that ERK1/2 and p38 MAPK mediate the effect of TGF-β on TIMP-3 expression. Specific activation of p38α and ERK1/2 by constitutively active mutants of MKK3b or MEK1, respectively, and simultaneous co-expression of Smad3 resulted in induction of TIMP-3 expression in the absence of TGF-β indicating that Smad3 co-operates with p38 and ERK1/2 in the induction of TIMP-3 expression. These results demonstrate the complex interplay between Smad3, p38α, and ERK1/2 signaling in the regulation of TIMP-3 gene expression in fibroblasts, which may play a role in inflammation, tissue repair, and fibrosis. 
 Extracellular matrix proteins expression profiling in chemoresistant variants of the A2780 ovarian cancer cell line. Ovarian cancer is the leading cause of death among gynaecological malignancies. Extracellular matrix (  ECM  ) can affect drug resistance by preventing the penetration of the drug into cancer cells and increased resistance to apoptosis. This study demonstrates alterations in the expression levels of   ECM   components and related genes in cisplatin-, doxorubicin-, topotecan-, and paclitaxel-resistant variants of the A2780 ovarian cancer cell line. Affymetrix Gene Chip Human Genome Array Strips were used for hybridisations. The genes that had altered expression levels in drug-resistant sublines were selected and filtered by scatter plots. The genes that were up- or downregulated more than fivefold were selected and listed. Among the investigated genes, 28 genes were upregulated, 10 genes were downregulated, and two genes were down- or upregulated depending on the cell line. Between upregulated genes 12 were upregulated very significantly--over 20-fold. These genes included COL1A2, COL12A1, COL21A1, LOX, TGFBI, LAMB1, EFEMP1, GPC3, SDC2, MGP, MMP3, and TIMP3. Four genes were very significantly downregulated: COL11A1, LAMA2, GPC6, and LUM. The expression profiles of investigated genes provide a preliminary insight into the relationship between drug resistance and the expression of   ECM   components. Identifying correlations between investigated genes and drug resistance will require further analysis. 
 Expression of TIMP3 mRNA is elevated in retinas affected by simplex retinitis pigmentosa. To explore the molecular and cellular mechanisms associated with photoreceptor death in retinitis pigmentosa (RP), we have investigated altered transcriptional activity in RP retinas by a differential cDNA screening approach. We identified a clone (K222) showing over-expression in simplex RP retinas compared with controls. K222 encodes a partial cDNA of the human tissue inhibitor of metalloproteinases-3 (TIMP3) gene, a member of a family of genes implicated in extracellular matrix (  ECM  ) remodelling. Increased expression of TIMP3 in degenerating RP retinas may reflect restructuring of the   ECM   architecture, and disruption of photoreceptor-matrix interactions could contribute to activation of apoptotic cell death processes. 
 Localization of the functional domains of human tissue inhibitor of metalloproteinases-3 and the effects of a Sorsby's fundus dystrophy mutation. A transient COS-7 cell expression system was used to investigate the functional domain arrangement of tissue inhibitor of metalloproteinases-3 (TIMP-3), specifically to assess the contribution of the amino- and carboxyl-terminal domains of the molecule to its matrix metalloproteinase (MMP) inhibitory and extracellular matrix (  ECM  ) binding properties. Wild type TIMP-3 was entirely localized to the   ECM   in both its glycosylated (27 kDa) and unglycosylated (24 kDa) forms. A COOH-terminally truncated TIMP-3 molecule was found to be a non-  ECM   bound MMP inhibitor, whereas a chimeric TIMP molecule, consisting of the NH2-terminal domain of TIMP-2 fused to the COOH-terminal domain of TIMP-3, displayed   ECM   binding, albeit with a lower affinity than the wild type TIMP-3 molecule. Thus the functional domain arrangement of TIMP-3 is analogous to that seen in TIMP-1 and -2, namely that the NH2-terminal domain is responsible for MMP inhibition whereas the COOH-terminal domain is most important in mediating the specific functions of the molecule. A mutant TIMP-3 in which serine 181 was changed to a cysteine, found in Sorsby's fundus dystrophy, a hereditary macular degenerative disease, was also expressed in COS-7 cells. This gave rise to an additional 48-kDa species (possibly a TIMP-3 dimer) that retained its ability to inhibit MMPs and localize to the   ECM  . These data favor the hypothesis that the TIMP-3 mutations seen in Sorsby's fundus dystrophy contribute to disease progression by accumulation of mutant protein rather than by the loss of functional TIMP-3. 
 The imbalance between TIMP3 and matrix-degrading enzymes plays an important role in intervertebral disc degeneration. It is well-known that one of the most important features of intervertebral disc degeneration (IDD) is the extracellular matrix (  ECM  ) degradation. Collagen and aggrecan are major components of   ECM  ; the degradation of   ECM   in intervertebral discs (IVDs) is closely related to the activities of collagenase and aggrecanase. TIMP-3 is the most efficient inhibitor of aggrecanase in IVD. However, only few studies focus on the potential relationship between TIMP-3 and IDD. In our study, we found TIMP-3 gene expression was decreased after stimulating with LPS in rat nucleus pulposus (NP) cells. Then we used a lentivirus vector to reconstruct rat NP cells which high expressed TIMP-3 gene (LV-TIMP3). The upregulation of MMPs and ADAMTSs induced by LPS was significantly inhibited in LV-TIMP3 cells. After overexpression of TIMP-3, the aggrecan breakdown caused by LPS was also reduced in both monolayer culture and three-dimension culture model. To further study the relation between TIMP-3 and IDD, we collected human NP tissue samples of different degenerative degrees. Real-time PCR and immunohistochemical staining showed that the expression of TIMP-3 was negatively correlated with the degree of intervertebral disc degeneration, while MMP-1 and ADAMTS-4 were markedly increased in degenerative IVD. Taken together, our results suggest that the imbalance between aggrecanase and TIMP-3 may play an important role in the pathogenesis of IDD and therefore be a potential therapeutic target for treating IDD. 
 Regulation of TIMP3 in diabetic nephropathy: a role for microRNAs. Diabetic nephropathy (DN) is the major cause of chronic kidney disease in developed countries and contributes significantly to increased morbidity and mortality among diabetic patients. Morphologically, DN is characterized by tubulo-interstitial fibrosis, thickening of the glomerular basement membrane and mesangial expansion mainly due to accumulation of extracellular matrix (  ECM  ).   ECM   turnover is regulated by metalloproteinases and tissue inhibitors of metalloproteinases (TIMPs) activities. In diabetic conditions, TIMP3 expression in kidney is strongly reduced, but the causes of this reduction are still unknown. The aim of this study was to elucidate at least one of these mechanisms which relies on differential expression of TIMP3-targeting microRNAs (miRs) in a hyperglycemic environment either in vitro (MES13 cell line) or in vivo (mouse kidney and human biopsies). Among the TIMP3-targeting miRs, miR-21 and miR-221 were significantly upregulated in kidneys from diabetic mice compared to control littermates, and in a mesangial cell line grown in high glucose conditions. In human samples, only miR-21 expression was increased in kidney biopsies from diabetic patients compared to healthy controls. The expression of miR-217, which targets TIMP3 indirectly through downregulation of SirT1, was also increased in diabetic kidney and MES13 cell line. In agreement with these result, SirT1 expression was reduced in mouse and human diabetic kidneys as well as in MES13 mesangial cell line. TIMP3 deficiency has recently emerged as a hallmark of DN in mouse and human. In this study, we demonstrated that this reduction is due, at least in part, to increased expression of certain TIMP3-targeting miRs in diabetic kidneys compared to healthy controls. Unveiling the post-transcriptional mechanisms responsible for TIMP3 downregulation in hyperglycemic conditions may orient toward the use of this protein as a possible therapeutic target in DN. 
 Alterations of ADAMTSs and TIMP-3 in human nucleus pulposus cells subjected to compressive load: Implications in the pathogenesis of human intervertebral disc degeneration. Intervertebral disc degeneration (IDD) pertains to the loss of extracellular matrix (  ECM  ), particularly the early loss of aggrecan, the turnover of which is regulated by ADAMTSs. Amongst the etiological factors of IDD, mechanical stress plays an important role in the physiological and pathological processes of nucleus pulposus (NP) cells. However, the role of ADAMTSs and their inhibitor in human NP cells under mechanical stress has not been elucidated to date. The purpose of this study was to investigate the role of ADAMTSs and TIMP-3 in NP cells under mechanical stress. Human NP cells isolated from non-degenerative and degenerative discs were subjected to dynamic compressive load. The expression of ADAMTSs, aggrecan, and TIMP-3 was detected by quantitative real-time PCR and/or Western blot. Consequently, the gene expression of ADAMTS-1, 4, and 5 increased significantly in loaded NP cells compared with not-loaded cells from either non-degenerative or degenerative discs, whereas the gene expression of aggrecan decreased significantly. Moreover, Western blot indicated increased protein levels of ADAMTSs-1, 4, and 5. However, the expression of TIMP-3 altered insignificantly. Together, this study is the first addressing the underlying mechanisms of compressive load as a contributing factor to IDD in terms of ADAMTSs. Our results suggest that compressive load leads to the increase in ADAMTS-1, 4, and 5 that contributes to the decrease of aggrecan and IDD via TIMP-3 independent machinery. 
  GeneRIFs  
 TIMP-3 interacts with the   ECM   via both its N- and C-terminal domains. The amino acids involved in   ECM   binding are all basic in nature: Lys-26, Lys-27, Lys-30, Lys-76 of the N-terminal domain and Arg-163, Lys-165 of the C-terminal domain 
  Summary  
 This gene belongs to the TIMP gene family. The proteins encoded by this gene family are inhibitors of the matrix metalloproteinases, a group of peptidases involved in degradation of the extracellular matrix (  ECM  ). Expression of this gene is induced in response to mitogenic stimulation and this netrin domain-containing protein is localized to the   ECM  . Mutations in this gene have been associated with the autosomal dominant disorder Sorsby's fundus dystrophy. [provided by RefSeq, Jul 2008] 
  [Ee]xtracellular [Mm]atrix  Component  
   extracellular matrix   
 proteinaceous   extracellular matrix   
  PubMed  
 Seven new loci associated with age-related macular degeneration. Age-related macular degeneration (AMD) is a common cause of blindness in older individuals. To accelerate the understanding of AMD biology and help design new therapies, we executed a collaborative genome-wide association study, including >17,100 advanced AMD cases and >60,000 controls of European and Asian ancestry. We identified 19 loci associated at P < 5 × 10(-8). These loci show enrichment for genes involved in the regulation of complement activity, lipid metabolism,   extracellular matri  x remodeling and angiogenesis. Our results include seven loci with associations reaching P < 5 × 10(-8) for the first time, near the genes COL8A1-FILIP1L, IER3-DDR1, SLC16A8, TGFBR1, RAD51B, ADAMTS9 and B3GALTL. A genetic risk score combining SNP genotypes from all loci showed similar ability to distinguish cases and controls in all samples examined. Our findings provide new directions for biological, genetic and therapeutic studies of AMD. 
 Overexpression of matrix metalloproteinases in lung tissue of patients with primary spontaneous pneumothorax. Although blebs and bullae are frequently found in the apexes of lungs of patients with primary spontaneous pneumothorax (PSP), its pathogens remain unclear.To examine the role of proteases [matrix metalloproteinase (MMP)-2, MMP-7 and MMP-9] and antiproteases [tissue inhibitors of metalloproteinase (TIMP)-1, TIMP-2, TIMP-3 and TIMP-4] in the pathogenesis of PSP.Fifty consecutive PSP patients who received standard surgical care were enrolled in the study. Lung tissues from 20 patients with stage I non-small cell lung cancer were used as a control. Immunohistochemistry (IHC), reverse transcription-polymerase chain reaction (RT-PCR) and gelatin zymography were used to evaluate the expression of MMP and TIMP in the lung tissue of patients with PSP.Overexpression of MMP-2, MMP-7 and MMP-9 was found in the afflicted lung by IHC, zymography and RT-PCR. By IHC, higher expression of MMP-2 and MMP-9 in PSP patients was identified in alveolar macrophages and type II pneumocytes (88 and 92% of patients in macrophages, and 72 and 70% of patients in type II pneumocytes, respectively). MMP-2, MMP-7 and MMP-9 expression in patients was higher in mesothelial cells (66, 76 and 76%). Overexpression of TIMP-2 was detected in the   extracellular matrix   around bullae and blebs. Expression levels of TIMP-1, TIMP-3 and TIMP-4 were negligible (<10% of cells) in both PSP patients and controls.MMP-2, MMP-9, MMP-7 and TIMP-2 were upregulated in PSP lesions. These results suggest that an imbalance between the expression of proteases and antiproteases may be involved in the pathogeneses of PSP. 
 Association of a high activity of matrix metalloproteinase-9 to low levels of tissue inhibitors of metalloproteinase-1 and -3 in human hepatitis B-viral hepatoma cells. Matrix metalloproteinases (MMPs) play a major role in the turnover of   extracellular matrix   (ECM) during cancer invasion and metastasis, and tissue inhibitors of metalloproteinases (TIMPs) control MMPs, thus maintaining a balanced ECM catabolism under physiological conditions. The aim of this study was to assess the behavior of some MMPs (FASEB J., 7, 1993, 1434; Cancer Metastasis Rev., 9(4) 1990, 289) and TIMPs (Biochem. Biophys. Res. Commun., 301, 2003, 1069; FASEB J., 7, 1993, 1434; Nature, 370, 1994, 61). Competitive RT-PCR, gelatin-substrate zymography, and ELISA techniques were used for quantification. The hepatitis B virus (HBV)-DNA-containing hepatocellular carcinoma cell lines, Hep3B, HepG2-HBV and HFF-T2 contain highly activated matrix metallproteinase-9 (MMP-9), which is rarely found in normal liver cell lines such as the Chang lines. MMP-9 activities of HFH-T2, HepG2-HBV and Hep3B were significantly higher than that of non-HBV-hepatocellular carcinoma SK-Hep1 and HepG2 (HCC origin, HBV not detected), as assayed by gelatin zymography. Low levels of TIMP-1 and TIMP-3 were observed in HFH-T2, HepG2-HBV and Hep3B, while the TIMP-2 level was high, as evidenced by reverse zymography. In contrast, 3 TIMP-1, -2 and -3 were largely detected in Chang, HepG2 and SK-Hep1 cells. To investigate the nature of the quantitative regulation of MMPs and TIMPs for these cell lines at the transcriptional levels, a reverse transcriptase-polymerase chain reaction (RT-PCR) was carried out. Not only MMP-9 mRNAs of HFH-T2, HepG2-HBV and Hep3B but also MMP-9 mRNA of SK-Hep1 and HepG2 were highly expressed with no major differences among these four cell lines. However, TIMP-1 and TIMP-3 mRNAs of HFH-T2, HepG2-HBV and Hep3B were markedly reduced, while those of SK-Hep1, HepG2 and Chang cells were maintained at high levels. Finally, an invasion assay using matrigel indicated in an increase in invasiveness in HFH-T2, HepG2-HBV and Hep3B cells, but a decrease in invasiveness of Chang, HepG2 and SK-Hep1 cells. These results indicate that the overexpression of MMP-9 mRNAs and the suppression of TIMP-1 and TIMP-3 in HFH-T2, HepG2-HBV and Hep3B were the result of HBV transfection. Based on these results, it is concluded that HBV affects the malignance of hepatocellular cancer by elevating MMP-9 activity, and suppressing TIMP-1 and TIMP-3. 
 Inhibition of histone deacetylase activity in human endometrial stromal cells promotes   extracellular matrix   remodelling and limits embryo invasion. Invasion of the trophoblast into the maternal decidua is regulated by both the trophoectoderm and the endometrial stroma, and entails the action of tissue remodeling enzymes. Trophoblast invasion requires the action of metalloproteinases (MMPs) to degrade   extracellular matrix   (ECM) proteins and in turn, decidual cells express tissue inhibitors of MMPs (TIMPs). The balance between these promoting and restraining factors is a key event for the successful outcome of pregnancy. Gene expression is post-transcriptionally regulated by histone deacetylases (HDACs) that unpacks condensed chromatin activating gene expression. In this study we analyze the effect of histone acetylation on the expression of tissue remodeling enzymes and activity of human endometrial stromal cells (hESCs) related to trophoblast invasion control. Treatment of hESCs with the HDAC inhibitor trichostatin A (TSA) increased the expression of TIMP-1 and TIMP-3 while decreased MMP-2, MMP-9 and uPA and have an inhibitory effect on trophoblast invasion. Moreover, histone acetylation is detected at the promoters of TIMP-1 and TIMP-3 genes in TSA-treated. In addition, in an in vitro decidualized hESCs model, the increase of TIMP-1 and TIMP-3 expression is associated with histone acetylation at the promoters of these genes. Our results demonstrate that histone acetylation disrupt the balance of ECM modulators provoking a restrain of trophoblast invasion. These findings are important as an epigenetic mechanism that can be used to control trophoblast invasion. 
 Tissue inhibitor of metalloproteinases-1, -2, and -3 polymorphisms in a white population with intracranial aneurysms. Remodeling of the   extracellular matrix   seems to be a crucial event in the pathogenesis of cerebral aneurysms. Matrix metalloproteinases are the most important degrading enzymes in the   extracellular matrix  . Their activity is controlled predominantly by tissue inhibitors of metalloproteinases (TIMPs). To investigate the possible impact of genetic variants within the genes encoding TIMP-1, -2, and -3, we conducted this case-control study.A study sample was analyzed that comprised 44 patients with intracranial aneurysms and 44, 41, and 40 controls for the analysis of TIMP-1, -2, and -3, respectively. Differences in genotype and allele frequencies of identified polymorphisms were determined. The entire coding regions and parts of the promoter sequences of the TIMP-1, -2, and -3 genes were with using the automated laser fluorescence technique.Nine polymorphisms were identified, 3 located in TIMP-1 (-19C>T, 261C>T, 372T>C), 4 in TIMP-2 (-621C>T, -596A>C, -261G>A, 303G>A), and 2 in TIMP-3 (249T>C, 261C>T), whereas -621C>T, -596A>C, and -261G>A of the TIMP-2 gene are newly identified polymorphisms. We detected no deviation from Hardy-Weinberg equilibrium in any of the groups. The C allele of the 372T>C polymorphism was more frequently found in female than in male controls (exact nominal P=0.0012). However, this finding could not be validated by analysis of a second sample of 113 controls (exact nominal P=1.0000). There were no differences in genotype and allele frequencies between any of the other groups.Our analysis of the entire coding region of 3 TIMPs, which are the main inhibitors of metalloproteinase activity in the   extracellular matrix  , failed to show an association between genetic polymorphisms and an intracranial aneurysm. These data do not support the hypothesis that genetic variants within these genes have an impact on aneurysm development in the white population. 
 The expression and regulation of ADAMTS-1, -4, -5, -9, and -15, and TIMP-3 by TGFbeta1 in prostate cells: relevance to the accumulation of versican. Benign prostatic hyperplasia (BPH) is characterized by a proportional increase in the size of the stromal compartment of the gland, involving alterations to   extracellular matrix   (ECM) components. Some of these changes have been associated with the activity and expression of transforming growth factor beta1 (TGFbeta1). Versican (chondroitin sulphate proteoglycan-2) is overexpressed in BPH and prostate cancer and potentially contributes to disease pathology. A sub-group of the ADAMTS lineage of metalloproteases possess versican-degrading properties and are potential regulators of proteoglycan accumulation associated with BPH. These enzymes have one major inhibitor in the ECM, tissue inhibitor of metalloproteinases (TIMP)-3.The effect of TGFbeta on mRNA expression in prostatic stromal cells was determined by real-time qRT-PCR using primers to ADAMTS-1, -4, -5, -9, -15, versican, and TIMP-3. MMP-inhibitory potential (TIMP activity) of conditioned medium was measured using a fluorometric peptide substrate.Prostatic stromal cell cultures consistently expressed ADAMTS-1, -4, -5, -9, -15 and TIMP-3, in contrast to PC3, DU145, and LNCaP cells which failed to express at least two ADAMTS transcripts. In stromal cells, TGFbeta1 decreased ADAMTS-1, -5, -9, and -15 transcripts and increased ADAMTS-4, versican, and TIMP-3. TGFbeta also increased TIMP activity in conditioned medium.The induction of versican expression by TGFbeta in BPH stromal cells is in agreement with histological studies. The negative effect of TGFbeta1 on ADAMTS-1, -5, -9, and -15 coupled with increases in their inhibitor, TIMP-3 may aid the accumulation of versican in the stromal compartment of the prostate in BPH and prostate cancer. 
 Identification of the   extracellular matrix   (ECM) binding motifs of tissue inhibitor of metalloproteinases (TIMP)-3 and effective transfer to TIMP-1. Tissue inhibitor of metalloproteinases (TIMPs) are the endogenous inhibitors of the zinc-dependent endopeptidases of the matrix metalloproteinase families. There are four mammalian TIMPs (TIMP-1 to -4) but only TIMP-3 is sequestered to the   extracellular matrix   (ECM). The molecular basis for the TIMP-3:ECM association has never been fully investigated until now. In this report, we identify the unique amino acid configuration that constitutes the basis of the ECM binding motif in TIMP-3. By systematically exchanging the subdomains of the TIMPs and exhaustive mutation of TIMP-3, we have identified the surface residues directly responsible for ECM association. Contrary to the accepted view, we have found that TIMP-3 interacts with the ECM via both its N- and C-terminal domains. The amino acids involved in ECM binding are all basic in nature: Lys-26, Lys-27, Lys-30, Lys-76 of the N-terminal domain and Arg-163, Lys-165 of the C-terminal domain. Replacement of these residues with glutamate (E) and glutamine (Q) (K26/27/30/76E + R163/K165Q) resulted in a soluble TIMP-3 devoid of ECM-adhering ability. Using the ECM binding motif derived from TIMP-3, we have also created a TIMP-1 mutant (K26/27/30 + K76 transplant) capable of ECM association. This is the first instance of TIMPs being intentionally rendered soluble or ECM-bound. The ability to prepare TIMPs in soluble or ECM-bound forms also opens new avenues for future TIMP research. 
 Genetic analysis of polymorphisms in biologically relevant candidate genes in patients with abdominal aortic aneurysms. Abdominal aortic aneurysms (AAAs) are characterized by histologic signs of chronic inflammation, destructive remodeling of   extracellular matrix  , and depletion of vascular smooth muscle cells. We investigated the process of   extracellular matrix   remodeling by performing a genetic association study with polymorphisms in the genes for matrix metalloproteinases (MMPs), tissue inhibitors of metalloproteinases (TIMPs), and structural   extracellular matrix   molecules in AAA. Our hypothesis was that genetic variations in one or more of these genes contribute to greater or lesser activity of these gene products, and thereby contribute to susceptibility for developing AAAs.DNA samples from 812 unrelated white subject (AAA, n = 387; controls, n = 425) were genotyped for 14 polymorphisms in 13 different candidate genes: MMP1(nt-1607), MMP2(nt-955), MMP3(nt-1612), MMP9(nt-1562), MMP10(nt+180), MMP12(nt-82), MMP13(nt-77), TIMP1(nt+434), TIMP1(rs2070584), TIMP2(rs2009196), TIMP3(nt-1296), TGFB1(nt-509), ELN(nt+422), and COL3A1(nt+581). Odds ratios and P values adjusted for gender and country of origin using logistic regression and stratified by family history of AAA were calculated to test for association between genotype and disease status. Haplotype analysis was carried out for the two TIMP1 polymorphisms in male subjects.Analyses with one polymorphism per test without interactions showed an association with the two TIMP1 gene polymorphisms (nt+434, P = .0047; rs2070584, P = .015) in male subjects without a family history of AAA. The association remained significant when analyzing TIMP1 haplotypes (chi 2 P = .014 and empirical P = .009). In addition, we found a significant interaction between the polymorphism and gender for MMP10 ( P = .037) in cases without a family history of AAA, as well as between the polymorphism and country of origin for ELN ( P = .0169) and TIMP3 ( P = .0023) in cases with a family history of AAA.These findings suggest that genetic variations in TIMP1, TIMP3, MMP10, and ELN genes may contribute to the pathogenesis of AAAs. Further work is needed to confirm the findings in an independent set of samples and to study the functional role of these variants in AAA. It is noteworthy that contrary to a previous study, we did not find an association between the MMP9 (nt-1562) polymorphism and AAA, suggesting genetic heterogeneity of the disease.Abdominal aortic aneurysms (AAAs) are an important cardiovascular disease, but the genetic and environmental risk factors, which contribute to individual's risk to develop an aneurysm, are poorly understood. Histologically, AAAs are characterized by signs of chronic inflammation, destructive remodeling of the   extracellular matrix  , and depletion of vascular smooth muscle cells. We hypothesized that genes involved in these events could harbor changes that make individuals more susceptible to developing aneurysms. This study identified significant genetic associations between DNA sequence changes in tissue inhibitor of metalloproteinase 1 (TIMP1), TIMP3, matrix metalloproteinase 10 (MMP10) and elastin (ELN) genes, and AAA. The results will require confirmation using an independent set of samples. After replication it is possible that these sequence changes in combination with other risk factors could be used in the future to identify individuals who are at increased risk for developing an AAA. 
 Proteomics characterization of extracellular space components in the human aorta. The vascular   extracellular matrix   (ECM) is essential for the structural integrity of the vessel wall and also serves as a substrate for the binding and retention of secreted products of vascular cells as well as molecules coming from the circulation. Although proteomics has been previously applied to vascular tissues, few studies have specifically targeted the vascular ECM and its associated proteins. Thus, its detailed composition remains to be characterized. In this study, we describe a methodology for the extraction of extracellular proteins from human aortas and their identification by proteomics. The approach is based on (a) effective decellularization to enrich for scarce extracellular proteins, (b) successful solubilization and deglycosylation of ECM proteins, and (c) relative estimation of protein abundance using spectral counting. Our three-step extraction approach resulted in the identification of 103 extracellular proteins of which one-third have never been reported in the proteomics literature of vascular tissues. In particular, three glycoproteins (podocan, sclerostin, and agrin) were identified for the first time in human aortas at the protein level. We also identified extracellular adipocyte enhancer-binding protein 1, the cartilage glycoprotein asporin, and a previously hypothetical protein, retinal pigment epithelium (RPE) spondin. Moreover, our methodology allowed us to screen for proteolysis in the aortic samples based on the identification of proteolytic enzymes and their corresponding degradation products. For instance, we were able to detect matrix metalloproteinase-9 by mass spectrometry and relate its presence to degradation of fibronectin in a clinical specimen. We expect this proteomics methodology to further our understanding of the composition of the vascular extracellular environment, shed light on ECM remodeling and degradation, and provide insights into important pathological processes, such as plaque rupture, aneurysm formation, and restenosis. 
 The role of EZH2 in the regulation of the activity of matrix metalloproteinases in prostate cancer cells. Degradation of the   extracellular matrix   (ECM), a critical step in cancer metastasis, is determined by the balance between MMPs (matrix metalloproteinases) and their inhibitors TIMPs (tissue inhibitors of metalloproteinases). In cancer cells, this balance is shifted towards MMPs, promoting ECM degradation. Here, we show that EZH2 plays an active role in this process by repressing the expression of TIMP2 and TIMP3 in prostate cancer cells. The TIMP genes are derepressed by knockdown of EZH2 expression in human prostate cancer cells but repressed by overexpression of EZH2 in benign human prostate epithelial cells. EZH2 catalyzes H3K27 trimethylation and subsequent DNA methylation of the TIMP gene promoters. Overexpression of EZH2 confers an invasive phenotype on benign prostate epithelial cells; however, this phenotype is suppressed by cooverexpression of TIMP3. EZH2 knockdown markedly reduces the proteolytic activity of MMP-9, thereby decreasing the invasive activity of prostate cancer cells. These results suggest that the transcriptional repression of the TIMP genes by EZH2 may be a major mechanism to shift the MMPs/TIMPs balance in favor of MMP activity and thus to promote ECM degradation and subsequent invasion of prostate cancer cells. 
 Promoted activation of matrix metalloproteinase (MMP)-2 in keloid fibroblasts and increased expression of MMP-2 in collagen bundle regions: implications for mechanisms of keloid progression. Keloid is characterized by excessive deposition of collagen, resulting from aberrant   extracellular matrix   (ECM) production and degradation. The aim was to investigate the role of matrix metalloproteinases (MMPs) and tissue inhibitor of metalloproteinases (TIMPs) in pathological wound healing in keloids.Semiquantitative analysis of 60 keloid tissue samples and 25 mature scar tissue samples demonstrated significantly increased expression of MMP-2, TIMP-2 and TIMP-3 in keloids compared with mature scars. Within keloid regions, MMP-2 expression was significantly higher in collagen bundle regions than in non-collagen bundle regions. Double immunofluorescence revealed that keloid fibroblasts between collagen bundles exhibited MMP-2, TIMP-2 and membrane-type 1 MMP (MT1-MMP) co-expression, whereas only MMP-2 expression was evident on the edge of collagen bundles. Western blot analysis and gelatin zymography of 13 keloid-derived fibroblasts (KFbs) and six normal skin dermal-derived fibroblasts (NFbs) demonstrated that unstimulated KFbs exhibited significantly increased MMP-2 activity and expression compared with NFbs under the same conditions.These results together indicate that MMP-2 activity can be promoted in keloid fibroblasts between collagen bundles in cooperation with TIMP-2 and MT1-MMP. This could contribute to remodelling of collagen bundle regions and invasion of fibroblasts into peripheral normal regions through promoted degradation of ECM. 
 Association of matrix metalloproteinase gene polymorphisms with refractive error in Amish and Ashkenazi families. Matrix metalloproteinases (MMPs) and tissue inhibitors of metalloproteinases (TIMPs) are involved in scleral   extracellular matrix   remodeling and have shown differential expression in experimental myopia. The genetic association of refractive error and polymorphisms in MMP and TIMP genes in Old Order Amish (AMISH) and Ashkenazi Jewish (ASHK) families was investigated.Individuals from 55 AMISH and 63 ASHK families participated in the study. Ascertainment was designed to enrich the families for myopia; the mean spherical equivalent (MSE) refractive error (SD) was -1.61 (2.72) D in the AMISH, and -3.56 (3.32) D in the ASHK. One hundred forty-six common haplotype tagging SNPs covering 14 MMP and 4 TIMP genes were genotyped in 358 AMISH and 535 ASHK participants. Association analyses of MSE and the spherical component of refraction (SPH) were performed separately for the AMISH and the ASHK. Bonferroni-corrected significance thresholds and local false discovery rates were used to account for multiple testing.After they were filtered for quality-control, 127 SNPs were included in the analyses. No polymorphisms showed statistically significant association to refraction in the ASHK (minimum P = 0.0132). In AMISH, two SNPs showed evidence of association with refractive phenotypes: rs1939008 (P = 0.00016 for SPH); and rs9928731 (P = 0.00026 for SPH). These markers were each estimated to explain <5% of the variance of SPH in the AMISH sample.Statistically significant genetic associations of ocular refraction to polymorphisms near MMP1 and within MMP2 were identified in the AMISH but not among the ASHK families. The results suggest that the MMP1 and MMP2 genes are involved in refractive variation in the AMISH. Genetic and/or environmental heterogeneity most likely contribute to differences in association results between ethnic groups. 
 Overexpression of matrix metalloproteinases in lung tissue of patients with primary spontaneous pneumothorax. Although blebs and bullae are frequently found in the apexes of lungs of patients with primary spontaneous pneumothorax (PSP), its pathogens remain unclear.To examine the role of proteases [matrix metalloproteinase (MMP)-2, MMP-7 and MMP-9] and antiproteases [tissue inhibitors of metalloproteinase (TIMP)-1, TIMP-2, TIMP-3 and TIMP-4] in the pathogenesis of PSP.Fifty consecutive PSP patients who received standard surgical care were enrolled in the study. Lung tissues from 20 patients with stage I non-small cell lung cancer were used as a control. Immunohistochemistry (IHC), reverse transcription-polymerase chain reaction (RT-PCR) and gelatin zymography were used to evaluate the expression of MMP and TIMP in the lung tissue of patients with PSP.Overexpression of MMP-2, MMP-7 and MMP-9 was found in the afflicted lung by IHC, zymography and RT-PCR. By IHC, higher expression of MMP-2 and MMP-9 in PSP patients was identified in alveolar macrophages and type II pneumocytes (88 and 92% of patients in macrophages, and 72 and 70% of patients in type II pneumocytes, respectively). MMP-2, MMP-7 and MMP-9 expression in patients was higher in mesothelial cells (66, 76 and 76%). Overexpression of TIMP-2 was detected in the   extracellular matrix   around bullae and blebs. Expression levels of TIMP-1, TIMP-3 and TIMP-4 were negligible (<10% of cells) in both PSP patients and controls.MMP-2, MMP-9, MMP-7 and TIMP-2 were upregulated in PSP lesions. These results suggest that an imbalance between the expression of proteases and antiproteases may be involved in the pathogeneses of PSP. 
 The role of EZH2 in the regulation of the activity of matrix metalloproteinases in prostate cancer cells. Degradation of the   extracellular matrix   (ECM), a critical step in cancer metastasis, is determined by the balance between MMPs (matrix metalloproteinases) and their inhibitors TIMPs (tissue inhibitors of metalloproteinases). In cancer cells, this balance is shifted towards MMPs, promoting ECM degradation. Here, we show that EZH2 plays an active role in this process by repressing the expression of TIMP2 and TIMP3 in prostate cancer cells. The TIMP genes are derepressed by knockdown of EZH2 expression in human prostate cancer cells but repressed by overexpression of EZH2 in benign human prostate epithelial cells. EZH2 catalyzes H3K27 trimethylation and subsequent DNA methylation of the TIMP gene promoters. Overexpression of EZH2 confers an invasive phenotype on benign prostate epithelial cells; however, this phenotype is suppressed by cooverexpression of TIMP3. EZH2 knockdown markedly reduces the proteolytic activity of MMP-9, thereby decreasing the invasive activity of prostate cancer cells. These results suggest that the transcriptional repression of the TIMP genes by EZH2 may be a major mechanism to shift the MMPs/TIMPs balance in favor of MMP activity and thus to promote ECM degradation and subsequent invasion of prostate cancer cells. 
 Inhibition of histone deacetylase activity in human endometrial stromal cells promotes   extracellular matrix   remodelling and limits embryo invasion. Invasion of the trophoblast into the maternal decidua is regulated by both the trophoectoderm and the endometrial stroma, and entails the action of tissue remodeling enzymes. Trophoblast invasion requires the action of metalloproteinases (MMPs) to degrade   extracellular matrix   (ECM) proteins and in turn, decidual cells express tissue inhibitors of MMPs (TIMPs). The balance between these promoting and restraining factors is a key event for the successful outcome of pregnancy. Gene expression is post-transcriptionally regulated by histone deacetylases (HDACs) that unpacks condensed chromatin activating gene expression. In this study we analyze the effect of histone acetylation on the expression of tissue remodeling enzymes and activity of human endometrial stromal cells (hESCs) related to trophoblast invasion control. Treatment of hESCs with the HDAC inhibitor trichostatin A (TSA) increased the expression of TIMP-1 and TIMP-3 while decreased MMP-2, MMP-9 and uPA and have an inhibitory effect on trophoblast invasion. Moreover, histone acetylation is detected at the promoters of TIMP-1 and TIMP-3 genes in TSA-treated. In addition, in an in vitro decidualized hESCs model, the increase of TIMP-1 and TIMP-3 expression is associated with histone acetylation at the promoters of these genes. Our results demonstrate that histone acetylation disrupt the balance of ECM modulators provoking a restrain of trophoblast invasion. These findings are important as an epigenetic mechanism that can be used to control trophoblast invasion. 
 Promoted activation of matrix metalloproteinase (MMP)-2 in keloid fibroblasts and increased expression of MMP-2 in collagen bundle regions: implications for mechanisms of keloid progression. Keloid is characterized by excessive deposition of collagen, resulting from aberrant   extracellular matrix   (ECM) production and degradation. The aim was to investigate the role of matrix metalloproteinases (MMPs) and tissue inhibitor of metalloproteinases (TIMPs) in pathological wound healing in keloids.Semiquantitative analysis of 60 keloid tissue samples and 25 mature scar tissue samples demonstrated significantly increased expression of MMP-2, TIMP-2 and TIMP-3 in keloids compared with mature scars. Within keloid regions, MMP-2 expression was significantly higher in collagen bundle regions than in non-collagen bundle regions. Double immunofluorescence revealed that keloid fibroblasts between collagen bundles exhibited MMP-2, TIMP-2 and membrane-type 1 MMP (MT1-MMP) co-expression, whereas only MMP-2 expression was evident on the edge of collagen bundles. Western blot analysis and gelatin zymography of 13 keloid-derived fibroblasts (KFbs) and six normal skin dermal-derived fibroblasts (NFbs) demonstrated that unstimulated KFbs exhibited significantly increased MMP-2 activity and expression compared with NFbs under the same conditions.These results together indicate that MMP-2 activity can be promoted in keloid fibroblasts between collagen bundles in cooperation with TIMP-2 and MT1-MMP. This could contribute to remodelling of collagen bundle regions and invasion of fibroblasts into peripheral normal regions through promoted degradation of ECM. 
 Genetic analysis of polymorphisms in biologically relevant candidate genes in patients with abdominal aortic aneurysms. Abdominal aortic aneurysms (AAAs) are characterized by histologic signs of chronic inflammation, destructive remodeling of   extracellular matrix  , and depletion of vascular smooth muscle cells. We investigated the process of   extracellular matrix   remodeling by performing a genetic association study with polymorphisms in the genes for matrix metalloproteinases (MMPs), tissue inhibitors of metalloproteinases (TIMPs), and structural   extracellular matrix   molecules in AAA. Our hypothesis was that genetic variations in one or more of these genes contribute to greater or lesser activity of these gene products, and thereby contribute to susceptibility for developing AAAs.DNA samples from 812 unrelated white subject (AAA, n = 387; controls, n = 425) were genotyped for 14 polymorphisms in 13 different candidate genes: MMP1(nt-1607), MMP2(nt-955), MMP3(nt-1612), MMP9(nt-1562), MMP10(nt+180), MMP12(nt-82), MMP13(nt-77), TIMP1(nt+434), TIMP1(rs2070584), TIMP2(rs2009196), TIMP3(nt-1296), TGFB1(nt-509), ELN(nt+422), and COL3A1(nt+581). Odds ratios and P values adjusted for gender and country of origin using logistic regression and stratified by family history of AAA were calculated to test for association between genotype and disease status. Haplotype analysis was carried out for the two TIMP1 polymorphisms in male subjects.Analyses with one polymorphism per test without interactions showed an association with the two TIMP1 gene polymorphisms (nt+434, P = .0047; rs2070584, P = .015) in male subjects without a family history of AAA. The association remained significant when analyzing TIMP1 haplotypes (chi 2 P = .014 and empirical P = .009). In addition, we found a significant interaction between the polymorphism and gender for MMP10 ( P = .037) in cases without a family history of AAA, as well as between the polymorphism and country of origin for ELN ( P = .0169) and TIMP3 ( P = .0023) in cases with a family history of AAA.These findings suggest that genetic variations in TIMP1, TIMP3, MMP10, and ELN genes may contribute to the pathogenesis of AAAs. Further work is needed to confirm the findings in an independent set of samples and to study the functional role of these variants in AAA. It is noteworthy that contrary to a previous study, we did not find an association between the MMP9 (nt-1562) polymorphism and AAA, suggesting genetic heterogeneity of the disease.Abdominal aortic aneurysms (AAAs) are an important cardiovascular disease, but the genetic and environmental risk factors, which contribute to individual's risk to develop an aneurysm, are poorly understood. Histologically, AAAs are characterized by signs of chronic inflammation, destructive remodeling of the   extracellular matrix  , and depletion of vascular smooth muscle cells. We hypothesized that genes involved in these events could harbor changes that make individuals more susceptible to developing aneurysms. This study identified significant genetic associations between DNA sequence changes in tissue inhibitor of metalloproteinase 1 (TIMP1), TIMP3, matrix metalloproteinase 10 (MMP10) and elastin (ELN) genes, and AAA. The results will require confirmation using an independent set of samples. After replication it is possible that these sequence changes in combination with other risk factors could be used in the future to identify individuals who are at increased risk for developing an AAA. 
 Tissue inhibitor of metalloproteinases-1, -2, and -3 polymorphisms in a white population with intracranial aneurysms. Remodeling of the   extracellular matrix   seems to be a crucial event in the pathogenesis of cerebral aneurysms. Matrix metalloproteinases are the most important degrading enzymes in the   extracellular matrix  . Their activity is controlled predominantly by tissue inhibitors of metalloproteinases (TIMPs). To investigate the possible impact of genetic variants within the genes encoding TIMP-1, -2, and -3, we conducted this case-control study.A study sample was analyzed that comprised 44 patients with intracranial aneurysms and 44, 41, and 40 controls for the analysis of TIMP-1, -2, and -3, respectively. Differences in genotype and allele frequencies of identified polymorphisms were determined. The entire coding regions and parts of the promoter sequences of the TIMP-1, -2, and -3 genes were with using the automated laser fluorescence technique.Nine polymorphisms were identified, 3 located in TIMP-1 (-19C>T, 261C>T, 372T>C), 4 in TIMP-2 (-621C>T, -596A>C, -261G>A, 303G>A), and 2 in TIMP-3 (249T>C, 261C>T), whereas -621C>T, -596A>C, and -261G>A of the TIMP-2 gene are newly identified polymorphisms. We detected no deviation from Hardy-Weinberg equilibrium in any of the groups. The C allele of the 372T>C polymorphism was more frequently found in female than in male controls (exact nominal P=0.0012). However, this finding could not be validated by analysis of a second sample of 113 controls (exact nominal P=1.0000). There were no differences in genotype and allele frequencies between any of the other groups.Our analysis of the entire coding region of 3 TIMPs, which are the main inhibitors of metalloproteinase activity in the   extracellular matrix  , failed to show an association between genetic polymorphisms and an intracranial aneurysm. These data do not support the hypothesis that genetic variants within these genes have an impact on aneurysm development in the white population. 
 Association of matrix metalloproteinase gene polymorphisms with refractive error in Amish and Ashkenazi families. Matrix metalloproteinases (MMPs) and tissue inhibitors of metalloproteinases (TIMPs) are involved in scleral   extracellular matrix   remodeling and have shown differential expression in experimental myopia. The genetic association of refractive error and polymorphisms in MMP and TIMP genes in Old Order Amish (AMISH) and Ashkenazi Jewish (ASHK) families was investigated.Individuals from 55 AMISH and 63 ASHK families participated in the study. Ascertainment was designed to enrich the families for myopia; the mean spherical equivalent (MSE) refractive error (SD) was -1.61 (2.72) D in the AMISH, and -3.56 (3.32) D in the ASHK. One hundred forty-six common haplotype tagging SNPs covering 14 MMP and 4 TIMP genes were genotyped in 358 AMISH and 535 ASHK participants. Association analyses of MSE and the spherical component of refraction (SPH) were performed separately for the AMISH and the ASHK. Bonferroni-corrected significance thresholds and local false discovery rates were used to account for multiple testing.After they were filtered for quality-control, 127 SNPs were included in the analyses. No polymorphisms showed statistically significant association to refraction in the ASHK (minimum P = 0.0132). In AMISH, two SNPs showed evidence of association with refractive phenotypes: rs1939008 (P = 0.00016 for SPH); and rs9928731 (P = 0.00026 for SPH). These markers were each estimated to explain <5% of the variance of SPH in the AMISH sample.Statistically significant genetic associations of ocular refraction to polymorphisms near MMP1 and within MMP2 were identified in the AMISH but not among the ASHK families. The results suggest that the MMP1 and MMP2 genes are involved in refractive variation in the AMISH. Genetic and/or environmental heterogeneity most likely contribute to differences in association results between ethnic groups. 
 Identification of the   extracellular matrix   (ECM) binding motifs of tissue inhibitor of metalloproteinases (TIMP)-3 and effective transfer to TIMP-1. Tissue inhibitor of metalloproteinases (TIMPs) are the endogenous inhibitors of the zinc-dependent endopeptidases of the matrix metalloproteinase families. There are four mammalian TIMPs (TIMP-1 to -4) but only TIMP-3 is sequestered to the   extracellular matrix   (ECM). The molecular basis for the TIMP-3:ECM association has never been fully investigated until now. In this report, we identify the unique amino acid configuration that constitutes the basis of the ECM binding motif in TIMP-3. By systematically exchanging the subdomains of the TIMPs and exhaustive mutation of TIMP-3, we have identified the surface residues directly responsible for ECM association. Contrary to the accepted view, we have found that TIMP-3 interacts with the ECM via both its N- and C-terminal domains. The amino acids involved in ECM binding are all basic in nature: Lys-26, Lys-27, Lys-30, Lys-76 of the N-terminal domain and Arg-163, Lys-165 of the C-terminal domain. Replacement of these residues with glutamate (E) and glutamine (Q) (K26/27/30/76E + R163/K165Q) resulted in a soluble TIMP-3 devoid of ECM-adhering ability. Using the ECM binding motif derived from TIMP-3, we have also created a TIMP-1 mutant (K26/27/30 + K76 transplant) capable of ECM association. This is the first instance of TIMPs being intentionally rendered soluble or ECM-bound. The ability to prepare TIMPs in soluble or ECM-bound forms also opens new avenues for future TIMP research. 
 The expression and regulation of ADAMTS-1, -4, -5, -9, and -15, and TIMP-3 by TGFbeta1 in prostate cells: relevance to the accumulation of versican. Benign prostatic hyperplasia (BPH) is characterized by a proportional increase in the size of the stromal compartment of the gland, involving alterations to   extracellular matrix   (ECM) components. Some of these changes have been associated with the activity and expression of transforming growth factor beta1 (TGFbeta1). Versican (chondroitin sulphate proteoglycan-2) is overexpressed in BPH and prostate cancer and potentially contributes to disease pathology. A sub-group of the ADAMTS lineage of metalloproteases possess versican-degrading properties and are potential regulators of proteoglycan accumulation associated with BPH. These enzymes have one major inhibitor in the ECM, tissue inhibitor of metalloproteinases (TIMP)-3.The effect of TGFbeta on mRNA expression in prostatic stromal cells was determined by real-time qRT-PCR using primers to ADAMTS-1, -4, -5, -9, -15, versican, and TIMP-3. MMP-inhibitory potential (TIMP activity) of conditioned medium was measured using a fluorometric peptide substrate.Prostatic stromal cell cultures consistently expressed ADAMTS-1, -4, -5, -9, -15 and TIMP-3, in contrast to PC3, DU145, and LNCaP cells which failed to express at least two ADAMTS transcripts. In stromal cells, TGFbeta1 decreased ADAMTS-1, -5, -9, and -15 transcripts and increased ADAMTS-4, versican, and TIMP-3. TGFbeta also increased TIMP activity in conditioned medium.The induction of versican expression by TGFbeta in BPH stromal cells is in agreement with histological studies. The negative effect of TGFbeta1 on ADAMTS-1, -5, -9, and -15 coupled with increases in their inhibitor, TIMP-3 may aid the accumulation of versican in the stromal compartment of the prostate in BPH and prostate cancer. 
 Tissue inhibitor of metalloproteinases-1, -2, and -3 polymorphisms in a white population with intracranial aneurysms. Remodeling of the   extracellular matrix   seems to be a crucial event in the pathogenesis of cerebral aneurysms. Matrix metalloproteinases are the most important degrading enzymes in the   extracellular matrix  . Their activity is controlled predominantly by tissue inhibitors of metalloproteinases (TIMPs). To investigate the possible impact of genetic variants within the genes encoding TIMP-1, -2, and -3, we conducted this case-control study.A study sample was analyzed that comprised 44 patients with intracranial aneurysms and 44, 41, and 40 controls for the analysis of TIMP-1, -2, and -3, respectively. Differences in genotype and allele frequencies of identified polymorphisms were determined. The entire coding regions and parts of the promoter sequences of the TIMP-1, -2, and -3 genes were with using the automated laser fluorescence technique.Nine polymorphisms were identified, 3 located in TIMP-1 (-19C>T, 261C>T, 372T>C), 4 in TIMP-2 (-621C>T, -596A>C, -261G>A, 303G>A), and 2 in TIMP-3 (249T>C, 261C>T), whereas -621C>T, -596A>C, and -261G>A of the TIMP-2 gene are newly identified polymorphisms. We detected no deviation from Hardy-Weinberg equilibrium in any of the groups. The C allele of the 372T>C polymorphism was more frequently found in female than in male controls (exact nominal P=0.0012). However, this finding could not be validated by analysis of a second sample of 113 controls (exact nominal P=1.0000). There were no differences in genotype and allele frequencies between any of the other groups.Our analysis of the entire coding region of 3 TIMPs, which are the main inhibitors of metalloproteinase activity in the   extracellular matrix  , failed to show an association between genetic polymorphisms and an intracranial aneurysm. These data do not support the hypothesis that genetic variants within these genes have an impact on aneurysm development in the white population. 
 Association of a high activity of matrix metalloproteinase-9 to low levels of tissue inhibitors of metalloproteinase-1 and -3 in human hepatitis B-viral hepatoma cells. Matrix metalloproteinases (MMPs) play a major role in the turnover of   extracellular matrix   (ECM) during cancer invasion and metastasis, and tissue inhibitors of metalloproteinases (TIMPs) control MMPs, thus maintaining a balanced ECM catabolism under physiological conditions. The aim of this study was to assess the behavior of some MMPs (FASEB J., 7, 1993, 1434; Cancer Metastasis Rev., 9(4) 1990, 289) and TIMPs (Biochem. Biophys. Res. Commun., 301, 2003, 1069; FASEB J., 7, 1993, 1434; Nature, 370, 1994, 61). Competitive RT-PCR, gelatin-substrate zymography, and ELISA techniques were used for quantification. The hepatitis B virus (HBV)-DNA-containing hepatocellular carcinoma cell lines, Hep3B, HepG2-HBV and HFF-T2 contain highly activated matrix metallproteinase-9 (MMP-9), which is rarely found in normal liver cell lines such as the Chang lines. MMP-9 activities of HFH-T2, HepG2-HBV and Hep3B were significantly higher than that of non-HBV-hepatocellular carcinoma SK-Hep1 and HepG2 (HCC origin, HBV not detected), as assayed by gelatin zymography. Low levels of TIMP-1 and TIMP-3 were observed in HFH-T2, HepG2-HBV and Hep3B, while the TIMP-2 level was high, as evidenced by reverse zymography. In contrast, 3 TIMP-1, -2 and -3 were largely detected in Chang, HepG2 and SK-Hep1 cells. To investigate the nature of the quantitative regulation of MMPs and TIMPs for these cell lines at the transcriptional levels, a reverse transcriptase-polymerase chain reaction (RT-PCR) was carried out. Not only MMP-9 mRNAs of HFH-T2, HepG2-HBV and Hep3B but also MMP-9 mRNA of SK-Hep1 and HepG2 were highly expressed with no major differences among these four cell lines. However, TIMP-1 and TIMP-3 mRNAs of HFH-T2, HepG2-HBV and Hep3B were markedly reduced, while those of SK-Hep1, HepG2 and Chang cells were maintained at high levels. Finally, an invasion assay using matrigel indicated in an increase in invasiveness in HFH-T2, HepG2-HBV and Hep3B cells, but a decrease in invasiveness of Chang, HepG2 and SK-Hep1 cells. These results indicate that the overexpression of MMP-9 mRNAs and the suppression of TIMP-1 and TIMP-3 in HFH-T2, HepG2-HBV and Hep3B were the result of HBV transfection. Based on these results, it is concluded that HBV affects the malignance of hepatocellular cancer by elevating MMP-9 activity, and suppressing TIMP-1 and TIMP-3. 
 Genetic analysis of polymorphisms in biologically relevant candidate genes in patients with abdominal aortic aneurysms. Abdominal aortic aneurysms (AAAs) are characterized by histologic signs of chronic inflammation, destructive remodeling of   extracellular matrix  , and depletion of vascular smooth muscle cells. We investigated the process of   extracellular matrix   remodeling by performing a genetic association study with polymorphisms in the genes for matrix metalloproteinases (MMPs), tissue inhibitors of metalloproteinases (TIMPs), and structural   extracellular matrix   molecules in AAA. Our hypothesis was that genetic variations in one or more of these genes contribute to greater or lesser activity of these gene products, and thereby contribute to susceptibility for developing AAAs.DNA samples from 812 unrelated white subject (AAA, n = 387; controls, n = 425) were genotyped for 14 polymorphisms in 13 different candidate genes: MMP1(nt-1607), MMP2(nt-955), MMP3(nt-1612), MMP9(nt-1562), MMP10(nt+180), MMP12(nt-82), MMP13(nt-77), TIMP1(nt+434), TIMP1(rs2070584), TIMP2(rs2009196), TIMP3(nt-1296), TGFB1(nt-509), ELN(nt+422), and COL3A1(nt+581). Odds ratios and P values adjusted for gender and country of origin using logistic regression and stratified by family history of AAA were calculated to test for association between genotype and disease status. Haplotype analysis was carried out for the two TIMP1 polymorphisms in male subjects.Analyses with one polymorphism per test without interactions showed an association with the two TIMP1 gene polymorphisms (nt+434, P = .0047; rs2070584, P = .015) in male subjects without a family history of AAA. The association remained significant when analyzing TIMP1 haplotypes (chi 2 P = .014 and empirical P = .009). In addition, we found a significant interaction between the polymorphism and gender for MMP10 ( P = .037) in cases without a family history of AAA, as well as between the polymorphism and country of origin for ELN ( P = .0169) and TIMP3 ( P = .0023) in cases with a family history of AAA.These findings suggest that genetic variations in TIMP1, TIMP3, MMP10, and ELN genes may contribute to the pathogenesis of AAAs. Further work is needed to confirm the findings in an independent set of samples and to study the functional role of these variants in AAA. It is noteworthy that contrary to a previous study, we did not find an association between the MMP9 (nt-1562) polymorphism and AAA, suggesting genetic heterogeneity of the disease.Abdominal aortic aneurysms (AAAs) are an important cardiovascular disease, but the genetic and environmental risk factors, which contribute to individual's risk to develop an aneurysm, are poorly understood. Histologically, AAAs are characterized by signs of chronic inflammation, destructive remodeling of the   extracellular matrix  , and depletion of vascular smooth muscle cells. We hypothesized that genes involved in these events could harbor changes that make individuals more susceptible to developing aneurysms. This study identified significant genetic associations between DNA sequence changes in tissue inhibitor of metalloproteinase 1 (TIMP1), TIMP3, matrix metalloproteinase 10 (MMP10) and elastin (ELN) genes, and AAA. The results will require confirmation using an independent set of samples. After replication it is possible that these sequence changes in combination with other risk factors could be used in the future to identify individuals who are at increased risk for developing an AAA. 
 Tissue inhibitor of metalloproteinase-3 is up-regulated by transforming growth factor-beta1 in vitro and expressed in fibroblastic foci in vivo in idiopathic pulmonary fibrosis. Idiopathic pulmonary fibrosis (IPF) is characterized by fibroblast expansion and   extracellular matrix   accumulation. However, the mechanisms involved in matrix remodeling have not been elucidated. In this study, the authors aimed to evaluate the expression of the tissue inhibitors of matrix metalloproteinases (TIMPs) in human fibroblasts and whole tissues from IPF and normal lungs. They also determined the role of mitogen-activated protein kinase (MAPK) in TIMP3 expression. TIMP1, TIMP2, and TIMP3 were highly expressed in lung fibroblasts. Transforming growth factor (TGF)-beta1, a profibrotic mediator, induced strong up-regulation of TIMP3 at the mRNA and protein levels. The authors examined whether the MAPK pathway was involved in TGF-beta1-induced TIMP3 expression. TGF-beta1 induced the phosphorylation of p38 and extracellular signal-regulated kinase (ERK)1/2. Biochemical blockade of p38 by SB203580, but not of the ERK MAPK pathway, inhibited the effect of this factor. The effect was also blocked by the tyrosine kinase inhibitor genistein and by antagonizing TGF-beta1 receptor type I (activin-linked kinase [ALK5]). In IPF tissues TIMP3 gene expression was significantly increased and the protein was localized to fibroblastic foci and   extracellular matrix  . Our findings suggest that TGF-beta1-induced TIMP3 may be an important mediator in lung fibrogenesis. 
   Extracellular matrix   remodeling genes polymorphisms and risk of chronic bronchitis and recurrent pneumonia in children. We investigated the association of matrix metalloproteinases, the disintegrin and metalloprotease 33 and the tissue and serum inhibitors of proteinase gene polymorphisms with severe chronic respiratory diseases in Tatar children. We analyzed the case-control data sample from a total of 592 Tatar individuals, consisting of 119 children with chronic bronchitis, 138 with recurrent pneumonia and 335 control children residing in Ufa (Russia). The percentage of heterozygous genotype for the MMP9 (2660A>G) was higher among healthy children (52.54% vs 36.13% in chronic bronchitis patients, P(adj)=0.0033, P(cor)=0.033, odds ratio (OR)=0.51; and 36.96% in recurrent pneumonia group, P(adj)=0.0034, P(cor)=0.034, OR=0.53). The MMP12 (-82A>G) locus was associated with chronic bronchitis in the additive model (P(adj)=0.0091, P(cor)=0.09, OR=0.45, β=-0.798). The relationship between the 6A6A genotype of MMP3 (-1171 5A>6A) (P(adj)=0.0013, P(cor)=0.013, OR=3.91) and the 6A-A haplotype of MMP3 (-1171 5A>6A) and MMP12 (-82A>G) and recurrent pneumonia were unraveled (Padj=0.001, P(cor)=0.01, OR=2.07). This haplotype was also associated with a higher risk of chronic bronchitis (P(adj)=0.0012, P(cor)=0.012, OR=2.15). The TIMP3 (-1296T>C) was associated with recurrent pneumonia in the dominant model (P(adj)=0.0031, P(cor)=0.031, OR=1.91). The MMP9, MMP3 and TIMP3 (tissue inhibitors of matrix metalloproteinases) polymorphisms and MMP3 and MMP12 haplotypes may play a substantial role in susceptibility to severe airway and lung injury in children with chronic bronchitis and recurrent pneumonia. 
 Novel mutation in the TIMP3 gene causes Sorsby fundus dystrophy. To determine the molecular basis of a retinopathy previously described as dominant macular subretinal neovascularization with peripheral retinal degeneration.The TIMP3 gene was analyzed in family members, and 4 mutation-positive patients were studied using psychophysics and electroretinography.Cosegregating with disease in the family was a single base pair change in the TIMP3 gene, altering a conserved tyrosine to cysteine at amino acid position 172 (Y172C). There was psychophysical and electroretinographic evidence of rod dysfunction greater than cone dysfunction. Dark adaptometry showed abnormalities with regional retinal variation in degree.The Y172C mutation in the TIMP3 gene is another cause of Sorsby fundus dystrophy. The expression of this form of the disease, as in other C-terminal TIMP3 mutations, is speculated to be secondary to mutant TIMP-3, causing a decreased turnover of the   extracellular matrix  .The molecular clarification of inherited retinal degeneration involving abnormal   extracellular matrix   turnover in and around Bruch's membrane should provide clues to the pathogenesis of not only these particular diseases but also forms of age-related macular degeneration. 
 TIMP-3 inhibits the procollagen N-proteinase ADAMTS-2. ADAMTS-2 is an extracellular metalloproteinase responsible for cleaving the N-propeptides of procollagens I-III; an activity necessary for the formation of collagenous ECM (  extracellular matrix  ). The four TIMPs (tissue inhibitors of metalloproteinases) regulate the activities of matrix metalloproteinases, which are involved in degrading ECM components. Here we delineate the abilities of the TIMPs to affect biosynthetic processing of procollagens. TIMP-1, -2 and -4 show no inhibitory activity towards ADAMTS-2, in addition none of the TIMPs showed inhibitory activity towards bone morphogenetic protein 1, which is responsible for cleaving procollagen C-propeptides. In contrast, TIMP-3 is demonstrated to inhibit ADAMTS-2 in vitro with apparent Ki values of 160 and 602 nM, in the presence of heparin or without respectively; and TIMP-3 is shown to inhibit procollagen processing by cells. 
 Differential regulation of extracellular tissue inhibitor of metalloproteinases-3 levels by cell membrane-bound and shed low density lipoprotein receptor-related protein 1. Tissue inhibitor of metalloproteinases-3 (TIMP-3) plays a key role in regulating   extracellular matrix   turnover by inhibiting matrix metalloproteinases (MMPs), adamalysins (ADAMs), and adamalysins with thrombospondin motifs (ADAMTSs). We demonstrate that levels of this physiologically important inhibitor can be regulated post-translationally by endocytosis. TIMP-3 was endocytosed and degraded by a number of cell types including chondrocytes, fibroblasts, and monocytes, and we found that the endocytic receptor low density lipoprotein receptor-related protein-1 (LRP-1) plays a major role in TIMP-3 internalization. However, the cellular uptake of TIMP-3 significantly slowed down after 10 h due to shedding of LRP-1 from the cell surface and formation of soluble LRP-1 (sLRP-1)-TIMP-3 complexes. Addition of TIMP-3 to HTB94 human chondrosarcoma cells increased the release of sLRP-1 fragments of 500, 215, 160, and 110 kDa into the medium in a concentration-dependent manner, and all of these fragments were able to bind to TIMP-3. TIMP-3 bound to sLRP-1, which was resistant to endocytosis, retained its inhibitory activity against metalloproteinases. Extracellular levels of sLRP-1 can thus increase the half-life of TIMP-3 in the extracellular space, controlling the bioavailability of TIMP-3 to inhibit metalloproteinases. 
 Ovarian expression, localization, and function of tissue inhibitor of metalloproteinase 3 (TIMP3) during the periovulatory period of the human menstrual cycle. Ovulation involves reorganization of the   extracellular matrix   of the follicle. This study examines the expression, localization, and potential function of the tissue inhibitor of metalloproteinase 3 (TIMP3) during ovulation in women. The dominant follicle of the menstrual cycle was collected at specified times throughout the ovulatory process: pre-, early, late, and postovulatory. For quantitative studies, the follicle was bisected; granulosa and theca cells were separated and collected. For immunohistochemistry (IHC), the intact follicle was embedded and TIMP3 was localized. Additionally, granulosa cells were collected from women undergoing in vitro fertilization and treated with increasing concentrations of recombinant TIMP3, and cell viability was assessed. Real-time PCR for TIMP3 mRNA revealed an increase in TIMP3 mRNA expression in granulosa cells from the early to the late ovulatory stage. Thecal TIMP3 mRNA expression was constitutive across the periovulatory period. TIMP3 protein was localized by IHC to the granulosa and theca cell layers in pre-, early, and late ovulatory follicles as well as to the vascular bed. The staining was most intense in the granulosa and theca cells in the late ovulatory group. Treatment of human granulosa-lutein cells with exogenous recombinant TIMP3 for 24 h decreased cell viability by 60%. Using human follicles collected throughout the periovulatory period of the menstrual cycle, we have demonstrated that TIMP3 mRNA expression increases and that TIMP3 protein is in the appropriate cellular layers to regulate proteolytic remodeling as the follicle progresses toward ovulation. In addition, we have shown that elevated levels of TIMP3 lead to decreased cell viability. 
 TIMP3 regulates osteosarcoma cell migration, invasion, and chemotherapeutic resistances. Tissue inhibitors of metalloproteinases (TIMPs) inhibit matrix metalloproteinases (MMPs) to limit degradation of the   extracellular matrix  . Low levels of TIMP3 have been demonstrated in cancer tissues at advanced clinical stages, with positive distant metastasis and chemotherapeutic resistance. We examined the role of TIMP3 in osteosarcoma (OS) cell invasiveness and chemoresistance. TIMP3 was overexpressed or knocked down in the human OS cell lines Saos2 and MG63. Cell migration and invasion capacities were then evaluated using Transwell assays, and resistance to cisplatin was assessed by CCK-8 assay and flow cytometry. Real-time PCR and western blotting were used to investigate activation of signaling pathways downstream of TIMP3. Overexpression of TIMP3 inhibited the migration and invasion of Saos2 and MG63 cells, while knockdown of TIMP3 had the opposite effect. Cell survival after exposure to cisplatin was inhibited by TIMP3 overexpression in both Saos2 and MG63 cells. Consistently, downregulation of TIMP3 gene expression significantly decreased the sensitivity of OS cells to cisplatin treatment. MMP1, MMP2, Bcl-2, and Akt1 were all downregulated following TIMP3 overexpression, while Bax and cleaved caspase-3 were upregulated. TIMP3 knockdown had opposite effects on the regulation of these genes. Taken together, our findings suggest TIMP3 as a new target for inhibition of OS progression and chemotherapeutic resistance. 
 Tissue inhibitors of matrix metalloproteinases in serum are cardiac biomarkers in Emery-Dreifuss muscular dystrophy. Tissue inhibitors of matrix metalloproteinases (TIMPs) are known to be involved in cardiovascular diseases. Hitherto, they have not been examined in dilated cardiomyopathy in the course of Emery-Dreifuss muscular dystrophy (EDMD).To define TIMPs in serum because they might help in defining cardiac dysfunction at the early cardiological stages of this disease and detect preclinical stages of cardiomyopathy.Twenty-five EDMD patients connected with lamin A/C (AD-EDMD) or emerin (X-EDMD) deficiency and 20 healthy age-matched controls were examined. The serum levels of the tissue inhibitors TIMP-1, -2, -3 were quantified using the ELISA sandwich immunoassay procedure with appropriate antibodies.Serum levels of TIMP-1 were normal in autosomal AD-EDMD and increased in the majority of X-linked EDMD. The level of TIMP-2 was decreased in 25%/21% of AD-EDMD/X-EDMD cases. TIMP-3 serum level was significantly reduced in all the examined patients. Receiver operating curves indicated that in terms of sensitivity and specificity characteristics the performance of TIMP-3 (less that of TIMP-2) makes them the best markers of cardiac involvement among the examined TIMPs.Evidence shows that the levels of TIMP-3, and in some cases also TIMP-2, are decreased in EDMD. The decrease might be associated with an adverse effect on matrix metalloproteinases and remodelling of the myocardial matrix. The specific decrease of TIMP-3 indicates that this biomarker might help in early detection of cardiac involvement in EDMD. Up-regulation of TIMP-1 in the majority of patients with X-EDMD indicates increased myocardial   extracellular matrix   turnover, early onset of tissue remodelling, and may contribute to arrhythmia, frequently occurring in this form of the disease. 
 Identification and characterization of human tissue inhibitor of metalloproteinase-3 and detection of three additional metalloproteinase inhibitor activities in   extracellular matrix  . We have identified and characterized a novel human tissue inhibitor of metalloproteinase (TIMP). It is found exclusively in the   extracellular matrix   of a large number of cultured human cells, including: primary embryonal kidney (293), neuroblastoma (SK-N-SH), normal whole embryo (FHs 173We), cervical carcinoma (HeLa S3), colon adenocarcinoma (Caco-2), ileocecal adenocarcinoma (HCT-8), fibrosarcomas (SW 684 and Hs 913T) and normal gingival fibroblasts (GF11 and 1292). It was not detected in the conditioned media from any of these cell lines. Its apparent molecular mass of 24-25 kDa, as determined by its migration on protease-substrate gels, is intermediate between TIMP-1 (28.5 kDa) and TIMP-2 (21 kDa). Like the latter two proteins, human TIMP-3 contains intrachain disulfide bonds and displays altered electrophoretic mobility in the presence of beta-mercaptoethanol. The N-terminal, amino acid sequence of the protein is identical to that of chicken TIMP-3 (ChIMP-3), and its amino acid composition is similar. The protein is not N-glycosylated, as determined by treatment with N-glycosidase-F. Finally, it is recognized by antisera raised against pure ChIMP-3 but not by anti-human TIMP-1 or anti-human TIMP-2 antibodies. Based on these properties, we propose that this protein is TIMP-3 and is the human counterpart of ChIMP-3 (Pavloff et al., J. Biol. Chem. 267: 17321-17326, 1992). Two additional inhibitors detected in the matrix of human cell lines, designated inhibitor of metalloproteinase (IMP)-a and IMP-b, migrate with apparent masses of 29 kDa and 30 kDa. Both are N-glycosylated. A fourth inhibitor activity, which is smaller in mass than TIMP-3 and is also pecifically located in the matrix, is detectable in some cell lines. 
 Genetic variants near TIMP3 and high-density lipoprotein-associated loci influence susceptibility to age-related macular degeneration. We executed a genome-wide association scan for age-related macular degeneration (AMD) in 2,157 cases and 1,150 controls. Our results validate AMD susceptibility loci near CFH (P < 10(-75)), ARMS2 (P < 10(-59)), C2/CFB (P < 10(-20)), C3 (P < 10(-9)), and CFI (P < 10(-6)). We compared our top findings with the Tufts/Massachusetts General Hospital genome-wide association study of advanced AMD (821 cases, 1,709 controls) and genotyped 30 promising markers in additional individuals (up to 7,749 cases and 4,625 controls). With these data, we identified a susceptibility locus near TIMP3 (overall P = 1.1 x 10(-11)), a metalloproteinase involved in degradation of the   extracellular matrix   and previously implicated in early-onset maculopathy. In addition, our data revealed strong association signals with alleles at two loci (LIPC, P = 1.3 x 10(-7); CETP, P = 7.4 x 10(-7)) that were previously associated with high-density lipoprotein cholesterol (HDL-c) levels in blood. Consistent with the hypothesis that HDL metabolism is associated with AMD pathogenesis, we also observed association with AMD of HDL-c-associated alleles near LPL (P = 3.0 x 10(-3)) and ABCA1 (P = 5.6 x 10(-4)). Multilocus analysis including all susceptibility loci showed that 329 of 331 individuals (99%) with the highest-risk genotypes were cases, and 85% of these had advanced AMD. Our studies extend the catalog of AMD associated loci, help identify individuals at high risk of disease, and provide clues about underlying cellular pathways that should eventually lead to new therapies. 
 TGF-beta-induced expression of tissue inhibitor of metalloproteinases-3 gene in chondrocytes is mediated by extracellular signal-regulated kinase pathway and Sp1 transcription factor. Transforming growth factor (TGF-beta1) is a potent inducer of chondrogenesis and stimulant of cartilage   extracellular matrix   (ECM) synthesis. Tissue inhibitor of metalloproteinases-3 (TIMP-3) is located in ECM and is the major inhibitor of matrix metalloproteinases (MMPs) and aggrecanase, the principal enzymes implicated in collagen and aggrecan degradation in arthritis. We investigated the role of extracellular-signal-regulated kinase (ERK)-mitogen-activated protein kinases (MAPK) and Sp1 transcription factor in TGF-beta-induced TIMP-3 gene in chondrocytes and chondrosarcoma cells. TGF-beta time-dependently induced a sustained phosphorylation of ERK-MAPKs in primary human or bovine chondrocytes. Inhibitors of this pathway, PD98059 and U0126, downregulated TGF-beta-induced expression of TIMP-3 RNA and protein. Since the ERKs can phosphorylate Sp1, and the promoter of human TIMP-3 gene contains four Sp1-binding sites, we investigated whether Sp1 is a downstream target of this pathway. Mithramycin and WP631, the agents that prevent binding of Sp1 to its consensus site, downregulated TGF-beta-inducible TIMP-3 expression. Indeed, mithramycin blocked TGF-beta-stimulated Sp1 binding activity. Transfection of cytomegalovirus (CMV) promoter-Sp1 plasmid increased TIMP-3 promoter (-940 to +376)-driven luciferase activity. Depletion of Sp1 by transfection of an antisense phosphorothioate oligonucleotide suppressed TGF-beta-induced TIMP-3 protein expression, while its sense homolog had no effect. These results suggest that activation of ERK-MAPK pathway and Sp1 transcription factor play a pivotal role in the induction of TIMP-3 by TGF-beta in chondrocytes. 
 Sorsby's fundus dystrophy tissue inhibitor of metalloproteinases-3 (TIMP-3) mutants have unimpaired matrix metalloproteinase inhibitory activities, but affect cell adhesion to the   extracellular matrix  . The TIMP family of matrix metalloproteinase inhibitors consists of four members, of which TIMP-1, -2 and -4 are secreted, freely diffusible proteins, whereas TIMP-3 is ECM-associated. Mutations in the TIMP3 gene have been linked to Sorsby's fundus dystrophy (SFD), an autosomal dominant inherited retinal degenerative disease that leads to blindness. The SFD mutations characterized result in introduction of an unpaired cysteine residue in the C-terminal domain of TIMP-3. We have expressed four SFD mutant TIMP-3 proteins in baby hamster kidney (BHK) cells and evaluated their characteristics alongside wild-type TIMP-3. Analysis of the mutant proteins (Ser156Cys, Gly167Cys, Tyr168Cys and Ser181Cys) by SDS-PAGE and reverse zymography revealed that each of the mutants retained gelatinase A and gelatinase B inhibitory activity, and were localized to the ECM. Association rate constants for Ser156Cys TIMP-3 with gelatinase-A, gelatinase-B, stromelysin-1 and collagenase-3 were only moderately reduced compared to wild-type TIMP-3. However, all of the mutants displayed aberrant protein-protein interactions, resulting in the presence of additional proteins or complexes in ECM preparations. Two of the mutants (Ser156Cys and Ser181Cys) showed a marked propensity to form multiple higher molecular-weight complexes that retained TIMP activity on reverse zymography. Expression of the SFD mutant TIMP-3 (and to a lesser extent, wild-type TIMP-3) proteins in BHK cells conferred increased cell adhesiveness to the ECM. Our findings indicate that the pathogenesis of Sorsby's fundus dystrophy cannot be attributed to a failure to localize SFD TIMP-3 proteins to the ECM or defects in MMP inhibition, but may involve the formation of aberrant TIMP-3-containing protein complexes and altered cell adhesion. 
 Gene expression analysis of drug-resistant MCF-7 cells: implications for relation to   extracellular matrix   proteins. Since multidrug resistance is a multifactorial phenomenon, a large-scale expression analysis of drug-resistant cells by using high-density oligonucleotide microarrays may provide information about new candidate genes contributing to resistance.   Extracellular matrix   (ECM) is responsible for many aspects of proliferation and invasive/metastatic behavior of tumor cells. This study demonstrates alterations in gene expression levels of several ECM components, matrix metalloproteinases (MMPs), adamalysins (ADAMs and ADAMTSs) and tissue inhibitors of metalloproteinases (TIMPs) in paclitaxel, docetaxel, vincristine and doxorubicin-resistant MCF-7 cells.Resistant MCF-7 cells were developed by stepwise selection of cells in increasing concentrations of drugs. Affymetrix GeneChip Human Genome U133 Plus 2.0 Array was used for hybridizations. Statistical significance was determined by independent sample t test. The genes having altered expression levels in drug-resistant sublines were selected and filtered by volcano plots.Genes up/downregulated more than twofolds were selected and listed. Expression of 25 genes encoding ECM proteins (including collagen, finronectin and syndecan) and integrin receptor subunits were found to be upregulated in drug-resistant cells. In addition, expression levels of, 13 genes encoding MMPs, ADAMs, ADAMTSs and TIMPs (including MMP1, MMP9, ADAM9 and TIMP3) were found to be altered in drug-resistant sublines when compared with sensitive MCF-7.Based on the expression analysis profiles, this report provides a preliminary insight into the relationship between drug resistance and ECM components, which are related to invasion and metastasis. Correlation of each specific ECM component with drug resistance requires further analysis. 
 Promoter variants in tissue inhibitor of metalloproteinase-3 (TIMP-3) protect against susceptibility in pigeon breeders' disease. Tissue inhibitors of metalloproteinases (TIMPs) play a major role in   extracellular matrix   turnover in the lung. However, in chronic lung disorders such as idiopathic pulmonary fibrosis (IPF) and pigeon breeders' disease (PBD), TIMPs may promote an adverse non-degradative environment. We hypothesised that polymorphisms in TIMP-3 could affect susceptibility to IPF and PBD.Two promoter variants, -915A>G and -1296T>C, were genotyped in 323 healthy subjects, 94 subjects with IPF, 115 with PBD, and 90 exposed to avian antigen but without PBD. The severity of fibrosis in lung tissue and the clinical outcome after 1 year was determined in the PBD group.The variants did not influence susceptibility to IPF, but the rare alleles of both variants appeared to be protective against susceptibility to PBD (odds ratio (OR) for carriage of at least one rare allele from either variant 0.48, 95% CI 0.30 to 0.76, p = 0.002). Haplotype analysis of positions -915 and -1296 estimated four haplotypes: *A*T, *G*T, *A*C and *G*C, respectively. Their frequencies differed overall between subjects with PBD and healthy subjects (p = 0.0049) and this was attributable primarily to the *G*C haplotype (OR 0.53, 95% CI 0.36 to 0.77, p = 0.001). The severity of fibrosis correlated with poorer outcome in the PBD group (r = 0.73, p<0.01) but no relationship was seen between the *G*C haplotype and outcome or fibrosis. However, PBD subjects with the *G*C haplotype did have proportionally fewer lymphocytes in their bronchoalveolar fluid than those with the common *A*T haplotype (p = 0.029).TIMP-3 variants appear to contribute to susceptibility to PBD. This may be through the inflammatory reaction rather than the fibrotic reaction. 
 Tissue inhibitor of matrix metalloproteinase-3 levels in the   extracellular matrix   of lung, kidney, and eye increase with age. Tissue inhibitor of matrix metalloproteinase-3 (TIMP-3) is an important regulator of matrix metalloproteinase activity in many types of disease, including atherosclerosis, neoplasia, and inflammatory conditions. Among TIMPs, TIMP-3 uniquely binds the   extracellular matrix   (ECM). We performed IHC staining on 17 tissue microarrays containing >1500 samples to determine the location of ECM TIMP-3 staining in a variety of predominantly vascular tissues. We found a unique pattern of TIMP-3 staining in the ECM of renal arterioles, small pulmonary vessels and parenchyma, and Bruch's membrane in the retina. There was no staining in larger caliber arteries including coronary and internal mammary arteries. TIMP-3 protein accumulation was found to be an age-dependent phenomenon, with staining appearing in all three tissues in early adulthood and becoming more robust among the elderly. These findings may help to explain the late onset of the TIMP-3-associated ocular diseases Sorsby fundus dystrophy and age-related macular degeneration and suggest a similar phenomenon could be at work in other age-related conditions. 
 The tissue inhibitor of metalloproteinases-3 gene in breast carcinoma: identification of multiple polyadenylation sites and a stromal pattern of expression. Tissue inhibitor of metalloproteinases-3 (TIMP3) is the third member of the TIMP family of proteins, believed to play a significant role in controlling   extracellular matrix   remodeling.Differential screening of a human breast carcinoma cDNA library using substracted and PCR-amplified cDNA probes identified a 4.6-kb TIMP3 cDNA, which was used for further cDNA library screenings, Northern blot hybridizations, and the synthesis of riboprobes for in situ RNA hybridization analyses.The 4.6-kb full-length TIMP3 cDNA contains 3.7 kb of 3'-untranslated sequence. Additional TIMP3 cDNAs subsequently identified were colinear with the original sequence, but revealed use of four different polyadenylation signals within the 3'-untranslated region, which accounted for the 4.6-, 2.7-, 2.5-, and 2.1-kb TIMP3 transcripts noted in this and in previous studies. In situ RNA hybridizations demonstrated that in breast carcinoma the TIMP3 gene was predominantly expressed by fibroblastic cells within the tumor stroma adjacent to cancer cells. TIMP3 transcripts were also strongly detected in fibroblastic decidual cells of pregnant endometrium.Modulating the length of the 3'-untranslated region may represent a mechanism by which TIMP3 gene expression is controlled in tissues. The strong expression of the TIMP3 gene by fibroblastic cells in breast carcinoma supports the importance of tumor stroma as a source of factors influencing human carcinoma growth and progression. 
 Polymorphisms and Plasma Levels of Tissue Inhibitor of Metalloproteinase-3: Impact on Genetic Susceptibility and Clinical Outcome of Oral Cancer. Oral cancer, the fourth most common cancer among men in Taiwan, is associated with environmental carcinogens. Tissue inhibitor of metalloproteinase-3 (TIMP3), a member of the TIMP family, is the only protein that binds to the   extracellular matrix   for suppressing cancer cell growth, angiogenesis, migration, and invasion. The association of TIMP3 polymorphism with oral cancer susceptibility, however, has not yet been reported. In this study, 1947 participants-1200 healthy male controls and 747 male patients with oral cancer-were recruited. Allelic discrimination of TIMP3 -1296 T > C (rs9619311), TIMP3 C > T (rs9862), and TIMP3 C > T (rs11547635) polymorphisms were assessed through real-time polymerase chain reaction. The authors discovered that individuals carrying the polymorphic rs9862 allele are more susceptible to oral cancer [odds ratio (OR), 1.5; 95% confidence interval (CI), 1.2-1.9; adjusted OR (AOR), 1.6; 95% CI, 1.2-2.1] after adjustment for betel quid chewing, alcohol, and tobacco consumption. Among 601 betel quid chewers, the TIMP3 polymorphism rs9862 T/T carriers had a 32.2-fold (95% CI, 20.2-51.3) increased oral cancer risk compared with those carrying C/C and not chewing betel quid. In addition, the authors observed a significant association between rs9862 variants and large tumors (OR, 1.5; 95% CI, 1.0-2.3) development. Moreover, TIMP3 plasma levels significantly increased in oral cancer patients who have large tumor or carry T allele rs9862 polymorphism. In conclusion, these results suggest that gene-environment interactions between the TIMP3 rs9862 polymorphisms and betel quid may alter oral cancer susceptibility and tumor growth in Taiwanese men. 
 Genomic organization of the human tissue inhibitor of metalloproteinases-3 (TIMP3) The tissue inhibitors of metalloproteinases (TIMPs) play a crucial role in the physiological turnover of the   extracellular matrix   (ECM) by tightly regulating matrix metalloproteinase (MMP) activities. Disturbances in the TIMP/MMP system have been implicated in many disease processes where loss of ECM integrity is a principal feature. More recently, we have shown that mutations in TIMP3 cause the autosomal dominant disorder Sorsby's fundus dystrophy (SFD). This is a macular degeneration disorder with characteristic ECM irregularities in Bruch's membrane. To further facilitate mutational analysis and to provide a basis for functional studies, we now report the genomic organization of the human TIMP3 gene. 
 Tissue inhibitor of metalloproteinases-3 (TIMP-3) is a binding partner of epithelial growth factor-containing fibulin-like   extracellular matrix   protein 1 (EFEMP1). Implications for macular degenerations. Tissue inhibitor of metalloproteinases-3 (TIMP-3) is a matrix-bound inhibitor of matrix metalloproteinases. Mutations in the Timp-3 gene cause Sorsby fundus dystrophy (SFD), a hereditary macular degenerative disease. The pathogenic mechanisms responsible for the disease phenotype are unknown. In an in vivo quest for binding partners of the TIMP-3 protein in the subretina, we identified epidermal growth factor-containing fibulin-like   extracellular matrix   protein 1 (EFEMP1, also known as fibulin 3) as a strong interacting protein. The COOH-terminal end of TIMP-3 was involved in the interaction. Interestingly, a missense mutation in EFEMP1 is responsible for another hereditary macular degenerative disease, Malattia Leventinese (ML). Both SFD and ML have strong similarities to age-related macular degeneration (AMD), a major cause of blindness in the elderly population of the Western hemisphere. Our results were supported by significant accumulation and expression overlap of both TIMP-3 and EFEMP1 between the retinal pigment epithelia and Bruch membrane in the eyes of ML and AMD patients. These results provide the first link between two different macular degenerative disease genes and imply the possibility of a common pathogenic mechanism behind different forms of macular degeneration. 
 Oxidised, glycated LDL selectively influences tissue inhibitor of metalloproteinase-3 gene expression and protein production in human retinal capillary pericytes. Matrix metalloproteinases (MMPs) and their natural inhibitors, tissue inhibitor of metalloproteinases (TIMPs), regulate important biological processes including the homeostasis of the   extracellular matrix  , proteolysis of cell surface proteins, proteinase zymogen activation, angiogenesis and inflammation. Studies have shown that their balance is altered in retinal microvascular tissues in diabetes. Since LDLs modified by oxidation/glycation are implicated in the pathogenesis of diabetic vascular complications, we examined the effects of modified LDL on the gene expression and protein production of MMPs and TIMPs in retinal pericytes.Quiescent human retinal pericytes were exposed to native LDL (N-LDL), glycated LDL (G-LDL) and heavily oxidised and glycated LDL (HOG-LDL) for 24 h. We studied the expression of the genes encoding MMPs and TIMPs mRNAs by analysis of microarray data and quantitative PCR, and protein levels by immunoblotting and ELISA.Microarray analysis showed that MMP1, MMP2, MMP11, MMP14 and MMP25 and TIMP1, TIMP2, TIMP3 and TIMP4 were expressed in pericytes. Of these, only TIMP3 mRNA showed altered regulation, being expressed at significantly lower levels in response to HOG- vs N-LDL. Quantitative PCR and immunoblotting of cell/matrix proteins confirmed the reduction in TIMP3 mRNA and protein in response to HOG-LDL. In contrast to cellular TIMP3 protein, analysis of secreted TIMP1, TIMP2, MMP1 and collagenase activity indicated no changes in their production in response to modified LDL. Combined treatment with N- and HOG-LDL restored TIMP3 mRNA expression to a level comparable with that after N-LDL alone.Among the genes encoding for MMPs and TIMPs expressed in retinal pericytes, TIMP3 is uniquely regulated by HOG-LDL. Reduced TIMP3 expression might contribute to microvascular abnormalities in diabetic retinopathy. 
 TIMP-3 expression associates with malignant behaviors and predicts favorable survival in HCC. The tissue inhibitors of metalloproteinases (TIMPs) are proteins that specifically inhibit the proteolytic activity of the matrix metalloproteinases (MMPs). TIMP-3, the only member of the TIMPs that can tightly bind to the   extracellular matrix  , has been identified as a unique tumor suppressor that demonstrates the ability to inhibit tumor angiogenesis, invasion, and metastasis. This study aimed to detect the expression of TIMP-3 in hepatocellular carcinoma (HCC) and investigate the association between TIMP-3 expression and its clinicopathological significance in HCC patients. In the current study, reverse transcription-polymerase chain reaction (RT-PCR) and Western blotting of HCC cell lines and one-step quantitative reverse transcription PCR (qPCR) and immunohistochemistry (IHC) analyses in HCC tissues were performed, to characterize the TIMP-3 expression. Kaplan-Meier survival and Cox regression analyses were utilized to evaluate the prognosis of 101 HCC patients. The results showed that the expression of TIMP-3 in HCC was significantly decreased relative to that of non-cancerous cells and tissues. Furthermore, the TIMP-3 expression was statistically associated with malignant behaviors of HCC, including portal vein invasion (p = 0.036) and lymph node metastasis (p = 0.030). Cox regression analysis revealed that TIMP-3 expression was an independent prognostic factor for disease-free survival (p = 0.039) and overall survival (p = 0.049). These data indicate that TIMP-3 expression is a valuable prognostic biomarker for HCC and that TIMP-3 expression suggests a favorable prognosis for HCC patients. 
 Transforming growth factor Beta1 induction of tissue inhibitor of metalloproteinases 3 in articular chondrocytes is mediated by reactive oxygen species. Transforming growth factor beta1 (TGF-beta1) stimulates cartilage   extracellular matrix   synthesis but, in excess, evokes synovial inflammation, hyperplasia, and osteophyte formation in arthritic joints. TGF-beta1 induces tissue inhibitor of metalloproteinases 3 (TIMP-3), an inhibitor of cartilage-damaging matrix metalloproteianases and aggrecanases. We investigated the role of reactive oxygen species (ROS) in TIMP-3 induction by TGF-beta1. In primary human and bovine chondrocytes, ROS scavenger and antioxidant N-acetylcysteine (NAC) inhibited TGF-beta1-induced TIMP-3 mRNA and protein increases. Ebselen and ascorbate also reduced this induction. TGF-beta1 time-dependently induced ROS production that was suppressed by NAC. Hydrogen peroxide, a ROS, induced TIMP-3 RNA. The TIMP-3 increase induced by TGF-beta1 was partly Smad2-dependent. TGF-beta1-stimulated Smad2 phosphorylation was inhibited by NAC. Reduced glutathione and L-cysteine also blocked Smad2 and TIMP-3 induction by TGF-beta1, whereas a nonthiol, N-acetylalanine, did not. Smad2 was not activated by H2O2. Smad2 phosphorylation was independent, and TIMP-3 expression was dependent, on new protein synthesis. TGF-beta-stimulated ERK and JNK phosphorylation was also inhibited by NAC. However, inhibitory actions of NAC were not mediated by ERK activation. Thus, ROS mediate TGF-beta1-induced TIMP-3 gene expression. Blocking TGF-beta1-induced gene expression by modulating cellular redox status with thiols can be potentially beneficial for treating arthritic and other disorders caused by excessive TGF-beta1. 
 Overexpression of the tissue inhibitor of metalloproteinase-3 during Xenopus embryogenesis affects head and axial tissue formation. Tissue inhibitors of metalloproteinases (TIMPs) modulate   extracellular matrix   remodeling during embryonic development and disease. TIMP-3 expression was examined during Xenopus laevis embryogenesis: TIMP-3 transcripts detected in the maternal pool of RNA increased at the mid-blastula transition, decreased dramatically during gastrulation and increased again during neurulation and axis elongation. Interestingly, the decrease during gastrulation was not seen in LiCl treated (dorsalized) embryos. Whole mount in situ hybridization of TIMP-3 using DIG-labeled RNA probes demonstrated that the transcripts were present in all dorsal tissues during embryogenesis, but were prominent only in head structures starting at stage 35. Overexpression of TIMP-3 through transgenesis and RNA injections led to developmental abnormalities and death. Both overexpression strategies resulted in post-gastrulation perturbation including those to neural and head structures, as well as truncated axes. However, RNA injections resulted in more severe early defects such as failure of neural tube closure, and transgenesis caused truncated axes and head abnormalities. No transgenic embryo expressing TIMP-3 survived past stage 40. 
 Tissue inhibitor of metalloproteinase-3 (TIMP-3) gene is methylated in the development of esophageal adenocarcinoma: loss of expression correlates with poor prognosis. Amongst involvement in diverse physiological and pathological processes, TIMP-3 may have an important role in tumour development, growth and metastasis by interaction with metalloproteases in the   extracellular matrix  . We studied the role and prognostic effect of TIMP-3 in esophageal adenocarcinoma (EADC). TIMP-3 gene methylation and TIMP-3 mRNA expression were analysed in 5 esophageal cell lines and 24 resected EADCs. TIMP-3 protein expression was examined in the 5 cell lines and 79 resected EADCs with known clinicopathological features. TIMP-3 methylation signal was only detected in the OE33 EADC cell line. In tissues, 0% of case-matched normal, 72% of BE and 90% of EADC were positive for methylation. TIMP-3 mRNA was detected in all the cell lines and normal, metaplastic and tumour tissues. TIMP-3 protein was localised to the cytoplasm in cell lines and tissues. Demethylating treatment of OE33 increased protein expression. At the invading edge of tumours, protein staining was equal to, or reduced, compared to normal tissues. Reduction of protein expression was associated with disease stage (p = 0.046) and poor patient survival (OR 2.1, 95% CI 1.2-3.5, p = 0.007). Mean survival time was halved in patients with reduced tumour TIMP-3 expression, from 49 to 24 months. These studies have demonstrated association between methylation of the TIMP-3 gene and BE and EADC. Reduced expression of TIMP-3 protein in EADC is associated with increased tumour invasiveness and reduced patient survival. 
 Early activation of matrix metalloproteinases underlies the exacerbated systolic and diastolic dysfunction in mice lacking TIMP3 following myocardial infarction.   Extracellular matrix   (ECM) remodeling is a critical aspect of cardiac remodeling following myocardial infarction. Tissue inhibitors of metalloproteinases (TIMPs) are physiological inhibitors of matrix metalloproteinases (MMPs) that degrade the ECM proteins. TIMP3 is highly expressed in the heart, and is markedly downregulated in patients with ischemic cardiomyopathy. We therefore examined the time- and region-dependent role of TIMP3 in the cardiac response to myocardial infarction (MI). TIMP3(-/-) and wild-type (WT) mice were subjected to MI by ligation of the left anterior descending artery. TIMP3(-/-)-MI mice exhibited a significantly compromised rate of survival compared with WT-MI mice, primarily due to increased left ventricular (LV) rupture, greater infarct expansion, exacerbated LV dilation, and greater systolic and diastolic dysfunction. Second harmonic generation imaging of unfixed and unstained hearts revealed greater collagen disarray and reduced density in the TIMP3(-/-) infarct myocardium compared with the WT group. Gelatinolytic and collagenolytic activities increased in TIMP3(-/-) compared with WT hearts at 1 day post-MI but not at 3 days or 1 wk post-MI. Neutrophil infiltration and inflammatory MMPs were significantly increased in the infarct and peri-infarct regions of TIMP3(-/-)-MI hearts. Treatment of TIMP3(-/-) mice with a broad-spectrum MMP inhibitor (PD-166793) for 2 days before and 2 days after MI markedly improved post-MI infarct expansion, LV rupture incident, LV dilation, and systolic dysfunction in these mice up to 1 wk post-MI. Our data demonstrate that the initial rise in proteolytic activities early post-MI is a triggering factor for subsequent LV adverse remodeling, LV rupture, and dilated cardiomyopathy. Hence, timing of treatments to improve cardiac response to MI may be critical in producing favorable outcome. 
 A novel tissue inhibitor of metalloproteinases-3 mutation reveals a common molecular phenotype in Sorsby's fundus dystrophy. Sorsby's fundus dystrophy (SFD) is a dominantly inherited degenerative disease of the retina that leads to loss of vision in middle age. It has been shown to be caused by mutations in the gene for tissue inhibitor of metalloproteinases-3 (TIMP-3). Five different mutations have previously been identified, all introducing an extra cysteine residue into exon 5 (which forms part of the C-terminal domain) of the TIMP-3 molecule; however, the significance of these mutations to the disease phenotype was unknown. In this report, we describe the expression of several of these mutated genes, together with a previously unreported novel TIMP-3 mutation from a family with SFD that results in truncation of most of the C-terminal domain of the molecule. Despite these differences, all of these molecules are expressed and exhibit characteristics of the normal protein, including inhibition of metalloproteinases and binding to the   extracellular matrix  . However, unlike wild-type TIMP-3, they all form dimers. These observations, together with the recent finding that expression of TIMP-3 is increased, rather than decreased, in eyes from patients with SFD, provides compelling evidence that dimerized TIMP-3 plays an active role in the disease process by accumulating in the eye. Increased expression of TIMP-3 is also observed in other degenerative retinal diseases, including the more severe forms of age-related macular degeneration, the most common cause of blindness in the elderly in developed countries. We hypothesize that overexpression of TIMP-3 may prove to be a critical step in the progression of a variety of degenerative retinopathies. 
 TGF-β-elicited induction of tissue inhibitor of metalloproteinases (TIMP)-3 expression in fibroblasts involves complex interplay between Smad3, p38α, and ERK1/2. Transforming growth factor-β (TGF-β) promo  tes extracellular ma  trix deposition by down-regulating the expression of matrix degrading proteinases and upregulating their inhibitors. Tissue inhibitor of metalloproteinases (TIMP)-3 is an ECM-associated specific inhibitor of matrix degrading metalloproteinases. Here, we have characterized the signaling pathways mediating TGF-β-induced expression of TIMP-3. Basal and TGF-β-induced TIMP-3 mRNA expression was abolished in Smad4-deficient mouse embryonic fibroblasts and restoring Smad4 expression rescued the response. Inhibition of Smad signaling by expression of Smad7 and dominant negative Smad3 completely abolished TGF-β-elicited expression of TIMP-3 in human fibroblasts, whereas overexpression of Smad3 enhanced it. Inhibition of extracellular signal-regulated kinase 1/2 (ERK1/2) activation with PD98059 and p38 mitogen-activated protein kinase activity by SB203580 resulted in suppression of TGF-β-induced TIMP-3 expression, indicating that ERK1/2 and p38 MAPK mediate the effect of TGF-β on TIMP-3 expression. Specific activation of p38α and ERK1/2 by constitutively active mutants of MKK3b or MEK1, respectively, and simultaneous co-expression of Smad3 resulted in induction of TIMP-3 expression in the absence of TGF-β indicating that Smad3 co-operates with p38 and ERK1/2 in the induction of TIMP-3 expression. These results demonstrate the complex interplay between Smad3, p38α, and ERK1/2 signaling in the regulation of TIMP-3 gene expression in fibroblasts, which may play a role in inflammation, tissue repair, and fibrosis. 
 Human tissue inhibitor of metalloproteinases 3 interacts with both the N- and C-terminal domains of gelatinases A and B. Regulation by polyanions. We compared the association constants of tissue inhibitor of metalloproteinases (TIMP)-3 with various matrix metalloproteinases with those for TIMP-1 and TIMP-2 using a continuous assay. TIMP-3 behaved more like TIMP-2 than TIMP-1, showing rapid association with gelatinases A and B. Experiments with the N-terminal domain of gelatinase A, the isolated C-terminal domain, or an inactive progelatinase A mutant showed that the hemopexin domain of gelatinase A makes an important contribution to the interaction with TIMP-3. The exchange of portions of the gelatinase A hemopexin domain with that of stromelysin revealed that residues 568-631 of gelatinase A were required for rapid association with TIMP-3. The N-terminal domain of gelatinase B alone also showed slower association with TIMP-3, again implying significant C-domain interactions. The isolation of complexes between TIMP-3 and progelatinases A and B on gelatin-agarose demonstrated that TIMP-3 binds to both proenzymes. We analyzed the effect of various polyanions on the inhibitory activity of TIMP-3 in our soluble assay. The association rate was increased by dextran sulfate, heparin, and heparan sulfate, but not by dermatan sulfate or hyaluronic acid. Because TIMP-3 is sequestered in the   extracellular matrix  , the presence of certain heparan sulfate proteoglycans could enhance its inhibitory capacity. 
   Extracellular matrix   proteins expression profiling in chemoresistant variants of the A2780 ovarian cancer cell line. Ovarian cancer is the leading cause of death among gynaecological malignancies.   Extracellular matrix   (ECM) can affect drug resistance by preventing the penetration of the drug into cancer cells and increased resistance to apoptosis. This study demonstrates alterations in the expression levels of ECM components and related genes in cisplatin-, doxorubicin-, topotecan-, and paclitaxel-resistant variants of the A2780 ovarian cancer cell line. Affymetrix Gene Chip Human Genome Array Strips were used for hybridisations. The genes that had altered expression levels in drug-resistant sublines were selected and filtered by scatter plots. The genes that were up- or downregulated more than fivefold were selected and listed. Among the investigated genes, 28 genes were upregulated, 10 genes were downregulated, and two genes were down- or upregulated depending on the cell line. Between upregulated genes 12 were upregulated very significantly--over 20-fold. These genes included COL1A2, COL12A1, COL21A1, LOX, TGFBI, LAMB1, EFEMP1, GPC3, SDC2, MGP, MMP3, and TIMP3. Four genes were very significantly downregulated: COL11A1, LAMA2, GPC6, and LUM. The expression profiles of investigated genes provide a preliminary insight into the relationship between drug resistance and the expression of ECM components. Identifying correlations between investigated genes and drug resistance will require further analysis. 
 Expression of TIMP3 mRNA is elevated in retinas affected by simplex retinitis pigmentosa. To explore the molecular and cellular mechanisms associated with photoreceptor death in retinitis pigmentosa (RP), we have investigated altered transcriptional activity in RP retinas by a differential cDNA screening approach. We identified a clone (K222) showing over-expression in simplex RP retinas compared with controls. K222 encodes a partial cDNA of the human tissue inhibitor of metalloproteinases-3 (TIMP3) gene, a member of a family of genes implicated in   extracellular matrix   (ECM) remodelling. Increased expression of TIMP3 in degenerating RP retinas may reflect restructuring of the ECM architecture, and disruption of photoreceptor-matrix interactions could contribute to activation of apoptotic cell death processes. 
 Integrin α7 binds tissue inhibitor of metalloproteinase 3 to suppress growth of prostate cancer cells. Integrin α7 (ITGA7) is a tumor-suppressor gene that is critical for suppressing the growth of malignant tumors; however, the mechanisms allowing ITGA7 to suppress the growth of cancer cells remain unclear. Herein, we show that ITGA7 binds to tissue inhibitor of metalloproteinase 3 (TIMP3) in prostate cancer cells. The ITGA7-TIMP3 binding led to a decreased protein level of tumor necrosis factor α, cytoplasmic translocation of NF-κB, and down-regulation of cyclin D1. These changes led to an accumulation of cells in G0/G1 and a dramatic suppression of cell growth. Knocking down TIMP3 or ITGA7/TIMP3 binding interference largely abrogated the signaling changes induced by ITGA7, whereas a mutant ITGA7 lacking TIMP3 binding activity had no tumor-suppressor activity. Interestingly, knocking down ITGA7 ligand laminin β1 enhanced ITGA7-TIMP3 signaling and the downstream tumor-suppressor activity, suggesting the existence of a counterbalancing role bet  ween extracellular m  atrix and integrin signaling. As a result, this report demonstrates a novel and critical signaling mechanism of ITGA7, through the TIMP3/NF-κB/cyclin D1 pathway. 
 Localization of the functional domains of human tissue inhibitor of metalloproteinases-3 and the effects of a Sorsby's fundus dystrophy mutation. A transient COS-7 cell expression system was used to investigate the functional domain arrangement of tissue inhibitor of metalloproteinases-3 (TIMP-3), specifically to assess the contribution of the amino- and carboxyl-terminal domains of the molecule to its matrix metalloproteinase (MMP) inhibitory and   extracellular matrix   (ECM) binding properties. Wild type TIMP-3 was entirely localized to the ECM in both its glycosylated (27 kDa) and unglycosylated (24 kDa) forms. A COOH-terminally truncated TIMP-3 molecule was found to be a non-ECM bound MMP inhibitor, whereas a chimeric TIMP molecule, consisting of the NH2-terminal domain of TIMP-2 fused to the COOH-terminal domain of TIMP-3, displayed ECM binding, albeit with a lower affinity than the wild type TIMP-3 molecule. Thus the functional domain arrangement of TIMP-3 is analogous to that seen in TIMP-1 and -2, namely that the NH2-terminal domain is responsible for MMP inhibition whereas the COOH-terminal domain is most important in mediating the specific functions of the molecule. A mutant TIMP-3 in which serine 181 was changed to a cysteine, found in Sorsby's fundus dystrophy, a hereditary macular degenerative disease, was also expressed in COS-7 cells. This gave rise to an additional 48-kDa species (possibly a TIMP-3 dimer) that retained its ability to inhibit MMPs and localize to the ECM. These data favor the hypothesis that the TIMP-3 mutations seen in Sorsby's fundus dystrophy contribute to disease progression by accumulation of mutant protein rather than by the loss of functional TIMP-3. 
 Cloning of the cDNA encoding human tissue inhibitor of metalloproteinases-3 (TIMP-3) and mapping of the TIMP3 gene to chromosome 22. The tissue inhibitors of metalloproteinases (TIMPs) are natural inhibitors of the matrix metalloproteinases, a group of zinc-binding endopeptidases involved in the degradation of the   extracellular matrix  . We have isolated overlapping cDNAs encoding a novel human TIMP, TIMP-3. The cDNAs contain a 591-bp-long open reading frame encoding 9 amino acid residues of the signal peptide and 188 residues of the mature TIMP-3 polypeptide. Both the nucleotide sequence and the conceptual translation product of the human TIMP3 cDNA have a high degree of similarity to ChIMP-3, a recently cloned metalloproteinase inhibitor in the chicken, and to the TIMP1 and TIMP2 gene products, including 12 conserved cysteinyl residues at the same relative positions. The TIMP3 gene is expressed in many tissues, with highest expression in the placenta. Upon hybridization with a panel of human-hamster somatic cell hybrid DNAs, the TIMP3 gene segregated with clones containing chromosome 22. Using in situ hybridization to human metaphase chromosomes, we have assigned the locus for the TIMP3 gene to the q12.1-q13.2 region of human chromosome 22. 
 TIMP-3 is expressed in the human retinal pigment epithelium. TIMP-3 is the most recent member of the tissue inhibitor of metalloproteinases (TIMP) family. In the present study, we describe the expression of TIMP-3 messenger RNA (mRNA) by the retinal pigment epithelium of the normal human eye (hRPE). In addition to the three predominant transcripts of approximately 5.1, 2.8, and 2.4 Kbp found in several other human tissues at adult and fetal stages. The hRPE also expresses two RNA species of 1.2 and 1.0 Kbp. Based on the sequence analysis of cDNA clones isolated from a hRPE cDNA library, the use of alternate polyadenylation signals could account for the expression of these smaller transcripts. The possibility of an alternative mechanism of regulation of the expression of TIMP-3 by the RPE is not discarded. The number of RNA transcripts specific for TIMP-3 per nanogram of poly A+ RNA was quantified by RT-PCR. 9.6 x 10(5) transcripts per nanogram of polyA +RNA were found at the adult stage and 1.2 x 10(6) transcripts per nanogram of polyA +RNA were detected at the fetal stage. These findings were supported by the predominant labeling in the RPE layer of retinal tissue sections in in situ hybridization experiments. All of these data support the hypothesis that the production of TIMP-3 by the RPE may be crucial for the maintenance of Bruch's membrane, the complex layer of   extracellular matrix   that provides a structural substrate for the RPE in the healthy retina and is perturbed during the ageing process and in Sorby's Fundus Dystrophy a inherited disease. 
 Tissue inhibitor of metalloproteinase-3 (TIMP-3) regulates hematopoiesis and bone formation in vivo. Tissue inhibitor of metalloproteinases-3 (TIMP-3) inhibits matrix metalloproteinases and membrane-bound sheddases. TIMP-3 is associated with the   extracellular matrix   and is expressed in highly remodeling tissues. TIMP-3 function in the hematopoietic system is unknown.We now report that TIMP-3 is highly expressed in the endosteal region of the bone marrow (BM), particularly by osteoblasts, endothelial and multipotent mesenchymal stromal cells which are all important cellular components of hematopoietic stem cell (HSC) niches, whereas its expression is very low in mature leukocytes and hematopoietic stem and progenitor cells. A possible role of TIMP-3 as an important niche component was further suggested by its down-regulation during granulocyte colony-stimulating factor-induced mobilization. To further investigate TIMP-3 function, mouse HSC were retrovirally transduced with human TIMP-3 and transplanted into lethally irradiated recipients. TIMP-3 overexpression resulted in decreased frequency of B and T lymphocytes and increased frequency of myeloid cells in blood and BM, increased Lineage-negative Sca-1(+)KIT(+) cell proliferation in vivo and in vitro and increased colony-forming cell trafficking to blood and spleen. Finally, over-expression of human TIMP-3 caused a late onset fatal osteosclerosis.Our results suggest that TIMP-3 regulates HSC proliferation, differentiation and trafficking in vivo, as well as bone and bone turn-over, and that TIMP-3 is expressed by stromal cells forming HSC niches within the BM. Thus, TIMP-3 may be an important HSC niche component regulating both hematopoiesis and bone remodeling. 
 MiR-21 enhances melanoma invasiveness via inhibition of tissue inhibitor of metalloproteinases 3 expression: in vivo effects of MiR-21 inhibitor. Metastatic melanoma is the most aggressive form of this cancer. It is important to understand factors that increase or decrease metastatic activity in order to more effectively research and implement treatments for melanoma. Increased cell invasion through the   extracellular matrix   is required for metastasis and is enhanced by matrix metalloproteinases (MMPs). Tissue inhibitor of metalloproteinases 3 (TIMP3) inhibits MMP activity. It was previously shown by our group that miR-21, a potential regulator of TIMP3, is over-expressed in cutaneous melanoma. It was therefore hypothesized that increased levels of miR-21 expression would lead to decreased expression of TIMP3 and thereby enhance the invasiveness of melanoma cells. miR-21 over-expression in the melanoma cell lines WM1552c, WM793b, A375 and MEL 39 was accomplished via transfection with pre-miR-21. Immunoblot analysis of miR-21-overexpressing cell lines revealed reduced expression of TIMP3 as compared to controls. This in turn led to a significant increase in the invasiveness of the radial growth phase cell line WM1552c and the vertical growth phase cell line WM793b (p < 0.05), but not in the metastatic cell lines A375 or MEL 39. The proliferation and migration of miR-21 over-expressing cell lines was not affected. Reduced expression of TIMP3 was achieved by siRNA knockdown and significantly enhanced invasion of melanoma cell lines, mimicking the effects of miR-21 over-expression. Treatment of tumor cells with a linked nucleic acid antagomir to miR-21 inhibited tumor growth and increased tumor expression of TIMP3 in vivo in 01B74 Athymic NCr-nu/nu mice. Intra-tumoral injections of anti-miR-21 produced similar effects. This data shows that increased expression of miR-21 enhanced the invasive potential of melanoma cell lines through TIMP3 inhibition. Therefore, inhibition of miR-21 in melanoma may reduce melanoma invasiveness. 
 Loss of TIMP3 enhances interstitial nephritis and fibrosis. The balance of matrix metalloproteinases (MMPs) and tissue inhibitors of matrix metalloproteinases (TIMPs) determines the integrity of the   extracellular matrix  . TIMP3 is the most highly expressed tissue inhibitor of metalloproteinase (TIMP) in the kidney, but its function in renal disease is incompletely understood. In this study, TIMP3-/- mice demonstrated an age-dependent chronic tubulointerstitial fibrosis. After unilateral ureteral obstruction (UUO), young TIMP3-/- mice exhibited increased renal injury (tubular atrophy, cortical and medullary thinning, and vascular damage) compared with wild-type mice. In addition, TIMP3-/- mice had greater interstitial fibrosis; increased synthesis and deposition of type I collagen; increased activation of fibroblasts; enhanced apoptosis; and greater activation of MMP2, but not MMP9, after UUO. TIMP3 deficiency also led to accelerated processing of TNFalpha, demonstrated by significantly higher TACE activity and greater soluble TNFalpha levels by 3 d after UUO. The additional deletion of TNFalpha markedly reduced inflammation, apoptosis, and induction of a number of MMPs. Moreover, inhibition of MMPs in TIMP3-/-/TNFalpha-/- mice further abrogated postobstructive injury and prevented tubulointerestitial fibrosis. In humans, TIMP3 expression increased in the renal arteries and proximal tubules of subjects with diabetic nephropathy or chronic allograft nephropathy. Taken together, these results provide evidence that TIMP3 is an important mediator of kidney injury, and regulating its activity may have therapeutic benefit for patients with kidney disease. 
 Differential TIMP3 expression affects tumor progression and angiogenesis in melanomas through regulation of directionally persistent endothelial cell migration. The angiogenic potential of solid tumors, or the ability to initiate neovasculature development from pre-existing host vessels, is facilitated by soluble factors secreted by tumor cells and involves breaching of   extracellular matrix   barriers, endothelial cell (EC) proliferation, migration and reassembly. We evaluated the angiogenic potential of human melanoma cell lines differing in their degree of aggressiveness, based on their ability to regulate directionally persistent EC migration. We observed that conditioned-medium (CM) of the aggressive melanoma cell line BLM induced a high effective migratory response in ECs, while CMs of Mel57 and 1F6 had an inhibitory effect. Further, the melanoma cell lines exhibited a varied expression profile of tissue inhibitor of metalloproteinase-3 (TIMP3), detectable in the CM. TIMP3 expression inversely correlated with aggressiveness of the melanoma cell line, and ability of the respective CMs to induce directed EC migration. Interestingly, TIMP3 expression was found to be silenced in the BLM cell line, concurrent with its role as a tumor suppressor. Treatment with recombinant human TIMP3 and CM of modified, TIMP3 expressing, BLM cells mitigated directional EC migration, while CM of TIMP3 silenced 1F6 cells induced directed EC migration. The functional implication of TIMP3 expression on tumor growth and angiogenic potential in melanoma was evaluated in vivo. We observed that TIMP3 expression reduced tumor growth, angiogenesis and macrophage infiltration of BLM tumors while silencing TIMP3 increased tumor growth and angiogenesis of 1F6 tumors. Taken together, our results demonstrate that TIMP3 expression correlates with inhibition of directionally persistent EC migration and adversely affects the angiogenic potential and growth of melanomas. 
 The gene structure of tissue inhibitor of metalloproteinases (TIMP)-3 and its inhibitory activities define the distinct TIMP gene family. Matrix metalloproteinases (MMPs) and tissue inhibitors of metalloproteinases (TIMPs) play a critical role in   extracellular matrix   homeostasis. We have previously cloned human and mouse TIMP-3 cDNAs and mapped their chromosomal loci (Apte, S. S., Mattei, M-G., and Olsen, B. R. (1994) Genomics 19, 86-90; Apte, S. S., Hayashi, K., Seldin, M. F., Mattei, M-G., Hayashi, M., and Olsen, B. R. (1994) Dev. Dynam. 200, 177-197); the identification of TIMP3 mutations in Sorsby's fundus dystrophy has underscored the functional importance of TIMP-3. We now report that TIMP-3 is encoded by five exons spanning over 30 kilobase pairs of mouse genomic DNA. In the attribution of protein domains to specific exons, as well as exon structures, the Timp-3 and Timp-1 genes are similar, confirming the common evolutionary origin of the TIMPs and defining a distinct gene family. We have expressed human and mouse TIMP-3 in mouse NSO myeloma cells. In each case, an N-glycosylated 27-kDa protein was generated, that, like TIMP-1 and TIMP-2, inhibited collagenase-1, stromelysin-1, and gelatinases A and B. TIMP-3 and TIMP-1 inhibition were quantitatively similar, implying that all TIMPs are equally efficient in MMP inhibition. Instead, differential regulation of the TIMP genes or divergent C-terminal protein sequences may underlie distinct biological functions for each TIMP. 
 Requirement of phosphatidylinositol 3-kinase/Akt signaling pathway for regulation of tissue inhibitor of metalloproteinases-3 gene expression by TGF-beta in human chondrocytes. Transforming growth factor beta (TGF-beta1) induces cartilage   extracellular matrix   synthesis and tissue inhibitor of metalloproteinases-3 (TIMP-3), an important natural inhibitor of matrix metalloproteinases, aggrecanases and TNF-alpha-converting enzyme, which are implicated in cartilage degradation and joint inflammation. This study tested the hypothesis that Akt/protein kinase B signaling pathway could mediate TGF-beta1 induction of TIMP-3 in human articular chondrocytes. TGF-beta activated phosphorylation of Akt in a delayed and sustained fashion that correlated with TIMP-3 mRNA induction. Phosphatidylinositol kinase (PI3K) inhibitors, Wortmannin and LY294002 and Akt inhibitor (NL-71-101) significantly inhibited TGF-beta-induced Akt phosphorylation, TIMP-3 expression, TIMP-3 promoter (-940 to +376)-driven luciferase activity and Sp1 transcription factor binding. PI3K p85, Akt and Sp1 small interfering RNA (siRNA)-driven knockdown of the respective gene products significantly suppressed TGF-beta-induced TIMP-3 gene expression. TGF-beta-stimulated phosphorylation of p70S6 Kinase and TIMP-3 protein induction was inhibited by rapamycin. Thus TGF-beta induces TIMP-3 gene expression in human chondrocytes partly through PI3K/Akt pathway and Sp1 transcription factor and by translational mechanisms via mammalian target of rapamycin (mTOR) signaling. TGF-beta induction of pro-survival Akt cascade and TIMP-3 may be related to strengthening of cartilage   extracellular matrix  , increased chondrocyte viability and maintenance of joint tissue integrity. 
 The imbalance between TIMP3 and matrix-degrading enzymes plays an important role in intervertebral disc degeneration. It is well-known that one of the most important features of intervertebral disc degeneration (IDD) is the   extracellular matrix   (ECM) degradation. Collagen and aggrecan are major components of ECM; the degradation of ECM in intervertebral discs (IVDs) is closely related to the activities of collagenase and aggrecanase. TIMP-3 is the most efficient inhibitor of aggrecanase in IVD. However, only few studies focus on the potential relationship between TIMP-3 and IDD. In our study, we found TIMP-3 gene expression was decreased after stimulating with LPS in rat nucleus pulposus (NP) cells. Then we used a lentivirus vector to reconstruct rat NP cells which high expressed TIMP-3 gene (LV-TIMP3). The upregulation of MMPs and ADAMTSs induced by LPS was significantly inhibited in LV-TIMP3 cells. After overexpression of TIMP-3, the aggrecan breakdown caused by LPS was also reduced in both monolayer culture and three-dimension culture model. To further study the relation between TIMP-3 and IDD, we collected human NP tissue samples of different degenerative degrees. Real-time PCR and immunohistochemical staining showed that the expression of TIMP-3 was negatively correlated with the degree of intervertebral disc degeneration, while MMP-1 and ADAMTS-4 were markedly increased in degenerative IVD. Taken together, our results suggest that the imbalance between aggrecanase and TIMP-3 may play an important role in the pathogenesis of IDD and therefore be a potential therapeutic target for treating IDD. 
 Multivalent pseudopeptides targeting cell surface nucleoproteins inhibit cancer cell invasion through tissue inhibitor of metalloproteinases 3 (TIMP-3) release. Blockage of the metastasis process remains a significant clinical challenge, requiring innovative therapeutic approaches. For this purpose, molecules that inhibit matrix metalloproteinases activity or induce the expression of their natural inhibitor, the tissue inhibitor of metalloproteinases (TIMPs), are potentially interesting. In a previous study, we have shown that synthetic ligands binding to cell surface nucleolin/nucleophosmin and known as HB 19 for the lead compound and NucAnt 6L (N6L) for the most potent analog, inhibit both tumor growth and angiogenesis. Furthermore, they prevent metastasis in a RET transgenic mice model which develops melanoma. Here, we investigated the effect of N6L on the invasion capacity of MDA-MB-435 melanoma cells. Our results show that the multivalent pseudopeptide N6L inhibited Matrigel invasion of MDA-MB-435 cells in a modified Boyden chamber model. This was associated with an increase in TIMP-3 in the cell culture medium without a change in TIMP-3 mRNA expression suggesting its release from cell surface and/or   extracellular matrix  . This may be explained by our demonstrated N6L interaction with sulfated glycosaminoglycans and consequently the controlled bioavailability of glycosaminoglycan-bound TIMP-3. The implication of TIMP-3 in N6L-induced inhibition of cell invasion was evidenced by siRNA silencing experiments showing that the loss of TIMP-3 expression abrogated the effect of N6L. The inhibition of tumor cell invasion by N6L demonstrated in this study, in addition to its previously established inhibitory effect on tumor growth and angiogenesis, suggests that N6L represents a promising anticancer drug candidate warranting further investigation. 
 Systematic search for mutations in the human tissue inhibitor of metalloproteinases-3 (TIMP-3) gene on chromosome 22 and association study with schizophrenia. Several linkage studies have suggested that chromosome 22q12-q13 is a putative region for schizophrenic genes. In this study, the human tissue inhibitor of metalloproteinase-3 (TIMP-3) gene was investigated as positional candidate gene for schizophrenia because of its regulatory function on   extracellular matrix   proteins, cell adhesion molecules, and neural cell adhesion molecules in the brain. We systematically searched for the nucleotide variants by sequencing all the exons and their flanking intronic sequences in a sample of Chinese schizophrenic patients from Taiwan. Two silent mutations in the exon 3 were identified: c.249T-->C at codon 83 (His) and c.261C-->T at codon 87 (Ser). However, no mutations causing amino acid alteration or aberrant splicing of transcripts were observed. Hence, it is unlikely that the TIMP-3 gene itself may play an important role in the genetic susceptibility to schizophrenia. Further case control association study revealed a significant difference of genotype distribution of the c.249T-->C between schizophrenic patients and control. This finding supports that 22q12 is a schizophrenia susceptible region, and it is likely that there might be other genetic mutations in the neighborhood of the TIMP-3 gene locus that may contribute to the susceptibility of schizophrenia. 
 TIMP-3 gene transfection suppresses invasive and metastatic capacity of human hepatocarcinoma cell line HCC-7721. Tissue inhibitor of metalloproteinases (TIMPs) can restrain the tumor growth by their protein property and matrix metalloproteinases (MMPs) inhibition. There are currently four known TIMP family members-TIMP-1, 2, 3, 4. We determined whether increasing levels of TIMP-3 expression could suppress the malignant phenotype of human hepatocarcinoma cell line HCC-7721.A recombinant expression vector, which contained full-length cDNA of human TIMP-3, was constructed and transfected into the human hepatocarcinoma cell line HCC-7721 by the lipofectamine technique. In vitro and in vivo tests such as Western blotting, immunohistochemistry as well as xenografting in nude mice were used to analyze expression levels of TIMP and MMP, and changes in malignant phenotype after the gene transfection.TIMP-3 expression in TIMP-3 gene-transfected HCC-7721 cells was upregulated as assessed by Western blotting. The ability of in vitro invasion through a Boyden chamber was significantly decreased compared to controls. Following subcutaneous injection into nude mice, the TIMP-3 transfected cells suppressed primary tumor growth, as characterized by reduced tumor weight, size and microvasculature as well as maintaining the   extracellular matrix  .The results suggest that upregulation of TIMP-3 expression in HCC-7721 cells inhibits invasion capacity in vitro as well as tumorigenic and metastatic potential in nude mice. 
 Mutations in the tissue inhibitor of metalloproteinases-3 (TIMP3) in patients with Sorsby's fundus dystrophy. The hereditary macular dystrophies are progressive degenerations of the central retina and contribute significantly to irreversible visual loss in developed countries. Among these disorders, Sorsby's fundus dystrophy (SFD), an autosomal dominant condition, provides an excellent mendelian model for the study of the genetically complex age-related macular degeneration (AMD), the most common maculopathy in the elderly. Recently, we mapped the SFD locus to 22q13-qter. This same region contains the gene for tissue inhibitor of metalloproteinases-3 (TIMP3), which is known to play a pivotal role in   extracellular matrix   remodeling. We have now identified point mutations in the TIMP3 gene in affected members of two SFD pedigrees. These mutations are predicted to disrupt the tertiary structure and thus the functional properties of the mature protein. 
 Evaluation of serum lipid concentrations and genetic variants at high-density lipoprotein metabolism loci and TIMP3 in age-related macular degeneration. To analyze the association between polymorphisms in the TIMP3 gene and genes of the high-density lipoprotein (HDL) metabolism and age-related macular degeneration (AMD), and evaluate serum lipid and lipoprotein levels in AMD patients compared with control individuals.Single nucleotide polymorphisms in or near the TIMP3, ABCA1, FADS1-3, CETP, LIPC, and LPL genes were genotyped. Serum levels of apolipoprotein B (ApoB), apolipoprotein A2, lipoprotein a, cholesterol, triglycerides, and HDL-cholesterol were determined.Significant associations were found between AMD and variants in ABCA1 and FADS1-3, and a nearly significant association in TIMP3. No significant associations were observed for variants in LPL, LIPC, and CETP. We also observed a significant elevation of ApoB levels in serum of AMD patients. Other lipids and lipoproteins were not significantly altered.These results confirm associations of AMD with variants near the TIMP3 gene and at loci involved in HDL metabolism. They further highlight a role of the   extracellular matrix   and the HDL metabolism in the pathogenesis of AMD. This study identified increased ApoB levels as a possible new serum biomarker for AMD. 
 Regulation of TIMP3 in diabetic nephropathy: a role for microRNAs. Diabetic nephropathy (DN) is the major cause of chronic kidney disease in developed countries and contributes significantly to increased morbidity and mortality among diabetic patients. Morphologically, DN is characterized by tubulo-interstitial fibrosis, thickening of the glomerular basement membrane and mesangial expansion mainly due to accumulation of   extracellular matrix   (ECM). ECM turnover is regulated by metalloproteinases and tissue inhibitors of metalloproteinases (TIMPs) activities. In diabetic conditions, TIMP3 expression in kidney is strongly reduced, but the causes of this reduction are still unknown. The aim of this study was to elucidate at least one of these mechanisms which relies on differential expression of TIMP3-targeting microRNAs (miRs) in a hyperglycemic environment either in vitro (MES13 cell line) or in vivo (mouse kidney and human biopsies). Among the TIMP3-targeting miRs, miR-21 and miR-221 were significantly upregulated in kidneys from diabetic mice compared to control littermates, and in a mesangial cell line grown in high glucose conditions. In human samples, only miR-21 expression was increased in kidney biopsies from diabetic patients compared to healthy controls. The expression of miR-217, which targets TIMP3 indirectly through downregulation of SirT1, was also increased in diabetic kidney and MES13 cell line. In agreement with these result, SirT1 expression was reduced in mouse and human diabetic kidneys as well as in MES13 mesangial cell line. TIMP3 deficiency has recently emerged as a hallmark of DN in mouse and human. In this study, we demonstrated that this reduction is due, at least in part, to increased expression of certain TIMP3-targeting miRs in diabetic kidneys compared to healthy controls. Unveiling the post-transcriptional mechanisms responsible for TIMP3 downregulation in hyperglycemic conditions may orient toward the use of this protein as a possible therapeutic target in DN. 
 MicroRNAs and potential target interactions in psoriasis. Psoriasis is a chronic inflammatory skin disease often seen in patients with a genetic susceptibility. MicroRNAs (miRNA) are endogenous, short RNA molecules that can bind to parts of mRNA target genes, thus inhibiting their translation and causing accelerated turnover or transcript degradation. MicroRNAs are important in the pathogenesis of human diseases such as immunological disorders, as they regulate a broad range of biological processes.We investigated miRNA-mRNA interactions in involved (PP) and non-involved (PN) psoriatic skin compared with healthy skin (NN).Biopsies were obtained from PP, PN and NN, the miRNA and mRNA expression was analyzed by microarray techniques and a subset of miRNAs and mRNAs were validated by q-RT-PCR. Novel target interactions in psoriasis were found using PubMed, miRBase and RNAhybrid. In addition, TIMP3 protein expression was studied in PP, PN and NN. Finally, the miR-221/2-TIMP3 target interaction was studied in primary human keratinocytes by endogenous overexpression of the miRNAs.We identified 42 upregulated miRNAs and 5 downregulated miRNAs in PP compared with NN, and only few deregulated miRNAs in PN compared with NN. Based on the miRNA and mRNA profiles miR-21, -205, -221 and -222 were found to have the following potential mRNA targets in psoriatic skin: PDCD4, TPM1, P57, C-KIT, RTN4, SHIP2, TIMP3, RECK and NFIB. The identified target mRNAs were likely to be involved in cellular growth, proliferation, apoptosis and degradation of the   extracellular matrix  . Finally we found that TIMP3 is downregulated in psoriatic skin. In vitro overexpression of miR-221 and miR-222 lead to degradation of TIMP3 resulting in decreased TIMP3 protein level.Our data indicate several novel important associations for miRNAs in psoriasis and in particular the miR-221/2-TIMP3 target interaction could among others play a role in the psoriasis pathogenesis. 
 Expression profiling reveals that methylation of TIMP3 is involved in uveal melanoma development. Uveal melanoma is associated with a high tumor-related mortality due to the propensity to develop metastases. The mechanisms that are responsible for malignant dissemination are largely unknown and need to be explored to facilitate diagnosis and treatment of metastases. To identify molecules involved in dissemination, we used cell lines derived from a primary uveal melanoma and 2 liver metastases from the same patient as a model for tumor progression. Using a microarray representing 1,176 genes, we identified 63 differentially expressed genes. Forty genes showed a higher expression and 23 showed a lower expression in the primary cell line compared to the metastasis cell lines. These genes are involved in processes like angiogenesis, apoptosis, macrophage stimulation, and   extracellular matrix   regulation. In contrast, the 2 liver metastasis cell lines produced nearly identical expression profiles. Demethylation of the primary melanoma cell line by 5-aza-2'deoxycytidine treatment revealed that 19 genes were suppressed by hypermethylation. An important finding was the 5-fold decreased expression of TIMP3 in the metastatic cell lines, a molecule involved in   extracellular matrix   degradation. We demonstrate in the cell lines that TIMP3 expression is regulated by methylation. These observations were confirmed in primary uveal melanoma and suggests a role for TIMP3 in uveal melanoma development. 
 Abnormal recruitment of   extracellular matrix   proteins by excess Notch3 ECD: a new pathomechanism in CADASIL. Cerebral autosomal dominant arteriopathy with subcortical infarcts and leukoencephalopathy, or CADASIL, one of the most common inherited small vessel diseases of the brain, is characterized by a progressive loss of vascular smooth muscle cells and   extracellular matrix   accumulation. The disease is caused by highly stereotyped mutations within the extracellular domain of the NOTCH3 receptor (Notch3(ECD)) that result in an odd number of cysteine residues. While CADASIL-associated NOTCH3 mutations differentially affect NOTCH3 receptor function and activity, they all are associated with early accumulation of Notch3(ECD)-containing aggregates in small vessels. We still lack mechanistic explanation to link NOTCH3 mutations with small vessel pathology. Herein, we hypothesized that excess Notch3(ECD) could recruit and sequester functionally important proteins within small vessels of the brain. We performed biochemical, nano-liquid chromatography-tandem mass spectrometry and immunohistochemical analyses, using cerebral and arterial tissue derived from patients with CADASIL and mouse models of CADASIL that exhibit vascular lesions in the end- and early-stage of the disease, respectively. Biochemical fractionation of brain and artery samples demonstrated that mutant Notch3(ECD) accumulates in disulphide cross-linked detergent-insoluble aggregates in mice and patients with CADASIL. Further proteomic and immunohistochemical analyses identified two functionally important   extracellular matrix   proteins, tissue inhibitor of metalloproteinases 3 (TIMP3) and vitronectin (VTN) that are sequestered into Notch3(ECD)-containing aggregates. Using cultured cells, we show that increased levels or aggregation of Notch3 enhances the formation of Notch3(ECD)-TIMP3 complex, promoting TIMP3 recruitment and accumulation. In turn, TIMP3 promotes complex formation including NOTCH3 and VTN. In vivo, brain vessels from mice and patients with CADASIL exhibit elevated levels of both insoluble cross-linked and soluble TIMP3 species. Moreover, reverse zymography assays show a significant elevation of TIMP3 activity in the brain vessels from mice and patients with CADASIL. Collectively, our findings lend support to a Notch3(ECD) cascade hypothesis in CADASIL disease pathology, which posits that aggregation/accumulation of Notch3(ECD) in the brain vessels is a central event, promoting the abnormal recruitment of functionally important   extracellular matrix   proteins that may ultimately cause multifactorial toxicity. Specifically, our results suggest a dysregulation of TIMP3 activity, which could contribute to mutant Notch3(ECD) toxicity by impairing   extracellular matrix   homeostasis in small vessels. 
 Reactive site mutations in tissue inhibitor of metalloproteinase-3 disrupt inhibition of matrix metalloproteinases but not tumor necrosis factor-alpha-converting enzyme. Tissue inhibitor of metalloproteinase-3 (TIMP-3) is a dual inhibitor of the matrix metalloproteinases (MMPs) and some adamalysins, two families of extracellular and cell surface metalloproteinases that function in   extracellular matrix   turnover and the shedding of cell surface proteins. The mechanism of inhibition of MMPs by TIMPs has been well characterized, and since the catalytic domains of MMPs and adamalysins are homologous, it was assumed that the interaction of TIMP-3 with adamalysins is closely similar. Here we report that the inhibition of the extracellular region of ADAM-17 (tumor necrosis factor alpha-converting enzyme (TACE)) by the inhibitory domain of TIMP-3 (N-TIMP-3) shows positive cooperativity. Also, mutations in the core of the MMP interaction surface of N-TIMP-3 dramatically reduce the binding affinity for MMPs but have little effect on the inhibitory activity for TACE. These results suggest that the mechanism of inhibition of ADAM-17 by TIMP-3 may be distinct from that for MMPs. The mutant proteins are also effective inhibitors of tumor necrosis factor alpha (TNF-alpha) release from phorbol ester-stimulated cells, indicating that they provide a lead for engineering TACE-specific inhibitors that may reduce side effects arising from MMP inhibition and are possibly useful for treatment of diseases associated with excessive TNF-alpha levels such as rheumatoid arthritis. 
 Alterations of ADAMTSs and TIMP-3 in human nucleus pulposus cells subjected to compressive load: Implications in the pathogenesis of human intervertebral disc degeneration. Intervertebral disc degeneration (IDD) pertains to the loss of   extracellular matrix   (ECM), particularly the early loss of aggrecan, the turnover of which is regulated by ADAMTSs. Amongst the etiological factors of IDD, mechanical stress plays an important role in the physiological and pathological processes of nucleus pulposus (NP) cells. However, the role of ADAMTSs and their inhibitor in human NP cells under mechanical stress has not been elucidated to date. The purpose of this study was to investigate the role of ADAMTSs and TIMP-3 in NP cells under mechanical stress. Human NP cells isolated from non-degenerative and degenerative discs were subjected to dynamic compressive load. The expression of ADAMTSs, aggrecan, and TIMP-3 was detected by quantitative real-time PCR and/or Western blot. Consequently, the gene expression of ADAMTS-1, 4, and 5 increased significantly in loaded NP cells compared with not-loaded cells from either non-degenerative or degenerative discs, whereas the gene expression of aggrecan decreased significantly. Moreover, Western blot indicated increased protein levels of ADAMTSs-1, 4, and 5. However, the expression of TIMP-3 altered insignificantly. Together, this study is the first addressing the underlying mechanisms of compressive load as a contributing factor to IDD in terms of ADAMTSs. Our results suggest that compressive load leads to the increase in ADAMTS-1, 4, and 5 that contributes to the decrease of aggrecan and IDD via TIMP-3 independent machinery. 
  Conserved Domains  
 cd03585: NTR_TIMP; NTR domain, TIMP subfamily; TIMPs, or tissue inibitors of metalloproteases, are essential regulators of   extracellular matrix   turnover and remodeling. They form complexes with matrix metalloproteases (MMPs) and inactivate them irreversibly by ... 
  GeneRIFs  
   Extracellular matrix   proteins expression profiling in chemoresistant variants of the A2780 ovarian cancer cell line. 
 Dysregulation of TIMP3 activity in CADASIL patients contributes to impairment of   extracellular matrix   homeostasis. 
 The TIMP3 modulate   extracellular matrix   remodeling during embryonic development and disease. 
 TIMP-3 is a binding partner of epithelial growth factor-containing fibulin-like   extracellular matrix   protein 1 (EFEMP1) 
 In idiopathic pulmonary fibrosis tissues TIMP3 gene expression was increased and the protein was localized to fibroblastic foci and   extracellular matrix  . Our findings suggest that TGF-beta1-induced TIMP3 may be an important mediator in lung fibrogenesis. 
 Our analysis of the entire coding region of TIMP-1, -2, and -3, which are the main inhibitors of metalloproteinase activity in the   extracellular matrix  , failed to show an association between genetic polymorphisms and an intracranial aneurysm 
  Summary  
 This gene belongs to the TIMP gene family. The proteins encoded by this gene family are inhibitors of the matrix metalloproteinases, a group of peptidases involved in degradation of the   extracellular matrix   (ECM). Expression of this gene is induced in response to mitogenic stimulation and this netrin domain-containing protein is localized to the ECM. Mutations in this gene have been associated with the autosomal dominant disorder Sorsby's fundus dystrophy. [provided by RefSeq, Jul 2008] 
  [Ii]nvasive  PubMed  
 miR-221&222 regulate TRAIL resistance and enhance tumorigenicity through PTEN and TIMP3 downregulation. Lung and liver cancers are among the most deadly types of cancer. Despite improvements in treatment over the past few decades, patient survival remains poor, underlining the need for development of targeted therapies. MicroRNAs represent a class of small RNAs frequently deregulated in human malignancies. We now report that miR-221&222 are overexpressed in aggressive non-small cell lung cancer and hepatocarcinoma cells, as compared with less   invasive   and/or normal lung and liver cells. We show that miR-221&222, by targeting PTEN and TIMP3 tumor suppressors, induce TRAIL resistance and enhance cellular migration through the activation of the AKT pathway and metallopeptidases. Finally, we demonstrate that the MET oncogene is involved in miR-221&222 activation through the c-Jun transcription factor. 
 Association of a high activity of matrix metalloproteinase-9 to low levels of tissue inhibitors of metalloproteinase-1 and -3 in human hepatitis B-viral hepatoma cells. Matrix metalloproteinases (MMPs) play a major role in the turnover of extracellular matrix (ECM) during cancer invasion and metastasis, and tissue inhibitors of metalloproteinases (TIMPs) control MMPs, thus maintaining a balanced ECM catabolism under physiological conditions. The aim of this study was to assess the behavior of some MMPs (FASEB J., 7, 1993, 1434; Cancer Metastasis Rev., 9(4) 1990, 289) and TIMPs (Biochem. Biophys. Res. Commun., 301, 2003, 1069; FASEB J., 7, 1993, 1434; Nature, 370, 1994, 61). Competitive RT-PCR, gelatin-substrate zymography, and ELISA techniques were used for quantification. The hepatitis B virus (HBV)-DNA-containing hepatocellular carcinoma cell lines, Hep3B, HepG2-HBV and HFF-T2 contain highly activated matrix metallproteinase-9 (MMP-9), which is rarely found in normal liver cell lines such as the Chang lines. MMP-9 activities of HFH-T2, HepG2-HBV and Hep3B were significantly higher than that of non-HBV-hepatocellular carcinoma SK-Hep1 and HepG2 (HCC origin, HBV not detected), as assayed by gelatin zymography. Low levels of TIMP-1 and TIMP-3 were observed in HFH-T2, HepG2-HBV and Hep3B, while the TIMP-2 level was high, as evidenced by reverse zymography. In contrast, 3 TIMP-1, -2 and -3 were largely detected in Chang, HepG2 and SK-Hep1 cells. To investigate the nature of the quantitative regulation of MMPs and TIMPs for these cell lines at the transcriptional levels, a reverse transcriptase-polymerase chain reaction (RT-PCR) was carried out. Not only MMP-9 mRNAs of HFH-T2, HepG2-HBV and Hep3B but also MMP-9 mRNA of SK-Hep1 and HepG2 were highly expressed with no major differences among these four cell lines. However, TIMP-1 and TIMP-3 mRNAs of HFH-T2, HepG2-HBV and Hep3B were markedly reduced, while those of SK-Hep1, HepG2 and Chang cells were maintained at high levels. Finally, an invasion assay using matrigel indicated in an increase in   invasive  ness in HFH-T2, HepG2-HBV and Hep3B cells, but a decrease in   invasive  ness of Chang, HepG2 and SK-Hep1 cells. These results indicate that the overexpression of MMP-9 mRNAs and the suppression of TIMP-1 and TIMP-3 in HFH-T2, HepG2-HBV and Hep3B were the result of HBV transfection. Based on these results, it is concluded that HBV affects the malignance of hepatocellular cancer by elevating MMP-9 activity, and suppressing TIMP-1 and TIMP-3. 
 Evaluation of candidate stromal epithelial cross-talk genes identifies association between risk of serous ovarian cancer and TERT, a cancer susceptibility "hot-spot". We hypothesized that variants in genes expressed as a consequence of interactions between ovarian cancer cells and the host micro-environment could contribute to cancer susceptibility. We therefore used a two-stage approach to evaluate common single nucleotide polymorphisms (SNPs) in 173 genes involved in stromal epithelial interactions in the Ovarian Cancer Association Consortium (OCAC). In the discovery stage, cases with epithelial ovarian cancer (n=675) and controls (n=1,162) were genotyped at 1,536 SNPs using an Illumina GoldenGate assay. Based on Positive Predictive Value estimates, three SNPs-PODXL rs1013368, ITGA6 rs13027811, and MMP3 rs522616-were selected for replication using TaqMan genotyping in up to 3,059 serous   invasive   cases and 8,905 controls from 16 OCAC case-control studies. An additional 18 SNPs with Pper-allele<0.05 in the discovery stage were selected for replication in a subset of five OCAC studies (n=1,233 serous   invasive   cases; n=3,364 controls). The discovery stage associations in PODXL, ITGA6, and MMP3 were attenuated in the larger replication set (adj. Pper-allele>or=0.5). However genotypes at TERT rs7726159 were associated with ovarian cancer risk in the smaller, five-study replication study (Pper-allele=0.03). Combined analysis of the discovery and replication sets for this TERT SNP showed an increased risk of serous ovarian cancer among non-Hispanic whites [adj. ORper-allele 1.14 (1.04-1.24) p=0.003]. Our study adds to the growing evidence that, like the 8q24 locus, the telomerase reverse transcriptase locus at 5p15.33, is a general cancer susceptibility locus. 
 The role of EZH2 in the regulation of the activity of matrix metalloproteinases in prostate cancer cells. Degradation of the extracellular matrix (ECM), a critical step in cancer metastasis, is determined by the balance between MMPs (matrix metalloproteinases) and their inhibitors TIMPs (tissue inhibitors of metalloproteinases). In cancer cells, this balance is shifted towards MMPs, promoting ECM degradation. Here, we show that EZH2 plays an active role in this process by repressing the expression of TIMP2 and TIMP3 in prostate cancer cells. The TIMP genes are derepressed by knockdown of EZH2 expression in human prostate cancer cells but repressed by overexpression of EZH2 in benign human prostate epithelial cells. EZH2 catalyzes H3K27 trimethylation and subsequent DNA methylation of the TIMP gene promoters. Overexpression of EZH2 confers an   invasive   phenotype on benign prostate epithelial cells; however, this phenotype is suppressed by cooverexpression of TIMP3. EZH2 knockdown markedly reduces the proteolytic activity of MMP-9, thereby decreasing the   invasive   activity of prostate cancer cells. These results suggest that the transcriptional repression of the TIMP genes by EZH2 may be a major mechanism to shift the MMPs/TIMPs balance in favor of MMP activity and thus to promote ECM degradation and subsequent invasion of prostate cancer cells. 
 The role of EZH2 in the regulation of the activity of matrix metalloproteinases in prostate cancer cells. Degradation of the extracellular matrix (ECM), a critical step in cancer metastasis, is determined by the balance between MMPs (matrix metalloproteinases) and their inhibitors TIMPs (tissue inhibitors of metalloproteinases). In cancer cells, this balance is shifted towards MMPs, promoting ECM degradation. Here, we show that EZH2 plays an active role in this process by repressing the expression of TIMP2 and TIMP3 in prostate cancer cells. The TIMP genes are derepressed by knockdown of EZH2 expression in human prostate cancer cells but repressed by overexpression of EZH2 in benign human prostate epithelial cells. EZH2 catalyzes H3K27 trimethylation and subsequent DNA methylation of the TIMP gene promoters. Overexpression of EZH2 confers an   invasive   phenotype on benign prostate epithelial cells; however, this phenotype is suppressed by cooverexpression of TIMP3. EZH2 knockdown markedly reduces the proteolytic activity of MMP-9, thereby decreasing the   invasive   activity of prostate cancer cells. These results suggest that the transcriptional repression of the TIMP genes by EZH2 may be a major mechanism to shift the MMPs/TIMPs balance in favor of MMP activity and thus to promote ECM degradation and subsequent invasion of prostate cancer cells. 
 Evaluation of candidate stromal epithelial cross-talk genes identifies association between risk of serous ovarian cancer and TERT, a cancer susceptibility "hot-spot". We hypothesized that variants in genes expressed as a consequence of interactions between ovarian cancer cells and the host micro-environment could contribute to cancer susceptibility. We therefore used a two-stage approach to evaluate common single nucleotide polymorphisms (SNPs) in 173 genes involved in stromal epithelial interactions in the Ovarian Cancer Association Consortium (OCAC). In the discovery stage, cases with epithelial ovarian cancer (n=675) and controls (n=1,162) were genotyped at 1,536 SNPs using an Illumina GoldenGate assay. Based on Positive Predictive Value estimates, three SNPs-PODXL rs1013368, ITGA6 rs13027811, and MMP3 rs522616-were selected for replication using TaqMan genotyping in up to 3,059 serous   invasive   cases and 8,905 controls from 16 OCAC case-control studies. An additional 18 SNPs with Pper-allele<0.05 in the discovery stage were selected for replication in a subset of five OCAC studies (n=1,233 serous   invasive   cases; n=3,364 controls). The discovery stage associations in PODXL, ITGA6, and MMP3 were attenuated in the larger replication set (adj. Pper-allele>or=0.5). However genotypes at TERT rs7726159 were associated with ovarian cancer risk in the smaller, five-study replication study (Pper-allele=0.03). Combined analysis of the discovery and replication sets for this TERT SNP showed an increased risk of serous ovarian cancer among non-Hispanic whites [adj. ORper-allele 1.14 (1.04-1.24) p=0.003]. Our study adds to the growing evidence that, like the 8q24 locus, the telomerase reverse transcriptase locus at 5p15.33, is a general cancer susceptibility locus. 
 miR-221&222 regulate TRAIL resistance and enhance tumorigenicity through PTEN and TIMP3 downregulation. Lung and liver cancers are among the most deadly types of cancer. Despite improvements in treatment over the past few decades, patient survival remains poor, underlining the need for development of targeted therapies. MicroRNAs represent a class of small RNAs frequently deregulated in human malignancies. We now report that miR-221&222 are overexpressed in aggressive non-small cell lung cancer and hepatocarcinoma cells, as compared with less   invasive   and/or normal lung and liver cells. We show that miR-221&222, by targeting PTEN and TIMP3 tumor suppressors, induce TRAIL resistance and enhance cellular migration through the activation of the AKT pathway and metallopeptidases. Finally, we demonstrate that the MET oncogene is involved in miR-221&222 activation through the c-Jun transcription factor. 
 Association of a high activity of matrix metalloproteinase-9 to low levels of tissue inhibitors of metalloproteinase-1 and -3 in human hepatitis B-viral hepatoma cells. Matrix metalloproteinases (MMPs) play a major role in the turnover of extracellular matrix (ECM) during cancer invasion and metastasis, and tissue inhibitors of metalloproteinases (TIMPs) control MMPs, thus maintaining a balanced ECM catabolism under physiological conditions. The aim of this study was to assess the behavior of some MMPs (FASEB J., 7, 1993, 1434; Cancer Metastasis Rev., 9(4) 1990, 289) and TIMPs (Biochem. Biophys. Res. Commun., 301, 2003, 1069; FASEB J., 7, 1993, 1434; Nature, 370, 1994, 61). Competitive RT-PCR, gelatin-substrate zymography, and ELISA techniques were used for quantification. The hepatitis B virus (HBV)-DNA-containing hepatocellular carcinoma cell lines, Hep3B, HepG2-HBV and HFF-T2 contain highly activated matrix metallproteinase-9 (MMP-9), which is rarely found in normal liver cell lines such as the Chang lines. MMP-9 activities of HFH-T2, HepG2-HBV and Hep3B were significantly higher than that of non-HBV-hepatocellular carcinoma SK-Hep1 and HepG2 (HCC origin, HBV not detected), as assayed by gelatin zymography. Low levels of TIMP-1 and TIMP-3 were observed in HFH-T2, HepG2-HBV and Hep3B, while the TIMP-2 level was high, as evidenced by reverse zymography. In contrast, 3 TIMP-1, -2 and -3 were largely detected in Chang, HepG2 and SK-Hep1 cells. To investigate the nature of the quantitative regulation of MMPs and TIMPs for these cell lines at the transcriptional levels, a reverse transcriptase-polymerase chain reaction (RT-PCR) was carried out. Not only MMP-9 mRNAs of HFH-T2, HepG2-HBV and Hep3B but also MMP-9 mRNA of SK-Hep1 and HepG2 were highly expressed with no major differences among these four cell lines. However, TIMP-1 and TIMP-3 mRNAs of HFH-T2, HepG2-HBV and Hep3B were markedly reduced, while those of SK-Hep1, HepG2 and Chang cells were maintained at high levels. Finally, an invasion assay using matrigel indicated in an increase in   invasive  ness in HFH-T2, HepG2-HBV and Hep3B cells, but a decrease in   invasive  ness of Chang, HepG2 and SK-Hep1 cells. These results indicate that the overexpression of MMP-9 mRNAs and the suppression of TIMP-1 and TIMP-3 in HFH-T2, HepG2-HBV and Hep3B were the result of HBV transfection. Based on these results, it is concluded that HBV affects the malignance of hepatocellular cancer by elevating MMP-9 activity, and suppressing TIMP-1 and TIMP-3. 
 TIMP3 regulates osteosarcoma cell migration, invasion, and chemotherapeutic resistances. Tissue inhibitors of metalloproteinases (TIMPs) inhibit matrix metalloproteinases (MMPs) to limit degradation of the extracellular matrix. Low levels of TIMP3 have been demonstrated in cancer tissues at advanced clinical stages, with positive distant metastasis and chemotherapeutic resistance. We examined the role of TIMP3 in osteosarcoma (OS) cell   invasive  ness and chemoresistance. TIMP3 was overexpressed or knocked down in the human OS cell lines Saos2 and MG63. Cell migration and invasion capacities were then evaluated using Transwell assays, and resistance to cisplatin was assessed by CCK-8 assay and flow cytometry. Real-time PCR and western blotting were used to investigate activation of signaling pathways downstream of TIMP3. Overexpression of TIMP3 inhibited the migration and invasion of Saos2 and MG63 cells, while knockdown of TIMP3 had the opposite effect. Cell survival after exposure to cisplatin was inhibited by TIMP3 overexpression in both Saos2 and MG63 cells. Consistently, downregulation of TIMP3 gene expression significantly decreased the sensitivity of OS cells to cisplatin treatment. MMP1, MMP2, Bcl-2, and Akt1 were all downregulated following TIMP3 overexpression, while Bax and cleaved caspase-3 were upregulated. TIMP3 knockdown had opposite effects on the regulation of these genes. Taken together, our findings suggest TIMP3 as a new target for inhibition of OS progression and chemotherapeutic resistance. 
 Overexpression of TACE and TIMP3 mRNA in head and neck cancer: association with tumour development and progression. TACE/ADAM17 is a transmembranous protease that cleaves membrane-bound growth factors like EGFR ligands. TACE-dependent proteolysis is regulated by its inhibitor, tissue inhibitor of metalloproteinases 3 (TIMP3). This study analyses the role of TACE and TIMP3 mRNA expression in squamous cell carcinomas of the head and neck (HNSCCs).We analysed TACE and TIMP3 mRNA expression in HNSCCs from 106 patients by RNA in situ hybridisation.TACE mRNA was upregulated in HNSCCs compared with dysplastic (P<0.05) and normal epithelia (P<0.001), with strong hybridisation signals in 21.9% of   invasive   tumour tissues and 4.5% of dysplasia. Elevated mRNA levels were accompanied by increased amounts of TACE protein in HNSCCs. TIMP3 mRNA expression in HNSCC-associated stroma was significantly higher than in the stroma adjacent to dysplastic or normal epithelia. Expression of TACE mRNA in HNSCCs was associated with tumour stage (P=0.019) and regional lymph node metastasis (P=0.009). Furthermore, levels of TACE mRNA in HNSCCs correlated with the expression of TIMP3 mRNA in HNSCC-associated stroma. Concomitantly, patients expressing high levels of TACE and TIMP3 mRNA showed significantly reduced overall survival compared with those with low mRNA levels.Our results indicate an important role of TACE and TIMP3 during development and progression of HNSCCs. 
 Gene expression analysis of drug-resistant MCF-7 cells: implications for relation to extracellular matrix proteins. Since multidrug resistance is a multifactorial phenomenon, a large-scale expression analysis of drug-resistant cells by using high-density oligonucleotide microarrays may provide information about new candidate genes contributing to resistance. Extracellular matrix (ECM) is responsible for many aspects of proliferation and   invasive  /metastatic behavior of tumor cells. This study demonstrates alterations in gene expression levels of several ECM components, matrix metalloproteinases (MMPs), adamalysins (ADAMs and ADAMTSs) and tissue inhibitors of metalloproteinases (TIMPs) in paclitaxel, docetaxel, vincristine and doxorubicin-resistant MCF-7 cells.Resistant MCF-7 cells were developed by stepwise selection of cells in increasing concentrations of drugs. Affymetrix GeneChip Human Genome U133 Plus 2.0 Array was used for hybridizations. Statistical significance was determined by independent sample t test. The genes having altered expression levels in drug-resistant sublines were selected and filtered by volcano plots.Genes up/downregulated more than twofolds were selected and listed. Expression of 25 genes encoding ECM proteins (including collagen, finronectin and syndecan) and integrin receptor subunits were found to be upregulated in drug-resistant cells. In addition, expression levels of, 13 genes encoding MMPs, ADAMs, ADAMTSs and TIMPs (including MMP1, MMP9, ADAM9 and TIMP3) were found to be altered in drug-resistant sublines when compared with sensitive MCF-7.Based on the expression analysis profiles, this report provides a preliminary insight into the relationship between drug resistance and ECM components, which are related to invasion and metastasis. Correlation of each specific ECM component with drug resistance requires further analysis. 
 TIMP3 regulates migration, invasion and in vivo tumorigenicity of thyroid tumor cells. Papillary thyroid carcinoma (PTC) arises from the thyroid follicular epithelium and represents the most frequent thyroid malignancy. PTC is associated with gene rearrangements generating RET/PTC and TRK oncogenes, and to the BRAFV600E activating point mutation. A role of tumor-suppressor genes in the pathogenesis of PTC has not been assessed yet. The tissue inhibitor of metalloproteinase-3 (TIMP3) gene, encoding a metalloproteinases inhibitor and capable of inhibiting growth, angiogenesis, invasion and metastasis of several cancers, was found to be silenced by promoter methylation in a consistent fraction of PTCs, in association with tumor aggressiveness and BRAFV600E mutation, thus suggesting an oncosuppressor role. To explore this possibility, in this study we performed gene expression and functional studies. Analysis of gene expression data produced in our laboratory as well as meta-analysis of publicly available data sets confirmed the downregulation of TIMP3 gene expression in PTC with respect to normal thyroid. The functional consequences of TIMP3 downregulation were investigated in the PTC-derived NIM1 cell line, in which the expression of TIMP3 is silenced. Restoration of TIMP3 expression by exposure to soluble TIMP3 protein or by complementary DNA transfection had no effect on the growth rate of NIM1 cells. Instead, it affected the adhesive, migratory and   invasive   capabilities of NIM1 cells by modulating several proteins involved in these processes. A striking effect was observed in vivo, as TIMP3 reduced the tumorigenicity of NIM1 cells by repressing angiogenesis and macrophage infiltration. Our data indicate that the loss of TIMP3 expression exerts a functional role in the pathogenesis of PTC. 
 Differences between scirrhous and non-scirrhous human gastric carcinomas from the aspect of proMMP-2 activation regulated by TIMP-3. Gastric carcinomas can be classified into scirrhous carcinomas (SC), i.e. 'linitis plastica' or Borrmann 4 gastric cancer, and non-scirrhous carcinomas (NSC). SC are characterized by diffuse   invasive   growth patterns with marked fibrosis, frequent peritoneal dissemination and lymph-node metastases and poor prognosis, while NSC show medullary growth patterns and common hematogenous metastases. To study the differences in local expression levels of matrix metalloproteinases (MMPs) and tissue inhibitors of metalloproteinases (TIMPs) between SC and NSC, we examined the expression of MMPs and TIMPs in human gastric carcinoma tissues by several methods including sandwich-enzyme immunoassay systems, gelatin zymography, reverse transcriptase-polymerase chain reaction (RT-PCR), real-time quantitative PCR, immunoblotting, immunohistochemistry and in situ zymography. Of the seven MMPs and two TIMPs tested, only proMMP-2 levels were remarkably higher in SC than in NSC (P < 0.01), and proMMP-2 activation ratio was significantly lower in SC than in NSC (P < 0.05). TIMP-3 mRNA levels were remarkably about 2-fold higher in SC than in NSC tissues (P < 0.01). TIMP-3 production in SC was confirmed by immunoblotting and TIMP-3 was immunolocalized to stromal fibroblasts in SC. TIMP-3 mRNA levels inversely correlated with proMMP-2 activation ratios, although the expression levels of MT1-MMP and MT2-MMP were not different in SC and NSC. By in situ zymography, gelatinolytic activity appeared to be weaker in SC than in NSC. All these data suggest that proMMP-2 activation is down-regulated by TIMP-3 expressed in scirrhous gastric carcinomas. Our findings may explain the differences in clinical behaviors of SC and NSC. 
 Tissue inhibitor of metalloproteinase-3 (TIMP-3) gene is methylated in the development of esophageal adenocarcinoma: loss of expression correlates with poor prognosis. Amongst involvement in diverse physiological and pathological processes, TIMP-3 may have an important role in tumour development, growth and metastasis by interaction with metalloproteases in the extracellular matrix. We studied the role and prognostic effect of TIMP-3 in esophageal adenocarcinoma (EADC). TIMP-3 gene methylation and TIMP-3 mRNA expression were analysed in 5 esophageal cell lines and 24 resected EADCs. TIMP-3 protein expression was examined in the 5 cell lines and 79 resected EADCs with known clinicopathological features. TIMP-3 methylation signal was only detected in the OE33 EADC cell line. In tissues, 0% of case-matched normal, 72% of BE and 90% of EADC were positive for methylation. TIMP-3 mRNA was detected in all the cell lines and normal, metaplastic and tumour tissues. TIMP-3 protein was localised to the cytoplasm in cell lines and tissues. Demethylating treatment of OE33 increased protein expression. At the invading edge of tumours, protein staining was equal to, or reduced, compared to normal tissues. Reduction of protein expression was associated with disease stage (p = 0.046) and poor patient survival (OR 2.1, 95% CI 1.2-3.5, p = 0.007). Mean survival time was halved in patients with reduced tumour TIMP-3 expression, from 49 to 24 months. These studies have demonstrated association between methylation of the TIMP-3 gene and BE and EADC. Reduced expression of TIMP-3 protein in EADC is associated with increased tumour   invasive  ness and reduced patient survival. 
 The Clinical Utility of TIMP3 Expression in ACTH-Secreting Pituitary Tumor. In recent years, the tissue inhibitor of metalloproteinase-3 (TIMP3) plays a pivotal role in tumorigenesis, while the role of TIMP3 in adrenocorticotrophic hormone (ACTH)-secreting pituitary adenomas remains unclear. In this study, 86 sporadic pituitary tumor specimens, including ACTH (40), GH (18), PRL-secreting (8), and non-functioining (20) and non-tumorous pituitary samples (n = 10) were available, and then, the mRNA and protein expression of TIMP3 was quantified by quantitative reverse transcriptase polymerase chain reaction (RT-PCR), western blotting, and immunohistochemistry, respectively. Our findings showed that TIMP3 expression was significantly correlated with Ki-67 expression and   the inva  siveness of pituitary adenomas. TIMP3 mRNA and protein expression were reduced in ACTH-secreting pituitary adenomas and the other three types of pituitary adenomas compared to adjacent non-tumorous pituitary tissues (all p < .01). On the other hand, the expression of TIMP3 was negatively correlated with tumor size and Ki-67 in ACTH-secreting pituitary adenomas. TIMP3 mRNA expression was significantly l  ower in   invasive pituitary adenomas than tha  t in non  invasive ones (1.92-fold, p < .05). TIMP3 protein levels were also significantly lower in the   majority   of invasive adenomas (1.41-fold, p < .05) Furthermore, TIMP3 mRNA and protein expression were significantly lower in pituitary giant adenomas than those in microadenomas (2.58-fold, p < .05). In conclusion, the expression of TIMP3 is low in pituitary adenomas including ACTH-secreting pituitary adenomas and negatively associated with tumor aggressiveness. TIMPs may play a potential role in the progression of ACTH-secreting pituitary adenomas and be usefu  l as a b  iomarker of invasiveness. 
 An immunohistochemical study of TIMP-3 expression in oesophageal squamous cell carcinoma. Tissue inhibitor of metalloproteinase-3 (TIMP-3) inhibits the activity of matrix metalloproteinase, which may play an important role in carcinoma invasion and metastasis. We have investigated the relationship between TIMP-3 reduction and clinicopathological factors in oesophageal squamous cell carcinoma (ESCC). We examined tissue specimens that had been removed from 90 patients with thoracic oesophageal cancer who had undergone surgery between 1983 and 2001. Immunohistochemical staining was performed by the standard streptavidin-biotin method. Immunostaining of TIMP-3 was seen in the cytoplasm of cancer cells and normal oesophageal epithelial cells, particularly in cells located in shallow areas of the tumour. TIMP-3 preserved (+), moderate (+/-), and reduced (-) cases accounted for 30, 27, and 33 of the 90 patients, respectively (33, 30, 37%). Significant correlations were observed between TIMP-3 expression and depth of tumour invasion (P=0.001), number of lymph node metastases (P=0.003), infiltrative growth pattern (P=0.003), and disease stage (P=0.005). The survival rates of patients with TIMP-3 (-) cancer were significantly lower than those of patients with TIMP-3 (+) and TIMP-3 (+/-) cancer (P=0.0003). The mean 5-year survival rates of patients with TIMP-3 (+), (+/-), and (-) were 50, 58, and 21%, respectively. In conclusion, decreased expression of TIMP-3 protein correlates with   invasive   activity and metastasis. This makes the prognosis for patients with cancer that has lost TIMP-3 significantly less favourable than that for patients with cancer that has maintained TIMP-3. 
 MiR-21 enhances melanoma   invasive  ness via inhibition of tissue inhibitor of metalloproteinases 3 expression: in vivo effects of MiR-21 inhibitor. Metastatic melanoma is the most aggressive form of this cancer. It is important to understand factors that increase or decrease metastatic activity in order to more effectively research and implement treatments for melanoma. Increased cell invasion through the extracellular matrix is required for metastasis and is enhanced by matrix metalloproteinases (MMPs). Tissue inhibitor of metalloproteinases 3 (TIMP3) inhibits MMP activity. It was previously shown by our group that miR-21, a potential regulator of TIMP3, is over-expressed in cutaneous melanoma. It was therefore hypothesized that increased levels of miR-21 expression would lead to decreased expression of TIMP3 and thereby enhance the   invasive  ness of melanoma cells. miR-21 over-expression in the melanoma cell lines WM1552c, WM793b, A375 and MEL 39 was accomplished via transfection with pre-miR-21. Immunoblot analysis of miR-21-overexpressing cell lines revealed reduced expression of TIMP3 as compared to controls. This in turn led to a significant increase in the   invasive  ness of the radial growth phase cell line WM1552c and the vertical growth phase cell line WM793b (p < 0.05), but not in the metastatic cell lines A375 or MEL 39. The proliferation and migration of miR-21 over-expressing cell lines was not affected. Reduced expression of TIMP3 was achieved by siRNA knockdown and significantly enhanced invasion of melanoma cell lines, mimicking the effects of miR-21 over-expression. Treatment of tumor cells with a linked nucleic acid antagomir to miR-21 inhibited tumor growth and increased tumor expression of TIMP3 in vivo in 01B74 Athymic NCr-nu/nu mice. Intra-tumoral injections of anti-miR-21 produced similar effects. This data shows that increased expression of miR-21 enhanced the   invasive   potential of melanoma cell lines through TIMP3 inhibition. Therefore, inhibition of miR-21 in melanoma may reduce melanoma   invasive  ness. 
 MicroRNA-21 regulates breast cancer invasion partly by targeting tissue inhibitor of metalloproteinase 3 expression. MicroRNAs are non-coding RNA molecules that posttranscriptionally regulate expression of target genes and have been implicated in the progress of cancer proliferation, differentiation and apoptosis. The aim of this study was to determine whether microRNA-21 (miR-21), a specific microRNA implicated in multiple aspects of carcinogenesis, impacts breast cancer invasion by regulating the tissue inhibitor of metalloproteinase 3 (TIMP3) gene.miR-21 expression was investigated in 32 matched breast cancer and normal breast tissues, and in four human breast cancer cell lines, by Taqman quantitative real-time PCR. Cell   invasive   ability was determined by matrigel invasion assay in vitro, in cells transfected with miR-21 or anti-miR-21 oligonucleotides. In addition, the regulation of tissue inhibitor of metalloproteinase 3 (TIMP3) by miR-21 was evaluated by western blotting and luciferase assays.Of the 32 paired samples analyzed, 25 breast cancer tissues displayed overexpression of miR-21 in comparison with matched normal breast epithelium. Additionally, incidence of lymph node metastasis closely correlated with miR-21 expression, suggesting a role for miR-21 in metastasis. Similarly, each of the four breast cancer cell lines analyzed overexpressed miR-21, to varied levels. Further, cells transfected with miR-21 showed significantly increased matrigel invasion compared with control cells, whereas transfection with anti-miR-21 significantly decreased cell invasion. Evaluation of TIMP3 protein levels, a peptidase involved in extarcellular matrix degredation, inversely correlated with miR-21 expression.As knockdown of miR-21 increased TIMP3 protein expression and luciferase reporter activity, our data suggests that miR-21 could promote invasion in breast cancer cells via its regulation of TIMP3. 
 Low tissue inhibitor of metalloproteinases 3 and high matrix metalloproteinase 14 levels defines a subpopulation of highly   invasive   foam-cell macrophages. An excess of metalloproteinases (MMPs) over tissue inhibitors of metalloproteinases (TIMPs) may favor atherosclerotic plaque rupture. We compared TIMP levels in nonfoamy and foam-cell macrophages (FCM) generated in vivo.In vivo generated rabbit FCM exhibited 84% reduced TIMP-3 protein compared to nonfoamy macrophages, and immunocytochemistry revealed a TIMP-3 negative subset (28%). Strikingly, only TIMP-3 negative FCM invaded a synthetic basement membrane, and invasion was inhibited by exogenous TIMP-3. TIMP-3 negative FCM also had increased proliferation and apoptosis rates compared to TIMP-3 positive cells, which were retarded by exogenous TIMP-3; this also reduced gelatinolytic activity. TIMP-3 negative FCM were found at the base of advanced rabbit plaques and in the rupture-prone shoulders of human plaques. To explain the actions of low TIMP-3 we observed a 26-fold increase in MT1-MMP (MMP-14) protein in FCM. Adding an MT1-MMP neutralizing antibody reduced foam-cell invasion, apoptosis, and gelatinolytic activity. Furthermore, MT1-MMP overexpressing and TIMP-3 negative FCM were found at the same locations in atherosclerotic plaques.These results demonstrate that TIMP-3 is downregulated in a distinct subpopulation of FCM which have increased MMP-14. These cells are highly   invasive   and have increased proliferation and apoptosis, all properties expected to destabilise atherosclerotic plaques. 
 Using urinary bFGF and TIMP3 levels to predict the presence of juvenile pilocytic astrocytoma and establish a distinct biomarker signature. OBJECTIVE The authors report the use of urinary biomarkers as a novel, non  invasive   technique to detect juvenile pilocytic astrocytomas (JPAs), capable of distinguishing JPAs from other CNS diseases, including other brain tumors. Preliminary screening of an array of tumors implicated proteases (including matrix metalloproteinases [MMPs]) and their inhibitors (tissue inhibitors of metalloproteinase [TIMPs]) as well as growth factors (including basic fibroblast growth factor [bFGF]) as candidate biomarkers. These data led the authors to hypothesize that tissue inhibitor of metalloproteinase 3 (TIMP3) and bFGF would represent high-probability candidates as JPA-specific biomarkers. METHODS Urine was collected from 107 patients, which included children with JPA (n = 21), medulloblastoma (n = 17), glioblastoma (n = 9), arteriovenous malformations (n = 25), moyamoya (n = 14), and age- and sex-matched controls (n = 21). Biomarker levels were quantified with enzyme-linked immunosorbent assay, tumor tissue expression was confirmed with immunohistochemical analysis, and longitudinal biomarker expression was correlated with imaging. Results were subjected to univariate and multivariate statistical analyses. RESULTS Using optimal urinary cutoff values of bFGF > 1.0 pg/μg and TIMP3 > 3.5 pg/μg, multiplexing bFGF and TIMP3 predicts JPA presence with 98% accuracy. Multiplexing bFGF and MMP13 distinguishes JPA from other brain tumor subtypes with up to 98% accuracy. Urinary biomarker expression correlated with both tumor immunohistochemistry and in vitro tumor levels. Urinary bFGF and TIMP3 decrease following successful tumor treatment and correlate with changes in tumor size. CONCLUSIONS This study identifies 2 urinary biomarkers-bFGF and TIMP3-that successfully detect one of the most common pediatric brain tumors with high accuracy. These data highlight potential benefits of urinary biomarkers and support their utility as diagnostic tools in the treatment of children with JPA. 
 Immunohistochemical analysis of TIMP-3 and MMP-9 in actinic keratosis, squamous cell carcinoma of the skin, and basal cell carcinoma. The expression of metalloproteinases and their inhibitors has been related to different   invasive   and metastatic potentials in cancer. This study aims to investigate the immunohistochemical expression of TIMP-3 and MMP-9 in samples of basal cell carcinoma (BCC), squamous cell carcinoma of the skin (SCC), and actinic keratosis (AK). Immunohistochemistry was performed to evaluate the expression of TIMP-3 and MMP-9 in samples of BCC (n=22), SCC (n=10), and AK (n=15). Ten fields of both tumor parenchyma and tumor stroma were photographed and counted in image software. The ratio of positive cells to total cells was used to quantify the staining. A higher expression of MMP-9 was found in tumor stroma of SCC compared to BCC and AK. No significant differences in TIMP-3 expression were observed among the groups. Considering the well-described differences between these neoplasms, these results provide additional evidence of the role of MMP-9 in tumor   invasive  ness of keratinocyte-derived tumors. 
 TIMP-3 gene transfection suppresses   invasive   and metastatic capacity of human hepatocarcinoma cell line HCC-7721. Tissue inhibitor of metalloproteinases (TIMPs) can restrain the tumor growth by their protein property and matrix metalloproteinases (MMPs) inhibition. There are currently four known TIMP family members-TIMP-1, 2, 3, 4. We determined whether increasing levels of TIMP-3 expression could suppress the malignant phenotype of human hepatocarcinoma cell line HCC-7721.A recombinant expression vector, which contained full-length cDNA of human TIMP-3, was constructed and transfected into the human hepatocarcinoma cell line HCC-7721 by the lipofectamine technique. In vitro and in vivo tests such as Western blotting, immunohistochemistry as well as xenografting in nude mice were used to analyze expression levels of TIMP and MMP, and changes in malignant phenotype after the gene transfection.TIMP-3 expression in TIMP-3 gene-transfected HCC-7721 cells was upregulated as assessed by Western blotting. The ability of in vitro invasion through a Boyden chamber was significantly decreased compared to controls. Following subcutaneous injection into nude mice, the TIMP-3 transfected cells suppressed primary tumor growth, as characterized by reduced tumor weight, size and microvasculature as well as maintaining the extracellular matrix.The results suggest that upregulation of TIMP-3 expression in HCC-7721 cells inhibits invasion capacity in vitro as well as tumorigenic and metastatic potential in nude mice. 
 Predicting   invasive   phenotype with CDH1, CDH13, CD44, and TIMP3 gene expression in primary breast cancer. We aimed to determine changes in the expression of the genes CDH1, CDH13, CD44, and TIMP3 to look for any relationship between them, HER2 and ESR1 expression at the RNA level, and the histopathological properties of tumors. We also analyzed the expression properties of double-negative (estrogen receptor [ER] and human epidermal growth factor receptor [HER2] both negative) breast tumors. Expression status was studied in fresh tissue at the mRNA level with quantitative PCR using hydrolysis probes. Sixty-two cancer patients and four normal controls were included in the study. When the tumor group was analyzed as a whole, the correlations of ESR1 with CDH1, CDH13, and TIMP3 were P < 0.05, P < 0.005, and P < 0.005, respectively. In ER-positive tumors, CDH1 and CDH13 were correlated directly (P < 0.005) when HER2 was correlated with CDH1, CDH13, and TIMP3 indirectly (P < 0.005, P < 0.005, and P < 0.05, respectively). CDH1 and CD44 had a strong indirect correlation (P < 0.005) in ER-negative tumors. There were significant differences in the expression levels of the CDH13, TIMP3, and CD44 genes (P < 0.005, P < 0.005, and P < 0.05, respectively) between the ER-positive and -negative groups. All four genes were found to be correlated with   invasive   properties in both ER-positive and -negative tumors. In double-negative tumor samples, only CD44 had a significant and strong correlation with stage, lymph node involvement, and metastasis (P < 0.05, P < 0.005, and P < 0.05, respectively). As a conclusion, a decrease in CDH1, CDH13, and TIMP3 expression levels with an increase in CD44 can be used as an indicator for invasion in both ER-positive and -negative breast tumors. In double-negative tumor tissues, CD44 can be considered a marker for aggressive properties. 
 MicroRNA-191 correlates with poor prognosis of colorectal carcinoma and plays multiple roles by targeting tissue inhibitor of metalloprotease 3. MicroRNA-191 (miR-191) is reported to be overexpressed in colorectal carcinoma (CRC), but the role of miR-191 in CRC progress remained unclear. This study demonstrated that High miR-191 expression was associated with clinical stage, lymph node metastasis, liver metastasis and depth of tumor invasion. Kaplan-Meier analysis indicated that patients with high miR-191 expression had a poor overall survival. Moreover, multivariate analysis showed that miR-191 was an independent prognostic factor in patients with CRC. Furthermore, we found that tissue inhibitor of metalloprotease 3 (TIMP3) was a direct target of miR-191 in colorectal cancer SW620 cells. TIMP3 downregulation mediated by miR-191 activated matrix metalloproteinases (MMPs) and thus promoted   invasive  ness of cancer cells. Anti-miR-191 could attenuate the   invasive  ness, suppress proliferation and induce apoptosis by restoring TIMP3 expression. Our results suggested that miR-191 might be a potential diagnostic and therapeutic target in patients with colorectal cancer. 
  GeneRIFs  
 This data shows that increased expression of miR-21 enhanced the   invasive   potential of melanoma cell lines through TIMP3 inhibition. 
 Anti-miR-191 could attenuate the   invasive  ness, suppress proliferation and induce apoptosis by restoring metalloprotease 3 expression 
 Decreased expression of TIMP-3 protein correlates with   invasive   activity and metastasis in esophageal squamous cell carcinoma 
 Reduced expression of TIMP-3 protein in esophageal adenocarcinoma is associated with increased tumour   invasive  ness 
  [Mm]etastasis  PubMed  
 Lysophosphatidic acid downregulates tissue inhibitor of metalloproteinases, which are negatively involved in lysophosphatidic acid-induced cell invasion. Ovarian cancer is a highly metastatic disease. Lysophosphatidic acid (LPA) levels are elevated in ascites from ovarian cancer patients, but its potential role in ovarian cancer   metastasis   has just begun to be revealed. In this work, we show that LPA stimulates invasion of primary ovarian cancer cells, but not ovarian epithelial or borderline ovarian tumor cells, although these benign cells indeed respond to LPA in cell migration. We have found that LPA downregulates tissue inhibitor of metalloproteinases (TIMPs). TIMP2 and TIMP3 play functional role in LPA-induced invasion as negative regulators. G(i) protein, phosphatidylinositol-3 kinase (PI3K), p38 mitogen-activated protein kinase (MAPK), cytosolic phospholipase A(2) and urokinase type plasminogen activator (uPA) are required for LPA-induced cells invasion. TIMP3 may affect two independent downstream targets, vascular endothelial growth factor receptor and p38 MAPK. In vivo, LPA stimulates tumor   metastasis   in an orthotopic ovarian tumor model, which can be inhibited by a PI3K inhibitor, LY294002. In summary, LPA is likely a key component for promoting ovarian   metastasis   in vivo. LPA downregulates TIMP3, which may have targets other than metalloproteinases. Our in vivo   metastasis   mouse model is useful for studying the efficacy of therapeutic regimes of ovarian cancer. 
 Association of a high activity of matrix metalloproteinase-9 to low levels of tissue inhibitors of metalloproteinase-1 and -3 in human hepatitis B-viral hepatoma cells. Matrix metalloproteinases (MMPs) play a major role in the turnover of extracellular matrix (ECM) during cancer invasion and   metastasis  , and tissue inhibitors of metalloproteinases (TIMPs) control MMPs, thus maintaining a balanced ECM catabolism under physiological conditions. The aim of this study was to assess the behavior of some MMPs (FASEB J., 7, 1993, 1434; Cancer   Metastasis   Rev., 9(4) 1990, 289) and TIMPs (Biochem. Biophys. Res. Commun., 301, 2003, 1069; FASEB J., 7, 1993, 1434; Nature, 370, 1994, 61). Competitive RT-PCR, gelatin-substrate zymography, and ELISA techniques were used for quantification. The hepatitis B virus (HBV)-DNA-containing hepatocellular carcinoma cell lines, Hep3B, HepG2-HBV and HFF-T2 contain highly activated matrix metallproteinase-9 (MMP-9), which is rarely found in normal liver cell lines such as the Chang lines. MMP-9 activities of HFH-T2, HepG2-HBV and Hep3B were significantly higher than that of non-HBV-hepatocellular carcinoma SK-Hep1 and HepG2 (HCC origin, HBV not detected), as assayed by gelatin zymography. Low levels of TIMP-1 and TIMP-3 were observed in HFH-T2, HepG2-HBV and Hep3B, while the TIMP-2 level was high, as evidenced by reverse zymography. In contrast, 3 TIMP-1, -2 and -3 were largely detected in Chang, HepG2 and SK-Hep1 cells. To investigate the nature of the quantitative regulation of MMPs and TIMPs for these cell lines at the transcriptional levels, a reverse transcriptase-polymerase chain reaction (RT-PCR) was carried out. Not only MMP-9 mRNAs of HFH-T2, HepG2-HBV and Hep3B but also MMP-9 mRNA of SK-Hep1 and HepG2 were highly expressed with no major differences among these four cell lines. However, TIMP-1 and TIMP-3 mRNAs of HFH-T2, HepG2-HBV and Hep3B were markedly reduced, while those of SK-Hep1, HepG2 and Chang cells were maintained at high levels. Finally, an invasion assay using matrigel indicated in an increase in invasiveness in HFH-T2, HepG2-HBV and Hep3B cells, but a decrease in invasiveness of Chang, HepG2 and SK-Hep1 cells. These results indicate that the overexpression of MMP-9 mRNAs and the suppression of TIMP-1 and TIMP-3 in HFH-T2, HepG2-HBV and Hep3B were the result of HBV transfection. Based on these results, it is concluded that HBV affects the malignance of hepatocellular cancer by elevating MMP-9 activity, and suppressing TIMP-1 and TIMP-3. 
 The role of EZH2 in the regulation of the activity of matrix metalloproteinases in prostate cancer cells. Degradation of the extracellular matrix (ECM), a critical step in cancer   metastasis  , is determined by the balance between MMPs (matrix metalloproteinases) and their inhibitors TIMPs (tissue inhibitors of metalloproteinases). In cancer cells, this balance is shifted towards MMPs, promoting ECM degradation. Here, we show that EZH2 plays an active role in this process by repressing the expression of TIMP2 and TIMP3 in prostate cancer cells. The TIMP genes are derepressed by knockdown of EZH2 expression in human prostate cancer cells but repressed by overexpression of EZH2 in benign human prostate epithelial cells. EZH2 catalyzes H3K27 trimethylation and subsequent DNA methylation of the TIMP gene promoters. Overexpression of EZH2 confers an invasive phenotype on benign prostate epithelial cells; however, this phenotype is suppressed by cooverexpression of TIMP3. EZH2 knockdown markedly reduces the proteolytic activity of MMP-9, thereby decreasing the invasive activity of prostate cancer cells. These results suggest that the transcriptional repression of the TIMP genes by EZH2 may be a major mechanism to shift the MMPs/TIMPs balance in favor of MMP activity and thus to promote ECM degradation and subsequent invasion of prostate cancer cells. 
 The role of EZH2 in the regulation of the activity of matrix metalloproteinases in prostate cancer cells. Degradation of the extracellular matrix (ECM), a critical step in cancer   metastasis  , is determined by the balance between MMPs (matrix metalloproteinases) and their inhibitors TIMPs (tissue inhibitors of metalloproteinases). In cancer cells, this balance is shifted towards MMPs, promoting ECM degradation. Here, we show that EZH2 plays an active role in this process by repressing the expression of TIMP2 and TIMP3 in prostate cancer cells. The TIMP genes are derepressed by knockdown of EZH2 expression in human prostate cancer cells but repressed by overexpression of EZH2 in benign human prostate epithelial cells. EZH2 catalyzes H3K27 trimethylation and subsequent DNA methylation of the TIMP gene promoters. Overexpression of EZH2 confers an invasive phenotype on benign prostate epithelial cells; however, this phenotype is suppressed by cooverexpression of TIMP3. EZH2 knockdown markedly reduces the proteolytic activity of MMP-9, thereby decreasing the invasive activity of prostate cancer cells. These results suggest that the transcriptional repression of the TIMP genes by EZH2 may be a major mechanism to shift the MMPs/TIMPs balance in favor of MMP activity and thus to promote ECM degradation and subsequent invasion of prostate cancer cells. 
 Lysophosphatidic acid downregulates tissue inhibitor of metalloproteinases, which are negatively involved in lysophosphatidic acid-induced cell invasion. Ovarian cancer is a highly metastatic disease. Lysophosphatidic acid (LPA) levels are elevated in ascites from ovarian cancer patients, but its potential role in ovarian cancer   metastasis   has just begun to be revealed. In this work, we show that LPA stimulates invasion of primary ovarian cancer cells, but not ovarian epithelial or borderline ovarian tumor cells, although these benign cells indeed respond to LPA in cell migration. We have found that LPA downregulates tissue inhibitor of metalloproteinases (TIMPs). TIMP2 and TIMP3 play functional role in LPA-induced invasion as negative regulators. G(i) protein, phosphatidylinositol-3 kinase (PI3K), p38 mitogen-activated protein kinase (MAPK), cytosolic phospholipase A(2) and urokinase type plasminogen activator (uPA) are required for LPA-induced cells invasion. TIMP3 may affect two independent downstream targets, vascular endothelial growth factor receptor and p38 MAPK. In vivo, LPA stimulates tumor   metastasis   in an orthotopic ovarian tumor model, which can be inhibited by a PI3K inhibitor, LY294002. In summary, LPA is likely a key component for promoting ovarian   metastasis   in vivo. LPA downregulates TIMP3, which may have targets other than metalloproteinases. Our in vivo   metastasis   mouse model is useful for studying the efficacy of therapeutic regimes of ovarian cancer. 
 Association of a high activity of matrix metalloproteinase-9 to low levels of tissue inhibitors of metalloproteinase-1 and -3 in human hepatitis B-viral hepatoma cells. Matrix metalloproteinases (MMPs) play a major role in the turnover of extracellular matrix (ECM) during cancer invasion and   metastasis  , and tissue inhibitors of metalloproteinases (TIMPs) control MMPs, thus maintaining a balanced ECM catabolism under physiological conditions. The aim of this study was to assess the behavior of some MMPs (FASEB J., 7, 1993, 1434; Cancer   Metastasis   Rev., 9(4) 1990, 289) and TIMPs (Biochem. Biophys. Res. Commun., 301, 2003, 1069; FASEB J., 7, 1993, 1434; Nature, 370, 1994, 61). Competitive RT-PCR, gelatin-substrate zymography, and ELISA techniques were used for quantification. The hepatitis B virus (HBV)-DNA-containing hepatocellular carcinoma cell lines, Hep3B, HepG2-HBV and HFF-T2 contain highly activated matrix metallproteinase-9 (MMP-9), which is rarely found in normal liver cell lines such as the Chang lines. MMP-9 activities of HFH-T2, HepG2-HBV and Hep3B were significantly higher than that of non-HBV-hepatocellular carcinoma SK-Hep1 and HepG2 (HCC origin, HBV not detected), as assayed by gelatin zymography. Low levels of TIMP-1 and TIMP-3 were observed in HFH-T2, HepG2-HBV and Hep3B, while the TIMP-2 level was high, as evidenced by reverse zymography. In contrast, 3 TIMP-1, -2 and -3 were largely detected in Chang, HepG2 and SK-Hep1 cells. To investigate the nature of the quantitative regulation of MMPs and TIMPs for these cell lines at the transcriptional levels, a reverse transcriptase-polymerase chain reaction (RT-PCR) was carried out. Not only MMP-9 mRNAs of HFH-T2, HepG2-HBV and Hep3B but also MMP-9 mRNA of SK-Hep1 and HepG2 were highly expressed with no major differences among these four cell lines. However, TIMP-1 and TIMP-3 mRNAs of HFH-T2, HepG2-HBV and Hep3B were markedly reduced, while those of SK-Hep1, HepG2 and Chang cells were maintained at high levels. Finally, an invasion assay using matrigel indicated in an increase in invasiveness in HFH-T2, HepG2-HBV and Hep3B cells, but a decrease in invasiveness of Chang, HepG2 and SK-Hep1 cells. These results indicate that the overexpression of MMP-9 mRNAs and the suppression of TIMP-1 and TIMP-3 in HFH-T2, HepG2-HBV and Hep3B were the result of HBV transfection. Based on these results, it is concluded that HBV affects the malignance of hepatocellular cancer by elevating MMP-9 activity, and suppressing TIMP-1 and TIMP-3. 
 HGF/c-Met overexpressions, but not met mutation, correlates with progression of non-small cell lung cancer. Hepatocyte Growth Factor (HGF) and its receptor c-Met are suggested to play an important role in progression of solid organ tumors by mediating cell motility, invasion and   metastasis  . Overexpression of HGF and c-Met have been shown in non-small-cell lung cancer (NSCLC). However, their role in tumor progression is not clearly defined. The aim of this study is to determine the role of HGF/c-Met pathway and its association with invasion related markers and clinicopathologic parameters in NSCLC. Immunohistochemical analysis was performed on 63 paraffin-embedded NSCLC tumor sections. The expressions of invasion related markers such as Matrix Metalloproteinases (MMPs) 2 and 9, Tissue Inhibitor Metalloproteinase (TIMP) 1 and 3 and RhoA were also examined. Co-expression of HGF/c-Met was significantly associated with lymph node invasion and TIMP-3 and RhoA overexpressions. There were positive correlation between TIMP-3 overexpression and advanced stage and negative correlation between RhoA overexpression and survival. DNA sequencing for Met mutations in both nonkinase and tyrosine kinase (TK) domain was established. A single nucleotide polymorphism (SNP) in sema domain and two SNPs in TK domain of c-Met were found. There was no statistically significant correlation between the presence of c-Met alterations and clinicopathologic parameters except shorter survival time in cases with two SNPs in TK domain. These results suggest that HGF/c-Met might exert their effects in tumor progression in association with RhoA and probably with TIMP-3. The blockade of the HGF/c-Met pathway with RhoA and/or TIMP-3 inhibitors may be an effective therapeutic target for NSCLC treatment. 
 Expression of TIMP-3 gene by construction of a eukaryotic cell expression vector and its role in reduction of   metastasis   in a human breast cancer cell line. The present study is aimed at studying the gene for TIMP-3, a mammalian tissue inhibitor, by constructing a recombinant eukaryotic cell vector for gene therapy in human breast cancer. We obtained the TIMP-3 gene from the human placent by RT-PCR. TIMP-3 gene was subcloned into pcDNA3.1 vetor from pMD18T vector by means of gene cloning to construct pcDNA3.1 recombinant vector. Human breast cancer cell line MDA-MB-453 was transfected with pcDNA3.1-TIMP3 recombinant vector using lipofectamine reagent. Then the expression of TIMP-3 and the effect on the   metastasis   of MDA-MB-453 were examined. The correct construction of pcDNA-TIMP3 was identified by means of restriction enzyme analysis, PCR amplication and nucleotide sequencing. Western blotting showed that the transfected cells were able to express TIMP-3, indicating that our construction of the pcDNA-TIMP3 eukaryotic expression vector was constructed successfully. Our experiments further indicated that the potential of   metastasis   was significantly reduced for the transfected cell line MDA-MB-453. 
 TIMP3 regulates osteosarcoma cell migration, invasion, and chemotherapeutic resistances. Tissue inhibitors of metalloproteinases (TIMPs) inhibit matrix metalloproteinases (MMPs) to limit degradation of the extracellular matrix. Low levels of TIMP3 have been demonstrated in cancer tissues at advanced clinical stages, with positive distant   metastasis   and chemotherapeutic resistance. We examined the role of TIMP3 in osteosarcoma (OS) cell invasiveness and chemoresistance. TIMP3 was overexpressed or knocked down in the human OS cell lines Saos2 and MG63. Cell migration and invasion capacities were then evaluated using Transwell assays, and resistance to cisplatin was assessed by CCK-8 assay and flow cytometry. Real-time PCR and western blotting were used to investigate activation of signaling pathways downstream of TIMP3. Overexpression of TIMP3 inhibited the migration and invasion of Saos2 and MG63 cells, while knockdown of TIMP3 had the opposite effect. Cell survival after exposure to cisplatin was inhibited by TIMP3 overexpression in both Saos2 and MG63 cells. Consistently, downregulation of TIMP3 gene expression significantly decreased the sensitivity of OS cells to cisplatin treatment. MMP1, MMP2, Bcl-2, and Akt1 were all downregulated following TIMP3 overexpression, while Bax and cleaved caspase-3 were upregulated. TIMP3 knockdown had opposite effects on the regulation of these genes. Taken together, our findings suggest TIMP3 as a new target for inhibition of OS progression and chemotherapeutic resistance. 
 Overexpression of TACE and TIMP3 mRNA in head and neck cancer: association with tumour development and progression. TACE/ADAM17 is a transmembranous protease that cleaves membrane-bound growth factors like EGFR ligands. TACE-dependent proteolysis is regulated by its inhibitor, tissue inhibitor of metalloproteinases 3 (TIMP3). This study analyses the role of TACE and TIMP3 mRNA expression in squamous cell carcinomas of the head and neck (HNSCCs).We analysed TACE and TIMP3 mRNA expression in HNSCCs from 106 patients by RNA in situ hybridisation.TACE mRNA was upregulated in HNSCCs compared with dysplastic (P<0.05) and normal epithelia (P<0.001), with strong hybridisation signals in 21.9% of invasive tumour tissues and 4.5% of dysplasia. Elevated mRNA levels were accompanied by increased amounts of TACE protein in HNSCCs. TIMP3 mRNA expression in HNSCC-associated stroma was significantly higher than in the stroma adjacent to dysplastic or normal epithelia. Expression of TACE mRNA in HNSCCs was associated with tumour stage (P=0.019) and regional lymph node   metastasis   (P=0.009). Furthermore, levels of TACE mRNA in HNSCCs correlated with the expression of TIMP3 mRNA in HNSCC-associated stroma. Concomitantly, patients expressing high levels of TACE and TIMP3 mRNA showed significantly reduced overall survival compared with those with low mRNA levels.Our results indicate an important role of TACE and TIMP3 during development and progression of HNSCCs. 
 Gene expression analysis of drug-resistant MCF-7 cells: implications for relation to extracellular matrix proteins. Since multidrug resistance is a multifactorial phenomenon, a large-scale expression analysis of drug-resistant cells by using high-density oligonucleotide microarrays may provide information about new candidate genes contributing to resistance. Extracellular matrix (ECM) is responsible for many aspects of proliferation and invasive/metastatic behavior of tumor cells. This study demonstrates alterations in gene expression levels of several ECM components, matrix metalloproteinases (MMPs), adamalysins (ADAMs and ADAMTSs) and tissue inhibitors of metalloproteinases (TIMPs) in paclitaxel, docetaxel, vincristine and doxorubicin-resistant MCF-7 cells.Resistant MCF-7 cells were developed by stepwise selection of cells in increasing concentrations of drugs. Affymetrix GeneChip Human Genome U133 Plus 2.0 Array was used for hybridizations. Statistical significance was determined by independent sample t test. The genes having altered expression levels in drug-resistant sublines were selected and filtered by volcano plots.Genes up/downregulated more than twofolds were selected and listed. Expression of 25 genes encoding ECM proteins (including collagen, finronectin and syndecan) and integrin receptor subunits were found to be upregulated in drug-resistant cells. In addition, expression levels of, 13 genes encoding MMPs, ADAMs, ADAMTSs and TIMPs (including MMP1, MMP9, ADAM9 and TIMP3) were found to be altered in drug-resistant sublines when compared with sensitive MCF-7.Based on the expression analysis profiles, this report provides a preliminary insight into the relationship between drug resistance and ECM components, which are related to invasion and   metastasis  . Correlation of each specific ECM component with drug resistance requires further analysis. 
 Quantitative detection of TIMP-3 promoter hypermethylation and its prognostic significance in esophageal squamous cell carcinoma. Tissue inhibitor of metalloproteinase-3 (TIMP-3) has an inhibitory effect on tumor development, growth and   metastasis   due to its interaction with matrix metalloproteinases (MMPs). We investigated the prognostic significance of TIMP-3 gene promoter methylation in esophageal squamous cell carcinoma (ESCC). TIMP-3 methylation was analyzed by MethyLight, a quantitative and methylation-specific PCR method. Hypermethylation was found in 9/51 (18%) surgically resected ESCC samples and was associated with reduced disease-free (p=0.0039) and overall survival (p=0.0047). Upon multivariate analysis, it was found to be an independent prognostic parameter for poor survival and was associated with earlier recurrence after surgery (p=0.0238), especially via pleural dissemination (p=0.001). Expression levels for TIMP-3 protein and for several MMPs were analyzed by Western blot analysis in 20 pairs of ESCC and adjacent normal tissue. The expression of MMP-2, -7 and -9 proteins in tumor tissue was significantly higher than in corresponding normal mucosa (p=0.0051, 0.0064 and 0.0004, respectively). In contrast, the expression of TIMP-3 protein in tumor tissue was significantly lower than in matched normal mucosa (p=0.0152). Tumors with TIMP-3 hypermethylation expressed lower levels of TIMP-3 protein compared to those without hypermethylation (p=0.0357). These results demonstrate that TIMP-3 hypermethylation is associated with lower TIMP-3 protein expression in ESCC and with poor patient survival due to a high frequency of recurrence by pleural dissemination. 
 TIMP3 regulates migration, invasion and in vivo tumorigenicity of thyroid tumor cells. Papillary thyroid carcinoma (PTC) arises from the thyroid follicular epithelium and represents the most frequent thyroid malignancy. PTC is associated with gene rearrangements generating RET/PTC and TRK oncogenes, and to the BRAFV600E activating point mutation. A role of tumor-suppressor genes in the pathogenesis of PTC has not been assessed yet. The tissue inhibitor of metalloproteinase-3 (TIMP3) gene, encoding a metalloproteinases inhibitor and capable of inhibiting growth, angiogenesis, invasion and   metastasis   of several cancers, was found to be silenced by promoter methylation in a consistent fraction of PTCs, in association with tumor aggressiveness and BRAFV600E mutation, thus suggesting an oncosuppressor role. To explore this possibility, in this study we performed gene expression and functional studies. Analysis of gene expression data produced in our laboratory as well as meta-analysis of publicly available data sets confirmed the downregulation of TIMP3 gene expression in PTC with respect to normal thyroid. The functional consequences of TIMP3 downregulation were investigated in the PTC-derived NIM1 cell line, in which the expression of TIMP3 is silenced. Restoration of TIMP3 expression by exposure to soluble TIMP3 protein or by complementary DNA transfection had no effect on the growth rate of NIM1 cells. Instead, it affected the adhesive, migratory and invasive capabilities of NIM1 cells by modulating several proteins involved in these processes. A striking effect was observed in vivo, as TIMP3 reduced the tumorigenicity of NIM1 cells by repressing angiogenesis and macrophage infiltration. Our data indicate that the loss of TIMP3 expression exerts a functional role in the pathogenesis of PTC. 
 Association of aberrant methylation of tumor suppressor genes with tumor aggressiveness and BRAF mutation in papillary thyroid cancer. The role of aberrant tumor suppressor gene methylation in the aggressiveness of papillary thyroid cancer (PTC) has not been documented. By showing promoter methylation-induced gene silencing in PTC-derived cell lines, we first demonstrated the functional consequence of methylation of several recently identified tumor suppressor genes, including those for tissue inhibitor of metalloproteinase-3 (TIMP3), SLC5A8, death-associated protein kinase (DAPK) and retinoic acid receptor beta2 (RARbeta2). We then investigated the role of methylation of these genes in the aggressiveness of PTC by examining the relationship of their aberrant methylation to clinicopathological characteristics and BRAF mutation in 231 primary PTC tumors. Methylation of TIMP3, SLC5A8 and DAPK was significantly associated with several aggressive features of PTC, including extrathyroidal invasion, lymph node   metastasis  , multifocality and advanced tumor stages. Methylation of these genes was also significantly associated with BRAF mutation in PTC, either individually or collectively in various combinations. Methylation of these genes, either individually or collectively, occurred more frequently in more aggressive classical and tall-cell PTC subtypes than in less aggressive follicular-variant PTC, with the latter known to infrequently harbor BRAF mutation. Several other tumor suppressor genes investigated were not methylated. These results suggest that aberrant methylation and hence silencing of TIMP3, SLC5A8, DAPK and RARbeta2, in association with BRAF mutation, may be an important step in PTC tumorigenesis and progression. 
 Genetic heterogeneity of breast cancer   metastasis   may be related to miR-21 regulation of TIMP-3 in translation. MicroRNAs are noncoding RNA molecules that posttranscriptionally regulated expression of target gene and implicate the progress of cancer proliferation, differentiation, and apoptosis. The aim of this study is to determine whether microRNA-21 (miR-21), a specific microRNA implicated in multiple aspects of carcinogenesis, promoted breast cancer   metastasis   by regulating the tissue inhibitor of metalloproteinase 3 (TIMP-3) gene.miR-21 of serum and tissue from 40 patients (30 patients with breast cancer) were detected by real-time quantitative reverse transcriptase polymerase chain reaction (RT-qPCR). TIMP-3 of tissue from the patient was tested by real-time RT-qPCR. Protein expression of TIMP-3 was evaluated by western blotting. Correlation analysis was performed between miR-21 and TIMP-3.Of the 40 samples from tissue and serum analyzed, the miR-21 expression was significantly higher in high invasion   metastasis   group (HIMG) that in low invasion   metastasis   group (LIMG); the latter was higher than that in normal group (NG). Additionally, the TIMP-3 expression was significantly lower in HIMG than in LIMG; the latter was lower than that in NG. There was significantly inverse correlation between miR-21 and TIMP-3 extracted from tissue.Our data suggest that miR-21 could promote   metastasis   in breast cancer via the regulation of TIMP3 translation, and there was consistency between miR-21 of serum and miR-21 in tissue. 
 TIMP-3 expression associates with malignant behaviors and predicts favorable survival in HCC. The tissue inhibitors of metalloproteinases (TIMPs) are proteins that specifically inhibit the proteolytic activity of the matrix metalloproteinases (MMPs). TIMP-3, the only member of the TIMPs that can tightly bind to the extracellular matrix, has been identified as a unique tumor suppressor that demonstrates the ability to inhibit tumor angiogenesis, invasion, and   metastasis  . This study aimed to detect the expression of TIMP-3 in hepatocellular carcinoma (HCC) and investigate the association between TIMP-3 expression and its clinicopathological significance in HCC patients. In the current study, reverse transcription-polymerase chain reaction (RT-PCR) and Western blotting of HCC cell lines and one-step quantitative reverse transcription PCR (qPCR) and immunohistochemistry (IHC) analyses in HCC tissues were performed, to characterize the TIMP-3 expression. Kaplan-Meier survival and Cox regression analyses were utilized to evaluate the prognosis of 101 HCC patients. The results showed that the expression of TIMP-3 in HCC was significantly decreased relative to that of non-cancerous cells and tissues. Furthermore, the TIMP-3 expression was statistically associated with malignant behaviors of HCC, including portal vein invasion (p = 0.036) and lymph node   metastasis   (p = 0.030). Cox regression analysis revealed that TIMP-3 expression was an independent prognostic factor for disease-free survival (p = 0.039) and overall survival (p = 0.049). These data indicate that TIMP-3 expression is a valuable prognostic biomarker for HCC and that TIMP-3 expression suggests a favorable prognosis for HCC patients. 
 Methylated TIMP-3 DNA in body fluids is an independent prognostic factor for gastric cancer. Fluid methylated DNA may be a suitable biomarker for cancer patients.To investigate whether circulating methylated tissue inhibitor of metalloproteinase 3 (TIMP-3) DNA in body fluids is a useful prognostic biomarker in gastric cancer (GC).TIMP-3 methylation was detected by real-time methylation-specific polymerase chain reaction in tumor tissues, paired preoperative peritoneal washes (PPWs), and paired serum samples from 92 GC patients.The frequency of TIMP-3 methylation was significantly elevated in GC tissues (63.04%; 58 of 92) compared with that in paired adjacent normal tissue (4.3%; 4 of 92) (P < .001). TIMP-3 methylation correlated closely with peritoneal   metastasis   and TNM stage (all P < .001). The frequency of TIMP-3 methylation in preoperative peritoneal washes and serum samples was 53.3% (49 of 92) and 58.7% (54 of 92), respectively. The Aζ values of the receiver operator characteristic curve for methylated TIMP-3 were 0.966 and 0.922 for serum and preoperative peritoneal washes, respectively, compared with those in GC tissues. The patients with elevated methylated TIMP-3 levels in body fluids had poorer disease-free survival rates than those without (all P < .001). Cox regression analysis showed that detection of methylated TIMP-3 DNA in body fluids was an independent risk factor for GC patients, with a remarkable decrease in disease-free survival 30 months after surgical resection of the gastric tumor.Presence of methylated TIMP-3 DNA in body fluids is a useful biomarker for predicting the progression and prognosis of GC patients. 
 Tissue inhibitor of metalloproteinase-3 (TIMP-3) gene is methylated in the development of esophageal adenocarcinoma: loss of expression correlates with poor prognosis. Amongst involvement in diverse physiological and pathological processes, TIMP-3 may have an important role in tumour development, growth and   metastasis   by interaction with metalloproteases in the extracellular matrix. We studied the role and prognostic effect of TIMP-3 in esophageal adenocarcinoma (EADC). TIMP-3 gene methylation and TIMP-3 mRNA expression were analysed in 5 esophageal cell lines and 24 resected EADCs. TIMP-3 protein expression was examined in the 5 cell lines and 79 resected EADCs with known clinicopathological features. TIMP-3 methylation signal was only detected in the OE33 EADC cell line. In tissues, 0% of case-matched normal, 72% of BE and 90% of EADC were positive for methylation. TIMP-3 mRNA was detected in all the cell lines and normal, metaplastic and tumour tissues. TIMP-3 protein was localised to the cytoplasm in cell lines and tissues. Demethylating treatment of OE33 increased protein expression. At the invading edge of tumours, protein staining was equal to, or reduced, compared to normal tissues. Reduction of protein expression was associated with disease stage (p = 0.046) and poor patient survival (OR 2.1, 95% CI 1.2-3.5, p = 0.007). Mean survival time was halved in patients with reduced tumour TIMP-3 expression, from 49 to 24 months. These studies have demonstrated association between methylation of the TIMP-3 gene and BE and EADC. Reduced expression of TIMP-3 protein in EADC is associated with increased tumour invasiveness and reduced patient survival. 
 Smad2/3/4 Pathway Contributes to TGF-β-Induced MiRNA-181b Expression to Promote Gastric Cancer   Metastasi  s by Targeting Timp3. Transforming growth factor beta (TGF-β) plays a major role in tumorigenesis. MicroRNA-181b (miRNA-181b) is a multifaceted miRNA that has been implicated in many cellular processes such as cell fate determination and cellular invasion. This study aimed to confirm the relationship of miRNA-181b and the TGF-β-Smad2/3/4 pathway with the induction of the epithelial-to-mesenchymal transition (EMT) in gastric cancer.This study investigated the ability of TGF-β to induce migration by wound healing and transwell invasion assays in human gastric cancer cell lines. miRNA expression was altered using miRNA-181b mimic and inhibitor in the same system. Expression of miRNA-181b, the hypothetical target gene Timp3 and EMT-related markers were analyzed by real-time real-time quantitative RT-PCR. Immunoblotting was used to investigate the levels of phospho-Smad2 and Smad4. Dual-luciferase reporter assays were performed to confirm the direct binding of miRNA-181b to Timp3.miRNA-181b was significantly upregulated in response to TGF-β treatment in gastric cancer cell lines. Overexpression of miR-181b mimic induced an in vitro EMT-like change to a phenotype similar to that following TGF-β treatment alone and was reversed by miRNA-181b inhibitor. Inhibition of TGF-β-Smad2/3 signaling with SD-208 significantly attenuated the upregulation of miRNA-181b. Knockdown of Smad4 in gastric cancer cells strongly attenuated the upregulation of miRNA-181b. Moreover, miR-181b was found to directly target the 3' untranslated region (3'UTR) of Timp3 mRNA affecting TGF-β-induced EMT.Our results elucidate a novel mechanism through which the TGF-β pathway regulates the EMT of gastric cancer cells by increasing the levels of miRNA-181b to target Timp3 via the Smad2/3/4-dependent pathway. These findings provide insights into the cellular and environmental factors regulating EMT, which may guide future studies on therapeutic strategies targeting these cells. 
 An immunohistochemical study of TIMP-3 expression in oesophageal squamous cell carcinoma. Tissue inhibitor of metalloproteinase-3 (TIMP-3) inhibits the activity of matrix metalloproteinase, which may play an important role in carcinoma invasion and   metastasis  . We have investigated the relationship between TIMP-3 reduction and clinicopathological factors in oesophageal squamous cell carcinoma (ESCC). We examined tissue specimens that had been removed from 90 patients with thoracic oesophageal cancer who had undergone surgery between 1983 and 2001. Immunohistochemical staining was performed by the standard streptavidin-biotin method. Immunostaining of TIMP-3 was seen in the cytoplasm of cancer cells and normal oesophageal epithelial cells, particularly in cells located in shallow areas of the tumour. TIMP-3 preserved (+), moderate (+/-), and reduced (-) cases accounted for 30, 27, and 33 of the 90 patients, respectively (33, 30, 37%). Significant correlations were observed between TIMP-3 expression and depth of tumour invasion (P=0.001), number of lymph node metastases (P=0.003), infiltrative growth pattern (P=0.003), and disease stage (P=0.005). The survival rates of patients with TIMP-3 (-) cancer were significantly lower than those of patients with TIMP-3 (+) and TIMP-3 (+/-) cancer (P=0.0003). The mean 5-year survival rates of patients with TIMP-3 (+), (+/-), and (-) were 50, 58, and 21%, respectively. In conclusion, decreased expression of TIMP-3 protein correlates with invasive activity and   metastasis  . This makes the prognosis for patients with cancer that has lost TIMP-3 significantly less favourable than that for patients with cancer that has maintained TIMP-3. 
 [Expressions and significance of TIMP-3 and mtp53 in non-small cell lung cancer]. Tissue inhibitor of metalloproteinase-3 (TIMP-3) can regulate tumor infiltration and   metastasis   through multiple channels and is likely associated with mutant-type p53 (mtp53). This study detected the expressions of TIMP-3 and mtp53 in non-small cell lung cancer (NSCLC) and lymph node   metastasis   using tissue microarray and evaluated their significance.TIMP-3 and mtp53 expressions were detected in 288 cases of NSCLC (NSCLC group), 106 cases of metastatic carcinoma in lymph nodes (  metastasis   group), and 24 cases of benign lesions in the bronchial mucosa epithelium (control group) by immunohistochemical staining (LSAB and Elivision).The expression of TIMP-3 in the NSCLC and   metastasis   groups was lower than that in the control group (P<0.001), but the reverse was true for the expression of mtp53 (P<0.001). TIMP-3 and mtp53 expressions differed between NSCLC with (P=0.015) and without (P=0.030) lymph node   metastasis  . TIMP-3 expression correlated with NSCLC grade (P=0.030), whereas mtp53 expression correlated with TNM stage (P=0.016) and NSCLC histological type (P=0.004). Moreover, the expressions of TIMP-3 and mtp53 were negative in NSCLC cases (P=0.008) and correlated with patient survival (P=0.011 and P=0.003, respectively).Low expression of TIMP-3 and high expression of mtp53 in NSCLC can promote tumor   metastasis   and inhibit each other. TIMP-3 and mtp53 are promising targets for studying the metastatic mechanism of NSCLC. 
 MiR-21 enhances melanoma invasiveness via inhibition of tissue inhibitor of metalloproteinases 3 expression: in vivo effects of MiR-21 inhibitor. Metastatic melanoma is the most aggressive form of this cancer. It is important to understand factors that increase or decrease metastatic activity in order to more effectively research and implement treatments for melanoma. Increased cell invasion through the extracellular matrix is required for   metastasis   and is enhanced by matrix metalloproteinases (MMPs). Tissue inhibitor of metalloproteinases 3 (TIMP3) inhibits MMP activity. It was previously shown by our group that miR-21, a potential regulator of TIMP3, is over-expressed in cutaneous melanoma. It was therefore hypothesized that increased levels of miR-21 expression would lead to decreased expression of TIMP3 and thereby enhance the invasiveness of melanoma cells. miR-21 over-expression in the melanoma cell lines WM1552c, WM793b, A375 and MEL 39 was accomplished via transfection with pre-miR-21. Immunoblot analysis of miR-21-overexpressing cell lines revealed reduced expression of TIMP3 as compared to controls. This in turn led to a significant increase in the invasiveness of the radial growth phase cell line WM1552c and the vertical growth phase cell line WM793b (p < 0.05), but not in the metastatic cell lines A375 or MEL 39. The proliferation and migration of miR-21 over-expressing cell lines was not affected. Reduced expression of TIMP3 was achieved by siRNA knockdown and significantly enhanced invasion of melanoma cell lines, mimicking the effects of miR-21 over-expression. Treatment of tumor cells with a linked nucleic acid antagomir to miR-21 inhibited tumor growth and increased tumor expression of TIMP3 in vivo in 01B74 Athymic NCr-nu/nu mice. Intra-tumoral injections of anti-miR-21 produced similar effects. This data shows that increased expression of miR-21 enhanced the invasive potential of melanoma cell lines through TIMP3 inhibition. Therefore, inhibition of miR-21 in melanoma may reduce melanoma invasiveness. 
 Promoter methylation and expression of TIMP3 gene in gastric cancer. Gastric carcinoma development is a multi-stage process that involves more than one gene. Aberrant changes in DNA methylation are considered as the third mechanism that leads to anti-oncogene inactivation, which plays an essential role in tumor development. In this study, we assessed the relationship among the aberrant methylation of the promoter CpG islands of tissue inhibitor of metalloproteinase 3 (TIMP3) gene, its protein expression, and the clinicopathological features of gastric adenocarcinoma.The methylation status of the promoter CpG islands and the protein expression of TIMP3 gene in tumors and adjacent normal mucosal tissues of 78 patients with gastric adenocarcinoma were detected by methylation-specific PCR (MSP) and immunohistochemistry.The CpG island methylation of TIMP3 was detected in tumor tissues, cancer-adjacent tissues, and lymph nodes with   metastasis  . In increasing order, the hypermethylation frequency of these tissues were 35.9% (28 of 78 non-neoplastic tissues), 85% (17 of 20 early-stage cases), 89.7% (52 of 58 progressive-stage cases), and 100% (78 of 78 metastatic lymph node). A marked difference was found between tumors and non-neoplastic tissues (P<0.05), but no difference existed among the subgroups of tumors (P>0.05). Immunohistochemistry analysis confirmed TIMP3 down-regulation in tumor tissues. The rate of TIMP3 gene expression was 100% in non-neoplastic tissues but apparently decreased to various extents at different stages, i.e., decreased to 30% (6/20) at the early stage, to 3.4% (2/58) at the progressive stage, and to 0% (0/78) in metastatic lymph nodes. Among the 70 tumor tissues with negative TIMP3 expression, 64 (91.4%) were hypermethylated and 6 were unmethylated (8.6%), indicating a significant association between hypermethylation and reduced or negative TIMP3 expression (P<0.01).The hypermethylation of the promoter region in CpG islands is the main mechanism of TIMP3 gene expression and may provide evidence for the molecular diagnosis and stage evaluation of gastric cancer.The virtual slides for this article can be found here: http://www.diagnosticpathology.diagnomx.eu/vs/1756134016954958. 
 MicroRNA-21 regulates breast cancer invasion partly by targeting tissue inhibitor of metalloproteinase 3 expression. MicroRNAs are non-coding RNA molecules that posttranscriptionally regulate expression of target genes and have been implicated in the progress of cancer proliferation, differentiation and apoptosis. The aim of this study was to determine whether microRNA-21 (miR-21), a specific microRNA implicated in multiple aspects of carcinogenesis, impacts breast cancer invasion by regulating the tissue inhibitor of metalloproteinase 3 (TIMP3) gene.miR-21 expression was investigated in 32 matched breast cancer and normal breast tissues, and in four human breast cancer cell lines, by Taqman quantitative real-time PCR. Cell invasive ability was determined by matrigel invasion assay in vitro, in cells transfected with miR-21 or anti-miR-21 oligonucleotides. In addition, the regulation of tissue inhibitor of metalloproteinase 3 (TIMP3) by miR-21 was evaluated by western blotting and luciferase assays.Of the 32 paired samples analyzed, 25 breast cancer tissues displayed overexpression of miR-21 in comparison with matched normal breast epithelium. Additionally, incidence of lymph node   metastasis   closely correlated with miR-21 expression, suggesting a role for miR-21 in   metastasis  . Similarly, each of the four breast cancer cell lines analyzed overexpressed miR-21, to varied levels. Further, cells transfected with miR-21 showed significantly increased matrigel invasion compared with control cells, whereas transfection with anti-miR-21 significantly decreased cell invasion. Evaluation of TIMP3 protein levels, a peptidase involved in extarcellular matrix degredation, inversely correlated with miR-21 expression.As knockdown of miR-21 increased TIMP3 protein expression and luciferase reporter activity, our data suggests that miR-21 could promote invasion in breast cancer cells via its regulation of TIMP3. 
 EZH2 regulates cancer cell migration through repressing TIMP-3 in non-small cell lung cancer. Histone methylations play important roles in human cancer   metastasis  . Enhancer of zeste homolog 2 (EZH2) is a key component of the polycomb repressor complex 2, which is responsible for histone H3K27 methylation. EZH2 is overexpressed in lung cancer and epigenetically silences tumor suppressor genes. Here, we showed that EZH2 was up-regulated in lung cancer and had a positive correlation with pathologic stage, nodal involvement in lung cancer patients. Moreover, overexpression of EZH2 was correlated with reduced tissue inhibitor of metalloproteinase-3 (TIMP-3) expression, which was shown to be negatively associated with tumor   metastasis  . Of note, overall survival time of patients with high EZH2/low TIMP-3 expression was significantly shorter than that of patients with low EZH2/high TIMP-3 (P = 0.031). RNA interfering and pharmacologic inhibition of EZH2 reduced histone H3 lysine 27 tri-methylation level and increased TIMP-3 expression level. Knockdown of EZH2 by siRNA significantly reduced A549 cancer cell migration. In contrast, reduction of TIMP-3 in A549 cells partially rescued EZH2 deficiency-induced loss of cell migration capacity. Taken together, our findings indicate that EZH2 accelerates cancer cell migration, in part, via the repression of TIMP-3 expression, suggesting a potential mechanism by which EZH2 promotes lung cancer progression and   metastasis  . 
 Characterization of gene methylation in human papillomavirus associated-head and neck squamous cell carcinoma. Head and neck squamous cell carcinoma (HNSCC) has become one of the most common forms of cancer worldwide. Hypermethylation-induced silencing of tumor-associated gene has been proposed as an important cofactor in cancer pathology. This paper aimed to characterize the gene methylation patterns in human papillomavirus (HPV) associated-HNSCC. TIMP3 and APC methylation status in neoplastic (N = 92) and non-neoplastic tissues (N = 92) of HNSCC as well as their association with HPV infection were investigated via methylation-specific PCR assays. Results indicated that methylation level of TIMP3 was markedly higher in HPV-positive tumors as compared with HPV-negative tumors. Both TIMP3 and APC methylation were associated with lymph node   metastasis   and higher clinical stage of tumors. Patients with methylation at TIMP3 or APC had worse prognoses as compared to those without these alterations. This is the first study that shows a possible linkage between HPV infection and APC methylation. Methylation patterns of tumor-related genes may contribute to different disease prognosis in HNSCC according to the HPV infection status. 
 Multivalent pseudopeptides targeting cell surface nucleoproteins inhibit cancer cell invasion through tissue inhibitor of metalloproteinases 3 (TIMP-3) release. Blockage of the   metastasis   process remains a significant clinical challenge, requiring innovative therapeutic approaches. For this purpose, molecules that inhibit matrix metalloproteinases activity or induce the expression of their natural inhibitor, the tissue inhibitor of metalloproteinases (TIMPs), are potentially interesting. In a previous study, we have shown that synthetic ligands binding to cell surface nucleolin/nucleophosmin and known as HB 19 for the lead compound and NucAnt 6L (N6L) for the most potent analog, inhibit both tumor growth and angiogenesis. Furthermore, they prevent   metastasis   in a RET transgenic mice model which develops melanoma. Here, we investigated the effect of N6L on the invasion capacity of MDA-MB-435 melanoma cells. Our results show that the multivalent pseudopeptide N6L inhibited Matrigel invasion of MDA-MB-435 cells in a modified Boyden chamber model. This was associated with an increase in TIMP-3 in the cell culture medium without a change in TIMP-3 mRNA expression suggesting its release from cell surface and/or extracellular matrix. This may be explained by our demonstrated N6L interaction with sulfated glycosaminoglycans and consequently the controlled bioavailability of glycosaminoglycan-bound TIMP-3. The implication of TIMP-3 in N6L-induced inhibition of cell invasion was evidenced by siRNA silencing experiments showing that the loss of TIMP-3 expression abrogated the effect of N6L. The inhibition of tumor cell invasion by N6L demonstrated in this study, in addition to its previously established inhibitory effect on tumor growth and angiogenesis, suggests that N6L represents a promising anticancer drug candidate warranting further investigation. 
 Predicting invasive phenotype with CDH1, CDH13, CD44, and TIMP3 gene expression in primary breast cancer. We aimed to determine changes in the expression of the genes CDH1, CDH13, CD44, and TIMP3 to look for any relationship between them, HER2 and ESR1 expression at the RNA level, and the histopathological properties of tumors. We also analyzed the expression properties of double-negative (estrogen receptor [ER] and human epidermal growth factor receptor [HER2] both negative) breast tumors. Expression status was studied in fresh tissue at the mRNA level with quantitative PCR using hydrolysis probes. Sixty-two cancer patients and four normal controls were included in the study. When the tumor group was analyzed as a whole, the correlations of ESR1 with CDH1, CDH13, and TIMP3 were P < 0.05, P < 0.005, and P < 0.005, respectively. In ER-positive tumors, CDH1 and CDH13 were correlated directly (P < 0.005) when HER2 was correlated with CDH1, CDH13, and TIMP3 indirectly (P < 0.005, P < 0.005, and P < 0.05, respectively). CDH1 and CD44 had a strong indirect correlation (P < 0.005) in ER-negative tumors. There were significant differences in the expression levels of the CDH13, TIMP3, and CD44 genes (P < 0.005, P < 0.005, and P < 0.05, respectively) between the ER-positive and -negative groups. All four genes were found to be correlated with invasive properties in both ER-positive and -negative tumors. In double-negative tumor samples, only CD44 had a significant and strong correlation with stage, lymph node involvement, and   metastasis   (P < 0.05, P < 0.005, and P < 0.05, respectively). As a conclusion, a decrease in CDH1, CDH13, and TIMP3 expression levels with an increase in CD44 can be used as an indicator for invasion in both ER-positive and -negative breast tumors. In double-negative tumor tissues, CD44 can be considered a marker for aggressive properties. 
 Clinicopathologic significance of BAG1 and TIMP3 expression in colon carcinoma. To explore the expression of BAG1 and tissue inhibitor of metalloproteinase 3 (TIMP3) in colon carcinoma and their correlation and clinicopathologic significance.SABC immunohistochemistry was used to detect the expression of BAG1 and TIMP3 in 80 colon carcinoma tissues and 20 normal colonic mucosa.Positive rate of BAG1 in colon carcinoma tissue (80%) was notably higher compared to normal colonic mucosa (10%) (P < 0.05). However, no significant difference was observed in positive rate of TIMP3 in colon carcinoma tissue (43.75%) as compared with normal colonic mucosa (60%) (P > 0.05). Expression of BAG1 and TIMP3 was strongly associated with colon carcinoma differentiation, Duke's staging, lymph node   metastasis   and survival rate (P < 0.05), but not associated with gender and age. Moreover, BAG1 expression was not correlated with TIMP3.Our results suggest that over-expression of BAG1 or attenuated expression of TIMP3 may play an important role in genesis and development of colon carcinoma. The protein expression levels of BAG1 and TIMP3 are related to the malignant degree, infiltration and   metastasis   of colon carcinoma. BAG1 and TIMP3 might be new biological parameters in predicting invasion and   metastasis   of colon carcinoma. 
 Expression profiling reveals that methylation of TIMP3 is involved in uveal melanoma development. Uveal melanoma is associated with a high tumor-related mortality due to the propensity to develop metastases. The mechanisms that are responsible for malignant dissemination are largely unknown and need to be explored to facilitate diagnosis and treatment of metastases. To identify molecules involved in dissemination, we used cell lines derived from a primary uveal melanoma and 2 liver metastases from the same patient as a model for tumor progression. Using a microarray representing 1,176 genes, we identified 63 differentially expressed genes. Forty genes showed a higher expression and 23 showed a lower expression in the primary cell line compared to the   metastasis   cell lines. These genes are involved in processes like angiogenesis, apoptosis, macrophage stimulation, and extracellular matrix regulation. In contrast, the 2 liver   metastasis   cell lines produced nearly identical expression profiles. Demethylation of the primary melanoma cell line by 5-aza-2'deoxycytidine treatment revealed that 19 genes were suppressed by hypermethylation. An important finding was the 5-fold decreased expression of TIMP3 in the metastatic cell lines, a molecule involved in extracellular matrix degradation. We demonstrate in the cell lines that TIMP3 expression is regulated by methylation. These observations were confirmed in primary uveal melanoma and suggests a role for TIMP3 in uveal melanoma development. 
 Expression of ADAMs (a disintegrin and metalloproteases) and TIMP-3 (tissue inhibitor of metalloproteinase-3) in human prostatic adenocarcinomas. A disintegrin and metalloproteases (ADAMs) are proteins that contain both a disintegrin and metalloprotease domain and have potential implications for the   metastasis   of human cancer cells via cell adhesion and protease activities. In this study, we analyzed the expression levels of ADAM-9, ADAM-10 and ADAM-17 (TNF-alpha converting enzyme, TACE), and TIMP-3 (tissue inhibitor of metalloproteinase-3) in human prostatic tumor cell lines as well as in clinical patient materials (BPH and tumor tissue samples). Human prostatic tumor cell lines (MDA PCa 2b, LNCaP-C33, -C51, -C81, -Pro5, -Ln3, -C4-2, PC3, and DU145) showed varied levels of expression for ADAM-9, -10 and -17 mRNA. A strong expression of ADAM-17/TACE was further revealed by Western blot analysis in prostatic tumor cell lines. In the case of clinical material, all the tumor samples (8/8) revealed the expression of ADAM-9, -10, and -17 compared to the specimens of benign prostatic hyperplasia (BPH) where 80% of (8/10) samples showed the expression of ADAM-9, 86% (6/7) of ADAM-10, and 30% (3/10) of ADAM-17/TACE. Interestingly, expression of a potent inhibitor of ADAM-17 (TIMP-3) was not detected in any prostatic tumor cell lines (0/9), while TIMP-3 expression was detected in 82% (9/11) of BPH samples. Androgen-sensitive LNCaP-C33 cells exhibited differences in ADAMs regulation by 5alpha-dihydrotestosterone (DHT), while such differences were not detectable in androgen-independent LNCaP-C81 cells. These results suggest that an inverse expression pattern of ADAM-17/TACE and TIMP-3, and the regulation of ADAMs with DHT might play an important role in the pathogenesis of prostate cancer. 
 MicroRNA-191 correlates with poor prognosis of colorectal carcinoma and plays multiple roles by targeting tissue inhibitor of metalloprotease 3. MicroRNA-191 (miR-191) is reported to be overexpressed in colorectal carcinoma (CRC), but the role of miR-191 in CRC progress remained unclear. This study demonstrated that High miR-191 expression was associated with clinical stage, lymph node   metastasis  , liver   metastasis   and depth of tumor invasion. Kaplan-Meier analysis indicated that patients with high miR-191 expression had a poor overall survival. Moreover, multivariate analysis showed that miR-191 was an independent prognostic factor in patients with CRC. Furthermore, we found that tissue inhibitor of metalloprotease 3 (TIMP3) was a direct target of miR-191 in colorectal cancer SW620 cells. TIMP3 downregulation mediated by miR-191 activated matrix metalloproteinases (MMPs) and thus promoted invasiveness of cancer cells. Anti-miR-191 could attenuate the invasiveness, suppress proliferation and induce apoptosis by restoring TIMP3 expression. Our results suggested that miR-191 might be a potential diagnostic and therapeutic target in patients with colorectal cancer. 
  GeneRIFs  
 Both TIMP3 and APC methylation were associated with lymph node   metastasis   and higher clinical stage of tumors. Patients with methylation at TIMP3 or APC had worse prognoses as compared to those without these alterations. 
 TIMP-3 expression was associated with malignant behaviors of hepatocellular carcinoma, including portal vein invasion and lymph node   metastasis  . TIMP-3 expression was an independent prognostic factor for disease-free survival and overall survival 
 miR-21 could promote   metastasis   in breast cancer via the regulation of TIMP3 translation 
 The expression of TIMP-3 in the non-small cell lung cancer and   metastasis   groups was downregulated. 
 Decreased expression of TIMP-3 protein correlates with invasive activity and   metastasis   in esophageal squamous cell carcinoma 
 role in reduction of   metastasis   in breast cancer cell line 
  [Cc]ell [Ii]nvasion  PubMed  
 Lysophosphatidic acid downregulates tissue inhibitor of metalloproteinases, which are negatively involved in lysophosphatidic acid-induced   cell invasion  . Ovarian cancer is a highly metastatic disease. Lysophosphatidic acid (LPA) levels are elevated in ascites from ovarian cancer patients, but its potential role in ovarian cancer metastasis has just begun to be revealed. In this work, we show that LPA stimulates invasion of primary ovarian cancer cells, but not ovarian epithelial or borderline ovarian tumor cells, although these benign cells indeed respond to LPA in cell migration. We have found that LPA downregulates tissue inhibitor of metalloproteinases (TIMPs). TIMP2 and TIMP3 play functional role in LPA-induced invasion as negative regulators. G(i) protein, phosphatidylinositol-3 kinase (PI3K), p38 mitogen-activated protein kinase (MAPK), cytosolic phospholipase A(2) and urokinase type plasminogen activator (uPA) are required for LPA-induced cells invasion. TIMP3 may affect two independent downstream targets, vascular endothelial growth factor receptor and p38 MAPK. In vivo, LPA stimulates tumor metastasis in an orthotopic ovarian tumor model, which can be inhibited by a PI3K inhibitor, LY294002. In summary, LPA is likely a key component for promoting ovarian metastasis in vivo. LPA downregulates TIMP3, which may have targets other than metalloproteinases. Our in vivo metastasis mouse model is useful for studying the efficacy of therapeutic regimes of ovarian cancer. 
 Lysophosphatidic acid downregulates tissue inhibitor of metalloproteinases, which are negatively involved in lysophosphatidic acid-induced   cell invasion  . Ovarian cancer is a highly metastatic disease. Lysophosphatidic acid (LPA) levels are elevated in ascites from ovarian cancer patients, but its potential role in ovarian cancer metastasis has just begun to be revealed. In this work, we show that LPA stimulates invasion of primary ovarian cancer cells, but not ovarian epithelial or borderline ovarian tumor cells, although these benign cells indeed respond to LPA in cell migration. We have found that LPA downregulates tissue inhibitor of metalloproteinases (TIMPs). TIMP2 and TIMP3 play functional role in LPA-induced invasion as negative regulators. G(i) protein, phosphatidylinositol-3 kinase (PI3K), p38 mitogen-activated protein kinase (MAPK), cytosolic phospholipase A(2) and urokinase type plasminogen activator (uPA) are required for LPA-induced cells invasion. TIMP3 may affect two independent downstream targets, vascular endothelial growth factor receptor and p38 MAPK. In vivo, LPA stimulates tumor metastasis in an orthotopic ovarian tumor model, which can be inhibited by a PI3K inhibitor, LY294002. In summary, LPA is likely a key component for promoting ovarian metastasis in vivo. LPA downregulates TIMP3, which may have targets other than metalloproteinases. Our in vivo metastasis mouse model is useful for studying the efficacy of therapeutic regimes of ovarian cancer. 
 MiR-21 down-regulation suppresses cell growth, invasion and induces cell apoptosis by targeting FASL, TIMP3, and RECK genes in esophageal carcinoma. miR-21 is overexpressed in esophageal squamous cell carcinoma (ESCC) and is thought to be correlated with the development of the cancer. The target gene of miR-21 including FASL, TIMP3 and RECK is revealed by researchers. miR-21 may be involved in the tumorgenesis of ESCC by targeting FASL, TIMP3 and RECK.The purpose of this study was to explore the mechanism of miR-21 in the development of ESCC.miR-21 expression in ESCC and the matched non-malignant adjacent tissues (NMATs) was examined by qRT-PCR. Cell growth, cell apoptosis and   cell invasion   ability of EC9706 and EC-1 cells was examined after the cells were transfected with miR-21 inhibitor. The potential target genes of miR-21 including FASL, TIMP3 and RECK were examined by western blot and Luciferase reporter assay.miR-21 expression was increased significantly in ESCC tissues compared with NMAT. miR-21 down-regulation inhibits cell growth,   cell invasion   and induces cells to apoptosis. FASL, TIMP3 and RECK are direct targets of miR-21.miR-21 down-regulation inhibits cell growth, invasion and induces cells to apoptosis by targeting FASL, TIMP3 and RECK genes. 
 ADAM17 co-purifies with TIMP-3 and modulates endothelial invasion responses in three-dimensional collagen matrices. In this study, we investigated potential mechanisms through which the known anti-angiogenic factor, tissue inhibitor of metalloproteinase-3 (TIMP-3) blocks angiogenesis. As a strategy to identify TIMP-3 binding proteins, we used tandem affinity purification, employing recombinant adenoviruses constructed to deliver TIMP-3 fused to C-terminal S and His tags (TIMP-3-S-His) or TIMP-1-S-His control to endothelial cells prior to extraction. Western blotting of final eluates revealed robust binding of A Disintegrin and Metalloproteinase (ADAM) 17 and a slight association of ADAM15 to TIMP-3, but not TIMP-1 control. To confirm a functional requirement for ADAM15 and 17 in mediating angiogenic events, a model of endothelial   cell invasion   was utilized. Silencing of ADAM17, but not ADAM15, expression using small interfering RNA (siRNA) interfered with invasion, resulting in decreased density of invading cells and decreased invasion distance. Stable EC lines expressing short hairpin RNA directed to ADAM17 were similarly inhibited. To confirm these results, dominant negative mutants (DeltaMPs) of ADAM10, ADAM15 or ADAM17 were delivered using recombinant lentiviruses. Expression of ADAM17 DeltaMP, but not ADAM10 or ADAM15 DeltaMP, decreased invasion density and distance. Further, time-lapse analyses revealed ADAM17 DeltaMP cells exhibited far greater numbers of protruding sprouts compared to control, suggesting an inability of extended processes to retract properly. Immunofluorescence analyses revealed ADAM17 localized to bifurcations in invading sprouts. These data jointly indicate a role for ADAM17 in modulating endothelial sprouting events during angiogenesis. 
 Loss of TIMP-3 promotes tumor invasion via elevated IL-6 production and predicts poor survival and relapse in HPV-infected non-small cell lung cancer. Human papillomavirus (HPV) 16/18 E6 oncoprotein is expressed in lung tumors and is associated with p53 inactivation. The tissue inhibitor of metalloproteinase 3 (TIMP-3) is essential for limiting inflammation; therefore, we expected that TIMP-3 loss might induce chronic inflammation, thereby promoting tumor malignancy as well as poor survival and relapse in patients with HPV-infected non-small cell lung cancer. In this study, the loss of TIMP-3 by loss of heterozygosity and/or promoter hypermethylation was more frequent in HPV16/18 E6-positive tumors than in E6-negative tumors. To explore the possible underlying mechanism, E6-negative TL4 and CL1-0 cells were transfected with an E6 cDNA plasmid. A marked decrease in TIMP-3 expression was caused by promoter hypermethylation via increased DNA (cytosine-5-)-methyltransferase 1 (DNMT1) expression. Mechanistic studies indicated that TIMP-3 loss promoted interleukin-6 (IL-6) production, which led to   cell invasion   and anchorage-independent growth on soft agar plates. Kaplan-Meier and Cox regression models showed that patients with low-TIMP-3/high-IL-6 tumors had shorter overall survival and relapse-free survival periods when compared with patients with high-TIMP-3/low-IL-6 tumors. In summary, loss of TIMP-3 may increase IL-6 production via the tumor necrosis factor α/nuclear factor κB axis, thereby promoting tumor malignancy and subsequent relapse and poor survival in patients with HPV-infected non-small cell lung cancer. 
 MiR-21 enhances melanoma invasiveness via inhibition of tissue inhibitor of metalloproteinases 3 expression: in vivo effects of MiR-21 inhibitor. Metastatic melanoma is the most aggressive form of this cancer. It is important to understand factors that increase or decrease metastatic activity in order to more effectively research and implement treatments for melanoma. Increased   cell invasion   through the extracellular matrix is required for metastasis and is enhanced by matrix metalloproteinases (MMPs). Tissue inhibitor of metalloproteinases 3 (TIMP3) inhibits MMP activity. It was previously shown by our group that miR-21, a potential regulator of TIMP3, is over-expressed in cutaneous melanoma. It was therefore hypothesized that increased levels of miR-21 expression would lead to decreased expression of TIMP3 and thereby enhance the invasiveness of melanoma cells. miR-21 over-expression in the melanoma cell lines WM1552c, WM793b, A375 and MEL 39 was accomplished via transfection with pre-miR-21. Immunoblot analysis of miR-21-overexpressing cell lines revealed reduced expression of TIMP3 as compared to controls. This in turn led to a significant increase in the invasiveness of the radial growth phase cell line WM1552c and the vertical growth phase cell line WM793b (p < 0.05), but not in the metastatic cell lines A375 or MEL 39. The proliferation and migration of miR-21 over-expressing cell lines was not affected. Reduced expression of TIMP3 was achieved by siRNA knockdown and significantly enhanced invasion of melanoma cell lines, mimicking the effects of miR-21 over-expression. Treatment of tumor cells with a linked nucleic acid antagomir to miR-21 inhibited tumor growth and increased tumor expression of TIMP3 in vivo in 01B74 Athymic NCr-nu/nu mice. Intra-tumoral injections of anti-miR-21 produced similar effects. This data shows that increased expression of miR-21 enhanced the invasive potential of melanoma cell lines through TIMP3 inhibition. Therefore, inhibition of miR-21 in melanoma may reduce melanoma invasiveness. 
 MicroRNA-21 regulates breast cancer invasion partly by targeting tissue inhibitor of metalloproteinase 3 expression. MicroRNAs are non-coding RNA molecules that posttranscriptionally regulate expression of target genes and have been implicated in the progress of cancer proliferation, differentiation and apoptosis. The aim of this study was to determine whether microRNA-21 (miR-21), a specific microRNA implicated in multiple aspects of carcinogenesis, impacts breast cancer invasion by regulating the tissue inhibitor of metalloproteinase 3 (TIMP3) gene.miR-21 expression was investigated in 32 matched breast cancer and normal breast tissues, and in four human breast cancer cell lines, by Taqman quantitative real-time PCR. Cell invasive ability was determined by matrigel invasion assay in vitro, in cells transfected with miR-21 or anti-miR-21 oligonucleotides. In addition, the regulation of tissue inhibitor of metalloproteinase 3 (TIMP3) by miR-21 was evaluated by western blotting and luciferase assays.Of the 32 paired samples analyzed, 25 breast cancer tissues displayed overexpression of miR-21 in comparison with matched normal breast epithelium. Additionally, incidence of lymph node metastasis closely correlated with miR-21 expression, suggesting a role for miR-21 in metastasis. Similarly, each of the four breast cancer cell lines analyzed overexpressed miR-21, to varied levels. Further, cells transfected with miR-21 showed significantly increased matrigel invasion compared with control cells, whereas transfection with anti-miR-21 significantly decreased   cell invasion  . Evaluation of TIMP3 protein levels, a peptidase involved in extarcellular matrix degredation, inversely correlated with miR-21 expression.As knockdown of miR-21 increased TIMP3 protein expression and luciferase reporter activity, our data suggests that miR-21 could promote invasion in breast cancer cells via its regulation of TIMP3. 
 Low tissue inhibitor of metalloproteinases 3 and high matrix metalloproteinase 14 levels defines a subpopulation of highly invasive foam-cell macrophages. An excess of metalloproteinases (MMPs) over tissue inhibitors of metalloproteinases (TIMPs) may favor atherosclerotic plaque rupture. We compared TIMP levels in nonfoamy and foam-cell macrophages (FCM) generated in vivo.In vivo generated rabbit FCM exhibited 84% reduced TIMP-3 protein compared to nonfoamy macrophages, and immunocytochemistry revealed a TIMP-3 negative subset (28%). Strikingly, only TIMP-3 negative FCM invaded a synthetic basement membrane, and invasion was inhibited by exogenous TIMP-3. TIMP-3 negative FCM also had increased proliferation and apoptosis rates compared to TIMP-3 positive cells, which were retarded by exogenous TIMP-3; this also reduced gelatinolytic activity. TIMP-3 negative FCM were found at the base of advanced rabbit plaques and in the rupture-prone shoulders of human plaques. To explain the actions of low TIMP-3 we observed a 26-fold increase in MT1-MMP (MMP-14) protein in FCM. Adding an MT1-MMP neutralizing antibody reduced foam-  cell invasion  , apoptosis, and gelatinolytic activity. Furthermore, MT1-MMP overexpressing and TIMP-3 negative FCM were found at the same locations in atherosclerotic plaques.These results demonstrate that TIMP-3 is downregulated in a distinct subpopulation of FCM which have increased MMP-14. These cells are highly invasive and have increased proliferation and apoptosis, all properties expected to destabilise atherosclerotic plaques. 
 Multivalent pseudopeptides targeting cell surface nucleoproteins inhibit cancer   cell invasion   through tissue inhibitor of metalloproteinases 3 (TIMP-3) release. Blockage of the metastasis process remains a significant clinical challenge, requiring innovative therapeutic approaches. For this purpose, molecules that inhibit matrix metalloproteinases activity or induce the expression of their natural inhibitor, the tissue inhibitor of metalloproteinases (TIMPs), are potentially interesting. In a previous study, we have shown that synthetic ligands binding to cell surface nucleolin/nucleophosmin and known as HB 19 for the lead compound and NucAnt 6L (N6L) for the most potent analog, inhibit both tumor growth and angiogenesis. Furthermore, they prevent metastasis in a RET transgenic mice model which develops melanoma. Here, we investigated the effect of N6L on the invasion capacity of MDA-MB-435 melanoma cells. Our results show that the multivalent pseudopeptide N6L inhibited Matrigel invasion of MDA-MB-435 cells in a modified Boyden chamber model. This was associated with an increase in TIMP-3 in the cell culture medium without a change in TIMP-3 mRNA expression suggesting its release from cell surface and/or extracellular matrix. This may be explained by our demonstrated N6L interaction with sulfated glycosaminoglycans and consequently the controlled bioavailability of glycosaminoglycan-bound TIMP-3. The implication of TIMP-3 in N6L-induced inhibition of   cell invasion   was evidenced by siRNA silencing experiments showing that the loss of TIMP-3 expression abrogated the effect of N6L. The inhibition of tumor   cell invasion   by N6L demonstrated in this study, in addition to its previously established inhibitory effect on tumor growth and angiogenesis, suggests that N6L represents a promising anticancer drug candidate warranting further investigation. 
  GeneRIFs  
 Multivalent pseudopeptides targeting cell surface nucleoproteins inhibit cancer   cell invasion   through tissue inhibitor of metalloproteinases 3 (TIMP-3) release. 
   
  
 CASP7 
 
  NCBI Gene  
  GeneRIF  
  Pubmed  
 
 
  Search Term  Rank  Fields  
  [Mm]igration  6  PubMed(2)  
  [Ee]xtracellular [Mm]atrix  8  PubMed(1)  
  [Ii]nvasive  10  PubMed(2); GeneRIFs(1)  
  [Mm]etastasis  12  PubMed(2)  
 
 
 hide/show details for CASP7 
  [Mm]igration  PubMed  
 Differential regulation of human YY1 and caspase 7 promoters by prohibitin through E2F1 and p53 binding sites. Prohibitin is a 30 kDa growth suppressive protein that has pleiotropic functions in the cell. Although prohibitin has been demonstrated to have potent transcriptional regulatory functions, it has also been proposed to facilitate protein folding in the mitochondria and promote cell   migration   in association with Raf-1. Our previous studies have shown that prohibitin physically interacts with the marked-box domain of E2F family members and represses their transcriptional activity; in contrast, prohibitin could bind to and enhance the transcriptional activity of p53. Here, we show that promoters of human YY1 (Yin and Yang 1) as well as caspase 7 genes are modulated by prohibitin. YY1 promoter activity was reduced upon overexpression of prohibitin, while it was enhanced when prohibitin was depleted by small interfering RNA techniques. The repressive effects of prohibitin on the YY1 promoter were mediated through E2F binding sites, as seen by mutational analysis and chromatin immunoprecipitation assays. Further, depletion of E2F1 prevented prohibitin from repressing the YY1 promoter. In contrast with YY1, prohibitin overexpression led to enhanced levels of caspase 7, whereas depletion of prohibitin reduced it. Interestingly, the caspase 7 promoter was found to have p53-binding sites and prohibitin activated this promoter through p53. These studies show that prohibitin can have diverse effects on the expression of different genes and the activity of various cellular promoters is affected by prohibitin. Further, it appears very likely that prohibitin carries out many of its cellular functions by affecting the transcription of different genes. 
 MicroRNA-224 is implicated in lung cancer pathogenesis through targeting caspase-3 and caspase-7. We recently reported that miR-224 was significantly up-regulated in non-small cell lung cancer (NSCLC) tissues, in particular in resected NSCLC metastasis. We further demonstrated that miR-224 functions as an oncogene in NSCLC by directly targeting TNFAIP1 and SMAD4. However, the biological functions of miR-224 in NSCLC are controversial and underlying mechanisms of miR-224 in the progression and metastasis of lung cancer remain to be further explored. Here we report that caspase3 (CASP3) and caspase7 (CASP7) are previously unidentified targets of miR-224 in NSCLC, and that miR-224 promotes lung cancer cells proliferation and   migration   in part by directly targeting CASP7 and down-regulating its expression. In addition, miR-224 attenuated TNF-α induced apoptosis by direct targeting of CASP3 resulting in reduction of cleaved PARP1 expression in lung cancer cells. Furthermore, the expression of miR-224 negatively correlates with the expression of CASP7 and CASP3 in tissue samples from patients with lung cancer. Finally, we found that activated NF-κB signaling is involved in the regulation of miR-224 expression in lung cancer. Our study provides new insight in understanding of oncogenic role of miR-224 in the lung cancer pathogenesis and suggests that NF-κB/miR-224/CASP3, 7 pathway could be a putative therapeutic target in lung cancer. 
  [Ee]xtracellular [Mm]atrix  PubMed  
 Cleavage of focal adhesion kinase by caspases during apoptosis. Apoptotic cells undergo characteristic morphological changes that include detachment of cell attachment from the substratum and loss of cell-cell interactions. Attachment of cells to the   extracellular matrix   and to other cells is mediated by integrins. The interactions of integrins with the   extracellular matrix   activates focal adhesion kinase (FAK) and suppresses apoptosis in diverse cell types. Members of the tumor necrosis family such as Fas and Apo-2L, also known as tumor necrosis factor-related apoptosis-inducing ligand (TRAIL), induce apoptosis in both suspension and adherent cells through the activation of caspases. These caspases, when activated, cleave substrates that are important for the maintenance of nuclear and membrane integrity. In this study, we show that FAK is sequentially cleaved into two different fragments early in Apo-2L-induced apoptosis. We also demonstrate that FAK cleavage is mediated by caspases and that FAK shows unique sensitivity to different caspases. Our results suggest that disruption of FAK may contribute to the morphological changes observed in apoptotic suspension and adherent cells. 
  [Ii]nvasive  PubMed  
 Phosphorylation of caspase-7 by p21-activated protein kinase (PAK) 2 inhibits chemotherapeutic drug-induced apoptosis of breast cancer cell lines. p21-activated kinase (PAK) 2, a member of the PAK family of serine/threonine protein kinases, plays an important role in physiological processes such as motility, survival, mitosis, and apoptosis. However, the role of PAK2 in resistance to chemotherapy is unclear. Here we report that PAK2 is highly expressed in human breast cancer cell lines and human breast   invasive   carcinoma tissue compared with a human non-tumorigenic mammary epithelial cell line and adjacent normal breast tissue, respectively. Interestingly, we found that PAK2 can bind with caspase-7 and phosphorylate caspase-7 at the Ser-30, Thr-173, and Ser-239 sites. Functionally, the phosphorylation of caspase-7 decreases its activity, thereby inhibiting cellular apoptosis. Our data indicate that highly expressed PAK2 mediates chemotherapeutic resistance in human breast   invasive   ductal carcinoma by negatively regulating caspase-7 activity. 
 Tumor necrosis factor-related apoptosis-inducing ligand (TRAIL) and paclitaxel have cooperative in vivo effects against glioblastoma multiforme cells. Tumor necrosis factor-related apoptosis-inducing ligand (TRAIL) in conjunction with microtubule-targeting agents may be a promising novel anticancer treatment strategy. In vitro studies have suggested that relatively low concentrations of TRAIL enhance the lethality of paclitaxel (Taxol) against human cancer cells. The increased efficacy may be due to the triggering of caspase activation, resulting in mitotic checkpoint abrogation and catastrophe. We show here that wild-type p53 protects cells from caspase-dependent death induced by this therapeutic combination in vitro. We have now also developed an imaging-based model system to test the in vivo efficacy of combined TRAIL and Taxol, in which tumor growth and treatment response can be monitored non  invasive  ly and in real-time. We further utilize bioluminescence, F18-fluorodeoxyglucose-positron emission tomography, and microscale computed tomography imaging to confirm the effects of combined treatment on tumors. These studies together provide the first in vivo confirmation that combined TRAIL plus paclitaxel results in better tumor control compared with either TRAIL or paclitaxel alone, and with no discernable increased normal tissue toxicity in the mouse. Interestingly, the in vivo antitumor response elicited by combined treatment was not affected by the p53 status of the tumor cells. These preclinical observations together suggest the therapeutic potential of combining TRAIL plus paclitaxel in cancer treatment, and support further preclinical and future clinical testing. 
  GeneRIFs  
 highly expressed PAK2 mediates chemotherapeutic resistance in human breast   invasive   ductal carcinoma by negatively regulating caspase-7 activity 
  [Mm]etastasis  PubMed  
 MicroRNA-106b-25 cluster expression is associated with early disease recurrence and targets caspase-7 and focal adhesion in human prostate cancer. The miR-106b-25 microRNA (miRNA) cluster is a candidate oncogene in human prostate cancer. Here, we report that miRNAs encoded by miR-106b-25 are upregulated in both primary tumors and distant   metastasis  . Moreover, increased tumor miR-106b expression was associated with disease recurrence and the combination of high miR-106b and low CASP7 (caspase-7) expressions in primary tumors was an independent predictor of early disease recurrence (adjusted hazard ratio=4.1; 95% confidence interval: 1.6-12.3). To identify yet unknown oncogenic functions of miR-106b, we overexpressed it in LNCaP human prostate cancer cells to examine miR-106b-induced global expression changes among protein-coding genes. The approach revealed that CASP7 is a direct target of miR-106b, which was confirmed by western blot analysis and a 3'-untranslated region reporter assay. Moreover, selected phenotypes induced by miR-106b knockdown in DU145 human prostate cancer cells did not develop when both miR-106b and CASP7 expression were inhibited. Further analyses showed that CASP7 is downregulated in primary prostate tumors and metastatic lesions across multiple data sets and is by itself associated with disease recurrence and disease-specific survival. Using bioinformatics, we also observed that miR-106b-25 may specifically influence focal adhesion-related pathways. This observation was experimentally examined using miR-106b-25-transduced 22Rv1 human prostate cancer cells. After infection with a miR-106b-25 lentiviral expression construct, 22Rv1 cells showed increased adhesion to basement membrane- and bone matrix-related filaments and enhanced soft agar growth. In summary, miR-106b-25 was found to be associated with prostate cancer progression and disease outcome and may do so by altering apoptosis- and focal adhesion-related pathways. 
 MicroRNA-224 is implicated in lung cancer pathogenesis through targeting caspase-3 and caspase-7. We recently reported that miR-224 was significantly up-regulated in non-small cell lung cancer (NSCLC) tissues, in particular in resected NSCLC   metastasis  . We further demonstrated that miR-224 functions as an oncogene in NSCLC by directly targeting TNFAIP1 and SMAD4. However, the biological functions of miR-224 in NSCLC are controversial and underlying mechanisms of miR-224 in the progression and   metastasis   of lung cancer remain to be further explored. Here we report that caspase3 (CASP3) and caspase7 (CASP7) are previously unidentified targets of miR-224 in NSCLC, and that miR-224 promotes lung cancer cells proliferation and migration in part by directly targeting CASP7 and down-regulating its expression. In addition, miR-224 attenuated TNF-α induced apoptosis by direct targeting of CASP3 resulting in reduction of cleaved PARP1 expression in lung cancer cells. Furthermore, the expression of miR-224 negatively correlates with the expression of CASP7 and CASP3 in tissue samples from patients with lung cancer. Finally, we found that activated NF-κB signaling is involved in the regulation of miR-224 expression in lung cancer. Our study provides new insight in understanding of oncogenic role of miR-224 in the lung cancer pathogenesis and suggests that NF-κB/miR-224/CASP3, 7 pathway could be a putative therapeutic target in lung cancer. 
   
  
 HECA 
 
  NCBI Gene  
  GeneRIF  
  Pubmed  
 
 
  Search Term  Rank  Fields  
  [Mm]igration  6  PubMed(1)  
 
 
 hide/show details for HECA 
  [Mm]igration  PubMed  
 The Human Homolog of Drosophila Headcase Acts as a Tumor Suppressor through Its Blocking Effect on the Cell Cycle in Hepatocellular Carcinoma. The molecular pathogenesis of hepatocellular carcinoma (HCC) is heterogeneous and extremely complex. Thus, for individual molecular targeted therapy, novel molecular markers are needed. The abnormal expression of the human homolog of Drosophila headcase (HECA homo) has been found in pancreatic, colorectal, and oral squamous cell carcinoma. Studies of oral squamous cell carcinoma have also demonstrated that the HECA homo protein can be negatively controlled by the Wnt-pathway and transcription factor 4 (TCF4) and can slow cell division by interacting with cyclins and CDKs. However, the role of HECA in HCC has not been reported elsewhere. Here, immunohistochemical analysis revealed that the downregulation of HECA homo protein occurred in 71.0% (66/93) of HCC cases and was positively correlated with a poorly differentiated grade, high serum AFP level, liver cirrhosis and large tumor size. The expression of HECA homo was detected in five live cell lines. In vitro, the overexpression of HECA homo in HepG2, Huh-7 and MHCC-97H cells could inhibit cell proliferation and colony formation and induce G1 phase arrest. In contrast, the downregulation of HECA homo could promote cell proliferation, colony formation and the cell cycle process. However, neither the overexpression nor downregulation of HECA homo in the three cell lines could affect cell   migration   or invasion. Collectively, HECA homo is regularly expressed in normal live cells, and the HECA homo protein level is heterogeneously altered in HCC, but the downregulation of HECA homo is more common and positively correlated with several malignant phenotypes. The HECA homo protein can slow cell proliferation to some extent primarily through its blocking effect on the cell cycle. Hence, the HECA homo protein may act as a tumor suppressor in HCC and might be a potential molecular marker for diagnostic classification and targeted therapy in HCC. 
   
  
 SLC30A1 
 
  NCBI Gene  
  GeneRIF  
  Pubmed  
 
 
  Search Term  Rank  Fields  
  [Ii]nvasive  10  PubMed(2)  
 
 
 hide/show details for SLC30A1 
  [Ii]nvasive  PubMed  
 Genetic susceptibility to distinct bladder cancer subphenotypes. Clinical, pathologic, and molecular evidence indicate that bladder cancer is heterogeneous with pathologic/molecular features that define distinct subphenotypes with different prognoses. It is conceivable that specific patterns of genetic susceptibility are associated with particular subphenotypes.To examine evidence for the contribution of germline genetic variation to bladder cancer heterogeneity.The Spanish Bladder Cancer/EPICURO Study is a case-control study based in 18 hospitals located in five areas in Spain. Cases were patients with a newly diagnosed, histologically confirmed, urothelial cell carcinoma of the bladder from 1998 to 2001. Case diagnoses were reviewed and uniformly classified by pathologists following the World Health Organisation/International Society of Urological Pathology 1999 criteria. Controls were hospital-matched patients (n=1149).A total of 1526 candidate variants in 423 candidate genes were analysed. Three distinct subphenotypes were defined according to stage and grade: low-grade nonmuscle   invasive   (n=586), high-grade nonmuscle   invasive   (n=219), and muscle   invasive   (n=246). The association between each variant and subphenotype was assessed by polytomous risk models adjusting for potential confounders. Heterogeneity in genetic susceptibility among subphenotypes was also tested.Two established bladder cancer susceptibility genotypes, NAT2 slow-acetylation and GSTM1-null, exhibited similar associations among the subphenotypes, as did VEGF-rs25648, which was previously identified in our study. Other variants conferred risks for specific tumour subphenotypes such as PMS2-rs6463524 and CD4-rs3213427 (respective heterogeneity p values of 0.006 and 0.004), which were associated with muscle-  invasive   tumours (per-allele odds ratios [95% confidence interval] of 0.56 [0.41-0.77] and 0.71 [0.57-0.88], respectively) but not with non-muscle-  invasive   tumours. Heterogeneity p values were not robust in multiple testing according to their false-discovery rate.These exploratory analyses suggest that genetic susceptibility loci might be related to the molecular/pathologic diversity of bladder cancer. Validation through large-scale replication studies and the study of additional genes and single nucleotide polymorphisms are required. 
 Genetic susceptibility to distinct bladder cancer subphenotypes. Clinical, pathologic, and molecular evidence indicate that bladder cancer is heterogeneous with pathologic/molecular features that define distinct subphenotypes with different prognoses. It is conceivable that specific patterns of genetic susceptibility are associated with particular subphenotypes.To examine evidence for the contribution of germline genetic variation to bladder cancer heterogeneity.The Spanish Bladder Cancer/EPICURO Study is a case-control study based in 18 hospitals located in five areas in Spain. Cases were patients with a newly diagnosed, histologically confirmed, urothelial cell carcinoma of the bladder from 1998 to 2001. Case diagnoses were reviewed and uniformly classified by pathologists following the World Health Organisation/International Society of Urological Pathology 1999 criteria. Controls were hospital-matched patients (n=1149).A total of 1526 candidate variants in 423 candidate genes were analysed. Three distinct subphenotypes were defined according to stage and grade: low-grade nonmuscle   invasive   (n=586), high-grade nonmuscle   invasive   (n=219), and muscle   invasive   (n=246). The association between each variant and subphenotype was assessed by polytomous risk models adjusting for potential confounders. Heterogeneity in genetic susceptibility among subphenotypes was also tested.Two established bladder cancer susceptibility genotypes, NAT2 slow-acetylation and GSTM1-null, exhibited similar associations among the subphenotypes, as did VEGF-rs25648, which was previously identified in our study. Other variants conferred risks for specific tumour subphenotypes such as PMS2-rs6463524 and CD4-rs3213427 (respective heterogeneity p values of 0.006 and 0.004), which were associated with muscle-  invasive   tumours (per-allele odds ratios [95% confidence interval] of 0.56 [0.41-0.77] and 0.71 [0.57-0.88], respectively) but not with non-muscle-  invasive   tumours. Heterogeneity p values were not robust in multiple testing according to their false-discovery rate.These exploratory analyses suggest that genetic susceptibility loci might be related to the molecular/pathologic diversity of bladder cancer. Validation through large-scale replication studies and the study of additional genes and single nucleotide polymorphisms are required. 
   
  
 GRINA 
 
  NCBI Gene  
  GeneRIF  
  Pubmed  
 
 
  Search Term  Rank  Fields  
  [Mm]etastasis  12  PubMed(1); GeneRIFs(1)  
 
 
 hide/show details for GRINA 
  [Mm]etastasis  PubMed  
 Transmembrane BAX inhibitor motif containing (TMBIM) family proteins perturbs a trans-Golgi network enzyme, Gb3 synthase, and reduces Gb3 biosynthesis. Globotriaosylceramide (Gb3) is a well known receptor for Shiga toxin (Stx), produced by enterohemorrhagic Escherichia coli and Shigella dysenteriae. The expression of Gb3 also affects several diseases, including cancer   metastasis   and Fabry disease, which prompted us to look for factors involved in its metabolism. In the present study, we isolated two cDNAs that conferred resistance to Stx-induced cell death in HeLa cells by expression cloning: ganglioside GM3 synthase and the COOH terminus region of glutamate receptor, ionotropic, N-methyl-D-asparate-associated protein 1 (GRINA), a member of the transmembrane BAX inhibitor motif containing (TMBIM) family. Overexpression of the truncated form, named GRINA-C, and some members of the full-length TMBIM family, including FAS inhibitory molecule 2 (FAIM2), reduced Gb3, and lactosylceramide was accumulated instead. The change of glycolipid composition was restored by overexpression of Gb3 synthase, suggesting that the synthase is affected by GRINA-C and FAIM2. Interestingly, the mRNA level of Gb3 synthase was unchanged. Rather, localization of the synthase as well as TGN46, a trans-Golgi network marker, was perturbed to form punctate structures, and degradation of the synthase in lysosomes was enhanced. Furthermore, GRINA-C was associated with Gb3 synthase. These observations may demonstrate a new type of posttranscriptional regulation of glycosyltransferases. 
  GeneRIFs  
 The tmbim3 may participate in cell death regulation by interacting with proteins of Bcl-2 family, promoting tumor   metastasis  , which is deduced from the evolutionary conservation of the membrane protein family containing multiple membrane spanning segments 
   
  
 KCNN2 
 
  NCBI Gene  
  GeneRIF  
  Pubmed  
 
 
  Search Term  Rank  Fields  
  [Mm]etastasis  12  PubMed(1)  
 
 
 hide/show details for KCNN2 
  [Mm]etastasis  PubMed  
 Ca2+-activated K+ channels in human melanoma cells are up-regulated by hypoxia involving hypoxia-inducible factor-1alpha and the von Hippel-Lindau protein. Under chronic hypoxia, tumour cells undergo adaptive changes involving hypoxia-inducible factors (HIFs). Here we report that ion currents mediated by Ca2+-activated K+ (K(Ca)) channels in human melanoma IGR1 cells are increased by chronic hypoxia (3% O2), as well as by hypoxia mimetics. This increase involves the HIF system as confirmed by overexpression of HIF-1alpha or the von Hippel-Lindau tumour suppressor gene. Under normoxic conditions the K(Ca) channels in IGR1 cells showed pharmacological characteristics of intermediate conductance K(Ca) subtype IK channels, whereas the subtype SK2 channels were up-regulated under hypoxia, shown with pharmacological tools and with mRNA analysis. Hypoxia increased cell proliferation, but the K(Ca) channel blockers apamin and charybdotoxin slowed down cell growth, particularly under hypoxic conditions. Similar results were obtained for the cell line IGR39 and for acutely isolated cells from a biopsy of a melanoma   metastasis  . Thus, up-regulation of K(Ca) channels may be a novel mechanism by which HIFs can contribute to the malignant phenotype of human tumour cells. 
   
  
 CNTN6 
 
  NCBI Gene  
  GeneRIF  
  Pubmed  
 
 
  Search Term  Rank  Fields  
  [Mm]igration  6  PubMed(1)  
 
 
 hide/show details for CNTN6 
  [Mm]igration  PubMed  
 GWAS identifies novel SLE susceptibility genes and explains the association of the HLA region. In a genome-wide association study (GWAS) of individuals of European ancestry afflicted with systemic lupus erythematosus (SLE) the extensive utilization of imputation, step-wise multiple regression, lasso regularization and increasing study power by utilizing false discovery rate instead of a Bonferroni multiple test correction enabled us to identify 13 novel non-human leukocyte antigen (HLA) genes and confirmed the association of four genes previously reported to be associated. Novel genes associated with SLE susceptibility included two transcription factors (EHF and MED1), two components of the NF-κB pathway (RASSF2 and RNF114), one gene involved in adhesion and endothelial   migratio  n (CNTN6) and two genes involved in antigen presentation (BIN1 and SEC61G). In addition, the strongly significant association of multiple single-nucleotide polymorphisms (SNPs) in the HLA region was assigned to HLA alleles and serotypes and deconvoluted into four primary signals. The novel SLE-associated genes point to new directions for both the diagnosis and treatment of this debilitating autoimmune disease. 
   
  
 CNTN4 
 
  NCBI Gene  
  GeneRIF  
  Pubmed  
 
 
  Search Term  Rank  Fields  
  [Mm]etastasis  12  PubMed(1)  
 
 
 hide/show details for CNTN4 
  [Mm]etastasis  PubMed  
 A genome-wide association study identifies SNP in DCC is associated with gallbladder cancer in the Japanese population. Gallbladder cancer (GC) is a relatively uncommon cancer with higher incidence in certain areas including Japan. Because of the difficulty in diagnosis, prognosis of GC is very poor. To identify genetic determinants of GC, we conducted a genome-wide association study (GWAS) in 41 GC patients and 866 controls. Association between each single-nucleotide polymorphism (SNP) with GC susceptibility was evaluated by multivariate logistic regression analysis conditioned on age and gender of subjects. SNPs that showed suggestive association (P<1 × 10(-4)) with GC were further examined in 30 cases and 898 controls. SNP rs7504990 in the DCC (deleted in colorectal cancer, 18q21.3) that encodes a netrin 1 receptor achieved a combined P-value of 7.46 × 10(-8) (OR=6.95; 95% CI=3.43-14.08). Subsequent imputation analysis identified multiple SNPs with similarly strong associations in an adjacent genomic region, where loss of heterozygosity was reported in GC and other cancers. Reduced expression of DCC was indicated to be associated with the poorly differentiated histological type, increased proliferation an  d metastas  is through loss of adhesiveness. However, due to the limited sample size investigated here, further replication study and functional analysis would be necessary to further confirm the result of the association. 
   
  
 CNTN1 
 
  NCBI Gene  
  GeneRIF  
  Pubmed  
 
 
  Search Term  Rank  Fields  
  [Mm]igration  6  PubMed(4); GeneRIFs(1)  
  ECM  7  PubMed(1)  
  [Ee]xtracellular [Mm]atrix  8  PubMed(5)  
  [Ii]nvasive  10  PubMed(4)  
  [Mm]etastasis  12  PubMed(9); GeneRIFs(4)  
  [Cc]ell [Ii]nvasion  13  PubMed(3)  
 
 
 hide/show details for CNTN1 
  [Mm]igration  PubMed  
 Significances of contactin-1 expression in human gastric cancer and knockdown of contactin-1 expression inhibits invasion and metastasis of MKN45 gastric cancer cells. Contactin-1 (CNTN-1) has been shown to promote cancer metastasis. Previously, we have reported that the expression of CNTN-1 was upregulated in gastric cancer tissues compared with adjacent normal tissues. Here, we investigated the significance of CNTN-1 expression and its underlying mechanism of metastasis mediated by epithelial-mesenchymal transition (EMT) in gastric cancer.The expressions of CNTN-1 and EMT-related proteins were assayed through immunohistochemical staining of pathological specimens from patients with gastric cancer. Other methods including reverse transcriptase polymerase chain reaction, Western blotting, stably transfected against CNTN-1 into MKN45 cells,   migration   and invasion assays in vitro and nude mouse tumorigenicity in vivo were also utilized.The results revealed that CNTN-1 expression was elevated and positively correlated with metastasis, EMT-related markers and poor prognosis in patients with gastric cancer. Moreover, CNTN-1 expression might associate with invasive ability to some extent in gastric cancer cell lines KATO-Ш, SGC7901 and MKN45. Knockdown of CNTN-1 expression in MKN45 cells using short hairpin RNA (shRNA) had notable effects on cell   migratio  n and invasion, rather than proliferation in vitro and in vivo. Furthermore, suppression of CNTN-1 expression altered EMT through inhibition of transcription factor Slug, rather than Snail.Our study demonstrated that the elevated CNTN-1 expression closely correlated with cancer metastasis and patient survival, and its functions seemed to be important in   migratio  n and invasion of gastric cancer cells via EMT alteration probably mediated by inhibition of Slug. CNTN-1 may be a potential therapeutic target for gastric cancer. 
 Contactin is expressed in human astrocytic gliomas and mediates repulsive effects. Contactin is a cell surface adhesion molecule that is normally expressed by neurons and oligodendrocytes. Particularly high levels of contactin are present during brain development. Using subtractive cloning, we identified contactin transcripts as overexpressed in glioblastomas compared with normal brain. We confirmed contactin overexpression in glioblastomas at the protein level, and localized contactin to the surface of glial fibrillary acidic protein (GFAP)-expressing glioblastoma cells. In contrast, normal astrocytes did not express contactin. Analyzing different types of astrocytic tumors, we detected an association between increasing malignancy grade and contactin expression. Functionally, contactin had repellent effects on glioma cells in vitro, as demonstrated by adhesion and   migration   assays. Overexpression of contactin by transfection into glioblastoma cells did not alter the proliferation rate or adhesion to various extracellular matrix proteins as well as adhesion to cells expressing the specific contactin ligand the protein tyrosine phosphatase zeta (PTPzeta). Our findings suggest that contactin has repellent effects on glioma cells to which it is presented as a ligand, but it does not alter the proliferative or adhesive capacities of cells that overexpress the molecule. The repulsive properties of contactin may be a key factor in glioma disaggregation, and may contribute to the diffuse infiltration pattern characteristic of glioma cells in human brain. 
 VEGF-C promotes the development of esophageal cancer via regulating CNTN-1 expression. Vascular endothelial growth factor C (VEGF-C) is a key regulator of angiogenesis and lymphangiogenesis. VEGF-C is also implicated in the development of esophageal cancer. We investigated the mRNA levels of VEGF-C and its receptors in 38 esophageal squamous cell carcinoma specimens (ESCCs) and matched adjacent normal esophageal tissues via real-time PCR. The mRNA levels of VEGF-C, VEGFR-2 and VEGFR-3 were significantly upregulated in ESCCs versus respective side normal tissues. To explore the influence of VEGF-C on esophageal cancer progression, the expression of VEGF-C was manipulated in esophageal cancer cell lines TE-1 and Eca-109. VEGF-C transcription, translation and secretion were significantly enhanced in cells stably transfected with a VEGF-C overexpression vector or attenuated in VEGF-C shRNA-transfected cell lines. In vitro, TE-1 cells stably transfected with a VEGF-C overexpression vector exhibited an increased rate of cell proliferation,   migration   and focus formation, whereas knockdown of VEGF-C inhibited cell proliferation,   migration   and focus formation. Similar results were obtained for Eca-109 cells. VEGF-C mediated biological function through transcription of CNTN-1, which is implicated in tumor invasion and metastasis. The expression of VEGF-C was correlated with that of CNTN-1 and cell proliferation and   migration   induced by VEGF-C were reversed by silencing of CNTN-1. In addition, nude mice inoculated with VEGF-C shRNA-transfected cells exhibited a significantly decreased tumor size in vivo via reduced VEGFR-2 and VEGFR-3 phosphorylation and microvessel formation. VEGF-C upregulation may be involved in esophageal tumor progression. Vector-based RNA interference (RNAi) targeting VEGF-C is a potential therapeutic method for human esophageal carcinoma. 
 [Under hypoxia condition contactin-1 regulates   migration   of MKN45 cells through RhoA pathway]. Recent studies have suggested that contactin-1 has a key role in cancer cell proliferation and   migration  , however the detailed mechanism of this process is still unclear. Here, human gastric cancer cell line MKN45 was employed. It was found that under hypoxia conditions contactin-1 mRNA and protein levels were both up-regulated by HIF-1alpha expression. Furthermore, although hypoxia increased the   migration   rate of MKN45 cells, contactin-1 (CNTN1) shRNA reversed this process. Meanwhile, RhoA V14 and RhoA V14N19 mutation constructs were employed, and it was found that constitutively active form of RhoA reversed the cell   migration   suppression induced by contactin-1 knockdown, while dominant-negative form of RhoA blocked hypoxia induced hyper  migration  . Apart from this, contactin-1 displayed the ability to phosphorylate the RhoA activator p115 RhoGEF. Thus, under hypoxia conditions, elevated HIF-1alpha seems to up-regulate contactin-1 expression and by this activate RhoA and facilitate   migration   of cancer cells. 
  GeneRIFs  
 under hypoxia conditions, elevated HIF-1alpha seems to up-regulate contactin-1 expression and by this activate RhoA and facilitate   migration   of cancer cells. 
  ECM  PubMed  
 Multi-ligand interactions with receptor-like protein tyrosine phosphatase beta: implications for intercellular signaling. Receptor-like protein tyrosine phosphatase beta (RPTP beta) shows structural and functional similarity to cell adhesion molecules (CAMs). It binds to several neuronal CAMs and extracellular matrix (  ECM  ) proteins that combine to form cell-recognition complexes. Here, the authors discuss the implications of such complexes for intercellular signaling, and the regulation of RPTP activity by cell-cell and cell-  ECM   contact. 
  [Ee]xtracellular [Mm]atrix  PubMed  
 Phosphacan short isoform, a novel non-proteoglycan variant of phosphacan/receptor protein tyrosine phosphatase-beta, interacts with neuronal receptors and promotes neurite outgrowth. Phosphacan, one of the principal proteoglycans in the   extracellular matrix   of the central nervous system, is implicated in neuron-glia interactions associated with neuronal differentiation and myelination. We report here the identification of a novel truncated form of phosphacan, phosphacan short isoform (PSI), that corresponds to the N-terminal carbonic anhydrase- and fibronectin type III-like domains and half of the spacer region. The novel cDNA transcript was isolated by screening of a neonatal brain cDNA expression library using a polyclonal antibody raised against phosphacan. Expression of this transcript in vivo was confirmed by Northern blot hybridization. Analysis of brain protein extracts reveals the presence of a 90-kDa glycosylated protein in the phosphate-buffered saline-insoluble 100000 x g fraction that reacts with antisera against both phosphacan and a recombinant PSI protein and that has the predicted N-terminal sequence. This protein is post-translationally modified with oligosaccharides, including the HNK-1 epitope, but, unlike phosphacan, it is not a proteoglycan. The expression of the PSI protein varies during central nervous system development in a fashion similar to that observed for phosphacan, being first detected around embryonic day 16 and then showing a dramatic increase in expression to plateau around the second week post-natal. Both the native and recombinant PSI protein can interact with the Ig cell adhesion molecules, F3/contactin and L1, and in neurite outgrowth assays, the PSI protein can promote outgrowth of cortical neurons when used as a coated substrate. Hence, the identification of this novel isoform of phosphacan/receptor protein tyrosine phosphatase-beta provides a new component in cell-cell and cell-  extracellular matrix   signaling events in which these proteins have been implicated. 
 Multi-ligand interactions with receptor-like protein tyrosine phosphatase beta: implications for intercellular signaling. Receptor-like protein tyrosine phosphatase beta (RPTP beta) shows structural and functional similarity to cell adhesion molecules (CAMs). It binds to several neuronal CAMs and   extracellular matrix   (ECM) proteins that combine to form cell-recognition complexes. Here, the authors discuss the implications of such complexes for intercellular signaling, and the regulation of RPTP activity by cell-cell and cell-ECM contact. 
 Contactin is expressed in human astrocytic gliomas and mediates repulsive effects. Contactin is a cell surface adhesion molecule that is normally expressed by neurons and oligodendrocytes. Particularly high levels of contactin are present during brain development. Using subtractive cloning, we identified contactin transcripts as overexpressed in glioblastomas compared with normal brain. We confirmed contactin overexpression in glioblastomas at the protein level, and localized contactin to the surface of glial fibrillary acidic protein (GFAP)-expressing glioblastoma cells. In contrast, normal astrocytes did not express contactin. Analyzing different types of astrocytic tumors, we detected an association between increasing malignancy grade and contactin expression. Functionally, contactin had repellent effects on glioma cells in vitro, as demonstrated by adhesion and migration assays. Overexpression of contactin by transfection into glioblastoma cells did not alter the proliferation rate or adhesion to various   extracellular matrix   proteins as well as adhesion to cells expressing the specific contactin ligand the protein tyrosine phosphatase zeta (PTPzeta). Our findings suggest that contactin has repellent effects on glioma cells to which it is presented as a ligand, but it does not alter the proliferative or adhesive capacities of cells that overexpress the molecule. The repulsive properties of contactin may be a key factor in glioma disaggregation, and may contribute to the diffuse infiltration pattern characteristic of glioma cells in human brain. 
 Signaling events following the interaction of the neuronal adhesion molecule F3 with the N-terminal domain of tenascin-R. Interaction between the   extracellular matrix   protein tenascin-R and the neuronal adhesion molecule F3 might be involved in the formation of neuronal networks. In this study, the fragment of tenascin-R comprising epithelial growth factor (EGF)-like repeats and the cysteine-rich NH2 terminal stretch (EGF-L), known to be inhibitory for growing neurites and repellent for growth cones, was used to investigate the signaling events following the F3/EGF-L interaction. We addressed this question using an in vitro test with F3-transfected Chinese hamster ovary (CHO) cells that allowed us to measure the kinetics, magnitude and specificity of the repellent effect resulting from the specific F3/EGF-L interaction. We showed that the repellent effect was counteracted by addition of the serine/threonine kinase and -phosphatase modulators (staurosporine, okadaic acid and H7) but not by modulators of tyrosine kinase or -phosphatases. This result indicates that the intracellular signals activated by the repellent effect involve a serine/threonine kinase pathway. Furthermore, the repellent effect of the EGF-L fragment for growth cones of cultured cerebellar neurons was also abolished by the identical modulators of serine/threonine kinase and -phosphatases. The inhibition of neurite outgrowth from hippocampal neurons by EGF-L was abolished in the presence of the serine threonine-kinase inhibitor H7. These results strongly suggest that the F3/tenascin-R interaction through EGF-L involves an intracellular activation of serine/ threonine kinase(s) in all F3-expressing cells tested. 
 Distinct effects of recombinant tenascin-R domains in neuronal cell functions and identification of the domain interacting with the neuronal recognition molecule F3/11. We have identified distinct domains of the rat   extracellular matrix   glycoprotein tenascin-R using recombinant fragments of the molecule that confer neuronal cell functions. In short-term adhesion assays (0.5 h), cerebellar neurons adhered best to the fragment representing the fibrinogen knob (FG), but also the fibronectin type III (FN) repeats 1-2 and 6-8. FG, FN1-2 and FN3-5 were the most repellent fragments for neuronal cell bodies. Neurites and growth cones were strongly repelled from areas coated with fragments containing the cysteine-rich stretch and the EGF-like domains (EGF-L), FN1-2, FN3-5 and FG. Polarization of morphology of hippocampal neurons was exclusively associated with FG, while EGF-L prevented neurite outgrowth altogether. The binding site of the neuronal receptor for tenascin-R, the immunoglobulin superfamily adhesion molecule F3/11, was localized to EGF-L. The combined observations show distinct, but also overlapping functions for the different tenascin-R domains. They further suggest the existence of multiple neuronal tenascin-R receptors which influence the response of neurons to their   extracellular matrix   environment. 
  [Ii]nvasive  PubMed  
 Significances of contactin-1 expression in human gastric cancer and knockdown of contactin-1 expression inhibits invasion and metastasis of MKN45 gastric cancer cells. Contactin-1 (CNTN-1) has been shown to promote cancer metastasis. Previously, we have reported that the expression of CNTN-1 was upregulated in gastric cancer tissues compared with adjacent normal tissues. Here, we investigated the significance of CNTN-1 expression and its underlying mechanism of metastasis mediated by epithelial-mesenchymal transition (EMT) in gastric cancer.The expressions of CNTN-1 and EMT-related proteins were assayed through immunohistochemical staining of pathological specimens from patients with gastric cancer. Other methods including reverse transcriptase polymerase chain reaction, Western blotting, stably transfected against CNTN-1 into MKN45 cells, migration and invasion assays in vitro and nude mouse tumorigenicity in vivo were also utilized.The results revealed that CNTN-1 expression was elevated and positively correlated with metastasis, EMT-related markers and poor prognosis in patients with gastric cancer. Moreover, CNTN-1 expression might associate with   invasive   ability to some extent in gastric cancer cell lines KATO-Ш, SGC7901 and MKN45. Knockdown of CNTN-1 expression in MKN45 cells using short hairpin RNA (shRNA) had notable effects on cell migration and invasion, rather than proliferation in vitro and in vivo. Furthermore, suppression of CNTN-1 expression altered EMT through inhibition of transcription factor Slug, rather than Snail.Our study demonstrated that the elevated CNTN-1 expression closely correlated with cancer metastasis and patient survival, and its functions seemed to be important in migration and invasion of gastric cancer cells via EMT alteration probably mediated by inhibition of Slug. CNTN-1 may be a potential therapeutic target for gastric cancer. 
 Contactin 1 as a potential biomarker promotes cell proliferation and invasion in thyroid cancer. Contactin 1 (CNTN1) as a member of the immunoglobulin superfamily plays important role in the development of nervous system. Recent studies find that elevated CNTN1 can promote the metastasis of cancer. However, the expression and function of CNTN1 in thyroid cancer are still unknown. Here, we firstly find CNTN1 is a new gene which can be regulated by RET/PTC3 (Ret proto-oncogene and Ret-activating protein ELE1) rearrangement gene and the protein level of CNTN1 is increasing in thyroid cancer. Besides this change is positively associated with the TNM stage and tumor size. Moreover, we confirm that knockdown of CNTN1 significantly inhibits the tumor proliferation,   invasive  ness and represses the expression of cyclin D1 (CCND1). In conclusion, CNTN1 will be a potential diagnosis biomarker and therapy target for thyroid cancer. 
 Diversity of contactin mRNA in human brain tumors. In order to address the molecular signature of human glioma, we investigated the polymorphism of 5'UTR of the mRNA of Contactin, an adhesion molecule which plays a role in the   invasive   behavior of these tumors. Contactin mRNA is identified by RT-PCR and a strategy based on rapid amplification of cDNA ends (RACE) reveals different 5'UTRs resulting from both an alternative use of two types of leader exons and a splicing mechanism within the 5'UTR. The spliced exon is an Alu-containing element specific to the primate lineage, thus indicating a recent evolution of regulatory processes specific to the simian line that occurs on this gene. Each 5'UTR exhibits different transcription/translation efficiencies and contains features that allow translation to occur independently of the classic cap-dependent mechanism. These data illustrate the complex regulation of Contactin expression in human brain tumors occurring at both transcriptional and translation levels. The different 5'UTRs are differentially expressed in diverse types of human tumors. Thus, the polymorphism occurring within the non-coding part of the Contactin mRNA reveals new opportunities to explore deregulation that occurs during the oncogenic process. 
 Knockdown of contactin-1 expression suppresses invasion and metastasis of lung adenocarcinoma. Numerous genetic changes are associated with cancer cell metastasis and invasion. In search for key regulators of invasion and metastasis, a panel of lung cancer cell lines with different   invasive   ability was screened. The gene for contactin-1 was found to play an essential role in tumor invasion and metastasis. Suppression of contactin-1 expression abolished the ability of lung adenocarcinoma cells to invade Matrigel in vitro as well as the polymerization of filamentous-actin and the formation of focal adhesion structures. Furthermore, knockdown of contactin-1 resulted in extensive inhibition of tumor metastasis and in increased survival in an animal model. RhoA but not Cdc42 or Rac1 was found to serve a critical role in contactin-1-mediated invasion and metastasis. Contactin-1-specific RNA interference resulted in loss of metastatic and   invasive   capacity in both in vitro and in vivo models. This loss was overturned by constitutive expression of the active form of RhoA. Contactin-1 was differentially expressed in tumor tissues, and its expression correlated with tumor stage, lymph node metastasis, and patient survival. Contactin-1 is proposed to function importantly in the invasion and metastasis of lung adenocarcinoma cells via RhoA-mediated mechanisms. 
  [Mm]etastasis  PubMed  
 Significances of contactin-1 expression in human gastric cancer and knockdown of contactin-1 expression inhibits invasion and   metastasis   of MKN45 gastric cancer cells. Contactin-1 (CNTN-1) has been shown to promote cancer   metastasis  . Previously, we have reported that the expression of CNTN-1 was upregulated in gastric cancer tissues compared with adjacent normal tissues. Here, we investigated the significance of CNTN-1 expression and its underlying mechanism of   metastasis   mediated by epithelial-mesenchymal transition (EMT) in gastric cancer.The expressions of CNTN-1 and EMT-related proteins were assayed through immunohistochemical staining of pathological specimens from patients with gastric cancer. Other methods including reverse transcriptase polymerase chain reaction, Western blotting, stably transfected against CNTN-1 into MKN45 cells, migration and invasion assays in vitro and nude mouse tumorigenicity in vivo were also utilized.The results revealed that CNTN-1 expression was elevated and positively correlated with   metastasis  , EMT-related markers and poor prognosis in patients with gastric cancer. Moreover, CNTN-1 expression might associate with invasive ability to some extent in gastric cancer cell lines KATO-Ш, SGC7901 and MKN45. Knockdown of CNTN-1 expression in MKN45 cells using short hairpin RNA (shRNA) had notable effects on cell migration and invasion, rather than proliferation in vitro and in vivo. Furthermore, suppression of CNTN-1 expression altered EMT through inhibition of transcription factor Slug, rather than Snail.Our study demonstrated that the elevated CNTN-1 expression closely correlated with cancer   metastasi  s and patient survival, and its functions seemed to be important in migration and invasion of gastric cancer cells via EMT alteration probably mediated by inhibition of Slug. CNTN-1 may be a potential therapeutic target for gastric cancer. 
 Increased sensitivity of human lung adenocarcinoma cells to cisplatin associated with downregulated contactin-1. Contactin-1 (CNTN-1), a glycosyl phosphatidylinositol anchor neural cell adhesion molecule (ACAM), is thought to function not only in nervous system development but also in the invasion and   metastasis   of several tumours. To investigate whether CNTN-1 is involved in multidrug resistance (MDR) in lung adenocarcinoma, CNTN-1 expression was compared between MDR human lung adenocarcinoma A549/cisplatin (A549/DDP) cells and its progenitor A549 cells. The comparison showed that CNTN-1 expression in A549/DDP cells was significantly higher than in A549 cells both at the mRNA level and the protein level. In order to confirm the physiological function of the abnormal expression, lentivirus-mediated short hairpin RNA (shRNA) was used to silence CNTN-1. Cell cytotoxicity assay and cell apoptosis assay revealed that silencing CNTN-1 both in A549 cells and in A549/DDP cells not only rendered cells more sensitive to cisplatin than the negative control, but also increased the cisplatin-induced apoptosis.   Metastasis   and invasion assays demonstrated that CNTN-1 knockdown reduced   metastasis   and invasion but did not affect A549 or A549/DDP cell proliferation. To investigate whether the abnormal expression of CNTN-1 is associated with characteristics of patients with non-small cell lung cancer (NSCLC), immunohistochemistry was used to detect CNTN-1 expression in 143 tissue samples from NSCLC patients and the results showed that the degree of CNTN-1 expression positively correlated with lymphatic invasion in patients with lung adenocarcinoma who received adjuvant cisplatin- or carboplatin-based treatment after surgery. Thus, we concluded that CNTN-1 is closely related with MDR of lung adenocarcinoma. Additionally, CNTN-1 is a novel marker to predict chemotherapeutic efficacy of patients with lung adenocarcinoma, especially with regard to cisplatin- or carboplatin-based regimens. 
 Contactin 1 as a potential biomarker promotes cell proliferation and invasion in thyroid cancer. Contactin 1 (CNTN1) as a member of the immunoglobulin superfamily plays important role in the development of nervous system. Recent studies find that elevated CNTN1 can promote the   metastasis   of cancer. However, the expression and function of CNTN1 in thyroid cancer are still unknown. Here, we firstly find CNTN1 is a new gene which can be regulated by RET/PTC3 (Ret proto-oncogene and Ret-activating protein ELE1) rearrangement gene and the protein level of CNTN1 is increasing in thyroid cancer. Besides this change is positively associated with the TNM stage and tumor size. Moreover, we confirm that knockdown of CNTN1 significantly inhibits the tumor proliferation, invasiveness and represses the expression of cyclin D1 (CCND1). In conclusion, CNTN1 will be a potential diagnosis biomarker and therapy target for thyroid cancer. 
 Contactin-1 reduces E-cadherin expression via activating AKT in lung cancer. Contactin-1 has been shown to promote cancer   metastasis  . However, the underlying mechanisms remain unclear. We report here that knockdown of contactin-1 in A549 lung cancer cells reduced A549 cell invasion and the cell's ability to grow in soft agar without affecting cell proliferation. Reduction of contactin-1 resulted in upregulation of E-cadherin, consistent with E-cadherin being inhibitive of cancer cell invasion. In an effort to investigate the mechanisms whereby contactin-1 reduces E-cadherin expression, we observed that contactin-1 plays a role in AKT activation, as knockdown of contactin-1 attenuated AKT activation. Additionally, inhibition of AKT activation significantly enhanced E-cadherin expression, an observation that mimics the situation observed in contactin-1 knockdown, suggesting that activation of AKT plays a role in contactin-1-mediated downregulation of E-cadherin. In addition, we were able to show that knockdown of contactin-1 did not further reduce A549 cell's invasion ability, when AKT activation was inhibited by an AKT inhibitor. To further support our findings, we overexpressed CNTN-1 in two CNTN-1 null breast cancer cell lines expressing E-cadherin. Upon overexpression, CNTN-1 reduced E-cadherin levels in one cell line and increased AKT activation in the other. Furthermore, in our study of 63 primary lung cancers, we observed 65% of primary lung cancers being contactin-1 positive and in these carcinomas, 61% were E-cadherin negative. Collectively, we provide evidence that contactin-1 plays a role in the downregulation of E-cadherin in lung cancer and that AKT activation contributes to this process. In a study of mechanisms responsible for contactin-1 to activate AKT, we demonstrated that knockdown of CNTN-1 in A549 cells did not enhance PTEN expression but upregulated PHLPP2, a phosphatase that dephosphorylates AKT. These observations thus suggest that contactin-1 enhances AKT activation in part by preventing PHLPP2-mediated AKT dephosphrorylation. 
 Contactin-1 (CNTN-1) overexpression is correlated with advanced clinical stage and lymph node   metastasis   in oesophageal squamous cell carcinomas. Oesophageal squamous cell carcinoma is one of the deadliest malignancies worldwide. Contactin-1, a neural adhesion molecule, is implicated in tumour invasion and   metastasis  . The purpose of this study was to investigate the expression of CNTN-1 in normal and cancerous oesophageal tissue, and the potential relevance to clinicopathological features.Thirty normal oesophageal tissue samples and 82 primary oesophageal squamous cell carcinoma tissue samples were included in this study. The expression levels of CNTN-1, VEGF-C and HIF-1α messenger RNA were determined using reverse transcriptase-polymerase chain reaction and quantitative real-time polymerase chain reaction. The expression of the CNTN-1 protein was measured using immunohistochemistry.The expression of CNTN-1 messenger RNA was significantly increased in the tumour tissue compared with the normal oesophageal tissue (P=0.001). The oesophageal squamous cell carcinoma tissue consistently showed higher CNTN-1 protein levels. The CNTN-1 expression correlated with the oesophageal squamous cell carcinoma stage (P=0.006), lymph node   metastasi  s (P=0.018) and lymphatic invasion (P=0.035). The messenger RNA level of CNTN-1 correlated significantly with those of VEGF-C and HIF-1α.The expression of CNTN-1 is upregulated in the oesophageal squamous cell carcinoma tissue and related to stage, lymph nod  e metastas  is and lymphatic invasion. Thus, CNTN-1 may be involved in the progression and pathogenesis of oesophageal squamous cell carcinoma. 
 Neural Cell Adhesion Protein CNTN1 Promotes the Metastatic Progression of Prostate Cancer. Prostate cancer   metastasis   is the main cause of disease-related mortality. Elucidating the mechanisms underlying prostate cancer   metastasis   is critical for effective therapeutic intervention. In this study, we performed gene-expression profiling of prostate cancer stem-like cells (PCSC) derived from DU145 human prostate cancer cells to identify factors involved in metastatic progression. Our studies revealed contactin 1 (CNTN1), a neural cell adhesion protein, to be a prostate cancer-promoting factor. CNTN1 knockdown reduced PCSC-mediated tumor initiation, whereas CNTN1 overexpression enhanced prostate cancer cell invasion in vitro and promoted xenograft tumor formation and lung   metastasis   in vivo. In addition, CNTN1 overexpression in DU145 cells and corresponding xenograft tumors resulted in elevated AKT activation and reduced E-cadherin (CDH1) expression. CNTN1 expression was not readily detected in normal prostate glands, but was clearly evident on prostate cancer cells in primary tumors and lymph node and bone metastases. Tumors from 637 patients expressing CNTN1 were associated with prostate cancer progression and worse biochemical recurrence-free survival following radical prostatectomy (P < 0.05). Collectively, our findings demonstrate that CNTN1 promotes prostate cancer progression and   metastasis  , prompting further investigation into the mechanisms that enable neural proteins to become aberrantly expressed in non-neural malignancies. 
 VEGF-C promotes the development of esophageal cancer via regulating CNTN-1 expression. Vascular endothelial growth factor C (VEGF-C) is a key regulator of angiogenesis and lymphangiogenesis. VEGF-C is also implicated in the development of esophageal cancer. We investigated the mRNA levels of VEGF-C and its receptors in 38 esophageal squamous cell carcinoma specimens (ESCCs) and matched adjacent normal esophageal tissues via real-time PCR. The mRNA levels of VEGF-C, VEGFR-2 and VEGFR-3 were significantly upregulated in ESCCs versus respective side normal tissues. To explore the influence of VEGF-C on esophageal cancer progression, the expression of VEGF-C was manipulated in esophageal cancer cell lines TE-1 and Eca-109. VEGF-C transcription, translation and secretion were significantly enhanced in cells stably transfected with a VEGF-C overexpression vector or attenuated in VEGF-C shRNA-transfected cell lines. In vitro, TE-1 cells stably transfected with a VEGF-C overexpression vector exhibited an increased rate of cell proliferation, migration and focus formation, whereas knockdown of VEGF-C inhibited cell proliferation, migration and focus formation. Similar results were obtained for Eca-109 cells. VEGF-C mediated biological function through transcription of CNTN-1, which is implicated in tumor invasion and   metastasis  . The expression of VEGF-C was correlated with that of CNTN-1 and cell proliferation and migration induced by VEGF-C were reversed by silencing of CNTN-1. In addition, nude mice inoculated with VEGF-C shRNA-transfected cells exhibited a significantly decreased tumor size in vivo via reduced VEGFR-2 and VEGFR-3 phosphorylation and microvessel formation. VEGF-C upregulation may be involved in esophageal tumor progression. Vector-based RNA interference (RNAi) targeting VEGF-C is a potential therapeutic method for human esophageal carcinoma. 
 Contactin 1 (CNTN1) expression associates with regional lymph node   metastasis   and is a novel predictor of prognosis in patients with oral squamous cell carcinoma. The contactin 1 (CNTN1) gene exerts oncogene‑like activities and its expression has been linked to several human malignancies. In this study, a possible association between CNTN1 expression and clinicopathological parameters and clinical outcomes in patients with oral squamous cell carcinoma (OSCC) was examined. CNTN1 protein expression was evaluated by immunohistochemistry in OSCC tissues of 45 patients. For the immunohistochemical assessment of CNTN1 expression, the cytoplasmic staining labeling index was analyzed using a semiquantitative score. The association between CNTN1 protein levels and clinicopathological factors was analyzed using the Mann-Whitney U test for categorical variables and the Kruskal-Wallis test for continuous variables. The effects of CNTN1 expression on overall and disease-free survival were assessed by using univariate survival analysis. The transcript levels of CNTN1 were detected in OSCC cell lines. In addition, specific siRNA against CNTN1 was applied to investigate the effect exerted by CNTN1 ablation on OSCC cell lines by proliferation and invasion assays in vitro. During follow-up, 16 patients (35.56%) had succumbed to OSCC; the median follow-up of patients was 5.0 years (range, 0.2-8.3). A high expression of CNTN1 was markedly associated with the regional lymph nod  e metastas  is of patients with OSCC (P=0.006). CNTN1 expression was significantly associated with overall survival of patients with OSCC (P=0.032; log-rank test) and disease-free survival of patients with OSCC (P=0.038; log-rank test). In addition, CNTN1 ablation notably suppressed the invasion potential of OSCC cell lines, but there was no significant change in the proliferation of OSCC cell lines by CNTN1 knockdown in vitro. The study supports CNTN1 as a novel predictor of regional lymph nod  e metastas  is in patients with OSCC and a prognostic marker for OSCC in patients. 
 Knockdown of contactin-1 expression suppresses invasion and   metastasis   of lung adenocarcinoma. Numerous genetic changes are associated with cancer cell   metastasis   and invasion. In search for key regulators of invasion and   metastasis  , a panel of lung cancer cell lines with different invasive ability was screened. The gene for contactin-1 was found to play an essential role in tumor invasion and   metastasis  . Suppression of contactin-1 expression abolished the ability of lung adenocarcinoma cells to invade Matrigel in vitro as well as the polymerization of filamentous-actin and the formation of focal adhesion structures. Furthermore, knockdown of contactin-1 resulted in extensive inhibition of tumor   metastasis   and in increased survival in an animal model. RhoA but not Cdc42 or Rac1 was found to serve a critical role in contactin-1-mediated invasion and   metastasis  . Contactin-1-specific RNA interference resulted in loss of metastatic and invasive capacity in both in vitro and in vivo models. This loss was overturned by constitutive expression of the active form of RhoA. Contactin-1 was differentially expressed in tumor tissues, and its expression correlated with tumor stage, lymph node   metastasis  , and patient survival. Contactin-1 is proposed to function importantly in the invasion and   metastasis   of lung adenocarcinoma cells via RhoA-mediated mechanisms. 
  GeneRIFs  
 High CNTN-1 expression is associated with   metastasis   in gastric cancer. 
 A high expression of CNTN1 was markedly associated with the regional lymph node   metastasis   of patients with oral squamous cell carcinoma. 
 VEGF-C mediated biological function through transcription of CNTN-1, which is implicated in tumor invasion and   metastasis  . 
 Contactin-1 is proposed to function importantly in the invasion and   metastasis   of lung adenocarcinoma cells via RhoA-mediated mechanisms. 
  [Cc]ell [Ii]nvasion  PubMed  
 Effect of lentivirus-mediated shRNA targeting VEGFR-3 on proliferation, apoptosis and invasion of gastric cancer cells. It has been reported that vascular endothelial growth factor receptor 3 (VEGFR-3) is highly expressed in most tumor tissues, including gastric cancer. However, the effects of VEGFR-3 knockdown on the proliferation, apoptosis and invasion of gastric cancer cells and downstream signaling molecules have not yet been well established. In the present study, four short hairpin RNA (shRNA) sequences targeting the VEGFR-3 gene (NM_002020) were designed and cloned into a lentiviral vector, pRNAT-U6.2/Lenti, to construct four recombinant lentiviral vectors. The vectors with the two highest interfering efficiencies were selected to be co-transfected with packaging vectors in HEK293T cells to assemble lentivirus particles. Results from Western blot analysis showed that the VEGFR-3 shRNA-4 lentivirus-infected group (sh#4) had the highest efficiency of gene silencing in the MKN45 cell line compared with the parental and control group. The sh#4 group significantly slowed cell proliferation, decreased the mean percentage of S-phase cells and increased the mean percentage of G1 phase cells, promoted cell apoptosis, and also significantly inhibited   cell invasion   of MKN45 compared with the other two groups. Furthermore, the expression of the anti-apoptotic factor Bcl-2 was significantly decreased in the sh#4 group compared to that of the other two groups. Moreover, results from qRT-PCR revealed that knockdown of VEGFR-3 with the shRNA lentiviral vector resulted in down-regulation of the downstream neural cell adhesion molecule contactin-1 (CNTN-1). In conclusion, the recombinant lentivirus particles were able to remarkably suppress VEGFR-3 expression, regulate the cell cycle, inhibit proliferation and induce apoptosis in the MKN45 cell lines. 
 Contactin-1 reduces E-cadherin expression via activating AKT in lung cancer. Contactin-1 has been shown to promote cancer metastasis. However, the underlying mechanisms remain unclear. We report here that knockdown of contactin-1 in A549 lung cancer cells reduced A549   cell invasion   and the cell's ability to grow in soft agar without affecting cell proliferation. Reduction of contactin-1 resulted in upregulation of E-cadherin, consistent with E-cadherin being inhibitive of cancer   cell invasion  . In an effort to investigate the mechanisms whereby contactin-1 reduces E-cadherin expression, we observed that contactin-1 plays a role in AKT activation, as knockdown of contactin-1 attenuated AKT activation. Additionally, inhibition of AKT activation significantly enhanced E-cadherin expression, an observation that mimics the situation observed in contactin-1 knockdown, suggesting that activation of AKT plays a role in contactin-1-mediated downregulation of E-cadherin. In addition, we were able to show that knockdown of contactin-1 did not further reduce A549 cell's invasion ability, when AKT activation was inhibited by an AKT inhibitor. To further support our findings, we overexpressed CNTN-1 in two CNTN-1 null breast cancer cell lines expressing E-cadherin. Upon overexpression, CNTN-1 reduced E-cadherin levels in one cell line and increased AKT activation in the other. Furthermore, in our study of 63 primary lung cancers, we observed 65% of primary lung cancers being contactin-1 positive and in these carcinomas, 61% were E-cadherin negative. Collectively, we provide evidence that contactin-1 plays a role in the downregulation of E-cadherin in lung cancer and that AKT activation contributes to this process. In a study of mechanisms responsible for contactin-1 to activate AKT, we demonstrated that knockdown of CNTN-1 in A549 cells did not enhance PTEN expression but upregulated PHLPP2, a phosphatase that dephosphorylates AKT. These observations thus suggest that contactin-1 enhances AKT activation in part by preventing PHLPP2-mediated AKT dephosphrorylation. 
 Neural Cell Adhesion Protein CNTN1 Promotes the Metastatic Progression of Prostate Cancer. Prostate cancer metastasis is the main cause of disease-related mortality. Elucidating the mechanisms underlying prostate cancer metastasis is critical for effective therapeutic intervention. In this study, we performed gene-expression profiling of prostate cancer stem-like cells (PCSC) derived from DU145 human prostate cancer cells to identify factors involved in metastatic progression. Our studies revealed contactin 1 (CNTN1), a neural cell adhesion protein, to be a prostate cancer-promoting factor. CNTN1 knockdown reduced PCSC-mediated tumor initiation, whereas CNTN1 overexpression enhanced prostate cancer   cell invasion   in vitro and promoted xenograft tumor formation and lung metastasis in vivo. In addition, CNTN1 overexpression in DU145 cells and corresponding xenograft tumors resulted in elevated AKT activation and reduced E-cadherin (CDH1) expression. CNTN1 expression was not readily detected in normal prostate glands, but was clearly evident on prostate cancer cells in primary tumors and lymph node and bone metastases. Tumors from 637 patients expressing CNTN1 were associated with prostate cancer progression and worse biochemical recurrence-free survival following radical prostatectomy (P < 0.05). Collectively, our findings demonstrate that CNTN1 promotes prostate cancer progression and metastasis, prompting further investigation into the mechanisms that enable neural proteins to become aberrantly expressed in non-neural malignancies. 
   
  
 ABCG4 
 
  NCBI Gene  
  GeneRIF  
  Pubmed  
 
 
  Search Term  Rank  Fields  
  [Mm]etastasis  12  PubMed(1)  
 
 
 hide/show details for ABCG4 
  [Mm]etastasis  PubMed  
 High ABCG4 Expression Is Associated with Poor Prognosis in Non-Small-Cell Lung Cancer Patients Treated with Cisplatin-Based Chemotherapy. ATP-binding cassette (ABC) transporters are associated with poor response to chemotherapy, and confer a poor prognosis in various malignancies. However, the association between the expression of the ABC sub-family G member 4 (ABCG4) and prognosis in patients with non-small-cell lung cancer (NSCLC) remains unclear. NSCLC tissue samples (n = 140) and normal lung tissue samples (n = 90) were resected from patients with stage II to IV NSCLC between May 2004 and May 2009. ABCG4 mRNA and protein expressions were detected by RT-PCR, western blot, and immunohistochemistry. Patients received four cycles of cisplatin-based post-surgery chemotherapy and were followed up until May 31st, 2014. ABCG4 positivity rate was higher in NSCLC than in normal lung tissues (48.6% vs. 0%, P<0.001) and ABCG4 expression was significantly associated with poor differentiation, higher tumor node   metastasis   (TNM) stage, and adenocarcinoma histological type (all P<0.001). Univariate (HR = 2.284, 95%CI: 1.570-3.324, P<0.001) and multivariate (HR = 2.236, 95%CI: 1.505-3.321, P<0.001) analyses showed that ABCG4 expression was an independent factor associated with a poor prognosis in NSCLC. Patients with ABCG4-positive NSCLC had shorter median survival than ABCG4-negative NSCLC (20.1 vs. 43.2 months, P<0.001). The prognostic significance of ABCG4 expression was apparent in stages III and IV NSCLC. In conclusion, high ABCG4 expression was associated with a poor prognosis in patients with NSCLC treated with cisplatin-based chemotherapy. 
   
  
 MMP14 
 
  NCBI Gene  
  GeneRIF  
  Pubmed  
 
 
  Search Term  Rank  Fields  
  [Ff]ilopodia  1  PubMed(2)  
  [Ii]nvadopodia  3  PubMed(9); GeneRIFs(4)  
  [Ii]nvadopodium  4  PubMed(1)  
  [Pp]odosome(s)?  5  PubMed(2)  
  [Mm]igration  6  Process(3); PubMed(124); GeneRIFs(31)  
  ECM  7  PubMed(39); GeneRIFs(4)  
  [Ee]xtracellular [Mm]atrix  8  Conserved Domains(1); Process(1); Component(1); Summary(1); PubMed(120); GeneRIFs(9)  
  [Ii]nvasive  10  PubMed(116); GeneRIFs(37)  
  [Mm]etastasize  11  PubMed(2)  
  [Mm]etastasis  12  PubMed(105); GeneRIFs(25); Summary(1)  
  [Cc]ell [Ii]nvasion  13  PubMed(62); GeneRIFs(9)  
 
 
 hide/show details for MMP14 
  [Ff]ilopodia  PubMed  
 Moesin regulates the motility of oral cancer cells via MT1-MMP and E-cadherin/p120-catenin adhesion complex. The present study aimed to clarify the role of Moesin in oral squamous cell carcinoma (OSCC) progression, especially in regulation of cell motility.Immunohistochemistry and western blotting were used to investigate the expression of Moesin, E-cadherin, p120-catenin and MT1-MMP in normal epithelia, dysplasia and OSCCs. Then, Moesin was knockdown by siRNA in OSCC cell lines, WSU-HN6 and CAL27, and the biological role of Moesin in cell adhesion and motility was evaluated by transwell system, cell spreading and aggregation assays. The interactions between Moesin, MT1-MMP and E-cadherin/p120-catenin complex were determined by co-immunoprecipitation and immunofluorescence.Moesin expression was found decreased in the membrane and increased in cytoplasm during the malignant transformation of oral epithelia, and cytoplasmic overexpression of Moesin correlated with nodal metastasis and poor prognosis of OSCCs. Furthermore, Moesin-silencing induced an increased cell-cell adhesion but decreased invasiveness, which was subsequently demonstrated might due to Moesin-mediated E-cadherin and p120-catenin interaction. Meantime, Moesin-silencing significantly down-regulated MT1-MMP expression, accompanied by reduced cell motility and impaired   filopodia   formation, which was also observed when MT1-MMP knockdown by RNAi or tissue inhibitor (TIMP2), indicating the involvement of MT1-MMP in Moesin-mediated cell motility. Finally, the relationship between Moesin, E-cadherin and MT1-MMP was confirmed in OSCC tissue samples.Taken together, our results indicate Moesin may regulate cell motility through its interactions with MT1-MMP and E-cadherin/p120-catenin adhesion complex and cytoplasmic expression of Moesin correlates with nodal metastasis and poor prognosis of OSCCs, indicating Moesin may be a potential candidate for targeted gene therapy for OSCCs. 
 Moesin regulates the motility of oral cancer cells via MT1-MMP and E-cadherin/p120-catenin adhesion complex. The present study aimed to clarify the role of Moesin in oral squamous cell carcinoma (OSCC) progression, especially in regulation of cell motility.Immunohistochemistry and western blotting were used to investigate the expression of Moesin, E-cadherin, p120-catenin and MT1-MMP in normal epithelia, dysplasia and OSCCs. Then, Moesin was knockdown by siRNA in OSCC cell lines, WSU-HN6 and CAL27, and the biological role of Moesin in cell adhesion and motility was evaluated by transwell system, cell spreading and aggregation assays. The interactions between Moesin, MT1-MMP and E-cadherin/p120-catenin complex were determined by co-immunoprecipitation and immunofluorescence.Moesin expression was found decreased in the membrane and increased in cytoplasm during the malignant transformation of oral epithelia, and cytoplasmic overexpression of Moesin correlated with nodal metastasis and poor prognosis of OSCCs. Furthermore, Moesin-silencing induced an increased cell-cell adhesion but decreased invasiveness, which was subsequently demonstrated might due to Moesin-mediated E-cadherin and p120-catenin interaction. Meantime, Moesin-silencing significantly down-regulated MT1-MMP expression, accompanied by reduced cell motility and impaired   filopodia   formation, which was also observed when MT1-MMP knockdown by RNAi or tissue inhibitor (TIMP2), indicating the involvement of MT1-MMP in Moesin-mediated cell motility. Finally, the relationship between Moesin, E-cadherin and MT1-MMP was confirmed in OSCC tissue samples.Taken together, our results indicate Moesin may regulate cell motility through its interactions with MT1-MMP and E-cadherin/p120-catenin adhesion complex and cytoplasmic expression of Moesin correlates with nodal metastasis and poor prognosis of OSCCs, indicating Moesin may be a potential candidate for targeted gene therapy for OSCCs. 
  [Ii]nvadopodia  PubMed  
 SNAP23, Syntaxin4, and vesicle-associated membrane protein 7 (VAMP7) mediate trafficking of membrane type 1-matrix metalloproteinase (MT1-MMP) during invadopodium formation and tumor cell invasion. Movement through the extracellular matrix (ECM) requires cells to degrade ECM components, primarily through the action of matrix metalloproteinases (MMPs). Membrane type 1-matrix metalloproteinase (MT1-MMP) has an essential role in matrix degradation and cell invasion and localizes to subcellular degradative structures termed   invadopodia  . Trafficking of MT1-MMP to   invadopodia   is required for the function of these structures, and here we examine the role of N-ethylmaleimide-sensitive factor-activating protein receptor (SNARE)-mediated membrane traffic in the transport of MT1-MMP to   invadopodia  . During invadopodium formation in MDA-MB-231 human breast cancer cells, increased association of SNAP23, Syntaxin4, and vesicle-associated membrane protein 7 (VAMP7) is detected by coimmunoprecipitation. Blocking the function of these SNAREs perturbs invadopodium-based ECM degradation and cell invasion. Increased level of SNAP23-Syntaxin4-VAMP7 interaction correlates with decreased Syntaxin4 phosphorylation. These results reveal an important role for SNARE-regulated trafficking of MT1-MMP to   invadopodia   during cellular invasion of ECM. 
 MicroRNA-133a regulates the mRNAs of two   invadopodia  -related proteins, FSCN1 and MMP14, in esophageal cancer. FSCN1 and matrix metalloproteinase 14 (MMP14) are both   invadopodia  -related proteins. We herein elucidate the tumourigenicity of these proteins and identify novel therapeutic agents in esophageal squamous cell carcinoma (ESCC).FSCN1 and MMP14 were evaluated by immunohistochemistry and quantitative PCR, and microRNA (miR)-133a was also evaluated by PCR in surgical ESCC specimens. The roles of FSCN1, MMP14 and miR-133a were established in ESCC cells.The expression of FSCN1 or MMP14 was an independent poor prognostic factor according to a multivariate analysis of immunohistochemistry, and their co-expression correlated with the poorest overall survival (OS) out of all the examined factors. Additionally, their mRNAs significantly correlated and both inversely correlated with miR-133a in surgical specimens. Transfection of a miR-133a mimic decreased the mRNA and protein levels of both FSCN1 and MMP14 in ESCC cells. The knockdown of FSCN1 or MMP14 and transfection of a miR-133a mimic inhibited the proliferation and invasion of ESCC cells. Patients with a lower miR-133a expression have a significantly poorer OS than those with a higher expression.The combined expression of FSCN1 and MMP14 is associated with a poor prognosis, and miR-133a, which regulates their mRNAs, can serve as a strong tumour suppressor of ESCC. 
 CDCP1 regulates the function of MT1-MMP and   invadopodia  -mediated invasion of cancer cells. Complement C1r/C1s, Uegf, Bmp1 (CUB) domain-containing protein 1 (CDCP1) is a transmembrane protein that regulates anchorage-independent growth and cancer cell migration and invasion. Expression of CDCP1 is detected in a number of cancer cell lines and tissues and is closely correlated with poor prognosis.   Invadopodia   are actin-based protrusions on the surface of invasive cancer cells that promote the degradation of the extracellular matrix (ECM) via localized proteolysis, which is mainly mediated by membrane type 1 matrix metalloproteinase (MT1-MMP). MT1-MMP is accumulated at   invadopodia   by targeted delivery via membrane trafficking. The present study shows that CDCP1 is required for ECM degradation by   invadopodia   in human breast cancer and melanoma cells. CDCP1 localized to caveolin-1-containing vesicular structures and lipid rafts and was detected in close proximity to   invadopodia  . Further biochemical analysis revealed that substantial amounts of CDCP1 existed in the Triton X-100 insoluble lipid raft fraction. CDCP1 was coimmunoprecipitated with MT1-MMP and colocalized with MT1-MMP at the vesicular structures. The siRNA-mediated knockdown of the CDCP1 expression markedly inhibited MT1-MMP-dependent ECM degradation and Matrigel invasion and reduced the accumulation of MT1-MMP at   invadopodia  , as shown by immunofluorescence analysis. These results indicate that CDCP1 is an essential regulator of the trafficking and function of MT1-MMP- and   invadopodia  -mediated invasion of cancer cells. 
 A novel protein associated with membrane-type 1 matrix metalloproteinase binds p27(kip1) and regulates RhoA activation, actin remodeling, and matrigel invasion. Pericellular proteolysis by membrane-type 1 matrix metalloproteinase (MT1-MMP) plays a pivotal role in tumor cell invasion. Localization of MT1-MMP at the invasion front of cells, e.g. on lamellipodia and   invadopodia  , has to be regulated in coordination with reorganization of the actin cytoskeleton. However, little is known about how such invasion-related actin structures are regulated at the sites where MT1-MMP localizes. During analysis of MT1-MMP-associated proteins, we identified a heretofore uncharacterized protein. This protein, which we call p27RF-Rho, enhances activation of RhoA by releasing it from inhibition by p27(kip1) and thereby regulates actin structures. p27(kip1) is a well known cell cycle regulator in the nucleus. In contrast, cytoplasmic p27(kip1) has been demonstrated to bind GDP-RhoA and inhibit GDP-GTP exchange mediated by guanine nucleotide exchange factors. p27RF-Rho binds p27(kip1) and prevents p27(kip1) from binding to RhoA, thereby freeing the latter for activation. Knockdown of p27RF-Rho expression renders cells resistant to RhoA activation stimuli, whereas overexpression of p27RF-Rho sensitizes cells to such stimulation. p27RF-Rho exhibits a punctate distribution in invasive human tumor cell lines. Stimulation of the cells with lysophosphatidic acid induces activation of RhoA and induces the formation of punctate actin structures within foci of p27RF-Rho localization. Some of the punctate actin structures co-localize with MT1-MMP and cortactin. Down-regulation of p27RF-Rho prevents both redistribution of actin into the punctate structures and tumor cell invasion. Thus, p27RF-Rho is a new potential target for cancer therapy development. 
 Lipid rafts and caveolin-1 are required for   invadopodia   formation and extracellular matrix degradation by human breast cancer cells.   Invadopodia   are ventral membrane protrusions through which invasive cancer cells degrade the extracellular matrix. They are thought to function in the migration of cancer cells through tissue barriers, which is necessary for cancer invasion and metastasis. Although many protein components of   invadopodia   have been identified, the organization and the role of membrane lipids in   invadopodia   are not well understood. In this study, the role of lipid rafts, which are cholesterol-enriched membrane microdomains, in the assembly and function of   invadopodia   in human breast cancer cells was investigated. Lipid rafts are enriched, internalized, and dynamically trafficked at   invadopodia   sites. Perturbation of lipid raft formation due to depleting or sequestering membrane cholesterol blocked the   invadopodia  -mediated degradation of the gelatin matrix. Caveolin-1 (Cav-1), a resident protein of lipid rafts and caveolae, accumulates at   invadopodia   and colocalizes with the internalized lipid raft membranes. Membrane type 1 matrix metalloproteinase (MT1-MMP), a matrix proteinase associated with   invadopodia  , is localized at lipid raft-enriched membrane fractions and cotrafficked and colocalized with Cav-1 at   invadopodia  . The small interfering RNA-mediated silencing of Cav-1 inhibited the   invadopodia  -mediated and MT1-MMP-dependent degradation of the gelatin matrix. Furthermore, Cav-1 and MT1-MMP are coexpressed in invasive human breast cancer cell lines that have an ability to form   invadopodia  . These results indicate that   invadopodia   are the sites where enrichment and trafficking of lipid rafts occur and that Cav-1 is an essential regulator of MT1-MMP function and   invadopodia  -mediated breast cancer cell invasion. 
 Neoplastic progression of the human breast cancer cell line G3S1 is associated with elevation of cytoskeletal dynamics and upregulation of MT1-MMP. The newly established breast cancer cell line G3S1, derived from EM-G3 breast cancer progenitors, was analyzed for functional changes related to neoplastic progression manifested by elevated invasiveness and enhanced capability to degrade gelatin. Degradation of gelatin and invasiveness of G3S1 cells was found to be dependent on the activity of matrix proteinases and actin cytoskeletal dynamics. Therefore, the expression and activity of these proteases was compared in G3S1 and EM-G3 cells. Despite enhanced capability of G3S1 cells to degrade gelatin, these cells exhibited lower levels of secreted extracellular matrix degrading proteases than parental EM-G3 cells. However, the expression of membrane-bound MT1-MMP was strongly elevated in G3S1 cells. While the degradation of gelatin was associated with   invadopodia  -like structures in both EM-G3 and G3S1 cells, the cytoskeletal remodeling dynamics was greatly elevated in G3S1 cells, suggesting that upregulation of MT1-MMP, together with elevation of cytoskeletal remodeling dynamics can effectively cause elevated invasiveness and enhanced matrix degrading capability in G3S1 cells. 
 YB-1 alters MT1-MMP trafficking and stimulates MCF-7 breast tumor invasion and metastasis. YB-1 is a member of the cold shock domain family, with complex roles in DNA structure, gene transcription and translation. YB-1 promotes chromosomal instability, and mammary gland transgenic expression induces tumors with 100% penetrance. YB-1 is linked to poor prognosis in breast carcinoma and is a strong predictor of relapse and disease-specific survival. Survival is directly tied to the extent of local invasion and distal metastasis, processes dependent upon the activity of the membrane type I-matrix metalloproteinase, MT1-MMP. Non-invasive MCF-7 breast adenocarcinoma cells were transfected with YB-1/EGFP. YB-1 protein was detected in the   invadopodia   of cells with a migratory phenotype. There was increased expression of MT1-MMP protein concentrated at the leading edges of motile cells, which were highly invasive in collagen three-dimensional culture. The rates of MT1-MMP protein endocytosis and recycling to the cell surface were elevated in clones expressing higher levels of YB-1 protein. Control MCF-7 cells formed nonfatal, non-invasive, differentiated adenocarcinomas in vivo. MCF-7 cells expressing a twofold increase in YB-1 formed highly anaplastic tumors with local invasion, pulmonary metastases and high lethality. We conclude that YB-1 contributes to the development of an invasive, metastatic breast carcinoma phenotype by enhanced presentation of MT1-MMP at the sites of cellular invasion. 
 TOM1L1 drives membrane delivery of MT1-MMP to promote ERBB2-induced breast cancer cell invasion. ERBB2 overexpression in human breast cancer leads to invasive carcinoma but the mechanism is not clearly understood. Here we report that TOM1L1 is co-amplified with ERBB2 and defines a subgroup of HER2(+)/ER(+) tumours with early metastatic relapse. TOM1L1 encodes a GAT domain-containing trafficking protein and is a SRC substrate that negatively regulates tyrosine kinase signalling. We demonstrate that TOM1L1 upregulation enhances the invasiveness of ERBB2-transformed cells. This pro-tumoural function does not involve SRC, but implicates membrane-bound membrane-type 1 MMP (MT1-MMP)-dependent activation of   invadopodia  , membrane protrusions specialized in extracellular matrix degradation. Mechanistically, ERBB2 elicits the indirect phosphorylation of TOM1L1 on Ser321. The phosphorylation event promotes GAT-dependent association of TOM1L1 with the sorting protein TOLLIP and trafficking of the metalloprotease MT1-MMP from endocytic compartments to   invadopodia   for tumour cell invasion. Collectively, these results show that TOM1L1 is an important element of an ERBB2-driven proteolytic invasive programme and that TOM1L1 amplification potentially enhances the metastatic progression of ERBB2-positive breast cancers. 
 MT1-MMP-dependent invasion is regulated by TI-VAMP/VAMP7. Proteolytic degradation of the extracellular matrix (ECM) is one intrinsic property of metastatic tumor cells to breach tissue barriers and to disseminate into different tissues. This process is initiated by the formation of   invadopodia  , which are actin-driven, finger-like membrane protrusions. Yet, little is known on how   invadopodia   are endowed with the functional machinery of proteolytic enzymes [1, 2]. The key protease MT1-MMP (membrane type 1-matrix metalloproteinase) confers proteolytic activity to   invadopodia   and thus invasion capacity of cancer cells [3-6]. Here, we report that MT1-MMP-dependent matrix degradation at   invadopodia   is regulated by the v-SNARE TI-VAMP/VAMP7, hence providing the molecular inventory mediating focal degradative activity of cancer cells. As observed by TIRF microscopy, MT1-MMP-mCherry and GFP-VAMP7 were simultaneously detected at proteolytic sites. Functional ablation of VAMP7 decreased the ability of breast cancer cells to degrade and invade in a MT1-MMP-dependent fashion. Moreover, the number of   invadopodia   was dramatically decreased in VAMP7- and MT1-MMP-depleted cells, indicative of a positive-feedback loop in which the protease as a cargo of VAMP7-targeted transport vesicles regulates maturation of   invadopodia  . Collectively, these data point to a specific role of VAMP7 in delivering MT1-MMP to sites of degradation, maintaining the functional machinery required for invasion. 
  GeneRIFs  
 TOM1L1 enhances ERBB2-induced cell invasiveness by promoting   invadopodia   formation and MT1-MMP trafficking to the plasma membrane. 
 Increased level of SNAP23-Syntaxin4-VAMP7 interaction correlates with decreased Syntaxin4 phosphorylation and trafficking of MT1-MMP to   invadopodia   during cellular invasion. 
 CDCP1 is an essential regulator of the trafficking and function of MT1-MMP- and   invadopodia  -mediated invasion of cancer cells. 
 Cav-1 is an essential regulator of MT1-MMP function and   invadopodia  -mediated breast cancer cell invasion 
  [Ii]nvadopodium  PubMed  
 SNAP23, Syntaxin4, and vesicle-associated membrane protein 7 (VAMP7) mediate trafficking of membrane type 1-matrix metalloproteinase (MT1-MMP) during   invadopodium   formation and tumor cell invasion. Movement through the extracellular matrix (ECM) requires cells to degrade ECM components, primarily through the action of matrix metalloproteinases (MMPs). Membrane type 1-matrix metalloproteinase (MT1-MMP) has an essential role in matrix degradation and cell invasion and localizes to subcellular degradative structures termed invadopodia. Trafficking of MT1-MMP to invadopodia is required for the function of these structures, and here we examine the role of N-ethylmaleimide-sensitive factor-activating protein receptor (SNARE)-mediated membrane traffic in the transport of MT1-MMP to invadopodia. During   invadopodium   formation in MDA-MB-231 human breast cancer cells, increased association of SNAP23, Syntaxin4, and vesicle-associated membrane protein 7 (VAMP7) is detected by coimmunoprecipitation. Blocking the function of these SNAREs perturbs   invadopodium  -based ECM degradation and cell invasion. Increased level of SNAP23-Syntaxin4-VAMP7 interaction correlates with decreased Syntaxin4 phosphorylation. These results reveal an important role for SNARE-regulated trafficking of MT1-MMP to invadopodia during cellular invasion of ECM. 
  [Pp]odosome(s)?  PubMed  
 A specific subset of RabGTPases controls cell surface exposure of MT1-MMP, extracellular matrix degradation and three-dimensional invasion of macrophages. The matrix metalloproteinase MT1-MMP has a major impact on invasive cell migration in both physiological and pathological settings such as immune cell extravasation or metastasis of cancer cells. Surface-associated MT1-MMP is able to cleave components of the extracellular matrix, which is a prerequisite for proteolytic invasive migration. However, current knowledge on the molecular mechanisms that regulate MT1-MMP trafficking to and from the cell surface is limited. We have identified three members of the RabGTPase family, Rab5a, Rab8a and Rab14, as crucial regulators of MT1-MMP trafficking and function in primary human macrophages. Both overexpressed and endogenous forms show prominent colocalisation with MT1-MMP-positive vesicles, whereas expression of mutant constructs, as well as siRNA-induced knockdown, reveal that these RabGTPases are crucial in the regulation of MT1-MMP surface exposure, contact of MT1-MMP-positive vesicles with   podosomes  , extracellular matrix degradation in two and three dimensions, as well as three-dimensional proteolytic invasion of macrophages. Collectively, our results identify Rab5a, Rab8a and Rab14 as major regulators of MT1-MMP trafficking and invasive migration of primary human macrophages, which could be promising potential targets for manipulation of immune cell invasion. 
 Metalloproteinase MT1-MMP islets act as memory devices for   podosome   reemergence.   Podosomes   are dynamic cell adhesions that are also sites of extracellular matrix degradation, through recruitment of matrix-lytic enzymes, particularly of matrix metalloproteinases. Using total internal reflection fluorescence microscopy, we show that the membrane-bound metalloproteinase MT1-MMP is enriched not only at   podosomes   but also at distinct "islets" embedded in the plasma membrane of primary human macrophages. MT1-MMP islets become apparent upon   podosome   dissolution and persist beyond   podosome   lifetime. Importantly, the majority of MT1-MMP islets are reused as sites of   podosome   reemergence. siRNA-mediated knockdown and recomplementation analyses show that islet formation is based on the cytoplasmic tail of MT1-MMP and its ability to bind the subcortical actin cytoskeleton. Collectively, our data reveal a previously unrecognized phase in the   podosome   life cycle and identify a structural function of MT1-MMP that is independent of its proteolytic activity. MT1-MMP islets thus act as cellular memory devices that enable efficient and localized reformation of   podosomes  , ensuring coordinated matrix degradation and invasion. 
  [Mm]igration  Process  
 astrocyte cell   migration   
 positive regulation of cell   migration   
 positive regulation of macrophage   migration   
  PubMed  
 Timp-2 binding with cellular MT1-MMP stimulates invasion-promoting MEK/ERK signaling in cancer cells. Both invasion-promoting MT1-MMP and its physiological inhibitor TIMP-2 play a significant role in tumorigenesis and are identified in the most aggressive cancers. Despite its antiproteolytic effects in vitro, clinical data suggest that TIMP-2 expression is positively associated with tumor recurrence, thus emphasizing the wide-ranging role of TIMP-2 in malignancies. To shed light on this role of TIMP-2, we report that low concentrations of TIMP-2, by interacting with MT1-MMP (a specific membrane receptor of TIMP-2), induce the MEK/ERK signaling cascade in fibrosarcoma HT1080 cells which express MT1-MMP naturally. TIMP-2 binding with cell surface-associated MT1-MMP stimulates phosphorylation of MEK1/2, which is upstream of ERK1/2, and the ERK1/2 substrate p90RSK. Consistent with volumes of literature, we confirmed that the activation of ERK stimulated cell   migration  . Both the transcriptional silencing of MT1-MMP and the inhibition of MEK1/2 reversed the signaling effects of TIMP-2/MT1-MMP while the active site-targeting MMP inhibitor GM6001 did not. Our data suggest that both the interactions of TIMP-2 with MT1-MMP, which activate the pro-migratory ERK signaling cascade,and the conventional inhibition of MT1-MMP's catalytic activity by TIMP-2, play a role in the invasion-promoting function of MT1-MMP. The TIMP-2-induced stimulation of ERK signaling in cancer cells explains the direct, as opposed to the inverse, association of TIMP-2 expression with poor prognosis in cancer. 
 Differential regulation of membrane type 1-matrix metalloproteinase activity by ERK 1/2- and p38 MAPK-modulated tissue inhibitor of metalloproteinases 2 expression controls transforming growth factor-beta1-induced pericellular collagenolysis. Acquisition of matrix metalloproteinase-2 (MMP-2) activity is temporally associated with increased   migration   and invasiveness of cancer cells. ProMMP-2 activation requires multimolecular complex assembly involving proMMP-2, membrane type 1-MMP (MT1-MMP, MMP-14), and tissue inhibitor of metalloproteinases-2 (TIMP-2). Because transforming growth factor-beta1 (TGF-beta1) promotes tumor invasion in advanced squamous cell carcinomas, the role of TGF-beta1 in the regulation of MMP activity in a cellular model of invasive oral squamous cell carcinoma was examined. Treatment of oral squamous cell carcinoma cells with TGF-beta1 promoted MMP-dependent cell scattering and collagen invasion, increased expression of MMP-2 and MT1-MMP, and enhanced MMP-2 activation. TGF-beta1 induced concomitant activation of ERK1/2 and p38 MAPK, and kinase inhibition studies revealed a negative regulatory role for ERK1/2 in modulating acquisition of MMP-2 activity. Thus, a reciprocal effect on proMMP-2 activation was observed whereupon blocking ERK1/2 phosphorylation promoted proMMP-2 activation and MT1-MMP activity, whereas inhibiting p38 MAPK activity decreased proteolytic potential. The cellular mechanism for the control of MT1-MMP catalytic activity involved concurrent reciprocal modulation of TIMP-2 expression by ERK1/2 and p38 MAPKs, such that inhibition of ERK1/2 phosphorylation decreased TIMP-2 production, and down-regulation of p38 MAPK activity enhanced TIMP-2 synthesis. Further, p38 MAPK inhibition promoted ERK1/2 phosphorylation, providing additional evidence for cross-talk between MAPK pathways. These observations demonstrate the complex reciprocal effects of ERK1/2 and p38 MAPK in the regulation of MMP activity, which could complicate the use of MAPK-specific inhibitors as therapeutic agents to down-regulate the biologic effects of TGF-beta1 on pericellular collagen degradation and tumor invasion. 
 Notch1 Autoactivation via Transcriptional Regulation of Furin, Which Sustains Notch1 Signaling by Processing Notch1-Activating Proteases ADAM10 and Membrane Type 1 Matrix Metalloproteinase. Notch1 is an evolutionarily conserved transmembrane receptor involved in melanoma growth. Notch1 is first cleaved by furin in the Golgi apparatus to produce the biologically active heterodimer. Following ligand binding, Notch1 is cleaved at the cell membrane by proteases such as ADAM10 and -17 and membrane type 1 matrix metalloproteinase (MT1-MMP), the latter of which we recently identified as a novel protease involved in Notch1 processing. The final cleavage is γ-secretase dependent and releases the active Notch intracellular domain (NIC). We now demonstrate that Notch1 directly regulates furin expression. Aside from activating Notch1, furin cleaves and activates several proteases, including MT1-MMP, ADAM10, and ADAM17. By chromatin immunoprecipitation and a reporter assay, we demonstrate that Notch1 binds at position -1236 of the furin promoter and drives furin expression. The Notch1-dependent enhancement of furin expression increases the activities of MT1-MMP and ADAM10 but not that of ADAM17, as demonstrated by short hairpin RNA (shRNA) knockdown of furin, and promotes the cleavage of Notch1 itself. These data highlight a novel positive-feedback loop whereby Notch1-dependent furin expression can induce Notch1 signaling by increasing Notch1 processing and by potentiating the activity of the proteases responsible for Notch1 activation. This leads to Notch1 signal amplification, which can promote melanoma tumor growth and progression, as demonstrated by the inhibition of cell   migratio  n and invasion upon furin inhibition downstream of Notch1. Disruption of such feedback signaling might represent an avenue for the treatment of melanoma. 
 Vinculin negatively regulates transcription of MT1-MMP through MEK/ERK pathway. Vinculin regulates a variety of cellular functions partly through stabilization of tumor suppressor PTEN. In order to study the role of vinculin in tumor progression other than PTEN stabilization, vinculin was knocked down in PTEN-deficient squamous cell carcinoma HSC-4 cells. Knockdown of vinculin induced phenotypical change by reducing cell-cell and cell-extracellular matrix adhesions, and enhanced MT1-MMP expression at transcription level and subsequent cell   migration  . Up-regulation of MT1-MMP transcription by vinculin knockdown was abrogated by ERK inhibition. These results suggest that vinculin negatively regulates malignant phenotype of tumor cells including MT1-MMP transcription through MEK/ERK pathway. 
 A novel high-content analysis tool reveals Rab8-driven cytoskeletal reorganization through Rho GTPases, calpain and MT1-MMP. Rab8 is a small Ras-related GTPase that regulates polarized membrane transport to the plasma membrane. Here, we developed a high-content analysis (HCA) tool to dissect Rab8-mediated actin and focal adhesion reorganization that revealed that Rab8 activation significantly induced Rac1 and Tiam1 to mediate cortical actin polymerization and RhoA-dependent stress fibre disassembly. Rab8 activation increased Rac1 activity, whereas its depletion activated RhoA, which led to reorganization of the actin cytoskeleton. Rab8 was also associated with focal adhesions, promoting their disassembly in a microtubule-dependent manner. This Rab8 effect involved calpain, MT1-MMP (also known as MMP14) and Rho GTPases. Moreover, we demonstrate the role of Rab8 in the cell   migration   process. Indeed, Rab8 is required for EGF-induced cell polarization and chemotaxis, as well as for the directional persistency of intrinsic cell motility. These data reveal that Rab8 drives cell motility by mechanisms both dependent and independent of Rho GTPases, thereby regulating the establishment of cell polarity, turnover of focal adhesions and actin cytoskeleton rearrangements, thus determining the directionality of cell   migration  . 
 ECM regulates MT1-MMP localization with beta1 or alphavbeta3 integrins at distinct cell compartments modulating its internalization and activity on human endothelial cells. Regulation of membrane-type 1 matrix metalloproteinase (MT1-MMP) by different extracellular matrices (ECMs) on human endothelial cells (ECs) has been investigated. First, MT1-MMP is found at the intercellular contacts of confluent ECs grown on beta1 integrin-dependent matrix such as type 1 collagen (COL I), fibronectin (FN), or fibrinogen (FG), but not on gelatin (GEL) or vitronectin (VN). The novel localization of MT1-MMP at cell-cell contacts is assessed by confocal videomicroscopy of MT1-MMP-GFP-transfected ECs. Moreover, MT1-MMP colocalizes with beta1 integrins at the intercellular contacts, whereas it is preferentially found with alphavbeta3 integrin at motility-associated structures on migrating ECs. In addition, clustered integrins recruit MT1-MMP and neutralizing anti-beta1 or anti-alphav integrin mAb displace MT1-MMP from its specific sites, pointing to a biochemical association that is finally demonstrated by coimmunoprecipitation assays. On the other hand, COL I, FN, or FG up-regulate cell surface MT1-MMP on confluent ECs by an impairment of its internalization, whereas expression and internalization are not modified on GEL or VN. In addition, MT1-MMP activity is diminished in confluent ECs on COL I, FN, or FG. Finally, MT1-MMP participates and cooperates with beta1 and alphavbeta3 integrins in the   migration   of ECs on different ECM. These data show a novel mechanism by which ECM regulates MT1-MMP association with beta1 or alphavbeta3 integrins at distinct cellular compartments, thus modulating its internalization, activity, and function on human ECs. 
 Analysis of the myosin-II-responsive focal adhesion proteome reveals a role for β-Pix in negative regulation of focal adhesion maturation. Focal adhesions undergo myosin-II-mediated maturation wherein they grow and change composition to modulate integrin signalling for cell   migratio  n, growth and differentiation. To determine how focal adhesion composition is affected by myosin II activity, we performed proteomic analysis of isolated focal adhesions and compared protein abundance in focal adhesions from cells with and without myosin II inhibition. We identified 905 focal adhesion proteins, 459 of which changed in abundance with myosin II inhibition, defining the myosin-II-responsive focal adhesion proteome. The abundance of 73% of the proteins in the myosin-II-responsive focal adhesion proteome was enhanced by contractility, including proteins involved in Rho-mediated focal adhesion maturation and endocytosis- and calpain-dependent focal adhesion disassembly. During myosin II inhibition, 27% of proteins in the myosin-II-responsive focal adhesion proteome, including proteins involved in Rac-mediated lamellipodial protrusion, were enriched in focal adhesions, establishing that focal adhesion protein recruitment is also negatively regulated by contractility. We focused on the Rac guanine nucleotide exchange factor β-Pix, documenting its role in the negative regulation of focal adhesion maturation and the promotion of lamellipodial protrusion and focal adhesion turnover to drive cel  l migrati  on. 
 Tissue inhibitor of metalloproteinases-2 binding to membrane-type 1 matrix metalloproteinase induces MAPK activation and cell growth by a non-proteolytic mechanism. Membrane-type 1 matrix metalloproteinase (MT1-MMP), a transmembrane proteinase with a short cytoplasmic domain and an extracellular catalytic domain, controls a variety of physiological and pathological processes through the proteolytic degradation of extracellular or transmembrane proteins. MT1-MMP forms a complex on the cell membrane with its physiological protein inhibitor, tissue inhibitor of metalloproteinases-2 (TIMP-2). Here we show that, in addition to extracellular proteolysis, MT1-MMP and TIMP-2 control cell proliferation and   migration   through a non-proteolytic mechanism. TIMP-2 binding to MT1-MMP induces activation of ERK1/2 by a mechanism that does not require the proteolytic activity and is mediated by the cytoplasmic tail of MT1-MMP. MT1-MMP-mediated activation of ERK1/2 up-regulates cell   migration   and proliferation in vitro independently of extracellular matrix proteolysis. Proteolytically inactive MT1-MMP promotes tumor growth in vivo, whereas proteolytically active MT1-MMP devoid of cytoplasmic tail does not have this effect. These findings illustrate a novel role for MT1-MMP-TIMP-2 interaction, which controls cell functions by a mechanism independent of extracellular matrix degradation. 
 TIMP-2 Interaction with MT1-MMP Activates the AKT Pathway and Protects Tumor Cells from Apoptosis. Membrane-type 1 matrix metalloproteinase (MT1-MMP), a transmembrane proteinase with an extracellular catalytic domain and a short cytoplasmic tail, degrades a variety of extracellular matrix (ECM) components. In addition, MT1-MMP activates intracellular signaling through proteolysis-dependent and independent mechanisms. We have previously shown that binding of tissue inhibitor of metalloproteinases-2 (TIMP-2) to MT1-MMP controls cell proliferation and   migration  , as well as tumor growth in vivo by activating the Ras-extracellular signal regulated kinase-1 and -2 (ERK1/2) pathway through a mechanism that requires the cytoplasmic but not the proteolytic domain of MT1-MMP. Here we show that in MT1-MMP expressing cells TIMP-2 also induces rapid and sustained activation of AKT in a dose- and time-dependent manner and by a mechanism independent of the proteolytic activity of MT1-MMP. Fibroblast growth factor receptor-1 mediates TIMP-2 induction of ERK1/2 but not of AKT activation; however, Ras activation is necessary to transduce the TIMP-2-activated signal to both the ERK1/2 and AKT pathways. ERK1/2 and AKT activation by TIMP-2 binding to MT1-MMP protects tumor cells from apoptosis induced by serum starvation. Conversely, TIMP-2 upregulates apoptosis induced by three-dimensional type I collagen in epithelial cancer cells. Thus, TIMP-2 interaction with MT1-MMP provides tumor cells with either pro- or anti-apoptotic signaling depending on the extracellular environment and apoptotic stimulus. 
 Norcantharidin enhances TIMP‑2 anti‑vasculogenic mimicry activity for human gallbladder cancers through downregulating MMP‑2 and MT1‑MMP. Vasculogenic mimicry (VM) is a tumor microcirculation pattern in highly aggressive gallbladder cancers (GBCs). We recently reported the anti‑VM activity of norcantharidin (NCTD) in highly aggressive GBC‑SD cells and xenografts. In this study, we further investigated that NCTD enhanced tissue inhibitor of matrix metalloproteinase‑2 (TIMP‑2) anti‑VM activity for GBCs and the underlying mechanisms. In vivo and in vitro experiments were performed to determine the effects of NCTD in combination with TIMP‑2 on tumor growth, host survival, VM formation, hemodynamic of GBC‑SD xenografts, and VM‑like networks and malignant phenotypes of GBC‑SD cells. Expression of matrix metalloproteinase (MMP)‑2 and membrane type 1‑MMP (MT1‑MMP) among human GBCs, GBC‑SD cells and xenografts were determined, respectively. The results showed that expression of MMP‑2 and MT1‑MMP in human GBCs, GBC‑SD cells and xenografts was significantly related to VM in GBCs; a shorter survival time of VM‑positive patients with high expression of MMP‑2 or MT1‑MMP compared to that of the patients with low expression. After treatment with NCTD+TIMP‑2, tumor growth, VM formation, VM hemodynamic of the xenografts in vivo were significantly inhibited as compared to control, NCTD or TIMP‑2 group, with a prolonged survival time of the xenograft mice (log‑rank test, P=0.0115); and these observations were confirmed by VM‑like networks by 3‑D matr  ices and   showed that proliferation, apoptosis, invasion, migration of GBC‑SD cells in vitro were markedly affected. Furthermore, expression of MMP‑2 and MT1‑MMP in VM formation of the xenografts in vivo and GBC‑SD cells in vitro was downregulated as compared to control, NCTD or TIMP‑2 group. Thus, we concluded that NCTD enhances TIMP‑2 antitumor and anti‑VM activities in GBCs through downregulating MMP‑2 and MT1‑MMP. 
 A novel high-content analysis tool reveals Rab8-driven cytoskeletal reorganization through Rho GTPases, calpain and MT1-MMP. Rab8 is a small Ras-related GTPase that regulates polarized membrane transport to the plasma membrane. Here, we developed a high-content analysis (HCA) tool to dissect Rab8-mediated actin and focal adhesion reorganization that revealed that Rab8 activation significantly induced Rac1 and Tiam1 to mediate cortical actin polymerization and RhoA-dependent stress fibre disassembly. Rab8 activation increased Rac1 activity, whereas its depletion activated RhoA, which led to reorganization of the actin cytoskeleton. Rab8 was also associated with focal adhesions, promoting their disassembly in a microtubule-dependent manner. This Rab8 effect involved calpain, MT1-MMP (also known as MMP14) and Rho GTPases. Moreover, we demonstrate the role of Rab8 in the cell   migration   process. Indeed, Rab8 is required for EGF-induced cell polarization and chemotaxis, as well as for the directional persistency of intrinsic cell motility. These data reveal that Rab8 drives cell motility by mechanisms both dependent and independent of Rho GTPases, thereby regulating the establishment of cell polarity, turnover of focal adhesions and actin cytoskeleton rearrangements, thus determining the directionality of cell   migration  . 
 TIMP-2 Interaction with MT1-MMP Activates the AKT Pathway and Protects Tumor Cells from Apoptosis. Membrane-type 1 matrix metalloproteinase (MT1-MMP), a transmembrane proteinase with an extracellular catalytic domain and a short cytoplasmic tail, degrades a variety of extracellular matrix (ECM) components. In addition, MT1-MMP activates intracellular signaling through proteolysis-dependent and independent mechanisms. We have previously shown that binding of tissue inhibitor of metalloproteinases-2 (TIMP-2) to MT1-MMP controls cell proliferation and   migration  , as well as tumor growth in vivo by activating the Ras-extracellular signal regulated kinase-1 and -2 (ERK1/2) pathway through a mechanism that requires the cytoplasmic but not the proteolytic domain of MT1-MMP. Here we show that in MT1-MMP expressing cells TIMP-2 also induces rapid and sustained activation of AKT in a dose- and time-dependent manner and by a mechanism independent of the proteolytic activity of MT1-MMP. Fibroblast growth factor receptor-1 mediates TIMP-2 induction of ERK1/2 but not of AKT activation; however, Ras activation is necessary to transduce the TIMP-2-activated signal to both the ERK1/2 and AKT pathways. ERK1/2 and AKT activation by TIMP-2 binding to MT1-MMP protects tumor cells from apoptosis induced by serum starvation. Conversely, TIMP-2 upregulates apoptosis induced by three-dimensional type I collagen in epithelial cancer cells. Thus, TIMP-2 interaction with MT1-MMP provides tumor cells with either pro- or anti-apoptotic signaling depending on the extracellular environment and apoptotic stimulus. 
 Notch1 Autoactivation via Transcriptional Regulation of Furin, Which Sustains Notch1 Signaling by Processing Notch1-Activating Proteases ADAM10 and Membrane Type 1 Matrix Metalloproteinase. Notch1 is an evolutionarily conserved transmembrane receptor involved in melanoma growth. Notch1 is first cleaved by furin in the Golgi apparatus to produce the biologically active heterodimer. Following ligand binding, Notch1 is cleaved at the cell membrane by proteases such as ADAM10 and -17 and membrane type 1 matrix metalloproteinase (MT1-MMP), the latter of which we recently identified as a novel protease involved in Notch1 processing. The final cleavage is γ-secretase dependent and releases the active Notch intracellular domain (NIC). We now demonstrate that Notch1 directly regulates furin expression. Aside from activating Notch1, furin cleaves and activates several proteases, including MT1-MMP, ADAM10, and ADAM17. By chromatin immunoprecipitation and a reporter assay, we demonstrate that Notch1 binds at position -1236 of the furin promoter and drives furin expression. The Notch1-dependent enhancement of furin expression increases the activities of MT1-MMP and ADAM10 but not that of ADAM17, as demonstrated by short hairpin RNA (shRNA) knockdown of furin, and promotes the cleavage of Notch1 itself. These data highlight a novel positive-feedback loop whereby Notch1-dependent furin expression can induce Notch1 signaling by increasing Notch1 processing and by potentiating the activity of the proteases responsible for Notch1 activation. This leads to Notch1 signal amplification, which can promote melanoma tumor growth and progression, as demonstrated by the inhibition of cell   migratio  n and invasion upon furin inhibition downstream of Notch1. Disruption of such feedback signaling might represent an avenue for the treatment of melanoma. 
 Norcantharidin enhances TIMP‑2 anti‑vasculogenic mimicry activity for human gallbladder cancers through downregulating MMP‑2 and MT1‑MMP. Vasculogenic mimicry (VM) is a tumor microcirculation pattern in highly aggressive gallbladder cancers (GBCs). We recently reported the anti‑VM activity of norcantharidin (NCTD) in highly aggressive GBC‑SD cells and xenografts. In this study, we further investigated that NCTD enhanced tissue inhibitor of matrix metalloproteinase‑2 (TIMP‑2) anti‑VM activity for GBCs and the underlying mechanisms. In vivo and in vitro experiments were performed to determine the effects of NCTD in combination with TIMP‑2 on tumor growth, host survival, VM formation, hemodynamic of GBC‑SD xenografts, and VM‑like networks and malignant phenotypes of GBC‑SD cells. Expression of matrix metalloproteinase (MMP)‑2 and membrane type 1‑MMP (MT1‑MMP) among human GBCs, GBC‑SD cells and xenografts were determined, respectively. The results showed that expression of MMP‑2 and MT1‑MMP in human GBCs, GBC‑SD cells and xenografts was significantly related to VM in GBCs; a shorter survival time of VM‑positive patients with high expression of MMP‑2 or MT1‑MMP compared to that of the patients with low expression. After treatment with NCTD+TIMP‑2, tumor growth, VM formation, VM hemodynamic of the xenografts in vivo were significantly inhibited as compared to control, NCTD or TIMP‑2 group, with a prolonged survival time of the xenograft mice (log‑rank test, P=0.0115); and these observations were confirmed by VM‑like networks by 3‑D matr  ices and   showed that proliferation, apoptosis, invasion, migration of GBC‑SD cells in vitro were markedly affected. Furthermore, expression of MMP‑2 and MT1‑MMP in VM formation of the xenografts in vivo and GBC‑SD cells in vitro was downregulated as compared to control, NCTD or TIMP‑2 group. Thus, we concluded that NCTD enhances TIMP‑2 antitumor and anti‑VM activities in GBCs through downregulating MMP‑2 and MT1‑MMP. 
 Vinculin negatively regulates transcription of MT1-MMP through MEK/ERK pathway. Vinculin regulates a variety of cellular functions partly through stabilization of tumor suppressor PTEN. In order to study the role of vinculin in tumor progression other than PTEN stabilization, vinculin was knocked down in PTEN-deficient squamous cell carcinoma HSC-4 cells. Knockdown of vinculin induced phenotypical change by reducing cell-cell and cell-extracellular matrix adhesions, and enhanced MT1-MMP expression at transcription level and subsequent cell   migration  . Up-regulation of MT1-MMP transcription by vinculin knockdown was abrogated by ERK inhibition. These results suggest that vinculin negatively regulates malignant phenotype of tumor cells including MT1-MMP transcription through MEK/ERK pathway. 
 Timp-2 binding with cellular MT1-MMP stimulates invasion-promoting MEK/ERK signaling in cancer cells. Both invasion-promoting MT1-MMP and its physiological inhibitor TIMP-2 play a significant role in tumorigenesis and are identified in the most aggressive cancers. Despite its antiproteolytic effects in vitro, clinical data suggest that TIMP-2 expression is positively associated with tumor recurrence, thus emphasizing the wide-ranging role of TIMP-2 in malignancies. To shed light on this role of TIMP-2, we report that low concentrations of TIMP-2, by interacting with MT1-MMP (a specific membrane receptor of TIMP-2), induce the MEK/ERK signaling cascade in fibrosarcoma HT1080 cells which express MT1-MMP naturally. TIMP-2 binding with cell surface-associated MT1-MMP stimulates phosphorylation of MEK1/2, which is upstream of ERK1/2, and the ERK1/2 substrate p90RSK. Consistent with volumes of literature, we confirmed that the activation of ERK stimulated cell   migration  . Both the transcriptional silencing of MT1-MMP and the inhibition of MEK1/2 reversed the signaling effects of TIMP-2/MT1-MMP while the active site-targeting MMP inhibitor GM6001 did not. Our data suggest that both the interactions of TIMP-2 with MT1-MMP, which activate the pro-migratory ERK signaling cascade,and the conventional inhibition of MT1-MMP's catalytic activity by TIMP-2, play a role in the invasion-promoting function of MT1-MMP. The TIMP-2-induced stimulation of ERK signaling in cancer cells explains the direct, as opposed to the inverse, association of TIMP-2 expression with poor prognosis in cancer. 
 Tissue inhibitor of metalloproteinases-2 binding to membrane-type 1 matrix metalloproteinase induces MAPK activation and cell growth by a non-proteolytic mechanism. Membrane-type 1 matrix metalloproteinase (MT1-MMP), a transmembrane proteinase with a short cytoplasmic domain and an extracellular catalytic domain, controls a variety of physiological and pathological processes through the proteolytic degradation of extracellular or transmembrane proteins. MT1-MMP forms a complex on the cell membrane with its physiological protein inhibitor, tissue inhibitor of metalloproteinases-2 (TIMP-2). Here we show that, in addition to extracellular proteolysis, MT1-MMP and TIMP-2 control cell proliferation and   migration   through a non-proteolytic mechanism. TIMP-2 binding to MT1-MMP induces activation of ERK1/2 by a mechanism that does not require the proteolytic activity and is mediated by the cytoplasmic tail of MT1-MMP. MT1-MMP-mediated activation of ERK1/2 up-regulates cell   migration   and proliferation in vitro independently of extracellular matrix proteolysis. Proteolytically inactive MT1-MMP promotes tumor growth in vivo, whereas proteolytically active MT1-MMP devoid of cytoplasmic tail does not have this effect. These findings illustrate a novel role for MT1-MMP-TIMP-2 interaction, which controls cell functions by a mechanism independent of extracellular matrix degradation. 
 Differential regulation of membrane type 1-matrix metalloproteinase activity by ERK 1/2- and p38 MAPK-modulated tissue inhibitor of metalloproteinases 2 expression controls transforming growth factor-beta1-induced pericellular collagenolysis. Acquisition of matrix metalloproteinase-2 (MMP-2) activity is temporally associated with increased   migration   and invasiveness of cancer cells. ProMMP-2 activation requires multimolecular complex assembly involving proMMP-2, membrane type 1-MMP (MT1-MMP, MMP-14), and tissue inhibitor of metalloproteinases-2 (TIMP-2). Because transforming growth factor-beta1 (TGF-beta1) promotes tumor invasion in advanced squamous cell carcinomas, the role of TGF-beta1 in the regulation of MMP activity in a cellular model of invasive oral squamous cell carcinoma was examined. Treatment of oral squamous cell carcinoma cells with TGF-beta1 promoted MMP-dependent cell scattering and collagen invasion, increased expression of MMP-2 and MT1-MMP, and enhanced MMP-2 activation. TGF-beta1 induced concomitant activation of ERK1/2 and p38 MAPK, and kinase inhibition studies revealed a negative regulatory role for ERK1/2 in modulating acquisition of MMP-2 activity. Thus, a reciprocal effect on proMMP-2 activation was observed whereupon blocking ERK1/2 phosphorylation promoted proMMP-2 activation and MT1-MMP activity, whereas inhibiting p38 MAPK activity decreased proteolytic potential. The cellular mechanism for the control of MT1-MMP catalytic activity involved concurrent reciprocal modulation of TIMP-2 expression by ERK1/2 and p38 MAPKs, such that inhibition of ERK1/2 phosphorylation decreased TIMP-2 production, and down-regulation of p38 MAPK activity enhanced TIMP-2 synthesis. Further, p38 MAPK inhibition promoted ERK1/2 phosphorylation, providing additional evidence for cross-talk between MAPK pathways. These observations demonstrate the complex reciprocal effects of ERK1/2 and p38 MAPK in the regulation of MMP activity, which could complicate the use of MAPK-specific inhibitors as therapeutic agents to down-regulate the biologic effects of TGF-beta1 on pericellular collagen degradation and tumor invasion. 
 ECM regulates MT1-MMP localization with beta1 or alphavbeta3 integrins at distinct cell compartments modulating its internalization and activity on human endothelial cells. Regulation of membrane-type 1 matrix metalloproteinase (MT1-MMP) by different extracellular matrices (ECMs) on human endothelial cells (ECs) has been investigated. First, MT1-MMP is found at the intercellular contacts of confluent ECs grown on beta1 integrin-dependent matrix such as type 1 collagen (COL I), fibronectin (FN), or fibrinogen (FG), but not on gelatin (GEL) or vitronectin (VN). The novel localization of MT1-MMP at cell-cell contacts is assessed by confocal videomicroscopy of MT1-MMP-GFP-transfected ECs. Moreover, MT1-MMP colocalizes with beta1 integrins at the intercellular contacts, whereas it is preferentially found with alphavbeta3 integrin at motility-associated structures on migrating ECs. In addition, clustered integrins recruit MT1-MMP and neutralizing anti-beta1 or anti-alphav integrin mAb displace MT1-MMP from its specific sites, pointing to a biochemical association that is finally demonstrated by coimmunoprecipitation assays. On the other hand, COL I, FN, or FG up-regulate cell surface MT1-MMP on confluent ECs by an impairment of its internalization, whereas expression and internalization are not modified on GEL or VN. In addition, MT1-MMP activity is diminished in confluent ECs on COL I, FN, or FG. Finally, MT1-MMP participates and cooperates with beta1 and alphavbeta3 integrins in the   migration   of ECs on different ECM. These data show a novel mechanism by which ECM regulates MT1-MMP association with beta1 or alphavbeta3 integrins at distinct cellular compartments, thus modulating its internalization, activity, and function on human ECs. 
 The Oncogenic Response to MiR-335 Is Associated with Cell Surface Expression of Membrane-Type 1 Matrix Metalloproteinase (MT1-MMP) Activity. MicroRNA miR-335 has been reported to have both tumor suppressor and oncogenic activities. In order to determine possible tissue and cell type differences in response to miR-335, we examined the effect of miR-335 on cell expression of MT1-MMP, a proteinase commonly expressed in tumors and associated with cell proliferation and   migration  . miR-335 increased cell surface expression of MT1-MMP in fibrosarcoma HT-1080 and benign prostate BPH-1 cells, but not in prostate LNCaP or breast MCF-7 tumor cells. miR-335 stimulated proliferation and cell   migration   in a wound healing in vitro assay in HT-1080, BPH-1, and U87 glioblastoma cells, cells which demonstrated significant cell surface expression of MT1-MMP. In contrast, miR-335 did not affect proliferation or   migration   in cells without a prominent plasma membrane associated MT1-MMP activity. Our data suggest that differences in response to miR-335 by tumor cells may lie in part in the mechanism of regulation of MT1-MMP production. 
 Decreased MT1-MMP in gastric cancer suppressed cell   migration   and invasion via regulating MMPs and EMT. Membrane type 1-matrix metalloproteinase (MT1-MMP) has been identified to play a significant role in several types of cancers, but little is known about the significance of MT1-MMP in gastric cancer patients. The purpose of this study is to investigate the involvement of MT1-MMP in tumor progression of gastric cancer. MT1-MMP expression levels were examined in gastric cancer tissues and cells, and normal gastric tissues and cells. The effects and molecular mechanisms of MT1-MMP expression on cell proliferation,   migration  , and invasion were also explored. In our results, MT1-MMP messenger RNA (mRNA) and protein expression levels were significantly increased in gastric cancer tissue. Moreover, the overexpression of MT1-MMP was positively associated with the status of clinical stage and lymph node metastasis through real-time PCR. Furthermore, knocking down MT1-MMP expression significantly suppressed the cell   migration   and invasion in vitro and regulated the expression of MMPs and epithelial-mesenchymal transition (EMT)-associated genes. In conclusions, our study demonstrates that MT1-MMP was overexpressed in gastric cancer tissue, and reduced expression of MT1-MMP suppressed cell   migration  , invasion, and through regulating the expression of MMPs and the process of EMT in gastric cancer. 
 Transforming growth factor-beta induces CD44 cleavage that promotes   migration   of MDA-MB-435s cells through the up-regulation of membrane type 1-matrix metalloproteinase. CD44, a transmembrane receptor for hyaluronic acid, is implicated in various adhesion-dependent cellular processes, including cell   migration  , tumor cell metastasis and invasion. Recent studies demonstrated that CD44 expressed in cancer cells can be proteolytically cleaved at the ectodomain by membrane type 1-matrix metalloproteinase (MT1-MMP) to form soluble CD44 and that CD44 cleavage plays a critical role in cancer cell   migration  . Here, we show that transforming growth factor-beta (TGF-beta), a multifunctional cytokine involved in cell proliferation, differentiation,   migration   and pathological processes, induces MT1-MMP expression in MDA-MB-435s cells. TGF-beta-induced MT1-MMP expression was blocked by the specific extracellular regulated kinase-1/2 (ERK1/2) inhibitor PD98059 and the specific phosphoinositide 3-OH kinase (PI3K) inhibitor LY294002. In addition, treatment with SP600125, an inhibitor for c-Jun NH(2)-terminal kinase (JNK), resulted in a significant inhibition of MT1-MMP production. These data suggest that ERK1/2, PI3K, and JNK likely play a role in TGF-beta-induced MT1-MMP expression. Interestingly, treatment of MDA-MB-435s cells with TGF-beta resulted in a colocalization of MT1-MMP and CD44 in the cell membrane and in an increased level of soluble CD44. Using an electric cell-substrate impedance sensing cell-electrode system, we demonstrated that TGF-beta treatment promotes MDA-MB-435s cell   migration  , involving MT1-MMP-mediated CD44 cleavage. MT1-MMP siRNA transfection-inhibited TGF-beta-induced cancer cell transendothelial   migration  . Thus, this study contributes to our understanding of molecular mechanisms that play a critical role in tumor cell invasion and metastasis. 
 MicroRNA-22 inhibits tumor growth and metastasis in gastric cancer by directly targeting MMP14 and Snail. MicroRNAs (miRNAs) deregulation is frequent in human gastric cancers (GCs), but the role of specific miRNAs involved in this disease remains elusive. MiR-22 was previously reported to act as tumor suppressors or oncogenes in diverse cancers. However, their accurate expression, function and mechanism in GC are largely unclear. Here, we found that the expression of miR-22 was significantly reduced in clinical GC tissues compared with paired adjacent normal tissues, and was significantly correlated with a more aggressive phenotype of GC in patients, and miR-22 low expression correlated with poor overall survival. The introduction of miR-22 markedly suppressed GC cell growth,   migration   and invasion, and inhibition of miR-22 promoted GC cell proliferation,   migration   and invasion in vitro. We further demonstrated that miR-22 acted as tumor suppressors through targeting extracellular matrix (ECM) remodeling member matrix metalloproteinase 14 (MMP14) and epithelial-to-mesenchymal transition (EMT) inducer Snail in GC. Moreover, ectopic expression of MMP14 or Snail restored inhibitory effects of miR-22 on cell   migration   and invasion in GC cells, and a negative relationship between the miR-22 expression and MMP14 or Snail mRNA levels was observed in GC. Finally, overexpression of miR-22 suppressed tumor growth, peritoneal dissemination and pulmonary metastasis in vivo. Taken together, we identified that miR-22 is a potent tumor suppressor in GC. MiR-22 downregulation promotes GC invasion and metastasis by upregulating MMP14 and Snail, and then inducing ECM remodeling and EMT. These findings provide a better understanding of the development and progression of GC and may be an important implication for future therapy of the GC. 
 Proteolysis of EphA2 Converts It from a Tumor Suppressor to an Oncoprotein. Eph receptor tyrosine kinases are considered candidate therapeutic targets in cancer, but they can exert opposing effects on cell growth. In the presence of its ligands, Eph receptor EphA2 suppresses signaling by other growth factor receptors, including ErbB, whereas ligand-independent activation of EphA2 augments ErbB signaling. To deploy EphA2-targeting drugs effectively in tumors, the anti-oncogenic ligand-dependent activation state of EphA2 must be discriminated from its oncogenic ligand-independent state. Because the molecular basis for the latter is little understood, we investigated how the activation state of EphA2 can be switched in tumor tissue. We found that ligand-binding domain of EphA2 is cleaved frequently by the membrane metalloproteinase MT1-MMP, a powerful modulator of the pericellular environment in tumor cells. EphA2 immunostaining revealed a significant loss of the N-terminal portion of EphA2 in areas of tumor tissue that expressed MT1-MMP. Moreover, EphA2 phosphorylation patterns that signify ligand-independent activation were observed specifically in these areas of tumor tissue. Mechanistic experiments revealed that processing of EphA2 by MT1-MMP promoted ErbB signaling, anchorage-independent growth, and cell   migration  . Conversely, expression of a proteolysis-resistant mutant of EphA2 prevented tumorigenesis and metastasis of human tumor xenografts in mice. Overall, our results showed how the proteolytic state of EphA2 in tumors determines its effector function and influences its status as a candidate biomarker for targeted therapy. 
 KIF1B promotes glioma   migration   and invasion via cell surface localization of MT1-MMP. Malignant glioma is notorious for its aggressiveness and poor prognosis, and the invasiveness of glioma cells is the major obstacle. Accumulating evidence indicates that kinesin superfamily proteins (KIFs) may play key roles in tumor invasiveness, but the mechanisms remained unresolved. Our previous study demonstrated that membrane type 1-matrix metalloproteinase (MT1-MMP) was involved in Kinesin family member 1B (KIF1B)-modulated invasion of gastric cancer cells. Therefore, the role of KIF1B in glioma cell invasion and its relationship with MT1-MMP were explored in the present study. We found that aberrantly increased expression of KIF1B was associated with worse WHO pathological classification and Karnofsky performance status (KPS), which also showed a trend towards worse prognosis. In the transwell assay, knockdown of KIF1B using siRNA repressed U87MG and A172 glioma cell   migration   and invasion. Silencing KIF1B inhibited expression of membranal MT1-MMP; however, the amount of MT1-MMP in the whole cell lysate was not affected. In conclusion, targeting KIF1B may be an option for anti-invasive therapies targeting glioma. 
 Membrane type 1-matrix metalloproteinase promotes human prostate cancer invasion and metastasis. Development of metastases requires cancer cells to breach underlying basement membrane, migrate through interstitial stroma and gain access to blood or lymphatic vessels. Membrane type 1-matrix metalloproteinase (MT1-MMP) has been linked with these processes. Expression of MT1-MMP in human prostate cancer correlates with the stage of this disseminated disease. The mechanism underlying this observation, however, still remains to be understood. To study the role of MT1-MMP in prostate cancer dissemination, endogenous and recombinant MT1-MMP expressed in human prostate cancer cell lines (DU-145 and LNCaP) were examined. Using FITC-labeled Matrigel, a soluble basement membrane extract coated coverslips, LNCaP cells stably expressing a chimera of MT1-MMP and Green Fluorescent Protein (MT1-GFP) degraded Matrigel and readily migrated over degraded substrates. The degradation of Matrigel by LNCaP cells expressing MT1-GFP was sensitive to MMP inhibitors, CT-1746 and TIMP-2, but not TIMP-1. Cell   migration   was dramatically enhanced by expression of MT1-MMP. By employing surgical orthotopic implantation of LNCaP cells stably expressing MT1-GFP into the prostate gland of immunodeficient mice, we demonstrated that MT1-MMP promotes lymph node and lung metastasis of prostate cancer cells. Together, these results emphasize the pivotal role of MT1-MMP in prostate cancer dissemination and confirm that MT1-MMP is a suitable target to prevent cancer metastasis. 
 E6/E7 oncoproteins of high risk HPV-16 upregulate MT1-MMP, MMP-2 and MMP-9 and promote the   migration   of cervical cancer cells. E6 and E7 of high risk human papillomavirus 16 (HPV16) were reported to correlate with the cervical cancer (CC). And the presence of matrix metalloproteinases (MMPs) has also been indicated to be associated with CC.The present study investigated the expression of MMPs (MT1-MMP, MMP-2 and MMP-9) in CC cells with HPV16-E6/E7 oncoprotein(s) negative or positive, and then determined the regulation of HPV16-E6/E7 oncoproteins on the expression of MMPs (MT1-MMP, MMP-2 and MMP-9) and the   migration   of cervical cancer Caski and SiHa cells with RNAi technology.It was demonstrated that the overexpression or the knockdown of HPV16-E6/E7 promoted or reduced MT1-MMP, MMP-2 and MMP-9 in CC cells. And the HPV16-E6, -E7 or -E6E7 influenced the   migration   of CC cells. The overexpression or the knockdown of them promoted or inhibited the   migration   of C33A or Caski/SiHa cells. Moreover, the chemical inhibition of MMP-2 or MMP-9 significantly reduced the   migration   of CC Caski or SiHa cells.Our results demonstrated that the E6-HPV16 or E7-HPV16 promoted the activity of MMP-2/9, and contributed to the   migration   of cervical cells. 
 Cleavage of syndecan-1 by membrane type matrix metalloproteinase-1 stimulates cell   migration  . The transmembrane heparan sulfate proteoglycan syndecan-1 was identified from a human placenta cDNA library by the expression cloning method as a gene product that interacts with membrane type matrix metalloproteinase-1 (MT1-MMP). Co-expression of MT1-MMP with syndecan-1 in HEK293T cells promoted syndecan-1 shedding, and concentration of cell-associated syndecan-1 was reduced. Treatment of cells with MMP inhibitor BB-94 or tissue inhibitor of MMP (TIMP)-2 but not TIMP-1 interfered with the syndecan-1 shedding promoted by MT1-MMP expression. In contrast, syndecan-1 shedding induced by 12-O-tetradecanoylphorbol-13-acetate treatment was inhibited by BB-94 but not by either TIMP-1 or TIMP-2. Shedding of syndecan-1 was also induced by MT3-MMP but not by other MT-MMPs. Recombinant syndecan-1 core protein was shown to be cleaved by recombinant MT1-MMP or MT3-MMP preferentially at the Gly245-Leu246 peptide bond. HT1080 fibrosarcoma cells stably transfected with the syndecan-1 cDNA (HT1080/SDC), which express endogenous MT1-MMP, spontaneously shed syndecan-1.   Migration   of HT1080/SDC cells on collagen-coated dishes was significantly slower than that of control HT1080 cells. Treatment of HT1080/SDC cells with BB-94 or TIMP-2 induced accumulation of syndecan-1 on the cell surface, concomitant with further retardation of cell   migration  . Substitution of Gly245 of syndecan-1 with Leu significantly reduced shedding from HT1080/SDC cells and cell   migration  . These results suggest that the shedding of syndecan-1 promoted by MT1-MMP through the preferential cleavage of Gly245-Leu246 peptide bond stimulates cell   migration  . 
 MT1-MMP regulates VEGF-A expression through a complex with VEGFR-2 and Src. Membrane-type-1 matrix metalloproteinase (MT1-MMP) is a zinc-dependent type-I transmembrane metalloproteinase involved in pericellular proteolysis,   migration   and invasion, with elevated levels correlating with a poor prognosis in cancer. MT1-MMP-mediated transcriptional regulation of genes in cancer cells can contribute to tumour growth, although this is poorly understood at a mechanistic level. In this study, we investigated the mechanism by which MT1-MMP regulates the expression of VEGF-A in breast cancer cells. We discovered that MT1-MMP regulates VEGFR-2 cell surface localisation and forms a complex with VEGFR-2 and Src that is dependent on the MT1-MMP hemopexin domain and independent of its catalytic activity. Although the localisation of VEGFR-2 was independent of the catalytic and intracellular domain of MT1-MMP, intracellular signalling dependent on VEGFR-2 activity leading to VEGF-A transcription still required the MT1-MMP catalytic and intracellular domain, including residues Y573, C574 and DKV582. However, there was redundancy in the function of the catalytic activity of MT1-MMP, as this could be substituted with MMP-2 or MMP-7 in cells expressing inactive MT1-MMP. The signalling cascade dependent on the MT1-MMP-VEGFR-2-Src complex activated Akt and mTOR, ultimately leading to increased VEGF-A transcription. 
 miR-34a inhibits   migration   and invasion of tongue squamous cell carcinoma via targeting MMP9 and MMP14. miR-34a is an important tumor suppressor gene in various cancer types. But little is known about the dysregulation of miR-34a in tongue squamous cell carcinoma (TSCC). In this study, we investigate the expression and potential role of miR-34a in TSCC.We evaluated miR-34a expression and its relationship with clinicopathological characters in 75 pairs of TSCC samples, and confirmed the role of miR-34a for predicting lymph node metastases from a further 15 pairs of paraffin-embedded TSCC specimens with stringent clinicopathological recruitment criteria using quantitative reverse transcription polymerase chain reaction (qRT-PCR). The effects of miR-34a on cell proliferation,   migration   and invasion were examined in TSCC cell lines using Cell Counting Kit-8 assay, wound healing assay and transwell assay, respectively. The effects of miR-34a on the expression of matrix metalloproteinase (MMP) 9 and 14 were detected by luciferase reporter assays and Western blot analysis. The expression of miR-34a, MMP9 and MMP14 were also confirmed in TSCC samples by in situ hybridization and immunohistochemistry.miR-34a expression in tumor tissues from TSCC patients with positive lymph node metastases was significantly lower than that with negative lymph node metastases. Overexpression of miR-34a significantly suppressed   migration   and invasion in TSCC cells and simultaneously inhibited the expression of MMP9 and MMP14 through targeting the coding region and the 3'untranslated region, respectively. Moreover, miR-34a expression in TSCC was inversely correlated with protein expression of MMP9 and MMP14 in the TSCC samples.miR-34a plays an important role in lymph node metastases of TSCC through targeting MMP9 and MMP14 and may have potential applications in prognosis prediction and gene therapy for lymph node metastases of TSCC patients. 
 MT1-MMP dependent repression of the tumor suppressor SPRY4 contributes to MT1-MMP driven melanoma cell motility. Metastatic melanoma is the deadliest of all skin cancers. Despite progress in diagnostics and treatment of melanoma, the prognosis for metastatic patients remains poor. We previously showed that Membrane-type 1 Matrix Metalloproteinase (MT1-MMP) is one of the drivers of melanoma metastasis. Classically, MT1-MMP regulates a verity of cellular functions including cell-to-cell interaction and cell-to-matrix communication. Recently, MT1-MMP has been found to also modulate gene expression. To specifically assess MT1-MMP dependent gene regulation in melanoma, microarray gene expression analysis was performed in a melanoma cell line whose metastatic properties depend on the activity of MT1-MMP. We identified the tumor suppressor gene SPRY4 as a new transcriptional target of MT1-MMP that is negatively regulated by the protease. Knockdown of MT1-MMP enhances SPRY4 expression at the mRNA and protein level. SPRY4 expression inversely correlates with that of MT1-MMP in melanoma samples and importantly, correlates with melanoma patient survival. SPRY4 modulates MT1-MMP dependent cell   migration   such that inhibition of SPRY4 rescues cell   migration   that has been impaired by MT1-MMP knock down. MT1-MMP decreases SPRY4 in part through an MMP2/RAC1 axis we previously show promotes cell motility downstream of MT1-MMP. These results identify the tumor suppressor SPRY4 as a novel molecular effector of MT1-MMP affecting melanoma cell motility. 
 MT1-MMP association with membrane lipid rafts facilitates G-CSF--induced hematopoietic stem/progenitor cell mobilization. Soluble matrix metalloproteinases (MMPs) facilitate the egress of hematopoietic stem/progenitor cells (HSPC) from the bone marrow (BM) during granulocyte colony-stimulating factor (G-CSF)-induced mobilization. Because membrane-type (MT)1-MMP, which is localized on the leading edge of migrating cells, activates the latent forms of soluble MMPs, we investigated its role in HSPC mobilization.We examined the effect of G-CSF on the expression of MT1-MMP and its activities (proMMP-2 activation and   migration  ) in hematopoietic cells. We also investigated the subcellular localization of MT1-MMP and the signaling pathways that regulate its expression and function in hematopoietic cells after exposure to G-CSF.We found that G-CSF increases MT1-MMP transcription and protein synthesis in hematopoietic cells; proMMP-2 activation in cocultures of HSPC with BM fibroblasts; chemoinvasion across reconstituted basement membrane Matrigel toward a stromal cell-derived factor-1 gradient, which is reduced by small interfering RNA silencing of MT1-MMP; and localization of MT1-MMP to membrane lipid rafts through a mechanism that is regulated by the phosphatidylinositol 3-kinase signaling pathway. Disruption of raft formation (by the cholesterol-sequestering agent methyl-beta-cyclodextrin) abrogated phosphatidylinositol 3-kinase phosphorylation and MT1-MMP incorporation into lipid rafts resulting in reduced proMMP-2 activation and HSPC   migration  .G-CSF-induced upregulation of MT1-MMP in hematopoietic cells and its enhanced incorporation into membrane lipid rafts contributes to proMMP-2 activation, which facilitates mobilization of HSPC. 
 MicroRNA-145 inhibits the growth, invasion, metastasis and angiogenesis of neuroblastoma cells through targeting hypoxia-inducible factor 2 alpha. Recent evidence shows that hypoxia-inducible factor 2 alpha (HIF-2α) may have critical roles in the growth and progression of neuroblastoma (NB) under non-hypoxic conditions. However, the underlying mechanisms and clinical potentials of normoxic HIF-2α expression in NB still remain largely unknown. In this study, HIF-2α immunostaining was identified in 26/42 NB tissues, which was correlated with clinicopathological features. In subtotal 20 NB cases, microRNA-145 (miR-145) was downregulated and inversely correlated with HIF-2α expression. Bioinformatics analysis revealed a putative miR-145 binding site in the 3'-untranslated region (3'-UTR) of HIF-2α messenger RNA (mRNA). Overexpression or knockdown of miR-145 responsively altered both the mRNA and protein levels of HIF-2α and its downstream genes, cyclin D1, matrix metalloproteinase 14 and vascular endothelial growth factor, in normoxically cultured NB cell lines SH-SY5Y and SK-N-SH. In a luciferase reporter system, miR-145 downregulated the luciferase activity of HIF-2α 3'-UTR, and these effects were abolished by a mutation in the putative miR-145-binding site. Overexpression of miR-145 suppressed the growth, invasion, metastasis and angiogenesis of SH-SY5Y and SK-N-SH cells in vitro and in vivo, while restoration of HIF-2α expression rescued the tumor cells from miR-145-mediated defects in these biological features. Furthermore, anti-miR-145 inhibitor rescued the HIF-2α knockdown-mediated repression on the   growth,   migration, invasion and angiogenesis of NB cells. These data indicate that miR-145 suppresses HIF-2α expression via the binding site in the 3'-UTR under normoxic conditions, thus inhibiting the aggressiveness and angiogenesis of NB. 
 Membrane-Type 1 Matrix Metalloproteinase Downregulates Fibroblast Growth Factor-2 Binding to the Cell Surface and Intracellular Signaling. Membrane-type 1 matrix metalloproteinase (MT1-MMP, MMP-14), a transmembrane proteinase with an extracellular catalytic domain and a short cytoplasmic tail, degrades extracellular matrix components and controls diverse cell functions through proteolytic and non-proteolytic interactions with extracellular, intracellular, and transmembrane proteins. Here we show that in tumor cells MT1-MMP downregulates fibroblast growth factor-2 (FGF-2) signaling by reducing the amount of FGF-2 bound to the cell surface with high and low affinity. FGF-2 induces weaker activation of ERK1/2 MAP kinase in MT1-MMP expressing cells than in cells devoid of MT1-MMP. This effect is abolished in cells that express proteolytically inactive MT1-MMP but persists in cells expressing MT1-MMP mutants devoid of hemopexin-like or cytoplasmic domain, showing that FGF-2 signaling is downregulated by MT1-MMP proteolytic activity. MT1-MMP expression results in downregulation of FGFR-1 and -4, and in decreased amount of cell surface-associated FGF-2. In addition, MT1-MMP strongly reduces the amount of FGF-2 bound to the cell surface with low affinity. Because FGF-2 association with low-affinity binding sites is a prerequisite for binding to its high-affinity receptors, downregulation of low-affinity binding to the cell surface results in decreased FGF-2 signaling. Consistent with this conclusion, FGF-2 induction of tumor cell   migration   and invasion in vitro is stronger in cells devoid of MT1- MMP than in MT1-MMP expressing cells. Thus, MT1-MMP controls FGF-2 signaling by a proteolytic mechanism that decreases the cell's biological response to FGF-2. 
 Non-destructive and selective imaging of the functionally active, pro-invasive membrane type-1 matrix metalloproteinase (MT1-MMP) enzyme in cancer cells. Proteolytic activity of cell surface-associated MT1-matrix metalloproteinase (MMP) (MMP-14) is directly related to cell   migration  , invasion, and metastasis. MT1-MMP is regulated as a proteinase by activation and conversion of the latent proenzyme into the active enzyme, and also via inhibition by tissue inhibitors of MMPs (TIMPs) and self-proteolysis. MT1-MMP is also regulated as a membrane protein through its internalization and recycling. Routine immunohistochemistry, flow cytometry, reverse transcription-PCR, and immunoblotting methodologies do not allow quantitative imaging and assessment of the cell-surface levels of the active, TIMP-free MT1-MMP enzyme. Here, we developed a fluorescent reporter prototype that targets the cellular active MT1-MMP enzyme alone. The reporter (MP-3653) represents a liposome tagged with a fluorochrome and functionalized with a PEG chain spacer linked to an inhibitory hydroxamate warhead. Our studies using the MP-3653 reporter and its inactive derivative demonstrated that MP-3653 can be efficiently used not only to visualize the trafficking of MT1-MMP through the cell compartment, but also to quantify the femtomolar range amounts of the cell surface-associated active MT1-MMP enzyme in multiple cancer cell types, including breast carcinoma, fibrosarcoma, and melanoma. Thus, the levels of the naturally expressed, fully functional, active cellular MT1-MMP enzyme are roughly equal to 1 × 10(5) molecules/cell, whereas these levels are in a 1 × 10(6) range in the cells with the enforced MT1-MMP expression. We suggest that the reporter we developed will contribute to the laboratory studies of MT1-MMP and then, ultimately, to the design of novel, more efficient prognostic approaches and personalized cancer therapies. 
 Calcium regulation of matrix metalloproteinase-mediated   migration   in oral squamous cell carcinoma cells. Activation of matrix metalloproteinase 2 (MMP-2) has been shown to play a significant role in the behavior of cancer cells, affecting both   migration   and invasion. The activation process requires multimolecular complex formation involving pro-MMP-2, membrane type 1-MMP (MT1-MMP), and tissue inhibitor of metalloproteinases-2 (TIMP-2). Because calcium is an important regulator of keratinocyte function, we evaluated the effect of calcium on MMP regulation in an oral squamous cell carcinoma line (SCC25). Increasing extracellular calcium (0.09-1.2 mm) resulted in a dose-dependent increase in MT1-MMP-dependent pro-MMP-2 activation. Despite the requirement for MT1-MMP in the activation process, no changes in MT1-MMP expression, cell surface localization, or endocytosis were apparent. However, increased generation of the catalytically inactive 43-kDa MT1-MMP autolysis product and decline in the TIMP-2 levels in conditioned media were observed. The decrease in TIMP-2 levels in the conditioned media was prevented by a broad spectrum MMP inhibitor, suggesting that calcium promotes recruitment of TIMP-2 to MT1-MMP on the cell surface. Despite the decline in soluble TIMP-2, no accumulation of TIMP-2 in cell lysates was seen. Blocking TIMP-2 degradation with bafilomycin A1 significantly increased cell-associated TIMP-2 levels in the presence of high calcium. These data suggest that the decline in TIMP-2 is because of increased calcium-mediated MT1-MMP-dependent degradation of TIMP-2. In functional studies, increasing calcium enhanced MMP-dependent cellular   migration   on laminin-5-rich matrix using an in vitro colony dispersion assay. Taken together, these results suggest that changes in extracellular calcium can regulate post-translational MMP dynamics and thus affect the cellular behavior of oral squamous cell carcinoma. 
 CD44 directs membrane-type 1 matrix metalloproteinase to lamellipodia by associating with its hemopexin-like domain. Membrane-type 1 matrix metalloproteinase (MT1- MMP) localizes at the front of migrating cells and degrades the extracellular matrix barrier during cancer invasion. However, it is poorly understood how the polarized distribution of MT1-MMP at the   migration   front is regulated. Here, we demonstrate that MT1-MMP forms a complex with CD44H via the hemopexin-like (PEX) domain. A mutant MT1-MMP lacking the PEX domain failed to bind CD44H and did not localize at the lamellipodia. The cytoplasmic tail of CD44H, which comprises interfaces that associate with the actin cytoskeleton, was important for its localization at lamellipodia. Overexpression of a CD44H mutant lacking the cytoplasmic tail also prevented MT1-MMP from localizing at the lamellipodia. Modulation of F-actin with cytochalasin D revealed that both CD44H and MT1-MMP co-localize closely with the actin cytoskeleton, dependent on the cytoplasmic tail of CD44H. Thus, CD44H appears to act as a linker that connects MT1-MMP to the actin cytoskeleton and to play a role in directing MT1-MMP to the   migration   front. The PEX domain of MT1-MMP was indispensable in promoting cell   migration   and CD44H shedding. 
 Membrane type 1 matrix metalloprotease cleaves laminin-10 and promotes prostate cancer cell   migration  . Disruption of the extracellular matrix by proteases is crucial for tumor invasion. Laminin-10 (Ln-10) has previously been identified as a substrate for cell   migration   and cell adhesion, and is present in the basal lamina (BL) of both normal prostate and prostate cancer. Here, we investigate a role for membrane type 1 matrix metalloprotease (MT1-MMP) in modifying this Ln-10-rich BL. MT1-MMP is a transmembrane member of the MMP family that has been demonstrated to be upregulated as prostate cancer progresses from normal to prostate intraepithelial neoplasia to invasive cancer, suggesting a role for MT1-MMP in the invasion of prostate cancer. We show that MT1-MMP cleaves the alpha5 chain of purified human Ln-10 from its 350-kDa form into 310-, 190-, 160-, and 45-kDa fragments. This cleavage causes a decrease in DU-145 prostate cancer cell adhesion to purified Ln-10, and an increase in trans  migration   of DU-145 cells through cleaved Ln-10. We also show that prostate cancer cells expressing membrane-bound MT1-MMP cleave the alpha5 chain of Ln-10. Ln alpha5-chain cleavage is also observed in human prostate cancer tissues. These findings suggest that prostate cancer cells expressing high levels of MT1-MMP have increased invasive potential through their ability to degrade and invade Ln-10 barriers. 
 Cleavage of metastasis suppressor gene product KiSS-1 protein/metastin by matrix metalloproteinases. A human placenta cDNA library was screened by the expression cloning method for gene products that interact with matrix metalloproteinases (MMPs), and we isolated a cDNA whose product formed a stable complex with pro-MMP-2 and pro-MMP-9. The cDNA encoded the metastasis suppressor gene KiSS-1. KiSS-1 protein was shown to form a complex with pro-MMP. KiSS-1 protein is known to be processed to peptide ligand of a G-protein-coupled receptor (hOT7T175) named metastin, and suppresses metastasis of tumors expressing the receptor. Active MMP-2, MMP-9, MT1-MMP, MT3-MMP and MT5-MMP cleaved the Gly118-Leu119 peptide bond of not only full-length KiSS-1 protein but also metastin decapeptide. Metastin decapeptide induced formation of focal adhesion and actin stress fibers in cells expressing the receptor, and digestion of metastin decapeptide by MMP abolished its ligand activity.   Migration   of HT1080 cells expressing hOT7T175 that harbor a high-level MMP activity was only slightly suppressed by either metastin decapeptide or MMP inhibitor BB-94 alone, but the combination of metastin decapeptide and BB-94 showed a synergistic effect in blocking cell   migration  . We propose that metastin could be used as an antimetastatic agent in combination with MMP inhibitor, or MMP-resistant forms of metastin could be developed and may also be efficacious. 
 p63/MT1-MMP axis is required for in situ to invasive transition in basal-like breast cancer. The transition of ductal carcinoma in situ (DCIS) to invasive breast carcinoma requires tumor cells to cross the basement membrane (BM). However, mechanisms underlying BM trans  migration   are poorly understood. Here, we report that expression of membrane-type 1 (MT1)-matrix metalloproteinase (MMP), a key component of the BM invasion program, increases during breast cancer progression at the in situ to invasive breast carcinoma transition. In the intraductal xenograft model, MT1-MMP is required for BM trans  migration   of MCF10DCIS.com breast adenocarcinoma cells and is overexpressed in cell clusters overlying focal BM disruptions and at the invasive tumor front. Mirrored upregulation of p63 and MT1-MMP is observed at the edge of MCF10DCIS.com xenograft tumors and p63 is required for induction of MT1-MMP-dependent invasive program in response to microenvironmental signals. Immunohistochemistry and analysis of public database reveal that p63 and MT1-MMP are upregulated in human basal-like breast tumors suggesting that p63/MT1-MMP axis contributes to progression of basal-like breast cancers with elevated p63 and MT1-MMP levels. 
 Up-regulation of vascular endothelial growth factor by membrane-type 1 matrix metalloproteinase stimulates human glioma xenograft growth and angiogenesis. Membrane-type (MT) 1 matrix metalloproteinase (MMP) is up-regulated in many tumor types and has been implicated in tumor progression and metastasis. MT1-MMP is critical for pericellular degradation of the extracellular matrix, thereby promoting tumor cell invasion and dissemination. To grow efficiently in vivo, tumor cells induce angiogenesis in both primary solid tumors and metastatic foci. The present study describes a functional link between the expression of MT1-MMP and vascular endothelial growth factor (VEGF) production in human glioma U251 xenografts in athymic mice. To investigate the effects of MT1-MMP on VEGF expression, U251 cells were stably transfected with MT1-MMP to generate the U-MT cell line overexpressing the enzyme. In vitro, the U-MT cells had an increased rate of proliferation and   migration   as well as the ability to activate the MMP-2 proenzyme and directionally remodel a three-dimensional collagen matrix. These findings suggested higher tumorigenicity of U-MT cells relative to the vector-control U-neo cells. In agreement with the in vitro data, U-MT xenografts in BALB/c nu/nu mice displayed markedly increased growth rates and elevated levels of angiogenesis. In contrast, U-neo cells formed small, minimally vascularized tumors. The elevated angiogenesis in U-MT xenografts was associated with an up-regulation of VEGF expression in tumor cells. In addition, U-MT cells in vitro secreted twice as much VEGF as the control cells. GM6001, a hydroxamate inhibitor of MMP activity, down-regulated the production of VEGF in U-MT cells to the levels observed in the U-neo control. Our results demonstrate that the enhanced tumorigenicity of glioma cells overexpressing MT1-MMP involves stimulation of angiogenesis through the up-regulation of VEGF production. 
 Endothelial cell lumen and vascular guidance tunnel formation requires MT1-MMP-dependent proteolysis in 3-dimensional collagen matrices. Here we show that endothelial cells (EC) require matrix type 1-metalloproteinase (MT1-MMP) for the formation of lumens and tube networks in 3-dimensional (3D) collagen matrices. A fundamental consequence of EC lumen formation is the generation of vascular guidance tunnels within collagen matrices through an MT1-MMP-dependent proteolytic process. Vascular guidance tunnels represent a conduit for EC motility within these spaces (a newly remodeled 2D matrix surface) to both assemble and remodel tube structures. Interestingly, it appears that twice as many tunnel spaces are created than are occupied by tube networks after several days of culture. After tunnel formation, these spaces represent a 2D migratory surface within 3D collagen matrices allowing for EC   migration   in an MMP-independent fashion. Blockade of EC lumenogenesis using inhibitors that interfere with the process (eg, integrin, MMP, PKC, Src) completely abrogates the formation of vascular guidance tunnels. Thus, the MT1-MMP-dependent proteolytic process that creates tunnel spaces is directly and functionally coupled to the signaling mechanisms required for EC lumen and tube network formation. In summary, a fundamental and previously unrecognized purpose of EC tube morphogenesis is to create networks of matrix conduits that are necessary for EC   migration   and tube remodeling events critical to blood vessel assembly. 
 VEGF stimulates HDAC7 phosphorylation and cytoplasmic accumulation modulating matrix metalloproteinase expression and angiogenesis. Histone acetylation/deacetylation plays an important role in the control of gene expression, tissue growth, and development. In particular, histone deacetylases 7 (HDAC7), a member of class IIa HDACs, is crucial in maintaining vascular integrity. However, whether HDAC7 is involved in the processes of vascular endothelial signaling and angiogenesis remains unclear. Here, we investigated the role of HDAC7 in vascular endothelial growth factor (VEGF) signaling and angiogenesis.We show for the first time that VEGF stimulated phosphorylation of HDAC7 at the sites of Ser178, Ser344, and Ser479 in a dose- and time-dependent manner, which leads to the cytoplasmic accumulation of HDAC7. Using pharmacological inhibitors, siRNA, and adenoviruses carrying dominant-negative mutants, we found that phospholipase Cgamma/protein kinase C/protein kinase D1 (PKD1)-dependent signal pathway mediated HDAC7 phosphorylation and cytoplasmic accumulation by VEGF. Infection of ECs with adenoviruses encoding a mutant of HDAC7 specifically deficient in PKD1-dependent phosphorylation inhibited VEGF-induced angiogenic gene expression, including matrix metalloproteinases MT1-matrix metalloproteinase (MMP) and MMP10. Moreover, HDAC7 and its targeting genes were involved in VEGF-stimulated endothelial cell   migration  , tube formation, and microvessel sprouting.Our results demonstrate that VEGF stimulates PKD1-dependent HDAC7 phosphorylation and cytoplasmic accumulation in endothelial cells modulating gene expression and angiogenesis. 
 Membrane type 1-matrix metalloproteinase is involved in the   migration   of human monocyte-derived dendritic cells. Dendritic cells (DC) are highly mobile APC. The trafficking of both immature and mature DC is crucial for their functions, which depends mainly on chemotactic attraction and matrix metalloproteinases (MMP) activity. MMP that are in a transmembrane form belong to membrane type (MT)-MMP, among which MT1-MMP has been shown to possess strong proteolytic activity that is capable of degrading extracellular matrix molecules. Although it is well established that MMP are zinc-dependent endopeptidases that collectively degrade most components of the extracellular matrix, relatively little is known about MT-MMP-mediated matrix degradation during DC   migration  . In this study, we showed that MT1-MMP was expressed in human monocyte-derived immature and mature DC by semi-quantitative reverse transcription PCR and western blotting analyses. Moreover, immunofluorescence microscopic studies showed that MT1-MMP was expressed on the membrane surface of DC. Blocking of MT1-MMP activity greatly reduced the invasion capacity of immature DC in Matrigel, whereas mature DC mobility was not affected. Taken together, our results show a novel functional link between MT1-MMP and DC motility and suggest that MT1-MMP may play an important role in modulating the   migration   of immature DC. 
 CDCP1 regulates the function of MT1-MMP and invadopodia-mediated invasion of cancer cells. Complement C1r/C1s, Uegf, Bmp1 (CUB) domain-containing protein 1 (CDCP1) is a transmembrane protein that regulates anchorage-independent growth and cancer cell   migration   and invasion. Expression of CDCP1 is detected in a number of cancer cell lines and tissues and is closely correlated with poor prognosis. Invadopodia are actin-based protrusions on the surface of invasive cancer cells that promote the degradation of the extracellular matrix (ECM) via localized proteolysis, which is mainly mediated by membrane type 1 matrix metalloproteinase (MT1-MMP). MT1-MMP is accumulated at invadopodia by targeted delivery via membrane trafficking. The present study shows that CDCP1 is required for ECM degradation by invadopodia in human breast cancer and melanoma cells. CDCP1 localized to caveolin-1-containing vesicular structures and lipid rafts and was detected in close proximity to invadopodia. Further biochemical analysis revealed that substantial amounts of CDCP1 existed in the Triton X-100 insoluble lipid raft fraction. CDCP1 was coimmunoprecipitated with MT1-MMP and colocalized with MT1-MMP at the vesicular structures. The siRNA-mediated knockdown of the CDCP1 expression markedly inhibited MT1-MMP-dependent ECM degradation and Matrigel invasion and reduced the accumulation of MT1-MMP at invadopodia, as shown by immunofluorescence analysis. These results indicate that CDCP1 is an essential regulator of the trafficking and function of MT1-MMP- and invadopodia-mediated invasion of cancer cells. 
 A novel and selective membrane type-1 matrix metalloproteinase (MT1-MMP) inhibitor reduces cancer cell motility and tumor growth. Matrix metalloproteinases (MMPs), and especially membrane type-1 matrix metalloproteinase (MT1-MMP, MMP-14), play a role in cancer progression and can have a prognostic value. Various synthetic broad-spectrum MMP inhibitors have been developed but have had little success in cancer patient treatment owing to side effects. Until recently, selective targeting of specific MMPs has not been possible due to lack of specific inhibitors. Here we have developed a selective MT1-MMP peptide-inhibitor GACFSIAHECGA, which did not affect the activities of many other MMPs including MMP-1, -2, -3, -7, -8, -9, -10, -11, -12, -13, -15, -17 or -20. In a fluorescent peptide cleavage assay it displayed an IC(50) value of 150 microM. The peptide effectively inhibited the   migration   and invasion of cancer cell lines in vitro. Furthermore, in vivo the peptide reduced the growth of tongue carcinoma xenografts and prolonged the survival of mice. Overall these results suggest that selective MT1-MMP inhibitors may have utility as anticancer agents. 
 Mechanism of metastasis by membrane type 1-matrix metalloproteinase in hepatocellular carcinoma. To investigate the precise role of membrane type 1-matrix metalloproteinase (MT1-MMP) in hepatocellular carcinoma (HCC) metastasis.Human HCC cells Hep3B with overexpression of MT1-MMP were established by stable transfection, and compared with control cells carrying the empty vector. Cells were examined in vivo for their differences in the metastatic ability of athymic nude mice, and analyzed in vitro for their differences in invasion ability by invasion chamber coated with Matrigel, adhesion towards collagen I and   migration   through culture chamber. Cell proliferation and apoptosis in adherent and suspension status were evaluated by MTT and flow cytometry analysis.We found that overexpression of MT1-MMP could increase intrahepatic metastasis in nude mice with orthotopic implantation of HCC cells (incidence of 100% [MT1-MMP transfectants] vs 40% [vector control transfectants], P<0.05). MT1-MMP could also enhance cell invasion through Matrigel (107.7 vs 39.3 cells/field, P<0.001), adhesion towards matrix (0.30 vs 0.12 absorbance unit at 540 nm, P<0.001), cell   migration   (89.3 vs 39.0 cells/field, P<0.001), and cell proliferation (24.3 vs 40.5 h/doubling, P<0.001). We also observed that MT1-MMP supported cell survival (71.4% vs 23.9%, P<0.001) with reduced apoptosis (43.7% vs 51.0%, P<0.05) in an attachment-free environment.MT1-MMP overexpression could enhance metastasis. In addition to its active role in matrix degradation during tumor invasion, MT1-MMP enhances tumor cell survival upon challenge of detachment, which is important during metastasis when cells enter the circulation. 
 The E-cadherin-repressed hNanos1 gene induces tumor cell invasion by upregulating MT1-MMP expression. In this study, we examined the role of the E-cadherin-repressed gene human Nanos1 (hNanos1) in tumor invasion process. First, our in vivo study revealed that hNanos1 mRNAs were overexpressed in invasive lung carcinomas. Moreover, hNanos1 was co-localized with MT1-MMP (membrane type 1-matrix metalloproteinase) in E-cadherin-negative invasive lung tumor clusters. Using an inducible Tet-on system, we showed that induction of hNanos1 expression in DLD1 cells increased their migratory and invasive abilities in a three-dimensional   migration   and in a modified Boyden chamber assay. Accordingly, we demonstrated that hNanos1 upregulated MT1-MMP expression at the mRNA and protein levels. Inversely, using an RNA interference strategy to inhibit hNanos1 expression in invasive Hs578T, BT549 and BZR cancer cells, we observed a downregulation of MT1-MMP mRNA and protein and concomitantly a decrease of the invasive capacities of tumor cells in a modified Boyden chamber assay. Taken together, our results demonstrate that hNanos1, by regulating MT1-MMP expression, plays an important role in the acquisition of invasive properties by epithelial tumor cells. 
 MT1-MMP Inhibits the Activity of Bst-2 via Their Cytoplasmic Domains Dependent Interaction. Bst-2 (bone marrow stromal cell antigen 2) is a type II membrane protein, and it acts as a tetherin to inhibit virion releasing from infectious cells. Membrane type-1 matrix metalloproteinase (MT1-MMP) is a protease. It plays a pivotal role in cellular growth and   migration   by activating proMMP-2 into active MMP2. Our results here elaborate that MT1-MMP inhibits the tetherin activity of Bst-2 by interacting with Bst-2, and the cytoplasmic domains of both Bst-2 and MT1-MMP play critical roles within this interaction. Based on our experimental data, the assays for virion release and co-immunoprecipitation have clearly demonstrated that the activity of Bst-2 is markedly inhibited by MT1-MMP via their interaction; and both the N-terminal domain of Bst-2 and the C-terminal domain of MT1-MMP are important in the interaction. Immunostaining and Confocal Microscopy assay shows that MT1-MMP interacts with Bst-2 to form granular particles trafficking into cytoplasm from membrane and, finally, results in Bst-2 and MT1-MMP both being inhibited. In addition, mutant experiments elucidate that the N-terminal domain of Bst-2 is not only important in relating to the activity of Bst-2 itself, but is important for inhibiting the MT1-MMP/proMMP2/MMP2 pathway. These findings suggest that MT1-MMP is a novel inhibitor of Bst-2 in MT1-MMP expressed cell lines and also indicate that both the N-terminal domain of Bst-2 and the C-terminal domain of MT1-MMP are crucial in down-regulation. 
 miR-150-5p inhibits hepatoma cell   migration   and invasion by targeting MMP14. Hepatocellular carcinoma (HCC) is one of the leading causes of cancer-related mortality worldwide. Despite progress in diagnostics and treatment of HCC, its prognosis remains poor because the molecular mechanisms underlying hepatocarcinogenesis are not well understood. In the study, we focused on identifying the role of miRNAs in HCC progression. miRNA microarray was used to analyze the differentially expressed miRNAs, and the results were validated by qPCR. We found that the miR-150-5p expression is down-regulated in HCC tissues compared with pair non-tumor tissues. miR-150-5p expression is also decreased in metastatic cancer tissues compared with pair primary tissues, indicating that miR-150-5p may be involved in HCC metastasis. Functionally, miR-150-5p inhibition significantly promotes hepatoma cell   migration   and invasion, whereas miR-150-5p overexpression suppresses cancer cell   migration   and invasion in vitro. The matrix metalloproteinase 14 (MMP14) is identified as a new target gene of miR-150-5p. miR-150-5p markedly inhibits MMP14 expression in hepatoma cells, and miR-150-5p expression is negative correlation with MMP14 expression in vivo. More important, re-expression of MMP14 in hepatoma cells partially reverses the effect of miR-150-5p in inhibiting cell invasion. 
 Dimerization of MT1-MMP during cellular invasion detected by fluorescence resonance energy transfer. Homodimerization of the membrane-bound collagenase MT1-MMP [membrane-type 1 MMP (matrix metalloproteinase)] is crucial for its collagenolytic activity. However, it is not clear whether this dimerization is regulated during cellular invasion into three-dimensional collagen matrices. To address this question, we established a fluorescence resonance energy transfer system to detect MT1-MMP dimerization and analysed the process in cells invading through three-dimensional collagen. Our data indicate that dimerization occurs dynamically and constantly at the leading edge of migrating cells, but not the trailing edge. We found that polarized dimerization was not due to ECM (extracellular matrix) attachment, but was rather controlled by reorganization of the actin cytoskeleton by the small GTPases, Cdc42 (cell division cycle 42) and Rac1. Our data indicate that cell-surface collagenolytic activity is regulated co-ordinately with cell   migration   events to enable penetration of the matrix physical barrier. 
 Overexpression of proto-oncogene FBI-1 activates membrane type 1-matrix metalloproteinase in association with adverse outcome in ovarian cancers. FBI-1 (factor that binds to the inducer of short transcripts of human immunodeficiency virus-1) is a member of the POK (POZ and Kruppel) family of transcription factors and play important roles in cellular differentiation and oncogenesis. Recent evidence suggests that FBI-1 is expressed at high levels in a subset of human lymphomas and some epithelial solid tumors. However, the function of FBI-1 in human ovarian cancers remains elusive.In this study, we investigated the role of FBI-1 in human ovarian cancers, in particularly, its function in cancer cell invasion via modulating membrane type 1-matrix metalloproteinase (MT1-MMP). Significantly higher FBI-1 protein and mRNA expression levels were demonstrated in ovarian cancers samples and cell lines compared with borderline tumors and benign cystadenomas. Increased FBI-1 mRNA expression was correlated significantly with gene amplification (P = 0.037). Moreover, higher FBI-1 expression was found in metastatic foci (P = 0.036) and malignant ascites (P = 0.021), and was significantly associated with advanced stage (P = 0.012), shorter overall survival (P = 0.032) and disease-free survival (P = 0.016). In vitro, overexpressed FBI-1 significantly enhanced cell   migration   and invasion both in OVCA 420 and SKOV-3 ovarian carcinoma cells, irrespective of p53 status, accompanied with elevated expression of MT1-MMP, but not MMP-2 or TIMP-2. Moreover, knockdown of MT1-MMP abolished FBI-1-mediated cell   migration   and invasion. Conversely, stable knockdown of FBI-1 remarkably reduced the motility of these cells with decreased expression of MT1-MMP. Promoter assay and chromatin immunoprecipitation study indicated that FBI-1 could directly interact with the promoter spanning ~600 bp of the 5'-flanking sequence of MT1-MMP and enhanced its expression in a dose-dependent manner. Furthermore, stable knockdown and ectopic expression of FBI-1 decreased and increased cell proliferation respectively in OVCA 420, but not in the p53 null SKOV-3 cells.Our results suggested an important role of FBI-1 in ovarian cancer cell proliferation, cell mobility, and invasiveness, and that FBI-1 can be a potential target of chemotherapy. 
 Sphingosine-1-phosphate induces the association of membrane-type 1 matrix metalloproteinase with p130Cas in endothelial cells. Membrane-type 1 matrix metalloproteinase (MT1-MMP) plays an important role in sphingosine-1-phosphate(S1P)-dependent   migration   of endothelial cells but the underlying mechanisms remain largely unknown. Herein, we show that S1P promotes the relocalization of MT1-MMP to peripheral actin-rich membrane ruffles that is coincident with its association with the adaptor protein p130Cas at the leading edge of migrating cells. Immunoprecipitation and confocal microscopy analyses suggest that this interaction required the tyrosine phosphorylation of p130Cas and also involves S1P-dependent phosphorylation of MT1-MMP within its cytoplasmic sequence. The interaction of MT1-MMP with p130Cas at the cell periphery suggests the existence of a close interplay between pericellular proteolysis and signaling pathways involved in EC   migration  . 
 Inhibition of matrix metalloproteinase 14 (MMP-14)-mediated cancer cell   migration  . Matrix metalloproteinases (MMPs) have been shown to be key players in both extracellular matrix remodeling and cell   migration   during cancer metastasis. MMP-14, a membrane-anchored MMP, in particular, is closely associated with these processes. The hemopexin (PEX) domain of MMP-14 has been proposed as the modulating region involved in the molecular cross-talk that initiates cell   migration   through homodimerization of MMP-14 as well as heterodimerization with the cell surface adhesion molecule CD44. In this study, minimal regions required for function within the PEX domain were investigated through a series of substitution mutations. Blades I and IV were found to be involved in cell   migration  . We found that blade IV is necessary for MMP-14 homodimerization and that blade I is required for CD44 MMP-14 heterodimerization. Cross-talk between MMP-14 and CD44 results in phosphorylation of EGF receptor and downstream activation of the MAPK and PI3K signaling pathways involved in cell   migration  . Based on these mutagenesis analyses, peptides mimicking the essential outermost strand motifs within the PEX domain of MMP-14 were designed. These synthetic peptides inhibit MMP-14-enhanced cell   migration   in a dose-dependent manner but have no effect on the function of other MMPs. Furthermore, these peptides interfere with cancer metastasis without affecting primary tumor growth. Thus, targeting the MMP-14 hemopexin domain represents a novel approach to inhibit MMP-14-mediated cancer dissemination. 
 Role of MT1-MMP in estrogen-mediated cellular processes of intimal hyperplasia. Hormone replacement therapy increases intimal hyperplasia (IH) following vascular intervention. Matrix metalloproteinases (MMPs) play a role in IH development. We have shown estrogen up-regulates MT1-MMP expression, a transmembrane protein that activates MMP-2, and increases vascular smooth muscle cell (VSMC) collagen invasion via increased MMP-2 activity. Here we hypothesize inhibition of MT1-MMP will prevent hormonally-stimulated increased MMP-2 activation and the downstream cellular processes of IH pathogenesis.VSMCs from a postmenopausal donor were transfected with MT1-MMP or negative control siRNAs, treated with estrogen (Est), analyzed by q-PCR, Western blot, zymography,   migration  , invasion, and proliferation assays.Est treatment of MT1-MMP silenced cells still resulted in increased MT1-MMP expression (C = 41% ± 4%; Est = 52% ± 2%; P < 0.05). Silencing of MT1-MMP decreased basal MMP-2 activity (nonsilenced = 100%; MT1-silenced = 87% ± 3%; P < 0.05) but had no effect on basal invasion or proliferation. Est treatment of MT1-MMP silenced cells still resulted in increased MMP-2 activity (C = 87% ± 3%; Est = 101% ± 4%; P < 0.05) and invasion (C = 89% ± 6%; Est = 109% ± 3%; P < 0.05) compared with MT1-MMP silenced control cells. However, silencing of MT1-MMP did inhibit Est- and serum-stimulated proliferation (C = 106% ± 18%; Est = 104% ± 16%; FBS = 121% ± 24%; P = NS).Silencing of MT1-MMP in aged VSMCs results in impaired but not complete inhibition of basal and Est-stimulated increases in MMP-2 activity. Other mechanisms appear to be playing a role in hormonally-regulated cellular processes of IH pathogenesis. Future studies will target other signaling cascades, with the goal of identifying mechanisms responsible for hormonally-modulated unbalanced MMPs. In vivo manipulation of the expression patterns of MT1-MMP will be examined for the prevention of IH in animal models of vascular disease. 
 Biochemical evidence of the interactions of membrane type-1 matrix metalloproteinase (MT1-MMP) with adenine nucleotide translocator (ANT): potential implications linking proteolysis with energy metabolism in cancer cells. Invasion-promoting MT1-MMP (membrane type-1 matrix metalloproteinase) is a key element in cell   migration   processes. To identify the proteins that interact and therefore co-precipitate with this proteinase from cancer cells, we used the proteolytically active WT (wild-type), the catalytically inert E240A and the C-end truncated (tailless; DeltaCT) MT1-MMP-FLAG constructs as baits. The identity of the pulled-down proteins was determined by LC-MS/MS (liquid chromatography tandem MS) and then confirmed by Western blotting using specific antibodies. We determined that, in breast carcinoma MCF cells (MCF-7 cells), ANT (adenine nucleotide translocator) efficiently interacted with the WT, E240A and DeltaCT constructs. The WT and E240A constructs also interacted with alpha-tubulin, an essential component of clathrin-mediated endocytosis. In turn, tubulin did not co-precipitate with the DeltaCT construct because of the inefficient endocytosis of the latter, thus suggesting a high level of selectivity of our test system. To corroborate these results, we then successfully used the ANT2-FLAG construct as a bait to pull-down MT1-MMP, which was naturally produced by fibrosarcoma HT1080 cells. We determined that the presence of the functionally inert catalytic domain alone was sufficient to cause the proteinase to interact with ANT2, thus indicating that there is a non-proteolytic mode of these interactions. Overall, it is tempting to hypothesize that by interacting with pro-invasive MT1-MMP, ANT plays a yet to be identified role in a coupling mechanism between energy metabolism and pericellular proteolysis in migrating cancer cells. 
 Chronic hypoxia inhibits MMP-2 activation and cellular invasion in human cardiac myofibroblasts. Cardiac myofibroblasts are pivotal to adaptive remodelling after myocardial infarction (MI). These normally quiescent cells invade and proliferate as a wound healing response, facilitated by activation of matrix metalloproteinases, particularly MMP-2. Following MI these reparative events occur under chronically hypoxic conditions yet the mechanisms by which hypoxia might modulate MMP-2 activation and cardiac myofibroblast invasion have not been investigated. Human cardiac myofibroblasts cultured in collagen-supplemented medium were exposed to normoxia (20% O(2)) or hypoxia (1% O(2)) for up to 48 h. Secreted levels of total and active MMP-2 were quantified using gelatin zymography, TIMP-2 and membrane-associated MT1-MMP were quantified with ELISA, whole cell MT1-MMP by immunoblotting and immunocytochemistry and MT1-MMP mRNA with real-time RT-PCR. Cellular invasion was assessed in modified Boyden chambers and   migration   by scratch wound assay. In the human cardiac myofibroblast, MT1-MMP was central to MMP-2 activation and activated MMP-2 necessary for invasion, confirmed by gene silencing. MMP-2 activation was substantially attenuated by hypoxia (P<0.001), paralleled by inhibition of myofibroblast invasion (P<0.05). In contrast,   migration   was independent of either MT1-MMP or MMP-2. Reduced membrane expression of MT1-MMP (P<0.05) was responsible for the hypoxic reduction of MMP-2 activation, with no change in either total MMP-2 or TIMP-2. In conclusion, hypoxia reduces MMP-2 activation and subsequent invasion of human cardiac myofibroblasts by reducing membrane expression of MT1-MMP and may delay healing after MI. Regulation of these MMPs remains an attractive target for therapeutic intervention. 
 Caveolin-1 inhibits membrane-type 1 matrix metalloproteinase activity. Membrane-type 1 matrix metalloproteinase (MT1-MMP) is a zinc-dependent proteinase found in cholesterol-rich lipid rafts on the plasma membrane. MT1-MMP hydrolyzes extracellular matrix (ECM) proteins, activates pro-matrix metalloproteinase-2 (proMMP-2) and plays an important role in ECM remodeling, cancer cell   migration   and metastasis. The role of caveolin-1, an integral protein of caveolae, in the activation of MT1-MMP remains largely unknown. Here, we show that the expression of caveolin-1 attenuates the activation of proMMP-2, reduces proteolytic cleavage of ECM and inhibits cell   migration  . We utilized the cytoplasmic tail domain deletion (DeltaCT) or the E240A mutant of MT1-MMP. Co-expression of caveolin-1 with the wild-type or the DeltaCT MT1-MMP decreased the proMMP-2 activation and inhibited collagen degradation and cell   migration  . Caveolin-1 had no effect on the catalytically inert E240A MT1-MMP. Our findings suggest that caveolin-1 is essential in the down-regulation of MT1-MMP activity by promoting internalization from the cell surface. 
 Multi-step pericellular proteolysis controls the transition from individual to collective cancer cell invasion. Invasive cell   migration   through tissue barriers requires pericellular remodelling of extracellular matrix (ECM) executed by cell-surface proteases, particularly membrane-type-1 matrix metalloproteinase (MT1-MMP/MMP-14). Using time-resolved multimodal microscopy, we show how invasive HT-1080 fibrosarcoma and MDA-MB-231 breast cancer cells coordinate mechanotransduction and fibrillar collagen remodelling by segregating the anterior force-generating leading edge containing beta1 integrin, MT1-MMP and F-actin from a posterior proteolytic zone executing fibre breakdown. During forward movement, sterically impeding fibres are selectively realigned into microtracks of single-cell calibre. Microtracks become expanded by multiple following cells by means of the large-scale degradation of lateral ECM interfaces, ultimately prompting transition towards collective invasion similar to that in vivo. Both ECM track widening and transition to multicellular invasion are dependent on MT1-MMP-mediated collagenolysis, shown by broad-spectrum protease inhibition and RNA interference. Thus, invasive   migration   and proteolytic ECM remodelling are interdependent processes that control tissue micropatterning and macropatterning and, consequently, individual and collective cell   migration  . 
 Membrane-type 1 matrix metalloproteinase regulates cell   migration   during zebrafish gastrulation: evidence for an interaction with non-canonical Wnt signaling. Key to invasiveness is the ability of tumor cells to modify the extracellular matrix, become motile, and engage in directed   migration   towards the vasculature. One significant protein associated with metastatic progression is membrane-type 1 matrix metalloproteinase (MT1-MMP/MMP14). How MMP14 activity is coordinated with other signaling pathways to regulate cell   migration   in vivo is largely unknown. Here we have used zebrafish embryogenesis as a model to understand the potential relationship between MMP14-dependent pericellular proteolysis, cell polarity, and motility. Knockdown of zebrafish Mmp14 function disrupted gastrulation convergence and extension cell movements and craniofacial morphogenesis. Using time-lapse imaging and morphometric analyses, we show that Mmp14 is required for proper cell polarity underlying the directed   migration   of mesodermal cells during gastrulation. We have identified a genetic interaction between mmp14 and non-canonical Wnt signaling, a pathway that also regulates cell polarity in embryonic tissues and is increasingly being linked with tumor cell   migration  . Finally, we demonstrate that Van Gogh-like 2, a key regulator of the non-canonical Wnt pathway, co-localizes with MMP14 and becomes redistributed towards the leading edge of polarized human cancer cells. Together, our results support the notion that pathways regulating pericellular proteolysis and cell polarity converge to promote efficient cell   migration  . 
 Neurokinin-1 receptor mediated breast cancer cell   migration   by increased expression of MMP-2 and MMP-14. Breast cancer (BC) is a common reason of cancer-associated death in female. To develop novel strategy of therapeutics, it is crucial to comprehensively understand the receptor status of BC cells on the surface and inner, because chemical messengers can bind the receptors and promote tumorigenesis. Compared with normal and benign samples, BC cell lines and malignant biopsies showed higher expression of neurokinin-1 receptor (NK1). In current work, we examined the role and mechanism of NK1 receptor signaling in BC cell   migration  . Human hemokinin-1 (hHK-1) was the peripheral agonist of NK1 receptor. Our results showed that by activating NK1 receptor, hHK-1 promoted the   migration   of BC cells. Gelatin zymography and WB experiment showed that hHK-1 enhanced the levels of MMP-2 and MMP-14; inhibition of these two MMPs blocked hHK-1-induced cell   migration  . We further explored the underlying mechanism. hHK-1 incuced the phosphorylation of ERK1/2, JNK and Akt through PKC or PKA pathway. The phosphorylation of these kinases further regulated the activation of transcriptional factor AP-1 and NF-κB. Inhibition of AP-1 and NF-κB reduced the up-regulation of MMP-2 and MMP-14 by hHK-1. Taken together, we showed NK1 receptor was an important regulator of human BC cel  l migrati  on and a potential target for BC treatment. 
 Hic-5 mediates the initiation of endothelial sprouting by regulating a key surface metalloproteinase. During angiogenesis, endothelial cells must coordinate matrix proteolysis with   migration  . Here, we tested whether the focal adhesion scaffold protein Hic-5 (also known as TGFB1I1) regulated endothelial sprouting in three dimensions. Hic-5 silencing reduced endothelial sprouting and lumen formation, and sprouting defects were rescued by the return of Hic-5 expression. Pro-angiogenic factors enhanced colocalization and complex formation between membrane type-1 matrix metalloproteinase (MT1-MMP, also known as MMP14) and Hic-5, but not between paxillin and MT1-MMP. The LIM2 and LIM3 domains of Hic-5 were necessary and sufficient for Hic-5 to form a complex with MT1-MMP. The degree of interaction between MT1-MMP and Hic-5 and the localization of the complex within detergent-resistant membrane fractions were enhanced during endothelial sprouting, and Hic-5 depletion lowered the surface levels of MT1-MMP. In addition, we observed that loss of Hic-5 partially reduced complex formation between MT1-MMP and focal adhesion kinase (FAK, also known as PTK2), suggesting that Hic-5 bridges MT1-MMP and FAK. Finally, Hic-5 LIM2-LIM3 deletion mutants reduced sprout initiation. Hic-5, MT1-MMP and FAK colocalized in angiogenic vessels during porcine pregnancy, supporting that this complex assembles during angiogenesis in vivo. Collectively, Hic-5 appears to enhance complex formation between MT1-MMP and FAK in activated endothelial cells, which likely coordinates matrix proteolysis and cell motility. 
 Induction of the MMP-14 gene in macrophages of the atherosclerotic plaque: role of SAF-1 in the induction process. Based on epidemiological and pathological studies, it is becoming increasingly clear that matrix metalloproteinases (MMPs) play an important role in the pathogenesis of atherosclerosis by participating in vascular remodeling, smooth muscle cell   migration  , and plaque disruption. MMP-14, because of its unique ability to cause pericellular degradation, its broad substrate specificity, its synthesis in an active form, and its ability to activate other matrix metalloproteinases, is recognized as a prominent member of this family. MMP-14 is detected at high levels in the atherosclerotic plaque. To understand the induction mechanism of MMP-14 under atherogenic conditions, we examined its expression pattern in response to oxidized low-density lipoproteins (ox-LDLs) that are believed to play an important role in atherogenesis. We report that in macrophages, ox-LDLs markedly elevate the levels of MMP-14 mRNA and protein. The cis-acting elements supporting this increase were identified to be present within -213 and -1 nucleotides of the MMP-14 promoter. DNase I protection assay revealed, within this region, two major elements, of which one serves as the DNA-binding site for SAF-1 transcription factor. Increased binding of SAF-1 to the MMP-14 promoter correlated with the transcriptional upregulation of MMP-14 gene. Furthermore, induction of endogenous MMP-14 gene, MMP-14 promoter driven reporter gene expression and MMP-2 processing activity during overexpression of SAF-1 and coexpression of SAF-1 and MMP-14 in the macrophages present in the atherosclerotic plaque implicate SAF-1 as a key regulator of MMP-14 gene induction in macrophage cells. 
 Proteolytic and non-proteolytic roles of membrane type-1 matrix metalloproteinase in malignancy. This manuscript provides an overview of the dynamic interactions which play an important role in regulating cancer cell functions. We describe and discuss, primarily, those interactions which involve membrane type-1 matrix metalloproteinase (MT1-MMP), its physiological inhibitor tissue inhibitor of metalloproteinases-2 (TIMP-2), furin-like proprotein convertases and the low density lipoprotein-related protein 1 (LRP1) signaling scavenger receptor. The interaction among these cellular proteins controls the efficiency of the activation of MT1-MMP and the unorthodox intracellular signaling which is generated by the catalytically inert complex of MT1-MMP with TIMP-2 and which plays a potentially important role in the   migration   of cancer cells. Our in-depth understanding of these cellular mechanisms may provide the key to solving the puzzling TIMP-2 paradox. This unsolved paradox arises from the fact that TIMP-2 is a powerful inhibitor of MMPs including MT1-MMP, but at the same time high levels of TIMP-2 positively correlate with an unfavorable prognosis in cancer patients. Solving the TIMP-2 paradox may lead to solving a similar PAI-1 paradox and produce a clearer understanding of the biochemical mechanisms which control the functionality of the urokinase-type plasminogen activator*urokinase receptor*plasminogen activator inhibitor type-1 (uPAR*uPA*PAI-1) system in cancer. 
 The dimer interface of the membrane type 1 matrix metalloproteinase hemopexin domain: crystal structure and biological functions. Homodimerization is an essential step for membrane type 1 matrix metalloproteinase (MT1-MMP) to activate proMMP-2 and to degrade collagen on the cell surface. To uncover the molecular basis of the hemopexin (Hpx) domain-driven dimerization of MT1-MMP, a crystal structure of the Hpx domain was solved at 1.7 Å resolution. Two interactions were identified as potential biological dimer interfaces in the crystal structure, and mutagenesis studies revealed that the biological dimer possesses a symmetrical interaction where blades II and III of molecule A interact with blades III and II of molecule B. The mutations of amino acids involved in the interaction weakened the dimer interaction of Hpx domains in solution, and incorporation of these mutations into the full-length enzyme significantly inhibited dimer-dependent functions on the cell surface, including proMMP-2 activation, collagen degradation, and invasion into the three-dimensional collagen matrix, whereas dimer-independent functions, including gelatin film degradation and two-dimensional cel  l migrati  on, were not affected. These results shed light on the structural basis of MT1-MMP dimerization that is crucial to promote cellular invasion. 
 MMP-14 mediated MMP-9 expression is involved in TGF-beta1-induced keratinocyte   migration  . The importance of expression of matrix metalloproteinase (MMP) in keratinocyte   migration   is well established, but its role remains unclear. Here we investigated the function of MMP-14 in TGF-beta1-induced keratinocyte   migration  . TGF-beta1 stimulated cell   migration   and the expression of MMP-2, -9 in HaCaT human keratinocyte cells. When we lowered MMP-14 mRNA with siRNA, cell   migration  , and MMP-9 expression decreased. Furthermore, the MMP-14 siRNA also reduced activation of JNK in response to TGF-beta1, and a JNK-specific inhibitor decreased both cell   migration   and MMP-9 expression. Taken together, these results suggest that TGF-beta1-induced HaCaT cell   migration   is mediated by MMP-14, which regulates MMP-9 expression via JNK signaling. 
 Regulation of vascular smooth muscle cell expression and function of matrix metalloproteinases is mediated by estrogen and progesterone exposure. Postmenopausal women receiving hormone replacement therapy (HRT) have been reported to have more adverse outcomes after vascular reconstructions, including increased intimal hyperplasia development and bypass graft failure. HRT may be affecting the pathway contributing to intimal hyperplasia. An important component of this pathway involves matrix metalloproteinases (MMPs), implicated in vascular remodeling due to their ability to degrade components of the extracellular matrix. We hypothesize that estrogen (Est) and progesterone (Prog) upregulate the MMP pathway in vascular smooth muscle cells (VSMCs) thereby increasing MMP activity and function.VSMCs were incubated with Est (5 ng/mL), Prog (50 ng/mL), Est + Prog combination (Est/Prog), and/or doxycycline (40 microg/mL; Doxy). Using reverse transcriptase polymerase chain reaction (RT-PCR) analysis we have previously shown membrane type 1-MMP (MT1-MMP) messenger ribonucleic acid (mRNA) levels are significantly increased by Est. Here, Western blot analyses indicated MT1-MMP and MMP-2 protein levels, not tissue inhibitor of MMP-2 (TIMP-2), were increased in response to Est and Est/Prog (P < .05 vs control). In-gel zymography revealed that Est and Est/Prog resulted in increased MMP-2 activity (hormone groups, P < .05 vs control) with no significant difference among the hormone groups. VSMC   migration   was increased by 45 +/- 14% in response to Est (P < .05 vs control), as measured using a modified Boyden chamber assay. Doxycycline significantly inhibited basal and Est/Prog-stimulated increases in MMP-2 activity (P < .05 vs control; P < .05 vs hormone groups), and partially blocked basal and hormonally stimulated   migration   (P < .05 vs control and Est).Estrogen and progesterone affects the MMP pathway by increasing MMP-2 enzymatic activity, possibly via the upregulation of MT1-MMP expression without a corresponding increase in TIMP expression. This increased collagenase activity increases VSMC motility and their ability to migrate through a collagen type IV lattice. Est/Prog upregulation of MT1-MMP may contribute to the adverse effect of HRT on vascular interventions. 
 Membrane type 1-matrix metalloproteinase is activated during   migration   of human endothelial cells and modulates endothelial motility and matrix remodeling. Matrix metalloproteinases are thought to play an important role in endothelial cell   migration   and matrix remodeling. We have used an in vitro wound healing   migration   model and newly generated anti-membrane type 1-matrix metalloproteinase (MT1-MMP) monoclonal antibodies (mAbs) to characterize the role of MT1-MMP during this process. First, the expression and shedding of MT1-MMP are up-regulated upon induction of   migration   in endothelial cells, as demonstrated by flow cytometry and Western blot analysis. Furthermore, MT1-MMP is concentrated at discrete areas in migrating endothelial cells, in contrast to the diffuse pattern observed in confluent cells. Interestingly,   migration   of endothelial cells results in the stimulation of MT1-MMP activity, as shown by its ability to process pro-MMP-2 and to degrade fibrinogen assessed by zymography. Moreover, MT1-MMP-mediated gelatin degradation is enriched at   migration   sites. mAbs generated against the MT1-MMP catalytic domain are shown to inhibit MT1-MMP enzymatic activity and to impair both phorbol 12-myristate 13-acetate-induced endothelial   migration   and invasion of collagen and fibrin gels. Furthermore, a reduction in the formation of capillary tubes in Matrigel is also observed when endothelial cells are pretreated with the blocking anti-MT1-MMP mAbs. Altogether, these data demonstrate that MT1-MMP plays an important role during endothelial cell   migration  , and its activity can modulate endothelial   migration  , invasion, and formation of capillary tubes during the angiogenic response. 
 miR‑133a suppresses cell proliferation  , migrati  on and invasion in human lung cancer by targeting MMP‑14. Lung cancer is the leading cause of cancer‑related mortality worldwide. Over half of lung cancer cases are diagnosed after metastasis, for which the median survival time is approximately 8 months. microRNAs (miRNAs), which are a class of single‑stranded endogenous non‑coding RNAs, are likely to be involved in most biological processes. miR‑133 plays roles in cardiac development and disease, and recent studies showed that miR‑133 is downregulated in various human malignancies, such as bladder and lung cancer. However, its detailed role in the processes of cancer remains to be determined. In the present study, we found that in the lung cancer cell lines A549 and NCI‑H1299 overexpression of miR‑133a suppressed c  ell proli  feration, migration and invasion. The miR‑133a‑induc  ed suppre  ssion of cell migration and invasion can be reversed by miR‑133a‑specific inhibitor. According to the mRNA sequence, matrix metalloproteinase (MMP)‑14, which is an important regulator of metastasis, is a predicted target of miR‑133a. This was confirmed by dual luciferase reporter assay. Moreover, miR‑133a overexpression decreases the mRNA and protein levels of MMP‑14. Collectively, these results suggest that miR‑133a may inhibit lung cancer metastasis by targeting MMP‑14 and may be used as an anti‑metastatic therapy in lung cancer patients. 
 Matrix metalloproteinase-14 is a mechanically regulated activator of secreted MMPs and invasion. Matrix metalloproteinases (MMPs) are extracellular matrix (ECM) degrading enzymes and have complex and specific regulation networks. This includes activation interactions, where one MMP family member activates another. ECM degradation and MMP activation can be initiated by several different stimuli including changes in ECM mechanical properties or intracellular contractility. These mechanical stimuli are known enhancers of metastatic potential. MMP-14 facilitates local ECM degradation and is well known as a major mediator of cell   migration  , angiogenesis and invasion. Recently, function blocking antibodies have been developed to specifically block MMP-14, providing a useful tool for research as well as therapeutic applications. Here we utilize a selective MMP-14 function blocking antibody to delineate the role of MMP-14 as an activator of other MMPs in response to changes in cellular contractility and ECM stiffness. Inhibition using function blocking antibodies reveals that MMP-14 activates soluble MMPs like MMP-2 and -9 under various mechanical stimuli in the pancreatic cancer cell line, Panc-1. In addition, inhibition of MMP-14 abates Panc-1 cell extension into 3D gels to levels seen with non-specific pan-MMP inhibitors at higher concentrations. This strengthens the case for MMP function blocking antibodies as more potent and specific MMP inhibition therapeutics. 
 microRNA-9 targets matrix metalloproteinase 14 to inhibit invasion, metastasis, and angiogenesis of neuroblastoma cells. Matrix metalloproteinase (MMP)-14 is the only membrane-anchored MMP that plays a critical role in tumor metastasis and angiogenesis. However, the mechanisms underlying MMP-14 expression in tumors still remain largely unknown. In this study, MMP-14 immunostaining was identified in 29/42 neuroblastoma tissues, which was correlated with clinicopathologic features and shorter patients' survival. In subtotal 20 neuroblastoma cases, microRNA 9 (miR-9) was downregulated and inversely correlated with MMP-14 expression. Bioinformatics analysis revealed a putative miR-9-binding site in the 3'-untranslated region (3'-UTR) of MMP-14 mRNA. Overexpression or knockdown of miR-9 responsively altered both the mRNA and protein levels of MMP-14 and its downstream gene, vascular endothelial growth factor, in cultured neuroblastoma cell lines SH-SY5Y and SK-N-SH. In an MMP-14 3'-UTR luciferase reporter system, miR-9 downregulated the luciferase activity, and these effects were abolished by a mutation in the putative miR-9-binding site. Overexpression of miR-9 suppressed the invasion, metastasis, and angiogenesis of SH-SY5Y and SK-N-SH cells in vitro and in vivo. In addition, the effects of miR-9 on MMP-14 expression, adhesion,   migration  , invasion, and angiogenesis were rescued by overexpression of MMP-14 in these cells. Furthermore, anti-miR-9 inhibitor or knockdown of MMP-14 respectively increased or inhibited the   migration  , invasion, and angiogenesis of neuroblastoma cells. These data indicate that miR-9 suppresses MMP-14 expression via the binding site in the 3'-UTR, thus inhibiting the invasion, metastasis, and angiogenesis of neuroblastoma. 
 Membrane-type matrix metalloproteinase-1 (MT1-MMP) is a processing enzyme for human laminin gamma 2 chain. Processing of the laminin-5 (Ln-5) gamma 2 chain by membrane-type-1 matrix metalloproteinases (MT1-MMP) promotes   migration   and invasion of epithelial and tumor cells. We previously demonstrated that MT1-MMP cleaves the rat gamma 2 chain at two sites, producing two major C-terminal fragments of 100 (gamma 2') and 80 (gamma 2 x) kDa and releasing a 30-kDa fragment containing epidermal growth factor (EGF)-like motifs (domain III (DIII) fragment). The DIII fragment bound the EGF receptor (EGF-R) and stimulated cell scattering and   migration  . However, it is not yet clear whether human Ln-5 is processed in a similar fashion to rat Ln-5 because one of the two MT1-MMP cleavage sites present in rat gamma 2 is not found in human gamma 2. To identify the exact cleavage site for MT1-MMP in human Ln-5, we purified both the whole molecule as well as a monomeric form of human gamma 2 that is frequently expressed by malignant tumor cells. Like rat Ln-5, both the monomer of gamma 2, as well as the gamma 2 derived from intact Ln-5, were cleaved by MT1-MMP in vitro, generating C-terminal gamma 2' (100 kDa) and gamma 2 x (85 kDa) fragments and releasing DIII fragments (25 and 27k Da). In addition to the conserved first cleavage site used to generate gamma 2', two adjacent cleavage sites (Gly(559)-Asp(560) and Gly(579)-Ser(580)) were found that could generate the gamma 2 x and DIII fragments. Two of the three EGF-like motifs present in the rat DIII fragment are present in the 27-kDa human fragment, and like the rat DIII, this fragment can promote breast carcinoma cell   migration   by engaging the EGF-R. These results suggest that MT1-MMP processing of Ln-5 in human tumors may stimulate the EGF-R, resulting in increased tumor cell scattering and   migration   that could possibly increase their metastatic potential. 
 Membrane type 1-matrix metalloproteinase is involved in   migration   of human monocytes and is regulated through their interaction with fibronectin or endothelium. Membrane type 1-matrix metalloproteinase (MT1-MMP) is involved in endothelial and tumor-cell   migration  , but its putative role in leukocyte   migration   has not been characterized yet. Here, we demonstrate that anti-MT1-MMP monoclonal antibody (mAb) impaired monocyte chemotactic protein-1 (MCP-1)-stimulated monocyte   migration   on fibronectin (FN), vascular cell adhesion molecule-1 (VCAM-1), and intercellular adhesion molecule-1 (ICAM-1). In addition, monocyte trans  migration   through tumor necrosis factor-alpha (TNF-alpha)-activated endothelium is also inhibited by anti-MT1-MMP mAb. Therefore, regulation of MT1-MMP in human peripheral blood monocytes was investigated. First, MT1-MMP clustering was observed at motility-associated membrane protrusions of MCP-1-stimulated monocytes migrating on FN, VCAM-1, or ICAM-1 and at the leading edge, together with profilin, of monocytes transmigrating through activated endothelial cells. In addition, up-regulation of MT1-MMP expression was induced in human monocytes upon attachment to FN in a manner dependent on alpha4beta1 and alpha5beta1 integrins. Binding of monocytes to TNF-alpha-activated human endothelial cells as well as to VCAM-1 or ICAM-1 also resulted in an increase of MT1-MMP expression. These findings correlated with an enhancement of MT1-MMP fibrinolytic activity in monocytes bound to FN, VCAM-1, or ICAM-1. Our data show that MT1-MMP is required during human monocyte   migration   and endothelial trans  migration   and that MT1-MMP localization, expression, and activity are regulated in monocytes upon contact with FN or endothelial ligands, pointing to a key role of MT1-MMP in monocyte recruitment during inflammation. 
 Membrane-type matrix metalloproteinase 1 regulates trophoblast functions and is reduced in fetal growth restriction. Fetal growth restriction (FGR) results from placental insufficiency to adequately supply the fetus. This insufficiency involves impaired cytotrophoblast functions, including reduced   migration   and invasion, proliferation, and syncytium formation. Membrane-type matrix metalloproteinase 1 (MT1-MMP) is a key enzyme in these cellular processes. MT1-MMP exists in various forms: a 63-kDa proenzyme is synthesized as primary translation product, which is cleaved into a 57-kDa membrane-anchored active form. We hypothesized that reduced placental MT1-MMP in FGR impairs trophoblast functions. MT1-MMP mRNA and active enzyme was quantified in placentas from FGR and age-matched control pregnancies. MT1-MMP protein was localized in first-trimester and term placentas. Putative MT1-MMP functions in trophoblasts were determined using two blocking antibodies for measuring   migration   and proliferation, as well as fusion of primary trophoblasts and trophoblast-derived cells. MT1-MMP was expressed predominantly in the syncytiotrophoblast and the villous and extravillous cytotrophoblasts. In FGR placentas, levels of MT1-MMP mRNA and of active MT1-MMP protein were reduced (-34.2%, P < 0.05, and -21.5%, P < 0.01, respectively), compared with age-matched controls. MT1-MMP-blocking antibodies diminished   migration  , proliferation, and trophoblast fusion. We conclude that reduced placental MT1-MMP in FGR may contribute to the impaired trophoblast functions associated with this pathology. 
 Testican 2 abrogates inhibition of membrane-type matrix metalloproteinases by other testican family proteins. Testican family proteins are putative extracellular heparan/chondroitin sulfate proteoglycans of unknown function. We identified recently N-Tes, which is a product of testican 3 splicing variant gene, as an inhibitor of membrane-type matrix metalloproteinases (MT-MMPs). The inhibitory function is common among testican family members except for testican 2, which was shown to uniquely abolish inhibition of MT1-MMP- or MT3-MMP-mediated pro-MMP-2 activation by other testican family members. Testican 2 inactivates N-Tes by binding to the COOH-terminal extracellular calcium-binding domain of N-Tes through its NH(2)-terminal unique domain as demonstrated by coimmunoprecipitation analysis, and, thus, testican 2 was unable to inactivate a N-Tes deletion mutant lacking the extracellular calcium-binding domain (N-Tes-Delta 122).   Migration   of U251 cells on collagen, which was dependent on MT1-MMP activity under serum-free condition, was inhibited by N-Tes or N-Tes-Delta 122 deposited on collagen. Testican 2 was not incorporated into collagen by itself, and was deposited only in the presence of N-Tes, suggesting that testican 2 binds to N-Tes deposited on collagen. Binding of testican 2 to N-Tes deposited on collagen allowed   migration   of cells expressing MT1-MMP. Unlike wild-type N-Tes, N-Tes-Delta 122 did not bind to testican 2, and, thus, expression of testican 2 did not recover cell   migration   blocked by N-Tes-Delta 122. In situ hybridization showed that neurons are a major source of all of the testican family members in the normal brain. The quantitative reverse transcription-PCR analysis demonstrated that all of the testican family members are expressed prominently in normal brain, and their expression levels decrease as tumor grade increases. The expression level of testican 2 was the highest among testican family members regardless of histological grade of astrocytic tumors. These results suggest that abundant distribution of testican 2 may contribute to glioma invasion by inactivating other testican family members including N-Tes, which all inhibit MT-MMPs. We propose that N-Tes-Delta 122, which is resistant to testican 2, may have therapeutic potential as a barrier against glioma invasion. 
 Lipid rafts and caveolin-1 are required for invadopodia formation and extracellular matrix degradation by human breast cancer cells. Invadopodia are ventral membrane protrusions through which invasive cancer cells degrade the extracellular matrix. They are thought to function in the   migration   of cancer cells through tissue barriers, which is necessary for cancer invasion and metastasis. Although many protein components of invadopodia have been identified, the organization and the role of membrane lipids in invadopodia are not well understood. In this study, the role of lipid rafts, which are cholesterol-enriched membrane microdomains, in the assembly and function of invadopodia in human breast cancer cells was investigated. Lipid rafts are enriched, internalized, and dynamically trafficked at invadopodia sites. Perturbation of lipid raft formation due to depleting or sequestering membrane cholesterol blocked the invadopodia-mediated degradation of the gelatin matrix. Caveolin-1 (Cav-1), a resident protein of lipid rafts and caveolae, accumulates at invadopodia and colocalizes with the internalized lipid raft membranes. Membrane type 1 matrix metalloproteinase (MT1-MMP), a matrix proteinase associated with invadopodia, is localized at lipid raft-enriched membrane fractions and cotrafficked and colocalized with Cav-1 at invadopodia. The small interfering RNA-mediated silencing of Cav-1 inhibited the invadopodia-mediated and MT1-MMP-dependent degradation of the gelatin matrix. Furthermore, Cav-1 and MT1-MMP are coexpressed in invasive human breast cancer cell lines that have an ability to form invadopodia. These results indicate that invadopodia are the sites where enrichment and trafficking of lipid rafts occur and that Cav-1 is an essential regulator of MT1-MMP function and invadopodia-mediated breast cancer cell invasion. 
 The planar cell polarity protein Van Gogh-Like 2 regulates tumor cell   migration   and matrix metalloproteinase-dependent invasion. Van Gogh-Like 2 (VANGL2) is a planar cell polarity protein essential for collective   migration   during embryonic development, yet its contribution to tumor cell motility and invasion are unknown. We report for the first time that loss of VANGL2 in human cancer cells promotes efficient collective and directed   migration   and matrix metalloproteinase (MMP)-dependent ECM invasion. We show that VANGL2 knockdown cells exhibit increased activation of secreted MMP2, higher levels of membrane-localized MMP14, and decreased cell-surface fibronectin. These important findings support the notion that planar cell polarity proteins act in coordination with known regulators of cancer cell   migration   to influence invasion and perhaps metastasis. 
 Relation between laminin-5 gamma 2 chain and cell surface metalloproteinase MT1-MMP in clear cell carcinoma of the ovary. Ovarian clear cell carcinoma (CCC) characteristically shows stromal accumulation of the basement membrane material laminin-5 (LN-5). LN-5 works not only as a substrate for cell anchorage but also as a stimulator for cell   migration  . Our previous study showed that CCC cell increased   migration   over excessive LN-5 in vitro. However, it remains unclear why LN-5 rather promotes cell   migration   than cell anchorage. A recent study has shown that the processing of the LN-5 gamma 2 chain by membrane type 1-matrix metalloproteinase (MT1-MMP) was responsible for increasing cell   migration   activity but reducing cell anchoring activity of LN-5. In the present study, 3 CCC cell lines were examined for LN-5 gamma 2 chain levels by Western blotting. Processed gamma 2 chain (105 kDa) was detected in the extracellular matrix of all 3 CCC cell lines in vitro. Immunocytochemically they expressed MT1-MMP on the cell surface. In surgical specimens, 22 of 30 (73%) CCCs showed membranous expression of MT1-MMP, and stromal accumulation of the LN-5 gamma 2 chain, especially in the area of papillary architecture. These results indicate that proteolytic processing of the LN-5 gamma 2 chain by MT1-MMP is involved in cell   migration   and subsequent architectural organization in ovarian CCC. 
 Interaction of MT1-MMP and laminin-5gamma2 chain correlates with metastasis and invasiveness in human esophageal squamous cell carcinoma. To gain insights into metastatic mechanisms in esophageal squamous cell carcinoma (ESCC), we established sublines (MLuB1 and MLuC1) with different capacity of spontaneous lung metastasis by subcutaneous injection of a human ESCC cell line (EC 9706) into nude mices. The incidence of the mice with lung metastasis produced by MLuC1 (87%) was significantly higher than that of MLuB1 (22%). The gene expression profiles of the two sublines were compared with cDNA arrays containing 5,000 known genes, and 47 genes were differentially expressed > or =2.0 fold. Laminin-5gamma2 chain (Ln-5gamma2) was one of the up-regulated genes in MLuC1 cells. Proteolytically processed forms of gamma2 are known to promote   migration   of a multitude of epithelial cells in vitro. Western-blotting analysis revealed that degraded fragments of Ln-5gamma2 and active form of membrane-type matrix metalloproteinase-1 (MT1-MMP) in MLuC1 was significantly higher than those in MLuB1. Expression of MT1-MMP was observed in 60 of 75 Ln-5gamma2-positive carcinoma tissues (80%). Co-expression of the two proteins was significantly associated with depth of invasion (P = 0.012). Moreover, proteolytic fragments of Ln-5gamma2 and active forms of MT1-MMP were frequently found in tumor tissues, whereas in the corresponding normal esophageal tissues there were only intact forms of gamma2 and MT1-MMP. siRNA-mediated silencing of MT1-MMP significantly reduced production of gamma2' and gamma2x in MLuC1 cells and inhibited cell   migration  . The results suggest that MT1-MMP is an enzyme responsible for Ln-5gamma2 cleavage in ESCC, and interaction between them may play a critical role in promoting invasion and metastasis of human ESCC. 
 Membrane-type 1 matrix metalloproteinase cytoplasmic tail-binding protein-1 is a new member of the Cupin superfamily. A possible multifunctional protein acting as an invasion suppressor down-regulated in tumors. Membrane-type 1 matrix metalloproteinase (MT1-MMP/MMP-14) is an enzyme that promotes tumor cell invasion in tissues. Although the proteolytic activity of MT1-MMP is indispensable for invasion, it is also regulated by functions of the cytoplasmic tail. In this study we obtained a new human gene whose product binds to the tail sequence in yeast. The product, MTCBP-1, is a 19-kDa protein that belongs to the newly proposed Cupin superfamily composed of proteins with diverse functions. MTCBP-1 expressed in cells formed a complex with MT1-MMP and co-localized at the membrane. It was also detected in both the cytoplasm and nucleus, where MT1-MMP does not exist. In human tumor cell lines MTCBP-1 expression was significantly low compared with non-transformed fibroblasts, and enforced expression of MTCBP-1 inhibited the activity of MT1-MMP in promoting cell   migration   and invasion. MTCBP-1 showed significant homology to the bacterial aci-reductone dioxygenase, which is an enzyme in methionine metabolism. The C-terminal part of MTCBP-1 is identical to Sip-L, which is reported to be important for human hepatitis C virus replication. Thus, MTCBP-1 may have multiple functions other than the regulation of MT1-MMP, which presumably depends on the subcellular compartment. 
 Vandetanib-induced inhibition of neuroblastoma cell   migration   and invasion is associated with downregulation of the SDF-1/CXCR4 axis and matrix metalloproteinase 14. Rearranged during transfection (RET) is widely expressed in neuroblastoma (NB) and partly contributes to high metastatic potential and survival of NB. The aim of the present study was to investigate whether vandetanib (a RET inhibitor) inhibits proliferation,   migration   and invasion of NB cells in vitro. The effects of vandetanib on the proliferation, apoptosis and cell cycle and on RET phosphorylation of SK-N-SH and SH-SY5Y cells were evaluated in vitro. The   migration   and invasion potential of vandetanib-treated NB cells were analyzed using Transwell cell   migration   and invasion assays, respectively. qPCR, western blotting and immunofluorescence were used to detect mRNA and protein levels in NB cells treated with vandetanib. Our data demonstrated that vandetanib inhibits the proliferation of SK-N-SH and SH-SY5Y cells and that this inhibition is mediated by the induction of G1 phase cell cycle arrest at lower concentrations and by apoptosis at higher concentrations. In the presence of vandetanib, the   migration   and invasion of two NB cell lines were markedly decreased compared with the control group (p<0.01). In addition, our data showed that the levels of C-X-C chemokine receptor type 4 (CXCR4) and matrix metalloproteinase 14 (MMP14) mRNA expression in NB cell lines treated with vandetanib were significantly lower than those in the cells that were treated with vehicle (p<0.01) and similar results were obtained for protein levels as determined by western blotting and immunofluorescence analysis. Vandetanib may inhibit the proliferation,   migration   and invasion of NB cells in vitro. The potential mechanisms for the inhibition of NB   migration   and invasion by vandetanib may partly be attributed to the ability of vandetanib to suppress the expression of CXCR4 and MMP14 in human NB cells. 
 Cleavage and functional loss of human apolipoprotein E by digestion of matrix metalloproteinase-14. By means of a degradomic approach applying proteomic techniques, we previously suggested that apolipoprotein E (apoE) is a substrate of matrix metalloproteinase-14 (MMP-14). Here we confirm that apoE is, in fact, a substrate of MMP-14 and also of MMP-7 and MMP-2 to a lesser extent. The 34 kDa apoE protein was initially processed by MMP-14 into fragments with molecular masses of 28, 23, 21, and 11 kDa. MMP-14 cleavage sites within the apoE protein were determined by C-terminal labeling of MMP-14-digested apoE fragments with isotope ((18)O/(16)O = 1:1) and identification of the doublet fragments or peptides showing 2 Da difference by MS, along with N-terminal sequencing of the fragments. It was determined that the primary MMP-14 cleavage sites were A(176)-I(177), P(183)-L(184), P(202)-L(203), and Q(249)-I(250). The MMP-14-mediated cleavage of apoE was consistent regardless of whether apoE existed in its lipid-bound or lipid-free form. Upon digestion with MMP-14, apoE loses its ability to suppress the platelet-derived growth factor-induced   migration   of rat vascular smooth muscle cells. Considering the important role of apoE for lipid metabolism and atherosclerosis protection, our findings suggest that MMP-14 plays an essential role for the development of hyperlipidemia and atherosclerosis as a result of degradation of apoE. 
 BST-2 binding with cellular MT1-MMP blocks cell growth and   migration   via decreasing MMP2 activity. MT1-MMP (membrane type 1-matrix metalloproteinase) plays important roles in cell growth and tumor invasion via mediating cleavage of MMP2/gelatinase A and a variety of substrates including type I collagen. BST-2 (bone marrow stromal cell antigen 2) is a membrane tetherin whose expression dramatically reduces the release of a broad range of enveloped viruses including HIV from infected cells. In this study, we provided evidence that both transient and IFN-α induced BST-2 could decrease the activity of MMP2 via binding to cellular MT1-MMP on its C-terminus and inhibiting its proteolytic activity; and finally block cell growth and   migratio  n. Zymography gel and Western blot experiments demonstrated that BST-2 decreased MMP2 activity, but no effect on the expression of MMP2 and MT1-MMP genes. Confocal and immunoprecipitation data showed that BST-2 co-localized and interacted with MT1-MMP. This interaction inhibited the proteolytic enzyme activity of MT1-MMP, and blocked the activation of proMMP2. Experimental results of C-terminus deletion mutant of MT1-MMP showed that activity of MMP2 was no change and also no interaction existed between the mutant and BST-2 after co-transfection with the mutant and BST-2. It meant that C-terminus of MT1-MMP played a key role in the interaction with BST-2. In addition, cell growth in 3D type I collagen gel lattice and cell   migratio  n were all inhibited by BST-2. Taken together, BST-2, as a membrane protein and a tetherin of enveloped viruses, was a novel inhibitor of MT1-MMP and could be considerable as an inhibitor of cancer cell growth and   migratio  n on clinic. 
 Androgens regulate MMPs and the cellular processes of intimal hyperplasia. Testosterone deficiency has been associated with an increased risk of vascular disease. Matrix metalloproteinases (MMPs) have been implicated in vascular remodeling. Our group has demonstrated an association between female hormones and MMP-modulated intimal hyperplasia. In the present study, we investigated testosterone in the modulation of MMPs and the cellular processes of intimal hyperplasia.Male vascular smooth muscle cells (VSMCs) were treated with a range of testosterone or dihydrotestosterone (DHT) concentrations (0.3-3000 nM). MMPs were assayed using quantitative polymerase chain reaction, Western blot analysis, and zymography. VSM  C migrati  on and proliferation were assayed using Boyden chamber and MTT assays.MT1-MMP gene expression was not affected by low DHT exposure but was downregulated at high levels (3000 nM = 85% ± 3%). TIMP-2 gene expression was downregulated at low DHT exposure (0.3 nM = 82% ± 4%, 3.0 nM = 82% ± 1%) but was not affected at high levels. MMP-2 enzymatic activity was increased at low DHT exposure (3.0 nM = 110% ± 4%) and decreased below basal levels at high doses (300 nM = 91% ± 7%, 3000 nM = 77% ± 8%). High concentrations of DHT d  ecreased   VSMC migration (3.0 nM = 72% ± 9%, 30 nM = 50% ± 6%, 300 nM = 47% ± 5%, 3000 nM = 53% ± 6%). Testos  terone al  so decreased migration but had less effect. The highest tested concentration of DHT and testosterone decreased the basal VSMC proliferation (3000 nM = 87% ± 3% and 87% ± 4% respectively).The DHT levels differentially affected the expression of regulatory isoforms responsible for the activation and inhibition of MMP-2, leading to an inverse relationship among the DHT levels,   MMP-2 act  ivity, and VSMC migration. In vivo studies will be used to examine testosterone deficiency and supplementation in MMP-modulated intimal hyperplasia in animal models of vascular disease. These studies are needed as a prerequisite to determining whether testosterone replacement in testosterone-deficient men should be evaluated for attenuation of atherosclerosis. 
 Cancer cell-associated MT1-MMP promotes blood vessel invasion and distant metastasis in triple-negative mammary tumors. Functional roles for the cancer cell-associated membrane type I matrix metalloproteinase (MT1-MMP) during early steps of the metastatic cascade in primary tumors remain unresolved. In an effort to determine its significance, we determined the in vivo effects of RNAi-mediated downregulation in mammary cancer cells on the   migration  , blood and lymphatic vessel invasion (LVI), and lymph node and lung metastasis. We also correlated the expression of cancer cell MT1-MMP with blood vessel invasion (BVI) in 102 breast cancer biopsies. MT1-MMP downregulation in cancer cells decreased lung metastasis without affecting primary tumor growth. The inhibition of lung metastasis correlated with reduced cancer cell   migration   and BVI. Furthermore, cancer cell-expressed MT1-MMP upregulated the expression of MT1-MMP in vascular endothelial cells, but did not affect MT1-MMP expression in lymphatic endothelial cells, LVI, or lymph node metastasis. Of clinical importance, we observed that elevated MT1-MMP expression correlated with BVI in biopsies from triple-negative breast cancers (TNBC), which have a poor prognosis and high incidence of distant metastasis, relative to other breast cancer subtypes. Together, our findings established that MT1-MMP activity in breast tumors is essential for BVI, but not LVI, and that MT1-MMP should be further explored as a predictor and therapeutic target of hematogenous metastasis in TNBC patients. 
 CD44 binding through the hemopexin-like domain is critical for its shedding by membrane-type 1 matrix metalloproteinase. Membrane-type 1 matrix metalloproteinase (MT1-MMP) is a potent modulator of pericellular environment through its proteolytic activity and promotes   migration  , invasion, and proliferation of tumor cells. During cell   migration  , MT1-MMP binds to CD44H, a major hyaluronan receptor, through the hemopexin-like (HPX) domain and localizes at the   migration   front. MT1-MMP is also responsible for shedding CD44H, which supports CD44H-mediated cell   migration  . In this study, we asked whether the binding of MT1-MMP to CD44H is a prerequisite step for the successive shedding. Deletion of the HPX domain deprived MT1-MMP of its shedding activity. Furthermore, disruption of the CD44H/MT1-MMP complex by overexpressing the HPX fragments resulted in inhibition of the shedding. Thus, the CD44H in the complex appears to be the direct substrate of MT1-MMP for shedding. Interestingly, other members of the MT-MMP family showed varied extents of CD44H shedding. Domain swapping between MT1-MMP and other MT-MMPs revealed that the ability of the HPX domains to bind CD44H is conserved among them. However, the shedding activity was different depending on the catalytic domains. The conserved binding ability of the HPX domains suggests that CD44H may act as a core molecule assembling multiple MT-MMPs on the cell surface. 
 Matrilysin shedding of syndecan-1 regulates chemokine mobilization and transepithelial efflux of neutrophils in acute lung injury. The influx of inflammatory cells to sites of injury is largely directed by signals from the epithelium, but how these cells form chemotactic gradients is not known. In matrilysin null mice, neutrophils remained confined in the interstitium of injured lungs and did not advance into the alveolar space. Impaired transepithelial   migration   was accompanied by a lack of both shed syndecan-1, a heparan sulfate proteoglycan, and KC, a CXC chemokine, in the alveolar fluid. KC was bound to shed syndecan-1, and it was not detected in the lavage of syndecan-1 null mice. In vitro, matrilysin cleaved syndecan-1 from the surface of cells. Thus, matrilysin-mediated shedding of syndecan-1/KC complexes from the mucosal surface directs and confines neutrophil influx to sites of injury. 
 Activated α2-macroglobulin induces Müller glial cel  l migrati  on by regulating MT1-MMP activity through LRP1. In retinal proliferative diseases, Müller glial cells (MGCs) acquire migratory abilities. However, the mechanisms that regulate th  is migrat  ion remain poorly understood. In addition, proliferative disorders associated with enhanced activities of matrix metalloprotease 2 (MMP-2) and MMP-9 also present increased levels of the protease inhibitor α2-macroglobulin (α2M) and its receptor, the low-density lipoprotein receptor-related protein 1 (LRP1). In the present work, we investigated whether the protease activated form of α2M, α2M*, and LRP1 are involved with the MGC migratory process. By performing wound-s  cratch mi  gration and zymography assays, we demonstrated that α2M* induc  ed cell m  igration and proMMP-2 activation in the human Müller glial cell line, MIO-M1. This induction was blocked when LRP1 and MT1-MMP were knocked down with siRNA techniques. Using fluorescence microscopy and biochemical procedures, we found that α2M* induced an increase in LRP1 and MT1-MMP accumulation in early endosomes, followed by endocytic recycling and intracellular distribution of MT1-MMP toward cellular protrusions. Moreover, Rab11-dominant negative mutant abrogated MT1-MMP recycling path  way, cell   migration, and proMMP-2 activation induced by α2M*. In conclusion, α2M*, through its receptor LRP1, induc  es cellul  ar migration of Müller glial cells by a mechanism that involves MT1-MMP intracellular traffic to the plasma membrane by a Rab11-dependent recycling pathway. 
 Matrix metalloproteinase processing of monocyte chemoattractant proteins generates CC chemokine receptor antagonists with anti-inflammatory properties in vivo. Monocyte chemoattractant protein (MCP)-3 is inactivated upon cleavage by the matrix metalloproteinase (MMP) gelatinase A (MMP-2). We investigated the susceptibility to proteolytic processing of the 4 human MCPs by 8 recombinant MMPs to determine whether MCP-3 is an isolated example or represents a general susceptibility of chemokines to proteolytic inactivation by these important inflammatory proteases. In addition to MMP-2, MCP-3 is efficiently cleaved by membrane type 1 (MT1)-MMP, the cellular activator of MMP-2, and by collagenase-1 and collagenase-3 (MMP-1, MMP-13) and stromelysin-1 (MMP-3). Specificity was shown by absence of cleavage by matrilysin (MMP-7) and the leukocytic MMPs neutrophil collagenase (MMP-8) and gelatinase B (MMP-9). The closely related chemokines MCP-1, MCP-2, and MCP-4 were not cleaved by MMP-2 or MT1-MMP, but were cleaved by MMP-1 and MMP-3 with varying efficiency. MCPs were typically cleaved between residues 4 and 5, but MCP-4 was further processed at Val7-Pro8. Synthetic MCP analogs corresponding to the MMP-cleaved forms bound CC chemokine receptor (CCR)-2 and CCR-3, but lacked chemoattractant activity in pre-B cells transfected with CCR-2 and CCR-3 or in THP-1 monocytic cells, a transformed leukemic cell line. Moreover, the truncated products of MCP-2 and MCP-4, like MCP-3, were potent antagonists of their cognate CC chemokine receptors in transwell cell   migration   assays in vitro. When they were injected 24 hours after the initiation of carrageenan-induced inflammation in rat paws, their in vivo antagonist activities were revealed by a greater than 66% reduction in inflammatory edema progression after 12 hours. We propose that MMPs have an important role in modulating inflammatory and immune responses by processing chemokines in wound healing and in disease. 
 Three-dimensional matrix fiber alignment modulates cell   migration   and MT1-MMP utility by spatially and temporally directing protrusions. Multiple attributes of the three-dimensional (3D) extracellular matrix (ECM) have been independently implicated as regulators of cell motility, including pore size, crosslink density, structural organization, and stiffness. However, these parameters cannot be independently varied within a complex 3D ECM protein network. We present an integrated, quantitative study of these parameters across a broad range of complex matrix configurations using self-assembling 3D collagen and show how each parameter relates to the others and to cell motility. Increasing collagen density resulted in a decrease and then an increase in both pore size and fiber alignment, which both correlated significantly with cell motility but not bulk matrix stiffness within the range tested. However, using the crosslinking enzyme Transglutaminase II to alter microstructure independently of density revealed that motility is most significantly predicted by fiber alignment. Cellular protrusion rate, protrusion orientation, speed of   migration  , and invasion distance showed coupled biphasic responses to increasing collagen density not predicted by 2D models or by stiffness, but instead by fiber alignment. The requirement of matrix metalloproteinase (MMP) activity was also observed to depend on microstructure, and a threshold of MMP utility was identified. Our results suggest that fiber topography guides protrusions and thereby MMP activity and motility. 
 Enhanced membrane-type 1 matrix metalloproteinase expression by hyaluronan oligosaccharides in breast cancer cells facilitates CD44 cleavage and tumor cell   migration  . Hyaluronan (HA), a component of the extracellular matrix, plays an important role in cell-cell adhesion and cell   migration  . Membrane type 1-matrix metalloproteinase (MT1‑MMP) is often expressed in invasive cancer cells. CD44, a transmembrane receptor for HA, is implicated in various adhesion-dependent cellular processes including cel  l migrati  on, tumor cell metastasis and invasion. Previous studies have shown that CD44 is highly expressed in cancer cells and may be proteolytically cleaved at the ectodomain by MT1-MMP; this process of inducing CD44 cleavage plays a critical role in cancer cel  l migrati  on. We hypothesized that HA modulates MT1-MMP expression to facilitate breast cancer cel  l migrati  on. Flow cytometry, real-time PCR, western blotting and immunofluorescence staining were used to quantify HA-induced MT1-MMP expression in breast cancer cells. In order to validate the relevance of cel  l migrati  on and HA-induced MT1-MMP, we analyzed the cel  l migrati  on via matrigel-coated transwell. We found that after HA oligosaccharide (6.5 kDA) stimulation, MT1-MMP expression in the membrane of breast cancer cells was increased. In response to HA oligosaccharide stimulation, significant upregulation of MT1-MMP mRNA occurred. Our data also provide evidence that HA oligosaccharide enhances MT1-MMP; the elevated expression of MT1-MMP confers enhanced CD44 cleavage and cel  l migrati  on. In conclusion, we have identified a new function of HA in the induction of MT1-MMP expression in breast cancer cell lines and CD44 cleavage to increase cel  l migrati  on during the invasion process. The HA oligosaccharide-induced MT1-MMP expression in breast cancer cells may be a critical step in the formation of metastatic colonies. 
 Membrane Type 1 Matrix Metalloproteinase Regulates Monocyte   Migration   and Collagen Destruction in Tuberculosis. Tuberculosis (TB) remains a global pandemic and drug resistance is rising. Multicellular granuloma formation is the pathological hallmark of Mycobacterium tuberculosis infection. The membrane type 1 matrix metalloproteinase (MT1-MMP or MMP-14) is a collagenase that is key in leukocyte   migration   and collagen destruction. In patients with TB, induced sputum MT1-MMP mRNA levels were increased 5.1-fold compared with matched controls and correlated positively with extent of lung infiltration on chest radiographs (r = 0.483; p < 0.05). M. tuberculosis infection of primary human monocytes increased MT1-MMP surface expression 31.7-fold and gene expression 24.5-fold. M. tuberculosis-infected monocytes degraded collagen matrix in an MT1-MMP-dependent manner, and MT1-MMP neutralization decreased collagen degradation by 73%. In human TB granulomas, MT1-MMP immunoreactivity was observed in macrophages throughout the granuloma. Monocyte-monocyte networks caused a 17.5-fold increase in MT1-MMP surface expression dependent on p38 MAPK and G protein-coupled receptor-dependent signaling. Monocytes migrating toward agarose beads impregnated with conditioned media from M. tuberculosis-infected monocytes expressed MT1-MMP. Neutralization of MT1-MMP activity decreased this M. tuberculosis network-dependent monocyte   migration   by 44%. Taken together, we demonstrate that MT1-MMP is central to two key elements of TB pathogenesis, causing collagen degradation and regulating monocyte   migration  . 
 MT1-MMP proinvasive activity is regulated by a novel Rab8-dependent exocytic pathway. MT1-matrix metalloproteinase (MT1-MMP) is one of the most critical factors in the invasion machinery of tumor cells. Subcellular localization to invasive structures is key for MT1-MMP proinvasive activity. However, the mechanism driving this polarized distribution remains obscure. We now report that polarized exocytosis of MT1-MMP occurs during MDA-MB-231 adenocarcinoma cell   migration   into collagen type I three-dimensional matrices. Polarized trafficking of MT1-MMP is triggered by beta1 integrin-mediated adhesion to collagen, and is required for protease localization at invasive structures. Localization of MT1-MMP within VSV-G/Rab8-positive vesicles, but not in Rab11/Tf/TfRc-positive compartment in invasive cells, suggests the involvement of the exocytic traffic pathway. Furthermore, constitutively active Rab8 mutants induce MT1-MMP exocytic traffic, collagen degradation and invasion, whereas Rab8- but not Rab11-knockdown inhibited these processes. Altogether, these data reveal a novel pathway of MT1-MMP redistribution to invasive structures, exocytic vesicle trafficking, which is crucial for its role in tumor cell invasiveness. Mechanistically, MT1-MMP delivery to invasive structures, and therefore its proinvasive activity, is regulated by Rab8 GTPase. 
 Membrane type-1 matrix metalloproteinase (MT1-MMP) processing of pro-alphav integrin regulates cross-talk between alphavbeta3 and alpha2beta1 integrins in breast carcinoma cells. We have recently demonstrated that in breast carcinoma MCF7 cells MT1-MMP processes the alphav, alpha3, and alpha5 integrin precursors generating the respective mature S-S-linked heavy and light alpha-chains. The precursor of alpha2 integrin subunit was found resistant to MT1-MMP proteolysis. The processing of the alphav subunit by MT1-MMP facilitated alphavbeta3-dependent adhesion, activation of FAK signaling pathway, and   migration   of MCF7 cells on vitronectin. To elucidate further the effects of MT1-MMP on cellular integrins, we examined the functional activity of alpha5beta1 and alpha2beta1 integrins in MCF7 cells expressing MT1-MMP. Either expression of MT1-MMP alone or its coexpression with alphavbeta3 failed to affect the functionality of alpha5beta1 integrin, and adhesion of cells to fibronectin. MT1-MMP, however, profoundly affected the cross-talk involving alphavbeta3 and alpha2beta1 integrins. In MT1-MMP-deficient cells, integrin alphavbeta3 suppressed the functional activity of the collagen-binding alpha2beta1 integrin receptor and diminished cell adhesion to type I collagen. Coexpression of MT1-MMP with integrin alphavbeta3 restored the functionality of alpha2beta1 integrin and, consequently, the ability of MCF7 cells to adhere efficiently to collagen. We conclude that the MT1-MMP-controlled cross-talk between alphavbeta3 and alpha2beta1 integrins supports binding of aggressive, MT1-MMP-, and alphavbeta3 integrin-expressing malignant cells on type I collagen, the most common substratum of the extracellular matrix. 
 Degenerate wave and capacitive coupling increase human MSC invasion and proliferation while reducing cytotoxicity in an in vitro wound healing model. Non-unions pose complications in fracture management that can be treated using electrical stimulation (ES). Bone marrow mesenchymal stem cells (BMMSCs) are essential in fracture healing; however, the effect of different clinical ES waveforms on BMMSCs cellular activities remains unknown. We compared the effects of direct current (DC), capacitive coupling (CC), pulsed electromagnetic field (PEMF) and degenerate wave (DW) on cellular activities including cytotoxicity, proliferation, cell-kinetics and apoptosis by stimulating human-BMMSCs 3 hours a day, up to 5 days. In addition,   migration   and invasion were assessed using fluorescence microscopy and by quantifying gene and protein expression. We found that DW had the greatest proliferative and least apoptotic and cytotoxic effects compared to other waveforms. DC, DW and CC stimulations resulted in a higher number of cells in S phase and G(2)/M phase as shown by cell cycle analysis. CC and DW caused more cells to invade collagen and showed increased MMP-2 and MT1-MMP expression. DC increased cellular   migration   in a scratch-wound assay and all ES waveforms enhanced expression of migratory genes with DC having the greatest effect. All ES treated cells showed similar progenitor potential as determined by MSC differentiation assay. All above findings were shown to be statistically significant (p<0.05). We conclude that ES can influence BMMSCs activities, especially DW and CC, which show greater invasion and higher cell proliferation compared to other types of ES. Application of DW or CC to the fracture site may help in the recruitment of BMMSCs to the wound that may enhance rate of bone healing at the fracture site. 
 Membrane Type 1 Matrix Metalloproteinase induces an epithelial to mesenchymal transition and cancer stem cell-like properties in SCC9 cells. Tissue invasion and metastasis are acquired abilities of cancer and related to the death in oral squamous cell carcinoma (OSCC). Emerging observations indicate that the epithelial-to-mesenchymal transition (EMT) is associated with tumor progression and the generation of cells with cancer stem cells (CSCs) properties. Membrane Type 1 Matrix Metalloproteinase (MT1-MMP) is a cell surface proteinase, which is involved in degrading extracellular matrix components that can promote tumor invasion and cell   migration  .In the current study, we utilized SCC9 cells stably transfected with an empty vector (SCC9-N) or a vector encoding human MT1-MMP (SCC9-M) to study the role of MT1-MMP in EMT development.Upon up-regulation of MT1-MMP, SCC9-M cells underwent EMT, in which they presented a fibroblast-like phenotype and had a decreased expression of epithelial markers (E-cadherin, cytokeratin18 and β-catenin) and an increased expression of mesenchymal markers (vimentin and fibronectin). We further demonstrated that MT1-MMP-induced morphologic changes increased the level of Twist and ZEB, and were dependent on repressing the transcription of E-cadherin. These activities resulted in low adhesive, high invasive abilities of the SCC9-M cells. Furthermore, MT1-MMP-induced transformed cells exhibited cancer stem cell (CSC)-like characteristics, such as low proliferation, self-renewal ability, resistance to chemotherapeutic drugs and apoptosis, and expression of CSCs surface markers.In conclusion, our study indicates that overexpression of MT1-MMP induces EMT and results in the acquisition of CSC-like properties in SCC9 cells. Our growing understanding of the mechanism regulating EMT may provide new targets against invasion and metastasis in OSCC. 
 NHE1 mediates   migration   and invasion of HeLa cells via regulating the expression and localization of MT1-MMP. Na(+)/H(+) exchanger 1 (NHE1), acting as an important regulator of intracellular pH (pH(i)) and extracellular pH (pH(e)), has been known to play a key role in the metastasis of many solid tumours. However, the exact mechanism underlying these processes, especially in cervical cancer, is still poorly understood. In the current study, we first showed that the inhibition of NHE1 activity by the specific inhibitor cariporide could suppress   migration   and invasion of HeLa cells in vitro. Moreover, cariporide also reversed the enhanced   migration   and invasion in HeLa cells by overexpressed membrane-type 1 matrix metalloproteinase (MT1-MMP). Subsequently, our results showed that NHE1 regulated the expression of MT1-MMP at both messenger RNA and protein levels as well as its localization. Meanwhile, we observed slight modification in the morphology of HeLa cell after treating with cariporide. The present work indicates that NHE1 mediates HeLa cell metastasis via regulating the expression and localization of MT1-MMP and provides a theoretical basis for the development of novel therapeutic strategies targeting cervical cancer. 
 Membrane-type 1-matrix metalloproteinase regulates intracellular adhesion molecule-1 (ICAM-1)-mediated monocyte trans  migration  . We examined the mechanism regulating intercellular cell adhesion molecule-1 (ICAM-1)-dependent monocyte transendothelial   migration  . Monocyte   migration   through endothelial cells expressing ICAM-1 alone was comparable to that of tumor necrosis factor-alpha-treated cells. Trans  migration   was reduced in ICAM-1 lacking the cytoplasmic tail and in tyrosine to alanine substitutions at Tyr-485 and Tyr-474. Tissue inhibitors of matrix metalloproteinases (TIMPs) -2 and -3 blocked trans  migration  , whereas TIMP-1 was ineffective. This profile suggested a role for membrane-type matrix metalloproteinases (MT-MMPs) in trans  migration  . Inhibitory antibodies and small interference RNA directed against MT1-MMP blocked trans  migration  , whereas overexpression of MT1-MMP in endothelial cells or monocytes promoted trans  migration  . MT1-MMP mediated the ectodomain cleavage of ICAM-1 that was blocked by TIMP-2 and -3. Overexpression of MT1-MMP rescued function in ICAM-1Y485A, and to a lesser extent in the cytoplasmic tail-deleted ICAM-1. In a binding assay, wild-type ICAM-1 bound to purified MT1-MMP while ICAM-1 mutants bound poorly. MT1-MMP co-localized with ICAM-1 at distinct structures in endothelial cells. MT1-MMP localization with cells expressing ICAM-1 mutations was reduced and diffused. These results indicate that the cytoplasmic tail of ICAM-1 regulates leukocyte trans  migration   through MT1-MMP interaction. 
 Expression of membrane type 1 matrix metalloproteinase in papillomavirus-positive cells: role of the human papillomavirus (HPV) 16 and HPV8 E7 gene products. Matrix metalloproteinases (MMPs) degrade extracellular matrix. They are involved in cellular proliferation,   migration  , angiogenesis, invasion and metastasis. MT-1 MMP, a membrane-bound MMP, is expressed in carcinomas of the uterine cervix in vivo. This type of cancer is associated with human papillomavirus (HPV) infection. Here it was shown that keratinocytes transformed with HPV16 or HPV18 in vitro, and HPV-positive cervical carcinoma cell lines, constitutively expressed MT-1 MMP. Expression of the E7 protein from the mucosal and cutaneous high-risk types HPV16 and HPV8, but not from the cutaneous low-risk type HPV1, was sufficient to induce MT-1 MMP expression in primary human keratinocytes and HaCaT cells. As a consequence, MMP-2 was activated. MT-1 MMP expression might play a role in the HPV life cycle by promoting proliferation of host cells and might contribute to their invasive phenotype during malignant progression. 
 Shedding of kidney injury molecule-1 by membrane-type 1 matrix metalloproteinase. Co-expression of membrane-type 1 matrix metalloproteinase (MT1-MMP) with kidney injury molecule-1 (KIM-1) in HEK293T cells resulted in cleavage and shedding of KIM-1 ectodomain. Analysis of cleavage products using KIM-1 mutants localized cleavage site at the juxtamembrane region. HT1080 cells were stably transfected with expression plasmid for KIM-1 or its mutant with deletion of the juxtamembrane region (Asp(261)-Gly(295)) to establish HT/KIM-1 or HT/ΔKIM-1 cells, respectively. KIM-1 protein appeared on cell surface at low level in HT/KIM-1 cells, and accumulated by the treatment with MMP inhibitor BB-94 or small interfering RNA (siRNA) to MT1-MMP, indicating that MT1-MMP is involved in cleavage and shedding of KIM-1. In contrast, HT/ΔKIM-1 cells expressed KIM-1 protein at high level regardless of BB-94 or siRNA treatment. Cells expressing high level KIM-1 protein exhibited phagocytosis of Escherichia coli and reduced cell adhesion and spreading on collagen-coated plate compared with KIM-1 negative cells. Control HT1080 and HT/KIM-1 cells showed significantly higher invasive growth in collagen gel, cel  l migrati  on on collagen-coated plate and liver metastasis in chick embryo than HT/ΔKIM-1 cells. These results suggest that KIM-1 negatively regulates cellular function mediated through interaction with collagen, and MT1-MMP abrogates it through the cleavage and shedding of KIM-1. 
 Angiostatin inhibits endothelial MMP-2 and MMP-14 expression: a hypoxia specific mechanism of action. Angiostatin is an angiogenesis inhibitor in part generated by and released from platelets. Since platelets upon thrombus formation can give rise to areas of hypoxia, we investigated the effects of angiostatin on endothelial cell   migration   and apoptosis during hypoxia. Human microvascular endothelial cells (HMVEC-L) were exposed to angiostatin under normoxic or hypoxic conditions. Apoptosis was measured by flow-cytometry. HMVEC-L   migration   was studied using a modified Boyden Chamber assay, in which   migration   is MMP-dependent. MMP-2, MMP-14, and VEGF levels were measured using immunoblot, Q-PCR and ELISA. During hypoxia HMVEC-L were protected from angiostatin-induced apoptosis due to increased hypoxia-induced VEGF expression. However, MMP-dependent   migration   of HMVEC-L was inhibited by angiostatin under hypoxic but not normoxic conditions. Angiostatin decreased MMP-2 at the gene and protein levels only in HMVEC-L exposed to hypoxia. A similar result was obtained for MMP-14. Higher angiostatin concentrations, as would be seen during thrombosis, induced HMVEC-L apoptosis, which was not rescued by VEGF. Under hypoxic conditions angiostatin's primary anti-angiogenic mechanism is likely inhibition of endothelial cell MMP-dependent endothelial cell   migration  . Only at higher concentrations does angiostatin induce endothelial cell death. This study identifies a novel angiostatin anti-angiogenesis mechanism that is only triggered under pathological-like conditions. 
 Methyl jasmonate abolishes the   migration  , invasion and angiogenesis of gastric cancer cells through down-regulation of matrix metalloproteinase 14. Recent evidence indicates that methyl jasmonate (MJ), a plant stress hormone, exhibits anti-cancer activity on human cancer cells. The aim of this study is to determine whether sub-cytotoxic MJ can abolish the   migration  , invasion and angiogenesis gastric cancer cells.Human gastric cancer cell lines SGC-7901 and MKN-45 were treated with diverse concentrations of MJ. Cell viability, proliferation,   migration  , invasion and angiogenesis capabilities of cancer cells were measured by MTT colorimetry, EdU incorporation, scratch assay, matrigel invasion assay, and tube formation assay. Gene expression was detected by western blot and real-time quantitative RT-PCR. Binding of transcription factor on gene promoter was detected by chromatin immunoprecipitation.Sub-cytotoxic (0.05 to 0.2 mM) MJ attenuated the   migration  , invasion and angiogenesis, but not the cell viability or proliferation, of gastric cancer cells in a time- and dose-dependent manner, with down-regulation of matrix metalloproteinase 14 (MMP-14) and its downstream gene vascular endothelial growth factor. Restoration of MMP-14 expression rescued the SGC-7901 and MKN-45 cells from sub-cytotoxic MJ-inhibited   migration  , invasion and angiogenesis. In addition, sub-cytotoxic MJ decreased the specificity protein 1 (Sp1) expression and binding on MMP-14 promoter, while restoration of Sp1 expression rescued the cancer cells from sub-cytotoxic MJ-mediated defects in MMP-14 expression,   migration  , invasion and angiogenesis.Sub-cytotoxic MJ attenuates the MMP-14 expression via decreasing the Sp1 expression and binding on MMP-14 promoter, thus inhibiting the   migration  , invasion and angiogenesis of gastric cancer cells. 
 Downregulation of Akt and FAK phosphorylation reduces invasion of glioblastoma cells by impairment of MT1-MMP shuttling to lamellipodia and downregulates MMPs expression. Human malignant glioblastomas are highly invasive tumors. Increased cell motility and degradation of the surrounding extracellular matrix are essential for tumor invasion. PI3K/Akt signaling pathway emerges as a common pathway regulating cellular proliferation,   migration   and invasion; however, its contribution to particular process and downstream cascades remain poorly defined. We have previously demonstrated that Cyclosporin A (CsA) affects glioblastoma invasion in organotypic brain slices and tumorigenicity in mice. Here we show that CsA impairs   migration   and invasion of human glioblastoma cells by downregulation of Akt phosphorylation. Interference with PI-3K/Akt signaling was crucial for CsA effect on invasion, because overexpression of constitutively active myr-Akt antagonized drug action. Furthermore, the drug was not effective in T98G glioblastoma cells with constitutively high level of phosphorylated Akt. CsA, comparably to pharmacological inhibitors of PI3K/Akt signaling (LY294002, A443654), reduced motility of glioblastoma cells, diminished MMP-2 gelatinolytic activity and MMP-2 and MT1-MMP expression. The latter effect was mimicked by overexpression of dominant negative Akt mutants. We demonstrate that CsA and LY294002 reduced MMP transcription partly via modulation of IκB phosphorylation and NFκB transcriptional activity. Those effects were not mediated by inhibition of calcineurin, a classical CsA target. Additionally, CsA reduced phosphorylation and activity of focal adhesion kinase that was associated with rapid morphological alterations, rearrangement of lamellipodia and impairment of MT1-MMP translocation to membrane protrusions. Our results document novel, Akt-dependent mechanisms of interference with motility/invasion of human glioblastoma cells: through a rapid modulation of cell adhesion and MT1-MMP translocation to membrane protrusions and delayed, partly NFκB-dependent, downregulation of MMP-2 and MT1-MMP expression. This article is part of a Special Issue entitled: 11th European Symposium on Calcium. 
 Phosphorylation of membrane type 1-matrix metalloproteinase (MT1-MMP) and its vesicle-associated membrane protein 7 (VAMP7)-dependent trafficking facilitate cell invasion and   migration  . In multicellular organisms, uncontrolled movement of cells can contribute to pathological conditions, such as multiple sclerosis and cancer. In highly aggressive tumors, the expression of matrix metalloproteinases (MMPs) is linked to the capacity of tumor cells to invade surrounding tissue and current research indicates that the membrane-anchored membrane type 1-matrix metalloproteinase (MT1-MMP) has a central role in this process. Endocytosis and trafficking of MT1-MMP are essential for its proper function, and here we examine the phosphorylation, internalization, and recycling of this enzyme, and the associated biochemical signaling in HeLa and HT-1080 fibrosarcoma cells. Activation of protein kinase C with phorbol 12-myristate 13-acetate resulted in phosphorylation of endogenous MT1-MMP at Thr(567) in vivo. Mutation of Thr(567) to alanine (to mimic non-phosphorylated MT1-MMP) reduced internalization of MT1-MMP, whereas mutation of Thr(567) to glutamic acid (to mimic phosphorylation) resulted in decreased levels of MT1-MMP on the cell surface. The endosomal trafficking and recycling of MT1-MMP was found to be dependent upon Rab7 and VAMP7, and blocking the function of these proteins reduced cell   migration   and invasion. Intracellular trafficking of MT1-MMP was observed to be coupled to the trafficking of integrin α5 and phosphorylation of ERK that coincided with this was dependent on phosphorylation of MT1-MMP. Together, these results reveal important roles for MT1-MMP phosphorylation and trafficking in both cell signaling and cell invasion. 
 A cancer cell metalloprotease triad regulates the basement membrane trans  migration   program. Carcinoma cells initiate the metastatic cascade by inserting invasive pseudopodia through breaches in the basement membrane (BM), a specialized barrier of cross-linked, extracellular matrix macromolecules that underlies epithelial cells and ensheaths blood vessels. While BM invasion is the sine qua non of the malignant phenotype, the molecular programs that underlie this process remain undefined. To identify genes that direct BM remodeling and trans  migration  , we coupled high-resolution electron microscopy with an ex vivo model of invasion that phenocopies the major steps observed during the transition of carcinoma in situ to frank malignancy. Herein, a triad of membrane-anchored proteases, termed membrane type-1, type-2, and type-3 metalloproteinases, are identified as the triggering agents that independently confer cancer cells with the ability to proteolytically efface the BM scaffolding, initiate the assembly of invasive pseudopodia, and propagate trans  migration  . These studies characterize the first series of gene products capable of orchestrating the entire BM remodeling program that distinguishes the carcinomatous phenotype. 
 Collagen I and thrombin activate MMP-2 by MMP-14-dependent and -independent pathways: implications for airway smooth muscle   migration  . Increased proinflammatory mediators and ECM deposition are key features of the airways in asthma. Matrix metalloproteinases (MMPs) are produced by airway smooth muscle (ASM) cells and have multiple roles in inflammation and tissue remodeling. We hypothesized that components of the asthmatic airway would stimulate MMP production and activation by ASM and contribute to airway remodeling. We measured human ASM-derived MMP mRNA, protein, and activity by real-time RT-PCR, zymography, Western blotting, and MMP activity assay. Collagen I and thrombin caused a synergistic increase in MMP-2 protein and total MMP activity but paradoxically decreased MMP-2 mRNA. Additionally, collagen I activated MMP-2 in culture supernatants independent of the cell surface. Together, collagen I and thrombin strongly enhanced MMP-14 mRNA and protein but had no effect individually, suggesting increased MMP-14, the activating protease for MMP-2, may be partially responsible for MMP-2 activation. Furthermore, collagen I reduced tissue inhibitor of metalloproteinase-2 protein (TIMP-2). We examined the role of MMPs in functions of ASM related to airway remodeling and found   migration   and proliferation were MMP dependent, whereas adhesion and apoptosis were not. Ilomastat inhibited   migration   by 25%, which was also inhibited by TIMPs 1-4 and increased by the MMP-2 activator thrombin. These in vitro findings suggest that the environment within the airways of patients with asthma enhances MMP-2 and -14 protein and activity by a complex interaction of transcriptional and posttranscriptional mechanisms, which may contribute to ASM   migration  . 
 Elastin-derived peptides enhance angiogenesis by promoting endothelial cell   migration   and tubulogenesis through upregulation of MT1-MMP. Elastin-derived peptides display a wide range of biological activities in a number of normal and transformed cells but their involvement in angiogenesis has not been reported. In the present study, we show that kappa-elastin and VGVAPG hexapeptide elastin motif accelerated angiogenesis in the chick chorio-allantoic membrane in an in vivo model. They also stimulated pseudotube formation from human vascular and microvascular endothelial cells in the matrigel and collagen models as well as cell   migration   in an in vitro wound healing assay. Confocal and scanning electron microscopy analyses revealed the main reorganization of actin filaments mediated by elastin-derived peptides and changes in cell shape that correlated with a decrease of the cell form factor determined by computerized image analysis. Such elastin-derived peptide effects were attributed to upregulation of proMT1-MMP and proMMP-2 expression and activation at both the mRNA and protein levels. Batimastat, an inhibitor of furin convertase and TIMP-2, but not TIMP-1, totally abolished the influence of elastin-derived peptides (EDPs) on cell   migration   and tubulogenesis, thus favoring the involvement of MT1-MMP in such processes. To assess its contribution to EDP-mediated angiogenesis further, we used a small interfering RNA (siRNA) approach for specifically silencing MT1-MMP in human microvascular endothelial cells. Four sets of 21 bp siRNA duplexes targeting MT1-MMP mRNA were synthesized by in vitro transcription. Two of them proved to inhibit MT1-MMP expression efficiently but did not affect MT2-, MT3- and MT5-MMP expression. Seventy-two hours after transfection with 25 nM siRNAs EDP-induced MT1-MMP expression at the mRNA and protein levels was decreased fourfold. In parallel, proMMP-2 activation was inhibited. A scrambled siRNA, used as a negative control, had no effect. Finally, the effect of elastin peptides on pseudotube formation in MT1-MMP-siRNA transfected cells was totally abolished. These data emphasise the crucial role of MT1-MMP in the elastin-induced angiogenic phenotype of endothelial cells. 
 Membrane-type 1 matrix metalloproteinase modulates focal adhesion stability and cell   migration  . Membrane-type 1 matrix metalloproteinase (MT1-MMP) plays an important role in extracellular matrix-induced cell   migration   and the activation of extracellular signal-regulated kinase (ERK). We showed here that transfection of the MT1-MMP gene into HeLa cells promoted fibronectin-induced cell   migration  , which was accompanied by fibronectin degradation and reduction of stable focal adhesions, which function as anchors for actin-stress fibers. MT1-MMP expression attenuated integrin clustering that was induced by adhesion of cells to fibronectin. The attenuation of integrin clustering was abrogated by MT1-MMP inhibition with a synthetic MMP inhibitor, BB94. When cultured on fibronectin, HT1080 cells, which endogenously express MT1-MMP, showed so-called motile morphology with well-organized focal adhesion formation, well-oriented actin-stress fiber formation, and the lysis of fibronectin through trails of cell   migration  . Inhibition of endogenous MT1-MMP by BB94 treatment or expression of the MT1-MMP carboxyl-terminal domain, which negatively regulates MT1-MMP activity, resulted in the suppression of fibronectin lysis and cell   migration  . BB94 treatment promoted stable focal adhesion formation concomitant with enhanced phosphorylation of tyrosine 397 of focal adhesion kinase (FAK) and reduced ERK activation. These results suggest that lysis of the extracellular matrix by MT1-MMP promotes focal adhesion turnover and subsequent ERK activation, which in turn stimulates cell   migration  . 
 The type I collagen induction of MT1-MMP-mediated MMP-2 activation is repressed by alphaVbeta3 integrin in human breast cancer cells. The influence of alphaVbeta3 integrin on MT1-MMP functionality was studied in human breast cancer cells of differing beta3 integrin status. Overexpression of beta3 integrin caused increased cell surface expression of alphaV integrin and increased cellular adhesion to extracellular matrix (ECM) substrates in BT-549, MDA-MB-231 and MCF-7 cells. beta3 integrin expression also enhanced the   migration   of breast cancer cells on ECM substrates and enhanced collagen gel contraction. In vivo, alphaVbeta3 cooperated with MT1-MMP to increase the growth of MCF-7 cells after orthotopic inoculation in immunocompromised mice, but had no influence on in vitro proliferation. Despite these stimulatory effects, overexpression of beta3 integrin suppressed the type I collagen (Col I) induced MMP-2 activation in all breast cancer cell lines analyzed. This was also evident in extracts from the MCF-7 tumors in vivo, where MMP-2 activation was stimulated by MT1-MMP transfection, but attenuated with beta3 integrin expression. Although our studies confirm important biological effects of alphaVbeta3 integrin on enhancing cell adhesion and   migration  , ECM remodeling and tumor growth, beta3 integrin caused reduced MMP-2 activation in response to Col I in vitro, which appears to be physiologically relevant, as it was also seen in tumor xenografts in vivo. The reduction of MMP-2 activation (and thus MT1-MMP activity) by alphaVbeta3 in response to Col I may be important in scenarios where cells which are activated for matrix degradation need to preserve some pericellular collagen, perhaps as a substrate for cell adhesion and   migration  , thus maintaining a balanced level of proteolysis required for efficient tumor growth. 
 Inhibition mechanism of membrane metalloprotease by an exosite-swiveling conformational antibody. Membrane type 1 metalloprotease (MT1-MMP) is a membrane-anchored, zinc-dependent protease. MT1-MMP is an important mediator of cell   migratio  n and invasion, and overexpression of this enzyme has been correlated with the malignancy of various tumor types. Therefore, modulators of MT1-MMP activity are proposed to possess therapeutic potential in numerous invasive diseases. Here we report the inhibition mode of MT1-MMP by LEM-2/15 antibody, which targets a surface epitope of MT1-MMP. Specifically, the crystal structures of Fab LEM-2/15 in complex with the MT1-MMP surface antigen suggest that conformational swiveling of the enzyme surface loop is required for effective binding and consequent inhibition of MT1-MMP activity on the cell membrane. This inhibition mechanism appears to be effective in controlling active MT1-MMP in endothelial cells and at the leading edge of migratory cancer cells. 
 A specific subset of RabGTPases controls cell surface exposure of MT1-MMP, extracellular matrix degradation and three-dimensional invasion of macrophages. The matrix metalloproteinase MT1-MMP has a major impact on invasive cell   migration   in both physiological and pathological settings such as immune cell extravasation or metastasis of cancer cells. Surface-associated MT1-MMP is able to cleave components of the extracellular matrix, which is a prerequisite for proteolytic invasive   migration  . However, current knowledge on the molecular mechanisms that regulate MT1-MMP trafficking to and from the cell surface is limited. We have identified three members of the RabGTPase family, Rab5a, Rab8a and Rab14, as crucial regulators of MT1-MMP trafficking and function in primary human macrophages. Both overexpressed and endogenous forms show prominent colocalisation with MT1-MMP-positive vesicles, whereas expression of mutant constructs, as well as siRNA-induced knockdown, reveal that these RabGTPases are crucial in the regulation of MT1-MMP surface exposure, contact of MT1-MMP-positive vesicles with podosomes, extracellular matrix degradation in two and three dimensions, as well as three-dimensional proteolytic invasion of macrophages. Collectively, our results identify Rab5a, Rab8a and Rab14 as major regulators of MT1-MMP trafficking and invasive   migration   of primary human macrophages, which could be promising potential targets for manipulation of immune cell invasion. 
 Molecular signature of MT1-MMP: transactivation of the downstream universal gene network in cancer. Invasion-promoting MT1-MMP is directly linked to tumorigenesis and metastasis. Our studies led us to identify those genes, the expression of which is universally linked to MT1-MMP in multiple tumor types. Genome-wide expression profiling of MT1-MMP-overexpressing versus MT1-MMP-silenced cancer cells and a further data mining analysis of the preexisting expression database of 190 human tumors of 14 cancer types led us to identify 11 genes, the expression of which correlated firmly and universally with that of MT1-MMP (P < 0.00001). These genes included regulators of energy metabolism (NNT), trafficking and membrane fusion (SLCO2A1 and ANXA7), signaling and transcription (NR3C1, JAG1, PI3K delta, and CK2 alpha), chromatin rearrangement (SMARCA1), cell division (STK38/NDR1), apoptosis (DAPK1), and mRNA splicing (SNRPB2). Our subsequent extensive analysis of cultured cells, tumor xenografts, and cancer patient biopsies supported our data mining. Our results suggest that transcriptional reprogramming of the specific downstream genes, which themselves are associated with tumorigenesis, represents a distinctive "molecular signature" of the proteolytically active MT1-MMP. We suggest that the transactivation activity of MT1-MMP contributes to the promigratory cell phenotype, which is induced by this tumorigenic proteinase. The activated downstream gene network then begins functioning in unison with MT1-MMP to rework the signaling, transport, cell division, energy metabolism, and other critical cell functions and to commit the cell to   migration  , invasion, and, consequently, tumorigenesis. 
 Identification of membrane-type 1 matrix metalloproteinase tyrosine phosphorylation in association with neuroblastoma progression. Neuroblastoma is a pediatric tumor of neural crest cells that is clinically characterized by its variable evolution, from spontaneous regression to malignancy. Despite many advances in neuroblastoma research, 60% of neuroblastoma, which are essentially metastatic cases, are associated with poor clinical outcome due to the lack of effectiveness of current therapeutic strategies. Membrane-type 1 matrix metalloproteinase (MT1-MMP, MMP-14), an enzyme involved in several steps in tumor progression, has previously been shown to be associated with poor clinical outcome for neuroblastoma. Based on our recent demonstration that MT1-MMP phosphorylation is involved in the growth of fibrosarcoma tumors, we examined the potential role of phosphorylated MT1-MMP in neuroblastoma progression.Tyrosine phosphorylated MT1-MMP was immunostained on tissue microarray samples from 55 patients with neuroblastoma detected by mass screening (known to be predominantly associated with favourable outcome), and from 234 patients with standard diagnosed neuroblastoma. In addition, the effects of a non phosphorylable version of MT1-MMP on neuroblastoma cell   migration   and proliferation were investigated within three-dimensional collagen matrices.Although there is no correlation between the extent of tyrosine phosphorylation of MT1-MMP (pMT1-MMP) and MYCN amplification or clinical stage, we observed greater phosphorylation of pMT1-MMP in standard neuroblastoma, while it is less evident in neuroblastoma from mass screening samples (P = 0.0006) or in neuroblastoma samples from patients younger than one year (P = 0.0002). In vitro experiments showed that overexpression of a non-phosphorylable version of MT1-MMP reduced MT1-MMP-mediated neuroblastoma cell   migration   and proliferation within a three-dimensional type I collagen matrix, suggesting a role for the phosphorylated enzyme in the invasive properties of neuroblastoma cells.Overall, these results suggest that tyrosine phosphorylated MT1-MMP plays an important role in neuroblastoma progression and that its expression is preferentially observed in tumor specimens from neuroblastoma patients showing poor clinical outcome. 
 Evidence of MTCBP-1 interaction with the cytoplasmic domain of MT1-MMP: Implications in the autophagy cell index of high-grade glioblastoma. Progression of astrocytic tumors is, in part, related to their dysregulated autophagy capacity. Recent evidence indicates that upstream autophagy signaling events can be triggered by MT1-MMP, a membrane-bound matrix metalloproteinase that contributes to the invasive phenotype of brain cancer cells. The signaling functions of MT1-MMP require its intracellular domain, and recent identification of MTCBP-1, a cytoplasmic 19 kDa protein involved in the inhibition of MT1-MMP-mediated cell   migration  , suggests that modulation of MT1-MMP cytoplasmic domain-mediated signaling may affect other carcinogenic processes. Using qPCR and screening of cDNA generated from brain tumor tissues of grades I, II, III, and IV, MT1-MMP gene expression was found to correlate with increased grade of tumors. Inversely, MTCBP-1 expression decreased with increasing grade of brain tumor. Confocal microscopy and fluorescence resonance energy transfer (FRET) analysis revealed that overexpressing a cytoplasmic-deleted MT1-MMP recombinant protein mutant prevented MTCBP-1 recruitment to the intracellular leaf of plasma membrane in U87 glioblastoma cells. The interaction between MTCBP-1 and the 20 amino acids peptide representing the MT1-MMP cytoplasmic domain was confirmed by surface plasmon resonance. Overexpression of a full-length Wt-MT1-MMP triggered acidic autophagy vesicle formation and autophagic puncta formation for green fluorescent microtubule-associated protein 1 light chain 3 (GFP-LC3). Autophagic vesicles and GFP-LC3 puncta formation were abrogated in the presence of MTCBP-1. Our data elucidate a new role for MTCBP-1 regulating the intracellular function of MT1-MMP-mediated autophagy. The inverse correlation between MTCBP-1 and MT1-MMP expression with brain tumor grades could also contribute to the decreased autophagic index observed in high-grade tumors. 
 Effect of lumican on the   migration   of human mesenchymal stem cells and endothelial progenitor cells: involvement of matrix metalloproteinase-14. Increasing number of evidence shows that soluble factors and extracellular matrix (ECM) components provide an optimal microenvironment controlling human bone marrow mesenchymal stem cell (MSC) functions. Successful in vivo administration of stem cells lies in their ability to migrate through ECM barriers and to differentiate along tissue-specific lineages, including endothelium. Lumican, a protein of the small leucine-rich proteoglycan (SLRP) family, was shown to impede cell   migration   and angiogenesis. The aim of the present study was to analyze the role of lumican in the control of MSC   migration   and transition to functional endothelial progenitor cell (EPC).Lumican inhibited tube-like structures formation on Matrigel® by MSC, but not EPC. Since matrix metalloproteinases (MMPs), in particular MMP-14, play an important role in remodelling of ECM and enhancing cell   migratio  n, their expression and activity were investigated in the cells grown on different ECM substrata. Lumican down-regulated the MMP-14 expression and activity in MSC, but not in EPC. Lumican inhibited MSC, but not EPC   migratio  n and invasion. The inhibition of MSC   migratio  n and invasion by lumican was reversed by MMP-14 overexpression.Altogether, our results suggest that lumican inhibits MSC tube-like structure formation and   migratio  n via mechanisms that involve a decrease of MMP-14 expression and activity. 
 Membrane type 1-matrix metalloproteinase induces epithelial-to-mesenchymal transition in esophageal squamous cell carcinoma: Observations from clinical and in vitro analyses. Membrane type 1-matrix metalloproteinase (MT1-MMP) is associated with enhanced tumorigenicity in many cancers. A recent study has revealed that MT1-MMP induces epithelial-to-mesenchymal transition (EMT) in prostate and breast cancer cells. However, its role in esophageal squamous cell carcinoma (ESCC) has not been studied. Here, we investigated the role of MT1-MMP in the dissemination of ESCC. Expression of MT1-MMP was detected by immunohistochemistry and tissue microarray in 88 Kazakh ESCC patients. Western blotting was performed to detect endogenous and overexpressed exogenous MT1-MMP in the Eca109 and Eca9706 cell lines, respectively. Transwell assay was used to estimate MT1-MMP-induced invasion and metastasis. EMT-associated proteins were detected by immunohistochemistry and western blotting. The associations between the expression of MT1-MMP and EMT-associated proteins with clinicopathologic parameters were analyzed. Overexpression of MT1-MMP was confirmed in Kazakh ESCC patients. MT1-MMP levels were found to be correlated with the depth of tumor infiltration. MT1-MMP induced EMT in ESCC both in vivo and in vitro, N-cadherin and Vimentin expression was upregulated upon MT1-MMP transfection into cells. However, E-cadherin was found to be downregulated. MT1-MMP-induced EMT led to increase   migration   and invasion in ESCC cell lines. In conclusion, our results suggest that MT1-MMP promotes ESCC invasion and metastasis. 
 Regulation of MT1-MMP activity by β-catenin in MDCK non-cancer and HT1080 cancer cells. Past studies on β-catenin in cancer cells focused on nuclear localized β-catenin and its involvement in the Wnt pathway. Our goal here was to investigate the function of β-catenin in both the cytoplasm and nucleus on the regulation of MT1-MMP expression and activity. We found that β-catenin in MDCK non-cancer cells inhibited the cell surface localization of MT1-MMP, and thus its proteolytic activity on pro-MMP2 activation, via direct interaction with the 18-amino-acid cytoplasmic tail of MT1-MMP in the cytoplasm. In contrast, β-catenin in HT1080 cancer cells enhanced the activity of MT1-MMP by entering the nucleus and activating transcription factor Tcf-4/Lef, and elevating the level of MT1-MMP protein. We also found that enhancement of cell growth in three-dimensional (3-D)/two-dimensional (2-D) type I collagen gels and of   cell mig  ration by MT1-MMP were inhibited by β-catenin in MDCK cells, whereas these functions were enhanced in HT1080 cells. In addition, regulation of MT1-MMP by β-catenin involved E-cadherin in MDCK cells and Wnt-3a in HT1080 cells. Taken together, our results present a differential effect of cytoplasmic and nuclear β-catenin on MT1-MMP activity in non-cancer cells versus cancer cells. These differences were most probably due to different subcellular locations and different involved pathways of β-catenin in these cells. 
 Extracellular matrix metalloproteinase inducer (CD147) and membrane type 1-matrix metalloproteinase are expressed on tissue macrophages in calcific aortic stenosis and induce trans  migration   in an artificial valve model. Matrix metalloproteinases participate in remodeling of extracellular matrix, which is central to the development of aortic stenosis. Synthesis of certain matrix metalloproteinases is induced by the glycoprotein extracellular matrix metalloproteinase inducer. We investigated whether extracellular matrix metalloproteinase inducer and membrane-type 1 matrix metalloproteinase are abundant in calcific aortic valve and their role in the pathogenesis of this condition.Sixteen patients who underwent surgery for aortic stenosis (n = 12) or heart transplantation for ischemic cardiomyopathy (n = 4) were reviewed. Expression of extracellular matrix metalloproteinase inducer and membrane-type 1 matrix metalloproteinase proteins was assessed by Western blot (n = 4 per group), immunohistochemistry for aortic stenosis (n = 12) and ischemic cardiomyopathy (n = 2), and in situ zymography (n = 3 per group). Functional relevance was investigated using an artificial valve model.Extracellular matrix metalloproteinase inducer and membrane-type 1 matrix metalloproteinase were abundant in all stenotic valves. Control valves did not stain for either protein. Double immunofluorescence colocalized extracellular matrix metalloproteinase inducer and membrane-type 1 matrix metalloproteinase to macrophages. On Western blotting, both proteins were more abundant in stenotic valves than in control valves. In situ zymography demonstrated greater gelatinolytic activity in stenotic valves than in control valves. Silencing of the extracellular matrix metalloproteinase inducer gene using small interfering RNA reduced   migration   of monocytes in an artificial valve model.Extracellular matrix metalloproteinase inducer and membrane-type 1 matrix metalloproteinase were demonstrated on macrophages in stenotic aortic valves, into which extracellular matrix metalloproteinase inducer may promote monocyte im  migration  . The latter protein may therefore represent a potential target to reduce the development of aortic stenosis. 
 Expression of membrane type-1 matrix metalloproteinase, MT1-MMP in human breast cancer and its impact on invasiveness of breast cancer cells. MT1-MMP (membrane type-1 matrix metalloproteinase), otherwise known as MMP14 is a proteolytic enzyme known to be involved in degradating extracellular matrix and assist progression of cancer invasion and progression. We investigated the impact of targeting the expression of MT1-MMP in breast cancer and its clinical relevance. Human breast cancer cell line MDA-MB-231 was used. Expression of MT1-MMP in the breast cancer cell line was manipulated by way of retroviral ribozyme transgene. The in vitro invasion, growth and cell   migration   were determined on cell lines transfected with either the transgene or control plasmid. Protein and message levels of MMP14 was also assessed using immunohistochemistry and real-time quantitative analysis, and correlated with clinical and pathological information of the patients. Retroviral ribozyme transgene to human MT1-MMP successfully knocked down the levels of MT1-MMP mRNA from MDA-MB-231 cells. Reduction of MT1-MMP from the breast cancer cells resulted in significant reduction of in vitro invasiveness and loss of response to an invasion stimulus, HGF, compared with control and wild-type cells. The invasion index for MT1-MMP knockdown cells were 13+/-3.1 (without HGF) and 16.4+/-2.3 (with HGF, p=0.14), and the index for transfection control cells 25.3+/-4.3 (without HGF) and 40.4+/-4.1 (with HGF, p=0.0049). Transfection with the transgenes did not change the rate of cell growth. In clinical breast cancer, MT1-MMP staining was both membranous and cytoplasmic. Tumour cells displayed stronger staining compared with normal mammary epithelial cells. Tumour tissues had a marginally higher levels of the MMP14 transcript (8.6+/-1.9), compared with normal tissues (4.7+/-1.4), p=0.13. No significant difference was observed between node positive and node negative tumours (9.0+/-2.2 vs 8.7+/-3.1, p=0.24). Marginally higher levels of the MMP14 transcript were seen in tumours which developed metastasis and local recurrence. However, tumours from patients who died of breast cancer related causes had significantly higher levels of the transcript, compared with tumours from patients who remained disease-free 10 years after initial surgery (12.2+/-2.5 vs 6.3+/-1.2, p=0.0091). MT1-MMP is a proteolytic enzyme that is pivotal in controlling the invasiveness of breast cancer cells. It is highly expressed in aggressive breast tumours and is associated with clinical outcome. The enzyme is a potential therapeutic target in breast cancer. 
 Epidermal growth factor receptor-mediated membrane type 1 matrix metalloproteinase endocytosis regulates the transition between invasive versus expansive growth of ovarian carcinoma cells in three-dimensional collagen. The epidermal growth factor receptor (EGFR) is overexpressed in ovarian carcinomas and promotes cellular responses that contribute to ovarian cancer pathobiology. In addition to modulation of mitogenic and motogenic behavior, emerging data identify EGFR activation as a novel mechanism for rapid modification of the cell surface proteome. The transmembrane collagenase membrane type 1 matrix metalloproteinase (MT1-MMP, MMP-14) is a major contributor to pericelluar proteolysis in the ovarian carcinoma microenvironment and is subjected to extensive posttranslational regulation. In the present study, the contribution of EGFR activation to control of MT1-MMP cell surface dynamics was investigated. Unstimulated ovarian cancer cells display caveolar colocalization of EGFR and MT1-MMP, whereas EGFR activation prompts internalization via distinct endocytic pathways. EGF treatment results in phosphorylation of the MT1-MMP cytoplasmic tail, and cells expressing a tyrosine mutated form of MT1-MMP (MT1-MMP-Y(573)F) exhibit defective MT1-MMP internalization. As a result of sustained cell surface MT1-MMP activity, a phenotypic epithelial-mesenchymal transition is observed, characterized by enhanced   migration   and collagen invasion, whereas growth within three-dimensional collagen gels is inhibited. These data support an EGFR-dependent mechanism for regulation of the transition between invasive and expansive growth of ovarian carcinoma cells via modulation of MT1-MMP cell surface dynamics. 
 PEA3 is necessary for optimal epidermal growth factor receptor-stimulated matrix metalloproteinase expression and invasion of ovarian tumor cells. Elevated expression of the epidermal growth factor (EGF) receptor (EGFR) is detected in human ovarian tumors and is associated with decreased recurrence-free and overall survival. EGFR activation affects tumor progression in part by promoting tumor invasion through the induction of prometastatic matrix metalloproteinases (MMP). PEA3, an ETS family transcription factor, is elevated in advanced and metastatic ovarian cancer and regulates MMPs in various cell types, therefore, we investigated whether PEA3 is required for the EGFR-dependent induction of MMP mRNA. MMP-9 and MMP-14 mRNA levels were selectively increased in response to EGFR activity in ovarian tumor cells. EGFR activation resulted in nuclear accumulation of PEA3 and direct binding of PEA3, but not the related protein ETS-1, to the endogenous MMP-9 and MMP-14 promoters. Furthermore, PEA3 overexpression was sufficient to induce MMP-9 and MMP-14 mRNA, tumor cell   migration  , and invasion, suggesting that PEA3 is an important contributor to the metastatic phenotype. Additionally, inhibition of PEA3 expression via short interfering RNA reduced the EGF induction of MMP-9 and MMP-14 gene expression by 92% and 50%, respectively, and impaired EGF-stimulated tumor cell invasion. These results suggest that PEA3 is regulated by EGFR and that the elevated PEA3 expression detected in human ovarian cancer may divert cells to a more invasive phenotype by regulating MMP-9 and MMP-14. 
 miR-181a-5p Inhibits Cancer Cell   Migration   and Angiogenesis via Downregulation of Matrix Metalloproteinase-14. Upregulation of matrix metalloproteinase MMP-14 (MT1-MMP) is associated with poor prognosis in cancer patients, but it is unclear how MMP-14 becomes elevated in tumors. Here, we show that miR-181a-5p is downregulated in aggressive human breast and colon cancers where its levels correlate inversely with MMP-14 expression. In clinical specimens, enhanced expression of MMP-14 was observed in cancer cells located at the invasive front of tumors where miR-181a-5p was downregulated relative to adjacent normal cells. Bioinformatics analyses defined a potential miR-181a-5p response element within the 3'-untranslated region of MMP-14 that was validated in reporter gene experiments. Ectopic miR-181a-5p reduced MMP-14 expression, whereas miR-181a-5p attenuation elevated MMP-14 expression. In support of a critical relationship between these two genes, miR-181a-5p-mediated reduction of MMP-14 levels was sufficient to decrease cancer cell   migration  , invasion, and activation of pro-MMP-2. Furthermore, this reduction in MMP-14 levels was sufficient to reduce in vivo invasion and angiogenesis in chick chorioallantoic membrane assays. Taken together, our results establish the regulation of MMP-14 in cancers by miR-181a-5p through a posttranscriptional mechanism, and they further suggest strategies to elevate miR-181a-5p to prevent cancer metastasis. 
 Neurokinin-1 receptor directly mediates glioma cell   migration   by up-regulation of matrix metalloproteinase-2 (MMP-2) and membrane type 1-matrix metalloproteinase (MT1-MMP). Neurokinin-1 receptor (NK1R) occurs naturally on human glioblastomas. Its activation mediates glioma cell proliferation. However, it is unknown whether NK1R is directly involved in tumor cell   migration  . In this study, we found human hemokinin-1 (hHK-1), via NK1R, dose-dependently promoted the   migration   of U-251 and U-87 cells. In addition, we showed that hHK-1 enhanced the activity of MMP-2 and the expression of MMP-2 and MT1-matrix metalloproteinase (MMP), which were responsible for cell   migration  , because neutralizing the MMPs with antibodies decreased cell   migration  . The involved mechanisms were then investigated. In U-251, hHK-1 induced significant calcium efflux; phospholipase C inhibitor U-73122 reduced the calcium mobilization, the up-regulation of MMP-2 and MT1-MMP, and the cell   migration   induced by hHK-1, which meant the   migration   effect of NK1R was mainly mediated through the G(q)-PLC pathway. We further demonstrated that hHK-1 boosted rapid phosphorylation of ERK, JNK, and Akt; inhibition of ERK and Akt effectively reduced MMP-2 induction by hHK-1. Meanwhile, inhibition of ERK, JNK, and Akt reduced the MT1-MMP induction. hHK-1 stimulated significant phosphorylation of p65 and c-JUN in U-251. Reporter gene assays indicated hHK-1 enhanced both AP-1 and NF-κB activity; inhibition of ERK, JNK, and Akt dose-dependently suppressed the NF-κB activity; only the inhibition of ERK significantly suppressed the AP-1 activity. Treatment with specific inhibitors for AP-1 or NF-κB strongly blocked the MMP up-regulation by hHK-1. Taken together, our data suggested NK1R was a potential regulator of human glioma ce  ll migrat  ion by the up-regulation of MMP-2 and MT1-MMP. 
 The tetraspanin CD81 protein increases melanoma cell motility by up-regulating metalloproteinase MT1-MMP expression through the pro-oncogenic Akt-dependent Sp1 activation signaling pathways. Despite the importance of multiple tetraspanin proteins in cancer invasion and metastasis, little is known about the role and significance of tetraspanin CD81 in these processes. In the present study, we examined CD81 effects on melanoma cell invasiveness and metastasis. Transfection of CD81 into melanoma cells lacking endogenous CD81 expression significantly enhanced the migrating, invasive, and metastatic abilities of melanoma cells. Interestingly, membrane type 1 matrix metalloproteinase (MT1-MMP) expression was found in CD81-expressing melanoma cells but not in CD81-deficient cells. siRNA knockdown of CD81 in melanoma cells with endogenous CD81 demonstrated decreased MT1-MMP levels and cell motility. Notably, CD81-induced cell   migration   was abrogated by antibody blocking and siRNA knockdown of MT1-MMP, indicating that MT1-MMP is responsible for CD81-stimulated melanoma cell   migration  . Promoter analysis revealed an essential role of the Sp1 transcription factor in CD81-induced MT1-MMP transcription. We also demonstrate that the Sp1-activating Akt pathway is involved in adhesion-dependent CD81 signaling to induce MT1-MMP expression and cell motility. Importantly, human skin cancer tissue specimens displayed a positive correlation of CD81 with MT1-MMP expression levels and a close association of CD81 with malignant melanomas. Taken together, these results strongly suggest that CD81 stimulates melanoma cell motility by inducing MT1-MMP expression through the Akt-dependent Sp1 activation signaling pathway, leading to increased melanoma invasion and metastasis. 
  GeneRIFs  
 Gelatin zymography and WB experiment showed that hHK-1 enhanced the levels of MMP-2 and MMP-14; inhibition of these two MMPs blocked hHK-1-induced cell   migration  . 
 E6-HPV16/E7-HPV16 promoted the activity of MT1-MMP and MMP-2/9, contributing to the   migration   of cervical cells and neoplasm invasiveness. 
 our study demonstrates that MT1-MMP was overexpressed in gastric cancer tissue, and reduced expression of MT1-MMP suppressed cell   migration  , invasion, and through regulating the expression of MMPs and the process of EMT in gastric cancer. 
 MT1-MMP is central to two key elements of Tuberculosis pathogenesis, causing collagen degradation and regulating monocyte   migration  . 
 Suppression of the expression of CXCR4 and MMP14 by vandetanib in human neuroblastoma cells inhibits cell   migration   and invasion. 
 Rab5a, Rab8a and Rab14 are major regulators of MT1-MMP trafficking and invasive   migration   of primary human macrophages. 
 Data indicate that alpha2-macroglobulin (alpha2M*), through its receptor LRP1, induces cellular   migration   of Muller glial cells by a mechanism that involves MT1-MMP intracellular traffic to the plasma membrane by a Rab11-dependent recycling pathway. 
 our findings have identified inhibition of MMP-2 and -14 expression and MMP-dependent   migration   as a novel mechanism by which angiostatin inhibits angiogenesis under pathological-like conditions. 
 Methyl jasmonate attenuates MMP-14 expression via decreasing the Sp1 expression and binding on MMP-14 promoter, thus inhibiting the   migration  , invasion and angiogenesis of gastric cancer cells. 
 lumican inhibits mesenchymal stem cell tube-like structure formation and   migration   via mechanisms that involve a decrease of MMP-14 expression and activity 
 study identified a new function of hyaluronan (HA)in induction of MT1-MMP expression in breast cancer cell lines and CD44 cleavage to increase cell   migration   during invasion process; HA oligosaccharide-induced MT1-MMP expression in breast cancer cells may be a critical step in the formation of metastatic colonies 
 NK1R is a potential regulator of human glioma cell   migration   by the up-regulation of MMP-2 and MT1-MMP 
 Cross-talk between MMP-14 and CD44 results in phosphorylation of EGF receptor and downstream activation of the MAPK and PI3K signaling pathways involved in cell   migration  . 
 Extracellular matrix metalloproteinase inducer and membrane-type 1 matrix metalloproteinase were demonstrated on macrophages in stenotic aortic valves, into which extracellular matrix metalloproteinase inducer may promote monocyte im  migration  . 
 knockdown of MT1-MMP abolished FBI-1-mediated cell   migration   and invasion. Conversely, stable knockdown of FBI-1 remarkably reduced the motility of these cells with decreased expression of MT1-MMP. 
 Van Gogh-Like 2 regulates tumor cell   migration   and matrix metalloproteinase-dependent invasion. 
 TGF-beta treatment promotes MDA-MB-435s cell   migration  , involving MT1-MMP-mediated CD44 cleavage. 
 proteolytic processing of the LN-5 gamma 2 chain by MT1-MMP is involved in cell   migration   and subsequent architectural organization in ovarian clear cell carcinoma 
 Estrogen and progeterone upregulate MT1-MMP/MMP-2 and alter cell   migration   in vascular smooth cells. 
 TGF-beta1-induced HaCaT cell   migration   is mediated by MMP-14, which regulates MMP-9 expression via JNK signaling 
 Data show that Mmp14 is required for proper cell polarity underlying the directed   migration   of mesodermal cells during gastrulation, and identify a genetic interaction between mmp14 and non-canonical Wnt signaling. 
 The interaction of MT1-MMP with p130Cas at the cell periphery suggests the existence of a close interplay between pericellular proteolysis and signaling pathways involved in endothelial cell   migration  . 
 the cytoplasmic tail of ICAM-1 regulates leukocyte trans  migration   through MT1-MMP interaction 
 These results suggest that lysis of the extracellular matrix by MT1-MMP promotes focal adhesion turnover and subsequent ERK activation, which in turn stimulates cell   migration  . 
 MT1-MMP processing of laminin-5 in human tumors may resulting in increased tumor cell scattering and   migration   
 MT1-MMP is required during human monocyte   migration   and endothelial trans  migration   
 Type-1 metalloproteinases, are identified as the triggering agents that independently confer cancer cells with the ability to proteolytically efface the BM scaffolding, initiate the assembly of invasive pseudopodia, and propagate trans  migration  . 
 demonstrated that tumor cell   migration   was dramatically enhanced by expression of MT1-MMP and that MT1-MMP promotes lymph node and lung metastasis of prostate cancer cells 
 Taken together, our results show a novel functional link between MT1-MMP and DC motility and suggest that MT1-MMP may play an important role in modulating the   migration   of immature DC. 
 shedding of syndecan-1 promoted by MT1-MMP through the preferential cleavage of Gly245-Leu246 peptide bond stimulates cell   migration   
 MT1-MMP plays an important role during endothelial cell   migration  , and its activity can modulate endothelial   migration  , invasion, and formation of capillary tubes during the angiogenic response. 
  ECM  PubMed  
 CLASPs link focal-adhesion-associated microtubule capture to localized exocytosis and adhesion site turnover. Turnover of integrin-based focal adhesions (FAs) with the extracellular matrix (  ECM  ) is essential for coordinated cell movement. In collectively migrating human keratinocytes, FAs assemble near the leading edge, grow and mature as a result of contractile forces and disassemble underneath the advancing cell body. We report that clustering of microtubule-associated CLASP1 and CLASP2 proteins around FAs temporally correlates with FA turnover. CLASPs and LL5β (also known as PHLDB2), which recruits CLASPs to FAs, facilitate FA disassembly. CLASPs are further required for FA-associated   EC  M degradation, and matrix metalloprotease inhibition slows FA disassembly similarly to CLASP or PHLDB2 (LL5β) depletion. Finally, CLASP-mediated microtubule tethering at FAs establishes an FA-directed transport pathway for delivery, docking and localized fusion of exocytic vesicles near FAs. We propose that CLASPs couple microtubule organization, vesicle transport and cell interactions with th  e E  CM, establishing a local secretion pathway that facilitates FA turnover by severing cell-matrix connections. 
   ECM   regulates MT1-MMP localization with beta1 or alphavbeta3 integrins at distinct cell compartments modulating its internalization and activity on human endothelial cells. Regulation of membrane-type 1 matrix metalloproteinase (MT1-MMP) by different extracellular matrices (  ECM  s) on human endothelial cells (ECs) has been investigated. First, MT1-MMP is found at the intercellular contacts of confluent ECs grown on beta1 integrin-dependent matrix such as type 1 collagen (COL I), fibronectin (FN), or fibrinogen (FG), but not on gelatin (GEL) or vitronectin (VN). The novel localization of MT1-MMP at cell-cell contacts is assessed by confocal videomicroscopy of MT1-MMP-GFP-transfected ECs. Moreover, MT1-MMP colocalizes with beta1 integrins at the intercellular contacts, whereas it is preferentially found with alphavbeta3 integrin at motility-associated structures on migrating ECs. In addition, clustered integrins recruit MT1-MMP and neutralizing anti-beta1 or anti-alphav integrin mAb displace MT1-MMP from its specific sites, pointing to a biochemical association that is finally demonstrated by coimmunoprecipitation assays. On the other hand, COL I, FN, or FG up-regulate cell surface MT1-MMP on confluent ECs by an impairment of its internalization, whereas expression and internalization are not modified on GEL or VN. In addition, MT1-MMP activity is diminished in confluent ECs on COL I, FN, or FG. Finally, MT1-MMP participates and cooperates with beta1 and alphavbeta3 integrins in the migration of ECs on different   ECM  . These data show a novel mechanism by which   ECM   regulates MT1-MMP association with beta1 or alphavbeta3 integrins at distinct cellular compartments, thus modulating its internalization, activity, and function on human ECs. 
 Regulation of endodermal differentiation of human embryonic stem cells through integrin-  ECM   interactions. Many cellular responses during development are regulated by interactions between integrin receptors and extracellular matrix proteins (  ECM  Ps). Although the majority of recent studies in human embryonic stem cell (hESC) differentiation have focused on the role of growth factors, such as FGF, TGFβ, and WNT, relatively little is known about the role of   EC  MP-integrin signaling in this process. Moreover, current strategies to direct hESC differentiation into various lineages are inefficient and have yet to produce functionally mature cells in vitro. This suggests that additional factors, such as   EC  MPs, are required for the efficient differentiation of hESCs. Using a high-throughput multifactorial cellular array technology, we investigated the effect of hundreds of   EC  MP combinations and concentrations on differentiation of several hPSC lines to definitive endoderm (DE), an early embryonic cell population fated to give rise to internal organs such as the lung, liver, pancreas, stomach, and intestine. From this screen we identified fibronectin (FN) and vitronectin (VTN) as   EC  MP components that promoted DE differentiation. Analysis of integrin expression revealed that differentiation toward DE led to an increase in FN-binding integrin α5 (ITGA5) and VTN-binding integrin αV (ITGAV). Conditional short hairpin RNA-mediated knockdown of ITGA5 and ITGAV disrupted hESC differentiation toward DE. Finally, fluorescence-based cell sorting for ITGA5 and ITGAV significantly enriched cells with gene expression signatures associated with DE, demonstrating that these cell surface proteins permit isolation and enrichment of DE from hESCs. These data provide evidence that FN and VTN promote endoderm differentiation of hESCs through interaction with ITGA5 and ITGAV, and th  at   ECMP-integrin interactions are required for hESC differentiation into functionally mature cells. 
 TIMP-2 Interaction with MT1-MMP Activates the AKT Pathway and Protects Tumor Cells from Apoptosis. Membrane-type 1 matrix metalloproteinase (MT1-MMP), a transmembrane proteinase with an extracellular catalytic domain and a short cytoplasmic tail, degrades a variety of extracellular matrix (  ECM  ) components. In addition, MT1-MMP activates intracellular signaling through proteolysis-dependent and independent mechanisms. We have previously shown that binding of tissue inhibitor of metalloproteinases-2 (TIMP-2) to MT1-MMP controls cell proliferation and migration, as well as tumor growth in vivo by activating the Ras-extracellular signal regulated kinase-1 and -2 (ERK1/2) pathway through a mechanism that requires the cytoplasmic but not the proteolytic domain of MT1-MMP. Here we show that in MT1-MMP expressing cells TIMP-2 also induces rapid and sustained activation of AKT in a dose- and time-dependent manner and by a mechanism independent of the proteolytic activity of MT1-MMP. Fibroblast growth factor receptor-1 mediates TIMP-2 induction of ERK1/2 but not of AKT activation; however, Ras activation is necessary to transduce the TIMP-2-activated signal to both the ERK1/2 and AKT pathways. ERK1/2 and AKT activation by TIMP-2 binding to MT1-MMP protects tumor cells from apoptosis induced by serum starvation. Conversely, TIMP-2 upregulates apoptosis induced by three-dimensional type I collagen in epithelial cancer cells. Thus, TIMP-2 interaction with MT1-MMP provides tumor cells with either pro- or anti-apoptotic signaling depending on the extracellular environment and apoptotic stimulus. 
 TIMP-2 Interaction with MT1-MMP Activates the AKT Pathway and Protects Tumor Cells from Apoptosis. Membrane-type 1 matrix metalloproteinase (MT1-MMP), a transmembrane proteinase with an extracellular catalytic domain and a short cytoplasmic tail, degrades a variety of extracellular matrix (  ECM  ) components. In addition, MT1-MMP activates intracellular signaling through proteolysis-dependent and independent mechanisms. We have previously shown that binding of tissue inhibitor of metalloproteinases-2 (TIMP-2) to MT1-MMP controls cell proliferation and migration, as well as tumor growth in vivo by activating the Ras-extracellular signal regulated kinase-1 and -2 (ERK1/2) pathway through a mechanism that requires the cytoplasmic but not the proteolytic domain of MT1-MMP. Here we show that in MT1-MMP expressing cells TIMP-2 also induces rapid and sustained activation of AKT in a dose- and time-dependent manner and by a mechanism independent of the proteolytic activity of MT1-MMP. Fibroblast growth factor receptor-1 mediates TIMP-2 induction of ERK1/2 but not of AKT activation; however, Ras activation is necessary to transduce the TIMP-2-activated signal to both the ERK1/2 and AKT pathways. ERK1/2 and AKT activation by TIMP-2 binding to MT1-MMP protects tumor cells from apoptosis induced by serum starvation. Conversely, TIMP-2 upregulates apoptosis induced by three-dimensional type I collagen in epithelial cancer cells. Thus, TIMP-2 interaction with MT1-MMP provides tumor cells with either pro- or anti-apoptotic signaling depending on the extracellular environment and apoptotic stimulus. 
   ECM   regulates MT1-MMP localization with beta1 or alphavbeta3 integrins at distinct cell compartments modulating its internalization and activity on human endothelial cells. Regulation of membrane-type 1 matrix metalloproteinase (MT1-MMP) by different extracellular matrices (  ECM  s) on human endothelial cells (ECs) has been investigated. First, MT1-MMP is found at the intercellular contacts of confluent ECs grown on beta1 integrin-dependent matrix such as type 1 collagen (COL I), fibronectin (FN), or fibrinogen (FG), but not on gelatin (GEL) or vitronectin (VN). The novel localization of MT1-MMP at cell-cell contacts is assessed by confocal videomicroscopy of MT1-MMP-GFP-transfected ECs. Moreover, MT1-MMP colocalizes with beta1 integrins at the intercellular contacts, whereas it is preferentially found with alphavbeta3 integrin at motility-associated structures on migrating ECs. In addition, clustered integrins recruit MT1-MMP and neutralizing anti-beta1 or anti-alphav integrin mAb displace MT1-MMP from its specific sites, pointing to a biochemical association that is finally demonstrated by coimmunoprecipitation assays. On the other hand, COL I, FN, or FG up-regulate cell surface MT1-MMP on confluent ECs by an impairment of its internalization, whereas expression and internalization are not modified on GEL or VN. In addition, MT1-MMP activity is diminished in confluent ECs on COL I, FN, or FG. Finally, MT1-MMP participates and cooperates with beta1 and alphavbeta3 integrins in the migration of ECs on different   ECM  . These data show a novel mechanism by which   ECM   regulates MT1-MMP association with beta1 or alphavbeta3 integrins at distinct cellular compartments, thus modulating its internalization, activity, and function on human ECs. 
 MicroRNA-22 inhibits tumor growth and metastasis in gastric cancer by directly targeting MMP14 and Snail. MicroRNAs (miRNAs) deregulation is frequent in human gastric cancers (GCs), but the role of specific miRNAs involved in this disease remains elusive. MiR-22 was previously reported to act as tumor suppressors or oncogenes in diverse cancers. However, their accurate expression, function and mechanism in GC are largely unclear. Here, we found that the expression of miR-22 was significantly reduced in clinical GC tissues compared with paired adjacent normal tissues, and was significantly correlated with a more aggressive phenotype of GC in patients, and miR-22 low expression correlated with poor overall survival. The introduction of miR-22 markedly suppressed GC cell growth, migration and invasion, and inhibition of miR-22 promoted GC cell proliferation, migration and invasion in vitro. We further demonstrated that miR-22 acted as tumor suppressors through targeting extracellular matrix (  ECM  ) remodeling member matrix metalloproteinase 14 (MMP14) and epithelial-to-mesenchymal transition (EMT) inducer Snail in GC. Moreover, ectopic expression of MMP14 or Snail restored inhibitory effects of miR-22 on cell migration and invasion in GC cells, and a negative relationship between the miR-22 expression and MMP14 or Snail mRNA levels was observed in GC. Finally, overexpression of miR-22 suppressed tumor growth, peritoneal dissemination and pulmonary metastasis in vivo. Taken together, we identified that miR-22 is a potent tumor suppressor in GC. MiR-22 downregulation promotes GC invasion and metastasis by upregulating MMP14 and Snail, and then inducing   ECM   remodeling and EMT. These findings provide a better understanding of the development and progression of GC and may be an important implication for future therapy of the GC. 
 Fluid shear stress promotes proprotein convertase-dependent activation of MT1-MMP. During angiogenesis, endothelial cells (ECs(1)) initiate new blood vessel growth and invade into the extracellular matrix (  ECM  ). Membrane type-1 matrix metalloproteinase (MT1-MMP) facilitates this process and translocates to the plasma membrane following activation to promote   ECM   cleavage. The N-terminal pro-domain within MT1-MMP must be processed for complete activity of the proteinase. This study investigated whether MT1-MMP activation was altered by sphingosine 1-phosphate (S1P) and wall shear stress (WSS), which combine to stimulate EC invasion in three dimensional (3D) collagen matrices. MT1-MMP was activated rapidly and completely by WSS but not S1P. Proprotein convertases (PCs) promoted MT1-MMP processing, prompting us to test whether WSS or S1P treatments increased PC activity. Like MT1-MMP, PC activity increased with WSS, while S1P had no effect. A pharmacological PC inhibitor completely blocked S1P- and WSS-induced EC invasion and MT1-MMP translocation to the plasma membrane. Further, a recombinant PC inhibitor reduced MT1-MMP activation and decreased lumen formation in invading ECs, a process known to be controlled by MT1-MMP. Thus, we conclude that PC and MT1-MMP activation are mechanosensitive events that are required for EC invasion into 3D collagen matrices. 
 MMP-14 is necessary but not sufficient for invasion of three-dimensional collagen by human muscle satellite cells. The twenty-five known matrix metalloproteases (MMPs) and their endogenous inhibitors, tissue inhibitors of metalloproteases (TIMPs), mediate cell invasion through the extracellular matrix (  ECM  ). In a comparative three-dimensional assay, we analyzed human and mouse satellite cells' competence to invade an artificial   ECM   (collagen I). We identified a single MMP that 1) is expressed by human muscle satellite cells; 2) is induced at the mRNA/protein level by adhesion to collagen I; and 3) is necessary for invasion into a collagen I matrix. Interestingly, murine satellite cells neither express this MMP, nor invade the collagen matrix. However, exogenous human MMP-14 is not sufficient to induce invasion of a collagen matrix by murine cells, emphasizing species differences. 
 SNAP23, Syntaxin4, and vesicle-associated membrane protein 7 (VAMP7) mediate trafficking of membrane type 1-matrix metalloproteinase (MT1-MMP) during invadopodium formation and tumor cell invasion. Movement through the extracellular matrix (  ECM  ) requires cells to degrade   ECM   components, primarily through the action of matrix metalloproteinases (MMPs). Membrane type 1-matrix metalloproteinase (MT1-MMP) has an essential role in matrix degradation and cell invasion and localizes to subcellular degradative structures termed invadopodia. Trafficking of MT1-MMP to invadopodia is required for the function of these structures, and here we examine the role of N-ethylmaleimide-sensitive factor-activating protein receptor (SNARE)-mediated membrane traffic in the transport of MT1-MMP to invadopodia. During invadopodium formation in MDA-MB-231 human breast cancer cells, increased association of SNAP23, Syntaxin4, and vesicle-associated membrane protein 7 (VAMP7) is detected by coimmunoprecipitation. Blocking the function of these SNAREs perturbs invadopodium-based   ECM   degradation and cell invasion. Increased level of SNAP23-Syntaxin4-VAMP7 interaction correlates with decreased Syntaxin4 phosphorylation. These results reveal an important role for SNARE-regulated trafficking of MT1-MMP to invadopodia during cellular invasion of   ECM  . 
 Metalloendoprotease cleavage triggers gelsolin amyloidogenesis. Amyloid diseases like Alzheimer's disease and familial amyloidosis of Finnish type (FAF) stem from endoproteolytic cleavage of a precursor protein to generate amyloidogenic peptides that accumulate as amyloid deposits in a tissue-specific manner. FAF patients deposit both 8 and 5 kDa peptides derived from mutant (D187Y/N) plasma gelsolin in the extracellular matrix (  ECM  ). The first of two aberrant sequential proteolytic events is executed by furin to yield a 68 kDa (C68) secreted fragment. We now identify the metalloprotease MT1-matrix metalloprotease (MMP), an integral membrane protein active in the   ECM  , as a protease that processes C68 to the amyloidogenic peptides. We further demonstrate that   ECM   components are capable of accelerating gelsolin amyloidogenesis. Proteolysis by MT1-MMP-like proteases proximal to the unique chemical environment of the   ECM   offers an explanation for the tissue-specific deposition observed in FAF and provides critical insight into new therapeutic strategies. 
 Collective epithelial cell invasion overcomes mechanical barriers of collagenous extracellular matrix by a narrow tube-like geometry and MMP14-dependent local softening. Collective cell invasion (CCI) through interstitial collagenous extracellular matrix (  ECM  ) is crucial to the initial stages of branching morphogenesis, and a hallmark of tissue repair and dissemination of certain tumors. The collagenous   ECM   acts as a mechanical barrier against CCI. However, the physical nature of this barrier and how it is overcome by cells remains incompletely understood. To address these questions, we performed theoretical and experimental analysis of mammary epithelial branching morphogenesis in 3D type I collagen (collagen-I) gels. We found that the mechanical resistance of collagen-I is largely due to its elastic rather than its viscous properties. We also identified two strategies utilized by mammary epithelial cells that can independently minimize   ECM   mechanical resistance during CCI. First, cells adopt a narrow tube-like geometry during invasion, which minimizes the elastic opposition from the   ECM   as revealed by theoretical modeling of the most frequent invasive shapes and sizes. Second, the stiffness of the collagenous   ECM   is reduced at invasive fronts due to its degradation by matrix metalloproteinases (MMPs), as indicated by direct measurements of collagen-I microelasticity by atomic force microscopy. Molecular techniques further specified that the membrane-bound MMP14 mediates degradation of collagen-I at invasive fronts. Thus, our findings reveal that MMP14 is necessary to efficiently reduce the physical restraints imposed by collagen-I during branching morphogenesis, and help our overall understanding of how forces are balanced between cells and their surrounding   ECM   to maintain collective geometry and mechanical stability during CCI. 
 Increasing extracellular matrix collagen level and MMP activity induces cyst development in polycystic kidney disease. Polycystic Kidney Disease (PKD) kidneys exhibit increased extracellular matrix (  ECM  ) collagen expression and metalloproteinases (MMPs) activity. We investigated the role of these increases on cystic disease progression in PKD kidneys.We examined the role of type I collagen (collagen I) and membrane bound type 1 MMP (MT1-MMP) on cyst development using both in vitro 3 dimensional (3D) collagen gel culture and in vivo PCK rat model of PKD.We found that collagen concentration is critical in controlling the morphogenesis of MDCK cells cultured in 3D gels. MDCK cells did not form 3D structures at collagen I concentrations lower than 1 mg/ml but began forming tubules when the concentration reaches 1 mg/ml. Significantly, these cells began to form cyst when collagen I concentration reached to 1.2 mg/ml, and the ratios of cyst to tubule structures increased as the collagen I concentration increased. These cells exclusively formed cyst structures at a collagen I concentration of 1.8 mg/ml or higher. Overexpression of MT1-MMP in MDCK cells significantly induced cyst growth in 3D collagen gel culture. Conversely, inhibition of MMPs activity with doxycycline, a FDA approved pan-MMPs inhibitor, dramatically slowed cyst growth. More importantly, the treatment of PCK rats with doxycycline significantly decreased renal tubule cell proliferation and markedly inhibited the cystic disease progression.Our data suggest that increased collagen expression and MMP activity in PKD kidneys may induce cyst formation and expansion. Our findings also suggest that MMPs may serve as a therapeutic target for the treatment of human PKD. 
 CDCP1 regulates the function of MT1-MMP and invadopodia-mediated invasion of cancer cells. Complement C1r/C1s, Uegf, Bmp1 (CUB) domain-containing protein 1 (CDCP1) is a transmembrane protein that regulates anchorage-independent growth and cancer cell migration and invasion. Expression of CDCP1 is detected in a number of cancer cell lines and tissues and is closely correlated with poor prognosis. Invadopodia are actin-based protrusions on the surface of invasive cancer cells that promote the degradation of the extracellular matrix (  ECM  ) via localized proteolysis, which is mainly mediated by membrane type 1 matrix metalloproteinase (MT1-MMP). MT1-MMP is accumulated at invadopodia by targeted delivery via membrane trafficking. The present study shows that CDCP1 is required for   ECM   degradation by invadopodia in human breast cancer and melanoma cells. CDCP1 localized to caveolin-1-containing vesicular structures and lipid rafts and was detected in close proximity to invadopodia. Further biochemical analysis revealed that substantial amounts of CDCP1 existed in the Triton X-100 insoluble lipid raft fraction. CDCP1 was coimmunoprecipitated with MT1-MMP and colocalized with MT1-MMP at the vesicular structures. The siRNA-mediated knockdown of the CDCP1 expression markedly inhibited MT1-MMP-dependent   ECM   degradation and Matrigel invasion and reduced the accumulation of MT1-MMP at invadopodia, as shown by immunofluorescence analysis. These results indicate that CDCP1 is an essential regulator of the trafficking and function of MT1-MMP- and invadopodia-mediated invasion of cancer cells. 
 Angiotensin II-induced MMP-2 activity and MMP-14 and basigin protein expression are mediated via the angiotensin II receptor type 1-mitogen-activated protein kinase 1 pathway in retinal pigment epithelium: implications for age-related macular degeneration. Accumulation of various lipid-rich extracellular matrix (  ECM  ) deposits under the retinal pigment epithelium (RPE) has been observed in eyes with age-related macular degeneration (AMD). RPE-derived matrix metalloproteinase (MMP)-2, MMP-14, and basigin (BSG) are major enzymes involved in the maintenance of   ECM   turnover. Hypertension (HTN) is a systemic risk factor for AMD. It has previously been reported that angiotensin II (Ang II), one of the most important hormones associated with HTN, increases MMP-2 activity and its key regulator, MMP-14, in RPE, inducing breakdown of the RPE basement membrane, which may lead to progression of sub-RPE deposits. Ang II exerts most of its actions by activating the mitogen-activated protein kinase (MAPK) signaling pathway. Herein is explored the MAPK signaling pathway as a potential key intracellular modulator of Ang II-induced increase in MMP-2 activity and MMP-14 and BSG protein expression. It was observed that Ang II stimulates phosphorylation of extracellular signal-regulated kinase (ERK) and p38 MAPK in RPE cells and ERK/p38 and Jun N-terminal kinase (JNK) in mice. These effects were mediated by Ang II type 1 receptors. Blockade of ERK or p38 MAPK abrogated the increase in MMP-2 activity and MMP-14 and BSG proteins in ARPE-19 cells. A better understanding of the molecular events by which Ang II induces   ECM   dysregulation is of critical importance to further define its contribution to the progression of sub-RPE deposits in AMD patients with HTN. 
 MT1-MMP releases latent TGF-beta1 from endothelial cell extracellular matrix via proteolytic processing of LTBP-1. Targeting of transforming growth factor beta (TGF-beta) to the extracellular matrix (  ECM  ) by latent TGF-beta binding proteins (LTBPs) regulates the availability of TGF-beta for interactions with endothelial cells during their quiescence and activation. However, the mechanisms which release TGF-beta complexes from the   ECM   need elucidation. We find here that morphological activation of endothelial cells by phorbol 12-myristate 13-acetate (PMA) resulted in membrane-type 1 matrix metalloproteinase (MT1-MMP) -mediated solubilization of latent TGF-beta complexes from the   ECM   by proteolytic processing of LTBP-1. These processes required the activities of PKC and ERK1/2 signaling pathways and were coupled with markedly increased MT1-MMP expression. The functional role of MT1-MMP in LTBP-1 release was demonstrated by gene silencing using lentiviral short-hairpin RNA as well as by the inhibition with tissue inhibitors of metalloproteinases, TIMP-2 and TIMP-3. Negligible effects of TIMP-1 and uPA/plasmin system inhibitors indicated that secreted MMPs or uPA/plasmin system did not contribute to the release of LTBP-1. Current results identify MT1-MMP-mediated proteolytic processing of   ECM  -bound LTBP-1 as a mechanism to release latent TGF-beta from the subendothelial matrix. 
 Dimerization of MT1-MMP during cellular invasion detected by fluorescence resonance energy transfer. Homodimerization of the membrane-bound collagenase MT1-MMP [membrane-type 1 MMP (matrix metalloproteinase)] is crucial for its collagenolytic activity. However, it is not clear whether this dimerization is regulated during cellular invasion into three-dimensional collagen matrices. To address this question, we established a fluorescence resonance energy transfer system to detect MT1-MMP dimerization and analysed the process in cells invading through three-dimensional collagen. Our data indicate that dimerization occurs dynamically and constantly at the leading edge of migrating cells, but not the trailing edge. We found that polarized dimerization was not due to   ECM   (extracellular matrix) attachment, but was rather controlled by reorganization of the actin cytoskeleton by the small GTPases, Cdc42 (cell division cycle 42) and Rac1. Our data indicate that cell-surface collagenolytic activity is regulated co-ordinately with cell migration events to enable penetration of the matrix physical barrier. 
 Single nucleotide polymorphisms and haplotypes of MMP-14 are associated with the risk and pathological development of oral cancer. Matrix metalloproteinase (MMP)-14 is one of the pericellular collagenases to degrade extracellular matrix (  ECM  ), which is involved to the modulation of susceptibility or clinicopathological features of a cancer. The contributions of MMP-14 on the susceptibility or clinicopathological features of certain cancers have been well documented, and the expression of MMP-14 in oral squamous cell carcinoma (OSCC) also has been observed. This study was designed to examine the association of MMP-14 gene polymorphisms with the susceptibility and clinicopathological development of OSCC.A total of 363 patients with OSCC and 506 healthy control subjects were recruited. Six single nucleotide polymorphisms (SNPs) of MMP-14 genes were analyzed by polymerase chain reaction-restriction fragment length polymorphism genotyping and haplotype-base analysis.MMP-14 +7096 TC/CC genotypes might lower the risk of OSCC, and MMP-14 +6767 GA/AA genotypes cause a poor clinical status in OSCC patients. The +6727 C: +6767 G: +7096 T: +8153 G haplotype and diplotype increased the risk for OSCC by 1.706-fold (95% confidence interval (CI) 1.383-2.105) and 2.276-fold (95% CI = 1.531-3.384), respectively, compared with the reference. The diplotype with at least one CGTG exhibited a high risk (adjusted odds ratio, 1.639; 95% CI, 1.005-2.673) for developing a poor clinicopathological diagnosis of OSCC compared with the others/other diplotype.The +7096 and +6767 polymorphic genotypes and haplotype +6727 C: +6767 G: +7096 T: +8153 G of MMP-14 gene might contribute to the prediction of susceptibility and pathological development of OSCC. 
 Rho-ROCK-myosin signaling mediates membrane type 1 matrix metalloproteinase-induced cellular aggregation of keratinocytes. Membrane type 1-matrix metalloproteinase (MT1-MMP, MMP14), which is associated with extracellular matrix (  ECM  ) breakdown in squamous cell carcinoma (SCC), promotes tumor formation and epithelial-mesenchymal transition. However, in this report we demonstrate that MT1-MMP, by cleaving the underlying   ECM  , causes cellular aggregation of keratinocytes and SCC cells. Treatment with an MMP inhibitor abrogated MT1-MMP-induced phenotypic changes, but decreasing E-cadherin expression did not affect MT1-MMP-induced cellular aggregation. As ROCK1/2 can regulate cell-cell and cell-  ECM   interaction, we examined its role in mediating MT1-MMP-induced phenotypic changes. Blocking ROCK1/2 expression or activity abrogated the cellular aggregation resulting from MT1-MMP expression. Additionally, blocking Rho and non-muscle myosin attenuated MT1-MMP-induced phenotypic changes. Moreover, SCC cells expressing only the catalytically active MT1-MMP protein demonstrated increased cellular aggregation and increased myosin II activity in vivo when injected subcutaneously into nude mice. Together, these results demonstrate that expression of MT1-MMP may be anti-tumorigenic in keratinocytes by promoting cellular aggregation. 
 Mayer-Rokitansky-Küster-Hauser syndrome discordance in monozygotic twins: matrix metalloproteinase 14, low-density lipoprotein receptor-related protein 10, extracellular matrix, and neoangiogenesis genes identified as candidate genes in a tissue-specific mosaicism. To find a potential underlying cause for Mayer-Rokitansky-Küster-Hauser syndrome (MRKHS) discordance in monozygotic twins.Prospective comparative study.University hospital.Our study genetically analyzed 5 MRKHS-discordant monozygotic twin pairs with the unique opportunity to include saliva and rudimentary uterine tissue.Blood, saliva, or rudimentary uterine tissue from five MRKHS-discordant twins was analyzed and compared between twin pairs as well as within the same individual where applicable. We used copy number variations (CNVs) to identify differences.CNVs in blood, rudimentary uterine tissue, and saliva, network analysis, and review of the literature.One duplication found in the affected twin included two genes, matrix metalloproteinase 14 (MMP14) and low-density lipoprotein receptor-related protein 10 (LRP10), which have known functions in the embryonic development of the uterus and endometrium. The duplicated region was detected in rudimentary uterine tissue from the same individual but not in saliva, making a tissue-specific mosaicism a possible explanation for twin discordance. Additional network analysis revealed important connections to differentially expressed genes from previous studies. These genes encode several molecules involved in extracellular matrix   (E  CM) remodeling and neoangiogenesis.MMP-14, LRP-10  , E  CM, and neoangiogenesis genes are identified as candidate genes in a tissue-specific mosaicism. The detected clusters provide evidence of deficient vascularization during uterine development and/or disturbed reorganization o  f E  CM components, potentially during müllerian duct elongation signaled by the embryologically relevant phosphatidylinositol 3-kinase/protein kinase B pathway. Therefore, we consider these genes to be new candidates in the manifestation of MRKHS. 
 Caveolin-1 inhibits membrane-type 1 matrix metalloproteinase activity. Membrane-type 1 matrix metalloproteinase (MT1-MMP) is a zinc-dependent proteinase found in cholesterol-rich lipid rafts on the plasma membrane. MT1-MMP hydrolyzes extracellular matrix (  ECM  ) proteins, activates pro-matrix metalloproteinase-2 (proMMP-2) and plays an important role in   ECM   remodeling, cancer cell migration and metastasis. The role of caveolin-1, an integral protein of caveolae, in the activation of MT1-MMP remains largely unknown. Here, we show that the expression of caveolin-1 attenuates the activation of proMMP-2, reduces proteolytic cleavage of   ECM   and inhibits cell migration. We utilized the cytoplasmic tail domain deletion (DeltaCT) or the E240A mutant of MT1-MMP. Co-expression of caveolin-1 with the wild-type or the DeltaCT MT1-MMP decreased the proMMP-2 activation and inhibited collagen degradation and cell migration. Caveolin-1 had no effect on the catalytically inert E240A MT1-MMP. Our findings suggest that caveolin-1 is essential in the down-regulation of MT1-MMP activity by promoting internalization from the cell surface. 
 Multi-step pericellular proteolysis controls the transition from individual to collective cancer cell invasion. Invasive cell migration through tissue barriers requires pericellular remodelling of extracellular matrix (  ECM  ) executed by cell-surface proteases, particularly membrane-type-1 matrix metalloproteinase (MT1-MMP/MMP-14). Using time-resolved multimodal microscopy, we show how invasive HT-1080 fibrosarcoma and MDA-MB-231 breast cancer cells coordinate mechanotransduction and fibrillar collagen remodelling by segregating the anterior force-generating leading edge containing beta1 integrin, MT1-MMP and F-actin from a posterior proteolytic zone executing fibre breakdown. During forward movement, sterically impeding fibres are selectively realigned into microtracks of single-cell calibre. Microtracks become expanded by multiple following cells by means of the large-scale degradation of lateral   ECM   interfaces, ultimately prompting transition towards collective invasion similar to that in vivo. Both   ECM   track widening and transition to multicellular invasion are dependent on MT1-MMP-mediated collagenolysis, shown by broad-spectrum protease inhibition and RNA interference. Thus, invasive migration and proteolytic   ECM   remodelling are interdependent processes that control tissue micropatterning and macropatterning and, consequently, individual and collective cell migration. 
 Matrix metalloproteinases 19 and 20 cleave aggrecan and cartilage oligomeric matrix protein (COMP). Matrix metalloproteinase (MMP)-19 and MMP-20 (enamelysin) are two recently discovered members of the MMP family. These enzymes are involved in the degradation of the various components of the extracellular matrix (  ECM  ) during development, haemostasis and pathological conditions. Whereas MMP-19 mRNA is found widely expressed in body tissues, including the synovium of normal and rheumatoid arthritic patients, MMP-20 expression is restricted to the enamel organ. In this study we investigated the ability of MMP-19 and MMP-20 to cleave two of the macromolecules characterising the cartilage   ECM  , namely aggrecan and the cartilage oligomeric matrix protein (COMP). Both MMPs hydrolysed aggrecan efficiently at the well-described MMP cleavage site between residues Asn(341) and Phe(342), as shown by Western blotting using neo-epitope antibodies. Furthermore, the two enzymes cleaved COMP in a distinctive manner, generating a major proteolytic product of 60 kDa. Our results suggest that MMP-19 may participate in the degradation of aggrecan and COMP in arthritic disease, whereas MMP-20, due to its unique expression pattern, may primarily be involved in the turnover of these molecules during tooth development. 
 Matrix metalloproteinase-14 is a mechanically regulated activator of secreted MMPs and invasion. Matrix metalloproteinases (MMPs) are extracellular matrix (  ECM  ) degrading enzymes and have complex and specific regulation networks. This includes activation interactions, where one MMP family member activates another.   ECM   degradation and MMP activation can be initiated by several different stimuli including changes in   ECM   mechanical properties or intracellular contractility. These mechanical stimuli are known enhancers of metastatic potential. MMP-14 facilitates local   ECM   degradation and is well known as a major mediator of cell migration, angiogenesis and invasion. Recently, function blocking antibodies have been developed to specifically block MMP-14, providing a useful tool for research as well as therapeutic applications. Here we utilize a selective MMP-14 function blocking antibody to delineate the role of MMP-14 as an activator of other MMPs in response to changes in cellular contractility and   ECM   stiffness. Inhibition using function blocking antibodies reveals that MMP-14 activates soluble MMPs like MMP-2 and -9 under various mechanical stimuli in the pancreatic cancer cell line, Panc-1. In addition, inhibition of MMP-14 abates Panc-1 cell extension into 3D gels to levels seen with non-specific pan-MMP inhibitors at higher concentrations. This strengthens the case for MMP function blocking antibodies as more potent and specific MMP inhibition therapeutics. 
 3-phosphoinositide-dependent protein kinase-1 (PDK1) promotes invasion and activation of matrix metalloproteinases. Metastasis is a major cause of morbidity and mortality in breast cancer with tumor cell invasion playing a crucial role in the metastatic process. PDK1 is a key molecule that couples PI3K to cell proliferation and survival signals in response to growth factor receptor activation, and is oncogenic when expressed in mouse mammary epithelial cells. We now present evidence showing that PDK1-expressing cells exhibit enhanced anchorage-dependent and -independent cell growth and are highly invasive when grown on Matrigel. These properties correlate with induction of MMP-2 activity, increased MT1-MMP expression and a unique gene expression profile.Invasion assays in Matrigel, MMP-2 zymogram analysis, gene microarray analysis and mammary isografts were used to characterize the invasive and proliferative function of cells expressing PDK1. Tissue microarray analysis of human breast cancers was used to measure PDK1 expression in invasive tumors by IHC.Enhanced invasion on Matrigel in PDK1-expressing cells was accompanied by increased MMP-2 activity resulting from stabilization against proteasomal degradation. Increased MMP-2 activity was accompanied by elevated levels of MT1-MMP, which is involved in generating active MMP-2. Gene microarray analysis identified increased expression of the   ECM  -associated genes decorin and type I procollagen, whose gene products are substrates of MT1-MMP. Mammary fat pad isografts of PDK1-expressing cells produced invasive adenocarcinomas. Tissue microarray analysis of human invasive breast cancer indicated that PDK1pSer241 was strongly expressed in 90% of samples.These results indicate that PDK1 serves as an important effector of mammary epithelial cell growth and invasion in the transformed phenotype. PDK1 mediates its effect in part by MT1-MMP induction, which in turn activates MMP-2 and modulates the   ECM   proteins decorin and collagen. The presence of increased PDK1 expression in the majority of invasive breast cancers suggests its importance in the metastatic process. 
 Secretion of active membrane type 1 matrix metalloproteinase (MMP-14) into extracellular space in microvesicular exosomes. Membrane type 1 matrix metalloproteinase (MT1-MMP, MMP14) is an efficient extracellular matrix (  ECM  ) degrading enzyme that plays important roles in tissue homeostasis and cell invasion. Like a number of type I membrane proteins, MT1-MMP can be internalized from the cell surface through early and recycling endosomes to late endosomes, and recycled to the plasma membrane. Late endosomes participate in the biogenesis of small (30-100 nm) vesicles, exosomes, which redirect plasma membrane proteins for extracellular secretion. We hypothesized that some of the endosomal MT1-MMP could be directed to exosomes for extracellular release. Using cultured human fibrosarcoma (HT-1080) and melanoma (G361) cells we provide evidence that both the full-length 60 kDa and the proteolytically processed 43 kDa forms of MT1-MMP are secreted in exosomes. The isolated exosomes were identified by their vesicular structure in electron microscopy and by exosomal marker proteins CD9 and tumor susceptibility gene (TSG101). Furthermore, exosomes contained beta1-integrin (CD29). The exosomes were able to activate pro-MMP-2 and degrade type 1 collagen and gelatin, suggesting that the exosomal MT1-MMP was functionally active. The targeting of MT1-MMP in exosomes represents a novel mechanism for cancer cells to secrete membrane type metalloproteolytic activity into the extracellular space. 
 The planar cell polarity protein Van Gogh-Like 2 regulates tumor cell migration and matrix metalloproteinase-dependent invasion. Van Gogh-Like 2 (VANGL2) is a planar cell polarity protein essential for collective migration during embryonic development, yet its contribution to tumor cell motility and invasion are unknown. We report for the first time that loss of VANGL2 in human cancer cells promotes efficient collective and directed migration and matrix metalloproteinase (MMP)-dependent   ECM   invasion. We show that VANGL2 knockdown cells exhibit increased activation of secreted MMP2, higher levels of membrane-localized MMP14, and decreased cell-surface fibronectin. These important findings support the notion that planar cell polarity proteins act in coordination with known regulators of cancer cell migration to influence invasion and perhaps metastasis. 
 MT1-MMP regulates the turnover and endocytosis of extracellular matrix fibronectin. The extracellular matrix (  ECM  ) is dynamically remodeled by cells during development, normal tissue homeostasis and in a variety of disease processes. We previously showed that fibronectin is an important regulator of   ECM   remodeling. The deposition and/or polymerization of fibronectin into the   ECM   controls the deposition and stability of other   ECM   molecules. In addition, agents that inhibit fibronectin polymerization promote the turnover of fibronectin fibrils and enhance   ECM   fibronectin endocytosis and intracellular degradation. Endocytosis of   ECM   fibronectin is regulated by β1 integrins, including α5β1 integrin. We have examined the role of extracellular proteases in regulati  ng   ECM fibronectin turnover. Our data show that membrane type matrix metalloproteinase 1 (MT1-MMP; also known as MMP14) is a crucial regulator of fibronectin turnover. Cells lacking MT1-MMP show reduced turnover and endocytosis   of   ECM fibronectin. MT1-MMP regulat  es   ECM fibronectin remodeling by promoting extracellular cleavage of fibronectin and by regulating α5β1-integrin endocytosis. Our data also show that fibronectin polymerization stabilizes fibronectin fibrils and inhi  bit  s ECM fibronectin endocytosis by inhibiting α5β1-integrin endocytosis. These data are the first to show t  hat   an ECM protein and its modifying enzyme can regulate integrin endocytosis. These data also show that integrin trafficking plays a major role in modu  lat  ing ECM fibronectin remodeling. The dual depende  nce   of ECM fibronectin turnover on extracellular proteolysis and endocytosis highlights the complex regulatory mechanisms that c  ont  rol ECM remodeling to ensure maintenance of proper tissue function. 
 Three-dimensional matrix fiber alignment modulates cell migration and MT1-MMP utility by spatially and temporally directing protrusions. Multiple attributes of the three-dimensional (3D) extracellular matrix (  ECM  ) have been independently implicated as regulators of cell motility, including pore size, crosslink density, structural organization, and stiffness. However, these parameters cannot be independently varied within a complex 3D   ECM   protein network. We present an integrated, quantitative study of these parameters across a broad range of complex matrix configurations using self-assembling 3D collagen and show how each parameter relates to the others and to cell motility. Increasing collagen density resulted in a decrease and then an increase in both pore size and fiber alignment, which both correlated significantly with cell motility but not bulk matrix stiffness within the range tested. However, using the crosslinking enzyme Transglutaminase II to alter microstructure independently of density revealed that motility is most significantly predicted by fiber alignment. Cellular protrusion rate, protrusion orientation, speed of migration, and invasion distance showed coupled biphasic responses to increasing collagen density not predicted by 2D models or by stiffness, but instead by fiber alignment. The requirement of matrix metalloproteinase (MMP) activity was also observed to depend on microstructure, and a threshold of MMP utility was identified. Our results suggest that fiber topography guides protrusions and thereby MMP activity and motility. 
 MT1-MMP regulates MMP-2 expression and angiogenesis-related functions in human umbilical vein endothelial cells. Membrane type 1 (MT1)-MMP is a member of matrix metalloproteinases (MMPs) that regulates extracellular matrix remodeling. In addition, MT1-MMP also serves as a multi-functional protein. However, the functional role of MT1-MMP in human endothelial cells remains unclear. In this study we use real-time PCR and Western blotting to demonstrate for the first time that MMP-2 expression is regulated by MT1-MMP in human endothelial cells. Moreover, MMP-2 activity is also modulated by MT1-MMP. In addition we found that endothelial cells,   ECM   adhesion and human endothelial cell tube formation, which are known to be regulated by MMP-2, are blocked by MT1-MMP siRNA. These results suggest that MT1-MMP plays an important role in regulating angiogenesis in human endothelial cells. 
 Molecular dissection of the structural machinery underlying the tissue-invasive activity of membrane type-1 matrix metalloproteinase. Membrane type-1 matrix metalloproteinase (MT1-MMP) drives cell invasion through three-dimensional (3-D) extracellular matrix (  ECM  ) barriers dominated by type I collagen or fibrin. Based largely on analyses of its impact on cell function under two-dimensional culture conditions, MT1-MMP is categorized as a multifunctional molecule with 1) a structurally distinct, N-terminal catalytic domain; 2) a C-terminal hemopexin domain that regulates substrate recognition as well as conformation; and 3) a type I transmembrane domain whose cytosolic tail controls protease trafficking and signaling cascades. The MT1-MMP domains that subserve cell trafficking through 3-D   ECM   barriers in vitro or in vivo, however, remain largely undefined. Herein, we demonstrate that collagen-invasive activity is not confined strictly to the catalytic, hemopexin, transmembrane, or cytosolic domain sequences of MT1-MMP. Indeed, even a secreted collagenase supports invasion when tethered to the cell surface in the absence of the MT1-MMP hemopexin, transmembrane, and cytosolic tail domains. By contrast, the ability of MT1-MMP to support fibrin-invasive activity diverges from collagenolytic potential, and alternatively, it requires the specific participation of MT-MMP catalytic and hemopexin domains. Hence, the tissue-invasive properties of MT1-MMP are unexpectedly embedded within distinct, but parsimonious, sequences that serve to tether the requisite matrix-degradative activity to the surface of migrating cells. 
 MT1-MMP-dependent invasion is regulated by TI-VAMP/VAMP7. Proteolytic degradation of the extracellular matrix (  ECM  ) is one intrinsic property of metastatic tumor cells to breach tissue barriers and to disseminate into different tissues. This process is initiated by the formation of invadopodia, which are actin-driven, finger-like membrane protrusions. Yet, little is known on how invadopodia are endowed with the functional machinery of proteolytic enzymes [1, 2]. The key protease MT1-MMP (membrane type 1-matrix metalloproteinase) confers proteolytic activity to invadopodia and thus invasion capacity of cancer cells [3-6]. Here, we report that MT1-MMP-dependent matrix degradation at invadopodia is regulated by the v-SNARE TI-VAMP/VAMP7, hence providing the molecular inventory mediating focal degradative activity of cancer cells. As observed by TIRF microscopy, MT1-MMP-mCherry and GFP-VAMP7 were simultaneously detected at proteolytic sites. Functional ablation of VAMP7 decreased the ability of breast cancer cells to degrade and invade in a MT1-MMP-dependent fashion. Moreover, the number of invadopodia was dramatically decreased in VAMP7- and MT1-MMP-depleted cells, indicative of a positive-feedback loop in which the protease as a cargo of VAMP7-targeted transport vesicles regulates maturation of invadopodia. Collectively, these data point to a specific role of VAMP7 in delivering MT1-MMP to sites of degradation, maintaining the functional machinery required for invasion. 
 Emerging concepts in the regulation of membrane-type 1 matrix metalloproteinase activity. Pericellular proteolysis mediated by membrane-type 1 matrix metalloproteinase (MT1-MMP) represents an essential component of the cellular machinery involved in the dissolution and penetration of   ECM   barriers by tumor cells. Although most studies on the proinvasive properties of MT1-MMP have focused on its unusually broad proteolytic activity towards several   ECM   components and cell surface receptors, recent evidence indicate that the cytoplasmic domain of the enzyme also actively participates in tumor cell invasion by regulating the cell surface localization of MT1-MMP as well as the activation of signal transduction cascades. The identification of the molecular events by which the intracellular domain of MT1-MMP links proteolysis of the surrounding matrix by the enzyme to modification of cell function may thus provide important new information on the mechanisms by which this enzyme controls the invasive behavior of neoplastic cells in vivo. 
 Collagen I and thrombin activate MMP-2 by MMP-14-dependent and -independent pathways: implications for airway smooth muscle migration. Increased proinflammatory mediators and   ECM   deposition are key features of the airways in asthma. Matrix metalloproteinases (MMPs) are produced by airway smooth muscle (ASM) cells and have multiple roles in inflammation and tissue remodeling. We hypothesized that components of the asthmatic airway would stimulate MMP production and activation by ASM and contribute to airway remodeling. We measured human ASM-derived MMP mRNA, protein, and activity by real-time RT-PCR, zymography, Western blotting, and MMP activity assay. Collagen I and thrombin caused a synergistic increase in MMP-2 protein and total MMP activity but paradoxically decreased MMP-2 mRNA. Additionally, collagen I activated MMP-2 in culture supernatants independent of the cell surface. Together, collagen I and thrombin strongly enhanced MMP-14 mRNA and protein but had no effect individually, suggesting increased MMP-14, the activating protease for MMP-2, may be partially responsible for MMP-2 activation. Furthermore, collagen I reduced tissue inhibitor of metalloproteinase-2 protein (TIMP-2). We examined the role of MMPs in functions of ASM related to airway remodeling and found migration and proliferation were MMP dependent, whereas adhesion and apoptosis were not. Ilomastat inhibited migration by 25%, which was also inhibited by TIMPs 1-4 and increased by the MMP-2 activator thrombin. These in vitro findings suggest that the environment within the airways of patients with asthma enhances MMP-2 and -14 protein and activity by a complex interaction of transcriptional and posttranscriptional mechanisms, which may contribute to ASM migration. 
 The type I collagen induction of MT1-MMP-mediated MMP-2 activation is repressed by alphaVbeta3 integrin in human breast cancer cells. The influence of alphaVbeta3 integrin on MT1-MMP functionality was studied in human breast cancer cells of differing beta3 integrin status. Overexpression of beta3 integrin caused increased cell surface expression of alphaV integrin and increased cellular adhesion to extracellular matrix (  ECM  ) substrates in BT-549, MDA-MB-231 and MCF-7 cells. beta3 integrin expression also enhanced the migration of breast cancer cells on   ECM   substrates and enhanced collagen gel contraction. In vivo, alphaVbeta3 cooperated with MT1-MMP to increase the growth of MCF-7 cells after orthotopic inoculation in immunocompromised mice, but had no influence on in vitro proliferation. Despite these stimulatory effects, overexpression of beta3 integrin suppressed the type I collagen (Col I) induced MMP-2 activation in all breast cancer cell lines analyzed. This was also evident in extracts from the MCF-7 tumors in vivo, where MMP-2 activation was stimulated by MT1-MMP transfection, but attenuated with beta3 integrin expression. Although our studies confirm important biological effects of alphaVbeta3 integrin on enhancing cell adhesion and migration,   ECM   remodeling and tumor growth, beta3 integrin caused reduced MMP-2 activation in response to Col I in vitro, which appears to be physiologically relevant, as it was also seen in tumor xenografts in vivo. The reduction of MMP-2 activation (and thus MT1-MMP activity) by alphaVbeta3 in response to Col I may be important in scenarios where cells which are activated for matrix degradation need to preserve some pericellular collagen, perhaps as a substrate for cell adhesion and migration, thus maintaining a balanced level of proteolysis required for efficient tumor growth. 
 Effect of lumican on the migration of human mesenchymal stem cells and endothelial progenitor cells: involvement of matrix metalloproteinase-14. Increasing number of evidence shows that soluble factors and extracellular matrix (  ECM  ) components provide an optimal microenvironment controlling human bone marrow mesenchymal stem cell (MSC) functions. Successful in vivo administration of stem cells lies in their ability to migrate through   ECM   barriers and to differentiate along tissue-specific lineages, including endothelium. Lumican, a protein of the small leucine-rich proteoglycan (SLRP) family, was shown to impede cell migration and angiogenesis. The aim of the present study was to analyze the role of lumican in the control of MSC migration and transition to functional endothelial progenitor cell (EPC).Lumican inhibited tube-like structures formation on Matrigel® by MSC, but not EPC. Since matrix metalloproteinases (MMPs), in particular MMP-14, play an important role in remodelling of   EC  M and enhancing cell migration, their expression and activity were investigated in the cells grown on different   EC  M substrata. Lumican down-regulated the MMP-14 expression and activity in MSC, but not in EPC. Lumican inhibited MSC, but not EPC migration and invasion. The inhibition of MSC migration and invasion by lumican was reversed by MMP-14 overexpression.Altogether, our results suggest that lumican inhibits MSC tube-like structure formation and migration via mechanisms that involve a decrease of MMP-14 expression and activity. 
 Cloning of a human gene potentially encoding a novel matrix metalloproteinase having a C-terminal transmembrane domain. Matrix metalloproteinases (MMPs) play key roles in tissue remodeling during physiological and pathological processes by degrading various extracellular matrix (  ECM  ) components. Although nine distinct MMPs have been characterized by cDNA cloning, there are thought to be more corresponding to the complexity of the   ECM  . MMP genes expressed in human tissues and cell lines were analyzed by the polymerase chain reaction (PCR) using degenerate primers that corresponded to the conserved amino acid (aa) sequences of the MMPs. One isolated complementary DNA (cDNA) fragment had sequence homology to the reported MMPs, but was otherwise unique. A human placenta cDNA library (Clontech) was screened using the fragment as a probe and a 3.4-kb cDNA fragment containing a long open reading frame (potentially encoding 582 aa) was isolated. The putative gene product had a common domain structure and the conserved sequence of a MMP, but it had a unique transmembrane (TM)-like structure at the C terminus. It should, therefore, be an TM protein, whereas all the other reported MMPs are secretory proteins. Thus, the gene is thought to be the first of a new subclass of MMPs whose products are potentially expressed on the cell surface. 
 LIM kinase1 modulates function of membrane type matrix metalloproteinase 1: implication in invasion of prostate cancer cells. LIM kinase 1 (LIMK1) is an actin and microtubule cytoskeleton modulatory protein that is overexpressed in a number of cancerous tissues and cells and also promotes invasion and metastasis of prostate and breast cancer cells. Membrane type matrix metalloproteinase 1 (MT1-MMP) is a critical modulator of extracellular matrix (  ECM  ) turnover through pericellular proteolysis and thus plays crucial roles in neoplastic cell invasion and metastasis. MT1-MMP and its substrates pro-MMP-2 and pro-MMP-9 are often overexpressed in a variety of cancers including prostate cancer and the expression levels correlate with the grade of malignancy in prostate cancer cells. The purpose of this study is to determine any functional relation between LIMK1 and MT1-MMP and its implication in cell invasion.Our results showed that treatment with the hydroxamate inhibitor of MT1-MMP, MMP-2 and MMP-9 ilomastat inhibited LIMK1-induced invasion of benign prostate epithelial cells. Over expression of LIMK1 resulted in increased collagenolytic activity of MMP-2, and secretion of pro-MMP2 and pro-MMP-9. Cells over expressing LIMK1 also exhibited increased expression of MT1-MMP, transcriptional activation and its localization to the plasma membrane. LIMK1 physically associates with MT1-MMP and is colocalized with it to the Golgi vesicles. We also noted increased expression of both MT1-MMP and LIMK1 in prostate tumor tissues.Our results provide new information on regulation of MT1-MMP function by LIMK1 and showed for the first time, involvement of MMPs in LIMK1 induced cell invasion. 
 Expression Levels of Myostatin and Matrix Metalloproteinase 14 mRNAs in Uterine Leiomyoma are Correlated With Dysmenorrhea. Uterine leiomyoma is the most common benign neoplasm of female reproductive system, found in about 50% of women in reproductive age. The mechanisms of leiomyoma growth include cell proliferation, which is modulated by growth factors, and deposition of extracellular matrix (  ECM  ). Activin A and myostatin are growth factors that play a role in proliferation of leiomyoma cells. Matrix metalloproteinases (MMPs) are known for their ability to remodel the   ECM   in different biological systems. The aim of this study was to evaluate the expression levels of activin βA-subunit, myostatin, and MMP14 messenger RNAs (mRNAs) in uterine leiomyomas and the possible correlation of these factors with clinical features of the disease. Matrix metalloproteinase 14 was highly expressed in uterine leiomyoma and correlated with myostatin and activin A mRNA expression. Moreover, MMP14 and myostatin mRNA expression correlated significantly and directly with the intensity of dysmenorrhea. Overall, the present findings showed that MMP14 mRNA is highly expressed in uterine leiomyoma, where it correlates with the molecular expression of growth factors and is further increased in cases of intense dysmenorrhea. 
  GeneRIFs  
 MiR-22 downregulation promotes GC invasion and metastasis by upregulating MMP14 and Snail, and then inducing   ECM   remodeling and EMT. 
 MT1-MMP-mediated proteolytic processing of   ECM  -bound LTBP-1 is a mechanism to release latent TGF-beta from the subendothelial matrix. 
 Data show a novel mechanism by which   ECM   regulates membrane-type 1 matrix metalloproteinase (MT1-MMP) association with beta1 or alphavbeta3 integrins, thus modulating its internalization, activity, and function on human endothelial cells. 
 PDK1 mediates mammary epithelial cell growth and invasion in part by MT1-MMP induction, which in turn activates MMP-2 and modulates the   ECM   proteins decorin and collagen 
  [Ee]xtracellular [Mm]atrix  Conserved Domains  
 cd04278: ZnMc_MMP; Zinc-dependent metalloprotease, matrix metalloproteinase (MMP) sub-family. MMPs are responsible for a great deal of pericellular proteolysis of   extracellular matrix   and cell surface molecules, playing crucial roles in morphogenesis, cell fate ... 
  Process  
   extracellular matrix   disassembly 
  Component  
   extracellular matrix   
  Summary  
 Proteins of the matrix metalloproteinase (MMP) family are involved in the breakdown of   extracellular matrix   in normal physiological processes, such as embryonic development, reproduction, and tissue remodeling, as well as in disease processes, such as arthritis and metastasis. Most MMP's are secreted as inactive proproteins which are activated when cleaved by extracellular proteinases. However, the protein encoded by this gene is a member of the membrane-type MMP (MT-MMP) subfamily; each member of this subfamily contains a potential transmembrane domain suggesting that these proteins are expressed at the cell surface rather than secreted. This protein activates MMP2 protein, and this activity may be involved in tumor invasion. [provided by RefSeq, Jul 2008] 
  PubMed  
 EMMPRIN-induced MMP-2 activation cascade in human cervical squamous cell carcinoma. Tumor progression and recurrence of cervical cancer is associated with upregulation of matrix metalloproteinase 2 (MMP-2). We evaluated the location, origin and activity of MMP-2 in cervical squamous cell carcinomas in comparison with MT1-MMP (MMP-14), TIMP-2 and   extracellular matrix   metalloproteinase inducer (EMMPRIN). Positive immunostaining for MMP-2 in malignant cells was detected in 83% of the patients. Two patterns of tumor cell MMP-2 staining were observed: either homogenous in all tumor cells or confined to the cells neighboring the stroma (tumor-border staining pattern, TBS). Fluorescence in situ zymography showed active MMP-2 mainly around tumor nodules displaying TBS. The MMP-2 staining of TBS tumors correlated significantly with the presence of TIMP-2 and MT1-MMP, proteins involved in docking MMP-2 to the cell surface and essential for MMP-2 activation. In situ mRNA hybridization in TBS tumors demonstrated more abundant presence of MMP-2 mRNA in neighboring myofibroblasts than in the adjacent tumor cells. Moreover, the TBS MMP-2 pattern correlated with the presence of EMMPRIN (p = 0.023), suggesting that tumor cells induce MMP-2 production in nearby stromal cells. This pro-MMP-2 could subsequently be activated on tumor cells via the presence of MT1-MMP and TIMP-2. The biological relevance of this locally activated MMP-2 was underscored by the observation that only the TBS pattern of MMP-2 significantly correlated with decreased survival. In conclusion, the colocalization of EMMPRIN, MT1-MMP and TIMP-2 in human cervical carcinomas seems to be involved in a specific distribution pattern of tumor cell bound MMP-2, which is related with local proteolytic activity and therefore might be associated with worse prognosis of the patients. 
 The influence of MMP-14, TIMP-2 and MMP-2 expression on breast cancer prognosis. Matrix metalloproteinase (MMP)-2 is very active at degrading   extracellular matrix  . It is under the influence of an activator, membrane type 1 MMP (MMP-14), and the tissue inhibitor of metalloproteases (TIMP)-2. We hypothesized that the individual expression of these three markers or their balance may help to predict breast cancer prognosis.MMP-2, MMP-14 and TIMP-2 expression has been evaluated by 35S mRNA in situ hybridization on paraffin material of 539 breast cancers without distant metastasis at diagnosis and with a median follow-up of 9.2 years.MMP-2 and MMP-14 mRNA was detected primarily in reactive stromal cells whereas TIMP-2 mRNA was expressed by both stromal and cancer cells. Of the three molecules, an adjusted Cox model revealed that high MMP-14 mRNA (> or = 10% cells) alone predicted a significantly shorter overall survival (p = 0.031) when adjusted for clinical factors (tumor size and number of involved lymph nodes). Prognostic significance was lost when further adjusted for Her-2/neu and urokinase-type plasminogen activator (p = 0.284). Furthermore, when all three components were analyzed together, the survival was worst for patients with high MMP-2/high MMP-14/low TIMP-2 (5 year survival = 60%) and best with low MMP-2/low MMP-14/high TIMP-2 (5 year survival = 74%), but the difference did not reach statistical significance (p = 0.3285).Of the MMP-14/TIMP-2/MMP-2 complex, MMP-14 was the factor most significantly associated with the outcome of breast cancer and was an independent factor of poor overall survival when adjusted for clinical prognostic factors, but not for certain ancillary markers. 
 CLASPs link focal-adhesion-associated microtubule capture to localized exocytosis and adhesion site turnover. Turnover of integrin-based focal adhesions (FAs) with the   extracellular matrix   (ECM) is essential for coordinated cell movement. In collectively migrating human keratinocytes, FAs assemble near the leading edge, grow and mature as a result of contractile forces and disassemble underneath the advancing cell body. We report that clustering of microtubule-associated CLASP1 and CLASP2 proteins around FAs temporally correlates with FA turnover. CLASPs and LL5β (also known as PHLDB2), which recruits CLASPs to FAs, facilitate FA disassembly. CLASPs are further required for FA-associated ECM degradation, and matrix metalloprotease inhibition slows FA disassembly similarly to CLASP or PHLDB2 (LL5β) depletion. Finally, CLASP-mediated microtubule tethering at FAs establishes an FA-directed transport pathway for delivery, docking and localized fusion of exocytic vesicles near FAs. We propose that CLASPs couple microtubule organization, vesicle transport and cell interactions with the ECM, establishing a local secretion pathway that facilitates FA turnover by severing cell-matrix connections. 
 Vinculin negatively regulates transcription of MT1-MMP through MEK/ERK pathway. Vinculin regulates a variety of cellular functions partly through stabilization of tumor suppressor PTEN. In order to study the role of vinculin in tumor progression other than PTEN stabilization, vinculin was knocked down in PTEN-deficient squamous cell carcinoma HSC-4 cells. Knockdown of vinculin induced phenotypical change by reducing cell-cell and cell-  extracellular matrix   adhesions, and enhanced MT1-MMP expression at transcription level and subsequent cell migration. Up-regulation of MT1-MMP transcription by vinculin knockdown was abrogated by ERK inhibition. These results suggest that vinculin negatively regulates malignant phenotype of tumor cells including MT1-MMP transcription through MEK/ERK pathway. 
 Unveiling the surface epitopes that render tissue inhibitor of metalloproteinase-1 inactive against membrane type 1-matrix metalloproteinase. Membrane type 1-matrix metalloproteinase (MT1-MMP) is a zinc-dependent, membrane-associated endoproteinase of the metzincin family. The enzyme regulates   extracellular matrix   remodeling and is capable of cleaving a wide variety of transmembrane proteins. The enzymatic activity of MT1-MMP is regulated by endogenous inhibitors, the tissue inhibitor of metalloproteinases (TIMP). To date, four variants of mammalian TIMP have been identified. Whereas TIMP-2-4 are potent inhibitors against MT1-MMP, TIMP-1 displays negligible inhibitory activity against the enzyme. The rationale for such selectivity is hitherto unknown. Here we identify the surface epitopes that render TIMP-1 inactive against MT1-MMP. We show that TIMP-1 can be transformed into an active inhibitor against MT1-MMP by the mutation of a single residue, namely threonine 98 to leucine (T98L). The resultant mutant displayed inhibitory characteristics of a typical slow, tight binding inhibitor. The potency of the mutant could be further enhanced by the introduction of valine 4 to alanine (V4A) and proline 6 to valine (P6V) mutations. Indeed, the inhibitory profile of the triple mutant (V4A/P6V/T98L) is indistinguishable from those of other TIMPs. Our findings suggest that threonine 98 is critical in initiating MMP binding and complex stabilization. Our findings also provide a potential mechanistic explanation for MMP-TIMP selectivity. 
 Tissue inhibitor of metalloproteinases-2 binding to membrane-type 1 matrix metalloproteinase induces MAPK activation and cell growth by a non-proteolytic mechanism. Membrane-type 1 matrix metalloproteinase (MT1-MMP), a transmembrane proteinase with a short cytoplasmic domain and an extracellular catalytic domain, controls a variety of physiological and pathological processes through the proteolytic degradation of extracellular or transmembrane proteins. MT1-MMP forms a complex on the cell membrane with its physiological protein inhibitor, tissue inhibitor of metalloproteinases-2 (TIMP-2). Here we show that, in addition to extracellular proteolysis, MT1-MMP and TIMP-2 control cell proliferation and migration through a non-proteolytic mechanism. TIMP-2 binding to MT1-MMP induces activation of ERK1/2 by a mechanism that does not require the proteolytic activity and is mediated by the cytoplasmic tail of MT1-MMP. MT1-MMP-mediated activation of ERK1/2 up-regulates cell migration and proliferation in vitro independently of   extracellular matrix   proteolysis. Proteolytically inactive MT1-MMP promotes tumor growth in vivo, whereas proteolytically active MT1-MMP devoid of cytoplasmic tail does not have this effect. These findings illustrate a novel role for MT1-MMP-TIMP-2 interaction, which controls cell functions by a mechanism independent of   extracellular matrix   degradation. 
 A genetic association study of maternal and fetal candidate genes that predispose to preterm prelabor rupture of membranes (PROM). We sought to determine whether maternal/fetal single-nucleotide polymorphisms (SNPs) in candidate genes are associated with preterm prelabor rupture of membranes (pPROM).A case-control study was conducted in patients with pPROM (225 mothers and 155 fetuses) and 599 mothers and 628 fetuses with a normal pregnancy; 190 candidate genes and 775 SNPs were studied. Single locus/haplotype association analyses were performed; false discovery rate was used to correct for multiple testing (q* = 0.15).First, a SNP in tissue inhibitor of metalloproteinase 2 in mothers was significantly associated with pPROM (odds ratio, 2.12; 95% confidence interval, 1.47-3.07; P = .000068), and this association remained significant after correction for multiple comparisons. Second, haplotypes for Alpha 3 type IV collagen isoform precursor in the mother were associated with pPROM (global P = .003). Third, multilocus analysis identified a 3-locus model, which included maternal SNPs in collagen type I alpha 2, defensin alpha 5 gene, and endothelin 1.DNA variants in a maternal gene involved in   extracellular matrix   metabolism doubled the risk of pPROM. 
 Regulation of endodermal differentiation of human embryonic stem cells through integrin-ECM interactions. Many cellular responses during development are regulated by interactions between integrin receptors and   extracellular matrix   proteins (ECMPs). Although the majority of recent studies in human embryonic stem cell (hESC) differentiation have focused on the role of growth factors, such as FGF, TGFβ, and WNT, relatively little is known about the role of ECMP-integrin signaling in this process. Moreover, current strategies to direct hESC differentiation into various lineages are inefficient and have yet to produce functionally mature cells in vitro. This suggests that additional factors, such as ECMPs, are required for the efficient differentiation of hESCs. Using a high-throughput multifactorial cellular array technology, we investigated the effect of hundreds of ECMP combinations and concentrations on differentiation of several hPSC lines to definitive endoderm (DE), an early embryonic cell population fated to give rise to internal organs such as the lung, liver, pancreas, stomach, and intestine. From this screen we identified fibronectin (FN) and vitronectin (VTN) as ECMP components that promoted DE differentiation. Analysis of integrin expression revealed that differentiation toward DE led to an increase in FN-binding integrin α5 (ITGA5) and VTN-binding integrin αV (ITGAV). Conditional short hairpin RNA-mediated knockdown of ITGA5 and ITGAV disrupted hESC differentiation toward DE. Finally, fluorescence-based cell sorting for ITGA5 and ITGAV significantly enriched cells with gene expression signatures associated with DE, demonstrating that these cell surface proteins permit isolation and enrichment of DE from hESCs. These data provide evidence that FN and VTN promote endoderm differentiation of hESCs through interaction with ITGA5 and ITGAV, and that ECMP-integrin interactions are required for hESC differentiation into functionally mature cells. 
 Identification of fetal and maternal single nucleotide polymorphisms in candidate genes that predispose to spontaneous preterm labor with intact membranes. The purpose of this study was to determine whether maternal/fetal single nucleotide polymorphisms (SNPs) in candidate genes are associated with spontaneous preterm labor/delivery.A genetic association study was conducted in 223 mothers and 179 fetuses (preterm labor with intact membranes who delivered <37 weeks of gestation [preterm birth (PTB)]), and 599 mothers and 628 fetuses (normal pregnancy); 190 candidate genes and 775 SNPs were studied. Single locus/haplotype association analyses were performed; the false discovery rate was used to correct for multiple testing.The strongest single locus associations with PTB were interleukin-6 receptor 1 (fetus; P=.000148) and tissue inhibitor of metalloproteinase 2 (mother; P=.000197), which remained significant after correction for multiple comparisons. Global haplotype analysis indicated an association between a fetal DNA variant in insulin-like growth factor F2 and maternal alpha 3 type IV collagen isoform 1 (global, P=.004 and .007, respectively).An SNP involved in controlling fetal inflammation (interleukin-6 receptor 1) and DNA variants in maternal genes encoding for proteins involved in   extracellular matrix   metabolism approximately doubled the risk of PTB. 
 Association of matrix metalloproteinase gene polymorphisms with refractive error in Amish and Ashkenazi families. Matrix metalloproteinases (MMPs) and tissue inhibitors of metalloproteinases (TIMPs) are involved in scleral   extracellular matrix   remodeling and have shown differential expression in experimental myopia. The genetic association of refractive error and polymorphisms in MMP and TIMP genes in Old Order Amish (AMISH) and Ashkenazi Jewish (ASHK) families was investigated.Individuals from 55 AMISH and 63 ASHK families participated in the study. Ascertainment was designed to enrich the families for myopia; the mean spherical equivalent (MSE) refractive error (SD) was -1.61 (2.72) D in the AMISH, and -3.56 (3.32) D in the ASHK. One hundred forty-six common haplotype tagging SNPs covering 14 MMP and 4 TIMP genes were genotyped in 358 AMISH and 535 ASHK participants. Association analyses of MSE and the spherical component of refraction (SPH) were performed separately for the AMISH and the ASHK. Bonferroni-corrected significance thresholds and local false discovery rates were used to account for multiple testing.After they were filtered for quality-control, 127 SNPs were included in the analyses. No polymorphisms showed statistically significant association to refraction in the ASHK (minimum P = 0.0132). In AMISH, two SNPs showed evidence of association with refractive phenotypes: rs1939008 (P = 0.00016 for SPH); and rs9928731 (P = 0.00026 for SPH). These markers were each estimated to explain <5% of the variance of SPH in the AMISH sample.Statistically significant genetic associations of ocular refraction to polymorphisms near MMP1 and within MMP2 were identified in the AMISH but not among the ASHK families. The results suggest that the MMP1 and MMP2 genes are involved in refractive variation in the AMISH. Genetic and/or environmental heterogeneity most likely contribute to differences in association results between ethnic groups. 
 TIMP-2 Interaction with MT1-MMP Activates the AKT Pathway and Protects Tumor Cells from Apoptosis. Membrane-type 1 matrix metalloproteinase (MT1-MMP), a transmembrane proteinase with an extracellular catalytic domain and a short cytoplasmic tail, degrades a variety of   extracellular matrix   (ECM) components. In addition, MT1-MMP activates intracellular signaling through proteolysis-dependent and independent mechanisms. We have previously shown that binding of tissue inhibitor of metalloproteinases-2 (TIMP-2) to MT1-MMP controls cell proliferation and migration, as well as tumor growth in vivo by activating the Ras-extracellular signal regulated kinase-1 and -2 (ERK1/2) pathway through a mechanism that requires the cytoplasmic but not the proteolytic domain of MT1-MMP. Here we show that in MT1-MMP expressing cells TIMP-2 also induces rapid and sustained activation of AKT in a dose- and time-dependent manner and by a mechanism independent of the proteolytic activity of MT1-MMP. Fibroblast growth factor receptor-1 mediates TIMP-2 induction of ERK1/2 but not of AKT activation; however, Ras activation is necessary to transduce the TIMP-2-activated signal to both the ERK1/2 and AKT pathways. ERK1/2 and AKT activation by TIMP-2 binding to MT1-MMP protects tumor cells from apoptosis induced by serum starvation. Conversely, TIMP-2 upregulates apoptosis induced by three-dimensional type I collagen in epithelial cancer cells. Thus, TIMP-2 interaction with MT1-MMP provides tumor cells with either pro- or anti-apoptotic signaling depending on the extracellular environment and apoptotic stimulus. 
 TIMP-2 Interaction with MT1-MMP Activates the AKT Pathway and Protects Tumor Cells from Apoptosis. Membrane-type 1 matrix metalloproteinase (MT1-MMP), a transmembrane proteinase with an extracellular catalytic domain and a short cytoplasmic tail, degrades a variety of   extracellular matrix   (ECM) components. In addition, MT1-MMP activates intracellular signaling through proteolysis-dependent and independent mechanisms. We have previously shown that binding of tissue inhibitor of metalloproteinases-2 (TIMP-2) to MT1-MMP controls cell proliferation and migration, as well as tumor growth in vivo by activating the Ras-extracellular signal regulated kinase-1 and -2 (ERK1/2) pathway through a mechanism that requires the cytoplasmic but not the proteolytic domain of MT1-MMP. Here we show that in MT1-MMP expressing cells TIMP-2 also induces rapid and sustained activation of AKT in a dose- and time-dependent manner and by a mechanism independent of the proteolytic activity of MT1-MMP. Fibroblast growth factor receptor-1 mediates TIMP-2 induction of ERK1/2 but not of AKT activation; however, Ras activation is necessary to transduce the TIMP-2-activated signal to both the ERK1/2 and AKT pathways. ERK1/2 and AKT activation by TIMP-2 binding to MT1-MMP protects tumor cells from apoptosis induced by serum starvation. Conversely, TIMP-2 upregulates apoptosis induced by three-dimensional type I collagen in epithelial cancer cells. Thus, TIMP-2 interaction with MT1-MMP provides tumor cells with either pro- or anti-apoptotic signaling depending on the extracellular environment and apoptotic stimulus. 
 Vinculin negatively regulates transcription of MT1-MMP through MEK/ERK pathway. Vinculin regulates a variety of cellular functions partly through stabilization of tumor suppressor PTEN. In order to study the role of vinculin in tumor progression other than PTEN stabilization, vinculin was knocked down in PTEN-deficient squamous cell carcinoma HSC-4 cells. Knockdown of vinculin induced phenotypical change by reducing cell-cell and cell-  extracellular matrix   adhesions, and enhanced MT1-MMP expression at transcription level and subsequent cell migration. Up-regulation of MT1-MMP transcription by vinculin knockdown was abrogated by ERK inhibition. These results suggest that vinculin negatively regulates malignant phenotype of tumor cells including MT1-MMP transcription through MEK/ERK pathway. 
 Tissue inhibitor of metalloproteinases-2 binding to membrane-type 1 matrix metalloproteinase induces MAPK activation and cell growth by a non-proteolytic mechanism. Membrane-type 1 matrix metalloproteinase (MT1-MMP), a transmembrane proteinase with a short cytoplasmic domain and an extracellular catalytic domain, controls a variety of physiological and pathological processes through the proteolytic degradation of extracellular or transmembrane proteins. MT1-MMP forms a complex on the cell membrane with its physiological protein inhibitor, tissue inhibitor of metalloproteinases-2 (TIMP-2). Here we show that, in addition to extracellular proteolysis, MT1-MMP and TIMP-2 control cell proliferation and migration through a non-proteolytic mechanism. TIMP-2 binding to MT1-MMP induces activation of ERK1/2 by a mechanism that does not require the proteolytic activity and is mediated by the cytoplasmic tail of MT1-MMP. MT1-MMP-mediated activation of ERK1/2 up-regulates cell migration and proliferation in vitro independently of   extracellular matrix   proteolysis. Proteolytically inactive MT1-MMP promotes tumor growth in vivo, whereas proteolytically active MT1-MMP devoid of cytoplasmic tail does not have this effect. These findings illustrate a novel role for MT1-MMP-TIMP-2 interaction, which controls cell functions by a mechanism independent of   extracellular matrix   degradation. 
 Identification of fetal and maternal single nucleotide polymorphisms in candidate genes that predispose to spontaneous preterm labor with intact membranes. The purpose of this study was to determine whether maternal/fetal single nucleotide polymorphisms (SNPs) in candidate genes are associated with spontaneous preterm labor/delivery.A genetic association study was conducted in 223 mothers and 179 fetuses (preterm labor with intact membranes who delivered <37 weeks of gestation [preterm birth (PTB)]), and 599 mothers and 628 fetuses (normal pregnancy); 190 candidate genes and 775 SNPs were studied. Single locus/haplotype association analyses were performed; the false discovery rate was used to correct for multiple testing.The strongest single locus associations with PTB were interleukin-6 receptor 1 (fetus; P=.000148) and tissue inhibitor of metalloproteinase 2 (mother; P=.000197), which remained significant after correction for multiple comparisons. Global haplotype analysis indicated an association between a fetal DNA variant in insulin-like growth factor F2 and maternal alpha 3 type IV collagen isoform 1 (global, P=.004 and .007, respectively).An SNP involved in controlling fetal inflammation (interleukin-6 receptor 1) and DNA variants in maternal genes encoding for proteins involved in   extracellular matrix   metabolism approximately doubled the risk of PTB. 
 A genetic association study of maternal and fetal candidate genes that predispose to preterm prelabor rupture of membranes (PROM). We sought to determine whether maternal/fetal single-nucleotide polymorphisms (SNPs) in candidate genes are associated with preterm prelabor rupture of membranes (pPROM).A case-control study was conducted in patients with pPROM (225 mothers and 155 fetuses) and 599 mothers and 628 fetuses with a normal pregnancy; 190 candidate genes and 775 SNPs were studied. Single locus/haplotype association analyses were performed; false discovery rate was used to correct for multiple testing (q* = 0.15).First, a SNP in tissue inhibitor of metalloproteinase 2 in mothers was significantly associated with pPROM (odds ratio, 2.12; 95% confidence interval, 1.47-3.07; P = .000068), and this association remained significant after correction for multiple comparisons. Second, haplotypes for Alpha 3 type IV collagen isoform precursor in the mother were associated with pPROM (global P = .003). Third, multilocus analysis identified a 3-locus model, which included maternal SNPs in collagen type I alpha 2, defensin alpha 5 gene, and endothelin 1.DNA variants in a maternal gene involved in   extracellular matrix   metabolism doubled the risk of pPROM. 
 Association of matrix metalloproteinase gene polymorphisms with refractive error in Amish and Ashkenazi families. Matrix metalloproteinases (MMPs) and tissue inhibitors of metalloproteinases (TIMPs) are involved in scleral   extracellular matrix   remodeling and have shown differential expression in experimental myopia. The genetic association of refractive error and polymorphisms in MMP and TIMP genes in Old Order Amish (AMISH) and Ashkenazi Jewish (ASHK) families was investigated.Individuals from 55 AMISH and 63 ASHK families participated in the study. Ascertainment was designed to enrich the families for myopia; the mean spherical equivalent (MSE) refractive error (SD) was -1.61 (2.72) D in the AMISH, and -3.56 (3.32) D in the ASHK. One hundred forty-six common haplotype tagging SNPs covering 14 MMP and 4 TIMP genes were genotyped in 358 AMISH and 535 ASHK participants. Association analyses of MSE and the spherical component of refraction (SPH) were performed separately for the AMISH and the ASHK. Bonferroni-corrected significance thresholds and local false discovery rates were used to account for multiple testing.After they were filtered for quality-control, 127 SNPs were included in the analyses. No polymorphisms showed statistically significant association to refraction in the ASHK (minimum P = 0.0132). In AMISH, two SNPs showed evidence of association with refractive phenotypes: rs1939008 (P = 0.00016 for SPH); and rs9928731 (P = 0.00026 for SPH). These markers were each estimated to explain <5% of the variance of SPH in the AMISH sample.Statistically significant genetic associations of ocular refraction to polymorphisms near MMP1 and within MMP2 were identified in the AMISH but not among the ASHK families. The results suggest that the MMP1 and MMP2 genes are involved in refractive variation in the AMISH. Genetic and/or environmental heterogeneity most likely contribute to differences in association results between ethnic groups. 
 EMMPRIN-induced MMP-2 activation cascade in human cervical squamous cell carcinoma. Tumor progression and recurrence of cervical cancer is associated with upregulation of matrix metalloproteinase 2 (MMP-2). We evaluated the location, origin and activity of MMP-2 in cervical squamous cell carcinomas in comparison with MT1-MMP (MMP-14), TIMP-2 and   extracellular matrix   metalloproteinase inducer (EMMPRIN). Positive immunostaining for MMP-2 in malignant cells was detected in 83% of the patients. Two patterns of tumor cell MMP-2 staining were observed: either homogenous in all tumor cells or confined to the cells neighboring the stroma (tumor-border staining pattern, TBS). Fluorescence in situ zymography showed active MMP-2 mainly around tumor nodules displaying TBS. The MMP-2 staining of TBS tumors correlated significantly with the presence of TIMP-2 and MT1-MMP, proteins involved in docking MMP-2 to the cell surface and essential for MMP-2 activation. In situ mRNA hybridization in TBS tumors demonstrated more abundant presence of MMP-2 mRNA in neighboring myofibroblasts than in the adjacent tumor cells. Moreover, the TBS MMP-2 pattern correlated with the presence of EMMPRIN (p = 0.023), suggesting that tumor cells induce MMP-2 production in nearby stromal cells. This pro-MMP-2 could subsequently be activated on tumor cells via the presence of MT1-MMP and TIMP-2. The biological relevance of this locally activated MMP-2 was underscored by the observation that only the TBS pattern of MMP-2 significantly correlated with decreased survival. In conclusion, the colocalization of EMMPRIN, MT1-MMP and TIMP-2 in human cervical carcinomas seems to be involved in a specific distribution pattern of tumor cell bound MMP-2, which is related with local proteolytic activity and therefore might be associated with worse prognosis of the patients. 
 Unveiling the surface epitopes that render tissue inhibitor of metalloproteinase-1 inactive against membrane type 1-matrix metalloproteinase. Membrane type 1-matrix metalloproteinase (MT1-MMP) is a zinc-dependent, membrane-associated endoproteinase of the metzincin family. The enzyme regulates   extracellular matrix   remodeling and is capable of cleaving a wide variety of transmembrane proteins. The enzymatic activity of MT1-MMP is regulated by endogenous inhibitors, the tissue inhibitor of metalloproteinases (TIMP). To date, four variants of mammalian TIMP have been identified. Whereas TIMP-2-4 are potent inhibitors against MT1-MMP, TIMP-1 displays negligible inhibitory activity against the enzyme. The rationale for such selectivity is hitherto unknown. Here we identify the surface epitopes that render TIMP-1 inactive against MT1-MMP. We show that TIMP-1 can be transformed into an active inhibitor against MT1-MMP by the mutation of a single residue, namely threonine 98 to leucine (T98L). The resultant mutant displayed inhibitory characteristics of a typical slow, tight binding inhibitor. The potency of the mutant could be further enhanced by the introduction of valine 4 to alanine (V4A) and proline 6 to valine (P6V) mutations. Indeed, the inhibitory profile of the triple mutant (V4A/P6V/T98L) is indistinguishable from those of other TIMPs. Our findings suggest that threonine 98 is critical in initiating MMP binding and complex stabilization. Our findings also provide a potential mechanistic explanation for MMP-TIMP selectivity. 
 The influence of MMP-14, TIMP-2 and MMP-2 expression on breast cancer prognosis. Matrix metalloproteinase (MMP)-2 is very active at degrading   extracellular matrix  . It is under the influence of an activator, membrane type 1 MMP (MMP-14), and the tissue inhibitor of metalloproteases (TIMP)-2. We hypothesized that the individual expression of these three markers or their balance may help to predict breast cancer prognosis.MMP-2, MMP-14 and TIMP-2 expression has been evaluated by 35S mRNA in situ hybridization on paraffin material of 539 breast cancers without distant metastasis at diagnosis and with a median follow-up of 9.2 years.MMP-2 and MMP-14 mRNA was detected primarily in reactive stromal cells whereas TIMP-2 mRNA was expressed by both stromal and cancer cells. Of the three molecules, an adjusted Cox model revealed that high MMP-14 mRNA (> or = 10% cells) alone predicted a significantly shorter overall survival (p = 0.031) when adjusted for clinical factors (tumor size and number of involved lymph nodes). Prognostic significance was lost when further adjusted for Her-2/neu and urokinase-type plasminogen activator (p = 0.284). Furthermore, when all three components were analyzed together, the survival was worst for patients with high MMP-2/high MMP-14/low TIMP-2 (5 year survival = 60%) and best with low MMP-2/low MMP-14/high TIMP-2 (5 year survival = 74%), but the difference did not reach statistical significance (p = 0.3285).Of the MMP-14/TIMP-2/MMP-2 complex, MMP-14 was the factor most significantly associated with the outcome of breast cancer and was an independent factor of poor overall survival when adjusted for clinical prognostic factors, but not for certain ancillary markers. 
 Targeting a single function of the multifunctional matrix metalloprotease MT1-MMP: impact on lymphangiogenesis. The group of matrix metalloproteases (MMPs) is responsible for multiple processes of   extracellular matrix   remodeling in the healthy body but also for matrix and tissue destruction during cancer invasion and metastasis. The understanding of the contributions from each individual MMP, both in healthy and pathological events, has been complicated by the lack of specific inhibitors and the fact that some of the potent MMPs are multifunctional enzymes. These factors have also hampered the setup of therapeutic strategies targeting MMP activity. A tempting target is the membrane-associated MT1-MMP, which has well-documented importance in matrix degradation but which takes part in more than one pathway in this regard. In this report, we describe the selective targeting of a single function of this enzyme by means of a specific monoclonal antibody against MT1-MMP, raised in an MT1-MMP knock-out mouse. The antibody blocks the enzyme ability to activate proMMP-2 without interfering with the collagenolytic function or the general proteolytic activity of MT1-MMP. Using this antibody, we have shown that the MT1-MMP-catalyzed activation of proMMP-2 is involved in the outgrowth of cultured lymphatic endothelial cells in a collagen matrix in vitro, as well as in lymphatic vessel sprouting assayed ex vivo. This is the first example of the complete inactivation of a single function of a multifunctional MMP and the use of this strategy to pursue its role. 
 Lack of Association between Membrane-Type 1 Matrix Metalloproteinase Expression and Clinically Relevant Molecular or Morphologic Tumor Characteristics at the Leading Edge of Invasive Colorectal Carcinoma. Colorectal cancer (CRC) is one of the leading causes of death from cancer in the western world, but tumor biology and clinical course show great interindividual variation. Molecular and morphologic tumor characteristics, such as KRAS/BRAF mutation status, mismatch repair (MMR) protein expression, tumor growth pattern, and tumor cell budding, have been shown to be of key therapeutic and/or prognostic relevance in CRC. Membrane-type 1 matrix metalloproteinase (MT1-MMP) is a membrane-anchored zinc-binding endopeptidase that is expressed at the leading edge of various invasive carcinomas and promotes tumor cell invasion through degradation of the   extracellular matrix  . The aim of this study was to investigate possible associations between MT1-MMP expression and molecular tumor characteristics as well as morphologic features of tumor aggressiveness in a consecutive series of 79 CRC tissue samples. However, although MT1-MMP was expressed in 41/79 samples (52%), there was no significant association between MT1-MMP expression and KRAS/BRAF mutation status, MMR protein expression, presence of lymphovascular invasion, tumor growth pattern, tumor-infiltrating lymphocytes, or tumor cell budding in our sample cohort (P > 0.05). Thus, we conclude that although MT1-MMP may play a role in CRC invasion, it is not of key relevance to the current models of CRC invasion and aggressiveness. 
 MT1-MMP promotes cell growth and ERK activation through c-Src and paxillin in three-dimensional collagen matrix. Membrane-type 1 matrix metalloproteinase (MT1-MMP) is essential for tumor invasion and growth. We show here that MT1-MMP induces extracellular signal-regulated kinase (ERK) activation in cancer cells cultured in collagen gel, which is indispensable for their proliferation. Inhibition of MT1-MMP by MMP inhibitor or small interfering RNA suppressed activation of focal adhesion kinase (FAK) and ERK in MT1-MMP-expressing cancer cells, which resulted in up-regulation of p21(WAF1) and suppression of cell growth in collagen gel. Cell proliferation was also abrogated by the inhibitor against ERK pathway without affecting FAK phosphorylation. MT1-MMP and integrin alpha(v)beta(3) were shown to be involved in c-Src activation, which induced FAK and ERK activation in collagen gel. These MT1-MMP-mediated signal transductions were paxillin dependent, as knockdown of paxillin reduced cell growth and ERK activation, and co-expression of MT1-MMP with paxillin induced ERK activation. The results suggest that MT1-MMP contributes to proliferation of cancer cells in the   extracellular matrix   by activating ERK through c-Src and paxillin. 
 Increased aggressiveness of human prostate PC-3 tumor cells expressing cell surface localized membrane type-1 matrix metalloproteinase (MT1-MMP). Membrane type-1 matrix metalloproteinase (MT1-MMP) is a multidomain transmembrane endopeptidase with a major role in physiological and pathological processes through proteolysis of   extracellular matrix   and other pericellular proteins. We examined cell surface function of MT1-MMP in PC-3 human prostate tumor cells selected for metastasis in nude mice (PC-3-LN4), or transfected with the full-length wild-type (WT) MT1-MMP or with the mutant form lacking the cytoplasmic tail (Delta C-MT1-MMP). Enhanced cell surface MT1-MMP was determined by fluorescence-activated cell sorting analysis and evidenced mechanistically by increased activation of proMMP-2 and invasion into type-I collagen gels. PC-3 cells overexpressing MT1-MMP grew faster than mock-transfected control cells subcutaneously in nude mice. MT1-MMP localized in caveolae, as judged by immunofluorescence microscopy and sucrose-gradient, detergent-resistant cell fractionation. Delta C-MT1-MMP was strongly associated with caveolae, whereas the WT form was present in both caveolae and noncaveolae fractions. The role of plasma membrane MT1-MMP was supported by localization of MT1-MMP by immunofluorescence microscopy at the cell surface of tumor cells in primary prostate cancers. Increased plasma membrane localization of MT1-MMP, either in caveolae or in other lipid raft structures, is a mechanism to localize this proteinase in contact with   extracellular matrix   and other pericellular proteins, the cleavage of which can facilitate prostate cancer cell invasion and metastasis. 
 Association of metalloproteinase gene polymorphisms with systemic sclerosis in the European Caucasian population. Systemic sclerosis (SSc) is classified among the complex genetic disorders and is characterized by massive   extracellular matrix   deposits. These may be due to overactivation of transforming growth factor ss that may be in part a result of abnormal remodeling of   extracellular matrix   and microfibrils. Metalloproteinases (MMP) are a family of proteolytic enzymes, and MMP 2, 9, and 14 contribute to the degradation of microfibrils. Our aim was to determine whether polymorphisms of the MMP2, MMP9, and MMP14 genes confer susceptibility to SSc in a large population.A case-control study was performed in 659 SSc patients and 511 healthy matched controls from a European Caucasian population. Six Tag single-nucleotide polymorphisms (SNP) of the MMP2 gene and 2 SNP of MMP9 and MMP14 genes were genotyped.All SNP were in Hardy-Weinberg equilibrium in the control population. There was no association between the MMP2, MMP9, and MMP14 variants we investigated and SSc for allelic and genotype frequencies. No association was observed for the different subphenotypes of SSc patients.Our results in a large cohort of European Caucasian SSc patients do not support that MMP2, MMP9, and MMP14 genes are involved in the genetic background of SSc. 
   Extracellular matrix   metalloproteinase inducer regulates matrix metalloproteinase activity in cardiovascular cells: implications in acute myocardial infarction. Matrix metalloproteinases (MMPs) are thought to promote progression of atherosclerosis and cardiovascular complications such as plaque rupture. It has been suggested that, on tumor cells, the extracellular MMP inducer (EMMPRIN) is involved in MMP synthesis by as yet unknown mechanisms. On cardiovascular cells, regulation of EMMPRIN in vivo or any functional relevance for MMP induction in vitro has not yet been studied. Thus, we studied EMMPRIN expression on monocytes in acute myocardial infarction (MI) and its potential relevance for MMP activation.In 20 patients with acute MI, surface expression of EMMPRIN was significantly enhanced on monocytes compared with in 20 patients with chronic stable angina. EMMPRIN upregulation was associated with increased expression of the membrane type 1 MMP (MT1-MMP) on monocytes (flow cytometry) as well as MMP-9 activity (gelatin zymography) in the plasma. At 6 months after successful revascularization, EMMPRIN, MT1-MMP, and MMP-9 had normalized. The secretion of MMP-9 by monocytes was induced by monocyte adhesion to immobilized recombinant EMMPRIN or to EMMPRIN-transfected Chinese hamster ovary cells. Moreover, adherent EMMPRIN-transfected monocytic cells stimulated MMP-2 activity of human vascular smooth muscle cells. Gene silencing of EMMPRIN by small-interfering RNA hindered lipopolysaccharide-induced monocyte secretion of MMP-9, indicating a predominant role of EMMPRIN in MMP-9 induction.EMMPRIN and MT1-MMP are upregulated on monocytes in acute MI. During cellular interactions, EMMPRIN stimulates MMP-9 in monocytes and MMP-2 in smooth muscle cells, indicating that EMMPRIN may display a key regulatory role for MMP activity in cardiovascular pathologies. 
 MicroRNA-22 inhibits tumor growth and metastasis in gastric cancer by directly targeting MMP14 and Snail. MicroRNAs (miRNAs) deregulation is frequent in human gastric cancers (GCs), but the role of specific miRNAs involved in this disease remains elusive. MiR-22 was previously reported to act as tumor suppressors or oncogenes in diverse cancers. However, their accurate expression, function and mechanism in GC are largely unclear. Here, we found that the expression of miR-22 was significantly reduced in clinical GC tissues compared with paired adjacent normal tissues, and was significantly correlated with a more aggressive phenotype of GC in patients, and miR-22 low expression correlated with poor overall survival. The introduction of miR-22 markedly suppressed GC cell growth, migration and invasion, and inhibition of miR-22 promoted GC cell proliferation, migration and invasion in vitro. We further demonstrated that miR-22 acted as tumor suppressors through targeting   extracellular matrix   (ECM) remodeling member matrix metalloproteinase 14 (MMP14) and epithelial-to-mesenchymal transition (EMT) inducer Snail in GC. Moreover, ectopic expression of MMP14 or Snail restored inhibitory effects of miR-22 on cell migration and invasion in GC cells, and a negative relationship between the miR-22 expression and MMP14 or Snail mRNA levels was observed in GC. Finally, overexpression of miR-22 suppressed tumor growth, peritoneal dissemination and pulmonary metastasis in vivo. Taken together, we identified that miR-22 is a potent tumor suppressor in GC. MiR-22 downregulation promotes GC invasion and metastasis by upregulating MMP14 and Snail, and then inducing ECM remodeling and EMT. These findings provide a better understanding of the development and progression of GC and may be an important implication for future therapy of the GC. 
 Fluid shear stress promotes proprotein convertase-dependent activation of MT1-MMP. During angiogenesis, endothelial cells (ECs(1)) initiate new blood vessel growth and invade into the   extracellular matrix   (ECM). Membrane type-1 matrix metalloproteinase (MT1-MMP) facilitates this process and translocates to the plasma membrane following activation to promote ECM cleavage. The N-terminal pro-domain within MT1-MMP must be processed for complete activity of the proteinase. This study investigated whether MT1-MMP activation was altered by sphingosine 1-phosphate (S1P) and wall shear stress (WSS), which combine to stimulate EC invasion in three dimensional (3D) collagen matrices. MT1-MMP was activated rapidly and completely by WSS but not S1P. Proprotein convertases (PCs) promoted MT1-MMP processing, prompting us to test whether WSS or S1P treatments increased PC activity. Like MT1-MMP, PC activity increased with WSS, while S1P had no effect. A pharmacological PC inhibitor completely blocked S1P- and WSS-induced EC invasion and MT1-MMP translocation to the plasma membrane. Further, a recombinant PC inhibitor reduced MT1-MMP activation and decreased lumen formation in invading ECs, a process known to be controlled by MT1-MMP. Thus, we conclude that PC and MT1-MMP activation are mechanosensitive events that are required for EC invasion into 3D collagen matrices. 
 Membrane-type I matrix metalloproteinase-dependent regulation of rheumatoid arthritis synoviocyte function. In rheumatoid arthritis, the coordinated expansion of the synoviocyte mass is coupled with a pathologic angiogenic response that leads to the destructive remodeling of articular as well as surrounding connective tissues. Although rheumatoid synoviocytes express a multiplicity of proteolytic enzymes, the primary effectors of cartilage, ligament, and tendon damage remain undefined. Herein, we demonstrate that human rheumatoid synoviocytes mobilize the membrane-anchored matrix metalloproteinase (MMP), membrane-type I MMP (MT1-MMP), to dissolve and invade type I and type II collagen-rich tissues. Though rheumatoid synoviocytes also express a series of secreted collagenases, these proteinases are ineffective in mediating collagenolytic activity in the presence of physiologic concentrations of plasma- or synovial fluid-derived antiproteinases. Furthermore, MT1-MMP not only directs the tissue-destructive properties of rheumatoid synoviocytes but also controls synoviocyte-initiated angiogenic responses in vivo. Together, these findings identify MT1-MMP as a master regulator of the pathologic   extracellular matrix   remodeling that characterizes rheumatoid arthritis as well as the coupled angiogenic response that maintains the aggressive phenotype of the advancing pannus. 
 Immunolocalization of membrane-type 1 MMP in human rheumatoid synovium tissues. Membrane-type 1 matrix metalloproteinase (MT1-MMP, also known as MMP14), the best characterized membrane-anchored MMP, is an important matrix-degrading proteinase that could digest a broad spectrum of   extracellular matrix   proteins and accelerate angiogenesis. We have previously reported that some MMPs involved in the angiogenesis and the pannus formation within the joint, leading to the erosion of articular cartilage and bone in the pathological process of rheumatoid arthritis (RA). In the present study, we used immunohistochemistry assay and con-focal scanning technique to study the detailed immunolocalization of MT1-MMP in human RA synovium tissues as well as the infiltrating immune cell subsets. Our results showed that the positive MT1-MMP immunostaining could be found in synoviocytes, vascular endothelial cells, infiltrating macrophages and monocytes in RA synovium tissues, while weak or negative immunostaining could be found in infiltrating T cells, B cells and NK cells, respectively. Moreover, the Ki-67(+) highly proliferating synoviocytes also showed higher MT1-MMP expression in RA synoviocytes. Thus, the aberrant expression of MT1-MMP in RA synoviocytes as well as infiltrating immune cells may contribute to the proliferation of the synoviocytes, and the angiogenesis and the pannus formation in RA pathological progression. 
 MMP-14 is necessary but not sufficient for invasion of three-dimensional collagen by human muscle satellite cells. The twenty-five known matrix metalloproteases (MMPs) and their endogenous inhibitors, tissue inhibitors of metalloproteases (TIMPs), mediate cell invasion through the   extracellular matrix   (ECM). In a comparative three-dimensional assay, we analyzed human and mouse satellite cells' competence to invade an artificial ECM (collagen I). We identified a single MMP that 1) is expressed by human muscle satellite cells; 2) is induced at the mRNA/protein level by adhesion to collagen I; and 3) is necessary for invasion into a collagen I matrix. Interestingly, murine satellite cells neither express this MMP, nor invade the collagen matrix. However, exogenous human MMP-14 is not sufficient to induce invasion of a collagen matrix by murine cells, emphasizing species differences. 
 Tetraspanin proteins regulate membrane type-1 matrix metalloproteinase-dependent pericellular proteolysis. Membrane type-1 matrix metalloproteinase (MT1-MMP) supports tumor cell invasion through   extracellular matrix   barriers containing fibrin, collagen, fibronectin, and other proteins. Here, we show that simultaneous knockdown of two or three members of the tetraspanin family (CD9, CD81, and TSPAN12) markedly decreases MT1-MMP proteolytic functions in cancer cells. Affected functions include fibronectin proteolysis, invasion and growth in three-dimensional fibrin and collagen gels, and MMP-2 activation. Tetraspanin proteins (CD9, CD81, and TSPAN2) selectively coimmunoprecipitate and colocalize with MT1-MMP. Although tetraspanins do not affect the initial biosynthesis of MT1-MMP, they do protect the newly synthesized protein from lysosomal degradation and support its delivery to the cell surface. Interfering with MT1-MMP-tetraspanin collaboration may be a useful therapeutic approach to limit cancer cell invasion and metastasis. 
 SNAP23, Syntaxin4, and vesicle-associated membrane protein 7 (VAMP7) mediate trafficking of membrane type 1-matrix metalloproteinase (MT1-MMP) during invadopodium formation and tumor cell invasion. Movement through the   extracellular matrix   (ECM) requires cells to degrade ECM components, primarily through the action of matrix metalloproteinases (MMPs). Membrane type 1-matrix metalloproteinase (MT1-MMP) has an essential role in matrix degradation and cell invasion and localizes to subcellular degradative structures termed invadopodia. Trafficking of MT1-MMP to invadopodia is required for the function of these structures, and here we examine the role of N-ethylmaleimide-sensitive factor-activating protein receptor (SNARE)-mediated membrane traffic in the transport of MT1-MMP to invadopodia. During invadopodium formation in MDA-MB-231 human breast cancer cells, increased association of SNAP23, Syntaxin4, and vesicle-associated membrane protein 7 (VAMP7) is detected by coimmunoprecipitation. Blocking the function of these SNAREs perturbs invadopodium-based ECM degradation and cell invasion. Increased level of SNAP23-Syntaxin4-VAMP7 interaction correlates with decreased Syntaxin4 phosphorylation. These results reveal an important role for SNARE-regulated trafficking of MT1-MMP to invadopodia during cellular invasion of ECM. 
 The tetraspanin network modulates MT1-MMP cell surface trafficking. The membrane-type 1 matrix metalloproteinase (MT1-MMP) drives fundamental physiological and pathophysiological processes. Among other substrates, MT1-MMP cleaves components of the   extracellular matrix   and activates other matrix-cleaving proteases such as MMP-2. Trafficking is a highly effective means to modulate MT1-MMP cell surface expression, and hence regulate its function. Here, we describe the complex interaction of MT1-MMP with tetraspanins, their effects on MT1-MMP intracellular trafficking and proteolytic function. Tetraspanins are credited as membrane organizers that form a network within the membrane to regulate the trafficking of associated proteins. In short, we found MT1-MMP to interact with the tetraspanin-associated EWI-2 protein by a yeast two-hybrid screen. Immunoprecipitation analysis confirmed this interaction and further revealed that MT1-MMP also stably interacts with distinct tetraspanins (CD9, CD37, CD53, CD63, CD81, and CD82) and the tetraspanin-like MAL protein. By using different MT1-MMP truncation constructs and mutants, we observed that all tetraspanins and MAL associated with the hemopexin domain of MT1-MMP. Moreover, this interaction was independent of O-glycosylation of MT1-MMP and exclusively occurred in the endoplasmic reticulum. Here, the respective subcellular compartment was identified by fitting the MT1-MMP interaction pattern to a model for post-translational processing of MT1-MMP. In addition, tetraspanins differentially affected the cell surface localization of MT1-MMP, its capacity to activate pro-MMP-2, and the collagen invasion capacity. Interestingly, the degree of tetraspanin-MT1-MMP association did not correlate with its impact on MT1-MMP function. Tetraspanins thus distinctly affect MT1-MMP subcellular localization and function, and may constitute an effective mechanism to control MT1-MMP-dependent proteolysis at the cell surface. 
 Membrane-Type 1 Matrix Metalloproteinase Downregulates Fibroblast Growth Factor-2 Binding to the Cell Surface and Intracellular Signaling. Membrane-type 1 matrix metalloproteinase (MT1-MMP, MMP-14), a transmembrane proteinase with an extracellular catalytic domain and a short cytoplasmic tail, degrades   extracellular matrix   components and controls diverse cell functions through proteolytic and non-proteolytic interactions with extracellular, intracellular, and transmembrane proteins. Here we show that in tumor cells MT1-MMP downregulates fibroblast growth factor-2 (FGF-2) signaling by reducing the amount of FGF-2 bound to the cell surface with high and low affinity. FGF-2 induces weaker activation of ERK1/2 MAP kinase in MT1-MMP expressing cells than in cells devoid of MT1-MMP. This effect is abolished in cells that express proteolytically inactive MT1-MMP but persists in cells expressing MT1-MMP mutants devoid of hemopexin-like or cytoplasmic domain, showing that FGF-2 signaling is downregulated by MT1-MMP proteolytic activity. MT1-MMP expression results in downregulation of FGFR-1 and -4, and in decreased amount of cell surface-associated FGF-2. In addition, MT1-MMP strongly reduces the amount of FGF-2 bound to the cell surface with low affinity. Because FGF-2 association with low-affinity binding sites is a prerequisite for binding to its high-affinity receptors, downregulation of low-affinity binding to the cell surface results in decreased FGF-2 signaling. Consistent with this conclusion, FGF-2 induction of tumor cell migration and invasion in vitro is stronger in cells devoid of MT1- MMP than in MT1-MMP expressing cells. Thus, MT1-MMP controls FGF-2 signaling by a proteolytic mechanism that decreases the cell's biological response to FGF-2. 
 CD44 directs membrane-type 1 matrix metalloproteinase to lamellipodia by associating with its hemopexin-like domain. Membrane-type 1 matrix metalloproteinase (MT1- MMP) localizes at the front of migrating cells and degrades the   extracellular matrix   barrier during cancer invasion. However, it is poorly understood how the polarized distribution of MT1-MMP at the migration front is regulated. Here, we demonstrate that MT1-MMP forms a complex with CD44H via the hemopexin-like (PEX) domain. A mutant MT1-MMP lacking the PEX domain failed to bind CD44H and did not localize at the lamellipodia. The cytoplasmic tail of CD44H, which comprises interfaces that associate with the actin cytoskeleton, was important for its localization at lamellipodia. Overexpression of a CD44H mutant lacking the cytoplasmic tail also prevented MT1-MMP from localizing at the lamellipodia. Modulation of F-actin with cytochalasin D revealed that both CD44H and MT1-MMP co-localize closely with the actin cytoskeleton, dependent on the cytoplasmic tail of CD44H. Thus, CD44H appears to act as a linker that connects MT1-MMP to the actin cytoskeleton and to play a role in directing MT1-MMP to the migration front. The PEX domain of MT1-MMP was indispensable in promoting cell migration and CD44H shedding. 
 Metalloendoprotease cleavage triggers gelsolin amyloidogenesis. Amyloid diseases like Alzheimer's disease and familial amyloidosis of Finnish type (FAF) stem from endoproteolytic cleavage of a precursor protein to generate amyloidogenic peptides that accumulate as amyloid deposits in a tissue-specific manner. FAF patients deposit both 8 and 5 kDa peptides derived from mutant (D187Y/N) plasma gelsolin in the   extracellular matrix   (ECM). The first of two aberrant sequential proteolytic events is executed by furin to yield a 68 kDa (C68) secreted fragment. We now identify the metalloprotease MT1-matrix metalloprotease (MMP), an integral membrane protein active in the ECM, as a protease that processes C68 to the amyloidogenic peptides. We further demonstrate that ECM components are capable of accelerating gelsolin amyloidogenesis. Proteolysis by MT1-MMP-like proteases proximal to the unique chemical environment of the ECM offers an explanation for the tissue-specific deposition observed in FAF and provides critical insight into new therapeutic strategies. 
 Membrane type 1 matrix metalloprotease cleaves laminin-10 and promotes prostate cancer cell migration. Disruption of the   extracellular matrix   by proteases is crucial for tumor invasion. Laminin-10 (Ln-10) has previously been identified as a substrate for cell migration and cell adhesion, and is present in the basal lamina (BL) of both normal prostate and prostate cancer. Here, we investigate a role for membrane type 1 matrix metalloprotease (MT1-MMP) in modifying this Ln-10-rich BL. MT1-MMP is a transmembrane member of the MMP family that has been demonstrated to be upregulated as prostate cancer progresses from normal to prostate intraepithelial neoplasia to invasive cancer, suggesting a role for MT1-MMP in the invasion of prostate cancer. We show that MT1-MMP cleaves the alpha5 chain of purified human Ln-10 from its 350-kDa form into 310-, 190-, 160-, and 45-kDa fragments. This cleavage causes a decrease in DU-145 prostate cancer cell adhesion to purified Ln-10, and an increase in transmigration of DU-145 cells through cleaved Ln-10. We also show that prostate cancer cells expressing membrane-bound MT1-MMP cleave the alpha5 chain of Ln-10. Ln alpha5-chain cleavage is also observed in human prostate cancer tissues. These findings suggest that prostate cancer cells expressing high levels of MT1-MMP have increased invasive potential through their ability to degrade and invade Ln-10 barriers. 
 Collective epithelial cell invasion overcomes mechanical barriers of collagenous   extracellular matrix   by a narrow tube-like geometry and MMP14-dependent local softening. Collective cell invasion (CCI) through interstitial collagenous   extracellular matrix   (ECM) is crucial to the initial stages of branching morphogenesis, and a hallmark of tissue repair and dissemination of certain tumors. The collagenous ECM acts as a mechanical barrier against CCI. However, the physical nature of this barrier and how it is overcome by cells remains incompletely understood. To address these questions, we performed theoretical and experimental analysis of mammary epithelial branching morphogenesis in 3D type I collagen (collagen-I) gels. We found that the mechanical resistance of collagen-I is largely due to its elastic rather than its viscous properties. We also identified two strategies utilized by mammary epithelial cells that can independently minimize ECM mechanical resistance during CCI. First, cells adopt a narrow tube-like geometry during invasion, which minimizes the elastic opposition from the ECM as revealed by theoretical modeling of the most frequent invasive shapes and sizes. Second, the stiffness of the collagenous ECM is reduced at invasive fronts due to its degradation by matrix metalloproteinases (MMPs), as indicated by direct measurements of collagen-I microelasticity by atomic force microscopy. Molecular techniques further specified that the membrane-bound MMP14 mediates degradation of collagen-I at invasive fronts. Thus, our findings reveal that MMP14 is necessary to efficiently reduce the physical restraints imposed by collagen-I during branching morphogenesis, and help our overall understanding of how forces are balanced between cells and their surrounding ECM to maintain collective geometry and mechanical stability during CCI. 
 RABGTPases in MT1-MMP trafficking and cell invasion: Physiology versus pathology. The matrix metalloproteinase MT1-MMP is a central regulator of cell invasion in both physiological and pathological settings, such as tissue surveillance by immune cells and cancer cell metastasis. MT1-MMP cleaves a plethora of intra- and extracellular proteins, including   extracellular matrix   proteins, matrix receptors, and also other MMPs, and thus enables modification of both the cell surface proteome and the pericellular environment. Despite its importance for cell invasion, the pathways regulating MT1-MMP exposure on the cell surface are largely unknown. Recently, our groups discovered that a specific subset of RABGTPases, most notably RAB5a, is critical for MT1-MMP trafficking in primary human macrophages and carcinoma cells. Here, we discuss and contrast our findings for both cell types, pointing out common features and differences in the RABGTPase-dependent trafficking of MT1-MMP in health and disease. 
 Expression of membrane-type-1-matrix metalloproteinase and metalloproteinase-2 in nonsmall cell lung carcinomas. For the metastasis and invasion of cancer cells, destruction of   extracellular matrix   is essential. In this process, collagen is broken down by some matrix metalloproteinases. Matrix metalloproteinase 2 (MMP2) is able to cleave type IV collagen, and membrane-type-1-matrix metalloproteinase (MT1-MMP) induces activation of proMMP2. We investigated the expressions of MT1-MMP and MMP2 and their relation to both clinicopathologic parameters and clinical outcome in non-small cell lung carcinomas (NSCLC). Eighty-nine specimens of NSCLC were examined using in situ hybridization and immunohistochemistry. Each metalloproteinase was expressed within the cytoplasm of tumor cells with or without stromal cells in NSCLC. Tumors in which tumor cells strongly stained for MT1-MMP mRNA or protein made up more than 50% of the tumor area were found in 44 and 26% of cases, respectively. The corresponding values for MMP-2 mRNA and protein, were 51 and 26%. Our analysis of clinicopathological findings revealed a significant positive relationship between MT1-MMP mRNA and p-M. The correlation between MMP2 protein-staining status and overall survival rate reached significance in the univariate analysis. However, an association was not demonstrated in the multivariate analysis. The detection of MT1-MMP and MMP2 is likely to be of limited value in informing the prognosis in NSCLC. 
 Pressure and Temperature Effects on the Activity and Structure of the Catalytic Domain of Human MT1-MMP. Membrane type 1-matrix metalloproteinase (MT1-MMP or MMP-14) is a zinc-transmembrane metalloprotease involved in the degradation of   extracellular matrix   and tumor invasion. While changes in solvation of MT1-MMP have been recently studied, little is known about the structural and energetic changes associated with MT1-MMP while interacting with substrates. Steady-state kinetic and thermodynamic data (including activation energies and activation volumes) were measured over a wide range of temperatures and pressures by means of a stopped-flow fluorescence technique. Complementary temperature- and pressure-dependent Fourier-transform infrared measurements provided corresponding structural information of the protein. MT1-MMP is stable and active over a wide range of temperatures (10-55 °C). A small conformational change was detected at 37 °C, which is responsible for the change in activity observed at the same temperature. Pressure decreases the enzymatic activity until complete inactivation occurs at 2 kbar. The inactivation is associated with changes in the rate-limiting step of the reaction caused by additional hydration of the active site upon compression and/or minor conformational changes in the active site region. Based on these data, an energy and volume diagram could be established for the various steps of the enzymatic reaction. 
 Expression of membrane type I-matrix metalloproteinase in oral squamous cell carcinoma. A local invasion and lymph node metastasis (LNM) of an oral squamous cell carcinoma (OSCC) has a poor prognosis, and involves the degradation of the   extracellular matrix   mediated by multiple proteolytic enzymes including membrane type I-matrix metalloproteinase (MT1-MMP). This study aimed to determine the role of MT1-MMP in OSCC, to evaluate the immunohistochemical expression of MT1-MMP with regard to the invasiveness and LNM of the OSCC, and to evaluate the major source of MT1-MMP mRNA and its protein using immunohistochemistry and in situ hybridization. MT1-MMP expression was examined in 46 OSCCs via immunohistochemistry and non-radioisotope in situ hybridization. The relationship between MT1-MMP expression and LNM, as well as the histological invasiveness, was statistically analyzed. The results showed that whereas 12 out of the 18 OSCCs (66.7%) with LNM showed moderate to strong MT1-MMP expression, only nine of the 28 OSCCs (32.1%) without LNM expressed MT1-MMP strongly. MT1-MMP expression was significantly higher with regard to LNM (P=0.022). As the invasion grade became stronger (from grade a to grade d), MT1-MMP was significantly more strongly expressed (P=0.033). These results suggest that MT1-MMP is primarily secreted in the OSCC cells and is involved in the invasiveness of the OSCC and LNM. Moreover, MT1-MMP combined with other markers may be used to predict the metastatic potential of an OSCC. 
 Up-regulation of vascular endothelial growth factor by membrane-type 1 matrix metalloproteinase stimulates human glioma xenograft growth and angiogenesis. Membrane-type (MT) 1 matrix metalloproteinase (MMP) is up-regulated in many tumor types and has been implicated in tumor progression and metastasis. MT1-MMP is critical for pericellular degradation of the   extracellular matrix  , thereby promoting tumor cell invasion and dissemination. To grow efficiently in vivo, tumor cells induce angiogenesis in both primary solid tumors and metastatic foci. The present study describes a functional link between the expression of MT1-MMP and vascular endothelial growth factor (VEGF) production in human glioma U251 xenografts in athymic mice. To investigate the effects of MT1-MMP on VEGF expression, U251 cells were stably transfected with MT1-MMP to generate the U-MT cell line overexpressing the enzyme. In vitro, the U-MT cells had an increased rate of proliferation and migration as well as the ability to activate the MMP-2 proenzyme and directionally remodel a three-dimensional collagen matrix. These findings suggested higher tumorigenicity of U-MT cells relative to the vector-control U-neo cells. In agreement with the in vitro data, U-MT xenografts in BALB/c nu/nu mice displayed markedly increased growth rates and elevated levels of angiogenesis. In contrast, U-neo cells formed small, minimally vascularized tumors. The elevated angiogenesis in U-MT xenografts was associated with an up-regulation of VEGF expression in tumor cells. In addition, U-MT cells in vitro secreted twice as much VEGF as the control cells. GM6001, a hydroxamate inhibitor of MMP activity, down-regulated the production of VEGF in U-MT cells to the levels observed in the U-neo control. Our results demonstrate that the enhanced tumorigenicity of glioma cells overexpressing MT1-MMP involves stimulation of angiogenesis through the up-regulation of VEGF production. 
 Increasing   extracellular matrix   collagen level and MMP activity induces cyst development in polycystic kidney disease. Polycystic Kidney Disease (PKD) kidneys exhibit increased   extracellular matrix   (ECM) collagen expression and metalloproteinases (MMPs) activity. We investigated the role of these increases on cystic disease progression in PKD kidneys.We examined the role of type I collagen (collagen I) and membrane bound type 1 MMP (MT1-MMP) on cyst development using both in vitro 3 dimensional (3D) collagen gel culture and in vivo PCK rat model of PKD.We found that collagen concentration is critical in controlling the morphogenesis of MDCK cells cultured in 3D gels. MDCK cells did not form 3D structures at collagen I concentrations lower than 1 mg/ml but began forming tubules when the concentration reaches 1 mg/ml. Significantly, these cells began to form cyst when collagen I concentration reached to 1.2 mg/ml, and the ratios of cyst to tubule structures increased as the collagen I concentration increased. These cells exclusively formed cyst structures at a collagen I concentration of 1.8 mg/ml or higher. Overexpression of MT1-MMP in MDCK cells significantly induced cyst growth in 3D collagen gel culture. Conversely, inhibition of MMPs activity with doxycycline, a FDA approved pan-MMPs inhibitor, dramatically slowed cyst growth. More importantly, the treatment of PCK rats with doxycycline significantly decreased renal tubule cell proliferation and markedly inhibited the cystic disease progression.Our data suggest that increased collagen expression and MMP activity in PKD kidneys may induce cyst formation and expansion. Our findings also suggest that MMPs may serve as a therapeutic target for the treatment of human PKD. 
 Transmembrane-deletion mutants of the membrane-type matrix metalloproteinase-1 process progelatinase A and express intrinsic matrix-degrading activity. Membrane-type matrix metalloproteinase-1 (MT-MMP-1) has been proposed to play a critical role in regulating the expression of tissue-invasive phenotypes in normal and neoplastic cells by directly or indirectly mediating the activation of progelatinase A. To begin characterizing MT-MMP-1 structure-function relationships, transmembrane-deletion mutants were constructed, and the processing of the zymogens as well as the enzymic activity of the mature proteinases was analyzed. We now demonstrate that pro-MT-MMP-1 mutants are efficiently processed to active proteinases following post-translational endoproteolysis immediately downstream of an Arg108-Arg-Lys-Arg basic motif by a proprotein convertase-dependent pathway. The secreted form of active MT-MMP-1 not only displays an N terminus identical with that described for the processed wild-type enzyme at Tyr112 (Strongin, A. Y., Collier, I., Bannikov, G., Marmer, B. L., Grants, G. A., and Goldberg, G. I. (1995) J. Biol. Chem. 270, 5331-5338), but also directly mediated progelatinase A activation via a two-step proteolytic cascade indistinguishable from that observed with intact cells. Furthermore, although the only function previously ascribed to MT-MMP-1 is its ability to act as a progelatinase A activator, purified transmembrane deletion mutants also expressed proteolytic activities against a wide range of   extracellular matrix   molecules. Given recent reports that MT-MMP-1 ectodomains may undergo intercellular transfer in vivo (Okada, A., Bellocq, J.-P., Rouyer, N., Chenard, M.-P., Rio, M.-C., Chambon, P., and Basset, P. (1995) Proc. Natl. Acad. Sci. U. S. A. 92, 2730-2734), our data suggest that soluble forms of the proteinase confer recipient cells with the ability to not only process progelatinase A, but also directly degrade   extracellular matrix   components. 
 Control of MT1-MMP transport by atypical PKC during breast-cancer progression. Dissemination of carcinoma cells requires the pericellular degradation of the   extracellular matrix  , which is mediated by membrane type 1-matrix metalloproteinase (MT1-MMP). In this article, we report a co-up-regulation and colocalization of MT1-MMP and atypical protein kinase C iota (aPKCι) in hormone receptor-negative breast tumors in association with a higher risk of metastasis. Silencing of aPKC in invasive breast-tumor cell lines impaired the delivery of MT1-MMP from late endocytic storage compartments to the surface and inhibited matrix degradation and invasion. We provide evidence that aPKCι, in association with MT1-MMP-containing endosomes, phosphorylates cortactin, which is present in F-actin-rich puncta on MT1-MMP-positive endosomes and regulates cortactin association with the membrane scission protein dynamin-2. Thus, cell line-based observations and clinical data reveal the concerted activity of aPKC, cortactin, and dynamin-2, which control the trafficking of MT1-MMP from late endosome to the plasma membrane and play an important role in the invasive potential of breast-cancer cells. 
 Specific blockade of the ERK pathway inhibits the invasiveness of tumor cells: down-regulation of matrix metalloproteinase-3/-9/-14 and CD44. Elevated expression of matrix metalloproteinases (MMPs) is associated with increased metastatic potential in many tumor cells. As activation of the ERK pathway has been linked to the expression of MMP-9, we examined a possible correlation between ERK activation, MMP-9 expression, and invasive phenotype in human tumor cells. Activation state of the ERK pathway in tumor cells was well correlated with the invasive phenotype, which was determined by the ability of cells to invade through reconstituted   extracellular matrix  . Elevated expression of MMP-9 as well as of MMP-3, MMP-14, and CD44 was observed in tumor cells in which constitutive activation of the ERK pathway is detected. Blockade of the ERK pathway by treatment with PD184352, a specific and powerful inhibitor of mitogen-activated protein (MAP) kinase/ERK kinase (MEK), suppressed the expression of MMP-3, MMP-9, MMP-14, and CD44, and inhibited markedly the invasiveness of tumor cells. These results imply that, in addition to anti-proliferative effects, specific blockade of the ERK pathway is expected to result in anti-metastatic effects in tumor cells. 
 Membrane type 1-matrix metalloproteinase is involved in the migration of human monocyte-derived dendritic cells. Dendritic cells (DC) are highly mobile APC. The trafficking of both immature and mature DC is crucial for their functions, which depends mainly on chemotactic attraction and matrix metalloproteinases (MMP) activity. MMP that are in a transmembrane form belong to membrane type (MT)-MMP, among which MT1-MMP has been shown to possess strong proteolytic activity that is capable of degrading   extracellular matrix   molecules. Although it is well established that MMP are zinc-dependent endopeptidases that collectively degrade most components of the   extracellular matrix  , relatively little is known about MT-MMP-mediated matrix degradation during DC migration. In this study, we showed that MT1-MMP was expressed in human monocyte-derived immature and mature DC by semi-quantitative reverse transcription PCR and western blotting analyses. Moreover, immunofluorescence microscopic studies showed that MT1-MMP was expressed on the membrane surface of DC. Blocking of MT1-MMP activity greatly reduced the invasion capacity of immature DC in Matrigel, whereas mature DC mobility was not affected. Taken together, our results show a novel functional link between MT1-MMP and DC motility and suggest that MT1-MMP may play an important role in modulating the migration of immature DC. 
 CDCP1 regulates the function of MT1-MMP and invadopodia-mediated invasion of cancer cells. Complement C1r/C1s, Uegf, Bmp1 (CUB) domain-containing protein 1 (CDCP1) is a transmembrane protein that regulates anchorage-independent growth and cancer cell migration and invasion. Expression of CDCP1 is detected in a number of cancer cell lines and tissues and is closely correlated with poor prognosis. Invadopodia are actin-based protrusions on the surface of invasive cancer cells that promote the degradation of the   extracellular matrix   (ECM) via localized proteolysis, which is mainly mediated by membrane type 1 matrix metalloproteinase (MT1-MMP). MT1-MMP is accumulated at invadopodia by targeted delivery via membrane trafficking. The present study shows that CDCP1 is required for ECM degradation by invadopodia in human breast cancer and melanoma cells. CDCP1 localized to caveolin-1-containing vesicular structures and lipid rafts and was detected in close proximity to invadopodia. Further biochemical analysis revealed that substantial amounts of CDCP1 existed in the Triton X-100 insoluble lipid raft fraction. CDCP1 was coimmunoprecipitated with MT1-MMP and colocalized with MT1-MMP at the vesicular structures. The siRNA-mediated knockdown of the CDCP1 expression markedly inhibited MT1-MMP-dependent ECM degradation and Matrigel invasion and reduced the accumulation of MT1-MMP at invadopodia, as shown by immunofluorescence analysis. These results indicate that CDCP1 is an essential regulator of the trafficking and function of MT1-MMP- and invadopodia-mediated invasion of cancer cells. 
 A proteomic approach to identify substrates of matrix metalloproteinase-14 in human plasma. Matrix metalloproteinases (MMPs) are a family of zinc-containing endopeptidases that proteolyze   extracellular matrix   components as well as a variety of functional proteins. Here we describe a "degradomics" method that efficiently identifies substrates of MMP-14 in a complex protein mixture, such as plasma. Plasma proteins were incubated in the presence or absence of the MMP-14 catalytic domain and displayed on two-dimensional (2-D) gels. After a comparison of the gels, we selected 40 protein spots that reproducibly showed disparities. Upon in-gel digestion, mass determination, and peptide mass fingerprinting, we identified 15 different proteins from 31 spots. These proteins included six known substrates and nine potential substrates of MMP-14. Among the latter, the purified forms of apolipoprotein A-I, apolipoprotein E, and plasma gelsolin were cleaved in vitro by MMP-14, confirming that each of them is a novel substrate of MMP-14. These results demonstrate that our method rapidly and selectively identifies MMP-14 substrates from human plasma proteins. This method would thus constitute a powerful tool for identifying the substrates of MMPs and other proteases in highly complex mixtures of proteins and would enhance our understanding of the biological roles of these enzymes. 
 Basal localization of MT1-MMP is essential for epithelial cell morphogenesis in 3D collagen matrix. The membrane-anchored collagenase membrane type 1 matrix metalloprotease (MT1-MMP) has been shown to play an essential role during epithelial tubulogenesis in 3D collagen matrices; however, its regulation during tubulogenesis is not understood. Here, we report that degradation of collagen in polarized epithelial cells is post-translationally regulated by changing the localization of MT1-MMP from the apical to the basal surface. MT1-MMP predominantly localizes at the apical surface in inert polarized epithelial cells, whereas treatment with HGF induced basal localization of MT1-MMP followed by collagen degradation. The basal localization of MT1-MMP requires the ectodomains of the enzyme because deletion of the MT-loop region or the hemopexin domain inhibited basal localization of the enzyme. TGFβ is a well-known inhibitor of tubulogenesis and our data indicate that its mechanism of inhibition is, at least in part, due to inhibition of MT1-MMP localization to the basal surface. Interestingly, however, the effect of TGFβ was found to be bi-phasic: at high doses it effectively inhibited basal localization of MT1-MMP, whereas at lower doses tubulogenesis and basal localization of MT1-MMP was promoted. Taken together, these data indicate that basal localization of MT1-MMP is a key factor promoting the degradation o  f extracellular matr  ix by polarized epithelial cells, and that this is an essential part of epithelial morphogenesis in 3D collagen. 
 Angiotensin II-induced MMP-2 activity and MMP-14 and basigin protein expression are mediated via the angiotensin II receptor type 1-mitogen-activated protein kinase 1 pathway in retinal pigment epithelium: implications for age-related macular degeneration. Accumulation of various lipid-rich   extracellular matrix   (ECM) deposits under the retinal pigment epithelium (RPE) has been observed in eyes with age-related macular degeneration (AMD). RPE-derived matrix metalloproteinase (MMP)-2, MMP-14, and basigin (BSG) are major enzymes involved in the maintenance of ECM turnover. Hypertension (HTN) is a systemic risk factor for AMD. It has previously been reported that angiotensin II (Ang II), one of the most important hormones associated with HTN, increases MMP-2 activity and its key regulator, MMP-14, in RPE, inducing breakdown of the RPE basement membrane, which may lead to progression of sub-RPE deposits. Ang II exerts most of its actions by activating the mitogen-activated protein kinase (MAPK) signaling pathway. Herein is explored the MAPK signaling pathway as a potential key intracellular modulator of Ang II-induced increase in MMP-2 activity and MMP-14 and BSG protein expression. It was observed that Ang II stimulates phosphorylation of extracellular signal-regulated kinase (ERK) and p38 MAPK in RPE cells and ERK/p38 and Jun N-terminal kinase (JNK) in mice. These effects were mediated by Ang II type 1 receptors. Blockade of ERK or p38 MAPK abrogated the increase in MMP-2 activity and MMP-14 and BSG proteins in ARPE-19 cells. A better understanding of the molecular events by which Ang II induces ECM dysregulation is of critical importance to further define its contribution to the progression of sub-RPE deposits in AMD patients with HTN. 
 Matrix metalloproteinase gene polymorphisms and bronchopulmonary dysplasia: identification of MMP16 as a new player in lung development. Alveolarization requires coordinated   extracellular matrix   remodeling, a process in which matrix metalloproteinases (MMPs) play an important role. We postulated that polymorphisms in MMP genes might affect MMP function in preterm lungs and thus influence the risk of bronchopulmonary dysplasia (BPD).Two hundred and eighty-four consecutive neonates with a gestational age of <28 weeks were included in this prospective study. Forty-five neonates developed BPD. Nine single-nucleotide polymorphisms (SNPs) were sought in the MMP2, MMP14 and MMP16 genes. After adjustment for birth weight and ethnic origin, the TT genotype of MMP16 C/T (rs2664352) and the GG genotype of MMP16 A/G (rs2664349) were found to protect from BPD. These genotypes were also associated with a smaller active fraction of MMP2 and with a 3-fold-lower MMP16 protein level in tracheal aspirates collected within 3 days after birth. Further evaluation of MMP16 expression during the course of normal human and rat lung development showed relatively low expression during the canalicular and saccular stages and a clear increase in both mRNA and protein levels during the alveolar stage. In two newborn rat models of arrested alveolarization the lung MMP16 mRNA level was less than 50% of normal.MMP16 may be involved in the development of lung alveoli. MMP16 polymorphisms appear to influence not only the pulmonary expression and function of MMP16 but also the risk of BPD in premature infants. 
 The transmembrane domain is essential for the microtubular trafficking of membrane type-1 matrix metalloproteinase (MT1-MMP). Membrane type-1 matrix metalloproteinase (MT1-MMP) degrades the   extracellular matrix  , initiates the activation pathway of soluble MMPs and regulates the functionality of cell adhesion signaling receptors, thus playing an important role in many cell functions. Intracellular transport mechanisms, currently incompletely understood, regulate the presentation of MT1-MMP at the cell surface. We have focused our efforts on identifying these mechanisms. To understand the transport of MT1-MMP across the cell, we used substitution and deletion mutants, the trafficking of which was examined using antibody uptake and Chariot delivery experiments. Our experiments have demonstrated that the microtubulin cytoskeleton and the centrosomes (the microtubulin cytoskeleton-organizing centers) are essential for the trafficking and the internalization of MT1-MMP. We determined that after reaching the plasma membrane, MT1-MMP is internalized in the Rab-4-positive recycling endosomes and the Rab-11-positive pericentrosomal recycling endosomes. The microtubular trafficking causes the protease to accumulate in the pericentrosomal region of the cell. We believe that the presence of the transmembrane domain is required for the microtubular vesicular trafficking of MT1-MMP because the soluble mutants are not presented at the cell surface and they are not delivered to the centrosomes. The observed transport mechanisms provide a vehicle for the intracellular targets and, accordingly, for an intracellular cleavage function of MT1-MMP in malignant cells, which routinely overexpress this protease. 
 Characterization of stromelysin 1 (MMP-3), matrilysin (MMP-7), and membrane type 1 matrix metalloproteinase (MT1-MMP) derived fibrin(ogen) fragments D-dimer and D-like monomer: NH2-terminal sequences of late-stage digest fragments. Matrix metalloproteinases (MMPs) participate in physiological remodeling of the   extracellular matrix  . Recently we determined that both fibrinogen (Fg) and cross-linked fibrin (XL-Fb) are substrates for selected MMPs. Specifically, XL-Fb clots were solubilized by MMP-3 (stromelysin 1) by cleavage at gamma Gly 404-Ala 405, resulting in a D-like monomer fragment. Similarly, MMP-7 (matrilysin) and MT1-MMP (membrane type 1 matrix metalloproteinase) solubilized XL-Fb clots. However, the molecular mass of fragment D-dimer, obtained after MMP-7 and MT1-MMP degradation of XL-Fb, is similar to that of fragment D-dimer from plasmin degradation ( approximately 186 kDa). In contrast, fragment D-like monomer, from MMP-3 degradation of both fibrinogen (Fg) and XL-Fb, is similar to fragment D from plasmin degradation of Fg ( approximately 94 kDa). Reduced chains from MMP-3, MMP-7, and MT1-MMP digests of Fg and XL-Fb were subjected to direct sequence analyses and D/D-dimer alpha-chain showed cleavage at both alpha Asp 97-Phe 98 and alpha Asn 102-Asn 103. Degradation of the beta-chain resulted in microheterogeneity of cleavage sites at beta Asp 123-Leu 124, beta Asn 137-Val 138, and beta Glu 141-Tyr 142, whereas all three enzymes cleaved the gamma-chain at gamma Thr 83-Leu 84. In both Fg and XL-Fb, several cleavage sites obtained by proteolysis with MMP-3, MMP-7, and MT1-MMP were found to be in very close proximity to those obtained by plasmin on these same substrates. That does not occur with other MMPs such as MMP-1, -2, and -9 and MT2-MMP. The degradation of XL-Fb by MMPs suggests both plasmin-dependent and independent mechanisms of fibrinolysis that might be relevant in inflammation, angiogenesis, arthritis, and atherosclerosis. 
 Structural differences of matrix metalloproteinases. Homology modeling and energy minimization of enzyme-substrate complexes. Matrix metalloproteinases are extracellular enzymes taking part in the remodeling of   extracellular matrix  . The structures of the catalytic domain of MMP1, MMP3, MMP7 and MMP8 are known, but structures of enzymes belonging to this family still remain to be determined. A general approach to the homology modeling of matrix metalloproteinases, exemplified by the modeling of MMP2, MMP9, MMP12 and MMP14 is described. The models were refined using an energy minimization procedure developed for matrix metalloproteinases. This procedure includes incorporation of parameters for zinc and calcium ions in the AMBER 4.1 force field, applying a non-bonded approach and a full ion charge representation. Energy minimization of the apoenzymes yielded structures with distorted active sites, while reliable three-dimensional structures of the enzymes containing a substrate in active site were obtained. The structural differences between the eight enzyme-substrate complexes were studied with particular emphasis on the active site, and possible sites for obtaining selectivity among the MMP's are discussed. Differences in the P1' pocket are well-documented and have been extensively exploited in inhibitor design. The present work indicates that selectivity could be further improved by considering the P2 pocket as well. 
 Golgi reassembly stacking protein 55 interacts with membrane-type (MT) 1-matrix metalloprotease (MMP) and furin and plays a role in the activation of the MT1-MMP zymogen. Membrane-type 1 matrix metalloproteinase (MT1-MMP) is a proteinase involved in the remodelling of   extracellular matrix   and the cleavage of a number of substrates. MT1-MMP is synthesized as a zymogen that requires intracellular post-translational cleavage to gain biological activity. Furin, a member of the pro-protein convertase family, has been implicated in the proteolytic removal of the MT1-MMP prodomain sequence. In the present study, we demonstrate a role for the peripheral Golgi matrix protein GRASP55 in the furin-dependent activation of MT1-MMP. MT1-MMP and furin were found to co-localize with Golgi reassembly stacking protein 55 (GRASP55). Further analysis revealed that GRASP55 associated with the cytoplasmic domain of both proteases and that the LLY(573) motif in the MT1-MMP intracellular domain was crucial for the interaction with GRASP55. Overexpression of GRASP55 was found to enhance the formation of a complex between MT1-MMP and furin. Finally, we report that disruption of the interaction between GRASP55 and furin led to a reduction in pro-MT1-MMP activation. Taken together, these data suggest that GRASP55 may function as an adaptor protein coupling MT1-MMP with furin, thus leading to the activation of the zymogen. 
 MT1-MMP releases latent TGF-beta1 from endothelial cell   extracellular matrix   via proteolytic processing of LTBP-1. Targeting of transforming growth factor beta (TGF-beta) to the   extracellular matrix   (ECM) by latent TGF-beta binding proteins (LTBPs) regulates the availability of TGF-beta for interactions with endothelial cells during their quiescence and activation. However, the mechanisms which release TGF-beta complexes from the ECM need elucidation. We find here that morphological activation of endothelial cells by phorbol 12-myristate 13-acetate (PMA) resulted in membrane-type 1 matrix metalloproteinase (MT1-MMP) -mediated solubilization of latent TGF-beta complexes from the ECM by proteolytic processing of LTBP-1. These processes required the activities of PKC and ERK1/2 signaling pathways and were coupled with markedly increased MT1-MMP expression. The functional role of MT1-MMP in LTBP-1 release was demonstrated by gene silencing using lentiviral short-hairpin RNA as well as by the inhibition with tissue inhibitors of metalloproteinases, TIMP-2 and TIMP-3. Negligible effects of TIMP-1 and uPA/plasmin system inhibitors indicated that secreted MMPs or uPA/plasmin system did not contribute to the release of LTBP-1. Current results identify MT1-MMP-mediated proteolytic processing of ECM-bound LTBP-1 as a mechanism to release latent TGF-beta from the subendothelial matrix. 
 Dimerization of MT1-MMP during cellular invasion detected by fluorescence resonance energy transfer. Homodimerization of the membrane-bound collagenase MT1-MMP [membrane-type 1 MMP (matrix metalloproteinase)] is crucial for its collagenolytic activity. However, it is not clear whether this dimerization is regulated during cellular invasion into three-dimensional collagen matrices. To address this question, we established a fluorescence resonance energy transfer system to detect MT1-MMP dimerization and analysed the process in cells invading through three-dimensional collagen. Our data indicate that dimerization occurs dynamically and constantly at the leading edge of migrating cells, but not the trailing edge. We found that polarized dimerization was not due to ECM (  extracellular matrix  ) attachment, but was rather controlled by reorganization of the actin cytoskeleton by the small GTPases, Cdc42 (cell division cycle 42) and Rac1. Our data indicate that cell-surface collagenolytic activity is regulated co-ordinately with cell migration events to enable penetration of the matrix physical barrier. 
 Single nucleotide polymorphisms and haplotypes of MMP-14 are associated with the risk and pathological development of oral cancer. Matrix metalloproteinase (MMP)-14 is one of the pericellular collagenases to degrade   extracellular matrix   (ECM), which is involved to the modulation of susceptibility or clinicopathological features of a cancer. The contributions of MMP-14 on the susceptibility or clinicopathological features of certain cancers have been well documented, and the expression of MMP-14 in oral squamous cell carcinoma (OSCC) also has been observed. This study was designed to examine the association of MMP-14 gene polymorphisms with the susceptibility and clinicopathological development of OSCC.A total of 363 patients with OSCC and 506 healthy control subjects were recruited. Six single nucleotide polymorphisms (SNPs) of MMP-14 genes were analyzed by polymerase chain reaction-restriction fragment length polymorphism genotyping and haplotype-base analysis.MMP-14 +7096 TC/CC genotypes might lower the risk of OSCC, and MMP-14 +6767 GA/AA genotypes cause a poor clinical status in OSCC patients. The +6727 C: +6767 G: +7096 T: +8153 G haplotype and diplotype increased the risk for OSCC by 1.706-fold (95% confidence interval (CI) 1.383-2.105) and 2.276-fold (95% CI = 1.531-3.384), respectively, compared with the reference. The diplotype with at least one CGTG exhibited a high risk (adjusted odds ratio, 1.639; 95% CI, 1.005-2.673) for developing a poor clinicopathological diagnosis of OSCC compared with the others/other diplotype.The +7096 and +6767 polymorphic genotypes and haplotype +6727 C: +6767 G: +7096 T: +8153 G of MMP-14 gene might contribute to the prediction of susceptibility and pathological development of OSCC. 
 Inhibition of matrix metalloproteinase 14 (MMP-14)-mediated cancer cell migration. Matrix metalloproteinases (MMPs) have been shown to be key players in both   extracellular matrix   remodeling and cell migration during cancer metastasis. MMP-14, a membrane-anchored MMP, in particular, is closely associated with these processes. The hemopexin (PEX) domain of MMP-14 has been proposed as the modulating region involved in the molecular cross-talk that initiates cell migration through homodimerization of MMP-14 as well as heterodimerization with the cell surface adhesion molecule CD44. In this study, minimal regions required for function within the PEX domain were investigated through a series of substitution mutations. Blades I and IV were found to be involved in cell migration. We found that blade IV is necessary for MMP-14 homodimerization and that blade I is required for CD44 MMP-14 heterodimerization. Cross-talk between MMP-14 and CD44 results in phosphorylation of EGF receptor and downstream activation of the MAPK and PI3K signaling pathways involved in cell migration. Based on these mutagenesis analyses, peptides mimicking the essential outermost strand motifs within the PEX domain of MMP-14 were designed. These synthetic peptides inhibit MMP-14-enhanced cell migration in a dose-dependent manner but have no effect on the function of other MMPs. Furthermore, these peptides interfere with cancer metastasis without affecting primary tumor growth. Thus, targeting the MMP-14 hemopexin domain represents a novel approach to inhibit MMP-14-mediated cancer dissemination. 
 Rho-ROCK-myosin signaling mediates membrane type 1 matrix metalloproteinase-induced cellular aggregation of keratinocytes. Membrane type 1-matrix metalloproteinase (MT1-MMP, MMP14), which is associated with   extracellular matrix   (ECM) breakdown in squamous cell carcinoma (SCC), promotes tumor formation and epithelial-mesenchymal transition. However, in this report we demonstrate that MT1-MMP, by cleaving the underlying ECM, causes cellular aggregation of keratinocytes and SCC cells. Treatment with an MMP inhibitor abrogated MT1-MMP-induced phenotypic changes, but decreasing E-cadherin expression did not affect MT1-MMP-induced cellular aggregation. As ROCK1/2 can regulate cell-cell and cell-ECM interaction, we examined its role in mediating MT1-MMP-induced phenotypic changes. Blocking ROCK1/2 expression or activity abrogated the cellular aggregation resulting from MT1-MMP expression. Additionally, blocking Rho and non-muscle myosin attenuated MT1-MMP-induced phenotypic changes. Moreover, SCC cells expressing only the catalytically active MT1-MMP protein demonstrated increased cellular aggregation and increased myosin II activity in vivo when injected subcutaneously into nude mice. Together, these results demonstrate that expression of MT1-MMP may be anti-tumorigenic in keratinocytes by promoting cellular aggregation. 
 Mayer-Rokitansky-Küster-Hauser syndrome discordance in monozygotic twins: matrix metalloproteinase 14, low-density lipoprotein receptor-related protein 10,   extracellular matri  x, and neoangiogenesis genes identified as candidate genes in a tissue-specific mosaicism. To find a potential underlying cause for Mayer-Rokitansky-Küster-Hauser syndrome (MRKHS) discordance in monozygotic twins.Prospective comparative study.University hospital.Our study genetically analyzed 5 MRKHS-discordant monozygotic twin pairs with the unique opportunity to include saliva and rudimentary uterine tissue.Blood, saliva, or rudimentary uterine tissue from five MRKHS-discordant twins was analyzed and compared between twin pairs as well as within the same individual where applicable. We used copy number variations (CNVs) to identify differences.CNVs in blood, rudimentary uterine tissue, and saliva, network analysis, and review of the literature.One duplication found in the affected twin included two genes, matrix metalloproteinase 14 (MMP14) and low-density lipoprotein receptor-related protein 10 (LRP10), which have known functions in the embryonic development of the uterus and endometrium. The duplicated region was detected in rudimentary uterine tissue from the same individual but not in saliva, making a tissue-specific mosaicism a possible explanation for twin discordance. Additional network analysis revealed important connections to differentially expressed genes from previous studies. These genes encode several molecules involved i  n extracellular matr  ix (ECM) remodeling and neoangiogenesis.MMP-14, LRP-10, ECM, and neoangiogenesis genes are identified as candidate genes in a tissue-specific mosaicism. The detected clusters provide evidence of deficient vascularization during uterine development and/or disturbed reorganization of ECM components, potentially during müllerian duct elongation signaled by the embryologically relevant phosphatidylinositol 3-kinase/protein kinase B pathway. Therefore, we consider these genes to be new candidates in the manifestation of MRKHS. 
 Caveolin-1 inhibits membrane-type 1 matrix metalloproteinase activity. Membrane-type 1 matrix metalloproteinase (MT1-MMP) is a zinc-dependent proteinase found in cholesterol-rich lipid rafts on the plasma membrane. MT1-MMP hydrolyzes   extracellular matrix   (ECM) proteins, activates pro-matrix metalloproteinase-2 (proMMP-2) and plays an important role in ECM remodeling, cancer cell migration and metastasis. The role of caveolin-1, an integral protein of caveolae, in the activation of MT1-MMP remains largely unknown. Here, we show that the expression of caveolin-1 attenuates the activation of proMMP-2, reduces proteolytic cleavage of ECM and inhibits cell migration. We utilized the cytoplasmic tail domain deletion (DeltaCT) or the E240A mutant of MT1-MMP. Co-expression of caveolin-1 with the wild-type or the DeltaCT MT1-MMP decreased the proMMP-2 activation and inhibited collagen degradation and cell migration. Caveolin-1 had no effect on the catalytically inert E240A MT1-MMP. Our findings suggest that caveolin-1 is essential in the down-regulation of MT1-MMP activity by promoting internalization from the cell surface. 
 Multi-step pericellular proteolysis controls the transition from individual to collective cancer cell invasion. Invasive cell migration through tissue barriers requires pericellular remodelling of   extracellular matrix   (ECM) executed by cell-surface proteases, particularly membrane-type-1 matrix metalloproteinase (MT1-MMP/MMP-14). Using time-resolved multimodal microscopy, we show how invasive HT-1080 fibrosarcoma and MDA-MB-231 breast cancer cells coordinate mechanotransduction and fibrillar collagen remodelling by segregating the anterior force-generating leading edge containing beta1 integrin, MT1-MMP and F-actin from a posterior proteolytic zone executing fibre breakdown. During forward movement, sterically impeding fibres are selectively realigned into microtracks of single-cell calibre. Microtracks become expanded by multiple following cells by means of the large-scale degradation of lateral ECM interfaces, ultimately prompting transition towards collective invasion similar to that in vivo. Both ECM track widening and transition to multicellular invasion are dependent on MT1-MMP-mediated collagenolysis, shown by broad-spectrum protease inhibition and RNA interference. Thus, invasive migration and proteolytic ECM remodelling are interdependent processes that control tissue micropatterning and macropatterning and, consequently, individual and collective cell migration. 
 Membrane-type 1 matrix metalloproteinase regulates cell migration during zebrafish gastrulation: evidence for an interaction with non-canonical Wnt signaling. Key to invasiveness is the ability of tumor cells to modify the   extracellular matrix  , become motile, and engage in directed migration towards the vasculature. One significant protein associated with metastatic progression is membrane-type 1 matrix metalloproteinase (MT1-MMP/MMP14). How MMP14 activity is coordinated with other signaling pathways to regulate cell migration in vivo is largely unknown. Here we have used zebrafish embryogenesis as a model to understand the potential relationship between MMP14-dependent pericellular proteolysis, cell polarity, and motility. Knockdown of zebrafish Mmp14 function disrupted gastrulation convergence and extension cell movements and craniofacial morphogenesis. Using time-lapse imaging and morphometric analyses, we show that Mmp14 is required for proper cell polarity underlying the directed migration of mesodermal cells during gastrulation. We have identified a genetic interaction between mmp14 and non-canonical Wnt signaling, a pathway that also regulates cell polarity in embryonic tissues and is increasingly being linked with tumor cell migration. Finally, we demonstrate that Van Gogh-like 2, a key regulator of the non-canonical Wnt pathway, co-localizes with MMP14 and becomes redistributed towards the leading edge of polarized human cancer cells. Together, our results support the notion that pathways regulating pericellular proteolysis and cell polarity converge to promote efficient cell migration. 
 Gene polymorphisms and antigen levels of matrix metalloproteinase-1 in type 2 diabetes mellitus coexisting with coronary heart disease. Diabetes mellitus is a major risk factor for coronary heart disease (CHD). Matrix metalloproteinases (MMPs) can play a pivotal role in the remodelling of   extracellular matrix   associated with the development of atherosclerosis. Therefore, the aim of the study was to compare the distribution of genotypes and frequency of alleles of two polymorphisms of the MMP-1 gene promoter, an A/G substitution and a 1G/2G insertion, in correlation with antigen levels of matrix metalloproteinase-1 (MMP-1) in type 2 diabetic patients with or without CHD as well as individuals with normal glucose level without CHD.Genotypes of 115 patients with type 2 diabetes mellitus (T2DM) and a subpopulation of 66 patients with coexisting CHD as well as 120 non-diabetic control subjects were determined by PCR-based restriction fragment length polymorphism (PCR-RFLP).We demonstrated that antigen levels of MMP-1 in the serum of diabetic patients were significantly higher than those of individuals with normal glucose metabolism (p <0.05). Elevated levels of MMP-1 positively correlated with CHD occurrence in T2DM patients (p <0.01). The distribution of genotypes revealed higher frequency of the 2G/2G polymorphism variant in diabetic patients with CHD [OR 5.76, 95% CI (1.24; 26.87)], thus suggesting its strong association with high level of MMP-1. In T2DM patients with coexisting CHD, a higher frequency of the 2G allele of 1G/2G [OR 1.74, CI 95% (1.01; 2.99)] and the G allele of A/G polymorphism [OR 2.15, 95% CI (1.22; 3.80)] was also found.Our results suggest that type 2 diabetes mellitus is linked with elevated blood level of MMP-1, and polymorphisms of the promoter region of its gene might be associated with CHD. 
 Matrix metalloproteinases 19 and 20 cleave aggrecan and cartilage oligomeric matrix protein (COMP). Matrix metalloproteinase (MMP)-19 and MMP-20 (enamelysin) are two recently discovered members of the MMP family. These enzymes are involved in the degradation of the various components of the   extracellular matrix   (ECM) during development, haemostasis and pathological conditions. Whereas MMP-19 mRNA is found widely expressed in body tissues, including the synovium of normal and rheumatoid arthritic patients, MMP-20 expression is restricted to the enamel organ. In this study we investigated the ability of MMP-19 and MMP-20 to cleave two of the macromolecules characterising the cartilage ECM, namely aggrecan and the cartilage oligomeric matrix protein (COMP). Both MMPs hydrolysed aggrecan efficiently at the well-described MMP cleavage site between residues Asn(341) and Phe(342), as shown by Western blotting using neo-epitope antibodies. Furthermore, the two enzymes cleaved COMP in a distinctive manner, generating a major proteolytic product of 60 kDa. Our results suggest that MMP-19 may participate in the degradation of aggrecan and COMP in arthritic disease, whereas MMP-20, due to its unique expression pattern, may primarily be involved in the turnover of these molecules during tooth development. 
 Expression and prognostic role of MMP2, MMP9, MMP13, and MMP14 matrix metalloproteinases in sinonasal and oral malignant melanomas. Sinonasal and oral malignant melanomas are rare malignancies accounting for less than 2% of all melanomas. Matrix metalloproteinases (MMPs) are proteolytic enzymes required for   extracellular matrix   degradation in a variety of physiological and pathologic processes including wound healing, embryogenesis, tumor invasion, and metastases. We studied the correlation between expression of MMPs, nucleolar diameter of melanoma cells, different clinical and histologic parameters, and patient's outcome. Seventeen cases of sinonasal and oral malignant melanoma were studied. The expression of MMP2, MMP9, MMP13, and MMP14 was assessed immunohistochemically on paraffinized sections and measured by computer morphometry as well as silver-stained nucleolar diameter. A significant correlation was found between MMP2 and MMP14 expression and patient's outcome. Greater overall survival was seen in patients with average MMP2 expression less than 8000 microm(2)/x20 high-power field (P = .016). In patients with negative MMP14 staining, survival rate by the end of the follow-up was 38% compared with patients with positive MMP14 staining where survival rate was 0 (P = .03). A correlation with age at onset was also found; patients younger than 66 years had better overall survival rates than patients aged 66 years or older (P = .03). The maximal nucleolar diameter (MaxND) was another parameter that significantly correlated with clinical outcome. Patients with MaxND of 8 microm or larger showed a significant worse prognosis compared with the group with MaxND less than 8 microm (P = .0009). Our pilot study demonstrates that MMP2, MMP14, MMP9, and MaxND might be used as prognostic markers in patients with sinonasal and oral malignant melanoma. 
 Macrophages produce TGF-beta-induced (beta-ig-h3) following ingestion of apoptotic cells and regulate MMP14 levels and collagen turnover in fibroblasts. Phagocytic clearance of apoptotic cells by macrophages is an essential part in the resolution of inflammation. It coincides with activation of repair mechanisms, including accumulation of   extracellular matrix  . A possible link between clearance of apoptotic debris and accumulation of   extracellular matrix   has not been investigated. Production of collagen was measured in primary fibroblasts cocultured with macrophages. Ingestion of apoptotic cells by monocyte-derived macrophages led to up-regulation of collagen. Direct contact between macrophages and fibroblasts was not required for collagen up-regulation. Macrophages produced TGF-beta following ingestion of apoptotic cells, but the levels of this cytokine were lower than those required for a significant up-regulation of collagen. Simultaneously, the levels of TGF-beta-induced (TGFBI), or keratoepithelin/BIGH3, mRNA and protein were increased. In contrast, primary alveolar macrophages stimulated collagen production without exposure to apoptotic cells; there was no further increase in the levels of TGFBI, mRNA or protein, or collagen after ingestion of apoptotic cells. Stimulation of fibroblasts with TGFBI down-regulated MMP14 levels, decreased DNA binding by p53, increased DNA binding by PU.1, and up-regulated collagen protein but not mRNA levels. Overexpression of MMP14 or p53, or small interfering RNA-mediated inhibition of PU.1 led to an increase in MMP14 and a decline in collagen levels, whereas small interfering RNA-mediated inhibition of MMP14 led to elevation of collagen levels. In conclusion, monocyte-derived but not alveolar macrophages produce TGFBI following ingestion of apoptotic cells, leading to the down-regulation of MMP14 levels in fibroblasts through a mechanism involving p53 and PU.1, and to subsequent accumulation of collagen. 
 Regulation of vascular smooth muscle cell expression and function of matrix metalloproteinases is mediated by estrogen and progesterone exposure. Postmenopausal women receiving hormone replacement therapy (HRT) have been reported to have more adverse outcomes after vascular reconstructions, including increased intimal hyperplasia development and bypass graft failure. HRT may be affecting the pathway contributing to intimal hyperplasia. An important component of this pathway involves matrix metalloproteinases (MMPs), implicated in vascular remodeling due to their ability to degrade components of the   extracellular matrix  . We hypothesize that estrogen (Est) and progesterone (Prog) upregulate the MMP pathway in vascular smooth muscle cells (VSMCs) thereby increasing MMP activity and function.VSMCs were incubated with Est (5 ng/mL), Prog (50 ng/mL), Est + Prog combination (Est/Prog), and/or doxycycline (40 microg/mL; Doxy). Using reverse transcriptase polymerase chain reaction (RT-PCR) analysis we have previously shown membrane type 1-MMP (MT1-MMP) messenger ribonucleic acid (mRNA) levels are significantly increased by Est. Here, Western blot analyses indicated MT1-MMP and MMP-2 protein levels, not tissue inhibitor of MMP-2 (TIMP-2), were increased in response to Est and Est/Prog (P < .05 vs control). In-gel zymography revealed that Est and Est/Prog resulted in increased MMP-2 activity (hormone groups, P < .05 vs control) with no significant difference among the hormone groups. VSMC migration was increased by 45 +/- 14% in response to Est (P < .05 vs control), as measured using a modified Boyden chamber assay. Doxycycline significantly inhibited basal and Est/Prog-stimulated increases in MMP-2 activity (P < .05 vs control; P < .05 vs hormone groups), and partially blocked basal and hormonally stimulated migration (P < .05 vs control and Est).Estrogen and progesterone affects the MMP pathway by increasing MMP-2 enzymatic activity, possibly via the upregulation of MT1-MMP expression without a corresponding increase in TIMP expression. This increased collagenase activity increases VSMC motility and their ability to migrate through a collagen type IV lattice. Est/Prog upregulation of MT1-MMP may contribute to the adverse effect of HRT on vascular interventions. 
 Crystal structure of the complex formed by the membrane type 1-matrix metalloproteinase with the tissue inhibitor of metalloproteinases-2, the soluble progelatinase A receptor. The proteolytic activity of matrix metalloproteinases (MMPs) towards   extracellular matrix   components is held in check by the tissue inhibitors of metalloproteinases (TIMPs). The binary complex of TIMP-2 and membrane-type-1 MMP (MT1-MMP) forms a cell surface located 'receptor' involved in pro-MMP-2 activation. We have solved the 2.75 A crystal structure of the complex between the catalytic domain of human MT1-MMP (cdMT1-MMP) and bovine TIMP-2. In comparison with our previously determined MMP-3-TIMP-1 complex, both proteins are considerably tilted to one another and show new features. CdMT1-MMP, apart from exhibiting the classical MMP fold, displays two large insertions remote from the active-site cleft that might be important for interaction with macromolecular substrates. The TIMP-2 polypeptide chain, as in TIMP-1, folds into a continuous wedge; the A-B edge loop is much more elongated and tilted, however, wrapping around the S-loop and the beta-sheet rim of the MT1-MMP. In addition, both C-terminal edge loops make more interactions with the target enzyme. The C-terminal acidic tail of TIMP-2 is disordered but might adopt a defined structure upon binding to pro-MMP-2; the Ser2 side-chain of TIMP-2 extends into the voluminous S1' specificity pocket of cdMT1-MMP, with its Ogamma pointing towards the carboxylate of the catalytic Glu240. The lower affinity of TIMP-1 for MT1-MMP compared with TIMP-2 might be explained by a reduced number of favourable interactions. 
 Visualization of polarized membrane type 1 matrix metalloproteinase activity in live cells by fluorescence resonance energy transfer imaging. Membrane type 1 matrix metalloproteinase (MT1-MMP) plays a critical role in cancer cell biology by proteolytically remodeling the   extracellular matrix  . Utilizing fluorescence resonance energy transfer (FRET) imaging, we have developed a novel biosensor, with its sensing element anchoring at the extracellular surface of cell membrane, to visualize MT1-MMP activity dynamically in live cells with subcellular resolution. Epidermal growth factor (EGF) induced significant FRET changes in cancer cells expressing MT1-MMP, but not in MT1-MMP-deficient cells. EGF-induced FRET changes in MT1-MMP-deficient cells could be restored after reconstituting with wild-type MT1-MMP, but not MMP-2, MMP-9, or inactive MT1-MMP mutants. Deletion of the transmembrane domain in the biosensor or treatment with tissue inhibitor of metalloproteinase-2, a cell-impermeable MT1-MMP inhibitor, abolished the EGF-induced FRET response, indicating that MT1-MMP acts at the cell surface to generate FRET changes. In response to EGF, active MT1-MMP was directed to the leading edge of migrating cells along micropatterned fibronectin stripes, in tandem with the local accumulation of the EGF receptor, via a process dependent upon an intact cytoskeletal network. Hence, the MT1-MMP biosensor provides a powerful tool for characterizing the molecular processes underlying the spatiotemporal regulation of this critical class of enzymes. 
 Low collagenase-1 (MMP-1) and MT1-MMP expression levels are favourable survival markers in advanced colorectal carcinoma.   Extracellular matrix   degradation is required for invasive growth and metastasis formation in colorectal carcinoma; therefore, we examined matrix metalloproteinases expression (MMP-1, MMP-13 and MT1-MMP) and apoptosis in tumours from 49 patients with advanced colorectal disease.MMP expression was determined immunohistochemically and apoptotic index (AI) was ascertained using the TUNEL assay.Low levels of MT1-MMP, MMP-1 and AI were found to be favourable markers significantly associated with longer survival. MT1-MMP expression levels below the median (</=14.0% of tumour cells) were associated with better overall survival (median 26.2 vs. 15.6 months, p = 0.02) and MMP-1 expression levels below the median (</=28.7% of tumour cells) correlated with longer survival following metastasis (median 21.5 vs. 13.3 months, p = 0.05). MT1-MMP, MMP-1 and AI were all found to have significant independent effects on survival. Interestingly, MMP-1 expression levels above the median were associated with distal disease of the colon (p = 0.02).These findings reveal that MT1-MMP and MMP-1 expression levels and AI are useful prognostic indicators in advanced colorectal carcinoma and suggest that markers of MMP expression might be used in identifying patients who would benefit from new treatment modalities involving MMP inhibitors. 
 Matrix metalloproteinase-14 is a mechanically regulated activator of secreted MMPs and invasion. Matrix metalloproteinases (MMPs) are   extracellular matrix   (ECM) degrading enzymes and have complex and specific regulation networks. This includes activation interactions, where one MMP family member activates another. ECM degradation and MMP activation can be initiated by several different stimuli including changes in ECM mechanical properties or intracellular contractility. These mechanical stimuli are known enhancers of metastatic potential. MMP-14 facilitates local ECM degradation and is well known as a major mediator of cell migration, angiogenesis and invasion. Recently, function blocking antibodies have been developed to specifically block MMP-14, providing a useful tool for research as well as therapeutic applications. Here we utilize a selective MMP-14 function blocking antibody to delineate the role of MMP-14 as an activator of other MMPs in response to changes in cellular contractility and ECM stiffness. Inhibition using function blocking antibodies reveals that MMP-14 activates soluble MMPs like MMP-2 and -9 under various mechanical stimuli in the pancreatic cancer cell line, Panc-1. In addition, inhibition of MMP-14 abates Panc-1 cell extension into 3D gels to levels seen with non-specific pan-MMP inhibitors at higher concentrations. This strengthens the case for MMP function blocking antibodies as more potent and specific MMP inhibition therapeutics. 
 Membrane-type matrix metalloproteinases 1 and 2 exhibit broad-spectrum proteolytic capacities comparable to many matrix metalloproteinases. Soluble proenzyme forms of the catalytic domains of membrane-type matrix metalloproteinases 1 and 2 (MT1-MMP and MT2-MMP) and a form of MT1-MMP containing the catalytic and hemopexin domains were expressed as soluble recombinant proteins. Purified, activated forms of the MT-MMP were shown to degrade fibronectin, tenascin, nidogen, aggrecan and perlecan. Only MT2-MMP showed activity against laminin. MT1-MMP retaining the hemopexin domain was able to specifically cleave native type-I and type-III collagens into the 3/4-1/4 fragments typical of the specific collagenases. The catalytic domain alone did not retain this activity. The MT-MMP did not degrade interleukin-1beta, but, similarly to many other MMP, could process a pro [tumor necrosis factor (TNF) alpha] fusion protein to release mature TNF. However, the latter was subsequently degraded into smaller fragments. These results demonstrate that, in addition to their ability to activate other MMP, such as progelatinase A/proMMP2 and procollagenase-3/proMMP13, MT-MMP degrade a number of   extracellular matrix   macromolecules. Their location at the surface of cells implies that they could play a significant role in the modulation of cell-matrix interactions. 
 Secretion of active membrane type 1 matrix metalloproteinase (MMP-14) into extracellular space in microvesicular exosomes. Membrane type 1 matrix metalloproteinase (MT1-MMP, MMP14) is an efficient   extracellular matrix   (ECM) degrading enzyme that plays important roles in tissue homeostasis and cell invasion. Like a number of type I membrane proteins, MT1-MMP can be internalized from the cell surface through early and recycling endosomes to late endosomes, and recycled to the plasma membrane. Late endosomes participate in the biogenesis of small (30-100 nm) vesicles, exosomes, which redirect plasma membrane proteins for extracellular secretion. We hypothesized that some of the endosomal MT1-MMP could be directed to exosomes for extracellular release. Using cultured human fibrosarcoma (HT-1080) and melanoma (G361) cells we provide evidence that both the full-length 60 kDa and the proteolytically processed 43 kDa forms of MT1-MMP are secreted in exosomes. The isolated exosomes were identified by their vesicular structure in electron microscopy and by exosomal marker proteins CD9 and tumor susceptibility gene (TSG101). Furthermore, exosomes contained beta1-integrin (CD29). The exosomes were able to activate pro-MMP-2 and degrade type 1 collagen and gelatin, suggesting that the exosomal MT1-MMP was functionally active. The targeting of MT1-MMP in exosomes represents a novel mechanism for cancer cells to secrete membrane type metalloproteolytic activity into the extracellular space. 
 Lipid rafts and caveolin-1 are required for invadopodia formation and   extracellular matrix   degradation by human breast cancer cells. Invadopodia are ventral membrane protrusions through which invasive cancer cells degrade the   extracellular matrix  . They are thought to function in the migration of cancer cells through tissue barriers, which is necessary for cancer invasion and metastasis. Although many protein components of invadopodia have been identified, the organization and the role of membrane lipids in invadopodia are not well understood. In this study, the role of lipid rafts, which are cholesterol-enriched membrane microdomains, in the assembly and function of invadopodia in human breast cancer cells was investigated. Lipid rafts are enriched, internalized, and dynamically trafficked at invadopodia sites. Perturbation of lipid raft formation due to depleting or sequestering membrane cholesterol blocked the invadopodia-mediated degradation of the gelatin matrix. Caveolin-1 (Cav-1), a resident protein of lipid rafts and caveolae, accumulates at invadopodia and colocalizes with the internalized lipid raft membranes. Membrane type 1 matrix metalloproteinase (MT1-MMP), a matrix proteinase associated with invadopodia, is localized at lipid raft-enriched membrane fractions and cotrafficked and colocalized with Cav-1 at invadopodia. The small interfering RNA-mediated silencing of Cav-1 inhibited the invadopodia-mediated and MT1-MMP-dependent degradation of the gelatin matrix. Furthermore, Cav-1 and MT1-MMP are coexpressed in invasive human breast cancer cell lines that have an ability to form invadopodia. These results indicate that invadopodia are the sites where enrichment and trafficking of lipid rafts occur and that Cav-1 is an essential regulator of MT1-MMP function and invadopodia-mediated breast cancer cell invasion. 
 Relation between laminin-5 gamma 2 chain and cell surface metalloproteinase MT1-MMP in clear cell carcinoma of the ovary. Ovarian clear cell carcinoma (CCC) characteristically shows stromal accumulation of the basement membrane material laminin-5 (LN-5). LN-5 works not only as a substrate for cell anchorage but also as a stimulator for cell migration. Our previous study showed that CCC cell increased migration over excessive LN-5 in vitro. However, it remains unclear why LN-5 rather promotes cell migration than cell anchorage. A recent study has shown that the processing of the LN-5 gamma 2 chain by membrane type 1-matrix metalloproteinase (MT1-MMP) was responsible for increasing cell migration activity but reducing cell anchoring activity of LN-5. In the present study, 3 CCC cell lines were examined for LN-5 gamma 2 chain levels by Western blotting. Processed gamma 2 chain (105 kDa) was detected in the   extracellular matrix   of all 3 CCC cell lines in vitro. Immunocytochemically they expressed MT1-MMP on the cell surface. In surgical specimens, 22 of 30 (73%) CCCs showed membranous expression of MT1-MMP, and stromal accumulation of the LN-5 gamma 2 chain, especially in the area of papillary architecture. These results indicate that proteolytic processing of the LN-5 gamma 2 chain by MT1-MMP is involved in cell migration and subsequent architectural organization in ovarian CCC. 
 Neoplastic progression of the human breast cancer cell line G3S1 is associated with elevation of cytoskeletal dynamics and upregulation of MT1-MMP. The newly established breast cancer cell line G3S1, derived from EM-G3 breast cancer progenitors, was analyzed for functional changes related to neoplastic progression manifested by elevated invasiveness and enhanced capability to degrade gelatin. Degradation of gelatin and invasiveness of G3S1 cells was found to be dependent on the activity of matrix proteinases and actin cytoskeletal dynamics. Therefore, the expression and activity of these proteases was compared in G3S1 and EM-G3 cells. Despite enhanced capability of G3S1 cells to degrade gelatin, these cells exhibited lower levels of secreted   extracellular matrix   degrading proteases than parental EM-G3 cells. However, the expression of membrane-bound MT1-MMP was strongly elevated in G3S1 cells. While the degradation of gelatin was associated with invadopodia-like structures in both EM-G3 and G3S1 cells, the cytoskeletal remodeling dynamics was greatly elevated in G3S1 cells, suggesting that upregulation of MT1-MMP, together with elevation of cytoskeletal remodeling dynamics can effectively cause elevated invasiveness and enhanced matrix degrading capability in G3S1 cells. 
 The hemopexin-like C-terminal domain of membrane type 1 matrix metalloproteinase regulates proteolysis of a multifunctional protein, gC1qR. Matrix metalloproteinases (MMPs) including membrane type 1 MMP (MT1-MMP) can degrade   extracellular matrix   and cell surface receptor molecules and have an essential function in malignancy. Recently, we established a functional link between MT1-MMP and the receptor of complement component 1q (gC1qR). The gC1qR is known as a compartment-specific regulator of diverse cellular and viral proteins. Once released by proliferating cells, soluble gC1qR may inhibit complement component 1q hemolytic activity and play important roles in vivo in assisting tumor cells to evade destruction by complement. Here, we report that gC1qR is susceptible to MT1-MMP proteolysis in vitro and in cell cultures. The major MT1-MMP cleavage site (Gly(79) down arrow Gln(80)) is localized within the structurally disordered loop connecting the beta(3) and the beta(4) strands of gC1qR. The recombinant MT1-MMP construct that included the catalytic domain but lacked the hemopexin-like domain lost the proteolytic capacity; however, it retained the ability to bind gC1qR. Inhibition of MT1-MMP activity by a hydroxamate inhibitor converted the protease into a cell surface receptor of gC1qR and promoted co-precipitation MT1-MMP with the soluble gC1qR protein. It is tempting to hypothesize that these novel mechanisms may play important roles in vivo and have to be taken into account in designing hydroxamate-based cancer therapy. 
 MT1-MMP silencing by an shRNA-armed glioma-targeted conditionally replicative adenovirus (CRAd) improves its anti-glioma efficacy in vitro and in vivo. MMP14 (MT1-MMP) is a cell membrane-associated proteinase of the   extracellular matrix  , whose biological roles vary from angiogenesis to cell proliferation and survival. We recently found a direct correlation between MMP14 expression levels in brain tumors of glioma patients and the disease progression. By using gene silencing as an experimental approach we found that MMP14 knockdown decreases production of pro-angiogenic factors such as VEGF and IL8 and thereby suppresses angiogenesis in glioma tumors. Although the clinical relevance of MMP14 down-regulation and its possible implications for glioma therapy in humans remain unclear, we observed a significant improvement in animal survival upon down-regulation of MMP14 in murine intracranial glioma xenografts infected with MMP14 shRNA-expressing CRAd. We further found that down-regulation of MMP14 in gliomas by combinational treatment with CRAd-S-5/3 and Marimastat, a chemical inhibitor of metalloproteinases, augments suppression of pro-angiogenic factors, caused by the replication-competent adenovirus. We also demonstrated that delivery of MMP14-targeting shRNA by a fiber-modified adenoviral vector to the glioma cells effectively suppresses their proliferation in vitro and in vivo. Thus our data indicate that inhibition of MMP14 expression in tumors in combination with glioma virotherapy could be effectively utilized to suppress angiogenesis and neovascularization of glioma tumors by decreasing production of pro-angiogenic factors. 
 TOM1L1 drives membrane delivery of MT1-MMP to promote ERBB2-induced breast cancer cell invasion. ERBB2 overexpression in human breast cancer leads to invasive carcinoma but the mechanism is not clearly understood. Here we report that TOM1L1 is co-amplified with ERBB2 and defines a subgroup of HER2(+)/ER(+) tumours with early metastatic relapse. TOM1L1 encodes a GAT domain-containing trafficking protein and is a SRC substrate that negatively regulates tyrosine kinase signalling. We demonstrate that TOM1L1 upregulation enhances the invasiveness of ERBB2-transformed cells. This pro-tumoural function does not involve SRC, but implicates membrane-bound membrane-type 1 MMP (MT1-MMP)-dependent activation of invadopodia, membrane protrusions specialized in   extracellular matrix   degradation. Mechanistically, ERBB2 elicits the indirect phosphorylation of TOM1L1 on Ser321. The phosphorylation event promotes GAT-dependent association of TOM1L1 with the sorting protein TOLLIP and trafficking of the metalloprotease MT1-MMP from endocytic compartments to invadopodia for tumour cell invasion. Collectively, these results show that TOM1L1 is an important element of an ERBB2-driven proteolytic invasive programme and that TOM1L1 amplification potentially enhances the metastatic progression of ERBB2-positive breast cancers. 
 ADAMTS1 cleaves aggrecan at multiple sites and is differentially inhibited by metalloproteinase inhibitors. ADAMTS1 is a secreted protein that belongs to the recently described ADAMTS (a disintegrin and metalloprotease with thrombospondin repeats) family of proteases. Evaluation of ADAMTS1 catalytic activity on a panel of   extracellular matrix   proteins showed a restrictive substrate specificity which includes some proteoglycans. Our results demonstrated that human ADAMTS1 cleaves aggrecan at a previously shown site by its mouse homolog, but we have also identified additional cleavage sites that ultimately confirm the classification of this protease as an 'aggrecanase'. Specificity of ADAMTS1 activity was further verified when a point mutation in the zinc-binding domain abolished its catalytic effects, and latency conferred by the prodomain was also demonstrated using a furin cleavage site mutant. Suppression of ADAMTS1 activity was accomplished with a specific monoclonal antibody and some metalloprotease inhibitors, including tissue inhibitor of metalloproteinases 2 and 3. Finally, we developed an activity assay using an artificial peptide substrate based on the interglobular domain cleavage site (E(373)-A) of rat aggrecan. 
 Membrane type 1 matrix metalloproteinase (MT1-MMP/MMP-14) cleaves and releases a 22-kDa   extracellular matrix   metalloproteinase inducer (EMMPRIN) fragment from tumor cells. Proteolytic shedding is an important step in the functional down-regulation and turnover of most membrane proteins at the cell surface.   Extracellular matrix   metalloproteinase inducer (EMMPRIN) is a multifunctional glycoprotein that has two Ig-like domains in its extracellular portion and functions in cell adhesion as an inducer of matrix metalloproteinase (MMP) expression in surrounding cells. Although the shedding of EMMPRIN is reportedly because of cleavage by metalloproteinases, the responsible proteases, cleavage sites, and stimulants are not yet known. In this study, we found that human tumor HT1080 and A431 cells shed a 22-kDa EMMPRIN fragment into the culture medium. The shedding was enhanced by phorbol 12-myristate 13-acetate and inhibited by TIMP-2 but not by TIMP-1, suggesting the involvement of membrane-type MMPs (MT-MMPs). Indeed, down-regulation of the MT1-MMP expression in A431 cells using small interfering RNA inhibited the shedding. The 22-kDa fragment was purified, and the C-terminal amino acid was determined. A synthetic peptide spanning the cutting site was cleaved by MT1-MMP in vitro. The cleavage site is located in the linker region connecting the two Ig-like domains. The N-terminal Ig-like domain is important for the MMP inducing activity of EMMPRIN and for cell-cell interactions, presumably through its ability to engage in homophilic interactions, and the 22-kDa fragment retained the ability to augment MMP-2 expression in human fibroblasts. Thus, the MT1-MMP-dependent cleavage eliminates the functional N-terminal domain of EMMPRIN from the cell surface, which is expected to down-regulate its function. At the same time, the released 22-kDa fragment may mediate the expression of MMPs in tumor tissues. 
 MT1-MMP regulates the turnover and endocytosis of   extracellular matrix   fibronectin. The   extracellular matrix   (ECM) is dynamically remodeled by cells during development, normal tissue homeostasis and in a variety of disease processes. We previously showed that fibronectin is an important regulator of ECM remodeling. The deposition and/or polymerization of fibronectin into the ECM controls the deposition and stability of other ECM molecules. In addition, agents that inhibit fibronectin polymerization promote the turnover of fibronectin fibrils and enhance ECM fibronectin endocytosis and intracellular degradation. Endocytosis of ECM fibronectin is regulated by β1 integrins, including α5β1 integrin. We have examined the role of extracellular proteases in regulating ECM fibronectin turnover. Our data show that membrane type matrix metalloproteinase 1 (MT1-MMP; also known as MMP14) is a crucial regulator of fibronectin turnover. Cells lacking MT1-MMP show reduced turnover and endocytosis of ECM fibronectin. MT1-MMP regulates ECM fibronectin remodeling by promoting extracellular cleavage of fibronectin and by regulating α5β1-integrin endocytosis. Our data also show that fibronectin polymerization stabilizes fibronectin fibrils and inhibits ECM fibronectin endocytosis by inhibiting α5β1-integrin endocytosis. These data are the first to show that an ECM protein and its modifying enzyme can regulate integrin endocytosis. These data also show that integrin trafficking plays a major role in modulating ECM fibronectin remodeling. The dual dependence of ECM fibronectin turnover on extracellular proteolysis and endocytosis highlights the complex regulatory mechanisms that control ECM remodeling to ensure maintenance of proper tissue function. 
 Three-dimensional matrix fiber alignment modulates cell migration and MT1-MMP utility by spatially and temporally directing protrusions. Multiple attributes of the three-dimensional (3D)   extracellular matrix   (ECM) have been independently implicated as regulators of cell motility, including pore size, crosslink density, structural organization, and stiffness. However, these parameters cannot be independently varied within a complex 3D ECM protein network. We present an integrated, quantitative study of these parameters across a broad range of complex matrix configurations using self-assembling 3D collagen and show how each parameter relates to the others and to cell motility. Increasing collagen density resulted in a decrease and then an increase in both pore size and fiber alignment, which both correlated significantly with cell motility but not bulk matrix stiffness within the range tested. However, using the crosslinking enzyme Transglutaminase II to alter microstructure independently of density revealed that motility is most significantly predicted by fiber alignment. Cellular protrusion rate, protrusion orientation, speed of migration, and invasion distance showed coupled biphasic responses to increasing collagen density not predicted by 2D models or by stiffness, but instead by fiber alignment. The requirement of matrix metalloproteinase (MMP) activity was also observed to depend on microstructure, and a threshold of MMP utility was identified. Our results suggest that fiber topography guides protrusions and thereby MMP activity and motility. 
 Enhanced membrane-type 1 matrix metalloproteinase expression by hyaluronan oligosaccharides in breast cancer cells facilitates CD44 cleavage and tumor cell migration. Hyaluronan (HA), a component of the   extracellular matrix  , plays an important role in cell-cell adhesion and cell migration. Membrane type 1-matrix metalloproteinase (MT1‑MMP) is often expressed in invasive cancer cells. CD44, a transmembrane receptor for HA, is implicated in various adhesion-dependent cellular processes including cell migration, tumor cell metastasis and invasion. Previous studies have shown that CD44 is highly expressed in cancer cells and may be proteolytically cleaved at the ectodomain by MT1-MMP; this process of inducing CD44 cleavage plays a critical role in cancer cell migration. We hypothesized that HA modulates MT1-MMP expression to facilitate breast cancer cell migration. Flow cytometry, real-time PCR, western blotting and immunofluorescence staining were used to quantify HA-induced MT1-MMP expression in breast cancer cells. In order to validate the relevance of cell migration and HA-induced MT1-MMP, we analyzed the cell migration via matrigel-coated transwell. We found that after HA oligosaccharide (6.5 kDA) stimulation, MT1-MMP expression in the membrane of breast cancer cells was increased. In response to HA oligosaccharide stimulation, significant upregulation of MT1-MMP mRNA occurred. Our data also provide evidence that HA oligosaccharide enhances MT1-MMP; the elevated expression of MT1-MMP confers enhanced CD44 cleavage and cell migration. In conclusion, we have identified a new function of HA in the induction of MT1-MMP expression in breast cancer cell lines and CD44 cleavage to increase cell migration during the invasion process. The HA oligosaccharide-induced MT1-MMP expression in breast cancer cells may be a critical step in the formation of metastatic colonies. 
 E1AF expression is closely correlated with malignant phenotype of tongue squamous cell carcinoma through activation of MT1-MMP gene promoters. E1AF is an ets-oncogene family transcription factor that has been shown to up-regulate multiple MMPs whereas MMP-2, a potent   extracellular matrix   degrading enzyme, is not up-regulated. We investigated the activation mechanism of MMP-2 in oral squamous cell carcinoma. Chloramphenicol acetyltransferase (CAT) assay was utilized to investigate whether E1AF is able to up-regulate membrane type-1 matrix metalloproteinase (MT1-MMP), which is known to activate MMP-2. Expression of the CAT reporter gene under the control of the MT1-MMP promoter was increased approximately 40-fold by co-transfection with the E1AF expression vector. Real-time RT-PCR study was carried out in 25 patients with tongue squamous cell carcinoma, and the mRNA expression level of E1AF and MT1-MMP was synergistically increased. These results indicate that E1AF positively regulates transcription from MT1-MMP genes, which plays an important role in invasion and metastasis of squamous cell carcinoma of the tongue by converting pro-MMP-2 into active-MMP-2. 
 Membrane type-1 matrix metalloproteinase (MT1-MMP) processing of pro-alphav integrin regulates cross-talk between alphavbeta3 and alpha2beta1 integrins in breast carcinoma cells. We have recently demonstrated that in breast carcinoma MCF7 cells MT1-MMP processes the alphav, alpha3, and alpha5 integrin precursors generating the respective mature S-S-linked heavy and light alpha-chains. The precursor of alpha2 integrin subunit was found resistant to MT1-MMP proteolysis. The processing of the alphav subunit by MT1-MMP facilitated alphavbeta3-dependent adhesion, activation of FAK signaling pathway, and migration of MCF7 cells on vitronectin. To elucidate further the effects of MT1-MMP on cellular integrins, we examined the functional activity of alpha5beta1 and alpha2beta1 integrins in MCF7 cells expressing MT1-MMP. Either expression of MT1-MMP alone or its coexpression with alphavbeta3 failed to affect the functionality of alpha5beta1 integrin, and adhesion of cells to fibronectin. MT1-MMP, however, profoundly affected the cross-talk involving alphavbeta3 and alpha2beta1 integrins. In MT1-MMP-deficient cells, integrin alphavbeta3 suppressed the functional activity of the collagen-binding alpha2beta1 integrin receptor and diminished cell adhesion to type I collagen. Coexpression of MT1-MMP with integrin alphavbeta3 restored the functionality of alpha2beta1 integrin and, consequently, the ability of MCF7 cells to adhere efficiently to collagen. We conclude that the MT1-MMP-controlled cross-talk between alphavbeta3 and alpha2beta1 integrins supports binding of aggressive, MT1-MMP-, and alphavbeta3 integrin-expressing malignant cells on type I collagen, the most common substratum of the   extracellular matrix  . 
 MMP-14 and MMP-2 are key metalloproteases in Dupuytren's disease fibroblast-mediated contraction. Dupuytren's disease (DD) is a common fibrotic condition of the palmar fascia, leading to deposition of collagen-rich cords and progressive flexion of the fingers. The molecular mechanisms underlying the disease are poorly understood. We have previously shown altered expression of   extracellular matrix  -degrading proteases (matrix metalloproteases, MMPs, and 'a disintegrin and metalloprotease domain with thrombospondin motifs', ADAMTS, proteases) in palmar fascia from DD patients compared to control and shown that the expression of a sub-set of these genes correlates with post-operative outcome. In the current study we used an in vitro model of collagen contraction to identify the specific proteases which mediate this effect. We measured the expression of all MMPs, ADAMTSs and their inhibitors in fibroblasts derived from the palmar fascia of DD patients, both in monolayer culture and in the fibroblast-populated collagen lattice (FPCL) model of cell-mediated contraction. Key proteases, previously identified in our tissue studies, were expressed in vitro and regulated by tension in the FPCL, including MMP1, 2, 3, 13 and 14. Knockdown of MMP2 and MMP14 (but not MMP1, 3 and 13) inhibited cell-mediated contraction, and knockdown of MMP14 inhibited proMMP-2 activation. Interestingly, whilst collagen is degraded during the FPCL assay, this is not altered upon knockdown of any of the proteases examined. We conclude that MMP-14 (via its ability to activate proMMP-2) and MMP-2 are key proteases in collagen contraction mediated by fibroblasts in DD patients. These proteases may be drug targets or act as biomarkers for disease progression. 
 MT1-MMP regulates MMP-2 expression and angiogenesis-related functions in human umbilical vein endothelial cells. Membrane type 1 (MT1)-MMP is a member of matrix metalloproteinases (MMPs) that regulates   extracellular matrix   remodeling. In addition, MT1-MMP also serves as a multi-functional protein. However, the functional role of MT1-MMP in human endothelial cells remains unclear. In this study we use real-time PCR and Western blotting to demonstrate for the first time that MMP-2 expression is regulated by MT1-MMP in human endothelial cells. Moreover, MMP-2 activity is also modulated by MT1-MMP. In addition we found that endothelial cells, ECM adhesion and human endothelial cell tube formation, which are known to be regulated by MMP-2, are blocked by MT1-MMP siRNA. These results suggest that MT1-MMP plays an important role in regulating angiogenesis in human endothelial cells. 
 Molecular dissection of the structural machinery underlying the tissue-invasive activity of membrane type-1 matrix metalloproteinase. Membrane type-1 matrix metalloproteinase (MT1-MMP) drives cell invasion through three-dimensional (3-D)   extracellular matrix   (ECM) barriers dominated by type I collagen or fibrin. Based largely on analyses of its impact on cell function under two-dimensional culture conditions, MT1-MMP is categorized as a multifunctional molecule with 1) a structurally distinct, N-terminal catalytic domain; 2) a C-terminal hemopexin domain that regulates substrate recognition as well as conformation; and 3) a type I transmembrane domain whose cytosolic tail controls protease trafficking and signaling cascades. The MT1-MMP domains that subserve cell trafficking through 3-D ECM barriers in vitro or in vivo, however, remain largely undefined. Herein, we demonstrate that collagen-invasive activity is not confined strictly to the catalytic, hemopexin, transmembrane, or cytosolic domain sequences of MT1-MMP. Indeed, even a secreted collagenase supports invasion when tethered to the cell surface in the absence of the MT1-MMP hemopexin, transmembrane, and cytosolic tail domains. By contrast, the ability of MT1-MMP to support fibrin-invasive activity diverges from collagenolytic potential, and alternatively, it requires the specific participation of MT-MMP catalytic and hemopexin domains. Hence, the tissue-invasive properties of MT1-MMP are unexpectedly embedded within distinct, but parsimonious, sequences that serve to tether the requisite matrix-degradative activity to the surface of migrating cells. 
 Membrane Type 1 Matrix Metalloproteinase induces an epithelial to mesenchymal transition and cancer stem cell-like properties in SCC9 cells. Tissue invasion and metastasis are acquired abilities of cancer and related to the death in oral squamous cell carcinoma (OSCC). Emerging observations indicate that the epithelial-to-mesenchymal transition (EMT) is associated with tumor progression and the generation of cells with cancer stem cells (CSCs) properties. Membrane Type 1 Matrix Metalloproteinase (MT1-MMP) is a cell surface proteinase, which is involved in degrading   extracellular matrix   components that can promote tumor invasion and cell migration.In the current study, we utilized SCC9 cells stably transfected with an empty vector (SCC9-N) or a vector encoding human MT1-MMP (SCC9-M) to study the role of MT1-MMP in EMT development.Upon up-regulation of MT1-MMP, SCC9-M cells underwent EMT, in which they presented a fibroblast-like phenotype and had a decreased expression of epithelial markers (E-cadherin, cytokeratin18 and β-catenin) and an increased expression of mesenchymal markers (vimentin and fibronectin). We further demonstrated that MT1-MMP-induced morphologic changes increased the level of Twist and ZEB, and were dependent on repressing the transcription of E-cadherin. These activities resulted in low adhesive, high invasive abilities of the SCC9-M cells. Furthermore, MT1-MMP-induced transformed cells exhibited cancer stem cell (CSC)-like characteristics, such as low proliferation, self-renewal ability, resistance to chemotherapeutic drugs and apoptosis, and expression of CSCs surface markers.In conclusion, our study indicates that overexpression of MT1-MMP induces EMT and results in the acquisition of CSC-like properties in SCC9 cells. Our growing understanding of the mechanism regulating EMT may provide new targets against invasion and metastasis in OSCC. 
 Expression of membrane type 1 matrix metalloproteinase in papillomavirus-positive cells: role of the human papillomavirus (HPV) 16 and HPV8 E7 gene products. Matrix metalloproteinases (MMPs) degrade   extracellular matrix  . They are involved in cellular proliferation, migration, angiogenesis, invasion and metastasis. MT-1 MMP, a membrane-bound MMP, is expressed in carcinomas of the uterine cervix in vivo. This type of cancer is associated with human papillomavirus (HPV) infection. Here it was shown that keratinocytes transformed with HPV16 or HPV18 in vitro, and HPV-positive cervical carcinoma cell lines, constitutively expressed MT-1 MMP. Expression of the E7 protein from the mucosal and cutaneous high-risk types HPV16 and HPV8, but not from the cutaneous low-risk type HPV1, was sufficient to induce MT-1 MMP expression in primary human keratinocytes and HaCaT cells. As a consequence, MMP-2 was activated. MT-1 MMP expression might play a role in the HPV life cycle by promoting proliferation of host cells and might contribute to their invasive phenotype during malignant progression. 
 MT1-MMP-dependent invasion is regulated by TI-VAMP/VAMP7. Proteolytic degradation of the   extracellular matrix   (ECM) is one intrinsic property of metastatic tumor cells to breach tissue barriers and to disseminate into different tissues. This process is initiated by the formation of invadopodia, which are actin-driven, finger-like membrane protrusions. Yet, little is known on how invadopodia are endowed with the functional machinery of proteolytic enzymes [1, 2]. The key protease MT1-MMP (membrane type 1-matrix metalloproteinase) confers proteolytic activity to invadopodia and thus invasion capacity of cancer cells [3-6]. Here, we report that MT1-MMP-dependent matrix degradation at invadopodia is regulated by the v-SNARE TI-VAMP/VAMP7, hence providing the molecular inventory mediating focal degradative activity of cancer cells. As observed by TIRF microscopy, MT1-MMP-mCherry and GFP-VAMP7 were simultaneously detected at proteolytic sites. Functional ablation of VAMP7 decreased the ability of breast cancer cells to degrade and invade in a MT1-MMP-dependent fashion. Moreover, the number of invadopodia was dramatically decreased in VAMP7- and MT1-MMP-depleted cells, indicative of a positive-feedback loop in which the protease as a cargo of VAMP7-targeted transport vesicles regulates maturation of invadopodia. Collectively, these data point to a specific role of VAMP7 in delivering MT1-MMP to sites of degradation, maintaining the functional machinery required for invasion. 
 Restricted expression of membrane type 1-matrix metalloproteinase by myofibroblasts adjacent to human breast cancer cells. The membrane type-1 matrix metalloproteinase (MT1-MMP), a protease originally identified in breast carcinoma, is characterized by its capacity to activate other MMPs (MMP-2 and MMP-13) and to degrade   extracellular matrix  . Our study was undertaken to localize and identify the MT1-MMP expressing cells in human breast adenocarcinomas. A textural analysis of images obtained by immunohistochemistry and in situ hybridization showed precisely the co-expression of alpha smooth muscle actin (alphaSM actin) and MT1-MMP in myofibroblasts. MT1-MMP expression is confined to myofibroblasts in close contact with tumor cells. In sharp contrast, the expression of MMP-2 was more widely distributed in both alphaSM actin positive and negative cells close to and at distance from cancer cell clusters. Our in vitro observations are consistent with the higher level of MT1-MMP expression and of MMP-2 activation observed in alphaSM actin positive fibroblasts derived from breast tumors, as compared to normal breast fibroblasts. Collectively, these results implicate myofibroblasts as major producer of MT1-MMP in breast cancer and emphasize the importance of stromal-epithelial cell interactions in their progression. 
 Structural analysis and promoter characterization of the human membrane-type matrix metalloproteinase-1 (MT1-MMP) gene. Membrane type-1 matrix metalloproteinase (MT1-MMP) degrades   extracellular matrix   components directly and indirectly by activation of other matrix metalloproteinases (MMPs). In the present study, we have isolated and characterized the human MT1-MMP gene and its promoter. The gene consists of 10 exons and nine introns spanning more than 10 kilobases (kb). The locations of two exon-intron splicing sites are distinct from the preserved positions among other known MMP genes. Primer extension and RNAse and S1 nuclease protection analyses indicated that there are four major and several minor transcription start sites. The 5'-flanking sequence of the gene contains putative regulatory elements, including one Sp-1 site and four CCAAT-boxes, whereas there is no TATA-box. The Sp-1 binding site was functional, as shown by gel shift and supershift analyses. Transfection studies with promoter constructs containing 0.1 to 7.2 kb of 5'-flanking sequence coupled to a luciferase reporter gene indicated that the promoter contains additional positive and negative regulatory sequences. Deletion of the Sp-1 binding site by site-directed mutagenesis reduced luciferase activity by about 90%, demonstrating the crucial role of this element in maintaining MT1-MMP transcription. Our findings indicate that the human MT1-MMP promoter has distinctive structural and functional features compared with other MMP genes, which may lead to a unique expression pattern and regulation during physiological and pathological processes. 
 Reflux induces DNA strand breaks and expression changes of MMP1+9+14 in a human miniorgan culture model. Gastroesophageal reflux disease has been implicated in the pathogenesis of adenocarcinoma of the oesophagus. The same applies to laryngopharyngeal reflux (LPR) and squamous cell cancer of the head and neck, but so far, this link has not been proven. The impact of low pH and bile acids has not been studied extensively in cells other than oesophageal cancer cell lines and tissue. The aims of this study were to investigate the pathogenic potential of reflux and its single components on the mucosa of the upper respiratory tract. We measured DNA stability in human miniorgan cultures (MOCs) and primary epithelial cell cultures (EpCs) in response to reflux by the alkaline comet assay. As matrix metalloproteinases (MMPs) are involved in   extracellular matrix   remodelling processes and may contribute to cancer progression, we studied the expression of MMP1, -9, and -14 in MOCs, EpC, UM-SCC-22B, and FADUDD. DNA strand breaks (DNA-SBs) increased significantly at low pH and after incubation with human or artificial gastric juice. Single incubation with glycochenodeoxycholic acid also showed a significant increase in DNA-SBs. In epithelial cell cultures, human gastric juice increased the number of DNA-SBs at pH 4.5 and 5.5. Artificial gastric juice significantly up regulated the gene expression of MMP9. Western blot analysis confirmed the results of gene expression analysis, but the up regulation of MMP1, -9, and -14 was donor-specific. Reflux has the ability to promote genomic instability and may contribute to micro environmental changes suitable for the initiation of malignancy. Further functional gene analysis may elucidate the role of laryngopharyngeal reflux in the development of head neck squamous cell carcinoma (HNSCC). 
 Membrane type-1 matrix metalloprotease-independent activation of pro-matrix metalloprotease-2 by proprotein convertases. Matrix metalloprotease-2 is implicated in many biological processes and degrades extracellular and non-  extracellular matrix   molecules. Matrix metalloprotease-2 maintains a latent state through a cysteine-zinc ion pairing which, when disrupted, results in full enzyme activation. This pairing can be disrupted by a conformational change or cleavage within the propeptide. The best known activation mechanism for pro-matrix metalloprotease-2 occurs via cleavage of the propeptide by membrane type-1 matrix metalloprotease. However, significant residual activation of pro-matrix metalloprotease-2 is seen in membrane type-1 matrix metalloprotease knockout mice and in fibroblasts treated with metalloprotease inhibitors. These findings indicate the presence of a membrane type-1 matrix metalloprotease-independent activation mechanism for pro-matrix metalloprotease-2 in vivo, which prompted us to explore an alternative activation mechanism for pro-matrix metalloprotese-2. In this study, we demonstrate membrane type-1 matrix metalloprotease-independent propeptide processing of matrix metalloprotease-2 in HEK293F and various tumor cell lines, and show that proprotein convertases can mediate the processing intracellularly as well as extracellularly. Furthermore, processed matrix metalloprotease-2 exhibits enzymatic activity that is enhanced by intermolecular autolytic cleavage. Thus, our experimental data, taken together with the broad expression of proprotein convertases, suggest that the proprotein convertase-mediated processing may be a general activation mechanism for pro-matrix metalloprotease-2 in vivo. 
 Downregulation of Akt and FAK phosphorylation reduces invasion of glioblastoma cells by impairment of MT1-MMP shuttling to lamellipodia and downregulates MMPs expression. Human malignant glioblastomas are highly invasive tumors. Increased cell motility and degradation of the surrounding   extracellular matrix   are essential for tumor invasion. PI3K/Akt signaling pathway emerges as a common pathway regulating cellular proliferation, migration and invasion; however, its contribution to particular process and downstream cascades remain poorly defined. We have previously demonstrated that Cyclosporin A (CsA) affects glioblastoma invasion in organotypic brain slices and tumorigenicity in mice. Here we show that CsA impairs migration and invasion of human glioblastoma cells by downregulation of Akt phosphorylation. Interference with PI-3K/Akt signaling was crucial for CsA effect on invasion, because overexpression of constitutively active myr-Akt antagonized drug action. Furthermore, the drug was not effective in T98G glioblastoma cells with constitutively high level of phosphorylated Akt. CsA, comparably to pharmacological inhibitors of PI3K/Akt signaling (LY294002, A443654), reduced motility of glioblastoma cells, diminished MMP-2 gelatinolytic activity and MMP-2 and MT1-MMP expression. The latter effect was mimicked by overexpression of dominant negative Akt mutants. We demonstrate that CsA and LY294002 reduced MMP transcription partly via modulation of IκB phosphorylation and NFκB transcriptional activity. Those effects were not mediated by inhibition of calcineurin, a classical CsA target. Additionally, CsA reduced phosphorylation and activity of focal adhesion kinase that was associated with rapid morphological alterations, rearrangement of lamellipodia and impairment of MT1-MMP translocation to membrane protrusions. Our results document novel, Akt-dependent mechanisms of interference with motility/invasion of human glioblastoma cells: through a rapid modulation of cell adhesion and MT1-MMP translocation to membrane protrusions and delayed, partly NFκB-dependent, downregulation of MMP-2 and MT1-MMP expression. This article is part of a Special Issue entitled: 11th European Symposium on Calcium. 
 Intradomain cleavage of inhibitory prodomain is essential to protumorigenic function of membrane type-1 matrix metalloproteinase (MT1-MMP) in vivo. Invasive cancers use pericellular proteolysis to breach the   extracellular matrix   and basement membrane barriers and invade the surrounding tissue. Proinvasive membrane type-1 matrix metalloproteinase (MT1-MMP) is the primary mediator of proteolytic events on the cancer cell surface. MT1-MMP is synthesized as a zymogen. The latency of MT1-MMP is maintained by its N-terminal inhibitory prodomain. In the course of MT1-MMP activation, the R(108)RKR(111) ↓ Y(112) prodomain sequence is processed by furin. The intact prodomain released by furin alone, however, is a potent inhibitor of the emerging MT1-MMP enzyme. Evidence suggests that the prodomain undergoes intradomain cleavage at the PGD ↓ L(50) site followed by the release of the degraded prodomain by furin cleavage that finalizes the two-step activation event. These cleavages, only if combined, cause the activation of MT1-MMP. The significance of the intradomain cleavage in the protumorigenic program of MT1-MMP, however, remained unidentified. To identify this important parameter, in our current study, we used the cells that expressed the wild-type prodomain-based fluorescent biosensor and the mutant biosensor with the inactivated PGD↓L(50) cleavage site (L50D mutant) and also the cells with the enforced expression of the wild-type and L50D mutant MT1-MMP. Using cell-based tests, orthotopic breast cancer xenografts in mice, and genome-wide transcriptional profiling of cultured cells and tumor xenografts, we demonstrated that the intradomain cleavage of the PGD ↓ L(50) sequence of the prodomain is essential for the protumorigenic function of MT1-MMP. Our results emphasize the importance of the intradomain cleavages resulting in the inactivation of the respective inhibitory prodomains not only for MT1-MMP but also for other MMP family members. 
 A cancer cell metalloprotease triad regulates the basement membrane transmigration program. Carcinoma cells initiate the metastatic cascade by inserting invasive pseudopodia through breaches in the basement membrane (BM), a specialized barrier of cross-linked,   extracellular matrix   macromolecules that underlies epithelial cells and ensheaths blood vessels. While BM invasion is the sine qua non of the malignant phenotype, the molecular programs that underlie this process remain undefined. To identify genes that direct BM remodeling and transmigration, we coupled high-resolution electron microscopy with an ex vivo model of invasion that phenocopies the major steps observed during the transition of carcinoma in situ to frank malignancy. Herein, a triad of membrane-anchored proteases, termed membrane type-1, type-2, and type-3 metalloproteinases, are identified as the triggering agents that independently confer cancer cells with the ability to proteolytically efface the BM scaffolding, initiate the assembly of invasive pseudopodia, and propagate transmigration. These studies characterize the first series of gene products capable of orchestrating the entire BM remodeling program that distinguishes the carcinomatous phenotype. 
 The hemopexin domain of membrane-type matrix metalloproteinase-1 (MT1-MMP) Is not required for its activation of proMMP2 on cell surface but is essential for MT1-MMP-mediated invasion in three-dimensional type I collagen. Membrane-type matrix metalloproteinase-1 (MT1-MMP) plays a key role in tumor invasion and metastasis by degrading the   extracellular matrix   and activating proMMP2. Here we show that the conserved hemopexin domain is required for MT1-MMP-mediated invasion and growth in three-dimensional type I collagen matrix but not proMMP2 activation. Deletion of the hemopexin domains in MT1-, MT2-, MT3-, MT5-, and MT6-MMP does not impair their abilities to activate proMMP2. In fact, hemopexin-less MT5- and MT6-MMP activate proMMP2 better than their wild type counterparts. On the other hand, hemopexin-less MT1-MMP fails to promote cell invasion into type I collagen but retains the capacity to enhance the growth of Madin-Darby canine kidney cells as cysts in three-dimensional collagen matrix. Moreover, the hemopexin domain is also required for MT1-MMP-mediated invasion/scattering of MCF-7 cells in three-dimensional collagen matrix. Because growth and invasion in a three-dimensional model may correlate with tumor invasiveness in vivo, our data suggest that the hemopexin domains of MT-MMPs should be targeted for the development of anti-cancer therapies by employing screening assays developed for three-dimensional models rather than their enzymatic activity toward proMMP2. 
 Membrane-type 1 matrix metalloproteinase modulates focal adhesion stability and cell migration. Membrane-type 1 matrix metalloproteinase (MT1-MMP) plays an important role in   extracellular matrix  -induced cell migration and the activation of extracellular signal-regulated kinase (ERK). We showed here that transfection of the MT1-MMP gene into HeLa cells promoted fibronectin-induced cell migration, which was accompanied by fibronectin degradation and reduction of stable focal adhesions, which function as anchors for actin-stress fibers. MT1-MMP expression attenuated integrin clustering that was induced by adhesion of cells to fibronectin. The attenuation of integrin clustering was abrogated by MT1-MMP inhibition with a synthetic MMP inhibitor, BB94. When cultured on fibronectin, HT1080 cells, which endogenously express MT1-MMP, showed so-called motile morphology with well-organized focal adhesion formation, well-oriented actin-stress fiber formation, and the lysis of fibronectin through trails of cell migration. Inhibition of endogenous MT1-MMP by BB94 treatment or expression of the MT1-MMP carboxyl-terminal domain, which negatively regulates MT1-MMP activity, resulted in the suppression of fibronectin lysis and cell migration. BB94 treatment promoted stable focal adhesion formation concomitant with enhanced phosphorylation of tyrosine 397 of focal adhesion kinase (FAK) and reduced ERK activation. These results suggest that lysis of the   extracellular matrix   by MT1-MMP promotes focal adhesion turnover and subsequent ERK activation, which in turn stimulates cell migration. 
 The type I collagen induction of MT1-MMP-mediated MMP-2 activation is repressed by alphaVbeta3 integrin in human breast cancer cells. The influence of alphaVbeta3 integrin on MT1-MMP functionality was studied in human breast cancer cells of differing beta3 integrin status. Overexpression of beta3 integrin caused increased cell surface expression of alphaV integrin and increased cellular adhesion to   extracellular matrix   (ECM) substrates in BT-549, MDA-MB-231 and MCF-7 cells. beta3 integrin expression also enhanced the migration of breast cancer cells on ECM substrates and enhanced collagen gel contraction. In vivo, alphaVbeta3 cooperated with MT1-MMP to increase the growth of MCF-7 cells after orthotopic inoculation in immunocompromised mice, but had no influence on in vitro proliferation. Despite these stimulatory effects, overexpression of beta3 integrin suppressed the type I collagen (Col I) induced MMP-2 activation in all breast cancer cell lines analyzed. This was also evident in extracts from the MCF-7 tumors in vivo, where MMP-2 activation was stimulated by MT1-MMP transfection, but attenuated with beta3 integrin expression. Although our studies confirm important biological effects of alphaVbeta3 integrin on enhancing cell adhesion and migration, ECM remodeling and tumor growth, beta3 integrin caused reduced MMP-2 activation in response to Col I in vitro, which appears to be physiologically relevant, as it was also seen in tumor xenografts in vivo. The reduction of MMP-2 activation (and thus MT1-MMP activity) by alphaVbeta3 in response to Col I may be important in scenarios where cells which are activated for matrix degradation need to preserve some pericellular collagen, perhaps as a substrate for cell adhesion and migration, thus maintaining a balanced level of proteolysis required for efficient tumor growth. 
 Role of MMP14 gene polymorphisms in susceptibility and pathological development to hepatocellular carcinoma. Early detection of hepatocellular carcinoma (HCC) is seldom available because of the lack of reliable markers. Matrix metalloproteinase (MMP) 14 is a cell surface proteinase that displays a broad spectrum of activity against   extracellular matrix   components and promotes the invasion/metastasis of cells. MMP14 is overexpressed in HCC, and the level is correlated with poor overall survival. The purpose of this study was to examine whether the MMP14 gene polymorphisms are associated with the susceptibility and clinicopathological development of HCC.A total of 135 patients with HCC and 496 healthy control subjects were recruited. Six single nucleotide polymorphisms (SNPs) of MMP14 genes were analyzed by polymerase chain reaction-restriction fragment length polymorphism (PCR-RFLP) genotyping and haplotype-base analysis.A significant (p < 0.05) lower risk for HCC was shown in the individuals with MMP14 +6767 G/A and +7096 C/C genotypes compared with those with corresponding wild-type homozygotes; high frequency for anti-hepatitis C virus and cirrhosis positive were shown in the HCC patients with MMP14 +7096 TC/CC genotype after adjusting for other confounding factors. The distribution frequency of -165 T: +221 T: +6727 C: +6767 G: +7096 T: +8153 G haplotype and diplotype was significantly higher in the HCC patients than healthy control subjects.The +6767 and +7096 polymorphic genotypes and haplotype -165 T: +221 T: +6727 C: +6767 G: +7096 T: +8153 G of MMP14 gene might contribute to the prediction of susceptibility and pathological development to HCC. 
 A specific subset of RabGTPases controls cell surface exposure of MT1-MMP,   extracellular matrix   degradation and three-dimensional invasion of macrophages. The matrix metalloproteinase MT1-MMP has a major impact on invasive cell migration in both physiological and pathological settings such as immune cell extravasation or metastasis of cancer cells. Surface-associated MT1-MMP is able to cleave components of the   extracellular matrix  , which is a prerequisite for proteolytic invasive migration. However, current knowledge on the molecular mechanisms that regulate MT1-MMP trafficking to and from the cell surface is limited. We have identified three members of the RabGTPase family, Rab5a, Rab8a and Rab14, as crucial regulators of MT1-MMP trafficking and function in primary human macrophages. Both overexpressed and endogenous forms show prominent colocalisation with MT1-MMP-positive vesicles, whereas expression of mutant constructs, as well as siRNA-induced knockdown, reveal that these RabGTPases are crucial in the regulation of MT1-MMP surface exposure, contact of MT1-MMP-positive vesicles with podosomes,   extracellular matrix   degradation in two and three dimensions, as well as three-dimensional proteolytic invasion of macrophages. Collectively, our results identify Rab5a, Rab8a and Rab14 as major regulators of MT1-MMP trafficking and invasive migration of primary human macrophages, which could be promising potential targets for manipulation of immune cell invasion. 
 Metalloproteinase MT1-MMP islets act as memory devices for podosome reemergence. Podosomes are dynamic cell adhesions that are also sites of   extracellular matrix   degradation, through recruitment of matrix-lytic enzymes, particularly of matrix metalloproteinases. Using total internal reflection fluorescence microscopy, we show that the membrane-bound metalloproteinase MT1-MMP is enriched not only at podosomes but also at distinct "islets" embedded in the plasma membrane of primary human macrophages. MT1-MMP islets become apparent upon podosome dissolution and persist beyond podosome lifetime. Importantly, the majority of MT1-MMP islets are reused as sites of podosome reemergence. siRNA-mediated knockdown and recomplementation analyses show that islet formation is based on the cytoplasmic tail of MT1-MMP and its ability to bind the subcortical actin cytoskeleton. Collectively, our data reveal a previously unrecognized phase in the podosome life cycle and identify a structural function of MT1-MMP that is independent of its proteolytic activity. MT1-MMP islets thus act as cellular memory devices that enable efficient and localized reformation of podosomes, ensuring coordinated matrix degradation and invasion. 
 Effect of lumican on the migration of human mesenchymal stem cells and endothelial progenitor cells: involvement of matrix metalloproteinase-14. Increasing number of evidence shows that soluble factors and   extracellular matrix   (ECM) components provide an optimal microenvironment controlling human bone marrow mesenchymal stem cell (MSC) functions. Successful in vivo administration of stem cells lies in their ability to migrate through ECM barriers and to differentiate along tissue-specific lineages, including endothelium. Lumican, a protein of the small leucine-rich proteoglycan (SLRP) family, was shown to impede cell migration and angiogenesis. The aim of the present study was to analyze the role of lumican in the control of MSC migration and transition to functional endothelial progenitor cell (EPC).Lumican inhibited tube-like structures formation on Matrigel® by MSC, but not EPC. Since matrix metalloproteinases (MMPs), in particular MMP-14, play an important role in remodelling of ECM and enhancing cell migration, their expression and activity were investigated in the cells grown on different ECM substrata. Lumican down-regulated the MMP-14 expression and activity in MSC, but not in EPC. Lumican inhibited MSC, but not EPC migration and invasion. The inhibition of MSC migration and invasion by lumican was reversed by MMP-14 overexpression.Altogether, our results suggest that lumican inhibits MSC tube-like structure formation and migration via mechanisms that involve a decrease of MMP-14 expression and activity. 
   Extracellular matrix   metalloproteinase inducer (CD147) and membrane type 1-matrix metalloproteinase are expressed on tissue macrophages in calcific aortic stenosis and induce transmigration in an artificial valve model. Matrix metalloproteinases participate in remodeling of   extracellular matrix  , which is central to the development of aortic stenosis. Synthesis of certain matrix metalloproteinases is induced by the glycoprotein   extracellular matrix   metalloproteinase inducer. We investigated whether   extracellular matrix   metalloproteinase inducer and membrane-type 1 matrix metalloproteinase are abundant in calcific aortic valve and their role in the pathogenesis of this condition.Sixteen patients who underwent surgery for aortic stenosis (n = 12) or heart transplantation for ischemic cardiomyopathy (n = 4) were reviewed. Expression of   extracellular matrix   metalloproteinase inducer and membrane-type 1 matrix metalloproteinase proteins was assessed by Western blot (n = 4 per group), immunohistochemistry for aortic stenosis (n = 12) and ischemic cardiomyopathy (n = 2), and in situ zymography (n = 3 per group). Functional relevance was investigated using an artificial valve model.  Extracellular matrix   metalloproteinase inducer and membrane-type 1 matrix metalloproteinase were abundant in all stenotic valves. Control valves did not stain for either protein. Double immunofluorescence colocalized   extracellular matrix   metalloproteinase inducer and membrane-type 1 matrix metalloproteinase to macrophages. On Western blotting, both proteins were more abundant in stenotic valves than in control valves. In situ zymography demonstrated greater gelatinolytic activity in stenotic valves than in control valves. Silencing of the   extracellular matrix   metalloproteinase inducer gene using small interfering RNA reduced migration of monocytes in an artificial valve model.  Extracellular matrix   metalloproteinase inducer and membrane-type 1 matrix metalloproteinase were demonstrated on macrophages in stenotic aortic valves, into which   extracellular matrix   metalloproteinase inducer may promote monocyte immigration. The latter protein may therefore represent a potential target to reduce the development of aortic stenosis. 
 Differential expression of   extracellular matrix   constituents and cell adhesion molecules between malignant pleural mesothelioma and mesothelial hyperplasia. Malignant pleural mesothelioma (MPM) is a highly aggressive neoplasm associated with asbestos exposure. Currently, the molecular mechanisms that induce MPM development are still unknown. The purpose of this study was to identify new molecular biomarkers for mesothelial carcinogenesis.We analyzed a panel of 84 genes involved in   extracellular matrix   remodeling and cell adhesion by polymerase chain reaction (PCR) array in 15 samples of epithelioid mesothelioma and 10 samples of reactive mesothelial hyperplasia (MH; 3 of 25 samples were inadequate for mRNA analysis). To validate the differentially expressed genes identified by PCR array, we analyzed 27 more samples by immunohistochemistry, in addition to the 25 samples already studied.Twenty-five genes were differentially expressed in MPM and MH by PCR array. Of these we studied matrix metalloproteinase 7 (MMP7), MMP14, CD44, and integrin, alpha3 expression by immunohistochemistry in 26 epithelioid MPM and 26 MH samples from the entire series of 52 cases. We observed higher MMP14 and integrin, alpha3 expression in MPM samples compared with MH samples (p = 0.000002 and p = 0.000002, respectively). Conversely, CD44 expression was low in most (57.7%) mesothelioma samples but only in 11.5% of the MH samples (p = 0.0013). As regards MMP7, we did not observe differential expression between MH and MPM samples.We have extensively studied genes involved in cell adhesion and   extracellular matrix   remodeling in MPM and MH samples, gaining new insight into the pathophysiology of mesothelioma. Moreover, our data suggest that these factors could be potential biomarkers for MPM. 
 MMP14 gene polymorphisms in chronic obstructive pulmonary disease. Proteinase/antiproteinase imbalance is a widely accepted theory for the pathogenesis of COPD. Among various proteinases, matrix metalloproteinases (MMPs) digest   extracellular matrix   of the lung and play significant roles in the development of COPD. Polymorphisms of an MMP that upregulate its activity may result in the degradation of the lung matrix. A case-control study was performed to investigate the association of polymorphisms of the MMP14 gene with COPD. Japanese subjects (96 COPD patients and 61 controls) and Egyptian subjects (106 COPD patients and 72 controls) were recruited. Each subject was genotyped for seven single nucleotide polymorphisms (SNPs) of the MMP14 gene; -165 G/T and -72 G/A in the promoter region, +221 C/T in exon 1, +6727 C/G and +6767 G/A in exon 5, +7096 T/C in exon 6, and +8153 G/A in exon 8. The distributions of the genotype frequencies of these SNPs were not significantly different between the COPD patients and the controls in either ethnic group after correction of multiple comparisons. In the haplotype analysis, however, the haplotype -165 T : +221 T : +6727 C : +7096 C had a significantly higher frequency in the Egyptian COPD group than the control group (pcorr = 0.0063). The haplotype of the MMP14 gene, -165 T : +221 T : +6727 C : +7096 C, might be involved in the pathogenesis of COPD. 
 Expression of membrane type-1 matrix metalloproteinase, MT1-MMP in human breast cancer and its impact on invasiveness of breast cancer cells. MT1-MMP (membrane type-1 matrix metalloproteinase), otherwise known as MMP14 is a proteolytic enzyme known to be involved in degradating   extracellular matrix   and assist progression of cancer invasion and progression. We investigated the impact of targeting the expression of MT1-MMP in breast cancer and its clinical relevance. Human breast cancer cell line MDA-MB-231 was used. Expression of MT1-MMP in the breast cancer cell line was manipulated by way of retroviral ribozyme transgene. The in vitro invasion, growth and cell migration were determined on cell lines transfected with either the transgene or control plasmid. Protein and message levels of MMP14 was also assessed using immunohistochemistry and real-time quantitative analysis, and correlated with clinical and pathological information of the patients. Retroviral ribozyme transgene to human MT1-MMP successfully knocked down the levels of MT1-MMP mRNA from MDA-MB-231 cells. Reduction of MT1-MMP from the breast cancer cells resulted in significant reduction of in vitro invasiveness and loss of response to an invasion stimulus, HGF, compared with control and wild-type cells. The invasion index for MT1-MMP knockdown cells were 13+/-3.1 (without HGF) and 16.4+/-2.3 (with HGF, p=0.14), and the index for transfection control cells 25.3+/-4.3 (without HGF) and 40.4+/-4.1 (with HGF, p=0.0049). Transfection with the transgenes did not change the rate of cell growth. In clinical breast cancer, MT1-MMP staining was both membranous and cytoplasmic. Tumour cells displayed stronger staining compared with normal mammary epithelial cells. Tumour tissues had a marginally higher levels of the MMP14 transcript (8.6+/-1.9), compared with normal tissues (4.7+/-1.4), p=0.13. No significant difference was observed between node positive and node negative tumours (9.0+/-2.2 vs 8.7+/-3.1, p=0.24). Marginally higher levels of the MMP14 transcript were seen in tumours which developed metastasis and local recurrence. However, tumours from patients who died of breast cancer related causes had significantly higher levels of the transcript, compared with tumours from patients who remained disease-free 10 years after initial surgery (12.2+/-2.5 vs 6.3+/-1.2, p=0.0091). MT1-MMP is a proteolytic enzyme that is pivotal in controlling the invasiveness of breast cancer cells. It is highly expressed in aggressive breast tumours and is associated with clinical outcome. The enzyme is a potential therapeutic target in breast cancer. 
 Proteolytic activation of heparin-binding EGF-like growth factor by membrane-type matrix metalloproteinase-1 in ovarian carcinoma cells. Increased expression of heparin-binding EGF-like growth factor (HB-EGF) and membrane-type matrix metalloproteinase-1 (MT1-MMP) is frequently associated with various types of malignant tumor. HB EGF-like growth factor has been reported to promote the malignant progression of ovarian carcinoma. Based on this finding, inhibition of HB-EGF activity with CRM197 is now under phase I clinical evaluation. On the other hand, MT1-MMP expressed in ovarian carcinoma cells is thought to promote invasion and growth of tumor cells by degrading the   extracellular matrix  . However, we recently demonstrated that co-expression of MT1-MMP and HB-EGF in gastric carcinoma cells leads to cleavage of HB-EGF within its N-terminal heparin-binding region, converting it into a potent heparin-independent growth factor. In this study, we evaluated the importance of regulation of HB-EGF by MT1-MMP in clinical samples of ovarian carcinoma. We detected co-expression of HB-EGF and MT1-MMP in clear cell ovarian carcinoma tissues, particularly at the invasion front and in tumor cells that had disseminated into the ascites, whereas HB-EGF alone was expressed in non-invasive borderline ovarian tumor tissue. Furthermore, a soluble HB-EGF fragment that corresponds to that processed by MT1-MMP was detected in malignant ascites obtained from patients with metastatic ovarian carcinoma. Ovarian carcinoma cells that express MT1-MMP and HB-EGF exhibited enhanced cell growth in a 3D-collagen matrix and anchorage-independent growth in suspension. These results indicate that MT1-MMP co-expressed with HB-EGF in ovarian carcinoma cells potentiates the activity of HB-EGF to promote invasive tumor growth and spreading in vivo. 
 Cloning of a human gene potentially encoding a novel matrix metalloproteinase having a C-terminal transmembrane domain. Matrix metalloproteinases (MMPs) play key roles in tissue remodeling during physiological and pathological processes by degrading various   extracellular matrix   (ECM) components. Although nine distinct MMPs have been characterized by cDNA cloning, there are thought to be more corresponding to the complexity of the ECM. MMP genes expressed in human tissues and cell lines were analyzed by the polymerase chain reaction (PCR) using degenerate primers that corresponded to the conserved amino acid (aa) sequences of the MMPs. One isolated complementary DNA (cDNA) fragment had sequence homology to the reported MMPs, but was otherwise unique. A human placenta cDNA library (Clontech) was screened using the fragment as a probe and a 3.4-kb cDNA fragment containing a long open reading frame (potentially encoding 582 aa) was isolated. The putative gene product had a common domain structure and the conserved sequence of a MMP, but it had a unique transmembrane (TM)-like structure at the C terminus. It should, therefore, be an TM protein, whereas all the other reported MMPs are secretory proteins. Thus, the gene is thought to be the first of a new subclass of MMPs whose products are potentially expressed on the cell surface. 
 A matrix metalloproteinase expressed on the surface of invasive tumour cells. Gelatinase A (type-IV collagenase; M(r) 72,000) is produced by tumour stroma cells and is believed to be crucial for their invasion and metastasis, acting by degrading   extracellular matrix   macro-molecules such as type IV collagen. An inactive precursor of gelatinase A (pro-gelatinase A) is secreted and activated in invasive tumour tissue as a result of proteolysis which is mediated by a fraction of tumour cell membrane that is sensitive to metalloproteinase inhibitors. Here we report the cloning of the complementary DNA encoding a new matrix metalloproteinase with a potential transmembrane domain. Expression of the gene product on the cell surface induces specific activation of pro-gelatinase A in vitro and enhances cellular invasion of the reconstituted basement membrane. Tumour cells of invasive lung carcinomas, which contain activated forms of gelatinase A, were found to express the transcript and the gene product. The new metalloproteinase may thus trigger invasion by tumour cells by activating pro-gelatinase A on the tumour cell surface. 
 LIM kinase1 modulates function of membrane type matrix metalloproteinase 1: implication in invasion of prostate cancer cells. LIM kinase 1 (LIMK1) is an actin and microtubule cytoskeleton modulatory protein that is overexpressed in a number of cancerous tissues and cells and also promotes invasion and metastasis of prostate and breast cancer cells. Membrane type matrix metalloproteinase 1 (MT1-MMP) is a critical modulator of   extracellular matrix   (ECM) turnover through pericellular proteolysis and thus plays crucial roles in neoplastic cell invasion and metastasis. MT1-MMP and its substrates pro-MMP-2 and pro-MMP-9 are often overexpressed in a variety of cancers including prostate cancer and the expression levels correlate with the grade of malignancy in prostate cancer cells. The purpose of this study is to determine any functional relation between LIMK1 and MT1-MMP and its implication in cell invasion.Our results showed that treatment with the hydroxamate inhibitor of MT1-MMP, MMP-2 and MMP-9 ilomastat inhibited LIMK1-induced invasion of benign prostate epithelial cells. Over expression of LIMK1 resulted in increased collagenolytic activity of MMP-2, and secretion of pro-MMP2 and pro-MMP-9. Cells over expressing LIMK1 also exhibited increased expression of MT1-MMP, transcriptional activation and its localization to the plasma membrane. LIMK1 physically associates with MT1-MMP and is colocalized with it to the Golgi vesicles. We also noted increased expression of both MT1-MMP and LIMK1 in prostate tumor tissues.Our results provide new information on regulation of MT1-MMP function by LIMK1 and showed for the first time, involvement of MMPs in LIMK1 induced cell invasion. 
 Expression Levels of Myostatin and Matrix Metalloproteinase 14 mRNAs in Uterine Leiomyoma are Correlated With Dysmenorrhea. Uterine leiomyoma is the most common benign neoplasm of female reproductive system, found in about 50% of women in reproductive age. The mechanisms of leiomyoma growth include cell proliferation, which is modulated by growth factors, and deposition of   extracellular matrix   (ECM). Activin A and myostatin are growth factors that play a role in proliferation of leiomyoma cells. Matrix metalloproteinases (MMPs) are known for their ability to remodel the ECM in different biological systems. The aim of this study was to evaluate the expression levels of activin βA-subunit, myostatin, and MMP14 messenger RNAs (mRNAs) in uterine leiomyomas and the possible correlation of these factors with clinical features of the disease. Matrix metalloproteinase 14 was highly expressed in uterine leiomyoma and correlated with myostatin and activin A mRNA expression. Moreover, MMP14 and myostatin mRNA expression correlated significantly and directly with the intensity of dysmenorrhea. Overall, the present findings showed that MMP14 mRNA is highly expressed in uterine leiomyoma, where it correlates with the molecular expression of growth factors and is further increased in cases of intense dysmenorrhea. 
  GeneRIFs  
 Results indicate that both macrophages and cancer cells use a similar strategy, namely RAB GTPase-driven trafficking circuitries, to adjust their surface levels of MT1-MMP and thus drive   extracellular matrix   degradation and invasion. 
 Basal localization of MT1-MMP is a key factor promoting the degradation of   extracellular matrix   by polarized epithelial cells. 
 MT1-MMP is involved in   extracellular matrix   fibronectin turnover and endocytosis. 
   Extracellular matrix   metalloproteinase inducer and membrane-type 1 matrix metalloproteinase were demonstrated on macrophages in stenotic aortic valves, into which   extracellular matrix   metalloproteinase inducer may promote monocyte immigration. 
 The results suggest that MT1-MMP contributes to proliferation of cancer cells in the   extracellular matrix   by activating ERK through c-Src and paxillin. 
 these findings identify MT1-MMP as a master regulator of the pathologic   extracellular matrix   remodeling that characterizes rheumatoid arthritis 
 Findings illustrate a novel role for MT1-MMP-TIMP-2 interaction, which controls cell functions by a mechanism independent of   extracellular matrix   degradation. 
 These results suggest that lysis of the   extracellular matrix   by MT1-MMP promotes focal adhesion turnover and subsequent ERK activation, which in turn stimulates cell migration. 
 MT1-MMP-controlled cross-talk between alphavbeta3 and alpha2beta1 integrins supports binding of aggressive, MT1-MMP-, and alphavbeta3 integrin-expressing malignant cells on type I collagen, the most common substratum of the   extracellular matrix   
  [Ii]nvasive  PubMed  
 Moesin regulates the motility of oral cancer cells via MT1-MMP and E-cadherin/p120-catenin adhesion complex. The present study aimed to clarify the role of Moesin in oral squamous cell carcinoma (OSCC) progression, especially in regulation of cell motility.Immunohistochemistry and western blotting were used to investigate the expression of Moesin, E-cadherin, p120-catenin and MT1-MMP in normal epithelia, dysplasia and OSCCs. Then, Moesin was knockdown by siRNA in OSCC cell lines, WSU-HN6 and CAL27, and the biological role of Moesin in cell adhesion and motility was evaluated by transwell system, cell spreading and aggregation assays. The interactions between Moesin, MT1-MMP and E-cadherin/p120-catenin complex were determined by co-immunoprecipitation and immunofluorescence.Moesin expression was found decreased in the membrane and increased in cytoplasm during the malignant transformation of oral epithelia, and cytoplasmic overexpression of Moesin correlated with nodal metastasis and poor prognosis of OSCCs. Furthermore, Moesin-silencing induced an increased cell-cell adhesion but decreased   invasive  ness, which was subsequently demonstrated might due to Moesin-mediated E-cadherin and p120-catenin interaction. Meantime, Moesin-silencing significantly down-regulated MT1-MMP expression, accompanied by reduced cell motility and impaired filopodia formation, which was also observed when MT1-MMP knockdown by RNAi or tissue inhibitor (TIMP2), indicating the involvement of MT1-MMP in Moesin-mediated cell motility. Finally, the relationship between Moesin, E-cadherin and MT1-MMP was confirmed in OSCC tissue samples.Taken together, our results indicate Moesin may regulate cell motility through its interactions with MT1-MMP and E-cadherin/p120-catenin adhesion complex and cytoplasmic expression of Moesin correlates with nodal metastasis and poor prognosis of OSCCs, indicating Moesin may be a potential candidate for targeted gene therapy for OSCCs. 
 Differential regulation of membrane type 1-matrix metalloproteinase activity by ERK 1/2- and p38 MAPK-modulated tissue inhibitor of metalloproteinases 2 expression controls transforming growth factor-beta1-induced pericellular collagenolysis. Acquisition of matrix metalloproteinase-2 (MMP-2) activity is temporally associated with increased migration and   invasive  ness of cancer cells. ProMMP-2 activation requires multimolecular complex assembly involving proMMP-2, membrane type 1-MMP (MT1-MMP, MMP-14), and tissue inhibitor of metalloproteinases-2 (TIMP-2). Because transforming growth factor-beta1 (TGF-beta1) promotes tumor invasion in advanced squamous cell carcinomas, the role of TGF-beta1 in the regulation of MMP activity in a cellular model of   invasive   oral squamous cell carcinoma was examined. Treatment of oral squamous cell carcinoma cells with TGF-beta1 promoted MMP-dependent cell scattering and collagen invasion, increased expression of MMP-2 and MT1-MMP, and enhanced MMP-2 activation. TGF-beta1 induced concomitant activation of ERK1/2 and p38 MAPK, and kinase inhibition studies revealed a negative regulatory role for ERK1/2 in modulating acquisition of MMP-2 activity. Thus, a reciprocal effect on proMMP-2 activation was observed whereupon blocking ERK1/2 phosphorylation promoted proMMP-2 activation and MT1-MMP activity, whereas inhibiting p38 MAPK activity decreased proteolytic potential. The cellular mechanism for the control of MT1-MMP catalytic activity involved concurrent reciprocal modulation of TIMP-2 expression by ERK1/2 and p38 MAPKs, such that inhibition of ERK1/2 phosphorylation decreased TIMP-2 production, and down-regulation of p38 MAPK activity enhanced TIMP-2 synthesis. Further, p38 MAPK inhibition promoted ERK1/2 phosphorylation, providing additional evidence for cross-talk between MAPK pathways. These observations demonstrate the complex reciprocal effects of ERK1/2 and p38 MAPK in the regulation of MMP activity, which could complicate the use of MAPK-specific inhibitors as therapeutic agents to down-regulate the biologic effects of TGF-beta1 on pericellular collagen degradation and tumor invasion. 
 Low tissue inhibitor of metalloproteinases 3 and high matrix metalloproteinase 14 levels defines a subpopulation of highly   invasive   foam-cell macrophages. An excess of metalloproteinases (MMPs) over tissue inhibitors of metalloproteinases (TIMPs) may favor atherosclerotic plaque rupture. We compared TIMP levels in nonfoamy and foam-cell macrophages (FCM) generated in vivo.In vivo generated rabbit FCM exhibited 84% reduced TIMP-3 protein compared to nonfoamy macrophages, and immunocytochemistry revealed a TIMP-3 negative subset (28%). Strikingly, only TIMP-3 negative FCM invaded a synthetic basement membrane, and invasion was inhibited by exogenous TIMP-3. TIMP-3 negative FCM also had increased proliferation and apoptosis rates compared to TIMP-3 positive cells, which were retarded by exogenous TIMP-3; this also reduced gelatinolytic activity. TIMP-3 negative FCM were found at the base of advanced rabbit plaques and in the rupture-prone shoulders of human plaques. To explain the actions of low TIMP-3 we observed a 26-fold increase in MT1-MMP (MMP-14) protein in FCM. Adding an MT1-MMP neutralizing antibody reduced foam-cell invasion, apoptosis, and gelatinolytic activity. Furthermore, MT1-MMP overexpressing and TIMP-3 negative FCM were found at the same locations in atherosclerotic plaques.These results demonstrate that TIMP-3 is downregulated in a distinct subpopulation of FCM which have increased MMP-14. These cells are highly   invasive   and have increased proliferation and apoptosis, all properties expected to destabilise atherosclerotic plaques. 
 [Membrane type 1 matrix metalloproteinase (MT1-MMP) and the regulators of its activity as   invasive   factors in squamous cell cervical carcinomas]. Membrane type 1 matrix metalloproteinase (MT1MMP) is one of matrix metalloproteinases (MMP), which play а key role in tumor invasion and metastasis. The aim of this study was to elucidate the peculiarities of expression of MT1MMP and endogenous regulators of its activity: the activator - furin and the inhibitor - TIMP-2, as   invasiv  e factors of squamous cell cervical carcinomas (SCC). The study was carried out using 11 specimens of SCC and 11 specimens of morphologically normal tissue adjacent to the tumor. It was shown that the increase of MT1-MMP and furin expression and low of TIMP-2 expression makes the main contribution to the destructive   (invasiv  e) potential of SCC. Moreover, substantial expression of MT1-MMP was registered in the specimens of morphologically normal adjoining to tumor tissue. This expression was found to make an additional contribution to the destructive potential of the cervical tumor. 
 NEDD9 depletion leads to MMP14 inactivation by TIMP2 and prevents invasion and metastasis. The scaffolding protein NEDD9 is an established prometastatic marker in several cancers. Nevertheless, the molecular mechanisms of NEDD9-driven metastasis in cancers remain ill-defined. Here, using a comprehensive breast cancer tissue microarray, it was shown that increased levels of NEDD9 protein significantly correlated with the transition from carcinoma in situ to   invasive   carcinoma. Similarly, it was shown that NEDD9 overexpression is a hallmark of highly   invasive   breast cancer cells. Moreover, NEDD9 expression is crucial for the protease-dependent mesenchymal invasion of cancer cells at the primary site but not at the metastatic site. Depletion of NEDD9 is sufficient to suppress invasion of tumor cells in vitro and in vivo, leading to decreased circulating tumor cells and lung metastases in xenograft models. Mechanistically, NEDD9 localized to   invasive   pseudopods and was required for local matrix degradation. Depletion of NEDD9 impaired invasion of cancer cells through inactivation of membrane-bound matrix metalloproteinase MMP14 by excess TIMP2 on the cell surface. Inactivation of MMP14 is accompanied by reduced collagenolytic activity of soluble metalloproteinases MMP2 and MMP9. Reexpression of NEDD9 is sufficient to restore the activity of MMP14 and the   invasive   properties of breast cancer cells in vitro and in vivo. Collectively, these findings uncover critical steps in NEDD9-dependent invasion of breast cancer cells.This study provides a mechanistic basis for potential therapeutic interventions to prevent metastasis. 
 Expression and role of metalloproteinase-2 and endogenous tissue regulator in ameloblastoma. Ameloblastoma is a frequently encountered odontogenic benign tumor characterized by local   invasive  ness and high risk of recurrence. Matrix metalloproteinase (MMP)-2 can degrade type IV collagen, one of the major components in the basement membrane, resulting in the promotion of tumor invasion, whereas it is under the influence of an activator, membrane type 1-MMP-14 and the tissue inhibitor of metalloproteases (TIMP)-2. The aim of this study was to investigate combinatorial role played by MMP-2, TIMP-2 and MMP-14 in ameloblastoma.Transcriptional expression of MMP-2, TIMP-2 and MMP-14 in tissue extracts of 42 ameloblastomas and 10 dental follicles was detected using reverse transcription-polymerase chain reaction, and compared difference in clinical type and local recurrence of ameloblastoma.MMP-2, TIMP-2 and MMP-14 mRNA were detected in all investigated ameloblastoma tissues. While MMP-2 mRNA was only expressed in eight of 10 odontotheca tissues and TIMP- 2 and MMP-14 were not found in all odontotheca tissues. The mRNA expression of MMP-2, TIMP-2 and MMP-14 were significantly higher in ameloblastoma tissues than in odontotheca tissues. The mRNA levels of TIMP-2 and MMP-14 were significantly higher in recurrent and solid/multicystic ameloblastoma tissues than in primary and unicystic ameloblastoma tissues respectively.Of the MMP-2/TIMP-2/MMP-14 complex, a high expression of MMP-2, TIMP-2 and MMP-14 mRNA levels may contribute to the local   invasive   characteristics of ameloblastoma, whereas the local   invasive   capacity of ameloblastoma is more likely related to a high transcriptional levels of TIMP-2 and MMP-14. 
 Downregulation of reversion-inducing cysteine-rich protein with Kazal motifs in malignant melanoma: inverse correlation with membrane-type 1-matrix metalloproteinase and tissue inhibitor of metalloproteinase 2. The   invasive   phenotype of many tumors is associated with an imbalance between the matrix metalloproteinases (MMPs) and their inhibitors, tissue inhibitors of metalloproteinases (TIMPs), and the membrane-anchored reversion-inducing cysteine-rich protein with Kazal motifs (RECK). RECK inhibits MMP-2, MMP-9, and MT1-MMP, and has been linked to patient survival and better prognosis in several types of tumors. However, despite the wide implication of these MMPs in melanoma establishment and progression, the role of RECK in this type of tumor is still unknown. Here, we analyzed the expression of RECK, TIMP1, TIMP2, TIMP3, MT1MMP, MMP2, and MMP9 in two publicly available melanoma microarray datasets and in a panel of human melanoma cell lines. We found that RECK is downregulated in malignant melanoma, accompanied by upregulation of MT1MMP and TIMP2. In both datasets, we observed that the group of samples displaying higher RECK levels show lower median expression levels of MT1MMP and TIMP2 and higher levels of TIMP3. When tested in a sample-wise manner, these correlations were statistically significant. Inverse correlations between RECK, MT1MMP, and TIMP2 were verified in a panel of human melanoma cell lines and in a further reduced model that includes a pair of matched primary tumor-derived and metastasis-derived cell lines. Taken together, our data indicate a consistent correlation between RECK, MT1MMP, and TIMP2 across different models of clinical samples and cell lines and suggest evidence of the potential use of this subset of genes as a gene signature for diagnosing melanoma. 
 MT1-MMP modulates melanoma cell dissemination and metastasis through activation of MMP2 and RAC1. Metastatic melanoma remains the deadliest of all skin cancers with a survival rate at five years of less than 15%. MT1-MMP is a membrane-associated matrix metalloproteinase that controls pericellular proteolysis and is an important, invasion-promoting, pro-tumorigenic MMP in cancer. We show that deregulation of MT1-MMP expression happens as early as the transition from nevus to primary melanoma and continues to increase during melanoma progression. Furthermore, MT1-MMP expression is associated with poor melanoma patient outcome, underscoring a pivotal role of MT1-MMP in melanoma pathogenesis. We demonstrate that MT1-MMP is directly required for melanoma cells to metastasize, as cells deprived of MT1-MMP fail to form distant metastasis in an orthotopic mouse melanoma model. We show that MT1-MMP affects cell invasion by activating its target MMP2. Importantly, we demonstrate, for the first time, that activation of MMP2 by MT1-MMP is required to sustain RAC1 activity and promote MT1-MMP-dependent cell motility. These data highlight a novel MT1-MMP/MMP2/RAC1 signaling axis in melanoma that may represent an intriguing molecular target for the treatment of   invasive   melanoma. 
 Activation ratio of MMP-2 and expression of MT1-MMP are correlated in thymic epithelial tumours. To study the relationships between the activation of pro-MMP-2 and the mRNA levels of MT1-MMP and TIMP-2 in thymic tissues and thymic epithelial tumours.We examined the mRNA expressions of MMP-2, MT1-MMP and TIMP-2 with real-time reverse transcription-polymerase chain reaction (RT-PCR). The gelatinolytic activity and MMP-2 activation ratio of thymic tissues, thymomas, and thymic carcinomas were determined with gelatin zymography. The cellular localisation of MMP-2 was detected with film in situ gelatin zymography. We then examined the mRNA expression of MMP-2 in the epithelial tumour cells or lymphocytes and stromal cells distant from the tumour (obtained from two   invasive   thymomas) using laser-capture microdissection (LCM).The mRNA levels of MMP-2, MT1-MMP and TIMP-2 were significantly increased from thymic tissue, Stage I-II, III-IV thymomas to thymic carcinomas (p<0.005). They were also significantly increased from AB-B1 (lymphocyte rich and mixed types), B2-B3 (cortical and predominantly polygonal cells types) thymomas to thymic carcinomas (p<0.05). The MMP-2 activation ratio also had the same tendency among the above groups (p<0.05) and was directly correlated to MT1-MMP and TIMP-2 mRNA expression (Spearman rank correlation: r = 0.8627, r = 0.8314; p<0.005). Film in situ zymography demonstrated that positive expression is mainly localised in tumour cell nests and adjacent stroma cells. LCM and real-time RT-PCR results confirmed that the expression of MMP-2 was higher in epithelial tumour cells than in lymphocytes and stromal cells.MMP-2, MT1-MMP and TIMP-2 mRNA expression levels were correlated with clinical stages and histological subtypes of thymic epithelial tumours. The activation of pro-MMP-2 might be mediated by MT1-MMP and TIMP-2 up-regulation. 
 Matrix metalloproteinase-2, membranous type 1 matrix metalloproteinase, and tissue inhibitor of metalloproteinase-2 expression in ectopic and eutopic endometrium. To investigate expression of matrix metalloproteinase-2 (MMP-2), membranous type 1 matrix metalloproteinase (MT1-MMP), and tissue inhibitor of metalloproteinase-2 (TIMP-2) in ectopic and eutopic endometrium from women with and without endometriosis throughout the menstrual cycle.Molecular studies in human tissue.Reproductive immunology laboratory of a university medical center.Fifty-three premenopausal woman (23 with endometriosis and 30 without endometriosis) undergoing laparoscopic surgery. Endometrium and ectopic endometriosis tissue were obtained at the time of surgery.Messenger RNA and protein expression from eutopic and ectopic endometrium was analyzed by using quantitative competitive polymerase chain reaction, zymography, and Western blot assay.Uterine endometrium from women with endometriosis expressed higher levels of MMP-2 and MT1-MMP and lower levels of TIMP-2 than did endometrium from normal women.Eutopic endometrium from patients with endometriosis may be more   invasive   and prone to peritoneal implantation because of greater expression of MMP-2 and MT1-MMP and lower expression of TIMP-2 messenger RNA, compared with endometrium from women without endometriosis. Thus, increased proteolytic activity may help to explain the   invasive   factors that result in endometriosis. 
 Evaluation of candidate stromal epithelial cross-talk genes identifies association between risk of serous ovarian cancer and TERT, a cancer susceptibility "hot-spot". We hypothesized that variants in genes expressed as a consequence of interactions between ovarian cancer cells and the host micro-environment could contribute to cancer susceptibility. We therefore used a two-stage approach to evaluate common single nucleotide polymorphisms (SNPs) in 173 genes involved in stromal epithelial interactions in the Ovarian Cancer Association Consortium (OCAC). In the discovery stage, cases with epithelial ovarian cancer (n=675) and controls (n=1,162) were genotyped at 1,536 SNPs using an Illumina GoldenGate assay. Based on Positive Predictive Value estimates, three SNPs-PODXL rs1013368, ITGA6 rs13027811, and MMP3 rs522616-were selected for replication using TaqMan genotyping in up to 3,059 serous   invasive   cases and 8,905 controls from 16 OCAC case-control studies. An additional 18 SNPs with Pper-allele<0.05 in the discovery stage were selected for replication in a subset of five OCAC studies (n=1,233 serous   invasive   cases; n=3,364 controls). The discovery stage associations in PODXL, ITGA6, and MMP3 were attenuated in the larger replication set (adj. Pper-allele>or=0.5). However genotypes at TERT rs7726159 were associated with ovarian cancer risk in the smaller, five-study replication study (Pper-allele=0.03). Combined analysis of the discovery and replication sets for this TERT SNP showed an increased risk of serous ovarian cancer among non-Hispanic whites [adj. ORper-allele 1.14 (1.04-1.24) p=0.003]. Our study adds to the growing evidence that, like the 8q24 locus, the telomerase reverse transcriptase locus at 5p15.33, is a general cancer susceptibility locus. 
 Moesin regulates the motility of oral cancer cells via MT1-MMP and E-cadherin/p120-catenin adhesion complex. The present study aimed to clarify the role of Moesin in oral squamous cell carcinoma (OSCC) progression, especially in regulation of cell motility.Immunohistochemistry and western blotting were used to investigate the expression of Moesin, E-cadherin, p120-catenin and MT1-MMP in normal epithelia, dysplasia and OSCCs. Then, Moesin was knockdown by siRNA in OSCC cell lines, WSU-HN6 and CAL27, and the biological role of Moesin in cell adhesion and motility was evaluated by transwell system, cell spreading and aggregation assays. The interactions between Moesin, MT1-MMP and E-cadherin/p120-catenin complex were determined by co-immunoprecipitation and immunofluorescence.Moesin expression was found decreased in the membrane and increased in cytoplasm during the malignant transformation of oral epithelia, and cytoplasmic overexpression of Moesin correlated with nodal metastasis and poor prognosis of OSCCs. Furthermore, Moesin-silencing induced an increased cell-cell adhesion but decreased   invasive  ness, which was subsequently demonstrated might due to Moesin-mediated E-cadherin and p120-catenin interaction. Meantime, Moesin-silencing significantly down-regulated MT1-MMP expression, accompanied by reduced cell motility and impaired filopodia formation, which was also observed when MT1-MMP knockdown by RNAi or tissue inhibitor (TIMP2), indicating the involvement of MT1-MMP in Moesin-mediated cell motility. Finally, the relationship between Moesin, E-cadherin and MT1-MMP was confirmed in OSCC tissue samples.Taken together, our results indicate Moesin may regulate cell motility through its interactions with MT1-MMP and E-cadherin/p120-catenin adhesion complex and cytoplasmic expression of Moesin correlates with nodal metastasis and poor prognosis of OSCCs, indicating Moesin may be a potential candidate for targeted gene therapy for OSCCs. 
 [Membrane type 1 matrix metalloproteinase (MT1-MMP) and the regulators of its activity as   invasive   factors in squamous cell cervical carcinomas]. Membrane type 1 matrix metalloproteinase (MT1MMP) is one of matrix metalloproteinases (MMP), which play а key role in tumor invasion and metastasis. The aim of this study was to elucidate the peculiarities of expression of MT1MMP and endogenous regulators of its activity: the activator - furin and the inhibitor - TIMP-2, as   invasiv  e factors of squamous cell cervical carcinomas (SCC). The study was carried out using 11 specimens of SCC and 11 specimens of morphologically normal tissue adjacent to the tumor. It was shown that the increase of MT1-MMP and furin expression and low of TIMP-2 expression makes the main contribution to the destructive   (invasiv  e) potential of SCC. Moreover, substantial expression of MT1-MMP was registered in the specimens of morphologically normal adjoining to tumor tissue. This expression was found to make an additional contribution to the destructive potential of the cervical tumor. 
 MT1-MMP modulates melanoma cell dissemination and metastasis through activation of MMP2 and RAC1. Metastatic melanoma remains the deadliest of all skin cancers with a survival rate at five years of less than 15%. MT1-MMP is a membrane-associated matrix metalloproteinase that controls pericellular proteolysis and is an important, invasion-promoting, pro-tumorigenic MMP in cancer. We show that deregulation of MT1-MMP expression happens as early as the transition from nevus to primary melanoma and continues to increase during melanoma progression. Furthermore, MT1-MMP expression is associated with poor melanoma patient outcome, underscoring a pivotal role of MT1-MMP in melanoma pathogenesis. We demonstrate that MT1-MMP is directly required for melanoma cells to metastasize, as cells deprived of MT1-MMP fail to form distant metastasis in an orthotopic mouse melanoma model. We show that MT1-MMP affects cell invasion by activating its target MMP2. Importantly, we demonstrate, for the first time, that activation of MMP2 by MT1-MMP is required to sustain RAC1 activity and promote MT1-MMP-dependent cell motility. These data highlight a novel MT1-MMP/MMP2/RAC1 signaling axis in melanoma that may represent an intriguing molecular target for the treatment of   invasive   melanoma. 
 Downregulation of reversion-inducing cysteine-rich protein with Kazal motifs in malignant melanoma: inverse correlation with membrane-type 1-matrix metalloproteinase and tissue inhibitor of metalloproteinase 2. The   invasive   phenotype of many tumors is associated with an imbalance between the matrix metalloproteinases (MMPs) and their inhibitors, tissue inhibitors of metalloproteinases (TIMPs), and the membrane-anchored reversion-inducing cysteine-rich protein with Kazal motifs (RECK). RECK inhibits MMP-2, MMP-9, and MT1-MMP, and has been linked to patient survival and better prognosis in several types of tumors. However, despite the wide implication of these MMPs in melanoma establishment and progression, the role of RECK in this type of tumor is still unknown. Here, we analyzed the expression of RECK, TIMP1, TIMP2, TIMP3, MT1MMP, MMP2, and MMP9 in two publicly available melanoma microarray datasets and in a panel of human melanoma cell lines. We found that RECK is downregulated in malignant melanoma, accompanied by upregulation of MT1MMP and TIMP2. In both datasets, we observed that the group of samples displaying higher RECK levels show lower median expression levels of MT1MMP and TIMP2 and higher levels of TIMP3. When tested in a sample-wise manner, these correlations were statistically significant. Inverse correlations between RECK, MT1MMP, and TIMP2 were verified in a panel of human melanoma cell lines and in a further reduced model that includes a pair of matched primary tumor-derived and metastasis-derived cell lines. Taken together, our data indicate a consistent correlation between RECK, MT1MMP, and TIMP2 across different models of clinical samples and cell lines and suggest evidence of the potential use of this subset of genes as a gene signature for diagnosing melanoma. 
 NEDD9 depletion leads to MMP14 inactivation by TIMP2 and prevents invasion and metastasis. The scaffolding protein NEDD9 is an established prometastatic marker in several cancers. Nevertheless, the molecular mechanisms of NEDD9-driven metastasis in cancers remain ill-defined. Here, using a comprehensive breast cancer tissue microarray, it was shown that increased levels of NEDD9 protein significantly correlated with the transition from carcinoma in situ to   invasive   carcinoma. Similarly, it was shown that NEDD9 overexpression is a hallmark of highly   invasive   breast cancer cells. Moreover, NEDD9 expression is crucial for the protease-dependent mesenchymal invasion of cancer cells at the primary site but not at the metastatic site. Depletion of NEDD9 is sufficient to suppress invasion of tumor cells in vitro and in vivo, leading to decreased circulating tumor cells and lung metastases in xenograft models. Mechanistically, NEDD9 localized to   invasive   pseudopods and was required for local matrix degradation. Depletion of NEDD9 impaired invasion of cancer cells through inactivation of membrane-bound matrix metalloproteinase MMP14 by excess TIMP2 on the cell surface. Inactivation of MMP14 is accompanied by reduced collagenolytic activity of soluble metalloproteinases MMP2 and MMP9. Reexpression of NEDD9 is sufficient to restore the activity of MMP14 and the   invasive   properties of breast cancer cells in vitro and in vivo. Collectively, these findings uncover critical steps in NEDD9-dependent invasion of breast cancer cells.This study provides a mechanistic basis for potential therapeutic interventions to prevent metastasis. 
 Evaluation of candidate stromal epithelial cross-talk genes identifies association between risk of serous ovarian cancer and TERT, a cancer susceptibility "hot-spot". We hypothesized that variants in genes expressed as a consequence of interactions between ovarian cancer cells and the host micro-environment could contribute to cancer susceptibility. We therefore used a two-stage approach to evaluate common single nucleotide polymorphisms (SNPs) in 173 genes involved in stromal epithelial interactions in the Ovarian Cancer Association Consortium (OCAC). In the discovery stage, cases with epithelial ovarian cancer (n=675) and controls (n=1,162) were genotyped at 1,536 SNPs using an Illumina GoldenGate assay. Based on Positive Predictive Value estimates, three SNPs-PODXL rs1013368, ITGA6 rs13027811, and MMP3 rs522616-were selected for replication using TaqMan genotyping in up to 3,059 serous   invasive   cases and 8,905 controls from 16 OCAC case-control studies. An additional 18 SNPs with Pper-allele<0.05 in the discovery stage were selected for replication in a subset of five OCAC studies (n=1,233 serous   invasive   cases; n=3,364 controls). The discovery stage associations in PODXL, ITGA6, and MMP3 were attenuated in the larger replication set (adj. Pper-allele>or=0.5). However genotypes at TERT rs7726159 were associated with ovarian cancer risk in the smaller, five-study replication study (Pper-allele=0.03). Combined analysis of the discovery and replication sets for this TERT SNP showed an increased risk of serous ovarian cancer among non-Hispanic whites [adj. ORper-allele 1.14 (1.04-1.24) p=0.003]. Our study adds to the growing evidence that, like the 8q24 locus, the telomerase reverse transcriptase locus at 5p15.33, is a general cancer susceptibility locus. 
 Expression and role of metalloproteinase-2 and endogenous tissue regulator in ameloblastoma. Ameloblastoma is a frequently encountered odontogenic benign tumor characterized by local   invasive  ness and high risk of recurrence. Matrix metalloproteinase (MMP)-2 can degrade type IV collagen, one of the major components in the basement membrane, resulting in the promotion of tumor invasion, whereas it is under the influence of an activator, membrane type 1-MMP-14 and the tissue inhibitor of metalloproteases (TIMP)-2. The aim of this study was to investigate combinatorial role played by MMP-2, TIMP-2 and MMP-14 in ameloblastoma.Transcriptional expression of MMP-2, TIMP-2 and MMP-14 in tissue extracts of 42 ameloblastomas and 10 dental follicles was detected using reverse transcription-polymerase chain reaction, and compared difference in clinical type and local recurrence of ameloblastoma.MMP-2, TIMP-2 and MMP-14 mRNA were detected in all investigated ameloblastoma tissues. While MMP-2 mRNA was only expressed in eight of 10 odontotheca tissues and TIMP- 2 and MMP-14 were not found in all odontotheca tissues. The mRNA expression of MMP-2, TIMP-2 and MMP-14 were significantly higher in ameloblastoma tissues than in odontotheca tissues. The mRNA levels of TIMP-2 and MMP-14 were significantly higher in recurrent and solid/multicystic ameloblastoma tissues than in primary and unicystic ameloblastoma tissues respectively.Of the MMP-2/TIMP-2/MMP-14 complex, a high expression of MMP-2, TIMP-2 and MMP-14 mRNA levels may contribute to the local   invasive   characteristics of ameloblastoma, whereas the local   invasive   capacity of ameloblastoma is more likely related to a high transcriptional levels of TIMP-2 and MMP-14. 
 Low tissue inhibitor of metalloproteinases 3 and high matrix metalloproteinase 14 levels defines a subpopulation of highly   invasive   foam-cell macrophages. An excess of metalloproteinases (MMPs) over tissue inhibitors of metalloproteinases (TIMPs) may favor atherosclerotic plaque rupture. We compared TIMP levels in nonfoamy and foam-cell macrophages (FCM) generated in vivo.In vivo generated rabbit FCM exhibited 84% reduced TIMP-3 protein compared to nonfoamy macrophages, and immunocytochemistry revealed a TIMP-3 negative subset (28%). Strikingly, only TIMP-3 negative FCM invaded a synthetic basement membrane, and invasion was inhibited by exogenous TIMP-3. TIMP-3 negative FCM also had increased proliferation and apoptosis rates compared to TIMP-3 positive cells, which were retarded by exogenous TIMP-3; this also reduced gelatinolytic activity. TIMP-3 negative FCM were found at the base of advanced rabbit plaques and in the rupture-prone shoulders of human plaques. To explain the actions of low TIMP-3 we observed a 26-fold increase in MT1-MMP (MMP-14) protein in FCM. Adding an MT1-MMP neutralizing antibody reduced foam-cell invasion, apoptosis, and gelatinolytic activity. Furthermore, MT1-MMP overexpressing and TIMP-3 negative FCM were found at the same locations in atherosclerotic plaques.These results demonstrate that TIMP-3 is downregulated in a distinct subpopulation of FCM which have increased MMP-14. These cells are highly   invasive   and have increased proliferation and apoptosis, all properties expected to destabilise atherosclerotic plaques. 
 Activation ratio of MMP-2 and expression of MT1-MMP are correlated in thymic epithelial tumours. To study the relationships between the activation of pro-MMP-2 and the mRNA levels of MT1-MMP and TIMP-2 in thymic tissues and thymic epithelial tumours.We examined the mRNA expressions of MMP-2, MT1-MMP and TIMP-2 with real-time reverse transcription-polymerase chain reaction (RT-PCR). The gelatinolytic activity and MMP-2 activation ratio of thymic tissues, thymomas, and thymic carcinomas were determined with gelatin zymography. The cellular localisation of MMP-2 was detected with film in situ gelatin zymography. We then examined the mRNA expression of MMP-2 in the epithelial tumour cells or lymphocytes and stromal cells distant from the tumour (obtained from two   invasive   thymomas) using laser-capture microdissection (LCM).The mRNA levels of MMP-2, MT1-MMP and TIMP-2 were significantly increased from thymic tissue, Stage I-II, III-IV thymomas to thymic carcinomas (p<0.005). They were also significantly increased from AB-B1 (lymphocyte rich and mixed types), B2-B3 (cortical and predominantly polygonal cells types) thymomas to thymic carcinomas (p<0.05). The MMP-2 activation ratio also had the same tendency among the above groups (p<0.05) and was directly correlated to MT1-MMP and TIMP-2 mRNA expression (Spearman rank correlation: r = 0.8627, r = 0.8314; p<0.005). Film in situ zymography demonstrated that positive expression is mainly localised in tumour cell nests and adjacent stroma cells. LCM and real-time RT-PCR results confirmed that the expression of MMP-2 was higher in epithelial tumour cells than in lymphocytes and stromal cells.MMP-2, MT1-MMP and TIMP-2 mRNA expression levels were correlated with clinical stages and histological subtypes of thymic epithelial tumours. The activation of pro-MMP-2 might be mediated by MT1-MMP and TIMP-2 up-regulation. 
 Differential regulation of membrane type 1-matrix metalloproteinase activity by ERK 1/2- and p38 MAPK-modulated tissue inhibitor of metalloproteinases 2 expression controls transforming growth factor-beta1-induced pericellular collagenolysis. Acquisition of matrix metalloproteinase-2 (MMP-2) activity is temporally associated with increased migration and   invasive  ness of cancer cells. ProMMP-2 activation requires multimolecular complex assembly involving proMMP-2, membrane type 1-MMP (MT1-MMP, MMP-14), and tissue inhibitor of metalloproteinases-2 (TIMP-2). Because transforming growth factor-beta1 (TGF-beta1) promotes tumor invasion in advanced squamous cell carcinomas, the role of TGF-beta1 in the regulation of MMP activity in a cellular model of   invasive   oral squamous cell carcinoma was examined. Treatment of oral squamous cell carcinoma cells with TGF-beta1 promoted MMP-dependent cell scattering and collagen invasion, increased expression of MMP-2 and MT1-MMP, and enhanced MMP-2 activation. TGF-beta1 induced concomitant activation of ERK1/2 and p38 MAPK, and kinase inhibition studies revealed a negative regulatory role for ERK1/2 in modulating acquisition of MMP-2 activity. Thus, a reciprocal effect on proMMP-2 activation was observed whereupon blocking ERK1/2 phosphorylation promoted proMMP-2 activation and MT1-MMP activity, whereas inhibiting p38 MAPK activity decreased proteolytic potential. The cellular mechanism for the control of MT1-MMP catalytic activity involved concurrent reciprocal modulation of TIMP-2 expression by ERK1/2 and p38 MAPKs, such that inhibition of ERK1/2 phosphorylation decreased TIMP-2 production, and down-regulation of p38 MAPK activity enhanced TIMP-2 synthesis. Further, p38 MAPK inhibition promoted ERK1/2 phosphorylation, providing additional evidence for cross-talk between MAPK pathways. These observations demonstrate the complex reciprocal effects of ERK1/2 and p38 MAPK in the regulation of MMP activity, which could complicate the use of MAPK-specific inhibitors as therapeutic agents to down-regulate the biologic effects of TGF-beta1 on pericellular collagen degradation and tumor invasion. 
 Matrix metalloproteinase-2, membranous type 1 matrix metalloproteinase, and tissue inhibitor of metalloproteinase-2 expression in ectopic and eutopic endometrium. To investigate expression of matrix metalloproteinase-2 (MMP-2), membranous type 1 matrix metalloproteinase (MT1-MMP), and tissue inhibitor of metalloproteinase-2 (TIMP-2) in ectopic and eutopic endometrium from women with and without endometriosis throughout the menstrual cycle.Molecular studies in human tissue.Reproductive immunology laboratory of a university medical center.Fifty-three premenopausal woman (23 with endometriosis and 30 without endometriosis) undergoing laparoscopic surgery. Endometrium and ectopic endometriosis tissue were obtained at the time of surgery.Messenger RNA and protein expression from eutopic and ectopic endometrium was analyzed by using quantitative competitive polymerase chain reaction, zymography, and Western blot assay.Uterine endometrium from women with endometriosis expressed higher levels of MMP-2 and MT1-MMP and lower levels of TIMP-2 than did endometrium from normal women.Eutopic endometrium from patients with endometriosis may be more   invasive   and prone to peritoneal implantation because of greater expression of MMP-2 and MT1-MMP and lower expression of TIMP-2 messenger RNA, compared with endometrium from women without endometriosis. Thus, increased proteolytic activity may help to explain the   invasive   factors that result in endometriosis. 
 Lack of Association between Membrane-Type 1 Matrix Metalloproteinase Expression and Clinically Relevant Molecular or Morphologic Tumor Characteristics at the Leading Edge of   Invasive   Colorectal Carcinoma. Colorectal cancer (CRC) is one of the leading causes of death from cancer in the western world, but tumor biology and clinical course show great interindividual variation. Molecular and morphologic tumor characteristics, such as KRAS/BRAF mutation status, mismatch repair (MMR) protein expression, tumor growth pattern, and tumor cell budding, have been shown to be of key therapeutic and/or prognostic relevance in CRC. Membrane-type 1 matrix metalloproteinase (MT1-MMP) is a membrane-anchored zinc-binding endopeptidase that is expressed at the leading edge of various   invasive   carcinomas and promotes tumor cell invasion through degradation of the extracellular matrix. The aim of this study was to investigate possible associations between MT1-MMP expression and molecular tumor characteristics as well as morphologic features of tumor aggressiveness in a consecutive series of 79 CRC tissue samples. However, although MT1-MMP was expressed in 41/79 samples (52%), there was no significant association between MT1-MMP expression and KRAS/BRAF mutation status, MMR protein expression, presence of lymphovascular invasion, tumor growth pattern, tumor-infiltrating lymphocytes, or tumor cell budding in our sample cohort (P > 0.05). Thus, we conclude that although MT1-MMP may play a role in CRC invasion, it is not of key relevance to the current models of CRC invasion and aggressiveness. 
 Expression of the reversion-inducing cysteine-rich protein with Kazal motifs and matrix metalloproteinase-14 in neuroblastoma and the role in tumour metastasis. Neuroblastoma is the most common malignant tumour in infancy; the reversion-inducing cysteine-rich protein with Kazal motifs gene (RECK) is a tumour suppressor gene. Previous studies show that RECK inhibits tumour invasion and metastasis through negative regulation of the matrix metalloproteinase (MMP)-2, MMP-9 and MMP-14. Therefore, we wanted to detect the expression of RECK and MMP-14 in neuroblastomas to assess the correlation between the expression levels of these proteins, and to investigate the roles in the metastasis and development of the tumour. PV-6000 immunohistochemistry method was used to detect the expression levels of RECK and MMP-14 in 36 samples of neuroblastoma tissue. Samples from paraffin wax-embedded specimens and the complete clinicopathological data of 36 neuroblastoma and 10 ganglioneuroma patients were collected. The rate of expression of the RECK protein in the neuroblastoma was low (16.7%). Furthermore, it reduced with the increase in the   invasive   depth and distant metastasis (P = 0.015; P < 0.05). The rate of expression of the MMP-14 protein in the neuroblastoma was high (58.3%) and increased with the increase in the extent of   invasive   depth and distant metastasis (P = 0.002; P < 0.05). The expression of the RECK protein correlated negatively with that of MMP-14 (r = -0.418; P < 0.05). Low levels of the RECK protein are expressed in the neuroblastoma, while the MMP-14 protein is expressed at high levels. The RECK and MMP-14 proteins may serve as markers in the estimation of the extent of metastasis and dissemination of the neuroblastoma. 
 CD44 regulates pancreatic cancer invasion through MT1-MMP. Pancreatic cancer is one of the deadliest human malignancies due to its early metastatic spread and resistance to therapy. The mechanisms regulating pancreatic cancer metastasis are so far poorly understood. Here, using both in vitro and in vivo approaches, it is demonstrated that CD44, a transmembrane glycoprotein expressed on a subset of pancreatic cancer cells, is required for the induction of epithelial-mesenchymal transition (EMT) and the activation of an   invasive   program in pancreatic cancer. Mechanistically, the transcription factor Snail1 (SNAI1), a regulator of the EMT program, is a downstream target of CD44 in primary pancreatic cancer cells and regulates membrane bound metalloproteinase (MMP14/MT1-MMP) expression. In turn, MT1-MMP expression is required for pancreatic cancer invasion. Thus, these data establish the CD44-Snail-MMP axis as a key regulator of the EMT program and of invasion in pancreatic cancer.This study sets the stage for CD44 and MT1-MMP as therapeutic targets in pancreatic cancer, for which small molecule or biologic inhibitors are available. Visual Overview: http://mcr.aacrjournals.org/content/early/2014/09/10/1541-7786.MCR-14-0076/F1.large.jpg. 
 EphA2 cleavage by MT1-MMP triggers single cancer cell invasion via homotypic cell repulsion. Changes in EphA2 signaling can affect cancer cell-cell communication and motility through effects on actomyosin contractility. However, the underlying cell-surface interactions and molecular mechanisms of how EphA2 mediates these effects have remained unclear. We demonstrate here that EphA2 and membrane-anchored membrane type-1 matrix metalloproteinase (MT1-MMP) were selectively up-regulated and coexpressed in   invasive   breast carcinoma cells, where, upon physical interaction in same cell-surface complexes, MT1-MMP cleaved EphA2 at its Fibronectin type-III domain 1. This cleavage, coupled with EphA2-dependent Src activation, triggered intracellular EphA2 translocation, as well as an increase in RhoA activity and cell junction disassembly, which suggests an overall repulsive effect between cells. Consistent with this, cleavage-prone EphA2-D359I mutant shifted breast carcinoma cell invasion from collective to rounded single-cell invasion within collagen and in vivo. Up-regulated MT1-MMP also codistributed with intracellular EphA2 in   invasive   cells within human breast carcinomas. These results reveal a new proteolytic regulatory mechanism of cell-cell signaling in cancer invasion. 
 Immunohistochemical demonstration of EphA2 processing by MT1-MMP in   invasive   cutaneous squamous cell carcinoma. Erythropoietin-producing hepatocellular receptor-2 (EphA2) overexpression is prevalent in many types of human cancers, and it has been reported that high EphA2 expression is correlated with malignancy. Recent studies revealed that processing of EphA2 by cleaving off the N-terminal portion by membrane-type 1 matrix metalloproteinase (MT1-MMP) promotes invasion via stimulation of Ras in cancer cells in vitro. The objectives of this study were to investigate the presence and role of EphA2 processing in cutaneous squamous cell carcinoma (SCC) tissues. EphA2 (C-terminal and N-terminal) and MT1-MMP expression patterns and levels were analyzed immunohistochemically in SCC (n = 70) and Bowen disease (BD; n = 20). Levels of MT1-MMP and EphA2 expression were evaluated using digital image analysis. Proximity between MT1-MMP and EphA2 in cancer cells and its effect on EphA2 processing were investigated using a combination of in situ proximity ligation assay (PLA) and Western blotting. Immunohistochemical analyses showed that levels of EphA2 N-terminal expression were significantly lower than those of EphA2 C-terminal expression in SCC, whereas levels of EphA2 C- and N-terminal expression were similar in BD. Western blotting showed processed EphA2 fragments in human SCC tissues. Expression levels of MT1-MMP, EphA2, and processed EphA2 fragments were higher in SCC than BD. Proximity between MT1-MMP and EphA2 in SCC was demonstrated by in situ PLA. Our results suggest possible involvement of MT1-MMP processing of EphA2   in inva  siveness of cutaneous SCC. 
 [Expression of MT1-MMP and RECK protein in human gastric carcinoma]. To examine the expression of membrane-type-1 matrix metalloproteinase (MT1-MMP) and reversion-inducing cysteine-rich protein with Kazal motifs (RECK) in gastric carcinoma, and investigate its clinical significance, at the same time analyze the correlation between MT1-MMP and RECK expression.MT1-MMP and RECK expression in surgically resected tissue samples of gastric carcinoma was examined by immunohistochemical method (two-step method) , and its correlation with clinicopathological factors was analyzed.Among the 44 gastric carcinoma samples, 37 (84.1%) were stained positive for MT1-MMP, and 31 (70.5%) for RECK. The expression of MT1-MMP was much higher in poorly differentiated gastric carcinoma samples than moderately and well-differentiated samples (P = 0.015). The expression level of MT1-MMP was associated with   invasive   depth of tumor cells (P = 0.007), but no difference between sex and lymph node metastasis. On the contrary, the well-differentiated samples showed higher expression of RECK than poorly and moderately differentiated gastric carcinoma samples (P = 0.006). The expression level of RECK did not correlate with sex, lymph node metastasis and   invasive   depth of tumor cells. RECK expression showed no relation to MT1-MMP expression in the gastric carcinoma.Overexpression of MT1-MMP in gastric carcinoma may play an important role during tumor differentiation and metastasis, the RECK protein may have positive effects on the tumor differentiation. 
 KIF1B promotes glioma migration and invasion via cell surface localization of MT1-MMP. Malignant glioma is notorious for its aggressiveness and poor prognosis, and the   invasive  ness of glioma cells is the major obstacle. Accumulating evidence indicates that kinesin superfamily proteins (KIFs) may play key roles in tumor   invasive  ness, but the mechanisms remained unresolved. Our previous study demonstrated that membrane type 1-matrix metalloproteinase (MT1-MMP) was involved in Kinesin family member 1B (KIF1B)-modulated invasion of gastric cancer cells. Therefore, the role of KIF1B in glioma cell invasion and its relationship with MT1-MMP were explored in the present study. We found that aberrantly increased expression of KIF1B was associated with worse WHO pathological classification and Karnofsky performance status (KPS), which also showed a trend towards worse prognosis. In the transwell assay, knockdown of KIF1B using siRNA repressed U87MG and A172 glioma cell migration and invasion. Silencing KIF1B inhibited expression of membranal MT1-MMP; however, the amount of MT1-MMP in the whole cell lysate was not affected. In conclusion, targeting KIF1B may be an option for anti-  invasive   therapies targeting glioma. 
 Expression of gelatinases (MMP-2, MMP-9) and gelatinase activator (MMP-14) in actinic keratosis and in in situ and   invasive   squamous cell carcinoma. Given the established role of matrix metalloproteinases (MMPs) in physiological processes in the skin, we investigated the expression of MMP-2, MMP-9, and MMP-14 to evaluate their role in the grading and development of atypical epithelial lesions. Immunohistochemistry was performed using antibodies against these MMPs in actinic keratosis (AK; n = 24), squamous cell carcinoma (SCC) in situ (SCCIS; n = 27), SCC well differentiated (SCCWD; n = 28), and SCC moderately to poorly differentiated (SCCMPD; n = 20). Tumoral and stromal expression was assessed by intensity (SI) and percentage positivity (PC). The mean of the total score, calculated by adding intensity and percentage positivity, was used for statistical analyses. In AK, SCCIS, SCCWD, and SCCMPD, mean tumoral MMP-2 expression was 3.33, 4.07, 4.46, and 3.40, respectively (P = NS for all) and stromal expression was 1.42, 3.26, 3.07, and 1.55 respectively (P < 0.05 for AK vs. SCCIS/SCCWD and SCCMPD vs. SCCIS/SCCWD); mean tumoral MMP-9 expression was 4.33, 4.11, 4.46, and 3.35, respectively, and stromal expression was 4.29, 4.41, 4.75, and 4.60, respectively (P = NS for all) and, mean tumoral MMP-14 expression was 1.58, 2.41, 0.32, and 0.35, respectively (P < 0.05 AK vs. SCCWD and SCCIS vs. SCCWD/SCCMPD) and stromal expression was 3.04, 3.52, 0.46, and 0.60, respectively (P < 0.05 for AK vs. SCCWD/SCCMPD). Only MMP-14 showed a statistically significant linear trend with decreasing values for tumoral and stromal expression with invasion suggesting that it might be of use as a prognosticator. Enhanced stromal MMP-2 expression in SCCIS and SCCWD relative to AK suggests that it may be of relevance to disease progression. 
 Gonadotropin-releasing hormone-II increases membrane type I metalloproteinase production via beta-catenin signaling in ovarian cancer cells. GnRH-II is produced by ovarian cancer cells and enhances their   invasive  ness in vitro. In our studies of OVCAR-3 and CaOV-3 ovarian cancer cell lines, GnRH-II treatment induced phosphorylation of Akt and glycogen synthase kinase (GSK)3β, as well as β-catenin accumulation in the nucleus, and the latter was reduced by small interfering RNA (siRNA)-mediated depletion of the GnRH receptor. The phosphatidylinositol 3 kinase (PI3K)/Akt pathway is involved in β-catenin-dependent signaling, and pretreatment of these human ovarian cancer cells with a PI3K/Akt inhibitor, LY294002, attenuated GnRH-II-stimulated phosphorylation of GSK3β and inhibited GnRH-II-induced invasion. It also attenuated GnRH-II induced trans-activation of a β-catenin-dependent reporter gene, most likely because GSK3β phosphorylation promotes translocation of β-catenin to the nucleus. Membrane type I matrix metalloproteinase (MT1-MMP) contributes to tumor progression directly, or by processing the latent MMP-2 zymogen, and is a known target of β-catenin signaling. When OVCAR-3 and CaOV-3 cells were treated with GnRH-II, MT1-MMP levels increased approximately 3-fold, whereas siRNA-mediated depletion of GnRH receptor or pretreatment with LY294002 abrogated this. In addition, lithium chloride, which increases GSK3β phosphorylation and the nuclear translocation of β-catenin, increased MT1-MMP levels in these ovarian cancer cells. By contrast, depletion of β-catenin by siRNA treatment abolished GnRH-II-induced MT1-MMP synthesis and red  uced the  ir invasive potential. Furthermore, siRNA-mediated reduction of MT1-MMP levels reduced GnRH-II-induced invasion in ovarian cancer cells. We therefore conclude that GnRH-II stimulates the PI3K/Akt pathway, and the phosphorylation of GSK3β, thereby enhancing the β-catenin-dependent up-regulation of MT1-MMP production, which contributes to ovarian cancer metastasis. 
 NEDD9/Arf6-dependent endocytic trafficking of matrix metalloproteinase 14: a novel mechanism for blocking mesenchymal cell invasion and metastasis of breast cancer. NEDD9 is an established marker of   invasive   and metastatic cancers. NEDD9 downregulation has been shown to dramatically reduce cell invasion and metastasis in multiple tumors. The mechanisms by which NEDD9 regulates invasion are largely unknown. In the current study, we have found that NEDD9 is required for matrix metalloproteinase 14 (MMP14) enzymatic recovery/recycling through the late endosomes to enable disengagement of tissue inhibitor of matrix metalloproteinase 2 (TIMP2) and tumor invasion. Depletion of NEDD9 decreases targeting of the MMP14/TIMP2 complex to late endosomes and increases trafficking of MMP14 from early/sorting endosomes back to the surface in a small GTPase ADP ribosylation factor-6 (Arf6)-dependent manner. NEDD9 directly binds to Arf6-GTPase-activating protein, ARAP3 and Arf6-effector GGA3, thereby facilitating the Arf6 inactivation required for MMP14/TIMP2 targeting to late endosomes. Re-expression of NEDD9 or a decrease in Arf6 activity is sufficient to restore MMP14 activity and the   invasive   properties of tumor cells. Importantly, NEDD9 inhibition by Vivo-Morpholinos, an antisense therapy, decreases primary tumor growth and metastasis in xenograft models of breast cancer. Collectively, our findings uncover a novel mechanism to control tumor-cell dissemination through NEDD9/Arf6-dependent regulation of MMP14/TIMP2 trafficking, and validate NEDD9 as a clinically relevant therapeutic target to treat metastatic cancer. 
 Non-destructive and selective imaging of the functionally active, pro-  invasive   membrane type-1 matrix metalloproteinase (MT1-MMP) enzyme in cancer cells. Proteolytic activity of cell surface-associated MT1-matrix metalloproteinase (MMP) (MMP-14) is directly related to cell migration, invasion, and metastasis. MT1-MMP is regulated as a proteinase by activation and conversion of the latent proenzyme into the active enzyme, and also via inhibition by tissue inhibitors of MMPs (TIMPs) and self-proteolysis. MT1-MMP is also regulated as a membrane protein through its internalization and recycling. Routine immunohistochemistry, flow cytometry, reverse transcription-PCR, and immunoblotting methodologies do not allow quantitative imaging and assessment of the cell-surface levels of the active, TIMP-free MT1-MMP enzyme. Here, we developed a fluorescent reporter prototype that targets the cellular active MT1-MMP enzyme alone. The reporter (MP-3653) represents a liposome tagged with a fluorochrome and functionalized with a PEG chain spacer linked to an inhibitory hydroxamate warhead. Our studies using the MP-3653 reporter and its inactive derivative demonstrated that MP-3653 can be efficiently used not only to visualize the trafficking of MT1-MMP through the cell compartment, but also to quantify the femtomolar range amounts of the cell surface-associated active MT1-MMP enzyme in multiple cancer cell types, including breast carcinoma, fibrosarcoma, and melanoma. Thus, the levels of the naturally expressed, fully functional, active cellular MT1-MMP enzyme are roughly equal to 1 × 10(5) molecules/cell, whereas these levels are in a 1 × 10(6) range in the cells with the enforced MT1-MMP expression. We suggest that the reporter we developed will contribute to the laboratory studies of MT1-MMP and then, ultimately, to the design of novel, more efficient prognostic approaches and personalized cancer therapies. 
 Leptin-promoted human extravillous trophoblast invasion is MMP14 dependent and requires the cross talk between Notch1 and PI3K/Akt signaling. The overexpression of leptin is a crucial feature for the maintenance of pregnancy. The effects of leptin on trophoblast invasion are important to its reproductive function, but the underlying mechanisms remain poorly understood. MMP14 is a member of matrix metalloproteinase (MMP) family that is closely involved in the invasion process. Here, we characterized the importance of MMP14 in the proinvasion effect of leptin on EVT cells and elucidated its molecular mechanisms. Transwell assay revealed that leptin promoted invasion of the immortalized EVT cell line HTR-8/SVneo in a dose- and time-related fashion. Further studies suggested that leptin enhanced HTR-8/SVneo cell invasion by up-regulating MMP14 expression and that knockdown of MMP14 by small interference RNA (siRNA) blocked the proinvasion effect of leptin. Notably, leptin promoted the expression of Notch1 receptor and activated its signaling in HTR-8/SVneo cells, and blocking this pathway by siRNA inhibited both leptin-enhanced MMP14 expression and   invasive  ness of HTR-8/SVneo cells. Such effects of Notch1 signaling were related with the activation of the PI3K/Akt pathway, which was significantly activated after leptin stimulation and was interfered by Notch1 signaling perturbation. Taken together, our observations suggest that leptin is an effective regulator of MMP14 expression, which consequently plays critical roles in invasion of EVT cells. The promoting effects of leptin on MMP14 require the cross talk between Notch1 and PI3K/Akt signaling pathways. 
 Membrane type 1 matrix metalloprotease cleaves laminin-10 and promotes prostate cancer cell migration. Disruption of the extracellular matrix by proteases is crucial for tumor invasion. Laminin-10 (Ln-10) has previously been identified as a substrate for cell migration and cell adhesion, and is present in the basal lamina (BL) of both normal prostate and prostate cancer. Here, we investigate a role for membrane type 1 matrix metalloprotease (MT1-MMP) in modifying this Ln-10-rich BL. MT1-MMP is a transmembrane member of the MMP family that has been demonstrated to be upregulated as prostate cancer progresses from normal to prostate intraepithelial neoplasia to   invasive   cancer, suggesting a role for MT1-MMP in the invasion of prostate cancer. We show that MT1-MMP cleaves the alpha5 chain of purified human Ln-10 from its 350-kDa form into 310-, 190-, 160-, and 45-kDa fragments. This cleavage causes a decrease in DU-145 prostate cancer cell adhesion to purified Ln-10, and an increase in transmigration of DU-145 cells through cleaved Ln-10. We also show that prostate cancer cells expressing membrane-bound MT1-MMP cleave the alpha5 chain of Ln-10. Ln alpha5-chain cleavage is also observed in human prostate cancer tissues. These findings suggest that prostate cancer cells expressing high levels of MT1-MMP have increased   invasive   potential through their ability to degrade and invade Ln-10 barriers. 
 Collective epithelial cell invasion overcomes mechanical barriers of collagenous extracellular matrix by a narrow tube-like geometry and MMP14-dependent local softening. Collective cell invasion (CCI) through interstitial collagenous extracellular matrix (ECM) is crucial to the initial stages of branching morphogenesis, and a hallmark of tissue repair and dissemination of certain tumors. The collagenous ECM acts as a mechanical barrier against CCI. However, the physical nature of this barrier and how it is overcome by cells remains incompletely understood. To address these questions, we performed theoretical and experimental analysis of mammary epithelial branching morphogenesis in 3D type I collagen (collagen-I) gels. We found that the mechanical resistance of collagen-I is largely due to its elastic rather than its viscous properties. We also identified two strategies utilized by mammary epithelial cells that can independently minimize ECM mechanical resistance during CCI. First, cells adopt a narrow tube-like geometry during invasion, which minimizes the elastic opposition from the ECM as revealed by theoretical modeling of the most frequent   invasive   shapes and sizes. Second, the stiffness of the collagenous ECM is reduced at   invasive   fronts due to its degradation by matrix metalloproteinases (MMPs), as indicated by direct measurements of collagen-I microelasticity by atomic force microscopy. Molecular techniques further specified that the membrane-bound MMP14 mediates degradation of collagen-I at   invasive   fronts. Thus, our findings reveal that MMP14 is necessary to efficiently reduce the physical restraints imposed by collagen-I during branching morphogenesis, and help our overall understanding of how forces are balanced between cells and their surrounding ECM to maintain collective geometry and mechanical stability during CCI. 
 p63/MT1-MMP axis is required for in situ to   invasive   transition in basal-like breast cancer. The transition of ductal carcinoma in situ (DCIS) to   invasive   breast carcinoma requires tumor cells to cross the basement membrane (BM). However, mechanisms underlying BM transmigration are poorly understood. Here, we report that expression of membrane-type 1 (MT1)-matrix metalloproteinase (MMP), a key component of the BM invasion program, increases during breast cancer progression at the in situ to   invasive   breast carcinoma transition. In the intraductal xenograft model, MT1-MMP is required for BM transmigration of MCF10DCIS.com breast adenocarcinoma cells and is overexpressed in cell clusters overlying focal BM disruptions and at the   invasive   tumor front. Mirrored upregulation of p63 and MT1-MMP is observed at the edge of MCF10DCIS.com xenograft tumors and p63 is required for induction of MT1-MMP-dependent   invasive   program in response to microenvironmental signals. Immunohistochemistry and analysis of public database reveal that p63 and MT1-MMP are upregulated in human basal-like breast tumors suggesting that p63/MT1-MMP axis contributes to progression of basal-like breast cancers with elevated p63 and MT1-MMP levels. 
 Increased serum level of membrane type 1-matrix metalloproteinase (MT1-MMP/MMP-14) in patients with breast cancer. Different types of matrix metalloproteinases, including membrane type 1 matrix metalloproteinase (MT1-MMP/MMP-14) can be easily detected in biological fluids and therefore may be contemplated as putative tumor markers. Although increased activity of MT1-MMP/MMP-14 have already been found in breast cancer, little is known about its circulating levels. The aim of the present study was therefore to evaluate serum levels of active form of membrane type 1 matrix metalloproteinase (MT1-MMP/MMP-14). A novel type of activity enzyme-linked immunosorbent assay was used to detect serum levels of MT1-MMP/MMP-14 in 18 patients with   invasive   ductal breast cancer and 11 healthy controls. In the breast cancer group of patients MT1-MMP/MMP-14 mean (+/-SD) concentration was 16.91+/-5.87 ng/ml which was significantly higher (p<0.0001) than the mean values obtained for the control i.e. 8.55+/-1.66 ng/ml.Higher levels of soluble form of MT1-MMP/MMP-14 could play a role in   invasive  ness and metastasis of breast cancer. Whether or not it has a potential as biochemical marker remains to be determined. 
 Shedding of the matrix metalloproteinases MMP-2, MMP-9, and MT1-MMP as membrane vesicle-associated components by endothelial cells. Production of matrix-degrading proteases, particularly matrix metalloproteinases (MMPs), by endothelial cells is a critical event during angiogenesis, the process of vessel neoformation that occurs in normal and pathological conditions. MMPs are known to be highly regulated at the level of synthesis and activation, however, little is known about the regulation of MMP secretion by endothelial cells. We found that cultured human umbilical vein endothelial cells shed vesicles (300 to 600 nm) originating from localized areas of the cell plasma membrane, as revealed by ultrastructural analysis. Normal and reverse zymography, Western blot, and immunogold analyses of the vesicles showed two gelatinases, MMP-2 and MMP-9, in both the active and proenzyme forms, the MT1-MMP proenzyme located on the external side of the vesicle membrane and the two inhibitors TIMP-1 and TIMP-2. Serum and the angiogenic factors, fibroblast growth factor-2 and vascular endothelial growth factor, stimulated the shedding of MMPs as vesicle components. Shedding the vesicle was rapid, as it was already completed after 4 hours. Addition of shed vesicles to human umbilical vein endothelial cells resulted in autocrine stimulation of invasion through a layer of reconstituted basement membrane (Matrigel) and cord formation on Matrigel. We conclude that endothelial cells shed MMP-containing vesicles and this may be a mechanism for regulating focalized proteolytic activity vital to   invasive   and morphogenic events during angiogenesis. 
 Expression of membrane type I-matrix metalloproteinase in oral squamous cell carcinoma. A local invasion and lymph node metastasis (LNM) of an oral squamous cell carcinoma (OSCC) has a poor prognosis, and involves the degradation of the extracellular matrix mediated by multiple proteolytic enzymes including membrane type I-matrix metalloproteinase (MT1-MMP). This study aimed to determine the role of MT1-MMP in OSCC, to evaluate the immunohistochemical expression of MT1-MMP with regard to the   invasive  ness and LNM of the OSCC, and to evaluate the major source of MT1-MMP mRNA and its protein using immunohistochemistry and in situ hybridization. MT1-MMP expression was examined in 46 OSCCs via immunohistochemistry and non-radioisotope in situ hybridization. The relationship between MT1-MMP expression and LNM, as well as the histological   invasive  ness, was statistically analyzed. The results showed that whereas 12 out of the 18 OSCCs (66.7%) with LNM showed moderate to strong MT1-MMP expression, only nine of the 28 OSCCs (32.1%) without LNM expressed MT1-MMP strongly. MT1-MMP expression was significantly higher with regard to LNM (P=0.022). As the invasion grade became stronger (from grade a to grade d), MT1-MMP was significantly more strongly expressed (P=0.033). These results suggest that MT1-MMP is primarily secreted in the OSCC cells and is involved in the   invasive  ness of the OSCC and LNM. Moreover, MT1-MMP combined with other markers may be used to predict the metastatic potential of an OSCC. 
 Regulation of membrane-type 1 matrix metalloproteinase expression by zonula occludens-2 in human lung cancer cells. During tumor invasion, tumor epithelial cells acquire migratory and   invasive   properties involving important phenotypic alterations. Among these changes, one can observe reorganization or a loss of cell-cell adhesion complexes such as tight junctions (TJs). TJs are composed of transmembrane proteins (occludin, claudins) linked to the actin cytoskeleton through cytoplasmic adaptor molecules including those of the zonula occludens family (ZO-1, -2, -3). We here evaluated the potential role of ZO-2 in the acquisition of   invasive   properties by tumor cells. In vivo, we showed a decrease of ZO-2 expression in bronchopulmonary cancers, with a preferential localization in the cytoplasm. In addition, in vitro, the localization of ZO-2 varied according to   invasive   properties of tumor cells, with a cytoplasmic localization correlating with invasion. In addition, we demonstrated that ZO-2 inhibition increases   invasive   and migrative capacities of   invasive   tumor cells. This was associated with an increase of MT1-MMP. These results suggest that ZO-2, besides its structural role in tight junction assembly, can act also as a repressor of tumor progression through its ability to reduce the expression of tumor-promoting genes in   invasive   tumor cells. 
 Transmembrane-deletion mutants of the membrane-type matrix metalloproteinase-1 process progelatinase A and express intrinsic matrix-degrading activity. Membrane-type matrix metalloproteinase-1 (MT-MMP-1) has been proposed to play a critical role in regulating the expression of tissue-  invasive   phenotypes in normal and neoplastic cells by directly or indirectly mediating the activation of progelatinase A. To begin characterizing MT-MMP-1 structure-function relationships, transmembrane-deletion mutants were constructed, and the processing of the zymogens as well as the enzymic activity of the mature proteinases was analyzed. We now demonstrate that pro-MT-MMP-1 mutants are efficiently processed to active proteinases following post-translational endoproteolysis immediately downstream of an Arg108-Arg-Lys-Arg basic motif by a proprotein convertase-dependent pathway. The secreted form of active MT-MMP-1 not only displays an N terminus identical with that described for the processed wild-type enzyme at Tyr112 (Strongin, A. Y., Collier, I., Bannikov, G., Marmer, B. L., Grants, G. A., and Goldberg, G. I. (1995) J. Biol. Chem. 270, 5331-5338), but also directly mediated progelatinase A activation via a two-step proteolytic cascade indistinguishable from that observed with intact cells. Furthermore, although the only function previously ascribed to MT-MMP-1 is its ability to act as a progelatinase A activator, purified transmembrane deletion mutants also expressed proteolytic activities against a wide range of extracellular matrix molecules. Given recent reports that MT-MMP-1 ectodomains may undergo intercellular transfer in vivo (Okada, A., Bellocq, J.-P., Rouyer, N., Chenard, M.-P., Rio, M.-C., Chambon, P., and Basset, P. (1995) Proc. Natl. Acad. Sci. U. S. A. 92, 2730-2734), our data suggest that soluble forms of the proteinase confer recipient cells with the ability to not only process progelatinase A, but also directly degrade extracellular matrix components. 
 Control of MT1-MMP transport by atypical PKC during breast-cancer progression. Dissemination of carcinoma cells requires the pericellular degradation of the extracellular matrix, which is mediated by membrane type 1-matrix metalloproteinase (MT1-MMP). In this article, we report a co-up-regulation and colocalization of MT1-MMP and atypical protein kinase C iota (aPKCι) in hormone receptor-negative breast tumors in association with a higher risk of metastasis. Silencing of aPKC in   invasiv  e breast-tumor cell lines impaired the delivery of MT1-MMP from late endocytic storage compartments to the surface and inhibited matrix degradation and invasion. We provide evidence that aPKCι, in association with MT1-MMP-containing endosomes, phosphorylates cortactin, which is present in F-actin-rich puncta on MT1-MMP-positive endosomes and regulates cortactin association with the membrane scission protein dynamin-2. Thus, cell line-based observations and clinical data reveal the concerted activity of aPKC, cortactin, and dynamin-2, which control the trafficking of MT1-MMP from late endosome to the plasma membrane and play an important role in th  e invasi  ve potential of breast-cancer cells. 
 Protein expression of MMP-2 and MT1-MMP in actinic keratosis, squamous cell carcinoma of the skin, and basal cell carcinoma. Squamous cell carcinoma (SCC) and basal cell carcinoma (BCC) are 2 skin neoplasms with distinct potentials to invasion and metastasis. Actinic keratosis (AK) is a precursor lesion of SCC. Immunohistochemistry was performed to evaluate the expression of MMP-2 and MT1-MMP in samples of BCC (n = 29), SCC (n = 12), and AK (n = 13). The ratio of positive cells to total cells was used to quantify the staining. Statistical significance was considered under the level P < .05. We found a higher expression of MMP-2 in tumor stroma and parenchyma of SCC as compared to BCC. The expression of this protein was also similar between SCC and its precursor actinic keratosis, and it was higher in the stroma of high-risk BCC when compared to low-risk BCC. MT1-MMP, which is an activator of MMP-2, was similarly expressed in all groups. Our results suggest that MMP-2 expression may contribute to the distinct   invasive   patterns seen in SCC and BCC. 
 Specific blockade of the ERK pathway inhibits the   invasive  ness of tumor cells: down-regulation of matrix metalloproteinase-3/-9/-14 and CD44. Elevated expression of matrix metalloproteinases (MMPs) is associated with increased metastatic potential in many tumor cells. As activation of the ERK pathway has been linked to the expression of MMP-9, we examined a possible correlation between ERK activation, MMP-9 expression, and   invasive   phenotype in human tumor cells. Activation state of the ERK pathway in tumor cells was well correlated with the   invasive   phenotype, which was determined by the ability of cells to invade through reconstituted extracellular matrix. Elevated expression of MMP-9 as well as of MMP-3, MMP-14, and CD44 was observed in tumor cells in which constitutive activation of the ERK pathway is detected. Blockade of the ERK pathway by treatment with PD184352, a specific and powerful inhibitor of mitogen-activated protein (MAP) kinase/ERK kinase (MEK), suppressed the expression of MMP-3, MMP-9, MMP-14, and CD44, and inhibited markedly the   invasive  ness of tumor cells. These results imply that, in addition to anti-proliferative effects, specific blockade of the ERK pathway is expected to result in anti-metastatic effects in tumor cells. 
 Distinct progression-associated expression of tumor and stromal MMPs in HaCaT skin SCCs correlates with onset of invasion. Matrix metalloproteinases (MMPs) are critically involved in tumor invasion and metastasis. However, failure of broad spectrum MMP inhibitors in clinical trials emphasizes the need for detailed analyses of the specific role of different MMPs in tumor malignancy. Using HaCaT-keratinocyte clones representing distinct stages in skin squamous cell carcinoma (SCC) progression, we demonstrate the expression of specific tumor and stroma-derived MMPs with the onset and maintenance of tumor invasion. Although MMP-9-positive leukocytes are present in benign and malignant tumor transplants at the onset of stromal activation and angiogenesis, mRNA expression of stroma-derived MMP-9 as well as MMP-2, -13 and -14 is exclusively found in enhanced malignant tumor transplants. Their expression initiates with the onset of invasion, whereas being absent in early non  invasive   stages of malignant transplants. In addition, a high expression of tumor-derived MMP-1, -2 and -14 contributes to malignant and   invasive   tumor growth. However, stroma-derived MMP-3 is exclusively restricted to very late-stage   invasive   and malignant transplants. The functional contribution of these proteases to   invasive   growth is supported by the gelatinolytic activity in the tumor transplants that again initiates with the onset of   invasive   growth suggesting a crucial role of MMP-2, -9, -13 and -14 for the establishment of a reactive stroma that promotes tumor invasion. These data demonstrate a complex cooperation of distinct tumor and stroma-derived MMPs in the establishment of malignant tumors and provide the basis for a more specific use of highly selective MMP inhibitors during distinct stages of tumor progression. 
 CDCP1 regulates the function of MT1-MMP and invadopodia-mediated invasion of cancer cells. Complement C1r/C1s, Uegf, Bmp1 (CUB) domain-containing protein 1 (CDCP1) is a transmembrane protein that regulates anchorage-independent growth and cancer cell migration and invasion. Expression of CDCP1 is detected in a number of cancer cell lines and tissues and is closely correlated with poor prognosis. Invadopodia are actin-based protrusions on the surface of   invasive   cancer cells that promote the degradation of the extracellular matrix (ECM) via localized proteolysis, which is mainly mediated by membrane type 1 matrix metalloproteinase (MT1-MMP). MT1-MMP is accumulated at invadopodia by targeted delivery via membrane trafficking. The present study shows that CDCP1 is required for ECM degradation by invadopodia in human breast cancer and melanoma cells. CDCP1 localized to caveolin-1-containing vesicular structures and lipid rafts and was detected in close proximity to invadopodia. Further biochemical analysis revealed that substantial amounts of CDCP1 existed in the Triton X-100 insoluble lipid raft fraction. CDCP1 was coimmunoprecipitated with MT1-MMP and colocalized with MT1-MMP at the vesicular structures. The siRNA-mediated knockdown of the CDCP1 expression markedly inhibited MT1-MMP-dependent ECM degradation and Matrigel invasion and reduced the accumulation of MT1-MMP at invadopodia, as shown by immunofluorescence analysis. These results indicate that CDCP1 is an essential regulator of the trafficking and function of MT1-MMP- and invadopodia-mediated invasion of cancer cells. 
 A novel protein associated with membrane-type 1 matrix metalloproteinase binds p27(kip1) and regulates RhoA activation, actin remodeling, and matrigel invasion. Pericellular proteolysis by membrane-type 1 matrix metalloproteinase (MT1-MMP) plays a pivotal role in tumor cell invasion. Localization of MT1-MMP at the invasion front of cells, e.g. on lamellipodia and invadopodia, has to be regulated in coordination with reorganization of the actin cytoskeleton. However, little is known about how such invasion-related actin structures are regulated at the sites where MT1-MMP localizes. During analysis of MT1-MMP-associated proteins, we identified a heretofore uncharacterized protein. This protein, which we call p27RF-Rho, enhances activation of RhoA by releasing it from inhibition by p27(kip1) and thereby regulates actin structures. p27(kip1) is a well known cell cycle regulator in the nucleus. In contrast, cytoplasmic p27(kip1) has been demonstrated to bind GDP-RhoA and inhibit GDP-GTP exchange mediated by guanine nucleotide exchange factors. p27RF-Rho binds p27(kip1) and prevents p27(kip1) from binding to RhoA, thereby freeing the latter for activation. Knockdown of p27RF-Rho expression renders cells resistant to RhoA activation stimuli, whereas overexpression of p27RF-Rho sensitizes cells to such stimulation. p27RF-Rho exhibits a punctate distribution in   invasive   human tumor cell lines. Stimulation of the cells with lysophosphatidic acid induces activation of RhoA and induces the formation of punctate actin structures within foci of p27RF-Rho localization. Some of the punctate actin structures co-localize with MT1-MMP and cortactin. Down-regulation of p27RF-Rho prevents both redistribution of actin into the punctate structures and tumor cell invasion. Thus, p27RF-Rho is a new potential target for cancer therapy development. 
 The E-cadherin-repressed hNanos1 gene induces tumor cell invasion by upregulating MT1-MMP expression. In this study, we examined the role of the E-cadherin-repressed gene human Nanos1 (hNanos1) in tumor invasion process. First, our in vivo study revealed that hNanos1 mRNAs were overexpressed in   invasive   lung carcinomas. Moreover, hNanos1 was co-localized with MT1-MMP (membrane type 1-matrix metalloproteinase) in E-cadherin-negative   invasive   lung tumor clusters. Using an inducible Tet-on system, we showed that induction of hNanos1 expression in DLD1 cells increased their migratory and   invasive   abilities in a three-dimensional migration and in a modified Boyden chamber assay. Accordingly, we demonstrated that hNanos1 upregulated MT1-MMP expression at the mRNA and protein levels. Inversely, using an RNA interference strategy to inhibit hNanos1 expression in   invasive   Hs578T, BT549 and BZR cancer cells, we observed a downregulation of MT1-MMP mRNA and protein and concomitantly a decrease of the   invasive   capacities of tumor cells in a modified Boyden chamber assay. Taken together, our results demonstrate that hNanos1, by regulating MT1-MMP expression, plays an important role in the acquisition of   invasive   properties by epithelial tumor cells. 
 The Wnt/planar cell polarity protein-tyrosine kinase-7 (PTK7) is a highly efficient proteolytic target of membrane type-1 matrix metalloproteinase: implications in cancer and embryogenesis. PTK7 is an essential component of the Wnt/planar cell polarity (PCP) pathway. We provide evidence that the Wnt/PCP pathway converges with pericellular proteolysis in both normal development and cancer. Here, we demonstrate that membrane type-1 matrix metalloproteinase (MT1-MMP), a key pro  invasive   proteinase, functions as a principal sheddase of PTK7. MT1-MMP directly cleaves the exposed PKP(621)↓LI sequence of the seventh Ig-like domain of the full-length membrane PTK7 and generates, as a result, an N-terminal, soluble PTK7 fragment (sPTK7). The enforced expression of membrane PTK7 in cancer cells leads to the actin cytoskeleton reorganization and the inhibition of cell invasion. MT1-MMP silencing and the analysis of the uncleavable L622D PTK7 mutant confirm the significance of MT1-MMP proteolysis of PTK7 in cell functions. Our data also demonstrate that a fine balance between the metalloproteinase activity and PTK7 levels is required for normal development of zebrafish (Danio rerio). Aberration of this balance by the proteinase inhibition or PTK7 silencing results in the PCP-dependent convergent extension defects in the zebrafish. Overall, our data suggest that the MT1-MMP-PTK7 axis plays an important role in both cancer cell invasion and normal embryogenesis in vertebrates. Further insight into these novel mechanisms may promote understanding of directional cell motility and lead to the identification of therapeutics to treat PCP-related developmental disorders and malignancy. 
 Epigenetic control of the invasion-promoting MT1-MMP/MMP-2/TIMP-2 axis in cancer cells. Membrane type-1 matrix metalloproteinase (MT1-MMP) is an activator of soluble MMP-2. The activity of both MMPs is regulated by their physiological inhibitor TIMP-2. An MT1-MMP/MMP-2/TIMP-2 axis plays a key role in the   invasive   behavior of many cell types. Despite its importance, epigenetic control of this pro-  invasive   axis is insufficiently studied, and, as a result, its modification in a rational and clinically beneficial manner is exceedingly difficult. Therefore, we performed an epigenetic analysis of the MT1-MMP, MMP-2, and TIMP-2 gene promoters in highly migratory glioblastoma cells and in low migratory breast carcinoma MCF-7 cells. We determined, for the first time, that the epigenetic control leading to the transcriptional silencing of both MMPs includes hypermethylation of the corresponding CpG regions and histone H3 lysine-27 trimethylation (H3K27me3). In turn, undermethylation of the CpG islands and low levels of histone H3 lysine-27 trimethylation are features of transcriptionally active MT1-MMP and MMP-2 genes in   invasive   cancer cells. Additional histone modifications we have analyzed, including H3ac and H3K4me2, are present in both transcriptionally active and inactive promoters of both MMPs. Histone H3 lysine-4 trimethylation is likely to play no significant role in regulating MT1-MMP and MMP-2. The pattern of epigenetic regulation of TIMP-2 was clearly distinct from that of MMPs and included the coordinated methylation and demethylation of the two CpG regions in the promoter. Our results suggest that the epigenetic control plays an important role in both the balanced regulation of the MT1-MMP/MMP-2/TIMP-2 axis and the   invasive   behavior in cancer cells. 
 Polarized MT1-MMP-CD44 interaction and CD44 cleavage during cell retraction reveal an essential role for MT1-MMP in CD44-mediated invasion. The adhesion molecule CD44 and the membrane-type matrix metalloproteinase MT1-MMP act coordinately in tumor cells to promote cell invasion through a yet unclear mechanism. We are interested in studying the interplay between CD44 and MT1-MMP in carcinoma cells embedded in HA containing three-dimensional collagen I matrices (3D HA-Col I) by time-lapse confocal microscopy imaging. Here we report the in vivo interaction between CD44 and MT1-MMP, revealed by fluorescence resonance energy transfer (FRET) microscopy. MT1-MMP interacts with CD44 preferentially at the trailing edge of the invading tumor cells during rear retraction and on membrane fragments released during the invasion process. A fluorescent biosensor designed to monitor the proteolytic processing of CD44 by live cell imaging demonstrates that cleavage of the CD44 extracellular domain is enriched in the retracting rear ends of   invasive   tumor cells. Invasion assays showed that MT1-MMP mediates CD44-dependent tumor-cell invasion, whereas CD44 is not essential for MT1-MMP-mediated invasion of 3D HA-Col I matrices. Together, our results support a role for MT1-MMP in cell retraction during CD44-mediated cell invasion. 
 mRNA expression of the five membrane-type matrix metalloproteinases MT1-MT5 in human prostatic cell lines and their down-regulation in human malignant prostatic tissue. The aim of this study was to assess the expression of membrane-type matrix metalloproteinases (MT-MMPs) 1-5 in the human prostatic cell lines BPH-1, LNCaP, DU 145, PC-3, in malignant and non-malignant prostatic tissue samples, and in epithelial cells cultured from these tissue samples.Matched malignant and non-malignant tissue specimens were obtained from 12 men with untreated prostate carcinoma after radical prostatectomy. Expression of mRNA for the five MT-MMPs was quantified by real-time PCR technique and normalized to the expression of the housekeeping gene glyceraldehyde-3-phosphate dehydrogenase (GAPDH).The expression of the five MT-MMPs was distinctly different not only between the prostate cell lines but also varied in the same cell line. There was a general higher expression of all MT-MMPs except for MT3-MMP in the androgen-insensitive cells DU 145 and PC-3 compared with that in the androgen-sensitive LNCaP cells. Their relatively high expression in the benign prostatic cell line BPH-1 and also in the primary cell cultures from malignant and non-malignant tissue samples argues against a simple association between MT-MMP expression and   invasive  ness. In malignant tissue samples and their corresponding cell cultures, the expression of most MT-MMPs was down-regulated in comparison to the normal counterparts. There was no correlation between tumor classification data and the MT-MMP expression results.In contrast to other carcinoma, the down-regulation of most MT-MMPs is typical for prostate carcinoma. It seems to occur mainly in epithelial cells and has to be examined as special characteristic of this tumor entity in further studies. 
 GI24 enhances tumor   invasive  ness by regulating cell surface membrane-type 1 matrix metalloproteinase. GI24, an immunoglobulin superfamily member, has been cloned from a placenta cDNA library as a gene product that promoted activation of matrix metalloproteinase (MMP)-2 mediated by membrane type (MT) 1-MMP. Co-expression of GI24 with MT1-MMP in HEK293T cells increased the cell-surface level of MT1-MMP concomitant with the cleavage of the GI24 at the juxtamembrane site to shed the extracellular domain. HT1080 fibrosarcoma cells stably transfected with the GI24 gene expressed a higher level of MT1-MMP and showed more   invasive   ability in collagen gel than the control cells. GI24 was cleaved in HT1080 cells, which was blocked by the administration of MMP inhibitor BB94 or transfection of small interfering RNA (siRNA) targeting MT1-MMP. GI24 expression is relatively high in some squamous carcinoma and hepatocarcinoma cell lines. Transfection of siRNA for GI24 into oral squamous carcinoma-derived HSC-4 cells, which express GI24 and MT1-MMP genes reduced the expression of not only GI24 but also MT1-MMP, and attenuated   invasive   growth in the collagen matrix. These results suggest that GI24 contributes to tumor-  invasive   growth in the collagen matrix by augmenting cell surface MT1-MMP. 
 Overexpression of proto-oncogene FBI-1 activates membrane type 1-matrix metalloproteinase in association with adverse outcome in ovarian cancers. FBI-1 (factor that binds to the inducer of short transcripts of human immunodeficiency virus-1) is a member of the POK (POZ and Kruppel) family of transcription factors and play important roles in cellular differentiation and oncogenesis. Recent evidence suggests that FBI-1 is expressed at high levels in a subset of human lymphomas and some epithelial solid tumors. However, the function of FBI-1 in human ovarian cancers remains elusive.In this study, we investigated the role of FBI-1 in human ovarian cancers, in particularly, its function in cancer cell invasion via modulating membrane type 1-matrix metalloproteinase (MT1-MMP). Significantly higher FBI-1 protein and mRNA expression levels were demonstrated in ovarian cancers samples and cell lines compared with borderline tumors and benign cystadenomas. Increased FBI-1 mRNA expression was correlated significantly with gene amplification (P = 0.037). Moreover, higher FBI-1 expression was found in metastatic foci (P = 0.036) and malignant ascites (P = 0.021), and was significantly associated with advanced stage (P = 0.012), shorter overall survival (P = 0.032) and disease-free survival (P = 0.016). In vitro, overexpressed FBI-1 significantly enhanced cell migration and invasion both in OVCA 420 and SKOV-3 ovarian carcinoma cells, irrespective of p53 status, accompanied with elevated expression of MT1-MMP, but not MMP-2 or TIMP-2. Moreover, knockdown of MT1-MMP abolished FBI-1-mediated cell migration and invasion. Conversely, stable knockdown of FBI-1 remarkably reduced the motility of these cells with decreased expression of MT1-MMP. Promoter assay and chromatin immunoprecipitation study indicated that FBI-1 could directly interact with the promoter spanning ~600 bp of the 5'-flanking sequence of MT1-MMP and enhanced its expression in a dose-dependent manner. Furthermore, stable knockdown and ectopic expression of FBI-1 decreased and increased cell proliferation respectively in OVCA 420, but not in the p53 null SKOV-3 cells.Our results suggested an important role of FBI-1 in ovarian cancer cell proliferation, cell mobility, and   invasive  ness, and that FBI-1 can be a potential target of chemotherapy. 
 Matrix metalloproteinase localisation by in situ-RT-PCR in archival human breast biopsy material. Utilising archival human breast cancer biopsy material we examined the stromal/epithelial interactions of several matrix metalloproteinases (MMPs) using in situ-RT-PCR (IS-RT-PCR). In breast cancer, the stromal/epithelial interactions that occur, and the site of production of these proteases, are central to understanding their role in   invasive   and metastatic processes. We examined MT1-MMP (MMP-14, membrane type-1-MMP), MMP-1 (interstitial collagenase) and MMP-3 (stromelysin-1) for their localisation profile in progressive breast cancer biopsy material (poorly differentiated   invasive   breast carcinoma (PDIBC),   invasive   breast carcinomas (IBC) and lymph node metastases (LNM)). Expression of MT1-MMP, MMP-1 and MMP-3 was observed in both the tumour epithelial and surrounding stromal cells in most tissue sections examined. MT1-MMP expression was predominantly localised to the tumour component in the pre-  invasive   lesions. MMP-1 gene expression was relatively well distributed between both tissue compartments, while MMP-3 demonstrated highest expression levels in the stromal tissue surrounding the epithelial tumour cells. The results demonstrate the ability to distinguish compartmental gene expression profiles using IS-RT-PCR. Further, we suggest a role for MT1-MMP in early tumour progression, expression of MMP-1 during metastasis and focal expression pattern of MMP-3 in areas of expansion. These expression profiles may provide markers for early breast cancer diagnoses and present potential therapeutic targets. 
 Development of PEGylated peptide probes conjugated with (18)F-labeled BODIPY for PET/optical imaging of MT1-MMP activity. Since the processing activity of the matrix metalloproteinase MT1-MMP regulates various cellular functions such as motility, invasion, growth, differentiation and apoptosis, precise in vivo evaluation of MT1-MMP activity in cancers can provide beneficial information for both basic and clinical studies. For this purpose, we designed a cleavable Positron Emission Tomography (PET)/optical imaging probe consisting of BODIPY650/665 and polyethylene glycol (PEG) conjugated to opposite ends of MT1-MMP substrate peptides. We used in vitro and in vivo fluorescence experiments to select suitable substrate peptide sequences and PEG sizes for the MT1-MMP probes and obtained an optimized structure referred to here as MBP-2k. Radiofluorinated MBP-2k ([(18)F]MBP-2k) was then successfully synthesized via an (18)F-(19)F isotopic exchange reaction in BODIPY650/665. After intravenous injection into mice with xenografted tumors, [(18)F]MBP-2k showed significantly higher accumulation in HT1080 tumors with high MT1-MMP activity than in A549 tumors that have low MT1-MMP activity. Moreover, PET images showed better contrast in HT1080 tumors. These results show that [(18)F]MBP-2k can be used as a hybrid PET/optical imaging agent and is a promising probe for non-  invasive   monitoring of MT1-MMP activity in cancers. This probe may also efficiently combine targeted tumor imaging with image-guided surgery that could be beneficial for patients in the future. 
 Tumor cell invasion of von Hippel Lindau renal cell carcinoma cells is mediated by membrane type-1 matrix metalloproteinase. Metastatic renal cell carcinoma (RCC) remains the leading cause of mortality in patients with clear cell RCC arising from mutations in the von Hippel Lindau (VHL) tumor suppressor. Successful RCC tumor suppression by VHL requires the negative regulation of hypoxia inducible factor alpha (HIF alpha) protein and its downstream targets. Thus, identification of HIF target genes responsible for RCC tumor progression will aid in the development of therapies for this disease. We previously identified membrane type-1 matrix metalloproteinase (MT1-MMP) as a transcriptional target of HIF-2alpha in RCC cells null for VHL and showed that MT1-MMP is overexpressed in these cells. MT1-MMP is a key regulator of tumor progression through its functions as a matrix-degrading enzyme, as well as its ability to cleave factors, such as adhesion molecules and other MMPs. The aim of this study was to investigate the contribution of MT1-MMP to the   invasive   potential of RCC cells using in vitro type I collagen degradation and invasion assays.We evaluated RCC cells wild-type (WT8) and null (pRc-9) for VHL for   invasive   characteristics and showed that the pRc-9 cells demonstrated a greater propensity for both invasion and degradation of a type I collagen matrix. Furthermore, overexpression of either HIF-2alpha or MT1-MMP in the poorly   invasive   cell line, WT8, promoted collagen degradation and invasion of these cells. Finally, using RNAi, we show that inhibition of MT1-MMP suppresses tumor cell invasion of RCC cells.Our results suggest that MT1-MMP is a major mediator of tumor cell   invasive  ness and type I collagen degradation by VHL RCC cells that express either MT1-MMP or HIF-2alpha. As such, MT1-MMP may represent a novel target for anti-invasion therapy for this disease. 
 Association of matrix metalloproteinase-1 gene polymorphism with glioblastoma multiforme in a northern Indian population. Matrix metalloproteinase-1 (MMP-1) is known to be involved in the pathogenesis of glioma. It damages the extra-cellular matrix to produce   invasive  ness in cancer tissue, and hence has a direct effect in cancer invasion. The study aims to explore the association of single nucleotide polymorphism of -1607 MMP-1 gene with susceptibility to glioblastoma multiforme (GBM) in northern Indian subjects. One hundred and ten GBM patients and 150 healthy controls were included in this study. 1607 MMP-1 gene was studied by PCR-RFLP; different genotypes being combinations of 1G and 2G allele (1G/1G, 1G/2G and 2G/2G). 2G/2G genotype was significantly associated with GBM patients (OR, 2.24; P = 0.016; 95% CI, 1.16-4.30) as compared to controls. Prevalence of the 2G allele of -1607 MMP-1 polymorphism was significantly greater in GBM patients as compared to controls (62.3 vs 48.3%, OR, 1.76; P = 0.002; 95% CI, 1.23-2.52). This study suggests that the 2G/2G genotype and 2G allele of -1607 MMP-1 polymorphism are associated with an increased susceptibility for developing GBM. 
 Biochemical evidence of the interactions of membrane type-1 matrix metalloproteinase (MT1-MMP) with adenine nucleotide translocator (ANT): potential implications linking proteolysis with energy metabolism in cancer cells. Invasion-promoting MT1-MMP (membrane type-1 matrix metalloproteinase) is a key element in cell migration processes. To identify the proteins that interact and therefore co-precipitate with this proteinase from cancer cells, we used the proteolytically active WT (wild-type), the catalytically inert E240A and the C-end truncated (tailless; DeltaCT) MT1-MMP-FLAG constructs as baits. The identity of the pulled-down proteins was determined by LC-MS/MS (liquid chromatography tandem MS) and then confirmed by Western blotting using specific antibodies. We determined that, in breast carcinoma MCF cells (MCF-7 cells), ANT (adenine nucleotide translocator) efficiently interacted with the WT, E240A and DeltaCT constructs. The WT and E240A constructs also interacted with alpha-tubulin, an essential component of clathrin-mediated endocytosis. In turn, tubulin did not co-precipitate with the DeltaCT construct because of the inefficient endocytosis of the latter, thus suggesting a high level of selectivity of our test system. To corroborate these results, we then successfully used the ANT2-FLAG construct as a bait to pull-down MT1-MMP, which was naturally produced by fibrosarcoma HT1080 cells. We determined that the presence of the functionally inert catalytic domain alone was sufficient to cause the proteinase to interact with ANT2, thus indicating that there is a non-proteolytic mode of these interactions. Overall, it is tempting to hypothesize that by interacting with pro-  invasive   MT1-MMP, ANT plays a yet to be identified role in a coupling mechanism between energy metabolism and pericellular proteolysis in migrating cancer cells. 
 Multi-step pericellular proteolysis controls the transition from individual to collective cancer cell invasion.   Invasive   cell migration through tissue barriers requires pericellular remodelling of extracellular matrix (ECM) executed by cell-surface proteases, particularly membrane-type-1 matrix metalloproteinase (MT1-MMP/MMP-14). Using time-resolved multimodal microscopy, we show how   invasive   HT-1080 fibrosarcoma and MDA-MB-231 breast cancer cells coordinate mechanotransduction and fibrillar collagen remodelling by segregating the anterior force-generating leading edge containing beta1 integrin, MT1-MMP and F-actin from a posterior proteolytic zone executing fibre breakdown. During forward movement, sterically impeding fibres are selectively realigned into microtracks of single-cell calibre. Microtracks become expanded by multiple following cells by means of the large-scale degradation of lateral ECM interfaces, ultimately prompting transition towards collective invasion similar to that in vivo. Both ECM track widening and transition to multicellular invasion are dependent on MT1-MMP-mediated collagenolysis, shown by broad-spectrum protease inhibition and RNA interference. Thus,   invasive   migration and proteolytic ECM remodelling are interdependent processes that control tissue micropatterning and macropatterning and, consequently, individual and collective cell migration. 
 MT1-MMP- and Cdc42-dependent signaling co-regulate cell invasion and tunnel formation in 3D collagen matrices. Complex signaling events control tumor invasion in three-dimensional (3D) extracellular matrices. Recent evidence suggests that cells utilize both matrix metalloproteinase (MMP)-dependent and MMP-independent means to traverse 3D matrices. Herein, we demonstrate that lysophosphatidic-acid-induced HT1080 cell invasion requires membrane-type-1 (MT1)-MMP-mediated collagenolysis to generate matrix conduits the width of a cellular nucleus. We define these spaces as single-cell invasion tunnels (SCITs). Once established, cells can migrate within SCITs in an MMP-independent manner. Endothelial cells, smooth muscle cells and fibroblasts also generate SCITs during   invasive   events, suggesting that SCIT formation represents a fundamental mechanism of cellular motility within 3D matrices. Coordinated cellular signaling events are required during SCIT formation. MT1-MMP, Cdc42 and its associated downstream effectors such as MRCK (myotonic dystrophy kinase-related Cdc42-binding kinase) and Pak4 (p21 protein-activated kinase 4), protein kinase Calpha and the Rho-associated coiled-coil-containing protein kinases (ROCK-1 and ROCK-2) coordinate signaling necessary for SCIT formation. Finally, we show that MT1-MMP and Cdc42 are fundamental components of a co-associated invasion-signaling complex that controls directed single-cell invasion of 3D collagen matrices. 
 Membrane-type 1 matrix metalloproteinase regulates cell migration during zebrafish gastrulation: evidence for an interaction with non-canonical Wnt signaling. Key to   invasive  ness is the ability of tumor cells to modify the extracellular matrix, become motile, and engage in directed migration towards the vasculature. One significant protein associated with metastatic progression is membrane-type 1 matrix metalloproteinase (MT1-MMP/MMP14). How MMP14 activity is coordinated with other signaling pathways to regulate cell migration in vivo is largely unknown. Here we have used zebrafish embryogenesis as a model to understand the potential relationship between MMP14-dependent pericellular proteolysis, cell polarity, and motility. Knockdown of zebrafish Mmp14 function disrupted gastrulation convergence and extension cell movements and craniofacial morphogenesis. Using time-lapse imaging and morphometric analyses, we show that Mmp14 is required for proper cell polarity underlying the directed migration of mesodermal cells during gastrulation. We have identified a genetic interaction between mmp14 and non-canonical Wnt signaling, a pathway that also regulates cell polarity in embryonic tissues and is increasingly being linked with tumor cell migration. Finally, we demonstrate that Van Gogh-like 2, a key regulator of the non-canonical Wnt pathway, co-localizes with MMP14 and becomes redistributed towards the leading edge of polarized human cancer cells. Together, our results support the notion that pathways regulating pericellular proteolysis and cell polarity converge to promote efficient cell migration. 
 NHE1 mediates MDA-MB-231 cells invasion through the regulation of MT1-MMP. Na⁺/H⁺ exchanger 1 (NHE1), an important regulator of intracellular pH (pH(i)) and extracellular pH (pH(e)), has been shown to play a key role in breast cancer metastasis. However, the exact mechanism by which NHE1 mediates breast cancer metastasis is not yet well known. We showed here that inhibition of NHE1 activity, with specific inhibitor Cariporide, could suppress MDA-MB-231 cells invasion as well as the activity and expression of MT1-MMP. Overexpression of MT1-MMP resulted in a distinguished increase in MDA-MB-231 ce  lls inva  siveness, but treatment with Cariporide reversed the MT1-MMP-mediated enhan  ced inva  siveness. To explore the role of MAPK signaling pathways in NHE1-mediated breast cancer metastasis, we compared the difference of constitutively phosphorylated ERK1/2, p38 MAPK and JNK in   non-inva  sive MCF-7 cells   and inva  sive MDA-MB-231 cells. Interestingly, we found that the phosphorylation levels of ERK1/2 and p38 MAPK in MDA-MB-231 cells were higher than in MCF-7 cells, but both MCF-7 cells and MDA-MB-231 cells expressed similar constitutively phosphorylated JNK. Treating MDA-MB-231 cells with Cariporide led to decreased phosphorylation level of both p38 MAPK and ERK1/2 in a time-dependent manner, but JNK activity was not influenced. Supplementation with MAPK inhibitor (MEK inhibitor PD98059, p38 MAPK inhibitor SB203580 and JNK inhibitor SP600125) or Cariporide all exhibited significant depression of MDA-MB-231 cells invasion and MT1-MMP expression. Furthermore, we co-treated MDA-MB-231 cells with MAPK inhibitor and Cariporide. The result showed that Cariporide synergistically suppressed invasion and MT1-MMP expression with MEK inhibitor and p38 MAPK inhibitor, but not be synergistic with the JNK inhibitor. These findings suggest that NHE1 mediates MDA-MB-231 cells invasion partly through regulating MT1-MMP in ERK1/2 and p38 MAPK signaling pathways dependent manner. 
 Influence of β-catenin small interfering RNA on human osteosarcoma cells. This study examined the effect of small interfering RNA-mediated β-catenin knockdown on the survival, invasion and chemosensitivity of human osteosarcoma cells (U2-OS cells). The siRNA against β-catenin was constructed and transfected into U2-OS cells. The expression of β-catenin was detected by qRT-PCR and Western blotting. Cell growth and apoptosis was detected in the presence or absence of doxorubicin by MTT and flow cytometry, respectively. Cell invasion ability was measured by transwell assay. The results showed that the transfection of β-catenin siRNA resulted in decreased expression of β-catenin, suppression of invasion and motility of U2-OS cells, reduced chemosensitivity to doxorubicin in vitro, and little change in cell growth and apoptosis. Additionally, down-regulated MT1-MMP expression was found after transfection. It was concluded that knockdown of β-catenin gene may decrea  se the i  nvasive ability of human osteosarcoma cells through down-regulated MT1-MMP expression, and the chemosensitivity of osteosarcoma cells against doxorubicin. 
 Suppression of membrane-type 1 matrix metalloproteinase (MMP)-mediated MMP-2 activation and tumor invasion by testican 3 and its splicing variant gene product, N-Tes. Using expression cloning to screen a human fetal kidney cDNA library for regulator(s) of pro-matrix metalloproteinase (MMP)-2 processing mediated by membrane-type (MT) 1 MMP, we isolated a cDNA whose product interfered with pro-MMP-2 activation. It encodes the NH(2)-terminal 313-amino acid region of a calcium-binding proteoglycan, testican 3, with a 3-amino acid substitution at the COOH terminus and thus was named N-Tes. N-Tes comprises a signal peptide, a unique domain, a follistatin-like domain, and a Ca(2+)-binding domain but lacks a COOH-terminal thyroglobulin domain and two putative glycosaminoglycan attachment sites of testican 3. Pro-MMP-2 activation by MT3-MMP was also inhibited by the coexpression of N-Tes. Immunoprecipitation analysis demonstrated direct interaction of N-Tes with either MT1-MMP or MT3-MMP. Expression of testican 1 or testican 3 but not testican 2 also inhibited pro-MMP-2 activation by either MT1-MMP or MT3-MMP. Deletion and substitution of amino acids residues in N-Tes revealed that the unique NH(2)-terminal domain of N-Tes is responsible for the inhibition of pro-MMP-2 activation by MT-MMPs. Expression of N-Tes and testican 3 was detected in normal brain but down-regulated in glioma tissues. Transfection of either the N-Tes or testican 3 gene into U251 glioma cells or Madin-Darby canine kidney cells transformed by erbB2 suppressed their   invasive   growth in collagen gel. These results suggest that both N-Tes and testican 3 would interfere with tumor invasion by inhibiting MT-MMPs. 
 mDia1 regulates breast cancer invasion by controlling membrane type 1-matrix metalloproteinase localization. Mammalian diaphanous-related formin 1 (mDia1) expression has been linked with progression of malignant cancers in various tissues. However, the precise molecular mechanism underlying mDia1-mediated invasion in cancer cells has not been fully elucidated. In this study, we found that mDia1 is upregulated in   invasive   breast cancer cells. Knockdown of mDia1 in   invasive   breast cancer profoundly reduced   invasive   activity by controlling cellular localization of membrane type 1-matrix metalloproteinase (MT1-MMP) through interaction with microtubule tracks. Gene silencing and ectopic expression of the active form of mDia1 showed that mDia1 plays a key role in the intracellular trafficking of MT1-MMP to the plasma membrane through microtubules. We also demonstrated that highly   invasive   breast cancer cells possessed   invasive   activity in a 3D culture system, which was significantly reduced upon silencing mDia1 or MT1-MMP. Furthermore, mDia1-deficient cells cultured in 3D matrix showed impaired expression of the cancer stem cell marker genes, CD44 and CD133. Collectively, our findings suggest that regulation of cellular trafficking and microtubule-mediated localization of MT1-MMP by mDia1 is likely important in breast cancer invasion through the expression of cancer stem cell genes. 
 Membrane type 1 matrix metalloproteinase is a crucial promoter of synovial invasion in human rheumatoid arthritis. A hallmark of rheumatoid arthritis (RA) is invasion of the synovial pannus into cartilage, and this process requires degradation of the collagen matrix. The aim of this study was to explore the role of one of the collagen-degrading matrix metalloproteinases (MMPs), membrane type 1 MMP (MT1-MMP), in synovial pannus   invasive  ness.The expression and localization of MT1-MMP in human RA pannus were investigated by Western blot analysis of primary synovial cells and immunohistochemical analysis of RA joint specimens. The functional role of MT1-MMP was analyzed by 3-dimensional (3-D) collagen invasion assays and a cartilage invasion assay in the presence or absence of tissue inhibitor of metalloproteinases 1 (TIMP-1), TIMP-2, or GM6001. The effect of adenoviral expression of a dominant-negative MT1-MMP construct lacking a catalytic domain was also examined.MT1-MMP was highly expressed at the pannus-cartilage junction in RA joints. Freshly isolated rheumatoid synovial tissue and isolated RA synovial fibroblasts invaded into a 3-D collagen matrix in an MT1-MMP-dependent manner. Invasion was blocked by TIMP-2 and GM6001 but not by TIMP-1. Invasion was also inhibited by the overexpression of a dominant-negative MT1-MMP, which inhibits collagenolytic activity and proMMP-2 activation by MT1-MMP on the cell surface. Synovial fibroblasts also invaded into cartilage in an MT1-MMP-dependent manner. This process was further enhanced by removing aggrecan from the cartilage matrix.MT1-MMP serves as an essential collagen-degrading proteinase during pannus invasion in human RA. Specific inhibition of MT1-MMP-dependent invasion may represent a novel therapeutic strategy for RA. 
 A role of MMP-14 in the regulation of   invasive  ness of nasopharyngeal carcinoma. Although matrix metalloproteinase 14 (MMP-14) has been shown to play a substantial role in the carcinogenesis of some types of cancer, its involvement in the pathogenesis of nasopharyngeal carcinoma (NPC) has not been reported. Here, we analyzed MMP-14 levels in the NPC specimens from patients and compared with the paired normal nasopharynx (NNP) tissues. We found that NPC had significantly higher messenger RNA (mRNA) and protein levels of MMP-14. Moreover, higher levels of MMP-14 correlated with more advanced status of clinical stage and lymphatic metastasis. In vitro, MMP-14 levels determined the potential of   invasive  ness of NPC cells, possibly through induction of EMT-associated genes. Our data thus highlight MMP-14 as a novel therapeutic target for NPC. 
 The hepatitis B virus X protein promotes tumor cell invasion by inducing membrane-type matrix metalloproteinase-1 and cyclooxygenase-2 expression. Hepatocellular carcinoma is strongly associated with chronic infection by the hepatitis B virus (HBV) and has poor prognosis due to intrahepatic metastasis. HBx is often the only HBV protein detected in hepatic tumor cells; however, its contribution to tumor invasion and metastasis has not been established so far. In this work, we show that HBx enhances tumor cell invasion, both in vivo and in vitro. The increased   invasive   capacity induced by HBx is mediated by an upregulation of membrane-type 1 matrix metalloproteinase (MT1-MMP) expression, which in turn activates matrix metalloproteinase-2. Induction of both MT1-MMP expression and cell invasion by HBx is dependent on cyclooxygenase-2 (COX-2) activity. In addition, HBx upregulates the expression of COX-2, which is mediated by the transcriptional activation of the COX-2 gene promoter in a nuclear factor of activated T cell-dependent (NF-AT-dependent) manner. These results demonstrate the ability of HBx to promote tumor cell invasion by a mechanism involving the upregulation of MT1-MMP and COX-2 and provide new insights into the mechanism of action of this viral protein and its involvement in tumor metastasis and recurrence of hepatocellular carcinoma. 
 Low collagenase-1 (MMP-1) and MT1-MMP expression levels are favourable survival markers in advanced colorectal carcinoma. Extracellular matrix degradation is required for   invasive   growth and metastasis formation in colorectal carcinoma; therefore, we examined matrix metalloproteinases expression (MMP-1, MMP-13 and MT1-MMP) and apoptosis in tumours from 49 patients with advanced colorectal disease.MMP expression was determined immunohistochemically and apoptotic index (AI) was ascertained using the TUNEL assay.Low levels of MT1-MMP, MMP-1 and AI were found to be favourable markers significantly associated with longer survival. MT1-MMP expression levels below the median (</=14.0% of tumour cells) were associated with better overall survival (median 26.2 vs. 15.6 months, p = 0.02) and MMP-1 expression levels below the median (</=28.7% of tumour cells) correlated with longer survival following metastasis (median 21.5 vs. 13.3 months, p = 0.05). MT1-MMP, MMP-1 and AI were all found to have significant independent effects on survival. Interestingly, MMP-1 expression levels above the median were associated with distal disease of the colon (p = 0.02).These findings reveal that MT1-MMP and MMP-1 expression levels and AI are useful prognostic indicators in advanced colorectal carcinoma and suggest that markers of MMP expression might be used in identifying patients who would benefit from new treatment modalities involving MMP inhibitors. 
 Gene expression of adhesion molecules and matrix metalloproteinases in endometriosis. Various types of cell adhesion molecules and matrix metalloproteinases (MMPs) seem to play an important role in the invasion process of endometriosis; however, limited investigation has focused on their gene expression in human peritoneal endometriotic lesions. A total of 63 endometriotic tissues were surgically obtained from 35 women with endometriosis, which included 43 pigmented and 20 non-pigmented lesions. Gene expression levels of E-cadherin, alpha- and beta-catenin, MMP-2, MMP-9 and membrane-type 1 (MT1)-MMP in these endometriotic lesions were compared with those in normal eutopic endometrium obtained from 12 women without endometriosis. MMP-2, MMP-9 and MT1-MMP mRNA expression in pigmented lesions was significantly higher than that in normal endometrium (p < 0.05), whereas E-cadherin, alpha- and beta-catenin mRNA expression was not suppressed in endometriotic lesions. There was a close correlation between MMP-2 or MT1-MMP and E-cadherin, alpha- or beta-catenin gene expression in 63 endometriotic tissues examined (p < 0.01). Immunohistochemical expression of E-cadherin, alpha- and beta-catenin in glandular epithelial cells was positive not only for all of seven cases with normal eutopic endometrium but also for 9 of 11 with ovarian endometriosis. MMP expression in ectopic endometrium was much greater than that in eutopic endometrium. These results suggest that endometriotic tissues expressing MMPs might be   invasive   and simultaneously possess cell-to-cell adhesion property in pelvic peritoneal foci. 
 Silencing of the MT1-MMP/ G6PT axis suppresses calcium mobilization by sphingosine-1-phosphate in glioblastoma cells. The contributions of membrane type-1 matrix metalloproteinase (MT1-MMP) and of the glucose-6-phosphate transporter (G6PT) in sphingosine-1-phosphate (S1P)-mediated Ca(2+) mobilization were assessed in glioblastoma cells. We show that gene silencing of MT1-MMP or G6PT decreased the extent of S1P-induced Ca(2+) mobilization, chemotaxis, and extracellular signal-related kinase phosphorylation. Chlorogenic acid and (-)-epigallocatechin-3-gallate, two diet-derived inhibitors of G6PT and of MT1-MMP, respectively, reduced S1P-mediated Ca(2+) mobilization. An intact MT1-MMP/G6PT signaling axis is thus required for efficient Ca(2+) mobilization in response to bioactive lipids such as S1P. Targeted inhibition of either MT1-MMP or G6PT may lead to reduced infiltrative and   invasive   properties of brain tumor cells. 
 3-phosphoinositide-dependent protein kinase-1 (PDK1) promotes invasion and activation of matrix metalloproteinases. Metastasis is a major cause of morbidity and mortality in breast cancer with tumor cell invasion playing a crucial role in the metastatic process. PDK1 is a key molecule that couples PI3K to cell proliferation and survival signals in response to growth factor receptor activation, and is oncogenic when expressed in mouse mammary epithelial cells. We now present evidence showing that PDK1-expressing cells exhibit enhanced anchorage-dependent and -independent cell growth and are highly   invasive   when grown on Matrigel. These properties correlate with induction of MMP-2 activity, increased MT1-MMP expression and a unique gene expression profile.Invasion assays in Matrigel, MMP-2 zymogram analysis, gene microarray analysis and mammary isografts were used to characterize the   invasive   and proliferative function of cells expressing PDK1. Tissue microarray analysis of human breast cancers was used to measure PDK1 expression in   invasive   tumors by IHC.Enhanced invasion on Matrigel in PDK1-expressing cells was accompanied by increased MMP-2 activity resulting from stabilization against proteasomal degradation. Increased MMP-2 activity was accompanied by elevated levels of MT1-MMP, which is involved in generating active MMP-2. Gene microarray analysis identified increased expression of the ECM-associated genes decorin and type I procollagen, whose gene products are substrates of MT1-MMP. Mammary fat pad isografts of PDK1-expressing cells produced   invasive   adenocarcinomas. Tissue microarray analysis of human   invasive   breast cancer indicated that PDK1pSer241 was strongly expressed in 90% of samples.These results indicate that PDK1 serves as an important effector of mammary epithelial cell growth and invasion in the transformed phenotype. PDK1 mediates its effect in part by MT1-MMP induction, which in turn activates MMP-2 and modulates the ECM proteins decorin and collagen. The presence of increased PDK1 expression in the majority of   invasive   breast cancers suggests its importance in the metastatic process. 
 Membrane-type 1 matrix metalloproteinase expression is regulated by zonula occludens-1 in human breast cancer cells. The acquisition of a migratory/  invasive   phenotype by tumor cells is characterized by the loss of cell-cell adhesion contacts and the expression of degradative properties. In this study, we examined the effect of the disorganization of occludin/zonula occludens (ZO)-1 tight junction (TJ) complexes on the expression of membrane-type 1 matrix metalloproteinase (MT1-MMP). We first compared the expression of MT1-MMP and the localization of occludin/ZO-1 complexes in breast tumor cell lines displaying various degrees of   invasive  ness. We showed that the expression of MT1-MMP in   invasive   breast tumor cell lines correlates with the absence of occludin and with a cytoplasmic localization of ZO-1. In contrast, non  invasive   cell lines displayed a membrane staining for both ZO-1 and occludin and did not express MT1-MMP. In vivo, cytoplasmic ZO-1 and MT1-MMP could be detected in   invasive   tumor clusters of human breast carcinomas. We then used RNA interference strategy to inhibit ZO-1 expression in   invasive   BT549 cells and to evaluate the effect of ZO-1 down-regulation on MT1-MMP expression. We observed that ZO-1 small interfering RNA transfection down-regulates MT1-MMP mRNAs and proteins and subsequently decreases the ability of tumor cells to invade a reconstituted basement membrane in a Boyden chamber assay. Inversely, transfection of expression vectors encoding wild-type ZO-1 or the NH2-terminal fragment of ZO-1 comprising the PSD95/DLG/ZO-1 domains in BT549 activated a human MT1-MMP promoter luciferase reporter construct and increased cell   invasive  ness. Such transfections concomitantly activated the beta-catenin/TCF/LEF pathway. Our results therefore show that ZO-1, besides its structural role in TJ assembly, can intervene in signaling events promoting tumor cell invasion. 
 Lipid rafts and caveolin-1 are required for invadopodia formation and extracellular matrix degradation by human breast cancer cells. Invadopodia are ventral membrane protrusions through which   invasive   cancer cells degrade the extracellular matrix. They are thought to function in the migration of cancer cells through tissue barriers, which is necessary for cancer invasion and metastasis. Although many protein components of invadopodia have been identified, the organization and the role of membrane lipids in invadopodia are not well understood. In this study, the role of lipid rafts, which are cholesterol-enriched membrane microdomains, in the assembly and function of invadopodia in human breast cancer cells was investigated. Lipid rafts are enriched, internalized, and dynamically trafficked at invadopodia sites. Perturbation of lipid raft formation due to depleting or sequestering membrane cholesterol blocked the invadopodia-mediated degradation of the gelatin matrix. Caveolin-1 (Cav-1), a resident protein of lipid rafts and caveolae, accumulates at invadopodia and colocalizes with the internalized lipid raft membranes. Membrane type 1 matrix metalloproteinase (MT1-MMP), a matrix proteinase associated with invadopodia, is localized at lipid raft-enriched membrane fractions and cotrafficked and colocalized with Cav-1 at invadopodia. The small interfering RNA-mediated silencing of Cav-1 inhibited the invadopodia-mediated and MT1-MMP-dependent degradation of the gelatin matrix. Furthermore, Cav-1 and MT1-MMP are coexpressed in   invasive   human breast cancer cell lines that have an ability to form invadopodia. These results indicate that invadopodia are the sites where enrichment and trafficking of lipid rafts occur and that Cav-1 is an essential regulator of MT1-MMP function and invadopodia-mediated breast cancer cell invasion. 
 Graded activation of the MEK1/MT1-MMP axis determines renal epithelial cell tumor phenotype. Activation of Raf/Ras/mitogen-activated protein kinase (MEK)/mitogen-activated protein kinase signaling and elevated expression of membrane type-1 matrix metalloproteinase (MT1-MMP) are associated with von Hippel-Lindau gene alterations in renal cell carcinoma. We postulated that the degree of MEK activation was related to graded expression of MT1-MMP and the resultant phenotype of renal epithelial tumors. Madin Darby canine kidney epithelial cells transfected with a MEK1 expression plasmid yielded populations with morphologic phenotypes ranging from epithelial, mixed epithelial/mesenchymal to mesenchymal. Clones were analyzed for MEK1 activity, MT1-MMP expression and extent of epithelial-mesenchymal transition. Phenotypes of the MDCK-MEK1 clones were evaluated in vivo with nu/nu mice. Tissue microarray of renal cell cancers was quantitatively assessed for expression of phosphorylated MEK1 and MT1-MMP proteins and correlations drawn to Fuhrman nuclear grade. Graded increases in the MEK signaling module were associated with graded induction of epithelial-mesenchymal transition of the MDCK cells and induction of MT1-MMP transcription and synthesis. Inhibition of MEK1 and MT1-MMP activity reversed the epithelial-mesenchymal transition. Tumors generated by epithelial, mixed epithelial/mesenchymal and mesenchymal MDCK clones demonstrated a gradient of phenotypes extending from well-differentiated, fully encapsulated non-  invasive   tumors to tumors with an anaplastic morphology, high Fuhrman nuclear score, neoangiogenesis and invasion. Tumor microarray demonstrated a statistically significant association between the extent of phosphorylated MEK1, MT1-MMP expression and nuclear grade. We conclude that graded increases in the MEK1 signaling module are correlated with M1-MMP expression, renal epithelial cell tumor phenotype,   invasive   activity and nuclear grade. Phosphorylated MEK1 and MT1-MMP may represent novel, and mechanistic, biomarkers for the assessment of renal cell carcinoma. 
 Interaction of MT1-MMP and laminin-5gamma2 chain correlates with metastasis and   invasive  ness in human esophageal squamous cell carcinoma. To gain insights into metastatic mechanisms in esophageal squamous cell carcinoma (ESCC), we established sublines (MLuB1 and MLuC1) with different capacity of spontaneous lung metastasis by subcutaneous injection of a human ESCC cell line (EC 9706) into nude mices. The incidence of the mice with lung metastasis produced by MLuC1 (87%) was significantly higher than that of MLuB1 (22%). The gene expression profiles of the two sublines were compared with cDNA arrays containing 5,000 known genes, and 47 genes were differentially expressed > or =2.0 fold. Laminin-5gamma2 chain (Ln-5gamma2) was one of the up-regulated genes in MLuC1 cells. Proteolytically processed forms of gamma2 are known to promote migration of a multitude of epithelial cells in vitro. Western-blotting analysis revealed that degraded fragments of Ln-5gamma2 and active form of membrane-type matrix metalloproteinase-1 (MT1-MMP) in MLuC1 was significantly higher than those in MLuB1. Expression of MT1-MMP was observed in 60 of 75 Ln-5gamma2-positive carcinoma tissues (80%). Co-expression of the two proteins was significantly associated with depth of invasion (P = 0.012). Moreover, proteolytic fragments of Ln-5gamma2 and active forms of MT1-MMP were frequently found in tumor tissues, whereas in the corresponding normal esophageal tissues there were only intact forms of gamma2 and MT1-MMP. siRNA-mediated silencing of MT1-MMP significantly reduced production of gamma2' and gamma2x in MLuC1 cells and inhibited cell migration. The results suggest that MT1-MMP is an enzyme responsible for Ln-5gamma2 cleavage in ESCC, and interaction between them may play a critical role in promoting invasion and metastasis of human ESCC. 
 Neoplastic progression of the human breast cancer cell line G3S1 is associated with elevation of cytoskeletal dynamics and upregulation of MT1-MMP. The newly established breast cancer cell line G3S1, derived from EM-G3 breast cancer progenitors, was analyzed for functional changes related to neoplastic progression manifested by elevated   invasive  ness and enhanced capability to degrade gelatin. Degradation of gelatin and   invasive  ness of G3S1 cells was found to be dependent on the activity of matrix proteinases and actin cytoskeletal dynamics. Therefore, the expression and activity of these proteases was compared in G3S1 and EM-G3 cells. Despite enhanced capability of G3S1 cells to degrade gelatin, these cells exhibited lower levels of secreted extracellular matrix degrading proteases than parental EM-G3 cells. However, the expression of membrane-bound MT1-MMP was strongly elevated in G3S1 cells. While the degradation of gelatin was associated with invadopodia-like structures in both EM-G3 and G3S1 cells, the cytoskeletal remodeling dynamics was greatly elevated in G3S1 cells, suggesting that upregulation of MT1-MMP, together with elevation of cytoskeletal remodeling dynamics can effectively cause elevated   invasive  ness and enhanced matrix degrading capability in G3S1 cells. 
 YB-1 alters MT1-MMP trafficking and stimulates MCF-7 breast tumor invasion and metastasis. YB-1 is a member of the cold shock domain family, with complex roles in DNA structure, gene transcription and translation. YB-1 promotes chromosomal instability, and mammary gland transgenic expression induces tumors with 100% penetrance. YB-1 is linked to poor prognosis in breast carcinoma and is a strong predictor of relapse and disease-specific survival. Survival is directly tied to the extent of local invasion and distal metastasis, processes dependent upon the activity of the membrane type I-matrix metalloproteinase, MT1-MMP. Non-  invasive   MCF-7 breast adenocarcinoma cells were transfected with YB-1/EGFP. YB-1 protein was detected in the invadopodia of cells with a migratory phenotype. There was increased expression of MT1-MMP protein concentrated at the leading edges of motile cells, which were highly   invasive   in collagen three-dimensional culture. The rates of MT1-MMP protein endocytosis and recycling to the cell surface were elevated in clones expressing higher levels of YB-1 protein. Control MCF-7 cells formed nonfatal, non-  invasive  , differentiated adenocarcinomas in vivo. MCF-7 cells expressing a twofold increase in YB-1 formed highly anaplastic tumors with local invasion, pulmonary metastases and high lethality. We conclude that YB-1 contributes to the development of an   invasive  , metastatic breast carcinoma phenotype by enhanced presentation of MT1-MMP at the sites of cellular invasion. 
 N-Acetylglucosaminyltransferase V triggers overexpression of MT1-MMP and reinforces the   invasive  /metastatic potential of cancer cells. N-Acetylglucosaminyltransferase V (GnT-V) is an enzyme that catalyzes the formation of a β1,6-N-acetylglucosamine (GlcNAc) side chain to a core mannosyl residue in N-linked glycoproteins. Besides its direct function of producing aberrant glycoproteins, it promotes cancer progression by its involvement in the stimulation of oncoproteins. Herein, we report that GnT-V guided the transcriptional activation of membrane-type matrix metalloproteinase-1 (MT1-MMP) in cancer cells. The activated MT1-MMP expression had dual effects on cancer progression. It not only promoted proteolytic activity for cancer cells per se, but also led to the activation of MMP-2. Consequently, the activation of the two MMPs triggered by GnT-V intensified the   invasiv  e potential. A quantitative analysis using clinical tissues revealed a relatively strong correlation between GnT-V overexpression and MT1-MMP upregulation. In this study, we report for the first time that GnT-V directs cancer progression by modulating MMPs in cancer. 
 Pancreatic cancer cells respond to type I collagen by inducing snail expression to promote membrane type 1 matrix metalloproteinase-dependent collagen invasion. Pancreatic ductal adenocarcinoma (PDAC) is characterized by pronounced fibrotic reaction composed primarily of type I collagen. Although type I collagen functions as a barrier to invasion, pancreatic cancer cells have been shown to respond to type I collagen by becoming more motile and   invasive  . Because epithelial-mesenchymal transition is also associated with cancer invasion, we examined the extent to which collagen modulated the expression of Snail, a well known regulator of epithelial-mesenchymal transition. Relative to cells grown on tissue culture plastic, PDAC cells grown in three-dimensional collagen gels induced Snail. Inhibiting the activity or expression of the TGF-β type I receptor abrogated collagen-induced Snail. Downstream of the receptor, we showed that Smad3 and Smad4 were critical for the induction of Snail by collagen. In contrast, Smad2 or ERK1/2 was not involved in collagen-mediated Snail expression. Overexpression of Snail in PDAC cells resulted in a robust membrane type 1-matrix metalloproteinase (MT1-MMP, MMP-14)-dependent invasion through collagen-coated transwell chambers. Snail-expressing PDAC cells also demonstrated MT1-MMP-dependent scattering in three-dimensional collagen gels. Mechanistically, Snail increased the expression of MT1-MMP through activation of ERK-MAPK signaling, and inhibiting ERK signaling in Snail-expressing cells blocked two-dimensional collagen invasion and attenuated scattering in three-dimensional collagen. To provide in vivo support for our findings that Snail can regulate MT1-MMP, we examined the expression of Snail and MT1-MMP in human PDAC tumors and found a statistically significant positive correlation between MT1-MMP and Snail in these tumors. Overall, our data demonstrate that pancreatic cancer cells increase Snail on encountering collagen-rich milieu and suggest that the desmoplastic reaction actively contributes to PDAC progression. 
 Cleavage of hepatocyte growth factor activator inhibitor-1 by membrane-type MMP-1 activates matriptase. Co-expression of membrane-type 1 (MT1)-MMP with hepatocyte growth factor activator inhibitor-1 (HAI-1) in HEK293T cells resulted in cleavage of HAI-1 to produce three fragments. Recombinant MT1-MMP was shown to cleave HAI-1 protein in vitro. Hepatocyte growth factor activator inhibitor-1 was initially identified as the cognate inhibitor of matriptase, a transmembrane serine protease that processes urokinase-type plasminogen activator (uPA). Co-expression of HAI-1 with matriptase suppressed matriptase protease activity, and co-expression of MT1-MMP with them resulted in recovery of matriptase activity by stimulating shedding of HAI-1 fragments. Matriptase protein was detected in squamous carcinoma-derived HSC-4 cells, however, matriptase protease activity was undetectable. Transfection of siRNA for HAI-1 enhanced serine protease activity, which was suppressed by cotransfection of matriptase siRNA. Collagen-gel culture or treatment with concanavalin A (ConA) of HSC-4 cells enhanced MT1-MMP activity, which induced shedding of HAI-1 fragments and conversely stimulated uPA activation by these cells. Serine protease activity, including uPA activation of cells treated with ConA, was abrogated by downregulation of either matriptase or MT1-MMP through the transfection of each siRNA. These results suggest that MT1-MMP induced by collagen-gel culture or ConA treatment causes cleavage and shedding of HAI-1 protein, which allows activation of matriptase in HSC-4 cells. HSC-4 cells showed a characteristic   invasive   growth by forming vacuole-like structures in collagen gel, which was suppressed by transfection of siRNA for either MT1-MMP or matriptase, suggesting that activation of matriptase through the cleavage of HAI-1 is one of the MT1-MMP multifunctions essential for   invasive   growth of HSC-4 cells. 
 Platelet activating factor inhibits the expression of matrix metalloproteinases and affects   invasive  ness and differentiation in a system of human neuroblastoma clones. Platelet Activating Factor (PAF), an inflammatory bioactive lipid, has been shown to be involved in the regulation of the activity of matrix metalloproteinases (MMPs). In view of the role played by MMPs in tumor cell   invasive  ness, we investigated whether PAF influences MMP activity in a system of neuroblastoma clones, the AA5 and AE12 cells, isolated from the human LaN1 neuroblastoma cell line. These clones were characterized by an inverse relationship between   invasive  ness and differentiative capacity and by the expression of specific cell surface PAF receptors. We found that the levels of mRNAs specific for MMP-2 and for MT1-MMP, the MMP-2 activator, were reduced in both clones treated with 300 nM PAF. These changes are consistent with the reduced secretion and activation of MMP-2 found in the neuroblastoma clones exposed to PAF. These effects were accompanied by an inhibition of   invasive  ness through Matrigel and by a promotion of differentiation, as revealed by an increased percentage of cells with neurites. The finding that both neuroblastoma clones exposed to the metalloproteinase inhibitors, BB3103 and 1,10-phenanthroline, increased their differentiative capacity and reduced their   invasive  ness through Matrigel, represents a further indication that PAF modulates differentiation and   invasive  ness by affecting the activity of MMPs. 
 TOM1L1 drives membrane delivery of MT1-MMP to promote ERBB2-induced breast cancer cell invasion. ERBB2 overexpression in human breast cancer leads to   invasive   carcinoma but the mechanism is not clearly understood. Here we report that TOM1L1 is co-amplified with ERBB2 and defines a subgroup of HER2(+)/ER(+) tumours with early metastatic relapse. TOM1L1 encodes a GAT domain-containing trafficking protein and is a SRC substrate that negatively regulates tyrosine kinase signalling. We demonstrate that TOM1L1 upregulation enhances the   invasive  ness of ERBB2-transformed cells. This pro-tumoural function does not involve SRC, but implicates membrane-bound membrane-type 1 MMP (MT1-MMP)-dependent activation of invadopodia, membrane protrusions specialized in extracellular matrix degradation. Mechanistically, ERBB2 elicits the indirect phosphorylation of TOM1L1 on Ser321. The phosphorylation event promotes GAT-dependent association of TOM1L1 with the sorting protein TOLLIP and trafficking of the metalloprotease MT1-MMP from endocytic compartments to invadopodia for tumour cell invasion. Collectively, these results show that TOM1L1 is an important element of an ERBB2-driven proteolytic   invasive   programme and that TOM1L1 amplification potentially enhances the metastatic progression of ERBB2-positive breast cancers. 
 The clinical relevance of stromal matrix metalloproteinase expression in ovarian cancer. Matrix metalloproteinases (MMP) are proteolytic enzymes implicated in cancer progression and metastasis. We sought to determine the role of epithelial (tumor cell-derived) and stromal (host-derived) expression of MMPs in predicting the clinical outcome of patients with epithelial ovarian cancer (EOC).MMP-2, MMP-9, and membrane type 1 (MT1)-MMP expression was evaluated using immunohistochemistry in 90   invasive   EOCs, and samples were scored for epithelial and stromal staining. Results were correlated with clinicopathologic characteristics using univariate and multivariate analyses.High expression of MMP-2, MMP-9, and MT1-MMP in tumor epithelium was detected in 54%, 97%, and 100% of cases, and in stromal compartments, in 38%, 70%, and 38% of cases, respectively. High stromal expression of MMP-2, MMP-9, and MT1-MMP was significantly associated with aggressive features such as high stage, high grade ascites, and positive lymph node status. Kaplan-Meier analysis showed that high epithelial and stromal expression of MMP-2, MMP-9, and MT1-MMP were each significantly associated with shorter disease-specific survival (DSS; P < 0.01). On tree-structured survival analysis, patients with strong epithelial MT1-MMP expression had the shortest DSS, whereas patients with moderate epithelial MT1-MMP and low stromal MMP-9 expression had the longest DSS (P < 0.01). On multivariate analysis, high stromal expression of MMP-9 (P = 0.01) and MT1-MMP (P = 0.04), strong epithelial MT1-MMP (P = 0.01) and high stage (P = 0.04) were independent predictors of poor DSS.Overexpression of stromal MMP-9 and MT1-MMP is independently associated with shorter DSS in EOC. Thus, host-derived MMPs are valuable predictors of clinical outcome in EOC. 
 Enhanced membrane-type 1 matrix metalloproteinase expression by hyaluronan oligosaccharides in breast cancer cells facilitates CD44 cleavage and tumor cell migration. Hyaluronan (HA), a component of the extracellular matrix, plays an important role in cell-cell adhesion and cell migration. Membrane type 1-matrix metalloproteinase (MT1‑MMP) is often expressed i  n invasi  ve cancer cells. CD44, a transmembrane receptor for HA, is implicated in various adhesion-dependent cellular processes including cell migration, tumor cell metastasis and invasion. Previous studies have shown that CD44 is highly expressed in cancer cells and may be proteolytically cleaved at the ectodomain by MT1-MMP; this process of inducing CD44 cleavage plays a critical role in cancer cell migration. We hypothesized that HA modulates MT1-MMP expression to facilitate breast cancer cell migration. Flow cytometry, real-time PCR, western blotting and immunofluorescence staining were used to quantify HA-induced MT1-MMP expression in breast cancer cells. In order to validate the relevance of cell migration and HA-induced MT1-MMP, we analyzed the cell migration via matrigel-coated transwell. We found that after HA oligosaccharide (6.5 kDA) stimulation, MT1-MMP expression in the membrane of breast cancer cells was increased. In response to HA oligosaccharide stimulation, significant upregulation of MT1-MMP mRNA occurred. Our data also provide evidence that HA oligosaccharide enhances MT1-MMP; the elevated expression of MT1-MMP confers enhanced CD44 cleavage and cell migration. In conclusion, we have identified a new function of HA in the induction of MT1-MMP expression in breast cancer cell lines and CD44 cleavage to increase cell migration during the invasion process. The HA oligosaccharide-induced MT1-MMP expression in breast cancer cells may be a critical step in the formation of metastatic colonies. 
 MT1-MMP pro  invasive   activity is regulated by a novel Rab8-dependent exocytic pathway. MT1-matrix metalloproteinase (MT1-MMP) is one of the most critical factors in the invasion machinery of tumor cells. Subcellular localization to   invasive   structures is key for MT1-MMP pro  invasive   activity. However, the mechanism driving this polarized distribution remains obscure. We now report that polarized exocytosis of MT1-MMP occurs during MDA-MB-231 adenocarcinoma cell migration into collagen type I three-dimensional matrices. Polarized trafficking of MT1-MMP is triggered by beta1 integrin-mediated adhesion to collagen, and is required for protease localization at   invasive   structures. Localization of MT1-MMP within VSV-G/Rab8-positive vesicles, but not in Rab11/Tf/TfRc-positive compartment in   invasive   cells, suggests the involvement of the exocytic traffic pathway. Furthermore, constitutively active Rab8 mutants induce MT1-MMP exocytic traffic, collagen degradation and invasion, whereas Rab8- but not Rab11-knockdown inhibited these processes. Altogether, these data reveal a novel pathway of MT1-MMP redistribution to   invasive   structures, exocytic vesicle trafficking, which is crucial for its role in tumor cell   invasive  ness. Mechanistically, MT1-MMP delivery to   invasive   structures, and therefore its pro  invasive   activity, is regulated by Rab8 GTPase. 
 Molecular dissection of the structural machinery underlying the tissue-  invasive   activity of membrane type-1 matrix metalloproteinase. Membrane type-1 matrix metalloproteinase (MT1-MMP) drives cell invasion through three-dimensional (3-D) extracellular matrix (ECM) barriers dominated by type I collagen or fibrin. Based largely on analyses of its impact on cell function under two-dimensional culture conditions, MT1-MMP is categorized as a multifunctional molecule with 1) a structurally distinct, N-terminal catalytic domain; 2) a C-terminal hemopexin domain that regulates substrate recognition as well as conformation; and 3) a type I transmembrane domain whose cytosolic tail controls protease trafficking and signaling cascades. The MT1-MMP domains that subserve cell trafficking through 3-D ECM barriers in vitro or in vivo, however, remain largely undefined. Herein, we demonstrate that collagen-  invasive   activity is not confined strictly to the catalytic, hemopexin, transmembrane, or cytosolic domain sequences of MT1-MMP. Indeed, even a secreted collagenase supports invasion when tethered to the cell surface in the absence of the MT1-MMP hemopexin, transmembrane, and cytosolic tail domains. By contrast, the ability of MT1-MMP to support fibrin-  invasive   activity diverges from collagenolytic potential, and alternatively, it requires the specific participation of MT-MMP catalytic and hemopexin domains. Hence, the tissue-  invasive   properties of MT1-MMP are unexpectedly embedded within distinct, but parsimonious, sequences that serve to tether the requisite matrix-degradative activity to the surface of migrating cells. 
 Membrane Type 1 Matrix Metalloproteinase induces an epithelial to mesenchymal transition and cancer stem cell-like properties in SCC9 cells. Tissue invasion and metastasis are acquired abilities of cancer and related to the death in oral squamous cell carcinoma (OSCC). Emerging observations indicate that the epithelial-to-mesenchymal transition (EMT) is associated with tumor progression and the generation of cells with cancer stem cells (CSCs) properties. Membrane Type 1 Matrix Metalloproteinase (MT1-MMP) is a cell surface proteinase, which is involved in degrading extracellular matrix components that can promote tumor invasion and cell migration.In the current study, we utilized SCC9 cells stably transfected with an empty vector (SCC9-N) or a vector encoding human MT1-MMP (SCC9-M) to study the role of MT1-MMP in EMT development.Upon up-regulation of MT1-MMP, SCC9-M cells underwent EMT, in which they presented a fibroblast-like phenotype and had a decreased expression of epithelial markers (E-cadherin, cytokeratin18 and β-catenin) and an increased expression of mesenchymal markers (vimentin and fibronectin). We further demonstrated that MT1-MMP-induced morphologic changes increased the level of Twist and ZEB, and were dependent on repressing the transcription of E-cadherin. These activities resulted in low adhesive, high   invasiv  e abilities of the SCC9-M cells. Furthermore, MT1-MMP-induced transformed cells exhibited cancer stem cell (CSC)-like characteristics, such as low proliferation, self-renewal ability, resistance to chemotherapeutic drugs and apoptosis, and expression of CSCs surface markers.In conclusion, our study indicates that overexpression of MT1-MMP induces EMT and results in the acquisition of CSC-like properties in SCC9 cells. Our growing understanding of the mechanism regulating EMT may provide new targets against invasion and metastasis in OSCC. 
 Expression of membrane type 1 matrix metalloproteinase in papillomavirus-positive cells: role of the human papillomavirus (HPV) 16 and HPV8 E7 gene products. Matrix metalloproteinases (MMPs) degrade extracellular matrix. They are involved in cellular proliferation, migration, angiogenesis, invasion and metastasis. MT-1 MMP, a membrane-bound MMP, is expressed in carcinomas of the uterine cervix in vivo. This type of cancer is associated with human papillomavirus (HPV) infection. Here it was shown that keratinocytes transformed with HPV16 or HPV18 in vitro, and HPV-positive cervical carcinoma cell lines, constitutively expressed MT-1 MMP. Expression of the E7 protein from the mucosal and cutaneous high-risk types HPV16 and HPV8, but not from the cutaneous low-risk type HPV1, was sufficient to induce MT-1 MMP expression in primary human keratinocytes and HaCaT cells. As a consequence, MMP-2 was activated. MT-1 MMP expression might play a role in the HPV life cycle by promoting proliferation of host cells and might contribute to their   invasive   phenotype during malignant progression. 
 Shedding of kidney injury molecule-1 by membrane-type 1 matrix metalloproteinase. Co-expression of membrane-type 1 matrix metalloproteinase (MT1-MMP) with kidney injury molecule-1 (KIM-1) in HEK293T cells resulted in cleavage and shedding of KIM-1 ectodomain. Analysis of cleavage products using KIM-1 mutants localized cleavage site at the juxtamembrane region. HT1080 cells were stably transfected with expression plasmid for KIM-1 or its mutant with deletion of the juxtamembrane region (Asp(261)-Gly(295)) to establish HT/KIM-1 or HT/ΔKIM-1 cells, respectively. KIM-1 protein appeared on cell surface at low level in HT/KIM-1 cells, and accumulated by the treatment with MMP inhibitor BB-94 or small interfering RNA (siRNA) to MT1-MMP, indicating that MT1-MMP is involved in cleavage and shedding of KIM-1. In contrast, HT/ΔKIM-1 cells expressed KIM-1 protein at high level regardless of BB-94 or siRNA treatment. Cells expressing high level KIM-1 protein exhibited phagocytosis of Escherichia coli and reduced cell adhesion and spreading on collagen-coated plate compared with KIM-1 negative cells. Control HT1080 and HT/KIM-1 cells showed significantly highe  r invasi  ve growth in collagen gel, cell migration on collagen-coated plate and liver metastasis in chick embryo than HT/ΔKIM-1 cells. These results suggest that KIM-1 negatively regulates cellular function mediated through interaction with collagen, and MT1-MMP abrogates it through the cleavage and shedding of KIM-1. 
 Conversion of stationary to   invasive   tumor initiating cells (TICs): role of hypoxia in membrane type 1-matrix metalloproteinase (MT1-MMP) trafficking. Emerging evidence has implicated the role of tumor initiating cells (TICs) in the process of cancer metastasis. The mechanism underlying the conversion of TICs from stationary to   invasive   remains to be characterized. In this report, we employed less   invasive   breast cancer TICs, SK-3rd, that displays CD44(high)/CD24(low) with high mammosphere-forming and tumorigenic capacities, to investigate the mechanism by which stationary TICs are converted to   invasive   TICs.   Invasive   ability of SK-3rd TICs was markedly enhanced when the cells were cultured under hypoxic conditions. Given the role of membrane type 1-matrix metalloproteinase (MT1-MMP) in cancer invasion/metastasis, we explored a possible involvement of MT1-MMP in hypoxia-induced TIC invasion. Silencing of MT1-MMP by a shRNA approach resulted in diminution of hypoxia-induced cell invasion in vitro and metastasis in vivo. Under hypoxic conditions, MT1-MMP redistributed from cytoplasmic storage pools to the cell surface of TICs, which coincides with the increased cell invasion. In addition, CD44, a cancer stem-like cell marker, inversely correlated with increased cell surface MT1-MMP. Interestingly, cell surface MT1-MMP gradually disappeared when the hypoxia-treated cells were switched to normoxia, suggesting the plasticity of TICs in response to oxygen content. Furthermore, we dissected the pathways leading to upregulated MT1-MMP in cytoplasmic storage pools under normoxic conditions, by demonstrating a cascade involving Twist1-miR10b-HoxD10 leading to enhanced MT1-MMP expression in SK-3rd TICs. These observations suggest that MT1-MMP is a key molecule capable of executing conversion of stationary TICs to   invasive   TICs under hypoxic conditions and thereby controlling metastasis. 
 Emerging concepts in the regulation of membrane-type 1 matrix metalloproteinase activity. Pericellular proteolysis mediated by membrane-type 1 matrix metalloproteinase (MT1-MMP) represents an essential component of the cellular machinery involved in the dissolution and penetration of ECM barriers by tumor cells. Although most studies on the pro  invasive   properties of MT1-MMP have focused on its unusually broad proteolytic activity towards several ECM components and cell surface receptors, recent evidence indicate that the cytoplasmic domain of the enzyme also actively participates in tumor cell invasion by regulating the cell surface localization of MT1-MMP as well as the activation of signal transduction cascades. The identification of the molecular events by which the intracellular domain of MT1-MMP links proteolysis of the surrounding matrix by the enzyme to modification of cell function may thus provide important new information on the mechanisms by which this enzyme controls the   invasive   behavior of neoplastic cells in vivo. 
 CLIC3 controls recycling of late endosomal MT1-MMP and dictates invasion and metastasis in breast cancer. Chloride intracellular channel 3 (CLIC3) drives   invasive  ness of pancreatic and ovarian cancer by acting in concert with Rab25 to regulate the recycling of α5β1 integrin from late endosomes to the plasma membrane. Here, we show that in two estrogen receptor (ER)-negative breast cancer cell lines, CLIC3 has little influence on integrin recycling, but controls trafficking of the pr  o-invasi  ve matrix metalloproteinase MT1-MMP (also known as MMP14). In MDA-MB-231 cells, MT1-MMP and CLIC3 are localized primarily to late endosomal/lysosomal compartments located above the plane of adhesion and near the nucleus. MT1-MMP is transferred from these late endosomes to sites of cell-matrix adhesion in a CLIC3-dependent fashion. Correspondingly, CLIC3-knockdown opposes MT1-MMP-dependen  t invasi  ve processes. These include the disruption of the basement membrane as acini formed from MCF10DCIS.com cells acquir  e invasi  ve characteristics in 3D culture, and the invasion of MDA-MB-231 cells into Matrigel or organotypic plugs of type I collagen. Consistent with this, expression of CLIC3 predicts poor prognosis in ER-negative breast cancer. The identification of MT1-MMP as a cargo of a CLIC3-regulated pathway that drives invasion highlights the importance of late endosomal sorting and trafficking in breast cancer. 
 Downregulation of Akt and FAK phosphorylation reduces invasion of glioblastoma cells by impairment of MT1-MMP shuttling to lamellipodia and downregulates MMPs expression. Human malignant glioblastomas are highly   invasive   tumors. Increased cell motility and degradation of the surrounding extracellular matrix are essential for tumor invasion. PI3K/Akt signaling pathway emerges as a common pathway regulating cellular proliferation, migration and invasion; however, its contribution to particular process and downstream cascades remain poorly defined. We have previously demonstrated that Cyclosporin A (CsA) affects glioblastoma invasion in organotypic brain slices and tumorigenicity in mice. Here we show that CsA impairs migration and invasion of human glioblastoma cells by downregulation of Akt phosphorylation. Interference with PI-3K/Akt signaling was crucial for CsA effect on invasion, because overexpression of constitutively active myr-Akt antagonized drug action. Furthermore, the drug was not effective in T98G glioblastoma cells with constitutively high level of phosphorylated Akt. CsA, comparably to pharmacological inhibitors of PI3K/Akt signaling (LY294002, A443654), reduced motility of glioblastoma cells, diminished MMP-2 gelatinolytic activity and MMP-2 and MT1-MMP expression. The latter effect was mimicked by overexpression of dominant negative Akt mutants. We demonstrate that CsA and LY294002 reduced MMP transcription partly via modulation of IκB phosphorylation and NFκB transcriptional activity. Those effects were not mediated by inhibition of calcineurin, a classical CsA target. Additionally, CsA reduced phosphorylation and activity of focal adhesion kinase that was associated with rapid morphological alterations, rearrangement of lamellipodia and impairment of MT1-MMP translocation to membrane protrusions. Our results document novel, Akt-dependent mechanisms of interference with motility/invasion of human glioblastoma cells: through a rapid modulation of cell adhesion and MT1-MMP translocation to membrane protrusions and delayed, partly NFκB-dependent, downregulation of MMP-2 and MT1-MMP expression. This article is part of a Special Issue entitled: 11th European Symposium on Calcium. 
 Intradomain cleavage of inhibitory prodomain is essential to protumorigenic function of membrane type-1 matrix metalloproteinase (MT1-MMP) in vivo.   Invasive   cancers use pericellular proteolysis to breach the extracellular matrix and basement membrane barriers and invade the surrounding tissue. Pro  invasive   membrane type-1 matrix metalloproteinase (MT1-MMP) is the primary mediator of proteolytic events on the cancer cell surface. MT1-MMP is synthesized as a zymogen. The latency of MT1-MMP is maintained by its N-terminal inhibitory prodomain. In the course of MT1-MMP activation, the R(108)RKR(111) ↓ Y(112) prodomain sequence is processed by furin. The intact prodomain released by furin alone, however, is a potent inhibitor of the emerging MT1-MMP enzyme. Evidence suggests that the prodomain undergoes intradomain cleavage at the PGD ↓ L(50) site followed by the release of the degraded prodomain by furin cleavage that finalizes the two-step activation event. These cleavages, only if combined, cause the activation of MT1-MMP. The significance of the intradomain cleavage in the protumorigenic program of MT1-MMP, however, remained unidentified. To identify this important parameter, in our current study, we used the cells that expressed the wild-type prodomain-based fluorescent biosensor and the mutant biosensor with the inactivated PGD↓L(50) cleavage site (L50D mutant) and also the cells with the enforced expression of the wild-type and L50D mutant MT1-MMP. Using cell-based tests, orthotopic breast cancer xenografts in mice, and genome-wide transcriptional profiling of cultured cells and tumor xenografts, we demonstrated that the intradomain cleavage of the PGD ↓ L(50) sequence of the prodomain is essential for the protumorigenic function of MT1-MMP. Our results emphasize the importance of the intradomain cleavages resulting in the inactivation of the respective inhibitory prodomains not only for MT1-MMP but also for other MMP family members. 
 A cancer cell metalloprotease triad regulates the basement membrane transmigration program. Carcinoma cells initiate the metastatic cascade by inserting   invasive   pseudopodia through breaches in the basement membrane (BM), a specialized barrier of cross-linked, extracellular matrix macromolecules that underlies epithelial cells and ensheaths blood vessels. While BM invasion is the sine qua non of the malignant phenotype, the molecular programs that underlie this process remain undefined. To identify genes that direct BM remodeling and transmigration, we coupled high-resolution electron microscopy with an ex vivo model of invasion that phenocopies the major steps observed during the transition of carcinoma in situ to frank malignancy. Herein, a triad of membrane-anchored proteases, termed membrane type-1, type-2, and type-3 metalloproteinases, are identified as the triggering agents that independently confer cancer cells with the ability to proteolytically efface the BM scaffolding, initiate the assembly of   invasive   pseudopodia, and propagate transmigration. These studies characterize the first series of gene products capable of orchestrating the entire BM remodeling program that distinguishes the carcinomatous phenotype. 
 Inhibition of MT1-MMP activity using functional antibody fragments selected against its hemopexin domain. The membrane associated MMP, MT1-MMP, is a critical pericellular protease involved in tumour cell invasion and angiogenesis and is highly up-regulated in numerous human cancers. It therefore represents an exciting new therapeutic cancer-specific target. We have generated recombinant human scFv antibodies against the non-catalytic, hemopexin domain of MT1-MMP that modulate its interactions with collagen. One of these is an effective inhibitor of the   invasive   capacity of cancer cells and of angiogenesis in model systems. This demonstrates that targeting sites outside the catalytic domain presents a potential novel approach to proteinase inhibition that could have applications in cancer therapeutics. 
 The hemopexin domain of membrane-type matrix metalloproteinase-1 (MT1-MMP) Is not required for its activation of proMMP2 on cell surface but is essential for MT1-MMP-mediated invasion in three-dimensional type I collagen. Membrane-type matrix metalloproteinase-1 (MT1-MMP) plays a key role in tumor invasion and metastasis by degrading the extracellular matrix and activating proMMP2. Here we show that the conserved hemopexin domain is required for MT1-MMP-mediated invasion and growth in three-dimensional type I collagen matrix but not proMMP2 activation. Deletion of the hemopexin domains in MT1-, MT2-, MT3-, MT5-, and MT6-MMP does not impair their abilities to activate proMMP2. In fact, hemopexin-less MT5- and MT6-MMP activate proMMP2 better than their wild type counterparts. On the other hand, hemopexin-less MT1-MMP fails to promote cell invasion into type I collagen but retains the capacity to enhance the growth of Madin-Darby canine kidney cells as cysts in three-dimensional collagen matrix. Moreover, the hemopexin domain is also required for MT1-MMP-mediated invasion/scattering of MCF-7 cells in three-dimensional collagen matrix. Because growth and invasion in a three-dimensional model may correlate with tumor   invasive  ness in vivo, our data suggest that the hemopexin domains of MT-MMPs should be targeted for the development of anti-cancer therapies by employing screening assays developed for three-dimensional models rather than their enzymatic activity toward proMMP2. 
 Up-regulation of angiopoietin-2, matrix metalloprotease-2, membrane type 1 metalloprotease, and laminin 5 gamma 2 correlates with the   invasive  ness of human glioma. Diffuse infiltration of malignant human glioma cells into surrounding brain structures occurs through the activation of multigenic programs. We recently showed that angiopoietin-2 (Ang2) induces glioma invasion through the activation of matrix metalloprotease-2 (MMP-2). Here, we report that up-regulation of Ang2, MMP-2, membrane type 1-MMP (MT1-MMP), and laminin 5 gamma 2 (LN 5 gamma 2) in tumor cells correlates with glioma invasion. Analyses of 57 clinical human glioma biopsies of World Health Organization grade I to IV tumors displaying a distinct   invasive   edge and 39 glioma specimens that only contain the central region of the tumor showed that Ang2, MMP-2, MT1-MMP, and LN 5 gamma 2 were co-overexpressed in   invasive   areas but not in the central regions of the glioma tissues. Statistical analyses revealed a significant link between the preferential expression of these molecules and   invasive  ness. Protein analyses of microdissected primary glioma tissue showed up-regulation and activation of MT1-MMP and LN 5 gamma 2 at the   invasive   edge of the tumors, supporting this observation. Concordantly, in human U87MG glioma xenografts engineered to express Ang2, increased expression of MT1-MMP and LN 5 gamma 2, along with MMP-2 up-regulation, in actively invading glioma cells was also evident. In cell culture, stimulation of glioma cells by overexpressing Ang2 or exposure to exogenous Ang2 promoted the expression and activation of MMP-2, MT1-MMP, and LN 5 gamma 2. These results suggest that up-regulation of Ang2, MMP-2, MT1-MMP, and LN 5 gamma 2 is associated with the   invasive  ness displayed by human gliomas and that induction of these molecules by Ang2 may be essential for glioma invasion. 
 Involvement of syntaxin 4 in the transport of membrane-type 1 matrix metalloproteinase to the plasma membrane in human gastric epithelial cells. Membrane-type 1 matrix metalloproteinase (MT1-MMP) localized on the plasma membrane plays a central role in various normal biological responses including tissue remodeling, wound heeling, and angiogenesis and in cancer cell invasion and metastasis, by functioning as a collagenase and activating other matrix metalloproteinases. In order to elucidate the molecular mechanism of the MT1-MMP targeted localization on the plasma membrane, we examined the participation of syntaxin proteins in MT1-MMP intracellular transport to the plasma membrane in human gastric epithelial AGS cells. Western blotting showed that syntaxin 3 and 4 proteins, which are known to function in intracellular transport towards the plasma membrane, were expressed in AGS cells. Immunocytochemistry revealed that transient transfection of AGS cells with dominant-negative mutant syntaxin 4 decreased plasma membrane MT1-MMP expression. In contrast, transient transfection with either dominant-negative mutant syntaxin 3 or 7 did not affect MT1-MMP localization on the plasma membrane. Cell surface biotinylation assay and Matrigel chamber assay demonstrated that stable transfection with dominant-negative mutant syntaxin 4 decreased the amount of MT1-MMP on the plasma membranes and inhibited the cell   invasive  ness. We suggest that syntaxin 4 is involved in the intracellular transport of MT1-MMP toward the plasma membrane. 
 MicroRNA-10b induces glioma cell invasion by modulating MMP-14 and uPAR expression via HOXD10. MicroRNAs are small endogenous noncoding RNAs, which modulate target gene expression by binding with target mRNA sequences in the 3'untranslated region (UTR) with an imperfect complementarity that inhibits the mRNA translation. Many microRNAs have been reported to function as tumor oncogenes or anti-oncogenes. Recently, more and more microRNAs have been reported to contribute to a tumor's   invasive   potential. Here, we show that microRNA-10b (miR-10b) was over-expressed in glioma samples and directly associated with the glioma's pathological grade and malignancy. We also found that miR-10b induced glioma cell invasion by modulating tumor invasion factors MMP-14 and uPAR expression via the direct target HOXD10. The miR-10b/HOXD10/MMP-14/uPAR signaling pathway might contribute to the invasion of glioma. Accordingly, glioma cells lost their   invasive   ability when treated with specific antisense oligonucleotides (miR-10b inhibitors), suggesting that miR-10b could be used as a new bio-target to cure glioma. 
 Inhibition mechanism of membrane metalloprotease by an exosite-swiveling conformational antibody. Membrane type 1 metalloprotease (MT1-MMP) is a membrane-anchored, zinc-dependent protease. MT1-MMP is an important mediator of cell migration and invasion, and overexpression of this enzyme has been correlated with the malignancy of various tumor types. Therefore, modulators of MT1-MMP activity are proposed to possess therapeutic potential in numerous   invasiv  e diseases. Here we report the inhibition mode of MT1-MMP by LEM-2/15 antibody, which targets a surface epitope of MT1-MMP. Specifically, the crystal structures of Fab LEM-2/15 in complex with the MT1-MMP surface antigen suggest that conformational swiveling of the enzyme surface loop is required for effective binding and consequent inhibition of MT1-MMP activity on the cell membrane. This inhibition mechanism appears to be effective in controlling active MT1-MMP in endothelial cells and at the leading edge of migratory cancer cells. 
 Fibroblast growth factor-1 transcriptionally induces membrane type-1 matrix metalloproteinase expression in prostate carcinoma cell line. We and others have shown that the matrix metalloproteinases, MT1-MMP is overexpressed in human prostate PIN lesions and   invasive   cancers compared to normal prostate epithelium. However, the mechanism for this overexpression is not understood. Evidence from our laboratory and others has indicated that fibroblast growth factors (FGFs) can regulate the expression of certain matrix metalloproteinase. In addition, human prostate fibroblasts are known to express certain FGFs, including FGF-1. The purpose of the work in this paper was to determine the mechanism involved in FGF-1 induced MT1-MMP expression in prostate carcinoma cells.We tested the ability of recombinant FGF-1 to induce MT1-MMP expression in prostate carcinoma cell line, LNCaP cells. We measured the MT1-MMP message by using Northern analyses and protein levels by Western analysis after FGF-1 treatment. Downstream signaling was investigated using dominant negative constructs for FGFR-1 and signal transducer and activator of transcription-3 (STAT3). Transient transfection was performed using reporter plasmids of the MT1-MMP gene promoter region (7.2 kb) linked to the firefly luciferase gene in the pGL3-Basic vector. For dominant negative studies FGFR-1 dominant negative plasmid in PCEP4 vector or STAT3 dominant negative plasmid in pCMV-1 vector was co-transfected with the MT1-MMP reporter plasmid.Recombinant FGF-1 significantly induced MT1-MMP expression in LNCaP prostate carcinoma cells. MT1-MMP message increased with FGF-1 treatment compared to that of untreated control LNCaP cells. Quantitation by digital image analysis revealed that this increase was twofold over untreated LNCaP cells. Treatment of pGL3-MT1-MMP-luciferase transfected cells with FGF-1 resulted in a twofold to fourfold increase in luciferase enzyme activity compared with untreated cells. Co-transfection of LNCaP with human MT1-MMP reporter construct and a dominant negative FGFR1 mutant showed that FGF-1-induced MT1-MMP expression in LNCaP cells was completely inhibited by the mutated FGFR-1, indicating that FGF receptor (FGFR) activation is necessary for induction of MT1-MMP. Further, expression of dominant negative STAT3 inhibited the FGF-1-induced transactivation of the human MT1-MMP 7.2-kb promoter.From these data, we conclude that FGF-1 induces MT1-MMP expression in prostate carcinoma cells through a transcriptional mechanism mediated through the FGFR and the transcription factor, STAT3. These results confirm earlier data indicating that acidic FGF and STAT3 are involved in the signaling leading to the expression of a MMP. Our findings support the idea that paracrine and autocrine factors play an important role in the regulation of MT1-MMP in human prostate carcinoma cells. 
 A specific subset of RabGTPases controls cell surface exposure of MT1-MMP, extracellular matrix degradation and three-dimensional invasion of macrophages. The matrix metalloproteinase MT1-MMP has a major impact on   invasive   cell migration in both physiological and pathological settings such as immune cell extravasation or metastasis of cancer cells. Surface-associated MT1-MMP is able to cleave components of the extracellular matrix, which is a prerequisite for proteolytic   invasive   migration. However, current knowledge on the molecular mechanisms that regulate MT1-MMP trafficking to and from the cell surface is limited. We have identified three members of the RabGTPase family, Rab5a, Rab8a and Rab14, as crucial regulators of MT1-MMP trafficking and function in primary human macrophages. Both overexpressed and endogenous forms show prominent colocalisation with MT1-MMP-positive vesicles, whereas expression of mutant constructs, as well as siRNA-induced knockdown, reveal that these RabGTPases are crucial in the regulation of MT1-MMP surface exposure, contact of MT1-MMP-positive vesicles with podosomes, extracellular matrix degradation in two and three dimensions, as well as three-dimensional proteolytic invasion of macrophages. Collectively, our results identify Rab5a, Rab8a and Rab14 as major regulators of MT1-MMP trafficking and   invasive   migration of primary human macrophages, which could be promising potential targets for manipulation of immune cell invasion. 
 Identification of membrane-type 1 matrix metalloproteinase tyrosine phosphorylation in association with neuroblastoma progression. Neuroblastoma is a pediatric tumor of neural crest cells that is clinically characterized by its variable evolution, from spontaneous regression to malignancy. Despite many advances in neuroblastoma research, 60% of neuroblastoma, which are essentially metastatic cases, are associated with poor clinical outcome due to the lack of effectiveness of current therapeutic strategies. Membrane-type 1 matrix metalloproteinase (MT1-MMP, MMP-14), an enzyme involved in several steps in tumor progression, has previously been shown to be associated with poor clinical outcome for neuroblastoma. Based on our recent demonstration that MT1-MMP phosphorylation is involved in the growth of fibrosarcoma tumors, we examined the potential role of phosphorylated MT1-MMP in neuroblastoma progression.Tyrosine phosphorylated MT1-MMP was immunostained on tissue microarray samples from 55 patients with neuroblastoma detected by mass screening (known to be predominantly associated with favourable outcome), and from 234 patients with standard diagnosed neuroblastoma. In addition, the effects of a non phosphorylable version of MT1-MMP on neuroblastoma cell migration and proliferation were investigated within three-dimensional collagen matrices.Although there is no correlation between the extent of tyrosine phosphorylation of MT1-MMP (pMT1-MMP) and MYCN amplification or clinical stage, we observed greater phosphorylation of pMT1-MMP in standard neuroblastoma, while it is less evident in neuroblastoma from mass screening samples (P = 0.0006) or in neuroblastoma samples from patients younger than one year (P = 0.0002). In vitro experiments showed that overexpression of a non-phosphorylable version of MT1-MMP reduced MT1-MMP-mediated neuroblastoma cell migration and proliferation within a three-dimensional type I collagen matrix, suggesting a role for the phosphorylated enzyme in the   invasive   properties of neuroblastoma cells.Overall, these results suggest that tyrosine phosphorylated MT1-MMP plays an important role in neuroblastoma progression and that its expression is preferentially observed in tumor specimens from neuroblastoma patients showing poor clinical outcome. 
 Membrane type 1 matrix metalloproteinase induces epithelial-to-mesenchymal transition in prostate cancer. By mining DNA microarray data bases at GenBank, we identified up-regulation of membrane type 1 matrix metalloproteinase (MT1-MMP) in human primary and metastatic prostate cancer specimens as compared with nonmalignant prostate tissues. To explore the role of up-regulated MT1-MMP in early stage cancer progression, we have employed a three-dimensional cell culture model. Minimally   invasive   human prostate cancer cells (LNCaP) were transfected with MT1-green fluorescent protein (GFP) chimeric cDNA as compared with GFP cDNA, and morphologic and phenotypic changes were characterized. GFP-expressing LNCaP cells formed multicellular spheroids with cuboidal-like epithelial morphology, whereas MT1-GFP-expressing cells displayed a fibroblast-like morphology and a scattered growth pattern in type I collagen gels. Cell morphologic changes were accompanied by decreased epithelial markers and enhanced mesenchymal markers, consistent with epithelial-to-mesenchymal transition. MT1-MMP-induced morphologic change and cell scattering were abrogated by target inhibition of either the catalytic domain or the hemopexin domain. We further demonstrated that MT1-MMP-induced phenotypic changes were dependent upon up-regulation of Wnt5a, which has been implicated in epithelial-to-mesenchymal transition. We conclude that MT1-MMP plays an important role in early cancer dissemination by converting epithelial cells to migratory mesenchymal-like cells. 
 Evidence of MTCBP-1 interaction with the cytoplasmic domain of MT1-MMP: Implications in the autophagy cell index of high-grade glioblastoma. Progression of astrocytic tumors is, in part, related to their dysregulated autophagy capacity. Recent evidence indicates that upstream autophagy signaling events can be triggered by MT1-MMP, a membrane-bound matrix metalloproteinase that contributes to the   invasive   phenotype of brain cancer cells. The signaling functions of MT1-MMP require its intracellular domain, and recent identification of MTCBP-1, a cytoplasmic 19 kDa protein involved in the inhibition of MT1-MMP-mediated cell migration, suggests that modulation of MT1-MMP cytoplasmic domain-mediated signaling may affect other carcinogenic processes. Using qPCR and screening of cDNA generated from brain tumor tissues of grades I, II, III, and IV, MT1-MMP gene expression was found to correlate with increased grade of tumors. Inversely, MTCBP-1 expression decreased with increasing grade of brain tumor. Confocal microscopy and fluorescence resonance energy transfer (FRET) analysis revealed that overexpressing a cytoplasmic-deleted MT1-MMP recombinant protein mutant prevented MTCBP-1 recruitment to the intracellular leaf of plasma membrane in U87 glioblastoma cells. The interaction between MTCBP-1 and the 20 amino acids peptide representing the MT1-MMP cytoplasmic domain was confirmed by surface plasmon resonance. Overexpression of a full-length Wt-MT1-MMP triggered acidic autophagy vesicle formation and autophagic puncta formation for green fluorescent microtubule-associated protein 1 light chain 3 (GFP-LC3). Autophagic vesicles and GFP-LC3 puncta formation were abrogated in the presence of MTCBP-1. Our data elucidate a new role for MTCBP-1 regulating the intracellular function of MT1-MMP-mediated autophagy. The inverse correlation between MTCBP-1 and MT1-MMP expression with brain tumor grades could also contribute to the decreased autophagic index observed in high-grade tumors. 
 Expression of membrane type-1 matrix metalloproteinase, MT1-MMP in human breast cancer and its impact on   invasive  ness of breast cancer cells. MT1-MMP (membrane type-1 matrix metalloproteinase), otherwise known as MMP14 is a proteolytic enzyme known to be involved in degradating extracellular matrix and assist progression of cancer invasion and progression. We investigated the impact of targeting the expression of MT1-MMP in breast cancer and its clinical relevance. Human breast cancer cell line MDA-MB-231 was used. Expression of MT1-MMP in the breast cancer cell line was manipulated by way of retroviral ribozyme transgene. The in vitro invasion, growth and cell migration were determined on cell lines transfected with either the transgene or control plasmid. Protein and message levels of MMP14 was also assessed using immunohistochemistry and real-time quantitative analysis, and correlated with clinical and pathological information of the patients. Retroviral ribozyme transgene to human MT1-MMP successfully knocked down the levels of MT1-MMP mRNA from MDA-MB-231 cells. Reduction of MT1-MMP from the breast cancer cells resulted in significant reduction of in vitro   invasive  ness and loss of response to an invasion stimulus, HGF, compared with control and wild-type cells. The invasion index for MT1-MMP knockdown cells were 13+/-3.1 (without HGF) and 16.4+/-2.3 (with HGF, p=0.14), and the index for transfection control cells 25.3+/-4.3 (without HGF) and 40.4+/-4.1 (with HGF, p=0.0049). Transfection with the transgenes did not change the rate of cell growth. In clinical breast cancer, MT1-MMP staining was both membranous and cytoplasmic. Tumour cells displayed stronger staining compared with normal mammary epithelial cells. Tumour tissues had a marginally higher levels of the MMP14 transcript (8.6+/-1.9), compared with normal tissues (4.7+/-1.4), p=0.13. No significant difference was observed between node positive and node negative tumours (9.0+/-2.2 vs 8.7+/-3.1, p=0.24). Marginally higher levels of the MMP14 transcript were seen in tumours which developed metastasis and local recurrence. However, tumours from patients who died of breast cancer related causes had significantly higher levels of the transcript, compared with tumours from patients who remained disease-free 10 years after initial surgery (12.2+/-2.5 vs 6.3+/-1.2, p=0.0091). MT1-MMP is a proteolytic enzyme that is pivotal in controlling the   invasive  ness of breast cancer cells. It is highly expressed in aggressive breast tumours and is associated with clinical outcome. The enzyme is a potential therapeutic target in breast cancer. 
 Epidermal growth factor receptor-mediated membrane type 1 matrix metalloproteinase endocytosis regulates the transition between   invasive   versus expansive growth of ovarian carcinoma cells in three-dimensional collagen. The epidermal growth factor receptor (EGFR) is overexpressed in ovarian carcinomas and promotes cellular responses that contribute to ovarian cancer pathobiology. In addition to modulation of mitogenic and motogenic behavior, emerging data identify EGFR activation as a novel mechanism for rapid modification of the cell surface proteome. The transmembrane collagenase membrane type 1 matrix metalloproteinase (MT1-MMP, MMP-14) is a major contributor to pericelluar proteolysis in the ovarian carcinoma microenvironment and is subjected to extensive posttranslational regulation. In the present study, the contribution of EGFR activation to control of MT1-MMP cell surface dynamics was investigated. Unstimulated ovarian cancer cells display caveolar colocalization of EGFR and MT1-MMP, whereas EGFR activation prompts internalization via distinct endocytic pathways. EGF treatment results in phosphorylation of the MT1-MMP cytoplasmic tail, and cells expressing a tyrosine mutated form of MT1-MMP (MT1-MMP-Y(573)F) exhibit defective MT1-MMP internalization. As a result of sustained cell surface MT1-MMP activity, a phenotypic epithelial-mesenchymal transition is observed, characterized by enhanced migration and collagen invasion, whereas growth within three-dimensional collagen gels is inhibited. These data support an EGFR-dependent mechanism for regulation of the transition between   invasive   and expansive growth of ovarian carcinoma cells via modulation of MT1-MMP cell surface dynamics. 
 PEA3 is necessary for optimal epidermal growth factor receptor-stimulated matrix metalloproteinase expression and invasion of ovarian tumor cells. Elevated expression of the epidermal growth factor (EGF) receptor (EGFR) is detected in human ovarian tumors and is associated with decreased recurrence-free and overall survival. EGFR activation affects tumor progression in part by promoting tumor invasion through the induction of prometastatic matrix metalloproteinases (MMP). PEA3, an ETS family transcription factor, is elevated in advanced and metastatic ovarian cancer and regulates MMPs in various cell types, therefore, we investigated whether PEA3 is required for the EGFR-dependent induction of MMP mRNA. MMP-9 and MMP-14 mRNA levels were selectively increased in response to EGFR activity in ovarian tumor cells. EGFR activation resulted in nuclear accumulation of PEA3 and direct binding of PEA3, but not the related protein ETS-1, to the endogenous MMP-9 and MMP-14 promoters. Furthermore, PEA3 overexpression was sufficient to induce MMP-9 and MMP-14 mRNA, tumor cell migration, and invasion, suggesting that PEA3 is an important contributor to the metastatic phenotype. Additionally, inhibition of PEA3 expression via short interfering RNA reduced the EGF induction of MMP-9 and MMP-14 gene expression by 92% and 50%, respectively, and impaired EGF-stimulated tumor cell invasion. These results suggest that PEA3 is regulated by EGFR and that the elevated PEA3 expression detected in human ovarian cancer may divert cells to a more   invasive   phenotype by regulating MMP-9 and MMP-14. 
 Proteolytic activation of heparin-binding EGF-like growth factor by membrane-type matrix metalloproteinase-1 in ovarian carcinoma cells. Increased expression of heparin-binding EGF-like growth factor (HB-EGF) and membrane-type matrix metalloproteinase-1 (MT1-MMP) is frequently associated with various types of malignant tumor. HB EGF-like growth factor has been reported to promote the malignant progression of ovarian carcinoma. Based on this finding, inhibition of HB-EGF activity with CRM197 is now under phase I clinical evaluation. On the other hand, MT1-MMP expressed in ovarian carcinoma cells is thought to promote invasion and growth of tumor cells by degrading the extracellular matrix. However, we recently demonstrated that co-expression of MT1-MMP and HB-EGF in gastric carcinoma cells leads to cleavage of HB-EGF within its N-terminal heparin-binding region, converting it into a potent heparin-independent growth factor. In this study, we evaluated the importance of regulation of HB-EGF by MT1-MMP in clinical samples of ovarian carcinoma. We detected co-expression of HB-EGF and MT1-MMP in clear cell ovarian carcinoma tissues, particularly at the invasion front and in tumor cells that had disseminated into the ascites, whereas HB-EGF alone was expressed in non-  invasive   borderline ovarian tumor tissue. Furthermore, a soluble HB-EGF fragment that corresponds to that processed by MT1-MMP was detected in malignant ascites obtained from patients with metastatic ovarian carcinoma. Ovarian carcinoma cells that express MT1-MMP and HB-EGF exhibited enhanced cell growth in a 3D-collagen matrix and anchorage-independent growth in suspension. These results indicate that MT1-MMP co-expressed with HB-EGF in ovarian carcinoma cells potentiates the activity of HB-EGF to promote   invasive   tumor growth and spreading in vivo. 
 miR-181a-5p Inhibits Cancer Cell Migration and Angiogenesis via Downregulation of Matrix Metalloproteinase-14. Upregulation of matrix metalloproteinase MMP-14 (MT1-MMP) is associated with poor prognosis in cancer patients, but it is unclear how MMP-14 becomes elevated in tumors. Here, we show that miR-181a-5p is downregulated in aggressive human breast and colon cancers where its levels correlate inversely with MMP-14 expression. In clinical specimens, enhanced expression of MMP-14 was observed in cancer cells located at the   invasive   front of tumors where miR-181a-5p was downregulated relative to adjacent normal cells. Bioinformatics analyses defined a potential miR-181a-5p response element within the 3'-untranslated region of MMP-14 that was validated in reporter gene experiments. Ectopic miR-181a-5p reduced MMP-14 expression, whereas miR-181a-5p attenuation elevated MMP-14 expression. In support of a critical relationship between these two genes, miR-181a-5p-mediated reduction of MMP-14 levels was sufficient to decrease cancer cell migration, invasion, and activation of pro-MMP-2. Furthermore, this reduction in MMP-14 levels was sufficient to reduce in vivo invasion and angiogenesis in chick chorioallantoic membrane assays. Taken together, our results establish the regulation of MMP-14 in cancers by miR-181a-5p through a posttranscriptional mechanism, and they further suggest strategies to elevate miR-181a-5p to prevent cancer metastasis. 
 A matrix metalloproteinase expressed on the surface of   invasive   tumour cells. Gelatinase A (type-IV collagenase; M(r) 72,000) is produced by tumour stroma cells and is believed to be crucial for their invasion and metastasis, acting by degrading extracellular matrix macro-molecules such as type IV collagen. An inactive precursor of gelatinase A (pro-gelatinase A) is secreted and activated in   invasive   tumour tissue as a result of proteolysis which is mediated by a fraction of tumour cell membrane that is sensitive to metalloproteinase inhibitors. Here we report the cloning of the complementary DNA encoding a new matrix metalloproteinase with a potential transmembrane domain. Expression of the gene product on the cell surface induces specific activation of pro-gelatinase A in vitro and enhances cellular invasion of the reconstituted basement membrane. Tumour cells of   invasive   lung carcinomas, which contain activated forms of gelatinase A, were found to express the transcript and the gene product. The new metalloproteinase may thus trigger invasion by tumour cells by activating pro-gelatinase A on the tumour cell surface. 
 The tetraspanin CD81 protein increases melanoma cell motility by up-regulating metalloproteinase MT1-MMP expression through the pro-oncogenic Akt-dependent Sp1 activation signaling pathways. Despite the importance of multiple tetraspanin proteins in cancer invasion and metastasis, little is known about the role and significance of tetraspanin CD81 in these processes. In the present study, we examined CD81 effects on melanoma cell   invasive  ness and metastasis. Transfection of CD81 into melanoma cells lacking endogenous CD81 expression significantly enhanced the migrating,   invasive  , and metastatic abilities of melanoma cells. Interestingly, membrane type 1 matrix metalloproteinase (MT1-MMP) expression was found in CD81-expressing melanoma cells but not in CD81-deficient cells. siRNA knockdown of CD81 in melanoma cells with endogenous CD81 demonstrated decreased MT1-MMP levels and cell motility. Notably, CD81-induced cell migration was abrogated by antibody blocking and siRNA knockdown of MT1-MMP, indicating that MT1-MMP is responsible for CD81-stimulated melanoma cell migration. Promoter analysis revealed an essential role of the Sp1 transcription factor in CD81-induced MT1-MMP transcription. We also demonstrate that the Sp1-activating Akt pathway is involved in adhesion-dependent CD81 signaling to induce MT1-MMP expression and cell motility. Importantly, human skin cancer tissue specimens displayed a positive correlation of CD81 with MT1-MMP expression levels and a close association of CD81 with malignant melanomas. Taken together, these results strongly suggest that CD81 stimulates melanoma cell motility by inducing MT1-MMP expression through the Akt-dependent Sp1 activation signaling pathway, leading to increased melanoma invasion and metastasis. 
  GeneRIFs  
 possible involvement of membrane-type 1 matrix metalloproteinase processing of erythropoietin-producing hepatocellular receptor-2in   invasive  ness of cutaneous cutaneous squamous cell carcinoma 
 Report design of PEGylated peptide probes conjugated with (18)F-labeled BODIPY to be used as a hybrid PET/optical imaging agent and for non-  invasive   monitoring of MT1-MMP activity in cancers. 
 Overexpression of MT1-MMP in HT-1080 cells does not significantly alter the   invasive   ability of these cells. 
 TOM1L1 enhances ERBB2-induced cell   invasive  ness by promoting invadopodia formation and MT1-MMP trafficking to the plasma membrane. 
 E6-HPV16/E7-HPV16 promoted the activity of MT1-MMP and MMP-2/9, contributing to the migration of cervical cells and neoplasm   invasive  ness. 
 Results establish a novel role for NEDD9 as a key regulator of Arf6-dependent trafficking of MMP14 and its   invasive   properties, highlighting the basic mechanism utilized by NEDD9 to support breast cancer cell invasion and metastasis. 
 Up-regulation of MT1-MMP and furin expression and low TIMP-2 expression make the main contribution to the   invasive  ness potential of squamous cell cervical carcinomas. 
 Rab5a, Rab8a and Rab14 are major regulators of MT1-MMP trafficking and   invasive   migration of primary human macrophages. 
 demonstrated that ZO-2 inhibition increases   invasive   and migrative capacities of   invasive   tumor cells. This was associated with an increase of MT1-MMP 
 Up-regulated MT1-MMP codistributes with and cleaves intracellular EphA2 in   invasive   cells within human breast carcinomas. 
 Knockdown of beta-catenin gene may decrease the   invasive   ability of human osteosarcoma cells through down-regulating MT1-MMP expression. 
 MMP-14 expression decreased with progression from actinic keratosis to in situ and   invasive   squamous cell carcinoma. 
 The observations suggest that MT1-MMP is a key molecule capable of executing conversion of stationary TICs to   invasive   tumor initiating cells under hypoxic conditions and thereby controlling metastasis. 
 The expression level of MT1-MMP was associated with   invasive   depth of tumor cells. 
 conclude that graded increases in the MEK1 signaling module are correlated with M1-MMP expression, renal epithelial cell tumor phenotype,   invasive   activity and nuclear grade 
 MT1-MMP co-expressed with HB-EGF in ovarian carcinoma cells potentiates the activity of HB-EGF to promote   invasive   tumor growth and spreading in vivo. 
 Results suggest that GI24 contributes to tumor-  invasive   growth in the collagen matrix by augmenting cell surface MT1-MMP. 
 YB-1 contributes to the development of an   invasive  , metastatic breast carcinoma phenotype by enhanced presentation of MT1-MMP at the sites of cellular invasion. 
 Of MMP-2/TIMP-2/MMP-14 complex, high expression of MMP-2, TIMP-2 & MMP-14 mRNA may contribute to local   invasive  ness of ameloblastoma; local   invasive   capacity of ameloblastoma more likely related to high transcription levels of TIMP-2 and MMP-14. 
 Upregulation of MT1-MMP, together with elevation of cytoskeletal remodeling dynamics can effectively cause elevated   invasive  ness and enhanced matrix degrading capability in breast cancer cells. 
 The identification of the molecular events by which the intracellular domain of MT1-MMP links proteolysis to modification of cell function may provide important information by which this enzyme controls the   invasive   behavior of neoplastic cells in vivo. 
 High expression of MMP-14 is associated with malignant and   invasive   skin squamous cell carcinoma growth. 
 Low Levels of MT1-MMP due to epigenetic silencing is associated with   invasive  ness in cancer. 
 the tissue-  invasive   properties of MT1-MMP are unexpectedly embedded within distinct, but parsimonious, sequences that serve to tether the requisite matrix-degradative activity to the surface of migrating cells 
 Targeted inhibition of either MT1-MMP or G6PT may lead to reduced infiltrative and   invasive   properties of brain tumor cells. 
 Data show how   invasive   tumor cells coordinate mechanotransduction and collagen remodelling by segregating the force-generating leading edge containing beta1 integrin, MT1-MMP and F-actin from a posterior proteolytic zone executing fibre breakdown. 
 Cytoplasmic ZO-1 and MT1-MMP could be detected in   invasive   tumor clusters of human breast carcinomas. 
 MT1-MMP is a major mediator of tumor cell   invasive  ness and type I collagen degradation by VHL RCC cells that express either MT1-MMP or HIF-2alpha 
 Levels of MMP14 mRNA were reduced in two neuroblastoma clones treated with 300 nM PAF, which was accompanied by an inhibition of   invasive  ness through Matrigel and by a promotion of differentiation. 
 prostate cancer cells expressing high levels of MT1-MMP have increased   invasive   potential through their ability to degrade and invade Ln-10 barriers 
 MT1-MMP is primarily secreted in the oral squamous cell carcinoma (OSCC) cells and is involved in the   invasive  ness of the OSCC and lymphatic metastasis. 
 Data demonstrate that blockade of the ERK pathway suppressed the expression of matrix metalloproteinases 3, 9, and 14, and CD44, and markedly inhibited the   invasive  ness of tumor cells. 
 Type-1 metalloproteinases, are identified as the triggering agents that independently confer cancer cells with the ability to proteolytically efface the BM scaffolding, initiate the assembly of   invasive   pseudopodia, and propagate transmigration. 
 MT1-MMP delivery to   invasive   structures, and therefore its pro  invasive   activity, is regulated by Rab8 GTPase. 
 shed as membrane vesicle associated components by endothelial cells and this may be a mechanism for regulating focalized proteolytic activity vital to   invasive   and morphogenic events during angiogenesis 
 These results suggest that PEA3 is regulated by EGFR and that the elevated PEA3 expression detected in human ovarian cancer may divert cells to a more   invasive   phenotype by regulating MMP-9 and MMP-14. 
 up-regulation of Ang2, MMP-2, MT1-MMP, and LN 5 gamma 2 is associated with the   invasive  ness displayed by human gliomas 
  [Mm]etastasize  PubMed  
 MT1-MMP modulates melanoma cell dissemination and metastasis through activation of MMP2 and RAC1. Metastatic melanoma remains the deadliest of all skin cancers with a survival rate at five years of less than 15%. MT1-MMP is a membrane-associated matrix metalloproteinase that controls pericellular proteolysis and is an important, invasion-promoting, pro-tumorigenic MMP in cancer. We show that deregulation of MT1-MMP expression happens as early as the transition from nevus to primary melanoma and continues to increase during melanoma progression. Furthermore, MT1-MMP expression is associated with poor melanoma patient outcome, underscoring a pivotal role of MT1-MMP in melanoma pathogenesis. We demonstrate that MT1-MMP is directly required for melanoma cells to   metastasize  , as cells deprived of MT1-MMP fail to form distant metastasis in an orthotopic mouse melanoma model. We show that MT1-MMP affects cell invasion by activating its target MMP2. Importantly, we demonstrate, for the first time, that activation of MMP2 by MT1-MMP is required to sustain RAC1 activity and promote MT1-MMP-dependent cell motility. These data highlight a novel MT1-MMP/MMP2/RAC1 signaling axis in melanoma that may represent an intriguing molecular target for the treatment of invasive melanoma. 
 MT1-MMP modulates melanoma cell dissemination and metastasis through activation of MMP2 and RAC1. Metastatic melanoma remains the deadliest of all skin cancers with a survival rate at five years of less than 15%. MT1-MMP is a membrane-associated matrix metalloproteinase that controls pericellular proteolysis and is an important, invasion-promoting, pro-tumorigenic MMP in cancer. We show that deregulation of MT1-MMP expression happens as early as the transition from nevus to primary melanoma and continues to increase during melanoma progression. Furthermore, MT1-MMP expression is associated with poor melanoma patient outcome, underscoring a pivotal role of MT1-MMP in melanoma pathogenesis. We demonstrate that MT1-MMP is directly required for melanoma cells to   metastasize  , as cells deprived of MT1-MMP fail to form distant metastasis in an orthotopic mouse melanoma model. We show that MT1-MMP affects cell invasion by activating its target MMP2. Importantly, we demonstrate, for the first time, that activation of MMP2 by MT1-MMP is required to sustain RAC1 activity and promote MT1-MMP-dependent cell motility. These data highlight a novel MT1-MMP/MMP2/RAC1 signaling axis in melanoma that may represent an intriguing molecular target for the treatment of invasive melanoma. 
  [Mm]etastasis  PubMed  
 Moesin regulates the motility of oral cancer cells via MT1-MMP and E-cadherin/p120-catenin adhesion complex. The present study aimed to clarify the role of Moesin in oral squamous cell carcinoma (OSCC) progression, especially in regulation of cell motility.Immunohistochemistry and western blotting were used to investigate the expression of Moesin, E-cadherin, p120-catenin and MT1-MMP in normal epithelia, dysplasia and OSCCs. Then, Moesin was knockdown by siRNA in OSCC cell lines, WSU-HN6 and CAL27, and the biological role of Moesin in cell adhesion and motility was evaluated by transwell system, cell spreading and aggregation assays. The interactions between Moesin, MT1-MMP and E-cadherin/p120-catenin complex were determined by co-immunoprecipitation and immunofluorescence.Moesin expression was found decreased in the membrane and increased in cytoplasm during the malignant transformation of oral epithelia, and cytoplasmic overexpression of Moesin correlated with nodal   metastasis   and poor prognosis of OSCCs. Furthermore, Moesin-silencing induced an increased cell-cell adhesion but decreased invasiveness, which was subsequently demonstrated might due to Moesin-mediated E-cadherin and p120-catenin interaction. Meantime, Moesin-silencing significantly down-regulated MT1-MMP expression, accompanied by reduced cell motility and impaired filopodia formation, which was also observed when MT1-MMP knockdown by RNAi or tissue inhibitor (TIMP2), indicating the involvement of MT1-MMP in Moesin-mediated cell motility. Finally, the relationship between Moesin, E-cadherin and MT1-MMP was confirmed in OSCC tissue samples.Taken together, our results indicate Moesin may regulate cell motility through its interactions with MT1-MMP and E-cadherin/p120-catenin adhesion complex and cytoplasmic expression of Moesin correlates with nodal   metastasis   and poor prognosis of OSCCs, indicating Moesin may be a potential candidate for targeted gene therapy for OSCCs. 
 The influence of MMP-14, TIMP-2 and MMP-2 expression on breast cancer prognosis. Matrix metalloproteinase (MMP)-2 is very active at degrading extracellular matrix. It is under the influence of an activator, membrane type 1 MMP (MMP-14), and the tissue inhibitor of metalloproteases (TIMP)-2. We hypothesized that the individual expression of these three markers or their balance may help to predict breast cancer prognosis.MMP-2, MMP-14 and TIMP-2 expression has been evaluated by 35S mRNA in situ hybridization on paraffin material of 539 breast cancers without distant   metastasis   at diagnosis and with a median follow-up of 9.2 years.MMP-2 and MMP-14 mRNA was detected primarily in reactive stromal cells whereas TIMP-2 mRNA was expressed by both stromal and cancer cells. Of the three molecules, an adjusted Cox model revealed that high MMP-14 mRNA (> or = 10% cells) alone predicted a significantly shorter overall survival (p = 0.031) when adjusted for clinical factors (tumor size and number of involved lymph nodes). Prognostic significance was lost when further adjusted for Her-2/neu and urokinase-type plasminogen activator (p = 0.284). Furthermore, when all three components were analyzed together, the survival was worst for patients with high MMP-2/high MMP-14/low TIMP-2 (5 year survival = 60%) and best with low MMP-2/low MMP-14/high TIMP-2 (5 year survival = 74%), but the difference did not reach statistical significance (p = 0.3285).Of the MMP-14/TIMP-2/MMP-2 complex, MMP-14 was the factor most significantly associated with the outcome of breast cancer and was an independent factor of poor overall survival when adjusted for clinical prognostic factors, but not for certain ancillary markers. 
 Differential localization of MT1-MMP in human prostate cancer tissue: role of IGF-1R in MT1-MMP expression. MT1-MMP is a metalloproteinase involved in prostate cancer   metastasis  . The IGF-1R is a tyrosine kinase receptor involved with tumor progression and   metastasis  . The purpose of this investigation was to examine MT1-MMP and IGF-1R expression and localization in prostate cancer tissues and explore the role of IGF-1R in regulating MT1-MMP in prostate cancer cell lines.Immunohistochemistry was utilized to study MT1-MMP and IGF-1R expression in human prostate tissues. IGF-1R regulation of MT1-MMP expression was determined by gene promoter analysis, quantitative RT-PCR and Western blot analysis following pharmacological inhibition of the receptor in PC-3N cells and treatment of LNCaP cells with androgen and IGF-1.MT1-MMP expression was high in the apical regions of the luminal cells in PIN and prostate cancer and less intense in the basalateral regions of benign tissues. IGF-1R was expressed primarily in the basal cells of normal glands and highly expressed in prostate cancer. Inhibition of IGF-1R in PC-3N cells decreased MT1-MMP expression and treatment of LNCaP cells with a synthetic androgen and IGF-1 increased MT1-MMP expression.These data demonstrate that MT1-MMP is highly expressed in the apical cytoplasmic regions of the luminal cells in PIN and prostate cancer when compared to basalateral cytoplasmic membrane staining in benign glands. Additionally, we demonstrate that IGF-1R is highly expressed in human prostate carcinoma. These findings suggest that MT1-MMP localization and IGF-1R expression in prostate carcinoma could be predictive biomarkers for aggressive disease and support IGF-1R as a promising therapeutic target to decrease processes of prostate cancer   metastasis  . 
 [Membrane type 1 matrix metalloproteinase (MT1-MMP) and the regulators of its activity as invasive factors in squamous cell cervical carcinomas]. Membrane type 1 matrix metalloproteinase (MT1MMP) is one of matrix metalloproteinases (MMP), which play а key role in tumor invasion and   metastasi  s. The aim of this study was to elucidate the peculiarities of expression of MT1MMP and endogenous regulators of its activity: the activator - furin and the inhibitor - TIMP-2, as invasive factors of squamous cell cervical carcinomas (SCC). The study was carried out using 11 specimens of SCC and 11 specimens of morphologically normal tissue adjacent to the tumor. It was shown that the increase of MT1-MMP and furin expression and low of TIMP-2 expression makes the main contribution to the destructive (invasive) potential of SCC. Moreover, substantial expression of MT1-MMP was registered in the specimens of morphologically normal adjoining to tumor tissue. This expression was found to make an additional contribution to the destructive potential of the cervical tumor. 
 NEDD9 depletion leads to MMP14 inactivation by TIMP2 and prevents invasion and   metastasis  . The scaffolding protein NEDD9 is an established prometastatic marker in several cancers. Nevertheless, the molecular mechanisms of NEDD9-driven   metastasis   in cancers remain ill-defined. Here, using a comprehensive breast cancer tissue microarray, it was shown that increased levels of NEDD9 protein significantly correlated with the transition from carcinoma in situ to invasive carcinoma. Similarly, it was shown that NEDD9 overexpression is a hallmark of highly invasive breast cancer cells. Moreover, NEDD9 expression is crucial for the protease-dependent mesenchymal invasion of cancer cells at the primary site but not at the metastatic site. Depletion of NEDD9 is sufficient to suppress invasion of tumor cells in vitro and in vivo, leading to decreased circulating tumor cells and lung metastases in xenograft models. Mechanistically, NEDD9 localized to invasive pseudopods and was required for local matrix degradation. Depletion of NEDD9 impaired invasion of cancer cells through inactivation of membrane-bound matrix metalloproteinase MMP14 by excess TIMP2 on the cell surface. Inactivation of MMP14 is accompanied by reduced collagenolytic activity of soluble metalloproteinases MMP2 and MMP9. Reexpression of NEDD9 is sufficient to restore the activity of MMP14 and the invasive properties of breast cancer cells in vitro and in vivo. Collectively, these findings uncover critical steps in NEDD9-dependent invasion of breast cancer cells.This study provides a mechanistic basis for potential therapeutic interventions to prevent   metastasis  . 
 Downregulation of reversion-inducing cysteine-rich protein with Kazal motifs in malignant melanoma: inverse correlation with membrane-type 1-matrix metalloproteinase and tissue inhibitor of metalloproteinase 2. The invasive phenotype of many tumors is associated with an imbalance between the matrix metalloproteinases (MMPs) and their inhibitors, tissue inhibitors of metalloproteinases (TIMPs), and the membrane-anchored reversion-inducing cysteine-rich protein with Kazal motifs (RECK). RECK inhibits MMP-2, MMP-9, and MT1-MMP, and has been linked to patient survival and better prognosis in several types of tumors. However, despite the wide implication of these MMPs in melanoma establishment and progression, the role of RECK in this type of tumor is still unknown. Here, we analyzed the expression of RECK, TIMP1, TIMP2, TIMP3, MT1MMP, MMP2, and MMP9 in two publicly available melanoma microarray datasets and in a panel of human melanoma cell lines. We found that RECK is downregulated in malignant melanoma, accompanied by upregulation of MT1MMP and TIMP2. In both datasets, we observed that the group of samples displaying higher RECK levels show lower median expression levels of MT1MMP and TIMP2 and higher levels of TIMP3. When tested in a sample-wise manner, these correlations were statistically significant. Inverse correlations between RECK, MT1MMP, and TIMP2 were verified in a panel of human melanoma cell lines and in a further reduced model that includes a pair of matched primary tumor-derived and   metastasis  -derived cell lines. Taken together, our data indicate a consistent correlation between RECK, MT1MMP, and TIMP2 across different models of clinical samples and cell lines and suggest evidence of the potential use of this subset of genes as a gene signature for diagnosing melanoma. 
 MT1-MMP modulates melanoma cell dissemination and   metastasis   through activation of MMP2 and RAC1. Metastatic melanoma remains the deadliest of all skin cancers with a survival rate at five years of less than 15%. MT1-MMP is a membrane-associated matrix metalloproteinase that controls pericellular proteolysis and is an important, invasion-promoting, pro-tumorigenic MMP in cancer. We show that deregulation of MT1-MMP expression happens as early as the transition from nevus to primary melanoma and continues to increase during melanoma progression. Furthermore, MT1-MMP expression is associated with poor melanoma patient outcome, underscoring a pivotal role of MT1-MMP in melanoma pathogenesis. We demonstrate that MT1-MMP is directly required for melanoma cells to metastasize, as cells deprived of MT1-MMP fail to form distant   metastasis   in an orthotopic mouse melanoma model. We show that MT1-MMP affects cell invasion by activating its target MMP2. Importantly, we demonstrate, for the first time, that activation of MMP2 by MT1-MMP is required to sustain RAC1 activity and promote MT1-MMP-dependent cell motility. These data highlight a novel MT1-MMP/MMP2/RAC1 signaling axis in melanoma that may represent an intriguing molecular target for the treatment of invasive melanoma. 
 Moesin regulates the motility of oral cancer cells via MT1-MMP and E-cadherin/p120-catenin adhesion complex. The present study aimed to clarify the role of Moesin in oral squamous cell carcinoma (OSCC) progression, especially in regulation of cell motility.Immunohistochemistry and western blotting were used to investigate the expression of Moesin, E-cadherin, p120-catenin and MT1-MMP in normal epithelia, dysplasia and OSCCs. Then, Moesin was knockdown by siRNA in OSCC cell lines, WSU-HN6 and CAL27, and the biological role of Moesin in cell adhesion and motility was evaluated by transwell system, cell spreading and aggregation assays. The interactions between Moesin, MT1-MMP and E-cadherin/p120-catenin complex were determined by co-immunoprecipitation and immunofluorescence.Moesin expression was found decreased in the membrane and increased in cytoplasm during the malignant transformation of oral epithelia, and cytoplasmic overexpression of Moesin correlated with nodal   metastasis   and poor prognosis of OSCCs. Furthermore, Moesin-silencing induced an increased cell-cell adhesion but decreased invasiveness, which was subsequently demonstrated might due to Moesin-mediated E-cadherin and p120-catenin interaction. Meantime, Moesin-silencing significantly down-regulated MT1-MMP expression, accompanied by reduced cell motility and impaired filopodia formation, which was also observed when MT1-MMP knockdown by RNAi or tissue inhibitor (TIMP2), indicating the involvement of MT1-MMP in Moesin-mediated cell motility. Finally, the relationship between Moesin, E-cadherin and MT1-MMP was confirmed in OSCC tissue samples.Taken together, our results indicate Moesin may regulate cell motility through its interactions with MT1-MMP and E-cadherin/p120-catenin adhesion complex and cytoplasmic expression of Moesin correlates with nodal   metastasis   and poor prognosis of OSCCs, indicating Moesin may be a potential candidate for targeted gene therapy for OSCCs. 
 [Membrane type 1 matrix metalloproteinase (MT1-MMP) and the regulators of its activity as invasive factors in squamous cell cervical carcinomas]. Membrane type 1 matrix metalloproteinase (MT1MMP) is one of matrix metalloproteinases (MMP), which play а key role in tumor invasion and   metastasi  s. The aim of this study was to elucidate the peculiarities of expression of MT1MMP and endogenous regulators of its activity: the activator - furin and the inhibitor - TIMP-2, as invasive factors of squamous cell cervical carcinomas (SCC). The study was carried out using 11 specimens of SCC and 11 specimens of morphologically normal tissue adjacent to the tumor. It was shown that the increase of MT1-MMP and furin expression and low of TIMP-2 expression makes the main contribution to the destructive (invasive) potential of SCC. Moreover, substantial expression of MT1-MMP was registered in the specimens of morphologically normal adjoining to tumor tissue. This expression was found to make an additional contribution to the destructive potential of the cervical tumor. 
 MT1-MMP modulates melanoma cell dissemination and   metastasis   through activation of MMP2 and RAC1. Metastatic melanoma remains the deadliest of all skin cancers with a survival rate at five years of less than 15%. MT1-MMP is a membrane-associated matrix metalloproteinase that controls pericellular proteolysis and is an important, invasion-promoting, pro-tumorigenic MMP in cancer. We show that deregulation of MT1-MMP expression happens as early as the transition from nevus to primary melanoma and continues to increase during melanoma progression. Furthermore, MT1-MMP expression is associated with poor melanoma patient outcome, underscoring a pivotal role of MT1-MMP in melanoma pathogenesis. We demonstrate that MT1-MMP is directly required for melanoma cells to metastasize, as cells deprived of MT1-MMP fail to form distant   metastasis   in an orthotopic mouse melanoma model. We show that MT1-MMP affects cell invasion by activating its target MMP2. Importantly, we demonstrate, for the first time, that activation of MMP2 by MT1-MMP is required to sustain RAC1 activity and promote MT1-MMP-dependent cell motility. These data highlight a novel MT1-MMP/MMP2/RAC1 signaling axis in melanoma that may represent an intriguing molecular target for the treatment of invasive melanoma. 
 Downregulation of reversion-inducing cysteine-rich protein with Kazal motifs in malignant melanoma: inverse correlation with membrane-type 1-matrix metalloproteinase and tissue inhibitor of metalloproteinase 2. The invasive phenotype of many tumors is associated with an imbalance between the matrix metalloproteinases (MMPs) and their inhibitors, tissue inhibitors of metalloproteinases (TIMPs), and the membrane-anchored reversion-inducing cysteine-rich protein with Kazal motifs (RECK). RECK inhibits MMP-2, MMP-9, and MT1-MMP, and has been linked to patient survival and better prognosis in several types of tumors. However, despite the wide implication of these MMPs in melanoma establishment and progression, the role of RECK in this type of tumor is still unknown. Here, we analyzed the expression of RECK, TIMP1, TIMP2, TIMP3, MT1MMP, MMP2, and MMP9 in two publicly available melanoma microarray datasets and in a panel of human melanoma cell lines. We found that RECK is downregulated in malignant melanoma, accompanied by upregulation of MT1MMP and TIMP2. In both datasets, we observed that the group of samples displaying higher RECK levels show lower median expression levels of MT1MMP and TIMP2 and higher levels of TIMP3. When tested in a sample-wise manner, these correlations were statistically significant. Inverse correlations between RECK, MT1MMP, and TIMP2 were verified in a panel of human melanoma cell lines and in a further reduced model that includes a pair of matched primary tumor-derived and   metastasis  -derived cell lines. Taken together, our data indicate a consistent correlation between RECK, MT1MMP, and TIMP2 across different models of clinical samples and cell lines and suggest evidence of the potential use of this subset of genes as a gene signature for diagnosing melanoma. 
 NEDD9 depletion leads to MMP14 inactivation by TIMP2 and prevents invasion and   metastasis  . The scaffolding protein NEDD9 is an established prometastatic marker in several cancers. Nevertheless, the molecular mechanisms of NEDD9-driven   metastasis   in cancers remain ill-defined. Here, using a comprehensive breast cancer tissue microarray, it was shown that increased levels of NEDD9 protein significantly correlated with the transition from carcinoma in situ to invasive carcinoma. Similarly, it was shown that NEDD9 overexpression is a hallmark of highly invasive breast cancer cells. Moreover, NEDD9 expression is crucial for the protease-dependent mesenchymal invasion of cancer cells at the primary site but not at the metastatic site. Depletion of NEDD9 is sufficient to suppress invasion of tumor cells in vitro and in vivo, leading to decreased circulating tumor cells and lung metastases in xenograft models. Mechanistically, NEDD9 localized to invasive pseudopods and was required for local matrix degradation. Depletion of NEDD9 impaired invasion of cancer cells through inactivation of membrane-bound matrix metalloproteinase MMP14 by excess TIMP2 on the cell surface. Inactivation of MMP14 is accompanied by reduced collagenolytic activity of soluble metalloproteinases MMP2 and MMP9. Reexpression of NEDD9 is sufficient to restore the activity of MMP14 and the invasive properties of breast cancer cells in vitro and in vivo. Collectively, these findings uncover critical steps in NEDD9-dependent invasion of breast cancer cells.This study provides a mechanistic basis for potential therapeutic interventions to prevent   metastasis  . 
 Differential localization of MT1-MMP in human prostate cancer tissue: role of IGF-1R in MT1-MMP expression. MT1-MMP is a metalloproteinase involved in prostate cancer   metastasis  . The IGF-1R is a tyrosine kinase receptor involved with tumor progression and   metastasis  . The purpose of this investigation was to examine MT1-MMP and IGF-1R expression and localization in prostate cancer tissues and explore the role of IGF-1R in regulating MT1-MMP in prostate cancer cell lines.Immunohistochemistry was utilized to study MT1-MMP and IGF-1R expression in human prostate tissues. IGF-1R regulation of MT1-MMP expression was determined by gene promoter analysis, quantitative RT-PCR and Western blot analysis following pharmacological inhibition of the receptor in PC-3N cells and treatment of LNCaP cells with androgen and IGF-1.MT1-MMP expression was high in the apical regions of the luminal cells in PIN and prostate cancer and less intense in the basalateral regions of benign tissues. IGF-1R was expressed primarily in the basal cells of normal glands and highly expressed in prostate cancer. Inhibition of IGF-1R in PC-3N cells decreased MT1-MMP expression and treatment of LNCaP cells with a synthetic androgen and IGF-1 increased MT1-MMP expression.These data demonstrate that MT1-MMP is highly expressed in the apical cytoplasmic regions of the luminal cells in PIN and prostate cancer when compared to basalateral cytoplasmic membrane staining in benign glands. Additionally, we demonstrate that IGF-1R is highly expressed in human prostate carcinoma. These findings suggest that MT1-MMP localization and IGF-1R expression in prostate carcinoma could be predictive biomarkers for aggressive disease and support IGF-1R as a promising therapeutic target to decrease processes of prostate cancer   metastasis  . 
 The influence of MMP-14, TIMP-2 and MMP-2 expression on breast cancer prognosis. Matrix metalloproteinase (MMP)-2 is very active at degrading extracellular matrix. It is under the influence of an activator, membrane type 1 MMP (MMP-14), and the tissue inhibitor of metalloproteases (TIMP)-2. We hypothesized that the individual expression of these three markers or their balance may help to predict breast cancer prognosis.MMP-2, MMP-14 and TIMP-2 expression has been evaluated by 35S mRNA in situ hybridization on paraffin material of 539 breast cancers without distant   metastasis   at diagnosis and with a median follow-up of 9.2 years.MMP-2 and MMP-14 mRNA was detected primarily in reactive stromal cells whereas TIMP-2 mRNA was expressed by both stromal and cancer cells. Of the three molecules, an adjusted Cox model revealed that high MMP-14 mRNA (> or = 10% cells) alone predicted a significantly shorter overall survival (p = 0.031) when adjusted for clinical factors (tumor size and number of involved lymph nodes). Prognostic significance was lost when further adjusted for Her-2/neu and urokinase-type plasminogen activator (p = 0.284). Furthermore, when all three components were analyzed together, the survival was worst for patients with high MMP-2/high MMP-14/low TIMP-2 (5 year survival = 60%) and best with low MMP-2/low MMP-14/high TIMP-2 (5 year survival = 74%), but the difference did not reach statistical significance (p = 0.3285).Of the MMP-14/TIMP-2/MMP-2 complex, MMP-14 was the factor most significantly associated with the outcome of breast cancer and was an independent factor of poor overall survival when adjusted for clinical prognostic factors, but not for certain ancillary markers. 
 Targeting a single function of the multifunctional matrix metalloprotease MT1-MMP: impact on lymphangiogenesis. The group of matrix metalloproteases (MMPs) is responsible for multiple processes of extracellular matrix remodeling in the healthy body but also for matrix and tissue destruction during cancer invasion and   metastasis  . The understanding of the contributions from each individual MMP, both in healthy and pathological events, has been complicated by the lack of specific inhibitors and the fact that some of the potent MMPs are multifunctional enzymes. These factors have also hampered the setup of therapeutic strategies targeting MMP activity. A tempting target is the membrane-associated MT1-MMP, which has well-documented importance in matrix degradation but which takes part in more than one pathway in this regard. In this report, we describe the selective targeting of a single function of this enzyme by means of a specific monoclonal antibody against MT1-MMP, raised in an MT1-MMP knock-out mouse. The antibody blocks the enzyme ability to activate proMMP-2 without interfering with the collagenolytic function or the general proteolytic activity of MT1-MMP. Using this antibody, we have shown that the MT1-MMP-catalyzed activation of proMMP-2 is involved in the outgrowth of cultured lymphatic endothelial cells in a collagen matrix in vitro, as well as in lymphatic vessel sprouting assayed ex vivo. This is the first example of the complete inactivation of a single function of a multifunctional MMP and the use of this strategy to pursue its role. 
 Prostate cancer-associated membrane type 1-matrix metalloproteinase: a pivotal role in bone response and intraosseous tumor growth. Membrane type 1-matrix metalloproteinase (MT1-MMP) is a major mediator of collagen I degradation. In human samples, we show that prostate cancer cells in skeletal metastases consistently express abundant MT1-MMP protein. Because prostate cancer bone   metastasis   requires remodeling of the collagen-rich bone matrix, we investigated the role of cancer cell-derived MT1-MMP in an experimental model of tumor-bone interaction. MT1-MMP-deficient LNCaP human prostate cancer cells were stably transfected with human wild-type MT1-MMP (MT1wt). Furthermore, endogenous MT1-MMP was down-regulated by small interfering RNA in DU145 human prostate cancer cells. Intratibial tumor injection in severe combined immunodeficient mice was used to simulate intraosseous growth of metastatic tumors. LNCaP-MT1wt cells produced larger osseous tumors than Neo control cells and induced osteolysis, whereas DU145 MT1-MMP-silenced transfectants induced osteogenic changes. In vitro assays showed that MT1wt overexpression enhanced collagen I degradation, whereas MT1-MMP-silencing did the opposite, suggesting that tumor-derived MT1-MMP may contribute directly to bone remodeling. LNCaP-MT1wt-derived conditioned medium stimulated in vitro multinucleated osteoclast formation. This effect was inhibited by osteoprotegerin, a decoy receptor for receptor activator of nuclear factor kappaB ligand, and by 4-[4-(methanesulfonamido) phenoxy] phenylsulfonyl methylthiirane, an MT1-MMP inhibitor. Our findings are consistent with the hypothesis that prostate cancer-associated MT1-MMP plays a direct and/or indirect role in bone matrix degradation, thus favoring intraosseous tumor expansion. 
 Expression of the reversion-inducing cysteine-rich protein with Kazal motifs and matrix metalloproteinase-14 in neuroblastoma and the role in tumour   metastasis  . Neuroblastoma is the most common malignant tumour in infancy; the reversion-inducing cysteine-rich protein with Kazal motifs gene (RECK) is a tumour suppressor gene. Previous studies show that RECK inhibits tumour invasion and   metastasis   through negative regulation of the matrix metalloproteinase (MMP)-2, MMP-9 and MMP-14. Therefore, we wanted to detect the expression of RECK and MMP-14 in neuroblastomas to assess the correlation between the expression levels of these proteins, and to investigate the roles in the   metastasis   and development of the tumour. PV-6000 immunohistochemistry method was used to detect the expression levels of RECK and MMP-14 in 36 samples of neuroblastoma tissue. Samples from paraffin wax-embedded specimens and the complete clinicopathological data of 36 neuroblastoma and 10 ganglioneuroma patients were collected. The rate of expression of the RECK protein in the neuroblastoma was low (16.7%). Furthermore, it reduced with the increase in the invasive depth and distant   metastasis   (P = 0.015; P < 0.05). The rate of expression of the MMP-14 protein in the neuroblastoma was high (58.3%) and increased with the increase in the extent of invasive depth and distant   metastasis   (P = 0.002; P < 0.05). The expression of the RECK protein correlated negatively with that of MMP-14 (r = -0.418; P < 0.05). Low levels of the RECK protein are expressed in the neuroblastoma, while the MMP-14 protein is expressed at high levels. The RECK and MMP-14 proteins may serve as markers in the estimation of the extent of   metastasis   and dissemination of the neuroblastoma. 
 Decreased MT1-MMP in gastric cancer suppressed cell migration and invasion via regulating MMPs and EMT. Membrane type 1-matrix metalloproteinase (MT1-MMP) has been identified to play a significant role in several types of cancers, but little is known about the significance of MT1-MMP in gastric cancer patients. The purpose of this study is to investigate the involvement of MT1-MMP in tumor progression of gastric cancer. MT1-MMP expression levels were examined in gastric cancer tissues and cells, and normal gastric tissues and cells. The effects and molecular mechanisms of MT1-MMP expression on cell proliferation, migration, and invasion were also explored. In our results, MT1-MMP messenger RNA (mRNA) and protein expression levels were significantly increased in gastric cancer tissue. Moreover, the overexpression of MT1-MMP was positively associated with the status of clinical stage and lymph node   metastasis   through real-time PCR. Furthermore, knocking down MT1-MMP expression significantly suppressed the cell migration and invasion in vitro and regulated the expression of MMPs and epithelial-mesenchymal transition (EMT)-associated genes. In conclusions, our study demonstrates that MT1-MMP was overexpressed in gastric cancer tissue, and reduced expression of MT1-MMP suppressed cell migration, invasion, and through regulating the expression of MMPs and the process of EMT in gastric cancer. 
 CD44 regulates pancreatic cancer invasion through MT1-MMP. Pancreatic cancer is one of the deadliest human malignancies due to its early metastatic spread and resistance to therapy. The mechanisms regulating pancreatic cancer   metastasis   are so far poorly understood. Here, using both in vitro and in vivo approaches, it is demonstrated that CD44, a transmembrane glycoprotein expressed on a subset of pancreatic cancer cells, is required for the induction of epithelial-mesenchymal transition (EMT) and the activation of an invasive program in pancreatic cancer. Mechanistically, the transcription factor Snail1 (SNAI1), a regulator of the EMT program, is a downstream target of CD44 in primary pancreatic cancer cells and regulates membrane bound metalloproteinase (MMP14/MT1-MMP) expression. In turn, MT1-MMP expression is required for pancreatic cancer invasion. Thus, these data establish the CD44-Snail-MMP axis as a key regulator of the EMT program and of invasion in pancreatic cancer.This study sets the stage for CD44 and MT1-MMP as therapeutic targets in pancreatic cancer, for which small molecule or biologic inhibitors are available. Visual Overview: http://mcr.aacrjournals.org/content/early/2014/09/10/1541-7786.MCR-14-0076/F1.large.jpg. 
 Direct activation of pro-matrix metalloproteinase-2 by leukolysin/membrane-type 6 matrix metalloproteinase/matrix metalloproteinase 25 at the asn(109)-Tyr bond. Leukolysin/membrane-type 6 matrix metalloproteinase (leukolysin/MT6-MMP), a glycosylphosphatidylinositol-anchored neutrophil matrix metalloproteinase, is also abnormally expressed in brain cancer tissues. Yet, little is known about its role in cancer progression. Here we show that MT6-MMP is capable of activating proMMP-2, an enzyme implicated in tumor invasion and   metastasis  . Although MT6-MMP is only 10% as active as MT5-MMP in mediating proMMP-2 activation, it generates a higher ratio of mature/intermediate forms of MMP-2 than MT5-MMP. Consistently, purified CAT of MT6-MMP converts proMMP-2 into mostly the mature form. Using the catalytically inactive mutant MMP-2EA (the E404A mutant of proMMP-2), which cannot autocatalytically mature from the intermediate form into the mature one, we show that MT6-MMP cleaves not only the known MT-MMP-processing site at Asn(66)-Leu but also the previously unsuspected Asn(109)-Tyr to yield a fully mature molecule. Despite their difference in mediating proMMP-2 activation in transfected cells, the CAT of MT6-MMP appears to be as efficient as that of MT5-MMP in cleaving proMMP-2EA in buffer, suggesting that its CAT is a strong proMMP-2 activator. Indeed, the CAT of MT6-MMP can partially substitute the CAT of prototypical MT1-MMP in mediating proMMP-2 activation. Taken these facts together, we conclude that MT6-MMP may participate in tumor invasion and   metastasis   by directly converting proMMP-2 into active form. 
 Increased aggressiveness of human prostate PC-3 tumor cells expressing cell surface localized membrane type-1 matrix metalloproteinase (MT1-MMP). Membrane type-1 matrix metalloproteinase (MT1-MMP) is a multidomain transmembrane endopeptidase with a major role in physiological and pathological processes through proteolysis of extracellular matrix and other pericellular proteins. We examined cell surface function of MT1-MMP in PC-3 human prostate tumor cells selected for   metastasis   in nude mice (PC-3-LN4), or transfected with the full-length wild-type (WT) MT1-MMP or with the mutant form lacking the cytoplasmic tail (Delta C-MT1-MMP). Enhanced cell surface MT1-MMP was determined by fluorescence-activated cell sorting analysis and evidenced mechanistically by increased activation of proMMP-2 and invasion into type-I collagen gels. PC-3 cells overexpressing MT1-MMP grew faster than mock-transfected control cells subcutaneously in nude mice. MT1-MMP localized in caveolae, as judged by immunofluorescence microscopy and sucrose-gradient, detergent-resistant cell fractionation. Delta C-MT1-MMP was strongly associated with caveolae, whereas the WT form was present in both caveolae and noncaveolae fractions. The role of plasma membrane MT1-MMP was supported by localization of MT1-MMP by immunofluorescence microscopy at the cell surface of tumor cells in primary prostate cancers. Increased plasma membrane localization of MT1-MMP, either in caveolae or in other lipid raft structures, is a mechanism to localize this proteinase in contact with extracellular matrix and other pericellular proteins, the cleavage of which can facilitate prostate cancer cell invasion and   metastasis  . 
 Transforming growth factor-beta induces CD44 cleavage that promotes migration of MDA-MB-435s cells through the up-regulation of membrane type 1-matrix metalloproteinase. CD44, a transmembrane receptor for hyaluronic acid, is implicated in various adhesion-dependent cellular processes, including cell migration, tumor cell   metastasis   and invasion. Recent studies demonstrated that CD44 expressed in cancer cells can be proteolytically cleaved at the ectodomain by membrane type 1-matrix metalloproteinase (MT1-MMP) to form soluble CD44 and that CD44 cleavage plays a critical role in cancer cell migration. Here, we show that transforming growth factor-beta (TGF-beta), a multifunctional cytokine involved in cell proliferation, differentiation, migration and pathological processes, induces MT1-MMP expression in MDA-MB-435s cells. TGF-beta-induced MT1-MMP expression was blocked by the specific extracellular regulated kinase-1/2 (ERK1/2) inhibitor PD98059 and the specific phosphoinositide 3-OH kinase (PI3K) inhibitor LY294002. In addition, treatment with SP600125, an inhibitor for c-Jun NH(2)-terminal kinase (JNK), resulted in a significant inhibition of MT1-MMP production. These data suggest that ERK1/2, PI3K, and JNK likely play a role in TGF-beta-induced MT1-MMP expression. Interestingly, treatment of MDA-MB-435s cells with TGF-beta resulted in a colocalization of MT1-MMP and CD44 in the cell membrane and in an increased level of soluble CD44. Using an electric cell-substrate impedance sensing cell-electrode system, we demonstrated that TGF-beta treatment promotes MDA-MB-435s cell migration, involving MT1-MMP-mediated CD44 cleavage. MT1-MMP siRNA transfection-inhibited TGF-beta-induced cancer cell transendothelial migration. Thus, this study contributes to our understanding of molecular mechanisms that play a critical role in tumor cell invasion and   metastasis  . 
 MicroRNA-22 inhibits tumor growth and   metastasis   in gastric cancer by directly targeting MMP14 and Snail. MicroRNAs (miRNAs) deregulation is frequent in human gastric cancers (GCs), but the role of specific miRNAs involved in this disease remains elusive. MiR-22 was previously reported to act as tumor suppressors or oncogenes in diverse cancers. However, their accurate expression, function and mechanism in GC are largely unclear. Here, we found that the expression of miR-22 was significantly reduced in clinical GC tissues compared with paired adjacent normal tissues, and was significantly correlated with a more aggressive phenotype of GC in patients, and miR-22 low expression correlated with poor overall survival. The introduction of miR-22 markedly suppressed GC cell growth, migration and invasion, and inhibition of miR-22 promoted GC cell proliferation, migration and invasion in vitro. We further demonstrated that miR-22 acted as tumor suppressors through targeting extracellular matrix (ECM) remodeling member matrix metalloproteinase 14 (MMP14) and epithelial-to-mesenchymal transition (EMT) inducer Snail in GC. Moreover, ectopic expression of MMP14 or Snail restored inhibitory effects of miR-22 on cell migration and invasion in GC cells, and a negative relationship between the miR-22 expression and MMP14 or Snail mRNA levels was observed in GC. Finally, overexpression of miR-22 suppressed tumor growth, peritoneal dissemination and pulmonary   metastasis   in vivo. Taken together, we identified that miR-22 is a potent tumor suppressor in GC. MiR-22 downregulation promotes GC invasion and   metastasis   by upregulating MMP14 and Snail, and then inducing ECM remodeling and EMT. These findings provide a better understanding of the development and progression of GC and may be an important implication for future therapy of the GC. 
 Proteolysis of EphA2 Converts It from a Tumor Suppressor to an Oncoprotein. Eph receptor tyrosine kinases are considered candidate therapeutic targets in cancer, but they can exert opposing effects on cell growth. In the presence of its ligands, Eph receptor EphA2 suppresses signaling by other growth factor receptors, including ErbB, whereas ligand-independent activation of EphA2 augments ErbB signaling. To deploy EphA2-targeting drugs effectively in tumors, the anti-oncogenic ligand-dependent activation state of EphA2 must be discriminated from its oncogenic ligand-independent state. Because the molecular basis for the latter is little understood, we investigated how the activation state of EphA2 can be switched in tumor tissue. We found that ligand-binding domain of EphA2 is cleaved frequently by the membrane metalloproteinase MT1-MMP, a powerful modulator of the pericellular environment in tumor cells. EphA2 immunostaining revealed a significant loss of the N-terminal portion of EphA2 in areas of tumor tissue that expressed MT1-MMP. Moreover, EphA2 phosphorylation patterns that signify ligand-independent activation were observed specifically in these areas of tumor tissue. Mechanistic experiments revealed that processing of EphA2 by MT1-MMP promoted ErbB signaling, anchorage-independent growth, and cell migration. Conversely, expression of a proteolysis-resistant mutant of EphA2 prevented tumorigenesis and   metastasis   of human tumor xenografts in mice. Overall, our results showed how the proteolytic state of EphA2 in tumors determines its effector function and influences its status as a candidate biomarker for targeted therapy. 
 [Expression of MT1-MMP and RECK protein in human gastric carcinoma]. To examine the expression of membrane-type-1 matrix metalloproteinase (MT1-MMP) and reversion-inducing cysteine-rich protein with Kazal motifs (RECK) in gastric carcinoma, and investigate its clinical significance, at the same time analyze the correlation between MT1-MMP and RECK expression.MT1-MMP and RECK expression in surgically resected tissue samples of gastric carcinoma was examined by immunohistochemical method (two-step method) , and its correlation with clinicopathological factors was analyzed.Among the 44 gastric carcinoma samples, 37 (84.1%) were stained positive for MT1-MMP, and 31 (70.5%) for RECK. The expression of MT1-MMP was much higher in poorly differentiated gastric carcinoma samples than moderately and well-differentiated samples (P = 0.015). The expression level of MT1-MMP was associated with invasive depth of tumor cells (P = 0.007), but no difference between sex and lymph node   metastasis  . On the contrary, the well-differentiated samples showed higher expression of RECK than poorly and moderately differentiated gastric carcinoma samples (P = 0.006). The expression level of RECK did not correlate with sex, lymph node   metastasis   and invasive depth of tumor cells. RECK expression showed no relation to MT1-MMP expression in the gastric carcinoma.Overexpression of MT1-MMP in gastric carcinoma may play an important role during tumor differentiation and   metastasis  , the RECK protein may have positive effects on the tumor differentiation. 
 Membrane type 1-matrix metalloproteinase promotes human prostate cancer invasion and   metastasis  . Development of metastases requires cancer cells to breach underlying basement membrane, migrate through interstitial stroma and gain access to blood or lymphatic vessels. Membrane type 1-matrix metalloproteinase (MT1-MMP) has been linked with these processes. Expression of MT1-MMP in human prostate cancer correlates with the stage of this disseminated disease. The mechanism underlying this observation, however, still remains to be understood. To study the role of MT1-MMP in prostate cancer dissemination, endogenous and recombinant MT1-MMP expressed in human prostate cancer cell lines (DU-145 and LNCaP) were examined. Using FITC-labeled Matrigel, a soluble basement membrane extract coated coverslips, LNCaP cells stably expressing a chimera of MT1-MMP and Green Fluorescent Protein (MT1-GFP) degraded Matrigel and readily migrated over degraded substrates. The degradation of Matrigel by LNCaP cells expressing MT1-GFP was sensitive to MMP inhibitors, CT-1746 and TIMP-2, but not TIMP-1. Cell migration was dramatically enhanced by expression of MT1-MMP. By employing surgical orthotopic implantation of LNCaP cells stably expressing MT1-GFP into the prostate gland of immunodeficient mice, we demonstrated that MT1-MMP promotes lymph node and lung   metastasis   of prostate cancer cells. Together, these results emphasize the pivotal role of MT1-MMP in prostate cancer dissemination and confirm that MT1-MMP is a suitable target to prevent cancer   metastasis  . 
 Atypical localization of membrane type 1-matrix metalloproteinase in the nucleus is associated with aggressive features of hepatocellular carcinoma. Membrane type 1-matrix metalloproteinase (MT1-MMP) is a versatile proteinase and recent studies indicated it could be internalized. Our earlier study found that it is overexpressed in hepatocellular carcinoma (HCC) and could promote intrahepatic   metastasis  . The present study was conducted to examine its subcellular localization and its clinicopathological significance in HCC after curative partial hepatectomy. Localization of MT1-MMP in 101 pairs of HCCs and their adjacent liver tissues, and 8 normal liver tissues was examined by the immunohistochemical method. MT1-MMP protein was localized at membrane and cytoplasm of hepatocytes in the normal and tumor adjacent liver tissues. In contrast, the HCCs were highly heterogeneous with variable degrees of membrane, cytoplasmic, and even nuclear staining. Interestingly, patients with presence of nuclear MT1-MMP were associated with poor overall survival (log-rank test, P=0.043) and large tumor size (>5 cm) (Fisher's exact test, P=0.031). Subcellular distribution was further demonstrated by Western blotting and immunofluorescence with Hep3B stable transfectant overexpressing MT1-MMP. Western blot analyses of subcellular fractions confirmed a differential partitioning of various post-translationally modified MT1-MMP in these fractions. Different antibodies corroborated the presence of MT1-MMP in the nuclear fraction. Concomitant nuclear presence of MMP2 with MT1-MMP further indicated its potential involvement in the nuclear functions. MT1-MMP co-localized with caveolin-1 at the perinuclear region, suggesting nuclear translocation of MT1-MMP via caveolae-mediated endocytosis. In summary, the association of nuclear MT1-MMP with aggressive tumor features including poor prognosis and large tumor expands its functional repertoire and further indicates a new functional role of MMPs within nuclei of tumor cells. 
 Gonadotropin-releasing hormone-II increases membrane type I metalloproteinase production via beta-catenin signaling in ovarian cancer cells. GnRH-II is produced by ovarian cancer cells and enhances their invasiveness in vitro. In our studies of OVCAR-3 and CaOV-3 ovarian cancer cell lines, GnRH-II treatment induced phosphorylation of Akt and glycogen synthase kinase (GSK)3β, as well as β-catenin accumulation in the nucleus, and the latter was reduced by small interfering RNA (siRNA)-mediated depletion of the GnRH receptor. The phosphatidylinositol 3 kinase (PI3K)/Akt pathway is involved in β-catenin-dependent signaling, and pretreatment of these human ovarian cancer cells with a PI3K/Akt inhibitor, LY294002, attenuated GnRH-II-stimulated phosphorylation of GSK3β and inhibited GnRH-II-induced invasion. It also attenuated GnRH-II induced trans-activation of a β-catenin-dependent reporter gene, most likely because GSK3β phosphorylation promotes translocation of β-catenin to the nucleus. Membrane type I matrix metalloproteinase (MT1-MMP) contributes to tumor progression directly, or by processing the latent MMP-2 zymogen, and is a known target of β-catenin signaling. When OVCAR-3 and CaOV-3 cells were treated with GnRH-II, MT1-MMP levels increased approximately 3-fold, whereas siRNA-mediated depletion of GnRH receptor or pretreatment with LY294002 abrogated this. In addition, lithium chloride, which increases GSK3β phosphorylation and the nuclear translocation of β-catenin, increased MT1-MMP levels in these ovarian cancer cells. By contrast, depletion of β-catenin by siRNA treatment abolished GnRH-II-induced MT1-MMP synthesis and reduced their invasive potential. Furthermore, siRNA-mediated reduction of MT1-MMP levels reduced GnRH-II-induced invasion in ovarian cancer cells. We therefore conclude that GnRH-II stimulates the PI3K/Akt pathway, and the phosphorylation of GSK3β, thereby enhancing the β-catenin-dependent up-regulation of MT1-MMP production, which contributes to ov  arian canc  er metastasis. 
 NEDD9/Arf6-dependent endocytic trafficking of matrix metalloproteinase 14: a novel mechanism for blocking mesenchymal cell invasion and   metastasis   of breast cancer. NEDD9 is an established marker of invasive and metastatic cancers. NEDD9 downregulation has been shown to dramatically reduce cell invasion and   metastasis   in multiple tumors. The mechanisms by which NEDD9 regulates invasion are largely unknown. In the current study, we have found that NEDD9 is required for matrix metalloproteinase 14 (MMP14) enzymatic recovery/recycling through the late endosomes to enable disengagement of tissue inhibitor of matrix metalloproteinase 2 (TIMP2) and tumor invasion. Depletion of NEDD9 decreases targeting of the MMP14/TIMP2 complex to late endosomes and increases trafficking of MMP14 from early/sorting endosomes back to the surface in a small GTPase ADP ribosylation factor-6 (Arf6)-dependent manner. NEDD9 directly binds to Arf6-GTPase-activating protein, ARAP3 and Arf6-effector GGA3, thereby facilitating the Arf6 inactivation required for MMP14/TIMP2 targeting to late endosomes. Re-expression of NEDD9 or a decrease in Arf6 activity is sufficient to restore MMP14 activity and the invasive properties of tumor cells. Importantly, NEDD9 inhibition by Vivo-Morpholinos, an antisense therapy, decreases primary tumor growth and   metastasis   in xenograft models of breast cancer. Collectively, our findings uncover a novel mechanism to control tumor-cell dissemination through NEDD9/Arf6-dependent regulation of MMP14/TIMP2 trafficking, and validate NEDD9 as a clinically relevant therapeutic target to treat metastatic cancer. 
 Tetraspanin proteins regulate membrane type-1 matrix metalloproteinase-dependent pericellular proteolysis. Membrane type-1 matrix metalloproteinase (MT1-MMP) supports tumor cell invasion through extracellular matrix barriers containing fibrin, collagen, fibronectin, and other proteins. Here, we show that simultaneous knockdown of two or three members of the tetraspanin family (CD9, CD81, and TSPAN12) markedly decreases MT1-MMP proteolytic functions in cancer cells. Affected functions include fibronectin proteolysis, invasion and growth in three-dimensional fibrin and collagen gels, and MMP-2 activation. Tetraspanin proteins (CD9, CD81, and TSPAN2) selectively coimmunoprecipitate and colocalize with MT1-MMP. Although tetraspanins do not affect the initial biosynthesis of MT1-MMP, they do protect the newly synthesized protein from lysosomal degradation and support its delivery to the cell surface. Interfering with MT1-MMP-tetraspanin collaboration may be a useful therapeutic approach to limit cancer cell invasion and   metastasis  . 
 [Gene expression of matrix metalloproteinase 14 in laryngeal squamous cell carcinoma]. To investigate the role of expression of matrix metallo-proteinase 14 (MMP14) in laryngeal carcinomas and the relationship between MMP14 expression and the laryngeal biological behavior.The gene expression differences of MMP14 between fresh laryngeal cancer tissues and their surrounding normal mucosa was analyzed by RT-PCR. The expression of MMP14 protein in paraffin-embedded tissues was determined immunohistochemically. All statistical analyses were performed by SPSS version 10.0.In the 33 cases of matched specimens, MMP14 gene expression was much higher in tumor tissues than that in surrounding ing normal tissues in 26 cases and lower in 2 cases, whereas in other 5 cases, no significant difference was observed between the cancer tissue and the surrounding normal tissue. MMP14 gene expression was not different in different stages in laryngeal glottic cancers, but correlated to the differentiation and lymph node   metastasis   (P < 0.05). There was correlation between MMP14 gene expression and the stage, differentiation and lymph node   metastasis   in the laryngeal supraglottic cancers (P < .05). MMP14 protein was localized predominantly in the carcinoma cell cytoplasm and in the stromal fibroblast cytoplasm, and weakly or not expressed in surrounding normal tissue. MMP14 protein expression was much higher in tumor tissue than that in surrounding normal tissue in most of the cases. In general, MMP14 protein expression was the same as MMP14 gene expression and related to the stage, differentiation and lymph node   metastasis   in the laryngeal cancers. There was no survival difference at 3, 5 and 7-year between the group with higher MMP14 protein expression in tumor tissues than surrounding normal tissues and the group with no difference of MMP14 protein expression (Log Rank, P=0.5535).The protein MMP14 may play role in laryngeal cancer invasion in a certain extent, but important role in lymph node   metastasis   of laryngeal cancer. The over-expression of MMP14 protein may be a marker for lymph node   metastasis   of laryngeal cancer. 
 Effect on the expression of MMP-2, MT-MMP in laryngeal carcinoma Hep-2 cell line by antisense glucose transporter-1. Glucose transporter protein-1 (Glut-1) is correlated with biological behaviors of malignant tumors. However, there was no evidence that overexpression of Glut-1 mechanistically lead to invasion or   metastasis   of cancer cells. We hypothesized that Glut-1 regulates the expression of membrane type 1-MMP (MT1-MMP) and matrix metalloproteinase-2 (MMP-2).Analysis of the expression of Glut-1, MMP-2, β-actin, and MT1-MMP was performed using RT-PCR. Expression of Glut-1 protein, MMP-2, and MT1-MMP was detected by Western blotting.At mRNA and protein levels, Glut-1 and MMP-2 were co-expressed in the Hep-2 laryngeal carcinoma cell line. After transfection, Glut-1 antisense oligodeoxynucleotide (AS-ODN) decreased the expression of MMP-2 mRNA and protein as well as Glut-1 mRNA and protein. Glut-1 AS-ODN also decreased the expression of MT1-MMP mRNA.Co-expression of Glut-1 and MMP-2 in Hep-2 laryngeal carcinoma cells and Glut-1 may regulate MMP-2 and MT1-MMP expression. 
 MT1-MMP dependent repression of the tumor suppressor SPRY4 contributes to MT1-MMP driven melanoma cell motility. Metastatic melanoma is the deadliest of all skin cancers. Despite progress in diagnostics and treatment of melanoma, the prognosis for metastatic patients remains poor. We previously showed that Membrane-type 1 Matrix Metalloproteinase (MT1-MMP) is one of the drivers of melanoma   metastasis  . Classically, MT1-MMP regulates a verity of cellular functions including cell-to-cell interaction and cell-to-matrix communication. Recently, MT1-MMP has been found to also modulate gene expression. To specifically assess MT1-MMP dependent gene regulation in melanoma, microarray gene expression analysis was performed in a melanoma cell line whose metastatic properties depend on the activity of MT1-MMP. We identified the tumor suppressor gene SPRY4 as a new transcriptional target of MT1-MMP that is negatively regulated by the protease. Knockdown of MT1-MMP enhances SPRY4 expression at the mRNA and protein level. SPRY4 expression inversely correlates with that of MT1-MMP in melanoma samples and importantly, correlates with melanoma patient survival. SPRY4 modulates MT1-MMP dependent cell migration such that inhibition of SPRY4 rescues cell migration that has been impaired by MT1-MMP knock down. MT1-MMP decreases SPRY4 in part through an MMP2/RAC1 axis we previously show promotes cell motility downstream of MT1-MMP. These results identify the tumor suppressor SPRY4 as a novel molecular effector of MT1-MMP affecting melanoma cell motility. 
 MicroRNA-145 inhibits the growth, invasion,   metastasis   and angiogenesis of neuroblastoma cells through targeting hypoxia-inducible factor 2 alpha. Recent evidence shows that hypoxia-inducible factor 2 alpha (HIF-2α) may have critical roles in the growth and progression of neuroblastoma (NB) under non-hypoxic conditions. However, the underlying mechanisms and clinical potentials of normoxic HIF-2α expression in NB still remain largely unknown. In this study, HIF-2α immunostaining was identified in 26/42 NB tissues, which was correlated with clinicopathological features. In subtotal 20 NB cases, microRNA-145 (miR-145) was downregulated and inversely correlated with HIF-2α expression. Bioinformatics analysis revealed a putative miR-145 binding site in the 3'-untranslated region (3'-UTR) of HIF-2α messenger RNA (mRNA). Overexpression or knockdown of miR-145 responsively altered both the mRNA and protein levels of HIF-2α and its downstream genes, cyclin D1, matrix metalloproteinase 14 and vascular endothelial growth factor, in normoxically cultured NB cell lines SH-SY5Y and SK-N-SH. In a luciferase reporter system, miR-145 downregulated the luciferase activity of HIF-2α 3'-UTR, and these effects were abolished by a mutation in the putative miR-145-binding site. Overexpression of miR-145 suppressed the growth, inv  asion, met  astasis and angiogenesis of SH-SY5Y and SK-N-SH cells in vitro and in vivo, while restoration of HIF-2α expression rescued the tumor cells from miR-145-mediated defects in these biological features. Furthermore, anti-miR-145 inhibitor rescued the HIF-2α knockdown-mediated repression on the growth, migration, invasion and angiogenesis of NB cells. These data indicate that miR-145 suppresses HIF-2α expression via the binding site in the 3'-UTR under normoxic conditions, thus inhibiting the aggressiveness and angiogenesis of NB. 
 Non-destructive and selective imaging of the functionally active, pro-invasive membrane type-1 matrix metalloproteinase (MT1-MMP) enzyme in cancer cells. Proteolytic activity of cell surface-associated MT1-matrix metalloproteinase (MMP) (MMP-14) is directly related to cell migration, invasion, and   metastasis  . MT1-MMP is regulated as a proteinase by activation and conversion of the latent proenzyme into the active enzyme, and also via inhibition by tissue inhibitors of MMPs (TIMPs) and self-proteolysis. MT1-MMP is also regulated as a membrane protein through its internalization and recycling. Routine immunohistochemistry, flow cytometry, reverse transcription-PCR, and immunoblotting methodologies do not allow quantitative imaging and assessment of the cell-surface levels of the active, TIMP-free MT1-MMP enzyme. Here, we developed a fluorescent reporter prototype that targets the cellular active MT1-MMP enzyme alone. The reporter (MP-3653) represents a liposome tagged with a fluorochrome and functionalized with a PEG chain spacer linked to an inhibitory hydroxamate warhead. Our studies using the MP-3653 reporter and its inactive derivative demonstrated that MP-3653 can be efficiently used not only to visualize the trafficking of MT1-MMP through the cell compartment, but also to quantify the femtomolar range amounts of the cell surface-associated active MT1-MMP enzyme in multiple cancer cell types, including breast carcinoma, fibrosarcoma, and melanoma. Thus, the levels of the naturally expressed, fully functional, active cellular MT1-MMP enzyme are roughly equal to 1 × 10(5) molecules/cell, whereas these levels are in a 1 × 10(6) range in the cells with the enforced MT1-MMP expression. We suggest that the reporter we developed will contribute to the laboratory studies of MT1-MMP and then, ultimately, to the design of novel, more efficient prognostic approaches and personalized cancer therapies. 
 Cleavage of   metastasis   suppressor gene product KiSS-1 protein/metastin by matrix metalloproteinases. A human placenta cDNA library was screened by the expression cloning method for gene products that interact with matrix metalloproteinases (MMPs), and we isolated a cDNA whose product formed a stable complex with pro-MMP-2 and pro-MMP-9. The cDNA encoded the   metastasis   suppressor gene KiSS-1. KiSS-1 protein was shown to form a complex with pro-MMP. KiSS-1 protein is known to be processed to peptide ligand of a G-protein-coupled receptor (hOT7T175) named metastin, and suppresses   metastasis   of tumors expressing the receptor. Active MMP-2, MMP-9, MT1-MMP, MT3-MMP and MT5-MMP cleaved the Gly118-Leu119 peptide bond of not only full-length KiSS-1 protein but also metastin decapeptide. Metastin decapeptide induced formation of focal adhesion and actin stress fibers in cells expressing the receptor, and digestion of metastin decapeptide by MMP abolished its ligand activity. Migration of HT1080 cells expressing hOT7T175 that harbor a high-level MMP activity was only slightly suppressed by either metastin decapeptide or MMP inhibitor BB-94 alone, but the combination of metastin decapeptide and BB-94 showed a synergistic effect in blocking cell migration. We propose that metastin could be used as an antimetastatic agent in combination with MMP inhibitor, or MMP-resistant forms of metastin could be developed and may also be efficacious. 
 RABGTPases in MT1-MMP trafficking and cell invasion: Physiology versus pathology. The matrix metalloproteinase MT1-MMP is a central regulator of cell invasion in both physiological and pathological settings, such as tissue surveillance by immune cells and cancer cell   metastasis  . MT1-MMP cleaves a plethora of intra- and extracellular proteins, including extracellular matrix proteins, matrix receptors, and also other MMPs, and thus enables modification of both the cell surface proteome and the pericellular environment. Despite its importance for cell invasion, the pathways regulating MT1-MMP exposure on the cell surface are largely unknown. Recently, our groups discovered that a specific subset of RABGTPases, most notably RAB5a, is critical for MT1-MMP trafficking in primary human macrophages and carcinoma cells. Here, we discuss and contrast our findings for both cell types, pointing out common features and differences in the RABGTPase-dependent trafficking of MT1-MMP in health and disease. 
 Expression of membrane-type-1-matrix metalloproteinase and metalloproteinase-2 in nonsmall cell lung carcinomas. For the   metastasis   and invasion of cancer cells, destruction of extracellular matrix is essential. In this process, collagen is broken down by some matrix metalloproteinases. Matrix metalloproteinase 2 (MMP2) is able to cleave type IV collagen, and membrane-type-1-matrix metalloproteinase (MT1-MMP) induces activation of proMMP2. We investigated the expressions of MT1-MMP and MMP2 and their relation to both clinicopathologic parameters and clinical outcome in non-small cell lung carcinomas (NSCLC). Eighty-nine specimens of NSCLC were examined using in situ hybridization and immunohistochemistry. Each metalloproteinase was expressed within the cytoplasm of tumor cells with or without stromal cells in NSCLC. Tumors in which tumor cells strongly stained for MT1-MMP mRNA or protein made up more than 50% of the tumor area were found in 44 and 26% of cases, respectively. The corresponding values for MMP-2 mRNA and protein, were 51 and 26%. Our analysis of clinicopathological findings revealed a significant positive relationship between MT1-MMP mRNA and p-M. The correlation between MMP2 protein-staining status and overall survival rate reached significance in the univariate analysis. However, an association was not demonstrated in the multivariate analysis. The detection of MT1-MMP and MMP2 is likely to be of limited value in informing the prognosis in NSCLC. 
 Increased serum level of membrane type 1-matrix metalloproteinase (MT1-MMP/MMP-14) in patients with breast cancer. Different types of matrix metalloproteinases, including membrane type 1 matrix metalloproteinase (MT1-MMP/MMP-14) can be easily detected in biological fluids and therefore may be contemplated as putative tumor markers. Although increased activity of MT1-MMP/MMP-14 have already been found in breast cancer, little is known about its circulating levels. The aim of the present study was therefore to evaluate serum levels of active form of membrane type 1 matrix metalloproteinase (MT1-MMP/MMP-14). A novel type of activity enzyme-linked immunosorbent assay was used to detect serum levels of MT1-MMP/MMP-14 in 18 patients with invasive ductal breast cancer and 11 healthy controls. In the breast cancer group of patients MT1-MMP/MMP-14 mean (+/-SD) concentration was 16.91+/-5.87 ng/ml which was significantly higher (p<0.0001) than the mean values obtained for the control i.e. 8.55+/-1.66 ng/ml.Higher levels of soluble form of MT1-MMP/MMP-14 could play a role in invasiveness and   metastasis   of breast cancer. Whether or not it has a potential as biochemical marker remains to be determined. 
 Clinicopathological and prognostic significance of α5β1-integrin and MMP-14 expressions in colorectal cancer. The purpose of this study was to evaluate the association of expression level of α5β1-integrin and MMP-14 with clinicopathologic features and prognosis in colorectal cancer (CRC). The expressions of α5β1-integrin and MMP-14 in normal colorectal mucosa and CRC tissue were detected with immunohistochemistry. We estimated the five-year survival rate by the Kaplan-Meier method. The positive expressions rates of α5β1-integrin and MMP-14 in CRC tissue were 60.6% and 63.3% respectively, and there were significant differences on their positive expression rates between in CRC tissue and in normal colorectal mucosa(P<0.05). The expression rates of α5β1-integrin and MMP-14 in patients with poor histological differentiation, l  ymph node   metastasis and high clinical staging were heightened. There was a significant difference (P<0.05) on the five-year survival rate for α5β1-integrin expression, which was 44.6% in positive groups and 75.5% in negative groups. And there was a significant difference (P<0.05) on the five-year survival rate for MMP-14 expression, which was 48.2% in positive group and 73.1% in negative group. The expression of α5β1-integrin and MMP-14 is correlated with the pr  ogression   and metastasis of CRC, and α5β1-integrin and MMP-14 may be used as prognostic markers in CRC. 
 Expression of membrane type I-matrix metalloproteinase in oral squamous cell carcinoma. A local invasion and lymph node   metastasis   (LNM) of an oral squamous cell carcinoma (OSCC) has a poor prognosis, and involves the degradation of the extracellular matrix mediated by multiple proteolytic enzymes including membrane type I-matrix metalloproteinase (MT1-MMP). This study aimed to determine the role of MT1-MMP in OSCC, to evaluate the immunohistochemical expression of MT1-MMP with regard to the invasiveness and LNM of the OSCC, and to evaluate the major source of MT1-MMP mRNA and its protein using immunohistochemistry and in situ hybridization. MT1-MMP expression was examined in 46 OSCCs via immunohistochemistry and non-radioisotope in situ hybridization. The relationship between MT1-MMP expression and LNM, as well as the histological invasiveness, was statistically analyzed. The results showed that whereas 12 out of the 18 OSCCs (66.7%) with LNM showed moderate to strong MT1-MMP expression, only nine of the 28 OSCCs (32.1%) without LNM expressed MT1-MMP strongly. MT1-MMP expression was significantly higher with regard to LNM (P=0.022). As the invasion grade became stronger (from grade a to grade d), MT1-MMP was significantly more strongly expressed (P=0.033). These results suggest that MT1-MMP is primarily secreted in the OSCC cells and is involved in the invasiveness of the OSCC and LNM. Moreover, MT1-MMP combined with other markers may be used to predict the metastatic potential of an OSCC. 
 Up-regulation of vascular endothelial growth factor by membrane-type 1 matrix metalloproteinase stimulates human glioma xenograft growth and angiogenesis. Membrane-type (MT) 1 matrix metalloproteinase (MMP) is up-regulated in many tumor types and has been implicated in tumor progression and   metastasis  . MT1-MMP is critical for pericellular degradation of the extracellular matrix, thereby promoting tumor cell invasion and dissemination. To grow efficiently in vivo, tumor cells induce angiogenesis in both primary solid tumors and metastatic foci. The present study describes a functional link between the expression of MT1-MMP and vascular endothelial growth factor (VEGF) production in human glioma U251 xenografts in athymic mice. To investigate the effects of MT1-MMP on VEGF expression, U251 cells were stably transfected with MT1-MMP to generate the U-MT cell line overexpressing the enzyme. In vitro, the U-MT cells had an increased rate of proliferation and migration as well as the ability to activate the MMP-2 proenzyme and directionally remodel a three-dimensional collagen matrix. These findings suggested higher tumorigenicity of U-MT cells relative to the vector-control U-neo cells. In agreement with the in vitro data, U-MT xenografts in BALB/c nu/nu mice displayed markedly increased growth rates and elevated levels of angiogenesis. In contrast, U-neo cells formed small, minimally vascularized tumors. The elevated angiogenesis in U-MT xenografts was associated with an up-regulation of VEGF expression in tumor cells. In addition, U-MT cells in vitro secreted twice as much VEGF as the control cells. GM6001, a hydroxamate inhibitor of MMP activity, down-regulated the production of VEGF in U-MT cells to the levels observed in the U-neo control. Our results demonstrate that the enhanced tumorigenicity of glioma cells overexpressing MT1-MMP involves stimulation of angiogenesis through the up-regulation of VEGF production. 
 Control of MT1-MMP transport by atypical PKC during breast-cancer progression. Dissemination of carcinoma cells requires the pericellular degradation of the extracellular matrix, which is mediated by membrane type 1-matrix metalloproteinase (MT1-MMP). In this article, we report a co-up-regulation and colocalization of MT1-MMP and atypical protein kinase C iota (aPKCι) in hormone receptor-negative breast tumors in association with a higher risk of   metastasi  s. Silencing of aPKC in invasive breast-tumor cell lines impaired the delivery of MT1-MMP from late endocytic storage compartments to the surface and inhibited matrix degradation and invasion. We provide evidence that aPKCι, in association with MT1-MMP-containing endosomes, phosphorylates cortactin, which is present in F-actin-rich puncta on MT1-MMP-positive endosomes and regulates cortactin association with the membrane scission protein dynamin-2. Thus, cell line-based observations and clinical data reveal the concerted activity of aPKC, cortactin, and dynamin-2, which control the trafficking of MT1-MMP from late endosome to the plasma membrane and play an important role in the invasive potential of breast-cancer cells. 
 Protein expression of MMP-2 and MT1-MMP in actinic keratosis, squamous cell carcinoma of the skin, and basal cell carcinoma. Squamous cell carcinoma (SCC) and basal cell carcinoma (BCC) are 2 skin neoplasms with distinct potentials to invasion and   metastasis  . Actinic keratosis (AK) is a precursor lesion of SCC. Immunohistochemistry was performed to evaluate the expression of MMP-2 and MT1-MMP in samples of BCC (n = 29), SCC (n = 12), and AK (n = 13). The ratio of positive cells to total cells was used to quantify the staining. Statistical significance was considered under the level P < .05. We found a higher expression of MMP-2 in tumor stroma and parenchyma of SCC as compared to BCC. The expression of this protein was also similar between SCC and its precursor actinic keratosis, and it was higher in the stroma of high-risk BCC when compared to low-risk BCC. MT1-MMP, which is an activator of MMP-2, was similarly expressed in all groups. Our results suggest that MMP-2 expression may contribute to the distinct invasive patterns seen in SCC and BCC. 
 Distinct progression-associated expression of tumor and stromal MMPs in HaCaT skin SCCs correlates with onset of invasion. Matrix metalloproteinases (MMPs) are critically involved in tumor invasion and   metastasis  . However, failure of broad spectrum MMP inhibitors in clinical trials emphasizes the need for detailed analyses of the specific role of different MMPs in tumor malignancy. Using HaCaT-keratinocyte clones representing distinct stages in skin squamous cell carcinoma (SCC) progression, we demonstrate the expression of specific tumor and stroma-derived MMPs with the onset and maintenance of tumor invasion. Although MMP-9-positive leukocytes are present in benign and malignant tumor transplants at the onset of stromal activation and angiogenesis, mRNA expression of stroma-derived MMP-9 as well as MMP-2, -13 and -14 is exclusively found in enhanced malignant tumor transplants. Their expression initiates with the onset of invasion, whereas being absent in early noninvasive stages of malignant transplants. In addition, a high expression of tumor-derived MMP-1, -2 and -14 contributes to malignant and invasive tumor growth. However, stroma-derived MMP-3 is exclusively restricted to very late-stage invasive and malignant transplants. The functional contribution of these proteases to invasive growth is supported by the gelatinolytic activity in the tumor transplants that again initiates with the onset of invasive growth suggesting a crucial role of MMP-2, -9, -13 and -14 for the establishment of a reactive stroma that promotes tumor invasion. These data demonstrate a complex cooperation of distinct tumor and stroma-derived MMPs in the establishment of malignant tumors and provide the basis for a more specific use of highly selective MMP inhibitors during distinct stages of tumor progression. 
 A large-scale study of MT1-MMP as a marker for isolated tumor cells in peripheral blood and bone marrow in gastric cancer cases. Recently, a marker for predicting   metastasis   or recurrence precisely in solid cancers has been focused on instead of the identification of isolated tumor cells detected by epithelial genes in circulating system. We identified a candidate marker in gastric cancer by microarray and validated it through a quantitative real-time reverse transcriptase-polymerase chain reaction (RT-PCR) assay.To identify   metastasis  -related genes, we performed cDNA microarray analysis of total RNA from whole bone marrow blood from six cases with   metastasis   and three cases without   metastasis  . We determined clinical significance of the identified gene by microarray analysis with quantitative real-time RT-PCR in bone marrow and peripheral blood from 810 cases of gastric cancer.We focused on membrane type 1 matrix metalloproteinase (MT1-MMP) as a candidate marker to predict distant   metastasis   among identified genes. MT1-MMP-positive expression in peripheral blood was associated with incidence of peritoneal dissemination, lymphatic permeation, vascular permeation, and lymph node   metastasis  . MT1-MMP-positive expression in bone marrow was also significantly related to the incidence of distant   metastasis   and peritoneal dissemination.The expression of MT1-MMP in peripheral blood from gastric cancer cases was a powerful indicator of distant   metastasis   especially for peritoneal dissemination. The presence of MT1-MMP-expressing cells in bone marrow indicated higher risk for distant   metastasis   in gastric cancer cases. 
 Mechanism of   metastasis   by membrane type 1-matrix metalloproteinase in hepatocellular carcinoma. To investigate the precise role of membrane type 1-matrix metalloproteinase (MT1-MMP) in hepatocellular carcinoma (HCC)   metastasis  .Human HCC cells Hep3B with overexpression of MT1-MMP were established by stable transfection, and compared with control cells carrying the empty vector. Cells were examined in vivo for their differences in the metastatic ability of athymic nude mice, and analyzed in vitro for their differences in invasion ability by invasion chamber coated with Matrigel, adhesion towards collagen I and migration through culture chamber. Cell proliferation and apoptosis in adherent and suspension status were evaluated by MTT and flow cytometry analysis.We found that overexpression of MT1-MMP could increase intrahepatic   metastasis   in nude mice with orthotopic implantation of HCC cells (incidence of 100% [MT1-MMP transfectants] vs 40% [vector control transfectants], P<0.05). MT1-MMP could also enhance cell invasion through Matrigel (107.7 vs 39.3 cells/field, P<0.001), adhesion towards matrix (0.30 vs 0.12 absorbance unit at 540 nm, P<0.001), cell migration (89.3 vs 39.0 cells/field, P<0.001), and cell proliferation (24.3 vs 40.5 h/doubling, P<0.001). We also observed that MT1-MMP supported cell survival (71.4% vs 23.9%, P<0.001) with reduced apoptosis (43.7% vs 51.0%, P<0.05) in an attachment-free environment.MT1-MMP overexpression could enhance   metastasis  . In addition to its active role in matrix degradation during tumor invasion, MT1-MMP enhances tumor cell survival upon challenge of detachment, which is important during   metastasis   when cells enter the circulation. 
 Membrane-type I matrix metalloproteinase-dependent ectodomain shedding of mucin16/ CA-125 on ovarian cancer cells modulates adhesion and invasion of peritoneal mesothelium. Mucin16 [MUC16/cancer antigen 125 (CA-125)], a high-molecular-weight glycoprotein expressed on the ovarian tumor cell surface, potentiates   metastasis   via selective binding to mesothelin on peritoneal mesothelial cells. Shed MUC16/CA-125 is detectable in sera from ovarian cancer patients. We investigated the potential role of membrane type 1 matrix metalloproteinase (MT1-MMP, MMP-14), a transmembrane collagenase highly expressed in ovarian cancer cells, in MUC16/CA-125 ectodomain shedding. An inverse correlation between MT1-MMP and MUC16 immunoreactivity was observed in human ovarian tumors and cells. Further, when MUC16-expressing OVCA433 cells were engineered to overexpress MT1-MMP, surface expression of MUC16/CA-125 was lost, whereas cells expressing the inactive E240A mutant retained surface MUC16/CA-125. As a functional consequence, decreased adhesion of cells expressing catalytically active MT1-MMP to three-dimensional meso-mimetic cultures and intact ex vivo peritoneal tissue explants was observed. Nevertheless, meso-mimetic invasion is enhanced in MT1-MMP-expressing cells. Together, these data support a model wherein acquisition of catalytically active MT1-MMP expression in ovarian cancer cells induces MUC16/CA-125 ectodomain shedding, reducing adhesion to meso-mimetic cultures and to intact peritoneal explants. However, proteolytic clearing of MUC16/CA-125, catalyzed by MT1-MMP, may then expose integrins for high-affinity cell binding to peritoneal tissues, thereby anchoring metastatic lesions for subsequent proliferation within the collagen-rich sub-mesothelial matrix. 
 Matrix Metalloproteinase 14 Overexpression Is Correlated with the Progression and Poor Prognosis of Nasopharyngeal Carcinoma. Matrix metalloproteinase 14 (MMP14) has been identified to play a significant role in several types of cancers, but little is known about the significance of MMP14 in nasopharyngeal carcinoma (NPC) patients. The aim of this study was to explore the association of MMP14 expression with clinicopathologic features and prognosis in NPC.MMP14 mRNA and protein expressions were examined in NPC and nasopharyngeal tissues through real-time PCR and immunohistochemistry. Meanwhile, the relationship of MMP14 expression levels with clinical features and prognosis of NPC patients was analyzed.MMP14 mRNA expression was markedly higher in NPC tissues than in nasopharyngeal epithelium tissues (p = 0.002). Using immunohistochemistry, staining for MMP14 protein was found in the normal nasopharyngeal epithelial cells and malignant epithelial cells, but increased expression of MMP14 was observed in NPC samples compared with normal nasopharyngeal epithelium samples (p = 0.027). In addition, high levels of MMP14 protein were positively correlated with the status of clinical stage (p = 0.009), N classification (p = 0.006), and distant   metastasis   (p = 0.005) of NPC patients. Patients with higher MMP14 expression had a significantly shorter overall survival time than did patients with low MMP14 expression. Multivariate analysis indicated that the level of MMP14 expression was an independent prognostic indicator (p < 0.001) for the survival of patients with NPC.MMP14 overexpression is a potentially unfavorable prognostic factor for NPC patients. 
 miR-150-5p inhibits hepatoma cell migration and invasion by targeting MMP14. Hepatocellular carcinoma (HCC) is one of the leading causes of cancer-related mortality worldwide. Despite progress in diagnostics and treatment of HCC, its prognosis remains poor because the molecular mechanisms underlying hepatocarcinogenesis are not well understood. In the study, we focused on identifying the role of miRNAs in HCC progression. miRNA microarray was used to analyze the differentially expressed miRNAs, and the results were validated by qPCR. We found that the miR-150-5p expression is down-regulated in HCC tissues compared with pair non-tumor tissues. miR-150-5p expression is also decreased in metastatic cancer tissues compared with pair primary tissues, indicating that miR-150-5p may be involved in HCC   metastasis  . Functionally, miR-150-5p inhibition significantly promotes hepatoma cell migration and invasion, whereas miR-150-5p overexpression suppresses cancer cell migration and invasion in vitro. The matrix metalloproteinase 14 (MMP14) is identified as a new target gene of miR-150-5p. miR-150-5p markedly inhibits MMP14 expression in hepatoma cells, and miR-150-5p expression is negative correlation with MMP14 expression in vivo. More important, re-expression of MMP14 in hepatoma cells partially reverses the effect of miR-150-5p in inhibiting cell invasion. 
 Glycosylation broadens the substrate profile of membrane type 1 matrix metalloproteinase. Membrane type 1 matrix metalloproteinase (MT1-MMP) is a collagenolytic enzyme that has been implicated in normal development and in pathological processes such as cancer   metastasis  . The activity of MT1-MMP is regulated extensively at the post-translational level, and the current data support the hypothesis that MT1-MMP activity is modulated by glycosylation. Enzymatic deglycosylation, site-directed mutagenesis, and lectin precipitation assays were used to demonstrate that MT1-MMP contains O-linked complex carbohydrates on the Thr(291), Thr(299), Thr(300), and/or Ser(301) residues in the proline-rich linker region. MT1-MMP glycoforms were detected in human cancer cell lines, suggesting that MT1-MMP activity may be regulated by differential glycosylation in vivo. Although the autolytic processing and interstitial collagenase activity of MT1-MMP were not impaired in glycosylation-deficient mutants, cell surface MT1-MMP-catalyzed activation of pro-matrix metalloproteinase-2 (proMMP-2) required proper glycosylation of MT1-MMP. The inability of carbohydrate-free MT1-MMP to activate proMMP-2 was not a result of defective MT1-MMP zymogen activation, aberrant protein stability, or inability of the mature enzyme to oligomerize. Rather, our data support a mechanism whereby glycosylation affects the recruitment of tissue inhibitor of metalloproteinases-2 (TIMP-2) to the cell surface, resulting in defective formation of the MT1-MMP/TIMP-2/proMMP-2 trimeric activation complex. These data provide evidence for an additional mechanism for post-translational control of MT1-MMP activity and suggest that glycosylation of MT1-MMP may regulate its substrate targeting. 
 Expression of RECK and MMPs in Hepatoblastoma and Neuroblastoma and Comparative Analysis on the Tumor   Metastasis  . To explore the expression of RECK and relevant matrix metalloproteinases (MMPs) in hepatoblastoma (HB) and neuroblastoma (NB) and their clinical significance in the tumor   metastasis  .Forty-five wax-stone samples of HB and 43 wax-stone samples of NB removed by surgical resection and confirmed by pathology in Linyi Yishui Central Hospital were selected. According to presence and absence of   metastasis  , both NB and HB samples were divided into metastatic group and non-metastatic group, namely NB metastatic group (n=28), NB non-metastatic group (n=15), HB metastatic group (n=15) and HB non-metastatic group (n=30). The expression of RECK, membrane type-1 matrix metalloproteinase (MT1-MMP) in HB tissue and RECK, MMP-14 in NB tissue was detected using immunohistochemical method, and the correlation between RECK and MT1-MMP, MMP-14 was analyzed.The metastatic rate of NB was dramatically higher than that of HB, with statistical significance (P=0.003). The positive rate of RECK expression in NB group (30.2%) was slightly lower than in HB group (40.0%), but no significant difference was presented (P=0.338). The positive rate of MMPs expression in NB metastatic group was evidently higher than in HB metastatic group (P=0.024). The results of Spearman correlation analysis revealed that the expression of RECK in HB and NB tissues had a significantly-negative correlation with MT1-MMP and MMP-14, respectively (r=-0.499, P=0.012; r=-0.636, P=0.000).In HB and NB tissues, RECK is expressed lowly, while relevant MMPs highly, and RECK inhibits the tumor invasion and   metastasis   through negative regulation of relevant MMPs. 
 KLF8 and FAK cooperatively enrich the active MMP14 on the cell surface required for the metastatic progression of breast cancer. Krüppel-like factor 8 (KLF8) regulates critical gene transcription associated with cancer. The underlying mechanisms, however, remain largely unidentified. We have recently demonstrated that KLF8 expression enhances the activity but not expression of matrix metalloproteinase-2 (MMP2), the target substrate of MMP14. Here, we report a novel KLF8 to MMP14 signaling that promotes human breast cancer invasion and   metastasi  s. Using cell lines for inducible expression and knockdown of KLF8, we demonstrate that KLF8 promotes MMP14 expression at the transcriptional level. Knocking down KLF8 expression inhibited the breast cancer cell invasion both in vitro and in vivo as well as the lung   metastasi  s in mice, which could be rescued by ectopic expression of MMP14. Promoter reporter assays and oligonucleotide and chromatin immunoprecipitations determined that KLF8 activates the human MMP14 gene promoter by both directly acting on the promoter and indirectly via promoting the nuclear translocation of β-catenin, the expression of T-cell factor-1 (TCF1) and subsequent activation of the promoter by the β-catenin/TCF1 complex. Inhibition of focal adhesion kinase (FAK) using pharmacological inhibitor, RNA interference or knockout showed that the cell surface presentation of active MMP14 downstream of KLF8 depends on FAK expression and activity. Taken together, this work identified novel signaling mechanisms by which KLF8 and FAK work together to promote the extracellular activity of MMP14 critical for breast canc  er metasta  sis. 
 Inhibition of matrix metalloproteinase 14 (MMP-14)-mediated cancer cell migration. Matrix metalloproteinases (MMPs) have been shown to be key players in both extracellular matrix remodeling and cell migration during cancer   metastasis  . MMP-14, a membrane-anchored MMP, in particular, is closely associated with these processes. The hemopexin (PEX) domain of MMP-14 has been proposed as the modulating region involved in the molecular cross-talk that initiates cell migration through homodimerization of MMP-14 as well as heterodimerization with the cell surface adhesion molecule CD44. In this study, minimal regions required for function within the PEX domain were investigated through a series of substitution mutations. Blades I and IV were found to be involved in cell migration. We found that blade IV is necessary for MMP-14 homodimerization and that blade I is required for CD44 MMP-14 heterodimerization. Cross-talk between MMP-14 and CD44 results in phosphorylation of EGF receptor and downstream activation of the MAPK and PI3K signaling pathways involved in cell migration. Based on these mutagenesis analyses, peptides mimicking the essential outermost strand motifs within the PEX domain of MMP-14 were designed. These synthetic peptides inhibit MMP-14-enhanced cell migration in a dose-dependent manner but have no effect on the function of other MMPs. Furthermore, these peptides interfere with cancer   metastasis   without affecting primary tumor growth. Thus, targeting the MMP-14 hemopexin domain represents a novel approach to inhibit MMP-14-mediated cancer dissemination. 
 Matrix metalloproteinase localisation by in situ-RT-PCR in archival human breast biopsy material. Utilising archival human breast cancer biopsy material we examined the stromal/epithelial interactions of several matrix metalloproteinases (MMPs) using in situ-RT-PCR (IS-RT-PCR). In breast cancer, the stromal/epithelial interactions that occur, and the site of production of these proteases, are central to understanding their role in invasive and metastatic processes. We examined MT1-MMP (MMP-14, membrane type-1-MMP), MMP-1 (interstitial collagenase) and MMP-3 (stromelysin-1) for their localisation profile in progressive breast cancer biopsy material (poorly differentiated invasive breast carcinoma (PDIBC), invasive breast carcinomas (IBC) and lymph node metastases (LNM)). Expression of MT1-MMP, MMP-1 and MMP-3 was observed in both the tumour epithelial and surrounding stromal cells in most tissue sections examined. MT1-MMP expression was predominantly localised to the tumour component in the pre-invasive lesions. MMP-1 gene expression was relatively well distributed between both tissue compartments, while MMP-3 demonstrated highest expression levels in the stromal tissue surrounding the epithelial tumour cells. The results demonstrate the ability to distinguish compartmental gene expression profiles using IS-RT-PCR. Further, we suggest a role for MT1-MMP in early tumour progression, expression of MMP-1 during   metastasis   and focal expression pattern of MMP-3 in areas of expansion. These expression profiles may provide markers for early breast cancer diagnoses and present potential therapeutic targets. 
 The chemokine receptor CXCR4 and the metalloproteinase MT1-MMP are mutually required during melanoma   metastasis   to lungs. Melanoma is the most aggressive skin cancer once   metastasis   begins; therefore, it is important to characterize the molecular players involved in melanoma dissemination. The chemokine receptor CXCR4 and the membrane-bound metalloproteinase MT1-MMP are expressed on melanoma cells and represent candidate molecules for the control of   metastasis  . Using human melanoma transfectants that either overexpress or silence CXCR4 or MT1-MMP, or that have a combination of overexpression and interference of these proteins, we show that CXCR4 and MT1-MMP coordinate their activities at different steps along melanoma cell   metastasis   into the lungs. Results from in vivo xenograft mouse models of melanoma lung colonization and mice survival and short-term, homing nested polymerase chain reaction experiments from lung samples indicated that CXCR4 is required at early phases of melanoma cell arrival in the lungs. In contrast, MT1-MMP is not needed for these initial steps but promotes subsequent invasion and dissemination of the tumor with CXCR4. Investigation of potential cross talk between CXCR4 and MT1-MMP revealed that MT1-MMP accumulates intracellularly after melanoma cell stimulation with the CXCR4 ligand CXCL12, and that this process involves the activation of the Rac-Erk1/2 pathway. Subsequent to cell contact with specific basement membrane proteins, MT1-MMP redistributes to the cell membrane in a phosphatidylinositol 3-kinase-dependent manner. These results suggest that combination therapies that target CXCR4 and MT1-MMP should improve the limitations of the current therapies for metastatic melanoma. 
 Caveolin-1 inhibits membrane-type 1 matrix metalloproteinase activity. Membrane-type 1 matrix metalloproteinase (MT1-MMP) is a zinc-dependent proteinase found in cholesterol-rich lipid rafts on the plasma membrane. MT1-MMP hydrolyzes extracellular matrix (ECM) proteins, activates pro-matrix metalloproteinase-2 (proMMP-2) and plays an important role in ECM remodeling, cancer cell migration and   metastasis  . The role of caveolin-1, an integral protein of caveolae, in the activation of MT1-MMP remains largely unknown. Here, we show that the expression of caveolin-1 attenuates the activation of proMMP-2, reduces proteolytic cleavage of ECM and inhibits cell migration. We utilized the cytoplasmic tail domain deletion (DeltaCT) or the E240A mutant of MT1-MMP. Co-expression of caveolin-1 with the wild-type or the DeltaCT MT1-MMP decreased the proMMP-2 activation and inhibited collagen degradation and cell migration. Caveolin-1 had no effect on the catalytically inert E240A MT1-MMP. Our findings suggest that caveolin-1 is essential in the down-regulation of MT1-MMP activity by promoting internalization from the cell surface. 
 NHE1 mediates MDA-MB-231 cells invasion through the regulation of MT1-MMP. Na⁺/H⁺ exchanger 1 (NHE1), an important regulator of intracellular pH (pH(i)) and extracellular pH (pH(e)), has been shown to play a key role in breast can  cer metast  asis. However, the exact mechanism by which NHE1 mediates breast can  cer metast  asis is not yet well known. We showed here that inhibition of NHE1 activity, with specific inhibitor Cariporide, could suppress MDA-MB-231 cells invasion as well as the activity and expression of MT1-MMP. Overexpression of MT1-MMP resulted in a distinguished increase in MDA-MB-231 cells invasiveness, but treatment with Cariporide reversed the MT1-MMP-mediated enhanced invasiveness. To explore the role of MAPK signaling pathways in NHE1-mediated breast can  cer metast  asis, we compared the difference of constitutively phosphorylated ERK1/2, p38 MAPK and JNK in non-invasive MCF-7 cells and invasive MDA-MB-231 cells. Interestingly, we found that the phosphorylation levels of ERK1/2 and p38 MAPK in MDA-MB-231 cells were higher than in MCF-7 cells, but both MCF-7 cells and MDA-MB-231 cells expressed similar constitutively phosphorylated JNK. Treating MDA-MB-231 cells with Cariporide led to decreased phosphorylation level of both p38 MAPK and ERK1/2 in a time-dependent manner, but JNK activity was not influenced. Supplementation with MAPK inhibitor (MEK inhibitor PD98059, p38 MAPK inhibitor SB203580 and JNK inhibitor SP600125) or Cariporide all exhibited significant depression of MDA-MB-231 cells invasion and MT1-MMP expression. Furthermore, we co-treated MDA-MB-231 cells with MAPK inhibitor and Cariporide. The result showed that Cariporide synergistically suppressed invasion and MT1-MMP expression with MEK inhibitor and p38 MAPK inhibitor, but not be synergistic with the JNK inhibitor. These findings suggest that NHE1 mediates MDA-MB-231 cells invasion partly through regulating MT1-MMP in ERK1/2 and p38 MAPK signaling pathways dependent manner. 
 A role of MMP-14 in the regulation of invasiveness of nasopharyngeal carcinoma. Although matrix metalloproteinase 14 (MMP-14) has been shown to play a substantial role in the carcinogenesis of some types of cancer, its involvement in the pathogenesis of nasopharyngeal carcinoma (NPC) has not been reported. Here, we analyzed MMP-14 levels in the NPC specimens from patients and compared with the paired normal nasopharynx (NNP) tissues. We found that NPC had significantly higher messenger RNA (mRNA) and protein levels of MMP-14. Moreover, higher levels of MMP-14 correlated with more advanced status of clinical stage and lymphatic   metastasis  . In vitro, MMP-14 levels determined the potential of invasiveness of NPC cells, possibly through induction of EMT-associated genes. Our data thus highlight MMP-14 as a novel therapeutic target for NPC. 
 The hepatitis B virus X protein promotes tumor cell invasion by inducing membrane-type matrix metalloproteinase-1 and cyclooxygenase-2 expression. Hepatocellular carcinoma is strongly associated with chronic infection by the hepatitis B virus (HBV) and has poor prognosis due to intrahepatic   metastasis  . HBx is often the only HBV protein detected in hepatic tumor cells; however, its contribution to tumor invasion and   metastasis   has not been established so far. In this work, we show that HBx enhances tumor cell invasion, both in vivo and in vitro. The increased invasive capacity induced by HBx is mediated by an upregulation of membrane-type 1 matrix metalloproteinase (MT1-MMP) expression, which in turn activates matrix metalloproteinase-2. Induction of both MT1-MMP expression and cell invasion by HBx is dependent on cyclooxygenase-2 (COX-2) activity. In addition, HBx upregulates the expression of COX-2, which is mediated by the transcriptional activation of the COX-2 gene promoter in a nuclear factor of activated T cell-dependent (NF-AT-dependent) manner. These results demonstrate the ability of HBx to promote tumor cell invasion by a mechanism involving the upregulation of MT1-MMP and COX-2 and provide new insights into the mechanism of action of this viral protein and its involvement in tumor   metastasis   and recurrence of hepatocellular carcinoma. 
 miR‑133a suppresses cell proliferation, migration and invasion in human lung cancer by targeting MMP‑14. Lung cancer is the leading cause of cancer‑related mortality worldwide. Over half of lung cancer cases are diagnosed   after meta  stasis, for which the median survival time is approximately 8 months. microRNAs (miRNAs), which are a class of single‑stranded endogenous non‑coding RNAs, are likely to be involved in most biological processes. miR‑133 plays roles in cardiac development and disease, and recent studies showed that miR‑133 is downregulated in various human malignancies, such as bladder and lung cancer. However, its detailed role in the processes of cancer remains to be determined. In the present study, we found that in the lung cancer cell lines A549 and NCI‑H1299 overexpression of miR‑133a suppressed cell proliferation, migration and invasion. The miR‑133a‑induced suppression of cell migration and invasion can be reversed by miR‑133a‑specific inhibitor. According to the mRNA sequence, matrix metalloproteinase (MMP)‑14, which   is an impo  rtant regulator of metastasis, is a predicted target of miR‑133a. This was confirmed by dual luciferase reporter assay. Moreover, miR‑133a overexpression decreases the mRNA and protein levels of MMP‑14. Collectively, these results suggest tha  t miR‑13  3a may inhibit lung cancer metastasis by targeting MMP‑14 and may be used as an anti‑metastatic therapy in lung cancer patients. 
 Low collagenase-1 (MMP-1) and MT1-MMP expression levels are favourable survival markers in advanced colorectal carcinoma. Extracellular matrix degradation is required for invasive growth and   metastasis   formation in colorectal carcinoma; therefore, we examined matrix metalloproteinases expression (MMP-1, MMP-13 and MT1-MMP) and apoptosis in tumours from 49 patients with advanced colorectal disease.MMP expression was determined immunohistochemically and apoptotic index (AI) was ascertained using the TUNEL assay.Low levels of MT1-MMP, MMP-1 and AI were found to be favourable markers significantly associated with longer survival. MT1-MMP expression levels below the median (</=14.0% of tumour cells) were associated with better overall survival (median 26.2 vs. 15.6 months, p = 0.02) and MMP-1 expression levels below the median (</=28.7% of tumour cells) correlated with longer survival following   metastasis   (median 21.5 vs. 13.3 months, p = 0.05). MT1-MMP, MMP-1 and AI were all found to have significant independent effects on survival. Interestingly, MMP-1 expression levels above the median were associated with distal disease of the colon (p = 0.02).These findings reveal that MT1-MMP and MMP-1 expression levels and AI are useful prognostic indicators in advanced colorectal carcinoma and suggest that markers of MMP expression might be used in identifying patients who would benefit from new treatment modalities involving MMP inhibitors. 
 MMP-14 overexpression correlates with poor prognosis in non-small cell lung cancer. Matrix metalloproteinase-14 (MMP-14) has been demonstrated to play an important role in tumor progression. The aim of this study was to analyze the correlation between MMP-14 expression and clinicopathologic features and its prognostic significance in non-small cell lung cancer (NSCLC). Immunohistochemical staining for MMP-14 protein was performed in 104 patients with NSCLC. High levels of MMP-14 protein were positively correlated with the status of clinical stage (I-II vs. III-IV; P < 0.001), N classification (N0-N1 vs. N2-N3; P < 0.001),   distant me  tastasis (no vs. yes; P = 0.014), and differentiated degree (high vs. low or undifferentiated; P = 0.001). The patients with higher MMP-14 expression of protein had shorter survival time than patients with low MMP-14 expression. Multivariate analysis indicated that the level of MMP-14 expression was an independent prognostic indicator (P < 0.001) for the survival of patients with NSCLC. In conclusion, MMP-14 is a potential unfavorable prognostic factor for patients with NSCLC. 
 microRNA-9 targets matrix metalloproteinase 14 to inhibit invasion,   metastasis  , and angiogenesis of neuroblastoma cells. Matrix metalloproteinase (MMP)-14 is the only membrane-anchored MMP that plays a critical role in tumor   metastasis   and angiogenesis. However, the mechanisms underlying MMP-14 expression in tumors still remain largely unknown. In this study, MMP-14 immunostaining was identified in 29/42 neuroblastoma tissues, which was correlated with clinicopathologic features and shorter patients' survival. In subtotal 20 neuroblastoma cases, microRNA 9 (miR-9) was downregulated and inversely correlated with MMP-14 expression. Bioinformatics analysis revealed a putative miR-9-binding site in the 3'-untranslated region (3'-UTR) of MMP-14 mRNA. Overexpression or knockdown of miR-9 responsively altered both the mRNA and protein levels of MMP-14 and its downstream gene, vascular endothelial growth factor, in cultured neuroblastoma cell lines SH-SY5Y and SK-N-SH. In an MMP-14 3'-UTR luciferase reporter system, miR-9 downregulated the luciferase activity, and these effects were abolished by a mutation in the putative miR-9-binding site. Overexpression of miR-9 suppressed the invasion,   metastasis  , and angiogenesis of SH-SY5Y and SK-N-SH cells in vitro and in vivo. In addition, the effects of miR-9 on MMP-14 expression, adhesion, migration, invasion, and angiogenesis were rescued by overexpression of MMP-14 in these cells. Furthermore, anti-miR-9 inhibitor or knockdown of MMP-14 respectively increased or inhibited the migration, invasion, and angiogenesis of neuroblastoma cells. These data indicate that miR-9 suppresses MMP-14 expression via the binding site in the 3'-UTR, thus inhibiting the invasion,   metastasis  , and angiogenesis of neuroblastoma. 
 miRNA-337-3p suppresses neuroblastoma progression by repressing the transcription of matrix metalloproteinase 14. Recent evidence shows the emerging roles of endogenous microRNAs (miRNAs) in repressing gene transcription. However, the miRNAs inhibiting the transcription of matrix metalloproteinase 14 (MMP-14), a membrane-anchored MMP crucial for the tumorigenesis and aggressiveness, still remain largely unknown. In this study, through mining computational algorithm program and genome-wide Argonaute profiling dataset, we identified one binding site of miRNA-337-3p (miR-337-3p) within the MMP-14 promoter. We demonstrated that miR-337-3p was under-expressed and inversely correlated with MMP-14 expression in clinical specimens and cell lines of neuroblastoma (NB), the most common extracranial solid tumor in childhood. Patients with high miR-337-3p expression had greater survival probability. miR-337-3p suppressed the promoter activity, nascent transcription, and expression of MMP-14, resulting in decreased levels of vascular endothelial growth factor, in cultured NB cell lines. Mechanistically, miR-337-3p recognized its binding site and recruited Argonaute 2 to facilitate the enrichment of repressive epigenetic markers and decrease the binding of RNA polymerase II and specificity protein 1 on the MMP-14 promoter. Gain- and loss-of-function studies demonstrated that miR-337-3p suppressed the growth, invasion,   metastasis  , and angiogenesis of NB cells in vitro and in vivo. In addition, restoration of MMP-14 expression rescued the NB cells from changes in these biological features. Taken together, these data indicate that miR-337-3p directly binds the MMP-14 promoter to repress its transcription, thus suppressing the progression of NB. 
 3-phosphoinositide-dependent protein kinase-1 (PDK1) promotes invasion and activation of matrix metalloproteinases.   Metastasis   is a major cause of morbidity and mortality in breast cancer with tumor cell invasion playing a crucial role in the metastatic process. PDK1 is a key molecule that couples PI3K to cell proliferation and survival signals in response to growth factor receptor activation, and is oncogenic when expressed in mouse mammary epithelial cells. We now present evidence showing that PDK1-expressing cells exhibit enhanced anchorage-dependent and -independent cell growth and are highly invasive when grown on Matrigel. These properties correlate with induction of MMP-2 activity, increased MT1-MMP expression and a unique gene expression profile.Invasion assays in Matrigel, MMP-2 zymogram analysis, gene microarray analysis and mammary isografts were used to characterize the invasive and proliferative function of cells expressing PDK1. Tissue microarray analysis of human breast cancers was used to measure PDK1 expression in invasive tumors by IHC.Enhanced invasion on Matrigel in PDK1-expressing cells was accompanied by increased MMP-2 activity resulting from stabilization against proteasomal degradation. Increased MMP-2 activity was accompanied by elevated levels of MT1-MMP, which is involved in generating active MMP-2. Gene microarray analysis identified increased expression of the ECM-associated genes decorin and type I procollagen, whose gene products are substrates of MT1-MMP. Mammary fat pad isografts of PDK1-expressing cells produced invasive adenocarcinomas. Tissue microarray analysis of human invasive breast cancer indicated that PDK1pSer241 was strongly expressed in 90% of samples.These results indicate that PDK1 serves as an important effector of mammary epithelial cell growth and invasion in the transformed phenotype. PDK1 mediates its effect in part by MT1-MMP induction, which in turn activates MMP-2 and modulates the ECM proteins decorin and collagen. The presence of increased PDK1 expression in the majority of invasive breast cancers suggests its importance in the metastatic process. 
 Lipid rafts and caveolin-1 are required for invadopodia formation and extracellular matrix degradation by human breast cancer cells. Invadopodia are ventral membrane protrusions through which invasive cancer cells degrade the extracellular matrix. They are thought to function in the migration of cancer cells through tissue barriers, which is necessary for cancer invasion and   metastasis  . Although many protein components of invadopodia have been identified, the organization and the role of membrane lipids in invadopodia are not well understood. In this study, the role of lipid rafts, which are cholesterol-enriched membrane microdomains, in the assembly and function of invadopodia in human breast cancer cells was investigated. Lipid rafts are enriched, internalized, and dynamically trafficked at invadopodia sites. Perturbation of lipid raft formation due to depleting or sequestering membrane cholesterol blocked the invadopodia-mediated degradation of the gelatin matrix. Caveolin-1 (Cav-1), a resident protein of lipid rafts and caveolae, accumulates at invadopodia and colocalizes with the internalized lipid raft membranes. Membrane type 1 matrix metalloproteinase (MT1-MMP), a matrix proteinase associated with invadopodia, is localized at lipid raft-enriched membrane fractions and cotrafficked and colocalized with Cav-1 at invadopodia. The small interfering RNA-mediated silencing of Cav-1 inhibited the invadopodia-mediated and MT1-MMP-dependent degradation of the gelatin matrix. Furthermore, Cav-1 and MT1-MMP are coexpressed in invasive human breast cancer cell lines that have an ability to form invadopodia. These results indicate that invadopodia are the sites where enrichment and trafficking of lipid rafts occur and that Cav-1 is an essential regulator of MT1-MMP function and invadopodia-mediated breast cancer cell invasion. 
 The planar cell polarity protein Van Gogh-Like 2 regulates tumor cell migration and matrix metalloproteinase-dependent invasion. Van Gogh-Like 2 (VANGL2) is a planar cell polarity protein essential for collective migration during embryonic development, yet its contribution to tumor cell motility and invasion are unknown. We report for the first time that loss of VANGL2 in human cancer cells promotes efficient collective and directed migration and matrix metalloproteinase (MMP)-dependent ECM invasion. We show that VANGL2 knockdown cells exhibit increased activation of secreted MMP2, higher levels of membrane-localized MMP14, and decreased cell-surface fibronectin. These important findings support the notion that planar cell polarity proteins act in coordination with known regulators of cancer cell migration to influence invasion and perhaps   metastasis  . 
 Interaction of MT1-MMP and laminin-5gamma2 chain correlates with   metastasis   and invasiveness in human esophageal squamous cell carcinoma. To gain insights into metastatic mechanisms in esophageal squamous cell carcinoma (ESCC), we established sublines (MLuB1 and MLuC1) with different capacity of spontaneous lung   metastasis   by subcutaneous injection of a human ESCC cell line (EC 9706) into nude mices. The incidence of the mice with lung   metastasis   produced by MLuC1 (87%) was significantly higher than that of MLuB1 (22%). The gene expression profiles of the two sublines were compared with cDNA arrays containing 5,000 known genes, and 47 genes were differentially expressed > or =2.0 fold. Laminin-5gamma2 chain (Ln-5gamma2) was one of the up-regulated genes in MLuC1 cells. Proteolytically processed forms of gamma2 are known to promote migration of a multitude of epithelial cells in vitro. Western-blotting analysis revealed that degraded fragments of Ln-5gamma2 and active form of membrane-type matrix metalloproteinase-1 (MT1-MMP) in MLuC1 was significantly higher than those in MLuB1. Expression of MT1-MMP was observed in 60 of 75 Ln-5gamma2-positive carcinoma tissues (80%). Co-expression of the two proteins was significantly associated with depth of invasion (P = 0.012). Moreover, proteolytic fragments of Ln-5gamma2 and active forms of MT1-MMP were frequently found in tumor tissues, whereas in the corresponding normal esophageal tissues there were only intact forms of gamma2 and MT1-MMP. siRNA-mediated silencing of MT1-MMP significantly reduced production of gamma2' and gamma2x in MLuC1 cells and inhibited cell migration. The results suggest that MT1-MMP is an enzyme responsible for Ln-5gamma2 cleavage in ESCC, and interaction between them may play a critical role in promoting invasion and   metastasis   of human ESCC. 
 YB-1 alters MT1-MMP trafficking and stimulates MCF-7 breast tumor invasion and   metastasis  . YB-1 is a member of the cold shock domain family, with complex roles in DNA structure, gene transcription and translation. YB-1 promotes chromosomal instability, and mammary gland transgenic expression induces tumors with 100% penetrance. YB-1 is linked to poor prognosis in breast carcinoma and is a strong predictor of relapse and disease-specific survival. Survival is directly tied to the extent of local invasion and distal   metastasis  , processes dependent upon the activity of the membrane type I-matrix metalloproteinase, MT1-MMP. Non-invasive MCF-7 breast adenocarcinoma cells were transfected with YB-1/EGFP. YB-1 protein was detected in the invadopodia of cells with a migratory phenotype. There was increased expression of MT1-MMP protein concentrated at the leading edges of motile cells, which were highly invasive in collagen three-dimensional culture. The rates of MT1-MMP protein endocytosis and recycling to the cell surface were elevated in clones expressing higher levels of YB-1 protein. Control MCF-7 cells formed nonfatal, non-invasive, differentiated adenocarcinomas in vivo. MCF-7 cells expressing a twofold increase in YB-1 formed highly anaplastic tumors with local invasion, pulmonary metastases and high lethality. We conclude that YB-1 contributes to the development of an invasive, metastatic breast carcinoma phenotype by enhanced presentation of MT1-MMP at the sites of cellular invasion. 
 Prognostic value of membrane type 1 and 2 matrix metalloproteinase expression and gelatinase A activity in bladder cancer. To analyze the behavior of matrix metalloproteinases (MMPs) in their active state in patients with bladder cancer.A retrospective study of 50 patients with localized bladder cancer who underwent tumor resection between June 2006 and June 2007 at the National Cancer Institute in Cairo, Egypt was carried out. Tissue samples were collected and the expression of membrane type 1 (MT1) and type 2 (MT2) MMPs was determined by Western blotting. Gelatinase A (MMP-2) activity was estimated by zymographic analysis in tissue samples of each patient and the values were correlated with clinical tumor stage and lymph node status.The behavior of MMP-2 showed statistical significance in 90% of tumor tissues compared with 22% of adjacent normal tissues (p<0.001). MT1-MMP was expressed in 88% of tumor tissues compared with 24% of normal tissues (p<0.001); MT2-MMP was expressed in 74% of tumor tissues compared with 12% of normal tissues (p<0.001). While there was a highly significant association between MMP-2 activity and MT1-MMP expression in tumor tissues (p<0.001), there was a moderately significant association between MMP-2 activity and MT2-MMP expression (p=0.018). The results also revealed an association between MT1-MMP and MT2-MMP expression in tumor tissues (p<0.001). MMP-2 activity and MT2-MMP expression in tumor tissues were statistically associated with high tumor stage (p=0.039 and p=0.014, respectively), while the expression of MT1-MMP showed no association with tumor stage (p=0.139).MMP-2 activity is associated with an increase in MT2-MMP expression and with lymph node   metastasis  . No association was found between MT1-MMP expression and lymph node   metastasis  . 
 Cancer cell-associated MT1-MMP promotes blood vessel invasion and distant   metastasis   in triple-negative mammary tumors. Functional roles for the cancer cell-associated membrane type I matrix metalloproteinase (MT1-MMP) during early steps of the metastatic cascade in primary tumors remain unresolved. In an effort to determine its significance, we determined the in vivo effects of RNAi-mediated downregulation in mammary cancer cells on the migration, blood and lymphatic vessel invasion (LVI), and lymph node and lung   metastasis  . We also correlated the expression of cancer cell MT1-MMP with blood vessel invasion (BVI) in 102 breast cancer biopsies. MT1-MMP downregulation in cancer cells decreased lung   metastasis   without affecting primary tumor growth. The inhibition of lung   metastasis   correlated with reduced cancer cell migration and BVI. Furthermore, cancer cell-expressed MT1-MMP upregulated the expression of MT1-MMP in vascular endothelial cells, but did not affect MT1-MMP expression in lymphatic endothelial cells, LVI, or lymph node   metastasis  . Of clinical importance, we observed that elevated MT1-MMP expression correlated with BVI in biopsies from triple-negative breast cancers (TNBC), which have a poor prognosis and high incidence of distant   metastasis  , relative to other breast cancer subtypes. Together, our findings established that MT1-MMP activity in breast tumors is essential for BVI, but not LVI, and that MT1-MMP should be further explored as a predictor and therapeutic target of hematogenous   metastasis   in TNBC patients. 
 The clinical relevance of stromal matrix metalloproteinase expression in ovarian cancer. Matrix metalloproteinases (MMP) are proteolytic enzymes implicated in cancer progression and   metastasis  . We sought to determine the role of epithelial (tumor cell-derived) and stromal (host-derived) expression of MMPs in predicting the clinical outcome of patients with epithelial ovarian cancer (EOC).MMP-2, MMP-9, and membrane type 1 (MT1)-MMP expression was evaluated using immunohistochemistry in 90 invasive EOCs, and samples were scored for epithelial and stromal staining. Results were correlated with clinicopathologic characteristics using univariate and multivariate analyses.High expression of MMP-2, MMP-9, and MT1-MMP in tumor epithelium was detected in 54%, 97%, and 100% of cases, and in stromal compartments, in 38%, 70%, and 38% of cases, respectively. High stromal expression of MMP-2, MMP-9, and MT1-MMP was significantly associated with aggressive features such as high stage, high grade ascites, and positive lymph node status. Kaplan-Meier analysis showed that high epithelial and stromal expression of MMP-2, MMP-9, and MT1-MMP were each significantly associated with shorter disease-specific survival (DSS; P < 0.01). On tree-structured survival analysis, patients with strong epithelial MT1-MMP expression had the shortest DSS, whereas patients with moderate epithelial MT1-MMP and low stromal MMP-9 expression had the longest DSS (P < 0.01). On multivariate analysis, high stromal expression of MMP-9 (P = 0.01) and MT1-MMP (P = 0.04), strong epithelial MT1-MMP (P = 0.01) and high stage (P = 0.04) were independent predictors of poor DSS.Overexpression of stromal MMP-9 and MT1-MMP is independently associated with shorter DSS in EOC. Thus, host-derived MMPs are valuable predictors of clinical outcome in EOC. 
 Increased expression of MMP14 correlates with the poor prognosis of Chinese patients with gastric cancer. The role of matrix metalloproteinase 14 (MMP14) has been identified to involve tumor progression and prognosis. The purpose of this study is to investigate the role of MMP14 in tumor progression and prognosis of gastric cancer. This study indicated that MMP14 mRNA and protein were overexpressed in gastric cancer tissue (P<0.001 and P=0.037, respectively) and significantly associated with clinical stage (P=0.005), lymph node   metastasis   (P=0.003), and distant   metastasis   (P=0.017). Moreover, we found that the overexpression of MMP14 was a significant predictor of poor prognosis for gastric cancer patients (P<0.001). Furthermore, we performed a meta-analysis which included 594 cases from 3 studies and showed that MMP14 overexpression was a significantly poor prognostic factor in Chinese patients with gastric cancer and HR (95% CI) was 2.17 (1.64-2.86). In conclusion, MMP14 plays an important role on gastric cancer progression and prognosis and acts as a convictive biomarker for prognostic prediction for Chinese patients with gastric cancer. 
 Epidermal growth factor receptor regulates MT1-MMP and MMP-2 synthesis in SiHa cells via both PI3-K/AKT and MAPK/ERK pathways. Matrix metalloproteinase 2 (MMP-2) and membrane type 1 matrix metalloproteinase (MT1-MMP) have been identified as important participants in tumor invasion,   metastasis  , and angiogenesis. Membrane type 1 matrix metalloproteinase has also been recognized as a major activator of MMP-2. The purpose of this study was to investigate epidermal growth factor (EGF) mediating signal pathways in the regulation of MMP-2 and MT1-MMP in SiHa cells, a cervical cancer cell line. We showed here that EGF induced the expression of MT1-MMP and inhibited the expression of MMP-2 at both the mRNA and protein levels. Membrane type 1 matrix metalloproteinase induction was blocked by mitogen-activated protein kinase or extracellular signal-regulated kinase inhibitors PD98059 and U0126 but not by phosphatidylinositol-3 kinase (PI3-K) inhibitors LY294002 and wortmannin. Interestingly, the mitogen-activated protein kinase or extracellular signal-regulated kinase inhibitors PD98059 and U0126 actually increased MMP-2 mRNA and protein synthesis, whereas the PI3-K inhibitors LY294002 and wortmannin further suppressed the expression of MMP-2. Our results suggest that EGF receptor up-regulated the expression of MT1-MMP and down-regulated the synthesis of MMP-2 through the mitogen-activated protein kinase/extracellular signal-regulated kinase pathway while concomitantly transmitting a mild positive regulatory signal to the expression of MMP-2 via the PI3-K/AKT pathway in SiHa cells. Furthermore, we found that EGF elevated the activity of MMP-2 in culture media. 
 Presence of active gelatinases in endometrial carcinoma and correlation of matrix metalloproteinase expression with increasing tumor grade and invasion. The actions of the extracellular-matrix degrading enzymes, matrix metalloproteinases (MMPs), are implicated in tumorigenesis. The cellular localization of MMP-2, MMP-9, membrane type 1 (MT1)-MMP, tissue inhibitors of metalloproteinases (TIMPs) 1-3, and the presence of active gelatinases were investigated in endometrial carcinoma.Endometrial carcinomas were grouped according to histologic grade (Grades 1-3), depth of myometrial invasion (0, < 50%, > 50%) and the presence of vascular/lymphatic invasion. Twenty-nine endometrial carcinoma biopsies were investigated immunohistochemically to determine the tissue localization of MMP-2 (gelatinase A), MMP-9 (gelatinase B), MT1-MMP, and TIMPs 1-3. In situ hybridization was performed to localize MMP-2 and MMP-9 mRNA. The presence of active gelatinases was assessed using in situ zymography.Epithelial tumor cells were the main site of MMP-2, MMP-9, and MT1-MMP protein. Variable stromal cell localization was also observed, particularly in areas adjacent to tumor nests. Semiquantitative analysis revealed increases in MMP-9 and MMP-2 but not MT1-MMP staining scores in tumor epithelial cells in the transition from histologic Grade 1 to Grades 2 and 3. Matrix metalloproteinase-9 and MT1-MMP staining scores in tumor cells were significantly associated with the presence of myometrial invasion and vascular/lymphatic invasion, while MMP-2 did not correlate with these factors. In addition, MT1-MMP was co-localized with MMP-2, supporting its role in the activation of proMMP-2. Tumor cells from all histologic grades stained intensely for TIMP-2 and TIMP-3 proteins, while variable stromal staining was observed. In Grade 1 carcinomas TIMP-1 was predominantly immunolocalized to the stromal compartment with variable tumor cell localization being observed in Grades 2 and 3 carcinomas. Matrix metalloproteinase-9 and MMP-2 mRNAs were predominantly observed in tumor epithelial cells as well as in the stroma to varying degrees. In situ zymography revealed active forms of gelatinases at the cellular surface and in association with tumor epithelial cells within endometrial carcinoma tissues.These data suggest that increasing expression of MMPs and endometrial carcinoma progression are closely related. Active gelatinases are present in endometrial carcinoma, resulting in alterations to the microenvironment that promote tumor invasion and   metastasis  . 
 Enhanced membrane-type 1 matrix metalloproteinase expression by hyaluronan oligosaccharides in breast cancer cells facilitates CD44 cleavage and tumor cell migration. Hyaluronan (HA), a component of the extracellular matrix, plays an important role in cell-cell adhesion and cell migration. Membrane type 1-matrix metalloproteinase (MT1‑MMP) is often expressed in invasive cancer cells. CD44, a transmembrane receptor for HA, is implicated in various adhesion-dependent cellular processes including cell migration, tumor cel  l metastas  is and invasion. Previous studies have shown that CD44 is highly expressed in cancer cells and may be proteolytically cleaved at the ectodomain by MT1-MMP; this process of inducing CD44 cleavage plays a critical role in cancer cell migration. We hypothesized that HA modulates MT1-MMP expression to facilitate breast cancer cell migration. Flow cytometry, real-time PCR, western blotting and immunofluorescence staining were used to quantify HA-induced MT1-MMP expression in breast cancer cells. In order to validate the relevance of cell migration and HA-induced MT1-MMP, we analyzed the cell migration via matrigel-coated transwell. We found that after HA oligosaccharide (6.5 kDA) stimulation, MT1-MMP expression in the membrane of breast cancer cells was increased. In response to HA oligosaccharide stimulation, significant upregulation of MT1-MMP mRNA occurred. Our data also provide evidence that HA oligosaccharide enhances MT1-MMP; the elevated expression of MT1-MMP confers enhanced CD44 cleavage and cell migration. In conclusion, we have identified a new function of HA in the induction of MT1-MMP expression in breast cancer cell lines and CD44 cleavage to increase cell migration during the invasion process. The HA oligosaccharide-induced MT1-MMP expression in breast cancer cells may be a critical step in the formation of metastatic colonies. 
 E1AF expression is closely correlated with malignant phenotype of tongue squamous cell carcinoma through activation of MT1-MMP gene promoters. E1AF is an ets-oncogene family transcription factor that has been shown to up-regulate multiple MMPs whereas MMP-2, a potent extracellular matrix degrading enzyme, is not up-regulated. We investigated the activation mechanism of MMP-2 in oral squamous cell carcinoma. Chloramphenicol acetyltransferase (CAT) assay was utilized to investigate whether E1AF is able to up-regulate membrane type-1 matrix metalloproteinase (MT1-MMP), which is known to activate MMP-2. Expression of the CAT reporter gene under the control of the MT1-MMP promoter was increased approximately 40-fold by co-transfection with the E1AF expression vector. Real-time RT-PCR study was carried out in 25 patients with tongue squamous cell carcinoma, and the mRNA expression level of E1AF and MT1-MMP was synergistically increased. These results indicate that E1AF positively regulates transcription from MT1-MMP genes, which plays an important role in invasion and   metastasis   of squamous cell carcinoma of the tongue by converting pro-MMP-2 into active-MMP-2. 
 Leptin-mediated regulation of MT1-MMP localization is KIF1B dependent and enhances gastric cancer cell invasion. Leptin overexpression is closely correlated with gastric cancer (GC) invasion, but its exact effect and the underlying mechanism in tumorigenesis remain poorly understood. Membrane type 1-matrix metalloproteinase (MT1-MMP), a surface-anchored 'master switch' proteinase, is overexpressed and plays crucial roles in tumor invasion. Here, we characterized the influence of leptin on the generation and surface localization of MT1-MMP in GC and elucidated its molecular mechanisms. Our results revealed that leptin promoted GC cell invasion in vitro by upregulating MT1-MMP expression. Furthermore, cell surface biotinylation assay and flow cytometry demonstrated that the surface expression of MT1-MMP was also enhanced by leptin, and knockdown of kinesin family member 1B (KIF1B, a microtubule plus end-directed monomeric motor protein) by small interference RNA inhibited this process. Notably, coimmunoprecipitation analysis indicated that leptin enhanced the interaction of MT1-MMP with KIF1B in a time-dependent manner, which consequently contributed to GC cell invasion. Moreover, leptin increased MT1-MMP or KIF1B expression by the protein kinase B (AKT) pathway and extracellular signal-regulated kinase 1/2 partially participated in this process. However, only AKT was implicated in the leptin-mediated membrane localization of MT1-MMP. Immunohistochemistry analysis revealed that leptin, MT1-MMP and KIF1B are overexpressed in GC tissues, and they positively correlated with clinical stage and lymph node   metastasis  . These observations indicate that this regulatory network exists in vivo. Taken together, our findings suggest that leptin is an effective intracellular stimulator of MT1-MMP and that leptin-enhanced cell surface localization of MT1-MMP is dependent on KIF1B, which consequently plays a critical role in GC invasion. 
 The expression of matrix metalloproteinases in intrahepatic cholangiocarcinoma, hilar (Klatskin tumor), middle and distal extrahepatic cholangiocarcinoma, gallbladder cancer, and ampullary carcinoma: role of matrix metalloproteinases in tumor progression and prognosis. Carcinomas of the biliary tree are rare tumors of the gastrointestinal tract, with an increasing incidence in recent years. Biliary neoplasms are classified into intra- and extrahepatic cholangiocarcinoma (Klatskin tumor, middle and distal extrahepatic tumors), gallbladder adenocarcinoma, and ampullary carcinoma. We aimed to determine the expression profile of matrix metalloproteinase (MMP)-2, MMP-9 and MMP-14 in the biliary neoplasms classified according to their localization and the relation with the prognosis.Ten gallbladder adenocarcinoma, 8 distal bile duct carcinomas (distal cholangiocarcinoma), 8 Klatskin tumors, 8 intrahepatic cholangiocarcinomas and 10 ampullary carcinomas were included in the study. The immunohistochemical expression of MMP-2, MMP-9 and MMP-14 was detected in the nontumoral, metaplastic, dysplastic and tumoral epithelia. The tumor differentiation, angiolymphatic and perineural invasion of the tumor, and presence of lymph node and distant   metastasis   were determined. Survey of the patients was noted from the patient follow-up data.The nontumoral epithelia of the gallbladder, intrahepatic ducts, and Klatskin tumor did not express MMP-2. MMP-2 expression was detected in the distal part of the biliary ducts, in 75% (6/18) of cases and in the nontumoral epithelia of the ampullary region in 50% (5/10) of cases. The metaplastic and dysplastic epithelia were positively stained in all of the gallbladder adenocarcinoma, distal cholangiocarcinoma and ampullary tumors. In the intrahepatic cholangiocarcinoma, the hepatocytes were positively stained but the infiltrative tumors were spared. Klatskin tumors were also not stained with MMP-2. The gallbladder adenocarcinoma, distal cholangiocarcinoma and ampullary carcinomas expressed MMP-2 in 30%, 37% and 40% of the cases, respectively. MMP-9 and MMP-14 were expressed in normal, metaplastic, and dysplastic epithelium and tumoral cells in all of the cases of the groups. Expressions of MMPs were higher in subjects with neural invasion, but there was no correlation between MMP expression and tumor differentiation or angiolymphatic invasion.When tumors of the biliary system are divided as intrahepatic and extrahepatic cholangiocarcinomas, MMP-2 expression was present in the extrahepatic cholangiocarcinomas including gallbladder carcinomas. Like the intrahepatic cholangiocarcinoma, Klatskin tumors also did not express MMP-2. This can be related with its characteristic growth pattern. MMP-9 and MMP- 14 were present in metaplasia, dysplasia carcinoma sequence in all of the bile tract tumors, suggesting that MMPs play an important role in carcinogenesis. The higher expression of the MMPs with neural invasion suggests the significant role of those tumors in the invasion activity. 
 Membrane Type 1 Matrix Metalloproteinase induces an epithelial to mesenchymal transition and cancer stem cell-like properties in SCC9 cells. Tissue invasion and   metastasis   are acquired abilities of cancer and related to the death in oral squamous cell carcinoma (OSCC). Emerging observations indicate that the epithelial-to-mesenchymal transition (EMT) is associated with tumor progression and the generation of cells with cancer stem cells (CSCs) properties. Membrane Type 1 Matrix Metalloproteinase (MT1-MMP) is a cell surface proteinase, which is involved in degrading extracellular matrix components that can promote tumor invasion and cell migration.In the current study, we utilized SCC9 cells stably transfected with an empty vector (SCC9-N) or a vector encoding human MT1-MMP (SCC9-M) to study the role of MT1-MMP in EMT development.Upon up-regulation of MT1-MMP, SCC9-M cells underwent EMT, in which they presented a fibroblast-like phenotype and had a decreased expression of epithelial markers (E-cadherin, cytokeratin18 and β-catenin) and an increased expression of mesenchymal markers (vimentin and fibronectin). We further demonstrated that MT1-MMP-induced morphologic changes increased the level of Twist and ZEB, and were dependent on repressing the transcription of E-cadherin. These activities resulted in low adhesive, high invasive abilities of the SCC9-M cells. Furthermore, MT1-MMP-induced transformed cells exhibited cancer stem cell (CSC)-like characteristics, such as low proliferation, self-renewal ability, resistance to chemotherapeutic drugs and apoptosis, and expression of CSCs surface markers.In conclusion, our study indicates that overexpression of MT1-MMP induces EMT and results in the acquisition of CSC-like properties in SCC9 cells. Our growing understanding of the mechanism regulating EMT may provide new targets against invasion and   metastasi  s in OSCC. 
 NHE1 mediates migration and invasion of HeLa cells via regulating the expression and localization of MT1-MMP. Na(+)/H(+) exchanger 1 (NHE1), acting as an important regulator of intracellular pH (pH(i)) and extracellular pH (pH(e)), has been known to play a key role in the   metastasis   of many solid tumours. However, the exact mechanism underlying these processes, especially in cervical cancer, is still poorly understood. In the current study, we first showed that the inhibition of NHE1 activity by the specific inhibitor cariporide could suppress migration and invasion of HeLa cells in vitro. Moreover, cariporide also reversed the enhanced migration and invasion in HeLa cells by overexpressed membrane-type 1 matrix metalloproteinase (MT1-MMP). Subsequently, our results showed that NHE1 regulated the expression of MT1-MMP at both messenger RNA and protein levels as well as its localization. Meanwhile, we observed slight modification in the morphology of HeLa cell after treating with cariporide. The present work indicates that NHE1 mediates HeLa cell   metastasis   via regulating the expression and localization of MT1-MMP and provides a theoretical basis for the development of novel therapeutic strategies targeting cervical cancer. 
 Hepatocyte nuclear factor 4 alpha promotes the invasion,   metastasis   and angiogenesis of neuroblastoma cells via targeting matrix metalloproteinase 14. Matrix metalloproteinase 14 (MMP-14) is the only membrane-anchored MMP that plays critical roles in tumorigenesis and aggressiveness. However, the regulatory mechanisms underlying the high MMP-14 expression in neuroblastoma (NB), a highly malignant tumor in childhood, still remain unclear. Herein, we applied an integrative approach to analyze the public datasets, and identified hepatocyte nuclear factor 4 alpha (HNF4α) as a crucial transcription factor facilitating the MMP-14 expression in NB. In clinical NB tissues, HNF4α was up-regulated and positively correlated with MMP-14 expression, and was an independent prognostic factor for unfavorable outcome of patients. Luciferase reporter and chromatin immunoprecipitation assays indicated that HNF4α directly targeted the binding site within the MMP-14 promoter to facilitate its transcription. Knockdown of HNF4α suppressed the invasi  on, metast  asis and angiogenesis of NB cells in vitro and in vivo. Conversely, ectopic expression of HNF4α promoted the invas  ion, metas  tasis and angiogenesis of NB cells. Importantly, restoration of MMP-14 expression prevented the tumor cells from HNF4α-mediated changes in these biological features. Taken together, HNF4α exhibits oncogenic activity that affects the aggressiveness and angiogenesis of NB through activating the transcription of MMP-14. 
 Limited independent prognostic value of MMP-14 and MMP-2 expression in ovarian cancer. In cancer, various MMPs play a role in progression and   metastasis   and their overexpression generally indicates a poor prognosis. MMP-14 is the main activator of MMP-2 and both molecules play a role in normal ovarian follicular development. Earlier reports indicated a prognostic value for both MMP-14 and MMP-2 in ovarian cancer. This study was designed to determine the prognostic value of MMP-14 and MMP-2 expression in ovarian cancer with data on long-term follow-up.Tumor samples of 94 consecutive ovarian cancer patients from one regional laboratory were evaluated. Clinical and survival data were collected and related to known prognostic factors, as well as to the expression of MMP-14 and MMP-2 as determined by semi-quantitative immunohistochemistry.Epithelial MMP-14 expression correlated with stromal MMP-14 expression (rho = .47, p < .01) and epithelial MMP-2 expression was found to correlate with both MMP-14 epithelial and stromal expression (rho = -.28, p < .01 respectively rho = -.21, p < .05). In univariable analysis of 64 advanced-staged tumours, no MMP parameter was significant for progression-free or overall survival. In multivariable analysis for PFS, stromal MMP-14 expression and epithelial MMP-2 expression remained in the model. For overall survival, no MMP parameter showed significance.We confirmed the correlation between epithelial and stromal MMP-14 expression and between epithelial MMP-2 and both epithelial and stromal MMP-14 expression. In this study with long-term follow-up, the independent prognostic value of MMP-14 and MMP-2 expression in ovarian cancer is limited to a role in PFS for stromal MMP-14 expression and epithelial MMP-2 expression. 
 Expression of membrane type 1 matrix metalloproteinase in papillomavirus-positive cells: role of the human papillomavirus (HPV) 16 and HPV8 E7 gene products. Matrix metalloproteinases (MMPs) degrade extracellular matrix. They are involved in cellular proliferation, migration, angiogenesis, invasion and   metastasis  . MT-1 MMP, a membrane-bound MMP, is expressed in carcinomas of the uterine cervix in vivo. This type of cancer is associated with human papillomavirus (HPV) infection. Here it was shown that keratinocytes transformed with HPV16 or HPV18 in vitro, and HPV-positive cervical carcinoma cell lines, constitutively expressed MT-1 MMP. Expression of the E7 protein from the mucosal and cutaneous high-risk types HPV16 and HPV8, but not from the cutaneous low-risk type HPV1, was sufficient to induce MT-1 MMP expression in primary human keratinocytes and HaCaT cells. As a consequence, MMP-2 was activated. MT-1 MMP expression might play a role in the HPV life cycle by promoting proliferation of host cells and might contribute to their invasive phenotype during malignant progression. 
 Shedding of kidney injury molecule-1 by membrane-type 1 matrix metalloproteinase. Co-expression of membrane-type 1 matrix metalloproteinase (MT1-MMP) with kidney injury molecule-1 (KIM-1) in HEK293T cells resulted in cleavage and shedding of KIM-1 ectodomain. Analysis of cleavage products using KIM-1 mutants localized cleavage site at the juxtamembrane region. HT1080 cells were stably transfected with expression plasmid for KIM-1 or its mutant with deletion of the juxtamembrane region (Asp(261)-Gly(295)) to establish HT/KIM-1 or HT/ΔKIM-1 cells, respectively. KIM-1 protein appeared on cell surface at low level in HT/KIM-1 cells, and accumulated by the treatment with MMP inhibitor BB-94 or small interfering RNA (siRNA) to MT1-MMP, indicating that MT1-MMP is involved in cleavage and shedding of KIM-1. In contrast, HT/ΔKIM-1 cells expressed KIM-1 protein at high level regardless of BB-94 or siRNA treatment. Cells expressing high level KIM-1 protein exhibited phagocytosis of Escherichia coli and reduced cell adhesion and spreading on collagen-coated plate compared with KIM-1 negative cells. Control HT1080 and HT/KIM-1 cells showed significantly higher invasive growth in collagen gel, cell migration on collagen-coated plate and live  r metastas  is in chick embryo than HT/ΔKIM-1 cells. These results suggest that KIM-1 negatively regulates cellular function mediated through interaction with collagen, and MT1-MMP abrogates it through the cleavage and shedding of KIM-1. 
 Conversion of stationary to invasive tumor initiating cells (TICs): role of hypoxia in membrane type 1-matrix metalloproteinase (MT1-MMP) trafficking. Emerging evidence has implicated the role of tumor initiating cells (TICs) in the process of cancer   metastasis  . The mechanism underlying the conversion of TICs from stationary to invasive remains to be characterized. In this report, we employed less invasive breast cancer TICs, SK-3rd, that displays CD44(high)/CD24(low) with high mammosphere-forming and tumorigenic capacities, to investigate the mechanism by which stationary TICs are converted to invasive TICs. Invasive ability of SK-3rd TICs was markedly enhanced when the cells were cultured under hypoxic conditions. Given the role of membrane type 1-matrix metalloproteinase (MT1-MMP) in cancer invasion/  metastasis  , we explored a possible involvement of MT1-MMP in hypoxia-induced TIC invasion. Silencing of MT1-MMP by a shRNA approach resulted in diminution of hypoxia-induced cell invasion in vitro and   metastasis   in vivo. Under hypoxic conditions, MT1-MMP redistributed from cytoplasmic storage pools to the cell surface of TICs, which coincides with the increased cell invasion. In addition, CD44, a cancer stem-like cell marker, inversely correlated with increased cell surface MT1-MMP. Interestingly, cell surface MT1-MMP gradually disappeared when the hypoxia-treated cells were switched to normoxia, suggesting the plasticity of TICs in response to oxygen content. Furthermore, we dissected the pathways leading to upregulated MT1-MMP in cytoplasmic storage pools under normoxic conditions, by demonstrating a cascade involving Twist1-miR10b-HoxD10 leading to enhanced MT1-MMP expression in SK-3rd TICs. These observations suggest that MT1-MMP is a key molecule capable of executing conversion of stationary TICs to invasive TICs under hypoxic conditions and thereby controlling   metastasis  . 
 Matrix metalloproteinase-14 is a negative prognostic marker for patients with gastric cancer. Matrix metalloproteinase-14 (MMP-14) has been considered to play an important role in invasion and   metastasis   of human solid tumor.The present study aimed to investigate the association of MMP-14 with overall survival in human gastric cancer.Gastric cancer and adjacent normal specimens were collected from 205 patients who had not received neoadjuvant chemotherapy. MMP-14 expression was investigated by immunohistochemistry assay and staining evaluation results were analyzed statistically in relation to overall survival of patients.MMP-14 expression proved to be increased in gastric cancer compared with that in normal tissues. It was also proved that MMP-14 expression was associated with tumor invasion,   metastasis  , and TNM stage while no correlations were detected between MMP-14 expression and age, sex, differentiation status, or Lauren's classification. Moreover, patients with gastric cancer of MMP-14-positive expression tend to have worse overall survival compared with those with MMP-14 negative expression.The present study confirmed the over-expression of MMP-14 in human gastric cancer and its association with tumor progression. It also provided the first evidence that MMP-14 expression in gastric cancer was an independent negative prognostic factor of patients. 
 CLIC3 controls recycling of late endosomal MT1-MMP and dictates invasion and   metastasis   in breast cancer. Chloride intracellular channel 3 (CLIC3) drives invasiveness of pancreatic and ovarian cancer by acting in concert with Rab25 to regulate the recycling of α5β1 integrin from late endosomes to the plasma membrane. Here, we show that in two estrogen receptor (ER)-negative breast cancer cell lines, CLIC3 has little influence on integrin recycling, but controls trafficking of the pro-invasive matrix metalloproteinase MT1-MMP (also known as MMP14). In MDA-MB-231 cells, MT1-MMP and CLIC3 are localized primarily to late endosomal/lysosomal compartments located above the plane of adhesion and near the nucleus. MT1-MMP is transferred from these late endosomes to sites of cell-matrix adhesion in a CLIC3-dependent fashion. Correspondingly, CLIC3-knockdown opposes MT1-MMP-dependent invasive processes. These include the disruption of the basement membrane as acini formed from MCF10DCIS.com cells acquire invasive characteristics in 3D culture, and the invasion of MDA-MB-231 cells into Matrigel or organotypic plugs of type I collagen. Consistent with this, expression of CLIC3 predicts poor prognosis in ER-negative breast cancer. The identification of MT1-MMP as a cargo of a CLIC3-regulated pathway that drives invasion highlights the importance of late endosomal sorting and trafficking in breast cancer. 
 Clinical relevance of cyclooxygenase-2 and matrix metalloproteinases (MMP-2 and MT1-MMP) in human breast cancer tissue. Breast cancer (BC) is the most common neoplasm among women in most developed countries, including Egypt. Elevated levels of certain proteins in human BC are associated with unfavorable prognosis and progressive stages of the disease. The aim of our study was to evaluate the protein expression profile and prognostic significance of cyclooxygenase-2 (COX-2), matrix metalloproteinase-2 (MMP-2), MMP-9 and membrane type 1-MMP (MT1-MMP) and their interaction in operable BC patients. The protein expression of COX-2, MMP-2 and MT1-MMP were evaluated by western blot technique, whereas enzymatic activity of MMP-2 and MMP-9 was determined by zymography in 47 breast cancer patients as well as normal adjacent tissues. Also, the correlation between these proteins and age, tumor size, LN stage, TNM stage, estrogen receptor, progesterone receptor, disease-free survival, and overall survival (OS) has been investigated. As compared to adjacent normal tissues, COX-2, MMP-2 and MT1-MMP were over-expressed in 43, 64, and 60 % of tumor tissues, respectively. In the same pattern, the activity of MMP-2 (62 %) and MMP-9 (45 %) was elevated in BC tissues. Multivariate analysis showed a positive correlation between the protein expression of COX-2, MMP-2, and MT1-MMP and the activity of MMP-2 and MMP-9 in BC patients. However, the enzymatic activity showed no correlation with clinicopathological features. This study confirms the preclinical evidence that COX-2 increased the expression of MT1-MMP, which in turn activates MMP-2. The lack of correlation with clinicopathological features, OS or disease-free survival ascertains the complexity of tumor progression and   metastasis   with many pro- and counter regulatory factors. 
 O-glycosylation regulates autolysis of cellular membrane type-1 matrix metalloproteinase (MT1-MMP). MT1-MMP is a key enzyme in cancer cell invasion and   metastasis  . The activity of cellular MT1-MMP is regulated by furin-like proprotein convertases, TIMPs, shedding, autoproteolysis, dimerization, exocytosis, endocytosis, and recycling. Our data demonstrate that, in addition to these already known mechanisms, MT1-MMP is regulated by O-glycosylation of its hinge region. Insignificant autolytic degradation is characteristic for naturally expressed, glycosylated, MT1-MMP. In turn, extensive autolytic degradation, which leads to the inactivation of the protease and the generation of its C-terminal membrane-tethered degraded species, is a feature of overexpressed MT1-MMP. We have determined that incomplete glycosylation stimulates extensive autocatalytic degradation and self-inactivation of MT1-MMP. Self-proteolysis commences during the secretory process of MT1-MMP through the cell compartment to the plasma membrane. The strongly negatively charged sialic acid is the most important functional moiety of the glycopart of MT1-MMP. We hypothesize that sialic acid of the O-glycosylation cassette restricts the access of the catalytic domain to the hinge region and to the autolytic cleavage site and protects MT1-MMP from autolysis. Overall, our results point out that there is a delicate balance between glycosylation and self-proteolysis of MT1-MMP in cancer cells and that when this balance is upset the catalytically potent MT1-MMP pool is self-proteolyzed. 
 The hemopexin domain of membrane-type matrix metalloproteinase-1 (MT1-MMP) Is not required for its activation of proMMP2 on cell surface but is essential for MT1-MMP-mediated invasion in three-dimensional type I collagen. Membrane-type matrix metalloproteinase-1 (MT1-MMP) plays a key role in tumor invasion and   metastasis   by degrading the extracellular matrix and activating proMMP2. Here we show that the conserved hemopexin domain is required for MT1-MMP-mediated invasion and growth in three-dimensional type I collagen matrix but not proMMP2 activation. Deletion of the hemopexin domains in MT1-, MT2-, MT3-, MT5-, and MT6-MMP does not impair their abilities to activate proMMP2. In fact, hemopexin-less MT5- and MT6-MMP activate proMMP2 better than their wild type counterparts. On the other hand, hemopexin-less MT1-MMP fails to promote cell invasion into type I collagen but retains the capacity to enhance the growth of Madin-Darby canine kidney cells as cysts in three-dimensional collagen matrix. Moreover, the hemopexin domain is also required for MT1-MMP-mediated invasion/scattering of MCF-7 cells in three-dimensional collagen matrix. Because growth and invasion in a three-dimensional model may correlate with tumor invasiveness in vivo, our data suggest that the hemopexin domains of MT-MMPs should be targeted for the development of anti-cancer therapies by employing screening assays developed for three-dimensional models rather than their enzymatic activity toward proMMP2. 
 The inactive 44-kDa processed form of membrane type 1 matrix metalloproteinase (MT1-MMP) enhances proteolytic activity via regulation of endocytosis of active MT1-MMP. Membrane type 1 (MT1) matrix metalloproteinase (MMP-14) is a membrane-tethered MMP considered to be a major mediator of pericellular proteolysis. MT1-MMP is regulated by a complex array of mechanisms, including processing and endocytosis that determine the pool of active proteases on the plasma membrane. Autocatalytic processing of active MT1-MMP generates an inactive membrane-tethered 44-kDa product (44-MT1) lacking the catalytic domain. This form preserves all other enzyme domains and is retained at the cell surface. Paradoxically, accumulation of the 44-kDa form has been associated with increased enzymatic activity. Here we report that expression of a recombinant 44-MT1 (Gly(285)-Val(582)) in HT1080 fibrosarcoma cells results in enhanced pro-MMP-2 activation, proliferation within a three-dimensional collagen I matrix, and tumor growth and lung   metastasis   in mice. Stimulation of pro-MMP-2 activation and growth in collagen I was also observed in other cell systems. Expression of 44-MT1 in HT1080 cells is associated with a delay in the rate of active MT1-MMP endocytosis resulting in higher levels of active enzyme at the cell surface. Consistently, deletion of the cytosolic domain obliterates the stimulatory effects of 44-MT1 on MT1-MMP activity. In contrast, deletion of the hinge turns the 44-MT1 form into a negative regulator of enzyme function in vitro and in vivo, suggesting a key role for the hinge region in the functional relationship between active and processed MT1-MMP. Together, these results suggest a novel role for the 44-kDa form of MT1-MMP generated during autocatalytic processing in maintaining the pool of active enzyme at the cell surface. 
 Involvement of syntaxin 4 in the transport of membrane-type 1 matrix metalloproteinase to the plasma membrane in human gastric epithelial cells. Membrane-type 1 matrix metalloproteinase (MT1-MMP) localized on the plasma membrane plays a central role in various normal biological responses including tissue remodeling, wound heeling, and angiogenesis and in cancer cell invasion and   metastasis  , by functioning as a collagenase and activating other matrix metalloproteinases. In order to elucidate the molecular mechanism of the MT1-MMP targeted localization on the plasma membrane, we examined the participation of syntaxin proteins in MT1-MMP intracellular transport to the plasma membrane in human gastric epithelial AGS cells. Western blotting showed that syntaxin 3 and 4 proteins, which are known to function in intracellular transport towards the plasma membrane, were expressed in AGS cells. Immunocytochemistry revealed that transient transfection of AGS cells with dominant-negative mutant syntaxin 4 decreased plasma membrane MT1-MMP expression. In contrast, transient transfection with either dominant-negative mutant syntaxin 3 or 7 did not affect MT1-MMP localization on the plasma membrane. Cell surface biotinylation assay and Matrigel chamber assay demonstrated that stable transfection with dominant-negative mutant syntaxin 4 decreased the amount of MT1-MMP on the plasma membranes and inhibited the cell invasiveness. We suggest that syntaxin 4 is involved in the intracellular transport of MT1-MMP toward the plasma membrane. 
 Role of MMP14 gene polymorphisms in susceptibility and pathological development to hepatocellular carcinoma. Early detection of hepatocellular carcinoma (HCC) is seldom available because of the lack of reliable markers. Matrix metalloproteinase (MMP) 14 is a cell surface proteinase that displays a broad spectrum of activity against extracellular matrix components and promotes the invasion/  metastasis   of cells. MMP14 is overexpressed in HCC, and the level is correlated with poor overall survival. The purpose of this study was to examine whether the MMP14 gene polymorphisms are associated with the susceptibility and clinicopathological development of HCC.A total of 135 patients with HCC and 496 healthy control subjects were recruited. Six single nucleotide polymorphisms (SNPs) of MMP14 genes were analyzed by polymerase chain reaction-restriction fragment length polymorphism (PCR-RFLP) genotyping and haplotype-base analysis.A significant (p < 0.05) lower risk for HCC was shown in the individuals with MMP14 +6767 G/A and +7096 C/C genotypes compared with those with corresponding wild-type homozygotes; high frequency for anti-hepatitis C virus and cirrhosis positive were shown in the HCC patients with MMP14 +7096 TC/CC genotype after adjusting for other confounding factors. The distribution frequency of -165 T: +221 T: +6727 C: +6767 G: +7096 T: +8153 G haplotype and diplotype was significantly higher in the HCC patients than healthy control subjects.The +6767 and +7096 polymorphic genotypes and haplotype -165 T: +221 T: +6727 C: +6767 G: +7096 T: +8153 G of MMP14 gene might contribute to the prediction of susceptibility and pathological development to HCC. 
 Matrix metalloproteinase-14 expression and its prognostic value in cervical carcinoma. The objective of the study was to evaluate the expression of matrix metalloproteinase-14 (MMP-14) in cervical carcinoma and correlate its expression with clinicopathological parameters, recurrence, and survival of the patients. The expressions of MMP-14 in normal cervical mucosa and cervical carcinoma tissue were detected with immunohistochemistry. Survival analysis by the Kaplan-Meier method was performed to assess prognostic significance. The positive expression rate of MMP-14 in cervical carcinoma tissue was 81.6 %(111/136), and there was significant difference on their positive expression rates between in cervical carcinoma tissue and in normal cervical mucosa(22.4 %)(13/58)(P < 0.05);The positive expression rates of MMP-14 in patients with poor histologic differentiation, lymph node   metastasis  , and recurrence group were heightened. Using Kaplan-Meier analysis, a comparison of survival curves of low versus high expressions of MMP-14 revealed a highly significant difference in human cervical carcinoma tissue (P < 0.05), which suggests that overexpression of MMP-14 is associated with a worse prognosis. The MMP-14 which promotes angiogenes is associated with lymph node   metastasis  , vascular invasion, and poor prognosis of cervical carcinoma. The current study shows that MMP-14 may be an independent prognostic factor for cervical carcinoma patients. 
 A specific subset of RabGTPases controls cell surface exposure of MT1-MMP, extracellular matrix degradation and three-dimensional invasion of macrophages. The matrix metalloproteinase MT1-MMP has a major impact on invasive cell migration in both physiological and pathological settings such as immune cell extravasation or   metastasis   of cancer cells. Surface-associated MT1-MMP is able to cleave components of the extracellular matrix, which is a prerequisite for proteolytic invasive migration. However, current knowledge on the molecular mechanisms that regulate MT1-MMP trafficking to and from the cell surface is limited. We have identified three members of the RabGTPase family, Rab5a, Rab8a and Rab14, as crucial regulators of MT1-MMP trafficking and function in primary human macrophages. Both overexpressed and endogenous forms show prominent colocalisation with MT1-MMP-positive vesicles, whereas expression of mutant constructs, as well as siRNA-induced knockdown, reveal that these RabGTPases are crucial in the regulation of MT1-MMP surface exposure, contact of MT1-MMP-positive vesicles with podosomes, extracellular matrix degradation in two and three dimensions, as well as three-dimensional proteolytic invasion of macrophages. Collectively, our results identify Rab5a, Rab8a and Rab14 as major regulators of MT1-MMP trafficking and invasive migration of primary human macrophages, which could be promising potential targets for manipulation of immune cell invasion. 
 Molecular signature of MT1-MMP: transactivation of the downstream universal gene network in cancer. Invasion-promoting MT1-MMP is directly linked to tumorigenesis and   metastasis  . Our studies led us to identify those genes, the expression of which is universally linked to MT1-MMP in multiple tumor types. Genome-wide expression profiling of MT1-MMP-overexpressing versus MT1-MMP-silenced cancer cells and a further data mining analysis of the preexisting expression database of 190 human tumors of 14 cancer types led us to identify 11 genes, the expression of which correlated firmly and universally with that of MT1-MMP (P < 0.00001). These genes included regulators of energy metabolism (NNT), trafficking and membrane fusion (SLCO2A1 and ANXA7), signaling and transcription (NR3C1, JAG1, PI3K delta, and CK2 alpha), chromatin rearrangement (SMARCA1), cell division (STK38/NDR1), apoptosis (DAPK1), and mRNA splicing (SNRPB2). Our subsequent extensive analysis of cultured cells, tumor xenografts, and cancer patient biopsies supported our data mining. Our results suggest that transcriptional reprogramming of the specific downstream genes, which themselves are associated with tumorigenesis, represents a distinctive "molecular signature" of the proteolytically active MT1-MMP. We suggest that the transactivation activity of MT1-MMP contributes to the promigratory cell phenotype, which is induced by this tumorigenic proteinase. The activated downstream gene network then begins functioning in unison with MT1-MMP to rework the signaling, transport, cell division, energy metabolism, and other critical cell functions and to commit the cell to migration, invasion, and, consequently, tumorigenesis. 
 Relation of MT1-MMP gene expression to outcomes in colorectal cancer. Matrix metalloproteinases are members of a large family of endopeptidases that participate in the extracellular-matrix degradation that accompanies cancer cell invasion,   metastasis   and angiogenesis. The membrane-type 1 matrix metalloproteinase (MT1-MMP) gene has been reported in various cancers and is associated with tumor invasion and   metastasis  . This study examined the relation of the relative expression of MT1-MMP gene to clinicopathological factors and outcomes in patients with colorectal cancer (CRC).We studied surgical specimens of cancer tissue and adjacent normal mucosa obtained from 202 patients with untreated CRC. The relative expression levels of MT1-MMP mRNA in cancer and in normal adjacent mucosa were measured by quantitative real-time reverse-transcriptase polymerase chain reaction.MT1-MMP gene expression was higher in cancer tissue than in adjacent normal mucosa. The level of MT1-MMP gene expression was not related to any clinicopathological factor. Overall survival at 5 years differed significantly between patients with high MT1-MMP gene expression and those with low expression.Overexpression of the MT1-MMP gene is considered a useful independent predictor of outcomes in patients with CRC. 
 Membrane type 1-matrix metalloproteinase induces epithelial-to-mesenchymal transition in esophageal squamous cell carcinoma: Observations from clinical and in vitro analyses. Membrane type 1-matrix metalloproteinase (MT1-MMP) is associated with enhanced tumorigenicity in many cancers. A recent study has revealed that MT1-MMP induces epithelial-to-mesenchymal transition (EMT) in prostate and breast cancer cells. However, its role in esophageal squamous cell carcinoma (ESCC) has not been studied. Here, we investigated the role of MT1-MMP in the dissemination of ESCC. Expression of MT1-MMP was detected by immunohistochemistry and tissue microarray in 88 Kazakh ESCC patients. Western blotting was performed to detect endogenous and overexpressed exogenous MT1-MMP in the Eca109 and Eca9706 cell lines, respectively. Transwell assay was used to estimate MT1-MMP-induced invasion and   metastasis  . EMT-associated proteins were detected by immunohistochemistry and western blotting. The associations between the expression of MT1-MMP and EMT-associated proteins with clinicopathologic parameters were analyzed. Overexpression of MT1-MMP was confirmed in Kazakh ESCC patients. MT1-MMP levels were found to be correlated with the depth of tumor infiltration. MT1-MMP induced EMT in ESCC both in vivo and in vitro, N-cadherin and Vimentin expression was upregulated upon MT1-MMP transfection into cells. However, E-cadherin was found to be downregulated. MT1-MMP-induced EMT led to increase migration and invasion in ESCC cell lines. In conclusion, our results suggest that MT1-MMP promotes ESCC invasion and   metastasis  . 
 Expression of membrane type-1 matrix metalloproteinase, MT1-MMP in human breast cancer and its impact on invasiveness of breast cancer cells. MT1-MMP (membrane type-1 matrix metalloproteinase), otherwise known as MMP14 is a proteolytic enzyme known to be involved in degradating extracellular matrix and assist progression of cancer invasion and progression. We investigated the impact of targeting the expression of MT1-MMP in breast cancer and its clinical relevance. Human breast cancer cell line MDA-MB-231 was used. Expression of MT1-MMP in the breast cancer cell line was manipulated by way of retroviral ribozyme transgene. The in vitro invasion, growth and cell migration were determined on cell lines transfected with either the transgene or control plasmid. Protein and message levels of MMP14 was also assessed using immunohistochemistry and real-time quantitative analysis, and correlated with clinical and pathological information of the patients. Retroviral ribozyme transgene to human MT1-MMP successfully knocked down the levels of MT1-MMP mRNA from MDA-MB-231 cells. Reduction of MT1-MMP from the breast cancer cells resulted in significant reduction of in vitro invasiveness and loss of response to an invasion stimulus, HGF, compared with control and wild-type cells. The invasion index for MT1-MMP knockdown cells were 13+/-3.1 (without HGF) and 16.4+/-2.3 (with HGF, p=0.14), and the index for transfection control cells 25.3+/-4.3 (without HGF) and 40.4+/-4.1 (with HGF, p=0.0049). Transfection with the transgenes did not change the rate of cell growth. In clinical breast cancer, MT1-MMP staining was both membranous and cytoplasmic. Tumour cells displayed stronger staining compared with normal mammary epithelial cells. Tumour tissues had a marginally higher levels of the MMP14 transcript (8.6+/-1.9), compared with normal tissues (4.7+/-1.4), p=0.13. No significant difference was observed between node positive and node negative tumours (9.0+/-2.2 vs 8.7+/-3.1, p=0.24). Marginally higher levels of the MMP14 transcript were seen in tumours which developed   metastasis   and local recurrence. However, tumours from patients who died of breast cancer related causes had significantly higher levels of the transcript, compared with tumours from patients who remained disease-free 10 years after initial surgery (12.2+/-2.5 vs 6.3+/-1.2, p=0.0091). MT1-MMP is a proteolytic enzyme that is pivotal in controlling the invasiveness of breast cancer cells. It is highly expressed in aggressive breast tumours and is associated with clinical outcome. The enzyme is a potential therapeutic target in breast cancer. 
 miR-181a-5p Inhibits Cancer Cell Migration and Angiogenesis via Downregulation of Matrix Metalloproteinase-14. Upregulation of matrix metalloproteinase MMP-14 (MT1-MMP) is associated with poor prognosis in cancer patients, but it is unclear how MMP-14 becomes elevated in tumors. Here, we show that miR-181a-5p is downregulated in aggressive human breast and colon cancers where its levels correlate inversely with MMP-14 expression. In clinical specimens, enhanced expression of MMP-14 was observed in cancer cells located at the invasive front of tumors where miR-181a-5p was downregulated relative to adjacent normal cells. Bioinformatics analyses defined a potential miR-181a-5p response element within the 3'-untranslated region of MMP-14 that was validated in reporter gene experiments. Ectopic miR-181a-5p reduced MMP-14 expression, whereas miR-181a-5p attenuation elevated MMP-14 expression. In support of a critical relationship between these two genes, miR-181a-5p-mediated reduction of MMP-14 levels was sufficient to decrease cancer cell migration, invasion, and activation of pro-MMP-2. Furthermore, this reduction in MMP-14 levels was sufficient to reduce in vivo invasion and angiogenesis in chick chorioallantoic membrane assays. Taken together, our results establish the regulation of MMP-14 in cancers by miR-181a-5p through a posttranscriptional mechanism, and they further suggest strategies to elevate miR-181a-5p to prevent cancer   metastasis  . 
 A matrix metalloproteinase expressed on the surface of invasive tumour cells. Gelatinase A (type-IV collagenase; M(r) 72,000) is produced by tumour stroma cells and is believed to be crucial for their invasion and   metastasis  , acting by degrading extracellular matrix macro-molecules such as type IV collagen. An inactive precursor of gelatinase A (pro-gelatinase A) is secreted and activated in invasive tumour tissue as a result of proteolysis which is mediated by a fraction of tumour cell membrane that is sensitive to metalloproteinase inhibitors. Here we report the cloning of the complementary DNA encoding a new matrix metalloproteinase with a potential transmembrane domain. Expression of the gene product on the cell surface induces specific activation of pro-gelatinase A in vitro and enhances cellular invasion of the reconstituted basement membrane. Tumour cells of invasive lung carcinomas, which contain activated forms of gelatinase A, were found to express the transcript and the gene product. The new metalloproteinase may thus trigger invasion by tumour cells by activating pro-gelatinase A on the tumour cell surface. 
 The tetraspanin CD81 protein increases melanoma cell motility by up-regulating metalloproteinase MT1-MMP expression through the pro-oncogenic Akt-dependent Sp1 activation signaling pathways. Despite the importance of multiple tetraspanin proteins in cancer invasion and   metastasis  , little is known about the role and significance of tetraspanin CD81 in these processes. In the present study, we examined CD81 effects on melanoma cell invasiveness and   metastasis  . Transfection of CD81 into melanoma cells lacking endogenous CD81 expression significantly enhanced the migrating, invasive, and metastatic abilities of melanoma cells. Interestingly, membrane type 1 matrix metalloproteinase (MT1-MMP) expression was found in CD81-expressing melanoma cells but not in CD81-deficient cells. siRNA knockdown of CD81 in melanoma cells with endogenous CD81 demonstrated decreased MT1-MMP levels and cell motility. Notably, CD81-induced cell migration was abrogated by antibody blocking and siRNA knockdown of MT1-MMP, indicating that MT1-MMP is responsible for CD81-stimulated melanoma cell migration. Promoter analysis revealed an essential role of the Sp1 transcription factor in CD81-induced MT1-MMP transcription. We also demonstrate that the Sp1-activating Akt pathway is involved in adhesion-dependent CD81 signaling to induce MT1-MMP expression and cell motility. Importantly, human skin cancer tissue specimens displayed a positive correlation of CD81 with MT1-MMP expression levels and a close association of CD81 with malignant melanomas. Taken together, these results strongly suggest that CD81 stimulates melanoma cell motility by inducing MT1-MMP expression through the Akt-dependent Sp1 activation signaling pathway, leading to increased melanoma invasion and   metastasis  . 
 LIM kinase1 modulates function of membrane type matrix metalloproteinase 1: implication in invasion of prostate cancer cells. LIM kinase 1 (LIMK1) is an actin and microtubule cytoskeleton modulatory protein that is overexpressed in a number of cancerous tissues and cells and also promotes invasion and   metastasis   of prostate and breast cancer cells. Membrane type matrix metalloproteinase 1 (MT1-MMP) is a critical modulator of extracellular matrix (ECM) turnover through pericellular proteolysis and thus plays crucial roles in neoplastic cell invasion and   metastasis  . MT1-MMP and its substrates pro-MMP-2 and pro-MMP-9 are often overexpressed in a variety of cancers including prostate cancer and the expression levels correlate with the grade of malignancy in prostate cancer cells. The purpose of this study is to determine any functional relation between LIMK1 and MT1-MMP and its implication in cell invasion.Our results showed that treatment with the hydroxamate inhibitor of MT1-MMP, MMP-2 and MMP-9 ilomastat inhibited LIMK1-induced invasion of benign prostate epithelial cells. Over expression of LIMK1 resulted in increased collagenolytic activity of MMP-2, and secretion of pro-MMP2 and pro-MMP-9. Cells over expressing LIMK1 also exhibited increased expression of MT1-MMP, transcriptional activation and its localization to the plasma membrane. LIMK1 physically associates with MT1-MMP and is colocalized with it to the Golgi vesicles. We also noted increased expression of both MT1-MMP and LIMK1 in prostate tumor tissues.Our results provide new information on regulation of MT1-MMP function by LIMK1 and showed for the first time, involvement of MMPs in LIMK1 induced cell invasion. 
  GeneRIFs  
 The results suggest that MT1-MMP promotes esophageal squamous cell carcinoma invasion and   metastasis  . 
 MiR-22 downregulation promotes GC invasion and   metastasis   by upregulating MMP14 and Snail, and then inducing ECM remodeling and EMT. 
 results indicate Moesin may regulate cell motility through its interactions with MT1-MMP and E-cadherin/p120-catenin adhesion complex and cytoplasmic expression of Moesin correlates with nodal   metastasis   and poor prognosis of OSCCs 
 higher levels of MMP-14 correlated with more advanced status of clinical stage and lymphatic   metastasis   
 High MMP14 expression is associated with   metastasis   in Hepatoblastoma and Neuroblastoma. 
 high levels of MMP14 protein were positively correlated with increased clinical stage,increased distant   metastasis   and shorter overall survival time than did patients with low MMP14 expression in nasopharyngeal carcinoma. 
 Results establish a novel role for NEDD9 as a key regulator of Arf6-dependent trafficking of MMP14 and its invasive properties, highlighting the basic mechanism utilized by NEDD9 to support breast cancer cell invasion and   metastasis  . 
 NHE1 mediates HeLa cell   metastasis   via regulating the expression and localization of MT1-MMP and provides a theoretical basis for the development of novel therapeutic strategies targeting cervical cancer. 
 CLIC3 controls trafficking of late endosomal MT1-MMP and dictates invasion and   metastasis   in breast cancer. 
 These results strongly suggest that CD81 stimulates melanoma cell motility by inducing MT1-MMP expression through the Akt-dependent Sp1 activation signaling pathway, leading to increased melanoma invasion and   metastasis  . 
 Report up-regulation of MT1-MMP and atypical protein kinase C in hormone receptor-negative breast tumors in association with a higher risk of   metastasis  . Silencing of aPKC impaired delivery of MT1-MMP from storage compartments and inhibited invasion. 
 High MMP14 expression is associated with   metastasis   of colorectal cancer. 
 MMP14 may play a role in laryngeal cancer invasion to a certain extent, but does play an important role in lymph node   metastasis   of laryngeal cancer. 
 miR-9 suppresses MMP-14 expression via the binding site in the 3'-UTR, thus inhibiting the invasion,   metastasis  , and angiogenesis of neuroblastoma. 
 The observations suggest that MT1-MMP is a key molecule capable of executing conversion of stationary TICs to invasive tumor initiating cells under hypoxic conditions and thereby controlling   metastasis  . 
 concluded that GnRHII stimulates PI3K/Akt pathway, and the phosphorylation of glycogen synthase kinase3beta, thereby enhancing the beta-catenin-dependent up-regulation of MT1-MMP production which contributes to ovarian cancer   metastasis   
 No association was found between MT1-MMP expression and lymph node   metastasis  . 
 RECK and MMP-14 proteins may serve as markers in the estimation of the extent of   metastasis   and dissemination of the neuroblastoma. 
 MT1-MMP-positive expression in bone marrow is associated with   metastasis   in gastric cancer 
 Data suggest a role for MT1-MMP in early tumour progression, expression of MMP-1 during   metastasis   and focal expression pattern of MMP-3 and the expression profiles may provide markers for early breast cancer diagnoses and potential therapeutic targets. 
 The results suggest that MT1-MMP is an enzyme responsible for Ln-5gamma2 cleavage in ESCC, and interaction between them may play a critical role in promoting invasion and   metastasis   of human E 
 E1AF positively regulates transcription from MT1-MMP genes, which plays an important role in invasion and   metastasis   of squamous cell carcinoma of the tongue by converting pro-MMP-2 into active-MMP-2 
 MT1-MMP is primarily secreted in the oral squamous cell carcinoma (OSCC) cells and is involved in the invasiveness of the OSCC and lymphatic   metastasis  . 
 Data suggest that membrane type 1-matrix metalloproteinase enhances tumor cell survival upon challenge of detachment, and its overexpression could enhance   metastasis  . 
 demonstrated that tumor cell migration was dramatically enhanced by expression of MT1-MMP and that MT1-MMP promotes lymph node and lung   metastasis   of prostate cancer cells 
  Summary  
 Proteins of the matrix metalloproteinase (MMP) family are involved in the breakdown of extracellular matrix in normal physiological processes, such as embryonic development, reproduction, and tissue remodeling, as well as in disease processes, such as arthritis and   metastasis  . Most MMP's are secreted as inactive proproteins which are activated when cleaved by extracellular proteinases. However, the protein encoded by this gene is a member of the membrane-type MMP (MT-MMP) subfamily; each member of this subfamily contains a potential transmembrane domain suggesting that these proteins are expressed at the cell surface rather than secreted. This protein activates MMP2 protein, and this activity may be involved in tumor invasion. [provided by RefSeq, Jul 2008] 
  [Cc]ell [Ii]nvasion  PubMed  
 Low tissue inhibitor of metalloproteinases 3 and high matrix metalloproteinase 14 levels defines a subpopulation of highly invasive foam-cell macrophages. An excess of metalloproteinases (MMPs) over tissue inhibitors of metalloproteinases (TIMPs) may favor atherosclerotic plaque rupture. We compared TIMP levels in nonfoamy and foam-cell macrophages (FCM) generated in vivo.In vivo generated rabbit FCM exhibited 84% reduced TIMP-3 protein compared to nonfoamy macrophages, and immunocytochemistry revealed a TIMP-3 negative subset (28%). Strikingly, only TIMP-3 negative FCM invaded a synthetic basement membrane, and invasion was inhibited by exogenous TIMP-3. TIMP-3 negative FCM also had increased proliferation and apoptosis rates compared to TIMP-3 positive cells, which were retarded by exogenous TIMP-3; this also reduced gelatinolytic activity. TIMP-3 negative FCM were found at the base of advanced rabbit plaques and in the rupture-prone shoulders of human plaques. To explain the actions of low TIMP-3 we observed a 26-fold increase in MT1-MMP (MMP-14) protein in FCM. Adding an MT1-MMP neutralizing antibody reduced foam-  cell invasion  , apoptosis, and gelatinolytic activity. Furthermore, MT1-MMP overexpressing and TIMP-3 negative FCM were found at the same locations in atherosclerotic plaques.These results demonstrate that TIMP-3 is downregulated in a distinct subpopulation of FCM which have increased MMP-14. These cells are highly invasive and have increased proliferation and apoptosis, all properties expected to destabilise atherosclerotic plaques. 
 MT1-MMP modulates melanoma cell dissemination and metastasis through activation of MMP2 and RAC1. Metastatic melanoma remains the deadliest of all skin cancers with a survival rate at five years of less than 15%. MT1-MMP is a membrane-associated matrix metalloproteinase that controls pericellular proteolysis and is an important, invasion-promoting, pro-tumorigenic MMP in cancer. We show that deregulation of MT1-MMP expression happens as early as the transition from nevus to primary melanoma and continues to increase during melanoma progression. Furthermore, MT1-MMP expression is associated with poor melanoma patient outcome, underscoring a pivotal role of MT1-MMP in melanoma pathogenesis. We demonstrate that MT1-MMP is directly required for melanoma cells to metastasize, as cells deprived of MT1-MMP fail to form distant metastasis in an orthotopic mouse melanoma model. We show that MT1-MMP affects   cell invasion   by activating its target MMP2. Importantly, we demonstrate, for the first time, that activation of MMP2 by MT1-MMP is required to sustain RAC1 activity and promote MT1-MMP-dependent cell motility. These data highlight a novel MT1-MMP/MMP2/RAC1 signaling axis in melanoma that may represent an intriguing molecular target for the treatment of invasive melanoma. 
 LOX-1-MT1-MMP axis is crucial for RhoA and Rac1 activation induced by oxidized low-density lipoprotein in endothelial cells. RhoA and Rac1 activation plays a key role in endothelial dysfunction. Lectin-like oxidized low-density lipoprotein receptor-1 (LOX-1) is a major receptor for oxidized low-density lipoprotein (ox-LDL) in endothelial cells (ECs). Membrane type 1 matrix metalloproteinase (MT1-MMP) has been shown to be involved in atherogenesis. This study was conducted to investigate the role of the LOX-1-MT1-MMP axis in RhoA and Rac1 activation in response to ox-LDL in ECs.Ox-LDL induced rapid RhoA and Rac1 activation as well as MT1-MMP activity in cultured human aortic ECs. Inhibition of LOX-1 prevented ox-LDL-dependent RhoA and Rac1 activation. Knockdown of MT1-MMP by small interfering RNA prevented ox-LDL-induced RhoA and Rac1 activation, indicating that MT1-MMP is upstream of RhoA and Rac1. Fluorescent immunostaining revealed the colocalization of LOX-1 and MT1-MMP, and the formation of a complex of LOX-1 with MT1-MMP was detected by immunoprecipitation. Blockade of LOX-1 or MT1-MMP prevented RhoA-dependent endothelial NO synthase protein downregulation and   cell invasion  , Rac1-mediated NADPH oxidase activity, and reactive oxygen species generation.The present study provides evidence that the LOX-1-MT1-MMP axis plays a crucial role in RhoA and Rac1 activation signalling pathways in ox-LDL stimulation, suggesting that this axis may be a promising target for treating endothelial dysfunction. 
 MT1-MMP modulates melanoma cell dissemination and metastasis through activation of MMP2 and RAC1. Metastatic melanoma remains the deadliest of all skin cancers with a survival rate at five years of less than 15%. MT1-MMP is a membrane-associated matrix metalloproteinase that controls pericellular proteolysis and is an important, invasion-promoting, pro-tumorigenic MMP in cancer. We show that deregulation of MT1-MMP expression happens as early as the transition from nevus to primary melanoma and continues to increase during melanoma progression. Furthermore, MT1-MMP expression is associated with poor melanoma patient outcome, underscoring a pivotal role of MT1-MMP in melanoma pathogenesis. We demonstrate that MT1-MMP is directly required for melanoma cells to metastasize, as cells deprived of MT1-MMP fail to form distant metastasis in an orthotopic mouse melanoma model. We show that MT1-MMP affects   cell invasion   by activating its target MMP2. Importantly, we demonstrate, for the first time, that activation of MMP2 by MT1-MMP is required to sustain RAC1 activity and promote MT1-MMP-dependent cell motility. These data highlight a novel MT1-MMP/MMP2/RAC1 signaling axis in melanoma that may represent an intriguing molecular target for the treatment of invasive melanoma. 
 LOX-1-MT1-MMP axis is crucial for RhoA and Rac1 activation induced by oxidized low-density lipoprotein in endothelial cells. RhoA and Rac1 activation plays a key role in endothelial dysfunction. Lectin-like oxidized low-density lipoprotein receptor-1 (LOX-1) is a major receptor for oxidized low-density lipoprotein (ox-LDL) in endothelial cells (ECs). Membrane type 1 matrix metalloproteinase (MT1-MMP) has been shown to be involved in atherogenesis. This study was conducted to investigate the role of the LOX-1-MT1-MMP axis in RhoA and Rac1 activation in response to ox-LDL in ECs.Ox-LDL induced rapid RhoA and Rac1 activation as well as MT1-MMP activity in cultured human aortic ECs. Inhibition of LOX-1 prevented ox-LDL-dependent RhoA and Rac1 activation. Knockdown of MT1-MMP by small interfering RNA prevented ox-LDL-induced RhoA and Rac1 activation, indicating that MT1-MMP is upstream of RhoA and Rac1. Fluorescent immunostaining revealed the colocalization of LOX-1 and MT1-MMP, and the formation of a complex of LOX-1 with MT1-MMP was detected by immunoprecipitation. Blockade of LOX-1 or MT1-MMP prevented RhoA-dependent endothelial NO synthase protein downregulation and   cell invasion  , Rac1-mediated NADPH oxidase activity, and reactive oxygen species generation.The present study provides evidence that the LOX-1-MT1-MMP axis plays a crucial role in RhoA and Rac1 activation signalling pathways in ox-LDL stimulation, suggesting that this axis may be a promising target for treating endothelial dysfunction. 
 Low tissue inhibitor of metalloproteinases 3 and high matrix metalloproteinase 14 levels defines a subpopulation of highly invasive foam-cell macrophages. An excess of metalloproteinases (MMPs) over tissue inhibitors of metalloproteinases (TIMPs) may favor atherosclerotic plaque rupture. We compared TIMP levels in nonfoamy and foam-cell macrophages (FCM) generated in vivo.In vivo generated rabbit FCM exhibited 84% reduced TIMP-3 protein compared to nonfoamy macrophages, and immunocytochemistry revealed a TIMP-3 negative subset (28%). Strikingly, only TIMP-3 negative FCM invaded a synthetic basement membrane, and invasion was inhibited by exogenous TIMP-3. TIMP-3 negative FCM also had increased proliferation and apoptosis rates compared to TIMP-3 positive cells, which were retarded by exogenous TIMP-3; this also reduced gelatinolytic activity. TIMP-3 negative FCM were found at the base of advanced rabbit plaques and in the rupture-prone shoulders of human plaques. To explain the actions of low TIMP-3 we observed a 26-fold increase in MT1-MMP (MMP-14) protein in FCM. Adding an MT1-MMP neutralizing antibody reduced foam-  cell invasion  , apoptosis, and gelatinolytic activity. Furthermore, MT1-MMP overexpressing and TIMP-3 negative FCM were found at the same locations in atherosclerotic plaques.These results demonstrate that TIMP-3 is downregulated in a distinct subpopulation of FCM which have increased MMP-14. These cells are highly invasive and have increased proliferation and apoptosis, all properties expected to destabilise atherosclerotic plaques. 
 Lack of Association between Membrane-Type 1 Matrix Metalloproteinase Expression and Clinically Relevant Molecular or Morphologic Tumor Characteristics at the Leading Edge of Invasive Colorectal Carcinoma. Colorectal cancer (CRC) is one of the leading causes of death from cancer in the western world, but tumor biology and clinical course show great interindividual variation. Molecular and morphologic tumor characteristics, such as KRAS/BRAF mutation status, mismatch repair (MMR) protein expression, tumor growth pattern, and tumor cell budding, have been shown to be of key therapeutic and/or prognostic relevance in CRC. Membrane-type 1 matrix metalloproteinase (MT1-MMP) is a membrane-anchored zinc-binding endopeptidase that is expressed at the leading edge of various invasive carcinomas and promotes tumor   cell invasion   through degradation of the extracellular matrix. The aim of this study was to investigate possible associations between MT1-MMP expression and molecular tumor characteristics as well as morphologic features of tumor aggressiveness in a consecutive series of 79 CRC tissue samples. However, although MT1-MMP was expressed in 41/79 samples (52%), there was no significant association between MT1-MMP expression and KRAS/BRAF mutation status, MMR protein expression, presence of lymphovascular invasion, tumor growth pattern, tumor-infiltrating lymphocytes, or tumor cell budding in our sample cohort (P > 0.05). Thus, we conclude that although MT1-MMP may play a role in CRC invasion, it is not of key relevance to the current models of CRC invasion and aggressiveness. 
 EphA2 cleavage by MT1-MMP triggers single cancer   cell invasion   via homotypic cell repulsion. Changes in EphA2 signaling can affect cancer cell-cell communication and motility through effects on actomyosin contractility. However, the underlying cell-surface interactions and molecular mechanisms of how EphA2 mediates these effects have remained unclear. We demonstrate here that EphA2 and membrane-anchored membrane type-1 matrix metalloproteinase (MT1-MMP) were selectively up-regulated and coexpressed in invasive breast carcinoma cells, where, upon physical interaction in same cell-surface complexes, MT1-MMP cleaved EphA2 at its Fibronectin type-III domain 1. This cleavage, coupled with EphA2-dependent Src activation, triggered intracellular EphA2 translocation, as well as an increase in RhoA activity and cell junction disassembly, which suggests an overall repulsive effect between cells. Consistent with this, cleavage-prone EphA2-D359I mutant shifted breast carcinoma   cell invasion   from collective to rounded single-  cell invasion   within collagen and in vivo. Up-regulated MT1-MMP also codistributed with intracellular EphA2 in invasive cells within human breast carcinomas. These results reveal a new proteolytic regulatory mechanism of cell-cell signaling in cancer invasion. 
 Increased aggressiveness of human prostate PC-3 tumor cells expressing cell surface localized membrane type-1 matrix metalloproteinase (MT1-MMP). Membrane type-1 matrix metalloproteinase (MT1-MMP) is a multidomain transmembrane endopeptidase with a major role in physiological and pathological processes through proteolysis of extracellular matrix and other pericellular proteins. We examined cell surface function of MT1-MMP in PC-3 human prostate tumor cells selected for metastasis in nude mice (PC-3-LN4), or transfected with the full-length wild-type (WT) MT1-MMP or with the mutant form lacking the cytoplasmic tail (Delta C-MT1-MMP). Enhanced cell surface MT1-MMP was determined by fluorescence-activated cell sorting analysis and evidenced mechanistically by increased activation of proMMP-2 and invasion into type-I collagen gels. PC-3 cells overexpressing MT1-MMP grew faster than mock-transfected control cells subcutaneously in nude mice. MT1-MMP localized in caveolae, as judged by immunofluorescence microscopy and sucrose-gradient, detergent-resistant cell fractionation. Delta C-MT1-MMP was strongly associated with caveolae, whereas the WT form was present in both caveolae and noncaveolae fractions. The role of plasma membrane MT1-MMP was supported by localization of MT1-MMP by immunofluorescence microscopy at the cell surface of tumor cells in primary prostate cancers. Increased plasma membrane localization of MT1-MMP, either in caveolae or in other lipid raft structures, is a mechanism to localize this proteinase in contact with extracellular matrix and other pericellular proteins, the cleavage of which can facilitate prostate cancer   cell invasion   and metastasis. 
 Transforming growth factor-beta induces CD44 cleavage that promotes migration of MDA-MB-435s cells through the up-regulation of membrane type 1-matrix metalloproteinase. CD44, a transmembrane receptor for hyaluronic acid, is implicated in various adhesion-dependent cellular processes, including cell migration, tumor cell metastasis and invasion. Recent studies demonstrated that CD44 expressed in cancer cells can be proteolytically cleaved at the ectodomain by membrane type 1-matrix metalloproteinase (MT1-MMP) to form soluble CD44 and that CD44 cleavage plays a critical role in cancer cell migration. Here, we show that transforming growth factor-beta (TGF-beta), a multifunctional cytokine involved in cell proliferation, differentiation, migration and pathological processes, induces MT1-MMP expression in MDA-MB-435s cells. TGF-beta-induced MT1-MMP expression was blocked by the specific extracellular regulated kinase-1/2 (ERK1/2) inhibitor PD98059 and the specific phosphoinositide 3-OH kinase (PI3K) inhibitor LY294002. In addition, treatment with SP600125, an inhibitor for c-Jun NH(2)-terminal kinase (JNK), resulted in a significant inhibition of MT1-MMP production. These data suggest that ERK1/2, PI3K, and JNK likely play a role in TGF-beta-induced MT1-MMP expression. Interestingly, treatment of MDA-MB-435s cells with TGF-beta resulted in a colocalization of MT1-MMP and CD44 in the cell membrane and in an increased level of soluble CD44. Using an electric cell-substrate impedance sensing cell-electrode system, we demonstrated that TGF-beta treatment promotes MDA-MB-435s cell migration, involving MT1-MMP-mediated CD44 cleavage. MT1-MMP siRNA transfection-inhibited TGF-beta-induced cancer cell transendothelial migration. Thus, this study contributes to our understanding of molecular mechanisms that play a critical role in tumor   cell invasion   and metastasis. 
 KIF1B promotes glioma migration and invasion via cell surface localization of MT1-MMP. Malignant glioma is notorious for its aggressiveness and poor prognosis, and the invasiveness of glioma cells is the major obstacle. Accumulating evidence indicates that kinesin superfamily proteins (KIFs) may play key roles in tumor invasiveness, but the mechanisms remained unresolved. Our previous study demonstrated that membrane type 1-matrix metalloproteinase (MT1-MMP) was involved in Kinesin family member 1B (KIF1B)-modulated invasion of gastric cancer cells. Therefore, the role of KIF1B in glioma   cell invasion   and its relationship with MT1-MMP were explored in the present study. We found that aberrantly increased expression of KIF1B was associated with worse WHO pathological classification and Karnofsky performance status (KPS), which also showed a trend towards worse prognosis. In the transwell assay, knockdown of KIF1B using siRNA repressed U87MG and A172 glioma cell migration and invasion. Silencing KIF1B inhibited expression of membranal MT1-MMP; however, the amount of MT1-MMP in the whole cell lysate was not affected. In conclusion, targeting KIF1B may be an option for anti-invasive therapies targeting glioma. 
 MMP-14 is necessary but not sufficient for invasion of three-dimensional collagen by human muscle satellite cells. The twenty-five known matrix metalloproteases (MMPs) and their endogenous inhibitors, tissue inhibitors of metalloproteases (TIMPs), mediate   cell invasion   through the extracellular matrix (ECM). In a comparative three-dimensional assay, we analyzed human and mouse satellite cells' competence to invade an artificial ECM (collagen I). We identified a single MMP that 1) is expressed by human muscle satellite cells; 2) is induced at the mRNA/protein level by adhesion to collagen I; and 3) is necessary for invasion into a collagen I matrix. Interestingly, murine satellite cells neither express this MMP, nor invade the collagen matrix. However, exogenous human MMP-14 is not sufficient to induce invasion of a collagen matrix by murine cells, emphasizing species differences. 
 NEDD9/Arf6-dependent endocytic trafficking of matrix metalloproteinase 14: a novel mechanism for blocking mesenchymal   cell invasion   and metastasis of breast cancer. NEDD9 is an established marker of invasive and metastatic cancers. NEDD9 downregulation has been shown to dramatically reduce   cell invasion   and metastasis in multiple tumors. The mechanisms by which NEDD9 regulates invasion are largely unknown. In the current study, we have found that NEDD9 is required for matrix metalloproteinase 14 (MMP14) enzymatic recovery/recycling through the late endosomes to enable disengagement of tissue inhibitor of matrix metalloproteinase 2 (TIMP2) and tumor invasion. Depletion of NEDD9 decreases targeting of the MMP14/TIMP2 complex to late endosomes and increases trafficking of MMP14 from early/sorting endosomes back to the surface in a small GTPase ADP ribosylation factor-6 (Arf6)-dependent manner. NEDD9 directly binds to Arf6-GTPase-activating protein, ARAP3 and Arf6-effector GGA3, thereby facilitating the Arf6 inactivation required for MMP14/TIMP2 targeting to late endosomes. Re-expression of NEDD9 or a decrease in Arf6 activity is sufficient to restore MMP14 activity and the invasive properties of tumor cells. Importantly, NEDD9 inhibition by Vivo-Morpholinos, an antisense therapy, decreases primary tumor growth and metastasis in xenograft models of breast cancer. Collectively, our findings uncover a novel mechanism to control tumor-cell dissemination through NEDD9/Arf6-dependent regulation of MMP14/TIMP2 trafficking, and validate NEDD9 as a clinically relevant therapeutic target to treat metastatic cancer. 
 Tetraspanin proteins regulate membrane type-1 matrix metalloproteinase-dependent pericellular proteolysis. Membrane type-1 matrix metalloproteinase (MT1-MMP) supports tumor   cell invasion   through extracellular matrix barriers containing fibrin, collagen, fibronectin, and other proteins. Here, we show that simultaneous knockdown of two or three members of the tetraspanin family (CD9, CD81, and TSPAN12) markedly decreases MT1-MMP proteolytic functions in cancer cells. Affected functions include fibronectin proteolysis, invasion and growth in three-dimensional fibrin and collagen gels, and MMP-2 activation. Tetraspanin proteins (CD9, CD81, and TSPAN2) selectively coimmunoprecipitate and colocalize with MT1-MMP. Although tetraspanins do not affect the initial biosynthesis of MT1-MMP, they do protect the newly synthesized protein from lysosomal degradation and support its delivery to the cell surface. Interfering with MT1-MMP-tetraspanin collaboration may be a useful therapeutic approach to limit cancer   cell invasion   and metastasis. 
 SNAP23, Syntaxin4, and vesicle-associated membrane protein 7 (VAMP7) mediate trafficking of membrane type 1-matrix metalloproteinase (MT1-MMP) during invadopodium formation and tumor   cell invasion  . Movement through the extracellular matrix (ECM) requires cells to degrade ECM components, primarily through the action of matrix metalloproteinases (MMPs). Membrane type 1-matrix metalloproteinase (MT1-MMP) has an essential role in matrix degradation and   cell invasion   and localizes to subcellular degradative structures termed invadopodia. Trafficking of MT1-MMP to invadopodia is required for the function of these structures, and here we examine the role of N-ethylmaleimide-sensitive factor-activating protein receptor (SNARE)-mediated membrane traffic in the transport of MT1-MMP to invadopodia. During invadopodium formation in MDA-MB-231 human breast cancer cells, increased association of SNAP23, Syntaxin4, and vesicle-associated membrane protein 7 (VAMP7) is detected by coimmunoprecipitation. Blocking the function of these SNAREs perturbs invadopodium-based ECM degradation and   cell invasion  . Increased level of SNAP23-Syntaxin4-VAMP7 interaction correlates with decreased Syntaxin4 phosphorylation. These results reveal an important role for SNARE-regulated trafficking of MT1-MMP to invadopodia during cellular invasion of ECM. 
 Calpain-mediated vimentin cleavage occurs upstream of MT1-MMP membrane translocation to facilitate endothelial sprout initiation. Endothelial cells normally line the vasculature and remain quiescent. However, these cells can be rapidly stimulated to undergo morphogenesis and initiate new blood vessel formation given the proper cues. This study reports a new mechanism for initiating angiogenic sprout formation that involves vimentin, the major intermediate filament protein in endothelial cells. Initial studies confirmed vimentin was required for sphingosine 1-phosphate (S1P)- and growth factor (GF)-induced endothelial   cell invasion  , and vimentin was cleaved by calpains during invasion. Calpains were predominantly activated by GF and were required for sprout initiation. Because others have reported membrane type 1-matrix metalloproteinase (MT1-MMP) is required for endothelial sprouting responses, we tested whether vimentin and calpain acted upstream of MT1-MMP. Both calpain and vimentin were required for successful MT1-MMP membrane translocation, which was stimulated by S1P. In addition, vimentin complexed with MT1-MMP in a manner that required both the cytoplasmic domain of MT1-MMP and calpain activation, which increased the soluble pool of vimentin in endothelial cells. Altogether, these data indicate that pro-angiogenic signals converge to activate calpain-dependent vimentin cleavage and increase vimentin solubility, which act upstream to facilitate MT1-MMP membrane translocation, resulting in successful endothelial sprout formation in three-dimensional collagen matrices. These findings help explain why S1P and GF synergize to stimulate robust sprouting in 3D collagen matrices. 
 Leptin-promoted human extravillous trophoblast invasion is MMP14 dependent and requires the cross talk between Notch1 and PI3K/Akt signaling. The overexpression of leptin is a crucial feature for the maintenance of pregnancy. The effects of leptin on trophoblast invasion are important to its reproductive function, but the underlying mechanisms remain poorly understood. MMP14 is a member of matrix metalloproteinase (MMP) family that is closely involved in the invasion process. Here, we characterized the importance of MMP14 in the proinvasion effect of leptin on EVT cells and elucidated its molecular mechanisms. Transwell assay revealed that leptin promoted invasion of the immortalized EVT cell line HTR-8/SVneo in a dose- and time-related fashion. Further studies suggested that leptin enhanced HTR-8/SVneo   cell invasion   by up-regulating MMP14 expression and that knockdown of MMP14 by small interference RNA (siRNA) blocked the proinvasion effect of leptin. Notably, leptin promoted the expression of Notch1 receptor and activated its signaling in HTR-8/SVneo cells, and blocking this pathway by siRNA inhibited both leptin-enhanced MMP14 expression and invasiveness of HTR-8/SVneo cells. Such effects of Notch1 signaling were related with the activation of the PI3K/Akt pathway, which was significantly activated after leptin stimulation and was interfered by Notch1 signaling perturbation. Taken together, our observations suggest that leptin is an effective regulator of MMP14 expression, which consequently plays critical roles in invasion of EVT cells. The promoting effects of leptin on MMP14 require the cross talk between Notch1 and PI3K/Akt signaling pathways. 
 Collective epithelial   cell invasion   overcomes mechanical barriers of collagenous extracellular matrix by a narrow tube-like geometry and MMP14-dependent local softening. Collective   cell invasion   (CCI) through interstitial collagenous extracellular matrix (ECM) is crucial to the initial stages of branching morphogenesis, and a hallmark of tissue repair and dissemination of certain tumors. The collagenous ECM acts as a mechanical barrier against CCI. However, the physical nature of this barrier and how it is overcome by cells remains incompletely understood. To address these questions, we performed theoretical and experimental analysis of mammary epithelial branching morphogenesis in 3D type I collagen (collagen-I) gels. We found that the mechanical resistance of collagen-I is largely due to its elastic rather than its viscous properties. We also identified two strategies utilized by mammary epithelial cells that can independently minimize ECM mechanical resistance during CCI. First, cells adopt a narrow tube-like geometry during invasion, which minimizes the elastic opposition from the ECM as revealed by theoretical modeling of the most frequent invasive shapes and sizes. Second, the stiffness of the collagenous ECM is reduced at invasive fronts due to its degradation by matrix metalloproteinases (MMPs), as indicated by direct measurements of collagen-I microelasticity by atomic force microscopy. Molecular techniques further specified that the membrane-bound MMP14 mediates degradation of collagen-I at invasive fronts. Thus, our findings reveal that MMP14 is necessary to efficiently reduce the physical restraints imposed by collagen-I during branching morphogenesis, and help our overall understanding of how forces are balanced between cells and their surrounding ECM to maintain collective geometry and mechanical stability during CCI. 
 RABGTPases in MT1-MMP trafficking and   cell invasion  : Physiology versus pathology. The matrix metalloproteinase MT1-MMP is a central regulator of   cell invasion   in both physiological and pathological settings, such as tissue surveillance by immune cells and cancer cell metastasis. MT1-MMP cleaves a plethora of intra- and extracellular proteins, including extracellular matrix proteins, matrix receptors, and also other MMPs, and thus enables modification of both the cell surface proteome and the pericellular environment. Despite its importance for   cell invasion  , the pathways regulating MT1-MMP exposure on the cell surface are largely unknown. Recently, our groups discovered that a specific subset of RABGTPases, most notably RAB5a, is critical for MT1-MMP trafficking in primary human macrophages and carcinoma cells. Here, we discuss and contrast our findings for both cell types, pointing out common features and differences in the RABGTPase-dependent trafficking of MT1-MMP in health and disease. 
 Up-regulation of vascular endothelial growth factor by membrane-type 1 matrix metalloproteinase stimulates human glioma xenograft growth and angiogenesis. Membrane-type (MT) 1 matrix metalloproteinase (MMP) is up-regulated in many tumor types and has been implicated in tumor progression and metastasis. MT1-MMP is critical for pericellular degradation of the extracellular matrix, thereby promoting tumor   cell invasion   and dissemination. To grow efficiently in vivo, tumor cells induce angiogenesis in both primary solid tumors and metastatic foci. The present study describes a functional link between the expression of MT1-MMP and vascular endothelial growth factor (VEGF) production in human glioma U251 xenografts in athymic mice. To investigate the effects of MT1-MMP on VEGF expression, U251 cells were stably transfected with MT1-MMP to generate the U-MT cell line overexpressing the enzyme. In vitro, the U-MT cells had an increased rate of proliferation and migration as well as the ability to activate the MMP-2 proenzyme and directionally remodel a three-dimensional collagen matrix. These findings suggested higher tumorigenicity of U-MT cells relative to the vector-control U-neo cells. In agreement with the in vitro data, U-MT xenografts in BALB/c nu/nu mice displayed markedly increased growth rates and elevated levels of angiogenesis. In contrast, U-neo cells formed small, minimally vascularized tumors. The elevated angiogenesis in U-MT xenografts was associated with an up-regulation of VEGF expression in tumor cells. In addition, U-MT cells in vitro secreted twice as much VEGF as the control cells. GM6001, a hydroxamate inhibitor of MMP activity, down-regulated the production of VEGF in U-MT cells to the levels observed in the U-neo control. Our results demonstrate that the enhanced tumorigenicity of glioma cells overexpressing MT1-MMP involves stimulation of angiogenesis through the up-regulation of VEGF production. 
 A novel protein associated with membrane-type 1 matrix metalloproteinase binds p27(kip1) and regulates RhoA activation, actin remodeling, and matrigel invasion. Pericellular proteolysis by membrane-type 1 matrix metalloproteinase (MT1-MMP) plays a pivotal role in tumor   cell invasion  . Localization of MT1-MMP at the invasion front of cells, e.g. on lamellipodia and invadopodia, has to be regulated in coordination with reorganization of the actin cytoskeleton. However, little is known about how such invasion-related actin structures are regulated at the sites where MT1-MMP localizes. During analysis of MT1-MMP-associated proteins, we identified a heretofore uncharacterized protein. This protein, which we call p27RF-Rho, enhances activation of RhoA by releasing it from inhibition by p27(kip1) and thereby regulates actin structures. p27(kip1) is a well known cell cycle regulator in the nucleus. In contrast, cytoplasmic p27(kip1) has been demonstrated to bind GDP-RhoA and inhibit GDP-GTP exchange mediated by guanine nucleotide exchange factors. p27RF-Rho binds p27(kip1) and prevents p27(kip1) from binding to RhoA, thereby freeing the latter for activation. Knockdown of p27RF-Rho expression renders cells resistant to RhoA activation stimuli, whereas overexpression of p27RF-Rho sensitizes cells to such stimulation. p27RF-Rho exhibits a punctate distribution in invasive human tumor cell lines. Stimulation of the cells with lysophosphatidic acid induces activation of RhoA and induces the formation of punctate actin structures within foci of p27RF-Rho localization. Some of the punctate actin structures co-localize with MT1-MMP and cortactin. Down-regulation of p27RF-Rho prevents both redistribution of actin into the punctate structures and tumor   cell invasion  . Thus, p27RF-Rho is a new potential target for cancer therapy development. 
 Mechanism of metastasis by membrane type 1-matrix metalloproteinase in hepatocellular carcinoma. To investigate the precise role of membrane type 1-matrix metalloproteinase (MT1-MMP) in hepatocellular carcinoma (HCC) metastasis.Human HCC cells Hep3B with overexpression of MT1-MMP were established by stable transfection, and compared with control cells carrying the empty vector. Cells were examined in vivo for their differences in the metastatic ability of athymic nude mice, and analyzed in vitro for their differences in invasion ability by invasion chamber coated with Matrigel, adhesion towards collagen I and migration through culture chamber. Cell proliferation and apoptosis in adherent and suspension status were evaluated by MTT and flow cytometry analysis.We found that overexpression of MT1-MMP could increase intrahepatic metastasis in nude mice with orthotopic implantation of HCC cells (incidence of 100% [MT1-MMP transfectants] vs 40% [vector control transfectants], P<0.05). MT1-MMP could also enhance   cell invasion   through Matrigel (107.7 vs 39.3 cells/field, P<0.001), adhesion towards matrix (0.30 vs 0.12 absorbance unit at 540 nm, P<0.001), cell migration (89.3 vs 39.0 cells/field, P<0.001), and cell proliferation (24.3 vs 40.5 h/doubling, P<0.001). We also observed that MT1-MMP supported cell survival (71.4% vs 23.9%, P<0.001) with reduced apoptosis (43.7% vs 51.0%, P<0.05) in an attachment-free environment.MT1-MMP overexpression could enhance metastasis. In addition to its active role in matrix degradation during tumor invasion, MT1-MMP enhances tumor cell survival upon challenge of detachment, which is important during metastasis when cells enter the circulation. 
 The E-cadherin-repressed hNanos1 gene induces tumor   cell invasion   by upregulating MT1-MMP expression. In this study, we examined the role of the E-cadherin-repressed gene human Nanos1 (hNanos1) in tumor invasion process. First, our in vivo study revealed that hNanos1 mRNAs were overexpressed in invasive lung carcinomas. Moreover, hNanos1 was co-localized with MT1-MMP (membrane type 1-matrix metalloproteinase) in E-cadherin-negative invasive lung tumor clusters. Using an inducible Tet-on system, we showed that induction of hNanos1 expression in DLD1 cells increased their migratory and invasive abilities in a three-dimensional migration and in a modified Boyden chamber assay. Accordingly, we demonstrated that hNanos1 upregulated MT1-MMP expression at the mRNA and protein levels. Inversely, using an RNA interference strategy to inhibit hNanos1 expression in invasive Hs578T, BT549 and BZR cancer cells, we observed a downregulation of MT1-MMP mRNA and protein and concomitantly a decrease of the invasive capacities of tumor cells in a modified Boyden chamber assay. Taken together, our results demonstrate that hNanos1, by regulating MT1-MMP expression, plays an important role in the acquisition of invasive properties by epithelial tumor cells. 
 The Wnt/planar cell polarity protein-tyrosine kinase-7 (PTK7) is a highly efficient proteolytic target of membrane type-1 matrix metalloproteinase: implications in cancer and embryogenesis. PTK7 is an essential component of the Wnt/planar cell polarity (PCP) pathway. We provide evidence that the Wnt/PCP pathway converges with pericellular proteolysis in both normal development and cancer. Here, we demonstrate that membrane type-1 matrix metalloproteinase (MT1-MMP), a key proinvasive proteinase, functions as a principal sheddase of PTK7. MT1-MMP directly cleaves the exposed PKP(621)↓LI sequence of the seventh Ig-like domain of the full-length membrane PTK7 and generates, as a result, an N-terminal, soluble PTK7 fragment (sPTK7). The enforced expression of membrane PTK7 in cancer cells leads to the actin cytoskeleton reorganization and the inhibition o  f cell invasi  on. MT1-MMP silencing and the analysis of the uncleavable L622D PTK7 mutant confirm the significance of MT1-MMP proteolysis of PTK7 in cell functions. Our data also demonstrate that a fine balance between the metalloproteinase activity and PTK7 levels is required for normal development of zebrafish (Danio rerio). Aberration of this balance by the proteinase inhibition or PTK7 silencing results in the PCP-dependent convergent extension defects in the zebrafish. Overall, our data suggest that the MT1-MMP-PTK7 axis plays an important role in both cance  r cell invasi  on and normal embryogenesis in vertebrates. Further insight into these novel mechanisms may promote understanding of directional cell motility and lead to the identification of therapeutics to treat PCP-related developmental disorders and malignancy. 
 Hypoxia promotes HO-8910PM ovarian cancer   cell invasion   via Snail-mediated MT1-MMP upregulation. The molecular mechanisms of ovarian cancer   cell invasion   under hypoxia remain unclear. Here we employed a 3D collagen model and chick chorioallantoic membrane (CAM) invasion assay to explore the influence of hypoxia on ovarian cancer   cell invasion  . Hypoxia (both 1% O2 and CoCl2 150 and 250 µM) induced HO-8910PM ovarian canc  er cell invas  ion in 3D collagen and collagenolysis determined by hydroxyproline. Pretreatment with a hypoxia inducible factor-1α inhibitor, YC-1, or MMP inhibitor, GM6001, significantly inhibited 3D collagen invasion and degradation and cell proliferation. Hypoxia stimulated both mRNA and protein expressions of membrane-type 1 matrix metalloproteinase (MT1-MMP) and promoted MT1-MMP translocation to the cell surface in an YC-1 sensitive manner. MT1-siRNA transfection inhibited hypoxia-induced invasion, proliferation, and collagen degradation of cells in 3D collagen. Hypoxia stimulated Snail mRNA and protein expression as well as translocation to nucleus in an YC-1 sensitive manner. Overexpression of Snail with a recombinant plasmid in HO-8910PM cells resulted in an enhanced invasion in 3D collagen. Transfection with Snail-specific siRNA significantly decreased MT1-MMP expression and 3D collagen invasion. Hypoxia-treated cells significantly broke the upper CAM surface of 11-day-old chick embryos and infiltrated interstitial tissue, completely blocked in the presence of YC-1 or GM6001, or after MT1-MMP siRNA or Snail siRNA transfection. Together, these data suggest that hypoxia promotes HO-8910PM ovarian cancer cell traffic through 3D matrix via Snail-mediated MT1-MMP upregulation, a possible molecular mechanism of ovarian can  cer cell inva  sion under hypoxia. 
 MT-LOOP-dependent localization of membrane type I matrix metalloproteinase (MT1-MMP) to the cell adhesion complexes promotes cancer   cell invasion  . Localization of membrane type I matrix metalloproteinase (MT1-MMP) to the leading edge is thought to be a crucial step during cancer   cell invasion  . However, its mechanisms and functional impact on cellular invasion have not been clearly defined. In this report, we have identified the MT-LOOP, a loop region in the catalytic domain of MT1-MMP ((163)PYAYIREG(170)), as an essential region for MT1-MMP to promote cellular invasion. Deletion of the MT-LOOP effectively inhibited functions of MT1-MMP on the cell surface, including proMMP-2 activation, degradation of gelatin and collagen films, and cellular invasion into a collagen matrix. This is not due to loss of the catalytic function of MT1-MMP but due to inefficient localization of the enzyme to β1-integrin-rich cell adhesion complexes at the plasma membrane. We also found that an antibody that specifically recognizes the MT-LOOP region of MT1-MMP (LOOPAb) inhibited MT1-MMP functions, fully mimicking the phenotype of the MT-LOOP deletion mutant. We therefore propose that the MT-LOOP region is an interface for molecular interactions that mediate enzyme localization to cell adhesion complexes and regulate MT1-MMP functions. Our findings have revealed a novel mechanism regulating MT1-MMP during cellular invasion and have identified the MT-LOOP as a potential exosite target region to develop selective MT1-MMP inhibitors. 
 Polarized MT1-MMP-CD44 interaction and CD44 cleavage during cell retraction reveal an essential role for MT1-MMP in CD44-mediated invasion. The adhesion molecule CD44 and the membrane-type matrix metalloproteinase MT1-MMP act coordinately in tumor cells to promote   cell invasion   through a yet unclear mechanism. We are interested in studying the interplay between CD44 and MT1-MMP in carcinoma cells embedded in HA containing three-dimensional collagen I matrices (3D HA-Col I) by time-lapse confocal microscopy imaging. Here we report the in vivo interaction between CD44 and MT1-MMP, revealed by fluorescence resonance energy transfer (FRET) microscopy. MT1-MMP interacts with CD44 preferentially at the trailing edge of the invading tumor cells during rear retraction and on membrane fragments released during the invasion process. A fluorescent biosensor designed to monitor the proteolytic processing of CD44 by live cell imaging demonstrates that cleavage of the CD44 extracellular domain is enriched in the retracting rear ends of invasive tumor cells. Invasion assays showed that MT1-MMP mediates CD44-dependent tumor-  cell invasion  , whereas CD44 is not essential for MT1-MMP-mediated invasion of 3D HA-Col I matrices. Together, our results support a role for MT1-MMP in cell retraction during CD44-mediated   cell invasion  . 
 miR-150-5p inhibits hepatoma cell migration and invasion by targeting MMP14. Hepatocellular carcinoma (HCC) is one of the leading causes of cancer-related mortality worldwide. Despite progress in diagnostics and treatment of HCC, its prognosis remains poor because the molecular mechanisms underlying hepatocarcinogenesis are not well understood. In the study, we focused on identifying the role of miRNAs in HCC progression. miRNA microarray was used to analyze the differentially expressed miRNAs, and the results were validated by qPCR. We found that the miR-150-5p expression is down-regulated in HCC tissues compared with pair non-tumor tissues. miR-150-5p expression is also decreased in metastatic cancer tissues compared with pair primary tissues, indicating that miR-150-5p may be involved in HCC metastasis. Functionally, miR-150-5p inhibition significantly promotes hepatoma cell migration and invasion, whereas miR-150-5p overexpression suppresses cancer cell migration and invasion in vitro. The matrix metalloproteinase 14 (MMP14) is identified as a new target gene of miR-150-5p. miR-150-5p markedly inhibits MMP14 expression in hepatoma cells, and miR-150-5p expression is negative correlation with MMP14 expression in vivo. More important, re-expression of MMP14 in hepatoma cells partially reverses the effect of miR-150-5p in inhibiting   cell invasion  . 
 A critical role for the membrane-type 1 matrix metalloproteinase in collagen phagocytosis. Degradation of collagen is important for the physiological remodeling of connective tissues during growth and development as well as in wound healing, inflammatory diseases, and cancer   cell invasion  . In remodeling adult tissues, degradation of collagen occurs primarily through a phagocytic pathway. However, although various steps in the phagocytic pathway have been characterized, the enzyme required to initially fragment collagen fibrils for subsequent phagocytosis has not been identified. We have used laser confocal microscopy, transmission electron microscopy, and biochemical assays to show that human fibroblasts initiate degradation of collagen through the collagenase activity of the membrane-bound metalloproteinase MT1-MMP. Degradation of natural and reconstituted collagen substrates correlated with the expression of MT1-MMP, which was localized at sites of collagen cleavage at the surface of the cells and also within the cells, whereas collagen degradation was abrogated when MT1-MMP expression was blocked by small interfering RNA treatment. In contrast to MT1-MMP, the gelatinolytic activity of MMP-2 was not required for collagen phagocytosis. These studies demonstrate a pivotal role of catalytically active MT1-MMP in preparing collagen fibrils for phagocytic degradation. 
 KLF8 and FAK cooperatively enrich the active MMP14 on the cell surface required for the metastatic progression of breast cancer. Krüppel-like factor 8 (KLF8) regulates critical gene transcription associated with cancer. The underlying mechanisms, however, remain largely unidentified. We have recently demonstrated that KLF8 expression enhances the activity but not expression of matrix metalloproteinase-2 (MMP2), the target substrate of MMP14. Here, we report a novel KLF8 to MMP14 signaling that promotes human breast cancer invasion and metastasis. Using cell lines for inducible expression and knockdown of KLF8, we demonstrate that KLF8 promotes MMP14 expression at the transcriptional level. Knocking down KLF8 expression inhibited the breast cancer   cell invasio  n both in vitro and in vivo as well as the lung metastasis in mice, which could be rescued by ectopic expression of MMP14. Promoter reporter assays and oligonucleotide and chromatin immunoprecipitations determined that KLF8 activates the human MMP14 gene promoter by both directly acting on the promoter and indirectly via promoting the nuclear translocation of β-catenin, the expression of T-cell factor-1 (TCF1) and subsequent activation of the promoter by the β-catenin/TCF1 complex. Inhibition of focal adhesion kinase (FAK) using pharmacological inhibitor, RNA interference or knockout showed that the cell surface presentation of active MMP14 downstream of KLF8 depends on FAK expression and activity. Taken together, this work identified novel signaling mechanisms by which KLF8 and FAK work together to promote the extracellular activity of MMP14 critical for breast cancer metastasis. 
 Overexpression of proto-oncogene FBI-1 activates membrane type 1-matrix metalloproteinase in association with adverse outcome in ovarian cancers. FBI-1 (factor that binds to the inducer of short transcripts of human immunodeficiency virus-1) is a member of the POK (POZ and Kruppel) family of transcription factors and play important roles in cellular differentiation and oncogenesis. Recent evidence suggests that FBI-1 is expressed at high levels in a subset of human lymphomas and some epithelial solid tumors. However, the function of FBI-1 in human ovarian cancers remains elusive.In this study, we investigated the role of FBI-1 in human ovarian cancers, in particularly, its function in cancer   cell invasion   via modulating membrane type 1-matrix metalloproteinase (MT1-MMP). Significantly higher FBI-1 protein and mRNA expression levels were demonstrated in ovarian cancers samples and cell lines compared with borderline tumors and benign cystadenomas. Increased FBI-1 mRNA expression was correlated significantly with gene amplification (P = 0.037). Moreover, higher FBI-1 expression was found in metastatic foci (P = 0.036) and malignant ascites (P = 0.021), and was significantly associated with advanced stage (P = 0.012), shorter overall survival (P = 0.032) and disease-free survival (P = 0.016). In vitro, overexpressed FBI-1 significantly enhanced cell migration and invasion both in OVCA 420 and SKOV-3 ovarian carcinoma cells, irrespective of p53 status, accompanied with elevated expression of MT1-MMP, but not MMP-2 or TIMP-2. Moreover, knockdown of MT1-MMP abolished FBI-1-mediated cell migration and invasion. Conversely, stable knockdown of FBI-1 remarkably reduced the motility of these cells with decreased expression of MT1-MMP. Promoter assay and chromatin immunoprecipitation study indicated that FBI-1 could directly interact with the promoter spanning ~600 bp of the 5'-flanking sequence of MT1-MMP and enhanced its expression in a dose-dependent manner. Furthermore, stable knockdown and ectopic expression of FBI-1 decreased and increased cell proliferation respectively in OVCA 420, but not in the p53 null SKOV-3 cells.Our results suggested an important role of FBI-1 in ovarian cancer cell proliferation, cell mobility, and invasiveness, and that FBI-1 can be a potential target of chemotherapy. 
 Tumor   cell invasion   of von Hippel Lindau renal cell carcinoma cells is mediated by membrane type-1 matrix metalloproteinase. Metastatic renal cell carcinoma (RCC) remains the leading cause of mortality in patients with clear cell RCC arising from mutations in the von Hippel Lindau (VHL) tumor suppressor. Successful RCC tumor suppression by VHL requires the negative regulation of hypoxia inducible factor alpha (HIF alpha) protein and its downstream targets. Thus, identification of HIF target genes responsible for RCC tumor progression will aid in the development of therapies for this disease. We previously identified membrane type-1 matrix metalloproteinase (MT1-MMP) as a transcriptional target of HIF-2alpha in RCC cells null for VHL and showed that MT1-MMP is overexpressed in these cells. MT1-MMP is a key regulator of tumor progression through its functions as a matrix-degrading enzyme, as well as its ability to cleave factors, such as adhesion molecules and other MMPs. The aim of this study was to investigate the contribution of MT1-MMP to the invasive potential of RCC cells using in vitro type I collagen degradation and invasion assays.We evaluated RCC cells wild-type (WT8) and null (pRc-9) for VHL for invasive characteristics and showed that the pRc-9 cells demonstrated a greater propensity for both invasion and degradation of a type I collagen matrix. Furthermore, overexpression of either HIF-2alpha or MT1-MMP in the poorly invasive cell line, WT8, promoted collagen degradation and invasion of these cells. Finally, using RNAi, we show that inhibition of MT1-MMP suppresses tumor   cell invasion   of RCC cells.Our results suggest that MT1-MMP is a major mediator of tumor cell invasiveness and type I collagen degradation by VHL RCC cells that express either MT1-MMP or HIF-2alpha. As such, MT1-MMP may represent a novel target for anti-invasion therapy for this disease. 
 Multi-step pericellular proteolysis controls the transition from individual to collective cancer   cell invasion  . Invasive cell migration through tissue barriers requires pericellular remodelling of extracellular matrix (ECM) executed by cell-surface proteases, particularly membrane-type-1 matrix metalloproteinase (MT1-MMP/MMP-14). Using time-resolved multimodal microscopy, we show how invasive HT-1080 fibrosarcoma and MDA-MB-231 breast cancer cells coordinate mechanotransduction and fibrillar collagen remodelling by segregating the anterior force-generating leading edge containing beta1 integrin, MT1-MMP and F-actin from a posterior proteolytic zone executing fibre breakdown. During forward movement, sterically impeding fibres are selectively realigned into microtracks of single-cell calibre. Microtracks become expanded by multiple following cells by means of the large-scale degradation of lateral ECM interfaces, ultimately prompting transition towards collective invasion similar to that in vivo. Both ECM track widening and transition to multicellular invasion are dependent on MT1-MMP-mediated collagenolysis, shown by broad-spectrum protease inhibition and RNA interference. Thus, invasive migration and proteolytic ECM remodelling are interdependent processes that control tissue micropatterning and macropatterning and, consequently, individual and collective cell migration. 
 MT1-MMP- and Cdc42-dependent signaling co-regulate   cell invasion   and tunnel formation in 3D collagen matrices. Complex signaling events control tumor invasion in three-dimensional (3D) extracellular matrices. Recent evidence suggests that cells utilize both matrix metalloproteinase (MMP)-dependent and MMP-independent means to traverse 3D matrices. Herein, we demonstrate that lysophosphatidic-acid-induced HT1080   cell invasion   requires membrane-type-1 (MT1)-MMP-mediated collagenolysis to generate matrix conduits the width of a cellular nucleus. We define these spaces as single-  cell invasion   tunnels (SCITs). Once established, cells can migrate within SCITs in an MMP-independent manner. Endothelial cells, smooth muscle cells and fibroblasts also generate SCITs during invasive events, suggesting that SCIT formation represents a fundamental mechanism of cellular motility within 3D matrices. Coordinated cellular signaling events are required during SCIT formation. MT1-MMP, Cdc42 and its associated downstream effectors such as MRCK (myotonic dystrophy kinase-related Cdc42-binding kinase) and Pak4 (p21 protein-activated kinase 4), protein kinase Calpha and the Rho-associated coiled-coil-containing protein kinases (ROCK-1 and ROCK-2) coordinate signaling necessary for SCIT formation. Finally, we show that MT1-MMP and Cdc42 are fundamental components of a co-associated invasion-signaling complex that controls directed single-  cell invasion   of 3D collagen matrices. 
 Influence of β-catenin small interfering RNA on human osteosarcoma cells. This study examined the effect of small interfering RNA-mediated β-catenin knockdown on the survival, invasion and chemosensitivity of human osteosarcoma cells (U2-OS cells). The siRNA against β-catenin was constructed and transfected into U2-OS cells. The expression of β-catenin was detected by qRT-PCR and Western blotting. Cell growth and apoptosis was detected in the presence or absence of doxorubicin by MTT and flow cytometry, respective  ly. Cell inva  sion ability was measured by transwell assay. The results showed that the transfection of β-catenin siRNA resulted in decreased expression of β-catenin, suppression of invasion and motility of U2-OS cells, reduced chemosensitivity to doxorubicin in vitro, and little change in cell growth and apoptosis. Additionally, down-regulated MT1-MMP expression was found after transfection. It was concluded that knockdown of β-catenin gene may decrease the invasive ability of human osteosarcoma cells through down-regulated MT1-MMP expression, and the chemosensitivity of osteosarcoma cells against doxorubicin. 
 MT1-MMP recognition by ERM proteins and its implication in CD44 shedding. Membrane type 1-matrix metalloproteinase (MT1-MMP) is a key enzyme involved in tumor   cell invasion   by shedding their cell-surface receptor CD44 anchored with F-actin through ezrin/radixin/moesin (ERM) proteins. We found the cytoplasmic tail of MT1-MMP directly binds the FERM domain of radixin, suggesting F-actin-based recruitment of MT1-MMP to CD44 for invasion. Our crystal structure shows that the central region of the MT1-MMP cytoplasmic tail binds subdomain A of the FERM domain, and makes an antiparallel β-β interaction with β2A-strand. This binding mode is distinct from the previously determined binding mode of CD44 to subdomain C. We showed that radixin simultaneously binds both MT1-MMP and CD44, indicating ERM protein-mediated colocalization of MT1-MMP and its substrate CD44 and anchoring to F-actin. Our study implies that ERM proteins contribute toward accelerated CD44 shedding by MT1-MMP through ERM protein-mediated interactions between their cytoplasmic tails. 
 The hepatitis B virus X protein promotes tumor   cell invasion   by inducing membrane-type matrix metalloproteinase-1 and cyclooxygenase-2 expression. Hepatocellular carcinoma is strongly associated with chronic infection by the hepatitis B virus (HBV) and has poor prognosis due to intrahepatic metastasis. HBx is often the only HBV protein detected in hepatic tumor cells; however, its contribution to tumor invasion and metastasis has not been established so far. In this work, we show that HBx enhances tumor   cell invasion  , both in vivo and in vitro. The increased invasive capacity induced by HBx is mediated by an upregulation of membrane-type 1 matrix metalloproteinase (MT1-MMP) expression, which in turn activates matrix metalloproteinase-2. Induction of both MT1-MMP expression and   cell invasion   by HBx is dependent on cyclooxygenase-2 (COX-2) activity. In addition, HBx upregulates the expression of COX-2, which is mediated by the transcriptional activation of the COX-2 gene promoter in a nuclear factor of activated T cell-dependent (NF-AT-dependent) manner. These results demonstrate the ability of HBx to promote tumor   cell invasion   by a mechanism involving the upregulation of MT1-MMP and COX-2 and provide new insights into the mechanism of action of this viral protein and its involvement in tumor metastasis and recurrence of hepatocellular carcinoma. 
 Modulation of the membrane type 1 matrix metalloproteinase cytoplasmic tail enhances tumor   cell invasion   and proliferation in three-dimensional collagen matrices. Increasing evidence suggests that the cytoplasmic tail of membrane type 1 matrix metalloproteinase (MT1-MMP) is subject to phosphorylation and that this modification may influence its enzymatic activity at the cell surface. In this study, phosphorylated MT1-MMP is detected using a phospho-specific antibody recognizing a protein kinase C consensus sequence (phospho-TXR), and a MT1-MMP tail peptide is phosphorylated by exogenous protein kinase C. To characterize the potential role of cytoplasmic residue Thr(567) in these processes, mutants that mimic a state of either constitutive (T567E) or defective phosphorylation (T567A) were expressed and analyzed for their functional effects on MT1-MMP activity and cellular behavior. Phospho-mimetic mutants of Thr(567) exhibit enhanced matrix invasion as well as more extensive growth within a three-dimensional type I collagen matrix. Together, these findings suggest that MT1-MMP surface action is regulated by phosphorylation at cytoplasmic tail residue Thr(567) and that this modification plays a critical role in processes that are linked to tumor progression. 
 3-phosphoinositide-dependent protein kinase-1 (PDK1) promotes invasion and activation of matrix metalloproteinases. Metastasis is a major cause of morbidity and mortality in breast cancer with tumor   cell invasion   playing a crucial role in the metastatic process. PDK1 is a key molecule that couples PI3K to cell proliferation and survival signals in response to growth factor receptor activation, and is oncogenic when expressed in mouse mammary epithelial cells. We now present evidence showing that PDK1-expressing cells exhibit enhanced anchorage-dependent and -independent cell growth and are highly invasive when grown on Matrigel. These properties correlate with induction of MMP-2 activity, increased MT1-MMP expression and a unique gene expression profile.Invasion assays in Matrigel, MMP-2 zymogram analysis, gene microarray analysis and mammary isografts were used to characterize the invasive and proliferative function of cells expressing PDK1. Tissue microarray analysis of human breast cancers was used to measure PDK1 expression in invasive tumors by IHC.Enhanced invasion on Matrigel in PDK1-expressing cells was accompanied by increased MMP-2 activity resulting from stabilization against proteasomal degradation. Increased MMP-2 activity was accompanied by elevated levels of MT1-MMP, which is involved in generating active MMP-2. Gene microarray analysis identified increased expression of the ECM-associated genes decorin and type I procollagen, whose gene products are substrates of MT1-MMP. Mammary fat pad isografts of PDK1-expressing cells produced invasive adenocarcinomas. Tissue microarray analysis of human invasive breast cancer indicated that PDK1pSer241 was strongly expressed in 90% of samples.These results indicate that PDK1 serves as an important effector of mammary epithelial cell growth and invasion in the transformed phenotype. PDK1 mediates its effect in part by MT1-MMP induction, which in turn activates MMP-2 and modulates the ECM proteins decorin and collagen. The presence of increased PDK1 expression in the majority of invasive breast cancers suggests its importance in the metastatic process. 
 Secretion of active membrane type 1 matrix metalloproteinase (MMP-14) into extracellular space in microvesicular exosomes. Membrane type 1 matrix metalloproteinase (MT1-MMP, MMP14) is an efficient extracellular matrix (ECM) degrading enzyme that plays important roles in tissue homeostasis and   cell invasion  . Like a number of type I membrane proteins, MT1-MMP can be internalized from the cell surface through early and recycling endosomes to late endosomes, and recycled to the plasma membrane. Late endosomes participate in the biogenesis of small (30-100 nm) vesicles, exosomes, which redirect plasma membrane proteins for extracellular secretion. We hypothesized that some of the endosomal MT1-MMP could be directed to exosomes for extracellular release. Using cultured human fibrosarcoma (HT-1080) and melanoma (G361) cells we provide evidence that both the full-length 60 kDa and the proteolytically processed 43 kDa forms of MT1-MMP are secreted in exosomes. The isolated exosomes were identified by their vesicular structure in electron microscopy and by exosomal marker proteins CD9 and tumor susceptibility gene (TSG101). Furthermore, exosomes contained beta1-integrin (CD29). The exosomes were able to activate pro-MMP-2 and degrade type 1 collagen and gelatin, suggesting that the exosomal MT1-MMP was functionally active. The targeting of MT1-MMP in exosomes represents a novel mechanism for cancer cells to secrete membrane type metalloproteolytic activity into the extracellular space. 
 Membrane-type 1 matrix metalloproteinase expression is regulated by zonula occludens-1 in human breast cancer cells. The acquisition of a migratory/invasive phenotype by tumor cells is characterized by the loss of cell-cell adhesion contacts and the expression of degradative properties. In this study, we examined the effect of the disorganization of occludin/zonula occludens (ZO)-1 tight junction (TJ) complexes on the expression of membrane-type 1 matrix metalloproteinase (MT1-MMP). We first compared the expression of MT1-MMP and the localization of occludin/ZO-1 complexes in breast tumor cell lines displaying various degrees of invasiveness. We showed that the expression of MT1-MMP in invasive breast tumor cell lines correlates with the absence of occludin and with a cytoplasmic localization of ZO-1. In contrast, noninvasive cell lines displayed a membrane staining for both ZO-1 and occludin and did not express MT1-MMP. In vivo, cytoplasmic ZO-1 and MT1-MMP could be detected in invasive tumor clusters of human breast carcinomas. We then used RNA interference strategy to inhibit ZO-1 expression in invasive BT549 cells and to evaluate the effect of ZO-1 down-regulation on MT1-MMP expression. We observed that ZO-1 small interfering RNA transfection down-regulates MT1-MMP mRNAs and proteins and subsequently decreases the ability of tumor cells to invade a reconstituted basement membrane in a Boyden chamber assay. Inversely, transfection of expression vectors encoding wild-type ZO-1 or the NH2-terminal fragment of ZO-1 comprising the PSD95/DLG/ZO-1 domains in BT549 activated a human MT1-MMP promoter luciferase reporter construct and increased cell invasiveness. Such transfections concomitantly activated the beta-catenin/TCF/LEF pathway. Our results therefore show that ZO-1, besides its structural role in TJ assembly, can intervene in signaling events promoting tumor   cell invasion  . 
 Lipid rafts and caveolin-1 are required for invadopodia formation and extracellular matrix degradation by human breast cancer cells. Invadopodia are ventral membrane protrusions through which invasive cancer cells degrade the extracellular matrix. They are thought to function in the migration of cancer cells through tissue barriers, which is necessary for cancer invasion and metastasis. Although many protein components of invadopodia have been identified, the organization and the role of membrane lipids in invadopodia are not well understood. In this study, the role of lipid rafts, which are cholesterol-enriched membrane microdomains, in the assembly and function of invadopodia in human breast cancer cells was investigated. Lipid rafts are enriched, internalized, and dynamically trafficked at invadopodia sites. Perturbation of lipid raft formation due to depleting or sequestering membrane cholesterol blocked the invadopodia-mediated degradation of the gelatin matrix. Caveolin-1 (Cav-1), a resident protein of lipid rafts and caveolae, accumulates at invadopodia and colocalizes with the internalized lipid raft membranes. Membrane type 1 matrix metalloproteinase (MT1-MMP), a matrix proteinase associated with invadopodia, is localized at lipid raft-enriched membrane fractions and cotrafficked and colocalized with Cav-1 at invadopodia. The small interfering RNA-mediated silencing of Cav-1 inhibited the invadopodia-mediated and MT1-MMP-dependent degradation of the gelatin matrix. Furthermore, Cav-1 and MT1-MMP are coexpressed in invasive human breast cancer cell lines that have an ability to form invadopodia. These results indicate that invadopodia are the sites where enrichment and trafficking of lipid rafts occur and that Cav-1 is an essential regulator of MT1-MMP function and invadopodia-mediated breast cancer   cell invasion  . 
 Membrane-type 1 matrix metalloproteinase cytoplasmic tail-binding protein-1 is a new member of the Cupin superfamily. A possible multifunctional protein acting as an invasion suppressor down-regulated in tumors. Membrane-type 1 matrix metalloproteinase (MT1-MMP/MMP-14) is an enzyme that promotes tumor   cell invasion   in tissues. Although the proteolytic activity of MT1-MMP is indispensable for invasion, it is also regulated by functions of the cytoplasmic tail. In this study we obtained a new human gene whose product binds to the tail sequence in yeast. The product, MTCBP-1, is a 19-kDa protein that belongs to the newly proposed Cupin superfamily composed of proteins with diverse functions. MTCBP-1 expressed in cells formed a complex with MT1-MMP and co-localized at the membrane. It was also detected in both the cytoplasm and nucleus, where MT1-MMP does not exist. In human tumor cell lines MTCBP-1 expression was significantly low compared with non-transformed fibroblasts, and enforced expression of MTCBP-1 inhibited the activity of MT1-MMP in promoting cell migration and invasion. MTCBP-1 showed significant homology to the bacterial aci-reductone dioxygenase, which is an enzyme in methionine metabolism. The C-terminal part of MTCBP-1 is identical to Sip-L, which is reported to be important for human hepatitis C virus replication. Thus, MTCBP-1 may have multiple functions other than the regulation of MT1-MMP, which presumably depends on the subcellular compartment. 
 TOM1L1 drives membrane delivery of MT1-MMP to promote ERBB2-induced breast cancer   cell invasion  . ERBB2 overexpression in human breast cancer leads to invasive carcinoma but the mechanism is not clearly understood. Here we report that TOM1L1 is co-amplified with ERBB2 and defines a subgroup of HER2(+)/ER(+) tumours with early metastatic relapse. TOM1L1 encodes a GAT domain-containing trafficking protein and is a SRC substrate that negatively regulates tyrosine kinase signalling. We demonstrate that TOM1L1 upregulation enhances the invasiveness of ERBB2-transformed cells. This pro-tumoural function does not involve SRC, but implicates membrane-bound membrane-type 1 MMP (MT1-MMP)-dependent activation of invadopodia, membrane protrusions specialized in extracellular matrix degradation. Mechanistically, ERBB2 elicits the indirect phosphorylation of TOM1L1 on Ser321. The phosphorylation event promotes GAT-dependent association of TOM1L1 with the sorting protein TOLLIP and trafficking of the metalloprotease MT1-MMP from endocytic compartments to invadopodia for tumour   cell invasion  . Collectively, these results show that TOM1L1 is an important element of an ERBB2-driven proteolytic invasive programme and that TOM1L1 amplification potentially enhances the metastatic progression of ERBB2-positive breast cancers. 
 Fluid shear stress and sphingosine 1-phosphate activate calpain to promote membrane type 1 matrix metalloproteinase (MT1-MMP) membrane translocation and endothelial invasion into three-dimensional collagen matrices. The vascular endothelium continually senses and responds to biochemical and mechanical stimuli to appropriately initiate angiogenesis. We have shown previously that fluid wall shear stress (WSS) and sphingosine 1-phosphate (S1P) cooperatively initiate the invasion of human umbilical vein endothelial cells into collagen matrices (Kang, H., Bayless, K. J., and Kaunas, R. (2008) Am. J. Physiol. Heart Circ. Physiol. 295, H2087-2097). Here, we investigated the role of calpains in the regulation of endothelial   cell invasion   in response to WSS and S1P. Calpain inhibition significantly decreased S1P- and WSS-induced invasion. Short hairpin RNA-mediated gene silencing demonstrated that calpain 1 and 2 were required for WSS and S1P-induced invasion. Also, S1P synergized with WSS to induce invasion and to activate calpains and promote calpain membrane localization. Calpain inhibition results in a cell morphology consistent with reduced matrix proteolysis. Membrane type 1-matrix metalloproteinase (MT1-MMP) has been shown by others to regulate endothelial   cell invasion  , prompting us to test whether calpain acted upstream of MT1-MMP. S1P and WSS synergistically activated MT1-MMP and induced cell membrane localization of MT1-MMP in a calpain-dependent manner. Calpain activation, MT1-MMP activation and MT1-MMP membrane localization were all maximal with 5.3 dynes/cm(2) WSS and S1P treatment, which correlated with maximal invasion responses. Our data show for the first time that 5.3 dynes/cm(2) WSS in the presence of S1P combine to activate calpains, which direct MT1-MMP membrane localization to initiate endothelial sprouting into three-dimensional collagen matrices. 
 Leptin-mediated regulation of MT1-MMP localization is KIF1B dependent and enhances gastric cancer   cell invasion  . Leptin overexpression is closely correlated with gastric cancer (GC) invasion, but its exact effect and the underlying mechanism in tumorigenesis remain poorly understood. Membrane type 1-matrix metalloproteinase (MT1-MMP), a surface-anchored 'master switch' proteinase, is overexpressed and plays crucial roles in tumor invasion. Here, we characterized the influence of leptin on the generation and surface localization of MT1-MMP in GC and elucidated its molecular mechanisms. Our results revealed that leptin promoted GC   cell invasion   in vitro by upregulating MT1-MMP expression. Furthermore, cell surface biotinylation assay and flow cytometry demonstrated that the surface expression of MT1-MMP was also enhanced by leptin, and knockdown of kinesin family member 1B (KIF1B, a microtubule plus end-directed monomeric motor protein) by small interference RNA inhibited this process. Notably, coimmunoprecipitation analysis indicated that leptin enhanced the interaction of MT1-MMP with KIF1B in a time-dependent manner, which consequently contributed to GC   cell invasion  . Moreover, leptin increased MT1-MMP or KIF1B expression by the protein kinase B (AKT) pathway and extracellular signal-regulated kinase 1/2 partially participated in this process. However, only AKT was implicated in the leptin-mediated membrane localization of MT1-MMP. Immunohistochemistry analysis revealed that leptin, MT1-MMP and KIF1B are overexpressed in GC tissues, and they positively correlated with clinical stage and lymph node metastasis. These observations indicate that this regulatory network exists in vivo. Taken together, our findings suggest that leptin is an effective intracellular stimulator of MT1-MMP and that leptin-enhanced cell surface localization of MT1-MMP is dependent on KIF1B, which consequently plays a critical role in GC invasion. 
 Molecular dissection of the structural machinery underlying the tissue-invasive activity of membrane type-1 matrix metalloproteinase. Membrane type-1 matrix metalloproteinase (MT1-MMP) drives   cell invasion   through three-dimensional (3-D) extracellular matrix (ECM) barriers dominated by type I collagen or fibrin. Based largely on analyses of its impact on cell function under two-dimensional culture conditions, MT1-MMP is categorized as a multifunctional molecule with 1) a structurally distinct, N-terminal catalytic domain; 2) a C-terminal hemopexin domain that regulates substrate recognition as well as conformation; and 3) a type I transmembrane domain whose cytosolic tail controls protease trafficking and signaling cascades. The MT1-MMP domains that subserve cell trafficking through 3-D ECM barriers in vitro or in vivo, however, remain largely undefined. Herein, we demonstrate that collagen-invasive activity is not confined strictly to the catalytic, hemopexin, transmembrane, or cytosolic domain sequences of MT1-MMP. Indeed, even a secreted collagenase supports invasion when tethered to the cell surface in the absence of the MT1-MMP hemopexin, transmembrane, and cytosolic tail domains. By contrast, the ability of MT1-MMP to support fibrin-invasive activity diverges from collagenolytic potential, and alternatively, it requires the specific participation of MT-MMP catalytic and hemopexin domains. Hence, the tissue-invasive properties of MT1-MMP are unexpectedly embedded within distinct, but parsimonious, sequences that serve to tether the requisite matrix-degradative activity to the surface of migrating cells. 
 Conversion of stationary to invasive tumor initiating cells (TICs): role of hypoxia in membrane type 1-matrix metalloproteinase (MT1-MMP) trafficking. Emerging evidence has implicated the role of tumor initiating cells (TICs) in the process of cancer metastasis. The mechanism underlying the conversion of TICs from stationary to invasive remains to be characterized. In this report, we employed less invasive breast cancer TICs, SK-3rd, that displays CD44(high)/CD24(low) with high mammosphere-forming and tumorigenic capacities, to investigate the mechanism by which stationary TICs are converted to invasive TICs. Invasive ability of SK-3rd TICs was markedly enhanced when the cells were cultured under hypoxic conditions. Given the role of membrane type 1-matrix metalloproteinase (MT1-MMP) in cancer invasion/metastasis, we explored a possible involvement of MT1-MMP in hypoxia-induced TIC invasion. Silencing of MT1-MMP by a shRNA approach resulted in diminution of hypoxia-induced   cell invasion   in vitro and metastasis in vivo. Under hypoxic conditions, MT1-MMP redistributed from cytoplasmic storage pools to the cell surface of TICs, which coincides with the increased   cell invasion  . In addition, CD44, a cancer stem-like cell marker, inversely correlated with increased cell surface MT1-MMP. Interestingly, cell surface MT1-MMP gradually disappeared when the hypoxia-treated cells were switched to normoxia, suggesting the plasticity of TICs in response to oxygen content. Furthermore, we dissected the pathways leading to upregulated MT1-MMP in cytoplasmic storage pools under normoxic conditions, by demonstrating a cascade involving Twist1-miR10b-HoxD10 leading to enhanced MT1-MMP expression in SK-3rd TICs. These observations suggest that MT1-MMP is a key molecule capable of executing conversion of stationary TICs to invasive TICs under hypoxic conditions and thereby controlling metastasis. 
 Emerging concepts in the regulation of membrane-type 1 matrix metalloproteinase activity. Pericellular proteolysis mediated by membrane-type 1 matrix metalloproteinase (MT1-MMP) represents an essential component of the cellular machinery involved in the dissolution and penetration of ECM barriers by tumor cells. Although most studies on the proinvasive properties of MT1-MMP have focused on its unusually broad proteolytic activity towards several ECM components and cell surface receptors, recent evidence indicate that the cytoplasmic domain of the enzyme also actively participates in tumor   cell invasion   by regulating the cell surface localization of MT1-MMP as well as the activation of signal transduction cascades. The identification of the molecular events by which the intracellular domain of MT1-MMP links proteolysis of the surrounding matrix by the enzyme to modification of cell function may thus provide important new information on the mechanisms by which this enzyme controls the invasive behavior of neoplastic cells in vivo. 
 The low density lipoprotein receptor-related protein LRP is regulated by membrane type-1 matrix metalloproteinase (MT1-MMP) proteolysis in malignant cells. We demonstrate that the presentation of LRP and the subsequent uptake of its ligands by malignant cells are both strongly regulated by MT1-MMP. Because LRP is essential for the clearance of multiple ligands, these findings have important implications for many pathophysiological processes including the pericellular proteolysis in neoplastic cells as well as the fate of the soluble matrix-degrading proteases such as MMP-2. MT1-MMP is a key protease in   cell invasion   and a physiological activator of MMP-2. Cellular LRP consists of a non-covalently associated 515-kDa extracellular alpha-chain (LRP-515) and an 85-kDa membrane-spanning beta-chain, and plays a dual role as a multifunctional endocytic receptor and a signaling molecule. Through the capture and uptake of several soluble proteases, LRP is involved in the regulation of matrix proteolysis. LRP-515 associates with the MT1-MMP catalytic domain and is highly susceptible to MT1-MMP proteolysis in vitro. Similar to MT1-MMP, the metalloproteinases MT2-MMP, MT3-MMP and MT4-MMP also degrade LRP. The N-terminal and C-terminal parts of the LRP-515 subunit are resistant and susceptible, respectively, to MT1-MMP proteolysis. In cells co-expressing LRP and MT1-MMP, the proteolytically competent protease decreases the levels of cellular LRP and releases its N-terminal portion in the extracellular milieu while the catalytically inert protease co-precipitates with LRP. These events implicate MT1-MMP, not only in the activation of MMP-2, but also in the mechanisms that control the subsequent fate of MMP-2 in cells and tissues. 
 An alternative processing of integrin alpha(v) subunit in tumor cells by membrane type-1 matrix metalloproteinase. Membrane type-1 matrix metalloproteinase (MT1-MMP) and alpha(v)beta(3) integrin are both essential to   cell invasion  . Maturation of integrin pro-alpha(v)chain (pro-alpha(v)) involves its cleavage by proprotein convertases (PC) to form the disulfide-bonded 125-kDa heavy and 25-kDa light alpha chains. Our report presents evidence of an alternative pathway of pro-alpha(v) processing involving MT1-MMP. In breast carcinoma MCF7 cells deficient in MT1-MMP, pro-alpha(v) is processed by a conventional furin-like PC, and the mature alpha(v) integrin subunit is represented by the 125-kDa heavy chain and the 25-kDa light chain commencing from the N-terminal Asp(891). In contrast, in cells co-expressing alpha(v)beta(3) and MT1-MMP, MT1-MMP functions as an integrin convertase. MT1-MMP specifically cleaves pro-alpha(v), generating a 115-kDa heavy chain with the truncated C terminus and a 25-kDa light chain commencing from the N-terminal Leu(892). PC-cleavable alpha(3) and alpha(5) but not the PC-resistant alpha(2) integrin subunit are also susceptible to MT1-MMP cleavage. These novel mechanisms involved in the processing of integrin alpha subunits underscore the significance and complexity of interactions between MT1-MMP and adhesion receptors and suggest that regulation of integrin functionality may be an important role of MT1-MMP in migrating tumor cells. 
 O-glycosylation regulates autolysis of cellular membrane type-1 matrix metalloproteinase (MT1-MMP). MT1-MMP is a key enzyme in cancer   cell invasion   and metastasis. The activity of cellular MT1-MMP is regulated by furin-like proprotein convertases, TIMPs, shedding, autoproteolysis, dimerization, exocytosis, endocytosis, and recycling. Our data demonstrate that, in addition to these already known mechanisms, MT1-MMP is regulated by O-glycosylation of its hinge region. Insignificant autolytic degradation is characteristic for naturally expressed, glycosylated, MT1-MMP. In turn, extensive autolytic degradation, which leads to the inactivation of the protease and the generation of its C-terminal membrane-tethered degraded species, is a feature of overexpressed MT1-MMP. We have determined that incomplete glycosylation stimulates extensive autocatalytic degradation and self-inactivation of MT1-MMP. Self-proteolysis commences during the secretory process of MT1-MMP through the cell compartment to the plasma membrane. The strongly negatively charged sialic acid is the most important functional moiety of the glycopart of MT1-MMP. We hypothesize that sialic acid of the O-glycosylation cassette restricts the access of the catalytic domain to the hinge region and to the autolytic cleavage site and protects MT1-MMP from autolysis. Overall, our results point out that there is a delicate balance between glycosylation and self-proteolysis of MT1-MMP in cancer cells and that when this balance is upset the catalytically potent MT1-MMP pool is self-proteolyzed. 
 Phosphorylation of membrane type 1-matrix metalloproteinase (MT1-MMP) and its vesicle-associated membrane protein 7 (VAMP7)-dependent trafficking facilitate   cell invasion   and migration. In multicellular organisms, uncontrolled movement of cells can contribute to pathological conditions, such as multiple sclerosis and cancer. In highly aggressive tumors, the expression of matrix metalloproteinases (MMPs) is linked to the capacity of tumor cells to invade surrounding tissue and current research indicates that the membrane-anchored membrane type 1-matrix metalloproteinase (MT1-MMP) has a central role in this process. Endocytosis and trafficking of MT1-MMP are essential for its proper function, and here we examine the phosphorylation, internalization, and recycling of this enzyme, and the associated biochemical signaling in HeLa and HT-1080 fibrosarcoma cells. Activation of protein kinase C with phorbol 12-myristate 13-acetate resulted in phosphorylation of endogenous MT1-MMP at Thr(567) in vivo. Mutation of Thr(567) to alanine (to mimic non-phosphorylated MT1-MMP) reduced internalization of MT1-MMP, whereas mutation of Thr(567) to glutamic acid (to mimic phosphorylation) resulted in decreased levels of MT1-MMP on the cell surface. The endosomal trafficking and recycling of MT1-MMP was found to be dependent upon Rab7 and VAMP7, and blocking the function of these proteins reduced cell migration and invasion. Intracellular trafficking of MT1-MMP was observed to be coupled to the trafficking of integrin α5 and phosphorylation of ERK that coincided with this was dependent on phosphorylation of MT1-MMP. Together, these results reveal important roles for MT1-MMP phosphorylation and trafficking in both cell signaling and   cell invasio  n. 
 Inhibition of MT1-MMP activity using functional antibody fragments selected against its hemopexin domain. The membrane associated MMP, MT1-MMP, is a critical pericellular protease involved in tumour   cell invasion   and angiogenesis and is highly up-regulated in numerous human cancers. It therefore represents an exciting new therapeutic cancer-specific target. We have generated recombinant human scFv antibodies against the non-catalytic, hemopexin domain of MT1-MMP that modulate its interactions with collagen. One of these is an effective inhibitor of the invasive capacity of cancer cells and of angiogenesis in model systems. This demonstrates that targeting sites outside the catalytic domain presents a potential novel approach to proteinase inhibition that could have applications in cancer therapeutics. 
 The hemopexin domain of membrane-type matrix metalloproteinase-1 (MT1-MMP) Is not required for its activation of proMMP2 on cell surface but is essential for MT1-MMP-mediated invasion in three-dimensional type I collagen. Membrane-type matrix metalloproteinase-1 (MT1-MMP) plays a key role in tumor invasion and metastasis by degrading the extracellular matrix and activating proMMP2. Here we show that the conserved hemopexin domain is required for MT1-MMP-mediated invasion and growth in three-dimensional type I collagen matrix but not proMMP2 activation. Deletion of the hemopexin domains in MT1-, MT2-, MT3-, MT5-, and MT6-MMP does not impair their abilities to activate proMMP2. In fact, hemopexin-less MT5- and MT6-MMP activate proMMP2 better than their wild type counterparts. On the other hand, hemopexin-less MT1-MMP fails to promote   cell invasion   into type I collagen but retains the capacity to enhance the growth of Madin-Darby canine kidney cells as cysts in three-dimensional collagen matrix. Moreover, the hemopexin domain is also required for MT1-MMP-mediated invasion/scattering of MCF-7 cells in three-dimensional collagen matrix. Because growth and invasion in a three-dimensional model may correlate with tumor invasiveness in vivo, our data suggest that the hemopexin domains of MT-MMPs should be targeted for the development of anti-cancer therapies by employing screening assays developed for three-dimensional models rather than their enzymatic activity toward proMMP2. 
 Involvement of syntaxin 4 in the transport of membrane-type 1 matrix metalloproteinase to the plasma membrane in human gastric epithelial cells. Membrane-type 1 matrix metalloproteinase (MT1-MMP) localized on the plasma membrane plays a central role in various normal biological responses including tissue remodeling, wound heeling, and angiogenesis and in cancer   cell invasion   and metastasis, by functioning as a collagenase and activating other matrix metalloproteinases. In order to elucidate the molecular mechanism of the MT1-MMP targeted localization on the plasma membrane, we examined the participation of syntaxin proteins in MT1-MMP intracellular transport to the plasma membrane in human gastric epithelial AGS cells. Western blotting showed that syntaxin 3 and 4 proteins, which are known to function in intracellular transport towards the plasma membrane, were expressed in AGS cells. Immunocytochemistry revealed that transient transfection of AGS cells with dominant-negative mutant syntaxin 4 decreased plasma membrane MT1-MMP expression. In contrast, transient transfection with either dominant-negative mutant syntaxin 3 or 7 did not affect MT1-MMP localization on the plasma membrane. Cell surface biotinylation assay and Matrigel chamber assay demonstrated that stable transfection with dominant-negative mutant syntaxin 4 decreased the amount of MT1-MMP on the plasma membranes and inhibited the cell invasiveness. We suggest that syntaxin 4 is involved in the intracellular transport of MT1-MMP toward the plasma membrane. 
 MicroRNA-10b induces glioma   cell invasion   by modulating MMP-14 and uPAR expression via HOXD10. MicroRNAs are small endogenous noncoding RNAs, which modulate target gene expression by binding with target mRNA sequences in the 3'untranslated region (UTR) with an imperfect complementarity that inhibits the mRNA translation. Many microRNAs have been reported to function as tumor oncogenes or anti-oncogenes. Recently, more and more microRNAs have been reported to contribute to a tumor's invasive potential. Here, we show that microRNA-10b (miR-10b) was over-expressed in glioma samples and directly associated with the glioma's pathological grade and malignancy. We also found that miR-10b induced glioma   cell invasion   by modulating tumor invasion factors MMP-14 and uPAR expression via the direct target HOXD10. The miR-10b/HOXD10/MMP-14/uPAR signaling pathway might contribute to the invasion of glioma. Accordingly, glioma cells lost their invasive ability when treated with specific antisense oligonucleotides (miR-10b inhibitors), suggesting that miR-10b could be used as a new bio-target to cure glioma. 
 A specific subset of RabGTPases controls cell surface exposure of MT1-MMP, extracellular matrix degradation and three-dimensional invasion of macrophages. The matrix metalloproteinase MT1-MMP has a major impact on invasive cell migration in both physiological and pathological settings such as immune cell extravasation or metastasis of cancer cells. Surface-associated MT1-MMP is able to cleave components of the extracellular matrix, which is a prerequisite for proteolytic invasive migration. However, current knowledge on the molecular mechanisms that regulate MT1-MMP trafficking to and from the cell surface is limited. We have identified three members of the RabGTPase family, Rab5a, Rab8a and Rab14, as crucial regulators of MT1-MMP trafficking and function in primary human macrophages. Both overexpressed and endogenous forms show prominent colocalisation with MT1-MMP-positive vesicles, whereas expression of mutant constructs, as well as siRNA-induced knockdown, reveal that these RabGTPases are crucial in the regulation of MT1-MMP surface exposure, contact of MT1-MMP-positive vesicles with podosomes, extracellular matrix degradation in two and three dimensions, as well as three-dimensional proteolytic invasion of macrophages. Collectively, our results identify Rab5a, Rab8a and Rab14 as major regulators of MT1-MMP trafficking and invasive migration of primary human macrophages, which could be promising potential targets for manipulation of immune   cell invasion  . 
 Relation of MT1-MMP gene expression to outcomes in colorectal cancer. Matrix metalloproteinases are members of a large family of endopeptidases that participate in the extracellular-matrix degradation that accompanies cancer   cell invasion  , metastasis and angiogenesis. The membrane-type 1 matrix metalloproteinase (MT1-MMP) gene has been reported in various cancers and is associated with tumor invasion and metastasis. This study examined the relation of the relative expression of MT1-MMP gene to clinicopathological factors and outcomes in patients with colorectal cancer (CRC).We studied surgical specimens of cancer tissue and adjacent normal mucosa obtained from 202 patients with untreated CRC. The relative expression levels of MT1-MMP mRNA in cancer and in normal adjacent mucosa were measured by quantitative real-time reverse-transcriptase polymerase chain reaction.MT1-MMP gene expression was higher in cancer tissue than in adjacent normal mucosa. The level of MT1-MMP gene expression was not related to any clinicopathological factor. Overall survival at 5 years differed significantly between patients with high MT1-MMP gene expression and those with low expression.Overexpression of the MT1-MMP gene is considered a useful independent predictor of outcomes in patients with CRC. 
 PEA3 is necessary for optimal epidermal growth factor receptor-stimulated matrix metalloproteinase expression and invasion of ovarian tumor cells. Elevated expression of the epidermal growth factor (EGF) receptor (EGFR) is detected in human ovarian tumors and is associated with decreased recurrence-free and overall survival. EGFR activation affects tumor progression in part by promoting tumor invasion through the induction of prometastatic matrix metalloproteinases (MMP). PEA3, an ETS family transcription factor, is elevated in advanced and metastatic ovarian cancer and regulates MMPs in various cell types, therefore, we investigated whether PEA3 is required for the EGFR-dependent induction of MMP mRNA. MMP-9 and MMP-14 mRNA levels were selectively increased in response to EGFR activity in ovarian tumor cells. EGFR activation resulted in nuclear accumulation of PEA3 and direct binding of PEA3, but not the related protein ETS-1, to the endogenous MMP-9 and MMP-14 promoters. Furthermore, PEA3 overexpression was sufficient to induce MMP-9 and MMP-14 mRNA, tumor cell migration, and invasion, suggesting that PEA3 is an important contributor to the metastatic phenotype. Additionally, inhibition of PEA3 expression via short interfering RNA reduced the EGF induction of MMP-9 and MMP-14 gene expression by 92% and 50%, respectively, and impaired EGF-stimulated tumor   cell invasion  . These results suggest that PEA3 is regulated by EGFR and that the elevated PEA3 expression detected in human ovarian cancer may divert cells to a more invasive phenotype by regulating MMP-9 and MMP-14. 
 Matrix Metallopeptidase 14 Plays an Important Role in Regulating Tumorigenic Gene Expression and Invasion Ability of HeLa Cells. The objectives of this study were to investigate the functional effect of matrix metallopeptidase 14 (MMP14) on   cell invasion   in cervical cancer cells (HeLa line) and to study the underlying molecular mechanisms.Expression vector of short hairpin RNA targeting MMP14 was treated in HeLa cells, and then, transfection efficiency was verified by a florescence microscope. Transwell assay was used to investigate   cell invasion   ability in HeLa cells. Quantitative polymerase chain reaction and Western blotting analysis were used to detect the expression of MMP14 and relative factors in messenger RNA and protein levels, respectively.Matrix metallopeptidase 14 short hairpin RNA expression vector transfection obviously decreased MMP14 expression in messenger RNA and protein levels. Down-regulation of MMP14 suppressed invasion ability of HeLa cells and reduced transforming growth factor β1 and vascular endothelial growth factor B expressions. Furthermore, MMP14 knockdown decreased bone sialoprotein and enhanced forkhead box protein L2 expression in both RNA and protein levels.Matrix metallopeptidase 14 plays an important role in regulating invasion of HeLa cells. Matrix metallopeptidase 14 knockdown contributes to attenuating the malignant phenotype of cervical cancer cell. 
 LIM kinase1 modulates function of membrane type matrix metalloproteinase 1: implication in invasion of prostate cancer cells. LIM kinase 1 (LIMK1) is an actin and microtubule cytoskeleton modulatory protein that is overexpressed in a number of cancerous tissues and cells and also promotes invasion and metastasis of prostate and breast cancer cells. Membrane type matrix metalloproteinase 1 (MT1-MMP) is a critical modulator of extracellular matrix (ECM) turnover through pericellular proteolysis and thus plays crucial roles in neoplastic   cell invasion   and metastasis. MT1-MMP and its substrates pro-MMP-2 and pro-MMP-9 are often overexpressed in a variety of cancers including prostate cancer and the expression levels correlate with the grade of malignancy in prostate cancer cells. The purpose of this study is to determine any functional relation between LIMK1 and MT1-MMP and its implication in   cell invasion  .Our results showed that treatment with the hydroxamate inhibitor of MT1-MMP, MMP-2 and MMP-9 ilomastat inhibited LIMK1-induced invasion of benign prostate epithelial cells. Over expression of LIMK1 resulted in increased collagenolytic activity of MMP-2, and secretion of pro-MMP2 and pro-MMP-9. Cells over expressing LIMK1 also exhibited increased expression of MT1-MMP, transcriptional activation and its localization to the plasma membrane. LIMK1 physically associates with MT1-MMP and is colocalized with it to the Golgi vesicles. We also noted increased expression of both MT1-MMP and LIMK1 in prostate tumor tissues.Our results provide new information on regulation of MT1-MMP function by LIMK1 and showed for the first time, involvement of MMPs in LIMK1 induced   cell invasion  . 
  GeneRIFs  
 hypoxia promotes HO-8910PM ovarian cancer cell traffic through 3D matrix via Snail-mediated MT1-MMP upregulation, a possible molecular mechanism of ovarian cancer   cell invasion   under hypoxia 
 Results establish a novel role for NEDD9 as a key regulator of Arf6-dependent trafficking of MMP14 and its invasive properties, highlighting the basic mechanism utilized by NEDD9 to support breast cancer   cell invasion   and metastasis. 
 Results reveal important roles for MT1-MMP phosphorylation and trafficking in both cell signaling and   cell invasion  . 
 miR-10b induced glioma   cell invasion   by modulating tumor invasion factors MMP-14 and uPAR expression via the direct target HOXD10 
 MT1-MMP and Cdc42 co-regulate tumor   cell invasion   in 3D collagen matrices. 
 Cav-1 is an essential regulator of MT1-MMP function and invadopodia-mediated breast cancer   cell invasion   
 These results support a role for MT1-MMP in cell retraction during CD44-mediated   cell invasion  . 
 The E-cadherin-repressed hNanos1 gene induces tumor   cell invasion   by upregulating MT1-MMP expression. 
 Results demonstrate the ability of hepatitis B virus X protein to promote tumor   cell invasion   by a mechanism involving the upregulation of membrane-type 1 matrix metalloproteinase (MT1-MMP) and cyclooxygenase-2 (COX-2). 
   
  
 MMP24 
 
  NCBI Gene  
  GeneRIF  
  Pubmed  
 
 
  Search Term  Rank  Fields  
  [Mm]igration  6  PubMed(3)  
  ECM  7  PubMed(1)  
  [Ee]xtracellular [Mm]atrix  8  PubMed(3); Component(1); Conserved Domains(1); Summary(1)  
  [Ii]nvasive  10  PubMed(3); GeneRIFs(1)  
  [Mm]etastasis  12  PubMed(1); Summary(1)  
 
 
 hide/show details for MMP24 
  [Mm]igration  PubMed  
 Cleavage of metastasis suppressor gene product KiSS-1 protein/metastin by matrix metalloproteinases. A human placenta cDNA library was screened by the expression cloning method for gene products that interact with matrix metalloproteinases (MMPs), and we isolated a cDNA whose product formed a stable complex with pro-MMP-2 and pro-MMP-9. The cDNA encoded the metastasis suppressor gene KiSS-1. KiSS-1 protein was shown to form a complex with pro-MMP. KiSS-1 protein is known to be processed to peptide ligand of a G-protein-coupled receptor (hOT7T175) named metastin, and suppresses metastasis of tumors expressing the receptor. Active MMP-2, MMP-9, MT1-MMP, MT3-MMP and MT5-MMP cleaved the Gly118-Leu119 peptide bond of not only full-length KiSS-1 protein but also metastin decapeptide. Metastin decapeptide induced formation of focal adhesion and actin stress fibers in cells expressing the receptor, and digestion of metastin decapeptide by MMP abolished its ligand activity.   Migration   of HT1080 cells expressing hOT7T175 that harbor a high-level MMP activity was only slightly suppressed by either metastin decapeptide or MMP inhibitor BB-94 alone, but the combination of metastin decapeptide and BB-94 showed a synergistic effect in blocking cell   migration  . We propose that metastin could be used as an antimetastatic agent in combination with MMP inhibitor, or MMP-resistant forms of metastin could be developed and may also be efficacious. 
 Expression of membrane-type 5 matrix metalloproteinase in human endometrium and endometriosis. The metalloproteinases (MMPs) are a family of proteolytic enzymes involved in tissue remodeling and cell   migration  . Endometrial tissue remodeling proceeds during the menstrual cycle and requires a temporary and spatially balanced expression of several different MMPs. Various members of the MMPs also seem to play an important role in the invasion process of endometriosis; however, so far only a limited number of studies have focused on membrane-associated MMPs.The present study investigated the expression of membrane-type 5 metalloproteinase (MT5-MMP) in the human endometrium and endometriotic lesions by microarray hybridization, real-time polymerase chain reaction (PCR) and immunofluorescence.Both the gene chip expression analyses as well as PCR indicated expression of MT5-MMP in normal human endometrium and strongly elevated transcript levels in most peritoneal endometriosis lesions analyzed. Moreover we detected enhanced MT5-MMP expression in the eutopic endometrium from patients suffering from endometriosis, further supporting a role of MT5-MMP in the formation of endometriosis. Immunohistochemical analysis was used to determine the intracellular localization and tissue distribution of MT5-MMP. While the MT5-MMP antigen expression could be clearly attributed to the membrane of epithelial cells, a highly complex differential immunohistochemical staining of MT5-MMP in the various compartments of endometrial tissue was observed. The strongest staining was seen in luminal epithelial cells, whereas endometrial glands frequently showed partial expression of MT5-MMP.Our microarray analysis and real-time PCR of MT5-MMP transcripts may point to an elevated tissue remodeling and cell   migration   in endometrium from endometriosis patients as implied by the function of related MMPs. 
 Mint-3 regulates the retrieval of the internalized membrane-type matrix metalloproteinase, MT5-MMP, to the plasma membrane by binding to its carboxyl end motif EWV. Membrane type matrix metalloproteinases (MT-MMPs) play a critical role in promoting cell growth and   migration   within the extracellular matrix by trafficking to specialized areas. Here we show that the carboxyl EWV motif of MT5-MMP serves as a retrieval signal for internalized MT5-MMP by interacting with Mint-3, a protein with two type III PDZ domains. Deletion of the EWV signal impairs the recycling of MT5-MMP without affecting its internalization, leading to decreased activity on the cell surface. A yeast two-hybrid screening identified Mint-3 as the EWV-binding protein. Mint-3 stimulates MT5-MMP activity when expressed at low levels in an EWV-dependent fashion, but inhibits its activity at higher levels independent of the EWV motif. siRNA-mediated knockdown of endogenous Mint-3 decreased MT5-MMP activity. Furthermore, Mint-3 significantly increased the level of MT5-MMP on the cell surface without affecting its synthesis and internalization. Therefore, Mints may be the adaptor proteins that regulate the trafficking of MT-MMPs. 
  ECM  PubMed  
 Upregulated expression of human membrane type-5 matrix metalloproteinase in kidneys from diabetic patients. Matrix metalloproteinases (MMPs) are a family of proteolytic enzymes that degrade the extracellular matrix (  ECM  ). The membrane-type matrix metalloproteinases (MT-MMPs) are a new family of MMPs that differ from other MMPs in that they have a transmembrane domain that anchors them to the cell surface. MT-MMPs have been shown to function as receptors and activators for other MMPs and to localize extracellular matrix proteolysis at the pericellular region. Here we report on mRNA and protein expression of the fifth human MT-MMP (MT5-MMP), a 64-kDa protein that is capable of converting pro-MMP-2 to its active form, in human kidney as well as its upregulation in diabetes. We also demonstrate upregulation of the active form of MMP-2 in kidney samples from patients with diabetes. Through immunohistochemistry, MT5-MMP expression was localized to the epithelial cells of the proximal and distal tubules, the collecting duct, and the loop of Henle. Furthermore, the tubular epithelial cells that expressed MT5-MMP were associated with tubular atrophy. Because renal tubular atrophy is a significant factor in the pathogenesis of diabetic nephropathy and renal failure and the molecular mechanisms regulating this process remain unknown, it is hypothesized that the elevated expression of MT5-MMP contributes to the activation of pro-MMP-2, which participates in the remodeling of the proximal and distal tubules as well as in the collecting duct. These results provide the first evidence of the expression of a MT-MMP in diabetes and suggest a novel role for MT5-MMP in the pathogenesis of renal tubular atrophy and end-stage renal disease. 
  [Ee]xtracellular [Mm]atrix  PubMed  
 Upregulated expression of human membrane type-5 matrix metalloproteinase in kidneys from diabetic patients. Matrix metalloproteinases (MMPs) are a family of proteolytic enzymes that degrade the   extracellular matrix   (ECM). The membrane-type matrix metalloproteinases (MT-MMPs) are a new family of MMPs that differ from other MMPs in that they have a transmembrane domain that anchors them to the cell surface. MT-MMPs have been shown to function as receptors and activators for other MMPs and to localize   extracellular matrix   proteolysis at the pericellular region. Here we report on mRNA and protein expression of the fifth human MT-MMP (MT5-MMP), a 64-kDa protein that is capable of converting pro-MMP-2 to its active form, in human kidney as well as its upregulation in diabetes. We also demonstrate upregulation of the active form of MMP-2 in kidney samples from patients with diabetes. Through immunohistochemistry, MT5-MMP expression was localized to the epithelial cells of the proximal and distal tubules, the collecting duct, and the loop of Henle. Furthermore, the tubular epithelial cells that expressed MT5-MMP were associated with tubular atrophy. Because renal tubular atrophy is a significant factor in the pathogenesis of diabetic nephropathy and renal failure and the molecular mechanisms regulating this process remain unknown, it is hypothesized that the elevated expression of MT5-MMP contributes to the activation of pro-MMP-2, which participates in the remodeling of the proximal and distal tubules as well as in the collecting duct. These results provide the first evidence of the expression of a MT-MMP in diabetes and suggest a novel role for MT5-MMP in the pathogenesis of renal tubular atrophy and end-stage renal disease. 
 Expression of the matrix metalloproteases 2, 14, 24, and 25 and tissue inhibitor 3 as potential molecular markers in advanced human gastric cancer. During progression of gastric cancer (GC), degradation of the   extracellular matrix   is mediated by the matrix metalloproteases (MMPs) and their tissue inhibitors (TIMPs): changes in the expression of these have been related to unfavorable prognosis in GC.To analyze the expression of certain MMPs and TIMPs in chronic superficial gastritis (SG) and GC.The expression of MMPs and TIMPs was determined using qRT-PCR; the expression was classified, using threshold cycle (C(T)) values, as very high (C(T) ≤ 25), high (C(T) = 26-30), moderate (C(T) = 31-35), low (C(T) = 36-39), or not detected (C(T) = 40). Strength of association was estimated between the proteins, which were detected by Western blot, and the risk of developing GC.We found a high expression of MMP1, MMP2, MMP14, TIMP1, and TIMP3; moderate one of MMP9 and MMP25, and low one of MMP13 and MMP24 in both tissues. In absolute mRNA levels, significant differences were found in expression of MMP2, MMP24, and MMP25, which are overexpressed in GC compared with SG. The presence of the proteins MMP-14 and TIMP-3 was associated with the risk of developing GC.We consider that MMP2, MMP24, and MMP25 and the proteins MMP-14 and TIMP-3 could be candidates for prognostic molecular markers in GC. 
 Mint-3 regulates the retrieval of the internalized membrane-type matrix metalloproteinase, MT5-MMP, to the plasma membrane by binding to its carboxyl end motif EWV. Membrane type matrix metalloproteinases (MT-MMPs) play a critical role in promoting cell growth and migration within the   extracellular matrix   by trafficking to specialized areas. Here we show that the carboxyl EWV motif of MT5-MMP serves as a retrieval signal for internalized MT5-MMP by interacting with Mint-3, a protein with two type III PDZ domains. Deletion of the EWV signal impairs the recycling of MT5-MMP without affecting its internalization, leading to decreased activity on the cell surface. A yeast two-hybrid screening identified Mint-3 as the EWV-binding protein. Mint-3 stimulates MT5-MMP activity when expressed at low levels in an EWV-dependent fashion, but inhibits its activity at higher levels independent of the EWV motif. siRNA-mediated knockdown of endogenous Mint-3 decreased MT5-MMP activity. Furthermore, Mint-3 significantly increased the level of MT5-MMP on the cell surface without affecting its synthesis and internalization. Therefore, Mints may be the adaptor proteins that regulate the trafficking of MT-MMPs. 
  Component  
 proteinaceous   extracellular matrix   
  Conserved Domains  
 cd04278: ZnMc_MMP; Zinc-dependent metalloprotease, matrix metalloproteinase (MMP) sub-family. MMPs are responsible for a great deal of pericellular proteolysis of   extracellular matrix   and cell surface molecules, playing crucial roles in morphogenesis, cell fate ... 
  Summary  
 This gene encodes a member of the peptidase M10 family of matrix metalloproteinases (MMPs). Proteins in this family are involved in the breakdown of   extracellular matrix   in normal physiological processes, such as embryonic development, reproduction, and tissue remodeling, as well as in disease processes, such as arthritis and metastasis. The encoded preproprotein is proteolytically processed to generate the mature protease. Unlike most MMPs, which are secreted, this protease is a member of the membrane-type MMP (MT-MMP) subfamily, contains a transmembrane domain and is expressed at the cell surface. Substrates of this protease include the proteins cadherin 2 and matrix metallopeptidase 2 (also known as 72 kDa type IV collagenase). [provided by RefSeq, Feb 2016] 
  [Ii]nvasive  PubMed  
 mRNA expression of the five membrane-type matrix metalloproteinases MT1-MT5 in human prostatic cell lines and their down-regulation in human malignant prostatic tissue. The aim of this study was to assess the expression of membrane-type matrix metalloproteinases (MT-MMPs) 1-5 in the human prostatic cell lines BPH-1, LNCaP, DU 145, PC-3, in malignant and non-malignant prostatic tissue samples, and in epithelial cells cultured from these tissue samples.Matched malignant and non-malignant tissue specimens were obtained from 12 men with untreated prostate carcinoma after radical prostatectomy. Expression of mRNA for the five MT-MMPs was quantified by real-time PCR technique and normalized to the expression of the housekeeping gene glyceraldehyde-3-phosphate dehydrogenase (GAPDH).The expression of the five MT-MMPs was distinctly different not only between the prostate cell lines but also varied in the same cell line. There was a general higher expression of all MT-MMPs except for MT3-MMP in the androgen-insensitive cells DU 145 and PC-3 compared with that in the androgen-sensitive LNCaP cells. Their relatively high expression in the benign prostatic cell line BPH-1 and also in the primary cell cultures from malignant and non-malignant tissue samples argues against a simple association between MT-MMP expression and   invasive  ness. In malignant tissue samples and their corresponding cell cultures, the expression of most MT-MMPs was down-regulated in comparison to the normal counterparts. There was no correlation between tumor classification data and the MT-MMP expression results.In contrast to other carcinoma, the down-regulation of most MT-MMPs is typical for prostate carcinoma. It seems to occur mainly in epithelial cells and has to be examined as special characteristic of this tumor entity in further studies. 
 mRNA expression of the five membrane-type matrix metalloproteinases MT1-MT5 in human prostatic cell lines and their down-regulation in human malignant prostatic tissue. The aim of this study was to assess the expression of membrane-type matrix metalloproteinases (MT-MMPs) 1-5 in the human prostatic cell lines BPH-1, LNCaP, DU 145, PC-3, in malignant and non-malignant prostatic tissue samples, and in epithelial cells cultured from these tissue samples.Matched malignant and non-malignant tissue specimens were obtained from 12 men with untreated prostate carcinoma after radical prostatectomy. Expression of mRNA for the five MT-MMPs was quantified by real-time PCR technique and normalized to the expression of the housekeeping gene glyceraldehyde-3-phosphate dehydrogenase (GAPDH).The expression of the five MT-MMPs was distinctly different not only between the prostate cell lines but also varied in the same cell line. There was a general higher expression of all MT-MMPs except for MT3-MMP in the androgen-insensitive cells DU 145 and PC-3 compared with that in the androgen-sensitive LNCaP cells. Their relatively high expression in the benign prostatic cell line BPH-1 and also in the primary cell cultures from malignant and non-malignant tissue samples argues against a simple association between MT-MMP expression and   invasive  ness. In malignant tissue samples and their corresponding cell cultures, the expression of most MT-MMPs was down-regulated in comparison to the normal counterparts. There was no correlation between tumor classification data and the MT-MMP expression results.In contrast to other carcinoma, the down-regulation of most MT-MMPs is typical for prostate carcinoma. It seems to occur mainly in epithelial cells and has to be examined as special characteristic of this tumor entity in further studies. 
 [Inhibitory effects of RNA interference on MMP-24 expression and   invasive  ness of ovarian cancer SKOV(3) cells]. To investigate the inhibitory effect of RNA interference (RNAi) on MMP-24 expression and   invasive  ness of ovarian cancer SKOV(3) cells.Two pairs of small interfering RNA (siRNA) specific to MMP-24 mRNA were designed and transfected into SKOV(3) cells. RT-PCR and Western blotting were used to detect the mRNA and protein expressions of MMP-24, and the cell   invasive  ness was assessed using an in vitro invasion test.After transfection with siRNA, the mRNA and protein expression levels of MMP-24 were obviously reduced in SKOV(3) cells, which also showed significantly decreased   invasive  ness in vitro.MMP-24 gene silencing by RNAi can suppress the   invasive  ness of ovarian cancer SKOV(3) cells in vitro, which may provide a new therapeutic approach of ovarian cancer. 
  GeneRIFs  
 MMP-24 gene silencing by RNAi can suppress the   invasive  ness of ovarian cancer SKOV(3) cells in vitro. 
  [Mm]etastasis  PubMed  
 Cleavage of   metastasis   suppressor gene product KiSS-1 protein/metastin by matrix metalloproteinases. A human placenta cDNA library was screened by the expression cloning method for gene products that interact with matrix metalloproteinases (MMPs), and we isolated a cDNA whose product formed a stable complex with pro-MMP-2 and pro-MMP-9. The cDNA encoded the   metastasis   suppressor gene KiSS-1. KiSS-1 protein was shown to form a complex with pro-MMP. KiSS-1 protein is known to be processed to peptide ligand of a G-protein-coupled receptor (hOT7T175) named metastin, and suppresses   metastasis   of tumors expressing the receptor. Active MMP-2, MMP-9, MT1-MMP, MT3-MMP and MT5-MMP cleaved the Gly118-Leu119 peptide bond of not only full-length KiSS-1 protein but also metastin decapeptide. Metastin decapeptide induced formation of focal adhesion and actin stress fibers in cells expressing the receptor, and digestion of metastin decapeptide by MMP abolished its ligand activity. Migration of HT1080 cells expressing hOT7T175 that harbor a high-level MMP activity was only slightly suppressed by either metastin decapeptide or MMP inhibitor BB-94 alone, but the combination of metastin decapeptide and BB-94 showed a synergistic effect in blocking cell migration. We propose that metastin could be used as an antimetastatic agent in combination with MMP inhibitor, or MMP-resistant forms of metastin could be developed and may also be efficacious. 
  Summary  
 This gene encodes a member of the peptidase M10 family of matrix metalloproteinases (MMPs). Proteins in this family are involved in the breakdown of extracellular matrix in normal physiological processes, such as embryonic development, reproduction, and tissue remodeling, as well as in disease processes, such as arthritis and   metastasis  . The encoded preproprotein is proteolytically processed to generate the mature protease. Unlike most MMPs, which are secreted, this protease is a member of the membrane-type MMP (MT-MMP) subfamily, contains a transmembrane domain and is expressed at the cell surface. Substrates of this protease include the proteins cadherin 2 and matrix metallopeptidase 2 (also known as 72 kDa type IV collagenase). [provided by RefSeq, Feb 2016] 
   
  
 RELA 
 
  NCBI Gene  
  GeneRIF  
  Pubmed  
 
 
  Search Term  Rank  Fields  
  [Mm]igration  6  PubMed(55); GeneRIFs(10)  
  ECM  7  PubMed(2)  
  [Ee]xtracellular [Mm]atrix  8  PubMed(16)  
  [Ii]nvasive  10  PubMed(29); GeneRIFs(4)  
  [Mm]etastasize  11  PubMed(2); GeneRIFs(1)  
  [Mm]etastasis  12  PubMed(62); GeneRIFs(10)  
  [Cc]ell [Ii]nvasion  13  PubMed(13); GeneRIFs(3)  
 
 
 hide/show details for RELA 
  [Mm]igration  PubMed  
 CARMA3 regulates the invasion,   migration  , and apoptosis of non-small cell lung cancer cells by activating NF-кB and suppressing the P38 MAPK signaling pathway. In our previous study, CARMA3 overexpression in lung cancer cells promoted cell proliferation and invasion; however, the mechanism underlying the role of CARMA3 in cancer cell invasion remained unclear. In the present study, knockdown of CARMA3 in A549 and H1299 cells suppressed cell invasion and   migratio  n, and downregulated matrix metalloprotease 9 expression at the protein and mRNA levels, as shown by Western blotting and real-time PCR. CARMA3 knockdown increased cell apoptosis, as shown by flow cytometry, increased the mRNA and protein expression levels of Bax and Caspase3, and downregulated Bcl-2 in A549 and H1299 cells. Phosphorylated P38 levels increased and NF-кB activation decreased following knockdown of CARMA3. SB203580, a P38 MAPK inhibitor, activated NF-кB, increased ce  ll migrat  ion, and inhibited cell apoptosis after knockdown of CARMA3 compared to knockdown of CARMA3 without SB203580. These findings indicate that CARMA3 may suppress the activation of the P38 MAPK signaling pathway to regulate invasio  n, migrat  ion and apoptosis of lung cancer cells by activating NF-кB (P65) in the nucleus. 
 microRNA-130a Promotes Human Keratinocyte Viability and   Migration   and Inhibits Apoptosis Through Direct Regulation of STK40-Mediated NF-κB Pathway and Indirect Regulation of SOX9-Meditated JNK/MAPK Pathway: A Potential Role in Psoriasis. Psoriasis is a chronic inflammatory skin disorder. The aim of this study was to determine a potential role of microRNA (miR)-130a in psoriasis, and underlying mechanism. Expression levels of miR-130a in psoriasis specimens and normal skin tissues were analyzed. MiR-130a mimic, inhibitor, miR-control, small interfering RNA (siRNA) specific serine/threonine kinase 40 (STK40), or sex-determining region Y chromosome-box 9 (SOX9) were transfected to human keratinocyte HaCaT cells, respectively. After transfection, the cell viability, apoptosis, and   migratio  n were determined. Luciferase reporter assay, quantitative reverse transcription-polymerase chain reaction, and western blot were performed to explore whether STK40 was a target of miR-130a. The effects of aberrant expressions of miR-130a, STK40, or SOX9 on key proteins of NF-κB and c-Jun N-terminal kinase (JNK)/mitogen-activated protein kinase (MAPK) pathway were assessed. The miR-130a levels were significantly higher in patients with psoriasis compared to the healthy controls (p < 0.01). Overexpressing miR-130a strikingly promoted HaCaT cell viabilit  y and mig  ration and inhibited apoptosis (p < 0.01 or p < 0.05). We confirmed that STK40 was a direct target of miR-130a, and STK40 was involved in miR-130a-induced cell functions. Overexpressing miR-130a significantly upregulated NF-κB p65, SOX9, p-c-Jun, p-JNK, and p-p38MAPK proteins and silencing miR-130a downregulated them. In addition, silencing STK40 alleviated the effects of anti-miR-130a on SOX9 expression. Furthermore, silencing SOX9 also decreased levels of p-c-Jun, p-JNK, and p-p38MAPK proteins. MiR-130a regulates human keratinocyte Ha  CaT viabi  lity, migration and apoptosis might be by direct regulation of STK40-mediated NF-κB pathway and by indirect regulation of SOX9-mediated downstream JNK/MAPK signaling pathway. 
 microRNA-130a Promotes Human Keratinocyte Viability and   Migration   and Inhibits Apoptosis Through Direct Regulation of STK40-Mediated NF-κB Pathway and Indirect Regulation of SOX9-Meditated JNK/MAPK Pathway: A Potential Role in Psoriasis. Psoriasis is a chronic inflammatory skin disorder. The aim of this study was to determine a potential role of microRNA (miR)-130a in psoriasis, and underlying mechanism. Expression levels of miR-130a in psoriasis specimens and normal skin tissues were analyzed. MiR-130a mimic, inhibitor, miR-control, small interfering RNA (siRNA) specific serine/threonine kinase 40 (STK40), or sex-determining region Y chromosome-box 9 (SOX9) were transfected to human keratinocyte HaCaT cells, respectively. After transfection, the cell viability, apoptosis, and   migratio  n were determined. Luciferase reporter assay, quantitative reverse transcription-polymerase chain reaction, and western blot were performed to explore whether STK40 was a target of miR-130a. The effects of aberrant expressions of miR-130a, STK40, or SOX9 on key proteins of NF-κB and c-Jun N-terminal kinase (JNK)/mitogen-activated protein kinase (MAPK) pathway were assessed. The miR-130a levels were significantly higher in patients with psoriasis compared to the healthy controls (p < 0.01). Overexpressing miR-130a strikingly promoted HaCaT cell viabilit  y and mig  ration and inhibited apoptosis (p < 0.01 or p < 0.05). We confirmed that STK40 was a direct target of miR-130a, and STK40 was involved in miR-130a-induced cell functions. Overexpressing miR-130a significantly upregulated NF-κB p65, SOX9, p-c-Jun, p-JNK, and p-p38MAPK proteins and silencing miR-130a downregulated them. In addition, silencing STK40 alleviated the effects of anti-miR-130a on SOX9 expression. Furthermore, silencing SOX9 also decreased levels of p-c-Jun, p-JNK, and p-p38MAPK proteins. MiR-130a regulates human keratinocyte Ha  CaT viabi  lity, migration and apoptosis might be by direct regulation of STK40-mediated NF-κB pathway and by indirect regulation of SOX9-mediated downstream JNK/MAPK signaling pathway. 
 CARMA3 regulates the invasion,   migration  , and apoptosis of non-small cell lung cancer cells by activating NF-кB and suppressing the P38 MAPK signaling pathway. In our previous study, CARMA3 overexpression in lung cancer cells promoted cell proliferation and invasion; however, the mechanism underlying the role of CARMA3 in cancer cell invasion remained unclear. In the present study, knockdown of CARMA3 in A549 and H1299 cells suppressed cell invasion and   migratio  n, and downregulated matrix metalloprotease 9 expression at the protein and mRNA levels, as shown by Western blotting and real-time PCR. CARMA3 knockdown increased cell apoptosis, as shown by flow cytometry, increased the mRNA and protein expression levels of Bax and Caspase3, and downregulated Bcl-2 in A549 and H1299 cells. Phosphorylated P38 levels increased and NF-кB activation decreased following knockdown of CARMA3. SB203580, a P38 MAPK inhibitor, activated NF-кB, increased ce  ll migrat  ion, and inhibited cell apoptosis after knockdown of CARMA3 compared to knockdown of CARMA3 without SB203580. These findings indicate that CARMA3 may suppress the activation of the P38 MAPK signaling pathway to regulate invasio  n, migrat  ion and apoptosis of lung cancer cells by activating NF-кB (P65) in the nucleus. 
 TGF-beta downregulates PTEN via activation of NF-kappaB in pancreatic cancer cells. TGF-beta utilizes receptor-activated SMAD signaling to mediate growth suppression; however, non-SMAD signaling that modulates the TGF-beta response in epithelial cells become apparent when the SMAD signaling is abrogated, a common occurrence in pancreatic cancers. Here, we examined whether TGF-beta utilized NF-kappaB to downregulate PTEN, a gene that is rarely mutated in pancreatic cancers. SMAD4-null BxPc3 and CAPAN-1 pancreatic cancer cells were treated with TGF-beta (10 ng/ml) and lysed, and cellular proteins were analyzed by Western blots using p-IkappaB, p65, and PTEN antibodies. PTEN promoter and NF-kappaB activities were assessed by PTEN-luc and p-NF-luc constructs, respectively. Dominant negative p-IkappaB-alpha-M (NF-kappaB superrepressor) was used to block activation of NF-kappaB. Cell motility was assessed by Boyden chamber   migration   assay. TGF-beta induced IkappaB-alpha phosphorylation followed by NF-kappaB p65 subunit nuclear translocation and increased NF-kappaB activity. IkappaB-alpha-M blocked TGF-beta-induced NF-kappaB activity, reversed downregulated PTEN promoter activity and PTEN expression, and prevented augmentation of cell motility induced by TGF-beta. SMAD4 restoration, but not knockdown of SMAD2 and/or 3, reversed TGF-beta-induced NF-kappaB activity. Thus TGF-beta suppresses PTEN in pancreatic cancer cells through NF-kappaB activation and enhances cell motility and invasiveness in a SMAD4-independent manner that can be counteracted when TGF-beta-SMAD signaling is restored. The TGF-beta/NF-kappaB/PTEN cascade may be a critical pathway for pancreatic cancer cells to proliferate and metastasize. 
 Stabilization of snail by NF-kappaB is required for inflammation-induced cell   migration   and invasion. The increased motility and invasiveness of tumor cells are reminiscent of epithelial-mesenchymal transition (EMT), which occurs during embryonic development, wound healing, and metastasis. In this study, we found that Snail is stabilized by the inflammatory cytokine TNFalpha through the activation of the NF-kappaB pathway. We demonstrated that NF-kappaB is required for the induction of COP9 signalosome 2 (CSN2), which, in turn, blocks the ubiquitination and degradation of Snail. Furthermore, we showed that the expression of Snail correlated with the activation of NF-kappaB in cancer cell lines and metastatic tumor samples. Knockdown of Snail expression inhibited cell   migration   and invasion induced by inflammatory cytokines and suppressed inflammation-mediated breast cancer metastasis. Our study provides a plausible mechanism for inflammation-induced metastasis. 
 CHFR, a potential tumor suppressor, downregulates interleukin-8 through the inhibition of NF-kappaB. The mitotic checkpoint gene CHFR (checkpoint with forkhead and ring finger domains) is silenced in various human cancers by promoter hypermethylation, suggesting that CHFR is a tumor suppressor. Here, we show that CHFR functions as a negative regulator of the nuclear factor-kappaB (NF-kappaB) pathway. Expression of CHFR inhibited NF-kappaB reporter activity, whereas knockdown of CHFR activated reporter activity. These activities are independent of its RING finger domain. Furthermore, we found that CHFR physically interacts with p65 in cells. Electrophoretic mobility shift assays (EMSAs) and ELISA-based NF-kappaB-binding assays showed that CHFR negatively regulated transcriptional activity of p65. In addition, our data show that interleukin (IL)-8 is significantly downregulated by CHFR, and that the   migration   of human endothelial cells is suppressed in culture medium conditioned from CHFR-expressing cancer cells. Using a xenograft model, we show that neovascularization is suppressed by adenovirus-mediated transfer of CHFR. These results indicate that expression of CHFR markedly reduces the expression of IL-8 through the inhibition of NF-kappaB. As the NF-kappaB signaling pathway plays a critical role in the development and progression of cancer, our findings show the functional relationship between epigenetic alteration and inflammation/angiogenesis in human cancer cells, thereby showing several potential targets for therapeutic intervention. 
 Silencing of Transient Receptor Potential Channel 4 Alleviates oxLDL-induced Angiogenesis in Human Coronary Artery Endothelial Cells by Inhibition of VEGF and NF-κB. Transient receptor potential channel 4 (TRPC4) plays central roles in endothelial cell function. The aim of this study was to investigate the silencing effects of TRPC4 on oxidized low-density lipoprotein (oxLDL)-induced angiogenesis in human coronary artery endothelial cells (HCAECs), as well as the underlying molecular mechanism involved in this process.HCAECs were transfected with small interfering RNA (siRNA) targeting TRPC4 (TRPC4-siRNA) or with a negative control (NC)-siRNA. The expression of TRPC4 was confirmed by real-time polymerase chain reaction (RT-PCR) and Western blotting. After the siRNA transfection, oxLDL was added to the medium. Cell proliferation,   migratio  n, and in vitro angiogenesis were determined by bromodeoxyuridine (BrdU) enzyme-linked immunosorbent assay (ELISA), Transwell assay and scratch-wound assay, respectively, and tube formation on Matrigel. Expression of vascular endothelial growth factor (VEGF) and nuclear factor (NF)-κB p65 were assessed by Western blotting.Both the mRNA and protein levels of TRPC4 were significantly reduced by transfection with TRPC4-siRNA compared to the control group or NC-siRNA group (P<0.05). Silencing of TRPC4 significantly decreased the cell proliferation  , migrati  on, and tube formation (all P<0.05). Furthermore, the expression levels of VEGF and NF-κB p65 were markedly lowered by silencing of TRPC4 in HCAECs.These results suggest that silencing of TRPC4 alleviates angiogenesis induced by oxLDL in HCAECs through inactivation of VEGF and NF-κB. Suppression of TRPC4 might be an alternative therapeutic strategy for atherosclerotic neovascularization. 
 ΔNp63 versatilely regulates a Broad NF-κB gene program and promotes squamous epithelial proliferation  , migrati  on, and inflammation. Head and neck squamous cell carcinoma (HNSCC) and many other epithelial malignancies exhibit increased proliferation, invasion, and inflammation, concomitant with aberrant nuclear activation of TP53 and NF-κB family members ΔNp63, cRel, and RelA. However, the mechanisms of cross-talk by which these transcription factors coordinate gene expression and the malignant phenotype remain elusive. In this study, we showed that ΔNp63 regulates a cohort of genes involved in cell growth, survival, adhesion, and inflammation, which substantially overlaps with the NF-κB transcriptome. ΔNp63 with cRel and/or RelA are recruited to form novel binding complexes on p63 or NF-κB/Rel sites of multitarget gene promoters. Overexpressed ΔNp63- or TNF-α-induced NF-κB and inflammatory cytokine interleukin-8 (IL-8) reporter activation depended on RelA/cRel regulatory binding sites. Depletion of RelA or ΔNp63 by small interfering RNA (siRNA) significantly inhibited NF-κB-specific, or TNF-α-induced IL-8 reporter activation. ΔNp63 siRNA significantly inhibited proliferation,   survival  , and migration by HNSCC cells in vitro. Consistent with these data, an increase in nuclear ΔNp63, accompanied by increased proliferation (Ki-67) and adhesion (β4 integrin) markers, and induced inflammatory cell infiltration was observed throughout HNSCC specimens, when compared with the basilar pattern of protein expression and minimal inflammation seen in nonmalignant mucosa. Furthermore, overexpression of ΔNp63α in squamous epithelial cells in transgenic mice leads to increased suprabasilar cRel, Ki-67, and cytokine expression, together with epidermal hyperplasia and diffuse inflammation, similar to HNSCC. Our study reveals ΔNp63 as a master transcription factor that, in coordination with NF-κB/Rels, orchestrates a broad gene program promoting epidermal hyperplasia, inflammation, and the malignant phenotype of HNSCC. 
 Down-modulation of Bis reduces the invasive ability of glioma cells induced by TPA, through NF-κB mediated activation of MMP-9. Bcl-2 interacting cell death suppressor (Bis) has been shown to have anti-apoptotic and anti-stress functions. Recently, increased Bis expression was reported to correlate with glioma aggressiveness. Here, we investigated the effect of Bis knockdown on the acquisition of the invasive phenotype of A172 glioma cells, induced by 12-O-Tetradecanoylphorbol-3-acetate (TPA), using a Transwell assay. Bis knockdown resulted in a significant decrease in the   migratio  n and invasion of A172 cells. Furthermore, Bis knockdown notably decreased TPA-induced matrix metalloproteinase-9 (MMP-9) activity and mRNA expression, as measured by zymography and quantitative real time PCR, respectively. A luciferase reporter assay indicated that Bis suppression significantly down-regulated NF-κB-driven transcription. Finally, we demonstrated that the rapid phosphorylation and subsequent degradation of IκB-α induced by TPA was remarkably delayed by Bis knockdown. These results suggest that Bis regulates the invasive ability of glioma cells elicited by TPA, by modulating NF-κB activation, and subsequent induction of MMP-9 mRNA. 
 Foxp3 downregulation in NSCLC mediates epithelial-mesenchymal transition via NF-κB signaling. Forkhead box P3 (Foxp3) is a member of forkhead box transcription factor family and it was identified as a tumor suppressor in various solid tumors. This study evaluated the expression of Foxp3 in non-small cell lung cancer (NSCLC) and investigated its role in epithelial‑mesenchymal transition (EMT) of cancer cells. qRT-PCR and western blot analysis were used for examining the expression of Foxp3 in NSCLC tissues and the non-tumor tissues. A tissue microarray was constructed and scored for evaluating the clinical significance of Foxp3 expression in NSCLC tissues. RNAi was employed for downregulating Foxp3 expression and cell proliferation was done with MTT assay. Transwell with or without basement membrane matrix was used for   cell migr  ation and invasion assay respectively. Foxp3 was found downregulated in NSCLC tissues compared with non-tumoral tissues; downregulation of Foxp3 predicted adverse tumor stage and overall survival; silencing of FOXP3 promoted the proliferat  ion, migr  ation and invasion ability of NSCLC cells and influenced the expression level of EMT-associated proteins. However, forced expression of Foxp3 could reverse this effect. Moreover, Foxp3 could interact with LMO2 and affect the expression level of TAL1, which was in accordance with the findings in T-cell acute lymphoblastic leukemia. By screening the signalling pathways, we observed an obvious upregulation of phosphorylated NF-κB in A549 and H520 cells after silencing of FOXP3. Our results suggest that Foxp3 suppressed NSCLC cell metastasis, at least partially, via NF-κB signaling. 
 Negative cross-talk between RelA and the glucocorticoid receptor: a possible mechanism for the antiinflammatory action of glucocorticoids. Glucocorticoids are efficient antiinflammatory agents, and their effects include transcriptional repression of several cytokines and adhesion molecules. Whereas glucocorticoids down-regulate the expression of genes relevant during inflammation, nuclear factor (NF)-kappa B/Rel proteins function as important positive regulators of these genes. The expression of intercellular adhesion molecule-1 (ICAM-1), which plays an essential role in recruitment and   migration   of leukocytes to sites of inflammation, is also down-regulated by glucocorticoids. We found that a functional NF-kappa B site in the ICAM-1 promoter, which can be activated by either 12-O-tetradecanoylphorbol-13-acetate or tumor necrosis factor-alpha (TNF alpha), is also the target for glucocorticoids. In this report we present evidence that the ligand-activated glucocorticoid receptor (GR) is able to repress RelA-mediated activation of the ICAM-1 NF-kappa B site. Conversely, transcriptional activation by GR via a glucocorticoid response element is specifically repressed by RelA, but not by other NF-kappa B/Rel family members. Mutational analysis of GR demonstrates that the DNA binding domain and the ligand binding domain are required for the functional repression of NF-kappa B activation. Despite the importance of the DNA binding domain, we found that the transcriptional repression of NF-kappa B, mediated by GR, is not caused by binding of GR to the ICAM-1 NF-kappa B element, but by a physical interaction between the GR and RelA protein. The repressive effect of GR on NF-kappa B-mediated activation was not shared by other steroid/thyroid receptors. Only the progesterone receptor, which belongs to the same subfamily as GR and which possesses high homology with GR, was able to repress NF-kappa B-mediated transcription. These studies highlight a possible molecular mechanism that can explain the antiinflammatory effects of glucocorticoid treatment during inflammation. 
 T cell factor-4 functions as a co-activator to promote NF-κB-dependent MMP-15 expression in lung carcinoma cells. Both TCF-4 and MMP-15 are closely linked to the development of lung cancer, while the regulatory role of TCF-4 in MMP-15 expression is still obscure. Here we found that expression of TCF-4 and MMP-15 was increased in lung cancer cells or tissues versus the normal ones. With gain-or loss-of -function studies, we demonstrated that TCF-4 positively regulated MMP-15 expression in mRNA and protein levels. With reporter gene assay, we found that TCF-4 regulated MMP-15 expression via a potential NF-κB binding element locating at -2833/-2824 in the mouse MMP-15 promoter. With ChIP and immunoblotting assays, we identified that TCF-4 functioned as a co-activator to potentiate the binding between p65 and MMP-15 promoter. Functionally, TCF-4 silence attenuated th  e migrati  on activity of LLC cells, while additional overexpression of MMP-15 rescued this effect in cell scratch test and transwel  l migrati  on assay. In xenograft model, TCF-4 silence-improved tumor lesions in lungs and survival time of LLC-tumor bearing mice were abolished by MMP-15 overexpression. In conclusion, we are the first to identify TCF-4 as a co-activator of NF-κB p65 to promote MMP-15 transcription and potentiate t  he migrat  ion activity of the lung cancer cells. Our findings shed light on the therapeutic strategies of this malignancy. 
 Small ubiquitin-related modifier 2/3 interacts with p65 and stabilizes it in the cytoplasm in HBV-associated hepatocellular carcinoma. SUMOylation, an important post-translational modification, associates with the development of hepatocellular carcinoma (HCC). p65, one of the most important subunits of NF-κB, is a key regulator in the development of HCC and has been reported to be SUMOylated by exogenous small ubiquitin-related modifier 3 (SUMO3) in HEK 293T cells. However, the relationship between p65 and SUMO2/3 in HCC remains unknown. This study was to investigate the interaction between p65 and SUMO2/3 and explore the potential roles involved in HCC.The expressions of p65 and SUMO2/3 in the liver tissues were detected by using immunohistochemistry. We performed double-labeled immunofluorescence and co-immunoprecipitation assay to verify the interaction between p65 and SUMO2/3. The extraction of nuclear and cytoplasmic proteins was performed, and the subcellular localization of p65 was detected. The proliferation and   migratio  n of hepatoma cells were observed using MTT, colony formation, and transwell assays.We found a strong SUMO2/3-positive immunoreactivity in the cytoplasm in the non-tumor tissues of HCC. However, SUMO2/3 level was down regulated in the tumor tissues as compared with the adjacent non-tumor tissues. In accordance with this finding, p65 was up regulated in the adjacent non-tumor tissues and almost localized in the cytoplasm. There was a close correlation between SUMO2/3 and p65 expressions in the liver tissues (R = 0.800, p = 0.006). The interaction between p65 and SUMO2/3 was verified by co-immunoprecipitation and double-labeled immunofluorescent assays. TNF-α (10 ng/ml) treatment for 30 min not only up regulated the cytoplasmic conjugated SUMO2/3, but also enhanced SUMO2/3-p65 interaction. Furthermore, we found that SUMO2/3 up regulated the cytoplasmic p65 protein level in a dose-dependent manner, but not affected its mRNA level. The increase of p65 protein by SUMO2/3 was abolished by MG132 treatment, a reversible inhibitor of proteasome. Meanwhile, TNF-α-induced increase of SUMO2/3-conjugated p65 was along with the reduction of the ubiquitin-conjugated p65. The further study showed that SUMO2/3 over-expression decreased the proliferative ability of hepatoma cells, but did no  t affect   the migration.SUMO2/3-p65 interaction may be a novel mechanism involved in the transformation from chronic hepatitis B to HCC via stabilizing cytoplasmic p65, which might shed light on understanding the tumorigenesis and development. 
 RelB, together with RelA, sustains cell survival and confers proteasome inhibitor sensitivity of chronic lymphocytic leukemia cells from bone marrow. Although the biological factors that contribute to the pathogenesis of chronic lymphocytic leukemia (CLL) remain widely unresolved, it has been suggested that dysregulated cell survival and proliferation are fundamental to this process. Constitutive classical nuclear factor kappa-light-chain-enhancer of activated B cells (NF-κB) activation protects CLL B-cells from cell death and plays a critical role in the acquisition of chemoresistance. RelB, representing the alternative NF-κB activity, functions specifically in lymphoid organogenesis and B-cell maturation. RelB indeed plays a tumor-supportive role and confers radiation resistance in tumors. However, the involvement of RelB in CLL has not been addressed. Here, we analyzed the NF-κB activation in 67 of CLL bone marrow (BM). Both the RelA and RelB activity were detected in CLL B-cells from BM, in spite of inevitable variability. Low RelB activity was linked to a favorable prognosis of CLL. T  he migrat  ion and adhesion abilities of CLL B-cells were not affected by the RelB activity. High RelB activity, together with the RelA activity, maintained basal survival of cells. The induction of RelA and RelB expression in the nucleus was responsible for better survival of CLL B-cells supported by bone marrow stromal cells. In addition, the presence of high RelB activity in CLL B-cells was correlated with sensitivity to proteasome inhibitor but not fludarabine. Taken together, we provided evidences that not only RelA but also RelB, subunits of NF-κB family, played an important role in the cellular behaviors of CLL cells from BM. The strength of RelB activity influenced the prognosis of CLL patients.RelB, with RelA activity, maintained the basal survival of CLL cells from BM. RelB, with RelA, conferred the proteasome inhibitor sensitivity of CLL cells. Induction of RelA and RelB was responsible for the better survival of CLL B-cells. The strength of RelB activity influenced the prognosis of CLL patients. 
 S100A4 promotes squamous cell laryngeal cancer Hep-2 cell invasion via NF-kB/MMP-9 signal. S100A4 is a member of the S100 family of calcium-binding proteins, which possesses a wide range of biological functions, such as regulation of angiogenesis, cell survival, motility, and invasion. Here, we demonstrate for the first time a major role of S100A4 in the cell invasion properties of the human laryngeal squamous carcinoma cells (LSCC) and evaluated the mechanism.Cultured human LSCC cell line Hep-2 was overexpressed by transfection of pcDNA3.1-S100A4 plasmid. For this, cellular Hep-2 expression was quantified by Western blot analysis. Moreover, cell invasion and   migration   assays were performed. Furthermore, the impact of the S100A4 on NF-kB activity and MMP-9 expression was detected.We found S100A4 potently promoted Hep-2 invasion, by increasing cell motility and matrix metalloproteinase-9 (MMP-9) production. The increase in MMP-9 production was mediated by activation of nuclear factor-kB transcriptional activity by S100A4. After MMP-9 and NF-kBp65 was inhibited by BB94 treatment and NF-kBp65 siRNA transfected, pcDNA3.1-S100A4 induced cell invasion and   migration   was decreased.Our findings thus establish S100A4 as a major factor in the invasive abilities of Hep-2 cells. 
 Melatonin inhibits MMP-9 transactivation and renal cell carcinoma metastasis by suppressing Akt-MAPKs pathway and NF-κB DNA-binding activity. Renal cell carcinoma (RCC) is the most lethal of all urological malignancies because of its potent metastasis potential. Melatonin exerts multiple tumor-suppressing activities through antiproliferative, proapoptotic, and anti-angiogenic actions and has been tested in clinical trials. However, the antimetastastic effect of melatonin and its underlying mechanism in RCC are unclear. In this study, we demonstrated that melatonin at the pharmacologic concentration (0.5-2 mm) considerably reduced the   migratio  n and invasion of RCC cells (Caki-1 and Achn). Furthermore, we found that melatonin suppressed metastasis of Caki-1 cells in spontaneous and experimental metastasis animal models. Mechanistic investigations revealed that melatonin transcriptionally inhibited MMP-9 by reducing p65- and p52-DNA-binding activities. Moreover, the Akt-mediated JNK1/2 and ERK1/2 signaling pathways were involved in melatonin-regulated MMP-9 transactivation and cell motility. Clinical samples revealed an inverse correlation between melatonin receptor 1A (MTNR1A) and MMP-9 expression in normal kidney and RCC tissues. In addition, a higher survival rate was found in MTNR1A(high) /MMP-9(low) patients than in MTNR1A(low) /MMP-9(high) patients. Overall, our results provide new insights into the role of melatonin-induced molecular regulation in suppressing RCC metastasis and suggest that melatonin has potential therapeutic applications for metastastic RCC. 
 Inactivation of M2 AChR/NF-κB signaling axis reverses epithelial-mesenchymal transition (EMT) and suppresses   migratio  n and invasion in non-small cell lung cancer (NSCLC). Non-neuronal cholinergic system is involved in lung physiology and lung cancer. However, the biochemical events downstream acetylcholine (ACh) receptor activation leading to carcinogenesis and tumor progression are not fully understood. Our previous work has shown that non-neuronal ACh acts as an autoparacrine growth factor to stimulate cell proliferation and promote epithelial-mesenchymal transition (EMT) in non-small cell lung cancer (NSCLC) via activation of M2 muscarinic receptor (M2R). The aim of the present study was to delineate the underlying mechanisms linking M2R and lung tumor progression, which may provide potential therapeutic targets to delay lung cancer progression. Inhibition of M2R by antagonist or siRNA suppresses NSCLC cell migratory and invasive capacities, reverses EMT and simultaneously inactivates PI3K/Akt, MAPK ERK and NF-κB p65. On the other hand, M2R activation stimulates NSCL  C migrati  on and invasion and promotes EMT via NF-κB p65 activation. Moreover, NF-κB p65 activation induced by M2R activation was partially inhibited by either Akt or ERK inhibitor. Taken together, these results demonstrated for the first time that NF-κB p65 activation is essential in NSCLC progression associated with non-neuronal cholinergic system. Our data suggest that M2R/ERK/Akt/NF-κB axis could be a potential target for NSCLC treatment. 
 Kaposi's sarcoma-associated herpesvirus-encoded latency-associated nuclear antigen reduces interleukin-8 expression in endothelial cells and impairs neutrophil chemotaxis by degrading nuclear p65. Latency-associated nuclear antigen 1 (LANA-1) of Kaposi's sarcoma-associated herpesvirus (KSHV) is the major viral latent protein and functions as a multifaceted protein. Here, we report that LANA-1 attenuates the endothelial response to tumor necrosis factor alpha (TNF-α) stimulation and inhibits consequent neutrophil chemotaxis. Reporter assays showed that LANA-1 constantly repressed nuclear factor (NF)-κB transactivity upon TNF-α stimulation. We also found that LANA-1 decreased nuclear p65 protein levels through enhancement of polyubiquitylation-mediated p65 degradation and that an elongin B/elongin C-cullin 5-LANA-1-p65 complex assembled by LANA-1 was responsible for this enhanced p65 degradation. In telomerase-immortalized human umbilical vein endothelial cells, LANA-1 was demonstrated to repress interleukin-8 expression, which was involved in neutrophil recruitment to the inflammatory site. Through an in vitro tr  ansmigrat  ion assay, we determined a suppressive effect of LANA-1 on neutrophil chemotaxis. Our work suggests that KSHV LANA-1 is a negative modulator of acute inflammation and sheds light on a new mechanism by which KSHV during the latent life cycle evades the host innate immune response. 
 Specific tumor-derived CCL2 mediated by pyruvate kinase M2 in colorectal cancer cells contributes to macrophage recruitment in tumor microenvironment. Development of colorectal cancer has been considered as a result of imbalance of pro- and anti-inflammatory intestinal microenvironment accompanied by macrophage recruitment. Despite macrophages are implicated in remodeling tumor microenvironment, the mechanism of macrophage recruitment is not fully elucidated yet. In this study, we reported clinical association of highly expressed pyruvate kinase M2 in colorectal cancer with macrophage attraction. The conditioned medium from Caco-2 and HT-29 cells with depleted pyruvate kinase M2 dramatically reduced macrophage recruitment, which is reversed by addition of, a critical chemotaxis factor to macrophage   migration  , rCCL2. Silencing of endogenous pyruvate kinase M2 markedly decreased CCL2 expression and secretion by real-time quantitative polymerase chain reaction and enzyme-linked immunosorbent assay. Endogenous pyruvate kinase M2 interacted with p65 and mediated nuclear factor-κB signaling pathway and mainly regulated phosphorylation of Ser276 on p65 nuclear factor-κB. In addition, inhibition of macrophage recruitment caused by pyruvate kinase M2 silencing was rescued by ectopic expression of p65. Interestingly, pyruvate kinase M2 highly expressed in colorectal cancer tissue, which is correction with macrophage distribution. Taken together, we revealed a novel mechanism of pyruvate kinase M2 in promoting colorectal cancer progression by recruitment of macrophages through p65 nuclear factor-κB-mediated expression of CCL2. 
 Morusin inhibits human cervical cancer stem cell growth and   migration   through attenuation of NF-κB activity and apoptosis induction. Cancer stem cells (CSCs) are believed to be responsible for tumor metastasis, recurrence, and high mortality of cancer patients due to their high tumorigenicity resistance to chemo-radiotherapy. Morusin possesses anti-cancer activity through attenuation of NF-κB activity, which is up-regulated in cancer stem cells. The purpose of this study is to confirm the growth an  d migrati  on inhibition effect of morusin on human cervical CSCs, and to clarify its partial mechanism of activity. Human cervical CSCs were enriched using non-adhesive culture system. Their stemness characteristics were identified with tumor sphere formation, self-renewal, toluidine blue staining  , migrati  on assays, RT-PCR analysis, and immunofluorescence staining of putative stem cell markers, Oct4, SOX2, and ALDH1; the epithelial-to-mesenchymal (EMT) transition markers and relevant transcription factors were evaluated with Western blotting. The growth an  d migrati  on inhibition effects of morusin on human cervical CSCs were tested by cell proliferation, tumor sphere formation, and transwell assay; apoptotic death of human cervical CSCs in response to morusin was measured with DAPI staining, apoptotic DNA fragmentation; NF-κBp65, Bcl-2, Bax, and caspase-3 protein expressions were detected through Western blotting. Under this non-adhesive culture system, typical tumor spheres appeared within 5-7 days, the tumor sphere formation, self-renewal, and c  ell migra  tion, expressions of putative stem cell markers, EMT markers, and relevant transcription factors of the tumor sphere cells were increased significantly. After morusin treatment, the proliferation, tumor sphere formation,   and migra  tion of human cervical CSCs were decreased significantly, DAPI-stained apoptotic cells increased, apoptotic DNA fragmentations formed evidently; the expression levels of NF-κBp65 and Bcl-2 decreased significantly, Bax, and caspase-3 increased significantly in a dose-dependent manner. Using the non-adhesive culture system, human cervical CSCs were enriched and expanded. Morusin has the potential to target and kill CSCs, and can inhibit human cervical growth   and migr  ation through NF-κB attenuation mediated apoptosis induction. 
 FOXP3 inhibits NF-κB activity and hence COX2 expression in gastric cancer cells. Gastric cancer remains the main cause of cancer related deaths all over the world, and upregulated COX2 is a key player in its development. The mechanism as to how COX2 is regulated during the gastric cancer development is largely unknown. In this study, we found that the expression of COX2 was closely correlated with NF-κB activity. Strikingly, NF-κB activity was not absolutely consistent with its nuclear localization. Especially, in some cancer cell lines, such as MKN28, there were abundant nuclear localized NF-κB, while NF-κB luciferase activity in this cell line was relatively low. Furthermore, FOXP3 was found to be abundantly expressed in these cells. When the nuclear localized NF-κB expression was adjusted with the expression of FOXP3, it then correlated well with NF-κB activity. Molecularly, increased FOXP3 expression can interact with NF-κB and thus repress its activity. Knockdown of FOXP3 could increase NF-κB activity, COX2 expression,   and cell   migration. Taken together, our study revealed that function of FOXP3 as a negative regulator of NF-κB activity and thus plays a tumor suppressor role by reducing cell metastasis. 
 Tanshinone IIA decreases the migratory ability of AGS cells by decreasing the protein expression of matrix metalloproteinases, nuclear factor κB-p65 and cyclooxygenase-2. During progression of gastric cancer, degradation of the extracellular matrix by matrix metalloproteinases (MMPs) has been associated with poor prognosis. Tanshinone IIA (Tan-IIA) exerts antitumor activity in a variety of human cancer cells. It is extracted from Danshen (Salviae miltiorrhizae radix), and induces apoptosis and inhibits the proliferation of gastric cancer cells. However, the molecular mechanisms underlying the inhibition of   migratio  n in gastric cancer by Tan-IIA have not been fully elucidated. In the present study, AGS cell   migratio  n ability was evaluated using a wound-healing assay. The protein expression levels of nuclear factor (NF)-κB-p65, cyclooxygenase (COX)-2, MMP-2, -7, and -9 and β-actin in AGS cells were measured by western blotting. The results demonstrated that AGS cells treated with Tan-IIA exhibit decreased protein expression levels of NF-κB-p65, COX-2, and MMP-2, -7 and -9. The results also indicate that Tan-IIA inhib  its migra  tion ability in a dose- and time-dependent manner. These findings demonstrate that Tan-IIA inhibits   the migra  tion ability of AGS human gastric cancer cells and that decreasing the protein expression of NF-κB-p65, COX-2, and MMP-2, -7 and -9 may be an underlying molecular mechanism. 
 Association of variants in genes encoding for macrophage-related functions with clinical outcome in patients with locoregional gastric cancer. Nuclear factor-kappaB (NF-κB) and CCL2/CCR2 chemokine axis play a central role in tumor progression such as stimulation of angiogenesis, acceleration of tumor invasion and   migratio  n, and suppression of innate immunosurveillance in the macrophage-related functions. There have been few reports regarding association of the macrophage function-related genes with the clinical outcome in gastric cancer. We hypothesized that variants in genes encoding for NF-κB and CCL2/CCR2 axis may predict prognosis in gastric cancer and tested whether the functional single-nucleotide polymorphisms (SNPs) will be associated with clinical outcome in patients with gastric cancer across two independent groups.This study enrolled two cohorts which consisted of 160 Japanese patients and 104 US patients with locoregional gastric cancer. Genomic DNA was analyzed for association of 11 SNPs in NFKB1, RELA, CCL2, and CCR2 with clinical outcome using PCR-based direct DNA sequencing.The univariable analysis showed four SNPs had significant association with clinical outcome in the Japanese cohort, NFKB1 rs230510 remained significant upon multivariable analysis. The patients with the A allele of the NFKB1 rs230510 had significantly longer overall survival (OS) compared with those with the T/T genotype in both the Japanese and US cohort in the univariable analysis. In contrast, genotypes with the T allele of CCL2 rs4586 were significantly associated with shorter OS compared with the C/C genotype in the US cohort [hazard ratio (HR) 2.43; P = 0.015] but longer OS in the Japanese cohort (HR 0.58; P = 0.021), resulting in the statistically significant opposite impact on OS (P = 0.001).Our study provides the first evidence that the NFKB1 rs230510 and CCL2 rs4586 are significantly associated with the clinical outcome in patients with locoregional gastric cancer. These results also suggest that the genetic predisposition of the host may dictate the immune-related component of the tumor for progression in gastric cancer. 
 The RASSF8 candidate tumor suppressor inhibits cell growth and regulates the Wnt and NF-kappaB signaling pathways. The Ras-assocation domain family (RASSF) of tumor suppressor proteins until recently contained six proteins named RASSF1-6. Recently, four novel family members, RASSF7-10, have been identified by homology searches for RA-domain-containing proteins. These additional RASSF members are divergent and structurally distinct from RASSF1-6, containing an N-terminal RA domain and lacking the Sav/RASSF/Hpo (SARAH) domain. Here, we show that RASSF8 is ubiquitously expressed throughout the murine embryo and in normal human adult tissues. Functionally, RNAi-mediated knockdown of RASSF8 in non-small-cell lung cancer (NSCLC) cell lines, increased anchorage-independent growth in soft agar and enhanced tumor growth in severe combined immunodeficiency (SCID) mice. Furthermore, EdU staining of RASSF8-depleted cells showed growth suppression in a manner dependent on contact inhibition. We show that endogenous RASSF8 is not only found in the nucleus, but is also membrane associated at sites of cell-cell adhesion, co-localizing with the adherens junction (AJ) component beta-catenin and binding to E-cadherin. Following RASSF8 depletion in two different lung cancer cell lines using alternative small interfering RNA (siRNA) sequences, we show that AJs are destabilized and E-cadherin is lost from the cell membrane. The AJ components beta-catenin and p65 are also lost from sites of cell-cell contact and are relocalized to the nucleus with a concomitant increase in beta-catenin-dependent and nuclear factor-kappaB (NF-kappaB)-dependent signaling following RASSF8 depletion. RASSF8 may also be required to maintain actin -cytoskeletal organization since immunofluorescence analysis shows a striking disorganization of the actin- cytoskeleton following RASSF8 depletion. Accordingly, scratch wound healing studies show increased cellular   migration   in RASSF8-deficient cells. These results implicate RASSF8 as a tumor suppressor gene that is essential for maintaining AJs function in epithelial cells and have a role in epithelial cell   migration  . 
 The tumor marker Fascin is strongly induced by the Tax oncoprotein of HTLV-1 through NF-kappaB signals. Oncogenic transformation of CD4(+) T cells by human T-cell lymphotropic virus type 1 (HTLV-1) is understood as the initial step to adult T-cell leukemia/lymphoma, a process that is mainly initiated by perturbation of cellular signaling by the viral Tax oncoprotein, a potent transcriptional regulator. In search of novel biomarkers with relevance to oncogenesis, we identified the tumor marker and actin-bundling protein Fascin (FSCN1) to be specifically and strongly up-regulated in both HTLV-1-transformed and adult T-cell leukemia/lymphoma patient-derived CD4(+) T cells. Fascin is important for   migration   and metastasis in various types of cancer. Here we report that a direct link can exist between a single viral oncoprotein and Fascin expression, as the viral oncoprotein Tax was sufficient to induce high levels of Fascin. Nuclear factor-κB signals were important for Tax-mediated transcriptional regulation of Fascin in T cells. This suggests that Fascin up-regulation by Tax contributes to the development of HTLV-1-associated pathogenesis. 
 Downregulation of MCT1 inhibits tumor growth, metastasis and enhances chemotherapeutic efficacy in osteosarcoma through regulation of the NF-κB pathway. Monocarboxylate transporter isoform 1 (MCT1) is an important member of the proton-linked MCT family and has been reported in an array of human cancer cell lines and primary human tumors. MCT1 expression is associated with developing a new therapeutic approach for cancer. In this study, we initially showed that MCT1 is expressed in a variety of human osteosarcoma cell lines. Moreover, we evaluated the therapeutic response of targeting MCT1 using shRNA or MCT1 inhibitor. Inhibiting MCT1 delayed tumor growth in vitro and in vivo, including in an orthotopic model of osteosarcoma. Targeting MCT1 greatly enhanced the sensitivity of human osteosarcoma cells to the chemotherapeutic drugs adriamycin (ADM). In addition, we observed that MCT1 knockdown significantly suppressed the metastatic activity of osteosarcoma, including wound healing, invasion and   migratio  n. Further mechanistic studies revealed that the antitumor effects of targeting MCT1 might be related to the NF-κB pathway. Immunochemistry assay showed that MCT1 was an independent positive prognostic marker in osteosarcoma patients. In conclusion, our data, for the first time, demonstrate that MCT1 inhibition has antitumor potential which is associated with the NF-κB pathway, and high MCT1 expression predicates poor overall survival in patients with osteosarcoma. 
 (-)-Epigallocatechin-3-gallate inhibits nasopharyngeal cancer stem cell self-renewal and   migration   and reverses the epithelial-mesenchymal transition via NF-κB p65 inactivation. The cancer stem cell (CSC) theory states that many types of cancer, including nasopharyngeal cancer (NPC), are initiated from and maintained by CSCs, which may be responsible for tumor relapse and resistance to therapy. It is imperative that nasopharyngeal cancer stem cells (NPCSCs) be specifically targeted to eradicate NPC and prevent recurrence. Epigallocatechin-3-gallate (EGCG) inhibits cancer progression by attenuating NF-κB p65 activity, which is upregulated in CSCs and plays an important role in epithelial-mesenchymal transition (EMT). The purpose of this study is to confirm the self-renewal an  d migrati  on inhibitory effects of EGCG toward NPCSCs and to clarify its mechanism of activity. We enriched and characterized NPCSCs by collecting spheroid-derived cells grown in serum-free medium (SFM) and examined the effects of EGCG on the characteristics of NPCSCs and studied the underlying mechanisms using soft agar colony assays, transwel  l migrati  on assays, reverse transcriptase polymerase chain reaction (RT-PCR), Western blot analysis, immunofluorescence staining, and xenograft studies. NPC spheroids enriched from NPC cell lines acquired CSC traits and underwent EMT. EGCG inhibited the NPCSCs' self-renewal an  d migrati  on and reversed EMT, and combined treatment with EGCG and cisplatin reduced the growth of CSC tumor xenografts. Moreover, EGCG inhibited NF-κB p65 activity by modulating the cellular localization of p65 and decreasing the transcriptional regulation of NF-κB p65 on Twist1 expression. NF-κB p65 is a novel therapeutic target in NPCSCs, and the inhibition of activated NF-κB p65 in CSCs by EGCG may offer an effective treatment for NPC. 
 Overexpression of response gene to complement 32 (RGC32) promotes cell invasion and induces epithelial-mesenchymal transition in lung cancer cells via the NF-κB signaling pathway. Response gene to complement 32 (RGC32) is a novel cellular protein that has been reported to be expressed aberrantly in multiple types of human tumors. However, the role of RGC32 in cancer is still controversial, and the molecular mechanisms by which RGC32 contributes to the development of cancer remain largely unknown. In the present study, we constructed a recombinant expression vector pCDNA3.1-RGC32 and transfected it into human lung cancer A549 cells. Stable transformanted cells were identified by real-time PCR and Western blot analysis. Functional analysis showed that forced overexpression of RGC32 increased invasive and   migratio  n capacities of lung cancer cells in vitro, and induced the acquisition of epithelial-mesenchymal transition (EMT) phenotype, as demonstrated by the spindle-like morphology, downregulation of E-cadherin, and upregulation of Vimentin, Fibronectin, Snail and Slug. Also, overexpression of RGC32 increased expression and activities of matrix metalloproteinase (MMP)-2 and MMP-9 in A549 cells. Furthermore, the downregulation of E-cadherin induced by RGC32 was remarkably attenuated by nuclear factor-κB (NF-κB) inhibitor BAY 11-7028 and small interfering RNA targeting NF-κB p65, suggesting a role of the NF-κB signaling pathway in RGC32-induced EMT. Taken together, our data suggest that RGC32 promotes   cell migr  ation and invasion and induces EMT in lung cancer cells via the NF-κB signaling pathway. 
 Molecular basis of nuclear factor-kappaB activation by astrocyte elevated gene-1. Malignant glioma is a consistently fatal brain cancer. The tumor invades the surrounding tissue, limiting complete surgical removal and thereby initiating recurrence. Identifying molecules critical for glioma invasion is essential to develop targeted, effective therapies. The expression of astrocyte elevated gene-1 (AEG-1) increases in malignant glioma and AEG-1 regulates in vitro invasion and   migration   of malignant glioma cells by activating the nuclear factor-kappaB (NF-kappaB) signaling pathway. The present studies elucidate the domains of AEG-1 important for mediating its function. Serial NH(2)-terminal and COOH-terminal deletion mutants were constructed and functional analysis revealed that the NH(2)-terminal 71 amino acids were essential for invasion,   migration  , and NF-kappaB-activating properties of AEG-1. The p65-interaction domain was identified between amino acids 101 to 205, indicating that p65 interaction alone is not sufficient to mediate AEG-1 function. Coimmunoprecipitation assays revealed that AEG-1 interacts with cyclic AMP-responsive element binding protein-binding protein (CBP), indicating that it might act as a bridging factor between NF-kappaB, CBP, and the basal transcription machinery. Chromatin immunoprecipitation assays showed that AEG-1 is associated with the NF-kappaB binding element in the interleukin-8 promoter. Thus, AEG-1 might function as a coactivator for NF-kappaB, consequently augmenting expression of genes necessary for invasion of glioma cells. In these contexts, AEG-1 represents a viable potential target for the therapy of malignant glioma. 
 Anti-rheumatic drug iguratimod (T-614) alleviates cancer-induced bone destruction via down-regulating interleukin-6 production in a nuclear factor-κB-dependent manner. Cytokines are believed to be involved in a "vicious circle" of progressive interactions in bone metastasis. Iguratimod is a novel anti-rheumatic drug which is reported to have the capability of anti-cytokines. In this study, a rat model was constructed to investigate the effect of iguratimod on bone metastasis and it was found that iguratimod alleviated cancer-induced bone destruction. To further explore whether an anti-tumor activity of iguratimod contributes to the effect of bone resorption suppression, two human breast cancer cell lines MDA-MB-231 and MCF-7 were studied. The effect of iguratimod on tumor proliferation was detected by CCK-8 assay and flow cytometry. The effects of iguratimod on   migratio  n and invasion of cancer cells were determined by wound-healing and Transwell assays. Results showed that high dose (30 μg/mL) iguratimod slightly suppressed the proliferation of cancer cells but failed to inhibit thei  r migrati  on and invasion capacity. Interestingly, iguratimod decreased the transcription level of IL-6 in MDA-MB-231 cells in a concentration-dependent manner. Moreover, iguratimod partially impaired NF-κB signaling by suppressing the phosphorylation of NF-κB p65 subunit. Our findings indicated that iguratimod may alleviate bone destruction by partially decreasing the expression of IL-6 in an NF-κB-dependent manner, while it has little effect on the tumor proliferation and invasion. 
 Expression of the constitutively activated RelA/NF-kappaB in human astrocytic tumors and the in vitro implication in the regulation of urokinase-type plasminogen activator,   migration  , and invasion. Although malignant gliomas are highly invasive tumors, a characteristic that contributes to the commonly observed therapeutic failures and local disease recurrences, the molecular events that regulate invasion in these tumors remain poorly understood. Because the transcription factor RelA/NF-kappaB has been shown to regulate invasion during several cellular processes, we have examined immunohistochemically expression of the constitutively activated RelA/NF-kappaB in tissues obtained from 49 astrocytic tumors [8 diffuse astrocytomas, 9 anaplastic astrocytomas (AAs) and 32 glioblastomas (GBMs)]. In addition, we examined the in vitro effects of antisense oligonucleotides and curcumin on the expression and activation of RelA/NF-kappaB, urokinase-type plasminogen activator (u-PA) expression,   migration  , and invasion in the T98G glioma cell line. Expression of the constitutively activated RelA/NF-kappaB was observed in 2 (25%) of 8 cases of diffuse astrocytomas, 5 (55.6%) of 9 cases of AAs, and 30 (93.8%) of 32 cases of GBMs. This expression was significantly correlated with the malignant potential in astrocytic tumors (P < 0.001). Moreover, antisense oligonucleotides and curcumin inhibited phorbol-12-myristate-13-acetate (PMA)-induced RelA/NF-kappaB expression or activation (or both), down-regulated u-PA expression, and reduced the   migration   and invasive potentials of T98G glioma cells. Thus, the expression of constitutively activated RelA/NF-kappaB is associated with malignancy potential in astrocytic tumors and may play a critical role in the regulation of u-PA expression and invasiveness in gliomas. RelA/NF-kappaB may therefore be an intriguing candidate for studies aimed at understanding and prevention of the invasiveness of gliomas. 
 Plexin-B1 activates NF-κB and IL-8 to promote a pro-angiogenic response in endothelial cells. The semaphorins and their receptors, the plexins, are proteins related to c-Met and the scatter factors that have been implicated in an expanding signal transduction network involving co-receptors, RhoA and Ras activation and deactivation, and phosphorylation events. Our previous work has demonstrated that Semaphorin 4D (Sema4D) acts through its receptor, Plexin-B1, on endothelial cells to promote angiogenesis in a RhoA and Akt-dependent manner. Since NF-κB has been linked to promotion of angiogenesis and can be activated by Akt in some contexts, we wanted to examine NF-κB in Sema4D treated cells to determine if there was biological significance for the pro-angiogenic phenotype observed in endothelium.Using RNA interference techniques, gel shifts and NF-κB reporter assays, we demonstrated NF-κB translocation to the nucleus in Sema4D treated endothelial cells occurring downstream of Plexin-B1. This response was necessary for endothelial   cell migr  ation and capillary tube formation and protected endothelial cells against apoptosis as well, but had no effect on cell proliferation. We dissected Plexin-B1 signaling with chimeric receptor constructs and discovered that the ability to activate NF-κB was dependent upon Plexin-B1 acting through Rho and Akt, but did not involve its role as a Ras inhibitor. Indeed, inhibition of Rho by C3 toxin and Akt by LY294002 blocked Sema4D-mediated endothelial   cell mig  ration and tubulogenesis. We also observed that Sema4D treatment of endothelial cells induced production of the NF-κB downstream target IL-8, a response necessary for angiogenesis. Finally, we could show through co-immunofluorescence for p65 and CD31 that Sema4D produced by tumor xenografts in nude mice activated NF-κB in vessels of the tumor stroma.These findings provide evidence that Sema4D/Plexin-B1-mediated NF-κB activation and IL-8 production is critical in the generation a pro-angiogenic phenotype in endothelial cells and suggests a new therapeutic target for the anti-angiogenic treatment of some cancers. 
 N-Myc-interacting protein (NMI) negatively regulates epithelial-mesenchymal transition by inhibiting the acetylation of NF-κB/p65. The epithelial-mesenchymal transition (EMT) plays an essential role in embryonic development, wound healing, tissue regeneration, organ fibrosis, and tumor progression. However, the mechanisms underlying this process are poorly understood. Many signaling pathways, including the NF-κB signaling pathway, trigger EMT during development and differentiation. In the present study, we report that N-Myc interactor (NMI) inhibits EMT progression by suppressing transcriptional activities of NF-κB in human gastric cancer cells. We show that the expression of NMI is significantly reduced in invasive gastric cancer cells and gastric cancer tissues. Overexpression of NMI inhibited ce  ll migrat  ion and invasion, and this inhibition was enhanced after TNF-α stimulation. Tumorigenicity assay in nude mice support the notion that NMI inhibits EMT in cancer cells. Mechanistically, NMI promotes the interaction between NF-κB/p65 and histone deacetylases (HDACs) and inhibits the acetylation and transcriptional activity of p65. The expression of p65 rescues NMI-mediated inhibition of EMT and the inhibition of the acetylation of p65 mediated by NMI is HDACs-dependent. Taken together, these findings suggest that NMI can suppress tumor invasion and metastasis by inhibiting NF-κB pathways, providing an alternative mechanism for EMT inhibition in stomach neoplasm. 
 [Construction and influence of human nuclear factor-κB p65 shRNA lentiviral  vector on malignant biological behavior of lung cancer cells]. Nuclear factor-κB is an important transcription factor and is closely associated with a variety of malignant tumors. The biological behavior of lung tumor cells can be reversed by inhibiting the expression of NF-κBp65 directly or indirectly. Nuclear factor-κBp65 gene shRNA recombinant plasmids were constructed and then infected with A549 cells. New stable cell lines were selected, and the abili  ty of mig  ration and adhesion was identified.Both scramble control sequence and interference sequence (shRNA) of human nuclear factor-κBp65 were designed and synthesized to build recombinant plasmids, with BamH I site at the 5' end and Xho I and EcoR I sites at the 3' end. A549 cells were infected, and stable transfection strains were selected by puromycin. Western blot and qRT-PCR methods were applied to assess the interference efficient of NF-κBp65 and the protein expression level of IκBα. Transwell and MTT assays were carried out to analyze the a  bility of   migration and adhesion of A549 cells separately.Recombinant plasmids were successfully built, and A549/NF-κB p65 scramble and A549/NF-κB p65 shRNA stable transfection strains were also successfully screened. Both mRNA and protein expression levels of NF-κBp65 showed that A549/NF-κBp65 shRNA cells decreased compared with A549/NF-κB p65 scramble cells and A549 cells, whereas the protein level of IκBα significantly   increase  d. Both migration and adhesion abilities were also reduced.In this study, both mRNA and protein expression levels of NF-κBp65 were effectively suppressed by RNA interference technique. NF-κBp65 inhibition can signif  icantly r  educe the migration and adhesion ability of A549 cells. 
 miR-508-3p concordantly silences NFKB1 and RELA to inactivate canonical NF-κB signaling in gastric carcinogenesis. NF-κB signaling pathway plays an important role in gastric carcinogenesis. The basic expression and functional role of NFKB1 and RELA (components of canonical NF-κB pathway) in gastric cancer (GC) have not been well elucidated. In this study, the role of NFKB1 and RELA in gastric tumorigenesis will be investigated and their regulation by microRNAs (miRNAs) will be deeply explored.The mRNA and protein expression of NFKB1 and RELA were investigated by qRT-PCR and Western blot in GC cell lines and primary tumors. The functional roles of NFKB1 and RELA in GC were demonstrated by MTT proliferation assay, monolayer colony formation, cell invasion a  nd migrat  ion, cell cycle analysis and in vivo study through siRNA mediated knockdown. Identification of NFKB1 as a direct target of tumor suppressor miRNA miR-508-3p was achieved by expression regulation assays together with dual luciferase activity experiments.NFKB1 and RELA were up-regulated in GC cell lines and primary tumors compared with normal gastric epithelium cells and their upregulation correlation with poor survival in GC. siRNA mediated knockdown of NFKB1 or RELA exhibited anti-oncogenic effect both in vitro and in vivo. NFKB1 was further revealed to be a direct target of miR-508-3p in gastric tumorigenesis and their expression showed negative correlation in primary GC samples. miR-508-3p was down-regulated in GC cells compared with normal gastric epithelium samples and its ectopic expression in GC cell lines also exerts tumor suppressor function. NFKB1 re-expression was found to partly abolish the tumor-suppressive effect of miR-508-3p in GC.All these findings supports that canonical NF-κB signaling pathway is activated in GC at least by the inactivation of miR-508-3p and this might have therapeutic potential in GC treatment. 
 Integrin-linked kinase overexpression promotes epithelial-mesenchymal transition via nuclear factor-κB signaling in colorectal cancer cells. To investigate the effect of integrin-linked kinase (ILK) on proliferation, metastasis, and invasion of the colorectal cancer cell line SW480.In this study, the colorectal cancer cell line SW480 was stably transfected with ILK plasmids, and small interfering RNA (siRNA) was used to knockdown expression of nuclear factor (NF)-κB/p65. Methylthiazole tetrazolium (MTT) assay was performed to measure proliferation, and the wound healin  g migrati  on assay and matrigel invasion assay were used to test the metastasis and invasion ability of SW480 cells. To explore the epithelial-mesenchymal transition (EMT) process, embryonic development, and the invasion and metastasis of tumors, the protein level of E-cadherin, vimentin, snail, and slug was detected by western blot. Immunofluorescence was also used to detect E-cadherin expression. Western blot was used to determine the level of phosphorylated-inhibitor of kappa B (IκB)a, inhibitor of gamma B (IγB)a, and nuclear factor kappa B (NF-κB) expressions and to explore the ILK signaling pathway.Western blot results revealed that ILK expression significantly increased when ILK was overexpressed in SW480 cells (P < 0.05). Proliferation, metastasis, and invasion ability were improved in the vector-ILK group compared to the vector group (P < 0.05). Immunofluorescence results revealed that E-cadherin fluorescence intensity decreased after ILK was overexpressed (P < 0.05). Western blot results revealed that the protein expression of E-cadherin was reduced, while vimentin, snail, and slug were upregulated when ILK was overexpressed in SW480 cells (P < 0.05). In order to determine the role of the NF-κB signaling pathway in ILK overexpression promoted EMT occurrence, we overexpressed ILK in SW480 cells and found that levels of NF-κB/p65 and cytoplasmic phosphorylated-IκBa were increased and that cytoplasmic IкBa levels were decreased compared to the control group (P < 0.05). Furthermore, NF-κB/p65 knockout revealed that E-cadherin was increased in the overexpressed ILK group.ILK overexpression improved the proliferation, metastasis, and invasion ability of SW480 cells, and this effect may be mediated by the NF-κB signaling pathway. 
 Melatonin induces cell apoptosis in Mia PaCa-2 cells via the suppression of nuclear factor-κB and activation of ERK and JNK: A novel therapeutic implication for pancreatic cancer. Melatonin is synthesized by the pineal gland and is released into the blood. In the last several years, some studies have shown that melatonin has anticancer properties; however, the mechanisms behind the antitumour traits are unclear, especially in pancreatic cancer. Therefore, in the present study, we investigated the antitumour effects of melatonin on the human pancreatic carcinoma cell line MIA PaCa‑2 and explored its biological mechanisms. MIA PaCa‑2 cells were treated with melatonin, and we used a CCK‑8 assay to evaluate the cell viability. We also used flow cytometry to observe cell apoptosis and western blot analysis to assess the protein expression. Our study found that melatonin inhibited cell viability, suppressed colony formation and reduce  d cell mi  gration and invasion and induced cell apoptosis in MIA PaCa‑2 cells. Our results showed that melatonin treatment inhibited NF‑κB p65 activation. Moreover, melatonin treatment activated the mitogen‑activated protein kinase pathways (c‑jun N‑terminal kinase and extracellular‑regulated kinase 1/2), which increased Bax protein expression and caspase‑3 cleavage and decreased Bcl‑2 protein expression. These new developments demonstrate that melatonin plays a potential role in anticancer treatment and may act as an effective therapeutic agent in the future. 
 Testes-specific protease 50 promotes cell invasion and metastasis by increasing NF-kappaB-dependent matrix metalloproteinase-9 expression. The high mortality in breast cancer is often associated with metastatic progression in patients. Previously we have demonstrated that testes-specific protease 50 (TSP50), an oncogene overexpressed in breast cancer samples, could promote cell proliferation and tumorigenesis. However, whether TSP50 also has a key role in cell invasion and cancer metastasis, and the mechanism underlying the process are still unclear. Here we found that TSP50 overexpression greatly promoted cell   migration  , invasion, adhesion and formation of the stellate structures in 3D culture system in vitro as well as lung metastasis in vivo. Conversely, TSP50 knockdown caused the opposite changes. Mechanistic studies revealed that NF-κB signaling pathway was required for TSP50-induced cell   migratio  n and metastasis, and further results indicated that TSP50 overexpression enhanced expression and secretion of MMP9, a target gene of NF-κB signaling. In addition, knockdown of MMP9 resulted in inhibition of cel  l migrati  on and invasion in vitro and lung metastasis in vivo. Most importantly, immunohistochemical staining of human breast cancer samples strongly showed that the coexpression of TSP50 and p65 as well as TSP50 and MMP9 were correlated with increased metastasis and poor survival. Furthermore, we found that some breast cancer diagnosis-associated features such as tumor size, tumor grade, estrogen receptors (ER) and progesterone receptors (PR) levels, were correlated well with TSP50/p65 and TSP50/MMP9 expression status. Taken together, this work identified the TSP50 activation of MMP9 as a novel signaling mechanism underlying human breast cancer invasion and metastasis. 
 Astrocyte elevated gene-1 is associated with metastasis in head and neck squamous cell carcinoma through p65 phosphorylation and upregulation of MMP1. The survival rate of head and neck squamous cell carcinoma (HNSCC) at advanced stage is poor, despite contemporary advances in treatment modalities. Recent studies have indicated that astrocyte elevated gene-1 (AEG-1), a single transmembrane protein without any known functional domains, is overexpressed in various malignancies and is implicated in both distant metastasis and poor survival.High expression of AEG-1 in HNSCC was positively correlated with regional lymph node metastasis and a poor 5-year survival rate. Knockdown of AEG-1 in HNSCC cell lines reduced their capacity for colony formation,   migration   and invasion. Furthermore, decreased tumor volume and metastatic foci were observed after knockdown of AEG-1 in subcutaneous xenografts and pulmonary metastasis assays in vivo, respectively. We also demonstrated that AEG-1 increased phosphorylation of the p65 subunit of NF-κB, and regulated the expression of MMP1 in HNSCC cells. Moreover, compromised phosphorylation of the p65 (RelA) subunit of NF-κB at serine 536 was observed upon silencing of AEG-1 in both HNSCC cell lines and clinical specimens.High expression of AEG-1 is associated with lymph node metastasis and its potentially associated mechanism is investigated. 
 NF-kappaB promotes breast cancer cell   migration   and metastasis by inducing the expression of the chemokine receptor CXCR4. Metastasis of cancer cells is a complex process involving multiple steps including invasion, angiogenesis, and trafficking of cancer cells through blood vessels, extravasations, organ-specific homing, and growth. While matrix metalloproteinases, urokinase-type plasminogen activator, and cytokines play a major role in invasion and angiogenesis, chemokines such as stromal derived factor-1alpha (SDF-1alpha) and their receptors such as CXCR4 are thought to play a critical role in motility, homing, and proliferation of cancer cells at specific metastatic sites. We and others have previously reported that the extracellular signal-activated transcription factor NF-kappaB up-regulates the expression of matrix metalloproteinases, urokinase-type plasminogen activator, and cytokines in highly metastatic breast cancer cell lines. In this report, we demonstrate that NF-kappaB regulates the motility of breast cancer cells by directly up-regulating the expression of CXCR4. Overexpression of the inhibitor of kappaB (IkappaB) in breast cancer cells with constitutive NF-kappaB activity resulted in reduced expression of CXCR4 and a corresponding loss of SDF-1alpha-mediated   migration   in vitro. Introduction of CXCR4 cDNA into IkappaB-expressing cells restored SDF-1alpha-mediated   migration  . Electrophoretic mobility shift assays and transient transfection assays revealed that the NF-kappaB subunits p65 and p50 bind directly to sequences within the -66 to +7 region of the CXCR4 promoter and activate transcription. We also show that the cell surface expression of CXCR4 and the SDF-1alpha-mediated   migration   are enhanced in breast cancer cells isolated from mammary fat pad xenografts compared with parental cells grown in culture. A further increase in CXCR4 cell surface expression and SDF-1alpha-mediated   migration   was observed with cancer cells that metastasized to the lungs. Taken together, these results implicate NF-kappaB in the   migration   and the organ-specific homing of metastatic breast cancer cells. 
 Suppression of the invasive potential of Glioblastoma cells by mTOR inhibitors involves modulation of NFκB and PKC-α signaling. Glioblastoma (GBM) is the most aggressive type of brain tumors in adults with survival period <1.5 years of patients. The role of mTOR pathway is documented in invasion an  d migrati  on, the features associated with aggressive phenotype in human GBM. However, most of the preclinical and clinical studies with mTOR inhibitors are focused on antiproliferative and cytotoxic activity in GBM. In this study, we demonstrate that mTOR inhibitors-rapamycin (RAP), temisirolimus (TEM), torin-1 (TOR) and PP242 suppress invasion an  d migrati  on induced by Tumor Necrosis Factor-α (TNFα) and tumor promoter, Phorbol 12-myristate 13-acetate (PMA) and also reduce the expression of the TNFα and IL1β suggesting their potential to regulate factors in microenvironment that support tumor progression. The mTOR inhibitors significantly decreased MMP-2 and MMP-9 mRNA, protein and activity that was enhanced by TNFα and PMA. The effect was mediated through reduction of Protein kinase C alpha (PKC-α) activity and downregulation of NFκB. TNFα- induced transcripts of NFκB targets -VEGF, pentraxin-3, cathepsin-B and paxillin, crucial in invasion were restored to basal level by these inhibitors. With limited therapeutic interventions currently available for GBM, our findings are significant and suggest that mTOR inhibitors may be explored as anti-invasive drugs for GBM treatment. 
 PinX1 serves as a potential prognostic indicator for clear cell renal cell carcinoma and inhibits its invasion and metastasis by suppressing MMP-2 via NF-κB-dependent transcription. PIN2/TRF1-interacting telomerase inhibitor 1 (PinX1) is a novel cloned gene which has been identified as a major haploinsufficient tumor suppressor essential for maintaining telomerase activity, the length of telomerase and chromosome stability. This study explored the clinical significance and biological function of PinX1 in human clear cell renal cell carcinoma (ccRCC). The clinical relevance of PinX1 in ccRCC was evaluated using tissue microarray and immunohistochemical staining in two independent human ccRCC cohorts. Our data demonstrated that PinX1 expression was dramatically decreased in ccRCC tissues compared with normal renal tissues and paired adjacent non-tumor tissues. Low PinX1 expression was significantly correlated with depth of invasion, lymph node metastasis and advanced TNM stage in patients, as well as with worse overall and disease-specific survival. Cox regression analysis revealed that PinX1 expression was an independent prognostic factor for ccRCC patients. Moreover, PinX1 inhibited the   migratio  n and invasion of ccRCC by suppressing MMP-2 expression and activity via NF-κB-dependent transcription in vitro. In vivo studies confirmed that PinX1 negatively regulated ccRCC metastasis and the expression of MMP-2 and NF-κB-p65. These findings indicate that PinX1 suppresses ccRCC metastasis and may serve as a ccRCC candidate clinical prognostic marker and a potential therapeutic target. 
 HDAC activity is required for p65/RelA-dependent repression of PPARdelta-mediated transactivation in human keratinocytes. Peroxisome proliferator-activated receptors (PPARs) play a key role in differentiation, inflammation,   migration  , and survival of epidermal keratinocytes. The NF-kappaB has long been known to play pivotal roles in immune and inflammatory responses, and furthermore NF-kappaB has been implicated in the regulation of epidermal homeostasis. Recent studies have established that p65/RelA is a potent repressor of PPARdelta-mediated transactivation in human keratinocytes. In this article we further investigate the molecular mechanisms dictating the NF-kappaB-dependent repression of PPARdelta in human keratinocytes. We demonstrate that repression is unique to p65/RelA, as no other member of the NF-kappaB family had an impact on PPARdelta-mediated transactivation. Interestingly, our results show that p65/RelA only represses PPARdelta-dependent transactivation when PPARdelta is bound to DNA via its DNA-binding domain. We show that repression is sensitive to inhibition of histone deacetylases (HDACs) by tricostatin A (TSA), suggesting that HDAC activity is indispensable for p65/RelA-mediated repression. Accordingly, we demonstrate that a ternary complex consisting of PPARdelta, p65/RelA, and HDAC1 is formed in vivo. Finally, we demonstrate that TSA relieves tumor necrosis factor-alpha (TNFalpha)-induced repression of PPARdelta-mediated transactivation of the PPARdelta target gene adipose differentiation-related protein (ADRP) indicating that cross-talk between PPARdelta and NF-kappaB is of biological significance in human keratinocytes. 
 microRNA-372 Suppresses   Migration   and Invasion by Targeting p65 in Human Prostate Cancer Cells. Prostate cancer (PCa) is one of the most prevalent malignant tumors. microRNAs (miRNAs) play an important role in cancer initiation, progression, and metastasis, and their roles in PCa are becoming more apparent. In this study, we found that microRNA-372 (miR-372) is downregulated in human PCa and inhibits the proliferation activity,   migration  , and invasion of DU145 cells. Subsequently, p65 is confirmed as a target of miR-372, and knockdown of p65 expression similarly resulted in decreased proliferation activity,   migration  , and invasion. CDK8, MMP-9, and prostate-specific antigen were involved in both these processes. Taken together, our results show evidence that miR-372 may function as a tumor suppressor gene by regulating p65 in PCa and may provide a strategy for blocking PCa metastasis. 
 [Effect of topotecan on expression of aquaporin protein 5 and nuclear factor-kappaB in ovarian cancer SKOV3 cells]. Overexpression of aquaporin protein 5 (AQP5) is associated with the metastasis,   migration   and angiogenesis of ovarian cancer, but its function in cell proliferation has not been described. This study was to explore the effects of topotecan (TPT) on the expression of AQP5, nuclear factor-kappaB (NF-kappaB) and its receptor IkappaBalpha in ovarian cancer SKOV3 cells.When SKOV3 cells were treated with TPT of various concentrations for different time, cell proliferation was measured by MTT assay, the expression of AQP5, NF-kappaB and IkappaBalpha was detected by Western blot.After SKOV3 cells were incubated with 0.4 microg/mL TPT for 24 h, the expression of AQP5, NF-kappaB p65 in cytoplasm and nuclei, and IkappaBalpha in cytoplasm were decreased, and remained at low levels till 72 h (P<0.05). When SKOV3 cells were treated with 0.2, 0.4, 0.6 and 0.8 microg/mL TPT for 24 h, the expression of AQP5 as well as NF-kappaB p65 and IkappaBalpha in nuclei were decreased (P<0.005). The protein level of AQP5 was decreased by 57.9% when cells were treated with 0.8 microg/mL TPT. AQP5 expression was negatively correlated to the proliferation inhibition rate of SKOV3 cells induced by TPT (r=-0.965, P<0.05), and positively correlated to NF-kappaB p65 and IkappaBalpha expression (r=0.903, 0.896, P<0.05).When inhibiting the proliferation of ovarian cancer cells, TPT may down-regulate the expression of AQP5 and NF-kappaB. 
 p75(NTR)-dependent activation of NF-κB regulates microRNA-503 transcription and pericyte-endothelial crosstalk in diabetes after limb ischaemia. The communication between vascular endothelial cells (ECs) and pericytes in the microvasculature is fundamental for vascular growth and homeostasis; however, these processes are disrupted by diabetes. Here we show that modulation of p75(NTR) expression in ECs exposed to high glucose activates transcription of miR-503, which negatively affects pericyte function. p75(NTR) activates NF-κB to bind the miR-503 promoter and upregulate miR-503 expression in ECs. NF-κB further induces activation of Rho kinase and shedding of endothelial microparticles carrying miR-503, which transfer miR-503 from ECs to vascular pericytes. The integrin-mediated uptake of miR-503 in the recipient pericytes reduces expression of EFNB2 and VEGFA, resulting in impair  ed migrat  ion and proliferation. We confirm operation of the above mechanisms in mouse models of diabetes, in which EC-derived miR-503 reduces pericyte coverage of capillaries, increased permeability and impaired post-ischaemic angiogenesis in limb muscles. Collectively, our data demonstrate that miR-503 regulates pericyte-endothelial crosstalk in microvascular diabetic complications. 
 Suppression of NF-kappaB-dependent gene expression by a hexamethylene bisacetamide-inducible protein HEXIM1 in human vascular smooth muscle cells. Neointima formation is a characteristic feature of atherosclerosis and post-angioplasty restenosis, in which various soluble factors and mechanical injury stimulate signalling pathways in vascular smooth muscle cells (VSMC), promoting their   migration   and proliferation, and the eventual formation of the neointima. The transcription factor NF-kappaB has been shown to play a pivotal role in this process. Hexamethylene bisacetamide, an inhibitor of VSMC proliferation, induces the mRNA expression of HEXIM1 (hexamethylene bisacetamide-inducible protein 1). However, the protein expression and function of HEXIM1 remain unknown.In the present study, we demonstrated that HEXIM1 localizes in the cytoplasm and nucleus, and its nuclear expression is restricted to discrete speckled areas. Treatment of VSMC with hexamethylene bisacetamide up-regulated HEXIM1 expression, not only in mRNA but also protein levels. Moreover, HEXIM1 is shown to suppress the transcriptional activity of NF-kappaB via its C-terminal leucine-rich domain. A glutathione-S-transferase pull down assay indicated that HEXIM1 interacts with the p65 subunit of NF-kappaB. In VSMC, treatment with hexamethylene bisacetamide resulted in a down-modulation of the transcription of NF-kappaB target genes.We may therefore conclude that HEXIM1 plays an inhibitory role in NF-kappaB-dependent gene expression in VSMC and is the candidate of a novel therapeutic target for inhibition of VSMC proliferation. 
 Differential regulation of MMPs by E2F1, Sp1 and NF-kappa B controls the small cell lung cancer invasive phenotype. E2F1 transcription factor plays a vital role in the regulation of diverse cellular processes including cell proliferation, apoptosis, invasion and metastasis. E2F1 overexpression has been demonstrated in small cell lung cancer (SCLC), and extensive metastasis in early phase is the most important feature of SCLC. In this study, we investigated the involvement of E2F1 in the process of invasion and metastasis in SCLC by regulating the expression of matrix metalloproteinases (MMPs).Immunohistochemistry was performed to evaluate the expression of E2F1 and MMPs in SCLC samples in a Chinese Han population. The impact of E2F1 on invasion and metastasis was observed by transwell and wound healing experiments with depletion of E2F1 by specific siRNA. The target genes regulated by E2F1 were identified by chromatin immunoprecipitation (ChIP)-to-sequence, and the expressions of target genes were detected by real time PCR and western blotting. The dual luciferase reporter system was performed to analyze the regulatory relationship between E2F1 and MMPs.E2F1 is an independent and adverse prognosis factor that is highly expressed in SCLC in a Chinese Han population. Knockdown of E2F1 by specific siRNA resulted in the downregulation of   migration   and invasion in SCLC. The expressions of MMP-9 and -16 in SCLC were higher than other MMPs, and their expressions were most significantly reduced after silencing E2F1. ChIP-to-sequence and promoter-based luciferase analysis demonstrated that E2F1 directly controlled MMP-16 expression via an E2F1 binding motif in the promoter. Although one E2F1 binding site was predicted in the MMP-9 promoter, luciferase analysis indicated that this binding site was not functionally required. Further study demonstrated that E2F1 transcriptionally controlled the expression of Sp1 and p65, which in turn enhanced the MMP-9 promoter activity in SCLC cells. The associations between E2F1, Sp1, p65, and MMP-9 were validated by immunohistochemistry staining in SCLC tumors.E2F1 acts as a transcriptional activator for MMPs and directly enhances MMP transcription by binding to E2F1 binding sequences in the promoter, or indirectly activates MMPs through enhanced Sp1 and NF-kappa B as a consequence of E2F1 activation in SCLC. 
 NF-κB and STAT3 cooperatively induce IL6 in starved cancer cells. A number of genes involved in tumorigenesis have been known to be controlled by signal transducer and activator of transcription 3 (STAT3) and NF-κB, either synergistically or individually. In starved cancer cells, we found that NF-κB was activated through endoplasmic reticulum stress signals, which depend on reactive oxygen species, cytosolic calcium and preserved translation of NF-κB p65 subunit, but independent of IκBα serine phosphorylation, thereby resulting in IL6 induction. STAT3 was required for proper induction of IL6 by NF-κB. They existed as identical nuclear complexes in proximal IL6 promoters, and STAT3 had critical roles in binding to IL6 promoters as well as nuclear retention of NF-κB. The conditioned media from starved cancer cells contained various secretory factors, such as IL6, IL9, VWF (von Willebrand factor), FREM1 (FRAS1 related extracellular matrix 1), SAA1 (serum amyloid A1), SDK1 (sidekick homolog 1) and ADAM12 (ADAM metallopeptidase domain 12), induced by NF-κB and STAT3 and promoted clonogenic capacities of cancer cells, and prolifera  tion and   migration of human umbilical vein endothelial cells. These results suggest novel survival strategies of cancer cells by which two oncogenic transcriptional factors, NF-κB and STAT3, are activated simultaneously by an intrinsic mechanism during stressful conditions of cancer cells, and they cooperatively induce various survival factors. 
 NF-κB-mediated transcriptional upregulation of TNFAIP2 by the Epstein-Barr virus oncoprotein, LMP1, promotes cell motility in nasopharyngeal carcinoma. Nasopharyngeal carcinoma (NPC), which is closely associated with Epstein-Barr virus (EBV), is a metastasis-prone epithelial cancer. We previously showed that tumor necrosis factor α-induced protein 2 (TNFAIP2) is highly expressed in NPC tumor tissues and is correlated with metastasis and poor survival in NPC patients. However, the underlying mechanism remains unclear. In this study, we demonstrate that the EBV oncoprotein, latent membrane protein 1 (LMP1), can transcriptionally induce TNFAIP2 expression via NF-κB. Quantitative RT-PCR and western blotting revealed that LMP1 induces TNFAIP2 expression through its C-terminal-activating region (CTAR2) domain, which is required for transduction of NF-κB (nuclear factor kappa-light-chain-enhancer of activated B cells) signaling. Inhibition of NF-κB activation or depletion of p65 (a component of NF-κB) by RNA interference abolished the LMP1-induced expression of TNFAIP2, whereas ectopic expression of p65 was sufficient to induce TNFAIP2 expression. Luciferase reporter assays showed that LMP1 transcriptionally induces TNFAIP2 expression through a newly identified NF-κB-binding site within the TNFAIP2 promoter (-3,869 to -3,860 bp). Immunohistochemical analysis of NPC biopsy specimens further revealed a significant correlation between the protein levels of TNFAIP2 and activated p65 (R=0.689, P<0.001), indicating that our findings are clinically relevant. Immunofluorescence microscopy and co-immunoprecipitation assays showed that TNFAIP2 associates with actin and is involved in the formation of actin-based membrane protrusions. Furthermore, t  ranswell   migration assays demonstrated that TNFAIP2 contributes to LMP1-induced cell motility. Collectively, these findings provide novel insights into the regulation of TNFAIP2 and its role in promoting NPC tumor progression. 
 PKD2 and PKD3 promote prostate cancer cell invasion by modulating NF-κB- and HDAC1-mediated expression and activation of uPA. Although protein kinase D3 (PKD3) has been shown to contribute to prostate cancer cell growth and survival, the role of PKD in prostate cancer cell motility remains unclear. Here, we show that PKD2 and PKD3 promote nuclear factor kappa B (NF-κB) signaling and urokinase-type plasminogen activator (uPA) expression/activation, which are crucial for prostate cancer cell invasion. Silencing of endogenous PKD2 and/or PKD3 markedly decreased prostate cancer cel  l migrati  on and invasion, reduced uPA and uPA receptor (uPAR) expression and increased plasminogen activator inhibitor-2 (PAI-2) expression. These results were further substantiated by the finding that PKD2 and PKD3 promoted the activity of uPA and matrix metalloproteinase 9 (MMP9). Furthermore, depletion of PKD2 and/or PKD3 decreased the level of binding of the p65 subunit of NF-κB to the promoter of the gene encoding uPA (PLAU), suppressing transcriptional activation of uPA. Endogenous PKD2 and PKD3 interacted with inhibitor of NF-κB (IκB) kinase β (IKKβ); PKD2 mainly regulated the phosphorylated IKK (pIKK)-phosphorylated IκB (pIκB)-IκB degradation cascade, p65 nuclear translocation, and phosphorylation of Ser276 on p65, whereas PKD3 was responsible for the phosphorylation of Ser536 on p65. Conversely, inhibition of uPA transactivation by PKD3 silencing was rescued by constitutive Ser536 p65 phosphorylation, and reduced tumor cell invasion resulting from PKD2 or PKD3 silencing was rescued by ectopic expression of p65. Interestingly, PKD3 interacted with histone deacetylase 1 (HDAC1), suppressing HDAC1 expression and decreasing its binding to the uPA promoter. Moreover, depletion of HDAC1 resulted in recovery of uPA transactivation in PKD3-knockdown cells. Taken together, these data suggest that PKD2 and PKD3 coordinate to promote prostate cancer cell invasion through p65 NF-κB- and HDAC1-mediated expression and activation of uPA. 
 Inhibiting valosin-containing protein suppresses osteosarcoma cell metastasis via AKT/nuclear factor of kappa B signaling pathway in vitro. The strategies of targeting valosin-containing protein (VCP) may have therapeutic potential for treating cancer metastasis. In this study, we aim to investigate the correlation of VCP protein expression in osteosarcoma (OS) tissues with pulmonary metastasis and its possible molecular mechanism.Expression of VCP in 60 OS specimens was detected by immunohistochemistry (IHC) and the relationship with metastasis was analyzed. An artificial micro ribonucleic acid, targeting VCP, was performed to silence the expression of VCP in U2-OS cells. Cell mobility was detected by wound healing and Transwell assays. Western blot and real-time polymerase chain reaction were performed to investigate the expression of VCP in U2-OS cells. Furthermore, the protein of pAKT (phosphorylated serine/threonine protein kinase) and nuclear factor of kappa B protein 65 were measured by western blot to evaluate the effect of silencing VCP on AKT/nuclear factor of kappa B (NF-kB) signaling pathway.The results showed that the expression level of VCP protein in cases with pulmonary metastases was significantly higher than that in those without metastasis (P = 0.004). The invasion and   migration   of U2-OS cells were suppressed by silencing VCP. Furthermore, silencing VCP could down-regulate the phosphorylation of AKT and nuclear transfer of NF-kB.Our findings suggested that inhibition of VCP could suppress OS cells invasion and   migration   through down-regulating AKT/NF-kB signaling pathway. 
 Down-regulation of the tumor suppressor PTEN by the tumor necrosis factor-alpha/nuclear factor-kappaB (NF-kappaB)-inducing kinase/NF-kappaB pathway is linked to a default IkappaB-alpha autoregulatory loop. The PTEN (phosphatase and tensin homolog deleted on chromosome ten) tumor suppressor gene affects multiple cellular processes including cell growth, proliferation, and cell   migration   by antagonizing phosphatidylinositol 3-kinase (PI3K). However, mechanisms by which PTEN expression is regulated have not been studied extensively. Similar to PTEN, tumor necrosis factor-alpha (TNF-alpha) affects a wide spectrum of diseases including inflammatory processes and cancer by acting as a mediator of apoptosis, inflammation, and immunity. In this study, we show that treatment of cancer cell lines with TNF-alpha decreases PTEN expression. In addition, overexpression of TNF-alpha downstream signaling targets, nuclear factor-kappaB (NF-kappaB)-inducing kinase (NIK) and p65 nuclear factor NF-kappaB, lowers PTEN expression, suggesting that TNF-alpha-induced down-regulation of PTEN is mediated through a TNF-alpha/NIK/NF-kappaB pathway. Down-regulation of PTEN by NIK/NF-kappaB results in activation of the PI3K/Akt pathway and augmentation of TNF-alpha-induced PI3K/Akt stimulation. Importantly, we demonstrate that this effect is associated with a lack of an inhibitor of kappaB (IkappaB)-alpha autoregulatory loop. Moreover, these findings suggest the interaction between PI3K/Akt and NF-kappaB via transcriptional regulation of PTEN and offer one possible explanation for increased tumorigenesis in systems in which NF-kappaB is chronically activated. In such a tumor system, these findings suggest a positive feedback loop whereby Akt activation of NF-kappaB further stimulates Akt via down-regulation of the PI3K inhibitor PTEN. 
 IKK-2 is required for TNF-alpha-induced invasion and proliferation of human mesenchymal stem cells. Mesenchymal stem cells (MSCs) can contribute to tissue repair by actively migrating to sites of tissue injury. However, the cellular and molecular mechanisms of MSC recruitment are largely unknown. The nuclear factor (NF)-kappaB pathway plays a pivotal role in regulating genes that influence cell   migration  , cell differentiation, inflammation, and proliferation. One of the major cytokines released at sites of injury is tumor necrosis factor-alpha (TNF-alpha), which is known to be a key regulator of the NF-kappaB pathway. Therefore, we hypothesized that TNF-alpha may lead to MSC invasion and proliferation by activation of the NF-kappaB pathway. TNF-receptor 1 and 2, NF-kappaB (p65), and IkappaB kinase 2 (IKK-2) are expressed in human MSCs (hMSCs). Stimulation of hMSCs with TNF-alpha caused a p65 translocation from the cytoplasm to nucleoplasm but did not change the expression profile of MSC markers. TNF-alpha strongly augmented the   migration   of hMSCs through the human extracellular matrix. Using lentiviral gene transfer, overexpressing a dominant-negative mutant of IKK-2 (dn-IKK-2) significantly blocked this effect. NF-kappaB target genes associated with   migration   (vascular cell adhesion molecule-1, CD44, and matrix metalloproteinase 9) were upregulated by TNF-alpha stimulation and blocked by dn-IKK-2. Moreover, using the bromodeoxyuridine assay, we showed that the inhibition of the NF-kappaB pathway caused a significant reduction in the basal proliferation rate. TNF-alpha stimulated the proliferation of hMSCs, whereas overexpression of dn-IKK-2 significantly blocked this effect. TNF-alpha led to the upregulated expression of the proliferation-associated gene cyclin D1. In conclusion, we demonstrated that the NF-kappaB pathway components, p65 and IKK-2, are expressed in hMSCs. Our data provide evidence that this signal transduction pathway is implicated in TNF-alpha-mediated invasion and proliferation of hMSCs. Therefore, hMSC recruitment to sites of tissue injury may, at least in part, be regulated by the NF-kappaB signal transduction pathway. 
  GeneRIFs  
 TCF-4 is a co-activator of NF-kappaB p65 that promotes MMP-15 transcription and potentiate the   migration   activity of the lung cancer cells 
 microRNA-372 suppresses   migration   and invasion by targeting p65 in human prostate cancer cells. 
 this study shows that the mTOR inhibitors suppression of invasion and   migration   of glioblastoma cells is mediated by reduction of PKC-alpha activity and downregulation of NFkappaB 
 These findings demonstrate that Tan-IIA inhibits the   migration   ability of AGS human gastric cancer cells and that decreasing the protein expression of NF-kappaB-p65, COX-2, and MMP-2, -7 and -9 may be an underlying molecular mechanism 
 Findings indicate that CARMA3 may suppress the activation of the P38 MAPK signaling pathway to regulate invasion,   migration   and apoptosis of lung cancer cells by activating NF-small ka, CyrillicB (P65) in the nucleus. 
 Inactivation of M2 AChR/NF-kappaB signaling axis reverses epithelial-mesenchymal transition and suppresses   migration   and invasion in non-small cell lung cancer. 
 NF-kappaBp65 inhibition significantly reduced the   migration   and adhesion ability of A549 cells. 
 These findings suggested that inhibition of VCP could suppress osteosarcoma cells invasion and   migration   through down-regulating AKT/NF-kB signaling pathway. 
 RGC32 promotes cell   migration   and invasion and induces epithelial-mesenchymal transition in lung cancer cells via the NF-kappaB signaling pathway. 
 NF-kappaB has a role in the   migration   and the organ-specific homing of metastatic breast cancer cells 
  ECM  PubMed  
 NF-κB regulates radioresistance mediated by β1-integrin in three-dimensional culture of breast cancer cells. β1-integrin induction enhances breast cancer cell survival after exposure to ionizing radiation (IR), but the mechanisms of this effect remain unclear. Although NF-κB initiates prosurvival signaling pathways post-IR, the molecular function of NF-κB with other key elements in radioresistance, particularly with respect to extracellular matrix-induced signaling, is not known. We discovered a typical NF-κB-binding site in the β1-integrin promoter region, indicating a possible regulatory role for NF-κB. Using three-dimensional laminin-rich extracellular matri  x (  3D lrECM) culture, we show that NF-κB is required for β1-integrin transactivation in T4-2 breast cancer cells post-IR. Inhibition of NF-κB reduced clonogenic survival and induced apoptosis and cytostasis in formed tumor colonies. In addition, T4-2 tumors with inhibition of NF-κB activity exhibit decreased growth in athymic mice, which was further reduced by IR with downregulated β1-integrin expression. Direct interactions between β1-integrin and NF-κB p65 were induced in nonmalignant breast epithelial cells, but not in malignant cells, indicating context-specific regulation. As β1-integrin also activates NF-κB, our findings reveal a novel forward feedback pathway that could be targeted to enhance therapy. 
 NF-κB regulates radioresistance mediated by β1-integrin in three-dimensional culture of breast cancer cells. β1-integrin induction enhances breast cancer cell survival after exposure to ionizing radiation (IR), but the mechanisms of this effect remain unclear. Although NF-κB initiates prosurvival signaling pathways post-IR, the molecular function of NF-κB with other key elements in radioresistance, particularly with respect to extracellular matrix-induced signaling, is not known. We discovered a typical NF-κB-binding site in the β1-integrin promoter region, indicating a possible regulatory role for NF-κB. Using three-dimensional laminin-rich extracellular matri  x (  3D lrECM) culture, we show that NF-κB is required for β1-integrin transactivation in T4-2 breast cancer cells post-IR. Inhibition of NF-κB reduced clonogenic survival and induced apoptosis and cytostasis in formed tumor colonies. In addition, T4-2 tumors with inhibition of NF-κB activity exhibit decreased growth in athymic mice, which was further reduced by IR with downregulated β1-integrin expression. Direct interactions between β1-integrin and NF-κB p65 were induced in nonmalignant breast epithelial cells, but not in malignant cells, indicating context-specific regulation. As β1-integrin also activates NF-κB, our findings reveal a novel forward feedback pathway that could be targeted to enhance therapy. 
  [Ee]xtracellular [Mm]atrix  PubMed  
 The androgen receptor induces integrin α6β1 to promote prostate tumor cell survival via NF-κB and Bcl-xL Independently of PI3K signaling. Recent studies indicate that androgen receptor (AR) signaling is critical for prostate cancer cell survival, even in castration-resistant disease wherein AR continues to function independently of exogenous androgens. Integrin-mediated adhesion to t  he extracellular mat  rix is also important for prostate cell survival. AR-positive prostate cancer cells express primarily integrin α6β1 and adhere to a laminin-rich matrix. In this study, we show that active nuclear-localized AR protects prostate cancer cells from death induced by phosphoinositide 3-kinase (PI3K) inhibition when cells adhere to laminin. Resistance to PI3K inhibition is mediated directly by an AR-dependent increase in integrin α6β1 mRNA transcription and protein expression. Subsequent signaling by integrin α6β1 in AR-expressing cells increased NF-κB activation and Bcl-xL expression. Blocking AR, integrin α6, NF-κB, or Bcl-xL concurrent with inhibition of PI3K was sufficient and necessary to trigger death of laminin-adherent AR-expressing cells. Taken together, these results define a novel integrin-dependent survival pathway in prostate cancer cells that is regulated by AR, independent of and parallel to the PI3K pathway. Our findings suggest that combined targeting of both the AR/α6β1 and PI3K pathways may effectively trigger prostate cancer cell death, enhancing the potential therapeutic value of PI3K inhibitors being evaluated in this setting. 
 Iron deficiency activates pro-inflammatory signaling in macrophages and foam cells via the p38 MAPK-NF-κB pathway. Major bleeding in patients with acute coronary syndrome (ACS) increases the risk of recurrent ACS and mortality. However, the mechanism involved is poorly understood. Bleeding induces iron deficiency. Iron deficiency enhances inflammation in other diseases. Thus, in this paper, the particular effect of iron deficiency on atherosclerotic plaque destabilization, especially the pro-inflammatory role of iron deficiency in atheroma and the mechanism involved were investigated  .Extracellular matri  x metalloproteinase inducer (EMMPRIN) and matrix metalloproteinase-9 (MMP-9) mRNA levels were investigated by RT-PCR. EMMPRIN and MMP-9 protein levels, nuclear factor (NF)-κB-p65 protein levels, peroxisome proliferator-activated receptor γ (PPARγ) protein levels, and mitogen-activated protein kinase (MAPK) phosphorylation were determined by western blotting. MMP-9 enzymatic activity was assayed by gelatin zymography.Iron deficiency enhanced EMMPRIN, MMP-9 production, and MMP-9 enzymatic activity in THP-1 derived macrophages and foam cells. Iron deficiency elicited the activation of NF-κB and p38 MAPK. By using the p38 inhibitor and NF-κB inhibitor, the study established that EMMPRIN and MMP-9 inductions by iron deficiency required the consecutive upstream activation of p38 MAPK and NF-κB. This pro-inflammatory action was not prevented by PPARγ agonist. Meanwhile, iron deficiency did not modulate PPARγ expression. Retinoid X receptor agonist suppressed the effects of iron deficiency on EMMPRIN , MMP-9, and NF-κB, but not on MAPK activation.Iron deficiency enhances atheroma inflammation through p38 MAPK-NF-κB-EMMPRIN/MMP-9 pathway. Our findings provide a potential mechanism for the association of major bleeding with recurrent ACS and mortality in patients with ACS. 
 NF-κB regulates radioresistance mediated by β1-integrin in three-dimensional culture of breast cancer cells. β1-integrin induction enhances breast cancer cell survival after exposure to ionizing radiation (IR), but the mechanisms of this effect remain unclear. Although NF-κB initiates prosurvival signaling pathways post-IR, the molecular function of NF-κB with other key elements in radioresistance, particularly with respec  t to extracellular m  atrix-induced signaling, is not known. We discovered a typical NF-κB-binding site in the β1-integrin promoter region, indicating a possible regulatory role for NF-κB. Using three-dimensional lamin  in-rich extracellula  r matrix (3D lrECM) culture, we show that NF-κB is required for β1-integrin transactivation in T4-2 breast cancer cells post-IR. Inhibition of NF-κB reduced clonogenic survival and induced apoptosis and cytostasis in formed tumor colonies. In addition, T4-2 tumors with inhibition of NF-κB activity exhibit decreased growth in athymic mice, which was further reduced by IR with downregulated β1-integrin expression. Direct interactions between β1-integrin and NF-κB p65 were induced in nonmalignant breast epithelial cells, but not in malignant cells, indicating context-specific regulation. As β1-integrin also activates NF-κB, our findings reveal a novel forward feedback pathway that could be targeted to enhance therapy. 
 NF-κB regulates radioresistance mediated by β1-integrin in three-dimensional culture of breast cancer cells. β1-integrin induction enhances breast cancer cell survival after exposure to ionizing radiation (IR), but the mechanisms of this effect remain unclear. Although NF-κB initiates prosurvival signaling pathways post-IR, the molecular function of NF-κB with other key elements in radioresistance, particularly with respec  t to extracellular m  atrix-induced signaling, is not known. We discovered a typical NF-κB-binding site in the β1-integrin promoter region, indicating a possible regulatory role for NF-κB. Using three-dimensional lamin  in-rich extracellula  r matrix (3D lrECM) culture, we show that NF-κB is required for β1-integrin transactivation in T4-2 breast cancer cells post-IR. Inhibition of NF-κB reduced clonogenic survival and induced apoptosis and cytostasis in formed tumor colonies. In addition, T4-2 tumors with inhibition of NF-κB activity exhibit decreased growth in athymic mice, which was further reduced by IR with downregulated β1-integrin expression. Direct interactions between β1-integrin and NF-κB p65 were induced in nonmalignant breast epithelial cells, but not in malignant cells, indicating context-specific regulation. As β1-integrin also activates NF-κB, our findings reveal a novel forward feedback pathway that could be targeted to enhance therapy. 
 Iron deficiency activates pro-inflammatory signaling in macrophages and foam cells via the p38 MAPK-NF-κB pathway. Major bleeding in patients with acute coronary syndrome (ACS) increases the risk of recurrent ACS and mortality. However, the mechanism involved is poorly understood. Bleeding induces iron deficiency. Iron deficiency enhances inflammation in other diseases. Thus, in this paper, the particular effect of iron deficiency on atherosclerotic plaque destabilization, especially the pro-inflammatory role of iron deficiency in atheroma and the mechanism involved were investigated  .Extracellular matri  x metalloproteinase inducer (EMMPRIN) and matrix metalloproteinase-9 (MMP-9) mRNA levels were investigated by RT-PCR. EMMPRIN and MMP-9 protein levels, nuclear factor (NF)-κB-p65 protein levels, peroxisome proliferator-activated receptor γ (PPARγ) protein levels, and mitogen-activated protein kinase (MAPK) phosphorylation were determined by western blotting. MMP-9 enzymatic activity was assayed by gelatin zymography.Iron deficiency enhanced EMMPRIN, MMP-9 production, and MMP-9 enzymatic activity in THP-1 derived macrophages and foam cells. Iron deficiency elicited the activation of NF-κB and p38 MAPK. By using the p38 inhibitor and NF-κB inhibitor, the study established that EMMPRIN and MMP-9 inductions by iron deficiency required the consecutive upstream activation of p38 MAPK and NF-κB. This pro-inflammatory action was not prevented by PPARγ agonist. Meanwhile, iron deficiency did not modulate PPARγ expression. Retinoid X receptor agonist suppressed the effects of iron deficiency on EMMPRIN , MMP-9, and NF-κB, but not on MAPK activation.Iron deficiency enhances atheroma inflammation through p38 MAPK-NF-κB-EMMPRIN/MMP-9 pathway. Our findings provide a potential mechanism for the association of major bleeding with recurrent ACS and mortality in patients with ACS. 
 Breast cancer metastasis suppressor 1 functions as a corepressor by enhancing histone deacetylase 1-mediated deacetylation of RelA/p65 and promoting apoptosis. The antiapoptotic transcription factor NF-kappaB is constitutively activated in many cancers and is important for cytokine-mediated progression and metastatic movement of tumors. Breast cancer metastasis suppressor 1 (BRMS1) is a metastasis suppressor gene whose mechanisms of action are poorly understood. In this report, we demonstrate that BRMS1 decreases the transactivation potential of RelA/p65 and ameliorates the expression of NF-kappaB-regulated antiapoptotic gene products. BRMS1 immunoprecipitates with the RelA/p65 subunit of NF-kappaB with protein-protein interactions occurring at the C terminus region of the rel homology domain but not at its known transactivation domains. Moreover, BRMS1 functions as a corepressor by promoting binding of HDAC1 to RelA/p65, where it deacetylates lysine K310 on RelA/p65, which suppresses RelA/p65 transcriptional activity. Selective small interfering RNA knockdown of BRMS1 confirms that chromatin-bound BRMS1 is required for deacetylation of RelA/p65, while enhancing chromatin occupancy of HDAC1 onto the NF-kappaB-regulated promoters cIAP2 and Bfl-1/A1. We observed in cells lacking BRMS1 a dramatic increase in cell viability after the loss of attachment from the   extracellular matrix  . Collectively, these results suggest that BRMS1 suppresses metastasis through its ability to function as a transcriptional corepressor of antiapoptotic genes regulated by NF-kappaB. 
 The elevated expression of osteopontin and NF-κB in human aortic aneurysms and its implication. The expression and significance of osteopontin (OPN) and NF-κB in patients with thoracic aortic aneurysm (TAA) and abdominal aortic aneurysm (AAA) were investigated. Thirteen TAA specimens, 20 AAA specimens and 6 normal aortic specimens were collected. The expression of OPN, nuclear factor-κB P65 (NF-κB P65), urokinase plasminogen activator (uPA), matrix metalloproteinase-2 (MMP-2) and matrix metalloproteinase-9 (MMP-9) were detected by using immunohistochemistry and Western blotting was employed to determine the expression of OPN and NF-κB P65. Immunohistochemical results showed that the expression of OPN, NF-κB P65, uPA, MMP-2 and MMP-9 was positive in all TAA and AAA specimens and negative in normal specimens, with the difference being statistically significant (P<0.05). There was no difference in the expression between TAA and AAA specimens (P>0.05). Correlation analysis revealed that there existed a positive correlation between the expression of OPN and that of NF-κB P65, uPA, MMP-2 and MMP-9 and between the expression of NF-κB P65 and that of uPA, MMP-2, MMP-9 (P<0.05). Western blotting demonstrated that OPN and NF-κB P65 were positive in AAA and TAA specimens, and negative in normal specimens with the differences being statistically significant (P<0.05). There were no statistically significant differences in the expression of OPN and NF-κB P65 between AAA and TAA specimens (P>0.05). It was concluded that OPN and NF-κB P65 were involved in the pathogenesis of TAA and AAA. OPN can up-regulate the expression of MMP and uPA via NF-κB signaling pathway thereby accelerating the deg  radation of extracel  lular matrix and playing an important role in the pathogenesis and development of TAA and AAA. 
 NF-kappaB induced by IL-1beta inhibits elastin transcription and myofibroblast phenotype. Interleukin (IL)-1beta released after lung injury regulates the production of   extracellular matrix   components. We found that IL-1beta treatment reduced the rate of elastin gene transcription by 74% in neonatal rat lung fibroblasts. Deletion analysis of the rat elastin promoter detected a cis-acting element located at -118 to -102 bp that strongly bound Sp1 and Sp3 but not nuclear factor (NF)-kappaB. This element mediated IL-1beta-induced inhibition of the elastin promoter. IL-1beta treatment did not affect the level of Sp1 but did induce translocation of the p65 subunit of NF-kappaB. Overexpression of p65 decreased elastin promoter activity and markedly reduced elastin mRNA. Immunoprecipitation studies indicated an interaction between the p65 subunit and Sp1 protein. Microarray analysis of mRNA isolated after overexpression of p65 or treatment with IL-1beta revealed downregulation of alpha-smooth muscle actin and calponin mRNAs. Expression of these genes is associated with the myofibroblast phenotype. These results indicate that IL-1beta activates the nuclear localization of NF-kappaB that subsequently interacts with Sp1 to downregulate elastin transcription and expression of the myofibroblast phenotype. 
 Tanshinone IIA decreases the migratory ability of AGS cells by decreasing the protein expression of matrix metalloproteinases, nuclear factor κB-p65 and cyclooxygenase-2. During progression of gastric cancer, degradation of the   extracellular matri  x by matrix metalloproteinases (MMPs) has been associated with poor prognosis. Tanshinone IIA (Tan-IIA) exerts antitumor activity in a variety of human cancer cells. It is extracted from Danshen (Salviae miltiorrhizae radix), and induces apoptosis and inhibits the proliferation of gastric cancer cells. However, the molecular mechanisms underlying the inhibition of migration in gastric cancer by Tan-IIA have not been fully elucidated. In the present study, AGS cell migration ability was evaluated using a wound-healing assay. The protein expression levels of nuclear factor (NF)-κB-p65, cyclooxygenase (COX)-2, MMP-2, -7, and -9 and β-actin in AGS cells were measured by western blotting. The results demonstrated that AGS cells treated with Tan-IIA exhibit decreased protein expression levels of NF-κB-p65, COX-2, and MMP-2, -7 and -9. The results also indicate that Tan-IIA inhibits migration ability in a dose- and time-dependent manner. These findings demonstrate that Tan-IIA inhibits the migration ability of AGS human gastric cancer cells and that decreasing the protein expression of NF-κB-p65, COX-2, and MMP-2, -7 and -9 may be an underlying molecular mechanism. 
 Integrin-linked kinase regulates melanoma angiogenesis by activating NF-κB/interleukin-6 signaling pathway. Integrin-linked kinase (ILK) is a highly conserved serine-threonine protein kinase involved in cell  -extracellular matri  x interactions, cytoskeletal organization and cell signaling. Overexpression of ILK in epithelial cells leads to anchorage-independent growth with increased cell cycle progression. Previously, we have shown that ILK upregulation strongly correlates with melanoma progression, invasion and inversely correlates with 5-year survival of melanoma patients. However, the molecular mechanism by which ILK enhances melanoma progression is currently unknown. In the present study, we found that proangiogenic molecule interleukin-6 (IL-6) is the downstream target of ILK in melanoma cells. ILK overexpression increased IL-6, whereas silencing of ILK suppressed IL-6 expression at both messenger RNA and protein levels. ILK also altered the activity and subcellular localization of nuclear factor-kappaB (NF-κB) subunit p65. We further found that ILK enhanced the IL-6 gene transcription by promoting the binding of NF-κB p65 to IL-6 promoter. Moreover, ILK overexpression in melanoma cells enhanced the tube-forming ability of endothelial cells in vitro and microvessel formation in vivo. ILK-induced tube and blood vessel formation of endothelial cells was significantly reduced upon IL-6 inhibition in ILK-overexpressing melanoma cells. To delineate the mechanism by which ILK-induced IL-6 production can enhance angiogenesis, further analysis of the downstream targets of IL-6 signaling showed an increased activity of the signal transducer and activator of transcription 3 (STAT3) in ILK-overexpressing cells. As STAT3 binds to vascular endothelial growth factor (VEGF) promoter, we found that VEGF levels were elevated in ILK-overexpressing cells and declined upon transfection of IL-6 small interfering RNA, suggesting that ILK may regulate VEGF expression through IL-6 pathway by activating STAT3. 
 Comparison of p38MAPK (mitogene activated protein kinase), p65 NFkappaB (nuclear factor kappa b) and EMMPRIN (  extracellular matrix   metalloproteinase inducer) expressions with tumor grade and stage of superficial and invasive bladder tumors. To identify the molecular mechanisms of bladder cancer invasion pathophysiology. To assess EMMPRIN, p65NFkappaB and p38MAPK expressions which play a role in signal transmission system of muscle and non muscle invasive bladder tumors. Fifty-seven patients with non muscle invasive tumors (mean age 65.2 +/- 16.1) and 34 patients with muscle invasive tumors (mean age 62.2 +/- 20.7) were included in this study. Normal tissue from the same patients' bladders were used as control group. Patients had either TUR or radical cystectomy and paraffin sections were prepared for immunohistochemistry. Expression density was evaluated semiquantitively according to tumor grade and invasion depth. Results were compared with Mann Whitney U, Wilcoxon W, Chi Square and variation analysis tests. MAPK and EMMPRIN expression was increased according to tumor grade (p < 0.05). These expressions were also significantly higher in muscle invasive tumors than in non muscle invasive ones (p < 0.05). In normal tissue samples of both TUR and radical cystectomy materials, EMMPRIN, NFkappaB and MAPK expressions were lower than in tumor samples (p < 0.05). NFkappaB wasn't related to tumor grade/stage (p > 0.05). It can be stated that MAPK and EMMPRIN expression is related to the grade of bladder tumor and that NFkappaB positivity is not related to the grade/stage of the disease. In future positivity of lymph nodes and visceral metastasis and survival must be assessed to define the relationship with the expressions in long term follow up studies involving a larger number of patients. 
 alpha6beta4 integrin activates Rac-dependent p21-activated kinase 1 to drive NF-kappaB-dependent resistance to apoptosis in 3D mammary acini. Malignant transformation and multidrug resistance are linked to resistance to apoptosis, yet the molecular mechanisms that mediate tumor survival remain poorly understood. Because the stroma can influence tumor behavior by regulating the tissue phenotype, we explored the role of   extracellular matrix   signaling and tissue organization in epithelial survival. We report that elevated (alpha6)beta4 integrin-dependent Rac-Pak1 signaling supports resistance to apoptosis in mammary acini by permitting stress-dependent activation of the p65 subunit of NF-kappaB through Pak1. We found that inhibiting Pak1 through expression of N17Rac or PID compromises NF-kappaB activation and renders mammary acini sensitive to death, but that resistance to apoptosis could be restored to these structures by overexpressing wild-type NF-kappaB p65. We also observed that acini expressing elevated levels of Pak1 can activate p65 and survive death treatments, even in the absence of activated Rac, yet will die if activation of NF-kappaB is simultaneously inhibited through expression of IkappaBalphaM. Thus, mammary tissues can resist apoptotic stimuli by activating NF-kappaB through alpha6beta4 integrin-dependent Rac-Pak1 signaling. Our data emphasize the importance of the   extracellular matrix   stroma in tissue survival and suggest that alpha6beta4 integrin-dependent Rac stimulation of Pak1 could be an important mechanism mediating apoptosis-resistance in some breast tumors. 
 NF-κB and STAT3 cooperatively induce IL6 in starved cancer cells. A number of genes involved in tumorigenesis have been known to be controlled by signal transducer and activator of transcription 3 (STAT3) and NF-κB, either synergistically or individually. In starved cancer cells, we found that NF-κB was activated through endoplasmic reticulum stress signals, which depend on reactive oxygen species, cytosolic calcium and preserved translation of NF-κB p65 subunit, but independent of IκBα serine phosphorylation, thereby resulting in IL6 induction. STAT3 was required for proper induction of IL6 by NF-κB. They existed as identical nuclear complexes in proximal IL6 promoters, and STAT3 had critical roles in binding to IL6 promoters as well as nuclear retention of NF-κB. The conditioned media from starved cancer cells contained various secretory factors, such as IL6, IL9, VWF (von Willebrand factor), FREM1 (FRAS1   related extracellula  r matrix 1), SAA1 (serum amyloid A1), SDK1 (sidekick homolog 1) and ADAM12 (ADAM metallopeptidase domain 12), induced by NF-κB and STAT3 and promoted clonogenic capacities of cancer cells, and proliferation and migration of human umbilical vein endothelial cells. These results suggest novel survival strategies of cancer cells by which two oncogenic transcriptional factors, NF-κB and STAT3, are activated simultaneously by an intrinsic mechanism during stressful conditions of cancer cells, and they cooperatively induce various survival factors. 
 The Changes in the Expression of NF-KB in a Degenerative Human Intervertebral Disc model. We aim at determining the changes in the expression of NF-kB signaling pathway in degenerative intervertebral discs. We collected normal and degenerated intervertebral discs tissues. The normal and degenerated cells were cultivated and their histopathology and immunofluoresence studies were used to observe the position of NF-kB p65 in the cell. We also treated the nucleus pulposus cells with inflammatory factors and inhibitors. Western blot was used to analyze the expression of different proteins. Real time fluorescence-based quantitative PCR was used for observation of NF-kB regulation of change in gene expression. Immunofluorescence showed that in the non-degenerative group the p65 was found in the cytoplasm of the nucleus pulposus cell while in the degenerated cell group the p65 protein was found in the nucleus of the cell. The expression of p65 increased with increase in the degree of degenerative change of the nucleus pulposus cell. RT-PCR showed that the expression of matrix metalloproteinases, aggrecanases and IL-6 was higher in the degenerative group. The amount of aggrecan and type II collagen was significantly decreased in the degenerative group. IL-1β was able to upregulate the activation of NF-kB and the expression of MMP-13 and ADAMTS-4 was also significantly increased. The effect of these proteins can be inhibited by the NF-kB inhibitor, BAY11-7082. The activation of the NK-kB signaling pathway in a degenerative intervertebral disc is gradually increased, regulating the over-expression of matrix-degrading enzymes. It plays an important role in the degradation of   extracellular matri  x. 
 Investigating NF-κB signaling in lung fibroblasts in 2D and 3D culture systems. Inflammatory respiratory diseases are amongst major global health challenges. Lung fibroblasts have been shown to play a key role in lung inflammatory responses. However, their exact role in initiation and maintenance of lung diseases has remained elusive partly due to the limited availability of physiologically relevant in vitro models. Therefore, developing new tools that enable investigating the molecular pathways (e.g. nuclear factor-kappa B (NF-κB) activation) that underpin inflammatory responses in fibroblasts could be a valuable resource for scientists working in this area of research.In order to investigate NF-κB activation in response to pro-inflammatory stimuli in real-time, we first developed two detection systems based on nuclear localization of NF-κB by immunostaining and luciferase reporter assay system. Furthermore using electrospun porous scaffolds, with similar geometry to human l  ung extracellular ma  trix, we developed 3D cultures of lung fibroblasts allowing comparing NF-κB activation in response to pro-inflammatory stimuli (i.e. TNF-α) in 2D and 3D. Our data clearly show that the magnitude of NF-κB activation in 2D cultures is substantially higher than 3D cultures. However, unlike 2D cultures, cells in the 3D model remained responsive to TNF-α at higher concentrations. The more subdued and wider dynamic range of NF-κB responses in 3D culture system was associated with a different expression pattern for TNF receptor I in 3D versus 2D cultures collectively reflecting a more in vivo like TNF receptor I expression and NF-κB activation pattern in the 3D system.Our data suggest that lung fibroblasts are actively involved in the pathogenesis of lung inflammation by activation of NF-κB signaling pathway. The 3D culture detection system provides a sensitive and biologically relevant tool for investigating different pro-inflammatory events involving lung fibroblasts. 
 IKK-2 is required for TNF-alpha-induced invasion and proliferation of human mesenchymal stem cells. Mesenchymal stem cells (MSCs) can contribute to tissue repair by actively migrating to sites of tissue injury. However, the cellular and molecular mechanisms of MSC recruitment are largely unknown. The nuclear factor (NF)-kappaB pathway plays a pivotal role in regulating genes that influence cell migration, cell differentiation, inflammation, and proliferation. One of the major cytokines released at sites of injury is tumor necrosis factor-alpha (TNF-alpha), which is known to be a key regulator of the NF-kappaB pathway. Therefore, we hypothesized that TNF-alpha may lead to MSC invasion and proliferation by activation of the NF-kappaB pathway. TNF-receptor 1 and 2, NF-kappaB (p65), and IkappaB kinase 2 (IKK-2) are expressed in human MSCs (hMSCs). Stimulation of hMSCs with TNF-alpha caused a p65 translocation from the cytoplasm to nucleoplasm but did not change the expression profile of MSC markers. TNF-alpha strongly augmented the migration of hMSCs through the human   extracellular matrix  . Using lentiviral gene transfer, overexpressing a dominant-negative mutant of IKK-2 (dn-IKK-2) significantly blocked this effect. NF-kappaB target genes associated with migration (vascular cell adhesion molecule-1, CD44, and matrix metalloproteinase 9) were upregulated by TNF-alpha stimulation and blocked by dn-IKK-2. Moreover, using the bromodeoxyuridine assay, we showed that the inhibition of the NF-kappaB pathway caused a significant reduction in the basal proliferation rate. TNF-alpha stimulated the proliferation of hMSCs, whereas overexpression of dn-IKK-2 significantly blocked this effect. TNF-alpha led to the upregulated expression of the proliferation-associated gene cyclin D1. In conclusion, we demonstrated that the NF-kappaB pathway components, p65 and IKK-2, are expressed in hMSCs. Our data provide evidence that this signal transduction pathway is implicated in TNF-alpha-mediated invasion and proliferation of hMSCs. Therefore, hMSC recruitment to sites of tissue injury may, at least in part, be regulated by the NF-kappaB signal transduction pathway. 
  [Ii]nvasive  PubMed  
 BRAFV600E mutation, TIMP-1 upregulation, and NF-κB activation: closing the loop on the papillary thyroid cancer trilogy. BRAF(V600E) is the most common mutation found in papillary thyroid carcinoma (PTC). Tissue inhibitor of metalloproteinases (TIMP-1) and nuclear factor (NF)-κB have been shown to play an important role in thyroid cancer. In particular, TIMP-1 binds its receptor CD63 on cell surface membrane and activates Akt signaling pathway, which is eventually responsible for its anti-apoptotic activity. The aim of our study was to evaluate whether interplay among these three factors exists and exerts a functional role in PTCs. To this purpose, 56 PTC specimens were analyzed for BRAF(V600E) mutation, TIMP-1 expression, and NF-κB activation. We found that BRAF(V600E) mutation occurs selectively in PTC nodules and is associated with hyperactivation of NF-κB and upregulation of both TIMP-1 and its receptor CD63. To assess the functional relationship among these factors, we first silenced BRAF gene in BCPAP cells, harboring BRAF(V600E) mutation. We found that silencing causes a marked decrease in TIMP-1 expression and NF-κB binding activity, as well as decre  ased inv  asiveness. After treatment with specific inhibitors of MAPK pathway, we found that only sorafenib was able to increase IκB-α and reduce both TIMP-1 expression and Akt phosphorylation in BCPAP cells, indicating that BRAF(V600E) activates NF-κB and this pathway is MEK-independent. Taken together, our findings demonstrate that BRAF(V600E) causes upregulation of TIMP-1 via NF-κB. TIMP-1 binds then its surface receptor CD63, leading eventually to Akt activation, which in turn confers antiapoptotic behavior and promotion of cell invasion. The recognition of this functional trilogy provides insight on how BRAF(V600E) determines cancer initiation, progress  ion, and   invasiveness in PTC, also identifying new therapeutic targets for the treatment of highly aggressive forms. 
 BRAFV600E mutation, TIMP-1 upregulation, and NF-κB activation: closing the loop on the papillary thyroid cancer trilogy. BRAF(V600E) is the most common mutation found in papillary thyroid carcinoma (PTC). Tissue inhibitor of metalloproteinases (TIMP-1) and nuclear factor (NF)-κB have been shown to play an important role in thyroid cancer. In particular, TIMP-1 binds its receptor CD63 on cell surface membrane and activates Akt signaling pathway, which is eventually responsible for its anti-apoptotic activity. The aim of our study was to evaluate whether interplay among these three factors exists and exerts a functional role in PTCs. To this purpose, 56 PTC specimens were analyzed for BRAF(V600E) mutation, TIMP-1 expression, and NF-κB activation. We found that BRAF(V600E) mutation occurs selectively in PTC nodules and is associated with hyperactivation of NF-κB and upregulation of both TIMP-1 and its receptor CD63. To assess the functional relationship among these factors, we first silenced BRAF gene in BCPAP cells, harboring BRAF(V600E) mutation. We found that silencing causes a marked decrease in TIMP-1 expression and NF-κB binding activity, as well as decre  ased inv  asiveness. After treatment with specific inhibitors of MAPK pathway, we found that only sorafenib was able to increase IκB-α and reduce both TIMP-1 expression and Akt phosphorylation in BCPAP cells, indicating that BRAF(V600E) activates NF-κB and this pathway is MEK-independent. Taken together, our findings demonstrate that BRAF(V600E) causes upregulation of TIMP-1 via NF-κB. TIMP-1 binds then its surface receptor CD63, leading eventually to Akt activation, which in turn confers antiapoptotic behavior and promotion of cell invasion. The recognition of this functional trilogy provides insight on how BRAF(V600E) determines cancer initiation, progress  ion, and   invasiveness in PTC, also identifying new therapeutic targets for the treatment of highly aggressive forms. 
 [Expressions of NF-κBp65 and IκBα in gestational trophoblastic disease and clinical significance]. To investigate the roles of nuclear factor κB p65 (NF-κBp65) and inhibitor of nuclear factor κB α (IκBα) in the development and metastasis of gestational trophoblastic neoplasia (GTN) by analyzing the expressions of NF-κBp65 and IκBα in normal early pregnancy villi and gestational trophoblastic diseases, and to reveal the relationship of NF-κBp65 and IκBα with age and clinical stage of GTN patients.The expressions of NF-κBp65 and IκBα were detected by immunohistochemistry in normal pregnancy villi (20 cases), hydatidiform mole  s (HM, 3  0 cases), invasive hydatidiform moles (IHM, 13 cases) and chorionic carcinomas (CCA, 15 cases).NF-κBp65 expression was statistically different (P<0.05) between normal pregnancy villi and IHM (P=0.013), normal pregnancy villi and CCA(P=0.018), HM and IHM(P=0.026), HM and CCA (P=0.035). Differences in IκBα expression were statistically significant between normal pregnancy villi and IHM, normal pregnancy villi and CCA, HM and IHM, HM and CCA (P<0.01). The expressions of NF-κBp65 and IκBα were correlated to clinical stage (P=0.043, 0.042, P<0.05), but not to patients' ages. Spearman correlation analysis revealed that there was a negative association between the protein expressions of NF-κBp65 and IκBα in GTN (r=-0.403, P=0.034, P<0.05).Up-regulated expression of NF-κBp65 and down-regulated expression of IκBα may be related to the development, invasion and metastasis of GTN. The expressions of NF-κBp65 and IκBα are negatively correlated in gestational trophoblastic tumor tissues. 
 Tim-3 facilitates osteosarcoma proliferation and metastasis through the NF-κB pathway and epithelial-mesenchymal transition. The aim of this study was to investigate the expression of T-cell immunoglobulin mucin domain molecule-3 (Tim-3) in osteosarcoma tissues, and analyze its effect on cell proliferation and metastasis in an osteosarcoma cell line. Tim-3 mRNA and protein expression in osteosarcoma tissue was detected by reverse transcriptase-polymerase chain reaction and immunohistochemistry, respectively. Additionally, the cell viability, apoptosis rate, and   invasiv  e ability of the osteosarcoma cell line MG-63 were tested using the methyl thiazolyl tetrazolium assay, Annexin V-propidium iodide flow cytometry, and a Transwell assay, respectively, following Tim-3 interference using small interfering RNA (siRNA). We also analyzed the expression of Snail, E-cadherin, vimentin, and nuclear factor (NF)-kB in the cells by western blot. We observed that Tim-3 mRNA and protein was significantly overexpressed in osteosarcoma tissues, compared to the adjacent normal tissue (P < 0.01). Moreover, MG-63 cells transfected with the Tim-3 siRNA presented lower cell viability, a greater number of apoptotic cells, and decreased   invasiv  e ability (P < 0.01), compared to control cells. Additionally, we observed a decrease in Snail and vimentin expression, an increase in the E-cadherin level, and an increase in NF-kB p65 phosphorylation (P < 0.01) in Tim-3 siRNA-transfected MG-63 cells. Based on these results, we concluded that Tim-3 is highly expressed in osteosarcoma tissue. Moreover, we speculated that interfering in Tim-3 expression could significantly suppress osteosarcoma cell (MG-63) proliferation and metastasis via the NF-kB/Snail signaling pathway and epithelial-mesenchymal transition. 
 TGF-beta downregulates PTEN via activation of NF-kappaB in pancreatic cancer cells. TGF-beta utilizes receptor-activated SMAD signaling to mediate growth suppression; however, non-SMAD signaling that modulates the TGF-beta response in epithelial cells become apparent when the SMAD signaling is abrogated, a common occurrence in pancreatic cancers. Here, we examined whether TGF-beta utilized NF-kappaB to downregulate PTEN, a gene that is rarely mutated in pancreatic cancers. SMAD4-null BxPc3 and CAPAN-1 pancreatic cancer cells were treated with TGF-beta (10 ng/ml) and lysed, and cellular proteins were analyzed by Western blots using p-IkappaB, p65, and PTEN antibodies. PTEN promoter and NF-kappaB activities were assessed by PTEN-luc and p-NF-luc constructs, respectively. Dominant negative p-IkappaB-alpha-M (NF-kappaB superrepressor) was used to block activation of NF-kappaB. Cell motility was assessed by Boyden chamber migration assay. TGF-beta induced IkappaB-alpha phosphorylation followed by NF-kappaB p65 subunit nuclear translocation and increased NF-kappaB activity. IkappaB-alpha-M blocked TGF-beta-induced NF-kappaB activity, reversed downregulated PTEN promoter activity and PTEN expression, and prevented augmentation of cell motility induced by TGF-beta. SMAD4 restoration, but not knockdown of SMAD2 and/or 3, reversed TGF-beta-induced NF-kappaB activity. Thus TGF-beta suppresses PTEN in pancreatic cancer cells through NF-kappaB activation and enhances cell motility and   invasive  ness in a SMAD4-independent manner that can be counteracted when TGF-beta-SMAD signaling is restored. The TGF-beta/NF-kappaB/PTEN cascade may be a critical pathway for pancreatic cancer cells to proliferate and metastasize. 
 Stabilization of snail by NF-kappaB is required for inflammation-induced cell migration and invasion. The increased motility and   invasive  ness of tumor cells are reminiscent of epithelial-mesenchymal transition (EMT), which occurs during embryonic development, wound healing, and metastasis. In this study, we found that Snail is stabilized by the inflammatory cytokine TNFalpha through the activation of the NF-kappaB pathway. We demonstrated that NF-kappaB is required for the induction of COP9 signalosome 2 (CSN2), which, in turn, blocks the ubiquitination and degradation of Snail. Furthermore, we showed that the expression of Snail correlated with the activation of NF-kappaB in cancer cell lines and metastatic tumor samples. Knockdown of Snail expression inhibited cell migration and invasion induced by inflammatory cytokines and suppressed inflammation-mediated breast cancer metastasis. Our study provides a plausible mechanism for inflammation-induced metastasis. 
 Nuclear factor-kappaB signaling and ezrin are essential for L1-mediated metastasis of colon cancer cells. Hyperactivation of beta-catenin-T-cell-factor (TCF)-regulated gene transcription is a hallmark of colorectal cancer (CRC). The cell-neural adhesion molecule L1CAM (hereafter referred to as L1) is a target of beta-catenin-TCF, exclusively expressed at the CRC   invasive   front in humans. L1 overexpression in CRC cells increases cell growth and motility, and promotes liver metastasis. Genes induced by L1 are also expressed in human CRC tissue but the mechanisms by which L1 confers metastasis are still unknown. We found that signaling by the nuclear factor kappaB (NF-kappaB) is essential, because inhibition of signaling by the inhibitor of kappaB super repressor (IkappaB-SR) blocked L1-mediated metastasis. Overexpression of the NF-kappaB p65 subunit was sufficient to increase CRC cell proliferation, motility and metastasis. Binding of the L1 cytodomain to ezrin - a cytoskeleton-crosslinking protein - is necessary for metastasis because when binding to L1 was interrupted or ezrin gene expression was suppressed with specific shRNA, metastasis did not occur. L1 and ezrin bound to and mediated the phosphorylation of IkappaB. We also observed a complex containing IkappaB, L1 and ezrin in the juxtamembrane region of CRC cells. Furthermore, we found that L1, ezrin and phosphorylated p65 are co-expressed at the   invasive   front in human CRC tissue, indicating that L1-mediated activation of NF-kappaB signaling involving ezrin is a major route of CRC progression. 
 Down-modulation of Bis reduces the   invasive   ability of glioma cells induced by TPA, through NF-κB mediated activation of MMP-9. Bcl-2 interacting cell death suppressor (Bis) has been shown to have anti-apoptotic and anti-stress functions. Recently, increased Bis expression was reported to correlate with glioma aggressiveness. Here, we investigated the effect of Bis knockdown on the acquisition of the   invasiv  e phenotype of A172 glioma cells, induced by 12-O-Tetradecanoylphorbol-3-acetate (TPA), using a Transwell assay. Bis knockdown resulted in a significant decrease in the migration and invasion of A172 cells. Furthermore, Bis knockdown notably decreased TPA-induced matrix metalloproteinase-9 (MMP-9) activity and mRNA expression, as measured by zymography and quantitative real time PCR, respectively. A luciferase reporter assay indicated that Bis suppression significantly down-regulated NF-κB-driven transcription. Finally, we demonstrated that the rapid phosphorylation and subsequent degradation of IκB-α induced by TPA was remarkably delayed by Bis knockdown. These results suggest that Bis regulates   the inva  sive ability of glioma cells elicited by TPA, by modulating NF-κB activation, and subsequent induction of MMP-9 mRNA. 
 S100A4 promotes squamous cell laryngeal cancer Hep-2 cell invasion via NF-kB/MMP-9 signal. S100A4 is a member of the S100 family of calcium-binding proteins, which possesses a wide range of biological functions, such as regulation of angiogenesis, cell survival, motility, and invasion. Here, we demonstrate for the first time a major role of S100A4 in the cell invasion properties of the human laryngeal squamous carcinoma cells (LSCC) and evaluated the mechanism.Cultured human LSCC cell line Hep-2 was overexpressed by transfection of pcDNA3.1-S100A4 plasmid. For this, cellular Hep-2 expression was quantified by Western blot analysis. Moreover, cell invasion and migration assays were performed. Furthermore, the impact of the S100A4 on NF-kB activity and MMP-9 expression was detected.We found S100A4 potently promoted Hep-2 invasion, by increasing cell motility and matrix metalloproteinase-9 (MMP-9) production. The increase in MMP-9 production was mediated by activation of nuclear factor-kB transcriptional activity by S100A4. After MMP-9 and NF-kBp65 was inhibited by BB94 treatment and NF-kBp65 siRNA transfected, pcDNA3.1-S100A4 induced cell invasion and migration was decreased.Our findings thus establish S100A4 as a major factor in the   invasive   abilities of Hep-2 cells. 
 Inactivation of M2 AChR/NF-κB signaling axis reverses epithelial-mesenchymal transition (EMT) and suppresses migration and invasion in non-small cell lung cancer (NSCLC). Non-neuronal cholinergic system is involved in lung physiology and lung cancer. However, the biochemical events downstream acetylcholine (ACh) receptor activation leading to carcinogenesis and tumor progression are not fully understood. Our previous work has shown that non-neuronal ACh acts as an autoparacrine growth factor to stimulate cell proliferation and promote epithelial-mesenchymal transition (EMT) in non-small cell lung cancer (NSCLC) via activation of M2 muscarinic receptor (M2R). The aim of the present study was to delineate the underlying mechanisms linking M2R and lung tumor progression, which may provide potential therapeutic targets to delay lung cancer progression. Inhibition of M2R by antagonist or siRNA suppresses NSCLC cell migratory and   invasiv  e capacities, reverses EMT and simultaneously inactivates PI3K/Akt, MAPK ERK and NF-κB p65. On the other hand, M2R activation stimulates NSCLC migration and invasion and promotes EMT via NF-κB p65 activation. Moreover, NF-κB p65 activation induced by M2R activation was partially inhibited by either Akt or ERK inhibitor. Taken together, these results demonstrated for the first time that NF-κB p65 activation is essential in NSCLC progression associated with non-neuronal cholinergic system. Our data suggest that M2R/ERK/Akt/NF-κB axis could be a potential target for NSCLC treatment. 
 MUC1 protein induces urokinase-type plasminogen activator (uPA) by forming a complex with NF-κB p65 transcription factor and binding to the uPA promoter, leading to enhanced   invasiv  eness of cancer cells. Mucin 1 (MUC1) is overexpressed in various human malignant tumors and its expression is correlated with a poor prognosis. MUC1 engages in signal transduction by interacting with receptors for growth and differentiation factors, which contributes to the growth and survival of cancer cells. However, the mechanism by which MUC1 promotes cancer cell invasion remains unclear. Microarray analysis revealed that expression of urokinase-type plasminogen activator (uPA) was elevated in MUC1-overexpressing cells. Furthermore, up- and down-modulation of MUC1 expression was clearly correlated with the change of uPA expression. An immunochemical study showed that the distribution of uPA coincided with that of MUC1 in various human cancer tissues. The MUC1 C-terminal domain (MUC1-CD) was associated with nuclear factor-κB (NF-κB) p65 in MUC1-expressing cells. Chromatin immunoprecipitation (ChIP) assays demonstrated that MUC1-CD existed with NF-κB p65 on the uPA promoter. Luciferase assays indicated that the uPA transcriptional activity was correlated with the level of MUC1 expression and that this MUC1-enhancing effect on the uPA transcription was abolished by introduction of mutations into the NF-κB binding sites on the uPA promoter. These results indicate that formation of the MUC1-CD and NF-κB p65 complex enhanced nuclear translocation of NF-κB p65 and subsequent occupancy of NF-κB binding region on the uPA promoter, leading to elevated transcription of uPA. We also demonstrated that uPA induced by MUC1 enhanced the matrix metalloproteinase (MMP)-2 and -9 activities, and consequently promoted cancer cell invasion. Thus, a MUC1 co-operating NF-κB signaling pathway plays a critical role in cancer cell invasion in MUC1-expressing cells. 
 NF-κBP65 promotes invasion and metastasis of oesophageal squamous cell cancer by regulating matrix metalloproteinase-9 and epithelial-to-mesenchymal transition. NF-κB has been recognized as one of the factors responsible for the development of cancer; however, the mechanism by which high expression of NF-κB contributes to the progression of human oesophageal squamous cell cancer (ESCC) is not fully understood. In our investigations, NF-κBP65 was overexpressed in human ESCC tissues, especially in ESCC tissues with deep invasion and lymph node metastasis. Suppression of NF-κBP65 by siRNA decreased the invasion and proliferation ability of EC9706 cells in vitro. Furthermore, siRNA-mediated NF-κBP65 knock-down could lead to the downregulation of MMP-9, a metastasis-related gene. Reduced E-cadherin is a hallma  rk of in  vasive carcinomas that have acquired epithelial-mesenchymal transition (EMT) phenotypes and Vimentin is another molecule that is used widely as a marker of the EMT. We found upregulation of E-cadherin expression and downregulation of Vimentin was induced by NF-κBP65 siRNA, which suggests that NF-κBP65 siRNA could inhibit the invasion and proliferation ability of ECSS through attenuating the expression of MMP-9 and EMT. Thus, ESCC NF-κBP65 could be a useful target for cancer prevention and therapy. 
 PRL-3 activates NF-κB signaling pathway by interacting with RAP1. Phosphatase of regenerating liver (PRL-3) promotes cancer metastasis through enhanced cell motility and   invasiv  eness, however its role in tumorigenesis remains unclear. Herein, we reported that PRL-3 interacts with telomere-related protein RAP1. PRL-3 promotes the cytosolic localization of RAP1, which is counteracted by silencing of PRL-3. Immunohistochemical staining of colon cancer tissue array (n=170) revealed that high level of PRL-3 associates with cytosolic localization of RAP1 (p=0.01). Microarray analysis showed that PRL-3 regulates expression of diverse genes and enhances phosphorylation of p65 subunit of NF-κB in a RAP1-dependent manner. Furthermore, PRL-3 transcriptionally activates RAP1 expression, which is counteracted by ablating p65. Therefore, our results demonstrate PRL-3 as a novel regulator of NF-κB signaling pathway through RAP1. 
 Comparison of p38MAPK (mitogene activated protein kinase), p65 NFkappaB (nuclear factor kappa b) and EMMPRIN (extracellular matrix metalloproteinase inducer) expressions with tumor grade and stage of superficial and   invasive   bladder tumors. To identify the molecular mechanisms of bladder cancer invasion pathophysiology. To assess EMMPRIN, p65NFkappaB and p38MAPK expressions which play a role in signal transmission system of muscle and non muscle   invasive   bladder tumors. Fifty-seven patients with non muscle   invasive   tumors (mean age 65.2 +/- 16.1) and 34 patients with muscle   invasive   tumors (mean age 62.2 +/- 20.7) were included in this study. Normal tissue from the same patients' bladders were used as control group. Patients had either TUR or radical cystectomy and paraffin sections were prepared for immunohistochemistry. Expression density was evaluated semiquantitively according to tumor grade and invasion depth. Results were compared with Mann Whitney U, Wilcoxon W, Chi Square and variation analysis tests. MAPK and EMMPRIN expression was increased according to tumor grade (p < 0.05). These expressions were also significantly higher in muscle   invasive   tumors than in non muscle   invasive   ones (p < 0.05). In normal tissue samples of both TUR and radical cystectomy materials, EMMPRIN, NFkappaB and MAPK expressions were lower than in tumor samples (p < 0.05). NFkappaB wasn't related to tumor grade/stage (p > 0.05). It can be stated that MAPK and EMMPRIN expression is related to the grade of bladder tumor and that NFkappaB positivity is not related to the grade/stage of the disease. In future positivity of lymph nodes and visceral metastasis and survival must be assessed to define the relationship with the expressions in long term follow up studies involving a larger number of patients. 
 Overexpression of response gene to complement 32 (RGC32) promotes cell invasion and induces epithelial-mesenchymal transition in lung cancer cells via the NF-κB signaling pathway. Response gene to complement 32 (RGC32) is a novel cellular protein that has been reported to be expressed aberrantly in multiple types of human tumors. However, the role of RGC32 in cancer is still controversial, and the molecular mechanisms by which RGC32 contributes to the development of cancer remain largely unknown. In the present study, we constructed a recombinant expression vector pCDNA3.1-RGC32 and transfected it into human lung cancer A549 cells. Stable transformanted cells were identified by real-time PCR and Western blot analysis. Functional analysis showed that forced overexpression of RGC32 increased   invasiv  e and migration capacities of lung cancer cells in vitro, and induced the acquisition of epithelial-mesenchymal transition (EMT) phenotype, as demonstrated by the spindle-like morphology, downregulation of E-cadherin, and upregulation of Vimentin, Fibronectin, Snail and Slug. Also, overexpression of RGC32 increased expression and activities of matrix metalloproteinase (MMP)-2 and MMP-9 in A549 cells. Furthermore, the downregulation of E-cadherin induced by RGC32 was remarkably attenuated by nuclear factor-κB (NF-κB) inhibitor BAY 11-7028 and small interfering RNA targeting NF-κB p65, suggesting a role of the NF-κB signaling pathway in RGC32-induced EMT. Taken together, our data suggest that RGC32 promotes cell migration and invasion and induces EMT in lung cancer cells via the NF-κB signaling pathway. 
 NGAL controls the metastatic potential of anaplastic thyroid carcinoma cells. We have previously identified neutrophil gelatinase-associated lipocalin (NGAL) as one of the genes mediating the oncogenic activity of nuclear factor-κB in human anaplastic thyroid carcinomas (ATCs).To further investigate the role of NGAL in thyroid cancer, we established NGAL knocked-down and NGAL overexpressing ATC cell lines.We found that the ability of NGAL knocked-down cells to degrade Matrigel in a transwell invasion assay and to form lung metastasis in nude mice was decreased. Because NGAL binds matrix metalloproteinase-9 (MMP-9), to form a macromolecular complex involved in the regulation of metastatic spread of cancer cells and given the strong expression of both genes in tissue specimens from human ATCs, we analyzed the MMP-9 enzymatic activity in NGAL-null ATC cells. Enzymatic immunoassays show that MMP-9 activity is reduced in NGAL-null ATC cells, even if its expression is not affected by NGAL inhibition. Ectopic expression of NGAL in an ATC cell line not expressing NGAL determines an increase of its metastatic property. The use of a mutated form of NGAL, unable to bind MMP-9, has no positive effect on the   invasiv  e potential of ATC cells and does not improve the MMP-9 enzymatic activity.Our results indicate NGAL as a novel target of nuclear factor-κB prometastatic activity in thyroid cancer through enhancement of MMP-9 enzymatic activity. 
 Dual knockdown of p65 and p50 subunits of NF-kappaB by siRNA inhibits the induction of inflammatory cytokines and significantly enhance apoptosis in human primary synoviocytes treated with tumor necrosis factor-alpha. In order to develop an anti-NF-kappaB siRNA as a novel class of anti-inflammatory drug, we have isolated a highly efficient siRNA targeting the p65 (RelA) subunit of NF-kappaB, hereafter named REL1096. To determine whether down-regulation of p65 by REL1096 can block the induction of inflammatory cytokines after treatment with tumor necrosis factor-alpha (TNF-alpha), human primary fibroblast-like synoviocytes were isolated from patients with rheumatoid arthritis. When treated with REL1096, the TNF-mediated induction of downstream target genes such as inflammatory cytokines, chemokines, and anti-apoptosis genes was drastically inhibited. To enhance the inhibitory effect of REL1096, cells were treated with siRNA targeting the p50 subunit of NF-kappaB together with REL1096. In addition to effective downregulation of inflammatory cytokines, knockdown of both p65 and p50 resulted in much more extensive apoptosis when compared to cells treated with either REL1096 or p50-siRNA alone. Thus, our results provide evidence for the potential use of siRNA targeting NF-kappaB as an effective means to treat rheumatoid arthritis. In addition to effective amelioration of synovial inflammation by downregulation of inflammatory cytokines, increased apoptosis by dual knockdown of p65 and p50 may prove advantageous in preventing   invasive  ness and destructiveness of hyperplastic synoviocytes. 
 Expression of the constitutively activated RelA/NF-kappaB in human astrocytic tumors and the in vitro implication in the regulation of urokinase-type plasminogen activator, migration, and invasion. Although malignant gliomas are highly   invasive   tumors, a characteristic that contributes to the commonly observed therapeutic failures and local disease recurrences, the molecular events that regulate invasion in these tumors remain poorly understood. Because the transcription factor RelA/NF-kappaB has been shown to regulate invasion during several cellular processes, we have examined immunohistochemically expression of the constitutively activated RelA/NF-kappaB in tissues obtained from 49 astrocytic tumors [8 diffuse astrocytomas, 9 anaplastic astrocytomas (AAs) and 32 glioblastomas (GBMs)]. In addition, we examined the in vitro effects of antisense oligonucleotides and curcumin on the expression and activation of RelA/NF-kappaB, urokinase-type plasminogen activator (u-PA) expression, migration, and invasion in the T98G glioma cell line. Expression of the constitutively activated RelA/NF-kappaB was observed in 2 (25%) of 8 cases of diffuse astrocytomas, 5 (55.6%) of 9 cases of AAs, and 30 (93.8%) of 32 cases of GBMs. This expression was significantly correlated with the malignant potential in astrocytic tumors (P < 0.001). Moreover, antisense oligonucleotides and curcumin inhibited phorbol-12-myristate-13-acetate (PMA)-induced RelA/NF-kappaB expression or activation (or both), down-regulated u-PA expression, and reduced the migration and   invasive   potentials of T98G glioma cells. Thus, the expression of constitutively activated RelA/NF-kappaB is associated with malignancy potential in astrocytic tumors and may play a critical role in the regulation of u-PA expression and   invasive  ness in gliomas. RelA/NF-kappaB may therefore be an intriguing candidate for studies aimed at understanding and prevention of the   invasive  ness of gliomas. 
 N-Myc-interacting protein (NMI) negatively regulates epithelial-mesenchymal transition by inhibiting the acetylation of NF-κB/p65. The epithelial-mesenchymal transition (EMT) plays an essential role in embryonic development, wound healing, tissue regeneration, organ fibrosis, and tumor progression. However, the mechanisms underlying this process are poorly understood. Many signaling pathways, including the NF-κB signaling pathway, trigger EMT during development and differentiation. In the present study, we report that N-Myc interactor (NMI) inhibits EMT progression by suppressing transcriptional activities of NF-κB in human gastric cancer cells. We show that the expression of NMI is significantly reduced   in invas  ive gastric cancer cells and gastric cancer tissues. Overexpression of NMI inhibited cell migration and invasion, and this inhibition was enhanced after TNF-α stimulation. Tumorigenicity assay in nude mice support the notion that NMI inhibits EMT in cancer cells. Mechanistically, NMI promotes the interaction between NF-κB/p65 and histone deacetylases (HDACs) and inhibits the acetylation and transcriptional activity of p65. The expression of p65 rescues NMI-mediated inhibition of EMT and the inhibition of the acetylation of p65 mediated by NMI is HDACs-dependent. Taken together, these findings suggest that NMI can suppress tumor invasion and metastasis by inhibiting NF-κB pathways, providing an alternative mechanism for EMT inhibition in stomach neoplasm. 
 Suppression of the   invasive   potential of Glioblastoma cells by mTOR inhibitors involves modulation of NFκB and PKC-α signaling. Glioblastoma (GBM) is the most aggressive type of brain tumors in adults with survival period <1.5 years of patients. The role of mTOR pathway is documented in invasion and migration, the features associated with aggressive phenotype in human GBM. However, most of the preclinical and clinical studies with mTOR inhibitors are focused on antiproliferative and cytotoxic activity in GBM. In this study, we demonstrate that mTOR inhibitors-rapamycin (RAP), temisirolimus (TEM), torin-1 (TOR) and PP242 suppress invasion and migration induced by Tumor Necrosis Factor-α (TNFα) and tumor promoter, Phorbol 12-myristate 13-acetate (PMA) and also reduce the expression of the TNFα and IL1β suggesting their potential to regulate factors in microenvironment that support tumor progression. The mTOR inhibitors significantly decreased MMP-2 and MMP-9 mRNA, protein and activity that was enhanced by TNFα and PMA. The effect was mediated through reduction of Protein kinase C alpha (PKC-α) activity and downregulation of NFκB. TNFα- induced transcripts of NFκB targets -VEGF, pentraxin-3, cathepsin-B and paxillin, crucial in invasion were restored to basal level by these inhibitors. With limited therapeutic interventions currently available for GBM, our findings are significant and suggest that mTOR inhibitors may be explor  ed as an  ti-invasive drugs for GBM treatment. 
 Vascular endothelial growth factor C enhances cervical cancer cell   invasive  ness via upregulation of galectin-3 protein. Vascular endothelial growth factor C (VEGF-C) promotes cervical cancer metastasis, while the detailed mechanism remains obscure. Recent evidence shows that galectin-3 (Gal-3), a glycan binding protein, interacts with the VEGF receptors and reinforces their signal transduction. In this study, we investigated the role of Gal-3 in VEGF-C-induced cervical cancer cell invasion. On cervical carcinoma cell line SiHa cells, silencing of Gal-3 expression with specific siRNA largely impaired VEGF-C-enhanced cell invasion. Treatment with VEGF-C for 12-48 h enhanced Gal-3 protein expression, which was inhibited by the addition of NF-κB inhibitor pyrrolidine dithiocarbamate (PDTC). Moreover, the silencing of NF-κB subunit p65 expression with specific siRNA attenuated VEGF-C-enhanced Gal-3 expression, suggesting that NF-κB is the key intermediate. Under VEGF-C stimulation, an enhanced interaction between VEGF receptor-3 (VEGF-R3) and Gal-3 was found, which may possibly lead to VEGF-R3 activation since exogenous Gal-3 induced VEGF-R3 phosphorylation in a dose- and time-dependent manner. In conclusion, our findings implied that VEGF-C enhanced cervical ca  ncer inv  asiveness via upregulation of Gal-3 protein through NF-κB pathway, which may shed light on potential therapeutic strategies for cervical cancer therapy. 
 A novel LZAP-binding protein, NLBP, inhibits cell invasion. LXXLL/leucine zipper-containing alternative reading frame (ARF)-binding protein (LZAP) was recently shown to function as a tumor suppressor through inhibition of the NF-kappaB signaling pathway. LZAP is also known as a negative regulator of cell invasion, and its expression was demonstrated to be reduced in several tumor tissues. However, the molecular mechanism of the negative effect of LZAP on cell invasion is unclear. In this study, we identify NLBP as a novel LZAP-binding protein using tandem affinity purification. We demonstrate the negative effects of NLBP on cell invasion and the NF-kappaB signaling pathway. NLBP expression was not detected in hepatocellular carcinoma cells with strong   invasive   activity, whereas its expression was detected in a hepatocellular carcinoma cell line with no   invasive   activity. We also demonstrate that these two proteins mutually affect the stability of each other by inhibiting ubiquitination of the other protein. Based on these results, we suggest that NLBP may act as a novel tumor suppressor by inhibiting cell invasion, blocking NF-kappaB signaling, and increasing stability of the LZAP protein. 
 Differential regulation of MMPs by E2F1, Sp1 and NF-kappa B controls the small cell lung cancer   invasive   phenotype. E2F1 transcription factor plays a vital role in the regulation of diverse cellular processes including cell proliferation, apoptosis, invasion and metastasis. E2F1 overexpression has been demonstrated in small cell lung cancer (SCLC), and extensive metastasis in early phase is the most important feature of SCLC. In this study, we investigated the involvement of E2F1 in the process of invasion and metastasis in SCLC by regulating the expression of matrix metalloproteinases (MMPs).Immunohistochemistry was performed to evaluate the expression of E2F1 and MMPs in SCLC samples in a Chinese Han population. The impact of E2F1 on invasion and metastasis was observed by transwell and wound healing experiments with depletion of E2F1 by specific siRNA. The target genes regulated by E2F1 were identified by chromatin immunoprecipitation (ChIP)-to-sequence, and the expressions of target genes were detected by real time PCR and western blotting. The dual luciferase reporter system was performed to analyze the regulatory relationship between E2F1 and MMPs.E2F1 is an independent and adverse prognosis factor that is highly expressed in SCLC in a Chinese Han population. Knockdown of E2F1 by specific siRNA resulted in the downregulation of migration and invasion in SCLC. The expressions of MMP-9 and -16 in SCLC were higher than other MMPs, and their expressions were most significantly reduced after silencing E2F1. ChIP-to-sequence and promoter-based luciferase analysis demonstrated that E2F1 directly controlled MMP-16 expression via an E2F1 binding motif in the promoter. Although one E2F1 binding site was predicted in the MMP-9 promoter, luciferase analysis indicated that this binding site was not functionally required. Further study demonstrated that E2F1 transcriptionally controlled the expression of Sp1 and p65, which in turn enhanced the MMP-9 promoter activity in SCLC cells. The associations between E2F1, Sp1, p65, and MMP-9 were validated by immunohistochemistry staining in SCLC tumors.E2F1 acts as a transcriptional activator for MMPs and directly enhances MMP transcription by binding to E2F1 binding sequences in the promoter, or indirectly activates MMPs through enhanced Sp1 and NF-kappa B as a consequence of E2F1 activation in SCLC. 
 Molecular basis of anti-inflammatory action of platelet-rich plasma on human chondrocytes: mechanisms of NF-κB inhibition via HGF. Loss of articular cartilage through injury or disease presents major clinical challenges also because cartilage has very poor regenerative capacity, giving rise to the development of biological approaches. As autologous blood product, platelet-rich plasma (PRP) provides a promising alternative to surgery by promoting safe and natural healing. Here we tested the possibility that PRP might be effective as an anti-inflammatory agent, providing an attractive basis for regeneration of articular cartilage, and two principal observations were done. First, activated PRP in chondrocytes reduced the transactivating activity of NF-κB, critical regulator of the inflammatory process, and decreased the expression of COX-2 and CXCR4 target genes. By analyzing a panel of cytokines with different biological significance, in activated PRP we observed increases in hepatocyte growth factor (HGF), interleukin-4 and tumor necrosis factor-α (TNF-α). HGF and TNF-α, by disrupting NF-κB-transactivating activity, were important for the anti-inflammatory function of activated PRP. The key molecular mechanisms involved in PRP-inhibitory effects on NF-κB activity were for HGF the enhanced cellular IkBα expression, that contributed to NF-κB-p65 subunit retention in the cytosol and nucleo-cytoplasmic shuttling, and for TNF-α the p50/50 DNA-binding causing inhibition of target-gene expression. Second, activated PRP in U937-monocytic cells reduced chemotaxis by inhibiting chemokine transactivation and CXCR4-receptor expression, thus possibly controlling local inflammation in cartilage. In conclusion, activated PRP is a promising biological therapeutic agent, as a scaffold   in micr  o-invasive articular cartilage regeneration, not only for its content of proliferative/differentiative growth factors, but also for the presence of anti-inflammatory agents including HGF. 
 Down-regulation of NF kappa B activation is an effective therapeutic modality in acquired platinum-resistant bladder cancer. No previous study has addressed the efficacy of NF-κB blockade when bladder tumors develop acquired resistance toward CDDP-treatments. We investigated the changes in NF-κB activation and therapeutic impact of NF-κB blockade by the novel NF-κB inhibitor dehydroxymethyl derivative of epoxyquinomicin (DHMEQ) in CDDP-resistant bladder cancer cells.Two hu  man inva  sive bladder cancer cell lines, T24 and T24PR, were used. The T24PR cell line was newly established as an acquired platinum-resistant subline by culturing in CDDP-containing medium for 6 months. Expression of intranuclear p65 protein in the fractionated two cell lines was determined by Western blotting analysis. DNA-binding activity of NF-κB was detected by electrophoretic mobility shift assay. The cytotoxic effects and induction of apoptosis were analyzed in vivo and in vitro.Intranuclear expression and DNA-binding activity of p65 were strongly enhanced in T24PR cells compared with those of T24 cells, and both were significantly suppressed by DHMEQ. Lowered cell viability and strong induction of apoptosis were observed by treatment with DHMEQ alone in these chemo-resistant cells compared with parent cells. As T24PR cells did not show dramatic cross-resistance to paclitaxel in the in vitro study, we next examined whether the combination of DHMEQ with paclitaxel could enhance the therapeutic effect of the paclitaxel treatment in T24PR tumors. Using mouse xenograft models, the mean volume of tumors treated with the combination of DHMEQ (2 mg/kg) and paclitaxel (10 mg/kg) was significantly smaller than those treated with paclitaxel alone (p < 0.05), and the reduction of tumor volume in mice treated with DHMEQ in combination with paclitaxel and paclitaxel alone as compared to vehicle control was 66.9% and 17.0%, respectively.There was a distinct change in the activation level of NF-κB between T24 and T24PR cells, suggesting strong nuclear localization of NF-κB could be a promising target after developing acquired platinum-resistance in bladder cancer. 
 Clinical significance of nuclear factor (NF)-kappaB levels in urothelial carcinoma of the urinary bladder. Nuclear factor (NF)-kappaB has been reported to be constitutively activated in various human neoplasms. However, its clinical significance in bladder urothelial carcinoma (UC) remains an unresolved issue. We conducted this study trying to elucidate the role of NFkappaB in bladder UC and its potential prognostic significance, by quantifying immunohistochemically the levels of p65/RelA expression in paraffin-embedded tissue from 116 patients. Some of the cases had previously been stained for cellular FLICE-like inhibitory protein (c-FLIP) and bcl-2. Seventy-four cases displayed concurrent cytoplasmic and nuclear immunoreactivity, whereas 18 only nuclear immunoexpression and 21 only cytoplasmic immunoexpression, and the remaining three cases were negative for p65/RelA. Nuclear p65/RelA expression was positively associated with tumour grade and T-category (p=0.0001 in both cases). In addition, cytoplasmic p65/RelA expression was lower in advanced T-category (p=0.0030). Moreover, p65/RelA nuclear expression was positively correlated with c-FLIP (p=0.0109) and bcl-2 (p=0.0452). p65/RelA nuclear expression adversely affected survival in both univariate and multivariate analysis in superficial (Ta-T1; p=0.0010 and p=0.0008) as well as in muscle-  invasive   carcinomas (T2-T4; p=0.0004 and p=0.0003). Our results demonstrate that NF-kappaB nuclear expression is correlated with histologic grade and T category in bladder UC. Moreover, NF-kappaB nuclear expression emerges as an independent prognosticator of adverse significance, conveying information beyond that obtained by standard clinicopathological prognosticators. 
 NF-κB-induced KIAA1199 promotes survival through EGFR signalling. Constitutive activation of EGFR- and NF-κB-dependent pathways is a hallmark of cancer, yet signalling proteins that connect both oncogenic cascades are poorly characterized. Here we define KIAA1199 as a BCL-3- and p65-dependent gene in transformed keratinocytes. KIAA1199 expression is enhanced on human papillomavirus (HPV) infection and is aberrantly expressed in clinical cases of cervical (pre)neoplastic lesions. Mechanistically, KIAA1199 binds Plexin A2 and protects from Semaphorin 3A-mediated cell death by promoting EGFR stability and signalling. Moreover, KIAA1199 is an EGFR-binding protein and KIAA1199 deficiency impairs EGF-dependent Src, MEK1 and ERK1/2 phosphorylations. Therefore, EGFR stability and signalling to downstream kinases requires KIAA1199. As such, KIAA1199 promotes EGF-mediated epithelial-mesenchymal transition (EMT). Taken together, our data define KIAA1199 as an oncogenic protein induced by HPV infection and constitutive NF-κB activity that transmits pro-survival a  nd invas  ive signals through EGFR signalling. 
 Up-regulation of cathepsin B expression and enhanced secretion in mitochondrial DNA-depleted osteosarcoma cells. mtDNA (mitochondrial DNA) mutations that impair oxidative phosphorylation can contribute to carcinogenesis through the increased production of reactive oxygen species and through the release of proteins involved in cell motility and invasion. On the other hand, many human cancers are associated with both the up-regulation and the increased secretion of several proteases and heparanase. In the present study, we tried to determine whether the depletion in mtDNA could modulate the expression and/or the secretion of some lysosomal hydrolases in the 143B osteosarcoma cells, as these mtDNA-depleted cells are characterized by a higher degree of   invasive  ness than the parental cells.In comparison with the parental cells, we measured a higher amount of procathepsin B in the conditioned culture medium of the 143B cells lacking mtDNA (rho(0) 143B cells), as well as a rise in the specific activity of intracellular cathepsin B. In addition, we observed an activation of the transcription factor NF-kappaB (nuclear factor kappaB) in the cells devoid of functional mitochondria. Finally, we demonstrated that the down-regulation of the NF-kappaB p65 subunit by RNA interference led to a reduction in cathepsin B expression in rho(0) 143B cells.The up-regulation of cathepsin B by NF-kappaB, followed by its secretion into the extracellular environment, might be partly responsible for the previously reported   invasive  ness of the mtDNA-depleted 143B osteosarcoma cells. 
 Antiestrogen resistance in breast cancer and the role of estrogen receptor signaling. Antiestrogens include agents such as tamoxifen, toremifene, raloxifene, and fulvestrant. Currently, tamoxifen is the only drug approved for use in breast cancer chemoprevention, and it remains the treatment of choice for most women with hormone receptor positive,   invasive   breast carcinoma. While antiestrogens have been available since the early 1970s, we still do not fully understand their mechanisms of action and resistance. Essentially, two forms of antiestrogen resistance occur: de novo resistance and acquired resistance. Absence of estrogen receptor (ER) expression is the most common de novo resistance mechanism, whereas a complete loss of ER expression is not common in acquired resistance. Antiestrogen unresponsiveness appears to be the major acquired resistance phenotype, with a switch to an antiestrogen-stimulated growth being a minor phenotype. Since antiestrogens compete with estrogens for binding to ER, clinical response to antiestrogens may be affected by exogenous estrogenic exposures. Such exposures include estrogenic hormone replacement therapies and dietary and environmental exposures that directly or indirectly increase a tumor's estrogenic environment. Whether antiestrogen resistance can be conferred by a switch from predominantly ERalpha to ERbeta expression remains unanswered, but predicting response to antiestrogen therapy requires only measurement of ERalpha expression. The role of altered receptor coactivator or corepressor expression in antiestrogen resistance also is unclear, and understanding their roles may be confounded by their ubiquitous expression and functional redundancy. We have proposed a gene network approach to exploring the mechanistic aspects of antiestrogen resistance. Using transcriptome and proteome analyses, we have begun to identify candidate genes that comprise one component of a larger, putative gene network. These candidate genes include NFkappaB, interferon regulatory factor-1, nucleophosmin, and the X-box binding protein-1. The network also may involve signaling through ras and MAPK, implicating crosstalk with growth factors and cytokines. Ultimately, signaling affects the expression/function of the proliferation and/or apoptotic machineries. 
  GeneRIFs  
 Data suggest VEGFC (vascular endothelial growth factor C) enhances cervical cancer   invasive  ness via up-regulation of galectin-3 via stimulation of NFkappaB/RELA pathway; galectin-3 activates VEGFR3 (vascular endothelial growth factor receptor-3). 
 Bis regulates the   invasive   ability of glioma cells elicited by TPA, by modulating p65 NF-kappaB activation, and subsequent induction of MMP-9 mRNA. 
 The expression of constitutively activated RelA/NF-kappaB is associated with malignancy potential in astrocytic tumors and may play a critical role in the regulation of u-PA expression and   invasive  ness in gliomas. 
 increased apoptosis by dual knockdown of p65 and p50 may prove advantageous in preventing   invasive  ness and destructiveness of hyperplastic synoviocytes 
  [Mm]etastasize  PubMed  
 TGF-beta downregulates PTEN via activation of NF-kappaB in pancreatic cancer cells. TGF-beta utilizes receptor-activated SMAD signaling to mediate growth suppression; however, non-SMAD signaling that modulates the TGF-beta response in epithelial cells become apparent when the SMAD signaling is abrogated, a common occurrence in pancreatic cancers. Here, we examined whether TGF-beta utilized NF-kappaB to downregulate PTEN, a gene that is rarely mutated in pancreatic cancers. SMAD4-null BxPc3 and CAPAN-1 pancreatic cancer cells were treated with TGF-beta (10 ng/ml) and lysed, and cellular proteins were analyzed by Western blots using p-IkappaB, p65, and PTEN antibodies. PTEN promoter and NF-kappaB activities were assessed by PTEN-luc and p-NF-luc constructs, respectively. Dominant negative p-IkappaB-alpha-M (NF-kappaB superrepressor) was used to block activation of NF-kappaB. Cell motility was assessed by Boyden chamber migration assay. TGF-beta induced IkappaB-alpha phosphorylation followed by NF-kappaB p65 subunit nuclear translocation and increased NF-kappaB activity. IkappaB-alpha-M blocked TGF-beta-induced NF-kappaB activity, reversed downregulated PTEN promoter activity and PTEN expression, and prevented augmentation of cell motility induced by TGF-beta. SMAD4 restoration, but not knockdown of SMAD2 and/or 3, reversed TGF-beta-induced NF-kappaB activity. Thus TGF-beta suppresses PTEN in pancreatic cancer cells through NF-kappaB activation and enhances cell motility and invasiveness in a SMAD4-independent manner that can be counteracted when TGF-beta-SMAD signaling is restored. The TGF-beta/NF-kappaB/PTEN cascade may be a critical pathway for pancreatic cancer cells to proliferate and   metastasize  . 
 NF-kappaB promotes breast cancer cell migration and metastasis by inducing the expression of the chemokine receptor CXCR4. Metastasis of cancer cells is a complex process involving multiple steps including invasion, angiogenesis, and trafficking of cancer cells through blood vessels, extravasations, organ-specific homing, and growth. While matrix metalloproteinases, urokinase-type plasminogen activator, and cytokines play a major role in invasion and angiogenesis, chemokines such as stromal derived factor-1alpha (SDF-1alpha) and their receptors such as CXCR4 are thought to play a critical role in motility, homing, and proliferation of cancer cells at specific metastatic sites. We and others have previously reported that the extracellular signal-activated transcription factor NF-kappaB up-regulates the expression of matrix metalloproteinases, urokinase-type plasminogen activator, and cytokines in highly metastatic breast cancer cell lines. In this report, we demonstrate that NF-kappaB regulates the motility of breast cancer cells by directly up-regulating the expression of CXCR4. Overexpression of the inhibitor of kappaB (IkappaB) in breast cancer cells with constitutive NF-kappaB activity resulted in reduced expression of CXCR4 and a corresponding loss of SDF-1alpha-mediated migration in vitro. Introduction of CXCR4 cDNA into IkappaB-expressing cells restored SDF-1alpha-mediated migration. Electrophoretic mobility shift assays and transient transfection assays revealed that the NF-kappaB subunits p65 and p50 bind directly to sequences within the -66 to +7 region of the CXCR4 promoter and activate transcription. We also show that the cell surface expression of CXCR4 and the SDF-1alpha-mediated migration are enhanced in breast cancer cells isolated from mammary fat pad xenografts compared with parental cells grown in culture. A further increase in CXCR4 cell surface expression and SDF-1alpha-mediated migration was observed with cancer cells that   metastasize  d to the lungs. Taken together, these results implicate NF-kappaB in the migration and the organ-specific homing of metastatic breast cancer cells. 
  GeneRIFs  
 The TGF-beta/NF-kappaB/PTEN cascade may be a critical pathway for pancreatic cancer cells to proliferate and   metastasize   
  [Mm]etastasis  PubMed  
 [The expression and clinical significance of SphK1 and nuclear factor-κB p65 in human colon carcinoma]. To investigate the expression of sphingosine kinase 1 (SphK1) and NF-κB in colon carcinoma tissues and their correlation with clinicopathologic features.Sixty-six paraffin-embedded colon carcinoma samples and 66 fresh colon carcinoma samples were tested using immunohistochemistry, RT-PCR and Western blot, respectively.In 66 fresh colon carcinoma samples, the positive rate of SphK1 and NF-κB mRNA expression were 84.85%(56/66) and 74.24% (49/66), while the positive rate of SphK1 and NF-κB protein detected by Western blot were 78.79% (52/66) and 69.70% (46/66). The positive rates were higher than those in the adjacent tissues [mRNA: 63.64% (42/66), 48.49% (32/66); protein: 57.58% (38/66), 45.45% (30/66)] and the normal mucosa [mRNA: 42.42% (28/66), 25.76% (17/66); protein: 36.36% (24/66), 24.24% (16/66)], with statistical significances (all P values < 0.05). The mean expressive levels of SphK1 and NF-κB mRNA and protein in colon carcinoma were both significantly higher than those in the adjacent tissues and the normal mucosa (mRNA: 0.55 ± 0.06 vs 0.35 ± 0.05 vs 0.25 ± 0.05, 0.75 ± 0.06 vs 0.43 ± 0.05 vs 0.30 ± 0.04; protein: 0.77 ± 0.05 vs 0.38 ± 0.06 vs 0.12 ± 0.03, 0.45 ± 0.08 vs 0.23 ± 0.05 vs 0.13 ± 0.03; all P values < 0.05). There was a close correlation between SphK1 and NF-κB expression levels (r = 0.459, P = 0.036). The results of immunohistochemistry were similar to those of RT-PCR and Western blot. Overexpression of SphK1 and NF-κB in colon carcinoma was related with depth of invasion, dist  ant and ly  mph node metastasis and Dukes' stages (all P values < 0.05). The expression of SphK1 was also related with differentiation (P < 0.05).Overexpression of SphK1 and NF-κB may be involved in the pathogenesis and progression of colon carcinoma. Moreover, SphK1 and NF-κB may be correlated w  ith the in  vasion and metastasis of colon carcinoma. 
 [The expression and clinical significance of SphK1 and nuclear factor-κB p65 in human colon carcinoma]. To investigate the expression of sphingosine kinase 1 (SphK1) and NF-κB in colon carcinoma tissues and their correlation with clinicopathologic features.Sixty-six paraffin-embedded colon carcinoma samples and 66 fresh colon carcinoma samples were tested using immunohistochemistry, RT-PCR and Western blot, respectively.In 66 fresh colon carcinoma samples, the positive rate of SphK1 and NF-κB mRNA expression were 84.85%(56/66) and 74.24% (49/66), while the positive rate of SphK1 and NF-κB protein detected by Western blot were 78.79% (52/66) and 69.70% (46/66). The positive rates were higher than those in the adjacent tissues [mRNA: 63.64% (42/66), 48.49% (32/66); protein: 57.58% (38/66), 45.45% (30/66)] and the normal mucosa [mRNA: 42.42% (28/66), 25.76% (17/66); protein: 36.36% (24/66), 24.24% (16/66)], with statistical significances (all P values < 0.05). The mean expressive levels of SphK1 and NF-κB mRNA and protein in colon carcinoma were both significantly higher than those in the adjacent tissues and the normal mucosa (mRNA: 0.55 ± 0.06 vs 0.35 ± 0.05 vs 0.25 ± 0.05, 0.75 ± 0.06 vs 0.43 ± 0.05 vs 0.30 ± 0.04; protein: 0.77 ± 0.05 vs 0.38 ± 0.06 vs 0.12 ± 0.03, 0.45 ± 0.08 vs 0.23 ± 0.05 vs 0.13 ± 0.03; all P values < 0.05). There was a close correlation between SphK1 and NF-κB expression levels (r = 0.459, P = 0.036). The results of immunohistochemistry were similar to those of RT-PCR and Western blot. Overexpression of SphK1 and NF-κB in colon carcinoma was related with depth of invasion, dist  ant and ly  mph node metastasis and Dukes' stages (all P values < 0.05). The expression of SphK1 was also related with differentiation (P < 0.05).Overexpression of SphK1 and NF-κB may be involved in the pathogenesis and progression of colon carcinoma. Moreover, SphK1 and NF-κB may be correlated w  ith the in  vasion and metastasis of colon carcinoma. 
 Immunohistochemical evaluation of midkine and nuclear factor-kappa B as diagnostic biomarkers for papillary thyroid cancer and synchronous   metastasis  . Midkine (MK) is a multifunctional cytokine identified to be a promising cancer biomarker. Nuclear factor-kappa B (NF-κB) is an important transcription factor that plays a pivotal role in tumorigenesis. We aimed to investigate values of MK and NF-κB as markers for diagnosis and synchronou  s metastas  is prediction in papillary thyroid cancer (PTC).76 cases of PTC and 70 cases of multi-nodular goiter (MNG) were retrieved. The PTC group was further divided into subgroup 1 (16 cases with synchronous metastases) and subgroup 2 (60 cases without metastases). A retrospective review of demographic and clinical information was performed. Immunohistochemistry of MK, NF-κB p65 and Ki-67 was performed on paraffin-embedded specimens and results were quantified. Diagnostic values of the parameters were conducted by receiver operating characteristic (ROC) curves. Protein levels of MK and NF-κB p65 were then confirmed by Western blot.Immunoreactivities of MK, NF-κB p65 and Ki-67 were significantly higher in the PTC group than in the MNG group with good differential diagnostic capabilities. Moreover, immunoreactivities of all three parameters were significantly higher in subgroup 1 than in subgroup 2 with good synchro  nous metas  tasis predictive efficacies. Western blot showed that MK and NF-κB p65 protein levels in lesions from subgroup 1 were significantly higher than those from subgroup 2, both of which were significantly higher than in MNG lesions.We discovered that MK and NF-κB immunohistochemistries can potentially be used for differential diagnosis between PTC and MNG, and for prediction of synchronous metastases. 
 Breast cancer   metastasis   suppressor 1 functions as a corepressor by enhancing histone deacetylase 1-mediated deacetylation of RelA/p65 and promoting apoptosis. The antiapoptotic transcription factor NF-kappaB is constitutively activated in many cancers and is important for cytokine-mediated progression and metastatic movement of tumors. Breast cancer   metastasis   suppressor 1 (BRMS1) is a   metastasis   suppressor gene whose mechanisms of action are poorly understood. In this report, we demonstrate that BRMS1 decreases the transactivation potential of RelA/p65 and ameliorates the expression of NF-kappaB-regulated antiapoptotic gene products. BRMS1 immunoprecipitates with the RelA/p65 subunit of NF-kappaB with protein-protein interactions occurring at the C terminus region of the rel homology domain but not at its known transactivation domains. Moreover, BRMS1 functions as a corepressor by promoting binding of HDAC1 to RelA/p65, where it deacetylates lysine K310 on RelA/p65, which suppresses RelA/p65 transcriptional activity. Selective small interfering RNA knockdown of BRMS1 confirms that chromatin-bound BRMS1 is required for deacetylation of RelA/p65, while enhancing chromatin occupancy of HDAC1 onto the NF-kappaB-regulated promoters cIAP2 and Bfl-1/A1. We observed in cells lacking BRMS1 a dramatic increase in cell viability after the loss of attachment from the extracellular matrix. Collectively, these results suggest that BRMS1 suppresses   metastasis   through its ability to function as a transcriptional corepressor of antiapoptotic genes regulated by NF-kappaB. 
 Activation of nuclear factor-κB contributes to growth and aggressiveness of papillary thyroid carcinoma. Nuclear factor-κB (NF-κB) is involved in proliferation, angiogenesis, a  nd metasta  sis in various malignancies; however, the role of NF-κB in papillary thyroid carcinoma (PTC) has not been fully elucidated. The purpose of this study was to elucidate the role and clinicopathological significance of the NF-κB signaling pathway in PTC. We investigated NF-κB RelA expression in 122 patients with conventional PTC by immunohistochemistry, and evaluated the correlation between RelA expression and clinicopathological parameters, including BRAF(V600E) mutation. Nuclear expression of NF-κB RelA, regardless of cytoplasmic expression, was identified in 91 of 122 PTCs (74.6%), and was more frequent in PTCs larger than 1cm (overt PTC) (P=0.001). There were significant differences in clinicopathological parameters, such as extrathyroidal extension (P=0.031),   nodal met  astasis (P=0.021) and BRAF(V600E) mutation (P=0.039), between NF-κB-positive and negative PTCs. Proliferation index was strongly associated with NF-κB activation (P=0.045) but not with BRAF(V600E) mutation (P=0.141). Taken together, our results suggest that NF-κB RelA activation contributes, at least in part, to tumor growth and aggressiveness of PTC after tumor transformation. The expression pattern of NF-κB may serve as a prognostic marker and a potential therapeutic target. 
 [Expressions of NF-κBp65 and IκBα in gestational trophoblastic disease and clinical significance]. To investigate the roles of nuclear factor κB p65 (NF-κBp65) and inhibitor of nuclear factor κB α (IκBα) in the develop  ment and m  etastasis of gestational trophoblastic neoplasia (GTN) by analyzing the expressions of NF-κBp65 and IκBα in normal early pregnancy villi and gestational trophoblastic diseases, and to reveal the relationship of NF-κBp65 and IκBα with age and clinical stage of GTN patients.The expressions of NF-κBp65 and IκBα were detected by immunohistochemistry in normal pregnancy villi (20 cases), hydatidiform moles (HM, 30 cases), invasive hydatidiform moles (IHM, 13 cases) and chorionic carcinomas (CCA, 15 cases).NF-κBp65 expression was statistically different (P<0.05) between normal pregnancy villi and IHM (P=0.013), normal pregnancy villi and CCA(P=0.018), HM and IHM(P=0.026), HM and CCA (P=0.035). Differences in IκBα expression were statistically significant between normal pregnancy villi and IHM, normal pregnancy villi and CCA, HM and IHM, HM and CCA (P<0.01). The expressions of NF-κBp65 and IκBα were correlated to clinical stage (P=0.043, 0.042, P<0.05), but not to patients' ages. Spearman correlation analysis revealed that there was a negative association between the protein expressions of NF-κBp65 and IκBα in GTN (r=-0.403, P=0.034, P<0.05).Up-regulated expression of NF-κBp65 and down-regulated expression of IκBα may be related to   the develo  pment, invasion and metastasis of GTN. The expressions of NF-κBp65 and IκBα are negatively correlated in gestational trophoblastic tumor tissues. 
 Tim-3 facilitates osteosarcoma proliferation and   metastasis   through the NF-κB pathway and epithelial-mesenchymal transition. The aim of this study was to investigate the expression of T-cell immunoglobulin mucin domain molecule-3 (Tim-3) in osteosarcoma tissues, and analyze its effect on cell proliferation and   metastasi  s in an osteosarcoma cell line. Tim-3 mRNA and protein expression in osteosarcoma tissue was detected by reverse transcriptase-polymerase chain reaction and immunohistochemistry, respectively. Additionally, the cell viability, apoptosis rate, and invasive ability of the osteosarcoma cell line MG-63 were tested using the methyl thiazolyl tetrazolium assay, Annexin V-propidium iodide flow cytometry, and a Transwell assay, respectively, following Tim-3 interference using small interfering RNA (siRNA). We also analyzed the expression of Snail, E-cadherin, vimentin, and nuclear factor (NF)-kB in the cells by western blot. We observed that Tim-3 mRNA and protein was significantly overexpressed in osteosarcoma tissues, compared to the adjacent normal tissue (P < 0.01). Moreover, MG-63 cells transfected with the Tim-3 siRNA presented lower cell viability, a greater number of apoptotic cells, and decreased invasive ability (P < 0.01), compared to control cells. Additionally, we observed a decrease in Snail and vimentin expression, an increase in the E-cadherin level, and an increase in NF-kB p65 phosphorylation (P < 0.01) in Tim-3 siRNA-transfected MG-63 cells. Based on these results, we concluded that Tim-3 is highly expressed in osteosarcoma tissue. Moreover, we speculated that interfering in Tim-3 expression could significantly suppress osteosarcoma cell (MG-63) proliferation and   metastasi  s via the NF-kB/Snail signaling pathway and epithelial-mesenchymal transition. 
 Impact of curcumin, raspberry extract, and neem leaf extract on rel protein-regulated cell death/radiosensitization in pancreatic cancer cells. Nuclear factor κB (NF-κB) plays an intrinsic role in promoting growth, angiogenesis, an  d metastas  is in pancreatic cancer (PC) and serves as a mechanism underlying therapeutic resistance. Accordingly, we investigated the efficacy of bioactive phytochemicals in inhibiting radiotherapy (RT)-induced NF-κB activity, signaling, and NF-κB-dependent regulation of cell death.Panc-1, BxPC-3, and MIA PaCa-2 cells exposed to 10 Gy (single high dose [SDR]) or 2 Gy/d for 5 days (fractionated radiation [FIR]) with or without curcumin (CUR), neem leaf extract (NLE), or black raspberry extract (RSE) were analyzed.Radiotherapy profoundly induced NF-κB-DNA-binding activity with relatively robust activation after FIR. Curcumin, NLE, and RSE significantly inhibited both constitutive and RT-induced NF-κB. Furthermore, quantitative polymerase chain reaction profiling of 88 NF-κB pathway molecules demonstrated that CUR, NLE, and RSE comprehensively, yet differentially inhibited FIR/SDR-induced genes. Functionally, CUR, NLE, and RSE markedly conferred RT-inhibited cell viability/survival, robustly activated caspase-3/7 activity, and subsequent cell death. More importantly, NF-κB overexpression and silencing studies demonstrate that these compounds potentiate RT-induced cell death by targeting RT-induced NF-κB.These data strongly imply that CUR, NLE, and RSE may serve as effective "deliverables" to potentiate RT in PC cure and further throw light that these phytochemicals-induced cell killing may involve selective regulation of RT-induced NF-κB. 
 Stabilization of snail by NF-kappaB is required for inflammation-induced cell migration and invasion. The increased motility and invasiveness of tumor cells are reminiscent of epithelial-mesenchymal transition (EMT), which occurs during embryonic development, wound healing, and   metastasis  . In this study, we found that Snail is stabilized by the inflammatory cytokine TNFalpha through the activation of the NF-kappaB pathway. We demonstrated that NF-kappaB is required for the induction of COP9 signalosome 2 (CSN2), which, in turn, blocks the ubiquitination and degradation of Snail. Furthermore, we showed that the expression of Snail correlated with the activation of NF-kappaB in cancer cell lines and metastatic tumor samples. Knockdown of Snail expression inhibited cell migration and invasion induced by inflammatory cytokines and suppressed inflammation-mediated breast cancer   metastasis  . Our study provides a plausible mechanism for inflammation-induced   metastasis  . 
 Nuclear factor-kappaB signaling and ezrin are essential for L1-mediated   metastasis   of colon cancer cells. Hyperactivation of beta-catenin-T-cell-factor (TCF)-regulated gene transcription is a hallmark of colorectal cancer (CRC). The cell-neural adhesion molecule L1CAM (hereafter referred to as L1) is a target of beta-catenin-TCF, exclusively expressed at the CRC invasive front in humans. L1 overexpression in CRC cells increases cell growth and motility, and promotes liver   metastasis  . Genes induced by L1 are also expressed in human CRC tissue but the mechanisms by which L1 confers   metastasis   are still unknown. We found that signaling by the nuclear factor kappaB (NF-kappaB) is essential, because inhibition of signaling by the inhibitor of kappaB super repressor (IkappaB-SR) blocked L1-mediated   metastasis  . Overexpression of the NF-kappaB p65 subunit was sufficient to increase CRC cell proliferation, motility and   metastasis  . Binding of the L1 cytodomain to ezrin - a cytoskeleton-crosslinking protein - is necessary for   metastasis   because when binding to L1 was interrupted or ezrin gene expression was suppressed with specific shRNA,   metastasis   did not occur. L1 and ezrin bound to and mediated the phosphorylation of IkappaB. We also observed a complex containing IkappaB, L1 and ezrin in the juxtamembrane region of CRC cells. Furthermore, we found that L1, ezrin and phosphorylated p65 are co-expressed at the invasive front in human CRC tissue, indicating that L1-mediated activation of NF-kappaB signaling involving ezrin is a major route of CRC progression. 
 [The relation of nuclear factor kappa B to angiogenesis and clinical outcomes in adenoid cystic carcinoma of salivary glands]. To evaluate the expression of nuclear factor kappa B (NF-kappaB) p65 in relation to angiogenesis microvessel density (MVD) and clinical outcomes in adenoid cystic carcinoma of salivary glands (ACCs).Immunohistochemical staining method was used to quantify the protein expression levels of NF-kappaB p65 in 80 surgically resected salivary gland ACC and normal salivary tissues. In all cases of ACCs, microvessel density was evaluated by counting any CD(34)-reactive endothelial cell or endothelial-cell cluster. The patients with ACCs were followed up from 1992 to 2002.NF-kappaB p65 was detected in the cytoplasm of normal and ACC cells, but was only in the nucleus of tumor cells; while there was no NF-kappaB p65 nuclear staining in all the controls. The mean value of MVD was (47.07 +/- 13.44), which had significant correlations with NF-kappaB p65 expression (P < 0.01). In three histological types of salivary gland ACC, the expressions of NF-kappaB and MVD were significantly higher in solid type than in cribriform type and tubular type (P < 0.01). NF-kappaB p65 expression and MVD were significantly correlated to clinical stage, tumor size, perineural and vascular invasion, recurrence and   metastasis   (P < 0.05), but there were no correlations between those three factors and patient age, gender and tumor site (P > 0.05). Multivariate analysis showed NF-kappaB (P < 0.05) expression, MVD (P < 0.01), histotypes (P < 0.01), and perineural invasion (P < 0.05) had an independent prognostic impact on overall survival.The expression of NF-kappaB p65 was related to MVD, and the correlation between those two factors and clinico-pathological factors and prognosis of ACCs are significant. 
 Expression of NF-kappaB and human telomerase reverse transcriptase in gastric cancer and precancerous lesions. To investigate the expression of NF-kappaBp65 protein and human telomerase reverse transcriptase (hTERT) and their correlation in gastric cancer and precancerous lesions.Forty-one patients with primary gastric cancer, 15 with dysplasia, 23 intestinal metaplasia and 10 with normal gastric mucosa were included in this study. Expression of NF-kappaBp65 protein, hTERT mRNA and protein were determined by immunohistochemistry and in situ hybridization.The rate of p65 expression in normal gastric mucosa, intestinal metaplasia, dysplasia and carcinoma was 0%, 34.78%, 53.33% and 60.98%, respectively, while the rate of hTERT mRNA expression was 10.00%, 39.13%, 66.67% and 85.37% and the rate of hTERT protein expression was 0%, 30.43%, 60.00% and 78.05%, respectively. All the three parameters were significantly increased in dysplasia and carcinoma compared to normal mucosa, while the expression levels were also significantly higher in carcinoma than in intestinal metaplasia (P<0.05). In gastric cancer tissues, nuclear staining rates of p65 and hTERT protein were both significantly associated with the degree of differentiation, lymph node   metastasis  , clinical stage and invasion depth (P<0.05). However, hTERT mRNA expression was only significantly associated with clinical stage. There was a positive correlation between p65 and hTERT mRNA (rs=0.661-0.752, P<0.01), and between hTERT protein and hTERT mRNA (rs=0.609-0.750, P<0.01).NF-kappaBp65 and hTERT expressions are upregulated at the early stage of gastric carcinogenesis. NF-kappaB activation may contribute to hTERT expression and thereby enhance telomerase activity, which represents an important step in carcinogenesis progress. 
 Nuclear NF-κB expression correlates with outcome among patients with head and neck squamous cell carcinoma treated with primary chemoradiation therapy. To examine whether nuclear NF-κB expression correlates with outcome in patients with head and neck squamous cell carcinoma (HNSCC) treated with primary chemoradiation therapy (CRT).Between 2007 and 2010, 101 patients with locally advanced primary HNSCC were treated with definitive simultaneous CRT. Pretreatment biopsy specimens were analyzed for NF-κB p65 (RelA) nuclear immunoreactivity. A sample was assigned to be positive with more than 5% positive nuclear expression. The predictive relevance of NF-κB and clinicopathologic factors for overall survival (OS), progression-free survival (PFS), local progression-free survival (LPFS),   and metast  asis-free survival (DMFS) was examined by univariate and multivariate analysis.No significant differences between the groups were observed with regard to age, sex, total radiation dose, fractionation mode, total chemotherapy applied, T stage or grading. Patients with p65 nuclear positive biopsy specimens showed significantly a higher rate of lymph n  ode metast  asis (cN2c or cN3 status, P=.034). Within a mean follow-up time of 25 months (range, 2.33-62.96 months) OS, PFS, and DMFS were significantly poorer in the p65 nuclear positive group (P=.008, P=.027, and P=.008, respectively). These correlations remained significant in multivariate analysis.NF-κB/p65 nuclear expression is associated with increased lymphatic and hematogenous tumor dissemination and decreased survival in HNSCC patients treated with primary CRT. Our results may foster further investigation of a predictive relevance of NF-κB/p65 and its role as a suitable target for a molecular-based targeted therapy in HNSCC cancer. 
 Expressions of nuclear factor kappaB, inducible nitric oxide synthase, and vascular endothelial growth factor in adenoid cystic carcinoma of salivary glands: correlations with the angiogenesis and clinical outcome. To evaluate the expressions of nuclear factor kappaB (NF-kappaB p65), inducible nitric oxide synthase enzyme (iNOS), and vascular endothelial growth factor (VEGF) in relation to angiogenesis (microvessel density, MVD) and clinical outcomes in adenoid cystic carcinoma (ACC) of salivary glands.Immunohistochemical staining was used to quantify the protein expression levels of NF-kappaB p65, iNOS, and VEGF in 80 surgically resected ACCs and 20 normal salivary tissues. In all cases of ACCs, MVD was evaluated by counting CD34-reactive endothelial cells or endothelial cell clusters.The nuclear localization of NF-kappaB p65 was only detected in ACC cells. Both iNOS and VEGF staining activities in ACCs were more significant than those in normal gland tissues (P < 0.01). MVD had significant correlations with NF-kappaB p65, iNOS, and VEGF expressions (P < 0.01). In three histologic types of ACCs, the NF-kappaB, iNOS, VEGF expressions, and MVD were significantly higher in solid type than in cribriform and tubular types (P < 0.01). The NF-kappaB, iNOS, VEGF expressions, and MVD were significantly correlated with clinical stage, tumor size, vascular invasion, recurrence, and   metastasis   (P < 0.05). Multivariate analysis showed NF-kappaB, iNOS and VEGF expression, MVD, solid histotype, and perineural invasion had an independent prognostic effect on overall survival.The expressions of NF-kappaB p65, iNOS, and VEGF were related with MVD. Clinical outcomes raised the possibility that the overexpression of these cytokines might contribute to tumor angiogenesis and have prognostic value in ACCs. 
 Foxp3 downregulation in NSCLC mediates epithelial-mesenchymal transition via NF-κB signaling. Forkhead box P3 (Foxp3) is a member of forkhead box transcription factor family and it was identified as a tumor suppressor in various solid tumors. This study evaluated the expression of Foxp3 in non-small cell lung cancer (NSCLC) and investigated its role in epithelial‑mesenchymal transition (EMT) of cancer cells. qRT-PCR and western blot analysis were used for examining the expression of Foxp3 in NSCLC tissues and the non-tumor tissues. A tissue microarray was constructed and scored for evaluating the clinical significance of Foxp3 expression in NSCLC tissues. RNAi was employed for downregulating Foxp3 expression and cell proliferation was done with MTT assay. Transwell with or without basement membrane matrix was used for cell migration and invasion assay respectively. Foxp3 was found downregulated in NSCLC tissues compared with non-tumoral tissues; downregulation of Foxp3 predicted adverse tumor stage and overall survival; silencing of FOXP3 promoted the proliferation, migration and invasion ability of NSCLC cells and influenced the expression level of EMT-associated proteins. However, forced expression of Foxp3 could reverse this effect. Moreover, Foxp3 could interact with LMO2 and affect the expression level of TAL1, which was in accordance with the findings in T-cell acute lymphoblastic leukemia. By screening the signalling pathways, we observed an obvious upregulation of phosphorylated NF-κB in A549 and H520 cells after silencing of FOXP3. Our results suggest that Foxp3 suppressed NSCLC   cell meta  stasis, at least partially, via NF-κB signaling. 
 A cytoplasmic NF-κB interacting long noncoding RNA blocks IκB phosphorylation and suppresses breast cance  r metastas  is. NF-κB is a critical link between inflammation and cancer, but whether long non-coding RNAs (lncRNAs) regulate its activation remains unknown. Here, we identify an NF-KappaB Interacting LncRNA (NKILA), which is upregulated by NF-κB, binds to NF-κB/IκB, and directly masks phosphorylation motifs of IκB, thereby inhibiting IKK-induced IκB phosphorylation and NF-κB activation. Unlike DNA that is dissociated from NF-κB by IκB, NKILA interacts with NF-κB/IκB to form a stable complex. Importantly, NKILA is essential to prevent over-activation of NF-κB pathway in inflammation-stimulated breast epithelial cells. Furthermore, low NKILA expression is associated with   breast can  cer metastasis and poor patient prognosis. Therefore, lncRNAs can directly interact with functional domains of signaling proteins, serving as a class of NF-κB modulators to   suppress c  ancer metastasis. 
 Melatonin inhibits MMP-9 transactivation and renal cell carcinoma   metastasis   by suppressing Akt-MAPKs pathway and NF-κB DNA-binding activity. Renal cell carcinoma (RCC) is the most lethal of all urological malignancies because of its potent   metastasi  s potential. Melatonin exerts multiple tumor-suppressing activities through antiproliferative, proapoptotic, and anti-angiogenic actions and has been tested in clinical trials. However, the antimetastastic effect of melatonin and its underlying mechanism in RCC are unclear. In this study, we demonstrated that melatonin at the pharmacologic concentration (0.5-2 mm) considerably reduced the migration and invasion of RCC cells (Caki-1 and Achn). Furthermore, we found that melatonin suppressed   metastasi  s of Caki-1 cells in spontaneous and experimental   metastasi  s animal models. Mechanistic investigations revealed that melatonin transcriptionally inhibited MMP-9 by reducing p65- and p52-DNA-binding activities. Moreover, the Akt-mediated JNK1/2 and ERK1/2 signaling pathways were involved in melatonin-regulated MMP-9 transactivation and cell motility. Clinical samples revealed an inverse correlation between melatonin receptor 1A (MTNR1A) and MMP-9 expression in normal kidney and RCC tissues. In addition, a higher survival rate was found in MTNR1A(high) /MMP-9(low) patients than in MTNR1A(low) /MMP-9(high) patients. Overall, our results provide new insights into the role of melatonin-induced molecular regulation in suppressing RCC   metastasi  s and suggest that melatonin has potential therapeutic applications for metastastic RCC. 
 Osteopontin expression correlates with nuclear factor-κB activation and apoptosis downregulation in clear cell renal cell carcinoma. Osteopontin (OPN) is a phosphoglycoprotein implicated in tumorigenesis and tumor cell   metastasi  s. Apoptosis inhibition is one of the mechanisms that contribute to development and progression of cancer, and might be initiated by OPN interaction with tumor cells. The aim of this study was to analyze the relation between OPN and nuclear factor-kappa B (NF-κB) expression in clear cell renal cell carcinoma (CCRCC), as well as their relation to apoptotic activity of tumor cells. Expression of OPN protein and p65 NF-κB subunit was analyzed immunohistochemically in 87 CCRCC samples, and compared mutually and with apoptotic index. Expression of OPN mRNA was analyzed using quantitative real-time PCR and compared with OPN and NF-κB protein expression in 22 CCRCC samples. Statistical analysis showed an association of p65 NF-κB with OPN mRNA (p=0.015) and protein (p<0.001). Also, we found an inverse relationship of OPN with NF-κB protein expression and apoptotic activity of tumor cells (p=0.006 and p=0.022, respectively). Our results indicate that p65 NF-κB signaling pathway may be involved in OPN-mediated CCRCC progression, partly by protecting tumor cells from apoptosis. Therefore, both molecules can constitute potential targets for therapeutic intervention in CCRCC. 
 [Expressions of NF-kappaB p65 in laryngeal carcinoma and normal mucous membrane and its clinical significance]. To evaluate expressions of NF-kappaB p65 in laryngeal carcinoma and normal mucous membrane and its clinical significance.The immunohistochemical method and the in situ hybridization were employed in detecting the NF-kappaB p65 expressions in surgical specimen from 48 laryngeal carcinoma and 20 normal mucous membrane.(1) The expression of NF kappaB p65 was higher than in normal laryngeal mucous membrane. (2) Positive expression of NF-kappaB p65 correlated with the development of distant   metastasis  .The results of the study show that the NF-kappaB p65 protein is a significant predictor of survival in laryngeal carcinoma patients. 
 Different cellular localization of NF-κB p65 expression as an indicator of different prognoses of stage I-III gastric cancer patients. Nuclear factor-κB p65 (NF-κB p65) may play a significant role as a biomarker in tumor progression a  nd metasta  sis. However, the correlation between cellular localization of NF-κB p65 expression and the prognosis of gastric cancer (GC) patients has not been studied. The present study was designed to investigate the location of NF-κB p65 expression in GC, and evaluate its correlation with clinicopathological parameters of GC patients.NF-κB p65 expressions in GC tissue and corresponding nonmalignant tissue from gastrectomy of 115 stage I-III GC patients were detected by immunohistochemistry. In addition, correlations between the staining results and the clinicopathologic features and survival of the GC patients were analyzed.The percentage of NF-κB p65 expression in GC tissue and the corresponding nonmalignant tissue was 73.9% and 46.80%, respectively. No significant correlation was found between NF-κB p65 expression and the clinicopathologic parameters. Cox univariate analysis indicated that both nuclear staining and cytoplasmic staining of NF-κB p65 expression correlated with the prognosis of GC patients (log-rank, p = 0.0182; p = 0.0144, respectively).High nuclear expression of NF-κB p65 is an independent prognostic marker predicting a better survival, while high cytoplasmic staining indicates a worse prognosis of GC patients. 
 Morusin inhibits human cervical cancer stem cell growth and migration through attenuation of NF-κB activity and apoptosis induction. Cancer stem cells (CSCs) are believed to be responsible for tumor   metastasi  s, recurrence, and high mortality of cancer patients due to their high tumorigenicity resistance to chemo-radiotherapy. Morusin possesses anti-cancer activity through attenuation of NF-κB activity, which is up-regulated in cancer stem cells. The purpose of this study is to confirm the growth and migration inhibition effect of morusin on human cervical CSCs, and to clarify its partial mechanism of activity. Human cervical CSCs were enriched using non-adhesive culture system. Their stemness characteristics were identified with tumor sphere formation, self-renewal, toluidine blue staining, migration assays, RT-PCR analysis, and immunofluorescence staining of putative stem cell markers, Oct4, SOX2, and ALDH1; the epithelial-to-mesenchymal (EMT) transition markers and relevant transcription factors were evaluated with Western blotting. The growth and migration inhibition effects of morusin on human cervical CSCs were tested by cell proliferation, tumor sphere formation, and transwell assay; apoptotic death of human cervical CSCs in response to morusin was measured with DAPI staining, apoptotic DNA fragmentation; NF-κBp65, Bcl-2, Bax, and caspase-3 protein expressions were detected through Western blotting. Under this non-adhesive culture system, typical tumor spheres appeared within 5-7 days, the tumor sphere formation, self-renewal, and cell migration, expressions of putative stem cell markers, EMT markers, and relevant transcription factors of the tumor sphere cells were increased significantly. After morusin treatment, the proliferation, tumor sphere formation, and migration of human cervical CSCs were decreased significantly, DAPI-stained apoptotic cells increased, apoptotic DNA fragmentations formed evidently; the expression levels of NF-κBp65 and Bcl-2 decreased significantly, Bax, and caspase-3 increased significantly in a dose-dependent manner. Using the non-adhesive culture system, human cervical CSCs were enriched and expanded. Morusin has the potential to target and kill CSCs, and can inhibit human cervical growth and migration through NF-κB attenuation mediated apoptosis induction. 
 Intraperitoneal administration of a small interfering RNA targeting nuclear factor-kappa B with paclitaxel successfully prolongs the survival of xenograft model mice with peritoneal   metastasis   of gastric cancer. Activation of nuclear factor-kappa B (NF-kappaB) has been detected in various malignant tumors, including gastric carcinoma, and is associated with tumor growth,   metastasis  , resistance to chemotherapeutic agents and poor prognosis. Therefore, NF-kappaB is a potential target for antitumor therapy. In this study, we used a small interfering RNA (siRNA) to knockdown NF-kappaB p65 expression and determined whether intraperitoneal administration of NF-kappaB p65 siRNA and paclitaxel was effective for treating peritoneal   metastasis   of gastric cancer. Western blot analysis revealed that NF-kappaB p65 expression was diminished by NF-kappaB p65 siRNA. Apoptotic cells were increased after transfection of NF-kappaB p65 siRNA compared with control siRNA in the treatment with paclitaxel. In a murine xenograft model, abundant fluorescence was observed on the surface of intraperitoneal nodules of gastric cancer after siRNA administration. Moreover, intraperitoneal administration of NF-kappaB p65 siRNA reduced NF-kappaB expression in intraperitoneal nodules of gastric cancer. Finally, mice treated by intraperitoneal administration of NF-kappaB p65 siRNA and paclitaxel survived for a significantly longer time than mice treated by intraperitoneal administration of paclitaxel alone (p = 0.0002). Taken together, the present results demonstrate that intraperitoneal administration of NF-kappaB p65 siRNA and paclitaxel inhibited cancer growth in mice with peritoneal   metastasis   of gastric cancer. Therefore, intraperitoneal administration of NF-kappaB p65 siRNA and paclitaxel may provide a breakthrough in the treatment of peritoneal   metastasis   of gastric cancer. 
 FOXP3 inhibits NF-κB activity and hence COX2 expression in gastric cancer cells. Gastric cancer remains the main cause of cancer related deaths all over the world, and upregulated COX2 is a key player in its development. The mechanism as to how COX2 is regulated during the gastric cancer development is largely unknown. In this study, we found that the expression of COX2 was closely correlated with NF-κB activity. Strikingly, NF-κB activity was not absolutely consistent with its nuclear localization. Especially, in some cancer cell lines, such as MKN28, there were abundant nuclear localized NF-κB, while NF-κB luciferase activity in this cell line was relatively low. Furthermore, FOXP3 was found to be abundantly expressed in these cells. When the nuclear localized NF-κB expression was adjusted with the expression of FOXP3, it then correlated well with NF-κB activity. Molecularly, increased FOXP3 expression can interact with NF-κB and thus repress its activity. Knockdown of FOXP3 could increase NF-κB activity, COX2 expression, and cell migration. Taken together, our study revealed that function of FOXP3 as a negative regulator of NF-κB activity and thus plays a tumor suppressor role by redu  cing cell   metastasis. 
 NF-κBP65 promotes invasion and   metastasi  s of oesophageal squamous cell cancer by regulating matrix metalloproteinase-9 and epithelial-to-mesenchymal transition. NF-κB has been recognized as one of the factors responsible for the development of cancer; however, the mechanism by which high expression of NF-κB contributes to the progression of human oesophageal squamous cell cancer (ESCC) is not fully understood. In our investigations, NF-κBP65 was overexpressed in human ESCC tissues, especially in ESCC tissues with deep invasion and lymph n  ode metast  asis. Suppression of NF-κBP65 by siRNA decreased the invasion and proliferation ability of EC9706 cells in vitro. Furthermore, siRNA-mediated NF-κBP65 knock-down could lead to the downregulation of MMP  -9, a meta  stasis-related gene. Reduced E-cadherin is a hallmark of invasive carcinomas that have acquired epithelial-mesenchymal transition (EMT) phenotypes and Vimentin is another molecule that is used widely as a marker of the EMT. We found upregulation of E-cadherin expression and downregulation of Vimentin was induced by NF-κBP65 siRNA, which suggests that NF-κBP65 siRNA could inhibit the invasion and proliferation ability of ECSS through attenuating the expression of MMP-9 and EMT. Thus, ESCC NF-κBP65 could be a useful target for cancer prevention and therapy. 
 Intracellular annexin A2 regulates NF-κB signaling by binding to the p50 subunit: implications for gemcitabine resistance in pancreatic cancer. Annexin A2 (ANXA2) expression is highly upregulated in many types of cancer. Although cell surface localization of ANXA2 has been reported to have a critical role in the progression and   metastasi  s of a variety of tumors, including pancreatic cancer, the biological role of intracellular ANXA2 is not fully understood. Herein the role of intracellular ANXA2 was investigated in a pancreatic cancer cell line. We first determined whether ANXA2 is involved in NF-κB signaling pathways. ANXA2 bound to the p50 subunit of NF-κB in a calcium-independent manner, and the ANXA2-p50 complex translocated into the nucleus. Furthermore, ANXA2 increased the transcriptional activity of NF-κB in both the resting and activated states and upregulated the transcription of several target genes downstream of NF-κB, including that encoding interleukin (IL)-6, which contributes to anti-apoptotic signaling. In Mia-Paca2 cells, we determined the effects of wild-type ANXA2 and an ANXA2 mutant, Y23A, which suppresses the cell surface localization, on upregulation of NF-κB transcriptional activity and secretion of IL-6. Both wild-type and Y23A ANXA2 induced anti-apoptotic effects in response to treatment with tumor necrosis factor-α or gemcitabine. Based on these results, we suggest that ANXA2 mediates resistance to gemcitabine by directly increasing the activity of NF-κB. Collectively, these data may provide additional information about the biological role of ANXA2 in pancreatic cancer and suggest that ANXA2 is a potential biomarker for the drug resistance phenotype and a candidate therapeutic target for the treatment of pancreatic cancer. 
 Small interfering RNA targeting of Recepteur d'Origine Nantais induces apoptosis via modulation of nuclear factor-kappaB and Bcl-2 family in gastric cancer cells. The abnormal accumulation and activation of the receptor tyrosine kinase, Recepteur d'Origine Nantais (RON), has been implicated in tumorigenesis and   metastasis   in epithelial tumors including gastric cancer. This study examined whether the sequence-specific small interfering RNA (siRNA) suppression of the RON expression could induce apoptotic cell death, and investigated the involved molecular mechanisms. Sequence-specific siRNA effectively suppressed the RON expression at both the mRNA and protein levels. Silencing of the RON expression significantly inhibited gastric cancer cell proliferation and induced apoptosis in a time-dependent manner. The induction of apoptosis was confirmed by the ladder-patterned DNA fragmentation, the presence of cleaved and condensed nuclear chromatin and the increased number of annexin V-positive cells. RON-targeted siRNA effectively inhibited the constitutive nuclear factor-kappaB (NF-kappaB) activation as revealed by an altered electrophoretic mobility shift. In agreement with this, silencing of the RON expression resulted in a decrease in the nuclear level of the p65 subunit of NF-kappaB. The transfection of siRNA, which blocked the RON expression, also caused a change in the ratio of Bax/Bcl-2 in a manner that favored apoptosis. The siRNA silencing of RON induced cytochrome c release and the activation of caspase-8 and caspase-9. These results indicate that RON-targeted siRNA could be therapeutically efficacious by inducing cell apoptosis through the modulation of the NF-kappaB and Bcl-2 family in gastric cancer cells. 
 The tumor marker Fascin is strongly induced by the Tax oncoprotein of HTLV-1 through NF-kappaB signals. Oncogenic transformation of CD4(+) T cells by human T-cell lymphotropic virus type 1 (HTLV-1) is understood as the initial step to adult T-cell leukemia/lymphoma, a process that is mainly initiated by perturbation of cellular signaling by the viral Tax oncoprotein, a potent transcriptional regulator. In search of novel biomarkers with relevance to oncogenesis, we identified the tumor marker and actin-bundling protein Fascin (FSCN1) to be specifically and strongly up-regulated in both HTLV-1-transformed and adult T-cell leukemia/lymphoma patient-derived CD4(+) T cells. Fascin is important for migration and   metastasis   in various types of cancer. Here we report that a direct link can exist between a single viral oncoprotein and Fascin expression, as the viral oncoprotein Tax was sufficient to induce high levels of Fascin. Nuclear factor-κB signals were important for Tax-mediated transcriptional regulation of Fascin in T cells. This suggests that Fascin up-regulation by Tax contributes to the development of HTLV-1-associated pathogenesis. 
 Downregulation of MCT1 inhibits tumor growth,   metastasis   and enhances chemotherapeutic efficacy in osteosarcoma through regulation of the NF-κB pathway. Monocarboxylate transporter isoform 1 (MCT1) is an important member of the proton-linked MCT family and has been reported in an array of human cancer cell lines and primary human tumors. MCT1 expression is associated with developing a new therapeutic approach for cancer. In this study, we initially showed that MCT1 is expressed in a variety of human osteosarcoma cell lines. Moreover, we evaluated the therapeutic response of targeting MCT1 using shRNA or MCT1 inhibitor. Inhibiting MCT1 delayed tumor growth in vitro and in vivo, including in an orthotopic model of osteosarcoma. Targeting MCT1 greatly enhanced the sensitivity of human osteosarcoma cells to the chemotherapeutic drugs adriamycin (ADM). In addition, we observed that MCT1 knockdown significantly suppressed the metastatic activity of osteosarcoma, including wound healing, invasion and migration. Further mechanistic studies revealed that the antitumor effects of targeting MCT1 might be related to the NF-κB pathway. Immunochemistry assay showed that MCT1 was an independent positive prognostic marker in osteosarcoma patients. In conclusion, our data, for the first time, demonstrate that MCT1 inhibition has antitumor potential which is associated with the NF-κB pathway, and high MCT1 expression predicates poor overall survival in patients with osteosarcoma. 
 [Expression of heparanase and nuclear factor kappa B in pancreatic adenocarcinoma]. To detect the expressions of heparanase and nuclear factor kappa B p65 (NF-kappaB p65) in pancreatic adenocarcinomas and analyze their relation to patients' prognosis and the regulatory mechanism of NF-kappaB on heparanase expression.Heparanase and NF-kappaB p65 proteins in the tumor and adjacent tissues were detected by immunohistochemistry in 48 patients with pancreatic adenocarcinoma and analyzed for their clinicopathological significance.Heparanase and NF-kappaB p65 proteins were found in 30 (62.5%) and 22 (45.9%) tumor specimens, respectively, a rate significantly higher than that in the adjacent tissues. High heparanase expression was closely related to advanced TNM stage (P=0.031), lymph node   metastasis   (P=0.003) and decreased 3-year postoperative survival (20.0% vs 0%, P=0.001). NF-kappaB p65 expression was associated with lymph node   metastasis   (P=0.017) and distant   metastasis   (P=0.031), but had a higher positive rate in heparanase-positive cases than in heparanase-negative cases (P=0.018). Multivariate analysis showed that neither heparanase nor NF-kappaB p65 was the independent prognostic factors.Heparanase is overexpressed in pancreatic adenocarcinomas in association with decreased postoperative survival. NF-kappaB may up-regulate heparanase expression and promote heparanase-dependent tumor invasion and   metastasis  . 
 PRL-3 activates NF-κB signaling pathway by interacting with RAP1. Phosphatase of regenerating liver (PRL-3) promotes cancer   metastasi  s through enhanced cell motility and invasiveness, however its role in tumorigenesis remains unclear. Herein, we reported that PRL-3 interacts with telomere-related protein RAP1. PRL-3 promotes the cytosolic localization of RAP1, which is counteracted by silencing of PRL-3. Immunohistochemical staining of colon cancer tissue array (n=170) revealed that high level of PRL-3 associates with cytosolic localization of RAP1 (p=0.01). Microarray analysis showed that PRL-3 regulates expression of diverse genes and enhances phosphorylation of p65 subunit of NF-κB in a RAP1-dependent manner. Furthermore, PRL-3 transcriptionally activates RAP1 expression, which is counteracted by ablating p65. Therefore, our results demonstrate PRL-3 as a novel regulator of NF-κB signaling pathway through RAP1. 
 Comparison of p38MAPK (mitogene activated protein kinase), p65 NFkappaB (nuclear factor kappa b) and EMMPRIN (extracellular matrix metalloproteinase inducer) expressions with tumor grade and stage of superficial and invasive bladder tumors. To identify the molecular mechanisms of bladder cancer invasion pathophysiology. To assess EMMPRIN, p65NFkappaB and p38MAPK expressions which play a role in signal transmission system of muscle and non muscle invasive bladder tumors. Fifty-seven patients with non muscle invasive tumors (mean age 65.2 +/- 16.1) and 34 patients with muscle invasive tumors (mean age 62.2 +/- 20.7) were included in this study. Normal tissue from the same patients' bladders were used as control group. Patients had either TUR or radical cystectomy and paraffin sections were prepared for immunohistochemistry. Expression density was evaluated semiquantitively according to tumor grade and invasion depth. Results were compared with Mann Whitney U, Wilcoxon W, Chi Square and variation analysis tests. MAPK and EMMPRIN expression was increased according to tumor grade (p < 0.05). These expressions were also significantly higher in muscle invasive tumors than in non muscle invasive ones (p < 0.05). In normal tissue samples of both TUR and radical cystectomy materials, EMMPRIN, NFkappaB and MAPK expressions were lower than in tumor samples (p < 0.05). NFkappaB wasn't related to tumor grade/stage (p > 0.05). It can be stated that MAPK and EMMPRIN expression is related to the grade of bladder tumor and that NFkappaB positivity is not related to the grade/stage of the disease. In future positivity of lymph nodes and visceral   metastasis   and survival must be assessed to define the relationship with the expressions in long term follow up studies involving a larger number of patients. 
 Cleaved caspase-3 and nuclear factor-kappaB p65 are prognostic factors in metastatic serous ovarian carcinoma. Tumor progression and treatment failure in ovarian carcinoma are frequently associated with   metastasis   to effusions. The present study analyzed the expression and clinical role of nuclear factor-kappaB p65, nuclear factor-kappaB inhibitor alpha, and parameters of apoptosis in serous carcinoma. Cleaved caspase-3 and caspase-8 levels and deoxyuridine triphosphate incorporation were measured in 65 effusions using flow cytometry. Effusions (n = 209) and corresponding primary carcinomas and solid metastases (n = 114) were immunohistochemically analyzed for nuclear factor-kappaB p65 and nuclear factor-kappaB inhibitor alpha expression. Effusions (n = 75) were further analyzed for nuclear factor-kappaB phospho-p65 (Ser536) levels using immunoblotting. Results were analyzed for association with anatomic site, clinicopathologic parameters, and survival. Caspase cleavage and deoxyuridine triphosphate incorporation were limited to less than 10% of cells in most effusions. Nuclear factor-kappaB p65 expression was frequently detected at all anatomic sites, with less frequent cytoplasmic nuclear factor-kappaB p65 and nuclear factor-kappaB inhibitor alpha expressions. Immunoblotting showed nuclear factor-kappaB p65 phosphorylation in 72 (96%) of 75 effusions. Higher than median cleaved caspase-3 levels correlated with improved overall and progression-free survival in univariate analysis of all patients (P = .024 and P = .046, respectively) and of those with postchemotherapy effusions (P = .042 and P = .036, respectively). Cleaved caspase-3 expression was an independent predictor of longer progression-free survival for patients with postchemotherapy effusions (P = .029). Nuclear factor-kappaB p65 expression correlated with poor progression-free survival for all patients (P = .048) and for those with postchemotherapy effusions (P = .025). Ovarian carcinoma cells in effusions undergo little apoptosis, but high levels of cleaved caspase-3 are associated with improved survival. Nuclear factor-kappaB p65 is frequently expressed in advanced-stage serous ovarian carcinoma, and its nuclear localization is associated with poor progression-free survival. 
 Activity and expression pattern of NF-κB /P65 in peripheral blood from hepatocellular carcinoma patients - link to hypoxia inducible factor -1α. Hepatocellular carcinoma is a complex and heterogeneous tumor with poor prognosis due to frequent intrahepatic spread and extrahepati  c metastas  is. The molecular mechanisms underlying HCC pathogenesis still remain obscure.We aimed to investigate the abundance and the DNA binding activity of nuclear factor kappa B/p65 subunit in peripheral blood mononuclear cells from patients with HCC and to assess its prognostic significance and association with hypoxia inducible factor one alpha (HIF-1α) in blood.This study was carried out on 40 patients classified equally into liver cirrhosis (group I) and HCC (group II), in addition to 20 healthy volunteers (group III). All groups were subjected to measurement of NF-κB /P65 subunit expression levels by real time-PCR, and DNA binding activity was evaluated by transcription factor binding immunoassay. Serum HIF-1α levels were estimated by enzyme-linked immunosorbent assay (ELISA). Significant increase of both the expression level and DNA binding activity of NF-κB /P65 subunit together with serum HIF-1 alpha levels was noted in HCC patients compared to liver cirrhosis and control subjects, with significant positive correlation with parameters for bad prognosis of HCC. In conclusion, NF-κB signaling is activated in HCC and associated with disease prognosis and with high circulating levels of HIF-1 alpha. 
 NGAL controls the metastatic potential of anaplastic thyroid carcinoma cells. We have previously identified neutrophil gelatinase-associated lipocalin (NGAL) as one of the genes mediating the oncogenic activity of nuclear factor-κB in human anaplastic thyroid carcinomas (ATCs).To further investigate the role of NGAL in thyroid cancer, we established NGAL knocked-down and NGAL overexpressing ATC cell lines.We found that the ability of NGAL knocked-down cells to degrade Matrigel in a transwell invasion assay and to form lung   metastasi  s in nude mice was decreased. Because NGAL binds matrix metalloproteinase-9 (MMP-9), to form a macromolecular complex involved in the regulation of metastatic spread of cancer cells and given the strong expression of both genes in tissue specimens from human ATCs, we analyzed the MMP-9 enzymatic activity in NGAL-null ATC cells. Enzymatic immunoassays show that MMP-9 activity is reduced in NGAL-null ATC cells, even if its expression is not affected by NGAL inhibition. Ectopic expression of NGAL in an ATC cell line not expressing NGAL determines an increase of its metastatic property. The use of a mutated form of NGAL, unable to bind MMP-9, has no positive effect on the invasive potential of ATC cells and does not improve the MMP-9 enzymatic activity.Our results indicate NGAL as a novel target of nuclear factor-κB prometastatic activity in thyroid cancer through enhancement of MMP-9 enzymatic activity. 
 Anti-rheumatic drug iguratimod (T-614) alleviates cancer-induced bone destruction via down-regulating interleukin-6 production in a nuclear factor-κB-dependent manner. Cytokines are believed to be involved in a "vicious circle" of progressive interactions in bone   metastasi  s. Iguratimod is a novel anti-rheumatic drug which is reported to have the capability of anti-cytokines. In this study, a rat model was constructed to investigate the effect of iguratimod on bone   metastasi  s and it was found that iguratimod alleviated cancer-induced bone destruction. To further explore whether an anti-tumor activity of iguratimod contributes to the effect of bone resorption suppression, two human breast cancer cell lines MDA-MB-231 and MCF-7 were studied. The effect of iguratimod on tumor proliferation was detected by CCK-8 assay and flow cytometry. The effects of iguratimod on migration and invasion of cancer cells were determined by wound-healing and Transwell assays. Results showed that high dose (30 μg/mL) iguratimod slightly suppressed the proliferation of cancer cells but failed to inhibit their migration and invasion capacity. Interestingly, iguratimod decreased the transcription level of IL-6 in MDA-MB-231 cells in a concentration-dependent manner. Moreover, iguratimod partially impaired NF-κB signaling by suppressing the phosphorylation of NF-κB p65 subunit. Our findings indicated that iguratimod may alleviate bone destruction by partially decreasing the expression of IL-6 in an NF-κB-dependent manner, while it has little effect on the tumor proliferation and invasion. 
 Proteasome inhibition increases recruitment of IκB kinase β (IKKβ), S536P-p65, and transcription factor EGR1 to interleukin-8 (IL-8) promoter, resulting in increased IL-8 production in ovarian cancer cells. Proinflammatory and pro-angiogenic chemokine interleukin-8 (IL-8, CXCL8) contributes to ovarian cancer progression through its induction of tumor cell proliferation, survival, angiogenesis, a  nd metasta  sis. Proteasome inhibition by bortezomib, which has been used as a frontline therapy in multiple myeloma, has shown only limited effectiveness in ovarian cancer and other solid tumors. However, the responsible mechanisms remain elusive. Here, we show that proteasome inhibition dramatically increases the IL-8 expression and release in ovarian cancer cells. The responsible mechanism involves an increased nuclear accumulation of IκB kinase β (IKKβ) and an increased recruitment of the nuclear IKKβ, p65-phosphorylated at Ser-536, and the transcription factor early growth response-1 (EGR-1) to the endogenous IL-8 promoter. Coimmunoprecipitation studies identified the nuclear EGR-1 associated with IKKβ and with p65, with preferential binding to S536P-p65. Both IKKβ activity and EGR-1 expression are required for the increased IL-8 expression induced by proteasome inhibition in ovarian cancer cells. Interestingly, in multiple myeloma cells the IL-8 release is not increased by bortezomib. Together, these data indicate that the increased IL-8 release may represent one of the underlying mechanisms responsible for the decreased effectiveness of proteasome inhibition in ovarian cancer treatment and identify IKKβ and EGR-1 as potential new targets in ovarian cancer combination therapies. 
 N-Myc-interacting protein (NMI) negatively regulates epithelial-mesenchymal transition by inhibiting the acetylation of NF-κB/p65. The epithelial-mesenchymal transition (EMT) plays an essential role in embryonic development, wound healing, tissue regeneration, organ fibrosis, and tumor progression. However, the mechanisms underlying this process are poorly understood. Many signaling pathways, including the NF-κB signaling pathway, trigger EMT during development and differentiation. In the present study, we report that N-Myc interactor (NMI) inhibits EMT progression by suppressing transcriptional activities of NF-κB in human gastric cancer cells. We show that the expression of NMI is significantly reduced in invasive gastric cancer cells and gastric cancer tissues. Overexpression of NMI inhibited cell migration and invasion, and this inhibition was enhanced after TNF-α stimulation. Tumorigenicity assay in nude mice support the notion that NMI inhibits EMT in cancer cells. Mechanistically, NMI promotes the interaction between NF-κB/p65 and histone deacetylases (HDACs) and inhibits the acetylation and transcriptional activity of p65. The expression of p65 rescues NMI-mediated inhibition of EMT and the inhibition of the acetylation of p65 mediated by NMI is HDACs-dependent. Taken together, these findings suggest that NMI can suppress tumor invasion   and metas  tasis by inhibiting NF-κB pathways, providing an alternative mechanism for EMT inhibition in stomach neoplasm. 
 Integrin-linked kinase overexpression promotes epithelial-mesenchymal transition via nuclear factor-κB signaling in colorectal cancer cells. To investigate the effect of integrin-linked kinase (ILK) on proliferation,   metastasi  s, and invasion of the colorectal cancer cell line SW480.In this study, the colorectal cancer cell line SW480 was stably transfected with ILK plasmids, and small interfering RNA (siRNA) was used to knockdown expression of nuclear factor (NF)-κB/p65. Methylthiazole tetrazolium (MTT) assay was performed to measure proliferation, and the wound healing migration assay and matrigel invasion assay were used to test th  e metastas  is and invasion ability of SW480 cells. To explore the epithelial-mesenchymal transition (EMT) process, embryonic development, and the invasion an  d metastas  is of tumors, the protein level of E-cadherin, vimentin, snail, and slug was detected by western blot. Immunofluorescence was also used to detect E-cadherin expression. Western blot was used to determine the level of phosphorylated-inhibitor of kappa B (IκB)a, inhibitor of gamma B (IγB)a, and nuclear factor kappa B (NF-κB) expressions and to explore the ILK signaling pathway.Western blot results revealed that ILK expression significantly increased when ILK was overexpressed in SW480 cells (P < 0.05). Proliferat  ion, metas  tasis, and invasion ability were improved in the vector-ILK group compared to the vector group (P < 0.05). Immunofluorescence results revealed that E-cadherin fluorescence intensity decreased after ILK was overexpressed (P < 0.05). Western blot results revealed that the protein expression of E-cadherin was reduced, while vimentin, snail, and slug were upregulated when ILK was overexpressed in SW480 cells (P < 0.05). In order to determine the role of the NF-κB signaling pathway in ILK overexpression promoted EMT occurrence, we overexpressed ILK in SW480 cells and found that levels of NF-κB/p65 and cytoplasmic phosphorylated-IκBa were increased and that cytoplasmic IкBa levels were decreased compared to the control group (P < 0.05). Furthermore, NF-κB/p65 knockout revealed that E-cadherin was increased in the overexpressed ILK group.ILK overexpression improved the proli  feration,   metastasis, and invasion ability of SW480 cells, and this effect may be mediated by the NF-κB signaling pathway. 
 Testes-specific protease 50 promotes cell invasion and   metastasis   by increasing NF-kappaB-dependent matrix metalloproteinase-9 expression. The high mortality in breast cancer is often associated with metastatic progression in patients. Previously we have demonstrated that testes-specific protease 50 (TSP50), an oncogene overexpressed in breast cancer samples, could promote cell proliferation and tumorigenesis. However, whether TSP50 also has a key role in cell invasion and cancer   metastasis  , and the mechanism underlying the process are still unclear. Here we found that TSP50 overexpression greatly promoted cell migration, invasion, adhesion and formation of the stellate structures in 3D culture system in vitro as well as lung   metastasis   in vivo. Conversely, TSP50 knockdown caused the opposite changes. Mechanistic studies revealed that NF-κB signaling pathway was required for TSP50-induced cell migration and   metastasi  s, and further results indicated that TSP50 overexpression enhanced expression and secretion of MMP9, a target gene of NF-κB signaling. In addition, knockdown of MMP9 resulted in inhibition of cell migration and invasion in vitro and lun  g metastas  is in vivo. Most importantly, immunohistochemical staining of human breast cancer samples strongly showed that the coexpression of TSP50 and p65 as well as TSP50 and MMP9 were correlated with increase  d metastas  is and poor survival. Furthermore, we found that some breast cancer diagnosis-associated features such as tumor size, tumor grade, estrogen receptors (ER) and progesterone receptors (PR) levels, were correlated well with TSP50/p65 and TSP50/MMP9 expression status. Taken together, this work identified the TSP50 activation of MMP9 as a novel signaling mechanism underlying human breast cancer invasion an  d metastas  is. 
 Astrocyte elevated gene-1 is associated with   metastasis   in head and neck squamous cell carcinoma through p65 phosphorylation and upregulation of MMP1. The survival rate of head and neck squamous cell carcinoma (HNSCC) at advanced stage is poor, despite contemporary advances in treatment modalities. Recent studies have indicated that astrocyte elevated gene-1 (AEG-1), a single transmembrane protein without any known functional domains, is overexpressed in various malignancies and is implicated in both distant   metastasis   and poor survival.High expression of AEG-1 in HNSCC was positively correlated with regional lymph node   metastasis   and a poor 5-year survival rate. Knockdown of AEG-1 in HNSCC cell lines reduced their capacity for colony formation, migration and invasion. Furthermore, decreased tumor volume and metastatic foci were observed after knockdown of AEG-1 in subcutaneous xenografts and pulmonary   metastasis   assays in vivo, respectively. We also demonstrated that AEG-1 increased phosphorylation of the p65 subunit of NF-κB, and regulated the expression of MMP1 in HNSCC cells. Moreover, compromised phosphorylation of the p65 (RelA) subunit of NF-κB at serine 536 was observed upon silencing of AEG-1 in both HNSCC cell lines and clinical specimens.High expression of AEG-1 is associated with lymph nod  e metastas  is and its potentially associated mechanism is investigated. 
 Identification of a p65 peptide that selectively inhibits NF-kappa B activation induced by various inflammatory stimuli and its role in down-regulation of NF-kappaB-mediated gene expression and up-regulation of apoptosis. Because of the critical role of the nuclear transcription factor NF-kappaB in inflammation, viral replication, carcinogenesis, antiapoptosis, invasion, and   metastasis  , specific inhibitors of this nuclear factor are being sought and tested as treatments. NF-kappaB activation is known to require p65 phosphorylation at serine residues 276, 529, and 536 before it undergoes nuclear translocation. Small protein domains, termed protein transduction domains (PTDs), which are able to penetrate cell membranes can be used to transport other proteins across the cell membrane. We have identified two peptides from the p65 subunit of NF-kappaB (P1 and P6 were from amino acid residues 271-282 and 525-537, respectively) that, when linked with a PTD derived from the third helix sequence of antennapedia, inhibited tumor necrosis factor (TNF)-induced NF-kappaB activation in vivo. Linkage to the PTD was not, however, required to suppress the binding of the p50-p65-heterodimer to the DNA in vitro. PTD-p65-P1 had no effect on TNF-induced AP-1 activation. PTD-p65-P1 suppressed NF-kappaB activation induced by lipopolysaccharide, interleukin-1, okadaic acid, phorbol 12-myristate 13-acetate, H(2)O(2), and cigarette smoke condensate as well as that induced by TNF. PTD-p65-P1 had no effect on TNF-induced inhibitory subunit of NF-kappaB(IkappaBalpha) phosphorylation, IkappaBalpha degradation, or IkappaBalpha kinase activation, but it blocked TNF-induced p65 phosphorylation and nuclear translocation. NF-kappaB-regulated reporter gene expression induced by TNF, TNF receptor 1, TNF receptor-associated death domain, TNF receptor-associated factor-2, NF-kappaB-inducing kinase, IkappaBalpha kinase, and p65 was also suppressed by these peptides. Suppression of NF-kappaB by PTD-p65-P1 enhanced the apoptosis induced by TNF and chemotherapeutic agents. Overall, our results demonstrate the identification of a p65 peptide that can selectively inhibit NF-kappaB activation induced by various inflammatory stimuli, down-regulate NF-kappaB-mediated gene expression, and up-regulate apoptosis. 
 NF-kappaB promotes breast cancer cell migration and   metastasis   by inducing the expression of the chemokine receptor CXCR4.   Metastasis   of cancer cells is a complex process involving multiple steps including invasion, angiogenesis, and trafficking of cancer cells through blood vessels, extravasations, organ-specific homing, and growth. While matrix metalloproteinases, urokinase-type plasminogen activator, and cytokines play a major role in invasion and angiogenesis, chemokines such as stromal derived factor-1alpha (SDF-1alpha) and their receptors such as CXCR4 are thought to play a critical role in motility, homing, and proliferation of cancer cells at specific metastatic sites. We and others have previously reported that the extracellular signal-activated transcription factor NF-kappaB up-regulates the expression of matrix metalloproteinases, urokinase-type plasminogen activator, and cytokines in highly metastatic breast cancer cell lines. In this report, we demonstrate that NF-kappaB regulates the motility of breast cancer cells by directly up-regulating the expression of CXCR4. Overexpression of the inhibitor of kappaB (IkappaB) in breast cancer cells with constitutive NF-kappaB activity resulted in reduced expression of CXCR4 and a corresponding loss of SDF-1alpha-mediated migration in vitro. Introduction of CXCR4 cDNA into IkappaB-expressing cells restored SDF-1alpha-mediated migration. Electrophoretic mobility shift assays and transient transfection assays revealed that the NF-kappaB subunits p65 and p50 bind directly to sequences within the -66 to +7 region of the CXCR4 promoter and activate transcription. We also show that the cell surface expression of CXCR4 and the SDF-1alpha-mediated migration are enhanced in breast cancer cells isolated from mammary fat pad xenografts compared with parental cells grown in culture. A further increase in CXCR4 cell surface expression and SDF-1alpha-mediated migration was observed with cancer cells that metastasized to the lungs. Taken together, these results implicate NF-kappaB in the migration and the organ-specific homing of metastatic breast cancer cells. 
 PinX1 serves as a potential prognostic indicator for clear cell renal cell carcinoma and inhibits its invasion and   metastasis   by suppressing MMP-2 via NF-κB-dependent transcription. PIN2/TRF1-interacting telomerase inhibitor 1 (PinX1) is a novel cloned gene which has been identified as a major haploinsufficient tumor suppressor essential for maintaining telomerase activity, the length of telomerase and chromosome stability. This study explored the clinical significance and biological function of PinX1 in human clear cell renal cell carcinoma (ccRCC). The clinical relevance of PinX1 in ccRCC was evaluated using tissue microarray and immunohistochemical staining in two independent human ccRCC cohorts. Our data demonstrated that PinX1 expression was dramatically decreased in ccRCC tissues compared with normal renal tissues and paired adjacent non-tumor tissues. Low PinX1 expression was significantly correlated with depth of invasion, lymph node   metastasi  s and advanced TNM stage in patients, as well as with worse overall and disease-specific survival. Cox regression analysis revealed that PinX1 expression was an independent prognostic factor for ccRCC patients. Moreover, PinX1 inhibited the migration and invasion of ccRCC by suppressing MMP-2 expression and activity via NF-κB-dependent transcription in vitro. In vivo studies confirmed that PinX1 negatively regulated ccRC  C metastas  is and the expression of MMP-2 and NF-κB-p65. These findings indicate that PinX1 suppresses ccR  CC metasta  sis and may serve as a ccRCC candidate clinical prognostic marker and a potential therapeutic target. 
 Vascular endothelial growth factor C enhances cervical cancer cell invasiveness via upregulation of galectin-3 protein. Vascular endothelial growth factor C (VEGF-C) promotes cervical cancer   metastasis  , while the detailed mechanism remains obscure. Recent evidence shows that galectin-3 (Gal-3), a glycan binding protein, interacts with the VEGF receptors and reinforces their signal transduction. In this study, we investigated the role of Gal-3 in VEGF-C-induced cervical cancer cell invasion. On cervical carcinoma cell line SiHa cells, silencing of Gal-3 expression with specific siRNA largely impaired VEGF-C-enhanced cell invasion. Treatment with VEGF-C for 12-48 h enhanced Gal-3 protein expression, which was inhibited by the addition of NF-κB inhibitor pyrrolidine dithiocarbamate (PDTC). Moreover, the silencing of NF-κB subunit p65 expression with specific siRNA attenuated VEGF-C-enhanced Gal-3 expression, suggesting that NF-κB is the key intermediate. Under VEGF-C stimulation, an enhanced interaction between VEGF receptor-3 (VEGF-R3) and Gal-3 was found, which may possibly lead to VEGF-R3 activation since exogenous Gal-3 induced VEGF-R3 phosphorylation in a dose- and time-dependent manner. In conclusion, our findings implied that VEGF-C enhanced cervical cancer invasiveness via upregulation of Gal-3 protein through NF-κB pathway, which may shed light on potential therapeutic strategies for cervical cancer therapy. 
 Co-expression of β-arrestin1 and NF-кB is associated with cancer progression and poor prognosis in lung adenocarcinoma. β-arrestin1 and NF-κB have been demonstrated to be associated with tumorigenesis, tumor progression,   and metast  asis. Thus far, there is nevertheless little study about these two molecules in lung adenocarcinoma. The aim of this study was to investigate the correlation between β-arrestin1 and NF-κB expression and the clinicopathological characteristics in lung adenocarcinoma. A total of 115 surgically resected lung adenocarcinoma patients were recruited for the study. Expression of β-arrestin1 and p65 were detected by immunohistochemistry (IHC) in lung adenocarcinoma tissue samples. Nuclear expression of β-arrestin1 and p65 were observed in 39.1 % (45/115) and 46.1 % (53/115) cases of lung adenocarcinoma, respectively. And high expression of β-arrestin1 had negative prognostic impact for overall survival (OS) and disease-free survival (DFS) (p = 0.034 and p = 0.033). In addition, overexpression of p65 indicated a significantly poor OS and DFS than those of lower-expression (p = 0.038 and p = 0.041). Furthermore, co-expression of nuclear β-arrestin1 and p65 correlated with poorer OS and DFS in lung adenocarcinoma patients. Multivariate analysis using the Cox regression model confirmed that co-expression of nuclear β-arrestin1 and p65 was an independent prognostic factor for tumor progression (p = 0.008). In conclusion, these data indicated that co-expression of nuclear β-arrestin1 and p65 was a novel predictor for worse prognosis in patients with lung adenocarcinoma. 
 Increased staining for phosphorylated AKT and nuclear factor-kappaB p65 and their relationship with prognosis in epithelial ovarian cancer. AKT plays an important role in malignant behavior of tumors. The purpose of the present study was to determine the expression of phosphorylated AKT (P-AKT) and nuclear factor-kappaB (NF-kappaB) p65 and their association with clinicopathological parameters and prognosis in epithelial ovarian tumor. On immunohistochemistry 115 samples of ovarian tissue that included 68 specimens of epithelial ovarian cancer, 12 of borderline tumor, 24 of epithelial benign tumor and 11 of normal ovary, were evaluated. Sixty-three patients with ovarian cancer were followed up from 7 to 68 months. The positive expression rate of P-AKT and NF-kappaB p65 were higher in epithelial ovarian cancer than in normal ovarian tissue (P<0.01). Elevated P-AKT or NF-kappaB p65 expression was significantly correlated with late clinical stage (P<0.05 and P<0.01) and poor histological differentiation (both P<0.01). P-AKT expression was significantly correlated with NF-kappaB p65 immunostaining (phi=0.272, P<0.05). Elevated expression of P-AKT was negatively correlated with the survival of ovarian cancer patients, but it was not an independent prognostic factor after multivariate analysis. Overexpression of P-AKT and NF-kappaB p65 were involved in the carcinogenesis and   metastasis   of ovarian cancer. P-AKT might contribute to the malignant transformation through NF-kappaBp65 upregulation. 
 microRNA-372 Suppresses Migration and Invasion by Targeting p65 in Human Prostate Cancer Cells. Prostate cancer (PCa) is one of the most prevalent malignant tumors. microRNAs (miRNAs) play an important role in cancer initiation, progression, and   metastasis  , and their roles in PCa are becoming more apparent. In this study, we found that microRNA-372 (miR-372) is downregulated in human PCa and inhibits the proliferation activity, migration, and invasion of DU145 cells. Subsequently, p65 is confirmed as a target of miR-372, and knockdown of p65 expression similarly resulted in decreased proliferation activity, migration, and invasion. CDK8, MMP-9, and prostate-specific antigen were involved in both these processes. Taken together, our results show evidence that miR-372 may function as a tumor suppressor gene by regulating p65 in PCa and may provide a strategy for blocking PCa   metastasis  . 
 [Effect of topotecan on expression of aquaporin protein 5 and nuclear factor-kappaB in ovarian cancer SKOV3 cells]. Overexpression of aquaporin protein 5 (AQP5) is associated with the   metastasis  , migration and angiogenesis of ovarian cancer, but its function in cell proliferation has not been described. This study was to explore the effects of topotecan (TPT) on the expression of AQP5, nuclear factor-kappaB (NF-kappaB) and its receptor IkappaBalpha in ovarian cancer SKOV3 cells.When SKOV3 cells were treated with TPT of various concentrations for different time, cell proliferation was measured by MTT assay, the expression of AQP5, NF-kappaB and IkappaBalpha was detected by Western blot.After SKOV3 cells were incubated with 0.4 microg/mL TPT for 24 h, the expression of AQP5, NF-kappaB p65 in cytoplasm and nuclei, and IkappaBalpha in cytoplasm were decreased, and remained at low levels till 72 h (P<0.05). When SKOV3 cells were treated with 0.2, 0.4, 0.6 and 0.8 microg/mL TPT for 24 h, the expression of AQP5 as well as NF-kappaB p65 and IkappaBalpha in nuclei were decreased (P<0.005). The protein level of AQP5 was decreased by 57.9% when cells were treated with 0.8 microg/mL TPT. AQP5 expression was negatively correlated to the proliferation inhibition rate of SKOV3 cells induced by TPT (r=-0.965, P<0.05), and positively correlated to NF-kappaB p65 and IkappaBalpha expression (r=0.903, 0.896, P<0.05).When inhibiting the proliferation of ovarian cancer cells, TPT may down-regulate the expression of AQP5 and NF-kappaB. 
 DJ-1 enhances cell survival through the binding of Cezanne, a negative regulator of NF-kappaB. Heightened DJ-1 (Park7) expression is associated with a reduction in chemotherapeutic-induced cell death and poor prognosis in several cancers, whereas the loss of DJ-1 function is found in a subgroup of Parkinson disease associated with neuronal death. This study describes a novel pathway by which DJ-1 modulates cell survival. Mass spectrometry shows that DJ-1 interacts with BBS1, CLCF1, MTREF, and Cezanne/OTUD7B/Za20d1. Among these, Cezanne is a known deubiquitination enzyme that inhibits NF-κB activity. DJ-1/Cezanne interaction is confirmed by co-immunoprecipitation of overexpressed and endogenous proteins, maps to the amino-terminal 70 residues of DJ-1, and leads to the inhibition of the deubiquitinating activity of Cezanne. Microarray profiling of shRNA-transduced cells shows that DJ-1 and Cezanne regulate IL-8 and ICAM-1 expression in opposing directions. Similarly, DJ-1 enhances NF-κB nuclear translocation and cell survival, whereas Cezanne reduces these outcomes. Analysis of mouse Park7(-/-) primary cells confirms the regulation of ICAM-1 by DJ-1 and Cezanne. As NF-κB is important in cellular survival and transformation, IL-8 functions as an angiogenic factor and pro-survival signal, and ICAM-1 has been implicated in tumor progression, invasion, a  nd metasta  sis; these data provide an additional modality by which DJ-1 controls cell survival and possibly tumor progression via interaction with Cezanne. 
 Potential biomarkers involving IKK/RelA signal in early stage non-small cell lung cancer. The clinical relevance of nuclear factor kappaB (NF-kappaB) and its regulatory molecules on prognosis of patient with early stages of non-small cell lung cancer (NSCLC), remains unclear. Therefore, we conducted biomarker analyses with survival in patients with stages I and II NSCLC. Tumor samples were collected from 88 patients with early-stage NSCLC (stages I, II). A minimum follow-up period of 5 years was required. RelA, phosphorylated I kappaB (pI kappaB alpha), pIKK alpha/beta were detected by immunostaining. NF-kappaB DNA binding activity was assessed by electrophoretic mobility shift assay. Association of clinical and pathologic variables (e.g. sex, age, pathologic stage) with relevant molecules was determined by Pearson's chi(2) test or Fisher's exact test. Survival analysis based on single expression of RelA, pI kappaB alpha, pIKK alpha/beta as well as composite expressions were evaluated using Cox proportional hazards regression models, and log rank test followed Kaplan-Meier estimates. RelA, pI kappaB alpha, pIKK alpha/beta were observed as increased expression in NSCLC tissues compared with adjacent normal tissues and normal lung tissues. These molecules were associated with tumor-node-  metastasis   stages, T stages and histological status, respectively. Among the molecules analyzed, RelA and pI kappaB alpha-positive were statistically significant predictors of patient death in the entire patient population adjusted by age, gender and smoking status; furthermore both RelA and pI kappaB alpha-positive was the strongest prognostic indicators of poor prognosis by univariate and multivariate analyses. Borderline positive correlations were observed between RelA and pI kappaB alpha or pIKK alpha/beta expression. In this cohort of early-stage NSCLC patients, molecular markers, especially composite application of multiple biomarkers (both nuclear RelA and cytoplasmic pI kappaB-alpha expression) that independently predict overall survival have been identified. 
 The curcumin analog EF24 targets NF-κB and miRNA-21, and has potent anticancer activity in vitro and in vivo. EF24 is a curcumin analog that has improved anticancer activity over curcumin, but its therapeutic potential and mechanism of action is unknown, which is important to address as curcumin targets multiple signaling pathways. EF24 inhibits the NF-κB but not the JAK-STAT signaling pathway in DU145 human prostate cancer cells and B16 murine melanoma cells. EF24 induces apoptosis in these cells apparently by inhibiting miR-21 expression, and also enhances the expression of several miR-21 target genes, PTEN and PDCD4. EF24 treatment significantly suppressed the growth of DU145 prostate cancer xenografts in immunocompromised mice and resulted in tumor regression. EF24 enhanced the expression of the miR-21 target PTEN in DU145 tumor tissue, but suppressed the expression of markers of proliferating cells (cyclin D1 and Ki67). In syngeneic mice injected with B16 cells, EF24 treatment inhibited the formation of lun  g metastas  is, prolonged animal survival, inhibited miR-21 expression and increased the expression of miR-21 target genes. Expression profiling of miRNAs regulated by EF24 in vitro and in vivo showed that the antitumor activity of EF24 reflected the enhanced expression of potential tumor suppressor miRNAs as well as the suppressed expression of oncogenic miRNAs, including miR-21. Taken together, our data suggest that EF24 is a potent anticancer agent and selectively targets NF-κB signaling and miRNA expression, indicating that EF24 has significant potential as a therapeutic agent in various cancers. 
 Tumor   metastasis   and the reciprocal regulation of heparanase gene expression by nuclear factor kappa B in human gastric carcinoma tissue. To investigate whether NF-kappaB is activated in human gastric carcinoma tissues and, if so, to study whether there is any correlation between NF-kappaB activity and heparanase expression in gastric carcinoma.NF-kappaB activation was assayed by immunohistochemical staining in formalin-fixed, paraffin-embedded specimens from 45 gastric carcinoma patients. Electrophoretic mobility shift assay (EMSA) method was used for nuclear protein from these fresh tissue specimens. Heparanase gene expression was quantified using quantitative RT-PCR.The nuclear translocation of RelA (marker of NF-kappaB activation) was significantly higher in tumor cells compared to adjacent and normal epithelial cells ((41.3+/-3.52)% vs (0.38+/-0.22) %, t = 10.993, P = 0.000<0.05; (41.3+/-3.52)% vs (0+/-0.31)%, t = 11.484, P = 0.000<0.05). NF-kappaB activation was correlated with tumor invasion-related clinicopathological features such as lymphatic invasion, pathological stage, and depth of invasion (Z = 2.148, P = 0.032<0.05; chi(2) = 8.758, P = 0.033<0.05; chi(2) = 18.531, P = 0.006<0.05). NF-kappaB activation was significantly correlated with expression of heparanase gene (r = 0.194, P = 0.046<0.05).NF-kappaB RelA (p65) activation was related with increased heparanase gene expression and correlated with poor clinicopathological characteristics in gastric cancers. This suggests NF-kappaB as a major controller of the metastatic phenotype through its reciprocal regulation of some   metastasis  -related genes. 
 Activation of the nuclear factor kappaB pathway by astrocyte elevated gene-1: implications for tumor progression and   metastasis  . Astrocyte elevated gene-1 (AEG-1) was initially identified as an HIV-1- and tumor necrosis factor alpha (TNF-alpha)-inducible transcript in primary human fetal astrocytes by a rapid subtraction hybridization approach. Interestingly, AEG-1 expression is elevated in subsets of breast cancer, glioblastoma multiforme and melanoma cells and AEG-1 cooperates with Ha-ras to promote transformation of immortalized melanocytes. Activation of the transcription factor nuclear factor kappaB (NF-kappaB), a TNF-alpha downstream signaling component, is associated with several human illnesses, including cancer, and NF-kappaB controls the expression of multiple genes involved in tumor progression and   metastasis  . We now document that AEG-1 is a significant positive regulator of NF-kappaB. Enhanced expression of AEG-1 via a replication-incompetent adenovirus (Ad.AEG-1) in HeLa cells markedly increased binding of the transcriptional activator p50/p65 complex of NF-kappaB. The NF-kappaB activation induced by AEG-1 corresponded with degradation of IkappaBalpha and nuclear translocation of p65 that resulted in the induction of NF-kappaB downstream genes. Infection with an adenovirus expressing the mt32IkappaBalpha superrepressor (Ad.IkappaBalpha-mt32), which prevents p65 nuclear translocation, inhibited AEG-1-induced enhanced agar cloning efficiency and increased matrigel invasion of HeLa cells. We also document that TNF-alpha treatment resulted in nuclear translocation of both AEG-1 and p65 wherein these two proteins physically interacted, suggesting a potential mechanism by which AEG-1 could activate NF-kappaB. Our findings suggest that activation of NF-kappaB by AEG-1 could represent a key molecular mechanism by which AEG-1 promotes anchorage-independent growth and invasion, two central features of the neoplastic phenotype. 
 Comprehensive Evaluation of Nuclear Factor-κΒ Expression Patterns in Non-Small Cell Lung Cancer. Nuclear factor (NF)-κB signalling is required for lung adenocarcinoma development in mice, and both of its subunits RelA and RelB were independently reported to be highly expressed in human non-small cell lung cancer (NSCLC). To comprehensively examine NF-κB expression in NSCLC, we analyzed serial sections of primary tumor samples from 77 well-documented patients (36 adenocarcinomas, 40 squamous cell carcinomas and 3 large cell carcinomas) for immunoreactivity of RelA, RelB, P50, and P52/P100. Tumor and intratumoral stroma areas were discriminated based on proliferating cell nuclear antigen immunoreactivity and inflammatory infiltration was assessed in intratumoral stroma areas. NF-κB immunoreactivity was quantified by intensity, extent, and nuclear localization and was cross-examined with tumor cell proliferation, inflammatory infiltration, and clinical-pathologic data. We found that the expression of the different NF-κB subunits was not concordant, warranting our integral approach. Overall, RelA, RelB, and P50 were expressed at higher levels compared with P52/P100. However, RelA and P50 were predominantly expressed in intratumoral stroma, but RelB in tumor cells. Importantly, tumor area RelA expression was correlated with the intensity of inflammatory infiltration, whereas RelB expression was identified in proliferating tumor cells. Using multiple logistic regression, we identified that tumor RelB expression was an independent predictor of lymph   node meta  stasis, and tumor P50 was an independent predictor of TNM6 stage IIB or higher, whereas tumor RelA was an independent predictor of inflammatory infiltration. We conclude that pathologic studies of NF-κB expression in cancer should include multiple pathway components. Utilizing such an approach, we identified intriguing associations between distinct NF-κB subunits and clinical and pathologic features of NSCLC. 
 Differential regulation of MMPs by E2F1, Sp1 and NF-kappa B controls the small cell lung cancer invasive phenotype. E2F1 transcription factor plays a vital role in the regulation of diverse cellular processes including cell proliferation, apoptosis, invasion and   metastasis  . E2F1 overexpression has been demonstrated in small cell lung cancer (SCLC), and extensive   metastasis   in early phase is the most important feature of SCLC. In this study, we investigated the involvement of E2F1 in the process of invasion and   metastasis   in SCLC by regulating the expression of matrix metalloproteinases (MMPs).Immunohistochemistry was performed to evaluate the expression of E2F1 and MMPs in SCLC samples in a Chinese Han population. The impact of E2F1 on invasion and   metastasis   was observed by transwell and wound healing experiments with depletion of E2F1 by specific siRNA. The target genes regulated by E2F1 were identified by chromatin immunoprecipitation (ChIP)-to-sequence, and the expressions of target genes were detected by real time PCR and western blotting. The dual luciferase reporter system was performed to analyze the regulatory relationship between E2F1 and MMPs.E2F1 is an independent and adverse prognosis factor that is highly expressed in SCLC in a Chinese Han population. Knockdown of E2F1 by specific siRNA resulted in the downregulation of migration and invasion in SCLC. The expressions of MMP-9 and -16 in SCLC were higher than other MMPs, and their expressions were most significantly reduced after silencing E2F1. ChIP-to-sequence and promoter-based luciferase analysis demonstrated that E2F1 directly controlled MMP-16 expression via an E2F1 binding motif in the promoter. Although one E2F1 binding site was predicted in the MMP-9 promoter, luciferase analysis indicated that this binding site was not functionally required. Further study demonstrated that E2F1 transcriptionally controlled the expression of Sp1 and p65, which in turn enhanced the MMP-9 promoter activity in SCLC cells. The associations between E2F1, Sp1, p65, and MMP-9 were validated by immunohistochemistry staining in SCLC tumors.E2F1 acts as a transcriptional activator for MMPs and directly enhances MMP transcription by binding to E2F1 binding sequences in the promoter, or indirectly activates MMPs through enhanced Sp1 and NF-kappa B as a consequence of E2F1 activation in SCLC. 
 Effects of Celastrol on growth inhibition of U937 leukemia cells through the regulation of the Notch1/NF-kappaB signaling pathway in vitro. Leukemia is a malignant tumor highly dependent on nuclear factor kappa-light-chain-enhancer of activated B cells (NF-kappaB), which is relevant for the occurrence,   metastasis  , proliferation, apoptosis, and drug resistance of tumor cells. Research has confirmed that the NF-kappaB family is one of the target genes in the Notch signaling pathway. This study investigated the effects of Celastrol on the apoptosis of U937 cells and the expression levels of Notch1 and NF-kappaB in these cells.U937 cells were treated with various concentrations Celastrol (0.5-16.0) micromol/L for 12-60 h. MTT assay was performed to examine the effect of Celastrol on growth inhibition of U937 cells. Cell apoptosis was detected through both Annexin-V FITC/PI double-labeled cytometry and transmission electron microscopy (TEM). Cell cycle regulation was studied by propidium iodide. Western blot analysis and reverse transcription-polymerase chain reaction (RT-PCR) technologies were applied to assess the expression level of Notch1 in U937 cells. Subcellular distributions of NF-kappaB/p65 were detected through confocal microscopy.Celastrol presented striking growth inhibition and apoptosis induction potency on U937 cells in vitro in a time- and dose-dependent manner. The IC50 value of Celastrol for 24 h was (6.21 +/- 0.242) micromol/L. Moreover, Celastrol induced apoptosis in U937 cells in a cell-cycle dependent manner, which means that Celastrol could arrest U937 cells in the G0/G1 phase. Through TEM, apoptotic bodies containing nuclear fragments were found in Celastrol-treated U937 cells. Overexpression of Notch1 was found in U937 cells, while Celastrol could downregulate it at both the protein and mRNA level in a dose-dependent manner, and expression of NF-kappaB decreased in nuclei and increased in the cytoplasm (P < 0.05).Celastrol inhibited cell proliferation and induced apoptosis in U937 cells in a concentration-dependent manner. The possible mechanism might be involved in the regulation of a survival signaling pathway, such as Notch or NF-kappaB. 
 NF-κB-mediated transcriptional upregulation of TNFAIP2 by the Epstein-Barr virus oncoprotein, LMP1, promotes cell motility in nasopharyngeal carcinoma. Nasopharyngeal carcinoma (NPC), which is closely associated with Epstein-Barr virus (EBV), is a   metastasi  s-prone epithelial cancer. We previously showed that tumor necrosis factor α-induced protein 2 (TNFAIP2) is highly expressed in NPC tumor tissues and is correlated wit  h metastas  is and poor survival in NPC patients. However, the underlying mechanism remains unclear. In this study, we demonstrate that the EBV oncoprotein, latent membrane protein 1 (LMP1), can transcriptionally induce TNFAIP2 expression via NF-κB. Quantitative RT-PCR and western blotting revealed that LMP1 induces TNFAIP2 expression through its C-terminal-activating region (CTAR2) domain, which is required for transduction of NF-κB (nuclear factor kappa-light-chain-enhancer of activated B cells) signaling. Inhibition of NF-κB activation or depletion of p65 (a component of NF-κB) by RNA interference abolished the LMP1-induced expression of TNFAIP2, whereas ectopic expression of p65 was sufficient to induce TNFAIP2 expression. Luciferase reporter assays showed that LMP1 transcriptionally induces TNFAIP2 expression through a newly identified NF-κB-binding site within the TNFAIP2 promoter (-3,869 to -3,860 bp). Immunohistochemical analysis of NPC biopsy specimens further revealed a significant correlation between the protein levels of TNFAIP2 and activated p65 (R=0.689, P<0.001), indicating that our findings are clinically relevant. Immunofluorescence microscopy and co-immunoprecipitation assays showed that TNFAIP2 associates with actin and is involved in the formation of actin-based membrane protrusions. Furthermore, transwell migration assays demonstrated that TNFAIP2 contributes to LMP1-induced cell motility. Collectively, these findings provide novel insights into the regulation of TNFAIP2 and its role in promoting NPC tumor progression. 
 Secretome of tumor-associated leukocytes augment epithelial-mesenchymal transition in positive lymph node breast cancer patients via activation of EGFR/Tyr845 and NF-κB/p65 signaling pathway. Epithelial-mesenchymal transition (EMT) is an essential process in breast cancer   metastasi  s. The aim of the present study was to determine the role of secretions of tumor-associated leukocytes (TALs) isolated from negative and positive lymph nodes (nLNs and pLNs, respectively) breast cancer patients in regulating EMT mechanism and the associated signaling pathways. We found an increased infiltration of TALs, which was associated with downregulation of E-cadherin and over-expression of vimentin in the breast carcinoma tissues of pLNs as compared to nLNs patients and normal breast tissues obtained from healthy volunteers during mammoplasty. Furthermore, TALs isolated from pLNs breast cancer patients secreted an elevated panel of cytokines by up to 2-5-fold when compared with those isolated from nLNs patients. Secretome of TALs of pLNs possessed higher TARC, IGF-1, IL-3, TNF-β, IL-5, G-CSF, IL-4, and IL-1α with more than a fivefold compared to those of nLNs. Using the human breast cancer cell lines MCF-7 and MDA-MB-231, we found that cytokines secreted by TALs isolated from nLNs and pLNs breast cancer patients promoted EMT via upregulation of TGF-β and vimentin and downregulation of E-cadherin at messenger RNA (mRNA) levels in both cell lines and at protein level in MCF-7. While TGF-β is over-expressed by MDA-MB-231 seeded in media conditioned by secretome of TALs isolated from nLNs and pLNs breast cancer patients. The downstream TGF-β signaling transcription factors, Snail, Slug, and Twist, known to be associated with EMT mechanism were over-expressed by MCF-7 and MDA-MB-231 seeded in media conditioned by secretome of TALs isolated from nLNs and pLNs breast cancer patients. Acquisition of EMT in MCF-7 cells is mechanistically attributed to the activation of EGFR((Tyr845)) and NF-κB/p65((Ser276)) signaling which are significantly highly expressed by MCF-7 cells seeded in media conditioned by secretome of TALs isolated from pLNs compared to nLNs patients. Overall, this study provides implications of secretome of TALs and activated EGFR((Tyr845)) and NF-κB/p65((Ser276)) in EMT process that may be considered a therapeutic strategy to inhibit lym  ph node me  tastasis in breast cancer patients. 
 Analysis of the TGFβ-induced program in primary airway epithelial cells shows essential role of NF-κB/RelA signaling network in type II epithelial mesenchymal transition. The airway epithelial cell plays a central role in coordinating the pulmonary response to injury and inflammation. Here, transforming growth factor-β (TGFβ) activates gene expression programs to induce stem cell-like properties, inhibit expression of differentiated epithelial adhesion proteins and express mesenchymal contractile proteins. This process is known as epithelial mesenchymal transition (EMT); although much is known about the role of EMT in cellu  lar metast  asis in an oncogene-transformed cell, less is known about Type II EMT, that occurring in normal epithelial cells. In this study, we applied next generation sequencing (RNA-Seq) in primary human airway epithelial cells to understand the gene program controlling Type II EMT and how cytokine-induced inflammation modifies it.Generalized linear modeling was performed on a two-factor RNA-Seq experiment of 6 treatments of telomerase immortalized human small airway epithelial cells (3 replicates). Using a stringent cut-off, we identified 3,478 differentially expressed genes (DEGs) in response to EMT. Unbiased transcription factor enrichment analysis identified three clusters of EMT regulators, one including SMADs/TP63 and another NF-κB/RelA. Surprisingly, we also observed 527 of the EMT DEGs were also regulated by the TNF-NF-κB/RelA pathway. This Type II EMT program was compared to Type III EMT in TGFβ stimulated A549 alveolar lung cancer cells, revealing significant functional differences. Moreover, we observe that Type II EMT modifies the outcome of the TNF program, reducing IFN signaling and enhancing integrin signaling. We confirmed experimentally that TGFβ-induced the NF-κB/RelA pathway by observing a 2-fold change in NF-κB/RelA nuclear translocation. A small molecule IKK inhibitor blocked TGFβ-induced core transcription factor (SNAIL1, ZEB1 and Twist1) and mesenchymal gene (FN1 and VIM) expression.These data indicate that NF-κB/RelA controls a SMAD-independent gene network whose regulation is required for initiation of Type II EMT. Type II EMT dramatically affects the induction and kinetics of TNF-dependent gene networks. 
 NF-κB participates in chemokine receptor 7-mediated cell survival in metastatic squamous cell carcinoma of the head and neck. Metastatic squamous cell carcinoma of the head and neck (SCCHN) has been shown to express chemokine receptor 7 (CCR7), which activates phosphoinositide-3 kinase/Akt/mammalian target of rapamycin (PI3K/Akt/mTOR) signal pathway to promote the invasion and survival of SCCHN cells. Since nuclear factor-kappa B (NF-κB) is shown to be the downstream signal molecule of PI3K/Akt in many tumors, we investigated whether it also exists in the CCR7 pathway in SCCHN, and the relationship between NF-κB and PI3K/Akt/mTOR, and the role it plays in SCCHN. We assayed the phosphorylation of the inhibitor of NF-κB (IκBα), the NF-κB DNA-binding capacity and location. The results showed that the interaction between CCR7 and the ligand for CCR7, CCL19, induces phosphorylation of IκBα, causes NF-κB to translocate to the nucleus and raises the DNA-binding capacity of NF-κB. The phosphorylation and DNA-binding capacity were abolished by the inhibition of CCR7, PI3K, Akt and mTOR. Further research demonstrated that inhibitors of NF-κB and CCR7-PI3K attenuate the survival of CCR7-mediated cells, causing decreased viability, increased apoptosis and increased cell cycle arrest in SCCHN cells. In clinical samples from 78 patients, immunohistochemical assay also showed that CCR7 and NF-κB are not only highly expressed in SCCHN, but also correlated with each other, and related t  o lymph no  de metastasis and clinical stage. Together, our data indicate that NF-κB is activated by CCR7 via PI3K/Akt/mTOR, and this signal pathway plays an important role in regulating the cell survival and prognosis of SCCHN. 
 Inhibiting valosin-containing protein suppresses osteosarcoma cell   metastasis   via AKT/nuclear factor of kappa B signaling pathway in vitro. The strategies of targeting valosin-containing protein (VCP) may have therapeutic potential for treating cancer   metastasis  . In this study, we aim to investigate the correlation of VCP protein expression in osteosarcoma (OS) tissues with pulmonary   metastasis   and its possible molecular mechanism.Expression of VCP in 60 OS specimens was detected by immunohistochemistry (IHC) and the relationship with   metastasis   was analyzed. An artificial micro ribonucleic acid, targeting VCP, was performed to silence the expression of VCP in U2-OS cells. Cell mobility was detected by wound healing and Transwell assays. Western blot and real-time polymerase chain reaction were performed to investigate the expression of VCP in U2-OS cells. Furthermore, the protein of pAKT (phosphorylated serine/threonine protein kinase) and nuclear factor of kappa B protein 65 were measured by western blot to evaluate the effect of silencing VCP on AKT/nuclear factor of kappa B (NF-kB) signaling pathway.The results showed that the expression level of VCP protein in cases with pulmonary metastases was significantly higher than that in those without   metastasis   (P = 0.004). The invasion and migration of U2-OS cells were suppressed by silencing VCP. Furthermore, silencing VCP could down-regulate the phosphorylation of AKT and nuclear transfer of NF-kB.Our findings suggested that inhibition of VCP could suppress OS cells invasion and migration through down-regulating AKT/NF-kB signaling pathway. 
 [Study of the expression of nuclear factor-kappa B, matrix metalloproteinase-3, -9 on nasopharyngeal carcinoma and their clinical significance]. To test the expression of NF-kappaB/p65, MMP-3 and MMP-9 in nasopharyngeal carcinoma (NPC), and analyze their relationship and the clinical significance.All samples were examined for the expression of NF-kappaB/p65, MMP-3 and MMP-9 by streptavidin-peroxidase immunohistochemistry. Chi2-test and Spearman-test were used to exam the correlation within them and the expression difference between nasopharyngeal carcinoma and normal nasopharyngeal mucosa.Positive expression rate of NF-kappaB/p65 in nasopharyngeal carcinoma was 78.0%; positive expressions rate of MMP-3 and MMP-9 in nasopharyngeal carcinoma were 75.6%. There was positive relationship between NF-kappaB/p65, MMP-3, MMP-9 and N stage. The expression of NF-kappaB/p65 showed a significant positive correlation with the expression of MMP-3 and MMP-9. MMP-3 also had a significant positive correlation with MMP-9.The result suggests that NF-kappaB/p65, MMP-3 and MMP-9 are highly expressed in nasopharyngeal carcinoma cells. Their expression is correlated with lymph nodes   metastasis  , but do not have relationship with sex, age, pathological classification and clinical stage. Each of them has positive correlation with the others. 
  GeneRIFs  
 By screening the signalling pathways, we observed an obvious upregulation of phosphorylated NF-kappaB in A549 and H520 cells after silencing of FOXP3. Our results suggest that Foxp3 suppressed NSCLC cell   metastasis  , at least partially, via NF-kappaB signaling. 
 Tim-3 facilitates osteosarcoma proliferation and   metastasis   through the NF-kappaB pathway and epithelial-mesenchymal transition. 
 ILK overexpression improved the proliferation,   metastasis  , and invasion ability of colorectal tumor cells, and this effect may be mediated by the NF-kappaB/rel/A signaling pathway 
 PinX1 negatively regulated ccRCC   metastasis   and the expression of MMP-2 and NF-kappaB-p65 
 P65 is involved in the regulation of MMP9 expression and other key proteins implicated in neoplasm   metastasis   and invasion. 
 NF-kappaB/p65, MMP-3 and MMP-9 are highly expressed in nasopharyngeal carcinoma cells. Their expression is correlated with lymph nodes   metastasis  , but are not related to sex, age, pathological classification and clinical stage. 
 Positive expression of NF-kappaB p65 correlated with the development of distant   metastasis   in laryngeal carcinoma. 
 Overexpression of the NF-kappaB p65 increased colon cancer cells cell proliferation, motility and   metastasis  . 
 Overexpression of P-AKT and NF-kappaB p65 were involved in the carcinogenesis and   metastasis   of ovarian cancer. 
 intraperitoneal administration of NF-kappaB p65 siRNA and paclitaxel may provide a breakthrough in the treatment of peritoneal   metastasis   of gastric cancer 
  [Cc]ell [Ii]nvasion  PubMed  
 BRAFV600E mutation, TIMP-1 upregulation, and NF-κB activation: closing the loop on the papillary thyroid cancer trilogy. BRAF(V600E) is the most common mutation found in papillary thyroid carcinoma (PTC). Tissue inhibitor of metalloproteinases (TIMP-1) and nuclear factor (NF)-κB have been shown to play an important role in thyroid cancer. In particular, TIMP-1 binds its receptor CD63 on cell surface membrane and activates Akt signaling pathway, which is eventually responsible for its anti-apoptotic activity. The aim of our study was to evaluate whether interplay among these three factors exists and exerts a functional role in PTCs. To this purpose, 56 PTC specimens were analyzed for BRAF(V600E) mutation, TIMP-1 expression, and NF-κB activation. We found that BRAF(V600E) mutation occurs selectively in PTC nodules and is associated with hyperactivation of NF-κB and upregulation of both TIMP-1 and its receptor CD63. To assess the functional relationship among these factors, we first silenced BRAF gene in BCPAP cells, harboring BRAF(V600E) mutation. We found that silencing causes a marked decrease in TIMP-1 expression and NF-κB binding activity, as well as decreased invasiveness. After treatment with specific inhibitors of MAPK pathway, we found that only sorafenib was able to increase IκB-α and reduce both TIMP-1 expression and Akt phosphorylation in BCPAP cells, indicating that BRAF(V600E) activates NF-κB and this pathway is MEK-independent. Taken together, our findings demonstrate that BRAF(V600E) causes upregulation of TIMP-1 via NF-κB. TIMP-1 binds then its surface receptor CD63, leading eventually to Akt activation, which in turn confers antiapoptotic behavior and prom  otion of cell   invasion. The recognition of this functional trilogy provides insight on how BRAF(V600E) determines cancer initiation, progression, and invasiveness in PTC, also identifying new therapeutic targets for the treatment of highly aggressive forms. 
 CARMA3 regulates the invasion, migration, and apoptosis of non-small cell lung cancer cells by activating NF-кB and suppressing the P38 MAPK signaling pathway. In our previous study, CARMA3 overexpression in lung cancer cells promoted cell proliferation and invasion; however, the mechanism underlying the role of CARMA3 in cancer   cell invasio  n remained unclear. In the present study, knockdown of CARMA3 in A549 and H1299 cells suppressed   cell invasio  n and migration, and downregulated matrix metalloprotease 9 expression at the protein and mRNA levels, as shown by Western blotting and real-time PCR. CARMA3 knockdown increased cell apoptosis, as shown by flow cytometry, increased the mRNA and protein expression levels of Bax and Caspase3, and downregulated Bcl-2 in A549 and H1299 cells. Phosphorylated P38 levels increased and NF-кB activation decreased following knockdown of CARMA3. SB203580, a P38 MAPK inhibitor, activated NF-кB, increased cell migration, and inhibited cell apoptosis after knockdown of CARMA3 compared to knockdown of CARMA3 without SB203580. These findings indicate that CARMA3 may suppress the activation of the P38 MAPK signaling pathway to regulate invasion, migration and apoptosis of lung cancer cells by activating NF-кB (P65) in the nucleus. 
 CARMA3 regulates the invasion, migration, and apoptosis of non-small cell lung cancer cells by activating NF-кB and suppressing the P38 MAPK signaling pathway. In our previous study, CARMA3 overexpression in lung cancer cells promoted cell proliferation and invasion; however, the mechanism underlying the role of CARMA3 in cancer   cell invasio  n remained unclear. In the present study, knockdown of CARMA3 in A549 and H1299 cells suppressed   cell invasio  n and migration, and downregulated matrix metalloprotease 9 expression at the protein and mRNA levels, as shown by Western blotting and real-time PCR. CARMA3 knockdown increased cell apoptosis, as shown by flow cytometry, increased the mRNA and protein expression levels of Bax and Caspase3, and downregulated Bcl-2 in A549 and H1299 cells. Phosphorylated P38 levels increased and NF-кB activation decreased following knockdown of CARMA3. SB203580, a P38 MAPK inhibitor, activated NF-кB, increased cell migration, and inhibited cell apoptosis after knockdown of CARMA3 compared to knockdown of CARMA3 without SB203580. These findings indicate that CARMA3 may suppress the activation of the P38 MAPK signaling pathway to regulate invasion, migration and apoptosis of lung cancer cells by activating NF-кB (P65) in the nucleus. 
 BRAFV600E mutation, TIMP-1 upregulation, and NF-κB activation: closing the loop on the papillary thyroid cancer trilogy. BRAF(V600E) is the most common mutation found in papillary thyroid carcinoma (PTC). Tissue inhibitor of metalloproteinases (TIMP-1) and nuclear factor (NF)-κB have been shown to play an important role in thyroid cancer. In particular, TIMP-1 binds its receptor CD63 on cell surface membrane and activates Akt signaling pathway, which is eventually responsible for its anti-apoptotic activity. The aim of our study was to evaluate whether interplay among these three factors exists and exerts a functional role in PTCs. To this purpose, 56 PTC specimens were analyzed for BRAF(V600E) mutation, TIMP-1 expression, and NF-κB activation. We found that BRAF(V600E) mutation occurs selectively in PTC nodules and is associated with hyperactivation of NF-κB and upregulation of both TIMP-1 and its receptor CD63. To assess the functional relationship among these factors, we first silenced BRAF gene in BCPAP cells, harboring BRAF(V600E) mutation. We found that silencing causes a marked decrease in TIMP-1 expression and NF-κB binding activity, as well as decreased invasiveness. After treatment with specific inhibitors of MAPK pathway, we found that only sorafenib was able to increase IκB-α and reduce both TIMP-1 expression and Akt phosphorylation in BCPAP cells, indicating that BRAF(V600E) activates NF-κB and this pathway is MEK-independent. Taken together, our findings demonstrate that BRAF(V600E) causes upregulation of TIMP-1 via NF-κB. TIMP-1 binds then its surface receptor CD63, leading eventually to Akt activation, which in turn confers antiapoptotic behavior and prom  otion of cell   invasion. The recognition of this functional trilogy provides insight on how BRAF(V600E) determines cancer initiation, progression, and invasiveness in PTC, also identifying new therapeutic targets for the treatment of highly aggressive forms. 
 Negative regulation of NF-κB by the ING4 tumor suppressor in breast cancer. Nuclear Factor kappa B (NF-κB) is a key mediator of normal immune response but contributes to aggressive cancer cell phenotypes when aberrantly activated. Here we present evidence that the Inhibitor of Growth 4 (ING4) tumor suppressor negatively regulates NF-κB in breast cancer. We surveyed primary breast tumor samples for ING4 protein expression using tissue microarrays and a newly generated antibody. We found that 34% of tumors expressed undetectable to low levels of the ING4 protein (n = 227). Tumors with low ING4 expression were frequently large in size, high grade, and lymph node positive, suggesting that down-regulation of ING4 may contribute to breast cancer progression. In the same tumor set, we found that low ING4 expression correlated with high levels of nuclear phosphorylated p65/RelA (p-p65), an activated form of NF-κB (p = 0.018). Fifty seven percent of ING4-low/p-p65-high tumors were lymph node-positive, indicating a high metastatic tendency of these tumors. Conversely, ectopic expression of ING4 inhibited p65/RelA phosphorylation in T47D and MCF7 breast cancer cells. In addition, ING4 suppressed   PMA-induced c  ell invasion and NF-κB-target gene expression in T47D cells, indicating that ING4 inhibited NF-κB activity in breast cancer cells. Supportive of the ING4 function in the regulation of NF-κB-target gene expression, we found that ING4 expression levels inversely correlated with the expression of NF-κB-target genes in primary breast tumors by analyzing public gene expression datasets. Moreover, low ING4 expression or high expression of the gene signature composed of a subset of ING4-repressed NF-κB-target genes was associated with reduced disease-free survival in breast cancer patients. Taken together, we conclude that ING4 negatively regulates NF-κB in breast cancer. Consequently, down-regulation of ING4 leads to activation of NF-κB, contributing to tumor progression and reduced disease-free patient survival in breast cancer. 
 S100A4 promotes squamous cell laryngeal cancer Hep-2   cell invasion   via NF-kB/MMP-9 signal. S100A4 is a member of the S100 family of calcium-binding proteins, which possesses a wide range of biological functions, such as regulation of angiogenesis, cell survival, motility, and invasion. Here, we demonstrate for the first time a major role of S100A4 in the   cell invasion   properties of the human laryngeal squamous carcinoma cells (LSCC) and evaluated the mechanism.Cultured human LSCC cell line Hep-2 was overexpressed by transfection of pcDNA3.1-S100A4 plasmid. For this, cellular Hep-2 expression was quantified by Western blot analysis. Moreover,   cell invasion   and migration assays were performed. Furthermore, the impact of the S100A4 on NF-kB activity and MMP-9 expression was detected.We found S100A4 potently promoted Hep-2 invasion, by increasing cell motility and matrix metalloproteinase-9 (MMP-9) production. The increase in MMP-9 production was mediated by activation of nuclear factor-kB transcriptional activity by S100A4. After MMP-9 and NF-kBp65 was inhibited by BB94 treatment and NF-kBp65 siRNA transfected, pcDNA3.1-S100A4 induced   cell invasion   and migration was decreased.Our findings thus establish S100A4 as a major factor in the invasive abilities of Hep-2 cells. 
 MUC1 protein induces urokinase-type plasminogen activator (uPA) by forming a complex with NF-κB p65 transcription factor and binding to the uPA promoter, leading to enhanced invasiveness of cancer cells. Mucin 1 (MUC1) is overexpressed in various human malignant tumors and its expression is correlated with a poor prognosis. MUC1 engages in signal transduction by interacting with receptors for growth and differentiation factors, which contributes to the growth and survival of cancer cells. However, the mechanism by which MUC1 promotes cancer   cell invasio  n remains unclear. Microarray analysis revealed that expression of urokinase-type plasminogen activator (uPA) was elevated in MUC1-overexpressing cells. Furthermore, up- and down-modulation of MUC1 expression was clearly correlated with the change of uPA expression. An immunochemical study showed that the distribution of uPA coincided with that of MUC1 in various human cancer tissues. The MUC1 C-terminal domain (MUC1-CD) was associated with nuclear factor-κB (NF-κB) p65 in MUC1-expressing cells. Chromatin immunoprecipitation (ChIP) assays demonstrated that MUC1-CD existed with NF-κB p65 on the uPA promoter. Luciferase assays indicated that the uPA transcriptional activity was correlated with the level of MUC1 expression and that this MUC1-enhancing effect on the uPA transcription was abolished by introduction of mutations into the NF-κB binding sites on the uPA promoter. These results indicate that formation of the MUC1-CD and NF-κB p65 complex enhanced nuclear translocation of NF-κB p65 and subsequent occupancy of NF-κB binding region on the uPA promoter, leading to elevated transcription of uPA. We also demonstrated that uPA induced by MUC1 enhanced the matrix metalloproteinase (MMP)-2 and -9 activities, and consequently promoted   cancer cell   invasion. Thus, a MUC1 co-operating NF-κB signaling pathway plays a critical role i  n cancer cell   invasion in MUC1-expressing cells. 
 Overexpression of response gene to complement 32 (RGC32) promotes   cell invasion   and induces epithelial-mesenchymal transition in lung cancer cells via the NF-κB signaling pathway. Response gene to complement 32 (RGC32) is a novel cellular protein that has been reported to be expressed aberrantly in multiple types of human tumors. However, the role of RGC32 in cancer is still controversial, and the molecular mechanisms by which RGC32 contributes to the development of cancer remain largely unknown. In the present study, we constructed a recombinant expression vector pCDNA3.1-RGC32 and transfected it into human lung cancer A549 cells. Stable transformanted cells were identified by real-time PCR and Western blot analysis. Functional analysis showed that forced overexpression of RGC32 increased invasive and migration capacities of lung cancer cells in vitro, and induced the acquisition of epithelial-mesenchymal transition (EMT) phenotype, as demonstrated by the spindle-like morphology, downregulation of E-cadherin, and upregulation of Vimentin, Fibronectin, Snail and Slug. Also, overexpression of RGC32 increased expression and activities of matrix metalloproteinase (MMP)-2 and MMP-9 in A549 cells. Furthermore, the downregulation of E-cadherin induced by RGC32 was remarkably attenuated by nuclear factor-κB (NF-κB) inhibitor BAY 11-7028 and small interfering RNA targeting NF-κB p65, suggesting a role of the NF-κB signaling pathway in RGC32-induced EMT. Taken together, our data suggest that RGC32 promotes cell migration and invasion and induces EMT in lung cancer cells via the NF-κB signaling pathway. 
 miR-508-3p concordantly silences NFKB1 and RELA to inactivate canonical NF-κB signaling in gastric carcinogenesis. NF-κB signaling pathway plays an important role in gastric carcinogenesis. The basic expression and functional role of NFKB1 and RELA (components of canonical NF-κB pathway) in gastric cancer (GC) have not been well elucidated. In this study, the role of NFKB1 and RELA in gastric tumorigenesis will be investigated and their regulation by microRNAs (miRNAs) will be deeply explored.The mRNA and protein expression of NFKB1 and RELA were investigated by qRT-PCR and Western blot in GC cell lines and primary tumors. The functional roles of NFKB1 and RELA in GC were demonstrated by MTT proliferation assay, monolayer colony formatio  n, cell invas  ion and migration, cell cycle analysis and in vivo study through siRNA mediated knockdown. Identification of NFKB1 as a direct target of tumor suppressor miRNA miR-508-3p was achieved by expression regulation assays together with dual luciferase activity experiments.NFKB1 and RELA were up-regulated in GC cell lines and primary tumors compared with normal gastric epithelium cells and their upregulation correlation with poor survival in GC. siRNA mediated knockdown of NFKB1 or RELA exhibited anti-oncogenic effect both in vitro and in vivo. NFKB1 was further revealed to be a direct target of miR-508-3p in gastric tumorigenesis and their expression showed negative correlation in primary GC samples. miR-508-3p was down-regulated in GC cells compared with normal gastric epithelium samples and its ectopic expression in GC cell lines also exerts tumor suppressor function. NFKB1 re-expression was found to partly abolish the tumor-suppressive effect of miR-508-3p in GC.All these findings supports that canonical NF-κB signaling pathway is activated in GC at least by the inactivation of miR-508-3p and this might have therapeutic potential in GC treatment. 
 Testes-specific protease 50 promotes   cell invasion   and metastasis by increasing NF-kappaB-dependent matrix metalloproteinase-9 expression. The high mortality in breast cancer is often associated with metastatic progression in patients. Previously we have demonstrated that testes-specific protease 50 (TSP50), an oncogene overexpressed in breast cancer samples, could promote cell proliferation and tumorigenesis. However, whether TSP50 also has a key role in   cell invasion   and cancer metastasis, and the mechanism underlying the process are still unclear. Here we found that TSP50 overexpression greatly promoted cell migration, invasion, adhesion and formation of the stellate structures in 3D culture system in vitro as well as lung metastasis in vivo. Conversely, TSP50 knockdown caused the opposite changes. Mechanistic studies revealed that NF-κB signaling pathway was required for TSP50-induced cell migration and metastasis, and further results indicated that TSP50 overexpression enhanced expression and secretion of MMP9, a target gene of NF-κB signaling. In addition, knockdown of MMP9 resulted in inhibition of cell migration and invasion in vitro and lung metastasis in vivo. Most importantly, immunohistochemical staining of human breast cancer samples strongly showed that the coexpression of TSP50 and p65 as well as TSP50 and MMP9 were correlated with increased metastasis and poor survival. Furthermore, we found that some breast cancer diagnosis-associated features such as tumor size, tumor grade, estrogen receptors (ER) and progesterone receptors (PR) levels, were correlated well with TSP50/p65 and TSP50/MMP9 expression status. Taken together, this work identified the TSP50 activation of MMP9 as a novel signaling mechanism underlying human breast cancer invasion and metastasis. 
 Vascular endothelial growth factor C enhances cervical cancer cell invasiveness via upregulation of galectin-3 protein. Vascular endothelial growth factor C (VEGF-C) promotes cervical cancer metastasis, while the detailed mechanism remains obscure. Recent evidence shows that galectin-3 (Gal-3), a glycan binding protein, interacts with the VEGF receptors and reinforces their signal transduction. In this study, we investigated the role of Gal-3 in VEGF-C-induced cervical cancer   cell invasion  . On cervical carcinoma cell line SiHa cells, silencing of Gal-3 expression with specific siRNA largely impaired VEGF-C-enhanced   cell invasion  . Treatment with VEGF-C for 12-48 h enhanced Gal-3 protein expression, which was inhibited by the addition of NF-κB inhibitor pyrrolidine dithiocarbamate (PDTC). Moreover, the silencing of NF-κB subunit p65 expression with specific siRNA attenuated VEGF-C-enhanced Gal-3 expression, suggesting that NF-κB is the key intermediate. Under VEGF-C stimulation, an enhanced interaction between VEGF receptor-3 (VEGF-R3) and Gal-3 was found, which may possibly lead to VEGF-R3 activation since exogenous Gal-3 induced VEGF-R3 phosphorylation in a dose- and time-dependent manner. In conclusion, our findings implied that VEGF-C enhanced cervical cancer invasiveness via upregulation of Gal-3 protein through NF-κB pathway, which may shed light on potential therapeutic strategies for cervical cancer therapy. 
 A novel LZAP-binding protein, NLBP, inhibits   cell invasion  . LXXLL/leucine zipper-containing alternative reading frame (ARF)-binding protein (LZAP) was recently shown to function as a tumor suppressor through inhibition of the NF-kappaB signaling pathway. LZAP is also known as a negative regulator of   cell invasion  , and its expression was demonstrated to be reduced in several tumor tissues. However, the molecular mechanism of the negative effect of LZAP on   cell invasion   is unclear. In this study, we identify NLBP as a novel LZAP-binding protein using tandem affinity purification. We demonstrate the negative effects of NLBP on   cell invasion   and the NF-kappaB signaling pathway. NLBP expression was not detected in hepatocellular carcinoma cells with strong invasive activity, whereas its expression was detected in a hepatocellular carcinoma cell line with no invasive activity. We also demonstrate that these two proteins mutually affect the stability of each other by inhibiting ubiquitination of the other protein. Based on these results, we suggest that NLBP may act as a novel tumor suppressor by inhibiting   cell invasion  , blocking NF-kappaB signaling, and increasing stability of the LZAP protein. 
 PKD2 and PKD3 promote prostate cancer   cell invasion   by modulating NF-κB- and HDAC1-mediated expression and activation of uPA. Although protein kinase D3 (PKD3) has been shown to contribute to prostate cancer cell growth and survival, the role of PKD in prostate cancer cell motility remains unclear. Here, we show that PKD2 and PKD3 promote nuclear factor kappa B (NF-κB) signaling and urokinase-type plasminogen activator (uPA) expression/activation, which are crucial for prostate cance  r cell invasi  on. Silencing of endogenous PKD2 and/or PKD3 markedly decreased prostate cancer cell migration and invasion, reduced uPA and uPA receptor (uPAR) expression and increased plasminogen activator inhibitor-2 (PAI-2) expression. These results were further substantiated by the finding that PKD2 and PKD3 promoted the activity of uPA and matrix metalloproteinase 9 (MMP9). Furthermore, depletion of PKD2 and/or PKD3 decreased the level of binding of the p65 subunit of NF-κB to the promoter of the gene encoding uPA (PLAU), suppressing transcriptional activation of uPA. Endogenous PKD2 and PKD3 interacted with inhibitor of NF-κB (IκB) kinase β (IKKβ); PKD2 mainly regulated the phosphorylated IKK (pIKK)-phosphorylated IκB (pIκB)-IκB degradation cascade, p65 nuclear translocation, and phosphorylation of Ser276 on p65, whereas PKD3 was responsible for the phosphorylation of Ser536 on p65. Conversely, inhibition of uPA transactivation by PKD3 silencing was rescued by constitutive Ser536 p65 phosphorylation, and redu  ced tumor cel  l invasion resulting from PKD2 or PKD3 silencing was rescued by ectopic expression of p65. Interestingly, PKD3 interacted with histone deacetylase 1 (HDAC1), suppressing HDAC1 expression and decreasing its binding to the uPA promoter. Moreover, depletion of HDAC1 resulted in recovery of uPA transactivation in PKD3-knockdown cells. Taken together, these data suggest that PKD2 and PKD3 coordinate to promote prosta  te cancer cel  l invasion through p65 NF-κB- and HDAC1-mediated expression and activation of uPA. 
  GeneRIFs  
 S100A4/NFkB/MMP-9 signaling pathway may play an important role during laryngeal squamous carcinoma   cell invasion  . 
 these data suggest that PKD2 and PKD3 coordinate to promote prostate cancer   cell invasion   through p65 NF-kappaB- and HDAC1-mediated expression and activation of uPA. 
 BRAF(V600E) causes upregulation of TIMP-1 via NF-kappaB. TIMP-1 binds then its surface receptor CD63, leading eventually to Akt activation, which in turn confers antiapoptotic behavior and promotion of   cell invasion  . 
   
  
 PLEKHG2 
 
  NCBI Gene  
  GeneRIF  
  Pubmed  
 
 
  Search Term  Rank  Fields  
  [Mm]igration  6  PubMed(3)  
 
 
 hide/show details for PLEKHG2 
  [Mm]igration  PubMed  
 Mutation of ARHGAP9 in patients with coronary spastic angina. Coronary artery spasm has an important function in the etiology of variant angina and other acute coronary syndromes. Abnormal activation of Rho-family GTPases has been observed in cardiovascular disorders, but the function of genetic variability in Rho-family GTPases remains to be evaluated in cardiovascular disorders. We examined the genetic variability of Rho-family GTPases and their regulators in coronary artery spasm. We performed a comprehensive candidate gene analysis of 67 single nucleotide polymorphisms with amino-acid substitution in Rho-family GTPases and their regulators in 103 unrelated Japanese patients with acetylcholine-induced coronary artery spasm and 102 control Japanese subjects without acetylcholine-induced coronary artery spasm. We noted an association of the single nucleotide polymorphism of ARHGAP9 (rs11544238, Ala370Ser) with coronary artery spasm (odds ratio =2.67). We found that ARHGAP9 inactivated Rac as RacGAP and that the mRNA level of ARHGAP9 was strongly detected in hematopoietic cells. ARHGAP9 negatively regulated cell   migration  . The Ala370Ser polymorphism counteracted ARHGAP9-reduced cell   migration  , spreading and adhesion. The Ala370Ser polymorphism in the ARHGAP9 gene is associated with coronary artery spasm. These data suggest that the polymorphism of ARHGAP9 has a critical function in the infiltration of hematopoietic cells into the endothelium and inflammation leading to endothelial dysfunction. 
 Mutation of ARHGAP9 in patients with coronary spastic angina. Coronary artery spasm has an important function in the etiology of variant angina and other acute coronary syndromes. Abnormal activation of Rho-family GTPases has been observed in cardiovascular disorders, but the function of genetic variability in Rho-family GTPases remains to be evaluated in cardiovascular disorders. We examined the genetic variability of Rho-family GTPases and their regulators in coronary artery spasm. We performed a comprehensive candidate gene analysis of 67 single nucleotide polymorphisms with amino-acid substitution in Rho-family GTPases and their regulators in 103 unrelated Japanese patients with acetylcholine-induced coronary artery spasm and 102 control Japanese subjects without acetylcholine-induced coronary artery spasm. We noted an association of the single nucleotide polymorphism of ARHGAP9 (rs11544238, Ala370Ser) with coronary artery spasm (odds ratio =2.67). We found that ARHGAP9 inactivated Rac as RacGAP and that the mRNA level of ARHGAP9 was strongly detected in hematopoietic cells. ARHGAP9 negatively regulated cell   migration  . The Ala370Ser polymorphism counteracted ARHGAP9-reduced cell   migration  , spreading and adhesion. The Ala370Ser polymorphism in the ARHGAP9 gene is associated with coronary artery spasm. These data suggest that the polymorphism of ARHGAP9 has a critical function in the infiltration of hematopoietic cells into the endothelium and inflammation leading to endothelial dysfunction. 
 PLEKHG2 promotes heterotrimeric G protein βγ-stimulated lymphocyt  e migrati  on via Rac and Cdc42 activation and actin polymerization. PLEKHG2 is a Dbl family Rho guanine nucleotide exchange factor (RhoGEF) whose gene was originally identified as being upregulated in a leukemia mouse model and was later shown to be activated by heterotrimeric G protein βγ (Gβγ) subunits. However, its function and activation mechanisms remain elusive. Here we show that, compared to its expression in primary human T cells, its expression is upregulated in several leukemia cell lines, including Jurkat T cells. Downregulation of PLEKHG2 in Jurkat T cells by small interfering RNAs (siRNAs) specifically inhibited Gβγ-stimulated Rac and Cdc42, but not RhoA, activation. Consequently, suppressing PLEKHG2 expression blocked actin polymerization and SDF1α-stimulated ly  mphocyte   migration. Additional studies indicate that Gβγ likely activates PLEKHG2, in part by binding the N terminus of PLEKHG2 to release an autoinhibition imposed by its C terminus, which interacts with a region encompassing the catalytic Dbl homology (DH) domain. As a result, overexpressing either the N terminus or the C terminus of PLEKHG2 blocked Gβγ-stimulated Rac and Cdc42 activation and prevented Jurkat T cells from forming membrane protrusions and migrating. Together, our studies have provided the first evidence for the endogenous function of PLEKHG2, which may serve as a key Gβγ-stimulated RhoGEF that regulates lymphocyte chemotaxis via Rac and Cdc42 activation and actin polymerization. 
   
  
 CD109 
 
  NCBI Gene  
  GeneRIF  
  Pubmed  
 
 
  Search Term  Rank  Fields  
  [Mm]igration  6  PubMed(3)  
  ECM  7  PubMed(1)  
  [Ee]xtracellular [Mm]atrix  8  PubMed(2)  
  [Ii]nvasive  10  PubMed(3); GeneRIFs(1)  
  [Mm]etastasis  12  PubMed(3); GeneRIFs(1)  
 
 
 hide/show details for CD109 
  [Mm]igration  PubMed  
 Quantitative proteomics identifies a Dab2/integrin module regulating cell   migration  . Clathrin-associated endocytic adapters recruit cargoes to coated pits as a first step in endocytosis. We developed an unbiased quantitative proteomics approach to identify and quantify glycoprotein cargoes for an endocytic adapter, Dab2. Surface levels of integrins beta1, alpha1, alpha2, and alpha3 but not alpha5 or alphav chains were specifically increased on Dab2-deficient HeLa cells. Dab2 colocalizes with integrin beta1 in coated pits that are dispersed over the cell surface, suggesting that it regulates bulk endocytosis of inactive integrins. Depletion of Dab2 inhibits cell   migration   and polarized movement of integrin beta1 and vinculin to the leading edge. By manipulating intracellular and surface integrin beta1 levels, we show that   migration   speed correlates with the intracellular integrin pool but not the surface level. Together, these results suggest that Dab2 internalizes integrins freely diffusing on the cell surface and that Dab2 regulates   migration  , perhaps by maintaining an internal pool of integrins that can be recycled to create new adhesions at the leading edge. 
 CD109 attenuates TGF-β1 signaling and enhances EGF signaling in SK-MG-1 human glioblastoma cells. CD109 is a glycosylphosphatidylinositol-anchored cell surface protein that is frequently detected in squamous cell carcinomas. CD109 is a negative regulator of TGF-β1 signaling in human keratinocytes, and the N-terminal fragment of CD109 secreted from cells after cleavage by the furin protease is important for modulating TGF-β1 signaling. Previously, we found that CD109 is expressed in human glioblastoma cells; however, the role of CD109 in glioblastoma cells is not established. Here, we describe the effects of CD109 in human glioblastoma cell lines. Three glioblastoma cell lines, SK-MG-1, U251MG and MG178, were tested and CD109 overexpression attenuated TGF-β1 signaling and enhanced EGF signaling in SK-MG-1, but not in U251MG or MG178. The N-terminal CD109 fragment in SK-MG-1 was hyperglycosylated compared with that in MG178 or U251MG. The conditioned medium of CD109-overexpressing SK-MG-1, containing the secreted N-terminal CD109, had a negative effect on TGF-β1 signaling in wild-type SK-MG-1 and MG178, whereas it did not show any effect on EGF signaling. In addition, cell surface CD109 interacts with EGF receptor in SK-MG-1 overexpressing CD109, and exhibited enhanced   cell migr  ation and invasion. These findings suggest that CD109 attenuates TGF-β1 signaling and enhances EGF signaling in SK-MG-1 cells and that the membrane-anchored CD109 may play major roles in the EGF signaling pathway. 
 Soluble CD109 binds TGF-β and antagonizes TGF-β signalling and responses. Transforming growth factor-β (TGF-β) is a multifunctional cytokine implicated in many diseases, including tissue fibrosis and cancer. TGF-β mediates diverse biological responses by signalling through type I and II TGF-β receptors (TβRI and TβRII). We have previously identified CD109, a glycosylphosphatidylinositol (GPI)-anchored protein, as a novel TGF-β co-receptor that negatively regulates TGF-β signalling and responses and demonstrated that membrane-anchored CD109 promotes TGF-β receptor degradation via a SMAD7/Smurf2-mediated mechanism. To determine whether CD109 released from the cell surface (soluble CD109 or sCD109) also acts as a TGF-β antagonist, we determined the efficacy of recombinant sCD109 to interact with TGF-β and inhibit TGF-β signalling and responses. Our results demonstrate that sCD109 binds TGF-β with high affinity as determined by surface plasmon resonance (SPR) and cell-based radioligand binding and affinity labelling competition assays. SPR detected slow dissociation kinetics between sCD109 and TGF-β at low concentrations, indicating a stable and effective interaction. In addition, sCD109 antagonizes TGF-β-induced Smad2/3 phosphorylation, trans  cription   and cell migration. Together, our results suggest that sCD109 can bind TGF-β, inhibit TGF-β binding to its receptors and decrease TGF-β signalling and TGF-β-induced cellular responses. 
  ECM  PubMed  
 CD109, a TGF-β co-receptor, attenuates extracellular matrix production in scleroderma skin fibroblasts. Scleroderma or systemic sclerosis (SSc) is a complex connective tissue disease characterized by fibrosis of skin and internal organs. Transforming growth factor beta (TGF-β) plays a key role in the pathogenesis of SSc fibrosis. We have previously identified CD109 as a novel TGF-β co-receptor that inhibits TGF-β signaling. The aim of the present study was to determine the role of CD109 in regulating extracellular matr  ix   (ECM) production in human SSc skin fibroblasts.CD109 expression was determined in skin tissue and cultured skin fibroblasts of SSc patients and normal healthy subjects, using immunofluorescence, western blot and RT-PCR. The effect of CD109   on   ECM synthesis was determined by blocking CD109 expression using CD109-specific siRNA or addition of recombinant CD109 protein, and analyzing the expression   of   ECM components by western blot.The expression of CD109 proteinis markedly increased in SSc skin tissue in vivo and in SSc skin fibroblasts in vitro as compared to their normal counterparts. Importantly, both SSc and normal skin fibroblasts transfected with CD109-specific siRNA display increased fibronectin, collagen type I and CCN2 protein levels and enhanced Smad2/3 phosphorylation compared with control siRNA transfectants. Furthermore, addition of recombinant CD109 protein decreases TGF-β1-induced fibronectin, collagen type I and CCN2 levels in SSc and normal fibroblasts.The upregulation of CD109 protein in SSc may represent an adaptation or consequence of aberrant TGF-β signaling in SSc. Our finding that CD109 is able to decrease exce  ssi  ve ECM production in SSc fibroblasts suggest that this molecule has potential therapeutic value for the treatment of SSc. 
  [Ee]xtracellular [Mm]atrix  PubMed  
 CD109, a TGF-β co-receptor, attenuates   extracellular matri  x production in scleroderma skin fibroblasts. Scleroderma or systemic sclerosis (SSc) is a complex connective tissue disease characterized by fibrosis of skin and internal organs. Transforming growth factor beta (TGF-β) plays a key role in the pathogenesis of SSc fibrosis. We have previously identified CD109 as a novel TGF-β co-receptor that inhibits TGF-β signaling. The aim of the present study was to determine the role of CD109 in regulat  ing extracellular ma  trix (ECM) production in human SSc skin fibroblasts.CD109 expression was determined in skin tissue and cultured skin fibroblasts of SSc patients and normal healthy subjects, using immunofluorescence, western blot and RT-PCR. The effect of CD109 on ECM synthesis was determined by blocking CD109 expression using CD109-specific siRNA or addition of recombinant CD109 protein, and analyzing the expression of ECM components by western blot.The expression of CD109 proteinis markedly increased in SSc skin tissue in vivo and in SSc skin fibroblasts in vitro as compared to their normal counterparts. Importantly, both SSc and normal skin fibroblasts transfected with CD109-specific siRNA display increased fibronectin, collagen type I and CCN2 protein levels and enhanced Smad2/3 phosphorylation compared with control siRNA transfectants. Furthermore, addition of recombinant CD109 protein decreases TGF-β1-induced fibronectin, collagen type I and CCN2 levels in SSc and normal fibroblasts.The upregulation of CD109 protein in SSc may represent an adaptation or consequence of aberrant TGF-β signaling in SSc. Our finding that CD109 is able to decrease excessive ECM production in SSc fibroblasts suggest that this molecule has potential therapeutic value for the treatment of SSc. 
 CD109-mediated degradation of TGF-β receptors and inhibition of TGF-β responses involve regulation of SMAD7 and Smurf2 localization and function. Transforming growth factor-β (TGF-β) is a multifunctional cytokine that regulates a wide variety of cellular processes including proliferation, differentiation,   and extracellular ma  trix deposition. Dysregulation of TGF-β signaling is associated with several diseases such as cancer and tissue fibrosis. TGF-β signals through two transmembrane proteins known as the type I (TGFBR1) and type II (TGFBR2) receptors. The levels of these receptors at the cell surface are tightly regulated by several mechanisms, including degradation following recruitment of the E3 ubiquitin ligase SMAD ubiquitination regulatory factor (Smurf) 2 by SMAD7. In addition, TGF-β co-receptors can modulate TGF-β signaling receptor activity in a cell-specific manner. We have previously identified a novel TGF-β co-receptor, CD109, a glycosyl phosphatidylinositol (GPI)-anchored protein that negatively regulates TGF-β signaling. Despite CD109's potential relevance as a regulator of TGF-β action in vivo, the mechanisms by which CD109 regulates TGF-β signaling are still incompletely understood. Previously, we have shown that CD109 downregulates TGF-β signaling by promoting TGF-β receptor localization into the lipid raft/caveolae compartment and by enhancing TGF-β receptor degradation. Here, we demonstrate that CD109 enhances SMAD7/Smurf2-mediated degradation of TGFBR1 in a ligand-dependent manner. Moreover, we show that CD109 regulates the localization and the association of SMAD7/Smurf2 with TGFBR1. Finally, we demonstrate that CD109's inhibitory effect on TGF-β signaling and responses require SMAD7 expression and Smurf2 ubiquitin ligase activity. Taken together, these results suggest that CD109 is an important regulator of SMAD7/Smurf2-mediated degradation of TGFBR1. 
  [Ii]nvasive  PubMed  
 Correlation of pathological grade and tumor stage of urothelial carcinomas with CD109 expression. Bladder cancer is one of the most common malignant diseases. Since a high-rate of recurrence is a serious problem for early stage urothelial carcinomas, new strategies for the management of recurrent urothelial carcinomas have been explored. CD109 is a glycosylphosphatidylinositol-anchored glycoprotein and is expressed in various cancer tissues, mainly squamous cell carcinomas. CD109 negatively controls transforming growth factor (TGF)-β/Smad signaling in vitro. In this study, we analyzed the clinical significance of CD109 expression in bladder cancer using immunohistochemistry. Of 156 urothelial carcinoma tissues, 69.9% were positive for CD109, whereas CD109 was not expressed in seven normal bladder epithelia. CD109 expression was significantly higher in non-muscle  -invasiv  e (pTa+pT1) or low-grade (G1+G2) tumors than in muscle  -invasiv  e (pT2-4) or high-grade (G3) tumors, and was associated with cancer-specific survival. Simultaneous immunostaining of CD109 and phosphorylated Smad2 showed an inverse immunoreactivity relationship between the two, suggesting that CD109 inhibits TGF-β/Smad signaling in tumor tissues. Interestingly, CD109 was found to be highly expressed in the basal layer of no  n-invasi  ve urothelial carcinomas, and the expression pattern was similar to that of CD44, a marker of cancer stem cells. These findings suggest that CD109 is involved in bladder tumorigenesis and is a potential target for cancer immunotherapy. 
 CD109, a new marker for myoepithelial cells of mammary, salivary, and lacrimal glands and prostate basal cells. The CD109 gene encodes a glycosylphosphatidylinositol (GPI)-anchored cell surface protein. Herein it is shown that CD109 is highly expressed in myoepithelial cells of mammary, salivary, and lacrimal glands; and in prostate basal cells. The anti-CD109 antibody generated by the authors was available for formalin-fixed paraffin section, and it strongly stained myoepithelial cells and basal cells but not ductal, acinar, and secretory cells in these glands. CD109 expression was negative in examined breast ductal carcinomas and prostate adenocarcinomas. These findings indicate that CD109 is a useful marker for the diagnosis of   invasive   breast and prostate carcinomas. 
 High expression of CD109 antigen regulates the phenotype of cancer stem-like cells/cancer-initiating cells in the novel epithelioid sarcoma cell line ESX and is related to poor prognosis of soft tissue sarcoma. Epithelioid sarcoma (ES) is a relatively rare, highly malignant soft tissue sarcoma. The mainstay of treatment is resection or amputation. Currently other therapeutic options available for this disease are limited. Therefore, a novel therapeutic option needs to be developed. In the present study, we established a new human ES cell line (ESX) and analyzed the characteristics of its cancer stem-like cells/cancer-initiating cells (CSCs/CICs) based on ALDH1 activity. We demonstrated that a subpopulation of ESX cells with high ALDH1 activity (ALDH(high) cells) correlated with enhanced clonogenic ability, sphere-formation ability, and   invasive  ness in vitro and showed higher tumorigenicity in vivo. Next, using gene expression profiling, we identified CD109, a GPI-anchored protein upregulated in the ALDH(high) cells. CD109 mRNA was highly expressed in various sarcoma cell lines, but weakly expressed in normal adult tissues. CD109-positive cells in ESX predominantly formed spheres in culture, whereas siCD109 reduced ALDH1 expression and inhibited the cell proliferation in vitro. Subsequently, we evaluated the expression of CD109 protein in 80 clinical specimens of soft tissue sarcoma. We found a strong correlation between CD109 protein expression and the prognosis (P = 0.009). In conclusion, CD109 might be a CSC/CIC marker in epithelioid sarcoma. Moreover, CD109 is a promising prognostic biomarker and a molecular target of cancer therapy for sarcomas including ES. 
  GeneRIFs  
 Findings indicate that CD109 is a useful marker for the diagnosis of   invasive   breast and prostate carcinomas. 
  [Mm]etastasis  PubMed  
 Mesotrypsin promotes malignant growth of breast cancer cells through shedding of CD109. Serine proteases have been implicated in many stages of cancer development, facilitating tumor cell growth, invasion, and   metastasis  , and naturally occurring serine protease inhibitors have shown promise as potential anticancer therapeutics. Optimal design of inhibitors as potential therapeutics requires the identification of the specific serine proteases involved in disease progression and the functional targets responsible for the tumor-promoting properties. Here, we use the HMT-3522 breast cancer progression series grown in 3D organotypic culture conditions to find that serine protease inhibitors cause morphological reversion of the malignant T4-2 cells, assessed by inhibition of proliferation and formation of acinar structures with polarization of basal markers, implicating serine protease activity in their malignant growth behavior. We identify PRSS3/mesotrypsin upregulation in T4-2 cells as compared to their nonmalignant progenitors, and show that knockdown of PRSS3 attenuates, and treatment with recombinant purified mesotrypsin enhances, the malignant growth phenotype. Using proteomic methods, we identify CD109 as the functional proteolytic target of mesotrypsin. Our study identifies a new mediator and effector of breast cancer growth and progression. 
 CD109 is a potential target for triple-negative breast cancer. The aim of this study is to explore the expression of CD109 in breast cancer stem cells and the relationship between CD109 protein and clinicopathological characteristics of breast cancer. CD44+/CD24- tumor cells (CSCs) were selected by flow cytometry. The protein expression of CD109 was analyzed by immunohistochemistry staining, and the relationship between CD109 and clinicopathological parameters of breast cancer was determined. CD109 positively regulated the proliferation of breast CSCs in vitro, and CD109 protein expression was significantly higher in triple-negative breast cancer (TNBC) compared to non-TNBC (63.78 vs. 3.71 %, P = 0.001). Moreover, CD109 protein expression was related to the histological grade of breast cancer (P = 0.015), whereas age (P = 0.731), tumor size (P = 0.995), clinical stage (P = 0.  644), and   lymph node metastasis (P = 0.924) were not. In the logistic regression model, histological grade (P = 0.001) and molecular type (P = 0.001) were significantly related to CD109 expression. The patients with high expression of CD109 protein had significantly poorer postoperative disease-specific survival than those with no or low expression of CD109 protein (P = 0.001). In the Cox regression, CD109 was an independent prognostic factor (P = 0.001). CD109 is highly expressed in TNBC and is a potential biomarker for the initiation, progression, and differentiation of breast cancer tumors. 
 Prognostic impact of CD109 expression in myxofibrosarcoma. CD109, a TGF-β co-receptor, is reported to be preferentially expressed during the early stages of tumorigenesis in several carcinoma types. Myxofibrosarcoma is one of the most common soft-tissue sarcomas found in elderly patients. This study aimed to elucidate the impact of CD109 expression in myxofibrosarcoma on prognosis and recurrence.Immunohistochemical staining for CD109 was performed on archival specimens from 37 patients. The Fisher exact test was used to evaluate association between CD109 expression and other clinicopathological features. Survival analysis was performed using Kaplan-Meier curves, and the prognostic significance was evaluated using the log-rank test. Multivariate analysis of factors was performed using Cox regression analysis.CD109 overexpression was significantly associated with surgical stage and distant   metastasi  s (P = 0.00499, and 0.011, respectively). The frequency of CD109 overexpression was approximately 10% and CD109 overexpression was significantly associated with decreased overall survival (P = 0.004). Five-year overall survival rates 77% and 0% for CD109-negative and CD109-positive patients, respectively. In multivariate analysis, CD109 overexpression was the only independent risk factor for poor outcome (P = 0.02; hazard ratio, 10.64; 95% confidence interval, 1.47-76.91).Immunohistochemical CD109 expression in myxofibrosarcoma was associated with poor prognosis. 
  GeneRIFs  
 CD109 overexpression was significantly associated with surgical stage, distant   metastasis  , and poor prognosis in myxofibrosarcoma. 
   
  
 ABCG1 
 
  NCBI Gene  
  GeneRIF  
  Pubmed  
 
 
  Search Term  Rank  Fields  
  [Ee]xtracellular [Mm]atrix  8  PubMed(1)  
 
 
 hide/show details for ABCG1 
  [Ee]xtracellular [Mm]atrix  PubMed  
 ABCG1-mediated generation of extracellular cholesterol microdomains. Previous studies have demonstrated that the ATP-binding cassette transporters (ABC)A1 and ABCG1 function in many aspects of cholesterol efflux from macrophages. In this current study, we continued our investigation of extracellular cholesterol microdomains that form during enrichment of macrophages with cholesterol. Human monocyte-derived macrophages and mouse bone marrow-derived macrophages, differentiated with macrophage colony-stimulating factor (M-CSF) or granulocyte macrophage colony-stimulation factor (GM-CSF), were incubated with acetylated LDL (AcLDL) to allow for cholesterol enrichment and processing. We utilized an anti-cholesterol microdomain monoclonal antibody to reveal pools of unesterified cholesterol, which were found both in the   extracellular matrix   and associated with the cell surface, that we show function in reverse cholesterol transport. Coincubation of AcLDL with 50 μg/ml apoA-I eliminated all extracellular and cell surface-associated cholesterol microdomains, while coincubation with the same concentration of HDL only removed   extracellular matri  x-associated cholesterol microdomains. Only at an HDL concentration of 200 µg/ml did HDL eliminate the cholesterol microdomains that were cell-surface associated. The deposition of cholesterol microdomains was inhibited by probucol, but it was increased by the liver X receptor (LXR) agonist TO901317, which upregulates ABCA1 and ABCG1. Extracellular cholesterol microdomains did not develop when ABCG1-deficient mouse bone marrow-derived macrophages were enriched with cholesterol. Our findings show that generation of extracellular cholesterol microdomains is mediated by ABCG1 and that reverse cholesterol transport occurs not only at the cell surface but also within the extracellular space. 
   
  
 COTL1 
 
  NCBI Gene  
  GeneRIF  
  Pubmed  
 
 
  Search Term  Rank  Fields  
  [Mm]igration  6  PubMed(1)  
  [Mm]etastasize  11  PubMed(1)  
  [Mm]etastasis  12  PubMed(2)  
 
 
 hide/show details for COTL1 
  [Mm]igration  PubMed  
 Coactosin-like 1 antagonizes cofilin to promote lamellipodial protrusion at the immune synapse. Actin depolymerizing factor-homology (ADF-H) family proteins regulate actin filament dynamics at multiple cellular locations. Herein, we have investigated the function of the ADF-H family member coactosin-like 1 (COTL1) in the regulation of actin dynamics at the T cell immune synapse (IS). We initially identified COTL1 in a genetic screen to identify novel regulators of T cell activation, and subsequently found that it associates with F-actin and localizes at the IS in response to TCR+CD28 stimulation. Live cell microscopy showed that depletion of COTL1 protein impaired T cell spreading in response to TCR ligation and abrogated lamellipodial protrusion at the T cell - B cell contact site, producing only a band of F-actin. Significantly, re-expression of wild type COTL1, but not a mutant deficient in F-actin binding could rescue these defects. In addition, COTL1 depletion reduced T cell   migration  . In vitro studies showed that COTL1 and cofilin compete with each other for binding to F-actin, and COTL1 protects F-actin from cofilin-mediated depolymerization. While depletion of cofilin enhanced F-actin assembly and lamellipodial protrusion at the IS, concurrent depletion of both COTL1 and cofilin restored lamellipodia formation. Taken together, our results suggest that COTL1 regulates lamellipodia dynamics in part by protecting F-actin from cofilin-mediated disassembly. 
  [Mm]etastasize  PubMed  
 Differential expression of PAI-RBP1, C1orf142, and COTL1 in non-small cell lung cancer cell lines with different tumor metastatic potential. Human non-small cell lung cancer (NSCLC) is one of the most common malignancies in the modern world. Its recurrence is mainly due to its ability to invade and   metastasize  . However, the precise mechanism for tumor development and metastasis is still not fully understood. To shed light on the development of lung cancer, the human giant cell lung carcinoma cell lines 95D with high metastatic potential and 95C with low metastatic potential were selected in this study. The 2 cell lines originated from the same parental cell and share a similar genetic background. In the current study, we identified 3 differentially expressed proteins in 95C and 95D cell lines, namely, PAI-RBP1, C1orf142, and COTL1, by using 2-dimensional electrophoresis proteomics analysis. We found that PAI-RBP1 and C1orf142 expression levels were higher in 95D than in 95C cells, whereas COTL1 expression level was lower in 95D when compared to 95C cells. We also confirmed these results by reverse transcription-polymerase chain reaction and immunoblotting analyses. The messenger RNA and protein levels of PAI-RBP1 and C1orf142 were much higher in 95D than in 95C cells, and COTL1 expression level was lower in 95D than in 95C cells. The PAI-RBP1 expression was assessed by immunohistochemistry in 70 NSCLC and 7 normal lung tissue samples from patients. PAI-RBP1 expression level was higher in tumor tissues (positive staining in 87.1% of cases [61/70]) than in normal tissues (positive staining in 14.3% of cases [1/7]). In conclusion, by studying protein expression in NSCLC cell lines with high and low metastasis as well as in human lung cancer tissues, we have identified 3 proteins, namely, PAI-RBP1, C1orf142, and COTL1, which were differentially expressed in NSCLC cell lines with different metastatic potential. In addition, we also found that PAI-RBP1 might contribute to NSCLC development. 
  [Mm]etastasis  PubMed  
 Proteomic analysis of human small cell lung cancer tissues: up-regulation of coactosin-like protein-1. Small cell lung cancer (SCLC) is the leading cause of cancer death, with a high propensity for aggressiveness and   metastasis   even in an early stage. Thus, identification of biomarkers as early diagnostics and treatment is needed. In this study, we investigated differentially regulated proteins between human SCLC tissues and normal bronchial epithelium by proteomic analysis using two-dimensional electrophoresis (2-DE) and MALDI-TOF mass spectrometry. Seven proteins and protein isoforms, including, γ-actin, tubulin α-1B, laminin B1, coactosin-like protein-1 (COTL-1), ubiquitin carboxyl-terminal esterase L1, ubiquitin-conjugating enzyme E2-25K, and carbonic anhydrase 1, were up-regulated more than 2 fold in SCLC tissues. In particular, up-regulated COTL-1 expression was validated by Western blot analysis, immunohistochemistry, and reverse transcription quantitative polymerase chain reaction (RT-qPCR). Moreover, most SCLC tissues (93%; 28/30) were COTL-1-positive in immunohistochemistry, whereas only 16% (10/64) of nonsmall cell lung cancer (NSLC) tissues were. Taken together, this SCLC proteomic data may help in establishing a human SCLC proteome database. COTL-1 may be a biomarker or a therapeutic target in SCLC patients. 
 Differential expression of PAI-RBP1, C1orf142, and COTL1 in non-small cell lung cancer cell lines with different tumor metastatic potential. Human non-small cell lung cancer (NSCLC) is one of the most common malignancies in the modern world. Its recurrence is mainly due to its ability to invade and metastasize. However, the precise mechanism for tumor development and   metastasis   is still not fully understood. To shed light on the development of lung cancer, the human giant cell lung carcinoma cell lines 95D with high metastatic potential and 95C with low metastatic potential were selected in this study. The 2 cell lines originated from the same parental cell and share a similar genetic background. In the current study, we identified 3 differentially expressed proteins in 95C and 95D cell lines, namely, PAI-RBP1, C1orf142, and COTL1, by using 2-dimensional electrophoresis proteomics analysis. We found that PAI-RBP1 and C1orf142 expression levels were higher in 95D than in 95C cells, whereas COTL1 expression level was lower in 95D when compared to 95C cells. We also confirmed these results by reverse transcription-polymerase chain reaction and immunoblotting analyses. The messenger RNA and protein levels of PAI-RBP1 and C1orf142 were much higher in 95D than in 95C cells, and COTL1 expression level was lower in 95D than in 95C cells. The PAI-RBP1 expression was assessed by immunohistochemistry in 70 NSCLC and 7 normal lung tissue samples from patients. PAI-RBP1 expression level was higher in tumor tissues (positive staining in 87.1% of cases [61/70]) than in normal tissues (positive staining in 14.3% of cases [1/7]). In conclusion, by studying protein expression in NSCLC cell lines with high and low   metastasis   as well as in human lung cancer tissues, we have identified 3 proteins, namely, PAI-RBP1, C1orf142, and COTL1, which were differentially expressed in NSCLC cell lines with different metastatic potential. In addition, we also found that PAI-RBP1 might contribute to NSCLC development. 
   
  
 IRAK4 
 
  NCBI Gene  
  GeneRIF  
  Pubmed  
 
 
  Search Term  Rank  Fields  
  [Mm]igration  6  Process(1)  
  [Ii]nvasive  10  PubMed(8); GeneRIFs(2); Summary(1)  
 
 
 hide/show details for IRAK4 
  [Mm]igration  Process  
 neutrophil   migration   
  [Ii]nvasive  PubMed  
 Rare variants in MYD88, IRAK4 and IKBKG and susceptibility to   invasive   pneumococcal disease: a population-based case-control study. Although rare variants within the Toll-like receptor signalling pathway genes have been found to underlie human primary immunodeficiencies associated with selective predisposition to   invasive   pneumococcal disease (IPD), the contribution of variants in these genes to IPD susceptibility at the population level remains unknown. Complete re-sequencing of IRAK4, MYD88 and IKBKG genes was undertaken in 164 IPD cases from the UK and 164 geographically-matched population-based controls. 233 single-nucleotide variants (SNVs) were identified, of which ten were in coding regions. Four rare coding variants were predicted to be deleterious, two variants in MYD88 and two in IRAK4. The predicted deleterious variants in MYD88 were observed as two heterozygote cases but not seen in controls. Frequencies of predicted deleterious IRAK4 SNVs were the same in cases and controls. Our findings suggest that rare, functional variants in MYD88, IRAK4 or IKBKG do not significantly contribute to IPD susceptibility in adults at the population level. 
 HIV induces both a down-regulation of IRAK-4 that impairs TLR signalling and an up-regulation of the antibiotic peptide dermcidin in monocytic cells. HIV-infected individuals have an increased risk of   invasive   bacterial infections, even at early clinical stages with relatively normal CD4(+) T-cell counts. The pathogenic mechanisms behind this are not fully understood. However, an increasing number of studies indicate that HIV may impair the innate immunity to bacteria by infecting key cells of the monocyte/macrophage lineage. In this study, the effects of HIV infection on the protein profile of undifferentiated monocyte-like THP-1 cells were examined by a mass spectrometric approach based on stable isotope labelling with amino acid in cell culture (SILAC). We identified 651 proteins, of which nine proteins were down-regulated and 17 proteins were up-regulated in HIV-infected THP-1 cells as compared to uninfected controls. Most remarkably, the IL-1 receptor associated kinase 4 (IRAK-4), which is essential for virtually all TLR signalling, was suppressed, whereas the precursor for the antibiotic peptide Dermcidin was up-regulated in HIV-infected cells. Upon stimulation of either TLR2 or TLR4, the HIV-infected THP-1 cells displayed reduced TNF-alpha secretion. The HIV-induced down-regulation of IRAK-4 was reconfirmed in monocyte-derived macrophage cell cultures. These data suggests that HIV may impair the TLR signalling cascade for pathogen recognition in cells of the monocyte/macrophage lineage and thus, may reduce the ability of the innate immune system to sense invading pathogens and initiate appropriate responses. 
 Inherited human IRAK-4 deficiency: an update. Interleukin-1 receptor-associated kinase-4 (IRAK-4), a member of the IRAK family, plays an essential role in Toll-like receptor- and IL-1 receptor-mediated signaling. We briefly review inherited human IRAK-4 deficiency, a recently described primary immunodeficiency leading to recurrent,   invasive  , pyogenic bacteria infection, and   invasive   pneumococcal disease in particular. 
 Staphylococcal pericarditis, and liver and paratracheal abscesses as presentations in two new cases of interleukin-1 receptor associated kinase 4 deficiency. A deficiency in the interleukin-1 receptor activated kinase 4 (IRAK-4) has recently been associated with severe recurrent, predominantly Gram-positive bacterial infections.Two unrelated Canadian children with unique presentations of IRAK-4 deficiency are described. Both children had multiple Gram-positive bacterial infections, specifically Staphylococcus aureus and Streptococcus pneumoniae. Although these microorganisms in patients with IRAK-4 deficiency commonly cause   invasive   infections, such as meningitis, arthritis, and sepsis, the sites of infection in our patients were unique. In the first patient, staphylococcal pericarditis and, on a separate occasion, staphylococcal liver abscesses with generalized peritonitis were presentations. In the second child, S. aureus infection caused submandibular and periauricular lymphadenitis with unsuspected paratracheal abscess as well. These severe infections were not accompanied by the expected constitutional symptoms or hematologic and acute phase responses despite findings of advanced infection on diagnostic imaging.Cytokine production [interleukin (IL)-6, IL-8, and tumor necrosis factor (TNF)-alpha] by whole blood leukocytes and adherent monocytes after stimulation with IL-1beta or various Toll-like receptor agonists [lipopolysaccharide, Poly I:C, S. aureus peptidoglycan (PGN)] was analyzed. IRAK-4 genes were sequenced by standard techniques.Failure by whole blood leukocytes to produce IL-6 or TNF-alpha in response to any of these stimuli was the most consistent finding. In striking contrast, IL-8 production in response to PGN was normal in both cases. Both patients had novel and heterozygous mutations and deletions in the IRAK-4 gene.Our results indicate that PGN-induced IL-6 production is via IRAK-4 dependent mechanisms, whereas IL-8 response to PGN is via IRAK-4 independent mechanisms. Patients with relatively silent but   invasive   bacterial infection should raise suspicion of IRAK-4 immunodeficiency. 
 IRAK-4 deficiency as a cause for familial fatal   invasive   infection by Streptococcus pneumoniae. In this Letter to the Editor we report the case of two siblings with fatal pneumococcal meningitis as the initial manifestation of IRAK-4 deficiency caused by previously undescribed mutations in IRAK4. The letter also highlights the importance of   invasive   pneumococcal infection as a critical 'red flag' warning of the potential for an underlying primary immunodeficiency and identifies some of the challenges in making the clinical diagnosis of IRAK-4 deficiency. 
 IRAK-4 and MyD88 deficiencies impair IgM responses against T-independent bacterial antigens. IRAK-4 and MyD88 deficiencies impair interleukin 1 receptor and Toll-like receptor (TLR) signaling and lead to heightened susceptibility to   invasive   bacterial infections. Individuals with these primary immunodeficiencies have fewer immunoglobulin M (IgM)(+)IgD(+)CD27(+) B cells, a population that resembles murine splenic marginal zone B cells that mount T-independent antibody responses against bacterial antigens. However, the significance of this B-cell subset in humans is poorly understood. Using both a 610 carbohydrate array and enzyme-linked immunosorbent assay, we found that patients with IRAK-4 and MyD88 deficiencies have reduced serum IgM, but not IgG antibody, recognizing T-independent bacterial antigens. Moreover, the quantity of specific IgM correlated with IgM(+)IgD(+)CD27(+) B-cell frequencies. As with mouse marginal zone B cells, human IgM(+)CD27(+) B cells activated by TLR7 or TLR9 agonists produced phosphorylcholine-specific IgM. Further linking splenic IgM(+)IgD(+)CD27(+) B cells with production of T-independent IgM, serum from splenectomized subjects, who also have few IgM(+)IgD(+)CD27(+) B cells, had reduced antibacterial IgM. IRAK-4 and MyD88 deficiencies impaired TLR-induced proliferation of this B-cell subset, suggesting a means by which loss of this activation pathway leads to reduced cell numbers. Thus, by bolstering the IgM(+)IgD(+)CD27(+) B-cell subset, IRAK-4 and MyD88 promote optimal T-independent IgM antibody responses against bacteria in humans. 
 Selective predisposition to bacterial infections in IRAK-4-deficient children: IRAK-4-dependent TLRs are otherwise redundant in protective immunity. Human interleukin (IL) 1 receptor-associated kinase 4 (IRAK-4) deficiency is a recently discovered primary immunodeficiency that impairs Toll/IL-1R immunity, except for the Toll-like receptor (TLR) 3- and TLR4-interferon (IFN)-alpha/beta pathways. The clinical and immunological phenotype remains largely unknown. We diagnosed up to 28 patients with IRAK-4 deficiency, tested blood TLR responses for individual leukocyte subsets, and TLR responses for multiple cytokines. The patients' peripheral blood mononuclear cells (PBMCs) did not induce the 11 non-IFN cytokines tested upon activation with TLR agonists other than the nonspecific TLR3 agonist poly(I:C). The patients' individual cell subsets from both myeloid (granulocytes, monocytes, monocyte-derived dendritic cells [MDDCs], myeloid DCs [MDCs], and plasmacytoid DCs) and lymphoid (B, T, and NK cells) lineages did not respond to the TLR agonists that stimulated control cells, with the exception of residual responses to poly(I:C) and lipopolysaccharide in MDCs and MDDCs. Most patients (22 out of 28; 79%) suffered from   invasive   pneumococcal disease, which was often recurrent (13 out of 22; 59%). Other infections were rare, with the exception of severe staphylococcal disease (9 out of 28; 32%). Almost half of the patients died (12 out of 28; 43%). No death and no   invasive   infection occurred in patients older than 8 and 14 yr, respectively. The IRAK-4-dependent TLRs and IL-1Rs are therefore vital for childhood immunity to pyogenic bacteria, particularly Streptococcus pneumoniae. Conversely, IRAK-4-dependent human TLRs appear to play a redundant role in protective immunity to most infections, at most limited to childhood immunity to some pyogenic bacteria. 
 Association of Polymorphisms in IRAK1, IRAK4 and MyD88, and Severe   Invasive   Pneumococcal Disease. Severe   invasive   pneumococcal disease (SIPD) has high morbidity and mortality, conditioned by pneumococcus and host factors, such as Toll-like receptors and their Toll-IL1R common signaling pathway. The objectives of this study are (1) to correlate single nucleotide polymorphisms (SNPs) involved in some Toll-IL1R signaling pathway proteins (IRAK1, IRAK4, IRAKM and MyD88) with SIPD by comparing patients versus healthy controls. (2) To determine whether these SNPs influence SIPD outcome.Case-control prospective observational study: 60 pediatric patients with IPD and systemic inflammatory response syndrome, and 120 healthy volunteers. Well-known immunodeficiencies were excluded.SNPs genotypes and alleles. Other variables: demographic, previous infections, and clinical, analytical and microbiological evolution data.We have detected significant disequilibrium of SNPs frequencies between SIPD patients and controls in rs1059701-CC (IRAK1; P = 0.0067), rs4251513-CC (IRAK4; P < 0.0001), rs1461567-T (IRAK4; P = 0.0158) and rs6853-AA (MyD88; P < 0.0001). SIPD patients showed significant association between: leukocytosis > 15,000/mmc and rs1059702-nonTT (IRAK1; P = 0.0460), pleuropneumonia and rs1624395-G (IRAKM; P = 0.0147), and rs1370128-C (IRAKM; P = 0.0055), sequelae, and rs4251513-nonGG (IRAK4; P = 0.0055), death and rs6853-nonAA (P = 0.0054) and rs6853-G (P = 0.0065; MyD88).This is the first study to show an association between SNPs in IRAK1, IRAK4 and MyD88, and the presence of SIPD. Our data showed that some SNPs may lead to a higher risk of developing SIPD while other are related with the outcome in SIPD patients. Following PIRO score (predisposition, insult, response, organ dysfunction), identifying SNPs predisposing to infectious diseases, such as SIPD might help stratify patients with severe infectious diseases and design specific treatments. 
  GeneRIFs  
 This is the first study to show an association between single nucleotide polymorphisms in IRAK1, IRAK4 and MyD88, and the presence of severe   invasive   pneumococcal disease. 
 Inherited human IRAK-4 deficiency, a recently described primary immunodeficiency leading to recurrent,   invasive  , pyogenic bacteria infection, and   invasive   pneumococcal disease in particular, is reviewed. 
  Summary  
 This gene encodes a kinase that activates NF-kappaB in both the Toll-like receptor (TLR) and T-cell receptor (TCR) signaling pathways. The protein is essential for most innate immune responses. Mutations in this gene result in IRAK4 deficiency and recurrent   invasive   pneumococcal disease. Multiple transcript variants encoding different isoforms have been found for this gene. [provided by RefSeq, Aug 2011] 
   
  
 DUSP10 
 
  NCBI Gene  
  GeneRIF  
  Pubmed  
 
 
  Search Term  Rank  Fields  
  [Mm]igration  6  PubMed(3); GeneRIFs(1)  
  [Mm]etastasis  12  PubMed(1)  
 
 
 hide/show details for DUSP10 
  [Mm]igration  PubMed  
 Polycyclic aromatic hydrocarbon (PAH)-mediated upregulation of hepatic microRNA-181 family promotes cancer cell   migration   by targeting MAPK phosphatase-5, regulating the activation of p38 MAPK. Growing evidence indicates that changes in microRNA (miRNA) expression in cancer induced by chemical carcinogens play an important role in cancer development and progression by regulating related genes. However, the mechanisms underlying miRNA involvement in hepatocarcinogenesis induced by polycyclic aromatic hydrocarbons (PAHs) remain unclear. Thus, the identification of aberrant miRNA expression during PAH-induced cancer cell   migration   will lead to a better understanding of the substantial role of miRNAs in cancer progression. In the present study, miRNA expression profiling showed significant upregulation of miR-181a, -181b, and -181d in human hepatocellular carcinoma cells (HepG2 line) exposed to benzo[a]anthracene (BA) and benzo[k]fluoranthene (BF). MAPK phosphatase-5 (MKP-5), a validated miR-181 target that deactivates MAPKs, was markedly suppressed while phosphorylation of p38 MAPK was increased after BA and BF exposure. The   migration   of HepG2 cells, observed using the scratch wound-healing assay, also increased in a dose-dependent manner. Depletion of miR-181 family members by miRNA inhibitors enhanced the expression of MKP-5 and suppressed the phosphorylation of p38 MAPK. Furthermore, the depletion of the miR-181 family inhibited cancer cell   migration  . Based on these results, we conclude that the miR-181 family plays a critical role in PAH-induced hepatocarcinogenesis by targeting MKP-5, resulting in the regulation of p38 MAPK activation. 
 Polycyclic aromatic hydrocarbon (PAH)-mediated upregulation of hepatic microRNA-181 family promotes cancer cell   migration   by targeting MAPK phosphatase-5, regulating the activation of p38 MAPK. Growing evidence indicates that changes in microRNA (miRNA) expression in cancer induced by chemical carcinogens play an important role in cancer development and progression by regulating related genes. However, the mechanisms underlying miRNA involvement in hepatocarcinogenesis induced by polycyclic aromatic hydrocarbons (PAHs) remain unclear. Thus, the identification of aberrant miRNA expression during PAH-induced cancer cell   migration   will lead to a better understanding of the substantial role of miRNAs in cancer progression. In the present study, miRNA expression profiling showed significant upregulation of miR-181a, -181b, and -181d in human hepatocellular carcinoma cells (HepG2 line) exposed to benzo[a]anthracene (BA) and benzo[k]fluoranthene (BF). MAPK phosphatase-5 (MKP-5), a validated miR-181 target that deactivates MAPKs, was markedly suppressed while phosphorylation of p38 MAPK was increased after BA and BF exposure. The   migration   of HepG2 cells, observed using the scratch wound-healing assay, also increased in a dose-dependent manner. Depletion of miR-181 family members by miRNA inhibitors enhanced the expression of MKP-5 and suppressed the phosphorylation of p38 MAPK. Furthermore, the depletion of the miR-181 family inhibited cancer cell   migration  . Based on these results, we conclude that the miR-181 family plays a critical role in PAH-induced hepatocarcinogenesis by targeting MKP-5, resulting in the regulation of p38 MAPK activation. 
 DUSP10 regulates intestinal epithelial cell growth and colorectal tumorigenesis. Dual specificity phosphatase 10 (DUSP10), also known as MAP kinase phosphatase 5 (MKP5), negatively regulates the activation of MAP kinases. Genetic polymorphisms and aberrant expression of this gene are associated with colorectal cancer (CRC) in humans. However, the role of DUSP10 in intestinal epithelial tumorigenesis is not clear. Here, we showed that DUSP10 knockout (KO) mice had increased intestinal epithelial cell (IEC) proliferation and   migration   and developed less severe colitis than wild-type (WT) mice in response to dextran sodium sulphate (DSS) treatment, which is associated with increased ERK1/2 activation and Krüppel-like factor 5 (KLF5) expression in IEC. In line with increased IEC proliferation, DUSP10 KO mice developed more colon tumours with increased severity compared with WT mice in response to administration of DSS and azoxymethane (AOM). Furthermore, survival analysis of CRC patients demonstrated that high DUSP10 expression in tumours was associated with significant improvement in survival probability. Overexpression of DUSP10 in Caco-2 and RCM-1 cells inhibited cell proliferation. Our study showed that DUSP10 negatively regulates IEC growth and acts as a suppressor for CRC. Therefore, it could be targeted for the development of therapies for colitis and CRC. 
  GeneRIFs  
 Depletion of miR-181 family members by miRNA inhibitors enhanced the expression of MKP-5 and suppressed the phosphorylation of p38 MAPK after exposure to PAHs, which promotes cancer cell   migration  . 
  [Mm]etastasis  PubMed  
 Large-scale genetic study in East Asians identifies six new loci associated with colorectal cancer risk. Known genetic loci explain only a small proportion of the familial relative risk of colorectal cancer (CRC). We conducted a genome-wide association study of CRC in East Asians with 14,963 cases and 31,945 controls and identified 6 new loci associated with CRC risk (P = 3.42 × 10(-8) to 9.22 × 10(-21)) at 10q22.3, 10q25.2, 11q12.2, 12p13.31, 17p13.3 and 19q13.2. Two of these loci map to genes (TCF7L2 and TGFB1) with established roles in colorectal tumorigenesis. Four other loci are located in or near genes involved in transcriptional regulation (ZMIZ1), genome maintenance (FEN1), fatty acid metabolism (FADS1 and FADS2), cancer cell motility an  d metastas  is (CD9), and cell growth and differentiation (NXN). We also found suggestive evidence for three additional loci associated with CRC risk near genome-wide significance at 8q24.11, 10q21.1 and 10q24.2. Furthermore, we replicated 22 previously reported CRC-associated loci. Our study provides insights into the genetic basis of CRC and suggests the involvement of new biological pathways. 
   
  
 ADAM12 
 
  NCBI Gene  
  GeneRIF  
  Pubmed  
 
 
  Search Term  Rank  Fields  
  [Ff]ilopodia  1  PubMed(1)  
  [Ii]nvadopodia  3  PubMed(2)  
  [Pp]odosome(s)?  5  PubMed(1)  
  [Mm]igration  6  PubMed(10); GeneRIFs(1)  
  ECM  7  PubMed(4)  
  [Ee]xtracellular [Mm]atrix  8  PubMed(13); GeneRIFs(1)  
  [Ii]nvasive  10  PubMed(11); GeneRIFs(2)  
  [Mm]etastasis  12  PubMed(8); GeneRIFs(3)  
  [Cc]ell [Ii]nvasion  13  PubMed(4); GeneRIFs(1)  
 
 
 hide/show details for ADAM12 
  [Ff]ilopodia  PubMed  
 Cysteine-rich domain of human ADAM 12 (meltrin alpha) supports tumor cell adhesion. The ADAMs (A disintegrin and metalloprotease) comprise a family of membrane-anchored cell surface proteins with a putative role in cell-cell and/or cell-matrix interactions. By immunostaining, ADAM 12 (meltrin alpha) was up-regulated in several human carcinomas and could be detected along the tumor cell membranes. Because of this intriguing staining pattern, we investigated whether human ADAM 12 supports tumor cell adhesion. Using an in vitro assay using recombinant polypeptides expressed in Escherichia coli, we examined the ability of individual domains of human ADAM 12 and ADAM 15 to support tumor cell adhesion. We found that the disintegrin-like domain of human ADAM 15 supported adhesion of alphavbeta3-expressing A375 melanoma cells. In the case of human ADAM 12, however, recombinant polypeptides of the cysteine-rich domain but not the disintegrin-like domain supported cell adhesion of a panel of carcinoma cell lines. On attachment to recombinant polypeptides from the cysteine-rich domain of human ADAM 12, most tumor cell lines, such as MDA-MB-231 breast carcinoma cells, were rounded and associated with numerous actin-containing   filopodia   and used a cell surface heparan sulfate proteoglycan to attach. Finally, we demonstrated that authentic full-length human ADAM 12 could bind to heparin Sepharose. Together these results suggest a novel role of the cysteine-rich domain of ADAM 12 -- that of supporting tumor cell adhesion. 
  [Ii]nvadopodia  PubMed  
 Extracellular engagement of ADAM12 induces clusters of   invadopodia   with localized ectodomain shedding activity.   Invadopodia   are dynamic actin structures at the cell surface that degrade extracellular matrix and act as sites of signal transduction. The biogenesis of   invadopodia  , including the mechanisms regulating their formation, composition, and turnover is not entirely understood. Here, we demonstrate that antibody ligation of ADAM12, a transmembrane disintegrin and metalloprotease, resulted in the rapid accumulation of   invadopodia   with extracellular matrix-degrading capacity in epithelial cells expressing the αvβ3 integrin and active c-Src kinase. The induction o  f invadopod  ia clusters required an intact c-Src interaction site in the ADAM12 cytoplasmic domain, but was independent of the catalytic activity of ADAM12. Caveolin-1 and transmembrane protease MMP14/MT1-MMP were both present in the ADAM12-induced clusters o  f invadopod  ia, and cholesterol depletion prevented their formation, suggesting that lipid-raft microdomains are involved in the process. Importantly, our data demonstrate that ADAM12-mediated ectodomain shedding of epidermal growth factor receptor ligands can occur within thes  e invadopod  ia. Such localized growth factor signalling offers an interesting novel biological concept highly relevant to the properties of carcinoma cells, which often show upregulated ADAM12 and β3 integrin expression, together with high levels of c-Src kinase activity. 
 Notch increases the shedding of HB-EGF by ADAM12 to potentiate   invadopodia   formation in hypoxia. Notch regulates cell-cell contact-dependent signaling and is activated by hypoxia, a microenvironmental condition that promotes cellular invasion during both normal physiology and disease. The mechanisms by which hypoxia and Notch regulate cellular invasion are not fully elucidated. In this paper, we show that, in cancer cells, hypoxia increased the levels and activity of the ADAM12 metalloprotease in a Notch signaling-dependent manner, leading to increased ectodomain shedding of the epidermal growth factor (EGF) receptor (EGFR) ligand heparin-binding EGF-like growth factor. Released HB-EGF induced the formation of   invadopodia  , cellular structures that aid cancer cell invasion. Thus, we describe a signaling pathway that couples cell contact-dependent signaling with the paracrine activation of the EGFR, indicating cross talk between the Notch and EGFR pathways in promoting cancer cell invasion. This signaling pathway might regulate the coordinated acquisition of invasiveness by neighboring cells and mediate the communication between normoxic and hypoxic areas of tumors to facilitate cancer cell invasion. 
  [Pp]odosome(s)?  PubMed  
 The adaptor protein fish associates with members of the ADAMs family and localizes to   podosomes   of Src-transformed cells. Fish is a scaffolding protein and Src substrate. It contains an amino-terminal Phox homology (PX) domain and five Src homology 3 (SH3) domains, as well as multiple motifs for binding both SH2 and SH3 domain-containing proteins. We have determined that the PX domain of Fish binds 3-phosphorylated phosphatidylinositols (including phosphatidylinositol 3-phosphate and phosphatidylinositol 3,4-bisphosphate). Consistent with this, a fusion protein of green fluorescent protein and the Fish PX domain localized to punctate structures similar to endosomes in normal fibroblasts. However, the full-length Fish protein was largely cytoplasmic, suggesting that its PX domain may not be able to make intermolecular interactions in unstimulated cells. In Src-transformed cells, we observed a dramatic re-localization of some Fish molecules to actin-rich structures called   podosomes  ; the PX domain was both necessary and sufficient to effect this translocation. We used a phage display screen with the fifth SH3 domain of Fish and isolated ADAM19 as a binding partner. Subsequent analyses in mammalian cells demonstrated that Fish interacts with several members of the ADAMs family, including ADAMs 12, 15, and 19. In Src-transformed cells, ADAM12 co-localized with Fish in   podosomes  . Because members of the ADAMs family have been implicated in growth factor processing, as well as cell adhesion and motility, Fish could be acting as an adaptor molecule that allows Src to impinge on these processes. 
  [Mm]igration  PubMed  
 Role of A disintegrin and metalloprotease-12 in neutrophil recruitment induced by airway epithelium. Among proteases, metalloproteases are implicated in tissue remodeling, as shown in numerous diseases including allergy. ADAMs (A Disintegrin And Metalloprotease) metalloproteases are implicated in physiologic processes such as cytokine and growth factor shedding, cell   migration  , adhesion, or repulsion. Our aim was to measure ADAM-12 expression in airway epithelium and to define its role during the allergic response. To raise this question, we analyzed the ADAM-12 expression ex vivo after allergen exposure in patients with allergic rhinitis and in vitro in cultured primary human airway epithelial cells (AEC). Clones of BEAS-2B cells transfected with the full-length form of ADAM-12 were generated to study the consequences of ADAM-12 up-regulation on AEC function. After allergen challenge, a strong increase of ADAM-12 expression was observed in airway epithelium from patients with allergic rhinitis but not from control subjects. In contrast with the other HB-epidermal growth factor sheddases, ADAM-10 and -17, TNF-alpha in vitro increased the expression of ADAM-12 by AEC, an effect amplified by IL-4 and IL-13. Up-regulation of ADAM-12 in AEC increased the expression of alpha3 and alpha4 integrins and to the modulation of cell   migration   on fibronectin but not on collagen. Moreover, overexpression of ADAM-12 in BEAS-2B enhanced the secretion of CXCL1 and CXCL8 and their capacity to recruit neutrophils. CD47 was strongly decreased by ADAM-12 overexpression, a process associated with a reduced adhesion of neutrophils. These effects were mainly dependent on epidermal growth factor receptor activation. In summary, ADAM-12 is produced during allergic reaction by AEC and might increase neutrophil recruitment within airway mucosa. 
 ADAM12: a potential target for the treatment of chronic wounds. Wound healing is a complex process involving multiple cellular events, including cell proliferation,   migration  , and tissue remodeling. A disintegrin and metalloprotease 12 (ADAM12) is a membrane-anchored metalloprotease, which has been implicated in activation-inactivation of growth factors that play an important role in wound healing, including heparin-binding epidermal growth factor (EGF)-like growth factor (HB-EGF) and insulin growth factor (IGF) binding proteins. Here, we report that expression of ADAM12 is fivefold upregulated in the nonhealing edge of chronic ulcers compared to healthy skin, based on microarrays of biopsies taken from five patients and from healthy controls (p = 0.013). The increase in ADAM12 expression in chronic ulcers was confirmed by quantitative real-time polymerase chain reaction (RT-PCR). Moreover, immunohistochemical analysis demonstrated a pronounced increase in the membranous and intracellular signal for ADAM12 in the epidermis of chronic wounds compared to healthy skin. These findings, coupled with our previous observations that lack of keratinocyte   migration   contributes to the pathogenesis of chronic ulcers, prompted us to evaluate how the absence of ADAM12 affects the   migration   of mouse keratinocytes. Skin explants from newborn ADAM12-/- or wild-type (WT) mice were used to quantify keratinocyte   migration   out of the explants over a period of 7 days. We found a statistically significant increase in the   migration   of ADAM12-/- keratinocytes compared to WT control (p = 0.0014) samples. Taken together, the upregulation of ADAM12 in chronic wounds and the increased   migration   of keratinocytes in the absence of ADAM12 suggest that ADAM12 is an important mediator of wound healing. We hypothesize that increased expression of ADAM12 in chronic wounds impairs wound healing through the inhibition of keratinocyte   migration   and that topical ADAM12 inhibitors may therefore prove useful for the treatment of chronic wounds. 
 Breast cancer-associated mutations in metalloprotease disintegrin ADAM12 interfere with the intracellular trafficking and processing of the protein. ADAM12 has recently emerged as a Candidate Cancer Gene in a comprehensive genetic analysis of human breast cancers. Three somatic mutations in ADAM12 were observed at significant frequencies in breast cancers: D301H, G479E and L792F. The first 2 of these mutations involve highly conserved residues in ADAM12, and our computational sequence analysis confirms that they may be cancer-related. We show that the corresponding mutations in mouse ADAM12 inhibit the proteolytic processing and activation of ADAM12 in NIH3T3, COS-7, CHO-K1 cells and in MCF-7 breast cancer cells. The D/H and G/E ADAM12 mutants exert a dominant-negative effect on the processing of the wild-type ADAM12. Immunofluorescence analysis and cell surface biotinylation experiments demonstrate that the D/H and G/E mutants are retained inside the cell and are not transported to the cell surface. Consequently, the D/H and G/E mutants, unlike the wild-type ADAM12, are not capable of shedding Delta-like l, a ligand for Notch receptor, at the cell surface, or of stimulating cell   migration  . Our results suggest that the breast cancer-associated mutations interfere with the intracellular trafficking of ADAM12 and result in loss of the functional ADAM12 at the cell surface. 
 ADAM-15 inhibits wound healing in human intestinal epithelial cell monolayers. The disintegrin metalloproteases (or ADAMs) are membrane-anchored glycoproteins that have been implicated in cell-cell or cell-matrix interactions and in proteolysis of molecules on the cell surface. The expression and/or the pathophysiological implications of ADAMs are not known in intestinal epithelial cells. Therefore, our aim was to investigate the expression and the role of ADAMs in intestinal epithelial cells. Expression of ADAMs was assessed by RT-PCR, Western blot analysis, and immunufluorescence experiments. Wound-healing experiments were performed by using the electric cell substrate impedence sensing technology. Our results showed that ADAMs-10, -12, and -15 mRNA are expressed in the colonic human cell lines Caco2-BBE and HT29-Cl.19A. An ADAM-15 complementary DNA cloned from Caco2-BBE poly(A)+ RNA, and encompassing the entire coding region, was found to be shorter and to present a different region encoding the cytoplasmic tail compared with ADAM-15 sequence deposited in the database. In Caco2-BBE cells and colonic epithelial cells, ADAM-15 protein was found in the apical, basolateral, and intracellular compartments. We also showed that the overexpression of ADAM-15 reduced cell   migration   in a wound-healing assay in Caco2-BBE monolayers. Our data show that 1) ADAM-15 is expressed in human intestinal epithelia, 2) a new variant of ADAM-15 is expressed in a human intestinal epithelial cell line, and 3) ADAM-15 is involved in intestinal epithelial cells wound-healing processes. Together, these results suggest that ADAM-15 may have important pathophysiological roles in intestinal cells. 
 A disintegrin and metalloproteinase 12 (ADAM12) localizes to invasive trophoblast, promotes cell invasion and directs column outgrowth in early placental development. During pregnancy, stromal- and vascular-remodeling trophoblasts serve critical roles in directing placental development acquiring pro-invasive characteristics. The A Disintegrin and Metalloproteinase (ADAM) family of multifunctional proteins direct cellular processes across multiple organ systems via their intrinsic catalytic, cell adhesive and intracellular signaling properties. ADAM12, existing as two distinct splice variants (ADAM12L and ADAM12S), is highly expressed in the human placenta and promotes cell   migration   and invasion in several tumor cell lines; however, its role in trophoblast biology is unknown. In this study, ADAM12 was localized to anchoring trophoblast columns in first trimester placentas and to highly invasive extracellular matrix-degrading trophoblasts in placental villous explants. The importance of ADAM12 in directing trophoblast invasion was tested using loss-of and gain-of-function strategies, where siRNA-directed knockdown of ADAM12 inhibited trophoblast cell invasion while over-expression promoted   migration   and invasion in two trophoblastic cell models. In placental villous explant cultures, siRNA-directed loss of ADAM12 significantly dampened trophoblast column outgrowth. Additionally, we provide functional evidence for the ADAM12S variant in promoting trophoblast invasion and column outgrowth through a mechanism requiring its catalytic activity. This is the first study to assign a function for ADAM12 in trophoblast biology, where ADAM12 may play a central role regulating the behavior of invasive trophoblast subsets in early pregnancy. This study also underlines the importance of ADAM12L and ADAM12S in directing cell motility in normal developmental processes outside of cancer, specifically highlighting a potentially important function of ADAM12S in directing early placental development. 
 ADAM-12 as a diagnostic marker for the proliferation,   migration   and invasion in patients with small cell lung cancer. Small cell lung cancer (SCLC) is highly aggressive and is characterized by malignant metastasis. Approximately 90% of patients die due to extensive metastasis. The extracellular matrix (ECM) is a natural barrier that can prevent cellular invasion and metastasis. Therefore, degradation of the ECM must take place in order for extensive metastasis to occur. A disintegrin and metalloprotease (ADAM) is a multi-domain protease that plays an important role in tumorigenesis, as well as tumor development, invasion and metastasis. However, there have been few reports on the expression and role of ADAMs in SCLC. In the current study, the expression and role of ADAMs in SCLC proliferation, invasion and metastasis was investigated. A total of 150 SCLC tissue samples were examined by immunohistochemistry for ADAMs expression. ADAM-12 was found to be abundantly expressed in 72.67% samples and other ADAMs were found to be expressed in 10% to 40% of samples. ADAM-12 levels in serum and urine, from 70 SCLC patients and 40 normal controls, were also measured using ELISA. ADAM-12 expression was significantly higher in SCLC patients than in healthy controls and in patients with extensive disease compared to those with more limited disease. Silencing the expression of ADAM-12 in H1688 cells through the use of specific siRNA significantly reduced cellular proliferation, invasion and metastasis. Supplementing the expression of ADAM-12-L or -S in H345 cells, significantly enhanced cellular proliferation, invasion and metastasis. Animal models with metastatic SCLC also exhibited increased expression of ADAM-12 along with enhanced invasion and metastasis. In brief, ADAM-12 is an independent prognostic factor and diagnostic marker, and is involved in the proliferation, invasion and metastasis of SCLC. 
 A positive feedback loop between HER2 and ADAM12 in human head and neck cancer cells increases   migration   and invasion. Increased activation of epidermal growth factor receptor (EGFR) family members such as HER2/Erbb2 can result in more aggressive disease, resistance to chemotherapy and reduced survival of head and neck squamous cell carcinoma (HNSCC) patients. In order to identify mechanisms through which these receptor tyrosine kinases accelerate tumor progression, the regulation of metalloprotease expression by EGFR family members was investigated in 11 squamous cell carcinoma (SCC) cell lines. HER2 expression was significantly correlated with ADAM12 (A Disintegrin And Metalloprotease 12) expression in these cell lines and was co-expressed in human head and neck cancers. Inhibition of HER2 or EGFR decreased ADAM12 transcripts whereas HER2 transfection upregulated ADAM12 expression. To understand the molecular mechanisms underlying HER2 regulation of ADAM12, we investigated the signaling pathways directing ADAM12 production in SCC cells. Inhibition of phosphatidyl inositol-3-kinase or mammalian target of rapamycin decreased ADAM12 transcripts in HER2-expressing SCC cells, whereas transfection with AKT increased ADAM12 mRNA. Experiments utilizing ADAM12 transfection or siRNA targeting of ADAM12 revealed that the protease increased both the   migration   and invasiveness of oral SCC cells. Surprisingly, ADAM12 also increased HER2 message, protein levels and activity through an Ets1-dependent mechanism. Collectively, these results reveal a novel positive activation loop between ADAM12 and HER2 that may contribute to HNSCC progression. 
 ADAM12 transmembrane and secreted isoforms promote breast tumor growth: a distinct role for ADAM12-S protein in tumor metastasis. Increased levels of ADAM12 have been reported in a variety of human cancers. We have previously reported that urinary ADAM12 is predictive of disease status in breast cancer patients and that ADAM12 protein levels in urine increase with progression of disease. On the basis of these findings, the goal of this study was to elucidate the contribution of ADAM12 in breast tumor growth and progression. Overexpression of both the ADAM12-L (transmembrane) and ADAM12-S (secreted) isoforms in human breast tumor cells resulted in a significantly higher rate of tumor take and increased tumor size. Cells expressing the enzymatically inactive form of the secreted isoform, ADAM12-S, had tumor take rates and tumor volumes similar to those of wild-type cells, suggesting that the tumor-promoting activity of ADAM12-S was a function of its proteolytic activity. Of the two isoforms, only the secreted isoform, ADAM12-S, enhanced the ability of tumor cells to migrate and invade in vitro and resulted in a higher incidence of local and distant metastasis in vivo. This stimulatory effect of ADAM12-S on   migration   and invasion was dependent on its catalytic activity. Expression of both ADAM12 isoforms was found to be significantly elevated in human malignant breast tissue. Taken together, our results suggest that ADAM12 overexpression results in increased tumor take, tumor size, and metastasis in vivo. These findings suggest that ADAM12 may represent a potential therapeutic target in breast cancer. 
 TM4SF3 promotes esophageal carcinoma metastasis via upregulating ADAM12m expression. Esophageal cancer is characterized by rapid clinical progression and poor prognosis due to adjacent tissue invasion and distant organs metastasis at a very early stage. TM4SF3 (transmembrane 4 superfamily 3), a member of tetraspanin family, has been reported as a metastasis associated gene in many types of tumors. Herein, we described new properties of TM4SF3 in tumor metastasis, which suggested that this gene might be involved in esophageal carcinoma metastasis. Western blotting revealed that TM4SF3 was overexpressed in 57.1% (8/14) of esophageal carcinomas and esophageal carcinoma cell lines with high-invasive potential. Exogenous expression of TM4SF3 in two low-invasive esophageal carcinoma cell lines, KYSE150 and EC9706, significantly promoted cell   migration   and invasion. Upregulating TM4SF3 expression in EC9706 cells promoted xenograft tumor invading into surrounding tissues, enhanced lung metastasis, and shortened the lifespan of mice (median survival EC9706-TM4SF3 106.5 days versus EC9706-Vector 169.0 days, P < 0.0001) in a spontaneous metastasis model. Further studies demonstrated that ADAM12m was upregulated by TM4SF3 overexpression in vitro and in vivo. Abrogating up-expression of ADAM12m by siRNA significantly suppressed TM4SF3-mediated invasion. Together, these data from our studies indicated that overexpression of TM4SF3 in esophageal cancer conferred advantage to the invasion and metastasis of this destructive disease. Upregulated expression of ADAM12m by TM4SF3 might play a key role in TM4SF3-mediated invasion and metastasis. TM4SF3 and ADAM12m might be potential targets of esophageal carcinoma for anti-metastasis therapy. 
 Cellular roles of ADAM12 in health and disease. ADAM12 belongs to the large family of ADAMs (a disintegrin and metalloproteases) and possesses extracellular metalloprotease and cell-binding functions, as well as intracellular signaling capacities. Interest in ADAM12 has increased recently because its expression is related to tumor progression and it is a potential biomarker for breast cancer. It is therefore important to understand ADAM12's functions. Many cellular roles for ADAM12 have been suggested. It is an active metalloprotease, and has been implicated in insulin-like growth factor (IGF) receptor signaling, through cleavage of IGF-binding proteins, and in epidermal growth factor receptor (EGFR) pathways, via ectodomain shedding of membrane-tethered EGFR ligands. These proteolytic events may regulate diverse cellular responses, such as altered cell differentiation, proliferation,   migration  , and invasion. ADAM12 may also regulate cell-cell and cell-extracellular matrix contacts through interactions with cell surface receptors - integrins and syndecans - potentially influencing the actin cytoskeleton. Moreover, ADAM12 interacts with several cytoplasmic signaling and adaptor molecules through its intracellular domain, thereby directly transmitting signals to or from the cell interior. These ADAM12-mediated cellular effects appear to be critical events in both biological and pathological processes. This review presents current knowledge on ADAM12 functions gained from in vitro and in vivo observations, describes ADAM12's role in both normal physiology and pathology, particularly in cancer, and discusses important areas for future investigation. 
  GeneRIFs  
 increased expression of ADAM12 in chronic wounds impairs wound healing through the inhibition of keratinocyte   migration  . 
  ECM  PubMed  
 Polymorphic variation within the ADAMTS2, ADAMTS14, ADAMTS5, ADAM12 and TIMP2 genes and the risk of Achilles tendon pathology: a genetic association study. Achilles tendon pathology (ATP) is a multifactorial condition for which genetic risk factors have been identified. The ADAMTS, ADAM12 and TIMP2 genes encode enzymes that are important regulators of tendon homeostasis. ADAMTS2 and ADAMTS14 proteins are procollagen N-propeptidases for pro-collagen type I, type II, and type III. ADAMTS2, like COL5A1, has been linked to Ehlers-Danlos syndrome. Variants within ADAMTS5 and ADAM12 have been associated with osteoarthritis. TIMP2, a metalloprotease inhibitor, maintains homeostasis in the   ECM   by inhibiting ADAM, ADAMTS and MMP functions. We sought to determine whether single nucleotide polymorphisms (SNPs) within the ADAMTS2, ADAMTS5, ADAMTS14, ADAM12 and TIMP2 genes were associated with the risk of ATP in two independent populations.213 (115 ATP cases and 98 asymptomatic controls) South African Caucasian participants and 209 (60 ATP cases and 149 asymptomatic controls) Australian Caucasian participants were recruited for this case-control genetic association study.All participants were genotyped using TaqMan technology for the ADAMTS2 rs1054480, ADAMTS5 rs226794, ADAMTS14 rs4747096, ADAM12 rs3740199, and TIMP2 rs4789932 SNPs.We report for the first time a significant (p=0.016) genotypic association between the TIMP2 rs4789932 variant and ATP in a combined Caucasian cohort. We also identify an interaction between the ADAMTS14 rs4747096 variant and age of onset of ATP (p=0.024).Our data show that DNA sequence variation within the TIMP2 gene is a risk factor for ATP in Caucasians. Furthermore, carriage of the ADAMTS14 rs4747096 GG variant appears to delay onset of the injury in the ATP group. 
 Polymorphic variation within the ADAMTS2, ADAMTS14, ADAMTS5, ADAM12 and TIMP2 genes and the risk of Achilles tendon pathology: a genetic association study. Achilles tendon pathology (ATP) is a multifactorial condition for which genetic risk factors have been identified. The ADAMTS, ADAM12 and TIMP2 genes encode enzymes that are important regulators of tendon homeostasis. ADAMTS2 and ADAMTS14 proteins are procollagen N-propeptidases for pro-collagen type I, type II, and type III. ADAMTS2, like COL5A1, has been linked to Ehlers-Danlos syndrome. Variants within ADAMTS5 and ADAM12 have been associated with osteoarthritis. TIMP2, a metalloprotease inhibitor, maintains homeostasis in the   ECM   by inhibiting ADAM, ADAMTS and MMP functions. We sought to determine whether single nucleotide polymorphisms (SNPs) within the ADAMTS2, ADAMTS5, ADAMTS14, ADAM12 and TIMP2 genes were associated with the risk of ATP in two independent populations.213 (115 ATP cases and 98 asymptomatic controls) South African Caucasian participants and 209 (60 ATP cases and 149 asymptomatic controls) Australian Caucasian participants were recruited for this case-control genetic association study.All participants were genotyped using TaqMan technology for the ADAMTS2 rs1054480, ADAMTS5 rs226794, ADAMTS14 rs4747096, ADAM12 rs3740199, and TIMP2 rs4789932 SNPs.We report for the first time a significant (p=0.016) genotypic association between the TIMP2 rs4789932 variant and ATP in a combined Caucasian cohort. We also identify an interaction between the ADAMTS14 rs4747096 variant and age of onset of ATP (p=0.024).Our data show that DNA sequence variation within the TIMP2 gene is a risk factor for ATP in Caucasians. Furthermore, carriage of the ADAMTS14 rs4747096 GG variant appears to delay onset of the injury in the ATP group. 
 EMMPRIN and ADAM12 in prostate cancer: preliminary results of a prospective study. Extracellular metalloproteinase inducer (EMMPRIN) and a disintegrin and metalloproteinase (ADAM12) play a major role in cancer invasion and metastasis owing to the fact that they are directly related to the cell microenvironment and extracellular matrix (  ECM  ) degradation. The aim of this study was to search for an answer to the question "whether the determination of EMMPRIN and ADAM12 values especially in urine may be helpful for the early diagnosis of prostate cancer without employing invasive methods" and also to check whether they may be useful for the determination of the patients with high metastasis risk. Peripheral blood and urine from 66 prostate cancer patients (40 local, 20 locally advanced, 6 metastatic) and 14 healthy controls were evaluated by enzyme-linked immunosorbent assay (ELISA) method. Serum EMMPRIN and ADAM12 values of the patients were seen to be statistically higher than the serum EMMPRIN and ADAM12 values of the healthy controls (p=0.01 and p=0.001, respectively). The urine ADAM12 levels were significantly higher in patients (p=0.013). No significant relationships were found between urine EMMPRIN values of the patients and the healthy controls (p>0.05). Positive correlation between urine EMMPRIN-urine ADAM12 tests was found in total patients group (r=0.683, p=0.001). Our preliminary results revealed that serum EMMPRIN and ADAM12 values and urine ADAM12 values may be useful markers in prostate cancer therapy. Due to the high correlation between these two tests, we are of the opinion that the use of urine ADAM12 in clinic may be sufficient and favorable together with prostate-specific antigen (PSA) for treatment. 
 ADAM-12 as a diagnostic marker for the proliferation, migration and invasion in patients with small cell lung cancer. Small cell lung cancer (SCLC) is highly aggressive and is characterized by malignant metastasis. Approximately 90% of patients die due to extensive metastasis. The extracellular matrix (  ECM  ) is a natural barrier that can prevent cellular invasion and metastasis. Therefore, degradation of the   ECM   must take place in order for extensive metastasis to occur. A disintegrin and metalloprotease (ADAM) is a multi-domain protease that plays an important role in tumorigenesis, as well as tumor development, invasion and metastasis. However, there have been few reports on the expression and role of ADAMs in SCLC. In the current study, the expression and role of ADAMs in SCLC proliferation, invasion and metastasis was investigated. A total of 150 SCLC tissue samples were examined by immunohistochemistry for ADAMs expression. ADAM-12 was found to be abundantly expressed in 72.67% samples and other ADAMs were found to be expressed in 10% to 40% of samples. ADAM-12 levels in serum and urine, from 70 SCLC patients and 40 normal controls, were also measured using ELISA. ADAM-12 expression was significantly higher in SCLC patients than in healthy controls and in patients with extensive disease compared to those with more limited disease. Silencing the expression of ADAM-12 in H1688 cells through the use of specific siRNA significantly reduced cellular proliferation, invasion and metastasis. Supplementing the expression of ADAM-12-L or -S in H345 cells, significantly enhanced cellular proliferation, invasion and metastasis. Animal models with metastatic SCLC also exhibited increased expression of ADAM-12 along with enhanced invasion and metastasis. In brief, ADAM-12 is an independent prognostic factor and diagnostic marker, and is involved in the proliferation, invasion and metastasis of SCLC. 
  [Ee]xtracellular [Mm]atrix  PubMed  
 RGD-independent binding of integrin alpha9beta1 to the ADAM-12 and -15 disintegrin domains mediates cell-cell interaction. ADAMs (a disintegrin and metalloproteases) mediate several important processes (e.g. tumor necrosis factor-alpha release, fertilization, and myoblast fusion). The ADAM disintegrin domains generally lack RGD motifs, and their receptors are virtually unknown. Here we show that integrin alpha(9)beta(1) specifically interacts with the recombinant ADAMs-12 and -15 disintegrin domains in an RGD-independent manner. We also show that interaction between ADAM-12 or -15 and alpha(9)beta(1) supports cell-cell interaction. Interestingly, the cation requirement and integrin activation status required for alpha(9)beta(1)/ADAM-mediated cell adhesion and cell-cell interaction is similar to those required for known integrin-  extracellular matrix   interaction. These results are quite different from recent reports that ADAM-2/alpha(6)beta(1) interaction during sperm/egg fusion requires an integrin activation status distinct from that for   extracellular matrix   interaction. These results suggest that alpha(9)beta(1) may be a major receptor for ADAMs that lack RGD motifs, and that, considering a wide distribution of ADAMs and alpha(9)beta(1), this interaction may be of potential biological and pathological significance. 
 Differential expression of ADAM (a disintegrin and metalloproteinase) genes between human first trimester villous and extravillous trophoblast cells. A disintegrin and metalloproteinases (ADAMs) are members of the metzincin family of zinc-dependent metalloproteinases that play pivotal roles in the proteolytic degradation of the   extracellular matrix   for cell invasion. Few studies have investigated the ADAM subtypes that are expressed in first trimester trophoblast cells. The purpose of this study was to elucidate the differential expression profiles of ADAMs between first trimester villous trophoblast cells (VTs) and extravillous trophoblast cells (EVTs). We isolated EVTs from explanted human first trimester chorionic villi and investigated the mRNA expression levels of five members of the ADAM family (ADAMTS1, ADAMTS2, ADAM10, ADAM12, and ADAM17) using real-time PCR. Chorionic villous tips were defined as first trimester VTs. Of the differentially expressed ADAM genes between first trimester VTs and EVTs, ADAMTS1 was expressed at a significantly higher level in EVTs than in VTs. In contrast, both ADAM10 and ADAM12 were expressed at significantly higher levels in VTs than in EVTs. No differences were found in the mRNA levels of ADAMTS2 and ADAM17 between the two cell types. Moreover, we demonstrated that in VTs, the expression level of ADAM12 was significantly downregulated in the late first trimester (10-13 gestational weeks) compared to the middle first trimester (7-8 weeks). These results suggest that first trimester trophoblast cells express ADAM genes in cell type- and gestational age-dependent manners. Our data provide additional insight into the functions of ADAMs in the human placenta. 
 Differential expression of ADAM (a disintegrin and metalloproteinase) genes between human first trimester villous and extravillous trophoblast cells. A disintegrin and metalloproteinases (ADAMs) are members of the metzincin family of zinc-dependent metalloproteinases that play pivotal roles in the proteolytic degradation of the   extracellular matrix   for cell invasion. Few studies have investigated the ADAM subtypes that are expressed in first trimester trophoblast cells. The purpose of this study was to elucidate the differential expression profiles of ADAMs between first trimester villous trophoblast cells (VTs) and extravillous trophoblast cells (EVTs). We isolated EVTs from explanted human first trimester chorionic villi and investigated the mRNA expression levels of five members of the ADAM family (ADAMTS1, ADAMTS2, ADAM10, ADAM12, and ADAM17) using real-time PCR. Chorionic villous tips were defined as first trimester VTs. Of the differentially expressed ADAM genes between first trimester VTs and EVTs, ADAMTS1 was expressed at a significantly higher level in EVTs than in VTs. In contrast, both ADAM10 and ADAM12 were expressed at significantly higher levels in VTs than in EVTs. No differences were found in the mRNA levels of ADAMTS2 and ADAM17 between the two cell types. Moreover, we demonstrated that in VTs, the expression level of ADAM12 was significantly downregulated in the late first trimester (10-13 gestational weeks) compared to the middle first trimester (7-8 weeks). These results suggest that first trimester trophoblast cells express ADAM genes in cell type- and gestational age-dependent manners. Our data provide additional insight into the functions of ADAMs in the human placenta. 
 ADAM12 and alpha9beta1 integrin are instrumental in human myogenic cell differentiation. Knowledge on molecular systems involved in myogenic precursor cell (mpc) fusion into myotubes is fragmentary. Previous studies have implicated the a disintegrin and metalloproteinase (ADAM) family in most mammalian cell fusion processes. ADAM12 is likely involved in fusion of murine mpc and human rhabdomyosarcoma cells, but it requires yet unknown molecular partners to launch myogenic cell fusion. ADAM12 was shown able to mediate cell-to-cell attachment through binding alpha9beta1 integrin. We report that normal human mpc express both ADAM12 and alpha9beta1 integrin during their differentiation. Expression of alpha9 parallels that of ADAM12 and culminates at time of fusion. alpha9 and ADAM12 coimmunoprecipitate and participate to mpc adhesion. Inhibition of ADAM12/alpha9beta1 integrin interplay, by either ADAM12 antisense oligonucleotides or blocking antibody to alpha9beta1, inhibited overall mpc fusion by 47-48%, with combination of both strategies increasing inhibition up to 62%. By contrast with blockade of vascular cell adhesion molecule-1/alpha4beta1, which also reduced fusion, exposure to ADAM12 antisense oligonucleotides or anti-alpha9beta1 antibody did not induce detachment of mpc from   extracellular matrix  , suggesting specific involvement of ADAM12-alpha9beta1 interaction in the fusion process. Evaluation of the fusion rate with regard to the size of myotubes showed that both ADAM12 antisense oligonucleotides and alpha9beta1 blockade inhibited more importantly formation of large (> or =5 nuclei) myotubes than that of small (2-4 nuclei) myotubes. We conclude that both ADAM12 and alpha9beta1 integrin are expressed during postnatal human myogenic differentiation and that their interaction is mainly operative in nascent myotube growth. 
 Lysophosphatidic acid-induced ADAM12 expression mediates human adipose tissue-derived mesenchymal stem cell-stimulated tumor growth. Lysophosphatidic acid (LPA) is involved in mesenchymal stem cell-stimulated tumor growth in vivo. However, the molecular mechanism by which mesenchymal stem cells promote tumorigenesis remains elusive. In the present study, we demonstrate that conditioned medium from A549 human lung adenocarcinoma cells (A549 CM) induced the expression of ADAM12, a disintegrin and metalloproteases family member, in human adipose tissue-derived mesenchymal stem cells (hASCs). A549 CM-stimulated ADAM12 expression was abrogated by pretreatment of hASCs with the LPA receptor 1 inhibitor Ki16425 or by small interfering RNA-mediated silencing of LPA receptor 1, suggesting a key role for the LPA-LPA receptor 1 signaling axis in A549 CM-stimulated ADAM12 expression. Silencing of ADAM12 expression using small interfering RNA or short hairpin RNA abrogated LPA-induced expression of both α-smooth muscle actin, a marker of carcinoma-associated fibroblasts, and ADAM12 in hASCs. Using a xenograft transplantation model of A549 cells, we demonstrated that silencing of ADAM12 inhibited the hASC-stimulated in vivo growth of A549 xenograft tumors and the differentiation of transplanted hASCs to α-smooth muscle actin-positive carcinoma-associated fibroblasts. LPA-conditioned medium from hASCs induced the adhesion of A549 cells and silencing of ADAM12 inhibited LPA-induced expression o  f extracellular matr  ix proteins, periostin and βig-h3, in hASCs and LPA-conditioned medium-stimulated adhesion of A549 cells. These results suggest a pivotal role for LPA-stimulated ADAM12 expression in tumor growth and the differentiation of hASCs to carcinoma-associated fibroblasts expressing α-smooth muscle actin, periostin, and βig-h3. 
 Extracellular engagement of ADAM12 induces clusters of invadopodia with localized ectodomain shedding activity. Invadopodia are dynamic actin structures at the cell surface that degrade   extracellular matrix   and act as sites of signal transduction. The biogenesis of invadopodia, including the mechanisms regulating their formation, composition, and turnover is not entirely understood. Here, we demonstrate that antibody ligation of ADAM12, a transmembrane disintegrin and metalloprotease, resulted in the rapid accumulation of invadopodia with   extracellular matrix  -degrading capacity in epithelial cells expressing the αvβ3 integrin and active c-Src kinase. The induction of invadopodia clusters required an intact c-Src interaction site in the ADAM12 cytoplasmic domain, but was independent of the catalytic activity of ADAM12. Caveolin-1 and transmembrane protease MMP14/MT1-MMP were both present in the ADAM12-induced clusters of invadopodia, and cholesterol depletion prevented their formation, suggesting that lipid-raft microdomains are involved in the process. Importantly, our data demonstrate that ADAM12-mediated ectodomain shedding of epidermal growth factor receptor ligands can occur within these invadopodia. Such localized growth factor signalling offers an interesting novel biological concept highly relevant to the properties of carcinoma cells, which often show upregulated ADAM12 and β3 integrin expression, together with high levels of c-Src kinase activity. 
 EMMPRIN and ADAM12 in prostate cancer: preliminary results of a prospective study. Extracellular metalloproteinase inducer (EMMPRIN) and a disintegrin and metalloproteinase (ADAM12) play a major role in cancer invasion and metastasis owing to the fact that they are directly related to the cell microenvironment and   extracellular matrix   (ECM) degradation. The aim of this study was to search for an answer to the question "whether the determination of EMMPRIN and ADAM12 values especially in urine may be helpful for the early diagnosis of prostate cancer without employing invasive methods" and also to check whether they may be useful for the determination of the patients with high metastasis risk. Peripheral blood and urine from 66 prostate cancer patients (40 local, 20 locally advanced, 6 metastatic) and 14 healthy controls were evaluated by enzyme-linked immunosorbent assay (ELISA) method. Serum EMMPRIN and ADAM12 values of the patients were seen to be statistically higher than the serum EMMPRIN and ADAM12 values of the healthy controls (p=0.01 and p=0.001, respectively). The urine ADAM12 levels were significantly higher in patients (p=0.013). No significant relationships were found between urine EMMPRIN values of the patients and the healthy controls (p>0.05). Positive correlation between urine EMMPRIN-urine ADAM12 tests was found in total patients group (r=0.683, p=0.001). Our preliminary results revealed that serum EMMPRIN and ADAM12 values and urine ADAM12 values may be useful markers in prostate cancer therapy. Due to the high correlation between these two tests, we are of the opinion that the use of urine ADAM12 in clinic may be sufficient and favorable together with prostate-specific antigen (PSA) for treatment. 
 A disintegrin and metalloproteinase 12 (ADAM12) localizes to invasive trophoblast, promotes cell invasion and directs column outgrowth in early placental development. During pregnancy, stromal- and vascular-remodeling trophoblasts serve critical roles in directing placental development acquiring pro-invasive characteristics. The A Disintegrin and Metalloproteinase (ADAM) family of multifunctional proteins direct cellular processes across multiple organ systems via their intrinsic catalytic, cell adhesive and intracellular signaling properties. ADAM12, existing as two distinct splice variants (ADAM12L and ADAM12S), is highly expressed in the human placenta and promotes cell migration and invasion in several tumor cell lines; however, its role in trophoblast biology is unknown. In this study, ADAM12 was localized to anchoring trophoblast columns in first trimester placentas and to highly invasive   extracellular matrix  -degrading trophoblasts in placental villous explants. The importance of ADAM12 in directing trophoblast invasion was tested using loss-of and gain-of-function strategies, where siRNA-directed knockdown of ADAM12 inhibited trophoblast cell invasion while over-expression promoted migration and invasion in two trophoblastic cell models. In placental villous explant cultures, siRNA-directed loss of ADAM12 significantly dampened trophoblast column outgrowth. Additionally, we provide functional evidence for the ADAM12S variant in promoting trophoblast invasion and column outgrowth through a mechanism requiring its catalytic activity. This is the first study to assign a function for ADAM12 in trophoblast biology, where ADAM12 may play a central role regulating the behavior of invasive trophoblast subsets in early pregnancy. This study also underlines the importance of ADAM12L and ADAM12S in directing cell motility in normal developmental processes outside of cancer, specifically highlighting a potentially important function of ADAM12S in directing early placental development. 
 ADAM 12 cleaves   extracellular matrix   proteins and correlates with cancer status and stage. ADAM 12 is a member of a family of disintegrin-containing metalloproteases that have been implicated in a variety of diseases including Alzheimer's disease, arthritis, and cancer. We purified ADAM 12 from the urine of breast cancer patients via Q-Sepharose anion exchange and gelatin-Sepharose affinity chromatography followed by protein identification by matrix-assisted laser desorption/ionization-time of flight mass spectrometry. Four peptides were identified that spanned the amino acid sequence of ADAM 12. Immunoblot analysis using ADAM 12-specific antibodies detected an approximately 68-kDa band identified as the mature form of ADAM 12. To characterize catalytic properties of ADAM 12, full-length ADAM 12-S was expressed in COS-7 cells and purified. Substrate specificity studies demonstrated that ADAM 12-S degrades gelatin, type IV collagen, and fibronectin but not type I collagen or casein. Gelatinase activity of ADAM 12 was completely abrogated by zinc chelators 1,10-phenanthroline and EDTA and was partially inhibited by the hydroxamate inhibitor Marimastat. Endogenous matrix metalloprotease inhibitor TIMP-3 inhibited activity. To validate our initial identification of this enzyme in human urine, 117 urine samples from breast cancer patients and controls were analyzed by immunoblot. The majority of samples from cancer patients were positive for ADAM 12 (67 of 71, sensitivity 0.94) compared with urine from controls in which ADAM 12 was detected with significantly lower frequency. Densitometric analyses of immunoblots demonstrated that ADAM 12 protein levels were higher in urine from breast cancer patients than in control urine. In addition, median levels of ADAM 12 in urine significantly increased with disease progression. These data demonstrate for the first time that ADAM 12 is a gelatinase, that it can be detected in breast cancer patient urine, and that increased urinary levels of this protein correlate with breast cancer progression. They further support the possibility that detection of urinary ADAM 12 may prove useful in the development of noninvasive diagnostic and prognostic tests for breast and perhaps other cancers. 
 ADAM-12 as a diagnostic marker for the proliferation, migration and invasion in patients with small cell lung cancer. Small cell lung cancer (SCLC) is highly aggressive and is characterized by malignant metastasis. Approximately 90% of patients die due to extensive metastasis. The   extracellular matrix   (ECM) is a natural barrier that can prevent cellular invasion and metastasis. Therefore, degradation of the ECM must take place in order for extensive metastasis to occur. A disintegrin and metalloprotease (ADAM) is a multi-domain protease that plays an important role in tumorigenesis, as well as tumor development, invasion and metastasis. However, there have been few reports on the expression and role of ADAMs in SCLC. In the current study, the expression and role of ADAMs in SCLC proliferation, invasion and metastasis was investigated. A total of 150 SCLC tissue samples were examined by immunohistochemistry for ADAMs expression. ADAM-12 was found to be abundantly expressed in 72.67% samples and other ADAMs were found to be expressed in 10% to 40% of samples. ADAM-12 levels in serum and urine, from 70 SCLC patients and 40 normal controls, were also measured using ELISA. ADAM-12 expression was significantly higher in SCLC patients than in healthy controls and in patients with extensive disease compared to those with more limited disease. Silencing the expression of ADAM-12 in H1688 cells through the use of specific siRNA significantly reduced cellular proliferation, invasion and metastasis. Supplementing the expression of ADAM-12-L or -S in H345 cells, significantly enhanced cellular proliferation, invasion and metastasis. Animal models with metastatic SCLC also exhibited increased expression of ADAM-12 along with enhanced invasion and metastasis. In brief, ADAM-12 is an independent prognostic factor and diagnostic marker, and is involved in the proliferation, invasion and metastasis of SCLC. 
 ADAM12 induces actin cytoskeleton and   extracellular matrix   reorganization during early adipocyte differentiation by regulating beta1 integrin function. Changes in cell shape are a morphological hallmark of differentiation. In this study we report that the expression of ADAM12, a disintegrin and metalloprotease, dramatically affects cell morphology in preadipocytes, changing them from a flattened, fibroblastic appearance to a more rounded shape. We showed that the highest levels of ADAM12 mRNA were detected in preadipocytes at the critical stage when preadipocytes become permissive for adipogenic differentiation. Furthermore, as assessed by immunostaining, ADAM12 was transiently expressed at the cell surface concomitant with the reduced activity of beta1 integrin. Co-immunoprecipitation studies indicated the formation of ADAM12/beta1 integrin complexes in these preadipocytes. Overexpression of ADAM12 at the cell surface of 3T3-L1 preadipocytes achieved by transient transfection or retroviral transduction led to the disappearance of the extensive network of actin stress fibers that are characteristic of these cells, and its reorganization into a cortical network located beneath the cell membrane. The cells became more rounded, exhibited fewer vinculin-positive focal adhesions, and adhered less efficiently to fibronectin in attachment assays. Moreover, ADAM12-expressing cells were more prone to apoptosis, which could be prevented by treating the cells with beta1-activating antibodies. A reduced and re-organized fibronectin-rich   extracellular matrix   accompanied these changes. In addition, beta1 integrin was more readily extracted with Triton X-100 from cells overexpressing ADAM12 than from control cells. Collectively, these results show that surface expression of ADAM12 impairs the function of beta1 integrins and, consequently, alters the organization of the actin cytoskeleton and   extracellular matrix  . These events may be necessary for early adipocyte differentiation. 
 ADAM12-S stimulates bone growth in transgenic mice by modulating chondrocyte proliferation and maturation. ADAM12-S transgenic mice exhibit a pronounced increase in the length of bones, such as femur, tibia, and vertebrae. The effect of ADAM12-S on longitudinal bone growth involves the modulation of chondrocyte proliferation and maturation, likely through proteolytic activities and altered cell-  extracellular matrix   interactions in the growth plate.The disintegrin and metalloprotease ADAM12 is expressed in both osteoblasts and osteoclasts, suggesting a regulatory role of ADAM12 in bone. However, thus far, no in vivo function of ADAM12 in the skeleton has been reported.Transgenic mice expressing the secreted form of human ADAM12, ADAM12-S, or a truncated metalloprotease-deficient form of ADAM12-S in the circulation were used to study the effects of ADAM12 on the skeleton. In addition, murine chondrocyte cultures were used to study the effect of ADAM12-S on cell-  extracellular matrix   interactions.ADAM12-S transgenic mice exhibit increased longitudinal bone growth. The increased bone length is progressive and age dependent, with a maximum increase of 17% seen in the femur from 6-month-old transgenic mice. The effect is gene dose dependent, being more pronounced in mice expressing higher levels of the transgene than in a lower-expressing line. Histological analysis revealed no alterations in the growth plate organization, but mean growth plate width was increased. Both the cellular incorporation of bromodeoxyuridine and the width of the collagen type X-positive hypertrophic zone were increased in the growth plate of ADAM12-S transgenic mice. Importantly, mice expressing a truncated form of ADAM12-S that lacked the pro- and metalloprotease domains showed no alterations in bone length, suggesting that protease activity is required for the ADAM12-S effect. In vitro studies showed that ADAM12-S inhibits chondrocyte adhesion to fibronectin and collagen type II.ADAM12-S stimulates bone growth in mice by modulating chondrocyte proliferation and maturation through mechanisms probably involving both metalloprotease and adhesion activities. 
 Cellular roles of ADAM12 in health and disease. ADAM12 belongs to the large family of ADAMs (a disintegrin and metalloproteases) and possesses extracellular metalloprotease and cell-binding functions, as well as intracellular signaling capacities. Interest in ADAM12 has increased recently because its expression is related to tumor progression and it is a potential biomarker for breast cancer. It is therefore important to understand ADAM12's functions. Many cellular roles for ADAM12 have been suggested. It is an active metalloprotease, and has been implicated in insulin-like growth factor (IGF) receptor signaling, through cleavage of IGF-binding proteins, and in epidermal growth factor receptor (EGFR) pathways, via ectodomain shedding of membrane-tethered EGFR ligands. These proteolytic events may regulate diverse cellular responses, such as altered cell differentiation, proliferation, migration, and invasion. ADAM12 may also regulate cell-cell and cell-  extracellular matrix   contacts through interactions with cell surface receptors - integrins and syndecans - potentially influencing the actin cytoskeleton. Moreover, ADAM12 interacts with several cytoplasmic signaling and adaptor molecules through its intracellular domain, thereby directly transmitting signals to or from the cell interior. These ADAM12-mediated cellular effects appear to be critical events in both biological and pathological processes. This review presents current knowledge on ADAM12 functions gained from in vitro and in vivo observations, describes ADAM12's role in both normal physiology and pathology, particularly in cancer, and discusses important areas for future investigation. 
  GeneRIFs  
 surface expression of ADAM12 alters the organization of the actin cytoskeleton and   extracellular matrix   
  [Ii]nvasive  PubMed  
 The disintegrin-metalloproteinases ADAM 10, 12 and 17 are upregulated in invading peripheral tumor cells of basal cell carcinomas. Members of the a disintegrin and metalloproteinase (ADAM) family are expressed in malignant tumors and participate in the pathogenesis of cancer. However, the presence of ADAM 10, 12, 17 and their role in basal cell carcinoma (BCC) have not been described. The purpose of this study was to investigate expression of ADAM 10, 12 and 17 in BCC.Expression of ADAM 10, 12 and 17 was analyzed by immunohistochemistry in skin tissues obtained from 25 patients with different types of BCC.Immunoreactivity of ADAM 10, 12 and 17 was increased at the peripheral tumor margin compared with central areas of BCC tumor cell nests. Immunoreactivity of ADAM 10 and 12 was increased in the deep margin of invading tumor cell nests in mixed BCC. Focally increased expression of ADAM 12 was detected in squamous differentiated tumor cells of nodular BCC. In addition, immunoreactivity of ADAM 17 was increased in superficial BCC.ADAM 10, 12 and 17 showed different expression pattern in BCC histologic subtypes, indicating their different role in the BCC pathogenesis. Overexpression of ADAM 10, 12 and 17 immunoreactivity in deep invasion area of BCC indicates that these three proteases may play an important role in the locally   invasive   and highly destructive growth behavior of BCC. Additionally, we suggest that ADAM 17 may play an important role in early development of BCC. 
 The disintegrin-metalloproteinases ADAM 10, 12 and 17 are upregulated in invading peripheral tumor cells of basal cell carcinomas. Members of the a disintegrin and metalloproteinase (ADAM) family are expressed in malignant tumors and participate in the pathogenesis of cancer. However, the presence of ADAM 10, 12, 17 and their role in basal cell carcinoma (BCC) have not been described. The purpose of this study was to investigate expression of ADAM 10, 12 and 17 in BCC.Expression of ADAM 10, 12 and 17 was analyzed by immunohistochemistry in skin tissues obtained from 25 patients with different types of BCC.Immunoreactivity of ADAM 10, 12 and 17 was increased at the peripheral tumor margin compared with central areas of BCC tumor cell nests. Immunoreactivity of ADAM 10 and 12 was increased in the deep margin of invading tumor cell nests in mixed BCC. Focally increased expression of ADAM 12 was detected in squamous differentiated tumor cells of nodular BCC. In addition, immunoreactivity of ADAM 17 was increased in superficial BCC.ADAM 10, 12 and 17 showed different expression pattern in BCC histologic subtypes, indicating their different role in the BCC pathogenesis. Overexpression of ADAM 10, 12 and 17 immunoreactivity in deep invasion area of BCC indicates that these three proteases may play an important role in the locally   invasive   and highly destructive growth behavior of BCC. Additionally, we suggest that ADAM 17 may play an important role in early development of BCC. 
 Urinary concentrations of ADAM 12 from breast cancer patients pre- and post-surgery vs. cancer-free controls: a clinical study for biomarker validation. The ADAMs (A Disintegrin and Metalloproteinases) are a family of multi-domain, zinc-dependent metalloproteinase enzymes. ADAM 12 has been previously associated with the onset and progression of breast cancer, and elevated levels of ADAM 12 have been previously found in the urine of breast cancer patients. Aims of the current study are: 1) establish the viability of urinary ADAM 12 as a diagnostic marker for breast cancer, and 2) explore the effects of surgical tumor removal on the levels of urinary ADAM 12.A total of 96 patients have been recruited for this study, including 50 patients diagnosed with cancer, and 46 age-matched controls. Commercially available ELISA kits for ADAM 12 were used to quantify the presence and concentration of this enzyme in the urine from cancer patients with ductal carcinoma in situ (DCIS) and   invasive   breast cancer (IBC) both prior to any treatment and approximately two weeks following surgery, as well as from controls.We find no statistically significant differences between the concentrations of ADAM 12 in the urine of breast cancer patients prior to treatment and that of their age-matched controls; however the concentration of ADAM 12, both alone and as a function of urine total protein, are significantly elevated following surgery (p < 0.0001). Patients who underwent a mastectomy have significantly higher urinary ADAM 12 concentrations than those who underwent a lumpectomy (significant at p = 0.0271).These findings suggest that urinary ADAM 12 may not correlate directly with the status and stage of breast cancer as previously thought; rather these increases may be a result of tissue injury and inflammation from biopsy and surgical resection. Results of this study may suggest a need for biomarkers to be evaluated carefully in the context of tissue damage. 
 Notch increases the shedding of HB-EGF by ADAM12 to potentiate invadopodia formation in hypoxia. Notch regulates cell-cell contact-dependent signaling and is activated by hypoxia, a microenvironmental condition that promotes cellular invasion during both normal physiology and disease. The mechanisms by which hypoxia and Notch regulate cellular invasion are not fully elucidated. In this paper, we show that, in cancer cells, hypoxia increased the levels and activity of the ADAM12 metalloprotease in a Notch signaling-dependent manner, leading to increased ectodomain shedding of the epidermal growth factor (EGF) receptor (EGFR) ligand heparin-binding EGF-like growth factor. Released HB-EGF induced the formation of invadopodia, cellular structures that aid cancer cell invasion. Thus, we describe a signaling pathway that couples cell contact-dependent signaling with the paracrine activation of the EGFR, indicating cross talk between the Notch and EGFR pathways in promoting cancer cell invasion. This signaling pathway might regulate the coordinated acquisition of   invasive  ness by neighboring cells and mediate the communication between normoxic and hypoxic areas of tumors to facilitate cancer cell invasion. 
 Urinary ADAM12 and MMP-9/NGAL complex detect the presence of gastric cancer. Although the early diagnosis of gastric cancer provides the opportunity for curative endoscopic resection, comprehensive screening endoscopy would be   invasive   and expensive. To date, there is a complete absence of clinically useful gastric cancer biomarkers. With the goal of discovering non  invasive   biomarkers for the early diagnosis of gastric cancer, we have conducted a case-control study using urine samples from individuals with gastric cancer versus healthy control samples. Of the enrolled 106 patients from September, 2012 to April, 2013, a cohort of 70 patients composed of 35 patients with gastric cancer and 35 age- and sex-matched healthy controls was analyzed. The gastric cancer group was composed of stage IA of 62.9% (22/35). The urinary levels of MMP-9/NGAL complex (uMMP-9/NGAL) and ADAM12 (uADAM12) were significantly higher in the gastric cancer group compared with the healthy control group as determined by monospecific ELISAs (uMMP-9/NGAL: median, 85 pg/mL vs. 0 pg/mL; P = 0.020; uADAM12: median, 3.35 ng/mL vs. 1.44 ng/mL; P < 0.001). Multivariate analysis demonstrated that both uMMP-9/NGAL and uADAM12 were significant, independent diagnostic biomarkers for gastric cancer. Moreover, MMP-9/NGAL activity was significantly elevated as determined by gelatin zymography. The combination of uMMP-9/NGAL with uADAM12 distinguished between control samples and gastric cancer samples with an AUC of 0.825 (P < 0.001) in an ROC analysis. Significantly, immunohistochemical analyses demonstrated a high coexpression of MMP-9 and NGAL (P < 0.001) and high expression of ADAM12 (P < 0.001) in gastric cancer tissues compared with adjacent normal tissues (N = 35). In summary, uMMP-9/NGAL and uADAM12 are potential non  invasive   biomarkers for gastric cancer, including early-stage disease. 
 Extravillous trophoblast-associated ADAM12 exerts pro-  invasive   properties, including induction of integrin beta 1-mediated cellular spreading. ADAM12, consisting of a membrane-bound (ADAM12L) and a secreted (ADAM12S) form, is expressed exclusively in regenerating and developing tissue as well as in certain cancer types. Strong ADAM12 expression levels have been noticed in the human placenta, and deregulated ADAM12S levels were associated with various pregnancy-related disorders including pre-eclampsia and intrauterine growth restriction. However, the role of ADAM12 in trophoblast motility has not been investigated so far. Hence, the present study aimed to investigate the specific function of the protease by using different primary trophoblast cell models. Immunofluorescence and Western blot analyses of first trimester placental tissue and differentiating primary first trimester cytotrophoblasts (CTBs) indicated strong upregulation of both of the ADAM12 isoforms during extravillous trophoblast differentiation. Functional assays involving short interfering RNA (siRNA)-mediated knockdown studies in primary CTBs and first trimester explant cultures revealed a significant repression of trophoblast motility upon partial loss of ADAM12. Conversely, isoform-specific overexpression in the ADAM12-negative trophoblast cell line SGHPL-5 enhanced the   invasive   capacity of these cells. We further confirmed proteolytic activity of trophoblast-derived ADAM12S by demonstrating its potential to degrade insulin-like growth factor-binding protein 3. Finally, we suggest that ADAM12S exerts its pro-migratory function in trophoblasts by inducing integrin beta 1-mediated cellular spreading. 
 EMMPRIN and ADAM12 in prostate cancer: preliminary results of a prospective study. Extracellular metalloproteinase inducer (EMMPRIN) and a disintegrin and metalloproteinase (ADAM12) play a major role in cancer invasion and metastasis owing to the fact that they are directly related to the cell microenvironment and extracellular matrix (ECM) degradation. The aim of this study was to search for an answer to the question "whether the determination of EMMPRIN and ADAM12 values especially in urine may be helpful for the early diagnosis of prostate cancer without employing   invasive   methods" and also to check whether they may be useful for the determination of the patients with high metastasis risk. Peripheral blood and urine from 66 prostate cancer patients (40 local, 20 locally advanced, 6 metastatic) and 14 healthy controls were evaluated by enzyme-linked immunosorbent assay (ELISA) method. Serum EMMPRIN and ADAM12 values of the patients were seen to be statistically higher than the serum EMMPRIN and ADAM12 values of the healthy controls (p=0.01 and p=0.001, respectively). The urine ADAM12 levels were significantly higher in patients (p=0.013). No significant relationships were found between urine EMMPRIN values of the patients and the healthy controls (p>0.05). Positive correlation between urine EMMPRIN-urine ADAM12 tests was found in total patients group (r=0.683, p=0.001). Our preliminary results revealed that serum EMMPRIN and ADAM12 values and urine ADAM12 values may be useful markers in prostate cancer therapy. Due to the high correlation between these two tests, we are of the opinion that the use of urine ADAM12 in clinic may be sufficient and favorable together with prostate-specific antigen (PSA) for treatment. 
 A disintegrin and metalloproteinase 12 (ADAM12) localizes to   invasive   trophoblast, promotes cell invasion and directs column outgrowth in early placental development. During pregnancy, stromal- and vascular-remodeling trophoblasts serve critical roles in directing placental development acquiring pro-  invasive   characteristics. The A Disintegrin and Metalloproteinase (ADAM) family of multifunctional proteins direct cellular processes across multiple organ systems via their intrinsic catalytic, cell adhesive and intracellular signaling properties. ADAM12, existing as two distinct splice variants (ADAM12L and ADAM12S), is highly expressed in the human placenta and promotes cell migration and invasion in several tumor cell lines; however, its role in trophoblast biology is unknown. In this study, ADAM12 was localized to anchoring trophoblast columns in first trimester placentas and to highly   invasive   extracellular matrix-degrading trophoblasts in placental villous explants. The importance of ADAM12 in directing trophoblast invasion was tested using loss-of and gain-of-function strategies, where siRNA-directed knockdown of ADAM12 inhibited trophoblast cell invasion while over-expression promoted migration and invasion in two trophoblastic cell models. In placental villous explant cultures, siRNA-directed loss of ADAM12 significantly dampened trophoblast column outgrowth. Additionally, we provide functional evidence for the ADAM12S variant in promoting trophoblast invasion and column outgrowth through a mechanism requiring its catalytic activity. This is the first study to assign a function for ADAM12 in trophoblast biology, where ADAM12 may play a central role regulating the behavior of   invasive   trophoblast subsets in early pregnancy. This study also underlines the importance of ADAM12L and ADAM12S in directing cell motility in normal developmental processes outside of cancer, specifically highlighting a potentially important function of ADAM12S in directing early placental development. 
 ADAM 12 cleaves extracellular matrix proteins and correlates with cancer status and stage. ADAM 12 is a member of a family of disintegrin-containing metalloproteases that have been implicated in a variety of diseases including Alzheimer's disease, arthritis, and cancer. We purified ADAM 12 from the urine of breast cancer patients via Q-Sepharose anion exchange and gelatin-Sepharose affinity chromatography followed by protein identification by matrix-assisted laser desorption/ionization-time of flight mass spectrometry. Four peptides were identified that spanned the amino acid sequence of ADAM 12. Immunoblot analysis using ADAM 12-specific antibodies detected an approximately 68-kDa band identified as the mature form of ADAM 12. To characterize catalytic properties of ADAM 12, full-length ADAM 12-S was expressed in COS-7 cells and purified. Substrate specificity studies demonstrated that ADAM 12-S degrades gelatin, type IV collagen, and fibronectin but not type I collagen or casein. Gelatinase activity of ADAM 12 was completely abrogated by zinc chelators 1,10-phenanthroline and EDTA and was partially inhibited by the hydroxamate inhibitor Marimastat. Endogenous matrix metalloprotease inhibitor TIMP-3 inhibited activity. To validate our initial identification of this enzyme in human urine, 117 urine samples from breast cancer patients and controls were analyzed by immunoblot. The majority of samples from cancer patients were positive for ADAM 12 (67 of 71, sensitivity 0.94) compared with urine from controls in which ADAM 12 was detected with significantly lower frequency. Densitometric analyses of immunoblots demonstrated that ADAM 12 protein levels were higher in urine from breast cancer patients than in control urine. In addition, median levels of ADAM 12 in urine significantly increased with disease progression. These data demonstrate for the first time that ADAM 12 is a gelatinase, that it can be detected in breast cancer patient urine, and that increased urinary levels of this protein correlate with breast cancer progression. They further support the possibility that detection of urinary ADAM 12 may prove useful in the development of non  invasive   diagnostic and prognostic tests for breast and perhaps other cancers. 
 A positive feedback loop between HER2 and ADAM12 in human head and neck cancer cells increases migration and invasion. Increased activation of epidermal growth factor receptor (EGFR) family members such as HER2/Erbb2 can result in more aggressive disease, resistance to chemotherapy and reduced survival of head and neck squamous cell carcinoma (HNSCC) patients. In order to identify mechanisms through which these receptor tyrosine kinases accelerate tumor progression, the regulation of metalloprotease expression by EGFR family members was investigated in 11 squamous cell carcinoma (SCC) cell lines. HER2 expression was significantly correlated with ADAM12 (A Disintegrin And Metalloprotease 12) expression in these cell lines and was co-expressed in human head and neck cancers. Inhibition of HER2 or EGFR decreased ADAM12 transcripts whereas HER2 transfection upregulated ADAM12 expression. To understand the molecular mechanisms underlying HER2 regulation of ADAM12, we investigated the signaling pathways directing ADAM12 production in SCC cells. Inhibition of phosphatidyl inositol-3-kinase or mammalian target of rapamycin decreased ADAM12 transcripts in HER2-expressing SCC cells, whereas transfection with AKT increased ADAM12 mRNA. Experiments utilizing ADAM12 transfection or siRNA targeting of ADAM12 revealed that the protease increased both the migration and   invasive  ness of oral SCC cells. Surprisingly, ADAM12 also increased HER2 message, protein levels and activity through an Ets1-dependent mechanism. Collectively, these results reveal a novel positive activation loop between ADAM12 and HER2 that may contribute to HNSCC progression. 
 TM4SF3 promotes esophageal carcinoma metastasis via upregulating ADAM12m expression. Esophageal cancer is characterized by rapid clinical progression and poor prognosis due to adjacent tissue invasion and distant organs metastasis at a very early stage. TM4SF3 (transmembrane 4 superfamily 3), a member of tetraspanin family, has been reported as a metastasis associated gene in many types of tumors. Herein, we described new properties of TM4SF3 in tumor metastasis, which suggested that this gene might be involved in esophageal carcinoma metastasis. Western blotting revealed that TM4SF3 was overexpressed in 57.1% (8/14) of esophageal carcinomas and esophageal carcinoma cell lines with high-  invasive   potential. Exogenous expression of TM4SF3 in two low-  invasive   esophageal carcinoma cell lines, KYSE150 and EC9706, significantly promoted cell migration and invasion. Upregulating TM4SF3 expression in EC9706 cells promoted xenograft tumor invading into surrounding tissues, enhanced lung metastasis, and shortened the lifespan of mice (median survival EC9706-TM4SF3 106.5 days versus EC9706-Vector 169.0 days, P < 0.0001) in a spontaneous metastasis model. Further studies demonstrated that ADAM12m was upregulated by TM4SF3 overexpression in vitro and in vivo. Abrogating up-expression of ADAM12m by siRNA significantly suppressed TM4SF3-mediated invasion. Together, these data from our studies indicated that overexpression of TM4SF3 in esophageal cancer conferred advantage to the invasion and metastasis of this destructive disease. Upregulated expression of ADAM12m by TM4SF3 might play a key role in TM4SF3-mediated invasion and metastasis. TM4SF3 and ADAM12m might be potential targets of esophageal carcinoma for anti-metastasis therapy. 
  GeneRIFs  
 ADAM12 localizes to   invasive   trophoblast, promotes cell invasion and directs column outgrowth in early placental development. 
 Overexpression of ADAM 10, 12 and 17 immunoreactivity in deep invasion area of BCC indicates that these three proteases may play an important role in the locally   invasive   and highly destructive growth behavior of basal cell carcinoma 
  [Mm]etastasis  PubMed  
 EMMPRIN and ADAM12 in prostate cancer: preliminary results of a prospective study. Extracellular metalloproteinase inducer (EMMPRIN) and a disintegrin and metalloproteinase (ADAM12) play a major role in cancer invasion and   metastasis   owing to the fact that they are directly related to the cell microenvironment and extracellular matrix (ECM) degradation. The aim of this study was to search for an answer to the question "whether the determination of EMMPRIN and ADAM12 values especially in urine may be helpful for the early diagnosis of prostate cancer without employing invasive methods" and also to check whether they may be useful for the determination of the patients with high   metastasis   risk. Peripheral blood and urine from 66 prostate cancer patients (40 local, 20 locally advanced, 6 metastatic) and 14 healthy controls were evaluated by enzyme-linked immunosorbent assay (ELISA) method. Serum EMMPRIN and ADAM12 values of the patients were seen to be statistically higher than the serum EMMPRIN and ADAM12 values of the healthy controls (p=0.01 and p=0.001, respectively). The urine ADAM12 levels were significantly higher in patients (p=0.013). No significant relationships were found between urine EMMPRIN values of the patients and the healthy controls (p>0.05). Positive correlation between urine EMMPRIN-urine ADAM12 tests was found in total patients group (r=0.683, p=0.001). Our preliminary results revealed that serum EMMPRIN and ADAM12 values and urine ADAM12 values may be useful markers in prostate cancer therapy. Due to the high correlation between these two tests, we are of the opinion that the use of urine ADAM12 in clinic may be sufficient and favorable together with prostate-specific antigen (PSA) for treatment. 
 Metalloproteinase-disintegrin ADAM12 is associated with a breast tumor-initiating cell phenotype. Members of the ADAM family of proteases have been associated with mammary tumorigenesis. Gene profiling of human breast tumors identified several intrinsic subtypes of breast cancer, which differ in terms of their basic biology, response to chemotherapy/radiation, preferential sites of   metastasis  , and overall patient survival. Whether or not the expression of individual ADAM proteases is linked to a particular subtype of breast cancer and whether the functions of these ADAMs are relevant to the cancer subtype have not been investigated. We analyzed several transcriptomic datasets and found that ADAM12L is specifically up-regulated in claudin-low tumors. These tumors are poorly differentiated, exhibit aggressive characteristics, have molecular signatures of epithelial-to-mesenchymal transition (EMT), and are rich in markers of breast tumor-initiating cells (BTICs). Consistently, we find that ADAM12L, but not the alternative splice variant ADAM12S, is a part of stromal, mammosphere, and EMT gene signatures, which are all associated with BTICs. In patients with estrogen receptor-negative tumors, high expression of ADAM12L, but not ADAM12S, is predictive of resistance to neoadjuvant chemotherapy. Using MCF10DCIS.com breast cancer cells, which express the endogenous ADAM12L and efficiently form mammospheres when plated at the density of single cell per well, we show that ADAM12L plays an important role in supporting mammosphere growth. We postulate that ADAM12L may serve as a novel marker and/or a novel therapeutic target in BTICs. 
 An essential role of metalloprotease-disintegrin ADAM12 in triple-negative breast cancer. In the absence of HER2 overexpression, triple-negative breast cancers (TNBCs) rely on signaling by epidermal growth factor receptor (EGFR/ErbB1/HER1) to convey growth signals and stimulate cell proliferation. Soluble EGF-like ligands are derived from their transmembrane precursors by ADAM proteases, but the identity of the ADAM that is primarily responsible for ligand release and activation of EGFR in TNBCs is not clear. Using publicly available gene expression data for patients with lymph node-negative breast tumors who did not receive systemic treatment, we show that ADAM12L is the only ADAM with an expression level significantly associated with decreased distant   metastasis  -free survival times. Similar effect was not observed for patients with ER-negative non-TNBCs. There was a positive correlation between ADAM12L and HB-EGF and EGFR in TNBCs, but not in ER-negative non-TNBCs. We further demonstrate that ectopic expression of ADAM12L increased EGFR phosphorylation in a mouse intraductal xenograft model of early breast cancer. Finally, we detect strong correlation between the level of anti-ADAM12L and anti-phospho-EGFR immunostaining in human breast tumors using tissue microarrays. These studies suggest that ADAM12L is the primary protease responsible for the activation of EGFR in early stage, lymph node-negative TNBCs. Thus, our results may provide novel insight into the biology of TNBC. 
 Epigenetic regulation by Z-DNA silencer function controls cancer-associated ADAM-12 expression in breast cancer: cross-talk between MeCP2 and NF1 transcription factor family. A disintegrin and metalloprotease domain-containing protein 12 (ADAM-12) is upregulated in many human cancers and promotes cancer   metastasis  . Increased urinary level of ADAM-12 in breast and bladder cancers correlates with disease progression. However, the mechanism of its induction in cancer remains less understood. Previously, we reported a Z-DNA-forming negative regulatory element (NRE) in ADAM-12 that functions as a transcriptional suppressor to maintain a low-level expression of ADAM-12 in most normal cells. We now report here that overexpression of ADAM-12 in triple-negative MDA-MB-231 breast cancer cells and breast cancer tumors is likely due to a marked loss of this Z-DNA-mediated transcriptional suppression function. We show that Z-DNA suppressor operates by interaction with methyl-CpG-binding protein, MeCP2, a prominent epigenetic regulator, and two members of the nuclear factor 1 family of transcription factors, NF1C and NF1X. While this tripartite interaction is highly prevalent in normal breast epithelial cells, both in vitro and in vivo, it is significantly lower in breast cancer cells. Western blot analysis has revealed significant differences in the levels of these 3 proteins between normal mammary epithelial and breast cancer cells. Furthermore, we show, by NRE mutation analysis, that interaction of these proteins with the NRE is necessary for effective suppressor function. Our findings unveil a new epigenetic regulatory process in which Z-DNA/MeCP2/NF1 interaction leads to transcriptional suppression, loss of which results in ADAM-12 overexpression in breast cancer cells. 
 ADAM-12 as a diagnostic marker for the proliferation, migration and invasion in patients with small cell lung cancer. Small cell lung cancer (SCLC) is highly aggressive and is characterized by malignant   metastasis  . Approximately 90% of patients die due to extensive   metastasis  . The extracellular matrix (ECM) is a natural barrier that can prevent cellular invasion and   metastasis  . Therefore, degradation of the ECM must take place in order for extensive   metastasis   to occur. A disintegrin and metalloprotease (ADAM) is a multi-domain protease that plays an important role in tumorigenesis, as well as tumor development, invasion and   metastasis  . However, there have been few reports on the expression and role of ADAMs in SCLC. In the current study, the expression and role of ADAMs in SCLC proliferation, invasion and   metastasis   was investigated. A total of 150 SCLC tissue samples were examined by immunohistochemistry for ADAMs expression. ADAM-12 was found to be abundantly expressed in 72.67% samples and other ADAMs were found to be expressed in 10% to 40% of samples. ADAM-12 levels in serum and urine, from 70 SCLC patients and 40 normal controls, were also measured using ELISA. ADAM-12 expression was significantly higher in SCLC patients than in healthy controls and in patients with extensive disease compared to those with more limited disease. Silencing the expression of ADAM-12 in H1688 cells through the use of specific siRNA significantly reduced cellular proliferation, invasion and   metastasis  . Supplementing the expression of ADAM-12-L or -S in H345 cells, significantly enhanced cellular proliferation, invasion and   metastasis  . Animal models with metastatic SCLC also exhibited increased expression of ADAM-12 along with enhanced invasion and   metastasis  . In brief, ADAM-12 is an independent prognostic factor and diagnostic marker, and is involved in the proliferation, invasion and   metastasis   of SCLC. 
 ADAM12 transmembrane and secreted isoforms promote breast tumor growth: a distinct role for ADAM12-S protein in tumor   metastasis  . Increased levels of ADAM12 have been reported in a variety of human cancers. We have previously reported that urinary ADAM12 is predictive of disease status in breast cancer patients and that ADAM12 protein levels in urine increase with progression of disease. On the basis of these findings, the goal of this study was to elucidate the contribution of ADAM12 in breast tumor growth and progression. Overexpression of both the ADAM12-L (transmembrane) and ADAM12-S (secreted) isoforms in human breast tumor cells resulted in a significantly higher rate of tumor take and increased tumor size. Cells expressing the enzymatically inactive form of the secreted isoform, ADAM12-S, had tumor take rates and tumor volumes similar to those of wild-type cells, suggesting that the tumor-promoting activity of ADAM12-S was a function of its proteolytic activity. Of the two isoforms, only the secreted isoform, ADAM12-S, enhanced the ability of tumor cells to migrate and invade in vitro and resulted in a higher incidence of local and distant   metastasis   in vivo. This stimulatory effect of ADAM12-S on migration and invasion was dependent on its catalytic activity. Expression of both ADAM12 isoforms was found to be significantly elevated in human malignant breast tissue. Taken together, our results suggest that ADAM12 overexpression results in increased tumor take, tumor size, and   metastasis   in vivo. These findings suggest that ADAM12 may represent a potential therapeutic target in breast cancer. 
 Transforming growth factor-beta1-mediated activation of NF-kappaB contributes to enhanced ADAM-12 expression in mammary carcinoma cells. A disintegrin and metalloproteinase-12 (ADAM-12), a member of multifunctional family of proteins, is upregulated in many cancers, including breast, lung, liver, prostate, gastric, and bladder. The multidomain structure, composed of a prodomain, a metalloproteinase, disintegrin-like, epidermal growth factor-like, cysteine-rich and transmembrane domains, and a cytoplasmic tail, allows ADAM-12 to promote matrix degradation, cell-cell adhesion, and intracellular signaling capacities and thereby to play a critical role in cancer growth and   metastasis  . Despite ample evidence linking increased ADAM-12 expression with cancer, the mechanisms controlling its upregulation are still unknown. In the present study, transforming growth factor-β1 (TGF-β1) is shown to increase ADAM-12 mRNA expression in MDA-MB-231 breast carcinoma cells. We have identified a promoter element responsible for TGF-β1-mediated ADAM-12 induction. We show interaction of NF-κB with ADAM-12 promoter and that high level of NF-κB activity in breast carcinoma cells results in the upregulation of ADAM-12 expression. Site-directed mutagenesis of the NF-κB element in ADAM-12 promoter and inhibition of NF-κB activity by Bay-11-7085 and MG-132 significantly reduced TGF-β1-mediated increase of ADAM-12 promoter-driven gene expression. Transfection of cells with a dominant-negative mutant form of IκBα (IκBαΔN), which inhibits activation of NF-κB, significantly reduced transcription from ADAM-12 promoter-reporter in TGF-β1-stimulated MDA-MB-231 cancer cells. In correlation, overexpression of NF-κB induced ADAM-12 expression in a dose-dependent manner. DNA-binding and ChIP assays indicated that p65 subunit of NF-κB binds to ADAM-12 promoter. Together, our study identified a cellular mechanism for induction of ADAM-12, which involves NF-κB and its activation by TGF-β1. 
 TM4SF3 promotes esophageal carcinoma   metastasis   via upregulating ADAM12m expression. Esophageal cancer is characterized by rapid clinical progression and poor prognosis due to adjacent tissue invasion and distant organs   metastasis   at a very early stage. TM4SF3 (transmembrane 4 superfamily 3), a member of tetraspanin family, has been reported as a   metastasis   associated gene in many types of tumors. Herein, we described new properties of TM4SF3 in tumor   metastasis  , which suggested that this gene might be involved in esophageal carcinoma   metastasis  . Western blotting revealed that TM4SF3 was overexpressed in 57.1% (8/14) of esophageal carcinomas and esophageal carcinoma cell lines with high-invasive potential. Exogenous expression of TM4SF3 in two low-invasive esophageal carcinoma cell lines, KYSE150 and EC9706, significantly promoted cell migration and invasion. Upregulating TM4SF3 expression in EC9706 cells promoted xenograft tumor invading into surrounding tissues, enhanced lung   metastasis  , and shortened the lifespan of mice (median survival EC9706-TM4SF3 106.5 days versus EC9706-Vector 169.0 days, P < 0.0001) in a spontaneous   metastasis   model. Further studies demonstrated that ADAM12m was upregulated by TM4SF3 overexpression in vitro and in vivo. Abrogating up-expression of ADAM12m by siRNA significantly suppressed TM4SF3-mediated invasion. Together, these data from our studies indicated that overexpression of TM4SF3 in esophageal cancer conferred advantage to the invasion and   metastasis   of this destructive disease. Upregulated expression of ADAM12m by TM4SF3 might play a key role in TM4SF3-mediated invasion and   metastasis  . TM4SF3 and ADAM12m might be potential targets of esophageal carcinoma for anti-  metastasis   therapy. 
  GeneRIFs  
 ADAM-12 is an independent prognostic factor and diagnostic marker, and is involved in the proliferation, invasion and   metastasis   of small cell lung cancer . 
 ADAM12 overexpression results in increased tumor take, tumor size, and   metastasis   in vivo. These findings suggest that ADAM12 may represent a potential therapeutic target in breast cancer. 
 Upregulated expression of ADAM12m by TM4SF3 might play a key role in TM4SF3-mediated invasion and   metastasis   of esophageal cancer 
  [Cc]ell [Ii]nvasion  PubMed  
 Differential expression of ADAM (a disintegrin and metalloproteinase) genes between human first trimester villous and extravillous trophoblast cells. A disintegrin and metalloproteinases (ADAMs) are members of the metzincin family of zinc-dependent metalloproteinases that play pivotal roles in the proteolytic degradation of the extracellular matrix for   cell invasion  . Few studies have investigated the ADAM subtypes that are expressed in first trimester trophoblast cells. The purpose of this study was to elucidate the differential expression profiles of ADAMs between first trimester villous trophoblast cells (VTs) and extravillous trophoblast cells (EVTs). We isolated EVTs from explanted human first trimester chorionic villi and investigated the mRNA expression levels of five members of the ADAM family (ADAMTS1, ADAMTS2, ADAM10, ADAM12, and ADAM17) using real-time PCR. Chorionic villous tips were defined as first trimester VTs. Of the differentially expressed ADAM genes between first trimester VTs and EVTs, ADAMTS1 was expressed at a significantly higher level in EVTs than in VTs. In contrast, both ADAM10 and ADAM12 were expressed at significantly higher levels in VTs than in EVTs. No differences were found in the mRNA levels of ADAMTS2 and ADAM17 between the two cell types. Moreover, we demonstrated that in VTs, the expression level of ADAM12 was significantly downregulated in the late first trimester (10-13 gestational weeks) compared to the middle first trimester (7-8 weeks). These results suggest that first trimester trophoblast cells express ADAM genes in cell type- and gestational age-dependent manners. Our data provide additional insight into the functions of ADAMs in the human placenta. 
 Differential expression of ADAM (a disintegrin and metalloproteinase) genes between human first trimester villous and extravillous trophoblast cells. A disintegrin and metalloproteinases (ADAMs) are members of the metzincin family of zinc-dependent metalloproteinases that play pivotal roles in the proteolytic degradation of the extracellular matrix for   cell invasion  . Few studies have investigated the ADAM subtypes that are expressed in first trimester trophoblast cells. The purpose of this study was to elucidate the differential expression profiles of ADAMs between first trimester villous trophoblast cells (VTs) and extravillous trophoblast cells (EVTs). We isolated EVTs from explanted human first trimester chorionic villi and investigated the mRNA expression levels of five members of the ADAM family (ADAMTS1, ADAMTS2, ADAM10, ADAM12, and ADAM17) using real-time PCR. Chorionic villous tips were defined as first trimester VTs. Of the differentially expressed ADAM genes between first trimester VTs and EVTs, ADAMTS1 was expressed at a significantly higher level in EVTs than in VTs. In contrast, both ADAM10 and ADAM12 were expressed at significantly higher levels in VTs than in EVTs. No differences were found in the mRNA levels of ADAMTS2 and ADAM17 between the two cell types. Moreover, we demonstrated that in VTs, the expression level of ADAM12 was significantly downregulated in the late first trimester (10-13 gestational weeks) compared to the middle first trimester (7-8 weeks). These results suggest that first trimester trophoblast cells express ADAM genes in cell type- and gestational age-dependent manners. Our data provide additional insight into the functions of ADAMs in the human placenta. 
 Notch increases the shedding of HB-EGF by ADAM12 to potentiate invadopodia formation in hypoxia. Notch regulates cell-cell contact-dependent signaling and is activated by hypoxia, a microenvironmental condition that promotes cellular invasion during both normal physiology and disease. The mechanisms by which hypoxia and Notch regulate cellular invasion are not fully elucidated. In this paper, we show that, in cancer cells, hypoxia increased the levels and activity of the ADAM12 metalloprotease in a Notch signaling-dependent manner, leading to increased ectodomain shedding of the epidermal growth factor (EGF) receptor (EGFR) ligand heparin-binding EGF-like growth factor. Released HB-EGF induced the formation of invadopodia, cellular structures that aid cancer   cell invasion  . Thus, we describe a signaling pathway that couples cell contact-dependent signaling with the paracrine activation of the EGFR, indicating cross talk between the Notch and EGFR pathways in promoting cancer   cell invasion  . This signaling pathway might regulate the coordinated acquisition of invasiveness by neighboring cells and mediate the communication between normoxic and hypoxic areas of tumors to facilitate cancer   cell invasion  . 
 A disintegrin and metalloproteinase 12 (ADAM12) localizes to invasive trophoblast, promotes   cell invasion   and directs column outgrowth in early placental development. During pregnancy, stromal- and vascular-remodeling trophoblasts serve critical roles in directing placental development acquiring pro-invasive characteristics. The A Disintegrin and Metalloproteinase (ADAM) family of multifunctional proteins direct cellular processes across multiple organ systems via their intrinsic catalytic, cell adhesive and intracellular signaling properties. ADAM12, existing as two distinct splice variants (ADAM12L and ADAM12S), is highly expressed in the human placenta and promotes cell migration and invasion in several tumor cell lines; however, its role in trophoblast biology is unknown. In this study, ADAM12 was localized to anchoring trophoblast columns in first trimester placentas and to highly invasive extracellular matrix-degrading trophoblasts in placental villous explants. The importance of ADAM12 in directing trophoblast invasion was tested using loss-of and gain-of-function strategies, where siRNA-directed knockdown of ADAM12 inhibited trophoblast   cell invasion   while over-expression promoted migration and invasion in two trophoblastic cell models. In placental villous explant cultures, siRNA-directed loss of ADAM12 significantly dampened trophoblast column outgrowth. Additionally, we provide functional evidence for the ADAM12S variant in promoting trophoblast invasion and column outgrowth through a mechanism requiring its catalytic activity. This is the first study to assign a function for ADAM12 in trophoblast biology, where ADAM12 may play a central role regulating the behavior of invasive trophoblast subsets in early pregnancy. This study also underlines the importance of ADAM12L and ADAM12S in directing cell motility in normal developmental processes outside of cancer, specifically highlighting a potentially important function of ADAM12S in directing early placental development. 
  GeneRIFs  
 ADAM12 localizes to invasive trophoblast, promotes   cell invasion   and directs column outgrowth in early placental development. 
   
  
 ADAM19 
 
  NCBI Gene  
  GeneRIF  
  Pubmed  
 
 
  Search Term  Rank  Fields  
  [Mm]igration  6  PubMed(5); GeneRIFs(1); Summary(1)  
  [Ee]xtracellular [Mm]atrix  8  PubMed(1)  
  [Ii]nvasive  10  PubMed(2)  
  [Mm]etastasis  12  PubMed(2)  
  [Cc]ell [Ii]nvasion  13  PubMed(1)  
 
 
 hide/show details for ADAM19 
  [Mm]igration  PubMed  
 Role of microRNA-30c targeting ADAM19 in colorectal cancer. MicroRNAs (miRNAs) are deregulated in a number of cancers including colorectal cancer. MiR-30c belongs to miR-30 family, and is involved in a variety of malignant diseases. In this study, we detected the expression of miR-30c in colon cancer cell lines and clinical colon cancer specimens. MiR-30c was shown to be dramatically down-regulated both in cell lines and cancer tissues. Additionally, miR-30c could inhibit cancer cell growth,   migration   and invasion in vitro. Consistently, stable over-expression of miR-30c inhibited the growth and lung metastasis of colon cancer cell xenografts in vivo. Furthermore, bioinformatics algorithm and luciferase reporter assay indicated ADAM19 as a direct target of miR-30c. Of interest, further experiments demonstrated that inhibition of ADAM19 by miR-30c partially mediated the anti-tumor effect of miR-30c. Overall, our study provides the new insight that miR-30c inhibited colon cancer cells via targeting ADAM19. Thus, miR-30c might serve as a promising therapeutic strategy for colon cancer treatment. 
 Genetic and cellular studies highlight that A Disintegrin and Metalloproteinase 19 is a protective biomarker in human prostate cancer. Prostate cancer is the second most frequently diagnosed cancer in men worldwide. Current treatments include surgery, androgen ablation and radiation. Introduction of more targeted therapies in prostate cancer, based on a detailed knowledge of the signalling pathways, aims to reduce side effects, leading to better clinical outcomes for the patient. ADAM19 (A Disintegrin And Metalloproteinase 19) is a transmembrane and soluble protein which can regulate cell phenotype through cell adhesion and proteolysis. ADAM19 has been positively associated with numerous diseases, but has not been shown to be a tumor suppressor in the pathogenesis of any human cancers. Our group sought to investigate the role of ADAM19 in human prostate cancer.ADAM19 mRNA and protein levels were assessed in well characterised human prostate cancer cohorts. ADAM19 expression was assessed in normal prostate epithelial cells (RWPE-1) and prostate cancer cells (LNCaP, PC3) using western blotting and immunocytochemistry. Proliferation assays were conducted in LNCaP cells in which ADAM19 was over-expressed. In vitro scratch assays were performed in PC3 cells over-expressing ADAM19.Immunohistochemical studies highlighted that ADAM19 protein levels were elevated in normal prostate tissue compared to prostate cancer biopsies. Results from the clinical cohorts demonstrated that high levels of ADAM19 in microarrays are positively associated with lower stage (p = 0.02591) and reduced relapse (p = 0.00277) of human prostate cancer. In vitro, ADAM19 expression was higher in RWPE-1 cells compared to LNCaP cells. In addition, human ADAM19 over-expression reduced LNCaP cell proliferation and PC3 cell   migration  .Taken together, our immunohistochemical and microarray results and cellular studies have shown for the first time that ADAM19 is a protective factor for human prostate cancer. Further, this study suggests that upregulation of ADAM19 expression could be of therapeutic potential in human prostate cancer. 
 MiR-153 inhibits   migration   and invasion of human non-small-cell lung cancer by targeting ADAM19. MiR-153 was reported to be dysregulated in some human cancers. However, the function and mechanism of miR-153 in lung cancer cells remains unknown. In this study, we investigated the role of miR-153 in human non-small-cell lung cancer (NSCLC). Using qRT-PCR, we demonstrated that miR-153 was significantly decreased in clinical NSCLC tissues and cell lines, and downregulation of miR-153 was significantly correlated with lymph node status. We further found that ectopic expression of miR-153 significantly inhibited the proliferation and   migration   and invasion of NSCLC cells in vitro, suggesting that miR-153 may be a novel tumor suppressor in NSCLC. Further integrated analysis revealed that ADAM19 is as a direct and functional target of miR-153. Luciferase reporter assay demonstrated that miR-153 directly targeted 3'UTR of ADAM19, and correlation analysis revealed an inverse correlation between miR-153 and ADAM19 mRNA levels in clinical NSCLC tissues. Knockdown of ADAM19 inhibited   migration   and invasion of NSCLC cells which was similar with effects of overexpression of miR-153, while overexpression of ADAM19 attenuated the function of miR-153 in NSCLC cells. Taken together, our results highlight the significance of miR-153 and ADAM19 in the development and progression of NSCLC. 
 Metalloproteinase disintegrins ADAM8 and ADAM19 are highly regulated in human primary brain tumors and their expression levels and activities are associated with invasiveness. Patients with primary brain tumors have bleak prognoses and there is an urgent desire to identify new markers for sensitive diagnosis and new therapeutic targets for effective treatment. A family of proteins, the disintegrin and metalloproteinases (ADAMs or adamalysins), are cell surface and extracellular multidomain proteins implicated in cell-cell signaling, cell adhesion, and cell   migration  . Their putative biological and pathological roles make them candidates for promoting tumor growth and malignancy. We investigated the expression levels of 12 cerebrally expressed ADAM genes in human primary brain tumors (astrocytoma WHO grade I-III, glioblastoma WHO grade IV, oligoastrocytoma WHO grade II and III, oligodendroglioma WHO grade II and III, ependymoma WHO grade II and III, and primitive neuroectodermal tumor WHO grade IV) using real-time PCR. The mRNAs of the five ADAMs 8, 12, 15, 17, and 19 were significantly upregulated. The ADAM8 and ADAM19 proteins were mainly located in tumor cells and in some tumors in endothelia of blood vessels. In brain tumor tissue, ADAM8 and ADAM19 undergo activation by prodomain removal resulting in active proteases. By using specific peptide substrates for ADAM8 and ADAM19, respectively, we demonstrated that the proteases exert enhanced proteolytic activity in those tumor specimens with the highest expression levels. In addition, expression levels and the protease activities of ADAM8 and ADAM19 correlated with invasive activity of glioma cells, indicating that ADAM8 and ADAM19 may play a significant role in tumor invasion that may be detrimental to patients survival. 
 MiR-145 suppressed human retinoblastoma cell proliferation and invasion by targeting ADAM19. MicroRNAs (miRNAs) play critical roles in retinoblastoma (RB) initiation and progression, aberrant expression of miR-145 had been frequently reported in cancer studies. However, the role and mechanism of its function in RB is still unclear. In this study, our data showed that miR-145 was downregulated in RB tissues and cell lines. Overexpression of miR-145 suppressed RB cell proliferation,   migration   and invasion in vitro. ADAM19 was identified as a direct target of miR-145. Silencing of ADAM19 significantly inhibited RB cell proliferation,   migration   and invasion. In addition, a reverse correlation between miR-145 and ADAM19 expression was noted in RB tissues. Taken together, these findings suggested that miR-145 functions as a tumor suppressor in RB by directly targeting ADAM19. miR-145 could be an anticancer therapeutic target for RB patients. 
  GeneRIFs  
 MiR-153 has a role in inhibiting   migration   and invasion of human non-small-cell lung cancer by targeting ADAM19 
  Summary  
 This gene encodes a member of the ADAM (a disintegrin and metalloprotease domain) family. Members of this family are membrane-anchored proteins structurally related to snake venom disintegrins and have been implicated in a variety of biological processes involving cell-cell and cell-matrix interactions, including fertilization, muscle development, and neurogenesis. This member is a type I transmembrane protein and serves as a marker for dendritic cell differentiation. It has been demonstrated to be an active metalloproteinase, which may be involved in normal physiological processes such as cell   migration  , cell adhesion, cell-cell and cell-matrix interactions, and signal transduction. It is proposed to play a role in pathological processes, such as cancer, inflammatory diseases, renal diseases, and Alzheimer's disease. [provided by RefSeq, May 2013] 
  [Ee]xtracellular [Mm]atrix  PubMed  
 ADAM19/adamalysin 19 structure, function, and role as a putative target in tumors and inflammatory diseases. A disintegrin and metalloproteinase 19 (ADAM19, or adamalysin 19) is a cell surface glycoprotein with a signal sequence, a prodomain, a metalloproteinase domain, a disintegrin domain, a cysteine-rich domain, a epidermal growth factor-like domain, a transmembrane domain, and a cytoplasmic domain. It is an endopeptidase that cleaves   extracellular matrix   proteins and sheds growth factors and cytokines such as neuregulins, heparin-binding epidermal growth factor, tumor necrosis factor (TNF)-alpha, and TNF-related activation-induced cytokine. The ADAM19 gene was cloned from human, monkey, and mouse. It is expressed in multiple organs and tissues including heart, lung, bones, brain, spleen, liver, skeletal muscle, kidney, and testes. ADAM19 plays essential roles in embryo implantation, cardiovascular morphogenesis, neurogenesis, and other developmental processes. It has constitutive alpha-secretase activity associated with processing Alzheimer's disease amyloid precursor protein (APP) to non-amyloidogenic fragments; thus, it is neuroprotective. Those observations indicate that inhibition of ADAM19 activity is undesirable during embryo development and morphogenesis, and during the development of Alzheimer's disease. On the contrary, in adults, ADAM19 is upregulated in human brain tumors such as astrocytoma and glioblastoma and is correlated with the invasiveness of glioma. It is also over-expressed by many human cancerous cell lines including cancers of the colon, ovary, lung, and brain. Abnormally high expression of ADAM19 is also linked to inflammation and fibrosis of the lung and kidney. Targeted inhibition of ADAM19 may be crucial for the treatment of certain types of tumors and inflammatory diseases. 
  [Ii]nvasive  PubMed  
 Metalloproteinase disintegrins ADAM8 and ADAM19 are highly regulated in human primary brain tumors and their expression levels and activities are associated with   invasive  ness. Patients with primary brain tumors have bleak prognoses and there is an urgent desire to identify new markers for sensitive diagnosis and new therapeutic targets for effective treatment. A family of proteins, the disintegrin and metalloproteinases (ADAMs or adamalysins), are cell surface and extracellular multidomain proteins implicated in cell-cell signaling, cell adhesion, and cell migration. Their putative biological and pathological roles make them candidates for promoting tumor growth and malignancy. We investigated the expression levels of 12 cerebrally expressed ADAM genes in human primary brain tumors (astrocytoma WHO grade I-III, glioblastoma WHO grade IV, oligoastrocytoma WHO grade II and III, oligodendroglioma WHO grade II and III, ependymoma WHO grade II and III, and primitive neuroectodermal tumor WHO grade IV) using real-time PCR. The mRNAs of the five ADAMs 8, 12, 15, 17, and 19 were significantly upregulated. The ADAM8 and ADAM19 proteins were mainly located in tumor cells and in some tumors in endothelia of blood vessels. In brain tumor tissue, ADAM8 and ADAM19 undergo activation by prodomain removal resulting in active proteases. By using specific peptide substrates for ADAM8 and ADAM19, respectively, we demonstrated that the proteases exert enhanced proteolytic activity in those tumor specimens with the highest expression levels. In addition, expression levels and the protease activities of ADAM8 and ADAM19 correlated with   invasive   activity of glioma cells, indicating that ADAM8 and ADAM19 may play a significant role in tumor invasion that may be detrimental to patients survival. 
 ADAM19/adamalysin 19 structure, function, and role as a putative target in tumors and inflammatory diseases. A disintegrin and metalloproteinase 19 (ADAM19, or adamalysin 19) is a cell surface glycoprotein with a signal sequence, a prodomain, a metalloproteinase domain, a disintegrin domain, a cysteine-rich domain, a epidermal growth factor-like domain, a transmembrane domain, and a cytoplasmic domain. It is an endopeptidase that cleaves extracellular matrix proteins and sheds growth factors and cytokines such as neuregulins, heparin-binding epidermal growth factor, tumor necrosis factor (TNF)-alpha, and TNF-related activation-induced cytokine. The ADAM19 gene was cloned from human, monkey, and mouse. It is expressed in multiple organs and tissues including heart, lung, bones, brain, spleen, liver, skeletal muscle, kidney, and testes. ADAM19 plays essential roles in embryo implantation, cardiovascular morphogenesis, neurogenesis, and other developmental processes. It has constitutive alpha-secretase activity associated with processing Alzheimer's disease amyloid precursor protein (APP) to non-amyloidogenic fragments; thus, it is neuroprotective. Those observations indicate that inhibition of ADAM19 activity is undesirable during embryo development and morphogenesis, and during the development of Alzheimer's disease. On the contrary, in adults, ADAM19 is upregulated in human brain tumors such as astrocytoma and glioblastoma and is correlated with the   invasive  ness of glioma. It is also over-expressed by many human cancerous cell lines including cancers of the colon, ovary, lung, and brain. Abnormally high expression of ADAM19 is also linked to inflammation and fibrosis of the lung and kidney. Targeted inhibition of ADAM19 may be crucial for the treatment of certain types of tumors and inflammatory diseases. 
  [Mm]etastasis  PubMed  
 [Expression and clinical significance of ADAM19 in endometrial carcinoma]. To investigate the expression of adamalysin19 (ADAM19) in human endometrial carcinoma and to explore its correlation with clinical behavior and pathological characters.Immunohistochemistry was conducted to detect the expression of ADAM19 in the endometrial tissue from 51 patients with endometrial carcinoma and 33 patients with normal endometrium.The results were analyzed with clinical data.Postive expression signals of ADAM19 were detected both in the endome-trial cancer tissue and normal endometrium. The expression level of ADAM19 in endometrial cancer tissue was significantly higher than that in normal endometrium (P<0.001).Those with lower histological grade or non-adenocarcinoma had a higher expression level(P=0.028 , P=0.042). In addition, ADAM19 in thecarcinoma with myometrial invasion had a higher expression trend than that without myometrial invasion (P=0.065). However, from the clinical data we found no correlation with surgical staging and   metastasis   of lymph nodes(P=0.328, P=0.529).The overexpression of ADAM19 suggests that ADAM19 correlates with the progress and prognosis of endometrial carcinoma, and may be implicated as a marker for the clinical behavior of endometrial carcinoma. 
 Role of microRNA-30c targeting ADAM19 in colorectal cancer. MicroRNAs (miRNAs) are deregulated in a number of cancers including colorectal cancer. MiR-30c belongs to miR-30 family, and is involved in a variety of malignant diseases. In this study, we detected the expression of miR-30c in colon cancer cell lines and clinical colon cancer specimens. MiR-30c was shown to be dramatically down-regulated both in cell lines and cancer tissues. Additionally, miR-30c could inhibit cancer cell growth, migration and invasion in vitro. Consistently, stable over-expression of miR-30c inhibited the growth and lung   metastasis   of colon cancer cell xenografts in vivo. Furthermore, bioinformatics algorithm and luciferase reporter assay indicated ADAM19 as a direct target of miR-30c. Of interest, further experiments demonstrated that inhibition of ADAM19 by miR-30c partially mediated the anti-tumor effect of miR-30c. Overall, our study provides the new insight that miR-30c inhibited colon cancer cells via targeting ADAM19. Thus, miR-30c might serve as a promising therapeutic strategy for colon cancer treatment. 
  [Cc]ell [Ii]nvasion  PubMed  
 Dynamic change of Adamalysin 19 (ADAM19) in human placentas and its effects on   cell invasion   and adhesion in human trophoblastic cells. Human ADAM19 is a recently identified member of the ADAM family. It is highly expressed in human placentas, but its dynamic change and function at the human feto-maternal interface during placentation remain to be elucidated. In this present study, the spatial and temporal expression and cellular localization of ADAM19 in normal human placentas were first demonstrated, and the effects of ADAM19 on trophoblast cell adhesion and invasion were further investigated by using a human choriocarcinoma cell line (JEG-3) as an in vitro model. The data demonstrated that ADAM19 was widely distributed in villous cytotrophoblast cells, syncytiotrophoblast cells, column trophoblasts, and villous capillary endothelial cells during early pregnancy. The mRNA and protein level of ADAM19 in placentas was high at gestational weeks 8-9, but diminished significantly at mid- and term pregnancy. In JEG-3 cells, the overexpression of ADAM19 led to diminished   cell invasion  , as well as increases in cell adhesiveness and the expression of E-cadherin, with no changes in beta-catenin expression observed. These data indicate that ADAM19 may participate in the coordinated regulation of human trophoblast cell behaviors during the process of placentation. 
   
  
 ZEB2 
 
  NCBI Gene  
  GeneRIF  
  Pubmed  
 
 
  Search Term  Rank  Fields  
  [Mm]igration  6  Process(2); PubMed(42); GeneRIFs(7)  
  [Ee]xtracellular [Mm]atrix  8  PubMed(2)  
  [Ii]nvasive  10  PubMed(17); GeneRIFs(2)  
  [Mm]etastasize  11  PubMed(3)  
  [Mm]etastasis  12  PubMed(49); GeneRIFs(13)  
  [Cc]ell [Ii]nvasion  13  PubMed(11); GeneRIFs(2)  
 
 
 hide/show details for ZEB2 
  [Mm]igration  Process  
 melanocyte   migration   
 neural crest cell   migration   
  PubMed  
 Downregulated microRNA-200a promotes EMT and tumor growth through the wnt/β-catenin pathway by targeting the E-cadherin repressors ZEB1/ZEB2 in gastric adenocarcinoma. In a previous study, we found that microRNA (miRNA)-200a suppresses Wnt/β-catenin signaling by interacting with β-catenin, thereby inhibiti  ng migrat  ion, invasion and proliferation. However, the mechanism involved in this suppression remains unclear. In the present study, we investigated the underlying mechanism of miR-200a regulation of epithelial-mesenchymal transition (EMT) in gastric carcinoma cells, and confirmed the tumor suppressor role of miR-200a in vivo. The expressions of miRNA-200a, -200b and -200c, identified by fluorescent in situ hybridization, were downregulated and inversely correlated with WHO grades of gastric adenocarcinoma (GA). The expression of the potential miR-200a target genes ZEB1 and ZEB2 was detected immunohistochemically. These examinations used the same tissue microarrays to analyze the relationships between miR-200a and potential target genes. The expression of miR-200a and ZEB1/ZEB2 in the same GA tissue microarrays was inversely related. Restored miR-200a expression inhibited tumor growth in nude mice harboring subcutaneous SGC7901 xenografts. The expression of N-cadherin, β-catenin, Twist1 and Snail2 decreased, and E-cadherin levels increased, when miR-200a was elevated, as tested by fluorescence microscopy and immunohistochemistry. Similar results were observed in vivo. We found upregulated miR-200a expression to increase E-cadherin and suppress the Wnt/β-catenin pathway by targeting ZEB1 and ZEB2 in GA, thus delaying tumor growth in vivo. The effect of miR-200a on Wnt/β-catenin signaling may provide a therapeutic target against EMT. 
 Androgen receptor as a regulator of ZEB2 expression and its implications in epithelial-to-mesenchymal transition in prostate cancer. Zinc finger E-box-binding protein 2 (ZEB2) is known to help mediate the epithelial-to-mesenchymal transition, and thereby it facilitates cancer metastasis. This study was initiated to explore whether ZEB2 expression differs in prostate cancer (PCa, n=7) and benign prostatic hyperplasia (BPH, n=7) tissues. In PCa tissues, the levels of both immunoreactive ZEB2 and androgen receptor (AR) were found to be significantly higher (P<0.05) when compared with BPH tissues. Co-regulation of AR and ZEB2 prompted us to investigate the role of androgenic stimuli in ZEB2 expression. ZEB2 expression was found to be significantly (P<0.05) upregulated after androgen stimulation and downregulated following AR silencing in LNCaP cells, an androgen-dependent PCa cell line. This finding suggested AR as a positive regulator of ZEB2 expression in androgen-dependent cells. Paradoxically, androgen-independent (AI) cell lines PC3 and DU145, known to possess low AR levels, showed significantly (P<0.05) higher expression of ZEB2 compared with LNCaP cells. Furthermore, forced expression of AR in PC3 (PC3-AR) and DU145 (DU-AR) cells led to reductions in ZEB2 expression, invasiveness, and   migration  . These cells also exhibited an increase in the levels of E-cadherin (a transcriptional target of ZEB2). Co-transfection of AR and ZEB2 cDNA constructs prevented the decline in invasiveness and   migration   to a significant extent. Additionally, ZEB2 downregulation was associated with an increase in miR200a/miR200b levels in PC3-AR cells and with a decrease in miR200a/miR200b levels in AR-silenced LNCaP cells. Thus, AR acts as a positive regulator of ZEB2 expression in androgen-dependent cells and as a negative regulator in AI PCa cells. 
 Forkhead box Q1 promotes hepatocellular carcinoma metastasis by transactivating ZEB2 and VersicanV1 expression. Forkhead box Q1 (FoxQ1) is a master regulator of tumor metastasis. However, the molecular mechanism of FoxQ1 in regulating hepatocellular carcinoma (HCC) metastasis remains unknown. Here we report a novel function for FoxQ1 in modifying the tumor microenvironment to promote HCC metastasis. FoxQ1 expression was an independent and significant risk factor for the recurrence and survival in two independent cohorts totaling 1,002 HCC patients. FoxQ1 induced epithelial-mesenchymal transition (EMT) through the transactivation of ZEB2 expression by directly binding to the ZEB2 promoter. Knockdown of ZEB2 decreased FoxQ1-enhanced HCC metastasis, whereas up-regulation of ZEB2 rescued the decreased metastasis induced by FoxQ1 knocking down. Additionally, serial deletion, site-directed mutagenesis, and a chromatin immunoprecipitation assays showed that VersicanV1, which promoted HCC metastasis and macrophage attraction, was a direct transcriptional target of FoxQ1. FoxQ1-induced VersicanV1 expression promoted the secretion of chemokine (C-C motif) ligand 2 (CCL2) from HCC cells. Chemotaxis assay showed that the culture media from FoxQ1-overexpressing HCC cells increased the migratory activity of the macrophages. Inhibition of VersicanV1 and CCL2 expression significantly inhibited FoxQ1-mediated macrophage   migration  . In animal studies, the up-regulation of FoxQ1 in HCC cells promoted HCC metastasis and intratumoral tumor associated macrophage (TAM) infiltration, whereas knockdown of VersicanV1 reduced FoxQ1-mediated HCC metastasis and intratumoral TAM infiltration. Depletion of macrophages using clodronate liposomes dramatically decreased FoxQ1-enhanced HCC metastasis. In human HCC tissues, FoxQ1 expression was positively correlated with ZEB2 and VersicanV1 expression and intratumoral TAM infiltration. Patients with positive coexpression of FoxQ1 and ZEB2, FoxQ1, and VersicanV1, or FoxQ1 and intratumoral TAMs were associated with poorer prognosis.FoxQ1 promotes HCC metastasis by transactivating ZEB2 and VersicanV1 expression, resulting in the induction of EMT and the recruitment of macrophage infiltration. 
 Directed   migration   of cortical interneurons depends on the cell-autonomous action of Sip1. GABAergic interneurons mainly originate in the medial ganglionic eminence (MGE) of the embryonic ventral telencephalon (VT) and migrate tangentially to the cortex, guided by membrane-bound and secreted factors. We found that Sip1 (Zfhx1b, Zeb2), a transcription factor enriched in migrating cortical interneurons, is required for their proper differentiation and correct guidance. The majority of Sip1 knockout interneurons fail to migrate to the neocortex and stall in the VT. RNA sequencing reveals that Sip1 knockout interneurons do not acquire a fully mature cortical interneuron identity and contain increased levels of the repulsive receptor Unc5b. Focal electroporation of Unc5b-encoding vectors in the MGE of wild-type brain slices disturbs   migration   to the neocortex, whereas reducing Unc5b levels in Sip1 knockout slices and brains rescues the   migration   defect. Our results reveal that Sip1, through tuning of Unc5b levels, is essential for cortical interneuron guidance. 
 MicroRNA-200b inhibits the growth and metastasis of glioma cells via targeting ZEB2. MicroRNAs (miRs) have been found to play important roles in mediating a variety of biological processes in human cancers, including tumor cell proliferation,   migration  , invasion, and epithelial-mesenchymal transition (EMT). In the present study, we aimed to investigate the putative role of miR‑200b in the progression of glioma. Real-time RT-PCR data showed that the miR‑200b levels were frequently reduced in primary glioma tissues (n=88) and cell lines, when compared to normal brain tissues (n=25). Moreover, decreased miR‑200b level was tightly associated with the malignant progression of glioma. Overexpression of miR‑200b significantly suppressed cell prolife  ration, m  igration, invasion and EMT in glioma U251 and U87 cells. Luciferase reporter assay data further identified ZEB2 as a direct target of miR‑200b, and the protein expression of ZEB2 was markedly reduced after overexpression of miR‑200b in U251 and U87 cells. Furthermore, restoration of ZEB2 effectively reversed the reduced expression of ZEB2, as well as the suppressive effects of miR‑200b overexpression on the p  roliferat  ion, migration, invasion and EMT in glioma U251 and U87 cells. Moreover, in vivo study showed that overexpression of miR‑200b significantly inhibited tumorigenesis as well as the tumor growth of glioma cells, and effectively protected nude mice from tumor-induced death. Taken together these findings suggest that miR‑200b has suppressive effects on t  he prolif  eration, migration, invasion and EMT of glioma cells, partly at least, via targeting ZEB2. Therefore, miR‑200b acts as a novel tumor suppressor in glioma, and thus may become a promising therapeutic candidate for glioma. 
 miR-590-3p suppresses cancer cell   migration  , invasion and epithelial-mesenchymal transition in glioblastoma multiforme by targeting ZEB1 and ZEB2. Invasion and   migration   of glioblastoma multiforme (GBM) is a multistep process and an important phenotype that causes this disease to invade surrounding tissues in the brain. The purpose of this study was to determine the role of miR-590-3p in regulation of epithelial mesenchymal transition (EMT) and metastasis of GBM cells. Expression levels of miR-590-3p in 15 GBM specimens with adjacent tissues and five GBM cell lines were assessed by quantitative RT-PCR. We found that miR-590-3p was down-regulated in detected GBM tissue samples and all of the GBM cell lines. In addition, Ectopic expression of miR-590-3p suppressed and miR-590-3p-in promoted EMT,   migration  , and invasion in U87MG and A172 cells. Bioinformatics coupled with luciferase and Western blot assays also revealed that miR-590-3p inhibited expression of ZEB1 and ZEB2, which are master regulators of tumor metastasis. Our study first indicates that miR-590-3p functions as a suppressor of GBM EMT and metastasis by targeting ZEB1 and ZEB2, and it may be a therapeutic target for metastatic GBM. 
 The miR-200 family inhibits epithelial-mesenchymal transition and cancer cell   migration   by direct targeting of E-cadherin transcriptional repressors ZEB1 and ZEB2. MicroRNAs are small non-coding RNA molecules that can regulate gene expression by interacting with multiple mRNAs and inducing either translation suppression or degradation of mRNA. Recently, several miRNAs were identified as either promoters or suppressors of metastasis. However, it is unclear in which step(s) of the multistep metastatic cascade these miRNAs play a defined functional role. To study the functional importance of miRNAs in epithelial-mesenchymal transition (EMT), a process thought to initiate metastasis by enhancing the motility of tumor cells, we used a well established in vitro EMT assay: transforming growth factor-beta-induced EMT in NMuMG murine mammary epithelial cells. We found that members of the miR-200 family, organized as two clusters in the genome, were repressed during EMT. Overexpression of each miRNA individually or as clusters in NMuMG cells hindered EMT by enhancing E-cadherin expression through direct targeting of ZEB1 and ZEB2, which encode transcriptional repressors of E-cadherin. In the 4TO7 mouse carcinoma cell line, which expresses low levels of endogenous E-cadherin and displays a mesenchymal phenotype, ectopic expression of the miR-200 family miRNAs significantly increased E-cadherin expression and altered cell morphology to an epithelial phenotype. Furthermore, ectopic expression of each miR-200 miRNA cluster significantly reduced the in vitro motility of 4TO7 cells in   migration   assays. These results suggested that loss of expression of the miR-200 family members may play a critical role in the repression of E-cadherin by ZEB1 and ZEB2 during EMT, thereby enhancing   migration   and invasion during cancer progression. 
 A developmentally regulated inducer of EMT, LBX1, contributes to breast cancer progression. Epithelial-to-mesenchymal transition (EMT) plays an important role during normal embryogenesis, and it has been implicated in cancer invasion and metastasis. Here, we report that Ladybird homeobox 1 (LBX1), a developmentally regulated homeobox gene, directs expression of the known EMT inducers ZEB1, ZEB2, Snail1, and transforming growth factor beta2 (TGFB2). In mammary epithelial cells, overexpression of LBX1 leads to morphological transformation, expression of mesenchymal markers, enhanced cell   migration  , increased CD44(high)/CD24(low) progenitor cell population, and tumorigenic cooperation with known oncogenes. In human breast cancer, LBX1 is up-regulated in the unfavorable estrogen receptor (ER)/progesterone (PR)/HER2 triple-negative basal-like subtype. Thus, aberrant expression of LBX1 may lead to the activation of a developmentally regulated EMT pathway in human breast cancer. 
 miRNA-205 is a candidate tumor suppressor that targets ZEB2 in renal cell carcinoma. This study aims to characterize the function of downregulated MicroRNA miR-205 in renal cell carcinoma (RCC), and show how the downstream zinc finger E-box-binding homeobox 2 (ZEB2) is negatively regulated by miR-205.The expression of miR-205 was detected in RCC and adjacent non-tumor tissues using real-time polymerase chain reaction (PCR). The expression of miR-205 and ZEB2 was detected in RCC cell lines using real-time PCR. The luciferase reporter assay was used to assess ZEB2 as a target of miR-205. Protein levels of ZEB2, E-cadherin, and vimentin were measured by western blot after overexpression of miR-205 in ACHN cells. In vivo functions of miR-205 in ACHN cells were measured by MTT assays,   migration   and invasion assays, and flow cytometry.MiR-205 was significantly downregulated in RCC samples and cell lines compared with matched non-tumor tissues and HK-2 cells, respectively. No significant difference was found in miR-205 expression between well differentiated and poorly to moderately differentiated groups or between phase I and phase II-III. ZEB2 was upregulated in RCC cell lines compared with expression in HK-2 cells. Upregulation of miR-205 expression caused the downregulation of ZEB2 and vimentin, and the upregulation of E-cadherin in ACHN cells. miR-205 also inhibited proliferation,   migration  , and invasion, and induced apoptosis of ACHN cells.miR-205 is a candidate tumor suppressor that targets ZEB2 in RCC. 
 Protein expression of ZEB2 in renal cell carcinoma and its prognostic significance in patient survival. ZEB2 has been reportedly shown to mediate the epithelial-to-mesenchymal transition (EMT) and disease aggressiveness in human tumors. However, the expression status of ZEB2 in renal cell carcinoma (RCC) and ZEB2's clinicopathologic/prognostic significance are poorly understood.In this study, tissue microarray, immunohistochemistry (IHC) and western blot analyses were utilized to investigate the ZEB2 expression status in RCC and adjacent renal tissue samples. In our study, samples from 116 RCC patients treated with radical nephrectomy were used as a training set to generate a ZEB2 optimal cut-point for patient outcome by receiver operating characteristic (ROC) analysis. For validation, the correlation of ZEB2 expression with the clinical characteristics and patient outcomes in another set (including 113 patients) was analyzed to validate the obtained cut-point. In the training and validation sets, high expression of ZEB2, defined by ROC analysis, predicted a poorer overall survival and progression-free survival, as evidenced by the univariate and multivariate analyses. In different subsets of overall patients, ZEB2 expression was also a prognostic indicator in patients with stage I/II, stage III/IV, grade 1/2 and grade 3/4 disease (P<0.05). Downregulation of ZEB2 by shRNA decreased the   migration   and invasion ability of 769-P cells in vitro. Furthermore, high ZEB2 expression was positively correlated with vimentin expression and inversely linked to E-cadherin expression in RCC.Our findings provide a basis for the concept that high ZEB2 expression in RCC may be important in the acquisition of an aggressive phenotype. This evidence suggests that ZEB2 overexpression (examined by IHC) is an independent biomarker for the poor prognosis of patients with RCC. 
 Fra-1/AP-1 induces EMT in mammary epithelial cells by modulating Zeb1/2 and TGFβ expression. Epithelial-to-mesenchymal transition (EMT) is essential for embryonic morphogenesis and wound healing and critical for tumour cell invasion and dissemination. The AP-1 transcription factor Fra-1 has been implicated in tumorigenesis and in tumour-associated EMT in human breast cancer. We observed a significant inverse correlation between Fra-1 mRNA expression and distant-metastasis-free survival in a large cohort of breast cancer patients derived from multiple array data sets. This unique correlation among Fos genes prompted us to assess the evolutionary conservation between Fra-1 functions in EMT of human and mouse cells. Ectopic expression of Fra-1 in fully polarized, non-tumourigenic, mouse mammary epithelial EpH4 cells induced a mesenchymal phenotype, characterized by a loss of epithelial and gain of mesenchymal markers. Proliferation, motility and invasiveness were also increased in the resulting EpFra1 cells, and the cells were tumourigenic and efficiently colonized the lung upon transplantation. Molecular analyses revealed increased expression of Tgfβ1 and the EMT-inducing transcription factors Zeb1, Zeb2 and Slug. Mechanistically, Fra-1 binds to the tgfb1 and zeb2 promoters and to an evolutionarily conserved region in the first intron of zeb1. Furthermore, increased activity of a zeb2 promoter reporter was detected in EpFra1 cells and shown to depend on AP-1-binding sites. Inhibiting TGFβ signalling in EpFra1 cells moderately increased the expression of epithelial markers, whereas silencing of zeb1 or zeb2 restored the epithelial phenotype and decreas  ed migrat  ion in vitro and tumorigenesis in vivo. Thus Fra-1 induces changes in the expression of genes encoding EMT-related transcription factors leading to the acquisition of mesenchymal, invasive and tumorigenic capacities by epithelial cells. This study defines a novel function of Fra-1/AP-1 in modulating tgfb1, zeb1 and zeb2 expression through direct binding to genomic regulatory regions, which establishes a basis for future in vivo genetic manipulations and preclinical studies using mouse models. 
 Down-regulation of miR-144 promotes thyroid cancer cell invasion by targeting ZEB1 and ZEB2. Thyroid cancer is the most common endocrine malignancy, and its incidence has increased rapidly worldwide. The molecular mechanisms underlying thyroid cancer tumorigenesis still need to be further investigated. MicroRNAs (miRNAs), short RNA molecules of approximately 22 nucleotides in length, play crucial roles in tumorigenesis. In the present study, we found that the expression of miR-144 was significantly down-regulated in thyroid cancer as compared with that in normal thyroid tissues, suggesting that miR-144 may be involved in thyroid cancer tumorigenesis. Moreover, our results showed that restoration of miR-144 in K1 and WRO thyroid cancer cells could suppress the invasion and   migration   capability of these cells. We also demonstrated that miR-144 suppressed the expression of ZEB1 and ZEB2, two E-cadherin suppressors, by directly binding to their 3'-untranslated regions. Furthermore, restoration of ZEB1 or ZEB2 partially rescued the miR-144-induced inhibition of cell invasion. These data suggest miR-144 function as a tumor suppressor in thyroid cancer. 
 miR-154 targeting ZEB2 in hepatocellular carcinoma functions as a potential tumor suppressor. MicroRNA-154 (miR-154) has been identified as a tumor suppressor in several types of human cancers; however, its clinical significance and function in human hepatocellular carcinoma (HCC) remain unclear. The aim of the present study was to analyze the clinical significance and cellular function of miR-154 in HCC patients. The data showed that miR-154 expression was consistently lower in HCC tissues and cell lines compared to that in matched tumor-adjacent tissues and a normal hepatic cell line, and its expression was negatively correlated with tumor differentiation (P<0.01), TNM stage (P<0.01) and lymph node metastasis (P<0.01). Restoration of miR-154 expression in HepG2 cells inhibited cell proliferation,   migration   and invasion, and induced apoptosis and cell arrest at the G1 phase in vitro, as well as suppressed tumor growth in a nude mouse model. Using a luciferase assay, we identified that miR-154 was able to target the 3'-untranslated region (3'UTR) of ZEB2 mRNA. Then, we revealed that miR-154 was able to reduce ZEB2 expression at the levels of mRNA and protein using qRT-PCR and western blot analysis. Notably, restoration of expression of ZEB2 weakened miR-154-mediated suppression of tumor progression. In conclusion, these results indicate that miR-154 functions as a tumor suppressor in HCC by suppressing ZEB2, suggesting that miR-154 may serve as a potential target for HCC. 
 Double-negative feedback loop between ZEB2 and miR-145 regulates epithelial-mesenchymal transition and stem cell properties in prostate cancer cells. The invasion and metastasis of tumors are triggered by an epithelial to mesenchymal transition (EMT), which is regulated by microRNAs (miRNAs). EMT also promotes malignant tumor progression and the maintenance of the stem cell property, which endows cancer cells with the capabilities of self-renewal and immortalized proliferation. The transcriptional repressor zinc-finger E-box binding homeobox 2 (ZEB2), as an EMT activator, might be an important promoter of metastasis in some tumors. Here, we report that ZEB2 directly represses the transcription of miR-145, which is a strong repressor of EMT. In turn, ZEB2 is also a direct target of miR-145. Further, our findings show that the downregulation of ZEB2 not only represses invasion,   migration  , EMT, and the stemness of prostate cancer (PCa) cells, but also suppresses the capability of PC-3 cells to invade bone in vivo. Importantly, the expression level of ZEB2 as revealed by immunohistochemical analysis is positively correlated to bone metastasis, the serum free PSA level, the total PSA level, and the Gleason score in PCa patients and is negatively correlated with miR-145 expression in primary PCa specimens. Thus, our findings demonstrate a double-negative feedback loop between ZEB2 and miR-145 and indicate that the ZEB2/miR-145 double-negative feedback loop plays a significant role in the control of EMT and stem cell properties during the bone metastasis of PCa cells. These results suggest that the double-negative feedback loop between ZEB2 and miR-145 contributes to PCa progression and metastasis and might have therapeutic relevance for the bone metastasis of PCa. 
 miR-200c modulates ovarian cancer cell metastasis potential by targeting zinc finger E-box-binding homeobox 2 (ZEB2) expression. This study was to investigate the effect of miR-200c on regulation of ovarian cancer cell metastasis potential and explore the underlying molecular events. qRT-PCR was used to analyze the level of miR-200c expression in 48 ovarian cancer and 30 normal ovarian tissue samples. pre-miR-200c was used to manipulate miR-200c expression in ovarian cancer cells for detection of changed phenotypes of tumor cells. Bioinformatics analysis was then used to predict target genes of miR-200c and GO and pathway analyses drew the miR-200c-related gene network. Luciferase reporter assay confirmed the target of miR-200c as ZEB2. Western blot was used to detect gene expressions in ovarian cancer cells. Level of miR-200c expression was much higher in ovarian cancer than in normal ovarian tissues, and miR-200c expression was inversely associated with advanced clinical stage and lymph node metastasis of ovarian cancer (p < 0.01). The database search predicted 186 miR-200c-targeting genes, and GO analysis showed that functions of these target genes were enriched in the protein binding and other biological processes. Furthermore, miR-200c expression inhibited ovarian cancer cell ES-2   migration   and invasion capacity by suppression of ZEB2 expression (p < 0.01). Overexpression of miR-200c regulated E-cadherin and vimentin expression in ovarian cancer cells. This study demonstrated high miR-200c expression in ovarian cancer tissues and ZEB2 as a targeting gene of miR-200c, which mediated the effects of miR-200c on regulation of ovarian cancer cell   migration   and invasion capacity and epithelial-to-mesenchymal transition. Thus, targeting of miR-200c or ZEB2 may serve as a potential therapeutic strategy for control of ovarian cancer. 
 Regulation of vimentin by SIP1 in human epithelial breast tumor cells. The expression of Smad interacting protein-1 (SIP1; ZEB2) and the de novo expression of vimentin are frequently involved in epithelial-to-mesenchymal transitions (EMTs) under both normal and pathological conditions. In the present study, we investigated the potential role of SIP1 in the regulation of vimentin during the EMT associated with breast tumor cell   migration   and invasion. Examining several breast tumor cell lines displaying various degrees of invasiveness, we found SIP1 and vimentin expression only in invasive cell lines. Also, using a model of cell   migration   with human mammary MCF10A cells, we showed that SIP1 is induced specifically in vimentin-positive migratory cells. Furthermore, transfection of SIP1 cDNA in MCF10A cells increased their vimentin expression both at the mRNA and protein levels and enhanced their migratory abilities in Boyden Chamber assays. Inversely, inhibition of SIP1 expression by RNAi strategies in BT-549 cells and MCF10A cells decreased vimentin expression. We also showed that SIP1 transfection did not activate the TOP-FLASH reporter system, suggesting that the beta-catenin/TCF pathway is not implicated in the regulation of vimentin by SIP1. Our results therefore implicate SIP1 in the regulation of vimentin observed in the EMT associated with breast tumor cell   migration  , a pathway that may contribute to the metastatic progression of breast cancer. 
 MicroRNA-141 inhibits   migration   of gastric cancer by targeting zinc finger E-box-binding homeobox 2. Human microRNA (miR)-141 is a member of the miR‑200 family, which has been reported to be downregulated in gastric cancer, and involved in the proliferation of gastric cancer cells. However, little is currently known regarding its role in th  e migrati  on of gastric cancer. The present study investigated the function of miR‑141 in gastric cancer c  ell migra  tion, and evaluated the contribution of zinc finger E‑box‑binding homeobox 1 and 2 (ZEB1/2) in miR‑14  1 mediate  d migration of gastric cancer cells. The expression levels of miR‑141 and its potential ZEB1/2 targets were examined by quantitative polymerase chain reaction (qPCR) and western blotting, respe  ctively.   The migration of SGC‑7901 and HGC‑27 gastric cancer cells, which had been transfected with an miRNA precursor, was   examined   by cell migration and wound healing assays. A luciferase activity assay was used to validate whether ZEB1/2 was a direct target of miR‑141. The results demonstrated that overexpression of miR‑141 ma  rkedly in  hibited the migration of gastric cancer cells in vitro. Forced overexpression of miR‑141 significantly reduced the luciferase activity of the 3'‑untranslated region of ZEB2 in gastric cancer cells. Furthermore, the mRNA and protein expression levels of ZEB2 were reduced in cells overexpressing miR‑141, whereas the protein expression levels of E‑cadherin were increased. In gastric tumor samples the expression levels of ZEB2 were inversely correlated with the expression of miR‑141. These results suggest that miR‑141 may be involved in the   inhibitio  n of gastric cancer cell migration, and that ZEB2 is a target gene of miR-141. 
 Overexpression of ZEB2 at the invasion front of colorectal cancer is an independent prognostic marker and regulates tumor invasion in vitro. Epithelial-to-mesenchymal transition (EMT) plays a pivotal role in tumor invasion and dissemination. EMT occurs predominantly at the tumor edge where it is induced by cytokines, the extracellular matrix environment, or hypoxia. In the tumor cell, it is further mediated by several transcription factors and microRNAs. The aim of this study was to explore the expression of EMT-associated genes at the invasive front in colorectal cancer and to evaluate their prognostic significance.We evaluated the expression of 13 EMT-associated genes at the invasion front of 30 colorectal liver metastases by quantitative real-time PCR. Immunostaining against zinc finger E-box-binding homeobox 2 (ZEB2) was carried out on 175 primary colorectal cancer specimens and 30 colorectal liver metastases and correlated to clinical and histopathologic data. DLD-1 cells were transfected with siRNA and subjected to   migration   and invasion assays.Gene expression analysis and immunohistochemistry showed an upregulation of ZEB2 at the invasion front in primary colorectal cancer and liver metastases. Overexpression of ZEB2 at the invasion front correlated significantly with tumor stage in primary colorectal cancer. Moreover, univariate and multivariate analysis revealed overexpression of ZEB2 at the invasion front as an independent prognostic marker for cancer-specific survival. Downregulation of ZEB2 by siRNA decreased the   migration   and invasion capacity of DLD-1 cells in vitro.Overexpression of ZEB2 at the invasion front correlates with tumor progression and predicts cancer-specific survival in primary colorectal cancer. Therefore, ZEB2 may be interesting as biomarker and potential target for treatment of colorectal cancer. 
 A miRNA signature associated with human metastatic medullary thyroid carcinoma. MicroRNAs (miRNAs) represent a class of small, non-coding RNAs that control gene expression by targeting mRNA and triggering either translational repression or RNA degradation. The objective of our study was to evaluate the involvement of miRNAs in human medullary thyroid carcinoma (MTC) and to identify the markers of metastatic cells and aggressive tumour behaviour. Using matched primary and metastatic tumour samples, we identified a subset of miRNAs aberrantly regulated in metastatic MTC. Deregulated miRNAs were confirmed by quantitative real-time PCR and validated by in situ hybridisation on a large independent set of primary and metastatic MTC samples. Our results uncovered ten miRNAs that were significantly expressed and deregulated in metastatic tumours: miR-10a, miR-200b/-200c, miR-7 and miR-29c were down-regulated and miR-130a, miR-138, miR-193a-3p, miR-373 and miR-498 were up-regulated. Bioinformatic approaches revealed potential miRNA targets and signals involved in metastatic MTC pathways.   Migration  , proliferation and invasion assays were performed in cell lines treated with miR-200 antagomirs to ascertain a direct role for this miRNA in MTC tumourigenesis. We show that the members of miR-200 family regulate the expression of E-cadherin by directly targeting ZEB1 and ZEB2 mRNA and through the enhanced expression of tumour growth factor β (TGFβ)-2 and TGFβ-1. Overall, the treated cells shifted to a mesenchymal phenotype, thereby acquiring an aggressive phenotype with increased motility and invasion. Our data identify a robust miRNA signature associated with metastatic MTC and distinct biological processes, e.g., TGFβ signalling pathway, providing new potential insights into the mechanisms of MTC metastasis. 
 MiRNA-205 modulates cellular invasion and   migration   via regulating zinc finger E-box binding homeobox 2 expression in esophageal squamous cell carcinoma cells. Esophageal squamous cell carcinoma (ESCC) is often diagnosed at later stages until they are incurable. MicroRNA (miR) is a small, non-coding RNA that negatively regulates gene expression mainly via translational repression. Accumulating evidence indicates that deregulation of miR is associated with human malignancies including ESCC. The aim of this study was to identify miR that could be specifically expressed and exert distinct biological actions in ESCC.Total RNA was extracted from ESCC cell lines, OE21 and TE10, and a non-malignant human esophageal squamous cell line, Het-1A, and subjected to microarray analysis. Expression levels of miR that showed significant differences between the 2 ESCC and Het-1A cells based on the comprehensive analysis were analyzed by the quantitative reverse transcriptase (RT)-PCR method. Then, functional analyses, including cellular proliferation, apoptosis and Matrigel invasion and the wound healing assay, for the specific miR were conducted. Using ESCC tumor samples and paired surrounding non-cancerous tissue obtained endoscopically, the association with histopathological differentiation was examined with quantitative RT-PCR.Based on the miR microarray analysis, there were 14 miRs that showed significant differences (more than 2-fold) in expression between the 2 ESCC cells and non-malignant Het-1A. Among the significantly altered miRs, miR-205 expression levels were exclusively higher in 5 ESCC cell lines examined than any other types of malignant cell lines and Het-1A. Thus, miR-205 could be a specific miR in ESCC. Modulation of miR-205 expression by transfection with its precursor or anti-miR-205 inhibitor did not affect ESCC cell proliferation and apoptosis, but miR-205 was found to be involved in cell invasion and   migration  . Western blot revealed that knockdown of miR-205 expression in ESCC cells substantially enhanced expression of zinc finger E-box binding homeobox 2, accompanied by reduction of E-cadherin, a regulator of epithelial mesenchymal transition. The miR-205 expression levels were not associated with histological differentiation of human ESCC.These results imply that miR-205 is an ESCC-specific miR that exerts tumor-suppressive activities with EMT inhibition by targeting ZEB2. 
 Direct interaction between miR-203 and ZEB2 suppresses epithelial-mesenchymal transition signaling and reduces lung adenocarcinoma chemoresistance. miR-203 is a tumor suppressor which participates in the pathogenesis of many tumors including lung adenocarcinoma. However, the role of miR-203 in suppressing chemotherapy resistance to cisplatin (cis-diamminedichloroplatinum; DDP) as well as its molecular mechanism is still to be determined in lung adenocarcinoma. In this study, we found that miR-203 decreased lung cancer cell   migration   and invasion, and that increased miR-203 expression sensitized lung adenocarcinoma cells to DDP in vitro Furthermore, ZEB2 was found to be a direct target of miR-203, which induces epithelial-mesenchymal transition (EMT) signal. Knock-down of ZEB2 significantly increased DDP chemosensitivity in lung adenocarcinoma. More interestingly, we also demonstrated that ZEB2 could directly bind to E-box of the miR-203 promoter and suppress its expression in lung adenocarcinoma. Our data reveal that miR-203 serves as a negative feedback by directly suppressing the upstream ZEB2 gene, which inhibits EMT signaling and reduces chemoresistance of DDP. Together, these results highlight a feedback loop between miR-203 and ZEB2, which participates in the pathogenesis of lung adenocarcinoma. 
 microRNA-148a dysregulation discriminates poor prognosis of hepatocellular carcinoma in association with USP4 overexpression. Hepatocellular carcinoma (HCC) is classified as a poor prognostic tumor, and becomes frequently aggressive. MicroRNAs emerge as key contributors to tumor progression. This study investigated whether miR-148a dysregulation differentiates poor prognosis of HCC, exploring new targets of miR-148a. miR-148a dysregulation discriminated not only the overall survival and recurrence free survival rates of HCC, but the microvascular invasion. In the human HCC samples, ubiquitin specific protease 4 (USP4) and sphingosine 1-phosphate receptor 1 (S1P1) were up-regulated as the new targets of miR-148a. USP4 and S1P1 were up-regulated in mesenchymal-type liver-tumor cells with miR-148a dysregulation, facilitating   migration   and proliferation of tumor cells. The inverse relationship between miR-148a and the identified targets was verified in a tumor xenograft model. In the analysis of human samples, the expression of USP4, but not S1P1, correlated with the decrease of miR-148a. In a heterotropic patient-derived HCC xenograft model, USP4 was also overexpressed in G1 and G2 tumors when miR-148a was dysregulated, reflecting the closer link between miR-148a and USP4 for a shift in the expansion phase of tumorgraft. In conclusion, miR-148a dysregulation affects the poor prognosis of HCC. Of the identified targets of miR-148a, USP4 overexpression may contribute to HCC progression towards more aggressive feature. 
 miR-139-5p suppresses cancer cell   migration   and invasion through targeting ZEB1 and ZEB2 in GBM. Invasion and   migration   of glioblastoma multiforme (GBM) is a multistep process and an important phenotype that causes this disease to invade surrounding tissues in the brain. Recent studies have highlighted that miRNAs play a pivotal role in controlling GBM cell plasticity. In this report, we used wound healing and transwell assays to identify a novel role of miR-139-5p in inhibition of GBM cell   migration   and invasion. Bioinformatics coupled with luciferase and Western blot assays also revealed that miR-139-5p inhibited expression of ZEB1 and ZEB2, which are master regulators of tumor metastasis. MiR-139-5p specifically interacts with the 3'-UTR regions of ZEB1 and ZEB2, attenuating their expression in GBM cells. To corroborate this finding, we rescued ZEB1 and ZEB2 expression and found partial but significant increases in miR-139-5p-suppressed invasion of GBM cells. The biological relevance of our study was validated by analyzing levels of miR-139-5p in GBM tissue. We found that its expression significantly downregulated compared to normal tissue and shorter overall survival rates in patients with lower miR-139-5p expression. These results confirm that miR-139-5p suppresses GBM   migration   and invasion and highlight its potential as a biomarker and therapeutic target for treating GBM. 
 miR-139-5p inhibits epithelial-mesenchymal transition,   migration   and invasion of hepatocellular carcinoma cells by targeting ZEB1 and ZEB2. Hepatocellular carcinoma (HCC) is the third most common cause of cancer deaths worldwide. miRNAs have been suggested to have important roles in HCC development. The purpose of this study was to determine the role of miR-139-5p in regulation of epithelial-mesenchymal transition (EMT) and metastasis of HCC cells. Expression levels of miR-139-5p in 49 HCC specimens with adjacent tissues and five HCC cell lines were assessed by quantitative RT-PCR. We found that miR-139-5p was down-regulated in 89.7% of the HCC tissue samples and all of the HCC cell lines. In addition, luciferase reporter assays validated direct binding of miR-139-5p to the 3' untranslated region of zinc finger E-box binding homeobox 1 (ZEB1) and ZEB2. Ectopic expression of miR-139-5p suppressed and miR-139-in promoted EMT,   migration  , and invasion in Hep3B and SMMC7721 cells. Furthermore, over-expression of ZEB1 and ZEB2 ablated the inhibitory effects of miR-139-5p on   migration   and invasion in HCC cells. Our study indicates that miR-139-5p functions as a suppressor of HCC EMT and metastasis by targeting ZEB1 and ZEB2, and it may be a therapeutic target for metastatic HCC. 
 MiR-132 suppresses the   migration   and invasion of lung cancer cells via targeting the EMT regulator ZEB2. MicroRNAs (miRNAs) are small, non-coding RNAs which can function as oncogenes or tumor suppressor genes in human cancers. Emerging evidence reveals that deregulation of miRNAs contributes to the human non-small cell lung cancer (NSCLC). In the present study, we demonstrated that the expression levels of miR-132 were dramatically decreased in examined NSCLC cell lines and clinical NSCLC tissue samples. Then, we found that introduction of miR-132 significantly suppressed the   migration   and invasion of lung cancer cells in vitro, suggesting that miR-132 may be a novel tumor suppressor. Further studies indicated that the EMT-related transcription factor ZEB2 was one direct target genes of miR-132, evidenced by the direct binding of miR-132 with the 3' untranslated region (3' UTR) of ZEB2. Further, miR-132 could decrease the expression of ZEB2 at the levels of mRNA and protein. Notably, the EMT marker E-cadherin or vimentin, a downstream of ZEB2, was also down-regulated or up-regulated upon miR-132 treatment. Additionally, over-expressing or silencing ZEB2 was able to elevate or inhibit the   migration   and invasion of lung cancer cells, parallel to the effect of miR-132 on the lung cancer cells. Meanwhile, knockdown of ZEB2 reversed the enhanced   migration   and invasion mediated by anti-miR-132. These results indicate that miR-132 suppresses the   migration   and invasion of NSCLC cells through targeting ZEB2 involving the EMT process. Thus, our finding provides new insight into the mechanism of NSCLC progression. Therapeutically, miR-132 may serve as a potential target in the treatment of human lung cancer. 
 MicroRNA-129-5p modulates epithelial-to-mesenchymal transition by targeting SIP1 and SOX4 during peritoneal dialysis. Peritoneal dialysis (PD) is the most readily feasible home-dialysis method for renal replacement therapy. However, repeated use of PD can lead to induction of mesothelial/epithelial-mesenchymal transition (MMT/EMT) and fibrosis, eventually leading to ultrafiltration failure and discontinuation of PD. MicroRNA-129-5p (miR-129-5p) is believed to be a potent downstream inhibitor of TGF-β1 in renal fibrosis, but the effect of miR-129-5p on MMT/EMT relevant to PD is unknown. In this study, as determined by microRNA array analysis and confirmed by northern blot analysis and real-time PCR, we demonstrate that miRNA-129-5p is decreased in mesothelial cells isolated from effluent of patients having PD for more than 6 months extending to several years compared with those who have undergone PD for less than 6 months. The decreased expression of miR-129-5p was accompanied with alterations in EMT-related genes and the expression of respective proteins in vivo. In addition, in in vitro studies we noted that the expression of E-cadherin and claudin-1 were significantly reduced with increased cell   migratio  n in HMrSV5, a human peritoneal mesothelial cell line (HPMC), treated with TGF-β1, whereas expression of vimentin, fibronectin and transcription factors SIP1 and SOX4 increased significantly, as assessed by real-time PCR, western blot analysis and immunofluorescence microscopy. Furthermore, alteration in EMT-related genes and proteins were reversed by overexpression of miR-129-5p. No effect was observed in cells treated with miR-negative control. Meanwhile, inhibition of SIP1 and SOX4 with their respective siRNA also could decrease the expression of EMT-related genes and protein levels in HPMCs induced with TGF-β1. Finally, we demonstrate that SIP1 can inhibit the promoter activity of E-cadherin while enhancing the promoter activity of vimentin. We also observed that miR-129-5p could directly target the 3'UTR of SIP1 and SOX4 genes, and repressed their post-transcriptional activities. These data suggest that there is a novel TGF-β1/miR-129-5p/SIP-1 or SOX4 pathway that has a significant role in MMT and fibrosis in the setting of PD. 
 ZEB2 promotes the metastasis of gastric cancer and modulates epithelial mesenchymal transition of gastric cancer cells. Invasion and metastasis are the hallmarks of advanced gastric cancer progression. Therefore, it is urgent to overcome metastasis in order to improve the survival of gastric cancer patients.This study aimed to examine the expression of ZEB2 in gastric cancer samples and analyze its correlation with clinicopathologic features. In addition, the molecular mechanism by which ZEB2 contributes to gastric cancer metastasis will be explored.ZEB2 expression in clinical gastric cancer samples was evaluated by immunohistochemical analysis. ZEB2 was knocked-down in HGC27 gastric cancer cells by shRNA and the effects on cell invasion and   migration   were examined by in vitro cell invasion and   migration   assays. The expression of epithelial marker E-cadherin, mesenchymal markers fibronecin and vimentin, and MMPs was detected by western blot analysis.The expression of ZEB2 was positively correlated with the depth of invasion, lymph node metastasis and TNM stage. In addition, patients with positive ZEB2 expression showed a significantly shorter overall survival time than did patients with negative ZEB2. shRNA mediated knockdown of ZEB2 resulted in reduced invasion and   migration   of HGC27 cells, along with the upregulation of E-cadherin and downregulation of fibronecin, vimentin, MMP2, and MMP9.ZEB2 expression is closely associated with the clinicopathological parameters of gastric cancer. ZEB2 promotes gastric cancer cell   migration   and invasion at least partly via the regulation of epithelial-mesenchymal transition. ZEB2 is a potential target for gene therapy of aggressive gastric cancer. 
 Langerhans cell maturation is accompanied by induction of N-cadherin and the transcriptional regulators of epithelial-mesenchymal transition ZEB1/2. Langerhans cells (LCs) are a unique subset of dendritic cells (DCs) that express epithelial adhesion molecules, allowing them to form contacts with epithelial cells and reside in epidermal/epithelial tissues. The dynamic regulation of epithelial adhesion plays a decisive role in the life cycle of LCs. It controls whether LCs remain immature and sessile within the epidermis or mature and egress to initiate immune responses. So far, the molecular machinery regulating epithelial adhesion molecules during LC maturation remains elusive. Here, we generated pure populations of immature human LCs in vitro to systematically probe for gene-expression changes during LC maturation. LCs down-regulate a set of epithelial genes including E-cadherin, while they upregulate the mesenchymal marker N-cadherin known to facilitate cell   migration  . In addition, N-cadherin is constitutively expressed by monocyte-derived DCs known to exhibit characteristics of both inflammatory-type and interstitial/dermal DCs. Moreover, the transcription factors ZEB1 and ZEB2 (ZEB is zinc-finger E-box-binding homeobox) are upregulated in migratory LCs. ZEB1 and ZEB2 have been shown to induce epithelial-to-mesenchymal transition (EMT) and invasive behavior in cancer cells undergoing metastasis. Our results provide the first hint that the molecular EMT machinery might facilitate LC mobilization. Moreover, our study suggests that N-cadherin plays a role during DC   migration  . 
 HOXB8 promotes tumor metastasis and the epithelial-mesenchymal transition via ZEB2 targets in gastric cancer. The homeobox B8 (HOXB8) functions as a sequence-specific transcription factor that is involved in development. Increased expression of this gene is associated with a wide variety of tumor; however, its function in gastric cancer has not been clarified. In the present study, the expression of HOXB8 in gastric cancer tissues and influence of HOXB8 on gastric cancer cellular were evaluated.The expression levels of HOXB8 mRNA in human gastric cancer tissues were analyzed through quantitative RT-PCR. To test the role of HOXB8 in gastric cancer metastasis, the cell transwell assay was performed. Microarray, ChIP-qPCR, and Western blot were used to explore the possible mechanism that HOXB8 promotes gastric cancer cells metastasis.In this study, we found that HOXB8 showed higher expression in metastatic tissues than no-metastatic tissues. Overexpression of HOXB8 can promote gastric cancer cells   migration   and invasion, while silencing HOXB8 leads to the opposite results. Overexpression of HOXB8 also increases the rate of metastasis in NCI-N87 mice, while silencing HOXB8 has the opposite results. Furthermore, HOXB8 promotes epithelial-mesenchymal transformation of AGS cells. We also found that ZEB2 can interact with HOXB8 and may be a downstream factor of HOXB8 by using microarray. Knockdown of ZEB2 can inhibit HOXB8-induced   migration   and invasion capacity, as well as the epithelial-mesenchymal transformation in gastric cancer cells.The results showed that HOXB8 plays an important role in the development and metastasis of gastric carcinoma. 
 MicroRNA-200b regulates cell proliferation, invasion, and   migration   by directly targeting ZEB2 in gastric carcinoma. The microRNA-200 (miR-200) family has been reported to induce epithelial differentiation and suppress epithelial-mesenchymal transition (EMT) by inhibiting translation of zinc finger E-box-binding homeobox (ZEB) 1 and 2 mRNAs in several types of cancers. This study aimed to clarify the role of miR-200b in regulating EMT and promoting cellular proliferation, invasion, and   migration   in gastric cancer.The relationships among the expression levels of miR-200b, ZEB1 and ZEB2, and E-cadherin mRNAs were analyzed by quantitative real-time reverse transcription-polymerase chain reaction in frozen tissue samples from 40 gastric cancer patients who underwent gastrectomy from 2008 to 2010. The effects of miR-200b on EMT in gastric cancer cells in vitro were also analyzed.Diffuse histologic type, depth of tumor, tumor size, lymph node metastasis, and lymphatic invasion were significantly higher in the low-miR-200b expression group compared with the high expression group. There was a strong correlation between the levels of miR-200b, and ZEB2 and E-cadherin mRNAs in gastric cancer patients. Upregulation of miR-200b in gastric cancer cells changed the cell morphology from fibroblast- to epithelial-like, associated with localization of E-cadherin to the plasma membrane. ZEB2 mRNA levels fell, while E-cadherin expression levels increased in gastric cells overexpressing miR-200b, associated with significantly reduced cellular proliferation, and inhibition of cellular   migration   and invasion.miR-200b regulates ZEB2 expression and thus controls metastasis in gastric cancer. 
 TMPRSS2/ERG promotes epithelial to mesenchymal transition through the ZEB1/ZEB2 axis in a prostate cancer model. Prostate cancer is the most common non-dermatologic malignancy in men in the Western world. Recently, a frequent chromosomal aberration fusing androgen regulated TMPRSS2 promoter and the ERG gene (TMPRSS2/ERG) was discovered in prostate cancer. Several studies demonstrated cooperation between TMPRSS2/ERG and other defective pathways in cancer progression. However, the unveiling of more specific pathways in which TMPRSS2/ERG takes part, requires further investigation. Using immortalized prostate epithelial cells we were able to show that TMPRSS2/ERG over-expressing cells undergo an Epithelial to Mesenchymal Transition (EMT), manifested by acquisition of mesenchymal morphology and markers as well as   migration   and invasion capabilities. These findings were corroborated in vivo, where the control cells gave rise to discrete nodules while the TMPRSS2/ERG-expressing cells formed malignant tumors, which expressed EMT markers. To further investigate the general transcription scheme induced by TMPRSS2/ERG, cells were subjected to a microarray analysis that revealed a distinct EMT expression program, including up-regulation of the EMT facilitators, ZEB1 and ZEB2, and down-regulation of the epithelial marker CDH1(E-Cadherin). A chromatin immunoprecipitation assay revealed direct binding of TMPRSS2/ERG to the promoter of ZEB1 but not ZEB2. However, TMPRSS2/ERG was able to bind the promoters of the ZEB2 modulators, IL1R2 and SPINT1. This set of experiments further illuminates the mechanism by which the TMPRSS2/ERG fusion affects prostate cancer progression and might assist in targeting TMPRSS2/ERG and its downstream targets in future drug design efforts. 
 Identification of a ZEB2-MITF-ZEB1 transcriptional network that controls melanogenesis and melanoma progression. Deregulation of signaling pathways that control differentiation, expansion and   migration   of neural crest-derived melanoblasts during normal development contributes also to melanoma progression and metastasis. Although several epithelial-to-mesenchymal (EMT) transcription factors, such as zinc finger E-box binding protein 1 (ZEB1) and ZEB2, have been implicated in neural crest cell biology, little is known about their role in melanocyte homeostasis and melanoma. Here we show that mice lacking Zeb2 in the melanocyte lineage exhibit a melanoblast   migration   defect and, unexpectedly, a severe melanocyte differentiation defect. Loss of Zeb2 in the melanocyte lineage results in a downregulation of the Microphthalmia-associated transcription factor (Mitf) and melanocyte differentiation markers concomitant with an upregulation of Zeb1. We identify a transcriptional signaling network in which the EMT transcription factor ZEB2 regulates MITF levels to control melanocyte differentiation. Moreover, our data are also relevant for human melanomagenesis as loss of ZEB2 expression is associated with reduced patient survival. 
 MicroRNA-200a inhibits CD133/1+ ovarian cancer stem cells   migration   and invasion by targeting E-cadherin repressor ZEB2. MicroRNA-200a (miR-200a) has been reported to be a prognostic marker and to play an important role in ovarian cancer progression. The aim of the study was to elucidate the mechanism of miR-200a involved in   migration   and invasion in CD133/1+ ovarian cancer stem cells (OCSCs).The expression of miR-200a between CD133/1+ and CD133/1- cells was performed using real-time PCR, and wound healing assay and matrigel invasion assay were used to detect   migration   and invasion of CD133/1+ cells, respectively, target gene regulated by miR-200a was detected using Dual Luciferase Reporter system, The expression levels of target gene were confirmed using real-time PCR and western blot.miR-200a was downregulated in CD133/1+ cells compared with CD133/1- cells, and overexpression of miR-200a significantly reduced CD133/1+ cells   migration   and invasion compare with negative control (NC) (p<0.05). The 3'-UTR of ZEB2 mRNA, a transcriptional repressor of E-cadherin, was found to be regulated directly by miR-200a. In addition, when miR-200a was overexpressed in CD133/1+ cells, the mRNA and protein levels of ZEB2 were both suppressed, which resulted in an increase in the E-cadherin expression level, suggesting that ZEB2 was a functionally important target of miR-200a in CD133/1+ cells.Our results suggest that loss of expression of miR-200a may play a critical role in the repression of E-cadherin by ZEB2, thereby enhancing   migration   and invasion in CD133/1+ cells. 
 miR-200a-mediated downregulation of ZEB2 and CTNNB1 differentially inhibits nasopharyngeal carcinoma cell growth,   migration   and invasion. Nasopharyngeal carcinoma (NPC), a highly metastatic and invasive malignant tumor originating from the nasopharynx, is widely prevalent in Southeast Asia, the Middle East and North Africa. Although viral, dietary and genetic factors have been implicated in NPC, the molecular basis of its pathogenesis is not well defined. Based on a recent microRNA (miRNA) microarray study showing miR-200 downregulation in NPC, we further investigated the role of miR-200a in NPC carcinogenesis. We found that the endogenous miR-200a expression level increases with the degree of differentiation in a panel of NPC cell lines, namely undifferentiated C666-1, high-differentiated CNE-1, and low-differentiated CNE-2 and HNE1 cells. By a series of gain-of-function and loss-of-function studies, we showed that over-expression of miR-200a inhibits C666-1 cell growth,   migration   and invasion, whereas its knock-down stimulates these processes in CNE-1 cells. In addition, we further identified ZEB2 and CTNNB1 as the functional downstream targets of miR-200a. Interestingly, knock-down of ZEB2 solely impeded NPC cell   migration   and invasion, whereas CTNNB1 suppression only inhibited NPC cell growth, suggesting that the inhibitory effects of miR-200a on NPC cell growth,   migration   and invasion are mediated by distinct targets and pathways. Our results reveal the important role of miR-200a as a regulatory factor of NPC carcinogenesis and a potential candidate for miRNA-based therapy against NPC. 
 MicroRNA-335 inhibits invasion and metastasis of colorectal cancer by targeting ZEB2. MicroRNAs have been suggested to play a vital role in regulate tumor progression and invasion. However, the expression of miR-335 in colorectal cancer (CRC) and its clinical significance are not known. Here, we report that miR-335 is a tumor suppressor by regulating expression of ZEB2. In this study, we showed that downregulated miR-335 levels in highly invasive CRC cell lines and tissues. Kaplan-Meier survival analysis indicated that patients with reduced miR-335 had a poor overall survival. Furthermore, enhancing the expression of miR-335 inhibited CRC cell   migration   and invasion in vitro and lung and liver metastasis in vivo, while silencing its expression resulted in increased   migration   and invasion. Additionally, we identified a novel miR-335 target, ZEB2, and the direct interaction between them was verified by 3'-untranslated region dual-luciferase reporter assay. In conclusion, our results demonstrate that miR-335 functions as a tumor suppressor and play a role in inhibiting metastasis of CRC cells through targeting ZEB2. These findings suggest that miR-335 may be useful as a new potential therapeutic target for CRC. 
 Zeb2: A multifunctional regulator of nervous system development. Zinc finger E-box binding homeobox (Zeb) 2 is a transcription factor, identified due its ability to bind Smad proteins, and consists of multiple functional domains which interact with a variety of transcriptional co-effectors. The complex nature of the Zeb2, both at its genetic and protein levels, underlie its multifunctional properties, with Zeb2 capable of acting individually or as part of a transcriptional complex to repress, and occasionally activate, target gene expression. This review introduces Zeb2 as an essential regulator of nervous system development. Zeb2 is expressed in the nervous system throughout its development, indicating its importance in neurogenic and gliogenic processes. Indeed, mutation of Zeb2 has dramatic neurological consequences both in animal models, and in humans with Mowat-Wilson syndrome, which results from heterozygous ZEB2 mutations. The mechanisms by which Zeb2 regulates the induction of the neuroectoderm (CNS primordium) and the neural crest (PNS primordium) are reviewed herein. We then describe how Zeb2 acts to direct the formation, delamination,   migration   and specification of neural crest cells. Zeb2 regulation of the development of a number of cerebral regions, including the neocortex and hippocampus, are then described. The diverse molecular mechanisms mediating Zeb2-directed development of various neuronal and glial populations are reviewed. The role of Zeb2 in spinal cord and enteric nervous system development is outlined, while its essential function in CNS myelination is also described. Finally, this review discusses how the neurodevelopmental defects of Zeb2 mutant mice delineate the developmental dysfunctions underpinning the multiple neurological defects observed in Mowat-Wilson syndrome patients. 
 microRNA-145 modulates epithelial-mesenchymal transition and suppresses proliferation,   migration   and invasion by targeting SIP1 in human cervical cancer cells. Previously, it has been reported that microRNA-145 (miR-145) is lowly expressed in human cervical cancers and that its putative tumour suppressive role may be attributed to epithelial-mesenchymal transition (EMT) regulation. Here, we aimed to assess whether miR-145 may affect EMT-associated markers/genes and suppress cervical cancer growth and motility, and to provide a mechanistic basis for these phenomena.The identification of the SMAD-interacting protein 1 (SIP1) mRNA as putative miR-145 target was investigated using a 3' untranslated region (3'UTR) luciferase assay and Western blotting, respectively. The functional effects of exogenous miR-145 expression, miR-145 suppression or siRNA-mediated SIP1 expression down-regulation in cervical cancer-derived C33A and SiHa cells were analysed using Western blotting, BrdU incorporation (proliferation), transwell   migratio  n and invasion assays. In addition, the expression levels of miR-145 and SIP1 were determined in primary human cervical cancer and non-cancer tissue samples using qRT-PCR.We found that miR-145 binds to the wild-type 3'UTR of SIP1, but not to its mutant counterpart, and that, through this binding, miR-145 can effectively down-regulate SIP1 expression. In addition, we found that exogenous miR-145 expression or siRNA-mediated down-regulation of SIP1 expression attenuates the proliferation,   migratio  n and invasion of C33A and SiHa cells and alters the expression of the EMT-associated markers CDH1, VIM and SNAI1, whereas inhibition of endogenous miR-145 expression elicited the opposite effects. The expression of miR-145 in cervical cancer tissue samples was found to be low, while that of SIP1 was found to be high compared to non-cancerous cervical tissues. An inverse expression correlation between the two was substantiated through the anlaysis of data deposited in the TCGA database.Our data indicate that low miR-145 expression levels in conjunction with elevated SIP1 expression levels may contribute to cervical cancer development. MiR-145-mediated regulation of SIP1 provides a novel mechanistic basis for its tumour suppressive mode of action in human cervical cancer cells. 
 MicroRNA-141 regulates Smad interacting protein 1 (SIP1) and inhibits   migration   and invasion of colorectal cancer cells. Colorectal cancer (CRC) is the third most common cancer in the world. Despite recent advances in diagnostics and treatment, prognosis for patients with advanced disease is still poor. microRNAs (miRNAs) are a class of endogenous, small noncoding RNA molecules which are crucial regulators of gene expression at the posttranscriptional level. miRNAs participate in many biological events and play an important role in various human diseases, including CRC.This study is to identify the role of miR-141 in   migration   and invasion of CRC cells.Expression of miR-141 and Smad interacting protein 1 (SIP1) were detected by real-time reverse-transcription polymerase chain reaction (RT-PCR) and Western blotting. The effect of miR-141 on   migration   and invasion of CRC cells was investigated using wound healing assay and Matrigel invasion assay in vitro. The regulation effect of miR-141 on SIP1 was evaluated by dual-luciferase reporter assay.We demonstrated that miR-141 levels correlate inversely with SIP1 protein levels as well as cell   migration   and invasion of CRC cells. SIP1 was identified as a functional target of miR-141.miR-141 regulates SIP1 to inhibit   migration   and invasion of CRC cells. miR-141 and SIP1 might be candidate therapeutic targets in CRC. 
 Role and prognostic significance of the epithelial-mesenchymal transition factor ZEB2 in ovarian cancer. ZEB2 is a key factor in epithelial-mesenchymal transition (EMT), a program controlling cell   migration   in embryonic development and adult tissue homeostasis. We demonstrated a role of ZEB2 in   migration   and anchorage-independent cell growth in ovarian cancer, as shown by ZEB2 silencing. We found that the RNA-binding protein HuR bound the 3'UTR of ZEB2 mRNA, acting as a positive regulator of ZEB2 protein expression. In Hey ovarian cell line, HuR silencing decreased ZEB2 and ZEB1 nuclear expression and impaired   migration  . In hypoglycemic conditions ZEB2 expression decreased, along with ZEB1, vimentin and cytoplasmic HuR, and a reduced cellular   migration   ability was observed. Analysis of ZEB2 and HuR expression in ovarian cancers revealed that nuclear ZEB2 is localized in tumor leading edge and co-localizes with cytoplasmic HuR. In a series of 143 ovarian cancer patients high expression of ZEB2 mRNA significantly correlated with a poor prognosis in term of both overall survival and progression- free survival. Moreover, at immunohistochemical evaluation, we found that prognostic significance of ZEB2 protein relies on its nuclear expression and co-localization with cytoplasmic HuR. In conclusion our findings indicated that nuclear ZEB2 may enhance progression of EMT transition and acquisition of an aggressive phenotype in ovarian cancer. 
 ZEB2 mediates multiple pathways regulating cell proliferation,   migration  , invasion, and apoptosis in glioma. The aim of the present study was to analyze the expression of Zinc finger E-box Binding homeobox 2 (ZEB2) in glioma and to explore the molecular mechanisms of ZEB2 that regulate cell proliferation,   migration  , invasion, and apoptosis.Expression of ZEB2 in 90 clinicopathologically characterized glioma patients was analyzed by immunohistochemistry. Furthermore, siRNA targeting ZEB2 was transfected into U251 and U87 glioma cell lines in vitro and proliferation,   migration  , invasion, and apoptosis were examined separately by MTT assay, Transwell chamber assay, flow cytometry, and western blot.The expression level of ZEB2 protein was significantly increased in glioma tissues compared to normal brain tissues (P<0.001). In addition, high levels of ZEB2 protein were positively correlated with pathology grade classification (P = 0.024) of glioma patients. Knockdown of ZEB2 by siRNA suppressed cell proliferation,   migration   and invasion, as well as induced cell apoptosis in glioma cells. Furthermore, ZEB2 downregulation was accompanied by decreased expression of CDK4/6, Cyclin D1, Cyclin E, E2F1, and c-myc, while p15 and p21 were upregulated. Lowered expression of ZEB2 enhanced E-cadherin levels but also inhibited β-Catenin, Vimentin, N-cadherin, and Snail expression. Several apoptosis-related regulators such as Caspase-3, Caspase-6, Caspase-9, and Cleaved-PARP were activated while PARP was inhibited after ZEB2 siRNA treatment.Overexpression of ZEB2 is an unfavorable factor that may facilitate glioma progression. Knockdown ZEB2 expression by siRNA suppressed cell proliferation,   migratio  n, invasion and promoted cell apoptosis in glioma cells. 
 Smad interacting protein 1 (SIP1) is associated with peritoneal carcinomatosis in intestinal type gastric cancer. Smad interacting protein 1 (SIP1) is an epithelial-mesenchymal transition (EMT)-inducible gene that plays a key role in tumor progression in various cancers. This study seeks to clarify the clinical and biological significance of SIP1 expression, especially in intestinal type gastric cancer. We analyzed the mRNA levels of SIP1 and other EMT regulators by real-time reverse transcription PCR in gastric tissue samples of 134 gastric cancer patients, and in five gastric cancer cell lines. SIP1 gene knockdown by siRNA transfection was performed to evaluate SIP1 function in gastric cancer cells. Expression of the SIP1 gene was significantly higher in cancerous tissue than in adjacent normal mucosa. Although the mRNA expression of the other EMT regulators tested (Snail, Slug, and Twist) was not correlated with clinicopathological factors, increased SIP1 expression was an independent prognostic factor and an independent risk factor for peritoneal dissemination. In addition, SIP1 expression was significantly positive and correlated with vimentin expression. For intestinal type gastric cancer in particular, elevated SIP1 expression was significantly correlated with peritoneal dissemination and poor prognosis (p < 0.05). In vitro, cell proliferation  , migrati  on, invasion, and resistance to anoikis were significantly inhibited in SIP1 siRNA-transfected MKN7 cells compared to control siRNA. SIP1 appears to play an important role in progression to peritoneal carcinomatosis and may be a therapeutic target for patients with intestinal type gastric cancer. 
 A Systematic Approach to Defining the microRNA Landscape in Metastasis. The microRNA (miRNA) landscape changes during the progression of cancer. We defined a metastasis-associated miRNA landscape using a systematic approach. We profiled and validated miRNA and mRNA expression in a unique series of human colorectal metastasis tissues together with their matched primary tumors and corresponding normal tissues. We identified an exclusive miRNA signature that is differentially expressed in metastases. Three of these miRNAs were identified as key drivers of an EMT-regulating network acting though a number of novel targets. These targets include SIAH1, SETD2, ZEB2, and especially FOXN3, which we demonstrated for the first time as a direct transcriptional suppressor of N-cadherin. The modulation of N-cadherin expression had significant impact on   migration  , invasion, and metastasis in two different in vivo models. The significant deregulation of the miRNAs defining the network was confirmed in an independent patient set as well as in a database of diverse malignancies derived from more than 6,000 patients. Our data define a novel metastasis-orchestrating network based on systematic hypothesis generation from metastasis tissues. 
  GeneRIFs  
 Knockdown of ZEB2 can inhibit HOXB8-induced   migration   and invasion capacity, as well as the epithelial-mesenchymal transformation in gastric cancer cells. The results showed that HOXB8 plays an important role in the development and metastasis of gastric carcinoma 
 restoration of miR-144 in K1 and WRO thyroid cancer cells suppressed invasion and   migration   capability of these cells; miR-144 also suppressed expression of ZEB1 and ZEB2, two E-cadherin suppressors, by directly binding to their 3'-untranslated regions. 
 These results indicate that miR-132 suppresses the   migration   and invasion of NSCLC cells through targeting ZEB2 involving the EMT process 
 ZEB2 expression is closely associated with the clinicopathological parameters of gastric cancer. ZEB2 promotes gastric cancer cell   migration   and invasion via the regulation of epithelial-mesenchymal transition. 
 Results suggest that loss of expression of miR-200a may play a critical role in the repression of E-cadherin by ZEB2, thereby enhancing   migration   and invasion in CD133/1+ cells. 
 loss of expression of the miR-200 family members may play a critical role in the repression of E-cadherin by ZEB1 and ZEB2 during EMT, thereby enhancing   migration   and invasion during cancer progression. 
 results therefore implicate SIP1 in the regulation of vimentin observed in the EMT associated with breast tumor cell   migration  , a pathway that may contribute to the metastatic progression of breast cancer 
  [Ee]xtracellular [Mm]atrix  PubMed  
 MicroRNA-192 in diabetic kidney glomeruli and its function in TGF-beta-induced collagen expression via inhibition of E-box repressors. Key features of diabetic nephropathy (DN) include the accumulation of   extracellular matrix   proteins such as collagen 1-alpha 1 and -2 (Col1a1 and -2). Transforming growth factor beta1 (TGF-beta), a key regulator of these   extracellular matrix   genes, is increased in mesangial cells (MC) in DN. By microarray profiling, we noted that TGF-beta increased Col1a2 mRNA in mouse MC (MMC) but also decreased mRNA levels of an E-box repressor, deltaEF1. TGF-beta treatment or short hairpin RNAs targeting deltaEF1 increased enhancer activity of upstream E-box elements in the Col1a2 gene. TGF-beta also decreased the expression of Smad-interacting protein 1 (SIP1), another E-box repressor similar to deltaEF1. Interestingly, we noted that SIP1 is a target of microRNA-192 (miR-192), a key miR highly expressed in the kidney. miR-192 levels also were increased by TGF-beta in MMC. TGF-beta treatment or transfection with miR-192 decreased endogenous SIP1 expression as well as reporter activity of a SIP1 3' UTR-containing luciferase construct in MMC. Conversely, a miR-192 inhibitor enhanced the luciferase activity, confirming SIP1 to be a miR-192 target. Furthermore, miR-192 synergized with deltaEF1 short hairpin RNAs to increase Col1a2 E-box-luc activity. Importantly, the in vivo relevance was noted by the observation that miR-192 levels were enhanced significantly in glomeruli isolated from streptozotocin-injected diabetic mice as well as diabetic db/db mice relative to corresponding nondiabetic controls, in parallel with increased TGF-beta and Col1a2 levels. These results uncover a role for miRs in the kidney and DN in controlling TGF-beta-induced Col1a2 expression by down-regulating E-box repressors. 
 Overexpression of ZEB2 at the invasion front of colorectal cancer is an independent prognostic marker and regulates tumor invasion in vitro. Epithelial-to-mesenchymal transition (EMT) plays a pivotal role in tumor invasion and dissemination. EMT occurs predominantly at the tumor edge where it is induced by cytokines, the   extracellular matrix   environment, or hypoxia. In the tumor cell, it is further mediated by several transcription factors and microRNAs. The aim of this study was to explore the expression of EMT-associated genes at the invasive front in colorectal cancer and to evaluate their prognostic significance.We evaluated the expression of 13 EMT-associated genes at the invasion front of 30 colorectal liver metastases by quantitative real-time PCR. Immunostaining against zinc finger E-box-binding homeobox 2 (ZEB2) was carried out on 175 primary colorectal cancer specimens and 30 colorectal liver metastases and correlated to clinical and histopathologic data. DLD-1 cells were transfected with siRNA and subjected to migration and invasion assays.Gene expression analysis and immunohistochemistry showed an upregulation of ZEB2 at the invasion front in primary colorectal cancer and liver metastases. Overexpression of ZEB2 at the invasion front correlated significantly with tumor stage in primary colorectal cancer. Moreover, univariate and multivariate analysis revealed overexpression of ZEB2 at the invasion front as an independent prognostic marker for cancer-specific survival. Downregulation of ZEB2 by siRNA decreased the migration and invasion capacity of DLD-1 cells in vitro.Overexpression of ZEB2 at the invasion front correlates with tumor progression and predicts cancer-specific survival in primary colorectal cancer. Therefore, ZEB2 may be interesting as biomarker and potential target for treatment of colorectal cancer. 
  [Ii]nvasive  PubMed  
 Androgen receptor as a regulator of ZEB2 expression and its implications in epithelial-to-mesenchymal transition in prostate cancer. Zinc finger E-box-binding protein 2 (ZEB2) is known to help mediate the epithelial-to-mesenchymal transition, and thereby it facilitates cancer metastasis. This study was initiated to explore whether ZEB2 expression differs in prostate cancer (PCa, n=7) and benign prostatic hyperplasia (BPH, n=7) tissues. In PCa tissues, the levels of both immunoreactive ZEB2 and androgen receptor (AR) were found to be significantly higher (P<0.05) when compared with BPH tissues. Co-regulation of AR and ZEB2 prompted us to investigate the role of androgenic stimuli in ZEB2 expression. ZEB2 expression was found to be significantly (P<0.05) upregulated after androgen stimulation and downregulated following AR silencing in LNCaP cells, an androgen-dependent PCa cell line. This finding suggested AR as a positive regulator of ZEB2 expression in androgen-dependent cells. Paradoxically, androgen-independent (AI) cell lines PC3 and DU145, known to possess low AR levels, showed significantly (P<0.05) higher expression of ZEB2 compared with LNCaP cells. Furthermore, forced expression of AR in PC3 (PC3-AR) and DU145 (DU-AR) cells led to reductions in ZEB2 expression,   invasive  ness, and migration. These cells also exhibited an increase in the levels of E-cadherin (a transcriptional target of ZEB2). Co-transfection of AR and ZEB2 cDNA constructs prevented the decline in   invasive  ness and migration to a significant extent. Additionally, ZEB2 downregulation was associated with an increase in miR200a/miR200b levels in PC3-AR cells and with a decrease in miR200a/miR200b levels in AR-silenced LNCaP cells. Thus, AR acts as a positive regulator of ZEB2 expression in androgen-dependent cells and as a negative regulator in AI PCa cells. 
 Fra-1/AP-1 induces EMT in mammary epithelial cells by modulating Zeb1/2 and TGFβ expression. Epithelial-to-mesenchymal transition (EMT) is essential for embryonic morphogenesis and wound healing and critical for tumour cell invasion and dissemination. The AP-1 transcription factor Fra-1 has been implicated in tumorigenesis and in tumour-associated EMT in human breast cancer. We observed a significant inverse correlation between Fra-1 mRNA expression and distant-metastasis-free survival in a large cohort of breast cancer patients derived from multiple array data sets. This unique correlation among Fos genes prompted us to assess the evolutionary conservation between Fra-1 functions in EMT of human and mouse cells. Ectopic expression of Fra-1 in fully polarized, non-tumourigenic, mouse mammary epithelial EpH4 cells induced a mesenchymal phenotype, characterized by a loss of epithelial and gain of mesenchymal markers. Proliferation, motility and   invasiv  eness were also increased in the resulting EpFra1 cells, and the cells were tumourigenic and efficiently colonized the lung upon transplantation. Molecular analyses revealed increased expression of Tgfβ1 and the EMT-inducing transcription factors Zeb1, Zeb2 and Slug. Mechanistically, Fra-1 binds to the tgfb1 and zeb2 promoters and to an evolutionarily conserved region in the first intron of zeb1. Furthermore, increased activity of a zeb2 promoter reporter was detected in EpFra1 cells and shown to depend on AP-1-binding sites. Inhibiting TGFβ signalling in EpFra1 cells moderately increased the expression of epithelial markers, whereas silencing of zeb1 or zeb2 restored the epithelial phenotype and decreased migration in vitro and tumorigenesis in vivo. Thus Fra-1 induces changes in the expression of genes encoding EMT-related transcription factors leading to the acquisition of mesenchyma  l, invas  ive and tumorigenic capacities by epithelial cells. This study defines a novel function of Fra-1/AP-1 in modulating tgfb1, zeb1 and zeb2 expression through direct binding to genomic regulatory regions, which establishes a basis for future in vivo genetic manipulations and preclinical studies using mouse models. 
 Regulation of vimentin by SIP1 in human epithelial breast tumor cells. The expression of Smad interacting protein-1 (SIP1; ZEB2) and the de novo expression of vimentin are frequently involved in epithelial-to-mesenchymal transitions (EMTs) under both normal and pathological conditions. In the present study, we investigated the potential role of SIP1 in the regulation of vimentin during the EMT associated with breast tumor cell migration and invasion. Examining several breast tumor cell lines displaying various degrees of   invasive  ness, we found SIP1 and vimentin expression only in   invasive   cell lines. Also, using a model of cell migration with human mammary MCF10A cells, we showed that SIP1 is induced specifically in vimentin-positive migratory cells. Furthermore, transfection of SIP1 cDNA in MCF10A cells increased their vimentin expression both at the mRNA and protein levels and enhanced their migratory abilities in Boyden Chamber assays. Inversely, inhibition of SIP1 expression by RNAi strategies in BT-549 cells and MCF10A cells decreased vimentin expression. We also showed that SIP1 transfection did not activate the TOP-FLASH reporter system, suggesting that the beta-catenin/TCF pathway is not implicated in the regulation of vimentin by SIP1. Our results therefore implicate SIP1 in the regulation of vimentin observed in the EMT associated with breast tumor cell migration, a pathway that may contribute to the metastatic progression of breast cancer. 
 A NOTCH3-mediated squamous cell differentiation program limits expansion of EMT-competent cells that express the ZEB transcription factors. Zinc finger E-box-binding (ZEB) proteins ZEB1 and ZEB2 are transcription factors essential in TGF-β-mediated senescence, epithelial-to-mesenchymal transition (EMT), and cancer stem cell functions. ZEBs are negatively regulated by members of the miR-200 microRNA family, but precisely how tumor cells expressing ZEBs emerge during   invasiv  e growth remains unknown. Here, we report that NOTCH3-mediated signaling prevents expansion of a unique subset of ZEB-expressing cells. ZEB expression was associated with the lack of cellular capability of undergoing NOTCH3-mediated squamous differentiation in human esophageal cells. Genetic inhibition of the Notch-mediated transcriptional activity by dominant-negative Mastermind-like 1 (DNMAML1) prevented squamous differentiation and induction of Notch target genes including NOTCH3. Moreover, DNMAML1-enriched EMT-competent cells exhibited robust upregulation of ZEBs, downregulation of the miR-200 family, and enhanced anchorage-independent growth and tumor formation in nude mice. RNA interference experiments suggested the involvement of ZEBs in anchorage-independent colony formation, invasion, and TGF-β-mediated EMT  . Invasi  ve growth and impaired squamous differentiation were recapitulated upon Notch inhibition by DNMAML1 in organotypic three-dimensional culture, a form of human tissue engineering. Together, our findings indicate that NOTCH3 is a key factor limiting the expansion of ZEB-expressing cells, providing novel mechanistic insights into the role of Notch signaling in the cell fate regulation and disease progression of esophageal squamous cancers. 
 Co-Expression of TWIST1 and ZEB2 in Oral Squamous Cell Carcinoma Is Associated with Poor Survival. Oral squamous cell carcinoma (OSCC) is an aggressive disease accounting for more than 260,000 cancer cases diagnosed and 128,000 deaths worldwide. A large majority of cancer deaths result from cancers that have metastasized beyond the primary tumor. The relationship between genetic changes and clinical outcome can reflect the biological events that promote cancer's aggressive behavior, and these can serve as molecular markers for improved patient management and survival. To this end, epithelial-mesenchymal transition (EMT) is a major process that promotes tumor invasion and metastasis, making EMT-related proteins attractive diagnostic biomarkers and therapeutic targets. In this study, we used immunohistochemistry to study the expression of a panel of transcription factors (TWIST1, SNAI1/2, ZEB1 and ZEB2) and other genes intimately related to EMT (CDH1 and LAMC2) at the   invasive   tumor front of OSCC tissues. The association between the expression of these proteins and clinico-pathological parameters were examined with Pearson Chi-square and correlation with survival was analyzed using Kaplan Meier analysis. Our results demonstrate that there was a significant differential expression of CDH1, LAMC2, SNAI1/2 and TWIST1 between OSCC and normal oral mucosa (NOM). Specifically, CDH1 loss was significantly associated with Broder's grading, while diffused LAMC2 was similarly associated with non-cohesive pattern of invasion. Notably, co-expression of TWIST1 and ZEB2 in OSCC was significantly associated with poorer overall survival, particularly in patients without detectable lymph node metastasis. This study demonstrates that EMT-related proteins are differentially expressed in OSCC and that the co-expression of TWIST1 and ZEB2 could be of clinical value in identifying patients with poor survival for appropriate patient management. 
 Overexpression of ZEB2 at the invasion front of colorectal cancer is an independent prognostic marker and regulates tumor invasion in vitro. Epithelial-to-mesenchymal transition (EMT) plays a pivotal role in tumor invasion and dissemination. EMT occurs predominantly at the tumor edge where it is induced by cytokines, the extracellular matrix environment, or hypoxia. In the tumor cell, it is further mediated by several transcription factors and microRNAs. The aim of this study was to explore the expression of EMT-associated genes at the   invasive   front in colorectal cancer and to evaluate their prognostic significance.We evaluated the expression of 13 EMT-associated genes at the invasion front of 30 colorectal liver metastases by quantitative real-time PCR. Immunostaining against zinc finger E-box-binding homeobox 2 (ZEB2) was carried out on 175 primary colorectal cancer specimens and 30 colorectal liver metastases and correlated to clinical and histopathologic data. DLD-1 cells were transfected with siRNA and subjected to migration and invasion assays.Gene expression analysis and immunohistochemistry showed an upregulation of ZEB2 at the invasion front in primary colorectal cancer and liver metastases. Overexpression of ZEB2 at the invasion front correlated significantly with tumor stage in primary colorectal cancer. Moreover, univariate and multivariate analysis revealed overexpression of ZEB2 at the invasion front as an independent prognostic marker for cancer-specific survival. Downregulation of ZEB2 by siRNA decreased the migration and invasion capacity of DLD-1 cells in vitro.Overexpression of ZEB2 at the invasion front correlates with tumor progression and predicts cancer-specific survival in primary colorectal cancer. Therefore, ZEB2 may be interesting as biomarker and potential target for treatment of colorectal cancer. 
 An autocrine TGF-beta/ZEB/miR-200 signaling network regulates establishment and maintenance of epithelial-mesenchymal transition. Epithelial-mesenchymal transition (EMT) is a form of cellular plasticity that is critical for embryonic development and tumor metastasis. A double-negative feedback loop involving the miR-200 family and ZEB (zinc finger E-box-binding homeobox) transcription factors has been postulated to control the balance between epithelial and mesenchymal states. Here we demonstrate using the epithelial Madin Darby canine kidney cell line model that, although manipulation of the ZEB/miR-200 balance is able to repeatedly switch cells between epithelial and mesenchymal states, the induction and maintenance of a stable mesenchymal phenotype requires the establishment of autocrine transforming growth factor-β (TGF-β) signaling to drive sustained ZEB expression. Furthermore, we show that prolonged autocrine TGF-β signaling induced reversible DNA methylation of the miR-200 loci with corresponding changes in miR-200 levels. Collectively, these findings demonstrate the existence of an autocrine TGF-β/ZEB/miR-200 signaling network that regulates plasticity between epithelial and mesenchymal states. We find a strong correlation between ZEBs and TGF-β and negative correlations between miR-200 and TGF-β and between miR-200 and ZEB  s, in in  vasive ductal carcinomas, consistent with an autocrine TGF-β/ZEB/miR-200 signaling network being active in breast cancers. 
 MicroRNA-153 functions as a tumor suppressor by targeting SET7 and ZEB2 in ovarian cancer cells. The overall goal of the present study was to find and validate unidentified miRNAs that regulate epithelial-mesenchymal transition (EMT) and proliferation in ovarian cancer. Furthermore, we demonstrate that the high expression of miR-153 in human epithelial ovarian cancer (EOC) is associated with better survival. The mean expression level of miR-153 in ovarian cancer was significantly lower than in the adjacent carcinoma tissue. In the present study, we report that miR-153 are negative regulators of SET7 and ZEB2, miR-153 regulates SET7/ZEB2 expression and promotes SET7/ZEB2 mRNA degradation. Further, confirmed by reporter assays, SET7/ZEB2 are downstream targets of miR-153 directly bound to the 3' untranslated region (3'-UTR). Clone formation and wound-healing assay as well as Transwell assay proved that silencing of SET7 or ZEB2 partially abolished the enhancement of cell proliferation and invasion induced by downregulated miR-153. SET7 and ZEB2 are negatively correlated with miR-153 expression in human ovarian cancer and indicated a worse survival. Considering the role of SET7 and ZEB2 in EOC, it is important to clarify how the expression of SET7 and ZEB2 are regulated. Based on our results miR-153 inhibits proliferation and suppresses EMT and the   invasive   potential of ovarian cancer cells through downregulation of SET7 and ZEB2, supporting the pursuit of miR-153 as a potential target for ovarian cancer intervention. 
 Direct repression of cyclin D1 by SIP1 attenuates cell cycle progression in cells undergoing an epithelial mesenchymal transition. Zinc finger transcription factors of the Snail/Slug and ZEB-1/SIP1 families control epithelial-mesenchymal transitions in development in cancer. Here, we studied SIP1-regulated mesenchymal conversion of epidermoid A431 cells. We found that concomitant with inducing   invasive   phenotype, SIP1 inhibited expression of cyclin D1 and induced hypophosphorylation of the Rb tumor suppressor protein. Repression of cyclin D1 was caused by direct binding of SIP1 to three sequence elements in the cyclin D1 gene promoter. By expressing exogenous cyclin D1 in A431/SIP1 cells and using RNA interference, we demonstrated that the repression of cyclin D1 gene by SIP1 was necessary and sufficient for Rb hypophosphorylation and accumulation of cells in G1 phase. A431 cells expressing SIP1 along with exogenous cyclin D1 were highly   invasive  , indicating that SIP1-regulated invasion is independent of attenuation of G1/S progression. However, in another epithelial-mesenchymal transition model, gradual mesenchymal conversion of A431 cells induced by a dominant negative mutant of E-cadherin produced no effect on the cell cycle. We suggest that impaired G1/S phase progression is a general feature of cells that have undergone EMT induced by transcription factors of the Snail/Slug and ZEB-1/SIP1 families. 
 Vimentin, zeb1 and Sip1 are up-regulated in triple-negative and basal-like breast cancers: association with an aggressive tumour phenotype. In epithelial-to-mesenchymal transition (EMT) epithelial cancer cells achieve mesenchymal features, essentially helping them to metastasize. There is some evidence that EMT could be increased in triple-negative (TNBC) or basal-like breast cancers, although more precise mechanisms considering e.g. EMT-regulating transcription factors are largely unknown. We assessed immunohistochemically vimentin (separately in in situ areas and in   invasive   cells) as an indicator of EMT, and also EMT-regulating transcription factors zeb1 (separately in stroma and tumour) and Sip1 (in nuclei and cytoplasm) in histological samples of 231 women with local or locally advanced   invasive   breast cancer. 51.1 % of patients had TNBC and 48.9 % oestrogen and progesterone receptor-positive and HER2 negative breast cancer. Basal-like breast cancers were defined as TNBC that also expressed epidermal growth factor receptor EGFR and/or cytokeratin 5/6. Vimentin expression i  n invasi  ve cells was higher in TNBCs (p = 9 × 10(-12)) compared to non-TNBC tumours. Vimentin (p = 2 × 10(-6)), nuclear Sip1 (p = 0.035) and zeb1 in stroma (p = 0.013) were overexpressed in basal-like cancers compared to non-basal-like TNBCs. In non-TNBC group findings between studied markers and clinicopathological factors were rare. However, in TNBC cases, vimenti  n expres  sion in invasive cells associated with poor differentiation (p = 0.00007), zeb1 expression in cancer cells with higher grade (p = 0.002), vascular invasion (p = 0.036) and larger T-class (p = 0.027), whereas stromal zeb1 associated with lymphatic vessel invasion (p = 0.036) and vascular invasion (p = 0.039). High nuclear Sip1 expression was prognostic for poor disease-free survival (p = 0.002) in the whole cohort. The current results emphasize the increased role of EMT in TNBC and especially in basal-like breast cancers. These observations also support the role of studied parameters in tumour progression. 
 Langerhans cell maturation is accompanied by induction of N-cadherin and the transcriptional regulators of epithelial-mesenchymal transition ZEB1/2. Langerhans cells (LCs) are a unique subset of dendritic cells (DCs) that express epithelial adhesion molecules, allowing them to form contacts with epithelial cells and reside in epidermal/epithelial tissues. The dynamic regulation of epithelial adhesion plays a decisive role in the life cycle of LCs. It controls whether LCs remain immature and sessile within the epidermis or mature and egress to initiate immune responses. So far, the molecular machinery regulating epithelial adhesion molecules during LC maturation remains elusive. Here, we generated pure populations of immature human LCs in vitro to systematically probe for gene-expression changes during LC maturation. LCs down-regulate a set of epithelial genes including E-cadherin, while they upregulate the mesenchymal marker N-cadherin known to facilitate cell migration. In addition, N-cadherin is constitutively expressed by monocyte-derived DCs known to exhibit characteristics of both inflammatory-type and interstitial/dermal DCs. Moreover, the transcription factors ZEB1 and ZEB2 (ZEB is zinc-finger E-box-binding homeobox) are upregulated in migratory LCs. ZEB1 and ZEB2 have been shown to induce epithelial-to-mesenchymal transition (EMT) and   invasive   behavior in cancer cells undergoing metastasis. Our results provide the first hint that the molecular EMT machinery might facilitate LC mobilization. Moreover, our study suggests that N-cadherin plays a role during DC migration. 
 ZEB2 promotes vasculogenic mimicry by TGF-β1 induced epithelial-to-mesenchymal transition in hepatocellular carcinoma. Zinc finger E-box binding homeobox 2 (ZEB2), an epithelial-mesenchymal transition (EMT) regulator, has been involved in invasion and metastasis of human tumor. Although EMT may be involved in vasculogenic mimicry (VM) formation, no reports describing the relation between ZEB2 and VM are available. We hypothesize that ZEB2 may promote VM formation in hepatocellular carcinoma (HCC).Paraffin-embedded tumor tissue samples from 92 patients were immunostained with anti-ZEB2 antibody. We found that the ZEB2 nuclear expression was significantly associated with VM formation and metastasis. Patients with VM and ZEB2 nuclear expression had a shorter survival period than those without expression. In vitro, ZEB2 overexpression significantly enhanced cell motility,   invasiv  eness, and VM formation of HepG2 cells. ZEB2 upregulation also increased VE-cadherin, Flt-1, and Flk-1 expression and activated MMPs. ZEB2 knockdown inhibited cell motility,   invasiv  eness, and VM formation in Bel7402 cells. ZEB2 knockdown also decreased VE-cadherin, Flt-1, and Flk-1 expression and MMP activity. In addition, EMT in HepG2 cells was induced by TGF-β1 treatment, and the kinetics of expression of EMT markers and regulators were assessed by Western blot analysis. The expression of ZEB2 increased significantly, and VM formation was promoted.ZEB2 can promote VM formation through the EMT pathway. Our findings may represent a novel therapeutic target in HCC. 
 The miR-200 family and miR-205 regulate epithelial to mesenchymal transition by targeting ZEB1 and SIP1. Epithelial to mesenchymal transition (EMT) facilitates tissue remodelling during embryonic development and is viewed as an essential early step in tumour metastasis. We found that all five members of the microRNA-200 family (miR-200a, miR-200b, miR-200c, miR-141 and miR-429) and miR-205 were markedly downregulated in cells that had undergone EMT in response to transforming growth factor (TGF)-beta or to ectopic expression of the protein tyrosine phosphatase Pez. Enforced expression of the miR-200 family alone was sufficient to prevent TGF-beta-induced EMT. Together, these microRNAs cooperatively regulate expression of the E-cadherin transcriptional repressors ZEB1 (also known as deltaEF1) and SIP1 (also known as ZEB2), factors previously implicated in EMT and tumour metastasis. Inhibition of the microRNAs was sufficient to induce EMT in a process requiring upregulation of ZEB1 and/or SIP1. Conversely, ectopic expression of these microRNAs in mesenchymal cells initiated mesenchymal to epithelial transition (MET). Consistent with their role in regulating EMT, expression of these microRNAs was found to be lost in   invasive   breast cancer cell lines with mesenchymal phenotype. Expression of the miR-200 family was also lost in regions of metaplastic breast cancer specimens lacking E-cadherin. These data suggest that downregulation of the microRNAs may be an important step in tumour progression. 
 Dysfunction of the Reciprocal Feedback Loop between GATA3- and ZEB2-Nucleated Repression Programs Contributes to Breast Cancer Metastasis. How loss-of-function of GATA3 contributes to the development of breast cancer is poorly understood. Here, we report that GATA3 nucleates a transcription repression program composed of G9A and MTA3-, but not MTA1- or MTA2-, constituted NuRD complex. Genome-wide analysis of the GATA3/G9A/NuRD(MTA3) targets identified a cohort of genes including ZEB2 that are critically involved in epithelial-to-mesenchymal transition and cell invasion. We demonstrate that the GATA3/G9A/NuRD(MTA3) complex inhibits the   invasive   potential of breast cancer cells in vitro and suppresses breast cancer metastasis in vivo. Strikingly, the expression of GATA3, G9A, and MTA3 is concurrently downregulated during breast cancer progression, leading to an elevated expression of ZEB2, which, in turn, represses the expression of G9A and MTA3 through the recruitment of G9A/NuRD(MTA1). 
 miR-200a-mediated downregulation of ZEB2 and CTNNB1 differentially inhibits nasopharyngeal carcinoma cell growth, migration and invasion. Nasopharyngeal carcinoma (NPC), a highly metastatic and   invasive   malignant tumor originating from the nasopharynx, is widely prevalent in Southeast Asia, the Middle East and North Africa. Although viral, dietary and genetic factors have been implicated in NPC, the molecular basis of its pathogenesis is not well defined. Based on a recent microRNA (miRNA) microarray study showing miR-200 downregulation in NPC, we further investigated the role of miR-200a in NPC carcinogenesis. We found that the endogenous miR-200a expression level increases with the degree of differentiation in a panel of NPC cell lines, namely undifferentiated C666-1, high-differentiated CNE-1, and low-differentiated CNE-2 and HNE1 cells. By a series of gain-of-function and loss-of-function studies, we showed that over-expression of miR-200a inhibits C666-1 cell growth, migration and invasion, whereas its knock-down stimulates these processes in CNE-1 cells. In addition, we further identified ZEB2 and CTNNB1 as the functional downstream targets of miR-200a. Interestingly, knock-down of ZEB2 solely impeded NPC cell migration and invasion, whereas CTNNB1 suppression only inhibited NPC cell growth, suggesting that the inhibitory effects of miR-200a on NPC cell growth, migration and invasion are mediated by distinct targets and pathways. Our results reveal the important role of miR-200a as a regulatory factor of NPC carcinogenesis and a potential candidate for miRNA-based therapy against NPC. 
 MicroRNA-335 inhibits invasion and metastasis of colorectal cancer by targeting ZEB2. MicroRNAs have been suggested to play a vital role in regulate tumor progression and invasion. However, the expression of miR-335 in colorectal cancer (CRC) and its clinical significance are not known. Here, we report that miR-335 is a tumor suppressor by regulating expression of ZEB2. In this study, we showed that downregulated miR-335 levels in highly   invasive   CRC cell lines and tissues. Kaplan-Meier survival analysis indicated that patients with reduced miR-335 had a poor overall survival. Furthermore, enhancing the expression of miR-335 inhibited CRC cell migration and invasion in vitro and lung and liver metastasis in vivo, while silencing its expression resulted in increased migration and invasion. Additionally, we identified a novel miR-335 target, ZEB2, and the direct interaction between them was verified by 3'-untranslated region dual-luciferase reporter assay. In conclusion, our results demonstrate that miR-335 functions as a tumor suppressor and play a role in inhibiting metastasis of CRC cells through targeting ZEB2. These findings suggest that miR-335 may be useful as a new potential therapeutic target for CRC. 
 ZEB2 upregulates integrin α5 expression through cooperation with Sp1 to induce invasion during epithelial-mesenchymal transition of human cancer cells. Epithelial-mesenchymal transition (EMT) is a process implicated in tumor invasion, metastasis, embryonic development and wound healing. ZEB2 is a transcription factor involved in EMT that represses E-cadherin transcription. Although E-cadherin downregulation is a major event during EMT and tumor progression, E-cadherin reduction is probably not sufficient for full   invasiv  eness. The mechanisms by which E-cadherin transcriptional repressors induce mesenchymal genes during EMT remain largely unknown. Here, we investigated the role of ZEB2 in the induction of integrin α5 during cancer EMT and its underlying mechanism. In human cancer cells, ZEB2 was found to directly upregulate integrin α5 transcription in a manner that is independent of the regulation of E-cadherin expression. Conversely, depletion of ZEB2 by small interfering RNA suppressed integrin α5 expression, leading to reduced invasion. Suppression of integrin α5 inhibited cancer cell invasion, suggesting an important role for integrin α5 in cancer progression. Furthermore, ZEB2 was found to activate the integrin α5 and vimentin promoters by interacting with and activating the transcription factor Sp1, suggesting that cooperation between ZEB2 and Sp1 represents a novel mechanism of mesenchymal gene activation during EMT. These findings increase our understanding of the pathways beyond E-cadherin reduction that regulate mesenchymal gene expression during EMT and cancer progression. 
  GeneRIFs  
 Based on our results miR-153 inhibits proliferation and suppresses EMT and the   invasive   potential of ovarian cancer cells through downregulation of SET7 and ZEB2, supporting the pursuit of miR-153 as a potential target for ovarian cancer intervention. 
 A431 cells expressing SIP1 along with exogenous cyclin D1 were highly   invasive   
  [Mm]etastasize  PubMed  
 Co-Expression of TWIST1 and ZEB2 in Oral Squamous Cell Carcinoma Is Associated with Poor Survival. Oral squamous cell carcinoma (OSCC) is an aggressive disease accounting for more than 260,000 cancer cases diagnosed and 128,000 deaths worldwide. A large majority of cancer deaths result from cancers that have   metastasize  d beyond the primary tumor. The relationship between genetic changes and clinical outcome can reflect the biological events that promote cancer's aggressive behavior, and these can serve as molecular markers for improved patient management and survival. To this end, epithelial-mesenchymal transition (EMT) is a major process that promotes tumor invasion and metastasis, making EMT-related proteins attractive diagnostic biomarkers and therapeutic targets. In this study, we used immunohistochemistry to study the expression of a panel of transcription factors (TWIST1, SNAI1/2, ZEB1 and ZEB2) and other genes intimately related to EMT (CDH1 and LAMC2) at the invasive tumor front of OSCC tissues. The association between the expression of these proteins and clinico-pathological parameters were examined with Pearson Chi-square and correlation with survival was analyzed using Kaplan Meier analysis. Our results demonstrate that there was a significant differential expression of CDH1, LAMC2, SNAI1/2 and TWIST1 between OSCC and normal oral mucosa (NOM). Specifically, CDH1 loss was significantly associated with Broder's grading, while diffused LAMC2 was similarly associated with non-cohesive pattern of invasion. Notably, co-expression of TWIST1 and ZEB2 in OSCC was significantly associated with poorer overall survival, particularly in patients without detectable lymph node metastasis. This study demonstrates that EMT-related proteins are differentially expressed in OSCC and that the co-expression of TWIST1 and ZEB2 could be of clinical value in identifying patients with poor survival for appropriate patient management. 
 Vimentin, zeb1 and Sip1 are up-regulated in triple-negative and basal-like breast cancers: association with an aggressive tumour phenotype. In epithelial-to-mesenchymal transition (EMT) epithelial cancer cells achieve mesenchymal features, essentially helping them to   metastasize  . There is some evidence that EMT could be increased in triple-negative (TNBC) or basal-like breast cancers, although more precise mechanisms considering e.g. EMT-regulating transcription factors are largely unknown. We assessed immunohistochemically vimentin (separately in in situ areas and in invasive cells) as an indicator of EMT, and also EMT-regulating transcription factors zeb1 (separately in stroma and tumour) and Sip1 (in nuclei and cytoplasm) in histological samples of 231 women with local or locally advanced invasive breast cancer. 51.1 % of patients had TNBC and 48.9 % oestrogen and progesterone receptor-positive and HER2 negative breast cancer. Basal-like breast cancers were defined as TNBC that also expressed epidermal growth factor receptor EGFR and/or cytokeratin 5/6. Vimentin expression in invasive cells was higher in TNBCs (p = 9 × 10(-12)) compared to non-TNBC tumours. Vimentin (p = 2 × 10(-6)), nuclear Sip1 (p = 0.035) and zeb1 in stroma (p = 0.013) were overexpressed in basal-like cancers compared to non-basal-like TNBCs. In non-TNBC group findings between studied markers and clinicopathological factors were rare. However, in TNBC cases, vimentin expression in invasive cells associated with poor differentiation (p = 0.00007), zeb1 expression in cancer cells with higher grade (p = 0.002), vascular invasion (p = 0.036) and larger T-class (p = 0.027), whereas stromal zeb1 associated with lymphatic vessel invasion (p = 0.036) and vascular invasion (p = 0.039). High nuclear Sip1 expression was prognostic for poor disease-free survival (p = 0.002) in the whole cohort. The current results emphasize the increased role of EMT in TNBC and especially in basal-like breast cancers. These observations also support the role of studied parameters in tumour progression. 
 SIP1 predicts progression and poor prognosis in pharyngeal squamous cell carcinoma. The epithelial-mesenchymal transition (EMT) is a crucial process in tumorigenesis that enables tumor cells to invade and   metastasize  . The transcription factors SIP1, SLUG, ZEB1, SNAI1, and TWIST are fundamental in regulating EMT. We investigated the relationships between several clinicopathological variables, prognosis, and SIP1, SLUG, or ZEB1 in a retrospective pharyngeal squamous cell carcinoma (PSCC) cohort.Immunohistochemistry was used to evaluate the expression of SIP1, SLUG, and ZEB1 in 108 tumor samples from a retrospective cohort of patients with PSCC.Tumors with positive epithelial SIP1 immunostaining were more advanced (SIII-IV, p=0.02) and had more lymph node metastases (p=0.04) than SIP1-negative tumors. Tumors with positive stromal staining of SIP1 relapsed more often than SIP1-negative tumors (p=0.007). Negative SIP1 immunoreactivity correlated significantly with better disease-specific survival (DSS) and better overall survival (OS) (p=0.012 and p=0.003 for epithelial reactivity, p=0.018 and p=0.003 for stromal reactivity, respectively). Lack of epithelial SIP1 expression remained an independent and favorable prognostic factor in a Cox proportional hazards model (p=0.046), together with high Karnofsky performance status score and low T class (p<0.001 for both). Co-expression of SNAI1, TWIST, and SIP1 in tumor epithelium predicted even shorter DSS than SIP1 expression alone (p<0.001) in the present study cohort.SIP1 is related to cancer progression and appears to be an independent prognostic factor in PSCC. 
  [Mm]etastasis  PubMed  
 Akt2/ZEB2 may be a biomarker for exfoliant cells in ascitic fluid in advanced grades of serous ovarian carcinoma. Ovarian cancers present a mild clinical course when diagnosed early but an aggressive pathway when diagnosed in the peri- and postmenopausal periods. However, the predictability of tumor progression is stochastic and is difficult to predict. In the present study, we hypothesized to examine the key pathways that are dysregulated to promote epithelial-mesenchymal transition in serous ovarian carcinoma. Examination of these steps would help to identify ascitic fluid with cells poised for   metastasis   or otherwise. We focused on examining the Akt2 expression, mainly because of its report as being overamplified in the aggressive variants of ovarian cancer, as well as TGFbeta-sensitivity of Akt2 that forms the key basis for   metastasis   initiation of most kinds of carcinoma. We obtained primary ovarian carcinoma samples as well as ascitic fluid and distantly metastatic ovarian carcinoma to examine the expression of Akt2. The results of the study demonstrated that in malignant exfoliated ovarian cancer cells, Smad4 expression was tremendously increased in the nuclei, suggesting nuclear translocation of Smad, which thereafter may have activated ZEB2, and thereafter genomically affected the expression of E-cadherin, myosin II, and vimentin, key components for initiating and sustaining   metastasis  . All of these may have been stimulated by increased cellular expression of Akt2 in metastatic variants of the serous ovarian carcinoma. The reliance on Akt2 and TGF beta signaling may also potentiate the case for Akt inhibitors or small molecule inhibitors of TGFbeta signaling like doxycycline as adjunct chemotherapy in serous ovarian carcinoma, especially the metastatic variants. 
 Akt2/ZEB2 may be a biomarker for exfoliant cells in ascitic fluid in advanced grades of serous ovarian carcinoma. Ovarian cancers present a mild clinical course when diagnosed early but an aggressive pathway when diagnosed in the peri- and postmenopausal periods. However, the predictability of tumor progression is stochastic and is difficult to predict. In the present study, we hypothesized to examine the key pathways that are dysregulated to promote epithelial-mesenchymal transition in serous ovarian carcinoma. Examination of these steps would help to identify ascitic fluid with cells poised for   metastasis   or otherwise. We focused on examining the Akt2 expression, mainly because of its report as being overamplified in the aggressive variants of ovarian cancer, as well as TGFbeta-sensitivity of Akt2 that forms the key basis for   metastasis   initiation of most kinds of carcinoma. We obtained primary ovarian carcinoma samples as well as ascitic fluid and distantly metastatic ovarian carcinoma to examine the expression of Akt2. The results of the study demonstrated that in malignant exfoliated ovarian cancer cells, Smad4 expression was tremendously increased in the nuclei, suggesting nuclear translocation of Smad, which thereafter may have activated ZEB2, and thereafter genomically affected the expression of E-cadherin, myosin II, and vimentin, key components for initiating and sustaining   metastasis  . All of these may have been stimulated by increased cellular expression of Akt2 in metastatic variants of the serous ovarian carcinoma. The reliance on Akt2 and TGF beta signaling may also potentiate the case for Akt inhibitors or small molecule inhibitors of TGFbeta signaling like doxycycline as adjunct chemotherapy in serous ovarian carcinoma, especially the metastatic variants. 
 LncRNA MALAT1 functions as a competing endogenous RNA to regulate ZEB2 expression by sponging miR-200s in clear cell kidney carcinoma. Long non-coding RNA (lncRNAs) play a critical role in the development of cancers. LncRNA   metastasis  -associated lung adenocarcinoma transcript 1(MALAT1) has recently been identified to be involved in tumorigenesis of several cancers such as lung cancer, bladder cancer and so on. Here, we found that MALAT1 exist a higher fold change (Tumor/Normal) in clear cell kidney carcinoma (KIRC) from The Cancer Genome Atlas (TCGA) Data Portal and a negative correlation with miR-200s family. We further demonstrated MALAT1 promote KIRC proliferation and   metastasis   through sponging miR-200s in vitro and in vivo. In addition, miR-200c can partly reverse the MALAT1's stimulation on proliferation and   metastasis   in KIRC. In summary we unveil a branch of the MALAT1/miR-200s/ZEB2 pathway that regulates the progression of KIRC. The inhibition of MALAT1 expression may be a promising strategy for KIRC therapy. 
 Androgen receptor as a regulator of ZEB2 expression and its implications in epithelial-to-mesenchymal transition in prostate cancer. Zinc finger E-box-binding protein 2 (ZEB2) is known to help mediate the epithelial-to-mesenchymal transition, and thereby it facilitates cancer   metastasis  . This study was initiated to explore whether ZEB2 expression differs in prostate cancer (PCa, n=7) and benign prostatic hyperplasia (BPH, n=7) tissues. In PCa tissues, the levels of both immunoreactive ZEB2 and androgen receptor (AR) were found to be significantly higher (P<0.05) when compared with BPH tissues. Co-regulation of AR and ZEB2 prompted us to investigate the role of androgenic stimuli in ZEB2 expression. ZEB2 expression was found to be significantly (P<0.05) upregulated after androgen stimulation and downregulated following AR silencing in LNCaP cells, an androgen-dependent PCa cell line. This finding suggested AR as a positive regulator of ZEB2 expression in androgen-dependent cells. Paradoxically, androgen-independent (AI) cell lines PC3 and DU145, known to possess low AR levels, showed significantly (P<0.05) higher expression of ZEB2 compared with LNCaP cells. Furthermore, forced expression of AR in PC3 (PC3-AR) and DU145 (DU-AR) cells led to reductions in ZEB2 expression, invasiveness, and migration. These cells also exhibited an increase in the levels of E-cadherin (a transcriptional target of ZEB2). Co-transfection of AR and ZEB2 cDNA constructs prevented the decline in invasiveness and migration to a significant extent. Additionally, ZEB2 downregulation was associated with an increase in miR200a/miR200b levels in PC3-AR cells and with a decrease in miR200a/miR200b levels in AR-silenced LNCaP cells. Thus, AR acts as a positive regulator of ZEB2 expression in androgen-dependent cells and as a negative regulator in AI PCa cells. 
 miR-132 inhibits colorectal cancer invasion and   metastasis   via directly targeting ZEB2. To investigate the biological role and underlying mechanism of miR-132 in colorectal cancer (CRC) progression and invasion.Quantitative RT-PCR analysis was used to examine the expression levels of miR-132 in five CRC cell lines (SW480, SW620, HCT116, HT29 and LoVo) and a normal colonic cell line NCM460, as well as in tumor tissues with or without metastases. The Kaplan-Meier method was used to analyze the prognostic significance of miR-132 in CRC patients. The biological effects of miR-132 were assessed in CRC cell lines using the transwell assay. Quantitative RT-PCR and western blot analyses were employed to evaluate the expression of miR-132 targets. The regulation of ZEB2 by miR-132 was confirmed using the luciferase activity assay.miR-132 was significantly down-regulated in the CRC cell lines compared with the normal colonic cell line (P < 0.05), as well as in the CRC tissues with distant metastases compared with the tissues without metastases (10.52 ± 4.69 vs 23.11 ± 7.84) (P < 0.001). Down-regulation of miR-132 was associated with tumor size (P = 0.016), distan  t metastas  is (P = 0.002), and TNM stage (P = 0.020) in CRC patients. Kaplan-Meier survival curve analysis indicated that patients with low expression of miR-132 tended to have worse disease-free survival than patients with high expression of miR-132 (P < 0.001). Moreover, ectopic expression of miR-132 markedly inhibited cell invasion (P < 0.05) and the epithelial-mesenchymal transition (EMT) in CRC cell lines. Further investigation revealed ZEB2, an EMT regulator, was a downstream target of miR-132.Our study indicated that miR-132 plays an important role in the invasion an  d metastas  is of CRC. 
 Forkhead box Q1 promotes hepatocellular carcinoma   metastasis   by transactivating ZEB2 and VersicanV1 expression. Forkhead box Q1 (FoxQ1) is a master regulator of tumor   metastasis  . However, the molecular mechanism of FoxQ1 in regulating hepatocellular carcinoma (HCC)   metastasis   remains unknown. Here we report a novel function for FoxQ1 in modifying the tumor microenvironment to promote HCC   metastasis  . FoxQ1 expression was an independent and significant risk factor for the recurrence and survival in two independent cohorts totaling 1,002 HCC patients. FoxQ1 induced epithelial-mesenchymal transition (EMT) through the transactivation of ZEB2 expression by directly binding to the ZEB2 promoter. Knockdown of ZEB2 decreased FoxQ1-enhanced HCC   metastasis  , whereas up-regulation of ZEB2 rescued the decreased   metastasis   induced by FoxQ1 knocking down. Additionally, serial deletion, site-directed mutagenesis, and a chromatin immunoprecipitation assays showed that VersicanV1, which promoted HCC   metastasis   and macrophage attraction, was a direct transcriptional target of FoxQ1. FoxQ1-induced VersicanV1 expression promoted the secretion of chemokine (C-C motif) ligand 2 (CCL2) from HCC cells. Chemotaxis assay showed that the culture media from FoxQ1-overexpressing HCC cells increased the migratory activity of the macrophages. Inhibition of VersicanV1 and CCL2 expression significantly inhibited FoxQ1-mediated macrophage migration. In animal studies, the up-regulation of FoxQ1 in HCC cells promoted HCC   metastasis   and intratumoral tumor associated macrophage (TAM) infiltration, whereas knockdown of VersicanV1 reduced FoxQ1-mediated HCC   metastasis   and intratumoral TAM infiltration. Depletion of macrophages using clodronate liposomes dramatically decreased FoxQ1-enhanced HCC   metastasis  . In human HCC tissues, FoxQ1 expression was positively correlated with ZEB2 and VersicanV1 expression and intratumoral TAM infiltration. Patients with positive coexpression of FoxQ1 and ZEB2, FoxQ1, and VersicanV1, or FoxQ1 and intratumoral TAMs were associated with poorer prognosis.FoxQ1 promotes HCC   metastasis   by transactivating ZEB2 and VersicanV1 expression, resulting in the induction of EMT and the recruitment of macrophage infiltration. 
 MicroRNA-200b inhibits the growth and   metastasis   of glioma cells via targeting ZEB2. MicroRNAs (miRs) have been found to play important roles in mediating a variety of biological processes in human cancers, including tumor cell proliferation, migration, invasion, and epithelial-mesenchymal transition (EMT). In the present study, we aimed to investigate the putative role of miR‑200b in the progression of glioma. Real-time RT-PCR data showed that the miR‑200b levels were frequently reduced in primary glioma tissues (n=88) and cell lines, when compared to normal brain tissues (n=25). Moreover, decreased miR‑200b level was tightly associated with the malignant progression of glioma. Overexpression of miR‑200b significantly suppressed cell proliferation, migration, invasion and EMT in glioma U251 and U87 cells. Luciferase reporter assay data further identified ZEB2 as a direct target of miR‑200b, and the protein expression of ZEB2 was markedly reduced after overexpression of miR‑200b in U251 and U87 cells. Furthermore, restoration of ZEB2 effectively reversed the reduced expression of ZEB2, as well as the suppressive effects of miR‑200b overexpression on the proliferation, migration, invasion and EMT in glioma U251 and U87 cells. Moreover, in vivo study showed that overexpression of miR‑200b significantly inhibited tumorigenesis as well as the tumor growth of glioma cells, and effectively protected nude mice from tumor-induced death. Taken together these findings suggest that miR‑200b has suppressive effects on the proliferation, migration, invasion and EMT of glioma cells, partly at least, via targeting ZEB2. Therefore, miR‑200b acts as a novel tumor suppressor in glioma, and thus may become a promising therapeutic candidate for glioma. 
 miR-590-3p suppresses cancer cell migration, invasion and epithelial-mesenchymal transition in glioblastoma multiforme by targeting ZEB1 and ZEB2. Invasion and migration of glioblastoma multiforme (GBM) is a multistep process and an important phenotype that causes this disease to invade surrounding tissues in the brain. The purpose of this study was to determine the role of miR-590-3p in regulation of epithelial mesenchymal transition (EMT) and   metastasis   of GBM cells. Expression levels of miR-590-3p in 15 GBM specimens with adjacent tissues and five GBM cell lines were assessed by quantitative RT-PCR. We found that miR-590-3p was down-regulated in detected GBM tissue samples and all of the GBM cell lines. In addition, Ectopic expression of miR-590-3p suppressed and miR-590-3p-in promoted EMT, migration, and invasion in U87MG and A172 cells. Bioinformatics coupled with luciferase and Western blot assays also revealed that miR-590-3p inhibited expression of ZEB1 and ZEB2, which are master regulators of tumor   metastasis  . Our study first indicates that miR-590-3p functions as a suppressor of GBM EMT and   metastasis   by targeting ZEB1 and ZEB2, and it may be a therapeutic target for metastatic GBM. 
 The miR-200 family inhibits epithelial-mesenchymal transition and cancer cell migration by direct targeting of E-cadherin transcriptional repressors ZEB1 and ZEB2. MicroRNAs are small non-coding RNA molecules that can regulate gene expression by interacting with multiple mRNAs and inducing either translation suppression or degradation of mRNA. Recently, several miRNAs were identified as either promoters or suppressors of   metastasis  . However, it is unclear in which step(s) of the multistep metastatic cascade these miRNAs play a defined functional role. To study the functional importance of miRNAs in epithelial-mesenchymal transition (EMT), a process thought to initiate   metastasis   by enhancing the motility of tumor cells, we used a well established in vitro EMT assay: transforming growth factor-beta-induced EMT in NMuMG murine mammary epithelial cells. We found that members of the miR-200 family, organized as two clusters in the genome, were repressed during EMT. Overexpression of each miRNA individually or as clusters in NMuMG cells hindered EMT by enhancing E-cadherin expression through direct targeting of ZEB1 and ZEB2, which encode transcriptional repressors of E-cadherin. In the 4TO7 mouse carcinoma cell line, which expresses low levels of endogenous E-cadherin and displays a mesenchymal phenotype, ectopic expression of the miR-200 family miRNAs significantly increased E-cadherin expression and altered cell morphology to an epithelial phenotype. Furthermore, ectopic expression of each miR-200 miRNA cluster significantly reduced the in vitro motility of 4TO7 cells in migration assays. These results suggested that loss of expression of the miR-200 family members may play a critical role in the repression of E-cadherin by ZEB1 and ZEB2 during EMT, thereby enhancing migration and invasion during cancer progression. 
 A developmentally regulated inducer of EMT, LBX1, contributes to breast cancer progression. Epithelial-to-mesenchymal transition (EMT) plays an important role during normal embryogenesis, and it has been implicated in cancer invasion and   metastasis  . Here, we report that Ladybird homeobox 1 (LBX1), a developmentally regulated homeobox gene, directs expression of the known EMT inducers ZEB1, ZEB2, Snail1, and transforming growth factor beta2 (TGFB2). In mammary epithelial cells, overexpression of LBX1 leads to morphological transformation, expression of mesenchymal markers, enhanced cell migration, increased CD44(high)/CD24(low) progenitor cell population, and tumorigenic cooperation with known oncogenes. In human breast cancer, LBX1 is up-regulated in the unfavorable estrogen receptor (ER)/progesterone (PR)/HER2 triple-negative basal-like subtype. Thus, aberrant expression of LBX1 may lead to the activation of a developmentally regulated EMT pathway in human breast cancer. 
 ZEB2/SIP1 as novel prognostic indicator in eyelid sebaceous gland carcinoma. Epithelial-mesenchymal transition (EMT) plays a pivotal role in tumor invasion and   metastasis   in various malignancies. ZEB2/SIP1 is an important EMT regulator and down-regulates E-cadherin expression. The present study was planned to explore status of EMT-associated markers ZEB2/SIP1 and E-cadherin in eyelid sebaceous gland carcinoma (SGC) and to correlate with clinicopathological high-risk features. Expressions of ZEB2 and E-cadherin were evaluated by immunohistochemistry in 65 cases of histopathologically proven eyelid SGC. The results were correlated with clinicopathological high-risk features and survival of the patients to determine the prognostic significance of ZEB2, E-cadherin, and various high-risk features. Cytoplasmic overexpression of ZEB2 and membranous loss of E-cadherin were seen in 68% and 66% of cases of eyelid SGC, respectively. ZEB2 overexpression was significantly associated with E-cadherin loss (P = .002). Overexpression of ZEB2 also showed significant association with lymph node   metastasis   (P = .046), orbital invasion (P = .049), large tumor size (P = .018), and advanced tumor stages (P = .036). Survival analysis revealed that patients with ZEB2 overexpression had poor survival. ZEB2 overexpression and orbital invasion were found to be independent prognostic indicators (univariate analysis). However, multivariate analysis showed that ZEB2 (hazard ratio, 0.094; 95% confidence interval, 00.012-0.709; P = .022) was the best poor prognostic indicator of eyelid SGC. Our study demonstrates the role of both ZEB2 and E-cadherin in the promotion of EMT in eyelid SGC. The outcome of this study also points toward ZEB2 as an independent prognostic marker as well as a potential therapeutic target in eyelid SGC. 
 Novel monoclonal antibodies detect Smad-interacting protein 1 (SIP1) in the cytoplasm of human cells from multiple tumor tissue arrays. Smad-interacting protein 1 (SIP1, also known as ZEB2) represses the transcription of E-cadherin and mediates epithelial-mesenchymal transition in development and tumor   metastasis  . Due to the lack of human SIP1-specific antibodies, its expression in human tumor tissues has not been studied in detail by immunohistochemistry. Hence, we generated two anti-SIP1 monoclonal antibodies, clones 1C6 and 6E5, with IgG1 and IgG2a isotypes, respectively. The specificity of these antibodies was shown by Western blotting studies using siRNA mediated downregulation of SIP1 and ZEB1 in a human osteosarcoma cell line. In the same context, we also compared them with 5 commercially available SIP1 antibodies. Antibody specificity was further verified in an inducible cell line system by immunofluorescence. By using both antibodies, we evaluated the tissue expression of SIP1 in paraffin-embedded tissue microarrays consisting of 22 normal and 101 tumoral tissues of kidney, colon, stomach, lung, esophagus, uterus, rectum, breast and liver. Interestingly, SIP1 predominantly displayed a cytoplasmic expression, while the nuclear localization of SIP1 was observed in only 6 cases. Strong expression of SIP1 was found in distal tubules of kidney, glandular epithelial cells of stomach and hepatocytes, implicating a co-expression of SIP1 and E-cadherin. Squamous epithelium of the esophagus and surface epithelium of colon and rectum were stained with moderate to weak intensity. Normal uterus, breast and lung tissues remained completely negative. By comparison with their normal tissues, we observed SIP1 overexpression in cancers of the kidney, breast, lung and uterus. However, SIP1 expression was found to be downregulated in tumors from colon, rectum, esophagus, liver and stomach tissues. Finally we did nuclear/cytoplasmic fractionation in 3 carcinoma cell lines and detected SIP1 in both fractions, nucleus being the dominant one. To our best knowledge, this is the first comprehensive immunohistochemical study of the expression of SIP1 in a series of human cancers. Our finding that SIP1 is not exclusively localized to nucleus suggests that the subcellular localization of SIP1 is regulated in normal and tumor tissues. These novel monoclonal antibodies may help elucidate the role of SIP1 in tumor development. 
 Fra-1/AP-1 induces EMT in mammary epithelial cells by modulating Zeb1/2 and TGFβ expression. Epithelial-to-mesenchymal transition (EMT) is essential for embryonic morphogenesis and wound healing and critical for tumour cell invasion and dissemination. The AP-1 transcription factor Fra-1 has been implicated in tumorigenesis and in tumour-associated EMT in human breast cancer. We observed a significant inverse correlation between Fra-1 mRNA expression and distant  -metastasi  s-free survival in a large cohort of breast cancer patients derived from multiple array data sets. This unique correlation among Fos genes prompted us to assess the evolutionary conservation between Fra-1 functions in EMT of human and mouse cells. Ectopic expression of Fra-1 in fully polarized, non-tumourigenic, mouse mammary epithelial EpH4 cells induced a mesenchymal phenotype, characterized by a loss of epithelial and gain of mesenchymal markers. Proliferation, motility and invasiveness were also increased in the resulting EpFra1 cells, and the cells were tumourigenic and efficiently colonized the lung upon transplantation. Molecular analyses revealed increased expression of Tgfβ1 and the EMT-inducing transcription factors Zeb1, Zeb2 and Slug. Mechanistically, Fra-1 binds to the tgfb1 and zeb2 promoters and to an evolutionarily conserved region in the first intron of zeb1. Furthermore, increased activity of a zeb2 promoter reporter was detected in EpFra1 cells and shown to depend on AP-1-binding sites. Inhibiting TGFβ signalling in EpFra1 cells moderately increased the expression of epithelial markers, whereas silencing of zeb1 or zeb2 restored the epithelial phenotype and decreased migration in vitro and tumorigenesis in vivo. Thus Fra-1 induces changes in the expression of genes encoding EMT-related transcription factors leading to the acquisition of mesenchymal, invasive and tumorigenic capacities by epithelial cells. This study defines a novel function of Fra-1/AP-1 in modulating tgfb1, zeb1 and zeb2 expression through direct binding to genomic regulatory regions, which establishes a basis for future in vivo genetic manipulations and preclinical studies using mouse models. 
 Double-negative feedback loop between long non-coding RNA TUG1 and miR-145 promotes epithelial to mesenchymal transition and radioresistance in human bladder cancer cells. LncRNAs have a critical role in the regulation of cellular processes such as cancer progression and   metastasis  . In the present study, we confirmed that TUG1 was overexpressed in bladder cancer tissues and established cell lines. Knockdown of TUG1 inhibited bladder cancer cell   metastasis   both in vitro and in vivo. Furthermore, we found that TUG1 promoted cancer cell invasion and radioresistance through inducing epithelial-to-mesenchymal transition (EMT). Interestingly, TUG1 decreased the expression of miR-145 and there was a reciprocal repression between TUG1 and miR-145 in an Argonaute2-dependent manner. ZEB2 was identified as a down-stream target of miR-145 and TUG1 exerted its function through the miR-145/ZEB2 axis. In summary, our data indicated that blocking TUG1 function may be an effective anti-cancer therapy. 
 miR-154 targeting ZEB2 in hepatocellular carcinoma functions as a potential tumor suppressor. MicroRNA-154 (miR-154) has been identified as a tumor suppressor in several types of human cancers; however, its clinical significance and function in human hepatocellular carcinoma (HCC) remain unclear. The aim of the present study was to analyze the clinical significance and cellular function of miR-154 in HCC patients. The data showed that miR-154 expression was consistently lower in HCC tissues and cell lines compared to that in matched tumor-adjacent tissues and a normal hepatic cell line, and its expression was negatively correlated with tumor differentiation (P<0.01), TNM stage (P<0.01) and lymph node   metastasis   (P<0.01). Restoration of miR-154 expression in HepG2 cells inhibited cell proliferation, migration and invasion, and induced apoptosis and cell arrest at the G1 phase in vitro, as well as suppressed tumor growth in a nude mouse model. Using a luciferase assay, we identified that miR-154 was able to target the 3'-untranslated region (3'UTR) of ZEB2 mRNA. Then, we revealed that miR-154 was able to reduce ZEB2 expression at the levels of mRNA and protein using qRT-PCR and western blot analysis. Notably, restoration of expression of ZEB2 weakened miR-154-mediated suppression of tumor progression. In conclusion, these results indicate that miR-154 functions as a tumor suppressor in HCC by suppressing ZEB2, suggesting that miR-154 may serve as a potential target for HCC. 
 Honokiol suppresses renal cancer cells'   metastasis   via dual-blocking epithelial-mesenchymal transition and cancer stem cell properties through modulating miR-141/ZEB2 signaling. Renal cell carcinoma (RCC) is associated with a high frequency of   metastasis   and only few therapies substantially prolong survival. Honokiol, isolated from Magnolia spp. bark, has been shown to exhibit pleiotropic anticancer effects in many cancer types. However, whether honokiol could suppress RCC   metastasis   has not been fully elucidated. In the present study, we found that honokiol suppressed renal cancer cells'   metastasis   via dual-blocking epithelial-mesenchymal transition (EMT) and cancer stem cell (CSC) properties. In addition, honokiol inhibited tumor growth in vivo. It was found that honokiol could upregulate miR-141, which targeted ZEB2 and modulated ZEB2 expression. Honokiol reversed EMT and suppressed CSC properties partly through the miR-141/ZEB2 axis. Our study suggested that honokiol may be a suitable therapeutic strategy for RCC treatment. 
 Double-negative feedback loop between ZEB2 and miR-145 regulates epithelial-mesenchymal transition and stem cell properties in prostate cancer cells. The invasion and   metastasis   of tumors are triggered by an epithelial to mesenchymal transition (EMT), which is regulated by microRNAs (miRNAs). EMT also promotes malignant tumor progression and the maintenance of the stem cell property, which endows cancer cells with the capabilities of self-renewal and immortalized proliferation. The transcriptional repressor zinc-finger E-box binding homeobox 2 (ZEB2), as an EMT activator, might be an important promoter of   metastasis   in some tumors. Here, we report that ZEB2 directly represses the transcription of miR-145, which is a strong repressor of EMT. In turn, ZEB2 is also a direct target of miR-145. Further, our findings show that the downregulation of ZEB2 not only represses invasion, migration, EMT, and the stemness of prostate cancer (PCa) cells, but also suppresses the capability of PC-3 cells to invade bone in vivo. Importantly, the expression level of ZEB2 as revealed by immunohistochemical analysis is positively correlated to bone   metastasis  , the serum free PSA level, the total PSA level, and the Gleason score in PCa patients and is negatively correlated with miR-145 expression in primary PCa specimens. Thus, our findings demonstrate a double-negative feedback loop between ZEB2 and miR-145 and indicate that the ZEB2/miR-145 double-negative feedback loop plays a significant role in the control of EMT and stem cell properties during the bone   metastasis   of PCa cells. These results suggest that the double-negative feedback loop between ZEB2 and miR-145 contributes to PCa progression and   metastasis   and might have therapeutic relevance for the bone   metastasis   of PCa. 
 miR-200c modulates ovarian cancer cell   metastasis   potential by targeting zinc finger E-box-binding homeobox 2 (ZEB2) expression. This study was to investigate the effect of miR-200c on regulation of ovarian cancer cell   metastasis   potential and explore the underlying molecular events. qRT-PCR was used to analyze the level of miR-200c expression in 48 ovarian cancer and 30 normal ovarian tissue samples. pre-miR-200c was used to manipulate miR-200c expression in ovarian cancer cells for detection of changed phenotypes of tumor cells. Bioinformatics analysis was then used to predict target genes of miR-200c and GO and pathway analyses drew the miR-200c-related gene network. Luciferase reporter assay confirmed the target of miR-200c as ZEB2. Western blot was used to detect gene expressions in ovarian cancer cells. Level of miR-200c expression was much higher in ovarian cancer than in normal ovarian tissues, and miR-200c expression was inversely associated with advanced clinical stage and lymph node   metastasis   of ovarian cancer (p < 0.01). The database search predicted 186 miR-200c-targeting genes, and GO analysis showed that functions of these target genes were enriched in the protein binding and other biological processes. Furthermore, miR-200c expression inhibited ovarian cancer cell ES-2 migration and invasion capacity by suppression of ZEB2 expression (p < 0.01). Overexpression of miR-200c regulated E-cadherin and vimentin expression in ovarian cancer cells. This study demonstrated high miR-200c expression in ovarian cancer tissues and ZEB2 as a targeting gene of miR-200c, which mediated the effects of miR-200c on regulation of ovarian cancer cell migration and invasion capacity and epithelial-to-mesenchymal transition. Thus, targeting of miR-200c or ZEB2 may serve as a potential therapeutic strategy for control of ovarian cancer. 
 Coexpression of hypoxia-inducible factor-2α, TWIST2, and SIP1 may correlate with invasion and   metastasi  s of salivary adenoid cystic carcinoma. Adenoid cystic carcinoma (ACC) of salivary gland is characterized by advanced local invasion and distant   metastasi  s. Intratumoral hypoxia was reported to be associated with epithelial-mesenchymal transition (EMT) regulators. The purpose of this study was to evaluate the relationship between hypoxia-inducible factor (HIF)-2α, TWIST2, and SIP1 expression and the invasion an  d metastas  is in ACC of salivary gland.In vitro we first detected the expression of HIF-2α, TWIST2, and SIP1 in two ACC cell lines by Western blot and real-time RT-PCR. Then, in vivo, a retrospective investigation of 121 patients with ACC from Department of Oral and Maxillofacial Surgery, West China Hospital of Stomatology, Sichuan University between 1996 and 2005 was carried out using immunohistochemistry to analyze the association between the expression of these three factors and clinical-pathological factors of patients.The protein and mRNA levels of HIF-2α, TWIST2, and SIP1 in the h  igh-metast  asis cell line (ACC-M) were much higher than those of the   low-metast  asis cell line (ACC-2). The positive expression of HIF-2α, TWIST2, and SIP1 (71.07%, 42.98%, and 38.02%, respectively) was associated with the perineural invasion, the local recurrence, and dis  tant metas  tasis of patients with ACC (P < 0.05). The patients with the positive coexpression of the three factors had a lower survival rate than those with the negative expression (P < 0.05).It is proposed that the elevated expression of HIF-2α, TWIST2, and SIP1 can contribute to invasio  n and meta  stasis of ACC, and there might be some correlation between the hypoxia microenvironment and EMT in ACC. 
 p53 regulates epithelial-mesenchymal transition through microRNAs targeting ZEB1 and ZEB2. p53 suppresses tumor progression and   metastasis  . Epithelial-mesenchymal transition (EMT) is a key process in tumor progression and   metastasis  . The transcription factors ZEB1 and ZEB2 promote EMT. Here, we show that p53 suppresses EMT by repressing expression of ZEB1 and ZEB2. By profiling 92 primary hepatocellular carcinomas (HCCs) and 9 HCC cell lines, we found that p53 up-regulates microRNAs (miRNAs), including miR-200 and miR-192 family members. The miR-200 family members transactivated by p53 then repress ZEB1/2 expression. p53-regulated miR-192 family members also repress ZEB2 expression. Inhibition or overexpression of the miRNAs affects p53-regulated EMT by altering ZEB1 and ZEB2 expression. Our findings indicate that p53 can regulate EMT, and that p53-regulated miRNAs are critical mediators of p53-regulated EMT. 
 Co-Expression of TWIST1 and ZEB2 in Oral Squamous Cell Carcinoma Is Associated with Poor Survival. Oral squamous cell carcinoma (OSCC) is an aggressive disease accounting for more than 260,000 cancer cases diagnosed and 128,000 deaths worldwide. A large majority of cancer deaths result from cancers that have metastasized beyond the primary tumor. The relationship between genetic changes and clinical outcome can reflect the biological events that promote cancer's aggressive behavior, and these can serve as molecular markers for improved patient management and survival. To this end, epithelial-mesenchymal transition (EMT) is a major process that promotes tumor invasion and   metastasis  , making EMT-related proteins attractive diagnostic biomarkers and therapeutic targets. In this study, we used immunohistochemistry to study the expression of a panel of transcription factors (TWIST1, SNAI1/2, ZEB1 and ZEB2) and other genes intimately related to EMT (CDH1 and LAMC2) at the invasive tumor front of OSCC tissues. The association between the expression of these proteins and clinico-pathological parameters were examined with Pearson Chi-square and correlation with survival was analyzed using Kaplan Meier analysis. Our results demonstrate that there was a significant differential expression of CDH1, LAMC2, SNAI1/2 and TWIST1 between OSCC and normal oral mucosa (NOM). Specifically, CDH1 loss was significantly associated with Broder's grading, while diffused LAMC2 was similarly associated with non-cohesive pattern of invasion. Notably, co-expression of TWIST1 and ZEB2 in OSCC was significantly associated with poorer overall survival, particularly in patients without detectable lymph node   metastasis  . This study demonstrates that EMT-related proteins are differentially expressed in OSCC and that the co-expression of TWIST1 and ZEB2 could be of clinical value in identifying patients with poor survival for appropriate patient management. 
 Epithelial-mesenchymal transition and mesenchymal-epithelial transition via regulation of ZEB-1 and ZEB-2 expression in pancreatic cancer. BACKGROUND AND OBJECTIES: Phenotypic plasticity of cancer cells via epithelial-mesenchymal transition (EMT) and mesenchymal-epithelial transition (MET) is essential for tumor progression and   metastasis  .Tissue samples were obtained from 76 pancreatic head cancers. We assessed the expression of E-cadherin, vimentin, ZEB-1, and ZEB-2 by immunohistochemical and immunofluorescence staining. Next, 147 metastatic lymph nodes from 45 pancreatic cancers with low expression of E-cadherin were obtained and divided into two categories according to the maximum diameter of the metastases: 2 mm or more and less than 2 mm.High expressions of ZEB-1 and ZEB-2 in the primary tumors were significantly associated with repression of E-cadherin (P = 0.0007), and poorer prognosis (P = 0.0322). Forty-three (29.3%) of the 147 metastatic tumors from pancreatic cancers with low expression of E-cadherin showed high E-cadherin expression. Cancer cells in the larger metastases showed high expression of E-cadherin (P = 0.0061) and low expression of ZEB-1 (P = 0.0170) and ZEB-2 (P = 0.0036) compared with those in the smaller metastases.In primary pancreatic tumors and metastatic lymph nodes, high and low expression of ZEB-1 and ZEB-2 was associated with mesenchymal and epithelial phenotype of cancer cells, respectively. 
 A miRNA signature associated with human metastatic medullary thyroid carcinoma. MicroRNAs (miRNAs) represent a class of small, non-coding RNAs that control gene expression by targeting mRNA and triggering either translational repression or RNA degradation. The objective of our study was to evaluate the involvement of miRNAs in human medullary thyroid carcinoma (MTC) and to identify the markers of metastatic cells and aggressive tumour behaviour. Using matched primary and metastatic tumour samples, we identified a subset of miRNAs aberrantly regulated in metastatic MTC. Deregulated miRNAs were confirmed by quantitative real-time PCR and validated by in situ hybridisation on a large independent set of primary and metastatic MTC samples. Our results uncovered ten miRNAs that were significantly expressed and deregulated in metastatic tumours: miR-10a, miR-200b/-200c, miR-7 and miR-29c were down-regulated and miR-130a, miR-138, miR-193a-3p, miR-373 and miR-498 were up-regulated. Bioinformatic approaches revealed potential miRNA targets and signals involved in metastatic MTC pathways. Migration, proliferation and invasion assays were performed in cell lines treated with miR-200 antagomirs to ascertain a direct role for this miRNA in MTC tumourigenesis. We show that the members of miR-200 family regulate the expression of E-cadherin by directly targeting ZEB1 and ZEB2 mRNA and through the enhanced expression of tumour growth factor β (TGFβ)-2 and TGFβ-1. Overall, the treated cells shifted to a mesenchymal phenotype, thereby acquiring an aggressive phenotype with increased motility and invasion. Our data identify a robust miRNA signature associated with metastatic MTC and distinct biological processes, e.g., TGFβ signalling pathway, providing new potential insights into the mechanisms of   MTC metast  asis. 
 An autocrine TGF-beta/ZEB/miR-200 signaling network regulates establishment and maintenance of epithelial-mesenchymal transition. Epithelial-mesenchymal transition (EMT) is a form of cellular plasticity that is critical for embryonic development and tumor   metastasis  . A double-negative feedback loop involving the miR-200 family and ZEB (zinc finger E-box-binding homeobox) transcription factors has been postulated to control the balance between epithelial and mesenchymal states. Here we demonstrate using the epithelial Madin Darby canine kidney cell line model that, although manipulation of the ZEB/miR-200 balance is able to repeatedly switch cells between epithelial and mesenchymal states, the induction and maintenance of a stable mesenchymal phenotype requires the establishment of autocrine transforming growth factor-β (TGF-β) signaling to drive sustained ZEB expression. Furthermore, we show that prolonged autocrine TGF-β signaling induced reversible DNA methylation of the miR-200 loci with corresponding changes in miR-200 levels. Collectively, these findings demonstrate the existence of an autocrine TGF-β/ZEB/miR-200 signaling network that regulates plasticity between epithelial and mesenchymal states. We find a strong correlation between ZEBs and TGF-β and negative correlations between miR-200 and TGF-β and between miR-200 and ZEBs, in invasive ductal carcinomas, consistent with an autocrine TGF-β/ZEB/miR-200 signaling network being active in breast cancers. 
 miR-139-5p suppresses cancer cell migration and invasion through targeting ZEB1 and ZEB2 in GBM. Invasion and migration of glioblastoma multiforme (GBM) is a multistep process and an important phenotype that causes this disease to invade surrounding tissues in the brain. Recent studies have highlighted that miRNAs play a pivotal role in controlling GBM cell plasticity. In this report, we used wound healing and transwell assays to identify a novel role of miR-139-5p in inhibition of GBM cell migration and invasion. Bioinformatics coupled with luciferase and Western blot assays also revealed that miR-139-5p inhibited expression of ZEB1 and ZEB2, which are master regulators of tumor   metastasis  . MiR-139-5p specifically interacts with the 3'-UTR regions of ZEB1 and ZEB2, attenuating their expression in GBM cells. To corroborate this finding, we rescued ZEB1 and ZEB2 expression and found partial but significant increases in miR-139-5p-suppressed invasion of GBM cells. The biological relevance of our study was validated by analyzing levels of miR-139-5p in GBM tissue. We found that its expression significantly downregulated compared to normal tissue and shorter overall survival rates in patients with lower miR-139-5p expression. These results confirm that miR-139-5p suppresses GBM migration and invasion and highlight its potential as a biomarker and therapeutic target for treating GBM. 
 miR-139-5p inhibits epithelial-mesenchymal transition, migration and invasion of hepatocellular carcinoma cells by targeting ZEB1 and ZEB2. Hepatocellular carcinoma (HCC) is the third most common cause of cancer deaths worldwide. miRNAs have been suggested to have important roles in HCC development. The purpose of this study was to determine the role of miR-139-5p in regulation of epithelial-mesenchymal transition (EMT) and   metastasis   of HCC cells. Expression levels of miR-139-5p in 49 HCC specimens with adjacent tissues and five HCC cell lines were assessed by quantitative RT-PCR. We found that miR-139-5p was down-regulated in 89.7% of the HCC tissue samples and all of the HCC cell lines. In addition, luciferase reporter assays validated direct binding of miR-139-5p to the 3' untranslated region of zinc finger E-box binding homeobox 1 (ZEB1) and ZEB2. Ectopic expression of miR-139-5p suppressed and miR-139-in promoted EMT, migration, and invasion in Hep3B and SMMC7721 cells. Furthermore, over-expression of ZEB1 and ZEB2 ablated the inhibitory effects of miR-139-5p on migration and invasion in HCC cells. Our study indicates that miR-139-5p functions as a suppressor of HCC EMT and   metastasis   by targeting ZEB1 and ZEB2, and it may be a therapeutic target for metastatic HCC. 
 A double-negative feedback loop between ZEB1-SIP1 and the microRNA-200 family regulates epithelial-mesenchymal transition. Epithelial to mesenchymal transition occurs during embryologic development to allow tissue remodeling and is proposed to be a key step in the   metastasis   of epithelial-derived tumors. The miR-200 family of microRNAs plays a major role in specifying the epithelial phenotype by preventing expression of the transcription repressors, ZEB1/deltaEF1 and SIP1/ZEB2. We show here that miR-200a, miR-200b, and the related miR-429 are all encoded on a 7.5-kb polycistronic primary miRNA (pri-miR) transcript. We show that the promoter for the pri-miR is located within a 300-bp segment located 4 kb upstream of miR-200b. This promoter region is sufficient to confer expression in epithelial cells and is repressed in mesenchymal cells by ZEB1 and SIP1 through their binding to a conserved pair of ZEB-type E-box elements located proximal to the transcription start site. These findings establish a double-negative feedback loop controlling ZEB1-SIP1 and miR-200 family expression that regulates cellular phenotype and has direct relevance to the role of these factors in tumor progression. 
 MicroRNA-200 family members and ZEB2 are associated with brain   metastasis   in gastric adenocarcinoma. Although the incidence of brain   metastasis   in gastric cancer is relatively low, its prevalence may increase with improved therapy and longer survival tumors. The molecular mechanisms underlying brain metastases are not well understood. To gain insight into the mechanism of brain   metastasis  , we studied differences in microRNA (miRNA) expression levels in 8 cases of matched primary gastric adenocarcinoma and brain metastatic adenocarcinoma using the Illumina microRNA microarray chip. We identified 6 upregulated and 2 downregulated miRNAs in all 8 cases simultaneously. Interestingly, 2 out of 8 miRNAs (hsa-miR‑141-3p and hsa-miR-200b-3p) belonged to the miR-200 family. Online microRNA database searching revealed that ZEB2 is the top-ranked target gene for hsa-miR141-3p and hsa-miR-200b-3p, prompting us to focus ZEB2 expression in brain metastatic adenocarcinoma. We confirmed that ZEB2 expression was markedly downregulated in some brain metastatic samples. In addition, decreased ZEB2 expression was noted by western blot analysis of 2 metastatic gastric adenocarcinoma cell types that were derived by in vivo selection following intracardiac injection of gastric cancer cell lines. In conclusion, we demonstrate that expression of miRNA-200 family members and ZEB2 are associated with brain metastases of gastric adenocarcinoma, not only in matched patient samples, but also in metastatic cell lines that were derived by in vivo selection. 
 ZEB2-Sp1 cooperation induces invasion by upregulating cadherin-11 and integrin α5 expression. Epithelial-mesenchymal transition (EMT) is a process implicated in invasion and   metastasi  s. EMT is characterized by repression of epithelial markers and induction of mesenchymal markers. ZEB2 is a transcriptional repressor of E-cadherin, leading to EMT. Previously, we have shown that ZEB2 directly upregulates integrin α5 transcription by cooperating with the transcription factor Sp1. In this study, we investigated the precise mechanism by which ZEB2 modulates invasion and EMT events and the role of Sp1 in ZEB2-induced invasion. We found that ZEB2 directly induced cadherin-11 transcription in an Sp1-dependent, but Smad- and E-box-independent, manner and repressed E-cadherin expression in an Sp1- and Smad-independent manner, leading to cadherin switch. Furthermore, ZEB2 upregulated Sp1 by enhancing Sp1 protein stability, and Sp1 was found to be critical for ZEB2-induced cancer cell invasion, mainly through induction of cadherin-11 and integrin α5. Expression levels of cadherin-11 and integrin α5 were interdependent and both modulated c-Jun N-terminal kinase-signaling activity and invasion. Immunofluorescence analysis showed that nuclear expression of ZEB2 was positively correlated with Sp1 expression in human colorectal cancers. Together, these findings demonstrate a previously unrecognized interplay between ZEB2, Sp1, cadherin-11 and integrin α5 that is, probably, significant in tumor progression   and metas  tasis. 
 ZEB2 promotes the   metastasis   of gastric cancer and modulates epithelial mesenchymal transition of gastric cancer cells. Invasion and   metastasis   are the hallmarks of advanced gastric cancer progression. Therefore, it is urgent to overcome   metastasis   in order to improve the survival of gastric cancer patients.This study aimed to examine the expression of ZEB2 in gastric cancer samples and analyze its correlation with clinicopathologic features. In addition, the molecular mechanism by which ZEB2 contributes to gastric cancer   metastasis   will be explored.ZEB2 expression in clinical gastric cancer samples was evaluated by immunohistochemical analysis. ZEB2 was knocked-down in HGC27 gastric cancer cells by shRNA and the effects on cell invasion and migration were examined by in vitro cell invasion and migration assays. The expression of epithelial marker E-cadherin, mesenchymal markers fibronecin and vimentin, and MMPs was detected by western blot analysis.The expression of ZEB2 was positively correlated with the depth of invasion, lymph node   metastasis   and TNM stage. In addition, patients with positive ZEB2 expression showed a significantly shorter overall survival time than did patients with negative ZEB2. shRNA mediated knockdown of ZEB2 resulted in reduced invasion and migration of HGC27 cells, along with the upregulation of E-cadherin and downregulation of fibronecin, vimentin, MMP2, and MMP9.ZEB2 expression is closely associated with the clinicopathological parameters of gastric cancer. ZEB2 promotes gastric cancer cell migration and invasion at least partly via the regulation of epithelial-mesenchymal transition. ZEB2 is a potential target for gene therapy of aggressive gastric cancer. 
 Langerhans cell maturation is accompanied by induction of N-cadherin and the transcriptional regulators of epithelial-mesenchymal transition ZEB1/2. Langerhans cells (LCs) are a unique subset of dendritic cells (DCs) that express epithelial adhesion molecules, allowing them to form contacts with epithelial cells and reside in epidermal/epithelial tissues. The dynamic regulation of epithelial adhesion plays a decisive role in the life cycle of LCs. It controls whether LCs remain immature and sessile within the epidermis or mature and egress to initiate immune responses. So far, the molecular machinery regulating epithelial adhesion molecules during LC maturation remains elusive. Here, we generated pure populations of immature human LCs in vitro to systematically probe for gene-expression changes during LC maturation. LCs down-regulate a set of epithelial genes including E-cadherin, while they upregulate the mesenchymal marker N-cadherin known to facilitate cell migration. In addition, N-cadherin is constitutively expressed by monocyte-derived DCs known to exhibit characteristics of both inflammatory-type and interstitial/dermal DCs. Moreover, the transcription factors ZEB1 and ZEB2 (ZEB is zinc-finger E-box-binding homeobox) are upregulated in migratory LCs. ZEB1 and ZEB2 have been shown to induce epithelial-to-mesenchymal transition (EMT) and invasive behavior in cancer cells undergoing   metastasis  . Our results provide the first hint that the molecular EMT machinery might facilitate LC mobilization. Moreover, our study suggests that N-cadherin plays a role during DC migration. 
 HOXB8 promotes tumor   metastasis   and the epithelial-mesenchymal transition via ZEB2 targets in gastric cancer. The homeobox B8 (HOXB8) functions as a sequence-specific transcription factor that is involved in development. Increased expression of this gene is associated with a wide variety of tumor; however, its function in gastric cancer has not been clarified. In the present study, the expression of HOXB8 in gastric cancer tissues and influence of HOXB8 on gastric cancer cellular were evaluated.The expression levels of HOXB8 mRNA in human gastric cancer tissues were analyzed through quantitative RT-PCR. To test the role of HOXB8 in gastric cancer   metastasis  , the cell transwell assay was performed. Microarray, ChIP-qPCR, and Western blot were used to explore the possible mechanism that HOXB8 promotes gastric cancer cells   metastasis  .In this study, we found that HOXB8 showed higher expression in metastatic tissues than no-metastatic tissues. Overexpression of HOXB8 can promote gastric cancer cells migration and invasion, while silencing HOXB8 leads to the opposite results. Overexpression of HOXB8 also increases the rate of   metastasis   in NCI-N87 mice, while silencing HOXB8 has the opposite results. Furthermore, HOXB8 promotes epithelial-mesenchymal transformation of AGS cells. We also found that ZEB2 can interact with HOXB8 and may be a downstream factor of HOXB8 by using microarray. Knockdown of ZEB2 can inhibit HOXB8-induced migration and invasion capacity, as well as the epithelial-mesenchymal transformation in gastric cancer cells.The results showed that HOXB8 plays an important role in the development and   metastasis   of gastric carcinoma. 
 ZEB2 promotes vasculogenic mimicry by TGF-β1 induced epithelial-to-mesenchymal transition in hepatocellular carcinoma. Zinc finger E-box binding homeobox 2 (ZEB2), an epithelial-mesenchymal transition (EMT) regulator, has been involved in invasion and   metastasi  s of human tumor. Although EMT may be involved in vasculogenic mimicry (VM) formation, no reports describing the relation between ZEB2 and VM are available. We hypothesize that ZEB2 may promote VM formation in hepatocellular carcinoma (HCC).Paraffin-embedded tumor tissue samples from 92 patients were immunostained with anti-ZEB2 antibody. We found that the ZEB2 nuclear expression was significantly associated with VM formation and   metastasi  s. Patients with VM and ZEB2 nuclear expression had a shorter survival period than those without expression. In vitro, ZEB2 overexpression significantly enhanced cell motility, invasiveness, and VM formation of HepG2 cells. ZEB2 upregulation also increased VE-cadherin, Flt-1, and Flk-1 expression and activated MMPs. ZEB2 knockdown inhibited cell motility, invasiveness, and VM formation in Bel7402 cells. ZEB2 knockdown also decreased VE-cadherin, Flt-1, and Flk-1 expression and MMP activity. In addition, EMT in HepG2 cells was induced by TGF-β1 treatment, and the kinetics of expression of EMT markers and regulators were assessed by Western blot analysis. The expression of ZEB2 increased significantly, and VM formation was promoted.ZEB2 can promote VM formation through the EMT pathway. Our findings may represent a novel therapeutic target in HCC. 
 MicroRNA-200b regulates cell proliferation, invasion, and migration by directly targeting ZEB2 in gastric carcinoma. The microRNA-200 (miR-200) family has been reported to induce epithelial differentiation and suppress epithelial-mesenchymal transition (EMT) by inhibiting translation of zinc finger E-box-binding homeobox (ZEB) 1 and 2 mRNAs in several types of cancers. This study aimed to clarify the role of miR-200b in regulating EMT and promoting cellular proliferation, invasion, and migration in gastric cancer.The relationships among the expression levels of miR-200b, ZEB1 and ZEB2, and E-cadherin mRNAs were analyzed by quantitative real-time reverse transcription-polymerase chain reaction in frozen tissue samples from 40 gastric cancer patients who underwent gastrectomy from 2008 to 2010. The effects of miR-200b on EMT in gastric cancer cells in vitro were also analyzed.Diffuse histologic type, depth of tumor, tumor size, lymph node   metastasis  , and lymphatic invasion were significantly higher in the low-miR-200b expression group compared with the high expression group. There was a strong correlation between the levels of miR-200b, and ZEB2 and E-cadherin mRNAs in gastric cancer patients. Upregulation of miR-200b in gastric cancer cells changed the cell morphology from fibroblast- to epithelial-like, associated with localization of E-cadherin to the plasma membrane. ZEB2 mRNA levels fell, while E-cadherin expression levels increased in gastric cells overexpressing miR-200b, associated with significantly reduced cellular proliferation, and inhibition of cellular migration and invasion.miR-200b regulates ZEB2 expression and thus controls   metastasis   in gastric cancer. 
 Expression of SIP1 in oral squamous cell carcinomas: implications for E-cadherin expression and tumor progression. Loss of E-cadherin expression allows carcinoma cells to liberate from the primary site and enhances invasion and   metastasis  . The genetic aberration of E-cadherin is a rare event in sporadic carcinomas, and transcription repressors are considered to take a central role in E-cadherin loss. However, expression of E-cadherin repressors is largely dependent on tissue and cell type. To identify the repressor expressed in oral squamous carcinomas, we compared the expression levels of E-cadherin and repressors by real-time RT-PCR. Among the repressors including SNAIL, SLUG, SIP1, E12 and E47, SIP1 was inversely correlated to E-cadherin (P < 0.05). Chromatin immunoprecipitation showed that SIP1 specifically bound to the E-cadherin promoter region. SIP1 expression was immuno-histochemically detected in 27.7% of 47 oral carcinomas, and SIP1-positive carcinomas did not express E-cadherin (P < 0.01). Thirteen patients with SIP1 staining showed a lower disease-specific survival rate (P < 0.05). Multivariate risk factor analysis demonstrated that SIP1 expression was an independent prognostic value for disease-specific overall survival (P < 0.05). These results suggest that SIP1 contributes to the loss of E-cadherin expression and that detection of SIP1 expression is a predictive and prognostic tool in clinical management of oral carcinomas. 
 Identification of a ZEB2-MITF-ZEB1 transcriptional network that controls melanogenesis and melanoma progression. Deregulation of signaling pathways that control differentiation, expansion and migration of neural crest-derived melanoblasts during normal development contributes also to melanoma progression and   metastasis  . Although several epithelial-to-mesenchymal (EMT) transcription factors, such as zinc finger E-box binding protein 1 (ZEB1) and ZEB2, have been implicated in neural crest cell biology, little is known about their role in melanocyte homeostasis and melanoma. Here we show that mice lacking Zeb2 in the melanocyte lineage exhibit a melanoblast migration defect and, unexpectedly, a severe melanocyte differentiation defect. Loss of Zeb2 in the melanocyte lineage results in a downregulation of the Microphthalmia-associated transcription factor (Mitf) and melanocyte differentiation markers concomitant with an upregulation of Zeb1. We identify a transcriptional signaling network in which the EMT transcription factor ZEB2 regulates MITF levels to control melanocyte differentiation. Moreover, our data are also relevant for human melanomagenesis as loss of ZEB2 expression is associated with reduced patient survival. 
 SIP1 protein protects cells from DNA damage-induced apoptosis and has independent prognostic value in bladder cancer. The epithelial-mesenchymal transition (EMT) contributes to cancer   metastasis  . Two ZEB family members, ZEB1 and ZEB2(SIP1), inhibit transcription of the E-cadherin gene and induce EMT in vitro. However, their relevance to human cancer is insufficiently studied. Here, we performed a comparative study of SIP1 and ZEB1 proteins in cancer cell lines and in one form of human malignancy, carcinoma of the bladder. Whereas ZEB1 protein was expressed in all E-cadherin-negative carcinoma cell lines, being in part responsible for the high motility of bladder cancer cells, SIP1 was hardly ever detectable in carcinoma cells in culture. However, SIP1 represented an independent factor of poor prognosis (P = 0.005) in a series of bladder cancer specimens obtained from patients treated with radiotherapy. In contrast, ZEB1 was rarely expressed in tumor tissues; and E-cadherin status did not correlate with the patients' survival. SIP1 protected cells from UV- and cisplatin-induced apoptosis in vitro but had no effect on the level of DNA damage. The anti-apoptotic effect of SIP1 was independent of either cell cycle arrest or loss of cell-cell adhesion and was associated with reduced phosphorylation of ATM/ATR targets in UV-treated cells. The prognostic value of SIP1 and its role in DNA damage response establish a link between genetic instability and   metastasis   and suggest a potential importance for this protein as a therapeutic target. In addition, we conclude that the nature of an EMT pathway rather than the deregulation of E-cadherin per se is critical for the progression of the disease and patients' survival. 
 Differences in the expression of epithelial-mesenchymal transition related molecules between primary tumors and pulmonary metastatic tumors in colorectal cancer. Epithelial-mesenchymal transition (EMT) is a key event in cancer   metastasis  . This study immunohistochemically examined the expression of EMT-related molecules in both primary colorectal cancer and pulmonary metastases, and analyzed the expression pattern.Ten patients with colorectal cancer that underwent surgical resections for both the primary tumor and metastatic pulmonary tumors were included. The expression status of EMT-related molecules was examined using immunohistochemical staining.Nine of the 10 cases maintained the expression of both E-cadherin and β-catenin in the primary site. The expression of E-cadherin and β-catenin in the pulmonary metastatic site was preserved in 10 and 12 out of 15 metastatic lesions, respectively. The EMT-related transcription factor, Twist, was positively expressed in all 10 cases, Smad interacting protein 1 (Sip1) in 9, Snail in 4 and Slug in 3 of the primary sites. On the other hand, staining for Twist, Sip1 and Snail at the metastatic pulmonary site, was negative in all 10 cases.The expression of EMT-related transcription factors in metastatic pulmonary tumors from colorectal cancer decreased in comparison to the primary tumors. These findings suggested that the expression status of EMT-related transcription factors might play an important role in the implantation of metastatic foci. 
 The miR-200 family and miR-205 regulate epithelial to mesenchymal transition by targeting ZEB1 and SIP1. Epithelial to mesenchymal transition (EMT) facilitates tissue remodelling during embryonic development and is viewed as an essential early step in tumour   metastasis  . We found that all five members of the microRNA-200 family (miR-200a, miR-200b, miR-200c, miR-141 and miR-429) and miR-205 were markedly downregulated in cells that had undergone EMT in response to transforming growth factor (TGF)-beta or to ectopic expression of the protein tyrosine phosphatase Pez. Enforced expression of the miR-200 family alone was sufficient to prevent TGF-beta-induced EMT. Together, these microRNAs cooperatively regulate expression of the E-cadherin transcriptional repressors ZEB1 (also known as deltaEF1) and SIP1 (also known as ZEB2), factors previously implicated in EMT and tumour   metastasis  . Inhibition of the microRNAs was sufficient to induce EMT in a process requiring upregulation of ZEB1 and/or SIP1. Conversely, ectopic expression of these microRNAs in mesenchymal cells initiated mesenchymal to epithelial transition (MET). Consistent with their role in regulating EMT, expression of these microRNAs was found to be lost in invasive breast cancer cell lines with mesenchymal phenotype. Expression of the miR-200 family was also lost in regions of metaplastic breast cancer specimens lacking E-cadherin. These data suggest that downregulation of the microRNAs may be an important step in tumour progression. 
 Dysfunction of the Reciprocal Feedback Loop between GATA3- and ZEB2-Nucleated Repression Programs Contributes to Breast Cancer   Metastasis  . How loss-of-function of GATA3 contributes to the development of breast cancer is poorly understood. Here, we report that GATA3 nucleates a transcription repression program composed of G9A and MTA3-, but not MTA1- or MTA2-, constituted NuRD complex. Genome-wide analysis of the GATA3/G9A/NuRD(MTA3) targets identified a cohort of genes including ZEB2 that are critically involved in epithelial-to-mesenchymal transition and cell invasion. We demonstrate that the GATA3/G9A/NuRD(MTA3) complex inhibits the invasive potential of breast cancer cells in vitro and suppresses breast cancer   metastasis   in vivo. Strikingly, the expression of GATA3, G9A, and MTA3 is concurrently downregulated during breast cancer progression, leading to an elevated expression of ZEB2, which, in turn, represses the expression of G9A and MTA3 through the recruitment of G9A/NuRD(MTA1). 
 Epithelial-mesenchymal transition transcription factor ZEB1/ZEB2 co-expression predicts poor prognosis and maintains tumor-initiating properties in head and neck cancer. Both epithelial-mesenchymal transition (EMT) and cancer stem cell (CSC) properties may be involved in   metastasis  , which contributes to the high mortality rate of patients with head and neck cancers (HNCs). However, the mechanisms through which the EMT transcription factors ZEB1 and ZEB2 regulate HNC are still unclear.Tumor initiating capability of HNC-CH133(+) cells with ZEB1/2 knockdown or co-overexpression was presented in vitro and in vivo.In the present study, we demonstrated that ZEB1/ZEB2 expression was significantly increased in HNC-CD133(+) CSC-like cells compared with HNC-CD133(-) cells. The small interfering RNA (siRNA)-mediated co-knockdown of ZEB1 and ZEB2 (siZEB1/2) in HNC-CH133(+) cells suppressed their CSC-like properties, including self-renewal ability, the expression of stemness markers, and drug resistance. In contrast, the co-overexpression of ZEB1/ZEB2 in HNC-CD133(-) cells enhanced their sphere-forming ability and increased the percentage of CD44-positive cells and side population cells. In vivo studies showed that the delivery of siZEB1/2 to xenograft tumors in nude mice reduced tumor growth and the rate of distant   metastasis  . In clinical samples, the levels of ZEB1/ZEB2 expression were low in local lesions but high in metastatic lymph nodes in HNC tissues. Patients with tumors that co-expressed ZEB1(high) and ZEB2(high) had especially poor survival rates.Therapies targeting ZEB1/ZEB2 in HNC-CD133(+) cells may provide a new approach for HNC therapy in the future. 
 MicroRNA-335 inhibits invasion and   metastasis   of colorectal cancer by targeting ZEB2. MicroRNAs have been suggested to play a vital role in regulate tumor progression and invasion. However, the expression of miR-335 in colorectal cancer (CRC) and its clinical significance are not known. Here, we report that miR-335 is a tumor suppressor by regulating expression of ZEB2. In this study, we showed that downregulated miR-335 levels in highly invasive CRC cell lines and tissues. Kaplan-Meier survival analysis indicated that patients with reduced miR-335 had a poor overall survival. Furthermore, enhancing the expression of miR-335 inhibited CRC cell migration and invasion in vitro and lung and liver   metastasis   in vivo, while silencing its expression resulted in increased migration and invasion. Additionally, we identified a novel miR-335 target, ZEB2, and the direct interaction between them was verified by 3'-untranslated region dual-luciferase reporter assay. In conclusion, our results demonstrate that miR-335 functions as a tumor suppressor and play a role in inhibiting   metastasis   of CRC cells through targeting ZEB2. These findings suggest that miR-335 may be useful as a new potential therapeutic target for CRC. 
 Ubiquitin ligase Cbl-b represses IGF-I-induced epithelial mesenchymal transition via ZEB2 and microRNA-200c regulation in gastric cancer cells. Insulin-like growth factor I (IGF-I) can induce epithelial mesenchymal transition (EMT) in many epithelial tumors; however, the molecular mechanism by which this occurs is not clearly understood. Additionally, little is known about the involvement of IGF-I in gastric cancer.Two gastric cancer cell lines were treated with IGF-I to induce EMT and levels of transcription factor ZEB2 and microRNA-200c (miR-200c) were measured. Cells were treated with Akt/ERK inhibitors to investigate the role of these pathways in IGF-I-mediated EMT. Transfection of shRNA plasmids was used to silence the ubiquitin ligase Cbl-b to assess its involvement in this process. The relationship between IGF-IR and Cbl-b expression, and the effect of IGF-IR and Cbl-b on   metastasis   were analyzed in primary gastric adenocarcinoma patients.IGF-I-induced gastric cancer cell EMT was accompanied by ZEB2 up-regulation. Furthermore, both Akt/ERK inhibitors and knockdown of Akt/ERK gene reversed IGF-I-induced ZEB2 up-regulation and EMT through up-regulation of miR-200c, suggesting the involvement of an Akt/ERK-miR-200c-ZEB2 axis in IGF-I-induced EMT. The ubiquitin ligase Cbl-b also ubiquitinated and degraded IGF-IR and inhibited the Akt/ERK-miR-200c-ZEB2 axis, leading to the repression of IGF-I-induced EMT. There was a significant negative correlation between the expression of IGF-IR and Cbl-b in gastric cancer patient tissues (r = -0.265, p < 0.05). More of patients with IGF-IR-positive expression and Cbl-b-negative expression were with lym  ph node me  tastasis (p < 0.001).Together, these findings demonstrate that the ubiquitin ligase Cbl-b represses IGF-I-induced EMT, likely through targeting IGF-IR for degradation and further inhibiting the Akt/ERK-miR-200c-ZEB2 axis in gastric cancer cells. 
 FOXA2 attenuates the epithelial to mesenchymal transition by regulating the transcription of E-cadherin and ZEB2 in human breast cancer. The Forkhead Box A2 (FOXA2) transcription factor is required for embryonic development and for normal functions of multiple adult tissues, in which the maintained expression of FOXA2 is usually related to preventing the progression of malignant transformation. In this study, we found that FOXA2 prevented the epithelial to mesenchymal transition (EMT) in human breast cancer. We observed a strong correlation between the expression levels of FOXA2 and the epithelial phenotype. Knockdown of FOXA2 promoted the mesenchymal phenotype, whereas stable overexpression of FOXA2 attenuated EMT in breast cancer cells. FOXA2 was found to endogenously bind to and stimulate the promoter of E-cadherin that is crucial for epithelial phenotype of the tumor cells. Meanwhile, FOXA2 prevented EMT of breast cancer cells by repressing the expression of EMT-related transcription factor ZEB2 through recruiting a transcriptional corepressor TLE3 to the ZEB2 promoter. The stable overexpression of FOXA2 abolished   metastasis   of breast cancer cells in vivo. This study confirmed that FOXA2 inhibited EMT in breast cancer cells by regulating the transcription of EMT-related genes such as E-cadherin and ZEB2. 
 Snail and SIP1 increase cancer invasion by upregulating MMP family in hepatocellular carcinoma cells. Loss of E-cadherin (E-cad) triggers invasion,   metastasis  , and dedifferentiation in various epithelial carcinomas. Recently, it has been reported that two transcription factors, Snail and SIP1 (Smad interacting protein 1), directly repress transcription of the E-cad gene by binding E-box on E-cad promoter. Our aim is to solve the molecular mechanism of Snail and SIP1 in hepatocellular carcinoma (HCC). We first showed an inverse correlation between E-cad and Snail/SIP 1 expression among five HCC lines with different phenotypes. The result indicated that undifferentiated, but not differentiated type expressed Snail/SIP1. Then, we established transfectants stably expressing Snail and SIP1 in two differentiated cells with E-cad expression. Suppressed expression of E-cad, morphologic change into fibroblastoid feature, and remarkable acceleration of invasion activity were observed in the transfectants. In reverse transcription-polymerase chain reaction series of genes relating to motility and invasion, we demonstrated striking evidence that matrix metalloproteinase (MMP-1), MMP-2, MMP-7, and MT1-MMP expressions were strongly upregulated by Snail. On the other hand, MMP-1, MMP-2, and MT1-MMP expressions were enhanced by SIP1 transfection, however, the intensity was weaker than that in Snail transfection. In conclusion, Snail or SIP1 expression may be induced during HCC progression, where Snail/SIP1 directly represses E-cad gene transcription and activates cancer invasion via the upregulation of the MMP gene family. 
 MicroRNA-200a suppresses metastatic potential of side population cells in human hepatocellular carcinoma by decreasing ZEB2. Although microRNA-200a (miR-200a) is frequently downregulated in cancer, its role in side population (SP) has not been investigated. In this study, 101 pairs of primary hepatocellular carcinoma (HCC) tissues and matched normal control tissues were analyzed for miR-200a expression and its clinicopathological value was determined. We found that miR-200a was downregulated in HCC/SP and this was associated   metastasis  . MiR-200a suppressed   metastasis   of SP cells. Overexpression of miR-200a in SP cells decreased   metastasis  -related markers and expression of ZEB2. The associations between miR-200a, SP cells and ZEB2 were validated in HCC. These findings reveal that miR-200a suppresses   metastasis   of SP cells by downregulating ZEB2. 
 Epithelial to mesenchymal transition is associated with shorter disease-free survival in hepatocellular carcinoma. Epithelial to mesenchymal transition (EMT) is involved in cancer cell invasion and   metastasis   as well as chemoresistance. Elucidation of EMT in hepatocellular carcinoma (HCC) might contribute to deeper understanding of its biology.Overall, 100 patients with HCC, who underwent resection, were analyzed. The messenger RNA (mRNA) expression of the epithelial marker E-cadherin and the mesenchymal marker Vimentin were measured, and the EMT status of each patient was determined as follows: Vimentin/E-cadherin <2 = Epithelial (E), Vimentin/E-cadherin ≥2 = Mesenchymal (M). The correlation between these values and clinicopathological factors and prognosis were analyzed statistically. Moreover, the expression of transcription factors involved in EMT (Twist-1, Snail, Slug, Zeb-1, and Zeb-2) were measured and the role of interleukin (IL)-6 in inducing EMT and chemoresistance was examined.Patients with a mesenchymal tumor were more prone to have an earlier recurrence than those with an epithelial tumor. EMT-inducing transcription factors were more highly expressed in mesenchymal tumors than in epithelial tumors, and Twist-1 and Zeb-2 were significantly overexpressed. α-Fetoprotein (AFP) values were significantly higher in patients with epithelial tumors, and AFP-expressing HCC cell lines were more responsive to sorafenib. IL-6 expression was significantly higher in mesenchymal tumors, and knockdown of IL-6 in mesenchymal HCC cell lines increased E-cadherin expression and sensitivity to sorafenib.Analysis of surgically resected tumors suggests that EMT is involved in early disease recurrence in HCC. Twist-1 and Zeb-2 might be important for inducing EMT, and IL-6 might be a potential therapeutic target for alleviating the chemoresistance of mesenchymal HCC tumors. 
 ZEB2 upregulates integrin α5 expression through cooperation with Sp1 to induce invasion during epithelial-mesenchymal transition of human cancer cells. Epithelial-mesenchymal transition (EMT) is a process implicated in tumor invasion,   metastasi  s, embryonic development and wound healing. ZEB2 is a transcription factor involved in EMT that represses E-cadherin transcription. Although E-cadherin downregulation is a major event during EMT and tumor progression, E-cadherin reduction is probably not sufficient for full invasiveness. The mechanisms by which E-cadherin transcriptional repressors induce mesenchymal genes during EMT remain largely unknown. Here, we investigated the role of ZEB2 in the induction of integrin α5 during cancer EMT and its underlying mechanism. In human cancer cells, ZEB2 was found to directly upregulate integrin α5 transcription in a manner that is independent of the regulation of E-cadherin expression. Conversely, depletion of ZEB2 by small interfering RNA suppressed integrin α5 expression, leading to reduced invasion. Suppression of integrin α5 inhibited cancer cell invasion, suggesting an important role for integrin α5 in cancer progression. Furthermore, ZEB2 was found to activate the integrin α5 and vimentin promoters by interacting with and activating the transcription factor Sp1, suggesting that cooperation between ZEB2 and Sp1 represents a novel mechanism of mesenchymal gene activation during EMT. These findings increase our understanding of the pathways beyond E-cadherin reduction that regulate mesenchymal gene expression during EMT and cancer progression. 
 A Systematic Approach to Defining the microRNA Landscape in   Metastasis  . The microRNA (miRNA) landscape changes during the progression of cancer. We defined a   metastasis  -associated miRNA landscape using a systematic approach. We profiled and validated miRNA and mRNA expression in a unique series of human colorectal   metastasis   tissues together with their matched primary tumors and corresponding normal tissues. We identified an exclusive miRNA signature that is differentially expressed in metastases. Three of these miRNAs were identified as key drivers of an EMT-regulating network acting though a number of novel targets. These targets include SIAH1, SETD2, ZEB2, and especially FOXN3, which we demonstrated for the first time as a direct transcriptional suppressor of N-cadherin. The modulation of N-cadherin expression had significant impact on migration, invasion, and   metastasis   in two different in vivo models. The significant deregulation of the miRNAs defining the network was confirmed in an independent patient set as well as in a database of diverse malignancies derived from more than 6,000 patients. Our data define a novel   metastasis  -orchestrating network based on systematic hypothesis generation from   metastasis   tissues. 
  GeneRIFs  
 Knockdown of ZEB2 can inhibit HOXB8-induced migration and invasion capacity, as well as the epithelial-mesenchymal transformation in gastric cancer cells. The results showed that HOXB8 plays an important role in the development and   metastasis   of gastric carcinoma 
 Findings reveal that miR-200a suppresses   metastasis   of SP cells by downregulating ZEB2. 
 co-expression of TWIST1 and ZEB2 in OSCC was significantly associated with poorer overall survival, particularly in patients without detectable lymph node   metastasis  . 
 Our study first indicates that miR-590-3p functions as a suppressor of GBM EMT and   metastasis   by targeting ZEB1 and ZEB2, and it may be a therapeutic target for metastatic GBM. 
 miRNA network regulates ZEB2 in in colorectal   metastasis   tissues. 
 These results suggest that the double-negative feedback loop between ZEB2 and miR-145 contributes to prostate cancer progression and   metastasis  . 
 ZEB2 was a target of miR-335 action in colorectal cancer   metastasis  . 
 miR-200c modulates ovarian cancer cell   metastasis   potential by targeting ZEB2 expression. 
 miR-132 plays an important role in colorectal cancer invasion and   metastasis   via directly targeting ZEB2 
 FoxQ1 promotes hepatocellular carcinoma   metastasis   by transactivating ZEB2 and VersicanV1 expression. 
 Elevated expression of SIP1 can contribute to invasion and   metastasis   of adenoid cystic carcinoma (ACC), and there might be some correlation between the hypoxia microenvironment and epithelial-mesenchymal transition in ACC. 
 The prognostic value of SIP1 and its role in DNA damage response establish a link between genetic instability and   metastasis  . 
 Data show that miR-200 and -205 microRNAs cooperatively regulate expression of the E-cadherin transcriptional repressors ZEB1 (also known as deltaEF1) and SIP1 (also known as ZEB2), factors previously implicated in EMT and tumour   metastasis  . 
  [Cc]ell [Ii]nvasion  PubMed  
 miR-132 inhibits colorectal cancer invasion and metastasis via directly targeting ZEB2. To investigate the biological role and underlying mechanism of miR-132 in colorectal cancer (CRC) progression and invasion.Quantitative RT-PCR analysis was used to examine the expression levels of miR-132 in five CRC cell lines (SW480, SW620, HCT116, HT29 and LoVo) and a normal colonic cell line NCM460, as well as in tumor tissues with or without metastases. The Kaplan-Meier method was used to analyze the prognostic significance of miR-132 in CRC patients. The biological effects of miR-132 were assessed in CRC cell lines using the transwell assay. Quantitative RT-PCR and western blot analyses were employed to evaluate the expression of miR-132 targets. The regulation of ZEB2 by miR-132 was confirmed using the luciferase activity assay.miR-132 was significantly down-regulated in the CRC cell lines compared with the normal colonic cell line (P < 0.05), as well as in the CRC tissues with distant metastases compared with the tissues without metastases (10.52 ± 4.69 vs 23.11 ± 7.84) (P < 0.001). Down-regulation of miR-132 was associated with tumor size (P = 0.016), distant metastasis (P = 0.002), and TNM stage (P = 0.020) in CRC patients. Kaplan-Meier survival curve analysis indicated that patients with low expression of miR-132 tended to have worse disease-free survival than patients with high expression of miR-132 (P < 0.001). Moreover, ectopic expression of miR-132 markedly inhibite  d cell invasi  on (P < 0.05) and the epithelial-mesenchymal transition (EMT) in CRC cell lines. Further investigation revealed ZEB2, an EMT regulator, was a downstream target of miR-132.Our study indicated that miR-132 plays an important role in the invasion and metastasis of CRC. 
 Fra-1/AP-1 induces EMT in mammary epithelial cells by modulating Zeb1/2 and TGFβ expression. Epithelial-to-mesenchymal transition (EMT) is essential for embryonic morphogenesis and wound healing and critical for tumour   cell invasio  n and dissemination. The AP-1 transcription factor Fra-1 has been implicated in tumorigenesis and in tumour-associated EMT in human breast cancer. We observed a significant inverse correlation between Fra-1 mRNA expression and distant-metastasis-free survival in a large cohort of breast cancer patients derived from multiple array data sets. This unique correlation among Fos genes prompted us to assess the evolutionary conservation between Fra-1 functions in EMT of human and mouse cells. Ectopic expression of Fra-1 in fully polarized, non-tumourigenic, mouse mammary epithelial EpH4 cells induced a mesenchymal phenotype, characterized by a loss of epithelial and gain of mesenchymal markers. Proliferation, motility and invasiveness were also increased in the resulting EpFra1 cells, and the cells were tumourigenic and efficiently colonized the lung upon transplantation. Molecular analyses revealed increased expression of Tgfβ1 and the EMT-inducing transcription factors Zeb1, Zeb2 and Slug. Mechanistically, Fra-1 binds to the tgfb1 and zeb2 promoters and to an evolutionarily conserved region in the first intron of zeb1. Furthermore, increased activity of a zeb2 promoter reporter was detected in EpFra1 cells and shown to depend on AP-1-binding sites. Inhibiting TGFβ signalling in EpFra1 cells moderately increased the expression of epithelial markers, whereas silencing of zeb1 or zeb2 restored the epithelial phenotype and decreased migration in vitro and tumorigenesis in vivo. Thus Fra-1 induces changes in the expression of genes encoding EMT-related transcription factors leading to the acquisition of mesenchymal, invasive and tumorigenic capacities by epithelial cells. This study defines a novel function of Fra-1/AP-1 in modulating tgfb1, zeb1 and zeb2 expression through direct binding to genomic regulatory regions, which establishes a basis for future in vivo genetic manipulations and preclinical studies using mouse models. 
 Double-negative feedback loop between long non-coding RNA TUG1 and miR-145 promotes epithelial to mesenchymal transition and radioresistance in human bladder cancer cells. LncRNAs have a critical role in the regulation of cellular processes such as cancer progression and metastasis. In the present study, we confirmed that TUG1 was overexpressed in bladder cancer tissues and established cell lines. Knockdown of TUG1 inhibited bladder cancer cell metastasis both in vitro and in vivo. Furthermore, we found that TUG1 promoted cancer   cell invasion   and radioresistance through inducing epithelial-to-mesenchymal transition (EMT). Interestingly, TUG1 decreased the expression of miR-145 and there was a reciprocal repression between TUG1 and miR-145 in an Argonaute2-dependent manner. ZEB2 was identified as a down-stream target of miR-145 and TUG1 exerted its function through the miR-145/ZEB2 axis. In summary, our data indicated that blocking TUG1 function may be an effective anti-cancer therapy. 
 Down-regulation of miR-144 promotes thyroid cancer   cell invasion   by targeting ZEB1 and ZEB2. Thyroid cancer is the most common endocrine malignancy, and its incidence has increased rapidly worldwide. The molecular mechanisms underlying thyroid cancer tumorigenesis still need to be further investigated. MicroRNAs (miRNAs), short RNA molecules of approximately 22 nucleotides in length, play crucial roles in tumorigenesis. In the present study, we found that the expression of miR-144 was significantly down-regulated in thyroid cancer as compared with that in normal thyroid tissues, suggesting that miR-144 may be involved in thyroid cancer tumorigenesis. Moreover, our results showed that restoration of miR-144 in K1 and WRO thyroid cancer cells could suppress the invasion and migration capability of these cells. We also demonstrated that miR-144 suppressed the expression of ZEB1 and ZEB2, two E-cadherin suppressors, by directly binding to their 3'-untranslated regions. Furthermore, restoration of ZEB1 or ZEB2 partially rescued the miR-144-induced inhibition of   cell invasion  . These data suggest miR-144 function as a tumor suppressor in thyroid cancer. 
 MicroRNA-138 Regulates Metastatic Potential of Bladder Cancer Through ZEB2. The cases of bladder cancer (BC) with poor prognosis largely result from the distal metastases of the primary tumor. Since microRNAs (miRNAs) play critical roles during cancer metastases, determination of the involved miRNAs in the regulation of the metastases of BC may provide novel therapeutic targets for BC treatment. Here, we aimed to study the role of miR-138 in regulation of BC   cell invasion   and metastases.We analyzed the levels of miR-138 and ZEB2, a key factor that regulates cancer   cell invasion  , in the BC specimens from the patients. We also studied the correlation between miR-138 and ZEB2. We performed bioinformatics analyses on the binding of miR-138 to the 3'-UTR of ZEB2 mRNA, and verified the biological effects of this binding through promoter luciferase reporter assay. The effects of miR-138-modification on BC   cell invasion   were evaluated in a transwell   cell invasion   assay and a scratch would healing assay.We found that the levels of miR-138 were significantly decreased and the levels of ZEB2 were significantly increased in BC specimens, compared to the paired normal bladder tissue. Metastatic BC appeared to contained lower levels of miR-138. Moreover, miR-138 and ZEB2 inversely correlated in BC specimens. Bioinformatics analyses showed that miR-138 targeted the 3'-UTR of ZEB2 mRNA to inhibit its translation. Furthermore, miR-138 overexpression inhibited ZEB2-mediated   cell invasion   and metastases, while miR-138 depletion increased ZEB2-mediated   cell invasion   and metastases in BC cells.Suppression of miR-138 in BC cells may promote ZEB2-mediated cancer invasion and metastases. Thus, miR-138 appears to be an intriguing therapeutic target to prevent metastases of BC. 
 MiRNA-205 modulates cellular invasion and migration via regulating zinc finger E-box binding homeobox 2 expression in esophageal squamous cell carcinoma cells. Esophageal squamous cell carcinoma (ESCC) is often diagnosed at later stages until they are incurable. MicroRNA (miR) is a small, non-coding RNA that negatively regulates gene expression mainly via translational repression. Accumulating evidence indicates that deregulation of miR is associated with human malignancies including ESCC. The aim of this study was to identify miR that could be specifically expressed and exert distinct biological actions in ESCC.Total RNA was extracted from ESCC cell lines, OE21 and TE10, and a non-malignant human esophageal squamous cell line, Het-1A, and subjected to microarray analysis. Expression levels of miR that showed significant differences between the 2 ESCC and Het-1A cells based on the comprehensive analysis were analyzed by the quantitative reverse transcriptase (RT)-PCR method. Then, functional analyses, including cellular proliferation, apoptosis and Matrigel invasion and the wound healing assay, for the specific miR were conducted. Using ESCC tumor samples and paired surrounding non-cancerous tissue obtained endoscopically, the association with histopathological differentiation was examined with quantitative RT-PCR.Based on the miR microarray analysis, there were 14 miRs that showed significant differences (more than 2-fold) in expression between the 2 ESCC cells and non-malignant Het-1A. Among the significantly altered miRs, miR-205 expression levels were exclusively higher in 5 ESCC cell lines examined than any other types of malignant cell lines and Het-1A. Thus, miR-205 could be a specific miR in ESCC. Modulation of miR-205 expression by transfection with its precursor or anti-miR-205 inhibitor did not affect ESCC cell proliferation and apoptosis, but miR-205 was found to be involved in   cell invasion   and migration. Western blot revealed that knockdown of miR-205 expression in ESCC cells substantially enhanced expression of zinc finger E-box binding homeobox 2, accompanied by reduction of E-cadherin, a regulator of epithelial mesenchymal transition. The miR-205 expression levels were not associated with histological differentiation of human ESCC.These results imply that miR-205 is an ESCC-specific miR that exerts tumor-suppressive activities with EMT inhibition by targeting ZEB2. 
 ZEB2-Sp1 cooperation induces invasion by upregulating cadherin-11 and integrin α5 expression. Epithelial-mesenchymal transition (EMT) is a process implicated in invasion and metastasis. EMT is characterized by repression of epithelial markers and induction of mesenchymal markers. ZEB2 is a transcriptional repressor of E-cadherin, leading to EMT. Previously, we have shown that ZEB2 directly upregulates integrin α5 transcription by cooperating with the transcription factor Sp1. In this study, we investigated the precise mechanism by which ZEB2 modulates invasion and EMT events and the role of Sp1 in ZEB2-induced invasion. We found that ZEB2 directly induced cadherin-11 transcription in an Sp1-dependent, but Smad- and E-box-independent, manner and repressed E-cadherin expression in an Sp1- and Smad-independent manner, leading to cadherin switch. Furthermore, ZEB2 upregulated Sp1 by enhancing Sp1 protein stability, and Sp1 was found to be critical for ZEB2-induced cance  r cell invasi  on, mainly through induction of cadherin-11 and integrin α5. Expression levels of cadherin-11 and integrin α5 were interdependent and both modulated c-Jun N-terminal kinase-signaling activity and invasion. Immunofluorescence analysis showed that nuclear expression of ZEB2 was positively correlated with Sp1 expression in human colorectal cancers. Together, these findings demonstrate a previously unrecognized interplay between ZEB2, Sp1, cadherin-11 and integrin α5 that is, probably, significant in tumor progression and metastasis. 
 ZEB2 promotes the metastasis of gastric cancer and modulates epithelial mesenchymal transition of gastric cancer cells. Invasion and metastasis are the hallmarks of advanced gastric cancer progression. Therefore, it is urgent to overcome metastasis in order to improve the survival of gastric cancer patients.This study aimed to examine the expression of ZEB2 in gastric cancer samples and analyze its correlation with clinicopathologic features. In addition, the molecular mechanism by which ZEB2 contributes to gastric cancer metastasis will be explored.ZEB2 expression in clinical gastric cancer samples was evaluated by immunohistochemical analysis. ZEB2 was knocked-down in HGC27 gastric cancer cells by shRNA and the effects on   cell invasion   and migration were examined by in vitro   cell invasion   and migration assays. The expression of epithelial marker E-cadherin, mesenchymal markers fibronecin and vimentin, and MMPs was detected by western blot analysis.The expression of ZEB2 was positively correlated with the depth of invasion, lymph node metastasis and TNM stage. In addition, patients with positive ZEB2 expression showed a significantly shorter overall survival time than did patients with negative ZEB2. shRNA mediated knockdown of ZEB2 resulted in reduced invasion and migration of HGC27 cells, along with the upregulation of E-cadherin and downregulation of fibronecin, vimentin, MMP2, and MMP9.ZEB2 expression is closely associated with the clinicopathological parameters of gastric cancer. ZEB2 promotes gastric cancer cell migration and invasion at least partly via the regulation of epithelial-mesenchymal transition. ZEB2 is a potential target for gene therapy of aggressive gastric cancer. 
 Dysfunction of the Reciprocal Feedback Loop between GATA3- and ZEB2-Nucleated Repression Programs Contributes to Breast Cancer Metastasis. How loss-of-function of GATA3 contributes to the development of breast cancer is poorly understood. Here, we report that GATA3 nucleates a transcription repression program composed of G9A and MTA3-, but not MTA1- or MTA2-, constituted NuRD complex. Genome-wide analysis of the GATA3/G9A/NuRD(MTA3) targets identified a cohort of genes including ZEB2 that are critically involved in epithelial-to-mesenchymal transition and   cell invasion  . We demonstrate that the GATA3/G9A/NuRD(MTA3) complex inhibits the invasive potential of breast cancer cells in vitro and suppresses breast cancer metastasis in vivo. Strikingly, the expression of GATA3, G9A, and MTA3 is concurrently downregulated during breast cancer progression, leading to an elevated expression of ZEB2, which, in turn, represses the expression of G9A and MTA3 through the recruitment of G9A/NuRD(MTA1). 
 Epithelial to mesenchymal transition is associated with shorter disease-free survival in hepatocellular carcinoma. Epithelial to mesenchymal transition (EMT) is involved in cancer   cell invasion   and metastasis as well as chemoresistance. Elucidation of EMT in hepatocellular carcinoma (HCC) might contribute to deeper understanding of its biology.Overall, 100 patients with HCC, who underwent resection, were analyzed. The messenger RNA (mRNA) expression of the epithelial marker E-cadherin and the mesenchymal marker Vimentin were measured, and the EMT status of each patient was determined as follows: Vimentin/E-cadherin <2 = Epithelial (E), Vimentin/E-cadherin ≥2 = Mesenchymal (M). The correlation between these values and clinicopathological factors and prognosis were analyzed statistically. Moreover, the expression of transcription factors involved in EMT (Twist-1, Snail, Slug, Zeb-1, and Zeb-2) were measured and the role of interleukin (IL)-6 in inducing EMT and chemoresistance was examined.Patients with a mesenchymal tumor were more prone to have an earlier recurrence than those with an epithelial tumor. EMT-inducing transcription factors were more highly expressed in mesenchymal tumors than in epithelial tumors, and Twist-1 and Zeb-2 were significantly overexpressed. α-Fetoprotein (AFP) values were significantly higher in patients with epithelial tumors, and AFP-expressing HCC cell lines were more responsive to sorafenib. IL-6 expression was significantly higher in mesenchymal tumors, and knockdown of IL-6 in mesenchymal HCC cell lines increased E-cadherin expression and sensitivity to sorafenib.Analysis of surgically resected tumors suggests that EMT is involved in early disease recurrence in HCC. Twist-1 and Zeb-2 might be important for inducing EMT, and IL-6 might be a potential therapeutic target for alleviating the chemoresistance of mesenchymal HCC tumors. 
 ZEB2 upregulates integrin α5 expression through cooperation with Sp1 to induce invasion during epithelial-mesenchymal transition of human cancer cells. Epithelial-mesenchymal transition (EMT) is a process implicated in tumor invasion, metastasis, embryonic development and wound healing. ZEB2 is a transcription factor involved in EMT that represses E-cadherin transcription. Although E-cadherin downregulation is a major event during EMT and tumor progression, E-cadherin reduction is probably not sufficient for full invasiveness. The mechanisms by which E-cadherin transcriptional repressors induce mesenchymal genes during EMT remain largely unknown. Here, we investigated the role of ZEB2 in the induction of integrin α5 during cancer EMT and its underlying mechanism. In human cancer cells, ZEB2 was found to directly upregulate integrin α5 transcription in a manner that is independent of the regulation of E-cadherin expression. Conversely, depletion of ZEB2 by small interfering RNA suppressed integrin α5 expression, leading to reduced invasion. Suppression of integrin α5 inhibited ca  ncer cell inv  asion, suggesting an important role for integrin α5 in cancer progression. Furthermore, ZEB2 was found to activate the integrin α5 and vimentin promoters by interacting with and activating the transcription factor Sp1, suggesting that cooperation between ZEB2 and Sp1 represents a novel mechanism of mesenchymal gene activation during EMT. These findings increase our understanding of the pathways beyond E-cadherin reduction that regulate mesenchymal gene expression during EMT and cancer progression. 
  GeneRIFs  
 Bioinformatics analyses showed that miR-138 targeted the 3'-UTR of ZEB2 mRNA to inhibit its translation. Furthermore, miR-138 overexpression inhibited ZEB2-mediated   cell invasion   and metastases, while miR-138 depletion increased ZEB2-mediated   cell invasion   and metastases in BC cells 
 Genome-wide analysis of the GATA3/G9A/NuRD(MTA3) targets identified a cohort of genes including ZEB2 that are critically involved in epithelial-to-mesenchymal transition and   cell invasion  . 
   
  
 ZEB1 
 
  NCBI Gene  
  GeneRIF  
  Pubmed  
 
 
  Search Term  Rank  Fields  
  [Ii]nvadopodia  3  PubMed(1)  
  [Mm]igration  6  PubMed(65); GeneRIFs(8)  
  ECM  7  PubMed(2); GeneRIFs(2)  
  [Ee]xtracellular [Mm]atrix  8  PubMed(3); GeneRIFs(1)  
  [Ii]nvasive  10  PubMed(71); GeneRIFs(17)  
  [Mm]etastasize  11  PubMed(2)  
  [Mm]etastasis  12  PubMed(114); GeneRIFs(21)  
  [Cc]ell [Ii]nvasion  13  PubMed(18); GeneRIFs(2)  
 
 
 hide/show details for ZEB1 
  [Ii]nvadopodia  PubMed  
 The ZEB1/miR-200c feedback loop regulates invasion via actin interacting proteins MYLK and TKS5. Epithelial to mesenchymal transition (EMT) is a developmental process which is aberrantly activated during cancer invasion and metastasis. Elevated expression of EMT-inducers like ZEB1 enables tumor cells to detach from the primary tumor and invade into the surrounding tissue. The main antagonist of ZEB1 in controlling EMT is the microRNA-200 family that is reciprocally linked to ZEB1 in a double negative feedback loop. Here, we further elucidate how the ZEB1/miR-200 feedback loop controls invasion of tumor cells. The process of EMT is attended by major changes in the actin cytoskeleton. Via in silico screening of genes encoding for actin interacting proteins, we identified two novel targets of miR-200c - TKS5 and MYLK (MLCK). Co-expression of both genes with ZEB1 was observed in several cancer cell lines as well as in breast cancer patients and correlated with low miR-200c levels. Depletion of TKS5 or MYLK in breast cancer cells reduced their invasive potential and their ability to form   invadopodia  . Whereas TKS5 is known to be a major component, we could identify MYLK as a novel player in   invadopodia   formation. In summary, TKS5 and MYLK represent two mediators of invasive behavior of cancer cells that are regulated by the ZEB1/miR-200 feedback loop. 
  [Mm]igration  PubMed  
 Downregulated microRNA-200a promotes EMT and tumor growth through the wnt/β-catenin pathway by targeting the E-cadherin repressors ZEB1/ZEB2 in gastric adenocarcinoma. In a previous study, we found that microRNA (miRNA)-200a suppresses Wnt/β-catenin signaling by interacting with β-catenin, thereby inhibiti  ng migrat  ion, invasion and proliferation. However, the mechanism involved in this suppression remains unclear. In the present study, we investigated the underlying mechanism of miR-200a regulation of epithelial-mesenchymal transition (EMT) in gastric carcinoma cells, and confirmed the tumor suppressor role of miR-200a in vivo. The expressions of miRNA-200a, -200b and -200c, identified by fluorescent in situ hybridization, were downregulated and inversely correlated with WHO grades of gastric adenocarcinoma (GA). The expression of the potential miR-200a target genes ZEB1 and ZEB2 was detected immunohistochemically. These examinations used the same tissue microarrays to analyze the relationships between miR-200a and potential target genes. The expression of miR-200a and ZEB1/ZEB2 in the same GA tissue microarrays was inversely related. Restored miR-200a expression inhibited tumor growth in nude mice harboring subcutaneous SGC7901 xenografts. The expression of N-cadherin, β-catenin, Twist1 and Snail2 decreased, and E-cadherin levels increased, when miR-200a was elevated, as tested by fluorescence microscopy and immunohistochemistry. Similar results were observed in vivo. We found upregulated miR-200a expression to increase E-cadherin and suppress the Wnt/β-catenin pathway by targeting ZEB1 and ZEB2 in GA, thus delaying tumor growth in vivo. The effect of miR-200a on Wnt/β-catenin signaling may provide a therapeutic target against EMT. 
 The miR-200 family inhibits epithelial-mesenchymal transition and cancer cell   migration   by direct targeting of E-cadherin transcriptional repressors ZEB1 and ZEB2. MicroRNAs are small non-coding RNA molecules that can regulate gene expression by interacting with multiple mRNAs and inducing either translation suppression or degradation of mRNA. Recently, several miRNAs were identified as either promoters or suppressors of metastasis. However, it is unclear in which step(s) of the multistep metastatic cascade these miRNAs play a defined functional role. To study the functional importance of miRNAs in epithelial-mesenchymal transition (EMT), a process thought to initiate metastasis by enhancing the motility of tumor cells, we used a well established in vitro EMT assay: transforming growth factor-beta-induced EMT in NMuMG murine mammary epithelial cells. We found that members of the miR-200 family, organized as two clusters in the genome, were repressed during EMT. Overexpression of each miRNA individually or as clusters in NMuMG cells hindered EMT by enhancing E-cadherin expression through direct targeting of ZEB1 and ZEB2, which encode transcriptional repressors of E-cadherin. In the 4TO7 mouse carcinoma cell line, which expresses low levels of endogenous E-cadherin and displays a mesenchymal phenotype, ectopic expression of the miR-200 family miRNAs significantly increased E-cadherin expression and altered cell morphology to an epithelial phenotype. Furthermore, ectopic expression of each miR-200 miRNA cluster significantly reduced the in vitro motility of 4TO7 cells in   migration   assays. These results suggested that loss of expression of the miR-200 family members may play a critical role in the repression of E-cadherin by ZEB1 and ZEB2 during EMT, thereby enhancing   migration   and invasion during cancer progression. 
 Fra-1/AP-1 induces EMT in mammary epithelial cells by modulating Zeb1/2 and TGFβ expression. Epithelial-to-mesenchymal transition (EMT) is essential for embryonic morphogenesis and wound healing and critical for tumour cell invasion and dissemination. The AP-1 transcription factor Fra-1 has been implicated in tumorigenesis and in tumour-associated EMT in human breast cancer. We observed a significant inverse correlation between Fra-1 mRNA expression and distant-metastasis-free survival in a large cohort of breast cancer patients derived from multiple array data sets. This unique correlation among Fos genes prompted us to assess the evolutionary conservation between Fra-1 functions in EMT of human and mouse cells. Ectopic expression of Fra-1 in fully polarized, non-tumourigenic, mouse mammary epithelial EpH4 cells induced a mesenchymal phenotype, characterized by a loss of epithelial and gain of mesenchymal markers. Proliferation, motility and invasiveness were also increased in the resulting EpFra1 cells, and the cells were tumourigenic and efficiently colonized the lung upon transplantation. Molecular analyses revealed increased expression of Tgfβ1 and the EMT-inducing transcription factors Zeb1, Zeb2 and Slug. Mechanistically, Fra-1 binds to the tgfb1 and zeb2 promoters and to an evolutionarily conserved region in the first intron of zeb1. Furthermore, increased activity of a zeb2 promoter reporter was detected in EpFra1 cells and shown to depend on AP-1-binding sites. Inhibiting TGFβ signalling in EpFra1 cells moderately increased the expression of epithelial markers, whereas silencing of zeb1 or zeb2 restored the epithelial phenotype and decreas  ed migrat  ion in vitro and tumorigenesis in vivo. Thus Fra-1 induces changes in the expression of genes encoding EMT-related transcription factors leading to the acquisition of mesenchymal, invasive and tumorigenic capacities by epithelial cells. This study defines a novel function of Fra-1/AP-1 in modulating tgfb1, zeb1 and zeb2 expression through direct binding to genomic regulatory regions, which establishes a basis for future in vivo genetic manipulations and preclinical studies using mouse models. 
 Down-regulation of miR-144 promotes thyroid cancer cell invasion by targeting ZEB1 and ZEB2. Thyroid cancer is the most common endocrine malignancy, and its incidence has increased rapidly worldwide. The molecular mechanisms underlying thyroid cancer tumorigenesis still need to be further investigated. MicroRNAs (miRNAs), short RNA molecules of approximately 22 nucleotides in length, play crucial roles in tumorigenesis. In the present study, we found that the expression of miR-144 was significantly down-regulated in thyroid cancer as compared with that in normal thyroid tissues, suggesting that miR-144 may be involved in thyroid cancer tumorigenesis. Moreover, our results showed that restoration of miR-144 in K1 and WRO thyroid cancer cells could suppress the invasion and   migration   capability of these cells. We also demonstrated that miR-144 suppressed the expression of ZEB1 and ZEB2, two E-cadherin suppressors, by directly binding to their 3'-untranslated regions. Furthermore, restoration of ZEB1 or ZEB2 partially rescued the miR-144-induced inhibition of cell invasion. These data suggest miR-144 function as a tumor suppressor in thyroid cancer. 
 A miRNA signature associated with human metastatic medullary thyroid carcinoma. MicroRNAs (miRNAs) represent a class of small, non-coding RNAs that control gene expression by targeting mRNA and triggering either translational repression or RNA degradation. The objective of our study was to evaluate the involvement of miRNAs in human medullary thyroid carcinoma (MTC) and to identify the markers of metastatic cells and aggressive tumour behaviour. Using matched primary and metastatic tumour samples, we identified a subset of miRNAs aberrantly regulated in metastatic MTC. Deregulated miRNAs were confirmed by quantitative real-time PCR and validated by in situ hybridisation on a large independent set of primary and metastatic MTC samples. Our results uncovered ten miRNAs that were significantly expressed and deregulated in metastatic tumours: miR-10a, miR-200b/-200c, miR-7 and miR-29c were down-regulated and miR-130a, miR-138, miR-193a-3p, miR-373 and miR-498 were up-regulated. Bioinformatic approaches revealed potential miRNA targets and signals involved in metastatic MTC pathways.   Migration  , proliferation and invasion assays were performed in cell lines treated with miR-200 antagomirs to ascertain a direct role for this miRNA in MTC tumourigenesis. We show that the members of miR-200 family regulate the expression of E-cadherin by directly targeting ZEB1 and ZEB2 mRNA and through the enhanced expression of tumour growth factor β (TGFβ)-2 and TGFβ-1. Overall, the treated cells shifted to a mesenchymal phenotype, thereby acquiring an aggressive phenotype with increased motility and invasion. Our data identify a robust miRNA signature associated with metastatic MTC and distinct biological processes, e.g., TGFβ signalling pathway, providing new potential insights into the mechanisms of MTC metastasis. 
 miR-139-5p inhibits epithelial-mesenchymal transition,   migration   and invasion of hepatocellular carcinoma cells by targeting ZEB1 and ZEB2. Hepatocellular carcinoma (HCC) is the third most common cause of cancer deaths worldwide. miRNAs have been suggested to have important roles in HCC development. The purpose of this study was to determine the role of miR-139-5p in regulation of epithelial-mesenchymal transition (EMT) and metastasis of HCC cells. Expression levels of miR-139-5p in 49 HCC specimens with adjacent tissues and five HCC cell lines were assessed by quantitative RT-PCR. We found that miR-139-5p was down-regulated in 89.7% of the HCC tissue samples and all of the HCC cell lines. In addition, luciferase reporter assays validated direct binding of miR-139-5p to the 3' untranslated region of zinc finger E-box binding homeobox 1 (ZEB1) and ZEB2. Ectopic expression of miR-139-5p suppressed and miR-139-in promoted EMT,   migration  , and invasion in Hep3B and SMMC7721 cells. Furthermore, over-expression of ZEB1 and ZEB2 ablated the inhibitory effects of miR-139-5p on   migration   and invasion in HCC cells. Our study indicates that miR-139-5p functions as a suppressor of HCC EMT and metastasis by targeting ZEB1 and ZEB2, and it may be a therapeutic target for metastatic HCC. 
 Langerhans cell maturation is accompanied by induction of N-cadherin and the transcriptional regulators of epithelial-mesenchymal transition ZEB1/2. Langerhans cells (LCs) are a unique subset of dendritic cells (DCs) that express epithelial adhesion molecules, allowing them to form contacts with epithelial cells and reside in epidermal/epithelial tissues. The dynamic regulation of epithelial adhesion plays a decisive role in the life cycle of LCs. It controls whether LCs remain immature and sessile within the epidermis or mature and egress to initiate immune responses. So far, the molecular machinery regulating epithelial adhesion molecules during LC maturation remains elusive. Here, we generated pure populations of immature human LCs in vitro to systematically probe for gene-expression changes during LC maturation. LCs down-regulate a set of epithelial genes including E-cadherin, while they upregulate the mesenchymal marker N-cadherin known to facilitate cell   migration  . In addition, N-cadherin is constitutively expressed by monocyte-derived DCs known to exhibit characteristics of both inflammatory-type and interstitial/dermal DCs. Moreover, the transcription factors ZEB1 and ZEB2 (ZEB is zinc-finger E-box-binding homeobox) are upregulated in migratory LCs. ZEB1 and ZEB2 have been shown to induce epithelial-to-mesenchymal transition (EMT) and invasive behavior in cancer cells undergoing metastasis. Our results provide the first hint that the molecular EMT machinery might facilitate LC mobilization. Moreover, our study suggests that N-cadherin plays a role during DC   migration  . 
 TMPRSS2/ERG promotes epithelial to mesenchymal transition through the ZEB1/ZEB2 axis in a prostate cancer model. Prostate cancer is the most common non-dermatologic malignancy in men in the Western world. Recently, a frequent chromosomal aberration fusing androgen regulated TMPRSS2 promoter and the ERG gene (TMPRSS2/ERG) was discovered in prostate cancer. Several studies demonstrated cooperation between TMPRSS2/ERG and other defective pathways in cancer progression. However, the unveiling of more specific pathways in which TMPRSS2/ERG takes part, requires further investigation. Using immortalized prostate epithelial cells we were able to show that TMPRSS2/ERG over-expressing cells undergo an Epithelial to Mesenchymal Transition (EMT), manifested by acquisition of mesenchymal morphology and markers as well as   migration   and invasion capabilities. These findings were corroborated in vivo, where the control cells gave rise to discrete nodules while the TMPRSS2/ERG-expressing cells formed malignant tumors, which expressed EMT markers. To further investigate the general transcription scheme induced by TMPRSS2/ERG, cells were subjected to a microarray analysis that revealed a distinct EMT expression program, including up-regulation of the EMT facilitators, ZEB1 and ZEB2, and down-regulation of the epithelial marker CDH1(E-Cadherin). A chromatin immunoprecipitation assay revealed direct binding of TMPRSS2/ERG to the promoter of ZEB1 but not ZEB2. However, TMPRSS2/ERG was able to bind the promoters of the ZEB2 modulators, IL1R2 and SPINT1. This set of experiments further illuminates the mechanism by which the TMPRSS2/ERG fusion affects prostate cancer progression and might assist in targeting TMPRSS2/ERG and its downstream targets in future drug design efforts. 
 miR-139-5p suppresses cancer cell   migration   and invasion through targeting ZEB1 and ZEB2 in GBM. Invasion and   migration   of glioblastoma multiforme (GBM) is a multistep process and an important phenotype that causes this disease to invade surrounding tissues in the brain. Recent studies have highlighted that miRNAs play a pivotal role in controlling GBM cell plasticity. In this report, we used wound healing and transwell assays to identify a novel role of miR-139-5p in inhibition of GBM cell   migration   and invasion. Bioinformatics coupled with luciferase and Western blot assays also revealed that miR-139-5p inhibited expression of ZEB1 and ZEB2, which are master regulators of tumor metastasis. MiR-139-5p specifically interacts with the 3'-UTR regions of ZEB1 and ZEB2, attenuating their expression in GBM cells. To corroborate this finding, we rescued ZEB1 and ZEB2 expression and found partial but significant increases in miR-139-5p-suppressed invasion of GBM cells. The biological relevance of our study was validated by analyzing levels of miR-139-5p in GBM tissue. We found that its expression significantly downregulated compared to normal tissue and shorter overall survival rates in patients with lower miR-139-5p expression. These results confirm that miR-139-5p suppresses GBM   migration   and invasion and highlight its potential as a biomarker and therapeutic target for treating GBM. 
 A developmentally regulated inducer of EMT, LBX1, contributes to breast cancer progression. Epithelial-to-mesenchymal transition (EMT) plays an important role during normal embryogenesis, and it has been implicated in cancer invasion and metastasis. Here, we report that Ladybird homeobox 1 (LBX1), a developmentally regulated homeobox gene, directs expression of the known EMT inducers ZEB1, ZEB2, Snail1, and transforming growth factor beta2 (TGFB2). In mammary epithelial cells, overexpression of LBX1 leads to morphological transformation, expression of mesenchymal markers, enhanced cell   migration  , increased CD44(high)/CD24(low) progenitor cell population, and tumorigenic cooperation with known oncogenes. In human breast cancer, LBX1 is up-regulated in the unfavorable estrogen receptor (ER)/progesterone (PR)/HER2 triple-negative basal-like subtype. Thus, aberrant expression of LBX1 may lead to the activation of a developmentally regulated EMT pathway in human breast cancer. 
 miR-590-3p suppresses cancer cell   migration  , invasion and epithelial-mesenchymal transition in glioblastoma multiforme by targeting ZEB1 and ZEB2. Invasion and   migration   of glioblastoma multiforme (GBM) is a multistep process and an important phenotype that causes this disease to invade surrounding tissues in the brain. The purpose of this study was to determine the role of miR-590-3p in regulation of epithelial mesenchymal transition (EMT) and metastasis of GBM cells. Expression levels of miR-590-3p in 15 GBM specimens with adjacent tissues and five GBM cell lines were assessed by quantitative RT-PCR. We found that miR-590-3p was down-regulated in detected GBM tissue samples and all of the GBM cell lines. In addition, Ectopic expression of miR-590-3p suppressed and miR-590-3p-in promoted EMT,   migration  , and invasion in U87MG and A172 cells. Bioinformatics coupled with luciferase and Western blot assays also revealed that miR-590-3p inhibited expression of ZEB1 and ZEB2, which are master regulators of tumor metastasis. Our study first indicates that miR-590-3p functions as a suppressor of GBM EMT and metastasis by targeting ZEB1 and ZEB2, and it may be a therapeutic target for metastatic GBM. 
 miR-590-3p suppresses cancer cell   migration  , invasion and epithelial-mesenchymal transition in glioblastoma multiforme by targeting ZEB1 and ZEB2. Invasion and   migration   of glioblastoma multiforme (GBM) is a multistep process and an important phenotype that causes this disease to invade surrounding tissues in the brain. The purpose of this study was to determine the role of miR-590-3p in regulation of epithelial mesenchymal transition (EMT) and metastasis of GBM cells. Expression levels of miR-590-3p in 15 GBM specimens with adjacent tissues and five GBM cell lines were assessed by quantitative RT-PCR. We found that miR-590-3p was down-regulated in detected GBM tissue samples and all of the GBM cell lines. In addition, Ectopic expression of miR-590-3p suppressed and miR-590-3p-in promoted EMT,   migration  , and invasion in U87MG and A172 cells. Bioinformatics coupled with luciferase and Western blot assays also revealed that miR-590-3p inhibited expression of ZEB1 and ZEB2, which are master regulators of tumor metastasis. Our study first indicates that miR-590-3p functions as a suppressor of GBM EMT and metastasis by targeting ZEB1 and ZEB2, and it may be a therapeutic target for metastatic GBM. 
 miR-139-5p suppresses cancer cell   migration   and invasion through targeting ZEB1 and ZEB2 in GBM. Invasion and   migration   of glioblastoma multiforme (GBM) is a multistep process and an important phenotype that causes this disease to invade surrounding tissues in the brain. Recent studies have highlighted that miRNAs play a pivotal role in controlling GBM cell plasticity. In this report, we used wound healing and transwell assays to identify a novel role of miR-139-5p in inhibition of GBM cell   migration   and invasion. Bioinformatics coupled with luciferase and Western blot assays also revealed that miR-139-5p inhibited expression of ZEB1 and ZEB2, which are master regulators of tumor metastasis. MiR-139-5p specifically interacts with the 3'-UTR regions of ZEB1 and ZEB2, attenuating their expression in GBM cells. To corroborate this finding, we rescued ZEB1 and ZEB2 expression and found partial but significant increases in miR-139-5p-suppressed invasion of GBM cells. The biological relevance of our study was validated by analyzing levels of miR-139-5p in GBM tissue. We found that its expression significantly downregulated compared to normal tissue and shorter overall survival rates in patients with lower miR-139-5p expression. These results confirm that miR-139-5p suppresses GBM   migration   and invasion and highlight its potential as a biomarker and therapeutic target for treating GBM. 
 Down-regulation of miR-144 promotes thyroid cancer cell invasion by targeting ZEB1 and ZEB2. Thyroid cancer is the most common endocrine malignancy, and its incidence has increased rapidly worldwide. The molecular mechanisms underlying thyroid cancer tumorigenesis still need to be further investigated. MicroRNAs (miRNAs), short RNA molecules of approximately 22 nucleotides in length, play crucial roles in tumorigenesis. In the present study, we found that the expression of miR-144 was significantly down-regulated in thyroid cancer as compared with that in normal thyroid tissues, suggesting that miR-144 may be involved in thyroid cancer tumorigenesis. Moreover, our results showed that restoration of miR-144 in K1 and WRO thyroid cancer cells could suppress the invasion and   migration   capability of these cells. We also demonstrated that miR-144 suppressed the expression of ZEB1 and ZEB2, two E-cadherin suppressors, by directly binding to their 3'-untranslated regions. Furthermore, restoration of ZEB1 or ZEB2 partially rescued the miR-144-induced inhibition of cell invasion. These data suggest miR-144 function as a tumor suppressor in thyroid cancer. 
 miR-139-5p inhibits epithelial-mesenchymal transition,   migration   and invasion of hepatocellular carcinoma cells by targeting ZEB1 and ZEB2. Hepatocellular carcinoma (HCC) is the third most common cause of cancer deaths worldwide. miRNAs have been suggested to have important roles in HCC development. The purpose of this study was to determine the role of miR-139-5p in regulation of epithelial-mesenchymal transition (EMT) and metastasis of HCC cells. Expression levels of miR-139-5p in 49 HCC specimens with adjacent tissues and five HCC cell lines were assessed by quantitative RT-PCR. We found that miR-139-5p was down-regulated in 89.7% of the HCC tissue samples and all of the HCC cell lines. In addition, luciferase reporter assays validated direct binding of miR-139-5p to the 3' untranslated region of zinc finger E-box binding homeobox 1 (ZEB1) and ZEB2. Ectopic expression of miR-139-5p suppressed and miR-139-in promoted EMT,   migration  , and invasion in Hep3B and SMMC7721 cells. Furthermore, over-expression of ZEB1 and ZEB2 ablated the inhibitory effects of miR-139-5p on   migration   and invasion in HCC cells. Our study indicates that miR-139-5p functions as a suppressor of HCC EMT and metastasis by targeting ZEB1 and ZEB2, and it may be a therapeutic target for metastatic HCC. 
 Fra-1/AP-1 induces EMT in mammary epithelial cells by modulating Zeb1/2 and TGFβ expression. Epithelial-to-mesenchymal transition (EMT) is essential for embryonic morphogenesis and wound healing and critical for tumour cell invasion and dissemination. The AP-1 transcription factor Fra-1 has been implicated in tumorigenesis and in tumour-associated EMT in human breast cancer. We observed a significant inverse correlation between Fra-1 mRNA expression and distant-metastasis-free survival in a large cohort of breast cancer patients derived from multiple array data sets. This unique correlation among Fos genes prompted us to assess the evolutionary conservation between Fra-1 functions in EMT of human and mouse cells. Ectopic expression of Fra-1 in fully polarized, non-tumourigenic, mouse mammary epithelial EpH4 cells induced a mesenchymal phenotype, characterized by a loss of epithelial and gain of mesenchymal markers. Proliferation, motility and invasiveness were also increased in the resulting EpFra1 cells, and the cells were tumourigenic and efficiently colonized the lung upon transplantation. Molecular analyses revealed increased expression of Tgfβ1 and the EMT-inducing transcription factors Zeb1, Zeb2 and Slug. Mechanistically, Fra-1 binds to the tgfb1 and zeb2 promoters and to an evolutionarily conserved region in the first intron of zeb1. Furthermore, increased activity of a zeb2 promoter reporter was detected in EpFra1 cells and shown to depend on AP-1-binding sites. Inhibiting TGFβ signalling in EpFra1 cells moderately increased the expression of epithelial markers, whereas silencing of zeb1 or zeb2 restored the epithelial phenotype and decreas  ed migrat  ion in vitro and tumorigenesis in vivo. Thus Fra-1 induces changes in the expression of genes encoding EMT-related transcription factors leading to the acquisition of mesenchymal, invasive and tumorigenic capacities by epithelial cells. This study defines a novel function of Fra-1/AP-1 in modulating tgfb1, zeb1 and zeb2 expression through direct binding to genomic regulatory regions, which establishes a basis for future in vivo genetic manipulations and preclinical studies using mouse models. 
 A miRNA signature associated with human metastatic medullary thyroid carcinoma. MicroRNAs (miRNAs) represent a class of small, non-coding RNAs that control gene expression by targeting mRNA and triggering either translational repression or RNA degradation. The objective of our study was to evaluate the involvement of miRNAs in human medullary thyroid carcinoma (MTC) and to identify the markers of metastatic cells and aggressive tumour behaviour. Using matched primary and metastatic tumour samples, we identified a subset of miRNAs aberrantly regulated in metastatic MTC. Deregulated miRNAs were confirmed by quantitative real-time PCR and validated by in situ hybridisation on a large independent set of primary and metastatic MTC samples. Our results uncovered ten miRNAs that were significantly expressed and deregulated in metastatic tumours: miR-10a, miR-200b/-200c, miR-7 and miR-29c were down-regulated and miR-130a, miR-138, miR-193a-3p, miR-373 and miR-498 were up-regulated. Bioinformatic approaches revealed potential miRNA targets and signals involved in metastatic MTC pathways.   Migration  , proliferation and invasion assays were performed in cell lines treated with miR-200 antagomirs to ascertain a direct role for this miRNA in MTC tumourigenesis. We show that the members of miR-200 family regulate the expression of E-cadherin by directly targeting ZEB1 and ZEB2 mRNA and through the enhanced expression of tumour growth factor β (TGFβ)-2 and TGFβ-1. Overall, the treated cells shifted to a mesenchymal phenotype, thereby acquiring an aggressive phenotype with increased motility and invasion. Our data identify a robust miRNA signature associated with metastatic MTC and distinct biological processes, e.g., TGFβ signalling pathway, providing new potential insights into the mechanisms of MTC metastasis. 
 Langerhans cell maturation is accompanied by induction of N-cadherin and the transcriptional regulators of epithelial-mesenchymal transition ZEB1/2. Langerhans cells (LCs) are a unique subset of dendritic cells (DCs) that express epithelial adhesion molecules, allowing them to form contacts with epithelial cells and reside in epidermal/epithelial tissues. The dynamic regulation of epithelial adhesion plays a decisive role in the life cycle of LCs. It controls whether LCs remain immature and sessile within the epidermis or mature and egress to initiate immune responses. So far, the molecular machinery regulating epithelial adhesion molecules during LC maturation remains elusive. Here, we generated pure populations of immature human LCs in vitro to systematically probe for gene-expression changes during LC maturation. LCs down-regulate a set of epithelial genes including E-cadherin, while they upregulate the mesenchymal marker N-cadherin known to facilitate cell   migration  . In addition, N-cadherin is constitutively expressed by monocyte-derived DCs known to exhibit characteristics of both inflammatory-type and interstitial/dermal DCs. Moreover, the transcription factors ZEB1 and ZEB2 (ZEB is zinc-finger E-box-binding homeobox) are upregulated in migratory LCs. ZEB1 and ZEB2 have been shown to induce epithelial-to-mesenchymal transition (EMT) and invasive behavior in cancer cells undergoing metastasis. Our results provide the first hint that the molecular EMT machinery might facilitate LC mobilization. Moreover, our study suggests that N-cadherin plays a role during DC   migration  . 
 Downregulated microRNA-200a promotes EMT and tumor growth through the wnt/β-catenin pathway by targeting the E-cadherin repressors ZEB1/ZEB2 in gastric adenocarcinoma. In a previous study, we found that microRNA (miRNA)-200a suppresses Wnt/β-catenin signaling by interacting with β-catenin, thereby inhibiti  ng migrat  ion, invasion and proliferation. However, the mechanism involved in this suppression remains unclear. In the present study, we investigated the underlying mechanism of miR-200a regulation of epithelial-mesenchymal transition (EMT) in gastric carcinoma cells, and confirmed the tumor suppressor role of miR-200a in vivo. The expressions of miRNA-200a, -200b and -200c, identified by fluorescent in situ hybridization, were downregulated and inversely correlated with WHO grades of gastric adenocarcinoma (GA). The expression of the potential miR-200a target genes ZEB1 and ZEB2 was detected immunohistochemically. These examinations used the same tissue microarrays to analyze the relationships between miR-200a and potential target genes. The expression of miR-200a and ZEB1/ZEB2 in the same GA tissue microarrays was inversely related. Restored miR-200a expression inhibited tumor growth in nude mice harboring subcutaneous SGC7901 xenografts. The expression of N-cadherin, β-catenin, Twist1 and Snail2 decreased, and E-cadherin levels increased, when miR-200a was elevated, as tested by fluorescence microscopy and immunohistochemistry. Similar results were observed in vivo. We found upregulated miR-200a expression to increase E-cadherin and suppress the Wnt/β-catenin pathway by targeting ZEB1 and ZEB2 in GA, thus delaying tumor growth in vivo. The effect of miR-200a on Wnt/β-catenin signaling may provide a therapeutic target against EMT. 
 TMPRSS2/ERG promotes epithelial to mesenchymal transition through the ZEB1/ZEB2 axis in a prostate cancer model. Prostate cancer is the most common non-dermatologic malignancy in men in the Western world. Recently, a frequent chromosomal aberration fusing androgen regulated TMPRSS2 promoter and the ERG gene (TMPRSS2/ERG) was discovered in prostate cancer. Several studies demonstrated cooperation between TMPRSS2/ERG and other defective pathways in cancer progression. However, the unveiling of more specific pathways in which TMPRSS2/ERG takes part, requires further investigation. Using immortalized prostate epithelial cells we were able to show that TMPRSS2/ERG over-expressing cells undergo an Epithelial to Mesenchymal Transition (EMT), manifested by acquisition of mesenchymal morphology and markers as well as   migration   and invasion capabilities. These findings were corroborated in vivo, where the control cells gave rise to discrete nodules while the TMPRSS2/ERG-expressing cells formed malignant tumors, which expressed EMT markers. To further investigate the general transcription scheme induced by TMPRSS2/ERG, cells were subjected to a microarray analysis that revealed a distinct EMT expression program, including up-regulation of the EMT facilitators, ZEB1 and ZEB2, and down-regulation of the epithelial marker CDH1(E-Cadherin). A chromatin immunoprecipitation assay revealed direct binding of TMPRSS2/ERG to the promoter of ZEB1 but not ZEB2. However, TMPRSS2/ERG was able to bind the promoters of the ZEB2 modulators, IL1R2 and SPINT1. This set of experiments further illuminates the mechanism by which the TMPRSS2/ERG fusion affects prostate cancer progression and might assist in targeting TMPRSS2/ERG and its downstream targets in future drug design efforts. 
 A developmentally regulated inducer of EMT, LBX1, contributes to breast cancer progression. Epithelial-to-mesenchymal transition (EMT) plays an important role during normal embryogenesis, and it has been implicated in cancer invasion and metastasis. Here, we report that Ladybird homeobox 1 (LBX1), a developmentally regulated homeobox gene, directs expression of the known EMT inducers ZEB1, ZEB2, Snail1, and transforming growth factor beta2 (TGFB2). In mammary epithelial cells, overexpression of LBX1 leads to morphological transformation, expression of mesenchymal markers, enhanced cell   migration  , increased CD44(high)/CD24(low) progenitor cell population, and tumorigenic cooperation with known oncogenes. In human breast cancer, LBX1 is up-regulated in the unfavorable estrogen receptor (ER)/progesterone (PR)/HER2 triple-negative basal-like subtype. Thus, aberrant expression of LBX1 may lead to the activation of a developmentally regulated EMT pathway in human breast cancer. 
 The miR-200 family inhibits epithelial-mesenchymal transition and cancer cell   migration   by direct targeting of E-cadherin transcriptional repressors ZEB1 and ZEB2. MicroRNAs are small non-coding RNA molecules that can regulate gene expression by interacting with multiple mRNAs and inducing either translation suppression or degradation of mRNA. Recently, several miRNAs were identified as either promoters or suppressors of metastasis. However, it is unclear in which step(s) of the multistep metastatic cascade these miRNAs play a defined functional role. To study the functional importance of miRNAs in epithelial-mesenchymal transition (EMT), a process thought to initiate metastasis by enhancing the motility of tumor cells, we used a well established in vitro EMT assay: transforming growth factor-beta-induced EMT in NMuMG murine mammary epithelial cells. We found that members of the miR-200 family, organized as two clusters in the genome, were repressed during EMT. Overexpression of each miRNA individually or as clusters in NMuMG cells hindered EMT by enhancing E-cadherin expression through direct targeting of ZEB1 and ZEB2, which encode transcriptional repressors of E-cadherin. In the 4TO7 mouse carcinoma cell line, which expresses low levels of endogenous E-cadherin and displays a mesenchymal phenotype, ectopic expression of the miR-200 family miRNAs significantly increased E-cadherin expression and altered cell morphology to an epithelial phenotype. Furthermore, ectopic expression of each miR-200 miRNA cluster significantly reduced the in vitro motility of 4TO7 cells in   migration   assays. These results suggested that loss of expression of the miR-200 family members may play a critical role in the repression of E-cadherin by ZEB1 and ZEB2 during EMT, thereby enhancing   migration   and invasion during cancer progression. 
 Delta-crystallin enhancer binding factor 1 controls the epithelial to mesenchymal transition phenotype and resistance to the epidermal growth factor receptor inhibitor erlotinib in human head and neck squamous cell carcinoma lines. Although the epidermal growth factor receptor (EGFR) is overexpressed in a majority of head and neck squamous cell carcinomas (HNSCC), only a minority of patients derive substantial clinical benefit from EGFR inhibitors. We initiated the present study to identify the mechanisms underlying erlotinib resistance in a panel of HNSCC cell lines.We used [(3)H]thymidine incorporation to characterize the heterogeneity of responsiveness to erlotinib-mediated growth inhibition in a panel of 27 human HNSCC cells. We characterized the molecular mechanisms involved in resistance using a representative subset of six erlotinib-sensitive and erlotinib-resistant HNSCC lines.Erlotinib had heterogeneous effects on DNA synthesis in HNSCC cells that correlated closely with molecular markers of epithelial to mesenchymal transition (EMT). Specifically, the drug-sensitive lines expressed high levels of E-cadherin and showed limited invasion and   migration   capabilities. In contrast, the erlotinib-resistant HNSCC lines expressed high levels of the E-cadherin repressor delta-crystallin enhancer binding factor 1 (deltaEF1; Zeb-1) and other mesenchymal markers and low levels of E-cadherin, and they were highly invasive and migratory. Small interfering RNA-mediated knockdown of deltaEF1 in the erlotinib-resistant cell lines (1386LN and UMSCC1) resulted in up-regulation of E-cadherin and increased sensitivity to erlotinib in an E-cadherin-dependent manner.DeltaEF1 controls the mesenchymal phenotype and drives erlotinib resistance in HNSCC cells. E-cadherin and deltaEF1 may prove to be useful markers in predicting EGFR inhibitor responsiveness. 
 Hypoxia enhances   migration   and invasion in glioblastoma by promoting a mesenchymal shift mediated by the HIF1α-ZEB1 axis. Glioblastoma (GBM) is the most common brain tumor in adults and the mesenchymal GBM subtype was reported to be the most malignant, presenting severe hypoxia and necrosis. Here, we investigated the possible role of a hypoxic microenvironment for inducing a mesenchymal and invasive phenotype. The exposure of non-mesenchymal SNB75 and U87 cells to hypoxia induced a strong change in cell morphology that was accompanied by enhanced invasive capacity and the acquisition of mesenchymal marker expression. Further analyses showed the induction of HIF1α and HIF2α by hypoxia and exposure to digoxin, a cardiac glycoside known to inhibit HIF1/2 expression, was able to prevent hypoxia-induced mesenchymal transition. ShRNA-mediated knockdown of HIF1α, and not HIF2α, prevented this transition, as well as the knockdown of the EMT transcription factor ZEB1. We provide further evidence for a hypoxia-induced mesenchymal shift in GBM primary material by showing co-localization of GLUT1, ZEB1 and the mesenchymal marker YKL40 in hypoxic regions of the tumor. Collectively, our results identify a HIF1α-ZEB1 signaling axis that promotes hypoxia induced mesenchymal shift and invasion in GBM in a cell line dependent fashion. 
 MiR-150 is associated with poor prognosis in esophageal squamous cell carcinoma via targeting the EMT inducer ZEB1. The association of microRNAs (miRs) with cancer progression has been established in many cancers including esophageal squamous cell carcinoma (ESCC). A public microarray database showed that the expression of miR-150 was lower in ESCC than in normal esophageal mucosa. Here, we focused on ZEB1, epithelial-mesenchymal-transition (EMT)-inducer, as a target gene of miR-150 based on in silico predictions. The purpose of this study was to clarify the clinicopathological significance of miR-150 in ESCC, and to investigate miR-150's EMT-regulatory ability. Quantitative RT-PCR was used to evaluate miR-150 expression in 108 curative resected ESCC samples to determine the clinicopathological significance. Moreover, we examined the in vitro and in vivo function of miR-150 via degradation of ZEB1. MiR-150 expression was significantly lower in cancer tissues compared to adjacent non-cancerous tissues (P < 0.001). Low expression of miR-150 in ESCC contributed to malignant potential, such as tumor depth, lymph node metastasis, lymphatic invasion, venous invasion, clinical staging, and poor prognosis (P < 0.05). In vitro assays showed that EMT-inducer-ZEB1 is a new direct target of miR-150. Moreover, miR-150 induced MET-like changes in TE-8 cells through ZEB1 degradation (e.g., E-cadherin expression, vimentin repression, epithelial morphology, and suppression of   migration   ability), and significantly inhibited tumorigenicity and tumor growth in a mouse xenograft model. Analysis of the regulation of ZEB1 by miR-150 could provide new insights into preventing metastasis and also suggests novel targeted therapeutic strategies in ESCC. 
 miR-1271 inhibits   migration  , invasion and epithelial-mesenchymal transition by targeting ZEB1 and TWIST1 in pancreatic cancer cells. Pancreatic cancer (PC) remains one of the most lethal types of cancer in adults. The purpose of this study was to determine the role of miR-1271 in regulation of epithelial mesenchymal transition (EMT) and metastasis of pancreatic cancer cells. miR-1271 was identified to be significantly down-regulated in PC tissues by miRNA array. Also, an increase of EMT-regulators ZEB1 and TWIST1 expression level is accompanied by a decrease of miR-1271. We showed that expression of miR-1271 was significantly down-regulated in PC tissues as compared with that in normal tissues. In addition, our results showed that miR-1271 expression levels were decreased while ZEB1 and TWIST1 expression levels were increased in detected PC cell lines. Moreover, ectopic expression of miR-1271 suppressed and antagomiR-1271 promoted proliferation,   migration  , and invasion in SW1990 and PANC-1 cells. Bioinformatics coupled with luciferase and Western blot assays also revealed that miR-1271 inhibited expression of ZEB1 and TWIST1, which are master regulators of tumor metastasis. Our study first indicates that miR-1271 functions as a suppressor in regulating of pancreatic cancer EMT by targeting ZEB1 and TWIST1, and it promise as a therapeutic target and prognostic marker for metastatic pancreatic cancer. 
 Identification of a PEAK1/ZEB1 signaling axis during TGFβ/fibronectin-induced EMT in breast cancer. Transforming Growth Factor beta (TGFβ) is the archetypal member of the TGFβ superfamily of ligands and has pleiotropic functions during normal development, adult tissue homeostasis and pathophysiological processes such as cancer. In epithelial cancers TGFβ signaling can either suppress tumor growth or promote metastasis via the induction of a well-characterized epithelial-mesenchymal transition (EMT) program. We recently reported that PEAK1 kinase mediates signaling cross talk between TGFβ receptors and integrin/Src/MAPK pathways and functions as a critical molecular regulator of TGFβ-induced breast cancer cell prolifera  tion, mig  ration, EMT and metastasis. Here, we examined the breast cancer cell contexts in which TGFβ induces both EMT and PEAK1, and discovered this event to be unique to oncogene-transformed mammary epithelial cells and triple-negative breast cancer cells. Using the Cancer BioPortal database, we identified PEAK1 co-expressors across multiple malignancies that are also common to the TGFβ response gene signature (TBRS). We then used the ScanSite database to identify predicted protein-protein binding partners of PEAK1 and the PEAK1-TBRS co-expressors. Analysis of the Cytoscape interactome and Babelomics-derived gene ontologies for a novel gene set including PEAK1, CRK, ZEB1, IL11 and COL4A1 enabled us to hypothesize that PEAK1 may be regulating TGFβ-induced EMT via its interaction with or regulation of these other genes. In this regard, we have demonstrated that PEAK1 is necessary for TGFβ to induce ZEB1-mediated EMT in the context of fibronectin/ITGB3 activation. These studies and future mechanistic studies will pave the way toward identifying the context in which TGFβ blockade may significantly improve breast cancer patient outcomes. 
 53BP1 suppresses epithelial-mesenchymal transition by downregulating ZEB1 through microRNA-200b/429 in breast cancer. Epithelial-mesenchymal transition (EMT) is an important mechanism of cancer invasion and metastasis. Although p53 binding protein 1 (53BP1) has been implicated in several biological processes, its function in EMT of human cancers has not yet been reported. Here, we show that 53BP1 negatively regulated EMT by modulating ZEB1 through targeting microRNA (miR)-200b and miR-429. Furthermore, 53BP1 promoted ZEB1-mediated upregulation of E-cadherin and also inhibited the expressions of mesenchymal markers, leading to increased   migration   and invasion in MDA-MB-231 breast cancer cells. Consistently, in MCF-7 breast cancer cells, low 53BP1 expression reduced E-cadherin expression, resulting in increased   migration   and invasion. These effects were reversed by miR-200b and miR-429 inhibition or overexpression. Sections of tumor xenograft model showed increased ZEB1 expression and decreased E-cadherin expression with the downregulation of 53BP1. In 18 clinical tissue samples, expression of 53BP1 was positively correlated with miR-200b and mir-429 and negatively correlated with ZEB1. It was also found that 53BP1 was associated with lymph node metastasis. Taken together, these results suggest that 53BP1 functioned as a tumor suppressor gene by its novel negative control of EMT through regulating the expression of miR-200b/429 and their target gene ZEB1. 
 GRHL2-miR-200-ZEB1 maintains the epithelial status of ovarian cancer through transcriptional regulation and histone modification. Epithelial-mesenchymal transition (EMT), a biological process by which polarized epithelial cells convert into a mesenchymal phenotype, has been implicated to contribute to the molecular heterogeneity of epithelial ovarian cancer (EOC). Here we report that a transcription factor--Grainyhead-like 2 (GRHL2) maintains the epithelial phenotype. EOC tumours with lower GRHL2 levels are associated with the Mes/Mesenchymal molecular subtype and a poorer overall survival. shRNA-mediated knockdown of GRHL2 in EOC cells with an epithelial phenotype results in EMT changes, with increased cell   migration  , invasion and motility. By ChIP-sequencing and gene expression microarray, microRNA-200b/a is identified as the direct transcriptional target of GRHL2 and regulates the epithelial status of EOC through ZEB1 and E-cadherin. Our study demonstrates that loss of GRHL2 increases the levels of histone mark H3K27me3 on promoters and GRHL2-binding sites at miR-200b/a and E-cadherin genes. These findings support GRHL2 as a pivotal gatekeeper of EMT in EOC via miR-200-ZEB1. 
 MicroRNA-23b functions as a tumor suppressor by regulating Zeb1 in bladder cancer. MicroRNAs (miRNAs) are small, non-coding RNAs that regulate gene expression by targeted repression of transcription and translation. In this study we show that miRNA-23b (miR-23b) acts as a tumor suppressor in bladder cancer. Quantitative real-time PCR analysis showed that miR-23b is significantly down-regulated in bladder cancer cell lines and tumor tissues compared to non-malignant cells and normal tissue samples. We also demonstrate that miR-23b expression has a potential to be diagnostic and prognostic biomarker in bladder cancer. High miR-23b expression is positively correlated with higher overall survival of bladder cancer patients as revealed by Kaplan-Meier analysis. ROC analysis showed that miR-23b expression can distinguish between normal and bladder cancer tissues. Further we elucidated the biological significance of miR-23b in bladder cancer. Over-expression of miR-23b in bladder cancer cells inhibited cell proliferation and impaired colony formation. Fluorescence activated cell sorting (FACS) analysis revealed that re-expression of miR-23b in bladder cancer cells induced G0/G1 cell cycle arrest and apoptosis while inhibiting cell   migration   and invasion. Luciferase reporter assays demonstrated that Zeb1, a crucial regulator of epithelial-to-mesenchymal transition (EMT), is a direct target of miR-23b in bladder cancer. These results show that loss of miR-23b confers a proliferative advantage and promotes bladder cancer cell   migration   and invasion. Furthermore, re-expression of miR-23b may be a beneficial therapeutic strategy for the treatment of human bladder cancer. 
 ABCB5-ZEB1 Axis Promotes Invasion and Metastasis in Breast Cancer Cells. ABCB5 belongs to the ATP-binding cassette (ABC) superfamily, which is recognized for playing a role in the failure of chemotherapy. ABCB5 has also been found to be overexpressed at the transcriptional level in a number of cancer subtypes, including breast cancer. However, the exact mechanism ABCB5 uses on cancer cell metastasis is still unclear. In the present study, we demonstrate that ABCB5 expression was increased in metastatic tissues when compared with nonmetastatic tissues. ABCB5 can significantly enhance metastasis and epithelial-mesenchymal transition (EMT), while knockdown of ABCB5 inhibited these processes. Microarray analysis indicated that ZEB1 may function as a downstream factor of ABCB5. Furthermore, the expression of ZEB1 in tissues is positively relevant to ABCB5 in breast cancer. Knocking down ZEB1 inhibits ABCB5 ectopic expression-induced   migration   and invasion, as well as EMT. Taken together, these results helped to realize the oncogene functions of ABCB5 in breast cancer cells and provided a new direction in treating breast cancer. 
 Porphyromonas gingivalis initiates a mesenchymal-like transition through ZEB1 in gingival epithelial cells. The oral anaerobe Porphyromonas gingivalis is associated with the development of cancers including oral squamous cell carcinoma (OSCC). Here, we show that infection of gingival epithelial cells with P. gingivalis induces expression and nuclear localization of the ZEB1 transcription factor, which controls epithelial-mesenchymal transition. P. gingivalis also caused an increase in ZEB1 expression as a dual species community with Fusobacterium nucleatum or Streptococcus gordonii. Increased ZEB1 expression was associated with elevated ZEB1 promoter activity and did not require suppression of the miR-200 family of microRNAs. P. gingivalis strains lacking the FimA fimbrial protein were attenuated in their ability to induce ZEB1 expression. ZEB1 levels correlated with an increase in expression of mesenchymal markers, including vimentin and MMP-9, and with enh  anced mig  ration of epithelial cells into matrigel. Knockdown of ZEB1 with siRNA prevented the P. gingivalis-induced increase in mesenchymal markers and epitheli  al cell m  igration. Oral infection of mice by P. gingivalis increased ZEB1 levels in gingival tissues, and intracellular P. gingivalis were detected by antibody staining in biopsy samples from OSCC. These findings indicate that FimA-driven ZEB1 expression could provide a mechanistic basis for a P. gingivalis contribution to OSCC. 
 MiR-205 promotes motility of ovarian cancer cells via targeting ZEB1. To investigate the clinical significance of microRNA-205 (miR-205) and zinc finger E-box binding homeobox 1 (ZEB1) in epithelial ovarian cancer (EOC) and the underlying mechanisms by which they are involved into tumorigenesis.Expression levels of miR-205 and ZEB1 mRNA in EOC tissues were detected by quantitative real-time PCR. Their associations with clinicopathological features of EOC patients were statistically analyzed. Luciferase reporter assay was used to confirm target associations between miR-205 and ZEB1. After that, the functions of miR-205-ZEB1 axis on cell   migration   and invasion were further determined by transwell assay in vitro.Expression levels of miR-205 (P=0.0001) and ZEB1 mRNA (P<0.0001) in clinical EOC tissues were significantly higher and lower than those in normal tissues, respectively. Interestingly, there was a negative correlation between miR-205 and ZEB1 mRNA expression in EOC tissues (P=0.01). Additionally, miR-205-upregulation and/or ZEB1-downregulation were significantly associated with high pathological grade and advanced clinical stage of EOC patients (all P<0.05). Meantime, luciferase reporter assays identified ZEB1 as a direct target of miR-205 in EOC cells. Moreover, miR-205 blockage inhibited, whereas miR-205 mimics promoted the motility of EOC cells in vitro. Importantly, all the alterations of the above cellular phenotypes by blocking or enhancing of miR-205 could be alleviated by subsequent suppression or re-introduction of its target ZEB1, respectively.MiR-205, acting as an oncogenic miRNA, may promote the clinical progression of EOC patients and enhance the cellular motility in vitro by directly and negatively regulating ZEB1, providing a potential therapeutic strategy for suppression of EOC metastasis. 
 TGF-β is an inducer of ZEB1-dependent mesenchymal transdifferentiation in glioblastoma that is associated with tumor invasion. Different molecular subtypes of glioblastoma (GBM) have been recently identified, of which the mesenchymal subtype is associated with worst prognoses. Here, we report that transforming growth factor-β (TGF-β) is able to induce a mesenchymal phenotype in GBM that involves activation of SMAD2 and ZEB1, a known transcriptional inducer of mesenchymal transition in epithelial cancers. TGF-β exposure of established and newly generated GBM cell lines was associated with morphological changes, enhanced mesenchymal marker expressi  on, migra  tion and invasion in vitro and in an orthotopic mouse model. TGF-β-induced mesenchymal differentiation and invasive behavior was prevented by chemical inhibition of TGF-β signaling as well as small interfering RNA (siRNA)-dependent silencing of ZEB1. Furthermore, TGF-β-responding and -nonresponding GBM neurospheres were identified in vitro. Interestingly, nonresponding cells displayed already high levels of pSMAD2 and ZEB1 that could not be suppressed by inhibition of TGF-β signaling, suggesting the involvement of yet unknown mechanisms. These different GBM neurospheres formed invasive tumors in mice as well as revealed mesenchymal marker expression in immunohistochemical analyses. Moreover, we also detected distinct zones with overlapping pSMAD2, elevated ZEB1 and mesenchymal marker expression in GBM patient material, suggestive of the induction of local, microenvironment-dependent mesenchymal differentiation. Overall, our findings indicate that GBM cells can acquire mesenchymal features associated with enhanced invasive potential following stimulation by secretory cytokines, such as TGF-β. This property of GBM contributes to heterogeneity in this tumor type and may blur the boundaries between the proposed transcriptional subtypes. Targeting TGF-β or downstream targets like ZEB1 might be of potential benefit in reducing the invasive phenotype of GBM in a subpopulation of patients. 
 Cordycepin (3'-deoxyadenosine) suppressed HMGA2, Twist1 and ZEB1-dependent melanoma invasion and metastasis by targeting miR-33b. Malignant melanoma, the most deadly form of skin cancer, has a high propensity for metastatic spread and is notoriously chemotherapy-resistant. Cordycepin, the active component of Cordyceps spp., has been identified to have anti-metastatic effect on tumor progression and thus possesses pharmacological and therapeutic potentials. However, the mechanisms of anti-metastatic effects of cordycepin at cellular levels remain elusive. We analyzed the effect of cordycepin on human melanoma miRNA expression profiles by miRNAarray and found that miR-33b was upregulated in highly-metastatic melanoma cell lines following cordycepin exposure. Cordycepin-mediated miR-33b expression was dependent on LXR-RXR heterodimer activation. miR-33b directly binds to HMGA2, Twist1 and ZEB1 3'-UTR to suppress their expression. The negative correlations between miR-33b levels and HMGA2, Twist1 or ZEB1 expression were detected in 72 patient melanoma tissue samples. By targeting HMGA2 and Twist1, miR-33b attenuated melanoma   migration   and invasiveness upon cordycepin exposure. miR-33b knockdown or ZEB1 overexpression reverted cordycepin-mediated mesenchymal-epithelial transition (MET), triggering the expression of N-cadherin. In spontaneous metastasis models, cordycepin suppressed tumor metastasis without altering primary tumor growth. We showed for the first time that targeting miRNA by cordycepin indicates a new mechanism of cordycepin-induced suppression of tumor metastasis and miR-33b/HMGA2/Twist1/ZEB1 axis plays critical roles in regulating melanoma dissemination. 
 MicroRNA-141 and microRNA-146b-5p inhibit the prometastatic mesenchymal characteristics through the RNA-binding protein AUF1 targeting the transcription factor ZEB1 and the protein kinase AKT. miR-141 and miR-146b-5p are two important tumor suppressor microRNAs, which control several cancer-related genes and processes. In the present report, we have shown that these microRNAs bind specific sites at the 3'-untranslated region (UTR) of the mRNA-binding protein AUF1, leading to its down-regulation. This inverse correlation between the levels of these microRNAs and AUF1 has been identified in various osteosarcoma cell lines. Additionally, we present clear evidence that AUF1 promotes mesenchymal features in osteosarcoma cells and that miR-141 and miR-146b-5p suppress this prometastatic process through AUF1 repression. Indeed, both microRNAs suppressed the invasion/  migration   and proliferation abilities of osteosarcoma cells through inhibiting the AKT protein kinase in an AUF1-dependent manner. We have also shown that AUF1 binds to and stabilizes the mRNA of the AKT activator phosphoinositide-dependent kinase-1 (PDK1). Furthermore, miR-141 and miR-146b-5p positively regulate the epithelial markers (E-cadherin and Epcam) and repress the mesenchymal markers (N-cadherin, Vimentin, Twist2, and ZEB1). These effects were mediated via the repression of the epithelial-to-mesenchymal inducer ZEB1 through targeting AUF1, which binds the 3'-UTR of the ZEB1 mRNA and reduces its turnover. These results indicate that at least some tumor suppressor functions of miR-141 and miR-146b-5p are mediated through the repression of the oncogenic potentials of AUF1. Therefore, these 3'-UTR-directed post-transcriptional gene expression regulators constitute promising new targets for diagnostic and/or therapeutic interventions. 
 Transcriptional activation of ZEB1 by Slug leads to cooperative regulation of the epithelial-mesenchymal transition-like phenotype in melanoma. The E-box-binding zinc finger transcription factors Slug and ZEB1 are important repressors of E-cadherin, contributing to epithelial-mesenchymal transition (EMT) in primary epithelial cancers. Activator or repressor status of EMT transcription factors defines consequences for tumorigenesis. We show that changes in expression levels of Slug in melanoma cell lines lead to concomitant alterations of ZEB1 expression. Electrophoretic mobility shift, luciferase reporter, and chromatin immunoprecipitation assays identified Slug as a direct transcriptional activator at E-boxes of the ZEB1 promoter. Transcriptional activation of ZEB1 was demonstrated to be specific for Slug, as EMT regulators Snail and Twist failed to influence ZEB1 expression. Slug and ZEB1 cooperatively repressed E-cadherin expression resulting in decreased adhesion to human keratinocytes, but promoted   migration   of melanoma cells. Our results show that the transcriptional activity of ZEB1 is increased by Slug, suggesting a hierarchical organized expression of EMT transcription factors through directed activation, triggering an EMT-like process in melanoma. 
 The microRNA-200/Zeb1 axis regulates ECM-dependent β1-integrin/FAK signaling, cancer cell invasion and metastasis through CRKL. Tumor cell metastasis is a complex process that has been mechanistically linked to the epithelial-mesenchymal transition (EMT). The double-negative feedback loop between the microRNA-200 family and the Zeb1 transcriptional repressor is a master EMT regulator, but there is incomplete understanding of how miR-200 suppresses invasion. Our recent efforts have focused on the tumor cell-matrix interactions essential to tumor cell activation. Herein we utilized both our Kras/p53 mutant mouse model and human lung cancer cell lines to demonstrate that upon miR-200 loss integrin β1-collagen I interactions drive 3D in vitr  o migrati  on/invasion and in vivo metastases. Zeb1-dependent EMT enhances tumor cell responsiveness to the ECM composition and activates FAK/Src pathway signaling by de-repression of the direct miR-200 target, CRKL. We demonstrate that CRKL serves as an adaptor molecule to facilitate focal adhesion formation, mediates outside-in signaling through Itgβ1 to drive cell invasion, and inside-out signaling that maintains tumor cell-matrix contacts required for cell invasion. Importantly, CRKL levels in pan-cancer TCGA analyses were predictive of survival and CRKL knockdown suppressed experimental metastases in vivo without affecting primary tumor growth. Our findings highlight the critical ECM-tumor cell interactions regulated by miR-200/Zeb1-dependent EMT that activate intracellular signaling pathways responsible for tumor cell invasion and metastasis. 
 Reciprocal regulation of ZEB1 and AR in triple negative breast cancer cells. Zinc-finger enhancer binding protein (ZEB1) is a transcription factor involved in the progression of cancer primarily through promoting epithelial to mesenchymal transition (EMT). ZEB1 represses the expression of E-cadherin by binding to E-box sequences in the promoter, thus decreasing epithelial differentiation. We show that ZEB1 and androgen receptor (AR) cross-talk in triple negative breast cancer cell lines. Chromatin immunoprecipitation analysis demonstrates that ZEB1 binds directly to the E-box located in the AR promoter. ZEB1 suppression by stably transfecting shRNA in a triple negative breast cancer cell line resulted in a decrease of AR mRNA, protein, and AR downstream targets. ZEB1 knockdown in triple negative breast cancer cells sensitized the cells to bicalutamide by reducing   migration   compared to the control cells. Conversely, blockade of AR signaling with bicalutamide resulted in a suppression of ZEB1 protein expression in two triple negative breast cancer cell lines. Furthermore, using a breast cancer tissue microarray, a majority of triple negative breast cancers exhibit positive staining for both ZEB1 and AR. Taken together, these results indicate that ZEB1 and AR regulate each other to promote cell   migration   in triple negative breast cancer cells. 
 miR-1236-3p represses the cell   migration   and invasion abilities by targeting ZEB1 in high-grade serous ovarian carcinoma. Ovarian cancer, particularly high-grade serous ovarian carcinoma (HG-SOC), is still the main cause of death among gynecological malignancies. However, the molecular mechanisms related to its malignant biological behavior are still unclear. Recent studies indicate that microRNAs (miRNAs) play an important role in tumor metastasis. Here, we report that miR-1236-3p expression was downregulated in HG-SOC when compared to that in normal fallopian tube tissue. Manipulation of miR-1236-3p significantly influenced the morphology,   migration   and invasion of ovarian cancer cell lines (A2780 and SKOV3). With dual-luciferase reporter assay, we demonstrated that miR‑1236-3p binds to the 3'UTR of zinc-finger E-box binding homeobox 1 (ZEB1) mRNA, and functions as a negative regulator of ZEB1. Furthermore, we revealed that manipulation of miR-1236-3p modulates ZEB1 expression and influences expression of its downstream genes E-cadherin and N-cadherin at both the mRNA and protein levels. We also found an inverse relationship between miR‑1236-3p and ZEB1 expression in the HG-SOC tissue samples. Taken together, our results indicate that miR-1236-3p regulates ovarian cancer metastasis by directly targeting ZEB1, and it may play an important role in the diagnosis and treatment of ovarian cancer. 
 Overexpression of zinc finger E-box binding homeobox factor 1 promotes tumor invasiveness and confers unfavorable prognosis in esophageal squamous cell carcinoma. Zinc finger E-box binding homeobox factor 1 (ZEB1), as a crucial mediator of "epithelial-mesenchymal transition," contributes to malignant progression of various epithelial tumors. However, its involvement in human esophageal squamous cell carcinoma (ESCC) remains unclear. In order to investigate the expression pattern of ZEB1 in ESCC tissues and evaluate its associations with tumor progression and patients' prognosis, 100 pairs of formalin-fixed and paraffin-embedded cancerous and adjacent noncancerous tissues from patients with ESCC were used to detect the expression pattern of ZEB1 by immunohistochemistry. Then, the association between ZEB1 expression, clinicopathological parameters, and prognosis of ESCC was examined. We also performed   migration   and invasion assays of small interfering RNA (siRNA)-targeted ZEB1-transfected cells in vitro. As a result, expression level of ZEB1 was significantly higher in ESCC tissues compared to that in adjacent noncancerous tissues (P < 0.001). High expression of ZEB1 was observed in 55.00 % (55/100) of ESCCs. In addition, high ZEB1 expression was found to be closely correlated with advanced tumor stage (P = 0.001), positive lymph node metastasis (P = 0.001), great tumor depth (P = 0.03), and high histologic grade (P = 0.008). Moreover, multivariate analysis showed that the status of ZEB1 expression was an independent predictor for overall survival in ESCC. Furthermore, knockdown of ZEB1 by transfection of siRN  A-ZEB1 ab  rogated the migration and invasion of ESCC cells in vitro. Taken together, our data offer the convincing evidence that ZEB1 may play a crucial role in promoting aggressive ESCC progression. ZEB1 may serve as an effective prognostic marker and a potential target for therapeutic intervention of ESCC. 
 miR-429 inhibits   migration   and invasion of breast cancer cells in vitro. Accumulating evidence indicates that microRNAs (miRNAs) are involved in regulating cancer invasion and metastasis, and an increasing number of research demonstrates that miRNAs can promote or inhibit cell motility depending on genetic background of different cancers and the microenvironment. In the present study, we established an in vivo bone metastasis model of breast cancer by injecting MDA-MB-231 cells into the left ventricle of nude mice, and then screened the differentially expressed miRNAs between parental and bone-metastatic MDA-MB-231 cells using miRNA array. The results revealed that decreased expression of miR-429 was probably involved in negatively regulating bone metastasis of breast cancer cells. On the other hand, overexpression of miR-429 in MDA-MB-231 cells remarkably suppressed invasion in vitro. We identified ZEB1 and CRKL as potential targets of miR-429 by analyzing combined results from in silico search and global expression array of the same RNA samples. Immunoblot assay confirmed that miR-429 reduced their expression at protein level. Taken together, our results offer an opportunity for further understanding of the recondite mechanisms underlying the bone metastasis of breast cancer. 
 MicroRNA-144 regulates proliferation, invasion, and apoptosis of cells in malignant solitary pulmonary nodule via zinc finger E-box-binding homeobox 1. This study was to investigate the expression of microRNA (miR)-144 in malignant solitary pulmonary nodule (SPN) tissues and peripheral blood, as well as the biological function of miR-144 in the occurrence and development of lung cancer.In this study, 39 malignant and 30 benign SPN patients were included. The expression of miR-144 was examined using quantitative real-time polymerase chain reaction. Receiver operating characteristic (ROC) curve was used to identify the clinical value of miR-144 in the early diagnosis of malignant SPN. MTT assay was performed to determine A549 cell proliferation and Transwell assay was used to detect changes in A549 cell invasion and   migration   ability. Flow cytometry was performed to monitor cell apoptosis, while Western blotting assay was used to measure protein expression levels. At last, dual-luciferase reporter assay was employed to test whether miR-144 regulates zinc finger E-box-binding homeobox 1 (ZEB1) gene expression.Expression of miR-144 was reduced in patients with malignant SPN. miR-144 had diagnostic value for malignant SPN. Proliferation of A549 cells was inhibited by miR-144. Invasion ability of A549 cells was reduced by miR-144. Apoptosis of A549 cells was promoted by miR-144. miR-144 induced A549 cell apoptosis by targeting ZEB1 protein. miR-144 regulated the expression of ZEB1 by interacting with its 3'-UTR region.Expression of miR-144 is reduced in malignant SPN tissues and peripheral blood, being of clinical value in the diagnosis of malignant SPN. miR-144 promotes the apoptosis of lung cancer cells, and inhibits the proliferation, invasion and   migration   of lung cancer by regulating ZEB1 gene. 
 ZEB1 Promotes Invasion in Human Fetal Neural Stem Cells and Hypoxic Glioma Neurospheres. Diffuse spread through brain parenchyma and the presence of hypoxic foci rimmed by neoplastic cells are two cardinal features of glioblastoma, and low oxygen is thought to drive movement of malignant gliomas in the core of the lesions. Transcription factors associated with epithelial-to-mesenchymal transition (EMT) have been linked to this invasion, and we found that hypoxia increased in vitro invasion up to fourfold in glioblastoma neurosphere lines and induced the expression of ZEB1. Immunohistochemical assessment of 295 surgical specimens consisting of various types of pediatric and adult brain cancers showed that ZEB1 expression was significantly higher in infiltrative lesions than less invasive tumors such as pilocytic astrocytoma and ependymoma. ZEB1 protein was also present in human fetal periventricular stem and progenitor cells and ZEB1 inhibition impaired   migration   of in vitro propagated human neural stem cells. The induction of ZEB1 protein in hypoxic glioblastoma neurospheres could be partially blocked by the HIF1alpha inhibitor digoxin. Targeting ZEB1 blocked hypoxia-augmented invasion of glioblastoma cells in addition to slowing them in normoxia. These data support the role for ZEB1 in invasive and high-grade brain tumors and suggest its key role in promoting invasion in the hypoxic tumor core as well as in the periphery. 
 Direct repression of MYB by ZEB1 suppresses proliferation and epithelial gene expression during epithelial-to-mesenchymal transition of breast cancer cells. Epithelial-to-mesenchymal transition (EMT) promotes cell   migration   and is important in metastasis. Cellular proliferation is often downregulated during EMT, and the reverse transition (MET) in metastases appears to be required for restoration of proliferation in secondary tumors. We studied the interplay between EMT and proliferation control by MYB in breast cancer cells.MYB, ZEB1, and CDH1 expression levels were manipulated by lentiviral small-hairpin RNA (shRNA)-mediated knockdown/overexpression, and verified with Western blotting, immunocytochemistry, and qRT-PCR. Proliferation was assessed with bromodeoxyuridine pulse labeling and flow cytometry, and sulforhodamine B assays. EMT was induced with epidermal growth factor for 9 days or by exposure to hypoxia (1% oxygen) for up to 5 days, and assessed with qRT-PCR, cell morphology, and colony morphology. Protein expression in human breast cancers was assessed with immunohistochemistry. ZEB1-MYB promoter binding and repression were determined with Chromatin Immunoprecipitation Assay and a luciferase reporter assay, respectively. Student paired t tests, Mann-Whitney, and repeated measures two-way ANOVA tests determined statistical significance (P < 0.05).Parental PMC42-ET cells displayed higher expression of ZEB1 and lower expression of MYB than did the PMC42-LA epithelial variant. Knockdown of ZEB1 in PMC42-ET and MDA-MB-231 cells caused increased expression of MYB and a transition to a more epithelial phenotype, which in PMC42-ET cells was coupled with increased proliferation. Indeed, we observed an inverse relation between MYB and ZEB1 expression in two in vitro EMT cell models, in matched human breast tumors and lymph node metastases, and in human breast cancer cell lines. Knockdown of MYB in PMC42-LA cells (MYBsh-LA) led to morphologic changes and protein expression consistent with an EMT. ZEB1 expression was raised in MYBsh-LA cells and significantly repressed in MYB-overexpressing MDA-MB-231 cells, which also showed reduced random   migration   and a shift from mesenchymal to epithelial colony morphology in two dimensional monolayer cultures. Finally, we detected binding of ZEB1 to MYB promoter in PMC42-ET cells, and ZEB1 overexpression repressed MYB promoter activity.This work identifies ZEB1 as a transcriptional repressor of MYB and suggests a reciprocal MYB-ZEB1 repressive relation, providing a mechanism through which proliferation and the epithelial phenotype may be coordinately modulated in breast cancer cells. 
 MicroRNA-based regulation of epithelial-hybrid-mesenchymal fate determination. Forward and backward transitions between epithelial and mesenchymal phenotypes play crucial roles in embryonic development and tissue repair. Aberrantly regulated transitions are also a hallmark of cancer metastasis. The genetic network that regulates these transitions appears to allow for the existence of a hybrid phenotype (epithelial/mesenchymal). Hybrid cells are endowed with mixed epithelial and mesenchymal characteristics, enabling specialized capabilities such as collective cell   migration  . Cell-fate determination between the three phenotypes is in fact regulated by a circuit composed of two highly interconnected chimeric modules--the miR-34/SNAIL and the miR-200/ZEB mutual-inhibition feedback circuits. Here, we used detailed modeling of microRNA-based regulation to study this core unit. More specifically, we investigated the functions of the two isolated modules and subsequently of the combined unit when the two modules are integrated into the full regulatory circuit. We found that miR-200/ZEB forms a tristable circuit that acts as a ternary switch, driven by miR-34/SNAIL, that is a monostable module that acts as a noise-buffering integrator of internal and external signals. We propose to associate the three stable states--(1,0), (high miR-200)/(low ZEB); (0,1), (low miR-200)/(high ZEB); and (1/2,1/2), (medium miR-200)/(medium ZEB)--with the epithelial, mesenchymal, and hybrid phenotypes, respectively. Our (1/2,1/2) state hypothesis is consistent with recent experimental studies (e.g., ZEB expression measurements in collectively migrating cells) and explains the lack of observed mesenchymal-to-hybrid transitions in metastatic cells and in induced pluripotent stem cells. Testable predictions of dynamic gene expression during complete and partial transitions are presented. 
 LincRNA-ROR promotes invasion, metastasis and tumor growth in pancreatic cancer through activating ZEB1 pathway. Pancreatic cancer (PC) remains one of the most lethal malignant tumors; early distant metastasis commonly results in poor prognosis. Recent studies confirmed the pivotal role of the long non-coding RNAs (lncRNAs) in tumorigenesis and metastasis of malignant tumors, including PC. However, little is known about the role of LincRNA-ROR (linc-ROR) in PC. In the present study, we found that linc-ROR was upregulated in PC tissues. Overexpression of linc-ROR promoted cells proliferation,   migration  , invasion and metastasis both in vitro and in a mouse model. Contrarily, knockdown of linc-ROR attenuated proliferation, invasion and distant metastasis. Mechanistically, we confirmed that linc-ROR up-regulates ZEB1 and then induces epithelial-mesenchymal transition (EMT), which promotes the aggressive biological behaviors of PC. Together, these results indicate that linc-ROR acts as an important regulator of ZEB1, can promote invasion and metastasis in PC, and may represent a novel therapeutic target. 
 Loss of MicroRNA-101 Promotes Epithelial to Mesenchymal Transition in Hepatocytes. Epithelial-to-mesenchymal transition (EMT) has been implicated in embryonic development and various pathological events. However, the involvement of microRNA in the process of EMT remains to be fully defined in hepatocyte. ZEB1 is a well-known transcriptional repressor of E-cadherin and plays a major role in triggering EMT during organ fibrosis and cancer cell metastasis. Computational microRNA target predictions detect a conserved sequence matching to miR-101 in the 3'UTR of ZEB1 mRNA. Our results confirm that miR-101 suppresses ZEB1 expression by targeting the predicted site of ZEB1 3'UTR. Subsequent investigations show that miR-101 is significantly downregulated in the cultured hepatocytes undergoing EMT and in the hepatocytes isolated from fibrotic liver. Along with the loss of miR-101, the ZEB1 expression increases simultaneously in hepatocytes. In addition, miR-101 levels in HCC cell lines are negatively associated with the ZEB1 productions and the metastatic potentials of tumor cells. Mechanistically, we demonstrate that miR-101 significantly inhibits the TGF-β1-induced EMT in hepatocytes, whereas inhibition of miR-101 promotes the EMT process as indicated by the changes of morphology, cell   migratio  n, and the expression profiles of EMT markers. In the fibrotic liver, ectopic expression of miR-101 can significantly downregulate ZEB1 in the hepatocyte and thereby reduces the mesenchymal marker expression. Moreover, miR-101 significantly inhibits the proliferation and   migratio  n of HCC cell. Our results demonstrate that miR-101 regulates HCC cell phenotype by upregulating the epithelial marker genes and suppressing the mesenchymal ones. 
 The effects of Micro-429 on inhibition of cervical cancer cells through targeting ZEB1 and CRKL. MicroRNA-429 (miR-429) has been suggested to inhibit epithelial-mesenchymal transition (EMT), mainly due to targeting of ZEB1 and ZEB2, which are repressors of the cell to cell contact protein, E-cadherin. In this study, we indicated that regulation of miR-429 in cervical cancer cells modulates cell   migration  , elongation, as well as transforming growth factor β (TGF-β)-induced stress fiber formation through regulating the cytoskeleton reorganization which is likely independent of the zinc finger E-box binding homeobox (ZEB)/E-cadherin axis. ZEB1 and Crk-like adapter protein (CRKL), as novel targets of miR-429 and direct regulators of the actin cytoskeleton were identified. Remarkably, expression levels of ZEB1 and CRKL were inversely associated with the level of miR-429 in cervical cancer cell lines. In addition, individual knockdown and over-expression of these targeting genes phenocopied the roles of miR-429 over-expression and inhibition on cell elongation  , migrati  on, stress fiber formation, and invasion. Targeting of ZEB1 by miR-429 led to a decreased expression and transcriptional activity of CRB3, regulated by interference with the translocation of the CRB3. This finally led to decreasing of the expression of Crumbs 3 (CRB3), which is needed for the formation of stress fiber and contractility. Therefore, miR-429 affects cervical cancer by modulating some EMT-related processes. And in this study, evidences were provided to support a role for miR-429 as a novel target suppressing invasion an  d migrati  on of human cervical cancer cells through modulation of its targeting genes ZEB1 and CRKL. Taken together, our data indicate that miR-429 plays a pivotal role in cervical cancer progression, which is a potential therapeutic target for patients. 
 Pien Tze Huang inhibits metastasis of human colorectal carcinoma cells via modulation of TGF-β1/ZEB/miR-200 signaling network. Tumor metastasis, a complex process involving the spread of malignant tumor cells from a primary tumor site to a distant organ, is a major cause of failure of cancer chemotherapy. Epithelial-mesenchymal transition (EMT) is a critical step for the initiation of cancer metastasis. The processes of EMT and metastasis are highly regulated by a double-negative feedback loop consisting of TGF-β1/ZEB pathway and miR-200 family, which therefore has become a promising target for cancer chemotherapy. Pien Tze Huang (PZH), a well-known traditional Chinese formula first prescribed in the Ming Dynasty, has been demonstrated to be clinically effective in the treatment of various types of human malignancy including colorectal cancer (CRC). Our published data proposed that PZH was able to induce apoptosis, inhibit cell proliferation and tumor angiogenesis, leading to the suppression of CRC growth in vitro and in vivo. To further elucidate the mode of action of PZH, in the present study we evaluated its effects on the metastatic capacities of human colorectal carcinoma HCT-8 cells and investigated the underlying molecular mechanisms. We found that PZH significantly inhibited th  e migrati  on and invasion of HCT-8 cells in a dose-dependent manner. In addition, PZH treatment inhibited the expression of key mediators of TGF-β1 signaling, such as TGF-β1, Smad2/3 and Smad4. Moreover, PZH treatment suppressed the expression of ZEB1 and ZEB2, two critical target genes of TGF-β1 pathway, leading to a decrease in the expression of mesenchymal marker N-cadherin and an increased expression of epithelial marker E-cadherin. Furthermore, PZH treatment upregulated the expression of miR-200a, miR-200b and miR-200c. Collectively, our findings in this study suggest that PZH can inhibit metastasis of colorectal cancer cells via modulating TGF-β1/ZEB/miR-200 signaling network, which might be one of the mechanisms whereby PZH exerts its anticancer function. 
 HIF-1α Promotes Epithelial-Mesenchymal Transition and Metastasis through Direct Regulation of ZEB1 in Colorectal Cancer. It is well recognized that hypoxia-inducible factor 1 alpha (HIF-1α) is involved in cancer metastasis, chemotherapy and poor prognosis. We previously found that deferoxamine, a hypoxia-mimetic agent, induces epithelial-mesenchymal transition (EMT) in colorectal cancer. Therefore, here we explored a new molecular mechanism for HIF-1α contributing to EMT and cancer metastasis through binding to ZEB1. In this study, we showed that overexpression of HIF-1α with adenovirus infection promoted EMT, cell invasion   and migra  tion in vitro and in vivo. On a molecular level, HIF-1α directly binding to the proximal promoter of ZEB1 via hypoxia response element (HRE) sites thus increasing the transactivity and expression of ZEB1. In addition, inhibition of ZEB1 was able to abrogate the HIF-1α-induced EMT and cell invasion. HIF-1α expression was highly correlated with the expression of ZEB1 in normal colorectal epithelium, primary and metastatic CRC tissues. Interestingly, both HIF-1α and ZEB1 were positively associated with Vimentin, an important mesenchymal marker of EMT, whereas negatively associated with E-cadherin expression. These findings suggest that HIF-1α enhances EMT and cancer metastasis by binding to ZEB1 promoter in CRC. HIF-1α and ZEB1 are both widely considered as tumor-initiating factors, but our results demonstrate that ZEB1 is a direct downstream of HIF-1α, suggesting a novel molecular mechanism for HIF-1α-inducing EMT and cancer metastasis. 
 MiR-10b Directly Targets ZEB1 and PIK3CA to Curb Adenomyotic Epithelial Cell Invasiveness via Upregulation of E-Cadherin and Inhibition of Akt Phosphorylation. Adenomyosis is a disease in which ectopic endometrial glands and stromal cells appear in the uterine myometrium. Despite its prevalence, the molecular mechanisms involved in the development of adenomyosis are largely unknown. The aim of this study was to investigate the role of miR-10b and its target genes ZEB1 and PIK3CA in adenomyosis.1387 miRNAs in human normal endometrium and ectopic endometrial lesions of adenomyosis using a microarray screen assay. The significant differential expression of 10 miRNAs was confirmed by qRT-PCR. The expression of miR-10b in endometrial epithelial cells isolated from normal endometrium and paired eutopic and ectopic endometrium of adenomyosis was measured by qRT-PCR. Subsequently, the targets of miR-10b were predicted by bioinformatics and confirmed using a luciferase assay, and the mRNA and protein expression of ZEB1 and PIK3CA were assessed in the endometrium or endometrial epithelial cells by qRT-PCR and western blotting or immunohistochemical analysis. Cell   migration   and cell invasion of endometrial epithelial cells with different treatments by Transwell assays. The expression of p-AKT, Akt and E-cadherin proteins was determined by Western blot analysis.MiR-10b expression was significantly downregulated in both adenomyotic lesions and adenomyotic epithelial cells. MiR-10b overexpression in adenomyotic epithelial cells inhibited cell   migration   and invasion. We then demonstrated that miR-10b directly targets the 3'-UTRs of ZEB1 and PIK3CA, and downregulates ZEB1 and PIK3CA in adenomyotic epithelial cells, leading to increased E-cadherin expression and decreased Akt phosphorylation.miR-10b directly targets ZEB1 and PIK3CA to curb adenomyotic epithelial cell invasiveness via upregulation of E-Cadherin and inhibition of Akt phosphorylation. 
 miR-655 Is an EMT-suppressive microRNA targeting ZEB1 and TGFBR2. Recently, the epithelial-to-mesenchymal transition (EMT) has been demonstrated to contribute to normal and disease processes including cancer progression. To explore EMT-suppressive microRNAs (miRNAs), we established a cell-based reporter system using a stable clone derived from a pancreatic cancer cell line, Panc1, transfected with a reporter construct containing a promoter sequence of CDH1/E-cadherin in the 5' upstream region of the ZsGreen1 reporter gene. Then, we performed function-based screening with 470 synthetic double-stranded RNAs (dsRNAs) mimicking human mature miRNAs using the system and identified miR-655 as a novel EMT-suppressive miRNA. Overexpression of miR-655 not only induced the upregulation of E-cadherin and downregulation of typical EMT-inducers but also suppressed   migration   and invasion of mesenchymal-like cancer cells accompanied by a morphological shift toward the epithelial phenotype. In addition, we found a significant correlation between miR-655 expression and a better prognosis in esophageal squamous cell carcinoma (ESCC). Moreover, ZEB1 and TGFBR2, which are essential components of the TGF-b signaling pathway, were identified as direct targets of miR-655, suggesting that the activation of the TGF-b-ZEB1-E-cadherin axis by aberrant downregulation of miR-655 may accelerate cancer progression. 
 ZEB1 enhances transendothelial   migration   and represses the epithelial phenotype of prostate cancer cells. Metastatic colonization involves cancer cell lodgment or adherence in the microvasculature and subsequent   migration   of those cells across the endothelium into a secondary organ site. To study this process further, we analyzed transendothelial   migration   of human PC-3 prostate cancer cells in vitro. We isolated a subpopulation of cells, TEM4-18, that crossed an endothelial barrier more efficiently, but surprisingly, were less invasive than parental PC-3 cells in other contexts in vitro. Importantly, TEM4-18 cells were more aggressive than PC-3 cells in a murine metastatic colonization model. Microarray and FACS analysis of these cells showed that the expression of many genes previously associated with leukocyte trafficking and cancer cell extravasation were either unchanged or down-regulated. Instead, TEM4-18 cells exhibited characteristic molecular markers of an epithelial-to-mesenchymal transition (EMT), including frank loss of E-cadherin expression and up-regulation of the E-cadherin repressor ZEB1. Silencing ZEB1 in TEM4-18 cells resulted in increased E-cadherin and reduced transendothelial   migration  . TEM4-18 cells also express N-cadherin, which was found to be necessary, but not sufficient for increased transendothelial   migration  . Our results extend the role of EMT in metastasis to transendothelial   migration   and implicate ZEB1 and N-cadherin in this process in prostate cancer cells. 
 Oridonin inhibition and miR‑200b‑3p/ZEB1 axis in human pancreatic cancer. The relationship among oridonin, miR-200b-3p and pancreatic cancer on epithelial-to-mesenchymal transition (EMT) was investigated for the molecular mechanism or signaling pathways on   the migra  tion in pancreatic cancer. BxPC-3 and PANC-1 cells were cultivated and the IC50 of oridonin in BxPC-3 and PANC-1 cells were obtained by the CCK-8 array. The expression of miR‑200b-3p was verified by using real-time PCR and its target gene was predicted. BxPC-3 and PANC-1 cells were treated with oridonin or transfected by miR-200b-3p, those cells were used for western blot assay, Transwell assay, ELISA, immunofluorescence staining, tumorigenesis assay in nude mice and immunohistochemical assay to verify the effects of oridonin or miR-200b-3p on pancreatic cancer. We found that oridonin inhibited the proliferation of BxPC-3 and PANC-1 cells in a dose-dependent manner. miR-200b-3p was downregulated by oridonin in BxPC-3 and PANC-1 cells. ZEB1 was a target gene for miR-200b-3p. Oridonin or overexpression of miR‑200b-3p can inhibit t  he cell m  igration in BxPC-3 and PANC-1 cells. miR-200b-3p can inhibit the EMT and oridonin can inhibit the expression of ZEB1, N-cadherin and fibronectin but not increase the expression of E-cadherin, while the cell adhesion molecules ICAM-1 and VCAM-1 were decreased by oridonin in BxPC-3 and PANC-1 cells and the cytoskeleton was altered by oridonin in PANC-1 cells compared with the control. In summary, the results demonstrate that miR‑200b-3p was able to inhibit the EMT of human pancreatic cancer in vivo and in vitro by targeted ZEB1. In vitro, oridonin had a certain e  ffect on   the migration in BxPC-3 and PANC-1 cells, but not though type III EMT by miR-200-3p/ZEB1 axis, and may be related to type Ⅱ EMT, tumor microenvironment or altering the cytoskeleton. In vivo, oridonin inh  ibited th  e cancer migration in the nude mouse model though inhibiting EMT. 
 ZEB1 coordinately regulates laminin-332 and {beta}4 integrin expression altering the invasive phenotype of prostate cancer cells. Metastasis involves the invasion of cancer cells across both the extracellular matrix and cellular barriers, and an evolving theme is that epithelial-to-mesenchymal transition (EMT) may mediate invasive cellular behavior. Previously, we isolated and analyzed a subpopulation of PC-3 prostate cancer cells, TEM4-18, and found that these cells both invaded an endothelial barrier more efficiently and exhibited enhanced metastatic colonization in vivo. Transendothelial   migration   of these cells depended on expression of ZEB1, a known regulator of EMT. Surprisingly, these cells were much less invasive than parental PC-3 cells in assays that involve matrix barriers. Here, we report that TEM4-18 cells express significantly reduced levels of two subunits of laminin-332 (β3 and γ2) and that exogenous laminin-332, or co-culture with laminin-332-expressing cells, rescues the in vitro invasion phenotype in these cells. Stable knockdown of ZEB1 in prostate cancer cells up-regulated LAMC2 and ITGB4 mRNA and protein and resulted in a concomitant increase in Transwel  l migrati  on. Using chromatin immunoprecipitation (ChIP), we show that ZEB1 directly interacts with the promoters of LAMC2 and ITGB4. These results provide a novel molecular basis for reduced laminin-332 observed in clinical prostate cancer specimens and demonstrate a context-dependent role for EMT in invasive cellular behavior. 
 ZEB1 limits adenoviral infectability by transcriptionally repressing the coxsackie virus and adenovirus receptor. We have previously reported that RAS-MEK (Cancer Res. 2003 May 1;63(9):2088-95) and TGF-β (Cancer Res. 2006 Feb 1;66(3):1648-57) signaling negatively regulate coxsackie virus and adenovirus receptor (CAR) cell-surface expression and adenovirus uptake. In the case of TGF-β, down-regulation of CAR occurred in context of epithelial-to-mesenchymal transition (EMT), a process associated with transcriptional repression of E-cadherin by, for instance, the E2 box-binding factors Snail, Slug, SIP1 or ZEB1. While EMT is crucial in embryonic development, it has been proposed to contribute to the formation of invasive and metastatic carcinomas by reducing cell-cell contacts and increasing cel  l migrati  on.Here, we show that ZEB1 represses CAR expression in both PANC-1 (pancreatic) and MDA-MB-231 (breast) human cancer cells. We demonstrate that ZEB1 physically associates with at least one of two closely spaced and conserved E2 boxes within the minimal CAR promoter here defined as genomic region -291 to -1 relative to the translational start ATG. In agreement with ZEB1's established role as a negative regulator of the epithelial phenotype, silencing its expression in MDA-MB-231 cells induced a partial Mesenchymal-to-Epithelial Transition (MET) characterized by increased levels of E-cadherin and CAR, and decreased expression of fibronectin. Conversely, knockdown of ZEB1 in PANC-1 cells antagonized both the TGF-β-induced down-regulation of E-cadherin and CAR and the reduction of adenovirus uptake. Interestingly, even though ZEB1 clearly contributes to the TGF-β-induced mesenchymal phenotype of PANC-1 cells, TGF-β did not seem to affect ZEB1's protein levels or subcellular localization. These findings suggest that TGF-β may inhibit CAR expression by regulating factor(s) that cooperate with ZEB1 to repress the CAR promoter, rather than by regulating ZEB1 expression levels. In addition to the negative E2 box-mediated regulation the minimal CAR promoter is positively regulated through conserved ETS and CRE elements.This report provides evidence that inhibition of ZEB1 may improve adenovirus uptake of cancer cells that have undergone EMT and for which ZEB1 is necessary to maintain the mesenchymal phenotype. Targeting of ZEB1 may reverse some aspects of EMT including the down-regulation of CAR. 
 HOXD9 promotes epithelial-mesenchymal transition and cancer metastasis by ZEB1 regulation in hepatocellular carcinoma. Hepatocellular carcinoma (HCC) is a common malignant tumor that severely threatens human health. The poor prognosis of HCC is mainly attributed to intrahepatic and extrahepatic metastases. HOXD9 proteins belong to a superfamily that regulates the development and control of many cellular processes, including proliferation, apoptosis, cell shape, and cell   migration  . HOXD9 can also function as an oncogene in several cancer cells. However, its biological function in human HCC requires further investigation. In this study, HOXD9 exhibited high expression in invasive HCC cells. HOXD9 overexpression can significantly enhance HCC cell   migration  , invasion, and metastasis, whereas silencing HOXD9 inhibits these processes. HOXD9 also promotes the epithelial-mesenchymal transition (EMT) of HCC cells. Microarray analysis suggests that ZEB1 can function as a downstream factor of HOXD9. HOXD9 can interact with the promoter region of ZEB1 and promotes ZEB1 expression. ZEB1 knockdown inhibits HOXD9-induced   migration   and invasion, as well as EMT in HCC cells. This study helps elucidates the oncogenic functions of HOXD9 in HCC. 
 MicroRNA-455 suppresses non-small cell lung cancer through targeting ZEB1. MicroRNA-455 (miRNA-455), which is downregulated in human cancer, potently mediates the multiple steps of carcinogenesis. However, the role of miR-455 in non-small cell lung cancer (NSCLC) carcinogenesis remains unclear. In present study, we determined the mature miRNA-455 expression in NSCLC tissues and cells by real-time PCR. Follow-up studies examined the effects of a miR-455 mimic (gain of function) on cell proliferation,   migration  , and invasion. Our data indicate that miR-455 was significantly down-regulated in NSCLC cell lines and tissues. In functional assays, overexpression of miR-455 suppressed the proliferation,   migration  , and invasion of NSCLC cell lines. Data from reporter assays showed that miR-455 directly binds to 3'UTR of ZEB1 and suppresses the endogenous level of ZEB1 protein expression. Furthermore, overexpression of ZEB1 reverses miR-455-suppressed malignant phenotype of NSCLC cells. Moreover, we found that upregulation of ZEB1 expression is inversely associated with miR-455 expression in NSCLC tissues. Taken together, miR-455 as an anti-oncogene in non-small cell lung cancer through up-regulation of ZEB1 and serve as a potential therapeutic target in NSCLC. 
 ABCG2/BCRP gene expression is related to epithelial-mesenchymal transition inducer genes in a papillary thyroid carcinoma cell line (TPC-1). Tumor malignancy is associated with the epithelial-mesenchymal transition (EMT) process and resistance to chemotherapy. However, little is known about the relationship between the EMT and the multidrug-resistance gene in thyroid tumor progression. We investigated whether the expression of the ABCG2/BCRP gene is associated with ZEB1 and other EMT inducer genes involved in tumor dedifferentiation. We established a subpopulation of cells that express the ABCG2/BCRP gene derived from the thyroid papillary carcinoma cell line (TPC-1), the so-called TPC-1 MITO-resistant subline. The most relevant findings in these TPC-1 selected cells were a statistically significant upregulation of ZEB1 and TWIST1 (35- and 15-fold change respectively), no changes in the relative expression of vimentin and SNAIL1, and no expression of E-cadherin. The TPC-1 MITO-resistant subline displayed a faster   migration   and greater invasive ability than parental cells in correlation with a significant upregulation of the survivin (BIRC5) gene (twofold change, P<0.05). The knockdown of ZEB1 promoted nuclear re-expression of E-cadherin, reduced expression of vimentin, N-cadherin, and BIRC5 genes, and reduced cell   migration   (P<0.05). Analysis of human thyroid carcinoma showed a slight overexpression of the ABCG2/BCRP at stages I and II (P<0.01), and a higher overexpression at stages III and IV (P<0.01). SNAIL1, TWIST1, and ZEB1 genes showed higher expression at stages III and IV than at stages I and II. E- and N-cadherin genes were upregulated at stages I and II of the disease (ninefold and tenfold change, respectively, P<0.01) but downregulated at stages III and IV (fourfold lower, P<0.01). These results could be a promising starting point for further study of the role of the ABCG2/BCRP gene in the progression of thyroid tumor. 
 A p21-ZEB1 complex inhibits epithelial-mesenchymal transition through the microRNA 183-96-182 cluster. The tumor suppressor p21 acts as a cell cycle inhibitor and has also been shown to regulate gene expression by functioning as a transcription corepressor. Here, we identified p21-regulated microRNAs (miRNAs) by sequencing small RNAs from isogenic p21(+/+) and p21(-/-) cells. Three abundant miRNA clusters, miR-200b-200a-429, miR-200c-141, and miR-183-96-182, were downregulated in p21-deficient cells. Consistent with the known function of the miR-200 family and p21 in inhibition of the epithelial-mesenchymal transition (EMT), we observed EMT upon loss of p21 in multiple model systems. To explore a role of the miR-183-96-182 cluster in EMT, we identified its genome-wide targets and found that miR-183 and miR-96 repressed common targets, including SLUG, ZEB1, ITGB1, and KLF4. Reintroduction of miR-200, miR-183, or miR-96 in p21(-/-) cells inhibited EMT, cell   migration  , and invasion. Conversely, antagonizing miR-200 and miR-183-96-182 cluster miRNAs in p21(+/+) cells increased invasion and elevated the levels of VIM, ZEB1, and SLUG mRNAs. Furthermore, we found that p21 forms a complex with ZEB1 at the miR-183-96-182 cluster promoter to inhibit transcriptional repression of this cluster by ZEB1, suggesting a reciprocal feedback loop. 
 Effect of down-regulated transcriptional repressor ZEB1 on the epithelial-mesenchymal transition of ovarian cancer cells. Progress has been made against early events of malignant transformation and drug resistance associated with epithelial ovarian cancer; uncontrolled metastases, however, still accounts for most patient deaths. The molecular mechanism that regulates the process of epithelial ovarian cancer metastases is not yet clearly understood. The purpose of this study was to investigate the effect of down-regulating the transcriptional repressor zinc-finger E-box-binding homeobox 1 (ZEB1) on an epithelial-mesenchymal transition (EMT) of human ovarian cancer SKOV3 cell line in vitro and in vivo.The human ovarian cancer cells SKOV3 and HO8910 were transfected with an expression vector-based small hairpin RNA (shRNA) targeting ZEB1 (shZEB1), and the stably transfected cells were selected. Colony-forming, wound-healing, and cellular   migration   assays were respectively used. The tumorigenicity of shZEB1-SKOV3 was also evaluated in mice.The shZEB1-SKOV3 and shZEB1-HO8910 cells showed a lower level of ZEB1 expression and weaker cell   migration   than the control cells. Moreover, down-regulating ZEB1 expression with shRNA in the cells enhanced the expression of miR-200c that acted as a tumor suppressor to inhibit the epithelial-mesenchymal transition of shZEB1-SKOV3 cells and to block shZEB1-SKOV3 cell metastasis in vivo. The shRNA-mediated down-regulation ZEB1 in SKOV3 cells significantly decreased the tumor growth in the xenograft mice.The shZEB1-mediated down-regulation of the ZEB1 expression in the SKOV3 cells may be considered for future clinical trials. 
 ΔNp63α attenuates tumor aggressiveness by suppressing miR-205/ZEB1-mediated epithelial-mesenchymal transition in cervical squamous cell carcinoma. Cervical cancer is one of the most common female cancers worldwide. Although the therapeutic outcomes of patients with early-stage cervical cancer have been significantly improved in the past decades, tumor metastasis and recurrence remain the major causes of cervical cancer-related deaths. In cervical squamous cell carcinoma (SCC), the aberrant activation of epithelial-mesenchymal transition (EMT), a crucial process in invasion and metastasis of epithelial cancer, could promote lymph nodal metastasis and recurrence, and predicts poor prognosis. In this study, we show that the expression levels of EMT markers, β-catenin and Vimentin, are associated with the p63 isoform ΔNp63α in SCC by using immunohistochemistry staining and analysis. Compared to the control SiHa cells (SiHa-NC), the expression of E-cadherin and β-catenin are upregulated, while Vimentin and ZEB1 are downregulated in the constructed SiHa cell line with stable ΔNp63α overexpression (SiHa-ΔNp63α). Bes  ides, the   migration and invasion abilities are also suppressed in SiHa-ΔNp63α cells with a typical epithelial morphology with cobblestone-like shape, suggesting that ΔNp63α is a vital EMT repressor in SCC cells. In addition, the involvement of miR-205/ZEB1 axis in the inhibition effect of ΔNp63α on EMT program is revealed by a miRNA array and confirmed by the subsequent transfection of the miR-205 mimic and antagomir. Moreover, SCC patients with low ΔNp63α expression and high EMT level show more frequent metastasis and recurrence as well as reduced overall survival. Therefore, EMT program and its vital repressor ΔNp63α could be used as biomarkers for tumor metastasis and recurrence in cervical cancer. 
 IL-32θ inhibits stemness and epithelial-mesenchymal transition of cancer stem cells via the STAT3 pathway in colon cancer. Interleukin (IL)-32 is a well-known cytokine associated with inflammation, virus infections and cancer. IL-32θ is a newly identified isoform of IL-32, whose function has yet to be elucidated. In this study, we investigated IL-32θ function in colon cancer stem cells. Using samples from colon cancer patients, we found that the expression of IL-32θ mRNAs was significantly suppressed in tumor regions. We investigated the effects of IL-32θ on colon cancer. Ectopic expression of IL-32θ attenuated inva  sion, mig  ration in vitro and in vivo tumorigenicity of colon cancer cells. IL-32θ inhibited epithelial-mesenchymal transition (EMT), resulting in the suppression of their migratory and invasive capabilities of HT29 colon cancer cells. In addition, IL-32θ altered various properties of CSCs, including sphere formation and expression of stemness related genes. IL-32θ directly bound to STAT3 and inhibited its nuclear translocation, leading to inhibited transcription of downstream factors, including Bmi1 and ZEB1. We showed that IL-32θ inhibited the STAT3-ZEB1 pathway and consequently inhibited key factors of stemness and EMT. Taken together, our findings reveal that IL-32θ can be a tumor suppressor, indicating that IL-32θ could possibly be used in therapies for colon cancer. 
 KDM4B promotes epithelial-mesenchymal transition through up-regulation of ZEB1 in pancreatic cancer. Lysine (K)-specific demethylase 4B (KDM4B) is a histone H3K9 demethylase and is reported to activate gene transcription through regulation of chromatin structures. Previous research has revealed that KDM4B plays special regulatory roles in colorectal, prostate and gastric cancers. However, its physiological role in pancreatic cancer remains largely unknown. In the present study, it is demonstrated KDM4B plays a crucial in epithelial-mesenchymal transition (EMT) in pancreatic cancer. siRNA mediated silencing of KDM4B inhibits cell   migration  , invasion and EMT. Moreover, KDM4B was demonstrated to epigenetically regulate the expression of ZEB1 in the TGF-β-induced EMT process. In tumor tissues of pancreatic cancer patient, the protein level of KDM4B was positively correlated with ZEB1. In conclusion, our results suggested that KDM4B is a key mediator in EMT process, and may serve as an important prognostic marker and therapeutic target for the metastatic progression of human pancreatic cancer. 
  GeneRIFs  
 n vitro, oridonin had a certain effect on the   migration   in BxPC-3 and PANC-1 cells, but not though type III EMT by miR-200-3p/ZEB1 axis, and may be related to type EMT, tumor microenvironment or altering the cytoskeleton. In vivo, oridonin inhibited the cancer   migration   in the nude mouse model though inhibiting EMT. 
 a role for miR-429 as a novel target suppressing invasion and   migration   of human cervical cancer cells through modulation of its targeting genes ZEB1 and CRKL, is reported. 
 miR-144 promotes the apoptosis of lung cancer cells, and inhibits the proliferation, invasion and   migration   of lung cancer by regulating ZEB1 gene. 
 restoration of miR-144 in K1 and WRO thyroid cancer cells suppressed invasion and   migration   capability of these cells; miR-144 also suppressed expression of ZEB1 and ZEB2, two E-cadherin suppressors, by directly binding to their 3'-untranslated regions. 
 The knockdown of ZEB1 promoted nuclear re-expression of E-cadherin, reduced expression of vimentin, N-cadherin, and BIRC5 genes, and reduced cell   migration  . 
 these results indicate that ZEB1 and AR regulate each other to promote cell   migration   in triple negative breast cancer cells 
 These results extend the role of epithelial-to-mesenchymal transition in metastasis to transendothelial   migration   and implicate ZEB1 and N-cadherin in this process in prostate cancer cells. 
 loss of expression of the miR-200 family members may play a critical role in the repression of E-cadherin by ZEB1 and ZEB2 during EMT, thereby enhancing   migration   and invasion during cancer progression. 
  ECM  PubMed  
 Involvement of ZEB1 and Snail1 in excessive production of extracellular matrix in Fuchs endothelial corneal dystrophy. Fuchs endothelial corneal dystrophy (FECD) due to corneal endothelial cell degeneration is a major cause of corneal transplantation. It is characterized by abnormal deposition of extracellular matrix (  ECM  ), such as corneal guttae, accompanied by a loss of endothelial cells. Although recent studies have revealed several genomic factors, the molecular pathophysiology of FECD has not yet been revealed. In this study, we establish a cellular in vitro model by using immortalized corneal endothelial cells obtained from late-onset FECD and control patients and examined the involvement of epithelial mesenchymal transition (EMT) on excessive   ECM   production. We demonstrate that the EMT-inducing genes ZEB1 and SNAI1 were highly expressed in corneal endothelial cells in FECD and were involved in excessive production of   ECM   proteins, such as type I collagen and fibronectin through the transforming growth factor (TGF)-β signaling pathway. Furthermore, we found that SB431542, a specific inhibitor of TGF-β type I ALK receptors, suppressed the expression of ZEB1 and Snail1 followed by reduced production o  f E  CM. These findings suggest that increased expression levels of ZEB1 and Snail1 in FECD cells were responsible for an increased responsiveness to TGF-β present in the aqueous humor and excessive production   of   ECM. In addition, these results suggest that the regulation of EMT-related genes by blocking the TGF-β signaling pathway may be a feasible therapeutic strategy for FECD. 
 The microRNA-200/Zeb1 axis regulates   ECM  -dependent β1-integrin/FAK signaling, cancer cell invasion and metastasis through CRKL. Tumor cell metastasis is a complex process that has been mechanistically linked to the epithelial-mesenchymal transition (EMT). The double-negative feedback loop between the microRNA-200 family and the Zeb1 transcriptional repressor is a master EMT regulator, but there is incomplete understanding of how miR-200 suppresses invasion. Our recent efforts have focused on the tumor cell-matrix interactions essential to tumor cell activation. Herein we utilized both our Kras/p53 mutant mouse model and human lung cancer cell lines to demonstrate that upon miR-200 loss integrin β1-collagen I interactions drive 3D in vitro migration/invasion and in vivo metastases. Zeb1-dependent EMT enhances tumor cell responsiveness to th  e E  CM composition and activates FAK/Src pathway signaling by de-repression of the direct miR-200 target, CRKL. We demonstrate that CRKL serves as an adaptor molecule to facilitate focal adhesion formation, mediates outside-in signaling through Itgβ1 to drive cell invasion, and inside-out signaling that maintains tumor cell-matrix contacts required for cell invasion. Importantly, CRKL levels in pan-cancer TCGA analyses were predictive of survival and CRKL knockdown suppressed experimental metastases in vivo without affecting primary tumor growth. Our findings highlight the critic  al   ECM-tumor cell interactions regulated by miR-200/Zeb1-dependent EMT that activate intracellular signaling pathways responsible for tumor cell invasion and metastasis. 
  GeneRIFs  
 Our findings highlight the critical   ECM  -tumor cell interactions regulated by miR-200/Zeb1-dependent EMT that activate intracellular signaling pathways responsible for tumor cell invasion and metastasis. 
 findings suggest that increased expression levels of ZEB1 and Snail1 in FECD cells were responsible for an increased responsiveness to TGF-beta present in the aqueous humor and excessive production of   ECM   
  [Ee]xtracellular [Mm]atrix  PubMed  
 Involvement of ZEB1 and Snail1 in excessive production of   extracellular matrix   in Fuchs endothelial corneal dystrophy. Fuchs endothelial corneal dystrophy (FECD) due to corneal endothelial cell degeneration is a major cause of corneal transplantation. It is characterized by abnormal deposition of   extracellular matrix   (ECM), such as corneal guttae, accompanied by a loss of endothelial cells. Although recent studies have revealed several genomic factors, the molecular pathophysiology of FECD has not yet been revealed. In this study, we establish a cellular in vitro model by using immortalized corneal endothelial cells obtained from late-onset FECD and control patients and examined the involvement of epithelial mesenchymal transition (EMT) on excessive ECM production. We demonstrate that the EMT-inducing genes ZEB1 and SNAI1 were highly expressed in corneal endothelial cells in FECD and were involved in excessive production of ECM proteins, such as type I collagen and fibronectin through the transforming growth factor (TGF)-β signaling pathway. Furthermore, we found that SB431542, a specific inhibitor of TGF-β type I ALK receptors, suppressed the expression of ZEB1 and Snail1 followed by reduced production of ECM. These findings suggest that increased expression levels of ZEB1 and Snail1 in FECD cells were responsible for an increased responsiveness to TGF-β present in the aqueous humor and excessive production of ECM. In addition, these results suggest that the regulation of EMT-related genes by blocking the TGF-β signaling pathway may be a feasible therapeutic strategy for FECD. 
 Inducible expression of TGFβ, snail and Zeb1 recapitulates EMT in vitro and in vivo in a NSCLC model. The progression of cancer from non-metastatic to metastatic is the critical transition in the course of the disease. The epithelial to mesenchymal transition (EMT) is a mechanism by which tumor cells acquire characteristics that improve metastatic efficiency. Targeting EMT processes in patients is therefore a potential strategy to block the transition to metastatic cancer and improve patient outcome. To develop models of EMT applicable to in vitro and in vivo settings, we engineered NCI-H358 non-small cell lung carcinoma cells to inducibly express three well-established drivers of EMT: activated transforming growth factor β (aTGFβ), Snail or Zeb1. We characterized the morphological, molecular and phenotypic changes induced by each of the drivers and compared the different end-states of EMT between the models. Both in vitro and in vivo, induction of the transgenes Snail and Zeb1 resulted in downregulation of epithelial markers and upregulation of mesenchymal markers, and reduced the ability of the cells to proliferate. Induced autocrine expression of aTGFβ caused marker and phenotypic changes consistent with EMT, a modest effect on growth rate, and a shift to a more invasive phenotype. In vivo, this manifested as tumor cell infiltration of the surrounding mouse stromal tissue. Overall, Snail and Zeb1 were sufficient to induce EMT in the cells, but aTGFβ induced a more complex EMT, in which change  s in extracellular m  atrix remodeling components were pronounced. 
 ZEB1 coordinately regulates laminin-332 and {beta}4 integrin expression altering the invasive phenotype of prostate cancer cells. Metastasis involves the invasion of cancer cells across both the   extracellular matrix   and cellular barriers, and an evolving theme is that epithelial-to-mesenchymal transition (EMT) may mediate invasive cellular behavior. Previously, we isolated and analyzed a subpopulation of PC-3 prostate cancer cells, TEM4-18, and found that these cells both invaded an endothelial barrier more efficiently and exhibited enhanced metastatic colonization in vivo. Transendothelial migration of these cells depended on expression of ZEB1, a known regulator of EMT. Surprisingly, these cells were much less invasive than parental PC-3 cells in assays that involve matrix barriers. Here, we report that TEM4-18 cells express significantly reduced levels of two subunits of laminin-332 (β3 and γ2) and that exogenous laminin-332, or co-culture with laminin-332-expressing cells, rescues the in vitro invasion phenotype in these cells. Stable knockdown of ZEB1 in prostate cancer cells up-regulated LAMC2 and ITGB4 mRNA and protein and resulted in a concomitant increase in Transwell migration. Using chromatin immunoprecipitation (ChIP), we show that ZEB1 directly interacts with the promoters of LAMC2 and ITGB4. These results provide a novel molecular basis for reduced laminin-332 observed in clinical prostate cancer specimens and demonstrate a context-dependent role for EMT in invasive cellular behavior. 
  GeneRIFs  
 Snail and Zeb1 were sufficient to induce epithelial to mesenchymal transition (EMT) in the cells, but activated TGFbeta induced a more complex EMT, in which changes in   extracellular matrix   remodeling components were pronounced. 
  [Ii]nvasive  PubMed  
 A NOTCH3-mediated squamous cell differentiation program limits expansion of EMT-competent cells that express the ZEB transcription factors. Zinc finger E-box-binding (ZEB) proteins ZEB1 and ZEB2 are transcription factors essential in TGF-β-mediated senescence, epithelial-to-mesenchymal transition (EMT), and cancer stem cell functions. ZEBs are negatively regulated by members of the miR-200 microRNA family, but precisely how tumor cells expressing ZEBs emerge during   invasiv  e growth remains unknown. Here, we report that NOTCH3-mediated signaling prevents expansion of a unique subset of ZEB-expressing cells. ZEB expression was associated with the lack of cellular capability of undergoing NOTCH3-mediated squamous differentiation in human esophageal cells. Genetic inhibition of the Notch-mediated transcriptional activity by dominant-negative Mastermind-like 1 (DNMAML1) prevented squamous differentiation and induction of Notch target genes including NOTCH3. Moreover, DNMAML1-enriched EMT-competent cells exhibited robust upregulation of ZEBs, downregulation of the miR-200 family, and enhanced anchorage-independent growth and tumor formation in nude mice. RNA interference experiments suggested the involvement of ZEBs in anchorage-independent colony formation, invasion, and TGF-β-mediated EMT  . Invasi  ve growth and impaired squamous differentiation were recapitulated upon Notch inhibition by DNMAML1 in organotypic three-dimensional culture, a form of human tissue engineering. Together, our findings indicate that NOTCH3 is a key factor limiting the expansion of ZEB-expressing cells, providing novel mechanistic insights into the role of Notch signaling in the cell fate regulation and disease progression of esophageal squamous cancers. 
 Fra-1/AP-1 induces EMT in mammary epithelial cells by modulating Zeb1/2 and TGFβ expression. Epithelial-to-mesenchymal transition (EMT) is essential for embryonic morphogenesis and wound healing and critical for tumour cell invasion and dissemination. The AP-1 transcription factor Fra-1 has been implicated in tumorigenesis and in tumour-associated EMT in human breast cancer. We observed a significant inverse correlation between Fra-1 mRNA expression and distant-metastasis-free survival in a large cohort of breast cancer patients derived from multiple array data sets. This unique correlation among Fos genes prompted us to assess the evolutionary conservation between Fra-1 functions in EMT of human and mouse cells. Ectopic expression of Fra-1 in fully polarized, non-tumourigenic, mouse mammary epithelial EpH4 cells induced a mesenchymal phenotype, characterized by a loss of epithelial and gain of mesenchymal markers. Proliferation, motility and   invasiv  eness were also increased in the resulting EpFra1 cells, and the cells were tumourigenic and efficiently colonized the lung upon transplantation. Molecular analyses revealed increased expression of Tgfβ1 and the EMT-inducing transcription factors Zeb1, Zeb2 and Slug. Mechanistically, Fra-1 binds to the tgfb1 and zeb2 promoters and to an evolutionarily conserved region in the first intron of zeb1. Furthermore, increased activity of a zeb2 promoter reporter was detected in EpFra1 cells and shown to depend on AP-1-binding sites. Inhibiting TGFβ signalling in EpFra1 cells moderately increased the expression of epithelial markers, whereas silencing of zeb1 or zeb2 restored the epithelial phenotype and decreased migration in vitro and tumorigenesis in vivo. Thus Fra-1 induces changes in the expression of genes encoding EMT-related transcription factors leading to the acquisition of mesenchyma  l, invas  ive and tumorigenic capacities by epithelial cells. This study defines a novel function of Fra-1/AP-1 in modulating tgfb1, zeb1 and zeb2 expression through direct binding to genomic regulatory regions, which establishes a basis for future in vivo genetic manipulations and preclinical studies using mouse models. 
 An autocrine TGF-beta/ZEB/miR-200 signaling network regulates establishment and maintenance of epithelial-mesenchymal transition. Epithelial-mesenchymal transition (EMT) is a form of cellular plasticity that is critical for embryonic development and tumor metastasis. A double-negative feedback loop involving the miR-200 family and ZEB (zinc finger E-box-binding homeobox) transcription factors has been postulated to control the balance between epithelial and mesenchymal states. Here we demonstrate using the epithelial Madin Darby canine kidney cell line model that, although manipulation of the ZEB/miR-200 balance is able to repeatedly switch cells between epithelial and mesenchymal states, the induction and maintenance of a stable mesenchymal phenotype requires the establishment of autocrine transforming growth factor-β (TGF-β) signaling to drive sustained ZEB expression. Furthermore, we show that prolonged autocrine TGF-β signaling induced reversible DNA methylation of the miR-200 loci with corresponding changes in miR-200 levels. Collectively, these findings demonstrate the existence of an autocrine TGF-β/ZEB/miR-200 signaling network that regulates plasticity between epithelial and mesenchymal states. We find a strong correlation between ZEBs and TGF-β and negative correlations between miR-200 and TGF-β and between miR-200 and ZEB  s, in in  vasive ductal carcinomas, consistent with an autocrine TGF-β/ZEB/miR-200 signaling network being active in breast cancers. 
 Vimentin, zeb1 and Sip1 are up-regulated in triple-negative and basal-like breast cancers: association with an aggressive tumour phenotype. In epithelial-to-mesenchymal transition (EMT) epithelial cancer cells achieve mesenchymal features, essentially helping them to metastasize. There is some evidence that EMT could be increased in triple-negative (TNBC) or basal-like breast cancers, although more precise mechanisms considering e.g. EMT-regulating transcription factors are largely unknown. We assessed immunohistochemically vimentin (separately in in situ areas and in   invasive   cells) as an indicator of EMT, and also EMT-regulating transcription factors zeb1 (separately in stroma and tumour) and Sip1 (in nuclei and cytoplasm) in histological samples of 231 women with local or locally advanced   invasive   breast cancer. 51.1 % of patients had TNBC and 48.9 % oestrogen and progesterone receptor-positive and HER2 negative breast cancer. Basal-like breast cancers were defined as TNBC that also expressed epidermal growth factor receptor EGFR and/or cytokeratin 5/6. Vimentin expression i  n invasi  ve cells was higher in TNBCs (p = 9 × 10(-12)) compared to non-TNBC tumours. Vimentin (p = 2 × 10(-6)), nuclear Sip1 (p = 0.035) and zeb1 in stroma (p = 0.013) were overexpressed in basal-like cancers compared to non-basal-like TNBCs. In non-TNBC group findings between studied markers and clinicopathological factors were rare. However, in TNBC cases, vimenti  n expres  sion in invasive cells associated with poor differentiation (p = 0.00007), zeb1 expression in cancer cells with higher grade (p = 0.002), vascular invasion (p = 0.036) and larger T-class (p = 0.027), whereas stromal zeb1 associated with lymphatic vessel invasion (p = 0.036) and vascular invasion (p = 0.039). High nuclear Sip1 expression was prognostic for poor disease-free survival (p = 0.002) in the whole cohort. The current results emphasize the increased role of EMT in TNBC and especially in basal-like breast cancers. These observations also support the role of studied parameters in tumour progression. 
 Langerhans cell maturation is accompanied by induction of N-cadherin and the transcriptional regulators of epithelial-mesenchymal transition ZEB1/2. Langerhans cells (LCs) are a unique subset of dendritic cells (DCs) that express epithelial adhesion molecules, allowing them to form contacts with epithelial cells and reside in epidermal/epithelial tissues. The dynamic regulation of epithelial adhesion plays a decisive role in the life cycle of LCs. It controls whether LCs remain immature and sessile within the epidermis or mature and egress to initiate immune responses. So far, the molecular machinery regulating epithelial adhesion molecules during LC maturation remains elusive. Here, we generated pure populations of immature human LCs in vitro to systematically probe for gene-expression changes during LC maturation. LCs down-regulate a set of epithelial genes including E-cadherin, while they upregulate the mesenchymal marker N-cadherin known to facilitate cell migration. In addition, N-cadherin is constitutively expressed by monocyte-derived DCs known to exhibit characteristics of both inflammatory-type and interstitial/dermal DCs. Moreover, the transcription factors ZEB1 and ZEB2 (ZEB is zinc-finger E-box-binding homeobox) are upregulated in migratory LCs. ZEB1 and ZEB2 have been shown to induce epithelial-to-mesenchymal transition (EMT) and   invasive   behavior in cancer cells undergoing metastasis. Our results provide the first hint that the molecular EMT machinery might facilitate LC mobilization. Moreover, our study suggests that N-cadherin plays a role during DC migration. 
 The miR-200 family and miR-205 regulate epithelial to mesenchymal transition by targeting ZEB1 and SIP1. Epithelial to mesenchymal transition (EMT) facilitates tissue remodelling during embryonic development and is viewed as an essential early step in tumour metastasis. We found that all five members of the microRNA-200 family (miR-200a, miR-200b, miR-200c, miR-141 and miR-429) and miR-205 were markedly downregulated in cells that had undergone EMT in response to transforming growth factor (TGF)-beta or to ectopic expression of the protein tyrosine phosphatase Pez. Enforced expression of the miR-200 family alone was sufficient to prevent TGF-beta-induced EMT. Together, these microRNAs cooperatively regulate expression of the E-cadherin transcriptional repressors ZEB1 (also known as deltaEF1) and SIP1 (also known as ZEB2), factors previously implicated in EMT and tumour metastasis. Inhibition of the microRNAs was sufficient to induce EMT in a process requiring upregulation of ZEB1 and/or SIP1. Conversely, ectopic expression of these microRNAs in mesenchymal cells initiated mesenchymal to epithelial transition (MET). Consistent with their role in regulating EMT, expression of these microRNAs was found to be lost in   invasive   breast cancer cell lines with mesenchymal phenotype. Expression of the miR-200 family was also lost in regions of metaplastic breast cancer specimens lacking E-cadherin. These data suggest that downregulation of the microRNAs may be an important step in tumour progression. 
 Fra-1/AP-1 induces EMT in mammary epithelial cells by modulating Zeb1/2 and TGFβ expression. Epithelial-to-mesenchymal transition (EMT) is essential for embryonic morphogenesis and wound healing and critical for tumour cell invasion and dissemination. The AP-1 transcription factor Fra-1 has been implicated in tumorigenesis and in tumour-associated EMT in human breast cancer. We observed a significant inverse correlation between Fra-1 mRNA expression and distant-metastasis-free survival in a large cohort of breast cancer patients derived from multiple array data sets. This unique correlation among Fos genes prompted us to assess the evolutionary conservation between Fra-1 functions in EMT of human and mouse cells. Ectopic expression of Fra-1 in fully polarized, non-tumourigenic, mouse mammary epithelial EpH4 cells induced a mesenchymal phenotype, characterized by a loss of epithelial and gain of mesenchymal markers. Proliferation, motility and   invasiv  eness were also increased in the resulting EpFra1 cells, and the cells were tumourigenic and efficiently colonized the lung upon transplantation. Molecular analyses revealed increased expression of Tgfβ1 and the EMT-inducing transcription factors Zeb1, Zeb2 and Slug. Mechanistically, Fra-1 binds to the tgfb1 and zeb2 promoters and to an evolutionarily conserved region in the first intron of zeb1. Furthermore, increased activity of a zeb2 promoter reporter was detected in EpFra1 cells and shown to depend on AP-1-binding sites. Inhibiting TGFβ signalling in EpFra1 cells moderately increased the expression of epithelial markers, whereas silencing of zeb1 or zeb2 restored the epithelial phenotype and decreased migration in vitro and tumorigenesis in vivo. Thus Fra-1 induces changes in the expression of genes encoding EMT-related transcription factors leading to the acquisition of mesenchyma  l, invas  ive and tumorigenic capacities by epithelial cells. This study defines a novel function of Fra-1/AP-1 in modulating tgfb1, zeb1 and zeb2 expression through direct binding to genomic regulatory regions, which establishes a basis for future in vivo genetic manipulations and preclinical studies using mouse models. 
 Langerhans cell maturation is accompanied by induction of N-cadherin and the transcriptional regulators of epithelial-mesenchymal transition ZEB1/2. Langerhans cells (LCs) are a unique subset of dendritic cells (DCs) that express epithelial adhesion molecules, allowing them to form contacts with epithelial cells and reside in epidermal/epithelial tissues. The dynamic regulation of epithelial adhesion plays a decisive role in the life cycle of LCs. It controls whether LCs remain immature and sessile within the epidermis or mature and egress to initiate immune responses. So far, the molecular machinery regulating epithelial adhesion molecules during LC maturation remains elusive. Here, we generated pure populations of immature human LCs in vitro to systematically probe for gene-expression changes during LC maturation. LCs down-regulate a set of epithelial genes including E-cadherin, while they upregulate the mesenchymal marker N-cadherin known to facilitate cell migration. In addition, N-cadherin is constitutively expressed by monocyte-derived DCs known to exhibit characteristics of both inflammatory-type and interstitial/dermal DCs. Moreover, the transcription factors ZEB1 and ZEB2 (ZEB is zinc-finger E-box-binding homeobox) are upregulated in migratory LCs. ZEB1 and ZEB2 have been shown to induce epithelial-to-mesenchymal transition (EMT) and   invasive   behavior in cancer cells undergoing metastasis. Our results provide the first hint that the molecular EMT machinery might facilitate LC mobilization. Moreover, our study suggests that N-cadherin plays a role during DC migration. 
 Vimentin, zeb1 and Sip1 are up-regulated in triple-negative and basal-like breast cancers: association with an aggressive tumour phenotype. In epithelial-to-mesenchymal transition (EMT) epithelial cancer cells achieve mesenchymal features, essentially helping them to metastasize. There is some evidence that EMT could be increased in triple-negative (TNBC) or basal-like breast cancers, although more precise mechanisms considering e.g. EMT-regulating transcription factors are largely unknown. We assessed immunohistochemically vimentin (separately in in situ areas and in   invasive   cells) as an indicator of EMT, and also EMT-regulating transcription factors zeb1 (separately in stroma and tumour) and Sip1 (in nuclei and cytoplasm) in histological samples of 231 women with local or locally advanced   invasive   breast cancer. 51.1 % of patients had TNBC and 48.9 % oestrogen and progesterone receptor-positive and HER2 negative breast cancer. Basal-like breast cancers were defined as TNBC that also expressed epidermal growth factor receptor EGFR and/or cytokeratin 5/6. Vimentin expression i  n invasi  ve cells was higher in TNBCs (p = 9 × 10(-12)) compared to non-TNBC tumours. Vimentin (p = 2 × 10(-6)), nuclear Sip1 (p = 0.035) and zeb1 in stroma (p = 0.013) were overexpressed in basal-like cancers compared to non-basal-like TNBCs. In non-TNBC group findings between studied markers and clinicopathological factors were rare. However, in TNBC cases, vimenti  n expres  sion in invasive cells associated with poor differentiation (p = 0.00007), zeb1 expression in cancer cells with higher grade (p = 0.002), vascular invasion (p = 0.036) and larger T-class (p = 0.027), whereas stromal zeb1 associated with lymphatic vessel invasion (p = 0.036) and vascular invasion (p = 0.039). High nuclear Sip1 expression was prognostic for poor disease-free survival (p = 0.002) in the whole cohort. The current results emphasize the increased role of EMT in TNBC and especially in basal-like breast cancers. These observations also support the role of studied parameters in tumour progression. 
 A NOTCH3-mediated squamous cell differentiation program limits expansion of EMT-competent cells that express the ZEB transcription factors. Zinc finger E-box-binding (ZEB) proteins ZEB1 and ZEB2 are transcription factors essential in TGF-β-mediated senescence, epithelial-to-mesenchymal transition (EMT), and cancer stem cell functions. ZEBs are negatively regulated by members of the miR-200 microRNA family, but precisely how tumor cells expressing ZEBs emerge during   invasiv  e growth remains unknown. Here, we report that NOTCH3-mediated signaling prevents expansion of a unique subset of ZEB-expressing cells. ZEB expression was associated with the lack of cellular capability of undergoing NOTCH3-mediated squamous differentiation in human esophageal cells. Genetic inhibition of the Notch-mediated transcriptional activity by dominant-negative Mastermind-like 1 (DNMAML1) prevented squamous differentiation and induction of Notch target genes including NOTCH3. Moreover, DNMAML1-enriched EMT-competent cells exhibited robust upregulation of ZEBs, downregulation of the miR-200 family, and enhanced anchorage-independent growth and tumor formation in nude mice. RNA interference experiments suggested the involvement of ZEBs in anchorage-independent colony formation, invasion, and TGF-β-mediated EMT  . Invasi  ve growth and impaired squamous differentiation were recapitulated upon Notch inhibition by DNMAML1 in organotypic three-dimensional culture, a form of human tissue engineering. Together, our findings indicate that NOTCH3 is a key factor limiting the expansion of ZEB-expressing cells, providing novel mechanistic insights into the role of Notch signaling in the cell fate regulation and disease progression of esophageal squamous cancers. 
 An autocrine TGF-beta/ZEB/miR-200 signaling network regulates establishment and maintenance of epithelial-mesenchymal transition. Epithelial-mesenchymal transition (EMT) is a form of cellular plasticity that is critical for embryonic development and tumor metastasis. A double-negative feedback loop involving the miR-200 family and ZEB (zinc finger E-box-binding homeobox) transcription factors has been postulated to control the balance between epithelial and mesenchymal states. Here we demonstrate using the epithelial Madin Darby canine kidney cell line model that, although manipulation of the ZEB/miR-200 balance is able to repeatedly switch cells between epithelial and mesenchymal states, the induction and maintenance of a stable mesenchymal phenotype requires the establishment of autocrine transforming growth factor-β (TGF-β) signaling to drive sustained ZEB expression. Furthermore, we show that prolonged autocrine TGF-β signaling induced reversible DNA methylation of the miR-200 loci with corresponding changes in miR-200 levels. Collectively, these findings demonstrate the existence of an autocrine TGF-β/ZEB/miR-200 signaling network that regulates plasticity between epithelial and mesenchymal states. We find a strong correlation between ZEBs and TGF-β and negative correlations between miR-200 and TGF-β and between miR-200 and ZEB  s, in in  vasive ductal carcinomas, consistent with an autocrine TGF-β/ZEB/miR-200 signaling network being active in breast cancers. 
 The miR-200 family and miR-205 regulate epithelial to mesenchymal transition by targeting ZEB1 and SIP1. Epithelial to mesenchymal transition (EMT) facilitates tissue remodelling during embryonic development and is viewed as an essential early step in tumour metastasis. We found that all five members of the microRNA-200 family (miR-200a, miR-200b, miR-200c, miR-141 and miR-429) and miR-205 were markedly downregulated in cells that had undergone EMT in response to transforming growth factor (TGF)-beta or to ectopic expression of the protein tyrosine phosphatase Pez. Enforced expression of the miR-200 family alone was sufficient to prevent TGF-beta-induced EMT. Together, these microRNAs cooperatively regulate expression of the E-cadherin transcriptional repressors ZEB1 (also known as deltaEF1) and SIP1 (also known as ZEB2), factors previously implicated in EMT and tumour metastasis. Inhibition of the microRNAs was sufficient to induce EMT in a process requiring upregulation of ZEB1 and/or SIP1. Conversely, ectopic expression of these microRNAs in mesenchymal cells initiated mesenchymal to epithelial transition (MET). Consistent with their role in regulating EMT, expression of these microRNAs was found to be lost in   invasive   breast cancer cell lines with mesenchymal phenotype. Expression of the miR-200 family was also lost in regions of metaplastic breast cancer specimens lacking E-cadherin. These data suggest that downregulation of the microRNAs may be an important step in tumour progression. 
 Delta-crystallin enhancer binding factor 1 controls the epithelial to mesenchymal transition phenotype and resistance to the epidermal growth factor receptor inhibitor erlotinib in human head and neck squamous cell carcinoma lines. Although the epidermal growth factor receptor (EGFR) is overexpressed in a majority of head and neck squamous cell carcinomas (HNSCC), only a minority of patients derive substantial clinical benefit from EGFR inhibitors. We initiated the present study to identify the mechanisms underlying erlotinib resistance in a panel of HNSCC cell lines.We used [(3)H]thymidine incorporation to characterize the heterogeneity of responsiveness to erlotinib-mediated growth inhibition in a panel of 27 human HNSCC cells. We characterized the molecular mechanisms involved in resistance using a representative subset of six erlotinib-sensitive and erlotinib-resistant HNSCC lines.Erlotinib had heterogeneous effects on DNA synthesis in HNSCC cells that correlated closely with molecular markers of epithelial to mesenchymal transition (EMT). Specifically, the drug-sensitive lines expressed high levels of E-cadherin and showed limited invasion and migration capabilities. In contrast, the erlotinib-resistant HNSCC lines expressed high levels of the E-cadherin repressor delta-crystallin enhancer binding factor 1 (deltaEF1; Zeb-1) and other mesenchymal markers and low levels of E-cadherin, and they were highly   invasive   and migratory. Small interfering RNA-mediated knockdown of deltaEF1 in the erlotinib-resistant cell lines (1386LN and UMSCC1) resulted in up-regulation of E-cadherin and increased sensitivity to erlotinib in an E-cadherin-dependent manner.DeltaEF1 controls the mesenchymal phenotype and drives erlotinib resistance in HNSCC cells. E-cadherin and deltaEF1 may prove to be useful markers in predicting EGFR inhibitor responsiveness. 
 Hypoxia enhances migration and invasion in glioblastoma by promoting a mesenchymal shift mediated by the HIF1α-ZEB1 axis. Glioblastoma (GBM) is the most common brain tumor in adults and the mesenchymal GBM subtype was reported to be the most malignant, presenting severe hypoxia and necrosis. Here, we investigated the possible role of a hypoxic microenvironment for inducing a mesenchymal and   invasiv  e phenotype. The exposure of non-mesenchymal SNB75 and U87 cells to hypoxia induced a strong change in cell morphology that was accompanied by enhanced   invasiv  e capacity and the acquisition of mesenchymal marker expression. Further analyses showed the induction of HIF1α and HIF2α by hypoxia and exposure to digoxin, a cardiac glycoside known to inhibit HIF1/2 expression, was able to prevent hypoxia-induced mesenchymal transition. ShRNA-mediated knockdown of HIF1α, and not HIF2α, prevented this transition, as well as the knockdown of the EMT transcription factor ZEB1. We provide further evidence for a hypoxia-induced mesenchymal shift in GBM primary material by showing co-localization of GLUT1, ZEB1 and the mesenchymal marker YKL40 in hypoxic regions of the tumor. Collectively, our results identify a HIF1α-ZEB1 signaling axis that promotes hypoxia induced mesenchymal shift and invasion in GBM in a cell line dependent fashion. 
 ZEB1 sensitizes lung adenocarcinoma to metastasis suppression by PI3K antagonism. Epithelial tumor cells that have undergone epithelial-to-mesenchymal transition (EMT) are typically prone to metastasis and drug resistance and contribute to a poor clinical outcome. The transcription factor ZEB1 is a known driver of EMT, and mediators of ZEB1 represent potential therapeutic targets for metastasis suppression. Here, we have shown that phosphatidylinositol 3-kinase-targeted (PI3K-targeted) therapy suppresses metastasis in a mouse model of Kras/Tp53-mutant lung adenocarcinoma that develops metastatic disease due to high expression of ZEB1. In lung adenocarcinoma cells from Kras/Tp53-mutant animals and human lung cancer cell lines, ZEB1 activated PI3K by derepressing miR-200 targets, including amphiregulin (AREG), betacellulin (BTC), and the transcription factor GATA6, which stimulated an EGFR/ERBB2 autocrine loop. Additionally, ZEB1-dependent derepression of the miR-200 and miR-183 target friend of GATA 2 (FOG2) enhanced GATA3-induced expression of the p110α catalytic subunit of PI3K. Knockdown of FOG2, p110α, and RHEB ameliorate  d invasi  ve and metastatic propensities of tumor cells. Surprisingly, FOG2 was not required for mesenchymal differentiation, suggesting that mesenchymal differentiation and invasion are distinct and separable processes. Together, these results indicate that ZEB1 sensitizes lung adenocarcinoma cells to metastasis suppression by PI3K-targeted therapy and suggest that treatments to selectively modify the metastatic behavior of mesenchymal tumor cells are feasible and may be of clinical value. 
 SIX1 promotes epithelial-mesenchymal transition in colorectal cancer through ZEB1 activation. Epithelial-mesenchymal transition (EMT) has a major role in cancer progression, as well as normal organ development and human pathology such as organ fibrosis and wound healing. Here, we performed a gene expression array specialized in EMT of colorectal cancer (CRC). From a comprehensive gene expression analysis using epithelial- and mesenchymal-like CRC cell lines, and following the ontology (GO) analysis, SIX1 gene was identified to be an EMT-related gene in CRC. Using SW480 cells stably transfected with a SIX1 expression construct and their control counterparts, we demonstrated that SIX1 overexpression represses CDH1 expression and promotes EMT in CRC. SIX1-induced CDH1 repression and EMT in CRC cells were correlated at least in part with posttranscriptional ZEB1 activation and miR-200-family transcriptional repression. In primary tumors of CRC, in accord with the functional findings, aberrant expression of SIX1 in cancer cells was observed at the disruption of the basement membrane and at the tumor   invasive   front, where tumor cells underwent EMT in vivo. Taken together, SIX1 overexpression is suggested to occur in carcinogenesis, and contribute to repression of CDH1 expression and promotion of EMT partly through repression of miR-200-family expression and activation of ZEB1 in CRC. 
 Knockdown of BAG3 induces epithelial-mesenchymal transition in thyroid cancer cells through ZEB1 activation. The process by which epithelial features are lost in favor of a mesenchymal phenotype is referred to as epithelial-mesenchymal transition (EMT). Most carcinomas use this mechanism to evade into neighboring tissues. Reduction or a loss of E-cadherin expression is a well-established hallmark of EMT. As a potent suppressor of E-cadherin, transcription factor ZEB1 is one of the key inducers of EMT, whose expression promotes tumorigenesis and metastasis of carcinomas. Bcl-2-associated athanogene 3 (BAG3) affects multifaceted cellular functions, including proliferation, apoptosis, cell adhesion and invasion, viral infection, and autophagy. Recently, we have reported a novel role of BAG3 implicated in EMT, while the mechanisms are poorly elucidated. The current study demonstrated that knockdown of BAG3 induced EMT, and increased cell migratory and   invasive  ness in thyroid cancer cells via transcriptional activation of ZEB1. We also found that BAG3 knockdown led to nuclear accumulation of β-catenin, which was responsible for the transcriptional activation of ZEB1. These results indicate BAG3 as a regulator of ZEB1 expression in EMT and as a regulator of metastasis in thyroid cancer cells, providing potential targets to prevent and/or treat thyroid cancer cell invasion and metastasis. 
 ZEB1 promotes epithelial-mesenchymal transition in cervical cancer metastasis. To investigate role of Zinc finger E-box binding homeobox 1 (ZEB1) in cervical cancer tissue (squamous cell carcinoma, SCC).Exploratory study.University hospital.Sixty patients with SCC, including stage CINIII (n = 10), IB1 (n = 10), IB2 (n = 10), IIA1 (n = 10), IIA2 (n = 10), and IIB (n = 10) were studied.Caski cells were transfected with recombinant shZEB1 lentivirus or shCtrl lentivirus to generate stable ZEB1-knockdown Caski cells.ZEB1 expression was analyzed by quantitative real-time polymerase chain reaction and immunohistochemistry in cervical cancer tissues. ZEB1 expression in Caski cells was down-regulated by short-hairpin RNA (shRNA) interference, and changes in ZEB1 expression corresponded with changes in the proliferation and migratory ability of Caski cells.Quantitative real-time polymerase chain reaction and immunohistochemistry results revealed that ZEB1 expression and the ratio of Vimentin to E-cadherin were high in 27 of 50 SCC patients and correlated with advanced International Federation of Gynecology and Obstetrics stage, tumor size >4 cm, and parametrial invasion. However, the expression of ZEB1 in cervical cancer tissue was independent of age and SCC antigen level. Transfection of ZEB1 shRNA in Caski cells significantly decreased the messenger RNA and protein expression of ZEB1, parallel with increased expression of the epithelial marker E-cadherin and decreased expression of the mesenchymal marker Vimentin. Furthermore, the proliferation and migratory ability of Caski cells were significantly lower in the transfected group than in the nontransfected control group.Down-regulation of ZEB1 expression may protect the   invasive   front of the tumors from converting to a mesenchymal phenotype by reducing the proliferation and motility of cervical cancer cells, suggesting that ZEB1 might be a potential therapeutic target for SCC. 
 Expression of the E-cadherin repressors Snail, Slug and Zeb1 in urothelial carcinoma of the urinary bladder: relation to stromal fibroblast activation and   invasive   behaviour of carcinoma cells. Epithelial-mesenchymal transition (EMT) is regulated by interaction of carcinoma and stromal cells and crucial for progression of urinary bladder carcinoma (UBC). Therefore, the influence of activated fibroblasts on the expression of E-cadherin repressors as well as EMT and invasion in UBC was investigated. A correlative analysis of the immunohistochemical expression of fibroblast (ASMA, S100A4, FAP, SDF1, PDGFRβ) and EMT (Snail, Slug, Zeb1, E-cadherin) markers was performed on 49 UBC cases of different stages. The impact of distinguishable growth factor stimulated fibroblasts on invasion, EMT, and E-cadherin repressor expression was investigated in an invasion model. In situ,   invasiv  eness was significantly correlated to the loss of membranous E-cadherin (E-cad_m) and increased Snail, Slug, Zeb1 in tumour cells, as well as to increased ASMA, S100A4, and PDGFRβ in stromal cells. A significant correlation to nodal metastasis could be evidenced for the loss of E-Cad_m, and for an increase in S100A4 and PDGFRβ. Comparison of stromal and EMT markers revealed significant correlations of ASMA to Snail and Slug; of S100A4 to the loss of E-cad_m and Zeb1; and of PDGFRβ to the loss of E-Cad_m, Slug and Zeb1. In vitro, TGFβ1 induced myofibroblasts were the strongest attractants, while aFGF or TGFβ1/aFGF stimulated fibroblasts were the most potent EMT inductors. As shown here for the first time, distinct sub-populations of fibroblasts are to various extents associated with EMT and tumour progression in UBC. These relevant findings might be the basis for the identification of new diagnostic markers and therapeutic targets selectively affecting tumour supporting CAF effects. 
 Expression of BMI1 and ZEB1 in epithelial-mesenchymal transition of tongue squamous cell carcinoma. Epithelial-mesenchymal transition (EMT) is a crucial event required for the invasion and progression of carcinogenesis, inducing stem-like properties in epithelial cells. In the present study, the expression of BMI1, which controls self-renewal in stem cells, as well as that of ZEB1, a transcription factor that regulates EMT, was evaluated for its role in EMT and the carcinogenic processes of tongue squamous cell carcinoma (TSCC). Collagen invasion assays using two TSCC cells and 64 tongue specimens (32 carcinomas and 32 dysplasias) were employed and analyzed in the present study. We assessed the protein and mRNA expression levels of BMI1, ZEB1, vimentin and E-cadherin in the two cell lines and tumor tissues. The protein and mRNA expression of BMI1 and ZEB1 occurred at the invasion of TSCC. The elevated levels of BMI1 and ZEB1 were accompanied by the downregulation of E-cadherin and upregulation of vimentin at the   invasive   front, indicative of EMT in vitro and in vivo. The results showed that BMI1 and ZEB1 are important factors in association with the promotion of EMT and invasion of TSCC. 
 Involvement of ZEB1 and E-cadherin in the invasion of lung squamous cell carcinoma. This study intended to investigate the expression of the ZEB1 and E-cadherin proteins in lung squamous cell carcinoma (LSCC) tissues and to examine the clinicopathological correlation between protein levels and LSCC. RT-PCR and Western blot were used to examine the expression of ZEB1 and E-cadherin mRNAs and proteins in LSCC tissues as well as in adjacent normal tissues, and then analyze the relationship between the clinicopathological characteristics and the expression changes of ZEB1 and E-cadherin mRNAs in LSCC. In addition, RNAi was used to knockdown the expression of the ZEB1 gene in Human HCC827 cells; subsequently, changes in the   invasive   ability of the resultant cells were studied. The positive rates of ZEB1 and E-cadherin mRNAs in LSCC tissues were 69.2 and 38.5 %, respectively. They differed significantly from the corresponding positive rates in the adjacent normal lung tissues (15.4 and 80.8 %, p < 0.05). There was a negative correlation between the protein levels of ZEB1 and E-cadherin in LSCC tissues (r = -0.714, p < 0.001); in addition, it was found that ZEB1 protein expression in LSCC tissues was significantly higher than that in the neighboring normal lung tissues (p < 0.05), and its expression was also significantly higher in patients with lymph node metastases and distant metastases compared to those patients without metastatic disease (p < 0.05). On the contrary, E-cadherin expression was significantly lower in LSCC tissues than that in the neighboring normal tissue (p < 0.05). It was lower in patients with lymph node metastasis and distant metastasis compared to patients without metastatic disease (p < 0.05). However, the expression of ZEB1 and E-cadherin was independent of gender, age, tumor size, or tumor differentiation level (p > 0.05). Transfection of ZEB1 siRNA into HCC827 cells significantly reduced the ZEB1 protein level (p < 0.01) and significantly elevated E-cadherin levels (p < 0.01). Moreover, significantly less ZEB1 siRNA-transfected cells migrated through Transwell chambers in the LSCC tissue than that in the control groups (untransfected or transfected with control siRNA, p < 0.01). The expression of the ZEB1 gene in LSCC tissues is downregulated with the expression of E-cadherin. On the other hand, the expression of siRNA against ZEB1 promotes E-cadherin expres  sion and   suppresses the invasive ability conferred by E-cadherin. In conclusion, our data suggested that overexpression of the ZEB1 gene is possibly associated with the occurrence, development, invasion of LSCC. 
 Induction of ZEB proteins by inactivation of RB protein is key determinant of mesenchymal phenotype of breast cancer. We previously showed that depletion of the retinoblastoma protein (RB) induces down-regulation of the adhesion molecule E-cadherin and thereby triggers the epithelial-mesenchymal transition. To further characterize the effect of RB inactivation on the phenotype of cancer cells, we have now examined RB expression in human breast cancer cell lines and clinical specimens. We found that RB-inactive cells exhibit a mesenchymal-like morphology and are highly   invasive  . We also found that ZEB proteins, transcriptional repressors of the E-cadherin gene, are markedly up-regulated in these cells in a manner sensitive to the miR-200 family of microRNAs. Moreover, depletion of ZEB in RB-inactive cells suppressed cell   invasive  ness and proliferation and induced epithelial marker expression. These results implicate ZEB in induction of the epithelial-mesenchymal transition, as well as in maintenance of the mesenchymal phenotype in RB-inactive cells. We also developed a screening program for inhibitors of ZEB1 expression and thereby identified several cyclin-dependent kinase inhibitors that blocked both ZEB1 expression and RB phosphorylation. Together, our findings suggest that RB inactivation contributes to tumor progression not only through loss of cell cycle control but also through up-regulation of ZEB expression and induction of an   invasive   phenotype. 
 Overexpression of ZEB1 associated with metastasis and invasion in patients with gastric carcinoma. The aim of this study was to investigate the expression of ZEB1 in gastric carcinoma, its correlation with the clinicopathology of gastric carcinoma, and the role of ZEB1 in invasion and metastasis in gastric carcinoma. ZEB1 expression was analyzed by immunohistochemistry and Western blot in 45 gastric carcinoma tissue samples that contained the adjacent gastric mucosa. The correlation between ZEB1 expression, the occurrence and development of gastric cancer, and clinical pathology was investigated. ZEB1 expression in the human gastric carcinoma cell line AGS was downregulated by RNA interference, and changes in ZEB1 expression corresponded with changes in the   invasive   and metastatic ability of AGS cells. Immunohistochemistry revealed that ZEB1 protein expression in gastric carcinoma tissues was significantly higher than in normal gastric mucosa tissues (p < 0.001). A lower degree of differentiation of gastric cancer (p = 0.009), a higher TNM (tumor, node, and metastasis) stage (p = 0.010), and a larger scope of invasion were correlated with higher expression of ZEB1 (p = 0.041, 0.002). However, the expression of ZEB1 in gastric carcinoma tissue was independent of gender, age, and tumor size (p > 0.05). Western blot results also showed that ZEB1 protein expression was significantly higher in gastric carcinoma tissue than in the adjacent normal gastric mucosa tissue (p = 0.008). A lower degree of differentiation of the gastric carcinoma correlated with a higher TNM stage, and a larger scope of invasion correlated with increased ZEB1 expression (p = 0.023). Transfection of ZEB1 siRNA in AGS cells significantly decreased the expression level of ZEB1 protein (p = 0.035). Furthermore, the number of cells that could pass through the Transwell chamber was significantly lower in the transfected group than in the non-transfected control group (p = 0.039), indicating that the suppression of ZEB1 expression could significantly reduce the   invasive   and metastatic ability of AGS cells (p = 0.005). Concluding, in gastric carcinoma tissue, overexpression of ZEB1 may be related to the occurrence and development as well as invasion and metastasis of gastric carcinoma. 
 ZEB1 represses E-cadherin and induces an EMT by recruiting the SWI/SNF chromatin-remodeling protein BRG1. Loss of E-cadherin is a key initial step in the transdifferentiation of epithelial cells to a mesenchymal phenotype, which occurs when tumor epithelial cells invade into surrounding tissues. Expression of the nuclear factor ZEB1 induces an epithelial-to-mesenchymal transition and confers a metastatic phenotype on carcinomas by repressing the E-cadherin gene at the transcriptional level. In this study, we show that ZEB1 interacts with the SWI/SNF chromatin-remodeling protein BRG1 to regulate E-cadherin independently of CtBP, its traditional co-repressor. Blocking the interaction between ZEB1 and BRG1 induces expression of E-cadherin and downregulation of the mesenchymal marker vimentin. ZEB1 and BRG1 colocalize in E-cadherin-negative cells from cancer lines and in the stroma of normal colon. Colocalization of ZEB1 and BRG1 in epithelial cells is only found in those de-differentiated cells characterized by nuclear beta-catenin staining at the   invasive   edge of the tumor. Our results identify ZEB1/BRG1 as a new transcriptional mechanism regulating E-cadherin expression and epithelial-to-mesenchymal transdifferentiation that may be involved during the initial stages of tumor invasion. 
 Heterogeneous expression of zinc-finger E-box-binding homeobox 1 plays a pivotal role in metastasis via regulation of miR-200c in epithelial-mesenchymal transition. Although epithelial-mesenchymal transition (EMT) has been implicated as the pivotal event in metastasis, there is insufficient evidence related to EMT in clinical settings. Intratumor heterogeneity may lead to underestimation of gene expression representing EMT. In the present study, we investigated the expression of EMT-associated genes and microRNAs in primary colorectal cancer while considering intratumor heterogeneity. One-hundred and thirty-three multiple spatially separated samples were obtained from 8 patients with metastatic colorectal cancers and 8 with non-metastatic colorectal cancers, from the tumor center (TC),   invasive   front (IF) and metastasis. Differences in gene and microRNA expression were investigated by microarray and quantitative reverse-transcription PCR. Gene expression microarray analysis detected 7920 sites showing differing levels of gene expression among the TC, IF and metastasis. Expression of the EMT-associated gene zinc-finger E-box-binding homeobox 1 (ZEB1) significantly increased in the IF (p<0.01). To exclude individual differences, the expression ratio between TC and IF in each tumor was applied to analysis. This approach enabled recognition of the activation of the VEGF and Wnt signaling pathways, which were involved in metastasis via promotion of EMT. While no activation of these pathways was seen at the TC, regardless of whether tumors were metastatic or non-metastatic, they were preferentially activated at the IF in metastatic tumors, where high ZEB1 expression was seen in connection with decreased miR-200c expression. Multiple sampling in a tumor revealed that heterogeneous ZEB1 expression induced by EMT-associated signaling pathways played a pivotal role in metastasis via regulation of miR-200c. 
 TGF-β is an inducer of ZEB1-dependent mesenchymal transdifferentiation in glioblastoma that is associated with tumor invasion. Different molecular subtypes of glioblastoma (GBM) have been recently identified, of which the mesenchymal subtype is associated with worst prognoses. Here, we report that transforming growth factor-β (TGF-β) is able to induce a mesenchymal phenotype in GBM that involves activation of SMAD2 and ZEB1, a known transcriptional inducer of mesenchymal transition in epithelial cancers. TGF-β exposure of established and newly generated GBM cell lines was associated with morphological changes, enhanced mesenchymal marker expression, migration and invasion in vitro and in an orthotopic mouse model. TGF-β-induced mesenchymal differentiation   and inv  asive behavior was prevented by chemical inhibition of TGF-β signaling as well as small interfering RNA (siRNA)-dependent silencing of ZEB1. Furthermore, TGF-β-responding and -nonresponding GBM neurospheres were identified in vitro. Interestingly, nonresponding cells displayed already high levels of pSMAD2 and ZEB1 that could not be suppressed by inhibition of TGF-β signaling, suggesting the involvement of yet unknown mechanisms. These different GBM neurospheres   formed   invasive tumors in mice as well as revealed mesenchymal marker expression in immunohistochemical analyses. Moreover, we also detected distinct zones with overlapping pSMAD2, elevated ZEB1 and mesenchymal marker expression in GBM patient material, suggestive of the induction of local, microenvironment-dependent mesenchymal differentiation. Overall, our findings indicate that GBM cells can acquire mesenchymal features associated with e  nhanced   invasive potential following stimulation by secretory cytokines, such as TGF-β. This property of GBM contributes to heterogeneity in this tumor type and may blur the boundaries between the proposed transcriptional subtypes. Targeting TGF-β or downstream targets like ZEB1 might be of potential benefit in red  ucing th  e invasive phenotype of GBM in a subpopulation of patients. 
 An Rb1-dependent amplification loop between Ets1 and Zeb1 is evident in thymocyte differentiation and   invasive   lung adenocarcinoma. Ras pathway mutation leads to induction and Erk phosphorylation and activation of the Ets1 transcription factor. Ets1 in turn induces cyclin E and cyclin dependent kinase (cdk) 2 to drive cell cycle progression. Ets1 also induces expression of the epithelial-mesenchymal transition (EMT) transcription factor Zeb1, and thereby links Ras mutation to EMT, which is thought to drive tumor invasion. Ras pathway mutations are detected by the Rb1 tumor suppression pathway, and mutation or inactivation of the Rb1 pathway is required for EMT.We examined linkage between Rb1, Ets1 and Zeb1. We found that an Rb1-E2F complex binds the Ets1 promoter and constitutively limits Ets1 expression. But, Rb1 repression of Zeb1 provides the major impact of Rb1 on Ets1 expression. We link Rb1 repression of Zeb1 to induction of miR-200 family members, which in turn target Ets1 mRNA. These findings suggest that Ets1 and Zeb1 comprise an amplification loop that is dependent upon miR-200 and regulated by Rb1. Thus, induction of Ets1 when the Rb1 pathway is lost may contribute to deregulated cell cycle progression through Ets1 induction of cyclin E and cdk2. Consistent with such an amplification loop, we correlate expression of Ets1 and Zeb1 in mouse and human lung adenocarcinoma. In addition we demonstrate that Ets1 expression in thymocytes is also dependent upon Zeb1.Taken together, our results provide evidence of an Rb1-dependent Ets1-Zeb1 amplification loop in thymocyte differentiation and tumor invasion. 
 Cordycepin (3'-deoxyadenosine) suppressed HMGA2, Twist1 and ZEB1-dependent melanoma invasion and metastasis by targeting miR-33b. Malignant melanoma, the most deadly form of skin cancer, has a high propensity for metastatic spread and is notoriously chemotherapy-resistant. Cordycepin, the active component of Cordyceps spp., has been identified to have anti-metastatic effect on tumor progression and thus possesses pharmacological and therapeutic potentials. However, the mechanisms of anti-metastatic effects of cordycepin at cellular levels remain elusive. We analyzed the effect of cordycepin on human melanoma miRNA expression profiles by miRNAarray and found that miR-33b was upregulated in highly-metastatic melanoma cell lines following cordycepin exposure. Cordycepin-mediated miR-33b expression was dependent on LXR-RXR heterodimer activation. miR-33b directly binds to HMGA2, Twist1 and ZEB1 3'-UTR to suppress their expression. The negative correlations between miR-33b levels and HMGA2, Twist1 or ZEB1 expression were detected in 72 patient melanoma tissue samples. By targeting HMGA2 and Twist1, miR-33b attenuated melanoma migration and   invasive  ness upon cordycepin exposure. miR-33b knockdown or ZEB1 overexpression reverted cordycepin-mediated mesenchymal-epithelial transition (MET), triggering the expression of N-cadherin. In spontaneous metastasis models, cordycepin suppressed tumor metastasis without altering primary tumor growth. We showed for the first time that targeting miRNA by cordycepin indicates a new mechanism of cordycepin-induced suppression of tumor metastasis and miR-33b/HMGA2/Twist1/ZEB1 axis plays critical roles in regulating melanoma dissemination. 
 Activation of canonical WNT/β-catenin signaling enhances in vitro motility of glioblastoma cells by activation of ZEB1 and other activators of epithelial-to-mesenchymal transition. Here we show that activation of the canonical WNT/β-catenin pathway increases the expression of stem cell genes and promotes the migratory an  d invasi  ve capacity of glioblastoma. Modulation of WNT signaling alters the expression of epithelial-to-mesenchymal transition activators, suggesting a role of this process in the regulation of glioma motility. Using immunohistochemistry in patient-derived glioblastoma samples we showed higher numbers of cells with intranuclear signal for β-catenin in the infiltrating edge of tumor compared to central tumor parenchyma. These findings suggest that canonical WNT/β-catenin pathway is a critical regulator of GBM invasion and may represent a potential therapeutic target. 
 Inhibition of CCN6 (Wnt-1-induced signaling protein 3) down-regulates E-cadherin in the breast epithelium through induction of snail and ZEB1. The cysteine-rich protein CCN6 [or Wnt-1-induced signaling protein 3 (WISP3)] exerts tumor-suppressive effects in aggressive inflammatory breast cancer. Loss of CCN6 is associated with poorly differentiated phenotypes and increased invasion. Here, we show that reduction of CCN6 expression occurs in 60% of   invasive   breast carcinomas and is associated with axillary lymph node metastases. Furthermore, low CCN6 expression in   invasive   carcinoma tissue samples correlates with reduced expression of E-cadherin. In vitro, RNA interference knockdown of CCN6 in two benign human mammary epithelial cell lines (HME and MCF10A) decreased expression of E-cadherin protein and mRNA and reduced activity of the E-cadherin promoter; this reduction was dependent on intact E-box elements. CCN6 knockdown in HME cells resulted in up-regulation of the E-cadherin transcriptional repressors Snail and ZEB1 and enhanced their recruitment and binding to the E-cadherin promoter as analyzed by chromatin immunoprecipitation assays. Small interfering RNA-mediated knockdown of ZEB1 or Snail blocked the down-regulation of E-cadherin caused by CCN6 inhibition. These data show, for the first time, that CCN6 expression is reduced or lost in a substantial number of   invasive   breast carcinomas and that CCN6 modulates transcriptional repressors of E-cadherin. Together, our results lead to a new hypothesis that Snail and ZEB1 are downstream of CCN6 and play a critical role in CCN6-mediated regulation of E-cadherin in breast cancer. 
 The transcription factor ZEB1 (deltaEF1) promotes tumour cell dedifferentiation by repressing master regulators of epithelial polarity. Epithelial to mesenchymal transition (EMT) is implicated in the progression of primary tumours towards metastasis and is likely caused by a pathological activation of transcription factors regulating EMT in embryonic development. To analyse EMT-causing pathways in tumourigenesis, we identified transcriptional targets of the E-cadherin repressor ZEB1 in   invasive   human cancer cells. We show that ZEB1 repressed multiple key determinants of epithelial differentiation and cell-cell adhesion, including the cell polarity genes Crumbs3, HUGL2 and Pals1-associated tight junction protein. ZEB1 associated with their endogenous promoters in vivo, and strongly repressed promotor activities in reporter assays. ZEB1 downregulation in undifferentiated cancer cells by RNA interference was sufficient to upregulate expression of these cell polarity genes on the RNA and protein level, to re-establish epithelial features and to impair cell motility in vitro. In human colorectal cancer, ZEB1 expression was limited to the tumour-host interface and was accompanied by loss of intercellular adhesion and tumour cell invasion. In   invasive   ductal and lobular breast cancer, upregulation of ZEB1 was stringently coupled to cancer cell dedifferentiation. Our data show that ZEB1 represents a key player in pathologic EMTs associated with tumour progression. 
 Zeb1-mediated T-cadherin repression increases the   invasive   potential of gallbladder cancer. Here, we report that the transcriptional regulator Zeb1 repressed the transcription of T-cadherin, to increase the   invasive   activity of gallbladder cancer cells. Zeb1 physically bound to the promoter of T-cadherin, repressed promoter activity in E-box-like sequence-dependent fashion, and suppressed T-cadherin expression. In gallbladder cancer tissues, Zeb1 was expressed at the cancer invasion front, whereas T-cadherin was exclusively expressed in non-  invasive   foci. Collagen gel invasion assay showed that T-cadherin was a negative regulator for gallbladder cancer invasion. These findings suggest that Zeb1 represses T-cadherin expression and thus increases the   invasive   activity of gallbladder cancer. 
 The ZEB1/miR-200c feedback loop regulates invasion via actin interacting proteins MYLK and TKS5. Epithelial to mesenchymal transition (EMT) is a developmental process which is aberrantly activated during cancer invasion and metastasis. Elevated expression of EMT-inducers like ZEB1 enables tumor cells to detach from the primary tumor and invade into the surrounding tissue. The main antagonist of ZEB1 in controlling EMT is the microRNA-200 family that is reciprocally linked to ZEB1 in a double negative feedback loop. Here, we further elucidate how the ZEB1/miR-200 feedback loop controls invasion of tumor cells. The process of EMT is attended by major changes in the actin cytoskeleton. Via in silico screening of genes encoding for actin interacting proteins, we identified two novel targets of miR-200c - TKS5 and MYLK (MLCK). Co-expression of both genes with ZEB1 was observed in several cancer cell lines as well as in breast cancer patients and correlated with low miR-200c levels. Depletion of TKS5 or MYLK in breast cancer cells reduced their   invasive   potential and their ability to form invadopodia. Whereas TKS5 is known to be a major component, we could identify MYLK as a novel player in invadopodia formation. In summary, TKS5 and MYLK represent two mediators of   invasive   behavior of cancer cells that are regulated by the ZEB1/miR-200 feedback loop. 
 Inhibition of ZEB1 reverses EMT and chemoresistance in docetaxel-resistant human lung adenocarcinoma cell line. Docetaxel has been used as one of the first-line chemotherapies in solid tumors including advanced non-small cell lung cancer (NSCLC). However, limited responses to chemotherapy are observed in clinic and the molecular mechanisms have not been fully understood. Emerging evidence suggests that epithelial-mesenchymal transition (EMT) plays an important role in the processes of tumor metastasis as well as resistance towards anticancer agents. In this study, it was observed that docetaxel-resistant human lung adenocarcinoma cell line (SPC-A1/DTX) was typical of mesenchymal phenotype. SPC-A1/DTX cell line has increased migratory and   invasive   capacity both in vitro and in vivo. Among the master EMT-inducing transcriptional factors, ZEB1 was found to be significantly increased in SPC-A1/DTX cell line. ZEB1 knockdown with RNA interference could reverse the EMT phenotype and inhibit the migratory ability of SPC-A1/DTX cells. Furthermore, inhibition of ZEB1 significantly enhanced the chemosensitivity of SPC-A1/DTX cells to docetaxel in vitro and in vivo and ectopic expression of ZEB1 increased the chemoresistance of SPC-A1 cells to docetaxel. All these results provide experimental evidence that ZEB1 might be an attractive target for the treatment of human NSCLC. 
 Overexpression of zinc finger E-box binding homeobox factor 1 promotes tumor   invasive  ness and confers unfavorable prognosis in esophageal squamous cell carcinoma. Zinc finger E-box binding homeobox factor 1 (ZEB1), as a crucial mediator of "epithelial-mesenchymal transition," contributes to malignant progression of various epithelial tumors. However, its involvement in human esophageal squamous cell carcinoma (ESCC) remains unclear. In order to investigate the expression pattern of ZEB1 in ESCC tissues and evaluate its associations with tumor progression and patients' prognosis, 100 pairs of formalin-fixed and paraffin-embedded cancerous and adjacent noncancerous tissues from patients with ESCC were used to detect the expression pattern of ZEB1 by immunohistochemistry. Then, the association between ZEB1 expression, clinicopathological parameters, and prognosis of ESCC was examined. We also performed migration and invasion assays of small interfering RNA (siRNA)-targeted ZEB1-transfected cells in vitro. As a result, expression level of ZEB1 was significantly higher in ESCC tissues compared to that in adjacent noncancerous tissues (P < 0.001). High expression of ZEB1 was observed in 55.00 % (55/100) of ESCCs. In addition, high ZEB1 expression was found to be closely correlated with advanced tumor stage (P = 0.001), positive lymph node metastasis (P = 0.001), great tumor depth (P = 0.03), and high histologic grade (P = 0.008). Moreover, multivariate analysis showed that the status of ZEB1 expression was an independent predictor for overall survival in ESCC. Furthermore, knockdown of ZEB1 by transfection of siRNA-ZEB1 abrogated the migration and invasion of ESCC cells in vitro. Taken together, our data offer the convincing evidence that ZEB1 may play a crucial role in promoting aggressive ESCC progression. ZEB1 may serve as an effective prognostic marker and a potential target for therapeutic intervention of ESCC. 
 The transcription factor ZEB1 (deltaEF1) represses Plakophilin 3 during human cancer progression. Plakophilin 3 (PKP3) belongs to the p120ctn family of armadillo-related proteins predominantly functioning in desmosome formation. Here we report that PKP3 is transcriptionally repressed by the E-cadherin repressor ZEB1 in metastatic cancer cells. ZEB1 physically associates with two conserved E-box elements in the PKP3 promoter and partially represses the activity of corresponding human and mouse PKP3 promoter fragments in reporter gene assays. In human tumours ZEB1 is upregulated in   invasive   cancer cells at the tumour-host interface, which is accompanied by downregulation of PKP3 expression levels. Hence, the transcriptional repression of PKP3 by ZEB1 contributes to ZEB1-mediated disintegration of intercellular adhesion and epithelial to mesenchymal transition. 
 Nuclear Snail1 and nuclear ZEB1 protein expression in   invasive   and intraductal human breast carcinomas. Snail1 and ZEB1 are transcriptional repressors that drive tumor initiation and metastasis in animal models. Snail1 and ZEB1 are frequently coexpressed in tumor cell lines, suggesting that these factors may cooperate to promote tumor progression. However, coexpression of these transcriptional repressors in primary human cancer specimens has not been investigated. Previous studies assessed expression in primary breast cancers of Snail1 messenger RNA, which does not reflect Snail1 activity because Snail1 is subject to posttranslational modifications that inhibit its nuclear localization/activity. In the current study, using breast tumor cell lines of known Snail1 and ZEB1 expression status, we developed immunohistochemistry protocols for detecting nuclear Snail1 and nuclear ZEB1 proteins. Using these protocols, we assessed nuclear Snail1 and nuclear ZEB1 expressions in primary human breast cancers of varying subtypes (n = 78). Nuclear Snail1 and estrogen receptor α expressions were inversely associated in primary breast cancers, and nuclear Snail1 was expressed in approximately 80% of triple-negative breast cancers (lacking estrogen receptor α, progesterone receptor, and human epidermal growth factor receptor 2 overexpression). In contrast, nuclear ZEB1 was expressed at a significantly lower frequency in these breast cancers. Notably, nuclear Snail1 protein was detected in 45% of ductal carcinoma in situ specimens (n = 29), raising the important possibility that nuclear Snail1 expression in early stage breast lesions may predict future development o  f invasi  ve breast cancer. Collectively, our studies demonstrate frequent expression of nuclear Snail1, but not nuclear ZEB1, i  n invasi  ve, triple-negative breast cancers as well as in intraductal carcinomas. 
 WASF3 regulates miR-200 inactivation by ZEB1 through suppression of KISS1 leading to increased   invasive  ness in breast cancer cells. The WASF3 gene promotes invasion and metastasis in breast cancer cells, which have undergone epithelial-to-mesenchyme transition (EMT). Overexpression of WASF3 in cells that do not show EMT increases their invasion potential as a result of increased ZEB1/2 levels, which specifically suppress the anti-invasion chromosome 1 miR-200a/200b/429 cluster. ZEB1/2 upregulation by WASF3 results from downregulation of KISS1, leading to the release of inhibition of nuclear factor (NF)κB by IκBα. We further show that ZEB1 expression is regulated by the NFκB transcription factor. Knockdown of WASF3 in breast cancer cells leads to reduced ZEB1 levels and increased miR-200 and E-cadherin levels, resulting in loss of invasion potential. The central regulation of this interactive pathway by WASF3 accounts for the increased invasion associated with increased WASF3 expression seen in aggressive breast cancer cells. WASF3, therefore, is a potential target to suppress invasion and metastasis in breast cancer cells. 
 Roles of STAT3 and ZEB1 proteins in E-cadherin down-regulation and human colorectal cancer epithelial-mesenchymal transition. The progression of colorectal carcinoma (CRC) to   invasive   and metastatic disease may involve localized occurrences of epithelial-mesenchymal transition (EMT). However, mechanisms of the EMT process in CRC progression are not fully understood. We previously showed that knockdown of signal transducer and activator of transcription 3 (STAT3) up-regulated E-cadherin (a key component in EMT progression) in CRC. In this study, we examined the roles of STAT3 in CRC EMT and ZEB1, an EMT inducer, in STAT3-induced down-regulation of E-cadherin. Knockdown of STAT3 significantly increased E-cadherin and decreased N-cadherin and vimentin expressions in highly   invasive   LoVo CRC cells. Meanwhile, overexpression of STAT3 significantly reduced E-cadherin and enhanced N-cadherin and vimentin expressions in weakly   invasive   SW1116 CRC cells. Activation of STAT3 significantly increased CRC cell   invasive  ness and resistance to apoptosis. Knockdown of STAT3 dramatically enhanced chemosensitivity of CRC cells to fluorouracil. STAT3 regulated ZEB1 expression in CRC cells, and the STAT3-induced decrease in E-cadherin and cell invasion depended on activation of ZEB1 in CRC cells. Additionally, pSTAT3(Tyr-705) and ZEB1 expressions were significantly correlated with TNM (tumor, lymph node, and metastasis stages) (p < 0.01). In conclusion, STAT3 may directly mediate EMT progression and regulate ZEB1 expression in CRC. ZEB1 may participate in STAT3-induced cell invasion and E-cadherin down-regulation in CRC cells. The expressions of pSTAT3(Tyr-705) and ZEB1 may be positively associated with CRC metastasis. Our data may provide potential targets to prevent and/or treat CRC invasion and metastasis. 
 ZEB1 Promotes Invasion in Human Fetal Neural Stem Cells and Hypoxic Glioma Neurospheres. Diffuse spread through brain parenchyma and the presence of hypoxic foci rimmed by neoplastic cells are two cardinal features of glioblastoma, and low oxygen is thought to drive movement of malignant gliomas in the core of the lesions. Transcription factors associated with epithelial-to-mesenchymal transition (EMT) have been linked to this invasion, and we found that hypoxia increased in vitro invasion up to fourfold in glioblastoma neurosphere lines and induced the expression of ZEB1. Immunohistochemical assessment of 295 surgical specimens consisting of various types of pediatric and adult brain cancers showed that ZEB1 expression was significantly higher in infiltrative lesions than less   invasive   tumors such as pilocytic astrocytoma and ependymoma. ZEB1 protein was also present in human fetal periventricular stem and progenitor cells and ZEB1 inhibition impaired migration of in vitro propagated human neural stem cells. The induction of ZEB1 protein in hypoxic glioblastoma neurospheres could be partially blocked by the HIF1alpha inhibitor digoxin. Targeting ZEB1 blocked hypoxia-augmented invasion of glioblastoma cells in addition to slowing them in normoxia. These data support the role for ZEB1 in   invasive   and high-grade brain tumors and suggest its key role in promoting invasion in the hypoxic tumor core as well as in the periphery. 
 Proteasome inhibitor-resistant cells cause EMT-induction via suppression of E-cadherin by miR-200 and ZEB1. Downregulation of E-cadherin (gene: CDH1) plays an important role in epithelial-mesenchymal transition (EMT), which is critical for normal development and disease states. As a result of long-term treatment of endometrial carcinoma Ishikawa cells with epoxomicin (EXM), the cells exhibited the phenotype for EXM-resistance (Ish/EXM cells). Moreover, CDH1 mRNA and its protein were suppressed and EMT was induced in Ish/EXM cells. Ish/EXM cells exhibited drug-resistance to other proteasome inhibitors, MG-132, PSI and PS-341 (Bortezomib). The proteasome inhibitor-resistant cells acquired   invasive  ness as a result of the chemotherapy. In Ish/EXM cells, E-cadherin was suppressed by upregulation of its transcriptional repressor ZEB1. Furthermore, expression of the miR-200 family (miR-200a, miR-200b, miR-200c and miR-141) found in Ishikawa cells was suppressed in Ish/EXM cells. Overexpression of the miR-200 family in Ish/EXM cells caused by transfection with the pre-miR-200 family induced downregulation of ZEB1 and enhanced expression of E-cadherin. Conversely, suppression of miR-200 expression in the Ishikawa cells by transfection with anti-miR-200 elevated the expression of ZEB1 and suppressed the expression of E-cadherin. These results suggest that acquirement of EXM-resistance in Ish/EXM cells induces up regulation of ZEB1 via suppression of the miR-200 family following suppression of E-cadherin. Since suppression of ZEB1 in Ish/EXM cells by treatment with its siRNA did not restore the miR-200 family expression, miR-200 family was placed upstream of ZEB1 to regulate the expression. 
 β-catenin/TCF4 complex induces the epithelial-to-mesenchymal transition (EMT)-activator ZEB1 to regulate tumor   invasiv  eness. In most carcinomas, invasion of malignant cells into surrounding tissues involves their molecular reprogramming as part of an epithelial-to-mesenchymal transition (EMT). Mutation of the APC gene in most colorectal carcinomas (CRCs) contributes to the nuclear translocation of the oncoprotein β-catenin that upon binding to T-cell and lymphoid enhancer (TCF-LEF) factors triggers an EMT and a p  roinvasi  ve gene expression profile. A key inducer of EMT is the ZEB1 transcription factor whose expression promotes tumorigenesis and metastasis in carcinomas. As inhibitor of the epithelial phenotype, ZEB1 is never present in the epithelium of normal colon or the tumor center of CRCs where β-catenin remains membranous. We show here that ZEB1 is expressed by epithelial cells in intestinal tumors from human patients (familial adenomatous polyposis) and mouse models (APC(Min/+)) with germline mutations of APC that result in nuclear accumulation of β-catenin. However, ZEB1 is not expressed in the epithelium of hereditary forms of CRCs that carry wild-type APC and where β-catenin is excluded from the nucleus (Lynch syndrome). We found that β-catenin/TCF4 binds directly to the ZEB1 promoter and activates its transcription. Knockdown of β-catenin and TCF4 in APC-mutated CRC cells inhibited endogenous ZEB1, whereas forced translocation of β-catenin to the nucleus in APC-wild-type CRC cells induced de novo expression of ZEB1. Upregulation of MT1-MMP and LAMC2 by β-catenin/TCF4 has been l  inked to   invasiveness in CRCs, and we show here that both proteins are activated by ZEB1 coexpressing with it in primary colorectal tumors with mutated APC. These results set ZEB1 as an effector of β-catenin/TCF4 signaling in EMT and tumor progression. 
 Inducible expression of TGFβ, snail and Zeb1 recapitulates EMT in vitro and in vivo in a NSCLC model. The progression of cancer from non-metastatic to metastatic is the critical transition in the course of the disease. The epithelial to mesenchymal transition (EMT) is a mechanism by which tumor cells acquire characteristics that improve metastatic efficiency. Targeting EMT processes in patients is therefore a potential strategy to block the transition to metastatic cancer and improve patient outcome. To develop models of EMT applicable to in vitro and in vivo settings, we engineered NCI-H358 non-small cell lung carcinoma cells to inducibly express three well-established drivers of EMT: activated transforming growth factor β (aTGFβ), Snail or Zeb1. We characterized the morphological, molecular and phenotypic changes induced by each of the drivers and compared the different end-states of EMT between the models. Both in vitro and in vivo, induction of the transgenes Snail and Zeb1 resulted in downregulation of epithelial markers and upregulation of mesenchymal markers, and reduced the ability of the cells to proliferate. Induced autocrine expression of aTGFβ caused marker and phenotypic changes consistent with EMT, a modest effect on growth rate, and a shift to a m  ore inva  sive phenotype. In vivo, this manifested as tumor cell infiltration of the surrounding mouse stromal tissue. Overall, Snail and Zeb1 were sufficient to induce EMT in the cells, but aTGFβ induced a more complex EMT, in which changes in extracellular matrix remodeling components were pronounced. 
 GLI2 cooperates with ZEB1 for transcriptional repression of CDH1 expression in human melanoma cells. In melanoma cells, high expression of the transcription factor GLI2 is associated with increased   invasive   potential and loss of E-cadherin expression, an event reminiscent of the epithelial-to-mesenchymal transition (EMT). Herein, we provide evidence that GLI2 represses E-cadherin gene (CDH1) expression in melanoma cells via distinct mechanisms, enhancing transcription of the EMT-activator ZEB1 and cooperative repression of CDH1 gene transcription via direct binding of both GLI2 and ZEB1 to two closely positioned Kruppel-like factor-binding sites within the CDH1 promoter. GLI2 silencing rescued CDH1 expression except in melanoma cell lines in which the CDH1 promoter was hypermethylated. Proximity ligation assays identified GLI2-ZEB1 complexes in melanoma cell nuclei, proportional to endogenous GLI2 and ZEB1 expression, and whose accumulation was enhanced by the classical EMT inducer TGF-β. These data identify GLI2 as a critical modulator of the cadherin switch in melanoma, a molecular process that is critical for metastatic spread of the disease. 
 Repression of E-cadherin by SNAIL, ZEB1, and TWIST in   invasive   ductal carcinomas of the breast: a cooperative effort? It has been suggested that down-regulation of E-cadherin in   invasive   breast ductal carcinomas is mediated by the aberrant expression of several of its transcriptional repressors, but their inhibitory role and clinical importance are not yet well established. We investigated gene and protein expression patterns of the E-cadherin repressors SNAIL, ZEB1, and TWIST in relation to clinicopathologic parameters, in a series of 88 patients with   invasive   breast ductal carcinomas. Up-regulation of SNAIL messenger RNA (P = .008) and down-regulation of TWIST (P = .022) were associated with triple-negative tumors, whereas ZEB1 gene expression was more frequent in hormone-positive tumors (P = .004). Loss of E-cadherin was found in 19% of the tumors, but it did not correlate with aberrant expression of any of the repressors investigated herein. Nonetheless, we found that ZEB-1 protein overexpression inversely correlated with high tumor grade (P = .018), nuclear grade (P = .002), and presence of lymph nodes (P = .001), and these data were consistent with the gene expression data for ZEB1. Clinically, down-regulation of ZEB1 messenger RNA was associated with poor overall survival (P = .011) and disease-free survival (P = .053), whereas patients with TWIST negative tumors had a worse overall survival (P = .008) and disease-free survival (P = .006). Our data indicate that deregulation of TWIST is somehow important in the aggressiveness of triple-negative carcinomas and poor patient outcome, whereas down-regulation of ZEB1 seems to play a role in tumor spread, metastases, and poor survival. 
 ZEB1 expression is correlated with tumor metastasis and reduced prognosis of breast carcinoma in Asian patients. Tumor metastasis is one of the key events leading to tumor relapse and poor prognosis. Nowadays, increasing evidences demonstrated that ZEB1 was implicated in human carcinogenesis. However, involvement of ZEB1 deregulation in tumorigenesis in Asian patients with breast carcinoma remains elusive. The present study included 102 Asian patients with breast carcinoma treated by surgery from January of 2005 to December of 2006, and the expression of ZEB1 was evaluated by immunohistochemistry. To further assess the prognostic value of ZEB1, Kaplan-Meier curves were constructed. In this study, elevated levels of ZEB1 expression was found in carcinomas with higher aggressive potential. We also correlated expression of ZEB1 with lymph node metastasis (P = 0.021), advanced clinical stage (P = 0.012) in all cases, and high tumor grade (P = 0.047) in   invasive   ductal carcinoma. Furthermore, our data suggested an elevated level of Ki-67 expression in cases with positive expression of ZEB1. Clinically, reduced overall survival and disease-free survival were observed in cases with positive ZEB1 expression than that in negative cases. Our results correlated ZEB1 with aggressive potentials of breast carcinoma and revealed a possibility for ZEB1 as a prognostic marker in breast carcinoma. 
 MiR-10b Directly Targets ZEB1 and PIK3CA to Curb Adenomyotic Epithelial Cell   Invasive  ness via Upregulation of E-Cadherin and Inhibition of Akt Phosphorylation. Adenomyosis is a disease in which ectopic endometrial glands and stromal cells appear in the uterine myometrium. Despite its prevalence, the molecular mechanisms involved in the development of adenomyosis are largely unknown. The aim of this study was to investigate the role of miR-10b and its target genes ZEB1 and PIK3CA in adenomyosis.1387 miRNAs in human normal endometrium and ectopic endometrial lesions of adenomyosis using a microarray screen assay. The significant differential expression of 10 miRNAs was confirmed by qRT-PCR. The expression of miR-10b in endometrial epithelial cells isolated from normal endometrium and paired eutopic and ectopic endometrium of adenomyosis was measured by qRT-PCR. Subsequently, the targets of miR-10b were predicted by bioinformatics and confirmed using a luciferase assay, and the mRNA and protein expression of ZEB1 and PIK3CA were assessed in the endometrium or endometrial epithelial cells by qRT-PCR and western blotting or immunohistochemical analysis. Cell migration and cell invasion of endometrial epithelial cells with different treatments by Transwell assays. The expression of p-AKT, Akt and E-cadherin proteins was determined by Western blot analysis.MiR-10b expression was significantly downregulated in both adenomyotic lesions and adenomyotic epithelial cells. MiR-10b overexpression in adenomyotic epithelial cells inhibited cell migration and invasion. We then demonstrated that miR-10b directly targets the 3'-UTRs of ZEB1 and PIK3CA, and downregulates ZEB1 and PIK3CA in adenomyotic epithelial cells, leading to increased E-cadherin expression and decreased Akt phosphorylation.miR-10b directly targets ZEB1 and PIK3CA to curb adenomyotic epithelial cell   invasive  ness via upregulation of E-Cadherin and inhibition of Akt phosphorylation. 
 Transcription factors zeb1, twist and snai1 in breast carcinoma. Epitheliomesenchymal transition (EMT) is the process where cancer cells attain fibroblastic features and are thus able to invade neighboring tissues. Transcriptional factors zeb1, snai1 and twist regulate EMT.We used immunohistochemistry to investigate the expression of zeb1, twist and snai1 in tumor and stromal compartments by in a large set of breast carcinomas. The results were compared with estrogen and progesterone receptor status, HER2 amplification, grade, histology, TNM status and survival of the patients.Nuclear expression for twist was seen in the epithelial tumor cell compartment in 3.6% and for snai1 in 3.1% of the cases while zeb1 was not detected at all in these areas. In contrast, the tumor stromal compartment showed nuclear zeb1 and twist expression in 75% and 52.4% of the cases, respectively. Although rare, nuclear expression of twist in the epithelial tumor cell compartment was associated with a poor outcome of the patients (p = 0.054 log rank, p = 0.013, Breslow, p = 0.025 Tarone-Ware). Expression of snai1, or expression of zeb1 or twist in the stromal compartment did not have any prognostic significance. Furthermore, none of these factors associated with the size of the tumors, nor with the presence of axillary or distant metastases. Expression of zeb1 and twist in the stromal compartment was positively associated with a positive estrogen or progesterone receptor status of the tumors. Stromal zeb1 expression was significantly lower in ductal in situ carcinomas than in   invasive   carcinomas (p = 0.020). Medullary carcinomas (p = 0.017) and mucinous carcinomas (p = 0.009) had a lower stromal expression of zeb1 than ductal carcinomas. Stromal twist expression was also lower in mucinous (p = 0.017) than in ductal carcinomas.Expression of transcriptional factors zeb1 and twist mainly occur in the stromal compartment of breast carcinomas, possibly representing two populations of cells; EMT transformed neoplastic cells and stromal fibroblastic cells undergoing activation of zeb1 and twist due to growth factors produced by the tumor. However, epithelial expression of twist was associated with a poor prognosis, hinting at its importance in the spread of breast carcinoma. 
 A transient, EMT-linked loss of basement membranes indicates metastasis and poor survival in colorectal cancer. Loss of the basement membrane (BM) is considered an important step toward tumor malignancy. However, the BM is still expressed in most typical colorectal adenocarcinomas; nevertheless, these tumors can invade and develop metastases. The aim of this study was to investigate the role, mechanisms, and clinical relevance of BM turnover in malignant colorectal cancer (CRC) progression.Expression of BM components and their transcriptional regulation and clinical relevance were investigated in human CRCs and cell lines.Our data show new aspects in BM turnover in CRCs with impact on malignant tumor progression: (1) The BM is still expressed in the main tumor mass of most colorectal adenocarcinomas, but selectively lost at   invasive   regions of the tumor in many cases. (2) Selective loss of the BM at the   invasive   front has high clinical and tumor biologic relevance for distant metastasis and survival. (3) The BM is reexpressed in metastases, indicating that its loss is transient and regulated by environmental factors. (4) This transient loss is not only due to proteolytic breakdown but to a down-regulated synthesis and linked to an epithelial-mesenchymal transition (EMT) in tumor cells, and, thereby, zinc-finger-enhancer protein 1 (ZEB1) is the crucial transcriptional repressor of BM components in CRCs.A transient BM loss at the   invasive   front is correlated with increased distant metastasis and poor patient survival, indicating its tumor biologic relevance and usefulness as a prognostic marker. Targeting ZEB1 might be a promising therapeutic option to prevent metastasis. 
 The dual function of PRMT1 in modulating epithelial-mesenchymal transition and cellular senescence in breast cancer cells through regulation of ZEB1. Although the involvement of protein arginine methyltransferase 1 (PRMT1) in tumorigenesis has been reported, its roles in breast cancer progression and metastasis has not been elucidated. Here we identified PRMT1 as a key regulator of the epithelial-mesenchymal transition (EMT) in breast cancer. We showed that the EMT program induced by PRMT1 endowed the human mammary epithelial cells with cancer stem cell properties. Moreover, PRMT1 promoted the migratory and   invasive   behaviors in breast cancer cells. We also demonstrated that abrogation of PRMT1 expression in breast cancer cells abated metastasis in vivo in mouse model. In addition, knockdown of PRMT1 arrested cell growth in G1 tetraploidy and induced cellular senescence. Mechanistically, PRMT1 impacted EMT process and cellular senescence by mediating the asymmetric dimethylation of arginine 3 of histone H4 (H4R3me2as) at the ZEB1 promoter to activate its transcription, indicating the essential roles of this epigenetic control both in EMT and in senescence. Thus, we unraveled a dual function of PRMT1 in modulation of both EMT and senescence via regulating ZEB1. This finding points to the potent value of PRMT1 as a dual therapeutic target for preventing metastasis and for inhibiting cancer cell growth in malignant breast cancer patients. 
 ZEB1 enhances transendothelial migration and represses the epithelial phenotype of prostate cancer cells. Metastatic colonization involves cancer cell lodgment or adherence in the microvasculature and subsequent migration of those cells across the endothelium into a secondary organ site. To study this process further, we analyzed transendothelial migration of human PC-3 prostate cancer cells in vitro. We isolated a subpopulation of cells, TEM4-18, that crossed an endothelial barrier more efficiently, but surprisingly, were less   invasive   than parental PC-3 cells in other contexts in vitro. Importantly, TEM4-18 cells were more aggressive than PC-3 cells in a murine metastatic colonization model. Microarray and FACS analysis of these cells showed that the expression of many genes previously associated with leukocyte trafficking and cancer cell extravasation were either unchanged or down-regulated. Instead, TEM4-18 cells exhibited characteristic molecular markers of an epithelial-to-mesenchymal transition (EMT), including frank loss of E-cadherin expression and up-regulation of the E-cadherin repressor ZEB1. Silencing ZEB1 in TEM4-18 cells resulted in increased E-cadherin and reduced transendothelial migration. TEM4-18 cells also express N-cadherin, which was found to be necessary, but not sufficient for increased transendothelial migration. Our results extend the role of EMT in metastasis to transendothelial migration and implicate ZEB1 and N-cadherin in this process in prostate cancer cells. 
 ZEB1 and TCF4 reciprocally modulate their transcriptional activities to regulate Wnt target gene expression. The canonical Wnt pathway (TCF4/β-catenin) has important roles during normal differentiation and in disease. Some Wnt functions depend on signaling gradients requiring the pathway to be tightly regulated. A key Wnt target is the transcription factor ZEB1 whose expression by cancer cells promotes tumor   invasiv  eness by repressing the expression of epithelial specification markers and activating mesenchymal genes, including a number of Wnt targets such as LAMC2 and uPA. The ability of ZEB1 to activate/repress its target genes depends on its recruitment of corepressors (CtBP, BRG1) or coactivators (p300) although conditions under which ZEB1 binds these cofactors are not elucidated. Here, we show that TCF4 and ZEB1 reciprocally modulate each other's transcriptional activity: ZEB1 enhances TCF4/β-catenin-mediated transcription and, in turn, Wnt signaling switches ZEB1 from a repressor into an activator. In colorectal cancer (CRC) cells with active Wnt signaling, ZEB1 enhances transcriptional activation of LAMC2 and uPA by TCF4/β-catenin. However, in CRC cells with inactive Wnt, ZEB1 represses both genes. Reciprocal modulation of ZEB1 and TCF4 activities involves their binding to DNA and mutual interaction. Wnt signaling turns ZEB1 into an activator by replacing binding of CtBP/BRG1 in favor of p300. Using a mouse model of Wnt-induced intestinal tumorigenesis, we found that downregulation of ZEB1 reduces the expression of LAMC2 in vivo. These results identify a mechanism through which Wnt and ZEB1 transcriptional activities are modulated, offering new approaches in cancer therapy. 
 Molecular regulation of ovarian cancer cell invasion. The molecular mechanism underlying ovarian cancer   invasive  ness and metastasis remains unclear. Since significant downregulation in microRNA 200 (miRNA200) family (miR200a, miR200b, and miR200c) has been reported in the   invasive   ovarian cancer cells, here, we used two human ovarian cancer cell lines, OVCAR3 and SKOV3, to study the molecular basis of miR200, matrix metalloproteinase 3 (MMP3) activation, and cancer   invasive  ness. We found that overexpression of either miR200 family member in OVCAR3 or SKOV3 cells significantly inhibited production and secretion of MMP3 and cancer   invasive  ness. Moreover, forced MMP3 expression abolished miR200-induced inhibition of ovarian cancer cell   invasive  ness, suggesting that miR200 family inhibited ovarian cell   invasive  ness via downregulating MMP3. Furthermore, ZEB1, a major target of miR200, was inhibited by miR200 overexpression. Forced ZEB1 expression abolished miR200-induced inhibition of ovarian cancer cell   invasive  ness, suggesting that ZEB1 is a direct target of miR200 for inhibiting ovarian cell   invasive  ness. Finally, phosphorylated SMAD3 (pSMAD3), a major partner of ZEB1, was efficiently inhibited by miR200, which could be restored by forced expression of ZEB1, but not by forced expression of MMP3, suggesting that ZEB1/pSMAD3 is signaling cascade upstream of MMP3 in this model. Taken together, our data suggest that miR200 family inhibited ovarian cancer cell   invasive  ness and metastasis by downregulating MMP3, possibly through ZEB1/pSMAD3. 
 ZEB1 expression in type I vs type II endometrial cancers: a marker of aggressive disease. Zinc-finger E-box-binding homeobox 1 (ZEB1) is a transcription factor containing two clusters of Kruppel-type zinc-fingers, by which it binds E-box-like sequences on target DNAs. A role for ZEB1 in tumor progression, specifically, epithelial to mesenchymal transitions, has recently been revealed. ZEB1 acts as a master repressor of E-cadherin and other epithelial markers. We previously demonstrated that ZEB1 is confined to the stromal compartment in normal endometrium and low-grade endometrial cancers. Here, we quantify ZEB1 protein expression in endometrial samples from 88 patients and confirm that it is expressed at significantly higher levels in the tumor-associated stroma of low-grade endometrioid adenocarcinomas (type I endometrial cancers) compared to hyperplastic or normal endometrium. In addition, as we previously reported, ZEB1 is aberrantly expressed in the epithelial-derived tumor cells of highly aggressive endometrial cancers, such as FIGO grade 3 endometrioid adenocarcinomas, uterine serous carcinomas, and malignant mixed Müllerian tumors (classified as type II endometrial cancers). We now demonstrate, in both human endometrial cancer specimens and cell lines, that when ZEB1 is inappropriately expressed in epithelial-derived tumor cells, E-cadherin expression is repressed, and that this inverse relationship correlates with increased migratory and   invasiv  e potential. Forced expression of ZEB1 in the nonmigratory, low-grade, relatively differentiated Ishikawa cell line renders them migratory. Conversely, reduction of ZEB1 in a highly migratory and aggressive type II cell line, Hec50co, results in reduced migratory capacity. Thus, ZEB1 may be a biomarker of aggressive endometrial cancers at high risk of recurrence. It may help identify women who would most benefit from chemotherapy. Furthermore, if expression of ZEB1 in type II endometrial cancers could be reversed, it might be exploited as therapy for these highly aggressive tumors. 
 ZEB1 coordinately regulates laminin-332 and {beta}4 integrin expression altering the   invasive   phenotype of prostate cancer cells. Metastasis involves the invasion of cancer cells across both the extracellular matrix and cellular barriers, and an evolving theme is that epithelial-to-mesenchymal transition (EMT) may mediate   invasive   cellular behavior. Previously, we isolated and analyzed a subpopulation of PC-3 prostate cancer cells, TEM4-18, and found that these cells both invaded an endothelial barrier more efficiently and exhibited enhanced metastatic colonization in vivo. Transendothelial migration of these cells depended on expression of ZEB1, a known regulator of EMT. Surprisingly, these cells were much less   invasive   than parental PC-3 cells in assays that involve matrix barriers. Here, we report that TEM4-18 cells express significantly reduced levels of two subunits of laminin-332 (β3 and γ2) and that exogenous laminin-332, or co-culture with laminin-332-expressing cells, rescues the in vitro invasion phenotype in these cells. Stable knockdown of ZEB1 in prostate cancer cells up-regulated LAMC2 and ITGB4 mRNA and protein and resulted in a concomitant increase in Transwell migration. Using chromatin immunoprecipitation (ChIP), we show that ZEB1 directly interacts with the promoters of LAMC2 and ITGB4. These results provide a novel molecular basis for reduced laminin-332 observed in clinical prostate cancer specimens and demonstrate a context-dependent role for EMT i  n invasi  ve cellular behavior. 
 Discoidin domain receptor 1 is a novel transcriptional target of ZEB1 in breast epithelial cells undergoing H-Ras-induced epithelial to mesenchymal transition. The epithelial-to-mesenchymal transition (EMT) process allows carcinoma cells to dissociate from the primary tumor thereby facilitating tumor cell invasion and metastasis. Ras-dependent hyperactive signaling is commonly associated with tumorigenesis, invasion, EMT, and metastasis. However, the downstream effectors by which Ras regulates EMT remain ill defined. In this study, we show that the H-Ras pathway leads to mesenchymal-like phenotypic changes in human breast epithelial cells by controlling the ZEB1/microRNA-200c axis. Moreover, H-Ras suppresses the expression of the discoidin domain receptor 1 (DDR1), a collagen receptor tyrosine kinase, via ZEB1, thus identifying ZEB1 as a novel transcriptional repressor of DDR1. Mutation studies on the putative promoter of the DDR1 gene revealed that bipartite Z- and E-box elements play a key role in transcriptional repression of DDR1 in Hs578T and MDA-MB-231 breast carcinoma cell lines by ZEB1. Furthermore, we found an inverse correlation between ZEB1 and DDR1 expression in various cancer cell lines and in human breast carcinoma tissues. Consistently, overexpression of DDR1 reduced the   invasive   phenotype of mesenchymal-like triple-negative breast cancer cells in 3D cultures and in vivo. Thus, ZEB1's role in maintenance of EMT in breast carcinoma cells is mediated in part by its ability to suppress DDR1 expression and consequently contribute to the activation of the   invasive   phenotype. Taken together, our results unveil a novel H-Ras/ZEB1/DDR1 network that contributes to breast cancer progression in triple-negative breast cancers. 
 ZEB1 limits adenoviral infectability by transcriptionally repressing the coxsackie virus and adenovirus receptor. We have previously reported that RAS-MEK (Cancer Res. 2003 May 1;63(9):2088-95) and TGF-β (Cancer Res. 2006 Feb 1;66(3):1648-57) signaling negatively regulate coxsackie virus and adenovirus receptor (CAR) cell-surface expression and adenovirus uptake. In the case of TGF-β, down-regulation of CAR occurred in context of epithelial-to-mesenchymal transition (EMT), a process associated with transcriptional repression of E-cadherin by, for instance, the E2 box-binding factors Snail, Slug, SIP1 or ZEB1. While EMT is crucial in embryonic development, it has been proposed to contribute to the formation o  f invasi  ve and metastatic carcinomas by reducing cell-cell contacts and increasing cell migration.Here, we show that ZEB1 represses CAR expression in both PANC-1 (pancreatic) and MDA-MB-231 (breast) human cancer cells. We demonstrate that ZEB1 physically associates with at least one of two closely spaced and conserved E2 boxes within the minimal CAR promoter here defined as genomic region -291 to -1 relative to the translational start ATG. In agreement with ZEB1's established role as a negative regulator of the epithelial phenotype, silencing its expression in MDA-MB-231 cells induced a partial Mesenchymal-to-Epithelial Transition (MET) characterized by increased levels of E-cadherin and CAR, and decreased expression of fibronectin. Conversely, knockdown of ZEB1 in PANC-1 cells antagonized both the TGF-β-induced down-regulation of E-cadherin and CAR and the reduction of adenovirus uptake. Interestingly, even though ZEB1 clearly contributes to the TGF-β-induced mesenchymal phenotype of PANC-1 cells, TGF-β did not seem to affect ZEB1's protein levels or subcellular localization. These findings suggest that TGF-β may inhibit CAR expression by regulating factor(s) that cooperate with ZEB1 to repress the CAR promoter, rather than by regulating ZEB1 expression levels. In addition to the negative E2 box-mediated regulation the minimal CAR promoter is positively regulated through conserved ETS and CRE elements.This report provides evidence that inhibition of ZEB1 may improve adenovirus uptake of cancer cells that have undergone EMT and for which ZEB1 is necessary to maintain the mesenchymal phenotype. Targeting of ZEB1 may reverse some aspects of EMT including the down-regulation of CAR. 
 E-cadherin, beta-catenin, and ZEB1 in malignant progression of cancer. The embryonic program 'epithelial-mesenchymal transition' (EMT) is activated during tumor invasion in disseminating cancer cells. Characteristic to these cells is a loss of E-cadherin expression, which can be mediated by EMT-inducing transcriptional repressors, e.g. ZEB1. Consequences of a loss of E-cadherin are an impairment of cell-cell adhesion, which allows detachment of cells, and nuclear localization of beta-catenin. In addition to an accumulation of cancer stem cells, nuclear beta-catenin induces a gene expression pattern favoring tumor invasion, and mounting evidence indicates multiple reciprocal interactions of E-cadherin and beta-catenin with EMT-inducing transcriptional repressors to stabilize an   invasive   mesenchymal phenotype of epithelial tumor cells. 
 HOXD9 promotes epithelial-mesenchymal transition and cancer metastasis by ZEB1 regulation in hepatocellular carcinoma. Hepatocellular carcinoma (HCC) is a common malignant tumor that severely threatens human health. The poor prognosis of HCC is mainly attributed to intrahepatic and extrahepatic metastases. HOXD9 proteins belong to a superfamily that regulates the development and control of many cellular processes, including proliferation, apoptosis, cell shape, and cell migration. HOXD9 can also function as an oncogene in several cancer cells. However, its biological function in human HCC requires further investigation. In this study, HOXD9 exhibited high expression in   invasive   HCC cells. HOXD9 overexpression can significantly enhance HCC cell migration, invasion, and metastasis, whereas silencing HOXD9 inhibits these processes. HOXD9 also promotes the epithelial-mesenchymal transition (EMT) of HCC cells. Microarray analysis suggests that ZEB1 can function as a downstream factor of HOXD9. HOXD9 can interact with the promoter region of ZEB1 and promotes ZEB1 expression. ZEB1 knockdown inhibits HOXD9-induced migration and invasion, as well as EMT in HCC cells. This study helps elucidates the oncogenic functions of HOXD9 in HCC. 
 ASPP2 controls epithelial plasticity and inhibits metastasis through β-catenin-dependent regulation of ZEB1. Epithelial to mesenchymal transition (EMT), and the reverse mesenchymal to epithelial transition (MET), are known examples of epithelial plasticity that are important in kidney development and cancer metastasis. Here we identify ASPP2, a haploinsufficient tumour suppressor, p53 activator and PAR3 binding partner, as a molecular switch of MET and EMT. ASPP2 contributes to MET in mouse kidney in vivo. Mechanistically, ASPP2 induces MET through its PAR3-binding amino-terminus, independently of p53 binding. ASPP2 prevents β-catenin from transactivating ZEB1, directly by forming an ASPP2-β-catenin-E-cadherin ternary complex and indirectly by inhibiting β-catenin's N-terminal phosphorylation to stabilize the β-catenin-E-cadherin complex. ASPP2 limits the   pro-inv  asive property of oncogenic RAS and inhibits tumour metastasis in vivo. Reduced ASPP2 expression results in EMT, and is associated with poor survival in hepatocellular carcinoma and breast cancer patients. Hence, ASPP2 is a key regulator of epithelial plasticity that connects cell polarity to the suppression of WNT signalling, EMT and tumour metastasis. 
 Wide expression of ZEB1 in sarcomatous component of spindle cell carcinoma of the esophagus. The pathogenesis of sarcomatous component in spindle cell carcinoma (SpCC) of the esophagus is unclear. To investigate the involvement of epithelial-mesenchymal transition (EMT) in sarcomatous differentiation, we performed immunohistochemistry for Slug, Twist, ZEB1, and ZEB2, transcription factors associated with EMT and E-cadherin, in 14 cases of SpCC of the esophagus. In order to verify the neoplastic nature of sarcomatous components, TP53 mutation status and protein expression were examined in each case. Nuclear ZEB1 expression was extensive in the sarcomatous component, greater than   invasive   front of carcinoma components (P < 0.0001). Membranous E-cadherin expression was mostly lost in sarcomatous cells in all cases (P < 0.0001). The p53 expression pattern was almost concordant between the two areas in all cases. TP53 mutation analysis revealed that seven cases harbored identical mutations in both components. One case had mutations only in the sarcomatous component. It is noteworthy that none of them harbored mutation in exon 5, unlike conventional esophageal squamous cell carcinoma. These findings show that ZEB1 are widely expressed in the sarcomatous area of SpCC of the esophagus, suggesting the involvement of EMT. The avoidance of exon 5 in terms of TP53 mutation may also be a feature of the tumor. 
 ABCG2/BCRP gene expression is related to epithelial-mesenchymal transition inducer genes in a papillary thyroid carcinoma cell line (TPC-1). Tumor malignancy is associated with the epithelial-mesenchymal transition (EMT) process and resistance to chemotherapy. However, little is known about the relationship between the EMT and the multidrug-resistance gene in thyroid tumor progression. We investigated whether the expression of the ABCG2/BCRP gene is associated with ZEB1 and other EMT inducer genes involved in tumor dedifferentiation. We established a subpopulation of cells that express the ABCG2/BCRP gene derived from the thyroid papillary carcinoma cell line (TPC-1), the so-called TPC-1 MITO-resistant subline. The most relevant findings in these TPC-1 selected cells were a statistically significant upregulation of ZEB1 and TWIST1 (35- and 15-fold change respectively), no changes in the relative expression of vimentin and SNAIL1, and no expression of E-cadherin. The TPC-1 MITO-resistant subline displayed a faster migration and greater   invasive   ability than parental cells in correlation with a significant upregulation of the survivin (BIRC5) gene (twofold change, P<0.05). The knockdown of ZEB1 promoted nuclear re-expression of E-cadherin, reduced expression of vimentin, N-cadherin, and BIRC5 genes, and reduced cell migration (P<0.05). Analysis of human thyroid carcinoma showed a slight overexpression of the ABCG2/BCRP at stages I and II (P<0.01), and a higher overexpression at stages III and IV (P<0.01). SNAIL1, TWIST1, and ZEB1 genes showed higher expression at stages III and IV than at stages I and II. E- and N-cadherin genes were upregulated at stages I and II of the disease (ninefold and tenfold change, respectively, P<0.01) but downregulated at stages III and IV (fourfold lower, P<0.01). These results could be a promising starting point for further study of the role of the ABCG2/BCRP gene in the progression of thyroid tumor. 
 Dual roles of the transcription factor grainyhead-like 2 (GRHL2) in breast cancer. Using a retrovirus-mediated cDNA expression cloning approach, we identified the grainyhead-like 2 (GRHL2) transcription factor as novel protooncogene. Overexpression of GRHL2 in NIH3T3 cells induced striking morphological changes, an increase in cell proliferation, anchorage-independent growth, and tumor growth in vivo. By combining a microarray analysis and a phylogenetic footprinting analysis with various biochemical assays, we identified the epidermal growth factor receptor family member Erbb3 as a novel GRHL2 target gene. In breast cancer cell lines, shRNA-mediated knockdown of GRHL2 expression or functional inactivation of GRHL2 using dominant negative GRHL2 proteins induces down-regulation of ERBB3 gene expression, a striking reduction in cell proliferation, and morphological and phenotypical alterations characteristic of an epithelial-to-mesenchymal transition (EMT), thus implying contradictory roles of GRHL2 in breast carcinogenesis. Interestingly, we could further demonstrate that expression of GRHL2 is directly suppressed by the transcription factor zinc finger enhancer-binding protein 1 (ZEB1), which in turn is a direct target for repression by GRHL2, suggesting that the EMT transcription factors GRHL2 and ZEB1 form a double negative regulatory feedback loop in breast cancer cells. Finally, a comprehensive immunohistochemical analysis of GRHL2 expression in primary breast cancers showed loss of GRHL2 expression at the   invasive   front of primary tumors. A pathophysiological relevance of GRHL2 in breast cancer metastasis is further demonstrated by our finding of a statistically significant association between loss of GRHL2 expression in primary breast cancers and lymph node metastasis. We thus demonstrate a crucial role of GRHL2 in breast carcinogenesis. 
 Down-regulation of TCF8 is involved in the leukemogenesis of adult T-cell leukemia/lymphoma. Adult T-cell leukemia/lymphoma (ATLL) is caused by latent human T-lymphotropic virus-1 (HTLV-1) infection. To clarify the molecular mechanism underlying leukemogenesis after viral infection, we precisely mapped 605 chromosomal breakpoints in 61 ATLL cases by spectral karyotyping and identified frequent chromosomal breakpoints in 10p11, 14q11, and 14q32. Single nucleotide polymorphism (SNP) array-comparative genomic hybridization (CGH), genetic, and expression analyses of the genes mapped within a common breakpoint cluster region in 10p11.2 revealed that in ATLL cells, transcription factor 8 (TCF8) was frequently disrupted by several mechanisms, including mainly epigenetic dysregulation. TCF8 mutant mice frequently developed   invasive   CD4(+) T-cell lymphomas in the thymus or in ascitic fluid in vivo. Down-regulation of TCF8 expression in ATLL cells in vitro was associated with resistance to transforming growth factor beta1 (TGF-beta1), a well-known characteristic of ATLL cells, suggesting that escape from TGF-beta1-mediated growth inhibition is important in the pathogenesis of ATLL. These findings indicate that TCF8 has a tumor suppressor role in ATLL. 
 The ZEB1 pathway links glioblastoma initiation, invasion and chemoresistance. Glioblastoma remains one of the most lethal types of cancer, and is the most common brain tumour in adults. In particular, tumour recurrence after surgical resection and radiation invariably occurs regardless of aggressive chemotherapy. Here, we provide evidence that the transcription factor ZEB1 (zinc finger E-box binding homeobox 1) exerts simultaneous influence over invasion, chemoresistance and tumourigenesis in glioblastoma. ZEB1 is preferentially expressed in   invasive   glioblastoma cells, where the ZEB1-miR-200 feedback loop interconnects these processes through the downstream effectors ROBO1, c-MYB and MGMT. Moreover, ZEB1 expression in glioblastoma patients is predictive of shorter survival and poor Temozolomide response. Our findings indicate that this regulator of epithelial-mesenchymal transition orchestrates key features of cancer stem cells in malignant glioma and identify ROBO1, OLIG2, CD133 and MGMT as novel targets of the ZEB1 pathway. Thus, ZEB1 is an important candidate molecule for glioblastoma recurrence, a marker of   invasive   tumour cells and a potential therapeutic target, along with its downstream effectors. 
 SET8 induces epithelial‑mesenchymal transition and enhances prostate cancer cell metastasis by cooperating with ZEB1. Mounting evidence suggested that histone H4K20-specific methyltransferase SET8 is required to maintain the malignant phenotype of various cancer types; however, the role of SET8 in mediating tumor metastasis in prostate cancer (PCa) has remained elusive. The present study demonstrated that small interfering RNA-mediated knockdown of SET8 inhibited th  e invasi  ve potential of the PCa cell line PC-3 in vitro. Knockdown of SET8 reduced sphere formation, downregulated E-cadherin and α-catenin, and upregulated N-cadherin and vimentin expression in CaP cells, while upregulation of SET8 expression with a recombinant plasmid had the opposite effect. Furthermore, SET8 was shown to be physically associated with the epithelial-mesenchymal transition (EMT) inducer zinc finger E-box-binding homeobox 1 (ZEB1) in PCa cell lines. Chromatin immunoprecipitation suggested that SET8 binds to the promoter of cell adhesion molecule E-cadherin and vimentin. Luciferase reporter assays suggested that E-cadherin and vimentin are direct targets of SET8; furthermore, loss- and gain-of function studies of SET8 and ZEB1 indicated that suppression of downstream E-cadherin and activation of vimentin are important mechanisms by which SET8 and ZEB1 cooperatively trigger metastasis. Furthermore, SET8-induced methylated H4K20 was indicated to exert a dual function in ZEB1-regulated gene expression. In conclusion, the present study revealed that SET8 and ZEB1 are functionally interdependent in promoting the EMT and enhancing t  he invas  ive potential of PCa cells in vitro. 
 Transcription factor 8 activates R-Ras to regulate angiogenesis. We have recently reported that transcription factor 8 (TCF8) negatively regulates pathological angiogenesis by regulating endothelial   invasive  ness by acting as a transcriptional attenuator of matrix metalloproteinase 1. TCF8 also modulates cell-matrix and cell-cell adhesion; however molecular mechanism of this TCF8 function remains obscure. Here, we provide evidence that TCF8 activates R-Ras, another class of angiogenic regulator, to suppress angiogenesis by a mechanism other than a transcriptional attenuator. Tube formation by human umbilical vein endothelial cells (HUVECs) facilitated by TCF8 suppression was significantly inhibited by the expression of constitutive active mutant of R-Ras. When we examined the mRNA expression levels of R-Ras regulators, no significant changes were observed to explain the R-Ras activation by TCF8. Interestingly, we found that TCF8 bound to CalDAG-GEFIII, an R-Ras activator, in the cytosol, indicating that TCF8 emanates signaling for R-Ras activation from cytosol to regulate angiogenesis negatively. 
 IL-32θ inhibits stemness and epithelial-mesenchymal transition of cancer stem cells via the STAT3 pathway in colon cancer. Interleukin (IL)-32 is a well-known cytokine associated with inflammation, virus infections and cancer. IL-32θ is a newly identified isoform of IL-32, whose function has yet to be elucidated. In this study, we investigated IL-32θ function in colon cancer stem cells. Using samples from colon cancer patients, we found that the expression of IL-32θ mRNAs was significantly suppressed in tumor regions. We investigated the effects of IL-32θ on colon cancer. Ectopic expression of IL-32θ attenuated invasion, migration in vitro and in vivo tumorigenicity of colon cancer cells. IL-32θ inhibited epithelial-mesenchymal transition (EMT), resulting in the suppression of their migrato  ry and i  nvasive capabilities of HT29 colon cancer cells. In addition, IL-32θ altered various properties of CSCs, including sphere formation and expression of stemness related genes. IL-32θ directly bound to STAT3 and inhibited its nuclear translocation, leading to inhibited transcription of downstream factors, including Bmi1 and ZEB1. We showed that IL-32θ inhibited the STAT3-ZEB1 pathway and consequently inhibited key factors of stemness and EMT. Taken together, our findings reveal that IL-32θ can be a tumor suppressor, indicating that IL-32θ could possibly be used in therapies for colon cancer. 
 MiR-200c suppresses TGF-β signaling and counteracts trastuzumab resistance and metastasis by targeting ZNF217 and ZEB1 in breast cancer. Resistance to trastuzumab and concomitantly distal metastasis are leading causes of mortality in HER2-positive breast cancers, the molecular basis of which remains largely unknown. Here, we generated trastuzumab-resistant breast cancer cells with increased tumorigenicity and   invasiv  eness compared with parental cells, and observed robust epithelial-mesenchymal transition (EMT) and consistently elevated TGF-β signaling in these cells. MiR-200c, which was the most significantly downregulated miRNA in trastuzumab-resistant cells, restored trastuzumab sensitivity and suppressed invasion of breast cancer cells by concurrently targeting ZNF217, a transcriptional activator of TGF-β, and ZEB1, a known mediator of TGF-β signaling. Given the reported backward inhibition of miR-200c by ZEB1, ZNF217 also exerts a feedback suppression of miR-200c via TGF-β/ZEB1 signaling. Restoration of miR-200c, silencing of ZEB1 or ZNF217 or blockade of TGF-β signaling increased trastuzumab sensitivity and suppr  essed in  vasiveness of breast cancer cells. Therefore, our study unraveled nested regulatory circuits of miR-200c/ZEB1 and miR-200c/ZNF217/TGF-β/ZEB1 in synergistically promoting trastuzumab resistance and metastasis of breast cancer cells. These findings provide novel insights into the common role of EMT and related molecular machinery in mediating the malignant phenotypes of breast cancers. 
 EMT-associated factors promote   invasive   properties of uveal melanoma cells. Transcription factors regulating the epithelial-to-mesenchymal transition (EMT) program contribute to carcinogenesis and metastasis in many tumors, including cutaneous melanoma. However, little is known about the role of EMT factors in the growth and metastatic dissemination of uveal melanoma cells. Here, we analyzed the expression and functions of the EMT factors ZEB1, Twist1, and Snail1 in uveal melanoma cell lines and primary tumors.ZEB1, Twist1, and Snail1 mRNA levels were measured using qPCR in five uveal melanoma cell lines and in 30 primary tumors. Gene expression was used to determine class 1 and class 2 signatures in the primary tumors. Short hairpin RNA was used to downregulate the expressions of the EMT factors; then, growth and transwell invasion assays were performed.ZEB1, Twist1, and Snail1 were expressed in all five uveal melanoma lines, with ZEB1 having the highest protein levels. ZEB1 mRNA was significantly elevated in highly metastatic class 2 primary tumors for which survival data were not available, whereas a high gene expression of Twist1 was associated with a worse prognosis in a separate tumor cohort analyzed by expression profiling. The genetic downregulation of ZEB1 in OCM1, OMM1, and 92.1 resulted in a more than 50% reduction in invasion, but only suppressed growth in OMM1 cells. Suppression of Twist1 in Mel290 and OMM1 reduced growth and invasion by more than 50%. The downregulation of Snail1 in the 92.1 cell line reduced invasion by 50%, but did not interfere with growth.The downregulation of ZEB1, Twist1, and Snail1 reduces the   invasive   properties of uveal melanoma cells, and the elevated mRNA levels of ZEB1 and Twist1 are associated with a more aggressive clinical phenotype in uveal melanoma samples. Therefore, these factors could represent new therapeutic targets in patients with ocular melanoma. 
 Zeb1 and Snail1 engage miR-200f transcriptional and epigenetic regulation during EMT. Cell plasticity is emerging as a key regulator of tumor progression and metastasis. During carcinoma dissemination epithelial cells undergo epithelial to mesenchymal transition (EMT) processes characterized by the acquisition of migratory/  invasive   properties, while the reverse, mesenchymal to epithelial transition (MET) process, is also essential for metastasis outgrowth. Different transcription factors, called EMT-TFs, including Snail, bHLH and Zeb families are drivers of the EMT branch of epithelial plasticity, and can be post-transcriptionally downregulated by several miRNAs, as the miR-200 family. The specific or redundant role of different EMT-TFs and their functional interrelations are not fully understood. To study the interplay between different EMT-TFs, comprehensive gain and loss-of-function studies of Snail1, Snail2 and/or Zeb1 factors were performed in the prototypical MDCK cell model system. We here describe that Snail1 and Zeb1 are mutually required for EMT induction while continuous Snail1 and Snail2 expression, but not Zeb1, is needed for maintenance of the mesenchymal phenotype in MDCK cells. In this model system, EMT is coordinated by Snail1 and Zeb1 through transcriptional and epigenetic downregulation of the miR-200 family. Interestingly, Snail1 is involved in epigenetic CpG DNA methylation of the miR-200 loci, essential to maintain the mesenchymal phenotype. The present results thus define a novel functional interplay between Snail and Zeb EMT-TFs in miR-200 family regulation providing a molecular link to their previous involvement in the generation of EMT process in vivo. 
  GeneRIFs  
 Results of heterogeneous expression of ZEB1 in the   invasive   front (IF) and tumor center (TC) showed the gene expression patterns for the IF different from those for the TC. Metastatic tumors exhibited higher expression of ZEB1 at the IF than non-metastatic tumors indicating that ZEB1 was involved in metastasis of colorectal neoplasm. 
 SET8 and ZEB1 are functionally interdependent in promoting the epithelial-mesenchymal transition and enhancing the   invasive   potential of prostate cancer cells in vitro. 
 TKS5 and MYLK represent two mediators of   invasive   behavior of cancer cells that are regulated by the ZEB1/miR-200 feedback loop 
 Results support the role for ZEB1 in   invasive   and high-grade brain tumors and suggest its key role in promoting invasion in the hypoxic tumor core as well as in the periphery 
 The downregulation of ZEB1, Twist1, and Snail1 reduces the   invasive   properties of uveal melanoma cells, and the elevated mRNA levels of ZEB1 and Twist1 are associated with a more aggressive clinical phenotype in uveal melanoma samples. 
 Results provides evidence that TCF4 and ZEB1 modulate each other's transcriptional activity in the regulation of tumor pro-  invasive   Wnt target genes. 
 Down-regulation of ZEB1 expression may protect the   invasive   front of the tumors from converting to a mesenchymal phenotype by reducing the proliferation and motility of cervical cancer cells. 
 Overexpression of zinc finger E-box binding homeobox factor 1 promotes tumor   invasive  ness and confers unfavorable prognosis in esophageal squamous cell carcinoma. 
 Down-regulation results in an inhibitory effect on the   invasive   and metastatic potential of epithelial ovarian cancer in vitro and in vivo by blocking the epithelial-mesenchymal transition process 
   Invasive  ness was significantly correlated to the increased Zeb1 in tumour cells, as well as to increased ASMA, S100A4, and PDGFRbeta in stromal cells. 
 ZEB1 is located on the   invasive   fronts exhibiting downregulation of the miR-200 family.TGF-beta induced ZEB1, reinforcing its role in epithelial-mesenchymal transition. ZEB1 knockdown prevented TGF-beta from inducing spindle-shaped cells & suppressing CDH1 
 RB inactivation contributes to tumor progression not only through loss of cell cycle control but also through up-regulation of ZEB expression and induction of an   invasive   phenotype. 
 beta-catenin/TCF4 complex induces the epithelial-to-mesenchymal transition (EMT)-activator ZEB1 to regulate tumor   invasive  ness. 
 Collectively, our studies demonstrate frequent expression of nuclear Snail1, but not nuclear ZEB1, in   invasive  , triple-negative breast cancers as well as in intraductal carcinomas. 
 down-regulation of ZEB1 seems to play a role in tumor spread, metastases, and poor survival in   invasive   ductal carcinomas of the breast. 
 ZEB1 coordinately regulates laminin-332 and {beta}4 integrin expression altering the   invasive   phenotype of prostate cancer cells 
 These findings suggest that Zeb1 represses T-cadherin expression and thus increases the   invasive   activity of gallbladder cancer. 
  [Mm]etastasize  PubMed  
 Vimentin, zeb1 and Sip1 are up-regulated in triple-negative and basal-like breast cancers: association with an aggressive tumour phenotype. In epithelial-to-mesenchymal transition (EMT) epithelial cancer cells achieve mesenchymal features, essentially helping them to   metastasize  . There is some evidence that EMT could be increased in triple-negative (TNBC) or basal-like breast cancers, although more precise mechanisms considering e.g. EMT-regulating transcription factors are largely unknown. We assessed immunohistochemically vimentin (separately in in situ areas and in invasive cells) as an indicator of EMT, and also EMT-regulating transcription factors zeb1 (separately in stroma and tumour) and Sip1 (in nuclei and cytoplasm) in histological samples of 231 women with local or locally advanced invasive breast cancer. 51.1 % of patients had TNBC and 48.9 % oestrogen and progesterone receptor-positive and HER2 negative breast cancer. Basal-like breast cancers were defined as TNBC that also expressed epidermal growth factor receptor EGFR and/or cytokeratin 5/6. Vimentin expression in invasive cells was higher in TNBCs (p = 9 × 10(-12)) compared to non-TNBC tumours. Vimentin (p = 2 × 10(-6)), nuclear Sip1 (p = 0.035) and zeb1 in stroma (p = 0.013) were overexpressed in basal-like cancers compared to non-basal-like TNBCs. In non-TNBC group findings between studied markers and clinicopathological factors were rare. However, in TNBC cases, vimentin expression in invasive cells associated with poor differentiation (p = 0.00007), zeb1 expression in cancer cells with higher grade (p = 0.002), vascular invasion (p = 0.036) and larger T-class (p = 0.027), whereas stromal zeb1 associated with lymphatic vessel invasion (p = 0.036) and vascular invasion (p = 0.039). High nuclear Sip1 expression was prognostic for poor disease-free survival (p = 0.002) in the whole cohort. The current results emphasize the increased role of EMT in TNBC and especially in basal-like breast cancers. These observations also support the role of studied parameters in tumour progression. 
 Vimentin, zeb1 and Sip1 are up-regulated in triple-negative and basal-like breast cancers: association with an aggressive tumour phenotype. In epithelial-to-mesenchymal transition (EMT) epithelial cancer cells achieve mesenchymal features, essentially helping them to   metastasize  . There is some evidence that EMT could be increased in triple-negative (TNBC) or basal-like breast cancers, although more precise mechanisms considering e.g. EMT-regulating transcription factors are largely unknown. We assessed immunohistochemically vimentin (separately in in situ areas and in invasive cells) as an indicator of EMT, and also EMT-regulating transcription factors zeb1 (separately in stroma and tumour) and Sip1 (in nuclei and cytoplasm) in histological samples of 231 women with local or locally advanced invasive breast cancer. 51.1 % of patients had TNBC and 48.9 % oestrogen and progesterone receptor-positive and HER2 negative breast cancer. Basal-like breast cancers were defined as TNBC that also expressed epidermal growth factor receptor EGFR and/or cytokeratin 5/6. Vimentin expression in invasive cells was higher in TNBCs (p = 9 × 10(-12)) compared to non-TNBC tumours. Vimentin (p = 2 × 10(-6)), nuclear Sip1 (p = 0.035) and zeb1 in stroma (p = 0.013) were overexpressed in basal-like cancers compared to non-basal-like TNBCs. In non-TNBC group findings between studied markers and clinicopathological factors were rare. However, in TNBC cases, vimentin expression in invasive cells associated with poor differentiation (p = 0.00007), zeb1 expression in cancer cells with higher grade (p = 0.002), vascular invasion (p = 0.036) and larger T-class (p = 0.027), whereas stromal zeb1 associated with lymphatic vessel invasion (p = 0.036) and vascular invasion (p = 0.039). High nuclear Sip1 expression was prognostic for poor disease-free survival (p = 0.002) in the whole cohort. The current results emphasize the increased role of EMT in TNBC and especially in basal-like breast cancers. These observations also support the role of studied parameters in tumour progression. 
  [Mm]etastasis  PubMed  
 A double-negative feedback loop between ZEB1-SIP1 and the microRNA-200 family regulates epithelial-mesenchymal transition. Epithelial to mesenchymal transition occurs during embryologic development to allow tissue remodeling and is proposed to be a key step in the   metastasis   of epithelial-derived tumors. The miR-200 family of microRNAs plays a major role in specifying the epithelial phenotype by preventing expression of the transcription repressors, ZEB1/deltaEF1 and SIP1/ZEB2. We show here that miR-200a, miR-200b, and the related miR-429 are all encoded on a 7.5-kb polycistronic primary miRNA (pri-miR) transcript. We show that the promoter for the pri-miR is located within a 300-bp segment located 4 kb upstream of miR-200b. This promoter region is sufficient to confer expression in epithelial cells and is repressed in mesenchymal cells by ZEB1 and SIP1 through their binding to a conserved pair of ZEB-type E-box elements located proximal to the transcription start site. These findings establish a double-negative feedback loop controlling ZEB1-SIP1 and miR-200 family expression that regulates cellular phenotype and has direct relevance to the role of these factors in tumor progression. 
 The miR-200 family inhibits epithelial-mesenchymal transition and cancer cell migration by direct targeting of E-cadherin transcriptional repressors ZEB1 and ZEB2. MicroRNAs are small non-coding RNA molecules that can regulate gene expression by interacting with multiple mRNAs and inducing either translation suppression or degradation of mRNA. Recently, several miRNAs were identified as either promoters or suppressors of   metastasis  . However, it is unclear in which step(s) of the multistep metastatic cascade these miRNAs play a defined functional role. To study the functional importance of miRNAs in epithelial-mesenchymal transition (EMT), a process thought to initiate   metastasis   by enhancing the motility of tumor cells, we used a well established in vitro EMT assay: transforming growth factor-beta-induced EMT in NMuMG murine mammary epithelial cells. We found that members of the miR-200 family, organized as two clusters in the genome, were repressed during EMT. Overexpression of each miRNA individually or as clusters in NMuMG cells hindered EMT by enhancing E-cadherin expression through direct targeting of ZEB1 and ZEB2, which encode transcriptional repressors of E-cadherin. In the 4TO7 mouse carcinoma cell line, which expresses low levels of endogenous E-cadherin and displays a mesenchymal phenotype, ectopic expression of the miR-200 family miRNAs significantly increased E-cadherin expression and altered cell morphology to an epithelial phenotype. Furthermore, ectopic expression of each miR-200 miRNA cluster significantly reduced the in vitro motility of 4TO7 cells in migration assays. These results suggested that loss of expression of the miR-200 family members may play a critical role in the repression of E-cadherin by ZEB1 and ZEB2 during EMT, thereby enhancing migration and invasion during cancer progression. 
 Fra-1/AP-1 induces EMT in mammary epithelial cells by modulating Zeb1/2 and TGFβ expression. Epithelial-to-mesenchymal transition (EMT) is essential for embryonic morphogenesis and wound healing and critical for tumour cell invasion and dissemination. The AP-1 transcription factor Fra-1 has been implicated in tumorigenesis and in tumour-associated EMT in human breast cancer. We observed a significant inverse correlation between Fra-1 mRNA expression and distant  -metastasi  s-free survival in a large cohort of breast cancer patients derived from multiple array data sets. This unique correlation among Fos genes prompted us to assess the evolutionary conservation between Fra-1 functions in EMT of human and mouse cells. Ectopic expression of Fra-1 in fully polarized, non-tumourigenic, mouse mammary epithelial EpH4 cells induced a mesenchymal phenotype, characterized by a loss of epithelial and gain of mesenchymal markers. Proliferation, motility and invasiveness were also increased in the resulting EpFra1 cells, and the cells were tumourigenic and efficiently colonized the lung upon transplantation. Molecular analyses revealed increased expression of Tgfβ1 and the EMT-inducing transcription factors Zeb1, Zeb2 and Slug. Mechanistically, Fra-1 binds to the tgfb1 and zeb2 promoters and to an evolutionarily conserved region in the first intron of zeb1. Furthermore, increased activity of a zeb2 promoter reporter was detected in EpFra1 cells and shown to depend on AP-1-binding sites. Inhibiting TGFβ signalling in EpFra1 cells moderately increased the expression of epithelial markers, whereas silencing of zeb1 or zeb2 restored the epithelial phenotype and decreased migration in vitro and tumorigenesis in vivo. Thus Fra-1 induces changes in the expression of genes encoding EMT-related transcription factors leading to the acquisition of mesenchymal, invasive and tumorigenic capacities by epithelial cells. This study defines a novel function of Fra-1/AP-1 in modulating tgfb1, zeb1 and zeb2 expression through direct binding to genomic regulatory regions, which establishes a basis for future in vivo genetic manipulations and preclinical studies using mouse models. 
 p53 regulates epithelial-mesenchymal transition through microRNAs targeting ZEB1 and ZEB2. p53 suppresses tumor progression and   metastasis  . Epithelial-mesenchymal transition (EMT) is a key process in tumor progression and   metastasis  . The transcription factors ZEB1 and ZEB2 promote EMT. Here, we show that p53 suppresses EMT by repressing expression of ZEB1 and ZEB2. By profiling 92 primary hepatocellular carcinomas (HCCs) and 9 HCC cell lines, we found that p53 up-regulates microRNAs (miRNAs), including miR-200 and miR-192 family members. The miR-200 family members transactivated by p53 then repress ZEB1/2 expression. p53-regulated miR-192 family members also repress ZEB2 expression. Inhibition or overexpression of the miRNAs affects p53-regulated EMT by altering ZEB1 and ZEB2 expression. Our findings indicate that p53 can regulate EMT, and that p53-regulated miRNAs are critical mediators of p53-regulated EMT. 
 Epithelial-mesenchymal transition and mesenchymal-epithelial transition via regulation of ZEB-1 and ZEB-2 expression in pancreatic cancer. BACKGROUND AND OBJECTIES: Phenotypic plasticity of cancer cells via epithelial-mesenchymal transition (EMT) and mesenchymal-epithelial transition (MET) is essential for tumor progression and   metastasis  .Tissue samples were obtained from 76 pancreatic head cancers. We assessed the expression of E-cadherin, vimentin, ZEB-1, and ZEB-2 by immunohistochemical and immunofluorescence staining. Next, 147 metastatic lymph nodes from 45 pancreatic cancers with low expression of E-cadherin were obtained and divided into two categories according to the maximum diameter of the metastases: 2 mm or more and less than 2 mm.High expressions of ZEB-1 and ZEB-2 in the primary tumors were significantly associated with repression of E-cadherin (P = 0.0007), and poorer prognosis (P = 0.0322). Forty-three (29.3%) of the 147 metastatic tumors from pancreatic cancers with low expression of E-cadherin showed high E-cadherin expression. Cancer cells in the larger metastases showed high expression of E-cadherin (P = 0.0061) and low expression of ZEB-1 (P = 0.0170) and ZEB-2 (P = 0.0036) compared with those in the smaller metastases.In primary pancreatic tumors and metastatic lymph nodes, high and low expression of ZEB-1 and ZEB-2 was associated with mesenchymal and epithelial phenotype of cancer cells, respectively. 
 A miRNA signature associated with human metastatic medullary thyroid carcinoma. MicroRNAs (miRNAs) represent a class of small, non-coding RNAs that control gene expression by targeting mRNA and triggering either translational repression or RNA degradation. The objective of our study was to evaluate the involvement of miRNAs in human medullary thyroid carcinoma (MTC) and to identify the markers of metastatic cells and aggressive tumour behaviour. Using matched primary and metastatic tumour samples, we identified a subset of miRNAs aberrantly regulated in metastatic MTC. Deregulated miRNAs were confirmed by quantitative real-time PCR and validated by in situ hybridisation on a large independent set of primary and metastatic MTC samples. Our results uncovered ten miRNAs that were significantly expressed and deregulated in metastatic tumours: miR-10a, miR-200b/-200c, miR-7 and miR-29c were down-regulated and miR-130a, miR-138, miR-193a-3p, miR-373 and miR-498 were up-regulated. Bioinformatic approaches revealed potential miRNA targets and signals involved in metastatic MTC pathways. Migration, proliferation and invasion assays were performed in cell lines treated with miR-200 antagomirs to ascertain a direct role for this miRNA in MTC tumourigenesis. We show that the members of miR-200 family regulate the expression of E-cadherin by directly targeting ZEB1 and ZEB2 mRNA and through the enhanced expression of tumour growth factor β (TGFβ)-2 and TGFβ-1. Overall, the treated cells shifted to a mesenchymal phenotype, thereby acquiring an aggressive phenotype with increased motility and invasion. Our data identify a robust miRNA signature associated with metastatic MTC and distinct biological processes, e.g., TGFβ signalling pathway, providing new potential insights into the mechanisms of   MTC metast  asis. 
 An autocrine TGF-beta/ZEB/miR-200 signaling network regulates establishment and maintenance of epithelial-mesenchymal transition. Epithelial-mesenchymal transition (EMT) is a form of cellular plasticity that is critical for embryonic development and tumor   metastasis  . A double-negative feedback loop involving the miR-200 family and ZEB (zinc finger E-box-binding homeobox) transcription factors has been postulated to control the balance between epithelial and mesenchymal states. Here we demonstrate using the epithelial Madin Darby canine kidney cell line model that, although manipulation of the ZEB/miR-200 balance is able to repeatedly switch cells between epithelial and mesenchymal states, the induction and maintenance of a stable mesenchymal phenotype requires the establishment of autocrine transforming growth factor-β (TGF-β) signaling to drive sustained ZEB expression. Furthermore, we show that prolonged autocrine TGF-β signaling induced reversible DNA methylation of the miR-200 loci with corresponding changes in miR-200 levels. Collectively, these findings demonstrate the existence of an autocrine TGF-β/ZEB/miR-200 signaling network that regulates plasticity between epithelial and mesenchymal states. We find a strong correlation between ZEBs and TGF-β and negative correlations between miR-200 and TGF-β and between miR-200 and ZEBs, in invasive ductal carcinomas, consistent with an autocrine TGF-β/ZEB/miR-200 signaling network being active in breast cancers. 
 miR-139-5p inhibits epithelial-mesenchymal transition, migration and invasion of hepatocellular carcinoma cells by targeting ZEB1 and ZEB2. Hepatocellular carcinoma (HCC) is the third most common cause of cancer deaths worldwide. miRNAs have been suggested to have important roles in HCC development. The purpose of this study was to determine the role of miR-139-5p in regulation of epithelial-mesenchymal transition (EMT) and   metastasis   of HCC cells. Expression levels of miR-139-5p in 49 HCC specimens with adjacent tissues and five HCC cell lines were assessed by quantitative RT-PCR. We found that miR-139-5p was down-regulated in 89.7% of the HCC tissue samples and all of the HCC cell lines. In addition, luciferase reporter assays validated direct binding of miR-139-5p to the 3' untranslated region of zinc finger E-box binding homeobox 1 (ZEB1) and ZEB2. Ectopic expression of miR-139-5p suppressed and miR-139-in promoted EMT, migration, and invasion in Hep3B and SMMC7721 cells. Furthermore, over-expression of ZEB1 and ZEB2 ablated the inhibitory effects of miR-139-5p on migration and invasion in HCC cells. Our study indicates that miR-139-5p functions as a suppressor of HCC EMT and   metastasis   by targeting ZEB1 and ZEB2, and it may be a therapeutic target for metastatic HCC. 
 Langerhans cell maturation is accompanied by induction of N-cadherin and the transcriptional regulators of epithelial-mesenchymal transition ZEB1/2. Langerhans cells (LCs) are a unique subset of dendritic cells (DCs) that express epithelial adhesion molecules, allowing them to form contacts with epithelial cells and reside in epidermal/epithelial tissues. The dynamic regulation of epithelial adhesion plays a decisive role in the life cycle of LCs. It controls whether LCs remain immature and sessile within the epidermis or mature and egress to initiate immune responses. So far, the molecular machinery regulating epithelial adhesion molecules during LC maturation remains elusive. Here, we generated pure populations of immature human LCs in vitro to systematically probe for gene-expression changes during LC maturation. LCs down-regulate a set of epithelial genes including E-cadherin, while they upregulate the mesenchymal marker N-cadherin known to facilitate cell migration. In addition, N-cadherin is constitutively expressed by monocyte-derived DCs known to exhibit characteristics of both inflammatory-type and interstitial/dermal DCs. Moreover, the transcription factors ZEB1 and ZEB2 (ZEB is zinc-finger E-box-binding homeobox) are upregulated in migratory LCs. ZEB1 and ZEB2 have been shown to induce epithelial-to-mesenchymal transition (EMT) and invasive behavior in cancer cells undergoing   metastasis  . Our results provide the first hint that the molecular EMT machinery might facilitate LC mobilization. Moreover, our study suggests that N-cadherin plays a role during DC migration. 
 miR-139-5p suppresses cancer cell migration and invasion through targeting ZEB1 and ZEB2 in GBM. Invasion and migration of glioblastoma multiforme (GBM) is a multistep process and an important phenotype that causes this disease to invade surrounding tissues in the brain. Recent studies have highlighted that miRNAs play a pivotal role in controlling GBM cell plasticity. In this report, we used wound healing and transwell assays to identify a novel role of miR-139-5p in inhibition of GBM cell migration and invasion. Bioinformatics coupled with luciferase and Western blot assays also revealed that miR-139-5p inhibited expression of ZEB1 and ZEB2, which are master regulators of tumor   metastasis  . MiR-139-5p specifically interacts with the 3'-UTR regions of ZEB1 and ZEB2, attenuating their expression in GBM cells. To corroborate this finding, we rescued ZEB1 and ZEB2 expression and found partial but significant increases in miR-139-5p-suppressed invasion of GBM cells. The biological relevance of our study was validated by analyzing levels of miR-139-5p in GBM tissue. We found that its expression significantly downregulated compared to normal tissue and shorter overall survival rates in patients with lower miR-139-5p expression. These results confirm that miR-139-5p suppresses GBM migration and invasion and highlight its potential as a biomarker and therapeutic target for treating GBM. 
 A developmentally regulated inducer of EMT, LBX1, contributes to breast cancer progression. Epithelial-to-mesenchymal transition (EMT) plays an important role during normal embryogenesis, and it has been implicated in cancer invasion and   metastasis  . Here, we report that Ladybird homeobox 1 (LBX1), a developmentally regulated homeobox gene, directs expression of the known EMT inducers ZEB1, ZEB2, Snail1, and transforming growth factor beta2 (TGFB2). In mammary epithelial cells, overexpression of LBX1 leads to morphological transformation, expression of mesenchymal markers, enhanced cell migration, increased CD44(high)/CD24(low) progenitor cell population, and tumorigenic cooperation with known oncogenes. In human breast cancer, LBX1 is up-regulated in the unfavorable estrogen receptor (ER)/progesterone (PR)/HER2 triple-negative basal-like subtype. Thus, aberrant expression of LBX1 may lead to the activation of a developmentally regulated EMT pathway in human breast cancer. 
 The miR-200 family and miR-205 regulate epithelial to mesenchymal transition by targeting ZEB1 and SIP1. Epithelial to mesenchymal transition (EMT) facilitates tissue remodelling during embryonic development and is viewed as an essential early step in tumour   metastasis  . We found that all five members of the microRNA-200 family (miR-200a, miR-200b, miR-200c, miR-141 and miR-429) and miR-205 were markedly downregulated in cells that had undergone EMT in response to transforming growth factor (TGF)-beta or to ectopic expression of the protein tyrosine phosphatase Pez. Enforced expression of the miR-200 family alone was sufficient to prevent TGF-beta-induced EMT. Together, these microRNAs cooperatively regulate expression of the E-cadherin transcriptional repressors ZEB1 (also known as deltaEF1) and SIP1 (also known as ZEB2), factors previously implicated in EMT and tumour   metastasis  . Inhibition of the microRNAs was sufficient to induce EMT in a process requiring upregulation of ZEB1 and/or SIP1. Conversely, ectopic expression of these microRNAs in mesenchymal cells initiated mesenchymal to epithelial transition (MET). Consistent with their role in regulating EMT, expression of these microRNAs was found to be lost in invasive breast cancer cell lines with mesenchymal phenotype. Expression of the miR-200 family was also lost in regions of metaplastic breast cancer specimens lacking E-cadherin. These data suggest that downregulation of the microRNAs may be an important step in tumour progression. 
 Epithelial-mesenchymal transition transcription factor ZEB1/ZEB2 co-expression predicts poor prognosis and maintains tumor-initiating properties in head and neck cancer. Both epithelial-mesenchymal transition (EMT) and cancer stem cell (CSC) properties may be involved in   metastasis  , which contributes to the high mortality rate of patients with head and neck cancers (HNCs). However, the mechanisms through which the EMT transcription factors ZEB1 and ZEB2 regulate HNC are still unclear.Tumor initiating capability of HNC-CH133(+) cells with ZEB1/2 knockdown or co-overexpression was presented in vitro and in vivo.In the present study, we demonstrated that ZEB1/ZEB2 expression was significantly increased in HNC-CD133(+) CSC-like cells compared with HNC-CD133(-) cells. The small interfering RNA (siRNA)-mediated co-knockdown of ZEB1 and ZEB2 (siZEB1/2) in HNC-CH133(+) cells suppressed their CSC-like properties, including self-renewal ability, the expression of stemness markers, and drug resistance. In contrast, the co-overexpression of ZEB1/ZEB2 in HNC-CD133(-) cells enhanced their sphere-forming ability and increased the percentage of CD44-positive cells and side population cells. In vivo studies showed that the delivery of siZEB1/2 to xenograft tumors in nude mice reduced tumor growth and the rate of distant   metastasis  . In clinical samples, the levels of ZEB1/ZEB2 expression were low in local lesions but high in metastatic lymph nodes in HNC tissues. Patients with tumors that co-expressed ZEB1(high) and ZEB2(high) had especially poor survival rates.Therapies targeting ZEB1/ZEB2 in HNC-CD133(+) cells may provide a new approach for HNC therapy in the future. 
 miR-590-3p suppresses cancer cell migration, invasion and epithelial-mesenchymal transition in glioblastoma multiforme by targeting ZEB1 and ZEB2. Invasion and migration of glioblastoma multiforme (GBM) is a multistep process and an important phenotype that causes this disease to invade surrounding tissues in the brain. The purpose of this study was to determine the role of miR-590-3p in regulation of epithelial mesenchymal transition (EMT) and   metastasis   of GBM cells. Expression levels of miR-590-3p in 15 GBM specimens with adjacent tissues and five GBM cell lines were assessed by quantitative RT-PCR. We found that miR-590-3p was down-regulated in detected GBM tissue samples and all of the GBM cell lines. In addition, Ectopic expression of miR-590-3p suppressed and miR-590-3p-in promoted EMT, migration, and invasion in U87MG and A172 cells. Bioinformatics coupled with luciferase and Western blot assays also revealed that miR-590-3p inhibited expression of ZEB1 and ZEB2, which are master regulators of tumor   metastasis  . Our study first indicates that miR-590-3p functions as a suppressor of GBM EMT and   metastasis   by targeting ZEB1 and ZEB2, and it may be a therapeutic target for metastatic GBM. 
 miR-590-3p suppresses cancer cell migration, invasion and epithelial-mesenchymal transition in glioblastoma multiforme by targeting ZEB1 and ZEB2. Invasion and migration of glioblastoma multiforme (GBM) is a multistep process and an important phenotype that causes this disease to invade surrounding tissues in the brain. The purpose of this study was to determine the role of miR-590-3p in regulation of epithelial mesenchymal transition (EMT) and   metastasis   of GBM cells. Expression levels of miR-590-3p in 15 GBM specimens with adjacent tissues and five GBM cell lines were assessed by quantitative RT-PCR. We found that miR-590-3p was down-regulated in detected GBM tissue samples and all of the GBM cell lines. In addition, Ectopic expression of miR-590-3p suppressed and miR-590-3p-in promoted EMT, migration, and invasion in U87MG and A172 cells. Bioinformatics coupled with luciferase and Western blot assays also revealed that miR-590-3p inhibited expression of ZEB1 and ZEB2, which are master regulators of tumor   metastasis  . Our study first indicates that miR-590-3p functions as a suppressor of GBM EMT and   metastasis   by targeting ZEB1 and ZEB2, and it may be a therapeutic target for metastatic GBM. 
 miR-139-5p suppresses cancer cell migration and invasion through targeting ZEB1 and ZEB2 in GBM. Invasion and migration of glioblastoma multiforme (GBM) is a multistep process and an important phenotype that causes this disease to invade surrounding tissues in the brain. Recent studies have highlighted that miRNAs play a pivotal role in controlling GBM cell plasticity. In this report, we used wound healing and transwell assays to identify a novel role of miR-139-5p in inhibition of GBM cell migration and invasion. Bioinformatics coupled with luciferase and Western blot assays also revealed that miR-139-5p inhibited expression of ZEB1 and ZEB2, which are master regulators of tumor   metastasis  . MiR-139-5p specifically interacts with the 3'-UTR regions of ZEB1 and ZEB2, attenuating their expression in GBM cells. To corroborate this finding, we rescued ZEB1 and ZEB2 expression and found partial but significant increases in miR-139-5p-suppressed invasion of GBM cells. The biological relevance of our study was validated by analyzing levels of miR-139-5p in GBM tissue. We found that its expression significantly downregulated compared to normal tissue and shorter overall survival rates in patients with lower miR-139-5p expression. These results confirm that miR-139-5p suppresses GBM migration and invasion and highlight its potential as a biomarker and therapeutic target for treating GBM. 
 miR-139-5p inhibits epithelial-mesenchymal transition, migration and invasion of hepatocellular carcinoma cells by targeting ZEB1 and ZEB2. Hepatocellular carcinoma (HCC) is the third most common cause of cancer deaths worldwide. miRNAs have been suggested to have important roles in HCC development. The purpose of this study was to determine the role of miR-139-5p in regulation of epithelial-mesenchymal transition (EMT) and   metastasis   of HCC cells. Expression levels of miR-139-5p in 49 HCC specimens with adjacent tissues and five HCC cell lines were assessed by quantitative RT-PCR. We found that miR-139-5p was down-regulated in 89.7% of the HCC tissue samples and all of the HCC cell lines. In addition, luciferase reporter assays validated direct binding of miR-139-5p to the 3' untranslated region of zinc finger E-box binding homeobox 1 (ZEB1) and ZEB2. Ectopic expression of miR-139-5p suppressed and miR-139-in promoted EMT, migration, and invasion in Hep3B and SMMC7721 cells. Furthermore, over-expression of ZEB1 and ZEB2 ablated the inhibitory effects of miR-139-5p on migration and invasion in HCC cells. Our study indicates that miR-139-5p functions as a suppressor of HCC EMT and   metastasis   by targeting ZEB1 and ZEB2, and it may be a therapeutic target for metastatic HCC. 
 Fra-1/AP-1 induces EMT in mammary epithelial cells by modulating Zeb1/2 and TGFβ expression. Epithelial-to-mesenchymal transition (EMT) is essential for embryonic morphogenesis and wound healing and critical for tumour cell invasion and dissemination. The AP-1 transcription factor Fra-1 has been implicated in tumorigenesis and in tumour-associated EMT in human breast cancer. We observed a significant inverse correlation between Fra-1 mRNA expression and distant  -metastasi  s-free survival in a large cohort of breast cancer patients derived from multiple array data sets. This unique correlation among Fos genes prompted us to assess the evolutionary conservation between Fra-1 functions in EMT of human and mouse cells. Ectopic expression of Fra-1 in fully polarized, non-tumourigenic, mouse mammary epithelial EpH4 cells induced a mesenchymal phenotype, characterized by a loss of epithelial and gain of mesenchymal markers. Proliferation, motility and invasiveness were also increased in the resulting EpFra1 cells, and the cells were tumourigenic and efficiently colonized the lung upon transplantation. Molecular analyses revealed increased expression of Tgfβ1 and the EMT-inducing transcription factors Zeb1, Zeb2 and Slug. Mechanistically, Fra-1 binds to the tgfb1 and zeb2 promoters and to an evolutionarily conserved region in the first intron of zeb1. Furthermore, increased activity of a zeb2 promoter reporter was detected in EpFra1 cells and shown to depend on AP-1-binding sites. Inhibiting TGFβ signalling in EpFra1 cells moderately increased the expression of epithelial markers, whereas silencing of zeb1 or zeb2 restored the epithelial phenotype and decreased migration in vitro and tumorigenesis in vivo. Thus Fra-1 induces changes in the expression of genes encoding EMT-related transcription factors leading to the acquisition of mesenchymal, invasive and tumorigenic capacities by epithelial cells. This study defines a novel function of Fra-1/AP-1 in modulating tgfb1, zeb1 and zeb2 expression through direct binding to genomic regulatory regions, which establishes a basis for future in vivo genetic manipulations and preclinical studies using mouse models. 
 A miRNA signature associated with human metastatic medullary thyroid carcinoma. MicroRNAs (miRNAs) represent a class of small, non-coding RNAs that control gene expression by targeting mRNA and triggering either translational repression or RNA degradation. The objective of our study was to evaluate the involvement of miRNAs in human medullary thyroid carcinoma (MTC) and to identify the markers of metastatic cells and aggressive tumour behaviour. Using matched primary and metastatic tumour samples, we identified a subset of miRNAs aberrantly regulated in metastatic MTC. Deregulated miRNAs were confirmed by quantitative real-time PCR and validated by in situ hybridisation on a large independent set of primary and metastatic MTC samples. Our results uncovered ten miRNAs that were significantly expressed and deregulated in metastatic tumours: miR-10a, miR-200b/-200c, miR-7 and miR-29c were down-regulated and miR-130a, miR-138, miR-193a-3p, miR-373 and miR-498 were up-regulated. Bioinformatic approaches revealed potential miRNA targets and signals involved in metastatic MTC pathways. Migration, proliferation and invasion assays were performed in cell lines treated with miR-200 antagomirs to ascertain a direct role for this miRNA in MTC tumourigenesis. We show that the members of miR-200 family regulate the expression of E-cadherin by directly targeting ZEB1 and ZEB2 mRNA and through the enhanced expression of tumour growth factor β (TGFβ)-2 and TGFβ-1. Overall, the treated cells shifted to a mesenchymal phenotype, thereby acquiring an aggressive phenotype with increased motility and invasion. Our data identify a robust miRNA signature associated with metastatic MTC and distinct biological processes, e.g., TGFβ signalling pathway, providing new potential insights into the mechanisms of   MTC metast  asis. 
 Langerhans cell maturation is accompanied by induction of N-cadherin and the transcriptional regulators of epithelial-mesenchymal transition ZEB1/2. Langerhans cells (LCs) are a unique subset of dendritic cells (DCs) that express epithelial adhesion molecules, allowing them to form contacts with epithelial cells and reside in epidermal/epithelial tissues. The dynamic regulation of epithelial adhesion plays a decisive role in the life cycle of LCs. It controls whether LCs remain immature and sessile within the epidermis or mature and egress to initiate immune responses. So far, the molecular machinery regulating epithelial adhesion molecules during LC maturation remains elusive. Here, we generated pure populations of immature human LCs in vitro to systematically probe for gene-expression changes during LC maturation. LCs down-regulate a set of epithelial genes including E-cadherin, while they upregulate the mesenchymal marker N-cadherin known to facilitate cell migration. In addition, N-cadherin is constitutively expressed by monocyte-derived DCs known to exhibit characteristics of both inflammatory-type and interstitial/dermal DCs. Moreover, the transcription factors ZEB1 and ZEB2 (ZEB is zinc-finger E-box-binding homeobox) are upregulated in migratory LCs. ZEB1 and ZEB2 have been shown to induce epithelial-to-mesenchymal transition (EMT) and invasive behavior in cancer cells undergoing   metastasis  . Our results provide the first hint that the molecular EMT machinery might facilitate LC mobilization. Moreover, our study suggests that N-cadherin plays a role during DC migration. 
 Epithelial-mesenchymal transition transcription factor ZEB1/ZEB2 co-expression predicts poor prognosis and maintains tumor-initiating properties in head and neck cancer. Both epithelial-mesenchymal transition (EMT) and cancer stem cell (CSC) properties may be involved in   metastasis  , which contributes to the high mortality rate of patients with head and neck cancers (HNCs). However, the mechanisms through which the EMT transcription factors ZEB1 and ZEB2 regulate HNC are still unclear.Tumor initiating capability of HNC-CH133(+) cells with ZEB1/2 knockdown or co-overexpression was presented in vitro and in vivo.In the present study, we demonstrated that ZEB1/ZEB2 expression was significantly increased in HNC-CD133(+) CSC-like cells compared with HNC-CD133(-) cells. The small interfering RNA (siRNA)-mediated co-knockdown of ZEB1 and ZEB2 (siZEB1/2) in HNC-CH133(+) cells suppressed their CSC-like properties, including self-renewal ability, the expression of stemness markers, and drug resistance. In contrast, the co-overexpression of ZEB1/ZEB2 in HNC-CD133(-) cells enhanced their sphere-forming ability and increased the percentage of CD44-positive cells and side population cells. In vivo studies showed that the delivery of siZEB1/2 to xenograft tumors in nude mice reduced tumor growth and the rate of distant   metastasis  . In clinical samples, the levels of ZEB1/ZEB2 expression were low in local lesions but high in metastatic lymph nodes in HNC tissues. Patients with tumors that co-expressed ZEB1(high) and ZEB2(high) had especially poor survival rates.Therapies targeting ZEB1/ZEB2 in HNC-CD133(+) cells may provide a new approach for HNC therapy in the future. 
 Epithelial-mesenchymal transition and mesenchymal-epithelial transition via regulation of ZEB-1 and ZEB-2 expression in pancreatic cancer. BACKGROUND AND OBJECTIES: Phenotypic plasticity of cancer cells via epithelial-mesenchymal transition (EMT) and mesenchymal-epithelial transition (MET) is essential for tumor progression and   metastasis  .Tissue samples were obtained from 76 pancreatic head cancers. We assessed the expression of E-cadherin, vimentin, ZEB-1, and ZEB-2 by immunohistochemical and immunofluorescence staining. Next, 147 metastatic lymph nodes from 45 pancreatic cancers with low expression of E-cadherin were obtained and divided into two categories according to the maximum diameter of the metastases: 2 mm or more and less than 2 mm.High expressions of ZEB-1 and ZEB-2 in the primary tumors were significantly associated with repression of E-cadherin (P = 0.0007), and poorer prognosis (P = 0.0322). Forty-three (29.3%) of the 147 metastatic tumors from pancreatic cancers with low expression of E-cadherin showed high E-cadherin expression. Cancer cells in the larger metastases showed high expression of E-cadherin (P = 0.0061) and low expression of ZEB-1 (P = 0.0170) and ZEB-2 (P = 0.0036) compared with those in the smaller metastases.In primary pancreatic tumors and metastatic lymph nodes, high and low expression of ZEB-1 and ZEB-2 was associated with mesenchymal and epithelial phenotype of cancer cells, respectively. 
 A developmentally regulated inducer of EMT, LBX1, contributes to breast cancer progression. Epithelial-to-mesenchymal transition (EMT) plays an important role during normal embryogenesis, and it has been implicated in cancer invasion and   metastasis  . Here, we report that Ladybird homeobox 1 (LBX1), a developmentally regulated homeobox gene, directs expression of the known EMT inducers ZEB1, ZEB2, Snail1, and transforming growth factor beta2 (TGFB2). In mammary epithelial cells, overexpression of LBX1 leads to morphological transformation, expression of mesenchymal markers, enhanced cell migration, increased CD44(high)/CD24(low) progenitor cell population, and tumorigenic cooperation with known oncogenes. In human breast cancer, LBX1 is up-regulated in the unfavorable estrogen receptor (ER)/progesterone (PR)/HER2 triple-negative basal-like subtype. Thus, aberrant expression of LBX1 may lead to the activation of a developmentally regulated EMT pathway in human breast cancer. 
 An autocrine TGF-beta/ZEB/miR-200 signaling network regulates establishment and maintenance of epithelial-mesenchymal transition. Epithelial-mesenchymal transition (EMT) is a form of cellular plasticity that is critical for embryonic development and tumor   metastasis  . A double-negative feedback loop involving the miR-200 family and ZEB (zinc finger E-box-binding homeobox) transcription factors has been postulated to control the balance between epithelial and mesenchymal states. Here we demonstrate using the epithelial Madin Darby canine kidney cell line model that, although manipulation of the ZEB/miR-200 balance is able to repeatedly switch cells between epithelial and mesenchymal states, the induction and maintenance of a stable mesenchymal phenotype requires the establishment of autocrine transforming growth factor-β (TGF-β) signaling to drive sustained ZEB expression. Furthermore, we show that prolonged autocrine TGF-β signaling induced reversible DNA methylation of the miR-200 loci with corresponding changes in miR-200 levels. Collectively, these findings demonstrate the existence of an autocrine TGF-β/ZEB/miR-200 signaling network that regulates plasticity between epithelial and mesenchymal states. We find a strong correlation between ZEBs and TGF-β and negative correlations between miR-200 and TGF-β and between miR-200 and ZEBs, in invasive ductal carcinomas, consistent with an autocrine TGF-β/ZEB/miR-200 signaling network being active in breast cancers. 
 p53 regulates epithelial-mesenchymal transition through microRNAs targeting ZEB1 and ZEB2. p53 suppresses tumor progression and   metastasis  . Epithelial-mesenchymal transition (EMT) is a key process in tumor progression and   metastasis  . The transcription factors ZEB1 and ZEB2 promote EMT. Here, we show that p53 suppresses EMT by repressing expression of ZEB1 and ZEB2. By profiling 92 primary hepatocellular carcinomas (HCCs) and 9 HCC cell lines, we found that p53 up-regulates microRNAs (miRNAs), including miR-200 and miR-192 family members. The miR-200 family members transactivated by p53 then repress ZEB1/2 expression. p53-regulated miR-192 family members also repress ZEB2 expression. Inhibition or overexpression of the miRNAs affects p53-regulated EMT by altering ZEB1 and ZEB2 expression. Our findings indicate that p53 can regulate EMT, and that p53-regulated miRNAs are critical mediators of p53-regulated EMT. 
 A double-negative feedback loop between ZEB1-SIP1 and the microRNA-200 family regulates epithelial-mesenchymal transition. Epithelial to mesenchymal transition occurs during embryologic development to allow tissue remodeling and is proposed to be a key step in the   metastasis   of epithelial-derived tumors. The miR-200 family of microRNAs plays a major role in specifying the epithelial phenotype by preventing expression of the transcription repressors, ZEB1/deltaEF1 and SIP1/ZEB2. We show here that miR-200a, miR-200b, and the related miR-429 are all encoded on a 7.5-kb polycistronic primary miRNA (pri-miR) transcript. We show that the promoter for the pri-miR is located within a 300-bp segment located 4 kb upstream of miR-200b. This promoter region is sufficient to confer expression in epithelial cells and is repressed in mesenchymal cells by ZEB1 and SIP1 through their binding to a conserved pair of ZEB-type E-box elements located proximal to the transcription start site. These findings establish a double-negative feedback loop controlling ZEB1-SIP1 and miR-200 family expression that regulates cellular phenotype and has direct relevance to the role of these factors in tumor progression. 
 The miR-200 family inhibits epithelial-mesenchymal transition and cancer cell migration by direct targeting of E-cadherin transcriptional repressors ZEB1 and ZEB2. MicroRNAs are small non-coding RNA molecules that can regulate gene expression by interacting with multiple mRNAs and inducing either translation suppression or degradation of mRNA. Recently, several miRNAs were identified as either promoters or suppressors of   metastasis  . However, it is unclear in which step(s) of the multistep metastatic cascade these miRNAs play a defined functional role. To study the functional importance of miRNAs in epithelial-mesenchymal transition (EMT), a process thought to initiate   metastasis   by enhancing the motility of tumor cells, we used a well established in vitro EMT assay: transforming growth factor-beta-induced EMT in NMuMG murine mammary epithelial cells. We found that members of the miR-200 family, organized as two clusters in the genome, were repressed during EMT. Overexpression of each miRNA individually or as clusters in NMuMG cells hindered EMT by enhancing E-cadherin expression through direct targeting of ZEB1 and ZEB2, which encode transcriptional repressors of E-cadherin. In the 4TO7 mouse carcinoma cell line, which expresses low levels of endogenous E-cadherin and displays a mesenchymal phenotype, ectopic expression of the miR-200 family miRNAs significantly increased E-cadherin expression and altered cell morphology to an epithelial phenotype. Furthermore, ectopic expression of each miR-200 miRNA cluster significantly reduced the in vitro motility of 4TO7 cells in migration assays. These results suggested that loss of expression of the miR-200 family members may play a critical role in the repression of E-cadherin by ZEB1 and ZEB2 during EMT, thereby enhancing migration and invasion during cancer progression. 
 The miR-200 family and miR-205 regulate epithelial to mesenchymal transition by targeting ZEB1 and SIP1. Epithelial to mesenchymal transition (EMT) facilitates tissue remodelling during embryonic development and is viewed as an essential early step in tumour   metastasis  . We found that all five members of the microRNA-200 family (miR-200a, miR-200b, miR-200c, miR-141 and miR-429) and miR-205 were markedly downregulated in cells that had undergone EMT in response to transforming growth factor (TGF)-beta or to ectopic expression of the protein tyrosine phosphatase Pez. Enforced expression of the miR-200 family alone was sufficient to prevent TGF-beta-induced EMT. Together, these microRNAs cooperatively regulate expression of the E-cadherin transcriptional repressors ZEB1 (also known as deltaEF1) and SIP1 (also known as ZEB2), factors previously implicated in EMT and tumour   metastasis  . Inhibition of the microRNAs was sufficient to induce EMT in a process requiring upregulation of ZEB1 and/or SIP1. Conversely, ectopic expression of these microRNAs in mesenchymal cells initiated mesenchymal to epithelial transition (MET). Consistent with their role in regulating EMT, expression of these microRNAs was found to be lost in invasive breast cancer cell lines with mesenchymal phenotype. Expression of the miR-200 family was also lost in regions of metaplastic breast cancer specimens lacking E-cadherin. These data suggest that downregulation of the microRNAs may be an important step in tumour progression. 
 ZEB1 sensitizes lung adenocarcinoma to   metastasis   suppression by PI3K antagonism. Epithelial tumor cells that have undergone epithelial-to-mesenchymal transition (EMT) are typically prone to   metastasis   and drug resistance and contribute to a poor clinical outcome. The transcription factor ZEB1 is a known driver of EMT, and mediators of ZEB1 represent potential therapeutic targets for   metastasis   suppression. Here, we have shown that phosphatidylinositol 3-kinase-targeted (PI3K-targeted) therapy suppresses   metastasis   in a mouse model of Kras/Tp53-mutant lung adenocarcinoma that develops metastatic disease due to high expression of ZEB1. In lung adenocarcinoma cells from Kras/Tp53-mutant animals and human lung cancer cell lines, ZEB1 activated PI3K by derepressing miR-200 targets, including amphiregulin (AREG), betacellulin (BTC), and the transcription factor GATA6, which stimulated an EGFR/ERBB2 autocrine loop. Additionally, ZEB1-dependent derepression of the miR-200 and miR-183 target friend of GATA 2 (FOG2) enhanced GATA3-induced expression of the p110α catalytic subunit of PI3K. Knockdown of FOG2, p110α, and RHEB ameliorated invasive and metastatic propensities of tumor cells. Surprisingly, FOG2 was not required for mesenchymal differentiation, suggesting that mesenchymal differentiation and invasion are distinct and separable processes. Together, these results indicate that ZEB1 sensitizes lung adenocarcinoma cells t  o metastas  is suppression by PI3K-targeted therapy and suggest that treatments to selectively modify the metastatic behavior of mesenchymal tumor cells are feasible and may be of clinical value. 
 MiR-150 is associated with poor prognosis in esophageal squamous cell carcinoma via targeting the EMT inducer ZEB1. The association of microRNAs (miRs) with cancer progression has been established in many cancers including esophageal squamous cell carcinoma (ESCC). A public microarray database showed that the expression of miR-150 was lower in ESCC than in normal esophageal mucosa. Here, we focused on ZEB1, epithelial-mesenchymal-transition (EMT)-inducer, as a target gene of miR-150 based on in silico predictions. The purpose of this study was to clarify the clinicopathological significance of miR-150 in ESCC, and to investigate miR-150's EMT-regulatory ability. Quantitative RT-PCR was used to evaluate miR-150 expression in 108 curative resected ESCC samples to determine the clinicopathological significance. Moreover, we examined the in vitro and in vivo function of miR-150 via degradation of ZEB1. MiR-150 expression was significantly lower in cancer tissues compared to adjacent non-cancerous tissues (P < 0.001). Low expression of miR-150 in ESCC contributed to malignant potential, such as tumor depth, lymph node   metastasis  , lymphatic invasion, venous invasion, clinical staging, and poor prognosis (P < 0.05). In vitro assays showed that EMT-inducer-ZEB1 is a new direct target of miR-150. Moreover, miR-150 induced MET-like changes in TE-8 cells through ZEB1 degradation (e.g., E-cadherin expression, vimentin repression, epithelial morphology, and suppression of migration ability), and significantly inhibited tumorigenicity and tumor growth in a mouse xenograft model. Analysis of the regulation of ZEB1 by miR-150 could provide new insights into preventing   metastasis   and also suggests novel targeted therapeutic strategies in ESCC. 
 Knockdown of BAG3 induces epithelial-mesenchymal transition in thyroid cancer cells through ZEB1 activation. The process by which epithelial features are lost in favor of a mesenchymal phenotype is referred to as epithelial-mesenchymal transition (EMT). Most carcinomas use this mechanism to evade into neighboring tissues. Reduction or a loss of E-cadherin expression is a well-established hallmark of EMT. As a potent suppressor of E-cadherin, transcription factor ZEB1 is one of the key inducers of EMT, whose expression promotes tumorigenesis and   metastasis   of carcinomas. Bcl-2-associated athanogene 3 (BAG3) affects multifaceted cellular functions, including proliferation, apoptosis, cell adhesion and invasion, viral infection, and autophagy. Recently, we have reported a novel role of BAG3 implicated in EMT, while the mechanisms are poorly elucidated. The current study demonstrated that knockdown of BAG3 induced EMT, and increased cell migratory and invasiveness in thyroid cancer cells via transcriptional activation of ZEB1. We also found that BAG3 knockdown led to nuclear accumulation of β-catenin, which was responsible for the transcriptional activation of ZEB1. These results indicate BAG3 as a regulator of ZEB1 expression in EMT and as a regulator of   metastasi  s in thyroid cancer cells, providing potential targets to prevent and/or treat thyroid cancer cell invasion and   metastasi  s. 
 A reciprocal repression between ZEB1 and members of the miR-200 family promotes EMT and invasion in cancer cells. The embryonic programme 'epithelial-mesenchymal transition' (EMT) is thought to promote malignant tumour progression. The transcriptional repressor zinc-finger E-box binding homeobox 1 (ZEB1) is a crucial inducer of EMT in various human tumours, and was recently shown to promote invasion and   metastasis   of tumour cells. Here, we report that ZEB1 directly suppresses transcription of microRNA-200 family members miR-141 and miR-200c, which strongly activate epithelial differentiation in pancreatic, colorectal and breast cancer cells. Notably, the EMT activators transforming growth factor beta2 and ZEB1 are the predominant targets downregulated by these microRNAs. These results indicate that ZEB1 triggers an microRNA-mediated feedforward loop that stabilizes EMT and promotes invasion of cancer cells. Alternatively, depending on the environmental trigger, this loop might switch and induce epithelial differentiation, and thus explain the strong intratumorous heterogeneity observed in many human cancers. 
 miR-128 modulates chemosensitivity and invasion of prostate cancer cells through targeting ZEB1. Recent reports strongly suggest the profound role of miRNAs in cancer therapeutic response and progression, including invasion and   metastasis  . The sensitivity to therapy and invasion is the major obstacle for successful treatment in prostate cancer. We aimed to investigate the regulative effect of miR-128/zinc-finger E-box-binding homeobox 1 axis on prostate cancer cell chemosensitivity and invasion.The miR-128 expression pattern of prostate cancer cell lines and tissues was detected by real-time reverse transcriptase-polymerase chain reaction, while the mRNA and protein expression levels of zinc-finger E-box-binding homeobox 1 were measured by real-time reverse transcriptase-polymerase chain reaction and western blot assay, respectively. Dual-luciferase reporter gene assay was used to find the direct target of miR-128. Furthermore, prostate cancer cells were treated with miR-128 mimic or zinc-finger E-box-binding homeobox 1-siRNA, and then the cells' chemosensitivity and invasion were detected by 3-(4,5-dimethylthiazol-2-yl)-2,5-diphenyltetrazolium bromide assay and transwell assay, respectively.We found miR-128 expression obviously decreased in prostate cancer tissues compared with paired normal tissues. Restored miR-128 expression sensitized prostate cancer cells to cisplatin and inhibited the invasion. Furthermore, there was an inverse expression pattern between miR-128 and zinc-finger E-box-binding homeobox 1 in prostate cancer cells and tissues, and zinc-finger E-box-binding homeobox 1 was identified as a direct target of miR-128 in prostate cancer. Knockdown of zinc-finger E-box-binding homeobox 1 expression efficiently sensitized prostate cancer cells to cisplatin and inhibited the invasion. However, ectopic zinc-finger E-box-binding homeobox 1 expression impaired the effects of miR-128 on chemosensitivity and invasion in prostate cancer cells.miR-128 functions as a potential cancer suppressor in prostate cancer progression and rational therapeutic strategies for prostate cancer would be developed based on miR-128/zinc-finger E-box-binding homeobox 1 axis. 
 An hTERT/ZEB1 complex directly regulates E-cadherin to promote epithelial-to-mesenchymal transition (EMT) in colorectal cancer. In human cancer, high telomerase expression is correlated with tumor aggressiveness and metastatic potential. Telomerase activation occurs through telomerase reverse transcriptase (hTERT) induction, which contributes to malignant transformation by stabilizing telomeres. Previous studies have shown that hTERT can promote tumor invasion and   metastasis   of gastric cancer, liver cancer and esophageal cancer. Epithelial-to-mesenchymal transition (EMT), a requirement for tumor invasion and   metastasis  , plays a key role in cancer progression. Although hTERT promotes EMT through Wnt signaling in several cancers, it is unknown if other signaling pathways are involved. In the present study, we found that hTERT and ZEB1 form a complex, which directly binds to the E-cadherin promoter, and then inhibits E-cadherin expression and promots EMT in colorectal cancer cells. hTERT overexpression in HCT116 and SW480 cells could induce E-cadherin down-regulation. However, E-cadherin expression was recovered when ZEB1 function was impaired even during hTERT overexpression. Taken together, our findings suggest that hTERT can promote cancer   metastasis   by stimulating EMT through the ZEB1 pathway and therefore inhibiting them may prevent cancer progression. 
 ZEB1 promotes epithelial-mesenchymal transition in cervical cancer   metastasis  . To investigate role of Zinc finger E-box binding homeobox 1 (ZEB1) in cervical cancer tissue (squamous cell carcinoma, SCC).Exploratory study.University hospital.Sixty patients with SCC, including stage CINIII (n = 10), IB1 (n = 10), IB2 (n = 10), IIA1 (n = 10), IIA2 (n = 10), and IIB (n = 10) were studied.Caski cells were transfected with recombinant shZEB1 lentivirus or shCtrl lentivirus to generate stable ZEB1-knockdown Caski cells.ZEB1 expression was analyzed by quantitative real-time polymerase chain reaction and immunohistochemistry in cervical cancer tissues. ZEB1 expression in Caski cells was down-regulated by short-hairpin RNA (shRNA) interference, and changes in ZEB1 expression corresponded with changes in the proliferation and migratory ability of Caski cells.Quantitative real-time polymerase chain reaction and immunohistochemistry results revealed that ZEB1 expression and the ratio of Vimentin to E-cadherin were high in 27 of 50 SCC patients and correlated with advanced International Federation of Gynecology and Obstetrics stage, tumor size >4 cm, and parametrial invasion. However, the expression of ZEB1 in cervical cancer tissue was independent of age and SCC antigen level. Transfection of ZEB1 shRNA in Caski cells significantly decreased the messenger RNA and protein expression of ZEB1, parallel with increased expression of the epithelial marker E-cadherin and decreased expression of the mesenchymal marker Vimentin. Furthermore, the proliferation and migratory ability of Caski cells were significantly lower in the transfected group than in the nontransfected control group.Down-regulation of ZEB1 expression may protect the invasive front of the tumors from converting to a mesenchymal phenotype by reducing the proliferation and motility of cervical cancer cells, suggesting that ZEB1 might be a potential therapeutic target for SCC. 
 Clinical implication of ZEB-1 and E-cadherin expression in hepatocellular carcinoma (HCC). While recent research has shown that expression of ZEB-1 in a variety of tumors has a crucial impact on patient survival, there is little information regarding ZEB-1 expression in hepatocellular carcinoma (HCC). This study investigated the co-expression of ZEB-1 and E-cadherin in HCC by immunohistochemistry and evaluated its association with clinical factors, including patient prognosis.A total of 108 patients with primary HCC treated by curative hepatectomy were enrolled. ZEB-1 expression was immunohistochemically categorized as positive if at least 1% cancer cells exhibited nuclear staining. E-cadherin expression was divided into preserved and reduced expression groups and correlations between ZEB-1 and E-cadherin expression and clinical factors were then evaluated.With respect to ZEB-1 expression, 23 patients were classified into the positive group and 85 into the negative group. Reduced E-cadherin expression was seen in 44 patients and preserved expression in the remaining 64 patients. ZEB-1 positivity was significantly associated with reduced expression of E-cadherin (p = 0.027). Moreover, significant associations were found between ZEB-1 expression and venous invasion and TNM stage. ZEB-1 positivity was associated with poorer prognosis (p = 0.025). Reduced E-cadherin expression was significantly associated with intra  hepatic me  tastasis and poorer prognosis (p = 0.047). In particular, patients with both ZEB-1 positivity and reduced E-cadherin expression had a poorer prognosis (p = 0.005). Regardless of E-cadherin status, ZEB-1 was not a significant prognostic factor by multivariate analysis. There was no statistical difference in overall survival when E-cadherin expression was reduced in the ZEB-1 positive group (p = 0.24).Positive ZEB-1 expression and loss of E-cadherin expression are correlated with poor prognosis in HCC patients and malignancy of ZEB-1 positive tumors involves EMT. 
 Expression of the ZEB1 (deltaEF1) transcription factor in human: additional insights. The zinc finger E-box binding transcription factor ZEB1 (deltaEF1/Nil-2-a/AREB6/zfhx1a/TCF8/zfhep/BZP) is emerging as an important regulator of the epithelial to mesenchymal transitions (EMT) required for development and cancer   metastasis  . ZEB1 promotes EMT by repressing genes contributing to the epithelial phenotype while activating those associated with the mesenchymal phenotype. TCF8 (zfhx1a), the gene encoding ZEB1, is induced by several potentially oncogenic ligands including TGF-beta, estrogen, and progesterone. TGF-beta appears to activate EMT, at least in part, by inducing ZEB1. However, our understanding of how ZEB1 contributes to signaling pathways elicited by estrogen and progesterone is quite limited, as is our understanding of its functional roles in normal adult tissues. To begin to address these questions, a human tissue mRNA array analysis was done. In adults, the highest ZEB1 mRNA expression is in bladder and uterus, whereas in the fetus highest expression is in lung, thymus, and heart. To further investigate the regulation of TCF8 by estrogen, ZEB1 mRNA was measured in ten estrogen-responsive cell lines, but it is only induced in the OV266 ovarian carcinoma line. Although high expression of ZEB1 mRNA is estrogen-dependent in normal human ovarian and endometrial biopsies, high expression is estrogen-independent in late stage ovarian and endometrial carcinomas, raising the possibility that deregulated expression promotes cancer progression. In contrast, TCF8 is at least partially deleted in 4 of 5 well-differentiated, grade I endometrial carcinomas, which may contribute to their non-aggressive phenotype. These data support the contention that high ZEB1 encourages gynecologic carcinoma progression. 
 Expression of the E-cadherin repressors Snail, Slug and Zeb1 in urothelial carcinoma of the urinary bladder: relation to stromal fibroblast activation and invasive behaviour of carcinoma cells. Epithelial-mesenchymal transition (EMT) is regulated by interaction of carcinoma and stromal cells and crucial for progression of urinary bladder carcinoma (UBC). Therefore, the influence of activated fibroblasts on the expression of E-cadherin repressors as well as EMT and invasion in UBC was investigated. A correlative analysis of the immunohistochemical expression of fibroblast (ASMA, S100A4, FAP, SDF1, PDGFRβ) and EMT (Snail, Slug, Zeb1, E-cadherin) markers was performed on 49 UBC cases of different stages. The impact of distinguishable growth factor stimulated fibroblasts on invasion, EMT, and E-cadherin repressor expression was investigated in an invasion model. In situ, invasiveness was significantly correlated to the loss of membranous E-cadherin (E-cad_m) and increased Snail, Slug, Zeb1 in tumour cells, as well as to increased ASMA, S100A4, and PDGFRβ in stromal cells. A significant correlation to noda  l metastas  is could be evidenced for the loss of E-Cad_m, and for an increase in S100A4 and PDGFRβ. Comparison of stromal and EMT markers revealed significant correlations of ASMA to Snail and Slug; of S100A4 to the loss of E-cad_m and Zeb1; and of PDGFRβ to the loss of E-Cad_m, Slug and Zeb1. In vitro, TGFβ1 induced myofibroblasts were the strongest attractants, while aFGF or TGFβ1/aFGF stimulated fibroblasts were the most potent EMT inductors. As shown here for the first time, distinct sub-populations of fibroblasts are to various extents associated with EMT and tumour progression in UBC. These relevant findings might be the basis for the identification of new diagnostic markers and therapeutic targets selectively affecting tumour supporting CAF effects. 
 miR-1271 inhibits migration, invasion and epithelial-mesenchymal transition by targeting ZEB1 and TWIST1 in pancreatic cancer cells. Pancreatic cancer (PC) remains one of the most lethal types of cancer in adults. The purpose of this study was to determine the role of miR-1271 in regulation of epithelial mesenchymal transition (EMT) and   metastasis   of pancreatic cancer cells. miR-1271 was identified to be significantly down-regulated in PC tissues by miRNA array. Also, an increase of EMT-regulators ZEB1 and TWIST1 expression level is accompanied by a decrease of miR-1271. We showed that expression of miR-1271 was significantly down-regulated in PC tissues as compared with that in normal tissues. In addition, our results showed that miR-1271 expression levels were decreased while ZEB1 and TWIST1 expression levels were increased in detected PC cell lines. Moreover, ectopic expression of miR-1271 suppressed and antagomiR-1271 promoted proliferation, migration, and invasion in SW1990 and PANC-1 cells. Bioinformatics coupled with luciferase and Western blot assays also revealed that miR-1271 inhibited expression of ZEB1 and TWIST1, which are master regulators of tumor   metastasi  s. Our study first indicates that miR-1271 functions as a suppressor in regulating of pancreatic cancer EMT by targeting ZEB1 and TWIST1, and it promise as a therapeutic target and prognostic marker for metastatic pancreatic cancer. 
 Overexpression of ZEB1 relates to   metastasis   and invasion in osteosarcoma. This study aimed to investigate the expression of ZEB1 in osteosarcoma tissues and to discuss the relationship between ZEB1 expression and osteosarcoma   metastasis  .Using RT-PCR and Western blotting, the mRNA and protein expressions of ZEB1 in the osteosarcoma and normal bone tissues were detected. Using the RNA interference technique, the expression of ZEB1 in the human osteosarcoma MG-63 cell line was downregulated, and the changes in the invasion of MG-63 cells were examined.The positive mRNA expression rate of ZEB1 in the osteosarcoma tissues was significantly higher than that in normal bone tissue (P < 0.05). The protein expression level of ZEB1 in the sarcoma tissues from patients with positive l  ung metast  asis was significantly higher than that from patients without l  ung metast  asis (P < 0.05). After the transfection of ZEB1 siRNA into the MG-63 cells, the protein expression of ZEB1 was significantly reduced (P < 0.05), and the number of cells that passed through the Transwell chamber was significantly lower than that in the non-transfected control group as well as the transfected control group (P < 0.05).The overexpression of ZEB1 in osteosarcoma may be related to the carcinogenesis and develop  ment as we  ll as metastasis and invasion of osteosarcoma. 
 Involvement of ZEB1 and E-cadherin in the invasion of lung squamous cell carcinoma. This study intended to investigate the expression of the ZEB1 and E-cadherin proteins in lung squamous cell carcinoma (LSCC) tissues and to examine the clinicopathological correlation between protein levels and LSCC. RT-PCR and Western blot were used to examine the expression of ZEB1 and E-cadherin mRNAs and proteins in LSCC tissues as well as in adjacent normal tissues, and then analyze the relationship between the clinicopathological characteristics and the expression changes of ZEB1 and E-cadherin mRNAs in LSCC. In addition, RNAi was used to knockdown the expression of the ZEB1 gene in Human HCC827 cells; subsequently, changes in the invasive ability of the resultant cells were studied. The positive rates of ZEB1 and E-cadherin mRNAs in LSCC tissues were 69.2 and 38.5 %, respectively. They differed significantly from the corresponding positive rates in the adjacent normal lung tissues (15.4 and 80.8 %, p < 0.05). There was a negative correlation between the protein levels of ZEB1 and E-cadherin in LSCC tissues (r = -0.714, p < 0.001); in addition, it was found that ZEB1 protein expression in LSCC tissues was significantly higher than that in the neighboring normal lung tissues (p < 0.05), and its expression was also significantly higher in patients with lymph node metastases and distant metastases compared to those patients without metastatic disease (p < 0.05). On the contrary, E-cadherin expression was significantly lower in LSCC tissues than that in the neighboring normal tissue (p < 0.05). It was lower in patients wi  th lymph n  ode metastasi  s and dist  ant metastasis compared to patients without metastatic disease (p < 0.05). However, the expression of ZEB1 and E-cadherin was independent of gender, age, tumor size, or tumor differentiation level (p > 0.05). Transfection of ZEB1 siRNA into HCC827 cells significantly reduced the ZEB1 protein level (p < 0.01) and significantly elevated E-cadherin levels (p < 0.01). Moreover, significantly less ZEB1 siRNA-transfected cells migrated through Transwell chambers in the LSCC tissue than that in the control groups (untransfected or transfected with control siRNA, p < 0.01). The expression of the ZEB1 gene in LSCC tissues is downregulated with the expression of E-cadherin. On the other hand, the expression of siRNA against ZEB1 promotes E-cadherin expression and suppresses the invasive ability conferred by E-cadherin. In conclusion, our data suggested that overexpression of the ZEB1 gene is possibly associated with the occurrence, development, invasion of LSCC. 
 Identification of a PEAK1/ZEB1 signaling axis during TGFβ/fibronectin-induced EMT in breast cancer. Transforming Growth Factor beta (TGFβ) is the archetypal member of the TGFβ superfamily of ligands and has pleiotropic functions during normal development, adult tissue homeostasis and pathophysiological processes such as cancer. In epithelial cancers TGFβ signaling can either suppress tumor growth or prom  ote metast  asis via the induction of a well-characterized epithelial-mesenchymal transition (EMT) program. We recently reported that PEAK1 kinase mediates signaling cross talk between TGFβ receptors and integrin/Src/MAPK pathways and functions as a critical molecular regulator of TGFβ-induced breast cancer cell proliferation, migration, EM  T and meta  stasis. Here, we examined the breast cancer cell contexts in which TGFβ induces both EMT and PEAK1, and discovered this event to be unique to oncogene-transformed mammary epithelial cells and triple-negative breast cancer cells. Using the Cancer BioPortal database, we identified PEAK1 co-expressors across multiple malignancies that are also common to the TGFβ response gene signature (TBRS). We then used the ScanSite database to identify predicted protein-protein binding partners of PEAK1 and the PEAK1-TBRS co-expressors. Analysis of the Cytoscape interactome and Babelomics-derived gene ontologies for a novel gene set including PEAK1, CRK, ZEB1, IL11 and COL4A1 enabled us to hypothesize that PEAK1 may be regulating TGFβ-induced EMT via its interaction with or regulation of these other genes. In this regard, we have demonstrated that PEAK1 is necessary for TGFβ to induce ZEB1-mediated EMT in the context of fibronectin/ITGB3 activation. These studies and future mechanistic studies will pave the way toward identifying the context in which TGFβ blockade may significantly improve breast cancer patient outcomes. 
 53BP1 suppresses epithelial-mesenchymal transition by downregulating ZEB1 through microRNA-200b/429 in breast cancer. Epithelial-mesenchymal transition (EMT) is an important mechanism of cancer invasion and   metastasis  . Although p53 binding protein 1 (53BP1) has been implicated in several biological processes, its function in EMT of human cancers has not yet been reported. Here, we show that 53BP1 negatively regulated EMT by modulating ZEB1 through targeting microRNA (miR)-200b and miR-429. Furthermore, 53BP1 promoted ZEB1-mediated upregulation of E-cadherin and also inhibited the expressions of mesenchymal markers, leading to increased migration and invasion in MDA-MB-231 breast cancer cells. Consistently, in MCF-7 breast cancer cells, low 53BP1 expression reduced E-cadherin expression, resulting in increased migration and invasion. These effects were reversed by miR-200b and miR-429 inhibition or overexpression. Sections of tumor xenograft model showed increased ZEB1 expression and decreased E-cadherin expression with the downregulation of 53BP1. In 18 clinical tissue samples, expression of 53BP1 was positively correlated with miR-200b and mir-429 and negatively correlated with ZEB1. It was also found that 53BP1 was associated with lymph node   metastasis  . Taken together, these results suggest that 53BP1 functioned as a tumor suppressor gene by its novel negative control of EMT through regulating the expression of miR-200b/429 and their target gene ZEB1. 
 ZEB1 promotes the progression and   metastasis   of cervical squamous cell carcinoma via the promotion of epithelial-mesenchymal transition. The process of epithelial-mesenchymal transition (EMT) clearly contributes to cancer   metastasis  . The aim of this study was to investigate the expression of the EMT-related transcription repressor ZEB1 and the expression of EMT-associated markers (E-cadherin, β-catenin and N-cadherin) in cervical squamous cell carcinoma. In addition, the role of ZEB1 and these EMT-associated markers in the progression and   metastasi  s of cervical squamous cell carcinoma was explored.The expression of ZEB1, E-cadherin, β-catenin and N-cadherin was evaluated in 81 specimens of cervical squamous cell carcinoma by immunohistochemistry; the clinicopathological significance of these markers was then analyzed.1) Of the 81 samples, 37 cases (45.7%) were positive for ZEB1, and nuclear expression of ZEB1 in tumor cells was positively associated with the differentiation status of the tumor tissue (P < 0.05), vascular invasion (P < 0.05) and lymph nod  e metastas  is (P < 0.05). 2) The loss of E-cadherin and β-catenin expression in tumor cells and the acquisition of N-cadherin expression were positively associated with the differentiation status of the tumor tissue (P < 0.05) and with the occurrence of vascular invasion (P < 0.05). 3) A significant negative correlation was observed between ZEB1 and E-cadherin expression (Spearman = -0.636, P < 0.05) and between ZEB1 and β-catenin expression (Spearman = -0.417, P < 0.05). Moreover, a significant positive correlation was observed between ZEB1 and N-cadherin expression (Spearman = 0.557, P < 0.05).These results emphasize the role of EMT in cervical squamous cell carcinoma. The upregulation of ZEB1 is associated with the abnormal expression of E-cadherin, β-catenin and N-cadherin, which might promote the progression   and metas  tasis of cervical squamous cell carcinoma. 
 Overexpression of ZEB1 associated with   metastasis   and invasion in patients with gastric carcinoma. The aim of this study was to investigate the expression of ZEB1 in gastric carcinoma, its correlation with the clinicopathology of gastric carcinoma, and the role of ZEB1 in invasion and   metastasis   in gastric carcinoma. ZEB1 expression was analyzed by immunohistochemistry and Western blot in 45 gastric carcinoma tissue samples that contained the adjacent gastric mucosa. The correlation between ZEB1 expression, the occurrence and development of gastric cancer, and clinical pathology was investigated. ZEB1 expression in the human gastric carcinoma cell line AGS was downregulated by RNA interference, and changes in ZEB1 expression corresponded with changes in the invasive and metastatic ability of AGS cells. Immunohistochemistry revealed that ZEB1 protein expression in gastric carcinoma tissues was significantly higher than in normal gastric mucosa tissues (p < 0.001). A lower degree of differentiation of gastric cancer (p = 0.009), a higher TNM (tumor, node, and   metastasis  ) stage (p = 0.010), and a larger scope of invasion were correlated with higher expression of ZEB1 (p = 0.041, 0.002). However, the expression of ZEB1 in gastric carcinoma tissue was independent of gender, age, and tumor size (p > 0.05). Western blot results also showed that ZEB1 protein expression was significantly higher in gastric carcinoma tissue than in the adjacent normal gastric mucosa tissue (p = 0.008). A lower degree of differentiation of the gastric carcinoma correlated with a higher TNM stage, and a larger scope of invasion correlated with increased ZEB1 expression (p = 0.023). Transfection of ZEB1 siRNA in AGS cells significantly decreased the expression level of ZEB1 protein (p = 0.035). Furthermore, the number of cells that could pass through the Transwell chamber was significantly lower in the transfected group than in the non-transfected control group (p = 0.039), indicating that the suppression of ZEB1 expression could significantly reduce the invasive and metastatic ability of AGS cells (p = 0.005). Concluding, in gastric carcinoma tissue, overexpression of ZEB1 may be related to the occurrence and development as well as invasion and   metastasis   of gastric carcinoma. 
 ZEB1 turns into a transcriptional activator by interacting with YAP1 in aggressive cancer types. Early dissemination,   metastasis   and therapy resistance are central hallmarks of aggressive cancer types and the leading cause of cancer-associated deaths. The EMT-inducing transcriptional repressor ZEB1 is a crucial stimulator of these processes, particularly by coupling the activation of cellular motility with stemness and survival properties. ZEB1 expression is associated with aggressive behaviour in many tumour types, but the potent effects cannot be solely explained by its proven function as a transcriptional repressor of epithelial genes. Here we describe a direct interaction of ZEB1 with the Hippo pathway effector YAP, but notably not with its paralogue TAZ. In consequence, ZEB1 switches its function to a transcriptional co-activator of a 'common ZEB1/YAP target gene set', thereby linking two pathways with similar cancer promoting effects. This gene set is a predictor of poor survival, therapy resistance and increased metastatic risk in breast cancer, indicating the clinical relevance of our findings. 
 Zeb1 regulates E-cadherin and Epcam (epithelial cell adhesion molecule) expression to control cell behavior in early zebrafish development. The ZEB1 transcription factor is best known as an inducer of epithelial-mesenchymal transitions (EMT) in cancer   metastasis  , acting through transcriptional repression of CDH1 (encoding E-cadherin) and the EMT-suppressing microRNA-200s (miR-200s). Here we analyze roles of the ZEB1 zebrafish orthologs, Zeb1a and Zeb1b, and of miR-200s in control of cell adhesion and morphogenesis during gastrulation and segmentation stages. Loss and gain of function analyses revealed that Zeb1 represses cdh1 expression to fine-tune adhesiveness of migrating deep blastodermal cells. Furthermore, Zeb1 acts as a repressor of epcam in the deep cells of the blastoderm and may contribute to control of epithelial integrity of enveloping layer cells, the outermost cells of the blastoderm. We found a similar ZEB1-dependent repression of EPCAM expression in human pancreatic and breast cancer cell lines, mediated through direct binding of ZEB1 to the EPCAM promoter. Thus, Zeb1 proteins employ several evolutionary conserved mechanisms to regulate cell-cell adhesion during development and cancer. 
 A Long Non-coding RNA Activated by Transforming Growth Factor-β is an Independent Prognostic Marker of Gastric Cancer. A recent study reported that long non-coding RNA activated by TGF-β (lncRNA-ATB) induced epithelial-mesenchymal transition (EMT) through the transforming growth factor-β (TGF-β)/miR-200s/ZEB axis in hepatocellular carcinoma. Herein, we focused on the clinical significance of lncRNA-ATB in gastric cancer (GC) patients.Quantitative reverse transcriptase polymerase chain reaction (qRT-PCR) was performed to examine expression of lncRNA-ATB, miR-200b, and miR-200c in GC tissues (n = 183). Patients were divided into high and low lncRNA-ATB expression groups using a cutoff of lncRNA-ATB/GAPDH ≥0.60 or <0.60 to determine the clinicopathological significance of lncRNA-ATB in GC. Moreover, we evaluated the expression of TGF-β, lncRNA-ATB, miR-200s, and ZEB1 in GC cell lines by qRT-PCR. GC cell lines were treated by recombinant TGF-β1 or TGF-β receptor inhibitor to examine morphologic changes and genetic alterations, such as lncRNA-ATB, miR-200s, and ZEB1 levels, with respect to the EMT phenotype.The high lncRNA-ATB group experienced a lower overall survival rate compared with the low lncRNA-ATB group, and multivariate analysis indicated that lncRNA-ATB was an independent prognostic factor (hazard ratio 3.50; 95 % CI 1.73-7.44; p = 0.0004). miR-200c levels were lower and ZEB1 levels were higher in the high lncRNA-ATB group than in the low lncRNA-ATB group. Treatment with TGF-β in GC cell lines resulted in morphological EMT changes, upregulation of lncRNA-ATB and ZEB1, and downregulation of miR-200c and CDH1. SB431542 reduced lncRNA-ATB expression.LncRNA-ATB plays an important role in EMT to promote inv  asion and   metastasis through the TGF-β/miR-200s/ZEB axis, resulting in a poor prognosis in GC. LncRNA-ATB is a novel biomarker of lncRNA, indicative of a poor prognosis in GC patients. 
 ZEB1 drives prometastatic actin cytoskeletal remodeling by downregulating miR-34a expression. Metastatic cancer is extremely difficult to treat, and the presence of metastases greatly reduces a cancer patient's likelihood of long-term survival. The ZEB1 transcriptional repressor promotes   metastasis   through downregulation of microRNAs (miRs) that are strong inducers of epithelial differentiation and inhibitors of stem cell factors. Given that each miR can target multiple genes with diverse functions, we posited that the prometastatic network controlled by ZEB1 extends beyond these processes. We tested this hypothesis using a mouse model of human lung adenocarcinoma   metastasis   driven by ZEB1, human lung carcinoma cells, and human breast carcinoma cells. Transcriptional profiling studies revealed that ZEB1 controls the expression of numerous oncogenic and tumor-suppressive miRs, including miR-34a. Ectopic expression of miR-34a decreased tumor cell invasion and   metastasis  , inhibited the formation of promigratory cytoskeletal structures, suppressed activation of the RHO GTPase family, and regulated a gene expression signature enriched in cytoskeletal functions and predictive of outcome in human lung adenocarcinomas. We identified several miR-34a target genes, including Arhgap1, which encodes a RHO GTPase activating protein that was required for tumor cell invasion. These findings demonstrate that ZEB1 drives prometastatic actin cytoskeletal remodeling by downregulating miR-34a expression and provide a compelling rationale to develop miR-34a as a therapeutic agent in lung cancer patients. 
 ABCB5-ZEB1 Axis Promotes Invasion and   Metastasis   in Breast Cancer Cells. ABCB5 belongs to the ATP-binding cassette (ABC) superfamily, which is recognized for playing a role in the failure of chemotherapy. ABCB5 has also been found to be overexpressed at the transcriptional level in a number of cancer subtypes, including breast cancer. However, the exact mechanism ABCB5 uses on cancer cell   metastasis   is still unclear. In the present study, we demonstrate that ABCB5 expression was increased in metastatic tissues when compared with nonmetastatic tissues. ABCB5 can significantly enhance   metastasis   and epithelial-mesenchymal transition (EMT), while knockdown of ABCB5 inhibited these processes. Microarray analysis indicated that ZEB1 may function as a downstream factor of ABCB5. Furthermore, the expression of ZEB1 in tissues is positively relevant to ABCB5 in breast cancer. Knocking down ZEB1 inhibits ABCB5 ectopic expression-induced migration and invasion, as well as EMT. Taken together, these results helped to realize the oncogene functions of ABCB5 in breast cancer cells and provided a new direction in treating breast cancer. 
 MiR-652 inhibits acidic microenvironment-induced epithelial-mesenchymal transition of pancreatic cancer cells by targeting ZEB1. Recent evidences suggest that the acidic microenvironment might facilitate epithelial mesenchymal transition (EMT) of tumor cells, while the effects of acidity on EMT of pancreatic cancer (PC) remain undefined. The present study demonstrated that acidity suppressed miR-652 expression, which further promoted EMT process by absenting inhibition on the transcriptional factor ZEB1 expression. At first, we found that acidity remarkably enhanced invasion ability of PC cells accompanying with increased mesenchymal and decreased epithelial markers. Meanwhile, miRNAs-microarray showed that miR-652, the potential regulator of ZEB1, was distinctly decreased in acidity-treated PC cells. Furthermore, restoration of miR-652 reversed acidity-induced EMT by inhibiting ZEB1 expression, while miR-652 inhibitor induced EMT in normal PC cells through promoting ZEB1 expression. Nevertheless, knockdown of ZEB1 significantly suppressed acidity-induced EMT in PC cells, but ZEB1 overexpression rescued the EMT which was inhibited by miR-652 overexpression. The in vivo results showed that the tumor growth and liver   metastasis   were remarkably retarded by both miR-652 overexpression and ZEB1 knockdown. The clinical samples further revealed that miR-652 was decreased in PC tissues and antagonistically correlated with ZEB1 expression, associating with late tumor stage, lymphatic invasion and   metastasis  . In conclusion, our study indicated a novel acidity/miR-652/ZEB1/EMT axis in the tumorigenesis of PC. 
 CUL4A induces epithelial-mesenchymal transition and promotes cancer   metastasis   by regulating ZEB1 expression. The ubiquitin ligase CUL4A has been implicated in tumorigenesis, but its contributions to progression and   metastasis   have not been evaluated. Here, we show that CUL4A is elevated in breast cancer as well as in ovarian, gastric, and colorectal tumors in which its expression level correlates positively with distant   metastasis  . CUL4A overexpression in normal or malignant human mammary epithelial cells increased their neoplastic properties in vitro and in vivo, markedly increasing epithelial-mesenchymal transition (EMT) and the metastatic capacity of malignant cells. In contrast, silencing CUL4A in aggressive breast cancer cells inhibited these processes. Mechanistically, we found that CUL4A modulated histone H3K4me3 at the promoter of the EMT regulatory gene ZEB1 in a manner associated with its transcription. ZEB1 silencing blocked CUL4A-driven proliferation, EMT, tumorigenesis, and   metastasis  . Furthermore, in human breast cancers, ZEB1 expression correlated positively with CUL4A expression and distant   metastasis  . Taken together, our findings reveal a pivotal role of CUL4A in regulating the metastatic behavior of breast cancer cells. 
 Biological roles and prognostic values of the epithelial-mesenchymal transition-mediating transcription factors Twist, ZEB1 and Slug in diffuse large B-cell lymphoma.   To evaluate the biological roles and prognostic significance of the epithelial-mesenchymal transition (EMT)-mediating transcription factors (TFs) Twist, ZEB1 and Slug in patients with diffuse large B-cell lymphoma (DLBCL). EMT has been shown to enhance solid tumou  r metastas  is, invasion, and proliferation.  Expression of Twist, ZEB1 and Slug was evaluated immunohistochemically in eight samples from reactive lymphoid tissues and in diagnostic samples from 102 DLBCL patients treated with curative intent with R-CHOP-type chemotherapy. ZEB1 and Slug expression correlated with adverse disease presentation. However, cytoplasmic Slug expression was linked to a favourable disease outcome, whereas nuclear expression of ZEB1 indicated an adverse outcome.  This study shows that an EMT-like process occurs in lymphomas. Of the TFs investigated, ZEB1 seems to be the main one associated with adverse clinical presentation and clinical outcome. Surprisingly, Slug expression in cytoplasm was linked to a favourable prognosis. Further studies are needed to evaluate whether inhibition of ZEB1 could serve as a therapeutic target. 
 MiR-205 promotes motility of ovarian cancer cells via targeting ZEB1. To investigate the clinical significance of microRNA-205 (miR-205) and zinc finger E-box binding homeobox 1 (ZEB1) in epithelial ovarian cancer (EOC) and the underlying mechanisms by which they are involved into tumorigenesis.Expression levels of miR-205 and ZEB1 mRNA in EOC tissues were detected by quantitative real-time PCR. Their associations with clinicopathological features of EOC patients were statistically analyzed. Luciferase reporter assay was used to confirm target associations between miR-205 and ZEB1. After that, the functions of miR-205-ZEB1 axis on cell migration and invasion were further determined by transwell assay in vitro.Expression levels of miR-205 (P=0.0001) and ZEB1 mRNA (P<0.0001) in clinical EOC tissues were significantly higher and lower than those in normal tissues, respectively. Interestingly, there was a negative correlation between miR-205 and ZEB1 mRNA expression in EOC tissues (P=0.01). Additionally, miR-205-upregulation and/or ZEB1-downregulation were significantly associated with high pathological grade and advanced clinical stage of EOC patients (all P<0.05). Meantime, luciferase reporter assays identified ZEB1 as a direct target of miR-205 in EOC cells. Moreover, miR-205 blockage inhibited, whereas miR-205 mimics promoted the motility of EOC cells in vitro. Importantly, all the alterations of the above cellular phenotypes by blocking or enhancing of miR-205 could be alleviated by subsequent suppression or re-introduction of its target ZEB1, respectively.MiR-205, acting as an oncogenic miRNA, may promote the clinical progression of EOC patients and enhance the cellular motility in vitro by directly and negatively regulating ZEB1, providing a potential therapeutic strategy for suppression of EOC   metastasis  . 
 Heterogeneous expression of zinc-finger E-box-binding homeobox 1 plays a pivotal role in   metastasis   via regulation of miR-200c in epithelial-mesenchymal transition. Although epithelial-mesenchymal transition (EMT) has been implicated as the pivotal event in   metastasis  , there is insufficient evidence related to EMT in clinical settings. Intratumor heterogeneity may lead to underestimation of gene expression representing EMT. In the present study, we investigated the expression of EMT-associated genes and microRNAs in primary colorectal cancer while considering intratumor heterogeneity. One-hundred and thirty-three multiple spatially separated samples were obtained from 8 patients with metastatic colorectal cancers and 8 with non-metastatic colorectal cancers, from the tumor center (TC), invasive front (IF) and   metastasis  . Differences in gene and microRNA expression were investigated by microarray and quantitative reverse-transcription PCR. Gene expression microarray analysis detected 7920 sites showing differing levels of gene expression among the TC, IF and   metastasis  . Expression of the EMT-associated gene zinc-finger E-box-binding homeobox 1 (ZEB1) significantly increased in the IF (p<0.01). To exclude individual differences, the expression ratio between TC and IF in each tumor was applied to analysis. This approach enabled recognition of the activation of the VEGF and Wnt signaling pathways, which were involved in   metastasis   via promotion of EMT. While no activation of these pathways was seen at the TC, regardless of whether tumors were metastatic or non-metastatic, they were preferentially activated at the IF in metastatic tumors, where high ZEB1 expression was seen in connection with decreased miR-200c expression. Multiple sampling in a tumor revealed that heterogeneous ZEB1 expression induced by EMT-associated signaling pathways played a pivotal role in   metastasis   via regulation of miR-200c. 
 Inhibition of TBK1 attenuates radiation-induced epithelial-mesenchymal transition of A549 human lung cancer cells via activation of GSK-3β and repression of ZEB1. Radiotherapy is an effective treatment method for lung cancer, particularly when the disease is at an advanced stage. However, previous researchers have observed that the majority of patients with conventional radiation therapy develop distant metastases and succumb to the disease. Thus, identifying and understanding novel pathways for the development of new therapeutic targets is a major goal in research on pulmonary neoplasms. Recent studies suggest that epithelial-mesenchymal transition (EMT) is the most important contributor to cancer   metastasi  s. Induction of this complex process requires endogenously produced microRNAs; specifically, downregulation of the miRNA-200c causes an induction of EMT. We recently identified the tank-binding kinase-1 (TBK1) as a downstream effector of the miR-200c-driven pathway, but the biological function of TBK1 in EMT remains unknown. In this study, we tested whether TBK1 has a role in radiation-induced EMT and identified associated potential mechanisms. Human alveolar type II epithelial carcinoma A549 cells were irradiated with (60)Co γ-rays. Western blotting revealed a time- and dose-dependent decrease in E-cadherin with a concomitant increase in vimentin after radiation, suggesting that the epithelial cells acquired a mesenchymal-like morphology. TBK1 siRNA significantly inhibited radiation-induced suppression of the epithelial marker E-cadherin and upregulation of the mesenchymal marker vimentin. The invasion and migratory potential of lung cancer cells upon radiation treatment was also reduced by TBK1 knockdown. Furthermore, radiation-induced EMT attenuated by TBK1 depletion was partially dependent on transcriptional factor ZEB1 expression. Finally, we found glycogen synthase kinase-3β (GSK-3β) is involved in regulation of radiation-induced EMT by TBK1. Thus, our findings reveal that TBK1 signaling regulates radiation-induced EMT by controlling GSK-3β phosphorylation and ZEB1 expression. TBK1 may therefore constitute a useful target for treatment of radiotherapy-ind  uced metas  tasis diseases. 
 Prognostic significance of Zinc finger E-box binding homeobox 1 (ZEB1) expression in cancer cells and cancer-associated fibroblasts in pancreatic head cancer. Pancreatic ductal adenocarcinoma (PDAC) is characterized by an aggressive biology and poor prognosis. Experimental evidence has suggested a role for the transcriptional repressor Zinc finger E-box binding homeobox 1 (ZEB1) in epithelial-mesenchymal transition, invasion, and   metastasis   in PDAC. ZEB1 expression has been observed in cancer cells as well as stromal fibroblasts. Our study aimed to evaluate the prognostic value of ZEB1 expression in PDAC tissue.Patient baseline and follow-up data were extracted from a prospectively maintained database. After clinicopathologic re-review, serial sliced tissue slides were immunostained for ZEB1, E-cadherin, vimentin, and pan-cytokeratin. ZEB1 expression in cancer cells and adjacent stromal fibroblasts was graded separately and correlated to routine histopathologic parameters and survival after resection.A total of 117 cases of PDAC were included in the study. High ZEB1 expression in cancer cells and in stromal cancer-associated fibroblasts was associated with poor prognosis. There was also a trend for poor prognosis with a lymph node ratio of greater than 0.10. In line with its role as an inducer of epithelial-mesenchymal transition, ZEB1 expression in cancer cells was positively correlated with Vimentin expression and negatively with E-Cadherin expression. In multivariate analysis, stromal ZEB1 expression grade was the only independent factor of survival after resection.Our data suggest that ZEB1 expression in cancer cells as well as in stromal fibroblasts are strong prognostic factors in PDAC. Stromal ZEB1 expression is identified for the first time as an independent predictor of survival after resection of PDAC. This observation suggests that therapies targeting ZEB1 and its downstream pathways could hit both cancer cells and supporting cancer-associated fibroblasts. 
 Cordycepin (3'-deoxyadenosine) suppressed HMGA2, Twist1 and ZEB1-dependent melanoma invasion and   metastasis   by targeting miR-33b. Malignant melanoma, the most deadly form of skin cancer, has a high propensity for metastatic spread and is notoriously chemotherapy-resistant. Cordycepin, the active component of Cordyceps spp., has been identified to have anti-metastatic effect on tumor progression and thus possesses pharmacological and therapeutic potentials. However, the mechanisms of anti-metastatic effects of cordycepin at cellular levels remain elusive. We analyzed the effect of cordycepin on human melanoma miRNA expression profiles by miRNAarray and found that miR-33b was upregulated in highly-metastatic melanoma cell lines following cordycepin exposure. Cordycepin-mediated miR-33b expression was dependent on LXR-RXR heterodimer activation. miR-33b directly binds to HMGA2, Twist1 and ZEB1 3'-UTR to suppress their expression. The negative correlations between miR-33b levels and HMGA2, Twist1 or ZEB1 expression were detected in 72 patient melanoma tissue samples. By targeting HMGA2 and Twist1, miR-33b attenuated melanoma migration and invasiveness upon cordycepin exposure. miR-33b knockdown or ZEB1 overexpression reverted cordycepin-mediated mesenchymal-epithelial transition (MET), triggering the expression of N-cadherin. In spontaneous   metastasis   models, cordycepin suppressed tumor   metastasis   without altering primary tumor growth. We showed for the first time that targeting miRNA by cordycepin indicates a new mechanism of cordycepin-induced suppression of tumor   metastasis   and miR-33b/HMGA2/Twist1/ZEB1 axis plays critical roles in regulating melanoma dissemination. 
 The transcription factor ZEB1 (deltaEF1) promotes tumour cell dedifferentiation by repressing master regulators of epithelial polarity. Epithelial to mesenchymal transition (EMT) is implicated in the progression of primary tumours towards   metastasis   and is likely caused by a pathological activation of transcription factors regulating EMT in embryonic development. To analyse EMT-causing pathways in tumourigenesis, we identified transcriptional targets of the E-cadherin repressor ZEB1 in invasive human cancer cells. We show that ZEB1 repressed multiple key determinants of epithelial differentiation and cell-cell adhesion, including the cell polarity genes Crumbs3, HUGL2 and Pals1-associated tight junction protein. ZEB1 associated with their endogenous promoters in vivo, and strongly repressed promotor activities in reporter assays. ZEB1 downregulation in undifferentiated cancer cells by RNA interference was sufficient to upregulate expression of these cell polarity genes on the RNA and protein level, to re-establish epithelial features and to impair cell motility in vitro. In human colorectal cancer, ZEB1 expression was limited to the tumour-host interface and was accompanied by loss of intercellular adhesion and tumour cell invasion. In invasive ductal and lobular breast cancer, upregulation of ZEB1 was stringently coupled to cancer cell dedifferentiation. Our data show that ZEB1 represents a key player in pathologic EMTs associated with tumour progression. 
 The microRNA-200/Zeb1 axis regulates ECM-dependent β1-integrin/FAK signaling, cancer cell invasion and   metastasi  s through CRKL. Tumor cell   metastasi  s is a complex process that has been mechanistically linked to the epithelial-mesenchymal transition (EMT). The double-negative feedback loop between the microRNA-200 family and the Zeb1 transcriptional repressor is a master EMT regulator, but there is incomplete understanding of how miR-200 suppresses invasion. Our recent efforts have focused on the tumor cell-matrix interactions essential to tumor cell activation. Herein we utilized both our Kras/p53 mutant mouse model and human lung cancer cell lines to demonstrate that upon miR-200 loss integrin β1-collagen I interactions drive 3D in vitro migration/invasion and in vivo metastases. Zeb1-dependent EMT enhances tumor cell responsiveness to the ECM composition and activates FAK/Src pathway signaling by de-repression of the direct miR-200 target, CRKL. We demonstrate that CRKL serves as an adaptor molecule to facilitate focal adhesion formation, mediates outside-in signaling through Itgβ1 to drive cell invasion, and inside-out signaling that maintains tumor cell-matrix contacts required for cell invasion. Importantly, CRKL levels in pan-cancer TCGA analyses were predictive of survival and CRKL knockdown suppressed experimental metastases in vivo without affecting primary tumor growth. Our findings highlight the critical ECM-tumor cell interactions regulated by miR-200/Zeb1-dependent EMT that activate intracellular signaling pathways responsible for tumor cell invasion a  nd metasta  sis. 
 The ZEB1/miR-200c feedback loop regulates invasion via actin interacting proteins MYLK and TKS5. Epithelial to mesenchymal transition (EMT) is a developmental process which is aberrantly activated during cancer invasion and   metastasis  . Elevated expression of EMT-inducers like ZEB1 enables tumor cells to detach from the primary tumor and invade into the surrounding tissue. The main antagonist of ZEB1 in controlling EMT is the microRNA-200 family that is reciprocally linked to ZEB1 in a double negative feedback loop. Here, we further elucidate how the ZEB1/miR-200 feedback loop controls invasion of tumor cells. The process of EMT is attended by major changes in the actin cytoskeleton. Via in silico screening of genes encoding for actin interacting proteins, we identified two novel targets of miR-200c - TKS5 and MYLK (MLCK). Co-expression of both genes with ZEB1 was observed in several cancer cell lines as well as in breast cancer patients and correlated with low miR-200c levels. Depletion of TKS5 or MYLK in breast cancer cells reduced their invasive potential and their ability to form invadopodia. Whereas TKS5 is known to be a major component, we could identify MYLK as a novel player in invadopodia formation. In summary, TKS5 and MYLK represent two mediators of invasive behavior of cancer cells that are regulated by the ZEB1/miR-200 feedback loop. 
 Inhibition of ZEB1 reverses EMT and chemoresistance in docetaxel-resistant human lung adenocarcinoma cell line. Docetaxel has been used as one of the first-line chemotherapies in solid tumors including advanced non-small cell lung cancer (NSCLC). However, limited responses to chemotherapy are observed in clinic and the molecular mechanisms have not been fully understood. Emerging evidence suggests that epithelial-mesenchymal transition (EMT) plays an important role in the processes of tumor   metastasis   as well as resistance towards anticancer agents. In this study, it was observed that docetaxel-resistant human lung adenocarcinoma cell line (SPC-A1/DTX) was typical of mesenchymal phenotype. SPC-A1/DTX cell line has increased migratory and invasive capacity both in vitro and in vivo. Among the master EMT-inducing transcriptional factors, ZEB1 was found to be significantly increased in SPC-A1/DTX cell line. ZEB1 knockdown with RNA interference could reverse the EMT phenotype and inhibit the migratory ability of SPC-A1/DTX cells. Furthermore, inhibition of ZEB1 significantly enhanced the chemosensitivity of SPC-A1/DTX cells to docetaxel in vitro and in vivo and ectopic expression of ZEB1 increased the chemoresistance of SPC-A1 cells to docetaxel. All these results provide experimental evidence that ZEB1 might be an attractive target for the treatment of human NSCLC. 
 ZEB1 expression in endometrial biopsy predicts lymph node metastases in patient with endometrial cancer. The purpose of this study was to analyze the expression of zinc-finger E-box-binding homeobox 1 (ZEB1) in endometrial biopsy and its correlation with preoperative characteristics, including lymph node metastases in patient with endometrial cancer.Using quantitative RT-PCR, ZEB1 expressions in endometrial biopsy from 452 patients were measured. The relationship between ZEB1 expression and preoperative characteristics was analyzed.ZEB1 expressions were significantly associated with subtype, grade, myometrial invasion, and lymph node metastases. Lymph node metastases could be identified with a sensitivity of 57.8% at specificity of 74.1% by ZEB1 expression in endometrial biopsy. Based on combination of preoperative characteristics and ZEB1 expression, lymph node metastases could be identified with a sensitivity of 62.1% at specificity of 96.2% prior to hysterectomy.ZEB1 expression in endometrial biopsy could help physicians to better predict the lymph node   metastasis   in patients with endometrial cancer prior to hysterectomy. 
 miR-1236-3p represses the cell migration and invasion abilities by targeting ZEB1 in high-grade serous ovarian carcinoma. Ovarian cancer, particularly high-grade serous ovarian carcinoma (HG-SOC), is still the main cause of death among gynecological malignancies. However, the molecular mechanisms related to its malignant biological behavior are still unclear. Recent studies indicate that microRNAs (miRNAs) play an important role in tumor   metastasis  . Here, we report that miR-1236-3p expression was downregulated in HG-SOC when compared to that in normal fallopian tube tissue. Manipulation of miR-1236-3p significantly influenced the morphology, migration and invasion of ovarian cancer cell lines (A2780 and SKOV3). With dual-luciferase reporter assay, we demonstrated that miR‑1236-3p binds to the 3'UTR of zinc-finger E-box binding homeobox 1 (ZEB1) mRNA, and functions as a negative regulator of ZEB1. Furthermore, we revealed that manipulation of miR-1236-3p modulates ZEB1 expression and influences expression of its downstream genes E-cadherin and N-cadherin at both the mRNA and protein levels. We also found an inverse relationship between miR‑1236-3p and ZEB1 expression in the HG-SOC tissue samples. Taken together, our results indicate that miR-1236-3p regulates ovarian can  cer metast  asis by directly targeting ZEB1, and it may play an important role in the diagnosis and treatment of ovarian cancer. 
 [Aberrant expressions of β-catenin and ZEB1 in bladder cancer and their significance]. To detect the expressions of β-catenin and zinc finger E-box binding homeobox 1 (ZEB1) in bladder cancer tissues, and analyze their correlation and significance in bladder cancer occurrence and progression.The study collected 79 specimens of bladder cancer. EnVision immunohistochemical staining was used to detect the expressions and distribution of β-catenin and ZEB1 protein. Their correlation was analyzed and their relationship with clinicopathological characteristics was investigated.There was a significant heterogeneity in the expression of β-catenin in bladder cancer tissues. It might be distributed in the cell membrane, cytoplasm or nucleus. In low-grade clinical and pathological urothelial carcinoma, β-catenin was mainly expressed in cell membrane and cytoplasm, but in high-grade clinical pathological bladder cancer tissues, it was mostly located in cell cytoplasm and nucleus. ZEB1 in bladder cancer tissues was mainly expressed in cell nucleus, and its expression was elevated with the increased tumor pathological grade and clinical stage. The expressions of the above two proteins were significantly correlated.Aberrant expressions of β-catenin and ZEB1 in bladder cancer tissues are relevant to bladder tumor differentiatio  n and meta  stasis, and the two expressions are evidently correlated. The two proteins can be simultaneously used as candidate targets for early diagnosis and prognosis prediction. 
 Overexpression of zinc finger E-box binding homeobox factor 1 promotes tumor invasiveness and confers unfavorable prognosis in esophageal squamous cell carcinoma. Zinc finger E-box binding homeobox factor 1 (ZEB1), as a crucial mediator of "epithelial-mesenchymal transition," contributes to malignant progression of various epithelial tumors. However, its involvement in human esophageal squamous cell carcinoma (ESCC) remains unclear. In order to investigate the expression pattern of ZEB1 in ESCC tissues and evaluate its associations with tumor progression and patients' prognosis, 100 pairs of formalin-fixed and paraffin-embedded cancerous and adjacent noncancerous tissues from patients with ESCC were used to detect the expression pattern of ZEB1 by immunohistochemistry. Then, the association between ZEB1 expression, clinicopathological parameters, and prognosis of ESCC was examined. We also performed migration and invasion assays of small interfering RNA (siRNA)-targeted ZEB1-transfected cells in vitro. As a result, expression level of ZEB1 was significantly higher in ESCC tissues compared to that in adjacent noncancerous tissues (P < 0.001). High expression of ZEB1 was observed in 55.00 % (55/100) of ESCCs. In addition, high ZEB1 expression was found to be closely correlated with advanced tumor stage (P = 0.001), positive ly  mph node m  etastasis (P = 0.001), great tumor depth (P = 0.03), and high histologic grade (P = 0.008). Moreover, multivariate analysis showed that the status of ZEB1 expression was an independent predictor for overall survival in ESCC. Furthermore, knockdown of ZEB1 by transfection of siRNA-ZEB1 abrogated the migration and invasion of ESCC cells in vitro. Taken together, our data offer the convincing evidence that ZEB1 may play a crucial role in promoting aggressive ESCC progression. ZEB1 may serve as an effective prognostic marker and a potential target for therapeutic intervention of ESCC. 
 Nuclear Snail1 and nuclear ZEB1 protein expression in invasive and intraductal human breast carcinomas. Snail1 and ZEB1 are transcriptional repressors that drive tumor initiation and   metastasis   in animal models. Snail1 and ZEB1 are frequently coexpressed in tumor cell lines, suggesting that these factors may cooperate to promote tumor progression. However, coexpression of these transcriptional repressors in primary human cancer specimens has not been investigated. Previous studies assessed expression in primary breast cancers of Snail1 messenger RNA, which does not reflect Snail1 activity because Snail1 is subject to posttranslational modifications that inhibit its nuclear localization/activity. In the current study, using breast tumor cell lines of known Snail1 and ZEB1 expression status, we developed immunohistochemistry protocols for detecting nuclear Snail1 and nuclear ZEB1 proteins. Using these protocols, we assessed nuclear Snail1 and nuclear ZEB1 expressions in primary human breast cancers of varying subtypes (n = 78). Nuclear Snail1 and estrogen receptor α expressions were inversely associated in primary breast cancers, and nuclear Snail1 was expressed in approximately 80% of triple-negative breast cancers (lacking estrogen receptor α, progesterone receptor, and human epidermal growth factor receptor 2 overexpression). In contrast, nuclear ZEB1 was expressed at a significantly lower frequency in these breast cancers. Notably, nuclear Snail1 protein was detected in 45% of ductal carcinoma in situ specimens (n = 29), raising the important possibility that nuclear Snail1 expression in early stage breast lesions may predict future development of invasive breast cancer. Collectively, our studies demonstrate frequent expression of nuclear Snail1, but not nuclear ZEB1, in invasive, triple-negative breast cancers as well as in intraductal carcinomas. 
 WASF3 regulates miR-200 inactivation by ZEB1 through suppression of KISS1 leading to increased invasiveness in breast cancer cells. The WASF3 gene promotes invasion and   metastasis   in breast cancer cells, which have undergone epithelial-to-mesenchyme transition (EMT). Overexpression of WASF3 in cells that do not show EMT increases their invasion potential as a result of increased ZEB1/2 levels, which specifically suppress the anti-invasion chromosome 1 miR-200a/200b/429 cluster. ZEB1/2 upregulation by WASF3 results from downregulation of KISS1, leading to the release of inhibition of nuclear factor (NF)κB by IκBα. We further show that ZEB1 expression is regulated by the NFκB transcription factor. Knockdown of WASF3 in breast cancer cells leads to reduced ZEB1 levels and increased miR-200 and E-cadherin levels, resulting in loss of invasion potential. The central regulation of this interactive pathway by WASF3 accounts for the increased invasion associated with increased WASF3 expression seen in aggressive breast cancer cells. WASF3, therefore, is a potential target to suppress invasion   and metast  asis in breast cancer cells. 
 Clinicopathological analysis of pleomorphic carcinoma of the lung: diffuse ZEB1 expression predicts poor survival. Pleomorphic carcinoma (PC) of the lung is a rare epithelial tumor. The clinicopathological characteristics and prognostic factors of PC are controversial. The information on the ZEB1 gene, which crucially impacts survival of patients with other malignant tumors, is limited for PC.Clinicopathological characteristics of 62 patients with PC were investigated in this study. Associations between immunohistochemical expression of ZEB1 and clinical factors, including patient prognosis, were examined. The patient population consisted of 51 (82.2%) men and 11 (17.8%) women, with a mean age of 65.5 years (range, 31-81 years).The overall survival rate of the 42 patients, for whom follow-up was available, was 68.3% at 5 years. Using TNM criteria, 7 (11.3%), 11 (17.7%), 3 (4.8%), 21 (33.8%), 15 (24.2%), 2 (3.2%), and 3 (4.8%) patients were classified under pathological stage IA, IB, IIA, IIB, IIIA, IIIB and IV carcinomas, respectively. Fifteen (24.1%) patients had tumors consisting entirely of spindle and giant cells (PC component). The other 47 (75.8%) cancers contained additional carcinoma components (i.e., adenocarcinoma (34/62, 54.8%), squamous cell carcinoma (7/62, 11.3%), adenosquamous carcinoma (4/62, 6.5%) and large cell carcinoma (2/62, 3.2%)). Four of 7 (57.1%) stage IA (<20mm) tumors consisted only of spindle and giant cells. ZEB1 expression was observed only in the PC component. Diffuse expression of ZEB1, was defined as positive nuclear staining in ≥75% of cancer cells, and was found in the PC component in 12 patients. Multivariate analysis revealed that lymph nod  e metastas  is, pleural invasion, and diffuse ZEB1 expression in the PC component predicted poorer disease-specific survival (p=0.007, 0.022, and 0.016, respectively).This is the first report to indicate that ZEB1 may be used as an immunohistochemical prognosticator of PC, which may be useful for histological assessment of PC in biopsy and surgical specimens. 
 The ubiquitin ligase Siah is a novel regulator of Zeb1 in breast cancer. Elucidating the mechanisms that underlie   metastasis   is of paramount importance to understanding tumor progression and to the development of novel therapeutics. Epithelial to Mesenchymal Transition (EMT) plays a vital role in tumor cell dissemination and is regulated by a core cassette of transcription factors. Despite recent advances, the molecular pathways that regulate the EMT program have not yet been fully delineated. We show that Siah ubiquitin ligases regulate Zeb1 protein, a key EMT transcription factor. The induction of EMT in breast cancer cells leads to the down-regulation of Siah, while the loss of Siah induces a mesenchymal phenotype, concurrent with an up-regulation of Zeb1. Overexpression of Siah in vitro mediates Zeb1 degradation, which can be blocked with a Siah peptide inhibitor. Thus, this work demonstrates that Siah is a novel regulator of EMT. This work is the first to identify a mechanism of post-translational regulation of the key Epithelial to Mesenchymal Transition transcription factor Zeb1. 
 miR-429 inhibits migration and invasion of breast cancer cells in vitro. Accumulating evidence indicates that microRNAs (miRNAs) are involved in regulating cancer invasion and   metastasis  , and an increasing number of research demonstrates that miRNAs can promote or inhibit cell motility depending on genetic background of different cancers and the microenvironment. In the present study, we established an in vivo bone   metastasis   model of breast cancer by injecting MDA-MB-231 cells into the left ventricle of nude mice, and then screened the differentially expressed miRNAs between parental and bone-metastatic MDA-MB-231 cells using miRNA array. The results revealed that decreased expression of miR-429 was probably involved in negatively regulating bone   metastasis   of breast cancer cells. On the other hand, overexpression of miR-429 in MDA-MB-231 cells remarkably suppressed invasion in vitro. We identified ZEB1 and CRKL as potential targets of miR-429 by analyzing combined results from in silico search and global expression array of the same RNA samples. Immunoblot assay confirmed that miR-429 reduced their expression at protein level. Taken together, our results offer an opportunity for further understanding of the recondite mechanisms underlying the bone   metastasis   of breast cancer. 
 The glycosyltransferase LARGE2 is repressed by Snail and ZEB1 in prostate cancer. Reductions in both expression of the dystroglycan core protein and functional glycosylation of the α-dystroglycan (αDG) subunit have been reported in a number of cancers and may contribute to disease progression. In the case of prostate cancer, one mechanism that contributes to αDG hypoglycosylation is transcriptional down-regulation of LARGE2 (GYLTY1B), a glycosyltransferase that produces the functional (laminin-binding) glycan on αDG, but the mechanism(s) underlying reduction of LARGE2 mRNA remain unclear. Here, we show that αDG hypoglycosylation is associated with epithelial-to-mesenchymal transition (EMT)-like status. We examined immunoreactivity for both functionally-glycosylated αDG and E-cadherin by flow cytometry and the relative expression of ZEB1 mRNA and the αDG glycosyltransferase LARGE2 mRNA in prostate and other cancer cell lines by quantitative RT-PCR. To study the role of ZEB1 and other transcription factors in the regulation of LARGE2, we employed overexpression and knockdown approaches. Snail- or ZEB1-driven EMT caused αDG hypoglycosylation by repressing expression of the LARGE2 mRNA, with both ZEB1-dependent and -independent mechanisms contributing to Snail-mediated LARGE2 repression. To examine the direct regulation of LARGE2 by Snail and ZEB1 we employed luciferase reporter and chromatin immunoprecipitation assays. Snail and ZEB1 were found to bind directly to the LARGE2 promoter, specifically to E/Z-box clusters. Furthermore, analysis of gene expression profiles of clinical samples in The Cancer Genome Atlas reveals negative correlation of LARGE2 and ZEB1 expression in various cancers. Collectively, our results suggest that LARGE2 is negatively regulated by Snail and/or ZEB1, revealing a mechanistic basis for αDG hypoglycosylation during prostate cancer progres  sion and m  etastasis. 
 The EMT-activator ZEB1 promotes tumorigenicity by repressing stemness-inhibiting microRNAs. Invasion and   metastasis   of carcinomas is promoted by the activation of the embryonic 'epithelial to mesenchymal transition' (EMT) program, which triggers cellular mobility and subsequent dissemination of tumour cells. We recently showed that the EMT-activator ZEB1 (zinc finger E-box binding homeobox 1) is a crucial promoter of   metastasis   and demonstrated that ZEB1 inhibits expression of the microRNA-200 (miR-200) family, whose members are strong inducers of epithelial differentiation. Here, we report that ZEB1 not only promotes tumour cell dissemination, but is also necessary for the tumour-initiating capacity of pancreatic and colorectal cancer cells. We show that ZEB1 represses expression of stemness-inhibiting miR-203 and that candidate targets of miR-200 family members are also stem cell factors, such as Sox2 and Klf4. Moreover, miR-200c, miR-203 and miR-183 cooperate to suppress expression of stem cell factors in cancer cells and mouse embryonic stem (ES) cells, as demonstrated for the polycomb repressor Bmi1. We propose that ZEB1 links EMT-activation and stemness-maintenance by suppressing stemness-inhibiting microRNAs (miRNAs) and thereby is a promoter of mobile, migrating cancer stem cells. Thus, targeting the ZEB1-miR-200 feedback loop might form the basis of a promising treatment for fatal tumours, such as pancreatic cancer. 
 The nuclear protein expression levels of SNAI1 and ZEB1 are involved in the progression and lymph node   metastasis   of cervical cancer via the epithelial-mesenchymal transition pathway. Growing evidence illustrates that aberrant activation of the epithelial-mesenchymal transition plays a key role in tumor cell invasion and   metastasis  . Transcription factors SNAI1 and ZEB1 regulate the epithelial-mesenchymal transition. To determine if SNAI1 and ZEB1 are involved in the   metastasis   of cervical cancer, we used immunohistochemistry to evaluate the expression of SNAI1, ZEB1, and vimentin in tumor and stromal compartments for a large set of cervical carcinoma samples. Results were evaluated using an H score (percentage × intensity). Of 70 samples, 64 cases (91.43%) were positive for SNAI1 expression. The median SNAI1 H score was 174.00 (range, 5-285). Sixty-seven cases (95.71%) were positive for ZEB1, with a median H score of 165.77 (range, 5-260). Nuclear expression of SNAI1 and ZEB1 in tumor cells was positively associated with International Federation of Gynecology and Obstetrics (FIGO) stages (P = .015 and P = .008, respectively) and lymph node   metastasi  s (P = .007 and P = .007, respectively); meanwhile, expression of vimentin in tumor cells was positively associated with lymph node   metastasi  s (P = .019). According to negative vimentin expression, nuclear expression of ZEB1 in tumor cells was positively associated with FIGO stages (P = .04). According to positive vimentin expression, nuclear expression of SNAI1 in tumor cells was positively associated with FIGO stages (P = .018) and pN (P = .029). In light of these findings, we propose that SNAI1 and ZEB1 have the potential to be used as a novel predictor of pelvic lymph node   metastasi  s and represent promising therapeutic targets in patients with cervical cancer. 
 Roles of STAT3 and ZEB1 proteins in E-cadherin down-regulation and human colorectal cancer epithelial-mesenchymal transition. The progression of colorectal carcinoma (CRC) to invasive and metastatic disease may involve localized occurrences of epithelial-mesenchymal transition (EMT). However, mechanisms of the EMT process in CRC progression are not fully understood. We previously showed that knockdown of signal transducer and activator of transcription 3 (STAT3) up-regulated E-cadherin (a key component in EMT progression) in CRC. In this study, we examined the roles of STAT3 in CRC EMT and ZEB1, an EMT inducer, in STAT3-induced down-regulation of E-cadherin. Knockdown of STAT3 significantly increased E-cadherin and decreased N-cadherin and vimentin expressions in highly invasive LoVo CRC cells. Meanwhile, overexpression of STAT3 significantly reduced E-cadherin and enhanced N-cadherin and vimentin expressions in weakly invasive SW1116 CRC cells. Activation of STAT3 significantly increased CRC cell invasiveness and resistance to apoptosis. Knockdown of STAT3 dramatically enhanced chemosensitivity of CRC cells to fluorouracil. STAT3 regulated ZEB1 expression in CRC cells, and the STAT3-induced decrease in E-cadherin and cell invasion depended on activation of ZEB1 in CRC cells. Additionally, pSTAT3(Tyr-705) and ZEB1 expressions were significantly correlated with TNM (tumor, lymph node, and   metastasis   stages) (p < 0.01). In conclusion, STAT3 may directly mediate EMT progression and regulate ZEB1 expression in CRC. ZEB1 may participate in STAT3-induced cell invasion and E-cadherin down-regulation in CRC cells. The expressions of pSTAT3(Tyr-705) and ZEB1 may be positively associated with CRC   metastasis  . Our data may provide potential targets to prevent and/or treat CRC invasion and   metastasis  . 
 Direct repression of MYB by ZEB1 suppresses proliferation and epithelial gene expression during epithelial-to-mesenchymal transition of breast cancer cells. Epithelial-to-mesenchymal transition (EMT) promotes cell migration and is important in   metastasis  . Cellular proliferation is often downregulated during EMT, and the reverse transition (MET) in metastases appears to be required for restoration of proliferation in secondary tumors. We studied the interplay between EMT and proliferation control by MYB in breast cancer cells.MYB, ZEB1, and CDH1 expression levels were manipulated by lentiviral small-hairpin RNA (shRNA)-mediated knockdown/overexpression, and verified with Western blotting, immunocytochemistry, and qRT-PCR. Proliferation was assessed with bromodeoxyuridine pulse labeling and flow cytometry, and sulforhodamine B assays. EMT was induced with epidermal growth factor for 9 days or by exposure to hypoxia (1% oxygen) for up to 5 days, and assessed with qRT-PCR, cell morphology, and colony morphology. Protein expression in human breast cancers was assessed with immunohistochemistry. ZEB1-MYB promoter binding and repression were determined with Chromatin Immunoprecipitation Assay and a luciferase reporter assay, respectively. Student paired t tests, Mann-Whitney, and repeated measures two-way ANOVA tests determined statistical significance (P < 0.05).Parental PMC42-ET cells displayed higher expression of ZEB1 and lower expression of MYB than did the PMC42-LA epithelial variant. Knockdown of ZEB1 in PMC42-ET and MDA-MB-231 cells caused increased expression of MYB and a transition to a more epithelial phenotype, which in PMC42-ET cells was coupled with increased proliferation. Indeed, we observed an inverse relation between MYB and ZEB1 expression in two in vitro EMT cell models, in matched human breast tumors and lymph node metastases, and in human breast cancer cell lines. Knockdown of MYB in PMC42-LA cells (MYBsh-LA) led to morphologic changes and protein expression consistent with an EMT. ZEB1 expression was raised in MYBsh-LA cells and significantly repressed in MYB-overexpressing MDA-MB-231 cells, which also showed reduced random migration and a shift from mesenchymal to epithelial colony morphology in two dimensional monolayer cultures. Finally, we detected binding of ZEB1 to MYB promoter in PMC42-ET cells, and ZEB1 overexpression repressed MYB promoter activity.This work identifies ZEB1 as a transcriptional repressor of MYB and suggests a reciprocal MYB-ZEB1 repressive relation, providing a mechanism through which proliferation and the epithelial phenotype may be coordinately modulated in breast cancer cells. 
 Mutant p53 gain-of-function induces epithelial-mesenchymal transition through modulation of the miR-130b-ZEB1 axis. The tumor suppressor gene p53 has been implicated in the regulation of epithelial-mesenchymal transition (EMT) and tumor   metastasis   by regulating microRNA (miRNA) expression. Here, we report that mutant p53 exerts oncogenic functions and promotes EMT in endometrial cancer (EC) by directly binding to the promoter of miR-130b (a negative regulator of ZEB1) and inhibiting its transcription. We transduced p53 mutants into p53-null EC cells, profiled the miRNA expression by miRNA microarray and identified miR-130b as a potential target of mutant p53. Ectopic expression of p53 mutants repressed the expression of miR-130b and triggered ZEB1-dependent EMT and cancer cell invasion. Loss of an endogenous p53 mutation increased the expression of miR-130b, which resulted in reduced ZEB1 expression and attenuation of the EMT phenotype. Furthermore, re-expression of miR-130b suppressed mutant p53-induced EMT and ZEB1 expression. Importantly, the expression of miR-130 was significantly reduced in EC tissues, and patients with higher expression levels of miR-130b survived longer. These data provide a novel understanding of the roles of p53 gain-of-function mutations in accelerating tumor progression and   metastasis   through modulation of the miR-130b-ZEB1 axis. 
 The transcriptional repressor ZEB1 promotes   metastasis   and loss of cell polarity in cancer. Invasion and   metastasis   are the hallmarks of malignant tumor progression and the main cause of death in cancer. The embryonic program "epithelial-mesenchymal transition" (EMT) is thought to trigger invasion by allowing tumor cell dissemination. Here, we describe that the EMT-inducing transcriptional repressor ZEB1 promotes colorectal cancer cell   metastasis   and loss of cell polarity. Thereby, ZEB1 suppresses the expression of cell polarity factors, in particular of Lgl2, which we found reduced in colorectal and breast cancers. We further show that retention of Lgl2 expression is critical for the epithelial phenotype and that its loss might be involved in   metastasis  . Thus, by linking EMT, loss of polarity, and   metastasis  , ZEB1 is a crucial promoter of malignant tumor progression. 
 β-catenin/TCF4 complex induces the epithelial-to-mesenchymal transition (EMT)-activator ZEB1 to regulate tumor invasiveness. In most carcinomas, invasion of malignant cells into surrounding tissues involves their molecular reprogramming as part of an epithelial-to-mesenchymal transition (EMT). Mutation of the APC gene in most colorectal carcinomas (CRCs) contributes to the nuclear translocation of the oncoprotein β-catenin that upon binding to T-cell and lymphoid enhancer (TCF-LEF) factors triggers an EMT and a proinvasive gene expression profile. A key inducer of EMT is the ZEB1 transcription factor whose expression promotes tumorigenesis an  d metastas  is in carcinomas. As inhibitor of the epithelial phenotype, ZEB1 is never present in the epithelium of normal colon or the tumor center of CRCs where β-catenin remains membranous. We show here that ZEB1 is expressed by epithelial cells in intestinal tumors from human patients (familial adenomatous polyposis) and mouse models (APC(Min/+)) with germline mutations of APC that result in nuclear accumulation of β-catenin. However, ZEB1 is not expressed in the epithelium of hereditary forms of CRCs that carry wild-type APC and where β-catenin is excluded from the nucleus (Lynch syndrome). We found that β-catenin/TCF4 binds directly to the ZEB1 promoter and activates its transcription. Knockdown of β-catenin and TCF4 in APC-mutated CRC cells inhibited endogenous ZEB1, whereas forced translocation of β-catenin to the nucleus in APC-wild-type CRC cells induced de novo expression of ZEB1. Upregulation of MT1-MMP and LAMC2 by β-catenin/TCF4 has been linked to invasiveness in CRCs, and we show here that both proteins are activated by ZEB1 coexpressing with it in primary colorectal tumors with mutated APC. These results set ZEB1 as an effector of β-catenin/TCF4 signaling in EMT and tumor progression. 
 MicroRNA-based regulation of epithelial-hybrid-mesenchymal fate determination. Forward and backward transitions between epithelial and mesenchymal phenotypes play crucial roles in embryonic development and tissue repair. Aberrantly regulated transitions are also a hallmark of cancer   metastasis  . The genetic network that regulates these transitions appears to allow for the existence of a hybrid phenotype (epithelial/mesenchymal). Hybrid cells are endowed with mixed epithelial and mesenchymal characteristics, enabling specialized capabilities such as collective cell migration. Cell-fate determination between the three phenotypes is in fact regulated by a circuit composed of two highly interconnected chimeric modules--the miR-34/SNAIL and the miR-200/ZEB mutual-inhibition feedback circuits. Here, we used detailed modeling of microRNA-based regulation to study this core unit. More specifically, we investigated the functions of the two isolated modules and subsequently of the combined unit when the two modules are integrated into the full regulatory circuit. We found that miR-200/ZEB forms a tristable circuit that acts as a ternary switch, driven by miR-34/SNAIL, that is a monostable module that acts as a noise-buffering integrator of internal and external signals. We propose to associate the three stable states--(1,0), (high miR-200)/(low ZEB); (0,1), (low miR-200)/(high ZEB); and (1/2,1/2), (medium miR-200)/(medium ZEB)--with the epithelial, mesenchymal, and hybrid phenotypes, respectively. Our (1/2,1/2) state hypothesis is consistent with recent experimental studies (e.g., ZEB expression measurements in collectively migrating cells) and explains the lack of observed mesenchymal-to-hybrid transitions in metastatic cells and in induced pluripotent stem cells. Testable predictions of dynamic gene expression during complete and partial transitions are presented. 
 LincRNA-ROR promotes invasion,   metastasis   and tumor growth in pancreatic cancer through activating ZEB1 pathway. Pancreatic cancer (PC) remains one of the most lethal malignant tumors; early distant   metastasis   commonly results in poor prognosis. Recent studies confirmed the pivotal role of the long non-coding RNAs (lncRNAs) in tumorigenesis and   metastasis   of malignant tumors, including PC. However, little is known about the role of LincRNA-ROR (linc-ROR) in PC. In the present study, we found that linc-ROR was upregulated in PC tissues. Overexpression of linc-ROR promoted cells proliferation, migration, invasion and   metastasis   both in vitro and in a mouse model. Contrarily, knockdown of linc-ROR attenuated proliferation, invasion and distant   metastasis  . Mechanistically, we confirmed that linc-ROR up-regulates ZEB1 and then induces epithelial-mesenchymal transition (EMT), which promotes the aggressive biological behaviors of PC. Together, these results indicate that linc-ROR acts as an important regulator of ZEB1, can promote invasion and   metastasis   in PC, and may represent a novel therapeutic target. 
 Loss of MicroRNA-101 Promotes Epithelial to Mesenchymal Transition in Hepatocytes. Epithelial-to-mesenchymal transition (EMT) has been implicated in embryonic development and various pathological events. However, the involvement of microRNA in the process of EMT remains to be fully defined in hepatocyte. ZEB1 is a well-known transcriptional repressor of E-cadherin and plays a major role in triggering EMT during organ fibrosis and cancer cell   metastasis  . Computational microRNA target predictions detect a conserved sequence matching to miR-101 in the 3'UTR of ZEB1 mRNA. Our results confirm that miR-101 suppresses ZEB1 expression by targeting the predicted site of ZEB1 3'UTR. Subsequent investigations show that miR-101 is significantly downregulated in the cultured hepatocytes undergoing EMT and in the hepatocytes isolated from fibrotic liver. Along with the loss of miR-101, the ZEB1 expression increases simultaneously in hepatocytes. In addition, miR-101 levels in HCC cell lines are negatively associated with the ZEB1 productions and the metastatic potentials of tumor cells. Mechanistically, we demonstrate that miR-101 significantly inhibits the TGF-β1-induced EMT in hepatocytes, whereas inhibition of miR-101 promotes the EMT process as indicated by the changes of morphology, cell migration, and the expression profiles of EMT markers. In the fibrotic liver, ectopic expression of miR-101 can significantly downregulate ZEB1 in the hepatocyte and thereby reduces the mesenchymal marker expression. Moreover, miR-101 significantly inhibits the proliferation and migration of HCC cell. Our results demonstrate that miR-101 regulates HCC cell phenotype by upregulating the epithelial marker genes and suppressing the mesenchymal ones. 
 Pien Tze Huang inhibits   metastasis   of human colorectal carcinoma cells via modulation of TGF-β1/ZEB/miR-200 signaling network. Tumor   metastasi  s, a complex process involving the spread of malignant tumor cells from a primary tumor site to a distant organ, is a major cause of failure of cancer chemotherapy. Epithelial-mesenchymal transition (EMT) is a critical step for the initiation of cancer   metastasi  s. The processes of EMT and   metastasi  s are highly regulated by a double-negative feedback loop consisting of TGF-β1/ZEB pathway and miR-200 family, which therefore has become a promising target for cancer chemotherapy. Pien Tze Huang (PZH), a well-known traditional Chinese formula first prescribed in the Ming Dynasty, has been demonstrated to be clinically effective in the treatment of various types of human malignancy including colorectal cancer (CRC). Our published data proposed that PZH was able to induce apoptosis, inhibit cell proliferation and tumor angiogenesis, leading to the suppression of CRC growth in vitro and in vivo. To further elucidate the mode of action of PZH, in the present study we evaluated its effects on the metastatic capacities of human colorectal carcinoma HCT-8 cells and investigated the underlying molecular mechanisms. We found that PZH significantly inhibited the migration and invasion of HCT-8 cells in a dose-dependent manner. In addition, PZH treatment inhibited the expression of key mediators of TGF-β1 signaling, such as TGF-β1, Smad2/3 and Smad4. Moreover, PZH treatment suppressed the expression of ZEB1 and ZEB2, two critical target genes of TGF-β1 pathway, leading to a decrease in the expression of mesenchymal marker N-cadherin and an increased expression of epithelial marker E-cadherin. Furthermore, PZH treatment upregulated the expression of miR-200a, miR-200b and miR-200c. Collectively, our findings in this study suggest that PZH can inh  ibit metas  tasis of colorectal cancer cells via modulating TGF-β1/ZEB/miR-200 signaling network, which might be one of the mechanisms whereby PZH exerts its anticancer function. 
 ZEB1 Upregulates VEGF Expression and Stimulates Angiogenesis in Breast Cancer. Although zinc finger E-box binding homeobox 1 (ZEB1) has been identified as a key factor in the regulation of breast cancer differentiation and   metastasis  , its potential role in modulating tumor angiogenesis has not been fully examined. Here, we present the novel finding that conditioned medium derived from ZEB1-expressing MDA-MB-231 cells significantly increased the capillary tube formation of human umbilical vein endothelial cells (HUVECs), whereas ZEB1 knockdown by RNA interference had the opposite effect. ZEB1 caused marked upregulation of the expression of vascular endothelial growth factor A (VEGFA) at both mRNA and protein levels. Pre-incubation of HUVECs with anti-VEGFA neutralized antibody attenuated ZEB1-mediated tube formation of HUVECs. In breast cancer tissues, expression of ZEB1 was positively correlated with those of VEGFA and CD31. At the molecular level, ZEB1 activated VEGFA transcription by increasing SP1 recruitment to its promoter, which was mediated via the activation of PI3K and p38 pathways. Using a nude mouse xenograft model, we demonstrated that elevated expression of ZEB1 promotes in vivo tumorigenesis and angiogenesis in breast cancer. Collectively, we found that ZEB1-expressing breast cancer cells increase VEGFA production and thus stimulate tumor growth and angiogenesis via a paracrine mechanism. 
 The ZEB1/miR-200 feedback loop controls Notch signalling in cancer cells. Notch signalling is important for development and tissue homeostasis and activated in many human cancers. Nevertheless, mutations in Notch pathway components are rare in solid tumours. ZEB1 is an activator of an epithelial-mesenchymal transition (EMT) and has crucial roles in tumour progression towards   metastasis  . ZEB1 and miR-200 family members repress expression of each other in a reciprocal feedback loop. Since miR-200 members target stem cell factors, ZEB1 indirectly induces stemness maintenance and associated drug resistance. Here, we link ZEB1 and its cancer promoting properties to Notch activation. We show that miR-200 members target Notch pathway components, such as Jagged1 (Jag1) and the mastermind-like coactivators Maml2 and Maml3, thereby mediating enhanced Notch activation by ZEB1. We further detected a coordinated upregulation of Jag1 and ZEB1, associated with reduced miR-200 expression in two aggressive types of human cancer, pancreatic adenocarcinoma and basal type of breast cancer. These findings explain increased Notch signalling in some types of cancers, where mutations in Notch pathway genes are rare. Moreover, they indicate an additional way how ZEB1 exerts its tumour progressing functions. 
 HIF-1α Promotes Epithelial-Mesenchymal Transition and   Metastasi  s through Direct Regulation of ZEB1 in Colorectal Cancer. It is well recognized that hypoxia-inducible factor 1 alpha (HIF-1α) is involved in cance  r metastas  is, chemotherapy and poor prognosis. We previously found that deferoxamine, a hypoxia-mimetic agent, induces epithelial-mesenchymal transition (EMT) in colorectal cancer. Therefore, here we explored a new molecular mechanism for HIF-1α contributing to EMT and canc  er metasta  sis through binding to ZEB1. In this study, we showed that overexpression of HIF-1α with adenovirus infection promoted EMT, cell invasion and migration in vitro and in vivo. On a molecular level, HIF-1α directly binding to the proximal promoter of ZEB1 via hypoxia response element (HRE) sites thus increasing the transactivity and expression of ZEB1. In addition, inhibition of ZEB1 was able to abrogate the HIF-1α-induced EMT and cell invasion. HIF-1α expression was highly correlated with the expression of ZEB1 in normal colorectal epithelium, primary and metastatic CRC tissues. Interestingly, both HIF-1α and ZEB1 were positively associated with Vimentin, an important mesenchymal marker of EMT, whereas negatively associated with E-cadherin expression. These findings suggest that HIF-1α enhances EMT an  d cancer m  etastasis by binding to ZEB1 promoter in CRC. HIF-1α and ZEB1 are both widely considered as tumor-initiating factors, but our results demonstrate that ZEB1 is a direct downstream of HIF-1α, suggesting a novel molecular mechanism for HIF-1α-inducing EMT   and cance  r metastasis. 
 ZEB1 expression is correlated with tumor   metastasis   and reduced prognosis of breast carcinoma in Asian patients. Tumor   metastasis   is one of the key events leading to tumor relapse and poor prognosis. Nowadays, increasing evidences demonstrated that ZEB1 was implicated in human carcinogenesis. However, involvement of ZEB1 deregulation in tumorigenesis in Asian patients with breast carcinoma remains elusive. The present study included 102 Asian patients with breast carcinoma treated by surgery from January of 2005 to December of 2006, and the expression of ZEB1 was evaluated by immunohistochemistry. To further assess the prognostic value of ZEB1, Kaplan-Meier curves were constructed. In this study, elevated levels of ZEB1 expression was found in carcinomas with higher aggressive potential. We also correlated expression of ZEB1 with lymph node   metastasis   (P = 0.021), advanced clinical stage (P = 0.012) in all cases, and high tumor grade (P = 0.047) in invasive ductal carcinoma. Furthermore, our data suggested an elevated level of Ki-67 expression in cases with positive expression of ZEB1. Clinically, reduced overall survival and disease-free survival were observed in cases with positive ZEB1 expression than that in negative cases. Our results correlated ZEB1 with aggressive potentials of breast carcinoma and revealed a possibility for ZEB1 as a prognostic marker in breast carcinoma. 
 Upregulation of long noncoding RNA ZEB1-AS1 promotes tumor   metastasis   and predicts poor prognosis in hepatocellular carcinoma. Hepatocellular carcinoma (HCC) is one of the leading causes of cancer-related mortality worldwide. Despite progress in diagnostics and treatment of HCC, its prognosis remains poor. Emerging studies showed that long noncoding RNAs (lncRNAs) have crucial regulatory roles in cancer biology. In the current study, differentially expressed lncRNAs between HCC and paired non-tumor tissues were identified using microarrays. The effects of a specific differentially expressed lncRNA (termed ZEB1-AS1) on tumor progression were investigated in vitro and in vivo. We found that ZEB1-AS1 is frequently upregulated in HCC samples, especially in metastatic tumor tissues. DNA methylation analysis shows a tumor-specific ZEB1-AS1 promoter hypomethylation. Aberrant methylation is tightly correlated with overexpression of ZEB1-AS1 in HCC. Patients with ZEB1-AS1 hypomethylation or with high ZEB1-AS1 expression have poor recurrence-free survival. Functionally, ZEB1-AS1 promotes tumor growth and   metastasis  , acts as an oncogene in HCC. The ZEB1-AS1 gene is located in physical contiguity with ZEB1 and positively regulates the ZEB1 expression. ZEB1 inhibition partially abrogates ZEB1-AS1-induced epithelial to mesenchymal transition (EMT) and cancer   metastasis  . Our results provide novel insights into the function of lncRNA-driven hepatocarcinogenesis, highlight the important role of ZEB1-AS1 and ZEB1 in HCC progression, and indicate that ZEB1-AS1 may be served as a valuable prognostic biomarker for HCC. 
   Metastasis   is regulated via microRNA-200/ZEB1 axis control of tumour cell PD-L1 expression and intratumoral immunosuppression. Immunosuppression of tumour-infiltrating lymphocytes (TIL) is a common feature of advanced cancer, but its biological basis has remained obscure. We demonstrate here a molecular link between epithelial-to-mesenchymal transition (EMT) and CD8(+) TIL immunosuppression, two key drivers of cancer progression. We show that microRNA-200 (miR-200), a cell-autonomous suppressor of EMT and   metastasis  , targets PD-L1. Moreover, ZEB1, an EMT activator and transcriptional repressor of miR-200, relieves miR-200 repression of PD-L1 on tumour cells, leading to CD8(+) T-cell immunosuppression and   metastasis  . These findings are supported by robust correlations between the EMT score, miR-200 levels and PD-L1 expression in multiple human lung cancer datasets. In addition to revealing a link between EMT and T-cell dysfunction, these findings also show that ZEB1 promotes   metastasis   through a heretofore unappreciated cell non-autonomous mechanism, and suggest that subgroups of patients in whom malignant progression is driven by EMT activators may respond to treatment with PD-L1 antagonists. 
 WNT7a induces E-cadherin in lung cancer cells. E-cadherin loss in cancer is associated with de-differentiation, invasion, and   metastasis  . Drosophila DE-cadherin is regulated by Wnt/beta-catenin signaling, although this has not been demonstrated in mammalian cells. We previously reported that expression of WNT7a, encoded on 3p25, was frequently downregulated in lung cancer, and that loss of E-cadherin or beta-catenin was a poor prognostic feature. Here we show that WNT7a both activates E-cadherin expression via a beta-catenin specific mechanism in lung cancer cells and is involved in a positive feedback loop. Li+, a GSK3 beta inhibitor, led to E-cadherin induction in an inositol-independent manner. Similarly, exposure to mWNT7a specifically induced free beta-catenin and E-cadherin. Among known transcriptional suppressors of E-cadherin, ZEB1 was uniquely correlated with E-cadherin loss in lung cancer cell lines, and its inhibition by RNA interference resulted in E-cadherin induction. Pharmacologic reversal of E-cadherin and WNT7a losses was achieved with Li+, histone deacetylase inhibition, or in some cases only with combined inhibitors. Our findings provide support that E-cadherin induction by WNT/beta-catenin signaling is an evolutionarily conserved pathway operative in lung cancer cells, and that loss of WNT7a expression may be important in lung cancer development or progression by its effects on E-cadherin. 
 A transient, EMT-linked loss of basement membranes indicates   metastasis   and poor survival in colorectal cancer. Loss of the basement membrane (BM) is considered an important step toward tumor malignancy. However, the BM is still expressed in most typical colorectal adenocarcinomas; nevertheless, these tumors can invade and develop metastases. The aim of this study was to investigate the role, mechanisms, and clinical relevance of BM turnover in malignant colorectal cancer (CRC) progression.Expression of BM components and their transcriptional regulation and clinical relevance were investigated in human CRCs and cell lines.Our data show new aspects in BM turnover in CRCs with impact on malignant tumor progression: (1) The BM is still expressed in the main tumor mass of most colorectal adenocarcinomas, but selectively lost at invasive regions of the tumor in many cases. (2) Selective loss of the BM at the invasive front has high clinical and tumor biologic relevance for distant   metastasis   and survival. (3) The BM is reexpressed in metastases, indicating that its loss is transient and regulated by environmental factors. (4) This transient loss is not only due to proteolytic breakdown but to a down-regulated synthesis and linked to an epithelial-mesenchymal transition (EMT) in tumor cells, and, thereby, zinc-finger-enhancer protein 1 (ZEB1) is the crucial transcriptional repressor of BM components in CRCs.A transient BM loss at the invasive front is correlated with increased distant   metastasis   and poor patient survival, indicating its tumor biologic relevance and usefulness as a prognostic marker. Targeting ZEB1 might be a promising therapeutic option to prevent   metastasis  . 
 Activation of miR200 by c-Myb depends on ZEB1 expression and miR200 promoter methylation. Tumor progression to   metastasis   is a complex, sequential process that requires proliferation, resistance to apoptosis, motility and invasion to colonize at distant sites. The acquisition of these features implies a phenotypic plasticity by tumor cells that must adapt to different conditions by modulating several signaling pathways (1) during the journey to the final site of   metastasis  . Several transcription factors and microRNA play a role in tumor progression, but less is known about the control of their expression during this process. Here, we demonstrate by ectopic expression and gene silencing that the proto-oncogene c-Myb activates the expression of the 5 members of miR200 family (miR200b, miR200a, miR429, miR200c and miR141) that are involved in the control of epithelial-mesenchymal transition (EMT) and   metastasis   in many types of cancers. Transcriptional activation of miR200 by c-Myb occurs through binding to myb binding sites located in the promoter regions of miR200 genes on human chromosomes 1 and 12. Furthermore, when c-Myb and the transcriptional repressor ZEB1 are co-expressed, as at the onset EMT, the repression by ZEB1 prevails over the activation by c-Myb, and the expression of miR200 is inhibited. We also demonstrate that during EMT induced by TGF-β, the promoters of miR200 genes are methylated, and their transcription is repressed regardless of the presence of repressors such as ZEB1 and activators such as c-Myb. Finally, we find a correlation between the expression of c-Myb and that of four out of 5 miR200 in a data set of 207 breast cancer patients. 
 The dual function of PRMT1 in modulating epithelial-mesenchymal transition and cellular senescence in breast cancer cells through regulation of ZEB1. Although the involvement of protein arginine methyltransferase 1 (PRMT1) in tumorigenesis has been reported, its roles in breast cancer progression and   metastasis   has not been elucidated. Here we identified PRMT1 as a key regulator of the epithelial-mesenchymal transition (EMT) in breast cancer. We showed that the EMT program induced by PRMT1 endowed the human mammary epithelial cells with cancer stem cell properties. Moreover, PRMT1 promoted the migratory and invasive behaviors in breast cancer cells. We also demonstrated that abrogation of PRMT1 expression in breast cancer cells abated   metastasis   in vivo in mouse model. In addition, knockdown of PRMT1 arrested cell growth in G1 tetraploidy and induced cellular senescence. Mechanistically, PRMT1 impacted EMT process and cellular senescence by mediating the asymmetric dimethylation of arginine 3 of histone H4 (H4R3me2as) at the ZEB1 promoter to activate its transcription, indicating the essential roles of this epigenetic control both in EMT and in senescence. Thus, we unraveled a dual function of PRMT1 in modulation of both EMT and senescence via regulating ZEB1. This finding points to the potent value of PRMT1 as a dual therapeutic target for preventing   metastasis   and for inhibiting cancer cell growth in malignant breast cancer patients. 
 A self-enforcing CD44s/ZEB1 feedback loop maintains EMT and stemness properties in cancer cells. Invasion and   metastasis   of carcinomas are often activated by induction of aberrant epithelial-mesenchymal transition (EMT). This is mainly driven by the transcription factor ZEB1, promoting tumor-initiating capacity correlated with increased expression of the putative stem cell marker CD44. However, the direct link between ZEB1, CD44 and tumourigenesis is still enigmatic. Remarkably, EMT-induced repression of ESRP1 controls alternative splicing of CD44, causing a shift in the expression from the variant CD44v to the standard CD44s isoform. We analyzed whether CD44 and ZEB1 regulate each other and show that ZEB1 controls CD44s splicing by repression of ESRP1 in breast and pancreatic cancer. Intriguingly, CD44s itself activates the expression of ZEB1, resulting in a self-sustaining ZEB1 and CD44s expression. Activation of this novel CD44s-ZEB1 regulatory loop has functional impact on tumor cells, as evident by increased tumor-sphere initiation capacity, drug-resistance and tumor recurrence. In summary, we identified a self-enforcing feedback loop that employs CD44s to activate ZEB1 expression. This renders tumor cell stemness independent of external stimuli, as ZEB1 downregulates ESRP1, further promoting CD44s isoform synthesis. 
 ZEB1 enhances transendothelial migration and represses the epithelial phenotype of prostate cancer cells. Metastatic colonization involves cancer cell lodgment or adherence in the microvasculature and subsequent migration of those cells across the endothelium into a secondary organ site. To study this process further, we analyzed transendothelial migration of human PC-3 prostate cancer cells in vitro. We isolated a subpopulation of cells, TEM4-18, that crossed an endothelial barrier more efficiently, but surprisingly, were less invasive than parental PC-3 cells in other contexts in vitro. Importantly, TEM4-18 cells were more aggressive than PC-3 cells in a murine metastatic colonization model. Microarray and FACS analysis of these cells showed that the expression of many genes previously associated with leukocyte trafficking and cancer cell extravasation were either unchanged or down-regulated. Instead, TEM4-18 cells exhibited characteristic molecular markers of an epithelial-to-mesenchymal transition (EMT), including frank loss of E-cadherin expression and up-regulation of the E-cadherin repressor ZEB1. Silencing ZEB1 in TEM4-18 cells resulted in increased E-cadherin and reduced transendothelial migration. TEM4-18 cells also express N-cadherin, which was found to be necessary, but not sufficient for increased transendothelial migration. Our results extend the role of EMT in   metastasis   to transendothelial migration and implicate ZEB1 and N-cadherin in this process in prostate cancer cells. 
 Molecular regulation of ovarian cancer cell invasion. The molecular mechanism underlying ovarian cancer invasiveness and   metastasis   remains unclear. Since significant downregulation in microRNA 200 (miRNA200) family (miR200a, miR200b, and miR200c) has been reported in the invasive ovarian cancer cells, here, we used two human ovarian cancer cell lines, OVCAR3 and SKOV3, to study the molecular basis of miR200, matrix metalloproteinase 3 (MMP3) activation, and cancer invasiveness. We found that overexpression of either miR200 family member in OVCAR3 or SKOV3 cells significantly inhibited production and secretion of MMP3 and cancer invasiveness. Moreover, forced MMP3 expression abolished miR200-induced inhibition of ovarian cancer cell invasiveness, suggesting that miR200 family inhibited ovarian cell invasiveness via downregulating MMP3. Furthermore, ZEB1, a major target of miR200, was inhibited by miR200 overexpression. Forced ZEB1 expression abolished miR200-induced inhibition of ovarian cancer cell invasiveness, suggesting that ZEB1 is a direct target of miR200 for inhibiting ovarian cell invasiveness. Finally, phosphorylated SMAD3 (pSMAD3), a major partner of ZEB1, was efficiently inhibited by miR200, which could be restored by forced expression of ZEB1, but not by forced expression of MMP3, suggesting that ZEB1/pSMAD3 is signaling cascade upstream of MMP3 in this model. Taken together, our data suggest that miR200 family inhibited ovarian cancer cell invasiveness and   metastasis   by downregulating MMP3, possibly through ZEB1/pSMAD3. 
 Expression of E-cadherin repressors SNAIL, ZEB1 and ZEB2 by tumour and stromal cells influences tumour-budding phenotype and suggests heterogeneity of stromal cells in pancreatic cancer. There is evidence that tumour-stroma interactions have a major role in the neoplastic progression of pancreatic ductal adenocarcinoma (PDAC). Tumour budding is thought to reflect the process of epithelial-mesenchymal transition (EMT); however, the relationship between tumour buds and EMT remains unclear. Here we characterize the tumour-budding- and stromal cells in PDAC at protein and mRNA levels concerning factors involved in EMT.mRNA in situ hybridisation and immunostaining for E-cadherin, β-catenin, SNAIL1, ZEB1, ZEB2, N-cadherin and TWIST1 were assessed in the main tumour, tumour buds and tumour stroma on multipunch tissue microarrays from 120 well-characterised PDACs and associated with the clinicopathological features, including peritumoural (PTB) and intratumoural (ITB) budding.Tumour-budding cells showed increased levels of ZEB1 (P<0.0001) and ZEB2 (P=0.0119) and reduced E-cadherin and β-catenin (P<0.0001, each) compared with the main tumour. Loss of membranous β-catenin in the main tumour (P=0.0009) and tumour buds (P=0.0053), without nuclear translocation, as well as increased SNAIL1 in tumour and stromal cells (P=0.0002, each) correlated with high PTB. ZEB1 overexpression in the main tumour-budding and stromal cells was associated with high ITB (P=0.0084; 0.0250 and 0.0029, respectively) and high PTB (P=0.0005; 0.0392 and 0.0007, respectively). ZEB2 overexpression in stromal cells correlated with higher pT stage (P=0.03), lymphatic invasion (P=0.0172) and lymph no  de metasta  sis (P=0.0152).In the tumour microenvironment of phenotypically aggressive PDAC, tumour-budding cells express EMT hallmarks at protein and mRNA levels underlining their EMT-type character and are surrounded by stromal cells expressing high levels of the E-cadherin repressors ZEB1, ZEB2 and SNAIL1, this being strongly associated with the tumour-budding phenotype. Moreover, our findings suggest the existence of subtypes of stromal cells in PDAC with phenotypical and functional heterogeneity. 
 miR-205 acts as a tumour radiosensitizer by targeting ZEB1 and Ubc13. Tumour cells associated with therapy resistance (radioresistance and drug resistance) are likely to give rise to local recurrence and distant metastatic relapse. Recent studies revealed microRNA (miRNA)-mediated regulation of   metastasis   and epithelial-mesenchymal transition; however, whether specific miRNAs regulate tumour radioresistance and can be exploited as radiosensitizing agents remains unclear. Here we find that miR-205 promotes radiosensitivity and is downregulated in radioresistant subpopulations of breast cancer cells, and that loss of miR-205 is highly associated with poor distant relapse-free survival in breast cancer patients. Notably, therapeutic delivery of miR-205 mimics via nanoliposomes can sensitize the tumour to radiation in a xenograft model. Mechanistically, radiation suppresses miR-205 expression through ataxia telangiectasia mutated (ATM) and zinc finger E-box binding homeobox 1 (ZEB1). Moreover, miR-205 inhibits DNA damage repair by targeting ZEB1 and the ubiquitin-conjugating enzyme Ubc13. These findings identify miR-205 as a radiosensitizing miRNA and reveal a new therapeutic strategy for radioresistant tumours. 
 ZEB1 coordinately regulates laminin-332 and {beta}4 integrin expression altering the invasive phenotype of prostate cancer cells.   Metastasis   involves the invasion of cancer cells across both the extracellular matrix and cellular barriers, and an evolving theme is that epithelial-to-mesenchymal transition (EMT) may mediate invasive cellular behavior. Previously, we isolated and analyzed a subpopulation of PC-3 prostate cancer cells, TEM4-18, and found that these cells both invaded an endothelial barrier more efficiently and exhibited enhanced metastatic colonization in vivo. Transendothelial migration of these cells depended on expression of ZEB1, a known regulator of EMT. Surprisingly, these cells were much less invasive than parental PC-3 cells in assays that involve matrix barriers. Here, we report that TEM4-18 cells express significantly reduced levels of two subunits of laminin-332 (β3 and γ2) and that exogenous laminin-332, or co-culture with laminin-332-expressing cells, rescues the in vitro invasion phenotype in these cells. Stable knockdown of ZEB1 in prostate cancer cells up-regulated LAMC2 and ITGB4 mRNA and protein and resulted in a concomitant increase in Transwell migration. Using chromatin immunoprecipitation (ChIP), we show that ZEB1 directly interacts with the promoters of LAMC2 and ITGB4. These results provide a novel molecular basis for reduced laminin-332 observed in clinical prostate cancer specimens and demonstrate a context-dependent role for EMT in invasive cellular behavior. 
 Discoidin domain receptor 1 is a novel transcriptional target of ZEB1 in breast epithelial cells undergoing H-Ras-induced epithelial to mesenchymal transition. The epithelial-to-mesenchymal transition (EMT) process allows carcinoma cells to dissociate from the primary tumor thereby facilitating tumor cell invasion and   metastasis  . Ras-dependent hyperactive signaling is commonly associated with tumorigenesis, invasion, EMT, and   metastasis  . However, the downstream effectors by which Ras regulates EMT remain ill defined. In this study, we show that the H-Ras pathway leads to mesenchymal-like phenotypic changes in human breast epithelial cells by controlling the ZEB1/microRNA-200c axis. Moreover, H-Ras suppresses the expression of the discoidin domain receptor 1 (DDR1), a collagen receptor tyrosine kinase, via ZEB1, thus identifying ZEB1 as a novel transcriptional repressor of DDR1. Mutation studies on the putative promoter of the DDR1 gene revealed that bipartite Z- and E-box elements play a key role in transcriptional repression of DDR1 in Hs578T and MDA-MB-231 breast carcinoma cell lines by ZEB1. Furthermore, we found an inverse correlation between ZEB1 and DDR1 expression in various cancer cell lines and in human breast carcinoma tissues. Consistently, overexpression of DDR1 reduced the invasive phenotype of mesenchymal-like triple-negative breast cancer cells in 3D cultures and in vivo. Thus, ZEB1's role in maintenance of EMT in breast carcinoma cells is mediated in part by its ability to suppress DDR1 expression and consequently contribute to the activation of the invasive phenotype. Taken together, our results unveil a novel H-Ras/ZEB1/DDR1 network that contributes to breast cancer progression in triple-negative breast cancers. 
 Clinicopathological significance of ZEB1 protein in patients with hepatocellular carcinoma. ZEB1, a member of the ZFH family of proteins (zinc-finger E-box binding homeobox), plays a central role in epithelial-mesenchymal transition (EMT) during carcinogenesis. In this study, we investigated the expression of ZEB1 in patients with hepatocellular carcinoma (HCC) and its clinical effects with underlying mechanisms.Expression levels of ZEB1 were assessed by Western blot in 5 HCC cell lines and in paired cancerous and noncancerous tissues from 110 patients with HCC. Short-hairpin RNA (shRNA) interference for ZEB1 was performed in MHCC-97H cell line.ZEB1 protein was detected at a relatively high level in metastatic human HCC cell lines (MHCC-97L and MHCC-97H) when compared with that in nonmetastatic HCC cell lines (Hep3B, PLC and Huh-7). ZEB1 was expressed at high levels in 72 of 110 HCC patients (65.4%) and correlated with advanced TNM stage, tumor size >5 cm, intrahepatic   metastasis  , vascular invasion, and frequent early recurrence. The results of multivariate analysis revealed that ZEB1 high expression was a significant prognostic factor for poor overall and disease-free survivals. Silencing ZEB1 resulted in significant suppression of motility of MHCC-97H cell line, which was accompanied with increased expression of the epithelial marker E-cadherin and decreased expression of the mesenchymal markers N-cadherin and vimentin. Furthermore, silencing ZEB1 prevented the spread of intrahepatic   metastasis   and increased overall survival in mouse orthotopic tumor models.This study shows that ZEB1 high expression was correlated with HCC malignant progression and subsequent poor patient survival by induction of EMT changes. 
 HOXD9 promotes epithelial-mesenchymal transition and cancer   metastasis   by ZEB1 regulation in hepatocellular carcinoma. Hepatocellular carcinoma (HCC) is a common malignant tumor that severely threatens human health. The poor prognosis of HCC is mainly attributed to intrahepatic and extrahepatic metastases. HOXD9 proteins belong to a superfamily that regulates the development and control of many cellular processes, including proliferation, apoptosis, cell shape, and cell migration. HOXD9 can also function as an oncogene in several cancer cells. However, its biological function in human HCC requires further investigation. In this study, HOXD9 exhibited high expression in invasive HCC cells. HOXD9 overexpression can significantly enhance HCC cell migration, invasion, and   metastasis  , whereas silencing HOXD9 inhibits these processes. HOXD9 also promotes the epithelial-mesenchymal transition (EMT) of HCC cells. Microarray analysis suggests that ZEB1 can function as a downstream factor of HOXD9. HOXD9 can interact with the promoter region of ZEB1 and promotes ZEB1 expression. ZEB1 knockdown inhibits HOXD9-induced migration and invasion, as well as EMT in HCC cells. This study helps elucidates the oncogenic functions of HOXD9 in HCC. 
 ZEB1 expression is associated with prognosis of intrahepatic cholangiocarcinoma. Intrahepatic cholangiocarcinoma (ICC) is one of the most aggressive malignant tumours, so the identification of molecular targets for ICC is an important issue. Zinc finger E-box binding homeobox 1 (ZEB1) is a key inducer of epithelial-mesenchymal transition (EMT). The aim of the present study was to clarify the clinical significance of ZEB1 in ICC and the associations between ZEB1 expression and EMT-related proteins.We immunohistochemically examined the expression of EMT-related proteins, namely ZEB1, vimentin and E-cadherin, in ICC specimens from 102 patients. The clinicopathological and prognostic values of these markers were evaluated.ZEB1 and vimentin were expressed in 46.1% and 43.1% of tumours, respectively, and E-cadherin expression was lost in 44.1% of tumours. ZEB1 expression showed a significant inverse correlation with E-cadherin expression (p=0.004) and a positive correlation with vimentin expression (p=0.022). Altered expression of ZEB1 was associated with aggressive tumour characteristics, including advanced tumour stage (p=0.037), undifferentiated-type histology (p=0.017), lymph node   metastasis   (p=0.024) and portal vein invasion (p=0.037). Moreover, overall survival rates were significantly lower for patients with high ZEB1 expression than for patients with low ZEB1 expression (p=0.027). Kaplan-Meier analysis also identified E-cadherin expression (p=0.041) and vimentin expression (p=0.049) as prognostic indicators for overall survival.ZEB1 expression is associated with tumour progression and poor prognosis in patients with ICC through positive correlations with vimentin and negative correlations with E-cadherin. ZEB1 expression is associated with a poor prognosis and might be an attractive target for the treatment of ICC. 
 ASPP2 controls epithelial plasticity and inhibits   metastasis   through β-catenin-dependent regulation of ZEB1. Epithelial to mesenchymal transition (EMT), and the reverse mesenchymal to epithelial transition (MET), are known examples of epithelial plasticity that are important in kidney development and cancer   metastasi  s. Here we identify ASPP2, a haploinsufficient tumour suppressor, p53 activator and PAR3 binding partner, as a molecular switch of MET and EMT. ASPP2 contributes to MET in mouse kidney in vivo. Mechanistically, ASPP2 induces MET through its PAR3-binding amino-terminus, independently of p53 binding. ASPP2 prevents β-catenin from transactivating ZEB1, directly by forming an ASPP2-β-catenin-E-cadherin ternary complex and indirectly by inhibiting β-catenin's N-terminal phosphorylation to stabilize the β-catenin-E-cadherin complex. ASPP2 limits the pro-invasive property of oncogenic RAS and inhibits tu  mour metas  tasis in vivo. Reduced ASPP2 expression results in EMT, and is associated with poor survival in hepatocellular carcinoma and breast cancer patients. Hence, ASPP2 is a key regulator of epithelial plasticity that connects cell polarity to the suppression of WNT signalling, EMT and tu  mour metas  tasis. 
 MicroRNA alteration and putative target genes in high-grade prostatic intraepithelial neoplasia and prostate cancer: STAT3 and ZEB1 are upregulated during prostate carcinogenesis. We aimed to identify alteration of cancer-related miRNAs in HGPIN and PCa, and to investigate the clinical implications of HGPIN as a precancerous lesion of PCa.Clinicopathologic analysis based on the status of HGPIN was performed in 388 patients who received radical prostatectomy between January 2005 and December 2008 in Severance Hospital. Among them, 10 paired HGPIN and PCa were prepared to perform miRNA microarray and quantitative real-time PCR. Fifty-two prostatectomy specimens were used to further validation of protein expression that was assessed by immunohistochemical staining (IHC) in matched non-neoplastic prostatic tissue (NPT), HGPIN, and PCa. Functional analysis was performed using a prostate normal cell line (RWPE-1) and two prostate cancer cell lines (LNCaP, PC-3) for comparison of expression of miR-155 and STAT3 mRNA before and after treatment of miR-155 mimetics/antagomir into each cell line.Patients with HGPIN had significantly less lymphovascular invasion, less lymph node   metastasis  , lower tumor volume, lower Gleason score, lower incidence of death, and longer overall survival compared to patients without HGPIN. MiR-155, miR-210, miR-153, and miR-200c were downregulated in HGPIN and PCa in common, compared to NPT. As putative target mRNAs, mRNA expression level of STAT3, ZEB1, and BACH1 was increased in PCa and HGPIN compared to NPT. mRNA expression level of ephrin-A3 was increased in PCa compared to NPT, and FGFRL1 was decreased in PCa compared to HGPIN and NPT. Protein expression assessed by IHC showed correlated results in STAT3, ZEB1, and ephrin-A3. Moreover, STAT3 and ZEB1 increased in a stepwise manner, from NPT to PCa. Treatment of miR-155 antagomir increased STAT3 mRNA expression in RWPE-1 cells, whereas treatment of miR-155 mimetics into PC-3 cells significantly decreased STAT3 expression.STAT3 and ZEB1 could be the key molecules altered at the early stages of carcinogenesis, especially in HGPIN. Prostate 76:937-947, 2016. © 2016 Wiley Periodicals, Inc. 
 Dual roles of the transcription factor grainyhead-like 2 (GRHL2) in breast cancer. Using a retrovirus-mediated cDNA expression cloning approach, we identified the grainyhead-like 2 (GRHL2) transcription factor as novel protooncogene. Overexpression of GRHL2 in NIH3T3 cells induced striking morphological changes, an increase in cell proliferation, anchorage-independent growth, and tumor growth in vivo. By combining a microarray analysis and a phylogenetic footprinting analysis with various biochemical assays, we identified the epidermal growth factor receptor family member Erbb3 as a novel GRHL2 target gene. In breast cancer cell lines, shRNA-mediated knockdown of GRHL2 expression or functional inactivation of GRHL2 using dominant negative GRHL2 proteins induces down-regulation of ERBB3 gene expression, a striking reduction in cell proliferation, and morphological and phenotypical alterations characteristic of an epithelial-to-mesenchymal transition (EMT), thus implying contradictory roles of GRHL2 in breast carcinogenesis. Interestingly, we could further demonstrate that expression of GRHL2 is directly suppressed by the transcription factor zinc finger enhancer-binding protein 1 (ZEB1), which in turn is a direct target for repression by GRHL2, suggesting that the EMT transcription factors GRHL2 and ZEB1 form a double negative regulatory feedback loop in breast cancer cells. Finally, a comprehensive immunohistochemical analysis of GRHL2 expression in primary breast cancers showed loss of GRHL2 expression at the invasive front of primary tumors. A pathophysiological relevance of GRHL2 in breast cancer   metastasis   is further demonstrated by our finding of a statistically significant association between loss of GRHL2 expression in primary breast cancers and lymph node   metastasis  . We thus demonstrate a crucial role of GRHL2 in breast carcinogenesis. 
 Effect of down-regulated transcriptional repressor ZEB1 on the epithelial-mesenchymal transition of ovarian cancer cells. Progress has been made against early events of malignant transformation and drug resistance associated with epithelial ovarian cancer; uncontrolled metastases, however, still accounts for most patient deaths. The molecular mechanism that regulates the process of epithelial ovarian cancer metastases is not yet clearly understood. The purpose of this study was to investigate the effect of down-regulating the transcriptional repressor zinc-finger E-box-binding homeobox 1 (ZEB1) on an epithelial-mesenchymal transition (EMT) of human ovarian cancer SKOV3 cell line in vitro and in vivo.The human ovarian cancer cells SKOV3 and HO8910 were transfected with an expression vector-based small hairpin RNA (shRNA) targeting ZEB1 (shZEB1), and the stably transfected cells were selected. Colony-forming, wound-healing, and cellular migration assays were respectively used. The tumorigenicity of shZEB1-SKOV3 was also evaluated in mice.The shZEB1-SKOV3 and shZEB1-HO8910 cells showed a lower level of ZEB1 expression and weaker cell migration than the control cells. Moreover, down-regulating ZEB1 expression with shRNA in the cells enhanced the expression of miR-200c that acted as a tumor suppressor to inhibit the epithelial-mesenchymal transition of shZEB1-SKOV3 cells and to block shZEB1-SKOV3 cell   metastasis   in vivo. The shRNA-mediated down-regulation ZEB1 in SKOV3 cells significantly decreased the tumor growth in the xenograft mice.The shZEB1-mediated down-regulation of the ZEB1 expression in the SKOV3 cells may be considered for future clinical trials. 
 δEF1 promotes osteolytic   metastasi  s of MDA-MB-231 breast cancer cells by regulating MMP-1 expression. Breast cancer   metastasi  s is supposed to involve several stages in which epithelial-mesenchymal transition (EMT) is regarded as the mechanistic basis for the behavior of cancer cells. Our recent studies have implicated that δEF1, a member of the zinc finger-homeodomain transcription factor family, is required for governing both breast cancer EMT and bone remodeling, highlighting a potential role of δEF1 in modulating bo  ne metasta  sis of breast cancer. In the present study, we further demonstrated that conditioned medium derived from δEF1-overexpressing MDA-MB-231 cells significantly induces osteoclast maturation and concurrently represses osteoblast differentiation and mineralization. On the contrary, conditioned medium derived from δEF1-interfered MDA-MB-231 cells exhibits an opposite effect, thus confirming the effect of δEF to mediate osteo  lytic meta  stasis of breast cancer. Furthermore, we found that, during this process, δEF1 remarkably up-regulates matrix metalloproteinase-1 (MMP-1) expression at both mRNA and protein levels in MDA-MB-231 cells. Importantly, the results of luciferase and CHIP assays indicated that δEF1 activates MMP-1 promoter activity through the AP-1 response element. Overexpression of δEF1 in MDA-MB-231 cells significantly increases the recruitment of the AP-1 components, c-Jun/c-Fos, to the endogenous MMP-1 promoter, which in effect could be mediated via the MAPK signaling pathway. In conclusion, these observations suggest a potent role of δEF1 to promote brea  st cancer   metastasis to bone by regulating secretion of growth factors in the tumor microenvironment. 
 ΔNp63α attenuates tumor aggressiveness by suppressing miR-205/ZEB1-mediated epithelial-mesenchymal transition in cervical squamous cell carcinoma. Cervical cancer is one of the most common female cancers worldwide. Although the therapeutic outcomes of patients with early-stage cervical cancer have been significantly improved in the past decades, tumo  r metastas  is and recurrence remain the major causes of cervical cancer-related deaths. In cervical squamous cell carcinoma (SCC), the aberrant activation of epithelial-mesenchymal transition (EMT), a crucial process in invasion an  d metastas  is of epithelial cancer, could promote lymph noda  l metastas  is and recurrence, and predicts poor prognosis. In this study, we show that the expression levels of EMT markers, β-catenin and Vimentin, are associated with the p63 isoform ΔNp63α in SCC by using immunohistochemistry staining and analysis. Compared to the control SiHa cells (SiHa-NC), the expression of E-cadherin and β-catenin are upregulated, while Vimentin and ZEB1 are downregulated in the constructed SiHa cell line with stable ΔNp63α overexpression (SiHa-ΔNp63α). Besides, the migration and invasion abilities are also suppressed in SiHa-ΔNp63α cells with a typical epithelial morphology with cobblestone-like shape, suggesting that ΔNp63α is a vital EMT repressor in SCC cells. In addition, the involvement of miR-205/ZEB1 axis in the inhibition effect of ΔNp63α on EMT program is revealed by a miRNA array and confirmed by the subsequent transfection of the miR-205 mimic and antagomir. Moreover, SCC patients with low ΔNp63α expression and high EMT level s  how more f  requent metastasis and recurrence as well as reduced overall survival. Therefore, EMT program and its vital repressor ΔNp63α could be used as b  iomarkers   for tumor metastasis and recurrence in cervical cancer. 
 SET8 induces epithelial‑mesenchymal transition and enhances prostate cancer cel  l metastas  is by cooperating with ZEB1. Mounting evidence suggested that histone H4K20-specific methyltransferase SET8 is required to maintain the malignant phenotype of various cancer types; however, the role of SET8 in mediating tumo  r metastas  is in prostate cancer (PCa) has remained elusive. The present study demonstrated that small interfering RNA-mediated knockdown of SET8 inhibited the invasive potential of the PCa cell line PC-3 in vitro. Knockdown of SET8 reduced sphere formation, downregulated E-cadherin and α-catenin, and upregulated N-cadherin and vimentin expression in CaP cells, while upregulation of SET8 expression with a recombinant plasmid had the opposite effect. Furthermore, SET8 was shown to be physically associated with the epithelial-mesenchymal transition (EMT) inducer zinc finger E-box-binding homeobox 1 (ZEB1) in PCa cell lines. Chromatin immunoprecipitation suggested that SET8 binds to the promoter of cell adhesion molecule E-cadherin and vimentin. Luciferase reporter assays suggested that E-cadherin and vimentin are direct targets of SET8; furthermore, loss- and gain-of function studies of SET8 and ZEB1 indicated that suppression of downstream E-cadherin and activation of vimentin are important mechanisms by which SET8 and ZEB1 cooperatively trigg  er metasta  sis. Furthermore, SET8-induced methylated H4K20 was indicated to exert a dual function in ZEB1-regulated gene expression. In conclusion, the present study revealed that SET8 and ZEB1 are functionally interdependent in promoting the EMT and enhancing the invasive potential of PCa cells in vitro. 
 MicroRNA-150 predicts a favorable prognosis in patients with epithelial ovarian cancer, and inhibits cell invasion and   metastasis   by suppressing transcriptional repressor ZEB1. MicroRNA (miR)-150 has been reported to be dramatically downregulated in human epithelial ovarian cancer (EOC) tissues and patients' serum compared to normal controls. This study aimed to investigate clinical significance and molecular mechanisms of miR-150 in EOC. In the current study, quantitative real-time PCR analysis showed that miR-150 was significantly downregulated in human EOC tissues compared to normal tissue samples. Then, we demonstrated the significant associations of miR-150 downregulation with aggressive clinicopathological features of EOC patients, including high clinical stage and pathological grade, and shorter overall and progression-free survivals. More importantly, the multivariate analysis identified miR-150 expression as an independent prognostic biomarker in EOC. After that, luciferase reporter assays demonstrated that Zinc Finger E-Box Binding Homeobox 1 (ZEB1), a crucial regulator of epithelial-to-mesenchymal transition (EMT), was a direct target of miR-150 in EOC cells. Moreover, we found that the ectopic expression of miR-150 could efficiently inhibit cell proliferation, invasion and   metastasis   by suppressing the expression of ZEB1. Furthermore, we also observed a significantly negative correlation between miR-150 and ZEB1 mRNA expression in EOC tissues (rs = -0.45, P<0.001). In conclusion, these findings offer the convincing evidence that aberrant expression of miR-150 may play a role in tumor progression and prognosis in patients with EOC. Moreover, our data reveal that miR-150 may function as a tumor suppressor and modulate EOC cell proliferation, and invasion by directly and negatively regulating ZEB1, implying the re-expression of miR-150 might be a potential therapeutic strategy for EOC. 
 MiR-200c suppresses TGF-β signaling and counteracts trastuzumab resistance and   metastasi  s by targeting ZNF217 and ZEB1 in breast cancer. Resistance to trastuzumab and concomitantly distal   metastasi  s are leading causes of mortality in HER2-positive breast cancers, the molecular basis of which remains largely unknown. Here, we generated trastuzumab-resistant breast cancer cells with increased tumorigenicity and invasiveness compared with parental cells, and observed robust epithelial-mesenchymal transition (EMT) and consistently elevated TGF-β signaling in these cells. MiR-200c, which was the most significantly downregulated miRNA in trastuzumab-resistant cells, restored trastuzumab sensitivity and suppressed invasion of breast cancer cells by concurrently targeting ZNF217, a transcriptional activator of TGF-β, and ZEB1, a known mediator of TGF-β signaling. Given the reported backward inhibition of miR-200c by ZEB1, ZNF217 also exerts a feedback suppression of miR-200c via TGF-β/ZEB1 signaling. Restoration of miR-200c, silencing of ZEB1 or ZNF217 or blockade of TGF-β signaling increased trastuzumab sensitivity and suppressed invasiveness of breast cancer cells. Therefore, our study unraveled nested regulatory circuits of miR-200c/ZEB1 and miR-200c/ZNF217/TGF-β/ZEB1 in synergistically promoting trastuzumab resistan  ce and met  astasis of breast cancer cells. These findings provide novel insights into the common role of EMT and related molecular machinery in mediating the malignant phenotypes of breast cancers. 
 EMT-associated factors promote invasive properties of uveal melanoma cells. Transcription factors regulating the epithelial-to-mesenchymal transition (EMT) program contribute to carcinogenesis and   metastasis   in many tumors, including cutaneous melanoma. However, little is known about the role of EMT factors in the growth and metastatic dissemination of uveal melanoma cells. Here, we analyzed the expression and functions of the EMT factors ZEB1, Twist1, and Snail1 in uveal melanoma cell lines and primary tumors.ZEB1, Twist1, and Snail1 mRNA levels were measured using qPCR in five uveal melanoma cell lines and in 30 primary tumors. Gene expression was used to determine class 1 and class 2 signatures in the primary tumors. Short hairpin RNA was used to downregulate the expressions of the EMT factors; then, growth and transwell invasion assays were performed.ZEB1, Twist1, and Snail1 were expressed in all five uveal melanoma lines, with ZEB1 having the highest protein levels. ZEB1 mRNA was significantly elevated in highly metastatic class 2 primary tumors for which survival data were not available, whereas a high gene expression of Twist1 was associated with a worse prognosis in a separate tumor cohort analyzed by expression profiling. The genetic downregulation of ZEB1 in OCM1, OMM1, and 92.1 resulted in a more than 50% reduction in invasion, but only suppressed growth in OMM1 cells. Suppression of Twist1 in Mel290 and OMM1 reduced growth and invasion by more than 50%. The downregulation of Snail1 in the 92.1 cell line reduced invasion by 50%, but did not interfere with growth.The downregulation of ZEB1, Twist1, and Snail1 reduces the invasive properties of uveal melanoma cells, and the elevated mRNA levels of ZEB1 and Twist1 are associated with a more aggressive clinical phenotype in uveal melanoma samples. Therefore, these factors could represent new therapeutic targets in patients with ocular melanoma. 
 Zeb1 and Snail1 engage miR-200f transcriptional and epigenetic regulation during EMT. Cell plasticity is emerging as a key regulator of tumor progression and   metastasis  . During carcinoma dissemination epithelial cells undergo epithelial to mesenchymal transition (EMT) processes characterized by the acquisition of migratory/invasive properties, while the reverse, mesenchymal to epithelial transition (MET) process, is also essential for   metastasis   outgrowth. Different transcription factors, called EMT-TFs, including Snail, bHLH and Zeb families are drivers of the EMT branch of epithelial plasticity, and can be post-transcriptionally downregulated by several miRNAs, as the miR-200 family. The specific or redundant role of different EMT-TFs and their functional interrelations are not fully understood. To study the interplay between different EMT-TFs, comprehensive gain and loss-of-function studies of Snail1, Snail2 and/or Zeb1 factors were performed in the prototypical MDCK cell model system. We here describe that Snail1 and Zeb1 are mutually required for EMT induction while continuous Snail1 and Snail2 expression, but not Zeb1, is needed for maintenance of the mesenchymal phenotype in MDCK cells. In this model system, EMT is coordinated by Snail1 and Zeb1 through transcriptional and epigenetic downregulation of the miR-200 family. Interestingly, Snail1 is involved in epigenetic CpG DNA methylation of the miR-200 loci, essential to maintain the mesenchymal phenotype. The present results thus define a novel functional interplay between Snail and Zeb EMT-TFs in miR-200 family regulation providing a molecular link to their previous involvement in the generation of EMT process in vivo. 
  GeneRIFs  
 Results of heterogeneous expression of ZEB1 in the invasive front (IF) and tumor center (TC) showed the gene expression patterns for the IF different from those for the TC. Metastatic tumors exhibited higher expression of ZEB1 at the IF than non-metastatic tumors indicating that ZEB1 was involved in   metastasis   of colorectal neoplasm. 
 Our findings highlight the critical ECM-tumor cell interactions regulated by miR-200/Zeb1-dependent EMT that activate intracellular signaling pathways responsible for tumor cell invasion and   metastasis  . 
 The upregulation of ZEB1 is associated with the abnormal expression of E-cadherin, beta-catenin and N-cadherin, which might promote the progression and   metastasis   of cervical squamous cell carcinoma. 
 Results indicate that linc-ROR acts as an important regulator of ZEB1, can promote invasion and   metastasis   in pancreatic cancer. 
 expression of ZEB1 and TWIST1, which are master regulators of tumor   metastasis  . Our study first indicates that miR-1271 functions as a suppressor in regulating of pancreatic cancer EMT by targeting ZEB1 and TWIST1. 
 HOXD9 promotes epithelial-mesenchymal transition and cancer   metastasis   by ZEB1 regulation in hepatocellular carcinoma 
 ZEB1 is a direct downstream of HIF-1alpha, suggesting a novel molecular mechanism for HIF-1alpha-inducing epithelial-mesenchymal transition and cancer   metastasis  . 
 Our study first indicates that miR-590-3p functions as a suppressor of GBM EMT and   metastasis   by targeting ZEB1 and ZEB2, and it may be a therapeutic target for metastatic GBM. 
 targeting miRNA by cordycepin indicates a new mechanism of cordycepin-induced suppression of tumor   metastasis  ; miR-33b/HMGA2/Twist1/ZEB1 axis plays critical roles in regulating melanoma dissemination 
 LARGE2 is negatively regulated by Snail and/or ZEB1, revealing a mechanistic basis for alphaDG hypoglycosylation during prostate cancer progression and   metastasis  . 
 our findings in this study suggest that PZH can inhibit   metastasis   of colorectal cancer cells via modulating TGF-beta1/ZEB/miR-200 signaling network, which might be one of the mechanisms whereby PZH exerts its anticancer function 
 our results indicate that miR-1236-3p regulates ovarian cancer   metastasis   by directly targeting ZEB1 
 These results indicate BAG3 as a regulator of ZEB1 expression in epithelial-mesenchymal transition and as a regulator of   metastasis   in thyroid cancer cells. 
 Study investigated nested regulatory circuits of miR-200c/ZEB1 and miR-200c/ZNF217/TGF-beta/ZEB1 in synergistically promoting trastuzumab resistance and   metastasis   of breast cancer cells. 
 ZEB1 sensitizes lung adenocarcinoma cells to   metastasis   suppression by PI3K-targeted therapy 
 The nuclear protein expression levels of SNAI1 and ZEB1 are involved in the progression and lymph node   metastasis   of cervical cancer via the epithelial-mesenchymal transition pathway. 
 These data provide a novel understanding of the roles of p53 gain-of-function mutations in accelerating tumor progression and   metastasis   through modulation of the miR-130b-ZEB1 axis. 
 overexpression of ZEB1 may be related to the occurrence and development as well as invasion and   metastasis   of gastric adenocarcinoma. 
 Overexpression of ZEB1 is associated with   metastasis   and invasion in osteosarcoma. 
 These results extend the role of epithelial-to-mesenchymal transition in   metastasis   to transendothelial migration and implicate ZEB1 and N-cadherin in this process in prostate cancer cells. 
 Data show that miR-200 and -205 microRNAs cooperatively regulate expression of the E-cadherin transcriptional repressors ZEB1 (also known as deltaEF1) and SIP1 (also known as ZEB2), factors previously implicated in EMT and tumour   metastasis  . 
  [Cc]ell [Ii]nvasion  PubMed  
 Fra-1/AP-1 induces EMT in mammary epithelial cells by modulating Zeb1/2 and TGFβ expression. Epithelial-to-mesenchymal transition (EMT) is essential for embryonic morphogenesis and wound healing and critical for tumour   cell invasio  n and dissemination. The AP-1 transcription factor Fra-1 has been implicated in tumorigenesis and in tumour-associated EMT in human breast cancer. We observed a significant inverse correlation between Fra-1 mRNA expression and distant-metastasis-free survival in a large cohort of breast cancer patients derived from multiple array data sets. This unique correlation among Fos genes prompted us to assess the evolutionary conservation between Fra-1 functions in EMT of human and mouse cells. Ectopic expression of Fra-1 in fully polarized, non-tumourigenic, mouse mammary epithelial EpH4 cells induced a mesenchymal phenotype, characterized by a loss of epithelial and gain of mesenchymal markers. Proliferation, motility and invasiveness were also increased in the resulting EpFra1 cells, and the cells were tumourigenic and efficiently colonized the lung upon transplantation. Molecular analyses revealed increased expression of Tgfβ1 and the EMT-inducing transcription factors Zeb1, Zeb2 and Slug. Mechanistically, Fra-1 binds to the tgfb1 and zeb2 promoters and to an evolutionarily conserved region in the first intron of zeb1. Furthermore, increased activity of a zeb2 promoter reporter was detected in EpFra1 cells and shown to depend on AP-1-binding sites. Inhibiting TGFβ signalling in EpFra1 cells moderately increased the expression of epithelial markers, whereas silencing of zeb1 or zeb2 restored the epithelial phenotype and decreased migration in vitro and tumorigenesis in vivo. Thus Fra-1 induces changes in the expression of genes encoding EMT-related transcription factors leading to the acquisition of mesenchymal, invasive and tumorigenic capacities by epithelial cells. This study defines a novel function of Fra-1/AP-1 in modulating tgfb1, zeb1 and zeb2 expression through direct binding to genomic regulatory regions, which establishes a basis for future in vivo genetic manipulations and preclinical studies using mouse models. 
 Down-regulation of miR-144 promotes thyroid cancer   cell invasion   by targeting ZEB1 and ZEB2. Thyroid cancer is the most common endocrine malignancy, and its incidence has increased rapidly worldwide. The molecular mechanisms underlying thyroid cancer tumorigenesis still need to be further investigated. MicroRNAs (miRNAs), short RNA molecules of approximately 22 nucleotides in length, play crucial roles in tumorigenesis. In the present study, we found that the expression of miR-144 was significantly down-regulated in thyroid cancer as compared with that in normal thyroid tissues, suggesting that miR-144 may be involved in thyroid cancer tumorigenesis. Moreover, our results showed that restoration of miR-144 in K1 and WRO thyroid cancer cells could suppress the invasion and migration capability of these cells. We also demonstrated that miR-144 suppressed the expression of ZEB1 and ZEB2, two E-cadherin suppressors, by directly binding to their 3'-untranslated regions. Furthermore, restoration of ZEB1 or ZEB2 partially rescued the miR-144-induced inhibition of   cell invasion  . These data suggest miR-144 function as a tumor suppressor in thyroid cancer. 
 Down-regulation of miR-144 promotes thyroid cancer   cell invasion   by targeting ZEB1 and ZEB2. Thyroid cancer is the most common endocrine malignancy, and its incidence has increased rapidly worldwide. The molecular mechanisms underlying thyroid cancer tumorigenesis still need to be further investigated. MicroRNAs (miRNAs), short RNA molecules of approximately 22 nucleotides in length, play crucial roles in tumorigenesis. In the present study, we found that the expression of miR-144 was significantly down-regulated in thyroid cancer as compared with that in normal thyroid tissues, suggesting that miR-144 may be involved in thyroid cancer tumorigenesis. Moreover, our results showed that restoration of miR-144 in K1 and WRO thyroid cancer cells could suppress the invasion and migration capability of these cells. We also demonstrated that miR-144 suppressed the expression of ZEB1 and ZEB2, two E-cadherin suppressors, by directly binding to their 3'-untranslated regions. Furthermore, restoration of ZEB1 or ZEB2 partially rescued the miR-144-induced inhibition of   cell invasion  . These data suggest miR-144 function as a tumor suppressor in thyroid cancer. 
 Fra-1/AP-1 induces EMT in mammary epithelial cells by modulating Zeb1/2 and TGFβ expression. Epithelial-to-mesenchymal transition (EMT) is essential for embryonic morphogenesis and wound healing and critical for tumour   cell invasio  n and dissemination. The AP-1 transcription factor Fra-1 has been implicated in tumorigenesis and in tumour-associated EMT in human breast cancer. We observed a significant inverse correlation between Fra-1 mRNA expression and distant-metastasis-free survival in a large cohort of breast cancer patients derived from multiple array data sets. This unique correlation among Fos genes prompted us to assess the evolutionary conservation between Fra-1 functions in EMT of human and mouse cells. Ectopic expression of Fra-1 in fully polarized, non-tumourigenic, mouse mammary epithelial EpH4 cells induced a mesenchymal phenotype, characterized by a loss of epithelial and gain of mesenchymal markers. Proliferation, motility and invasiveness were also increased in the resulting EpFra1 cells, and the cells were tumourigenic and efficiently colonized the lung upon transplantation. Molecular analyses revealed increased expression of Tgfβ1 and the EMT-inducing transcription factors Zeb1, Zeb2 and Slug. Mechanistically, Fra-1 binds to the tgfb1 and zeb2 promoters and to an evolutionarily conserved region in the first intron of zeb1. Furthermore, increased activity of a zeb2 promoter reporter was detected in EpFra1 cells and shown to depend on AP-1-binding sites. Inhibiting TGFβ signalling in EpFra1 cells moderately increased the expression of epithelial markers, whereas silencing of zeb1 or zeb2 restored the epithelial phenotype and decreased migration in vitro and tumorigenesis in vivo. Thus Fra-1 induces changes in the expression of genes encoding EMT-related transcription factors leading to the acquisition of mesenchymal, invasive and tumorigenic capacities by epithelial cells. This study defines a novel function of Fra-1/AP-1 in modulating tgfb1, zeb1 and zeb2 expression through direct binding to genomic regulatory regions, which establishes a basis for future in vivo genetic manipulations and preclinical studies using mouse models. 
 Knockdown of BAG3 induces epithelial-mesenchymal transition in thyroid cancer cells through ZEB1 activation. The process by which epithelial features are lost in favor of a mesenchymal phenotype is referred to as epithelial-mesenchymal transition (EMT). Most carcinomas use this mechanism to evade into neighboring tissues. Reduction or a loss of E-cadherin expression is a well-established hallmark of EMT. As a potent suppressor of E-cadherin, transcription factor ZEB1 is one of the key inducers of EMT, whose expression promotes tumorigenesis and metastasis of carcinomas. Bcl-2-associated athanogene 3 (BAG3) affects multifaceted cellular functions, including proliferation, apoptosis, cell adhesion and invasion, viral infection, and autophagy. Recently, we have reported a novel role of BAG3 implicated in EMT, while the mechanisms are poorly elucidated. The current study demonstrated that knockdown of BAG3 induced EMT, and increased cell migratory and invasiveness in thyroid cancer cells via transcriptional activation of ZEB1. We also found that BAG3 knockdown led to nuclear accumulation of β-catenin, which was responsible for the transcriptional activation of ZEB1. These results indicate BAG3 as a regulator of ZEB1 expression in EMT and as a regulator of metastasis in thyroid cancer cells, providing potential targets to prevent and/or treat thyroid cancer   cell invasio  n and metastasis. 
 ZEB1 drives prometastatic actin cytoskeletal remodeling by downregulating miR-34a expression. Metastatic cancer is extremely difficult to treat, and the presence of metastases greatly reduces a cancer patient's likelihood of long-term survival. The ZEB1 transcriptional repressor promotes metastasis through downregulation of microRNAs (miRs) that are strong inducers of epithelial differentiation and inhibitors of stem cell factors. Given that each miR can target multiple genes with diverse functions, we posited that the prometastatic network controlled by ZEB1 extends beyond these processes. We tested this hypothesis using a mouse model of human lung adenocarcinoma metastasis driven by ZEB1, human lung carcinoma cells, and human breast carcinoma cells. Transcriptional profiling studies revealed that ZEB1 controls the expression of numerous oncogenic and tumor-suppressive miRs, including miR-34a. Ectopic expression of miR-34a decreased tumor   cell invasion   and metastasis, inhibited the formation of promigratory cytoskeletal structures, suppressed activation of the RHO GTPase family, and regulated a gene expression signature enriched in cytoskeletal functions and predictive of outcome in human lung adenocarcinomas. We identified several miR-34a target genes, including Arhgap1, which encodes a RHO GTPase activating protein that was required for tumor   cell invasion  . These findings demonstrate that ZEB1 drives prometastatic actin cytoskeletal remodeling by downregulating miR-34a expression and provide a compelling rationale to develop miR-34a as a therapeutic agent in lung cancer patients. 
 The transcription factor ZEB1 (deltaEF1) promotes tumour cell dedifferentiation by repressing master regulators of epithelial polarity. Epithelial to mesenchymal transition (EMT) is implicated in the progression of primary tumours towards metastasis and is likely caused by a pathological activation of transcription factors regulating EMT in embryonic development. To analyse EMT-causing pathways in tumourigenesis, we identified transcriptional targets of the E-cadherin repressor ZEB1 in invasive human cancer cells. We show that ZEB1 repressed multiple key determinants of epithelial differentiation and cell-cell adhesion, including the cell polarity genes Crumbs3, HUGL2 and Pals1-associated tight junction protein. ZEB1 associated with their endogenous promoters in vivo, and strongly repressed promotor activities in reporter assays. ZEB1 downregulation in undifferentiated cancer cells by RNA interference was sufficient to upregulate expression of these cell polarity genes on the RNA and protein level, to re-establish epithelial features and to impair cell motility in vitro. In human colorectal cancer, ZEB1 expression was limited to the tumour-host interface and was accompanied by loss of intercellular adhesion and tumour   cell invasion  . In invasive ductal and lobular breast cancer, upregulation of ZEB1 was stringently coupled to cancer cell dedifferentiation. Our data show that ZEB1 represents a key player in pathologic EMTs associated with tumour progression. 
 The microRNA-200/Zeb1 axis regulates ECM-dependent β1-integrin/FAK signaling, cancer   cell invasio  n and metastasis through CRKL. Tumor cell metastasis is a complex process that has been mechanistically linked to the epithelial-mesenchymal transition (EMT). The double-negative feedback loop between the microRNA-200 family and the Zeb1 transcriptional repressor is a master EMT regulator, but there is incomplete understanding of how miR-200 suppresses invasion. Our recent efforts have focused on the tumor cell-matrix interactions essential to tumor cell activation. Herein we utilized both our Kras/p53 mutant mouse model and human lung cancer cell lines to demonstrate that upon miR-200 loss integrin β1-collagen I interactions drive 3D in vitro migration/invasion and in vivo metastases. Zeb1-dependent EMT enhances tumor cell responsiveness to the ECM composition and activates FAK/Src pathway signaling by de-repression of the direct miR-200 target, CRKL. We demonstrate that CRKL serves as an adaptor molecule to facilitate focal adhesion formation, mediates outside-in signaling through Itgβ1 to dri  ve cell invas  ion, and inside-out signaling that maintains tumor cell-matrix contacts required f  or cell invas  ion. Importantly, CRKL levels in pan-cancer TCGA analyses were predictive of survival and CRKL knockdown suppressed experimental metastases in vivo without affecting primary tumor growth. Our findings highlight the critical ECM-tumor cell interactions regulated by miR-200/Zeb1-dependent EMT that activate intracellular signaling pathways responsible for tum  or cell invas  ion and metastasis. 
 MicroRNA-144 regulates proliferation, invasion, and apoptosis of cells in malignant solitary pulmonary nodule via zinc finger E-box-binding homeobox 1. This study was to investigate the expression of microRNA (miR)-144 in malignant solitary pulmonary nodule (SPN) tissues and peripheral blood, as well as the biological function of miR-144 in the occurrence and development of lung cancer.In this study, 39 malignant and 30 benign SPN patients were included. The expression of miR-144 was examined using quantitative real-time polymerase chain reaction. Receiver operating characteristic (ROC) curve was used to identify the clinical value of miR-144 in the early diagnosis of malignant SPN. MTT assay was performed to determine A549 cell proliferation and Transwell assay was used to detect changes in A549   cell invasion   and migration ability. Flow cytometry was performed to monitor cell apoptosis, while Western blotting assay was used to measure protein expression levels. At last, dual-luciferase reporter assay was employed to test whether miR-144 regulates zinc finger E-box-binding homeobox 1 (ZEB1) gene expression.Expression of miR-144 was reduced in patients with malignant SPN. miR-144 had diagnostic value for malignant SPN. Proliferation of A549 cells was inhibited by miR-144. Invasion ability of A549 cells was reduced by miR-144. Apoptosis of A549 cells was promoted by miR-144. miR-144 induced A549 cell apoptosis by targeting ZEB1 protein. miR-144 regulated the expression of ZEB1 by interacting with its 3'-UTR region.Expression of miR-144 is reduced in malignant SPN tissues and peripheral blood, being of clinical value in the diagnosis of malignant SPN. miR-144 promotes the apoptosis of lung cancer cells, and inhibits the proliferation, invasion and migration of lung cancer by regulating ZEB1 gene. 
 The nuclear protein expression levels of SNAI1 and ZEB1 are involved in the progression and lymph node metastasis of cervical cancer via the epithelial-mesenchymal transition pathway. Growing evidence illustrates that aberrant activation of the epithelial-mesenchymal transition plays a key role in tumor   cell invasion   and metastasis. Transcription factors SNAI1 and ZEB1 regulate the epithelial-mesenchymal transition. To determine if SNAI1 and ZEB1 are involved in the metastasis of cervical cancer, we used immunohistochemistry to evaluate the expression of SNAI1, ZEB1, and vimentin in tumor and stromal compartments for a large set of cervical carcinoma samples. Results were evaluated using an H score (percentage × intensity). Of 70 samples, 64 cases (91.43%) were positive for SNAI1 expression. The median SNAI1 H score was 174.00 (range, 5-285). Sixty-seven cases (95.71%) were positive for ZEB1, with a median H score of 165.77 (range, 5-260). Nuclear expression of SNAI1 and ZEB1 in tumor cells was positively associated with International Federation of Gynecology and Obstetrics (FIGO) stages (P = .015 and P = .008, respectively) and lymph node metastasis (P = .007 and P = .007, respectively); meanwhile, expression of vimentin in tumor cells was positively associated with lymph node metastasis (P = .019). According to negative vimentin expression, nuclear expression of ZEB1 in tumor cells was positively associated with FIGO stages (P = .04). According to positive vimentin expression, nuclear expression of SNAI1 in tumor cells was positively associated with FIGO stages (P = .018) and pN (P = .029). In light of these findings, we propose that SNAI1 and ZEB1 have the potential to be used as a novel predictor of pelvic lymph node metastasis and represent promising therapeutic targets in patients with cervical cancer. 
 Roles of STAT3 and ZEB1 proteins in E-cadherin down-regulation and human colorectal cancer epithelial-mesenchymal transition. The progression of colorectal carcinoma (CRC) to invasive and metastatic disease may involve localized occurrences of epithelial-mesenchymal transition (EMT). However, mechanisms of the EMT process in CRC progression are not fully understood. We previously showed that knockdown of signal transducer and activator of transcription 3 (STAT3) up-regulated E-cadherin (a key component in EMT progression) in CRC. In this study, we examined the roles of STAT3 in CRC EMT and ZEB1, an EMT inducer, in STAT3-induced down-regulation of E-cadherin. Knockdown of STAT3 significantly increased E-cadherin and decreased N-cadherin and vimentin expressions in highly invasive LoVo CRC cells. Meanwhile, overexpression of STAT3 significantly reduced E-cadherin and enhanced N-cadherin and vimentin expressions in weakly invasive SW1116 CRC cells. Activation of STAT3 significantly increased CRC cell invasiveness and resistance to apoptosis. Knockdown of STAT3 dramatically enhanced chemosensitivity of CRC cells to fluorouracil. STAT3 regulated ZEB1 expression in CRC cells, and the STAT3-induced decrease in E-cadherin and   cell invasion   depended on activation of ZEB1 in CRC cells. Additionally, pSTAT3(Tyr-705) and ZEB1 expressions were significantly correlated with TNM (tumor, lymph node, and metastasis stages) (p < 0.01). In conclusion, STAT3 may directly mediate EMT progression and regulate ZEB1 expression in CRC. ZEB1 may participate in STAT3-induced   cell invasion   and E-cadherin down-regulation in CRC cells. The expressions of pSTAT3(Tyr-705) and ZEB1 may be positively associated with CRC metastasis. Our data may provide potential targets to prevent and/or treat CRC invasion and metastasis. 
 Mutant p53 gain-of-function induces epithelial-mesenchymal transition through modulation of the miR-130b-ZEB1 axis. The tumor suppressor gene p53 has been implicated in the regulation of epithelial-mesenchymal transition (EMT) and tumor metastasis by regulating microRNA (miRNA) expression. Here, we report that mutant p53 exerts oncogenic functions and promotes EMT in endometrial cancer (EC) by directly binding to the promoter of miR-130b (a negative regulator of ZEB1) and inhibiting its transcription. We transduced p53 mutants into p53-null EC cells, profiled the miRNA expression by miRNA microarray and identified miR-130b as a potential target of mutant p53. Ectopic expression of p53 mutants repressed the expression of miR-130b and triggered ZEB1-dependent EMT and cancer   cell invasion  . Loss of an endogenous p53 mutation increased the expression of miR-130b, which resulted in reduced ZEB1 expression and attenuation of the EMT phenotype. Furthermore, re-expression of miR-130b suppressed mutant p53-induced EMT and ZEB1 expression. Importantly, the expression of miR-130 was significantly reduced in EC tissues, and patients with higher expression levels of miR-130b survived longer. These data provide a novel understanding of the roles of p53 gain-of-function mutations in accelerating tumor progression and metastasis through modulation of the miR-130b-ZEB1 axis. 
 HIF-1α Promotes Epithelial-Mesenchymal Transition and Metastasis through Direct Regulation of ZEB1 in Colorectal Cancer. It is well recognized that hypoxia-inducible factor 1 alpha (HIF-1α) is involved in cancer metastasis, chemotherapy and poor prognosis. We previously found that deferoxamine, a hypoxia-mimetic agent, induces epithelial-mesenchymal transition (EMT) in colorectal cancer. Therefore, here we explored a new molecular mechanism for HIF-1α contributing to EMT and cancer metastasis through binding to ZEB1. In this study, we showed that overexpression of HIF-1α with adenovirus infection promoted E  MT, cell inva  sion and migration in vitro and in vivo. On a molecular level, HIF-1α directly binding to the proximal promoter of ZEB1 via hypoxia response element (HRE) sites thus increasing the transactivity and expression of ZEB1. In addition, inhibition of ZEB1 was able to abrogate the HIF-1α-induced EM  T and cell in  vasion. HIF-1α expression was highly correlated with the expression of ZEB1 in normal colorectal epithelium, primary and metastatic CRC tissues. Interestingly, both HIF-1α and ZEB1 were positively associated with Vimentin, an important mesenchymal marker of EMT, whereas negatively associated with E-cadherin expression. These findings suggest that HIF-1α enhances EMT and cancer metastasis by binding to ZEB1 promoter in CRC. HIF-1α and ZEB1 are both widely considered as tumor-initiating factors, but our results demonstrate that ZEB1 is a direct downstream of HIF-1α, suggesting a novel molecular mechanism for HIF-1α-inducing EMT and cancer metastasis. 
 MiR-10b Directly Targets ZEB1 and PIK3CA to Curb Adenomyotic Epithelial Cell Invasiveness via Upregulation of E-Cadherin and Inhibition of Akt Phosphorylation. Adenomyosis is a disease in which ectopic endometrial glands and stromal cells appear in the uterine myometrium. Despite its prevalence, the molecular mechanisms involved in the development of adenomyosis are largely unknown. The aim of this study was to investigate the role of miR-10b and its target genes ZEB1 and PIK3CA in adenomyosis.1387 miRNAs in human normal endometrium and ectopic endometrial lesions of adenomyosis using a microarray screen assay. The significant differential expression of 10 miRNAs was confirmed by qRT-PCR. The expression of miR-10b in endometrial epithelial cells isolated from normal endometrium and paired eutopic and ectopic endometrium of adenomyosis was measured by qRT-PCR. Subsequently, the targets of miR-10b were predicted by bioinformatics and confirmed using a luciferase assay, and the mRNA and protein expression of ZEB1 and PIK3CA were assessed in the endometrium or endometrial epithelial cells by qRT-PCR and western blotting or immunohistochemical analysis. Cell migration and   cell invasion   of endometrial epithelial cells with different treatments by Transwell assays. The expression of p-AKT, Akt and E-cadherin proteins was determined by Western blot analysis.MiR-10b expression was significantly downregulated in both adenomyotic lesions and adenomyotic epithelial cells. MiR-10b overexpression in adenomyotic epithelial cells inhibited cell migration and invasion. We then demonstrated that miR-10b directly targets the 3'-UTRs of ZEB1 and PIK3CA, and downregulates ZEB1 and PIK3CA in adenomyotic epithelial cells, leading to increased E-cadherin expression and decreased Akt phosphorylation.miR-10b directly targets ZEB1 and PIK3CA to curb adenomyotic epithelial cell invasiveness via upregulation of E-Cadherin and inhibition of Akt phosphorylation. 
 Integral role of transcription factor 8 in the negative regulation of tumor angiogenesis. Angiogenesis is involved in various physiologic and pathological conditions, including tumor growth, and is tightly regulated by the orchestration of proangiogenic and antiangiogenic factors. Inhibition of vascular endothelial growth factor (VEGF), the best-established antiangiogenic treatment in cancer, has shown some effectiveness; however, the identification of novel regulators, whose function is independent of VEGF, is required to achieve better outcomes. Here, we show that transcription factor 8 (TCF8) is up-regulated in endothelial cells during angiogenesis, acting as a negative regulator. Furthermore, TCF8 is specifically expressed in the endothelium of tumor vessels. Tcf8-heterozygous knockout mice are more permissive than wild-type mice to the formation of tumor blood vessels in s.c. implanted melanoma, which seems to contribute to the more aggressive growth and the lung metastases of the tumor in mutant mice. Suppression of TCF8 facilitates angiogenesis in both in vitro and ex vivo models, and displays comprehensive cellular phenotypes, including enhanced   cell invasion  , impaired cell adhesion, and increased cell monolayer permeability due to, at least partly, MMP1 overexpression, attenuation of focal adhesion formation, and insufficient VE-cadherin recruitment, respectively. Taken together, our findings define a novel, integral role for TCF8 in the regulation of pathologic angiogenesis, and propose TCF8 as a target for therapeutic intervention in cancer. 
 Molecular regulation of ovarian cancer   cell invasion  . The molecular mechanism underlying ovarian cancer invasiveness and metastasis remains unclear. Since significant downregulation in microRNA 200 (miRNA200) family (miR200a, miR200b, and miR200c) has been reported in the invasive ovarian cancer cells, here, we used two human ovarian cancer cell lines, OVCAR3 and SKOV3, to study the molecular basis of miR200, matrix metalloproteinase 3 (MMP3) activation, and cancer invasiveness. We found that overexpression of either miR200 family member in OVCAR3 or SKOV3 cells significantly inhibited production and secretion of MMP3 and cancer invasiveness. Moreover, forced MMP3 expression abolished miR200-induced inhibition of ovarian cancer cell invasiveness, suggesting that miR200 family inhibited ovarian cell invasiveness via downregulating MMP3. Furthermore, ZEB1, a major target of miR200, was inhibited by miR200 overexpression. Forced ZEB1 expression abolished miR200-induced inhibition of ovarian cancer cell invasiveness, suggesting that ZEB1 is a direct target of miR200 for inhibiting ovarian cell invasiveness. Finally, phosphorylated SMAD3 (pSMAD3), a major partner of ZEB1, was efficiently inhibited by miR200, which could be restored by forced expression of ZEB1, but not by forced expression of MMP3, suggesting that ZEB1/pSMAD3 is signaling cascade upstream of MMP3 in this model. Taken together, our data suggest that miR200 family inhibited ovarian cancer cell invasiveness and metastasis by downregulating MMP3, possibly through ZEB1/pSMAD3. 
 Discoidin domain receptor 1 is a novel transcriptional target of ZEB1 in breast epithelial cells undergoing H-Ras-induced epithelial to mesenchymal transition. The epithelial-to-mesenchymal transition (EMT) process allows carcinoma cells to dissociate from the primary tumor thereby facilitating tumor   cell invasion   and metastasis. Ras-dependent hyperactive signaling is commonly associated with tumorigenesis, invasion, EMT, and metastasis. However, the downstream effectors by which Ras regulates EMT remain ill defined. In this study, we show that the H-Ras pathway leads to mesenchymal-like phenotypic changes in human breast epithelial cells by controlling the ZEB1/microRNA-200c axis. Moreover, H-Ras suppresses the expression of the discoidin domain receptor 1 (DDR1), a collagen receptor tyrosine kinase, via ZEB1, thus identifying ZEB1 as a novel transcriptional repressor of DDR1. Mutation studies on the putative promoter of the DDR1 gene revealed that bipartite Z- and E-box elements play a key role in transcriptional repression of DDR1 in Hs578T and MDA-MB-231 breast carcinoma cell lines by ZEB1. Furthermore, we found an inverse correlation between ZEB1 and DDR1 expression in various cancer cell lines and in human breast carcinoma tissues. Consistently, overexpression of DDR1 reduced the invasive phenotype of mesenchymal-like triple-negative breast cancer cells in 3D cultures and in vivo. Thus, ZEB1's role in maintenance of EMT in breast carcinoma cells is mediated in part by its ability to suppress DDR1 expression and consequently contribute to the activation of the invasive phenotype. Taken together, our results unveil a novel H-Ras/ZEB1/DDR1 network that contributes to breast cancer progression in triple-negative breast cancers. 
 MicroRNA-150 predicts a favorable prognosis in patients with epithelial ovarian cancer, and inhibits   cell invasion   and metastasis by suppressing transcriptional repressor ZEB1. MicroRNA (miR)-150 has been reported to be dramatically downregulated in human epithelial ovarian cancer (EOC) tissues and patients' serum compared to normal controls. This study aimed to investigate clinical significance and molecular mechanisms of miR-150 in EOC. In the current study, quantitative real-time PCR analysis showed that miR-150 was significantly downregulated in human EOC tissues compared to normal tissue samples. Then, we demonstrated the significant associations of miR-150 downregulation with aggressive clinicopathological features of EOC patients, including high clinical stage and pathological grade, and shorter overall and progression-free survivals. More importantly, the multivariate analysis identified miR-150 expression as an independent prognostic biomarker in EOC. After that, luciferase reporter assays demonstrated that Zinc Finger E-Box Binding Homeobox 1 (ZEB1), a crucial regulator of epithelial-to-mesenchymal transition (EMT), was a direct target of miR-150 in EOC cells. Moreover, we found that the ectopic expression of miR-150 could efficiently inhibit cell proliferation, invasion and metastasis by suppressing the expression of ZEB1. Furthermore, we also observed a significantly negative correlation between miR-150 and ZEB1 mRNA expression in EOC tissues (rs = -0.45, P<0.001). In conclusion, these findings offer the convincing evidence that aberrant expression of miR-150 may play a role in tumor progression and prognosis in patients with EOC. Moreover, our data reveal that miR-150 may function as a tumor suppressor and modulate EOC cell proliferation, and invasion by directly and negatively regulating ZEB1, implying the re-expression of miR-150 might be a potential therapeutic strategy for EOC. 
  GeneRIFs  
 Our findings highlight the critical ECM-tumor cell interactions regulated by miR-200/Zeb1-dependent EMT that activate intracellular signaling pathways responsible for tumor   cell invasion   and metastasis. 
 Upregulation in ZEB1 is associated with ovarian cancer   cell invasion  . 
   
  
 PRDX6 
 
  NCBI Gene  
  GeneRIF  
  Pubmed  
 
 
  Search Term  Rank  Fields  
  [Mm]igration  6  PubMed(1)  
  ECM  7  PubMed(1)  
  [Ee]xtracellular [Mm]atrix  8  PubMed(1)  
  [Ii]nvasive  10  PubMed(4); GeneRIFs(1)  
  [Mm]etastasis  12  PubMed(5); GeneRIFs(1)  
  [Cc]ell [Ii]nvasion  13  PubMed(1); GeneRIFs(1)  
 
 
 hide/show details for PRDX6 
  [Mm]igration  PubMed  
 Keratinocyte growth factor and glucocorticoid induction of human peroxiredoxin 6 gene expression occur by independent mechanisms that are synergistic. Peroxiredoxin 6 (Prdx6), a 1-cys Prdx has both peroxidase and phospholipase A2 activities, protecting against oxidative stress and regulating pulmonary surfactant phospholipid metabolism. This study determined the mechanism by which keratinocyte growth factor (KGF) and the glucocorticoid analogue, dexamethasone (Dex), induce increased Prdx6 expression.Transcriptional activation by KGF in both A549 lung adenocarcinoma cells and rat lung alveolar epithelial type II (ATII) cells utilizes an antioxidant response element (ARE), located between 357 and 349 nucleotides before the PRDX6 translational start, that is also necessary for upregulation of the human PRDX6 promoter in response to oxidative stress. Activation is mediated by binding of the transcription factor, Nrf2, to the ARE as shown by experiments using siRNA against Nrf2 and by transfecting ATII cells isolated from lungs of Nrf2 null mice. KGF triggers the   migration   of Nrf2 from cytoplasm to nucleus where it binds to the PRDX6 promoter as shown by chromatin immunoprecipitation assays. Activation of transcription by Dex occurs through a glucocorticoid response element located about 750 nucleotides upstream of the PRDX6 translational start.This study demonstrates that KGF can activate an ARE in a promoter without reactive oxygen species involvement and that KGF and Dex can synergistically activate the PRDX6 promoter and protect cells from oxidative stress.These two different activators work through different DNA elements. Their combined effect on transcription of the reporter gene is synergistic; however, at the protein level, the combined effect is additive and protects cells from oxidative damage. 
  ECM  PubMed  
 PRDX6 attenuates oxidative stress- and TGFbeta-induced abnormalities of human trabecular meshwork cells. Oxidative stress and TGFbeta-induced disturbance of cells and tissues are implicated in initiation and progression of pathophysiology of cells/tissues. Using primary human trabecular meshwork (TM) cells from normal and glaucomatous subjects, this study demonstrated that peroxiredoxin (PRDX) 6, an antioxidant, offsets the deleterious effects of oxidative stress on TM cells by optimizing ROS and TGFbeta levels. An analysis of glaucomatous TM cells revealed a reduced expression of PRDX6 mRNA and protein. Biochemical assays disclosed enhanced levels of ROS, as well as high levels of TGFbetas and these cells expressed elevated extracellular matrix (  ECM  ) and Tsp1 proteins with reduced MMP2; conditions implicated in the pathophysiology of glaucoma. Non-glaucomatous TM cells exposed to TGFbetas/ROS showed similar features as in glaucomatous cells. The abnormalities induced were reversed by delivery of PRDX6. The data provide evidence that oxidative stress-induced abnormality in TM may be related to reduced PRDX6 expression and provide a foundation for antioxidant-based therapeutics for treating glaucoma. 
  [Ee]xtracellular [Mm]atrix  PubMed  
 PRDX6 attenuates oxidative stress- and TGFbeta-induced abnormalities of human trabecular meshwork cells. Oxidative stress and TGFbeta-induced disturbance of cells and tissues are implicated in initiation and progression of pathophysiology of cells/tissues. Using primary human trabecular meshwork (TM) cells from normal and glaucomatous subjects, this study demonstrated that peroxiredoxin (PRDX) 6, an antioxidant, offsets the deleterious effects of oxidative stress on TM cells by optimizing ROS and TGFbeta levels. An analysis of glaucomatous TM cells revealed a reduced expression of PRDX6 mRNA and protein. Biochemical assays disclosed enhanced levels of ROS, as well as high levels of TGFbetas and these cells expressed elevated   extracellular matrix   (ECM) and Tsp1 proteins with reduced MMP2; conditions implicated in the pathophysiology of glaucoma. Non-glaucomatous TM cells exposed to TGFbetas/ROS showed similar features as in glaucomatous cells. The abnormalities induced were reversed by delivery of PRDX6. The data provide evidence that oxidative stress-induced abnormality in TM may be related to reduced PRDX6 expression and provide a foundation for antioxidant-based therapeutics for treating glaucoma. 
  [Ii]nvasive  PubMed  
 Peroxiredoxin 6 promotes lung cancer cell invasion by inducing urokinase-type plasminogen activator via p38 kinase, phosphoinositide 3-kinase, and Akt. The peroxiredoxin family of peroxidase has six mammalian members (Prx 1-6). Considering their frequent up-regulation in cancer cells, Prxs may contribute to cancer cells' survival in face of oxidative stress. Here, we show that Prx 6 promotes the   invasive  ness of lung cancer cells, accompanied by an increase in the activity of phosphoinositide 3-kinase (PI3K), the phosphorylation of p38 kinase and Akt, and the protein levels of uPA. Functional studies reveal that these components support Prx 6-induced invasion in the sequence p38 kinase/PI3K, Akt, and uPA. The findings provide a new understanding of the action of Prx 6 in cancer. 
 [Expressions of peroxiredoxin 1, peroxiredoxin 6 and GFAP in human brain astrocytoma and their clinical significance]. To characterize the expressions of peroxiredoxin 1 (Prx1), peroxiredoxin 6 (Prx6) and glial fibrillary acidic protein (GFAP) in human brain astrocytoma and explore their clinical significance.The protein and mRNA expression levels of Prx1, Prx6 and GFAP in human brain astrocytoma and normal brain tissue specimens were determined by Western blotting, RT-PCR and immunohistochemistry.The protein and mRNA expressions of Prx1 and Prx6 increased significantly in the order of normal brain tissue, grade II astrocytoma, grade III astrocytoma and grade IV astrocytoma (P<0.05). The protein and mRNA expressions of GFAP decreased significantly in grade III and IV astrocytoma compared with those in grade II astrocytoma and normal brain tissues (P<0.05).Prx1 and Prx6 may play important roles in the invasion and malignant development of human brain astrocytoma, and may serve as biomarkers for evaluating the   invasive  ness, malignancy and prognosis of the tumor as well as potential molecular targets in astrocytoma therapy. 
 Phospholipase A2 activity of peroxiredoxin 6 promotes invasion and metastasis of lung cancer cells. Peroxiredoxins (PRDX) are a family of thiol-dependent peroxidases. Among the six mammalian members of this family, PRDX6 is the only protein that additionally exhibits phospholipase A(2) (PLA(2)) activity. The physiologic role of this interesting PRDX6 feature is largely unknown at present. In this study, we show that PRDX6 increases the metastatic potential of lung cancer cells. Functional analyses of the enzymatic activities of PRDX6, using specific pharmacologic inhibitors and mutagenesis studies, reveal that both peroxidase and PLA(2) activities are required for metastasis. Specifically, peroxidase activity facilitates the growth of cancer cells, and PLA(2) activity promotes   invasive  ness. Further investigation of the latter event discloses that PLA(2) activity promotes accumulation of arachidonic acid, which, in turn, induces the   invasive   pathway involving p38 kinase, phosphoinositide 3-kinase, Akt, and urokinase-type plasminogen activator. This study is the first to define the functions of the enzymatic activities of PRDX6 in metastasis and to show the involvement of arachidonic acid in PRDX6 action in intact cells. These novel findings provide a significant step toward elucidating the role of PRDX6 in cancer and the mechanism of its action. Mol Cancer Ther; 9(4); 825-32. (c)2010 AACR. 
 Identification of the functional role of peroxiredoxin 6 in the progression of breast cancer. The molecular mechanisms involved in breast cancer metastasis still remain unclear to date. In our previous study, differential expression of peroxiredoxin 6 was found between the highly metastatic MDA-MB-435HM cells and their parental counterparts, MDA-MB-435 cells. In this study, we investigated the effects of peroxiredoxin 6 on the proliferation and metastatic potential of human breast cancer cells and their potential mechanism.Expression of peroxiredoxin 6 in the highly metastatic MDA-MB-231HM cells was investigated by RT-PCR, real-time PCR and western blot. A recombinant expression plasmid of the human peroxiredoxin 6 gene was constructed and transfected into MDA-MB-231 and MDA-MB-435 cells. The effects of peroxiredoxin 6 on the proliferation and invasion of MDA-MB-231 and MDA-MB-435 cells were investigated by the Cell Counting Kit-8 method, colony-formation assay, adhesion assay, flow cytometry and invasion assay in vitro. miRNA was used to downregulate the expression of peroxiredoxin 6. Genes related to the invasion and metastasis of cancer were determined by RT-PCR, real-time PCR and western blot. The tumorigenicity and spontaneously metastatic capability regulated by peroxiredoxin 6 were determined using an orthotopic xenograft tumor model in athymic mice.Overexpression of peroxiredoxin 6 in MDA-MB-231HM cells compared with their parental counterparts was confirmed. Upregulation of peroxiredoxin 6 enhanced the in vitro proliferation and invasion of breast cancer cells. The enhancement was associated with decreasing levels of tissue inhibitor of matrix metalloproteinase (TIMP)-2 and increasing levels of the urokinase-type plasminogen activator receptor (uPAR), Ets-1 (E26 transformation-specific-1), matrix metalloproteinase (MMP)-9 and RhoC (ras homolog gene family, member C) expression. The results were further demonstrated by RNA interference experiments in vitro. In an in vivo study, we also demonstrated that peroxiredoxin 6-transfected breast cancer cells grew much faster and had more pulmonary metastases than control cells. By contrast, peroxiredoxin 6 knockdown breast cancer cells grew more slowly and had fewer pulmonary metastases. Effects similar to those of peroxiredoxin 6 on the uPAR, Ets-1, MMP-9, RhoC and TIMP-2 expression observed in in vitro studies were found in the in vivo study.Overexpression of peroxiredoxin 6 leads to a more   invasive   phenotype and metastatic potential in human breast cancer, at least in part, through regulation of the levels of uPAR, Ets-1, MMP-9, RhoC and TIMP-2 expression. 
  GeneRIFs  
 Overexpression of peroxiredoxin 6 leads to a more   invasive   phenotype and metastatic potential in human breast cancer, at least in part, through regulation of the levels of uPAR, Ets-1, MMP-9, RhoC and TIMP-2 expression. 
  [Mm]etastasis  PubMed  
 Proteome analysis of schizophrenia patients Wernicke's area reveals an energy metabolism dysregulation. Schizophrenia is likely to be a consequence of DNA alterations that, together with environmental factors, will lead to protein expression differences and the ultimate establishment of the illness. The superior temporal gyrus is implicated in schizophrenia and executes functions such as the processing of speech, language skills and sound processing.We performed an individual comparative proteome analysis using two-dimensional gel electrophoresis of 9 schizophrenia and 6 healthy control patients' left posterior superior temporal gyrus (Wernicke's area - BA22p) identifying by mass spectrometry several protein expression alterations that could be related to the disease.Our analysis revealed 11 downregulated and 14 upregulated proteins, most of them related to energy metabolism. Whereas many of the identified proteins have been previously implicated in schizophrenia, such as fructose-bisphosphate aldolase C, creatine kinase and neuron-specific enolase, new putative disease markers were also identified such as dihydrolipoyl dehydrogenase, tropomyosin 3, breast cancer   metastasis  -suppressor 1, heterogeneous nuclear ribonucleoproteins C1/C2 and phosphate carrier protein, mitochondrial precursor. Besides, the differential expression of peroxiredoxin 6 (PRDX6) and glial fibrillary acidic protein (GFAP) were confirmed by western blot in schizophrenia prefrontal cortex.Our data supports a dysregulation of energy metabolism in schizophrenia as well as suggests new markers that may contribute to a better understanding of this complex disease. 
 [Expression of peroxiredoxin 6 in gastric cancer and its clinical significance]. To elucidate the significance of anti-oxidant protein peroxiredoxin 6 (Prx6) expression in gastric cancer (GC) tissue.Eighty-six GC tissues and 69 para-cancer tissues from surgically resected specimens were included. And the clinico-pathological data were collected by reviewing medical records. Tissue microarray and immunohistochemistry (IHC) were used to detect Prx6 protein expression in tissues.Prx6 protein was predominantly expressed in cytoplasm. Its expression rate in GC tissues (32.6%, n = 28) was significantly lower than that in para-cancer normal tissues (94.2%, n = 65, P < 0.05). And its protein overexpression rate in well and moderately-differentiated GC tissues was significantly higher than that in lowly-differentiated ones (39.6% (21/53) vs 6.1% (2/33), P < 0.05). Prx6 expression in gastric cancer tissues was not significantly related to age and sex of patients, lesion site, depth of invasion, clinical staging, vascular invasion and liver   metastasis  .The level of Prx6 protein is lower in GC tissues than that in normal para-cancer ones. And it is significantly correlated with the differentiation degree of GC. 
 PRDX1 and PRDX6 are repressed in papillary thyroid carcinomas via BRAF V600E-dependent and -independent mechanisms. Many clinical studies highlight the dichotomous role of PRDXs in human cancers, where they can exhibit strong tumor-suppressive or tumor-promoting functions. Recent evidence suggests that lower expression of PRDXs correlates with cancer progression in colorectal cancer (CRC) or in esophageal squamous carcinoma. In the thyroid, increased levels of PRDX1 has been described in follicular adenomas and carcinomas, as well as in thyroiditis, while reduced levels of PRDX6 has been found in follicular adenomas. We studied the expression of PRDX1 and PRDX6, in a series of thyroid tissue samples, covering different thyroid diseases, including 13 papillary thyroid carcinomas (PTCs). Our results show that PRDX1 and PRDX6 are significantly reduced in all PTCs compared to normal tissues, to non-neoplastic tissue (MNG) or follicular neoplasms. This reduction is strongly evident in PTCs harboring BRAF V600E (31% of our cases). Using TPC-1 and BCPAP and FRTL-5 cell lines, we demonstrate for the first time that the presence of BRAF V600E is responsible of the hypoexpression of PRDX1 and PRDX6 both at mRNA and protein levels. Finally, independently of BRAF status, we observe an interesting correlation between the tumor size, the presence of lymph node   metastasis   and the lowest PRDX1 and PRDX6 levels. Therefore, these data indicate that PRDX1 and PRDX6 expression not only may play a key role in papillary thyroid carcinogenesis via a BRAF V600E-dependent mechanism, but their determination could be considered as potential tumor marker for indicating tumor progression in PTCs, independently of BRAF status. 
 Phospholipase A2 activity of peroxiredoxin 6 promotes invasion and   metastasis   of lung cancer cells. Peroxiredoxins (PRDX) are a family of thiol-dependent peroxidases. Among the six mammalian members of this family, PRDX6 is the only protein that additionally exhibits phospholipase A(2) (PLA(2)) activity. The physiologic role of this interesting PRDX6 feature is largely unknown at present. In this study, we show that PRDX6 increases the metastatic potential of lung cancer cells. Functional analyses of the enzymatic activities of PRDX6, using specific pharmacologic inhibitors and mutagenesis studies, reveal that both peroxidase and PLA(2) activities are required for   metastasis  . Specifically, peroxidase activity facilitates the growth of cancer cells, and PLA(2) activity promotes invasiveness. Further investigation of the latter event discloses that PLA(2) activity promotes accumulation of arachidonic acid, which, in turn, induces the invasive pathway involving p38 kinase, phosphoinositide 3-kinase, Akt, and urokinase-type plasminogen activator. This study is the first to define the functions of the enzymatic activities of PRDX6 in   metastasis   and to show the involvement of arachidonic acid in PRDX6 action in intact cells. These novel findings provide a significant step toward elucidating the role of PRDX6 in cancer and the mechanism of its action. Mol Cancer Ther; 9(4); 825-32. (c)2010 AACR. 
 Identification of the functional role of peroxiredoxin 6 in the progression of breast cancer. The molecular mechanisms involved in breast cancer   metastasis   still remain unclear to date. In our previous study, differential expression of peroxiredoxin 6 was found between the highly metastatic MDA-MB-435HM cells and their parental counterparts, MDA-MB-435 cells. In this study, we investigated the effects of peroxiredoxin 6 on the proliferation and metastatic potential of human breast cancer cells and their potential mechanism.Expression of peroxiredoxin 6 in the highly metastatic MDA-MB-231HM cells was investigated by RT-PCR, real-time PCR and western blot. A recombinant expression plasmid of the human peroxiredoxin 6 gene was constructed and transfected into MDA-MB-231 and MDA-MB-435 cells. The effects of peroxiredoxin 6 on the proliferation and invasion of MDA-MB-231 and MDA-MB-435 cells were investigated by the Cell Counting Kit-8 method, colony-formation assay, adhesion assay, flow cytometry and invasion assay in vitro. miRNA was used to downregulate the expression of peroxiredoxin 6. Genes related to the invasion and   metastasis   of cancer were determined by RT-PCR, real-time PCR and western blot. The tumorigenicity and spontaneously metastatic capability regulated by peroxiredoxin 6 were determined using an orthotopic xenograft tumor model in athymic mice.Overexpression of peroxiredoxin 6 in MDA-MB-231HM cells compared with their parental counterparts was confirmed. Upregulation of peroxiredoxin 6 enhanced the in vitro proliferation and invasion of breast cancer cells. The enhancement was associated with decreasing levels of tissue inhibitor of matrix metalloproteinase (TIMP)-2 and increasing levels of the urokinase-type plasminogen activator receptor (uPAR), Ets-1 (E26 transformation-specific-1), matrix metalloproteinase (MMP)-9 and RhoC (ras homolog gene family, member C) expression. The results were further demonstrated by RNA interference experiments in vitro. In an in vivo study, we also demonstrated that peroxiredoxin 6-transfected breast cancer cells grew much faster and had more pulmonary metastases than control cells. By contrast, peroxiredoxin 6 knockdown breast cancer cells grew more slowly and had fewer pulmonary metastases. Effects similar to those of peroxiredoxin 6 on the uPAR, Ets-1, MMP-9, RhoC and TIMP-2 expression observed in in vitro studies were found in the in vivo study.Overexpression of peroxiredoxin 6 leads to a more invasive phenotype and metastatic potential in human breast cancer, at least in part, through regulation of the levels of uPAR, Ets-1, MMP-9, RhoC and TIMP-2 expression. 
  GeneRIFs  
 This study is the first to define the functions of the enzymatic activities of PRDX6 in   metastasis   and to show the involvement of arachidonic acid in PRDX6 action in intact cells. 
  [Cc]ell [Ii]nvasion  PubMed  
 Peroxiredoxin 6 promotes lung cancer   cell invasion   by inducing urokinase-type plasminogen activator via p38 kinase, phosphoinositide 3-kinase, and Akt. The peroxiredoxin family of peroxidase has six mammalian members (Prx 1-6). Considering their frequent up-regulation in cancer cells, Prxs may contribute to cancer cells' survival in face of oxidative stress. Here, we show that Prx 6 promotes the invasiveness of lung cancer cells, accompanied by an increase in the activity of phosphoinositide 3-kinase (PI3K), the phosphorylation of p38 kinase and Akt, and the protein levels of uPA. Functional studies reveal that these components support Prx 6-induced invasion in the sequence p38 kinase/PI3K, Akt, and uPA. The findings provide a new understanding of the action of Prx 6 in cancer. 
  GeneRIFs  
 promotes lung cancer   cell invasion   by inducing urokinase-type plasminogen activator via p38 kinase, phosphoinositide 3-kinase, and Ak 
   
  
 ARHGEF6 
 
  NCBI Gene  
  GeneRIF  
  Pubmed  
 
 
  Search Term  Rank  Fields  
  [Pp]odosome(s)?  5  PubMed(1); GeneRIFs(1)  
  [Mm]igration  6  PubMed(8); GeneRIFs(1)  
  [Ee]xtracellular [Mm]atrix  8  PubMed(2)  
  [Ii]nvasive  10  PubMed(1)  
  [Mm]etastasis  12  PubMed(1)  
 
 
 hide/show details for ARHGEF6 
  [Pp]odosome(s)?  PubMed  
 PAK4 and alphaPIX determine   podosome   size and number in macrophages through localized actin regulation.   Podosomes   are actin-rich adhesion structures typical for monocytic cells and are implicated in migration and invasion. Major modes of   podosome   regulation include RhoGTPase signaling and actin regulatory pathways. However, it is not clearly understood how these signals induce highly localized changes in   podosome   formation and dynamics. Here, we show that the RhoGTPase effector PAK4, a member of the p21 associated kinase family, and its regulator alphaPIX (PAK-interacting exchange factor), are central to   podosome   formation in primary human macrophages. Immunofluorescence, biochemical and microarray data indicate that PAK4 acts as physiological regulator of   podosomes   in this system. Accordingly, transfection of a specific shRNA, as well as expression of PAK4 truncation mutants, resulted in reduced numbers of   podosomes   per cell. Moreover, expression of kinase active or inactive PAK4 mutants enhanced or reduced the size of individual   podosomes  , respectively, indicating a modulatory influence of PAK4 kinase activity on   podosome   size. Similar to the results gained with PAK4, cellular/overexpressed PIX was shown to be closely associated with   podosomes  . Moreover, both overexpression of alphaPIX wt and a mutant lacking the SH3 domain led to coalescence of   podosomes  . In sum, we propose that PAK4 and alphaPIX can induce highly localized changes in actin dynamics and thereby regulate size and number of   podosomes   in primary human macrophages. 
  GeneRIFs  
 PAK4 and alphaPIX can induce highly localized changes in actin dynamics and thereby regulate size and number of   podosomes   in primary human macrophages. 
  [Mm]igration  PubMed  
 Mutation of ARHGAP9 in patients with coronary spastic angina. Coronary artery spasm has an important function in the etiology of variant angina and other acute coronary syndromes. Abnormal activation of Rho-family GTPases has been observed in cardiovascular disorders, but the function of genetic variability in Rho-family GTPases remains to be evaluated in cardiovascular disorders. We examined the genetic variability of Rho-family GTPases and their regulators in coronary artery spasm. We performed a comprehensive candidate gene analysis of 67 single nucleotide polymorphisms with amino-acid substitution in Rho-family GTPases and their regulators in 103 unrelated Japanese patients with acetylcholine-induced coronary artery spasm and 102 control Japanese subjects without acetylcholine-induced coronary artery spasm. We noted an association of the single nucleotide polymorphism of ARHGAP9 (rs11544238, Ala370Ser) with coronary artery spasm (odds ratio =2.67). We found that ARHGAP9 inactivated Rac as RacGAP and that the mRNA level of ARHGAP9 was strongly detected in hematopoietic cells. ARHGAP9 negatively regulated cell   migration  . The Ala370Ser polymorphism counteracted ARHGAP9-reduced cell   migration  , spreading and adhesion. The Ala370Ser polymorphism in the ARHGAP9 gene is associated with coronary artery spasm. These data suggest that the polymorphism of ARHGAP9 has a critical function in the infiltration of hematopoietic cells into the endothelium and inflammation leading to endothelial dysfunction. 
 Cyclic Nucleotide-dependent Protein Kinases Target ARHGAP17 and ARHGEF6 Complexes in Platelets. Endothelial cells release prostacyclin (PGI2) and nitric oxide (NO) to inhibit platelet functions. PGI2 and NO effects are mediated by cyclic nucleotides, cAMP- and cGMP-dependent protein kinases (PKA, PKG), and largely unknown PKA and PKG substrate proteins. The small G-protein Rac1 plays a key role in platelets and was suggested to be a target of cyclic nucleotide signaling. We confirm that PKA and PKG activation reduces Rac1-GTP levels. Screening for potential mediators of this effect resulted in the identification of the Rac1-specific GTPase-activating protein ARHGAP17 and the guanine nucleotide exchange factor ARHGEF6 as new PKA and PKG substrates in platelets. We mapped the PKA/PKG phosphorylation sites to serine 702 on ARHGAP17 using Phos-tag gels and to serine 684 on ARHGEF6. We show that ARHGAP17 binds to the actin-regulating CIP4 protein in platelets and that Ser-702 phosphorylation interferes with this interaction. Reduced CIP4 binding results in enhanced inhibition of cell   migration   by ARHGAP17. Furthermore, we show that ARHGEF6 is constitutively linked to GIT1, a GAP of Arf family small G proteins, and that ARHGEF6 phosphorylation enables binding of the 14-3-3 adaptor protein to the ARHGEF6/GIT1 complex. PKA and PKG induced rearrangement of ARHGAP17- and ARHGEF6-associated protein complexes might contribute to Rac1 regulation and platelet inhibition. 
 N-α-acetyltransferase 10 protein suppresses cancer cell metastasis by binding PIX proteins and inhibiting Cdc42/Rac1 activity. N-α-acetyltransferase 10 protein, Naa10p, is an N-acetyltransferase known to be involved in cell cycle control. We found that Naa10p was expressed lower in varieties of malignancies with lymph node metastasis compared with non-lymph node metastasis. Higher Naa10p expression correlates the survival of lung cancer patients. Naa10p significantly suppresse  d migrati  on, tumor growth, and metastasis independent of its enzymatic activity. Instead, Naa10p binds to the GIT-binding domain of PIX, thereby preventing the formation of the GIT-PIX-Paxillin complex, resulting in reduced intrinsic Cdc42/Rac1 activity and decreased cel  l migrati  on. Forced expression of PIX in Naa10-transfected tumor cells restored th  e migrati  on and metastasis ability. We suggest that Naa10p functions as a tumor metastasis suppressor by disrupting the migratory complex, PIX-GIT- Paxillin, in cancer cells. 
 Cyclic Nucleotide-dependent Protein Kinases Target ARHGAP17 and ARHGEF6 Complexes in Platelets. Endothelial cells release prostacyclin (PGI2) and nitric oxide (NO) to inhibit platelet functions. PGI2 and NO effects are mediated by cyclic nucleotides, cAMP- and cGMP-dependent protein kinases (PKA, PKG), and largely unknown PKA and PKG substrate proteins. The small G-protein Rac1 plays a key role in platelets and was suggested to be a target of cyclic nucleotide signaling. We confirm that PKA and PKG activation reduces Rac1-GTP levels. Screening for potential mediators of this effect resulted in the identification of the Rac1-specific GTPase-activating protein ARHGAP17 and the guanine nucleotide exchange factor ARHGEF6 as new PKA and PKG substrates in platelets. We mapped the PKA/PKG phosphorylation sites to serine 702 on ARHGAP17 using Phos-tag gels and to serine 684 on ARHGEF6. We show that ARHGAP17 binds to the actin-regulating CIP4 protein in platelets and that Ser-702 phosphorylation interferes with this interaction. Reduced CIP4 binding results in enhanced inhibition of cell   migration   by ARHGAP17. Furthermore, we show that ARHGEF6 is constitutively linked to GIT1, a GAP of Arf family small G proteins, and that ARHGEF6 phosphorylation enables binding of the 14-3-3 adaptor protein to the ARHGEF6/GIT1 complex. PKA and PKG induced rearrangement of ARHGAP17- and ARHGEF6-associated protein complexes might contribute to Rac1 regulation and platelet inhibition. 
 Mutation of ARHGAP9 in patients with coronary spastic angina. Coronary artery spasm has an important function in the etiology of variant angina and other acute coronary syndromes. Abnormal activation of Rho-family GTPases has been observed in cardiovascular disorders, but the function of genetic variability in Rho-family GTPases remains to be evaluated in cardiovascular disorders. We examined the genetic variability of Rho-family GTPases and their regulators in coronary artery spasm. We performed a comprehensive candidate gene analysis of 67 single nucleotide polymorphisms with amino-acid substitution in Rho-family GTPases and their regulators in 103 unrelated Japanese patients with acetylcholine-induced coronary artery spasm and 102 control Japanese subjects without acetylcholine-induced coronary artery spasm. We noted an association of the single nucleotide polymorphism of ARHGAP9 (rs11544238, Ala370Ser) with coronary artery spasm (odds ratio =2.67). We found that ARHGAP9 inactivated Rac as RacGAP and that the mRNA level of ARHGAP9 was strongly detected in hematopoietic cells. ARHGAP9 negatively regulated cell   migration  . The Ala370Ser polymorphism counteracted ARHGAP9-reduced cell   migration  , spreading and adhesion. The Ala370Ser polymorphism in the ARHGAP9 gene is associated with coronary artery spasm. These data suggest that the polymorphism of ARHGAP9 has a critical function in the infiltration of hematopoietic cells into the endothelium and inflammation leading to endothelial dysfunction. 
 c-Cbl regulates αPix-mediated cell   migratio  n and invasion. c-Cbl, a RING-type ubiquitin E3 ligase, down-regulates receptor tyrosine kinases, including EGF receptor, and inhibits cell proliferation. Moreover, c-Cbl mutations are frequently found in patients with myeloid neoplasm. Therefore, c-Cbl is known as a tumor suppressor. αPix is expressed only in highly proliferative and mobile cells, including immune cells, and up-regulated in certain invasive tumors, such as glioblastoma multiforme. Here, we showed that c-Cbl serves as an ubiquitin E3 ligase for proteasome-mediated degradation of αPix, but not βPix. Remarkably, the rat C6 and human A172 glioma cells were unable to express c-Cbl, which leads to a dramatic accumulation of αPix. Depletion of αPix by shRNA markedly reduced the ability of the glioma cells to migrate and invade, whereas complementation of shRNA-insensitive αPix promoted it. These results indicate that c-Cbl negatively regulates αPix-mediat  ed cell m  igration and invasion and the lack of c-Cbl in the C6 and A172 glioma cells is responsible for their malignant behavior. 
 alphaPIX nucleotide exchange factor is activated by interaction with phosphatidylinositol 3-kinase. p21-activated kinase (PAK) is a common effector protein of the small GTPases Cdc42 and Rac, leading to the activation of downstream mitogen activated protein kinases. PAK also mediates polarized cytoskeletal changes induced by these GTPases. The recently identified PAK-interacting exchange factor (PIX) acts as a guanine nucleotide exchange factor on Rac, and colocalizes with PAK in a focal complex, but little is known about the associated signaling cascades, including upstream activators of PIX. In this study, we show that one of the isoforms of PIX, alphaPIX, is activated by signaling cascades from the platelet-derived growth factor (PDGF) receptor and EphB2 receptor, and from integrin-induced signaling through phosphatidylinositol 3-kinase (PI3-kinase). alphaPIX is activated by forming a complex with these receptors either via association with PAK and Nck, or direct association with the p85 regulatory subunit of PI3-kinase. Synthetic phosphoinositide and membrane targeted PI3-kinase augmented the alphaPIX activity in vivo. In Xenopus, aggregates of mesodermal cells derived from embryos microinjected with alphaPIX significantly increased the peripheral spreading on fibronectin substrate in response to PDGF through PI3-kinase. These results indicate that alphaPIX is activated by PI3-kinase, and is involved in the receptor mediated signaling leading to the activation of the kinase activity of PAK, and the   migration   of mesodermal cells on extracellular matrix. 
 PAK4 and alphaPIX determine podosome size and number in macrophages through localized actin regulation. Podosomes are actin-rich adhesion structures typical for monocytic cells and are implicated in   migration   and invasion. Major modes of podosome regulation include RhoGTPase signaling and actin regulatory pathways. However, it is not clearly understood how these signals induce highly localized changes in podosome formation and dynamics. Here, we show that the RhoGTPase effector PAK4, a member of the p21 associated kinase family, and its regulator alphaPIX (PAK-interacting exchange factor), are central to podosome formation in primary human macrophages. Immunofluorescence, biochemical and microarray data indicate that PAK4 acts as physiological regulator of podosomes in this system. Accordingly, transfection of a specific shRNA, as well as expression of PAK4 truncation mutants, resulted in reduced numbers of podosomes per cell. Moreover, expression of kinase active or inactive PAK4 mutants enhanced or reduced the size of individual podosomes, respectively, indicating a modulatory influence of PAK4 kinase activity on podosome size. Similar to the results gained with PAK4, cellular/overexpressed PIX was shown to be closely associated with podosomes. Moreover, both overexpression of alphaPIX wt and a mutant lacking the SH3 domain led to coalescence of podosomes. In sum, we propose that PAK4 and alphaPIX can induce highly localized changes in actin dynamics and thereby regulate size and number of podosomes in primary human macrophages. 
  GeneRIFs  
 c-Cbl negatively regulates alphaPix-mediated cell   migration   and invasion; the lack of c-Cbl in C6 and A172 glioma cells is responsible for their malignant behavior 
  [Ee]xtracellular [Mm]atrix  PubMed  
 AlphaPIX associates with calpain 4, the small subunit of calpain, and has a dual role in integrin-mediated cell spreading. Binding of integrins to the   extracellular matrix   results in actin cytoskeletal rearrangements, e.g. during cell spreading, by regulating the activity of Rho GTP-ases. We have shown previously that alphaPIX (Cool-2 or ARHGEF6), a Rac1/Cdc42-specific guanine nucleotide exchange factor (GEF), binds to beta-parvin/affixin and colocalizes with integrin-linked kinase in actively spreading cells, suggesting that alphaPIX is involved in integrin-induced signaling leading to activation of Rac1/Cdc42. Here we report calpain 4, the small subunit of the proteases mu-calpain and m-calpain, as a novel binding partner of alphaPIX. This association was identified by the CytoTrap system and confirmed by coimmunoprecipitation and glutathione S-transferase pull-down assays. The alphaPIX triple domain SH3-DH-PH was found to be required for calpain 4 binding. During integrin-dependent spreading of CHO-K1 cells, alphaPIX colocalized with mu- and m-calpain, integrin-linked kinase, and beta1 integrin in early integrin-containing clusters. Overexpression of alphaPIX wild type but not the GEF-deficient mutant (L386R/L387S) resulted in enhanced formation of characteristic cellular protrusions during cell spreading, suggesting that alphaPIX GEF activity is necessary for this specific actin cytoskeletal reorganization. The calpain inhibitors calpeptin and calpain inhibitor IV significantly inhibited integrin-dependent cell spreading. However, concomitant overexpression of alphaPIX wild type or the L386R/L387S mutant restored cell spreading. Together, these data suggest that alphaPIX is a component of early integrin clusters and plays a dual role in integrin-dependent cell spreading. Whereas alphaPIX GEF activity contributes to enhanced formation of cellular protrusions, the GEF-independent association with calpain 4 leads to induction of a yet unknown signaling cascade resulting in cell spreading. 
 alphaPIX nucleotide exchange factor is activated by interaction with phosphatidylinositol 3-kinase. p21-activated kinase (PAK) is a common effector protein of the small GTPases Cdc42 and Rac, leading to the activation of downstream mitogen activated protein kinases. PAK also mediates polarized cytoskeletal changes induced by these GTPases. The recently identified PAK-interacting exchange factor (PIX) acts as a guanine nucleotide exchange factor on Rac, and colocalizes with PAK in a focal complex, but little is known about the associated signaling cascades, including upstream activators of PIX. In this study, we show that one of the isoforms of PIX, alphaPIX, is activated by signaling cascades from the platelet-derived growth factor (PDGF) receptor and EphB2 receptor, and from integrin-induced signaling through phosphatidylinositol 3-kinase (PI3-kinase). alphaPIX is activated by forming a complex with these receptors either via association with PAK and Nck, or direct association with the p85 regulatory subunit of PI3-kinase. Synthetic phosphoinositide and membrane targeted PI3-kinase augmented the alphaPIX activity in vivo. In Xenopus, aggregates of mesodermal cells derived from embryos microinjected with alphaPIX significantly increased the peripheral spreading on fibronectin substrate in response to PDGF through PI3-kinase. These results indicate that alphaPIX is activated by PI3-kinase, and is involved in the receptor mediated signaling leading to the activation of the kinase activity of PAK, and the migration of mesodermal cells on   extracellular matrix  . 
  [Ii]nvasive  PubMed  
 c-Cbl regulates αPix-mediated cell migration and invasion. c-Cbl, a RING-type ubiquitin E3 ligase, down-regulates receptor tyrosine kinases, including EGF receptor, and inhibits cell proliferation. Moreover, c-Cbl mutations are frequently found in patients with myeloid neoplasm. Therefore, c-Cbl is known as a tumor suppressor. αPix is expressed only in highly proliferative and mobile cells, including immune cells, and up-regulated in certai  n invasi  ve tumors, such as glioblastoma multiforme. Here, we showed that c-Cbl serves as an ubiquitin E3 ligase for proteasome-mediated degradation of αPix, but not βPix. Remarkably, the rat C6 and human A172 glioma cells were unable to express c-Cbl, which leads to a dramatic accumulation of αPix. Depletion of αPix by shRNA markedly reduced the ability of the glioma cells to migrate and invade, whereas complementation of shRNA-insensitive αPix promoted it. These results indicate that c-Cbl negatively regulates αPix-mediated cell migration and invasion and the lack of c-Cbl in the C6 and A172 glioma cells is responsible for their malignant behavior. 
  [Mm]etastasis  PubMed  
 N-α-acetyltransferase 10 protein suppresses cancer cell   metastasi  s by binding PIX proteins and inhibiting Cdc42/Rac1 activity. N-α-acetyltransferase 10 protein, Naa10p, is an N-acetyltransferase known to be involved in cell cycle control. We found that Naa10p was expressed lower in varieties of malignancies with lymph nod  e metastas  is compared with non-lymph nod  e metastas  is. Higher Naa10p expression correlates the survival of lung cancer patients. Naa10p significantly suppressed migration, tumor growth, an  d metastas  is independent of its enzymatic activity. Instead, Naa10p binds to the GIT-binding domain of PIX, thereby preventing the formation of the GIT-PIX-Paxillin complex, resulting in reduced intrinsic Cdc42/Rac1 activity and decreased cell migration. Forced expression of PIX in Naa10-transfected tumor cells restored the migration an  d metastas  is ability. We suggest that Naa10p functions as a tumo  r metastas  is suppressor by disrupting the migratory complex, PIX-GIT- Paxillin, in cancer cells. 
   
  
 ARHGEF7 
 
  NCBI Gene  
  GeneRIF  
  Pubmed  
 
 
  Search Term  Rank  Fields  
  [Ii]nvadopodia  3  PubMed(1); GeneRIFs(1)  
  [Pp]odosome(s)?  5  PubMed(1)  
  [Mm]igration  6  Process(1); PubMed(22); GeneRIFs(6)  
  [Ee]xtracellular [Mm]atrix  8  PubMed(5)  
  [Ii]nvasive  10  PubMed(2)  
  [Mm]etastasis  12  PubMed(4)  
 
 
 hide/show details for ARHGEF7 
  [Ii]nvadopodia  PubMed  
 Hypoxia-induced   invadopodia   formation: a role for β-PIX. During tumour progression, oxygen tension in the microenvironment surrounding tumour cells is reduced, resulting in hypoxia. It is well established that cancer cells resist the negative effects of hypoxia by inducing angiogenesis predominantly via the activity of transcription factor hypoxia-inducible factor-1 (HIF-1). However, more recently HIF-1α has also been linked to increased invasive potential, although the molecular mechanisms remain to be defined. Invasive cancer cells are thought to employ membrane protrusions, terme  d invadopod  ia, to achieve matrix degradation. While man  y invadopod  ia components have been identified, signalling pathways that link extracellular stimuli t  o invadopod  ia formation remain largely unknown. Indeed, the relationship betwee  n invadopod  ia formation and HIF-1α has not been explored. We now report that HIF-1α is a driver   of invadop  odia formation. Furthermore, we have identified an important, direct and novel link between the Rho family activator β-PIX, HIF-1�  � and invad  opodia formation. Indeed, we find that β-PIX expression is essenti  al for inva  dopodia formation. In conclusion, we identify a new HIF-1α mechanistic pathway and suggest that β-PIX is a novel downstream signalling mediato  r during in  vadopodia formation. 
  GeneRIFs  
 BETA-PIX is a novel downstream signalling mediator during   invadopodia   formation. 
  [Pp]odosome(s)?  PubMed  
 Bin2 is a membrane sculpting N-BAR protein that influences leucocyte   podosomes  , motility and phagocytosis. Cell motility, adhesion and phagocytosis are controlled by actin and membrane remodelling processes. Bridging integrator-2 (Bin2) also called Breast cancer-associated protein 1 (BRAP1) is a predicted N-BAR domain containing protein with unknown function that is highly expressed in leucocytic cells. In the present study we solved the structure of Bin2 BAR domain and studied its membrane binding and bending properties in vitro and in vivo. Live-cell imaging experiments showed that Bin2 is associated with actin rich structures on the plasma membrane, where it was targeted through its N-BAR domain. Pull-down experiments and immunoprecipitations showed that Bin2 C-terminus bound SH3 domain containing proteins such as Endophilin A2 and α-PIX. siRNA of endogenous protein led to decreased cell migration, increased phagocytosis and reduced   podosom  e density and dynamics. In contrast, overexpression of Bin2 led to decreased phagocytosis and increased   podosom  e density and dynamics. We conclude that Bin2 is a membrane-sculpting protein that influences   podosom  e formation, motility and phagocytosis in leucocytes. Further understanding of this protein may be key to understand the behaviour of leucocytes under physiological and pathological conditions. 
  [Mm]igration  Process  
 positive regulation of fibroblast   migration   
  PubMed  
 GIT2 represses Crk- and Rac1-regulated cell spreading and Cdc42-mediated focal adhesion turnover. G protein-coupled receptor kinase interactors (GITs) regulate focal adhesion (FA) turnover, cell spreading, and motility through direct interaction with paxillin and the Rac-exchange factor Pak-interacting exchange factor beta (betaPIX). However, it is not clear whether GITs function to activate or repress motility or if the predominant GIT forms, GIT1 and GIT2, serve distinct or redundant roles. Here we demonstrate an obligatory role for endogenous GIT2 in repression of lamellipodial extension and FA turnover by Rac1- and Cdc42-dependent signaling pathways, respectively. Moreover, we show that the SH2-SH3 adaptor protein Crk is an essential target of GIT2 inhibition. Unexpectedly, we find that betaPIX is dispensable for the effects elicited by knockdown of GIT2. Finally, we show that loss of GIT2 is sufficient to induce   migration   of the nontransformed epithelial cell line MCF10A. These results suggest that inactivation of GIT2 function is a required step for induction of cell motility and that GIT2 may be a target of oncogenic signaling pathways that regulate cell   migration  . 
 N-α-acetyltransferase 10 protein suppresses cancer cell metastasis by binding PIX proteins and inhibiting Cdc42/Rac1 activity. N-α-acetyltransferase 10 protein, Naa10p, is an N-acetyltransferase known to be involved in cell cycle control. We found that Naa10p was expressed lower in varieties of malignancies with lymph node metastasis compared with non-lymph node metastasis. Higher Naa10p expression correlates the survival of lung cancer patients. Naa10p significantly suppresse  d migrati  on, tumor growth, and metastasis independent of its enzymatic activity. Instead, Naa10p binds to the GIT-binding domain of PIX, thereby preventing the formation of the GIT-PIX-Paxillin complex, resulting in reduced intrinsic Cdc42/Rac1 activity and decreased cel  l migrati  on. Forced expression of PIX in Naa10-transfected tumor cells restored th  e migrati  on and metastasis ability. We suggest that Naa10p functions as a tumor metastasis suppressor by disrupting the migratory complex, PIX-GIT- Paxillin, in cancer cells. 
 Analysis of the myosin-II-responsive focal adhesion proteome reveals a role for β-Pix in negative regulation of focal adhesion maturation. Focal adhesions undergo myosin-II-mediated maturation wherein they grow and change composition to modulate integrin signalling for cell   migratio  n, growth and differentiation. To determine how focal adhesion composition is affected by myosin II activity, we performed proteomic analysis of isolated focal adhesions and compared protein abundance in focal adhesions from cells with and without myosin II inhibition. We identified 905 focal adhesion proteins, 459 of which changed in abundance with myosin II inhibition, defining the myosin-II-responsive focal adhesion proteome. The abundance of 73% of the proteins in the myosin-II-responsive focal adhesion proteome was enhanced by contractility, including proteins involved in Rho-mediated focal adhesion maturation and endocytosis- and calpain-dependent focal adhesion disassembly. During myosin II inhibition, 27% of proteins in the myosin-II-responsive focal adhesion proteome, including proteins involved in Rac-mediated lamellipodial protrusion, were enriched in focal adhesions, establishing that focal adhesion protein recruitment is also negatively regulated by contractility. We focused on the Rac guanine nucleotide exchange factor β-Pix, documenting its role in the negative regulation of focal adhesion maturation and the promotion of lamellipodial protrusion and focal adhesion turnover to drive cel  l migrati  on. 
 Podocalyxin EBP50 ezrin molecular complex enhances the metastatic potential of renal cell carcinoma through recruiting Rac1 guanine nucleotide exchange factor ARHGEF7. Podocalyxin was initially identified in glomerular podocytes to critically maintain the structural and functional integrity of the glomerular ultrafiltrative apparatus. Lately, it has emerged as a malignant marker in tumors arising from a variety of tissue origins. By immunohistochemistry, we identified that 9.6% of renal cell carcinoma patients overexpress this protein. This subset of patients had significantly shorter disease-specific and overall survivals, and, importantly, we established podocalyxin overexpression as an independent prognostic factor for latent distant metastasis with multivariate analysis. Podocalyxin down-regulation by small interfering RNA led to defective   migration   in model renal tubular cells, which was corrected by re-expression of podocalyxin. The activity of the small GTPase Rac1, a well-characterized modulator of cell   migration  , was diminished by podocalyxin knock-down. Conversely, podocalyxin overexpression in human embryonic kidney cells up-regulated Rac1 activity, which depended on a complex formed by podocalyxin, ERM-binding phosphoprotein 50, ezrin, and ARHGEF7, a Rac1 activator. Therefore, podocalyxin can serve as a biomarker to identify renal cell carcinoma patients with higher metastatic potential for more aggressive intervention at earlier clinical stages. 
 Targeting and activation of Rac1 are mediated by the exchange factor beta-Pix. Rho guanosine triphosphatases (GTPases) are critical regulators of cytoskeletal dynamics and control complex functions such as cell adhesion, spreading,   migration  , and cell division. It is generally accepted that localized GTPase activation is required for the proper initiation of downstream signaling events, although the molecular mechanisms that control targeting of Rho GTPases are unknown. In this study, we show that the Rho GTPase Rac1, via a proline stretch in its COOH terminus, binds directly to the SH3 domain of the Cdc42/Rac activator beta-Pix (p21-activated kinase [Pak]-interacting exchange factor). The interaction with beta-Pix is nucleotide independent and is necessary and sufficient for Rac1 recruitment to membrane ruffles and to focal adhesions. In addition, the Rac1-beta-Pix interaction is required for Rac1 activation by beta-Pix as well as for Rac1-mediated spreading. Finally, using cells deficient for the beta-Pix-binding kinase Pak1, we show that Pak1 regulates the Rac1-beta-Pix interaction and controls cell spreading and adhesion-induced Rac1 activation. These data provide a model for the intracellular targeting and localized activation of Rac1 through its exchange factor beta-Pix. 
 Analysis of the myosin-II-responsive focal adhesion proteome reveals a role for β-Pix in negative regulation of focal adhesion maturation. Focal adhesions undergo myosin-II-mediated maturation wherein they grow and change composition to modulate integrin signalling for cell   migratio  n, growth and differentiation. To determine how focal adhesion composition is affected by myosin II activity, we performed proteomic analysis of isolated focal adhesions and compared protein abundance in focal adhesions from cells with and without myosin II inhibition. We identified 905 focal adhesion proteins, 459 of which changed in abundance with myosin II inhibition, defining the myosin-II-responsive focal adhesion proteome. The abundance of 73% of the proteins in the myosin-II-responsive focal adhesion proteome was enhanced by contractility, including proteins involved in Rho-mediated focal adhesion maturation and endocytosis- and calpain-dependent focal adhesion disassembly. During myosin II inhibition, 27% of proteins in the myosin-II-responsive focal adhesion proteome, including proteins involved in Rac-mediated lamellipodial protrusion, were enriched in focal adhesions, establishing that focal adhesion protein recruitment is also negatively regulated by contractility. We focused on the Rac guanine nucleotide exchange factor β-Pix, documenting its role in the negative regulation of focal adhesion maturation and the promotion of lamellipodial protrusion and focal adhesion turnover to drive cel  l migrati  on. 
 Podocalyxin EBP50 ezrin molecular complex enhances the metastatic potential of renal cell carcinoma through recruiting Rac1 guanine nucleotide exchange factor ARHGEF7. Podocalyxin was initially identified in glomerular podocytes to critically maintain the structural and functional integrity of the glomerular ultrafiltrative apparatus. Lately, it has emerged as a malignant marker in tumors arising from a variety of tissue origins. By immunohistochemistry, we identified that 9.6% of renal cell carcinoma patients overexpress this protein. This subset of patients had significantly shorter disease-specific and overall survivals, and, importantly, we established podocalyxin overexpression as an independent prognostic factor for latent distant metastasis with multivariate analysis. Podocalyxin down-regulation by small interfering RNA led to defective   migration   in model renal tubular cells, which was corrected by re-expression of podocalyxin. The activity of the small GTPase Rac1, a well-characterized modulator of cell   migration  , was diminished by podocalyxin knock-down. Conversely, podocalyxin overexpression in human embryonic kidney cells up-regulated Rac1 activity, which depended on a complex formed by podocalyxin, ERM-binding phosphoprotein 50, ezrin, and ARHGEF7, a Rac1 activator. Therefore, podocalyxin can serve as a biomarker to identify renal cell carcinoma patients with higher metastatic potential for more aggressive intervention at earlier clinical stages. 
 Targeting and activation of Rac1 are mediated by the exchange factor beta-Pix. Rho guanosine triphosphatases (GTPases) are critical regulators of cytoskeletal dynamics and control complex functions such as cell adhesion, spreading,   migration  , and cell division. It is generally accepted that localized GTPase activation is required for the proper initiation of downstream signaling events, although the molecular mechanisms that control targeting of Rho GTPases are unknown. In this study, we show that the Rho GTPase Rac1, via a proline stretch in its COOH terminus, binds directly to the SH3 domain of the Cdc42/Rac activator beta-Pix (p21-activated kinase [Pak]-interacting exchange factor). The interaction with beta-Pix is nucleotide independent and is necessary and sufficient for Rac1 recruitment to membrane ruffles and to focal adhesions. In addition, the Rac1-beta-Pix interaction is required for Rac1 activation by beta-Pix as well as for Rac1-mediated spreading. Finally, using cells deficient for the beta-Pix-binding kinase Pak1, we show that Pak1 regulates the Rac1-beta-Pix interaction and controls cell spreading and adhesion-induced Rac1 activation. These data provide a model for the intracellular targeting and localized activation of Rac1 through its exchange factor beta-Pix. 
 Scrib regulates PAK activity during the cell   migration   process. Genetic studies have highlighted the key role of Scrib in the development of Metazoans. Deficiency in Scrib impairs many aspects of cell polarity and cell movement although the mechanisms involved remain unclear. In mammals, Scrib belongs to a protein complex containing betaPIX, an exchange factor for Rac/Cdc42, and GIT1, a GTPase activating protein for ARF6 implicated in receptor recycling and exocytosis. Here we show that the Scrib complex associates with PAK, a serine-threonine kinase family crucial for cell   migration  . PAK colocalizes with members of the Scrib complex at the leading edge of heregulin-treated T47D breast cancer cells. We demonstrate that the Scrib complex is required for epithelial cells and primary mouse embryonic fibroblasts to efficiently respond to chemoattractant cues. In Scrib-deficient cells, the pool of cortical PAK is decreased, thereby precluding its proper activation by Rac. Loss of Scrib also impairs the polarized distribution of active Rac at the leading edge and compromises the regulated activation of the GTPase in T47D cells and mouse embryonic fibroblasts. These data underscore the role of Scrib in cell   migration   and show the strong impact of Scrib in the function of PAK and Rac, two key molecules implicated in this process. 
 Identification of MYO18A as a novel interacting partner of the PAK2/betaPIX/GIT1 complex and its potential function in modulating epithelial cell   migration  . The p21-activated kinase (PAK) 2 is known to be involved in numerous biological functions, including the regulation of actin reorganization and cell motility. To better understand the mechanisms underlying this regulation, we herein used a proteomic approach to identify PAK2-interacting proteins in human epidermoid carcinoma A431 cells. We found that MYO18A, an emerging member of the myosin superfamily, is a novel PAK2 binding partner. Using a siRNA knockdown strategy and in vitro binding assay, we discovered that MYO18A binds to PAK2 through the betaPIX/GIT1 complex. Under normal conditions, MYO18A and PAK2 colocalized in lamellipodia and membrane ruffles. Interestingly, knockdown of MYO18A in cells did not prevent formation of the PAK2/betaPIX/GIT1 complex, but rather apparently changed its localization to focal adhesions. Moreover, MYO18A-depleted cells showed dramatic changes in morphology and actin stress fiber and membrane ruffle formation and displayed increases in the number and size of focal adhesions.   Migration   assays revealed that MYO18A-depleted cells had decreased cell motility, and reexpression of MYO18A restored their   migration   ability. Collectively, our findings indicate that MYO18A is a novel binding partner of the PAK2/betaPIX/GIT1 complex and suggest that MYO18A may play an important role in regulating epithelial cell   migration   via affecting multiple cell machineries. 
 MCC, a new interacting protein for Scrib, is required for cell   migration   in epithelial cells. To further characterize the molecular events supporting the tumor suppressor activity of Scrib in mammals, we aim to identify new binding partners. We isolated MCC, a recently identified binding partner for beta-catenin, as a new interacting protein for Scrib. MCC interacts with both Scrib and the NHERF1/NHERF2/Ezrin complex in a PDZ-dependent manner. In T47D cells, MCC and Scrib proteins colocalize at the cell membrane and reduced expression of MCC results in impaired cell   migration  . By contrast to Scrib, MCC inhibits cell directed   migration   independently of Rac1, Cdc42 and PAK activation. Altogether, these results identify MCC as a potential scaffold protein regulating cell movement and able to bind Scrib, beta-catenin and NHERF1/2. 
 betaPIX controls cell motility and neurite extension by regulating the distribution of GIT1. Cell motility entails the reorganization of the cytoskeleton and membrane trafficking for effective protrusion. GIT1/p95-APP1 is a member of a family of GTPase-activating proteins for ARF GTPases that affect endocytosis, adhesion and   migration  . GIT1 associates with paxillin and a complex including the Rac/Cdc42 exchanging factors PIX/Cool and the kinase PAK. In this study, we show that overexpression of betaPIX induces the accumulation of endogenous and overexpressed GIT1 at large structures similar to those induced by an ArfGAP-defective mutant of GIT1 (p95-C2). Immunohistochemical analysis and immunoelectron microscopy reveal that these structures include the endogenous transferrin receptor. Time-lapse analysis during motogenic stimuli shows that the formation and perinuclear accumulation of the p95-C2-positive structures is paralleled by inhibition of lamellipodium formation and cell retraction. Both dimerization and a functional SH3 domain of betaPIX are required for the accumulation of GIT1 in fibroblasts, which is prevented by the monomeric PIX-PG-DeltaLZ. This mutant also prevents the formation of endocytic aggregates and inhibition of neurite outgrowth in retinal neurons expressing p95-C2. Our results indicate that betaPIX is an important regulator of the subcellular distribution of GIT1, and suggest that alteration in the level of expression of the complex affects the endocytic compartment and cell motility. 
 Paxillin LD4 motif binds PAK and PIX through a novel 95-kD ankyrin repeat, ARF-GAP protein: A role in cytoskeletal remodeling. Paxillin is a focal adhesion adaptor protein involved in the integration of growth factor- and adhesion-mediated signal transduction pathways. Repeats of a leucine-rich sequence named paxillin LD motifs (Brown M.C., M.S. Curtis, and C.E. Turner. 1998. Nature Struct. Biol. 5:677-678) have been implicated in paxillin binding to focal adhesion kinase (FAK) and vinculin. Here we demonstrate that the individual paxillin LD motifs function as discrete and selective protein binding interfaces. A novel scaffolding function is described for paxillin LD4 in the binding of a complex of proteins containing active p21 GTPase-activated kinase (PAK), Nck, and the guanine nucleotide exchange factor, PIX. The association of this complex with paxillin is mediated by a new 95-kD protein, p95PKL (paxillin-kinase linker), which binds directly to paxillin LD4 and PIX. This protein complex also binds to Hic-5, suggesting a conservation of LD function across the paxillin superfamily. Cloning of p95PKL revealed a multidomain protein containing an NH2-terminal ARF-GAP domain, three ankyrin-like repeats, a potential calcium-binding EF hand, calmodulin-binding IQ motifs, a myosin homology domain, and two paxillin-binding subdomains (PBS). Green fluorescent protein- (GFP-) tagged p95PKL localized to focal adhesions/complexes in CHO.K1 cells. Overexpression in neuroblastoma cells of a paxillin LD4 deletion mutant inhibited lamellipodia formation in response to insulin-like growth fac- tor-1. Microinjection of GST-LD4 into NIH3T3 cells significantly decreased cell   migration   into a wound. These data implicate paxillin as a mediator of p21 GTPase-regulated actin cytoskeletal reorganization through the recruitment to nascent focal adhesion structures of an active PAK/PIX complex potentially via interactions with p95PKL. 
 Bin2 is a membrane sculpting N-BAR protein that influences leucocyte podosomes, motility and phagocytosis. Cell motility, adhesion and phagocytosis are controlled by actin and membrane remodelling processes. Bridging integrator-2 (Bin2) also called Breast cancer-associated protein 1 (BRAP1) is a predicted N-BAR domain containing protein with unknown function that is highly expressed in leucocytic cells. In the present study we solved the structure of Bin2 BAR domain and studied its membrane binding and bending properties in vitro and in vivo. Live-cell imaging experiments showed that Bin2 is associated with actin rich structures on the plasma membrane, where it was targeted through its N-BAR domain. Pull-down experiments and immunoprecipitations showed that Bin2 C-terminus bound SH3 domain containing proteins such as Endophilin A2 and α-PIX. siRNA of endogenous protein led to decreased cell   migratio  n, increased phagocytosis and reduced podosome density and dynamics. In contrast, overexpression of Bin2 led to decreased phagocytosis and increased podosome density and dynamics. We conclude that Bin2 is a membrane-sculpting protein that influences podosome formation, motility and phagocytosis in leucocytes. Further understanding of this protein may be key to understand the behaviour of leucocytes under physiological and pathological conditions. 
 An extracellular-matrix-specific GEF-GAP interaction regulates Rho GTPase crosstalk for 3D collagen   migration  . Rho-family GTPases govern distinct types of cell   migration   on different extracellular matrix proteins in tissue culture or three-dimensional (3D) matrices. We searched for mechanisms selectively regulating 3D cell   migration   in different matrix environments and discovered a form of Cdc42-RhoA crosstalk governing cell   migration   through a specific pair of GTPase activator and inhibitor molecules. We first identified βPix, a guanine nucleotide exchange factor (GEF), as a specific regulator of   migratio  n in 3D collagen using an affinity-precipitation-based GEF screen. Knockdown of βPix specifically blocks cel  l migrati  on in fibrillar collagen microenvironments, leading to hyperactive cellular protrusion accompanied by increased collagen matrix contraction. Live FRET imaging and RNAi knockdown linked this βPix knockdown phenotype to loss of polarized Cdc42 but not Rac1 activity, accompanied by enhanced, de-localized RhoA activity. Mechanistically, collagen phospho-regulates βPix, leading to its association with srGAP1, a GTPase-activating protein (GAP), needed to suppress RhoA activity. Our results reveal a matrix-specific pathway controll  ing migra  tion involving a GEF-GAP interaction of βPix with srGAP1 that is critical for maintaining suppressive crosstalk between Cdc42 and RhoA during 3D coll  agen migr  ation. 
 Direct GSK-3β inhibition enhances mesenchymal stromal cell   migratio  n by increasing expression of β-PIX and CXCR4. Mesenchymal stromal cells (MSCs) are emerging as candidate cells for the treatment of neurological diseases because of their neural replacement, neuroprotective, and neurotrophic effects. However, the majority of MSCs transplanted by various routes fail to reach the site of injury, and they have demonstrated only minimal therapeutic benefit in clinical trials. Therefore, enhancing th  e migrati  on of MSCs to target sites is essential for this therapeutic strategy to be effective. In this study, we assessed whether inhibition of glycogen synthase kinase-3β (GSK-3β) increases   the migra  tion capacity of MSCs during ex vivo expansion. Human bone marrow MSCs (hBM-MSCs) were cultured with various GSK-3β inhibitors (LiCl, SB-415286, and AR-A014418). Usi  ng a migr  ation assay kit, we found that the motility of hBM-MSCs was significantly enhanced by GSK-3β inhibition. Western blot analysis revealed increased leve  ls of mig  ration-related signaling proteins such as phospho-GSK-3β, β-catenin, phospho-c-Raf, phospho-extracellular signal-regulated kinase (ERK), phospho-β-PAK-interacting exchange factor (PIX), and CXC chemokine receptor 4 (CXCR4). In addition, real-time polymerase chain reaction demonstrated increased expression of matrix metalloproteinase-2 (MMP-2), membrane-type MMP-1 (MT1-MMP), and β-PIX. In the reverse approach, treatment with β-PIX shRNA or CXCR4 inhibitor (AMD 3100) reduc  ed hBM-MS  C migration. These findings suggest that inhibition of GSK-3β during ex vivo expansion of hBM-MSCs may en  hance the  ir migration capacity by increasing expression of β-catenin, phospho-c-Raf, phospho-ERK, and β-PIX and the subsequent up-regulation of CXCR4.   Enhancing   the migration capacity of hBM-MSCs by treating these cells with GSK-3β inhibitors may increase their therapeutic potential. 
 BetaPIX and GIT1 regulate HGF-induced lamellipodia formation and WAVE2 transport. Formation of lamellipodia is the first step during cell   migration  , and involves actin reassembly at the leading edge of migrating cells through the membrane transport of WAVE2. However, the factors that regulate WAVE2 transport to the cell periphery for initiating lamellipodia formation have not been elucidated. We report here that in human breast cancer MDA-MB-231 cells, the hepatocyte growth factor (HGF) induced the association between the constitutive complex of betaPIX and GIT1 with WAVE2, which was concomitant with the induction of lamellipodia formation and WAVE2 transport. Although depletion of betaPIX by RNA interference abrogated the HGF-induced WAVE2 transport and lamellipodia formation, GIT1 depletion caused HGF-independent WAVE2 transport and lamellipodia formation. Collectively, we suggest that betaPIX releases cells from the GIT1-mediated suppression of HGF-independent responses and recruits GIT1 to WAVE2, thereby facilitating HGF-induced WAVE2 transport and lamellipodia formation. 
 Sorting nexin 27 protein regulates trafficking of a p21-activated kinase (PAK) interacting exchange factor (β-Pix)-G protein-coupled receptor kinase interacting protein (GIT) complex via a PDZ domain interaction. Sorting nexin 27 (SNX27) is a 62-kDa protein localized to early endosomes and known to regulate the intracellular trafficking of ion channels and receptors. In addition to a PX domain, SNX27 is the only sorting family member that contains a PDZ domain. To identify novel SNX27-PDZ binding partners, we performed a proteomic screen in mouse principal kidney cortical collecting duct cells using a GST-SNX27 fusion construct as bait. We found that β-Pix (p21-activated kinase-interactive exchange factor), a guanine nucleotide exchange factor for the Rho family of small GTPases known to regulate cell motility directly interacted with SNX27. The association of β-Pix and SNX27 is specific for β-Pix isoforms terminating in the type-1 PDZ binding motif (ETNL). In the same screen we also identified Git1/2 as a potential SNX27 interacting protein. The interaction between SNX27 and Git1/2 is indirect and mediated by β-Pix. Furthermore, we show recruitment of the β-Pix·Git complex to endosomal sites in a SNX27-dependent manner. Fi  nally, mi  gration assays revealed that depletion of SNX27 from HeLa and mouse principal kidney cortical collecting duct cells significantly decreases cell motility. We propose a model by which SNX27 regulates trafficking of β-Pix to focal adhesions and thereby influences cell motility. 
 Paxillin kinase linker (PKL) regulates Vav2 signaling during cell spreading and   migration  . The Rho family of GTPases plays an important role in coordinating dynamic changes in the cell   migration   machinery after integrin engagement with the extracellular matrix. Rho GTPases are activated by guanine nucleotide exchange factors (GEFs) and negatively regulated by GTPase-activating proteins (GAPs). However, the mechanisms by which GEFs and GAPs are spatially and temporally regulated are poorly understood. Here the activity of the proto-oncogene Vav2, a GEF for Rac1, RhoA, and Cdc42, is shown to be regulated by a phosphorylation-dependent interaction with the ArfGAP PKL (GIT2). PKL is required for Vav2 activation downstream of integrin engagement and epidermal growth factor (EGF) stimulation. In turn, Vav2 regulates the subsequent redistribution of PKL and the Rac1 GEF β-PIX to focal adhesions after EGF stimulation, suggesting a feedforward signaling loop that coordinates PKL-dependent Vav2 activation and PKL localization. Of interest, Vav2 is required for the efficient localization of PKL and β-PIX to the leading edge of migrating cells, and knockdown of Vav2 results in a decrease in directional persistence and polarization in migrating cells, suggesting a coordination between PKL/Vav2 signaling and PKL/β-PIX signaling during ce  ll migrat  ion. 
 Arhgef7 promotes activation of the Hippo pathway core kinase Lats. The Hippo pathway regulates tissue growth and organ size, and inactivation contributes to cancer. Signals flow through Mst/Lats kinases, which phosphorylate and promote cytoplasmic localization of the transcriptional regulators Yap and Taz to inhibit transcription. Here, we identify the multidomain-containing guanine nucleotide exchange factor (GEF) Arhgef7, or βPix, as a positive Hippo pathway regulator. We show that βPix, which localizes to the cytoplasm, binds both Lats and Yap/Taz and thereby promotes Lats-mediated phosphorylation of Yap/Taz in a GEF-independent manner. βPix is required downstream of both cell density sensing and actin cytoskeletal rearrangements, and we demonstrate that loss of βPix expression in normal mammary epithelial cells strongly reduces Yap/Taz phosphorylation, promotes nuclear localization and increases target gene expression. Conversely, increased expression of βPIX in breast cancer cell lines re-couples the Hippo kinase cassette to Yap/Taz, promoting localization of Yap/Taz to the cytoplasm and inhibiting   cell migr  ation and proliferation. These studies thus define βPix as a key component that links the Hippo kinase cassette to Yap/Taz in response to multiple upstream Hippo pathway activators. 
 β-PIX is critical for transplanted mesenchymal stromal cell   migratio  n. Bone marrow-derived mesenchymal stromal cells (MSCs) have been used successfully as a source of stem cells for treating neurodegenerative diseases. However, for reasons that are not clear, autologous MSC transplants have not yielded successful results in human trials. To test one possible reason, we compared the migratory ability of MSCs from amyotrophic lateral sclerosis (ALS) patients with those of healthy controls. We found that MSCs derived from ALS patients (ALS-MSCs) had a reduced ability to migrate, which may explain why autologous transplantation is not successful. We also found that expression of one of the intracellular factors implicated in   migratio  n, β-PIX, was significantly reduced in ALS-MSCs compared with healthy stem cells. Restoration of β-PIX expression by genetic manipulation restored the migratory ability of ALS-MSCs, and inhibition of β-PIX expression with shRNA reduced   the migra  tion of healthy MSCs. We suggest that transplantation of allogeneic or genetically modified autologous stem cells might be a more promising strategy for ALS patients than transplantation of autologous stem cells. 
 β-PIX controls intracellular viscoelasticity to regulate lung cancer cell   migratio  n. Cancer metastasis occurs via a progress involving abnormal cell   migratio  n. Cell   migratio  n, a dynamic physical process, is controlled by the cytoskeletal system, which includes the dynamics of actin organization and cellular adhesive organelles, focal adhesions (FAs). However, it is not known whether the organization of actin cytoskeletal system has a regulatory role in the physiologically relevant aspects of cancer metastasis. In the present studies, it was found that lung adenocarcinoma cells isolated from the secondary lung cancer of the lymph nodes, H1299 cells, show specific dynamics in terms of the actin cytoskeleton and FAs. This results in a higher level of mobility and this is regulated by an immature FA component, β-PIX (PAK-interacting exchange factor-β). In H1299 cells, β-PIX's activity was found not to be down-regulated by sequestration onto stress fibres, as the cells did not bundle actin filaments into stress fibres. Thus, β-PIX mainly remained localized at FAs, which allowed maturation of nascent adhesions into focal complexes; this resulted in actin polymerization, increased actin network integrity, changes in the intracellular microrheology at the peripheral of the cell, and cell polarity, which in turn regulated   cell migr  ation. Perturbation of β-PIX caused an inhibition of   cell mig  ration, incl  uding mig  ration velocity, accumulated distance and directional persistence. Our results demonstrate the importance of β-PIX to the regulation of high mobility of lung adenocarcinoma cell line H1299 and that this occurs via regulation of FA dynamics, changes in actin cytoskeleton organization and cell polarity. 
  GeneRIFs  
 Conversely, increased expression of betaPIX in breast cancer cell lines re-couples the Hippo kinase cassette to Yap/Taz, promoting localization of Yap/Taz to the cytoplasm and inhibiting cell   migration   and proliferation. 
 The interaction of betaPix with srGAP1 is critical for maintaining suppressive crosstalk between Cdc42 and RhoA during 3D collagen   migration  . 
 Inhibition of glycogen synthase kinase (GSK)-3beta increases the   migration   capacity of mesenchymal stromal cells during ex vivo expansion. 
 Data suggest a coordination between paxillin kinase linker (PKL)/Vav2 signaling and PKL/beta-PIX signaling during cell   migration  . 
 Results describe the role of beta-Pix in the negative regulation of focal adhesion maturation and the promotion of lamellipodial protrusion and focal adhesion turnover to drive cell   migration  . 
 MYO18A is a novel binding partner of the PAK2/betaPIX/GIT1 complex and suggest that MYO18A may play an important role in regulating epithelial cell   migration   via affecting multiple cell machineries. 
  [Ee]xtracellular [Mm]atrix  PubMed  
 Altered adhesive structures and their relation to RhoGTPase activation in merlin-deficient Schwannoma. Schwannomas are Schwann cell tumors of the nervous system that occur spontaneously and in patients with neurofibromatosis 2 (NF2) and lack the tumor suppressor merlin. Merlin is known to bind paxillin, beta1 integrin and focal adhesion kinase, members of focal contacts, multi-protein complexes that mediate cell adhesion to the   extracellular matrix  . Moreover, merlin-deficient Schwannomas show pathological adhesion to the   extracellular matrix   making the characterization of focal contacts indispensable. Using our Schwannoma in vitro model of human primary Schwann and Schwannoma cells, we here show that Schwannoma cells display an increased number of mature and stable focal contacts. In addition to an involvement of RhoA signaling via the Rho kinase ROCK, Rac1 plays a significant role in the pathological adhesion of Schwannoma cells. The Rac1 guanine exchange factor- beta-Pix, localizes to focal contacts in human primary Schwannoma cells, and we show that part of the Rac1 activation, an effect of merlin-deficiency, occurs at the level of focal contacts in human primary Schwannoma cells. Our results help explaining the pathological adhesion of Schwannoma cells, further strengthen the importance of RhoGTPase signaling in Schwannoma development, and suggest that merlin's role in tumor suppression is linked to focal contacts. 
 Altered adhesive structures and their relation to RhoGTPase activation in merlin-deficient Schwannoma. Schwannomas are Schwann cell tumors of the nervous system that occur spontaneously and in patients with neurofibromatosis 2 (NF2) and lack the tumor suppressor merlin. Merlin is known to bind paxillin, beta1 integrin and focal adhesion kinase, members of focal contacts, multi-protein complexes that mediate cell adhesion to the   extracellular matrix  . Moreover, merlin-deficient Schwannomas show pathological adhesion to the   extracellular matrix   making the characterization of focal contacts indispensable. Using our Schwannoma in vitro model of human primary Schwann and Schwannoma cells, we here show that Schwannoma cells display an increased number of mature and stable focal contacts. In addition to an involvement of RhoA signaling via the Rho kinase ROCK, Rac1 plays a significant role in the pathological adhesion of Schwannoma cells. The Rac1 guanine exchange factor- beta-Pix, localizes to focal contacts in human primary Schwannoma cells, and we show that part of the Rac1 activation, an effect of merlin-deficiency, occurs at the level of focal contacts in human primary Schwannoma cells. Our results help explaining the pathological adhesion of Schwannoma cells, further strengthen the importance of RhoGTPase signaling in Schwannoma development, and suggest that merlin's role in tumor suppression is linked to focal contacts. 
 An extracellular-matrix-specific GEF-GAP interaction regulates Rho GTPase crosstalk for 3D collagen migration. Rho-family GTPases govern distinct types of cell migration on different   extracellular matrix   proteins in tissue culture or three-dimensional (3D) matrices. We searched for mechanisms selectively regulating 3D cell migration in different matrix environments and discovered a form of Cdc42-RhoA crosstalk governing cell migration through a specific pair of GTPase activator and inhibitor molecules. We first identified βPix, a guanine nucleotide exchange factor (GEF), as a specific regulator of migration in 3D collagen using an affinity-precipitation-based GEF screen. Knockdown of βPix specifically blocks cell migration in fibrillar collagen microenvironments, leading to hyperactive cellular protrusion accompanied by increased collagen matrix contraction. Live FRET imaging and RNAi knockdown linked this βPix knockdown phenotype to loss of polarized Cdc42 but not Rac1 activity, accompanied by enhanced, de-localized RhoA activity. Mechanistically, collagen phospho-regulates βPix, leading to its association with srGAP1, a GTPase-activating protein (GAP), needed to suppress RhoA activity. Our results reveal a matrix-specific pathway controlling migration involving a GEF-GAP interaction of βPix with srGAP1 that is critical for maintaining suppressive crosstalk between Cdc42 and RhoA during 3D collagen migration. 
 Regulation of SPIN90 phosphorylation and interaction with Nck by ERK and cell adhesion. SPIN90 is a widely expressed Nck-binding protein that contains one Src homology 3 (SH3) domain, three Pro-rich motifs, and a serine/threonine-rich region, and is known to participate in sarcomere assembly during cardiac myocyte differentiation. We used in vitro binding assays and yeast two-hybrid screening analysis to identify Nck, betaPIX, Wiscott-Aldrich syndrome protein (WASP), and ERK1 as SPIN90-binding proteins. It appears that betaPIX, WASP, and SPIN90 form a complex that interacts with Nck in a manner dependent upon cell adhesion to   extracellular matrix  . The betaPIX.WASP.SPIN90.Nck interaction was abolished in suspended and cytochalasin D-treated cells, but was recovered when cells were replated on fibronectin-coated dishes. The SPIN90.betaPIX.WASP complex was stable, even in suspended cells, suggesting SPIN90 serves as an adaptor molecule to recruit other proteins to Nck at focal adhesions. In addition, we found that overexpression of the SPIN90 SH3 domain or Pro-rich region, respectively, abolished SPIN90.Nck and SPIN90.betaPIX interactions, resulting in detachment of cells from   extracellular matrix  . SPIN90 was phosphorylated by ERK1, which was, itself, activated by cell adhesion and platelet-derived growth factor. Such phosphorylation of SPIN90 likely promotes the interaction of the SPIN90.betaPIX.WASP complex and Nck. It thus appears that the interaction of the betaPIX.WASP.SPIN90 complex with Nck is crucial for stable cell adhesion and can be dynamically modulated by SPIN90 phosphorylation that is dependent on cell adhesion and ERK activation. 
 Paxillin kinase linker (PKL) regulates Vav2 signaling during cell spreading and migration. The Rho family of GTPases plays an important role in coordinating dynamic changes in the cell migration machinery after integrin engagement with the   extracellular matrix  . Rho GTPases are activated by guanine nucleotide exchange factors (GEFs) and negatively regulated by GTPase-activating proteins (GAPs). However, the mechanisms by which GEFs and GAPs are spatially and temporally regulated are poorly understood. Here the activity of the proto-oncogene Vav2, a GEF for Rac1, RhoA, and Cdc42, is shown to be regulated by a phosphorylation-dependent interaction with the ArfGAP PKL (GIT2). PKL is required for Vav2 activation downstream of integrin engagement and epidermal growth factor (EGF) stimulation. In turn, Vav2 regulates the subsequent redistribution of PKL and the Rac1 GEF β-PIX to focal adhesions after EGF stimulation, suggesting a feedforward signaling loop that coordinates PKL-dependent Vav2 activation and PKL localization. Of interest, Vav2 is required for the efficient localization of PKL and β-PIX to the leading edge of migrating cells, and knockdown of Vav2 results in a decrease in directional persistence and polarization in migrating cells, suggesting a coordination between PKL/Vav2 signaling and PKL/β-PIX signaling during cell migration. 
  [Ii]nvasive  PubMed  
 Activated Cdc42 sequesters c-Cbl and prevents EGF receptor degradation. Cdc42 is a Ras-related protein that has been implicated in the control of normal cell growth, and when improperly regulated, in cellular transformation and   invasive  ness. A variety of extracellular stimuli, including epidermal growth factor (EGF), activate Cdc42. Here, we show that activation of Cdc42 protects the EGF receptor from the negative regulatory activity of the c-Cbl ubiquitin ligase. Activated Cdc42 binds to p85Cool-1 (for cloned-out-of-library)/beta-Pix (for Pak-interactive exchange factor), a protein that directly associates with c-Cbl. This inhibits the binding of Cbl by the EGF receptor and thus prevents Cbl from catalyzing receptor ubiquitination. The role played by Cdc42 in regulating the timing of EGF receptor-Cbl interactions is underscored by the fact that constitutively active Cdc42(F28L), by persistently blocking the binding of Cbl to these receptors, leads to their aberrant accumulation and sustained EGF-stimulated ERK activation, thus resulting in cellular transformation. 
 Hypoxia-induced invadopodia formation: a role for β-PIX. During tumour progression, oxygen tension in the microenvironment surrounding tumour cells is reduced, resulting in hypoxia. It is well established that cancer cells resist the negative effects of hypoxia by inducing angiogenesis predominantly via the activity of transcription factor hypoxia-inducible factor-1 (HIF-1). However, more recently HIF-1α has also been linked to increase  d invasi  ve potential, although the molecular mechanisms remain to be defined  . Invasi  ve cancer cells are thought to employ membrane protrusions, termed invadopodia, to achieve matrix degradation. While many invadopodia components have been identified, signalling pathways that link extracellular stimuli to invadopodia formation remain largely unknown. Indeed, the relationship between invadopodia formation and HIF-1α has not been explored. We now report that HIF-1α is a driver of invadopodia formation. Furthermore, we have identified an important, direct and novel link between the Rho family activator β-PIX, HIF-1α and invadopodia formation. Indeed, we find that β-PIX expression is essential for invadopodia formation. In conclusion, we identify a new HIF-1α mechanistic pathway and suggest that β-PIX is a novel downstream signalling mediator during invadopodia formation. 
  [Mm]etastasis  PubMed  
 N-α-acetyltransferase 10 protein suppresses cancer cell   metastasi  s by binding PIX proteins and inhibiting Cdc42/Rac1 activity. N-α-acetyltransferase 10 protein, Naa10p, is an N-acetyltransferase known to be involved in cell cycle control. We found that Naa10p was expressed lower in varieties of malignancies with lymph nod  e metastas  is compared with non-lymph nod  e metastas  is. Higher Naa10p expression correlates the survival of lung cancer patients. Naa10p significantly suppressed migration, tumor growth, an  d metastas  is independent of its enzymatic activity. Instead, Naa10p binds to the GIT-binding domain of PIX, thereby preventing the formation of the GIT-PIX-Paxillin complex, resulting in reduced intrinsic Cdc42/Rac1 activity and decreased cell migration. Forced expression of PIX in Naa10-transfected tumor cells restored the migration an  d metastas  is ability. We suggest that Naa10p functions as a tumo  r metastas  is suppressor by disrupting the migratory complex, PIX-GIT- Paxillin, in cancer cells. 
 Podocalyxin EBP50 ezrin molecular complex enhances the metastatic potential of renal cell carcinoma through recruiting Rac1 guanine nucleotide exchange factor ARHGEF7. Podocalyxin was initially identified in glomerular podocytes to critically maintain the structural and functional integrity of the glomerular ultrafiltrative apparatus. Lately, it has emerged as a malignant marker in tumors arising from a variety of tissue origins. By immunohistochemistry, we identified that 9.6% of renal cell carcinoma patients overexpress this protein. This subset of patients had significantly shorter disease-specific and overall survivals, and, importantly, we established podocalyxin overexpression as an independent prognostic factor for latent distant   metastasis   with multivariate analysis. Podocalyxin down-regulation by small interfering RNA led to defective migration in model renal tubular cells, which was corrected by re-expression of podocalyxin. The activity of the small GTPase Rac1, a well-characterized modulator of cell migration, was diminished by podocalyxin knock-down. Conversely, podocalyxin overexpression in human embryonic kidney cells up-regulated Rac1 activity, which depended on a complex formed by podocalyxin, ERM-binding phosphoprotein 50, ezrin, and ARHGEF7, a Rac1 activator. Therefore, podocalyxin can serve as a biomarker to identify renal cell carcinoma patients with higher metastatic potential for more aggressive intervention at earlier clinical stages. 
 Podocalyxin EBP50 ezrin molecular complex enhances the metastatic potential of renal cell carcinoma through recruiting Rac1 guanine nucleotide exchange factor ARHGEF7. Podocalyxin was initially identified in glomerular podocytes to critically maintain the structural and functional integrity of the glomerular ultrafiltrative apparatus. Lately, it has emerged as a malignant marker in tumors arising from a variety of tissue origins. By immunohistochemistry, we identified that 9.6% of renal cell carcinoma patients overexpress this protein. This subset of patients had significantly shorter disease-specific and overall survivals, and, importantly, we established podocalyxin overexpression as an independent prognostic factor for latent distant   metastasis   with multivariate analysis. Podocalyxin down-regulation by small interfering RNA led to defective migration in model renal tubular cells, which was corrected by re-expression of podocalyxin. The activity of the small GTPase Rac1, a well-characterized modulator of cell migration, was diminished by podocalyxin knock-down. Conversely, podocalyxin overexpression in human embryonic kidney cells up-regulated Rac1 activity, which depended on a complex formed by podocalyxin, ERM-binding phosphoprotein 50, ezrin, and ARHGEF7, a Rac1 activator. Therefore, podocalyxin can serve as a biomarker to identify renal cell carcinoma patients with higher metastatic potential for more aggressive intervention at earlier clinical stages. 
 β-PIX controls intracellular viscoelasticity to regulate lung cancer cell migration. Cancer   metastasi  s occurs via a progress involving abnormal cell migration. Cell migration, a dynamic physical process, is controlled by the cytoskeletal system, which includes the dynamics of actin organization and cellular adhesive organelles, focal adhesions (FAs). However, it is not known whether the organization of actin cytoskeletal system has a regulatory role in the physiologically relevant aspects of cancer   metastasi  s. In the present studies, it was found that lung adenocarcinoma cells isolated from the secondary lung cancer of the lymph nodes, H1299 cells, show specific dynamics in terms of the actin cytoskeleton and FAs. This results in a higher level of mobility and this is regulated by an immature FA component, β-PIX (PAK-interacting exchange factor-β). In H1299 cells, β-PIX's activity was found not to be down-regulated by sequestration onto stress fibres, as the cells did not bundle actin filaments into stress fibres. Thus, β-PIX mainly remained localized at FAs, which allowed maturation of nascent adhesions into focal complexes; this resulted in actin polymerization, increased actin network integrity, changes in the intracellular microrheology at the peripheral of the cell, and cell polarity, which in turn regulated cell migration. Perturbation of β-PIX caused an inhibition of cell migration, including migration velocity, accumulated distance and directional persistence. Our results demonstrate the importance of β-PIX to the regulation of high mobility of lung adenocarcinoma cell line H1299 and that this occurs via regulation of FA dynamics, changes in actin cytoskeleton organization and cell polarity. 
   
  
 ETV6 
 
  NCBI Gene  
  GeneRIF  
  Pubmed  
 
 
  Search Term  Rank  Fields  
  [Mm]igration  6  PubMed(2); GeneRIFs(1)  
  [Ii]nvasive  10  PubMed(3); GeneRIFs(1)  
  [Mm]etastasize  11  PubMed(1)  
  [Mm]etastasis  12  PubMed(1)  
 
 
 hide/show details for ETV6 
  [Mm]igration  PubMed  
 PAX5/TEL acts as a transcriptional repressor causing down-modulation of CD19, enhances   migration   to CXCL12, and confers survival advantage in pre-BI cells. PAX5 is a transcription factor essential for B-cell development. Recently, it has been found as a frequent target of aberrancies in childhood acute lymphoblastic leukemia (ALL; 30% of B cell ALL cases), showing monoallelic loss, point mutations, or chromosomal translocations. The role of these aberrancies is still poorly understood. We previously cloned the PAX5/TEL fusion gene in a patient affected by B-cell precursor ALL with a t(9;12) translocation. This is the first report investigating the molecular and functional roles of PAX5/TEL protein in vitro from murine wild-type pre-BI cells. We showed that PAX5/TEL protein acts as an aberrant transcription factor with repressor function, recruiting mSin3A, down-regulating B220, CD19, BLNK, MB-1, FLT3, and mu heavy chain expression, thus suggesting a block on B-cell differentiation. In a PAX5-deficient context, the presence of PAX5/TEL did not replace PAX5 functions. PAX5/TEL protein enhances cell   migration   towards CXCL12, with the overexpression of CXCR4. Moreover, the presence of the fusion gene overcomes interleukin-7 withdrawal and interferes with transforming growth factor-beta1 pathway, inducing resistance and conferring cells an advantage in proliferation and survival. Thus, in vitro, the PAX5/TEL protein has a dominant effect on wild-type PAX5, interferes with the process of B-cell differentiation and   migration  , and induces resistance to apoptosis. Taken together, these phenomena likely represent key events in the process of B-cell transformation. 
 ETV6/RUNX1 transcript is a target of RNA-binding protein IGF2BP1 in t(12;21)(p13;q22)-positive acute lymphoblastic leukemia. The oncofetal RNA-binding protein IGF2BP1 (IGF2 mRNA binding protein 1) is overexpressed in a subset of cancers and promotes cell cycle,   migration   and aggressive phenotype by regulating post-transcriptionally a number of key mRNAs (e. g, ACTB, CD44, CTNNB1, KRAS, MAPK4, MYC, PTEN and others). IGF2BP1 is also overexpressed in t(12;21)(p13;q22)-positive acute lymphoblastic leukemia (ALL), but the biological significance of this phenomenon has not been addressed so far. We have identified leukemia fusion gene ETV6/RUNX1 mRNA to be highly enriched in immunoprecipitated fraction of endogenous IGF2BP1 from a model cell line REH and t(12;21)(p13;q22)-positive ALL samples. Furthermore, downregulation of IGF2BP1 by two-fold has resulted in a corresponding decrease of ETV6/RUNX1 mRNA validating this transcript as a target of IGF2BP1 protein in t(12;21)(p13;q22)-positive ALL. These data infer that IGF2BP1 is a potent regulator of ETV6/RUNX1 mRNA stability and potentially link this evolutionary-highly conserved protein to cell transformation events in ETV6/RUNX1-mediated leukemogenesis of t(12;21)(p13;q22)-positive ALL. 
  GeneRIFs  
 The PAX5/TEL protein has a dominant effect on wild-type PAX5, interferes with the process of B-cell differentiation and   migration  , and induces resistance to apoptosis. 
  [Ii]nvasive  PubMed  
 Secretory breast carcinomas with ETV6-NTRK3 fusion gene belong to the basal-like carcinoma spectrum. Secretory breast carcinomas (<0.15% of breast tumors) are associated with a characteristic morphology and a favorable prognosis. Remarkably, this entity is the only epithelial tumor of the breast with a balanced translocation, t(12;15), that creates an ETV6-NTRK3 gene fusion encoding chimeric tyrosine kinase also encountered in cellular mesoblastic nephroma and infantile fibrosarcoma. The aim of this study was to determine the phenotypic class (ie luminal A/B, ERBB2, basal-like) of secretory breast carcinoma. A series of six secretory breast carcinomas were identified in our files. The ETV6 rearrangement was confirmed in all cases by fluorescence in situ hybridization. Immunophenotype was assessed with anti-ER, PR, ERBB2, KIT, EGFR, E-cadherin, vimentin, PS100, smooth muscle actin, basal (CK5/6 and 14), luminal cytokeratins (CK8/18) and p63 antibodies. In situ and   invasive   components shared the same immunoprofile and were ER, PR, ERBB2 negative with expression of basal cytokeratins. ETV6 gene alterations were present in both in situ and   invasive   components, highlighting their genetic similarities. The immunoprofile data (triple-negative with expression of basal markers) showed that secretory breast carcinomas with ETV6-NTRK3 fusion gene belong to the phenotypic basal-like spectrum of breast carcinomas. These results support the hypothesis that secretory breast carcinomas have immunohistochemical and genetic features that distinguish them from other basal-like tumors of the breast. 
 ETV6 gene rearrangements in   invasive   breast carcinoma. The ETV6/TEL gene encodes a transcription factor frequently rearranged in several types of cancer. We looked for ETV6 rearrangements in   invasive   breast cancer using fluorescence in situ hybridization (FISH) of BAC probes on sections of tissue microarrays containing 632 tumor samples. Of these samples, signal of sufficient quality for screening by FISH was obtained for 356. Five cases (one lobular, one nontypical secretory, one mixed, and two ductal carcinomas) showed ETV6 rearrangement. 
 Mammary analogue secretory carcinoma of salivary glands: a clinicopathologic and molecular study including 2 cases harboring ETV6-X fusion. Mammary analogue secretory carcinoma (MASC) is a recently described low-grade carcinoma with morphologic and genetic similarity, including ETV6-NTRK3 fusion, to secretory carcinoma of the breast. ETV6 is frequently involved in other epithelial and nonepithelial tumors, and many fusion partners of ETV6 have been reported. In the present study, 14 Japanese MASC cases were clinicopathologically and molecularly analyzed. The median age of the patients was 39 years, and the male:female ratio was 6:8. All cases showed histopathologic findings compatible with those previously described for MASC and harbored an ETV6 split as visualized by fluorescence in situ hybridization. Two cases showed thick fibrous septa and   invasive   features including vascular or perineural tumor involvement, findings that are rare in MASC. In addition, in these 2 cases, non-NTRK3 genes appeared to fuse with ETV6 (ETV6-X fusion). NTRK1 and NTRK2, both members of the NTRK family, were not involved. Of the 14 MASC cases, the ETV6-NTRK3 fusion transcript was positive in 6 cases, and the relative expression level of the ETV6-NTRK3 fusion transcript was variable, ranging from 1 to 5.8. Results of the present study of MASC suggest that (1) ETV6 occasionally fuses with unknown non-NTRK3 genes, (2) ETV6-X cases might have an   invasive   histology, (3) for molecular diagnosis of MASC, fluorescence in situ hybridization to detect ETV6 splits is the method of choice, and (4) the expression level of the ETV6-NTRK3 fusion transcript is considerably variable. These findings provide a novel insight into the oncogenesis, histopathology, diagnosis, treatment, and prognosis of this newly recognized carcinoma. 
  GeneRIFs  
 ETV6 rearrangements in   invasive   breast cancer using fluorescence in situ hybridization (FISH) 
  [Mm]etastasize  PubMed  
 The antiapoptotic gene survivin is highly expressed in human chondrosarcoma and promotes drug resistance in chondrosarcoma cells in vitro. Chondrosarcoma is virtually resistant to chemotherapy and radiation therapy. Survivin, the smallest member of the inhibitor of apoptosis protein family, is a critical factor for tumor progression and resistance to conventional therapeutic approaches in a wide range of malignancies. However, the role of survivin in chondrosarcoma has not been well studied. We examined the importance of survivin gene expression in chondrosarcoma and analysed its influences on proliferation, apoptosis and resistance to chemotherapy in vitro.Resected chondrosarcoma specimens from which paraffin-embedded tissues could be extracted were available from 12 patients. In vitro experiments were performed in human chondrosarcoma cell lines SW1353 and Hs819.T. Immunohistochemistry, immunoblot, quantitative PCR, RNA interference, gene-overexpression and analyses of cell proliferation and apoptosis were performed.Expression of survivin protein was detected in all chondrosarcoma specimens analyzed, while undetectable in adult human cartilage. RNA interference targeting survivin resulted in a G2/M-arrest of the cell cycle and led to increased rates of apoptosis in chondrosarcoma cells in vitro. Overexpression of survivin resulted in pronounced resistance to doxorubicin treatment.These findings indicate that survivin plays a role in the pathogenesis and pronounced chemoresistance of high grade chondrosarcoma. Survivin antagonizing therapeutic strategies may lead to new treatment options in unresectable and   metastasize  d chondrosarcoma. 
  [Mm]etastasis  PubMed  
 Primary Cutaneous Mammary Analog Secretory Carcinoma With ETV6-NTRK3 Translocation. Mammary analog secretory carcinoma (MASC) is a recently described tumor of the salivary glands named for its morphological and molecular similarity to secretory carcinoma of the breast. Many primary carcinomas arising from the adnexal glands also share similar morphology to those arising from the breast. Brandt et al first described primary cutaneous MASC in 2009 and since then only 2 other cases have been reported. Herein, we describe a long-standing mass on the arm of an otherwise healthy 40-year-old female. Histologic examination revealed a circumscribed but unencapsulated, nodular tumor composed of bland epithelial cells arranged in solid and microcystic growth patterns. The cells showed vacuolated cytoplasm and round to oval nuclei with vesicular chromatin. Intraluminal homogenous eosinophilic secretions were present. Mitotic figures were not identified. The tumor cells stained positive for CK8/18, CK7, and S100 but were negative for other markers performed, including estrogen receptor, progesterone receptor, HER2/neu, paired box 8 (PAX8), and thyroid transcription factor 1 (TTF1). As the patient clinically had no other masses or known carcinomas, a diagnosis of primary cutaneous MASC was rendered. The ETV6-NTRK3 fusion transcript was subsequently detected by reverse transcriptase polymerase chain reaction amplification, further supporting the diagnosis. We present this case to review the histologic features of MASC and highlight the importance of recognizing this lesion not only as a possible cutaneous   metastasis   but also as a primary cutaneous tumor. 
   
  
 KEAP1 
 
  NCBI Gene  
  GeneRIF  
  Pubmed  
 
 
  Search Term  Rank  Fields  
  [Mm]igration  6  PubMed(4); GeneRIFs(1)  
  [Ii]nvasive  10  PubMed(6)  
  [Mm]etastasis  12  PubMed(9); GeneRIFs(3)  
 
 
 hide/show details for KEAP1 
  [Mm]igration  PubMed  
 The subcellular localization and activity of cortactin is regulated by acetylation and interaction with Keap1. Cortactin is an F-actin-binding protein that localizes to the cell cortex, where the actin remodeling that is required for cell   migration   occurs. We found that cortactin shuttled between the cytoplasm and the nucleus under basal conditions. We identified Kelch-like ECH-associated protein 1 (Keap1), a cytosolic protein that is involved in oxidant stress responses, as a binding partner of cortactin that promoted the cortical localization of cortactin and cell   migration  . The ability of cortactin to promote cell   migration   is regulated by various posttranslational modifications, including acetylation. We showed that the acetylated form of cortactin was mainly localized to the nucleus and that acetylation of cortactin decreased cell   migration   by inhibiting the binding of cortactin to Keap1. Our findings reveal that Keap1 regulates cell   migration   by affecting the subcellular localization and activity of cortactin independently of its role in oxidant stress responses. 
 Subcellular localization and cytoplasmic complex status of endogenous Keap1. Keap1 acts as a sensor for oxidative/electrophilic stress, an adaptor for Cullin-3-based ubiquitin ligase, and a regulator of Nrf2 activity through the interaction with Nrf2 Neh2 domain. However, the mechanism(s) of Nrf2   migration   into the nucleus in response to stress remains largely unknown due to the lack of a reliable antibody for the detection of endogenous Keap1 molecule. Here, we report the generation of a new monoclonal antibody for the detection of endogenous Keap1 molecules. Immunocytochemical analysis of mouse embryonic fibroblasts with the antibody revealed that under normal, unstressed condition, Keap1 is localized primarily in the cytoplasm with minimal amount in the nucleus and endoplasmic reticulum. This subcellular localization profile of Keap1 appears unchanged after treatment of cells with diethyl maleate, an electrophile, and/or Leptomycin B, a nuclear export inhibitor. Subcellular fractionation analysis of mouse liver cells showed similar results. No substantial change in the subcellular distribution profile could be observed in cells isolated from butylated hydroxyanisole-treated mice. Analyses of sucrose density gradient centrifugation of mouse liver cells indicated that Keap1 appears to form multiprotein complexes in the cytoplasm. These results demonstrate that endogenous Keap1 remains mostly in the cytoplasm, and electrophiles promote nuclear accumulation of Nrf2 without altering the subcellular localization of Keap1. 
 Keap1-Nrf2 Interaction Suppresses Cell Motility in Lung Adenocarcinomas by Targeting the S100P Protein. Kelch-like ECH-associated protein 1 (Keap1) is an E3 ligase participated in the cellular defense response against oxidative stress through nuclear factor erythroid-2-related factor 2 (Nrf2). However, the role of Keap1 in regulating cancer motility is still controversial. We investigated the contribution of the Keap1-Nrf2 axis in the progression of non-small cell lung cancer (NSCLC).The expression of Keap1 and Nrf2 was examined via immunohistochemistry, real-time PCR, and Western blot analysis in a cohort of NSCLC tissues and cells. A series of in vivo and in vitro assays was performed to elucidate the contribution of the Keap1-Nrf2 axis in lung cancer mobility and progression.Keap1 expression was decreased in specimens from NSCLC patients with lymph node metastasis compared with patients without metastasis. Higher Keap1 expression levels were correlated with the survival of NSCLC patients. Moreover, manipulation of Keap1 expression affected cell   migration  /invasion abilities. Depletion of Nrf2 relieved the   migration   promotion imposed by Keap1 suppression. Mechanistic investigations found that S100P was downregulated in both Keap1-overexpressing and Nrf2-knockdown NSCLC cells. Overexpression of Keap1 and knockdown of Nrf2 both suppressed S100P expression in NSCLC cells. Knockdown of S100P inhibited cell   migration   in highly invasive NSCLC cells and also relieved the   migration   promotion imposed by Keap1 suppression in weakly invasive NSCLC cells.Our findings suggest that Keap1 functions as a suppressor of tumor metastasis by targeting the Nrf2/S100P pathway in NSCLC cells. In addition, overexpression of Keap1 may be a novel NSCLC treatment strategy and/or useful biomarker for predicting NSCLC progression. 
 Aberrant RSPO3-LGR4 signaling in Keap1-deficient lung adenocarcinomas promotes tumor aggressiveness. The four R-spondins (RSPO1-4) and their three related receptors LGR4, 5 and 6 (LGR4-6) have emerged as a major ligand-receptor system with critical roles in development and stem cell survival through modulation of Wnt signaling. Recurrent, gain-of-expression gene fusions of RSPO2 (to EIF3E) and RSPO3 (to PTPRK) occur in a subset of human colorectal cancer. However, the exact roles and mechanisms of the RSPO-LGR system in oncogenesis remain largely unknown. We found that RSPO3 is aberrantly expressed at high levels in approximately half of Keap1-mutated lung adenocarcinomas (ADs). This high RSPO3 expression is driven by a combination of demethylation of its own promoter region and deficiency in Keap1 instead of gene fusion as in colon cancer. Patients with RSPO3-high tumors (~9%, 36/412) displayed much poorer survival than the rest of the cohort (median survival of 28 vs 163 months, log-rank test P<0.0001). Knockdown (KD) of RSPO3, LGR4 or their signaling mediator IQGAP1 in lung cancer cell lines with Keap1 deficiency and high RSPO3-LGR4 expression led to reduction in cell proliferation and   migration   in vitro, and KD of LGR4 or IQGAP1 resulted in decrease in tumor growth and metastasis in vivo. These findings suggest that aberrant RSPO3-LGR4 signaling potentially acts as a driving mechanism in the aggressiveness of Keap1-deficient lung ADs. 
  GeneRIFs  
 findings reveal that Keap1 regulates cell   migration   by affecting the subcellular localization and activity of cortactin independently of its role in oxidant stress responses. 
  [Ii]nvasive  PubMed  
 KEAP1 Genetic Polymorphisms Associate with Breast Cancer Risk and Survival Outcomes. Defective oxidative stress response may increase cancer susceptibility. In tumors, these rescue mechanisms may cause chemo- and radioresistance impacting patient outcome. We previously showed that genetic variation in the nuclear factor erythroid 2-related factor 2 (NFE2L2) is associated with breast cancer risk and prognosis. Here we further studied this pathway by investigating Kelch-like ECH-associated protein 1 (KEAP1).Five tagging SNPs in the KEAP1 gene were genotyped in 996 breast cancer cases and 880 controls from two Finnish case-control sets. KEAP1 protein expression was studied in 373   invasive   breast cancer tumors.rs34197572 genotype TT was associated with increased risk of breast cancer in the KBCP samples [P = 1.8×10(-4); OR, 7.314; confidence interval (CI), 2.185-24.478]. rs11085735 allele A was associated with lower KEAP1 protein expression (P = 0.040; OR,= 3.545) and high nuclear NRF2 expression (P = 0.009; OR, 2.445) and worse survival in all   invasiv  e cases (P = 0.023; HR, 1.634). When including treatment data, rs11085735 was associated with recurrence-free survival (RFS; P = 0.020; HR, 1.545) and breast cancer-specific survival (P = 0.016; HR, 1.683) and rs34197572 with overall survival (P = 0.045; HR, 1.304). rs11085735 associated with RFS also among tamoxifen-treated cases (P = 0.003; HR, 3.517). Among radiotherapy-treated cases, overall survival was associated with rs34197572 (P = 0.018; HR, 1.486) and rs8113472 (P = 0.025; HR, 1.455). RFS was associated with rs9676881 (P = 0.024; HR, 1.452) and rs1048290 (P = 0.020; HR, 1.468) among all   invasiv  e cases and among estrogen receptor (ER)-positive tamoxifen-treated cases (P = 0.018; HR, 2.407 and P = 0.015; HR, 2.476, respectively).The present findings suggest that the investigated SNPs have effects related to oxidative stress induced by cancer treatment, supporting involvement of the NRF2/KEAP1 pathway in breast cancer susceptibility and patient outcome. 
 Oxidative stress and counteracting mechanisms in hormone receptor positive, triple-negative and basal-like breast carcinomas. Triple-negative breast cancer (TNBC) and basal-like breast cancer (BLBC) are breast cancer subtypes with an especially poor prognosis. 8-Hydroxydeoxyguanosine (8-OHdG) is a widely used marker of oxidative stress and the redox-state-regulating enzymes peroxiredoxins (PRDXs) are efficient at depressing excessive reactive oxygen species. NF-E2-related factor 2 (Nrf2) and Kelch-like ECH-associated protein 1 (Keap1) are redox-sensitive transcription factors that regulate PRDX expression. This is the first study to assess oxidative stress and or cell redox state-regulating enzymes in TNBC and BLBC.We assessed immunohistochemical expression of 8-OHdG, Nrf2, Keap1, PRDX III and PRDX IV in 79 women with   invasive   ductal breast carcinomas. Of these tumors, 37 represented TNBC (grade II-III tumors with total lack of ER, PR and human epidermal growth factor receptor 2 [HER2] expression). Control cases (n = 42) were ER-positive, PR-positive and HER2-negative. Of the 37 TNBCs, 31 had BLBC phenotype (TNBC with expression of cytokeratin 5/6 or epidermal growth factor receptor 1).Patients with TNBC had worse breast cancer-specific survival (BCSS) than the control group (p = 0.015). Expression of 8-OHdG was significantly lower in TNBC than in the non-TNBC group (p < 0.005). 8-OHdG immunostaining was associated with better BCSS (p = 0.01), small tumor size (p < 0.0001) and low grade (p < 0.0005). Keap1 overexpression was observed in the TNBC cohort (p = 0.001) and Keap1-positive patients had worse BCSS than Keap1-negative women (p = 0.014). PRDX IV was overexpressed in the TNBC vs. the non-TNBC group (p = 0.022).Cellular redox state markers may be promising targets when elucidating the pathogenesis of TNBC. 
 TrkB Promotes Breast Cancer Metastasis via Suppression of Runx3 and Keap1 Expression. In metastatic breast cancer, the acquisition of malignant traits has been associated with the increased rate of cell growth and division, mobility, resistance to chemotherapy, and   invasive  ness. While screening for the key regulators of cancer metastasis, we observed that neurotrophin receptor TrkB is frequently overexpressed in breast cancer patients and breast cancer cell lines. Additionally, we demonstrate that TrkB expression and clinical breast tumor pathological phenotypes show significant correlation. Moreover, TrkB expression was significantly upregulated in basal-like, claudin-low, and metaplastic breast cancers from a published microarray database and in patients with triple-negative breast cancer, which is associated with a higher risk of   invasive   recurrence. Interestingly, we identified a new TrkB-regulated functional network that is important for the tumorigenicity and metastasis of breast cancer. We demonstrated that TrkB plays a key role in regulation of the tumor suppressors Runx3 and Keap1. A markedly increased expression of Runx3 and Keap1 was observed upon knockdown of TrkB, treatment with a TrkB inhibitor, and in TrkB kinase dead mutants. Additionally, the inhibition of PI3K/AKT activation significantly induced Runx3 and Keap1 expression. Furthermore, we showed that TrkB enhances metastatic potential and induces proliferation. These observations suggest that TrkB plays a key role in tumorigenicity and metastasis of breast cancer cells through suppression of Runx3 or Keap1 and that it is a promising target for future intervention strategies for preventing tumor metastasis and cancer chemoprevention. 
 Keap1-Nrf2 Interaction Suppresses Cell Motility in Lung Adenocarcinomas by Targeting the S100P Protein. Kelch-like ECH-associated protein 1 (Keap1) is an E3 ligase participated in the cellular defense response against oxidative stress through nuclear factor erythroid-2-related factor 2 (Nrf2). However, the role of Keap1 in regulating cancer motility is still controversial. We investigated the contribution of the Keap1-Nrf2 axis in the progression of non-small cell lung cancer (NSCLC).The expression of Keap1 and Nrf2 was examined via immunohistochemistry, real-time PCR, and Western blot analysis in a cohort of NSCLC tissues and cells. A series of in vivo and in vitro assays was performed to elucidate the contribution of the Keap1-Nrf2 axis in lung cancer mobility and progression.Keap1 expression was decreased in specimens from NSCLC patients with lymph node metastasis compared with patients without metastasis. Higher Keap1 expression levels were correlated with the survival of NSCLC patients. Moreover, manipulation of Keap1 expression affected cell migration/invasion abilities. Depletion of Nrf2 relieved the migration promotion imposed by Keap1 suppression. Mechanistic investigations found that S100P was downregulated in both Keap1-overexpressing and Nrf2-knockdown NSCLC cells. Overexpression of Keap1 and knockdown of Nrf2 both suppressed S100P expression in NSCLC cells. Knockdown of S100P inhibited cell migration in highly   invasive   NSCLC cells and also relieved the migration promotion imposed by Keap1 suppression in weakly   invasive   NSCLC cells.Our findings suggest that Keap1 functions as a suppressor of tumor metastasis by targeting the Nrf2/S100P pathway in NSCLC cells. In addition, overexpression of Keap1 may be a novel NSCLC treatment strategy and/or useful biomarker for predicting NSCLC progression. 
 An immunohistochemical study of NFE2L2, KEAP1 and 8-hydroxy-2'-deoxyguanosine and the EMT markers SNAI2, ZEB1 and TWIST1 in metastatic melanoma. Little is known regarding the role of redox balance regulators in metastatic melanomas, but there is some evidence for a link between epithelial-to-mesenchymal transition (EMT) and cellular redox status.We compared the immunohistochemical expression of nuclear factor erythroid-2-related factor 2 (NFE2L2), Kelch-like ECH-associated protein 1 (KEAP1), 8-hydroxy-2'-deoxyguanosine (8-OHdG), TWIST1, SNAI2 and ZEB1 between primary melanomas and metastases in a cohort of 23 nevi, 66 malignant melanomas and 22 metastases.Nuclear NFE2L2 expression was higher (p=0.003) and cytoplasmic KEAP1 lower (p=0.026) in metastatic lesions than at primary sites. Nuclear NFE2L2 expression was associated with the presence of distant metastases (p=0.040) and with nuclear TWIST1 expression (p=0.002). Patients having both NFE2L2 and TWIST1 expression in nuclei had an extremely poor prognosis (p=0.0003). In multivariate analysis nuclear TWIST1 expression was an independent predictor of a poorer prognosis (HR 2.99, 95% CI 1.17-7.69; p=0.023) and the   invasiv  e TWIST1/ZEB1 phenotype showed poorer melanoma-specific survival (HR 7.28, 95% CI 2.23-23.77; p=0.001). Nuclear expression of 8-OHdG (p=0.001) was lower at metastatic sites than in primary lesions.EMT signalling and the KEAP1/NFE2L2-axis are likely to be involved in metastatic spread of malignant melanoma and also appear to have potential interactions. 
 Aberrant Keap1 methylation in breast cancer and association with clinicopathological features. Keap1 (Kelch-like ECH-associated protein 1) is an adaptor protein that mediates the ubiquitination/degradation of genes regulating cell survival and apoptosis under oxidative stress conditions. We determined methylation status of the KEAP1 promoter in 102 primary breast cancers, 14 pre-  invasive   lesions, 38 paired normal breast tissues and 6 normal breast from reductive mammoplasty by quantitative methylation specific PCR (QMSP). Aberrant promoter methylation was detected in 52 out of the 102 primary breast cancer cases (51%) and 10 out of 14 pre-  invasive   lesions (71%). No mutations of the KEAP1 gene were identified in the 20 breast cancer cases analyzed by fluorescence based direct sequencing. Methylation was more frequent in the subgroup of patients identified as ER positive-HER2 negative tumors (66.7%) as compared with triple-negative breast cancers (35%) (p = 0.05, Chi-square test). The impact of the interactions between Er, PgR, Her2 expression and KEAP1 methylation on mortality was investigated by RECPAM multivariable statistical analysis, identifying four prognostic classes at different mortality risks. Triple-negative breast cancer patients with KEAP1 methylation had higher mortality risk than patients without triple-negative breast cancer (HR = 14.73, 95%CI: 3.65-59.37). Both univariable and multivariable COX regressions analyses showed that KEAP1 methylation was associated with a better progression free survival in patients treated with epirubicin/cyclophosfamide and docetaxel as sequential chemotherapy (HR = 0.082; 95%CI: 0.007-0.934). These results indicate that aberrant promoter methylation of the KEAP1 gene is involved in breast cancerogenesis. In addition, identifying patients with KEAP1 epigenetic abnormalities may contribute to disease progression prediction in breast cancer patients. 
  [Mm]etastasis  PubMed  
 Expression of LC3, LAMP2, KEAP1 and NRF2 in Salivary Adenoid Cystic Carcinoma. Salivary Adenoid Cystic Carcinoma (SACC) is a tumor characterized by inevitable local progression and terminal hematogenous   metastasis  . This study aimed to investigate the expression of LC3, LAMP2, KEAP1 and NRF2 in SACC. Human salivary gland tissue microarray which contains 74 SACC, 12 pleomorphic adenoma and 18 normal salivary gland specimens. High expression of LC3, LAMP2, KEAP1 and NRF2 were found in SACC patients, and LC3, LAMP2, KEAP1 and NRF2 expression were significantly higher in SACC than as compared with pleomorphic adenoma and (or) normal salivary gland. The expression of NRF2 was correlated with pathological type of human SACC (P < 0.05). Moreover, the high-expression of KEAP1 had significant correlations with LC3 (P < 0.001, R = 0.3195), and LAMP2 (P < 0.001, R = 0.3346) and NRF2 (P < 0.05, R = 0.2246) by using the Pearson correlation coefficient test. Our findings demonstrated that up-regulation of LC3, LAMP2, KEAP1 and NRF2 were associated with carcinogenesis and progression of SACC patients, suggesting that they may be useful molecular targets in salivary adenoid cystic carcinoma. 
 Expression and correlation of NRF2, KEAP1, NQO-1 and HO-1 in advanced squamous cell carcinoma of the larynx and their association with clinicopathologic features. The present study aimed specifically investigate the expression and correlation of kelch-like ECH-associated protein‑1 (KEAP1), nuclear factor (erythroid-derived 2)‑like 2 (NRF2), quinone oxidoreductase‑1 (NQO‑1) and heme oxygenase‑1 (HO‑1) in laryngeal cancer and their association with clinicopathological features. A total of 33 paired human fresh advanced laryngeal cancer and adjacent normal specimens were collected following total laryngectomy. Immunohistochemical and immunoblotting analysis were performed to evaluate the expression of KEAP1, NRF2, NQO‑1 and HO‑1. All of the patients clinicopathologic features were collected. Immunohistochemistry indicated that NRF2‑positive staining was as high as 79% (26/33) in the cancer samples and predominantly located in the nuclei, whereas positive expression of KEAP1, NQO‑1 and HO‑1 was detected in 70% (23/33), 76% (25/33) and 82% (27/33) of cancer tissues, respectively, and primarily expressed in the cytoplasm. The corresponding adjacent normal samples produced almost no or weak expression of the proteins. There were significant differences in protein expression between the two groups (P<0.01). Immunoblotting analysis also demonstrated that their expression levels were higher in cancer tissue compared with adjacent normal tissue. Notably, KEAP1, NQO‑1 and HO‑1 expression was positively correlated with nuclear NRF2 in cancer tissues, whereas they had no correlation with age, tumor stage (clinical stage III and IV  ), tumor s  ize and lymph node metastasis. In conclusion, increased expression of NRF2, KEAP1, NQO‑1 and HO‑1 were common in advanced laryngeal cancer. Evaluation of these factors may have important clinical significance for the diagnosis and treatment of this disease. 
 Expression and clinical significance of Kelch-like epichlorohydrin-associated protein 1 in breast cancer. Our objective was to explore the expression and clinical significance of Kelch-like epichlorohydrin-associated protein 1 (Keap1) in breast cancer tissue. Eighty-one breast cancer patients having undergone surgical treatment in our hospital between March 2002 and December 2008 were enrolled in this study. Normal tissue adjacent to tumors was used for the control samples. Diagnoses for all patients were confirmed by postoperative pathological examination. Immunohistochemical assays were used to measure the expression of Keap1 protein in breast cancer tissue and adjacent normal tissue, and its clinical significance was explored. We observed that 24.6% breast cancer tissue samples were positive for Keap1, a significantly lower proportion than that seen with adjacent normal tissue specimens (80.2%; P < 0.05). The presence of Keap1 expression did not correlate with age, tumor size, pathological classification, or degree of differentiation. However, it was found to be significantly associated with tumor-node-  metastasis   stage and the presence of lymphatic   metastasis  . Kaplan-Meier survival analysis showed a remarkably higher five-year survival rate among patients with positive Keap1 expression than in those lacking detectable levels of the protein (P = 0.032). Keap1 expression is significantly decreased in breast cancer tissue; therefore, the early detection of its expression might have great significance in determining prognosis for breast cancer patients. 
 [Keap1 expression for predicting the chemoresistance and prognosis of advanced non-small cell lung cancer]. It has been proven that Kelch-like ECH-associated protein 1 (Keap1) expression was correlated with the chemoresistance of platinum drugs. The aim of this study is to investigate the protein expression levels of Keap1 in non-small cell lung cancer (NSCLC) patients as well as to correlate its expression with the response rate (RR), progression-free survival (PFS), and overall survival (OS) of patients treated with platinum-based first-line chemotherapy.The immunohistochemical analysis of Keap1 expression was performed using tumor samples from 50 patients with stage III or IV NSCLC.The high expression ratio of Keap1 was 26.0%. The Keap1 expression was significantly correlated with the RR and PFS after platinum-based chemotherapy (P<0.05) but not with the gender, age, smoking, pathology type, differentiation,   metastasis  , and OS (P>0.05). The PFS was significantly longer in patients with high Keap1 expression than in those with low/negative expression (P=0.002). Furthermore, the level of Keap1 expression was an independent predictive factor of PFS after platinum-based first-line chemotherapy (P=0.007).The expression of Keap1 was significantly correlated with the RR and PFS of platinum-based first-line chemotherapy. Therefore, Keap1 may be a useful biomarker for predicting the chemosensitivity of patients with advanced-stage NSCLC. 
 Cancer Cell Growth Is Differentially Affected by Constitutive Activation of NRF2 by KEAP1 Deletion and Pharmacological Activation of NRF2 by the Synthetic Triterpenoid, RTA 405. Synthetic triterpenoids are antioxidant inflammation modulators (AIMs) that exhibit broad anticancer activity. AIMs bind to KEAP1 and inhibit its ability to promote NRF2 degradation. As a result, NRF2 increases transcription of genes that restore redox balance and reduce inflammation. AIMs inhibit tumor growth and   metastasis   by increasing NRF2 activity in the tumor microenvironment and by modulating the activity of oncogenic signaling pathways, including NF-κB, in tumor cells. Accumulating evidence suggests that KEAP1 loss or mutation--which results in high levels of sustained NRF2 activity--may promote cancer growth and increase chemoresistance. Loss of KEAP1 also increases the levels of other oncogenic proteins, including IKKβ and BCL2. The apparent survival advantage provided to some tumor cells by loss of functional KEAP1 raises the question of whether pharmacological inhibition of KEAP1 could promote tumor growth. To address this issue, we characterized the basal levels of KEAP1 and NRF2 in a panel of human tumor cell lines and profiled the activity of an AIM, RTA 405. We found that in tumor cell lines with low or mutant KEAP1, and in Keap1-/- murine embryonic fibroblasts, multiple KEAP1 targets including NRF2, IKKβ, and BCL2 were elevated. Keap1-/- murine embryonic fibroblasts also had higher rates of proliferation and colony formation than their wild-type counterparts. In cells with functional KEAP1, RTA 405 increased NRF2 levels, but not IKKβ or BCL2 levels, and did not increase cell proliferation or survival. Moreover, RTA 405 inhibited growth at similar concentrations in cells with different basal NRF2 activity levels and in cells with wild-type or mutant KRAS. Finally, pre-treatment with RTA 405 did not protect tumor cells from doxorubicin- or cisplatin-mediated growth inhibition. Collectively, these data demonstrate that pharmacological activation of NRF2 by AIMs is distinct from genetic activation and does not provide a growth or survival advantage to tumor cells. 
 TrkB Promotes Breast Cancer   Metastasis   via Suppression of Runx3 and Keap1 Expression. In metastatic breast cancer, the acquisition of malignant traits has been associated with the increased rate of cell growth and division, mobility, resistance to chemotherapy, and invasiveness. While screening for the key regulators of cancer   metastasis  , we observed that neurotrophin receptor TrkB is frequently overexpressed in breast cancer patients and breast cancer cell lines. Additionally, we demonstrate that TrkB expression and clinical breast tumor pathological phenotypes show significant correlation. Moreover, TrkB expression was significantly upregulated in basal-like, claudin-low, and metaplastic breast cancers from a published microarray database and in patients with triple-negative breast cancer, which is associated with a higher risk of invasive recurrence. Interestingly, we identified a new TrkB-regulated functional network that is important for the tumorigenicity and   metastasis   of breast cancer. We demonstrated that TrkB plays a key role in regulation of the tumor suppressors Runx3 and Keap1. A markedly increased expression of Runx3 and Keap1 was observed upon knockdown of TrkB, treatment with a TrkB inhibitor, and in TrkB kinase dead mutants. Additionally, the inhibition of PI3K/AKT activation significantly induced Runx3 and Keap1 expression. Furthermore, we showed that TrkB enhances metastatic potential and induces proliferation. These observations suggest that TrkB plays a key role in tumorigenicity and   metastasis   of breast cancer cells through suppression of Runx3 or Keap1 and that it is a promising target for future intervention strategies for preventing tumor   metastasis   and cancer chemoprevention. 
 Keap1-Nrf2 Interaction Suppresses Cell Motility in Lung Adenocarcinomas by Targeting the S100P Protein. Kelch-like ECH-associated protein 1 (Keap1) is an E3 ligase participated in the cellular defense response against oxidative stress through nuclear factor erythroid-2-related factor 2 (Nrf2). However, the role of Keap1 in regulating cancer motility is still controversial. We investigated the contribution of the Keap1-Nrf2 axis in the progression of non-small cell lung cancer (NSCLC).The expression of Keap1 and Nrf2 was examined via immunohistochemistry, real-time PCR, and Western blot analysis in a cohort of NSCLC tissues and cells. A series of in vivo and in vitro assays was performed to elucidate the contribution of the Keap1-Nrf2 axis in lung cancer mobility and progression.Keap1 expression was decreased in specimens from NSCLC patients with lymph node   metastasis   compared with patients without   metastasis  . Higher Keap1 expression levels were correlated with the survival of NSCLC patients. Moreover, manipulation of Keap1 expression affected cell migration/invasion abilities. Depletion of Nrf2 relieved the migration promotion imposed by Keap1 suppression. Mechanistic investigations found that S100P was downregulated in both Keap1-overexpressing and Nrf2-knockdown NSCLC cells. Overexpression of Keap1 and knockdown of Nrf2 both suppressed S100P expression in NSCLC cells. Knockdown of S100P inhibited cell migration in highly invasive NSCLC cells and also relieved the migration promotion imposed by Keap1 suppression in weakly invasive NSCLC cells.Our findings suggest that Keap1 functions as a suppressor of tumor   metastasis   by targeting the Nrf2/S100P pathway in NSCLC cells. In addition, overexpression of Keap1 may be a novel NSCLC treatment strategy and/or useful biomarker for predicting NSCLC progression. 
 Aberrant RSPO3-LGR4 signaling in Keap1-deficient lung adenocarcinomas promotes tumor aggressiveness. The four R-spondins (RSPO1-4) and their three related receptors LGR4, 5 and 6 (LGR4-6) have emerged as a major ligand-receptor system with critical roles in development and stem cell survival through modulation of Wnt signaling. Recurrent, gain-of-expression gene fusions of RSPO2 (to EIF3E) and RSPO3 (to PTPRK) occur in a subset of human colorectal cancer. However, the exact roles and mechanisms of the RSPO-LGR system in oncogenesis remain largely unknown. We found that RSPO3 is aberrantly expressed at high levels in approximately half of Keap1-mutated lung adenocarcinomas (ADs). This high RSPO3 expression is driven by a combination of demethylation of its own promoter region and deficiency in Keap1 instead of gene fusion as in colon cancer. Patients with RSPO3-high tumors (~9%, 36/412) displayed much poorer survival than the rest of the cohort (median survival of 28 vs 163 months, log-rank test P<0.0001). Knockdown (KD) of RSPO3, LGR4 or their signaling mediator IQGAP1 in lung cancer cell lines with Keap1 deficiency and high RSPO3-LGR4 expression led to reduction in cell proliferation and migration in vitro, and KD of LGR4 or IQGAP1 resulted in decrease in tumor growth and   metastasis   in vivo. These findings suggest that aberrant RSPO3-LGR4 signaling potentially acts as a driving mechanism in the aggressiveness of Keap1-deficient lung ADs. 
 Expression of the Nrf2 and Keap1 proteins and their clinical significance in osteosarcoma. To investigate the expression and clinical significance of nuclear factor (erythroid-derived 2)-like 2 (Nrf2) and Kelch-like ECH-associated protein 1 (Keap1) in osteosarcoma tissue.The data of 102 osteosarcoma patients who underwent surgical treatment at our hospital from June 2000 to March 2009 were collected. The expression levels of the Nrf2 and Keap1 proteins in osteosarcoma tissue and normal peritumour tissues were detected by immunohistochemistry, and the relationship between the expression level and the clinical and pathological features as well as the prognosis was explored.The nuclear expression rate of Nrf2 was 77.5% in osteosarcoma tissue, which was significantly higher than the rate in normal peritumour bone tissue (9.8%) (P < 0.05). The expression rate of the Keap1 protein in osteosarcoma tissue was 13.7%, which was significantly lower than the rate in normal peritumour tissue (80.4%). In addition, Nrf2/Keap1 expression was unrelated to patient gender and age, tumour site, and histological type and was related to   metastasis   and patient response to chemotherapy (P < 0.05). The five-year survival rate was significantly lower in patients with positive Nrf2 expression than in those with negative Nrf2 expression (p = 0.023), and it was significantly higher in patients with positive Keap1 expression than in those with negative Keap1 expression (P = 0.018).The expression of Nrf2-Keap1 is abnormal in osteosarcoma tissue and shows significant clinical relevance for determining the prognosis of osteosarcoma. 
  GeneRIFs  
 the expression of NRF2, KEAP1, NQO-1 and HO-1 are increased significantly in advanced laryngeal squamous cell carcinoma, compared with the adjacent normal mucosa. Remarkable relevance exists between high expression of KEAP1, NQO-1, HO-1 and nuclear NRF2. their expression levels were independent of age, tumor stage (clinical stage III and IV), tumor size and lymph node   metastasis  . 
 Results show up-regulation of TrkB and down-regulation of Runx3 and Keap1 in breast cancer cells and suggest that TrkB plays a key role in tumorigenicity and   metastasis   of breast cancer cells through suppression of Runx3 or Keap1. 
 Keap1 functions as a suppressor of tumor   metastasis   by targeting the Nrf2/S100P pathway in NSCLC cells. 
   
  
 BAG3 
 
  NCBI Gene  
  GeneRIF  
  Pubmed  
 
 
  Search Term  Rank  Fields  
  [Ff]ilopodia  1  PubMed(2)  
  [Mm]igration  6  PubMed(15); GeneRIFs(6)  
  [Ee]xtracellular [Mm]atrix  8  PubMed(1); GeneRIFs(1)  
  [Ii]nvasive  10  PubMed(11); GeneRIFs(2)  
  [Mm]etastasis  12  PubMed(10); GeneRIFs(2)  
  [Cc]ell [Ii]nvasion  13  PubMed(4); GeneRIFs(1)  
 
 
 hide/show details for BAG3 
  [Ff]ilopodia  PubMed  
 BAG3 regulates motility and adhesion of epithelial cancer cells. BAG3 protein binds to and regulates Hsp70 chaperone activity. The BAG3 protein contains a WW domain and a proline-rich region with SH3-binding motifs, suggesting that it may interact with proteins relevant to signal transduction, recruiting Hsp70 to signaling complexes and altering cell responses. BAG3 overexpression has been observed in human cancers. We show here that homozygous BAG3-deficient mouse embryonic fibroblasts (MEF) exhibit delayed formation of   filopodia   and focal adhesion complexes when freshly plated. BAG3-deficient MEFs show reduced cell motility in culture. We observed that endogenous BAG3 protein is highly expressed in many human epithelial cancer cell lines, especially adenocarcinomas. Gene transfer-mediated overexpression of BAG3 increased motility of Cos7 cell and several human cancer cell lines, including breast cancer MCF7 and prostate cancer DU145 and ALVA31 cell lines. Conversely, reduction of BAG3 protein by RNA interference (RNAi) decreased cell motility in four of four epithelial tumor lines tested. We observed an influence of BAG3 on cell adhesion in culture. In Cos7 kidney epithelial cells, BAG3 protein partially colocalizes with actin at the leading edge of migrating cells, wherein active actin polymerization and nucleation occur. RNAi-mediated reductions in BAG3 expression were associated with decreased Rac1 activity, suggesting a role for BAG3 in regulating this small GTPase involved in actin-cytoskeleton dynamics. In mice, RNAi-mediated reductions in BAG3 in a human tumor xenograft suppressed invasion and metastasis in vivo. Thus, the high levels of BAG3 protein seen in some epithelial cancer cell lines may be relevant to mechanisms of tumor invasion and metastasis. 
 CAIR-1/BAG-3 modulates cell adhesion and migration by downregulating activity of focal adhesion proteins. CAIR-1/BAG-3 is a stress and survival protein that has been shown to bind SH3 domain-containing proteins through its proline-rich (PXXP) domain. Because stress and survival pathways are active during invasion and metastasis, we hypothesized that CAIR-1 is a regulator of signaling pathways that modulate cell adhesion and migration. MDA-435 human breast carcinoma cells were stably transfected with full-length CAIR-1 (FL) or a proline-rich domain deleted mutant (dPXXP). FL cells migrated poorly through collagen IV-coated filters to serum (14% of control, p=0.0004), whereas migration of dPXXP cells was more robust (228%, p=0.00001). Adhesion to collagen IV-coated surfaces was reduced in FL cells and augmented in dPXXP cells (FL 64%, p=0.03; dPXXP 138%, p=0.01). Rhodamine-phalloidin staining highlighted more stress fibers and thicker   filopodia  l protrusions in dPXXP cells. Fewer focal adhesions were also seen in FL cells. A reduction in tyrosine phosphorylation of focal adhesion kinase (FAK) and paxillin occurred in FL cells under these conditions. In contrast, increased FAK and paxillin phosphorylation was documented in dPXXP cells. Differential FAK phosphorylation occurred at the major autophosphorylation site Y(397) and Src phosphorylation site Y(861). Concordant with these findings, there was decreased interaction between FAK and its downstream partners p(130)Cas and Crk observed in FL cells but not in dPXXP cells. These results collectively indicate that CAIR-1 may negatively regulate adhesion, focal adhesion assembly, signaling, and migration via its PXXP domain. 
  [Mm]igration  PubMed  
 BAG3 is upregulated by c-Jun and stabilizes JunD. BAG3 plays a regulatory role in a number of cellular processes, including cell proliferation, apoptosis, adhesion and   migration  , epithelial-mesenchymal transition (EMT), autophagy activation, and virus infection. The AP-1 transcription factors are implicated in a variety of important biological processes including cell differentiation, proliferation, apoptosis and oncogenesis. Recently, it has been reported that AP-1 protein c-Jun inhibits autophagy and enhances apoptotic cell death mediated by starvation. However, the molecular mechanisms remain unclear. For the first time, the current study demonstrated that serum starvation downregulated BAG3 at the transcriptional level via c-Jun. In addition, the current study reported that BAG3 stabilized JunD mRNA, which was, at least in part, responsible for the promotion of serum starvation mediated-growth inhibition by BAG3. 
 Down-modulation of Bis reduces the invasive ability of glioma cells induced by TPA, through NF-κB mediated activation of MMP-9. Bcl-2 interacting cell death suppressor (Bis) has been shown to have anti-apoptotic and anti-stress functions. Recently, increased Bis expression was reported to correlate with glioma aggressiveness. Here, we investigated the effect of Bis knockdown on the acquisition of the invasive phenotype of A172 glioma cells, induced by 12-O-Tetradecanoylphorbol-3-acetate (TPA), using a Transwell assay. Bis knockdown resulted in a significant decrease in the   migratio  n and invasion of A172 cells. Furthermore, Bis knockdown notably decreased TPA-induced matrix metalloproteinase-9 (MMP-9) activity and mRNA expression, as measured by zymography and quantitative real time PCR, respectively. A luciferase reporter assay indicated that Bis suppression significantly down-regulated NF-κB-driven transcription. Finally, we demonstrated that the rapid phosphorylation and subsequent degradation of IκB-α induced by TPA was remarkably delayed by Bis knockdown. These results suggest that Bis regulates the invasive ability of glioma cells elicited by TPA, by modulating NF-κB activation, and subsequent induction of MMP-9 mRNA. 
 Down-modulation of Bis reduces the invasive ability of glioma cells induced by TPA, through NF-κB mediated activation of MMP-9. Bcl-2 interacting cell death suppressor (Bis) has been shown to have anti-apoptotic and anti-stress functions. Recently, increased Bis expression was reported to correlate with glioma aggressiveness. Here, we investigated the effect of Bis knockdown on the acquisition of the invasive phenotype of A172 glioma cells, induced by 12-O-Tetradecanoylphorbol-3-acetate (TPA), using a Transwell assay. Bis knockdown resulted in a significant decrease in the   migratio  n and invasion of A172 cells. Furthermore, Bis knockdown notably decreased TPA-induced matrix metalloproteinase-9 (MMP-9) activity and mRNA expression, as measured by zymography and quantitative real time PCR, respectively. A luciferase reporter assay indicated that Bis suppression significantly down-regulated NF-κB-driven transcription. Finally, we demonstrated that the rapid phosphorylation and subsequent degradation of IκB-α induced by TPA was remarkably delayed by Bis knockdown. These results suggest that Bis regulates the invasive ability of glioma cells elicited by TPA, by modulating NF-κB activation, and subsequent induction of MMP-9 mRNA. 
 BAG3 is upregulated by c-Jun and stabilizes JunD. BAG3 plays a regulatory role in a number of cellular processes, including cell proliferation, apoptosis, adhesion and   migration  , epithelial-mesenchymal transition (EMT), autophagy activation, and virus infection. The AP-1 transcription factors are implicated in a variety of important biological processes including cell differentiation, proliferation, apoptosis and oncogenesis. Recently, it has been reported that AP-1 protein c-Jun inhibits autophagy and enhances apoptotic cell death mediated by starvation. However, the molecular mechanisms remain unclear. For the first time, the current study demonstrated that serum starvation downregulated BAG3 at the transcriptional level via c-Jun. In addition, the current study reported that BAG3 stabilized JunD mRNA, which was, at least in part, responsible for the promotion of serum starvation mediated-growth inhibition by BAG3. 
 BAG3 regulates cell proliferation,   migration  , and invasion in human colorectal cancer. Bcl2-associated athanogene 3 (BAG3) has been reported to be elevated in various tumors. However, it is unclear whether BAG3 has a functional role in the initiation and progression of colorectal cancer (CRC). Here, we collected CRC samples and cell lines to validate the pathway by using gene and protein assays. RT-PCR showed that the expression of BAG3 mRNA in CRC tissues was obviously higher than that in non-tumor tissues (p < 0.001). Immunohistochemical analysis showed that immunoreactivity of BAG3 was found in most CRC tissues and strongly correlated with TNM stage (p = 0.001), differentiation (p = 0.003), and metastasis (p = 0.010). Low expression of BAG3 in HCT-8 significantly reduced cellular   prolifer  ation, migration, and invasion. The analysis of in vitro cell showed that HCT-8 cells were exposed to si-BAG3, and its growth was inhibited depending on modulation of cell cycle G1/S checkpoints and cell cycle regulators, involving cyclin D1, cyclin A2, and cyclin B1. Furthermore, suppression of the epithelial-mesenchymal transition (EMT) by si-BAG3 is linked to the decreased expression of E-cadherin and the increased expression of N-cadherin, vimentin, and MMP9. In conclusion, in the present study, we demonstrated that BAG3 overexpression plays a critical role in cell   prolifer  ation, migration, and invasion of colorectal cancer. Our data suggests targeted inhibition of BAG3 may be useful for patients with CRC. 
 BAG3-mediated Mcl-1 stabilization contributes to drug resistance via interaction with USP9X in ovarian cancer. Paclitaxel in combination with carboplatin improves survival among patients with susceptible ovarian cancers, but no strategy has been established against resistant ovarian cancers. BAG3 (Bcl-2-associated athanogene 3) is one of six BAG family proteins, which are involved in such cellular processes as proliferation,   migration   and apoptosis. In addition, expression of BAG3 with Mcl-1, a Bcl-2 family protein, reportedly associates with resistance to chemotherapy. Our aim in this study was to evaluate the functional role of BAG3 and Mcl-1 in ovarian cancer chemoresistance and explore possible new targets for treatment. We found that combined expression of BAG3 and Mcl-1 was significantly associated with a poor prognosis in ovarian cancer patients. In vitro, BAG3 knockdown in ES2 clear ovarian cancer cells significantly increased the efficacy of paclitaxel in combination with the Mcl-1 antagonist MIM1, with or without the Bcl-2 family antagonist ABT737. Moreover, BAG3 was found to positively regulate Mcl-1 levels by binding to and inhibiting USP9X. Our data show that BAG3 and Mcl-1 are key mediators of resistance to chemotherapy in ovarian cancer. In BAG3 knockdown ES2 clear ovarian cancer cells, combination with ABT737 and MIM1 enhanced the efficacy of paclitaxel. These results suggest that inhibiting BAG3 in addition to anti-apoptotic Bcl-2 family proteins may be a useful therapeutic strategy for the treatment of chemoresistant ovarian cancers. 
 BAG3 regulates epithelial-mesenchymal transition and angiogenesis in human hepatocellular carcinoma. Bcl2-associated athanogene 3 (BAG3) protein is a co-chaperone of heat-shock protein (Hsp) 70 and may regulate major physiological and pathophysiological processes. However, few reports have examined the role of BAG3 in human hepatocellular carcinoma (HCC). In this study, we show that BAG3 regulates epithelial-mesenchymal transition (EMT) and angiogenesis in HCC. BAG3 was overexpressed in HCC tissues and cell lines. BAG3 knockdown resulted in reduction in   migration   and invasion of HCC cells, which was linked to reversion of EMT by increasing E-cadherin expression and decreasing N-cadherin, vimentin and slug expression, as well as suppressing matrix metalloproteinase 2 (MMP-2) expression. In a xenograft tumorigenicity model, BAG3 knockdown effectively inhibited tumor growth and metastasis through reduction in CD34 and VEGF expression and reversal of the EMT pathway. In conclusion, BAG3 is associated with the invasiveness and angiogenesis in HCC, and the BAG3 gene may be a novel therapeutic approach against HCC. 
 PKCδ-mediated phosphorylation of BAG3 at Ser187 site induces epithelial-mesenchymal transition and enhances invasiveness in thyroid cancer FRO cells. Protein kinase C delta (PKCδ) is a serine (Ser)/threonine kinase, which regulates numerous cellular processes, including proliferation, differentiation  , migrati  on and apoptosis. In the current study, Chinese hamster ovary cells were transfected with either a constitutively activated PKCδ or a dominant negative PKCδ, phosphoprotein enrichment, two-dimensional difference gel electrophoresis and mass spectrometry was combined to globally identified candidates of PKCδ cascade. We found that Bcl-2 associated athanogene 3 (BAG3) was one of the targets of PKCδ cascade, and BAG3 interacted with PKCδ in vivo. In addition, we clarified that BAG3 was phosphorylate at Ser187 site in a PKCδ-dependent manner in vivo. BAG3 has been implicated in multiple cellular functions, including proliferation, differentiation, apo  ptosis, m  igration, invasion, macroautophagy and so on. We generated wild-type (WT)-, Ser187Ala (S187A)- or Ser187Asp (S187D)-BAG3 stably expressing FRO cells, and noticed that phosphorylation state of BAG3 influenced FRO morphology. Finally, for the first time, we showed that BAG3 was implicated in epithelial-mesenchymal transition (EMT) procedure, and phosphorylation state at Ser187 site had a critical role in EMT regulation by BAG3. Collectively, the current study indicates that BAG3 is a novel substrate of PKCδ, and PKCδ-mediated phosphorylation of BAG3 is implicated in EMT and invasiveness of thyroid cancer cells. 
 Overexpressed BAG3 is a potential therapeutic target in chronic lymphocytic leukemia. Bcl-2-associated athanogene 3 (BAG3), a member of BAG family, is shown to sustain cell survival and underlie resistance to chemotherapy in human neoplastic cells. We aimed to determine the exact role and underlying mechanisms of BAG3 in human chronic lymphocytic leukemia (CLL). One hundred human CLL samples and 20 normal B-cell samples from healthy controls were collected. We measured the BAG3 expression in these cells and explored its relationship with known prognostic factors for CLL. The roles of BAG3 in cell apoptosis and   migration   were evaluated by small interfering RNA-mediated knockdown of BAG3 in primary CLL cells. We showed that BAG3 expression level was increased in CLL cells compared with normal B cells. Moreover, BAG3 expression was particularly upregulated in CD38 positive, unmutated immunoglobulin heavy-chain patients and those with lymphadenopathy and/or splenomegaly. Importantly, patients with increased BAG3 expression level have poor overall survival in subgroups with positive ZAP-70 or those without any "p53 abnormality". In addition, knocking down of BAG3 expression resulted in increased apoptotic ratio and decreased   migration   in primary CLL cells. Our data indicate that BAG3 is a marker of poor prognostic in specific subgroups of CLL patients and may be a potential therapeutic target for this disease. 
 Bis Expression in Patients with Surgically Resected Lung Cancer and its Clinical Significance. Bis, also known as BAG3, has been identified as a Bcl-2-interacting protein that enhances cellular anti-apoptotic activity. It is involved in cellular differentiation, angiogenesis,   migration  , and invasion in various tumors. The purpose of this study was to investigate the Bis expression pattern, and the clinical significance thereof, in patients with resected lung cancer.We studied 121 lung cancer patients who underwent curative surgical resection. Patient clinicopathological characteristics were reviewed retrospectively from medical records, including tumor recurrence and survival. The expression of Bis protein in lung cancer tissues was evaluated by immunohistochemical staining and was assessed using a four-tiered intensity score system (negative, weak, moderate, strong). Enhanced Bis expression at the periphery of a tumor facing the adjacent nontumor region was referred as "marginal activity."Although Bis expression was higher in squamous cell carcinoma than in adenocarcinoma, marginal activity was higher in adenocarcinoma than in squamous cell carcinoma. All of the small cell carcinomas and lung cancer with neuroendocrine differentiation examined were negative for Bis expression. Compared with stage I lung cancer, patients with stage II and IIIA lung cancer exhibited higher Bis protein levels in lung tissues. Recurrence and survival rates did not differ significantly according to Bis expression intensity score or marginal activity.Our study demonstrated that Bis expression differed according to the histological type and pathological stage of the lung cancer. Further studies are needed to assess its use as a biomarker and its role in the molecular pathogenesis of lung cancer. 
 Cellular mechanotransduction relies on tension-induced and chaperone-assisted autophagy. Mechanical tension is an ever-present physiological stimulus essential for the development and homeostasis of locomotory, cardiovascular, respiratory, and urogenital systems. Tension sensing contributes to stem cell differentiation, immune cell recruitment, and tumorigenesis. Yet, how mechanical signals are transduced inside cells remains poorly understood. Here, we identify chaperone-assisted selective autophagy (CASA) as a tension-induced autophagy pathway essential for mechanotransduction in muscle and immune cells. The CASA complex, comprised of the molecular chaperones Hsc70 and HspB8 and the cochaperone BAG3, senses the mechanical unfolding of the actin-crosslinking protein filamin. Together with the chaperone-associated ubiquitin ligase CHIP, the complex initiates the ubiquitin-dependent autophagic sorting of damaged filamin to lysosomes for degradation. Autophagosome formation during CASA depends on an interaction of BAG3 with synaptopodin-2 (SYNPO2). This interaction is mediated by the BAG3 WW domain and facilitates cooperation with an autophagosome membrane fusion complex. BAG3 also utilizes its WW domain to engage in YAP/TAZ signaling. Via this pathway, BAG3 stimulates filamin transcription to maintain actin anchoring and crosslinking under mechanical tension. By integrating tension sensing, autophagosome formation, and transcription regulation during mechanotransduction, the CASA machinery ensures tissue homeostasis and regulates fundamental cellular processes such as adhesion,   migration  , and proliferation. 
 High expression of BAG3 predicts a poor prognosis in human medulloblastoma. Bcl2-associated athanogene 3 (BAG3), a co-chaperone of the heat shock protein (Hsp) 70, regulates various physiological and pathological processes. However, its role in human medulloblastoma has not been clarified. First of all, the expression of BAG3 was examined in formalin-fixed, paraffin-embedded specimens by immunohistochemical staining. And then, the prognostic role of BAG3 was analyzed in 51 medulloblastoma samples. Finally, the roles of BAG3 in the proliferation,   migration  , and invasion of Daoy medulloblastoma cell were investigated using a specific short hairpin RNA (shRNA). The expression of BAG3 in medulloblastoma tissues was higher than nontumorous samples. Furthermore, BAG3 overexpression significantly correlated with poor prognosis of patients with medulloblastoma. The overall survival and tumor-free survival in patients with BAG3 low expression were higher than high expression. Univariate and multivariate analysis showed that BAG3 overexpression was an independent prognostic marker for medulloblastoma. After the BAG3 knockdown, the Daoy cells exhibited decreased the ability to proliferate and form neurosphere. The preliminary mechanism study showed that overexpression of BAG3 might facilitate the cell cycle transition from G1 to S phase by modulating the cyclin-dependent kinase 2 (CDK2) and cyclin E expression. Additionally, we found that BAG3 might enhance the medulloblastoma cell migratory and invasive ability. In summary, BAG3 overexpression may regulate the survival and invasive properties of medulloblastoma and may serve as a potential therapy target for medulloblastoma. 
 The co-chaperone BAG3 interacts with the cytosolic chaperonin CCT: new hints for actin folding. It has been recently hypothesized that BAG3 protein, a co-chaperone of Hsp70/Hsc70, is involved in the regulation of several cell processes, such as apoptosis, autophagy and cell motility. Following the identification of Hsc70/Hsp70, further BAG3 molecular partners such as PLC-gamma and HspB8 were likewise identified, thus contributing to the characterization of the mechanisms and the biological roles carried out by this versatile protein. By using a His-tagged BAG3 protein as bait, we fished out and identified the cytosolic chaperonin CCT, a new unreported BAG3 partner. The interaction between BAG3 and CCT was confirmed and characterized by co-immunoprecipitation experiments and surface plasmon resonance techniques. Furthermore, our analyses showed a slower CCT association and a faster dissociation with a truncated form of BAG3 containing the BAG domain, thus indicating that other protein regions are essential for a high-affinity interaction. ATP or ADP does not seem to significantly influence the chaperonin binding to BAG3 protein. On the other hand, our experiments showed that BAG3 silencing by small interfering RNA slowed down cell   migration   and influence the availability of correctly folded monomeric actin, analyzed by DNAse I binding assays and latrunculin A depolymerization studies. To our knowledge, this is the first report showing a biologically relevant interaction between the chaperonin CCT and BAG3 protein, thus suggesting interesting involvement in the folding processes regulated by CCT. 
 BAG3-dependent noncanonical autophagy induced by proteasome inhibition in HepG2 cells. Emerging lines of evidence have shown that blockade of ubiquitin-proteasome system (UPS) activates autophagy. The molecular players that regulate the relationship between them remain to be elucidated. Bcl-2 associated athanogene 3 (BAG3) is a member of the BAG co-chaperone family that regulates the ATPase activity of heat shock protein 70 (HSP70) chaperone family. Studies on BAG3 have demonstrated that it plays multiple roles in physiological and pathological processes, including antiapoptotic activity, signal transduction, regulatory role in virus infection, cell adhesion and   migration  . Recent studies have attracted much attention on its role in initiation of autophagy. The current study, for the first time, demonstrates that proteasome inhibitors elicit noncanonical autophagy, which was not suppressed by inhibitors of class III phosphatidylinositol 3-kinase (PtdIns3K) or shRNA against Beclin 1 (BECN1). In addition, we demonstrate that BAG3 is ascribed to activation of autophagy elicited by proteasome inhibitors and MAPK8/9/10 (also known as JNK1/2/3 respectively) activation is also implicated via upregulation of BAG3. Moreover, we found that noncanonical autophagy mediated by BAG3 suppresses responsiveness of HepG2 cells to proteasome inhibitors. 
 CAIR-1/BAG-3 modulates cell adhesion and   migration   by downregulating activity of focal adhesion proteins. CAIR-1/BAG-3 is a stress and survival protein that has been shown to bind SH3 domain-containing proteins through its proline-rich (PXXP) domain. Because stress and survival pathways are active during invasion and metastasis, we hypothesized that CAIR-1 is a regulator of signaling pathways that modulate cell adhesion and   migration  . MDA-435 human breast carcinoma cells were stably transfected with full-length CAIR-1 (FL) or a proline-rich domain deleted mutant (dPXXP). FL cells migrated poorly through collagen IV-coated filters to serum (14% of control, p=0.0004), whereas   migration   of dPXXP cells was more robust (228%, p=0.00001). Adhesion to collagen IV-coated surfaces was reduced in FL cells and augmented in dPXXP cells (FL 64%, p=0.03; dPXXP 138%, p=0.01). Rhodamine-phalloidin staining highlighted more stress fibers and thicker filopodial protrusions in dPXXP cells. Fewer focal adhesions were also seen in FL cells. A reduction in tyrosine phosphorylation of focal adhesion kinase (FAK) and paxillin occurred in FL cells under these conditions. In contrast, increased FAK and paxillin phosphorylation was documented in dPXXP cells. Differential FAK phosphorylation occurred at the major autophosphorylation site Y(397) and Src phosphorylation site Y(861). Concordant with these findings, there was decreased interaction between FAK and its downstream partners p(130)Cas and Crk observed in FL cells but not in dPXXP cells. These results collectively indicate that CAIR-1 may negatively regulate adhesion, focal adhesion assembly, signaling, and   migration   via its PXXP domain. 
  GeneRIFs  
 in the present study, we demonstrated that BAG3 overexpression plays a critical role in cell proliferation,   migration  , and invasion of colorectal cancer. Our data suggests targeted inhibition of BAG3 may be useful for patients with colorectal cancer 
 Bis knockdown resulted in a significant decrease in the   migration   and invasion of A172 glioma cells. Furthermore, Bis knockdown notably decreased TPA-induced matrix metalloproteinase-9 (MMP-9) activity and mRNA expression. 
 BAG3 was overexpressed in hepatocellular carcinoma. BAG3 knockdown resulted in reduction in   migration   and invasion of cells, which was linked to reversion of epithelial-mesenchymal transition. 
 Data suggest that BAG3 expression is increased in chronic lymphocytic leukemia (CLL) cells compared with normal B-cells; knocking down BAG3 expression results in increased apoptosis and decreased cell   migration   in primary CLL cells. 
 BAG3 balances autophagic degradation and YAP/TAZ-mediated transcription of filamin in mechanically strained cells and tissues. BAG3 uses its WW domain to associate with SYNPO2 during autophagosome formation or to bind the YAP/TAZ-inhibitors LATS1/2 and AMOTL1/2 during transcription regulation. This dual function is essential for tissue maintenance and for cell adhesion,   migration   and proliferation in mammals. 
 Results indicate that CAIR-1 may negatively regulate adhesion, focal adhesion assembly, signaling, and   migration   via its PXXP domain. 
  [Ee]xtracellular [Mm]atrix  PubMed  
 Involvement of JNK and NF-κB pathways in lipopolysaccharide (LPS)-induced BAG3 expression in human monocytic cells. Lipopolysaccharide (LPS) is an outer-membrane glycolipid component of Gram-negative bacteria known for its fervent ability to activate monocytic cells and for its potent proinflammatory capabilities. Bcl-2-associated athanogene 3 (BAG3) is a survival protein that has been shown to be stimulated during cell response to stressful conditions, such as exposure to high temperature, heavy metals, proteasome inhibition, and human immunodeficiency virus 1 (HIV-1) infection. In addition, BAG3 regulates replication of Varicella-Zoster Virus (VZV) and Herpes Simplex Virus (HSV) replication, suggesting that BAG3 could participate in the host response to infection. In the current study, we found that LPS increased the expression of BAG3 in a dose- and time-dependent manner. Actinomycin D completely blocked the LPS-induced BAG3 accumulation, as well as LPS activated the proximal promoter of BAG3 gene, supported that the induction by LPS occurred at the level of gene transcription. LPS-induced BAG3 expression was blocked by JNK or NF-κB inhibition, suggesting that JNK and NF-κB pathways participated in BAG3 induction by LPS. In addition, we also found that induction of BAG3 was implicated in monocytic cell adhesion   to extracellular mat  rix induced by LPS. Overall, the data support that BAG3 is induced by LPS via JNK and NF-κB-dependent signals, and involved in monocytic c  ell-extracellular ma  trix interaction, suggesting that BAG3 may have a role in the host response to LPS stimulation. 
  GeneRIFs  
 the data support that BAG3 is induced by LPS via JNK and NF-kappaB-dependent signals, and involved in monocytic cell-  extracellular matrix   interaction, suggesting that BAG3 may have a role in the host response to LPS stimulation 
  [Ii]nvasive  PubMed  
 Knockdown of BAG3 induces epithelial-mesenchymal transition in thyroid cancer cells through ZEB1 activation. The process by which epithelial features are lost in favor of a mesenchymal phenotype is referred to as epithelial-mesenchymal transition (EMT). Most carcinomas use this mechanism to evade into neighboring tissues. Reduction or a loss of E-cadherin expression is a well-established hallmark of EMT. As a potent suppressor of E-cadherin, transcription factor ZEB1 is one of the key inducers of EMT, whose expression promotes tumorigenesis and metastasis of carcinomas. Bcl-2-associated athanogene 3 (BAG3) affects multifaceted cellular functions, including proliferation, apoptosis, cell adhesion and invasion, viral infection, and autophagy. Recently, we have reported a novel role of BAG3 implicated in EMT, while the mechanisms are poorly elucidated. The current study demonstrated that knockdown of BAG3 induced EMT, and increased cell migratory and   invasive  ness in thyroid cancer cells via transcriptional activation of ZEB1. We also found that BAG3 knockdown led to nuclear accumulation of β-catenin, which was responsible for the transcriptional activation of ZEB1. These results indicate BAG3 as a regulator of ZEB1 expression in EMT and as a regulator of metastasis in thyroid cancer cells, providing potential targets to prevent and/or treat thyroid cancer cell invasion and metastasis. 
 Down-modulation of Bis reduces the   invasive   ability of glioma cells induced by TPA, through NF-κB mediated activation of MMP-9. Bcl-2 interacting cell death suppressor (Bis) has been shown to have anti-apoptotic and anti-stress functions. Recently, increased Bis expression was reported to correlate with glioma aggressiveness. Here, we investigated the effect of Bis knockdown on the acquisition of the   invasiv  e phenotype of A172 glioma cells, induced by 12-O-Tetradecanoylphorbol-3-acetate (TPA), using a Transwell assay. Bis knockdown resulted in a significant decrease in the migration and invasion of A172 cells. Furthermore, Bis knockdown notably decreased TPA-induced matrix metalloproteinase-9 (MMP-9) activity and mRNA expression, as measured by zymography and quantitative real time PCR, respectively. A luciferase reporter assay indicated that Bis suppression significantly down-regulated NF-κB-driven transcription. Finally, we demonstrated that the rapid phosphorylation and subsequent degradation of IκB-α induced by TPA was remarkably delayed by Bis knockdown. These results suggest that Bis regulates   the inva  sive ability of glioma cells elicited by TPA, by modulating NF-κB activation, and subsequent induction of MMP-9 mRNA. 
 Knockdown of BAG3 induces epithelial-mesenchymal transition in thyroid cancer cells through ZEB1 activation. The process by which epithelial features are lost in favor of a mesenchymal phenotype is referred to as epithelial-mesenchymal transition (EMT). Most carcinomas use this mechanism to evade into neighboring tissues. Reduction or a loss of E-cadherin expression is a well-established hallmark of EMT. As a potent suppressor of E-cadherin, transcription factor ZEB1 is one of the key inducers of EMT, whose expression promotes tumorigenesis and metastasis of carcinomas. Bcl-2-associated athanogene 3 (BAG3) affects multifaceted cellular functions, including proliferation, apoptosis, cell adhesion and invasion, viral infection, and autophagy. Recently, we have reported a novel role of BAG3 implicated in EMT, while the mechanisms are poorly elucidated. The current study demonstrated that knockdown of BAG3 induced EMT, and increased cell migratory and   invasive  ness in thyroid cancer cells via transcriptional activation of ZEB1. We also found that BAG3 knockdown led to nuclear accumulation of β-catenin, which was responsible for the transcriptional activation of ZEB1. These results indicate BAG3 as a regulator of ZEB1 expression in EMT and as a regulator of metastasis in thyroid cancer cells, providing potential targets to prevent and/or treat thyroid cancer cell invasion and metastasis. 
 Down-modulation of Bis reduces the   invasive   ability of glioma cells induced by TPA, through NF-κB mediated activation of MMP-9. Bcl-2 interacting cell death suppressor (Bis) has been shown to have anti-apoptotic and anti-stress functions. Recently, increased Bis expression was reported to correlate with glioma aggressiveness. Here, we investigated the effect of Bis knockdown on the acquisition of the   invasiv  e phenotype of A172 glioma cells, induced by 12-O-Tetradecanoylphorbol-3-acetate (TPA), using a Transwell assay. Bis knockdown resulted in a significant decrease in the migration and invasion of A172 cells. Furthermore, Bis knockdown notably decreased TPA-induced matrix metalloproteinase-9 (MMP-9) activity and mRNA expression, as measured by zymography and quantitative real time PCR, respectively. A luciferase reporter assay indicated that Bis suppression significantly down-regulated NF-κB-driven transcription. Finally, we demonstrated that the rapid phosphorylation and subsequent degradation of IκB-α induced by TPA was remarkably delayed by Bis knockdown. These results suggest that Bis regulates   the inva  sive ability of glioma cells elicited by TPA, by modulating NF-κB activation, and subsequent induction of MMP-9 mRNA. 
 BAG3 increases the   invasive  ness of uterine corpus carcinoma cells by suppressing miR-29b and enhancing MMP2 expression. Approximately 30% of uterine corpus carcinomas are diagnosed at an advanced stage and have a poor prognosis. Our previous study indicated that BCL2-associated athanogene 3 (BAG3) enhances matrix metalloproteinase-2 (MMP2) expression and binds to MMP2 to positively regulate the process of cell invasion in ovarian cancer cells. Recently, altered miRNA expression patterns were observed in several groups of patients with endometrial cancers. One of the altered miRNAs, miR-29b, reportedly reduces tumor   invasive  ness by suppressing MMP2 expression. Our aim in the present study was to examine the relationships among BAG3, miR-29b and MMP2 in endometrioid adenocarcinoma cells. We found that BAG3 suppresses miR-29b expression and enhances MMP2 expression, which in turn increases cell motility and   invasive  ness. Moreover, restoration of miR-29b through BAG3 knockdown reduced MMP2 expression, as well as cell motility and   invasive  ness. Collectively, our findings indicate that BAG3 enhances MMP2 expression by suppressing miR-29b, thereby increasing the metastatic potential of endometrioid adenocarcinomas. 
 BAG3 (BCL2-associated athanogene 3) interacts with MMP-2 to positively regulate invasion by ovarian carcinoma cells. BAG3 (BCL2-associated athanogene 3) is a member of the BAG family proteins, which interact with and regulate Hsp70. Our aim in the present study was to correlate BAG3 expression in ovarian cancer with invasion and progression-free survival. We found that BAG3 interacts with MMP2 in cultured ovarian cancer cells, and that knockdown of BAG3 expression downregulated MMP2 expression and diminished cell motility and   invasive  ness. The incidence of BAG3 positivity was significantly higher at advanced clinical stages of ovarian cancer than at early stages. We suggest that BAG3 binds to MMP2 to positively regulate the process of cell invasion. 
 BAG3 regulates epithelial-mesenchymal transition and angiogenesis in human hepatocellular carcinoma. Bcl2-associated athanogene 3 (BAG3) protein is a co-chaperone of heat-shock protein (Hsp) 70 and may regulate major physiological and pathophysiological processes. However, few reports have examined the role of BAG3 in human hepatocellular carcinoma (HCC). In this study, we show that BAG3 regulates epithelial-mesenchymal transition (EMT) and angiogenesis in HCC. BAG3 was overexpressed in HCC tissues and cell lines. BAG3 knockdown resulted in reduction in migration and invasion of HCC cells, which was linked to reversion of EMT by increasing E-cadherin expression and decreasing N-cadherin, vimentin and slug expression, as well as suppressing matrix metalloproteinase 2 (MMP-2) expression. In a xenograft tumorigenicity model, BAG3 knockdown effectively inhibited tumor growth and metastasis through reduction in CD34 and VEGF expression and reversal of the EMT pathway. In conclusion, BAG3 is associated with the   invasive  ness and angiogenesis in HCC, and the BAG3 gene may be a novel therapeutic approach against HCC. 
 PKCδ-mediated phosphorylation of BAG3 at Ser187 site induces epithelial-mesenchymal transition and enhances   invasiv  eness in thyroid cancer FRO cells. Protein kinase C delta (PKCδ) is a serine (Ser)/threonine kinase, which regulates numerous cellular processes, including proliferation, differentiation, migration and apoptosis. In the current study, Chinese hamster ovary cells were transfected with either a constitutively activated PKCδ or a dominant negative PKCδ, phosphoprotein enrichment, two-dimensional difference gel electrophoresis and mass spectrometry was combined to globally identified candidates of PKCδ cascade. We found that Bcl-2 associated athanogene 3 (BAG3) was one of the targets of PKCδ cascade, and BAG3 interacted with PKCδ in vivo. In addition, we clarified that BAG3 was phosphorylate at Ser187 site in a PKCδ-dependent manner in vivo. BAG3 has been implicated in multiple cellular functions, including proliferation, differentiation, apoptosis, migration, invasion, macroautophagy and so on. We generated wild-type (WT)-, Ser187Ala (S187A)- or Ser187Asp (S187D)-BAG3 stably expressing FRO cells, and noticed that phosphorylation state of BAG3 influenced FRO morphology. Finally, for the first time, we showed that BAG3 was implicated in epithelial-mesenchymal transition (EMT) procedure, and phosphorylation state at Ser187 site had a critical role in EMT regulation by BAG3. Collectively, the current study indicates that BAG3 is a novel substrate of PKCδ, and PKCδ-mediated phosphorylation of BAG3 is implicated i  n EMT an  d invasiveness of thyroid cancer cells. 
 High expression of BAG3 predicts a poor prognosis in human medulloblastoma. Bcl2-associated athanogene 3 (BAG3), a co-chaperone of the heat shock protein (Hsp) 70, regulates various physiological and pathological processes. However, its role in human medulloblastoma has not been clarified. First of all, the expression of BAG3 was examined in formalin-fixed, paraffin-embedded specimens by immunohistochemical staining. And then, the prognostic role of BAG3 was analyzed in 51 medulloblastoma samples. Finally, the roles of BAG3 in the proliferation, migration, and invasion of Daoy medulloblastoma cell were investigated using a specific short hairpin RNA (shRNA). The expression of BAG3 in medulloblastoma tissues was higher than nontumorous samples. Furthermore, BAG3 overexpression significantly correlated with poor prognosis of patients with medulloblastoma. The overall survival and tumor-free survival in patients with BAG3 low expression were higher than high expression. Univariate and multivariate analysis showed that BAG3 overexpression was an independent prognostic marker for medulloblastoma. After the BAG3 knockdown, the Daoy cells exhibited decreased the ability to proliferate and form neurosphere. The preliminary mechanism study showed that overexpression of BAG3 might facilitate the cell cycle transition from G1 to S phase by modulating the cyclin-dependent kinase 2 (CDK2) and cyclin E expression. Additionally, we found that BAG3 might enhance the medulloblastoma cell migratory and   invasive   ability. In summary, BAG3 overexpression may regulate the survival and   invasive   properties of medulloblastoma and may serve as a potential therapy target for medulloblastoma. 
 BAG3 protein delocalisation in prostate carcinoma. Despite the progressive increase of early diagnosis, a subset of prostate cancers show a metastasizing and lethal course, not always predictable upon the traditional prognostic parameters. The object of this study was to investigate the role of the survival co-chaperone protein BAG3 as a new prognostic marker for prostate cancer. BAG3 was detected by immunohistochemistry in 55 specimens of surgically removed prostate carcinomas and in 15 surgical specimens of non-neoplastic prostate tissues. Results were compared with clinic-pathological data and outcome of patients and statistically evaluated. BAG3 resulted expressed in all the cases: Non-neoplastic prostate tissue showed a cytoplasmatic staining with apical reinforcement, a finding which appears consistent with the reported connection of the protein with the membrane focal cell-adhesion complexes. In prostate carcinomas, BAG3 showed a progressive decrease of the expression level from well- to low-differentiated carcinoma, coupled with the loss of polarisation of the signal in metastasizing cases. These results indicate that BAG3 intra-cytoplasmic delocalisation is a specific feature of cancer versus non-neoplastic prostate and a candidate new marker for prediction of prostate cancer   invasive  ness and behaviour. 
 The anti-apoptotic BAG3 protein is expressed in lung carcinomas and regulates small cell lung carcinoma (SCLC) tumor growth. BAG3, member the HSP70 co-chaperones family, has been shown to play a relevant role in the survival, growth and   invasive  ness of different tumor types. In this study, we investigate the expression of BAG3 in 66 specimens from different lung tumors and the role of this protein in small cell lung cancer (SCLC) tumor growth. Normal lung tissue did not express BAG3 while we detected the expression of BAG3 by immunohistochemistry in all the 13 squamous cell carcinomas, 13 adenocarcinomas and 4 large cell carcinomas. Furthermore, we detected BAG3 expression in 22 of the 36 SCLCs analyzed. The role on SCLC cell survival was determined by down-regulating BAG3 levels in two human SCLC cell lines, i.e. H69 and H446, in vitro and measuring cisplatin induced apoptosis. Indeed down-regulation of BAG3 determines increased cell death and sensitizes cells to cisplatin treatment. The effect of BAG3 down-regulation on tumor growth was also investigated in an in vivo xenograft model by treating mice with an adenovirus expressing a specific bag3 siRNA. Treatment with bag3 siRNA-Ad significantly reduced tumor growth and improved animal survival. In conclusion we show that a subset of SCLCs over express BAG3 that exerts an anti-apoptotic effect resulting in resistance to chemotherapy. 
  GeneRIFs  
 BAG3 is a novel substrate of PKCdelta, and PKCdelta-mediated phosphorylation of BAG3 is implicated in EMT and   invasive  ness of thyroid cancer cells. 
 BAG3 intra-cytoplasmic delocalisation is a specific feature of cancer versus non-neoplastic prostate and a candidate new marker for prediction of prostate cancer   invasive  ness and behaviour. 
  [Mm]etastasis  PubMed  
 Knockdown of BAG3 induces epithelial-mesenchymal transition in thyroid cancer cells through ZEB1 activation. The process by which epithelial features are lost in favor of a mesenchymal phenotype is referred to as epithelial-mesenchymal transition (EMT). Most carcinomas use this mechanism to evade into neighboring tissues. Reduction or a loss of E-cadherin expression is a well-established hallmark of EMT. As a potent suppressor of E-cadherin, transcription factor ZEB1 is one of the key inducers of EMT, whose expression promotes tumorigenesis and   metastasis   of carcinomas. Bcl-2-associated athanogene 3 (BAG3) affects multifaceted cellular functions, including proliferation, apoptosis, cell adhesion and invasion, viral infection, and autophagy. Recently, we have reported a novel role of BAG3 implicated in EMT, while the mechanisms are poorly elucidated. The current study demonstrated that knockdown of BAG3 induced EMT, and increased cell migratory and invasiveness in thyroid cancer cells via transcriptional activation of ZEB1. We also found that BAG3 knockdown led to nuclear accumulation of β-catenin, which was responsible for the transcriptional activation of ZEB1. These results indicate BAG3 as a regulator of ZEB1 expression in EMT and as a regulator of   metastasi  s in thyroid cancer cells, providing potential targets to prevent and/or treat thyroid cancer cell invasion and   metastasi  s. 
 Knockdown of BAG3 induces epithelial-mesenchymal transition in thyroid cancer cells through ZEB1 activation. The process by which epithelial features are lost in favor of a mesenchymal phenotype is referred to as epithelial-mesenchymal transition (EMT). Most carcinomas use this mechanism to evade into neighboring tissues. Reduction or a loss of E-cadherin expression is a well-established hallmark of EMT. As a potent suppressor of E-cadherin, transcription factor ZEB1 is one of the key inducers of EMT, whose expression promotes tumorigenesis and   metastasis   of carcinomas. Bcl-2-associated athanogene 3 (BAG3) affects multifaceted cellular functions, including proliferation, apoptosis, cell adhesion and invasion, viral infection, and autophagy. Recently, we have reported a novel role of BAG3 implicated in EMT, while the mechanisms are poorly elucidated. The current study demonstrated that knockdown of BAG3 induced EMT, and increased cell migratory and invasiveness in thyroid cancer cells via transcriptional activation of ZEB1. We also found that BAG3 knockdown led to nuclear accumulation of β-catenin, which was responsible for the transcriptional activation of ZEB1. These results indicate BAG3 as a regulator of ZEB1 expression in EMT and as a regulator of   metastasi  s in thyroid cancer cells, providing potential targets to prevent and/or treat thyroid cancer cell invasion and   metastasi  s. 
 BAG3 regulates cell proliferation, migration, and invasion in human colorectal cancer. Bcl2-associated athanogene 3 (BAG3) has been reported to be elevated in various tumors. However, it is unclear whether BAG3 has a functional role in the initiation and progression of colorectal cancer (CRC). Here, we collected CRC samples and cell lines to validate the pathway by using gene and protein assays. RT-PCR showed that the expression of BAG3 mRNA in CRC tissues was obviously higher than that in non-tumor tissues (p < 0.001). Immunohistochemical analysis showed that immunoreactivity of BAG3 was found in most CRC tissues and strongly correlated with TNM stage (p = 0.001), differentiation (p =   0.003), an  d metastasis (p = 0.010). Low expression of BAG3 in HCT-8 significantly reduced cellular proliferation, migration, and invasion. The analysis of in vitro cell showed that HCT-8 cells were exposed to si-BAG3, and its growth was inhibited depending on modulation of cell cycle G1/S checkpoints and cell cycle regulators, involving cyclin D1, cyclin A2, and cyclin B1. Furthermore, suppression of the epithelial-mesenchymal transition (EMT) by si-BAG3 is linked to the decreased expression of E-cadherin and the increased expression of N-cadherin, vimentin, and MMP9. In conclusion, in the present study, we demonstrated that BAG3 overexpression plays a critical role in cell proliferation, migration, and invasion of colorectal cancer. Our data suggests targeted inhibition of BAG3 may be useful for patients with CRC. 
 BAG3 regulates epithelial-mesenchymal transition and angiogenesis in human hepatocellular carcinoma. Bcl2-associated athanogene 3 (BAG3) protein is a co-chaperone of heat-shock protein (Hsp) 70 and may regulate major physiological and pathophysiological processes. However, few reports have examined the role of BAG3 in human hepatocellular carcinoma (HCC). In this study, we show that BAG3 regulates epithelial-mesenchymal transition (EMT) and angiogenesis in HCC. BAG3 was overexpressed in HCC tissues and cell lines. BAG3 knockdown resulted in reduction in migration and invasion of HCC cells, which was linked to reversion of EMT by increasing E-cadherin expression and decreasing N-cadherin, vimentin and slug expression, as well as suppressing matrix metalloproteinase 2 (MMP-2) expression. In a xenograft tumorigenicity model, BAG3 knockdown effectively inhibited tumor growth and   metastasis   through reduction in CD34 and VEGF expression and reversal of the EMT pathway. In conclusion, BAG3 is associated with the invasiveness and angiogenesis in HCC, and the BAG3 gene may be a novel therapeutic approach against HCC. 
 BAG3 promotes pancreatic ductal adenocarcinoma growth by activating stromal macrophages. The incidence and death rate of pancreatic ductal adenocarcinoma (PDAC) have increased in recent years, therefore the identification of novel targets for treatment is extremely important. Interactions between cancer and stromal cells are critically involved in tumour formation and development of   metastasis  . Here we report that PDAC cells secrete BAG3, which binds and activates macrophages, inducing their activation and the secretion of PDAC supporting factors. We also identify IFITM-2 as a BAG3 receptor and show that it signals through PI3K and the p38 MAPK pathways. Finally, we show that the use of an anti-BAG3 antibody results in reduced tumour growth and prevents   metastasis   formation in three different mouse models. In conclusion, we identify a paracrine loop involved in PDAC growth and metastatic spreading, and show that an anti-BAG3 antibody has therapeutic potential. 
 Bag-3 expression is involved in pathogenesis and progression of colorectal carcinomas. Bcl-2-associated athanogene 3 (Bag-3) belongs to a member of the Hsc70 binding co-chaperone Bag-family proteins and has critical roles in protein homeostasis, cell survival, actin organization, cell adhesion, cell motility and tumor   metastasis  . To clarify the role of Bag-3 in colorectal carcinogenesis and subsequent development, its expression was examined by immunohistochemistry (IHC) and in situ hybridization (ISH) on tissue microarrays containing colorectal carcinomas, adenomas, non-neoplastic mucosa (NNM) and metastatic carcinomas in lymph node and liver. Colorectal carcinoma tissue and cell lines were studied for Bag-3 expression by RT-PCR, Western blot and immunofluorescence. The results demonstrated that Bag3 was distinctly expressed in Colo201, Colo205, DLD-1, HCT-15, HCT-116, HT-29, KM-12, SW480, SW620, and WiDr at both mRNA and protein levels. Carcinoma showed stronger Bag-3 expression than adjacent NNM by IHC and Western blot (P<0.05), while its mRNA had the opposite by real-time PCR and ISH (P<0.05). Metastatic carcinoma more frequently expressed Bag-3 mRNA in lymph node and liver than in primary carcinoma (P<0.05). Immunohistochemically, Bag-3 expression was seen to gradually decrease from carcinoma, adenoma to NNM (P<0.05). There was a positive correlation between Bag-3 expression and TNM staging and GRP94 expression (P<0.05), but no relationship to patient age or sex, tumor size, depth of invasion, lymphatic or venous invasion, lymph node   metastasis  , differentiation or prognosis of colorectal carcinomas (P<0.05). Our study indicated that aberrant Bag-3 expression might be involved in colorectal adenoma-adenocarcinoma sequence and subsequent progression. 
 Bcl2-associated athanogene 3 interactome analysis reveals a new role in modulating proteasome activity. Bcl2-associated athanogene 3 (BAG3), a member of the BAG family of co-chaperones, plays a critical role in regulating apoptosis, development, cell motility, autophagy, and tumor   metastasis   and in mediating cell adaptive responses to stressful stimuli. BAG3 carries a BAG domain, a WW domain, and a proline-rich repeat (PXXP), all of which mediate binding to different partners. To elucidate BAG3's interaction network at the molecular level, we employed quantitative immunoprecipitation combined with knockdown and human proteome microarrays to comprehensively profile the BAG3 interactome in humans. We identified a total of 382 BAG3-interacting proteins with diverse functions, including transferase activity, nucleic acid binding, transcription factors, proteases, and chaperones, suggesting that BAG3 is a critical regulator of diverse cellular functions. In addition, we characterized interactions between BAG3 and some of its newly identified partners in greater detail. In particular, bioinformatic analysis revealed that the BAG3 interactome is strongly enriched in proteins functioning within the proteasome-ubiquitination process and that compose the proteasome complex itself, suggesting that a critical biological function of BAG3 is associated with the proteasome. Functional studies demonstrated that BAG3 indeed interacts with the proteasome and modulates its activity, sustaining cell survival and underlying resistance to therapy through the down-modulation of apoptosis. Taken as a whole, this study expands our knowledge of the BAG3 interactome, provides a valuable resource for understanding how BAG3 affects different cellular functions, and demonstrates that biologically relevant data can be harvested using this kind of integrated approach. 
 BAG3 regulates motility and adhesion of epithelial cancer cells. BAG3 protein binds to and regulates Hsp70 chaperone activity. The BAG3 protein contains a WW domain and a proline-rich region with SH3-binding motifs, suggesting that it may interact with proteins relevant to signal transduction, recruiting Hsp70 to signaling complexes and altering cell responses. BAG3 overexpression has been observed in human cancers. We show here that homozygous BAG3-deficient mouse embryonic fibroblasts (MEF) exhibit delayed formation of filopodia and focal adhesion complexes when freshly plated. BAG3-deficient MEFs show reduced cell motility in culture. We observed that endogenous BAG3 protein is highly expressed in many human epithelial cancer cell lines, especially adenocarcinomas. Gene transfer-mediated overexpression of BAG3 increased motility of Cos7 cell and several human cancer cell lines, including breast cancer MCF7 and prostate cancer DU145 and ALVA31 cell lines. Conversely, reduction of BAG3 protein by RNA interference (RNAi) decreased cell motility in four of four epithelial tumor lines tested. We observed an influence of BAG3 on cell adhesion in culture. In Cos7 kidney epithelial cells, BAG3 protein partially colocalizes with actin at the leading edge of migrating cells, wherein active actin polymerization and nucleation occur. RNAi-mediated reductions in BAG3 expression were associated with decreased Rac1 activity, suggesting a role for BAG3 in regulating this small GTPase involved in actin-cytoskeleton dynamics. In mice, RNAi-mediated reductions in BAG3 in a human tumor xenograft suppressed invasion and   metastasis   in vivo. Thus, the high levels of BAG3 protein seen in some epithelial cancer cell lines may be relevant to mechanisms of tumor invasion and   metastasis  . 
 BAG3 directly associates with guanine nucleotide exchange factor of Rap1, PDZGEF2, and regulates cell adhesion. BAG3, a member of the Hsc70 binding co-chaperone BAG-family proteins, has critical roles in regulating actin organization, cell adhesion, cell motility and tumor   metastasis  . The PDZ domain containing guanine nucleotide exchange factor 2 (PDZGEF2) was cloned as a BAG3-interacting protein. PDZGEF2 induces activation of Rap1 and increases integrin-mediated cell adhesion. The PPDY motif at the C-terminus of PDZGEF2 binds to the WW domain of BAG3 in vitro and in vivo. BAG3 deletion mutant lacking the WW domain lose its cell adhesion and motility activity. Gene knockdown of PDZGEF2 leads to the loss of cell adhesion on fibronectin-coated plates while BAG3 overexpression increases cell adhesion in Cos7 cells, but not in PDZGEF2 gene knockdown cells indicating that PDZGEF2 is a critical partner for BAG3 in regulating cell adhesion. 
 CAIR-1/BAG-3 modulates cell adhesion and migration by downregulating activity of focal adhesion proteins. CAIR-1/BAG-3 is a stress and survival protein that has been shown to bind SH3 domain-containing proteins through its proline-rich (PXXP) domain. Because stress and survival pathways are active during invasion and   metastasis  , we hypothesized that CAIR-1 is a regulator of signaling pathways that modulate cell adhesion and migration. MDA-435 human breast carcinoma cells were stably transfected with full-length CAIR-1 (FL) or a proline-rich domain deleted mutant (dPXXP). FL cells migrated poorly through collagen IV-coated filters to serum (14% of control, p=0.0004), whereas migration of dPXXP cells was more robust (228%, p=0.00001). Adhesion to collagen IV-coated surfaces was reduced in FL cells and augmented in dPXXP cells (FL 64%, p=0.03; dPXXP 138%, p=0.01). Rhodamine-phalloidin staining highlighted more stress fibers and thicker filopodial protrusions in dPXXP cells. Fewer focal adhesions were also seen in FL cells. A reduction in tyrosine phosphorylation of focal adhesion kinase (FAK) and paxillin occurred in FL cells under these conditions. In contrast, increased FAK and paxillin phosphorylation was documented in dPXXP cells. Differential FAK phosphorylation occurred at the major autophosphorylation site Y(397) and Src phosphorylation site Y(861). Concordant with these findings, there was decreased interaction between FAK and its downstream partners p(130)Cas and Crk observed in FL cells but not in dPXXP cells. These results collectively indicate that CAIR-1 may negatively regulate adhesion, focal adhesion assembly, signaling, and migration via its PXXP domain. 
  GeneRIFs  
 These results indicate BAG3 as a regulator of ZEB1 expression in epithelial-mesenchymal transition and as a regulator of   metastasis   in thyroid cancer cells. 
 High levels of BAG3 protein seen in some epithelial cancer cell lines may be relevant to mechanisms of tumor invasion and   metastasis  . 
  [Cc]ell [Ii]nvasion  PubMed  
 Knockdown of BAG3 induces epithelial-mesenchymal transition in thyroid cancer cells through ZEB1 activation. The process by which epithelial features are lost in favor of a mesenchymal phenotype is referred to as epithelial-mesenchymal transition (EMT). Most carcinomas use this mechanism to evade into neighboring tissues. Reduction or a loss of E-cadherin expression is a well-established hallmark of EMT. As a potent suppressor of E-cadherin, transcription factor ZEB1 is one of the key inducers of EMT, whose expression promotes tumorigenesis and metastasis of carcinomas. Bcl-2-associated athanogene 3 (BAG3) affects multifaceted cellular functions, including proliferation, apoptosis, cell adhesion and invasion, viral infection, and autophagy. Recently, we have reported a novel role of BAG3 implicated in EMT, while the mechanisms are poorly elucidated. The current study demonstrated that knockdown of BAG3 induced EMT, and increased cell migratory and invasiveness in thyroid cancer cells via transcriptional activation of ZEB1. We also found that BAG3 knockdown led to nuclear accumulation of β-catenin, which was responsible for the transcriptional activation of ZEB1. These results indicate BAG3 as a regulator of ZEB1 expression in EMT and as a regulator of metastasis in thyroid cancer cells, providing potential targets to prevent and/or treat thyroid cancer   cell invasio  n and metastasis. 
 Knockdown of BAG3 induces epithelial-mesenchymal transition in thyroid cancer cells through ZEB1 activation. The process by which epithelial features are lost in favor of a mesenchymal phenotype is referred to as epithelial-mesenchymal transition (EMT). Most carcinomas use this mechanism to evade into neighboring tissues. Reduction or a loss of E-cadherin expression is a well-established hallmark of EMT. As a potent suppressor of E-cadherin, transcription factor ZEB1 is one of the key inducers of EMT, whose expression promotes tumorigenesis and metastasis of carcinomas. Bcl-2-associated athanogene 3 (BAG3) affects multifaceted cellular functions, including proliferation, apoptosis, cell adhesion and invasion, viral infection, and autophagy. Recently, we have reported a novel role of BAG3 implicated in EMT, while the mechanisms are poorly elucidated. The current study demonstrated that knockdown of BAG3 induced EMT, and increased cell migratory and invasiveness in thyroid cancer cells via transcriptional activation of ZEB1. We also found that BAG3 knockdown led to nuclear accumulation of β-catenin, which was responsible for the transcriptional activation of ZEB1. These results indicate BAG3 as a regulator of ZEB1 expression in EMT and as a regulator of metastasis in thyroid cancer cells, providing potential targets to prevent and/or treat thyroid cancer   cell invasio  n and metastasis. 
 BAG3 increases the invasiveness of uterine corpus carcinoma cells by suppressing miR-29b and enhancing MMP2 expression. Approximately 30% of uterine corpus carcinomas are diagnosed at an advanced stage and have a poor prognosis. Our previous study indicated that BCL2-associated athanogene 3 (BAG3) enhances matrix metalloproteinase-2 (MMP2) expression and binds to MMP2 to positively regulate the process of   cell invasion   in ovarian cancer cells. Recently, altered miRNA expression patterns were observed in several groups of patients with endometrial cancers. One of the altered miRNAs, miR-29b, reportedly reduces tumor invasiveness by suppressing MMP2 expression. Our aim in the present study was to examine the relationships among BAG3, miR-29b and MMP2 in endometrioid adenocarcinoma cells. We found that BAG3 suppresses miR-29b expression and enhances MMP2 expression, which in turn increases cell motility and invasiveness. Moreover, restoration of miR-29b through BAG3 knockdown reduced MMP2 expression, as well as cell motility and invasiveness. Collectively, our findings indicate that BAG3 enhances MMP2 expression by suppressing miR-29b, thereby increasing the metastatic potential of endometrioid adenocarcinomas. 
 BAG3 (BCL2-associated athanogene 3) interacts with MMP-2 to positively regulate invasion by ovarian carcinoma cells. BAG3 (BCL2-associated athanogene 3) is a member of the BAG family proteins, which interact with and regulate Hsp70. Our aim in the present study was to correlate BAG3 expression in ovarian cancer with invasion and progression-free survival. We found that BAG3 interacts with MMP2 in cultured ovarian cancer cells, and that knockdown of BAG3 expression downregulated MMP2 expression and diminished cell motility and invasiveness. The incidence of BAG3 positivity was significantly higher at advanced clinical stages of ovarian cancer than at early stages. We suggest that BAG3 binds to MMP2 to positively regulate the process of   cell invasion  . 
  GeneRIFs  
 The incidence of BAG3 positivity was significantly higher at advanced clinical stages of ovarian cancer than at early stages. It is suggected BAG3 binds to MMP2 to positively regulate the process of   cell invasion  . 
   
  
 SERPINI1 
 
  NCBI Gene  
  GeneRIF  
  Pubmed  
 
 
  Search Term  Rank  Fields  
  [Mm]igration  6  PubMed(1)  
  [Mm]etastasis  12  PubMed(1)  
 
 
 hide/show details for SERPINI1 
  [Mm]igration  PubMed  
 Neuroserpin regulates neurite outgrowth in nerve growth factor-treated PC12 cells. Neuroserpin is a serine protease inhibitor widely expressed in the developing and adult nervous systems and implicated in the regulation of proteases involved in processes such as synaptic plasticity, neuronal   migration   and axogenesis. We have analysed the effect of neuroserpin on growth factor-induced neurite outgrowth in PC12 cells. We show that small changes in neuroserpin expression result in changes to the number of cells extending neurites and total neurite length following NGF treatment. Increased expression of neuroserpin resulted in a decrease in the number of cells extending neurites and a reduction in total free neurite length whereas reduced levels of neuroserpin led to a small increase in the number of neurite extending cells and a significant increase in total free neurite length compared to the parent cell line. Neuroserpin also altered the response of PC12 cells to bFGF and EGF treatment. Neuroserpin was localised to dense cored secretory vesicles in PC12 cells but was unable to complex with its likely enzyme target, tissue plasminogen activator at the acidic pH found in these vesicles. These data suggest that modulation of neuroserpin levels at the extending neurite growth cone may play an important role in regulating axonal growth. 
  [Mm]etastasis  PubMed  
 Update of the human and mouse SERPIN gene superfamily. The serpin family comprises a structurally similar, yet functionally diverse, set of proteins. Named originally for their function as serine proteinase inhibitors, many of its members are not inhibitors but rather chaperones, involved in storage, transport, and other roles. Serpins are found in genomes of all kingdoms, with 36 human protein-coding genes and five pseudogenes. The mouse has 60 Serpin functional genes, many of which are orthologous to human SERPIN genes and some of which have expanded into multiple paralogous genes. Serpins are found in tissues throughout the body; whereas most are extracellular, there is a class of intracellular serpins. Serpins appear to have roles in inflammation, immune function, tumorigenesis, blood clotting, dementia, and cancer   metastasis  . Further characterization of these proteins will likely reveal potential biomarkers and therapeutic targets for disease. 
   
  
 RAB11FIP4 
 
  NCBI Gene  
  GeneRIF  
  Pubmed  
 
 
  Search Term  Rank  Fields  
  [Mm]igration  6  PubMed(1)  
  [Ii]nvasive  10  PubMed(2)  
  [Mm]etastasis  12  PubMed(1); GeneRIFs(1)  
 
 
 hide/show details for RAB11FIP4 
  [Mm]igration  PubMed  
 Hypoxia upregulates Rab11-family interacting protein 4 through HIF-1α to promote the metastasis of hepatocellular carcinoma. Hypoxic microenvironment is a powerful driving force for the invasion and metastasis of hepatocellular carcinoma (HCC). Hypoxia-inducible factor 1α (HIF-1α), as a crucial regulator of transcriptional responses to hypoxia, induces the expression of multiple target genes involved in different steps of HCC metastatic process. It is critical to find target genes associated with metastasis under hypoxia for shedding new light on molecular mechanism of HCC metastasis. In this study, we uncovered that hypoxia could induce the upregulation of Rab11-family interacting protein 4 (Rab11-FIP4) and activation of Rab11-FIP4 promoter by HIF-1α. The overexpression of Rab11-FIP4 significantly enhanced the mobility and invasiveness of HCC cells in vitro, also contributed to distant lung metastasis in vivo, whereas silencing of Rab11-FIP4 decreased the ability   of migra  tion and invasion in HCC cells in vitro and suppressed lung metastasis in vivo. Rab11-FIP4 facilitated HCC metastasis through the phosphorylation of PRAS40, which was regulated by mTOR. Furthermore, the expression level of Rab11-FIP4 was significantly increased in HCC tissues and high expression of Rab11-FIP4 was closely correlated with vascular invasion and poor prognosis in HCC patients. A markedly positive correlation between the expression of Rab11-FIP4 and HIF-1α was observed in HCC tissues and combination of Rab11-FIP4 and HIF-1α was a more valuable predictor of poor prognosis for HCC patients. In conclusion, Rab11-FIP4 is a target gene of HIF-1α and has a pro-metastatic role in HCC, suggesting that Rab11-FIP4 may be a promising candidate target for HCC treatment. 
  [Ii]nvasive  PubMed  
 Comparison of tear protein levels in breast cancer patients and healthy controls using a de novo proteomic approach. Non  invasive   biomarkers are urgently needed for early detection of breast cancer since the risk of recurrence, morbidity and mortality are closely related to disease stage at the time of primary surgery. In the past decade, many proteomics-based approaches were developed that utilize the protein profiling of human body fluids or identification of putative biomarkers to obtain more knowledge on the effects of cancer emergence and progression. Herein, we report on an analysis of proteins in the tear fluid from breast carcinoma patients and healthy women using a de novo proteomic approach and 25 mixed samples from each group. This study included 25 patients with primary   invasiv  e breast carcinoma and 25 age-matched healthy controls. We performed a MALDI-TOF-TOF-driven semi-quantitative comparison of tear protein levels in cancer (CA) and control (CTRL) using a de novo approach in pooled samples. Over 150 proteins in the tear fluid of CTRL and CA were identified. Using an in-house-developed algorithm we found more than 20 proteins distinctly upregulated or downregulated in the CTRL and CA groups. We identified several proteins that had modified expression in breast cancer patients. These proteins are involved in host immune system pathways (e.g., C1Q1 or S100A8) and different metabolic cascades (ALDH3A or TPI). Further validation of the results in an independent population combined with individual protein profiling of participants is needed to confirm the specificity of our findings and may lead to a better understanding of the pathological mechanism of breast cancer. 
